# Genome-wide association studies of brain imaging phenotypes in UK Biobank

**DOI:** 10.1038/s41586-018-0571-7

**Published:** 2018-10-10

**Authors:** Lloyd T. Elliott, Kevin Sharp, Fidel Alfaro-Almagro, Sinan Shi, Karla L. Miller, Gwenaëlle Douaud, Jonathan Marchini, Stephen M. Smith

**Affiliations:** 1 Department of Statistics, University of Oxford, Oxford, UK; 2 Centre for Functional MRI of the Brain (FMRIB), Wellcome Centre for Integrative Neuroimaging, University of Oxford, Oxford, UK; 3 The Wellcome Centre for Human Genetics, University of Oxford, Oxford, UK

**Keywords:** Genome-wide Association Studies, Single Nucleotide Polymorphisms (SNPs), Phenome-wide Association Study (PheWAS), Genetic Correlation Analysis, GWAS Results, Genetics of the nervous system, Quantitative trait, Genome-wide association studies

## Abstract

The genetic architecture of brain structure and function is largely unknown. To investigate this, we carried out genome-wide association studies of 3,144 functional and structural brain imaging phenotypes from UK Biobank (discovery dataset 8,428 subjects). Here we show that many of these phenotypes are heritable. We identify 148 clusters of associations between single nucleotide polymorphisms and imaging phenotypes that replicate at *P* < 0.05, when we would expect 21 to replicate by chance. Notable significant, interpretable associations include: iron transport and storage genes, related to magnetic susceptibility of subcortical brain tissue; extracellular matrix and epidermal growth factor genes, associated with white matter micro-structure and lesions; genes that regulate mid-line axon development, associated with organization of the pontine crossing tract; and overall 17 genes involved in development, pathway signalling and plasticity. Our results provide insights into the genetic architecture of the brain that are relevant to neurological and psychiatric disorders, brain development and ageing.

## Abstract (web summary)

Genome-wide association studies of brain imaging data from 8,428 individuals in UK Biobank show that many of the 3,144 traits studied are heritable, and genes associated with individual phenotypes are identified.

## Main

Brain structure and function vary between individuals and can be measured non-invasively using magnetic resonance imaging (MRI). The effects of neurological and psychiatric disorders such as Alzheimer’s disease, Parkinson’s disease, schizophrenia, bipolar disorder and autism can be seen in MRI data^1^. MRI can therefore provide intermediate endophenotypes that can be used to assess the genetic architecture of such disorders.

Structural MRI measures of brain anatomy include tissue and structure volumes, such as total grey matter volume and hippocampal volume, while other MRI modalities allow the mapping of different biological markers such as venous vasculature, microbleeds and aspects of white matter microstructure. Brain function is typically measured using task-based functional MRI (fMRI), in which subjects perform tasks or experience sensory stimuli; task-based fMRI uses imaging sensitive to local changes in blood oxygenation and flow caused by brain activity in grey matter. Brain connectivity can be divided into functional connectivity, where spontaneous temporal synchronizations between brain regions are measured using fMRI with subjects scanned at rest, and structural connectivity, measured using diffusion MRI (dMRI), which images the physical connections between brain regions based on how water molecules diffuse within white matter tracts. For those not familiar with the neuroimaging field, we have provided a glossary in Supplementary Note 1.

A new resource for relating neuroimaging to genetics is UK Biobank, a rich, long-term prospective epidemiological study of 500,000 volunteers^2^. Participants were 40–69 years old at recruitment, with one aim being to acquire as rich data as possible before disease onset. Identification of disease risk factors and early markers will increase over time with emerging clinical outcomes^3^. A brain and body imaging extension will scan 100,000 participants by 2020, with brain imaging including three structural modalities, resting and task-based fMRI, and diffusion MRI^4^ (Supplementary Table 1). An automated image processing pipeline removes artefacts and renders images comparable across modalities and participants; it also generates thousands of image-derived phenotypes (IDPs), distinct measures of brain structure and function^5^. Example IDPs include the volume of grey matter in distinct brain regions, and measures of functional and structural connectivity between specific pairs of brain areas. The combination of large subject numbers with multimodal imaging data acquired using homogeneous hardware and software is a unique feature of UK Biobank.

Another key component of the UK Biobank resource has been the collection of genome-wide genetic data using a purpose-designed genotyping array. A custom quality control, phasing and imputation pipeline was developed to address the challenges specific to the experimental design, scale, and diversity of the UK Biobank dataset. The genetic data were publicly released in July 2017 and consist of about 96 million genetic variants in almost 500,000 participants^6^.

Joint analysis of the genetic and brain imaging datasets produced by UK Biobank presents a unique opportunity for uncovering the genetic bases of brain structure and function, including genetic factors that are related to brain development, ageing and disease. In this study, we carried out genome-wide association studies (GWASs) for 3,144 IDPs, covering the entire brain and including ‘multimodal’ information on grey matter volume, area and thickness, white matter connections and functional connectivity, at 11,734,353 single-nucleotide polymorphisms (SNPs) in up to 8,428 individuals with both genetic and brain imaging data. We used two separate sets of data from UK Biobank to evaluate replication of significant genetic associations from the discovery phase. We also carried out multi-trait GWAS, SNP heritability analysis, genetic correlation analysis of IDPs with brain-related traits and an analysis of enrichment of genomic regions with different functions. Previous large-scale GWAS imaging studies have focused on narrower ranges of phenotypes including studies of: grey matter volume in seven subcortical regions by combining data across more than fifty studies^7,8^; whole-brain grey matter volumes and thicknesses by combining data from 59 acquisition sites^9^; and white matter connectivity in healthy young adult twins^10^. We expect that the homogeneous image acquisition and genetic data assay in UK Biobank will boost the power of our study.

The UK Biobank has approval from the North West Multi-centre Research Ethics Committee (MREC) to obtain and disseminate data and samples from the participants (http://www.ukbiobank.ac.uk/ethics/), and these ethical regulations cover the work in this study. Written informed consent was obtained from all participants.

All results are available on the Oxford Brain Imaging Genetics (BIG) web browser (http://big.stats.ox.ac.uk/), which allows users to browse associations by SNP, gene or phenotype. This was built from the PheWeb code base (https://github.com/statgen/pheweb/) and extended to allow easier searching of phenotypes. In addition to the brain IDP GWAS results, the browser also includes GWAS results from more than 2,500 other traits and diseases.

## Heritability and genetic correlations of IDPs

**Fig. 1:**
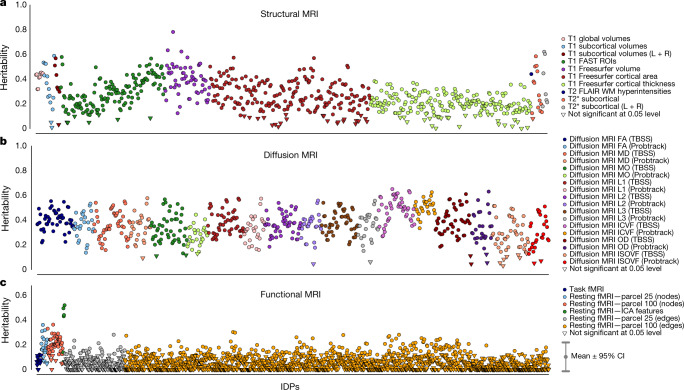
Estimated heritability of IDPs. Estimated heritability (*y*-axis) of all of the IDPs analysed (*n* = 8,428 subjects; see Methods for heritability calculation details). IDPs were split into three broad groups. **a**, Structural MRI. **b**, Diffusion MRI. **c**, Functional MRI. Points are coloured according to IDP groups. Circles and inverted triangles, respectively, are used to identify IDPs that do and do not have heritability significantly different from 0 at the 5% significance level. The mean 95% confidence interval (CI) error bar size is indicated at the bottom right.

Figure 1 shows the estimated SNP heritability (*h*^2^) of all IDPs and whether *h*^2^ is significantly different from 0 at the nominal 5% significance level (Supplementary Table 2, Supplementary Fig. 1). Out of 3,144 IDPs, 1,578 show significant SNP heritability. Of the structural MRI IDPs, volumetric measures are the most heritable and cortical thicknesses the least. Of the diffusion MRI measures, the tractography-based IDPs show lower heritability than the tract-skeleton-based IDPs. The resting-state fMRI functional connectivity edges show the lowest levels of SNP heritability, with just 235 of 1,771 IDPs being significant, which is consistent with additive heritability estimates from twin studies of network edges from fMRI and magnetoencephalography in the Human Connectome Project^11^. However, four of the six resting fMRI features identified by independent component analysis (ICA; estimated as data-driven reductions of this full set of fMRI edges) are much more highly heritable. By contrast, most of the resting-state node amplitude IDPs show significant evidence of SNP heritability; the task-related fMRI IDPs do not.

We found lower levels of SNP heritability for subcortical volumes than previously estimated in twin studies^12–14^ (Supplementary Fig. 2). This is typical of many traits in the literature^15^ and may result from upward bias in twin study estimates due to gene–gene and gene–environment interactions^16,17^, or downward bias of SNP heritability due to uncaptured rare genetic variation. We also compared the GWAS results for seven subcortical volumes with those obtained by the ENIGMA consortium (http://enigma.ini.usc.edu/research/download-enigma-gwas-results/), via a genetic correlation analysis (Supplementary Table 3). There was a strong correlation between the studies, suggesting that there were no major differences in how these phenotypes were measured. In all cases a perfect genetic correlation of 1 lies within the 95% confidence intervals.

Supplementary Fig. 3 shows the genetic correlations, together with the raw phenotype correlations, for several groups of analysed IDPs. There is a range of both strong and weak, positive and negative genetic correlations between the IDPs.

## Significant associations between IDPs and SNPs

In all analyses we estimated genetic effects with respect to the number of copies of the non-reference allele. Using a minor allele frequency filter of 1% and a –log_10_(*P* value) threshold of 7.5, we found 1,262 significant associations between SNPs and the 3,144 IDPs. These associations spanned all classes of IDPs, except task-related fMRI (Supplementary Table 4), with the swMRI T2* group showing a relatively large number of associations. The –log_10_(*P* value) threshold of 7.5 controls for the number of tests carried out across SNPs and accounts for the correlation structure between genetic variants. Of these 1,262 associations, 844 and 455 replicated at the 5% significance level using our two smaller replication datasets (see Methods and Supplementary Table 5). Some associated genetic loci overlapped across IDPs; we estimate that there are approximately 427 distinct associated genetic regions (clusters). One hundred and forty-eight of these clusters have a lead SNP that replicates at the 5% level in our replication set of 3,456 participants, and 91 below a 5% false discovery rate (FDR) threshold. We would expect about 21 of the lead SNPs in the 148 clusters to replicate under a null hypothesis of no association.

At a threshold of −log_10_(*P*) > 11, which additionally corrects for all 3,144 GWAS carried out (see Methods), we found 368 significant associations between genetic regions and distinct IDPs (Supplementary Table 6, Supplementary Fig. 4). These associations with 78 unique SNPs can be grouped together into 38 distinct clusters by grouping across IDPs (Extended Data Table 1). Taking our lead SNP in each of the 38 regions, we found that all 38 had *P* < 0.05 in our replication set of 3,456 participants, and all 38 were significant at 5% FDR. We found no appreciable change in these GWAS results when we included a set of potential body confound measures in addition to the main set of imaging confound measures (see Methods and Supplementary Fig. 5). We also carried out a winner’s curse corrected post-hoc power analysis that agreed well with the results of our replication studies (Supplementary Note 2).

Supplementary Figs. 6 and 7 provide genome-wide association plots (also known as Manhattan plots) and QQ-plots for all 3,144 IDPs and the subset of IDPs listed in Extended Data Table 1, respectively. Having identified a SNP as being associated with a given IDP, it can be useful then to explore the association with all other IDPs via a PheWAS (phenome-wide association study) plot. Supplementary Fig. 8 shows the PheWAS plots for all 78 SNPs listed in Supplementary Table 6 with −log_10_(*P*) > 11. The Oxford Brain Imaging Genetics (BIG) web browser (http://big.stats.ox.ac.uk/) allows researchers to view the PheWAS for any SNP of interest. We found that 4 of the 78 SNPs were associated (*P* < 0.05/3,144; that is, −log_10_(*P*) > 4.79) with all 3 classes of structural, dMRI and functional measures, and these were all SNPs in cluster 31 of Extended Data Table 1 (Supplementary Fig. 8, pages 62–65). This genetic locus is associated with the volume of the precuneus and cuneus, dMRI measures for the forceps major (a fibre bundle that connects the left and right cuneus), and two functional connections (parcellation 100 edges 614 and 619, which connect the precuneus to other cognitive networks). Supplementary Fig. 9 illustrates the sharing of association signal across IDPs at the 615 unique SNPs listed in Supplementary Table 5. Supplementary Fig. 10 shows the relationship between the number of associations found and the estimated SNP heritability for each IDP.

Overall, our results clearly replicate the majority of the loci identified by the ENIGMA consortium in two separate GWASs of seven brain subcortical volume IDPs in up to 13,171 subjects^7^, and of hippocampal volume in 33,536 subjects (although not all reached genome-wide significance, probably owing to the smaller sample size in our study; Supplementary Fig. 11). We also replicate an association between volume of white matter hyperintensities (‘lesions’) and SNPs in *TRIM47* (for example, rs3744017, *P* = 1.4 × 10^−12^, cluster 37)^18^.

It can be challenging to interpret precisely the function of SNPs identified in a GWAS. Most of the SNPs in the 38 loci in Extended Data Table 1 are either in genes, including 7 missense SNPs and 2 SNPs in untranslated regions (UTRs), or in high linkage disequilibrium with SNPs that are themselves in the genes of interest, and many are significant expression quantitative trait loci (eQTLs) in the GTEx database^19^. In total, we found 17 genetic loci that can be linked to genes that broadly contribute to brain development, patterning and plasticity (out of the 38 clusters reported in Extended Data Table 1; for more details, see Supplementary Note 3). Below we focus on some of the most compelling examples.

A major source of cross-subject differences seen in T2* data are microscopic variations in magnetic field, often associated with iron deposition in ageing and pathology^20^. We identified many associations between T2* in the caudate nucleus, putamen and pallidum and SNPs in genes (*TF*, rs4428180, *P* = 2.23 × 10^−22^; *HFE*, rs1800562, *P* = 6.6 × 10^−20^; *SLC25A37*, rs35469695, *P* = 2.22 × 10^−12^) or near genes (*FTH1*, rs11230859, *P* = 2.31 × 10^−17^) that are known to affect iron transport and storage, or neurodegeneration with brain iron accumulation (NBIA)^21^ (*COASY*, rs668799, *P* = 1.43 × 10^−17^). In addition, we identified four SNPs that either encode or are eQTLs of genes involved in transport of nutrients and minerals: *SLC44A5* (rs76934732, *P* = 8.51 × 10^−13^), *SLC39A8* (also known as *ZIP8*; rs13107325, *P* = 1.04 × 10^−42^), *SLC20A2* (rs2923405, *P* = 3.31 × 10^−17^) and *SLC39A12* (also known as *ZIP12*; rs10764176, *P* = 3.3 × 10^−21^). For more details, see Supplementary Note 3.

**Fig. 2:**
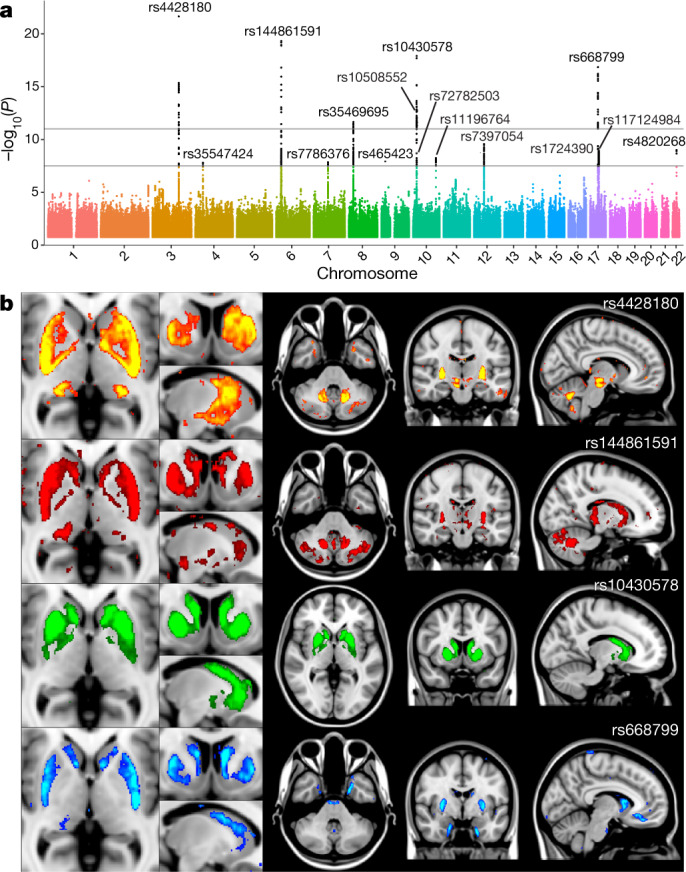
Manhattan plot and spatial mapping of the associations between T2* in the putamen and four SNPs. **a**, The Manhattan plot relates to the original GWAS for the IDP T2* in the bilateral putamen. The lower grey line indicates the –log_10_(*P* value) threshold of 7.5 and the upper line the threshold of 11 (see main text). **b**, The spatial maps show that the four SNPs (one per row) most strongly associated with T2* in the putamen have distinct voxelwise patterns of effect across the whole brain: the effect of rs4428180 (*TF*) is found in the dorsal putamen and body of the caudate nucleus, but also in the right subthalamic nucleus and substantia nigra, red nucleus, lateral geniculate nucleus of the thalamus and dentate nucleus; rs144861591 (*HFE*) in the dorsal striatum, subthalamic nucleus, dentate nucleus and Crus I/II of the cerebellum; rs10430578 (*SLC39A12*) in the whole dorsal striatum and pallidum; and rs668799 (*COASY*) in the whole dorsal striatum, subgenual cingulate cortex and entorhinal cortex. The standard MNI152 T1 image is used as background for the spatial maps (left is right). All group difference images (colour overlays) are thresholded at a T2* difference of 0.6 ms. These voxelwise SNP association maps were calculated from the discovery sample of 8,428 subjects (see main text).

**Extended Data Fig. 1:**
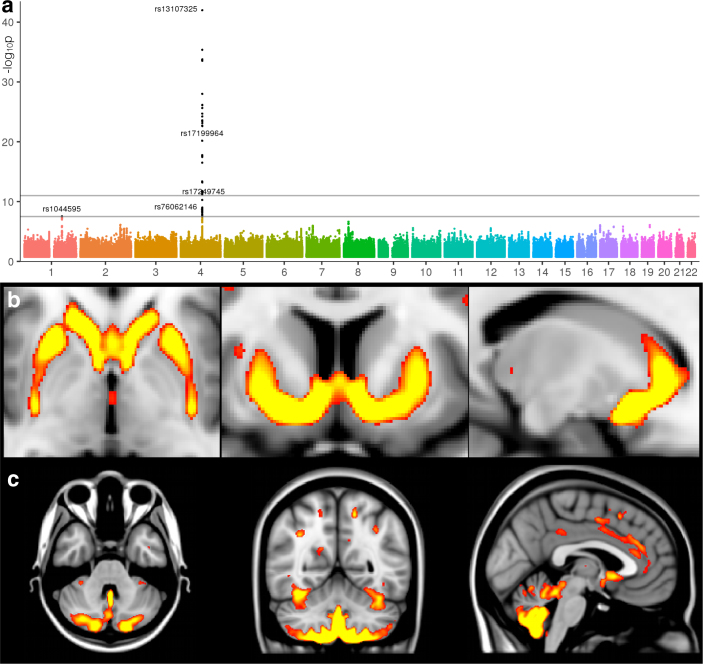
Manhattan plot and spatial mapping of the associations between grey matter volume and rs13107325 (*SLC39A8*). **a**, The Manhattan plot relates to the original GWAS for the IDP of grey matter volume in the left ventral striatum. **b**, **c**, Spatial mapping of rs13107325 against voxelwise local grey matter volume (grey matter was averaged across all 1,181 subjects with one copy of the non-reference allele, and the average from all 7,215 subjects that had zero copies was subtracted from that, for display in colour here; the difference was thresholded at 0.015 (unitless relative measure of local grey matter volume)). The maps show that the effect of rs13107325 is found more generally bilaterally in the ventral caudate, putamen, ventral striatum, anterior cingulate cortex, and with a strong cerebellar contribution (lobules VI–X), particularly in the prefrontal-projecting Crus I/II, which are selectively expanded in humans.

Interrogating images at a voxel-wise level can provide further insight about the detailed spatial localization of SNP associations and can possibly identify additional associated areas not already well captured by IDPs (while keeping in mind the statistical dangers of potential circularity^22^). For instance, by looking at the difference between the average T2* image from subjects with no copies versus one copy of the rs4428180 (*TF*) non-reference allele, we found effects of this SNP not just in the putamen and pallidum, but also in additional, smaller regions of subcortical structures not included as IDPs (Fig. 2). We similarly created in Fig. 2 the voxelwise differences associated with three additional SNPs, from the most significant GWAS associations with T2* in the putamen as seen in the Manhattan plot. This approach also allowed us to observe grey matter volume effects across the entire brain associated with rs13107325 (*SLC39A8*; Extended Data Fig. 1), which has been linked in previous (non-imaging) GWASs to intelligence^23^, schizophrenia^24^, blood pressure^25^ and higher risk of cardiovascular death^26^. These effects could now be observed in a relevant brain region, the anterior cingulate cortex, which has multifaceted roles including in fluid intelligence^27^, schizophrenia^28^ and modulating autonomic states of cardiovascular arousal^29^.

**Fig. 3:**
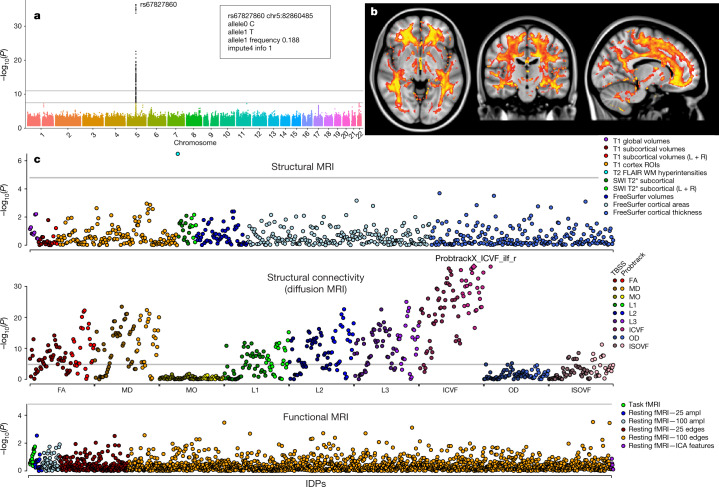
Manhattan plot, spatial mapping and PheWAS plot relating to the association between the dMRI ICVF measure and rs67827860 (*VCAN*). **a**, The Manhattan plot relates to the original IDP GWAS with the strongest association (ICVF in the right inferior longitudinal fasciculus using tractography, associated with rs67827860). The ICVF parameter, estimated from the NODDI modelling^36^, aims to quantify predominantly intra-axonal water in white matter, by estimating where water diffusion is restricted. Summary details of SNP rs67827860 are given in the top right box. The lower grey line indicates the –log_10_(*P* value) threshold of 7.5 and the upper line the threshold of 11. **b**, Spatial mapping of rs67827860 against voxelwise ICVF in white matter (ICVF was averaged across all 4,957 subjects with zero copies of the non-reference allele, and the average from all 2,304 subjects that had one copy was subtracted from that, for display in colour here; the difference was thresholded at 0.005 (unitless fractional measure)). Unlike the examples of (spatially) very focal effects in T2* and grey matter volume in Fig. 2 and Extended Data Fig. 1, the effects of this SNP are extremely widespread across most of the white matter tracts (associated with 45 out of the 199 IDPs in cluster 11, Supplementary Table 5). **c**, The PheWAS plot for SNP rs67827860 shows the association (−log_10_(*P*)) on the *y*-axis for the SNP with each of the 3,144 IDPs. The IDPs are arranged on the *x*-axis in the three panels: structural MRI IDPs (top), dMRI IDPs (middle) and fMRI IDPs (bottom). Points are coloured to delineate subgroups of IDPs. Grey lines show the Bonferroni multiple testing threshold of 4.79. In addition to the IDP of white matter hyperintensities volume, there is a notable association with numerous dMRI IDPs (especially diffusion tensor-derived measures of fractional anisotropy, mean diffusivity and L1, L2 and L3 eigenvalues of the diffusion tensor, as well as additional ICVF measures).

Notably, three SNPs related to our white matter IDPs were in genes or eQTLs of genes encoding three proteins of the extracellular matrix (ECM): rs2365715 (*P* = 5.38 × 10^−12^), an eQTL of *BCAN*, is associated with one dMRI microstructural measure in the genu of the corpus callosum; rs3762515 (*P* = 4.27 × 10^−13^), in the 5′ UTR of *EFEMP1*, with the volume of white matter lesions; and rs67827860 (*P* = 4.06 × 10^−37^, Fig. 3), located in an intron of *VCAN*, with multiple dMRI measures of most white matter tracts (199 IDPs in total). Overall, the vast majority of forebrain white matter-related dMRI IDPs were associated with SNPs related to genes that encode proteins involved in the extracellular matrix and epidermal growth factor signalling. These proteins have key roles in synaptic plasticity and myelin repair, and are associated with multiple sclerosis, stroke, amyotrophic lateral sclerosis and major depressive disorder (Supplementary Note 3).

**Fig. 4:**
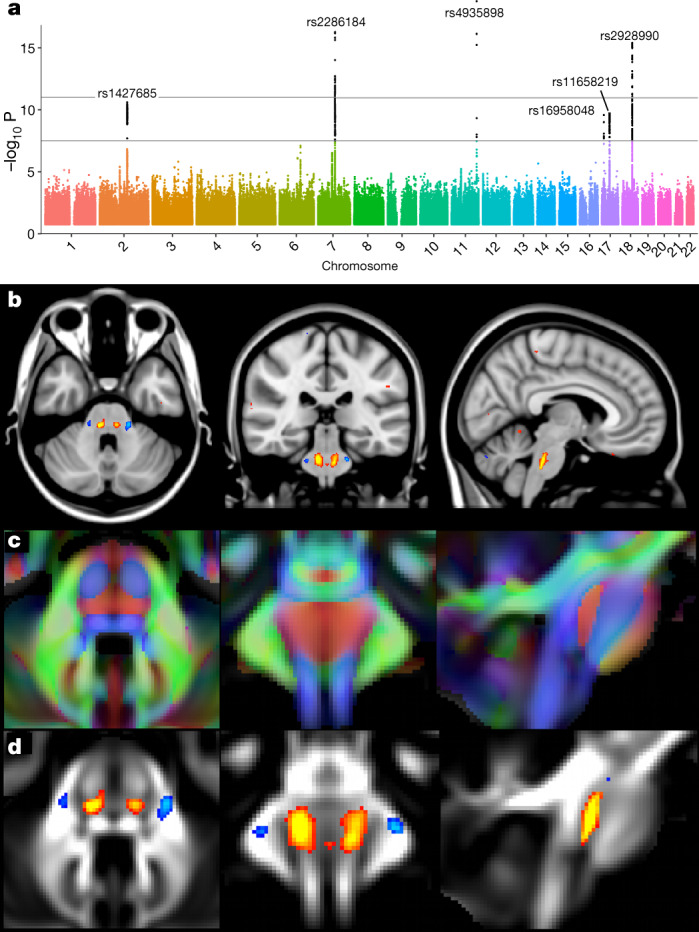
Manhattan plot and spatial mapping of the association between the dMRI tensor mode measure and SNP rs4935898 (*ROBO3*). **a**, The Manhattan plot relates to the original GWAS for the IDP of tensor mode in the crossing pontine tract associated with rs4935898. **b**–**d**, Tensor mode was averaged across all 6,807 subjects with approximately zero copies of the non-reference allele, and the average from all 703 subjects that had approximately one copy was subtracted from that, for display in red/yellow–blue/light blue here, thresholded at 0.05 (**b**, **d**). **b**, Results are shown overlaid on the MNI152 T1 structural image; by contrast, background in **c** and **d** is the UK Biobank average fractional anisotropy image, which shows clear tract structure within the brainstem. **c**, Orientation of the fibre tracts (in red, running left to right). The spatial distribution (not shown) for the effects of rs2286184 (*SEMA3D*) on tensor mode is almost identical to that of rs4935898, being again extremely spatially specific, with no extended effect elsewhere in the brain. These voxelwise SNP association maps were calculated from the discovery sample of 8,428 subjects (see main text).

Two additional examples further illustrate meaningful correspondences between the locations of our brain IDPs and significantly associated genes. First, the volume of the fourth ventricle, which develops from the central cavity of the neural tube, was found to be significantly associated with a SNP in, and eQTL of, *ALDH1A2* (rs2642636, *P* = 5.2 × 10^−16^). This gene encodes an enzyme that facilitates posterior organ development and prevents human neural tube defects, including spina bifida^30^. Second, we found two SNPs associated with dMRI IDPs of the crossing pontine tract (the part of the pontocerebellar fibre bundle that arises from the pontine nuclei and decussates across the brain midline to project to the contralateral cerebellar cortex) in genes that regulate axon guidance and fasciculation during development (*SEMA3D*, rs2286184, *P* = 5.31 × 10^−17^ and *ROBO3*, rs4935898 (missense), *P* = 1.76 × 10^−19^; Fig. 4). The exact location of our IDP in the crossing fibres of the pons coincides with the function of *ROBO3*, which is specifically required for axons to cross the midline in the hindbrain (pons, medulla oblongata and cerebellum); mutations in *ROBO3* result in horizontal gaze palsy, a disorder in which the corticospinal and somatosensory axons fail to cross the midline in the medulla^31^. Notably, all three significant associations with the IDP of the crossing pontine tract were found using the tensor mode of anisotropy (MO), a measure that is particularly useful in regions of crossing fibres^32^.

## Multi-phenotype association tests

One alternative strategy for analysing large numbers of IDPs is to use multi-trait tests that fit joint models of associations to groups of IDPs. Such approaches can use estimates of genetic correlation to boost power. In addition, by analysing *P* traits in one GWAS, these tests can avoid the need to correct for multiple genome-wide scans. We used a multi-trait test (see Methods) to analyse 23 groups of IDPs with up to 243 IDPs per group. These IDP groups were chosen to cover the majority of the IDP classes with significant IDP correlations in each grouping (Supplementary Table 7). Supplementary Fig. 12 shows the Manhattan plots for these genome-wide scans. Overall, across these 23 groups, we found 278 SNPs at about 160 loci associated with –log_10_(*P*) > 7.5 (Supplementary Table 8). Of these 278 SNPs, 170 survived a correction for 23 scans with –log_10_(*P*) > 8.86 and 138 of these 170 SNPs had a *P* value < 0.05 in the larger replication set of 3,456 samples. There can be large differences in *P* values between the multi-trait tests and the individual IDP tests (Supplementary Fig. 13), especially when taking account of the smaller number of tests carried out by the multi-trait approach (Supplementary Fig. 14). We found 25 loci that showed both a significant and replicated multi-trait association for an IDP group, while showing no genome-wide significance in the flanking region for any individual IDP in the corresponding group (Supplementary Table 9, Supplementary Note 3).

Three of these loci showed associations with the dMRI tensor mode of anisotropy measures (rs62073157, *P* = 4.07 × 10^−11^; rs35884657, *P* = 1.04 × 10^−9^; rs9939914, *P* = 1.15 × 10^−11^) and all were eQTLs of microtubule-related genes (*MAPT*, *TUBA1B* and *TUBB3*, respectively). The extended *MAPT* region has been repeatedly associated with Alzheimer’s and Parkinson’s diseases, frontotemporal dementia, and progressive supranuclear palsy (Supplementary Note 3).

Another example of the value of multi-trait testing can be seen in the association between IDPs of global brain volume measurements and an SNP located between *BANK1* and *SLC39A8*, which was previously identified in a GWAS of schizophrenia^33^ (rs35518360, *P* = 4.07 × 10^−12^). This locus is also part of a multimodal cluster from our single-trait GWAS that includes subcortical and cerebellar grey matter volumes, pallidum T2* and dMRI in midbrain white matter tracts (cluster 10 in Supplementary Table 6). The multi-trait test thus made it possible to uncover this additional association between global brain volume measurement and this locus, which might prove relevant for better understanding observations of smaller brain volume in (particularly first episode or drug-naive) patients with schizophrenia^34^.

## Genetic correlation with clinically relevant traits

We measured the genetic correlation between a subset of heritable IDPs and ten neurodegenerative, psychiatric and personality traits (see Methods). We found suggestive evidence of genetic correlation for amyotrophic lateral sclerosis (ALS), schizophrenia and stroke, mainly with dMRI measures in white matter tracts (Supplementary Fig. 15). Supplementary Table 10 contains genetic correlation estimates for all IDP–trait combinations; see Supplementary Note 4 for further details.

## Partitioning heritability by functional annotation

**Extended Data Fig. 2:**
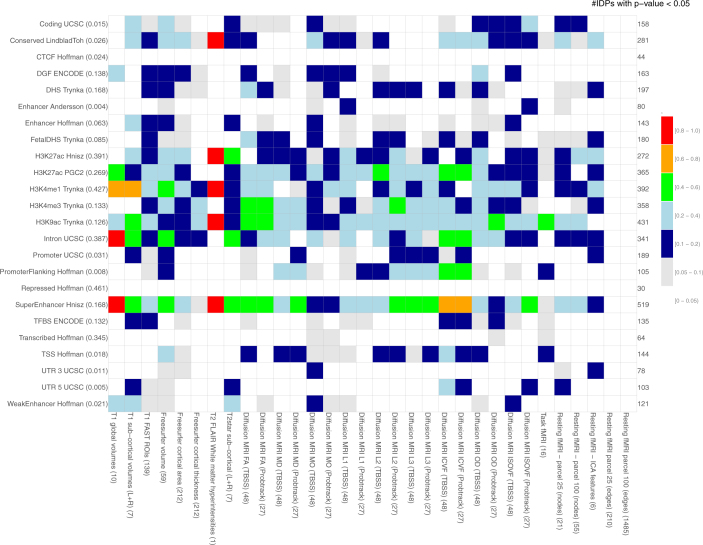
Partitioning of heritability by functional category. The plot shows the proportion of IDPs in each of the 23 IDP groupings (*x*-axis) that show a nominal enrichment *P* value <0.05 (two-sided tests, uncorrected *P* values, see Methods) for the 24 functional categories (*y*-axis). The total number of such IDPs for each category is given on the right edge of the plot. The number of IDPs in each IDP group is given in parentheses in the *x*-axis labels. The proportion of the genome annotated by each functional category is given in parentheses in the *y*-axis labels.

We applied a statistical approach that partitions the additive genetic heritability of a set of common variants for each of the 3,144 IDPs according to 24 functional annotations of the genome^35^. Extended Data Fig. 2 summarizes which functional annotations show enrichment stratified by 23 groups of IDPs (Supplementary Table 11). We find that regions of the genome annotated as super enhancers and several histone modifications show enrichment across many of the structural and diffusion IDP groups. Regions of the genome enriched for trimethylation of lysine 27 on histone H3 (H3K27me3) (and indicating strong evidence for silenced genes) show depletion of heritability across many of the IDP classes (Supplementary Fig. 16). IDP groups such as T1 subcortical volumes, dMRI fractional anisotropy (FA) and intracellular volume fraction (ICVF) show the strongest evidence of enrichment across multiple categories. The resting fMRI connectivity edge IDPs show no elevated enrichment, consistent with these traits showing low heritability (Fig. 1). Supplementary Fig. 17 shows this partitioning analysis for each IDP.

## Conclusions

Bringing together researchers with backgrounds in brain imaging and genetic association was key to this work. We have uncovered a large number of associations at the nominal level of GWAS significance (−log_10_(*P*) > 7.5) and at a more stringent threshold (−log_10_(*P*) > 11) designed to (probably over-conservatively) control for the number of IDPs tested. Our use of multi-trait tests uncovered further novel loci. We find associations with all the main IDP groups except the task fMRI measures (despite these measures containing usable signal, for example having unique cognitive associations^4^).

We mainly found associations between MRI measures and genes involved in brain development and plasticity, as well as genes contributing to the transport of iron, nutrients and minerals (Supplementary Note 3). The genes linked to brain development and plasticity tended to be related to mental health disorders, including major depression disorder and schizophrenia, whereas those that encoded iron-related proteins tended to be related to neurodegenerative disorders, such as amyotrophic lateral sclerosis, Parkinson’s disease and Alzheimer’s disease. We also uncovered enrichments of functional annotations for many of the structural and diffusion IDPs.

A valuable aspect of this work has been to link the associated SNPs back to spatial properties of the voxel-level brain imaging data. For example, we have linked SNPs associated with IDPs to both highly spatially localized and widely spatially distributed effects, restricting these voxelwise analyses to the same imaging modality from which the original phenotypic association was found (though of course other modalities could also be tested in the same way). In addition, looking at PheWAS plots has been useful when working with so many phenotypes. It has allowed us to investigate the overall patterns of association and has led to the identification of SNP associations that span multiple modalities.

We used two additional sets of 930 and 3,456 samples to replicate a large number of the associations uncovered at the discovery phase. Over coming years, the number of UK Biobank participants for whom imaging data are available will increase to 100,000, allowing more complete discovery of the genetic basis of human brain structure, function and connectivity. Combining the discovery and replication samples is also likely to lead to novel associations, as will the use of methods that can analyse the huge IDP × SNP matrix of summary statistics of association. A potential avenue of research will involve attempts to uncover causal pathways that link genetic variants to IDPs and then to a range of neurological, psychiatric and developmental disorders.

## Methods

### Imaging data and derived phenotypes

The UK Biobank brain imaging protocol consists of six distinct modalities covering structural, diffusion and functional imaging, summarized in Supplementary Table 1. For this study, we primarily used data from the February 2017 release of ~10,000 participants’ imaging data (and an additional ~5,000 subjects’ data released in January 2018 provided the larger replication sample).

The raw data from these six modalities have been processed for UK Biobank to create a set of IDPs^4,5^. These are available from UK Biobank, and it is these IDPs from the 2017–2018 data releases that we used in this study.

In addition to the IDPs directly available from UK Biobank, we created two extra sets of IDPs. First, we used FreeSurfer v6.0.0^37,38^ (https://surfer.nmr.mgh.harvard.edu) to model the cortical surface (inner and outer 2D surfaces of cortical grey matter), as well as modelling several subcortical structures. We used both the T1 and T2 FLAIR images as inputs to the FreeSurfer modelling (or just the T1 when the T2 was not available). FreeSurfer estimates a large number of structural phenotypes, including volumes of subcortical structures, surface area of parcels identified on the cortical surface, and grey matter cortical thickness within these areas. The areas are defined by mapping an atlas containing a canonical cortical parcellation onto an individual subject’s cortical surface model, thus achieving a parcellation of that surface. Here we used two atlases in common use with FreeSurfer: the Desikan-Killiany–Tourville atlas (denoted DKT^39^) and the Destrieux atlas (denoted a2009s^40^). The DKT parcellation is gyrus-based, whereas Destrieux aims to model both gyri and sulci based on the curvature of the surface. Cortical thickness is averaged across each parcel from each atlas, and the cortical area of each parcel is estimated, to create two IDPs for each parcel. Finally, subcortical volumes are estimated, to create a set of volumetric IDPs.

Second, we applied a dimension reduction approach to the large number of functional connectivity IDPs. Functional connectivity IDPs represent the network edges between many distinct pairs of brain regions, comprising in total 1,695 distinct region-pair brain connections (http://www.fmrib.ox.ac.uk/ukbiobank/). In addition to this being a very large number of IDPs from which to interpret association results, these individual IDPs tend to be substantially noisier than most of the other, more structural, IDPs. Hence, while we did carry out GWAS for each of these 1,695 connectivity IDPs, we also reduced the full set of connectivity IDPs into just six new summary IDPs using data-driven feature identification. We performed this dimensionality reduction by applying ICA^41^, applied to all functional connectivity IDPs from all subjects, to find linear combinations of IDPs that are independent between the different features (ICA components) identified^42^. We carried out the ICA feature estimation without any use of the genetic data, and we maximized independence between component IDP weights (as opposed to subject weights). We used split-half reproducibility (across subjects) to optimize both the initial dimensionality reduction (14 eigenvectors from a singular value decomposition was found to be optimal) and also the final number of ICA components (6 ICA components was optimal, with reproducibility of ICA weight vectors greater than *r* = 0.9). The resulting six ICA features were then treated as new IDPs, representing six independent sets (or, more accurately, linear combinations) of the original functional connectivity IDPs. These six new IDPs were added into the GWAS analyses. The six ICA features explain 4.9% of the total variance in the full set of network connection features, and are visualized in Supplementary Fig. 18. More details of the ICA analysis of the resting state data, together with browsing functionality of the highlighted brain regions can be found on the FMRIB UK Biobank Resource web page (http://www.fmrib.ox.ac.uk/ukbiobank/).

We organized all 3,144 IDPs into 9 groups (Supplementary Table 12), each with a distinct pattern of missing values (not all subjects have usable, high-quality data from all modalities^4^). For the GWAS in this study we did not try to impute missing IDPs owing to the low levels of correlation observed across groups.

The distributions of IDP values varied considerably between phenotype classes, with some phenotypes exhibiting substantial skew (Supplementary Fig. 19) that would probably invalidate the assumptions of the linear regression used to test for association. To ameliorate this, we quantile-normalized each of the IDPs before association testing. This transformation also helped to avoid undue influence of outlier values. We also (separately) tested an alternative process in which an outlier removal process was applied to the untransformed IDPs; this gave very similar results for almost all association tests, but was found to reduce the significance of a very small number of associations. This possible alternative method for IDP preprocessing was therefore not followed through (data not shown).

No statistical methods were used to predetermine sample size. The experiments were not randomized and the investigators were not blinded to allocation during experiments and outcome assessment.

### Genetic data processing

We used the imputed genetic dataset made available by UK Biobank in its July 2017 release^6^. This consists of >92 million autosomal variants imputed from the Haplotype Reference Consortium (HRC) reference panel^43^ and a merged UK10K + 1000 Genomes reference panel. We first identified a set of 12,623 participants who had also been imaged by UK Biobank. We then applied filters to remove variants with minor allele frequency (MAF) below 0.1% and with an imputation information score below 0.3, which reduced the number of SNPs to 18,174,817. We then kept only those samples (subjects) estimated to have recent British ancestry using the sample quality control information provided centrally by UK Biobank^6^ (using the variable *in.white.British.ancestry.subset* in the file ukb_sqc_v2.txt); population structure can be a serious confound to genetic association studies^44^, and this type of sample filtering is standard. This reduced the number of samples to 8,522. The UK Biobank dataset contains a number of close relatives (third cousins or closer). We therefore created a subset of 8,428 nominally unrelated subjects following procedures similar to those described previously^6^. After running GWAS on all the (SNP) variants in the 8,428 samples we applied three further variant filters to remove variants with a Hardy–Weinberg equilibrium *P* value <10^−7^, remove variants with MAF <0.1% and keep only those variants in the HRC reference panel. This resulted in a dataset with 11,734,353 SNPs.

We used two separate datasets to replicate the associated variants found in this study. The first set of 930 subjects was a subset of the 1,279 subjects with imaging data that we did not use for the main GWAS, who had primarily been excluded because they were not in the recent British ancestry subset. An examination of these samples according the genetic principal components (PCs) revealed that many of those samples are mostly of European ancestry (Supplementary Fig. 20). We selected 930 samples with a first genetic PC <14 from Supplementary Fig. 20 and these constituted the replication sample. In January 2018 a further tranche of 4,588 samples with imaging data was released by UK Biobank. Of these subjects, we selected 3,956 subjects that both had genetic data available and also had been imaged in the same imaging centre as the discovery sample. We applied the same pre-processing pipeline as for the discovery set. We then restricted this to 3,456 subjects that were of recent British ancestry and replication tests were then conducted on these 3,456 subjects.

### Potential confounds for brain IDP GWAS

There are a number of potential confounding variables when carrying out GWASs of brain IDPs. We used three sets of covariates in our analyses relating to (a) imaging confounds (b) measures of genetic ancestry, and (c) non-brain imaging body measures.

We identified a set of variables that were likely to represent imaging confounds, for example those associated with biases in noise or signal level, corruption of data by head motion or overall head size changes. For many of these we generated various versions (for example, using quantile normalization and also outlier removal, to generate two versions of a given variable, as well as including the squares of these to help model nonlinear effects of the potential confounds). This was done in order to generate a rich set of covariates and hence reduce as much as possible potential confounding effects on analyses such as the GWAS, which are particularly of concern when the subject numbers are so high^4,45^.

Age and sex are can be variables of biological interest, but can also be sources of imaging confounds, and here were included in the confound regressors. Head motion is summarized from resting and task-based fMRI as the mean displacement (in mm) between one time point and the next, averaged over all time points and across the brain. Head motion can be a confounding factor for all modalities and not just those comprising timeseries of volumes, but is readily estimable only from the timeseries modalities. Nevertheless, the amount of head motion is expected to be reasonably similar across all modalities (for example, correlation between head motion in resting and task fMRI is *r* = 0.52) and so it is worth using fMRI-derived head motion estimates as confound regressors for all modalities.

The exact location of the head and the radio-frequency receiver coil in the scanner can affect data quality and IDPs. To help to account for variations in position in different scanned participants, several variables have been generated that describe aspects of the positioning (see http://biobank.ctsu.ox.ac.uk/showcase/field.cgi?id=25756, http://biobank.ctsu.ox.ac.uk/showcase/field.cgi?id=25757, http://biobank.ctsu.ox.ac.uk/showcase/field.cgi?id=25758, and http://biobank.ctsu.ox.ac.uk/showcase/field.cgi?id=25759). The intention is that these can be useful as ‘confound variables’; for example, these might be regressed out of brain IDPs before carrying out correlations between IDPs and non-imaging variables. TablePosition is the *Z*-position of the coil (and the scanner table on which the coil sits) within the scanner (the *Z* axis points down the centre of the magnet). BrainCoGZ is somewhat similar, being the *Z*-position of the centre of the brain within the scanner (derived from the brain mask estimated from the T1-weighted structural image). BrainCoGX is the *X*-position (left–right) of the centre of the brain mask within the scanner. BrainBackY is the *Y*-position (front–back relative to the head) of the back of brain mask within the scanner.

UK Biobank brain imaging aims to maintain as fixed an acquisition protocol as possible during the 5–6 years that the scanning of 100,000 participants will take. There have been a number of minor software upgrades (the imaging study seeks to minimize any major hardware or software changes). Detailed descriptions of every protocol change, along with thorough investigations of the effects of these on the resulting data, will be the subject of a future paper. Here, we attempted to model any long-term (over scan date) changes or drifts in the imaging protocol or software or hardware performance, by generating a number of data-driven confounds. The first step was to form a temporary working version of the full subjects × IDPs matrix with outliers limited (see below) and no missing data, using a variant of low-rank matrix imputation with soft thresholding on the eigenvalues^46^. Next, the data were temporally regularized (approximate scale factor of several months with respect to scan date, see https://biobank.ctsu.ox.ac.uk/showcase/field.cgi?id=53, Instance 2) with spline-based smoothing. We then applied PCA and kept the top 10 components, to generate a basis set that reflects the primary modes of slowly changing drifts in the data.

To describe the full set of imaging confounds we use a notation where subscript *i* indicates quantile normalization of variables, and *m* indicates median-based outlier removal (discarding values greater than five times the median absolute deviation from the overall median). If no subscript is included, no normalization or outlier removal was carried out. Certain combinations of normalization and powers were not included, either because of very high redundancy with existing combinations, or because a particular combination was not well-behaved. The full set of variables used to create the confounds matrix are: *a*, age at time of scanning, demeaned (cross-subject mean subtracted); *s*, sex, demeaned; *q*, four confounds relating to the position of the radio-frequency coil and the head in the scanner (see above), all demeaned; *d*, ten drift confounds (see above); *m*, two measures of head motion (one from resting fMRI, one from task-based fMRI); and *h*, volumetric scaling factor needed to normalize for head size^47^.

The full matrix of imaging confounds is then:

$$[a\hspace{5pt}{a}^{2}\hspace{5pt}a\times s\hspace{5pt}{a}^{2}\times s\hspace{5pt}{a}_{i}\hspace{5pt}{a}_{i}^{2}\hspace{5pt}{a}_{i}\times s\hspace{5pt}{a}_{i}^{2}\times s\hspace{5pt}{m}_{m}\hspace{5pt}{m}_{m}^{2}\hspace{5pt}{h}_{m}\hspace{5pt}{q}_{m}\hspace{5pt}{q}_{m}^{2}\hspace{5pt}{d}_{m}\hspace{5pt}{m}_{i}\hspace{5pt}{h}_{i}\hspace{5pt}{q}_{i}\hspace{5pt}{q}_{i}^{2}\hspace{5pt}{d}_{i}]$$

Any missing values in this matrix are set to zero after all columns have had their mean subtracted. This results in a full-rank matrix of 53 columns (ratio of maximum to minimum eigenvalues is 42.6). Additional discussion on the dangers and interpretation of imaging confounds in big imaging data studies, particularly in the context of disease studies, has been published^45^.

Genetic ancestry is a well-known potential confound in GWAS. We ameliorated this by filtering out samples that were not of recent British ancestry. However, a set of 40 genetic principal components (PCs) has been provided by UK Biobank^6^, and we used these PCs as covariates in all of our analyses. The matrix of imaging confounds, together with a matrix of 40 genetic principal components, was regressed out of each IDP before the analyses reported here.

There exist a number of substantial correlations between IDPs and non-genetic variables collected on the UK Biobank subjects^4^. We therefore also carried out some analyses involving variables relating to blood pressure (diastolic and systolic), height, weight, head bone mineral density, head bone mineral content and two principal components from the broader set of bone mineral variables available (https://biobank.ctsu.ox.ac.uk/crystal/docs/DXA_explan_doc.pdf). Supplementary Fig. 21 shows the association of these eight variables against the IDPs and shows significant associations. These are variables that are likely to have a genetic basis, at least in part. Genetic variants associated with these variables might then produce false positive associations for IDPs. To investigate this possibility, we ran GWASs for these eight traits (conditioned on the imaging confounds and genetic PCs) (Supplementary Fig. 22). We also ran a parallel set of IDP GWASs with these ‘body confounds’ regressed out of the IDPs.

### Heritability and genetic correlation of IDPs

We used a linear mixed model implemented in the SBAT (sparse Bayesian association test) software (https://jmarchini.org/sbat/) to calculate additive genetic heritabilities for the *P* = 3,144 traits. To estimate genetic correlations we used a multi-trait mixed model. If *Y* is an *N* × *P* matrix of *P* phenotypes (columns) measured on *N* individuals (rows) then we use the model:

1 $$Y=U+\varepsilon $$

where *U* is an *N* × *P* matrix of random effects and *ε* is an *N* × *P* matrix of residuals, and these are modelled using Matrix normal distributions as follows:

$$U \sim MN\left(0,K,B\right)$$

$$\varepsilon  \sim MN\left(0,{I}_{N},E\right)$$

In this model, *K* is the *N* × *N* kinship matrix between individuals, *B* is the *P* × *P* matrix of genetic covariances between phenotypes and *E* is the *P* × *P* matrix of residual covariances between phenotypes. We estimate the covariance matrices *B* and *E* using a new C++ implementation of an EM algorithm^48^ included in the SBAT software (https://jmarchini.org/sbat/).

For the marginal heritabilities and genetic correlation analysis we used a realized relationship matrix (RRM) for the kinship matrix (*K*). This RRM was calculated from the 8,428 nominally unrelated individuals using fastLMM (https://github.com/MicrosoftGenomics/FaST-LMM). We used the subset of imputed SNPs that were both assayed by the genotyping chips and included in the HRC reference panel, and so will essentially be hard-called genotypes. In addition, all SNPs with duplicate rsids (reference SNP cluster IDs) were removed. PLINK (http://www.cog-genomics.org/plink/2.0/) was used for file conversion before input into fastLMM.

To estimate genetic correlations, we fit the model to several of the groupings of IDPs detailed in Supplementary Table 12. The estimated covariance matrices *B* and *E* were used to estimate the genetic correlation of pairs of IDPs. The genetic correlation between the *i*th and *j*th IDPs in a jointly analysed group of IDPs is estimated as

$${r}_{ij}=\frac{{B}_{ij}}{\sqrt{{B}_{ii}{B}_{jj}}}$$

### Multi-trait association tests

We used a multi-trait mixed model to test each SNP for association with different groupings of traits (Supplementary Table 7). The model has the form *Y* = *Gα* + *U* + *ε*, where *G* is an *N* × 1 vector of SNP dosages and *α* is a 1 × *P* vector of effect sizes. We fit the model using estimates of *B* and *E* from the ‘null’ model with *α* = 0 and a leave one chromosome out (LOCO) approach for RRM calculation. We ran this test on the main set of 8,428 samples and on the replication samples. For the replication analysis we used the estimates of *B* and *E* from the main set of 8,428 samples. This test was implemented in SBAT software.

### Genetic association of IDPs

We used BGENIE v1.2 (https://jmarchini.org/bgenie/) to carry out GWASs of imputed variants against each of the processed IDPs. This program was designed to carry out the large number of IDP GWAS required in this analysis. It avoids repeated reading of the genetic data file for each IDP and uses efficient linear algebra libraries and threading to achieve good performance. The program has already been used by several studies to analyse genetic data from the UK Biobank^49,50^. We fit an additive model of association at each variant, using expected genotype count (dosage) from the imputed genetic data. We ran associated tests on the main set of 8,428 samples and the replication samples.

### Identifying associated genetic loci

Most GWAS analyse only one or a few different phenotypes, and often uncover just a handful of associated genetic loci, which can be interrogated in detail. Owing to the large number of associations uncovered in this study, we developed an automated method to identify, distinguish and count individual associated loci from the 3,144 GWASs (one GWAS for each IDP). For each GWAS we first identified all variants with –log_10_(*P*) > 7.5. We applied an iterative process that starts by identifying the most strongly associated variant, storing it as a lead variant, and then removing it, and all variants within 0.25 cM from the list of variants (equivalent to approximately 250 kb in physical distance). The process was then repeated until the list of variants was empty. We applied this process to each GWAS using two filters on MAF: (a) MAF > 0.1%, and (b) MAF > 1%. We grouped associated lead SNPs across phenotypes into clusters. This process first grouped SNPs within 0.25 cM of each other, and this mostly produced sensible clusters, but some hand curation was used to merge or split clusters based on visual inspection of cluster plots and levels of linkage disequilibrium between SNPs. For some clusters in Extended Data Table 1, we report coding SNPs that were found to be in high linkage disequilibrium with the lead SNPs.

### Accounting for multiple IDPs

We adjusted the genome-wide significance threshold (−log_10_(*P*) > 7.5) by a Bonferroni factor (–log_10_(3,144) = 3.5) that accounts for the number of IDPs tested, giving a threshold of –log_10_(*P*) > 11. This assumes (incorrectly) that the IDPs are independent and so is likely to be conservative, but we preferred to be cautious when analysing so many IDPs.

### Genetic correlation analysis

We used linkage disequilibrium score regression^51^ to estimate the genetic correlation between the IDPs studied in our analysis and ten disease-, personality- or brain-related traits. We gathered summary statistics for GWASs of the neuroticism personality trait (https://www.thessgac.org/data), autism spectrum (https://www.med.unc.edu/pgc/) and sleep duration (http://www.t2diabetesgenes.org/data/) and also seven disease traits: attention deficit hyperactivity disorder, schizophrenia, major depressive disorder and bipolar disorder (https://www.med.unc.edu/pgc/), Alzheimer’s disease (http://web.pasteur-lille.fr/en/recherche/u744/igap/igap_download.php), stroke (PMC4818561 from http://cerebrovascularportal.org/informational/downloads) and amyotrophic lateral sclerosis (http://databrowser.projectmine.com/). The number of samples in each of these studies and the DOIs for the corresponding studies are provided in Supplementary Table 13.

For each IDP–trait pair, we used the LDSCORE regression software (v1.0.0; https://github.com/bulik/ldsc) to compute the genetic correlation between the IDP and the trait, with linkage disequilibrium measurements taken from the 1000 Genomes Project (provided by the maintainers of the LDSCORE regression software). We filtered the SNPs to include only those with imputation INFO ≥ 0.9 and MAF ≥ 0.1%. Only INFO scores for major depressive disorder, schizophrenia and attention deficit hyperactivity disorder were provided by the source studies, and so for these three analyses we applied the INFO threshold to both the SNPs from our study and also the source study. For the remaining six studies, an INFO filter was applied to the SNPs from our own study. Owing to low levels of heritability of the functional edge IDPs, all of these were removed from this analysis. As calculation of genetic correlation between traits only really makes sense if both traits are themselves heritable, we only used those IDPs with *z*-scores for significantly non-zero heritability greater than 4. In total, we used 897 IDPs. To account for correlations between IDPs, we used the raw phenotype correlation matrix to simulate *z*-scores (and associated tail probabilities) using samples from a multivariate normal distribution with that same correlation matrix.

### Analysis of enrichment of functional categories

We used the LDSCORE regression software to carry out the heritability enrichment partitioning analysis into different functional categories (https://github.com/bulik/ldsc). We used 24 functional categories: coding, UTR, promoter, intron, histone marks H3K4me1, H3K4me3, H3K9ac5 and two versions of H3K27ac, open chromatin DNase I hypersensitivity site (DHS) regions, combined chromHMM/Segway predictions, regions conserved in mammals, super-enhancers and active enhancers from the FANTOM5 panel of samples. For each IDP, the enrichment of each functional category was summarized as the proportion of *h*^2^ explained by the category divided by the proportion of common variants in the category. For each IDP and each annotation we used the two-sided enrichment *P* value as reported by the LDSCORE regression software. We labelled those *P* values as enriched or depleted depending on whether the enrichment estimate was greater or less than 1. We stratified these *P* values accordingly into 23 groups of IDPs.

### Code availability

Most of the software and code used in this study are publicly available, including custom Matlab scripts used to prepare IDPs for GWAS (http://www.fmrib.ox.ac.uk/ukbiobank/gwaspaper/). Pre-compiled binaries for the latest version of BGENIE and SBAT are available at https://jmarchini.org/software/. This software is currently licensed free for use by researchers at academic institutions. Commercial organizations wishing to use these packages must enquire about a licence from the University of Oxford. Brain image processing was largely carried out with FSL (FMRIB’s Software Library, https://fsl.fmrib.ox.ac.uk/fsl/fslwiki) and further Matlab-based preparation of IDPs and imaging confounds utilized code from FSLNets (https://fsl.fmrib.ox.ac.uk/fsl/fslwiki/FSLNets).

### Reporting summary

Further information on research design is available in the Nature Research Reporting Summary linked to this paper.

## Online content

Any methods, additional references, Nature Research reporting summaries, source data, statements of data availability and associated accession codes are available at 10.1038/s41586-018-0571-7.

## Supplementary information

Supplementary Information This file contains Supplementary Notes. It includes a glossary of MRI terms, winner’s curse correction of post-hoc power analysis of replication studies, further detail for SNP associations with imaging phenotypes, and genetic correlation with neurodegenerative, psychiatric and personality traits.

Reporting Summary

Supplementary Figures This file contains Supplementary Figures S1-S22.

Supplementary Tables This file contains Supplementary Tables S1-S13.

## Figures and Tables

**Extended Data Table 1:**
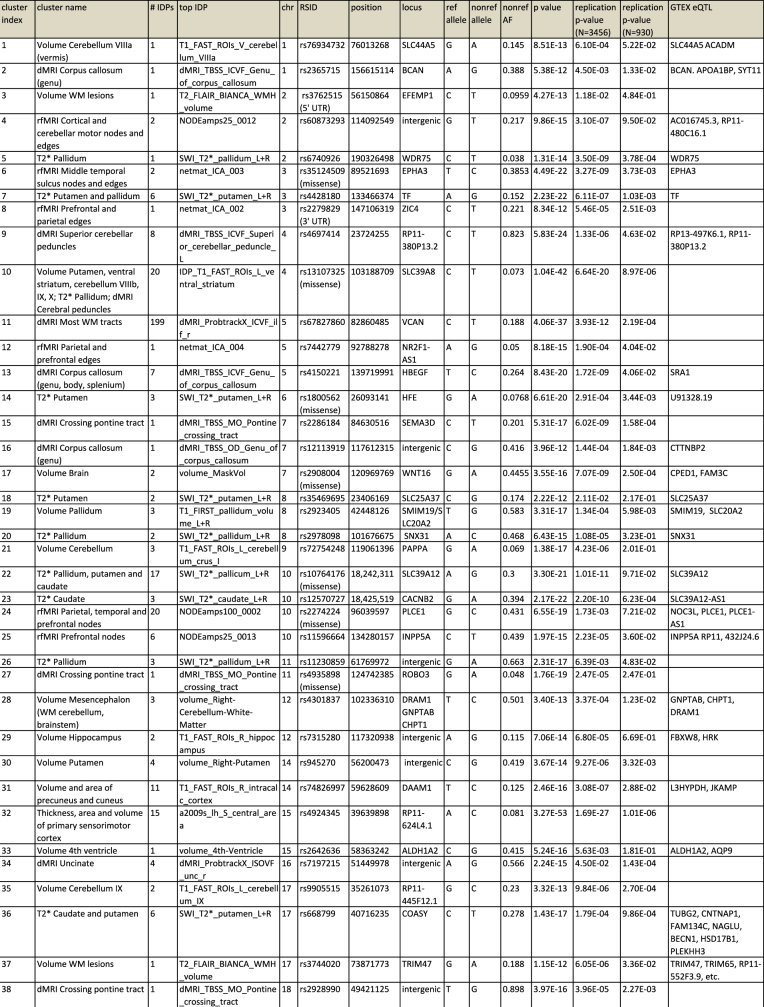
Summary of most highly associated SNP–IDP clusters

The table summarizes the 38 clusters of SNP–IDP associations (*n* = 8,428 subjects, see main text and Methods for details). For each cluster, the most significant association between an SNP and an IDP is detailed by the chromosome, rsID, base-pair position, SNP alleles, non-reference allele frequency, *P* value in the discovery sample and the replication *P* values. The locus column details a gene if the SNP is in that gene. If we found a coding SNP or eQTL in high linkage disequilibrium with the lead SNP, then this is reported instead.

## Data Availability

The full set of GWAS results from this study is available on the Oxford BIG web browser (http://big.stats.ox.ac.uk/), which allows users to browse associations by SNP, gene or phenotype.
