# Supplementary material for: Genome-wide association studies of brain imaging phenotypes in UK Biobank
Source: Nature. 2018 Oct 10;562(7726):210–6. doi: 10.1038/s41586-018-0571-7 (PMC6786974; doi:10.1038/s41586-018-0571-7)
Supplement: Supplementary file 1 — This file contains Supplementary Notes. It includes a glossary of MRI terms, winner’s curse correction of post-hoc power analysis of replication studies, further detail for SNP associations with imaging phenotypes, and genetic correlation with neurodegenerative, psychiatric and personality traits. [file 41586_2018_571_MOESM1_ESM.pdf]

nature

SUPPLEMENTARY INFORMATION

https://doi.org/10.1038/s41586-018-0571-7

In the format provided by the authors and unedited.

Genome-wide association studies of brain imaging phenotypes in UK Biobank

Lloyd T. Elliott[1], Kevin Sharp[1], Fidel Alfaro-Almagro[2], Sinan Shi[1], Karla L. Miller[2], Gwenaëlle Douaud[2], Jonathan Marchini[1,3,4]* & Stephen M. Smith[2,4]*

[1]Department of Statistics, University of Oxford, Oxford, UK. [2]Centre for Functional MRI of the Brain (FMRIB), Wellcome Centre for Integrative Neuroimaging, University of Oxford, Oxford, UK. [3]The Wellcome Centre for Human Genetics, University of Oxford, Oxford, UK. [4]These authors jointly supervised this work: Jonathan Marchini, Stephen Smith. *e-mail: marchini@stats.ox.ac.uk; steve@fmrib.ox.ac.uk

© 2018 Springer Nature Limited. All rights reserved.

Supplementary Note 1 : Glossary of MRI terms

dMRI (diffusion MRI): An imaging modality that captures how water molecules diffuse within tissue. This modality can be analyzed for tracing white matter pathways (tractography), as well as for microstructural features (tensor and NODDI models).

FA (fractional anisotropy): A tensor-based metric of microstructure reflecting the variation of water diffusion as a function of orientation. In white matter, high FA typically reflects strong diffusion along the tract, and is often interpreted as a proxy for tract integrity.

fMRI (functional MRI): An imaging method that detects brain activity indirectly based on changes in local blood oxygenation. Brain activity can either be induced by a stimulus or cognitive task, or be measured as spontaneous co-fluctuations between regions.

ICA (independent component analysis): A data-driven decomposition used (for example) in fMRI for parcellating the brain, usually into a set of spatially-independent maps, each with a single common timecourse. More generally, ICA reduces data to a number of independent features that often are more biophysically meaningful than one finds from simpler methods such as principal component analysis.

ICVF (intracellular volume fraction): See NODDI.

Mode of anisotropy: A tensor-based metric of microstructure that differentiates between voxels containing single fibres (with cigar-like diffusion, MO>0) from multiple fibres (with disc-like diffusion, MO<0), both of which result in high FA.

NODDI: A biophysical model of the diffusion MRI signal that aims to separate 'free' water (isotropic volume fraction, ISOVF) from restricted water (intracellular volume fraction, ICVF). NODDI also estimates the dispersion of 'neurite' fibres in the ICVF compartment.

swMRI (susceptibility-weighted MRI): An imaging modality driven by differences in tissue magnetic susceptibility due to (e.g.) tissue iron or lipids. Here, swMRI data is analysed to calculate the speed of MRI signal decay, characterized by the time constant T2*.

T2*: See swMRI.

T2 FLAIR (T2 fluid-attenuated inversion recovery): An imaging modality where free water signal is suppressed and some pathological tissues have high signal ('hyperintensities').

Tensor (or, diffusion tensor): A signal model approximating the diffusion of water in tissue as a three-dimensional ellipse, from which metrics that are relevant to tissue microstructure can be derived (e.g. fractional anisotropy).

Tract skeleton: A spatial map corresponding to the center of major white matter pathways. dMRI data is often analysed on a skeleton to reduce misalignment confounds.

Tractography: Identification of white matter pathways from dMRI data, in which the direction of fastest water diffusion is tracked voxel-by-voxel from a seed region of interest.

Voxel: A three-dimensional pixel. "Voxel-wise" maps involve every voxel in the image, in comparison to region-of-interest analyses that pool many voxels.

Supplementary Note 2: Winners' curse correction of post-hoc power analysis of replication studies

In order to investigate the numbers of replicated loci observed in our study we carried out a Winner's Curse correct power analysis. The power of a study is the probability of rejecting the null hypothesis given that the alternate hypothesis is true (*i.e.*, the true positive rate, or sensitivity). By linearity of expectation, the sum of the powers of each of the SNP associations is an estimate of the number of replications we should expect in a replication cohort, under the assumption that all of the associations are true. For example, in our discovery cohort we identified 1,262 associations at a *–log10 p*-value threshold of 7.5 (see Supplementary Table 5), and in the *N* = 930 replication cohort we replicated 455 of these associations at nominal significance. A power analysis can determine whether or not these 455 replications are more than we should expect, because it allows us to estimate (or 'predict') the number of replications we would see in a 'best case' scenario in which all the associations are true. This power analysis is based on *[1]*.

For a study that employs a linear model with significance level α, if the true value of the effect size is β and if the true value of the standard error is σ, then the power of a study with a cohort of size *N* is:

$$p(\beta\,, \sigma, N, \alpha) = f\left(g\left(1 - {}^{\alpha}\!/_{2}\,; N-2\right);\ N-2, {}^{|\beta|}\!/_{\sigma}\right).$$

Equation 1

Here $f(x; d, c)$ is the distribution function of a non-central Student-t distribution with *d* degrees of freedom and non-centrality parameter *c* evaluated at *x*, and $g(x; d)$ is the quantile function of a Student-t distribution with *d* degrees of freedom evaluated at *x [2]*.

To examine the power of a replication cohort, we may use Equation 1 and substitute the effect size found in the discovery cohort. However, this is a biased estimate of the true effect size. The bias arises from the fact that we are conditioning on the event that the association was found to be significant at level α in the discovery cohort. There is some fluctuation in effect size due to sampling noise, and so conditioning on significance will bias the sampling noise towards the tails of the distribution on the effect size. This is known as the Winner's Curse *[3]*. Methods have been provided for correcting this bias *[1]*, and we describe those methods here as they are relevant, and we adapt them to our replication paradigm. In *[1]*, the following approximation of the likelihood of the effect size is provided, conditioned on the observations of the study and conditioned on the truth of the alternate hypothesis:

$$L\left(\beta' \middle| \hat{\beta}, \hat{\sigma}, N, H_1, \alpha\right) \approx \frac{\varphi(\beta'; \hat{\beta}, \hat{\sigma})}{p(\beta', \hat{\sigma}, N, \alpha)}.$$

Equation 2

Here $\varphi(x; \mu, \sigma)$ is the density function of a normal distribution with mean μ and standard deviation σ evaluated at *x*. This likelihood, as a function of $(\beta', \hat{\beta})$ is supported on the set:

$$\left\{ (\beta', \hat{\beta}) \ : \mathrm{sign}(\beta') = \mathrm{sign}(\hat{\beta}),\ {|\hat{\beta}|}\big/_{\hat{\sigma}} > \ g\left(1 - {}^{\alpha}\!/_{2}\,; N-2\right) \right\}.$$

Equation 3

And so for the *i*-th association in our study, if we observe an effect size of $\widehat{\beta_i}$ and a standard error of $\widehat{\sigma_i}$ in the discovery cohort, and if the size of the discovery cohort is N^d, and if the significance level of the discovery is α, then in *[1]* the winners' curse corrected effect size is β_i^w and is given as follows:

$$\beta_i^w = \underset{\beta'}{\mathrm{argmin}}\, L\left(\beta' \middle| \widehat{\beta}_i, \widehat{\sigma}_i, N^d, H_1, \alpha\right).$$

Equation 4

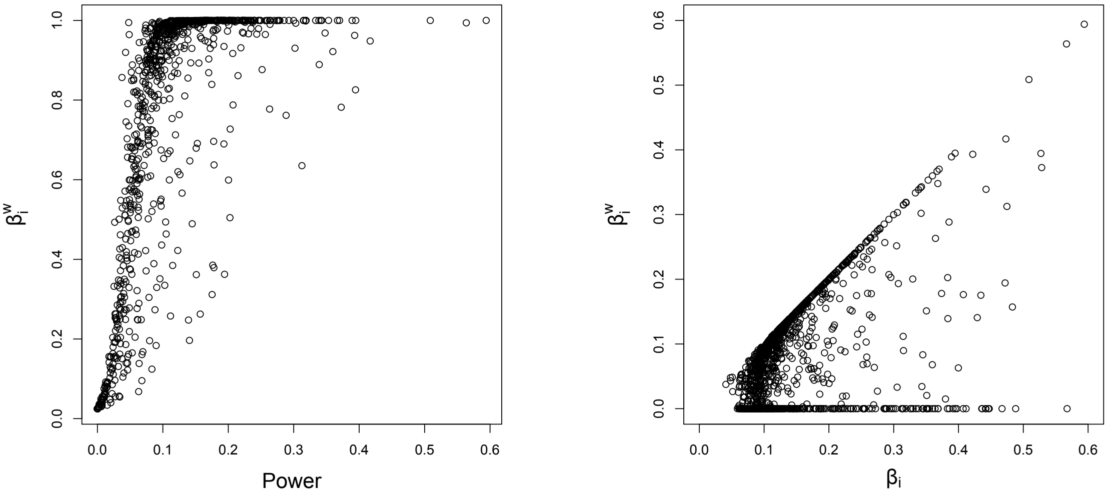

Supplementary Note 3; Figure 1: Winners' curse corrected effect sizes. Left) The y-axis shows the winners' curse corrected effect sizes for the 1,262 associations reported in Supplementary Table 5 and the x-axis shows the power to replicate the association in the N = 3,456 replication cohort. Right) The action of the winners' curse correction is shown; the x-axis shows the effect sizes before correction, and the y-axis shows the effect sizes after correction. Zeros in the corrected effect sizes indicate loss of significance in the discovery cohort.

As in *[2]*, We compute these winners' curse corrected effect sizes β_i^w using Nelder—Mead optimization *[4]*. The action of this correction is shown above in Supplementary Note 3; Figure 1. Using these winners' curse corrected effect sizes, we then compute an unbiased estimate of the expected number of associations that replicate at nominal significance in the replication cohort:

$$E[\#\mathrm{replications}|H_1] = \textstyle\sum_i p(\beta_i^w, \hat{\sigma}_i^r, N^r, 0.05).$$

Equation 5

Here $\hat{\sigma}_i^r$ is the observed standard error in the replication cohort, and N^r is the size of the replication cohort. Thus, Equation 5 provides a post-hoc winners' curse corrected power analysis to examine the expected number of replicated clusters.

Condition	*Predicted replications*	*Actual replications*
N = *930*	486	455
N = *3,456*	740	844
N = *3,456, singleton clusters*	72	68
N = *3,456, clusters*	161	148

Supplementary Note 3; Table 1: Predicted replications from post-hoc winners' curse corrected power analysis compared to actual number of replications in various replication conditions. The number of replications we actually find are less than what would be predicted under the alternate hypothesis for all conditions except N = 3,456.

Using Equation 5, we calculated that the expected number of replications under the alternate hypothesis among the 1,262 associations listed in Supplementary Table 5, and found that for the N = 930 replication cohort, we expected 486 replications (we found 455 in the experiment). And, for the N = 3,456 replication cohort, we expected 740 replications (and we found 844 in the experiment).

While the number of replicated associations we found for N = 3,456 is in excess of the expectation, we note that linkage disequilibrium among SNPs and correlation among phenotypes act to increase the variance of the estimate of this expectation. Associations that are all in the same "cluster" tend to all replicate or fail to replicate more or less together. To understand this variance, we restricted Equation 5 to sum only over the 314 "singleton" clusters — clusters that contained only a single association, and therefore exhibit less correlation. We calculate that in the N = 3,456 replication cohort, the expected number of replicated singleton clusters was 72 (we found 68 in the experiment).

We also used Equation 5 to calculate the expected number of replicated clusters in the N = 3,456 replication cohort. Assuming independence among the associations, the expected number of replicated clusters is as follows (a 'noisy or'):

$$E[\#\text{replications}] = \sum_j 1 - \prod_{i \in C_j}\left(1 - p(\beta_i^w, \hat{\sigma}_i^r, N^r, 0.05)\right).$$

Equation 6

Here C_j is the set of indices of the associations assigned to the j-th cluster. We calculated that the expected number of replicated clusters was 161 (and we found 148 in the experiment). So, even though the number of replications we find in the raw associations in the N = 3,456 replication cohort is larger than what is predicted by post-hoc winners' curse corrected power analysis; when we aggregate over clusters, or examine singleton clusters only, or examine the N = 930 replication cohort, we replicate fewer associations than what is predicted by post-hoc winners' curse corrected power analysis. These results are tabulated in Supplementary Note 3; Table 1. This analysis therefore suggests that our results are not outside of the realm of what could be expected through a post-hoc power analysis of the replication cohorts.

References

1. R. Xiao and M. Boehnke. *Quantifying and correcting for the winner's curse in quantitative-trait association studies*. Genetic Epidemiology. **35**(5), 133-138 (2011)
2. T. Hastie, R. Tibshirani and J. Friedman. *The Elements of Statistical Learning*. Springer. (2001)
3. R. H. Thaler. *Anomalies: The Winner's Curse*. Journal of Economic Perspectives. **2**(1), 191-202 (1988)
4. J. A. Nelder and R. Mead. *A simplex method for function minimization*. Computer Journal. **7**(4), 308-313 (1965)

Supplementary Note 3: Further detail for SNP associations with single imaging phenotypes and multiple-phenotypes

A. SNPs linked to genes that contribute broadly to brain development, patterning and plasticity

Beside *SEMA3D* and *ROBO3*, *BCAN* and *VCAN* have also been involved in axon guidance and signalling pathways in neurons[1]. Similarly, a SNP in *EPHA3* was associated in our GWAS with cluster 6, which included many rfMRI functional connections between the middle temporal sulcus and mainly prefrontal and parietal brain areas (rs35124509 (missense), P=4.49E-22). *EPHA3* mediates the regulation of cell migration and axon guidance[2], and regulates trans-axonal signalling[3]. The other relevant findings are: one SNP is an eQTL of *WDR75*, which codes for a protein that reduces the expression of homeobox *NANOG*[4] and was associated with T2* in the pallidum (rs6740926, P_{min}=1.31E-14, cluster 5); one SNP in 3' UTR of *ZIC4*, whose loss can lead to cerebellar malformations and was associated with multiple rfMRI connections mainly between prefrontal, cerebellar and parietal areas (rs2279829, P=8.34E-12, cluster 8); one SNP in *ZIP8* (see main text) which plays a role in brain development via release from choroid plexus; one SNP in *NR2F1-AS1* (*COUP-TF1*), a master regulator which interacts with *PAX6* (rs7442779, P=8.18E-15, cluster 12); one SNP in *HBEGF* which codes for a protein that stimulates neurogenesis in proliferative zones of the adult brain (see main text); one SNP in *WNT16* (rs2908004 (missense), P=3.5E-16, cluster 17); one SNP in *DAAM1*, which is involved in cell polarity and whose duplication is associated with cerebral palsy (rs74826997, P=2.5E-16, cluster 31); one SNP in *ZIP12* (see main text), whose knockdown delays neural tube closure and causes severe neural tube defects[5]; and SNPs in *EFEMP1*, *ALDH1A2* and *COASY* (see main text). Finally, another SNP in *PLCE1*, which codes for a protein that regulates various processes affecting cell growth, differentiation, and gene expression, was associated with amplitudes in various resting-state networks (top SNP rs2274224 (missense), P=6.55E-19, associated with the salience network, cluster 24), as well as body water composition and blood pressure in UK Biobank participants (which can be looked up using the Oxford BIG Browser www.big.stats.ox.ac.uk). Of note, two of the SNPs in cluster 24, associated respectively with

resting-state nodes in the precuneus and parietal lobule, were also found to be associated with migraine in 2 previous GWAS [6](rs11187838, P=3.05E-15, and rs10786156, P=4.57E-12).

B. SNPs linked to genes that relate to transport and storage of iron, nutrients and minerals

A major source of cross-subject differences seen in T2* data is microscopic variations in magnetic field, often associated with iron deposition in ageing and pathology.[7] We identified many associations between T2* in caudate, putamen and pallidum and SNPs in genes (*TF*, rs4428180, P_{min}=2.23E-22, cluster 7; *HFE,* rs1800562 (missense) P_{min}=6.6E-20, cluster 14; *SLC25A37*, rs35469695, P_{min}=2.22E-12, cluster 18) or near genes (*FTH1*, rs11230859, P_{min}=2.31E-17, cluster 26) known to affect iron transport and storage, as well as neurodegeneration with brain iron accumulation (NBIA)[8] (*COASY*, rs668799, P_{min}=1.43E-17, cluster 36). In particular, a SNP in *HFE* (rs1800562) is associated with haemoglobin levels,[9] iron status biomarkers[10] and LDL cholesterol[11]. In addition to *TF*, which transports iron from the intestine, and *SLC25A37*, a mitochondrial iron transporter, we identified four further SNPs that are either coding SNPs for, or eQTLs of, genes involved in transport of nutrients and minerals: *SLC44A5* (rs76934732, P=8.51E-13, cluster 1), *SLC39A8*/*ZIP8* (rs13107325 (missense) P_{min}=1.04E-42, cluster 10), *SLC20A2* (rs2923405, P_{min}=3.31E-17, cluster 19) and *SLC39A12*/*ZIP12* (rs10764176 (missense), P_{min}=3.3E-21, cluster 22).

C. SNPs linked to genes that relate to extended changes in white matter - the extracellular matrix, epidermal growth factor and white matter lesions

Three SNPs related to our white matter IDPs were in genes or eQTLs of genes coding for three proteins of the extracellular matrix (ECM). The first SNP (rs2365715, P=5.38E-12, cluster 2), an eQTL of *BCAN,* is associated with one dMRI microstructural measure in the genu of the corpus callosum. The second SNP (rs3762515, P=4.27E-13, cluster 3), in the 5’ UTR of *EFEMP1*, is associated with the volume of white matter lesions. Finally, the third SNP (rs67827860, P_{min}=4.06E-37, cluster 11, Fig. 3), located in an intron of *VCAN*, is in a cluster associated with multiple dMRI measures of most of the brain white matter tracts (199

IDPs in total). *BCAN* and *VCAN* in particular both code for chondroitin sulfate proteoglycans of the ECM, which are especially important for synaptic plasticity[12] and myelin repair.[13] *VCAN* is, for instance, increased in association with astrocytosis in multiple sclerosis[14], while both *BCAN* and *VCAN* are differentially regulated following spinal cord injury[15]. *BCAN*, *EFEMP1* and *VCAN* have been further associated in three separate GWAS with stroke[16], site of onset of amyotrophic lateral sclerosis[17] and major depressive disorder[18], respectively. Furthermore, *EFEMP1* is characterised by tandem arrays of epidermal growth factor (EGF)-like domains, and we also identified a strong association between the whole of the corpus callosum (genu, body and splenium) and a SNP in *HBEGF* (rs4150221, P_{min}=8.43E-20, cluster 13), a heparin-binding EGF-like growth factor. Similarly to *BCAN* and *VCAN*, *HBEGF* plays an important role in oligodendrocyte development and helps recovering WM injury in preterm babies[19]. Remarkably, this means that the vast majority of forebrain WM-related dMRI IDPs are associated in this study with SNPs related to genes coding for proteins involved either in the extracellular matrix, the epidermal growth factor, or both.

D. Novel loci discovered by multi-phenotype associations tests

In the multi-phenotype association tests, we found 25 loci that showed both a significant and replicated multi-trait association for an IDP group, while showing no genome-wide significance in the flanking region for any individual IDP in the corresponding grouping (Supplementary Table 9). Three of these loci show associations with the dMRI MO measures (rs62073157, P=4.07E-11; rs35884657, P=1.04E-09; rs9939914, P=1.15E-11) and all are eQTLs of microtubule related genes *MAPT, TUBA1B* and *TUBB3* respectively. The first SNP rs62073157 resides in a long stretch of LD (43.4-44.9Mb) on chromosome 17, known to contain a common inversion polymorphism[20]. This extended *MAPT* (encoding for Microtubule Associated Protein Tau) region has been repeatedly associated with several neurodegenerative disorders, such as Alzheimer's disease, where it has been shown to modulate the age of onset [21] and to be associated with *APOE* e4- alleles[22], fronto-temporal dementia[23] and progressive supranuclear palsy[24]. Notably, a locus in this *MAPT* region also shows overlap between Alzheimer's and Parkinson's diseases[25]. Mutations in tubulin genes have been shown to correlate strongly with multiple cortical and subcortical abnormalities[26].

Another example of the value of the multi-trait testing can be seen in the association between IDPs of global brain volume measurements and a SNP located between *BANK1* and *ZIP8*, previously identified in a GWAS of schizophrenia[27] (rs35518360, P=4.07E-12). This locus is also part of a multi-modal cluster from our single-trait GWAS that includes subcortical and cerebellar grey matter volumes, pallidum T2* and dMRI in midbrain white matter tracts (cluster 10 in Supplementary Table 6). The multi-trait test thus made it possible to uncover this additional association between global brain volume measurement and this locus, which might prove relevant in better understanding observations of smaller brain volume in (first episode/drug-naïve) schizophrenic patients [28].

Another locus (rs112651271, p=3.23E-11) is associated with a dMRI IDP group encompassing all measurements collected in major white matter tracts. This SNP lies 150Kb upstream of *EDNRA*, which plays a role in potent and long-lasting vasoconstriction, and (likely related to this), has been linked to hypertension and migraine, as well as intracranial aneurysm [29].

The multi-trait analysis also uncovered an association with SNPs in the *IL34* gene (rs12928124, p=1.31E-10) and FreeSurfer brain volume IDPs. IL-34 is a ligand of the CSF-1 receptor (CSF-1R) that regulates CNS microglial development and has been shown to regulate cortical development in mice[30]. Il-34 has also been shown to promote clearance of soluble oligomeric amyloid-β, which mediates synaptic dysfunction and neuronal damage in Alzheimer's disease[31].

E. Iron, cardiovascular traits and brain development in brain disorders

Of those genes involved in neurodegenerative disorders that we identified in our single-IDP association analysis, most mainly code for iron-related proteins. While *TF* and *HFE* might play a relevant role for iron mobilisation and regulation in neurodegenerative disorders such as Parkinson's disease, Creutzfeldt-Jakob disease, amyotrophic lateral sclerosis and Alzheimer's disease[32,33], *SLC25A37* shows increased expression in Alzheimer's and Friedreich's ataxia[34] and mutations in *COASY* are associated with neurodegeneration with brain iron accumulation[8].

One notable exception is in an LD region encompassing significant SNPs in both *MRC1* and *ZIP12* (cluster 22), which has been linked to neurodegenerative/neuropsychiatric disorders and cardiovascular processes (as opposed to iron-related processes). SNPs in *MRC1* have been shown in a GWAS to be associated with risk of cardiovascular disease[35] and *MRC1* expression is increased in a model of Alzheimer's disease[36], while *ZIP12* demonstrates altered expression in the cortex of subjects with schizophrenia[37]. Our significant SNPs in *ZIP8* (cluster 10) show a similar overlap, and the *ZIP8* hit has been found to be associated both with schizophrenia and Parkinson's disease[6], as well cardiovascular death[38].

Similarly to *ZIP8* and *ZIP12*, of those genes related to mental health disorders identified both in the single-IDP and multi-trait analyses, most are strongly involved in brain development and plasticity. This is the case of *VCAN*, for which SNPs have been associated in a GWAS with major depressive disorder [18], *SEMA3D* and *DAAM1*, which might both contribute to schizophrenia[39,40], *ROBO3,* that may be associated with autism[41] and *CTTNBP2*, for which disruption is related to autism[42], and whos knockdown reduces the density and size of dendritic spines in neurons (rs12113919, eQTL of *CTTNBP2*, P=3.96E-12, cluster 16). Interestingly, this latter SNP was associated here with one dMRI measure in the corpus callosum, a white matter tract that has been shown in dMRI meta-analyses to be the most consistently disrupted tract in autism[43,44].

References

1. Ohtake, Y., Wong, D., Abdul-Muneer, P. M., Selzer, M. E. & Li, S. Two PTP receptors mediate CSPG inhibition by convergent and divergent signaling pathways in neurons. *Sci Rep* **6,** srep37152 (2016).
2. Shi, G., Yue, G. & Zhou, R. EphA3 Functions are Regulated by Collaborating Phosphotyrosine Residues. *Cell research* **20,** 1263–1275 (2010).
3. Gallarda, B. W. *et al.* Segregation of Axial Motor and Sensory Pathways via Heterotypic Trans-Axonal Signaling. *Science* **320,** 233–236 (2008).
4. You, K. T., Park, J. & Kim, V. N. Role of the small subunit processome in the maintenance of pluripotent stem cells. *Genes Dev.* **29,** 2004–2009 (2015).
5. Chowanadisai, W., Graham, D. M., Keen, C. L., Rucker, R. B. & Messerli, M. A. Neurulation and neurite extension require the zinc transporter ZIP12 (slc39a12). *Proc. Natl. Acad. Sci. U.S.A.* **110,** 9903–9908 (2013).
6. Pickrell, J. K. *et al.* Detection and interpretation of shared genetic influences on 42 human traits. *Nat. Genet.* (2016). doi:10.1038/ng.3570
7. Duyn, J. MR susceptibility imaging. *J. Magn. Reson.* **229,** 198–207 (2013).
8. Dusi, S. *et al.* Exome sequence reveals mutations in CoA synthase as a cause of neurodegeneration with brain iron accumulation. *Am. J. Hum. Genet.* **94,** 11–22

(2014).
9. Wheeler, E. *et al.* Impact of common genetic determinants of Hemoglobin A1c on type 2 diabetes risk and diagnosis in ancestrally diverse populations: A transethnic genome-wide meta-analysis. *PLOS Medicine* **14,** e1002383 (2017).
10. Benyamin, B. *et al.* Novel loci affecting iron homeostasis and their effects in individuals at risk for hemochromatosis. *Nature Communications* **5,** 4926 (2014).
11. Consortium, G. L. G. *et al.* Discovery and refinement of loci associated with lipid levels. *Nat. Genet.* **45,** 1274–1283 (2013).
12. Dityatev, A., Schachner, M. & Sonderegger, P. The dual role of the extracellular matrix in synaptic plasticity and homeostasis. *Nature Reviews Neuroscience* **11,** 735–746 (2010).
13. Lau, L. W., Cua, R., Keough, M. B., Haylock-Jacobs, S. & Yong, V. W. Pathophysiology of the brain extracellular matrix: a new target for remyelination. *Nature Reviews Neuroscience* **14,** 722–729 (2013).
14. Sobel, R. A. & Ahmed, A. S. White Matter Extracellular Matrix Chondroitin Sulfate/Dermatan Sulfate Proteoglycans in Multiple Sclerosis. *J Neuropathol Exp Neurol* **60,** 1198–1207 (2001).
15. Shih, C.-H., Lacagnina, M., Leuer-Bisciotti, K. & Pröschel, C. Astroglial-Derived Periostin Promotes Axonal Regeneration after Spinal Cord Injury. *J. Neurosci.* **34,** 2438–2443 (2014).
16. Matarin, M. *et al.* A genome-wide genotyping study in patients with ischaemic stroke: initial analysis and data release. *The Lancet Neurology* **6,** 414–420 (2007).
17. Clark, J. A., Yeaman, E. J., Blizzard, C. A., Chuckowree, J. A. & Dickson, T. C. A Case for Microtubule Vulnerability in Amyotrophic Lateral Sclerosis: Altered Dynamics During Disease. *Frontiers in Cellular Neuroscience* **10,** 2910 (2016).
18. Lewis, C. M. *et al.* Genome-Wide Association Study of Major Recurrent Depression in the U.K. Population. *American Journal of Psychiatry* **167,** 949–957 (2010).
19. Scafidi, J. *et al.* Intranasal epidermal growth factor treatment rescues neonatal brain injury. *Nature* **506,** 230–234 (2013).
20. Stefansson, H. *et al.* A common inversion under selection in Europeans. *Nat. Genet.* **37,** 129–137 (2005).
21. Kauwe, J. S. K. *et al.* Variation in MAPT is associated with cerebrospinal fluid tau levels in the presence of amyloid-beta deposition. *Proc. Natl. Acad. Sci. U.S.A.* **105,** 8050–8054 (2008).
22. Jun, G. *et al.* A novel Alzheimer disease locus located near the gene encoding tau protein. *Mol. Psychiatry* **21,** 108–117 (2016).
23. Baker, M. *et al.* Mutations in progranulin cause tau-negative frontotemporal dementia linked to chromosome 17. *Nature* **442,** 916–919 (2006).
24. Höglinger, G. U. *et al.* Identification of common variants influencing risk of the tauopathy progressive supranuclear palsy. *Nat. Genet.* **43,** 699–705 (2011).
25. Desikan, R. S. *et al.* Genetic overlap between Alzheimer‘s disease and Parkinson’s disease at the MAPT locus. *Mol. Psychiatry* **20,** 1588–1595 (2015).
26. Mutch, C. A. *et al.* Disorders of Microtubule Function in Neurons: Imaging Correlates. *American Journal of Neuroradiology* **37,** 528–535 (2016).
27. Schizophrenia Working Group of the Psychiatric Genomics Consortium. Biological insights from 108 schizophrenia-associated genetic loci. *Nature* **511,** 421–427 (2014).
28. Haijma, S. V. *et al.* Brain volumes in schizophrenia: a meta-analysis in over 18 000 subjects. *Schizophr Bull* **39,** 1129–1138 (2013).
29. Low, S.-K. *et al.* Genome-wide association study for intracranial aneurysm in the Japanese population identifies three candidate susceptible loci and a functional genetic

variant at EDNRA. *Hum. Mol. Genet.* **21,** 2102–2110 (2012).
30. Nandi, S. *et al.* The CSF-1 receptor ligands IL-34 and CSF-1 exhibit distinct developmental brain expression patterns and regulate neural progenitor cell maintenance and maturation. *Developmental Biology* **367,** 100–113 (2012).
31. Mizuno, T. *et al.* Interleukin-34 selectively enhances the neuroprotective effects of microglia to attenuate oligomeric amyloid-β neurotoxicity. *Am. J. Pathol.* **179,** 2016–2027 (2011).
32. Nandar, W. & Connor, J. R. HFE gene variants affect iron in the brain. *J. Nutr.* **141,** 729S–739S (2011).
33. Leitner, D. F. & Connor, J. R. Functional roles of transferrin in the brain. *Biochimica et Biophysica Acta (BBA) - General Subjects* **1820,** 393–402 (2012).
34. Gao, G. & Chang, Y.-Z. Mitochondrial ferritin in the regulation of brain iron homeostasis and neurodegenerative diseases. *Frontiers in Pharmacology* **5,** 19 (2014).
35. Middelberg, R. P. S. *et al.* Genetic variants in LPL, OASL and TOMM40/APOE-C1-C2-C4 genes are associated with multiple cardiovascular-related traits. *BMC Med. Genet.* **12,** 123 (2011).
36. Srinivasan, K. *et al.* Untangling the brain's neuroinflammatory and neurodegenerative transcriptional responses. *Nature Communications* **7,** 11295 (2016).
37. Scarr, E. *et al.* Increased cortical expression of the zinc transporter SLC39A12 suggests a breakdown in zinc cellular homeostasis as part of the pathophysiology of schizophrenia. *npj Schizophrenia* **2,** npjschz20162 (2016).
38. Johansson, A. *et al.* Genome-wide association and Mendelian randomization study of NT-proBNP in patients with acute coronary syndrome. *Hum. Mol. Genet.* **25,** 1447–1456 (2016).
39. Fujii, T. *et al.* Possible association of the semaphorin 3D gene (SEMA3D) with schizophrenia. *J Psychiatr Res* **45,** 47–53 (2011).
40. Panaccione, I. *et al.* Neurodevelopment in schizophrenia: the role of the wnt pathways. *Curr Neuropharmacol* **11,** 535–558 (2013).
41. Anitha, A. *et al.* Genetic analyses of roundabout (ROBO) axon guidance receptors in autism. *Am. J. Med. Genet. B Neuropsychiatr. Genet.* **147B,** 1019–1027 (2008).
42. Iossifov, I. *et al.* De novo gene disruptions in children on the autistic spectrum. *Neuron* **74,** 285–299 (2012).
43. Aoki, Y., Abe, O., Nippashi, Y. & Yamasue, H. Comparison of white matter integrity between autism spectrum disorder subjects and typically developing individuals: a meta-analysis of diffusion tensor imaging tractography studies. *Mol Autism* **4,** 25 (2013).
44. Di, X., Azeez, A., Li, X., Haque, E. & Biswal, B. B. Disrupted focal white matter integrity in autism spectrum disorder: A voxel-based meta-analysis of diffusion tensor imaging studies. *Prog. Neuropsychopharmacol. Biol. Psychiatry* **82,** 242–248 (2018).

Supplementary Note 4: Genetic correlation with neurodegenerative, psychiatric and personality traits

We measured the genetic correlation (hence also co-heritability) between a subset of heritable IDPs and 10 neurodegenerative, psychiatric and personality traits (see Methods). We found suggestive evidence of elevated levels of non-zero genetic correlation for amyotrophic lateral sclerosis (ALS), schizophrenia and stroke, mainly with dMRI measures in white matter tracts (Supplementary Fig. 15). The strongest genetic correlation for ALS ($P<10^{-3}$) was found in the genu of the corpus callosum (with a co-heritability of 0.63). This is in line with consistent findings of corpus callosum involvement in this degenerative disorder [1]. Correlations found in schizophrenia with the tapetum ($P<10^{-3}$) were likely due to partial volume effects, given that the next most strongly associated IDPs reflect ventricular and thalamic volume, which are some of the most robust volumetric findings in this mental health disorder [2]; hence it is interesting to see the genetic input into this volumetric disease association. While more modest correlations in stroke were observed, it was across a wide range of dMRI IDPs, with the strongest genetic correlations ($P<10^{-2}$) in the corona radiata, internal capsule and thalamic radiations, white matter tracts that follow the probabilistic distribution of stroke.[3] Supplementary Table 10 contains genetic correlation estimates for all IDP/trait combinations with nominal p-value < 0.01, to highlight which IDPs occur in the tails of these distributions. However, in line with previous observations,[4] we also found evidence that the LDSCORE regression approach for estimating genetic correlation seems best suited to pairs of traits both of which are heritable and polygenic in genetic aetiology. For example, the deflated p-value distribution for the correlation of IDPs with Alzheimer's is driven by the large *APOE* association for Alzheimer's disease on chromosome 19.

References

1. Douaud, G., Filippini, N., Knight, S., Talbot, K. & Turner, M. R. Integration of structural and functional magnetic resonance imaging in amyotrophic lateral sclerosis. *Brain* **134,** 3470–3479 (2011).
2. Haijma, S. V. *et al*. Brain volumes in schizophrenia: a meta-analysis in over 18 000 subjects. *Schizophr Bull* **39,** 1129–1138 (2013).
3. Meyer, S. *et al*. Voxel-based lesion-symptom mapping of stroke lesions underlying somatosensory deficits. *Neuroimage Clin* **10,** 257–266 (2016).
4. Bulik-Sullivan, B. *et al*. An atlas of genetic correlations across human diseases and traits. *Nat. Genet.* **47,** 1236–1241 (2015).
