# Supplementary material for: Genome-wide association studies of brain imaging phenotypes in UK Biobank
Source: Nature. 2018 Oct 10;562(7726):210–6. doi: 10.1038/s41586-018-0571-7 (PMC6786974; doi:10.1038/s41586-018-0571-7)
Supplement: Supplementary file 2 — Reporting Summary [file 41586_2018_571_MOESM2_ESM.pdf]

nature research

Corresponding author(s): Jonathan Marchini
Stephen Smith

Reporting Summary

Nature Research wishes to improve the reproducibility of the work that we publish. This form provides structure for consistency and transparency in reporting. For further information on Nature Research policies, see Authors & Referees and the Editorial Policy Checklist.

Statistical parameters

When statistical analyses are reported, confirm that the following items are present in the relevant location (e.g. figure legend, table legend, main text, or Methods section).

n/a	Confirmed	
☐	☒	The exact sample size (*n*) for each experimental group/condition, given as a discrete number and unit of measurement
☐	☒	An indication of whether measurements were taken from distinct samples or whether the same sample was measured repeatedly
☐	☒	The statistical test(s) used AND whether they are one- or two-sided<br>*Only common tests should be described solely by name; describe more complex techniques in the Methods section.*
☐	☒	A description of all covariates tested
☐	☒	A description of any assumptions or corrections, such as tests of normality and adjustment for multiple comparisons
☐	☒	A full description of the statistics including central tendency (e.g. means) or other basic estimates (e.g. regression coefficient) AND variation (e.g. standard deviation) or associated estimates of uncertainty (e.g. confidence intervals)
☐	☒	For null hypothesis testing, the test statistic (e.g. *F*, *t*, *r*) with confidence intervals, effect sizes, degrees of freedom and *P* value noted<br>*Give P values as exact values whenever suitable.*
☐	☒	For Bayesian analysis, information on the choice of priors and Markov chain Monte Carlo settings
☐	☒	For hierarchical and complex designs, identification of the appropriate level for tests and full reporting of outcomes
☐	☒	Estimates of effect sizes (e.g. Cohen's *d*, Pearson's *r*), indicating how they were calculated
☐	☒	Clearly defined error bars<br>*State explicitly what error bars represent (e.g. SD, SE, CI)*

Our web collection on statistics for biologists may be useful.

Software and code

Policy information about availability of computer code

Data collection

There isn't a clear distinction between software used for data collection vs data analysis, so all software is listed here.
All software is described in the text - the list is:
Oxford BIG server http://big.stats.ox.ac.uk/
BGENIE v1.2 https://jmarchini.org/bgenie/
SBAT https://jmarchini.org/sbat/
fastLMM https://github.com/MicrosoftGenomics/FaST-LMM
PLINK v2.0 http://www.cog-genomics.org/plink/2.0/
LDSCORE v1.0.0 regression software https://github.com/bulik/ldsc
PheWeb https://github.com/statgen/pheweb/
FSL v5.0 https://fsl.fmrib.ox.ac.uk/fsl/fslwiki/
FSLNets v0.6 https://fsl.fmrib.ox.ac.uk/fsl/fslwiki/FSLNets
FreeSurfer v6.0.0 https://surfer.nmr.mgh.harvard.edu
Matlab code for working with IDPs is available at http://www.fmrib.ox.ac.uk/ukbiobank/gwaspaper/

Data analysis

Please see above.

For manuscripts utilizing custom algorithms or software that are central to the research but not yet described in published literature, software must be made available to editors/reviewers upon request. We strongly encourage code deposition in a community repository (e.g. GitHub). See the Nature Research guidelines for submitting code & software for further information.

Data

Policy information about availability of data

All manuscripts must include a data availability statement. This statement should provide the following information, where applicable:

- Accession codes, unique identifiers, or web links for publicly available datasets
- A list of figures that have associated raw data
- A description of any restrictions on data availability

Data Availability

Full details of availability of source data and results data are covered in the main text and are re-summarised here. The data used in this work was obtained from UK Biobank under Data Access Application 8107 and (as with all UK Biobank data) are available to any bona fide researcher upon data access application to UK Biobank.

A custom quality control, phasing and imputation pipeline was developed to address the challenges specific to the experimental design, scale, and diversity of the UK Biobank dataset. The genetic data was publicly released in July 2017 and consists of ~96 million genetic variants in ~500,000 participants.

The UK Biobank Brain imaging protocol consists of 6 distinct modalities covering structural, diffusion and functional imaging, summarised in Supplementary Table 1. For this study, we primarily used data from the February 2017 release of ~10,000 participants' imaging data (and an additional ~5,000 subjects' data released in January 2018 provided the larger replication sample). The raw data from these 6 modalities has been processed for UK Biobank to create a set of imaging derived phenotypes (IDPs). These are available from UK Biobank, and it is these IDPs from the 2017/18 data releases that we used in this study.

The full set of GWAS results from this study are available on the Oxford Brain Imaging Genetics (BIG) web browser, that allows users to browse associations by SNP, gene or phenotype.

For the genetic correlation analysis we used summary statistic data from several GWAS of brain related conditions as follows: the ISGC Cerebrovascular Disease Knowledge Portal, International Genomics of Alzheimer's Project (IGAP), the Project MinE GWAS Consortium, the Social Science Genetic Association Consortium (SSGAC), the University of Exeter research group on Type 2 Diabetes, Obesity, Growth & Reproductive Ageing Genetics, the Psychiatric Genomics Consortium (PGC) and the ENIGMA consortium.

Field-specific reporting

Please select the best fit for your research. If you are not sure, read the appropriate sections before making your selection.

☒ Life sciences ☐ Behavioural & social sciences

For a reference copy of the document with all sections, see nature.com/authors/policies/ReportingSummary-flat.pdf

Life sciences

Study design

All studies must disclose on these points even when the disclosure is negative.

Sample size

Please see next question for sample size. No power calculation was needed in advance and we used all samples available (see below).

Data exclusions

We used the imputed genetic dataset made available by UK Biobank in its July 2017 release[6]. This consists of >92 million autosomal variants imputed from the Haplotype Reference Consortium (HRC) reference panel[79] and a merged UK10K + 1000 Genomes reference panel. We first identified a set of 12,623 participants who had also been imaged by UK Biobank. We then applied filters to remove variants with minor allele frequency (MAF) below 0.1% and with an imputation information score below 0.3, which reduced the number of SNPs to 18,174,817. We then kept only those samples (subjects) estimated to have recent British ancestry using the sample quality control information provided centrally by UK Biobank[6] (using the variable in.white.British.ancestry.subset in the file ukb_sqc_v2.txt); population structure can be a serious confound to genetic association studies[80], and this type of sample filtering is standard. This reduced the number of samples to 8,522. The UK Biobank dataset contains a number of close relatives (3rd cousin or closer). We therefore created a subset of 8,428 nominally unrelated subjects following similar procedures in Bycroft et al. (2017). After running GWAS on all the (SNP) variants in the 8,428 samples we applied three further variant filters to remove variants with a HWE (Hardy-Weinberg equilibrium) p-value less than 10-7, remove variants with MAF<0.1% and to keep only those variants in the HRC reference panel. This resulted in a dataset with 11,734,353 SNPs. Subjects were aged 40-69y at the point of original recruitment and 45-79y at the time of MRI scanning; 52% female.

Replication

Two successful replication-sample analyses were carried out:

In all analyses we estimated genetic effects with respect to the number of copies of the non-reference allele. In the discovery dataset, using a minor allele frequency filter of 1% and a –log10 p-value threshold of 7.5, we found 1,262 significant associations between SNPs and the 3,144 IDPs. These associations span all classes of IDPs, except task fMRI (Supplementary Table 4), with the swMRI T2* group showing a relatively large number of associations. The –log10 p-value threshold of 7.5 controls for the number of tests carried out across SNPs and accounts for the correlation structure between genetic variants. 844 and 455 of these 1,262 associations replicated at the 5% significance level using our two smaller replication datasets (Methods and Supplementary Table 5). Some associated genetic loci overlap across IDPs; we estimate that there are approximately 427 distinct associated genetic regions ("clusters"), and 148 of these "clusters" have a lead SNP that replicates at the 5% level in our replication set of 3,456 participants, and 91 below a 5% False Discovery Rate (FDR) threshold. We would expect ~21 of the lead SNPs in the 148 clusters to replicate under a null hypothesis of no association.

At a threshold of -log10 p-value > 11, which additionally corrects for all 3,144 GWAS carried out (see Methods), we find 368 significant associations between genetic regions and distinct IDPs (Supplementary Table 6, Supplementary Fig. 4). These associations with 78 unique SNPs can be grouped together into 38 distinct clusters by grouping across IDPs (Extended Data Table 1). Taking our lead SNP in each of the 38 regions, we find that all 38 have p<0.05 in our replication set of 3,456 participants, and all 38 are significant at 5% FDR. We found no appreciable change in these GWAS results when we included a set of potential body confound measures in addition to the main set of imaging confound measures (see Methods and Supplementary Fig. 5). We also carried out a Winner's Curse corrected post-hoc power analysis that agrees well with the results of our replication studies. (Supplementary Note 2).

Randomization

UK Biobank is an observational prospective epidemiological study, and the GWAS and heritability analyses in our study use all available subjects that fulfil the criteria described above. Hence there is no equivalent process of randomization that comes into this analysis (this is not a controlled randomised study).

Blinding

For exactly the same reasons (this is not a controlled randomised study), there is no step equivalent to blinding involved.

Materials & experimental systems

Policy information about availability of materials

n/a	Involved in the study
☒	☐ Unique materials
☒	☐ Antibodies
☒	☐ Eukaryotic cell lines
☒	☐ Research animals
☐	☒ Human research participants

Human research participants

Policy information about studies involving human research participants

Population characteristics

We used the imputed genetic dataset made available by UK Biobank in its July 2017 release[6]. This consists of >92 million autosomal variants imputed from the Haplotype Reference Consortium (HRC) reference panel[79] and a merged UK10K + 1000 Genomes reference panel. We first identified a set of 12,623 participants who had also been imaged by UK Biobank. We then applied filters to remove variants with minor allele frequency (MAF) below 0.1% and with an imputation information score below 0.3, which reduced the number of SNPs to 18,174,817. We then kept only those samples (subjects) estimated to have recent British ancestry using the sample quality control information provided centrally by UK Biobank[6] (using the variable in.white.British.ancestry.subset in the file ukb_sqc_v2.txt); population structure can be a serious confound to genetic association studies[80], and this type of sample filtering is standard. This reduced the number of samples to 8,522. The UK Biobank dataset contains a number of close relatives (3rd cousin or closer). We therefore created a subset of 8,428 nominally unrelated subjects following similar procedures in Bycroft et al. (2017). After running GWAS on all the (SNP) variants in the 8,428 samples we applied three further variant filters to remove variants with a HWE (Hardy-Weinberg equilibrium) p-value less than 10-7, remove variants with MAF<0.1% and to keep only those variants in the HRC reference panel. This resulted in a dataset with 11,734,353 SNPs. Subjects were aged 40-69y at the point of original recruitment and 45-79y at the time of MRI scanning; 52% female.

Method-specific reporting

n/a	Involved in the study
☒	☐ ChIP-seq
☒	☐ Flow cytometry
☐	☒ Magnetic resonance imaging

Magnetic resonance imaging

Experimental design

Design type	Please see "Methods" for full details. Our analyses include data from Structural MRI (T1 and T2FLAIR), susceptibility-weighted MRI, diffusion MRI, task functional MRI and resting-state functional MRI.
Design specifications	MRI data processing (to generate imaging-derived phenotypes) was done previously and is full described in references 4 (Miller) and 5 (Alfaro-Almagro).
Behavioral performance measures	Behavioral performance in the MRI scanner was not used in this study.

Acquisition

Imaging type(s)	Please see "Methods" for full details. Our analyses include data from Structural MRI (T1 and T2FLAIR), susceptibility-weighted MRI, diffusion MRI, task functional MRI and resting-state functional MRI.
Field strength	3T
Sequence & imaging parameters	MRI data acquisition for these 6 modalities covers several pages of full detail, which is fully provided previously in reference 4 (Miller).
Area of acquisition	Siemens' auto-align was used to include the full brain in the imaged field-of-view; this was checked (and corrected if necessary) by the radiographer.

Diffusion MRI ☒ Used ☐ Not used

Parameters: Please see above for information about full details. Summary: 100 distinct directions spread over two b shells (1000 and 2000). 2mm isotropic voxels.

Preprocessing

Preprocessing software	See above (covered previously in full detail in Miller and in Alfaro-Almagro).
Normalization	See above (covered previously in full detail in Miller and in Alfaro-Almagro).
Normalization template	See above (covered previously in full detail in Miller and in Alfaro-Almagro).
Noise and artifact removal	See above (covered previously in full detail in Miller and in Alfaro-Almagro).
Volume censoring	See above (covered previously in full detail in Miller and in Alfaro-Almagro). No volume censoring.

Statistical modeling & inference

Model type and settings	See above (covered previously in full detail in Miller and in Alfaro-Almagro).
Effect(s) tested	See above (covered previously in full detail in Miller and in Alfaro-Almagro).

Specify type of analysis: ☒ Whole brain ☐ ROI-based ☐ Both

Statistic type for inference (See Eklund et al. 2016)	Inference was not carried out when generating IDPs, but within this study inference was applied at the level of the combined imaging-genetics modelling (see above).
Correction	See above (Statistic type for inference).

Models & analysis

n/a	Involved in the study
☐	☒ Functional and/or effective connectivity
☒	☐ Graph analysis
☐	☒ Multivariate modeling or predictive analysis

Functional and/or effective connectivity	Partial correlation.
Multivariate modeling and predictive analysis	New features were generated using independent component analysis applied to partial correlation edge strengths - see text around line ~500 in main paper.
