# Supplementary material for: Genome-wide association studies of brain imaging phenotypes in UK Biobank
Source: Nature. 2018 Oct 10;562(7726):210–6. doi: 10.1038/s41586-018-0571-7 (PMC6786974; doi:10.1038/s41586-018-0571-7)
Supplement: Supplementary file 3 — This file contains Supplementary Figures S1-S22. [file 41586_2018_571_MOESM3_ESM.zip › Figure-S7.pdf]

IDP GWAS Manhattan Plots

List of Figures

1 T1 FAST ROIs R intracalc cortex . . . 1
2 T1 FAST ROIs R hippocampus . . . 2
3 T1 FAST ROIs R ventral striatum . . . 3
4 T1 FAST ROIs L cerebellum crus I . . . 4
5 T1 FAST ROIs V cerebellum VIIIa . . . 5
6 T1 FAST ROIs L cerebellum IX . . . 6
7 T2 FLAIR BIANCA WMH volume . . . 7
8 dMRI TBSS MO Pontine crossing tract . . . 8
9 dMRI TBSS ICVF Genu of corpus callosum . . . 9
10 dMRI TBSS ICVF Splenium of corpus callosum . . . 10
11 dMRI TBSS ICVF Superior cerebellar peduncle L . . . 11
12 dMRI TBSS OD Genu of corpus callosum . . . 12
13 dMRI ProbtrackX ICVF ilf r . . . 13
14 dMRI ProbtrackX ISOVF unc r . . . 14
15 NETMAT amplitudes (25) 05 . . . 15
16 NETMAT amplitudes (25) 12 . . . 16
17 NETMAT amplitudes (25) 13 . . . 17
18 T1 FIRST left plus right pallidum volume . . . 18
19 SWI T2star left plus right caudate . . . 19
20 SWI T2star left plus right putamen . . . 20
21 SWI T2star left plus right pallidum . . . 21
22 FreeSurfer volume Left-Cerebellum-White-Matter . . . 22
23 FreeSurfer volume 4th-Ventricle . . . 23
24 FreeSurfer volume Right-Putamen . . . 24
25 FreeSurfer volume MaskVol . . . 25
26 FreeSurfer a2009s rh S central area . . . 26
27 NETMAT edge ICA 2 . . . 27
28 NETMAT edge ICA 3 . . . 28
29 NETMAT edge ICA 4 . . . 29

Figure 1: T1 FAST ROIs R intracalc cortex

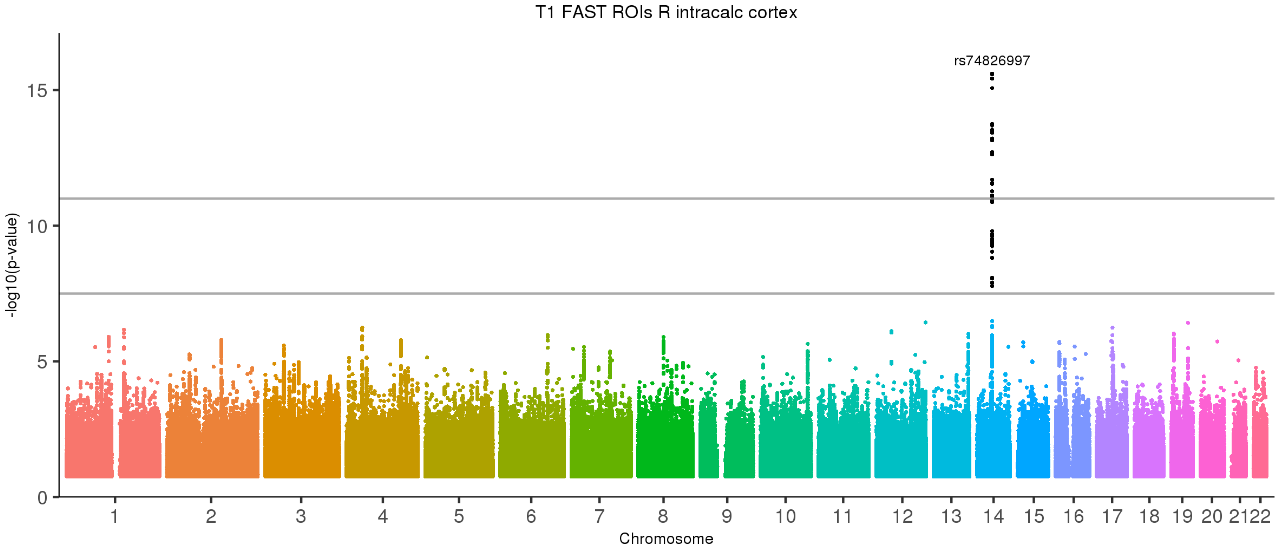

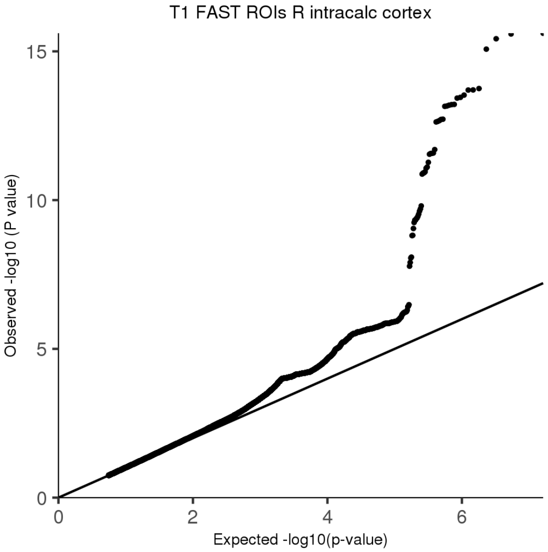

back to list of plots

Figure 2: T1 FAST ROIs R hippocampus

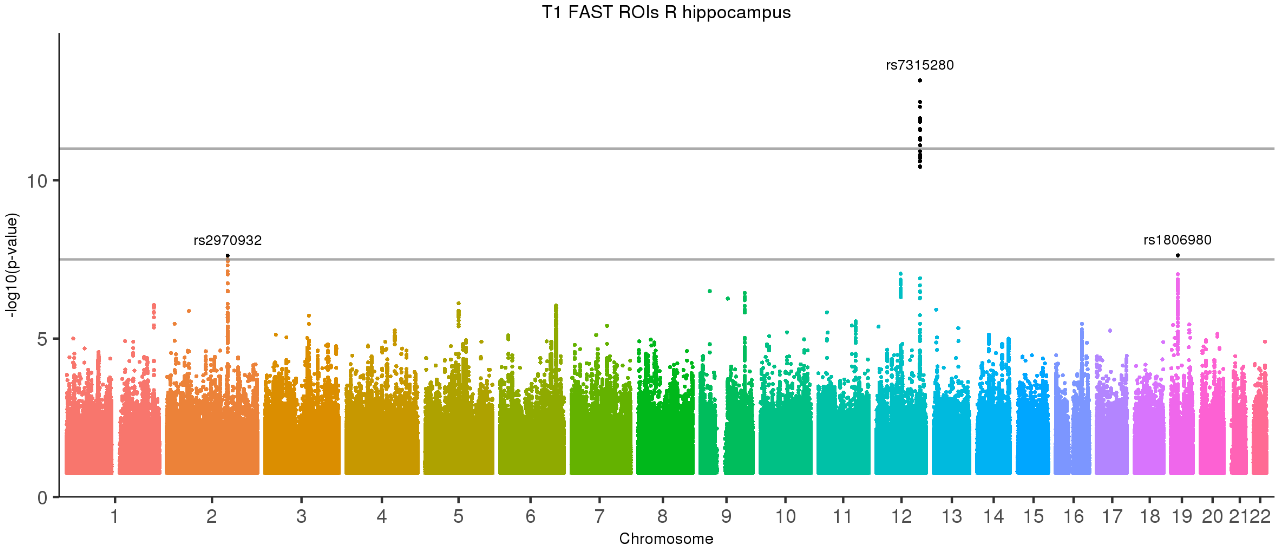

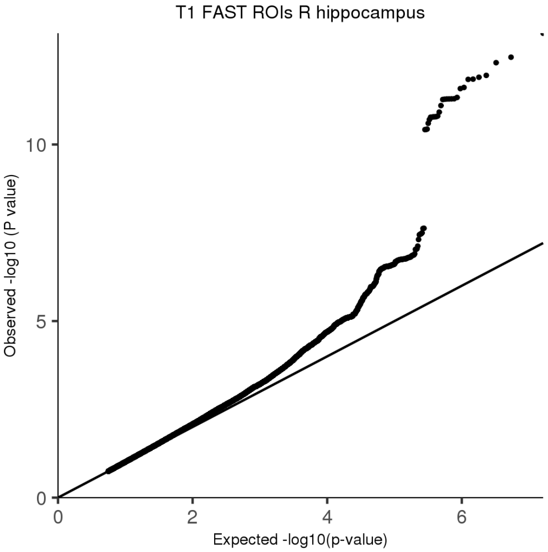

back to list of plots

Figure 3: T1 FAST ROIs R ventral striatum

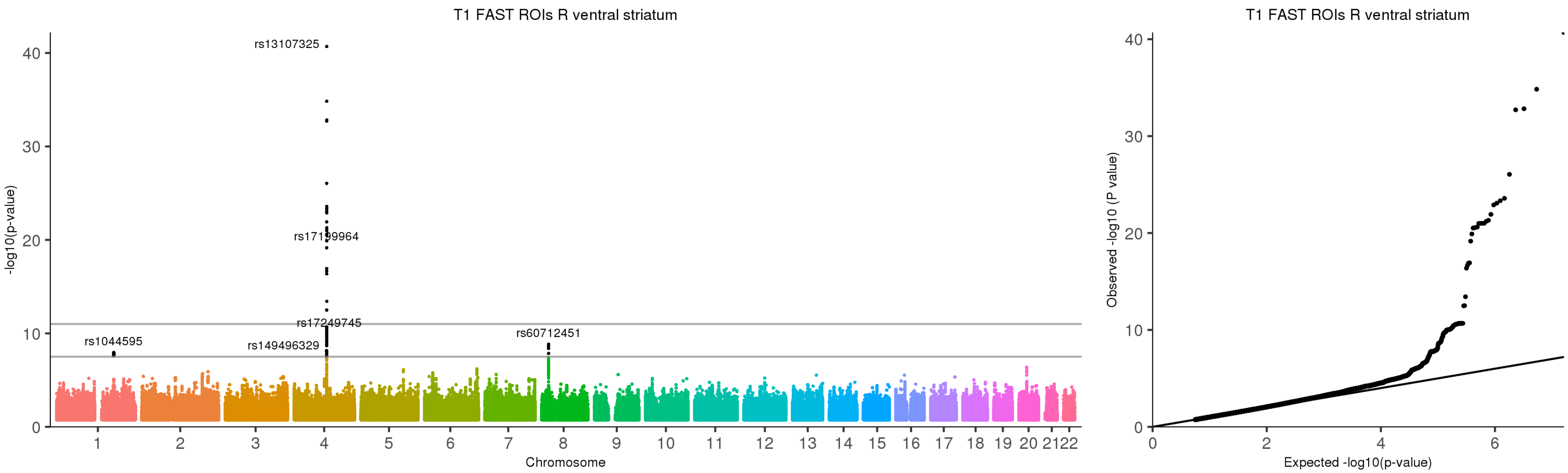

back to list of plots

Figure 4: T1 FAST ROIs L cerebellum crus I

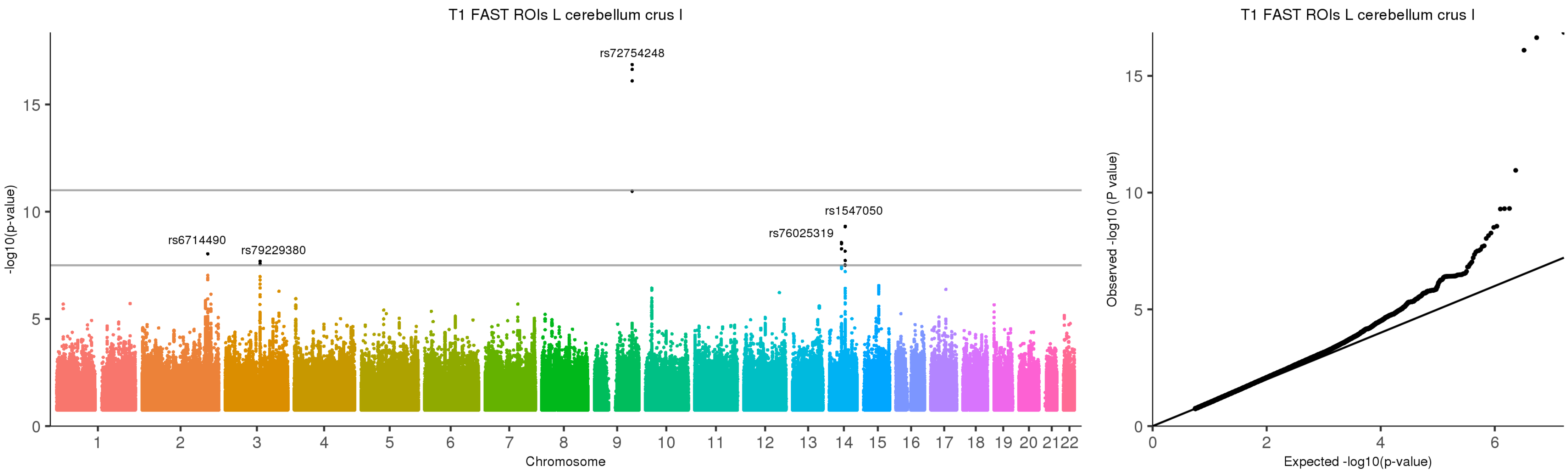

back to list of plots

Figure 5: T1 FAST ROIs V cerebellum VIIIa

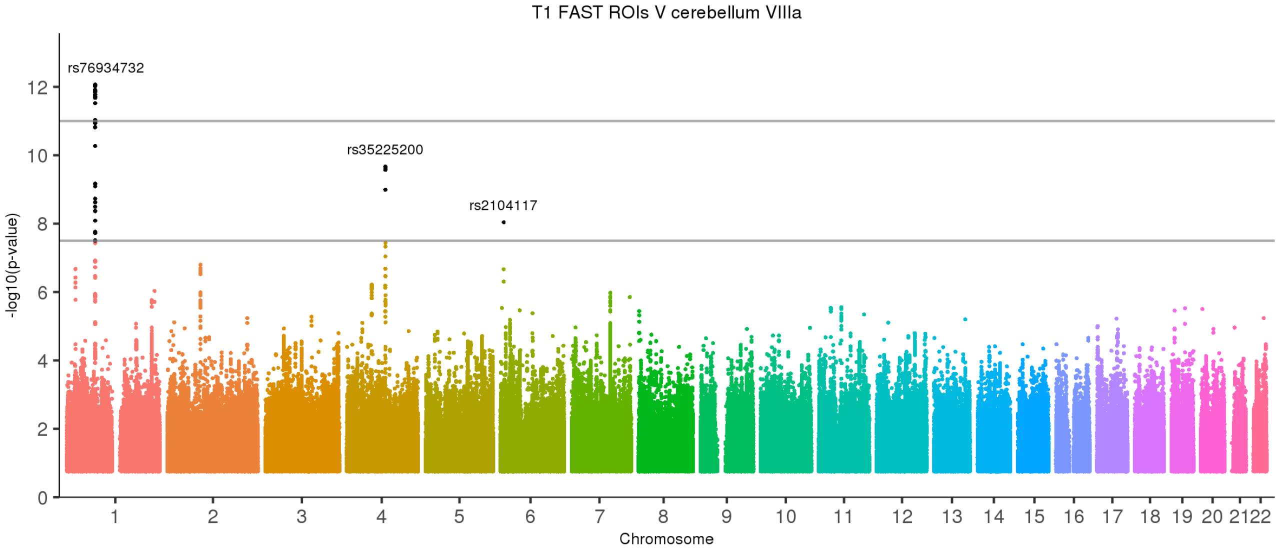

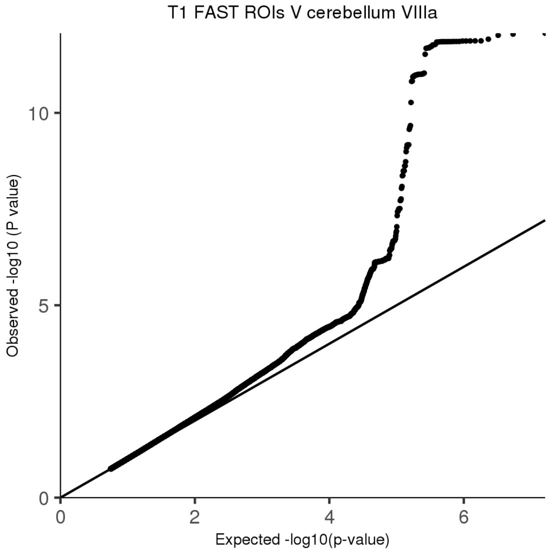

back to list of plots

Figure 6: T1 FAST ROIs L cerebellum IX

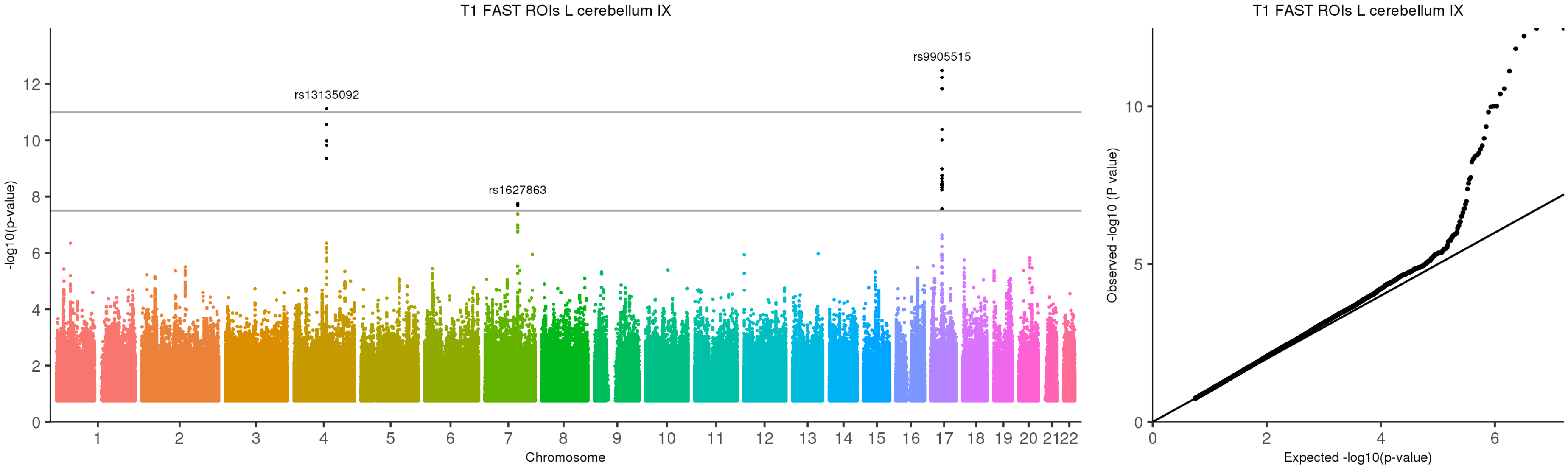

back to list of plots

Figure 7: T2 FLAIR BIANCA WMH volume

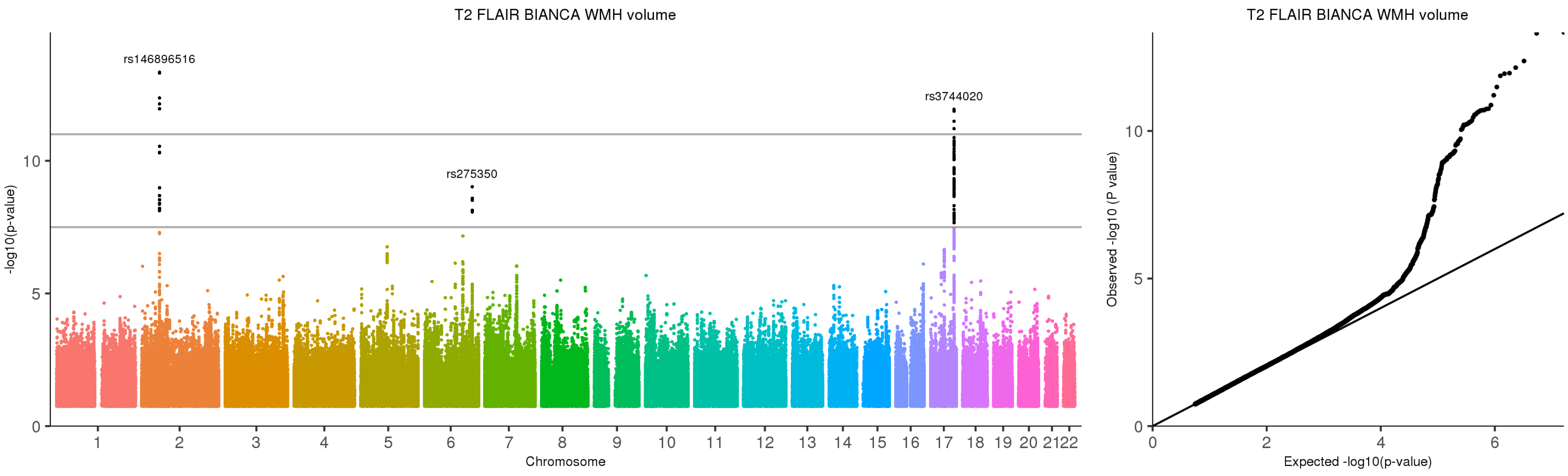

back to list of plots

Figure 8: dMRI TBSS MO Pontine crossing tract

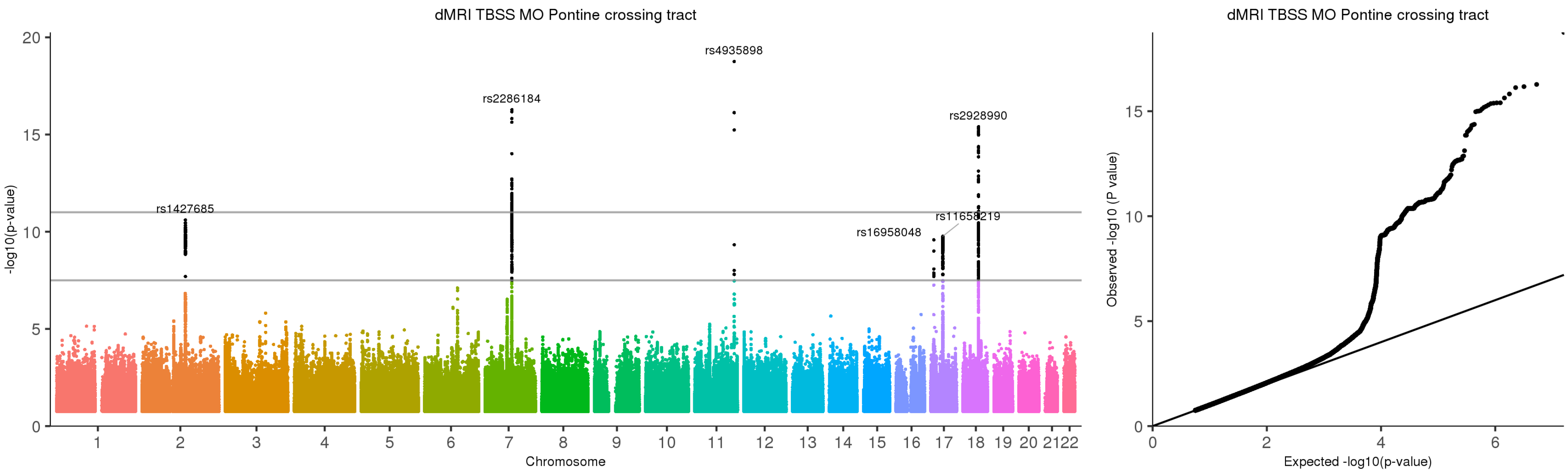

back to list of plots

Figure 9: dMRI TBSS ICVF Genu of corpus callosum

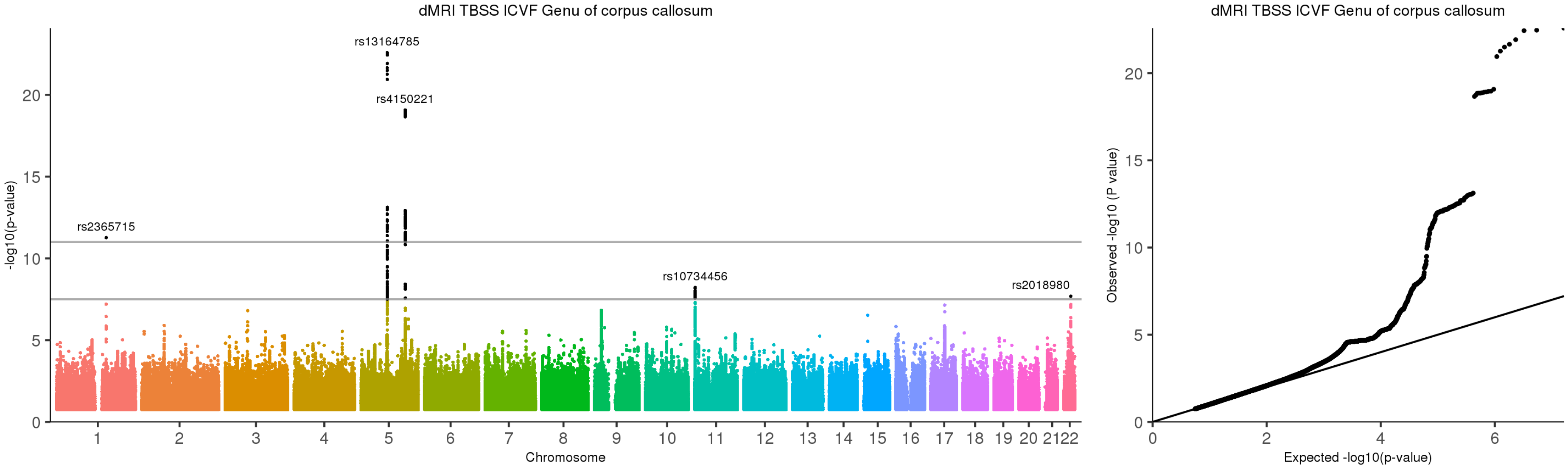

back to list of plots

Figure 10: dMRI TBSS ICVF Splenium of corpus callosum

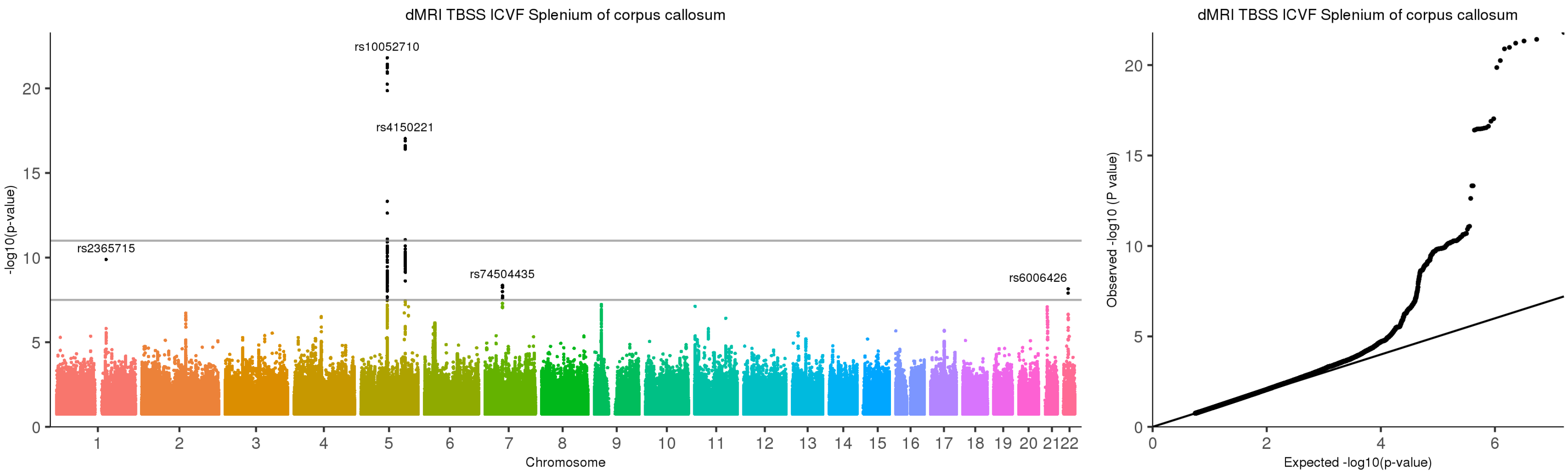

back to list of plots

Figure 11: dMRI TBSS ICVF Superior cerebellar peduncle L

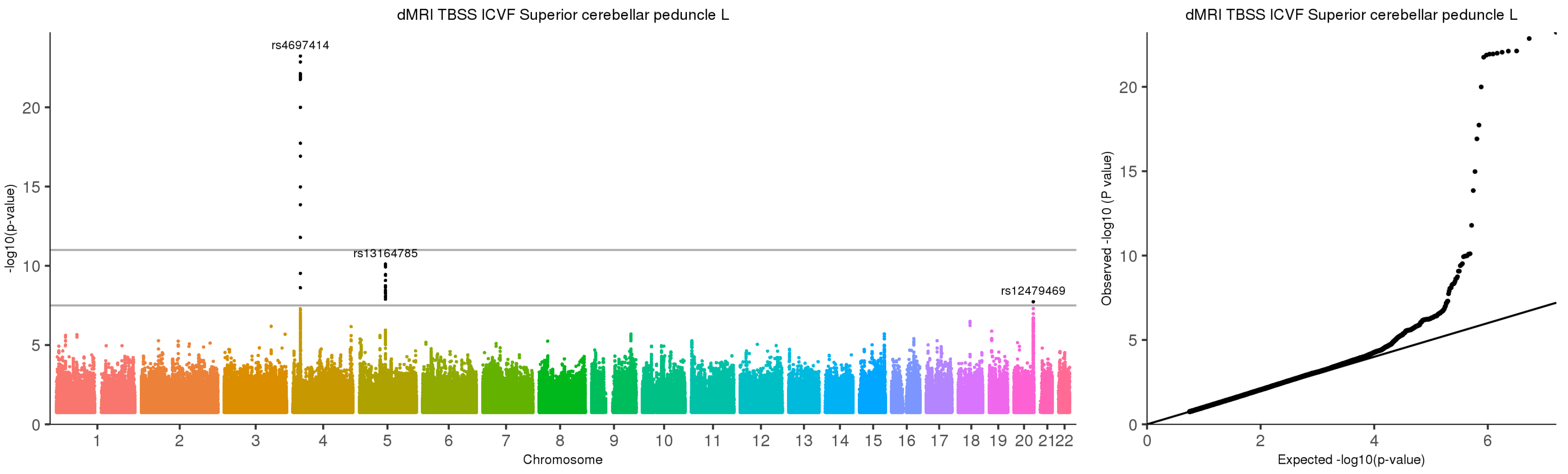

back to list of plots

Figure 12: dMRI TBSS OD Genu of corpus callosum

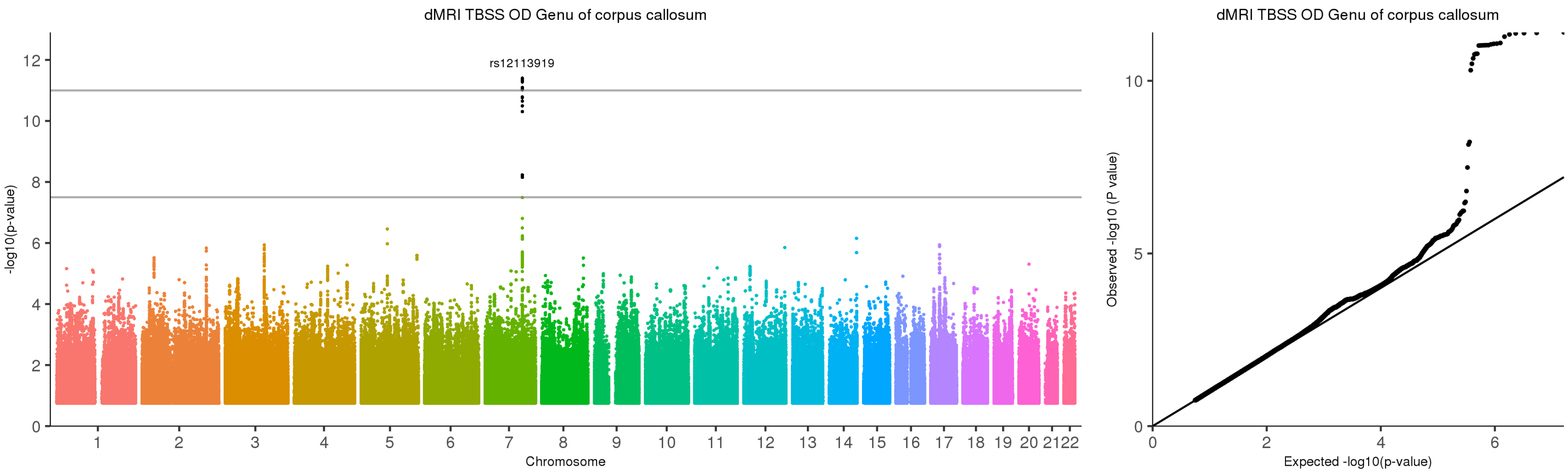

back to list of plots

Figure 13: dMRI ProbtrackX ICVF ilf r

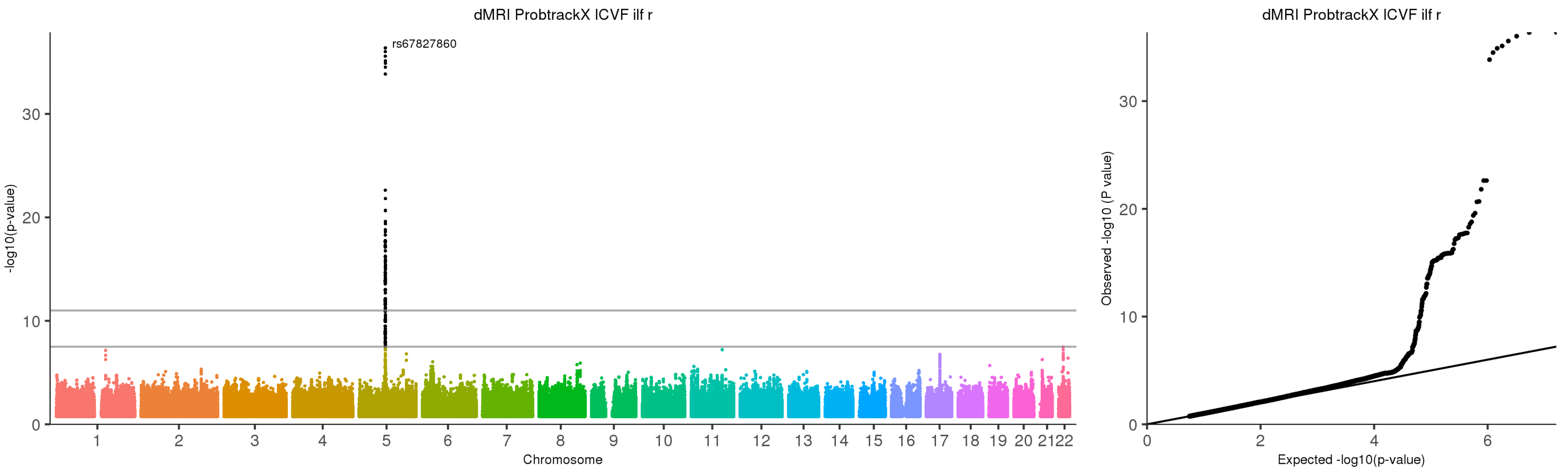

back to list of plots

Figure 14: dMRI ProbtrackX ISOVF unc r

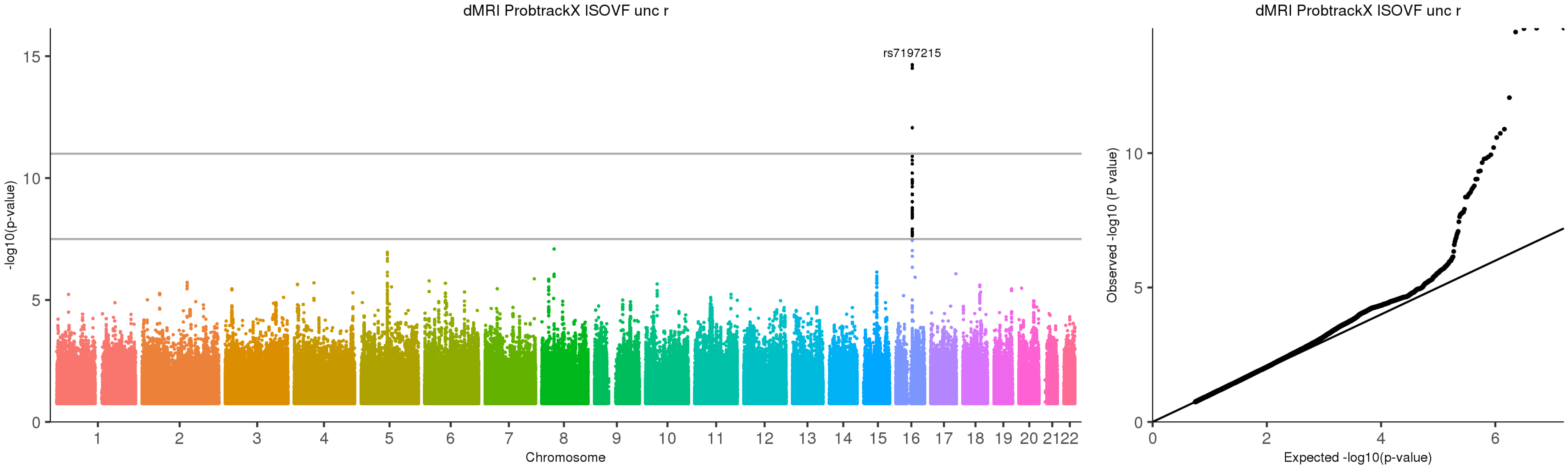

back to list of plots

Figure 15: NETMAT amplitudes (25) 05

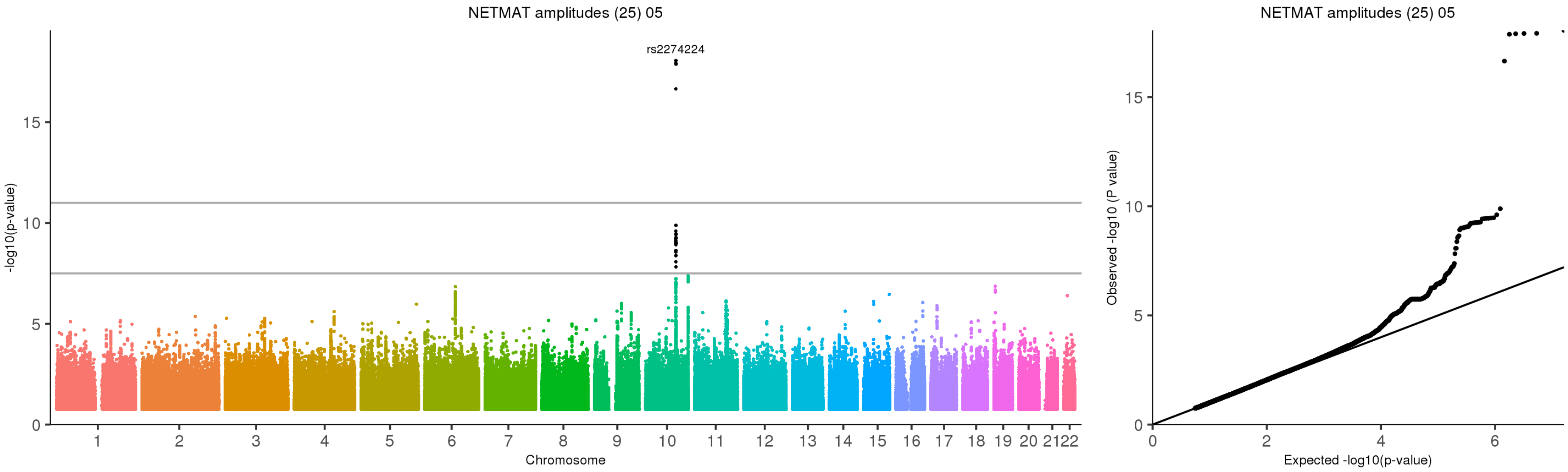

back to list of plots

Figure 16: NETMAT amplitudes (25) 12

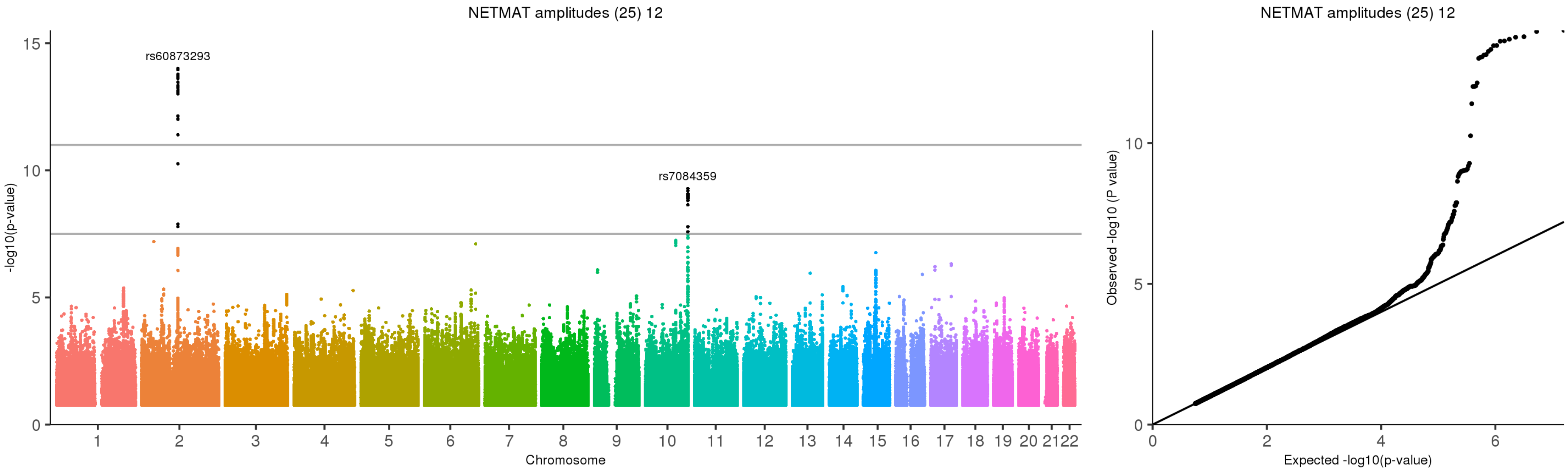

back to list of plots

Figure 17: NETMAT amplitudes (25) 13

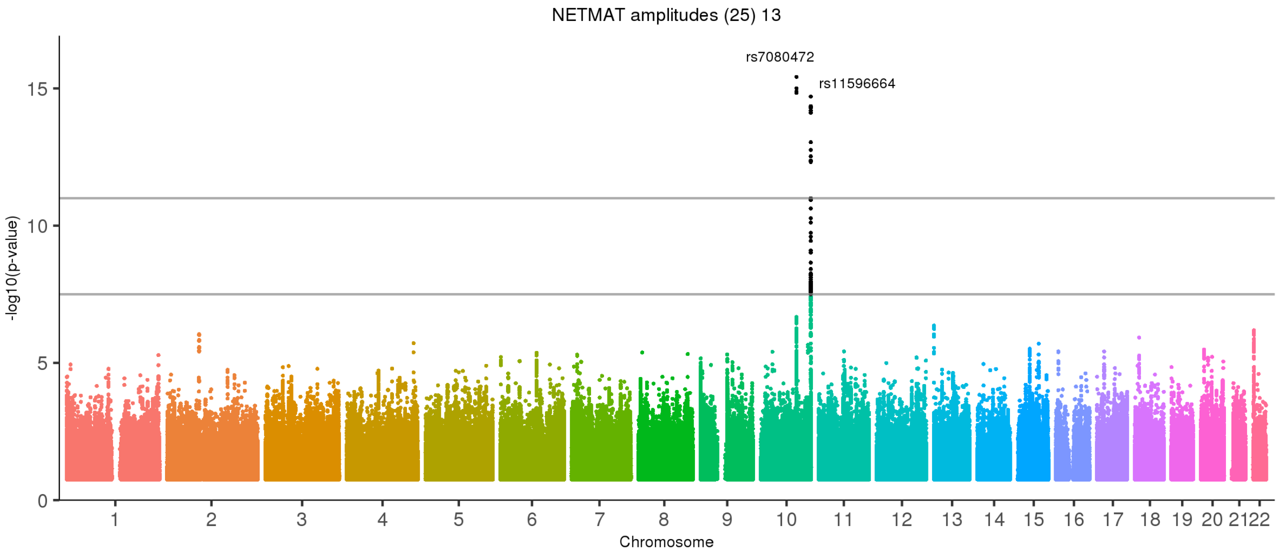

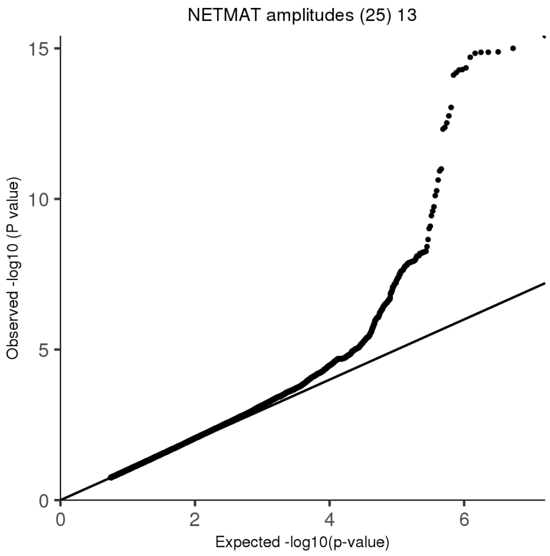

back to list of plots

Figure 18: T1 FIRST left plus right pallidum volume

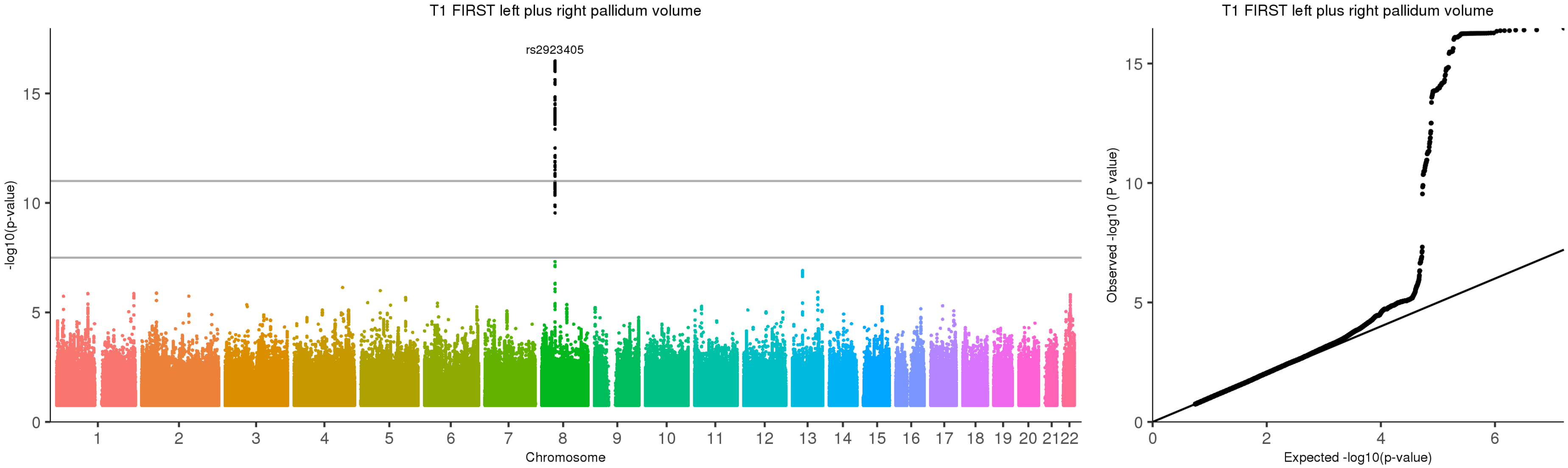

back to list of plots

Figure 19: SWI T2star left plus right caudate

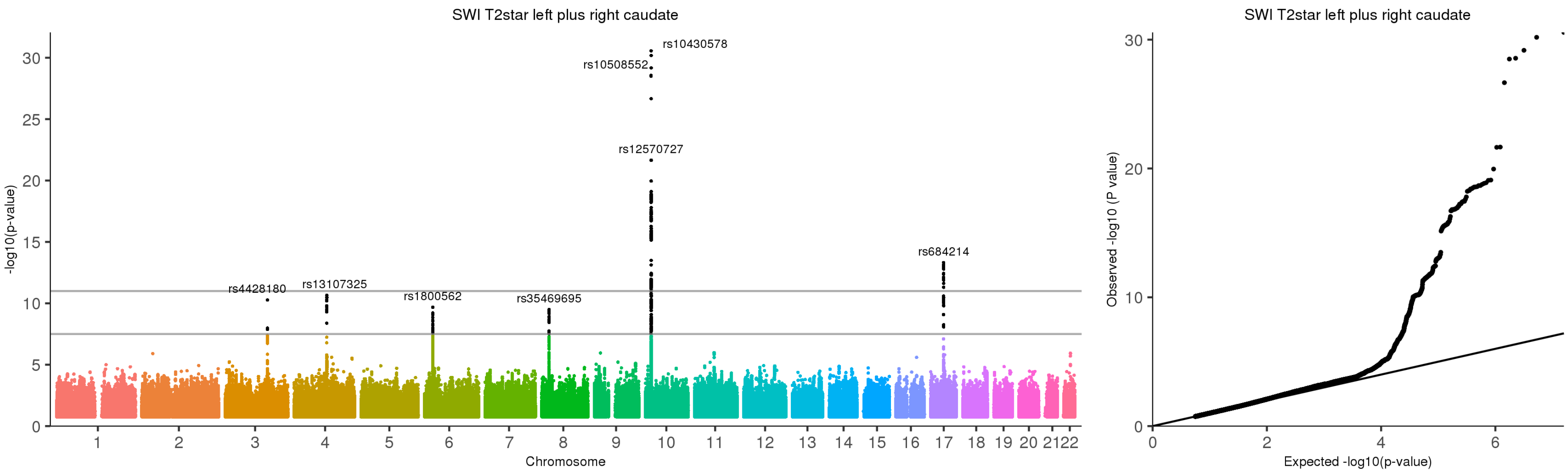

back to list of plots

Figure 20: SWI T2star left plus right putamen

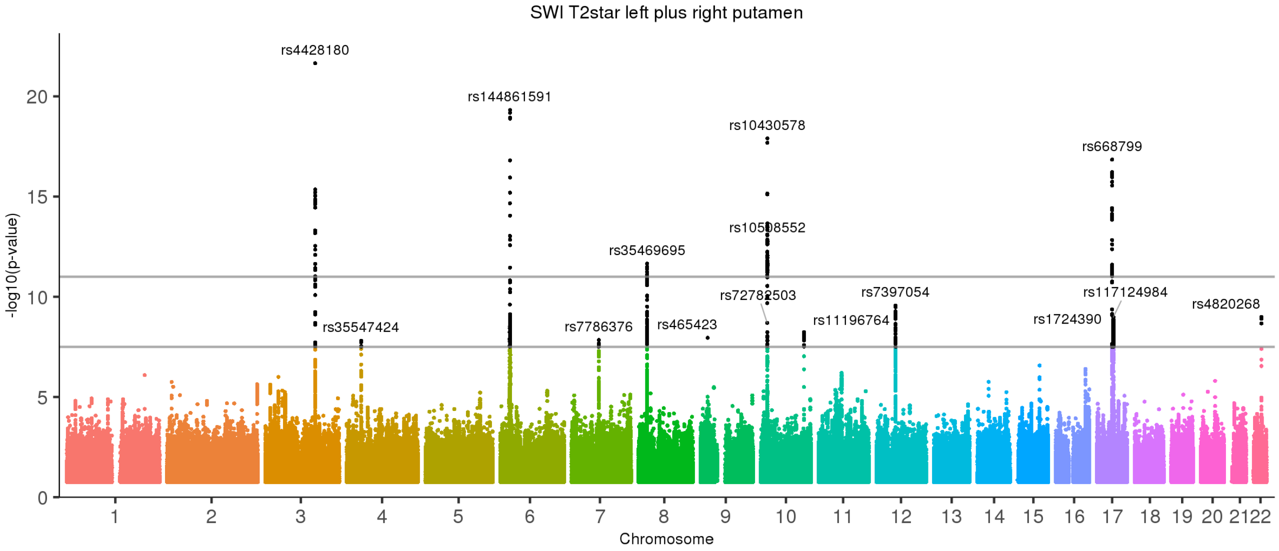

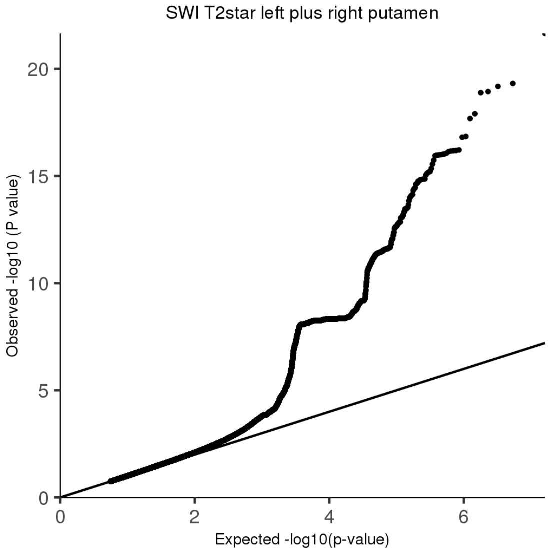

back to list of plots

Figure 21: SWI T2star left plus right pallidum

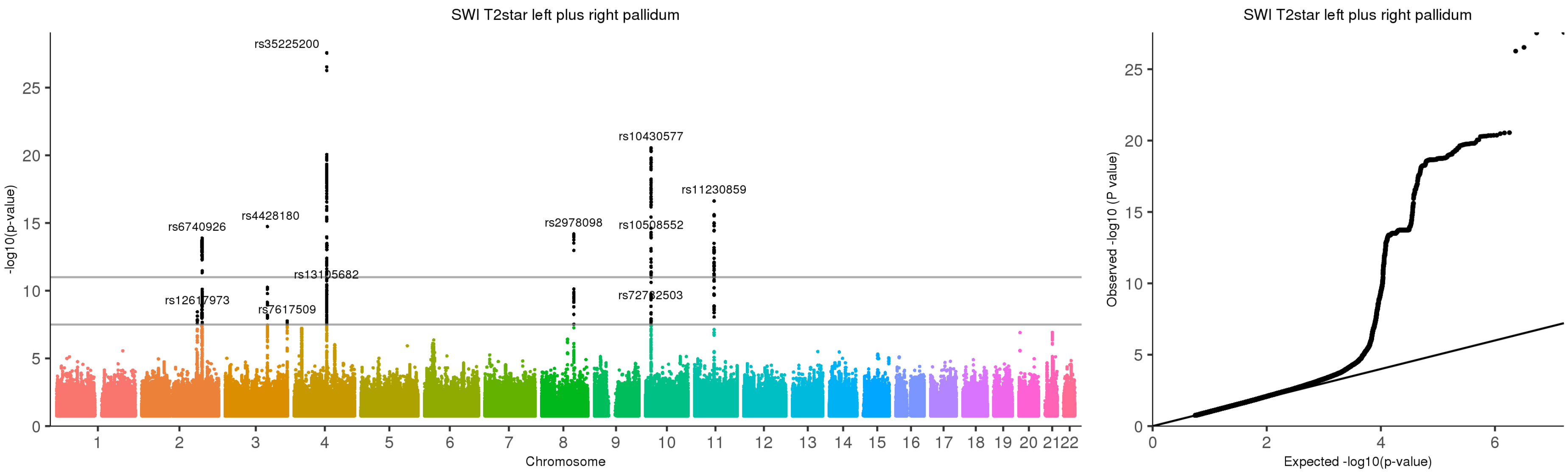

back to list of plots

Figure 22: FreeSurfer volume Left-Cerebellum-White-Matter

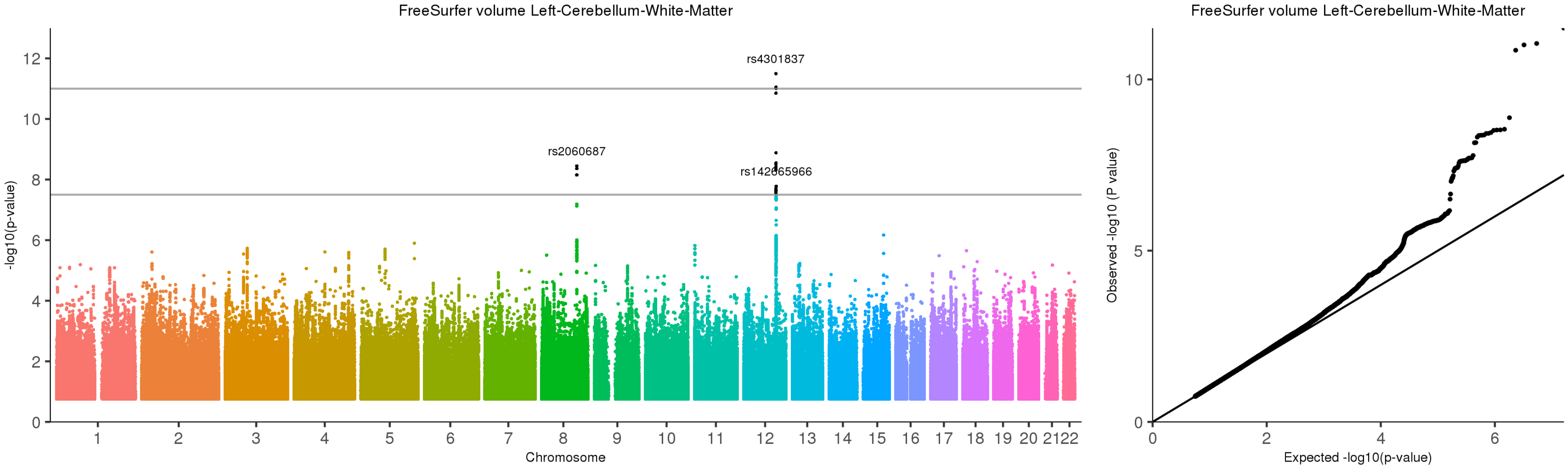

back to list of plots

Figure 23: FreeSurfer volume 4th-Ventricle

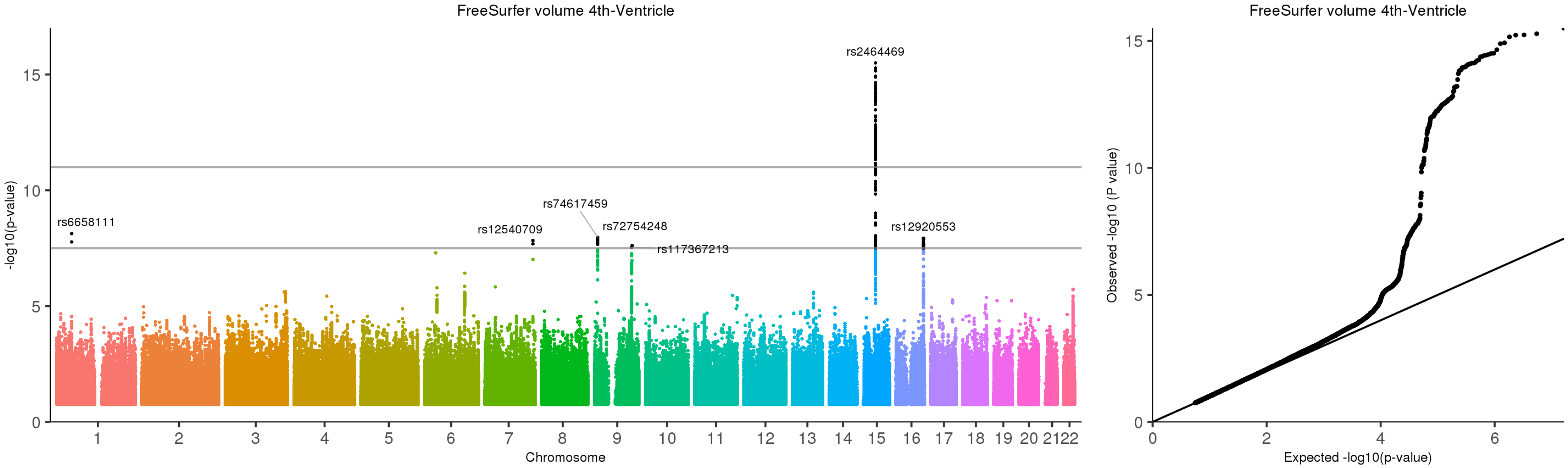

back to list of plots

Figure 24: FreeSurfer volume Right-Putamen

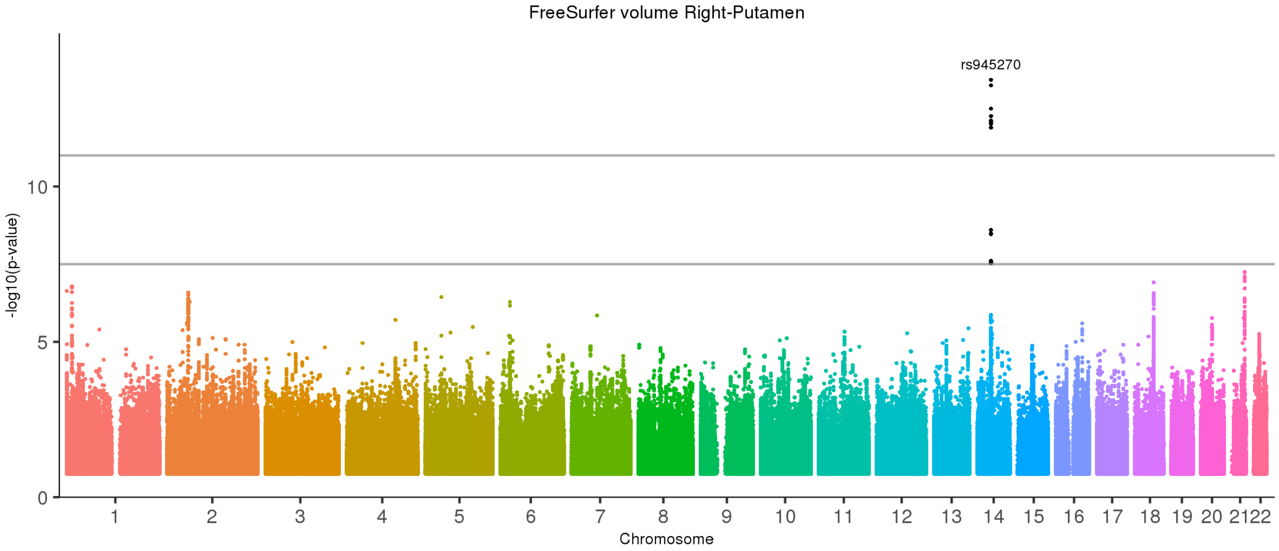

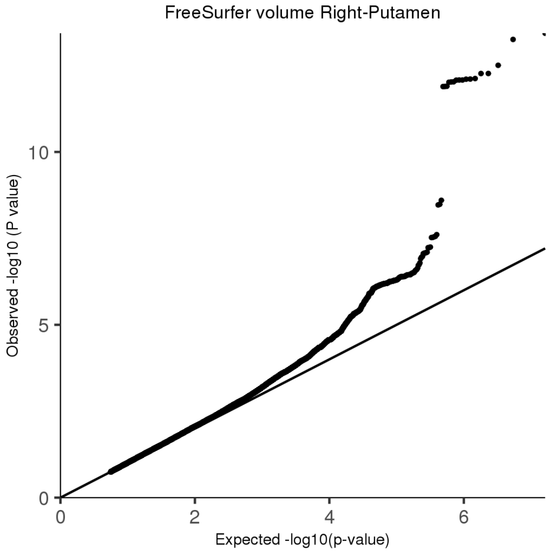

back to list of plots

Figure 25: FreeSurfer volume MaskVol

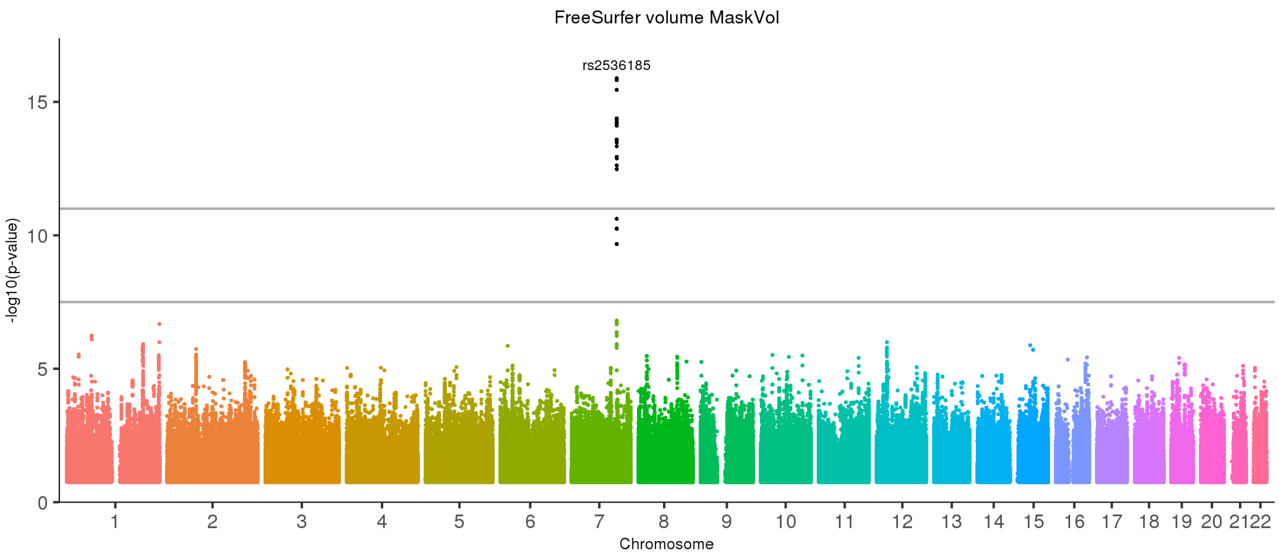

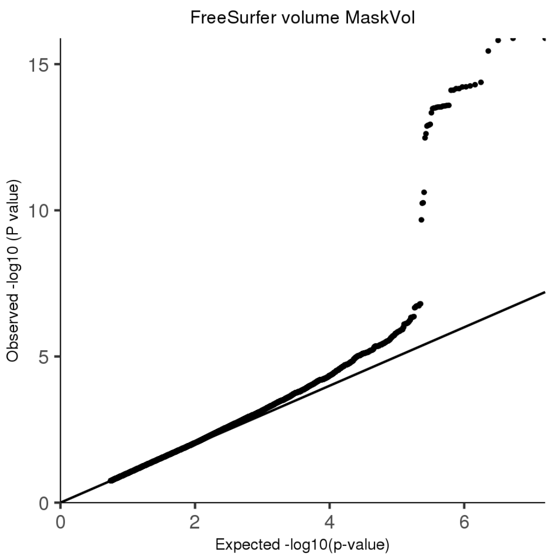

back to list of plots

Figure 26: FreeSurfer a2009s rh S central area

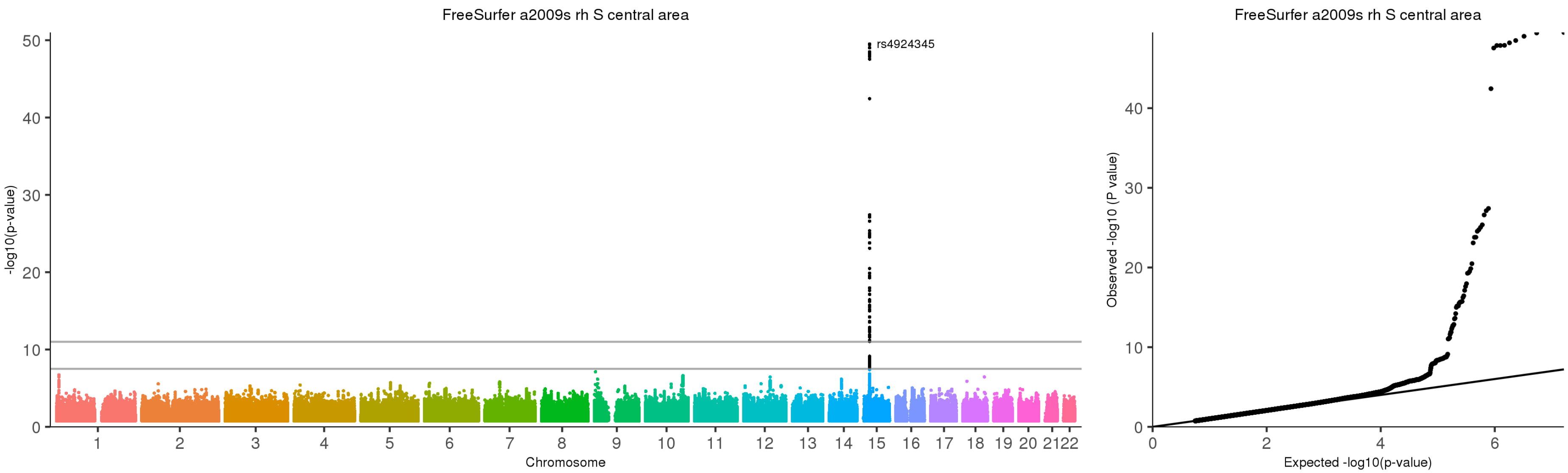

back to list of plots

Figure 27: NETMAT edge ICA 2

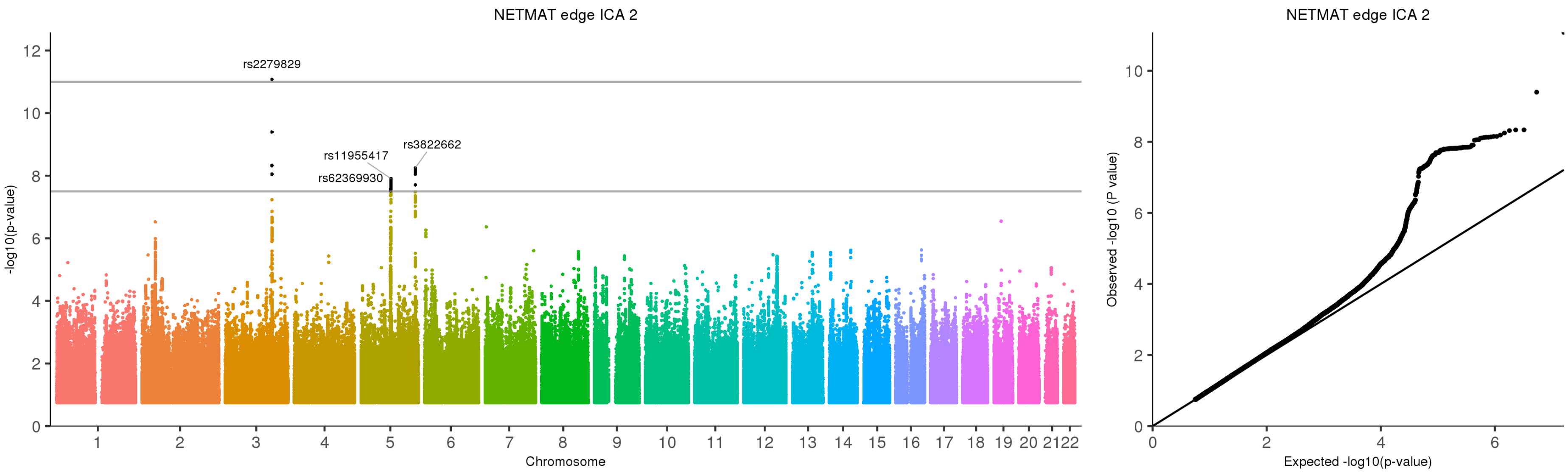

back to list of plots

Figure 28: NETMAT edge ICA 3

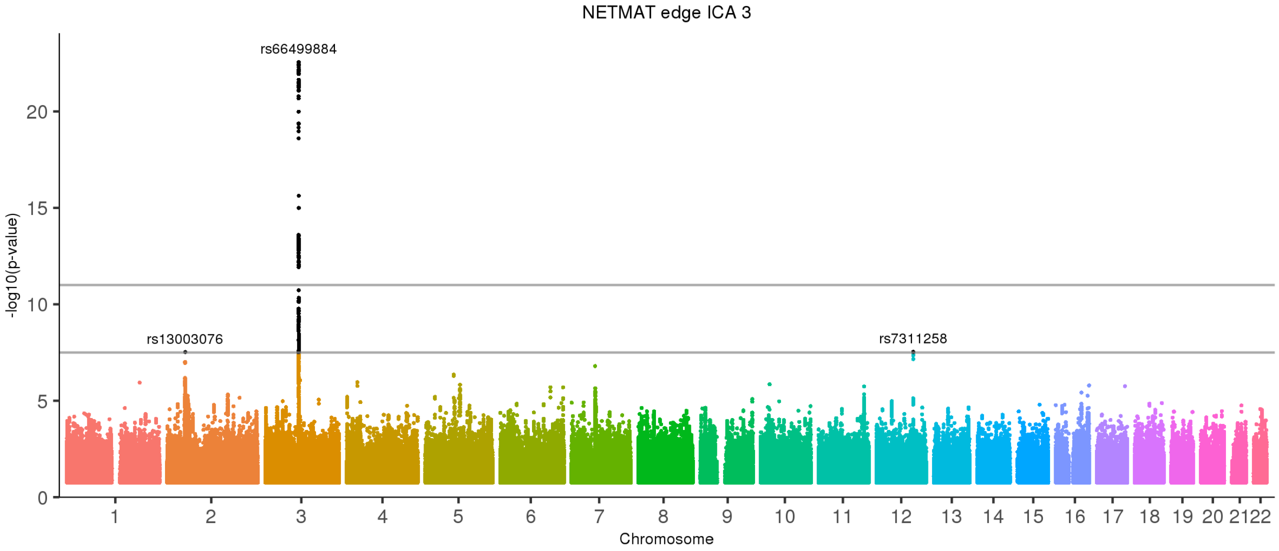

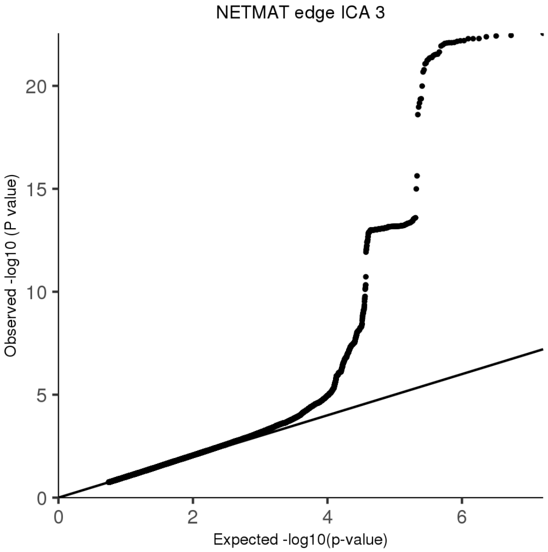

back to list of plots

Figure 29: NETMAT edge ICA 4

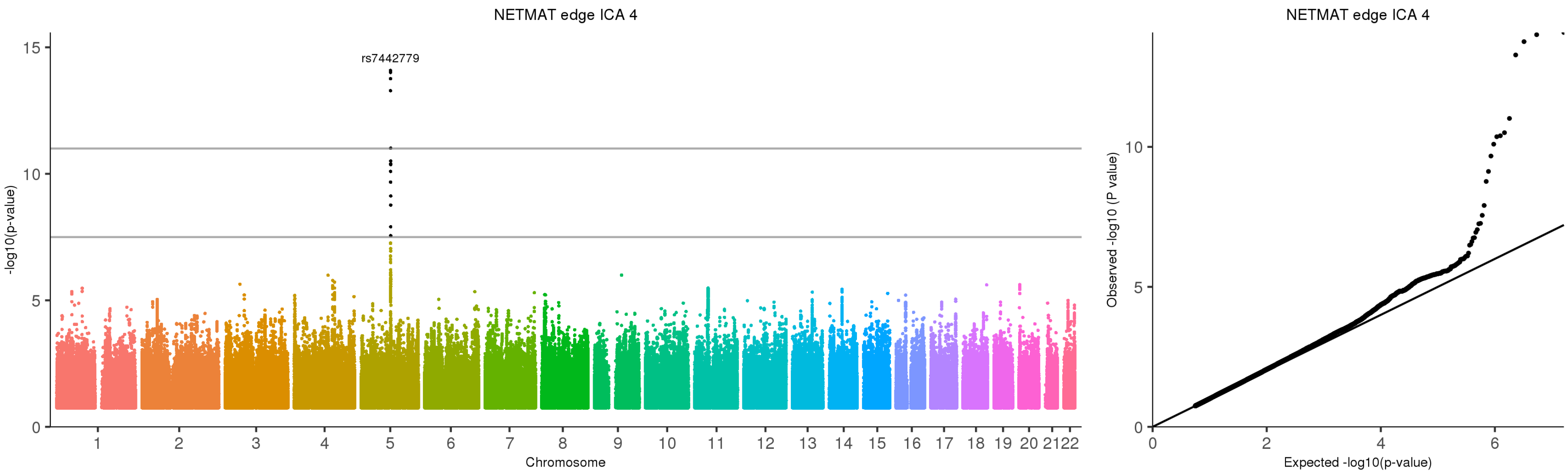

back to list of plots
