# Supplementary material for: Genome-wide association studies of brain imaging phenotypes in UK Biobank
Source: Nature. 2018 Oct 10;562(7726):210–6. doi: 10.1038/s41586-018-0571-7 (PMC6786974; doi:10.1038/s41586-018-0571-7)
Supplement: Supplementary file 3 — This file contains Supplementary Figures S1-S22. [file 41586_2018_571_MOESM3_ESM.zip › Figure-S8.pdf]

Supplementary Figure 8

Each of the subsequent pages shows a PheWAS plot for each one of the 78 SNPs with unique rsIDs in **Supplementary Table 6**. The association (-$\log_{10}$ p-value on the y-axis) for the SNP is shown with each of the 3,144 IDPs. The IDPs are arranged on the x-axis in the three panels : (top) Structural MRI IDPs, (middle) Structural connectivity/micro-structure dMRI IDPs, (bottom) functional MRI IDPs. Points are coloured to delineate subgroups of IDPs and detailed in the legends. Summary details of each SNP are given in the top right box. The grey line shows the Bonferroni multiple testing threshhold of 4.8.

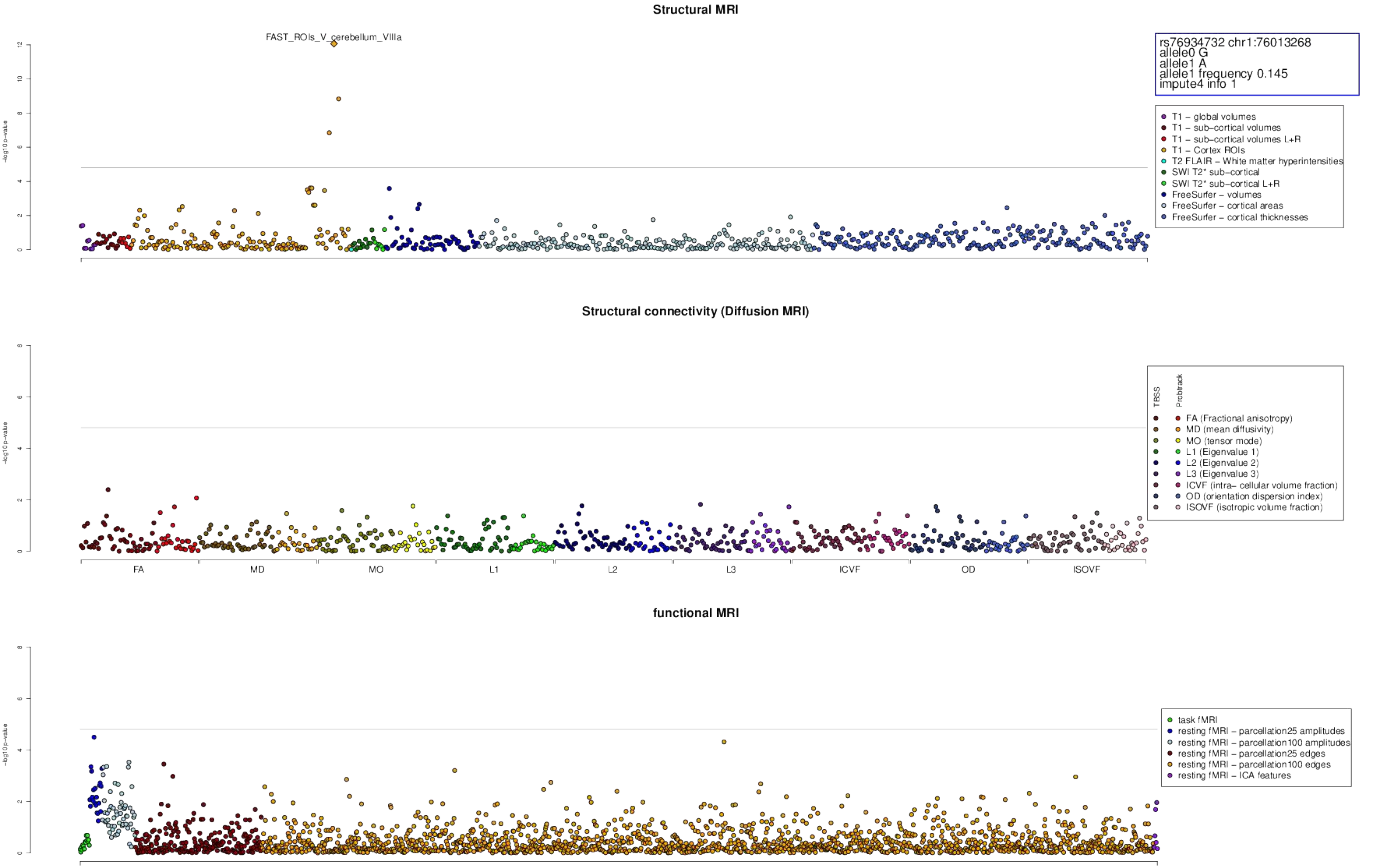

Structural MRI
FAST_ROIs_V_cerebellum_VIIIa
-log10 p-value
rs76934732 chr1:76013268
allele0 G
allele1 A
allele1 frequency 0.145
impute4 info 1
T1 – global volumes
T1 – sub-cortical volumes
T1 – sub-cortical volumes L+R
T1 – Cortex ROIs
T2 FLAIR – White matter hyperintensities
SWI T2* sub-cortical
SWI T2* sub-cortical L+R
FreeSurfer – volumes
FreeSurfer – cortical areas
FreeSurfer – cortical thicknesses
Structural connectivity (Diffusion MRI)
-log10 p-value
TBSS
Probtrack
FA (Fractional anisotropy)
MD (mean diffusivity)
MO (tensor mode)
L1 (Eigenvalue 1)
L2 (Eigenvalue 2)
L3 (Eigenvalue 3)
ICVF (intra- cellular volume fraction)
OD (orientation dispersion index)
ISOVF (isotropic volume fraction)
FA
MD
MO
L1
L2
L3
ICVF
OD
ISOVF
functional MRI
-log10 p-value
task fMRI
resting fMRI – parcellation25 amplitudes
resting fMRI – parcellation100 amplitudes
resting fMRI – parcellation25 edges
resting fMRI – parcellation100 edges
resting fMRI – ICA features

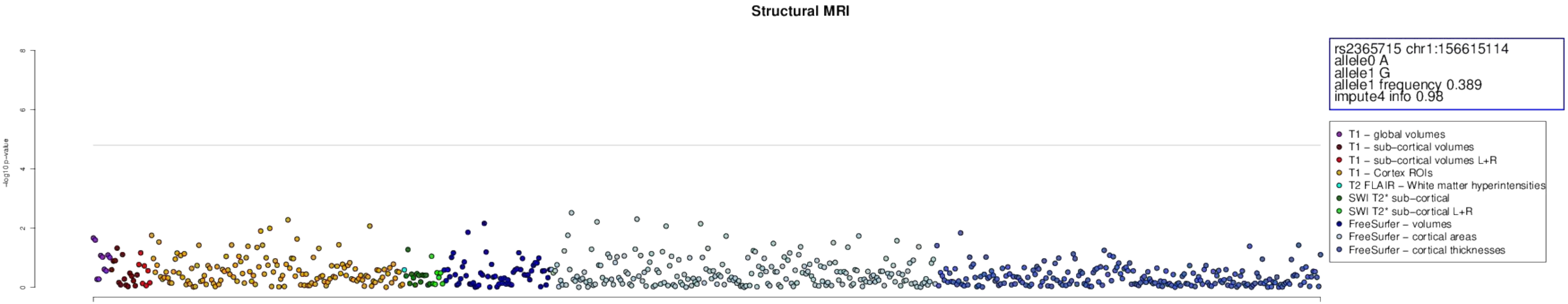
Structural MRI
-log10 p-value
rs2365715 chr1:156615114
allele0 A
allele1 G
allele1 frequency 0.389
impute4 info 0.98
T1 – global volumes
T1 – sub-cortical volumes
T1 – sub-cortical volumes L+R
T1 – Cortex ROIs
T2 FLAIR – White matter hyperintensities
SWI T2* sub-cortical
SWI T2* sub-cortical L+R
FreeSurfer – volumes
FreeSurfer – cortical areas
FreeSurfer – cortical thicknesses

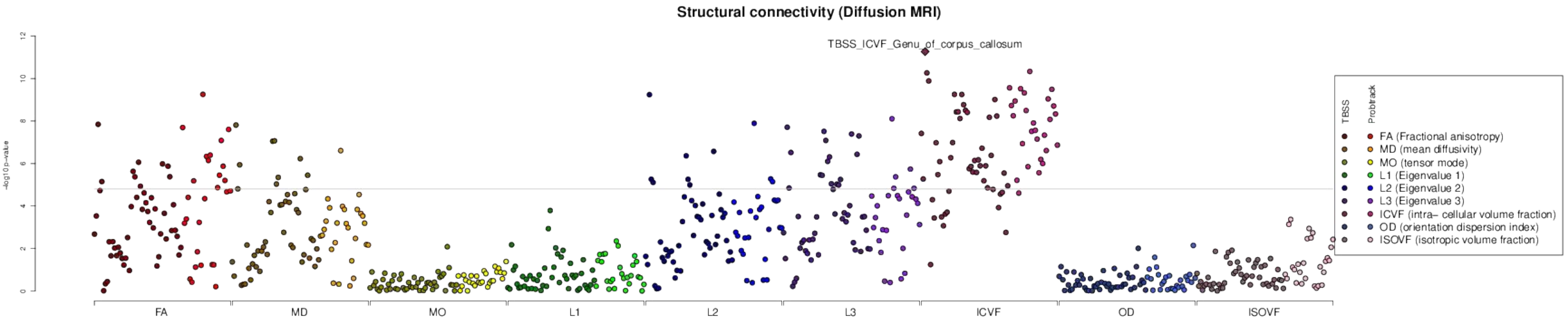
Structural connectivity (Diffusion MRI)
-log10 p-value
TBSS_ICVF_Genu_of_corpus_callosum
TBSS
Probtrack
FA (Fractional anisotropy)
MD (mean diffusivity)
MO (tensor mode)
L1 (Eigenvalue 1)
L2 (Eigenvalue 2)
L3 (Eigenvalue 3)
ICVF (intra- cellular volume fraction)
OD (orientation dispersion index)
ISOVF (isotropic volume fraction)
FA
MD
MO
L1
L2
L3
ICVF
OD
ISOVF

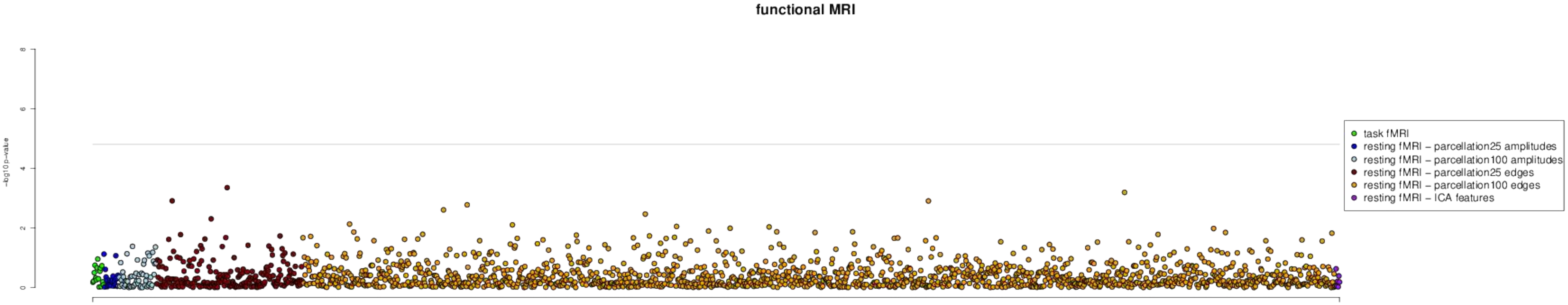
functional MRI
-log10 p-value
task fMRI
resting fMRI – parcellation25 amplitudes
resting fMRI – parcellation100 amplitudes
resting fMRI – parcellation25 edges
resting fMRI – parcellation100 edges
resting fMRI – ICA features

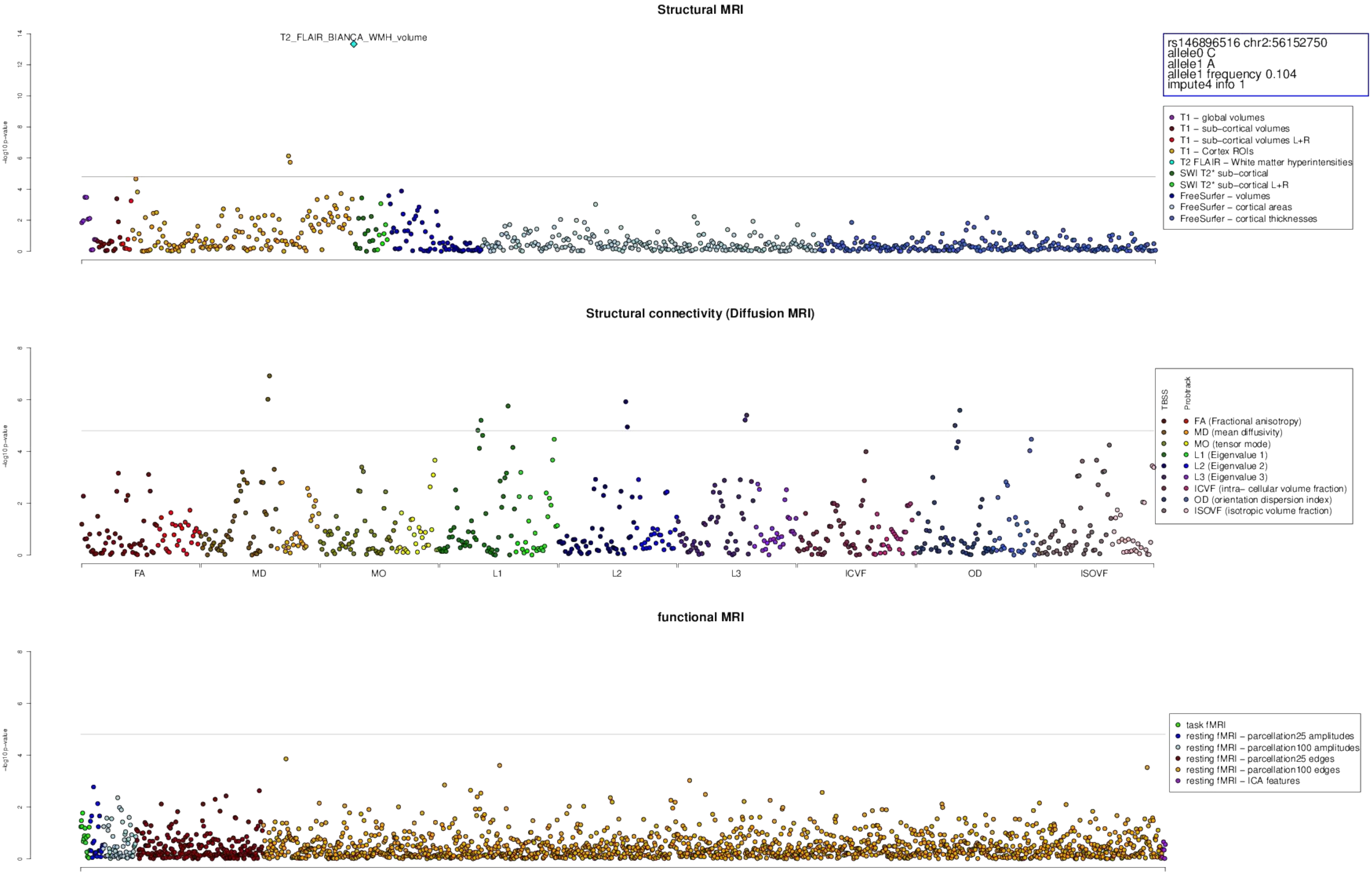
Structural MRI
T2_FLAIR_BIANCA_WMH_volume
-log10 p-value
rs146896516 chr2:56152750
allele0 C
allele1 A
allele1 frequency 0.104
impute4 info 1
T1 – global volumes
T1 – sub-cortical volumes
T1 – sub-cortical volumes L+R
T1 – Cortex ROIs
T2 FLAIR – White matter hyperintensities
SWI T2* sub-cortical
SWI T2* sub-cortical L+R
FreeSurfer – volumes
FreeSurfer – cortical areas
FreeSurfer – cortical thicknesses
Structural connectivity (Diffusion MRI)
TBSS
Probtrack
FA (Fractional anisotropy)
MD (mean diffusivity)
MO (tensor mode)
L1 (Eigenvalue 1)
L2 (Eigenvalue 2)
L3 (Eigenvalue 3)
ICVF (intra- cellular volume fraction)
OD (orientation dispersion index)
ISOVF (isotropic volume fraction)
FA
MD
MO
L1
L2
L3
ICVF
OD
ISOVF
functional MRI
task fMRI
resting fMRI – parcellation25 amplitudes
resting fMRI – parcellation100 amplitudes
resting fMRI – parcellation25 edges
resting fMRI – parcellation100 edges
resting fMRI – ICA features

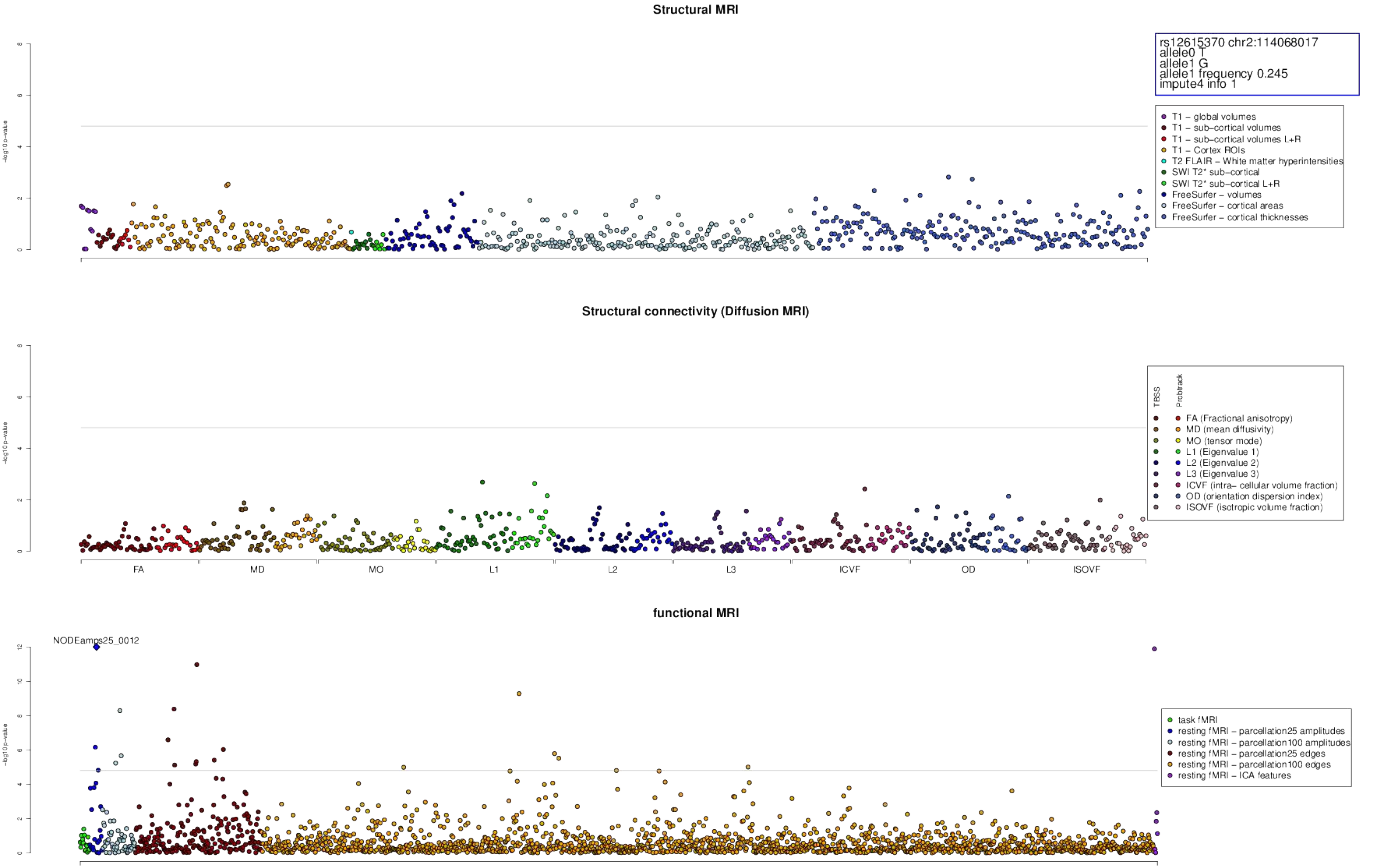

Structural MRI
rs12615370 chr2:114068017
allele0 T
allele1 G
allele1 frequency 0.245
impute4 info 1
T1 – global volumes
T1 – sub–cortical volumes
T1 – sub–cortical volumes L+R
T1 – Cortex ROIs
T2 FLAIR – White matter hyperintensities
SWI T2* sub–cortical
SWI T2* sub–cortical L+R
FreeSurfer – volumes
FreeSurfer – cortical areas
FreeSurfer – cortical thicknesses
–log10 p–value
Structural connectivity (Diffusion MRI)
TBSS
Probtrack
FA (Fractional anisotropy)
MD (mean diffusivity)
MO (tensor mode)
L1 (Eigenvalue 1)
L2 (Eigenvalue 2)
L3 (Eigenvalue 3)
ICVF (intra– cellular volume fraction)
OD (orientation dispersion index)
ISOVF (isotropic volume fraction)
FA
MD
MO
L1
L2
L3
ICVF
OD
ISOVF
functional MRI
NODEamps25_0012
task fMRI
resting fMRI – parcellation25 amplitudes
resting fMRI – parcellation100 amplitudes
resting fMRI – parcellation25 edges
resting fMRI – parcellation100 edges
resting fMRI – ICA features

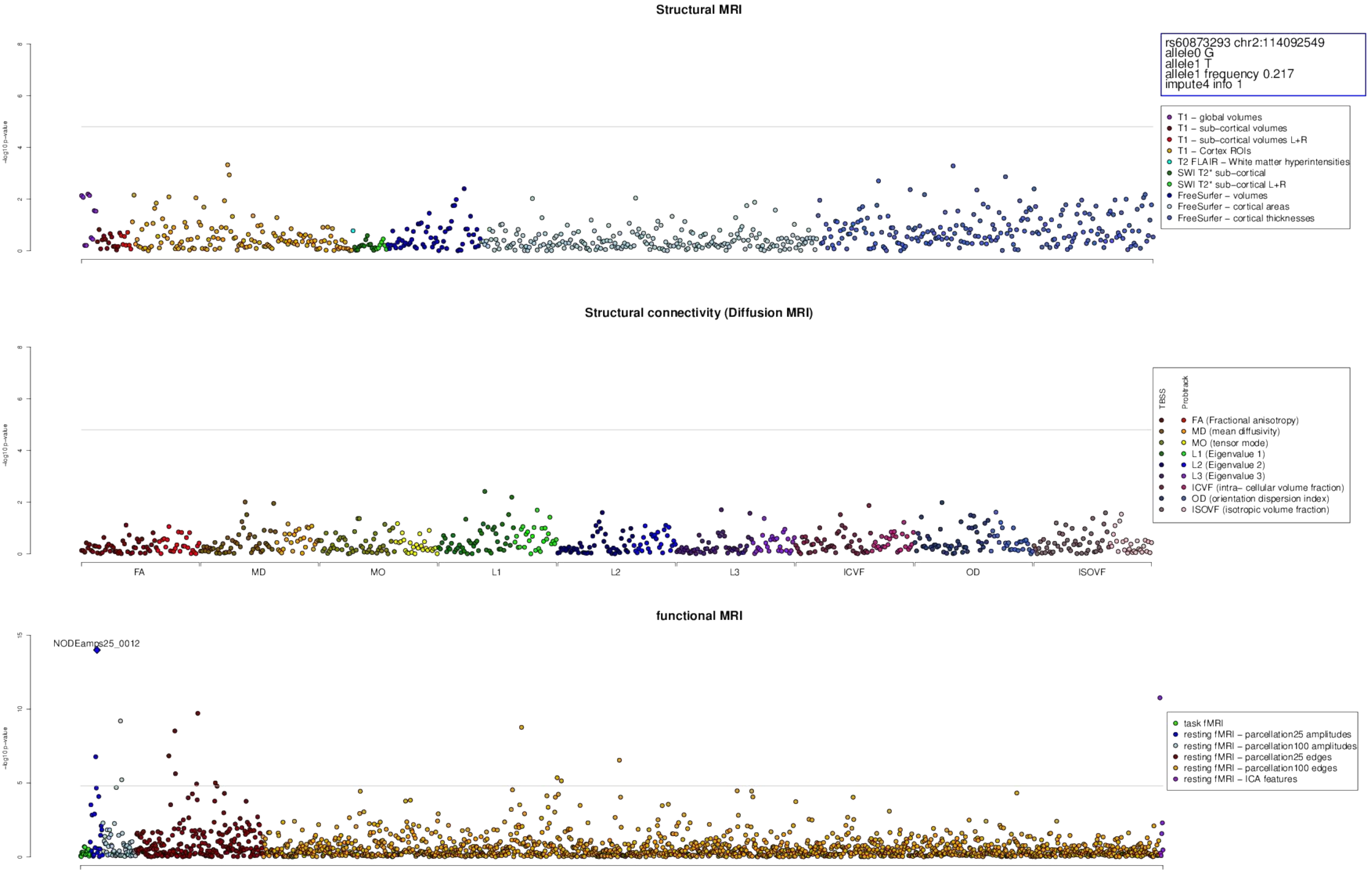
Structural MRI
rs60873293 chr2:114092549
allele0 G
allele1 T
allele1 frequency 0.217
impute4 info 1
T1 – global volumes
T1 – sub–cortical volumes
T1 – sub–cortical volumes L+R
T1 – Cortex ROIs
T2 FLAIR – White matter hyperintensities
SWI T2* sub–cortical
SWI T2* sub–cortical L+R
FreeSurfer – volumes
FreeSurfer – cortical areas
FreeSurfer – cortical thicknesses
-log10 p-value
Structural connectivity (Diffusion MRI)
TBSS
Probtrack
FA (Fractional anisotropy)
MD (mean diffusivity)
MO (tensor mode)
L1 (Eigenvalue 1)
L2 (Eigenvalue 2)
L3 (Eigenvalue 3)
ICVF (intra– cellular volume fraction)
OD (orientation dispersion index)
ISOVF (isotropic volume fraction)
FA
MD
MO
L1
L2
L3
ICVF
OD
ISOVF
functional MRI
NODEamps25_0012
task fMRI
resting fMRI – parcellation25 amplitudes
resting fMRI – parcellation100 amplitudes
resting fMRI – parcellation25 edges
resting fMRI – parcellation100 edges
resting fMRI – ICA features

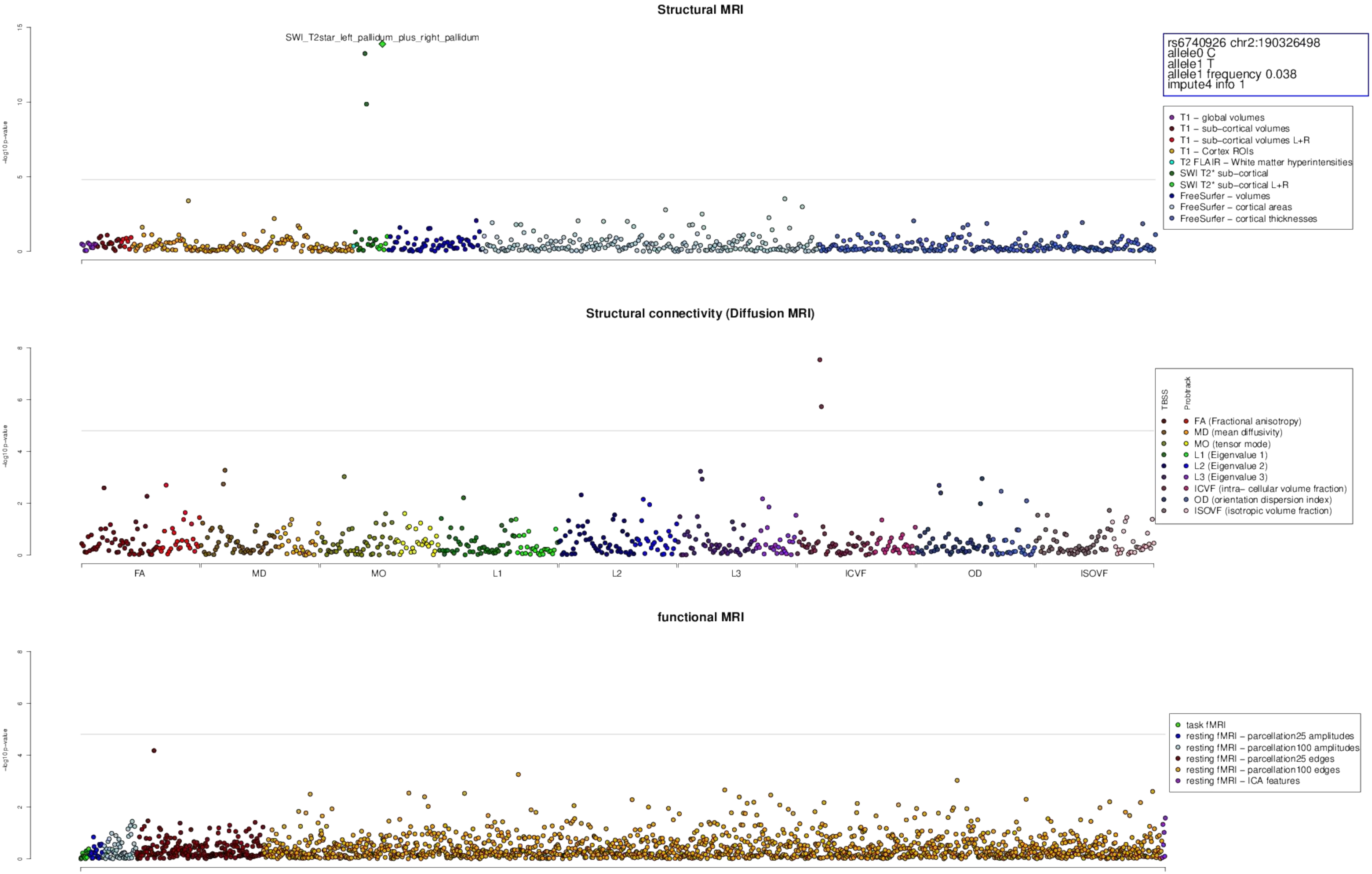
Structural MRI
SWI_T2star_left_pallidum_plus_right_pallidum
-log10 p-value
rs6740926 chr2:190326498
allele0 C
allele1 T
allele1 frequency 0.038
impute4 info 1
T1 – global volumes
T1 – sub-cortical volumes
T1 – sub-cortical volumes L+R
T1 – Cortex ROIs
T2 FLAIR – White matter hyperintensities
SWI T2* sub-cortical
SWI T2* sub-cortical L+R
FreeSurfer – volumes
FreeSurfer – cortical areas
FreeSurfer – cortical thicknesses
Structural connectivity (Diffusion MRI)
TBSS
Probtrack
FA (Fractional anisotropy)
MD (mean diffusivity)
MO (tensor mode)
L1 (Eigenvalue 1)
L2 (Eigenvalue 2)
L3 (Eigenvalue 3)
ICVF (intra- cellular volume fraction)
OD (orientation dispersion index)
ISOVF (isotropic volume fraction)
FA
MD
MO
L1
L2
L3
ICVF
OD
ISOVF
functional MRI
task fMRI
resting fMRI – parcellation25 amplitudes
resting fMRI – parcellation100 amplitudes
resting fMRI – parcellation25 edges
resting fMRI – parcellation100 edges
resting fMRI – ICA features

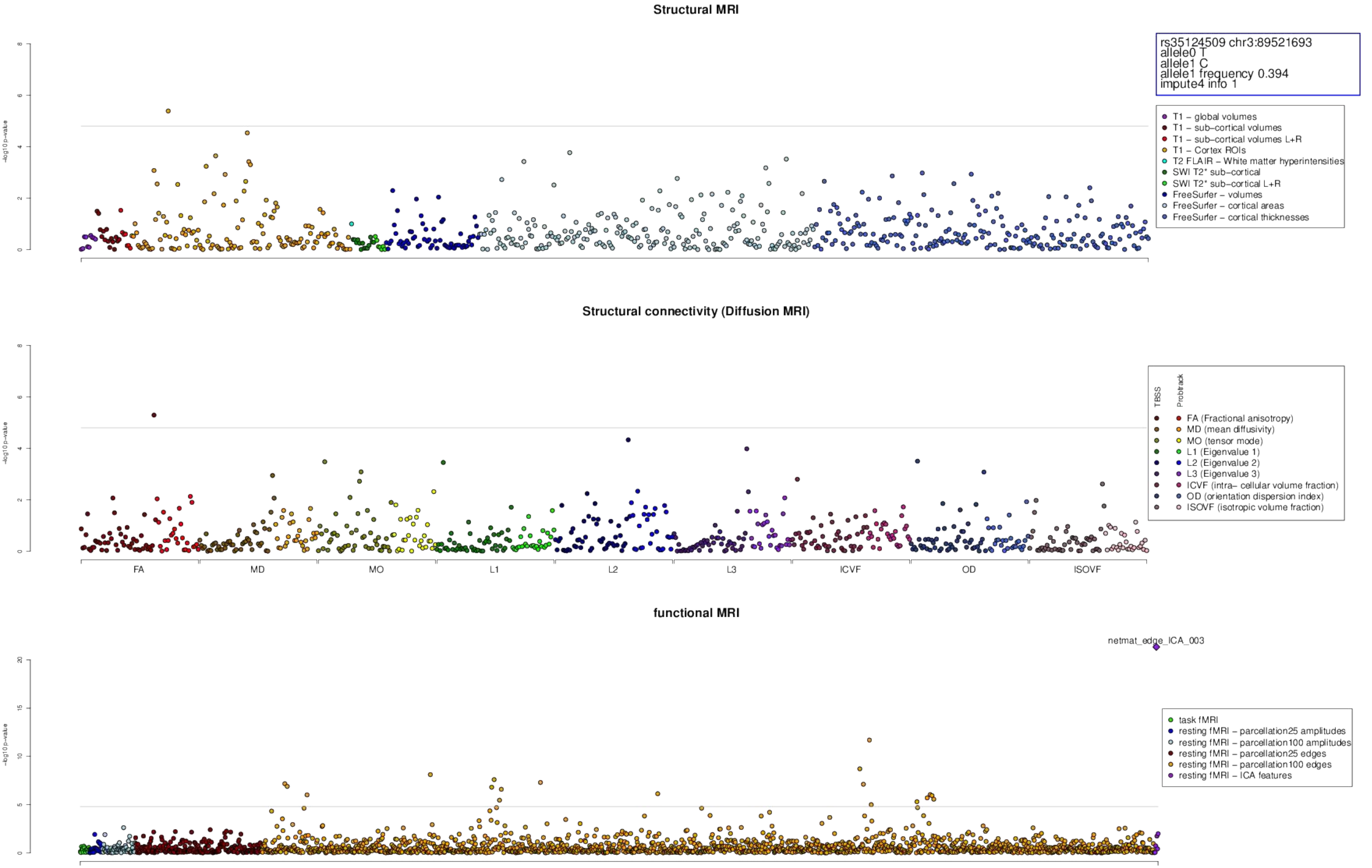
Structural MRI
rs35124509 chr3:89521693
allele0 T
allele1 C
allele1 frequency 0.394
impute4 info 1
T1 – global volumes
T1 – sub-cortical volumes
T1 – sub-cortical volumes L+R
T1 – Cortex ROIs
T2 FLAIR – White matter hyperintensities
SWI T2* sub-cortical
SWI T2* sub-cortical L+R
FreeSurfer – volumes
FreeSurfer – cortical areas
FreeSurfer – cortical thicknesses
-log10 p-value
Structural connectivity (Diffusion MRI)
TBSS
Probtrack
FA (Fractional anisotropy)
MD (mean diffusivity)
MO (tensor mode)
L1 (Eigenvalue 1)
L2 (Eigenvalue 2)
L3 (Eigenvalue 3)
ICVF (intra- cellular volume fraction)
OD (orientation dispersion index)
ISOVF (isotropic volume fraction)
FA
MD
MO
L1
L2
L3
ICVF
OD
ISOVF
functional MRI
netmat_edge_ICA_003
task fMRI
resting fMRI – parcellation25 amplitudes
resting fMRI – parcellation100 amplitudes
resting fMRI – parcellation25 edges
resting fMRI – parcellation100 edges
resting fMRI – ICA features

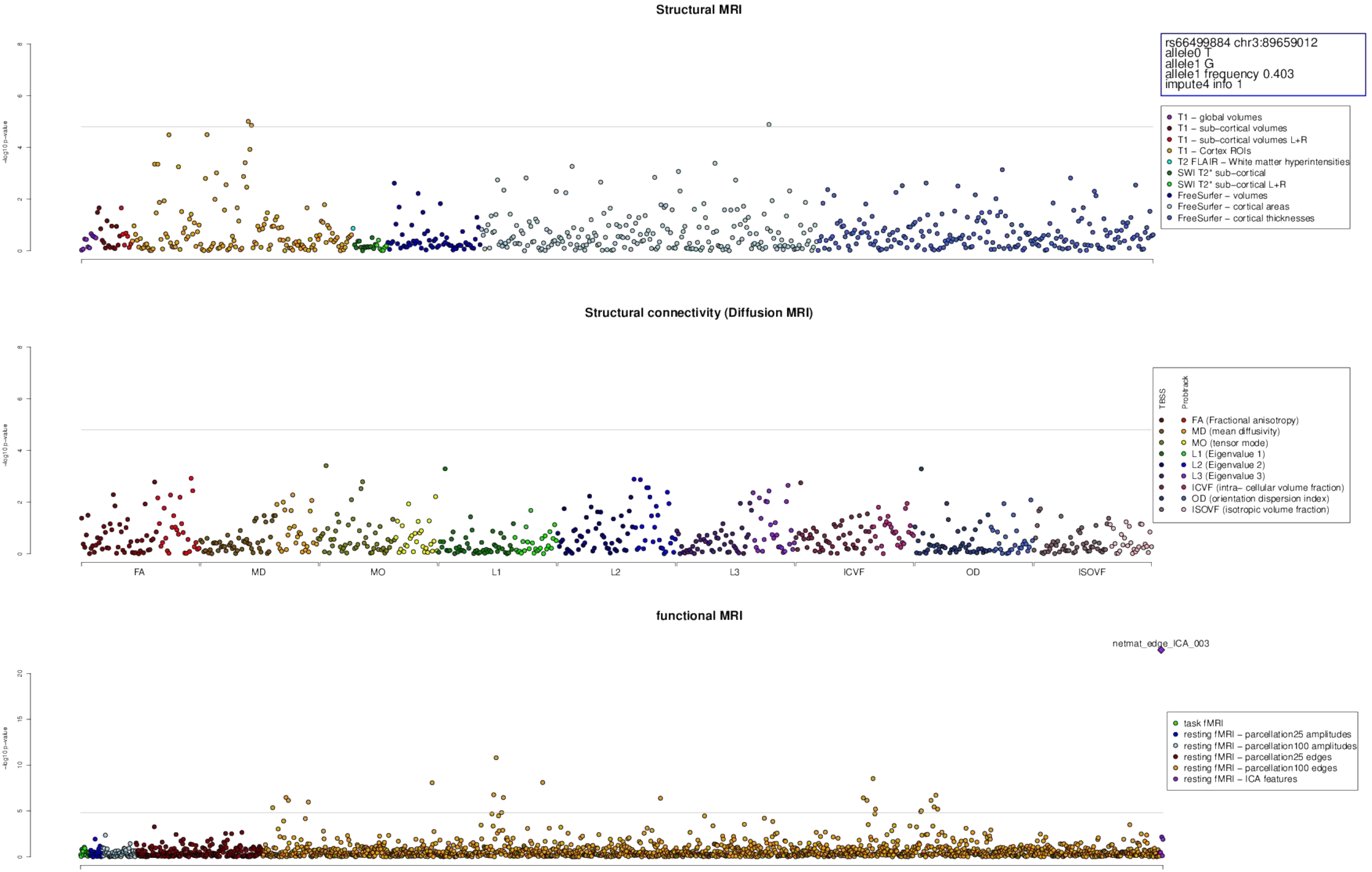

Structural MRI
rs66499884 chr3:89659012
allele0 T
allele1 G
allele1 frequency 0.403
impute4 info 1
T1 – global volumes
T1 – sub–cortical volumes
T1 – sub–cortical volumes L+R
T1 – Cortex ROIs
T2 FLAIR – White matter hyperintensities
SWI T2* sub–cortical
SWI T2* sub–cortical L+R
FreeSurfer – volumes
FreeSurfer – cortical areas
FreeSurfer – cortical thicknesses
-log10 p-value
Structural connectivity (Diffusion MRI)
TBSS
Probtrack
FA (Fractional anisotropy)
MD (mean diffusivity)
MO (tensor mode)
L1 (Eigenvalue 1)
L2 (Eigenvalue 2)
L3 (Eigenvalue 3)
ICVF (intra– cellular volume fraction)
OD (orientation dispersion index)
ISOVF (isotropic volume fraction)
FA
MD
MO
L1
L2
L3
ICVF
OD
ISOVF
functional MRI
netmat_edge_ICA_003
task fMRI
resting fMRI – parcellation25 amplitudes
resting fMRI – parcellation100 amplitudes
resting fMRI – parcellation25 edges
resting fMRI – parcellation100 edges
resting fMRI – ICA features

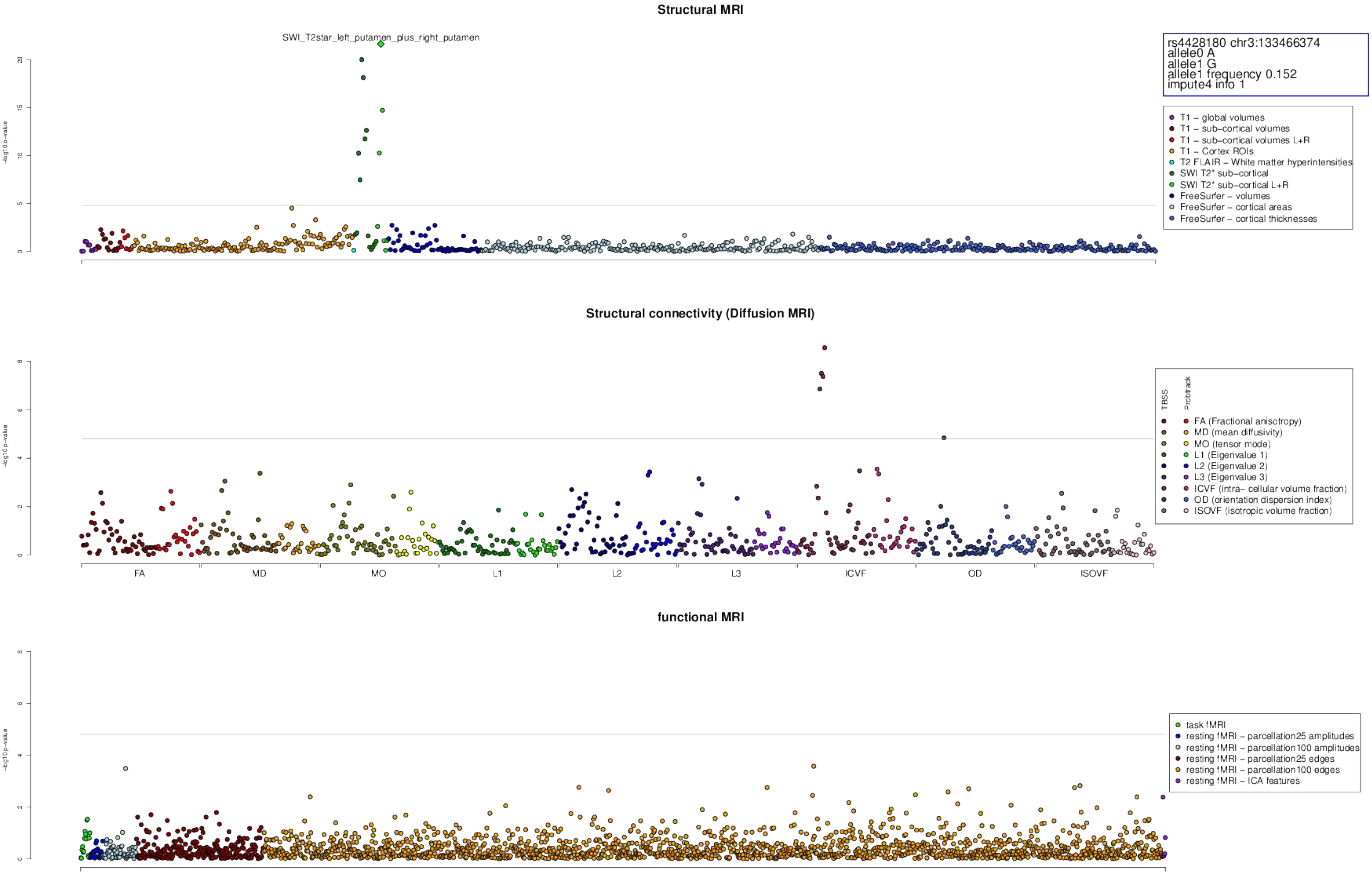

Structural MRI
SWI_T2star_left_putamen_plus_right_putamen
-log10 p-value
rs4428180 chr3:133466374
allele0 A
allele1 G
allele1 frequency 0.152
impute4 info 1
T1 – global volumes
T1 – sub-cortical volumes
T1 – sub-cortical volumes L+R
T1 – Cortex ROIs
T2 FLAIR – White matter hyperintensities
SWI T2* sub-cortical
SWI T2* sub-cortical L+R
FreeSurfer – volumes
FreeSurfer – cortical areas
FreeSurfer – cortical thicknesses
Structural connectivity (Diffusion MRI)
-log10 p-value
TBSS
Probtrack
FA (Fractional anisotropy)
MD (mean diffusivity)
MO (tensor mode)
L1 (Eigenvalue 1)
L2 (Eigenvalue 2)
L3 (Eigenvalue 3)
ICVF (intra- cellular volume fraction)
OD (orientation dispersion index)
ISOVF (isotropic volume fraction)
FA
MD
MO
L1
L2
L3
ICVF
OD
ISOVF
functional MRI
-log10 p-value
task fMRI
resting fMRI – parcellation25 amplitudes
resting fMRI – parcellation100 amplitudes
resting fMRI – parcellation25 edges
resting fMRI – parcellation100 edges
resting fMRI – ICA features

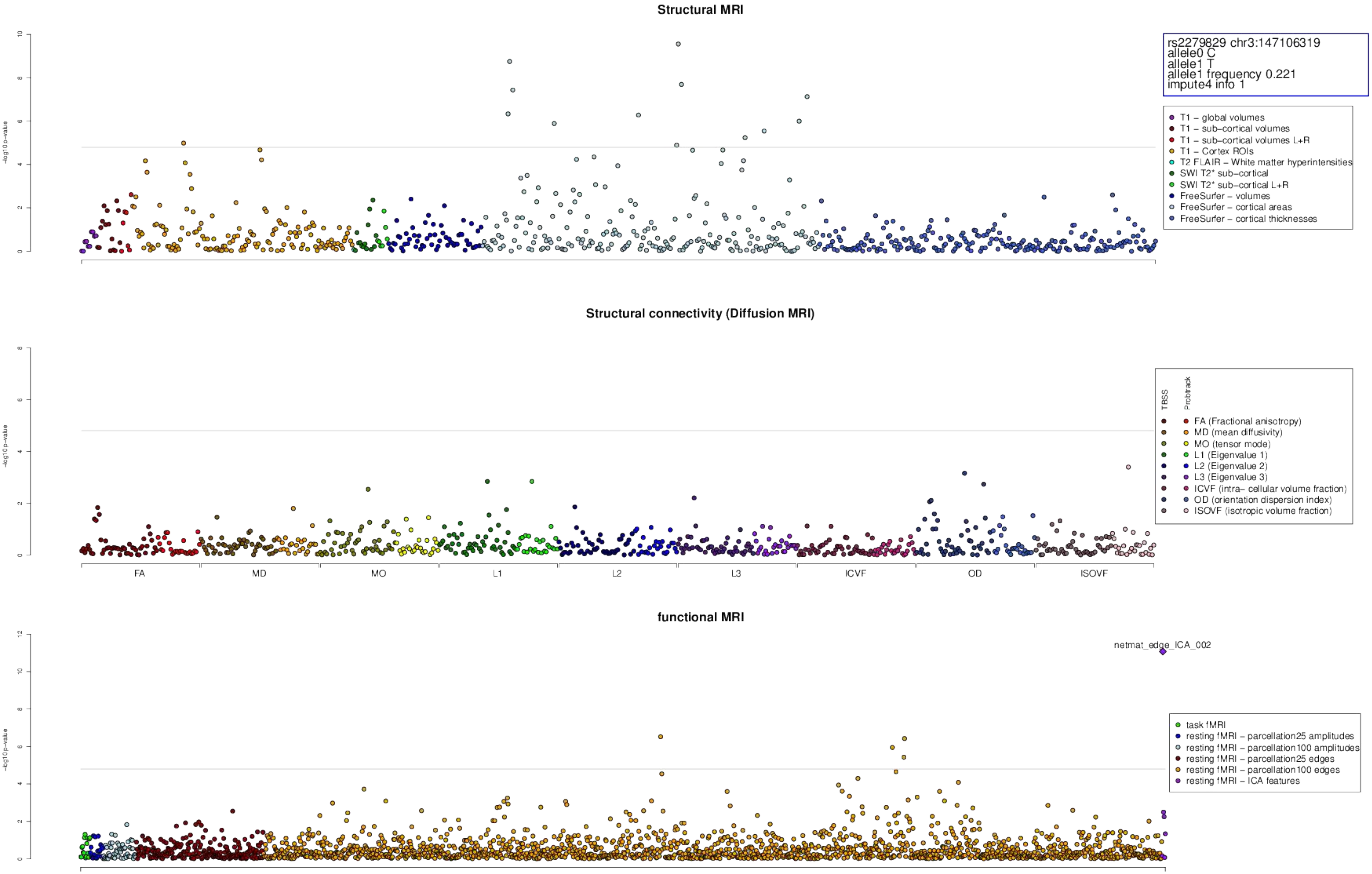
Structural MRI
-log10 p-value
rs2279829 chr3:147106319
allele0 C
allele1 T
allele1 frequency 0.221
impute4 info 1
T1 – global volumes
T1 – sub-cortical volumes
T1 – sub-cortical volumes L+R
T1 – Cortex ROIs
T2 FLAIR – White matter hyperintensities
SWI T2* sub-cortical
SWI T2* sub-cortical L+R
FreeSurfer – volumes
FreeSurfer – cortical areas
FreeSurfer – cortical thicknesses
Structural connectivity (Diffusion MRI)
TBSS
Probtrack
FA (Fractional anisotropy)
MD (mean diffusivity)
MO (tensor mode)
L1 (Eigenvalue 1)
L2 (Eigenvalue 2)
L3 (Eigenvalue 3)
ICVF (intra- cellular volume fraction)
OD (orientation dispersion index)
ISOVF (isotropic volume fraction)
FA
MD
MO
L1
L2
L3
ICVF
OD
ISOVF
functional MRI
netmat_edge_ICA_002
task fMRI
resting fMRI – parcellation25 amplitudes
resting fMRI – parcellation100 amplitudes
resting fMRI – parcellation25 edges
resting fMRI – parcellation100 edges
resting fMRI – ICA features

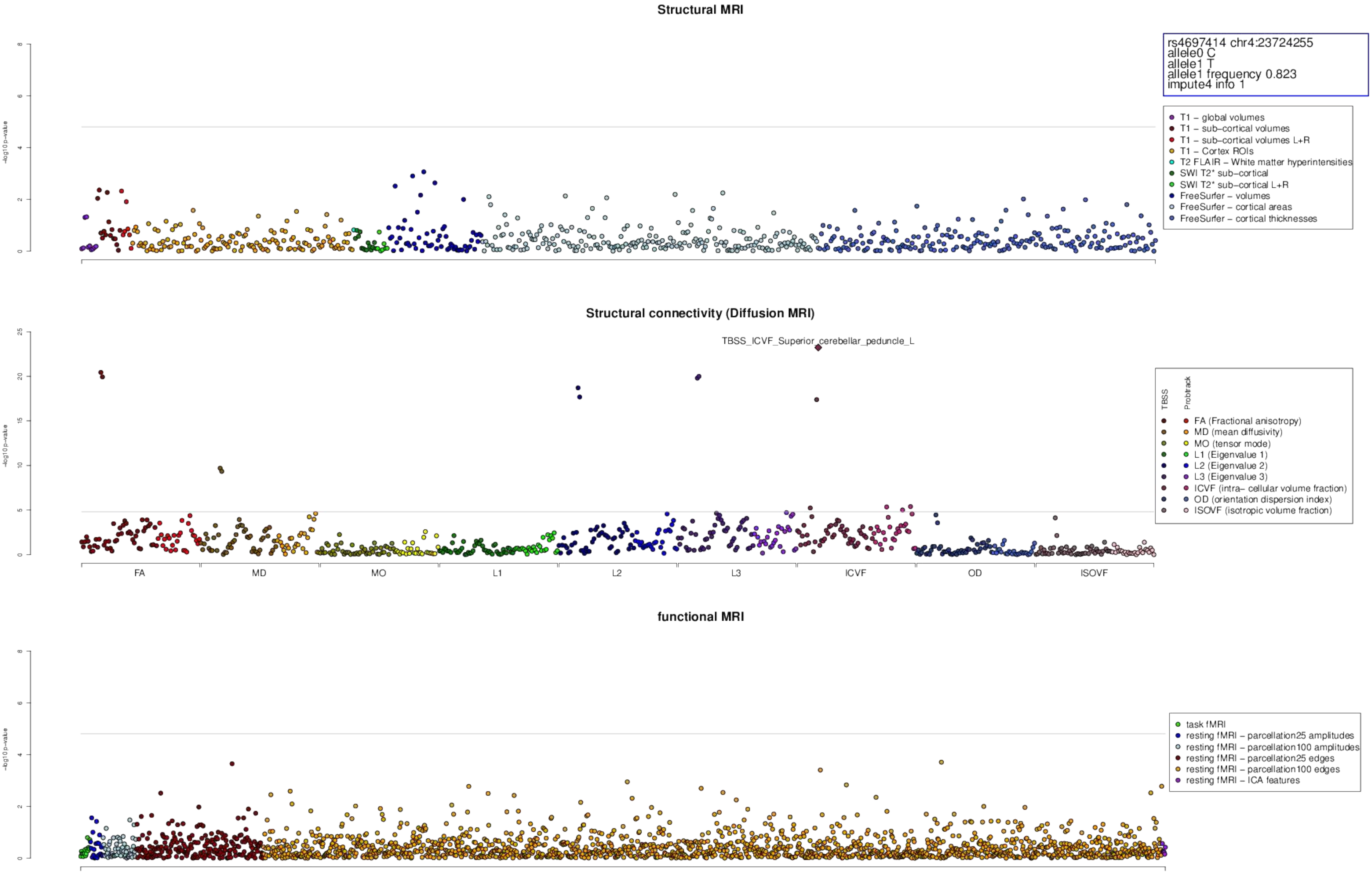
Structural MRI
rs4697414 chr4:23724255
allele0 C
allele1 T
allele1 frequency 0.823
impute4 info 1
T1 – global volumes
T1 – sub–cortical volumes
T1 – sub–cortical volumes L+R
T1 – Cortex ROIs
T2 FLAIR – White matter hyperintensities
SWI T2* sub–cortical
SWI T2* sub–cortical L+R
FreeSurfer – volumes
FreeSurfer – cortical areas
FreeSurfer – cortical thicknesses
–log10 p–value
Structural connectivity (Diffusion MRI)
TBSS_ICVF_Superior_cerebellar_peduncle_L
TBSS
Probtrack
FA (Fractional anisotropy)
MD (mean diffusivity)
MO (tensor mode)
L1 (Eigenvalue 1)
L2 (Eigenvalue 2)
L3 (Eigenvalue 3)
ICVF (intra– cellular volume fraction)
OD (orientation dispersion index)
ISOVF (isotropic volume fraction)
FA
MD
MO
L1
L2
L3
ICVF
OD
ISOVF
functional MRI
task fMRI
resting fMRI – parcellation25 amplitudes
resting fMRI – parcellation100 amplitudes
resting fMRI – parcellation25 edges
resting fMRI – parcellation100 edges
resting fMRI – ICA features

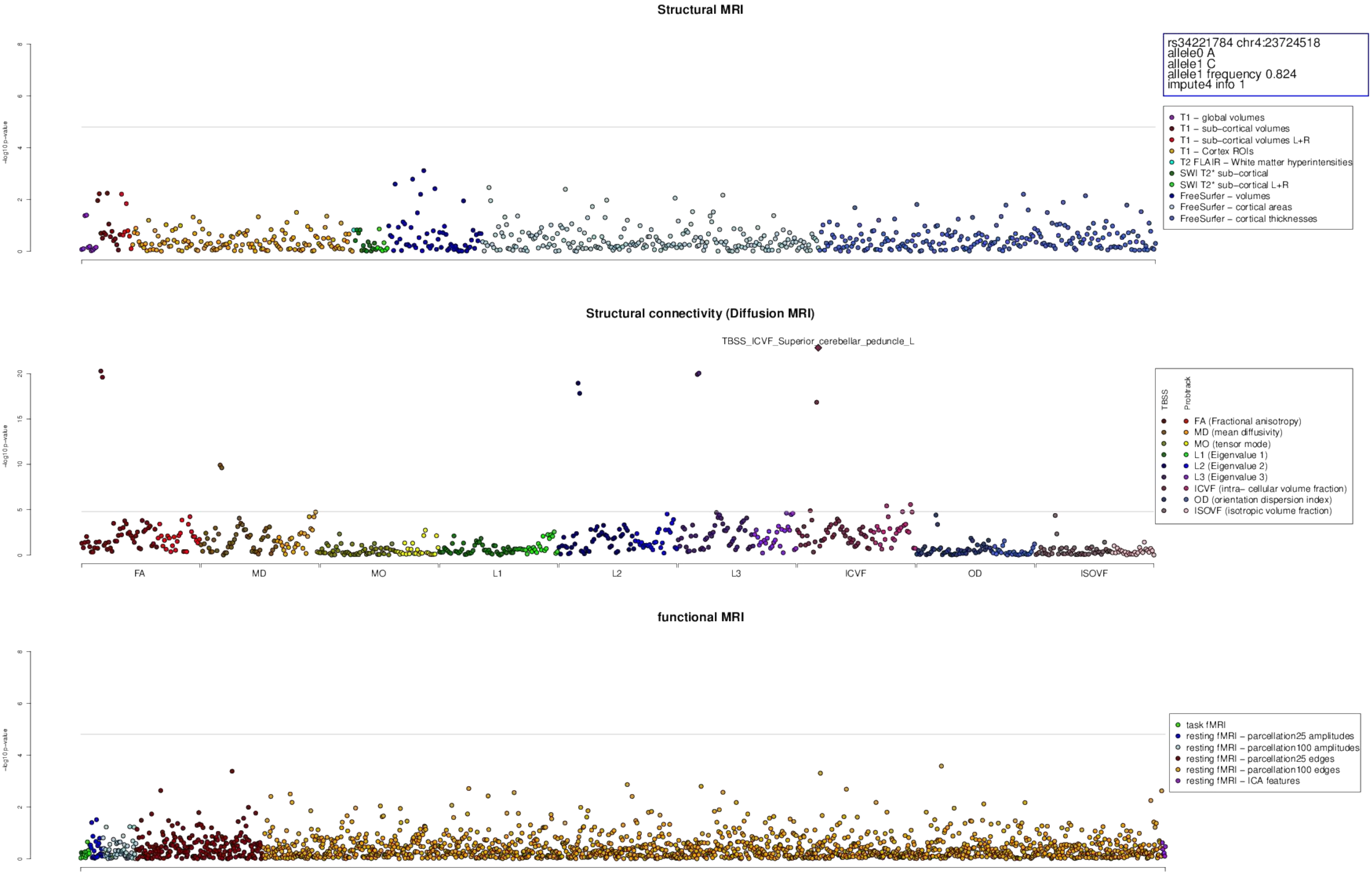

Structural MRI
rs34221784 chr4:23724518
allele0 A
allele1 C
allele1 frequency 0.824
impute4 info 1
T1 – global volumes
T1 – sub-cortical volumes
T1 – sub-cortical volumes L+R
T1 – Cortex ROIs
T2 FLAIR – White matter hyperintensities
SWI T2* sub-cortical
SWI T2* sub-cortical L+R
FreeSurfer – volumes
FreeSurfer – cortical areas
FreeSurfer – cortical thicknesses
-log10 p-value
0
2
4
6
8
Structural connectivity (Diffusion MRI)
TBSS_ICVF_Superior_cerebellar_peduncle_L
TBSS
Probtrack
FA (Fractional anisotropy)
MD (mean diffusivity)
MO (tensor mode)
L1 (Eigenvalue 1)
L2 (Eigenvalue 2)
L3 (Eigenvalue 3)
ICVF (intra- cellular volume fraction)
OD (orientation dispersion index)
ISOVF (isotropic volume fraction)
-log10 p-value
0
5
10
15
20
FA
MD
MO
L1
L2
L3
ICVF
OD
ISOVF
functional MRI
task fMRI
resting fMRI – parcellation25 amplitudes
resting fMRI – parcellation100 amplitudes
resting fMRI – parcellation25 edges
resting fMRI – parcellation100 edges
resting fMRI – ICA features
-log10 p-value
0
2
4
6
8

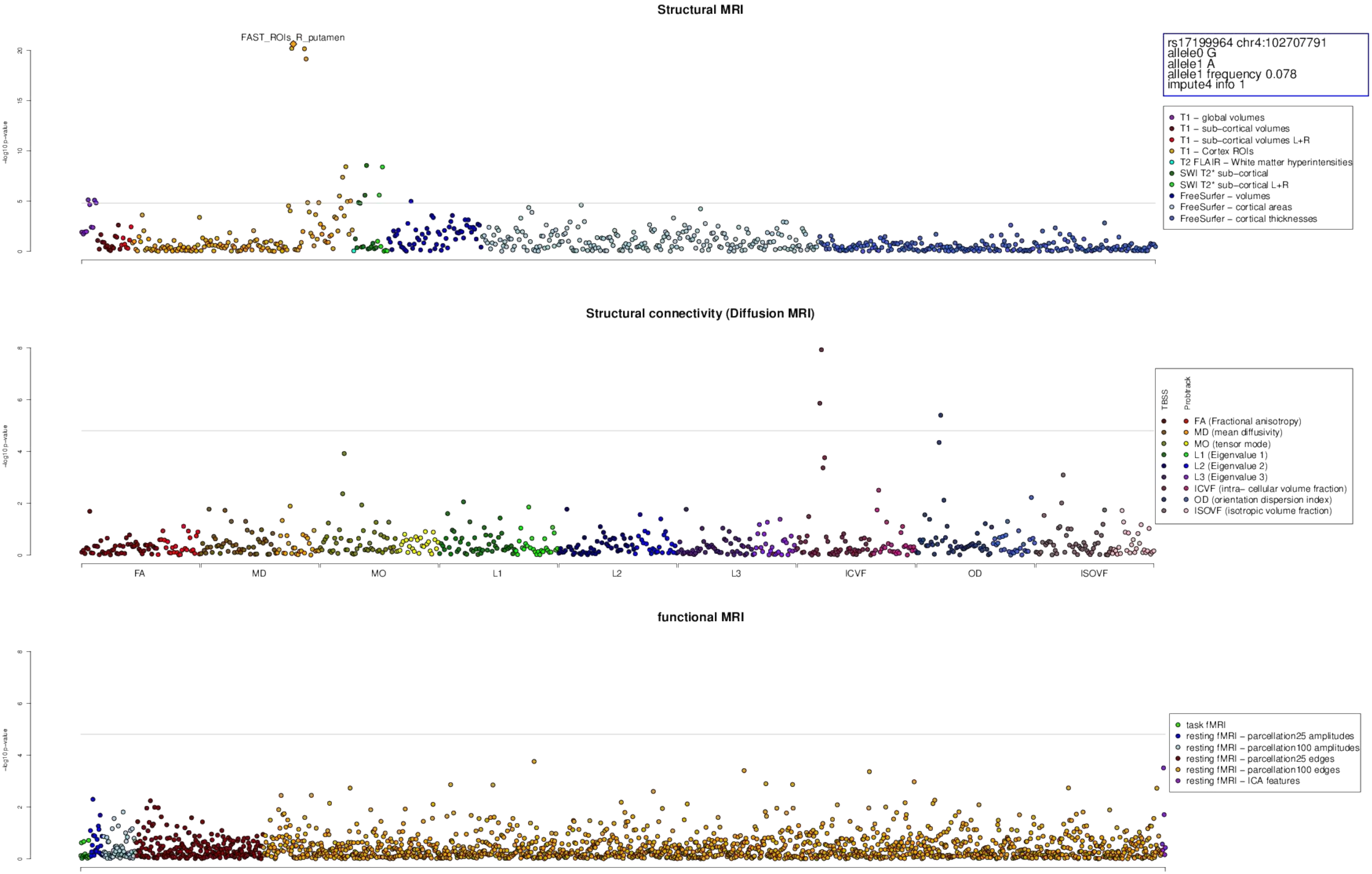

Structural MRI
FAST_ROIs_R_putamen
-log10 p-value
0
5
10
15
20
rs17199964 chr4:102707791
allele0 G
allele1 A
allele1 frequency 0.078
impute4 info 1
T1 – global volumes
T1 – sub-cortical volumes
T1 – sub-cortical volumes L+R
T1 – Cortex ROIs
T2 FLAIR – White matter hyperintensities
SWI T2* sub-cortical
SWI T2* sub-cortical L+R
FreeSurfer – volumes
FreeSurfer – cortical areas
FreeSurfer – cortical thicknesses
Structural connectivity (Diffusion MRI)
-log10 p-value
0
2
4
6
8
TBSS
Probtrack
FA (Fractional anisotropy)
MD (mean diffusivity)
MO (tensor mode)
L1 (Eigenvalue 1)
L2 (Eigenvalue 2)
L3 (Eigenvalue 3)
ICVF (intra– cellular volume fraction)
OD (orientation dispersion index)
ISOVF (isotropic volume fraction)
FA
MD
MO
L1
L2
L3
ICVF
OD
ISOVF
functional MRI
-log10 p-value
0
2
4
6
8
task fMRI
resting fMRI – parcellation25 amplitudes
resting fMRI – parcellation100 amplitudes
resting fMRI – parcellation25 edges
resting fMRI – parcellation100 edges
resting fMRI – ICA features

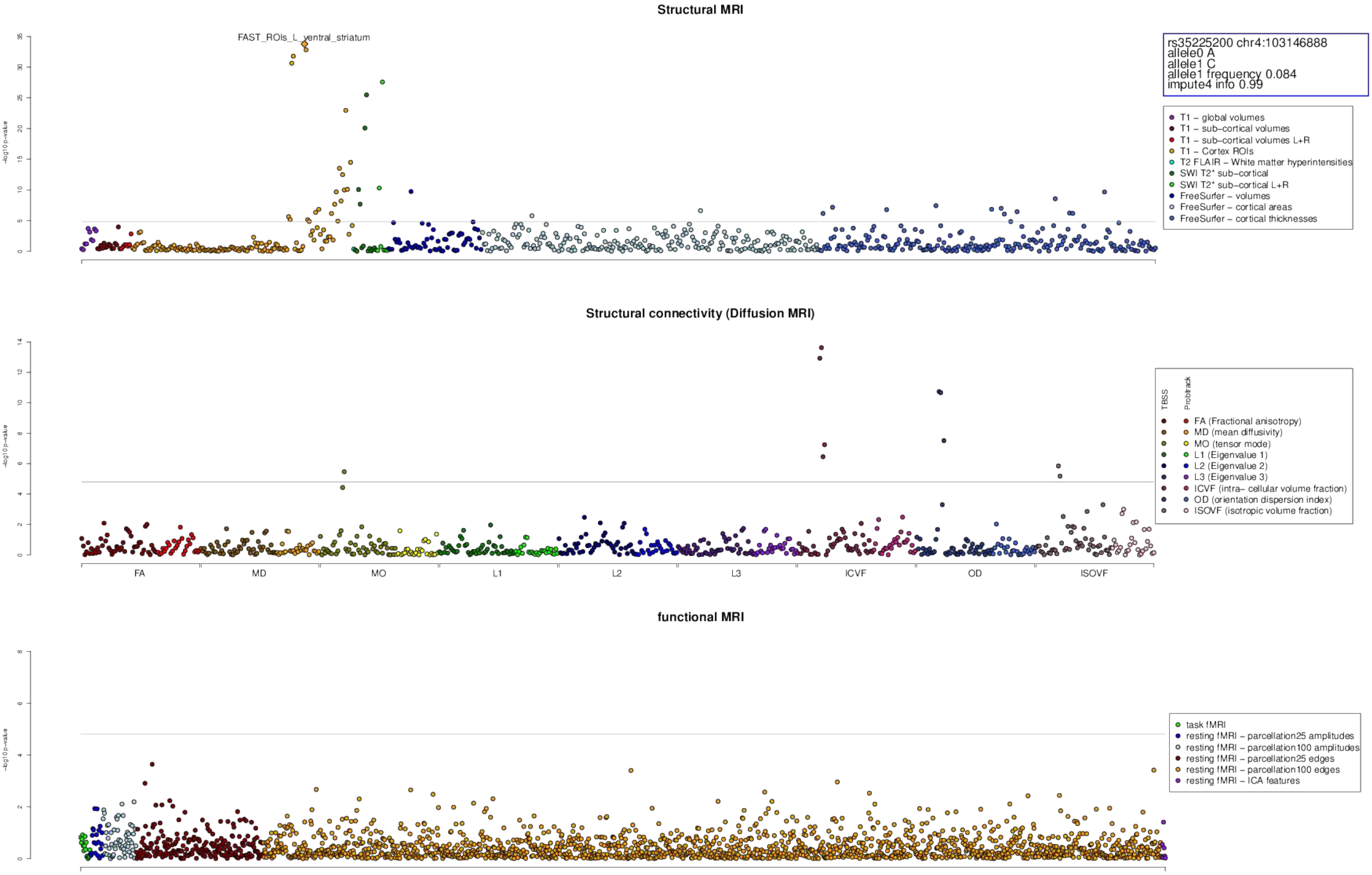
Structural MRI
FAST_ROIs_L_ventral_striatum
rs35225200 chr4:103146888
allele0 A
allele1 C
allele1 frequency 0.084
impute4 info 0.99
T1 – global volumes
T1 – sub-cortical volumes
T1 – sub-cortical volumes L+R
T1 – Cortex ROIs
T2 FLAIR – White matter hyperintensities
SWI T2* sub-cortical
SWI T2* sub-cortical L+R
FreeSurfer – volumes
FreeSurfer – cortical areas
FreeSurfer – cortical thicknesses
-log10 p-value
Structural connectivity (Diffusion MRI)
TBSS
Probtrack
FA (Fractional anisotropy)
MD (mean diffusivity)
MO (tensor mode)
L1 (Eigenvalue 1)
L2 (Eigenvalue 2)
L3 (Eigenvalue 3)
ICVF (intra- cellular volume fraction)
OD (orientation dispersion index)
ISOVF (isotropic volume fraction)
FA
MD
MO
L1
L2
L3
ICVF
OD
ISOVF
functional MRI
task fMRI
resting fMRI – parcellation25 amplitudes
resting fMRI – parcellation100 amplitudes
resting fMRI – parcellation25 edges
resting fMRI – parcellation100 edges
resting fMRI – ICA features

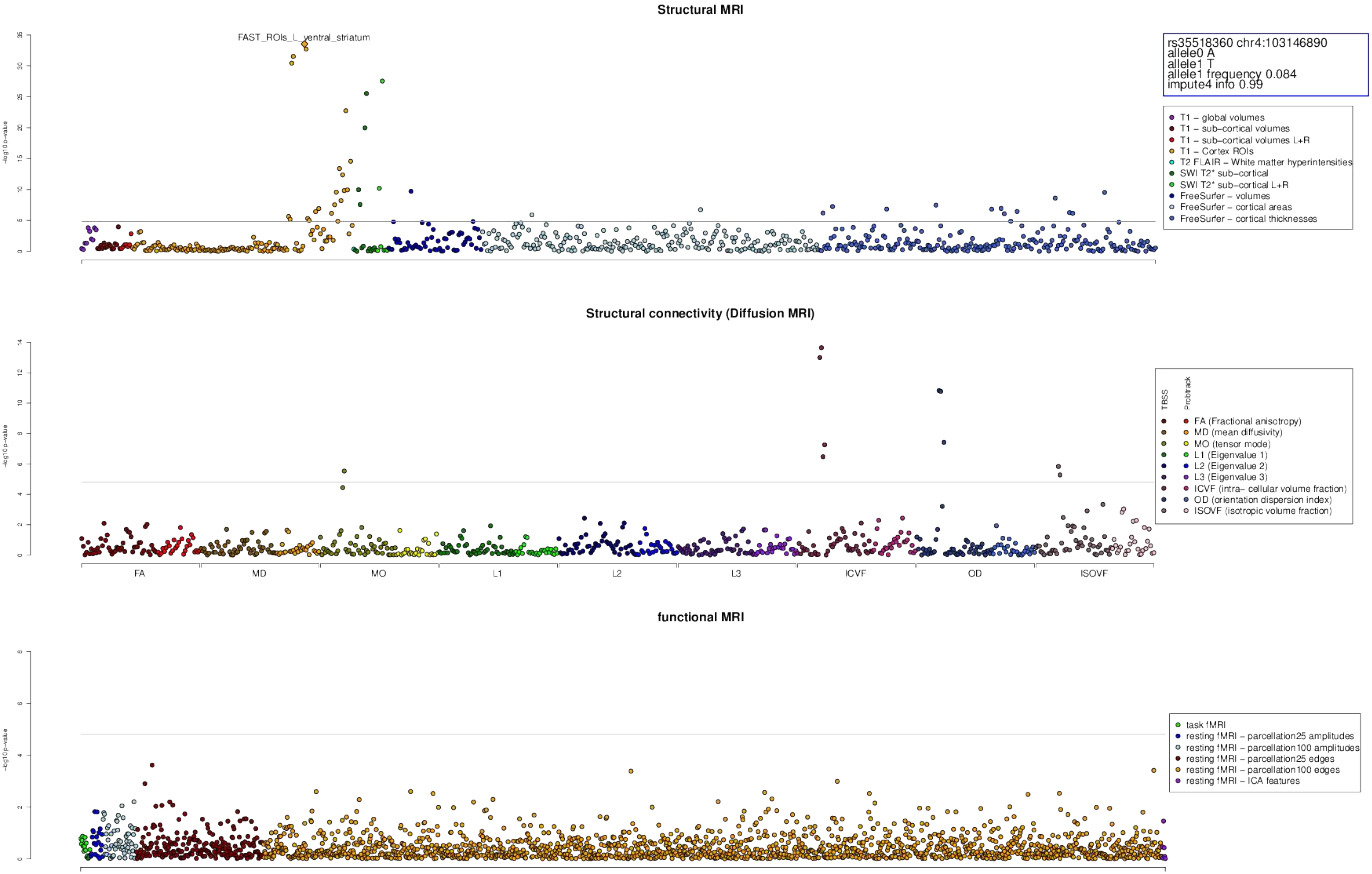

Structural MRI
FAST_ROIs_L_ventral_striatum
rs35518360 chr4:103146890
allele0 A
allele1 T
allele1 frequency 0.084
impute4 info 0.99
T1 – global volumes
T1 – sub-cortical volumes
T1 – sub-cortical volumes L+R
T1 – Cortex ROIs
T2 FLAIR – White matter hyperintensities
SWI T2* sub-cortical
SWI T2* sub-cortical L+R
FreeSurfer – volumes
FreeSurfer – cortical areas
FreeSurfer – cortical thicknesses
-log10 p-value
Structural connectivity (Diffusion MRI)
TBSS
Probtrack
FA (Fractional anisotropy)
MD (mean diffusivity)
MO (tensor mode)
L1 (Eigenvalue 1)
L2 (Eigenvalue 2)
L3 (Eigenvalue 3)
ICVF (intra– cellular volume fraction)
OD (orientation dispersion index)
ISOVF (isotropic volume fraction)
FA
MD
MO
L1
L2
L3
ICVF
OD
ISOVF
functional MRI
task fMRI
resting fMRI – parcellation25 amplitudes
resting fMRI – parcellation100 amplitudes
resting fMRI – parcellation25 edges
resting fMRI – parcellation100 edges
resting fMRI – ICA features

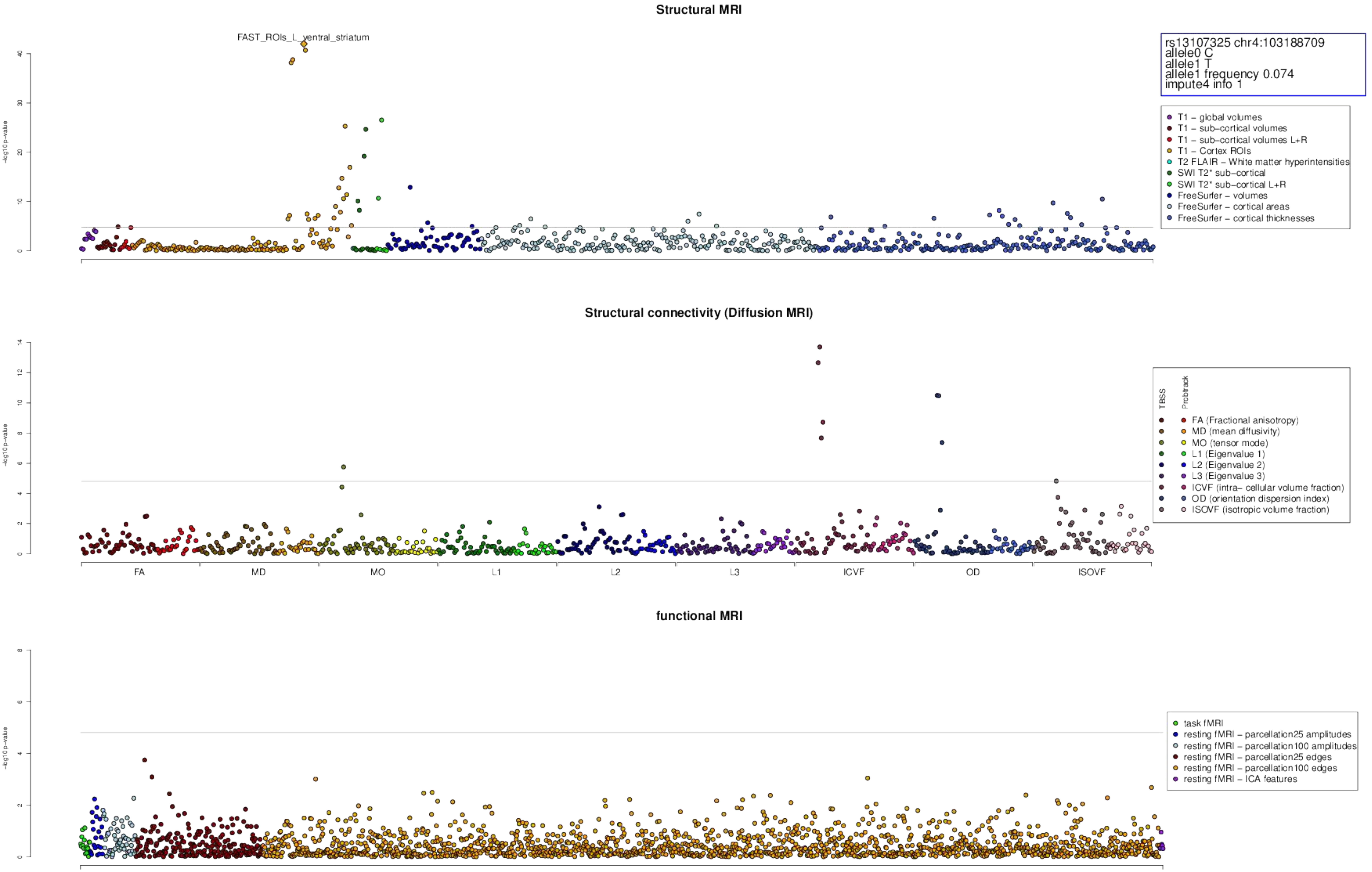
Structural MRI
FAST_ROIs_L_ventral_striatum
rs13107325 chr4:103188709
allele0 C
allele1 T
allele1 frequency 0.074
impute4 info 1
T1 – global volumes
T1 – sub-cortical volumes
T1 – sub-cortical volumes L+R
T1 – Cortex ROIs
T2 FLAIR – White matter hyperintensities
SWI T2* sub-cortical
SWI T2* sub-cortical L+R
FreeSurfer – volumes
FreeSurfer – cortical areas
FreeSurfer – cortical thicknesses
-log10 p-value
Structural connectivity (Diffusion MRI)
TBSS
Probtrack
FA (Fractional anisotropy)
MD (mean diffusivity)
MO (tensor mode)
L1 (Eigenvalue 1)
L2 (Eigenvalue 2)
L3 (Eigenvalue 3)
ICVF (intra- cellular volume fraction)
OD (orientation dispersion index)
ISOVF (isotropic volume fraction)
FA MD MO L1 L2 L3 ICVF OD ISOVF
functional MRI
task fMRI
resting fMRI – parcellation25 amplitudes
resting fMRI – parcellation100 amplitudes
resting fMRI – parcellation25 edges
resting fMRI – parcellation100 edges
resting fMRI – ICA features

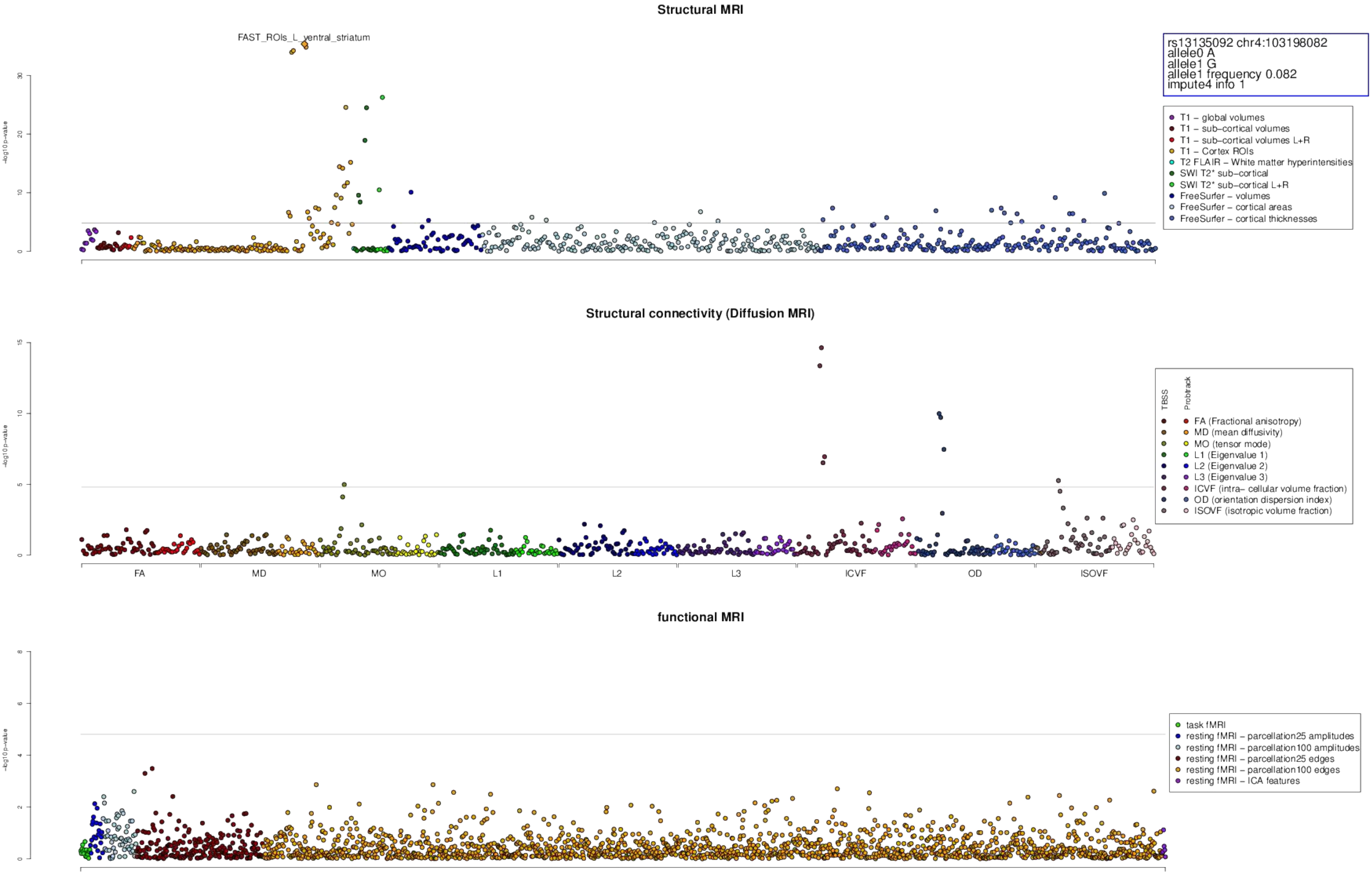

Structural MRI
FAST_ROIs_L_ventral_striatum
rs13135092 chr4:103198082
allele0 A
allele1 G
allele1 frequency 0.082
impute4 info 1
T1 – global volumes
T1 – sub–cortical volumes
T1 – sub–cortical volumes L+R
T1 – Cortex ROIs
T2 FLAIR – White matter hyperintensities
SWI T2* sub–cortical
SWI T2* sub–cortical L+R
FreeSurfer – volumes
FreeSurfer – cortical areas
FreeSurfer – cortical thicknesses
–log10 p–value
Structural connectivity (Diffusion MRI)
TBSS
Probtrack
FA (Fractional anisotropy)
MD (mean diffusivity)
MO (tensor mode)
L1 (Eigenvalue 1)
L2 (Eigenvalue 2)
L3 (Eigenvalue 3)
ICVF (intra– cellular volume fraction)
OD (orientation dispersion index)
ISOVF (isotropic volume fraction)
FA
MD
MO
L1
L2
L3
ICVF
OD
ISOVF
functional MRI
task fMRI
resting fMRI – parcellation25 amplitudes
resting fMRI – parcellation100 amplitudes
resting fMRI – parcellation25 edges
resting fMRI – parcellation100 edges
resting fMRI – ICA features

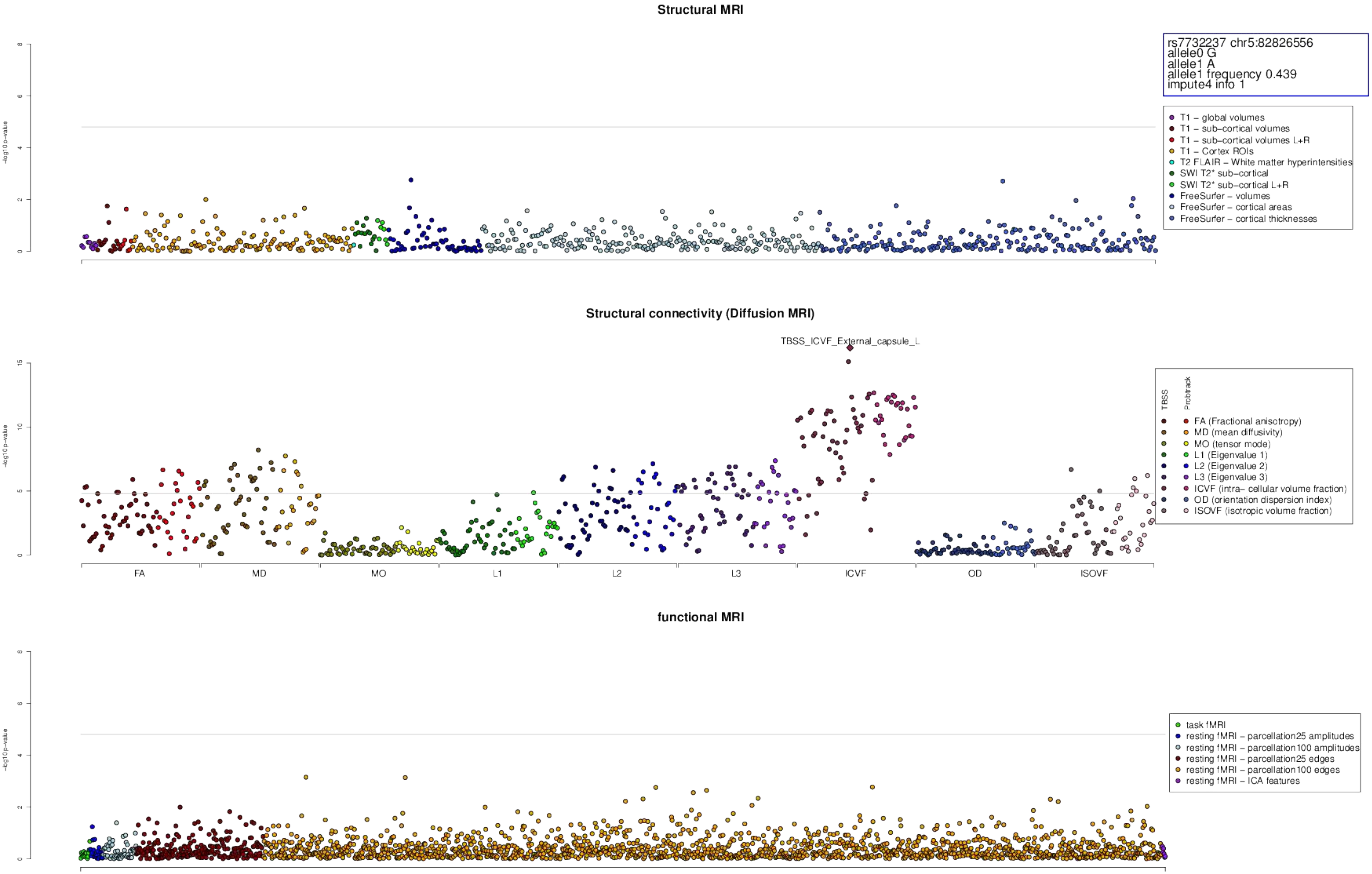

Structural MRI
rs7732237 chr5:82826556
allele0 G
allele1 A
allele1 frequency 0.439
impute4 info 1
T1 – global volumes
T1 – sub-cortical volumes
T1 – sub-cortical volumes L+R
T1 – Cortex ROIs
T2 FLAIR – White matter hyperintensities
SWI T2* sub-cortical
SWI T2* sub-cortical L+R
FreeSurfer – volumes
FreeSurfer – cortical areas
FreeSurfer – cortical thicknesses
-log10 p-value
Structural connectivity (Diffusion MRI)
TBSS_ICVF_External_capsule_L
TBSS
Probtrack
FA (Fractional anisotropy)
MD (mean diffusivity)
MO (tensor mode)
L1 (Eigenvalue 1)
L2 (Eigenvalue 2)
L3 (Eigenvalue 3)
ICVF (intra- cellular volume fraction)
OD (orientation dispersion index)
ISOVF (isotropic volume fraction)
FA
MD
MO
L1
L2
L3
ICVF
OD
ISOVF
functional MRI
task fMRI
resting fMRI – parcellation25 amplitudes
resting fMRI – parcellation100 amplitudes
resting fMRI – parcellation25 edges
resting fMRI – parcellation100 edges
resting fMRI – ICA features

Structural MRI

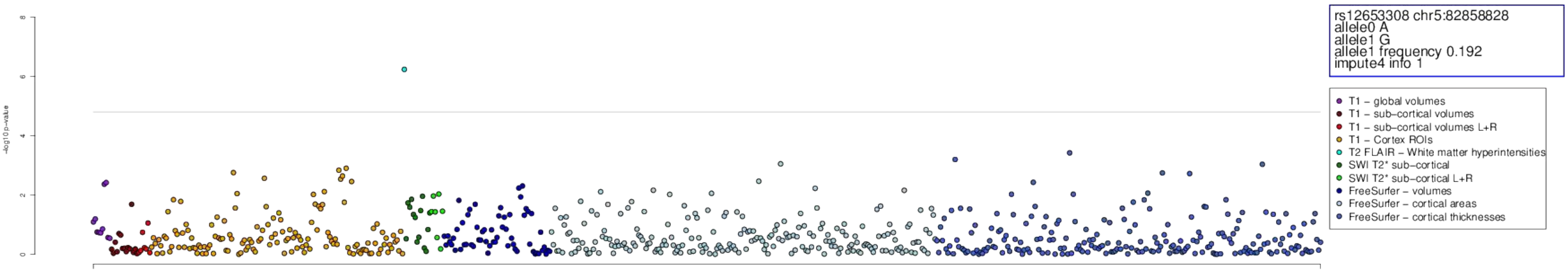

Structural connectivity (Diffusion MRI)

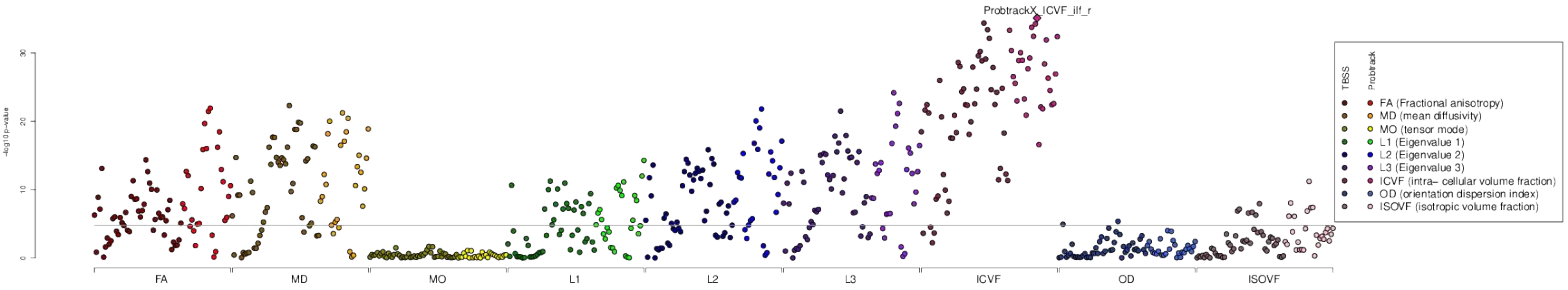

functional MRI

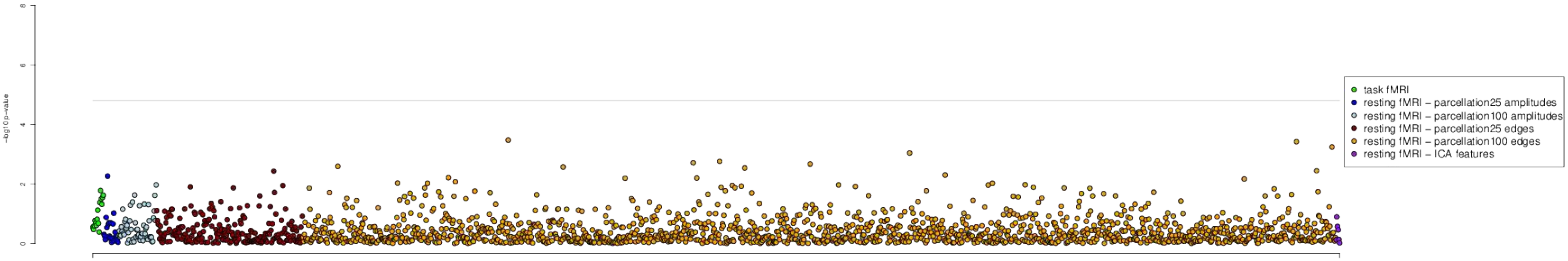

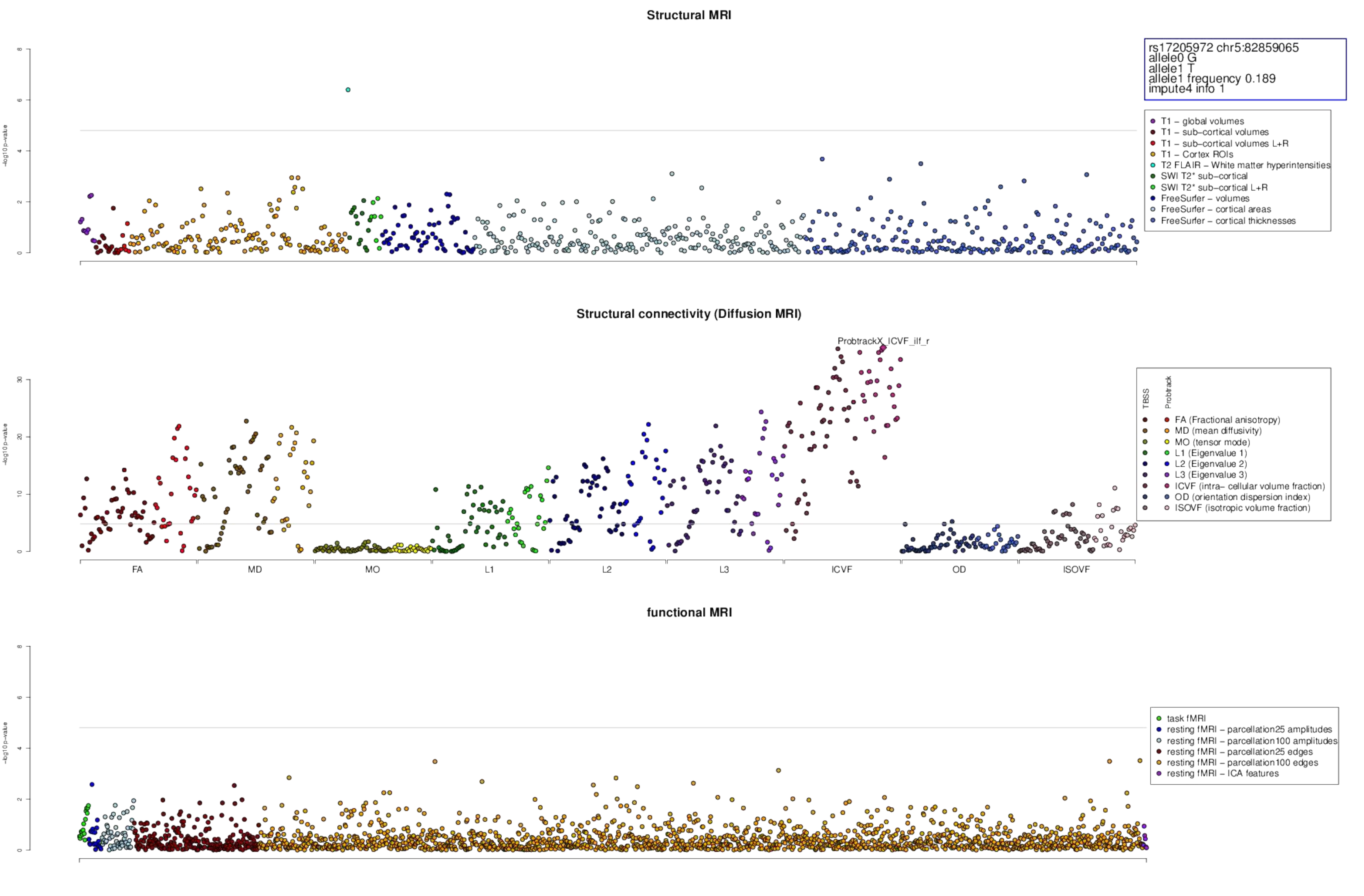

Structural MRI
rs17205972 chr5:82859065
allele0 G
allele1 T
allele1 frequency 0.189
impute4 info 1
T1 – global volumes
T1 – sub-cortical volumes
T1 – sub-cortical volumes L+R
T1 – Cortex ROIs
T2 FLAIR – White matter hyperintensities
SWI T2* sub-cortical
SWI T2* sub-cortical L+R
FreeSurfer – volumes
FreeSurfer – cortical areas
FreeSurfer – cortical thicknesses
-log10 p-value
Structural connectivity (Diffusion MRI)
ProbtrackX_ICVF_ilf_r
TBSS
Probtrack
FA (Fractional anisotropy)
MD (mean diffusivity)
MO (tensor mode)
L1 (Eigenvalue 1)
L2 (Eigenvalue 2)
L3 (Eigenvalue 3)
ICVF (intra- cellular volume fraction)
OD (orientation dispersion index)
ISOVF (isotropic volume fraction)
FA
MD
MO
L1
L2
L3
ICVF
OD
ISOVF
functional MRI
task fMRI
resting fMRI – parcellation25 amplitudes
resting fMRI – parcellation100 amplitudes
resting fMRI – parcellation25 edges
resting fMRI – parcellation100 edges
resting fMRI – ICA features

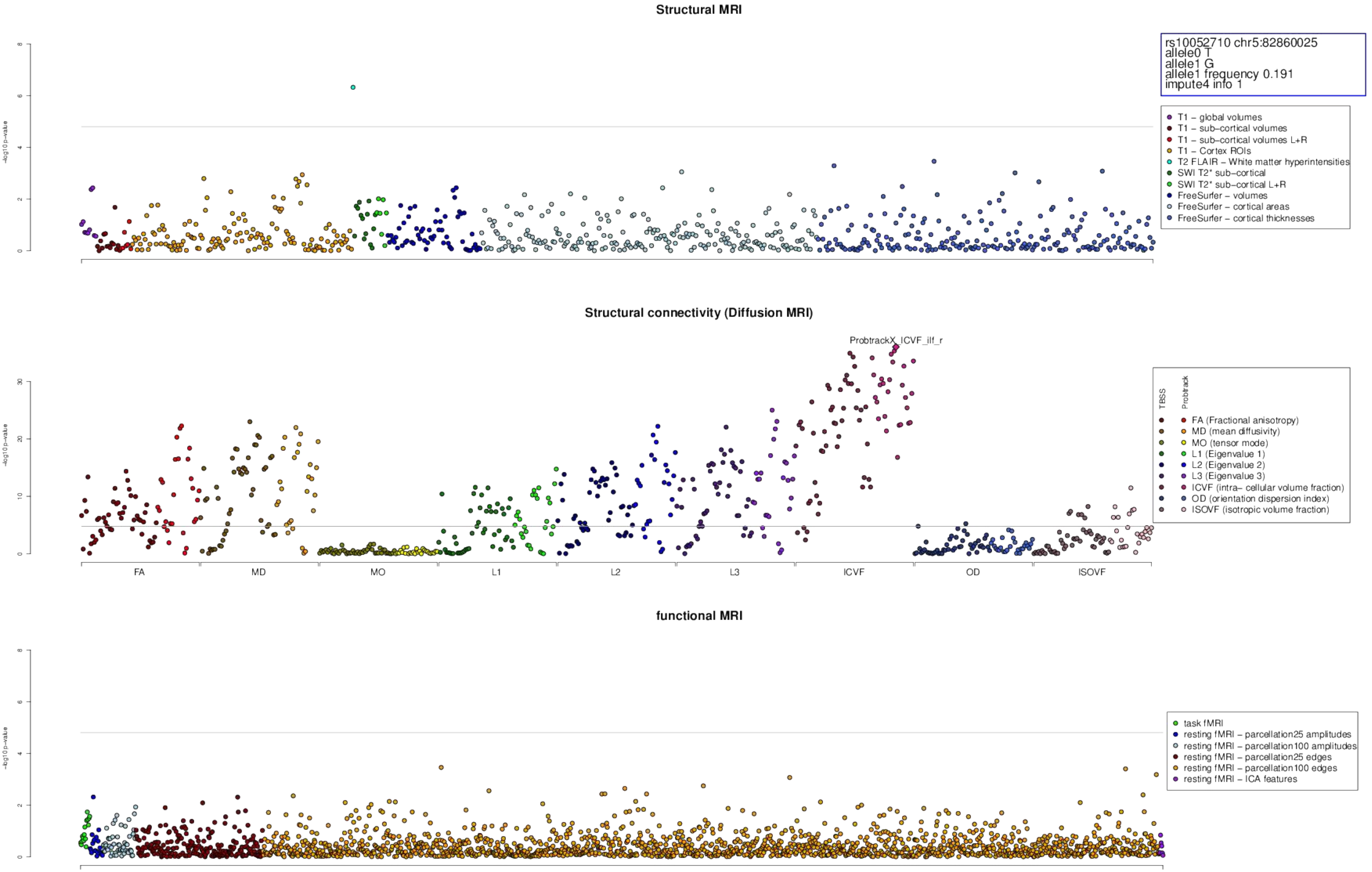

Structural MRI
rs10052710 chr5:82860025
allele0 T
allele1 G
allele1 frequency 0.191
impute4 info 1
T1 – global volumes
T1 – sub–cortical volumes
T1 – sub–cortical volumes L+R
T1 – Cortex ROIs
T2 FLAIR – White matter hyperintensities
SWI T2* sub–cortical
SWI T2* sub–cortical L+R
FreeSurfer – volumes
FreeSurfer – cortical areas
FreeSurfer – cortical thicknesses
–log10 p–value
Structural connectivity (Diffusion MRI)
ProbtrackX_ICVF_ilf_r
TBSS
Probtrack
FA (Fractional anisotropy)
MD (mean diffusivity)
MO (tensor mode)
L1 (Eigenvalue 1)
L2 (Eigenvalue 2)
L3 (Eigenvalue 3)
ICVF (intra– cellular volume fraction)
OD (orientation dispersion index)
ISOVF (isotropic volume fraction)
FA
MD
MO
L1
L2
L3
ICVF
OD
ISOVF
functional MRI
task fMRI
resting fMRI – parcellation25 amplitudes
resting fMRI – parcellation100 amplitudes
resting fMRI – parcellation25 edges
resting fMRI – parcellation100 edges
resting fMRI – ICA features

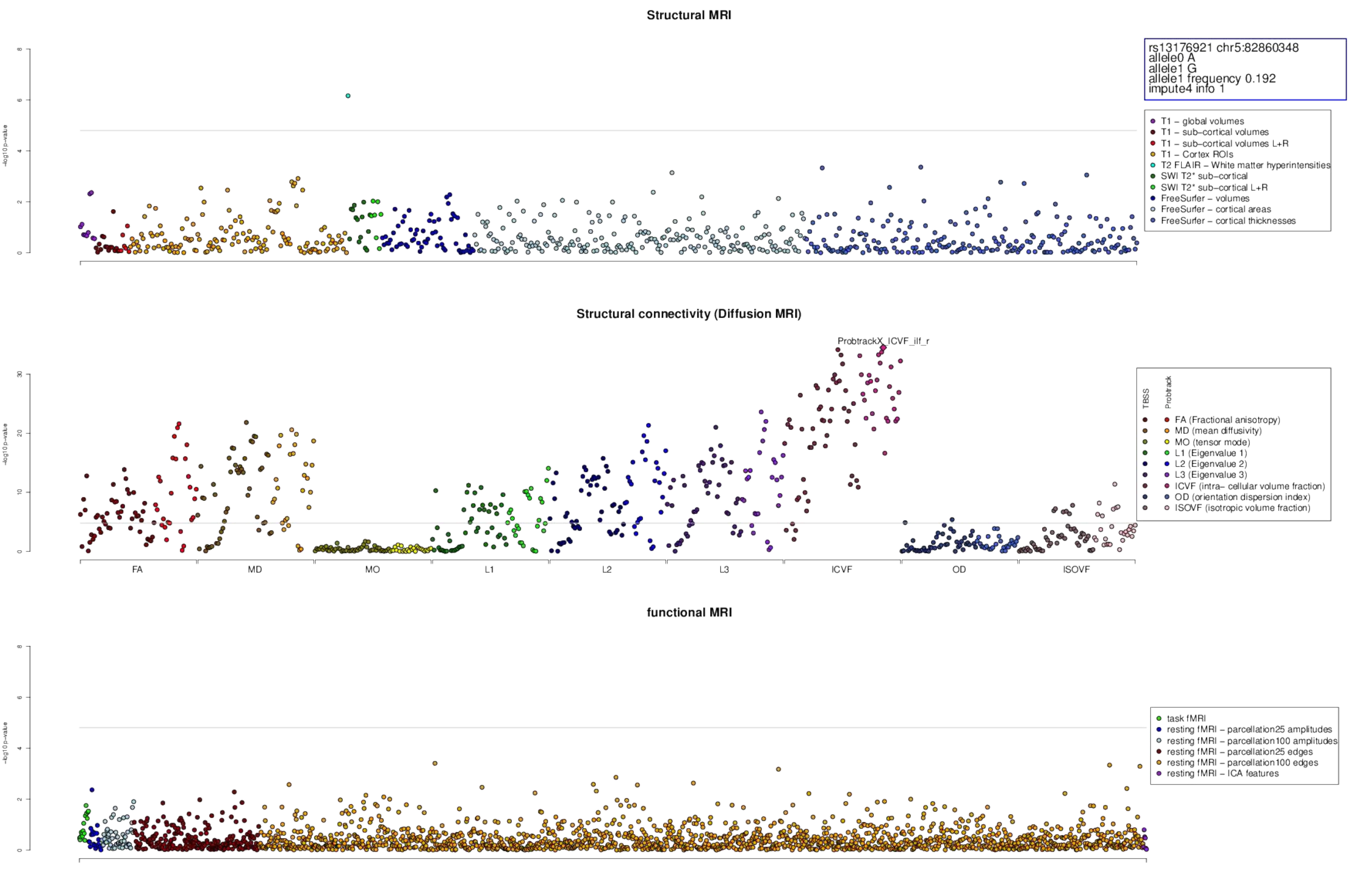
Structural MRI
rs13176921 chr5:82860348
allele0 A
allele1 G
allele1 frequency 0.192
impute4 info 1
T1 – global volumes
T1 – sub–cortical volumes
T1 – sub–cortical volumes L+R
T1 – Cortex ROIs
T2 FLAIR – White matter hyperintensities
SWI T2* sub–cortical
SWI T2* sub–cortical L+R
FreeSurfer – volumes
FreeSurfer – cortical areas
FreeSurfer – cortical thicknesses
–log10 p–value
Structural connectivity (Diffusion MRI)
ProbtrackX_ICVF_ilf_r
TBSS
Probtrack
FA (Fractional anisotropy)
MD (mean diffusivity)
MO (tensor mode)
L1 (Eigenvalue 1)
L2 (Eigenvalue 2)
L3 (Eigenvalue 3)
ICVF (intra– cellular volume fraction)
OD (orientation dispersion index)
ISOVF (isotropic volume fraction)
FA
MD
MO
L1
L2
L3
ICVF
OD
ISOVF
functional MRI
task fMRI
resting fMRI – parcellation25 amplitudes
resting fMRI – parcellation100 amplitudes
resting fMRI – parcellation25 edges
resting fMRI – parcellation100 edges
resting fMRI – ICA features

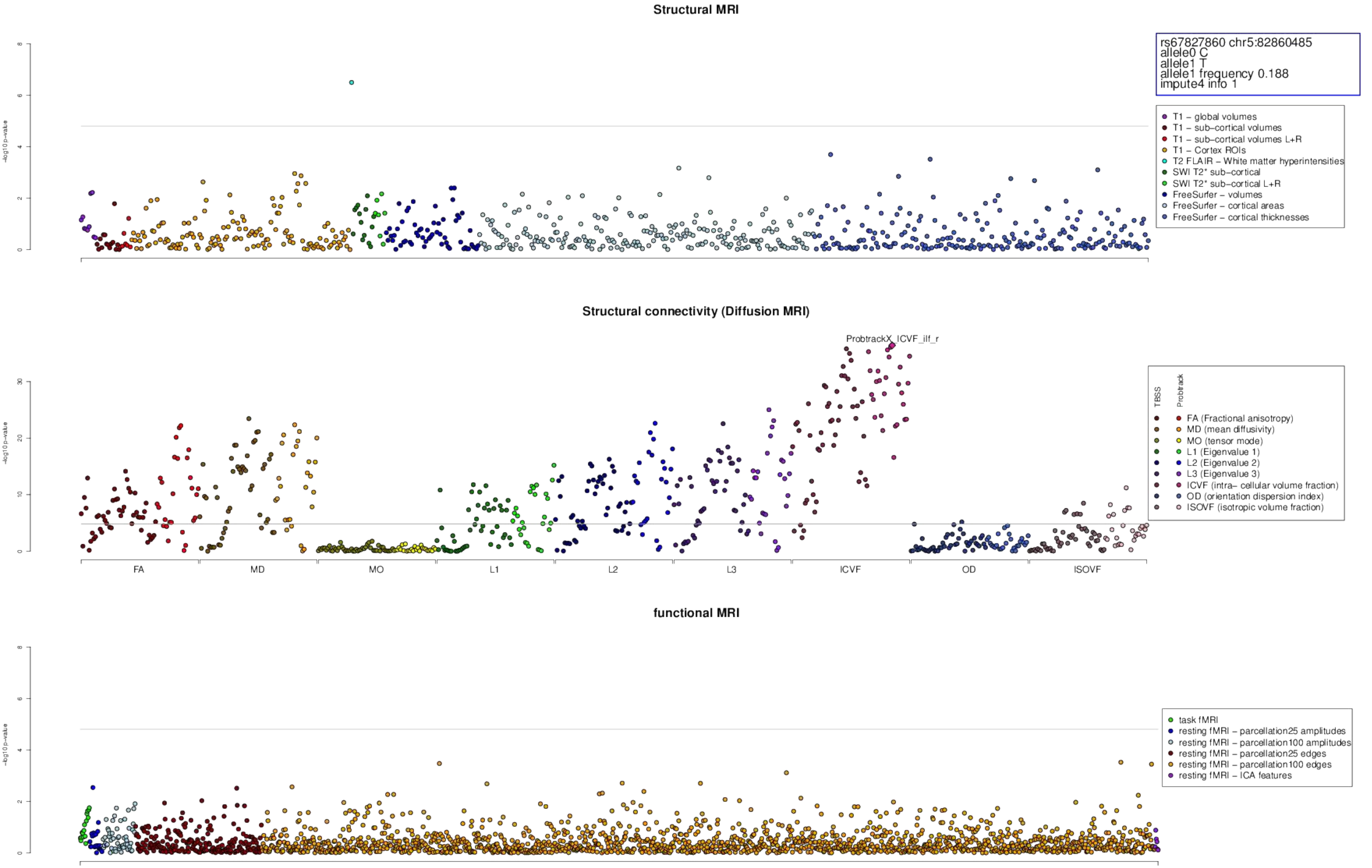

Structural MRI
rs67827860 chr5:82860485
allele0 C
allele1 T
allele1 frequency 0.188
impute4 info 1
T1 – global volumes
T1 – sub–cortical volumes
T1 – sub–cortical volumes L+R
T1 – Cortex ROIs
T2 FLAIR – White matter hyperintensities
SWI T2* sub–cortical
SWI T2* sub–cortical L+R
FreeSurfer – volumes
FreeSurfer – cortical areas
FreeSurfer – cortical thicknesses
-log10 p-value
Structural connectivity (Diffusion MRI)
ProbtrackX_ICVF_ilf_r
TBSS
Probtrack
FA (Fractional anisotropy)
MD (mean diffusivity)
MO (tensor mode)
L1 (Eigenvalue 1)
L2 (Eigenvalue 2)
L3 (Eigenvalue 3)
ICVF (intra– cellular volume fraction)
OD (orientation dispersion index)
ISOVF (isotropic volume fraction)
FA
MD
MO
L1
L2
L3
ICVF
OD
ISOVF
functional MRI
task fMRI
resting fMRI – parcellation25 amplitudes
resting fMRI – parcellation100 amplitudes
resting fMRI – parcellation25 edges
resting fMRI – parcellation100 edges
resting fMRI – ICA features

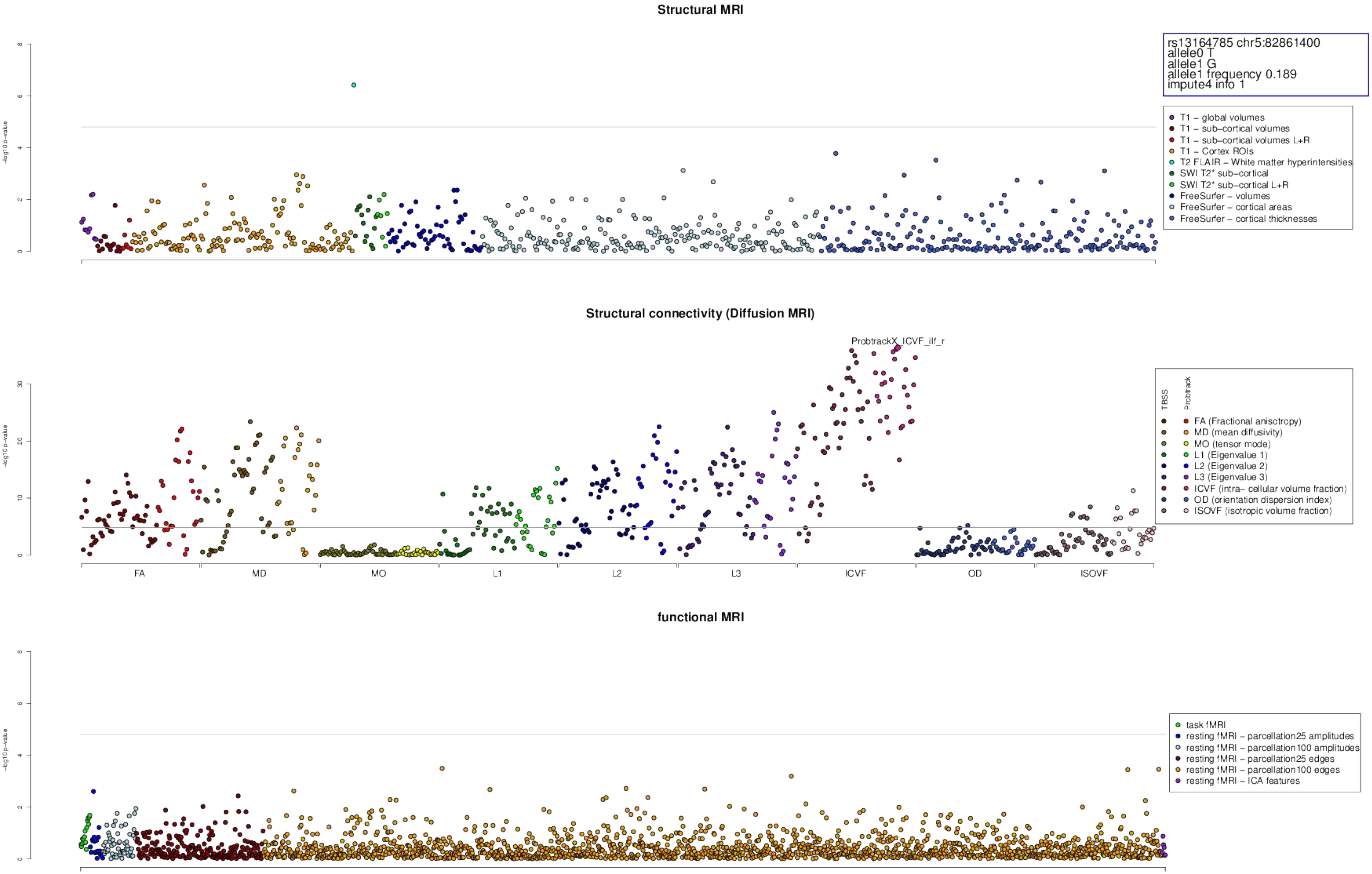
Structural MRI
rs13164785 chr5:82861400
allele0 T
allele1 G
allele1 frequency 0.189
impute4 info 1
T1 – global volumes
T1 – sub–cortical volumes
T1 – sub–cortical volumes L+R
T1 – Cortex ROIs
T2 FLAIR – White matter hyperintensities
SWI T2* sub–cortical
SWI T2* sub–cortical L+R
FreeSurfer – volumes
FreeSurfer – cortical areas
FreeSurfer – cortical thicknesses
–log10 p–value
Structural connectivity (Diffusion MRI)
ProbtrackX_ICVF_ilf_r
TBSS
Probtrack
FA (Fractional anisotropy)
MD (mean diffusivity)
MO (tensor mode)
L1 (Eigenvalue 1)
L2 (Eigenvalue 2)
L3 (Eigenvalue 3)
ICVF (intra– cellular volume fraction)
OD (orientation dispersion index)
ISOVF (isotropic volume fraction)
FA
MD
MO
L1
L2
L3
ICVF
OD
ISOVF
functional MRI
task fMRI
resting fMRI – parcellation25 amplitudes
resting fMRI – parcellation100 amplitudes
resting fMRI – parcellation25 edges
resting fMRI – parcellation100 edges
resting fMRI – ICA features

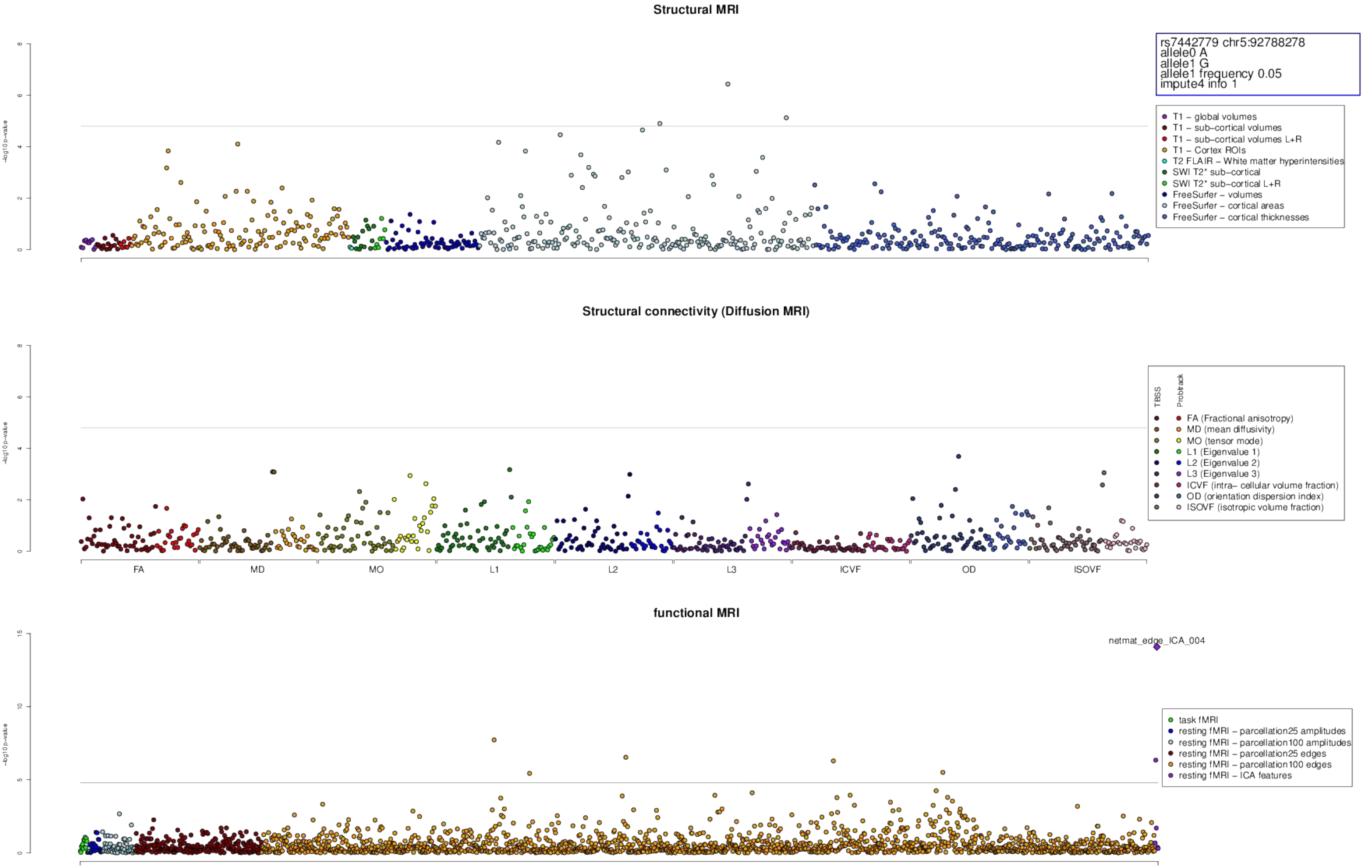

Structural MRI
rs7442779 chr5:92788278
allele0 A
allele1 G
allele1 frequency 0.05
impute4 info 1
T1 – global volumes
T1 – sub–cortical volumes
T1 – sub–cortical volumes L+R
T1 – Cortex ROIs
T2 FLAIR – White matter hyperintensities
SWI T2* sub–cortical
SWI T2* sub–cortical L+R
FreeSurfer – volumes
FreeSurfer – cortical areas
FreeSurfer – cortical thicknesses
-log10 p-value
Structural connectivity (Diffusion MRI)
TBSS
Probtrack
FA (Fractional anisotropy)
MD (mean diffusivity)
MO (tensor mode)
L1 (Eigenvalue 1)
L2 (Eigenvalue 2)
L3 (Eigenvalue 3)
ICVF (intra– cellular volume fraction)
OD (orientation dispersion index)
ISOVF (isotropic volume fraction)
FA
MD
MO
L1
L2
L3
ICVF
OD
ISOVF
functional MRI
netmat_edge_ICA_004
task fMRI
resting fMRI – parcellation25 amplitudes
resting fMRI – parcellation100 amplitudes
resting fMRI – parcellation25 edges
resting fMRI – parcellation100 edges
resting fMRI – ICA features

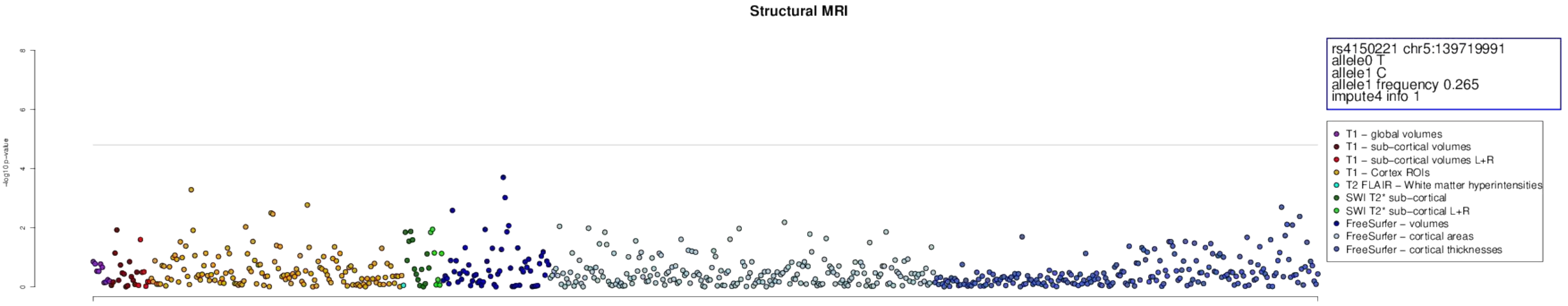
Structural MRI
rs4150221 chr5:139719991
allele0 T
allele1 C
allele1 frequency 0.265
impute4 info 1
T1 – global volumes
T1 – sub–cortical volumes
T1 – sub–cortical volumes L+R
T1 – Cortex ROIs
T2 FLAIR – White matter hyperintensities
SWI T2* sub–cortical
SWI T2* sub–cortical L+R
FreeSurfer – volumes
FreeSurfer – cortical areas
FreeSurfer – cortical thicknesses
-log10 p-value

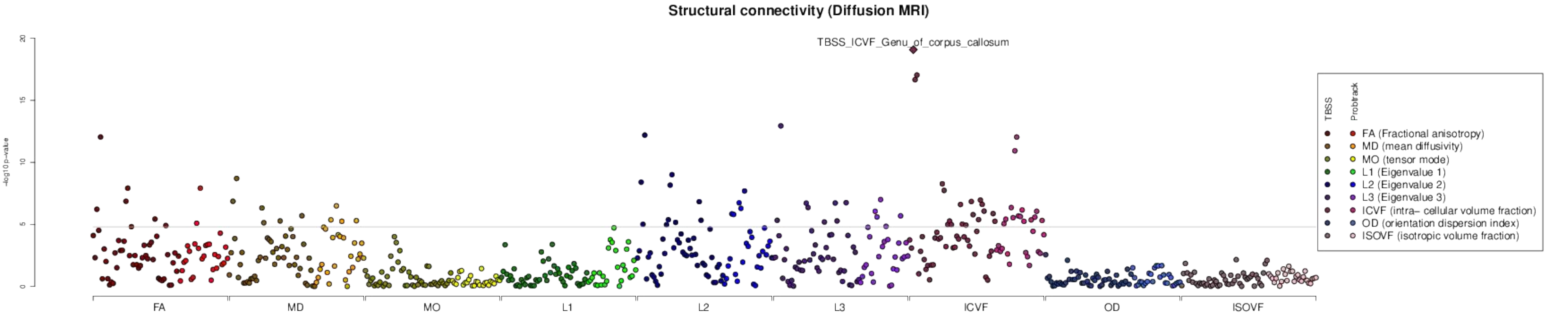
Structural connectivity (Diffusion MRI)
TBSS_ICVF_Genu_of_corpus_callosum
TBSS
Probtrack
FA (Fractional anisotropy)
MD (mean diffusivity)
MO (tensor mode)
L1 (Eigenvalue 1)
L2 (Eigenvalue 2)
L3 (Eigenvalue 3)
ICVF (intra– cellular volume fraction)
OD (orientation dispersion index)
ISOVF (isotropic volume fraction)
FA
MD
MO
L1
L2
L3
ICVF
OD
ISOVF
-log10 p-value

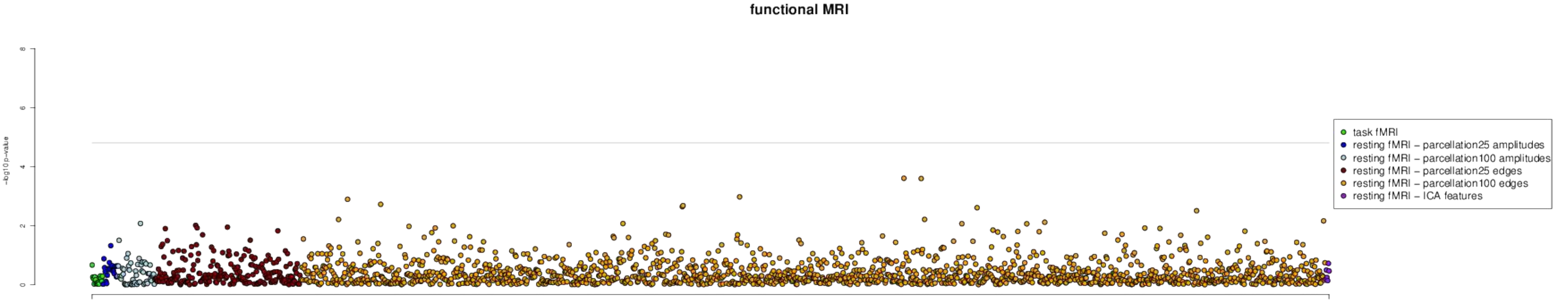
functional MRI
task fMRI
resting fMRI – parcellation25 amplitudes
resting fMRI – parcellation100 amplitudes
resting fMRI – parcellation25 edges
resting fMRI – parcellation100 edges
resting fMRI – ICA features
-log10 p-value

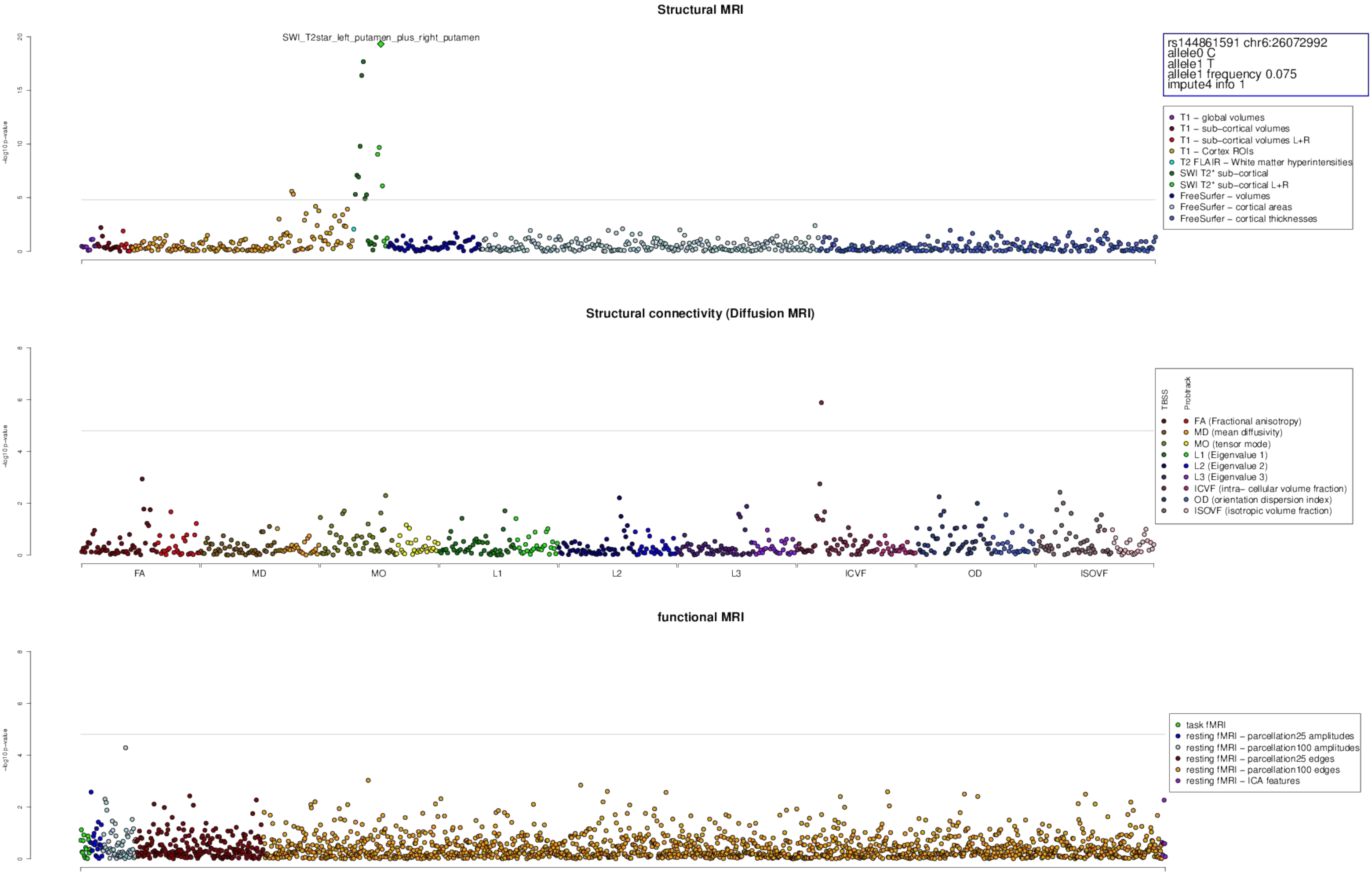
Structural MRI
SWI_T2star_left_putamen_plus_right_putamen
-log10 p-value
rs144861591 chr6:26072992
allele0 C
allele1 T
allele1 frequency 0.075
impute4 info 1
T1 – global volumes
T1 – sub-cortical volumes
T1 – sub-cortical volumes L+R
T1 – Cortex ROIs
T2 FLAIR – White matter hyperintensities
SWI T2* sub-cortical
SWI T2* sub-cortical L+R
FreeSurfer – volumes
FreeSurfer – cortical areas
FreeSurfer – cortical thicknesses
Structural connectivity (Diffusion MRI)
-log10 p-value
TBSS
Probtrack
FA (Fractional anisotropy)
MD (mean diffusivity)
MO (tensor mode)
L1 (Eigenvalue 1)
L2 (Eigenvalue 2)
L3 (Eigenvalue 3)
ICVF (intra- cellular volume fraction)
OD (orientation dispersion index)
ISOVF (isotropic volume fraction)
FA
MD
MO
L1
L2
L3
ICVF
OD
ISOVF
functional MRI
-log10 p-value
task fMRI
resting fMRI – parcellation25 amplitudes
resting fMRI – parcellation100 amplitudes
resting fMRI – parcellation25 edges
resting fMRI – parcellation100 edges
resting fMRI – ICA features

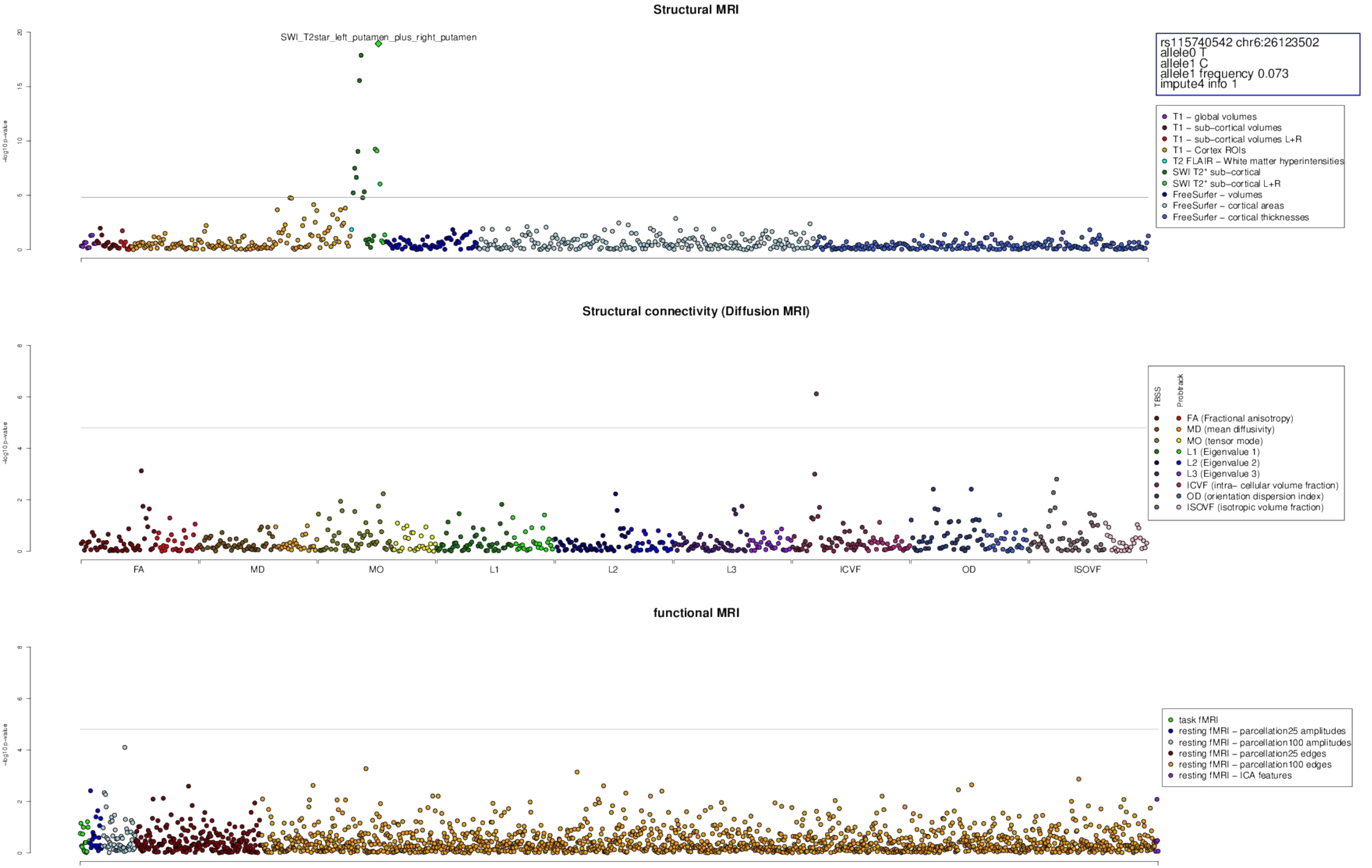
Structural MRI
SWI_T2star_left_putamen_plus_right_putamen
rs115740542 chr6:26123502
allele0 T
allele1 C
allele1 frequency 0.073
impute4 info 1
T1 – global volumes
T1 – sub–cortical volumes
T1 – sub–cortical volumes L+R
T1 – Cortex ROIs
T2 FLAIR – White matter hyperintensities
SWI T2* sub–cortical
SWI T2* sub–cortical L+R
FreeSurfer – volumes
FreeSurfer – cortical areas
FreeSurfer – cortical thicknesses
–log10 p–value
Structural connectivity (Diffusion MRI)
TBSS
Probtrack
FA (Fractional anisotropy)
MD (mean diffusivity)
MO (tensor mode)
L1 (Eigenvalue 1)
L2 (Eigenvalue 2)
L3 (Eigenvalue 3)
ICVF (intra– cellular volume fraction)
OD (orientation dispersion index)
ISOVF (isotropic volume fraction)
FA
MD
MO
L1
L2
L3
ICVF
OD
ISOVF
functional MRI
task fMRI
resting fMRI – parcellation25 amplitudes
resting fMRI – parcellation100 amplitudes
resting fMRI – parcellation25 edges
resting fMRI – parcellation100 edges
resting fMRI – ICA features

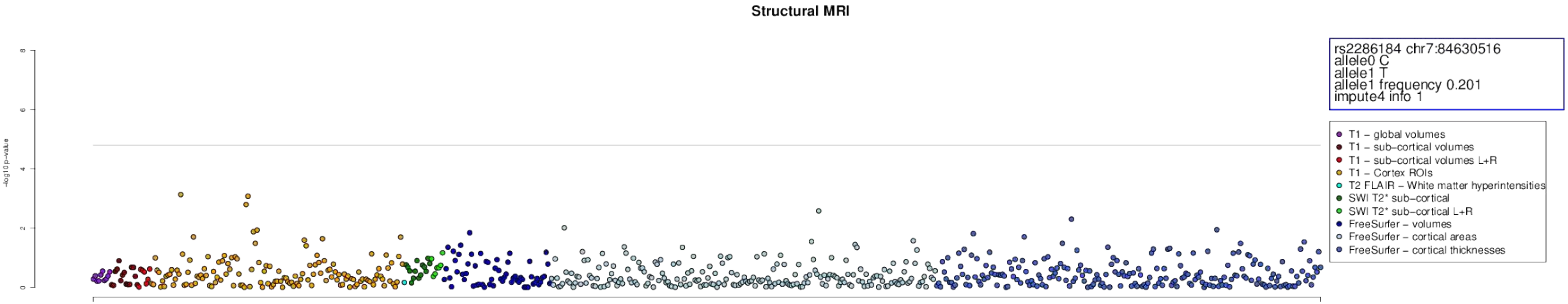
Structural MRI
-log10 p-value
rs2286184 chr7:84630516
allele0 C
allele1 T
allele1 frequency 0.201
impute4 info 1
T1 – global volumes
T1 – sub-cortical volumes
T1 – sub-cortical volumes L+R
T1 – Cortex ROIs
T2 FLAIR – White matter hyperintensities
SWI T2* sub-cortical
SWI T2* sub-cortical L+R
FreeSurfer – volumes
FreeSurfer – cortical areas
FreeSurfer – cortical thicknesses

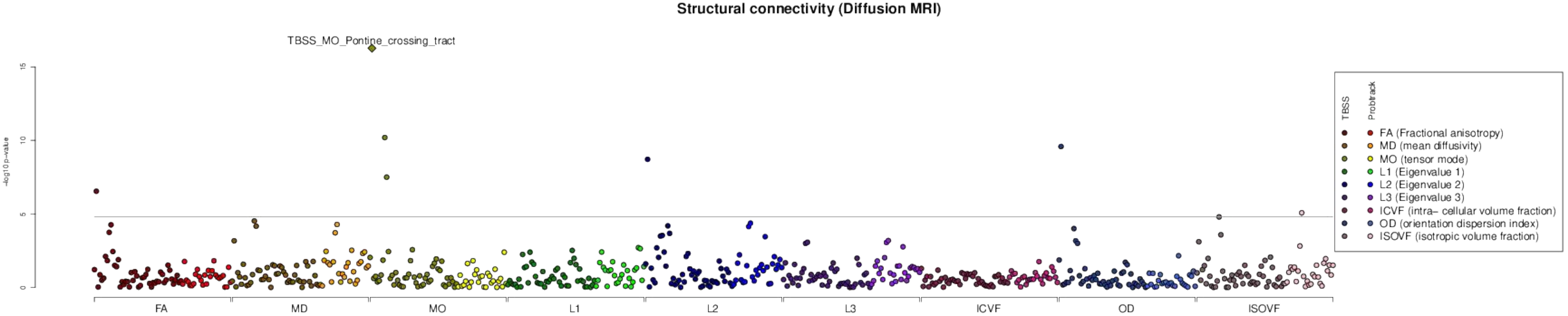
Structural connectivity (Diffusion MRI)
TBSS_MO_Pontine_crossing_tract
-log10 p-value
TBSS
Probtrack
FA (Fractional anisotropy)
MD (mean diffusivity)
MO (tensor mode)
L1 (Eigenvalue 1)
L2 (Eigenvalue 2)
L3 (Eigenvalue 3)
ICVF (intra- cellular volume fraction)
OD (orientation dispersion index)
ISOVF (isotropic volume fraction)
FA
MD
MO
L1
L2
L3
ICVF
OD
ISOVF

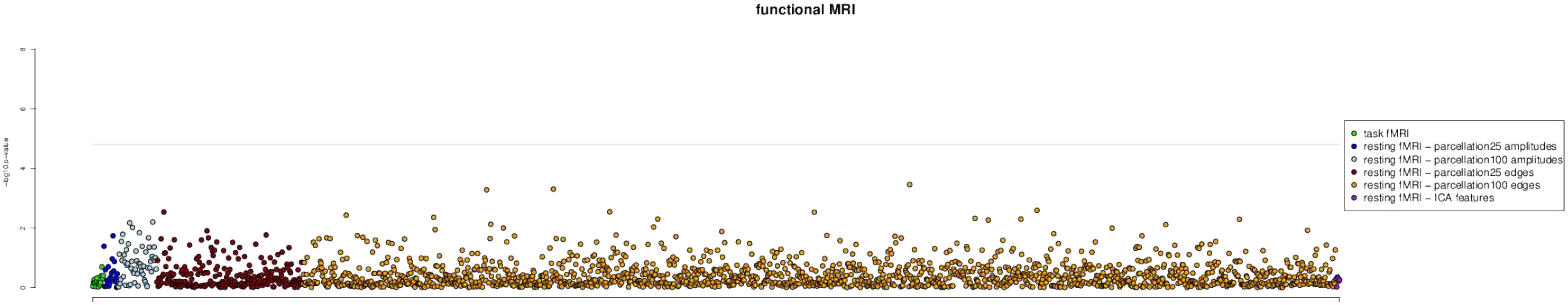
functional MRI
-log10 p-value
task fMRI
resting fMRI – parcellation25 amplitudes
resting fMRI – parcellation100 amplitudes
resting fMRI – parcellation25 edges
resting fMRI – parcellation100 edges
resting fMRI – ICA features

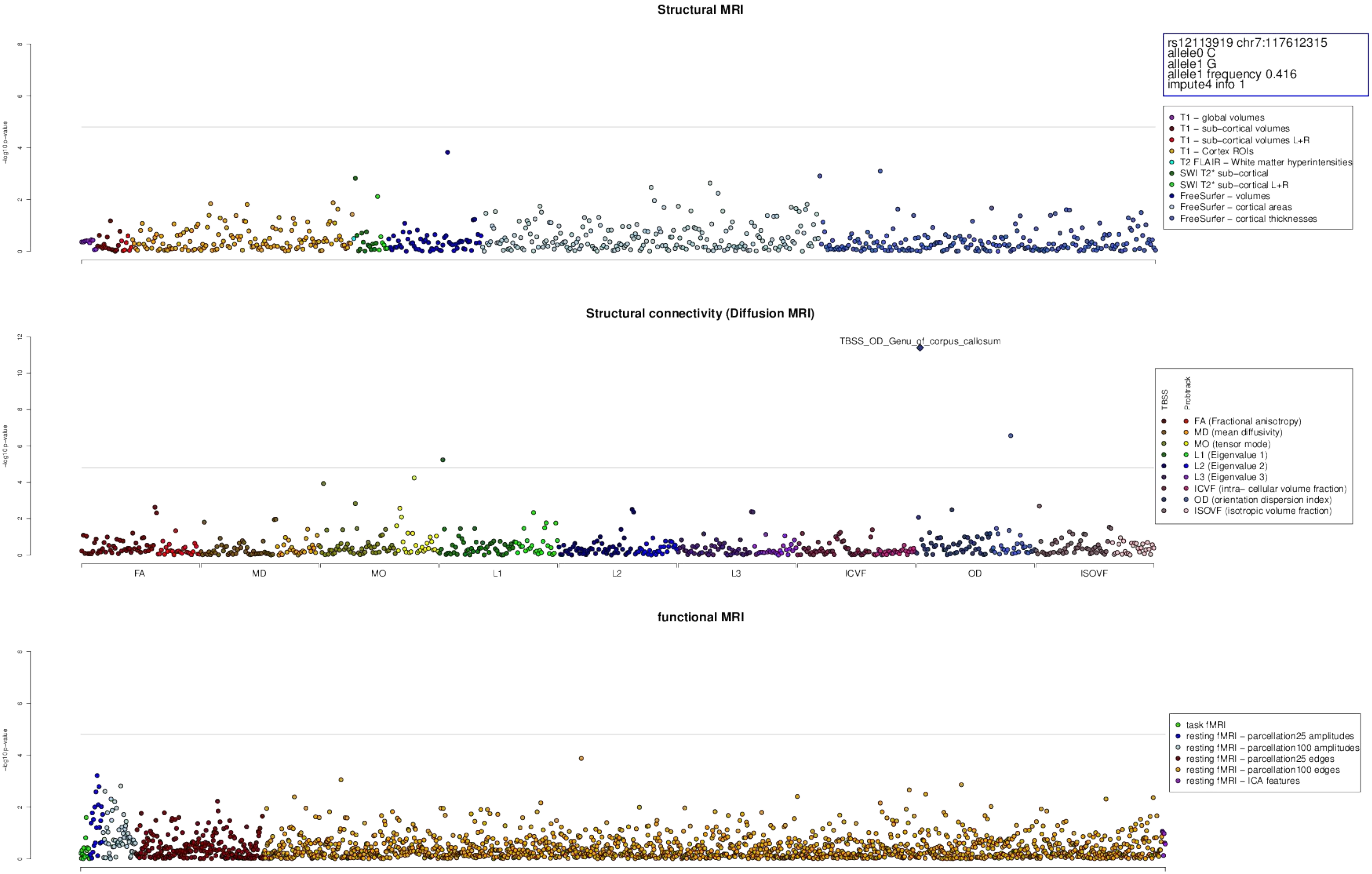
Structural MRI
-log10 p-value
rs12113919 chr7:117612315
allele0 C
allele1 G
allele1 frequency 0.416
impute4 info 1
T1 – global volumes
T1 – sub-cortical volumes
T1 – sub-cortical volumes L+R
T1 – Cortex ROIs
T2 FLAIR – White matter hyperintensities
SWI T2* sub-cortical
SWI T2* sub-cortical L+R
FreeSurfer – volumes
FreeSurfer – cortical areas
FreeSurfer – cortical thicknesses
Structural connectivity (Diffusion MRI)
TBSS_OD_Genu_of_corpus_callosum
TBSS
Probtrack
FA (Fractional anisotropy)
MD (mean diffusivity)
MO (tensor mode)
L1 (Eigenvalue 1)
L2 (Eigenvalue 2)
L3 (Eigenvalue 3)
ICVF (intra- cellular volume fraction)
OD (orientation dispersion index)
ISOVF (isotropic volume fraction)
FA
MD
MO
L1
L2
L3
ICVF
OD
ISOVF
functional MRI
task fMRI
resting fMRI – parcellation25 amplitudes
resting fMRI – parcellation100 amplitudes
resting fMRI – parcellation25 edges
resting fMRI – parcellation100 edges
resting fMRI – ICA features

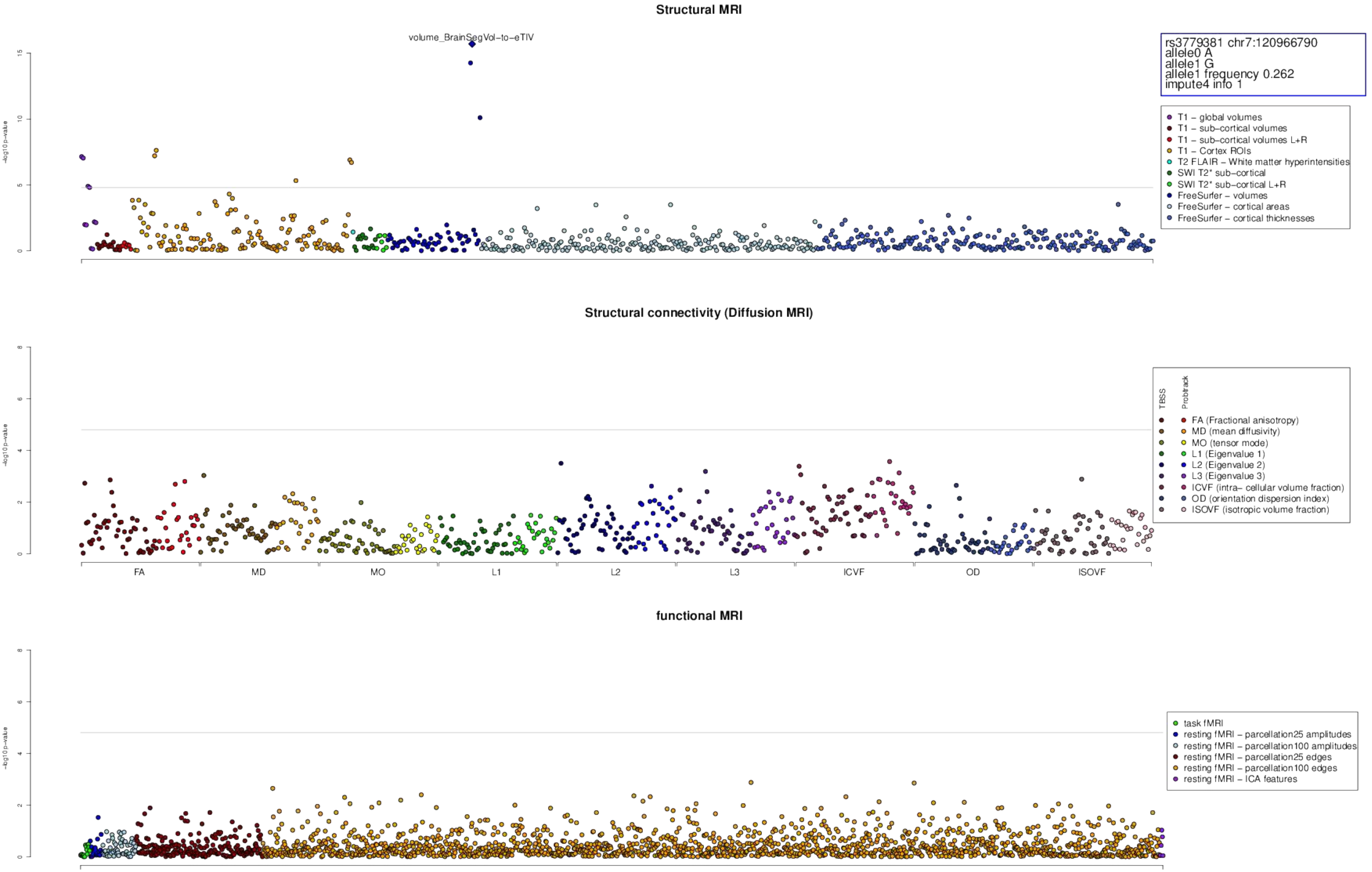

Structural MRI
volume_BrainSegVol-to-eTIV
rs3779381 chr7:120966790
allele0 A
allele1 G
allele1 frequency 0.262
impute4 info 1
T1 – global volumes
T1 – sub–cortical volumes
T1 – sub–cortical volumes L+R
T1 – Cortex ROIs
T2 FLAIR – White matter hyperintensities
SWI T2* sub–cortical
SWI T2* sub–cortical L+R
FreeSurfer – volumes
FreeSurfer – cortical areas
FreeSurfer – cortical thicknesses
-log10 p-value
Structural connectivity (Diffusion MRI)
TBSS
Probtrack
FA (Fractional anisotropy)
MD (mean diffusivity)
MO (tensor mode)
L1 (Eigenvalue 1)
L2 (Eigenvalue 2)
L3 (Eigenvalue 3)
ICVF (intra– cellular volume fraction)
OD (orientation dispersion index)
ISOVF (isotropic volume fraction)
FA
MD
MO
L1
L2
L3
ICVF
OD
ISOVF
functional MRI
task fMRI
resting fMRI – parcellation25 amplitudes
resting fMRI – parcellation100 amplitudes
resting fMRI – parcellation25 edges
resting fMRI – parcellation100 edges
resting fMRI – ICA features

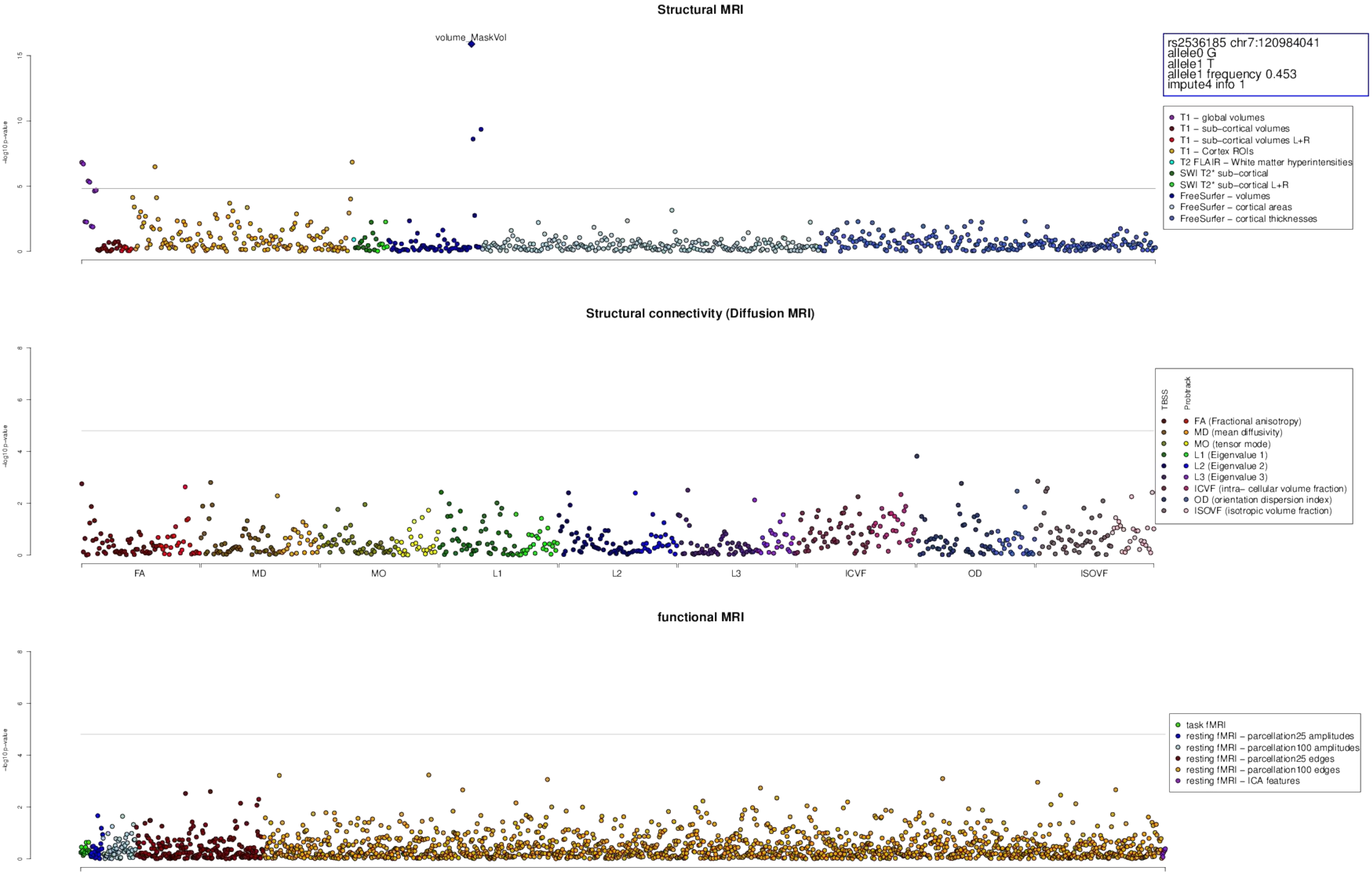
Structural MRI
volume_MaskVol
rs2536185 chr7:120984041
allele0 G
allele1 T
allele1 frequency 0.453
impute4 info 1
T1 – global volumes
T1 – sub–cortical volumes
T1 – sub–cortical volumes L+R
T1 – Cortex ROIs
T2 FLAIR – White matter hyperintensities
SWI T2* sub–cortical
SWI T2* sub–cortical L+R
FreeSurfer – volumes
FreeSurfer – cortical areas
FreeSurfer – cortical thicknesses
-log10 p-value
Structural connectivity (Diffusion MRI)
TBSS
Probtrack
FA (Fractional anisotropy)
MD (mean diffusivity)
MO (tensor mode)
L1 (Eigenvalue 1)
L2 (Eigenvalue 2)
L3 (Eigenvalue 3)
ICVF (intra– cellular volume fraction)
OD (orientation dispersion index)
ISOVF (isotropic volume fraction)
FA
MD
MO
L1
L2
L3
ICVF
OD
ISOVF
functional MRI
task fMRI
resting fMRI – parcellation25 amplitudes
resting fMRI – parcellation100 amplitudes
resting fMRI – parcellation25 edges
resting fMRI – parcellation100 edges
resting fMRI – ICA features

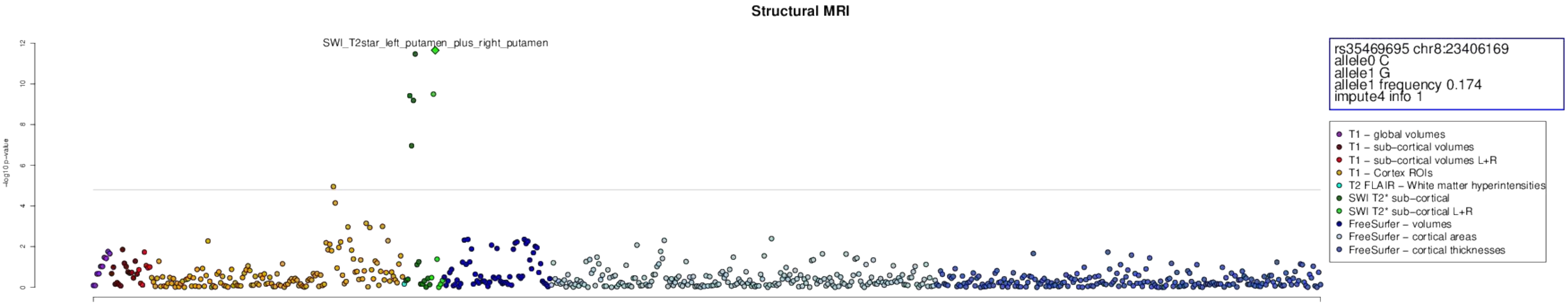

Structural MRI
SWI_T2star_left_putamen_plus_right_putamen
-log10 p-value
rs35469695 chr8:23406169
allele0 C
allele1 G
allele1 frequency 0.174
impute4 info 1
T1 – global volumes
T1 – sub-cortical volumes
T1 – sub-cortical volumes L+R
T1 – Cortex ROIs
T2 FLAIR – White matter hyperintensities
SWI T2* sub-cortical
SWI T2* sub-cortical L+R
FreeSurfer – volumes
FreeSurfer – cortical areas
FreeSurfer – cortical thicknesses

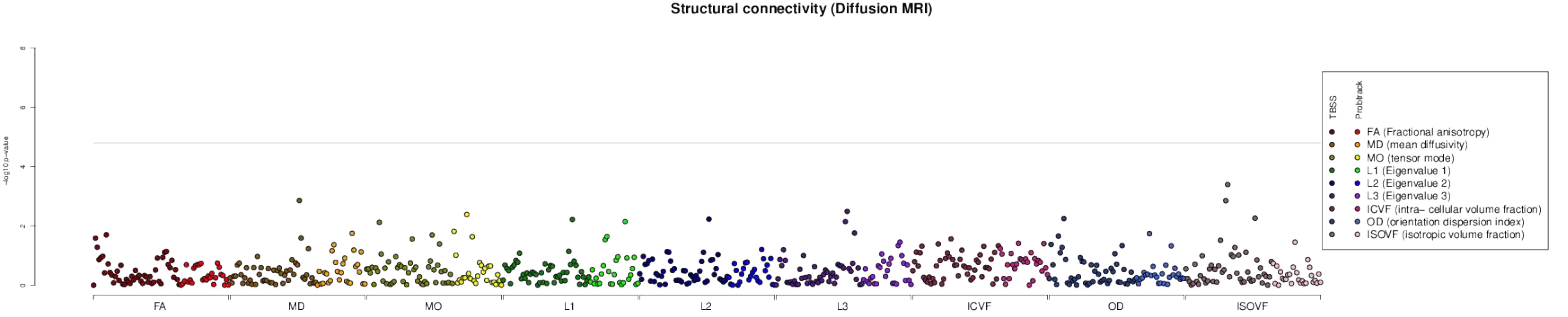

Structural connectivity (Diffusion MRI)
-log10 p-value
TBSS
Probtrack
FA (Fractional anisotropy)
MD (mean diffusivity)
MO (tensor mode)
L1 (Eigenvalue 1)
L2 (Eigenvalue 2)
L3 (Eigenvalue 3)
ICVF (intra- cellular volume fraction)
OD (orientation dispersion index)
ISOVF (isotropic volume fraction)
FA
MD
MO
L1
L2
L3
ICVF
OD
ISOVF

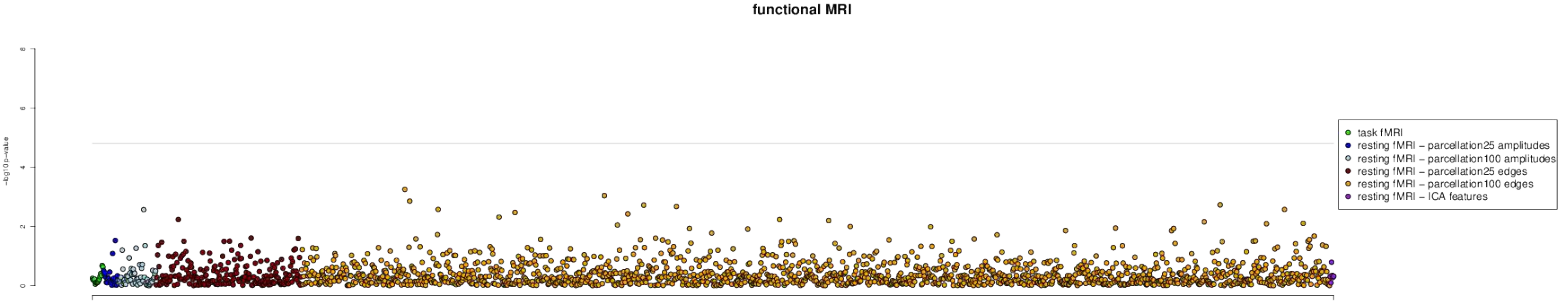

functional MRI
-log10 p-value
task fMRI
resting fMRI – parcellation25 amplitudes
resting fMRI – parcellation100 amplitudes
resting fMRI – parcellation25 edges
resting fMRI – parcellation100 edges
resting fMRI – ICA features

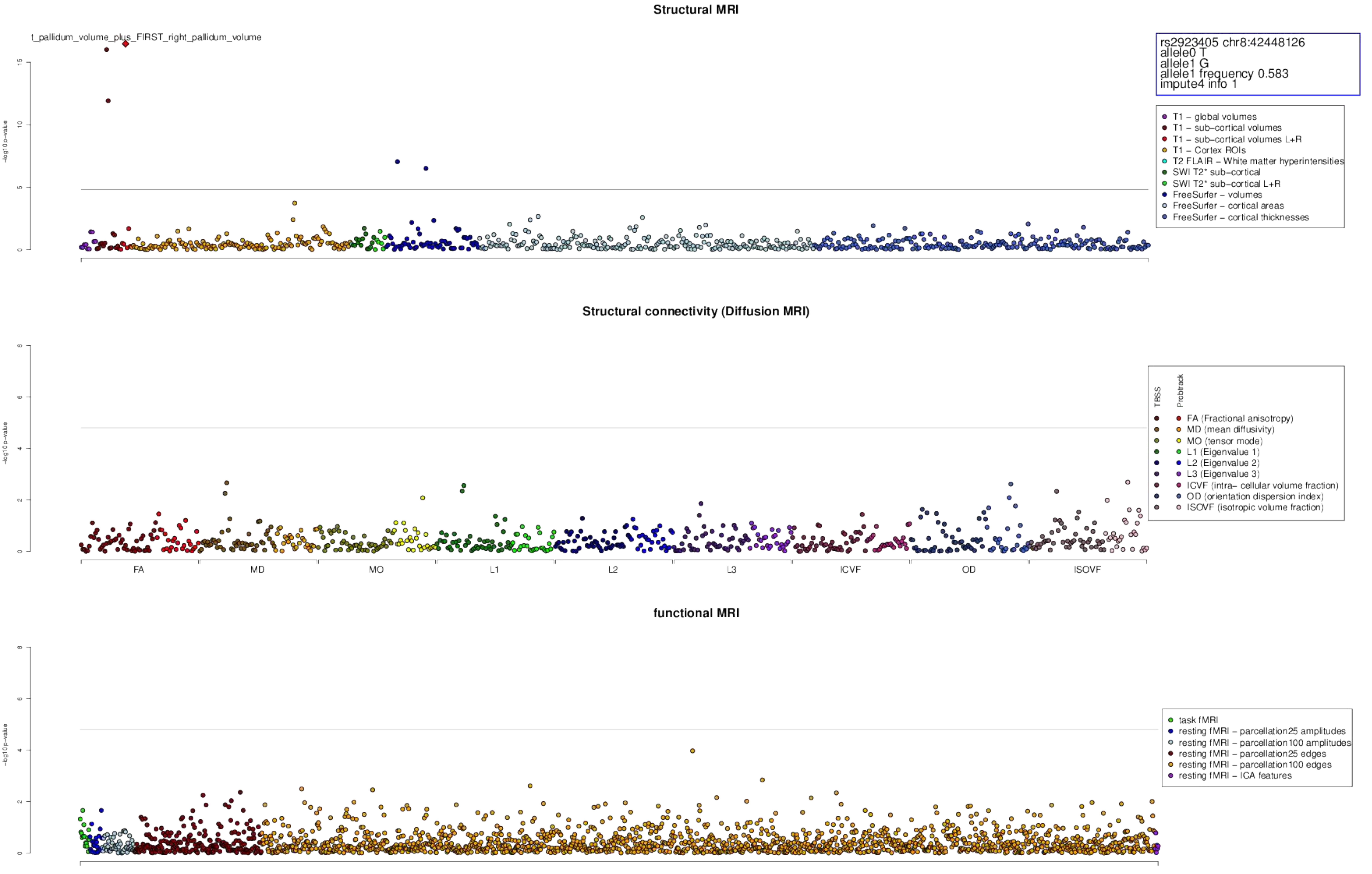
Structural MRI
t_pallidum_volume_plus_FIRST_right_pallidum_volume
rs2923405 chr8:42448126
allele0 T
allele1 G
allele1 frequency 0.583
impute4 info 1
T1 – global volumes
T1 – sub–cortical volumes
T1 – sub–cortical volumes L+R
T1 – Cortex ROIs
T2 FLAIR – White matter hyperintensities
SWI T2* sub–cortical
SWI T2* sub–cortical L+R
FreeSurfer – volumes
FreeSurfer – cortical areas
FreeSurfer – cortical thicknesses
-log10 p-value
Structural connectivity (Diffusion MRI)
TBSS
Probtrack
FA (Fractional anisotropy)
MD (mean diffusivity)
MO (tensor mode)
L1 (Eigenvalue 1)
L2 (Eigenvalue 2)
L3 (Eigenvalue 3)
ICVF (intra– cellular volume fraction)
OD (orientation dispersion index)
ISOVF (isotropic volume fraction)
FA
MD
MO
L1
L2
L3
ICVF
OD
ISOVF
functional MRI
task fMRI
resting fMRI – parcellation25 amplitudes
resting fMRI – parcellation100 amplitudes
resting fMRI – parcellation25 edges
resting fMRI – parcellation100 edges
resting fMRI – ICA features

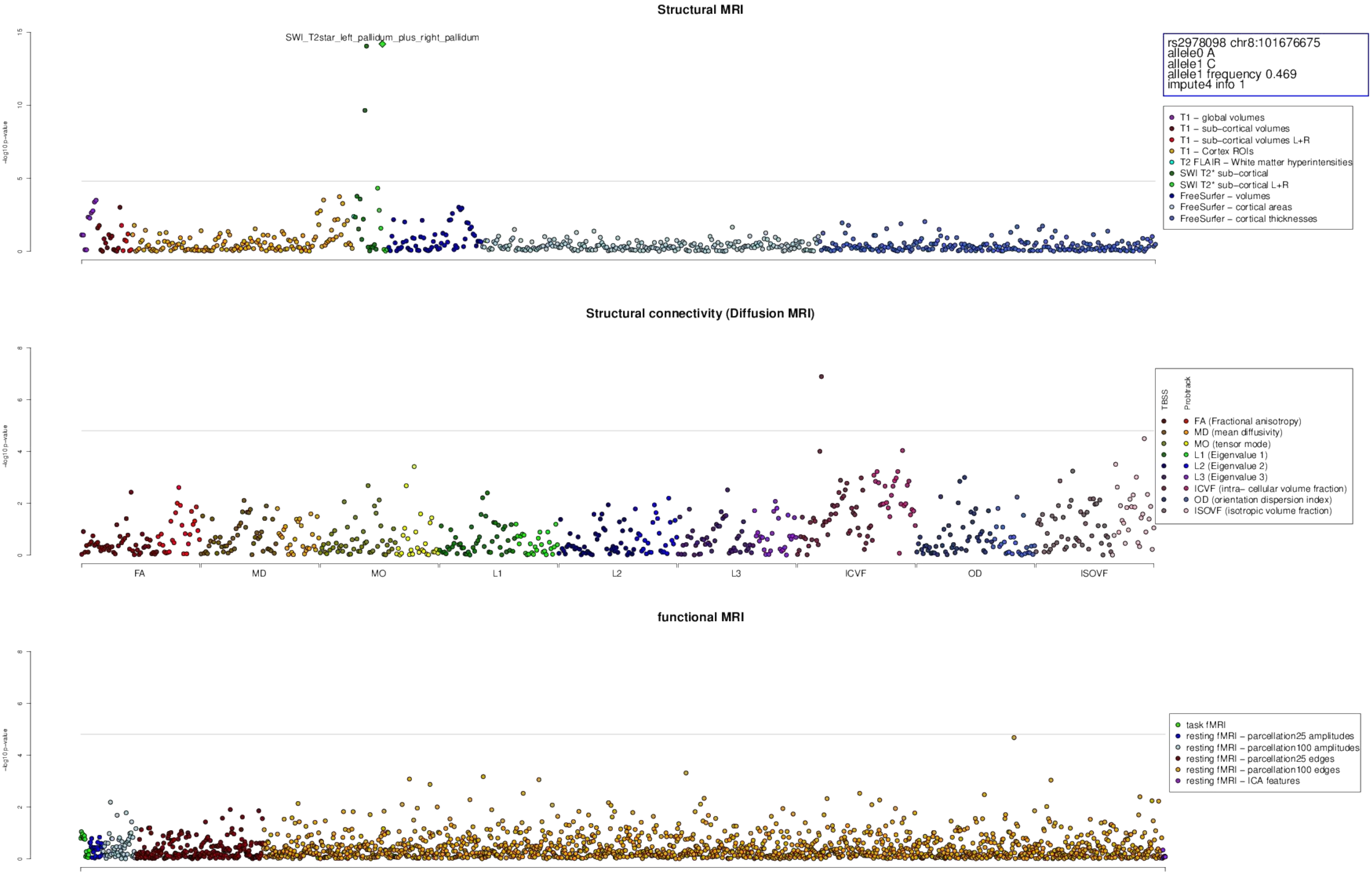
Structural MRI
SWI_T2star_left_pallidum_plus_right_pallidum
rs2978098 chr8:101676675
allele0 A
allele1 C
allele1 frequency 0.469
impute4 info 1
T1 – global volumes
T1 – sub–cortical volumes
T1 – sub–cortical volumes L+R
T1 – Cortex ROIs
T2 FLAIR – White matter hyperintensities
SWI T2* sub–cortical
SWI T2* sub–cortical L+R
FreeSurfer – volumes
FreeSurfer – cortical areas
FreeSurfer – cortical thicknesses
-log10 p-value
Structural connectivity (Diffusion MRI)
TBSS
Probtrack
FA (Fractional anisotropy)
MD (mean diffusivity)
MO (tensor mode)
L1 (Eigenvalue 1)
L2 (Eigenvalue 2)
L3 (Eigenvalue 3)
ICVF (intra– cellular volume fraction)
OD (orientation dispersion index)
ISOVF (isotropic volume fraction)
FA
MD
MO
L1
L2
L3
ICVF
OD
ISOVF
functional MRI
task fMRI
resting fMRI – parcellation25 amplitudes
resting fMRI – parcellation100 amplitudes
resting fMRI – parcellation25 edges
resting fMRI – parcellation100 edges
resting fMRI – ICA features

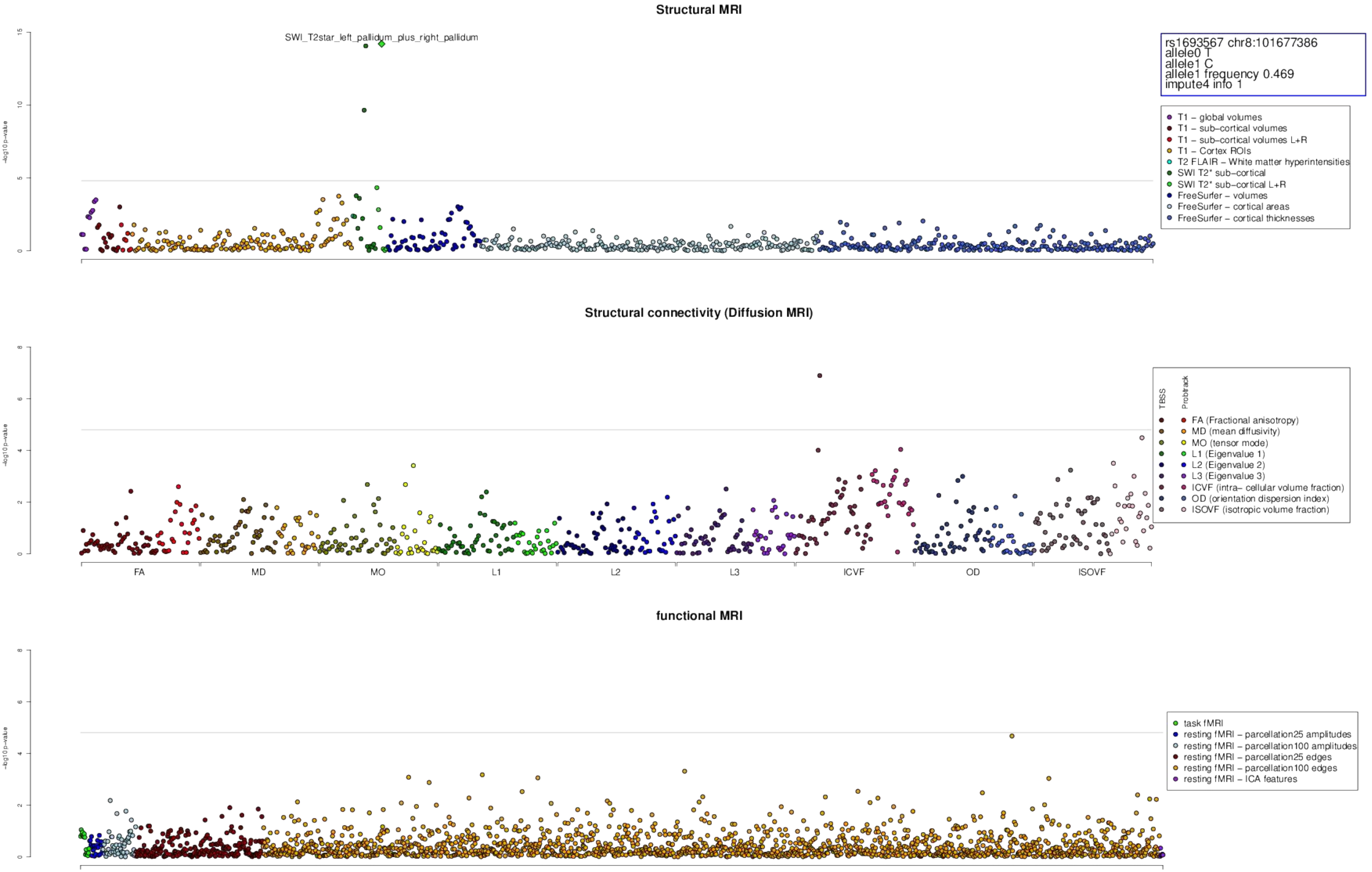

Structural MRI
SWI_T2star_left_pallidum_plus_right_pallidum
rs1693567 chr8:101677386
allele0 T
allele1 C
allele1 frequency 0.469
impute4 info 1
T1 – global volumes
T1 – sub-cortical volumes
T1 – sub-cortical volumes L+R
T1 – Cortex ROIs
T2 FLAIR – White matter hyperintensities
SWI T2* sub-cortical
SWI T2* sub-cortical L+R
FreeSurfer – volumes
FreeSurfer – cortical areas
FreeSurfer – cortical thicknesses
-log10 p-value
Structural connectivity (Diffusion MRI)
TBSS
Probtrack
FA (Fractional anisotropy)
MD (mean diffusivity)
MO (tensor mode)
L1 (Eigenvalue 1)
L2 (Eigenvalue 2)
L3 (Eigenvalue 3)
ICVF (intra- cellular volume fraction)
OD (orientation dispersion index)
ISOVF (isotropic volume fraction)
FA
MD
MO
L1
L2
L3
ICVF
OD
ISOVF
functional MRI
task fMRI
resting fMRI – parcellation25 amplitudes
resting fMRI – parcellation100 amplitudes
resting fMRI – parcellation25 edges
resting fMRI – parcellation100 edges
resting fMRI – ICA features

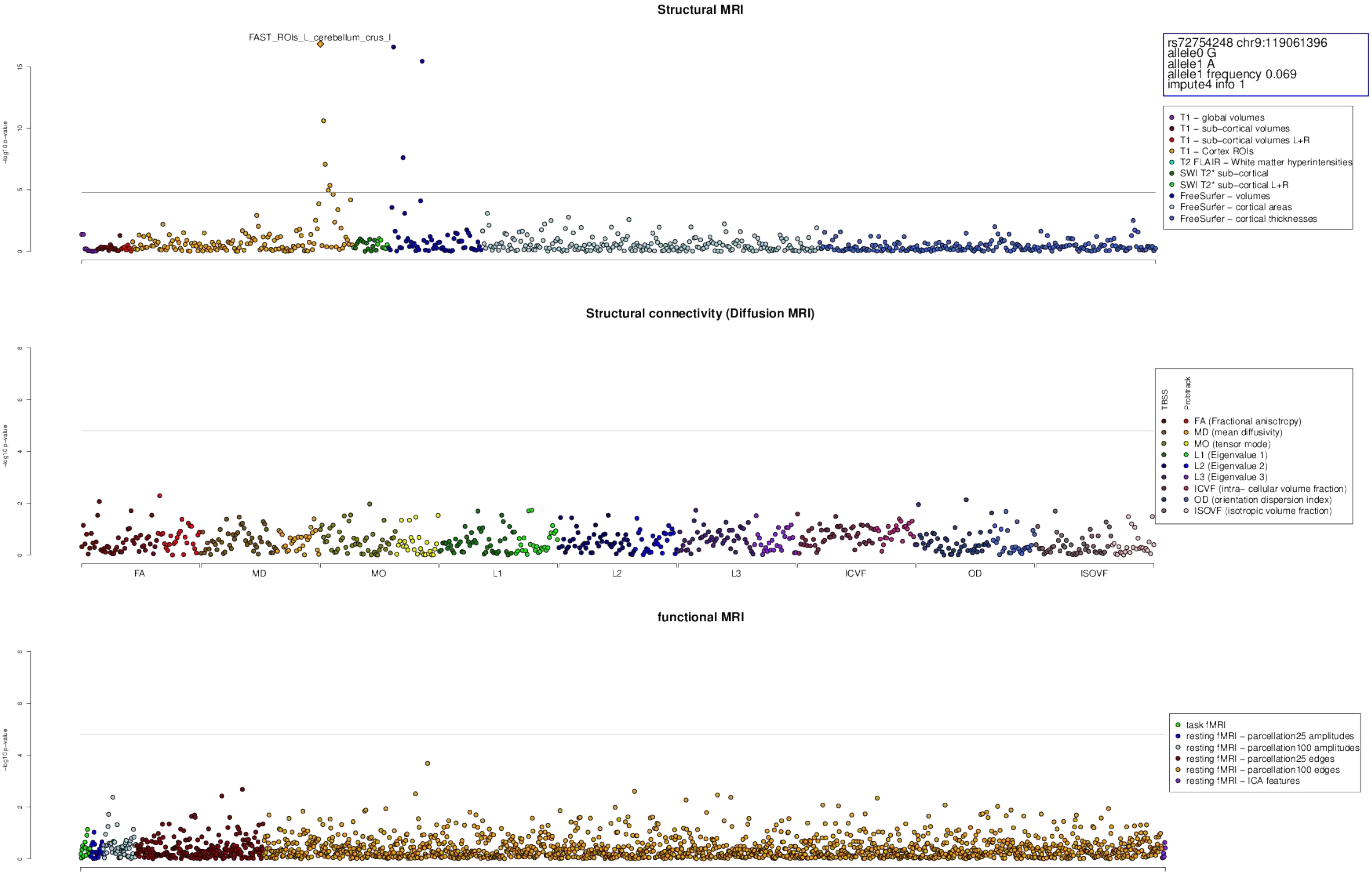
Structural MRI
FAST_ROIs_L_cerebellum_crus_I
rs72754248 chr9:119061396
allele0 G
allele1 A
allele1 frequency 0.069
impute4 info 1
T1 – global volumes
T1 – sub-cortical volumes
T1 – sub-cortical volumes L+R
T1 – Cortex ROIs
T2 FLAIR – White matter hyperintensities
SWI T2* sub-cortical
SWI T2* sub-cortical L+R
FreeSurfer – volumes
FreeSurfer – cortical areas
FreeSurfer – cortical thicknesses
-log10 p-value
Structural connectivity (Diffusion MRI)
TBSS
Probtrack
FA (Fractional anisotropy)
MD (mean diffusivity)
MO (tensor mode)
L1 (Eigenvalue 1)
L2 (Eigenvalue 2)
L3 (Eigenvalue 3)
ICVF (intra- cellular volume fraction)
OD (orientation dispersion index)
ISOVF (isotropic volume fraction)
FA
MD
MO
L1
L2
L3
ICVF
OD
ISOVF
functional MRI
task fMRI
resting fMRI – parcellation25 amplitudes
resting fMRI – parcellation100 amplitudes
resting fMRI – parcellation25 edges
resting fMRI – parcellation100 edges
resting fMRI – ICA features

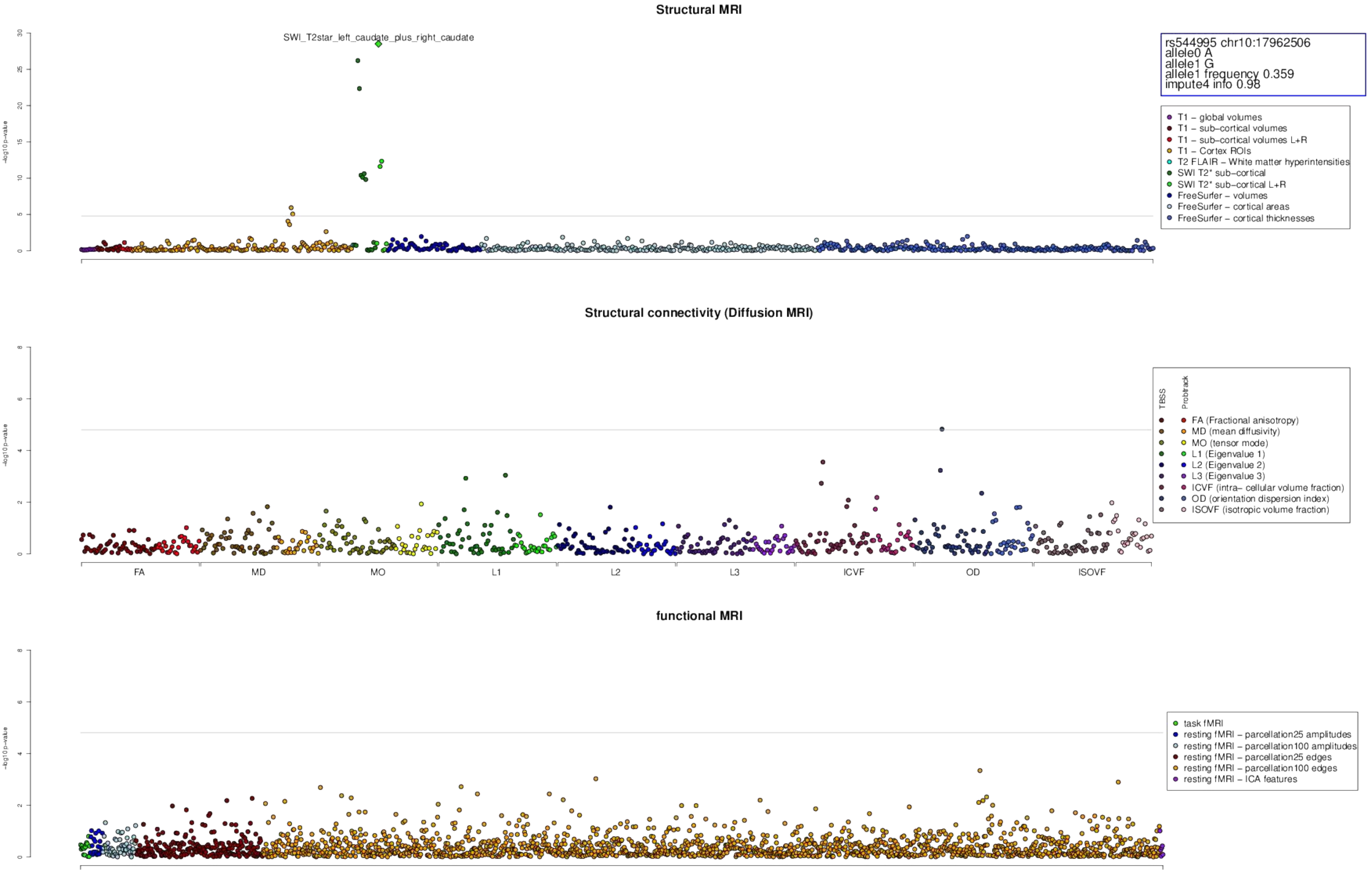
Structural MRI
SWI_T2star_left_caudate_plus_right_caudate
rs544995 chr10:17962506
allele0 A
allele1 G
allele1 frequency 0.359
impute4 info 0.98
T1 – global volumes
T1 – sub–cortical volumes
T1 – sub–cortical volumes L+R
T1 – Cortex ROIs
T2 FLAIR – White matter hyperintensities
SWI T2* sub–cortical
SWI T2* sub–cortical L+R
FreeSurfer – volumes
FreeSurfer – cortical areas
FreeSurfer – cortical thicknesses
–log10 p–value
Structural connectivity (Diffusion MRI)
TBSS
Probtrack
FA (Fractional anisotropy)
MD (mean diffusivity)
MO (tensor mode)
L1 (Eigenvalue 1)
L2 (Eigenvalue 2)
L3 (Eigenvalue 3)
ICVF (intra– cellular volume fraction)
OD (orientation dispersion index)
ISOVF (isotropic volume fraction)
FA
MD
MO
L1
L2
L3
ICVF
OD
ISOVF
functional MRI
task fMRI
resting fMRI – parcellation25 amplitudes
resting fMRI – parcellation100 amplitudes
resting fMRI – parcellation25 edges
resting fMRI – parcellation100 edges
resting fMRI – ICA features

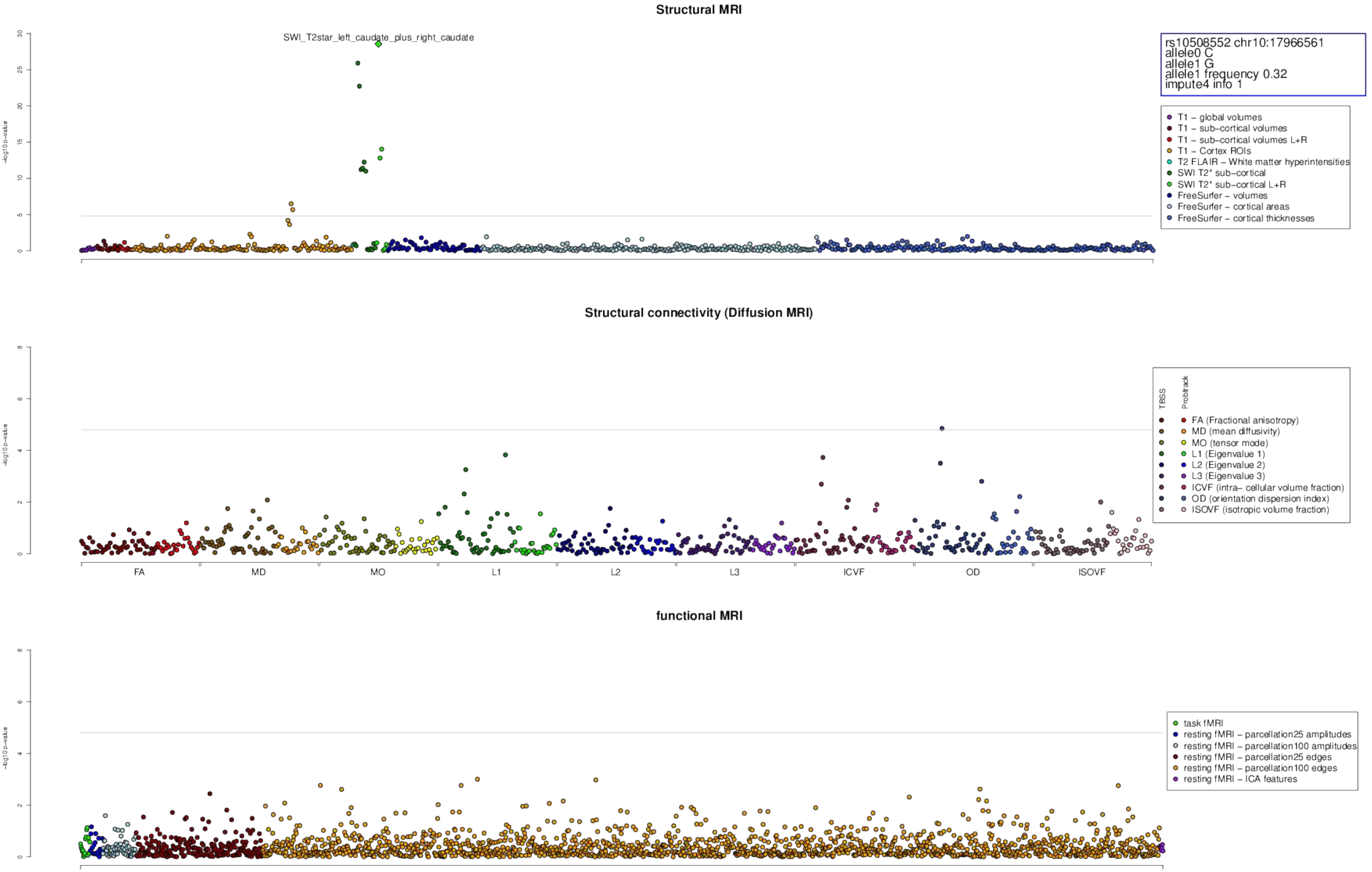

Structural MRI
SWI_T2star_left_caudate_plus_right_caudate
-log10 p-value
rs10508552 chr10:17966561
allele0 C
allele1 G
allele1 frequency 0.32
impute4 info 1
T1 – global volumes
T1 – sub-cortical volumes
T1 – sub-cortical volumes L+R
T1 – Cortex ROIs
T2 FLAIR – White matter hyperintensities
SWI T2* sub-cortical
SWI T2* sub-cortical L+R
FreeSurfer – volumes
FreeSurfer – cortical areas
FreeSurfer – cortical thicknesses
Structural connectivity (Diffusion MRI)
-log10 p-value
TBSS
Probtrack
FA (Fractional anisotropy)
MD (mean diffusivity)
MO (tensor mode)
L1 (Eigenvalue 1)
L2 (Eigenvalue 2)
L3 (Eigenvalue 3)
ICVF (intra- cellular volume fraction)
OD (orientation dispersion index)
ISOVF (isotropic volume fraction)
FA
MD
MO
L1
L2
L3
ICVF
OD
ISOVF
functional MRI
-log10 p-value
task fMRI
resting fMRI – parcellation25 amplitudes
resting fMRI – parcellation100 amplitudes
resting fMRI – parcellation25 edges
resting fMRI – parcellation100 edges
resting fMRI – ICA features

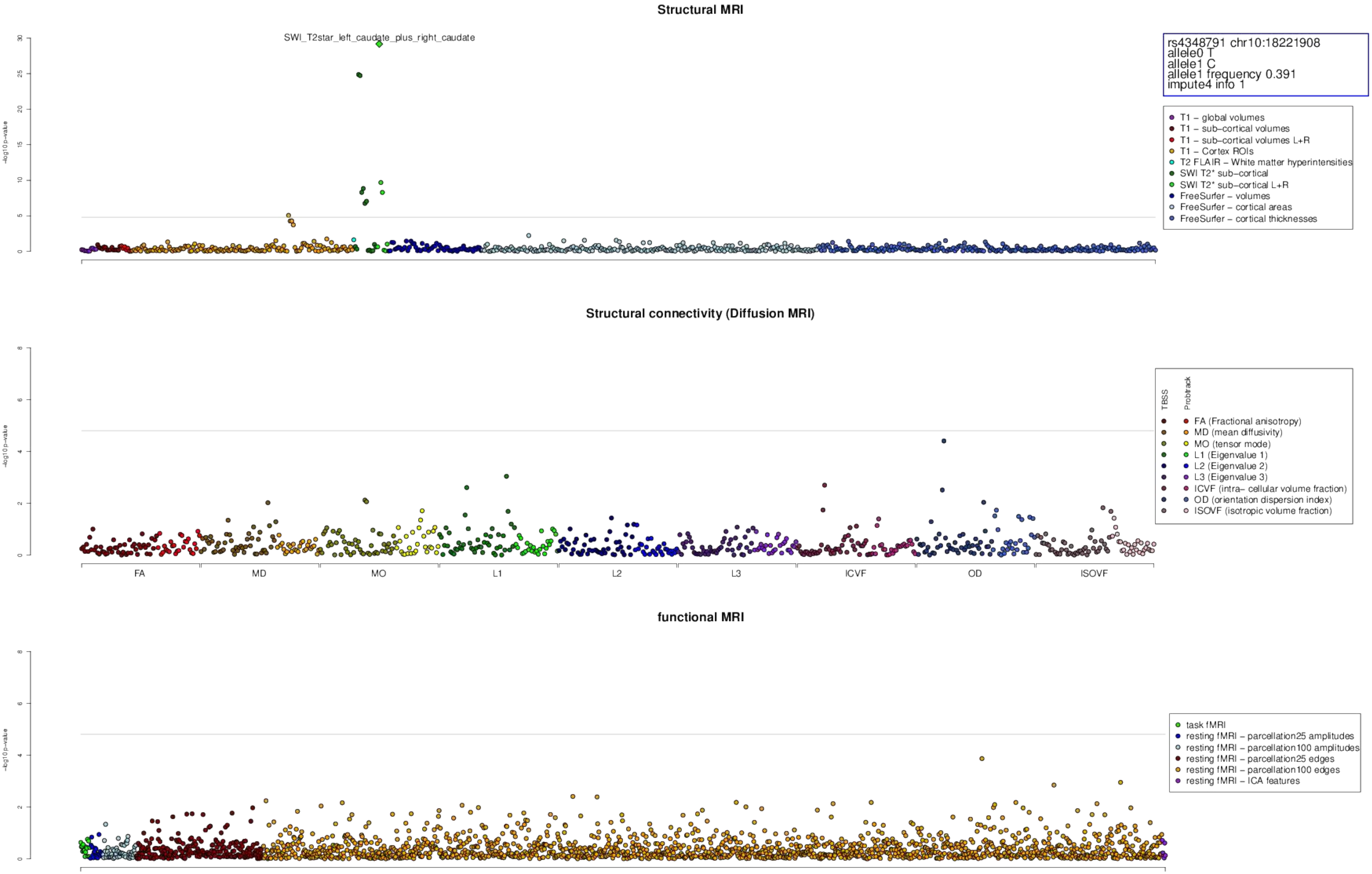
Structural MRI
SWI_T2star_left_caudate_plus_right_caudate
-log10 p-value
rs4348791 chr10:18221908
allele0 T
allele1 C
allele1 frequency 0.391
impute4 info 1
T1 – global volumes
T1 – sub-cortical volumes
T1 – sub-cortical volumes L+R
T1 – Cortex ROIs
T2 FLAIR – White matter hyperintensities
SWI T2* sub-cortical
SWI T2* sub-cortical L+R
FreeSurfer – volumes
FreeSurfer – cortical areas
FreeSurfer – cortical thicknesses
Structural connectivity (Diffusion MRI)
-log10 p-value
TBSS
Probtrack
FA (Fractional anisotropy)
MD (mean diffusivity)
MO (tensor mode)
L1 (Eigenvalue 1)
L2 (Eigenvalue 2)
L3 (Eigenvalue 3)
ICVF (intra- cellular volume fraction)
OD (orientation dispersion index)
ISOVF (isotropic volume fraction)
FA
MD
MO
L1
L2
L3
ICVF
OD
ISOVF
functional MRI
-log10 p-value
task fMRI
resting fMRI – parcellation25 amplitudes
resting fMRI – parcellation100 amplitudes
resting fMRI – parcellation25 edges
resting fMRI – parcellation100 edges
resting fMRI – ICA features

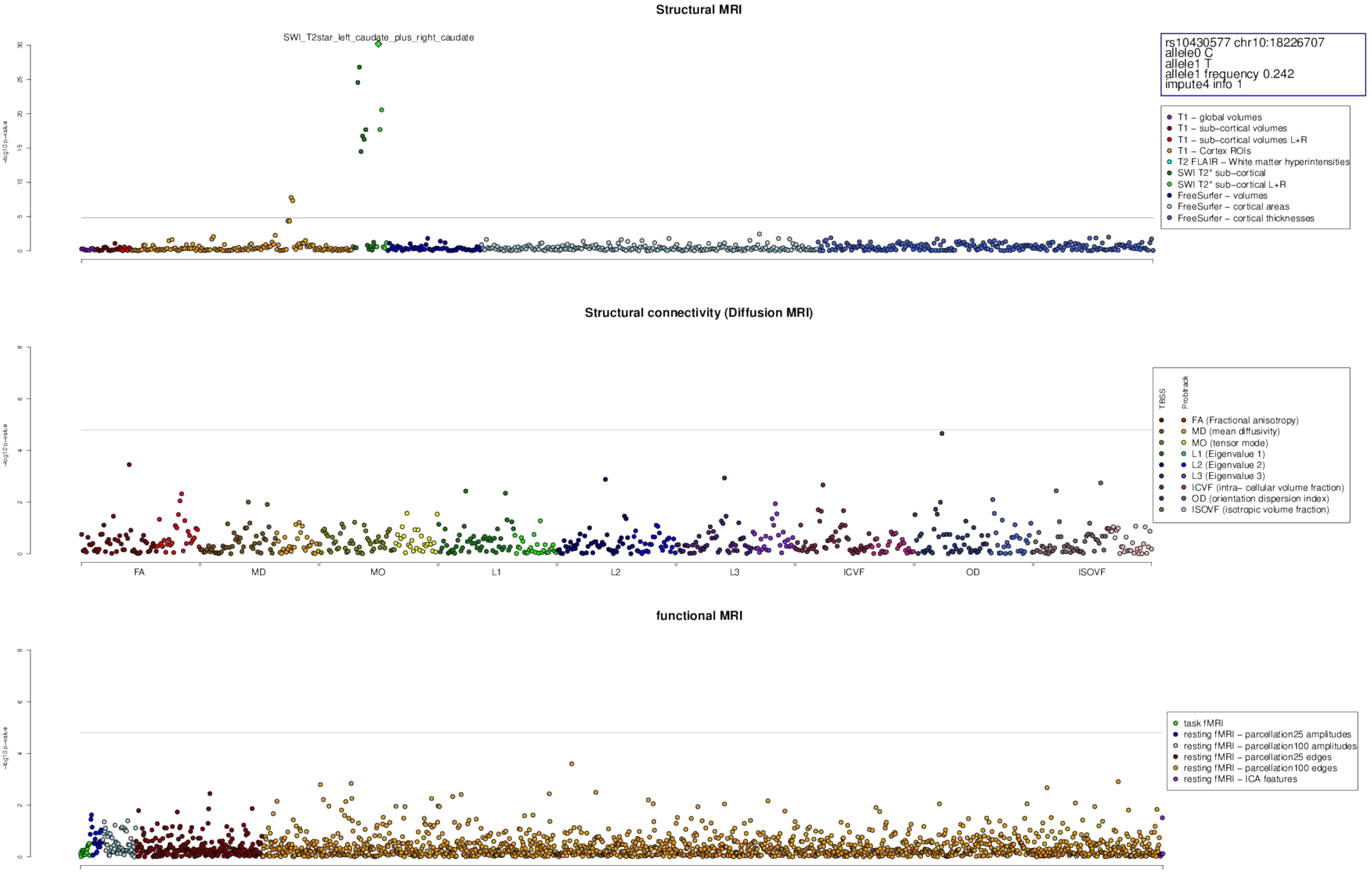

Structural MRI
SWI_T2star_left_caudate_plus_right_caudate
rs10430577 chr10:18226707
allele0 C
allele1 T
allele1 frequency 0.242
impute4 info 1
T1 – global volumes
T1 – sub–cortical volumes
T1 – sub–cortical volumes L+R
T1 – Cortex ROIs
T2 FLAIR – White matter hyperintensities
SWI T2* sub–cortical
SWI T2* sub–cortical L+R
FreeSurfer – volumes
FreeSurfer – cortical areas
FreeSurfer – cortical thicknesses
Structural connectivity (Diffusion MRI)
TBSS
Probtrack
FA (Fractional anisotropy)
MD (mean diffusivity)
MO (tensor mode)
L1 (Eigenvalue 1)
L2 (Eigenvalue 2)
L3 (Eigenvalue 3)
ICVF (intra– cellular volume fraction)
OD (orientation dispersion index)
ISOVF (isotropic volume fraction)
FA
MD
MO
L1
L2
L3
ICVF
OD
ISOVF
functional MRI
task fMRI
resting fMRI – parcellation25 amplitudes
resting fMRI – parcellation100 amplitudes
resting fMRI – parcellation25 edges
resting fMRI – parcellation100 edges
resting fMRI – ICA features

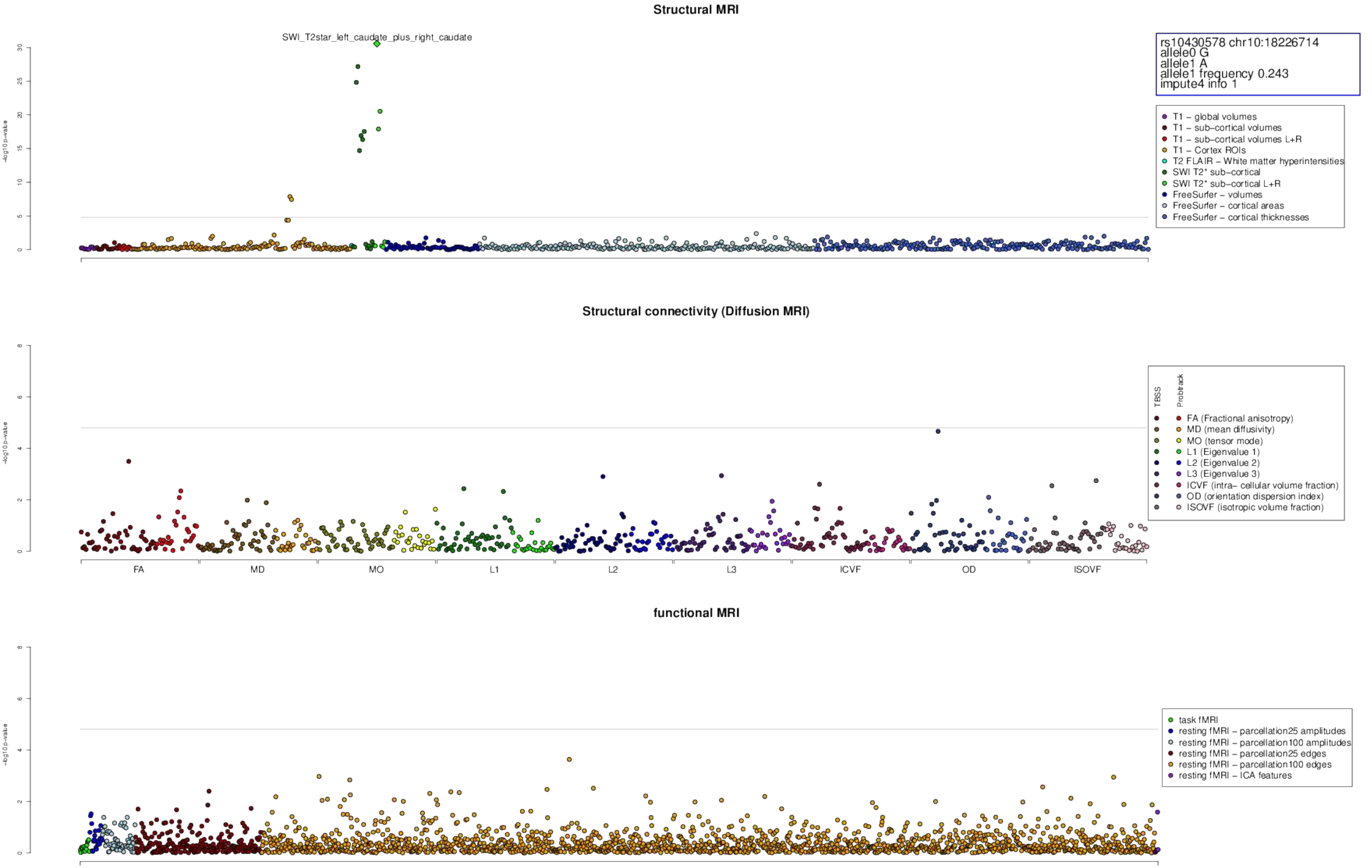
Structural MRI
SWI_T2star_left_caudate_plus_right_caudate
rs10430578 chr10:18226714
allele0 G
allele1 A
allele1 frequency 0.243
impute4 info 1
T1 – global volumes
T1 – sub-cortical volumes
T1 – sub-cortical volumes L+R
T1 – Cortex ROIs
T2 FLAIR – White matter hyperintensities
SWI T2* sub-cortical
SWI T2* sub-cortical L+R
FreeSurfer – volumes
FreeSurfer – cortical areas
FreeSurfer – cortical thicknesses
-log10 p-value
Structural connectivity (Diffusion MRI)
TBSS
Probtrack
FA (Fractional anisotropy)
MD (mean diffusivity)
MO (tensor mode)
L1 (Eigenvalue 1)
L2 (Eigenvalue 2)
L3 (Eigenvalue 3)
ICVF (intra- cellular volume fraction)
OD (orientation dispersion index)
ISOVF (isotropic volume fraction)
FA
MD
MO
L1
L2
L3
ICVF
OD
ISOVF
functional MRI
task fMRI
resting fMRI – parcellation25 amplitudes
resting fMRI – parcellation100 amplitudes
resting fMRI – parcellation25 edges
resting fMRI – parcellation100 edges
resting fMRI – ICA features

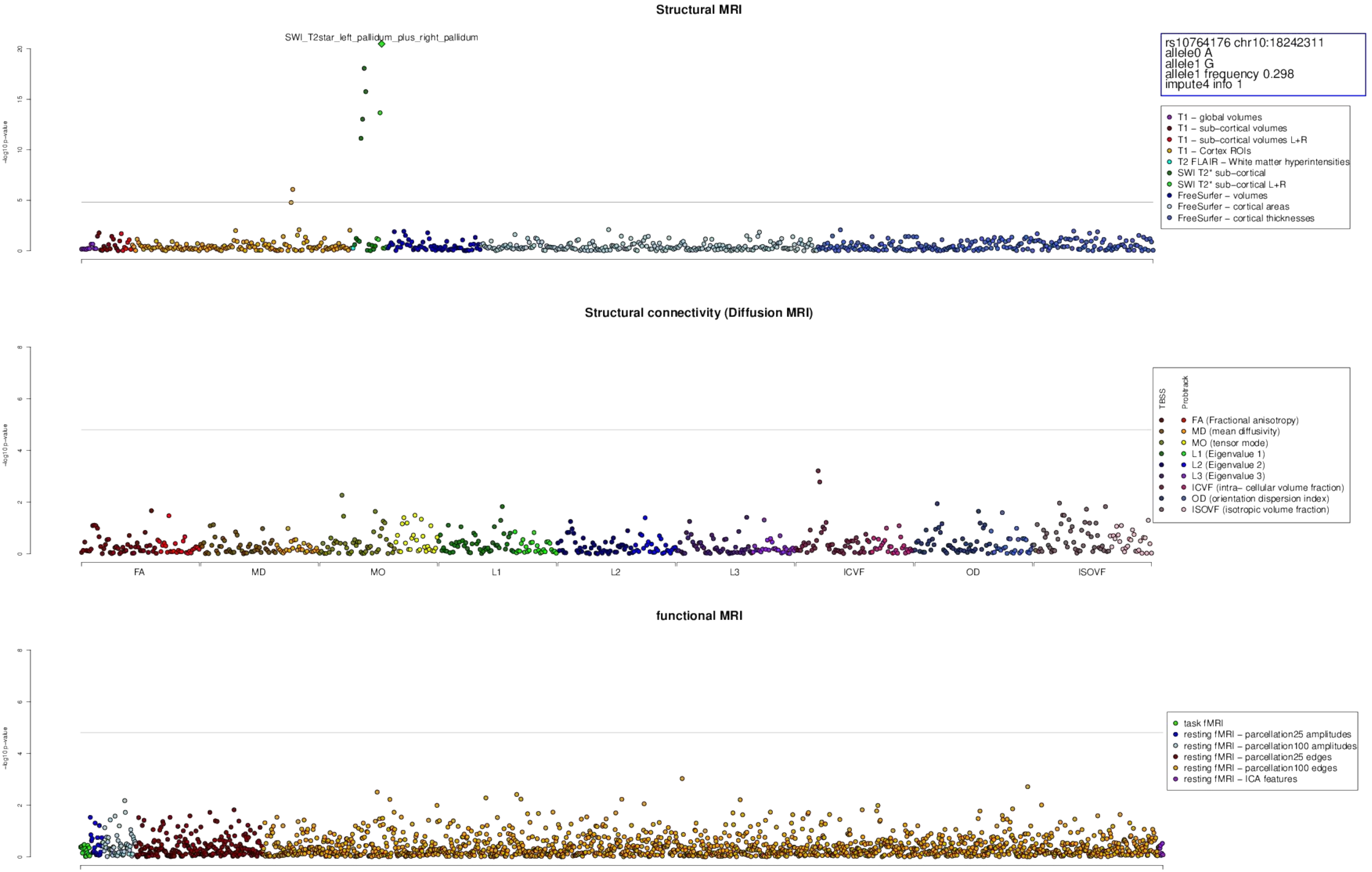
Structural MRI
SWI_T2star_left_pallidum_plus_right_pallidum
-log10 p-value
rs10764176 chr10:18242311
allele0 A
allele1 G
allele1 frequency 0.298
impute4 info 1
T1 – global volumes
T1 – sub-cortical volumes
T1 – sub-cortical volumes L+R
T1 – Cortex ROIs
T2 FLAIR – White matter hyperintensities
SWI T2* sub-cortical
SWI T2* sub-cortical L+R
FreeSurfer – volumes
FreeSurfer – cortical areas
FreeSurfer – cortical thicknesses
Structural connectivity (Diffusion MRI)
-log10 p-value
TBSS
Probtrack
FA (Fractional anisotropy)
MD (mean diffusivity)
MO (tensor mode)
L1 (Eigenvalue 1)
L2 (Eigenvalue 2)
L3 (Eigenvalue 3)
ICVF (intra- cellular volume fraction)
OD (orientation dispersion index)
ISOVF (isotropic volume fraction)
FA
MD
MO
L1
L2
L3
ICVF
OD
ISOVF
functional MRI
-log10 p-value
task fMRI
resting fMRI – parcellation25 amplitudes
resting fMRI – parcellation100 amplitudes
resting fMRI – parcellation25 edges
resting fMRI – parcellation100 edges
resting fMRI – ICA features

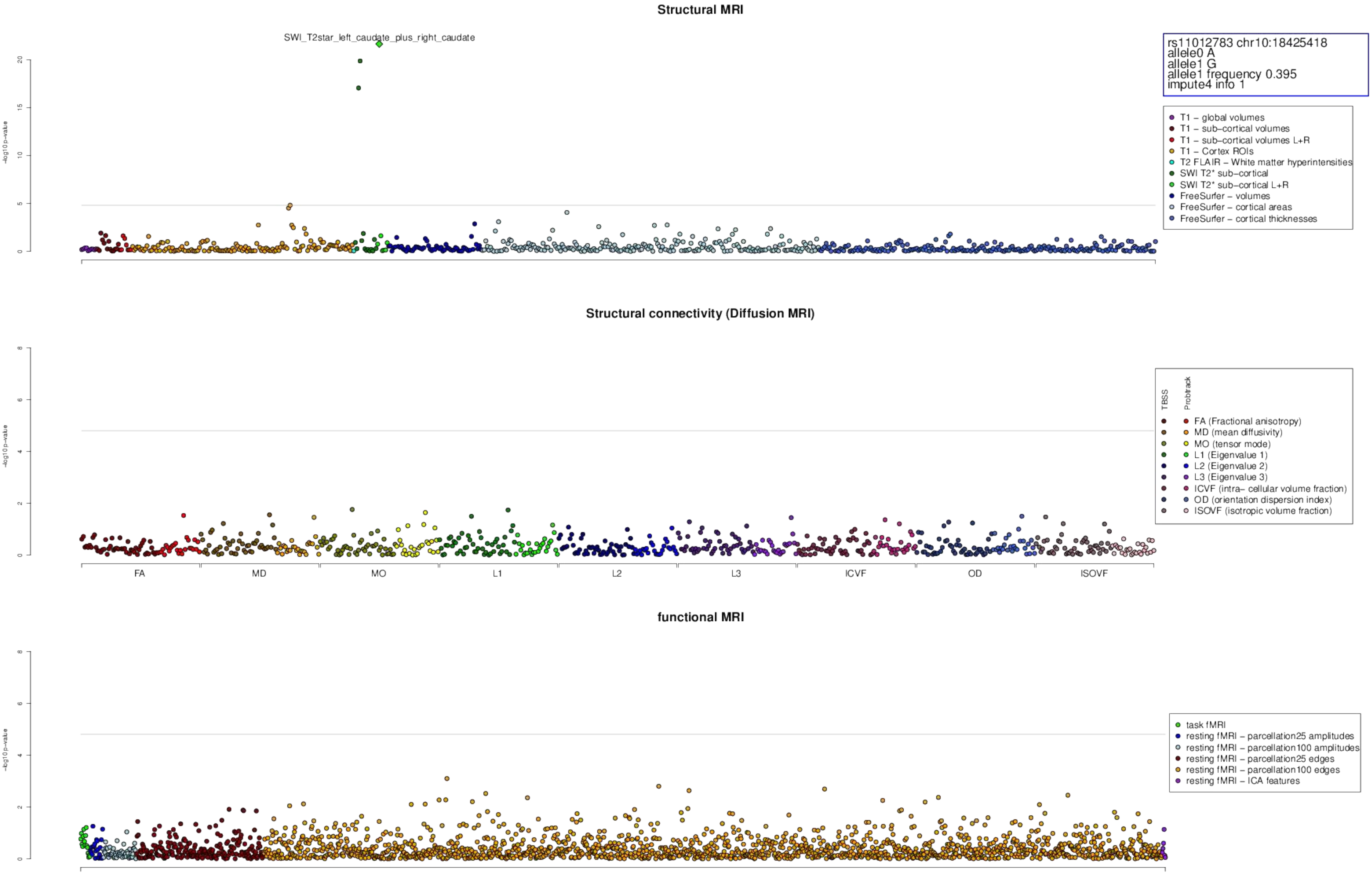

Structural MRI
SWI_T2star_left_caudate_plus_right_caudate
rs11012783 chr10:18425418
allele0 A
allele1 G
allele1 frequency 0.395
impute4 info 1
T1 – global volumes
T1 – sub–cortical volumes
T1 – sub–cortical volumes L+R
T1 – Cortex ROIs
T2 FLAIR – White matter hyperintensities
SWI T2* sub–cortical
SWI T2* sub–cortical L+R
FreeSurfer – volumes
FreeSurfer – cortical areas
FreeSurfer – cortical thicknesses
–log10 p–value
Structural connectivity (Diffusion MRI)
TBSS
Probtrack
FA (Fractional anisotropy)
MD (mean diffusivity)
MO (tensor mode)
L1 (Eigenvalue 1)
L2 (Eigenvalue 2)
L3 (Eigenvalue 3)
ICVF (intra– cellular volume fraction)
OD (orientation dispersion index)
ISOVF (isotropic volume fraction)
FA
MD
MO
L1
L2
L3
ICVF
OD
ISOVF
functional MRI
task fMRI
resting fMRI – parcellation25 amplitudes
resting fMRI – parcellation100 amplitudes
resting fMRI – parcellation25 edges
resting fMRI – parcellation100 edges
resting fMRI – ICA features

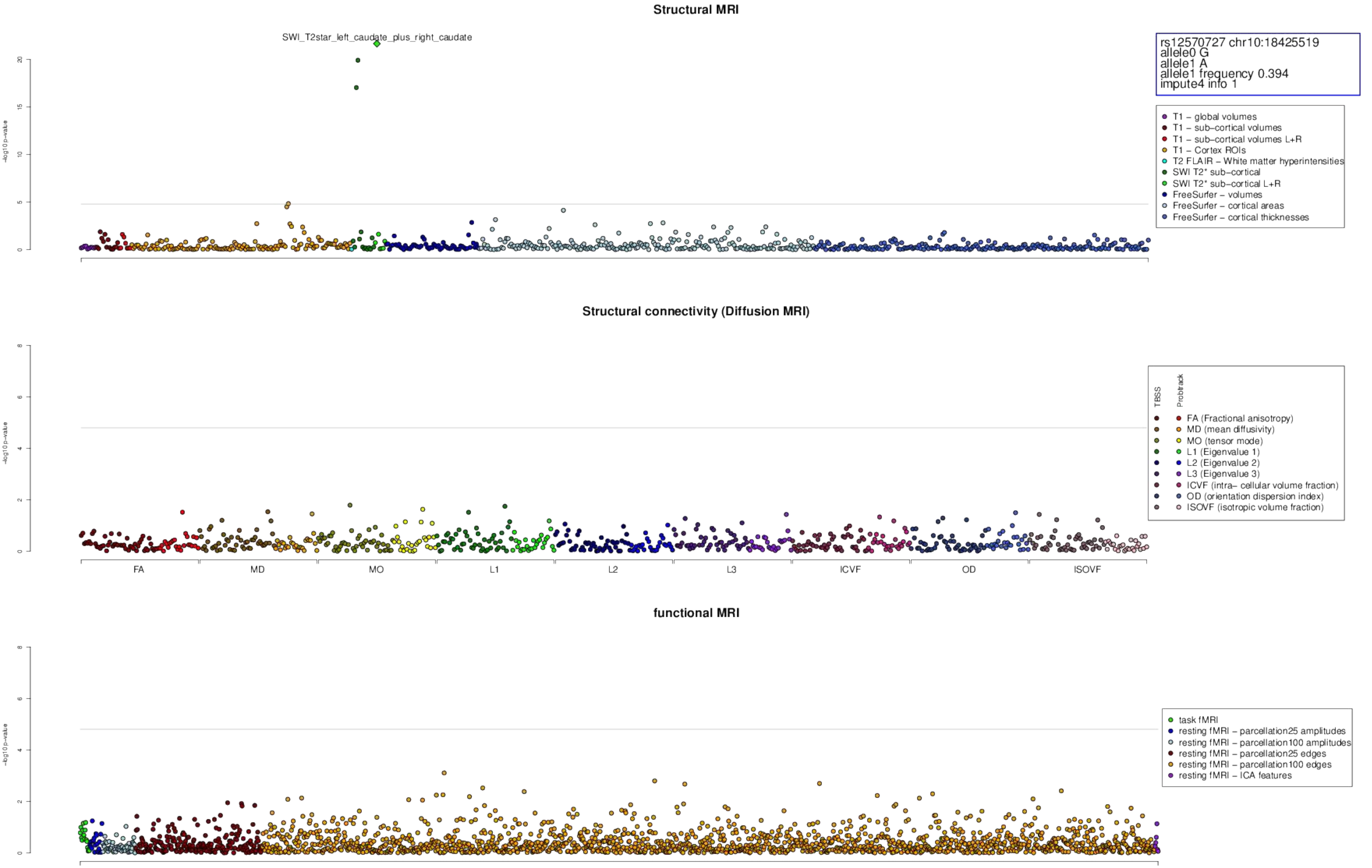
Structural MRI
SWI_T2star_left_caudate_plus_right_caudate
rs12570727 chr10:18425519
allele0 G
allele1 A
allele1 frequency 0.394
impute4 info 1
T1 – global volumes
T1 – sub–cortical volumes
T1 – sub–cortical volumes L+R
T1 – Cortex ROIs
T2 FLAIR – White matter hyperintensities
SWI T2* sub–cortical
SWI T2* sub–cortical L+R
FreeSurfer – volumes
FreeSurfer – cortical areas
FreeSurfer – cortical thicknesses
–log10 p–value
Structural connectivity (Diffusion MRI)
TBSS
Probtrack
FA (Fractional anisotropy)
MD (mean diffusivity)
MO (tensor mode)
L1 (Eigenvalue 1)
L2 (Eigenvalue 2)
L3 (Eigenvalue 3)
ICVF (intra– cellular volume fraction)
OD (orientation dispersion index)
ISOVF (isotropic volume fraction)
FA
MD
MO
L1
L2
L3
ICVF
OD
ISOVF
functional MRI
task fMRI
resting fMRI – parcellation25 amplitudes
resting fMRI – parcellation100 amplitudes
resting fMRI – parcellation25 edges
resting fMRI – parcellation100 edges
resting fMRI – ICA features

Structural MRI

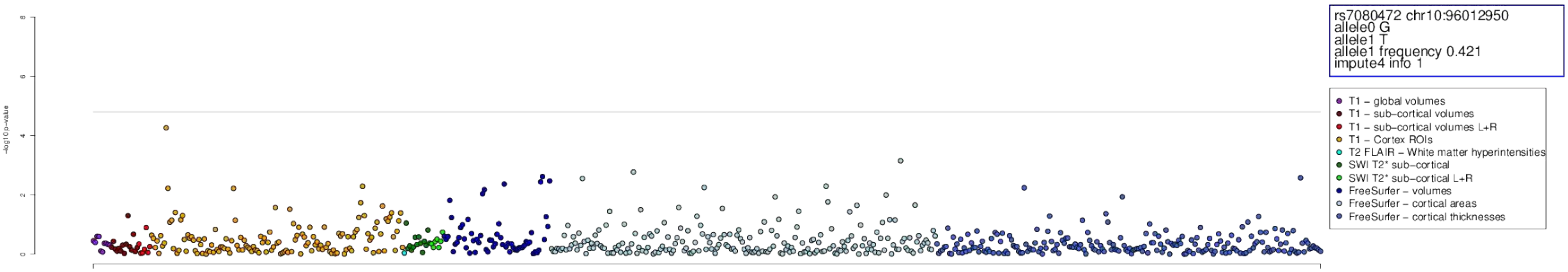

Structural connectivity (Diffusion MRI)

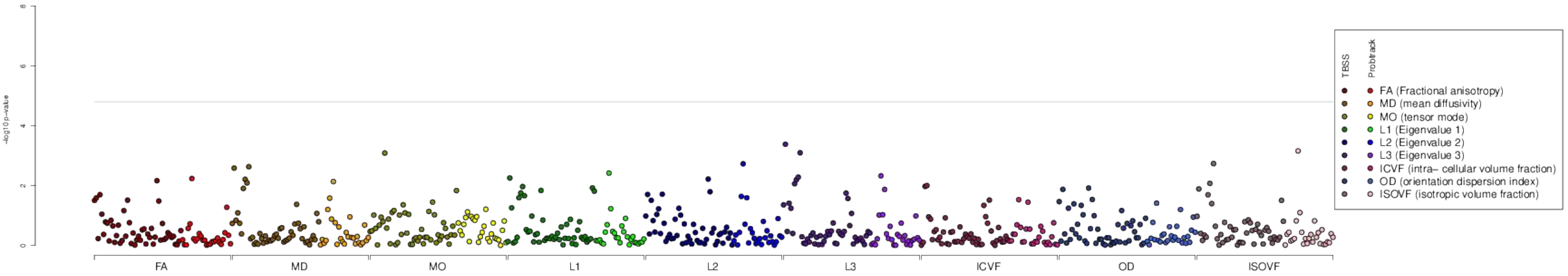

functional MRI

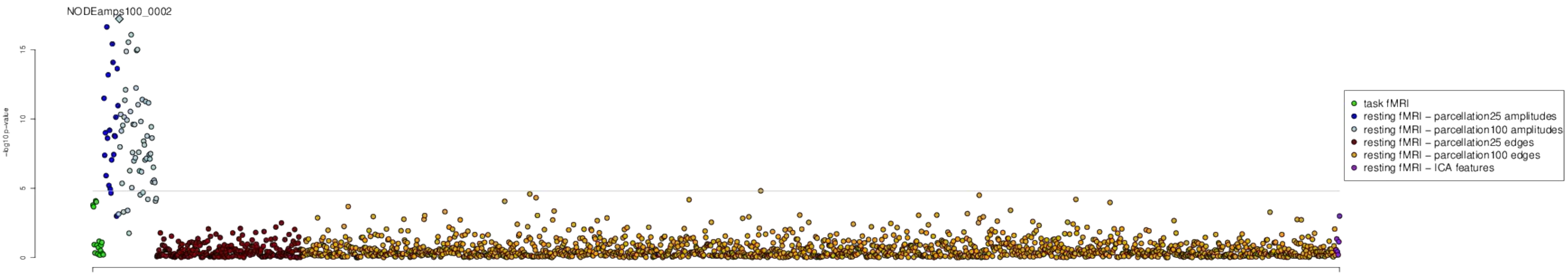

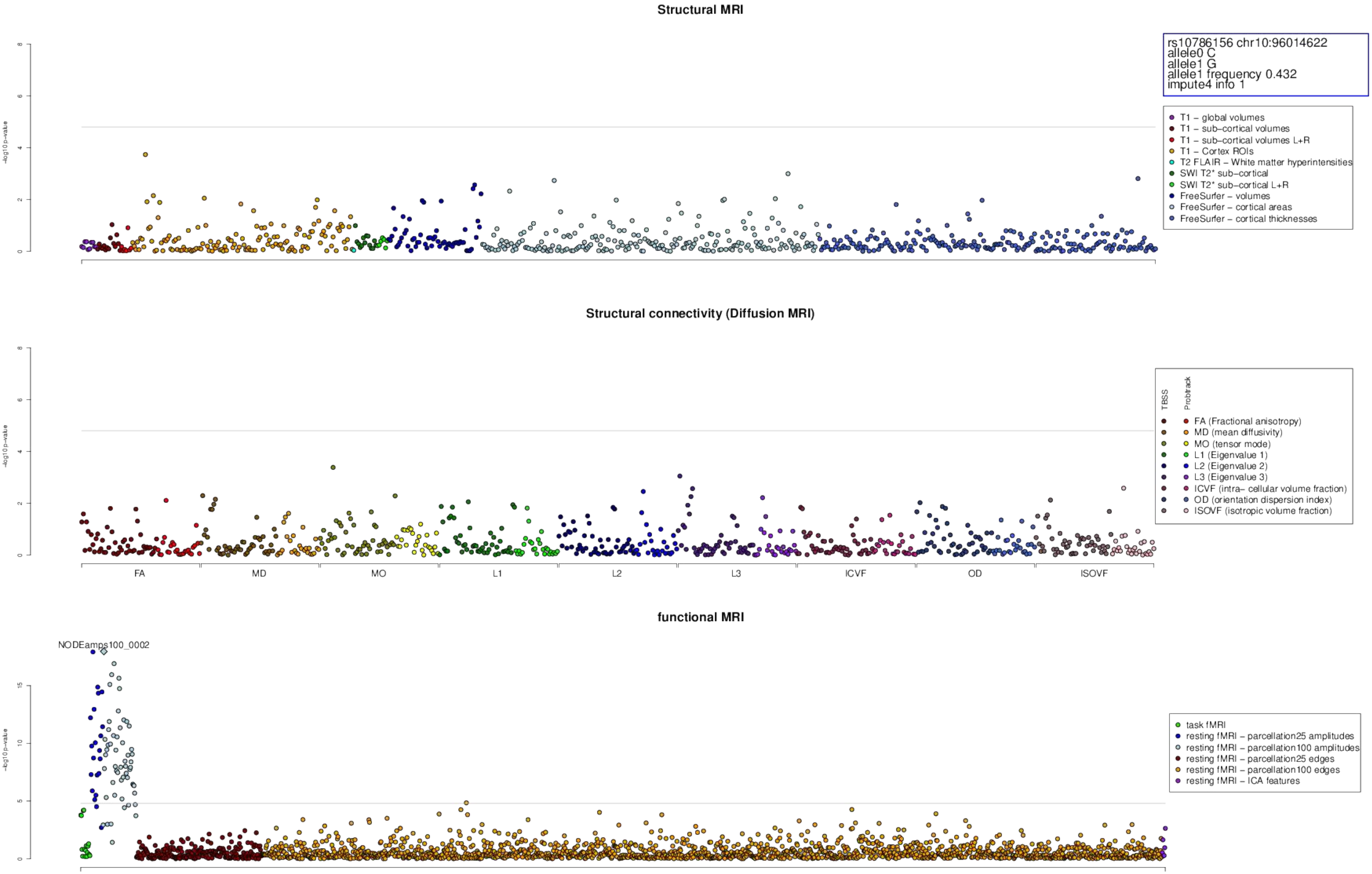
Structural MRI
rs10786156 chr10:96014622
allele0 C
allele1 G
allele1 frequency 0.432
impute4 info 1
T1 – global volumes
T1 – sub-cortical volumes
T1 – sub-cortical volumes L+R
T1 – Cortex ROIs
T2 FLAIR – White matter hyperintensities
SWI T2* sub-cortical
SWI T2* sub-cortical L+R
FreeSurfer – volumes
FreeSurfer – cortical areas
FreeSurfer – cortical thicknesses
-log10 p-value
Structural connectivity (Diffusion MRI)
TBSS
Probtrack
FA (Fractional anisotropy)
MD (mean diffusivity)
MO (tensor mode)
L1 (Eigenvalue 1)
L2 (Eigenvalue 2)
L3 (Eigenvalue 3)
ICVF (intra– cellular volume fraction)
OD (orientation dispersion index)
ISOVF (isotropic volume fraction)
FA
MD
MO
L1
L2
L3
ICVF
OD
ISOVF
functional MRI
NODEamps100_0002
task fMRI
resting fMRI – parcellation25 amplitudes
resting fMRI – parcellation100 amplitudes
resting fMRI – parcellation25 edges
resting fMRI – parcellation100 edges
resting fMRI – ICA features

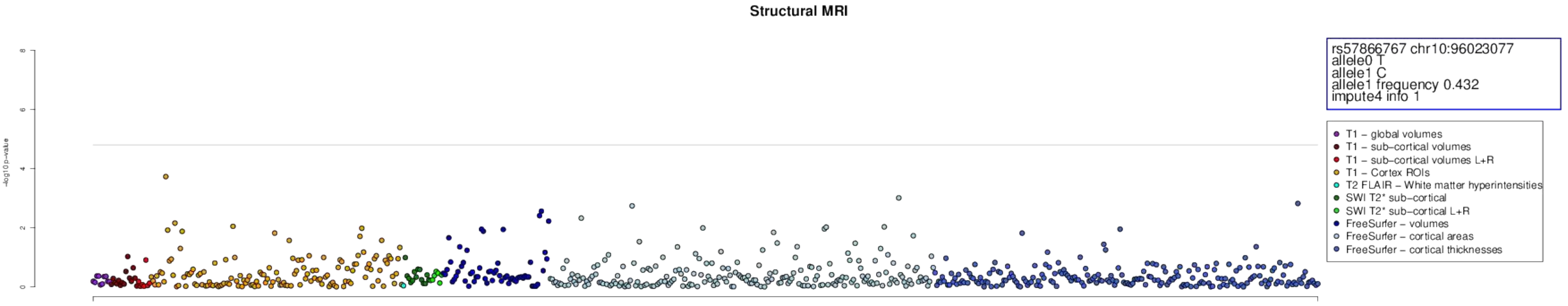
Structural MRI
rs57866767 chr10:96023077
allele0 T
allele1 C
allele1 frequency 0.432
impute4 info 1
T1 – global volumes
T1 – sub-cortical volumes
T1 – sub-cortical volumes L+R
T1 – Cortex ROIs
T2 FLAIR – White matter hyperintensities
SWI T2* sub-cortical
SWI T2* sub-cortical L+R
FreeSurfer – volumes
FreeSurfer – cortical areas
FreeSurfer – cortical thicknesses
-log10 p-value

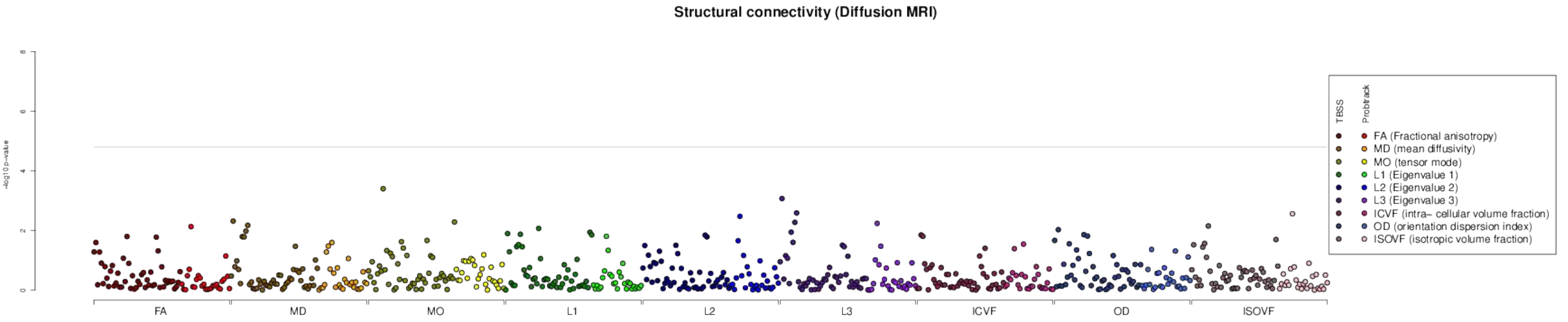
Structural connectivity (Diffusion MRI)
TBSS
Probtrack
FA (Fractional anisotropy)
MD (mean diffusivity)
MO (tensor mode)
L1 (Eigenvalue 1)
L2 (Eigenvalue 2)
L3 (Eigenvalue 3)
ICVF (intra- cellular volume fraction)
OD (orientation dispersion index)
ISOVF (isotropic volume fraction)
FA
MD
MO
L1
L2
L3
ICVF
OD
ISOVF
-log10 p-value

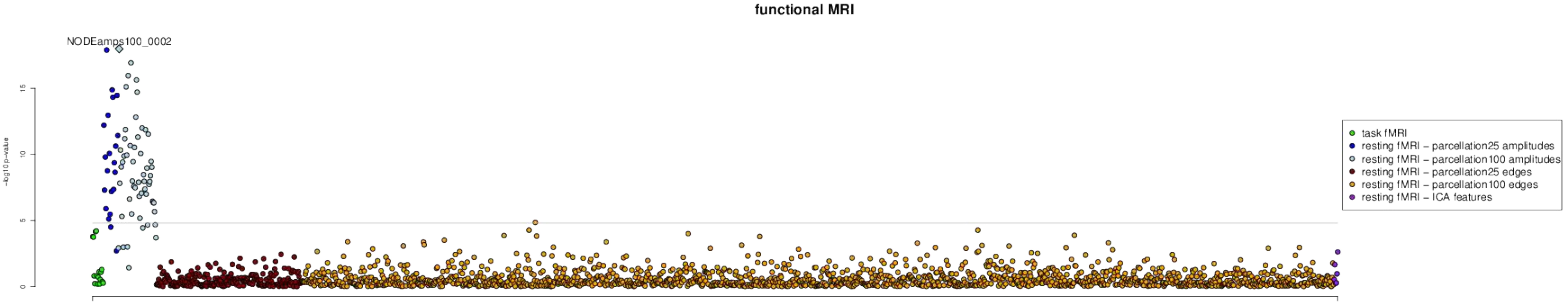
functional MRI
NODEamps100_0002
task fMRI
resting fMRI – parcellation25 amplitudes
resting fMRI – parcellation100 amplitudes
resting fMRI – parcellation25 edges
resting fMRI – parcellation100 edges
resting fMRI – ICA features
-log10 p-value

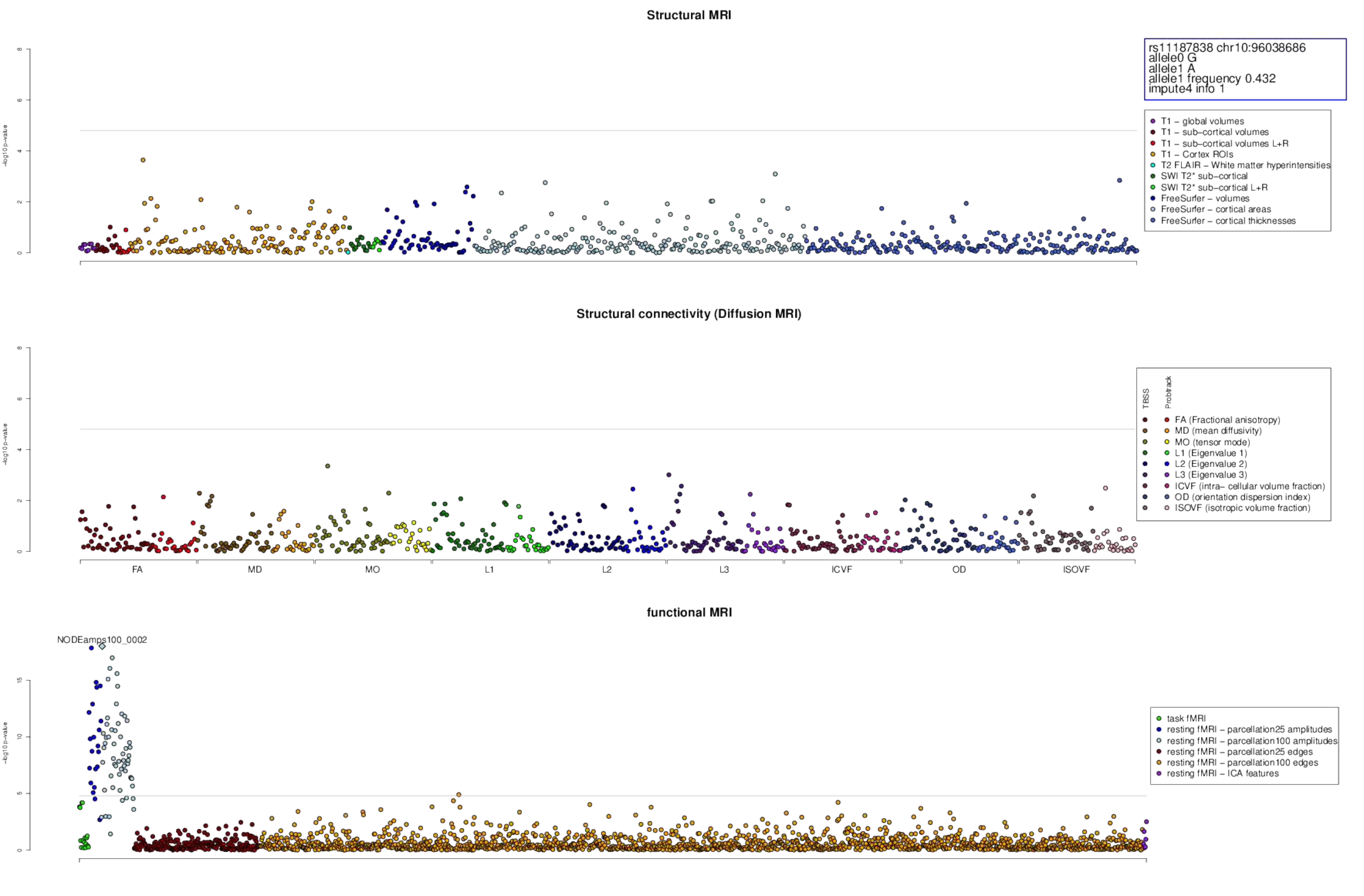

Structural MRI
rs11187838 chr10:96038686
allele0 G
allele1 A
allele1 frequency 0.432
impute4 info 1
T1 – global volumes
T1 – sub–cortical volumes
T1 – sub–cortical volumes L+R
T1 – Cortex ROIs
T2 FLAIR – White matter hyperintensities
SWI T2* sub–cortical
SWI T2* sub–cortical L+R
FreeSurfer – volumes
FreeSurfer – cortical areas
FreeSurfer – cortical thicknesses
-log10 p-value
Structural connectivity (Diffusion MRI)
TBSS
Probtrack
FA (Fractional anisotropy)
MD (mean diffusivity)
MO (tensor mode)
L1 (Eigenvalue 1)
L2 (Eigenvalue 2)
L3 (Eigenvalue 3)
ICVF (intra– cellular volume fraction)
OD (orientation dispersion index)
ISOVF (isotropic volume fraction)
FA
MD
MO
L1
L2
L3
ICVF
OD
ISOVF
functional MRI
NODEamps100_0002
task fMRI
resting fMRI – parcellation25 amplitudes
resting fMRI – parcellation100 amplitudes
resting fMRI – parcellation25 edges
resting fMRI – parcellation100 edges
resting fMRI – ICA features

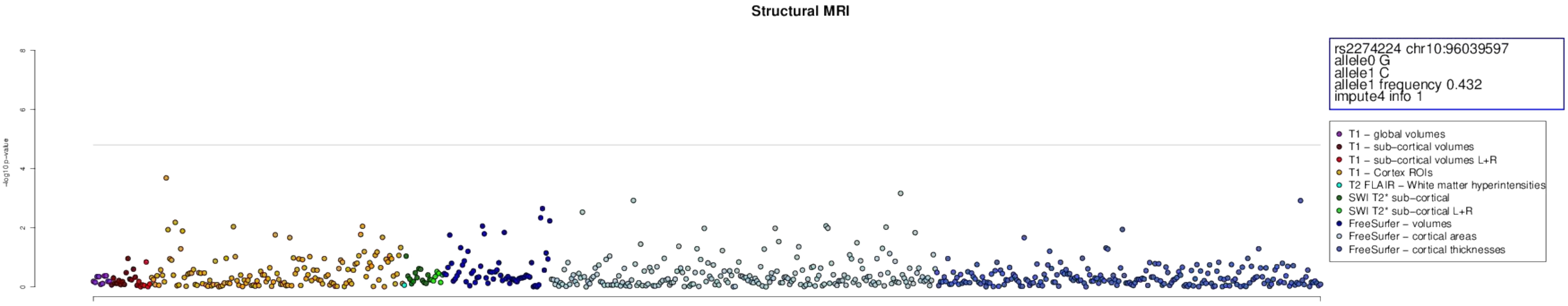
Structural MRI
-log10 p-value
rs2274224 chr10:96039597
allele0 G
allele1 C
allele1 frequency 0.432
impute4 info 1
T1 – global volumes
T1 – sub-cortical volumes
T1 – sub-cortical volumes L+R
T1 – Cortex ROIs
T2 FLAIR – White matter hyperintensities
SWI T2* sub-cortical
SWI T2* sub-cortical L+R
FreeSurfer – volumes
FreeSurfer – cortical areas
FreeSurfer – cortical thicknesses

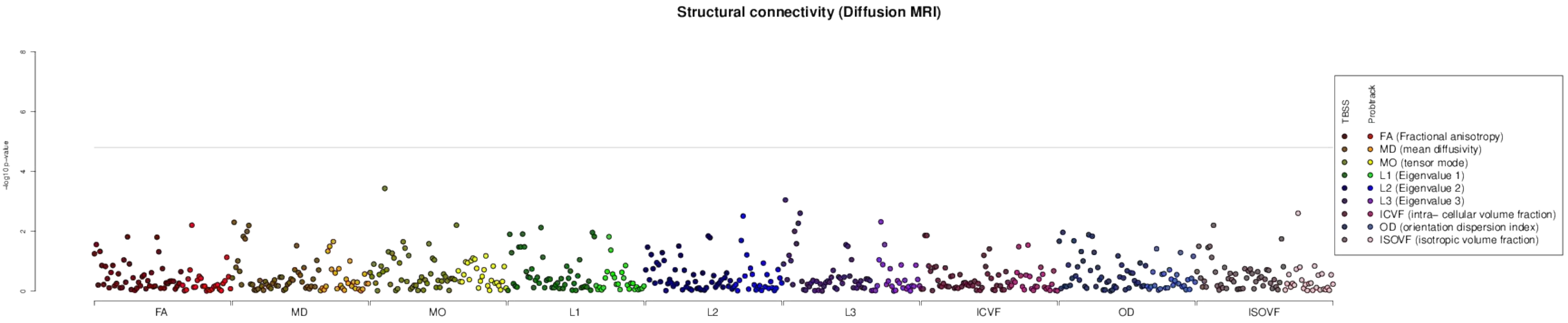
Structural connectivity (Diffusion MRI)
-log10 p-value
TBSS
Probtrack
FA (Fractional anisotropy)
MD (mean diffusivity)
MO (tensor mode)
L1 (Eigenvalue 1)
L2 (Eigenvalue 2)
L3 (Eigenvalue 3)
ICVF (intra- cellular volume fraction)
OD (orientation dispersion index)
ISOVF (isotropic volume fraction)
FA
MD
MO
L1
L2
L3
ICVF
OD
ISOVF

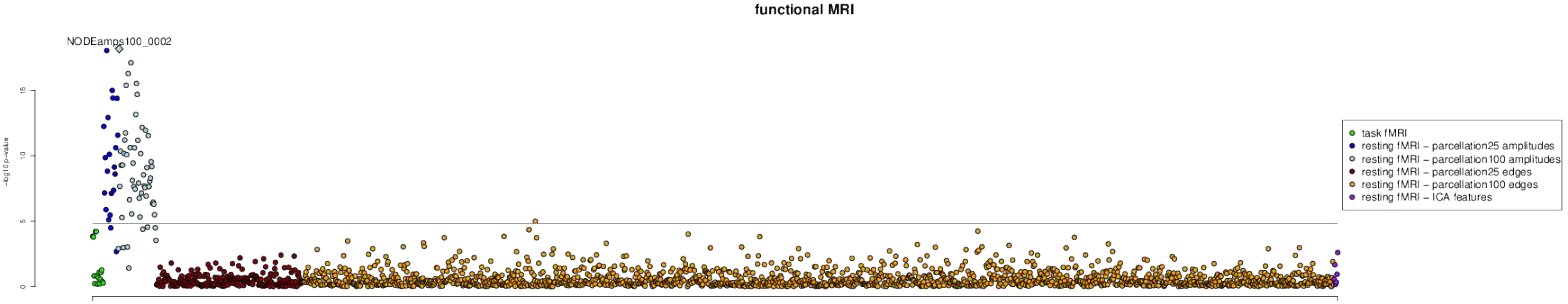
functional MRI
NODEamps100_0002
-log10 p-value
task fMRI
resting fMRI – parcellation25 amplitudes
resting fMRI – parcellation100 amplitudes
resting fMRI – parcellation25 edges
resting fMRI – parcellation100 edges
resting fMRI – ICA features

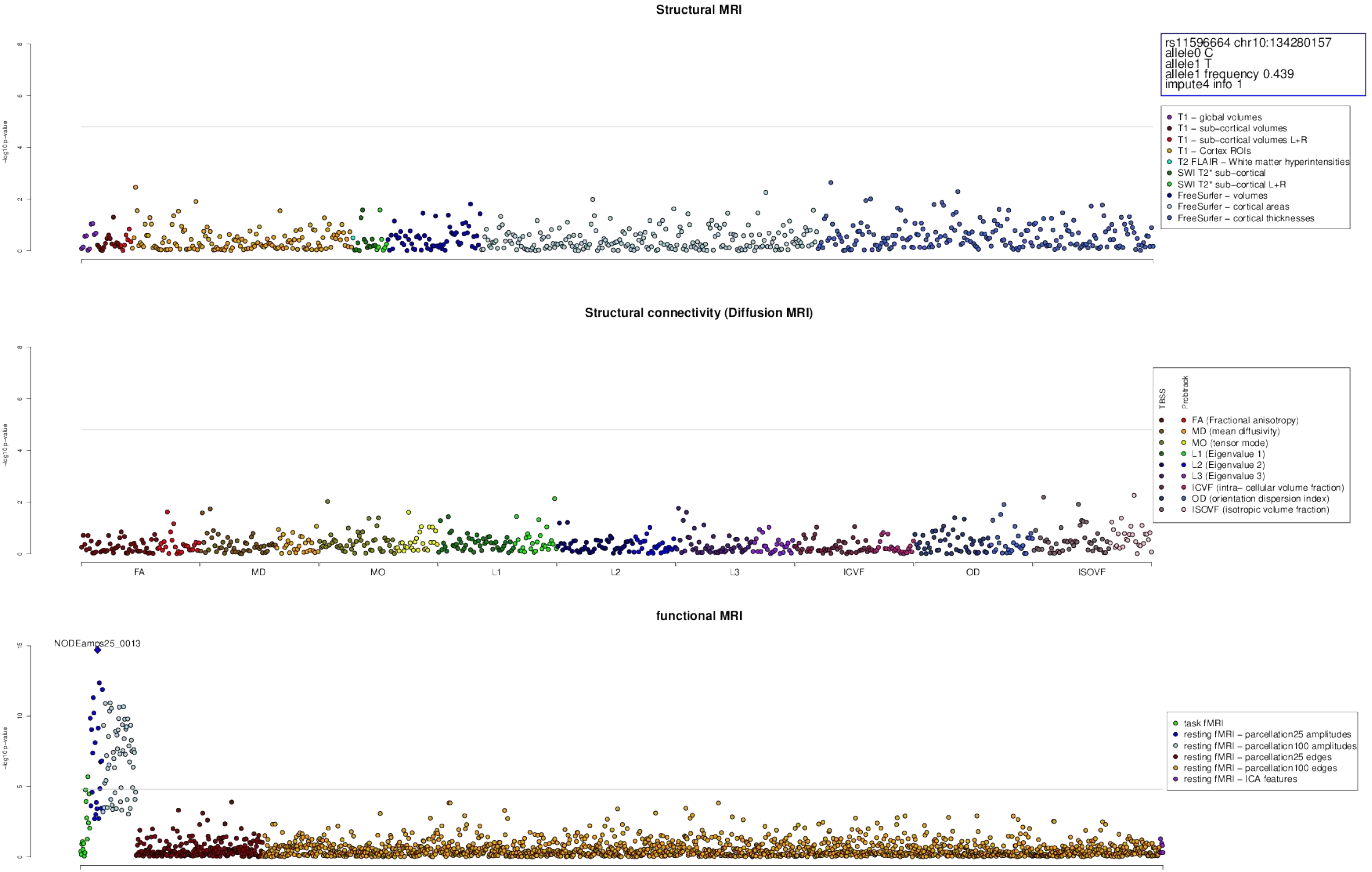

Structural MRI
rs11596664 chr10:134280157
allele0 C
allele1 T
allele1 frequency 0.439
impute4 info 1
T1 – global volumes
T1 – sub-cortical volumes
T1 – sub-cortical volumes L+R
T1 – Cortex ROIs
T2 FLAIR – White matter hyperintensities
SWI T2* sub-cortical
SWI T2* sub-cortical L+R
FreeSurfer – volumes
FreeSurfer – cortical areas
FreeSurfer – cortical thicknesses
-log10 p-value
Structural connectivity (Diffusion MRI)
TBSS
Probtrack
FA (Fractional anisotropy)
MD (mean diffusivity)
MO (tensor mode)
L1 (Eigenvalue 1)
L2 (Eigenvalue 2)
L3 (Eigenvalue 3)
ICVF (intra- cellular volume fraction)
OD (orientation dispersion index)
ISOVF (isotropic volume fraction)
FA
MD
MO
L1
L2
L3
ICVF
OD
ISOVF
functional MRI
NODEamps25_0013
task fMRI
resting fMRI – parcellation25 amplitudes
resting fMRI – parcellation100 amplitudes
resting fMRI – parcellation25 edges
resting fMRI – parcellation100 edges
resting fMRI – ICA features

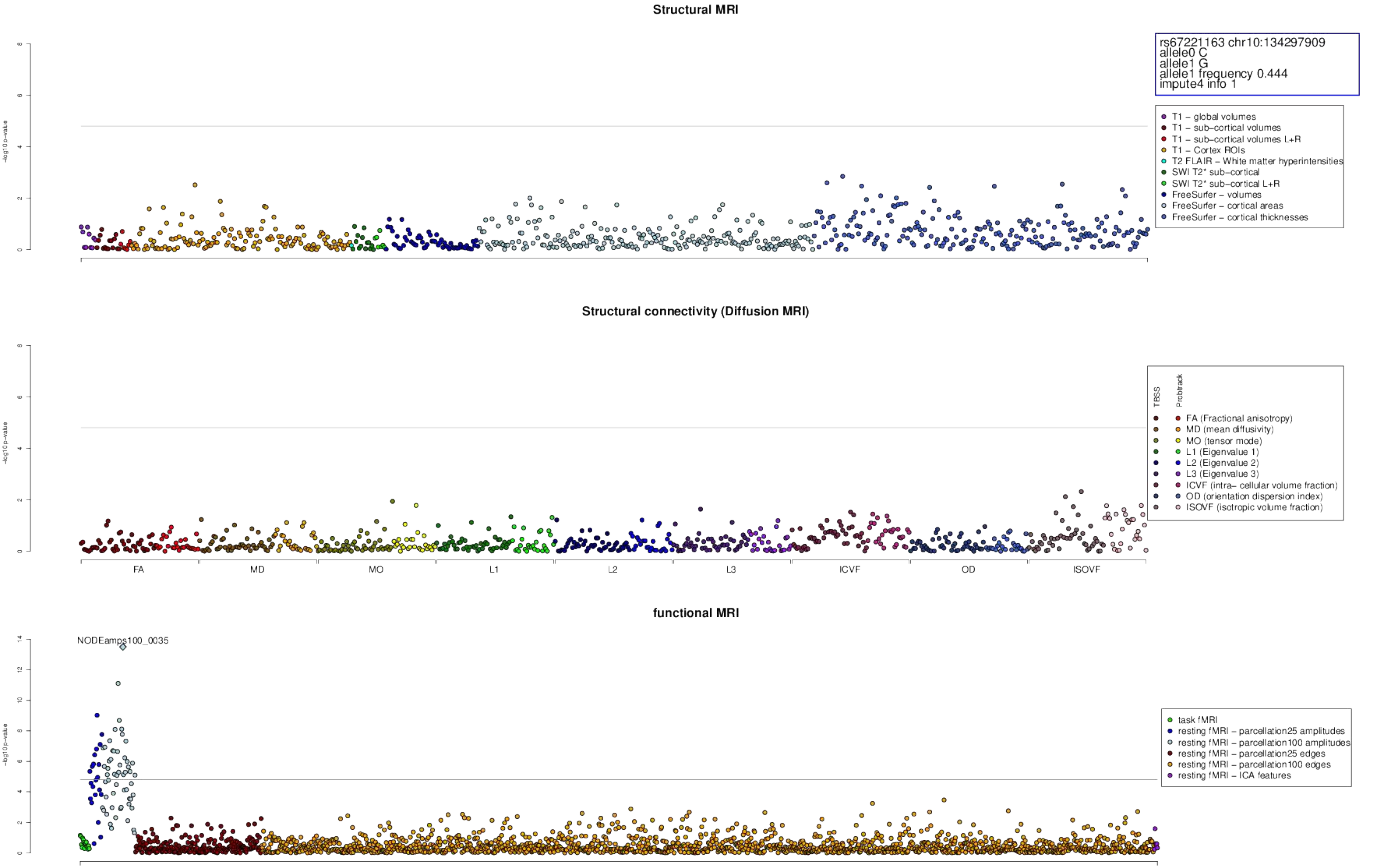
Structural MRI
rs67221163 chr10:134297909
allele0 C
allele1 G
allele1 frequency 0.444
impute4 info 1
T1 – global volumes
T1 – sub–cortical volumes
T1 – sub–cortical volumes L+R
T1 – Cortex ROIs
T2 FLAIR – White matter hyperintensities
SWI T2* sub–cortical
SWI T2* sub–cortical L+R
FreeSurfer – volumes
FreeSurfer – cortical areas
FreeSurfer – cortical thicknesses
Structural connectivity (Diffusion MRI)
TBSS
Probtrack
FA (Fractional anisotropy)
MD (mean diffusivity)
MO (tensor mode)
L1 (Eigenvalue 1)
L2 (Eigenvalue 2)
L3 (Eigenvalue 3)
ICVF (intra– cellular volume fraction)
OD (orientation dispersion index)
ISOVF (isotropic volume fraction)
FA
MD
MO
L1
L2
L3
ICVF
OD
ISOVF
functional MRI
NODEamps100_0035
task fMRI
resting fMRI – parcellation25 amplitudes
resting fMRI – parcellation100 amplitudes
resting fMRI – parcellation25 edges
resting fMRI – parcellation100 edges
resting fMRI – ICA features
–log10 p–value

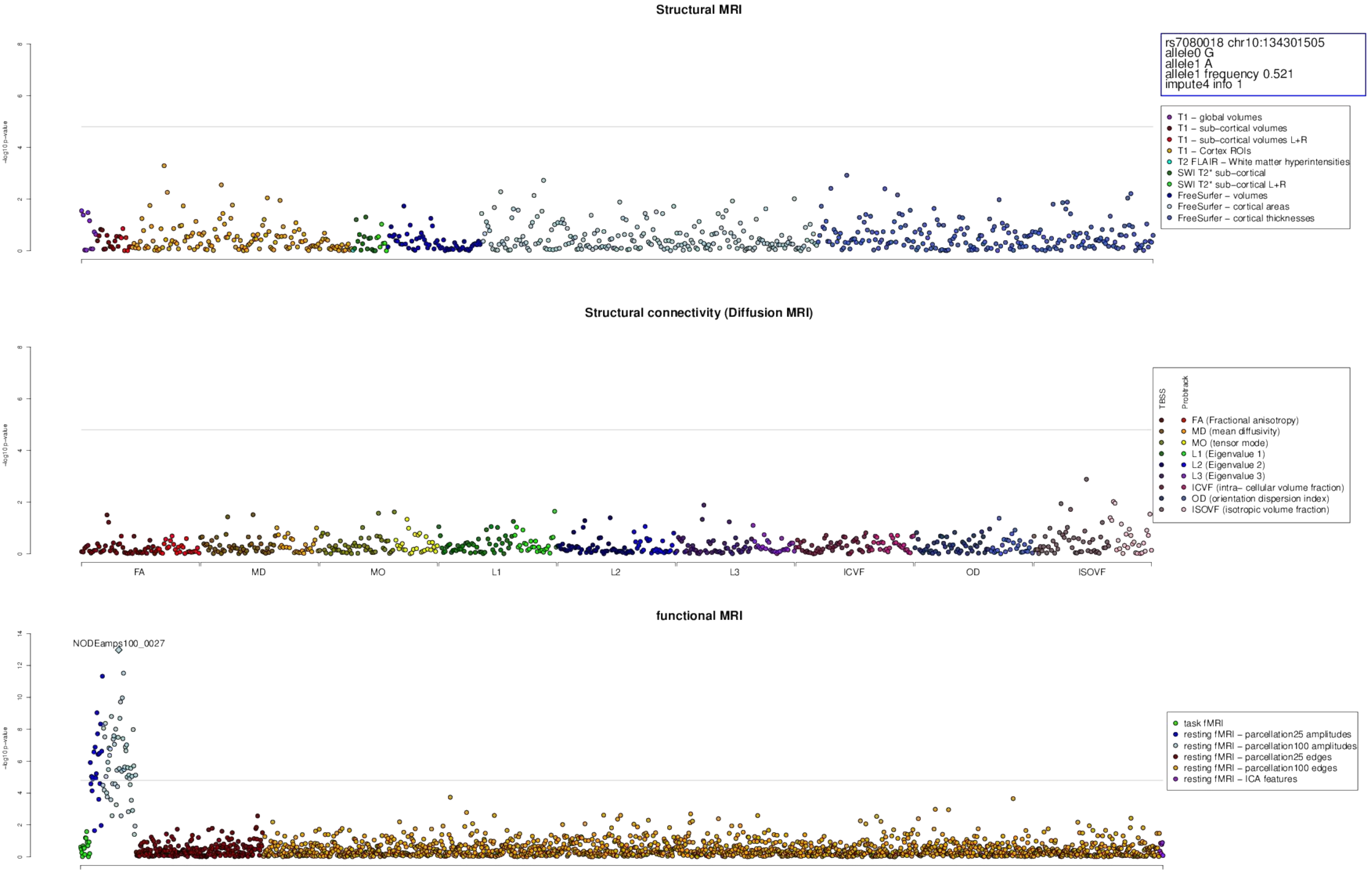
Structural MRI
rs7080018 chr10:134301505
allele0 G
allele1 A
allele1 frequency 0.521
impute4 info 1
T1 – global volumes
T1 – sub–cortical volumes
T1 – sub–cortical volumes L+R
T1 – Cortex ROIs
T2 FLAIR – White matter hyperintensities
SWI T2* sub–cortical
SWI T2* sub–cortical L+R
FreeSurfer – volumes
FreeSurfer – cortical areas
FreeSurfer – cortical thicknesses
–log10 p–value
Structural connectivity (Diffusion MRI)
TBSS
Probtrack
FA (Fractional anisotropy)
MD (mean diffusivity)
MO (tensor mode)
L1 (Eigenvalue 1)
L2 (Eigenvalue 2)
L3 (Eigenvalue 3)
ICVF (intra– cellular volume fraction)
OD (orientation dispersion index)
ISOVF (isotropic volume fraction)
FA
MD
MO
L1
L2
L3
ICVF
OD
ISOVF
functional MRI
NODEamps100_0027
task fMRI
resting fMRI – parcellation25 amplitudes
resting fMRI – parcellation100 amplitudes
resting fMRI – parcellation25 edges
resting fMRI – parcellation100 edges
resting fMRI – ICA features

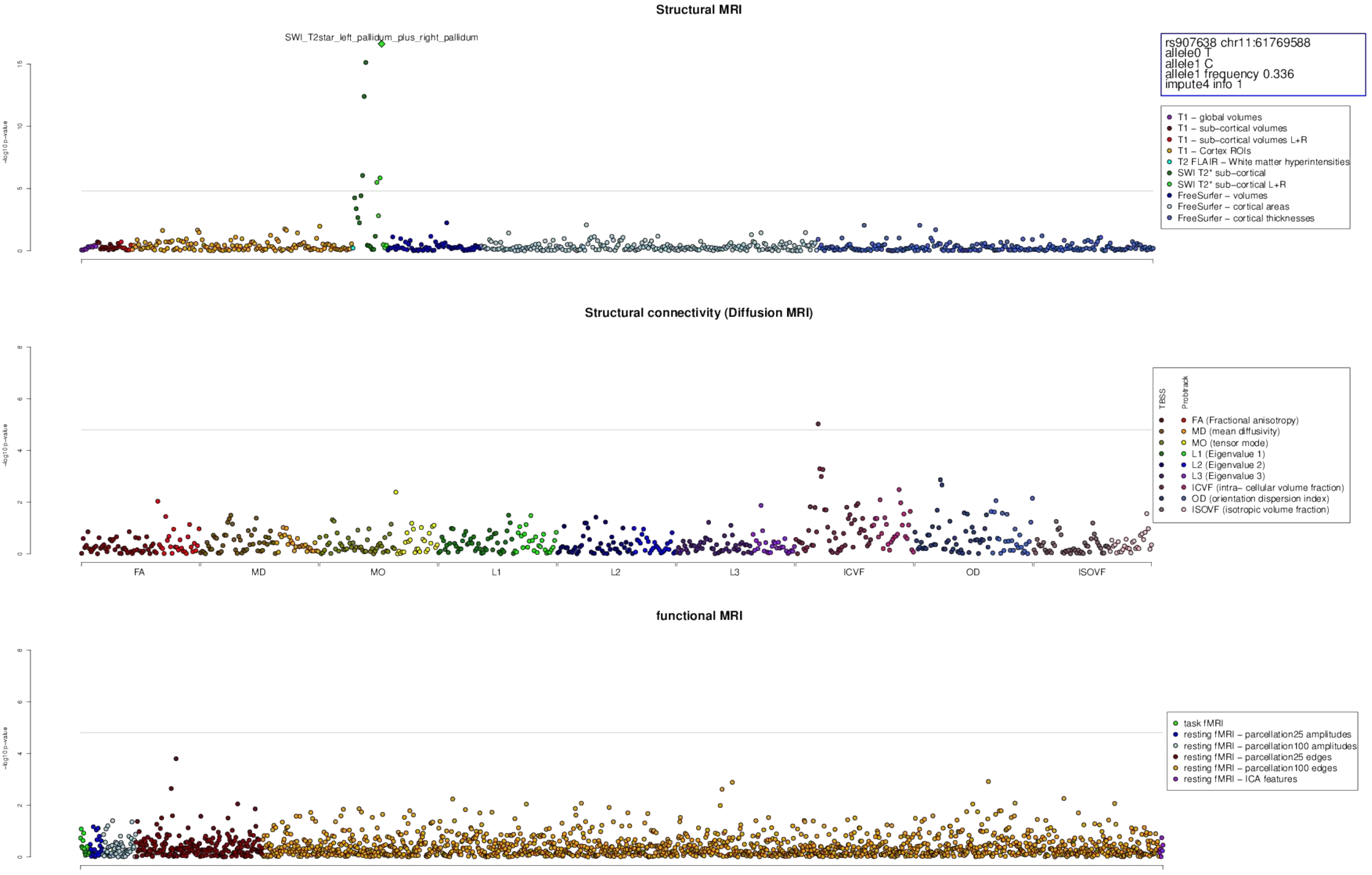

Structural MRI
SWI_T2star_left_pallidum_plus_right_pallidum
rs907638 chr11:61769588
allele0 T
allele1 C
allele1 frequency 0.336
impute4 info 1
T1 – global volumes
T1 – sub–cortical volumes
T1 – sub–cortical volumes L+R
T1 – Cortex ROIs
T2 FLAIR – White matter hyperintensities
SWI T2* sub–cortical
SWI T2* sub–cortical L+R
FreeSurfer – volumes
FreeSurfer – cortical areas
FreeSurfer – cortical thicknesses
-log10 p-value
Structural connectivity (Diffusion MRI)
TBSS
Probtrack
FA (Fractional anisotropy)
MD (mean diffusivity)
MO (tensor mode)
L1 (Eigenvalue 1)
L2 (Eigenvalue 2)
L3 (Eigenvalue 3)
ICVF (intra– cellular volume fraction)
OD (orientation dispersion index)
ISOVF (isotropic volume fraction)
FA
MD
MO
L1
L2
L3
ICVF
OD
ISOVF
functional MRI
task fMRI
resting fMRI – parcellation25 amplitudes
resting fMRI – parcellation100 amplitudes
resting fMRI – parcellation25 edges
resting fMRI – parcellation100 edges
resting fMRI – ICA features

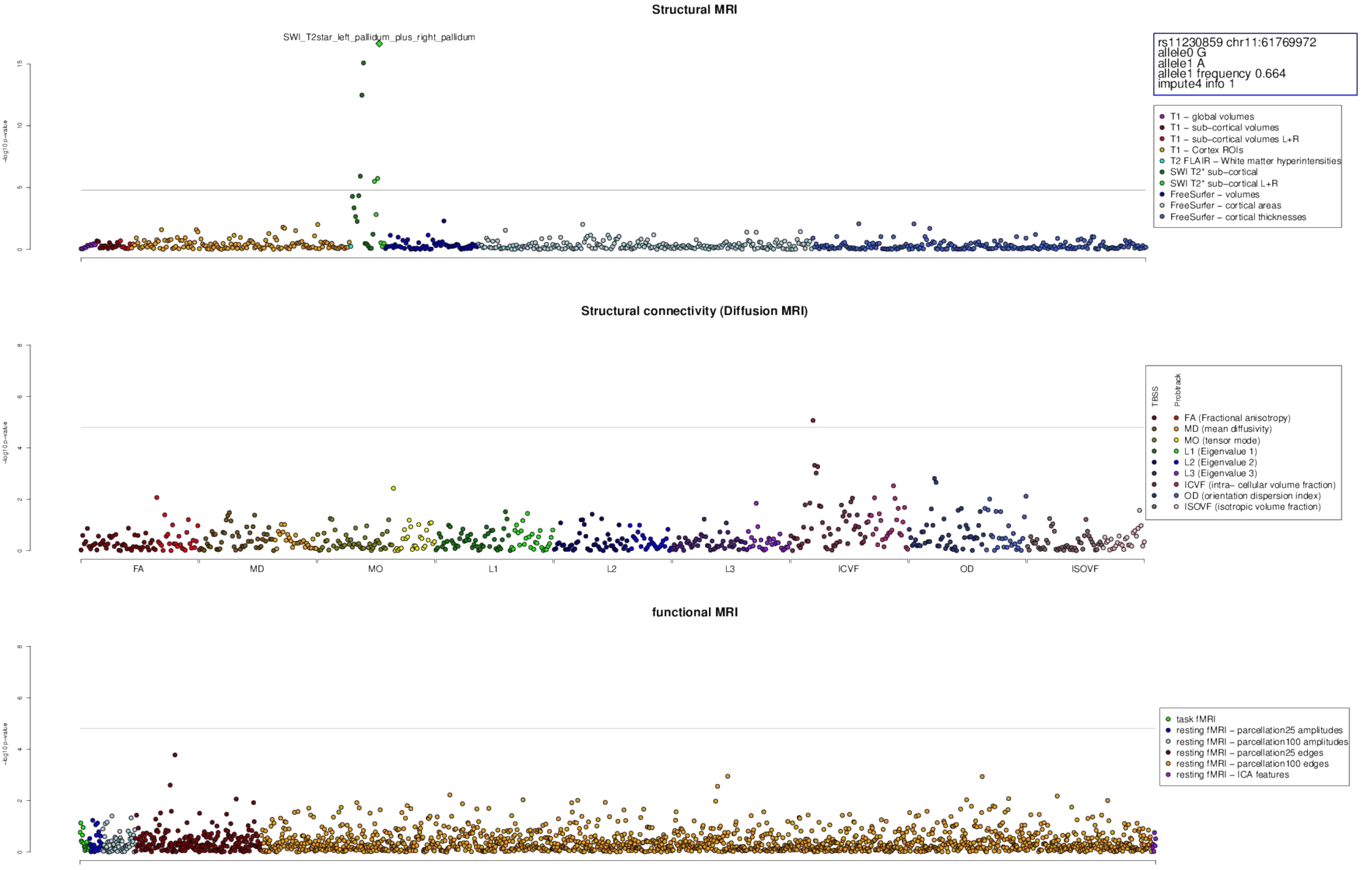

Structural MRI
SWI_T2star_left_pallidum_plus_right_pallidum
rs11230859 chr11:61769972
allele0 G
allele1 A
allele1 frequency 0.664
impute4 info 1
T1 – global volumes
T1 – sub-cortical volumes
T1 – sub-cortical volumes L+R
T1 – Cortex ROIs
T2 FLAIR – White matter hyperintensities
SWI T2* sub-cortical
SWI T2* sub-cortical L+R
FreeSurfer – volumes
FreeSurfer – cortical areas
FreeSurfer – cortical thicknesses
-log10 p-value
Structural connectivity (Diffusion MRI)
TBSS
Probtrack
FA (Fractional anisotropy)
MD (mean diffusivity)
MO (tensor mode)
L1 (Eigenvalue 1)
L2 (Eigenvalue 2)
L3 (Eigenvalue 3)
ICVF (intra- cellular volume fraction)
OD (orientation dispersion index)
ISOVF (isotropic volume fraction)
FA
MD
MO
L1
L2
L3
ICVF
OD
ISOVF
functional MRI
task fMRI
resting fMRI – parcellation25 amplitudes
resting fMRI – parcellation100 amplitudes
resting fMRI – parcellation25 edges
resting fMRI – parcellation100 edges
resting fMRI – ICA features

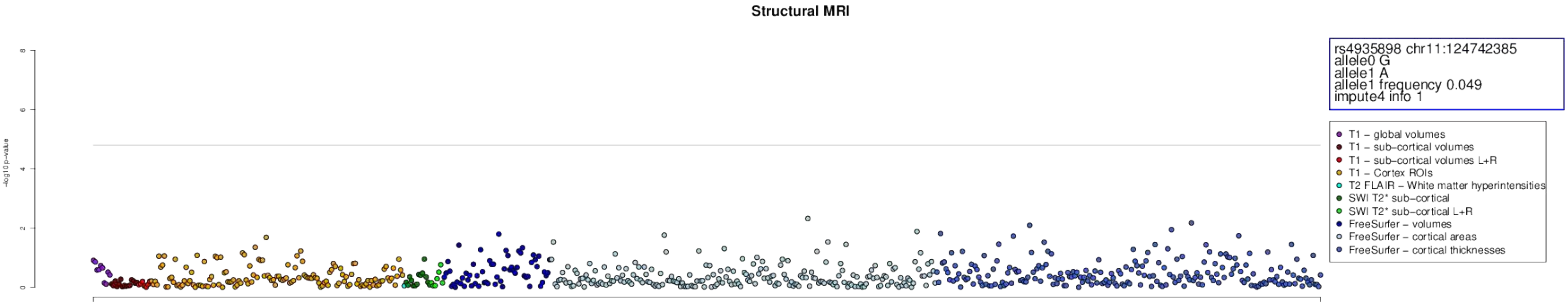
Structural MRI
-log10 p-value
0
2
4
6
8
rs4935898 chr11:124742385
allele0 G
allele1 A
allele1 frequency 0.049
impute4 info 1
T1 – global volumes
T1 – sub-cortical volumes
T1 – sub-cortical volumes L+R
T1 – Cortex ROIs
T2 FLAIR – White matter hyperintensities
SWI T2* sub-cortical
SWI T2* sub-cortical L+R
FreeSurfer – volumes
FreeSurfer – cortical areas
FreeSurfer – cortical thicknesses

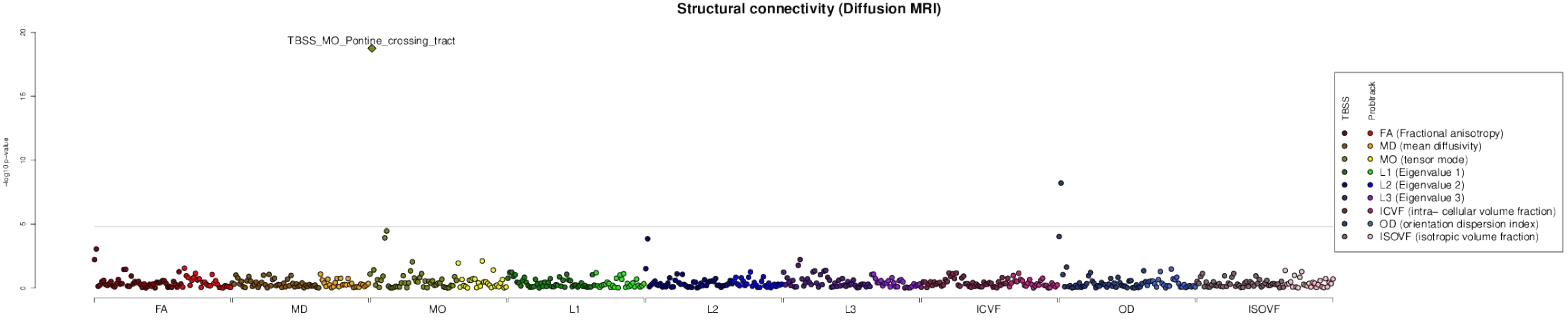
Structural connectivity (Diffusion MRI)
-log10 p-value
0
5
10
15
20
TBSS_MO_Pontine_crossing_tract
TBSS
Probtrack
FA (Fractional anisotropy)
MD (mean diffusivity)
MO (tensor mode)
L1 (Eigenvalue 1)
L2 (Eigenvalue 2)
L3 (Eigenvalue 3)
ICVF (intra- cellular volume fraction)
OD (orientation dispersion index)
ISOVF (isotropic volume fraction)
FA
MD
MO
L1
L2
L3
ICVF
OD
ISOVF

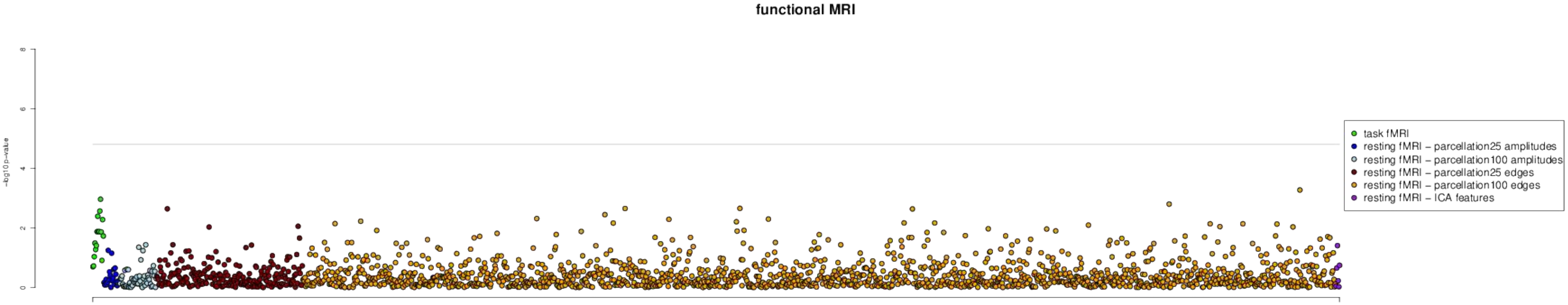
functional MRI
-log10 p-value
0
2
4
6
8
task fMRI
resting fMRI – parcellation25 amplitudes
resting fMRI – parcellation100 amplitudes
resting fMRI – parcellation25 edges
resting fMRI – parcellation100 edges
resting fMRI – ICA features

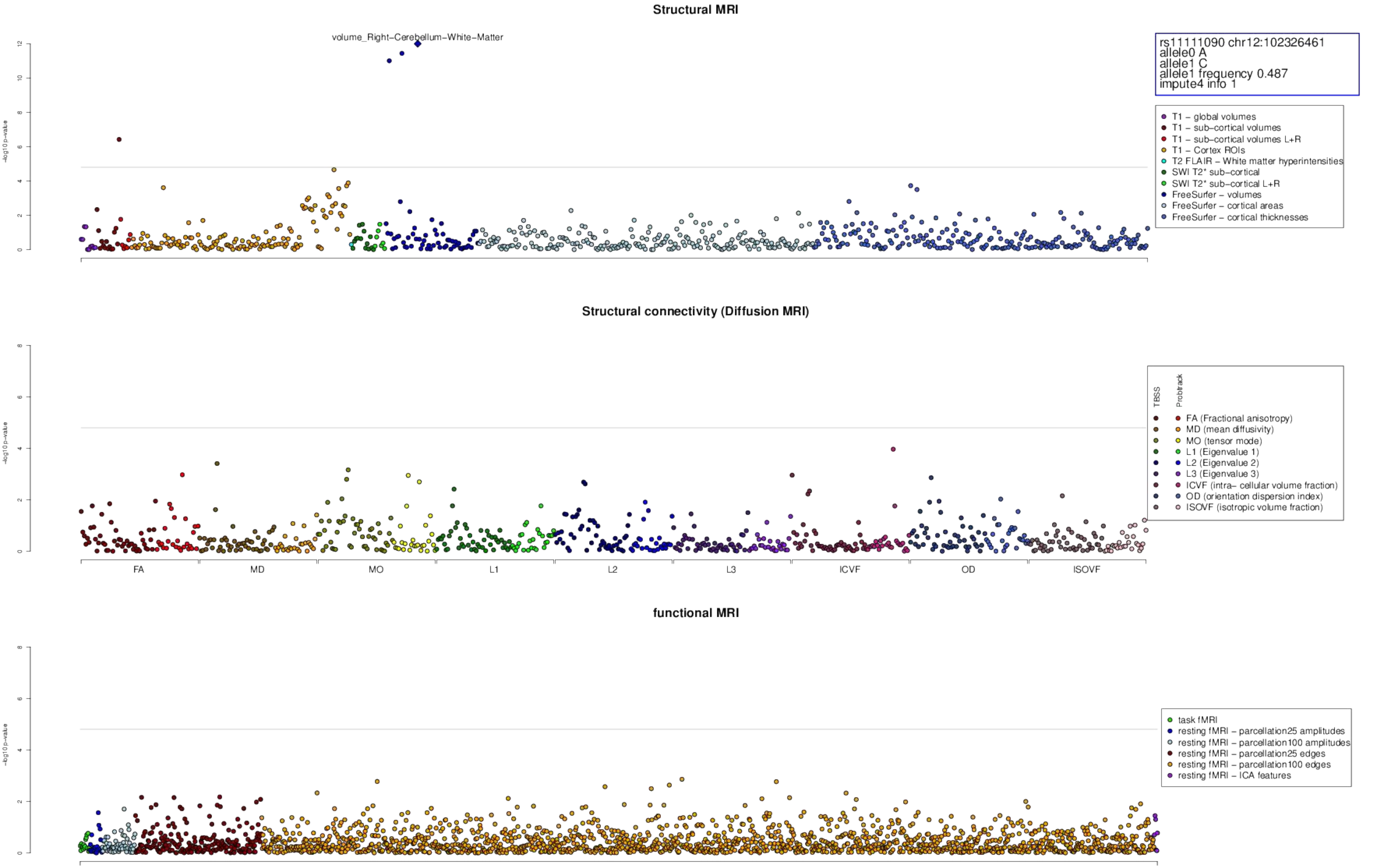
Structural MRI
volume_Right-Cerebellum-White-Matter
rs11111090 chr12:102326461
allele0 A
allele1 C
allele1 frequency 0.487
impute4 info 1
T1 – global volumes
T1 – sub-cortical volumes
T1 – sub-cortical volumes L+R
T1 – Cortex ROIs
T2 FLAIR – White matter hyperintensities
SWI T2* sub-cortical
SWI T2* sub-cortical L+R
FreeSurfer – volumes
FreeSurfer – cortical areas
FreeSurfer – cortical thicknesses
-log10 p-value
Structural connectivity (Diffusion MRI)
TBSS
Probtrack
FA (Fractional anisotropy)
MD (mean diffusivity)
MO (tensor mode)
L1 (Eigenvalue 1)
L2 (Eigenvalue 2)
L3 (Eigenvalue 3)
ICVF (intra- cellular volume fraction)
OD (orientation dispersion index)
ISOVF (isotropic volume fraction)
FA
MD
MO
L1
L2
L3
ICVF
OD
ISOVF
functional MRI
task fMRI
resting fMRI – parcellation25 amplitudes
resting fMRI – parcellation100 amplitudes
resting fMRI – parcellation25 edges
resting fMRI – parcellation100 edges
resting fMRI – ICA features

Structural MRI

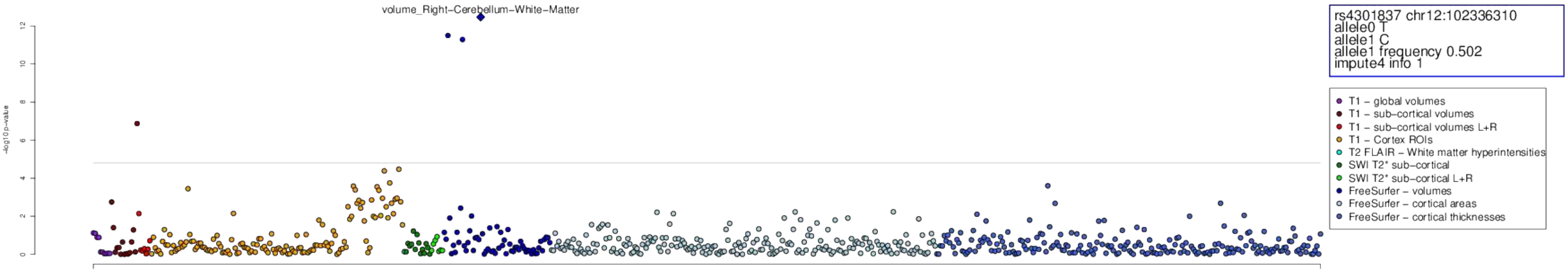

Structural connectivity (Diffusion MRI)

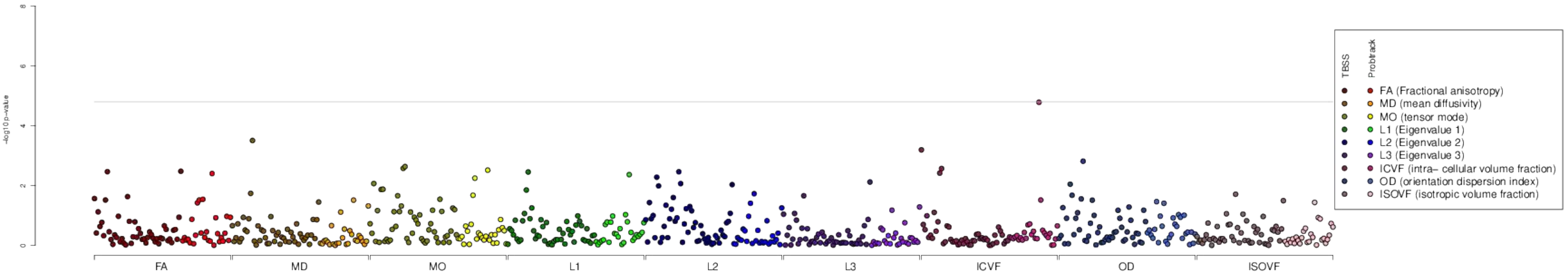

functional MRI

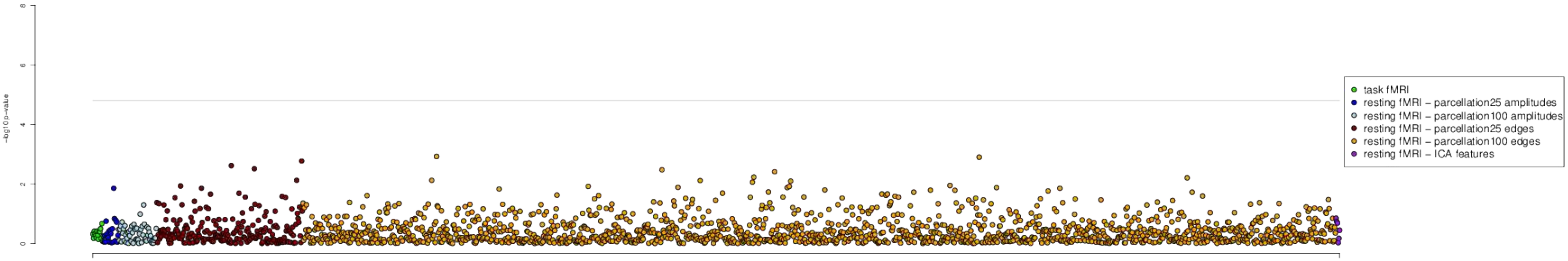

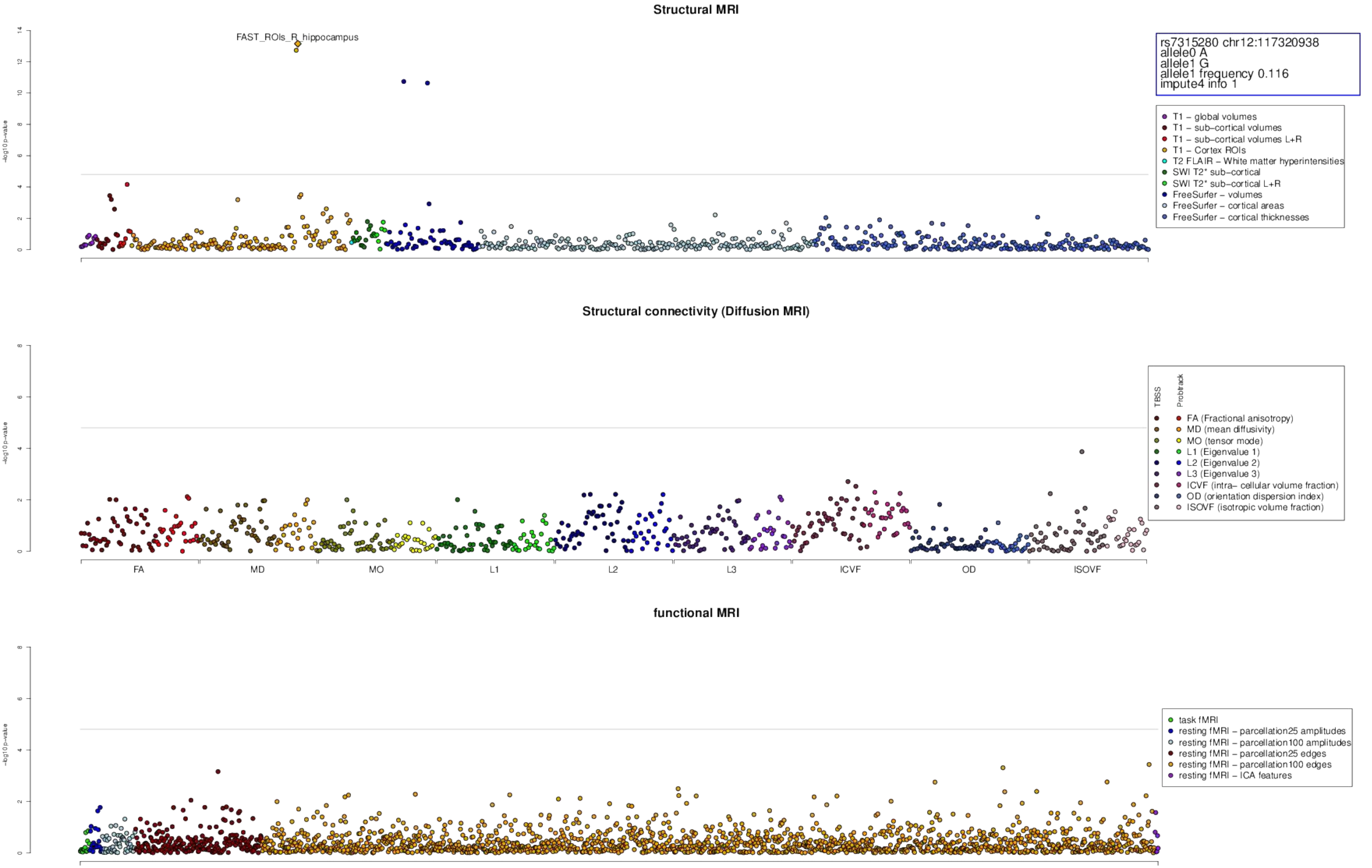

Structural MRI
FAST_ROIs_R_hippocampus
rs7315280 chr12:117320938
allele0 A
allele1 G
allele1 frequency 0.116
impute4 info 1
T1 – global volumes
T1 – sub-cortical volumes
T1 – sub-cortical volumes L+R
T1 – Cortex ROIs
T2 FLAIR – White matter hyperintensities
SWI T2* sub-cortical
SWI T2* sub-cortical L+R
FreeSurfer – volumes
FreeSurfer – cortical areas
FreeSurfer – cortical thicknesses
-log10 p-value
Structural connectivity (Diffusion MRI)
TBSS
Probtrack
FA (Fractional anisotropy)
MD (mean diffusivity)
MO (tensor mode)
L1 (Eigenvalue 1)
L2 (Eigenvalue 2)
L3 (Eigenvalue 3)
ICVF (intra– cellular volume fraction)
OD (orientation dispersion index)
ISOVF (isotropic volume fraction)
FA
MD
MO
L1
L2
L3
ICVF
OD
ISOVF
functional MRI
task fMRI
resting fMRI – parcellation25 amplitudes
resting fMRI – parcellation100 amplitudes
resting fMRI – parcellation25 edges
resting fMRI – parcellation100 edges
resting fMRI – ICA features

Structural MRI

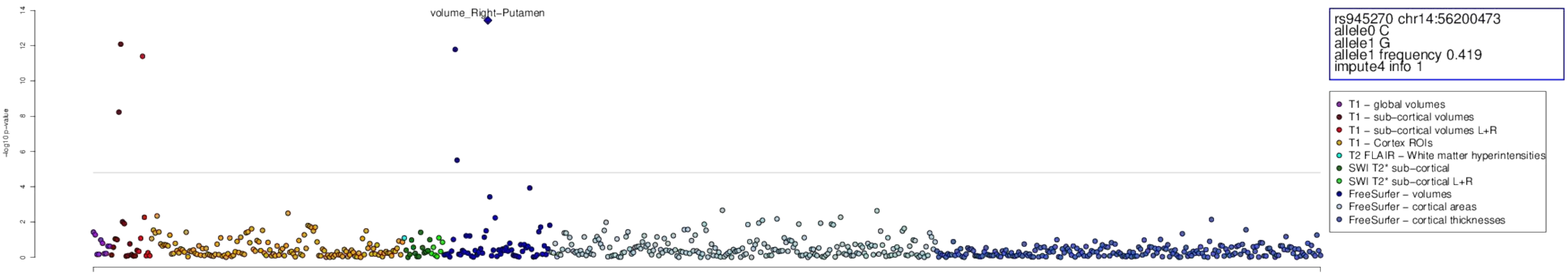

Structural connectivity (Diffusion MRI)

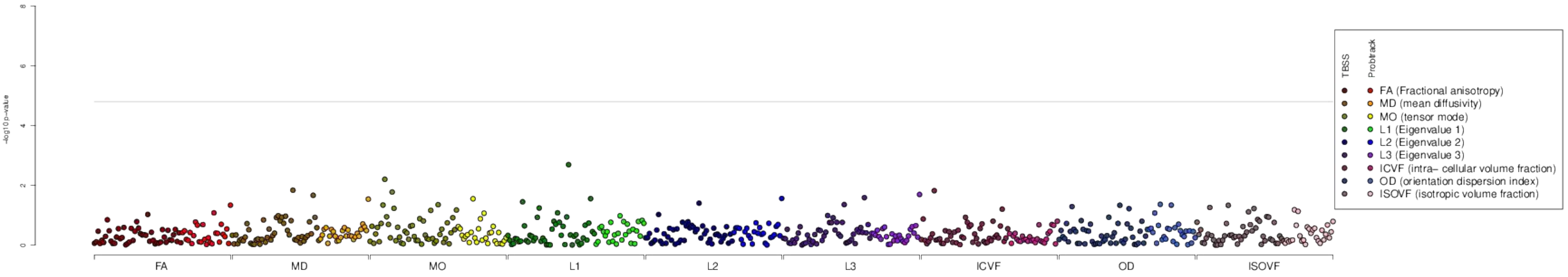

functional MRI

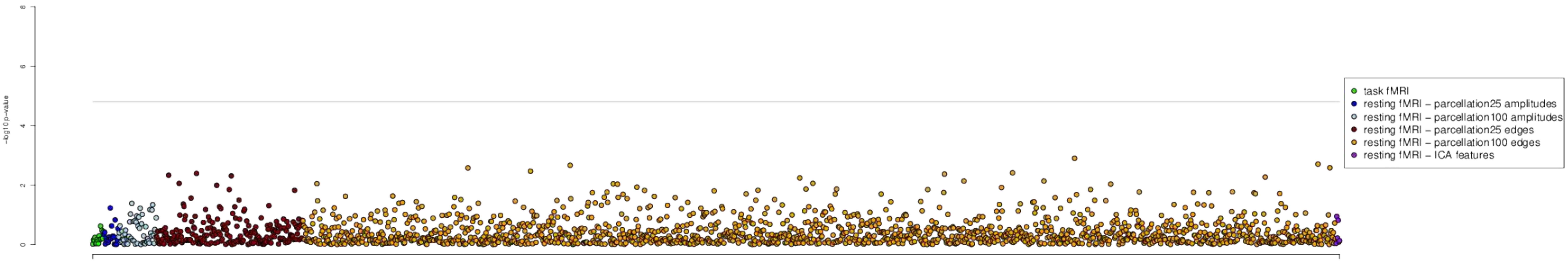

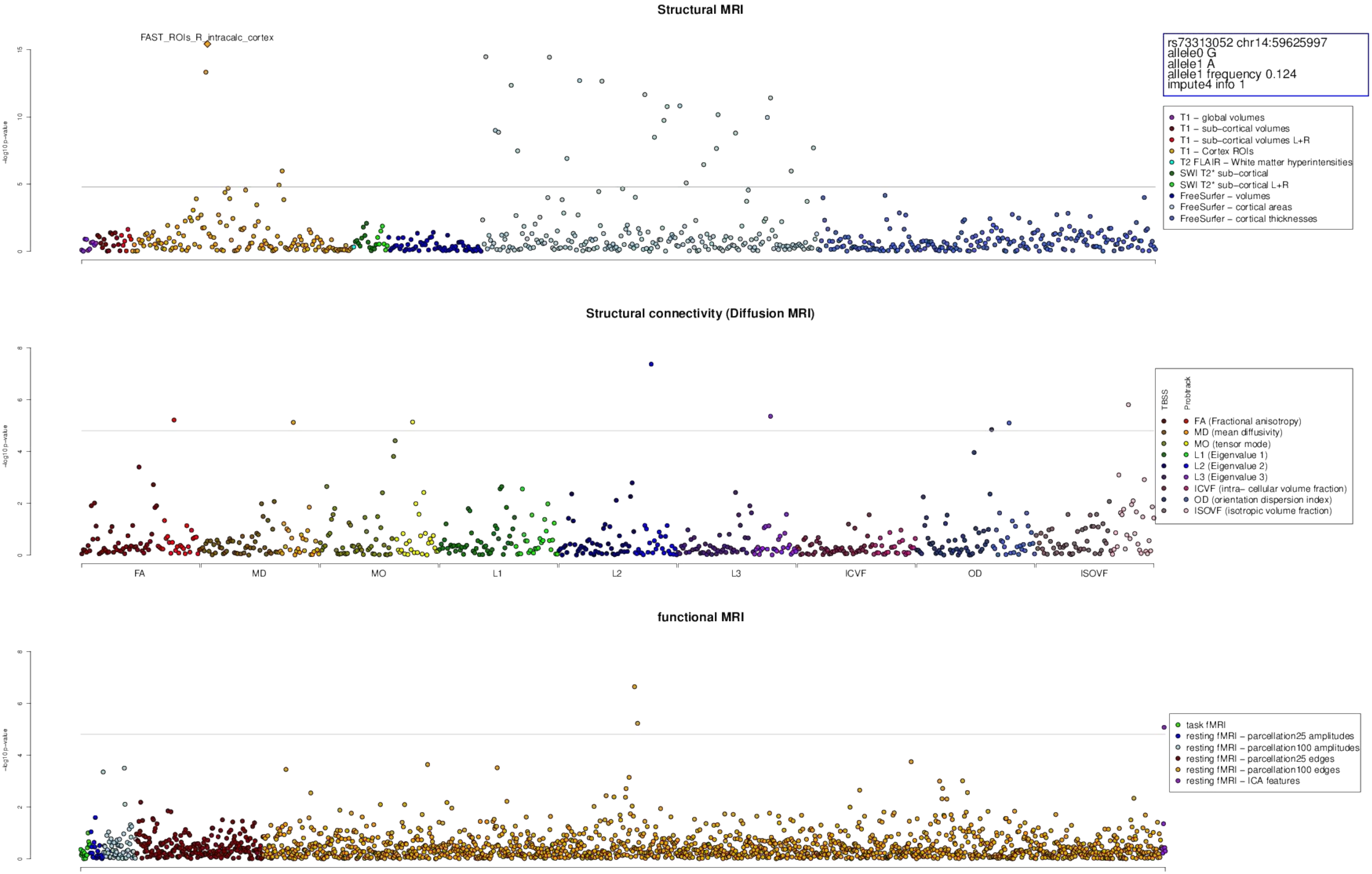

Structural MRI
FAST_ROIs_R_intracalc_cortex
rs73313052 chr14:59625997
allele0 G
allele1 A
allele1 frequency 0.124
impute4 info 1
T1 – global volumes
T1 – sub-cortical volumes
T1 – sub-cortical volumes L+R
T1 – Cortex ROIs
T2 FLAIR – White matter hyperintensities
SWI T2* sub-cortical
SWI T2* sub-cortical L+R
FreeSurfer – volumes
FreeSurfer – cortical areas
FreeSurfer – cortical thicknesses
-log10 p-value
Structural connectivity (Diffusion MRI)
TBSS
Probtrack
FA (Fractional anisotropy)
MD (mean diffusivity)
MO (tensor mode)
L1 (Eigenvalue 1)
L2 (Eigenvalue 2)
L3 (Eigenvalue 3)
ICVF (intra- cellular volume fraction)
OD (orientation dispersion index)
ISOVF (isotropic volume fraction)
FA
MD
MO
L1
L2
L3
ICVF
OD
ISOVF
functional MRI
task fMRI
resting fMRI – parcellation25 amplitudes
resting fMRI – parcellation100 amplitudes
resting fMRI – parcellation25 edges
resting fMRI – parcellation100 edges
resting fMRI – ICA features

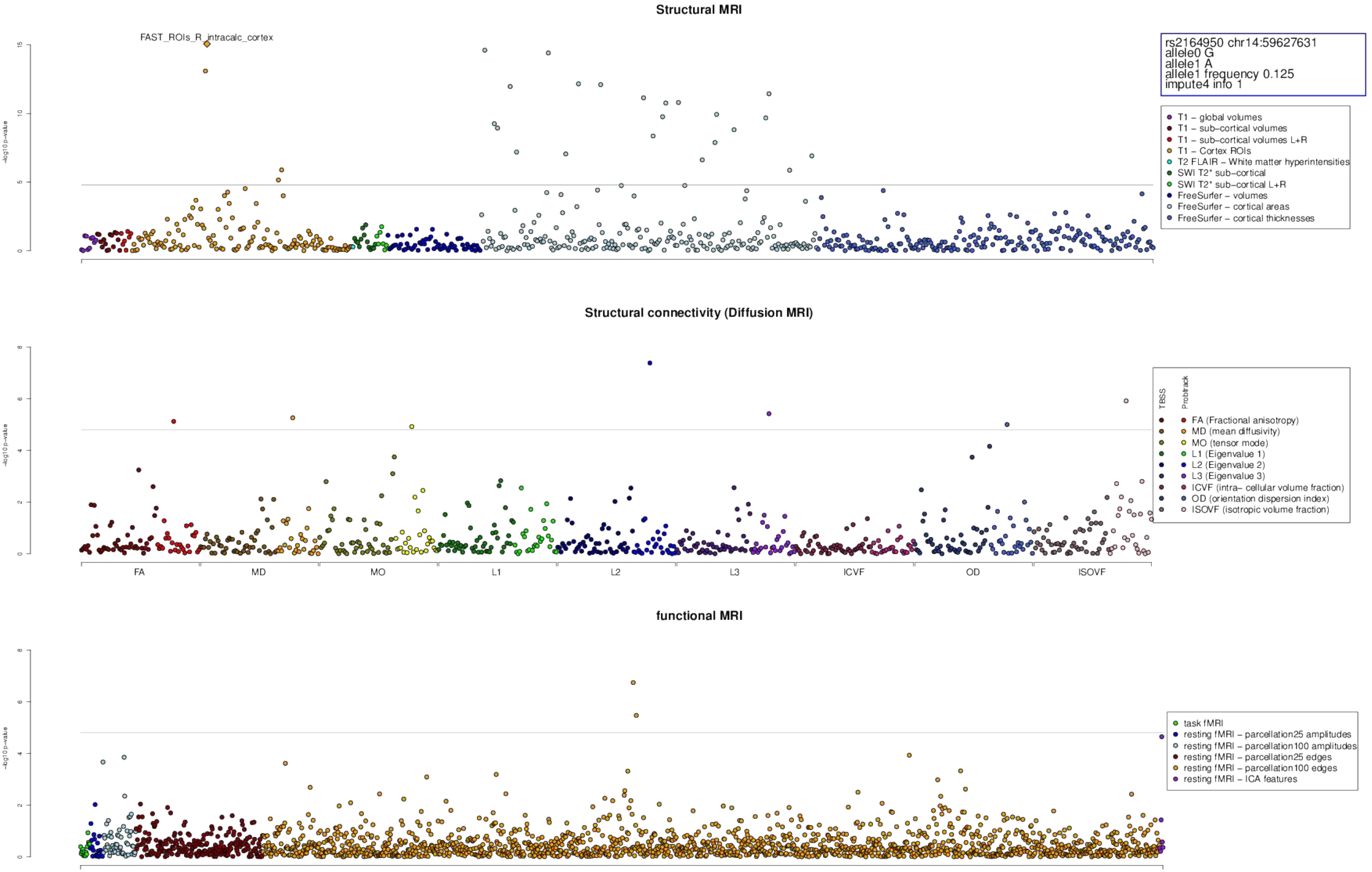
Structural MRI
FAST_ROIs_R_intracalc_cortex
-log10 p-value
rs2164950 chr14:59627631
allele0 G
allele1 A
allele1 frequency 0.125
impute4 info 1
T1 – global volumes
T1 – sub-cortical volumes
T1 – sub-cortical volumes L+R
T1 – Cortex ROIs
T2 FLAIR – White matter hyperintensities
SWI T2* sub-cortical
SWI T2* sub-cortical L+R
FreeSurfer – volumes
FreeSurfer – cortical areas
FreeSurfer – cortical thicknesses
Structural connectivity (Diffusion MRI)
-log10 p-value
TBSS
Probtrack
FA (Fractional anisotropy)
MD (mean diffusivity)
MO (tensor mode)
L1 (Eigenvalue 1)
L2 (Eigenvalue 2)
L3 (Eigenvalue 3)
ICVF (intra- cellular volume fraction)
OD (orientation dispersion index)
ISOVF (isotropic volume fraction)
FA
MD
MO
L1
L2
L3
ICVF
OD
ISOVF
functional MRI
-log10 p-value
task fMRI
resting fMRI – parcellation25 amplitudes
resting fMRI – parcellation100 amplitudes
resting fMRI – parcellation25 edges
resting fMRI – parcellation100 edges
resting fMRI – ICA features

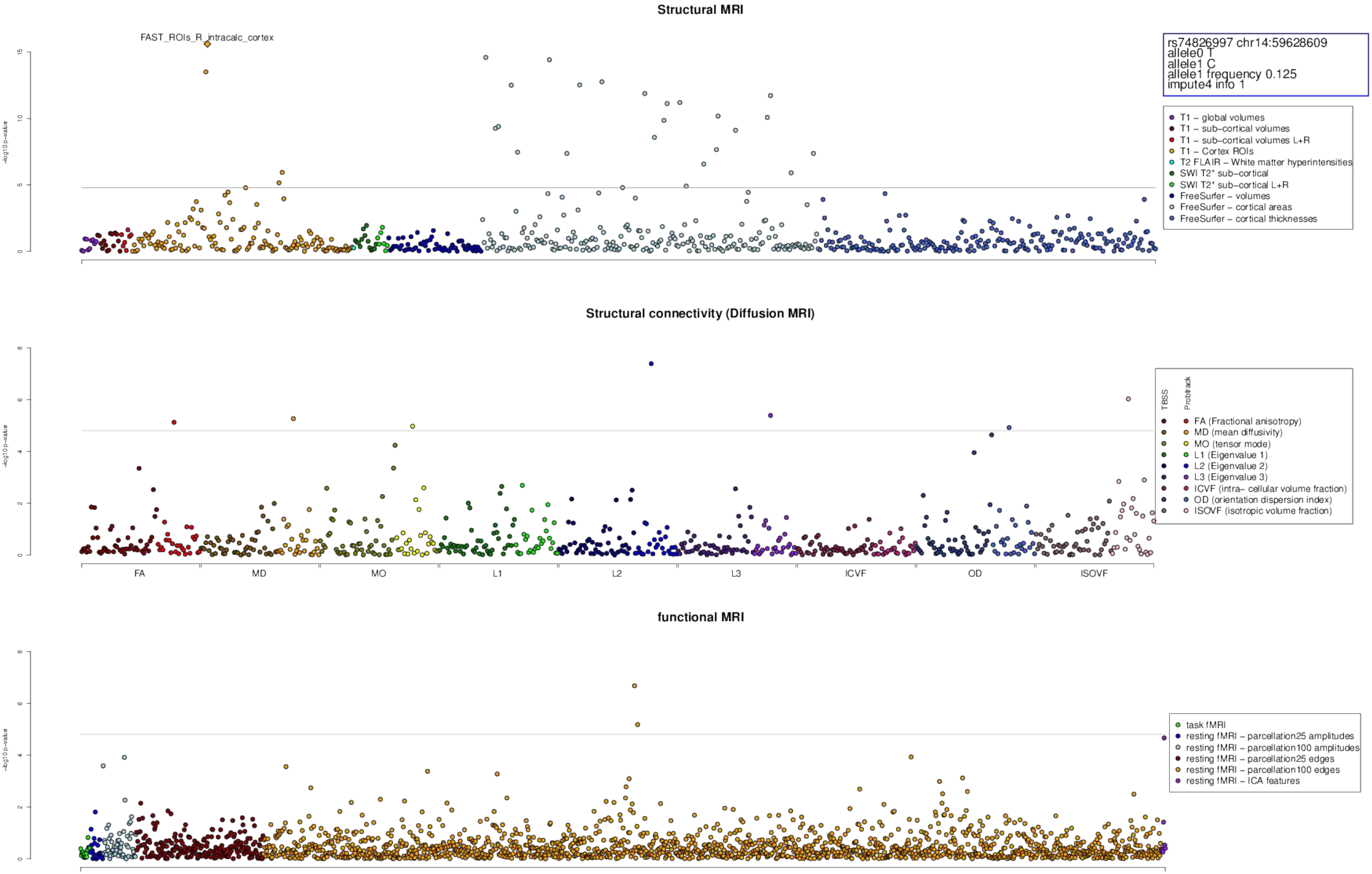

Structural MRI
FAST_ROIs_R_intracalc_cortex
rs74826997 chr14:59628609
allele0 T
allele1 C
allele1 frequency 0.125
impute4 info 1
T1 – global volumes
T1 – sub-cortical volumes
T1 – sub-cortical volumes L+R
T1 – Cortex ROIs
T2 FLAIR – White matter hyperintensities
SWI T2* sub-cortical
SWI T2* sub-cortical L+R
FreeSurfer – volumes
FreeSurfer – cortical areas
FreeSurfer – cortical thicknesses
-log10 p-value
Structural connectivity (Diffusion MRI)
TBSS
Probtrack
FA (Fractional anisotropy)
MD (mean diffusivity)
MO (tensor mode)
L1 (Eigenvalue 1)
L2 (Eigenvalue 2)
L3 (Eigenvalue 3)
ICVF (intra- cellular volume fraction)
OD (orientation dispersion index)
ISOVF (isotropic volume fraction)
FA
MD
MO
L1
L2
L3
ICVF
OD
ISOVF
functional MRI
task fMRI
resting fMRI – parcellation25 amplitudes
resting fMRI – parcellation100 amplitudes
resting fMRI – parcellation25 edges
resting fMRI – parcellation100 edges
resting fMRI – ICA features

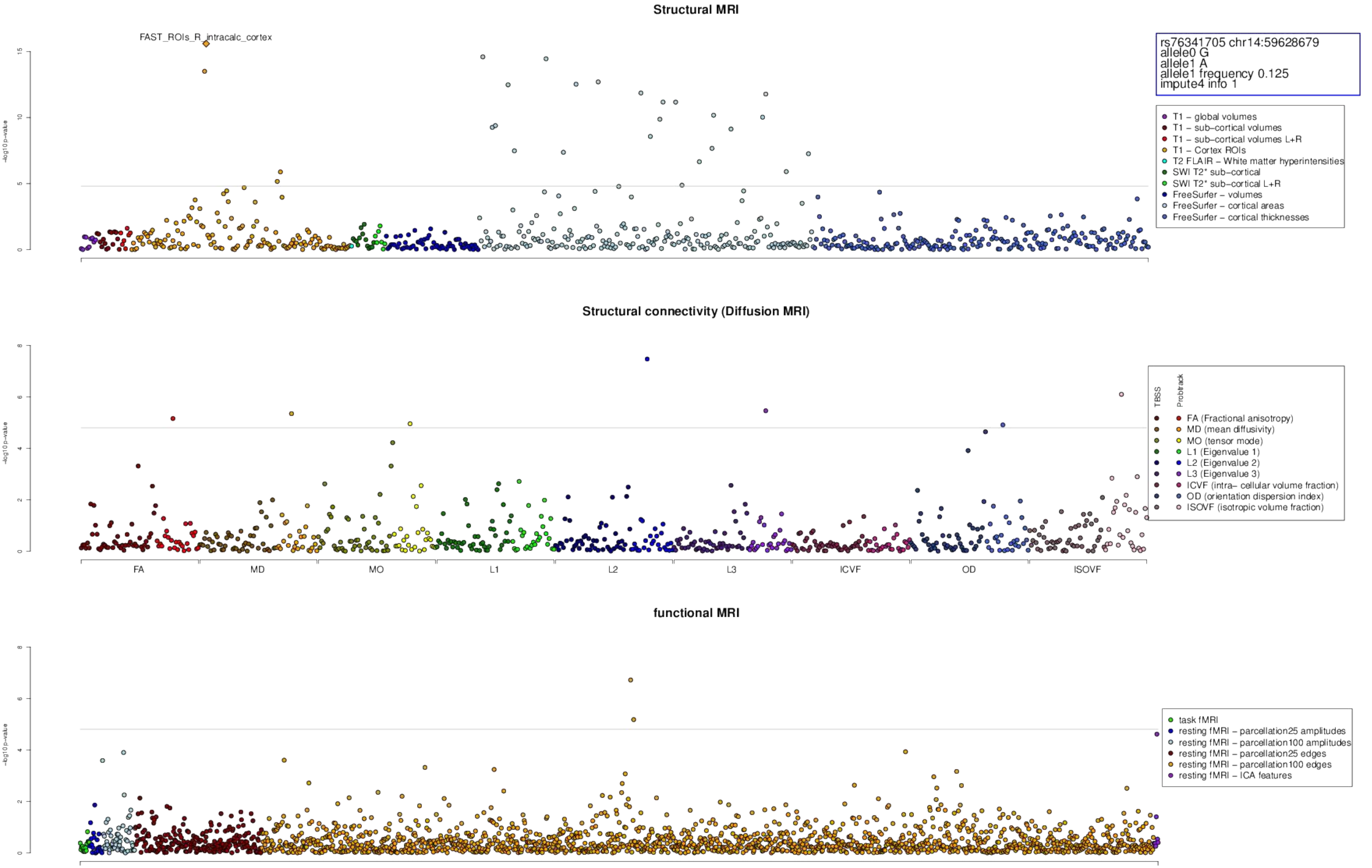
Structural MRI
FAST_ROIs_R_intracalc_cortex
rs76341705 chr14:59628679
allele0 G
allele1 A
allele1 frequency 0.125
impute4 info 1
T1 – global volumes
T1 – sub-cortical volumes
T1 – sub-cortical volumes L+R
T1 – Cortex ROIs
T2 FLAIR – White matter hyperintensities
SWI T2* sub-cortical
SWI T2* sub-cortical L+R
FreeSurfer – volumes
FreeSurfer – cortical areas
FreeSurfer – cortical thicknesses
-log10 p-value
Structural connectivity (Diffusion MRI)
TBSS
Probtrack
FA (Fractional anisotropy)
MD (mean diffusivity)
MO (tensor mode)
L1 (Eigenvalue 1)
L2 (Eigenvalue 2)
L3 (Eigenvalue 3)
ICVF (intra- cellular volume fraction)
OD (orientation dispersion index)
ISOVF (isotropic volume fraction)
FA
MD
MO
L1
L2
L3
ICVF
OD
ISOVF
functional MRI
task fMRI
resting fMRI – parcellation25 amplitudes
resting fMRI – parcellation100 amplitudes
resting fMRI – parcellation25 edges
resting fMRI – parcellation100 edges
resting fMRI – ICA features

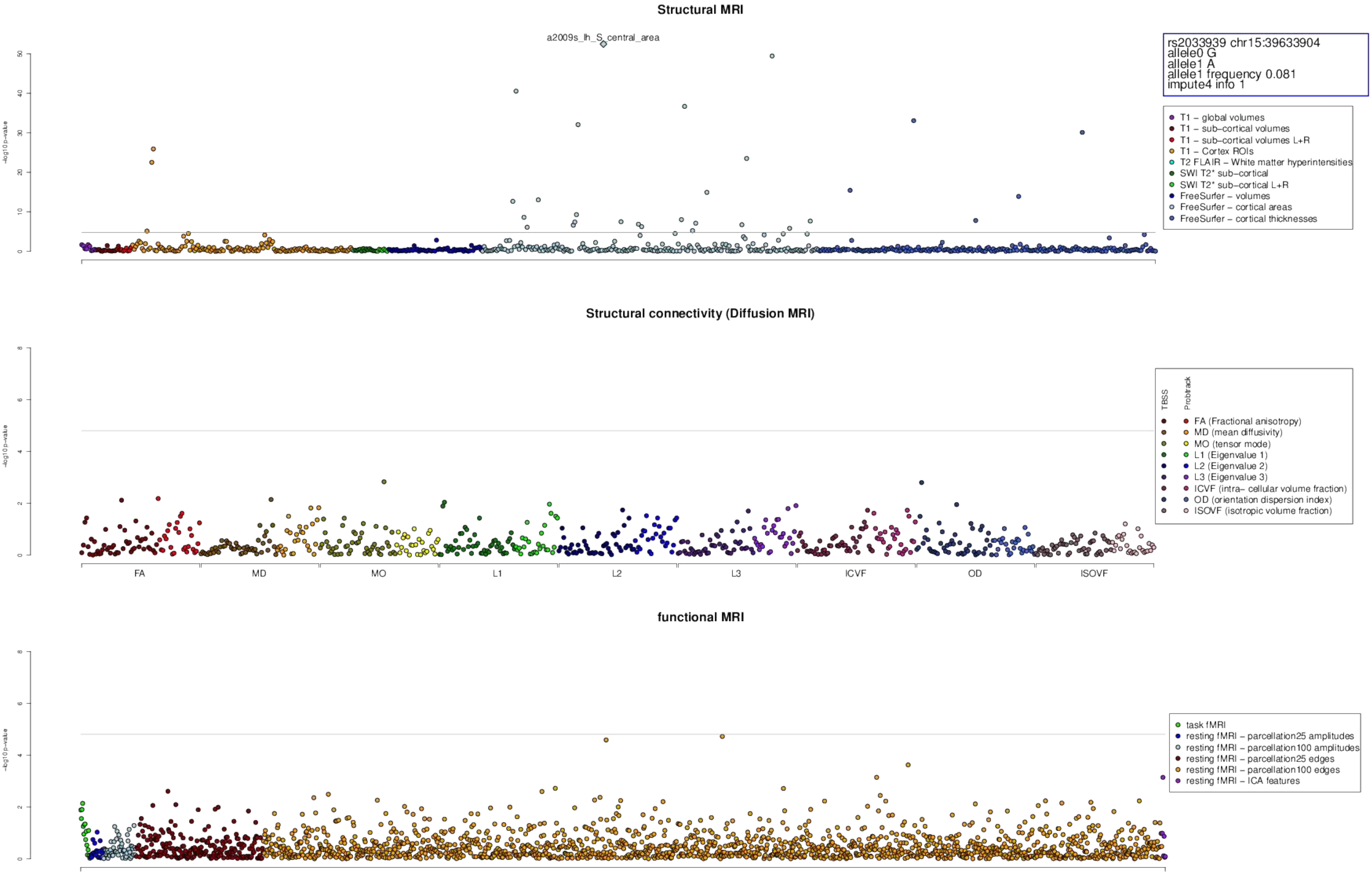

Structural MRI
a2009s_lh_S_central_area
rs2033939 chr15:39633904
allele0 G
allele1 A
allele1 frequency 0.081
impute4 info 1
T1 – global volumes
T1 – sub-cortical volumes
T1 – sub-cortical volumes L+R
T1 – Cortex ROIs
T2 FLAIR – White matter hyperintensities
SWI T2* sub-cortical
SWI T2* sub-cortical L+R
FreeSurfer – volumes
FreeSurfer – cortical areas
FreeSurfer – cortical thicknesses
Structural connectivity (Diffusion MRI)
TBSS
Probtrack
FA (Fractional anisotropy)
MD (mean diffusivity)
MO (tensor mode)
L1 (Eigenvalue 1)
L2 (Eigenvalue 2)
L3 (Eigenvalue 3)
ICVF (intra- cellular volume fraction)
OD (orientation dispersion index)
ISOVF (isotropic volume fraction)
FA
MD
MO
L1
L2
L3
ICVF
OD
ISOVF
functional MRI
task fMRI
resting fMRI – parcellation25 amplitudes
resting fMRI – parcellation100 amplitudes
resting fMRI – parcellation25 edges
resting fMRI – parcellation100 edges
resting fMRI – ICA features

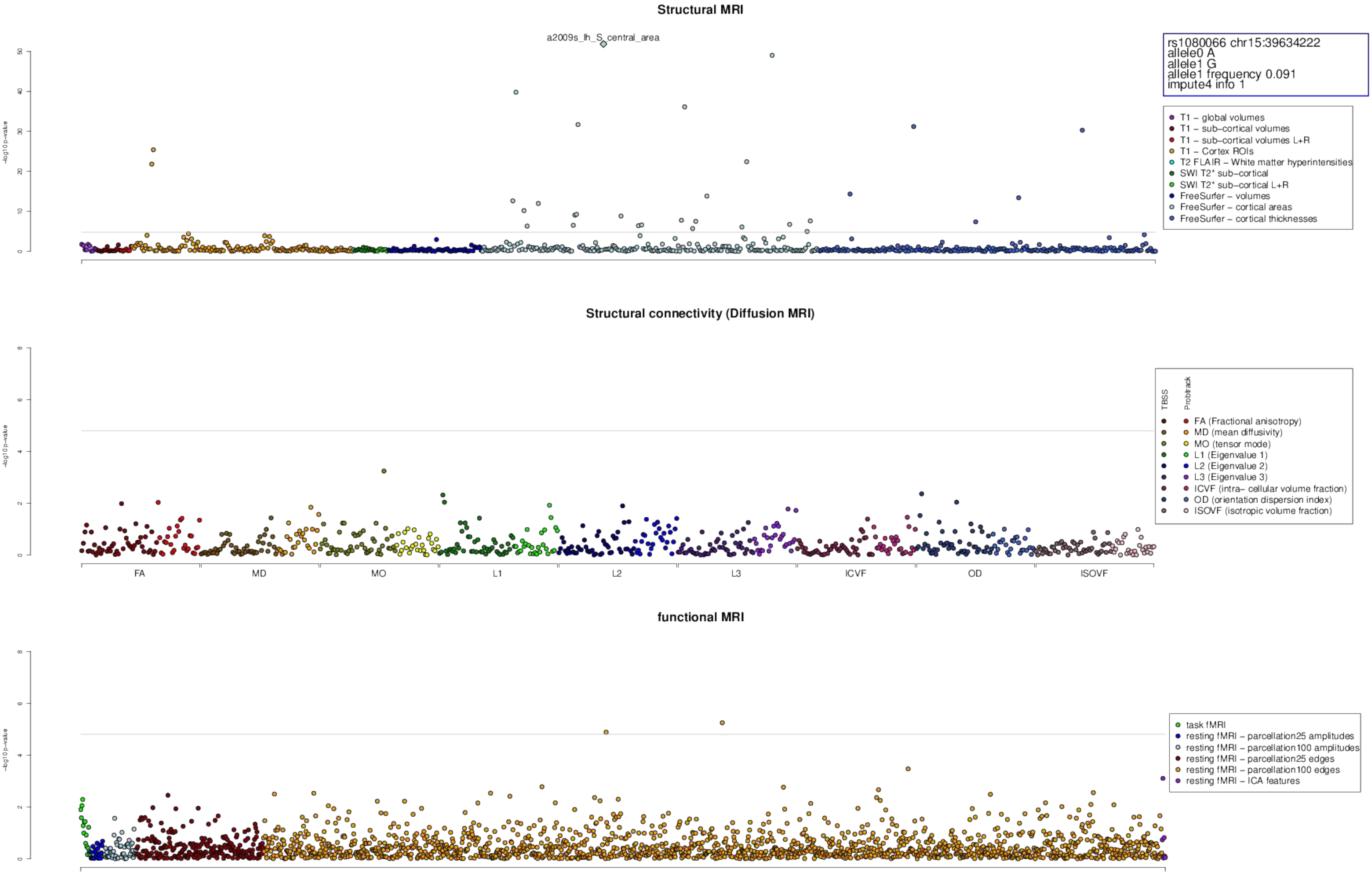

Structural MRI
a2009s_lh_S_central_area
-log10 p-value
0
10
20
30
40
50
rs1080066 chr15:39634222
allele0 A
allele1 G
allele1 frequency 0.091
impute4 info 1
T1 – global volumes
T1 – sub-cortical volumes
T1 – sub-cortical volumes L+R
T1 – Cortex ROIs
T2 FLAIR – White matter hyperintensities
SWI T2* sub-cortical
SWI T2* sub-cortical L+R
FreeSurfer – volumes
FreeSurfer – cortical areas
FreeSurfer – cortical thicknesses
Structural connectivity (Diffusion MRI)
-log10 p-value
0
2
4
6
8
TBSS
Probtrack
FA (Fractional anisotropy)
MD (mean diffusivity)
MO (tensor mode)
L1 (Eigenvalue 1)
L2 (Eigenvalue 2)
L3 (Eigenvalue 3)
ICVF (intra- cellular volume fraction)
OD (orientation dispersion index)
ISOVF (isotropic volume fraction)
FA
MD
MO
L1
L2
L3
ICVF
OD
ISOVF
functional MRI
-log10 p-value
0
2
4
6
8
task fMRI
resting fMRI – parcellation25 amplitudes
resting fMRI – parcellation100 amplitudes
resting fMRI – parcellation25 edges
resting fMRI – parcellation100 edges
resting fMRI – ICA features

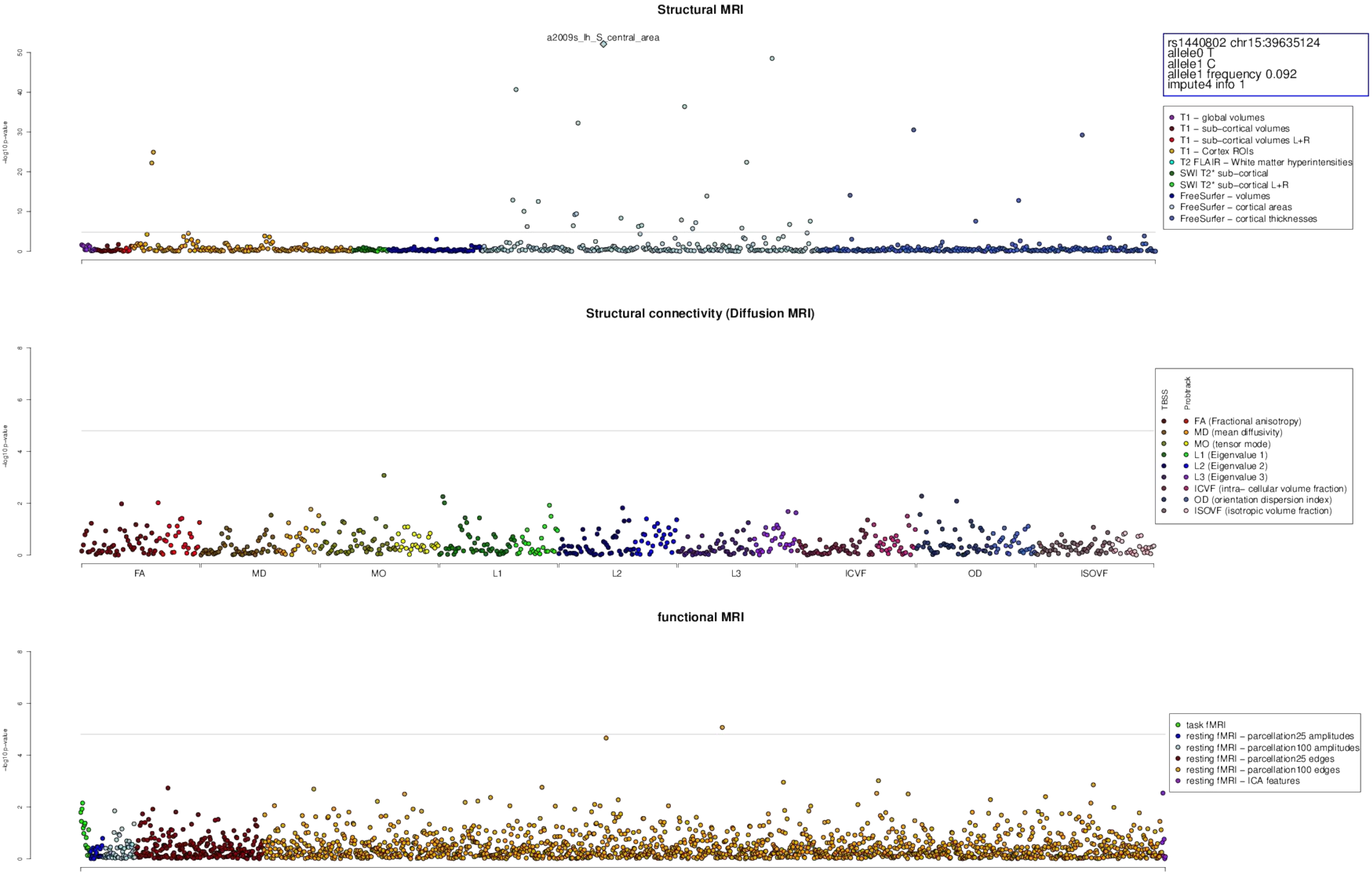
Structural MRI
a2009s_lh_S_central_area
rs1440802 chr15:39635124
allele0 T
allele1 C
allele1 frequency 0.092
impute4 info 1
T1 – global volumes
T1 – sub-cortical volumes
T1 – sub-cortical volumes L+R
T1 – Cortex ROIs
T2 FLAIR – White matter hyperintensities
SWI T2* sub-cortical
SWI T2* sub-cortical L+R
FreeSurfer – volumes
FreeSurfer – cortical areas
FreeSurfer – cortical thicknesses
-log10 p-value
Structural connectivity (Diffusion MRI)
TBSS
Probtrack
FA (Fractional anisotropy)
MD (mean diffusivity)
MO (tensor mode)
L1 (Eigenvalue 1)
L2 (Eigenvalue 2)
L3 (Eigenvalue 3)
ICVF (intra- cellular volume fraction)
OD (orientation dispersion index)
ISOVF (isotropic volume fraction)
FA
MD
MO
L1
L2
L3
ICVF
OD
ISOVF
functional MRI
task fMRI
resting fMRI – parcellation25 amplitudes
resting fMRI – parcellation100 amplitudes
resting fMRI – parcellation25 edges
resting fMRI – parcellation100 edges
resting fMRI – ICA features

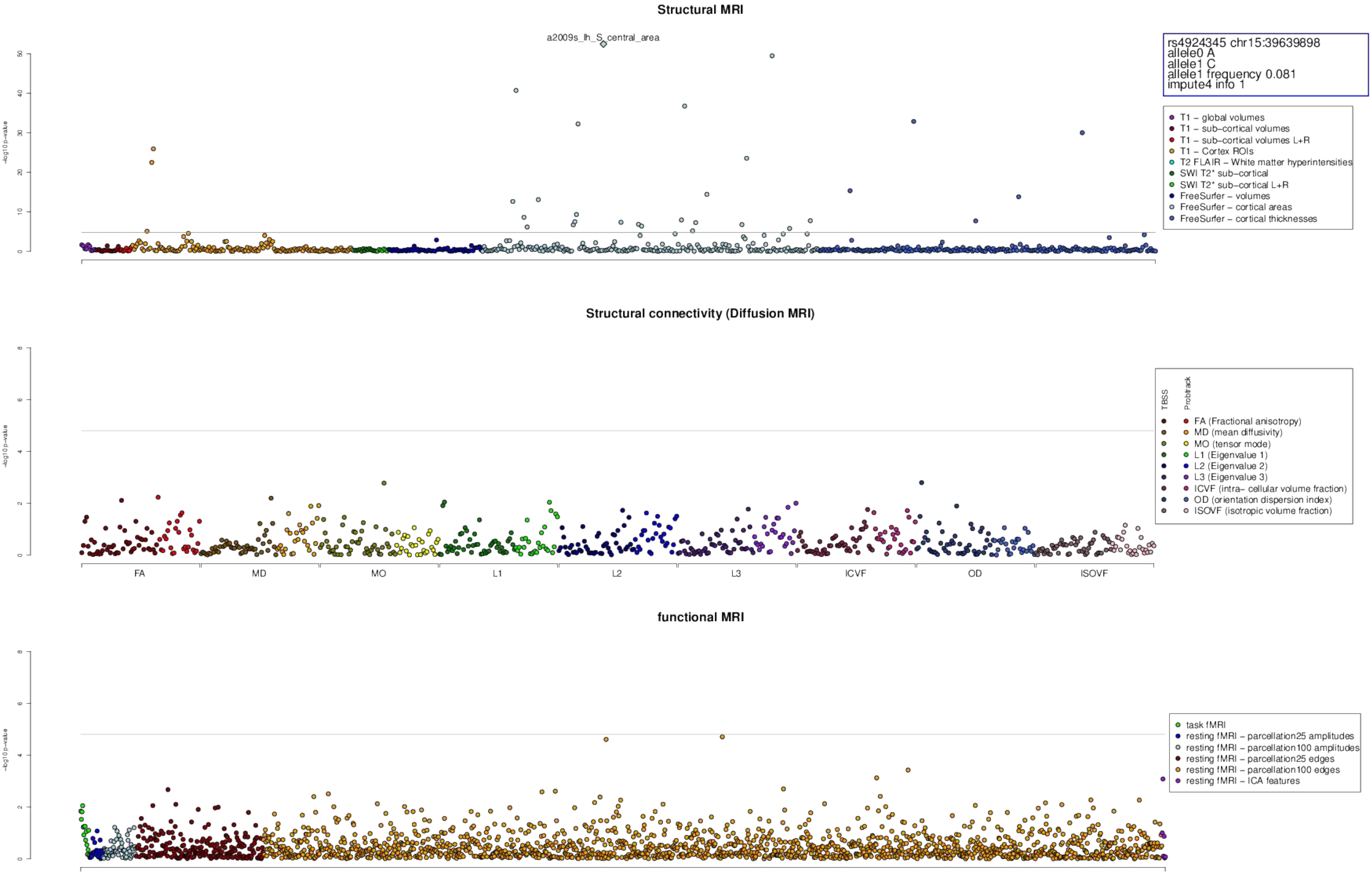

Structural MRI
a2009s_lh_S_central_area
rs4924345 chr15:39639898
allele0 A
allele1 C
allele1 frequency 0.081
impute4 info 1
T1 – global volumes
T1 – sub-cortical volumes
T1 – sub-cortical volumes L+R
T1 – Cortex ROIs
T2 FLAIR – White matter hyperintensities
SWI T2* sub-cortical
SWI T2* sub-cortical L+R
FreeSurfer – volumes
FreeSurfer – cortical areas
FreeSurfer – cortical thicknesses
-log10 p-value
Structural connectivity (Diffusion MRI)
TBSS
Probtrack
FA (Fractional anisotropy)
MD (mean diffusivity)
MO (tensor mode)
L1 (Eigenvalue 1)
L2 (Eigenvalue 2)
L3 (Eigenvalue 3)
ICVF (intra- cellular volume fraction)
OD (orientation dispersion index)
ISOVF (isotropic volume fraction)
FA
MD
MO
L1
L2
L3
ICVF
OD
ISOVF
functional MRI
task fMRI
resting fMRI – parcellation25 amplitudes
resting fMRI – parcellation100 amplitudes
resting fMRI – parcellation25 edges
resting fMRI – parcellation100 edges
resting fMRI – ICA features

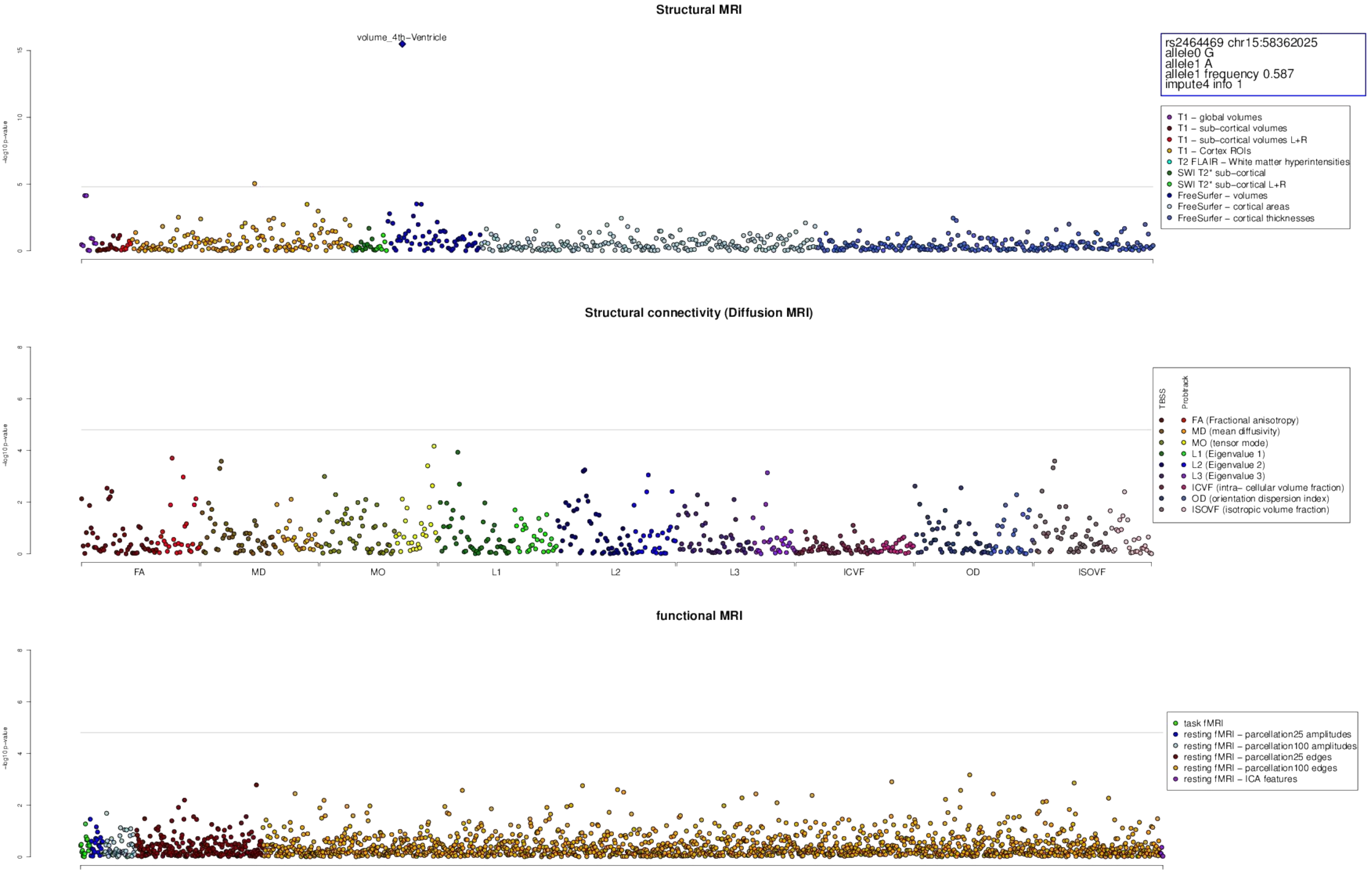

Structural MRI
volume_4th-Ventricle
rs2464469 chr15:58362025
allele0 G
allele1 A
allele1 frequency 0.587
impute4 info 1
T1 – global volumes
T1 – sub-cortical volumes
T1 – sub-cortical volumes L+R
T1 – Cortex ROIs
T2 FLAIR – White matter hyperintensities
SWI T2* sub-cortical
SWI T2* sub-cortical L+R
FreeSurfer – volumes
FreeSurfer – cortical areas
FreeSurfer – cortical thicknesses
-log10 p-value
Structural connectivity (Diffusion MRI)
TBSS
Probtrack
FA (Fractional anisotropy)
MD (mean diffusivity)
MO (tensor mode)
L1 (Eigenvalue 1)
L2 (Eigenvalue 2)
L3 (Eigenvalue 3)
ICVF (intra- cellular volume fraction)
OD (orientation dispersion index)
ISOVF (isotropic volume fraction)
FA
MD
MO
L1
L2
L3
ICVF
OD
ISOVF
functional MRI
task fMRI
resting fMRI – parcellation25 amplitudes
resting fMRI – parcellation100 amplitudes
resting fMRI – parcellation25 edges
resting fMRI – parcellation100 edges
resting fMRI – ICA features

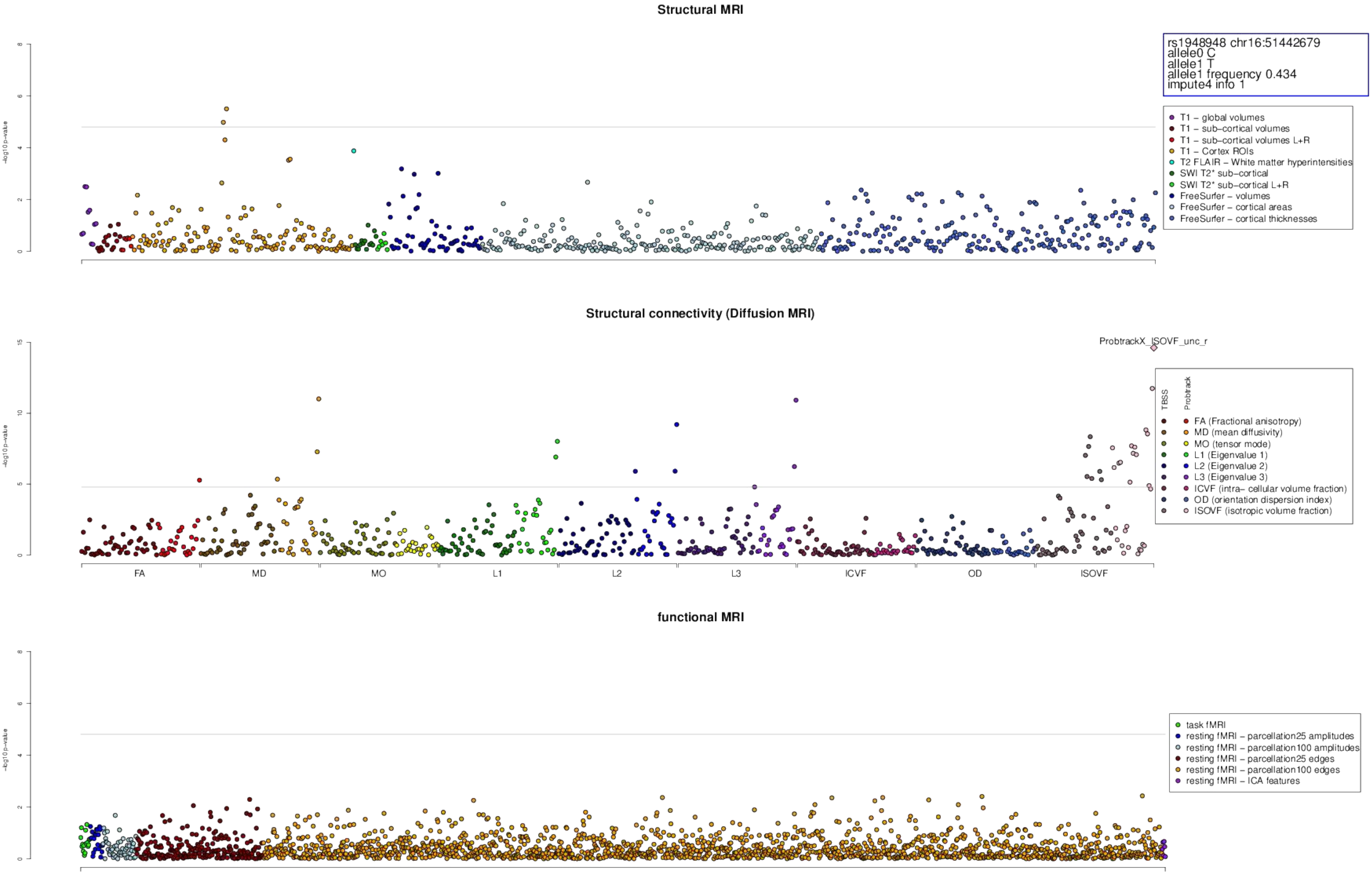
Structural MRI
rs1948948 chr16:51442679
allele0 C
allele1 T
allele1 frequency 0.434
impute4 info 1
T1 – global volumes
T1 – sub-cortical volumes
T1 – sub-cortical volumes L+R
T1 – Cortex ROIs
T2 FLAIR – White matter hyperintensities
SWI T2* sub-cortical
SWI T2* sub-cortical L+R
FreeSurfer – volumes
FreeSurfer – cortical areas
FreeSurfer – cortical thicknesses
-log10 p-value
Structural connectivity (Diffusion MRI)
ProbtrackX_ISOVF_unc_r
TBSS
Probtrack
FA (Fractional anisotropy)
MD (mean diffusivity)
MO (tensor mode)
L1 (Eigenvalue 1)
L2 (Eigenvalue 2)
L3 (Eigenvalue 3)
ICVF (intra- cellular volume fraction)
OD (orientation dispersion index)
ISOVF (isotropic volume fraction)
FA
MD
MO
L1
L2
L3
ICVF
OD
ISOVF
functional MRI
task fMRI
resting fMRI – parcellation25 amplitudes
resting fMRI – parcellation100 amplitudes
resting fMRI – parcellation25 edges
resting fMRI – parcellation100 edges
resting fMRI – ICA features

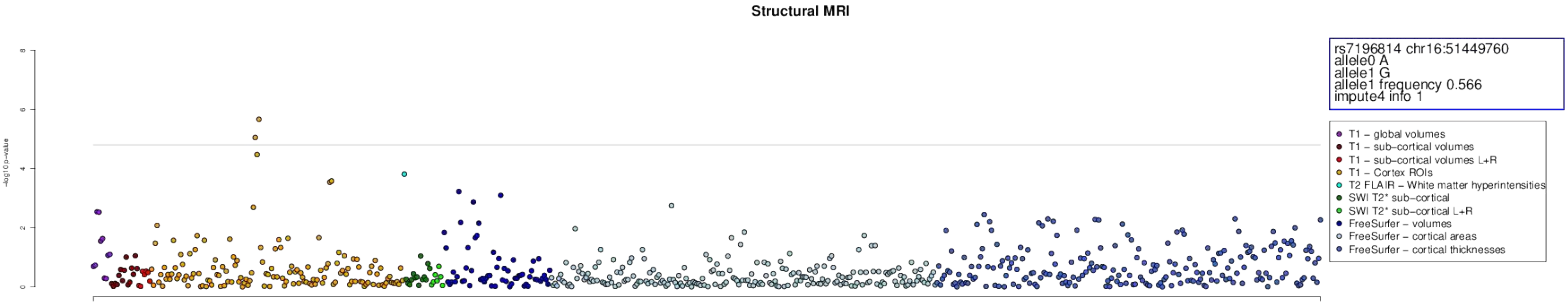

Structural MRI
-log10 p-value
rs7196814 chr16:51449760
allele0 A
allele1 G
allele1 frequency 0.566
impute4 info 1
T1 – global volumes
T1 – sub-cortical volumes
T1 – sub-cortical volumes L+R
T1 – Cortex ROIs
T2 FLAIR – White matter hyperintensities
SWI T2* sub-cortical
SWI T2* sub-cortical L+R
FreeSurfer – volumes
FreeSurfer – cortical areas
FreeSurfer – cortical thicknesses

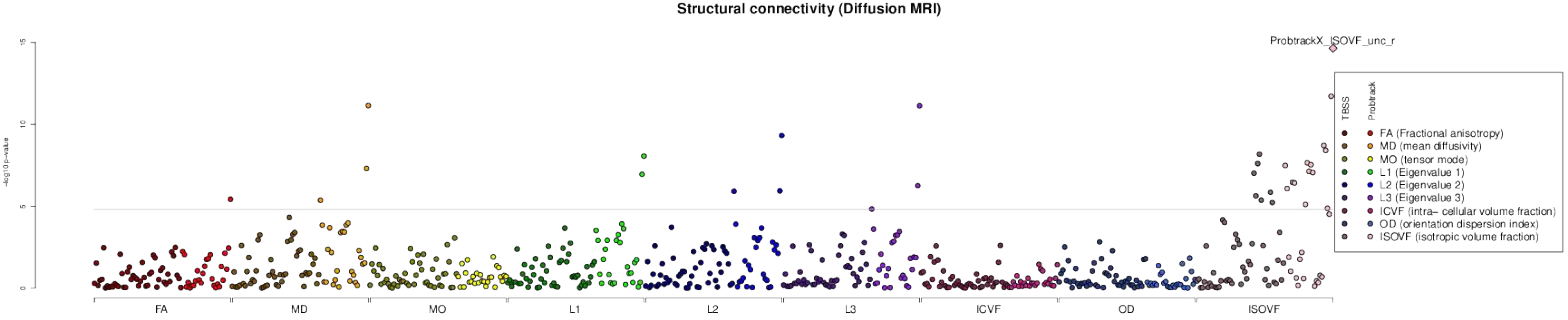

Structural connectivity (Diffusion MRI)
-log10 p-value
ProbtrackX_ISOVF_unc_r
TBSS
Probtrack
FA (Fractional anisotropy)
MD (mean diffusivity)
MO (tensor mode)
L1 (Eigenvalue 1)
L2 (Eigenvalue 2)
L3 (Eigenvalue 3)
ICVF (intra- cellular volume fraction)
OD (orientation dispersion index)
ISOVF (isotropic volume fraction)
FA
MD
MO
L1
L2
L3
ICVF
OD
ISOVF

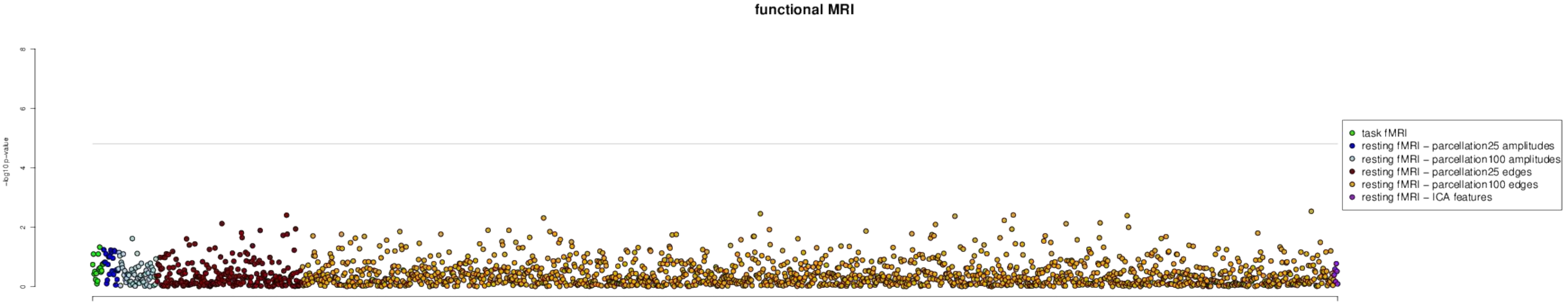

functional MRI
-log10 p-value
task fMRI
resting fMRI – parcellation25 amplitudes
resting fMRI – parcellation100 amplitudes
resting fMRI – parcellation25 edges
resting fMRI – parcellation100 edges
resting fMRI – ICA features

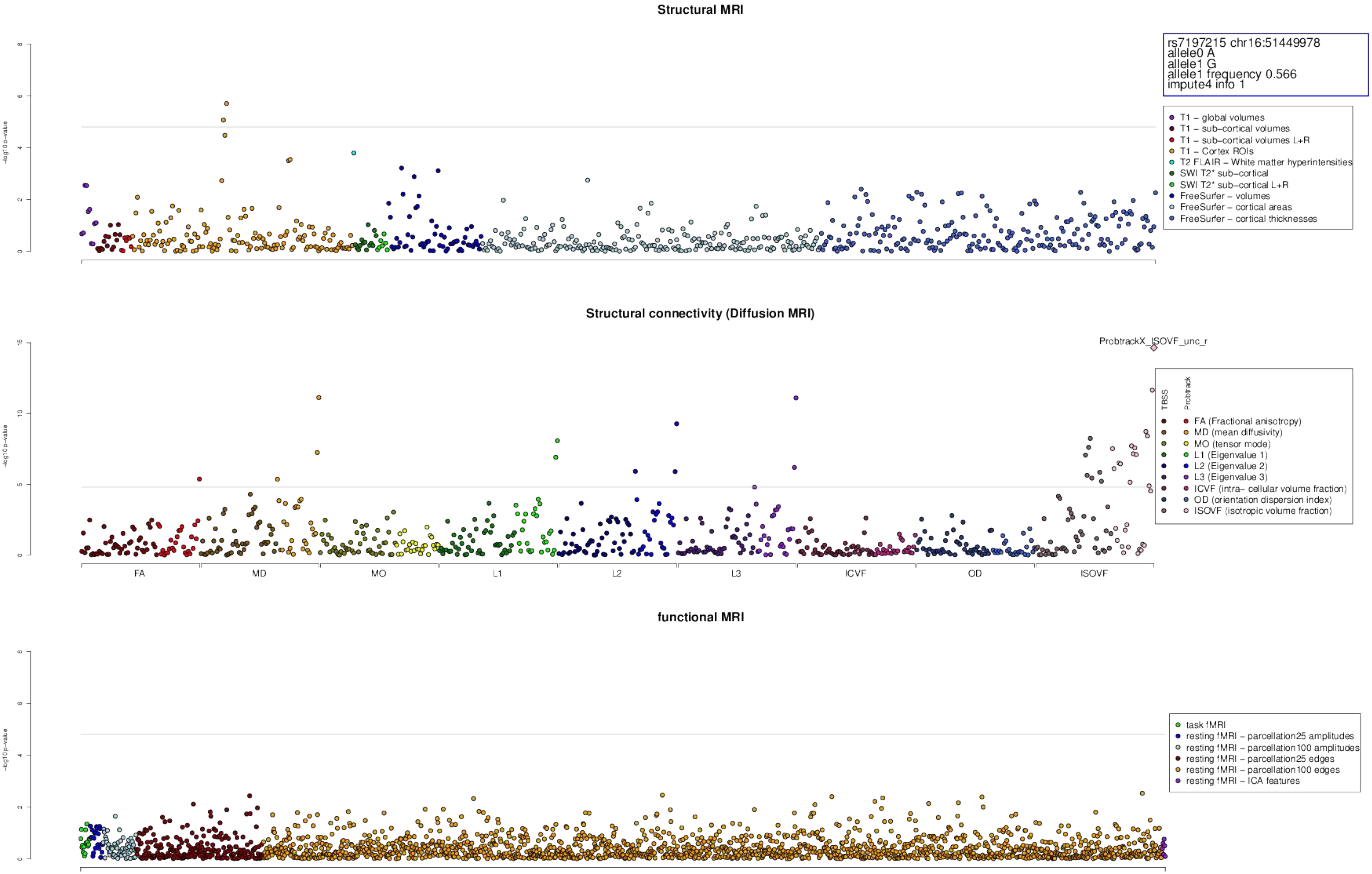
Structural MRI
-log10 p-value
rs7197215 chr16:51449978
allele0 A
allele1 G
allele1 frequency 0.566
impute4 info 1
T1 – global volumes
T1 – sub-cortical volumes
T1 – sub-cortical volumes L+R
T1 – Cortex ROIs
T2 FLAIR – White matter hyperintensities
SWI T2* sub-cortical
SWI T2* sub-cortical L+R
FreeSurfer – volumes
FreeSurfer – cortical areas
FreeSurfer – cortical thicknesses
Structural connectivity (Diffusion MRI)
ProbtrackX_ISOVF_unc_r
TBSS
Probtrack
FA (Fractional anisotropy)
MD (mean diffusivity)
MO (tensor mode)
L1 (Eigenvalue 1)
L2 (Eigenvalue 2)
L3 (Eigenvalue 3)
ICVF (intra– cellular volume fraction)
OD (orientation dispersion index)
ISOVF (isotropic volume fraction)
FA
MD
MO
L1
L2
L3
ICVF
OD
ISOVF
functional MRI
task fMRI
resting fMRI – parcellation25 amplitudes
resting fMRI – parcellation100 amplitudes
resting fMRI – parcellation25 edges
resting fMRI – parcellation100 edges
resting fMRI – ICA features

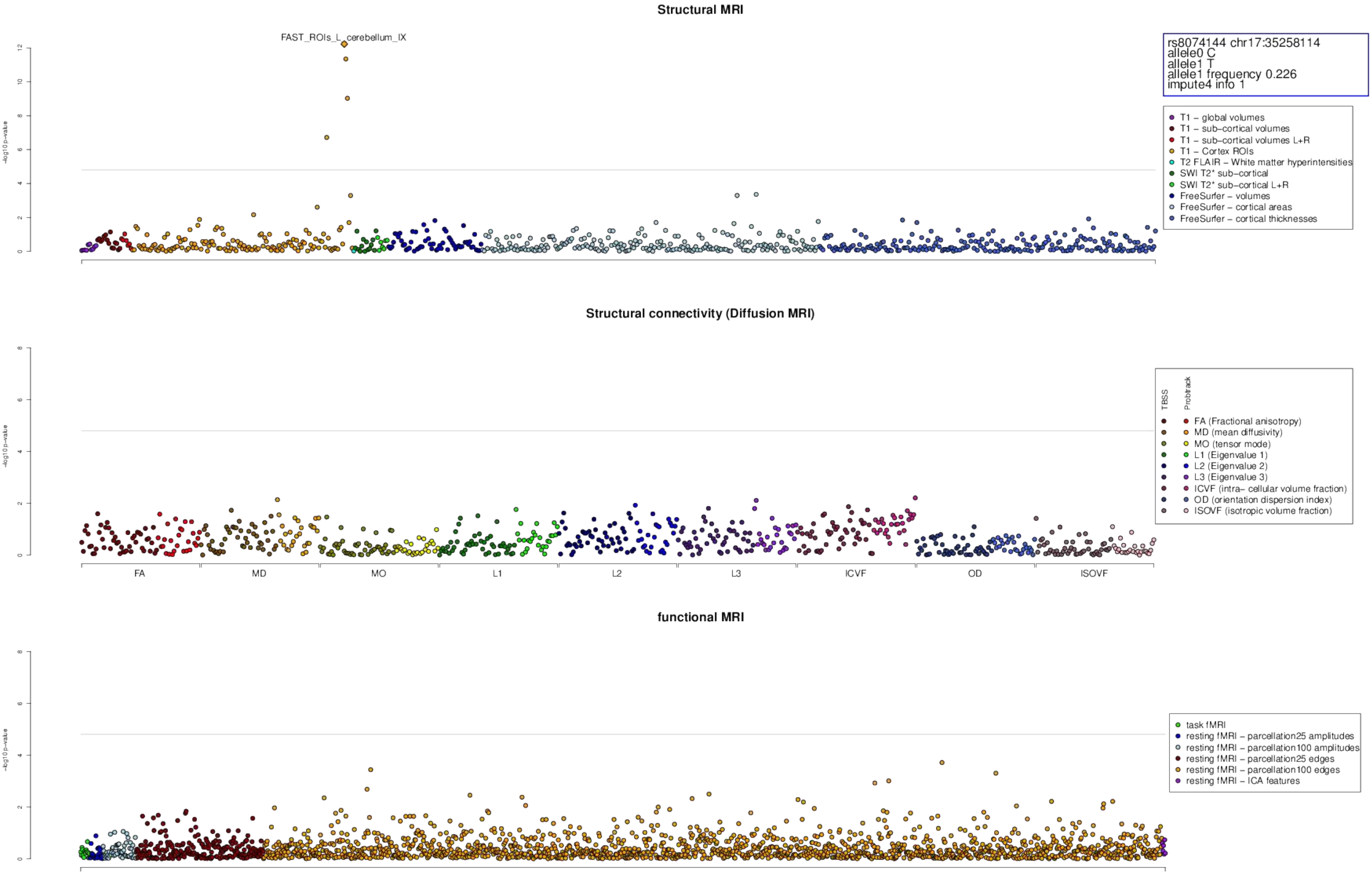
Structural MRI
FAST_ROIs_L_cerebellum_IX
-log10 p-value
rs8074144 chr17:35258114
allele0 C
allele1 T
allele1 frequency 0.226
impute4 info 1
T1 – global volumes
T1 – sub-cortical volumes
T1 – sub-cortical volumes L+R
T1 – Cortex ROIs
T2 FLAIR – White matter hyperintensities
SWI T2* sub-cortical
SWI T2* sub-cortical L+R
FreeSurfer – volumes
FreeSurfer – cortical areas
FreeSurfer – cortical thicknesses
Structural connectivity (Diffusion MRI)
TBSS
Probtrack
FA (Fractional anisotropy)
MD (mean diffusivity)
MO (tensor mode)
L1 (Eigenvalue 1)
L2 (Eigenvalue 2)
L3 (Eigenvalue 3)
ICVF (intra- cellular volume fraction)
OD (orientation dispersion index)
ISOVF (isotropic volume fraction)
FA
MD
MO
L1
L2
L3
ICVF
OD
ISOVF
functional MRI
task fMRI
resting fMRI – parcellation25 amplitudes
resting fMRI – parcellation100 amplitudes
resting fMRI – parcellation25 edges
resting fMRI – parcellation100 edges
resting fMRI – ICA features

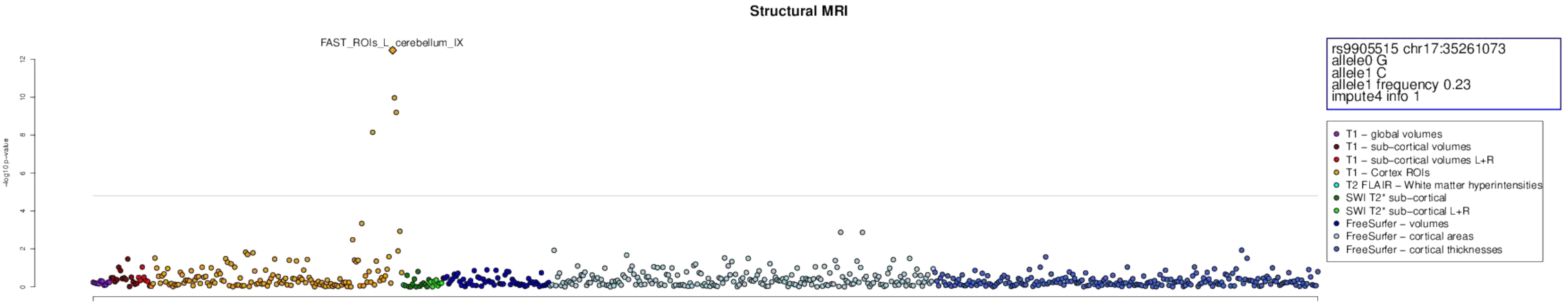

Structural MRI
FAST_ROIs_L_cerebellum_IX
rs9905515 chr17:35261073
allele0 G
allele1 C
allele1 frequency 0.23
impute4 info 1
T1 – global volumes
T1 – sub-cortical volumes
T1 – sub-cortical volumes L+R
T1 – Cortex ROIs
T2 FLAIR – White matter hyperintensities
SWI T2* sub-cortical
SWI T2* sub-cortical L+R
FreeSurfer – volumes
FreeSurfer – cortical areas
FreeSurfer – cortical thicknesses
-log10 p-value

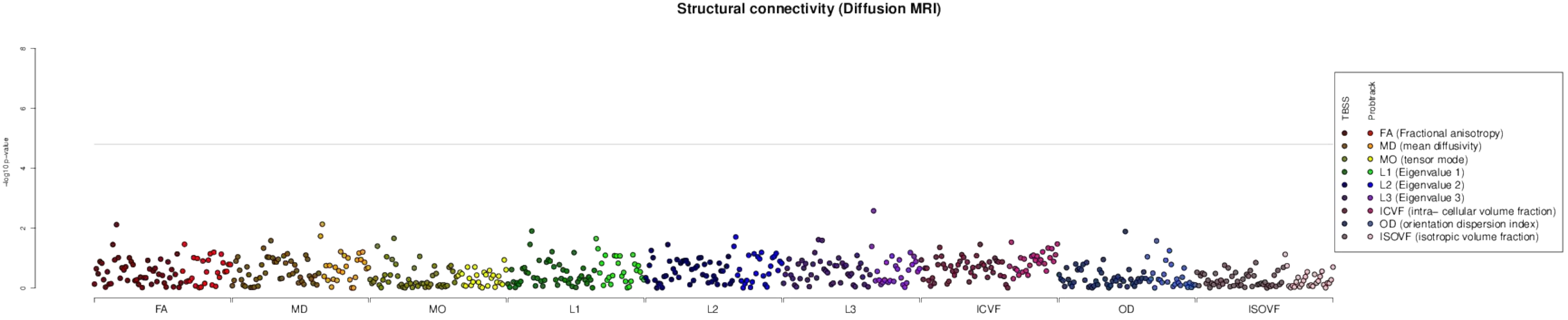

Structural connectivity (Diffusion MRI)
TBSS
Probtrack
FA (Fractional anisotropy)
MD (mean diffusivity)
MO (tensor mode)
L1 (Eigenvalue 1)
L2 (Eigenvalue 2)
L3 (Eigenvalue 3)
ICVF (intra- cellular volume fraction)
OD (orientation dispersion index)
ISOVF (isotropic volume fraction)
FA
MD
MO
L1
L2
L3
ICVF
OD
ISOVF
-log10 p-value

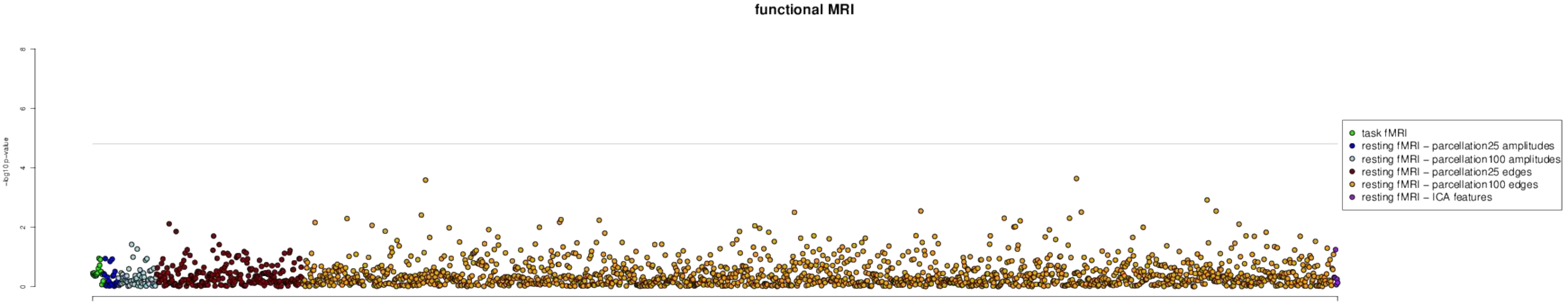

functional MRI
task fMRI
resting fMRI – parcellation25 amplitudes
resting fMRI – parcellation100 amplitudes
resting fMRI – parcellation25 edges
resting fMRI – parcellation100 edges
resting fMRI – ICA features
-log10 p-value

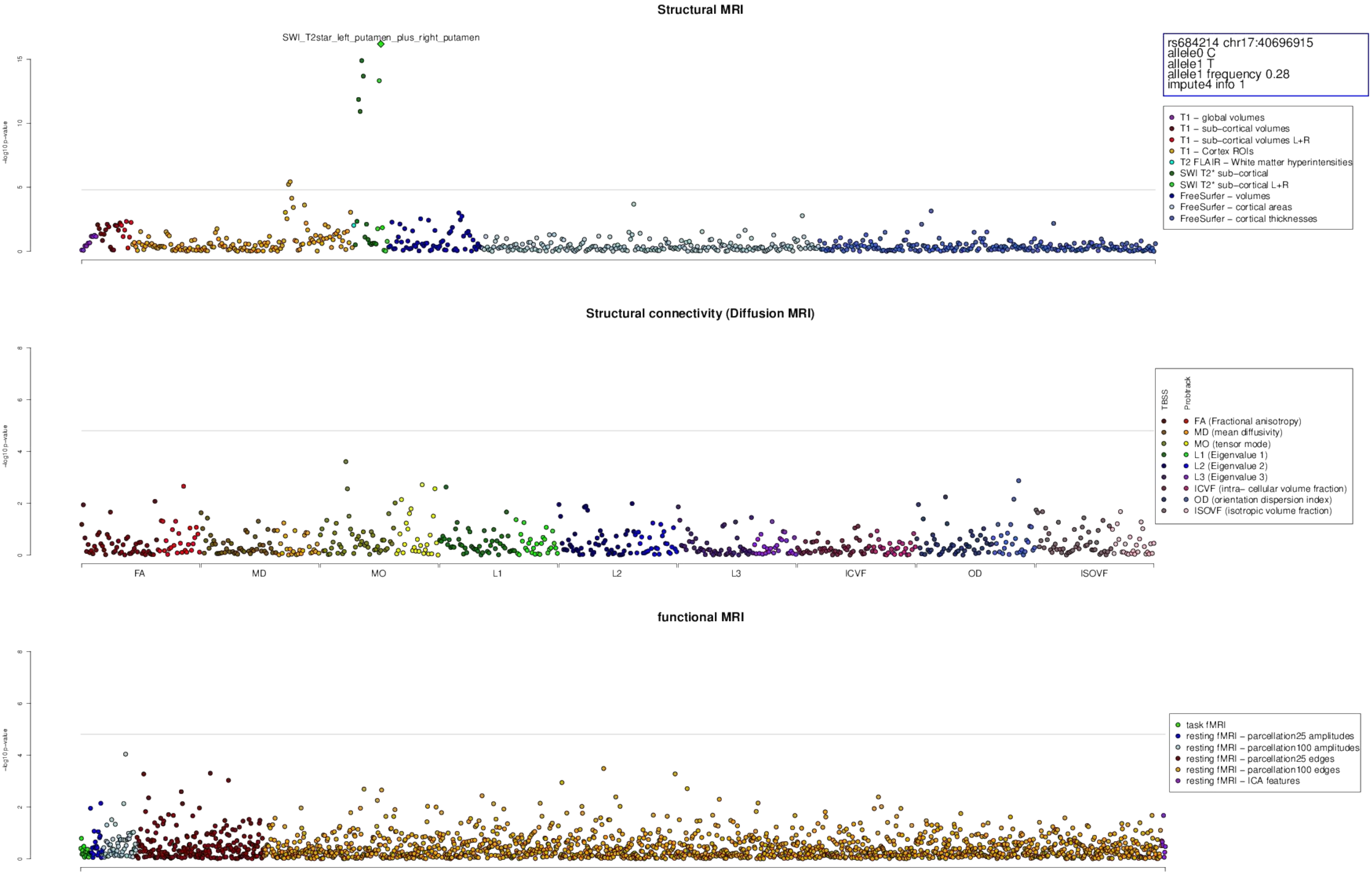

Structural MRI
SWI_T2star_left_putamen_plus_right_putamen
rs684214 chr17:40696915
allele0 C
allele1 T
allele1 frequency 0.28
impute4 info 1
T1 – global volumes
T1 – sub-cortical volumes
T1 – sub-cortical volumes L+R
T1 – Cortex ROIs
T2 FLAIR – White matter hyperintensities
SWI T2* sub-cortical
SWI T2* sub-cortical L+R
FreeSurfer – volumes
FreeSurfer – cortical areas
FreeSurfer – cortical thicknesses
-log10 p-value
Structural connectivity (Diffusion MRI)
TBSS
Probtrack
FA (Fractional anisotropy)
MD (mean diffusivity)
MO (tensor mode)
L1 (Eigenvalue 1)
L2 (Eigenvalue 2)
L3 (Eigenvalue 3)
ICVF (intra- cellular volume fraction)
OD (orientation dispersion index)
ISOVF (isotropic volume fraction)
FA
MD
MO
L1
L2
L3
ICVF
OD
ISOVF
functional MRI
task fMRI
resting fMRI – parcellation25 amplitudes
resting fMRI – parcellation100 amplitudes
resting fMRI – parcellation25 edges
resting fMRI – parcellation100 edges
resting fMRI – ICA features

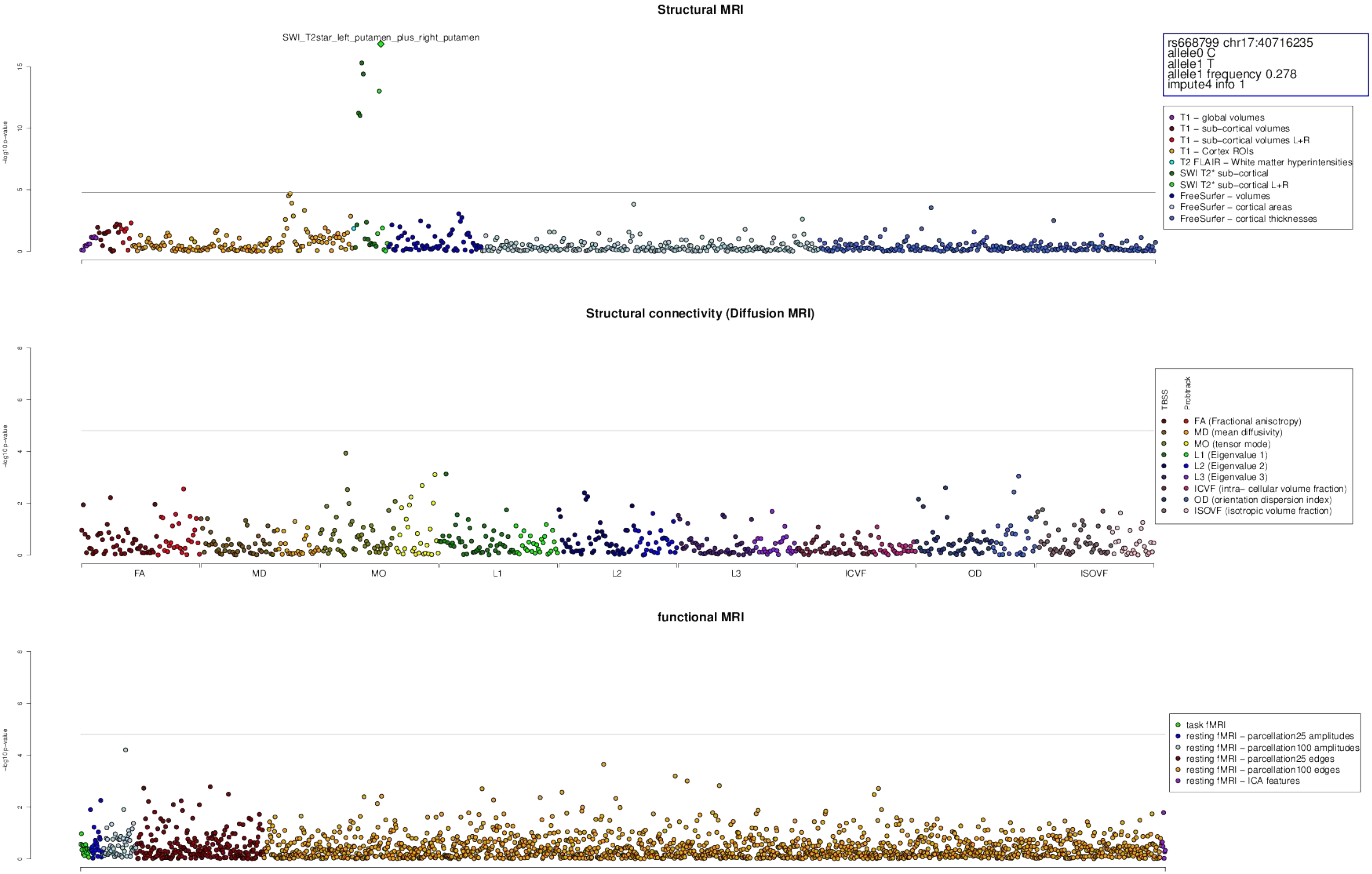

Structural MRI
SWI_T2star_left_putamen_plus_right_putamen
rs668799 chr17:40716235
allele0 C
allele1 T
allele1 frequency 0.278
impute4 info 1
T1 – global volumes
T1 – sub-cortical volumes
T1 – sub-cortical volumes L+R
T1 – Cortex ROIs
T2 FLAIR – White matter hyperintensities
SWI T2* sub-cortical
SWI T2* sub-cortical L+R
FreeSurfer – volumes
FreeSurfer – cortical areas
FreeSurfer – cortical thicknesses
-log10 p-value
Structural connectivity (Diffusion MRI)
TBSS
Probtrack
FA (Fractional anisotropy)
MD (mean diffusivity)
MO (tensor mode)
L1 (Eigenvalue 1)
L2 (Eigenvalue 2)
L3 (Eigenvalue 3)
ICVF (intra- cellular volume fraction)
OD (orientation dispersion index)
ISOVF (isotropic volume fraction)
FA
MD
MO
L1
L2
L3
ICVF
OD
ISOVF
functional MRI
task fMRI
resting fMRI – parcellation25 amplitudes
resting fMRI – parcellation100 amplitudes
resting fMRI – parcellation25 edges
resting fMRI – parcellation100 edges
resting fMRI – ICA features

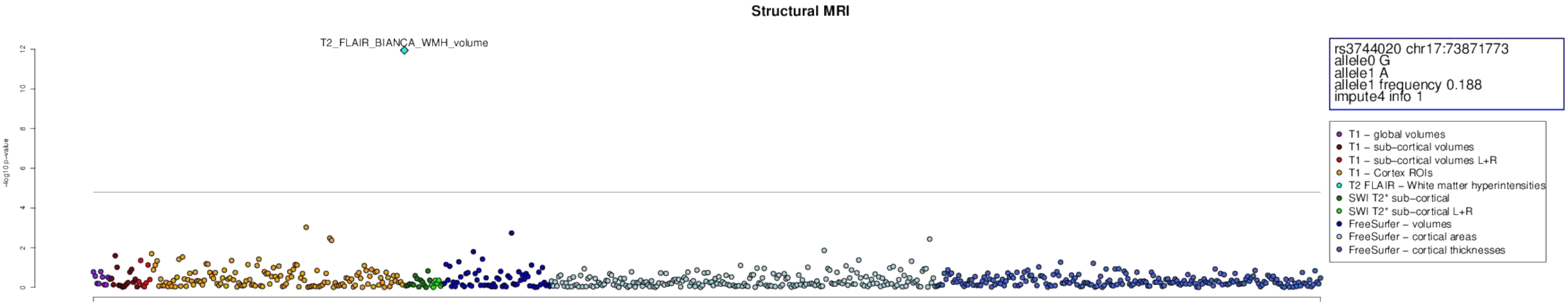
Structural MRI
T2_FLAIR_BIANCA_WMH_volume
-log10 p-value
rs3744020 chr17:73871773
allele0 G
allele1 A
allele1 frequency 0.188
impute4 info 1
T1 – global volumes
T1 – sub-cortical volumes
T1 – sub-cortical volumes L+R
T1 – Cortex ROIs
T2 FLAIR – White matter hyperintensities
SWI T2* sub-cortical
SWI T2* sub-cortical L+R
FreeSurfer – volumes
FreeSurfer – cortical areas
FreeSurfer – cortical thicknesses

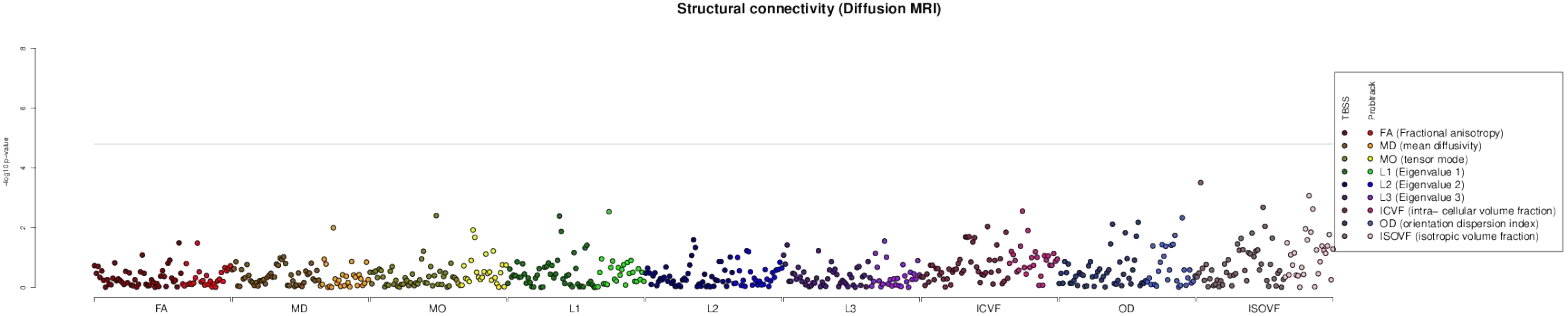
Structural connectivity (Diffusion MRI)
-log10 p-value
TBSS
Probtrack
FA (Fractional anisotropy)
MD (mean diffusivity)
MO (tensor mode)
L1 (Eigenvalue 1)
L2 (Eigenvalue 2)
L3 (Eigenvalue 3)
ICVF (intra- cellular volume fraction)
OD (orientation dispersion index)
ISOVF (isotropic volume fraction)
FA
MD
MO
L1
L2
L3
ICVF
OD
ISOVF

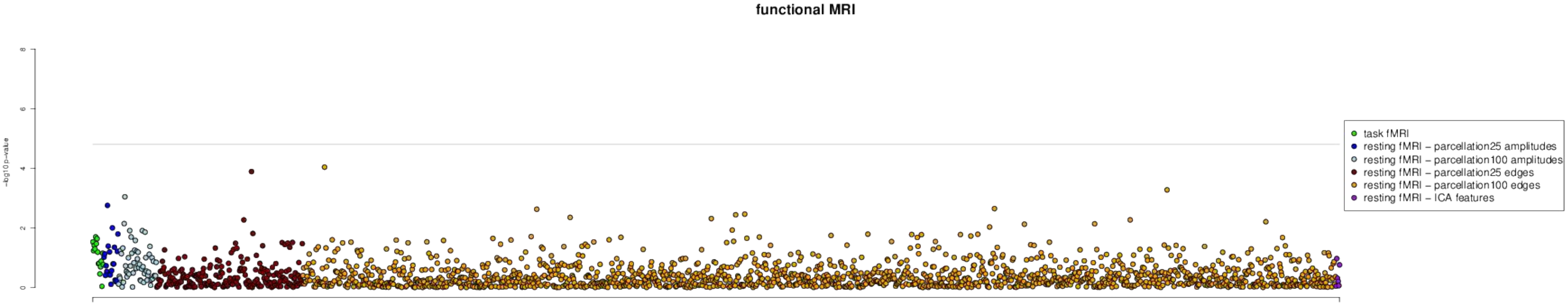
functional MRI
-log10 p-value
task fMRI
resting fMRI – parcellation25 amplitudes
resting fMRI – parcellation100 amplitudes
resting fMRI – parcellation25 edges
resting fMRI – parcellation100 edges
resting fMRI – ICA features

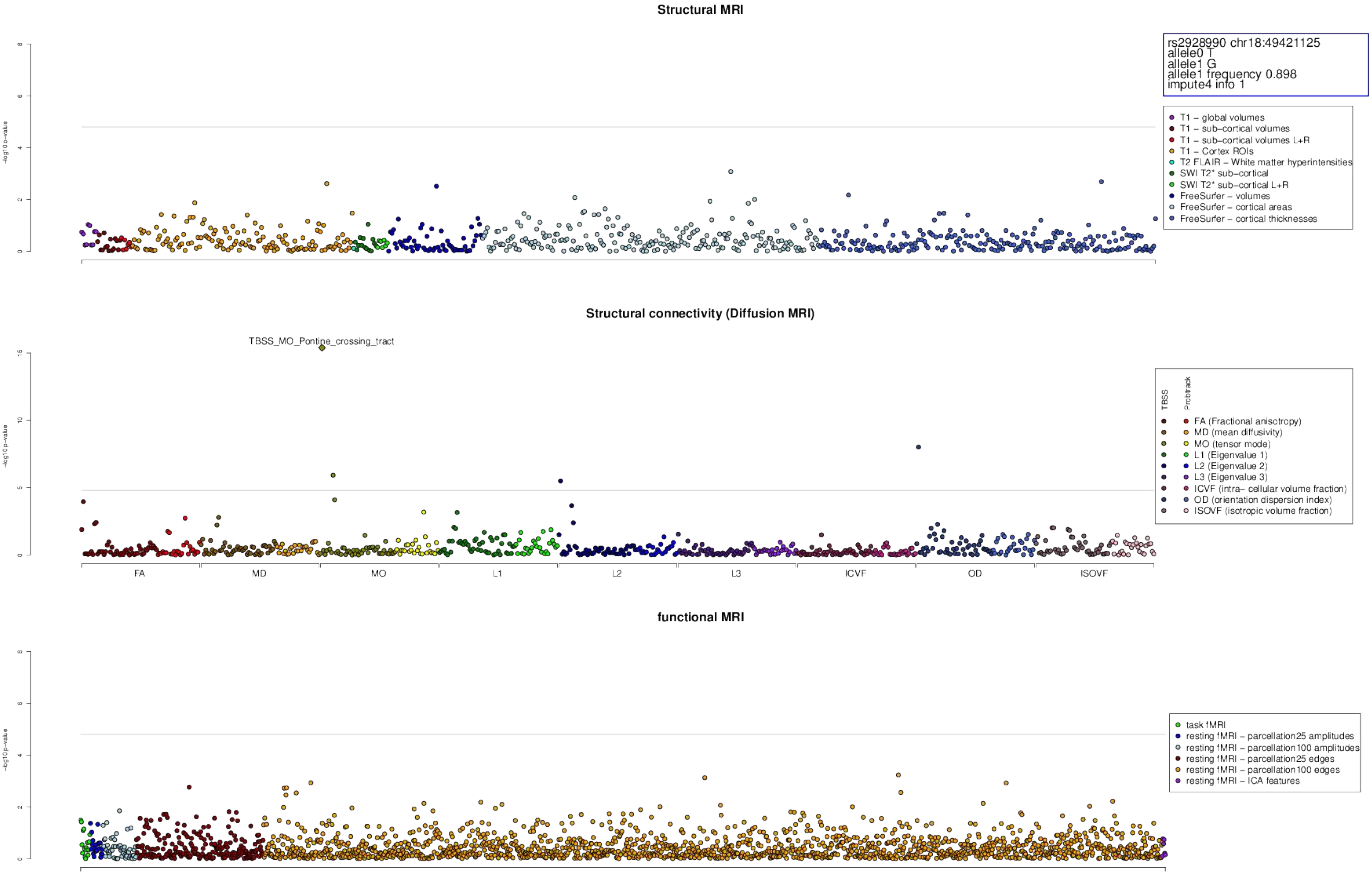
Structural MRI
rs2928990 chr18:49421125
allele0 T
allele1 G
allele1 frequency 0.898
impute4 info 1
-log10 p-value
T1 – global volumes
T1 – sub-cortical volumes
T1 – sub-cortical volumes L+R
T1 – Cortex ROIs
T2 FLAIR – White matter hyperintensities
SWI T2* sub-cortical
SWI T2* sub-cortical L+R
FreeSurfer – volumes
FreeSurfer – cortical areas
FreeSurfer – cortical thicknesses
Structural connectivity (Diffusion MRI)
TBSS_MO_Pontine_crossing_tract
TBSS
Probtrack
FA (Fractional anisotropy)
MD (mean diffusivity)
MO (tensor mode)
L1 (Eigenvalue 1)
L2 (Eigenvalue 2)
L3 (Eigenvalue 3)
ICVF (intra- cellular volume fraction)
OD (orientation dispersion index)
ISOVF (isotropic volume fraction)
FA
MD
MO
L1
L2
L3
ICVF
OD
ISOVF
functional MRI
task fMRI
resting fMRI – parcellation25 amplitudes
resting fMRI – parcellation100 amplitudes
resting fMRI – parcellation25 edges
resting fMRI – parcellation100 edges
resting fMRI – ICA features
