# Supplementary material for: Genome-wide association studies of brain imaging phenotypes in UK Biobank
Source: Nature. 2018 Oct 10;562(7726):210–6. doi: 10.1038/s41586-018-0571-7 (PMC6786974; doi:10.1038/s41586-018-0571-7)
Supplement: Supplementary file 3 — This file contains Supplementary Figures S1-S22. [file 41586_2018_571_MOESM3_ESM.zip › Figure-S9.pdf]

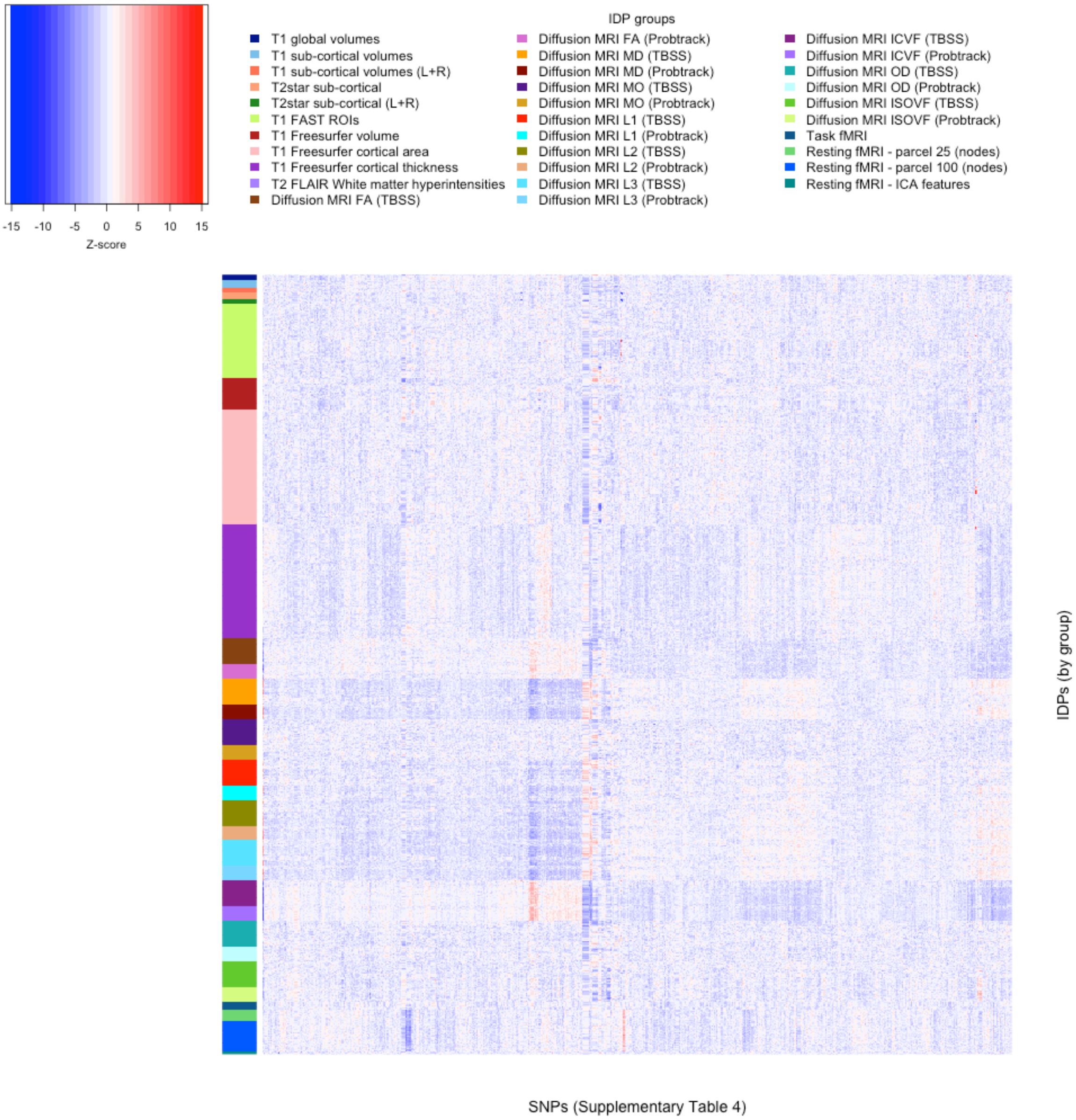

Supplementary Figure 9 : Comparison of Z-scores for associated SNPs across traits – The main rectangle is a heatmap. Columns correspond to the 615 unique SNPs listed in **Supplementary Table 5** with association $-\log_{10}$ p-value > 7.5. Rows correspond to IDPs excluding the resting fMRI parcel edges. Each element has a colour determined by the z-score for the association of the corresponding SNP-IDP pair as indicated by the colour key in the top left corner. In constructing this mapping, z-scores were binned coarsely into bins of size 1 (maximum difference between the smallest and largest z-scores within a bin). SNPs have been clustered according to Euclidean distance between the vectors of z-scores. IDPs are grouped in the same way as elsewhere in the paper, but clustered within each group. Rows are labelled by the colour of the appropriate IDP group. The corresponding descriptions of the groups are shown in the legend (labelled IDP groups) at the top of the figure. Even without a full clustering of the rows, some structure is apparent.
