# Supplementary material for: Genome-wide association studies of brain imaging phenotypes in UK Biobank
Source: Nature. 2018 Oct 10;562(7726):210–6. doi: 10.1038/s41586-018-0571-7 (PMC6786974; doi:10.1038/s41586-018-0571-7)
Supplement: Supplementary file 3 — This file contains Supplementary Figures S1-S22. [file 41586_2018_571_MOESM3_ESM.zip › Figure-S10.pdf]

Number of hits per IDP, by heritability

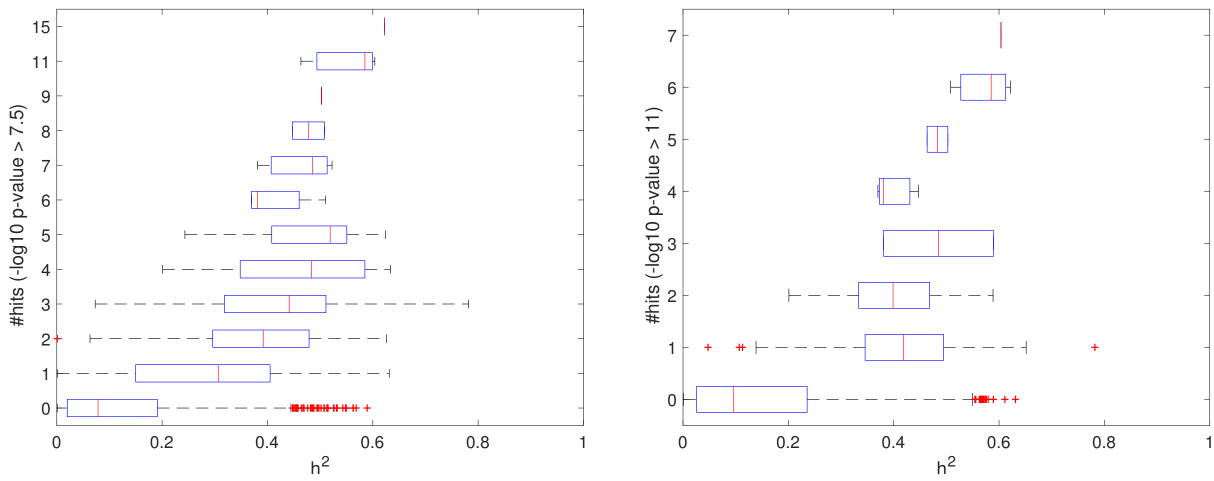

Supplementary Figure 10 – Comparison of number of associated loci (y-axis) and SNP-heritability (x-axis) for each IDP at two different significance thresholds : -log10(P)>7.5 (left) and -log10(P)>11 (right). N = 8,428 subjects, see Methods for calculation details. Boxplots show: median is the red line; ends of the box mark 25th and 75th percentiles; whiskers show most extreme values not considered outliers; outliers are shown with a red cross and are values lying more than 1.5x the interquartile range outside of the interquartile box.
