# Supplementary material for: Genome-wide association studies of brain imaging phenotypes in UK Biobank
Source: Nature. 2018 Oct 10;562(7726):210–6. doi: 10.1038/s41586-018-0571-7 (PMC6786974; doi:10.1038/s41586-018-0571-7)
Supplement: Supplementary file 3 — This file contains Supplementary Figures S1-S22. [file 41586_2018_571_MOESM3_ESM.zip › Figure-S11.pdf]

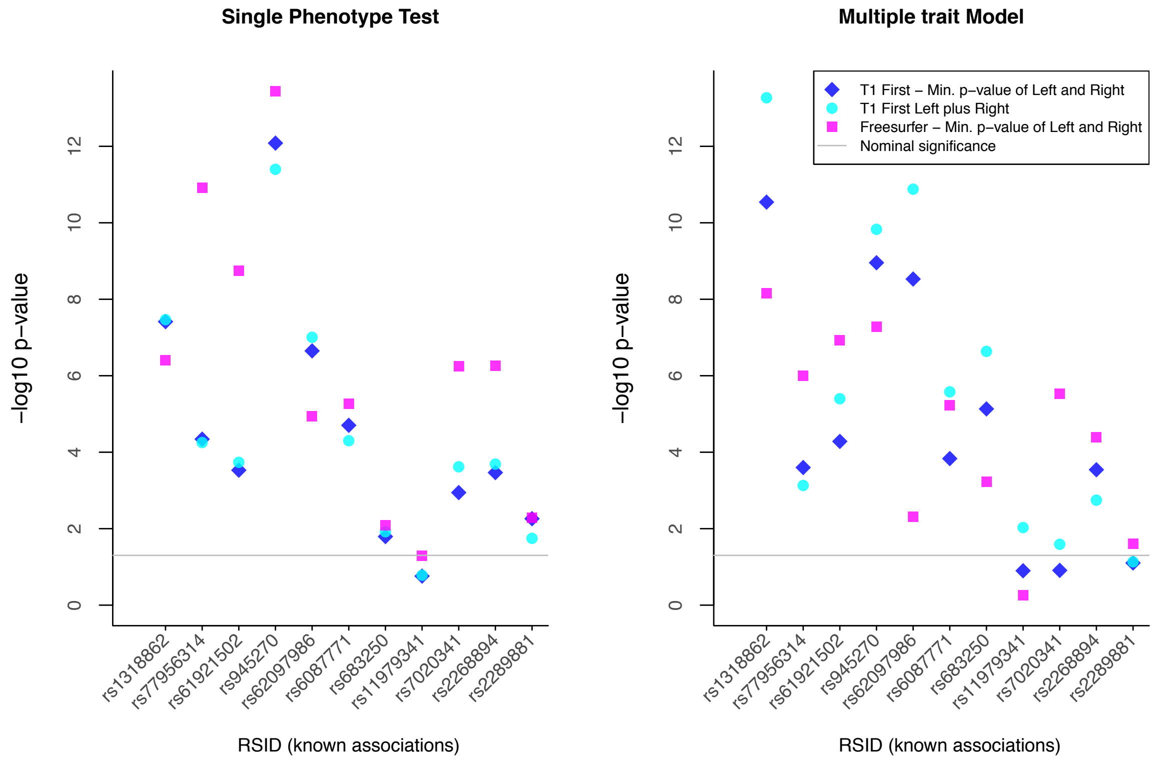

Supplementary Figure 11 – These figures show $-\log_{10}$ p-values obtained in the UKBiobank discovery sample for known associations of SNPs with volumes of subcortical structures. Each coloured dot corresponds to a SNP-phenotype association. The vertical axes correspond to negative $\log_{10}$ p-values in our discovery sample. The horizontal axes show lead SNPs for 11 associations reported by Hibar et al. 2015 and Hibar et al. 2017. These represent the seven associations that were replicated by Hibar et al. 2015 (using ~ 12000 discovery samples) and an additional four associations reported by Hibar et al. 2017 (using ~25000 discovery samples). The left hand figure shows results for tests of association between a SNP and the volume of the appropriate single subcortical structure; the right hand figure shows results for a multi-trait test of association between a SNP and an appropriate group of phenotypes. Different coloured dots denote results obtained under different protocols: blue and magenta correspond to the minimum p-value for association with the left or right subcortical structure as determined by T1 FIRST and Freesurfer respectively; cyan - corresponds to associations with combined T1 FIRST measurements of the left and right structures. All 11 associations achieve nominal significance (p-value < 0.05) under at least one protocol. Hibar et al. 2015 and 2017 both reported results of a GWAS meta-analysis for association with volume measurements of entire structures for which a variety of protocols had been employed.
