# Supplementary material for: Genome-wide association studies of brain imaging phenotypes in UK Biobank
Source: Nature. 2018 Oct 10;562(7726):210–6. doi: 10.1038/s41586-018-0571-7 (PMC6786974; doi:10.1038/s41586-018-0571-7)
Supplement: Supplementary file 3 — This file contains Supplementary Figures S1-S22. [file 41586_2018_571_MOESM3_ESM.zip › Figure-S12.pdf]

Supplementary Figure 12

Each of the subsequent pages shows a GWAS Manhattan plot for the multi-trait GWAS carried out on the 23 groupings of IDPs described in **Supplementary Table 7**.

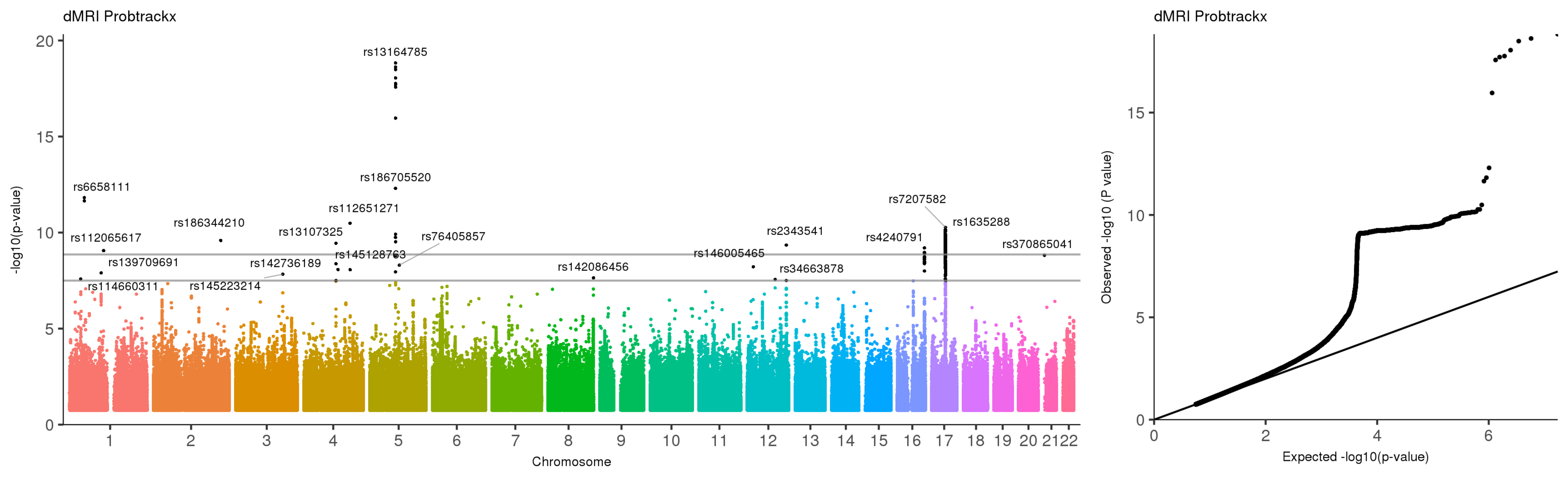
dMRI Probtrackx
rs13164785
rs186705520
rs6658111
rs112651271
rs186344210
rs13107325
rs76405857
rs112065617
rs145128763
rs139709691
rs142736189
rs114660311
rs145223214
rs142086456
rs146005465
rs2343541
rs34663878
rs7207582
rs1635288
rs4240791
rs370865041
-log10(p-value)
Chromosome
1 2 3 4 5 6 7 8 9 10 11 12 13 14 15 16 17 18 19 20 21 22
0 5 10 15 20
dMRI Probtrackx
Observed -log10 (P value)
Expected -log10(p-value)
0 2 4 6
0 5 10 15

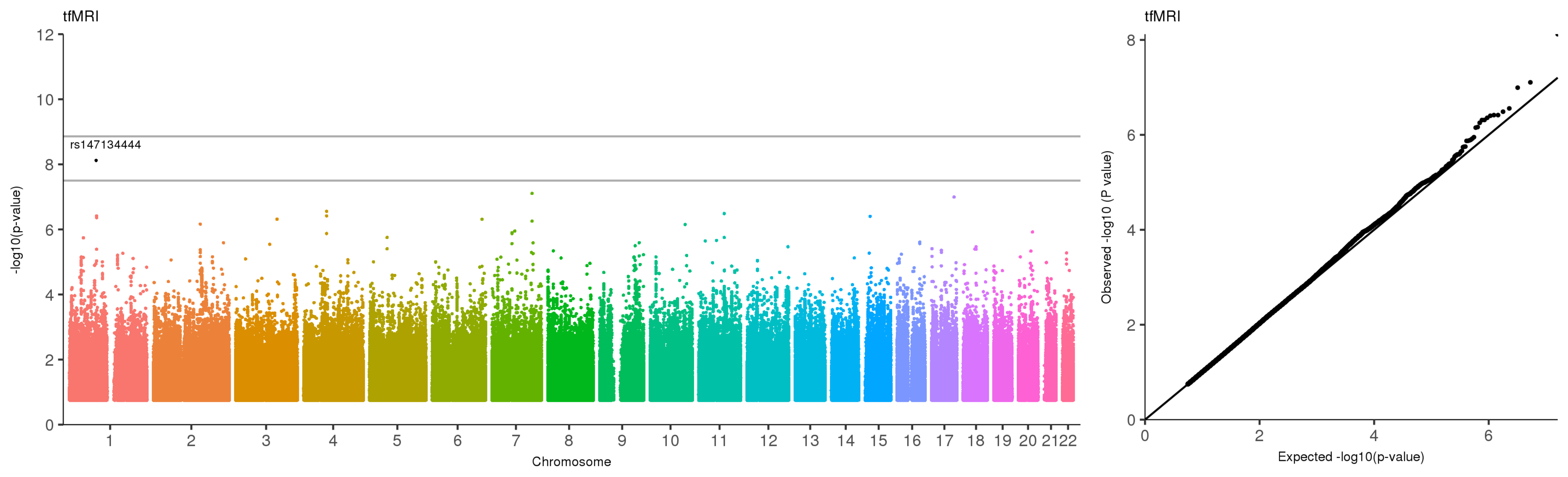
tfMRI
rs147134444
-log10(p-value)
Chromosome
1
2
3
4
5
6
7
8
9
10
11
12
13
14
15
16
17
18
19
20
21
22
0
2
4
6
8
10
12
tfMRI
Observed -log10 (P value)
Expected -log10(p-value)

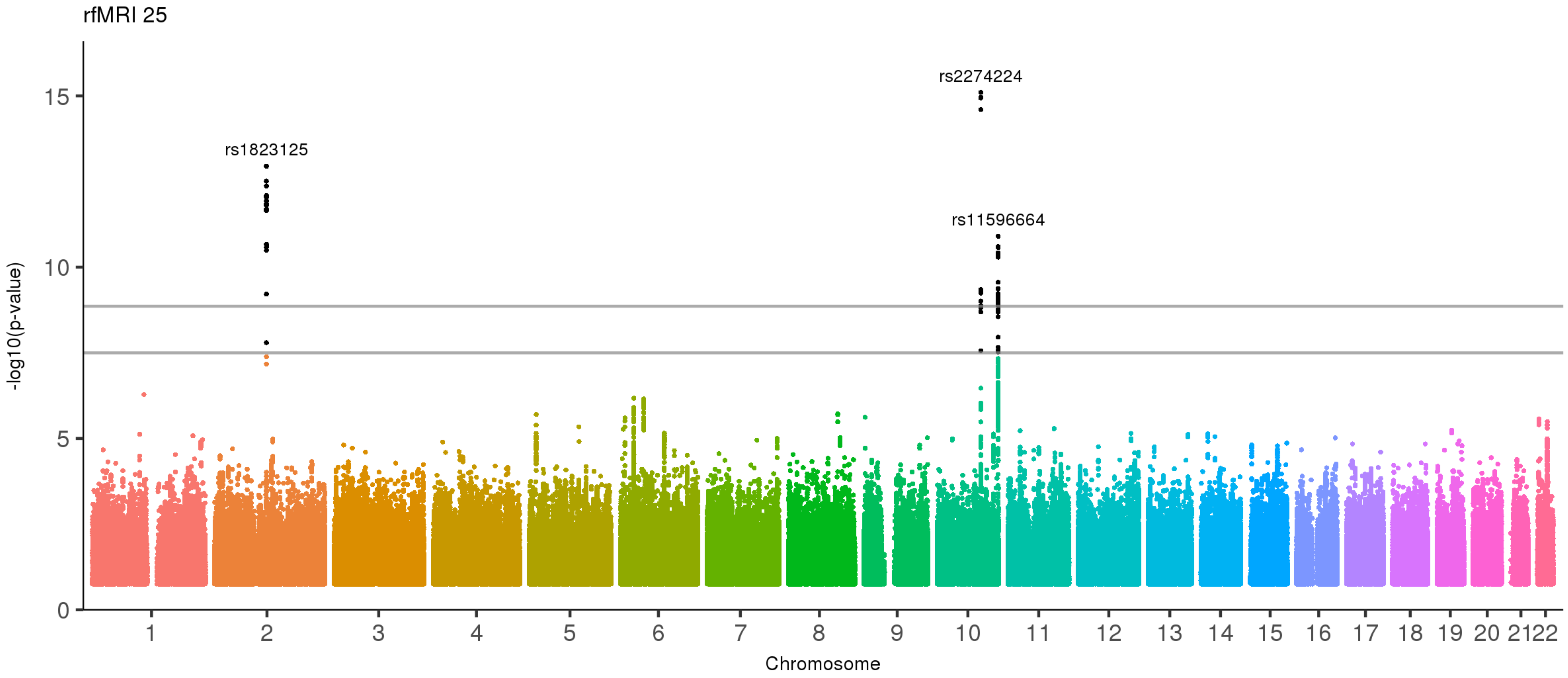
rfMRI 25
rs2274224
rs1823125
rs11596664
-log10(p-value)
15
10
5
0
1
2
3
4
5
6
7
8
9
10
11
12
13
14
15
16
17
18
19
20
21
22
Chromosome

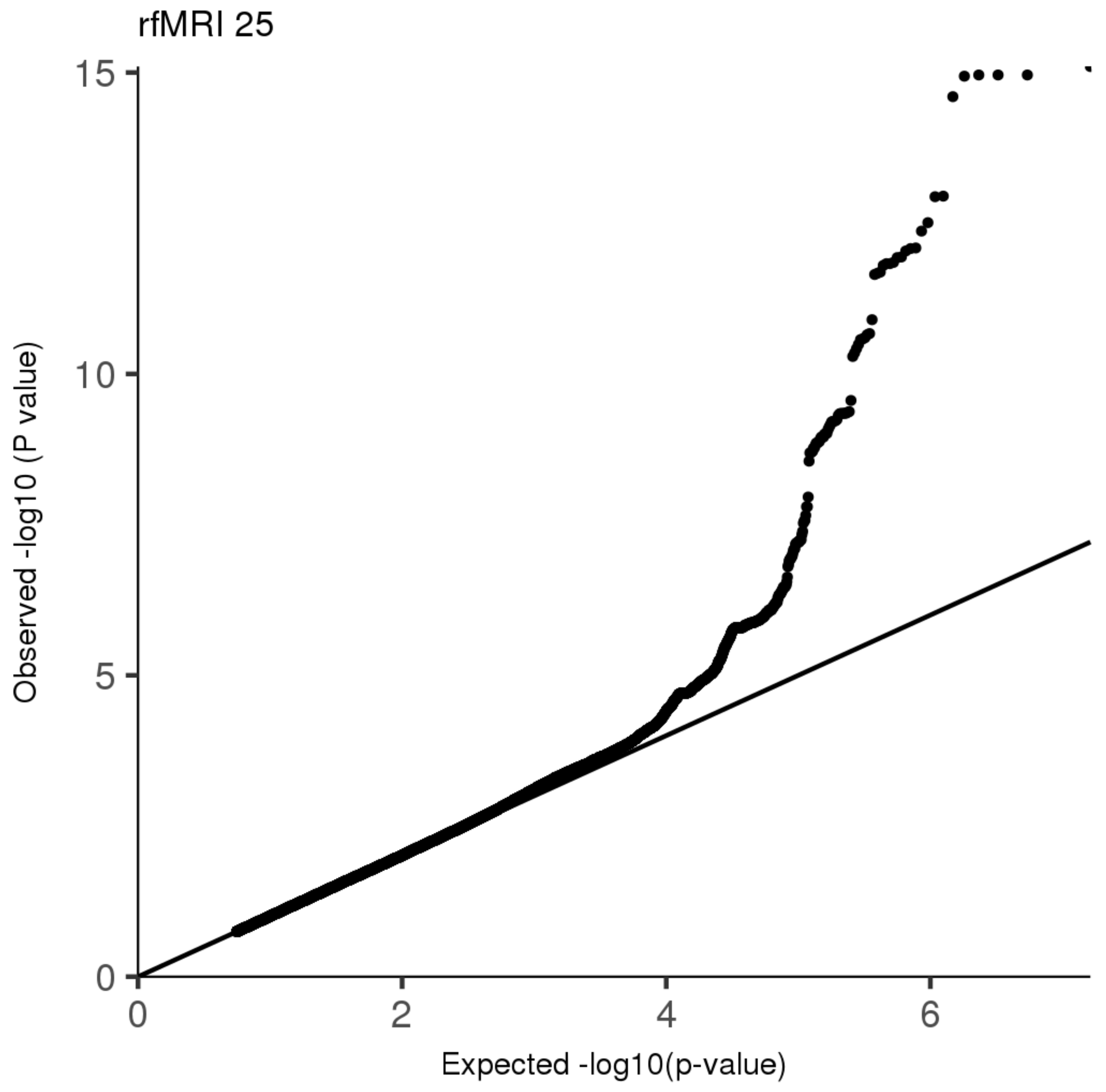
rfMRI 25
Observed -log10 (P value)
15
10
5
0
2
4
6
Expected -log10(p-value)

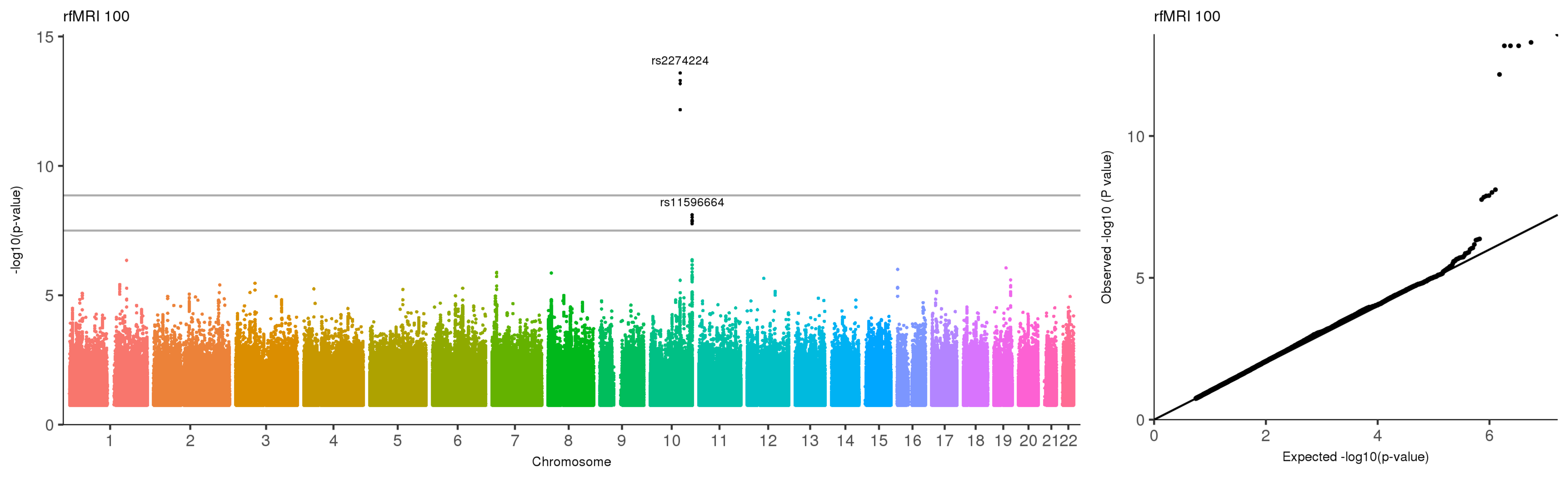
rfMRI 100
rs2274224
rs11596664
-log10(p-value)
Chromosome
rfMRI 100
Observed -log10 (P value)
Expected -log10(p-value)

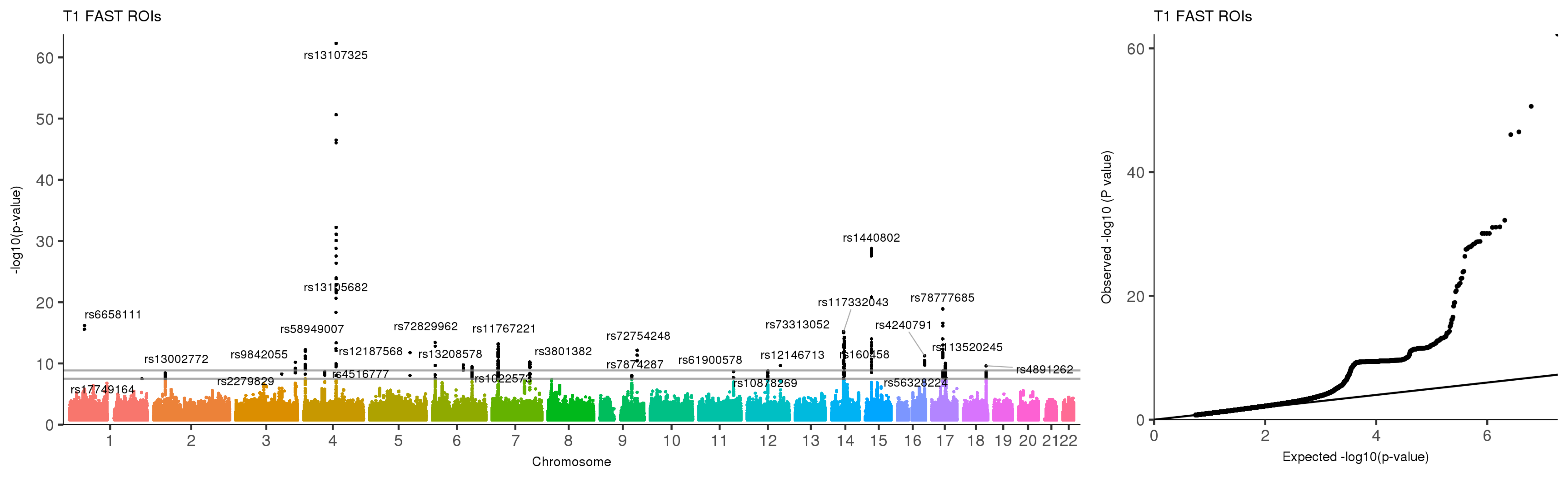

T1 FAST ROIs
-log10(p-value)
Chromosome
rs13107325
rs13105682
rs6658111
rs58949007
rs12187568
rs4516777
rs72829962
rs13208578
rs11767221
rs10225732
rs3801382
rs72754248
rs7874287
rs61900578
rs12146713
rs10878269
rs73313052
rs117332043
rs160458
rs1440802
rs4240791
rs78777685
rs113520245
rs56328224
rs4891262
rs13002772
rs9842055
rs2279829
rs17749164
T1 FAST ROIs
Observed -log10 (P value)
Expected -log10(p-value)

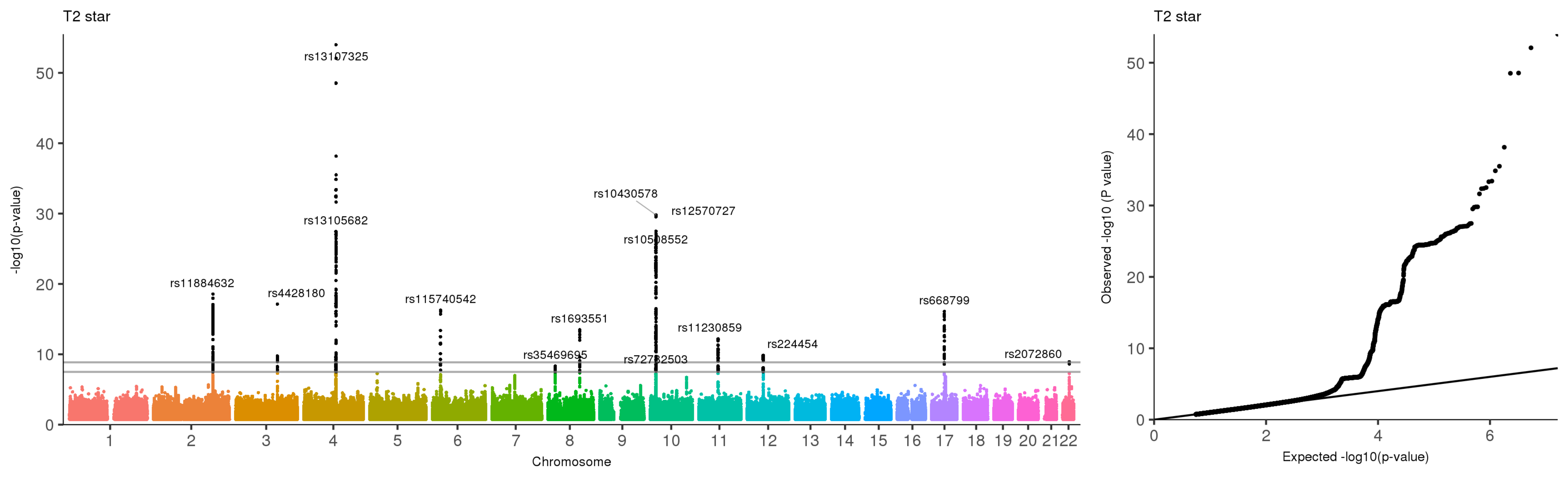
T2 star
rs13107325
rs13105682
rs11884632
rs4428180
rs115740542
rs35469695
rs1693551
rs10430578
rs12570727
rs10508552
rs72732503
rs11230859
rs224454
rs668799
rs2072860
-log10(p-value)
Chromosome
1 2 3 4 5 6 7 8 9 10 11 12 13 14 15 16 17 18 19 20 21 22
T2 star
Observed -log10 (P value)
Expected -log10(p-value)

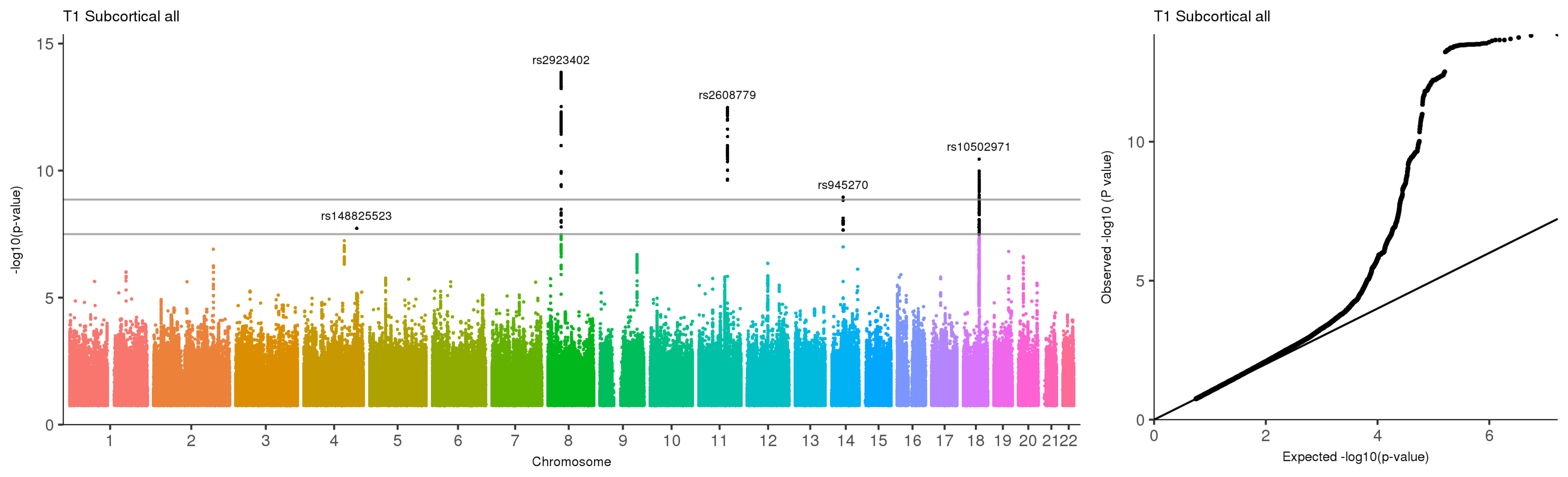

T1 Subcortical all
-log10(p-value)
Chromosome
rs2923402
rs2608779
rs10502971
rs945270
rs148825523
0
5
10
15
1
2
3
4
5
6
7
8
9
10
11
12
13
14
15
16
17
18
19
20
21
22
T1 Subcortical all
Observed -log10 (P value)
Expected -log10(p-value)
0
2
4
6
0
5
10

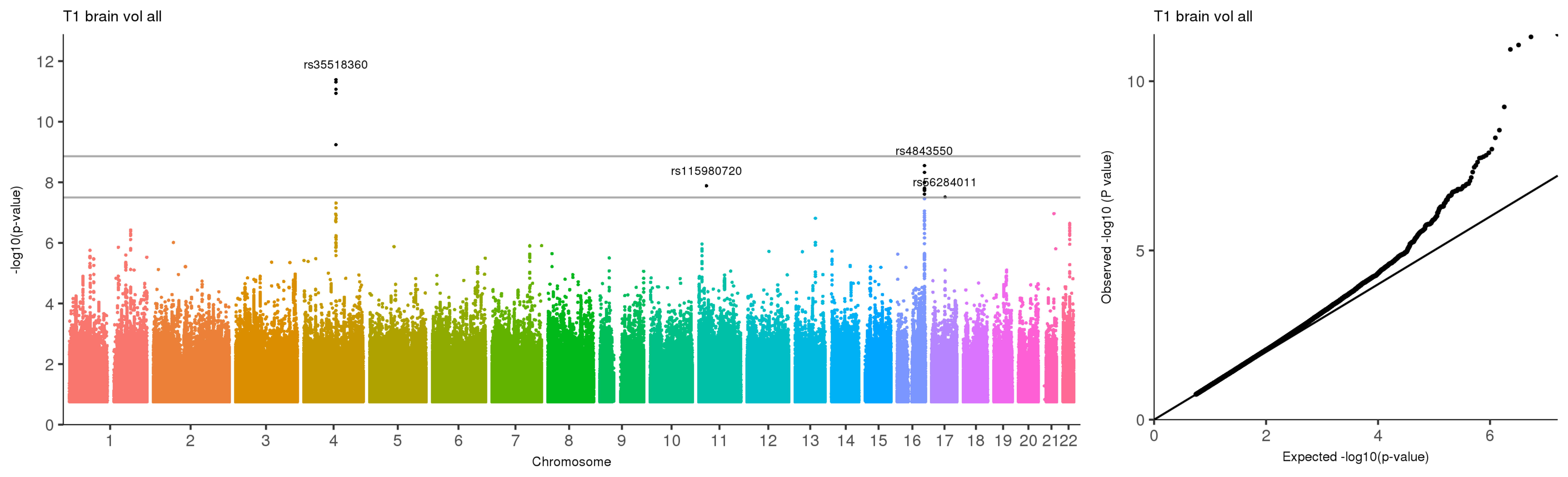
T1 brain vol all
rs35518360
rs4843550
rs115980720
rs66284011
-log10(p-value)
Chromosome
1 2 3 4 5 6 7 8 9 10 11 12 13 14 15 16 17 18 19 20 21 22
0 2 4 6 8 10 12
T1 brain vol all
Observed -log10 (P value)
Expected -log10(p-value)
0 5 10
0 2 4 6

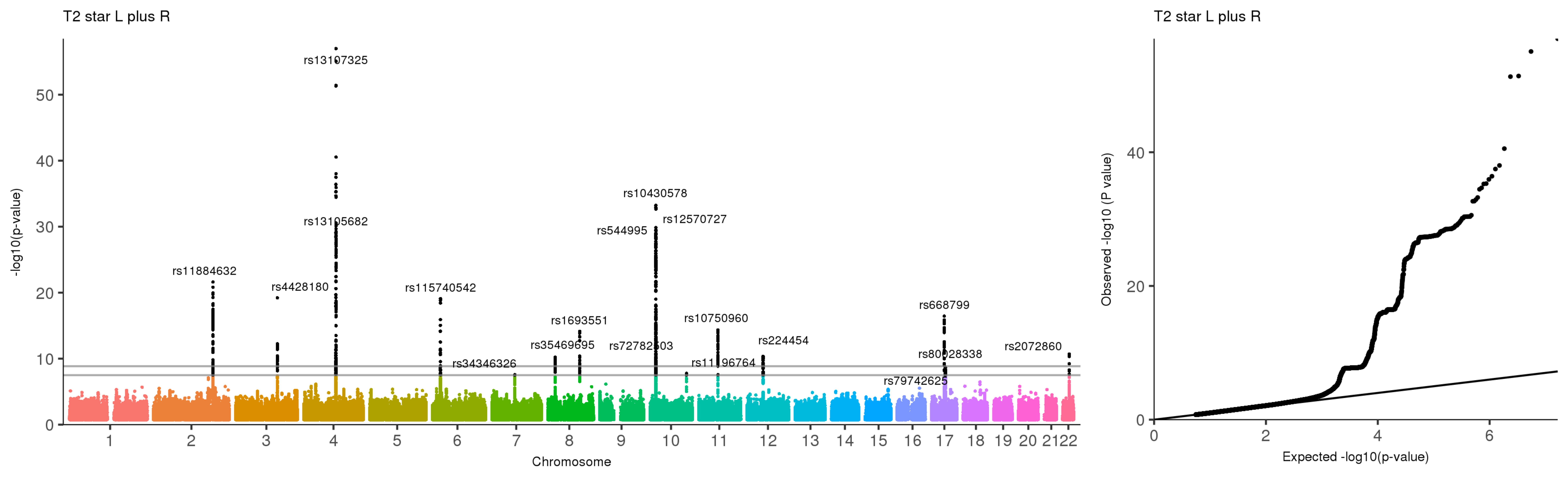
T2 star L plus R
-log10(p-value)
Chromosome
rs13107325
rs13105682
rs11884632
rs4428180
rs115740542
rs34346326
rs35469695
rs1693551
rs72782503
rs10430578
rs544995
rs12570727
rs10750960
rs11196764
rs224454
rs668799
rs80028338
rs79742625
rs2072860
T2 star L plus R
Observed -log10 (P value)
Expected -log10(p-value)

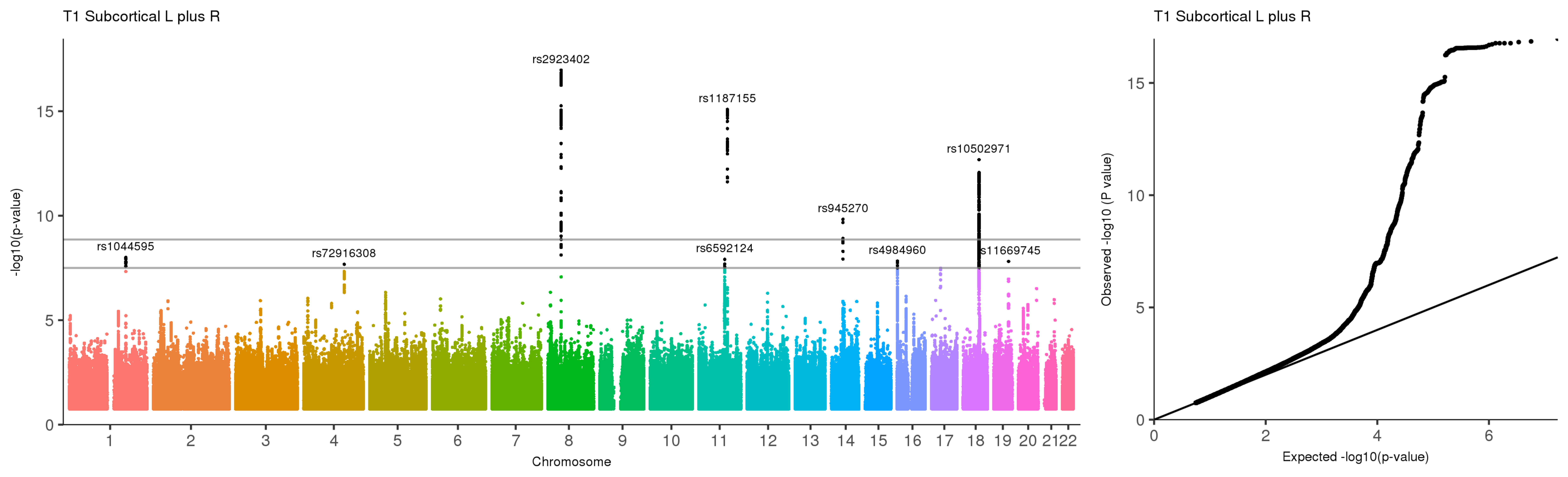
T1 Subcortical L plus R
rs2923402
rs1187155
rs10502971
rs945270
rs1044595
rs72916308
rs6592124
rs4984960
s11669745
-log10(p-value)
Chromosome
1
2
3
4
5
6
7
8
9
10
11
12
13
14
15
16
17
18
19
20
21
22
T1 Subcortical L plus R
Observed -log10 (P value)
Expected -log10(p-value)

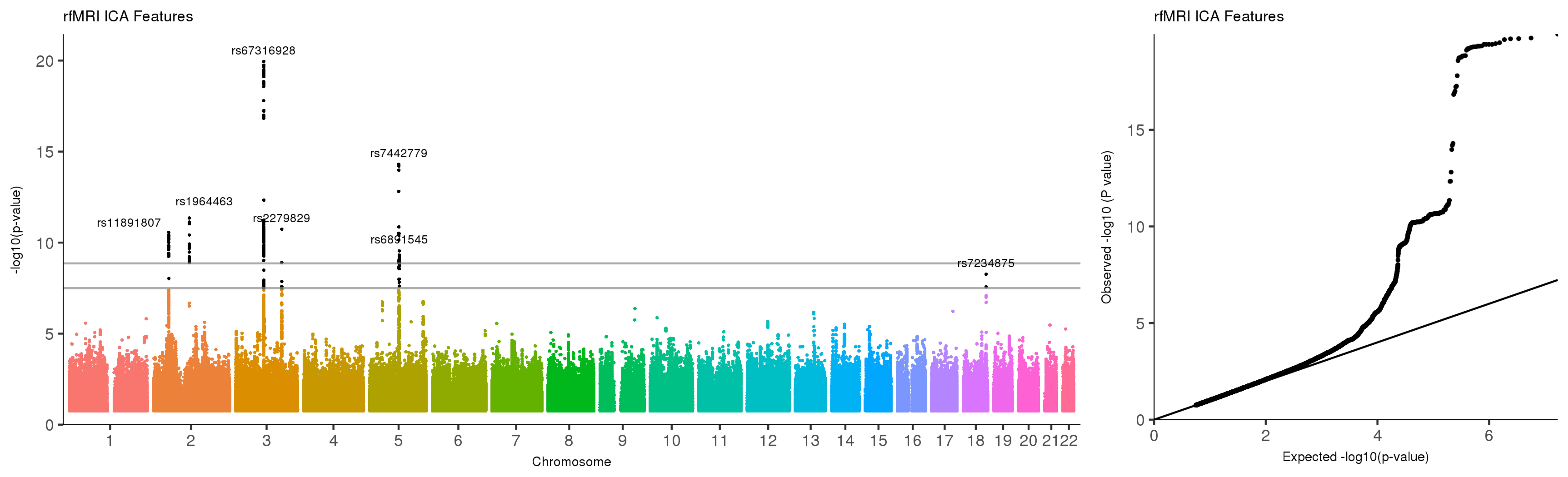
rfMRI ICA Features
rs67316928
rs7442779
rs1964463
rs11891807
rs2279829
rs6891545
rs7234875
-log10(p-value)
Chromosome
1
2
3
4
5
6
7
8
9
10
11
12
13
14
15
16
17
18
19
20
21
22
0
5
10
15
20
rfMRI ICA Features
Observed -log10 (P value)
Expected -log10(p-value)
0
2
4
6
0
5
10
15

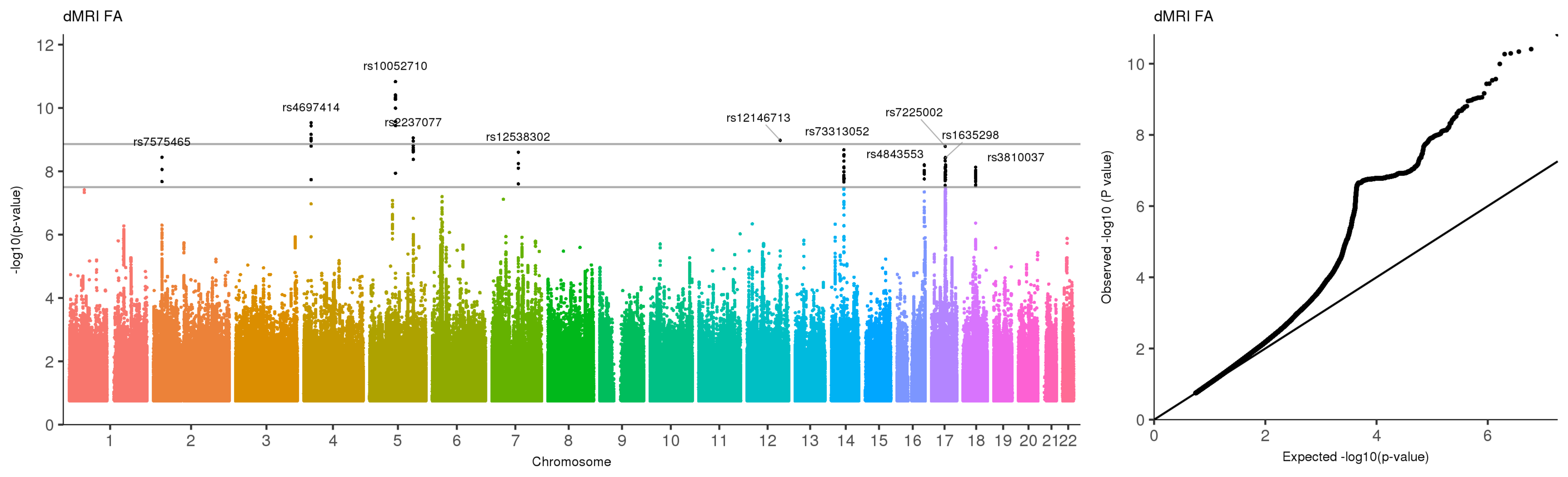
dMRI FA
rs10052710
rs4697414
rs2237077
rs7575465
rs12538302
rs12146713
rs73313052
rs7225002
rs1635298
rs4843553
rs3810037
-log10(p-value)
Chromosome
1
2
3
4
5
6
7
8
9
10
11
12
13
14
15
16
17
18
19
20
21
22
dMRI FA
Observed -log10 (P value)
Expected -log10(p-value)

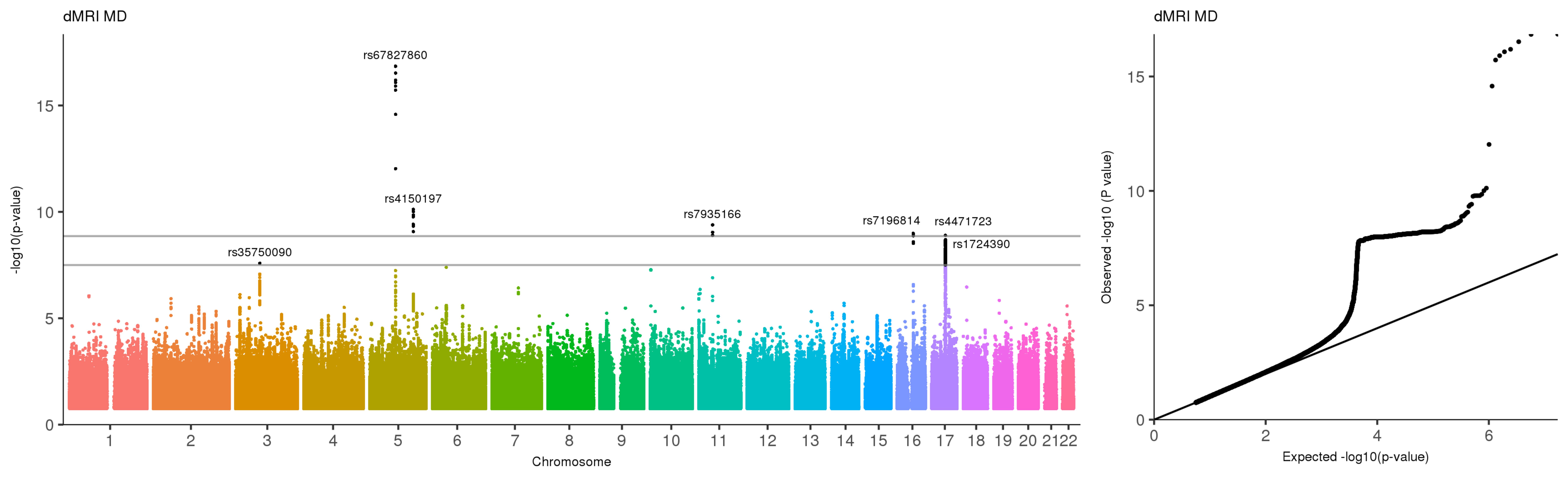
dMRI MD
rs67827860
rs4150197
rs7935166
rs7196814
rs4471723
rs1724390
rs35750090
-log10(p-value)
Chromosome
dMRI MD
Observed -log10 (P value)
Expected -log10(p-value)

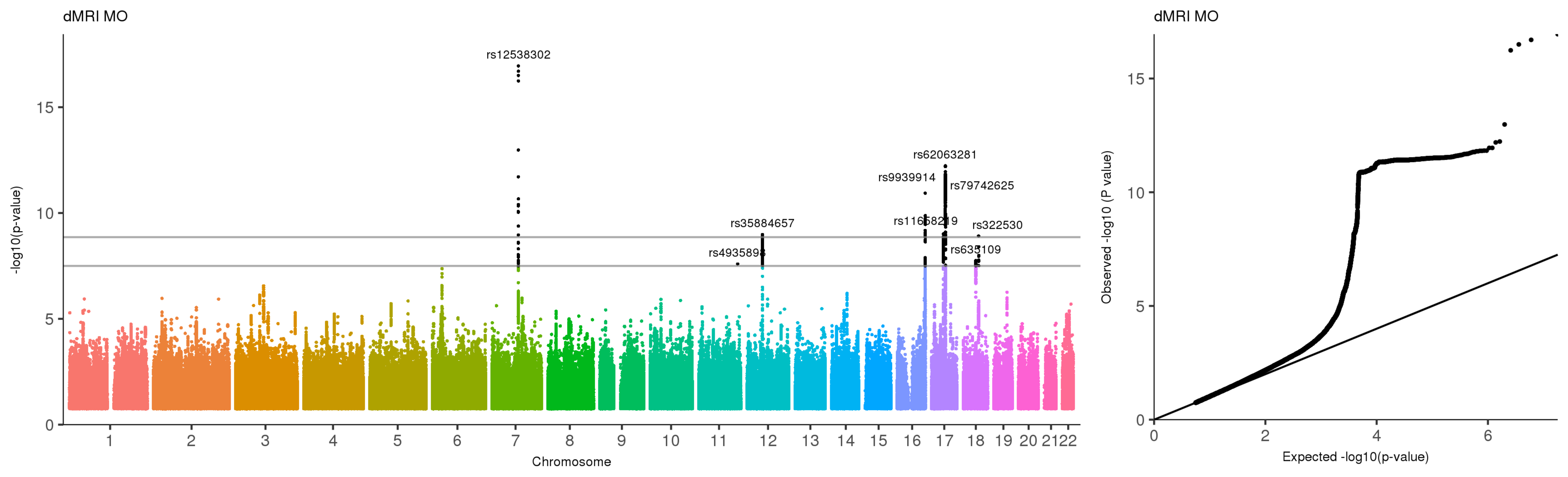
dMRI MO
rs12538302
rs62063281
rs9939914
rs79742625
rs35884657
rs11658219
rs322530
rs4935898
rs635109
-log10(p-value)
0
5
10
15
1
2
3
4
5
6
7
8
9
10
11
12
13
14
15
16
17
18
19
20
21
22
Chromosome
dMRI MO
Observed -log10 (P value)
Expected -log10(p-value)
0
2
4
6
0
5
10
15

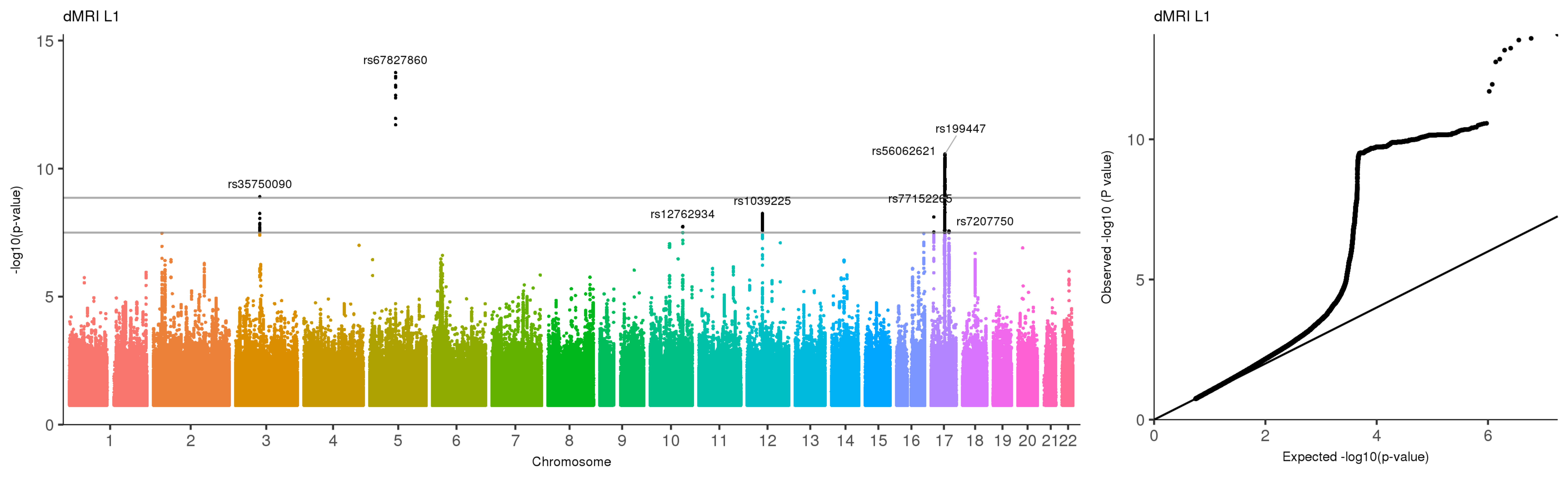
dMRI L1
rs67827860
rs35750090
rs12762934
rs1039225
rs56062621
rs199447
rs77152265
rs7207750
-log10(p-value)
Chromosome
1
2
3
4
5
6
7
8
9
10
11
12
13
14
15
16
17
18
19
20
21
22
0
5
10
15
dMRI L1
Observed -log10 (P value)
Expected -log10(p-value)
0
2
4
6
0
5
10

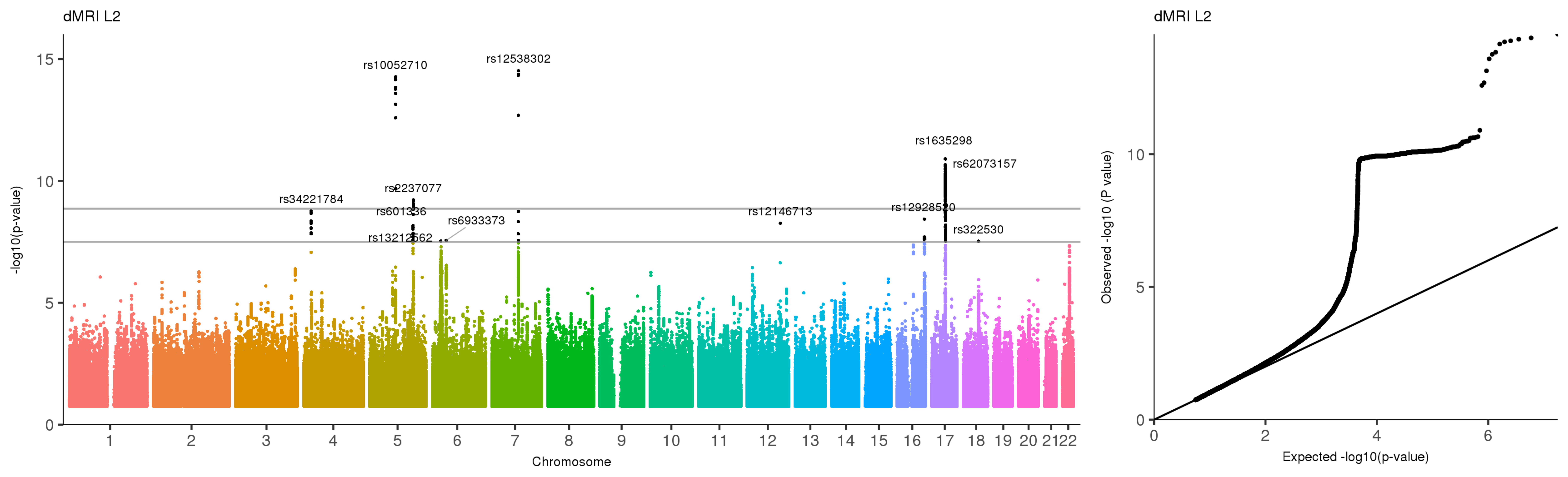

dMRI L2
rs10052710
rs12538302
rs1635298
rs62073157
rs2237077
rs34221784
rs601336
rs6933373
rs12146713
rs12928520
rs322530
rs13212562
-log10(p-value)
Chromosome
dMRI L2
Observed -log10 (P value)
Expected -log10(p-value)

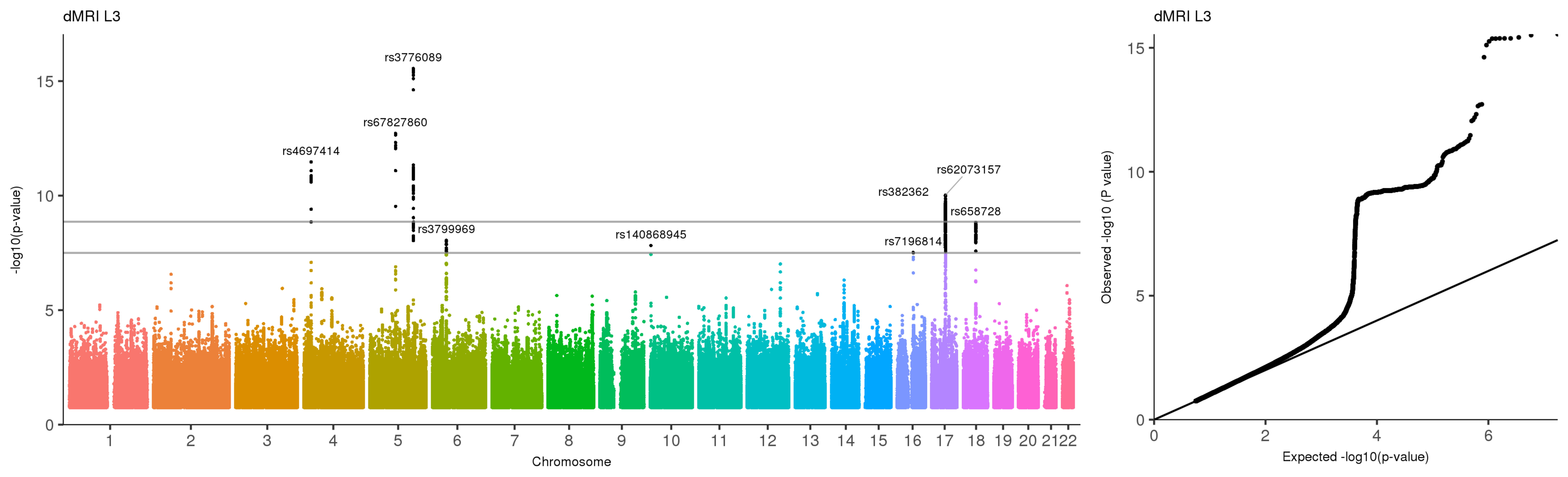
dMRI L3
rs3776089
rs67827860
rs4697414
rs3799969
rs140868945
rs62073157
rs382362
rs658728
rs7196814
-log10(p-value)
Chromosome
1 2 3 4 5 6 7 8 9 10 11 12 13 14 15 16 17 18 19 20 21 22
0 5 10 15
dMRI L3
Observed -log10 (P value)
Expected -log10(p-value)
0 2 4 6
0 5 10 15

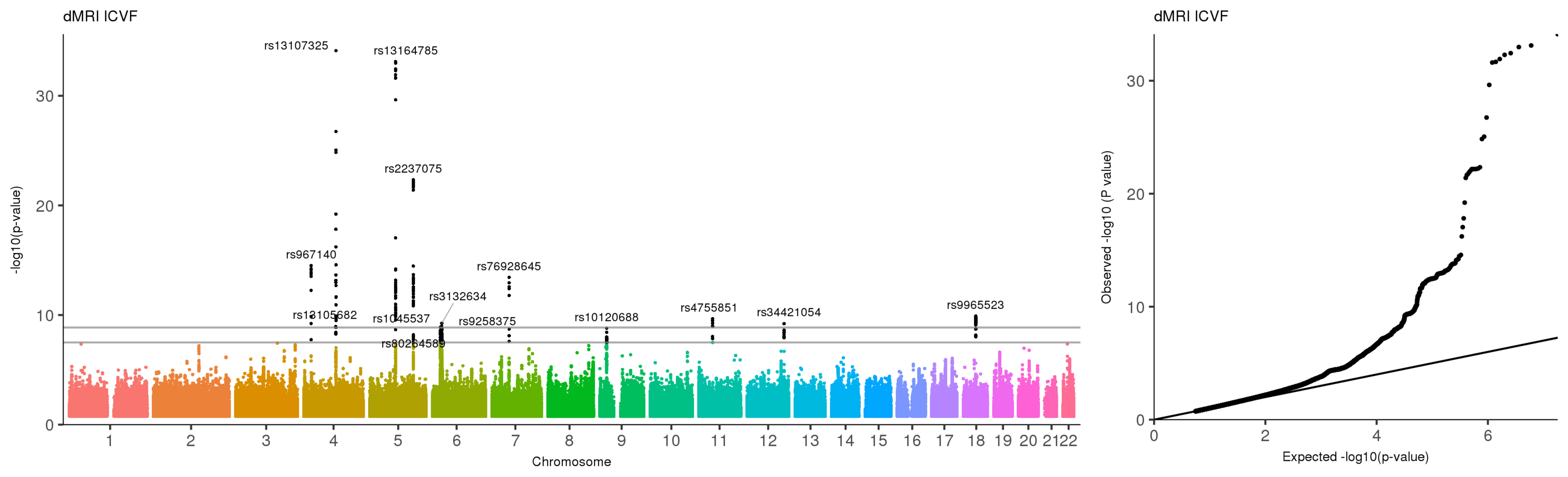
dMRI ICVF
-log10(p-value)
rs13107325
rs13164785
rs2237075
rs967140
rs76928645
rs3132634
rs13105682
rs1045537
rs9258375
rs10120688
rs4755851
rs34421054
rs9965523
rs80264589
Chromosome
dMRI ICVF
Observed -log10 (P value)
Expected -log10(p-value)

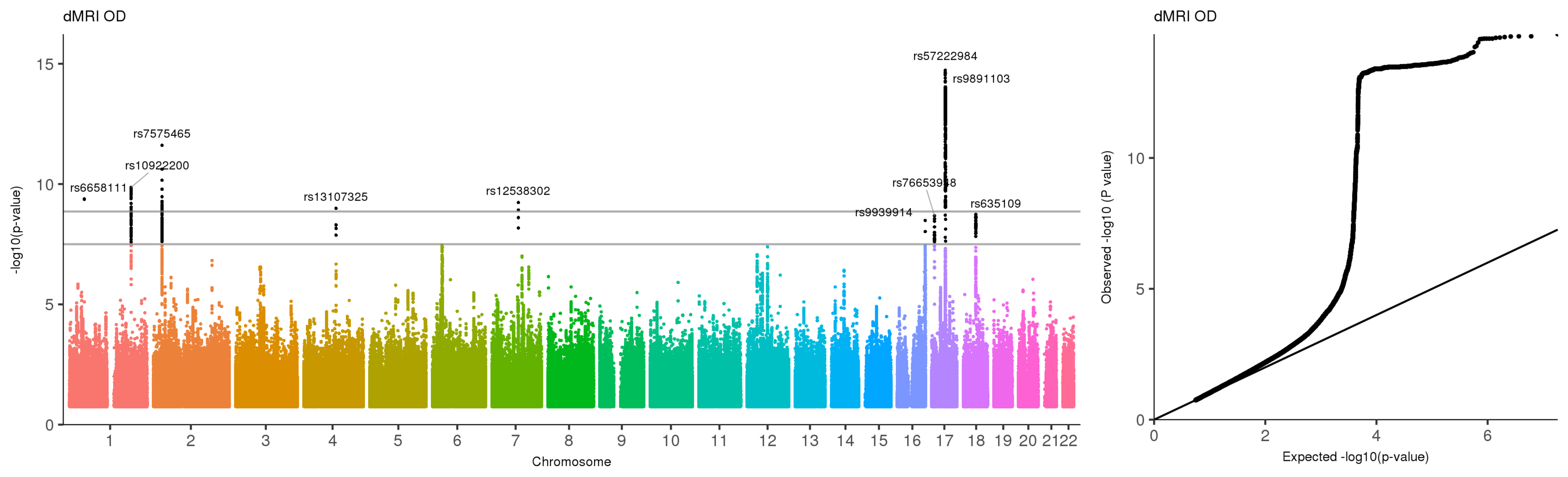
dMRI OD
rs57222984
rs9891103
rs7575465
rs10922200
rs6658111
rs13107325
rs12538302
rs76653948
rs9939914
rs635109
-log10(p-value)
Chromosome
dMRI OD
Observed -log10 (P value)
Expected -log10(p-value)

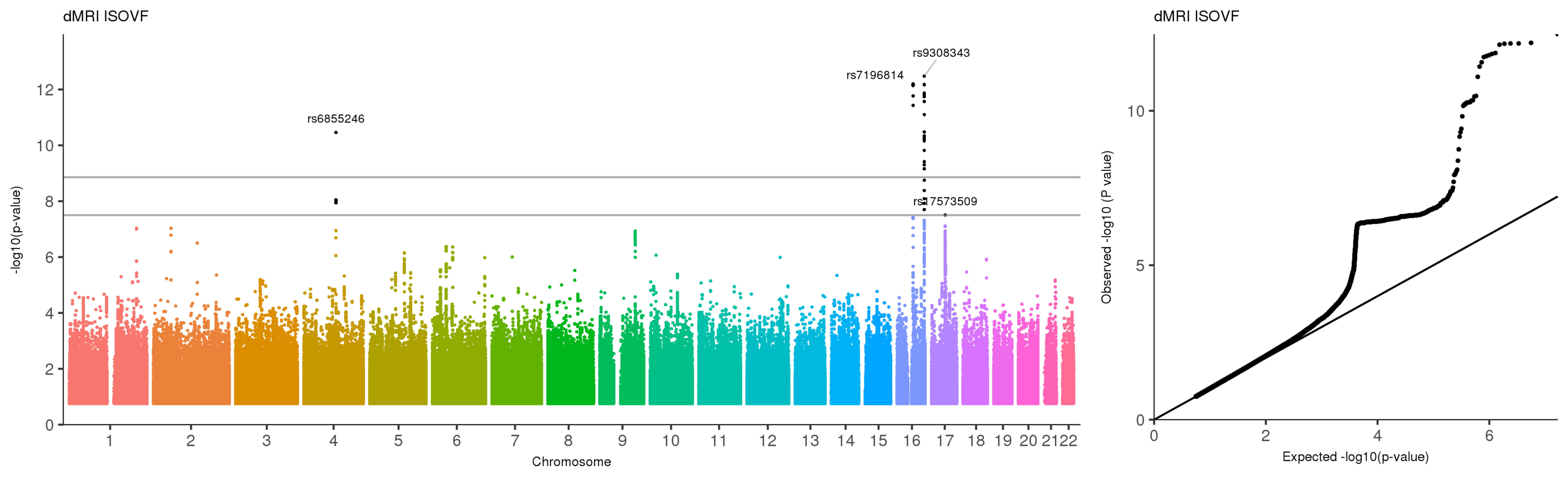

dMRI ISOVF
rs9308343
rs7196814
rs6855246
rs17573509
-log10(p-value)
Chromosome
1 2 3 4 5 6 7 8 9 10 11 12 13 14 15 16 17 18 19 20 21 22
dMRI ISOVF
Observed -log10 (P value)
Expected -log10(p-value)

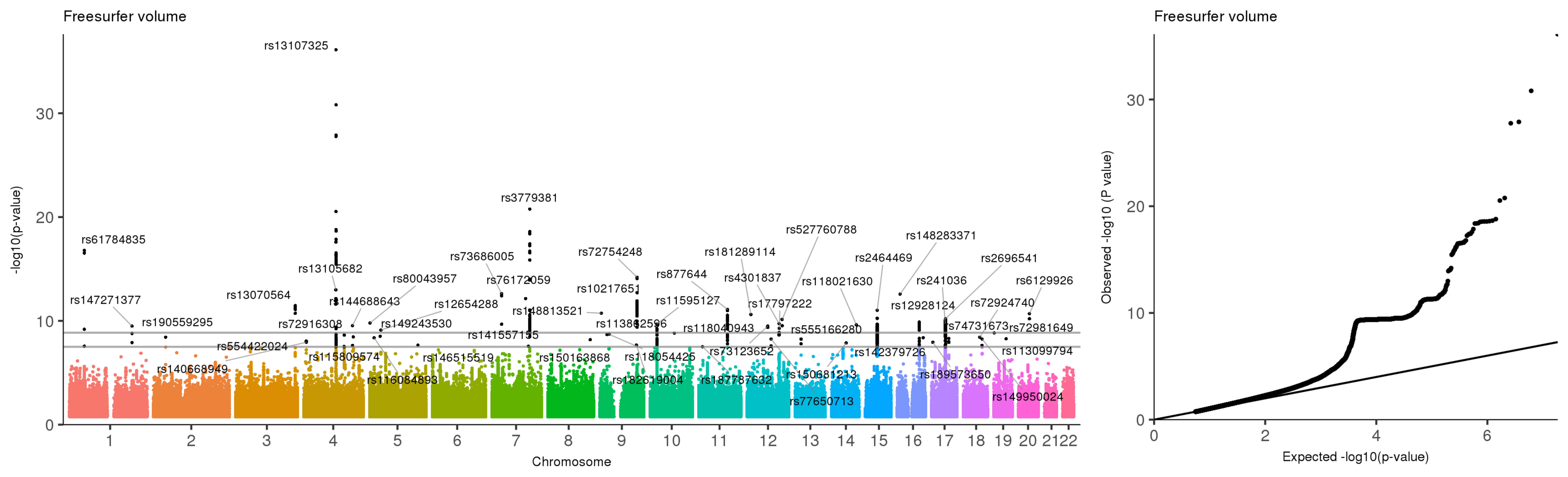
Freesurfer volume
-log10(p-value)
Chromosome
rs13107325
rs61784835
rs147271377
rs190559295
rs13070564
rs13105682
rs144688643
rs72916308
rs554422024
rs140668949
rs115809574
rs116084893
rs80043957
rs149243530
rs12654288
rs146515519
rs73686005
rs76172059
rs3779381
rs141557155
rs148813521
rs150163868
rs72754248
rs10217651
rs113882596
rs118054425
rs182619004
rs877644
rs11595127
rs118040943
rs73123652
rs187787632
rs181289114
rs4301837
rs17797222
rs527760788
rs118021630
rs555166280
rs150681213
rs77650713
rs2464469
rs142379726
rs148283371
rs12928124
rs241036
rs189573650
rs2696541
rs74731673
rs72924740
rs6129926
rs72981649
rs113099794
rs149950024
Observed -log10 (P value)
Expected -log10(p-value)

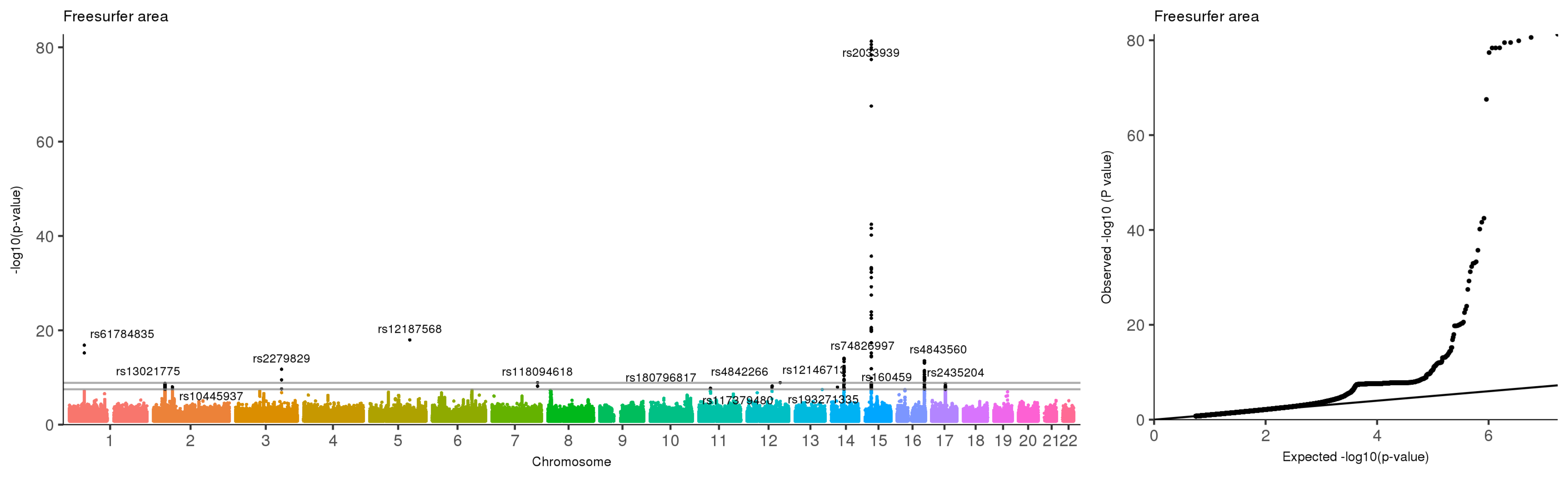
Freesurfer area
-log10(p-value)
Chromosome
rs61784835
rs13021775
rs10445937
rs2279829
rs12187568
rs118094618
rs180796817
rs4842266
rs117379480
rs12146713
rs193271335
rs74826997
rs2033939
rs160459
rs4843560
rs2435204
Freesurfer area
Observed -log10 (P value)
Expected -log10(p-value)

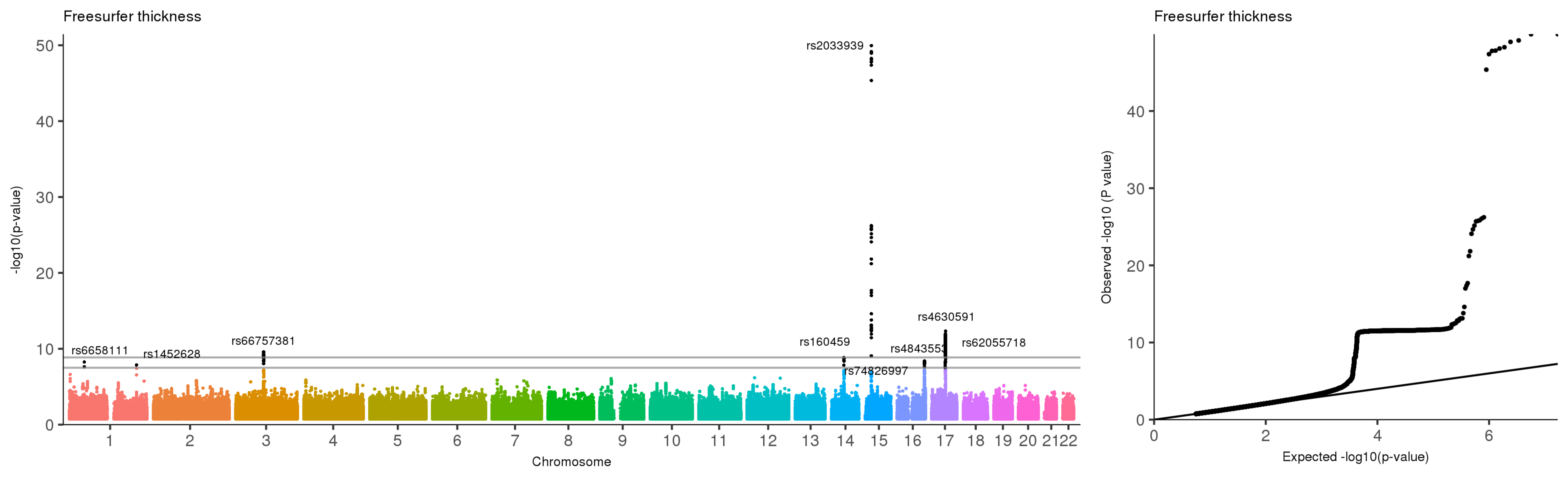
Freesurfer thickness
-log10(p-value)
Chromosome
rs2033939
rs4630591
rs66757381
rs6658111
rs1452628
rs160459
rs48435
rs62055718
rs74826997
Freesurfer thickness
Observed -log10 (P value)
Expected -log10(p-value)
