# Supplementary material for: Genome-wide association studies of brain imaging phenotypes in UK Biobank
Source: Nature. 2018 Oct 10;562(7726):210–6. doi: 10.1038/s41586-018-0571-7 (PMC6786974; doi:10.1038/s41586-018-0571-7)

Supplementary Figure 13 : Comparison of –log10 p values obtained from multi-trait and single IDP association tests. The two figures below compare -$\log_{10}$ p values for associations identified by one method with the maximum “comparable” -$\log_{10}$ p values obtained by the other method. **Left hand figure:** each point corresponds to an association between an IDP group and a SNP with multi-trait -$\log_{10}$ p value > 7.5 as detailed in supplementary table 8. Points are colour coded according to the MAF of the lead SNP for the multi-trait association as indicated in the legend at the top of the figure. Multi-trait -$\log_{10}$ p values are plotted on the horizontal axis. The vertical axis corresponds to the maximum -$\log_{10}$ p value obtained for an association between any single IDP in that group and any SNP within a flanking region of $\pm$ 250Kb of the lead SNP for the multi-trait association. **Right hand figure:** each point represents an association between a single IDP and a SNP with single IDP -$\log_{10}$ p value > 7.5 as detailed in supplementary table 5. Where the same SNP was the lead SNP for associations with multiple IDPs in the same group, only the strongest of these associations (maximum -$\log_{10}$ p value) is plotted. Points are colour coded according to the MAF of this SNP. The vertical axis corresponds to the single IDP -$\log_{10}$ p values. For each point, the horizontal axis corresponds to the maximum -$\log_{10}$ p value obtained for an association between the group to which the IDP belongs and any SNP within a flanking region of $\pm$ 250Kb around the lead SNP for the single IDP association.

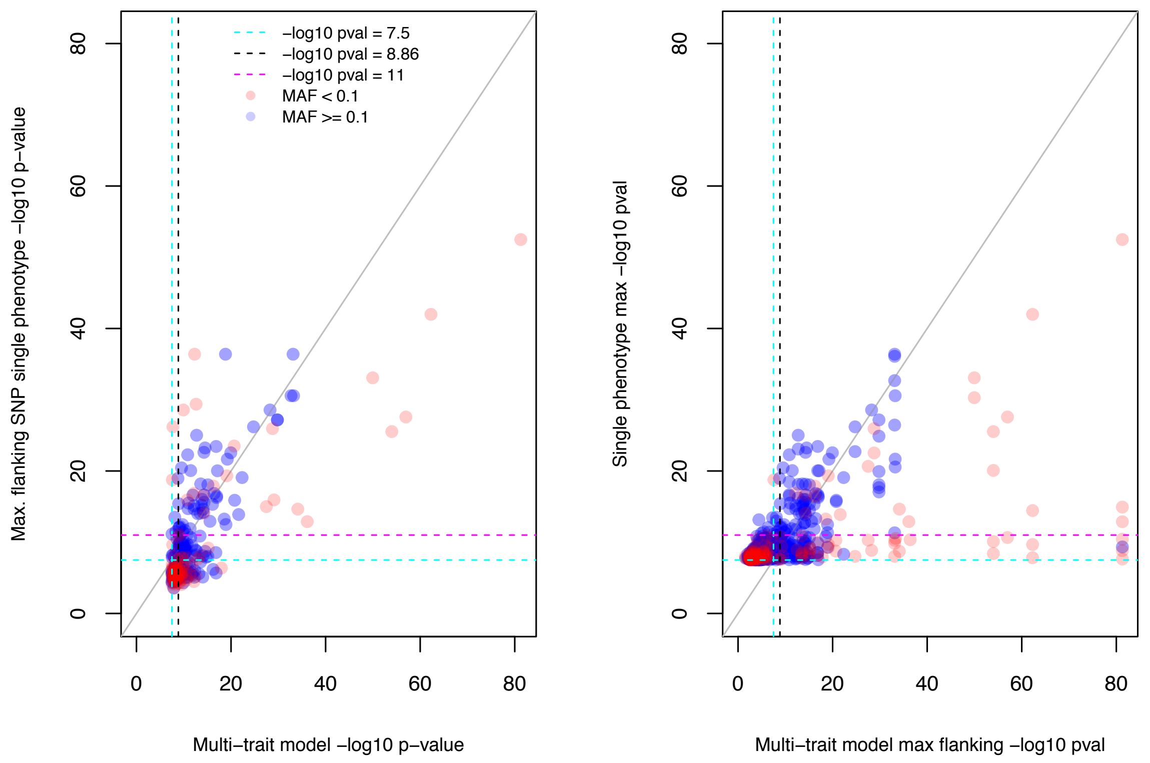

Supplement: Supplementary file 3 — This file contains Supplementary Figures S1-S22. [file 41586_2018_571_MOESM3_ESM.zip › Figure-S13.pdf]
