# Supplementary material for: Genome-wide association studies of brain imaging phenotypes in UK Biobank
Source: Nature. 2018 Oct 10;562(7726):210–6. doi: 10.1038/s41586-018-0571-7 (PMC6786974; doi:10.1038/s41586-018-0571-7)
Supplement: Supplementary file 3 — This file contains Supplementary Figures S1-S22. [file 41586_2018_571_MOESM3_ESM.zip › Figure-S14.pdf]

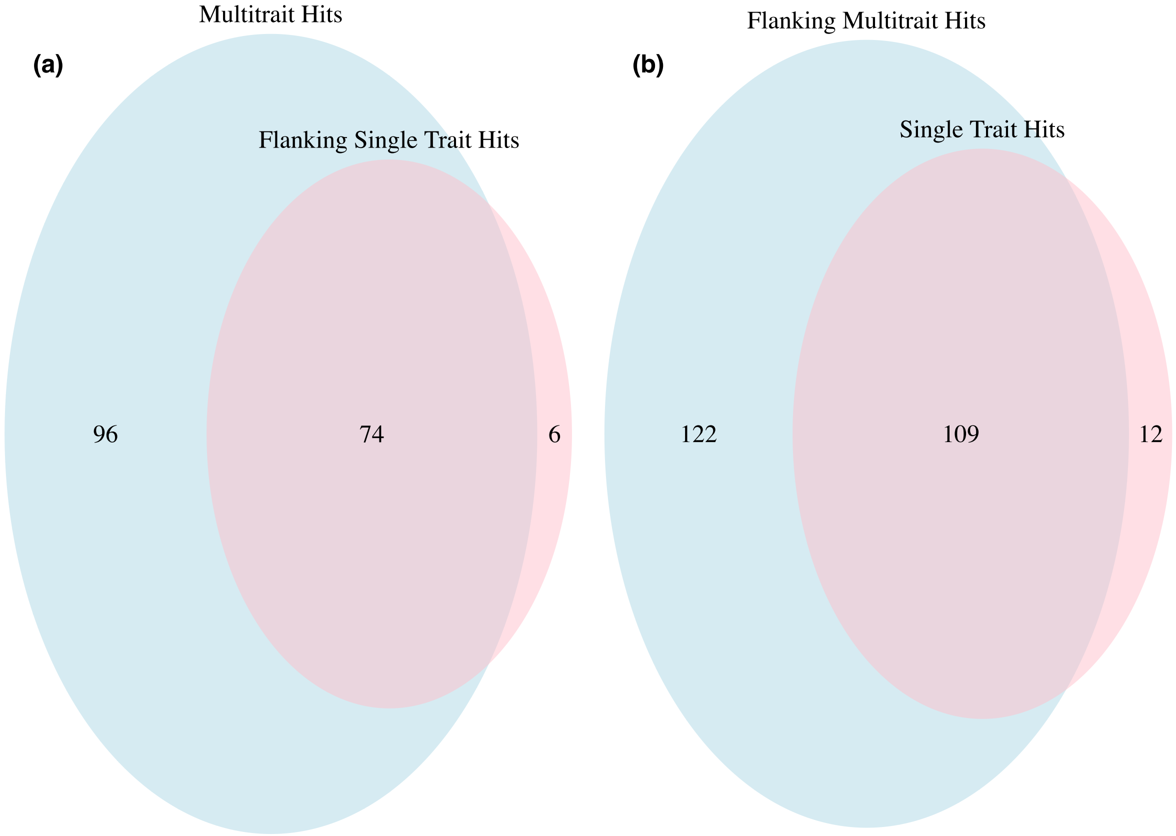

Supplementary Figure 14: Overlap of significantly associated loci obtained from multi-trait and single IDP association tests. The two figures above compare associations identified by one method with those indicated by the maximum "comparable" -log10 p values obtained by the other method. In the left hand **figure (a)**, the full set is chosen from associations for which *the lead SNP at the locus showed a multi-trait association* with -log10 p value > 7.5 (as detailed in Supplementary Table 8). The strength of the corresponding single phenotype association at each locus with any IDP in the relevant group was taken to be the maximum within a flanking region of ± 250Kb of the lead SNP of multi-trait association. The Venn diagram shows the relationship between two subsets of these associations: those with -log10 p value > 8.86 for multi-trait association and those with -log10 p value > 11 for a single phenotype association. Of the 170 significant multi-trait associations, 138 replicated at nominal significance in our larger replication cohort (Supplementary Table 8). While 96 of the significant multi-trait associations are not significant under single phenotype tests, 71 of these replicated in our larger replication analysis (Supplementary Table 8). Furthermore, 25 of these loci with replicated associations show no genome-wide significance (-log10 p value < 7.5) in the flanking region for any individual IDP in the corresponding IDP group (Supplementary Table 9).

In the right hand **figure (b)**, the full set is chosen from loci for which an association between *a single IDP and a SNP* had -log10 p value > 7.5 (as detailed in Supplementary Table 5). Where the same SNP was the lead SNP for associations with multiple IDPs in the same group, only the strongest of these associations (maximum -log10 p value) was considered. The corresponding multi-trait associations in this case correspond to the maximum -log10 p value obtained for an association between the group to which the appropriate IDP belongs and any SNP within a flanking region of ±250Kb around the lead SNP for the single IDP association. As in **figure (a),** the Venn diagram shows the relationship between two subsets of these associations: Multi-trait Hits are those with -log10 p value > 8.86. Single Trait Hits

are those with -log10 p value > 11.
