# Supplementary material for: Genome-wide association studies of brain imaging phenotypes in UK Biobank
Source: Nature. 2018 Oct 10;562(7726):210–6. doi: 10.1038/s41586-018-0571-7 (PMC6786974; doi:10.1038/s41586-018-0571-7)

Supplementary Figure 15 : **Genetic correlation QQ-plots between IDPs and 9 brain related conditions**. Quantile/quantile plots for genetic correlation p-values for 1,451 IDPs with each of 10 disease, personality or brain related conditions (see **Supplementary Table 10**). For each trait, the – log10 p-values for each of the 1,451 IDPs (y-axis) are plotted against their expected value (x-axis). Only IDPs with heritability z-score > 4 were included, and all netmat edge IDPs were excluded due to low levels of heritability. Expected p-values were calculated via simulation based on the raw correlation matrix of the IDPs.

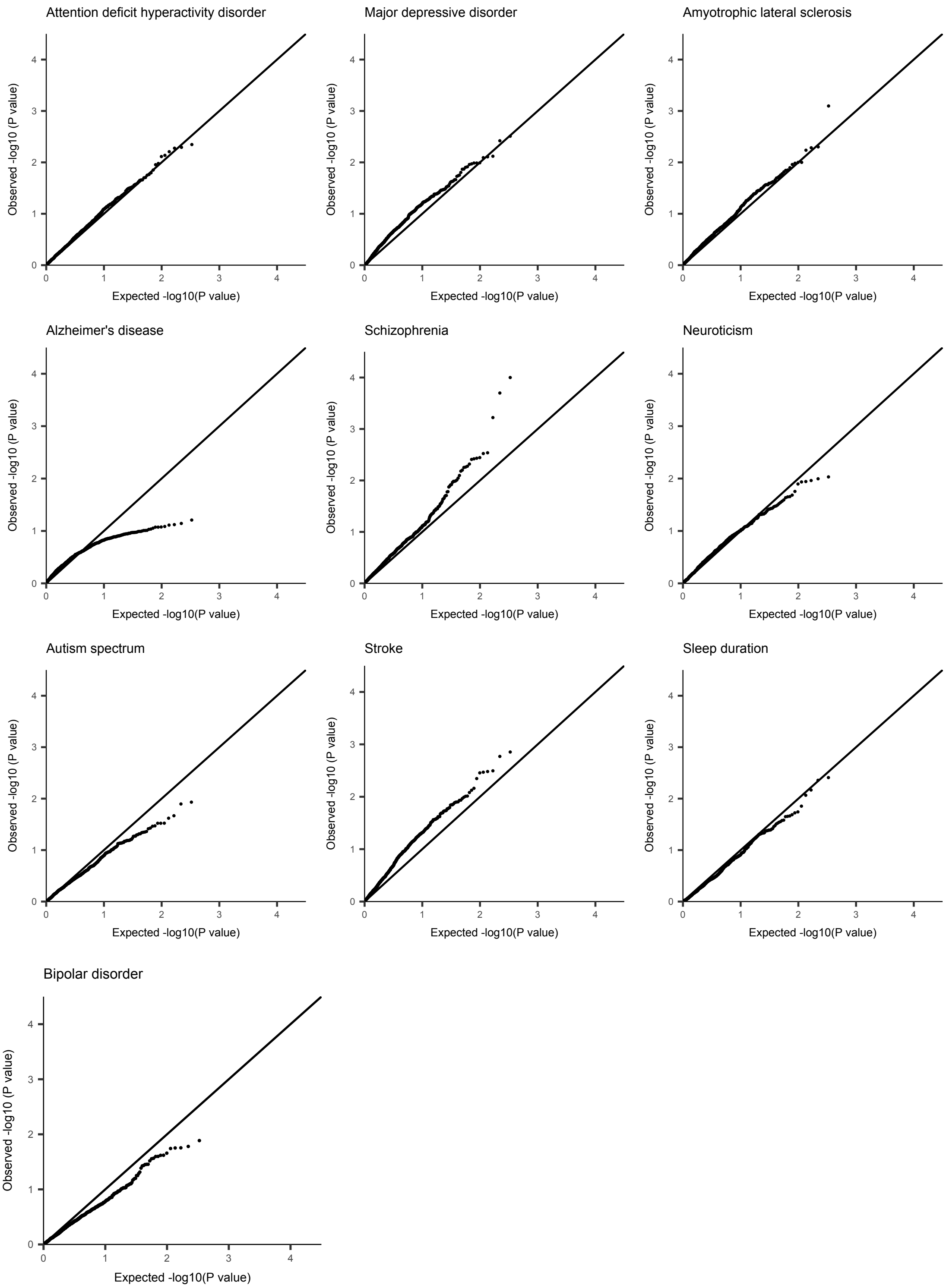

Supplement: Supplementary file 3 — This file contains Supplementary Figures S1-S22. [file 41586_2018_571_MOESM3_ESM.zip › Figure-S15.pdf]
