# Supplementary material for: Genome-wide association studies of brain imaging phenotypes in UK Biobank
Source: Nature. 2018 Oct 10;562(7726):210–6. doi: 10.1038/s41586-018-0571-7 (PMC6786974; doi:10.1038/s41586-018-0571-7)

Supplementary Figure 16: Partitioning of heritability by functional category. This figure is a companion to **Figure 6** of the main text. The figure shows the proportion of IDPs in each of the 23 IDP groupings (x-axis) that show a nominal *depletion* p-value < 0.05 (two-sided tests, uncorrected p-values, see Methods) for the 24 functional categories (y-axis). The total number of such IDPs for each category is given on the right hand edge of the plot. The number of IDPs in each IDP group is listed in brackets in the x-axis labels. The proportion of the genome annotated by each functional category is listed in brackets in the y-axis labels.

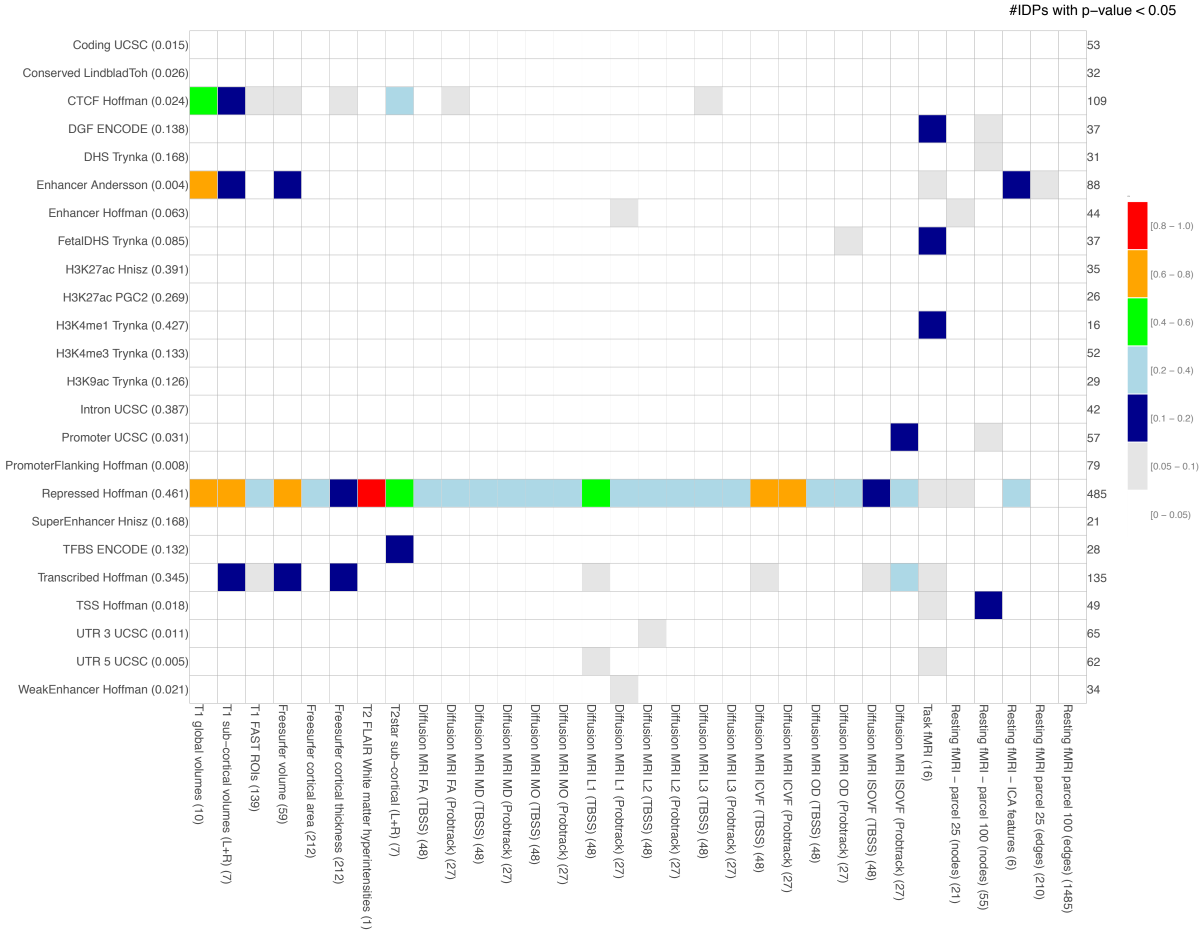

Supplement: Supplementary file 3 — This file contains Supplementary Figures S1-S22. [file 41586_2018_571_MOESM3_ESM.zip › Figure-S16.pdf]
