# Supplementary material for: Genome-wide association studies of brain imaging phenotypes in UK Biobank
Source: Nature. 2018 Oct 10;562(7726):210–6. doi: 10.1038/s41586-018-0571-7 (PMC6786974; doi:10.1038/s41586-018-0571-7)

Supplementary Figure 18

Functional connectivity ICA features

The methodology for generating these new features (in effect new IDPs) is described in the main text Methods. Feeding into ICA all functional connectivity networks from both group-ICA dimensionalities and from all subjects resulted in 6 split-half-reproducible components – the 6 new connectivity features.

Below we show these 6 features, displaying the 24 network connections (edges) with the highest ICA weights from each group-ICA dimensionality (level of functional brain parcellation).

Each network edge is depicted by summary images for a given pair of functional nodes; the network edge is the connectivity between these two nodes. The population average connection is indicated by the bar connecting the two nodes forming the connection (red indicates positive mean correlation, blue negative, and the width of the bar indicates the connection strength). These are partial correlation edge weights, and it is not expected that any triplet of connections (between any triplet of nodes) would need to all have the same sign. The coloured number is the (signed) weight for a given network edge in the corresponding ICA component; the higher the (absolute) value, the more an edge is involved in a given component.

Figure S18.1. Functional connectivity ICA feature #1.
Top: Functional edges and ICA weights from the d=25 group-ICA parcellation.
Bottom: Functional edges and ICA weights from the d=100 group-ICA parcellation.

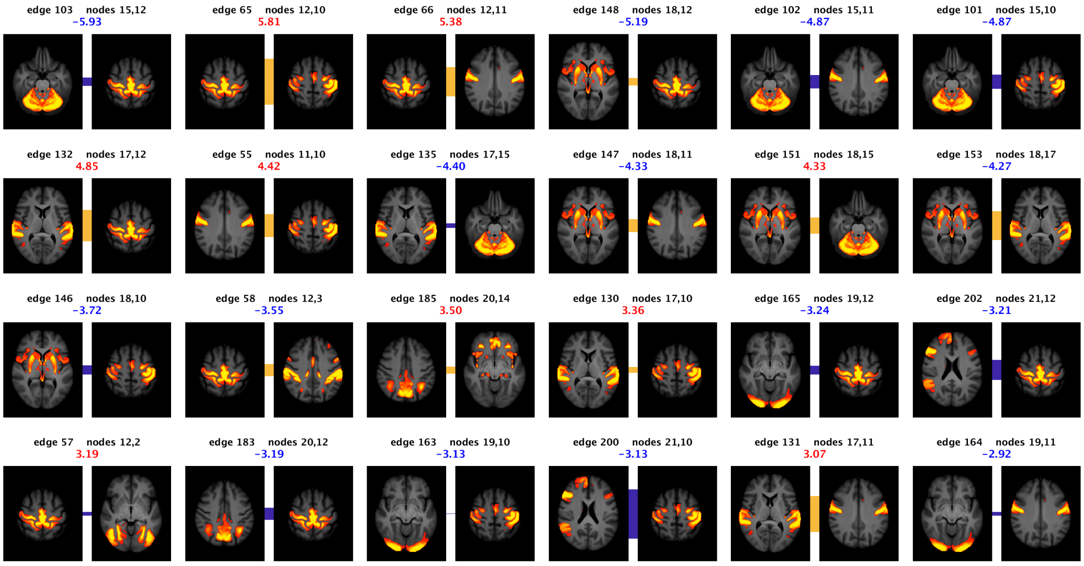

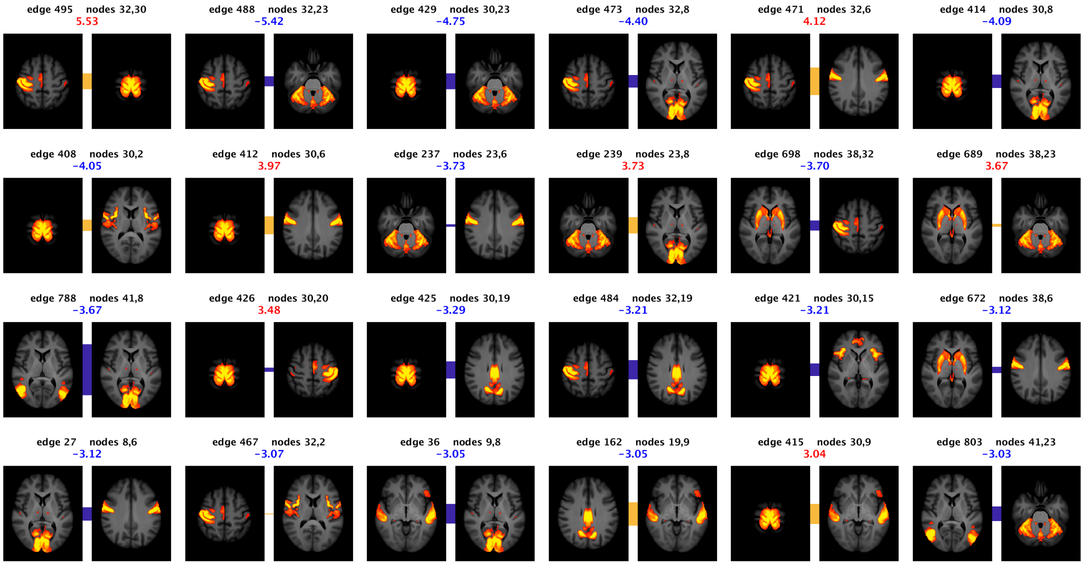

Figure S18.2. Functional connectivity ICA feature #2.
Top: Functional edges and ICA weights from the d=25 group-ICA parcellation.
Bottom: Functional edges and ICA weights from the d=100 group-ICA parcellation.

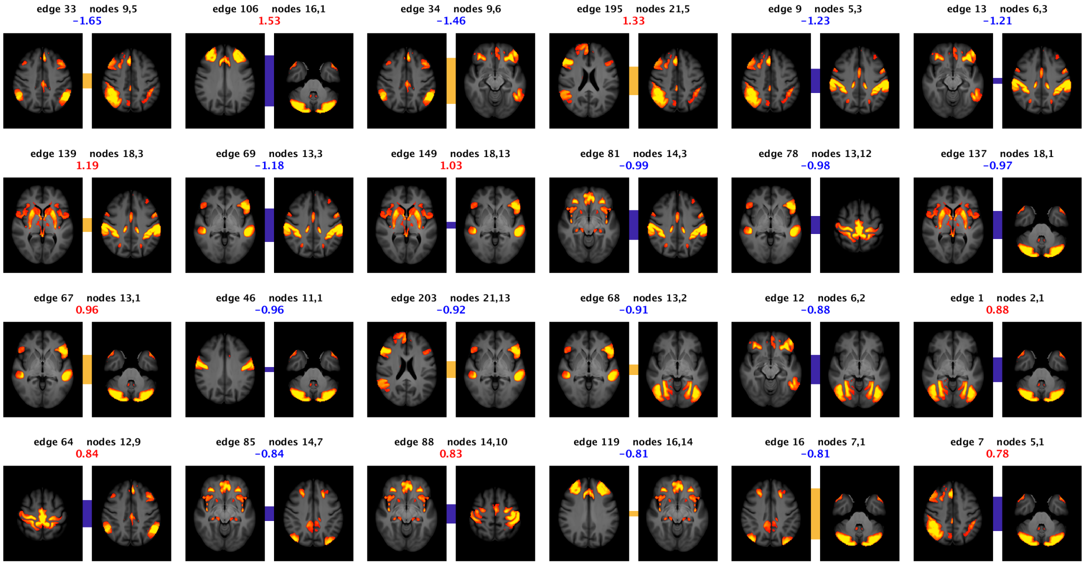

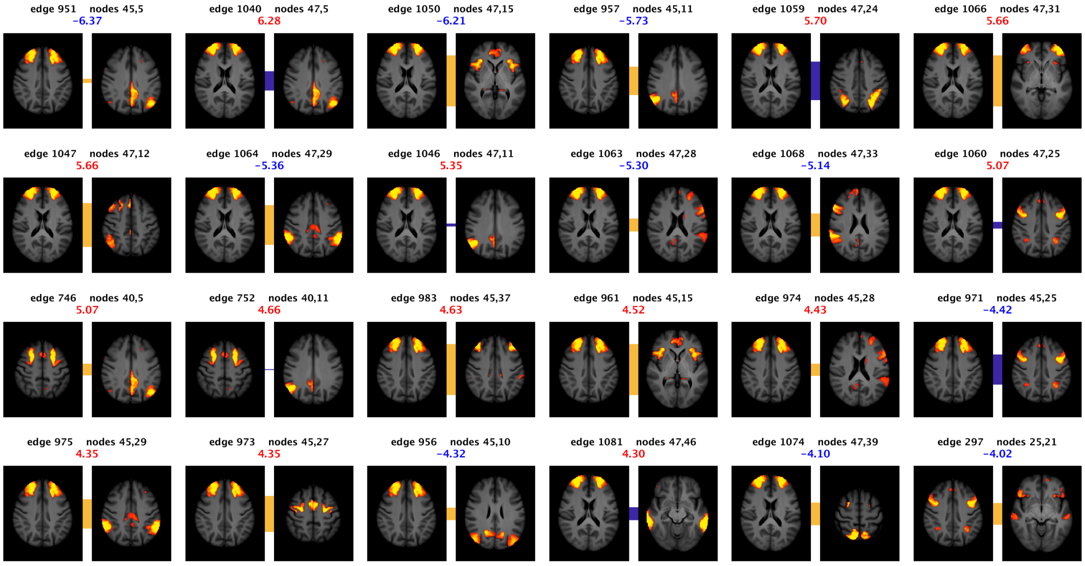

Figure S18.3. Functional connectivity ICA feature #3.
Top: Functional edges and ICA weights from the d=25 group-ICA parcellation.
Bottom: Functional edges and ICA weights from the d=100 group-ICA parcellation.

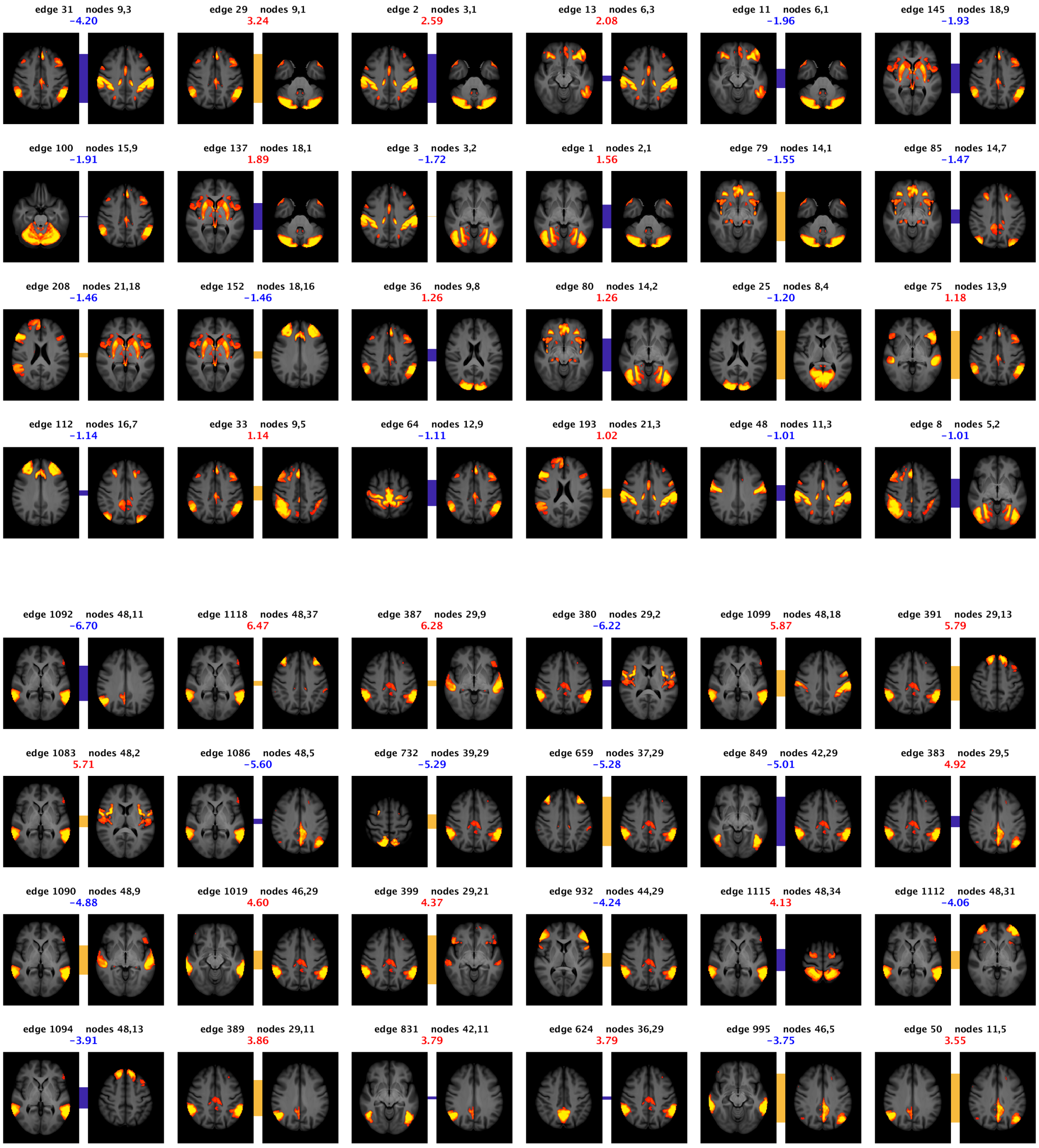

Figure S18.4. Functional connectivity ICA feature #4.
Top: Functional edges and ICA weights from the d=25 group-ICA parcellation.
Bottom: Functional edges and ICA weights from the d=100 group-ICA parcellation.

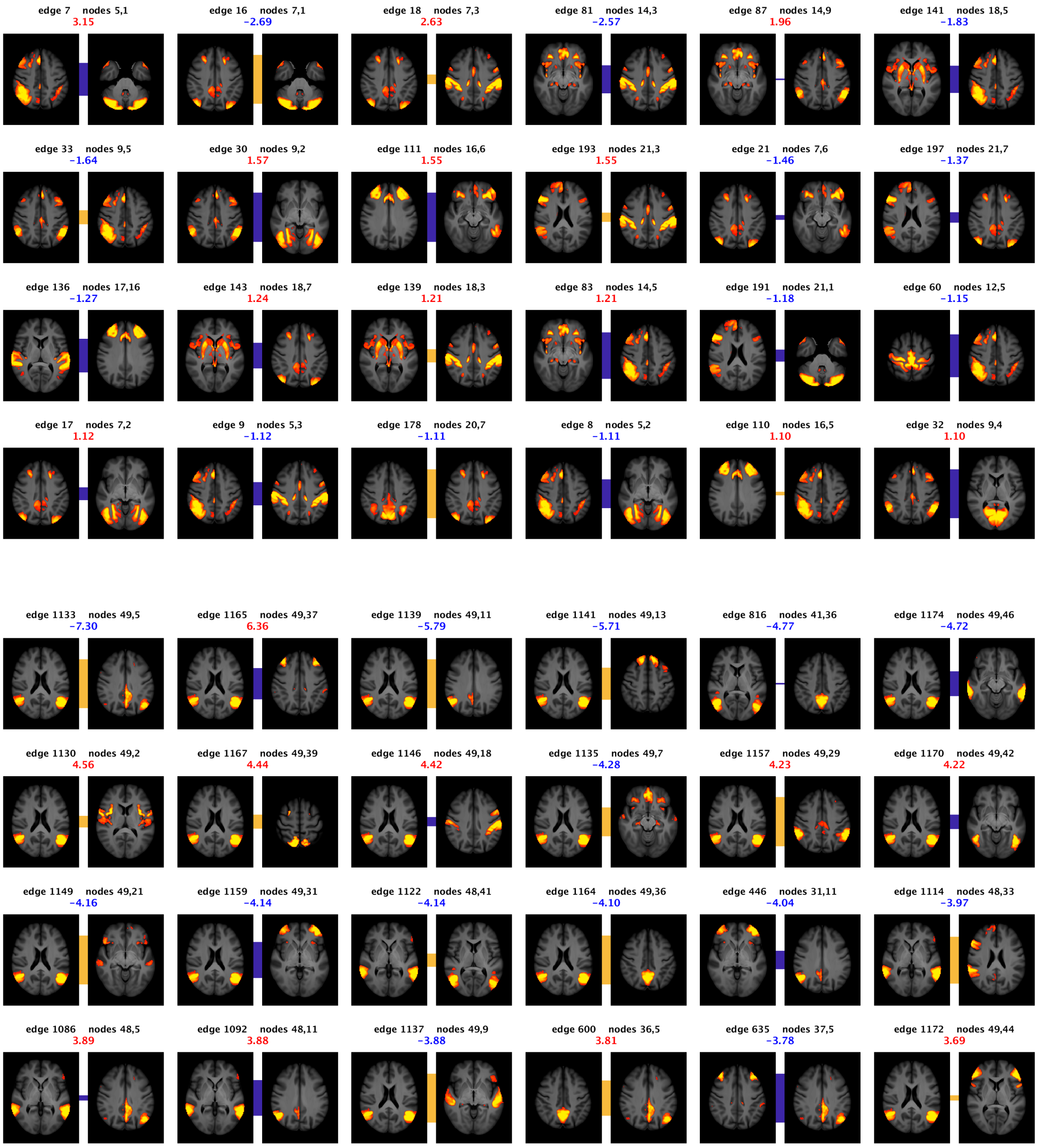

Figure S18.5. Functional connectivity ICA feature #5.
Top: Functional edges and ICA weights from the d=25 group-ICA parcellation.
Bottom: Functional edges and ICA weights from the d=100 group-ICA parcellation.

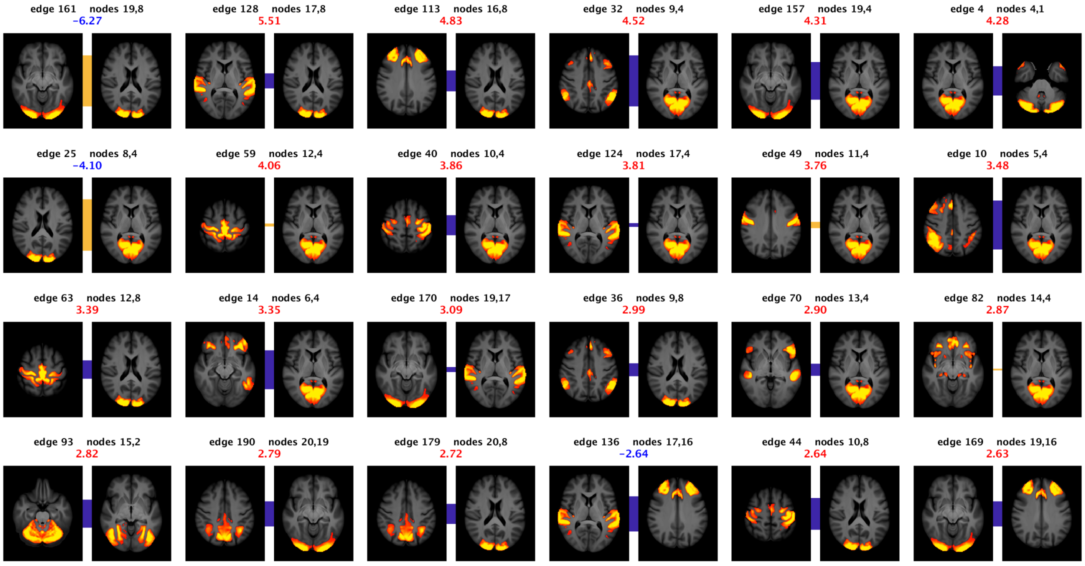

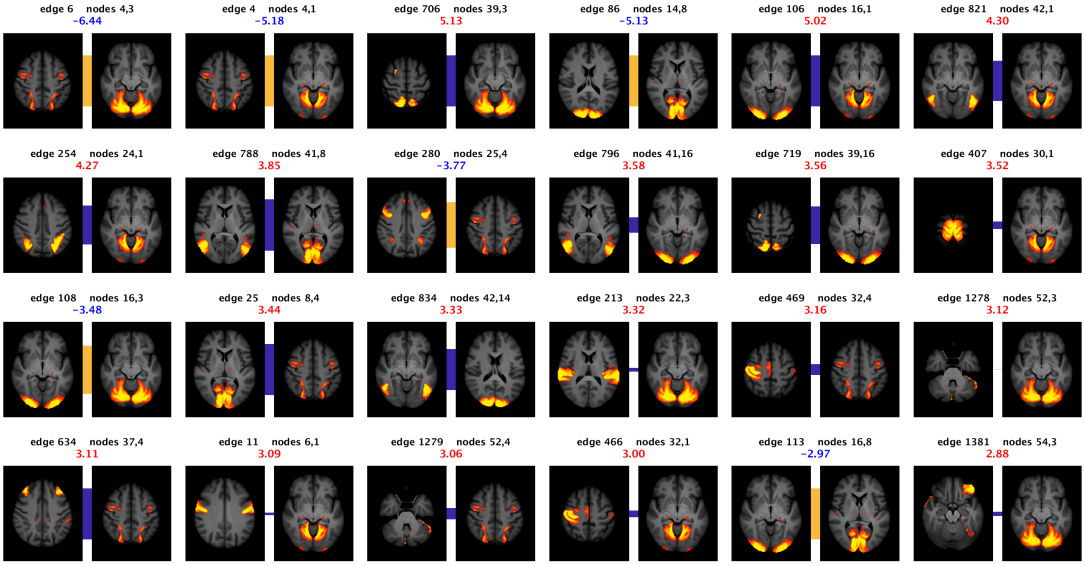

Figure S18.6. Functional connectivity ICA feature #6.
Top: Functional edges and ICA weights from the d=25 group-ICA parcellation.
Bottom: Functional edges and ICA weights from the d=100 group-ICA parcellation.

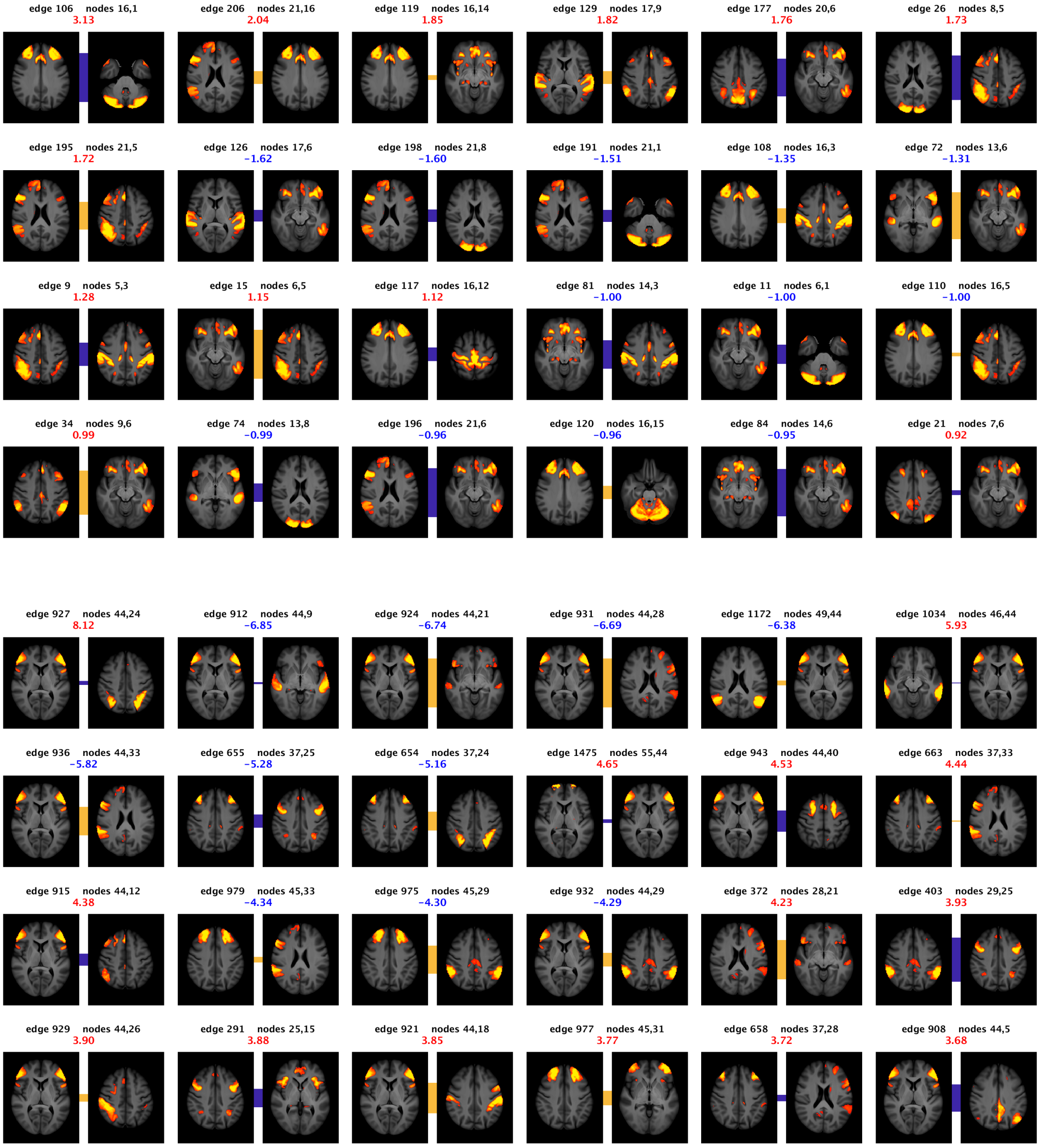

Supplement: Supplementary file 3 — This file contains Supplementary Figures S1-S22. [file 41586_2018_571_MOESM3_ESM.zip › Figure-S18.pdf]
