# Supplementary material for: Genome-wide association studies of brain imaging phenotypes in UK Biobank
Source: Nature. 2018 Oct 10;562(7726):210–6. doi: 10.1038/s41586-018-0571-7 (PMC6786974; doi:10.1038/s41586-018-0571-7)
Supplement: Supplementary file 3 — This file contains Supplementary Figures S1-S22. [file 41586_2018_571_MOESM3_ESM.zip › Figure-S19.pdf]

Supplementary Figure 19

<u>Non-Gaussian IDP distributions</u>

In order to investigate the extent to which some IDPs' distributions were non-Gaussian, we estimated the skewness and kurtosis for all IDPs, and show the 10 most extreme examples. Before these calculations, for each IDP, highly extreme outliers were removed (greater than 15 times median absolute deviation from the median), and all IDPs were then normalised to zero mean and unit standard deviation. Skewness and kurtosis were then estimated for all IDPs. Kurtosis was found to be highly correlated to the magnitude of the skew (r=0.88), so we show distributions of IDPs selected on the basis of having the most strongly positive and negative skewness.

	**skew**	**kurt**	**IDP name**
1	-2.3	9.3	dMRI TBSS MO Fornix
2	-2.3	10.3	dMRI TBSS MO Superior fronto-occipital fasciculus R
3	-2.1	8.7	dMRI TBSS MO Superior fronto-occipital fasciculus L
4	-2.0	8.8	dMRI TBSS MO Tapetum R
5	-1.8	8.3	dMRI TBSS MO Tapetum L
6	2.1	8.7	dMRI TBSS L2 Superior fronto-occipital fasciculus L
7	2.2	7.3	dMRI TBSS OD Fornix
8	2.2	8.4	T2 FLAIR BIANCA WMH volume
9	2.3	10.7	T1 FAST ROIs R pallidum
10	2.4	10.9	T1 FAST ROIs L pallidum

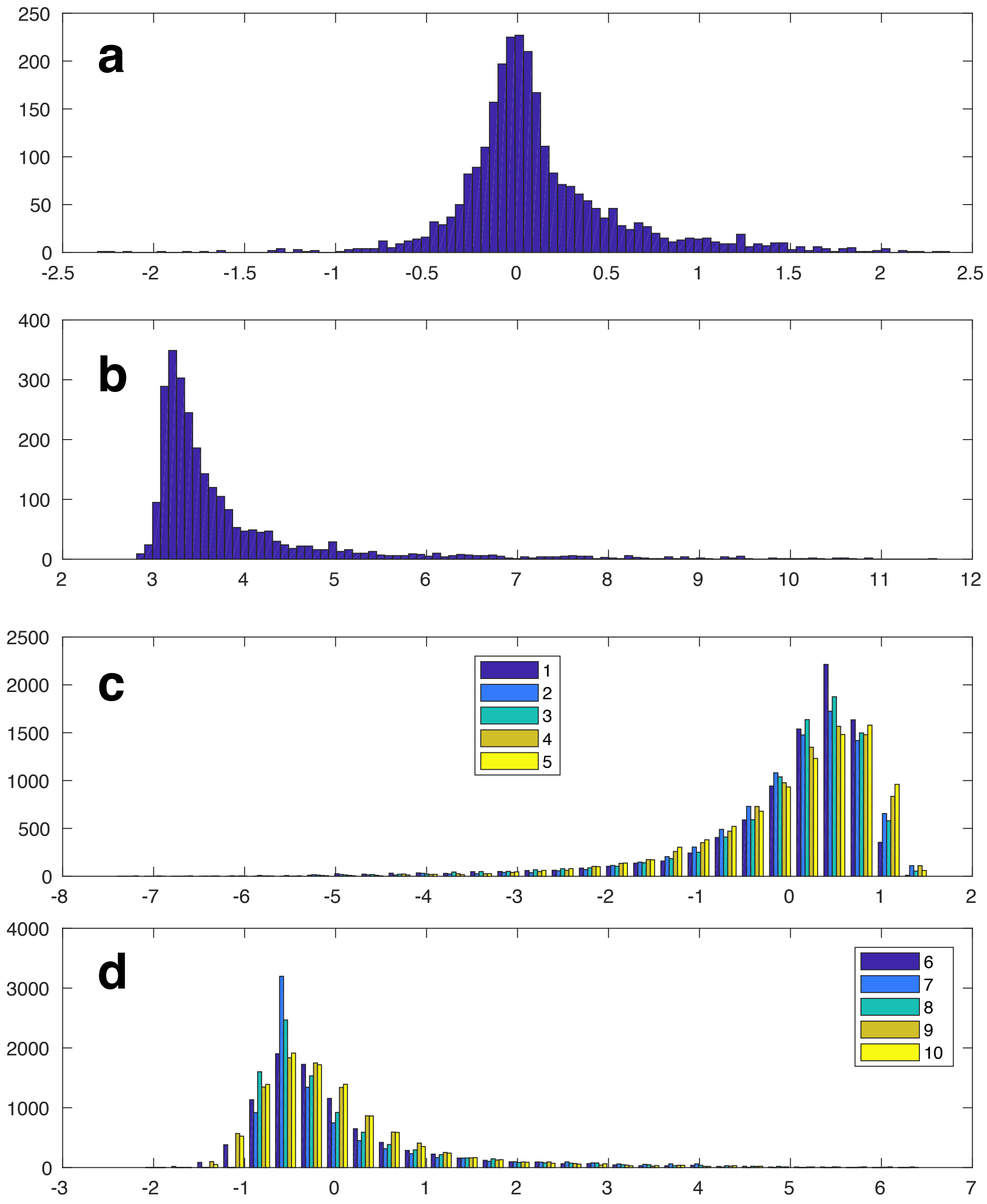

Supplementary Figure 19. Summary plots of the distributions of IDPs. a. Summary of skewness in the IDPs. For each IDP, its skewness is estimated, and the histogram shows the distribution of this across all IDPs. **b.** Summary of kurtosis in the IDPs. **c.** Distribution of all IDP values for the 5 IDPs with the most strongly negative skewness; the histograms are across all subjects. **d.** Distribution of IDP values for the 5 IDPs with the most strongly positive skewness.
