# Supplementary material for: Genome-wide association studies of brain imaging phenotypes in UK Biobank
Source: Nature. 2018 Oct 10;562(7726):210–6. doi: 10.1038/s41586-018-0571-7 (PMC6786974; doi:10.1038/s41586-018-0571-7)
Supplement: Supplementary file 3 — This file contains Supplementary Figures S1-S22. [file 41586_2018_571_MOESM3_ESM.zip › Figure-S20.pdf]

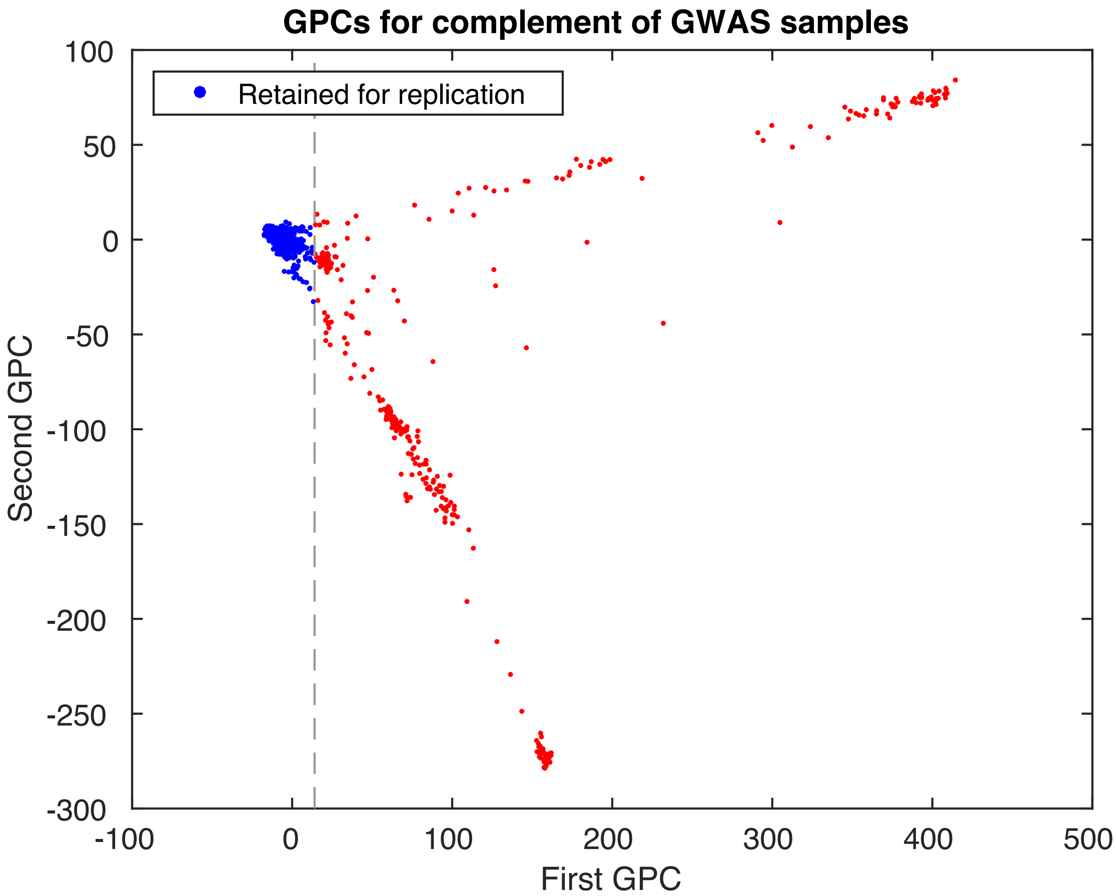

Supplementary Figure 20 : Identification of replication samples. The plot shows the 1st and 2nd genetic data principal component scores (GPCs) for the 1,279 samples that were left out of the main discovery sample set. The cluster of points around the (0,0) point corresponds to participants with predominantly European ancestry. We set a threshold at 14 for the 1st GPC to identify a set of European samples.
