# Supplementary material for: Genome-wide association studies of brain imaging phenotypes in UK Biobank
Source: Nature. 2018 Oct 10;562(7726):210–6. doi: 10.1038/s41586-018-0571-7 (PMC6786974; doi:10.1038/s41586-018-0571-7)
Supplement: Supplementary file 3 — This file contains Supplementary Figures S1-S22. [file 41586_2018_571_MOESM3_ESM.zip › Figure-S21.pdf]

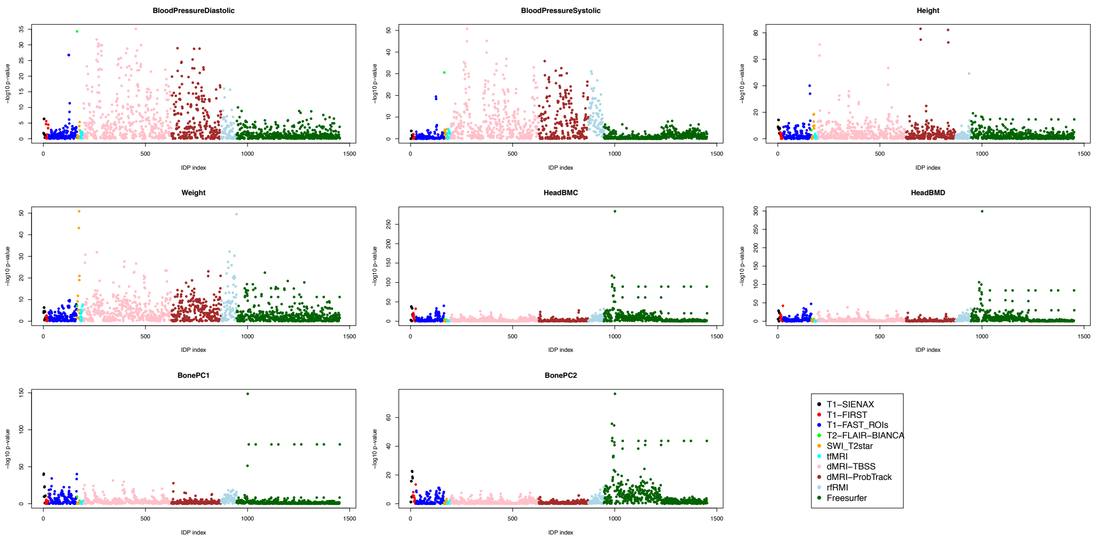

Supplementary Figure 21. Association of IDPs with Body Confounds. Each plot shows the association of a body confound measure with all of the IDPs except the resting fMRI parcellation edges. Analyses were linear regressions, two-sided tests, p-values uncorrected; see Methods for details.
