# Supplementary material for: Genome-wide association studies of brain imaging phenotypes in UK Biobank
Source: Nature. 2018 Oct 10;562(7726):210–6. doi: 10.1038/s41586-018-0571-7 (PMC6786974; doi:10.1038/s41586-018-0571-7)
Supplement: Supplementary file 3 — This file contains Supplementary Figures S1-S22. [file 41586_2018_571_MOESM3_ESM.zip › Figure-S22.pdf]

Supplementary Figure 22

Each of the subsequent pages shows a GWAS Manhattan plot (left) and QQ-plot (right) for the 8 different body confound measures.

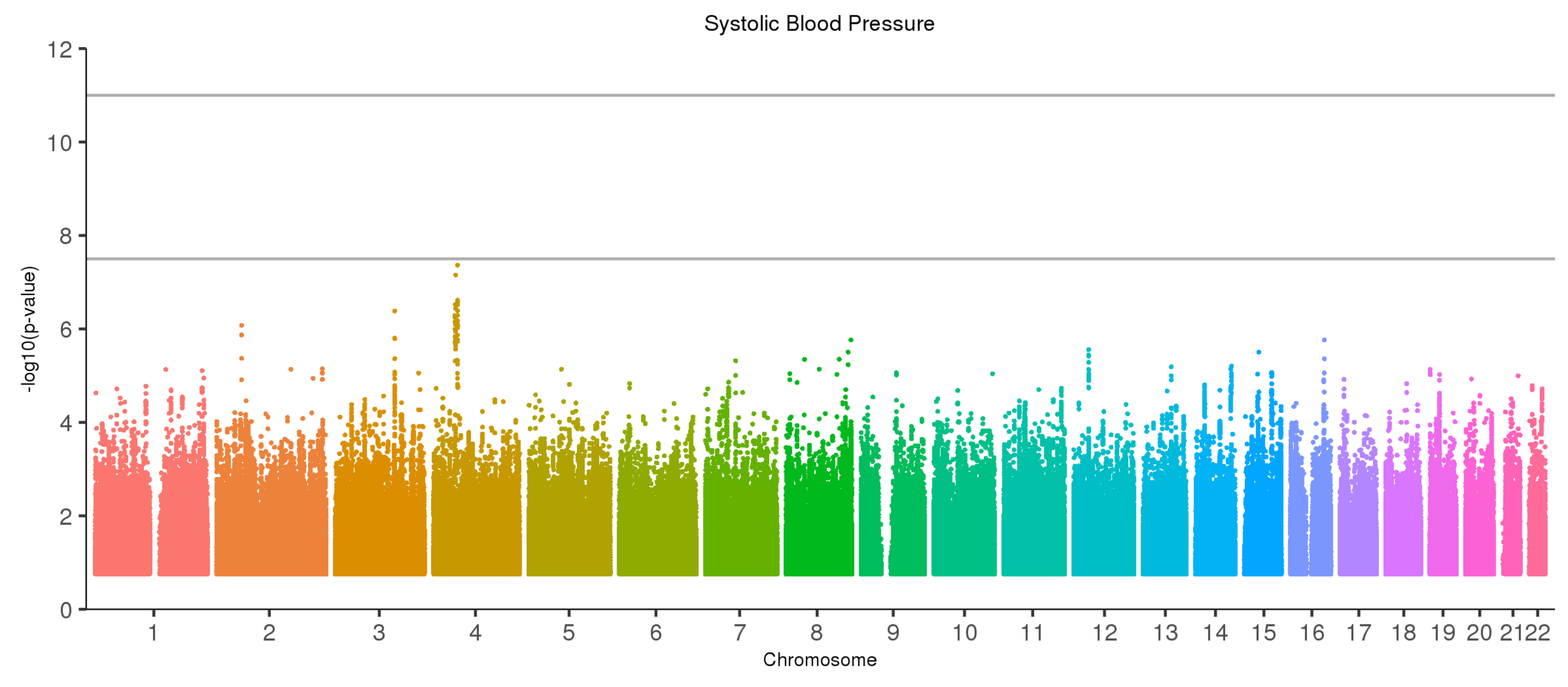

Systolic Blood Pressure
-log10(p-value)
0
2
4
6
8
10
12
1
2
3
4
5
6
7
8
9
10
11
12
13
14
15
16
17
18
19
20
21
22
Chromosome

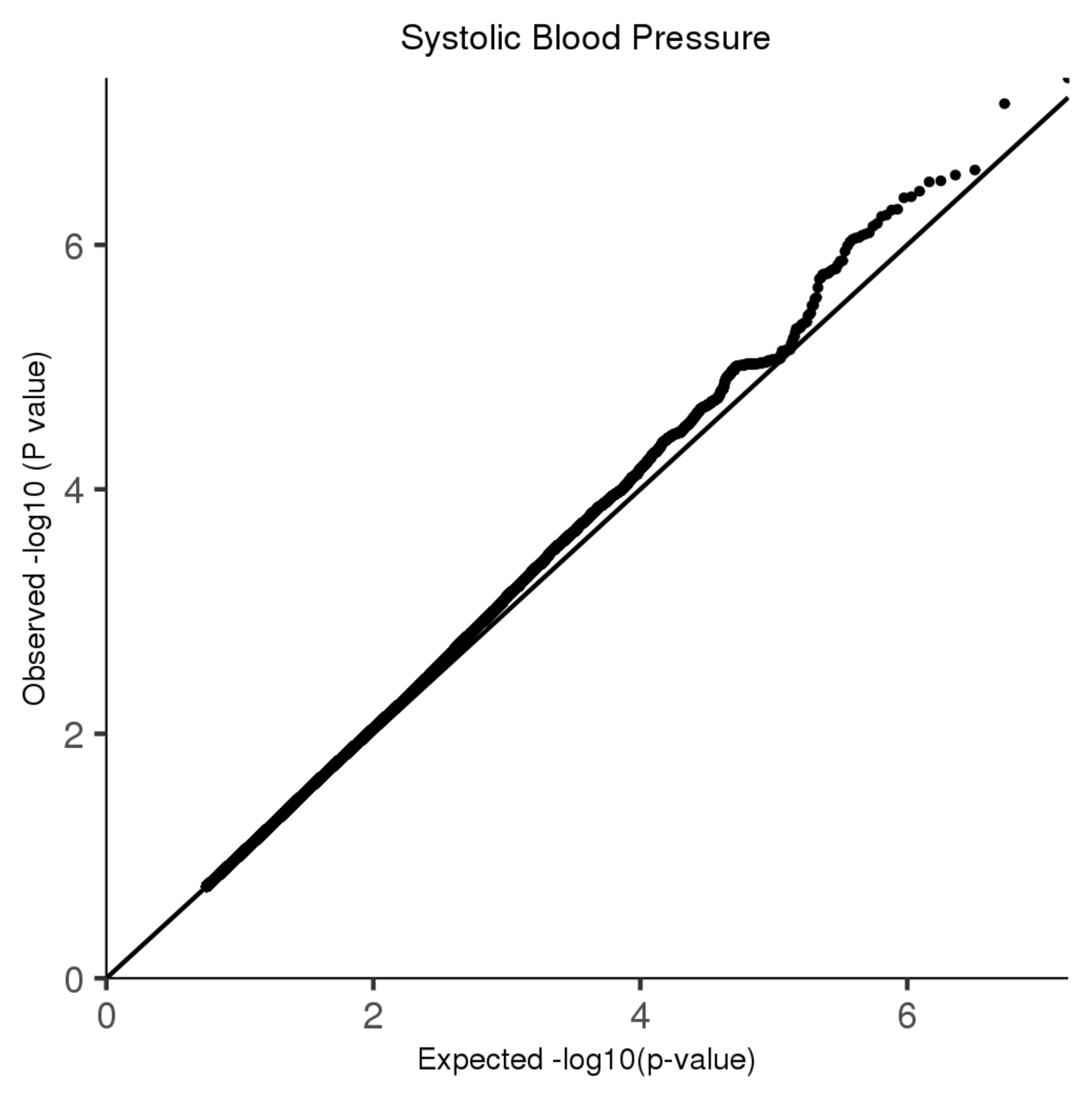

Systolic Blood Pressure
Observed -log10 (P value)
0
2
4
6
0
2
4
6
Expected -log10(p-value)

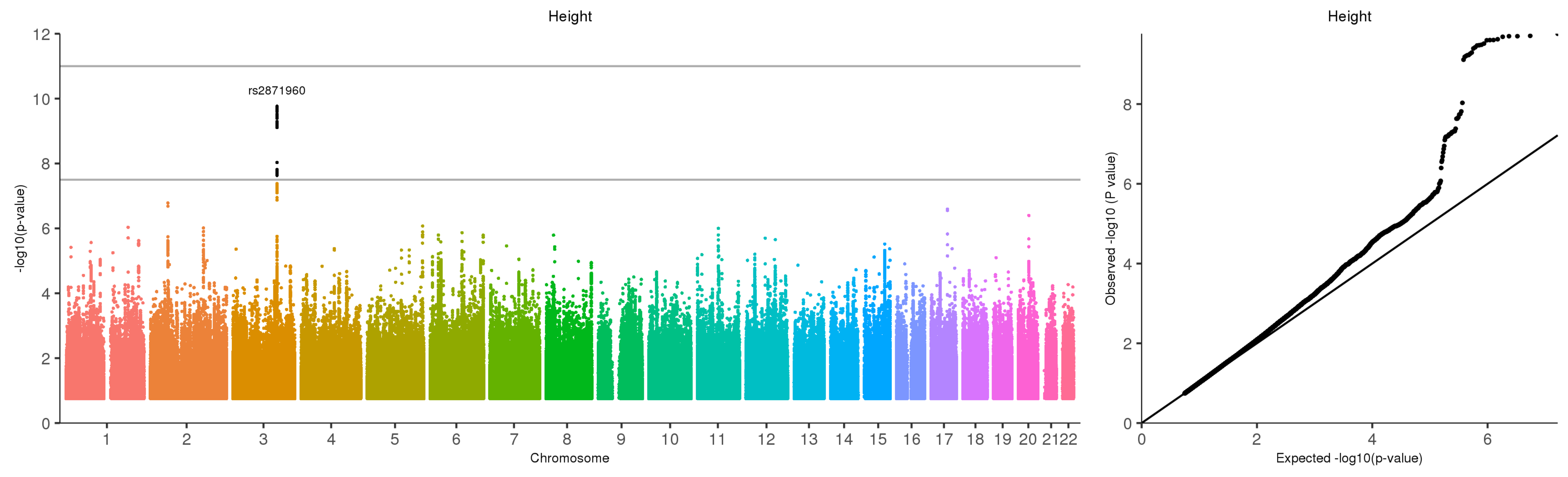
Height
rs2871960
-log10(p-value)
Chromosome
1
2
3
4
5
6
7
8
9
10
11
12
13
14
15
16
17
18
19
20
21
22
0
2
4
6
8
10
12
Height
Observed -log10 (P value)
Expected -log10(p-value)

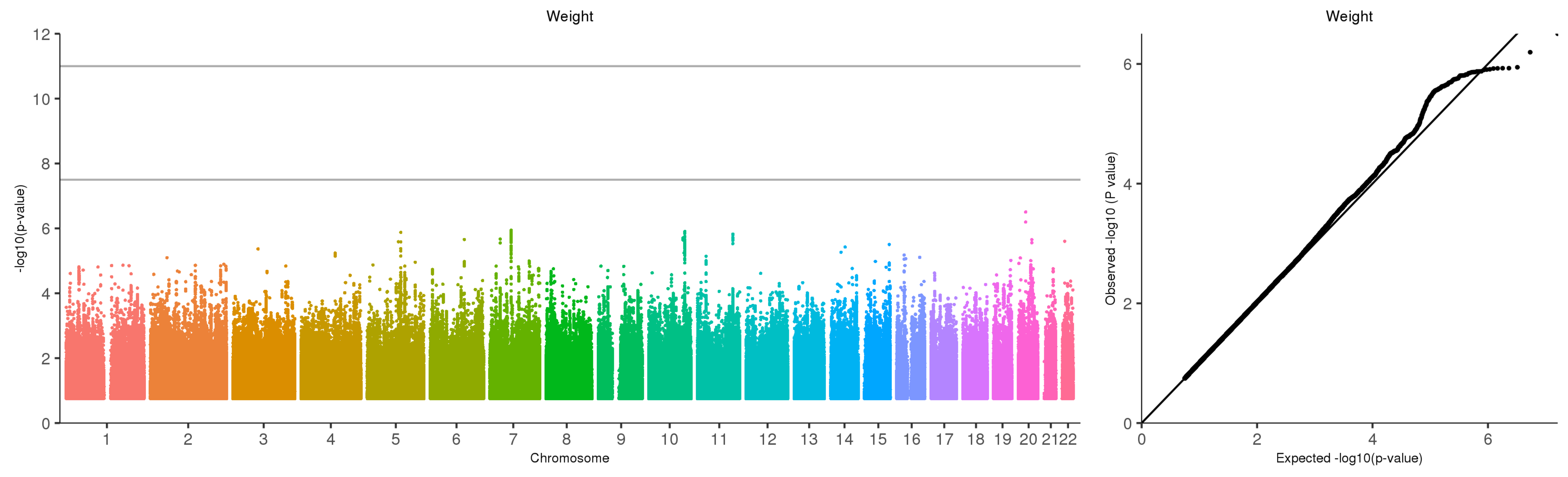

Weight
-log10(p-value)
Chromosome
1 2 3 4 5 6 7 8 9 10 11 12 13 14 15 16 17 18 19 20 21 22
0 2 4 6 8 10 12
Weight
Observed -log10 (P value)
Expected -log10(p-value)
0 2 4 6

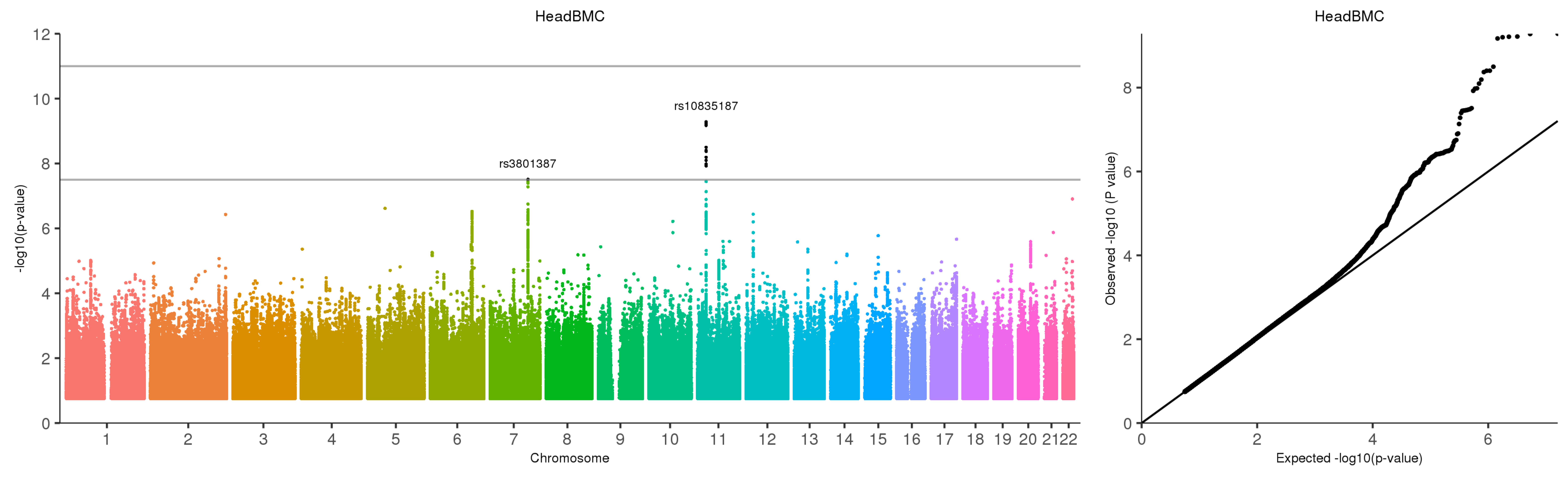
HeadBMC
rs10835187
rs3801387
-log10(p-value)
Chromosome
1
2
3
4
5
6
7
8
9
10
11
12
13
14
15
16
17
18
19
20
21
22
0
2
4
6
8
10
12
HeadBMC
Observed -log10 (P value)
Expected -log10(p-value)

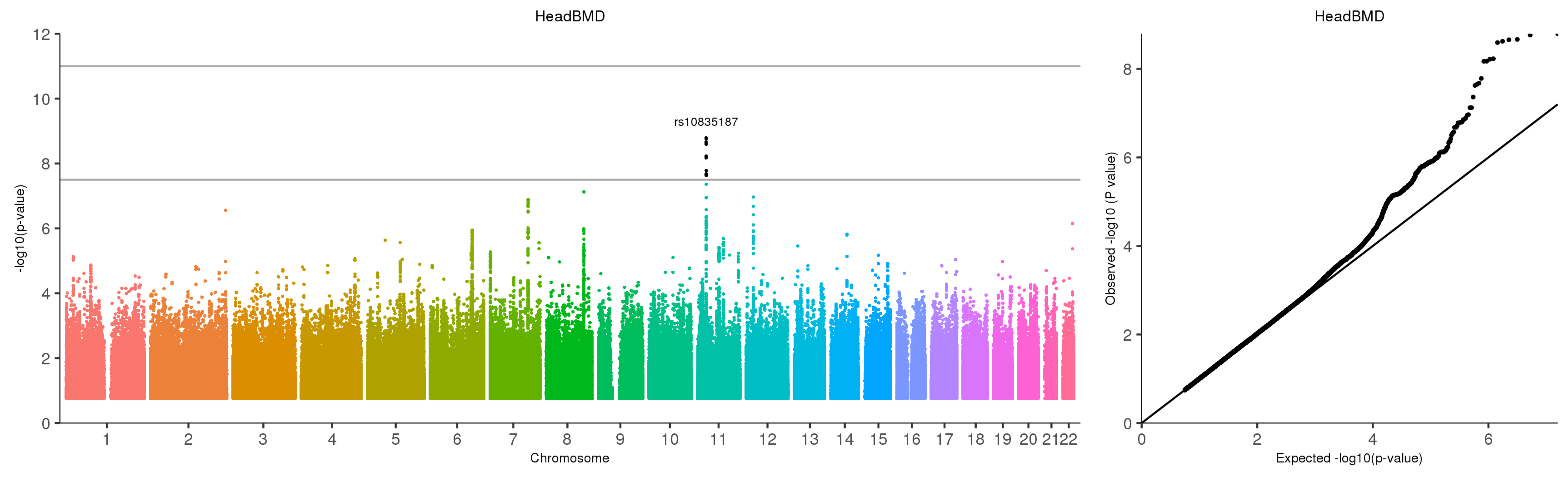
HeadBMD
rs10835187
-log10(p-value)
Chromosome
1 2 3 4 5 6 7 8 9 10 11 12 13 14 15 16 17 18 19 20 21 22
0 2 4 6 8 10 12
HeadBMD
Observed -log10 (P value)
Expected -log10(p-value)
0 2 4 6 8
0 2 4 6

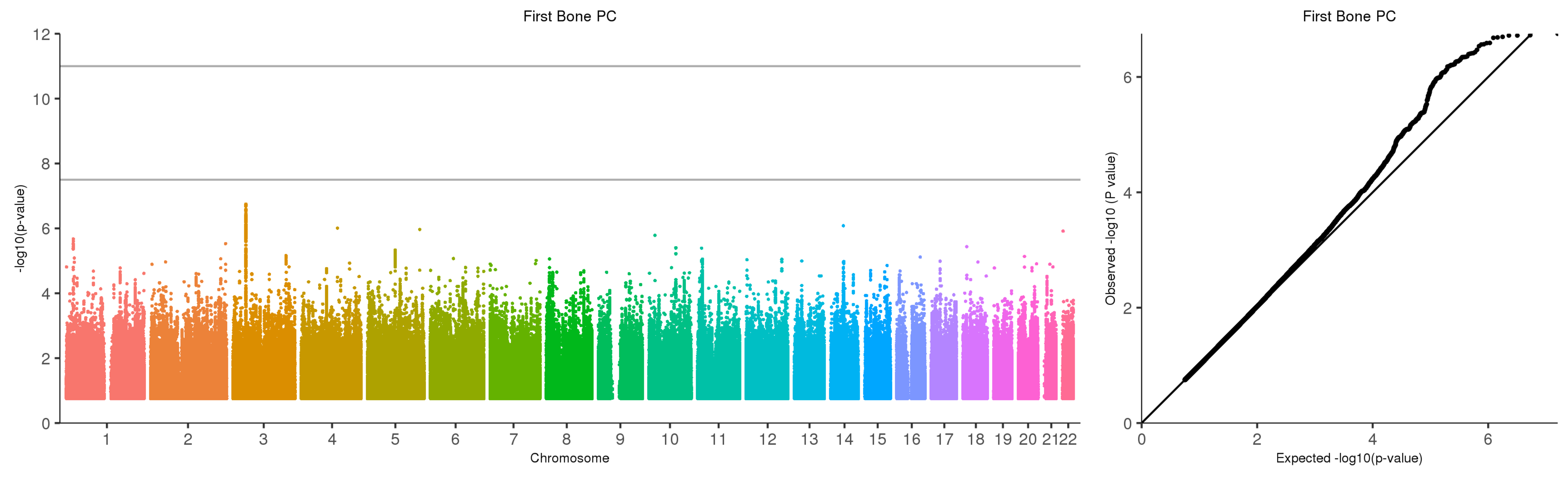

First Bone PC
-log10(p-value)
Chromosome
1
2
3
4
5
6
7
8
9
10
11
12
13
14
15
16
17
18
19
20
21
22
0
2
4
6
8
10
12
First Bone PC
Observed -log10 (P value)
Expected -log10(p-value)
0
2
4
6

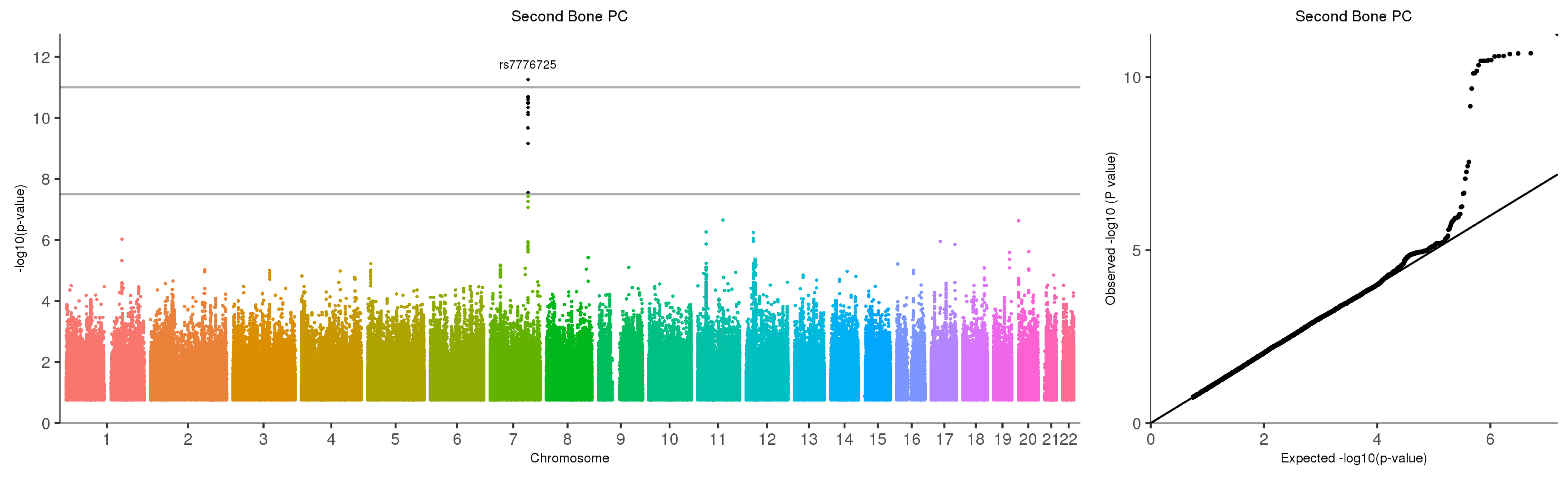

Second Bone PC
rs7776725
-log10(p-value)
Chromosome
1 2 3 4 5 6 7 8 9 10 11 12 13 14 15 16 17 18 19 20 21 22
0 2 4 6 8 10 12
Second Bone PC
Observed -log10 (P value)
Expected -log10(p-value)
0 5 10
0 2 4 6
