# Supplementary material for: Genome-wide association studies of brain imaging phenotypes in UK Biobank
Source: Nature. 2018 Oct 10;562(7726):210–6. doi: 10.1038/s41586-018-0571-7 (PMC6786974; doi:10.1038/s41586-018-0571-7)
Supplement: Supplementary file 3 — This file contains Supplementary Figures S1-S22. [file 41586_2018_571_MOESM3_ESM.zip › Figure-S1.pdf]

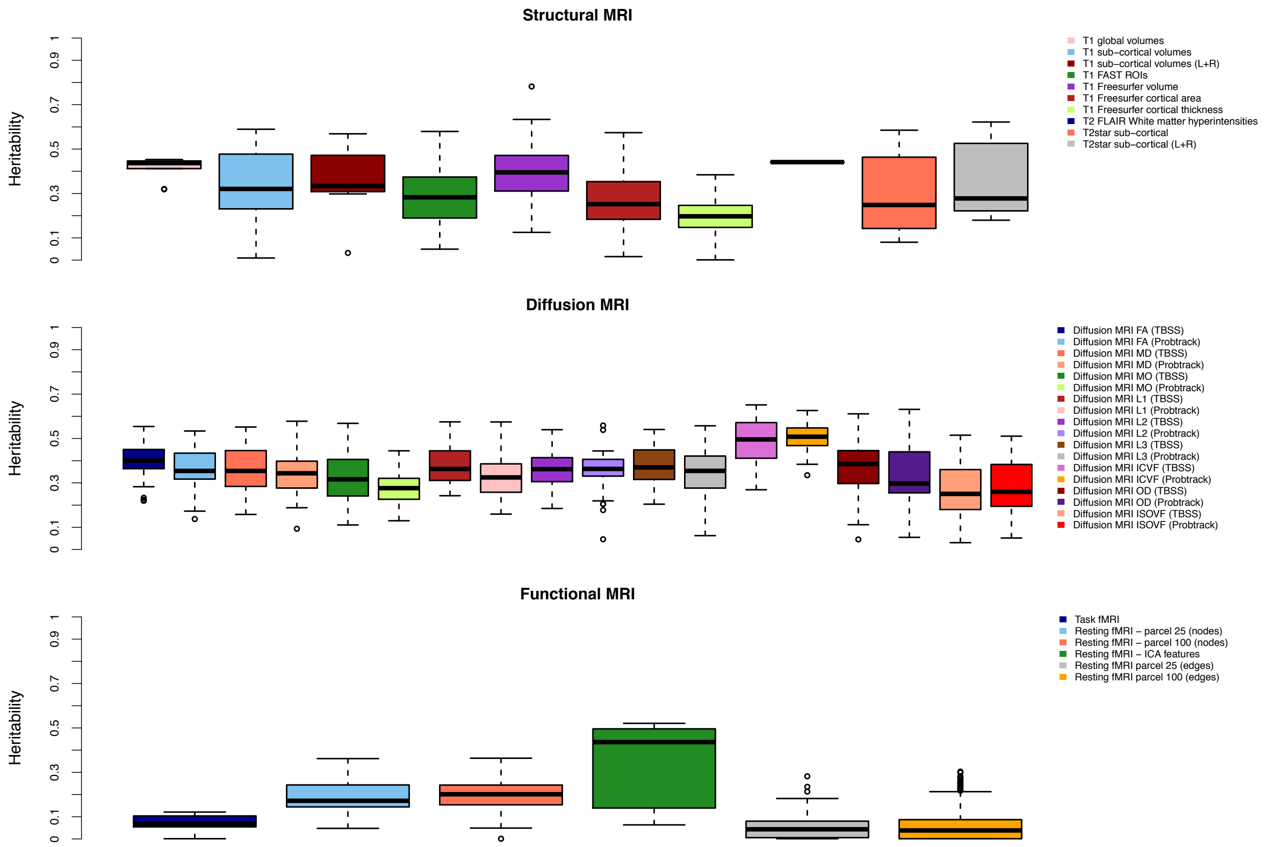

Supplementary Figure 1: **Estimated heritability of IDPs**. Boxplots of estimated heritability (y-axis) for different IDP groups analysed (N = 8,428 subjects, see Methods for heritability calculation details). IDPs have been split into three broad groups: Structural MRI (top), Diffusion MRI (middle) and Functional MRI (bottom). Boxplots are colored according to IDP groups. Boxplots show: median is the thick black line; ends of the box mark 25th and 75th percentiles; whiskers show most extreme values not considered outliers; outliers are shown with a circle and are values lying more than 1.5x the interquartile range outside of the interquartile box
