# Supplementary material for: Genome-wide association studies of brain imaging phenotypes in UK Biobank
Source: Nature. 2018 Oct 10;562(7726):210–6. doi: 10.1038/s41586-018-0571-7 (PMC6786974; doi:10.1038/s41586-018-0571-7)
Supplement: Supplementary file 3 — This file contains Supplementary Figures S1-S22. [file 41586_2018_571_MOESM3_ESM.zip › Figure-S2.pdf]

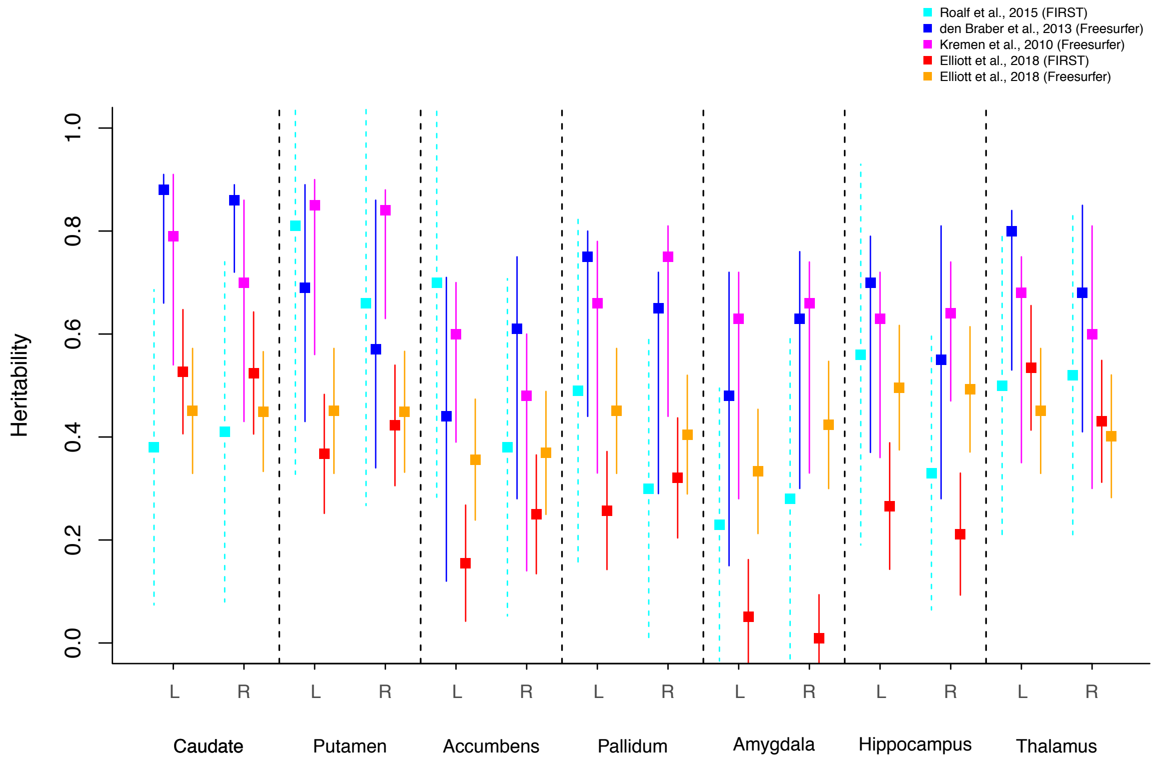

Supplementary Figure 2 : Forest plot comparing heritability estimates for the sub-cortical volumes. Estimates that we report using both FIRST (red) and Freesurfer (yellow) segmentations are compared to those from three previous studies: Roalf et al., 2015 (a study incorporating 190 individuals from 32 multiplex-multigenerational families with schizophrenia and 249 healthy volunteers) (cyan), den Braber et al., 2013 (a twin study incorporating 176 MZ and 88 DZ twin-pairs) (blue) and Kremen et al., 2010 (another twin study comprising 474 middle-aged male twins from the Vietnam Era Twin Study of Aging) (magenta). Coloured squares denote reported estimates and lines indicate the corresponding 95% confidence intervals. For the Roalf et al., 2015 study, only p values were reported, so the confidence intervals shown were estimated from these.
