# Supplementary material for: Genome-wide association studies of brain imaging phenotypes in UK Biobank
Source: Nature. 2018 Oct 10;562(7726):210–6. doi: 10.1038/s41586-018-0571-7 (PMC6786974; doi:10.1038/s41586-018-0571-7)
Supplement: Supplementary file 3 — This file contains Supplementary Figures S1-S22. [file 41586_2018_571_MOESM3_ESM.zip › Figure-S3.pdf]

Supplementary Figure 3

Each of the subsequent pages shows the estimated genetic correlation matrix (left) and the raw phenotypic correlation matrix for different subsets of IDPs. The genetic correlation matrices where calculated using the multi-trait mixed model approach described in the main text using the SBAT software. In some cases, phenotypes were pruned to remove pairs of IDPs with raw correlation greater that 0.9. The intention of these plots is to provide an overview of the levels of raw and genetic correlations that exist between the different IDPs.

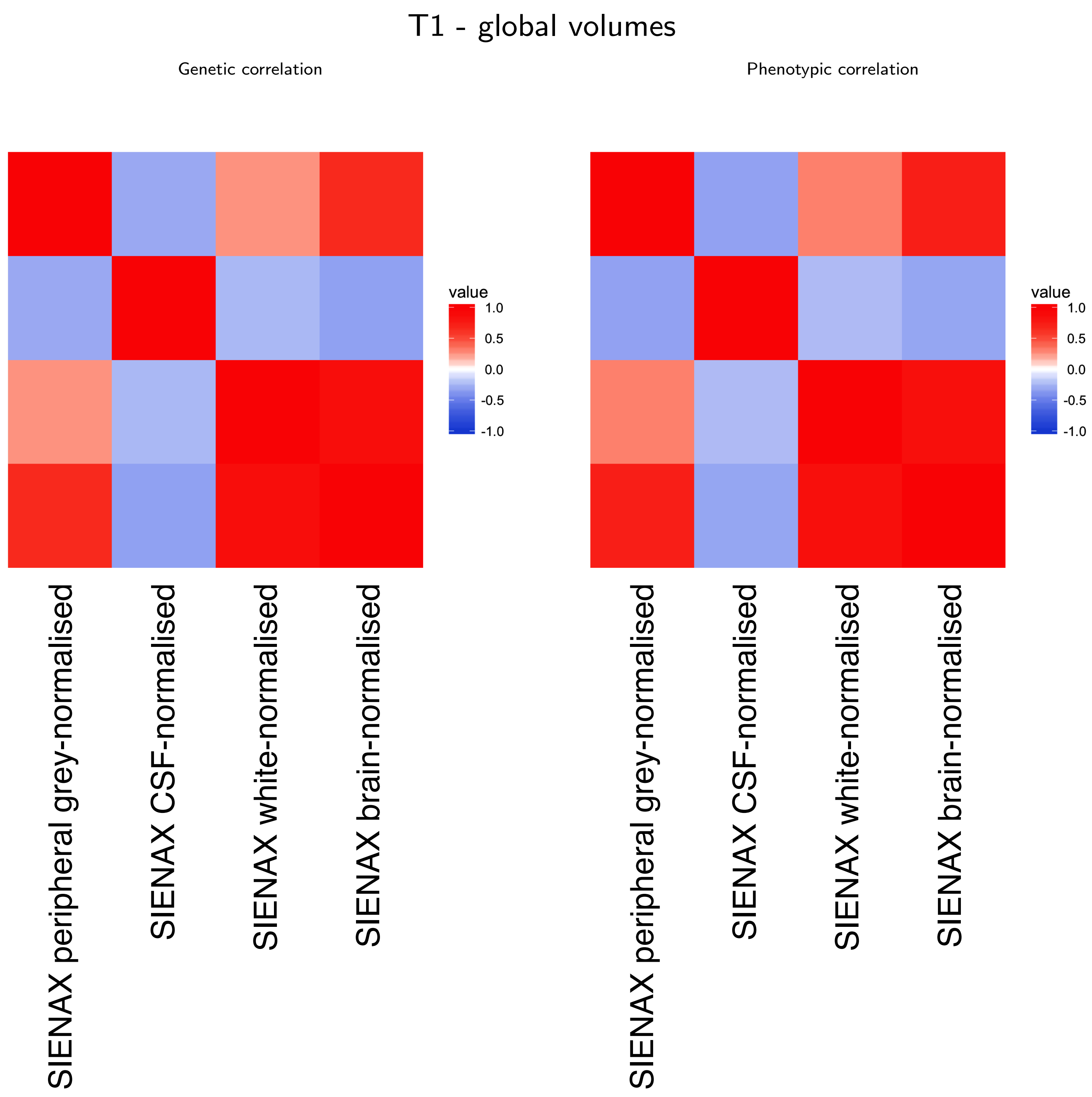
T1 - global volumes
Genetic correlation
Phenotypic correlation
value
1.0
0.5
0.0
-0.5
-1.0
SIENAX peripheral grey-normalised
SIENAX CSF-normalised
SIENAX white-normalised
SIENAX brain-normalised

T1 - sub-cortical volumes

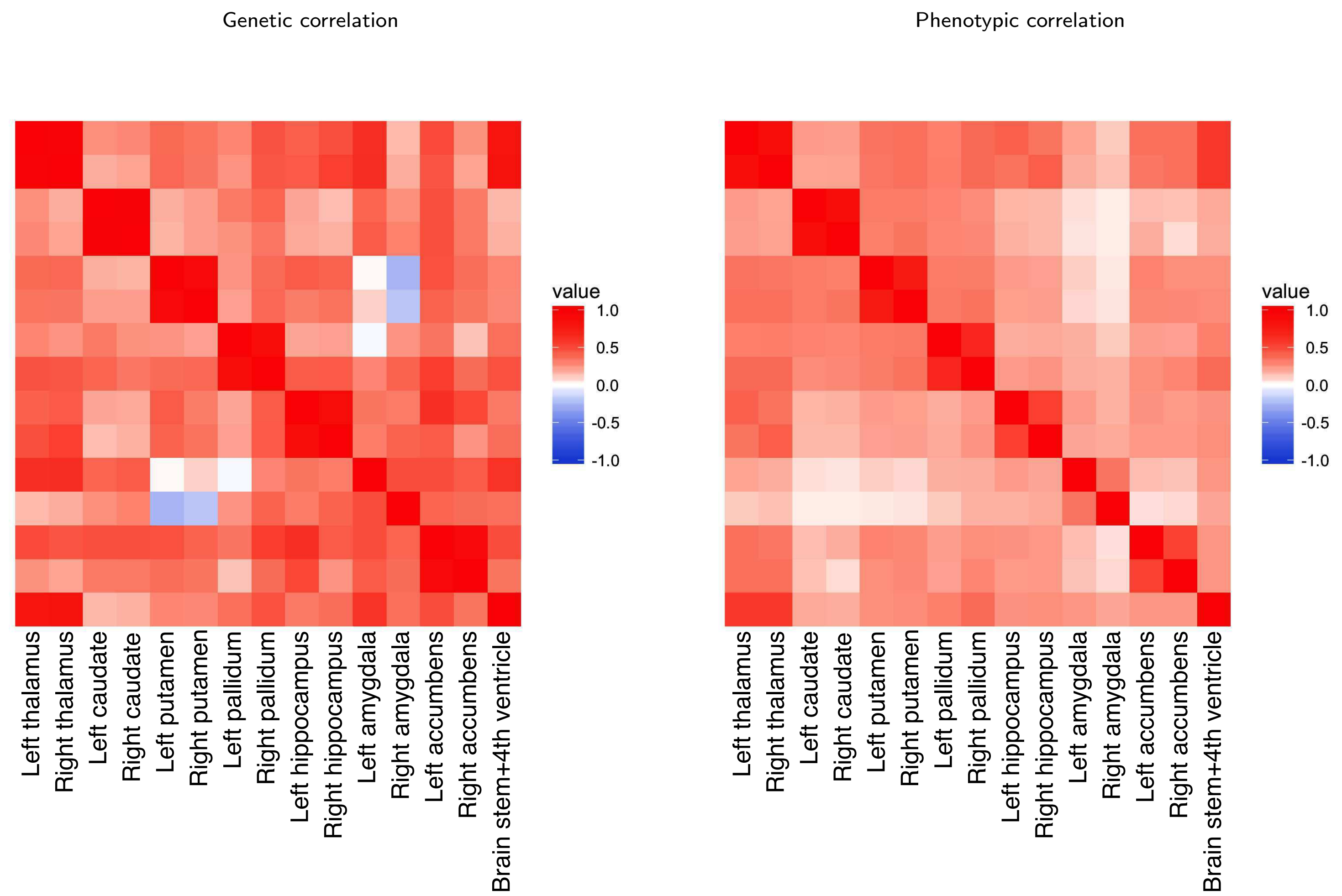

T1 - Cortex ROIs

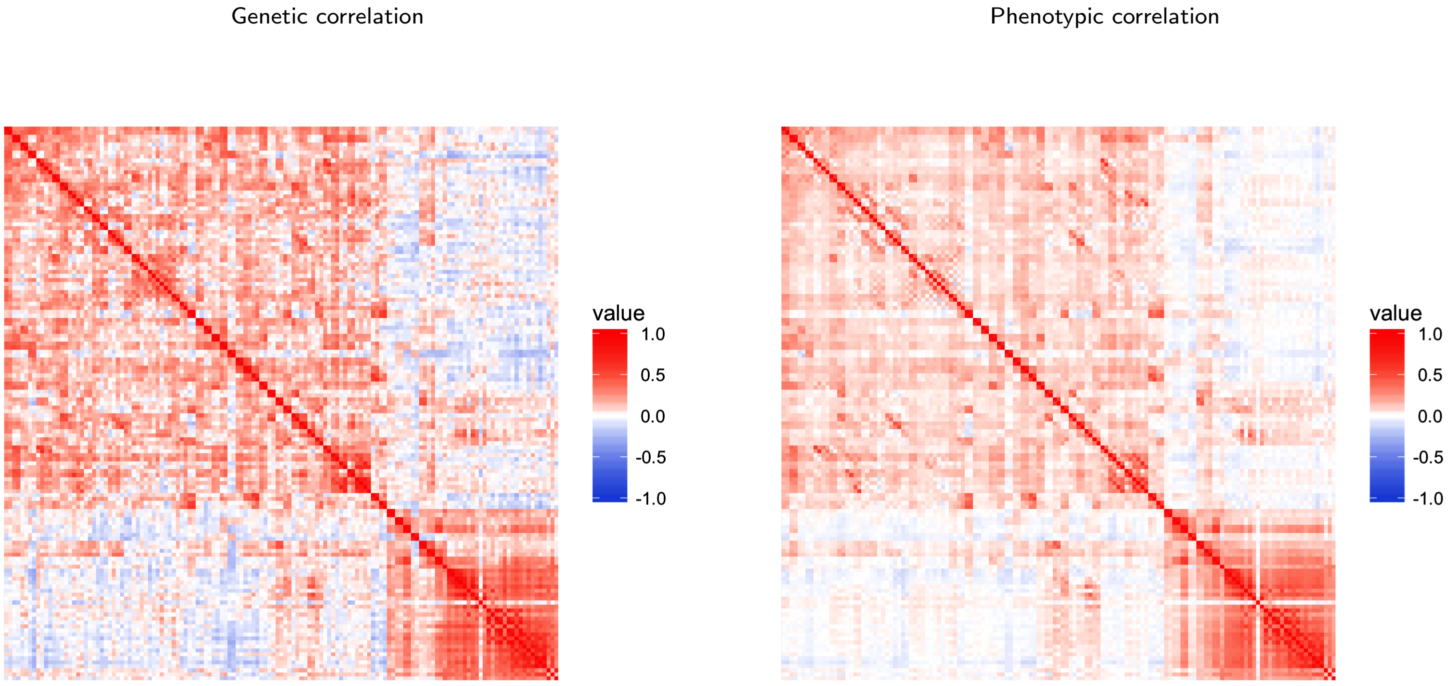

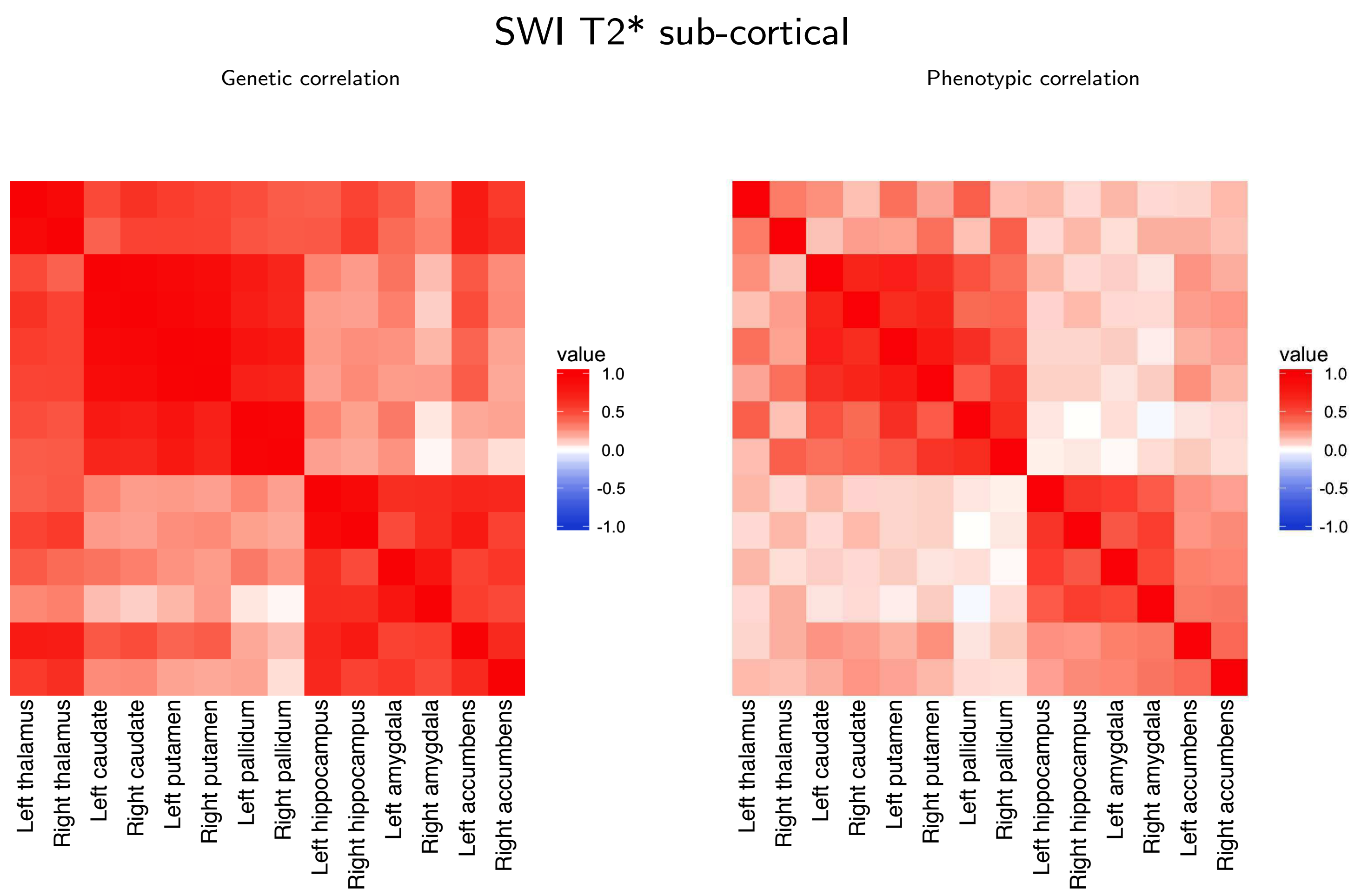
SWI T2* sub-cortical
Genetic correlation
Phenotypic correlation
value
1.0
0.5
0.0
-0.5
-1.0
Left thalamus
Right thalamus
Left caudate
Right caudate
Left putamen
Right putamen
Left pallidum
Right pallidum
Left hippocampus
Right hippocampus
Left amygdala
Right amygdala
Left accumbens
Right accumbens

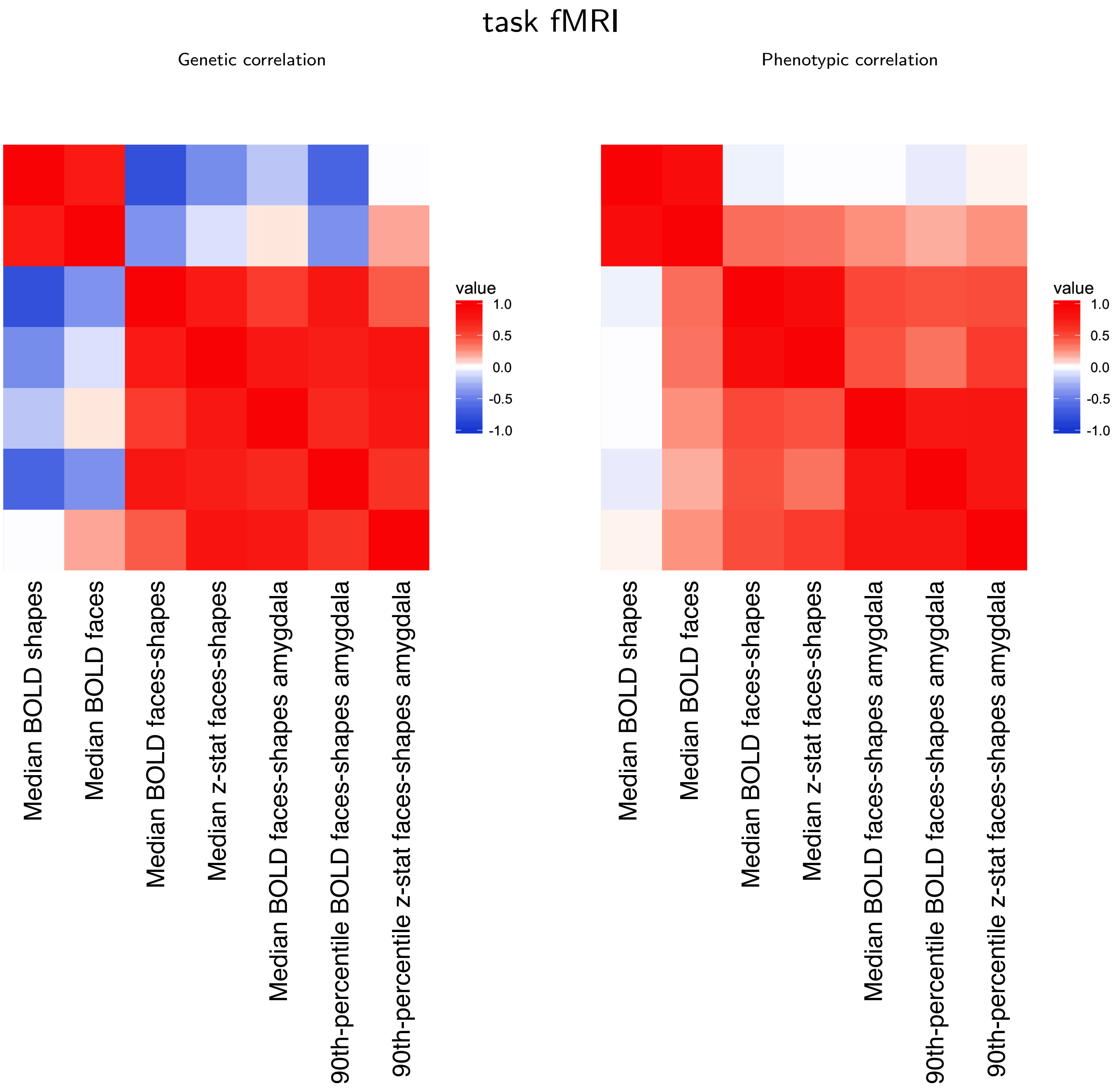
task fMRI
Genetic correlation
Phenotypic correlation
value
1.0
0.5
0.0
-0.5
-1.0
Median BOLD shapes
Median BOLD faces
Median BOLD faces-shapes
Median z-stat faces-shapes
Median BOLD faces-shapes amygdala
90th-percentile BOLD faces-shapes amygdala
90th-percentile z-stat faces-shapes amygdala

Diffusion MRI - TBSS

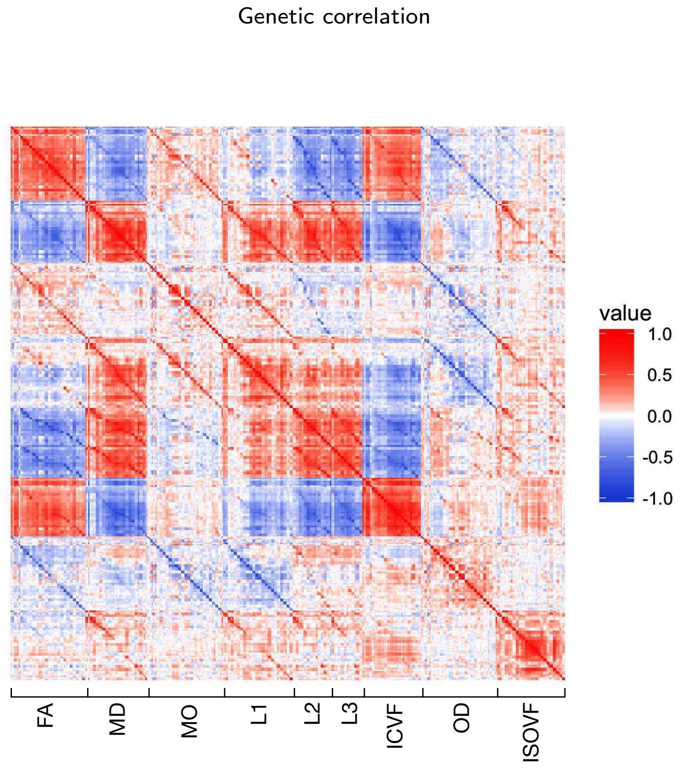

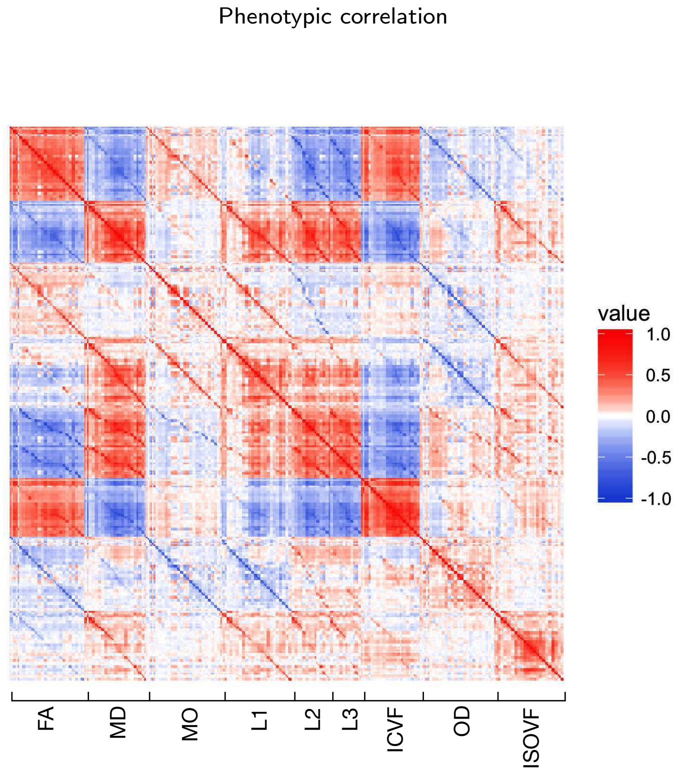

Diffusion MRI - ProbtrackX

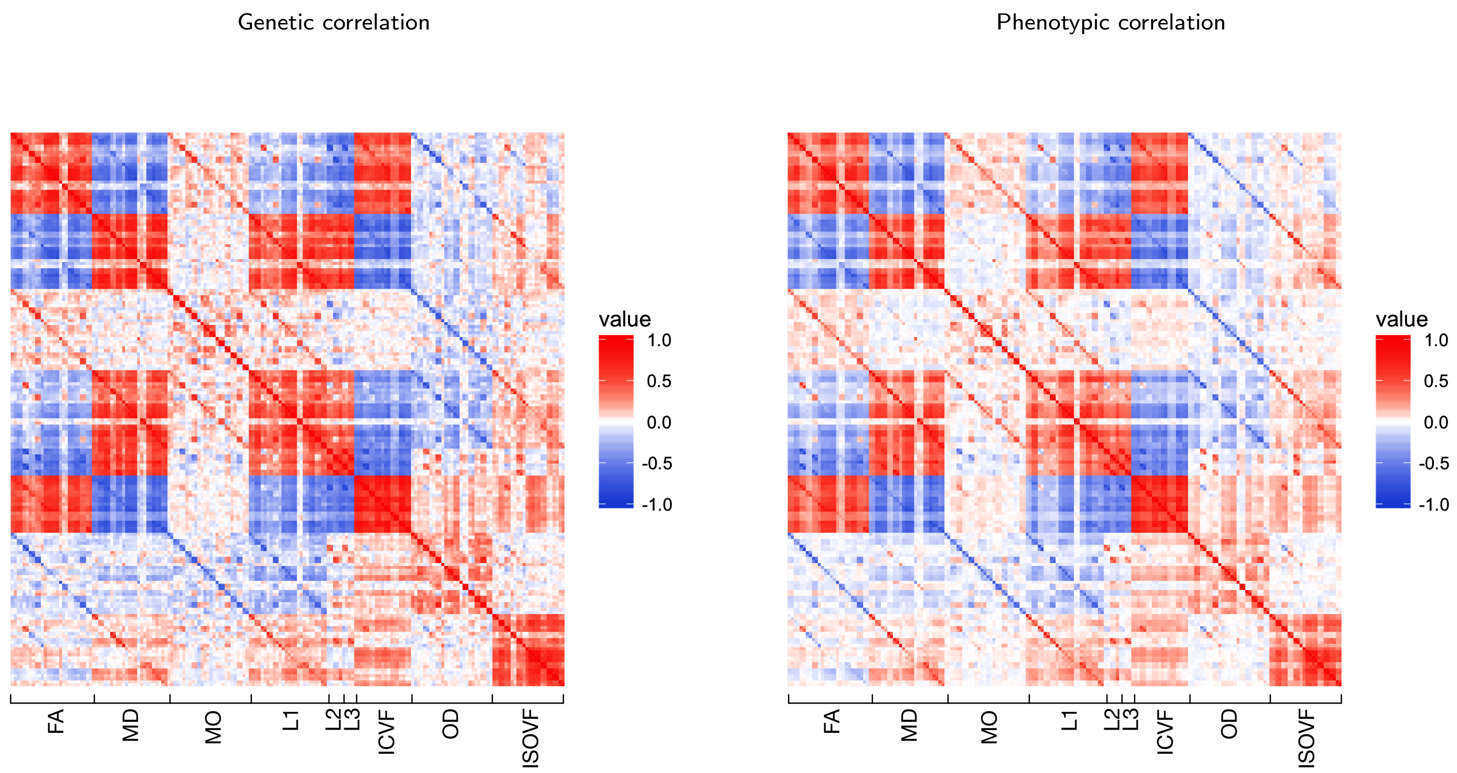

resting fMRI - parcellation25 amplitudes

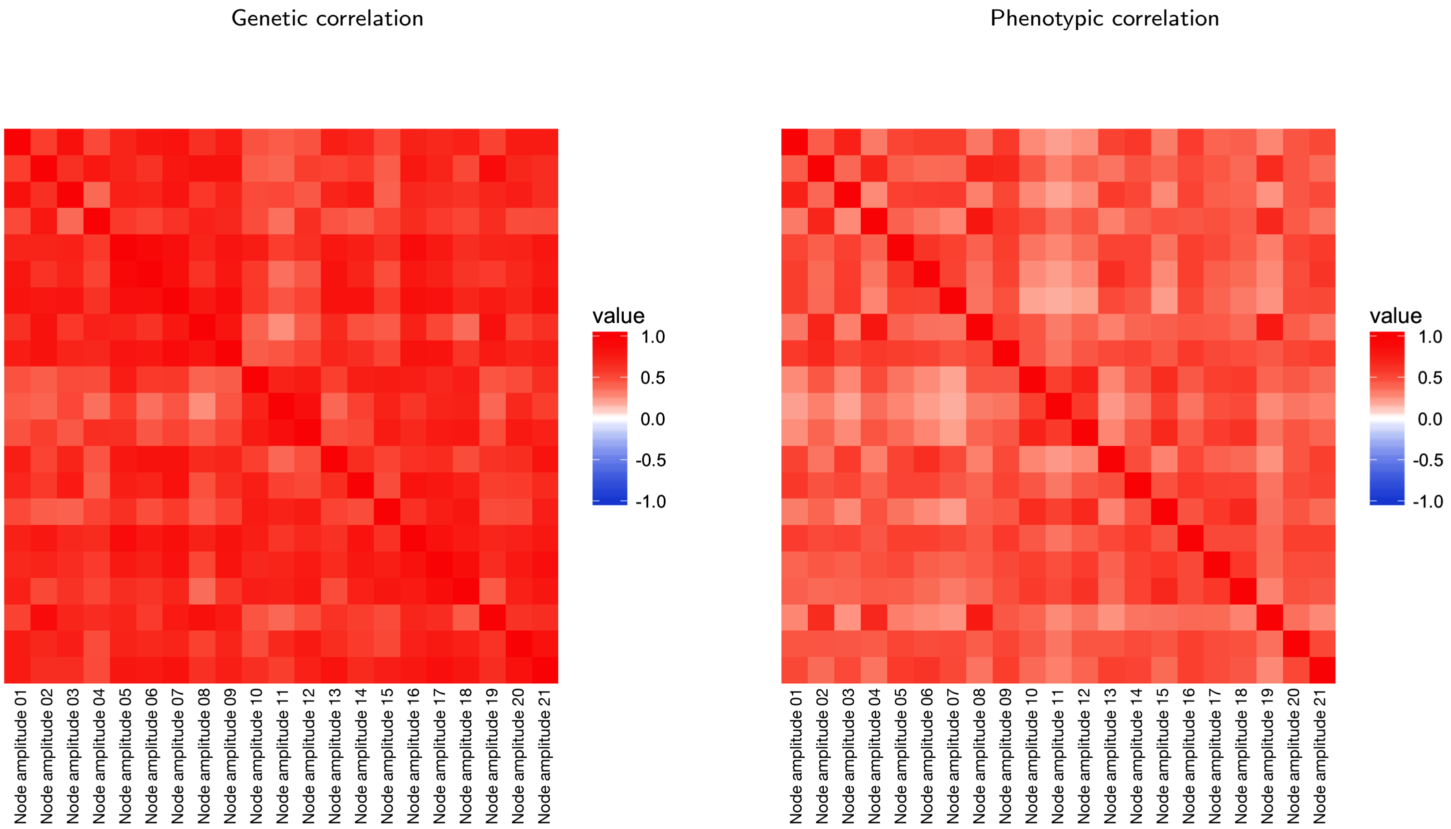

resting fMRI - parcellation100 amplitudes

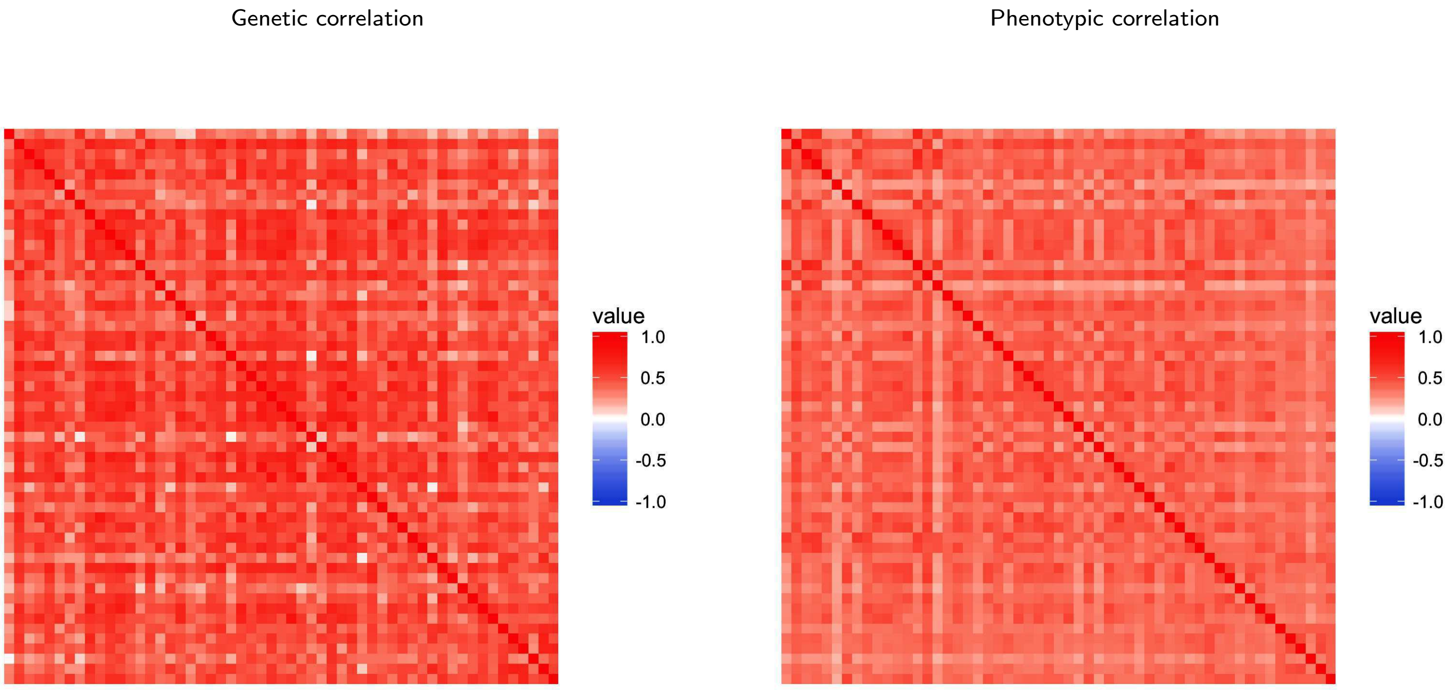

resting fMRI - parcellation25 edges

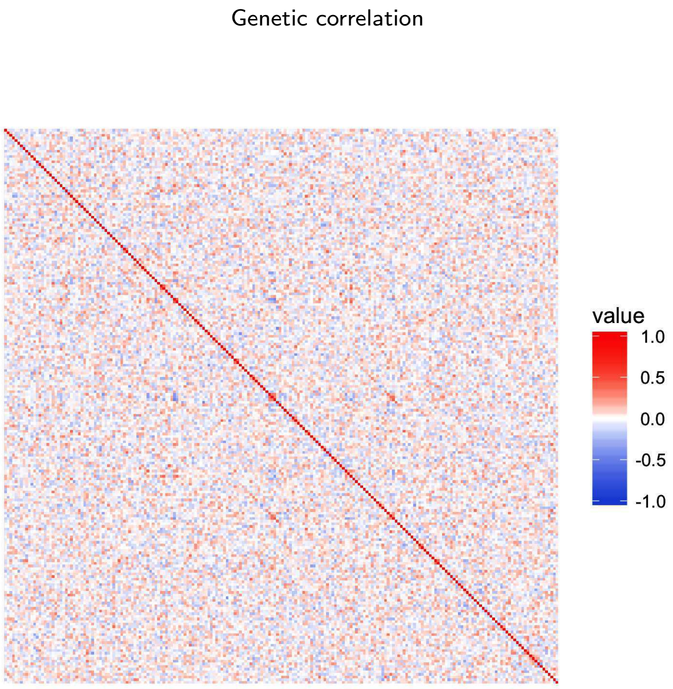

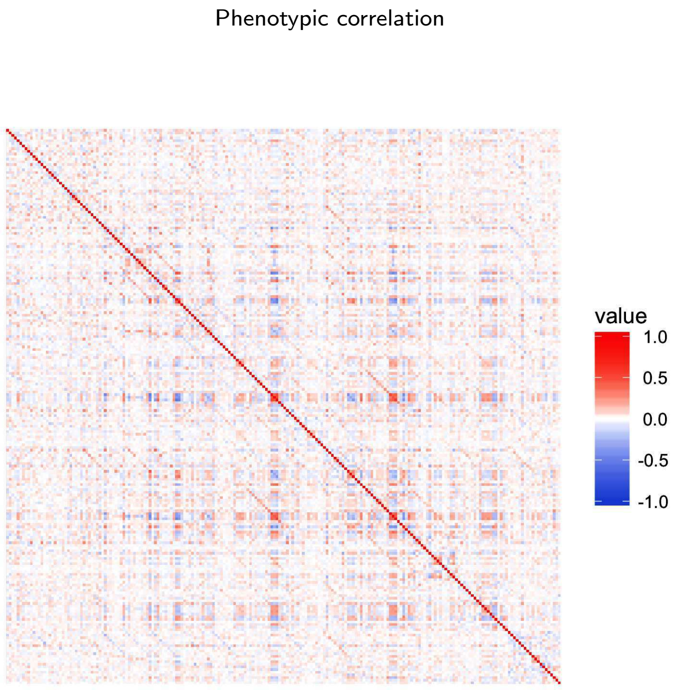

T1 - sub-cortical volumes L+R

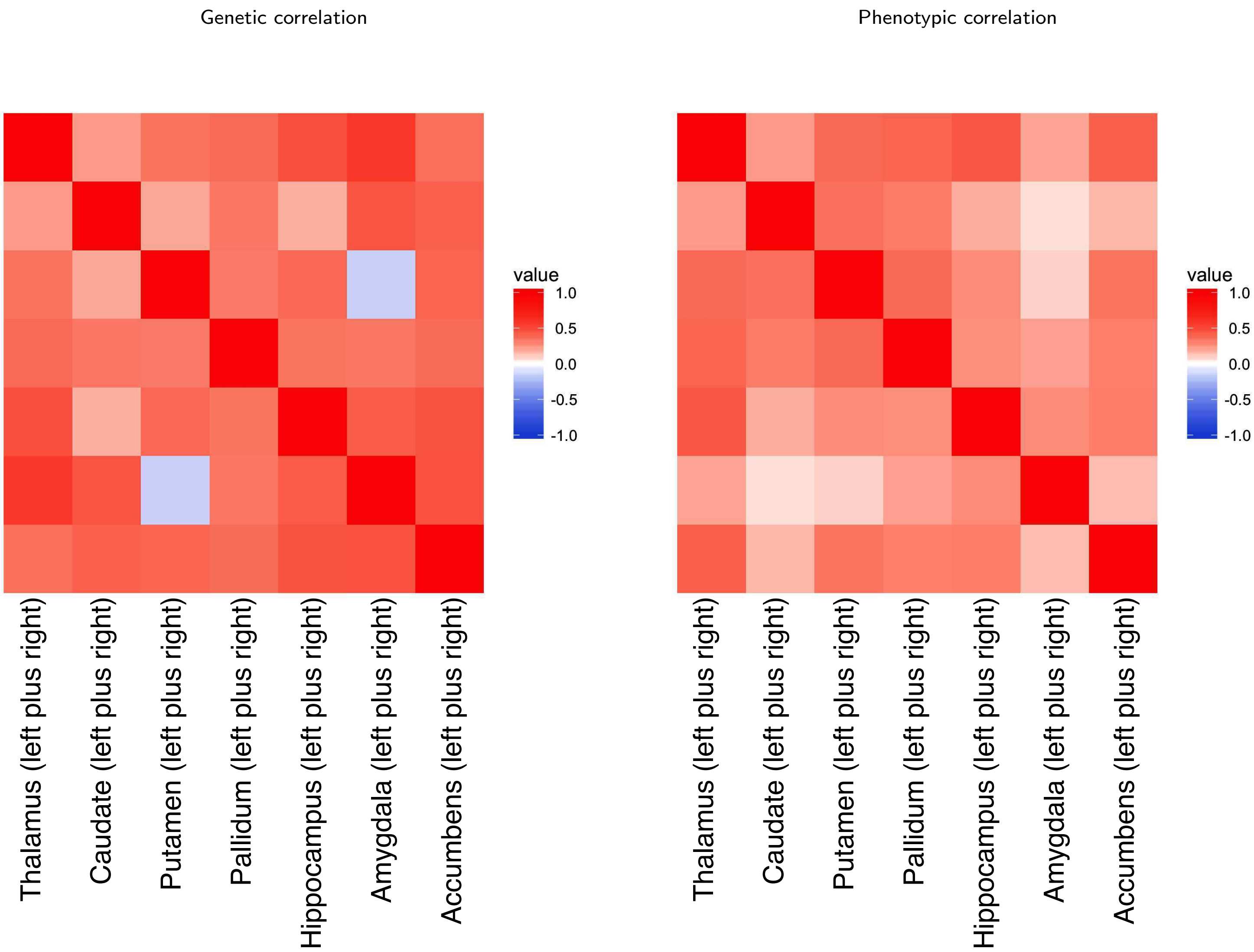

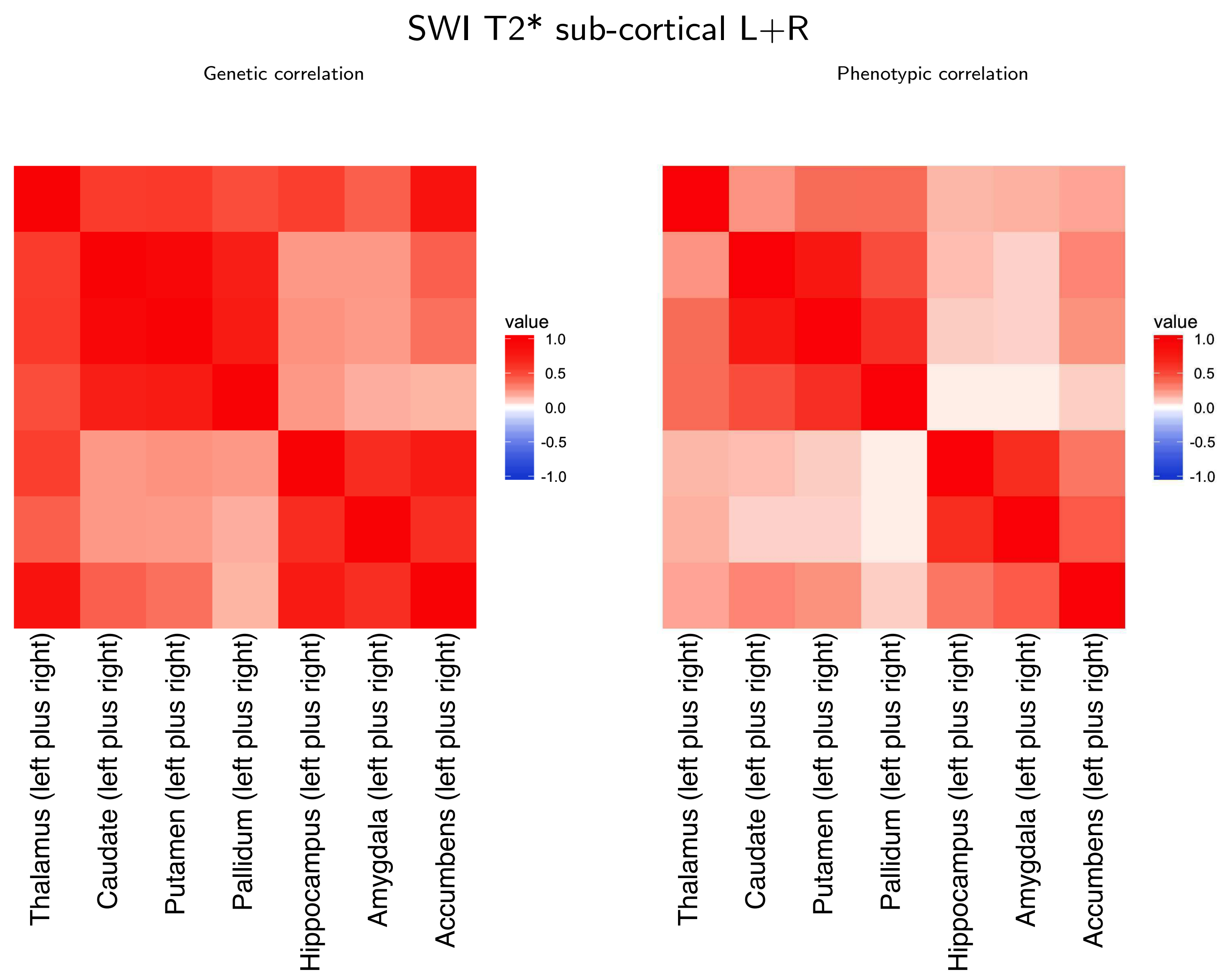

SWI T2* sub-cortical L+R
Genetic correlation
Phenotypic correlation
value
1.0
0.5
0.0
-0.5
-1.0
Thalamus (left plus right)
Caudate (left plus right)
Putamen (left plus right)
Pallidum (left plus right)
Hippocampus (left plus right)
Amygdala (left plus right)
Accumbens (left plus right)

FreeSurfer

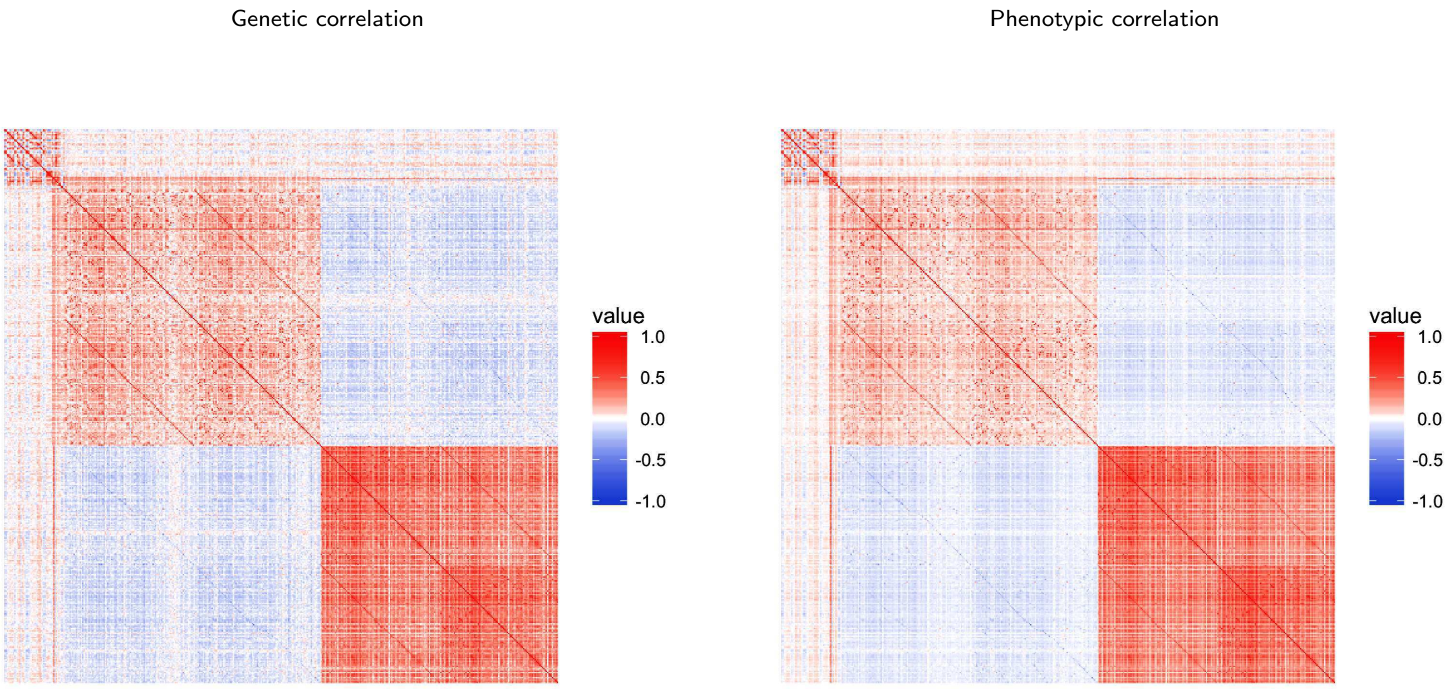
Genetic correlation
Phenotypic correlation
value
1.0
0.5
0.0
-0.5
-1.0

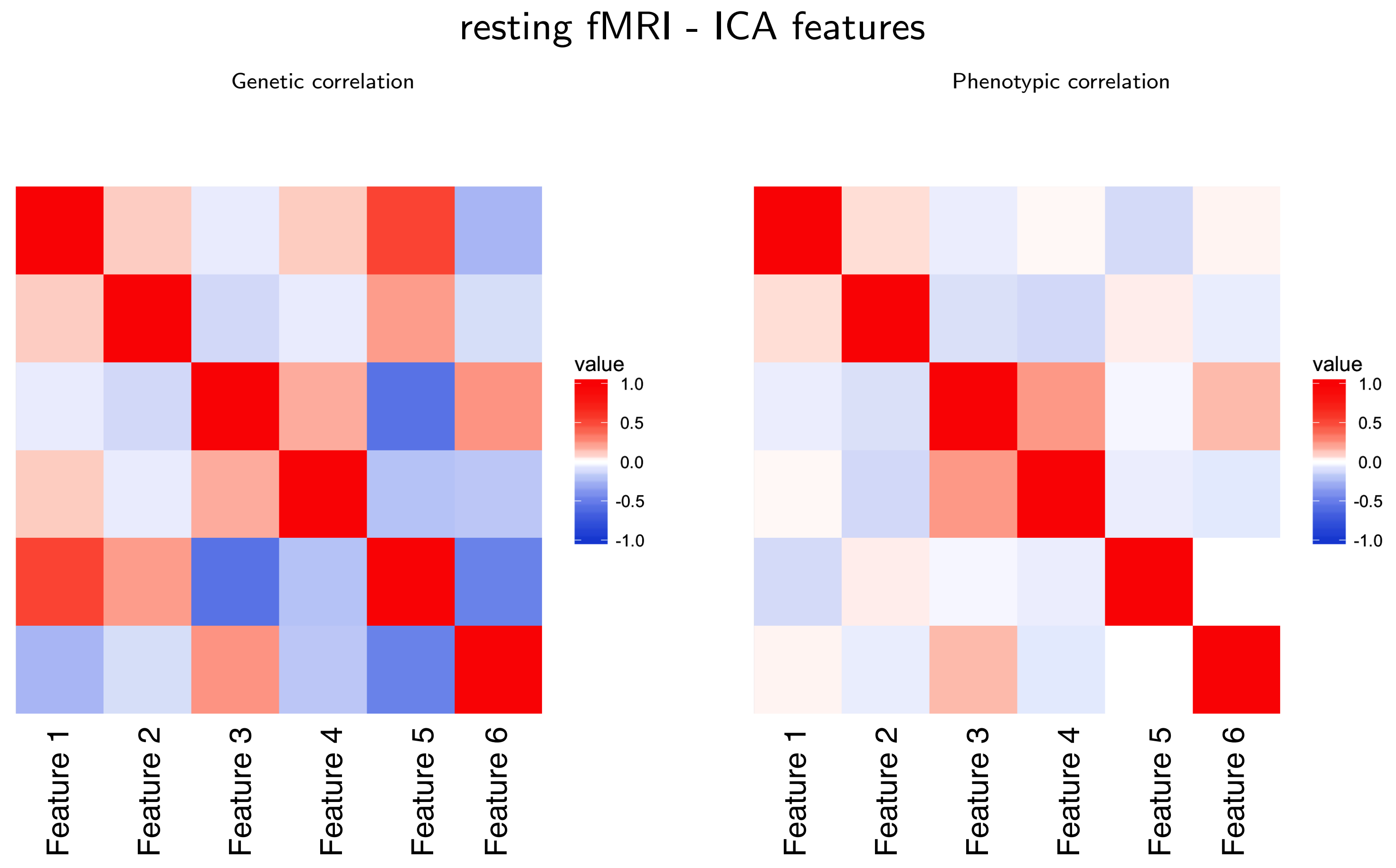
resting fMRI - ICA features
Genetic correlation
Phenotypic correlation
value
1.0
0.5
0.0
-0.5
-1.0
Feature 1
Feature 2
Feature 3
Feature 4
Feature 5
Feature 6
