# Supplementary material for: Genome-wide association studies of brain imaging phenotypes in UK Biobank
Source: Nature. 2018 Oct 10;562(7726):210–6. doi: 10.1038/s41586-018-0571-7 (PMC6786974; doi:10.1038/s41586-018-0571-7)
Supplement: Supplementary file 3 — This file contains Supplementary Figures S1-S22. [file 41586_2018_571_MOESM3_ESM.zip › Figure-S4.pdf]

## Supplementary Figure 4

Each of the subsequent pages shows the local association plots for the 368 SNP-IDP associations listed in **Supplementary Table 6**. In the top panel SNPs are plotted by their positions on the chromosome against association with the IDP (−log10 P value) on the left *y* axis. Points are coloured by their local linkage disequilibrium (LD) pattern with the focal SNP (purple diamond). Below the main plot are two tracks that show existing GWAS associations, and position and orientation of local genes.

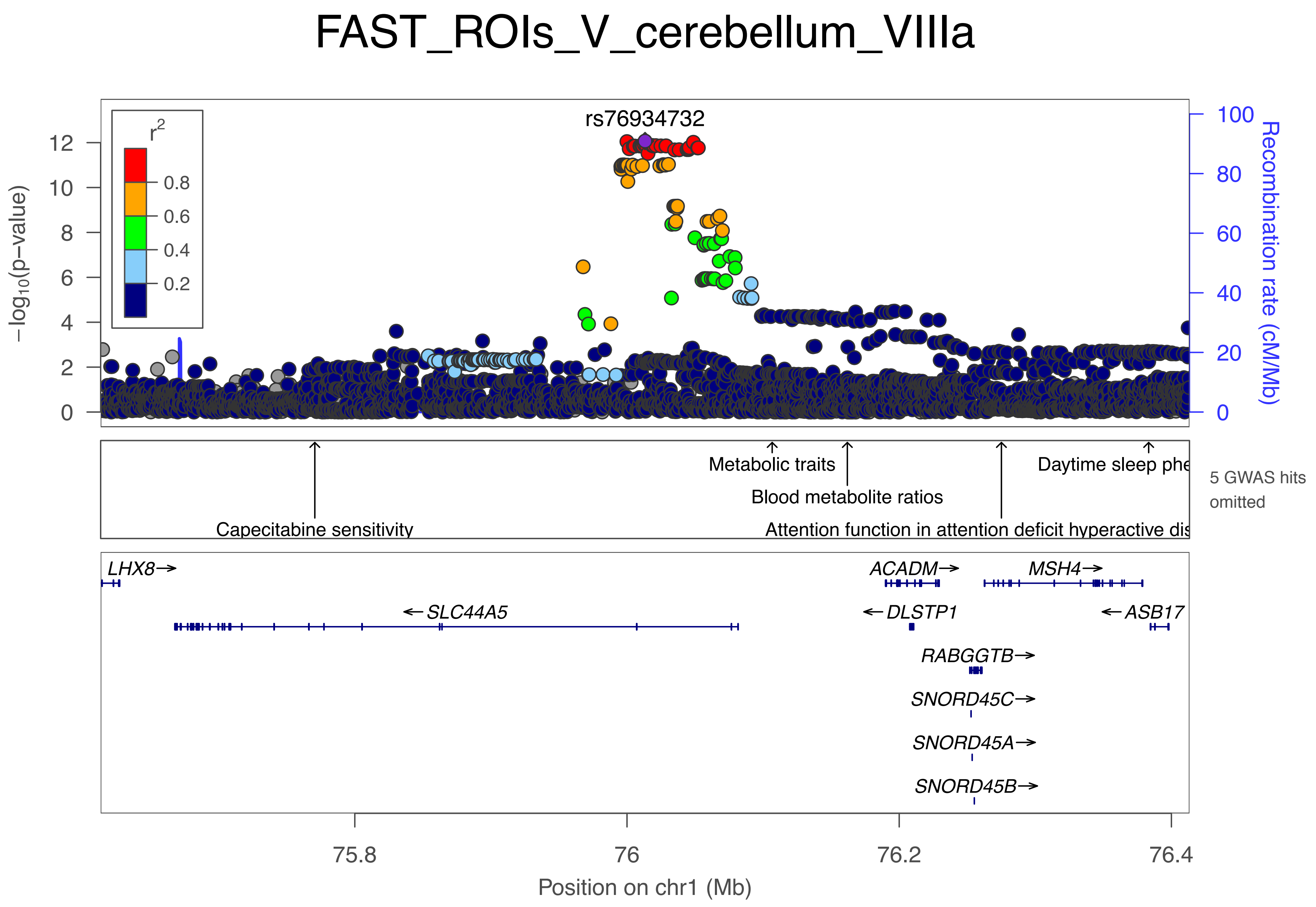

FAST_ROIs_V_cerebellum_VIIIa
rs76934732
r2
0.8
0.6
0.4
0.2
−log10(p−value)
Recombination rate (cM/Mb)
Metabolic traits
Blood metabolite ratios
Daytime sleep phe
Capecitabine sensitivity
Attention function in attention deficit hyperactive dis
5 GWAS hits omitted
LHX8
SLC44A5
ACADM
DLSTP1
MSH4
ASB17
RABGGTB
SNORD45C
SNORD45A
SNORD45B
75.8
76
76.2
76.4
Position on chr1 (Mb)

| | |
|---|---|
| date: | Thu Aug 17 17:58:46 2017 |
| build: | hg19 |
| display range: | chr1:75613268−76413268 [75613268−76413268] |
| hilite range: | 0 – 0 [ 0 – 0 ] |
| reference SNP: | chr1:76013268 |
| number of SNPs plotted: | 3686 |
| min P.value: | 8.51E−13 [chr1:76013268] |
| max P.value: | 10E−1 [chr1:75725482] |
| omitted GWAS Hits: | chr1:76.383297−Daytime sleep phenotypes, NA |
| omitted GWAS Hits: | NA, NA |

Make more plots at http://csg.sph.umich.edu/locuszoom/

| | |
|---|---|
| date: | Thu Aug 17 17:58:46 2017 |
| build: | hg19 |
| display range: | chr1:75613268−76413268 [75613268−76413268] |
| hilite range: | 0 – 0 [ 0 – 0 ] |
| reference SNP: | chr1:76013268 |
| number of SNPs plotted: | 3686 |
| min P.value: | 8.51E−13 [chr1:76013268] |
| max P.value: | 10E−1 [chr1:75725482] |
| omitted GWAS Hits: | chr1:76.383297−Daytime sleep phenotypes, NA |
| omitted GWAS Hits: | NA, NA |

Make more plots at http://csg.sph.umich.edu/locuszoom/

GWAS Catalog SNPs in Region

| chr | pos (Mb) | trait | snp |
|---|---|---|---|
| 1 | 75.77062 | Capecitabine sensitivity | rs1249675 |
| 1 | 75.91383 | Whole−brain volume (Alzheimer's disease interaction) | rs1857353 |
| 1 | 76.10667 | Metabolic traits | rs211718 |
| 1 | 76.13595 | Acylcarnitine levels | rs7552404 |
| 1 | 76.13595 | Blood metabolite levels | rs7552404 |
| 1 | 76.16189 | Blood metabolite ratios | rs4949874 |
| 1 | 76.16862 | Daytime sleep phenotypes | rs815306 |
| 1 | 76.21633 | Blood metabolite levels | rs11161521 |
| 1 | 76.27510 | Attention function in attention deficit hyperactive disorder | rs1144333 |
| 1 | 76.38330 | Daytime sleep phenotypes | rs10782582 |

# TBSS_ICVF_Genu_of_corpus_callosum

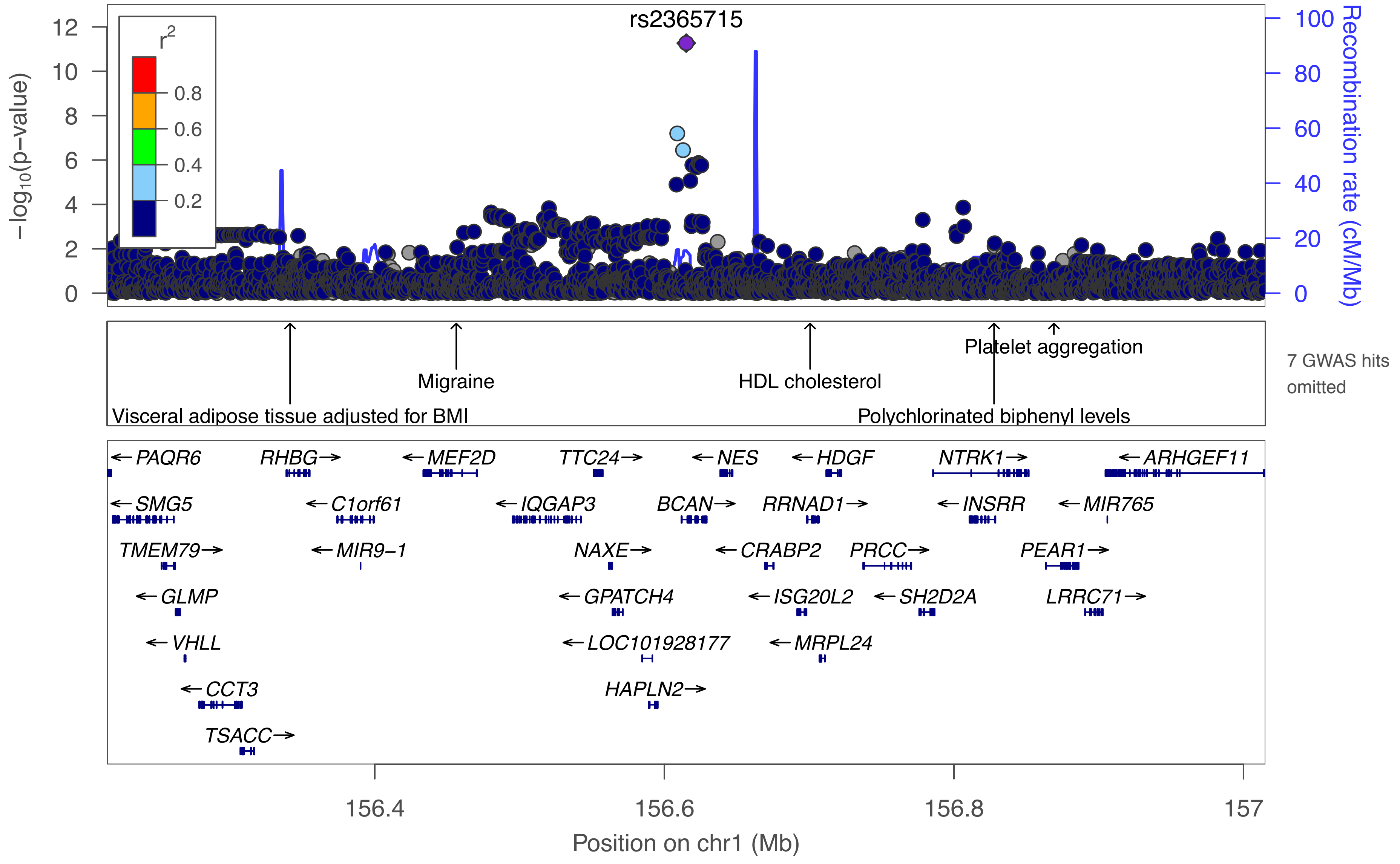

| | |
|---|---|
| date: | Thu Aug 17 18:11:30 2017 |
| build: | hg19 |
| display range: | chr1:156215114−157015114 [156215114−157015114] |
| hilite range: | 0 – 0 [ 0 – 0 ] |
| reference SNP: | chr1:156615114 |
| number of SNPs plotted: | 3160 |
| min P.value: | 5.38E−12 [chr1:156615114] |
| max P.value: | 10E−1 [chr1:156796697] |
| omitted GWAS Hits: | chr1:156.700651−HDL cholesterol, chr1:156.827703−Polychlorinated biphenyl levels |
| omitted GWAS Hits: | NA, NA |
| omitted GWAS Hits: | NA |

Make more plots at http://csg.sph.umich.edu/locuszoom/

GWAS Catalog SNPs in Region

| chr | pos (Mb) | trait | snp |
|---|---|---|---|
| 1 | 156.2555 | Glycated hemoglobin levels | rs6684514 |
| 1 | 156.2555 | Mean corpuscular hemoglobin concentration | rs6684514 |
| 1 | 156.3415 | Visceral adipose tissue adjusted for BMI | rs7525133 |
| 1 | 156.3565 | Subcutaneous adipose tissue | rs6686886 |
| 1 | 156.4462 | Migraine | rs2274316 |
| 1 | 156.4462 | Migraine – clinic–based | rs2274316 |
| 1 | 156.4507 | Migraine | rs1925950 |
| 1 | 156.4563 | Migraine | rs3790455 |
| 1 | 156.4565 | Resting heart rate | rs1171563 |
| 1 | 156.7007 | HDL cholesterol | rs12145743 |
| 1 | 156.8277 | Polychlorinated biphenyl levels | rs115699453 |
| 1 | 156.8690 | Platelet aggregation | rs12566888 |

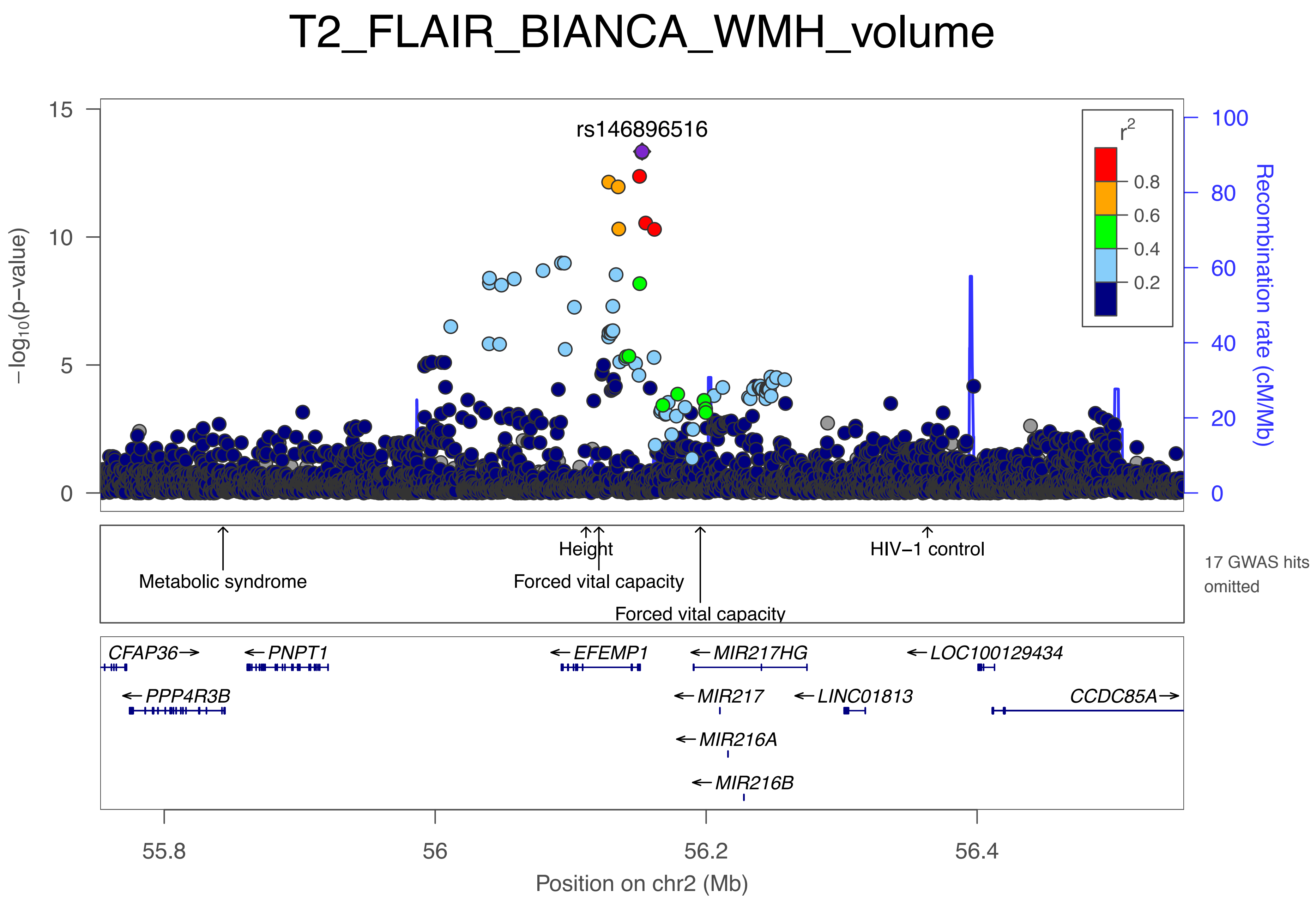

T2_FLAIR_BIANCA_WMH_volume
rs146896516
−log10(p−value)
Recombination rate (cM/Mb)
r2
0.8
0.6
0.4
0.2
15
10
5
0
100
80
60
40
20
Metabolic syndrome
Height
Forced vital capacity
Forced vital capacity
HIV−1 control
17 GWAS hits omitted
CFAP36
PNPT1
EFEMP1
MIR217HG
LOC100129434
PPP4R3B
MIR217
LINC01813
CCDC85A
MIR216A
MIR216B
55.8
56
56.2
56.4
Position on chr2 (Mb)

| | |
|---|---|
| date: | Thu Aug 17 18:04:38 2017 |
| build: | hg19 |
| display range: | chr2:55752750−56552750 [55752750−56552750] |
| hilite range: | 0 – 0 [ 0 – 0 ] |
| reference SNP: | chr2:56152750 |
| number of SNPs plotted: | 4119 |
| min P.value: | 4.58E−14 [chr2:56152750] |
| max P.value: | 10E−1 [chr2:56359853] |
| omitted GWAS Hits: | NA, NA |
| omitted GWAS Hits: | NA, NA |
| omitted GWAS Hits: | NA, NA |
| omitted GWAS Hits: | NA, NA |
| omitted GWAS Hits: | NA, NA |
| omitted GWAS Hits: | NA, NA |

Make more plots at http://csg.sph.umich.edu/locuszoom/

GWAS Catalog SNPs in Region

| chr | pos (Mb) | trait | snp |
|---|---|---|---|
| 2 | 55.84341 | Metabolic syndrome | rs782590 |
| 2 | 55.97373 | IgG glycosylation | rs2163237 |
| 2 | 56.00890 | Amyotrophic lateral sclerosis | rs7577894 |
| 2 | 56.01221 | Inguinal hernia | rs2009262 |
| 2 | 56.02587 | 3−hydroxy−1−methylpropylmercapturic acid levels in smokers | rs74760234 |
| 2 | 56.06718 | Height | rs4146922 |
| 2 | 56.08954 | Height | rs1367226 |
| 2 | 56.09689 | Height | rs3791679 |
| 2 | 56.09689 | Waist circumference adjusted for body mass index | rs3791679 |
| 2 | 56.10833 | Optic cup area | rs1346786 |
| 2 | 56.11131 | Height | rs3791675 |
| 2 | 56.12085 | Forced vital capacity | rs1430193 |
| 2 | 56.13510 | White matter hyperintensity burden | rs78857879 |
| 2 | 56.15656 | Post bronchodilator FEV1/FVC ratio | rs180765220 |
| 2 | 56.18293 | Post bronchodilator FEV1/FVC ratio | rs190486114 |
| 2 | 56.19147 | Schizophrenia | rs2868985 |
| 2 | 56.19570 | Forced vital capacity | rs62164511 |
| 2 | 56.19715 | Post bronchodilator FEV1/FVC ratio | rs186819721 |
| 2 | 56.19780 | Post bronchodilator FEV1/FVC ratio | rs138402956 |
| 2 | 56.21767 | Post bronchodilator FEV1/FVC ratio | rs182751789 |
| 2 | 56.25550 | Pelvic organ prolapse (moderate/severe) | rs3850352 |

# GWAS Catalog SNPs in Region

| chr | pos (Mb) | trait | snp |
|---|---|---|---|
| 2 | 56.36338 | HIV−1 control | rs6751715 |

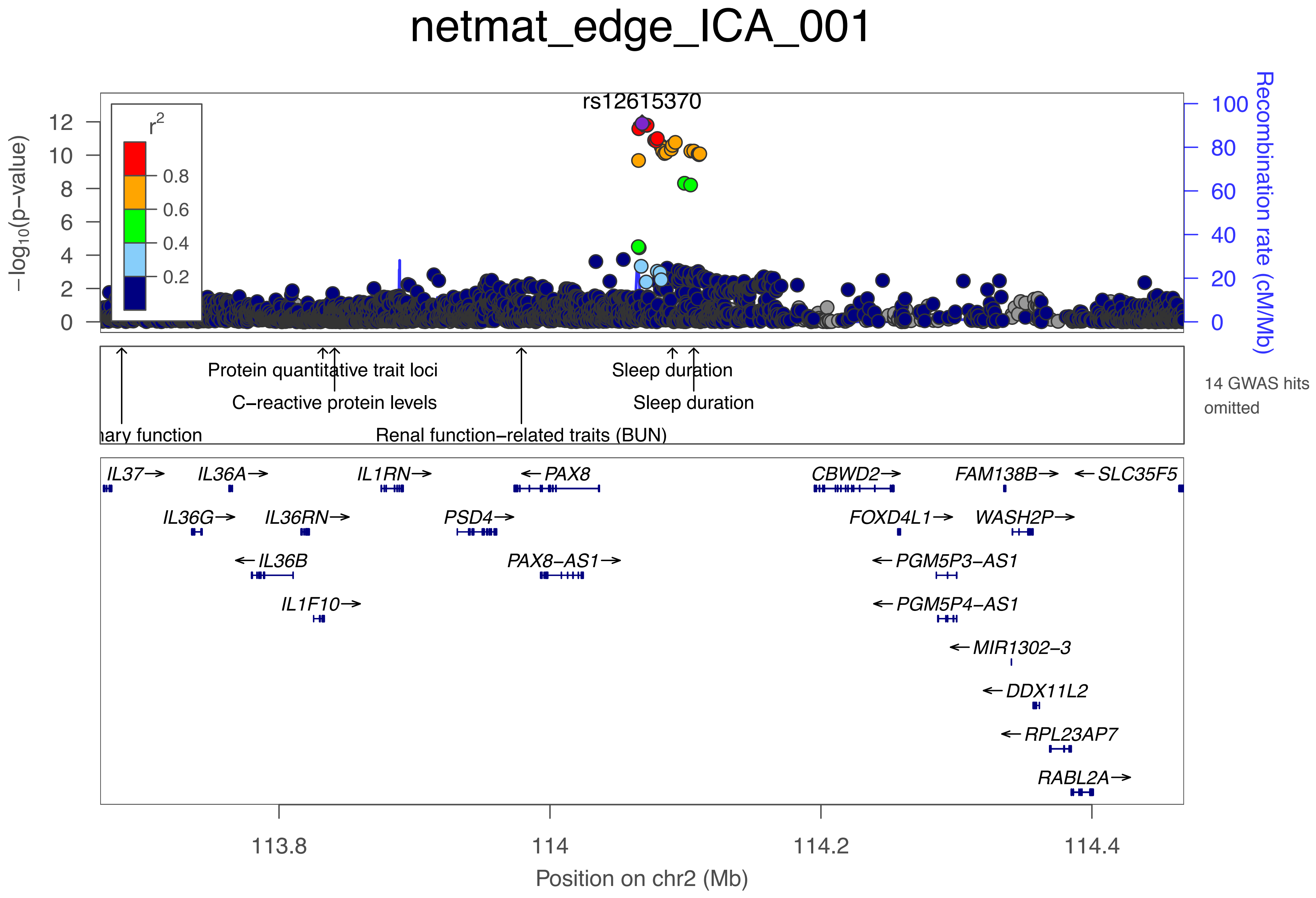

netmat_edge_ICA_001
rs12615370
r2
0.8
0.6
0.4
0.2
−log10(p−value)
Recombination rate (cM/Mb)
Protein quantitative trait loci
C−reactive protein levels
nary function
Renal function−related traits (BUN)
Sleep duration
Sleep duration
14 GWAS hits omitted
IL37→
IL36A→
IL1RN→
←PAX8
CBWD2→
FAM138B→
←SLC35F5
IL36G→
IL36RN→
PSD4→
FOXD4L1→
WASH2P→
←IL36B
PAX8−AS1→
←PGM5P3−AS1
IL1F10→
←PGM5P4−AS1
←MIR1302−3
←DDX11L2
←RPL23AP7
RABL2A→
113.8
114
114.2
114.4
Position on chr2 (Mb)

| | |
|---|---|
| date: | Sat Aug 19 17:32:05 2017 |
| build: | hg19 |
| display range: | chr2:113668017−114468017 [113668017−114468017] |
| hilite range: | 0 – 0 [ 0 – 0 ] |
| reference SNP: | chr2:114068017 |
| number of SNPs plotted: | 3145 |
| min P.value: | 1.26E−12 [chr2:114068017] |
| max P.value: | 10E−1 [chr2:114446440] |
| omitted GWAS Hits: | chr2:113.97894−Renal function−related traits (BUN), chr2:114.090412−Sleep duration |
| omitted GWAS Hits: | NA, NA |
| omitted GWAS Hits: | NA, NA |
| omitted GWAS Hits: | NA, NA |
| omitted GWAS Hits: | NA, NA |

Make more plots at http://csg.sph.umich.edu/locuszoom/

GWAS Catalog SNPs in Region

| chr | pos (Mb) | trait | snp |
|---|---|---|---|
| 2 | 113.6839 | Pulmonary function | rs10864907 |
| 2 | 113.6914 | Response to amphetamines | rs12467847 |
| 2 | 113.8323 | Protein quantitative trait loci | rs6761276 |
| 2 | 113.8323 | Inflammatory biomarkers | rs6743376 |
| 2 | 113.8378 | C−reactive protein levels or LDL−cholesterol levels (pleiotropy) | rs12711751 |
| 2 | 113.8381 | C−reactive protein levels or triglyceride levels (pleiotropy) | rs13409360 |
| 2 | 113.8410 | C−reactive protein levels | rs6734238 |
| 2 | 113.8410 | Fibrinogen | rs6734238 |
| 2 | 113.8410 | C−reactive protein | rs6734238 |
| 2 | 113.8410 | White blood cell count | rs6734238 |
| 2 | 113.8410 | Fibrinogen levels | rs6734238 |
| 2 | 113.8410 | C−reactive protein levels or total cholesterol levels (pleiotropy) | rs6734238 |
| 2 | 113.8476 | Stroke | rs11681884 |
| 2 | 113.8904 | Obesity−related traits | rs4252023 |
| 2 | 113.9729 | Mucinous ovarian carcinoma | rs752590 |
| 2 | 113.9789 | Renal function−related traits (BUN) | rs11123170 |
| 2 | 114.0164 | Mucinous ovarian carcinoma | rs72831838 |
| 2 | 114.0904 | Sleep duration | rs1823125 |
| 2 | 114.1061 | Sleep duration | rs62158211 |
| 2 | 114.1061 | Sleep duration (oversleepers vs undersleepers) | rs62158211 |

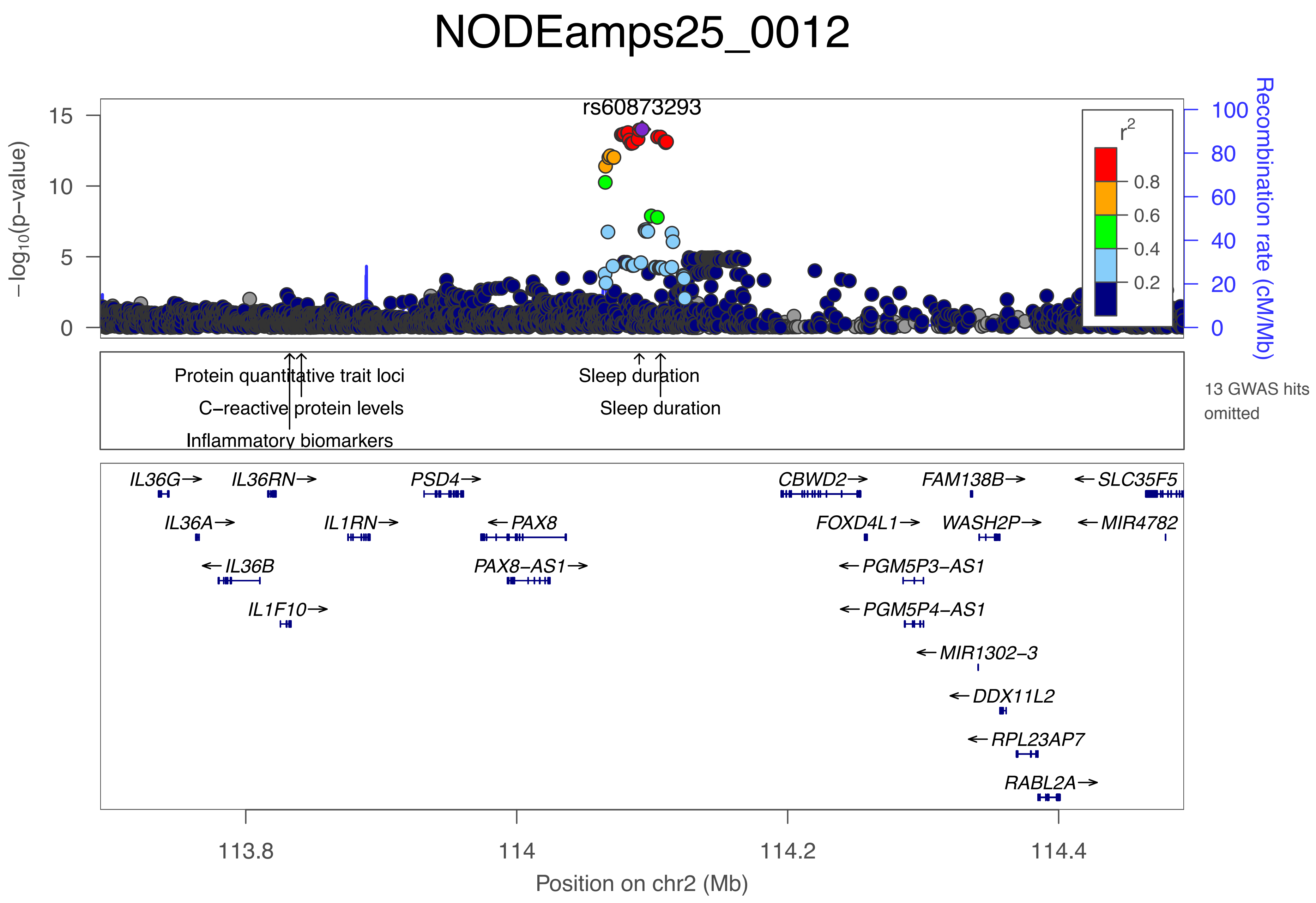
NODEamps25_0012
rs60873293
−log10(p−value)
Recombination rate (cM/Mb)
r2
0.8
0.6
0.4
0.2
Protein quantitative trait loci
C−reactive protein levels
Inflammatory biomarkers
Sleep duration
Sleep duration
13 GWAS hits omitted
IL36G→
IL36RN→
PSD4→
CBWD2→
FAM138B→
←SLC35F5
IL36A→
IL1RN→
←PAX8
FOXD4L1→
WASH2P→
←MIR4782
←IL36B
PAX8−AS1→
←PGM5P3−AS1
IL1F10→
←PGM5P4−AS1
←MIR1302−3
←DDX11L2
←RPL23AP7
RABL2A→
113.8
114
114.2
114.4
Position on chr2 (Mb)

| | |
|---|---|
| date: | Thu Aug 17 18:02:20 2017 |
| build: | hg19 |
| display range: | chr2:113692549−114492549 [113692549−114492549] |
| hilite range: | 0 – 0 [ 0 – 0 ] |
| reference SNP: | chr2:114092549 |
| number of SNPs plotted: | 3067 |
| min P.value: | 9.86E−15 [chr2:114092549] |
| max P.value: | 10E−1 [chr2:113762224] |
| omitted GWAS Hits: | chr2:114.090412−Sleep duration, chr2:114.106139−Sleep duration |
| omitted GWAS Hits: | NA, NA |
| omitted GWAS Hits: | NA, NA |
| omitted GWAS Hits: | NA, NA |
| omitted GWAS Hits: | NA |

GWAS Catalog SNPs in Region

| chr | pos (Mb) | trait | snp |
|---|---|---|---|
| 2 | 113.8323 | Protein quantitative trait loci | rs6761276 |
| 2 | 113.8323 | Inflammatory biomarkers | rs6743376 |
| 2 | 113.8378 | C−reactive protein levels or LDL−cholesterol levels (pleiotropy) | rs12711751 |
| 2 | 113.8381 | C−reactive protein levels or triglyceride levels (pleiotropy) | rs13409360 |
| 2 | 113.8410 | C−reactive protein levels | rs6734238 |
| 2 | 113.8410 | Fibrinogen | rs6734238 |
| 2 | 113.8410 | C−reactive protein | rs6734238 |
| 2 | 113.8410 | White blood cell count | rs6734238 |
| 2 | 113.8410 | Fibrinogen levels | rs6734238 |
| 2 | 113.8410 | C−reactive protein levels or total cholesterol levels (pleiotropy) | rs6734238 |
| 2 | 113.8476 | Stroke | rs11681884 |
| 2 | 113.8904 | Obesity−related traits | rs4252023 |
| 2 | 113.9729 | Mucinous ovarian carcinoma | rs752590 |
| 2 | 113.9789 | Renal function−related traits (BUN) | rs11123170 |
| 2 | 114.0164 | Mucinous ovarian carcinoma | rs72831838 |
| 2 | 114.0904 | Sleep duration | rs1823125 |
| 2 | 114.1061 | Sleep duration | rs62158211 |
| 2 | 114.1061 | Sleep duration (oversleepers vs undersleepers) | rs62158211 |

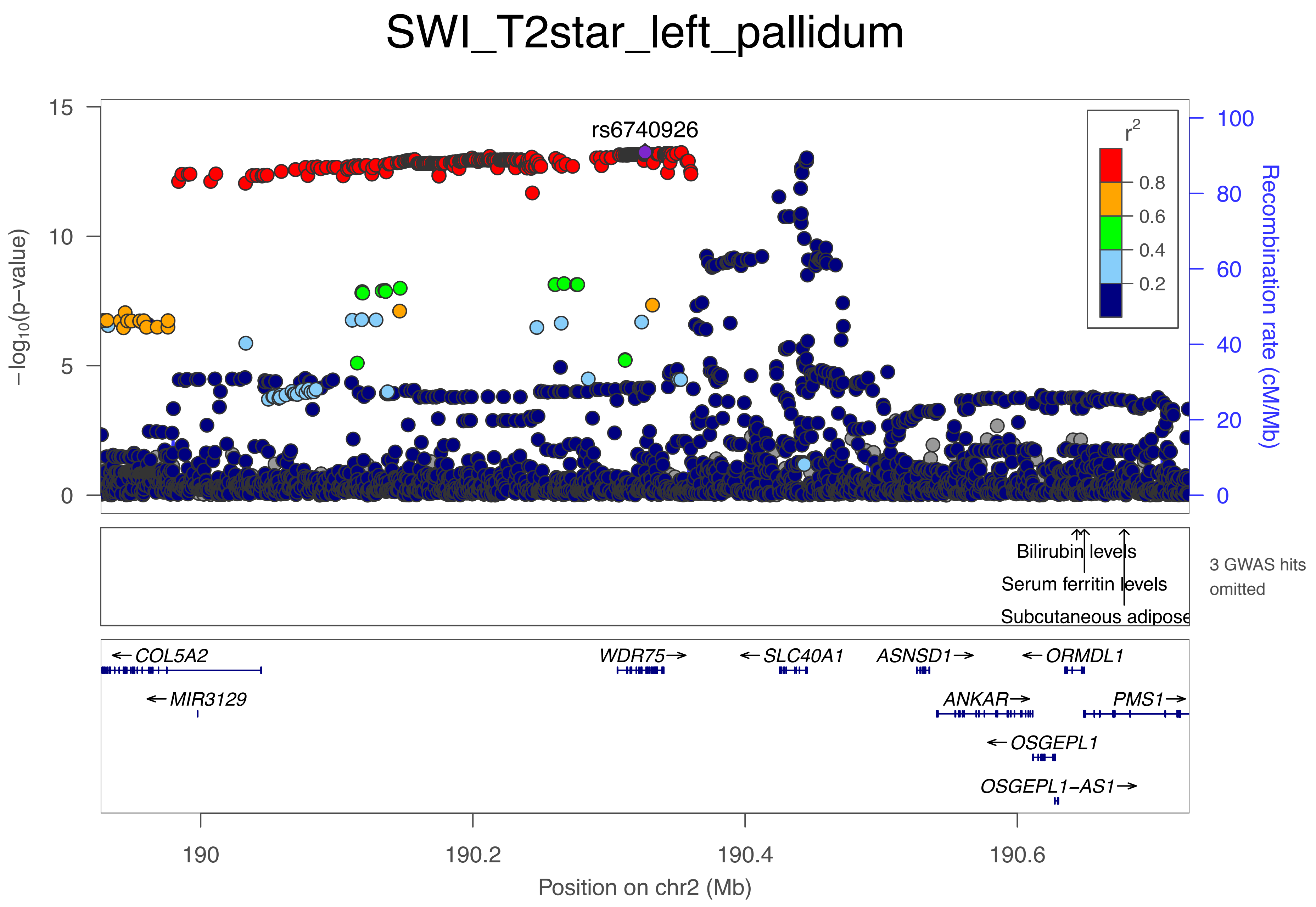
SWI_T2star_left_pallidum
rs6740926
−log10(p−value)
15
10
5
0
100
80
60
40
20
0
Recombination rate (cM/Mb)
r2
0.8
0.6
0.4
0.2
Bilirubin levels
Serum ferritin levels
Subcutaneous adipose
3 GWAS hits omitted
←COL5A2
←MIR3129
WDR75→
←SLC40A1
ASNSD1→
←ORMDL1
ANKAR→
PMS1→
←OSGEPL1
OSGEPL1−AS1→
190
190.2
190.4
190.6
Position on chr2 (Mb)

| | |
|---|---|
| date: | Thu Aug 17 18:10:54 2017 |
| build: | hg19 |
| display range: | chr2:189926498−190726498 [189926498−190726498] |
| hilite range: | 0 – 0 [ 0 – 0 ] |
| reference SNP: | chr2:190326498 |
| number of SNPs plotted: | 3013 |
| min P.value: | 5.68E−14 [chr2:190326498] |
| max P.value: | 9.99E−1 [chr2:190307272] |
| omitted GWAS Hits: | NA, NA |

Make more plots at http://csg.sph.umich.edu/locuszoom/

GWAS Catalog SNPs in Region

| chr | pos (Mb) | trait | snp |
|---|---|---|---|
| 2 | 190.3787 | Iron status biomarkers (ferritin levels) | rs744653 |
| 2 | 190.3787 | Iron status biomarkers (transferrin levels) | rs744653 |
| 2 | 190.6072 | Obesity−related traits | rs12053254 |
| 2 | 190.6436 | Bilirubin levels | rs7606224 |
| 2 | 190.6493 | Serum ferritin levels | rs5742933 |
| 2 | 190.6785 | Subcutaneous adipose tissue | rs5743030 |

# SWI_T2star_left_pallidum_plus_right_pallidum

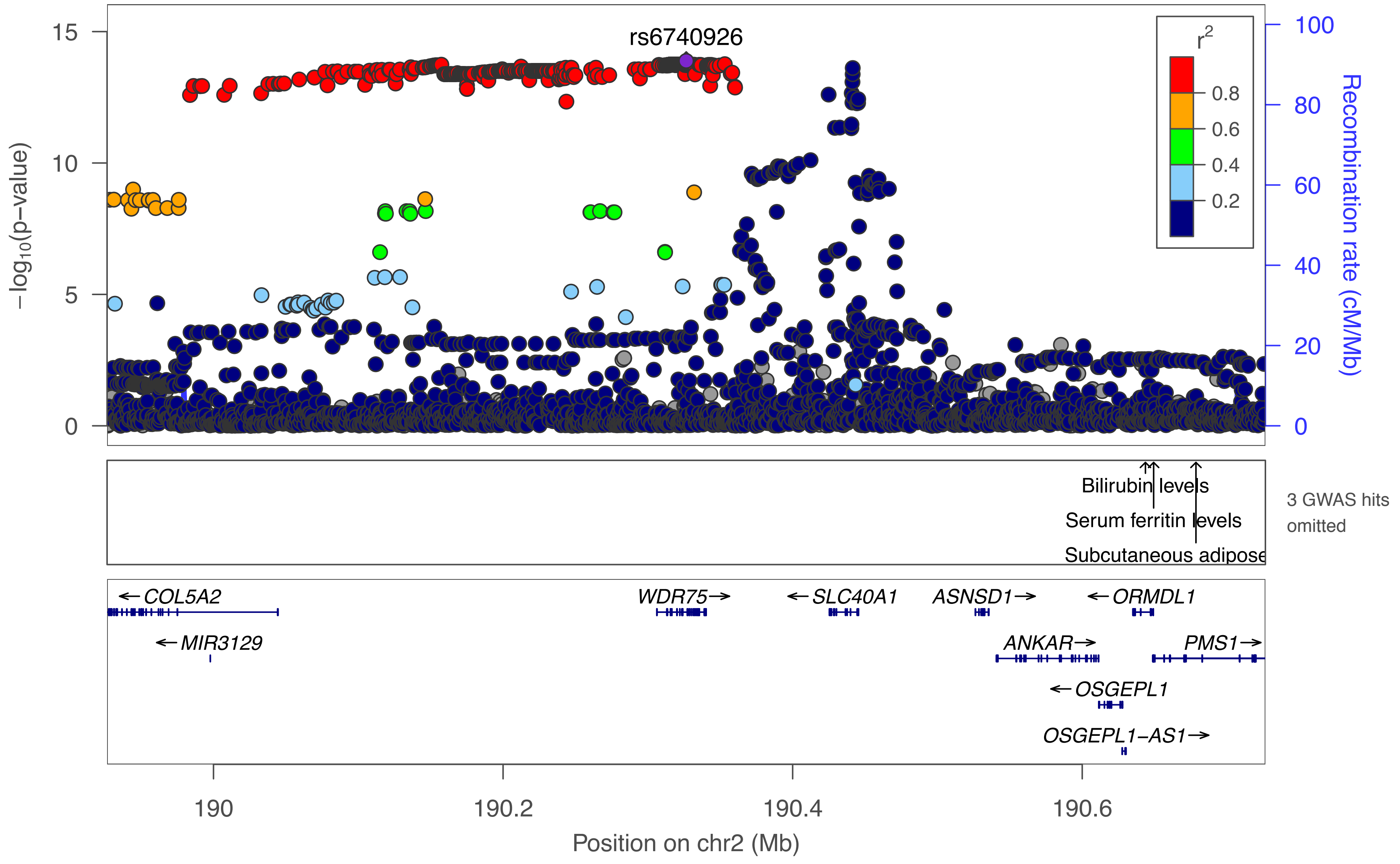

| | |
|---|---|
| date: | Thu Aug 17 18:10:54 2017 |
| build: | hg19 |
| display range: | chr2:189926498−190726498 [189926498−190726498] |
| hilite range: | 0 – 0 [ 0 – 0 ] |
| reference SNP: | chr2:190326498 |
| number of SNPs plotted: | 3013 |
| min P.value: | 1.31E−14 [chr2:190326498] |
| max P.value: | 10E−1 [chr2:190039575] |
| omitted GWAS Hits: | NA, NA |

Make more plots at http://csg.sph.umich.edu/locuszoom/

GWAS Catalog SNPs in Region

| chr | pos (Mb) | trait | snp |
|---|---|---|---|
| 2 | 190.3787 | Iron status biomarkers (ferritin levels) | rs744653 |
| 2 | 190.3787 | Iron status biomarkers (transferrin levels) | rs744653 |
| 2 | 190.6072 | Obesity–related traits | rs12053254 |
| 2 | 190.6436 | Bilirubin levels | rs7606224 |
| 2 | 190.6493 | Serum ferritin levels | rs5742933 |
| 2 | 190.6785 | Subcutaneous adipose tissue | rs5743030 |

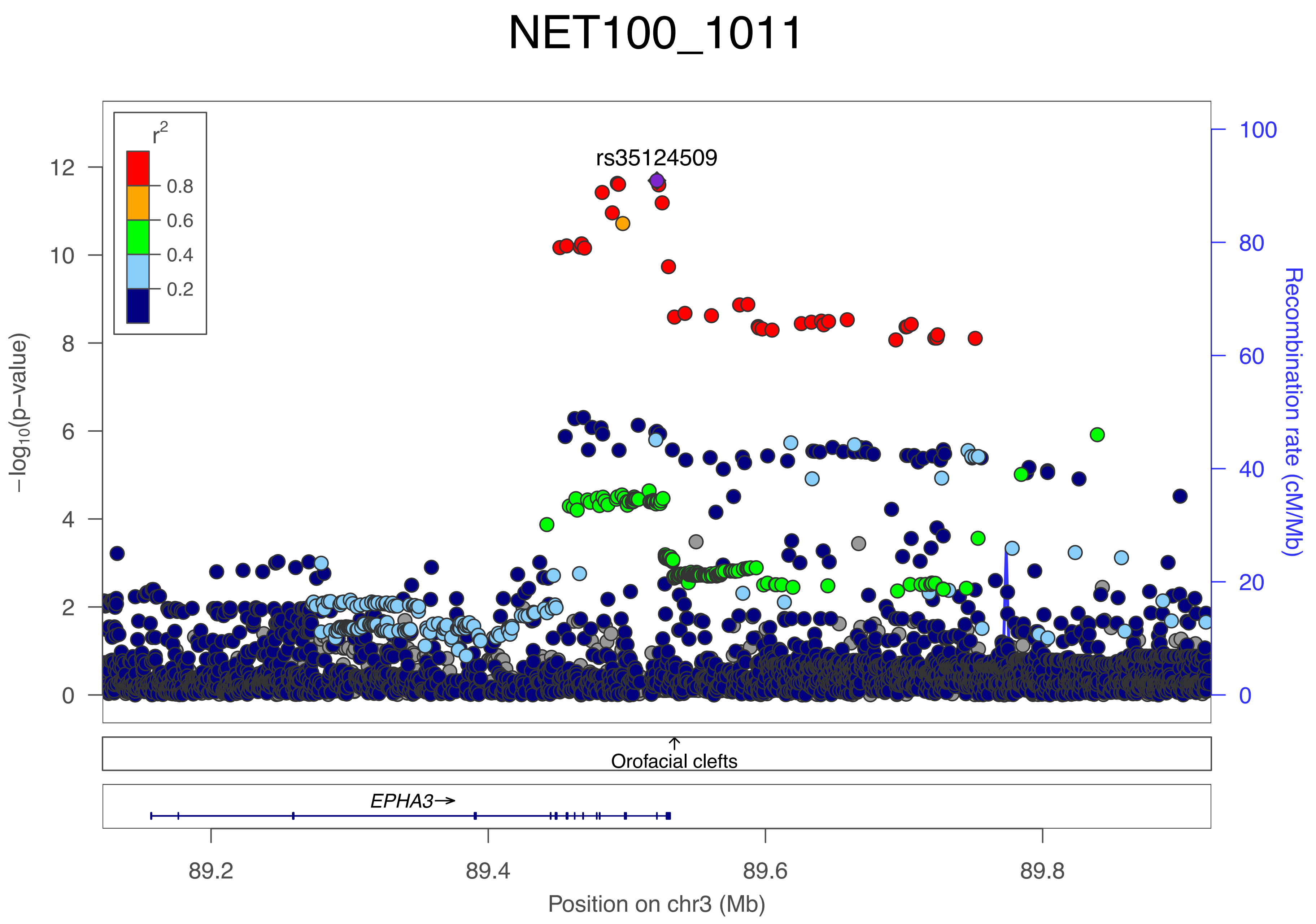
NET100_1011
rs35124509
r2
0.8
0.6
0.4
0.2
−log10(p−value)
Recombination rate (cM/Mb)
Orofacial clefts
EPHA3→
89.2
89.4
89.6
89.8
Position on chr3 (Mb)

| | |
|---|---|
| date: | Thu Aug 17 17:59:19 2017 |
| build: | hg19 |
| display range: | chr3:89121693–89921693 [89121693–89921693] |
| hilite range: | 0 – 0 [ 0 – 0 ] |
| reference SNP: | chr3:89521693 |
| number of SNPs plotted: | 3517 |
| min P.value: | 2.04E–12 [chr3:89521693] |
| max P.value: | 10E–1 [chr3:89562907] |

Make more plots at http://csg.sph.umich.edu/locuszoom/

GWAS Catalog SNPs in Region

| chr | pos (Mb) | trait | snp |
|---|---|---|---|
| 3 | 89.53438 | Orofacial clefts | rs7632427 |

# netmat_edge_ICA_003

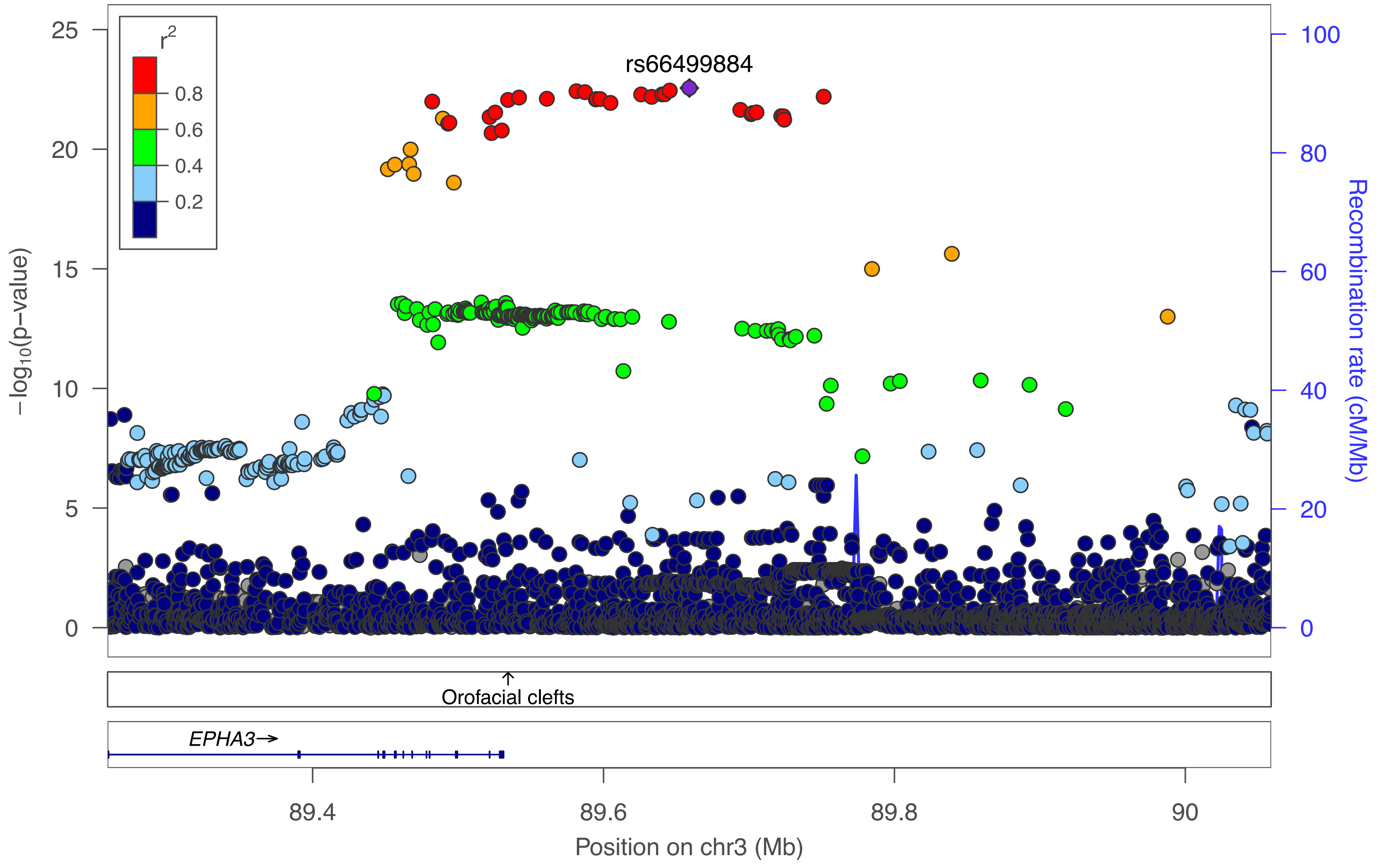

| | |
|---|---|
| date: | Sat Aug 19 17:37:26 2017 |
| build: | hg19 |
| display range: | chr3:89259012−90059012 [89259012−90059012] |
| hilite range: | 0 – 0  [ 0 – 0 ] |
| reference SNP: | chr3:89659012 |
| number of SNPs plotted: | 3873 |
| min P.value: | 2.77E−23 [chr3:89659012] |
| max P.value: | 10E−1 [chr3:90006653] |

Make more plots at http://csg.sph.umich.edu/locuszoom/

GWAS Catalog SNPs in Region

| chr | pos (Mb) | trait | snp |
|---|---|---|---|
| 3 | 89.53438 | Orofacial clefts | rs7632427 |

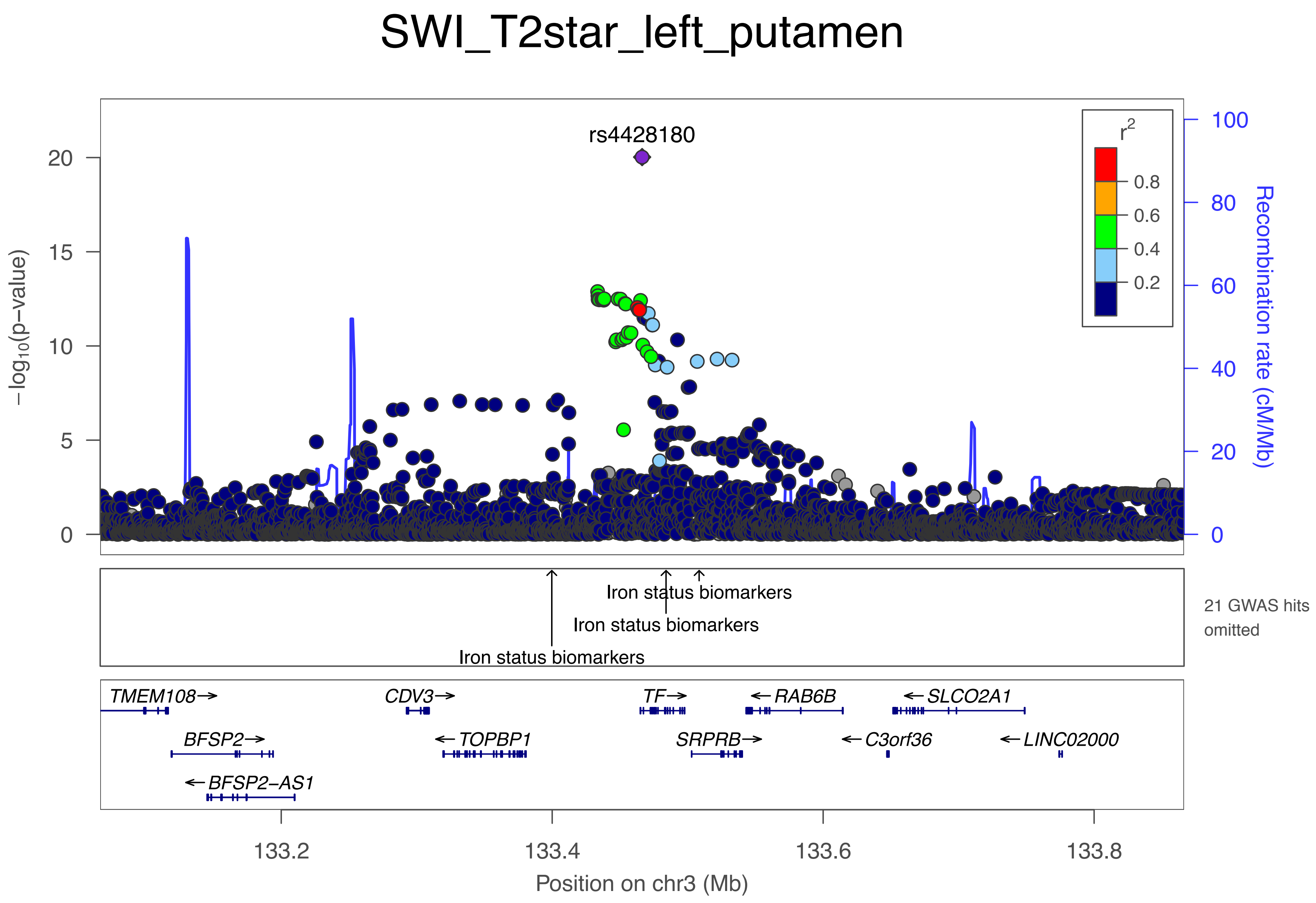

SWI_T2star_left_putamen
rs4428180
r2
0.8
0.6
0.4
0.2
−log10(p−value)
Recombination rate (cM/Mb)
Iron status biomarkers
Iron status biomarkers
Iron status biomarkers
21 GWAS hits omitted
TMEM108
CDV3
TF
RAB6B
SLCO2A1
BFSP2
TOPBP1
SRPRB
C3orf36
LINC02000
BFSP2−AS1
133.2
133.4
133.6
133.8
Position on chr3 (Mb)

| | |
|---|---|
| date: | Thu Aug 17 18:24:22 2017 |
| build: | hg19 |
| display range: | chr3:133066374−133866374 [133066374−133866374] |
| hilite range: | 0 – 0 [ 0 – 0 ] |
| reference SNP: | chr3:133466374 |
| number of SNPs plotted: | 3859 |
| min P.value: | 9.44E−21 [chr3:133466374] |
| max P.value: | 10E−1 [chr3:133574407] |
| omitted GWAS Hits: | NA, NA |
| omitted GWAS Hits: | NA, NA |
| omitted GWAS Hits: | NA, NA |
| omitted GWAS Hits: | NA, NA |
| omitted GWAS Hits: | NA, NA |
| omitted GWAS Hits: | NA, NA |
| omitted GWAS Hits: | NA, NA |

Make more plots at http://csg.sph.umich.edu/locuszoom/

GWAS Catalog SNPs in Region

| chr | pos (Mb) | trait | snp |
|---|---|---|---|
| 3 | 133.2609 | Bulimia nervosa | rs11708304 |
| 3 | 133.3997 | Iron status biomarkers | rs2718812 |
| 3 | 133.4106 | Autism spectrum disorder−related traits | rs1867503 |
| 3 | 133.4107 | Iron status biomarkers | rs1867504 |
| 3 | 133.4309 | Alzheimer disease and age of onset | rs190500289 |
| 3 | 133.4575 | Iron status biomarkers | rs9872999 |
| 3 | 133.4635 | Iron status biomarkers (transferrin levels) | rs8177179 |
| 3 | 133.4757 | Iron status biomarkers | rs1799852 |
| 3 | 133.4757 | Iron status biomarkers (transferrin saturation) | rs1799852 |
| 3 | 133.4758 | Alcohol consumption (transferrin glycosylation) | rs1799899 |
| 3 | 133.4777 | Iron status biomarkers (transferrin saturation) | rs8177240 |
| 3 | 133.4777 | Iron status biomarkers (transferrin levels) | rs8177240 |
| 3 | 133.4777 | Iron status biomarkers (iron levels) | rs8177240 |
| 3 | 133.4802 | Iron status biomarkers | rs8177253 |
| 3 | 133.4840 | Iron status biomarkers | rs3811647 |
| 3 | 133.4840 | Hepcidin levels | rs3811647 |
| 3 | 133.4840 | Alcohol consumption (transferrin glycosylation) | rs3811647 |
| 3 | 133.4840 | Hereditary hemochromatosis−related traits (HFE mutation homozygotes) | rs3811647 |
| 3 | 133.4944 | Alcohol consumption (transferrin glycosylation) | rs1049296 |
| 3 | 133.5085 | Iron status biomarkers | rs1830084 |
| 3 | 133.5371 | Alcohol consumption (transferrin glycosylation) | rs1534166 |

GWAS Catalog SNPs in Region

| chr | pos (Mb) | trait | snp |
|---|---|---|---|
| 3 | 133.5893 | Immune reponse to smallpox (secreted IL−1beta) | rs9835973 |
| 3 | 133.5971 | Post bronchodilator FEV1/FVC ratio | rs140798517 |
| 3 | 133.6289 | Post bronchodilator FEV1/FVC ratio | rs143087368 |

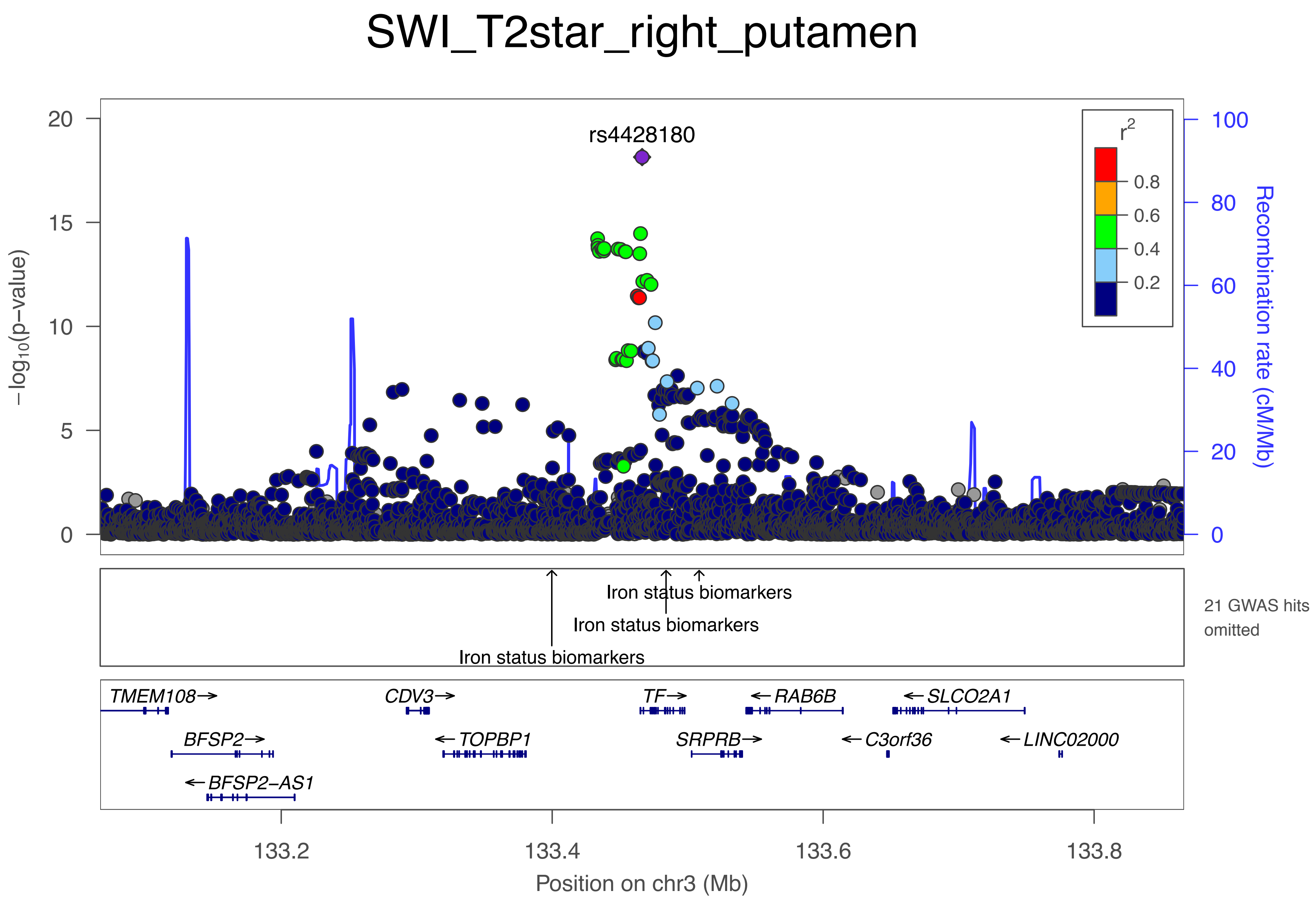

SWI_T2star_right_putamen
rs4428180
−log10(p−value)
Recombination rate (cM/Mb)
r2
0.8
0.6
0.4
0.2
0
5
10
15
20
0
20
40
60
80
100
Iron status biomarkers
Iron status biomarkers
Iron status biomarkers
21 GWAS hits omitted
TMEM108→
CDV3→
TF→
←RAB6B
←SLCO2A1
BFSP2→
←TOPBP1
SRPRB→
←C3orf36
←LINC02000
←BFSP2−AS1
133.2
133.4
133.6
133.8
Position on chr3 (Mb)

| | |
|---|---|
| date: | Thu Aug 17 18:24:22 2017 |
| build: | hg19 |
| display range: | chr3:133066374−133866374 [133066374−133866374] |
| hilite range: | 0 – 0 [ 0 – 0 ] |
| reference SNP: | chr3:133466374 |
| number of SNPs plotted: | 3859 |
| min P.value: | 7.24E−19 [chr3:133466374] |
| max P.value: | 10E−1 [chr3:133178432] |
| omitted GWAS Hits: | NA, NA |
| omitted GWAS Hits: | NA, NA |
| omitted GWAS Hits: | NA, NA |
| omitted GWAS Hits: | NA, NA |
| omitted GWAS Hits: | NA, NA |
| omitted GWAS Hits: | NA, NA |
| omitted GWAS Hits: | NA, NA |

Make more plots at http://csg.sph.umich.edu/locuszoom/

GWAS Catalog SNPs in Region

| chr | pos (Mb) | trait | snp |
|---|---|---|---|
| 3 | 133.2609 | Bulimia nervosa | rs11708304 |
| 3 | 133.3997 | Iron status biomarkers | rs2718812 |
| 3 | 133.4106 | Autism spectrum disorder–related traits | rs1867503 |
| 3 | 133.4107 | Iron status biomarkers | rs1867504 |
| 3 | 133.4309 | Alzheimer disease and age of onset | rs190500289 |
| 3 | 133.4575 | Iron status biomarkers | rs9872999 |
| 3 | 133.4635 | Iron status biomarkers (transferrin levels) | rs8177179 |
| 3 | 133.4757 | Iron status biomarkers | rs1799852 |
| 3 | 133.4757 | Iron status biomarkers (transferrin saturation) | rs1799852 |
| 3 | 133.4758 | Alcohol consumption (transferrin glycosylation) | rs1799899 |
| 3 | 133.4777 | Iron status biomarkers (transferrin saturation) | rs8177240 |
| 3 | 133.4777 | Iron status biomarkers (transferrin levels) | rs8177240 |
| 3 | 133.4777 | Iron status biomarkers (iron levels) | rs8177240 |
| 3 | 133.4802 | Iron status biomarkers | rs8177253 |
| 3 | 133.4840 | Iron status biomarkers | rs3811647 |
| 3 | 133.4840 | Hepcidin levels | rs3811647 |
| 3 | 133.4840 | Alcohol consumption (transferrin glycosylation) | rs3811647 |
| 3 | 133.4840 | Hereditary hemochromatosis–related traits (HFE mutation homozygotes) | rs3811647 |
| 3 | 133.4944 | Alcohol consumption (transferrin glycosylation) | rs1049296 |
| 3 | 133.5085 | Iron status biomarkers | rs1830084 |
| 3 | 133.5371 | Alcohol consumption (transferrin glycosylation) | rs1534166 |

GWAS Catalog SNPs in Region

| chr | pos (Mb) | trait | snp |
|---|---|---|---|
| 3 | 133.5893 | Immune reponse to smallpox (secreted IL−1beta) | rs9835973 |
| 3 | 133.5971 | Post bronchodilator FEV1/FVC ratio | rs140798517 |
| 3 | 133.6289 | Post bronchodilator FEV1/FVC ratio | rs143087368 |

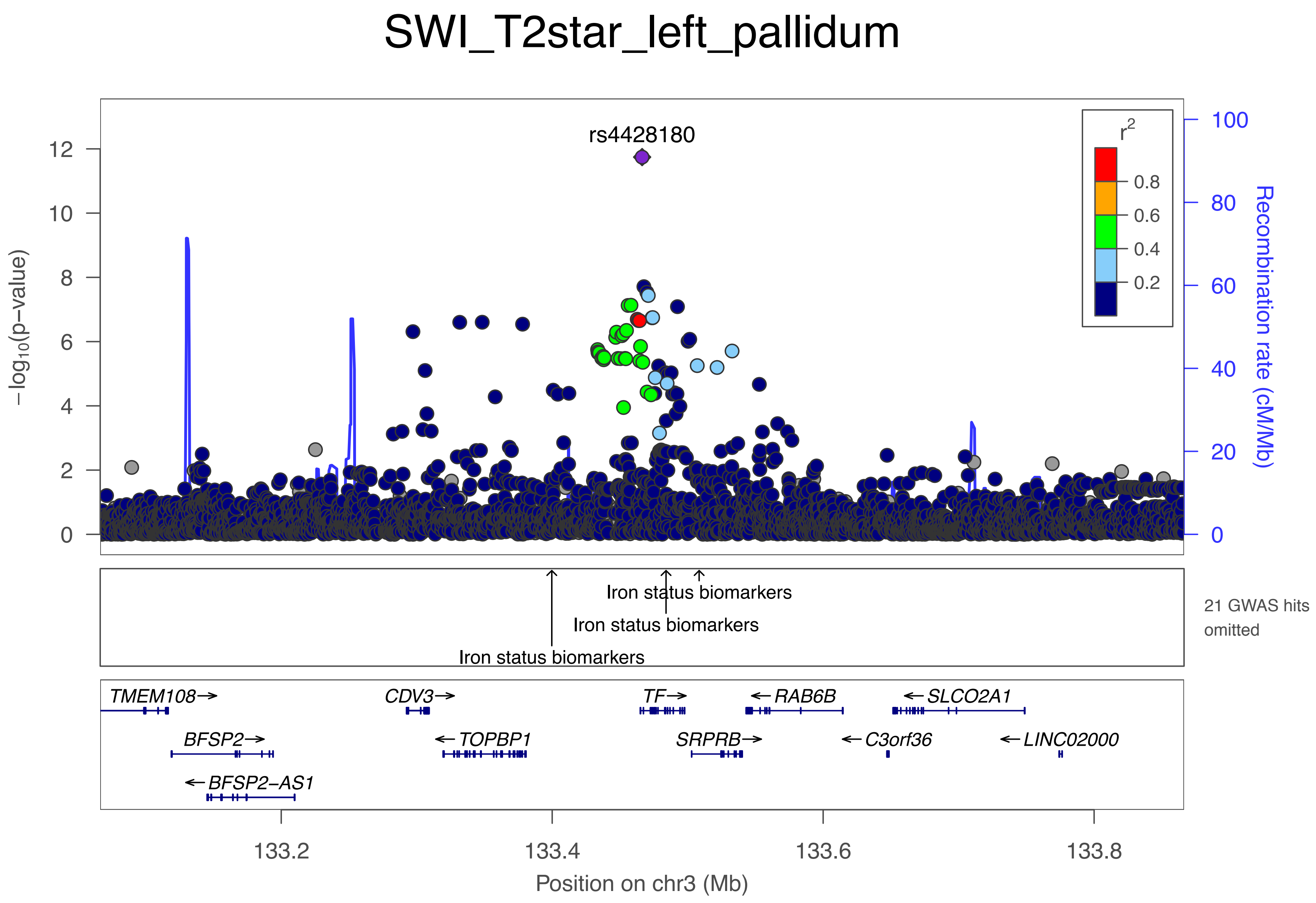

SWI_T2star_left_pallidum
rs4428180
r2
0.8
0.6
0.4
0.2
100
80
60
40
20
0
Recombination rate (cM/Mb)
12
10
8
6
4
2
0
−log10(p−value)
Iron status biomarkers
Iron status biomarkers
Iron status biomarkers
21 GWAS hits omitted
TMEM108
CDV3
TF
RAB6B
SLCO2A1
BFSP2
TOPBP1
SRPRB
C3orf36
LINC02000
BFSP2−AS1
133.2
133.4
133.6
133.8
Position on chr3 (Mb)

| | |
|---|---|
| date: | Thu Aug 17 18:24:22 2017 |
| build: | hg19 |
| display range: | chr3:133066374−133866374 [133066374−133866374] |
| hilite range: | 0 – 0 [ 0 – 0 ] |
| reference SNP: | chr3:133466374 |
| number of SNPs plotted: | 3859 |
| min P.value: | 1.8E−12 [chr3:133466374] |
| max P.value: | 9.99E−1 [chr3:133593610] |
| omitted GWAS Hits: | NA, NA |
| omitted GWAS Hits: | NA, NA |
| omitted GWAS Hits: | NA, NA |
| omitted GWAS Hits: | NA, NA |
| omitted GWAS Hits: | NA, NA |
| omitted GWAS Hits: | NA, NA |
| omitted GWAS Hits: | NA, NA |

Make more plots at http://csg.sph.umich.edu/locuszoom/

GWAS Catalog SNPs in Region

| chr | pos (Mb) | trait | snp |
|---|---|---|---|
| 3 | 133.2609 | Bulimia nervosa | rs11708304 |
| 3 | 133.3997 | Iron status biomarkers | rs2718812 |
| 3 | 133.4106 | Autism spectrum disorder−related traits | rs1867503 |
| 3 | 133.4107 | Iron status biomarkers | rs1867504 |
| 3 | 133.4309 | Alzheimer disease and age of onset | rs190500289 |
| 3 | 133.4575 | Iron status biomarkers | rs9872999 |
| 3 | 133.4635 | Iron status biomarkers (transferrin levels) | rs8177179 |
| 3 | 133.4757 | Iron status biomarkers | rs1799852 |
| 3 | 133.4757 | Iron status biomarkers (transferrin saturation) | rs1799852 |
| 3 | 133.4758 | Alcohol consumption (transferrin glycosylation) | rs1799899 |
| 3 | 133.4777 | Iron status biomarkers (transferrin saturation) | rs8177240 |
| 3 | 133.4777 | Iron status biomarkers (transferrin levels) | rs8177240 |
| 3 | 133.4777 | Iron status biomarkers (iron levels) | rs8177240 |
| 3 | 133.4802 | Iron status biomarkers | rs8177253 |
| 3 | 133.4840 | Iron status biomarkers | rs3811647 |
| 3 | 133.4840 | Hepcidin levels | rs3811647 |
| 3 | 133.4840 | Alcohol consumption (transferrin glycosylation) | rs3811647 |
| 3 | 133.4840 | Hereditary hemochromatosis−related traits (HFE mutation homozygotes) | rs3811647 |
| 3 | 133.4944 | Alcohol consumption (transferrin glycosylation) | rs1049296 |
| 3 | 133.5085 | Iron status biomarkers | rs1830084 |
| 3 | 133.5371 | Alcohol consumption (transferrin glycosylation) | rs1534166 |

GWAS Catalog SNPs in Region

| chr | pos (Mb) | trait | snp |
|---|---|---|---|
| 3 | 133.5893 | Immune reponse to smallpox (secreted IL−1beta) | rs9835973 |
| 3 | 133.5971 | Post bronchodilator FEV1/FVC ratio | rs140798517 |
| 3 | 133.6289 | Post bronchodilator FEV1/FVC ratio | rs143087368 |

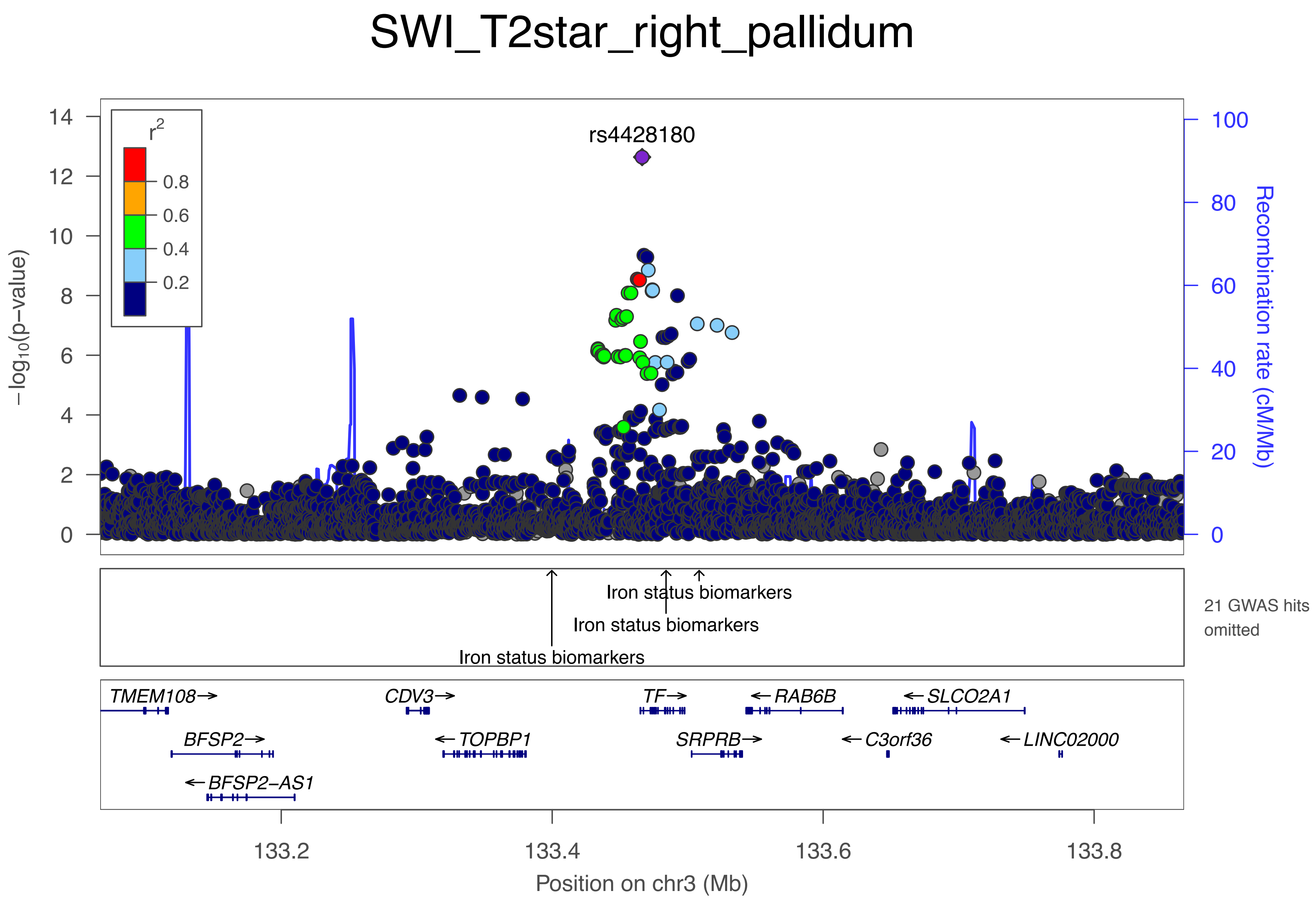
SWI_T2star_right_pallidum
rs4428180
r2
0.8
0.6
0.4
0.2
−log10(p−value)
Recombination rate (cM/Mb)
Iron status biomarkers
Iron status biomarkers
Iron status biomarkers
21 GWAS hits omitted
TMEM108
CDV3
TF
RAB6B
SLCO2A1
BFSP2
TOPBP1
SRPRB
C3orf36
LINC02000
BFSP2−AS1
133.2
133.4
133.6
133.8
Position on chr3 (Mb)

| | |
|---|---|
| date: | Thu Aug 17 18:24:22 2017 |
| build: | hg19 |
| display range: | chr3:133066374−133866374 [133066374−133866374] |
| hilite range: | 0 – 0 [ 0 – 0 ] |
| reference SNP: | chr3:133466374 |
| number of SNPs plotted: | 3859 |
| min P.value: | 2.3E−13 [chr3:133466374] |
| max P.value: | 10E−1 [chr3:133463823] |
| omitted GWAS Hits: | NA, NA |
| omitted GWAS Hits: | NA, NA |
| omitted GWAS Hits: | NA, NA |
| omitted GWAS Hits: | NA, NA |
| omitted GWAS Hits: | NA, NA |
| omitted GWAS Hits: | NA, NA |
| omitted GWAS Hits: | NA, NA |

Make more plots at http://csg.sph.umich.edu/locuszoom/

GWAS Catalog SNPs in Region

| chr | pos (Mb) | trait | snp |
|---|---|---|---|
| 3 | 133.2609 | Bulimia nervosa | rs11708304 |
| 3 | 133.3997 | Iron status biomarkers | rs2718812 |
| 3 | 133.4106 | Autism spectrum disorder−related traits | rs1867503 |
| 3 | 133.4107 | Iron status biomarkers | rs1867504 |
| 3 | 133.4309 | Alzheimer disease and age of onset | rs190500289 |
| 3 | 133.4575 | Iron status biomarkers | rs9872999 |
| 3 | 133.4635 | Iron status biomarkers (transferrin levels) | rs8177179 |
| 3 | 133.4757 | Iron status biomarkers | rs1799852 |
| 3 | 133.4757 | Iron status biomarkers (transferrin saturation) | rs1799852 |
| 3 | 133.4758 | Alcohol consumption (transferrin glycosylation) | rs1799899 |
| 3 | 133.4777 | Iron status biomarkers (transferrin saturation) | rs8177240 |
| 3 | 133.4777 | Iron status biomarkers (transferrin levels) | rs8177240 |
| 3 | 133.4777 | Iron status biomarkers (iron levels) | rs8177240 |
| 3 | 133.4802 | Iron status biomarkers | rs8177253 |
| 3 | 133.4840 | Iron status biomarkers | rs3811647 |
| 3 | 133.4840 | Hepcidin levels | rs3811647 |
| 3 | 133.4840 | Alcohol consumption (transferrin glycosylation) | rs3811647 |
| 3 | 133.4840 | Hereditary hemochromatosis−related traits (HFE mutation homozygotes) | rs3811647 |
| 3 | 133.4944 | Alcohol consumption (transferrin glycosylation) | rs1049296 |
| 3 | 133.5085 | Iron status biomarkers | rs1830084 |
| 3 | 133.5371 | Alcohol consumption (transferrin glycosylation) | rs1534166 |

GWAS Catalog SNPs in Region

| chr | pos (Mb) | trait | snp |
|---|---|---|---|
| 3 | 133.5893 | Immune reponse to smallpox (secreted IL−1beta) | rs9835973 |
| 3 | 133.5971 | Post bronchodilator FEV1/FVC ratio | rs140798517 |
| 3 | 133.6289 | Post bronchodilator FEV1/FVC ratio | rs143087368 |

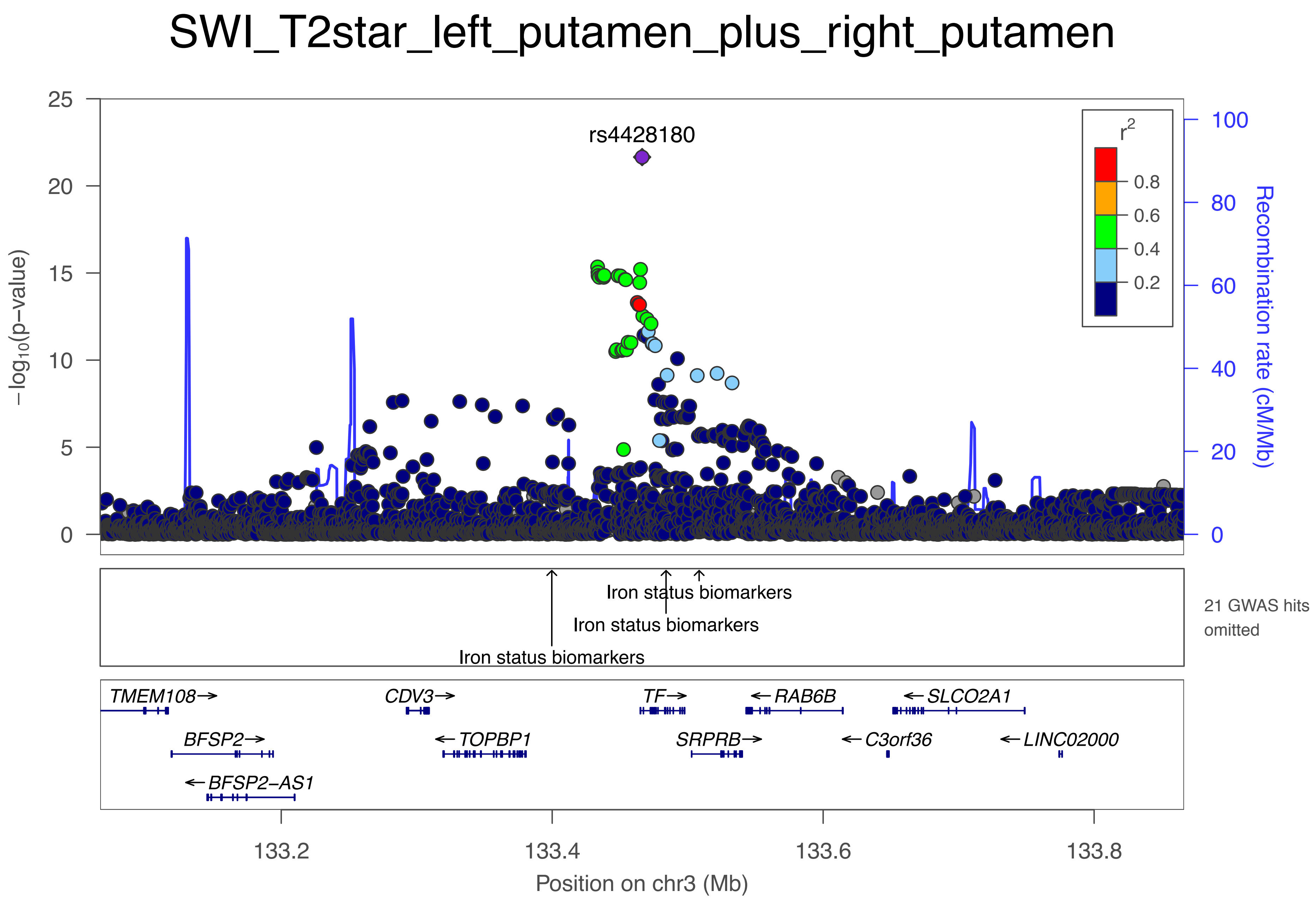
SWI_T2star_left_putamen_plus_right_putamen
rs4428180
$-\log_{10}$(p−value)
Recombination rate (cM/Mb)
$r^2$
0.8
0.6
0.4
0.2
Iron status biomarkers
Iron status biomarkers
Iron status biomarkers
21 GWAS hits omitted
TMEM108→
CDV3→
TF→
←RAB6B
←SLCO2A1
BFSP2→
←TOPBP1
SRPRB→
←C3orf36
←LINC02000
←BFSP2−AS1
133.2
133.4
133.6
133.8
Position on chr3 (Mb)

| | |
|---|---|
| date: | Thu Aug 17 18:24:22 2017 |
| build: | hg19 |
| display range: | chr3:133066374−133866374 [133066374−133866374] |
| hilite range: | 0 – 0 [ 0 – 0 ] |
| reference SNP: | chr3:133466374 |
| number of SNPs plotted: | 3859 |
| min P.value: | 2.23E−22 [chr3:133466374] |
| max P.value: | 10E−1 [chr3:133297659] |
| omitted GWAS Hits: | NA, NA |
| omitted GWAS Hits: | NA, NA |
| omitted GWAS Hits: | NA, NA |
| omitted GWAS Hits: | NA, NA |
| omitted GWAS Hits: | NA, NA |
| omitted GWAS Hits: | NA, NA |
| omitted GWAS Hits: | NA, NA |

Make more plots at http://csg.sph.umich.edu/locuszoom/

GWAS Catalog SNPs in Region

| chr | pos (Mb) | trait | snp |
|---|---|---|---|
| 3 | 133.2609 | Bulimia nervosa | rs11708304 |
| 3 | 133.3997 | Iron status biomarkers | rs2718812 |
| 3 | 133.4106 | Autism spectrum disorder−related traits | rs1867503 |
| 3 | 133.4107 | Iron status biomarkers | rs1867504 |
| 3 | 133.4309 | Alzheimer disease and age of onset | rs190500289 |
| 3 | 133.4575 | Iron status biomarkers | rs9872999 |
| 3 | 133.4635 | Iron status biomarkers (transferrin levels) | rs8177179 |
| 3 | 133.4757 | Iron status biomarkers | rs1799852 |
| 3 | 133.4757 | Iron status biomarkers (transferrin saturation) | rs1799852 |
| 3 | 133.4758 | Alcohol consumption (transferrin glycosylation) | rs1799899 |
| 3 | 133.4777 | Iron status biomarkers (transferrin saturation) | rs8177240 |
| 3 | 133.4777 | Iron status biomarkers (transferrin levels) | rs8177240 |
| 3 | 133.4777 | Iron status biomarkers (iron levels) | rs8177240 |
| 3 | 133.4802 | Iron status biomarkers | rs8177253 |
| 3 | 133.4840 | Iron status biomarkers | rs3811647 |
| 3 | 133.4840 | Hepcidin levels | rs3811647 |
| 3 | 133.4840 | Alcohol consumption (transferrin glycosylation) | rs3811647 |
| 3 | 133.4840 | Hereditary hemochromatosis−related traits (HFE mutation homozygotes) | rs3811647 |
| 3 | 133.4944 | Alcohol consumption (transferrin glycosylation) | rs1049296 |
| 3 | 133.5085 | Iron status biomarkers | rs1830084 |
| 3 | 133.5371 | Alcohol consumption (transferrin glycosylation) | rs1534166 |

GWAS Catalog SNPs in Region

| chr | pos (Mb) | trait | snp |
|---|---|---|---|
| 3 | 133.5893 | Immune reponse to smallpox (secreted IL−1beta) | rs9835973 |
| 3 | 133.5971 | Post bronchodilator FEV1/FVC ratio | rs140798517 |
| 3 | 133.6289 | Post bronchodilator FEV1/FVC ratio | rs143087368 |

# SWI_T2star_left_pallidum_plus_right_pallidum

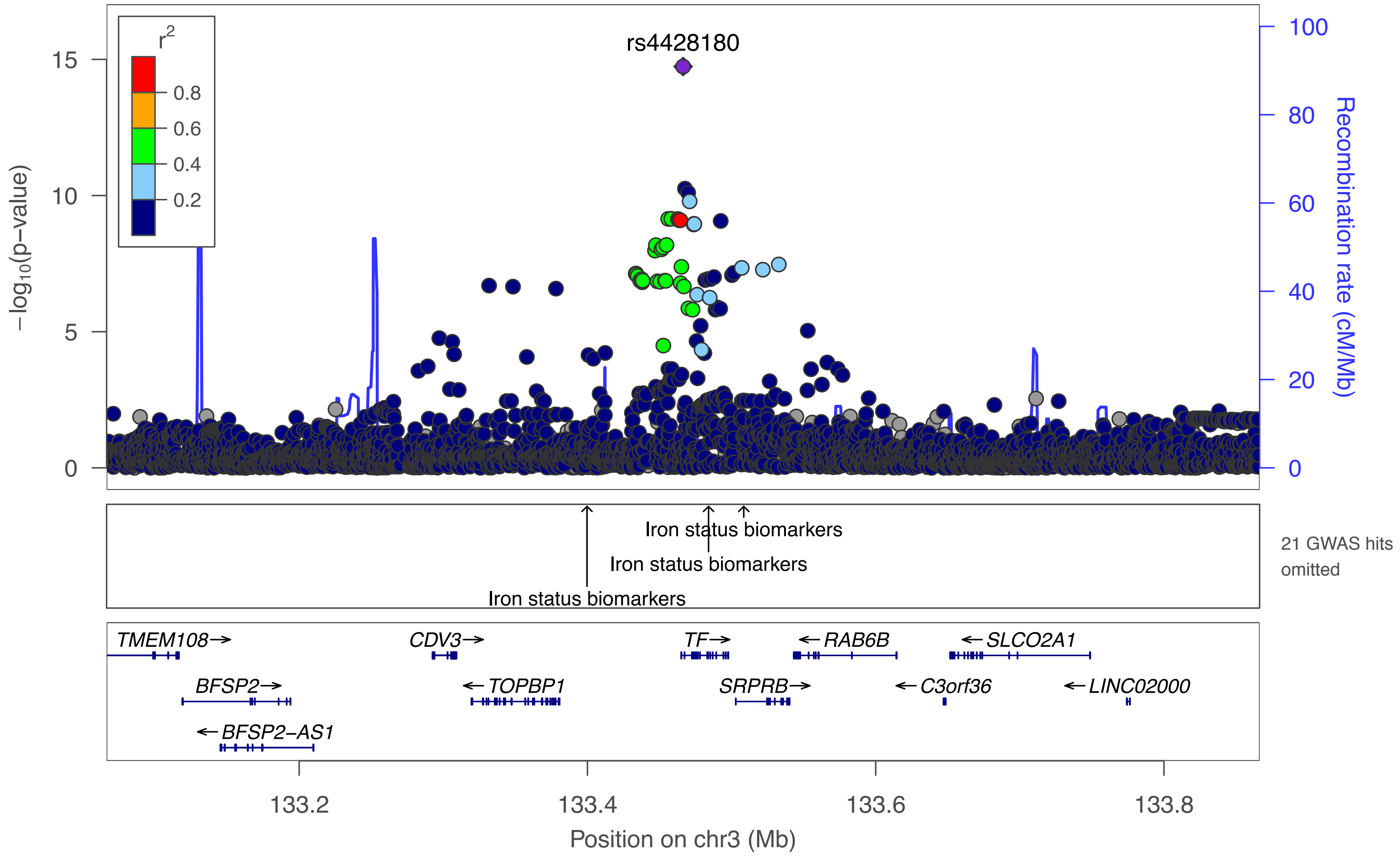

| | |
|---|---|
| date: | Thu Aug 17 18:24:22 2017 |
| build: | hg19 |
| display range: | chr3:133066374−133866374 [133066374−133866374] |
| hilite range: | 0 – 0 [ 0 – 0 ] |
| reference SNP: | chr3:133466374 |
| number of SNPs plotted: | 3859 |
| min P.value: | 1.83E−15 [chr3:133466374] |
| max P.value: | 9.99E−1 [chr3:133253919] |
| omitted GWAS Hits: | NA, NA |
| omitted GWAS Hits: | NA, NA |
| omitted GWAS Hits: | NA, NA |
| omitted GWAS Hits: | NA, NA |
| omitted GWAS Hits: | NA, NA |
| omitted GWAS Hits: | NA, NA |
| omitted GWAS Hits: | NA, NA |

Make more plots at http://csg.sph.umich.edu/locuszoom/

GWAS Catalog SNPs in Region

| chr | pos (Mb) | trait | snp |
|---|---|---|---|
| 3 | 133.2609 | Bulimia nervosa | rs11708304 |
| 3 | 133.3997 | Iron status biomarkers | rs2718812 |
| 3 | 133.4106 | Autism spectrum disorder−related traits | rs1867503 |
| 3 | 133.4107 | Iron status biomarkers | rs1867504 |
| 3 | 133.4309 | Alzheimer disease and age of onset | rs190500289 |
| 3 | 133.4575 | Iron status biomarkers | rs9872999 |
| 3 | 133.4635 | Iron status biomarkers (transferrin levels) | rs8177179 |
| 3 | 133.4757 | Iron status biomarkers | rs1799852 |
| 3 | 133.4757 | Iron status biomarkers (transferrin saturation) | rs1799852 |
| 3 | 133.4758 | Alcohol consumption (transferrin glycosylation) | rs1799899 |
| 3 | 133.4777 | Iron status biomarkers (transferrin saturation) | rs8177240 |
| 3 | 133.4777 | Iron status biomarkers (transferrin levels) | rs8177240 |
| 3 | 133.4777 | Iron status biomarkers (iron levels) | rs8177240 |
| 3 | 133.4802 | Iron status biomarkers | rs8177253 |
| 3 | 133.4840 | Iron status biomarkers | rs3811647 |
| 3 | 133.4840 | Hepcidin levels | rs3811647 |
| 3 | 133.4840 | Alcohol consumption (transferrin glycosylation) | rs3811647 |
| 3 | 133.4840 | Hereditary hemochromatosis−related traits (HFE mutation homozygotes) | rs3811647 |
| 3 | 133.4944 | Alcohol consumption (transferrin glycosylation) | rs1049296 |
| 3 | 133.5085 | Iron status biomarkers | rs1830084 |
| 3 | 133.5371 | Alcohol consumption (transferrin glycosylation) | rs1534166 |

GWAS Catalog SNPs in Region

| chr | pos (Mb) | trait | snp |
|---|---|---|---|
| 3 | 133.5893 | Immune reponse to smallpox (secreted IL−1beta) | rs9835973 |
| 3 | 133.5971 | Post bronchodilator FEV1/FVC ratio | rs140798517 |
| 3 | 133.6289 | Post bronchodilator FEV1/FVC ratio | rs143087368 |

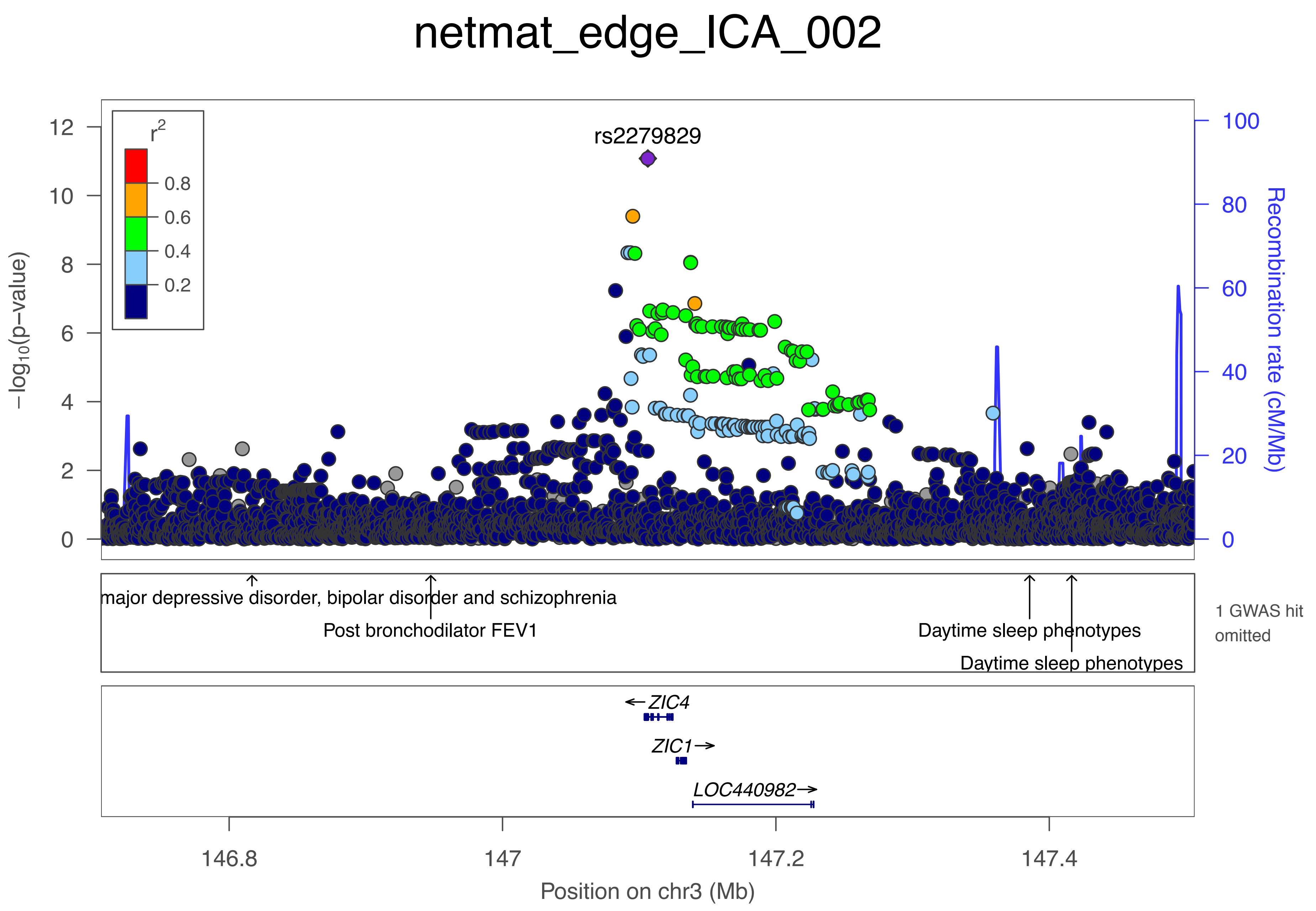
netmat_edge_ICA_002
rs2279829
r2
0.8
0.6
0.4
0.2
−log10(p−value)
Recombination rate (cM/Mb)
major depressive disorder, bipolar disorder and schizophrenia
Post bronchodilator FEV1
Daytime sleep phenotypes
Daytime sleep phenotypes
1 GWAS hit omitted
ZIC4
ZIC1
LOC440982
146.8
147
147.2
147.4
Position on chr3 (Mb)

| | |
|---|---|
| date: | Sat Aug 19 17:32:05 2017 |
| build: | hg19 |
| display range: | chr3:146706319−147506319 [146706319−147506319] |
| hilite range: | 0 – 0 [ 0 – 0 ] |
| reference SNP: | chr3:147106319 |
| number of SNPs plotted: | 3210 |
| min P.value: | 8.34E−12 [chr3:147106319] |
| max P.value: | 9.99E−1 [chr3:147143227] |
| omitted GWAS Hits: | NA |

Make more plots at http://csg.sph.umich.edu/locuszoom/

GWAS Catalog SNPs in Region

| chr | pos (Mb) | trait | snp |
|---|---|---|---|
| 3 | 146.8167 | Functional impairment in major depressive disorder, bipolar disorder and schizophrenia | rs4681346 |
| 3 | 146.9291 | Adult asthma | rs78558203 |
| 3 | 146.9475 | Post bronchodilator FEV1 | rs189504960 |
| 3 | 147.3856 | Daytime sleep phenotypes | rs2319326 |
| 3 | 147.4164 | Daytime sleep phenotypes | rs73151930 |

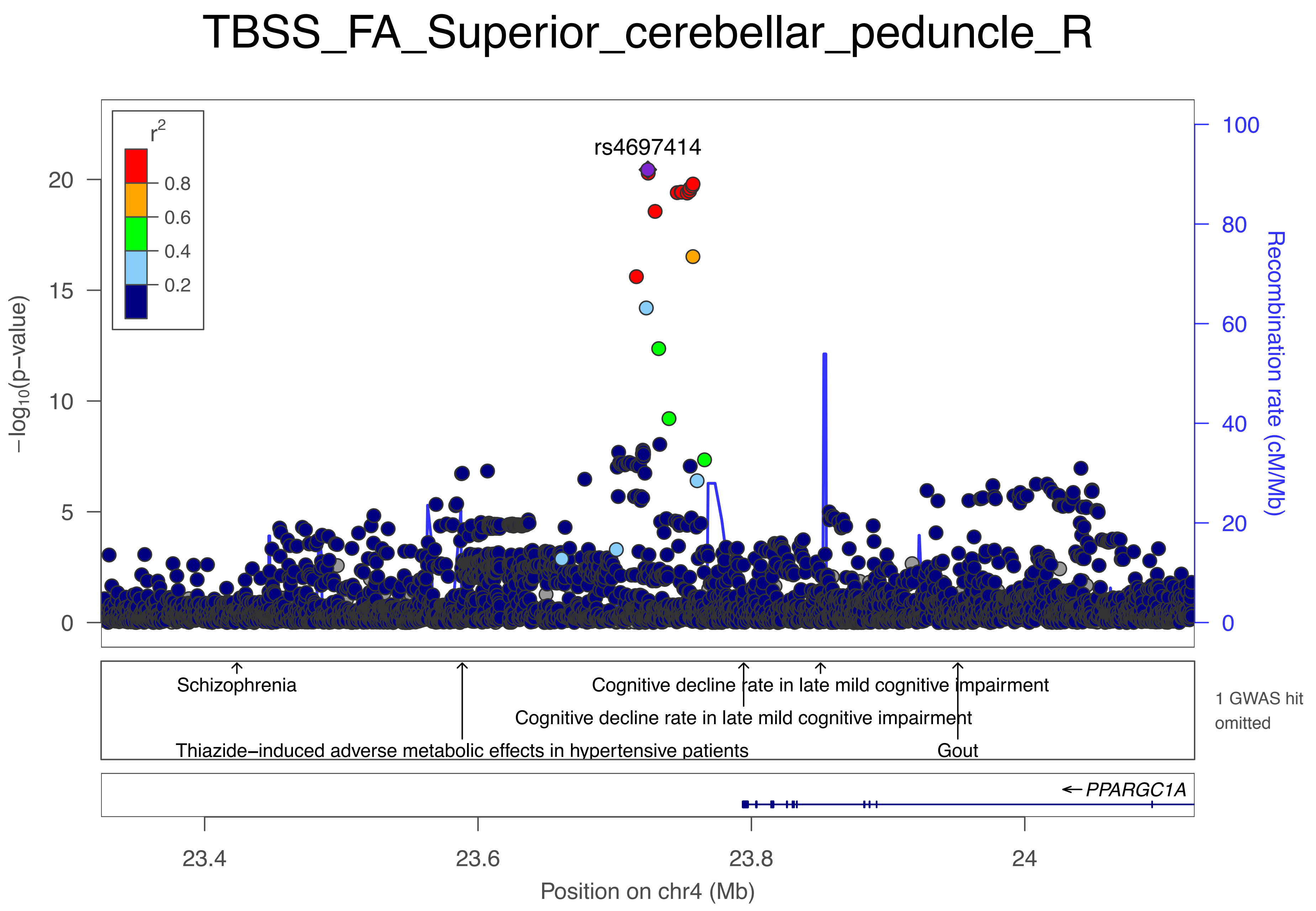

TBSS_FA_Superior_cerebellar_peduncle_R
rs4697414
r2
0.8
0.6
0.4
0.2
−log10(p−value)
Recombination rate (cM/Mb)
0
5
10
15
20
0
20
40
60
80
100
Schizophrenia
Cognitive decline rate in late mild cognitive impairment
Cognitive decline rate in late mild cognitive impairment
Thiazide−induced adverse metabolic effects in hypertensive patients
Gout
1 GWAS hit omitted
← PPARGC1A
23.4
23.6
23.8
24
Position on chr4 (Mb)

| | |
|---|---|
| date: | Thu Aug 17 18:08:11 2017 |
| build: | hg19 |
| display range: | chr4:23324255−24124255 [23324255−24124255] |
| hilite range: | 0 – 0 [ 0 – 0 ] |
| reference SNP: | chr4:23724255 |
| number of SNPs plotted: | 4219 |
| min P.value: | 3.62E−21 [chr4:23724255] |
| max P.value: | 10E−1 [chr4:23936858] |
| omitted GWAS Hits: | NA |

Make more plots at http://csg.sph.umich.edu/locuszoom/

GWAS Catalog SNPs in Region

| chr | pos (Mb) | trait | snp |
|---|---|---|---|
| 4 | 23.42360 | Schizophrenia | rs215411 |
| 4 | 23.58846 | Thiazide–induced adverse metabolic effects in hypertensive patients | rs1511453 |
| 4 | 23.79434 | Cognitive decline rate in late mild cognitive impairment | rs41359445 |
| 4 | 23.85057 | Cognitive decline rate in late mild cognitive impairment | rs188794202 |
| 4 | 23.95102 | Gout | rs12501032 |
| 4 | 23.95102 | Resting heart rate | rs12501032 |

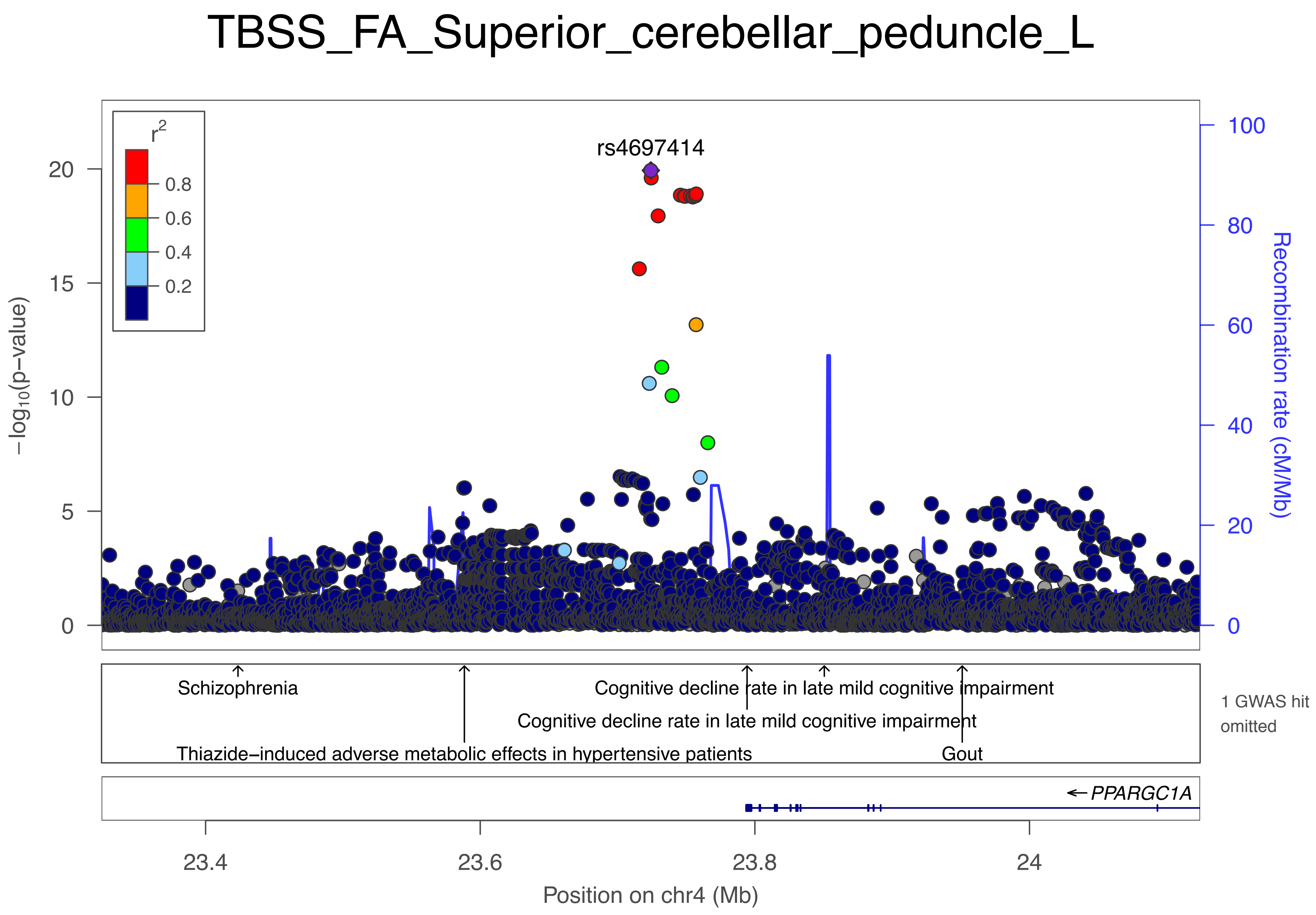
TBSS_FA_Superior_cerebellar_peduncle_L
rs4697414
r2
0.8
0.6
0.4
0.2
−log10(p−value)
Recombination rate (cM/Mb)
Schizophrenia
Cognitive decline rate in late mild cognitive impairment
Cognitive decline rate in late mild cognitive impairment
Thiazide−induced adverse metabolic effects in hypertensive patients
Gout
1 GWAS hit omitted
PPARGC1A
23.4
23.6
23.8
24
Position on chr4 (Mb)

| | |
|---|---|
| date: | Thu Aug 17 18:08:11 2017 |
| build: | hg19 |
| display range: | chr4:23324255−24124255 [23324255−24124255] |
| hilite range: | 0 – 0 [ 0 – 0 ] |
| reference SNP: | chr4:23724255 |
| number of SNPs plotted: | 4219 |
| min P.value: | 1.16E−20 [chr4:23724255] |
| max P.value: | 10E−1 [chr4:23475489] |
| omitted GWAS Hits: | NA |

Make more plots at http://csg.sph.umich.edu/locuszoom/

GWAS Catalog SNPs in Region

| chr | pos (Mb) | trait | snp |
|---|---|---|---|
| 4 | 23.42360 | Schizophrenia | rs215411 |
| 4 | 23.58846 | Thiazide−induced adverse metabolic effects in hypertensive patients | rs1511453 |
| 4 | 23.79434 | Cognitive decline rate in late mild cognitive impairment | rs41359445 |
| 4 | 23.85057 | Cognitive decline rate in late mild cognitive impairment | rs188794202 |
| 4 | 23.95102 | Gout | rs12501032 |
| 4 | 23.95102 | Resting heart rate | rs12501032 |

# TBSS_ICVF_Superior_cerebellar_peduncle_R

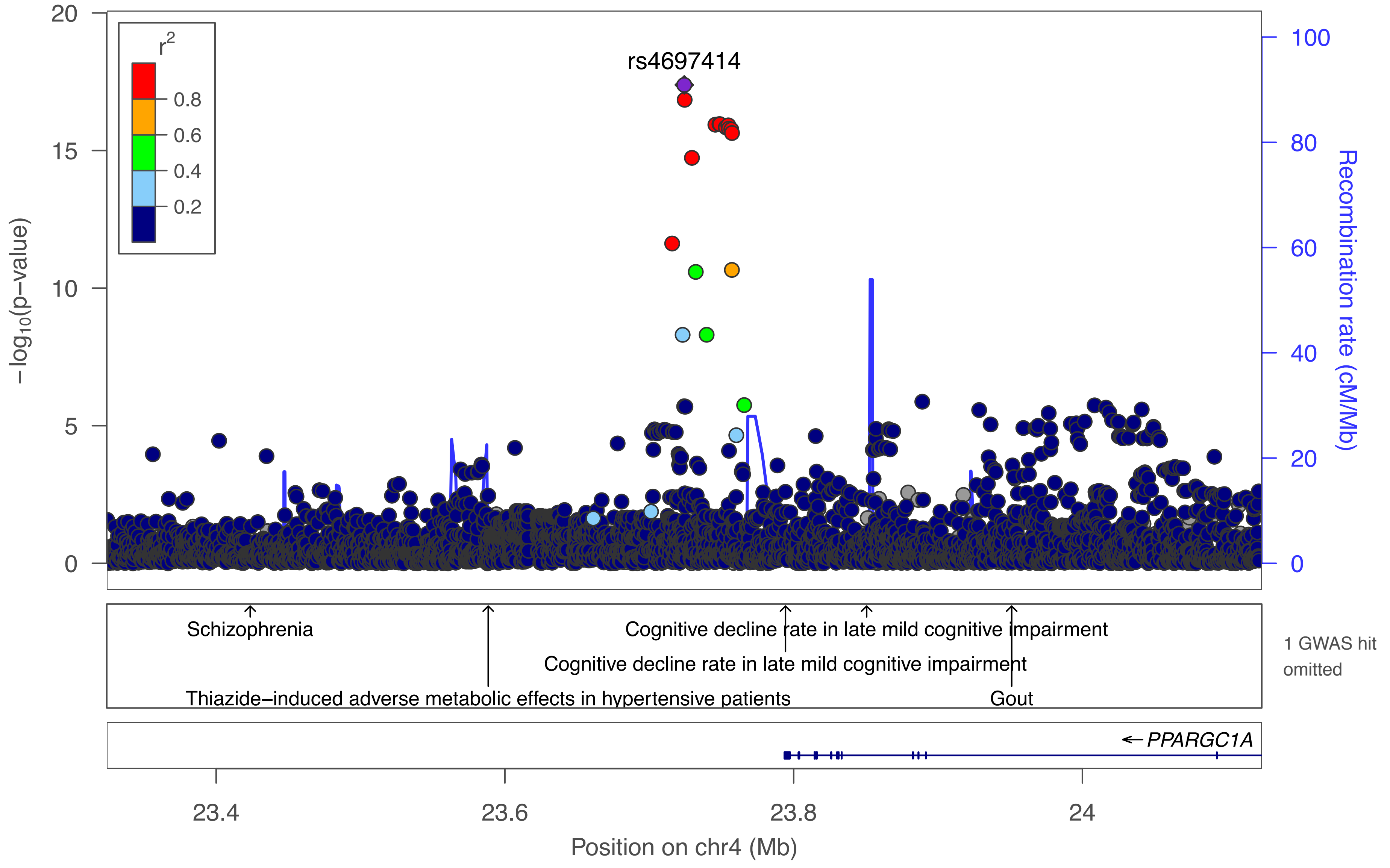

| | |
|---|---|
| date: | Thu Aug 17 18:08:11 2017 |
| build: | hg19 |
| display range: | chr4:23324255−24124255 [23324255−24124255] |
| hilite range: | 0 – 0 [ 0 – 0 ] |
| reference SNP: | chr4:23724255 |
| number of SNPs plotted: | 4219 |
| min P.value: | 4.12E−18 [chr4:23724255] |
| max P.value: | 10E−1 [chr4:23551534] |
| omitted GWAS Hits: | NA |

Make more plots at http://csg.sph.umich.edu/locuszoom/

GWAS Catalog SNPs in Region

| chr | pos (Mb) | trait | snp |
|---|---|---|---|
| 4 | 23.42360 | Schizophrenia | rs215411 |
| 4 | 23.58846 | Thiazide−induced adverse metabolic effects in hypertensive patients | rs1511453 |
| 4 | 23.79434 | Cognitive decline rate in late mild cognitive impairment | rs41359445 |
| 4 | 23.85057 | Cognitive decline rate in late mild cognitive impairment | rs188794202 |
| 4 | 23.95102 | Gout | rs12501032 |
| 4 | 23.95102 | Resting heart rate | rs12501032 |

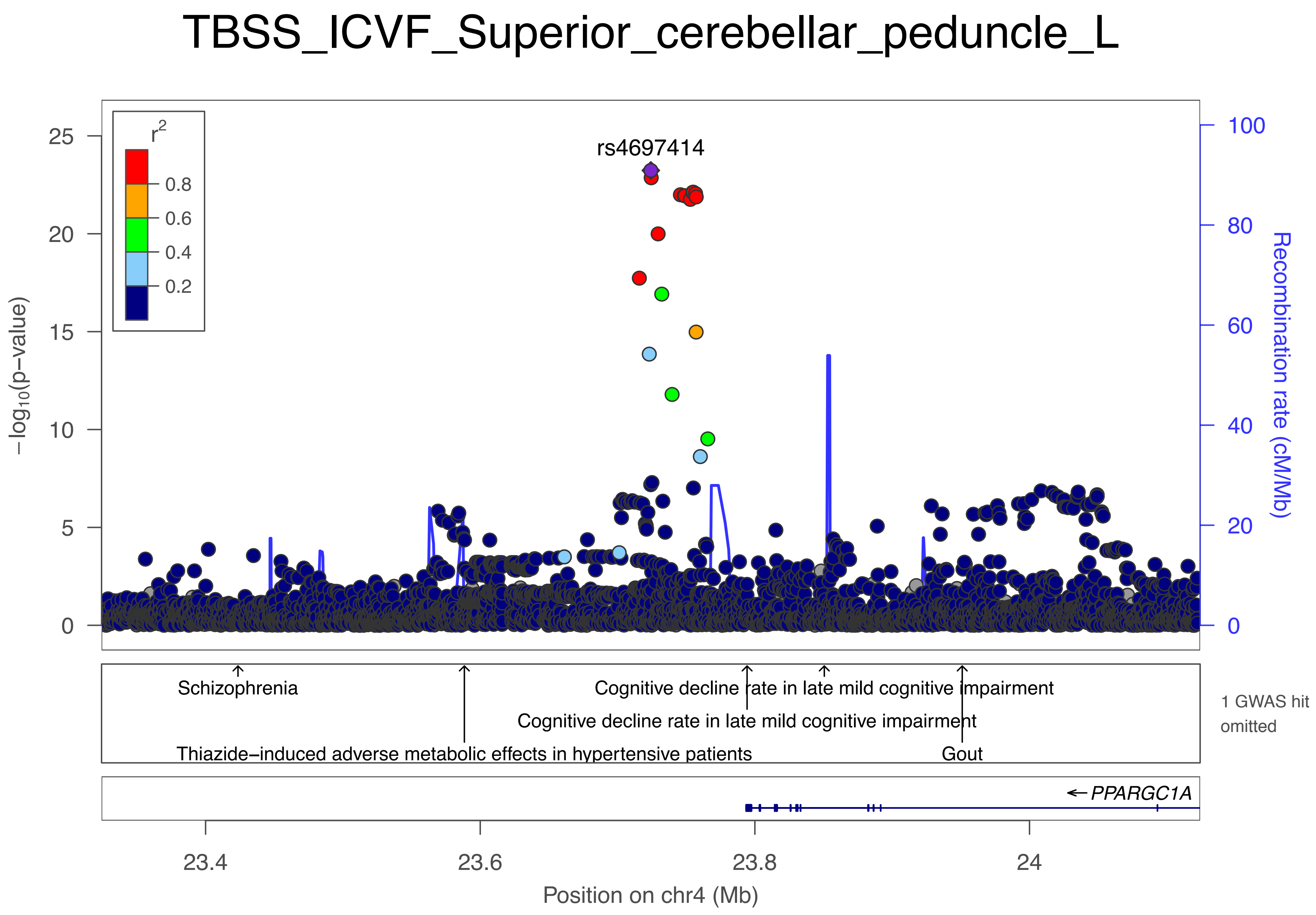

TBSS_ICVF_Superior_cerebellar_peduncle_L
rs4697414
r2
0.8
0.6
0.4
0.2
25
20
15
10
5
0
$-\log_{10}$(p−value)
100
80
60
40
20
0
Recombination rate (cM/Mb)
Schizophrenia
Cognitive decline rate in late mild cognitive impairment
Cognitive decline rate in late mild cognitive impairment
Thiazide−induced adverse metabolic effects in hypertensive patients
Gout
1 GWAS hit omitted
← PPARGC1A
23.4
23.6
23.8
24
Position on chr4 (Mb)

| | |
|---|---|
| date: | Thu Aug 17 18:08:11 2017 |
| build: | hg19 |
| display range: | chr4:23324255−24124255 [23324255−24124255] |
| hilite range: | 0 – 0 [ 0 – 0 ] |
| reference SNP: | chr4:23724255 |
| number of SNPs plotted: | 4219 |
| min P.value: | 5.83E−24 [chr4:23724255] |
| max P.value: | 10E−1 [chr4:23483330] |
| omitted GWAS Hits: | NA |

Make more plots at http://csg.sph.umich.edu/locuszoom/

GWAS Catalog SNPs in Region

| chr | pos (Mb) | trait | snp |
|---|---|---|---|
| 4 | 23.42360 | Schizophrenia | rs215411 |
| 4 | 23.58846 | Thiazide−induced adverse metabolic effects in hypertensive patients | rs1511453 |
| 4 | 23.79434 | Cognitive decline rate in late mild cognitive impairment | rs41359445 |
| 4 | 23.85057 | Cognitive decline rate in late mild cognitive impairment | rs188794202 |
| 4 | 23.95102 | Gout | rs12501032 |
| 4 | 23.95102 | Resting heart rate | rs12501032 |

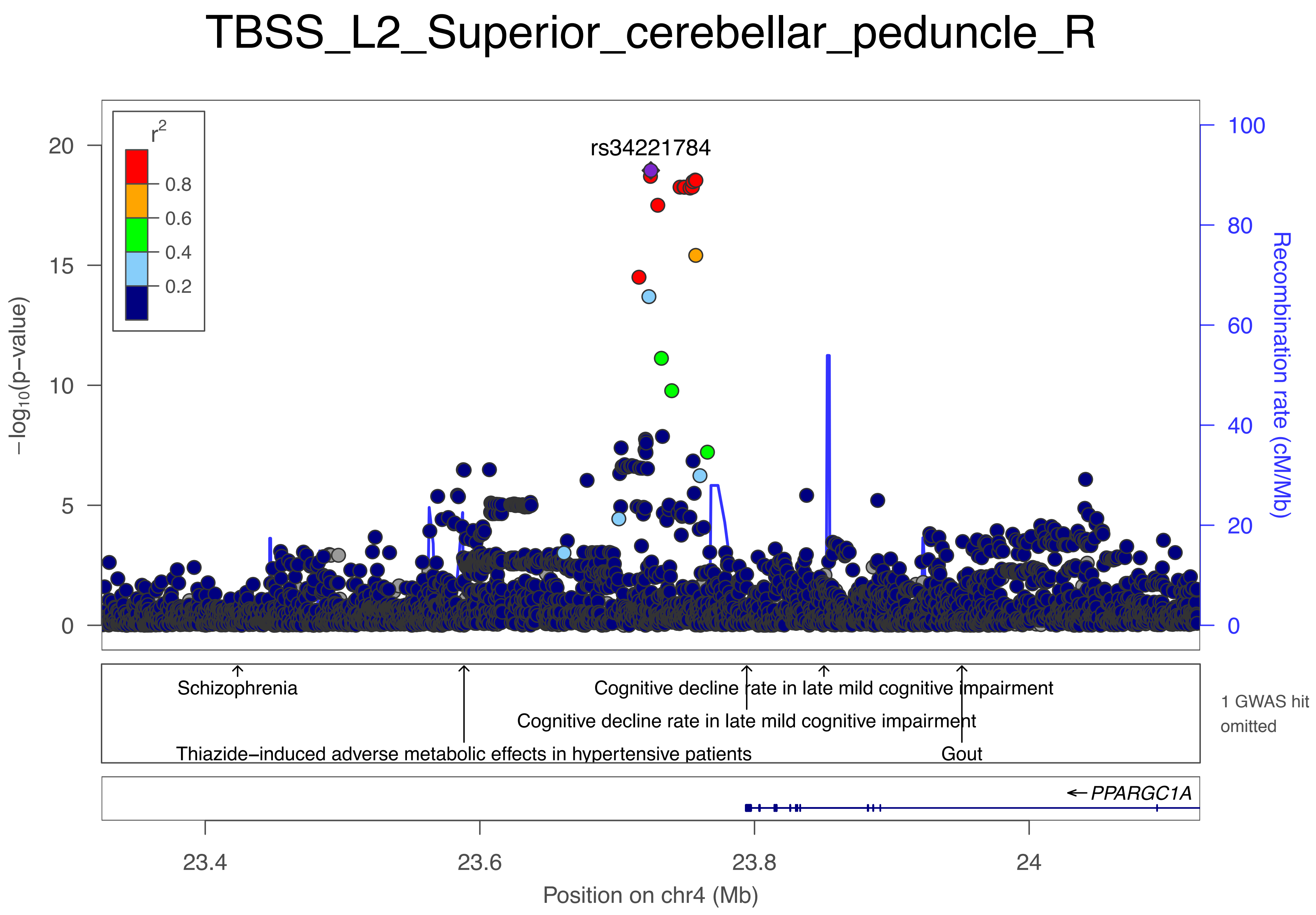
TBSS_L2_Superior_cerebellar_peduncle_R
rs34221784
r2
0.8
0.6
0.4
0.2
−log10(p−value)
20
15
10
5
0
Recombination rate (cM/Mb)
100
80
60
40
20
0
Schizophrenia
Cognitive decline rate in late mild cognitive impairment
Cognitive decline rate in late mild cognitive impairment
Thiazide−induced adverse metabolic effects in hypertensive patients
Gout
1 GWAS hit omitted
← PPARGC1A
23.4
23.6
23.8
24
Position on chr4 (Mb)

| | |
|---|---|
| date: | Thu Aug 17 18:08:11 2017 |
| build: | hg19 |
| display range: | chr4:23324518−24124518 [23324518−24124518] |
| hilite range: | 0 – 0 [ 0 – 0 ] |
| reference SNP: | chr4:23724518 |
| number of SNPs plotted: | 4218 |
| min P.value: | 1.12E−19 [chr4:23724518] |
| max P.value: | 10E−1 [chr4:23342785] |
| omitted GWAS Hits: | NA |

Make more plots at http://csg.sph.umich.edu/locuszoom/

GWAS Catalog SNPs in Region

| chr | pos (Mb) | trait | snp |
|---|---|---|---|
| 4 | 23.42360 | Schizophrenia | rs215411 |
| 4 | 23.58846 | Thiazide−induced adverse metabolic effects in hypertensive patients | rs1511453 |
| 4 | 23.79434 | Cognitive decline rate in late mild cognitive impairment | rs41359445 |
| 4 | 23.85057 | Cognitive decline rate in late mild cognitive impairment | rs188794202 |
| 4 | 23.95102 | Gout | rs12501032 |
| 4 | 23.95102 | Resting heart rate | rs12501032 |

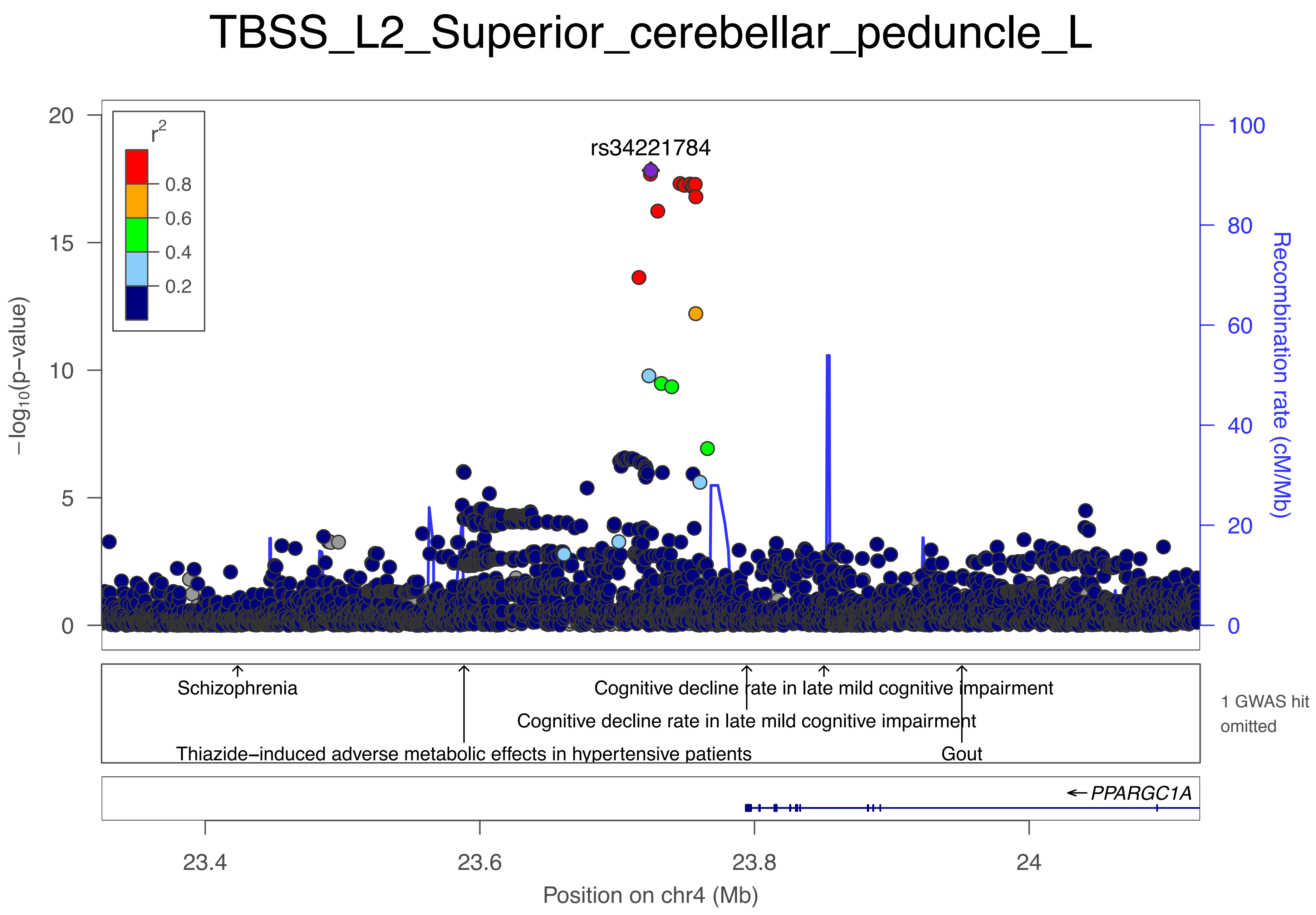

TBSS_L2_Superior_cerebellar_peduncle_L
r2
0.8
0.6
0.4
0.2
rs34221784
20
15
10
5
0
−log10(p−value)
100
80
60
40
20
0
Recombination rate (cM/Mb)
Schizophrenia
Cognitive decline rate in late mild cognitive impairment
Cognitive decline rate in late mild cognitive impairment
Thiazide−induced adverse metabolic effects in hypertensive patients
Gout
1 GWAS hit omitted
← PPARGC1A
23.4
23.6
23.8
24
Position on chr4 (Mb)

| | |
|---|---|
| date: | Thu Aug 17 18:08:12 2017 |
| build: | hg19 |
| display range: | chr4:23324518−24124518 [23324518−24124518] |
| hilite range: | 0 – 0 [ 0 – 0 ] |
| reference SNP: | chr4:23724518 |
| number of SNPs plotted: | 4218 |
| min P.value: | 1.49E−18 [chr4:23724518] |
| max P.value: | 10E−1 [chr4:23878838] |
| omitted GWAS Hits: | NA |

Make more plots at http://csg.sph.umich.edu/locuszoom/

GWAS Catalog SNPs in Region

| chr | pos (Mb) | trait | snp |
|---|---|---|---|
| 4 | 23.42360 | Schizophrenia | rs215411 |
| 4 | 23.58846 | Thiazide–induced adverse metabolic effects in hypertensive patients | rs1511453 |
| 4 | 23.79434 | Cognitive decline rate in late mild cognitive impairment | rs41359445 |
| 4 | 23.85057 | Cognitive decline rate in late mild cognitive impairment | rs188794202 |
| 4 | 23.95102 | Gout | rs12501032 |
| 4 | 23.95102 | Resting heart rate | rs12501032 |

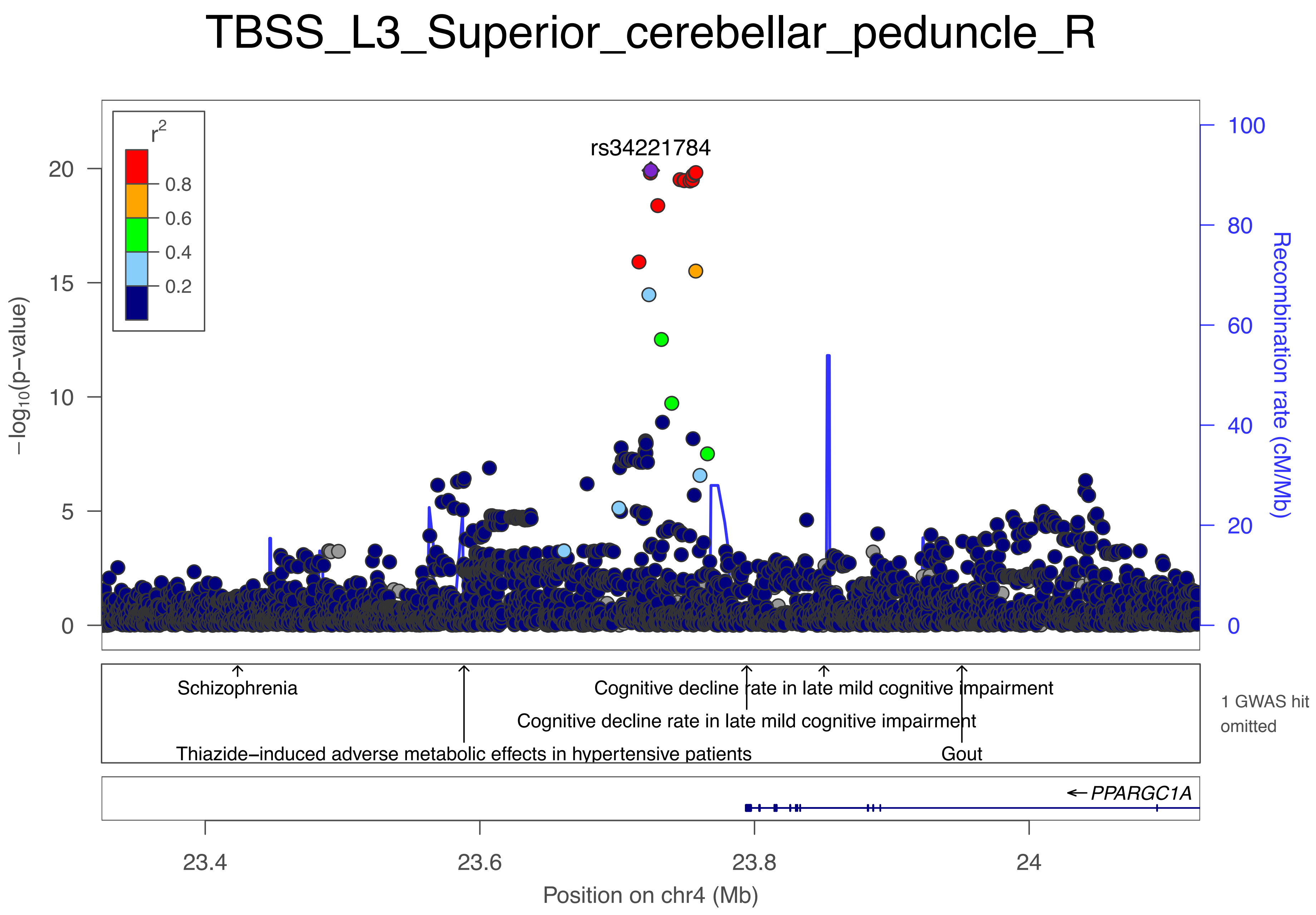

TBSS_L3_Superior_cerebellar_peduncle_R
rs34221784
r²
0.8
0.6
0.4
0.2
−log10(p−value)
Recombination rate (cM/Mb)
Schizophrenia
Cognitive decline rate in late mild cognitive impairment
Cognitive decline rate in late mild cognitive impairment
Thiazide−induced adverse metabolic effects in hypertensive patients
Gout
1 GWAS hit omitted
PPARGC1A
23.4
23.6
23.8
24
Position on chr4 (Mb)

| | |
|---|---|
| date: | Thu Aug 17 18:08:12 2017 |
| build: | hg19 |
| display range: | chr4:23324518−24124518 [23324518−24124518] |
| hilite range: | 0 – 0 [ 0 – 0 ] |
| reference SNP: | chr4:23724518 |
| number of SNPs plotted: | 4218 |
| min P.value: | 1.21E−20 [chr4:23724518] |
| max P.value: | 10E−1 [chr4:23326313] |
| omitted GWAS Hits: | NA |

Make more plots at http://csg.sph.umich.edu/locuszoom/

GWAS Catalog SNPs in Region

| chr | pos (Mb) | trait | snp |
|---|---|---|---|
| 4 | 23.42360 | Schizophrenia | rs215411 |
| 4 | 23.58846 | Thiazide−induced adverse metabolic effects in hypertensive patients | rs1511453 |
| 4 | 23.79434 | Cognitive decline rate in late mild cognitive impairment | rs41359445 |
| 4 | 23.85057 | Cognitive decline rate in late mild cognitive impairment | rs188794202 |
| 4 | 23.95102 | Gout | rs12501032 |
| 4 | 23.95102 | Resting heart rate | rs12501032 |

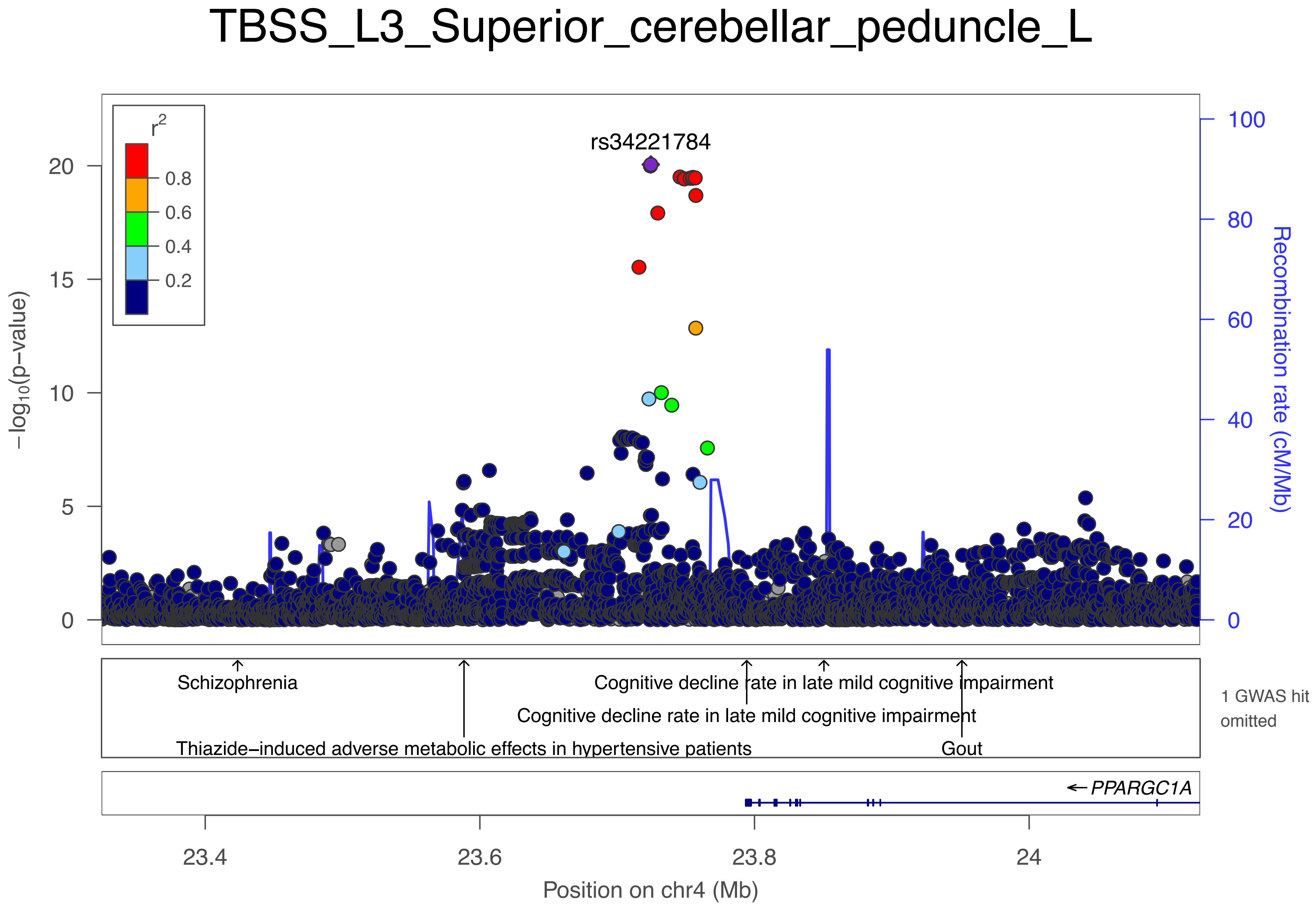
TBSS_L3_Superior_cerebellar_peduncle_L
r2
0.8
0.6
0.4
0.2
rs34221784
−log10(p−value)
Recombination rate (cM/Mb)
Schizophrenia
Cognitive decline rate in late mild cognitive impairment
Cognitive decline rate in late mild cognitive impairment
Thiazide−induced adverse metabolic effects in hypertensive patients
Gout
1 GWAS hit omitted
PPARGC1A
Position on chr4 (Mb)

| | |
|---|---|
| date: | Thu Aug 17 18:08:12 2017 |
| build: | hg19 |
| display range: | chr4:23324518−24124518 [23324518−24124518] |
| hilite range: | 0 – 0 [ 0 – 0 ] |
| reference SNP: | chr4:23724518 |
| number of SNPs plotted: | 4218 |
| min P.value: | 8.83E−21 [chr4:23724518] |
| max P.value: | 9.99E−1 [chr4:23515759] |
| omitted GWAS Hits: | NA |

Make more plots at http://csg.sph.umich.edu/locuszoom/

GWAS Catalog SNPs in Region

| chr | pos (Mb) | trait | snp |
|---|---|---|---|
| 4 | 23.42360 | Schizophrenia | rs215411 |
| 4 | 23.58846 | Thiazide–induced adverse metabolic effects in hypertensive patients | rs1511453 |
| 4 | 23.79434 | Cognitive decline rate in late mild cognitive impairment | rs41359445 |
| 4 | 23.85057 | Cognitive decline rate in late mild cognitive impairment | rs188794202 |
| 4 | 23.95102 | Gout | rs12501032 |
| 4 | 23.95102 | Resting heart rate | rs12501032 |

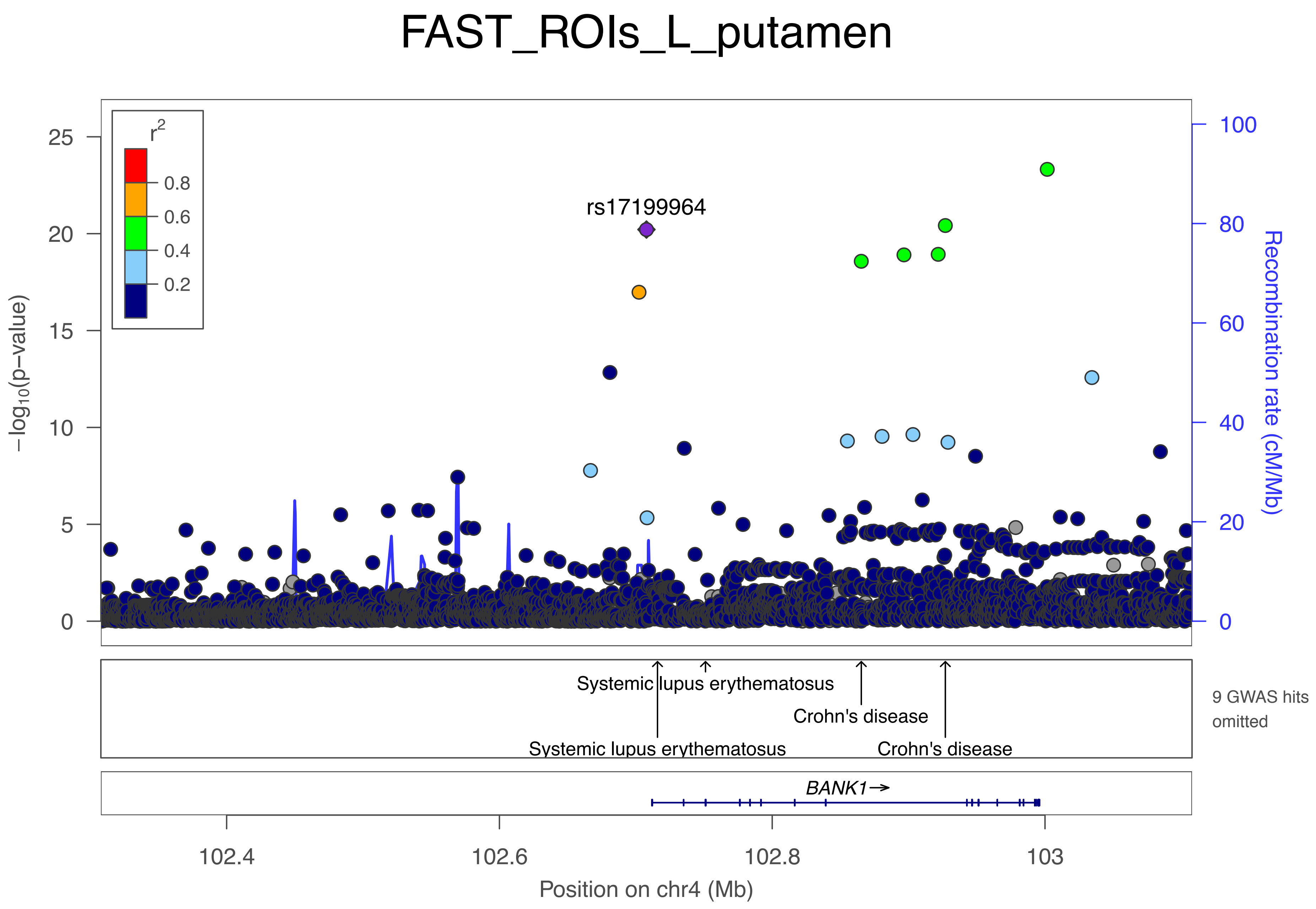
FAST_ROIs_L_putamen
r2
0.8
0.6
0.4
0.2
rs17199964
25
20
15
10
5
0
−log10(p−value)
100
80
60
40
20
0
Recombination rate (cM/Mb)
Systemic lupus erythematosus
Crohn's disease
Systemic lupus erythematosus
Crohn's disease
9 GWAS hits omitted
BANK1→
102.4
102.6
102.8
103
Position on chr4 (Mb)

| | |
|---|---|
| date: | Thu Aug 17 18:09:36 2017 |
| build: | hg19 |
| display range: | chr4:102307791−103107791 [102307791−103107791] |
| hilite range: | 0 – 0 [ 0 – 0 ] |
| reference SNP: | chr4:102707791 |
| number of SNPs plotted: | 3927 |
| min P.value: | 4.78E−24 [chr4:103001649] |
| max P.value: | 10E−1 [chr4:102430974] |
| omitted GWAS Hits: | chr4:102.926923−Crohn's disease, NA |
| omitted GWAS Hits: | NA, NA |
| omitted GWAS Hits: | NA, NA |

Make more plots at http://csg.sph.umich.edu/locuszoom/

GWAS Catalog SNPs in Region

| trait |
| --- |
| C–reactive protein and white blood cell count |
| Acute urticaria and angioedema (non–steroidal anti–inflammatory drug–induced) |
| Blood pressure |
| utism spectrum disorder, attention deficit–hyperactivity disorder, bipolar disorder, major depressive disorder, and schizophrenia (combin |
| Systemic lupus erythematosus |
| Systemic lupus erythematosus |
| Chronic lymphocytic leukemia |
| Systemic lupus erythematosus |
| Schizophrenia |
| Crohn's disease |
| Inflammatory bowel disease |
| Crohn's disease |
| Alzheimer disease and age of onset |

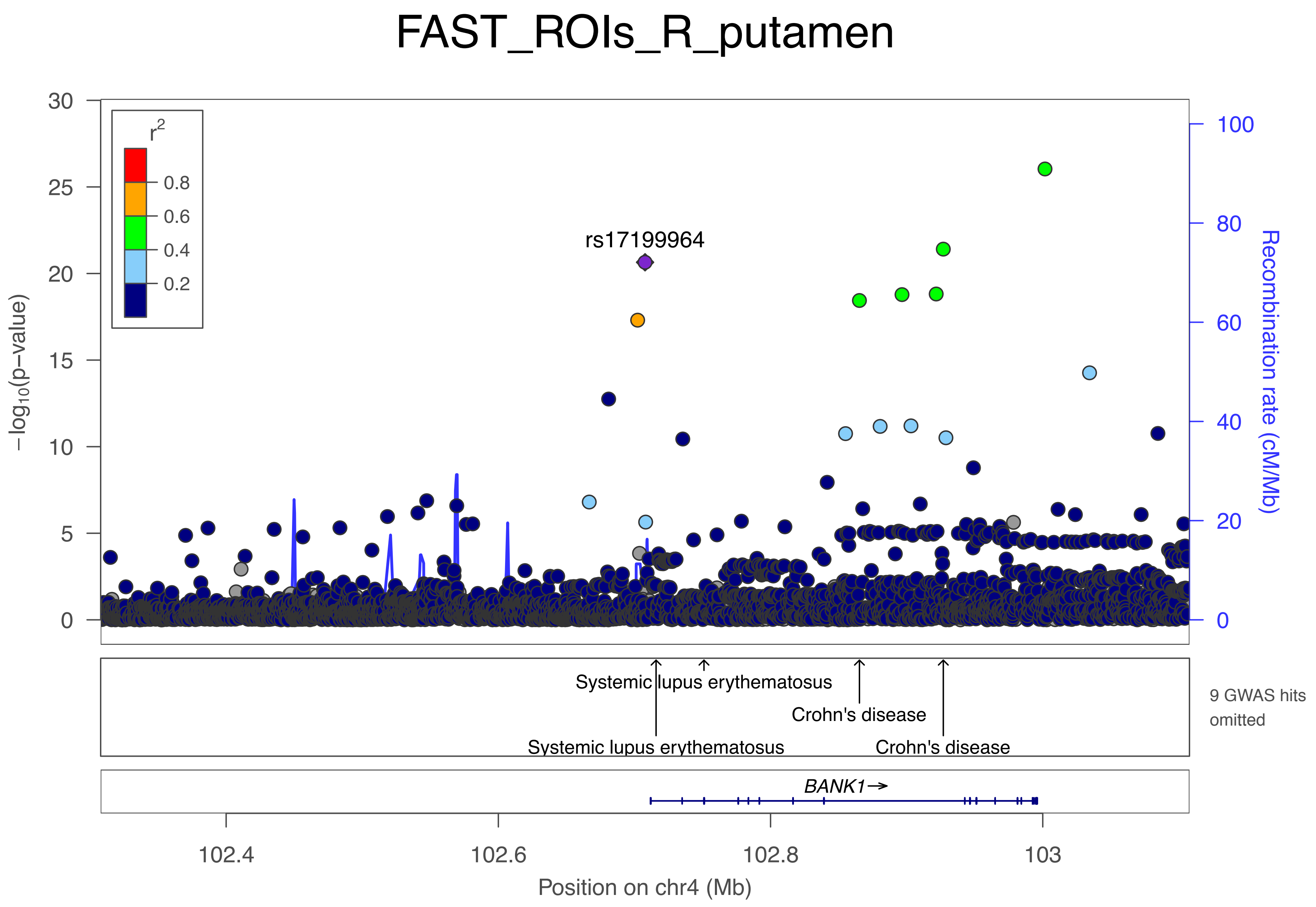

FAST_ROIs_R_putamen
r2
0.8
0.6
0.4
0.2
rs17199964
−log10(p−value)
Recombination rate (cM/Mb)
Systemic lupus erythematosus
Crohn's disease
Systemic lupus erythematosus
Crohn's disease
9 GWAS hits omitted
BANK1→
102.4
102.6
102.8
103
Position on chr4 (Mb)

| | |
|---|---|
| date: | Thu Aug 17 18:09:36 2017 |
| build: | hg19 |
| display range: | chr4:102307791−103107791 [102307791−103107791] |
| hilite range: | 0 – 0 [ 0 – 0 ] |
| reference SNP: | chr4:102707791 |
| number of SNPs plotted: | 3927 |
| min P.value: | 9.16E−27 [chr4:103001649] |
| max P.value: | 10E−1 [chr4:102530982] |
| omitted GWAS Hits: | chr4:102.926923−Crohn's disease, NA |
| omitted GWAS Hits: | NA, NA |
| omitted GWAS Hits: | NA, NA |

Make more plots at http://csg.sph.umich.edu/locuszoom/

GWAS Catalog SNPs in Region

| trait |
|---|
| C–reactive protein and white blood cell count |
| Acute urticaria and angioedema (non–steroidal anti–inflammatory drug–induced) |
| Blood pressure |
| utism spectrum disorder, attention deficit–hyperactivity disorder, bipolar disorder, major depressive disorder, and schizophrenia (combin |
| Systemic lupus erythematosus |
| Systemic lupus erythematosus |
| Chronic lymphocytic leukemia |
| Systemic lupus erythematosus |
| Schizophrenia |
| Crohn's disease |
| Inflammatory bowel disease |
| Crohn's disease |
| Alzheimer disease and age of onset |

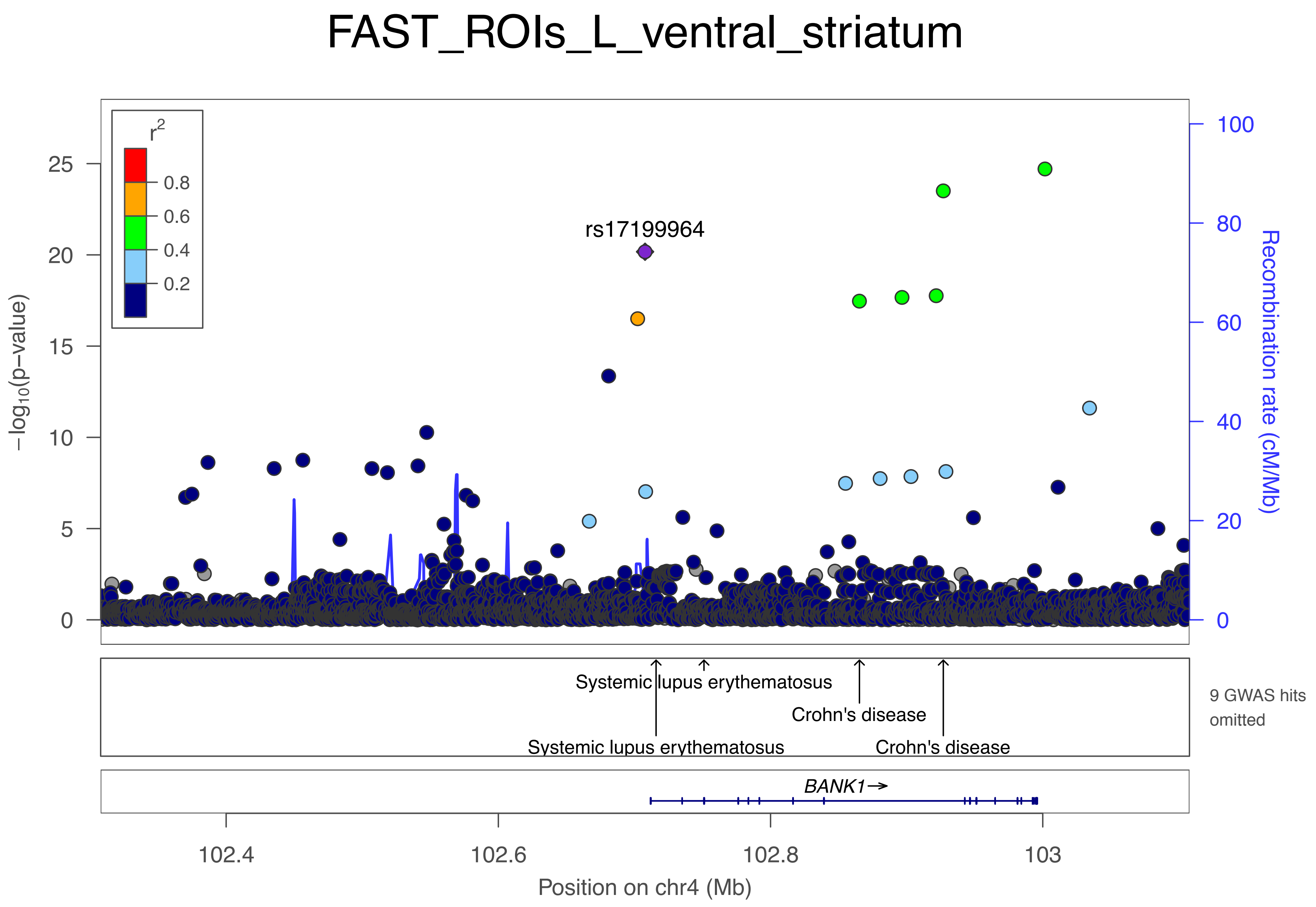
FAST_ROIs_L_ventral_striatum
r2
0.8
0.6
0.4
0.2
rs17199964
−log10(p−value)
Recombination rate (cM/Mb)
Systemic lupus erythematosus
Crohn's disease
Systemic lupus erythematosus
Crohn's disease
9 GWAS hits omitted
BANK1→
102.4
102.6
102.8
103
Position on chr4 (Mb)

| | |
|---|---|
| date: | Thu Aug 17 18:09:36 2017 |
| build: | hg19 |
| display range: | chr4:102307791−103107791 [102307791−103107791] |
| hilite range: | 0 – 0 [ 0 – 0 ] |
| reference SNP: | chr4:102707791 |
| number of SNPs plotted: | 3927 |
| min P.value: | 1.95E−25 [chr4:103001649] |
| max P.value: | 9.99E−1 [chr4:103023170] |
| omitted GWAS Hits: | chr4:102.926923−Crohn's disease, NA |
| omitted GWAS Hits: | NA, NA |
| omitted GWAS Hits: | NA, NA |

Make more plots at http://csg.sph.umich.edu/locuszoom/

GWAS Catalog SNPs in Region

| trait |
|---|
| C–reactive protein and white blood cell count |
| Acute urticaria and angioedema (non–steroidal anti–inflammatory drug–induced) |
| Blood pressure |
| utism spectrum disorder, attention deficit–hyperactivity disorder, bipolar disorder, major depressive disorder, and schizophrenia (combin |
| Systemic lupus erythematosus |
| Systemic lupus erythematosus |
| Chronic lymphocytic leukemia |
| Systemic lupus erythematosus |
| Schizophrenia |
| Crohn's disease |
| Inflammatory bowel disease |
| Crohn's disease |
| Alzheimer disease and age of onset |

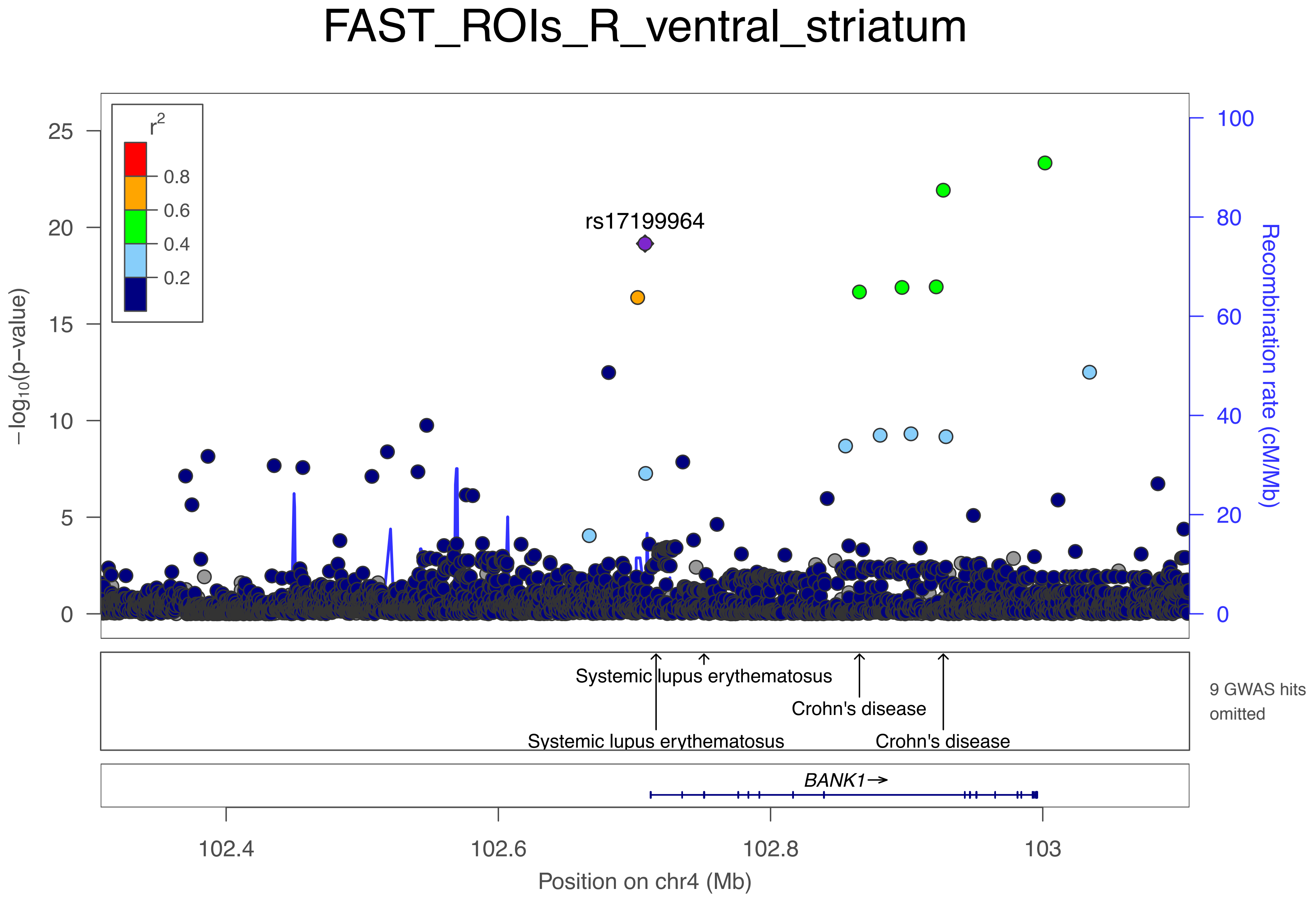

FAST_ROIs_R_ventral_striatum
r2
0.8
0.6
0.4
0.2
rs17199964
−log10(p−value)
Recombination rate (cM/Mb)
Systemic lupus erythematosus
Crohn's disease
Systemic lupus erythematosus
Crohn's disease
9 GWAS hits omitted
BANK1→
102.4
102.6
102.8
103
Position on chr4 (Mb)

| | |
|---|---|
| date: | Thu Aug 17 18:09:36 2017 |
| build: | hg19 |
| display range: | chr4:102307791−103107791 [102307791−103107791] |
| hilite range: | 0 – 0 [ 0 – 0 ] |
| reference SNP: | chr4:102707791 |
| number of SNPs plotted: | 3927 |
| min P.value: | 4.62E−24 [chr4:103001649] |
| max P.value: | 9.99E−1 [chr4:102412860] |
| omitted GWAS Hits: | chr4:102.926923−Crohn's disease, NA |
| omitted GWAS Hits: | NA, NA |
| omitted GWAS Hits: | NA, NA |

Make more plots at http://csg.sph.umich.edu/locuszoom/

GWAS Catalog SNPs in Region

| trait |
| --- |
| C–reactive protein and white blood cell count |
| Acute urticaria and angioedema (non–steroidal anti–inflammatory drug–induced) |
| Blood pressure |
| utism spectrum disorder, attention deficit–hyperactivity disorder, bipolar disorder, major depressive disorder, and schizophrenia (combin |
| Systemic lupus erythematosus |
| Systemic lupus erythematosus |
| Chronic lymphocytic leukemia |
| Systemic lupus erythematosus |
| Schizophrenia |
| Crohn's disease |
| Inflammatory bowel disease |
| Crohn's disease |
| Alzheimer disease and age of onset |

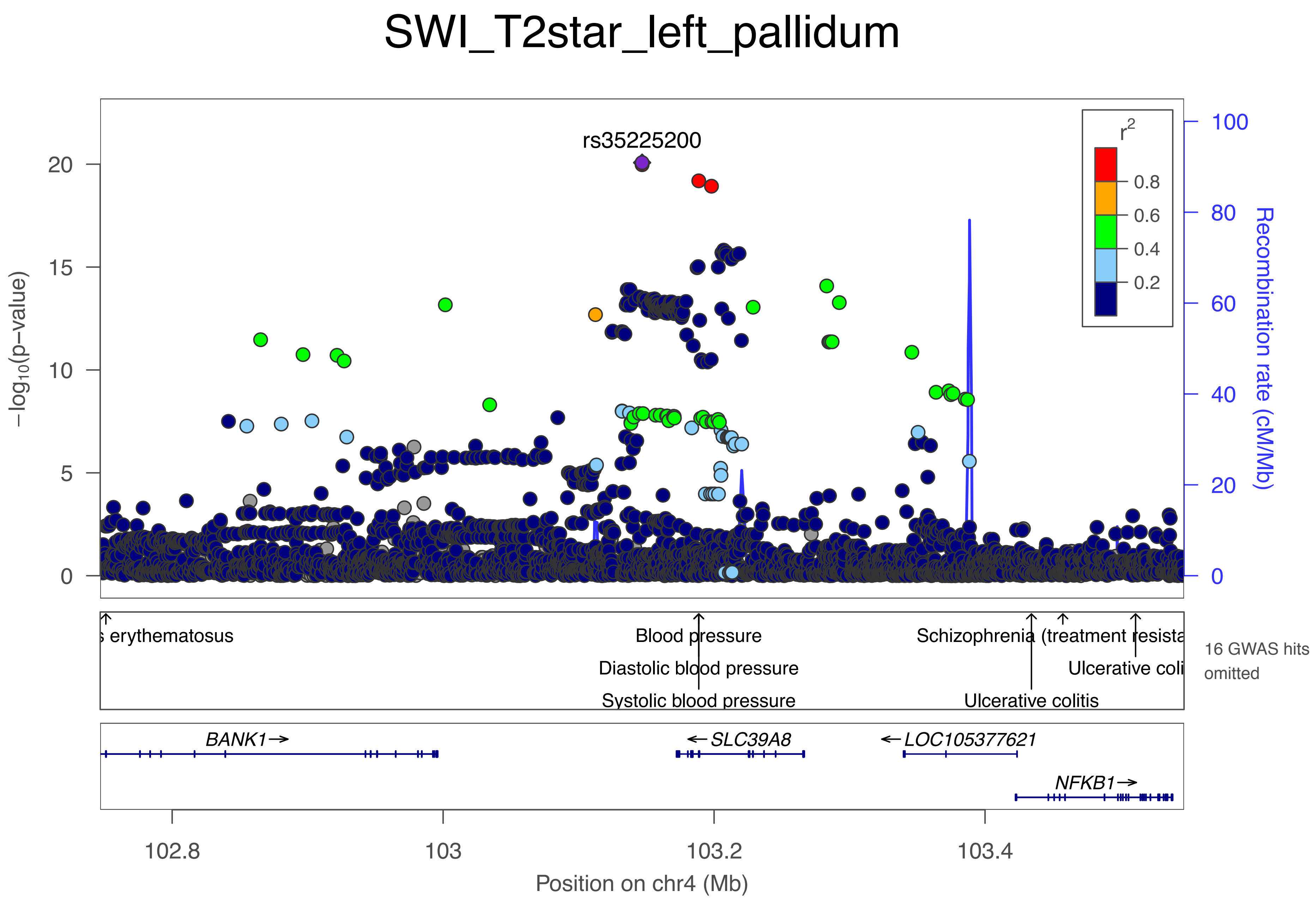
SWI_T2star_left_pallidum
rs35225200
−log10(p−value)
Recombination rate (cM/Mb)
r2
0.8
0.6
0.4
0.2
s erythematosus
Blood pressure
Diastolic blood pressure
Systolic blood pressure
Schizophrenia (treatment resista
Ulcerative coli
Ulcerative colitis
16 GWAS hits omitted
BANK1→
←SLC39A8
←LOC105377621
NFKB1→
102.8
103
103.2
103.4
Position on chr4 (Mb)

| | |
|---|---|
| date: | Thu Aug 17 18:09:15 2017 |
| build: | hg19 |
| display range: | chr4:102746888−103546888 [102746888−103546888] |
| hilite range: | 0 – 0 [ 0 – 0 ] |
| reference SNP: | chr4:103146888 |
| number of SNPs plotted: | 3536 |
| min P.value: | 8.41E−21 [chr4:103146888] |
| max P.value: | 10E−1 [chr4:103285398] |
| omitted GWAS Hits: | chr4:103.457418−Schizophrenia (treatment resistant), chr4:103.511114−Ulcerative colitis |
| omitted GWAS Hits: | NA, NA |
| omitted GWAS Hits: | NA, NA |
| omitted GWAS Hits: | NA, NA |
| omitted GWAS Hits: | NA, NA |
| omitted GWAS Hits: | NA |

Make more plots at http://csg.sph.umich.edu/locuszoom/

GWAS Catalog SNPs in Region

| chr | pos (Mb) | trait | snp |
|---|---|---|---|
| 4 | 102.7511 | Systemic lupus erythematosus | rs10516487 |
| 4 | 102.8416 | Schizophrenia | rs13119516 |
| 4 | 102.8653 | Crohn's disease | rs13126505 |
| 4 | 102.8653 | Inflammatory bowel disease | rs13126505 |
| 4 | 102.9269 | Crohn's disease | rs34592089 |
| 4 | 103.0542 | Alzheimer disease and age of onset | rs115641191 |
| 4 | 103.1469 | Schizophrenia | rs35518360 |
| 4 | 103.1887 | Blood pressure | rs13107325 |
| 4 | 103.1887 | Diastolic blood pressure | rs13107325 |
| 4 | 103.1887 | Systolic blood pressure | rs13107325 |
| 4 | 103.1887 | Hypertension | rs13107325 |
| 4 | 103.1887 | HDL cholesterol | rs13107325 |
| 4 | 103.1887 | Body mass index | rs13107325 |
| 4 | 103.1887 | Schizophrenia | rs13107325 |
| 4 | 103.1887 | NT−proBNP levels in acute coronary syndrome | rs13107325 |
| 4 | 103.1887 | Childhood body mass index | rs13107325 |
| 4 | 103.1981 | HDL cholesterol | rs13135092 |
| 4 | 103.2048 | Sitting height ratio | rs233817 |
| 4 | 103.2557 | Erythrocyte cadmium concentration in never smokers | rs7664683 |
| 4 | 103.3497 | Homeostasis model assessment of beta−cell function (interaction) | rs6533014 |
| 4 | 103.4343 | Ulcerative colitis | rs3774937 |

GWAS Catalog SNPs in Region

| chr | pos (Mb) | trait | snp |
|---|---|---|---|
| 4 | 103.4574 | Schizophrenia (treatment resistant) | rs230529 |
| 4 | 103.5111 | Ulcerative colitis | rs3774959 |

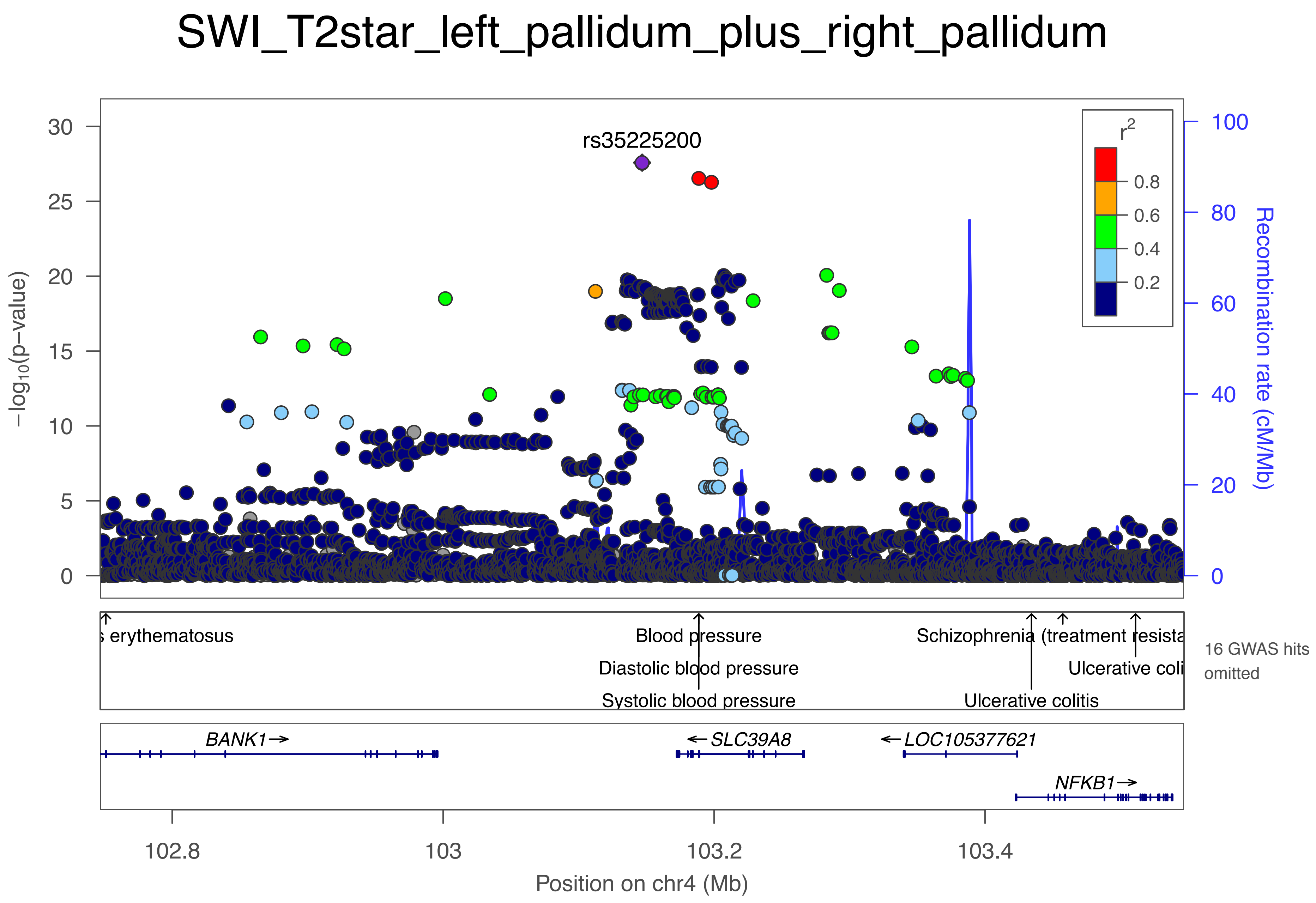
SWI_T2star_left_pallidum_plus_right_pallidum
rs35225200
r2
0.8
0.6
0.4
0.2
30
25
20
15
10
5
0
−log10(p−value)
100
80
60
40
20
0
Recombination rate (cM/Mb)
s erythematosus
Blood pressure
Diastolic blood pressure
Systolic blood pressure
Schizophrenia (treatment resista
Ulcerative coli
Ulcerative colitis
16 GWAS hits omitted
BANK1→
←SLC39A8
←LOC105377621
NFKB1→
102.8
103
103.2
103.4
Position on chr4 (Mb)

| | |
|---|---|
| date: | Thu Aug 17 18:09:15 2017 |
| build: | hg19 |
| display range: | chr4:102746888−103546888 [102746888−103546888] |
| hilite range: | 0 – 0 [ 0 – 0 ] |
| reference SNP: | chr4:103146888 |
| number of SNPs plotted: | 3536 |
| min P.value: | 2.64E−28 [chr4:103146888] |
| max P.value: | 9.99E−1 [chr4:103280897] |
| omitted GWAS Hits: | chr4:103.457418−Schizophrenia (treatment resistant), chr4:103.511114−Ulcerative colitis |
| omitted GWAS Hits: | NA, NA |
| omitted GWAS Hits: | NA, NA |
| omitted GWAS Hits: | NA, NA |
| omitted GWAS Hits: | NA, NA |
| omitted GWAS Hits: | NA |

Make more plots at http://csg.sph.umich.edu/locuszoom/

GWAS Catalog SNPs in Region

| chr | pos (Mb) | trait | snp |
|---|---|---|---|
| 4 | 102.7511 | Systemic lupus erythematosus | rs10516487 |
| 4 | 102.8416 | Schizophrenia | rs13119516 |
| 4 | 102.8653 | Crohn's disease | rs13126505 |
| 4 | 102.8653 | Inflammatory bowel disease | rs13126505 |
| 4 | 102.9269 | Crohn's disease | rs34592089 |
| 4 | 103.0542 | Alzheimer disease and age of onset | rs115641191 |
| 4 | 103.1469 | Schizophrenia | rs35518360 |
| 4 | 103.1887 | Blood pressure | rs13107325 |
| 4 | 103.1887 | Diastolic blood pressure | rs13107325 |
| 4 | 103.1887 | Systolic blood pressure | rs13107325 |
| 4 | 103.1887 | Hypertension | rs13107325 |
| 4 | 103.1887 | HDL cholesterol | rs13107325 |
| 4 | 103.1887 | Body mass index | rs13107325 |
| 4 | 103.1887 | Schizophrenia | rs13107325 |
| 4 | 103.1887 | NT−proBNP levels in acute coronary syndrome | rs13107325 |
| 4 | 103.1887 | Childhood body mass index | rs13107325 |
| 4 | 103.1981 | HDL cholesterol | rs13135092 |
| 4 | 103.2048 | Sitting height ratio | rs233817 |
| 4 | 103.2557 | Erythrocyte cadmium concentration in never smokers | rs7664683 |
| 4 | 103.3497 | Homeostasis model assessment of beta−cell function (interaction) | rs6533014 |
| 4 | 103.4343 | Ulcerative colitis | rs3774937 |

GWAS Catalog SNPs in Region

| chr | pos (Mb) | trait | snp |
|---|---|---|---|
| 4 | 103.4574 | Schizophrenia (treatment resistant) | rs230529 |
| 4 | 103.5111 | Ulcerative colitis | rs3774959 |

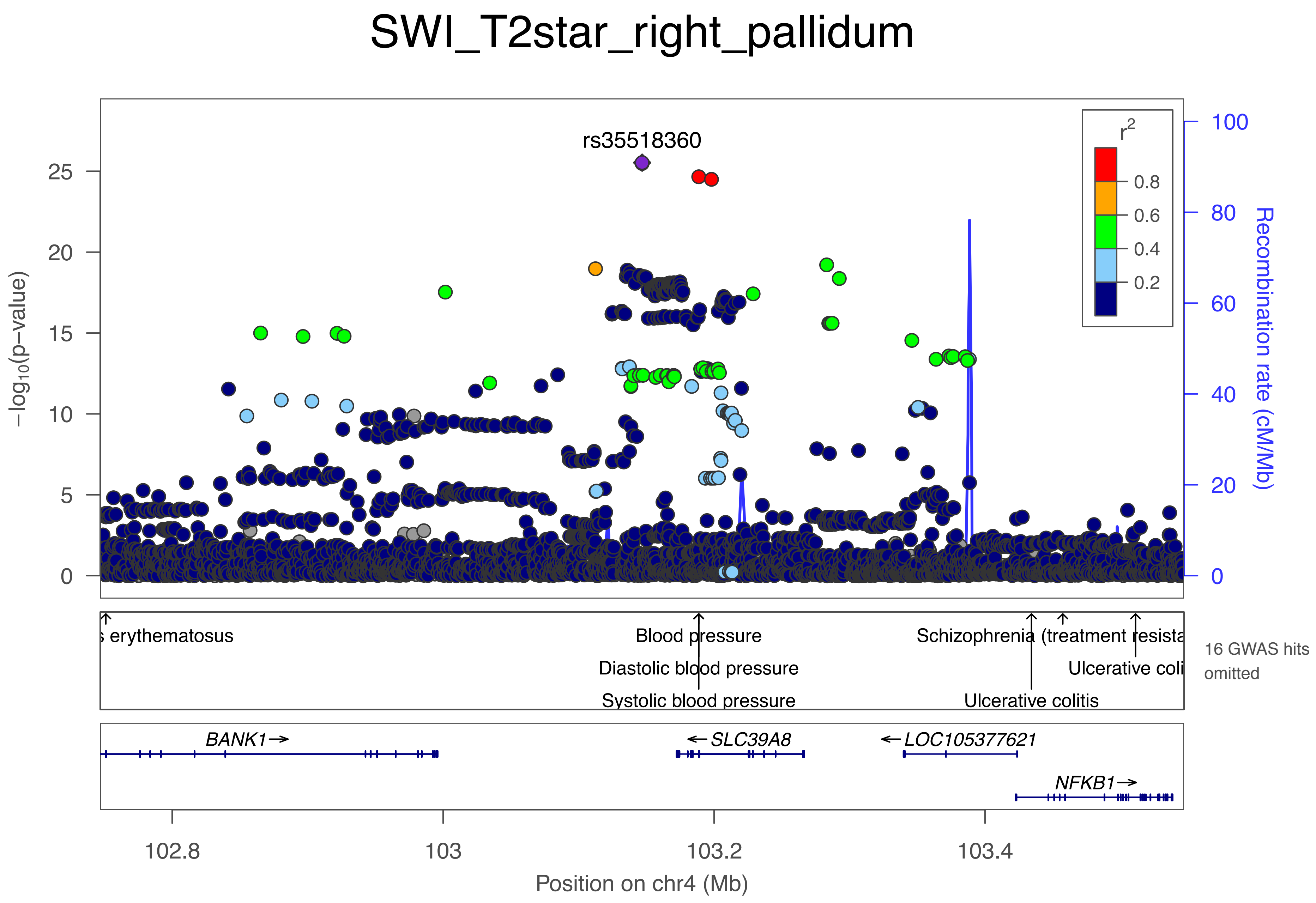
SWI_T2star_right_pallidum
rs35518360
−log10(p−value)
Recombination rate (cM/Mb)
r2
0.8
0.6
0.4
0.2
s erythematosus
Blood pressure
Diastolic blood pressure
Systolic blood pressure
Schizophrenia (treatment resista
Ulcerative coli
Ulcerative colitis
16 GWAS hits omitted
BANK1→
←SLC39A8
←LOC105377621
NFKB1→
102.8
103
103.2
103.4
Position on chr4 (Mb)

| | |
|---|---|
| date: | Thu Aug 17 18:09:15 2017 |
| build: | hg19 |
| display range: | chr4:102746890−103546890 [102746890−103546890] |
| hilite range: | 0 – 0 [ 0 – 0 ] |
| reference SNP: | chr4:103146890 |
| number of SNPs plotted: | 3536 |
| min P.value: | 2.96E−26 [chr4:103146890] |
| max P.value: | 10E−1 [chr4:102941039] |
| omitted GWAS Hits: | chr4:103.457418−Schizophrenia (treatment resistant), chr4:103.511114−Ulcerative colitis |
| omitted GWAS Hits: | NA, NA |
| omitted GWAS Hits: | NA, NA |
| omitted GWAS Hits: | NA, NA |
| omitted GWAS Hits: | NA, NA |
| omitted GWAS Hits: | NA |

Make more plots at http://csg.sph.umich.edu/locuszoom/

GWAS Catalog SNPs in Region

| chr | pos (Mb) | trait | snp |
|---|---|---|---|
| 4 | 102.7511 | Systemic lupus erythematosus | rs10516487 |
| 4 | 102.8416 | Schizophrenia | rs13119516 |
| 4 | 102.8653 | Crohn's disease | rs13126505 |
| 4 | 102.8653 | Inflammatory bowel disease | rs13126505 |
| 4 | 102.9269 | Crohn's disease | rs34592089 |
| 4 | 103.0542 | Alzheimer disease and age of onset | rs115641191 |
| 4 | 103.1469 | Schizophrenia | rs35518360 |
| 4 | 103.1887 | Blood pressure | rs13107325 |
| 4 | 103.1887 | Diastolic blood pressure | rs13107325 |
| 4 | 103.1887 | Systolic blood pressure | rs13107325 |
| 4 | 103.1887 | Hypertension | rs13107325 |
| 4 | 103.1887 | HDL cholesterol | rs13107325 |
| 4 | 103.1887 | Body mass index | rs13107325 |
| 4 | 103.1887 | Schizophrenia | rs13107325 |
| 4 | 103.1887 | NT−proBNP levels in acute coronary syndrome | rs13107325 |
| 4 | 103.1887 | Childhood body mass index | rs13107325 |
| 4 | 103.1981 | HDL cholesterol | rs13135092 |
| 4 | 103.2048 | Sitting height ratio | rs233817 |
| 4 | 103.2557 | Erythrocyte cadmium concentration in never smokers | rs7664683 |
| 4 | 103.3497 | Homeostasis model assessment of beta−cell function (interaction) | rs6533014 |
| 4 | 103.4343 | Ulcerative colitis | rs3774937 |

GWAS Catalog SNPs in Region

| chr | pos (Mb) | trait | snp |
|---|---|---|---|
| 4 | 103.4574 | Schizophrenia (treatment resistant) | rs230529 |
| 4 | 103.5111 | Ulcerative colitis | rs3774959 |

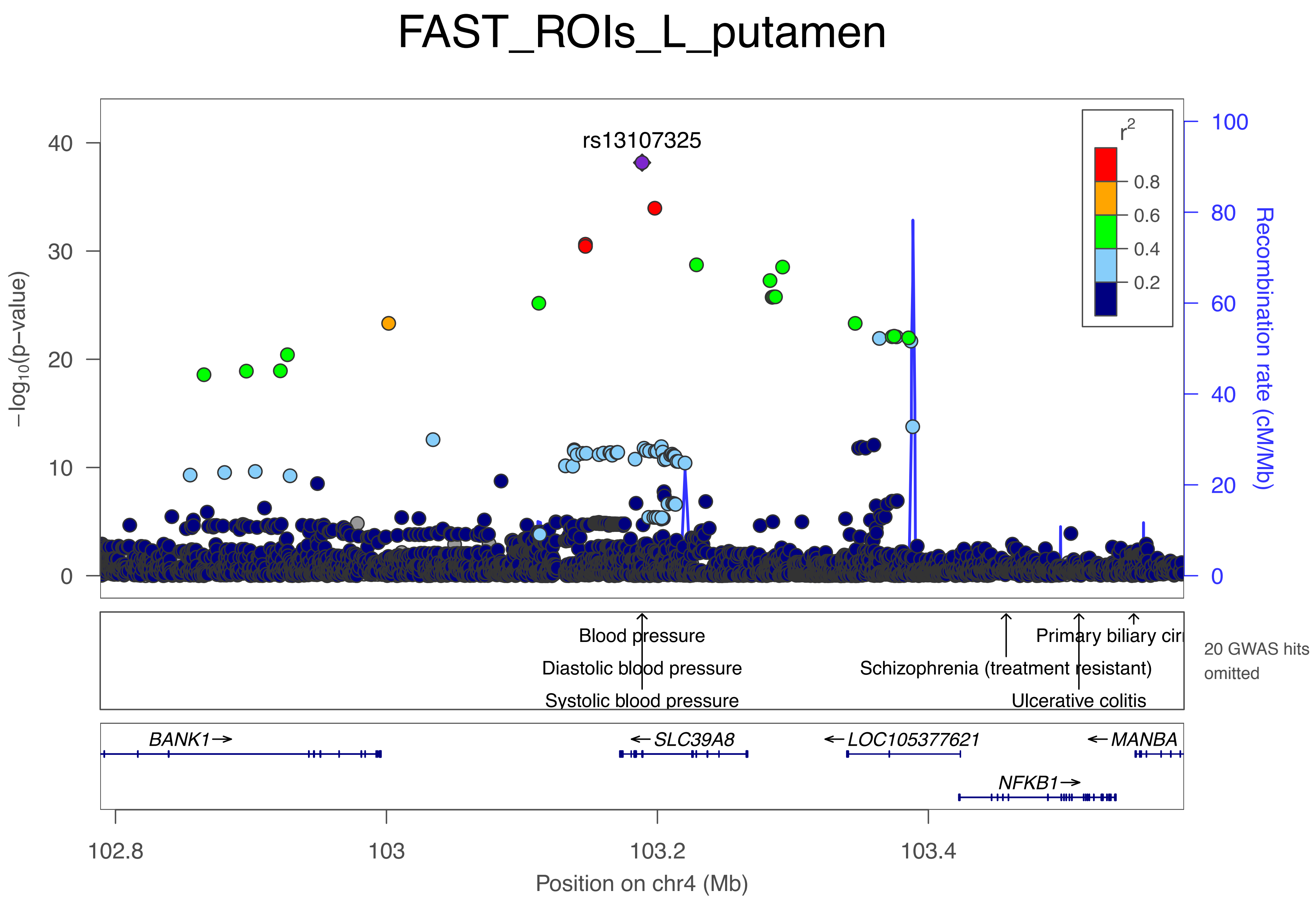
FAST_ROIs_L_putamen
rs13107325
−log10(p−value)
Recombination rate (cM/Mb)
r2
0.8
0.6
0.4
0.2
Blood pressure
Diastolic blood pressure
Systolic blood pressure
Primary biliary cir
Schizophrenia (treatment resistant)
Ulcerative colitis
20 GWAS hits omitted
BANK1
SLC39A8
LOC105377621
MANBA
NFKB1
102.8
103
103.2
103.4
Position on chr4 (Mb)

| | |
|---|---|
| date: | Thu Aug 17 18:01:59 2017 |
| build: | hg19 |
| display range: | chr4:102788709−103588709 [102788709−103588709] |
| hilite range: | 0 – 0 [ 0 – 0 ] |
| reference SNP: | chr4:103188709 |
| number of SNPs plotted: | 3534 |
| min P.value: | 6.79E−39 [chr4:103188709] |
| max P.value: | 9.96E−1 [chr4:103346519] |
| omitted GWAS Hits: | chr4:103.551603−Primary biliary cirrhosis, chr4:103.457418−Schizophrenia (treatment resistan |
| omitted GWAS Hits: | NA, NA |
| omitted GWAS Hits: | NA, NA |
| omitted GWAS Hits: | NA, NA |
| omitted GWAS Hits: | NA, NA |
| omitted GWAS Hits: | NA, NA |
| omitted GWAS Hits: | NA, NA |

Make more plots at http://csg.sph.umich.edu/locuszoom/

GWAS Catalog SNPs in Region

| chr | pos (Mb) | trait | snp |
|---|---|---|---|
| 4 | 102.8416 | Schizophrenia | rs13119516 |
| 4 | 102.8653 | Crohn's disease | rs13126505 |
| 4 | 102.8653 | Inflammatory bowel disease | rs13126505 |
| 4 | 102.9269 | Crohn's disease | rs34592089 |
| 4 | 103.0542 | Alzheimer disease and age of onset | rs115641191 |
| 4 | 103.1469 | Schizophrenia | rs35518360 |
| 4 | 103.1887 | Blood pressure | rs13107325 |
| 4 | 103.1887 | Diastolic blood pressure | rs13107325 |
| 4 | 103.1887 | Systolic blood pressure | rs13107325 |
| 4 | 103.1887 | Hypertension | rs13107325 |
| 4 | 103.1887 | HDL cholesterol | rs13107325 |
| 4 | 103.1887 | Body mass index | rs13107325 |
| 4 | 103.1887 | Schizophrenia | rs13107325 |
| 4 | 103.1887 | NT−proBNP levels in acute coronary syndrome | rs13107325 |
| 4 | 103.1887 | Childhood body mass index | rs13107325 |
| 4 | 103.1981 | HDL cholesterol | rs13135092 |
| 4 | 103.2048 | Sitting height ratio | rs233817 |
| 4 | 103.2557 | Erythrocyte cadmium concentration in never smokers | rs7664683 |
| 4 | 103.3497 | Homeostasis model assessment of beta−cell function (interaction) | rs6533014 |
| 4 | 103.4343 | Ulcerative colitis | rs3774937 |
| 4 | 103.4574 | Schizophrenia (treatment resistant) | rs230529 |

GWAS Catalog SNPs in Region

| chr | pos (Mb) | trait | snp |
|---|---|---|---|
| 4 | 103.5111 | Ulcerative colitis | rs3774959 |
| 4 | 103.5516 | Primary biliary cirrhosis | rs7665090 |
| 4 | 103.5527 | Primary biliary cirrhosis | rs1054037 |
| 4 | 103.5617 | Glomerular filtration rate (creatinine) | rs228611 |
| 4 | 103.5786 | Multiple sclerosis | rs228614 |

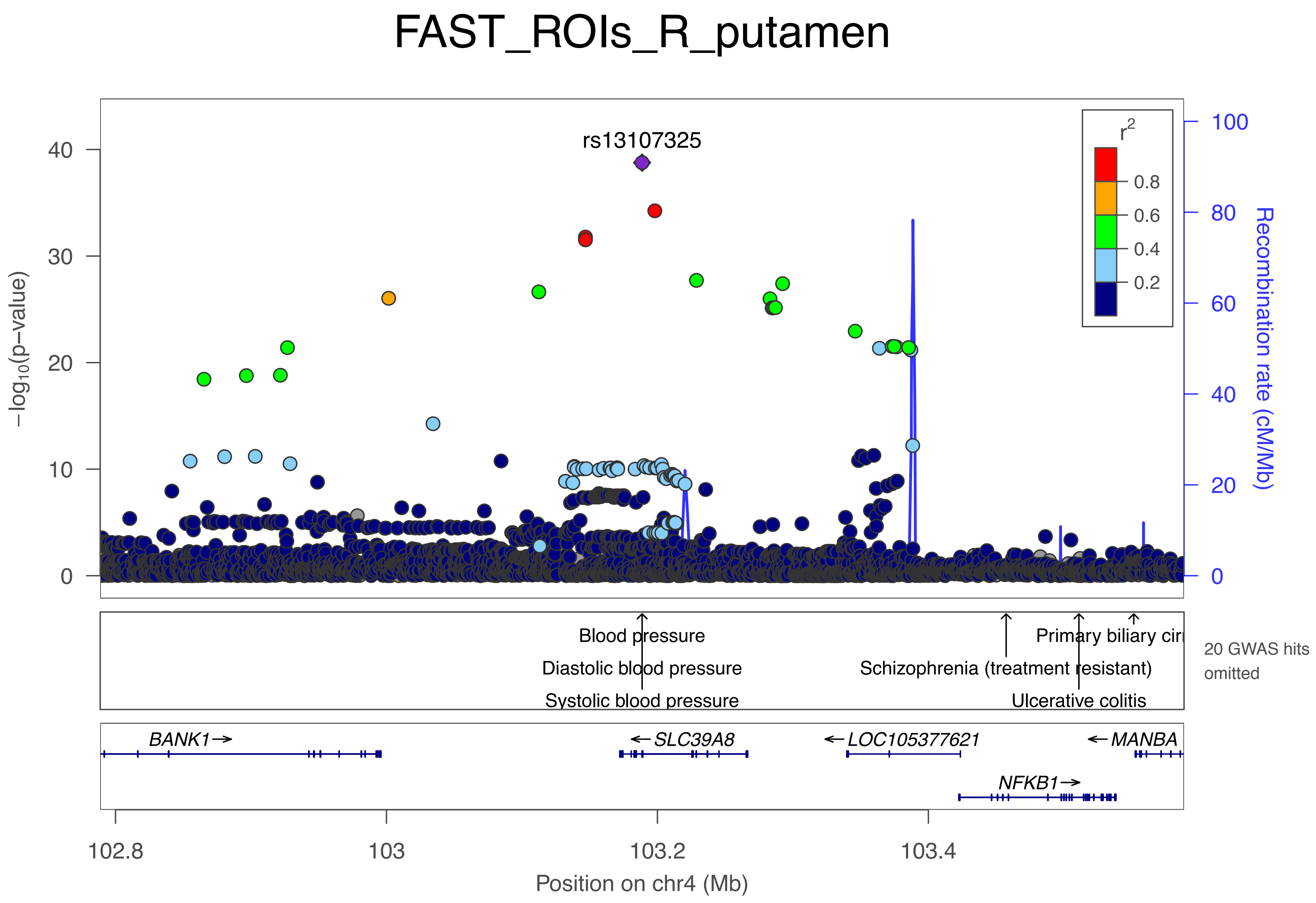
FAST_ROIs_R_putamen
rs13107325
−log10(p−value)
Recombination rate (cM/Mb)
r2
0.8
0.6
0.4
0.2
Blood pressure
Diastolic blood pressure
Systolic blood pressure
Primary biliary cir
Schizophrenia (treatment resistant)
Ulcerative colitis
20 GWAS hits omitted
BANK1→
←SLC39A8
←LOC105377621
←MANBA
NFKB1→
102.8
103
103.2
103.4
Position on chr4 (Mb)

| | |
|---|---|
| date: | Thu Aug 17 18:01:59 2017 |
| build: | hg19 |
| display range: | chr4:102788709−103588709 [102788709−103588709] |
| hilite range: | 0 – 0 [ 0 – 0 ] |
| reference SNP: | chr4:103188709 |
| number of SNPs plotted: | 3534 |
| min P.value: | 1.71E−39 [chr4:103188709] |
| max P.value: | 10E−1 [chr4:103194366] |
| omitted GWAS Hits: | chr4:103.551603−Primary biliary cirrhosis, chr4:103.457418−Schizophrenia (treatment resistan |
| omitted GWAS Hits: | NA, NA |
| omitted GWAS Hits: | NA, NA |
| omitted GWAS Hits: | NA, NA |
| omitted GWAS Hits: | NA, NA |
| omitted GWAS Hits: | NA, NA |
| omitted GWAS Hits: | NA, NA |

Make more plots at http://csg.sph.umich.edu/locuszoom/

GWAS Catalog SNPs in Region

| chr | pos (Mb) | trait | snp |
|---|---|---|---|
| 4 | 102.8416 | Schizophrenia | rs13119516 |
| 4 | 102.8653 | Crohn's disease | rs13126505 |
| 4 | 102.8653 | Inflammatory bowel disease | rs13126505 |
| 4 | 102.9269 | Crohn's disease | rs34592089 |
| 4 | 103.0542 | Alzheimer disease and age of onset | rs115641191 |
| 4 | 103.1469 | Schizophrenia | rs35518360 |
| 4 | 103.1887 | Blood pressure | rs13107325 |
| 4 | 103.1887 | Diastolic blood pressure | rs13107325 |
| 4 | 103.1887 | Systolic blood pressure | rs13107325 |
| 4 | 103.1887 | Hypertension | rs13107325 |
| 4 | 103.1887 | HDL cholesterol | rs13107325 |
| 4 | 103.1887 | Body mass index | rs13107325 |
| 4 | 103.1887 | Schizophrenia | rs13107325 |
| 4 | 103.1887 | NT−proBNP levels in acute coronary syndrome | rs13107325 |
| 4 | 103.1887 | Childhood body mass index | rs13107325 |
| 4 | 103.1981 | HDL cholesterol | rs13135092 |
| 4 | 103.2048 | Sitting height ratio | rs233817 |
| 4 | 103.2557 | Erythrocyte cadmium concentration in never smokers | rs7664683 |
| 4 | 103.3497 | Homeostasis model assessment of beta−cell function (interaction) | rs6533014 |
| 4 | 103.4343 | Ulcerative colitis | rs3774937 |
| 4 | 103.4574 | Schizophrenia (treatment resistant) | rs230529 |

GWAS Catalog SNPs in Region

| chr | pos (Mb) | trait | snp |
|---|---|---|---|
| 4 | 103.5111 | Ulcerative colitis | rs3774959 |
| 4 | 103.5516 | Primary biliary cirrhosis | rs7665090 |
| 4 | 103.5527 | Primary biliary cirrhosis | rs1054037 |
| 4 | 103.5617 | Glomerular filtration rate (creatinine) | rs228611 |
| 4 | 103.5786 | Multiple sclerosis | rs228614 |

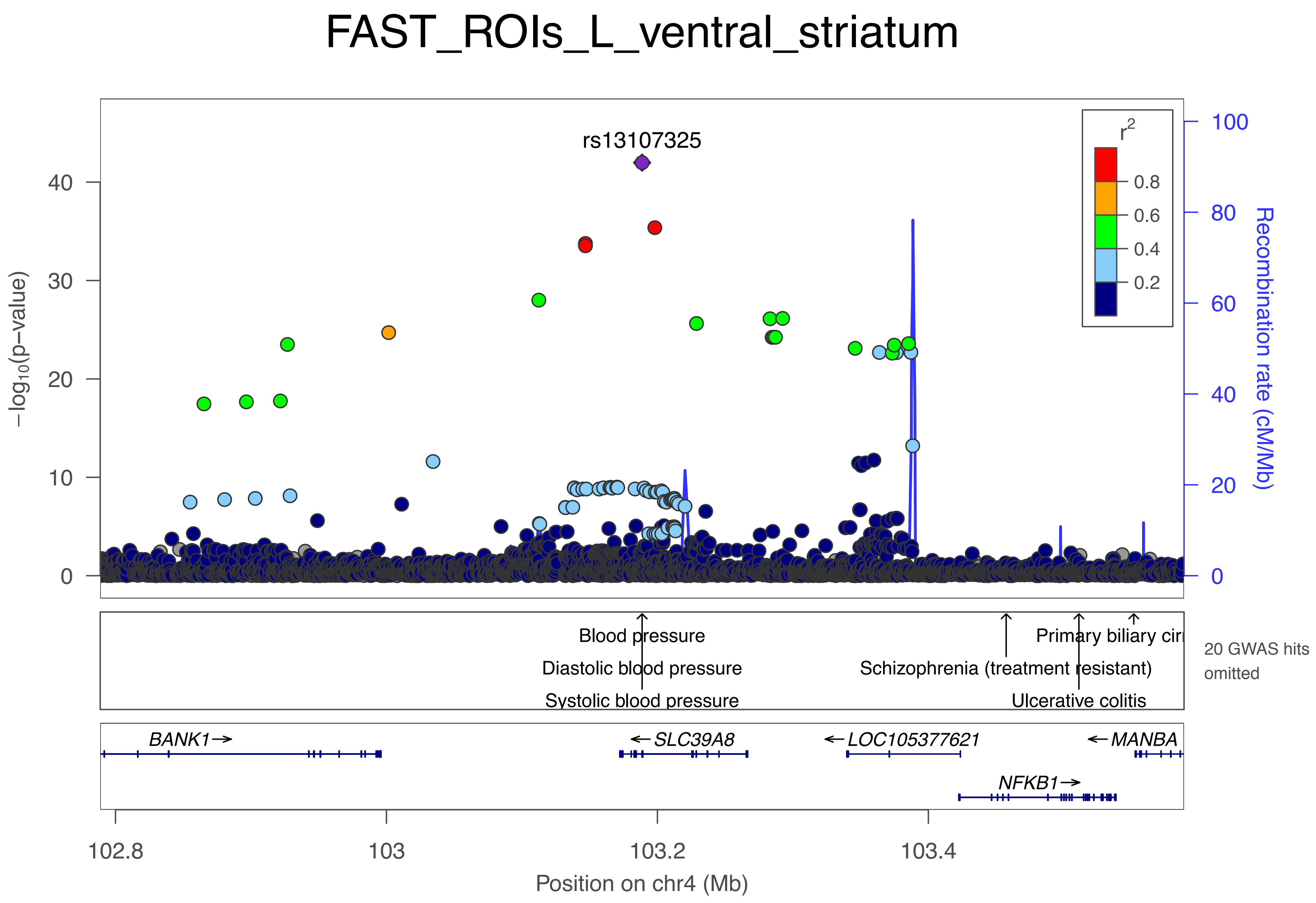
FAST_ROIs_L_ventral_striatum
rs13107325
r2
0.8
0.6
0.4
0.2
−log10(p−value)
40
30
20
10
0
100
80
60
40
20
0
Recombination rate (cM/Mb)
Blood pressure
Diastolic blood pressure
Systolic blood pressure
Primary biliary cir
Schizophrenia (treatment resistant)
Ulcerative colitis
20 GWAS hits omitted
BANK1→
←SLC39A8
←LOC105377621
←MANBA
NFKB1→
102.8
103
103.2
103.4
Position on chr4 (Mb)

| | |
|---|---|
| date: | Thu Aug 17 18:01:59 2017 |
| build: | hg19 |
| display range: | chr4:102788709−103588709 [102788709−103588709] |
| hilite range: | 0 – 0 [ 0 – 0 ] |
| reference SNP: | chr4:103188709 |
| number of SNPs plotted: | 3534 |
| min P.value: | 1.04E−42 [chr4:103188709] |
| max P.value: | 9.99E−1 [chr4:103178948] |
| omitted GWAS Hits: | chr4:103.551603−Primary biliary cirrhosis, chr4:103.457418−Schizophrenia (treatment resistan |
| omitted GWAS Hits: | NA, NA |
| omitted GWAS Hits: | NA, NA |
| omitted GWAS Hits: | NA, NA |
| omitted GWAS Hits: | NA, NA |
| omitted GWAS Hits: | NA, NA |
| omitted GWAS Hits: | NA, NA |

Make more plots at http://csg.sph.umich.edu/locuszoom/

GWAS Catalog SNPs in Region

| chr | pos (Mb) | trait | snp |
|---|---|---|---|
| 4 | 102.8416 | Schizophrenia | rs13119516 |
| 4 | 102.8653 | Crohn's disease | rs13126505 |
| 4 | 102.8653 | Inflammatory bowel disease | rs13126505 |
| 4 | 102.9269 | Crohn's disease | rs34592089 |
| 4 | 103.0542 | Alzheimer disease and age of onset | rs115641191 |
| 4 | 103.1469 | Schizophrenia | rs35518360 |
| 4 | 103.1887 | Blood pressure | rs13107325 |
| 4 | 103.1887 | Diastolic blood pressure | rs13107325 |
| 4 | 103.1887 | Systolic blood pressure | rs13107325 |
| 4 | 103.1887 | Hypertension | rs13107325 |
| 4 | 103.1887 | HDL cholesterol | rs13107325 |
| 4 | 103.1887 | Body mass index | rs13107325 |
| 4 | 103.1887 | Schizophrenia | rs13107325 |
| 4 | 103.1887 | NT−proBNP levels in acute coronary syndrome | rs13107325 |
| 4 | 103.1887 | Childhood body mass index | rs13107325 |
| 4 | 103.1981 | HDL cholesterol | rs13135092 |
| 4 | 103.2048 | Sitting height ratio | rs233817 |
| 4 | 103.2557 | Erythrocyte cadmium concentration in never smokers | rs7664683 |
| 4 | 103.3497 | Homeostasis model assessment of beta−cell function (interaction) | rs6533014 |
| 4 | 103.4343 | Ulcerative colitis | rs3774937 |
| 4 | 103.4574 | Schizophrenia (treatment resistant) | rs230529 |

GWAS Catalog SNPs in Region

| chr | pos (Mb) | trait | snp |
|---|---|---|---|
| 4 | 103.5111 | Ulcerative colitis | rs3774959 |
| 4 | 103.5516 | Primary biliary cirrhosis | rs7665090 |
| 4 | 103.5527 | Primary biliary cirrhosis | rs1054037 |
| 4 | 103.5617 | Glomerular filtration rate (creatinine) | rs228611 |
| 4 | 103.5786 | Multiple sclerosis | rs228614 |

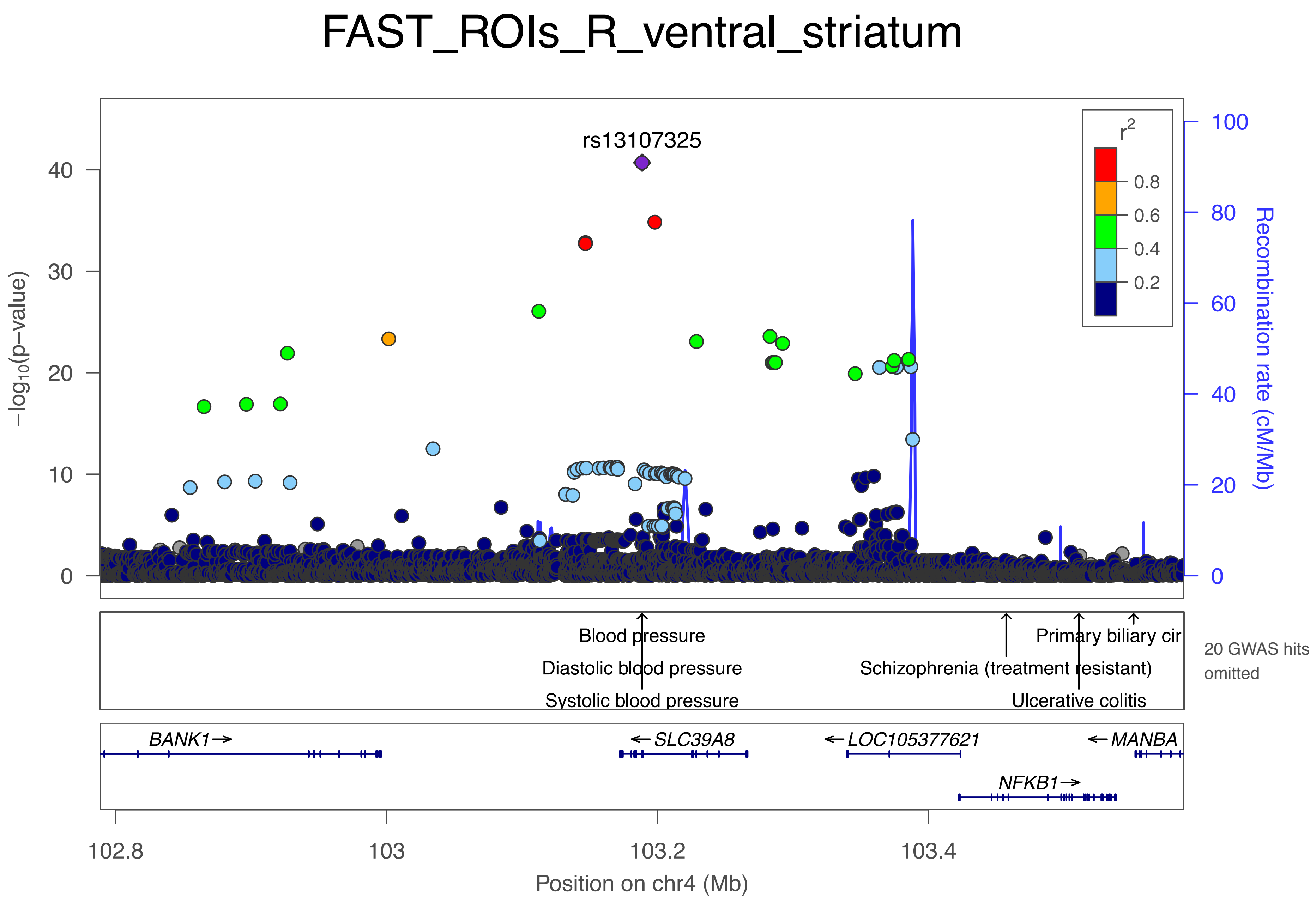

FAST_ROIs_R_ventral_striatum
rs13107325
−log10(p−value)
0
10
20
30
40
r2
0.8
0.6
0.4
0.2
100
80
60
40
20
0
Recombination rate (cM/Mb)
Blood pressure
Diastolic blood pressure
Systolic blood pressure
Primary biliary cir
Schizophrenia (treatment resistant)
Ulcerative colitis
20 GWAS hits omitted
BANK1→
←SLC39A8
←LOC105377621
←MANBA
NFKB1→
102.8
103
103.2
103.4
Position on chr4 (Mb)

| | |
|---|---|
| date: | Thu Aug 17 18:01:59 2017 |
| build: | hg19 |
| display range: | chr4:102788709−103588709 [102788709−103588709] |
| hilite range: | 0 – 0 [ 0 – 0 ] |
| reference SNP: | chr4:103188709 |
| number of SNPs plotted: | 3534 |
| min P.value: | 2.01E−41 [chr4:103188709] |
| max P.value: | 9.99E−1 [chr4:103457585] |
| omitted GWAS Hits: | chr4:103.551603−Primary biliary cirrhosis, chr4:103.457418−Schizophrenia (treatment resistan |
| omitted GWAS Hits: | NA, NA |
| omitted GWAS Hits: | NA, NA |
| omitted GWAS Hits: | NA, NA |
| omitted GWAS Hits: | NA, NA |
| omitted GWAS Hits: | NA, NA |
| omitted GWAS Hits: | NA, NA |

Make more plots at http://csg.sph.umich.edu/locuszoom/

GWAS Catalog SNPs in Region

| chr | pos (Mb) | trait | snp |
|---|---|---|---|
| 4 | 102.8416 | Schizophrenia | rs13119516 |
| 4 | 102.8653 | Crohn's disease | rs13126505 |
| 4 | 102.8653 | Inflammatory bowel disease | rs13126505 |
| 4 | 102.9269 | Crohn's disease | rs34592089 |
| 4 | 103.0542 | Alzheimer disease and age of onset | rs115641191 |
| 4 | 103.1469 | Schizophrenia | rs35518360 |
| 4 | 103.1887 | Blood pressure | rs13107325 |
| 4 | 103.1887 | Diastolic blood pressure | rs13107325 |
| 4 | 103.1887 | Systolic blood pressure | rs13107325 |
| 4 | 103.1887 | Hypertension | rs13107325 |
| 4 | 103.1887 | HDL cholesterol | rs13107325 |
| 4 | 103.1887 | Body mass index | rs13107325 |
| 4 | 103.1887 | Schizophrenia | rs13107325 |
| 4 | 103.1887 | NT−proBNP levels in acute coronary syndrome | rs13107325 |
| 4 | 103.1887 | Childhood body mass index | rs13107325 |
| 4 | 103.1981 | HDL cholesterol | rs13135092 |
| 4 | 103.2048 | Sitting height ratio | rs233817 |
| 4 | 103.2557 | Erythrocyte cadmium concentration in never smokers | rs7664683 |
| 4 | 103.3497 | Homeostasis model assessment of beta−cell function (interaction) | rs6533014 |
| 4 | 103.4343 | Ulcerative colitis | rs3774937 |
| 4 | 103.4574 | Schizophrenia (treatment resistant) | rs230529 |

GWAS Catalog SNPs in Region

| chr | pos (Mb) | trait | snp |
|---|---|---|---|
| 4 | 103.5111 | Ulcerative colitis | rs3774959 |
| 4 | 103.5516 | Primary biliary cirrhosis | rs7665090 |
| 4 | 103.5527 | Primary biliary cirrhosis | rs1054037 |
| 4 | 103.5617 | Glomerular filtration rate (creatinine) | rs228611 |
| 4 | 103.5786 | Multiple sclerosis | rs228614 |

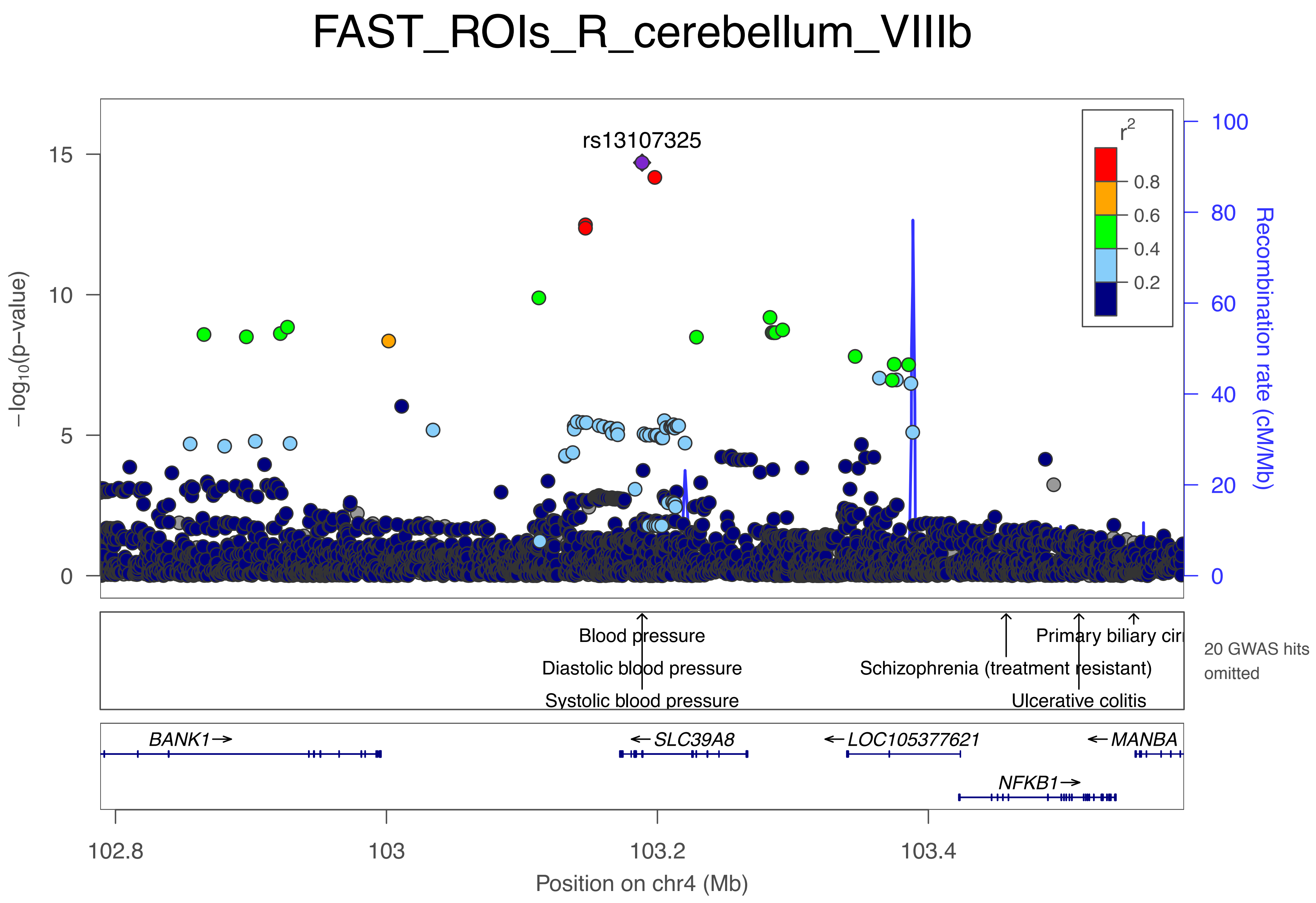
FAST_ROIs_R_cerebellum_VIIIb
rs13107325
r²
0.8
0.6
0.4
0.2
−log10(p−value)
Recombination rate (cM/Mb)
Blood pressure
Diastolic blood pressure
Systolic blood pressure
Primary biliary cir
Schizophrenia (treatment resistant)
Ulcerative colitis
20 GWAS hits omitted
BANK1→
←SLC39A8
←LOC105377621
←MANBA
NFKB1→
102.8
103
103.2
103.4
Position on chr4 (Mb)

| | |
|---|---|
| date: | Thu Aug 17 18:01:59 2017 |
| build: | hg19 |
| display range: | chr4:102788709−103588709 [102788709−103588709] |
| hilite range: | 0 – 0 [ 0 – 0 ] |
| reference SNP: | chr4:103188709 |
| number of SNPs plotted: | 3534 |
| min P.value: | 2E−15 [chr4:103188709] |
| max P.value: | 9.99E−1 [chr4:103203467] |
| omitted GWAS Hits: | chr4:103.551603−Primary biliary cirrhosis, chr4:103.457418−Schizophrenia (treatment resistan |
| omitted GWAS Hits: | NA, NA |
| omitted GWAS Hits: | NA, NA |
| omitted GWAS Hits: | NA, NA |
| omitted GWAS Hits: | NA, NA |
| omitted GWAS Hits: | NA, NA |
| omitted GWAS Hits: | NA, NA |

Make more plots at http://csg.sph.umich.edu/locuszoom/

GWAS Catalog SNPs in Region

| chr | pos (Mb) | trait | snp |
|---|---|---|---|
| 4 | 102.8416 | Schizophrenia | rs13119516 |
| 4 | 102.8653 | Crohn's disease | rs13126505 |
| 4 | 102.8653 | Inflammatory bowel disease | rs13126505 |
| 4 | 102.9269 | Crohn's disease | rs34592089 |
| 4 | 103.0542 | Alzheimer disease and age of onset | rs115641191 |
| 4 | 103.1469 | Schizophrenia | rs35518360 |
| 4 | 103.1887 | Blood pressure | rs13107325 |
| 4 | 103.1887 | Diastolic blood pressure | rs13107325 |
| 4 | 103.1887 | Systolic blood pressure | rs13107325 |
| 4 | 103.1887 | Hypertension | rs13107325 |
| 4 | 103.1887 | HDL cholesterol | rs13107325 |
| 4 | 103.1887 | Body mass index | rs13107325 |
| 4 | 103.1887 | Schizophrenia | rs13107325 |
| 4 | 103.1887 | NT−proBNP levels in acute coronary syndrome | rs13107325 |
| 4 | 103.1887 | Childhood body mass index | rs13107325 |
| 4 | 103.1981 | HDL cholesterol | rs13135092 |
| 4 | 103.2048 | Sitting height ratio | rs233817 |
| 4 | 103.2557 | Erythrocyte cadmium concentration in never smokers | rs7664683 |
| 4 | 103.3497 | Homeostasis model assessment of beta−cell function (interaction) | rs6533014 |
| 4 | 103.4343 | Ulcerative colitis | rs3774937 |
| 4 | 103.4574 | Schizophrenia (treatment resistant) | rs230529 |

GWAS Catalog SNPs in Region

| chr | pos (Mb) | trait | snp |
|---|---|---|---|
| 4 | 103.5111 | Ulcerative colitis | rs3774959 |
| 4 | 103.5516 | Primary biliary cirrhosis | rs7665090 |
| 4 | 103.5527 | Primary biliary cirrhosis | rs1054037 |
| 4 | 103.5617 | Glomerular filtration rate (creatinine) | rs228611 |
| 4 | 103.5786 | Multiple sclerosis | rs228614 |

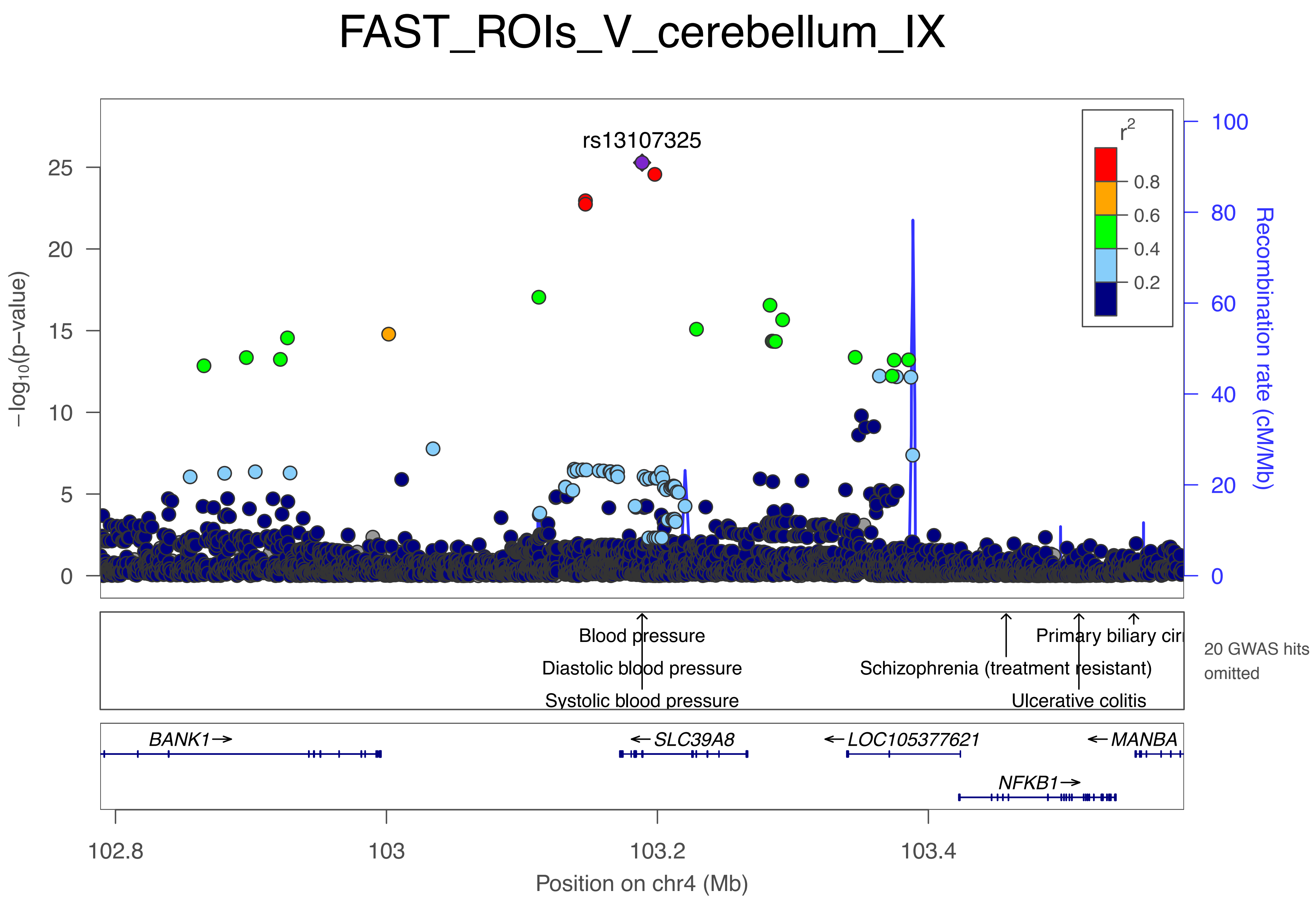

FAST_ROIs_V_cerebellum_IX
rs13107325
−log10(p−value)
Recombination rate (cM/Mb)
r2
0.8
0.6
0.4
0.2
Blood pressure
Diastolic blood pressure
Systolic blood pressure
Primary biliary cir
Schizophrenia (treatment resistant)
Ulcerative colitis
20 GWAS hits omitted
BANK1
SLC39A8
LOC105377621
MANBA
NFKB1
102.8
103
103.2
103.4
Position on chr4 (Mb)

| | |
|---|---|
| date: | Thu Aug 17 18:01:59 2017 |
| build: | hg19 |
| display range: | chr4:102788709−103588709 [102788709−103588709] |
| hilite range: | 0 – 0 [ 0 – 0 ] |
| reference SNP: | chr4:103188709 |
| number of SNPs plotted: | 3534 |
| min P.value: | 5.21E−26 [chr4:103188709] |
| max P.value: | 10E−1 [chr4:103507703] |
| omitted GWAS Hits: | chr4:103.551603−Primary biliary cirrhosis, chr4:103.457418−Schizophrenia (treatment resistan |
| omitted GWAS Hits: | NA, NA |
| omitted GWAS Hits: | NA, NA |
| omitted GWAS Hits: | NA, NA |
| omitted GWAS Hits: | NA, NA |
| omitted GWAS Hits: | NA, NA |
| omitted GWAS Hits: | NA, NA |

Make more plots at http://csg.sph.umich.edu/locuszoom/

GWAS Catalog SNPs in Region

| chr | pos (Mb) | trait | snp |
|---|---|---|---|
| 4 | 102.8416 | Schizophrenia | rs13119516 |
| 4 | 102.8653 | Crohn's disease | rs13126505 |
| 4 | 102.8653 | Inflammatory bowel disease | rs13126505 |
| 4 | 102.9269 | Crohn's disease | rs34592089 |
| 4 | 103.0542 | Alzheimer disease and age of onset | rs115641191 |
| 4 | 103.1469 | Schizophrenia | rs35518360 |
| 4 | 103.1887 | Blood pressure | rs13107325 |
| 4 | 103.1887 | Diastolic blood pressure | rs13107325 |
| 4 | 103.1887 | Systolic blood pressure | rs13107325 |
| 4 | 103.1887 | Hypertension | rs13107325 |
| 4 | 103.1887 | HDL cholesterol | rs13107325 |
| 4 | 103.1887 | Body mass index | rs13107325 |
| 4 | 103.1887 | Schizophrenia | rs13107325 |
| 4 | 103.1887 | NT−proBNP levels in acute coronary syndrome | rs13107325 |
| 4 | 103.1887 | Childhood body mass index | rs13107325 |
| 4 | 103.1981 | HDL cholesterol | rs13135092 |
| 4 | 103.2048 | Sitting height ratio | rs233817 |
| 4 | 103.2557 | Erythrocyte cadmium concentration in never smokers | rs7664683 |
| 4 | 103.3497 | Homeostasis model assessment of beta−cell function (interaction) | rs6533014 |
| 4 | 103.4343 | Ulcerative colitis | rs3774937 |
| 4 | 103.4574 | Schizophrenia (treatment resistant) | rs230529 |

GWAS Catalog SNPs in Region

| chr | pos (Mb) | trait | snp |
|---|---|---|---|
| 4 | 103.5111 | Ulcerative colitis | rs3774959 |
| 4 | 103.5516 | Primary biliary cirrhosis | rs7665090 |
| 4 | 103.5527 | Primary biliary cirrhosis | rs1054037 |
| 4 | 103.5617 | Glomerular filtration rate (creatinine) | rs228611 |
| 4 | 103.5786 | Multiple sclerosis | rs228614 |

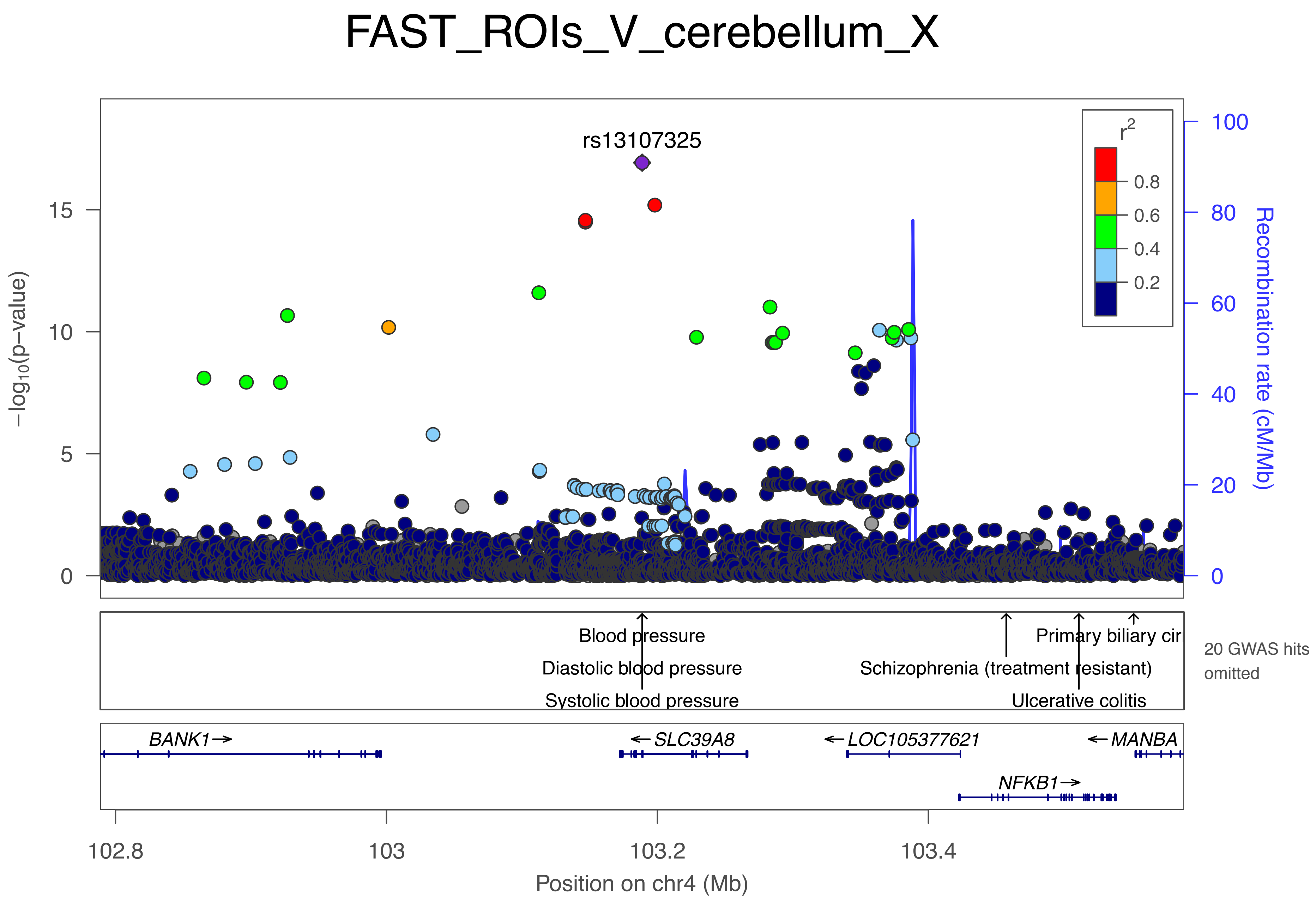
FAST_ROIs_V_cerebellum_X
rs13107325
−log10(p−value)
Recombination rate (cM/Mb)
r2
0.8
0.6
0.4
0.2
0
5
10
15
20
40
60
80
100
Blood pressure
Diastolic blood pressure
Systolic blood pressure
Primary biliary cir
Schizophrenia (treatment resistant)
Ulcerative colitis
20 GWAS hits omitted
BANK1→
←SLC39A8
←LOC105377621
←MANBA
NFKB1→
102.8
103
103.2
103.4
Position on chr4 (Mb)

| | |
|---|---|
| date: | Thu Aug 17 18:01:59 2017 |
| build: | hg19 |
| display range: | chr4:102788709−103588709 [102788709−103588709] |
| hilite range: | 0 – 0 [ 0 – 0 ] |
| reference SNP: | chr4:103188709 |
| number of SNPs plotted: | 3534 |
| min P.value: | 1.18E−17 [chr4:103188709] |
| max P.value: | 9.99E−1 [chr4:103322301] |
| omitted GWAS Hits: | chr4:103.551603−Primary biliary cirrhosis, chr4:103.457418−Schizophrenia (treatment resistan |
| omitted GWAS Hits: | NA, NA |
| omitted GWAS Hits: | NA, NA |
| omitted GWAS Hits: | NA, NA |
| omitted GWAS Hits: | NA, NA |
| omitted GWAS Hits: | NA, NA |
| omitted GWAS Hits: | NA, NA |

Make more plots at http://csg.sph.umich.edu/locuszoom/

GWAS Catalog SNPs in Region

| chr | pos (Mb) | trait | snp |
|---|---|---|---|
| 4 | 102.8416 | Schizophrenia | rs13119516 |
| 4 | 102.8653 | Crohn's disease | rs13126505 |
| 4 | 102.8653 | Inflammatory bowel disease | rs13126505 |
| 4 | 102.9269 | Crohn's disease | rs34592089 |
| 4 | 103.0542 | Alzheimer disease and age of onset | rs115641191 |
| 4 | 103.1469 | Schizophrenia | rs35518360 |
| 4 | 103.1887 | Blood pressure | rs13107325 |
| 4 | 103.1887 | Diastolic blood pressure | rs13107325 |
| 4 | 103.1887 | Systolic blood pressure | rs13107325 |
| 4 | 103.1887 | Hypertension | rs13107325 |
| 4 | 103.1887 | HDL cholesterol | rs13107325 |
| 4 | 103.1887 | Body mass index | rs13107325 |
| 4 | 103.1887 | Schizophrenia | rs13107325 |
| 4 | 103.1887 | NT−proBNP levels in acute coronary syndrome | rs13107325 |
| 4 | 103.1887 | Childhood body mass index | rs13107325 |
| 4 | 103.1981 | HDL cholesterol | rs13135092 |
| 4 | 103.2048 | Sitting height ratio | rs233817 |
| 4 | 103.2557 | Erythrocyte cadmium concentration in never smokers | rs7664683 |
| 4 | 103.3497 | Homeostasis model assessment of beta−cell function (interaction) | rs6533014 |
| 4 | 103.4343 | Ulcerative colitis | rs3774937 |
| 4 | 103.4574 | Schizophrenia (treatment resistant) | rs230529 |

GWAS Catalog SNPs in Region

| chr | pos (Mb) | trait | snp |
|---|---|---|---|
| 4 | 103.5111 | Ulcerative colitis | rs3774959 |
| 4 | 103.5516 | Primary biliary cirrhosis | rs7665090 |
| 4 | 103.5527 | Primary biliary cirrhosis | rs1054037 |
| 4 | 103.5617 | Glomerular filtration rate (creatinine) | rs228611 |
| 4 | 103.5786 | Multiple sclerosis | rs228614 |

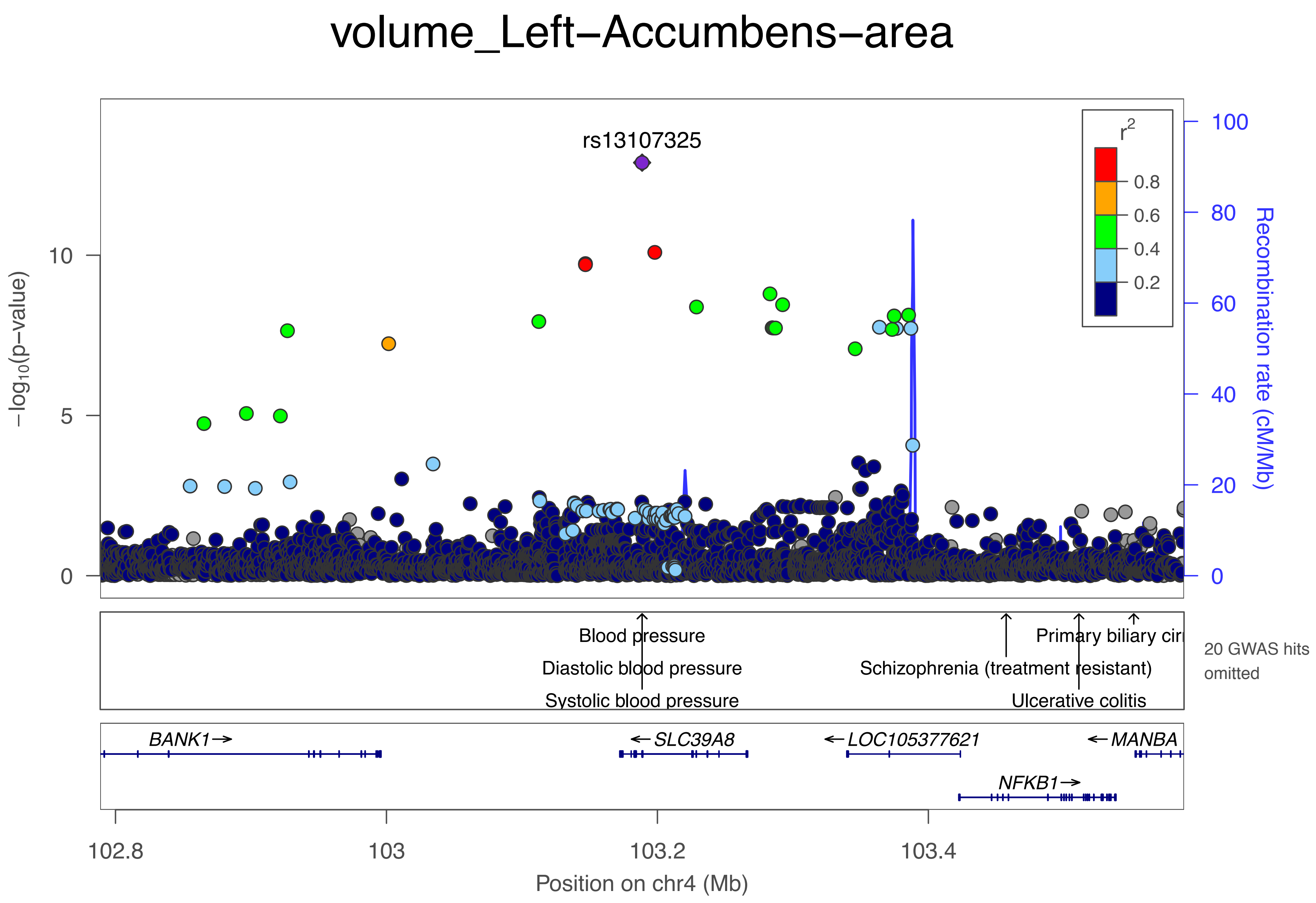

volume_Left−Accumbens−area
rs13107325
−log10(p−value)
Recombination rate (cM/Mb)
r2
0.8
0.6
0.4
0.2
Blood pressure
Diastolic blood pressure
Systolic blood pressure
Primary biliary cir
Schizophrenia (treatment resistant)
Ulcerative colitis
20 GWAS hits omitted
BANK1→
←SLC39A8
←LOC105377621
←MANBA
NFKB1→
102.8
103
103.2
103.4
Position on chr4 (Mb)

| | |
|---|---|
| date: | Thu Aug 17 18:01:59 2017 |
| build: | hg19 |
| display range: | chr4:102788709−103588709 [102788709−103588709] |
| hilite range: | 0 – 0 [ 0 – 0 ] |
| reference SNP: | chr4:103188709 |
| number of SNPs plotted: | 3534 |
| min P.value: | 1.29E−13 [chr4:103188709] |
| max P.value: | 10E−1 [chr4:103244676] |
| omitted GWAS Hits: | chr4:103.551603−Primary biliary cirrhosis, chr4:103.457418−Schizophrenia (treatment resistan |
| omitted GWAS Hits: | NA, NA |
| omitted GWAS Hits: | NA, NA |
| omitted GWAS Hits: | NA, NA |
| omitted GWAS Hits: | NA, NA |
| omitted GWAS Hits: | NA, NA |
| omitted GWAS Hits: | NA, NA |

Make more plots at http://csg.sph.umich.edu/locuszoom/

GWAS Catalog SNPs in Region

| chr | pos (Mb) | trait | snp |
|---|---|---|---|
| 4 | 102.8416 | Schizophrenia | rs13119516 |
| 4 | 102.8653 | Crohn's disease | rs13126505 |
| 4 | 102.8653 | Inflammatory bowel disease | rs13126505 |
| 4 | 102.9269 | Crohn's disease | rs34592089 |
| 4 | 103.0542 | Alzheimer disease and age of onset | rs115641191 |
| 4 | 103.1469 | Schizophrenia | rs35518360 |
| 4 | 103.1887 | Blood pressure | rs13107325 |
| 4 | 103.1887 | Diastolic blood pressure | rs13107325 |
| 4 | 103.1887 | Systolic blood pressure | rs13107325 |
| 4 | 103.1887 | Hypertension | rs13107325 |
| 4 | 103.1887 | HDL cholesterol | rs13107325 |
| 4 | 103.1887 | Body mass index | rs13107325 |
| 4 | 103.1887 | Schizophrenia | rs13107325 |
| 4 | 103.1887 | NT−proBNP levels in acute coronary syndrome | rs13107325 |
| 4 | 103.1887 | Childhood body mass index | rs13107325 |
| 4 | 103.1981 | HDL cholesterol | rs13135092 |
| 4 | 103.2048 | Sitting height ratio | rs233817 |
| 4 | 103.2557 | Erythrocyte cadmium concentration in never smokers | rs7664683 |
| 4 | 103.3497 | Homeostasis model assessment of beta−cell function (interaction) | rs6533014 |
| 4 | 103.4343 | Ulcerative colitis | rs3774937 |
| 4 | 103.4574 | Schizophrenia (treatment resistant) | rs230529 |

GWAS Catalog SNPs in Region

| chr | pos (Mb) | trait | snp |
|---|---|---|---|
| 4 | 103.5111 | Ulcerative colitis | rs3774959 |
| 4 | 103.5516 | Primary biliary cirrhosis | rs7665090 |
| 4 | 103.5527 | Primary biliary cirrhosis | rs1054037 |
| 4 | 103.5617 | Glomerular filtration rate (creatinine) | rs228611 |
| 4 | 103.5786 | Multiple sclerosis | rs228614 |

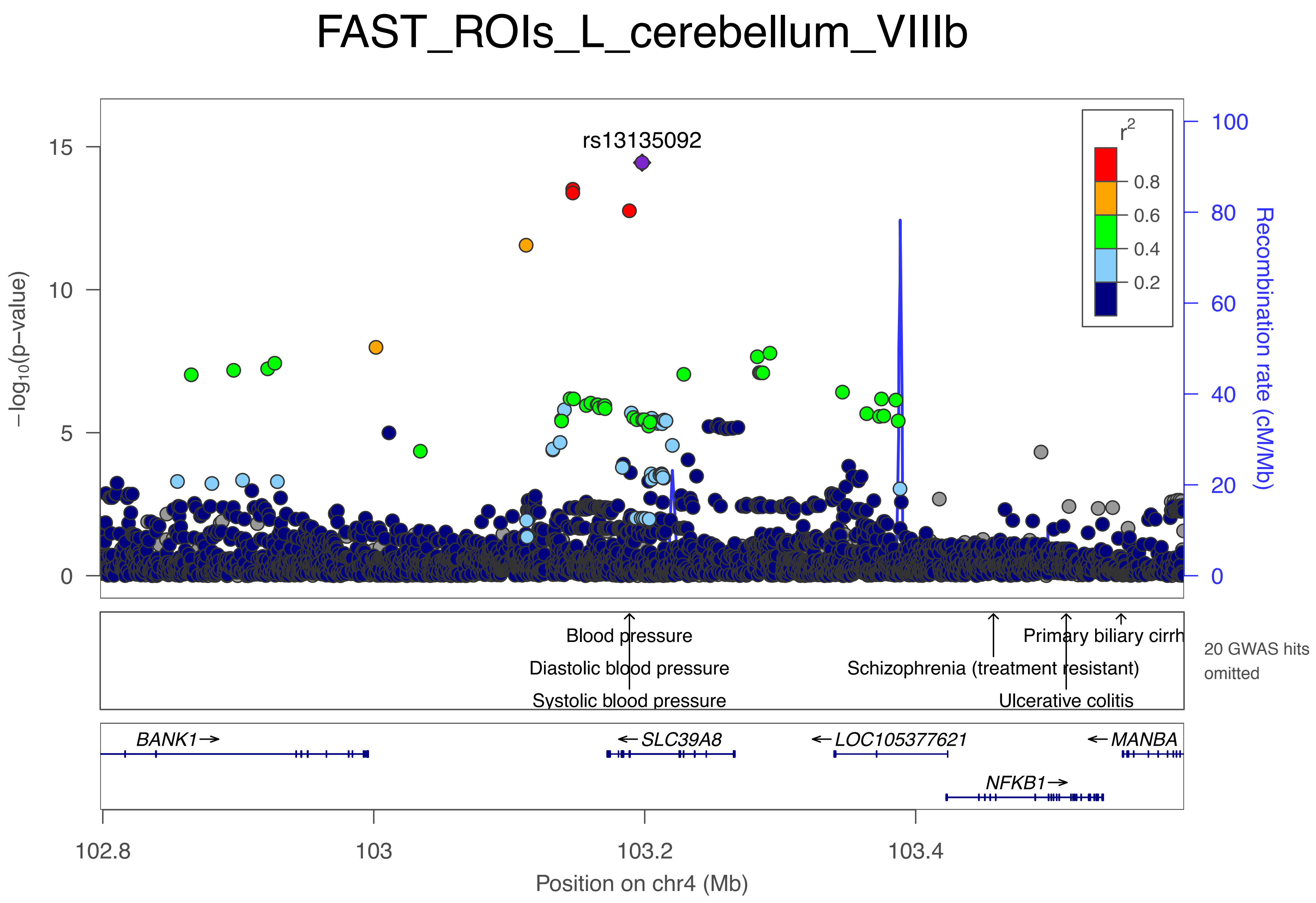
FAST_ROIs_L_cerebellum_VIIIb
rs13135092
r2
0.8
0.6
0.4
0.2
15
10
5
0
−log10(p−value)
100
80
60
40
20
0
Recombination rate (cM/Mb)
Blood pressure
Diastolic blood pressure
Systolic blood pressure
Primary biliary cirrh
Schizophrenia (treatment resistant)
Ulcerative colitis
20 GWAS hits omitted
BANK1→
←SLC39A8
←LOC105377621
←MANBA
NFKB1→
102.8
103
103.2
103.4
Position on chr4 (Mb)

| | |
|---|---|
| date: | Thu Aug 17 18:02:44 2017 |
| build: | hg19 |
| display range: | chr4:102798082−103598082 [102798082−103598082] |
| hilite range: | 0 – 0 [ 0 – 0 ] |
| reference SNP: | chr4:103198082 |
| number of SNPs plotted: | 3541 |
| min P.value: | 3.61E−15 [chr4:103198082] |
| max P.value: | 9.99E−1 [chr4:102907145] |
| omitted GWAS Hits: | chr4:103.551603−Primary biliary cirrhosis, chr4:103.457418−Schizophrenia (treatment resistan |
| omitted GWAS Hits: | NA, NA |
| omitted GWAS Hits: | NA, NA |
| omitted GWAS Hits: | NA, NA |
| omitted GWAS Hits: | NA, NA |
| omitted GWAS Hits: | NA, NA |
| omitted GWAS Hits: | NA, NA |

Make more plots at http://csg.sph.umich.edu/locuszoom/

GWAS Catalog SNPs in Region

| chr | pos (Mb) | trait | snp |
|---|---|---|---|
| 4 | 102.8416 | Schizophrenia | rs13119516 |
| 4 | 102.8653 | Crohn's disease | rs13126505 |
| 4 | 102.8653 | Inflammatory bowel disease | rs13126505 |
| 4 | 102.9269 | Crohn's disease | rs34592089 |
| 4 | 103.0542 | Alzheimer disease and age of onset | rs115641191 |
| 4 | 103.1469 | Schizophrenia | rs35518360 |
| 4 | 103.1887 | Blood pressure | rs13107325 |
| 4 | 103.1887 | Diastolic blood pressure | rs13107325 |
| 4 | 103.1887 | Systolic blood pressure | rs13107325 |
| 4 | 103.1887 | Hypertension | rs13107325 |
| 4 | 103.1887 | HDL cholesterol | rs13107325 |
| 4 | 103.1887 | Body mass index | rs13107325 |
| 4 | 103.1887 | Schizophrenia | rs13107325 |
| 4 | 103.1887 | NT−proBNP levels in acute coronary syndrome | rs13107325 |
| 4 | 103.1887 | Childhood body mass index | rs13107325 |
| 4 | 103.1981 | HDL cholesterol | rs13135092 |
| 4 | 103.2048 | Sitting height ratio | rs233817 |
| 4 | 103.2557 | Erythrocyte cadmium concentration in never smokers | rs7664683 |
| 4 | 103.3497 | Homeostasis model assessment of beta−cell function (interaction) | rs6533014 |
| 4 | 103.4343 | Ulcerative colitis | rs3774937 |
| 4 | 103.4574 | Schizophrenia (treatment resistant) | rs230529 |

GWAS Catalog SNPs in Region

| chr | pos (Mb) | trait | snp |
|---|---|---|---|
| 4 | 103.5111 | Ulcerative colitis | rs3774959 |
| 4 | 103.5516 | Primary biliary cirrhosis | rs7665090 |
| 4 | 103.5527 | Primary biliary cirrhosis | rs1054037 |
| 4 | 103.5617 | Glomerular filtration rate (creatinine) | rs228611 |
| 4 | 103.5786 | Multiple sclerosis | rs228614 |

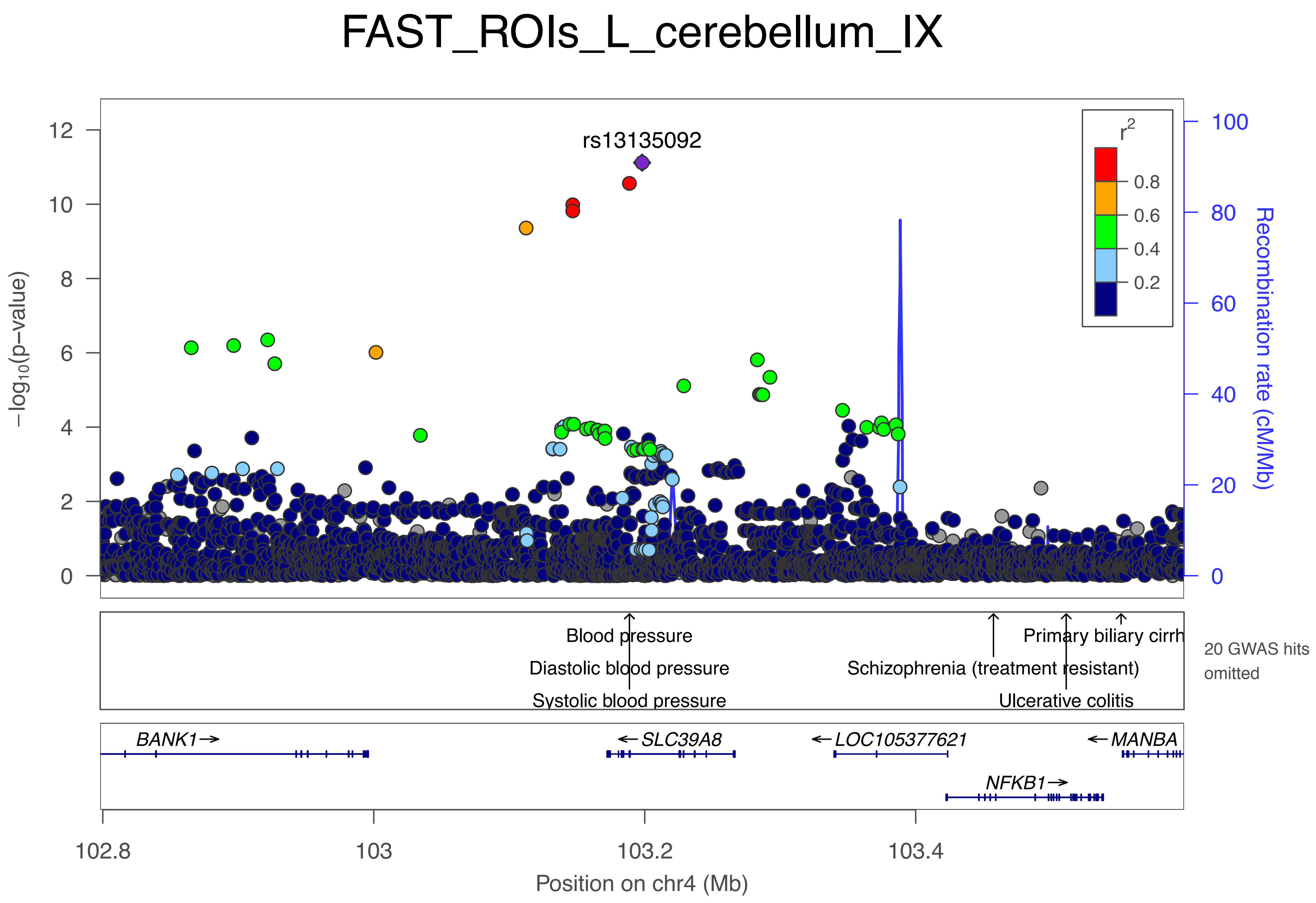

FAST_ROIs_L_cerebellum_IX
rs13135092
−log10(p−value)
Recombination rate (cM/Mb)
r2
0.8
0.6
0.4
0.2
12
10
8
6
4
2
0
100
80
60
40
20
0
Blood pressure
Diastolic blood pressure
Systolic blood pressure
Primary biliary cirrh
Schizophrenia (treatment resistant)
Ulcerative colitis
20 GWAS hits omitted
BANK1→
←SLC39A8
←LOC105377621
←MANBA
NFKB1→
102.8
103
103.2
103.4
Position on chr4 (Mb)

| | |
|---|---|
| date: | Thu Aug 17 18:02:44 2017 |
| build: | hg19 |
| display range: | chr4:102798082−103598082 [102798082−103598082] |
| hilite range: | 0 − 0 [ 0 − 0 ] |
| reference SNP: | chr4:103198082 |
| number of SNPs plotted: | 3541 |
| min P.value: | 7.6E−12 [chr4:103198082] |
| max P.value: | 10E−1 [chr4:103087990] |
| omitted GWAS Hits: | chr4:103.551603−Primary biliary cirrhosis, chr4:103.457418−Schizophrenia (treatment resistan |
| omitted GWAS Hits: | NA, NA |
| omitted GWAS Hits: | NA, NA |
| omitted GWAS Hits: | NA, NA |
| omitted GWAS Hits: | NA, NA |
| omitted GWAS Hits: | NA, NA |
| omitted GWAS Hits: | NA, NA |

Make more plots at http://csg.sph.umich.edu/locuszoom/

GWAS Catalog SNPs in Region

| chr | pos (Mb) | trait | snp |
|---|---|---|---|
| 4 | 102.8416 | Schizophrenia | rs13119516 |
| 4 | 102.8653 | Crohn's disease | rs13126505 |
| 4 | 102.8653 | Inflammatory bowel disease | rs13126505 |
| 4 | 102.9269 | Crohn's disease | rs34592089 |
| 4 | 103.0542 | Alzheimer disease and age of onset | rs115641191 |
| 4 | 103.1469 | Schizophrenia | rs35518360 |
| 4 | 103.1887 | Blood pressure | rs13107325 |
| 4 | 103.1887 | Diastolic blood pressure | rs13107325 |
| 4 | 103.1887 | Systolic blood pressure | rs13107325 |
| 4 | 103.1887 | Hypertension | rs13107325 |
| 4 | 103.1887 | HDL cholesterol | rs13107325 |
| 4 | 103.1887 | Body mass index | rs13107325 |
| 4 | 103.1887 | Schizophrenia | rs13107325 |
| 4 | 103.1887 | NT−proBNP levels in acute coronary syndrome | rs13107325 |
| 4 | 103.1887 | Childhood body mass index | rs13107325 |
| 4 | 103.1981 | HDL cholesterol | rs13135092 |
| 4 | 103.2048 | Sitting height ratio | rs233817 |
| 4 | 103.2557 | Erythrocyte cadmium concentration in never smokers | rs7664683 |
| 4 | 103.3497 | Homeostasis model assessment of beta−cell function (interaction) | rs6533014 |
| 4 | 103.4343 | Ulcerative colitis | rs3774937 |
| 4 | 103.4574 | Schizophrenia (treatment resistant) | rs230529 |

GWAS Catalog SNPs in Region

| chr | pos (Mb) | trait | snp |
|---|---|---|---|
| 4 | 103.5111 | Ulcerative colitis | rs3774959 |
| 4 | 103.5516 | Primary biliary cirrhosis | rs7665090 |
| 4 | 103.5527 | Primary biliary cirrhosis | rs1054037 |
| 4 | 103.5617 | Glomerular filtration rate (creatinine) | rs228611 |
| 4 | 103.5786 | Multiple sclerosis | rs228614 |

# FAST_ROIs_R_cerebellum_IX

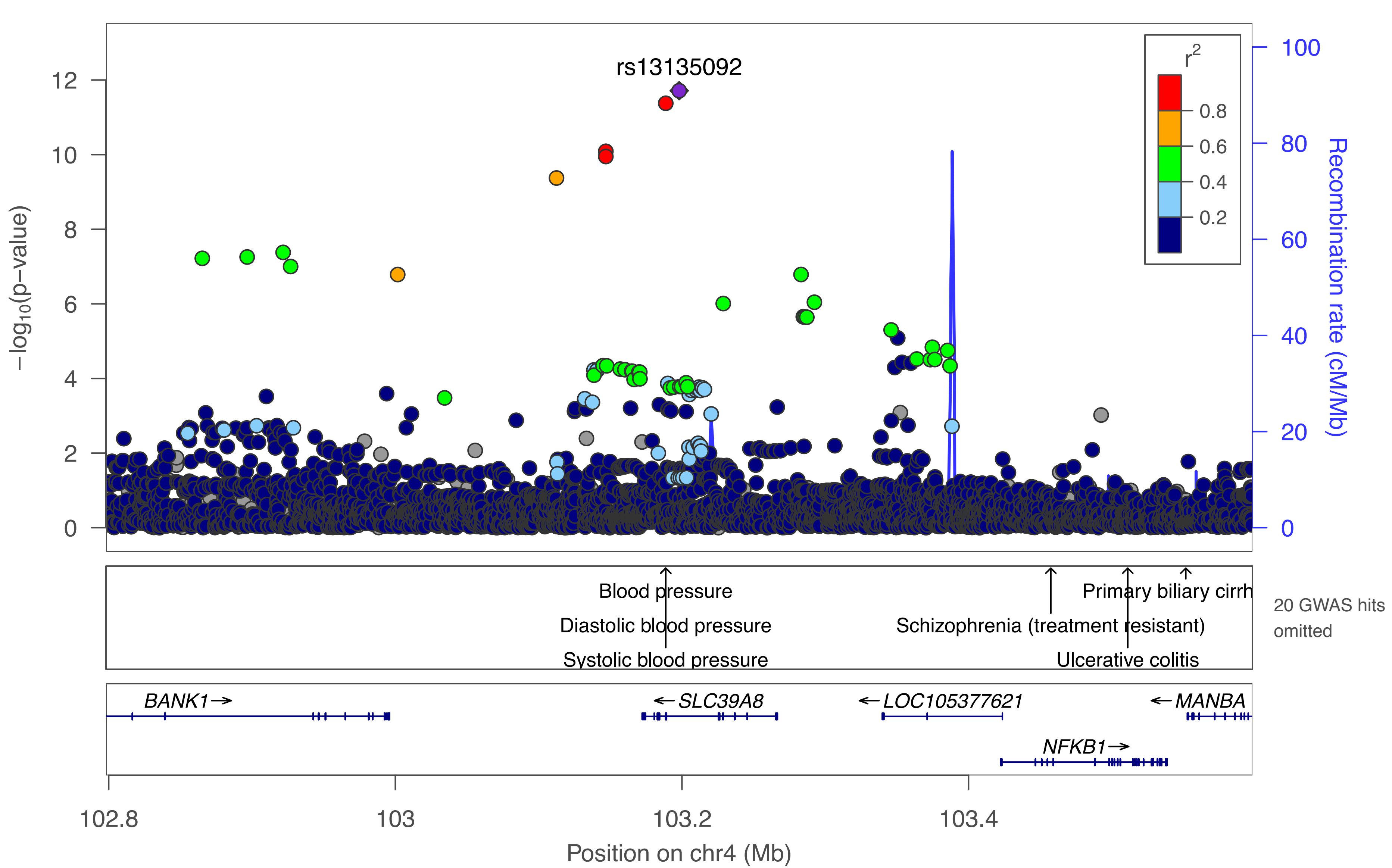

| | |
|---|---|
| date: | Thu Aug 17 18:09:02 2017 |
| build: | hg19 |
| display range: | chr4:102798082−103598082 [102798082−103598082] |
| hilite range: | 0 – 0 [ 0 – 0 ] |
| reference SNP: | chr4:103198082 |
| number of SNPs plotted: | 3541 |
| min P.value: | 1.94E−12 [chr4:103198082] |
| max P.value: | 10E−1 [chr4:103070765] |
| omitted GWAS Hits: | chr4:103.551603−Primary biliary cirrhosis, chr4:103.457418−Schizophrenia (treatment resistan |
| omitted GWAS Hits: | NA, NA |
| omitted GWAS Hits: | NA, NA |
| omitted GWAS Hits: | NA, NA |
| omitted GWAS Hits: | NA, NA |
| omitted GWAS Hits: | NA, NA |
| omitted GWAS Hits: | NA, NA |

Make more plots at http://csg.sph.umich.edu/locuszoom/

GWAS Catalog SNPs in Region

| chr | pos (Mb) | trait | snp |
|---|---|---|---|
| 4 | 102.8416 | Schizophrenia | rs13119516 |
| 4 | 102.8653 | Crohn's disease | rs13126505 |
| 4 | 102.8653 | Inflammatory bowel disease | rs13126505 |
| 4 | 102.9269 | Crohn's disease | rs34592089 |
| 4 | 103.0542 | Alzheimer disease and age of onset | rs115641191 |
| 4 | 103.1469 | Schizophrenia | rs35518360 |
| 4 | 103.1887 | Blood pressure | rs13107325 |
| 4 | 103.1887 | Diastolic blood pressure | rs13107325 |
| 4 | 103.1887 | Systolic blood pressure | rs13107325 |
| 4 | 103.1887 | Hypertension | rs13107325 |
| 4 | 103.1887 | HDL cholesterol | rs13107325 |
| 4 | 103.1887 | Body mass index | rs13107325 |
| 4 | 103.1887 | Schizophrenia | rs13107325 |
| 4 | 103.1887 | NT−proBNP levels in acute coronary syndrome | rs13107325 |
| 4 | 103.1887 | Childhood body mass index | rs13107325 |
| 4 | 103.1981 | HDL cholesterol | rs13135092 |
| 4 | 103.2048 | Sitting height ratio | rs233817 |
| 4 | 103.2557 | Erythrocyte cadmium concentration in never smokers | rs7664683 |
| 4 | 103.3497 | Homeostasis model assessment of beta−cell function (interaction) | rs6533014 |
| 4 | 103.4343 | Ulcerative colitis | rs3774937 |
| 4 | 103.4574 | Schizophrenia (treatment resistant) | rs230529 |

GWAS Catalog SNPs in Region

| chr | pos (Mb) | trait | snp |
|---|---|---|---|
| 4 | 103.5111 | Ulcerative colitis | rs3774959 |
| 4 | 103.5516 | Primary biliary cirrhosis | rs7665090 |
| 4 | 103.5527 | Primary biliary cirrhosis | rs1054037 |
| 4 | 103.5617 | Glomerular filtration rate (creatinine) | rs228611 |
| 4 | 103.5786 | Multiple sclerosis | rs228614 |

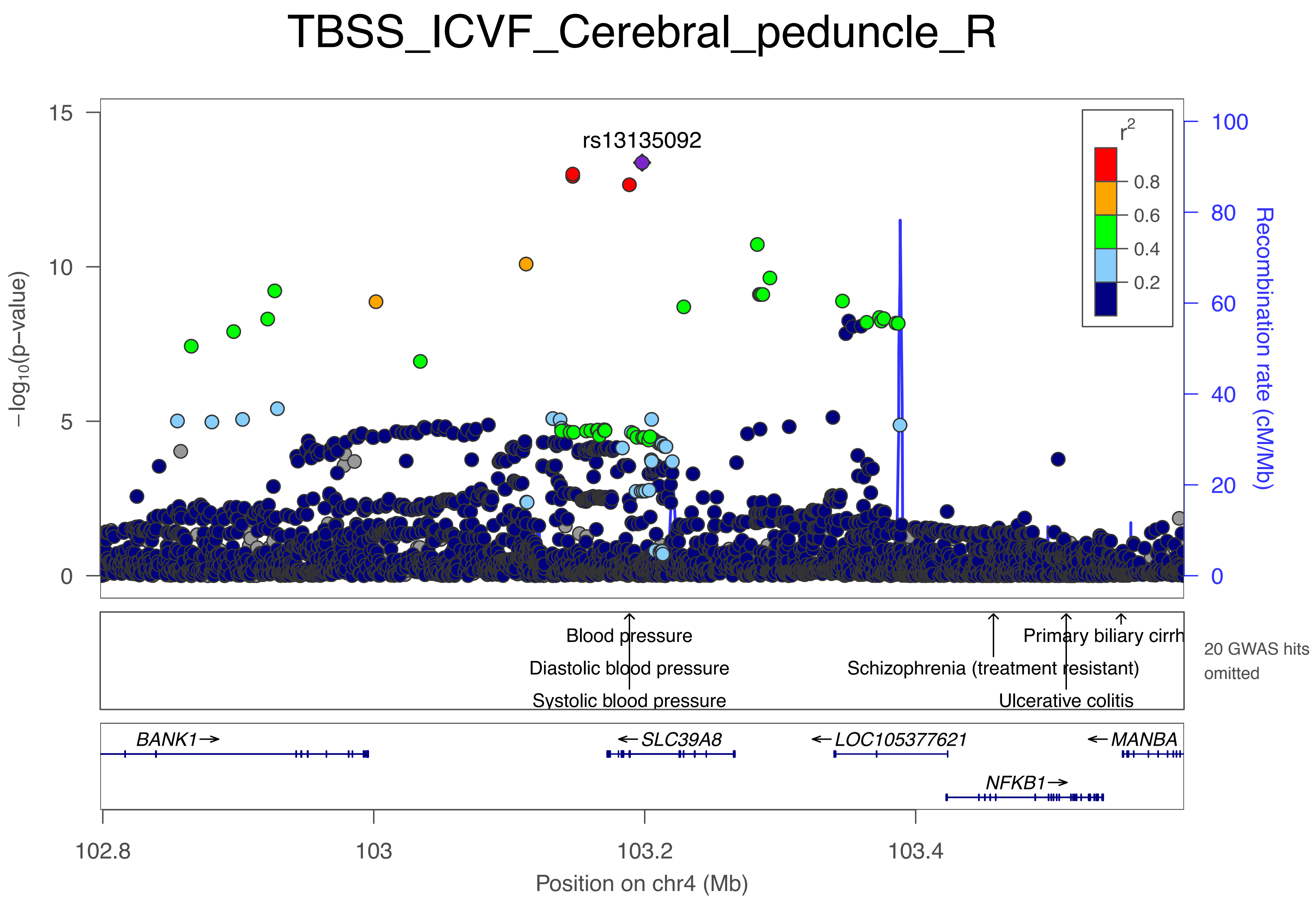

TBSS_ICVF_Cerebral_peduncle_R
rs13135092
−log10(p−value)
Recombination rate (cM/Mb)
r2
0.8
0.6
0.4
0.2
Blood pressure
Diastolic blood pressure
Systolic blood pressure
Schizophrenia (treatment resistant)
Primary biliary cirrh
Ulcerative colitis
20 GWAS hits omitted
BANK1
SLC39A8
LOC105377621
MANBA
NFKB1
102.8
103
103.2
103.4
Position on chr4 (Mb)

| | |
|---|---|
| date: | Thu Aug 17 18:09:02 2017 |
| build: | hg19 |
| display range: | chr4:102798082−103598082 [102798082−103598082] |
| hilite range: | 0 – 0 [ 0 – 0 ] |
| reference SNP: | chr4:103198082 |
| number of SNPs plotted: | 3541 |
| min P.value: | 4.26E−14 [chr4:103198082] |
| max P.value: | 10E−1 [chr4:102865446] |
| omitted GWAS Hits: | chr4:103.551603−Primary biliary cirrhosis, chr4:103.457418−Schizophrenia (treatment resistan |
| omitted GWAS Hits: | NA, NA |
| omitted GWAS Hits: | NA, NA |
| omitted GWAS Hits: | NA, NA |
| omitted GWAS Hits: | NA, NA |
| omitted GWAS Hits: | NA, NA |
| omitted GWAS Hits: | NA, NA |

Make more plots at http://csg.sph.umich.edu/locuszoom/

GWAS Catalog SNPs in Region

| chr | pos (Mb) | trait | snp |
|---|---|---|---|
| 4 | 102.8416 | Schizophrenia | rs13119516 |
| 4 | 102.8653 | Crohn's disease | rs13126505 |
| 4 | 102.8653 | Inflammatory bowel disease | rs13126505 |
| 4 | 102.9269 | Crohn's disease | rs34592089 |
| 4 | 103.0542 | Alzheimer disease and age of onset | rs115641191 |
| 4 | 103.1469 | Schizophrenia | rs35518360 |
| 4 | 103.1887 | Blood pressure | rs13107325 |
| 4 | 103.1887 | Diastolic blood pressure | rs13107325 |
| 4 | 103.1887 | Systolic blood pressure | rs13107325 |
| 4 | 103.1887 | Hypertension | rs13107325 |
| 4 | 103.1887 | HDL cholesterol | rs13107325 |
| 4 | 103.1887 | Body mass index | rs13107325 |
| 4 | 103.1887 | Schizophrenia | rs13107325 |
| 4 | 103.1887 | NT−proBNP levels in acute coronary syndrome | rs13107325 |
| 4 | 103.1887 | Childhood body mass index | rs13107325 |
| 4 | 103.1981 | HDL cholesterol | rs13135092 |
| 4 | 103.2048 | Sitting height ratio | rs233817 |
| 4 | 103.2557 | Erythrocyte cadmium concentration in never smokers | rs7664683 |
| 4 | 103.3497 | Homeostasis model assessment of beta−cell function (interaction) | rs6533014 |
| 4 | 103.4343 | Ulcerative colitis | rs3774937 |
| 4 | 103.4574 | Schizophrenia (treatment resistant) | rs230529 |

GWAS Catalog SNPs in Region

| chr | pos (Mb) | trait | snp |
|---|---|---|---|
| 4 | 103.5111 | Ulcerative colitis | rs3774959 |
| 4 | 103.5516 | Primary biliary cirrhosis | rs7665090 |
| 4 | 103.5527 | Primary biliary cirrhosis | rs1054037 |
| 4 | 103.5617 | Glomerular filtration rate (creatinine) | rs228611 |
| 4 | 103.5786 | Multiple sclerosis | rs228614 |

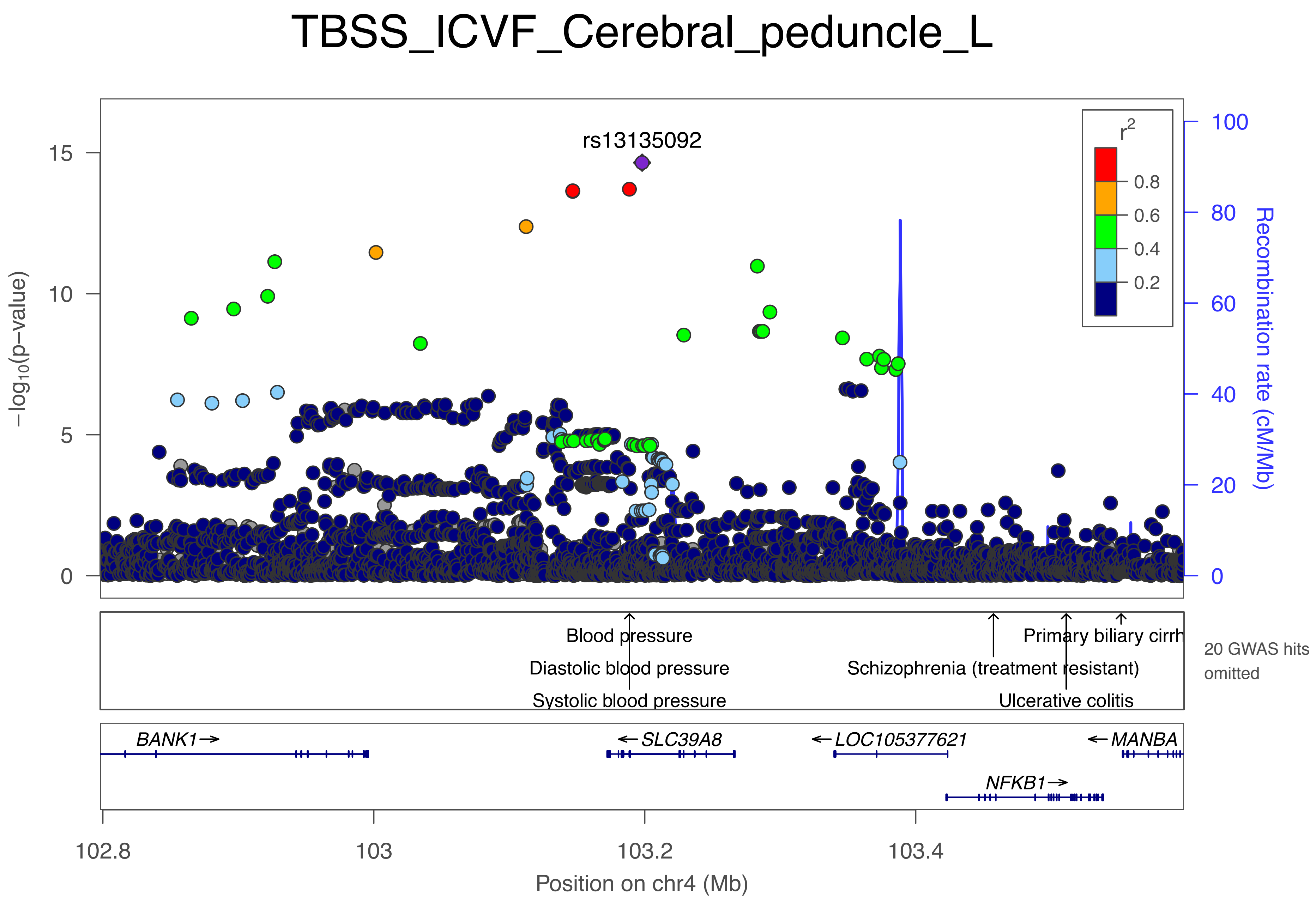

TBSS_ICVF_Cerebral_peduncle_L
rs13135092
r2
0.8
0.6
0.4
0.2
−log10(p−value)
Recombination rate (cM/Mb)
Blood pressure
Diastolic blood pressure
Systolic blood pressure
Primary biliary cirrh
Schizophrenia (treatment resistant)
Ulcerative colitis
20 GWAS hits omitted
BANK1
SLC39A8
LOC105377621
MANBA
NFKB1
102.8
103
103.2
103.4
Position on chr4 (Mb)

| | |
|---|---|
| date: | Thu Aug 17 18:09:02 2017 |
| build: | hg19 |
| display range: | chr4:102798082−103598082 [102798082−103598082] |
| hilite range: | 0 – 0 [ 0 – 0 ] |
| reference SNP: | chr4:103198082 |
| number of SNPs plotted: | 3541 |
| min P.value: | 2.26E−15 [chr4:103198082] |
| max P.value: | 10E−1 [chr4:103322304] |
| omitted GWAS Hits: | chr4:103.551603−Primary biliary cirrhosis, chr4:103.457418−Schizophrenia (treatment resistan |
| omitted GWAS Hits: | NA, NA |
| omitted GWAS Hits: | NA, NA |
| omitted GWAS Hits: | NA, NA |
| omitted GWAS Hits: | NA, NA |
| omitted GWAS Hits: | NA, NA |
| omitted GWAS Hits: | NA, NA |

Make more plots at http://csg.sph.umich.edu/locuszoom/

GWAS Catalog SNPs in Region

| chr | pos (Mb) | trait | snp |
|---|---|---|---|
| 4 | 102.8416 | Schizophrenia | rs13119516 |
| 4 | 102.8653 | Crohn's disease | rs13126505 |
| 4 | 102.8653 | Inflammatory bowel disease | rs13126505 |
| 4 | 102.9269 | Crohn's disease | rs34592089 |
| 4 | 103.0542 | Alzheimer disease and age of onset | rs115641191 |
| 4 | 103.1469 | Schizophrenia | rs35518360 |
| 4 | 103.1887 | Blood pressure | rs13107325 |
| 4 | 103.1887 | Diastolic blood pressure | rs13107325 |
| 4 | 103.1887 | Systolic blood pressure | rs13107325 |
| 4 | 103.1887 | Hypertension | rs13107325 |
| 4 | 103.1887 | HDL cholesterol | rs13107325 |
| 4 | 103.1887 | Body mass index | rs13107325 |
| 4 | 103.1887 | Schizophrenia | rs13107325 |
| 4 | 103.1887 | NT−proBNP levels in acute coronary syndrome | rs13107325 |
| 4 | 103.1887 | Childhood body mass index | rs13107325 |
| 4 | 103.1981 | HDL cholesterol | rs13135092 |
| 4 | 103.2048 | Sitting height ratio | rs233817 |
| 4 | 103.2557 | Erythrocyte cadmium concentration in never smokers | rs7664683 |
| 4 | 103.3497 | Homeostasis model assessment of beta−cell function (interaction) | rs6533014 |
| 4 | 103.4343 | Ulcerative colitis | rs3774937 |
| 4 | 103.4574 | Schizophrenia (treatment resistant) | rs230529 |

GWAS Catalog SNPs in Region

| chr | pos (Mb) | trait | snp |
|---|---|---|---|
| 4 | 103.5111 | Ulcerative colitis | rs3774959 |
| 4 | 103.5516 | Primary biliary cirrhosis | rs7665090 |
| 4 | 103.5527 | Primary biliary cirrhosis | rs1054037 |
| 4 | 103.5617 | Glomerular filtration rate (creatinine) | rs228611 |
| 4 | 103.5786 | Multiple sclerosis | rs228614 |

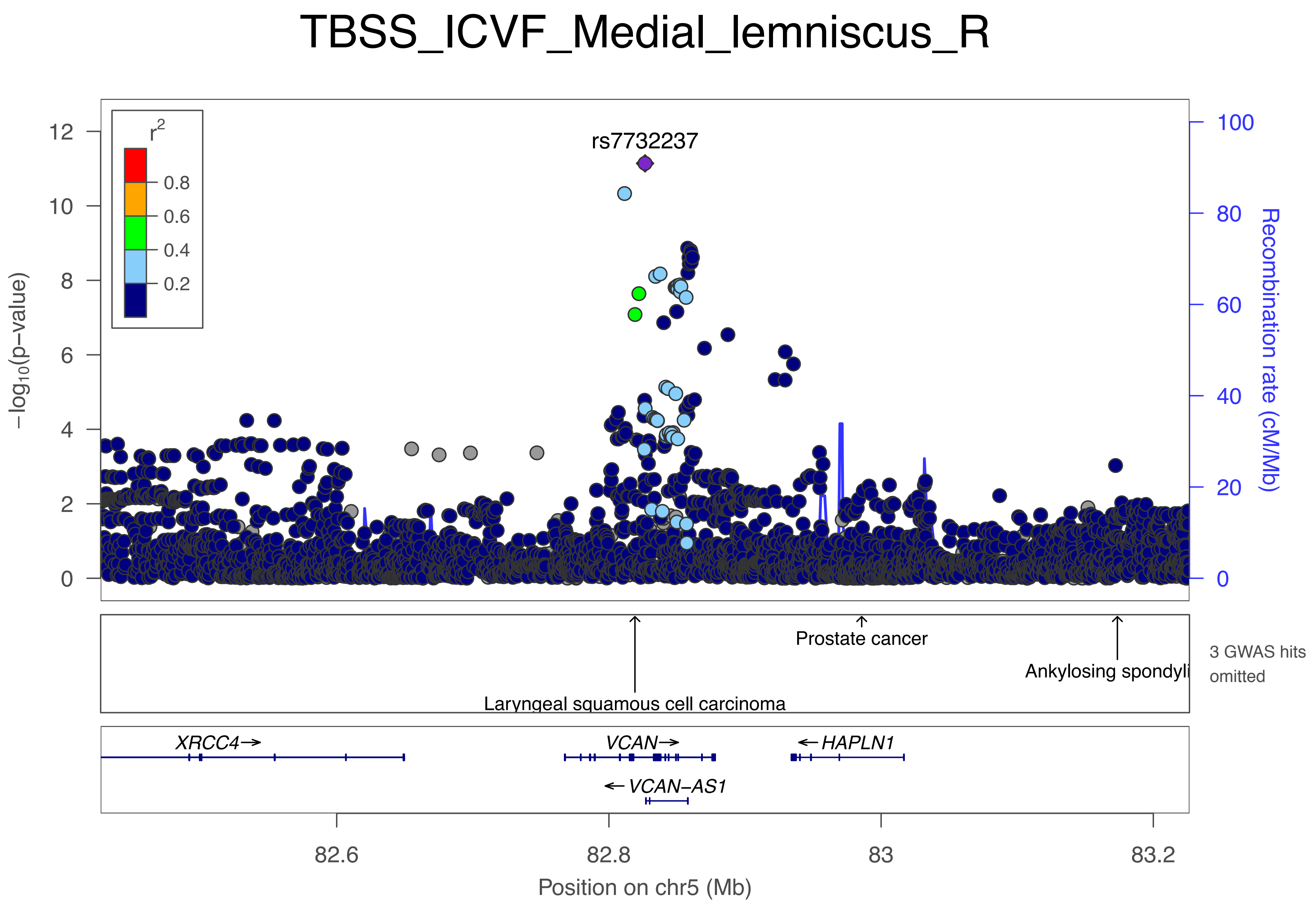
TBSS_ICVF_Medial_lemniscus_R
r2
0.8
0.6
0.4
0.2
rs7732237
−log10(p−value)
Recombination rate (cM/Mb)
Prostate cancer
Ankylosing spondyli
Laryngeal squamous cell carcinoma
3 GWAS hits omitted
XRCC4
VCAN
HAPLN1
VCAN−AS1
82.6
82.8
83
83.2
Position on chr5 (Mb)

| | |
|---|---|
| date: | Thu Aug 17 18:03:31 2017 |
| build: | hg19 |
| display range: | chr5:82426556−83226556 [82426556−83226556] |
| hilite range: | 0 – 0 [ 0 – 0 ] |
| reference SNP: | chr5:82826556 |
| number of SNPs plotted: | 3442 |
| min P.value: | 7.23E−12 [chr5:82826556] |
| max P.value: | 9.98E−1 [chr5:82951780] |
| omitted GWAS Hits: | NA, NA |

Make more plots at http://csg.sph.umich.edu/locuszoom/

GWAS Catalog SNPs in Region

| chr | pos (Mb) | trait | snp |
|---|---|---|---|
| 5 | 82.81912 | Laryngeal squamous cell carcinoma | rs310518 |
| 5 | 82.84549 | Diisocyanate–induced asthma | rs3852186 |
| 5 | 82.88991 | Major depressive disorder | rs310501 |
| 5 | 82.96073 | Visceral fat | rs3846635 |
| 5 | 82.98574 | Prostate cancer | rs4466137 |
| 5 | 83.17359 | Ankylosing spondylitis | rs4552569 |

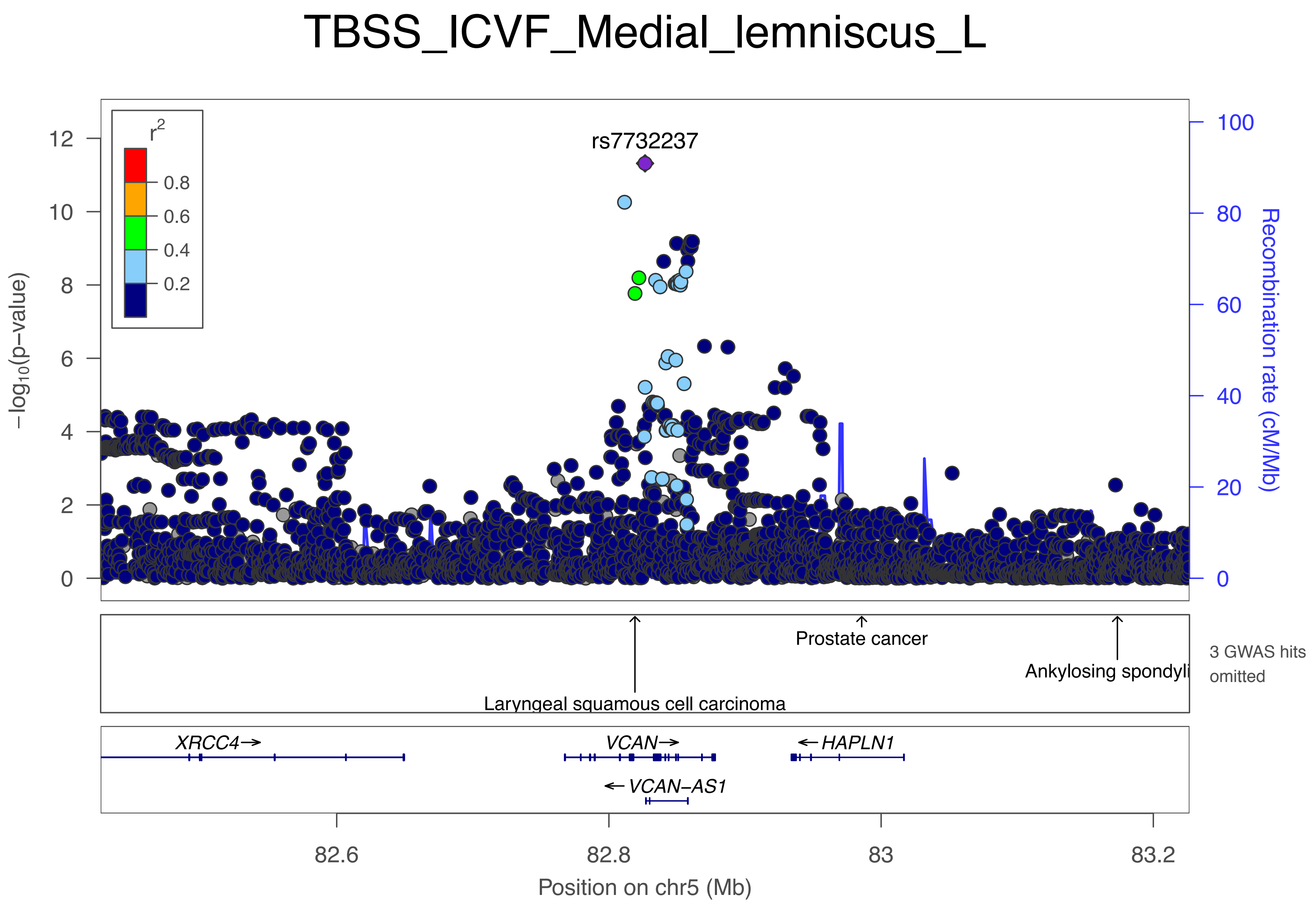

TBSS_ICVF_Medial_lemniscus_L
rs7732237
r2
0.8
0.6
0.4
0.2
12
10
8
6
4
2
0
−log10(p−value)
100
80
60
40
20
0
Recombination rate (cM/Mb)
Prostate cancer
Ankylosing spondyli
Laryngeal squamous cell carcinoma
3 GWAS hits
omitted
XRCC4→
VCAN→
←HAPLN1
←VCAN−AS1
82.6
82.8
83
83.2
Position on chr5 (Mb)

| | |
|---|---|
| date: | Thu Aug 17 18:03:31 2017 |
| build: | hg19 |
| display range: | chr5:82426556−83226556 [82426556−83226556] |
| hilite range: | 0 – 0 [ 0 – 0 ] |
| reference SNP: | chr5:82826556 |
| number of SNPs plotted: | 3442 |
| min P.value: | 4.82E−12 [chr5:82826556] |
| max P.value: | 10E−1 [chr5:82749904] |
| omitted GWAS Hits: | NA, NA |

Make more plots at http://csg.sph.umich.edu/locuszoom/

GWAS Catalog SNPs in Region

| chr | pos (Mb) | trait | snp |
|---|---|---|---|
| 5 | 82.81912 | Laryngeal squamous cell carcinoma | rs310518 |
| 5 | 82.84549 | Diisocyanate–induced asthma | rs3852186 |
| 5 | 82.88991 | Major depressive disorder | rs310501 |
| 5 | 82.96073 | Visceral fat | rs3846635 |
| 5 | 82.98574 | Prostate cancer | rs4466137 |
| 5 | 83.17359 | Ankylosing spondylitis | rs4552569 |

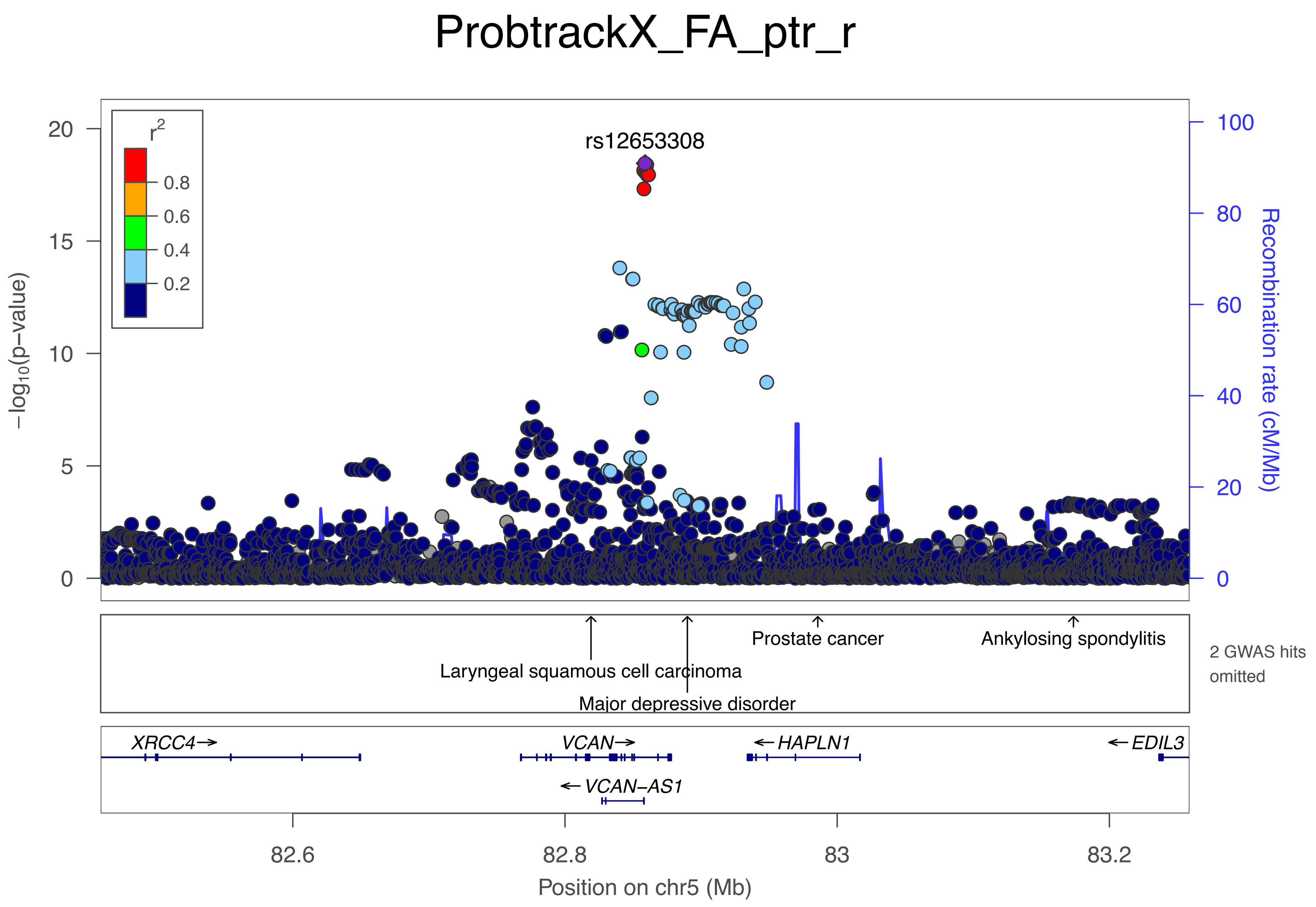

ProbtrackX_FA_ptr_r
rs12653308
r2
0.8
0.6
0.4
0.2
−log10(p−value)
Recombination rate (cM/Mb)
Prostate cancer
Ankylosing spondylitis
Laryngeal squamous cell carcinoma
Major depressive disorder
2 GWAS hits omitted
XRCC4→
VCAN→
←HAPLN1
←EDIL3
←VCAN−AS1
82.6
82.8
83
83.2
Position on chr5 (Mb)

| | |
|---|---|
| date: | Thu Aug 17 17:52:01 2017 |
| build: | hg19 |
| display range: | chr5:82458828−83258828 [82458828−83258828] |
| hilite range: | 0 – 0  [ 0 – 0 ] |
| reference SNP: | chr5:82858828 |
| number of SNPs plotted: | 3398 |
| min P.value: | 3.5E−19 [chr5:82858828] |
| max P.value: | 10E−1 [chr5:82813266] |
| omitted GWAS Hits: | NA, NA |

Make more plots at http://csg.sph.umich.edu/locuszoom/

GWAS Catalog SNPs in Region

| chr | pos (Mb) | trait | snp |
|---|---|---|---|
| 5 | 82.81912 | Laryngeal squamous cell carcinoma | rs310518 |
| 5 | 82.84549 | Diisocyanate–induced asthma | rs3852186 |
| 5 | 82.88991 | Major depressive disorder | rs310501 |
| 5 | 82.96073 | Visceral fat | rs3846635 |
| 5 | 82.98574 | Prostate cancer | rs4466137 |
| 5 | 83.17359 | Ankylosing spondylitis | rs4552569 |

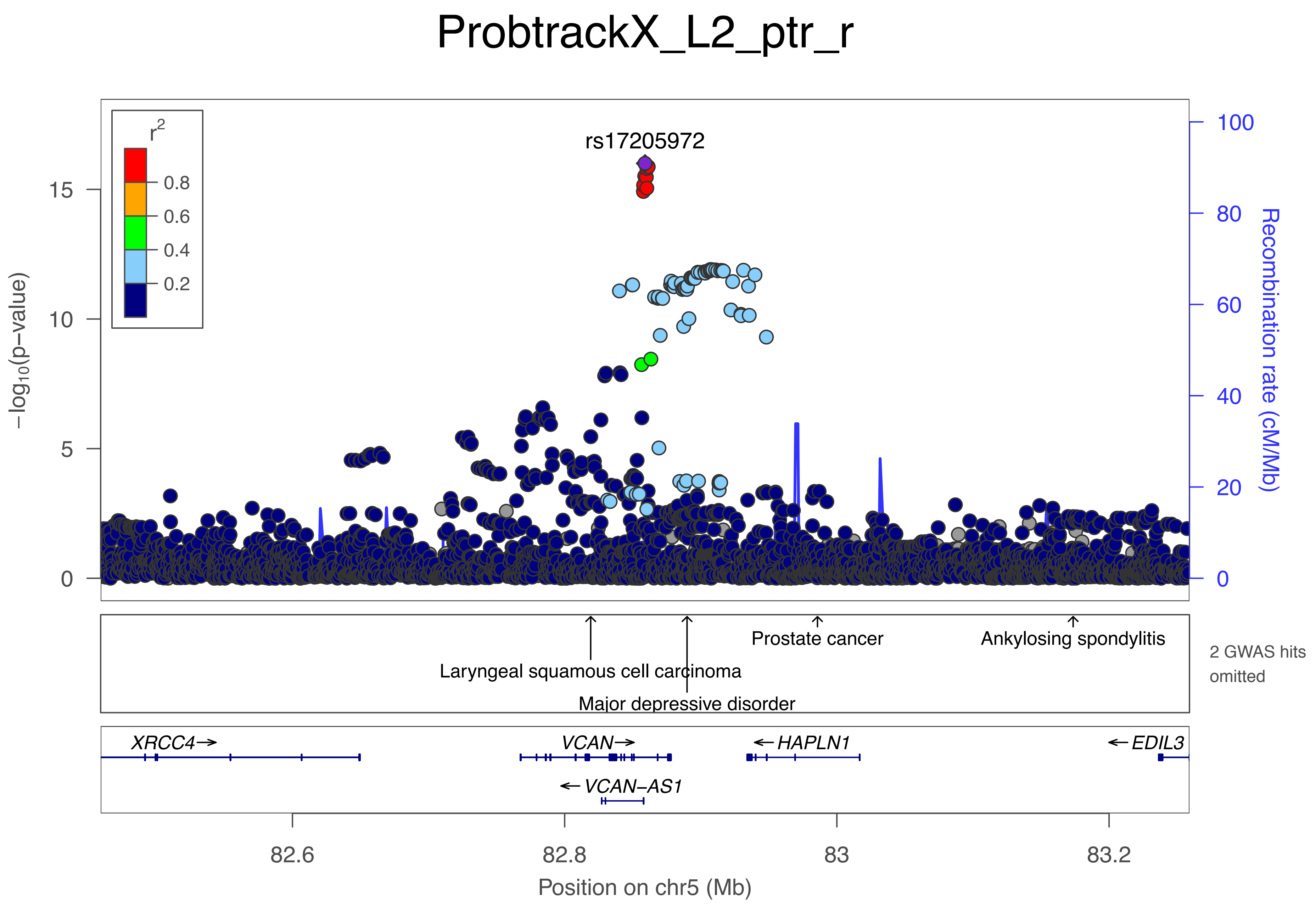
ProbtrackX_L2_ptr_r
rs17205972
r2
0.8
0.6
0.4
0.2
−log10(p−value)
15
10
5
0
100
80
60
40
20
0
Recombination rate (cM/Mb)
Prostate cancer
Ankylosing spondylitis
Laryngeal squamous cell carcinoma
Major depressive disorder
2 GWAS hits omitted
XRCC4→
VCAN→
← HAPLN1
← EDIL3
← VCAN−AS1
82.6
82.8
83
83.2
Position on chr5 (Mb)

| | |
|---|---|
| date: | Thu Aug 17 17:52:01 2017 |
| build: | hg19 |
| display range: | chr5:82459065−83259065 [82459065−83259065] |
| hilite range: | 0 – 0 [ 0 – 0 ] |
| reference SNP: | chr5:82859065 |
| number of SNPs plotted: | 3396 |
| min P.value: | 9.93E−17 [chr5:82859065] |
| max P.value: | 10E−1 [chr5:83027085] |
| omitted GWAS Hits: | NA, NA |

Make more plots at http://csg.sph.umich.edu/locuszoom/

GWAS Catalog SNPs in Region

| chr | pos (Mb) | trait | snp |
|---|---|---|---|
| 5 | 82.81912 | Laryngeal squamous cell carcinoma | rs310518 |
| 5 | 82.84549 | Diisocyanate–induced asthma | rs3852186 |
| 5 | 82.88991 | Major depressive disorder | rs310501 |
| 5 | 82.96073 | Visceral fat | rs3846635 |
| 5 | 82.98574 | Prostate cancer | rs4466137 |
| 5 | 83.17359 | Ankylosing spondylitis | rs4552569 |

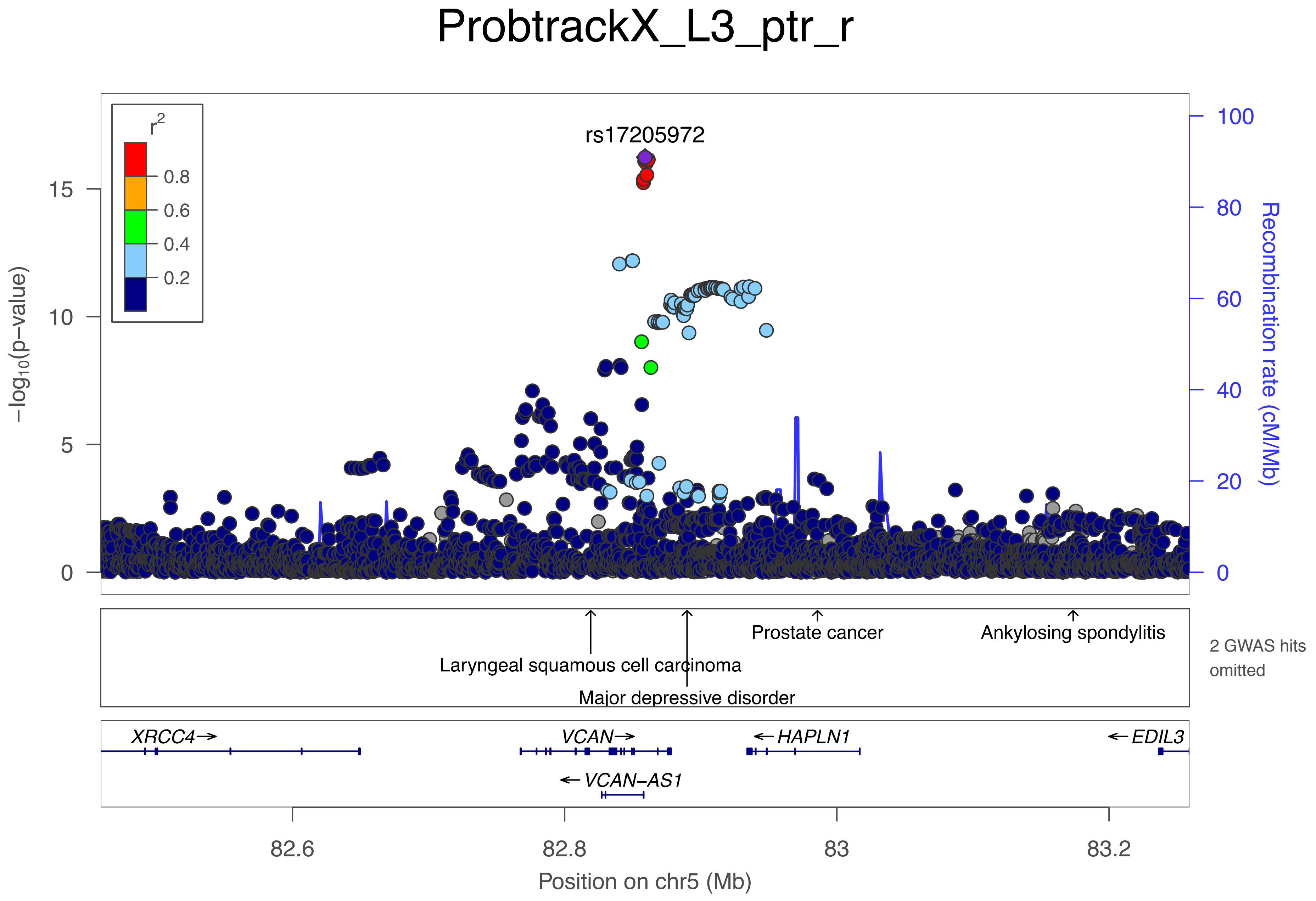

ProbtrackX_L3_ptr_r
r2
0.8
0.6
0.4
0.2
rs17205972
15
10
5
0
−log10(p−value)
100
80
60
40
20
0
Recombination rate (cM/Mb)
Prostate cancer
Ankylosing spondylitis
Laryngeal squamous cell carcinoma
Major depressive disorder
2 GWAS hits omitted
XRCC4→
VCAN→
←HAPLN1
←EDIL3
←VCAN−AS1
82.6
82.8
83
83.2
Position on chr5 (Mb)

| | |
|---|---|
| date: | Thu Aug 17 17:52:01 2017 |
| build: | hg19 |
| display range: | chr5:82459065−83259065 [82459065−83259065] |
| hilite range: | 0 – 0  [ 0 – 0 ] |
| reference SNP: | chr5:82859065 |
| number of SNPs plotted: | 3396 |
| min P.value: | 5.85E−17 [chr5:82859065] |
| max P.value: | 10E−1 [chr5:82513881] |
| omitted GWAS Hits: | NA, NA |

Make more plots at http://csg.sph.umich.edu/locuszoom/

GWAS Catalog SNPs in Region

| chr | pos (Mb) | trait | snp |
|---|---|---|---|
| 5 | 82.81912 | Laryngeal squamous cell carcinoma | rs310518 |
| 5 | 82.84549 | Diisocyanate–induced asthma | rs3852186 |
| 5 | 82.88991 | Major depressive disorder | rs310501 |
| 5 | 82.96073 | Visceral fat | rs3846635 |
| 5 | 82.98574 | Prostate cancer | rs4466137 |
| 5 | 83.17359 | Ankylosing spondylitis | rs4552569 |

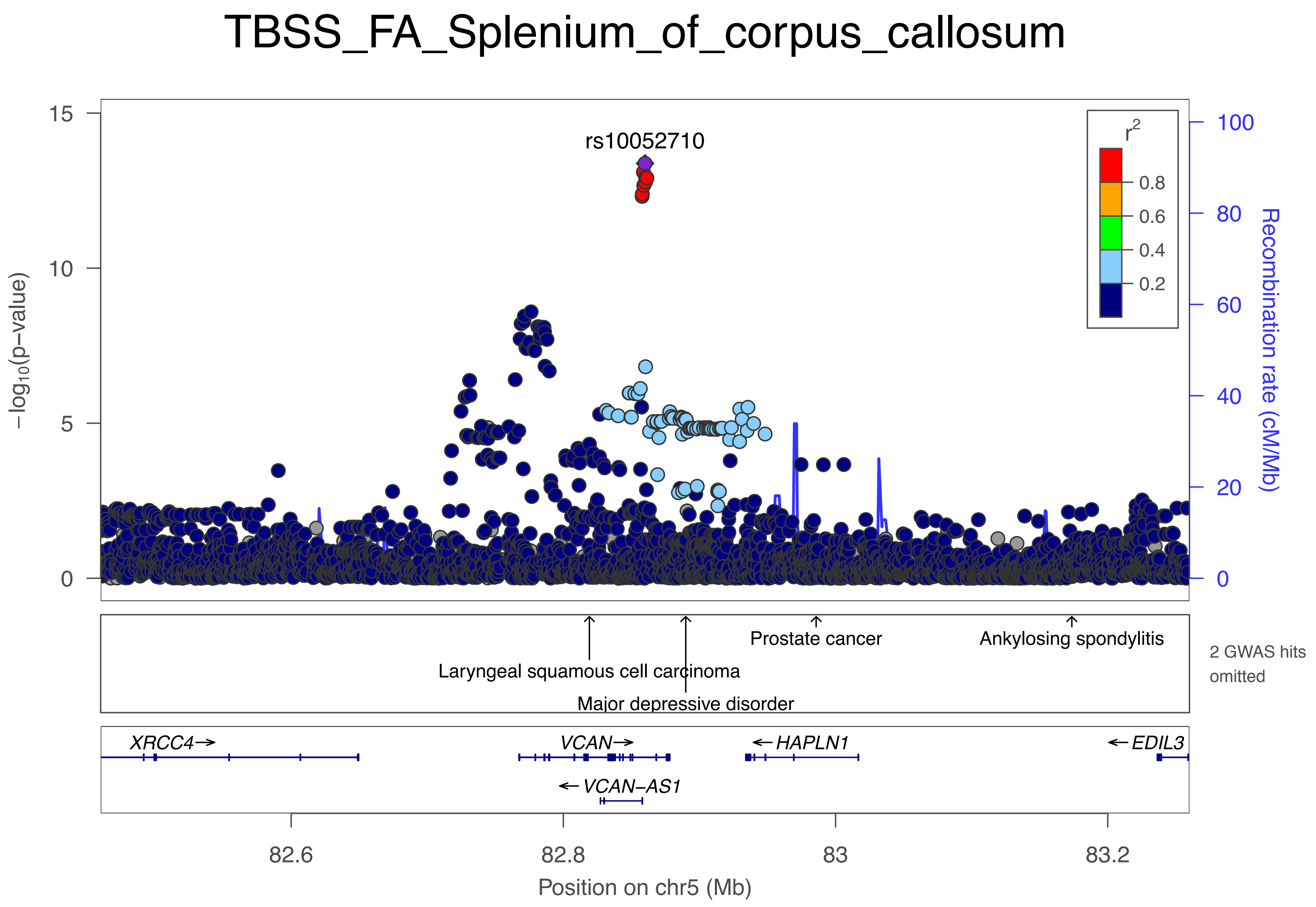
TBSS_FA_Splenium_of_corpus_callosum
rs10052710
−log10(p−value)
Recombination rate (cM/Mb)
r2
0.8
0.6
0.4
0.2
Laryngeal squamous cell carcinoma
Major depressive disorder
Prostate cancer
Ankylosing spondylitis
2 GWAS hits omitted
XRCC4
VCAN
HAPLN1
EDIL3
VCAN−AS1
82.6
82.8
83
83.2
Position on chr5 (Mb)

| | |
|---|---|
| date: | Thu Aug 17 17:52:01 2017 |
| build: | hg19 |
| display range: | chr5:82460025−83260025 [82460025−83260025] |
| hilite range: | 0 – 0 [ 0 – 0 ] |
| reference SNP: | chr5:82860025 |
| number of SNPs plotted: | 3392 |
| min P.value: | 4.17E−14 [chr5:82860025] |
| max P.value: | 10E−1 [chr5:82950639] |
| omitted GWAS Hits: | NA, NA |

Make more plots at http://csg.sph.umich.edu/locuszoom/

GWAS Catalog SNPs in Region

| chr | pos (Mb) | trait | snp |
|---|---|---|---|
| 5 | 82.81912 | Laryngeal squamous cell carcinoma | rs310518 |
| 5 | 82.84549 | Diisocyanate–induced asthma | rs3852186 |
| 5 | 82.88991 | Major depressive disorder | rs310501 |
| 5 | 82.96073 | Visceral fat | rs3846635 |
| 5 | 82.98574 | Prostate cancer | rs4466137 |
| 5 | 83.17359 | Ankylosing spondylitis | rs4552569 |

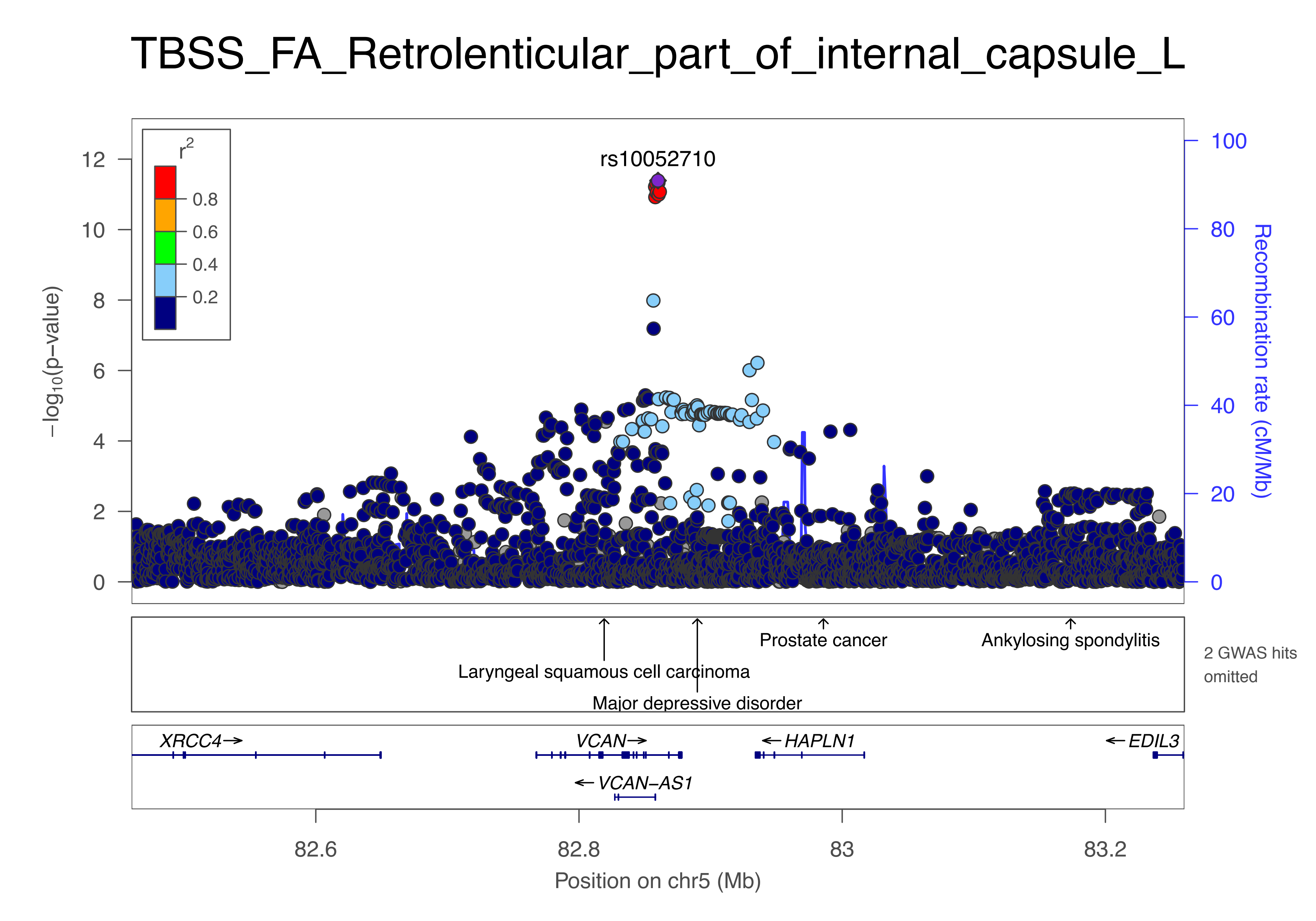

TBSS_FA_Retrolenticular_part_of_internal_capsule_L
rs10052710
r2
0.8
0.6
0.4
0.2
−log10(p−value)
0
2
4
6
8
10
12
100
80
60
40
20
0
Recombination rate (cM/Mb)
Laryngeal squamous cell carcinoma
Major depressive disorder
Prostate cancer
Ankylosing spondylitis
2 GWAS hits omitted
XRCC4
VCAN
VCAN−AS1
HAPLN1
EDIL3
82.6
82.8
83
83.2
Position on chr5 (Mb)

| | |
|---|---|
| date: | Thu Aug 17 17:52:01 2017 |
| build: | hg19 |
| display range: | chr5:82460025–83260025 [82460025–83260025] |
| hilite range: | 0 – 0 [ 0 – 0 ] |
| reference SNP: | chr5:82860025 |
| number of SNPs plotted: | 3392 |
| min P.value: | 4.06E–12 [chr5:82860025] |
| max P.value: | 10E–1 [chr5:82936267] |
| omitted GWAS Hits: | NA, NA |

Make more plots at http://csg.sph.umich.edu/locuszoom/

GWAS Catalog SNPs in Region

| chr | pos (Mb) | trait | snp |
|---|---|---|---|
| 5 | 82.81912 | Laryngeal squamous cell carcinoma | rs310518 |
| 5 | 82.84549 | Diisocyanate−induced asthma | rs3852186 |
| 5 | 82.88991 | Major depressive disorder | rs310501 |
| 5 | 82.96073 | Visceral fat | rs3846635 |
| 5 | 82.98574 | Prostate cancer | rs4466137 |
| 5 | 83.17359 | Ankylosing spondylitis | rs4552569 |

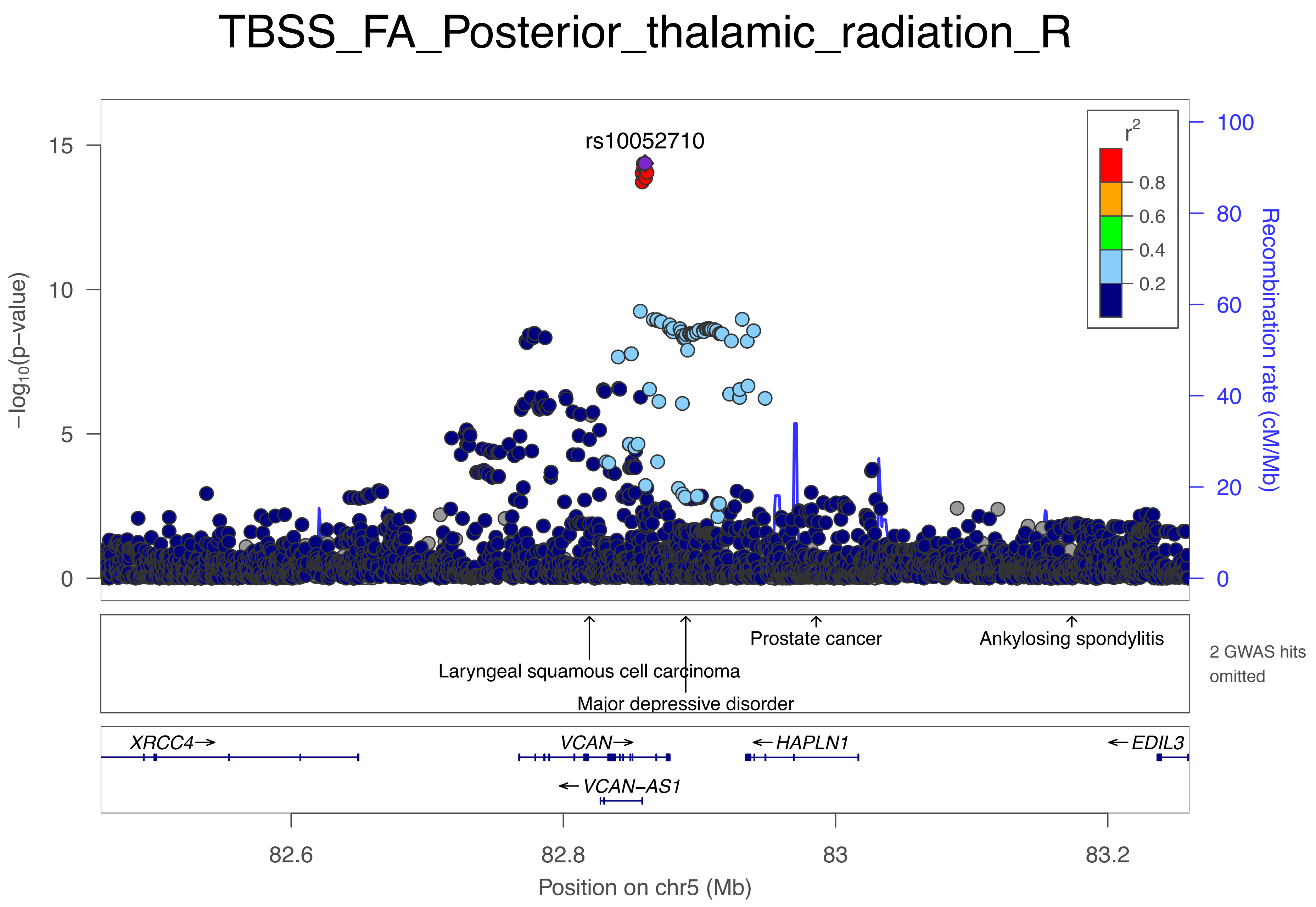

TBSS_FA_Posterior_thalamic_radiation_R
rs10052710
r2
0.8
0.6
0.4
0.2
−log10(p−value)
Recombination rate (cM/Mb)
Laryngeal squamous cell carcinoma
Major depressive disorder
Prostate cancer
Ankylosing spondylitis
2 GWAS hits omitted
XRCC4
VCAN
VCAN−AS1
HAPLN1
EDIL3
82.6
82.8
83
83.2
Position on chr5 (Mb)

| | |
|---|---|
| date: | Thu Aug 17 17:52:01 2017 |
| build: | hg19 |
| display range: | chr5:82460025−83260025 [82460025−83260025] |
| hilite range: | 0 – 0 [ 0 – 0 ] |
| reference SNP: | chr5:82860025 |
| number of SNPs plotted: | 3392 |
| min P.value: | 4.29E−15 [chr5:82860025] |
| max P.value: | 10E−1 [chr5:83220371] |
| omitted GWAS Hits: | NA, NA |

Make more plots at http://csg.sph.umich.edu/locuszoom/

GWAS Catalog SNPs in Region

| chr | pos (Mb) | trait | snp |
|---|---|---|---|
| 5 | 82.81912 | Laryngeal squamous cell carcinoma | rs310518 |
| 5 | 82.84549 | Diisocyanate–induced asthma | rs3852186 |
| 5 | 82.88991 | Major depressive disorder | rs310501 |
| 5 | 82.96073 | Visceral fat | rs3846635 |
| 5 | 82.98574 | Prostate cancer | rs4466137 |
| 5 | 83.17359 | Ankylosing spondylitis | rs4552569 |

# TBSS_FA_Posterior_thalamic_radiation_L

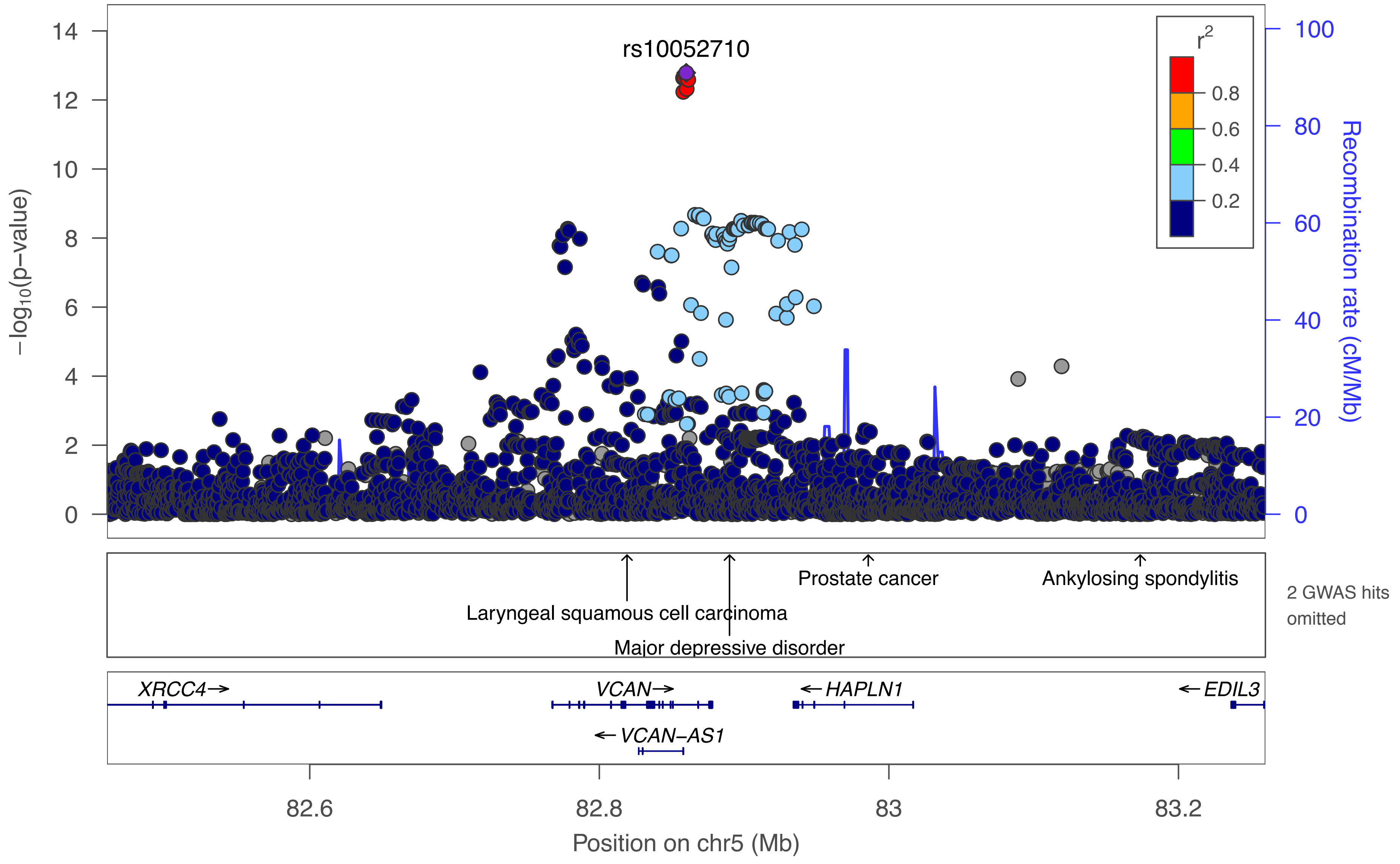

| | |
|---|---|
| date: | Thu Aug 17 17:52:01 2017 |
| build: | hg19 |
| display range: | chr5:82460025−83260025 [82460025−83260025] |
| hilite range: | 0 – 0 [ 0 – 0 ] |
| reference SNP: | chr5:82860025 |
| number of SNPs plotted: | 3392 |
| min P.value: | 1.63E−13 [chr5:82860025] |
| max P.value: | 10E−1 [chr5:82974539] |
| omitted GWAS Hits: | NA, NA |

Make more plots at http://csg.sph.umich.edu/locuszoom/

GWAS Catalog SNPs in Region

| chr | pos (Mb) | trait | snp |
|---|---|---|---|
| 5 | 82.81912 | Laryngeal squamous cell carcinoma | rs310518 |
| 5 | 82.84549 | Diisocyanate–induced asthma | rs3852186 |
| 5 | 82.88991 | Major depressive disorder | rs310501 |
| 5 | 82.96073 | Visceral fat | rs3846635 |
| 5 | 82.98574 | Prostate cancer | rs4466137 |
| 5 | 83.17359 | Ankylosing spondylitis | rs4552569 |

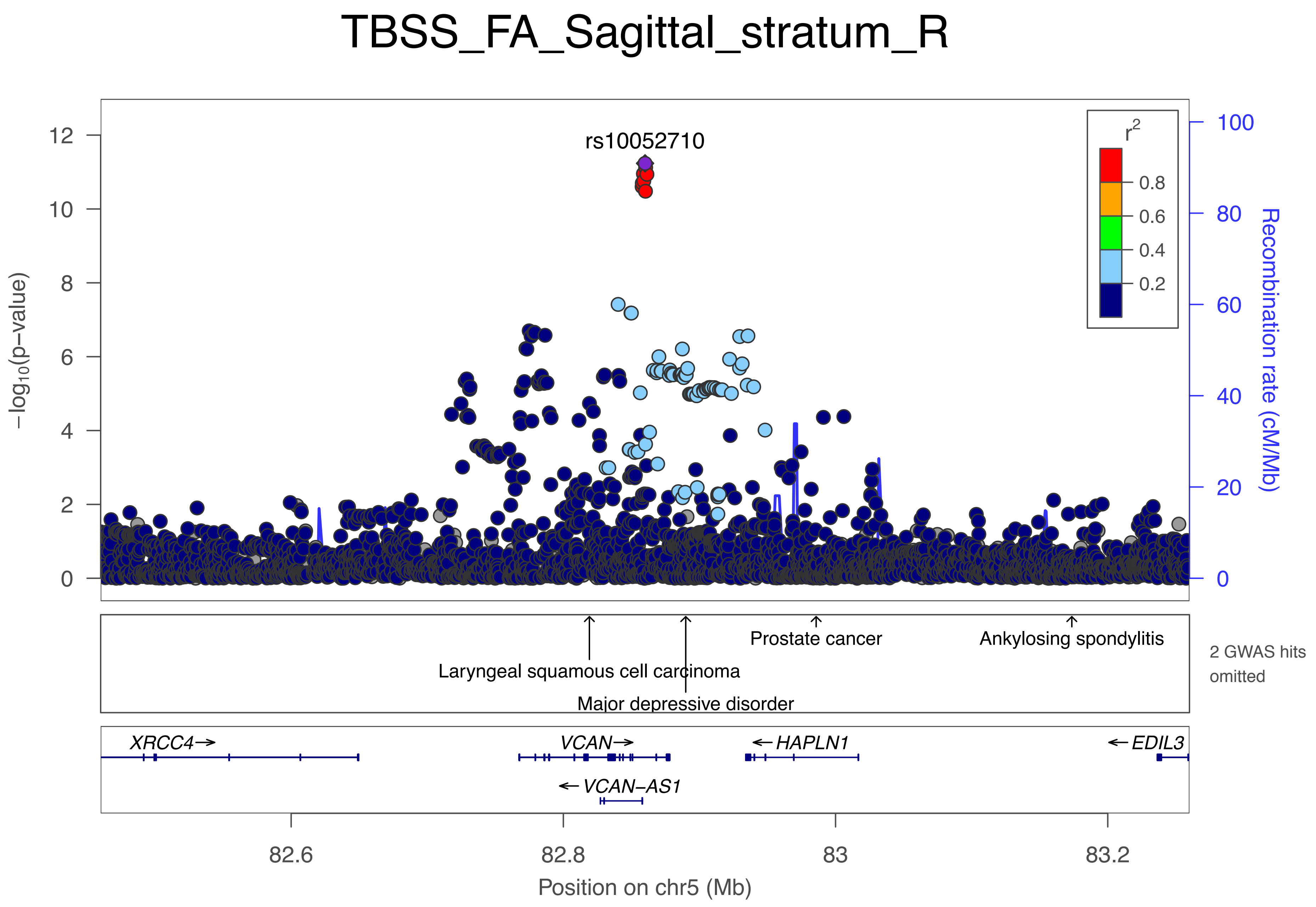
TBSS_FA_Sagittal_stratum_R
rs10052710
−log10(p−value)
Recombination rate (cM/Mb)
r2
0.8
0.6
0.4
0.2
Laryngeal squamous cell carcinoma
Major depressive disorder
Prostate cancer
Ankylosing spondylitis
2 GWAS hits omitted
XRCC4
VCAN
VCAN−AS1
HAPLN1
EDIL3
82.6
82.8
83
83.2
Position on chr5 (Mb)

| | |
|---|---|
| date: | Thu Aug 17 17:52:01 2017 |
| build: | hg19 |
| display range: | chr5:82460025−83260025 [82460025−83260025] |
| hilite range: | 0 – 0 [ 0 – 0 ] |
| reference SNP: | chr5:82860025 |
| number of SNPs plotted: | 3392 |
| min P.value: | 5.82E−12 [chr5:82860025] |
| max P.value: | 10E−1 [chr5:82777519] |
| omitted GWAS Hits: | NA, NA |

Make more plots at http://csg.sph.umich.edu/locuszoom/

GWAS Catalog SNPs in Region

| chr | pos (Mb) | trait | snp |
|---|---|---|---|
| 5 | 82.81912 | Laryngeal squamous cell carcinoma | rs310518 |
| 5 | 82.84549 | Diisocyanate–induced asthma | rs3852186 |
| 5 | 82.88991 | Major depressive disorder | rs310501 |
| 5 | 82.96073 | Visceral fat | rs3846635 |
| 5 | 82.98574 | Prostate cancer | rs4466137 |
| 5 | 83.17359 | Ankylosing spondylitis | rs4552569 |

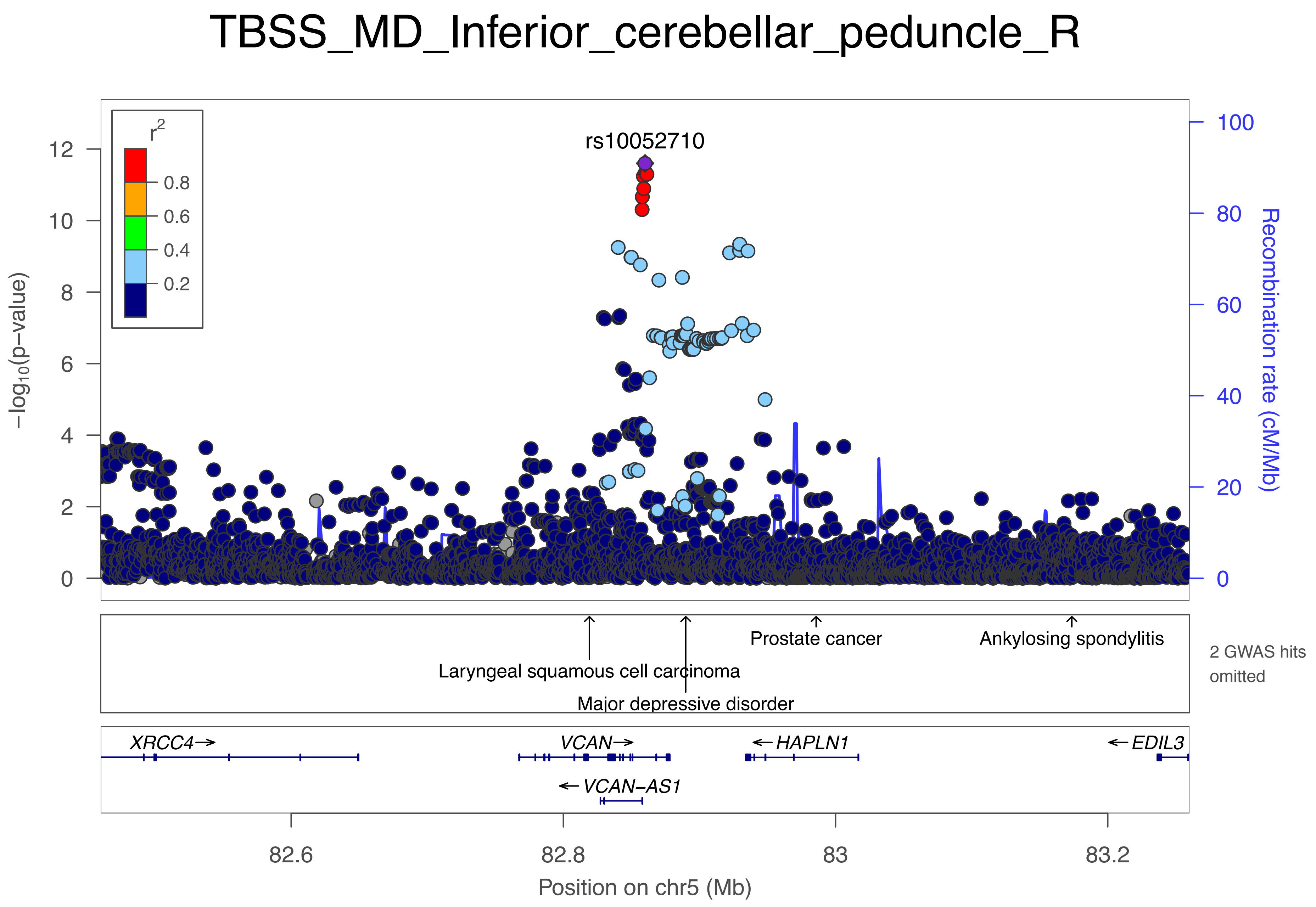

TBSS_MD_Inferior_cerebellar_peduncle_R
rs10052710
r²
0.8
0.6
0.4
0.2
−log10(p−value)
Recombination rate (cM/Mb)
0
2
4
6
8
10
12
20
40
60
80
100
Laryngeal squamous cell carcinoma
Major depressive disorder
Prostate cancer
Ankylosing spondylitis
2 GWAS hits omitted
XRCC4→
VCAN→
←HAPLN1
←EDIL3
←VCAN−AS1
82.6
82.8
83
83.2
Position on chr5 (Mb)

| | |
|---|---|
| date: | Thu Aug 17 17:52:01 2017 |
| build: | hg19 |
| display range: | chr5:82460025−83260025 [82460025−83260025] |
| hilite range: | 0 – 0 [ 0 – 0 ] |
| reference SNP: | chr5:82860025 |
| number of SNPs plotted: | 3392 |
| min P.value: | 2.52E−12 [chr5:82860025] |
| max P.value: | 10E−1 [chr5:82607038] |
| omitted GWAS Hits: | NA, NA |

Make more plots at http://csg.sph.umich.edu/locuszoom/

GWAS Catalog SNPs in Region

| chr | pos (Mb) | trait | snp |
|---|---|---|---|
| 5 | 82.81912 | Laryngeal squamous cell carcinoma | rs310518 |
| 5 | 82.84549 | Diisocyanate–induced asthma | rs3852186 |
| 5 | 82.88991 | Major depressive disorder | rs310501 |
| 5 | 82.96073 | Visceral fat | rs3846635 |
| 5 | 82.98574 | Prostate cancer | rs4466137 |
| 5 | 83.17359 | Ankylosing spondylitis | rs4552569 |

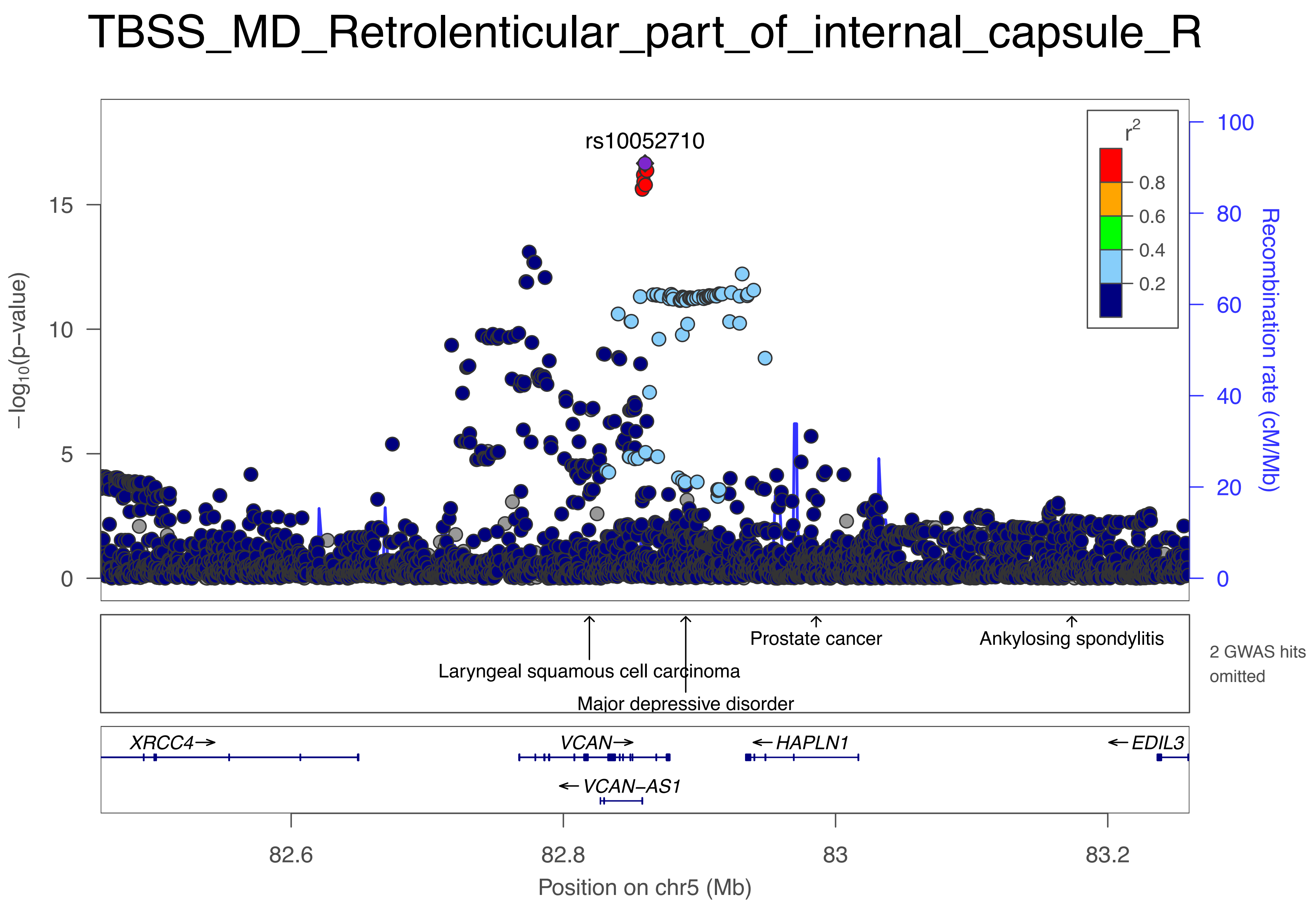

TBSS_MD_Retrolenticular_part_of_internal_capsule_R
rs10052710
−log10(p−value)
Recombination rate (cM/Mb)
r2
0.8
0.6
0.4
0.2
Laryngeal squamous cell carcinoma
Major depressive disorder
Prostate cancer
Ankylosing spondylitis
2 GWAS hits omitted
XRCC4
VCAN
VCAN−AS1
HAPLN1
EDIL3
82.6
82.8
83
83.2
Position on chr5 (Mb)

| | |
|---|---|
| date: | Thu Aug 17 17:52:01 2017 |
| build: | hg19 |
| display range: | chr5:82460025−83260025 [82460025−83260025] |
| hilite range: | 0 – 0 [ 0 – 0 ] |
| reference SNP: | chr5:82860025 |
| number of SNPs plotted: | 3392 |
| min P.value: | 2.2E−17 [chr5:82860025] |
| max P.value: | 10E−1 [chr5:83082527] |
| omitted GWAS Hits: | NA, NA |

Make more plots at http://csg.sph.umich.edu/locuszoom/

GWAS Catalog SNPs in Region

| chr | pos (Mb) | trait | snp |
|---|---|---|---|
| 5 | 82.81912 | Laryngeal squamous cell carcinoma | rs310518 |
| 5 | 82.84549 | Diisocyanate–induced asthma | rs3852186 |
| 5 | 82.88991 | Major depressive disorder | rs310501 |
| 5 | 82.96073 | Visceral fat | rs3846635 |
| 5 | 82.98574 | Prostate cancer | rs4466137 |
| 5 | 83.17359 | Ankylosing spondylitis | rs4552569 |

# TBSS_MD_Retrolenticular_part_of_internal_capsule_L

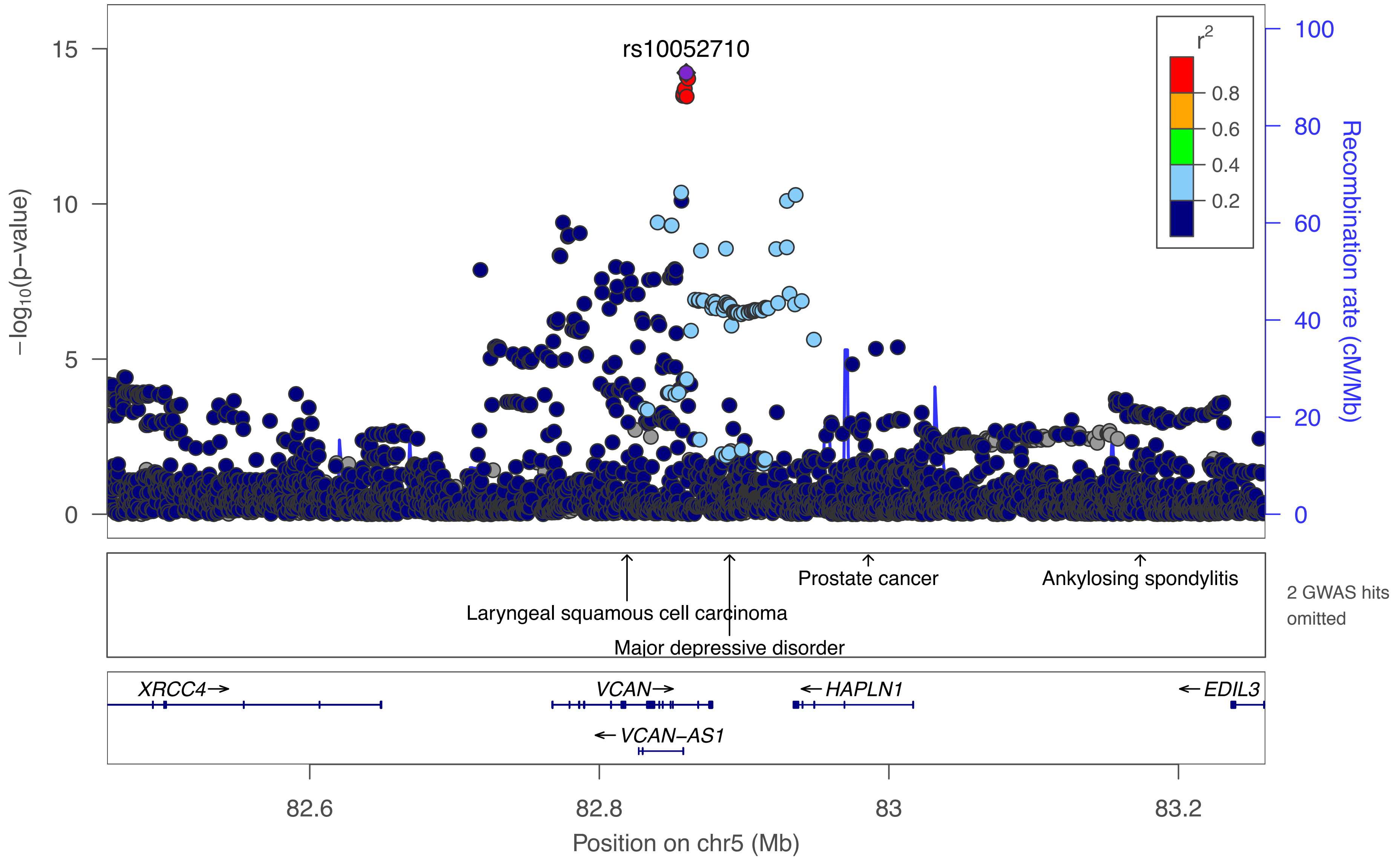

| | |
|---|---|
| date: | Thu Aug 17 17:52:01 2017 |
| build: | hg19 |
| display range: | chr5:82460025–83260025 [82460025–83260025] |
| hilite range: | 0 – 0 [ 0 – 0 ] |
| reference SNP: | chr5:82860025 |
| number of SNPs plotted: | 3392 |
| min P.value: | 5.96E–15 [chr5:82860025] |
| max P.value: | 9.99E–1 [chr5:82720045] |
| omitted GWAS Hits: | NA, NA |

Make more plots at http://csg.sph.umich.edu/locuszoom/

GWAS Catalog SNPs in Region

| chr | pos (Mb) | trait | snp |
|---|---|---|---|
| 5 | 82.81912 | Laryngeal squamous cell carcinoma | rs310518 |
| 5 | 82.84549 | Diisocyanate−induced asthma | rs3852186 |
| 5 | 82.88991 | Major depressive disorder | rs310501 |
| 5 | 82.96073 | Visceral fat | rs3846635 |
| 5 | 82.98574 | Prostate cancer | rs4466137 |
| 5 | 83.17359 | Ankylosing spondylitis | rs4552569 |

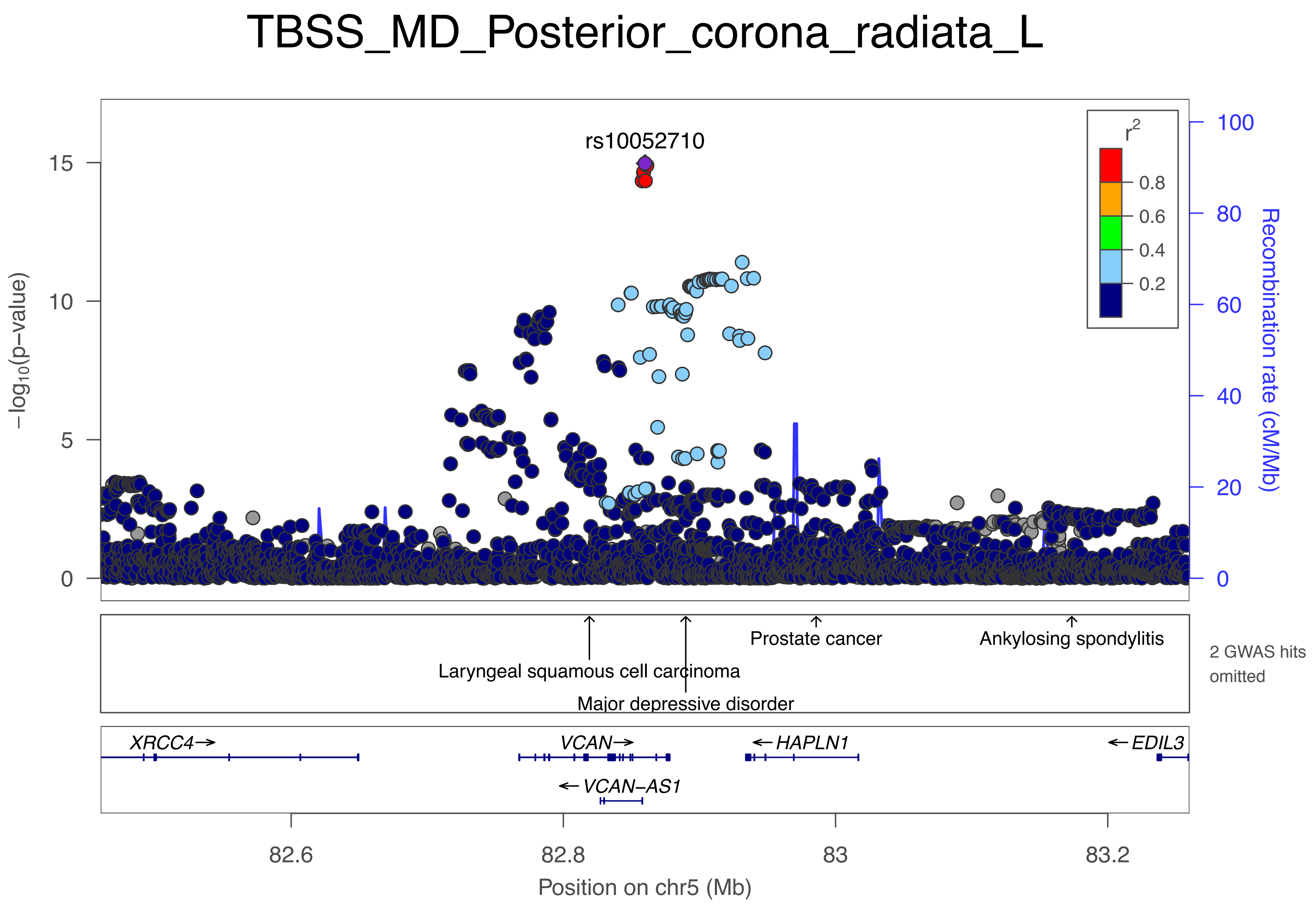

TBSS_MD_Posterior_corona_radiata_L
rs10052710
−log10(p−value)
Recombination rate (cM/Mb)
r2
0.8
0.6
0.4
0.2
Prostate cancer
Ankylosing spondylitis
Laryngeal squamous cell carcinoma
Major depressive disorder
2 GWAS hits omitted
XRCC4→
VCAN→
←HAPLN1
←EDIL3
←VCAN−AS1
82.6
82.8
83
83.2
Position on chr5 (Mb)

date: Thu Aug 17 17:52:01 2017

build: hg19

display range: chr5:82460025−83260025 [82460025−83260025]

hilite range: 0 – 0  [ 0 – 0 ]

reference SNP: chr5:82860025

number of SNPs plotted: 3392

min P.value: 1.07E−15 [chr5:82860025]

max P.value: 10E−1 [chr5:83072612]

omitted GWAS Hits: NA, NA

Make more plots at http://csg.sph.umich.edu/locuszoom/

GWAS Catalog SNPs in Region

| chr | pos (Mb) | trait | snp |
|---|---|---|---|
| 5 | 82.81912 | Laryngeal squamous cell carcinoma | rs310518 |
| 5 | 82.84549 | Diisocyanate–induced asthma | rs3852186 |
| 5 | 82.88991 | Major depressive disorder | rs310501 |
| 5 | 82.96073 | Visceral fat | rs3846635 |
| 5 | 82.98574 | Prostate cancer | rs4466137 |
| 5 | 83.17359 | Ankylosing spondylitis | rs4552569 |

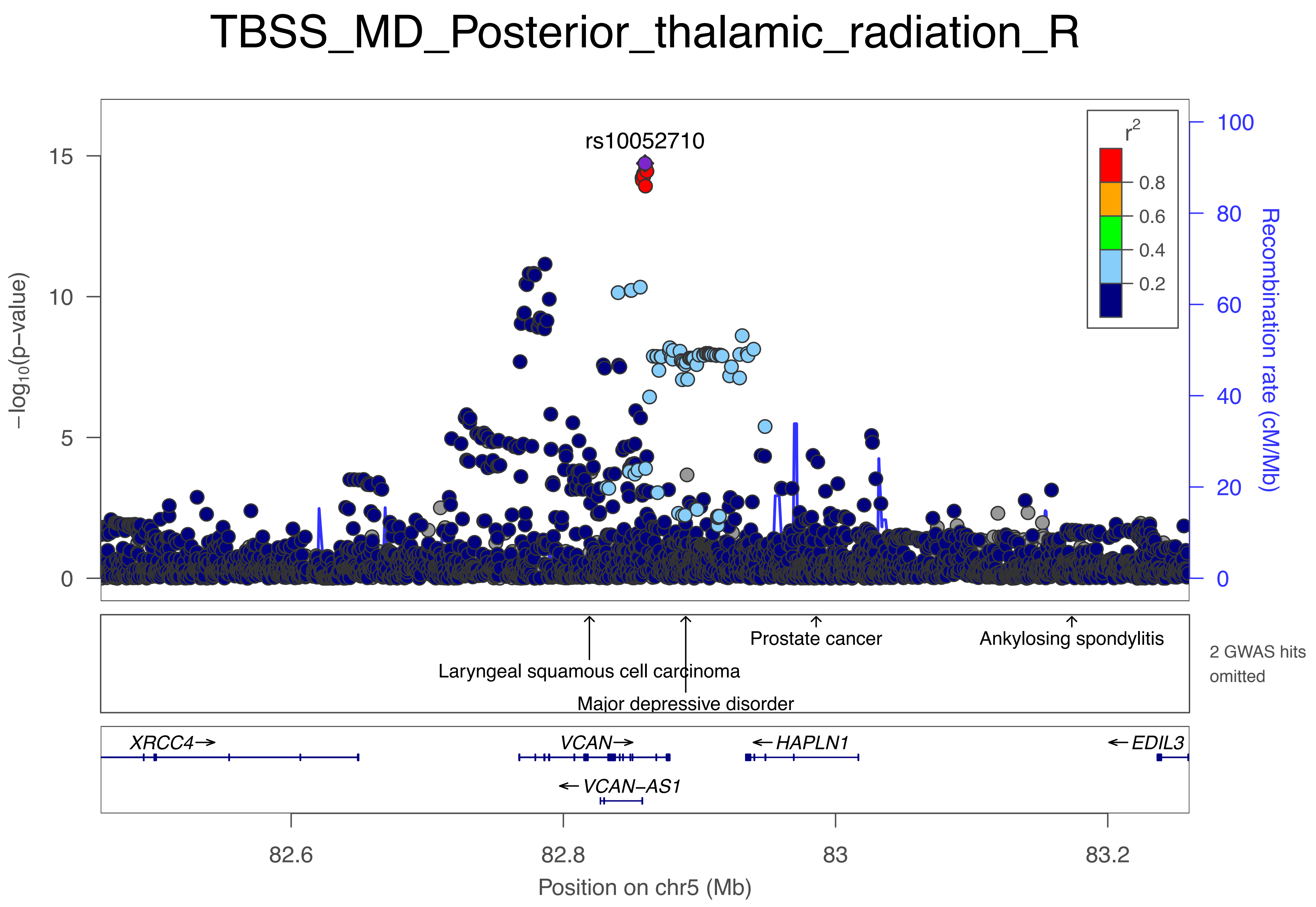

TBSS_MD_Posterior_thalamic_radiation_R
rs10052710
r2
0.8
0.6
0.4
0.2
100
80
60
40
20
0
Recombination rate (cM/Mb)
15
10
5
0
−log10(p−value)
Laryngeal squamous cell carcinoma
Major depressive disorder
Prostate cancer
Ankylosing spondylitis
2 GWAS hits omitted
XRCC4→
VCAN→
←HAPLN1
←EDIL3
←VCAN−AS1
82.6
82.8
83
83.2
Position on chr5 (Mb)

| | |
|---|---|
| date: | Thu Aug 17 17:52:01 2017 |
| build: | hg19 |
| display range: | chr5:82460025−83260025 [82460025−83260025] |
| hilite range: | 0 – 0 [ 0 – 0 ] |
| reference SNP: | chr5:82860025 |
| number of SNPs plotted: | 3392 |
| min P.value: | 1.85E−15 [chr5:82860025] |
| max P.value: | 10E−1 [chr5:82472156] |
| omitted GWAS Hits: | NA, NA |

Make more plots at http://csg.sph.umich.edu/locuszoom/

GWAS Catalog SNPs in Region

| chr | pos (Mb) | trait | snp |
|---|---|---|---|
| 5 | 82.81912 | Laryngeal squamous cell carcinoma | rs310518 |
| 5 | 82.84549 | Diisocyanate–induced asthma | rs3852186 |
| 5 | 82.88991 | Major depressive disorder | rs310501 |
| 5 | 82.96073 | Visceral fat | rs3846635 |
| 5 | 82.98574 | Prostate cancer | rs4466137 |
| 5 | 83.17359 | Ankylosing spondylitis | rs4552569 |

# TBSS_MD_Superior_longitudinal_fasciculus_R

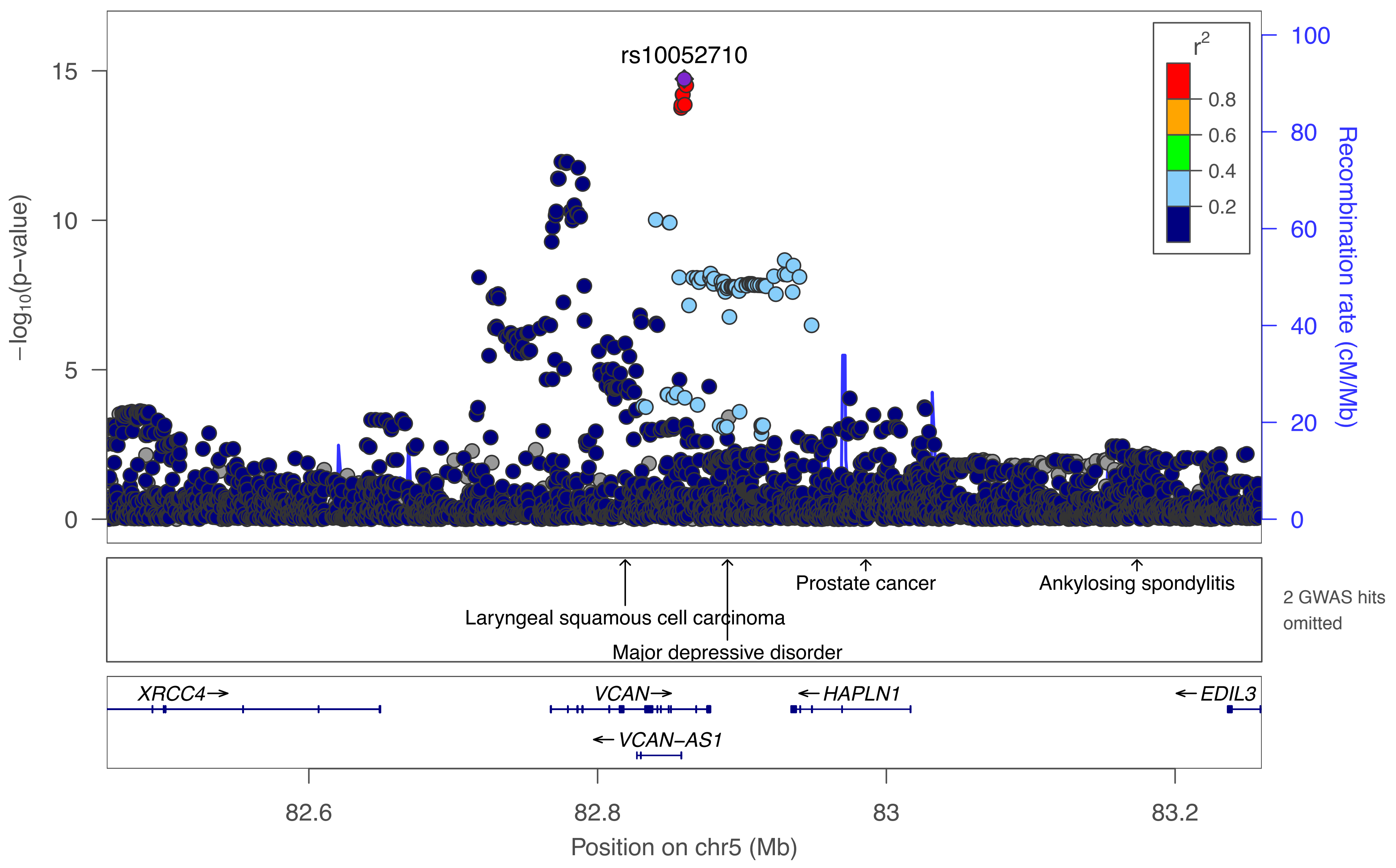

| | |
|---|---|
| date: | Thu Aug 17 17:52:01 2017 |
| build: | hg19 |
| display range: | chr5:82460025−83260025 [82460025−83260025] |
| hilite range: | 0 – 0  [ 0 – 0 ] |
| reference SNP: | chr5:82860025 |
| number of SNPs plotted: | 3392 |
| min P.value: | 1.87E−15 [chr5:82860025] |
| max P.value: | 9.99E−1 [chr5:82609092] |
| omitted GWAS Hits: | NA, NA |

Make more plots at http://csg.sph.umich.edu/locuszoom/

GWAS Catalog SNPs in Region

| chr | pos (Mb) | trait | snp |
|---|---|---|---|
| 5 | 82.81912 | Laryngeal squamous cell carcinoma | rs310518 |
| 5 | 82.84549 | Diisocyanate–induced asthma | rs3852186 |
| 5 | 82.88991 | Major depressive disorder | rs310501 |
| 5 | 82.96073 | Visceral fat | rs3846635 |
| 5 | 82.98574 | Prostate cancer | rs4466137 |
| 5 | 83.17359 | Ankylosing spondylitis | rs4552569 |

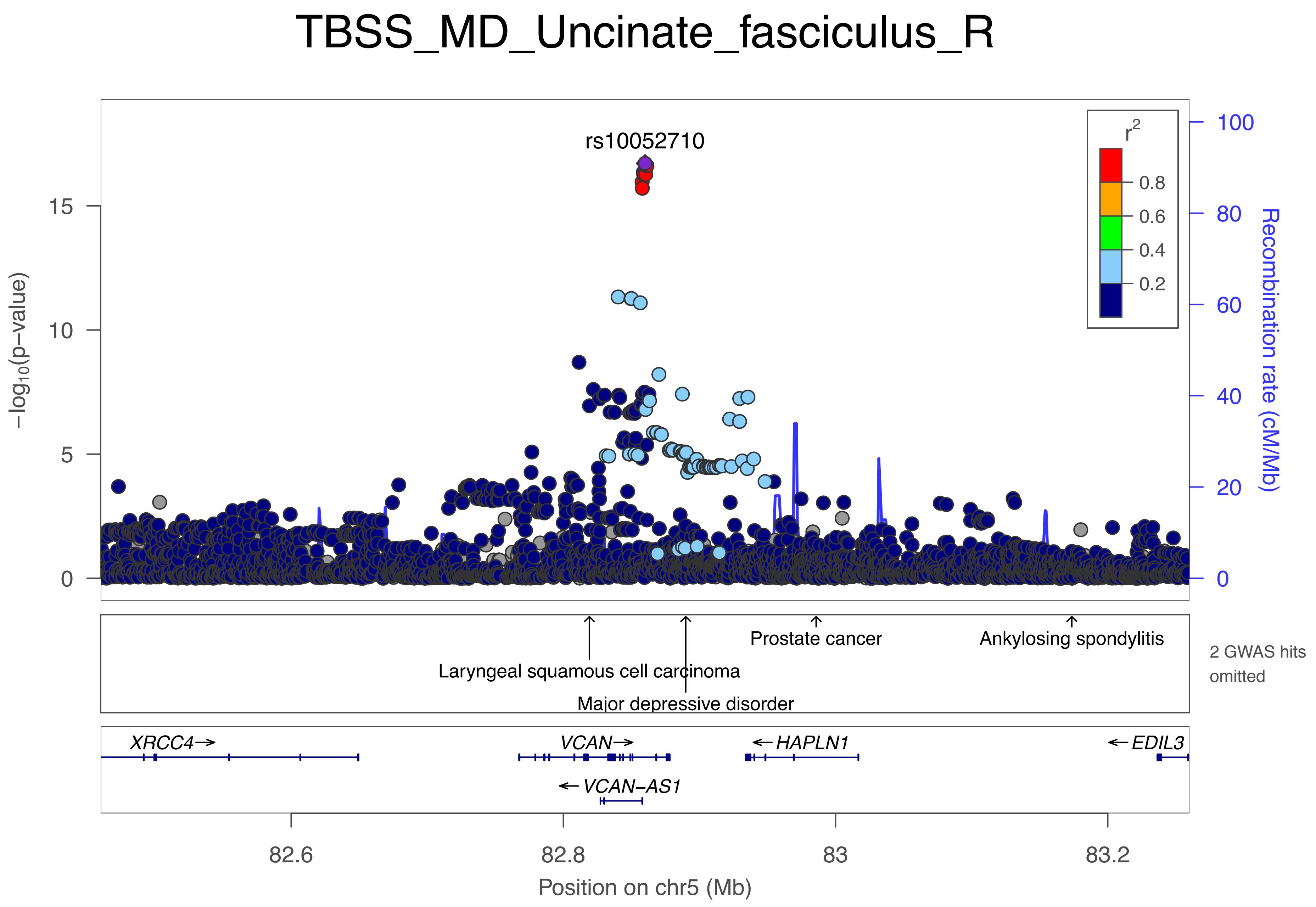
TBSS_MD_Uncinate_fasciculus_R
rs10052710
−log10(p−value)
Recombination rate (cM/Mb)
r²
0.8
0.6
0.4
0.2
Laryngeal squamous cell carcinoma
Major depressive disorder
Prostate cancer
Ankylosing spondylitis
2 GWAS hits omitted
XRCC4→
VCAN→
←HAPLN1
←EDIL3
←VCAN−AS1
82.6
82.8
83
83.2
Position on chr5 (Mb)

| | |
|---|---|
| date: | Thu Aug 17 18:07:35 2017 |
| build: | hg19 |
| display range: | chr5:82460025−83260025 [82460025−83260025] |
| hilite range: | 0 – 0 [ 0 – 0 ] |
| reference SNP: | chr5:82860025 |
| number of SNPs plotted: | 3392 |
| min P.value: | 1.95E−17 [chr5:82860025] |
| max P.value: | 9.99E−1 [chr5:82999322] |
| omitted GWAS Hits: | NA, NA |

Make more plots at http://csg.sph.umich.edu/locuszoom/

GWAS Catalog SNPs in Region

| chr | pos (Mb) | trait | snp |
|---|---|---|---|
| 5 | 82.81912 | Laryngeal squamous cell carcinoma | rs310518 |
| 5 | 82.84549 | Diisocyanate–induced asthma | rs3852186 |
| 5 | 82.88991 | Major depressive disorder | rs310501 |
| 5 | 82.96073 | Visceral fat | rs3846635 |
| 5 | 82.98574 | Prostate cancer | rs4466137 |
| 5 | 83.17359 | Ankylosing spondylitis | rs4552569 |

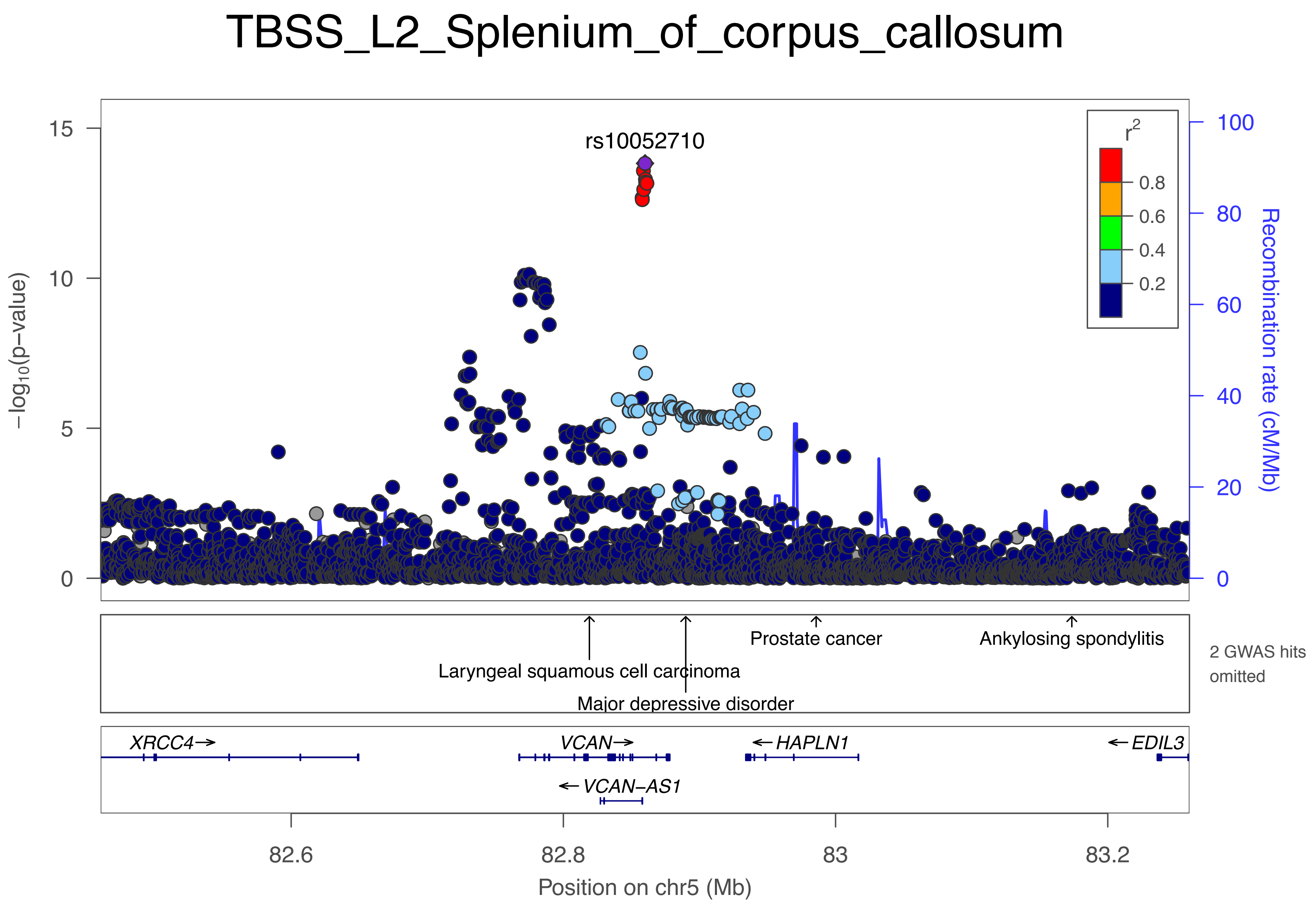
TBSS_L2_Splenium_of_corpus_callosum
rs10052710
r2
0.8
0.6
0.4
0.2
15
10
5
0
−log10(p−value)
100
80
60
40
20
0
Recombination rate (cM/Mb)
Prostate cancer
Ankylosing spondylitis
Laryngeal squamous cell carcinoma
Major depressive disorder
2 GWAS hits omitted
XRCC4→
VCAN→
←HAPLN1
←EDIL3
←VCAN−AS1
82.6
82.8
83
83.2
Position on chr5 (Mb)

| | |
|---|---|
| date: | Thu Aug 17 18:07:35 2017 |
| build: | hg19 |
| display range: | chr5:82460025−83260025 [82460025−83260025] |
| hilite range: | 0 – 0 [ 0 – 0 ] |
| reference SNP: | chr5:82860025 |
| number of SNPs plotted: | 3392 |
| min P.value: | 1.49E−14 [chr5:82860025] |
| max P.value: | 10E−1 [chr5:83008479] |
| omitted GWAS Hits: | NA, NA |

Make more plots at http://csg.sph.umich.edu/locuszoom/

GWAS Catalog SNPs in Region

| chr | pos (Mb) | trait | snp |
|---|---|---|---|
| 5 | 82.81912 | Laryngeal squamous cell carcinoma | rs310518 |
| 5 | 82.84549 | Diisocyanate–induced asthma | rs3852186 |
| 5 | 82.88991 | Major depressive disorder | rs310501 |
| 5 | 82.96073 | Visceral fat | rs3846635 |
| 5 | 82.98574 | Prostate cancer | rs4466137 |
| 5 | 83.17359 | Ankylosing spondylitis | rs4552569 |

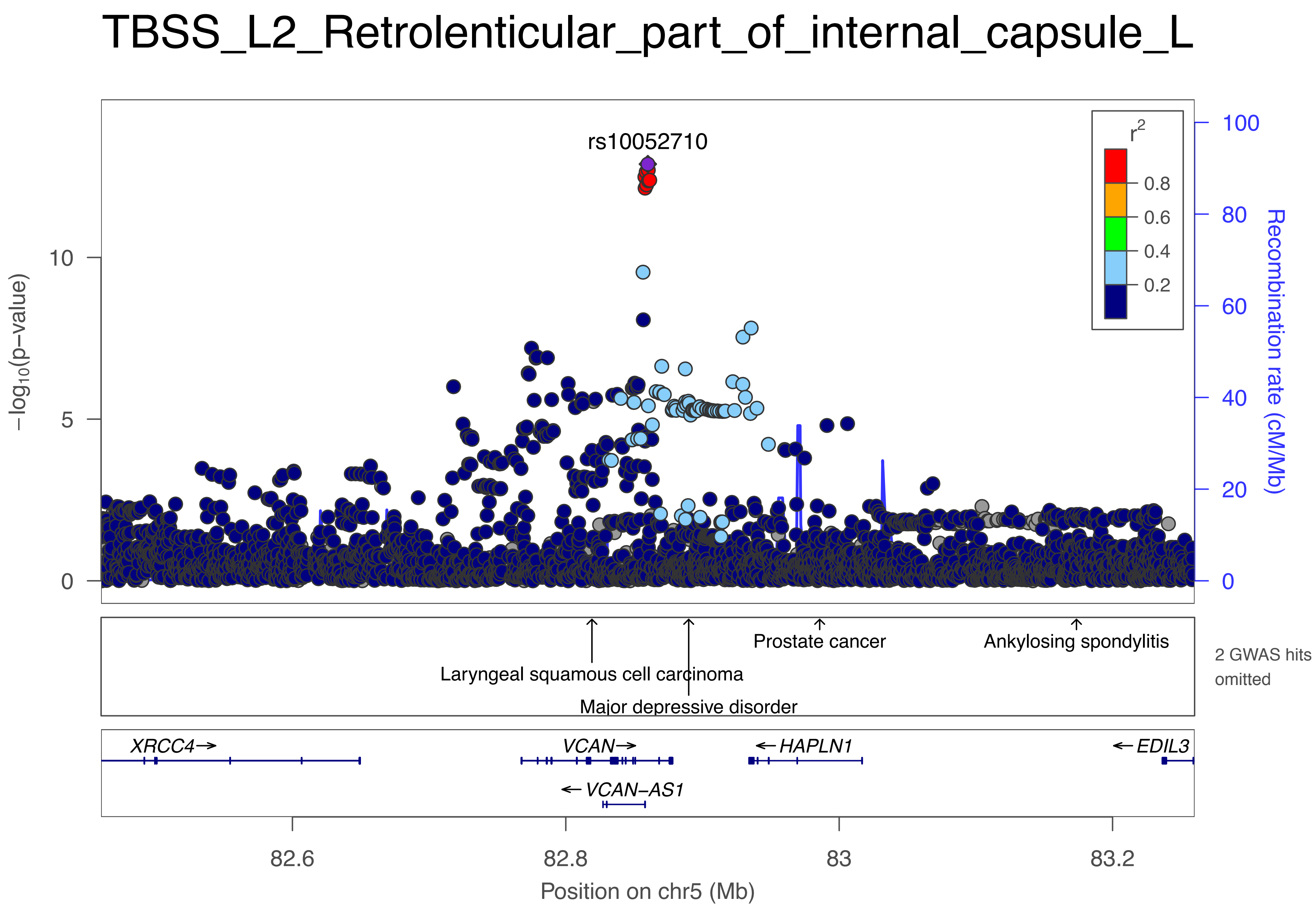

TBSS_L2_Retrolenticular_part_of_internal_capsule_L
rs10052710
r²
0.8
0.6
0.4
0.2
−log₁₀(p−value)
10
5
0
100
80
60
40
20
0
Recombination rate (cM/Mb)
Laryngeal squamous cell carcinoma
Major depressive disorder
Prostate cancer
Ankylosing spondylitis
2 GWAS hits omitted
XRCC4→
VCAN→
←HAPLN1
←EDIL3
←VCAN−AS1
82.6
82.8
83
83.2
Position on chr5 (Mb)

| | |
|---|---|
| date: | Thu Aug 17 17:52:01 2017 |
| build: | hg19 |
| display range: | chr5:82460025−83260025 [82460025−83260025] |
| hilite range: | 0 – 0 [ 0 – 0 ] |
| reference SNP: | chr5:82860025 |
| number of SNPs plotted: | 3392 |
| min P.value: | 1.3E−13 [chr5:82860025] |
| max P.value: | 10E−1 [chr5:82460502] |
| omitted GWAS Hits: | NA, NA |

Make more plots at http://csg.sph.umich.edu/locuszoom/

GWAS Catalog SNPs in Region

| chr | pos (Mb) | trait | snp |
|---|---|---|---|
| 5 | 82.81912 | Laryngeal squamous cell carcinoma | rs310518 |
| 5 | 82.84549 | Diisocyanate–induced asthma | rs3852186 |
| 5 | 82.88991 | Major depressive disorder | rs310501 |
| 5 | 82.96073 | Visceral fat | rs3846635 |
| 5 | 82.98574 | Prostate cancer | rs4466137 |
| 5 | 83.17359 | Ankylosing spondylitis | rs4552569 |

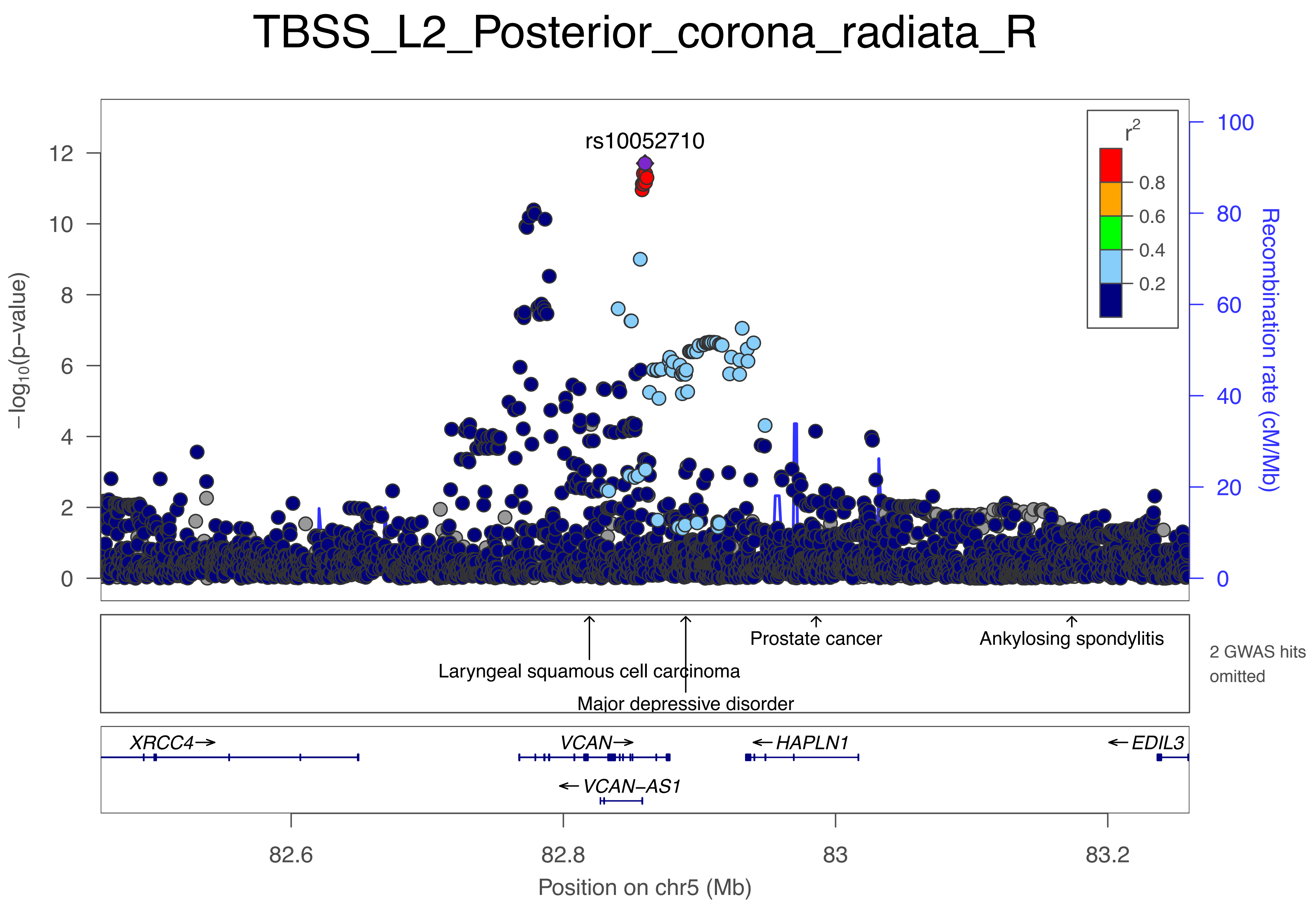
TBSS_L2_Posterior_corona_radiata_R
rs10052710
−log10(p−value)
Recombination rate (cM/Mb)
r2
0.8
0.6
0.4
0.2
Laryngeal squamous cell carcinoma
Major depressive disorder
Prostate cancer
Ankylosing spondylitis
2 GWAS hits omitted
XRCC4
VCAN
VCAN−AS1
HAPLN1
EDIL3
82.6
82.8
83
83.2
Position on chr5 (Mb)

| | |
|---|---|
| date: | Thu Aug 17 17:52:01 2017 |
| build: | hg19 |
| display range: | chr5:82460025−83260025 [82460025−83260025] |
| hilite range: | 0 – 0 [ 0 – 0 ] |
| reference SNP: | chr5:82860025 |
| number of SNPs plotted: | 3392 |
| min P.value: | 1.97E−12 [chr5:82860025] |
| max P.value: | 10E−1 [chr5:82913727] |
| omitted GWAS Hits: | NA, NA |

Make more plots at http://csg.sph.umich.edu/locuszoom/

GWAS Catalog SNPs in Region

| chr | pos (Mb) | trait | snp |
|---|---|---|---|
| 5 | 82.81912 | Laryngeal squamous cell carcinoma | rs310518 |
| 5 | 82.84549 | Diisocyanate–induced asthma | rs3852186 |
| 5 | 82.88991 | Major depressive disorder | rs310501 |
| 5 | 82.96073 | Visceral fat | rs3846635 |
| 5 | 82.98574 | Prostate cancer | rs4466137 |
| 5 | 83.17359 | Ankylosing spondylitis | rs4552569 |

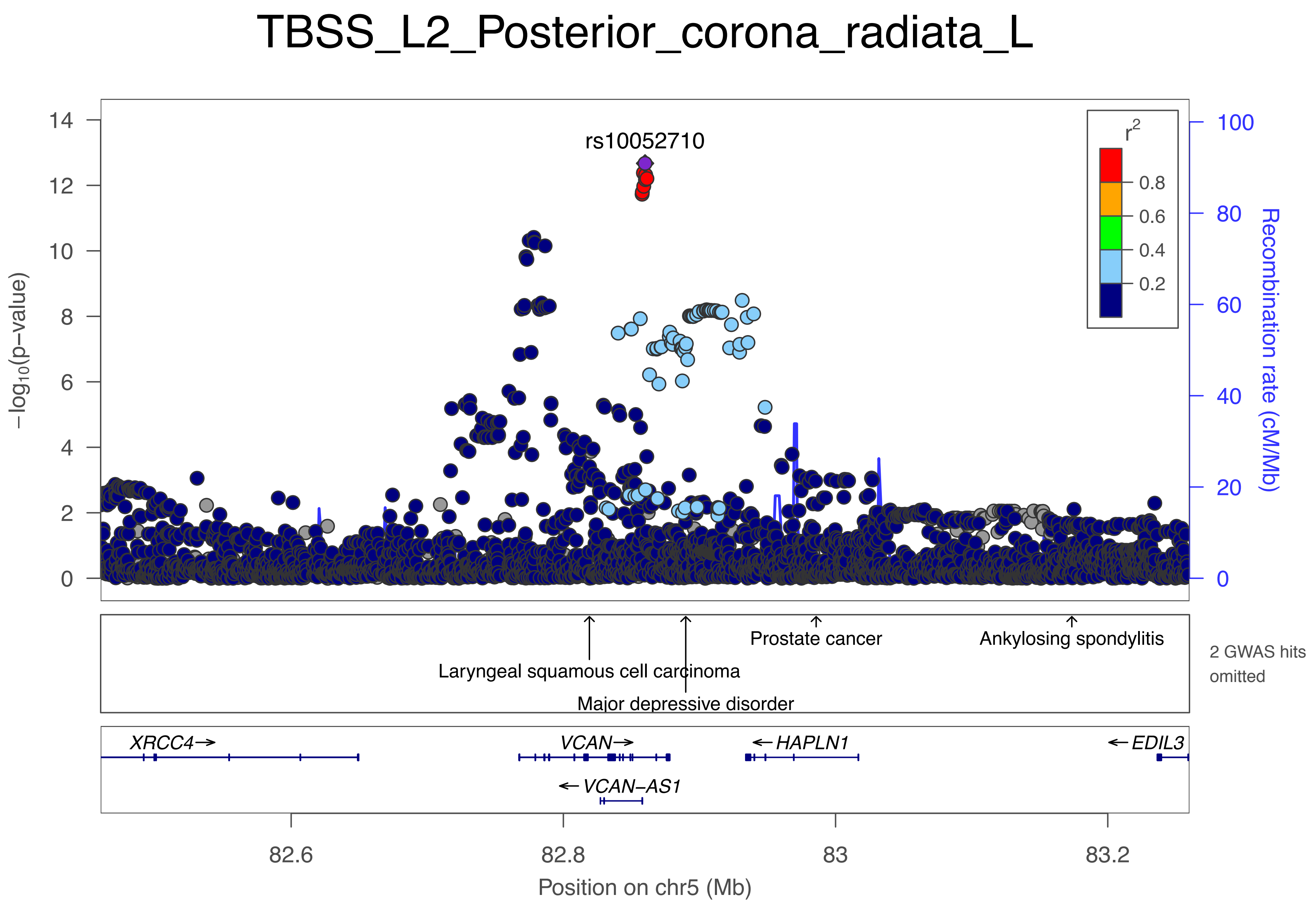
TBSS_L2_Posterior_corona_radiata_L
rs10052710
−log10(p−value)
Recombination rate (cM/Mb)
r2
0.8
0.6
0.4
0.2
Laryngeal squamous cell carcinoma
Major depressive disorder
Prostate cancer
Ankylosing spondylitis
2 GWAS hits omitted
XRCC4
VCAN
VCAN−AS1
HAPLN1
EDIL3
82.6
82.8
83
83.2
Position on chr5 (Mb)

| | |
|---|---|
| date: | Thu Aug 17 17:52:01 2017 |
| build: | hg19 |
| display range: | chr5:82460025−83260025 [82460025−83260025] |
| hilite range: | 0 – 0  [ 0 – 0 ] |
| reference SNP: | chr5:82860025 |
| number of SNPs plotted: | 3392 |
| min P.value: | 2.13E−13 [chr5:82860025] |
| max P.value: | 10E−1 [chr5:82723169] |
| omitted GWAS Hits: | NA, NA |

Make more plots at http://csg.sph.umich.edu/locuszoom/

GWAS Catalog SNPs in Region

| chr | pos (Mb) | trait | snp |
|---|---|---|---|
| 5 | 82.81912 | Laryngeal squamous cell carcinoma | rs310518 |
| 5 | 82.84549 | Diisocyanate–induced asthma | rs3852186 |
| 5 | 82.88991 | Major depressive disorder | rs310501 |
| 5 | 82.96073 | Visceral fat | rs3846635 |
| 5 | 82.98574 | Prostate cancer | rs4466137 |
| 5 | 83.17359 | Ankylosing spondylitis | rs4552569 |

# TBSS_L2_Posterior_thalamic_radiation_R

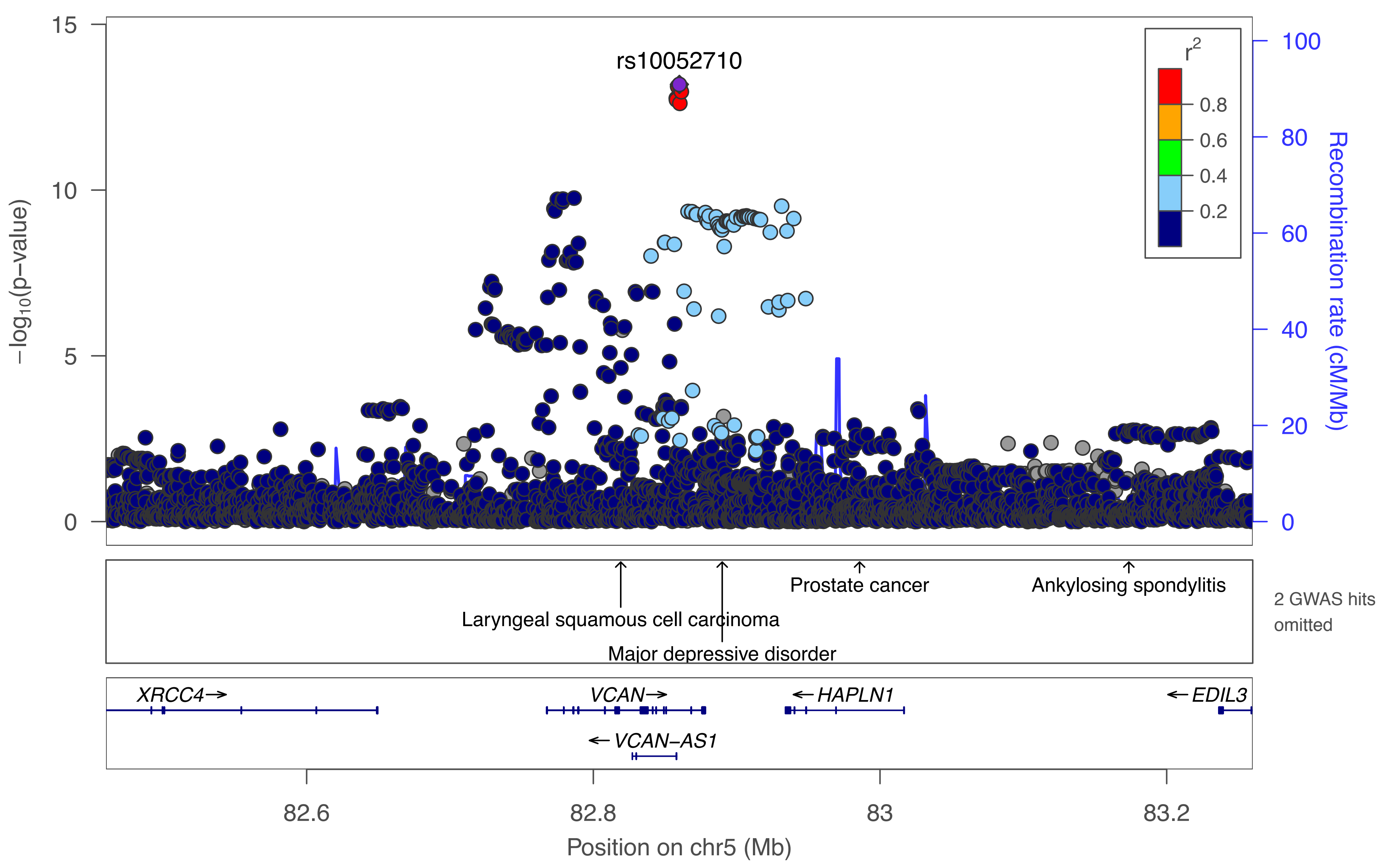

| | |
|---|---|
| date: | Thu Aug 17 17:52:01 2017 |
| build: | hg19 |
| display range: | chr5:82460025−83260025 [82460025−83260025] |
| hilite range: | 0 – 0 [ 0 – 0 ] |
| reference SNP: | chr5:82860025 |
| number of SNPs plotted: | 3392 |
| min P.value: | 6.5E−14 [chr5:82860025] |
| max P.value: | 10E−1 [chr5:82942774] |
| omitted GWAS Hits: | NA, NA |

Make more plots at http://csg.sph.umich.edu/locuszoom/

GWAS Catalog SNPs in Region

| chr | pos (Mb) | trait | snp |
|---|---|---|---|
| 5 | 82.81912 | Laryngeal squamous cell carcinoma | rs310518 |
| 5 | 82.84549 | Diisocyanate–induced asthma | rs3852186 |
| 5 | 82.88991 | Major depressive disorder | rs310501 |
| 5 | 82.96073 | Visceral fat | rs3846635 |
| 5 | 82.98574 | Prostate cancer | rs4466137 |
| 5 | 83.17359 | Ankylosing spondylitis | rs4552569 |

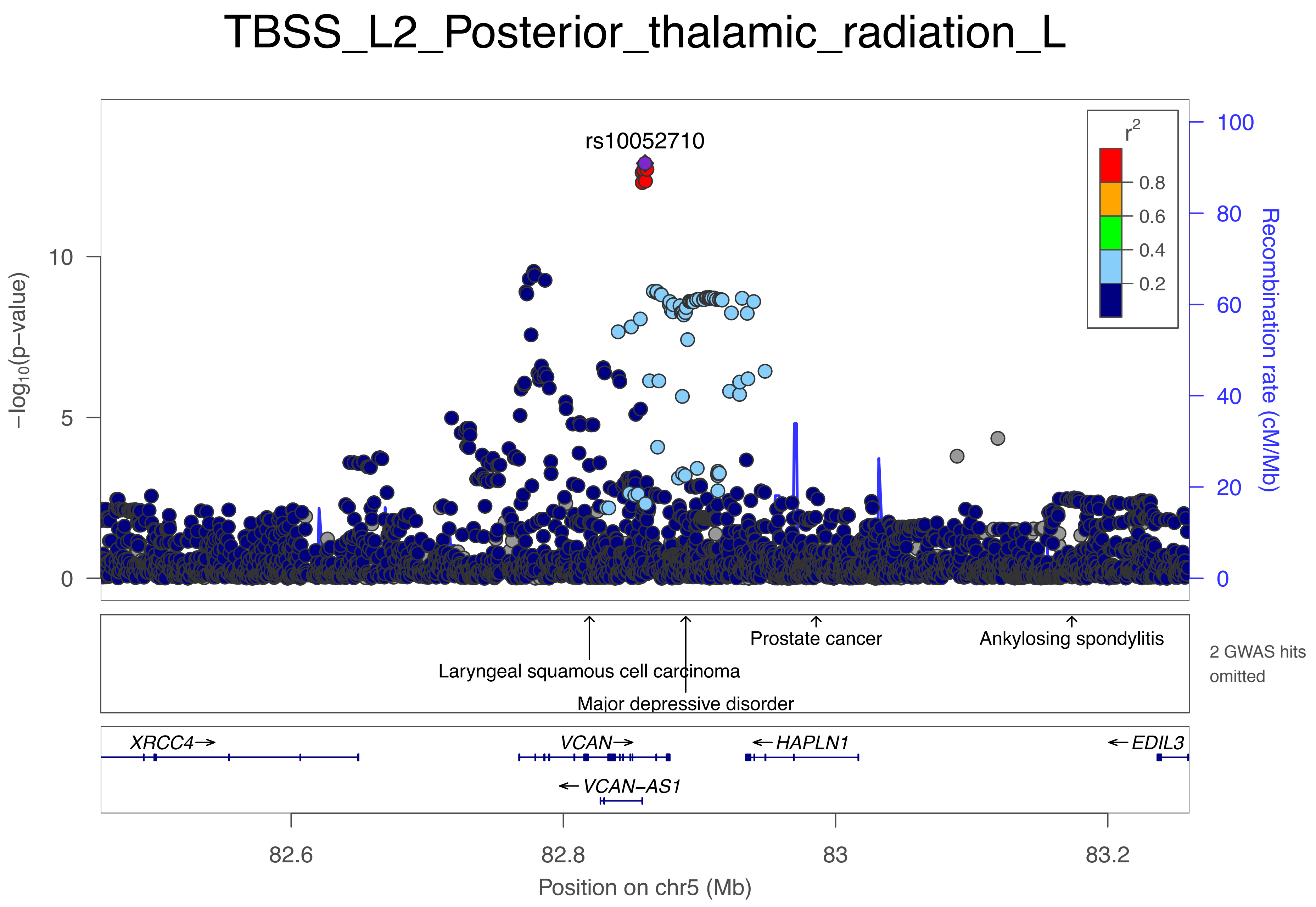
TBSS_L2_Posterior_thalamic_radiation_L
rs10052710
r2
0.8
0.6
0.4
0.2
−log10(p−value)
10
5
0
100
80
60
40
20
0
Recombination rate (cM/Mb)
Laryngeal squamous cell carcinoma
Major depressive disorder
Prostate cancer
Ankylosing spondylitis
2 GWAS hits omitted
XRCC4→
VCAN→
←VCAN−AS1
←HAPLN1
←EDIL3
82.6
82.8
83
83.2
Position on chr5 (Mb)

| | |
|---|---|
| date: | Thu Aug 17 17:52:01 2017 |
| build: | hg19 |
| display range: | chr5:82460025−83260025 [82460025−83260025] |
| hilite range: | 0 – 0 [ 0 – 0 ] |
| reference SNP: | chr5:82860025 |
| number of SNPs plotted: | 3392 |
| min P.value: | 1.26E−13 [chr5:82860025] |
| max P.value: | 9.99E−1 [chr5:82741980] |
| omitted GWAS Hits: | NA, NA |

Make more plots at http://csg.sph.umich.edu/locuszoom/

GWAS Catalog SNPs in Region

| chr | pos (Mb) | trait | snp |
|---|---|---|---|
| 5 | 82.81912 | Laryngeal squamous cell carcinoma | rs310518 |
| 5 | 82.84549 | Diisocyanate–induced asthma | rs3852186 |
| 5 | 82.88991 | Major depressive disorder | rs310501 |
| 5 | 82.96073 | Visceral fat | rs3846635 |
| 5 | 82.98574 | Prostate cancer | rs4466137 |
| 5 | 83.17359 | Ankylosing spondylitis | rs4552569 |

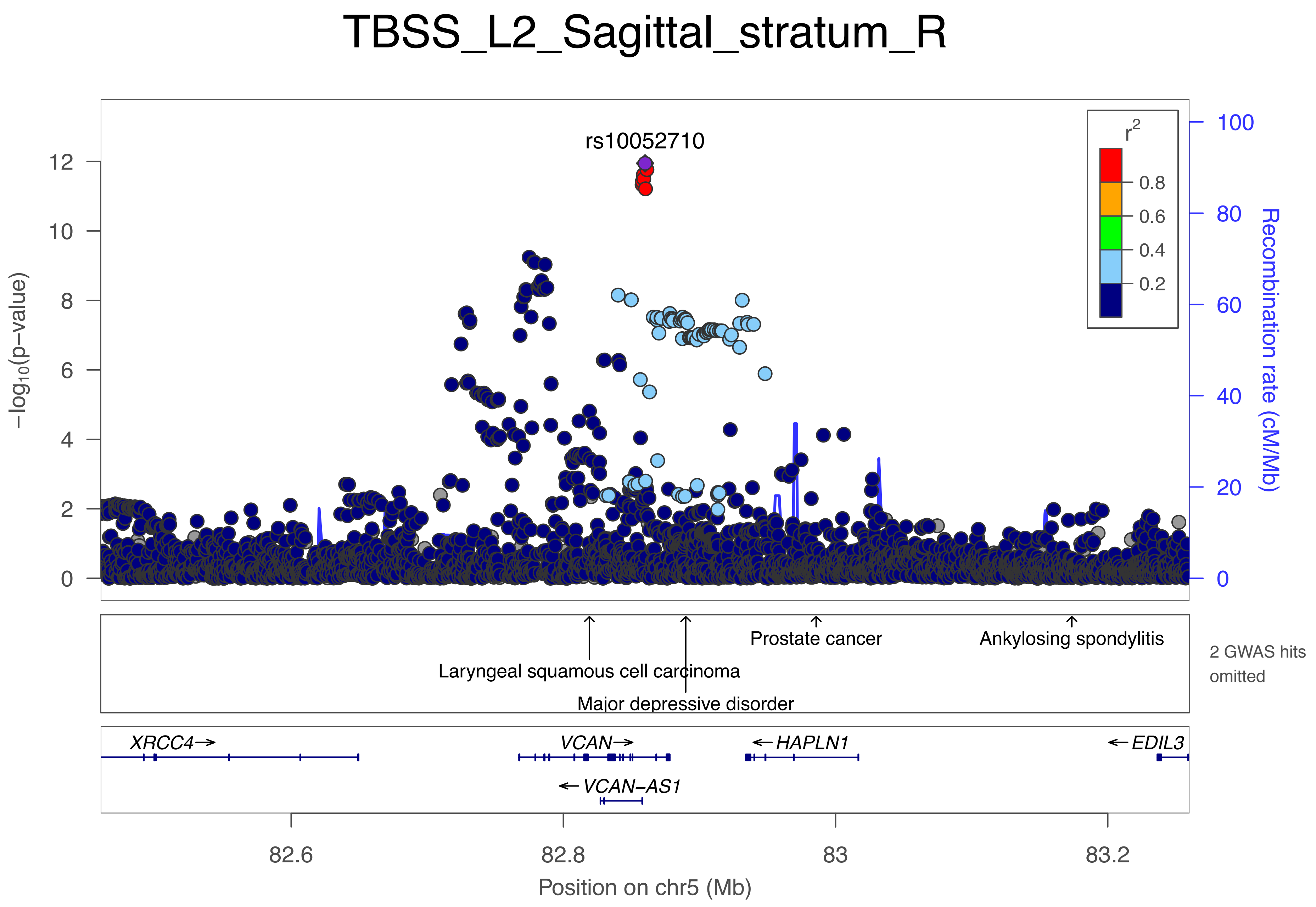

TBSS_L2_Sagittal_stratum_R
rs10052710
−log10(p−value)
Recombination rate (cM/Mb)
r2
0.8
0.6
0.4
0.2
Laryngeal squamous cell carcinoma
Major depressive disorder
Prostate cancer
Ankylosing spondylitis
2 GWAS hits omitted
XRCC4
VCAN
VCAN−AS1
HAPLN1
EDIL3
82.6
82.8
83
83.2
Position on chr5 (Mb)

date: Thu Aug 17 17:52:01 2017

build: hg19

display range: chr5:82460025−83260025 [82460025−83260025]

hilite range: 0 – 0 [ 0 – 0 ]

reference SNP: chr5:82860025

number of SNPs plotted: 3392

min P.value: 1.13E−12 [chr5:82860025]

max P.value: 9.99E−1 [chr5:82802571]

omitted GWAS Hits: NA, NA

Make more plots at http://csg.sph.umich.edu/locuszoom/

GWAS Catalog SNPs in Region

| chr | pos (Mb) | trait | snp |
|---|---|---|---|
| 5 | 82.81912 | Laryngeal squamous cell carcinoma | rs310518 |
| 5 | 82.84549 | Diisocyanate–induced asthma | rs3852186 |
| 5 | 82.88991 | Major depressive disorder | rs310501 |
| 5 | 82.96073 | Visceral fat | rs3846635 |
| 5 | 82.98574 | Prostate cancer | rs4466137 |
| 5 | 83.17359 | Ankylosing spondylitis | rs4552569 |

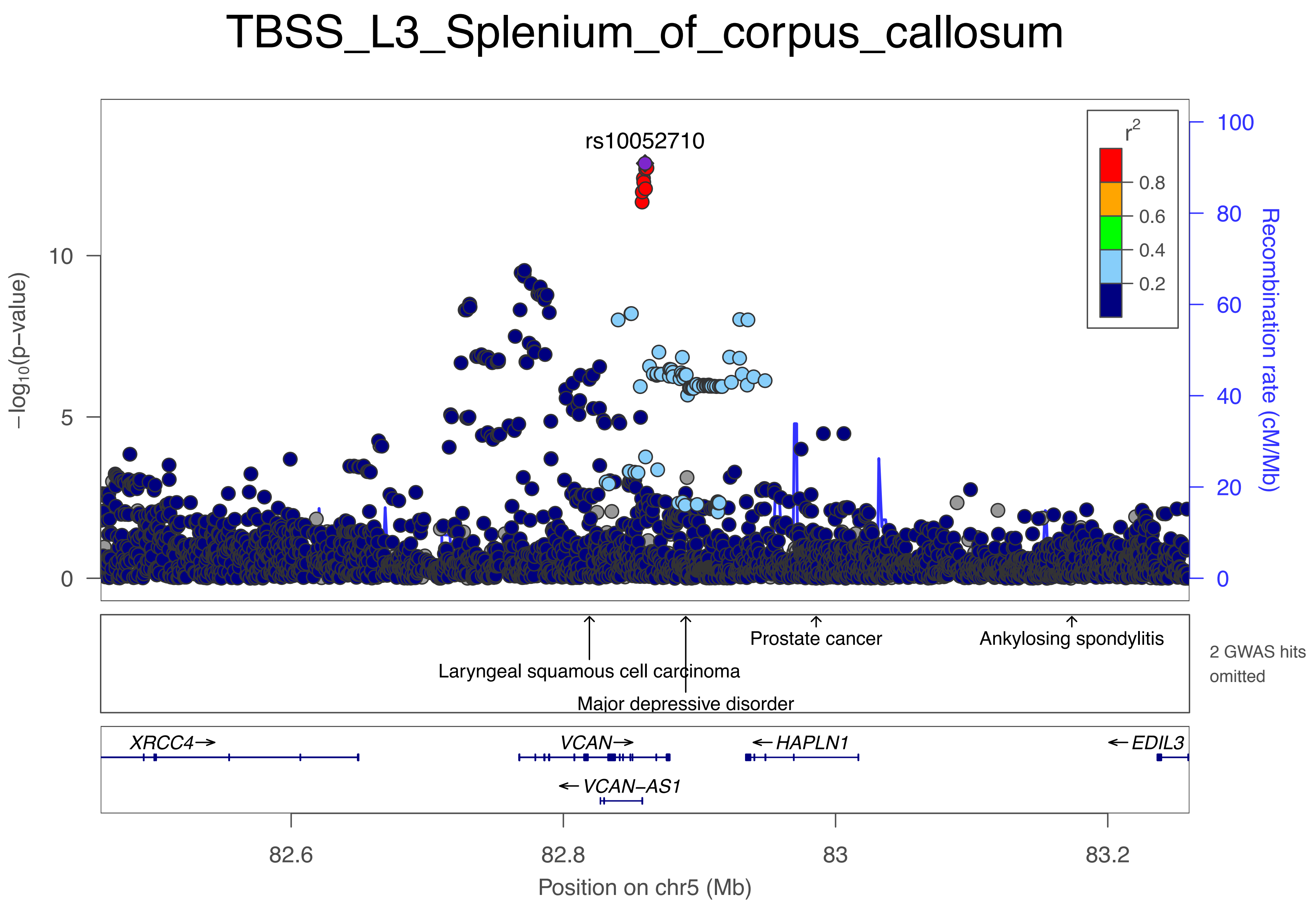

TBSS_L3_Splenium_of_corpus_callosum
rs10052710
r2
0.8
0.6
0.4
0.2
−log10(p−value)
Recombination rate (cM/Mb)
Prostate cancer
Ankylosing spondylitis
Laryngeal squamous cell carcinoma
Major depressive disorder
2 GWAS hits omitted
XRCC4
VCAN
HAPLN1
EDIL3
VCAN−AS1
82.6
82.8
83
83.2
Position on chr5 (Mb)

| | |
|---|---|
| date: | Thu Aug 17 17:52:01 2017 |
| build: | hg19 |
| display range: | chr5:82460025−83260025 [82460025−83260025] |
| hilite range: | 0 – 0 [ 0 – 0 ] |
| reference SNP: | chr5:82860025 |
| number of SNPs plotted: | 3392 |
| min P.value: | 1.38E−13 [chr5:82860025] |
| max P.value: | 10E−1 [chr5:83007358] |
| omitted GWAS Hits: | NA, NA |

Make more plots at http://csg.sph.umich.edu/locuszoom/

GWAS Catalog SNPs in Region

| chr | pos (Mb) | trait | snp |
|---|---|---|---|
| 5 | 82.81912 | Laryngeal squamous cell carcinoma | rs310518 |
| 5 | 82.84549 | Diisocyanate–induced asthma | rs3852186 |
| 5 | 82.88991 | Major depressive disorder | rs310501 |
| 5 | 82.96073 | Visceral fat | rs3846635 |
| 5 | 82.98574 | Prostate cancer | rs4466137 |
| 5 | 83.17359 | Ankylosing spondylitis | rs4552569 |

# TBSS_L3_Retrolenticular_part_of_internal_capsule_R

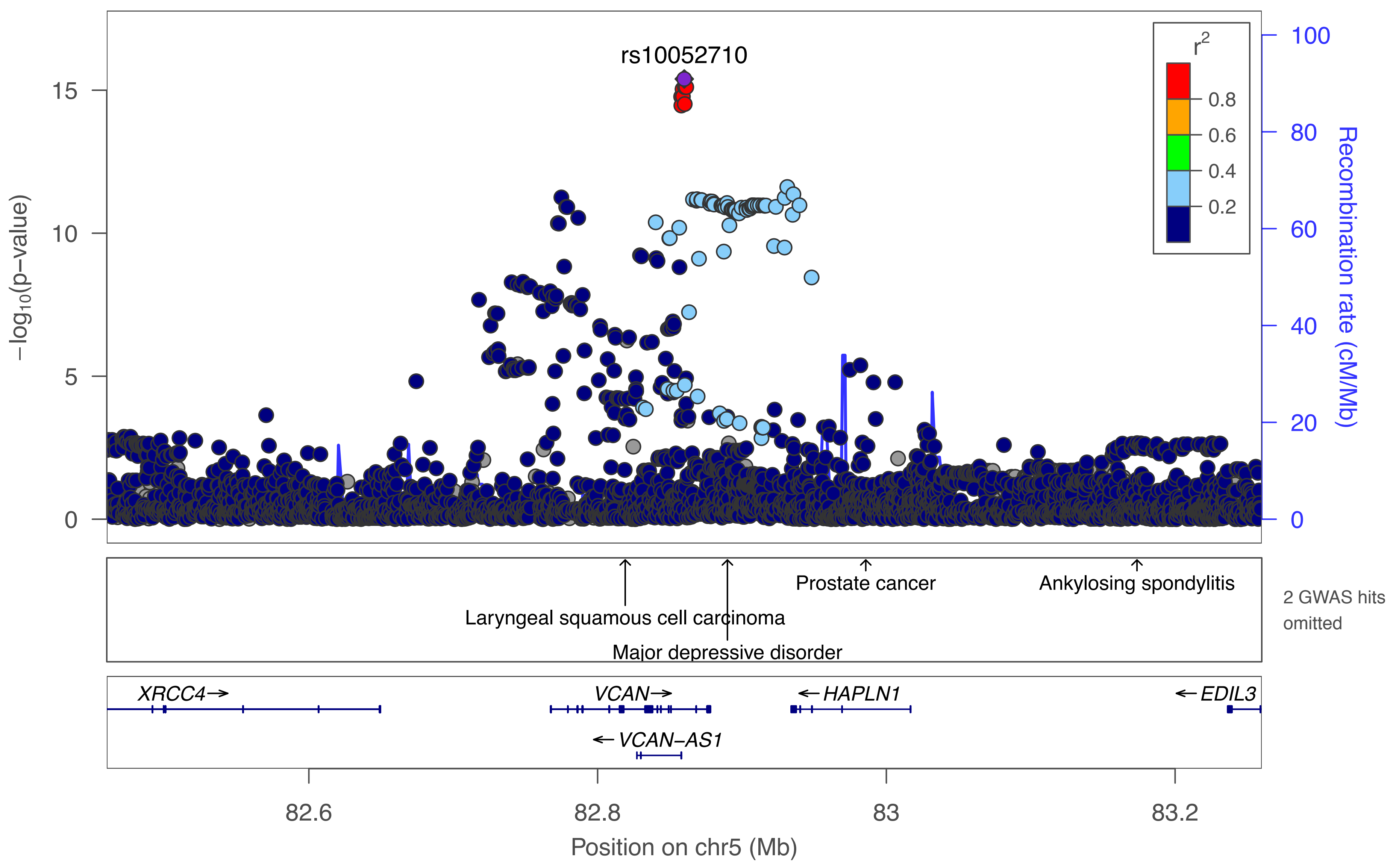

| | |
|---|---|
| date: | Thu Aug 17 17:52:01 2017 |
| build: | hg19 |
| display range: | chr5:82460025−83260025 [82460025−83260025] |
| hilite range: | 0 – 0 [ 0 – 0 ] |
| reference SNP: | chr5:82860025 |
| number of SNPs plotted: | 3392 |
| min P.value: | 4.06E−16 [chr5:82860025] |
| max P.value: | 9.99E−1 [chr5:82762095] |
| omitted GWAS Hits: | NA, NA |

Make more plots at http://csg.sph.umich.edu/locuszoom/

GWAS Catalog SNPs in Region

| chr | pos (Mb) | trait | snp |
|---|---|---|---|
| 5 | 82.81912 | Laryngeal squamous cell carcinoma | rs310518 |
| 5 | 82.84549 | Diisocyanate–induced asthma | rs3852186 |
| 5 | 82.88991 | Major depressive disorder | rs310501 |
| 5 | 82.96073 | Visceral fat | rs3846635 |
| 5 | 82.98574 | Prostate cancer | rs4466137 |
| 5 | 83.17359 | Ankylosing spondylitis | rs4552569 |

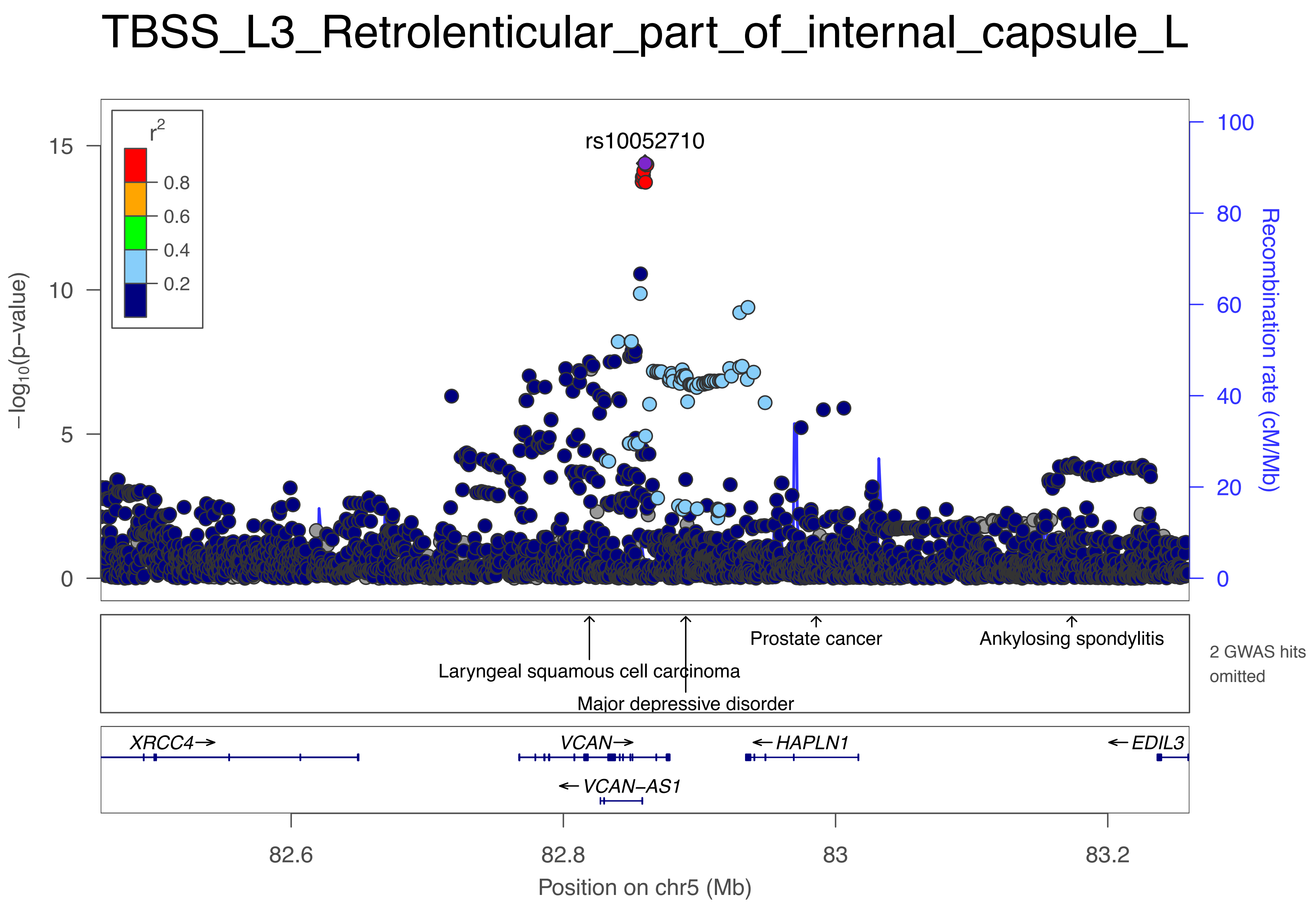
TBSS_L3_Retrolenticular_part_of_internal_capsule_L
r2
0.8
0.6
0.4
0.2
rs10052710
15
10
5
0
−log10(p−value)
100
80
60
40
20
0
Recombination rate (cM/Mb)
Laryngeal squamous cell carcinoma
Major depressive disorder
Prostate cancer
Ankylosing spondylitis
2 GWAS hits omitted
XRCC4→
VCAN→
←VCAN−AS1
←HAPLN1
←EDIL3
82.6
82.8
83
83.2
Position on chr5 (Mb)

| | |
|---|---|
| date: | Thu Aug 17 17:52:01 2017 |
| build: | hg19 |
| display range: | chr5:82460025−83260025 [82460025−83260025] |
| hilite range: | 0 – 0 [ 0 – 0 ] |
| reference SNP: | chr5:82860025 |
| number of SNPs plotted: | 3392 |
| min P.value: | 4.1E−15 [chr5:82860025] |
| max P.value: | 10E−1 [chr5:82566020] |
| omitted GWAS Hits: | NA, NA |

Make more plots at http://csg.sph.umich.edu/locuszoom/

GWAS Catalog SNPs in Region

| chr | pos (Mb) | trait | snp |
|---|---|---|---|
| 5 | 82.81912 | Laryngeal squamous cell carcinoma | rs310518 |
| 5 | 82.84549 | Diisocyanate–induced asthma | rs3852186 |
| 5 | 82.88991 | Major depressive disorder | rs310501 |
| 5 | 82.96073 | Visceral fat | rs3846635 |
| 5 | 82.98574 | Prostate cancer | rs4466137 |
| 5 | 83.17359 | Ankylosing spondylitis | rs4552569 |

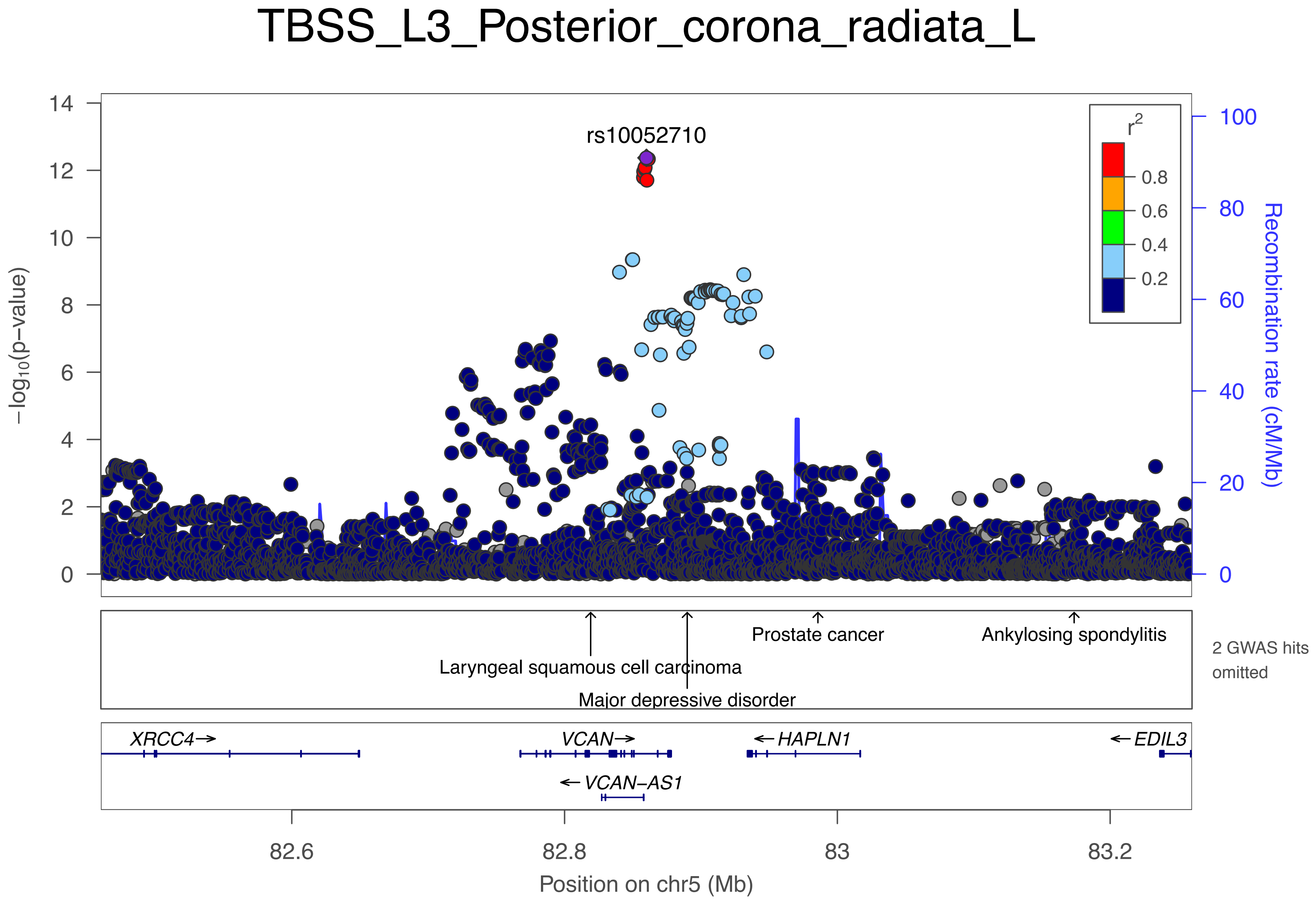

TBSS_L3_Posterior_corona_radiata_L
rs10052710
−log10(p−value)
Recombination rate (cM/Mb)
r2
0.8
0.6
0.4
0.2
Laryngeal squamous cell carcinoma
Major depressive disorder
Prostate cancer
Ankylosing spondylitis
2 GWAS hits omitted
XRCC4
VCAN
VCAN−AS1
HAPLN1
EDIL3
82.6
82.8
83
83.2
Position on chr5 (Mb)

date: Thu Aug 17 17:52:01 2017

build: hg19

display range: chr5:82460025−83260025 [82460025−83260025]

hilite range: 0 – 0  [ 0 – 0 ]

reference SNP: chr5:82860025

number of SNPs plotted: 3392

min P.value: 4.26E−13 [chr5:82860025]

max P.value: 10E−1 [chr5:83078656]

omitted GWAS Hits: NA, NA

Make more plots at http://csg.sph.umich.edu/locuszoom/

GWAS Catalog SNPs in Region

| chr | pos (Mb) | trait | snp |
| --- | --- | --- | --- |
| 5 | 82.81912 | Laryngeal squamous cell carcinoma | rs310518 |
| 5 | 82.84549 | Diisocyanate–induced asthma | rs3852186 |
| 5 | 82.88991 | Major depressive disorder | rs310501 |
| 5 | 82.96073 | Visceral fat | rs3846635 |
| 5 | 82.98574 | Prostate cancer | rs4466137 |
| 5 | 83.17359 | Ankylosing spondylitis | rs4552569 |

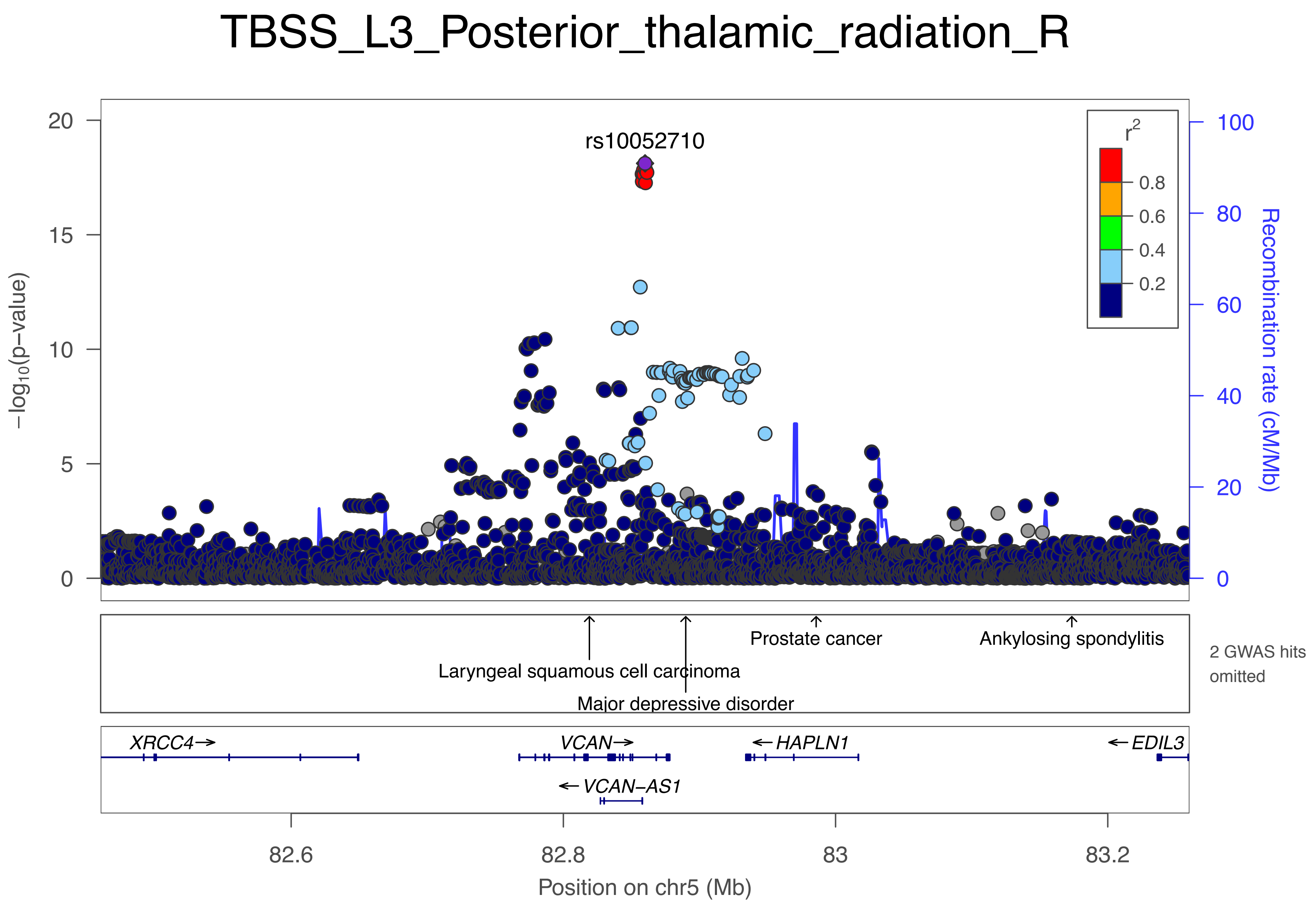

TBSS_L3_Posterior_thalamic_radiation_R
rs10052710
20
15
10
5
0
−log10(p−value)
r2
0.8
0.6
0.4
0.2
100
80
60
40
20
0
Recombination rate (cM/Mb)
Laryngeal squamous cell carcinoma
Major depressive disorder
Prostate cancer
Ankylosing spondylitis
2 GWAS hits omitted
XRCC4
VCAN
VCAN−AS1
HAPLN1
EDIL3
82.6
82.8
83
83.2
Position on chr5 (Mb)

| | |
|---|---|
| date: | Thu Aug 17 17:52:01 2017 |
| build: | hg19 |
| display range: | chr5:82460025−83260025 [82460025−83260025] |
| hilite range: | 0 – 0 [ 0 – 0 ] |
| reference SNP: | chr5:82860025 |
| number of SNPs plotted: | 3392 |
| min P.value: | 7.64E−19 [chr5:82860025] |
| max P.value: | 10E−1 [chr5:82502188] |
| omitted GWAS Hits: | NA, NA |

Make more plots at http://csg.sph.umich.edu/locuszoom/

GWAS Catalog SNPs in Region

| chr | pos (Mb) | trait | snp |
|---|---|---|---|
| 5 | 82.81912 | Laryngeal squamous cell carcinoma | rs310518 |
| 5 | 82.84549 | Diisocyanate–induced asthma | rs3852186 |
| 5 | 82.88991 | Major depressive disorder | rs310501 |
| 5 | 82.96073 | Visceral fat | rs3846635 |
| 5 | 82.98574 | Prostate cancer | rs4466137 |
| 5 | 83.17359 | Ankylosing spondylitis | rs4552569 |

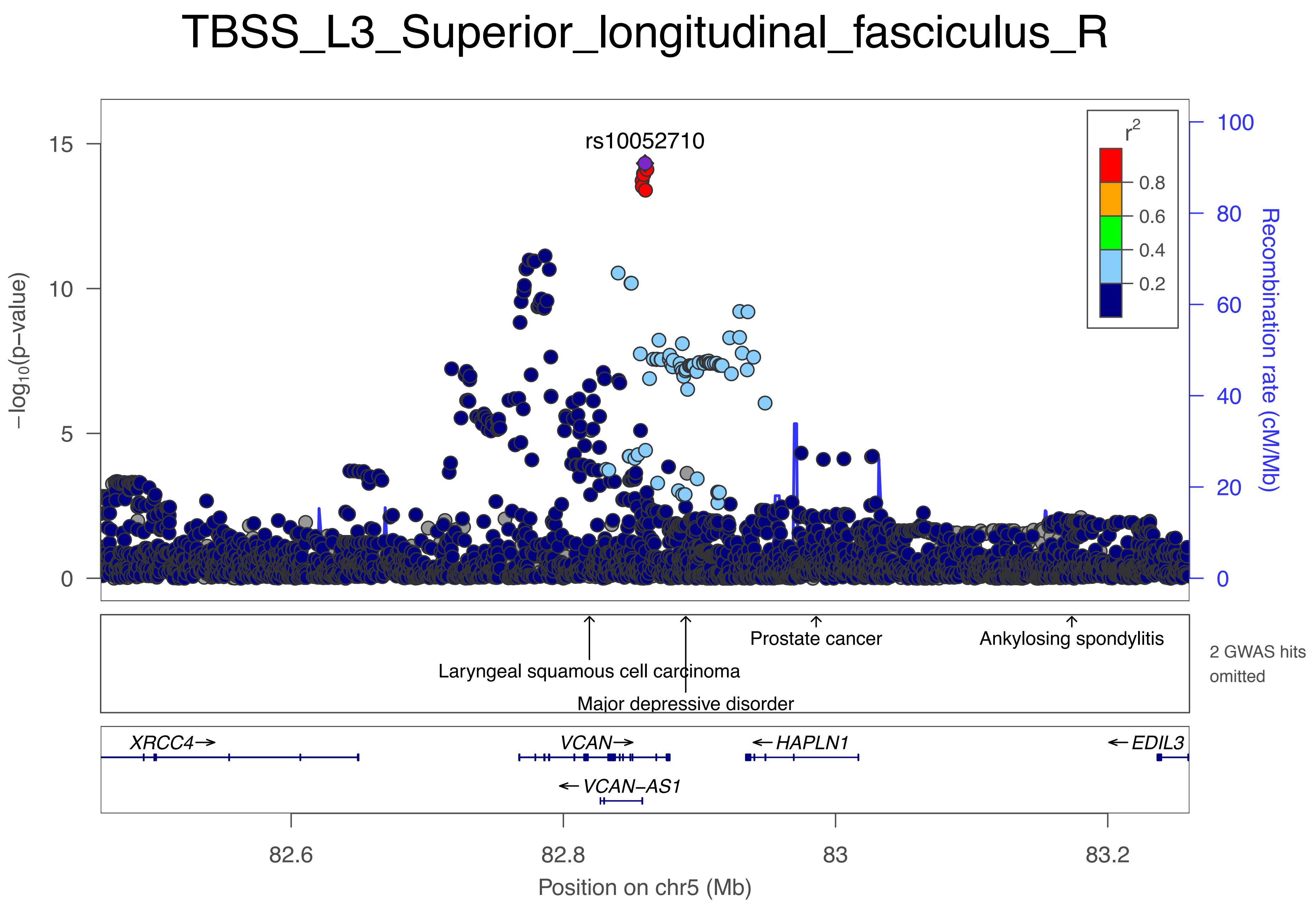

TBSS_L3_Superior_longitudinal_fasciculus_R
rs10052710
−log10(p−value)
Recombination rate (cM/Mb)
r2
0.8
0.6
0.4
0.2
Laryngeal squamous cell carcinoma
Major depressive disorder
Prostate cancer
Ankylosing spondylitis
2 GWAS hits omitted
XRCC4
VCAN
HAPLN1
EDIL3
VCAN−AS1
82.6
82.8
83
83.2
Position on chr5 (Mb)

| | |
|---|---|
| date: | Thu Aug 17 17:52:01 2017 |
| build: | hg19 |
| display range: | chr5:82460025−83260025 [82460025−83260025] |
| hilite range: | 0 – 0 [ 0 – 0 ] |
| reference SNP: | chr5:82860025 |
| number of SNPs plotted: | 3392 |
| min P.value: | 4.76E−15 [chr5:82860025] |
| max P.value: | 10E−1 [chr5:82521618] |
| omitted GWAS Hits: | NA, NA |

Make more plots at http://csg.sph.umich.edu/locuszoom/

GWAS Catalog SNPs in Region

| chr | pos (Mb) | trait | snp |
|---|---|---|---|
| 5 | 82.81912 | Laryngeal squamous cell carcinoma | rs310518 |
| 5 | 82.84549 | Diisocyanate–induced asthma | rs3852186 |
| 5 | 82.88991 | Major depressive disorder | rs310501 |
| 5 | 82.96073 | Visceral fat | rs3846635 |
| 5 | 82.98574 | Prostate cancer | rs4466137 |
| 5 | 83.17359 | Ankylosing spondylitis | rs4552569 |

# TBSS_ICVF_Middle_cerebellar_peduncle

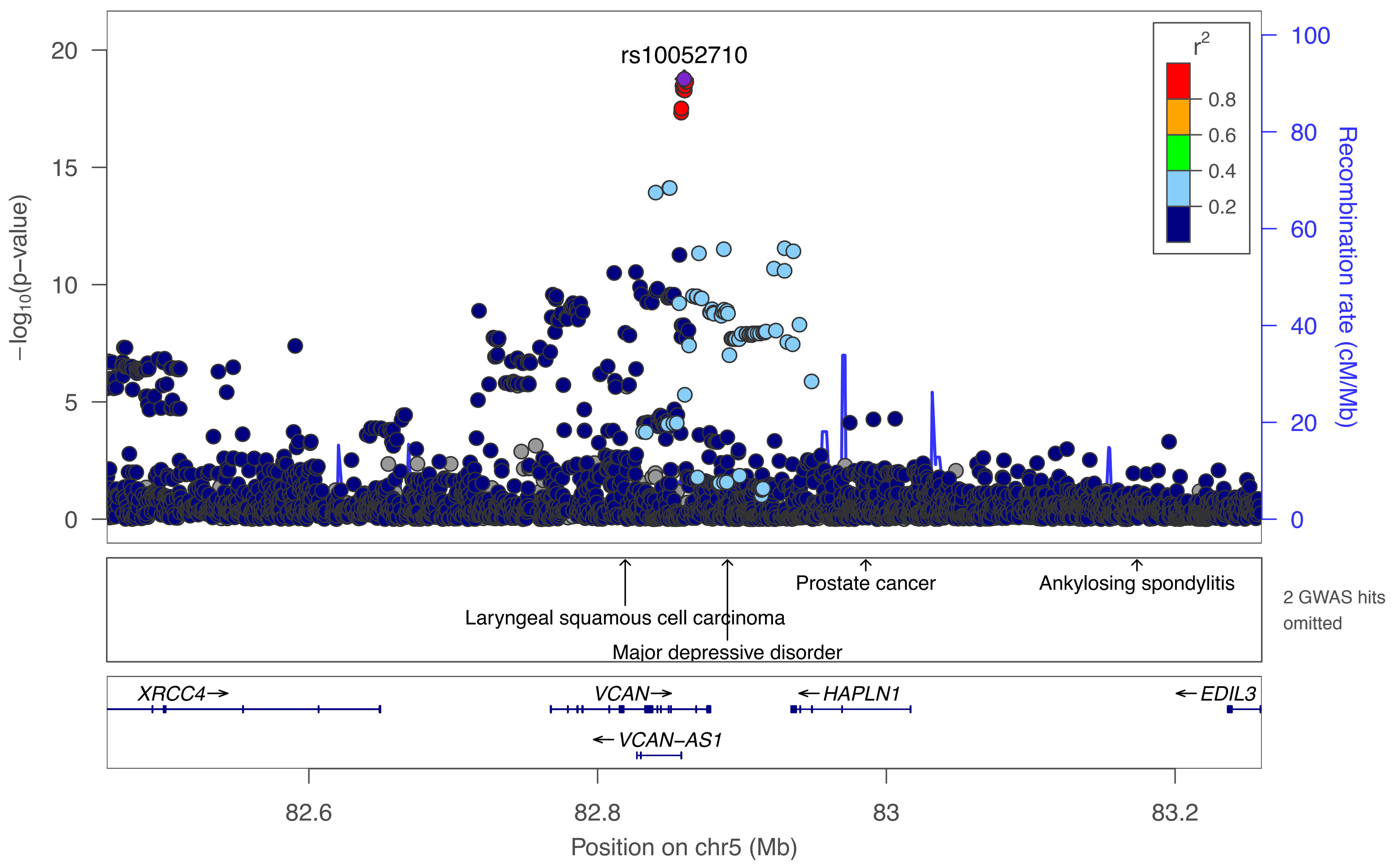

| | |
|---|---|
| date: | Thu Aug 17 17:52:01 2017 |
| build: | hg19 |
| display range: | chr5:82460025–83260025 [82460025–83260025] |
| hilite range: | 0 – 0  [ 0 – 0 ] |
| reference SNP: | chr5:82860025 |
| number of SNPs plotted: | 3392 |
| min P.value: | 1.71E–19 [chr5:82860025] |
| max P.value: | 9.99E–1 [chr5:82907161] |
| omitted GWAS Hits: | NA, NA |

Make more plots at http://csg.sph.umich.edu/locuszoom/

GWAS Catalog SNPs in Region

| chr | pos (Mb) | trait | snp |
| --- | --- | --- | --- |
| 5 | 82.81912 | Laryngeal squamous cell carcinoma | rs310518 |
| 5 | 82.84549 | Diisocyanate–induced asthma | rs3852186 |
| 5 | 82.88991 | Major depressive disorder | rs310501 |
| 5 | 82.96073 | Visceral fat | rs3846635 |
| 5 | 82.98574 | Prostate cancer | rs4466137 |
| 5 | 83.17359 | Ankylosing spondylitis | rs4552569 |

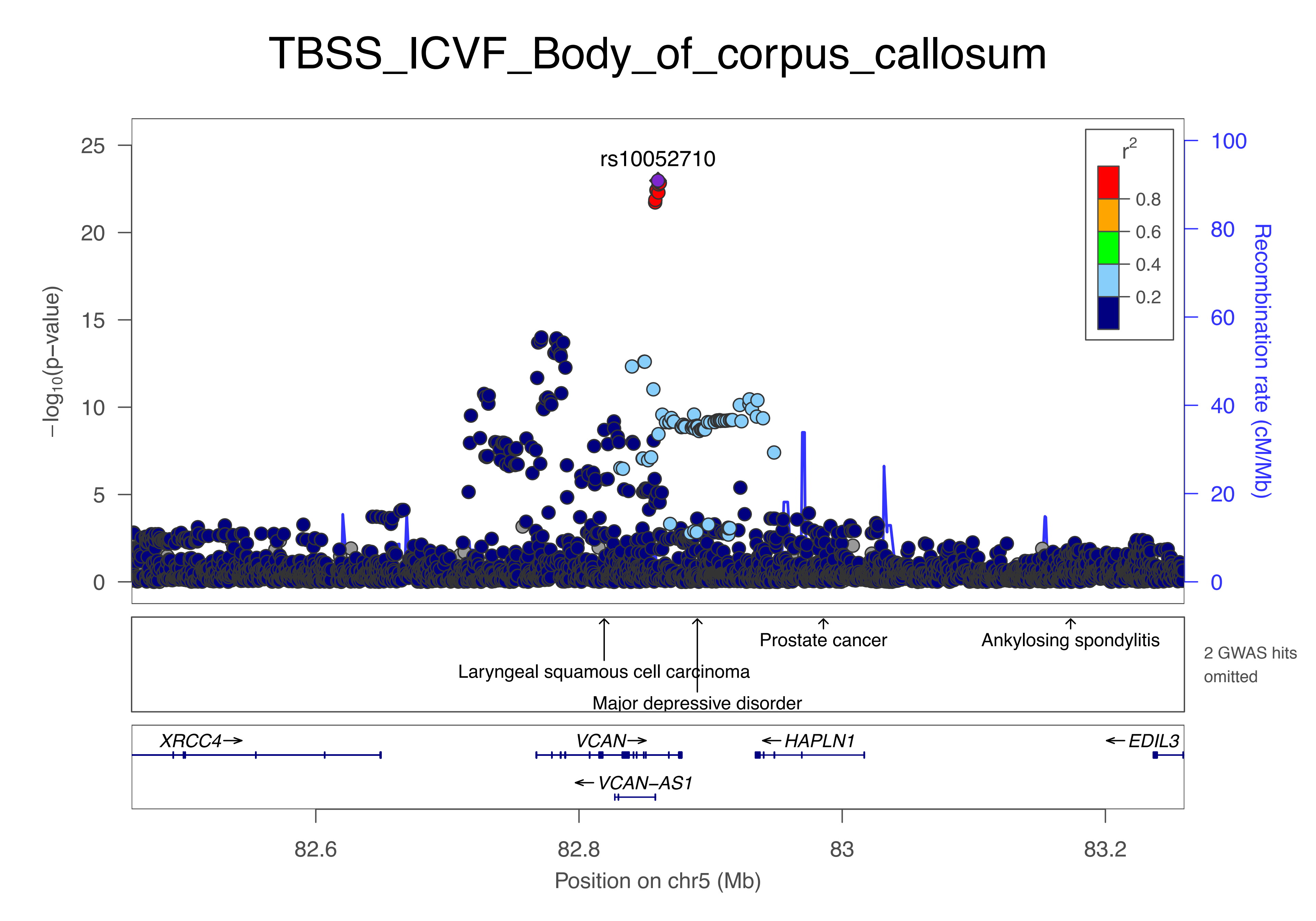

TBSS_ICVF_Body_of_corpus_callosum
rs10052710
25
20
15
10
5
0
−log10(p−value)
100
80
60
40
20
0
Recombination rate (cM/Mb)
r2
0.8
0.6
0.4
0.2
Prostate cancer
Ankylosing spondylitis
Laryngeal squamous cell carcinoma
Major depressive disorder
2 GWAS hits omitted
XRCC4→
VCAN→
←HAPLN1
←EDIL3
←VCAN−AS1
82.6
82.8
83
83.2
Position on chr5 (Mb)

| | |
|---|---|
| date: | Thu Aug 17 17:52:01 2017 |
| build: | hg19 |
| display range: | chr5:82460025–83260025 [82460025–83260025] |
| hilite range: | 0 – 0 [ 0 – 0 ] |
| reference SNP: | chr5:82860025 |
| number of SNPs plotted: | 3392 |
| min P.value: | 1.06E–23 [chr5:82860025] |
| max P.value: | 10E–1 [chr5:82477097] |
| omitted GWAS Hits: | NA, NA |

Make more plots at http://csg.sph.umich.edu/locuszoom/

GWAS Catalog SNPs in Region

| chr | pos (Mb) | trait | snp |
|---|---|---|---|
| 5 | 82.81912 | Laryngeal squamous cell carcinoma | rs310518 |
| 5 | 82.84549 | Diisocyanate–induced asthma | rs3852186 |
| 5 | 82.88991 | Major depressive disorder | rs310501 |
| 5 | 82.96073 | Visceral fat | rs3846635 |
| 5 | 82.98574 | Prostate cancer | rs4466137 |
| 5 | 83.17359 | Ankylosing spondylitis | rs4552569 |

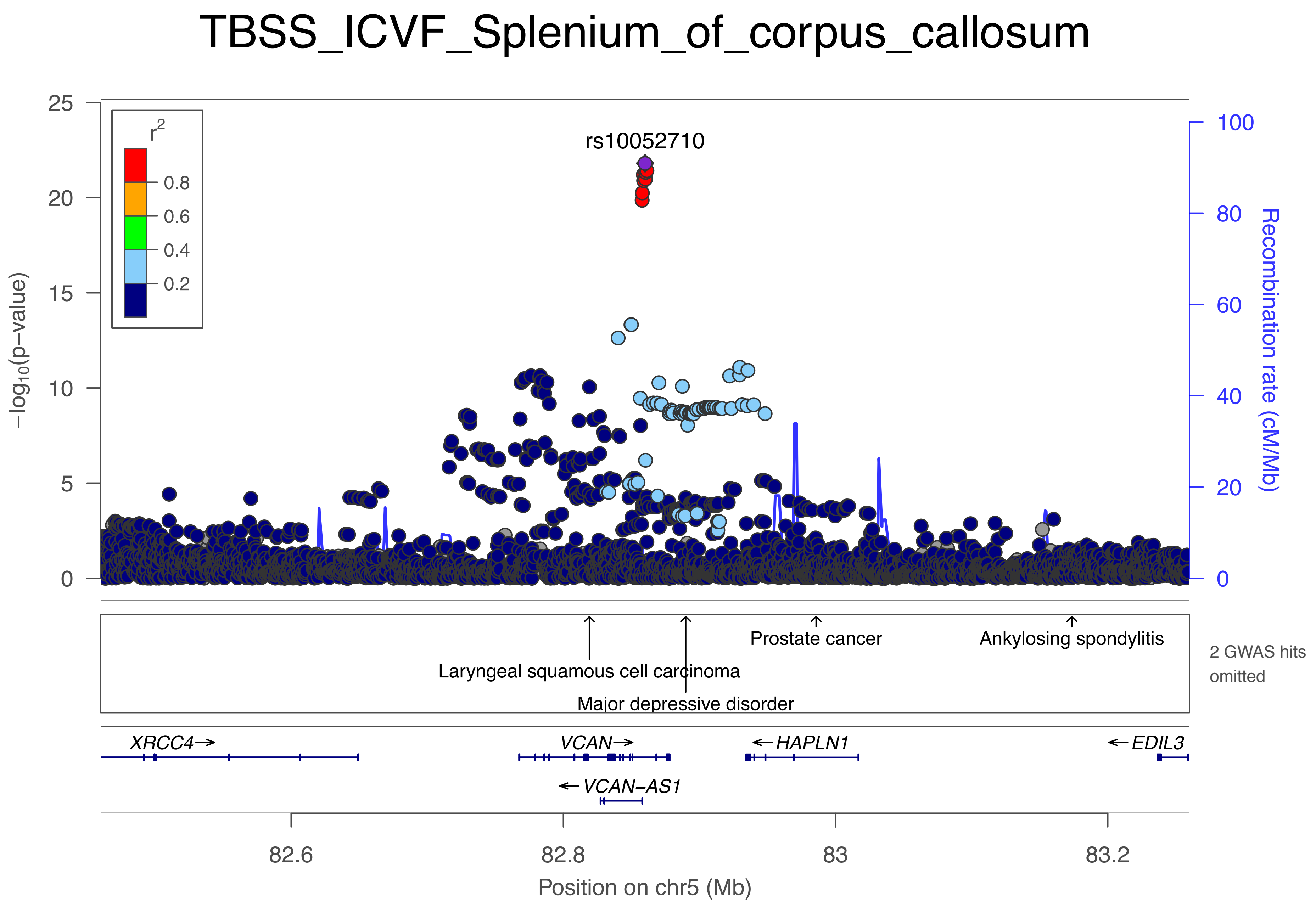
TBSS_ICVF_Splenium_of_corpus_callosum
rs10052710
r2
0.8
0.6
0.4
0.2
−log10(p−value)
Recombination rate (cM/Mb)
25
20
15
10
5
0
100
80
60
40
20
0
Laryngeal squamous cell carcinoma
Major depressive disorder
Prostate cancer
Ankylosing spondylitis
2 GWAS hits omitted
XRCC4→
VCAN→
←HAPLN1
←EDIL3
←VCAN−AS1
82.6
82.8
83
83.2
Position on chr5 (Mb)

| | |
|---|---|
| date: | Thu Aug 17 17:52:01 2017 |
| build: | hg19 |
| display range: | chr5:82460025–83260025 [82460025–83260025] |
| hilite range: | 0 – 0 [ 0 – 0 ] |
| reference SNP: | chr5:82860025 |
| number of SNPs plotted: | 3392 |
| min P.value: | 1.57E–22 [chr5:82860025] |
| max P.value: | 9.99E–1 [chr5:83072612] |
| omitted GWAS Hits: | NA, NA |

Make more plots at http://csg.sph.umich.edu/locuszoom/

GWAS Catalog SNPs in Region

| chr | pos (Mb) | trait | snp |
|---|---|---|---|
| 5 | 82.81912 | Laryngeal squamous cell carcinoma | rs310518 |
| 5 | 82.84549 | Diisocyanate–induced asthma | rs3852186 |
| 5 | 82.88991 | Major depressive disorder | rs310501 |
| 5 | 82.96073 | Visceral fat | rs3846635 |
| 5 | 82.98574 | Prostate cancer | rs4466137 |
| 5 | 83.17359 | Ankylosing spondylitis | rs4552569 |

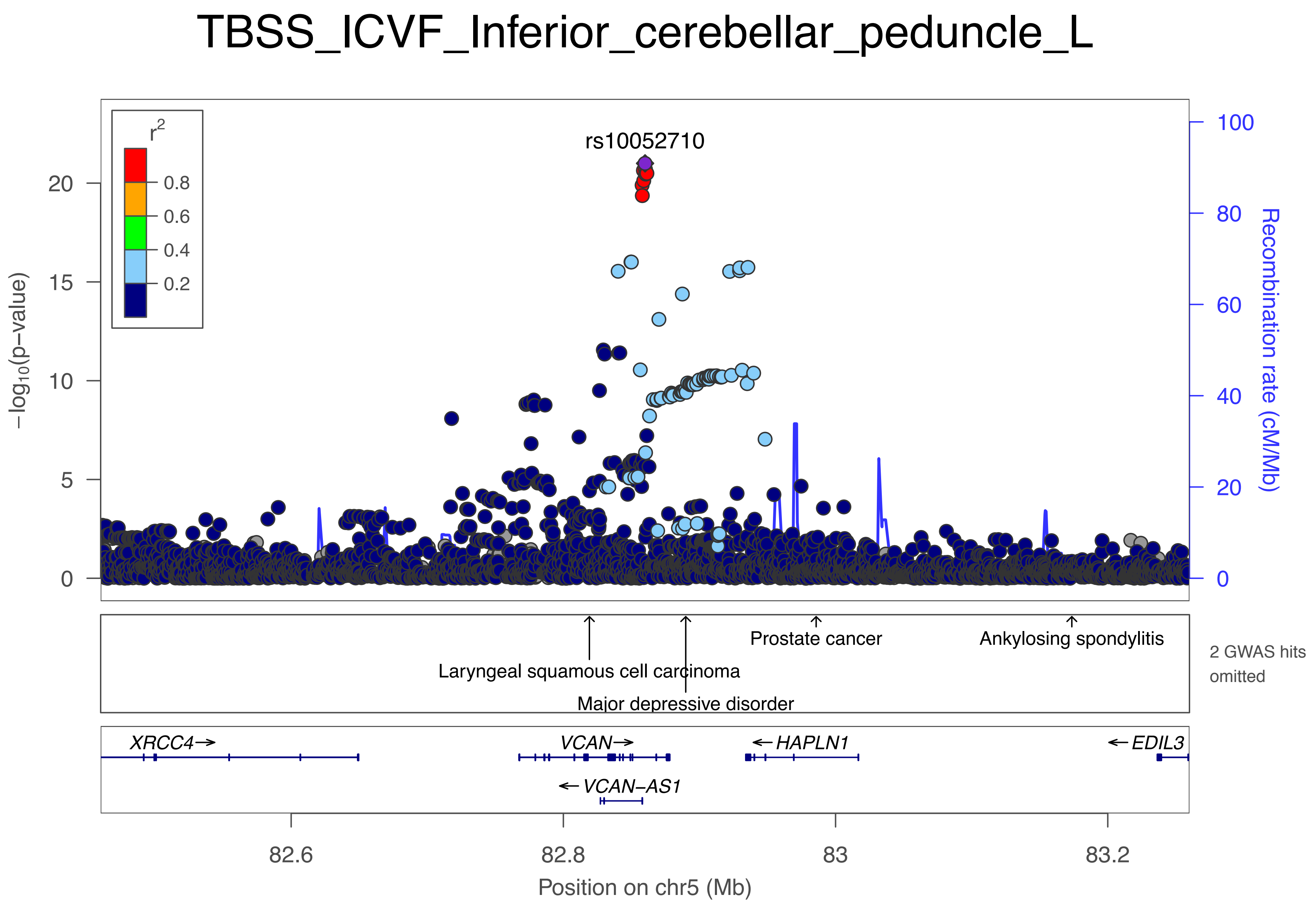
TBSS_ICVF_Inferior_cerebellar_peduncle_L
rs10052710
r2
0.8
0.6
0.4
0.2
−log10(p−value)
Recombination rate (cM/Mb)
Prostate cancer
Ankylosing spondylitis
Laryngeal squamous cell carcinoma
Major depressive disorder
2 GWAS hits omitted
XRCC4
VCAN
HAPLN1
EDIL3
VCAN−AS1
82.6
82.8
83
83.2
Position on chr5 (Mb)

| | |
|---|---|
| date: | Thu Aug 17 17:52:01 2017 |
| build: | hg19 |
| display range: | chr5:82460025−83260025 [82460025−83260025] |
| hilite range: | 0 – 0 [ 0 – 0 ] |
| reference SNP: | chr5:82860025 |
| number of SNPs plotted: | 3392 |
| min P.value: | 1E−21 [chr5:82860025] |
| max P.value: | 10E−1 [chr5:82568930] |
| omitted GWAS Hits: | NA, NA |

Make more plots at http://csg.sph.umich.edu/locuszoom/

GWAS Catalog SNPs in Region

| chr | pos (Mb) | trait | snp |
|---|---|---|---|
| 5 | 82.81912 | Laryngeal squamous cell carcinoma | rs310518 |
| 5 | 82.84549 | Diisocyanate–induced asthma | rs3852186 |
| 5 | 82.88991 | Major depressive disorder | rs310501 |
| 5 | 82.96073 | Visceral fat | rs3846635 |
| 5 | 82.98574 | Prostate cancer | rs4466137 |
| 5 | 83.17359 | Ankylosing spondylitis | rs4552569 |

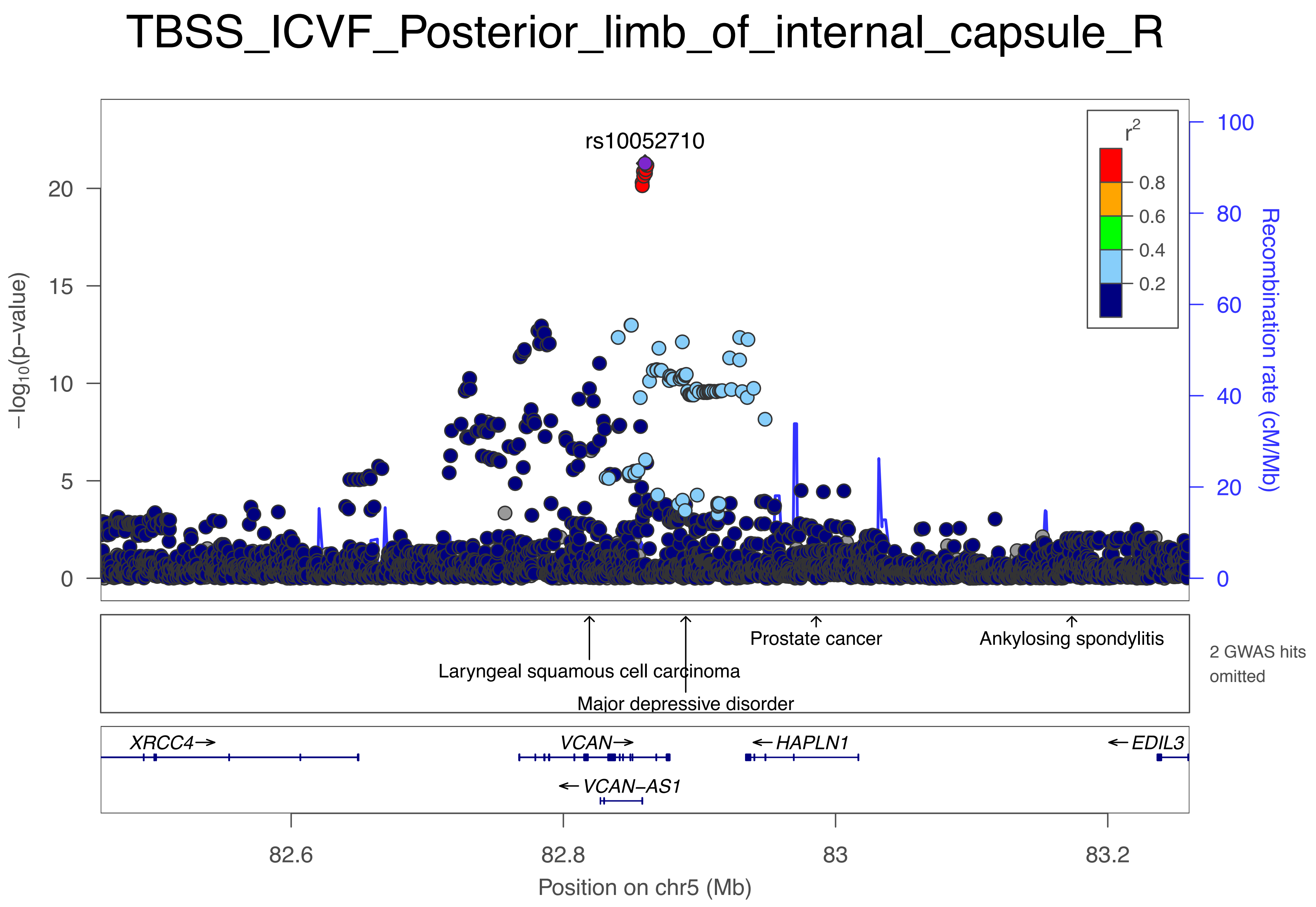
TBSS_ICVF_Posterior_limb_of_internal_capsule_R
rs10052710
r²
0.8
0.6
0.4
0.2
−log10(p−value)
0
5
10
15
20
Recombination rate (cM/Mb)
0
20
40
60
80
100
Laryngeal squamous cell carcinoma
Major depressive disorder
Prostate cancer
Ankylosing spondylitis
2 GWAS hits omitted
XRCC4
VCAN
VCAN−AS1
HAPLN1
EDIL3
82.6
82.8
83
83.2
Position on chr5 (Mb)

| | |
|---|---|
| date: | Thu Aug 17 17:52:01 2017 |
| build: | hg19 |
| display range: | chr5:82460025–83260025 [82460025–83260025] |
| hilite range: | 0 – 0 [ 0 – 0 ] |
| reference SNP: | chr5:82860025 |
| number of SNPs plotted: | 3392 |
| min P.value: | 5.21E–22 [chr5:82860025] |
| max P.value: | 9.99E–1 [chr5:83125143] |
| omitted GWAS Hits: | NA, NA |

Make more plots at http://csg.sph.umich.edu/locuszoom/

GWAS Catalog SNPs in Region

| chr | pos (Mb) | trait | snp |
|---|---|---|---|
| 5 | 82.81912 | Laryngeal squamous cell carcinoma | rs310518 |
| 5 | 82.84549 | Diisocyanate–induced asthma | rs3852186 |
| 5 | 82.88991 | Major depressive disorder | rs310501 |
| 5 | 82.96073 | Visceral fat | rs3846635 |
| 5 | 82.98574 | Prostate cancer | rs4466137 |
| 5 | 83.17359 | Ankylosing spondylitis | rs4552569 |

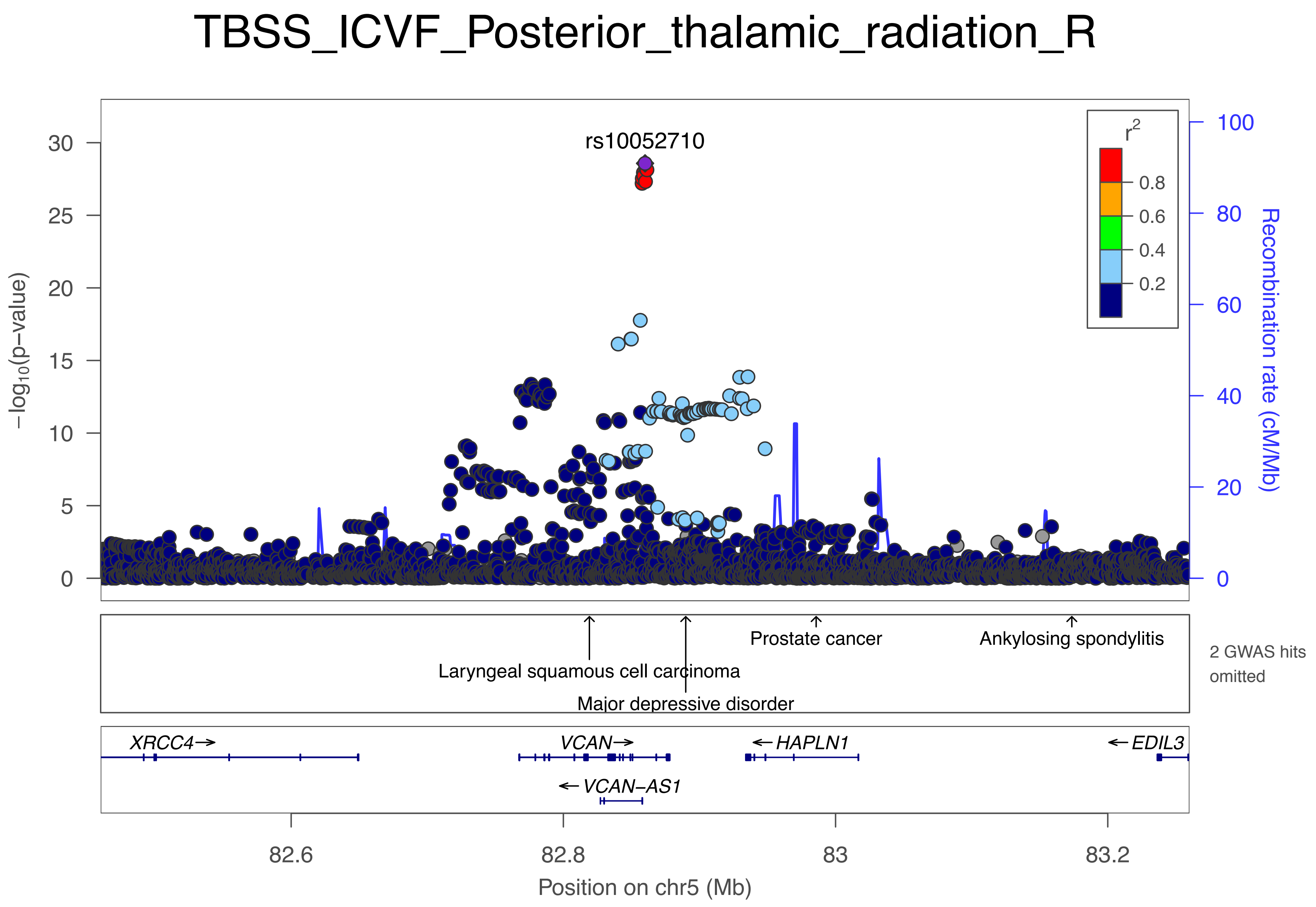
TBSS_ICVF_Posterior_thalamic_radiation_R
rs10052710
−log10(p−value)
Recombination rate (cM/Mb)
r2
0.8
0.6
0.4
0.2
Laryngeal squamous cell carcinoma
Major depressive disorder
Prostate cancer
Ankylosing spondylitis
2 GWAS hits omitted
XRCC4→
VCAN→
←VCAN−AS1
←HAPLN1
←EDIL3
82.6
82.8
83
83.2
Position on chr5 (Mb)

| | |
|---|---|
| date: | Thu Aug 17 17:52:01 2017 |
| build: | hg19 |
| display range: | chr5:82460025−83260025 [82460025−83260025] |
| hilite range: | 0 – 0  [ 0 – 0 ] |
| reference SNP: | chr5:82860025 |
| number of SNPs plotted: | 3392 |
| min P.value: | 2.65E−29 [chr5:82860025] |
| max P.value: | 10E−1 [chr5:82775906] |
| omitted GWAS Hits: | NA, NA |

Make more plots at http://csg.sph.umich.edu/locuszoom/

GWAS Catalog SNPs in Region

| chr | pos (Mb) | trait | snp |
|---|---|---|---|
| 5 | 82.81912 | Laryngeal squamous cell carcinoma | rs310518 |
| 5 | 82.84549 | Diisocyanate–induced asthma | rs3852186 |
| 5 | 82.88991 | Major depressive disorder | rs310501 |
| 5 | 82.96073 | Visceral fat | rs3846635 |
| 5 | 82.98574 | Prostate cancer | rs4466137 |
| 5 | 83.17359 | Ankylosing spondylitis | rs4552569 |

# TBSS_ICVF_Posterior_thalamic_radiation_L

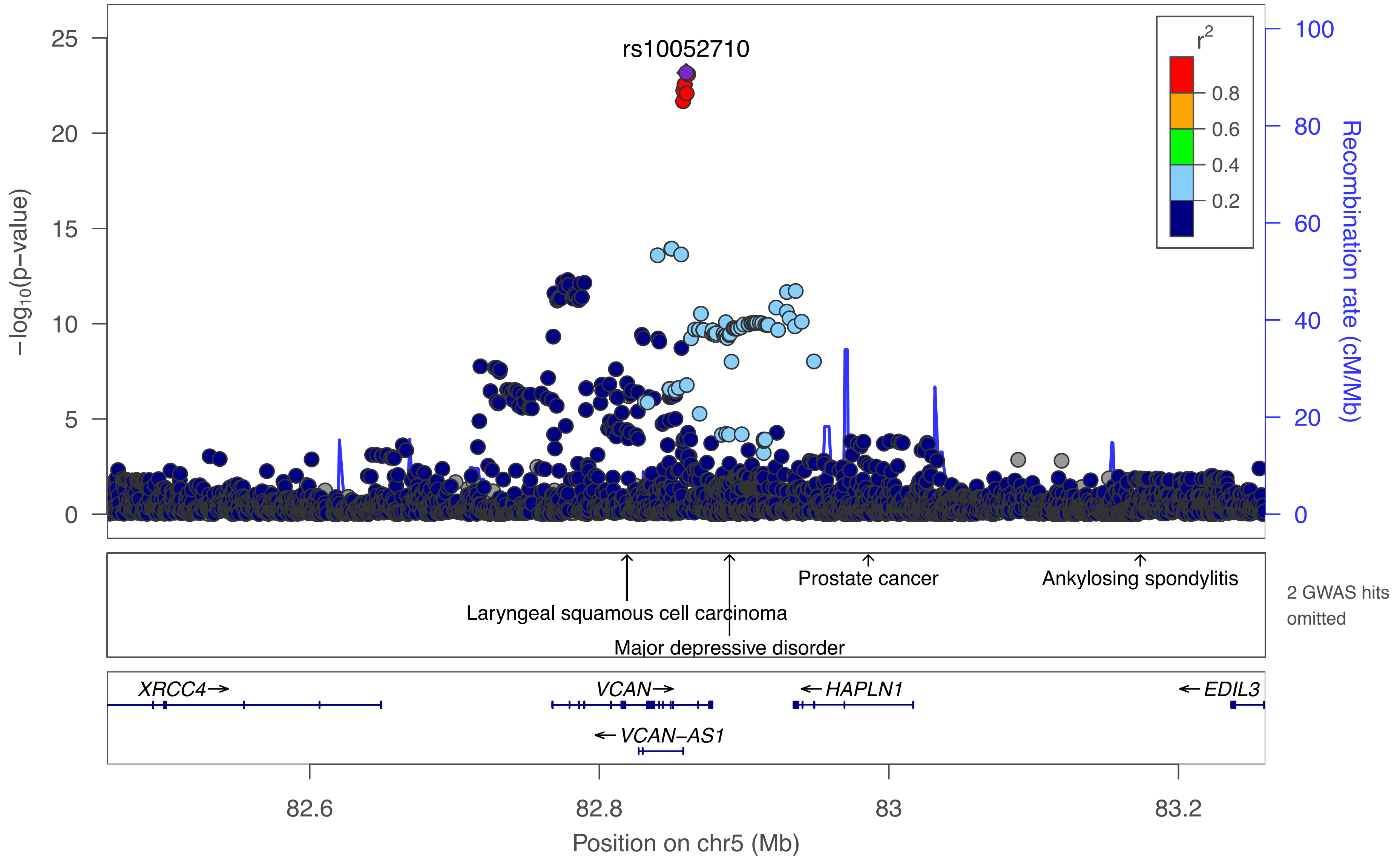

| | |
|---|---|
| date: | Thu Aug 17 17:52:01 2017 |
| build: | hg19 |
| display range: | chr5:82460025−83260025 [82460025−83260025] |
| hilite range: | 0 – 0 [ 0 – 0 ] |
| reference SNP: | chr5:82860025 |
| number of SNPs plotted: | 3392 |
| min P.value: | 6.75E−24 [chr5:82860025] |
| max P.value: | 10E−1 [chr5:82640336] |
| omitted GWAS Hits: | NA, NA |

Make more plots at http://csg.sph.umich.edu/locuszoom/

GWAS Catalog SNPs in Region

| chr | pos (Mb) | trait | snp |
|---|---|---|---|
| 5 | 82.81912 | Laryngeal squamous cell carcinoma | rs310518 |
| 5 | 82.84549 | Diisocyanate−induced asthma | rs3852186 |
| 5 | 82.88991 | Major depressive disorder | rs310501 |
| 5 | 82.96073 | Visceral fat | rs3846635 |
| 5 | 82.98574 | Prostate cancer | rs4466137 |
| 5 | 83.17359 | Ankylosing spondylitis | rs4552569 |

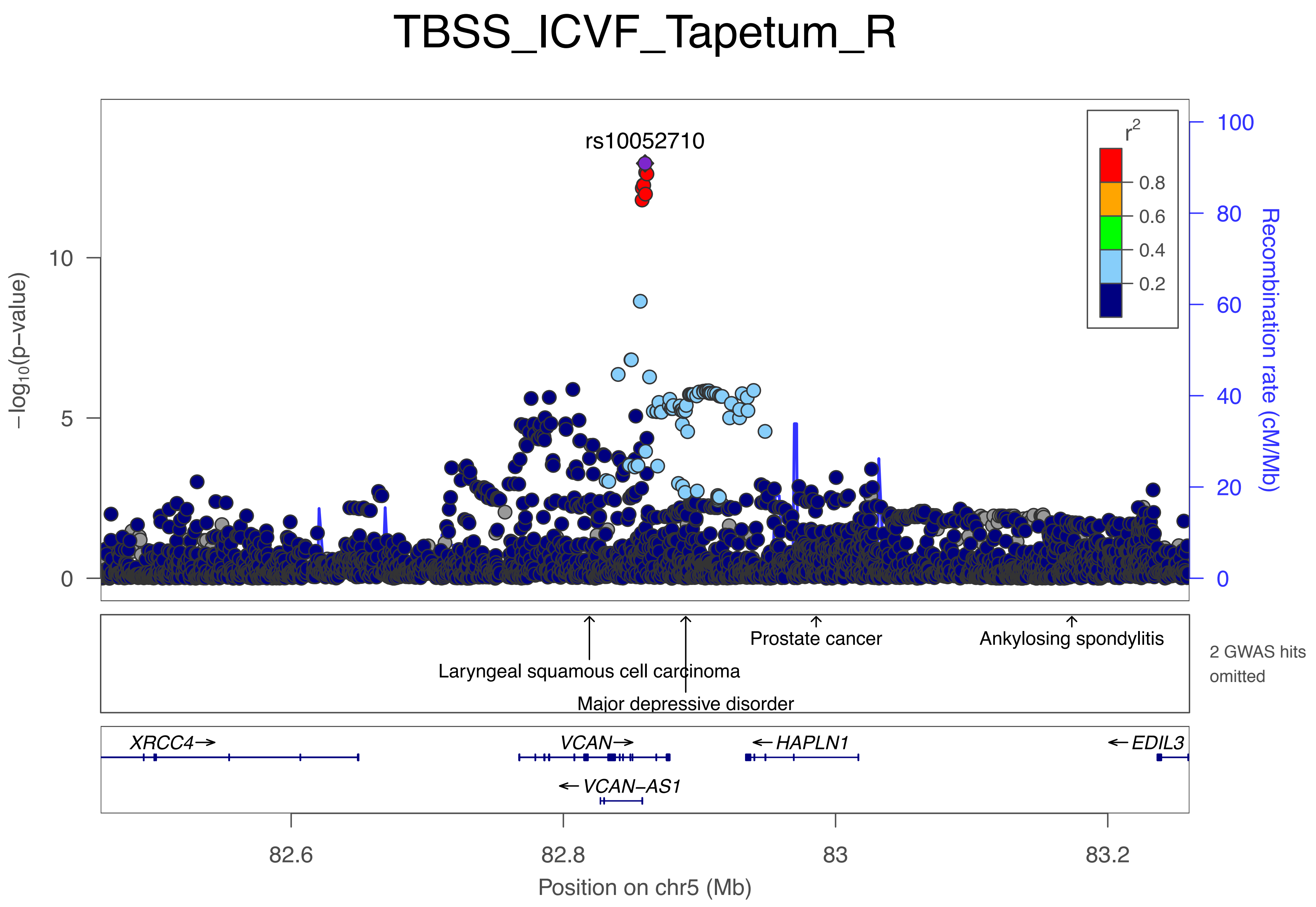
TBSS_ICVF_Tapetum_R
rs10052710
r²
0.8
0.6
0.4
0.2
100
80
60
40
20
0
Recombination rate (cM/Mb)
−log₁₀(p−value)
10
5
0
Laryngeal squamous cell carcinoma
Major depressive disorder
Prostate cancer
Ankylosing spondylitis
2 GWAS hits omitted
XRCC4→
VCAN→
←HAPLN1
←EDIL3
←VCAN−AS1
82.6
82.8
83
83.2
Position on chr5 (Mb)

| | |
|---|---|
| date: | Thu Aug 17 17:52:01 2017 |
| build: | hg19 |
| display range: | chr5:82460025−83260025 [82460025−83260025] |
| hilite range: | 0 – 0 [ 0 – 0 ] |
| reference SNP: | chr5:82860025 |
| number of SNPs plotted: | 3392 |
| min P.value: | 1.14E−13 [chr5:82860025] |
| max P.value: | 10E−1 [chr5:83136574] |
| omitted GWAS Hits: | NA, NA |

Make more plots at http://csg.sph.umich.edu/locuszoom/

GWAS Catalog SNPs in Region

| chr | pos (Mb) | trait | snp |
|---|---|---|---|
| 5 | 82.81912 | Laryngeal squamous cell carcinoma | rs310518 |
| 5 | 82.84549 | Diisocyanate–induced asthma | rs3852186 |
| 5 | 82.88991 | Major depressive disorder | rs310501 |
| 5 | 82.96073 | Visceral fat | rs3846635 |
| 5 | 82.98574 | Prostate cancer | rs4466137 |
| 5 | 83.17359 | Ankylosing spondylitis | rs4552569 |

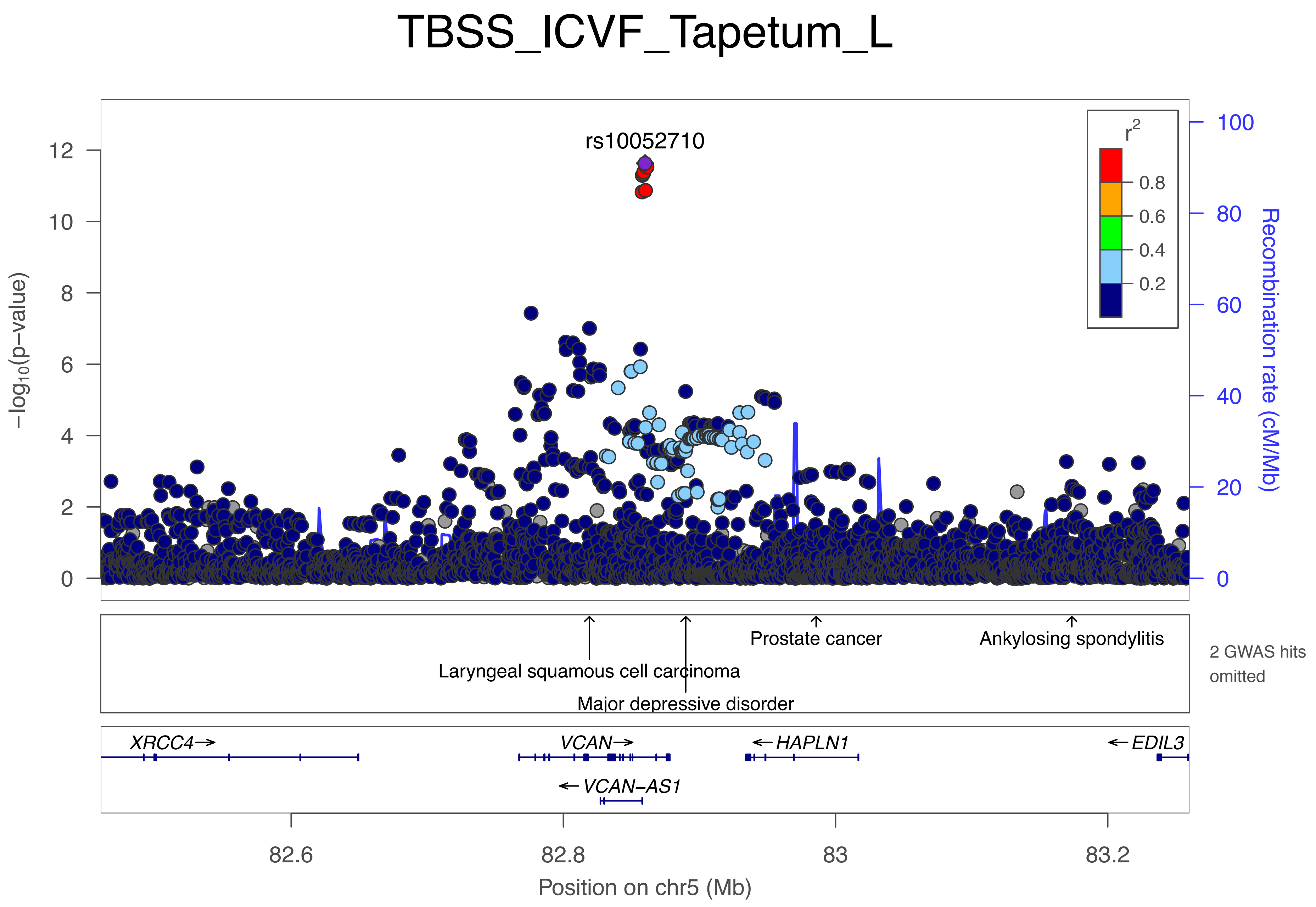
TBSS_ICVF_Tapetum_L
rs10052710
−log10(p−value)
Recombination rate (cM/Mb)
r2
0.8
0.6
0.4
0.2
Laryngeal squamous cell carcinoma
Major depressive disorder
Prostate cancer
Ankylosing spondylitis
2 GWAS hits omitted
XRCC4→
VCAN→
←VCAN−AS1
←HAPLN1
←EDIL3
82.6
82.8
83
83.2
Position on chr5 (Mb)

| | |
|---|---|
| date: | Thu Aug 17 17:52:01 2017 |
| build: | hg19 |
| display range: | chr5:82460025−83260025 [82460025−83260025] |
| hilite range: | 0 – 0 [ 0 – 0 ] |
| reference SNP: | chr5:82860025 |
| number of SNPs plotted: | 3392 |
| min P.value: | 2.35E−12 [chr5:82860025] |
| max P.value: | 10E−1 [chr5:82885093] |
| omitted GWAS Hits: | NA, NA |

Make more plots at http://csg.sph.umich.edu/locuszoom/

GWAS Catalog SNPs in Region

| chr | pos (Mb) | trait | snp |
|---|---|---|---|
| 5 | 82.81912 | Laryngeal squamous cell carcinoma | rs310518 |
| 5 | 82.84549 | Diisocyanate–induced asthma | rs3852186 |
| 5 | 82.88991 | Major depressive disorder | rs310501 |
| 5 | 82.96073 | Visceral fat | rs3846635 |
| 5 | 82.98574 | Prostate cancer | rs4466137 |
| 5 | 83.17359 | Ankylosing spondylitis | rs4552569 |

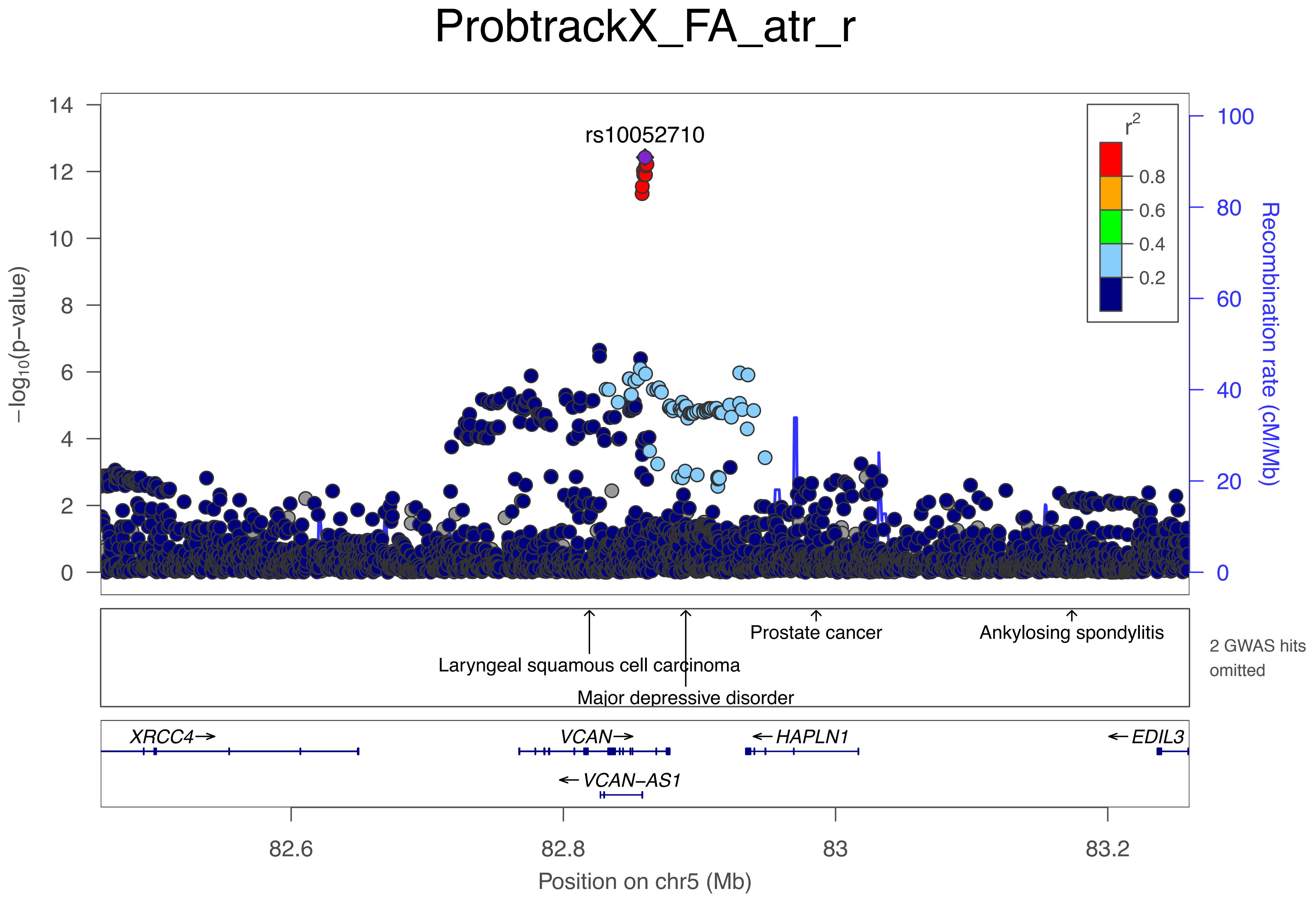

ProbtrackX_FA_atr_r
rs10052710
r2
0.8
0.6
0.4
0.2
14
12
10
8
6
4
2
0
−log10(p−value)
100
80
60
40
20
0
Recombination rate (cM/Mb)
Prostate cancer
Ankylosing spondylitis
Laryngeal squamous cell carcinoma
Major depressive disorder
2 GWAS hits omitted
XRCC4→
VCAN→
← HAPLN1
← EDIL3
← VCAN−AS1
82.6
82.8
83
83.2
Position on chr5 (Mb)

| | |
|---|---|
| date: | Thu Aug 17 17:52:01 2017 |
| build: | hg19 |
| display range: | chr5:82460025−83260025 [82460025−83260025] |
| hilite range: | 0 – 0 [ 0 – 0 ] |
| reference SNP: | chr5:82860025 |
| number of SNPs plotted: | 3392 |
| min P.value: | 3.76E−13 [chr5:82860025] |
| max P.value: | 10E−1 [chr5:82831442] |
| omitted GWAS Hits: | NA, NA |

Make more plots at http://csg.sph.umich.edu/locuszoom/

GWAS Catalog SNPs in Region

| chr | pos (Mb) | trait | snp |
|---|---|---|---|
| 5 | 82.81912 | Laryngeal squamous cell carcinoma | rs310518 |
| 5 | 82.84549 | Diisocyanate–induced asthma | rs3852186 |
| 5 | 82.88991 | Major depressive disorder | rs310501 |
| 5 | 82.96073 | Visceral fat | rs3846635 |
| 5 | 82.98574 | Prostate cancer | rs4466137 |
| 5 | 83.17359 | Ankylosing spondylitis | rs4552569 |

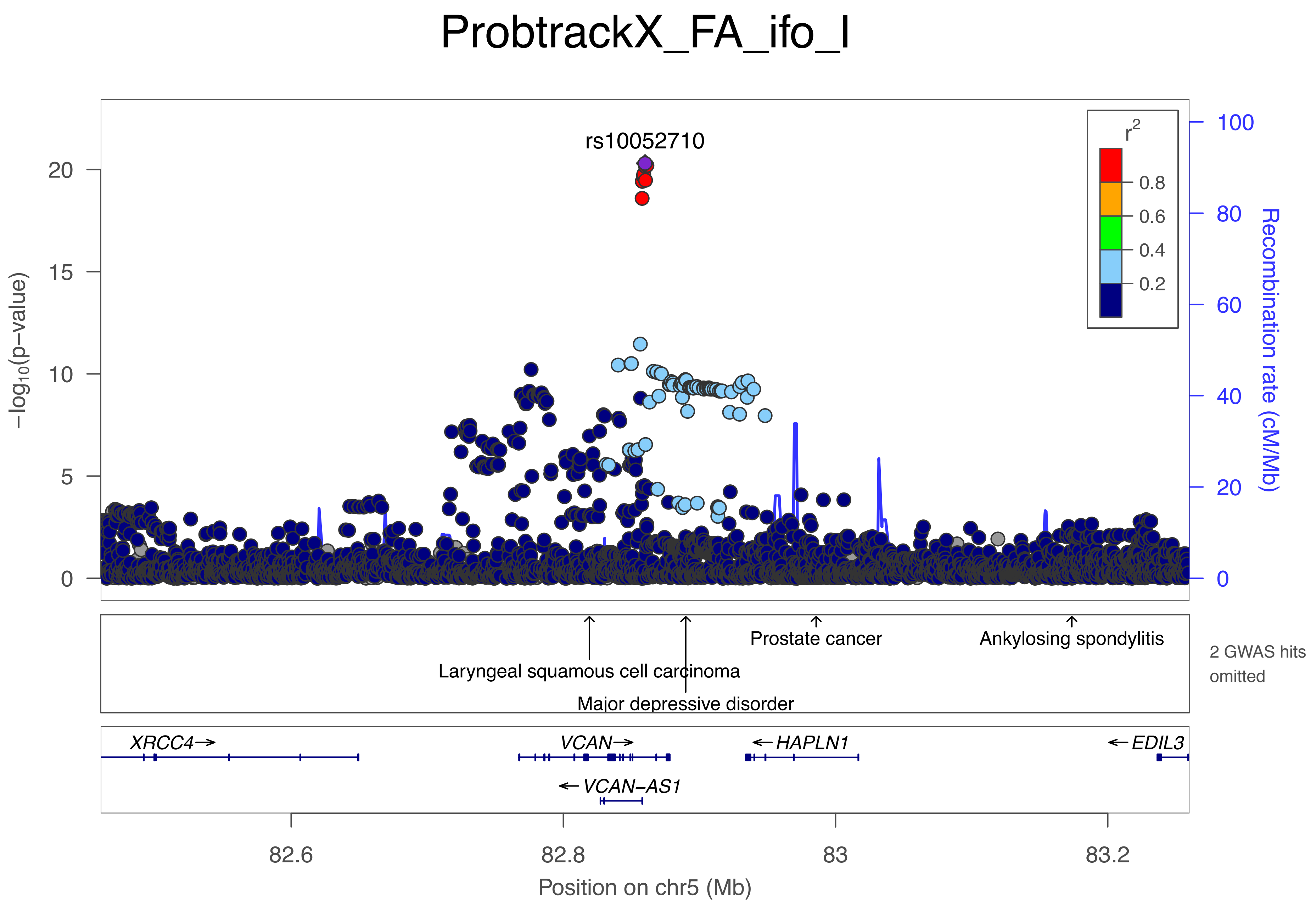
ProbtrackX_FA_ifo_l
rs10052710
−log10(p−value)
Recombination rate (cM/Mb)
r2
0.8
0.6
0.4
0.2
0
5
10
15
20
100
80
60
40
20
Prostate cancer
Ankylosing spondylitis
Laryngeal squamous cell carcinoma
Major depressive disorder
2 GWAS hits omitted
XRCC4→
VCAN→
←HAPLN1
←EDIL3
←VCAN−AS1
82.6
82.8
83
83.2
Position on chr5 (Mb)

| | |
|---|---|
| date: | Thu Aug 17 17:52:01 2017 |
| build: | hg19 |
| display range: | chr5:82460025−83260025 [82460025−83260025] |
| hilite range: | 0 – 0 [ 0 – 0 ] |
| reference SNP: | chr5:82860025 |
| number of SNPs plotted: | 3392 |
| min P.value: | 5E−21 [chr5:82860025] |
| max P.value: | 10E−1 [chr5:82749099] |
| omitted GWAS Hits: | NA, NA |

Make more plots at http://csg.sph.umich.edu/locuszoom/

GWAS Catalog SNPs in Region

| chr | pos (Mb) | trait | snp |
| --- | --- | --- | --- |
| 5 | 82.81912 | Laryngeal squamous cell carcinoma | rs310518 |
| 5 | 82.84549 | Diisocyanate–induced asthma | rs3852186 |
| 5 | 82.88991 | Major depressive disorder | rs310501 |
| 5 | 82.96073 | Visceral fat | rs3846635 |
| 5 | 82.98574 | Prostate cancer | rs4466137 |
| 5 | 83.17359 | Ankylosing spondylitis | rs4552569 |

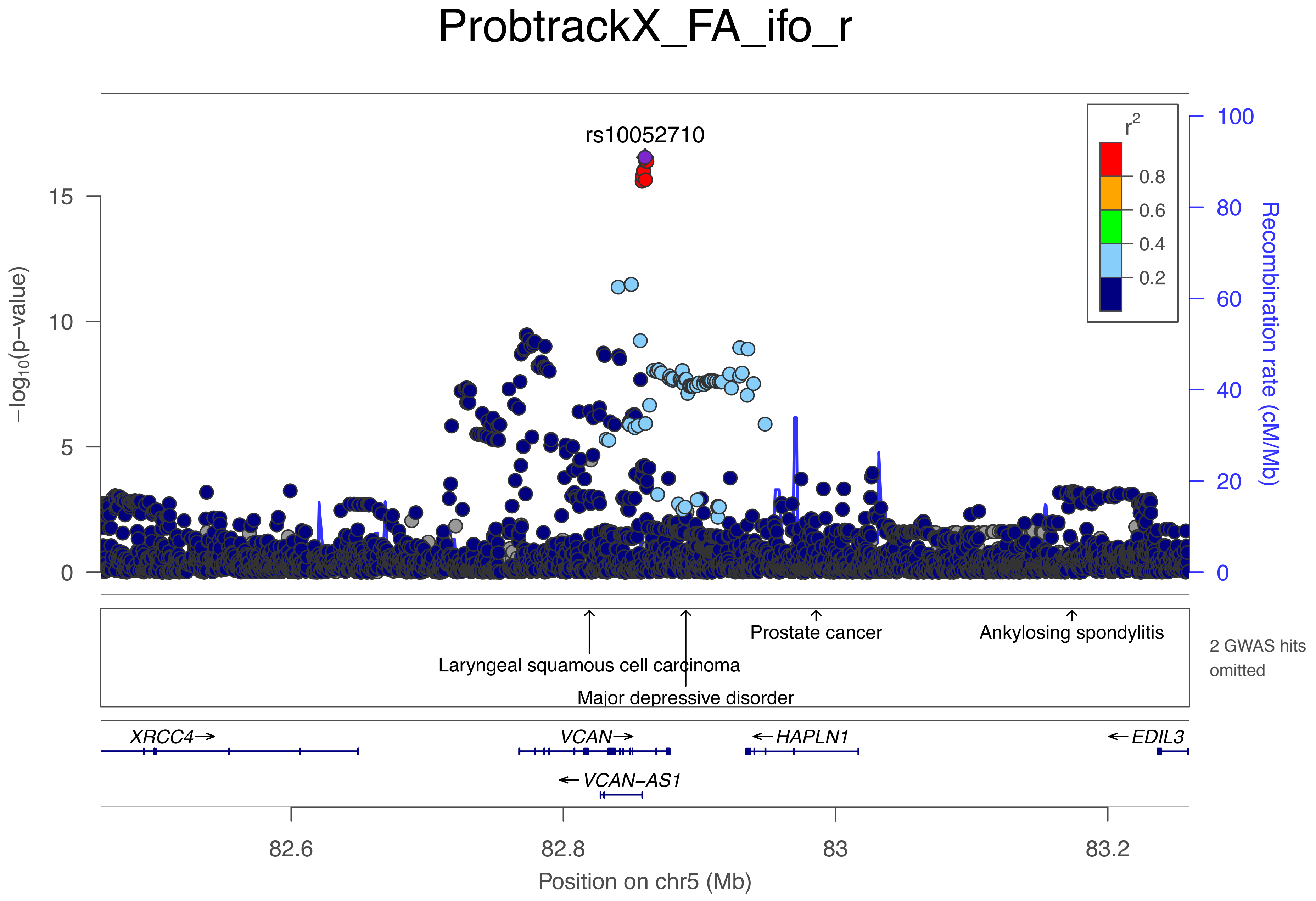

ProbtrackX_FA_ifo_r
rs10052710
−log10(p−value)
15
10
5
0
r2
0.8
0.6
0.4
0.2
100
80
60
40
20
0
Recombination rate (cM/Mb)
Prostate cancer
Ankylosing spondylitis
Laryngeal squamous cell carcinoma
Major depressive disorder
2 GWAS hits omitted
XRCC4→
VCAN→
←HAPLN1
←EDIL3
←VCAN−AS1
82.6
82.8
83
83.2
Position on chr5 (Mb)

| | |
|---|---|
| date: | Thu Aug 17 17:52:01 2017 |
| build: | hg19 |
| display range: | chr5:82460025−83260025 [82460025−83260025] |
| hilite range: | 0 – 0  [ 0 – 0 ] |
| reference SNP: | chr5:82860025 |
| number of SNPs plotted: | 3392 |
| min P.value: | 2.88E−17 [chr5:82860025] |
| max P.value: | 9.99E−1 [chr5:82845489] |
| omitted GWAS Hits: | NA, NA |

Make more plots at http://csg.sph.umich.edu/locuszoom/

GWAS Catalog SNPs in Region

| chr | pos (Mb) | trait | snp |
|---|---|---|---|
| 5 | 82.81912 | Laryngeal squamous cell carcinoma | rs310518 |
| 5 | 82.84549 | Diisocyanate–induced asthma | rs3852186 |
| 5 | 82.88991 | Major depressive disorder | rs310501 |
| 5 | 82.96073 | Visceral fat | rs3846635 |
| 5 | 82.98574 | Prostate cancer | rs4466137 |
| 5 | 83.17359 | Ankylosing spondylitis | rs4552569 |

# ProbtrackX_FA_ilf_l

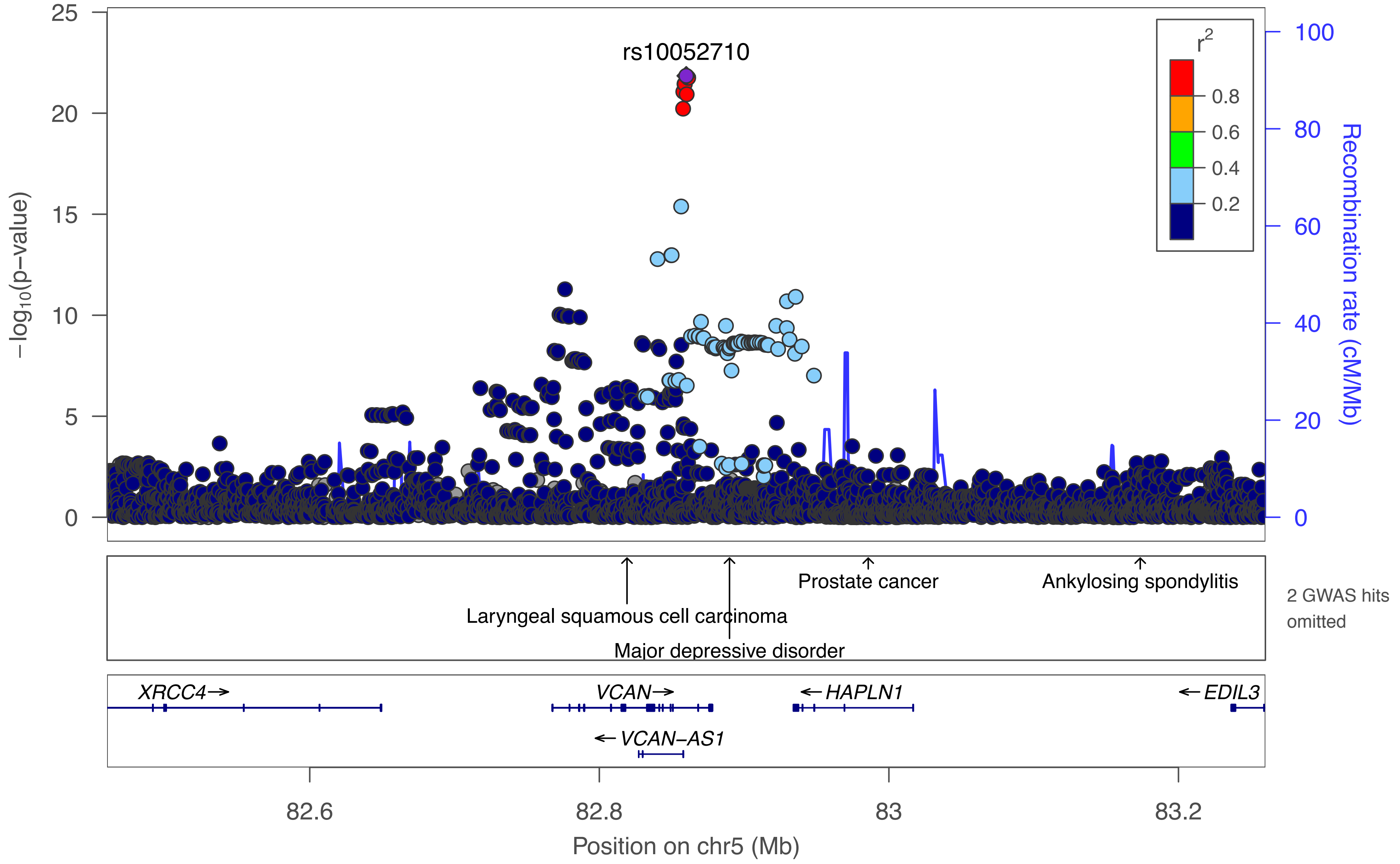

| | |
|---|---|
| date: | Thu Aug 17 17:52:01 2017 |
| build: | hg19 |
| display range: | chr5:82460025−83260025 [82460025−83260025] |
| hilite range: | 0 – 0 [ 0 – 0 ] |
| reference SNP: | chr5:82860025 |
| number of SNPs plotted: | 3392 |
| min P.value: | 1.41E−22 [chr5:82860025] |
| max P.value: | 10E−1 [chr5:83158123] |
| omitted GWAS Hits: | NA, NA |

Make more plots at http://csg.sph.umich.edu/locuszoom/

GWAS Catalog SNPs in Region

| chr | pos (Mb) | trait | snp |
| --- | --- | --- | --- |
| 5 | 82.81912 | Laryngeal squamous cell carcinoma | rs310518 |
| 5 | 82.84549 | Diisocyanate−induced asthma | rs3852186 |
| 5 | 82.88991 | Major depressive disorder | rs310501 |
| 5 | 82.96073 | Visceral fat | rs3846635 |
| 5 | 82.98574 | Prostate cancer | rs4466137 |
| 5 | 83.17359 | Ankylosing spondylitis | rs4552569 |

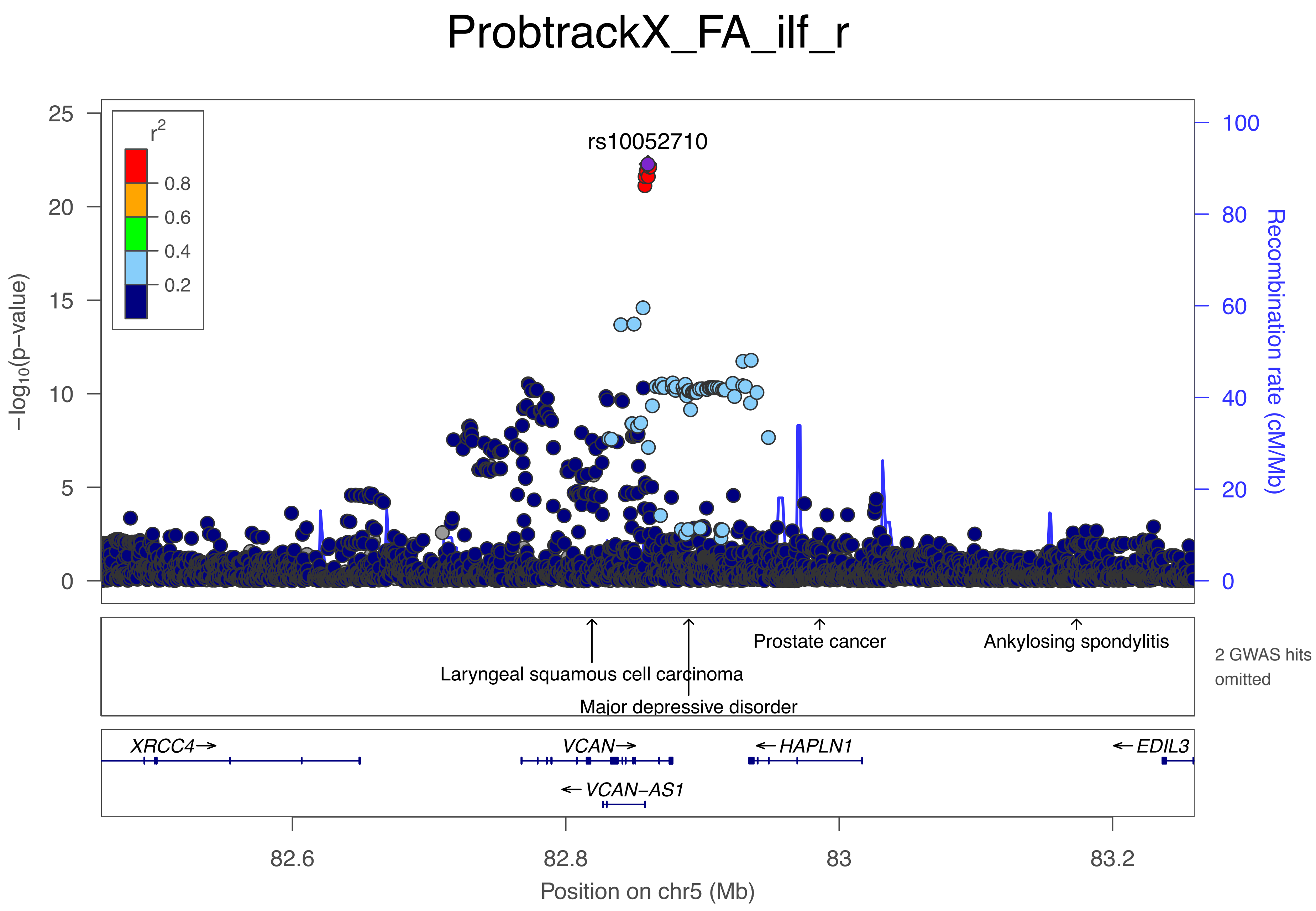
ProbtrackX_FA_ilf_r
rs10052710
r²
0.8
0.6
0.4
0.2
−log10(p−value)
Recombination rate (cM/Mb)
Laryngeal squamous cell carcinoma
Major depressive disorder
Prostate cancer
Ankylosing spondylitis
2 GWAS hits omitted
XRCC4→
VCAN→
←HAPLN1
←EDIL3
←VCAN−AS1
82.6
82.8
83
83.2
Position on chr5 (Mb)

| | |
|---|---|
| date: | Thu Aug 17 17:52:01 2017 |
| build: | hg19 |
| display range: | chr5:82460025−83260025 [82460025−83260025] |
| hilite range: | 0 – 0 [ 0 – 0 ] |
| reference SNP: | chr5:82860025 |
| number of SNPs plotted: | 3392 |
| min P.value: | 5.3E−23 [chr5:82860025] |
| max P.value: | 10E−1 [chr5:83162320] |
| omitted GWAS Hits: | NA, NA |

Make more plots at http://csg.sph.umich.edu/locuszoom/

GWAS Catalog SNPs in Region

| chr | pos (Mb) | trait | snp |
|---|---|---|---|
| 5 | 82.81912 | Laryngeal squamous cell carcinoma | rs310518 |
| 5 | 82.84549 | Diisocyanate–induced asthma | rs3852186 |
| 5 | 82.88991 | Major depressive disorder | rs310501 |
| 5 | 82.96073 | Visceral fat | rs3846635 |
| 5 | 82.98574 | Prostate cancer | rs4466137 |
| 5 | 83.17359 | Ankylosing spondylitis | rs4552569 |

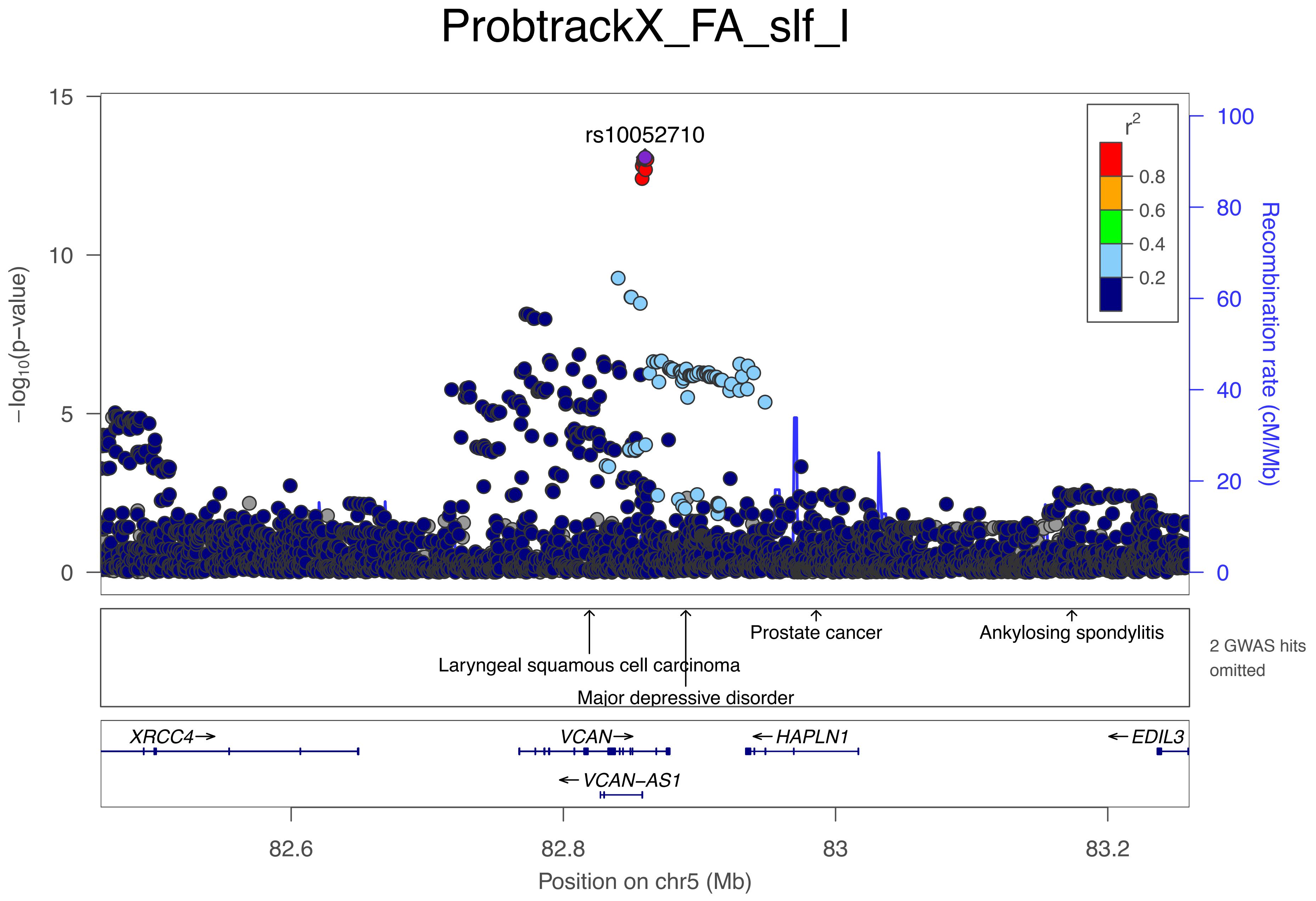

ProbtrackX_FA_slf_l
rs10052710
−log10(p−value)
Recombination rate (cM/Mb)
r2
0.8
0.6
0.4
0.2
Laryngeal squamous cell carcinoma
Major depressive disorder
Prostate cancer
Ankylosing spondylitis
2 GWAS hits omitted
XRCC4→
VCAN→
←HAPLN1
←EDIL3
←VCAN−AS1
82.6
82.8
83
83.2
Position on chr5 (Mb)

| | |
|---|---|
| date: | Thu Aug 17 17:52:01 2017 |
| build: | hg19 |
| display range: | chr5:82460025−83260025 [82460025−83260025] |
| hilite range: | 0 – 0 [ 0 – 0 ] |
| reference SNP: | chr5:82860025 |
| number of SNPs plotted: | 3392 |
| min P.value: | 8.36E−14 [chr5:82860025] |
| max P.value: | 10E−1 [chr5:82857173] |
| omitted GWAS Hits: | NA, NA |

Make more plots at http://csg.sph.umich.edu/locuszoom/

# GWAS Catalog SNPs in Region

| chr | pos (Mb) | trait | snp |
|---|---|---|---|
| 5 | 82.81912 | Laryngeal squamous cell carcinoma | rs310518 |
| 5 | 82.84549 | Diisocyanate–induced asthma | rs3852186 |
| 5 | 82.88991 | Major depressive disorder | rs310501 |
| 5 | 82.96073 | Visceral fat | rs3846635 |
| 5 | 82.98574 | Prostate cancer | rs4466137 |
| 5 | 83.17359 | Ankylosing spondylitis | rs4552569 |

# ProbtrackX_FA_slf_r

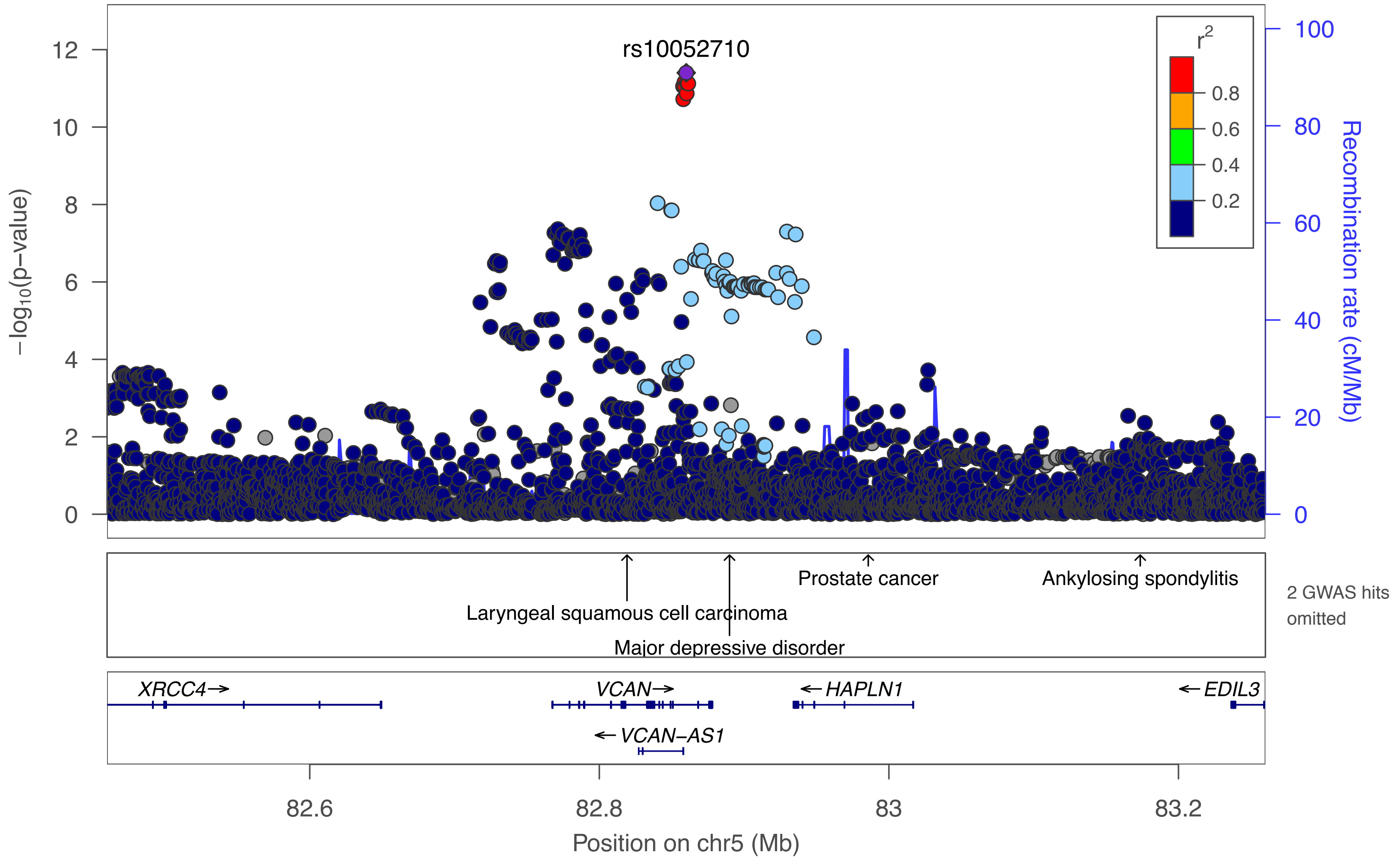

| | |
|---|---|
| date: | Thu Aug 17 17:52:01 2017 |
| build: | hg19 |
| display range: | chr5:82460025−83260025 [82460025−83260025] |
| hilite range: | 0 – 0 [ 0 – 0 ] |
| reference SNP: | chr5:82860025 |
| number of SNPs plotted: | 3392 |
| min P.value: | 3.96E−12 [chr5:82860025] |
| max P.value: | 10E−1 [chr5:82959109] |
| omitted GWAS Hits: | NA, NA |

Make more plots at http://csg.sph.umich.edu/locuszoom/

GWAS Catalog SNPs in Region

| chr | pos (Mb) | trait | snp |
|---|---|---|---|
| 5 | 82.81912 | Laryngeal squamous cell carcinoma | rs310518 |
| 5 | 82.84549 | Diisocyanate–induced asthma | rs3852186 |
| 5 | 82.88991 | Major depressive disorder | rs310501 |
| 5 | 82.96073 | Visceral fat | rs3846635 |
| 5 | 82.98574 | Prostate cancer | rs4466137 |
| 5 | 83.17359 | Ankylosing spondylitis | rs4552569 |

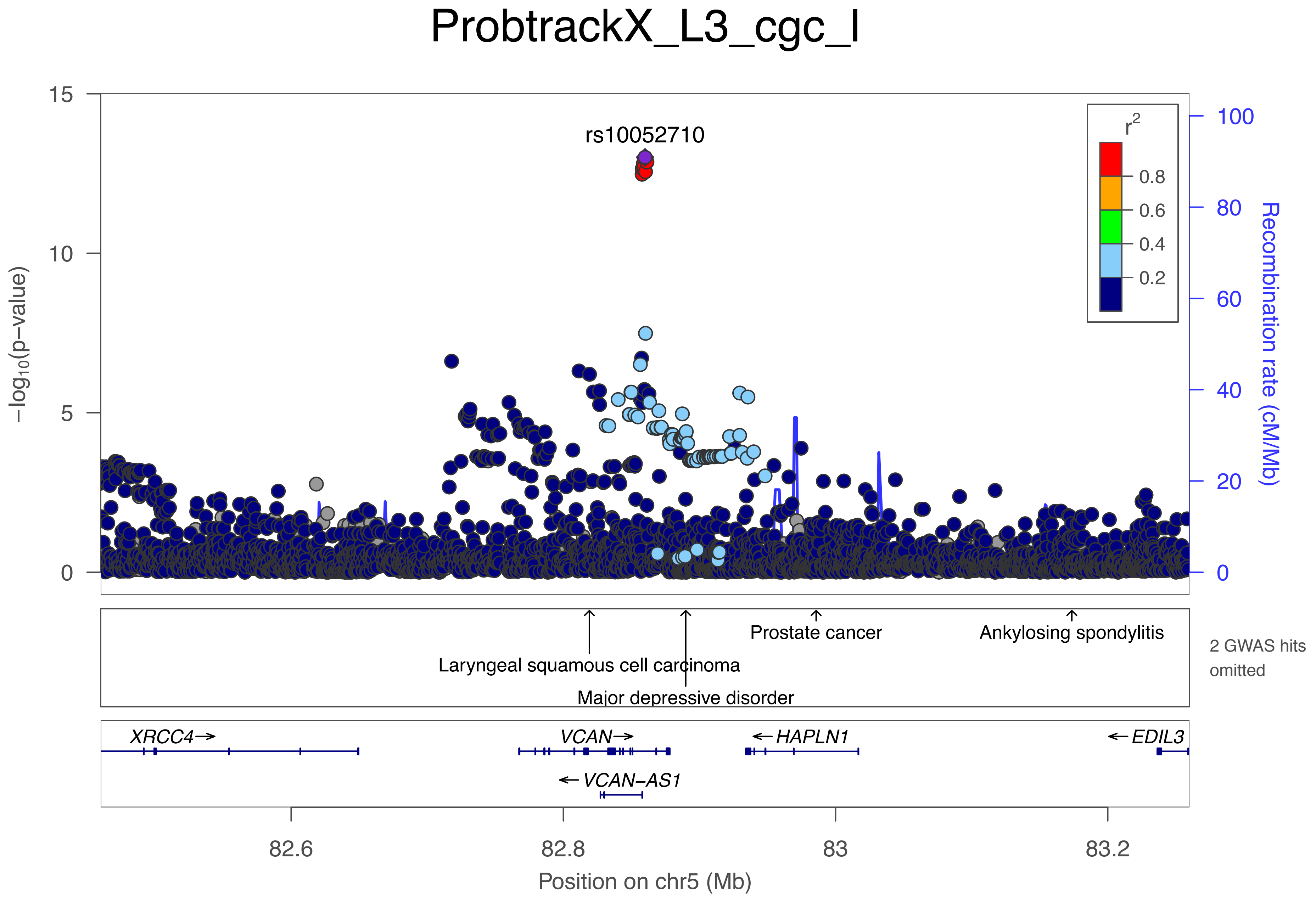

ProbtrackX_L3_cgc_l
rs10052710
r2
0.8
0.6
0.4
0.2
15
10
5
0
−log10(p−value)
100
80
60
40
20
0
Recombination rate (cM/Mb)
Laryngeal squamous cell carcinoma
Major depressive disorder
Prostate cancer
Ankylosing spondylitis
2 GWAS hits omitted
XRCC4→
VCAN→
←HAPLN1
←EDIL3
←VCAN−AS1
82.6
82.8
83
83.2
Position on chr5 (Mb)

| | |
|---|---|
| date: | Thu Aug 17 17:52:01 2017 |
| build: | hg19 |
| display range: | chr5:82460025−83260025 [82460025−83260025] |
| hilite range: | 0 – 0 [ 0 – 0 ] |
| reference SNP: | chr5:82860025 |
| number of SNPs plotted: | 3392 |
| min P.value: | 9.86E−14 [chr5:82860025] |
| max P.value: | 10E−1 [chr5:82990933] |
| omitted GWAS Hits: | NA, NA |

Make more plots at http://csg.sph.umich.edu/locuszoom/

# GWAS Catalog SNPs in Region

| chr | pos (Mb) | trait | snp |
|---|---|---|---|
| 5 | 82.81912 | Laryngeal squamous cell carcinoma | rs310518 |
| 5 | 82.84549 | Diisocyanate−induced asthma | rs3852186 |
| 5 | 82.88991 | Major depressive disorder | rs310501 |
| 5 | 82.96073 | Visceral fat | rs3846635 |
| 5 | 82.98574 | Prostate cancer | rs4466137 |
| 5 | 83.17359 | Ankylosing spondylitis | rs4552569 |

# ProbtrackX_L3_cgc_r

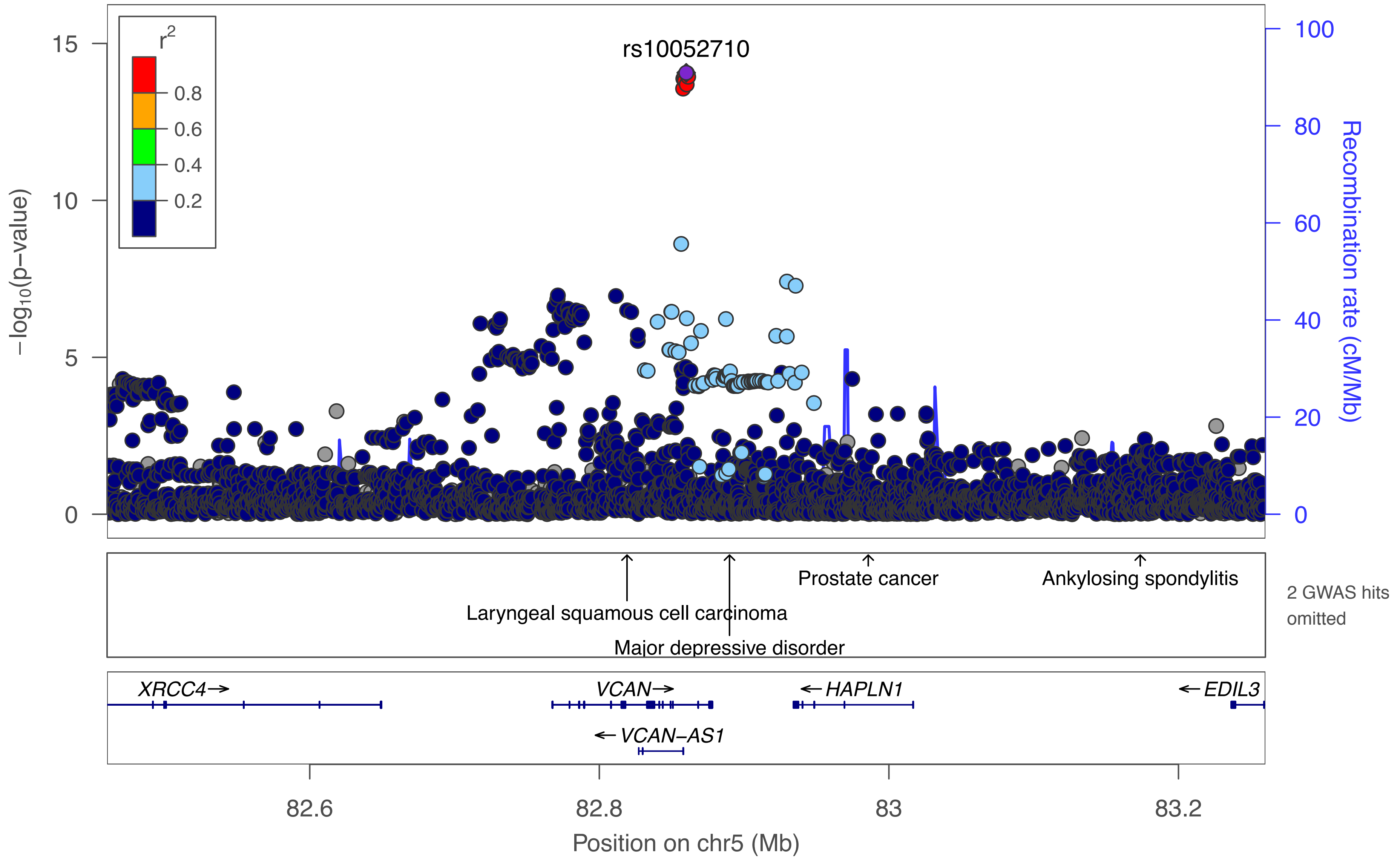

| | |
|---|---|
| date: | Thu Aug 17 17:52:01 2017 |
| build: | hg19 |
| display range: | chr5:82460025–83260025 [82460025–83260025] |
| hilite range: | 0 – 0 [ 0 – 0 ] |
| reference SNP: | chr5:82860025 |
| number of SNPs plotted: | 3392 |
| min P.value: | 8.61E–15 [chr5:82860025] |
| max P.value: | 9.99E–1 [chr5:82750346] |
| omitted GWAS Hits: | NA, NA |

Make more plots at http://csg.sph.umich.edu/locuszoom/

GWAS Catalog SNPs in Region

| chr | pos (Mb) | trait | snp |
|---|---|---|---|
| 5 | 82.81912 | Laryngeal squamous cell carcinoma | rs310518 |
| 5 | 82.84549 | Diisocyanate–induced asthma | rs3852186 |
| 5 | 82.88991 | Major depressive disorder | rs310501 |
| 5 | 82.96073 | Visceral fat | rs3846635 |
| 5 | 82.98574 | Prostate cancer | rs4466137 |
| 5 | 83.17359 | Ankylosing spondylitis | rs4552569 |

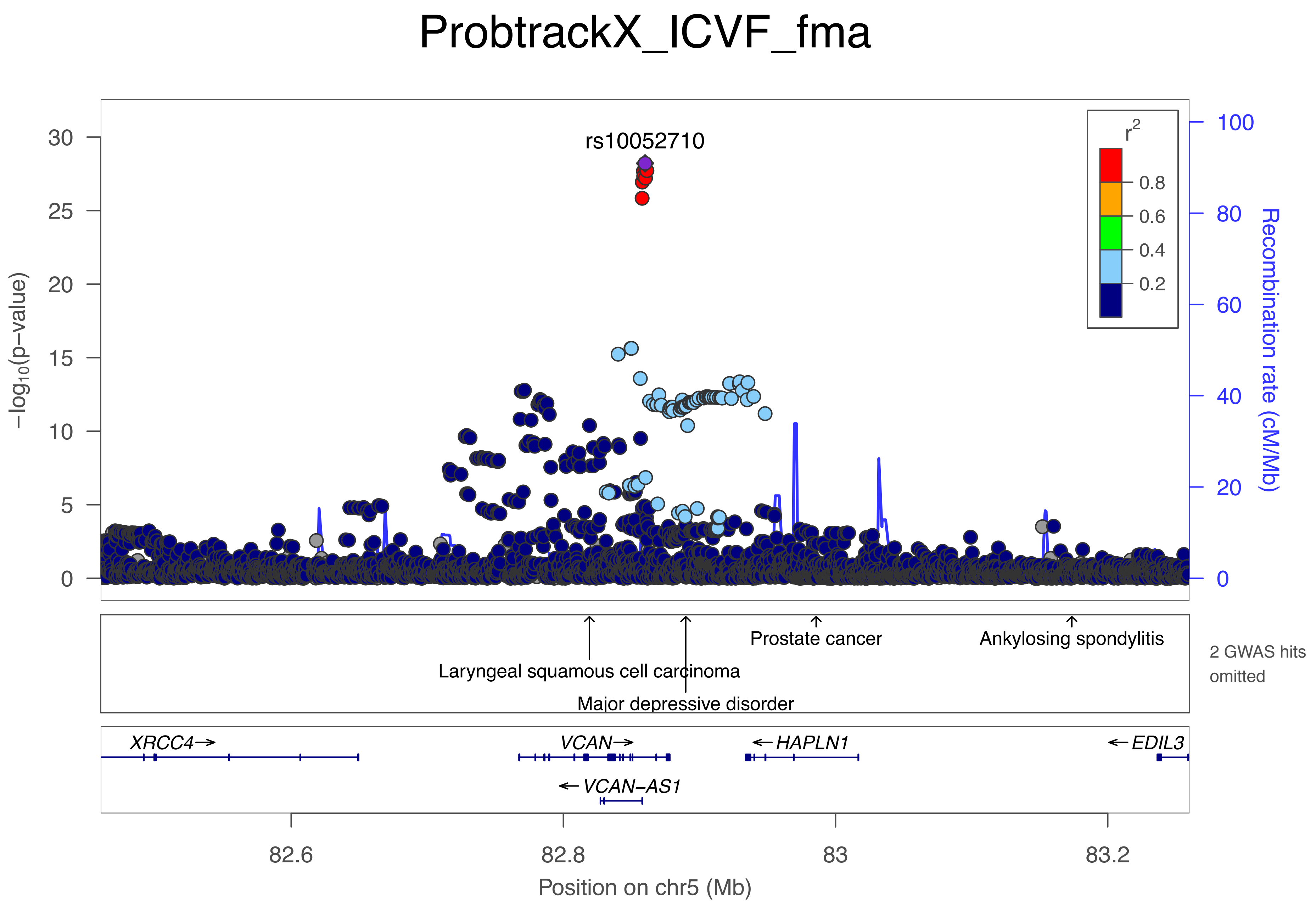

ProbtrackX_ICVF_fma
rs10052710
r2
0.8
0.6
0.4
0.2
30
25
20
15
10
5
0
−log10(p−value)
100
80
60
40
20
0
Recombination rate (cM/Mb)
Laryngeal squamous cell carcinoma
Major depressive disorder
Prostate cancer
Ankylosing spondylitis
2 GWAS hits omitted
XRCC4→
VCAN→
←VCAN−AS1
←HAPLN1
←EDIL3
82.6
82.8
83
83.2
Position on chr5 (Mb)

| | |
|---|---|
| date: | Thu Aug 17 17:52:01 2017 |
| build: | hg19 |
| display range: | chr5:82460025–83260025 [82460025–83260025] |
| hilite range: | 0 – 0 [ 0 – 0 ] |
| reference SNP: | chr5:82860025 |
| number of SNPs plotted: | 3392 |
| min P.value: | 6.15E–29 [chr5:82860025] |
| max P.value: | 9.99E–1 [chr5:82973987] |
| omitted GWAS Hits: | NA, NA |

Make more plots at http://csg.sph.umich.edu/locuszoom/

GWAS Catalog SNPs in Region

| chr | pos (Mb) | trait | snp |
|---|---|---|---|
| 5 | 82.81912 | Laryngeal squamous cell carcinoma | rs310518 |
| 5 | 82.84549 | Diisocyanate–induced asthma | rs3852186 |
| 5 | 82.88991 | Major depressive disorder | rs310501 |
| 5 | 82.96073 | Visceral fat | rs3846635 |
| 5 | 82.98574 | Prostate cancer | rs4466137 |
| 5 | 83.17359 | Ankylosing spondylitis | rs4552569 |

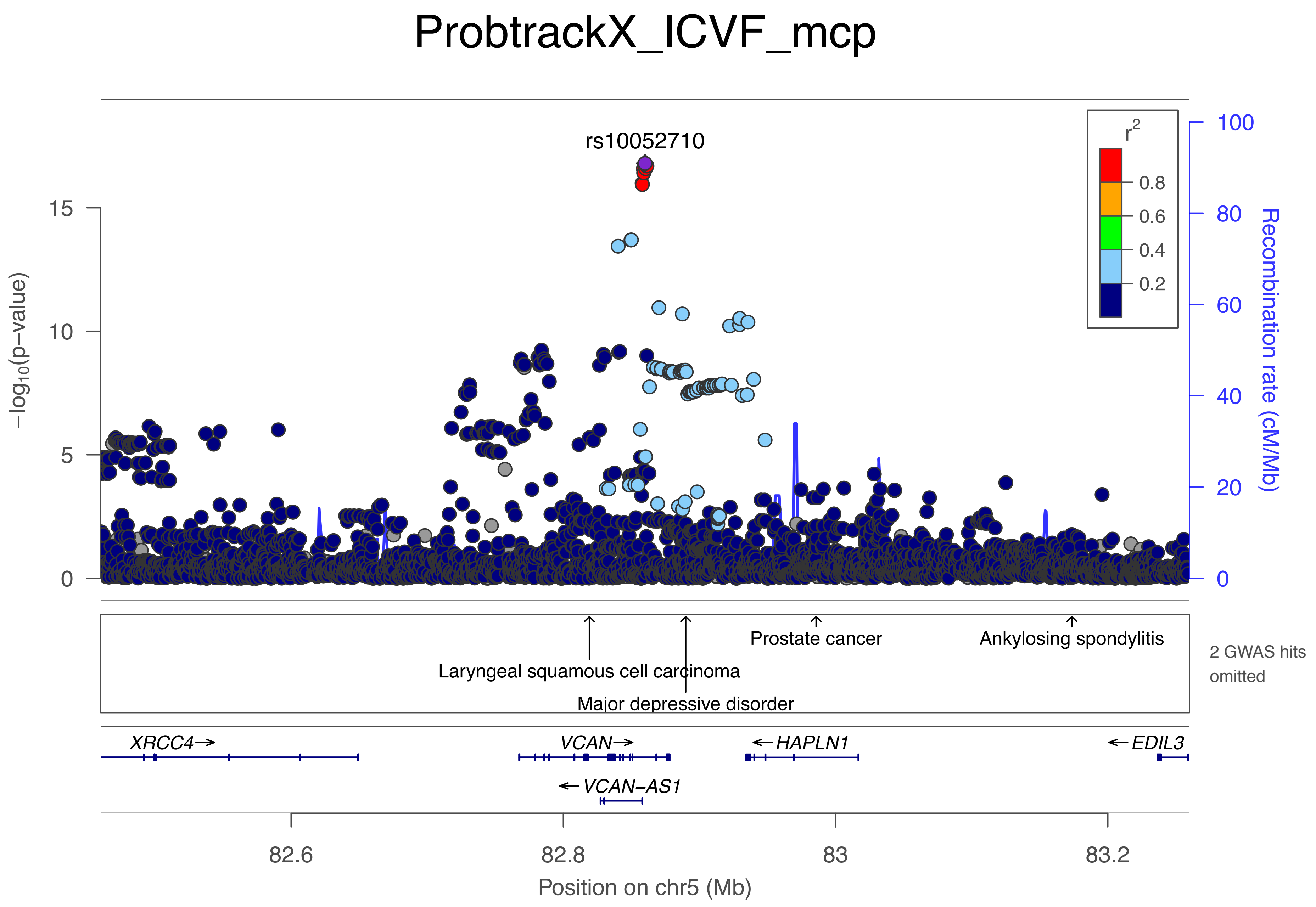
ProbtrackX_ICVF_mcp
rs10052710
−log10(p−value)
Recombination rate (cM/Mb)
r2
0.8
0.6
0.4
0.2
0
5
10
15
20
40
60
80
100
Laryngeal squamous cell carcinoma
Major depressive disorder
Prostate cancer
Ankylosing spondylitis
2 GWAS hits omitted
XRCC4
VCAN
VCAN−AS1
HAPLN1
EDIL3
82.6
82.8
83
83.2
Position on chr5 (Mb)

| | |
|---|---|
| date: | Thu Aug 17 17:52:01 2017 |
| build: | hg19 |
| display range: | chr5:82460025−83260025 [82460025−83260025] |
| hilite range: | 0 – 0 [ 0 – 0 ] |
| reference SNP: | chr5:82860025 |
| number of SNPs plotted: | 3392 |
| min P.value: | 1.6E−17 [chr5:82860025] |
| max P.value: | 10E−1 [chr5:82574349] |
| omitted GWAS Hits: | NA, NA |

Make more plots at http://csg.sph.umich.edu/locuszoom/

GWAS Catalog SNPs in Region

| chr | pos (Mb) | trait | snp |
|---|---|---|---|
| 5 | 82.81912 | Laryngeal squamous cell carcinoma | rs310518 |
| 5 | 82.84549 | Diisocyanate–induced asthma | rs3852186 |
| 5 | 82.88991 | Major depressive disorder | rs310501 |
| 5 | 82.96073 | Visceral fat | rs3846635 |
| 5 | 82.98574 | Prostate cancer | rs4466137 |
| 5 | 83.17359 | Ankylosing spondylitis | rs4552569 |

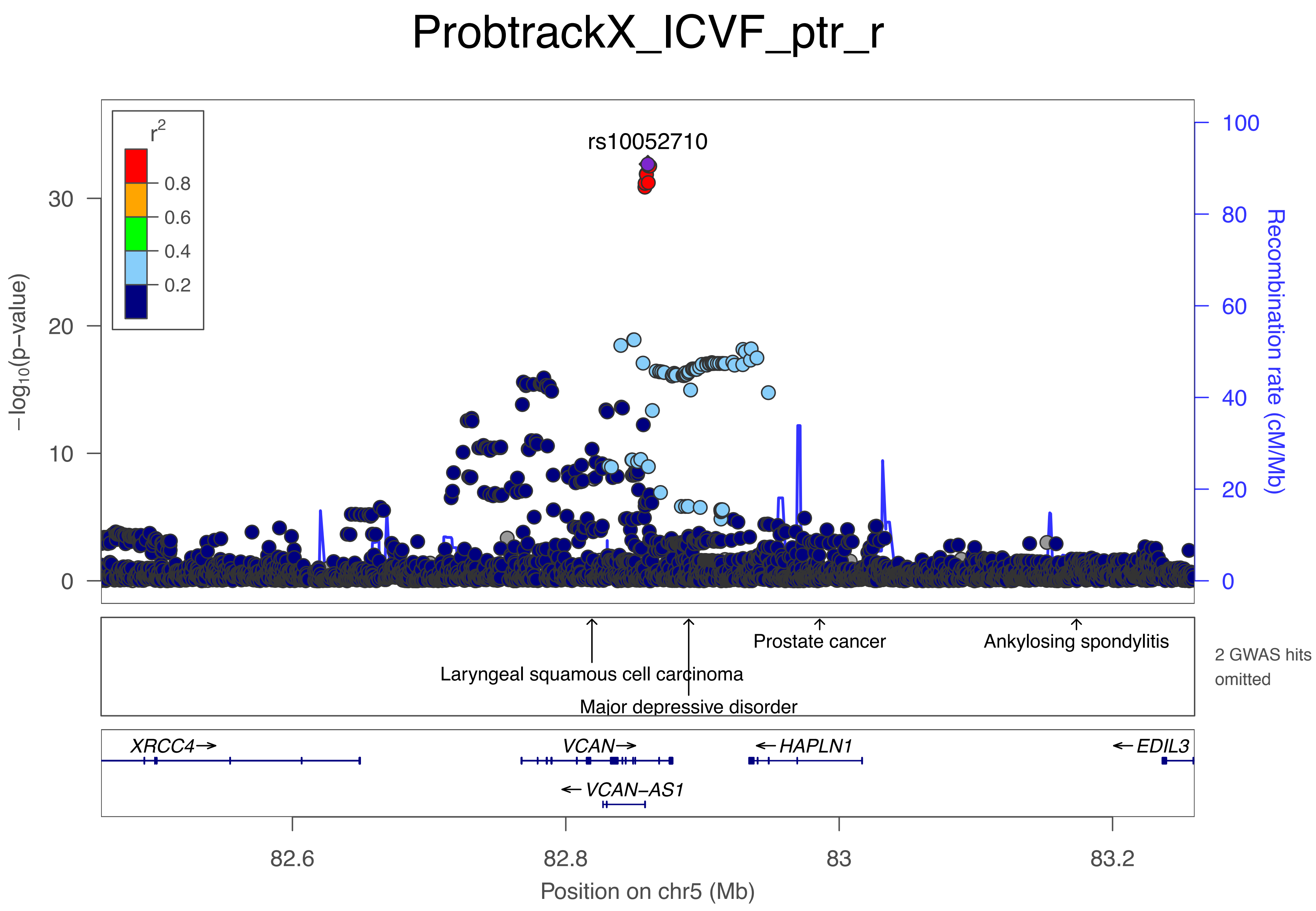
ProbtrackX_ICVF_ptr_r
r2
0.8
0.6
0.4
0.2
rs10052710
−log10(p−value)
30
20
10
0
100
80
60
40
20
0
Recombination rate (cM/Mb)
Laryngeal squamous cell carcinoma
Major depressive disorder
Prostate cancer
Ankylosing spondylitis
2 GWAS hits omitted
XRCC4→
VCAN→
←VCAN−AS1
←HAPLN1
←EDIL3
82.6
82.8
83
83.2
Position on chr5 (Mb)

| | |
|---|---|
| date: | Thu Aug 17 17:52:01 2017 |
| build: | hg19 |
| display range: | chr5:82460025–83260025 [82460025–83260025] |
| hilite range: | 0 – 0 [ 0 – 0 ] |
| reference SNP: | chr5:82860025 |
| number of SNPs plotted: | 3392 |
| min P.value: | 2.06E–33 [chr5:82860025] |
| max P.value: | 10E–1 [chr5:83098999] |
| omitted GWAS Hits: | NA, NA |

Make more plots at http://csg.sph.umich.edu/locuszoom/

GWAS Catalog SNPs in Region

| chr | pos (Mb) | trait | snp |
|---|---|---|---|
| 5 | 82.81912 | Laryngeal squamous cell carcinoma | rs310518 |
| 5 | 82.84549 | Diisocyanate–induced asthma | rs3852186 |
| 5 | 82.88991 | Major depressive disorder | rs310501 |
| 5 | 82.96073 | Visceral fat | rs3846635 |
| 5 | 82.98574 | Prostate cancer | rs4466137 |
| 5 | 83.17359 | Ankylosing spondylitis | rs4552569 |

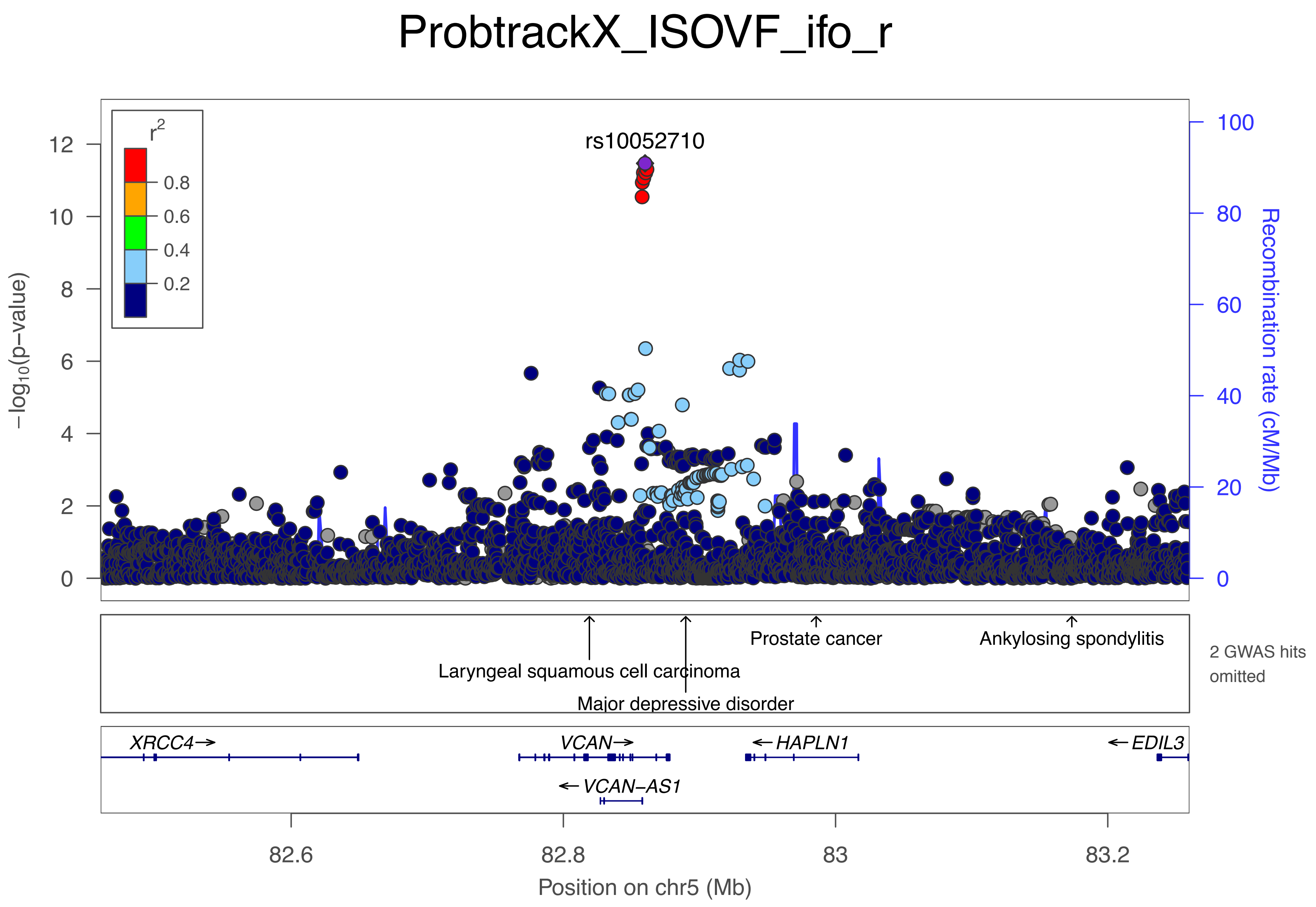

ProbtrackX_ISOVF_ifo_r
rs10052710
r2
0.8
0.6
0.4
0.2
−log10(p−value)
Recombination rate (cM/Mb)
Laryngeal squamous cell carcinoma
Major depressive disorder
Prostate cancer
Ankylosing spondylitis
2 GWAS hits omitted
XRCC4
VCAN
VCAN−AS1
HAPLN1
EDIL3
82.6
82.8
83
83.2
Position on chr5 (Mb)

| | |
|---|---|
| date: | Thu Aug 17 17:52:01 2017 |
| build: | hg19 |
| display range: | chr5:82460025−83260025 [82460025−83260025] |
| hilite range: | 0 – 0 [ 0 – 0 ] |
| reference SNP: | chr5:82860025 |
| number of SNPs plotted: | 3392 |
| min P.value: | 3.4E−12 [chr5:82860025] |
| max P.value: | 10E−1 [chr5:82463578] |
| omitted GWAS Hits: | NA, NA |

Make more plots at http://csg.sph.umich.edu/locuszoom/

GWAS Catalog SNPs in Region

| chr | pos (Mb) | trait | snp |
|---|---|---|---|
| 5 | 82.81912 | Laryngeal squamous cell carcinoma | rs310518 |
| 5 | 82.84549 | Diisocyanate–induced asthma | rs3852186 |
| 5 | 82.88991 | Major depressive disorder | rs310501 |
| 5 | 82.96073 | Visceral fat | rs3846635 |
| 5 | 82.98574 | Prostate cancer | rs4466137 |
| 5 | 83.17359 | Ankylosing spondylitis | rs4552569 |

# TBSS_ICVF_Inferior_cerebellar_peduncle_R

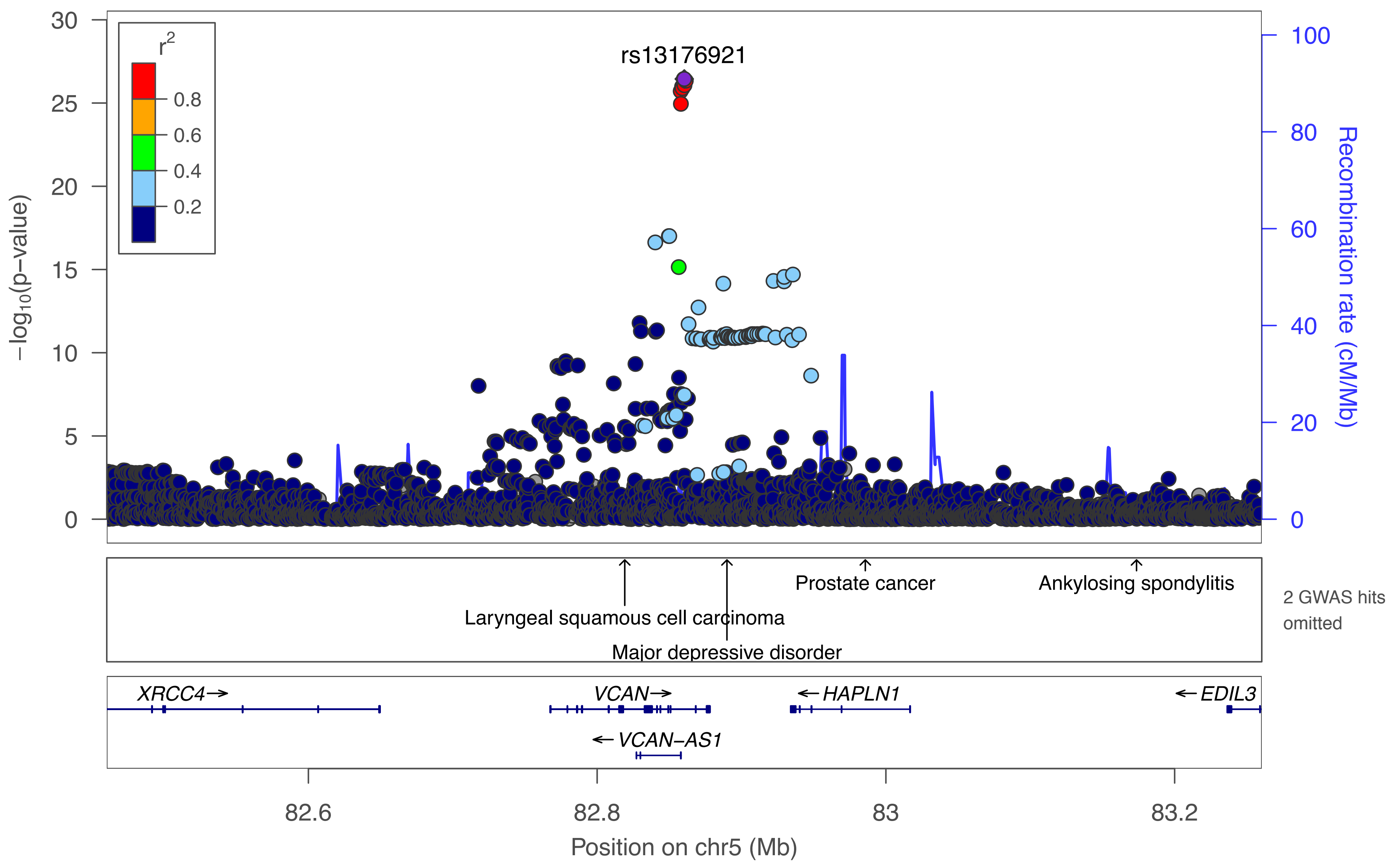

| | |
|---|---|
| date: | Thu Aug 17 17:52:01 2017 |
| build: | hg19 |
| display range: | chr5:82460348−83260348 [82460348−83260348] |
| hilite range: | 0 – 0 [ 0 – 0 ] |
| reference SNP: | chr5:82860348 |
| number of SNPs plotted: | 3392 |
| min P.value: | 3.59E−27 [chr5:82860025] |
| max P.value: | 9.99E−1 [chr5:83023489] |
| omitted GWAS Hits: | NA, NA |

Make more plots at http://csg.sph.umich.edu/locuszoom/

GWAS Catalog SNPs in Region

| chr | pos (Mb) | trait | snp |
|---|---|---|---|
| 5 | 82.81912 | Laryngeal squamous cell carcinoma | rs310518 |
| 5 | 82.84549 | Diisocyanate–induced asthma | rs3852186 |
| 5 | 82.88991 | Major depressive disorder | rs310501 |
| 5 | 82.96073 | Visceral fat | rs3846635 |
| 5 | 82.98574 | Prostate cancer | rs4466137 |
| 5 | 83.17359 | Ankylosing spondylitis | rs4552569 |

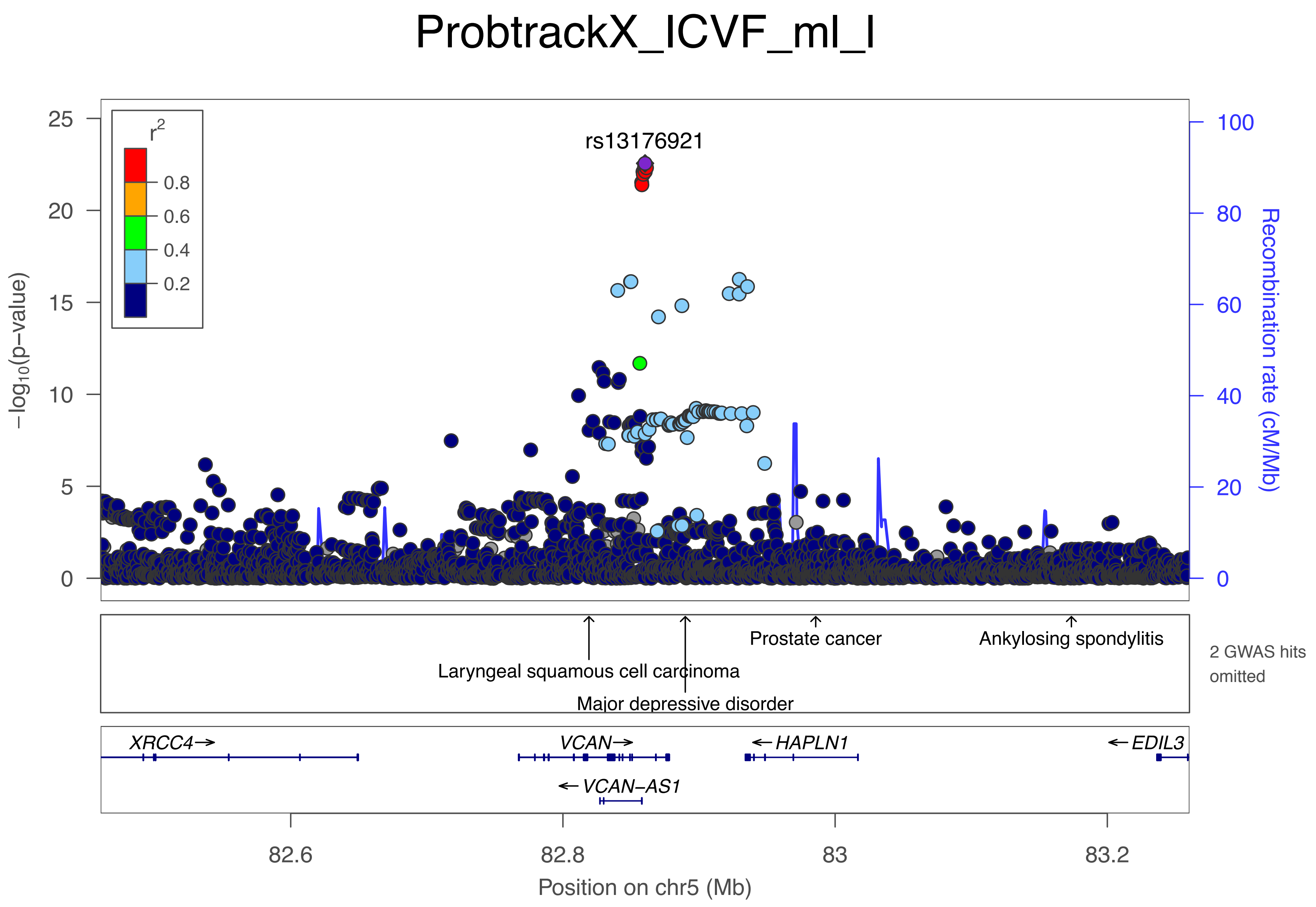
ProbtrackX_ICVF_ml_l
rs13176921
r2
0.8
0.6
0.4
0.2
−log10(p−value)
Recombination rate (cM/Mb)
Prostate cancer
Ankylosing spondylitis
Laryngeal squamous cell carcinoma
Major depressive disorder
2 GWAS hits omitted
XRCC4→
VCAN→
← HAPLN1
← EDIL3
← VCAN−AS1
82.6
82.8
83
83.2
Position on chr5 (Mb)

| | |
|---|---|
| date: | Thu Aug 17 17:52:01 2017 |
| build: | hg19 |
| display range: | chr5:82460348−83260348 [82460348−83260348] |
| hilite range: | 0 – 0 [ 0 – 0 ] |
| reference SNP: | chr5:82860348 |
| number of SNPs plotted: | 3392 |
| min P.value: | 2.77E−23 [chr5:82860348] |
| max P.value: | 9.99E−1 [chr5:83193306] |
| omitted GWAS Hits: | NA, NA |

Make more plots at http://csg.sph.umich.edu/locuszoom/

GWAS Catalog SNPs in Region

| chr | pos (Mb) | trait | snp |
|---|---|---|---|
| 5 | 82.81912 | Laryngeal squamous cell carcinoma | rs310518 |
| 5 | 82.84549 | Diisocyanate–induced asthma | rs3852186 |
| 5 | 82.88991 | Major depressive disorder | rs310501 |
| 5 | 82.96073 | Visceral fat | rs3846635 |
| 5 | 82.98574 | Prostate cancer | rs4466137 |
| 5 | 83.17359 | Ankylosing spondylitis | rs4552569 |

# TBSS_MD_Posterior_corona_radiata_R

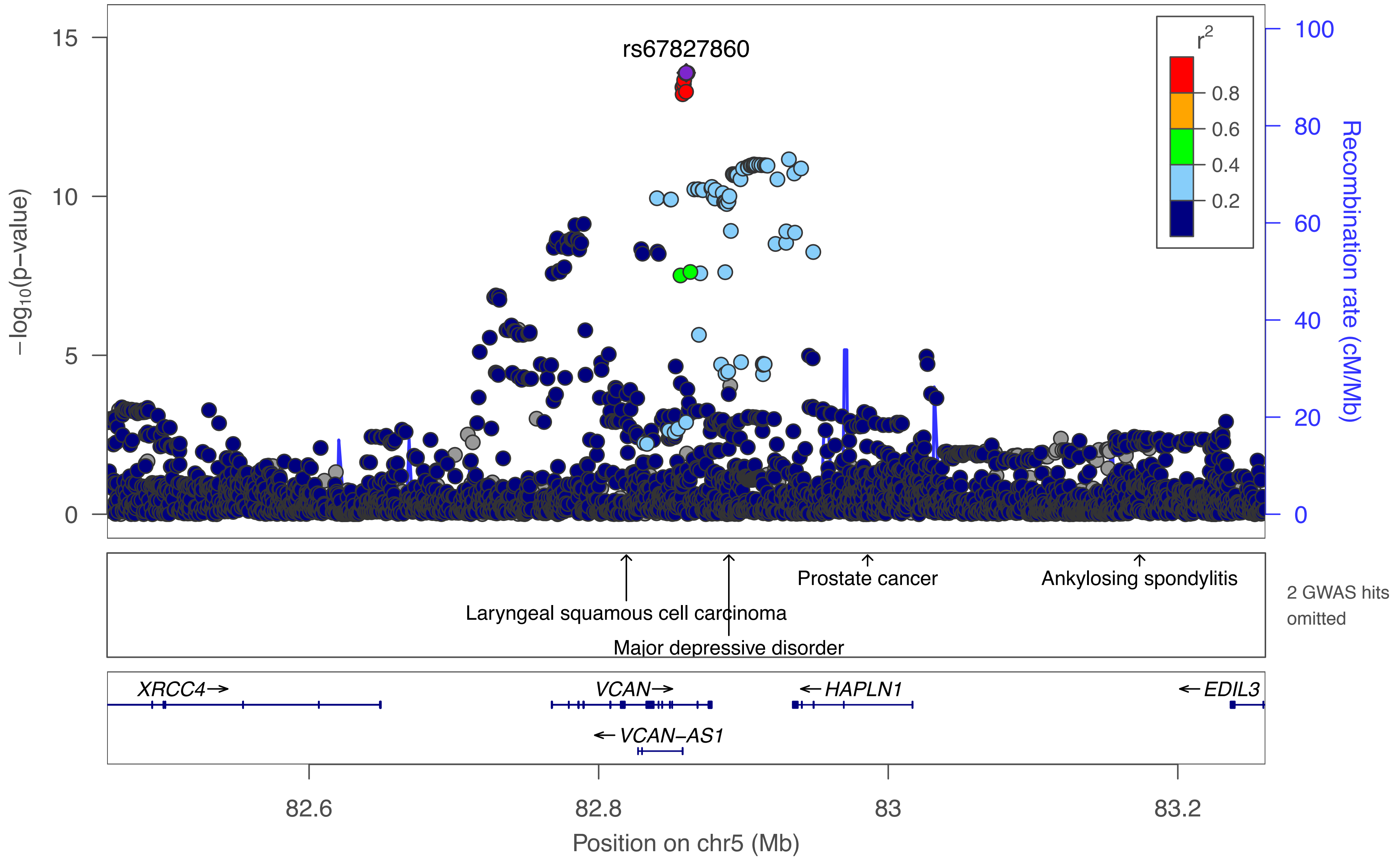

| | |
|---|---|
| date: | Thu Aug 17 17:52:01 2017 |
| build: | hg19 |
| display range: | chr5:82460485−83260485 [82460485−83260485] |
| hilite range: | 0 − 0 [ 0 − 0 ] |
| reference SNP: | chr5:82860485 |
| number of SNPs plotted: | 3393 |
| min P.value: | 1.3E−14 [chr5:82860485] |
| max P.value: | 9.99E−1 [chr5:83098673] |
| omitted GWAS Hits: | NA, NA |

Make more plots at http://csg.sph.umich.edu/locuszoom/

GWAS Catalog SNPs in Region

| chr | pos (Mb) | trait | snp |
|---|---|---|---|
| 5 | 82.81912 | Laryngeal squamous cell carcinoma | rs310518 |
| 5 | 82.84549 | Diisocyanate–induced asthma | rs3852186 |
| 5 | 82.88991 | Major depressive disorder | rs310501 |
| 5 | 82.96073 | Visceral fat | rs3846635 |
| 5 | 82.98574 | Prostate cancer | rs4466137 |
| 5 | 83.17359 | Ankylosing spondylitis | rs4552569 |

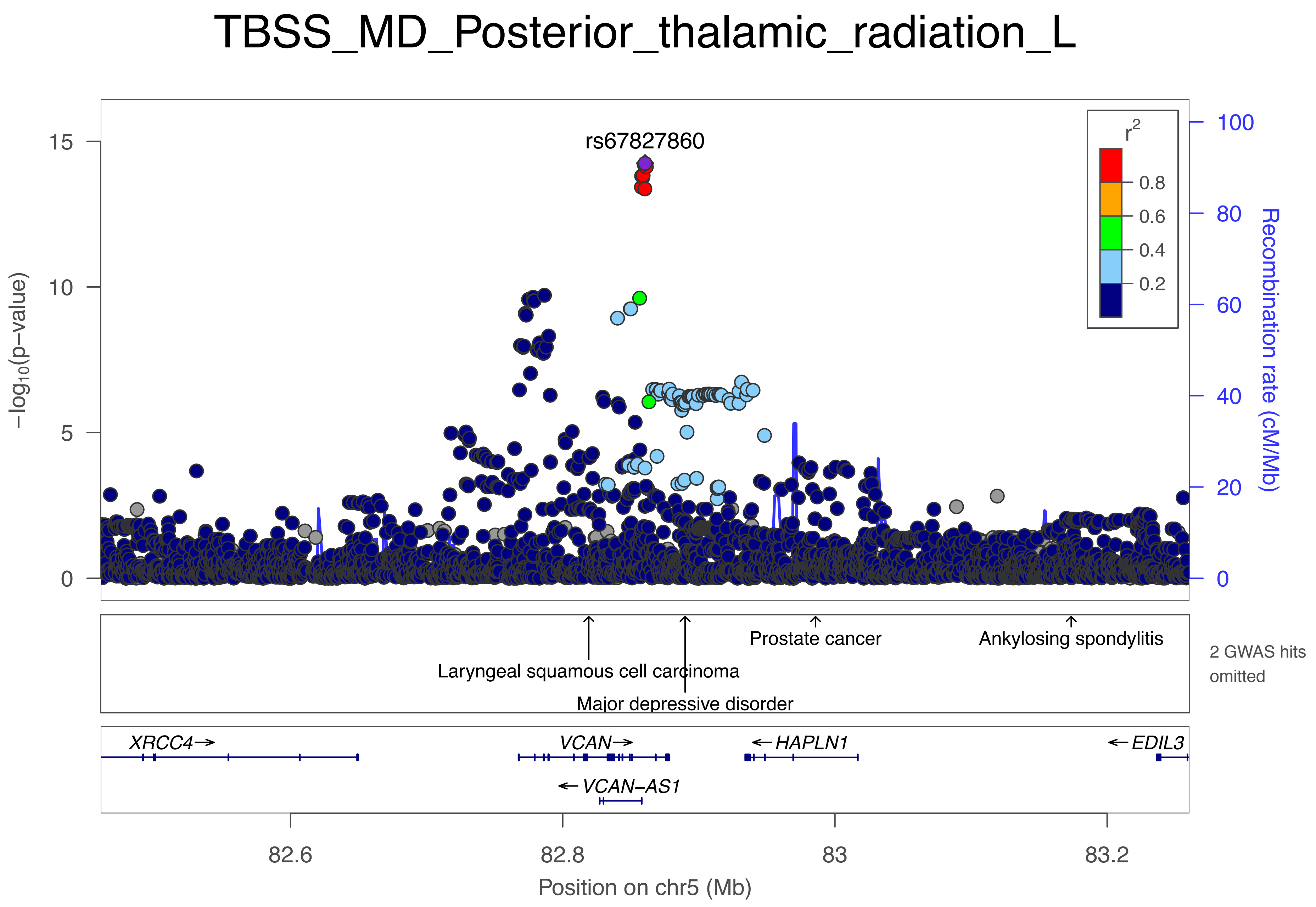
TBSS_MD_Posterior_thalamic_radiation_L
rs67827860
−log10(p−value)
Recombination rate (cM/Mb)
r2
0.8
0.6
0.4
0.2
Laryngeal squamous cell carcinoma
Major depressive disorder
Prostate cancer
Ankylosing spondylitis
2 GWAS hits omitted
XRCC4
VCAN
VCAN−AS1
HAPLN1
EDIL3
82.6
82.8
83
83.2
Position on chr5 (Mb)

| | |
|---|---|
| date: | Thu Aug 17 17:52:01 2017 |
| build: | hg19 |
| display range: | chr5:82460485−83260485 [82460485−83260485] |
| hilite range: | 0 – 0 [ 0 – 0 ] |
| reference SNP: | chr5:82860485 |
| number of SNPs plotted: | 3393 |
| min P.value: | 5.71E−15 [chr5:82860485] |
| max P.value: | 10E−1 [chr5:82936060] |
| omitted GWAS Hits: | NA, NA |

Make more plots at http://csg.sph.umich.edu/locuszoom/

GWAS Catalog SNPs in Region

| chr | pos (Mb) | trait | snp |
|---|---|---|---|
| 5 | 82.81912 | Laryngeal squamous cell carcinoma | rs310518 |
| 5 | 82.84549 | Diisocyanate−induced asthma | rs3852186 |
| 5 | 82.88991 | Major depressive disorder | rs310501 |
| 5 | 82.96073 | Visceral fat | rs3846635 |
| 5 | 82.98574 | Prostate cancer | rs4466137 |
| 5 | 83.17359 | Ankylosing spondylitis | rs4552569 |

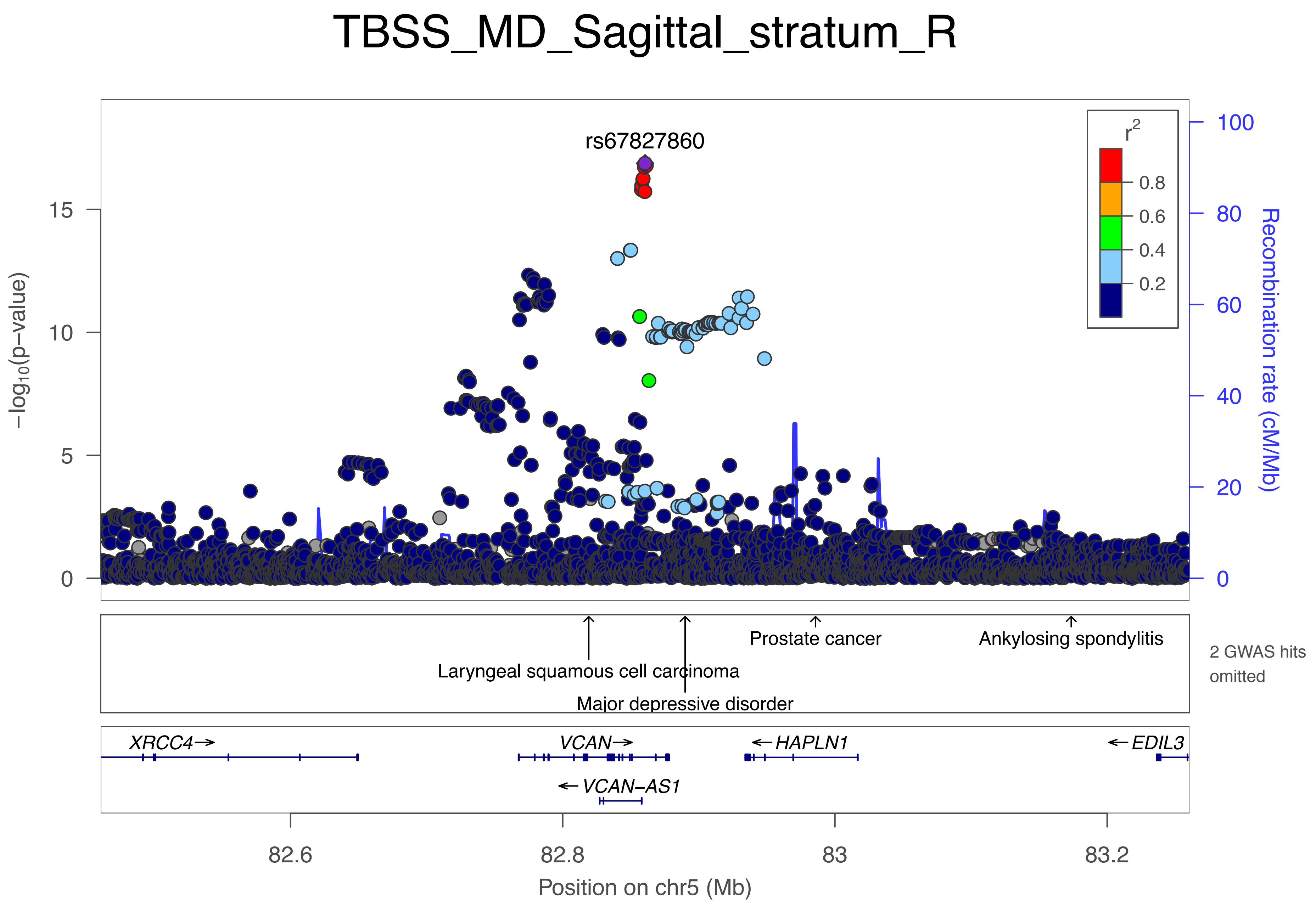

TBSS_MD_Sagittal_stratum_R
rs67827860
−log10(p−value)
Recombination rate (cM/Mb)
r2
0.8
0.6
0.4
0.2
0
5
10
15
0
20
40
60
80
100
Laryngeal squamous cell carcinoma
Major depressive disorder
Prostate cancer
Ankylosing spondylitis
2 GWAS hits omitted
XRCC4→
VCAN→
←VCAN−AS1
←HAPLN1
←EDIL3
82.6
82.8
83
83.2
Position on chr5 (Mb)

| | |
|---|---|
| date: | Thu Aug 17 17:52:01 2017 |
| build: | hg19 |
| display range: | chr5:82460485–83260485 [82460485–83260485] |
| hilite range: | 0 – 0 [ 0 – 0 ] |
| reference SNP: | chr5:82860485 |
| number of SNPs plotted: | 3393 |
| min P.value: | 1.35E–17 [chr5:82860485] |
| max P.value: | 10E–1 [chr5:83115806] |
| omitted GWAS Hits: | NA, NA |

Make more plots at http://csg.sph.umich.edu/locuszoom/

GWAS Catalog SNPs in Region

| chr | pos (Mb) | trait | snp |
|---|---|---|---|
| 5 | 82.81912 | Laryngeal squamous cell carcinoma | rs310518 |
| 5 | 82.84549 | Diisocyanate–induced asthma | rs3852186 |
| 5 | 82.88991 | Major depressive disorder | rs310501 |
| 5 | 82.96073 | Visceral fat | rs3846635 |
| 5 | 82.98574 | Prostate cancer | rs4466137 |
| 5 | 83.17359 | Ankylosing spondylitis | rs4552569 |

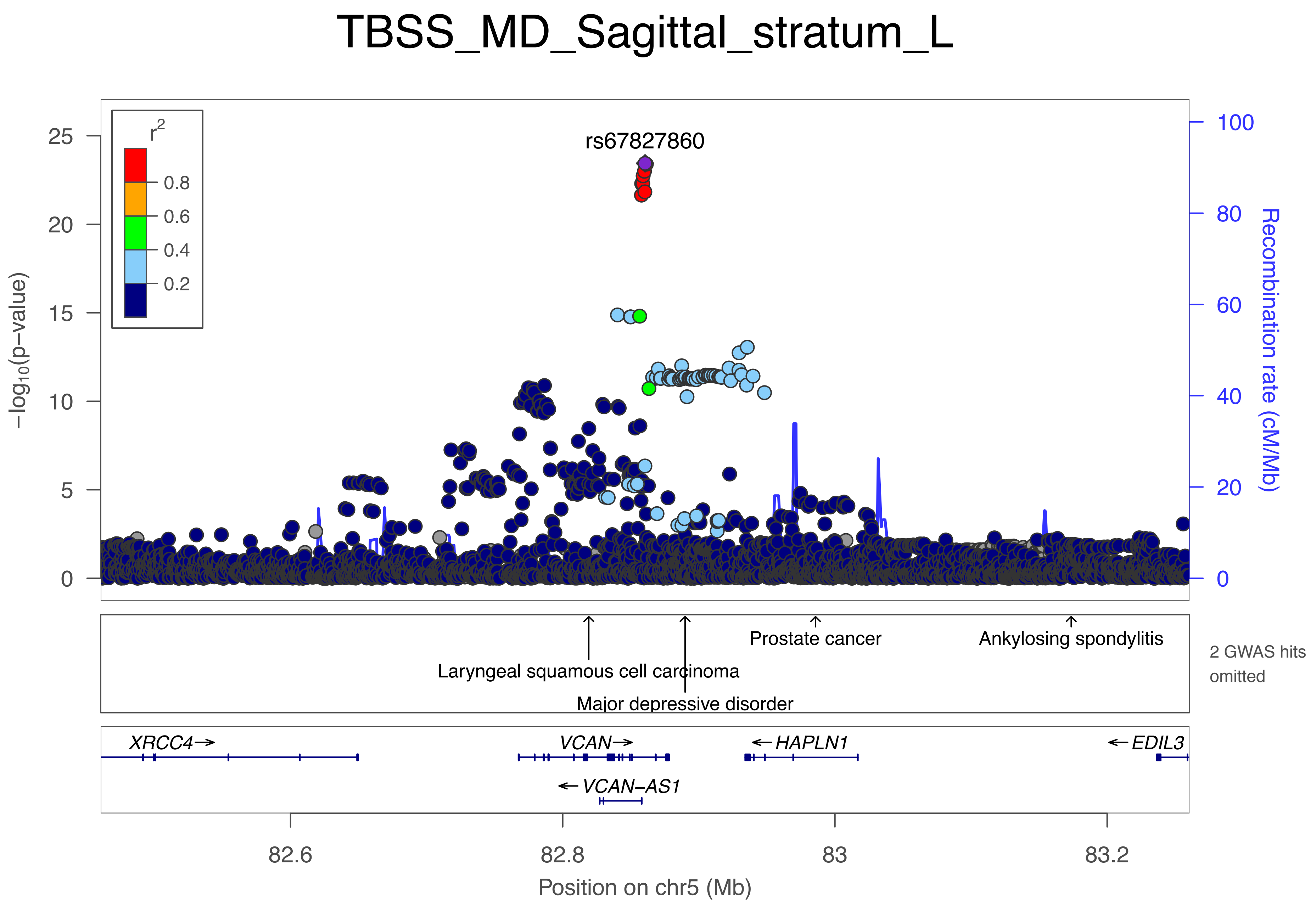

TBSS_MD_Sagittal_stratum_L
rs67827860
r2
0.8
0.6
0.4
0.2
25
20
15
10
5
0
−log10(p−value)
100
80
60
40
20
0
Recombination rate (cM/Mb)
Laryngeal squamous cell carcinoma
Major depressive disorder
Prostate cancer
Ankylosing spondylitis
2 GWAS hits omitted
XRCC4→
VCAN→
←VCAN−AS1
←HAPLN1
←EDIL3
82.6
82.8
83
83.2
Position on chr5 (Mb)

| | |
|---|---|
| date: | Thu Aug 17 17:52:01 2017 |
| build: | hg19 |
| display range: | chr5:82460485–83260485 [82460485–83260485] |
| hilite range: | 0 – 0 [ 0 – 0 ] |
| reference SNP: | chr5:82860485 |
| number of SNPs plotted: | 3393 |
| min P.value: | 3.62E–24 [chr5:82860485] |
| max P.value: | 9.99E–1 [chr5:82659709] |
| omitted GWAS Hits: | NA, NA |

Make more plots at http://csg.sph.umich.edu/locuszoom/

GWAS Catalog SNPs in Region

| chr | pos (Mb) | trait | snp |
|---|---|---|---|
| 5 | 82.81912 | Laryngeal squamous cell carcinoma | rs310518 |
| 5 | 82.84549 | Diisocyanate–induced asthma | rs3852186 |
| 5 | 82.88991 | Major depressive disorder | rs310501 |
| 5 | 82.96073 | Visceral fat | rs3846635 |
| 5 | 82.98574 | Prostate cancer | rs4466137 |
| 5 | 83.17359 | Ankylosing spondylitis | rs4552569 |

# TBSS_MD_Cingulum_hippocampus_R

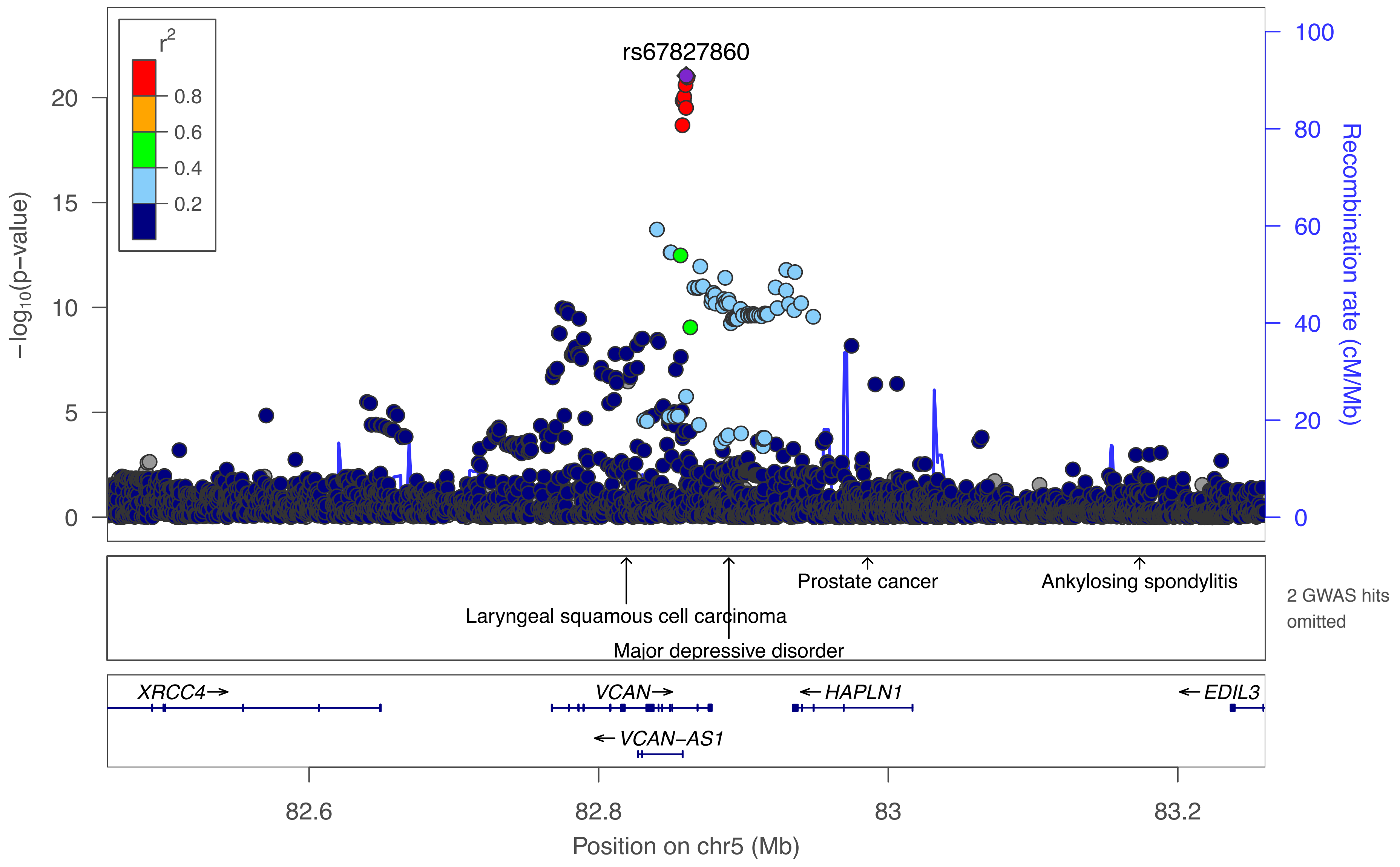

| | |
|---|---|
| date: | Thu Aug 17 17:52:01 2017 |
| build: | hg19 |
| display range: | chr5:82460485–83260485 [82460485–83260485] |
| hilite range: | 0 – 0 [ 0 – 0 ] |
| reference SNP: | chr5:82860485 |
| number of SNPs plotted: | 3393 |
| min P.value: | 9.14E–22 [chr5:82860485] |
| max P.value: | 10E–1 [chr5:82966054] |
| omitted GWAS Hits: | NA, NA |

Make more plots at http://csg.sph.umich.edu/locuszoom/

GWAS Catalog SNPs in Region

| chr | pos (Mb) | trait | snp |
|---|---|---|---|
| 5 | 82.81912 | Laryngeal squamous cell carcinoma | rs310518 |
| 5 | 82.84549 | Diisocyanate–induced asthma | rs3852186 |
| 5 | 82.88991 | Major depressive disorder | rs310501 |
| 5 | 82.96073 | Visceral fat | rs3846635 |
| 5 | 82.98574 | Prostate cancer | rs4466137 |
| 5 | 83.17359 | Ankylosing spondylitis | rs4552569 |

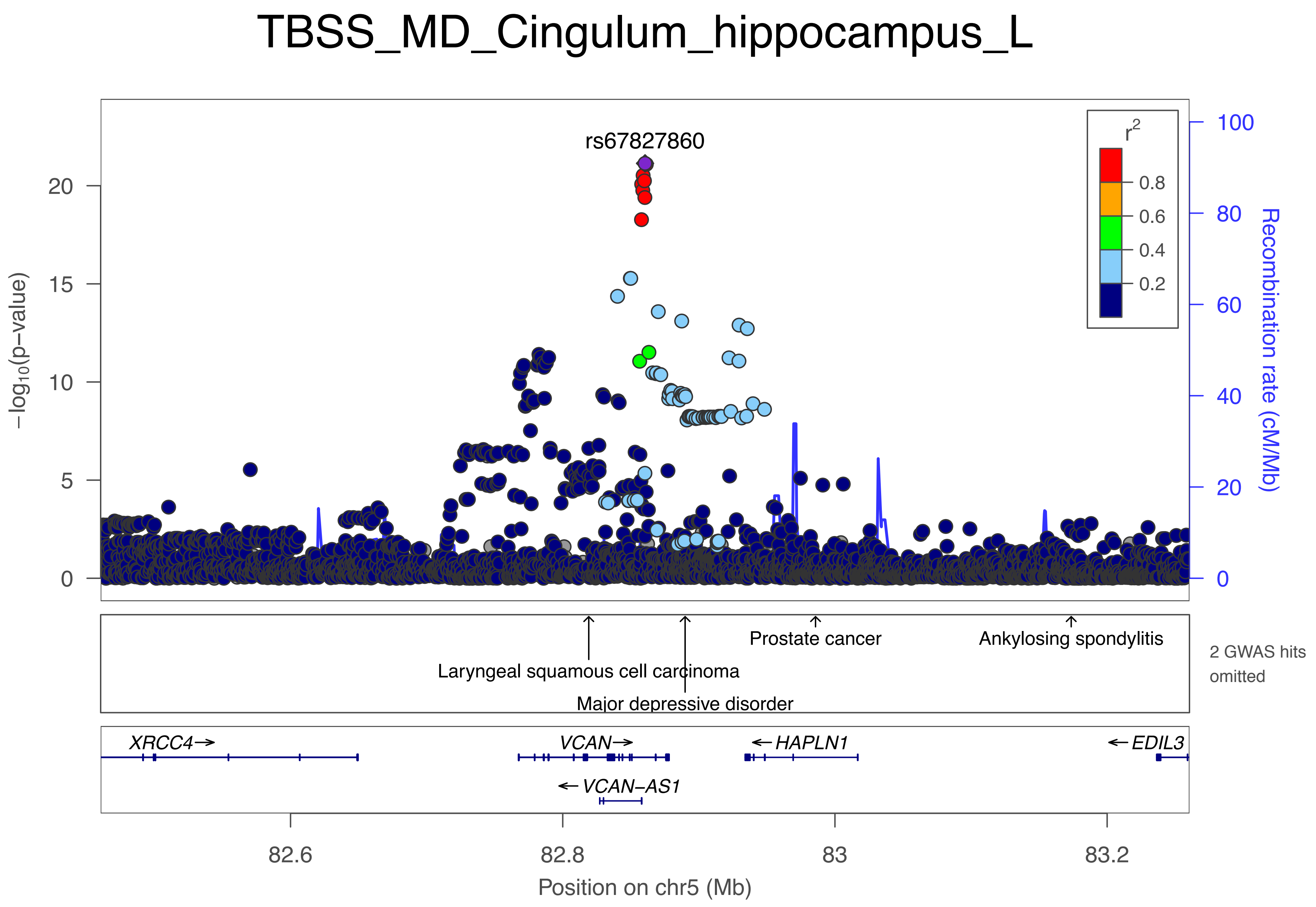

TBSS_MD_Cingulum_hippocampus_L
rs67827860
−log10(p−value)
0
5
10
15
20
r2
0.8
0.6
0.4
0.2
100
80
60
40
20
0
Recombination rate (cM/Mb)
Laryngeal squamous cell carcinoma
Major depressive disorder
Prostate cancer
Ankylosing spondylitis
2 GWAS hits omitted
XRCC4→
VCAN→
←HAPLN1
←EDIL3
←VCAN−AS1
82.6
82.8
83
83.2
Position on chr5 (Mb)

| | |
|---|---|
| date: | Thu Aug 17 17:52:01 2017 |
| build: | hg19 |
| display range: | chr5:82460485−83260485 [82460485−83260485] |
| hilite range: | 0 – 0 [ 0 – 0 ] |
| reference SNP: | chr5:82860485 |
| number of SNPs plotted: | 3393 |
| min P.value: | 7.26E−22 [chr5:82860485] |
| max P.value: | 10E−1 [chr5:83178988] |
| omitted GWAS Hits: | NA, NA |

Make more plots at http://csg.sph.umich.edu/locuszoom/

# GWAS Catalog SNPs in Region

| chr | pos (Mb) | trait | snp |
|---|---|---|---|
| 5 | 82.81912 | Laryngeal squamous cell carcinoma | rs310518 |
| 5 | 82.84549 | Diisocyanate–induced asthma | rs3852186 |
| 5 | 82.88991 | Major depressive disorder | rs310501 |
| 5 | 82.96073 | Visceral fat | rs3846635 |
| 5 | 82.98574 | Prostate cancer | rs4466137 |
| 5 | 83.17359 | Ankylosing spondylitis | rs4552569 |

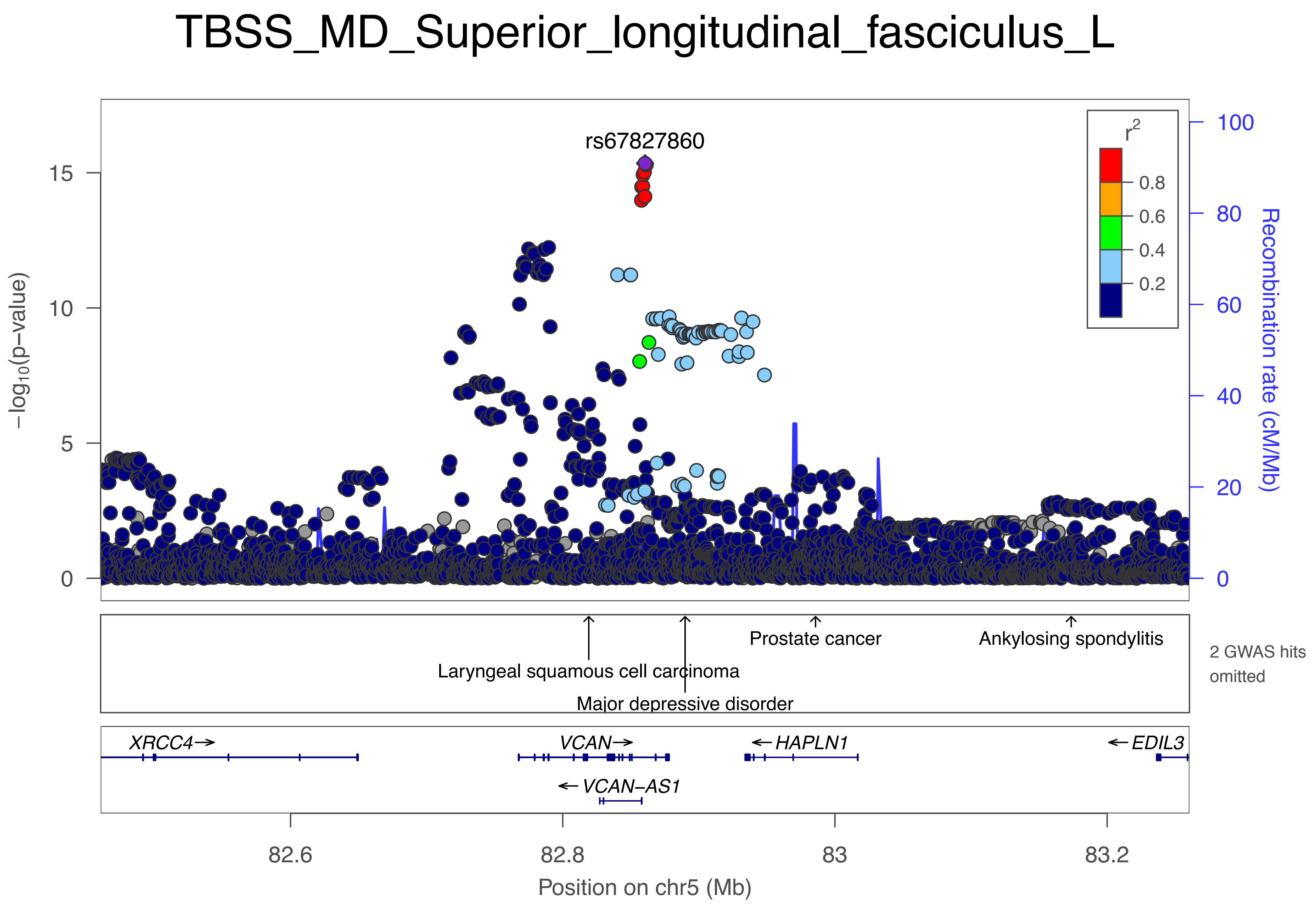

TBSS_MD_Superior_longitudinal_fasciculus_L
rs67827860
$-\log_{10}$(p−value)
15
10
5
0
r²
0.8
0.6
0.4
0.2
100
80
60
40
20
0
Recombination rate (cM/Mb)
Prostate cancer
Ankylosing spondylitis
Laryngeal squamous cell carcinoma
Major depressive disorder
2 GWAS hits omitted
XRCC4→
VCAN→
←HAPLN1
←EDIL3
←VCAN−AS1
82.6
82.8
83
83.2
Position on chr5 (Mb)

| | |
|---|---|
| date: | Thu Aug 17 17:52:01 2017 |
| build: | hg19 |
| display range: | chr5:82460485−83260485 [82460485−83260485] |
| hilite range: | 0 – 0 [ 0 – 0 ] |
| reference SNP: | chr5:82860485 |
| number of SNPs plotted: | 3393 |
| min P.value: | 4.5E−16 [chr5:82860485] |
| max P.value: | 10E−1 [chr5:83165955] |
| omitted GWAS Hits: | NA, NA |

Make more plots at http://csg.sph.umich.edu/locuszoom/

GWAS Catalog SNPs in Region

| chr | pos (Mb) | trait | snp |
|---|---|---|---|
| 5 | 82.81912 | Laryngeal squamous cell carcinoma | rs310518 |
| 5 | 82.84549 | Diisocyanate–induced asthma | rs3852186 |
| 5 | 82.88991 | Major depressive disorder | rs310501 |
| 5 | 82.96073 | Visceral fat | rs3846635 |
| 5 | 82.98574 | Prostate cancer | rs4466137 |
| 5 | 83.17359 | Ankylosing spondylitis | rs4552569 |

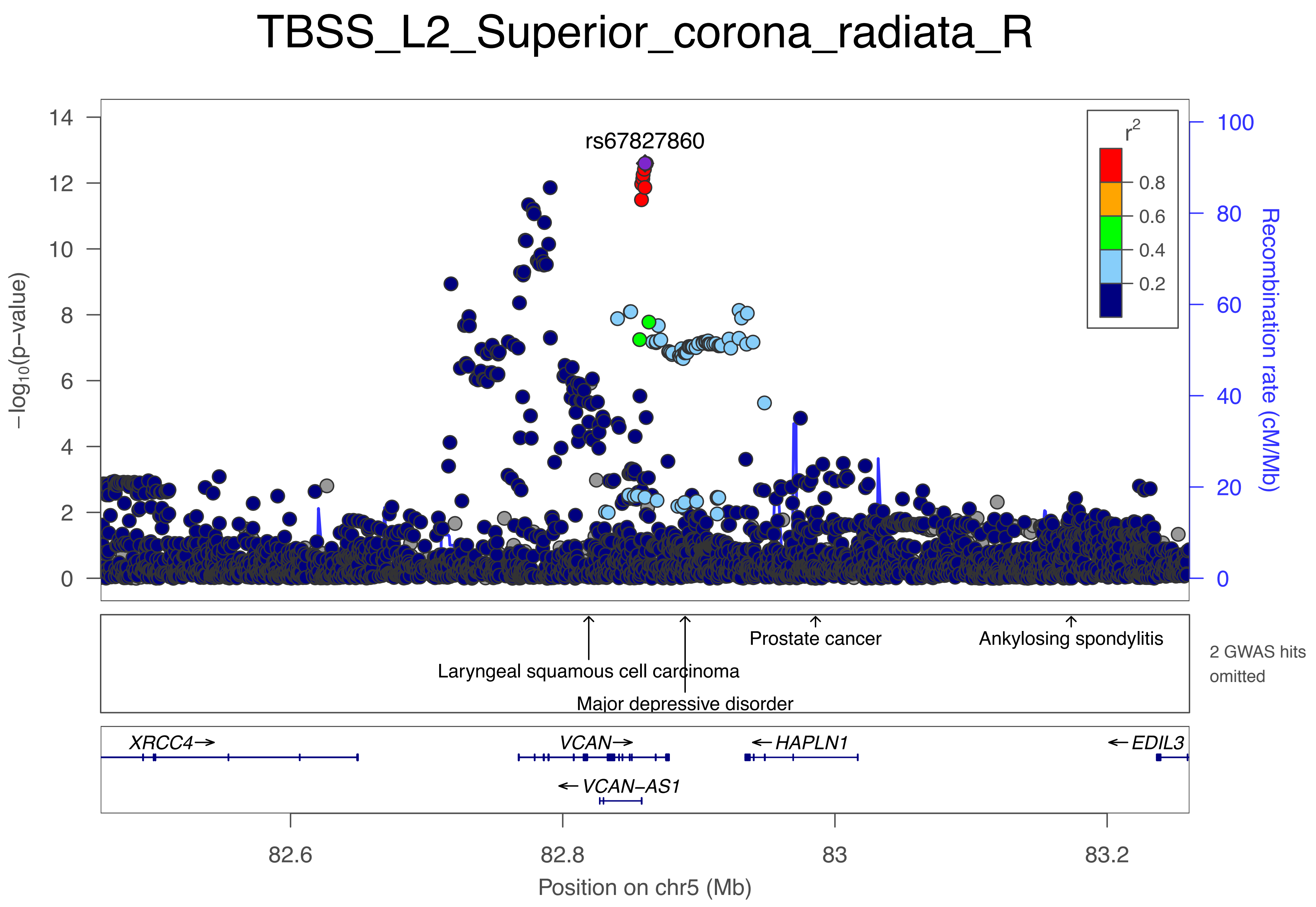
TBSS_L2_Superior_corona_radiata_R
rs67827860
−log10(p−value)
Recombination rate (cM/Mb)
r2
0.8
0.6
0.4
0.2
0
2
4
6
8
10
12
14
0
20
40
60
80
100
Prostate cancer
Ankylosing spondylitis
Laryngeal squamous cell carcinoma
Major depressive disorder
2 GWAS hits omitted
XRCC4
VCAN
HAPLN1
EDIL3
VCAN−AS1
82.6
82.8
83
83.2
Position on chr5 (Mb)

| | |
|---|---|
| date: | Thu Aug 17 17:52:01 2017 |
| build: | hg19 |
| display range: | chr5:82460485−83260485 [82460485−83260485] |
| hilite range: | 0 – 0 [ 0 – 0 ] |
| reference SNP: | chr5:82860485 |
| number of SNPs plotted: | 3393 |
| min P.value: | 2.53E−13 [chr5:82860485] |
| max P.value: | 10E−1 [chr5:83115847] |
| omitted GWAS Hits: | NA, NA |

Make more plots at http://csg.sph.umich.edu/locuszoom/

GWAS Catalog SNPs in Region

| chr | pos (Mb) | trait | snp |
|---|---|---|---|
| 5 | 82.81912 | Laryngeal squamous cell carcinoma | rs310518 |
| 5 | 82.84549 | Diisocyanate–induced asthma | rs3852186 |
| 5 | 82.88991 | Major depressive disorder | rs310501 |
| 5 | 82.96073 | Visceral fat | rs3846635 |
| 5 | 82.98574 | Prostate cancer | rs4466137 |
| 5 | 83.17359 | Ankylosing spondylitis | rs4552569 |

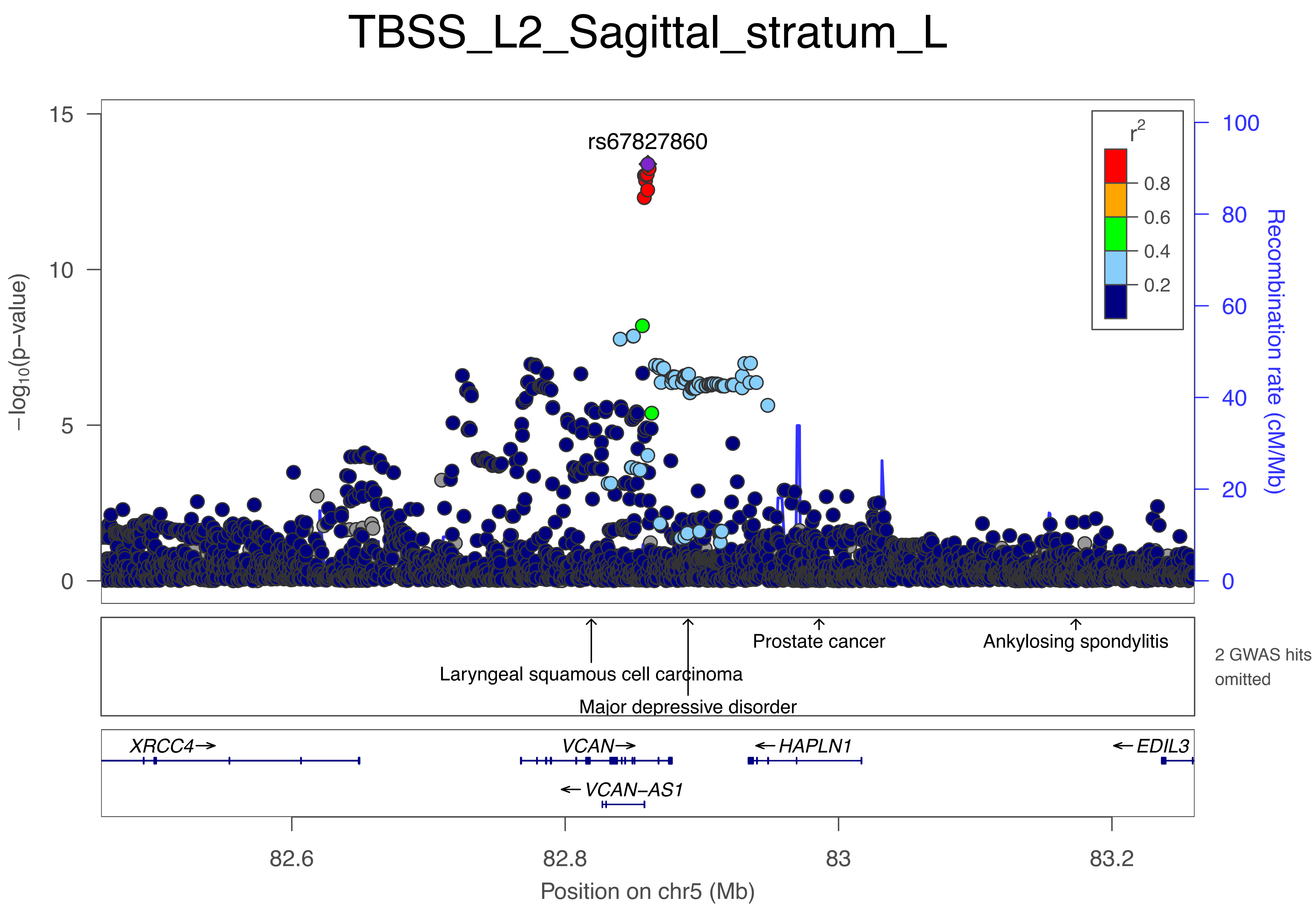
TBSS_L2_Sagittal_stratum_L
rs67827860
−log10(p−value)
Recombination rate (cM/Mb)
r2
0.8
0.6
0.4
0.2
Laryngeal squamous cell carcinoma
Major depressive disorder
Prostate cancer
Ankylosing spondylitis
2 GWAS hits omitted
XRCC4
VCAN
VCAN−AS1
HAPLN1
EDIL3
82.6
82.8
83
83.2
Position on chr5 (Mb)

| | |
|---|---|
| date: | Thu Aug 17 17:52:01 2017 |
| build: | hg19 |
| display range: | chr5:82460485−83260485 [82460485−83260485] |
| hilite range: | 0 – 0 [ 0 – 0 ] |
| reference SNP: | chr5:82860485 |
| number of SNPs plotted: | 3393 |
| min P.value: | 4.1E−14 [chr5:82860485] |
| max P.value: | 10E−1 [chr5:82973210] |
| omitted GWAS Hits: | NA, NA |

Make more plots at http://csg.sph.umich.edu/locuszoom/

GWAS Catalog SNPs in Region

| chr | pos (Mb) | trait | snp |
|---|---|---|---|
| 5 | 82.81912 | Laryngeal squamous cell carcinoma | rs310518 |
| 5 | 82.84549 | Diisocyanate–induced asthma | rs3852186 |
| 5 | 82.88991 | Major depressive disorder | rs310501 |
| 5 | 82.96073 | Visceral fat | rs3846635 |
| 5 | 82.98574 | Prostate cancer | rs4466137 |
| 5 | 83.17359 | Ankylosing spondylitis | rs4552569 |

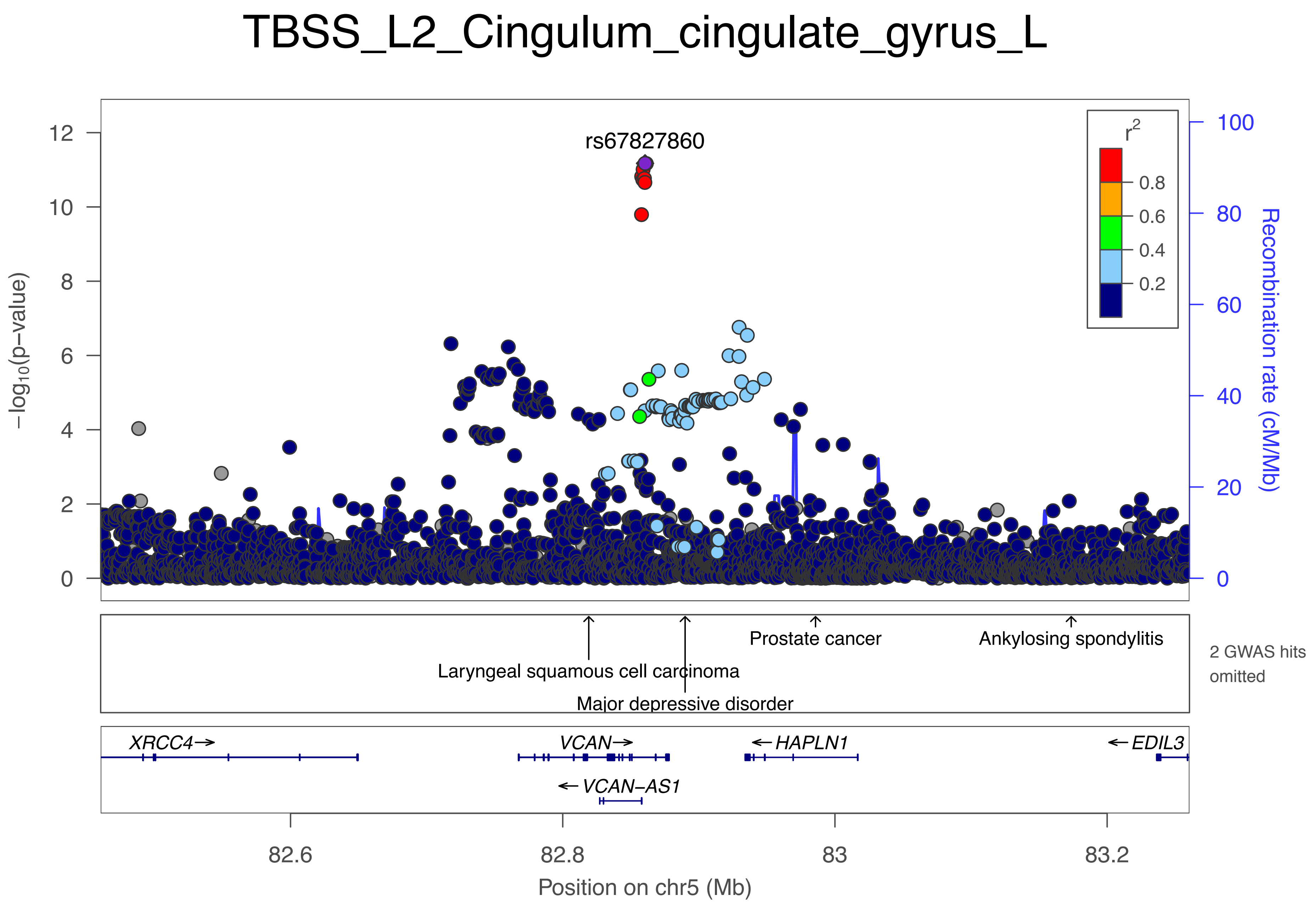
TBSS_L2_Cingulum_cingulate_gyrus_L
rs67827860
−log10(p−value)
Recombination rate (cM/Mb)
r2
0.8
0.6
0.4
0.2
Laryngeal squamous cell carcinoma
Major depressive disorder
Prostate cancer
Ankylosing spondylitis
2 GWAS hits omitted
XRCC4
VCAN
VCAN−AS1
HAPLN1
EDIL3
82.6
82.8
83
83.2
Position on chr5 (Mb)

| | |
|---|---|
| date: | Thu Aug 17 17:52:01 2017 |
| build: | hg19 |
| display range: | chr5:82460485–83260485 [82460485–83260485] |
| hilite range: | 0 – 0 [ 0 – 0 ] |
| reference SNP: | chr5:82860485 |
| number of SNPs plotted: | 3393 |
| min P.value: | 6.71E–12 [chr5:82860485] |
| max P.value: | 9.98E–1 [chr5:82730502] |
| omitted GWAS Hits: | NA, NA |

Make more plots at http://csg.sph.umich.edu/locuszoom/

GWAS Catalog SNPs in Region

| chr | pos (Mb) | trait | snp |
|---|---|---|---|
| 5 | 82.81912 | Laryngeal squamous cell carcinoma | rs310518 |
| 5 | 82.84549 | Diisocyanate–induced asthma | rs3852186 |
| 5 | 82.88991 | Major depressive disorder | rs310501 |
| 5 | 82.96073 | Visceral fat | rs3846635 |
| 5 | 82.98574 | Prostate cancer | rs4466137 |
| 5 | 83.17359 | Ankylosing spondylitis | rs4552569 |

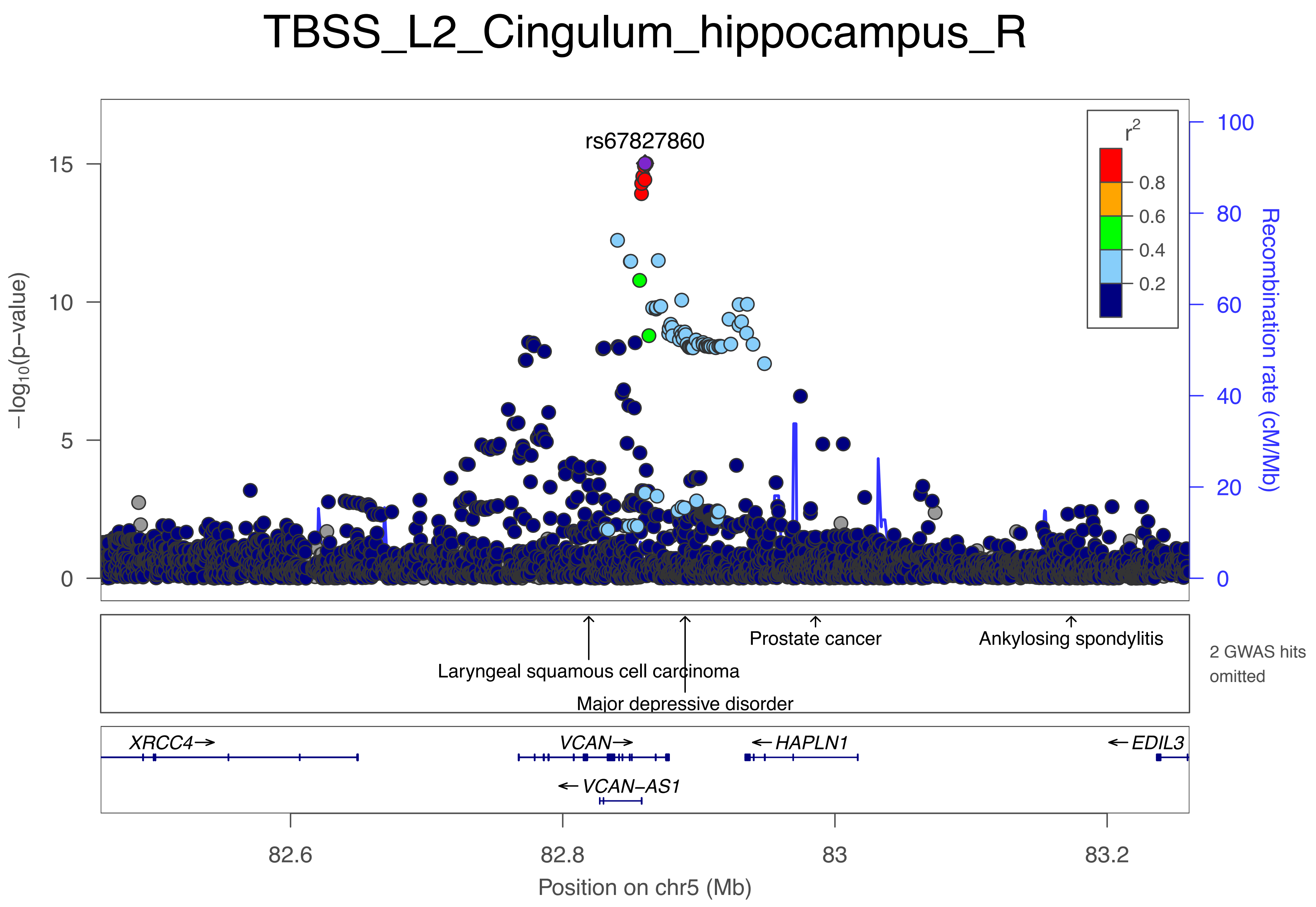

TBSS_L2_Cingulum_hippocampus_R
rs67827860
−log10(p−value)
15
10
5
0
r2
0.8
0.6
0.4
0.2
100
80
60
40
20
0
Recombination rate (cM/Mb)
Prostate cancer
Ankylosing spondylitis
Laryngeal squamous cell carcinoma
Major depressive disorder
2 GWAS hits omitted
XRCC4→
VCAN→
←HAPLN1
←EDIL3
←VCAN−AS1
82.6
82.8
83
83.2
Position on chr5 (Mb)

| | |
|---|---|
| date: | Thu Aug 17 17:52:01 2017 |
| build: | hg19 |
| display range: | chr5:82460485−83260485 [82460485−83260485] |
| hilite range: | 0 – 0 [ 0 – 0 ] |
| reference SNP: | chr5:82860485 |
| number of SNPs plotted: | 3393 |
| min P.value: | 9.57E−16 [chr5:82860485] |
| max P.value: | 9.99E−1 [chr5:82830595] |
| omitted GWAS Hits: | NA, NA |

Make more plots at http://csg.sph.umich.edu/locuszoom/

GWAS Catalog SNPs in Region

| chr | pos (Mb) | trait | snp |
|---|---|---|---|
| 5 | 82.81912 | Laryngeal squamous cell carcinoma | rs310518 |
| 5 | 82.84549 | Diisocyanate–induced asthma | rs3852186 |
| 5 | 82.88991 | Major depressive disorder | rs310501 |
| 5 | 82.96073 | Visceral fat | rs3846635 |
| 5 | 82.98574 | Prostate cancer | rs4466137 |
| 5 | 83.17359 | Ankylosing spondylitis | rs4552569 |

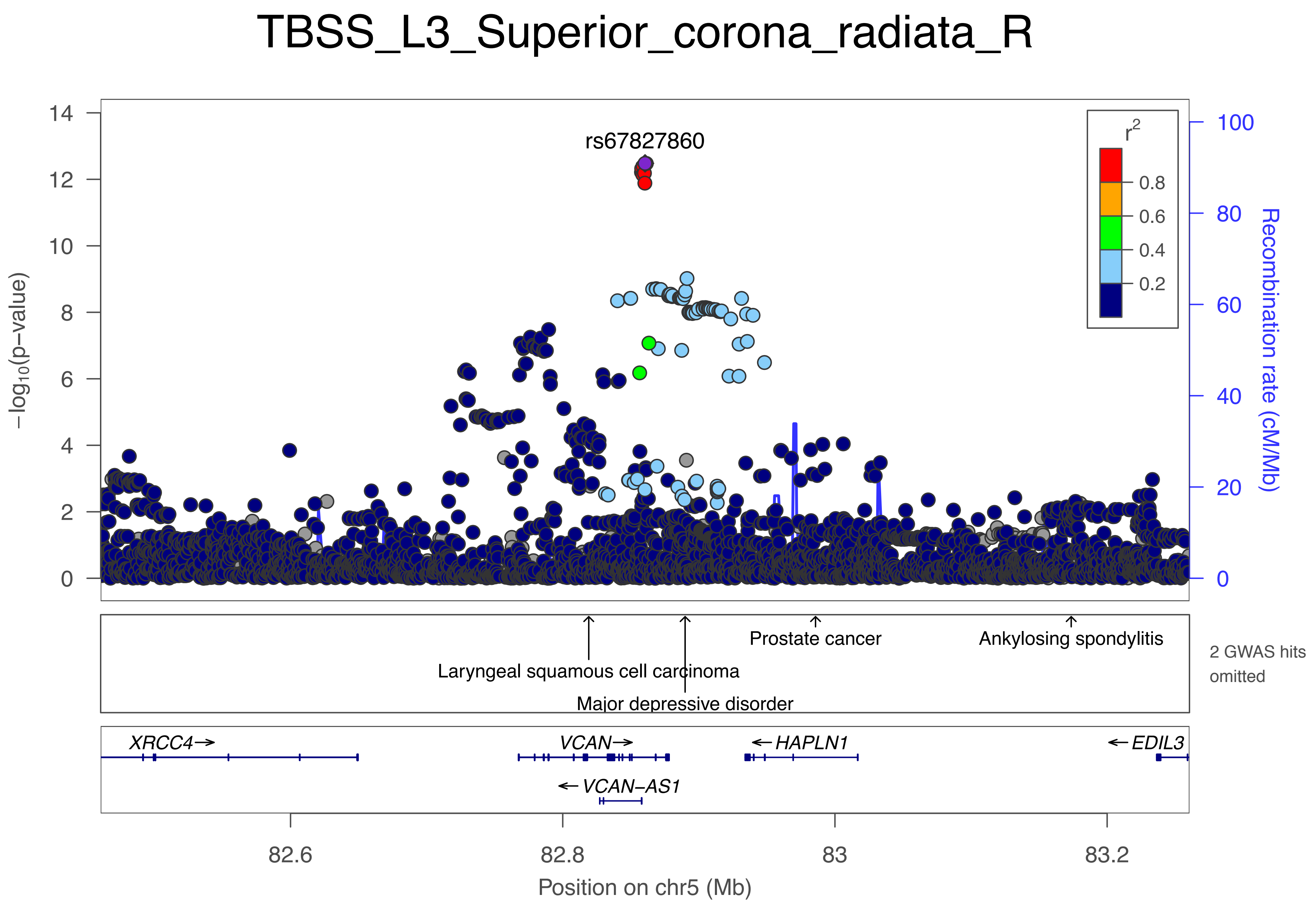

TBSS_L3_Superior_corona_radiata_R
rs67827860
−log10(p−value)
Recombination rate (cM/Mb)
r2
0.8
0.6
0.4
0.2
Prostate cancer
Ankylosing spondylitis
Laryngeal squamous cell carcinoma
Major depressive disorder
2 GWAS hits omitted
XRCC4→
VCAN→
←HAPLN1
←EDIL3
←VCAN−AS1
82.6
82.8
83
83.2
Position on chr5 (Mb)

| | |
|---|---|
| date: | Thu Aug 17 17:52:01 2017 |
| build: | hg19 |
| display range: | chr5:82460485−83260485 [82460485−83260485] |
| hilite range: | 0 – 0  [ 0 – 0 ] |
| reference SNP: | chr5:82860485 |
| number of SNPs plotted: | 3393 |
| min P.value: | 3.31E−13 [chr5:82860485] |
| max P.value: | 10E−1 [chr5:82867968] |
| omitted GWAS Hits: | NA, NA |

Make more plots at http://csg.sph.umich.edu/locuszoom/

GWAS Catalog SNPs in Region

| chr | pos (Mb) | trait | snp |
| --- | --- | --- | --- |
| 5 | 82.81912 | Laryngeal squamous cell carcinoma | rs310518 |
| 5 | 82.84549 | Diisocyanate–induced asthma | rs3852186 |
| 5 | 82.88991 | Major depressive disorder | rs310501 |
| 5 | 82.96073 | Visceral fat | rs3846635 |
| 5 | 82.98574 | Prostate cancer | rs4466137 |
| 5 | 83.17359 | Ankylosing spondylitis | rs4552569 |

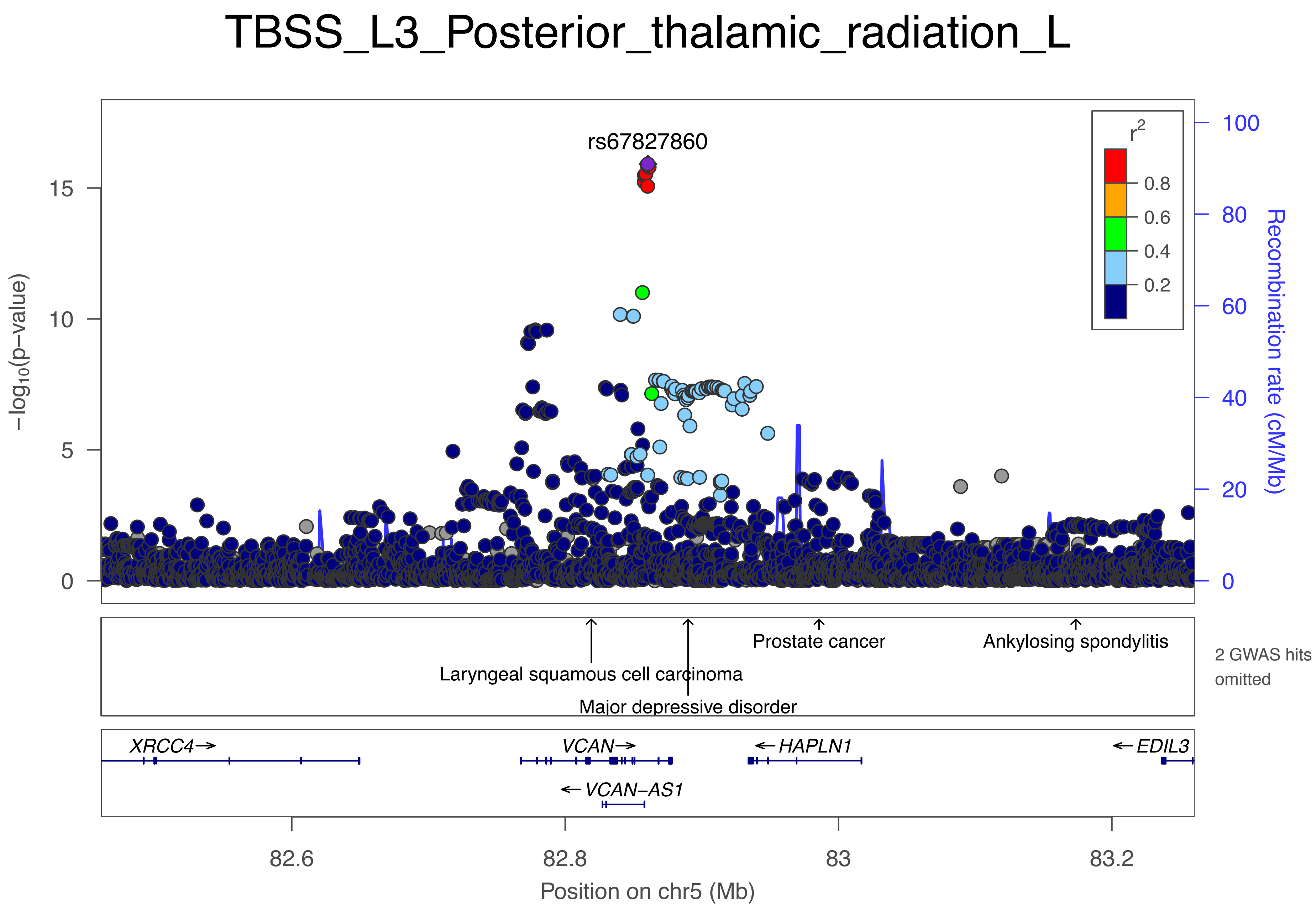
TBSS_L3_Posterior_thalamic_radiation_L
rs67827860
−log10(p−value)
15
10
5
0
r2
0.8
0.6
0.4
0.2
100
80
60
40
20
0
Recombination rate (cM/Mb)
Laryngeal squamous cell carcinoma
Major depressive disorder
Prostate cancer
Ankylosing spondylitis
2 GWAS hits omitted
XRCC4
VCAN
VCAN−AS1
HAPLN1
EDIL3
82.6
82.8
83
83.2
Position on chr5 (Mb)

| | |
|---|---|
| date: | Thu Aug 17 17:52:01 2017 |
| build: | hg19 |
| display range: | chr5:82460485−83260485 [82460485−83260485] |
| hilite range: | 0 – 0 [ 0 – 0 ] |
| reference SNP: | chr5:82860485 |
| number of SNPs plotted: | 3393 |
| min P.value: | 1.22E−16 [chr5:82860485] |
| max P.value: | 10E−1 [chr5:83097880] |
| omitted GWAS Hits: | NA, NA |

Make more plots at http://csg.sph.umich.edu/locuszoom/

GWAS Catalog SNPs in Region

| chr | pos (Mb) | trait | snp |
|---|---|---|---|
| 5 | 82.81912 | Laryngeal squamous cell carcinoma | rs310518 |
| 5 | 82.84549 | Diisocyanate–induced asthma | rs3852186 |
| 5 | 82.88991 | Major depressive disorder | rs310501 |
| 5 | 82.96073 | Visceral fat | rs3846635 |
| 5 | 82.98574 | Prostate cancer | rs4466137 |
| 5 | 83.17359 | Ankylosing spondylitis | rs4552569 |

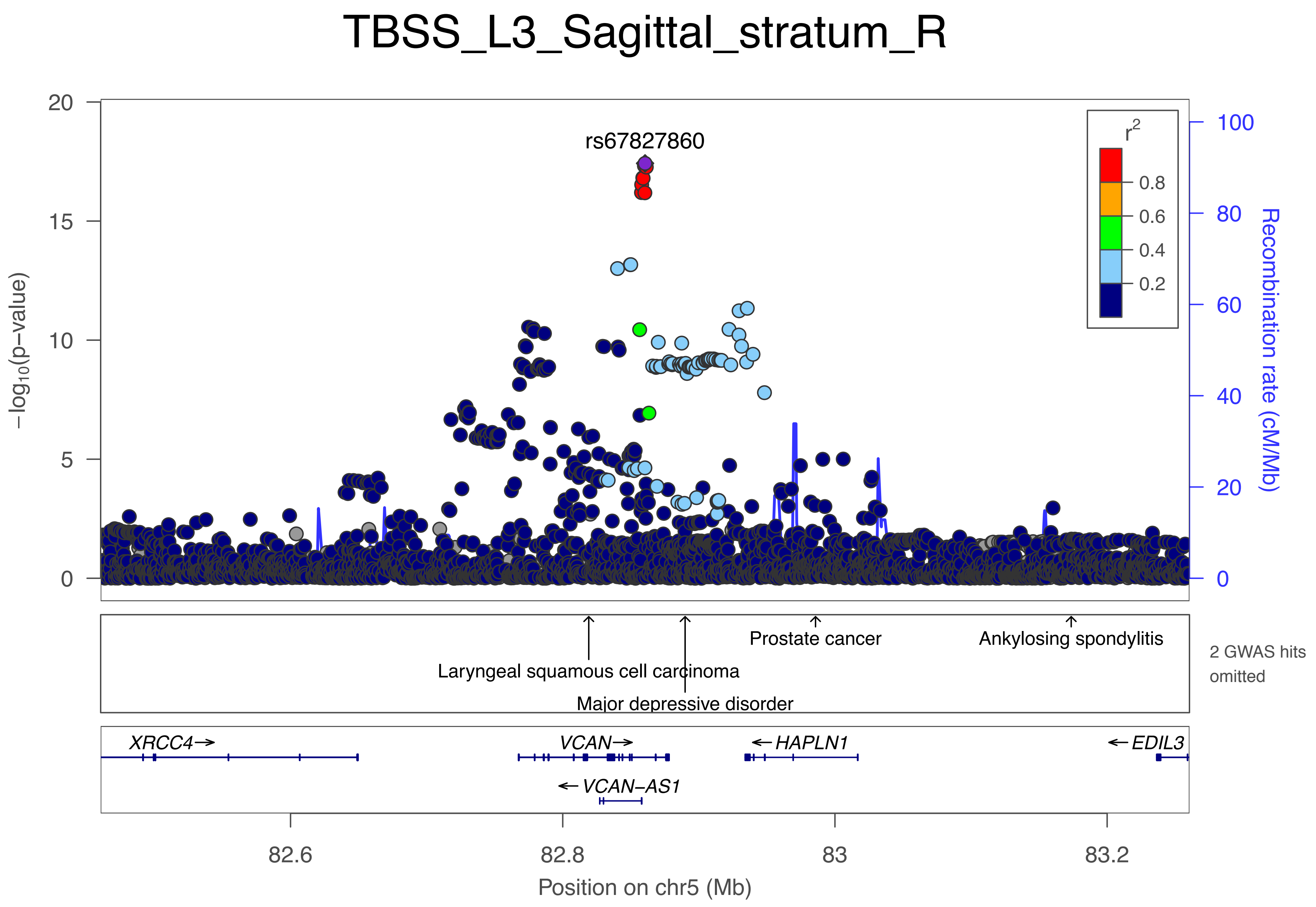

TBSS_L3_Sagittal_stratum_R
rs67827860
$-\log_{10}$(p−value)
Recombination rate (cM/Mb)
$r^2$
0.8
0.6
0.4
0.2
Prostate cancer
Ankylosing spondylitis
Laryngeal squamous cell carcinoma
Major depressive disorder
2 GWAS hits omitted
XRCC4→
VCAN→
←HAPLN1
←EDIL3
←VCAN−AS1
82.6
82.8
83
83.2
Position on chr5 (Mb)

| | |
|---|---|
| date: | Thu Aug 17 17:52:01 2017 |
| build: | hg19 |
| display range: | chr5:82460485−83260485 [82460485−83260485] |
| hilite range: | 0 – 0 [ 0 – 0 ] |
| reference SNP: | chr5:82860485 |
| number of SNPs plotted: | 3393 |
| min P.value: | 3.78E−18 [chr5:82860485] |
| max P.value: | 9.99E−1 [chr5:82495239] |
| omitted GWAS Hits: | NA, NA |

Make more plots at http://csg.sph.umich.edu/locuszoom/

GWAS Catalog SNPs in Region

| chr | pos (Mb) | trait | snp |
|---|---|---|---|
| 5 | 82.81912 | Laryngeal squamous cell carcinoma | rs310518 |
| 5 | 82.84549 | Diisocyanate–induced asthma | rs3852186 |
| 5 | 82.88991 | Major depressive disorder | rs310501 |
| 5 | 82.96073 | Visceral fat | rs3846635 |
| 5 | 82.98574 | Prostate cancer | rs4466137 |
| 5 | 83.17359 | Ankylosing spondylitis | rs4552569 |

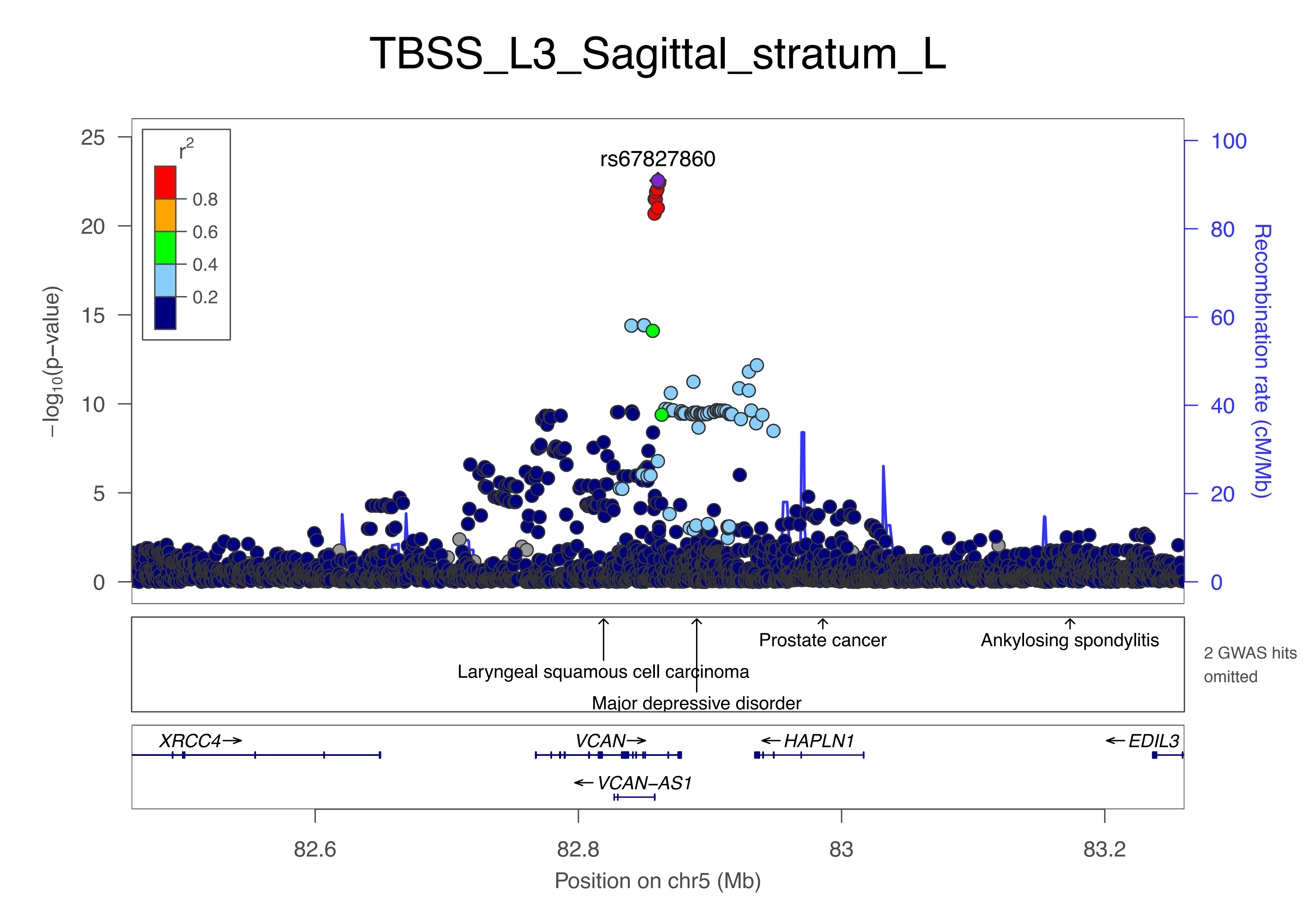
TBSS_L3_Sagittal_stratum_L
rs67827860
r2
0.8
0.6
0.4
0.2
−log10(p−value)
Recombination rate (cM/Mb)
Prostate cancer
Ankylosing spondylitis
Laryngeal squamous cell carcinoma
Major depressive disorder
2 GWAS hits omitted
XRCC4
VCAN
HAPLN1
EDIL3
VCAN−AS1
82.6
82.8
83
83.2
Position on chr5 (Mb)

| | |
|---|---|
| date: | Thu Aug 17 17:52:01 2017 |
| build: | hg19 |
| display range: | chr5:82460485−83260485 [82460485−83260485] |
| hilite range: | 0 – 0 [ 0 – 0 ] |
| reference SNP: | chr5:82860485 |
| number of SNPs plotted: | 3393 |
| min P.value: | 2.86E−23 [chr5:82860485] |
| max P.value: | 10E−1 [chr5:82466270] |
| omitted GWAS Hits: | NA, NA |

Make more plots at http://csg.sph.umich.edu/locuszoom/

GWAS Catalog SNPs in Region

| chr | pos (Mb) | trait | snp |
|---|---|---|---|
| 5 | 82.81912 | Laryngeal squamous cell carcinoma | rs310518 |
| 5 | 82.84549 | Diisocyanate–induced asthma | rs3852186 |
| 5 | 82.88991 | Major depressive disorder | rs310501 |
| 5 | 82.96073 | Visceral fat | rs3846635 |
| 5 | 82.98574 | Prostate cancer | rs4466137 |
| 5 | 83.17359 | Ankylosing spondylitis | rs4552569 |

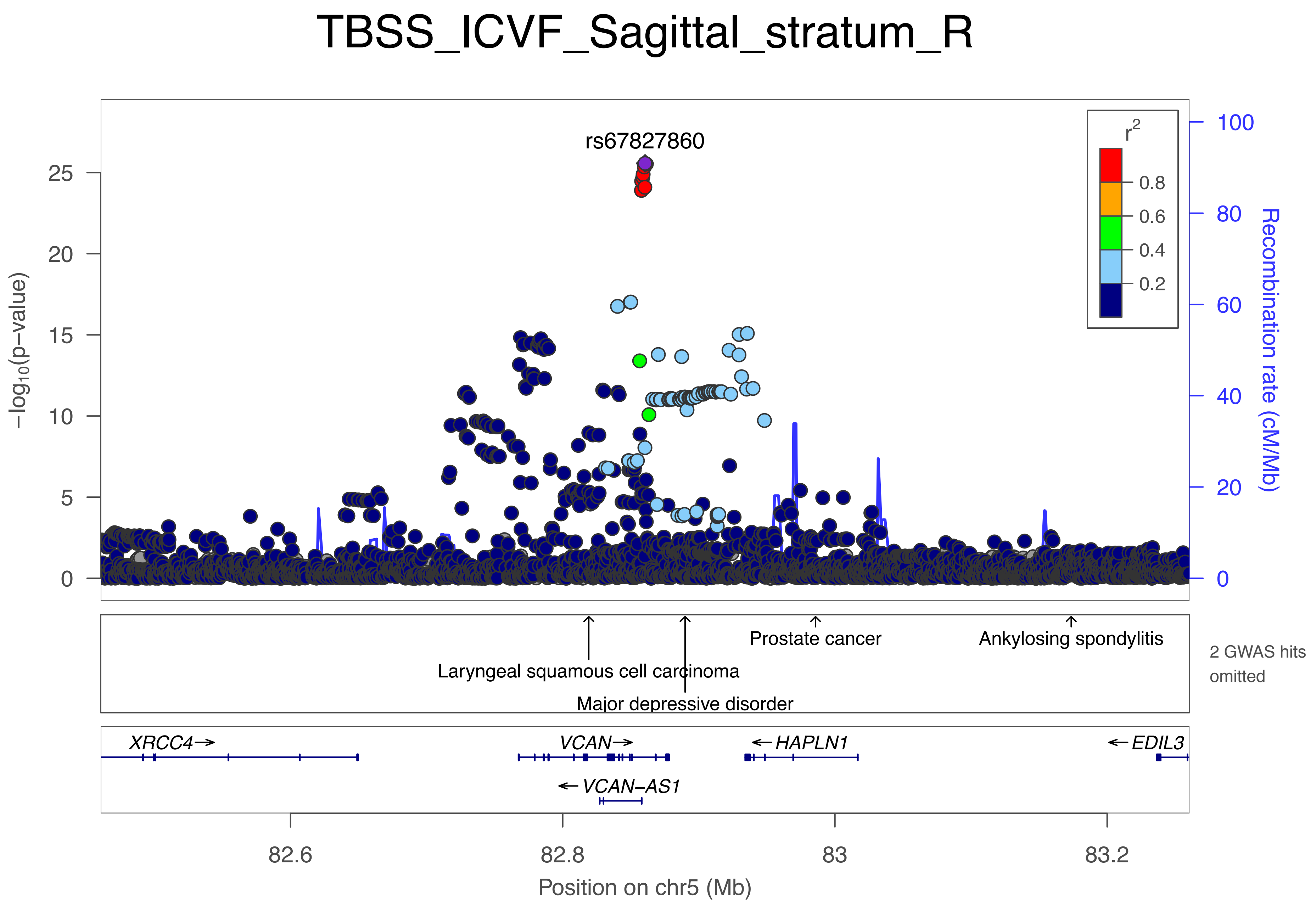
TBSS_ICVF_Sagittal_stratum_R
rs67827860
r2
0.8
0.6
0.4
0.2
−log10(p−value)
Recombination rate (cM/Mb)
Laryngeal squamous cell carcinoma
Major depressive disorder
Prostate cancer
Ankylosing spondylitis
2 GWAS hits omitted
XRCC4→
VCAN→
←VCAN−AS1
←HAPLN1
←EDIL3
82.6
82.8
83
83.2
Position on chr5 (Mb)

| | |
|---|---|
| date: | Thu Aug 17 17:52:01 2017 |
| build: | hg19 |
| display range: | chr5:82460485–83260485 [82460485–83260485] |
| hilite range: | 0 – 0 [ 0 – 0 ] |
| reference SNP: | chr5:82860485 |
| number of SNPs plotted: | 3393 |
| min P.value: | 2.69E–26 [chr5:82860485] |
| max P.value: | 9.99E–1 [chr5:82487589] |
| omitted GWAS Hits: | NA, NA |

Make more plots at http://csg.sph.umich.edu/locuszoom/

# GWAS Catalog SNPs in Region

| chr | pos (Mb) | trait | snp |
|---|---|---|---|
| 5 | 82.81912 | Laryngeal squamous cell carcinoma | rs310518 |
| 5 | 82.84549 | Diisocyanate–induced asthma | rs3852186 |
| 5 | 82.88991 | Major depressive disorder | rs310501 |
| 5 | 82.96073 | Visceral fat | rs3846635 |
| 5 | 82.98574 | Prostate cancer | rs4466137 |
| 5 | 83.17359 | Ankylosing spondylitis | rs4552569 |

# TBSS_ICVF_Fornix_cres+Stria_terminalis_R

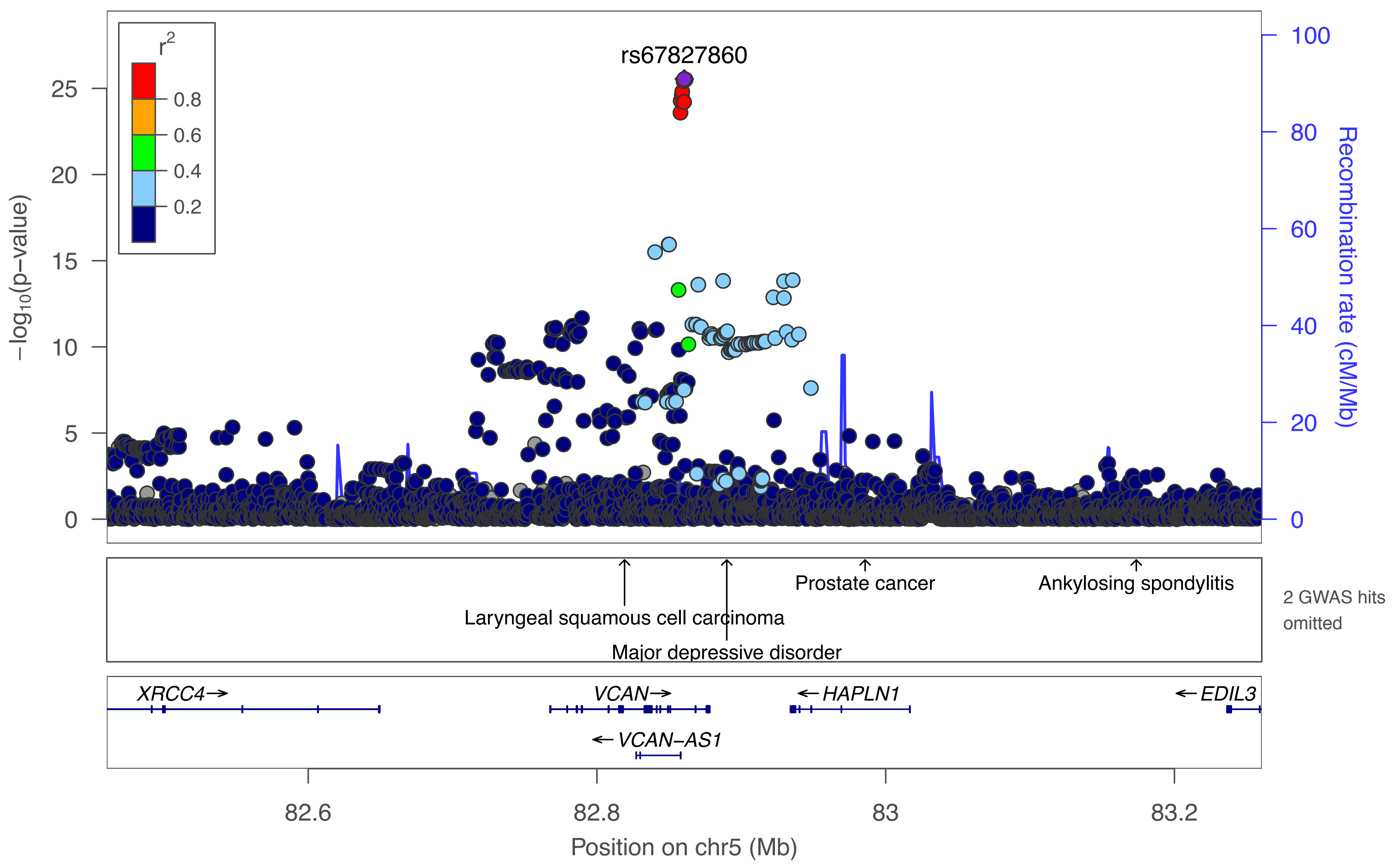

| | |
|---|---|
| date: | Thu Aug 17 17:52:01 2017 |
| build: | hg19 |
| display range: | chr5:82460485–83260485 [82460485–83260485] |
| hilite range: | 0 – 0 [ 0 – 0 ] |
| reference SNP: | chr5:82860485 |
| number of SNPs plotted: | 3393 |
| min P.value: | 2.84E–26 [chr5:82860485] |
| max P.value: | 10E–1 [chr5:83056492] |
| omitted GWAS Hits: | NA, NA |

Make more plots at http://csg.sph.umich.edu/locuszoom/

GWAS Catalog SNPs in Region

| chr | pos (Mb) | trait | snp |
|---|---|---|---|
| 5 | 82.81912 | Laryngeal squamous cell carcinoma | rs310518 |
| 5 | 82.84549 | Diisocyanate–induced asthma | rs3852186 |
| 5 | 82.88991 | Major depressive disorder | rs310501 |
| 5 | 82.96073 | Visceral fat | rs3846635 |
| 5 | 82.98574 | Prostate cancer | rs4466137 |
| 5 | 83.17359 | Ankylosing spondylitis | rs4552569 |

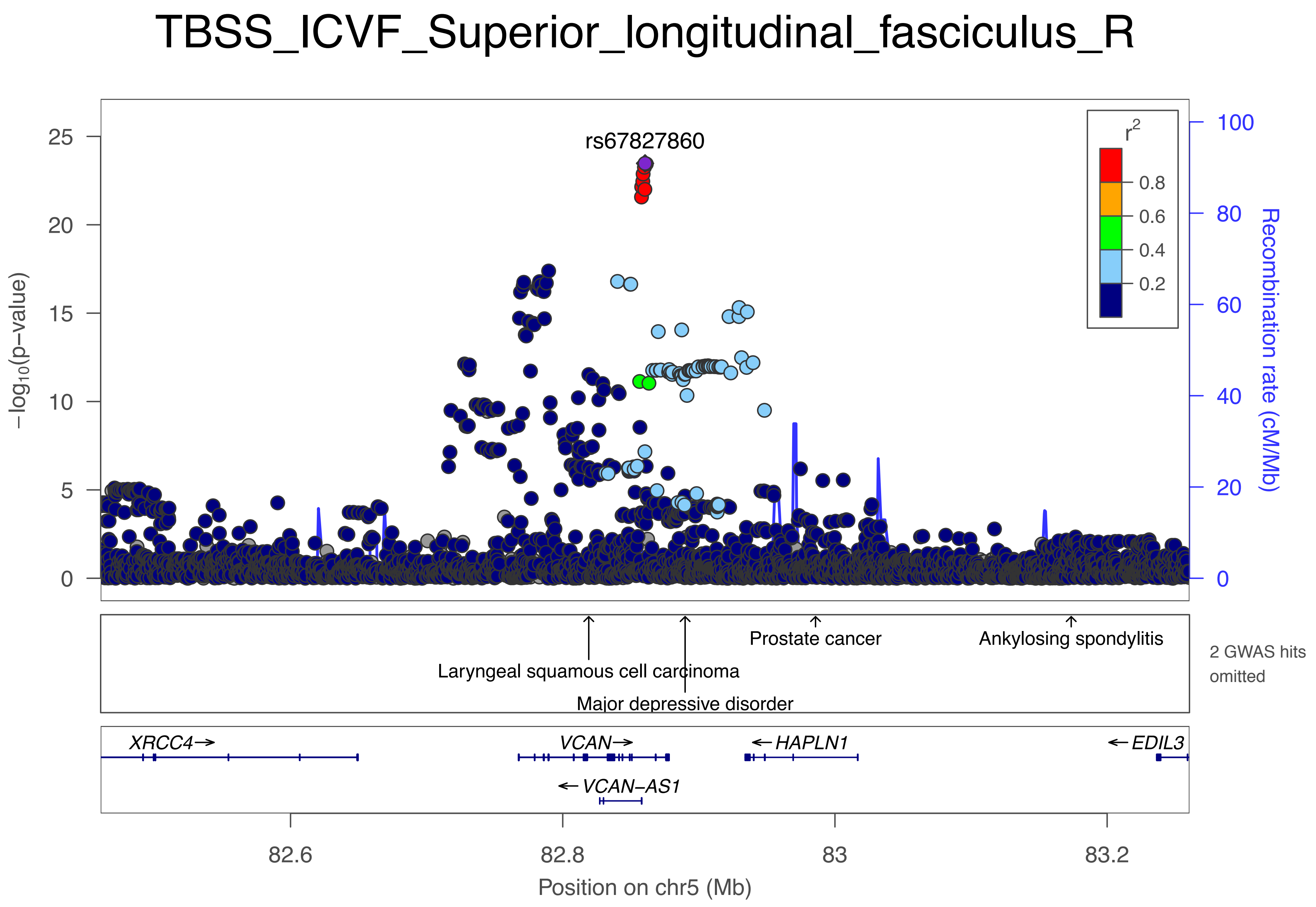

TBSS_ICVF_Superior_longitudinal_fasciculus_R
rs67827860
−log10(p−value)
25
20
15
10
5
0
100
80
60
40
20
0
Recombination rate (cM/Mb)
r2
0.8
0.6
0.4
0.2
Laryngeal squamous cell carcinoma
Major depressive disorder
Prostate cancer
Ankylosing spondylitis
2 GWAS hits omitted
XRCC4→
VCAN→
←HAPLN1
←EDIL3
←VCAN−AS1
82.6
82.8
83
83.2
Position on chr5 (Mb)

| | |
|---|---|
| date: | Thu Aug 17 17:52:01 2017 |
| build: | hg19 |
| display range: | chr5:82460485−83260485 [82460485−83260485] |
| hilite range: | 0 – 0  [ 0 – 0 ] |
| reference SNP: | chr5:82860485 |
| number of SNPs plotted: | 3393 |
| min P.value: | 3.36E−24 [chr5:82860485] |
| max P.value: | 10E−1 [chr5:82921389] |
| omitted GWAS Hits: | NA, NA |

Make more plots at http://csg.sph.umich.edu/locuszoom/

GWAS Catalog SNPs in Region

| chr | pos (Mb) | trait | snp |
|---|---|---|---|
| 5 | 82.81912 | Laryngeal squamous cell carcinoma | rs310518 |
| 5 | 82.84549 | Diisocyanate–induced asthma | rs3852186 |
| 5 | 82.88991 | Major depressive disorder | rs310501 |
| 5 | 82.96073 | Visceral fat | rs3846635 |
| 5 | 82.98574 | Prostate cancer | rs4466137 |
| 5 | 83.17359 | Ankylosing spondylitis | rs4552569 |

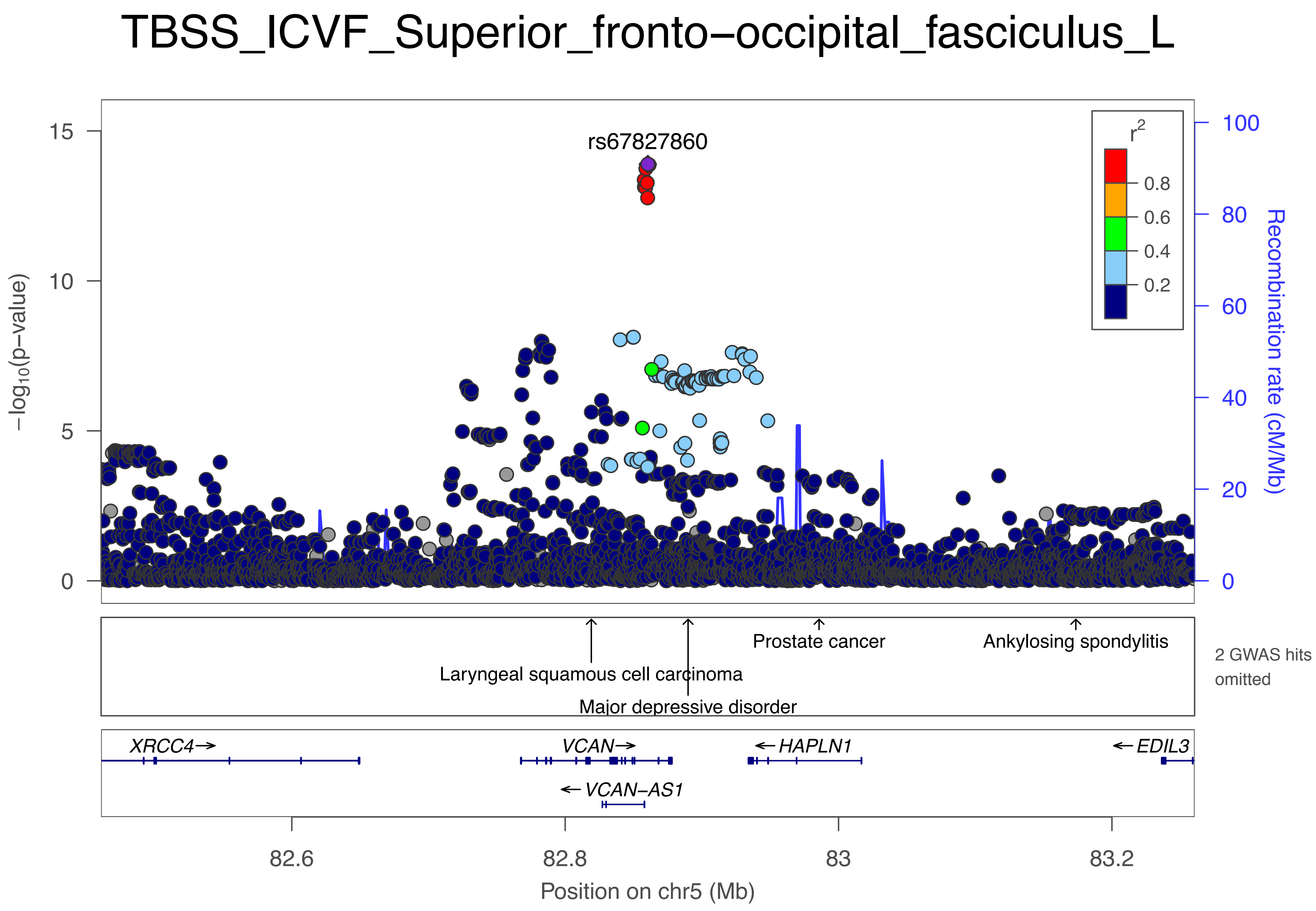
TBSS_ICVF_Superior_fronto−occipital_fasciculus_L
rs67827860
−log10(p−value)
15
10
5
0
100
80
60
40
20
0
Recombination rate (cM/Mb)
r2
0.8
0.6
0.4
0.2
Laryngeal squamous cell carcinoma
Major depressive disorder
Prostate cancer
Ankylosing spondylitis
2 GWAS hits omitted
XRCC4
VCAN
VCAN−AS1
HAPLN1
EDIL3
82.6
82.8
83
83.2
Position on chr5 (Mb)

| | |
|---|---|
| date: | Thu Aug 17 17:52:01 2017 |
| build: | hg19 |
| display range: | chr5:82460485−83260485 [82460485−83260485] |
| hilite range: | 0 – 0 [ 0 – 0 ] |
| reference SNP: | chr5:82860485 |
| number of SNPs plotted: | 3393 |
| min P.value: | 1.28E−14 [chr5:82860485] |
| max P.value: | 10E−1 [chr5:83117229] |
| omitted GWAS Hits: | NA, NA |

Make more plots at http://csg.sph.umich.edu/locuszoom/

GWAS Catalog SNPs in Region

| chr | pos (Mb) | trait | snp |
|---|---|---|---|
| 5 | 82.81912 | Laryngeal squamous cell carcinoma | rs310518 |
| 5 | 82.84549 | Diisocyanate–induced asthma | rs3852186 |
| 5 | 82.88991 | Major depressive disorder | rs310501 |
| 5 | 82.96073 | Visceral fat | rs3846635 |
| 5 | 82.98574 | Prostate cancer | rs4466137 |
| 5 | 83.17359 | Ankylosing spondylitis | rs4552569 |

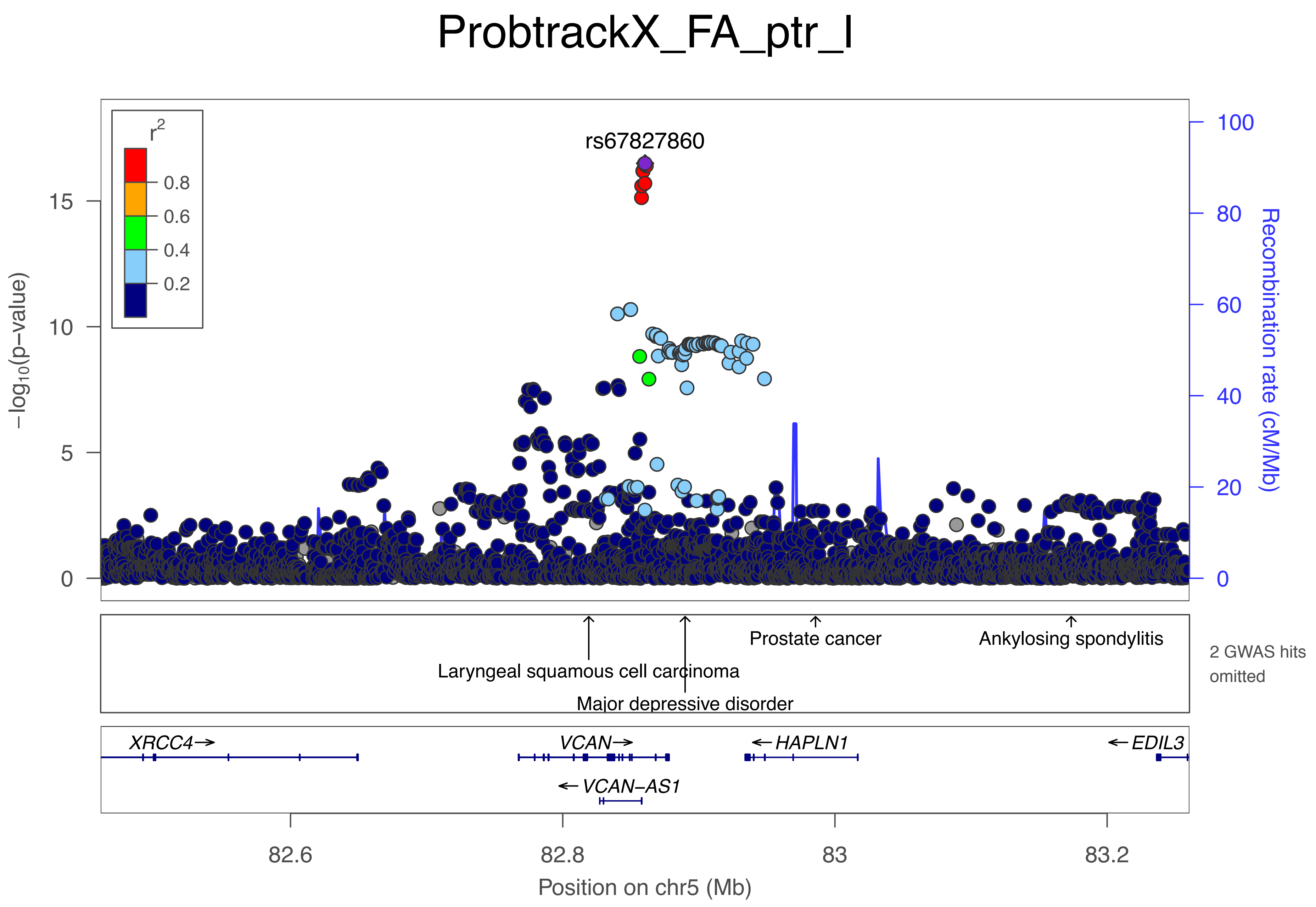

ProbtrackX_FA_ptr_l
r2
0.8
0.6
0.4
0.2
rs67827860
0
5
10
15
−log10(p−value)
0
20
40
60
80
100
Recombination rate (cM/Mb)
Laryngeal squamous cell carcinoma
Major depressive disorder
Prostate cancer
Ankylosing spondylitis
2 GWAS hits omitted
XRCC4→
VCAN→
←HAPLN1
←EDIL3
←VCAN−AS1
82.6
82.8
83
83.2
Position on chr5 (Mb)

| | |
|---|---|
| date: | Thu Aug 17 17:52:01 2017 |
| build: | hg19 |
| display range: | chr5:82460485−83260485 [82460485−83260485] |
| hilite range: | 0 – 0 [ 0 – 0 ] |
| reference SNP: | chr5:82860485 |
| number of SNPs plotted: | 3393 |
| min P.value: | 3.21E−17 [chr5:82860485] |
| max P.value: | 10E−1 [chr5:82867318] |
| omitted GWAS Hits: | NA, NA |

Make more plots at http://csg.sph.umich.edu/locuszoom/

GWAS Catalog SNPs in Region

| chr | pos (Mb) | trait | snp |
|---|---|---|---|
| 5 | 82.81912 | Laryngeal squamous cell carcinoma | rs310518 |
| 5 | 82.84549 | Diisocyanate–induced asthma | rs3852186 |
| 5 | 82.88991 | Major depressive disorder | rs310501 |
| 5 | 82.96073 | Visceral fat | rs3846635 |
| 5 | 82.98574 | Prostate cancer | rs4466137 |
| 5 | 83.17359 | Ankylosing spondylitis | rs4552569 |

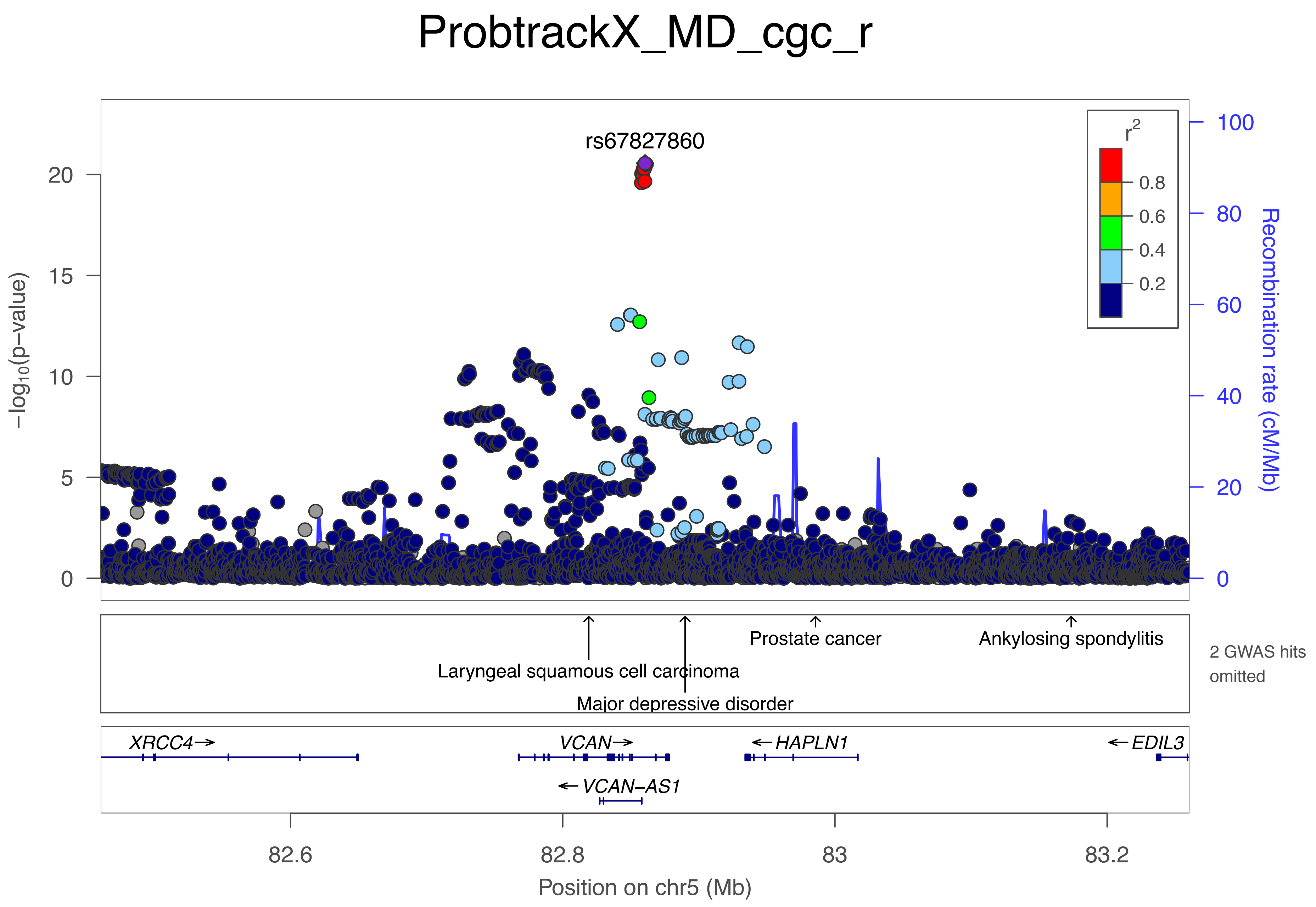

ProbtrackX_MD_cgc_r
rs67827860
−log10(p−value)
Recombination rate (cM/Mb)
r2
0.8
0.6
0.4
0.2
0
5
10
15
20
0
20
40
60
80
100
Laryngeal squamous cell carcinoma
Major depressive disorder
Prostate cancer
Ankylosing spondylitis
2 GWAS hits omitted
XRCC4→
VCAN→
←VCAN−AS1
←HAPLN1
←EDIL3
82.6
82.8
83
83.2
Position on chr5 (Mb)

| | |
|---|---|
| date: | Thu Aug 17 17:52:01 2017 |
| build: | hg19 |
| display range: | chr5:82460485–83260485 [82460485–83260485] |
| hilite range: | 0 – 0 [ 0 – 0 ] |
| reference SNP: | chr5:82860485 |
| number of SNPs plotted: | 3393 |
| min P.value: | 2.81E–21 [chr5:82860485] |
| max P.value: | 10E–1 [chr5:82750729] |
| omitted GWAS Hits: | NA, NA |

Make more plots at http://csg.sph.umich.edu/locuszoom/

GWAS Catalog SNPs in Region

| chr | pos (Mb) | trait | snp |
|---|---|---|---|
| 5 | 82.81912 | Laryngeal squamous cell carcinoma | rs310518 |
| 5 | 82.84549 | Diisocyanate–induced asthma | rs3852186 |
| 5 | 82.88991 | Major depressive disorder | rs310501 |
| 5 | 82.96073 | Visceral fat | rs3846635 |
| 5 | 82.98574 | Prostate cancer | rs4466137 |
| 5 | 83.17359 | Ankylosing spondylitis | rs4552569 |

# ProbtrackX_MD_ifo_l

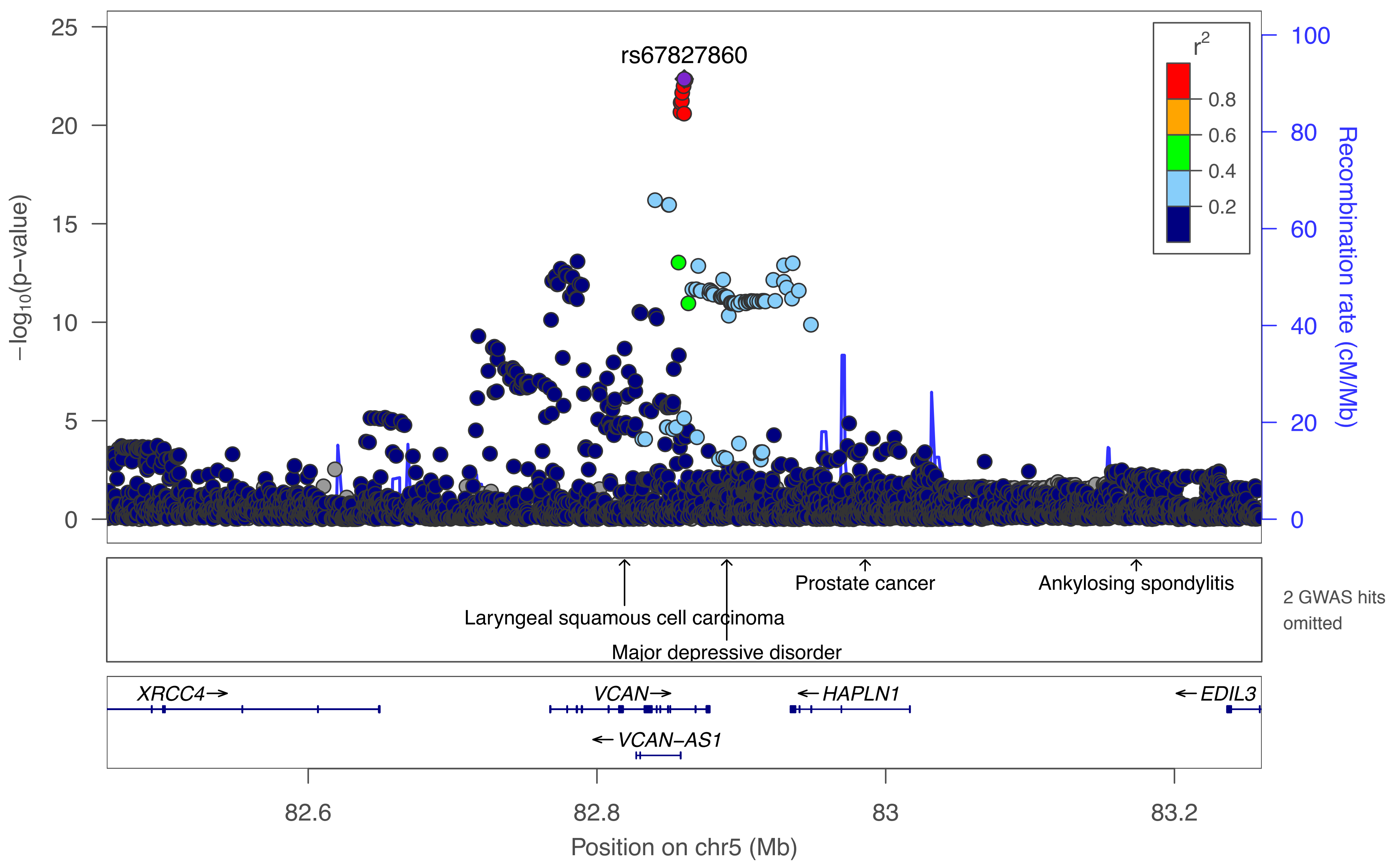

| | |
|---|---|
| date: | Thu Aug 17 17:52:01 2017 |
| build: | hg19 |
| display range: | chr5:82460485–83260485 [82460485–83260485] |
| hilite range: | 0 – 0 [ 0 – 0 ] |
| reference SNP: | chr5:82860485 |
| number of SNPs plotted: | 3393 |
| min P.value: | 4.47E–23 [chr5:82860485] |
| max P.value: | 10E–1 [chr5:82609713] |
| omitted GWAS Hits: | NA, NA |

Make more plots at http://csg.sph.umich.edu/locuszoom/

GWAS Catalog SNPs in Region

| chr | pos (Mb) | trait | snp |
|---|---|---|---|
| 5 | 82.81912 | Laryngeal squamous cell carcinoma | rs310518 |
| 5 | 82.84549 | Diisocyanate–induced asthma | rs3852186 |
| 5 | 82.88991 | Major depressive disorder | rs310501 |
| 5 | 82.96073 | Visceral fat | rs3846635 |
| 5 | 82.98574 | Prostate cancer | rs4466137 |
| 5 | 83.17359 | Ankylosing spondylitis | rs4552569 |

# ProbtrackX_MD_ifo_r

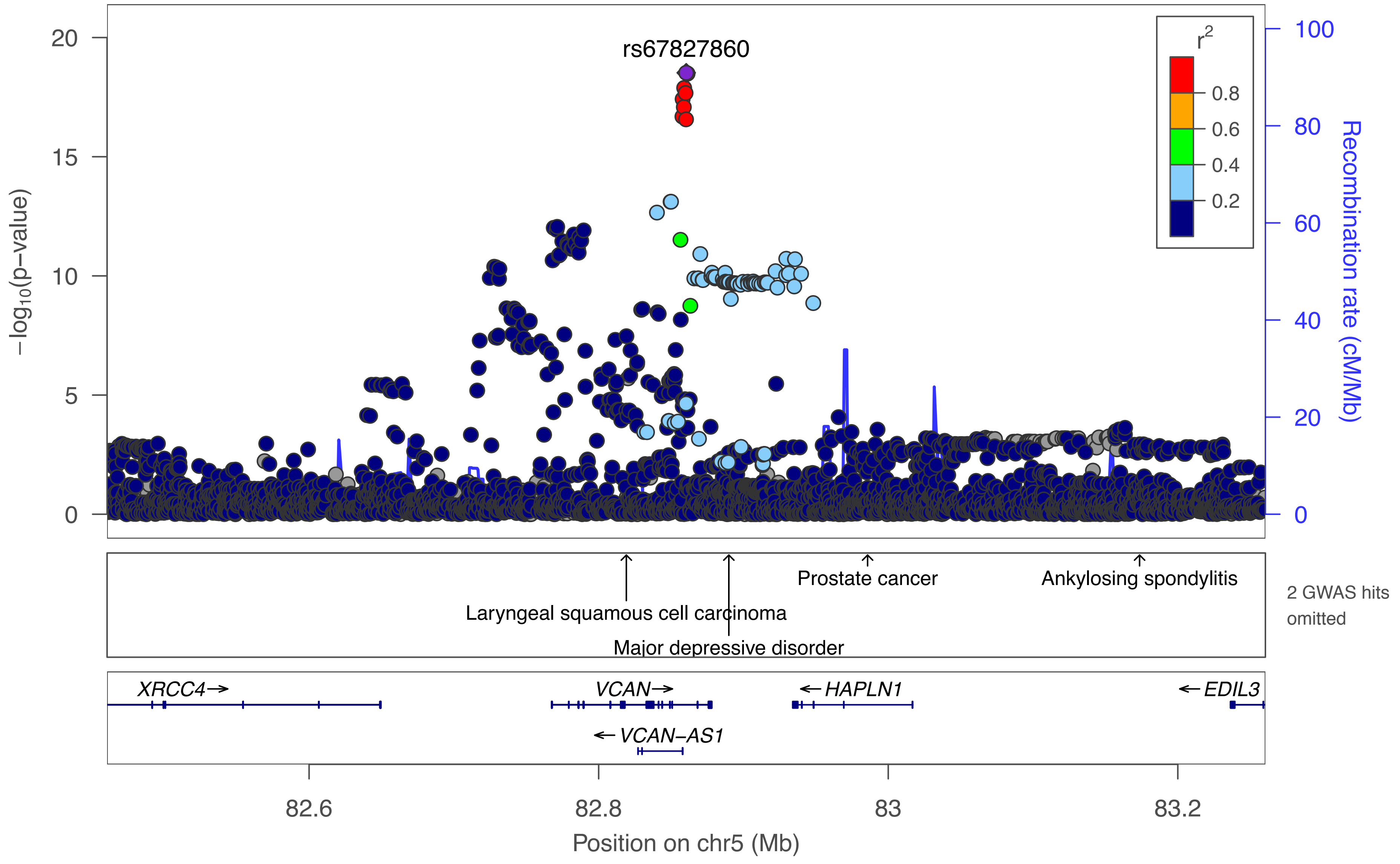

| | |
|---|---|
| date: | Thu Aug 17 17:52:01 2017 |
| build: | hg19 |
| display range: | chr5:82460485–83260485 [82460485–83260485] |
| hilite range: | 0 – 0 [ 0 – 0 ] |
| reference SNP: | chr5:82860485 |
| number of SNPs plotted: | 3393 |
| min P.value: | 3.01E–19 [chr5:82860485] |
| max P.value: | 10E–1 [chr5:82923125] |
| omitted GWAS Hits: | NA, NA |

Make more plots at http://csg.sph.umich.edu/locuszoom/

GWAS Catalog SNPs in Region

| chr | pos (Mb) | trait | snp |
|---|---|---|---|
| 5 | 82.81912 | Laryngeal squamous cell carcinoma | rs310518 |
| 5 | 82.84549 | Diisocyanate–induced asthma | rs3852186 |
| 5 | 82.88991 | Major depressive disorder | rs310501 |
| 5 | 82.96073 | Visceral fat | rs3846635 |
| 5 | 82.98574 | Prostate cancer | rs4466137 |
| 5 | 83.17359 | Ankylosing spondylitis | rs4552569 |

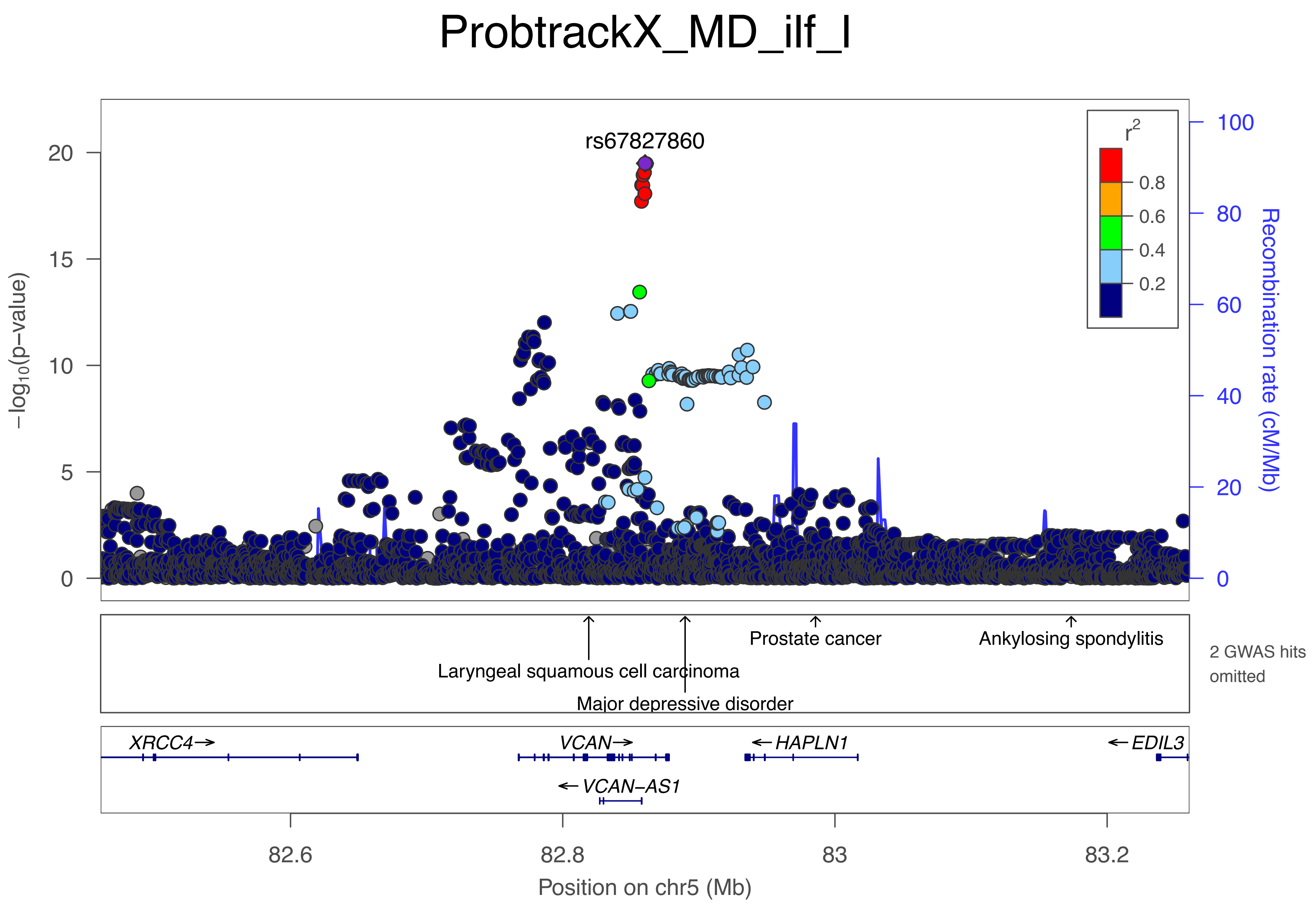
ProbtrackX_MD_ilf_l
rs67827860
$-\log_{10}$(p−value)
Recombination rate (cM/Mb)
r²
0.8
0.6
0.4
0.2
Laryngeal squamous cell carcinoma
Major depressive disorder
Prostate cancer
Ankylosing spondylitis
2 GWAS hits omitted
XRCC4→
VCAN→
←HAPLN1
←EDIL3
←VCAN−AS1
82.6
82.8
83
83.2
Position on chr5 (Mb)

| | |
|---|---|
| date: | Thu Aug 17 17:52:01 2017 |
| build: | hg19 |
| display range: | chr5:82460485–83260485 [82460485–83260485] |
| hilite range: | 0 – 0 [ 0 – 0 ] |
| reference SNP: | chr5:82860485 |
| number of SNPs plotted: | 3393 |
| min P.value: | 3.23E–20 [chr5:82860485] |
| max P.value: | 10E–1 [chr5:83083067] |
| omitted GWAS Hits: | NA, NA |

Make more plots at http://csg.sph.umich.edu/locuszoom/

GWAS Catalog SNPs in Region

| chr | pos (Mb) | trait | snp |
|---|---|---|---|
| 5 | 82.81912 | Laryngeal squamous cell carcinoma | rs310518 |
| 5 | 82.84549 | Diisocyanate–induced asthma | rs3852186 |
| 5 | 82.88991 | Major depressive disorder | rs310501 |
| 5 | 82.96073 | Visceral fat | rs3846635 |
| 5 | 82.98574 | Prostate cancer | rs4466137 |
| 5 | 83.17359 | Ankylosing spondylitis | rs4552569 |

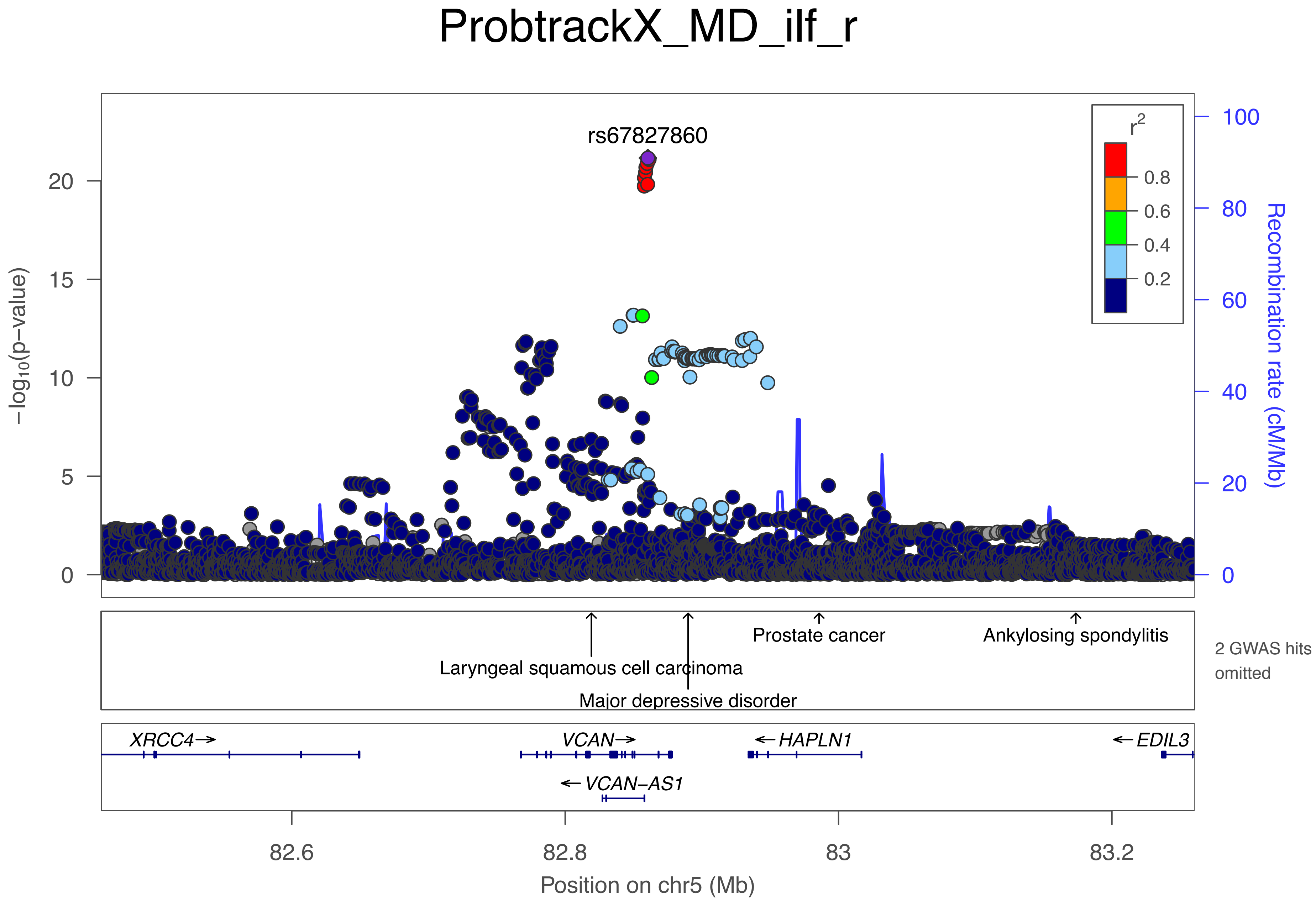
ProbtrackX_MD_ilf_r
rs67827860
r2
0.8
0.6
0.4
0.2
−log10(p−value)
Recombination rate (cM/Mb)
0
5
10
15
20
0
20
40
60
80
100
Laryngeal squamous cell carcinoma
Major depressive disorder
Prostate cancer
Ankylosing spondylitis
2 GWAS hits omitted
XRCC4→
VCAN→
←VCAN−AS1
←HAPLN1
←EDIL3
82.6
82.8
83
83.2
Position on chr5 (Mb)

| | |
|---|---|
| date: | Thu Aug 17 17:52:01 2017 |
| build: | hg19 |
| display range: | chr5:82460485−83260485 [82460485−83260485] |
| hilite range: | 0 – 0 [ 0 – 0 ] |
| reference SNP: | chr5:82860485 |
| number of SNPs plotted: | 3393 |
| min P.value: | 6.93E−22 [chr5:82860485] |
| max P.value: | 9.99E−1 [chr5:82524547] |
| omitted GWAS Hits: | NA, NA |

Make more plots at http://csg.sph.umich.edu/locuszoom/

GWAS Catalog SNPs in Region

| chr | pos (Mb) | trait | snp |
|---|---|---|---|
| 5 | 82.81912 | Laryngeal squamous cell carcinoma | rs310518 |
| 5 | 82.84549 | Diisocyanate–induced asthma | rs3852186 |
| 5 | 82.88991 | Major depressive disorder | rs310501 |
| 5 | 82.96073 | Visceral fat | rs3846635 |
| 5 | 82.98574 | Prostate cancer | rs4466137 |
| 5 | 83.17359 | Ankylosing spondylitis | rs4552569 |

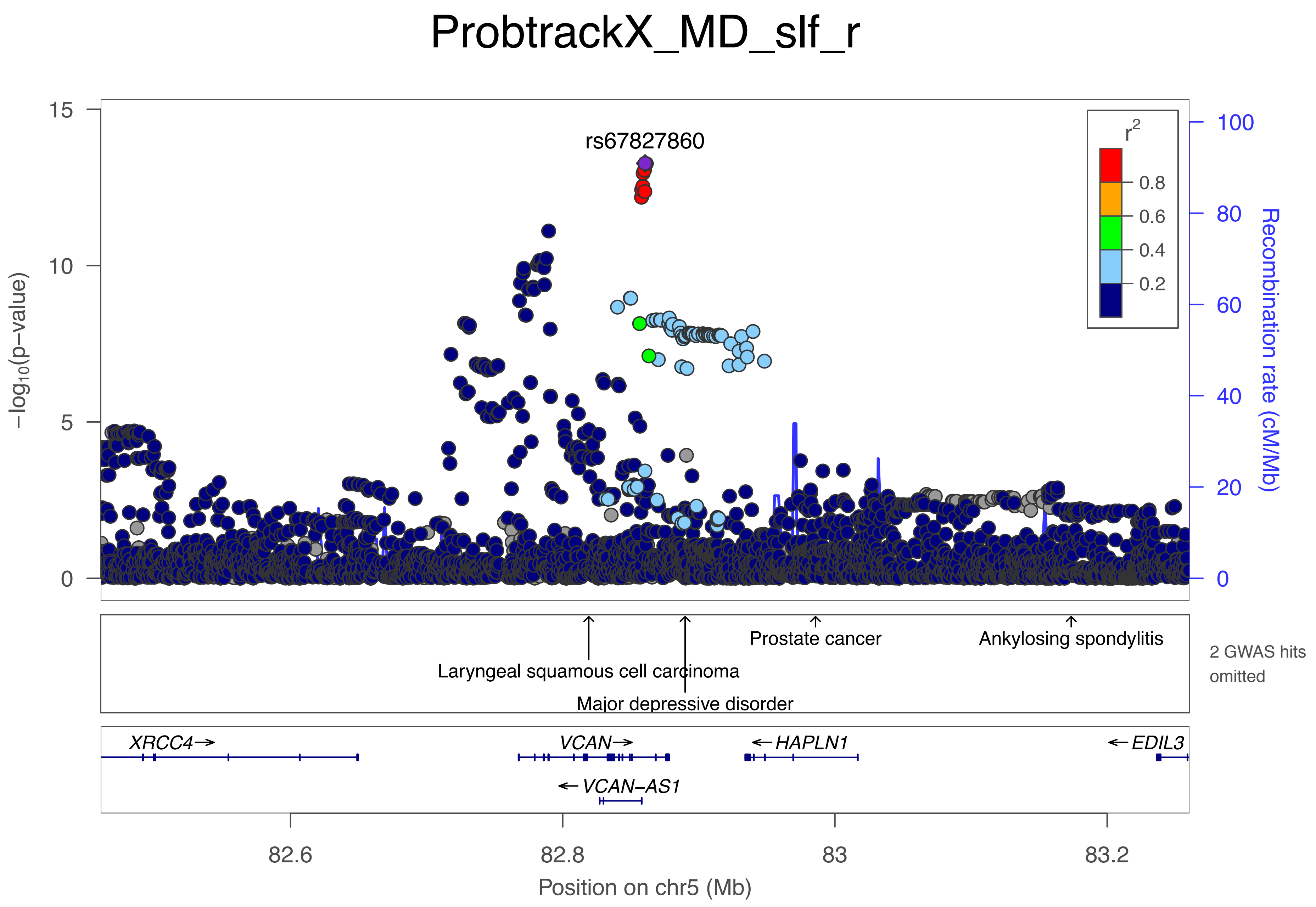
ProbtrackX_MD_slf_r
rs67827860
−log10(p−value)
Recombination rate (cM/Mb)
r2
0.8
0.6
0.4
0.2
Prostate cancer
Ankylosing spondylitis
Laryngeal squamous cell carcinoma
Major depressive disorder
2 GWAS hits omitted
XRCC4→
VCAN→
←HAPLN1
←EDIL3
←VCAN−AS1
82.6
82.8
83
83.2
Position on chr5 (Mb)

| | |
|---|---|
| date: | Thu Aug 17 17:52:01 2017 |
| build: | hg19 |
| display range: | chr5:82460485–83260485 [82460485–83260485] |
| hilite range: | 0 – 0 [ 0 – 0 ] |
| reference SNP: | chr5:82860485 |
| number of SNPs plotted: | 3393 |
| min P.value: | 5.38E–14 [chr5:82860485] |
| max P.value: | 10E–1 [chr5:83201241] |
| omitted GWAS Hits: | NA, NA |

Make more plots at http://csg.sph.umich.edu/locuszoom/

GWAS Catalog SNPs in Region

| chr | pos (Mb) | trait | snp |
|---|---|---|---|
| 5 | 82.81912 | Laryngeal squamous cell carcinoma | rs310518 |
| 5 | 82.84549 | Diisocyanate–induced asthma | rs3852186 |
| 5 | 82.88991 | Major depressive disorder | rs310501 |
| 5 | 82.96073 | Visceral fat | rs3846635 |
| 5 | 82.98574 | Prostate cancer | rs4466137 |
| 5 | 83.17359 | Ankylosing spondylitis | rs4552569 |

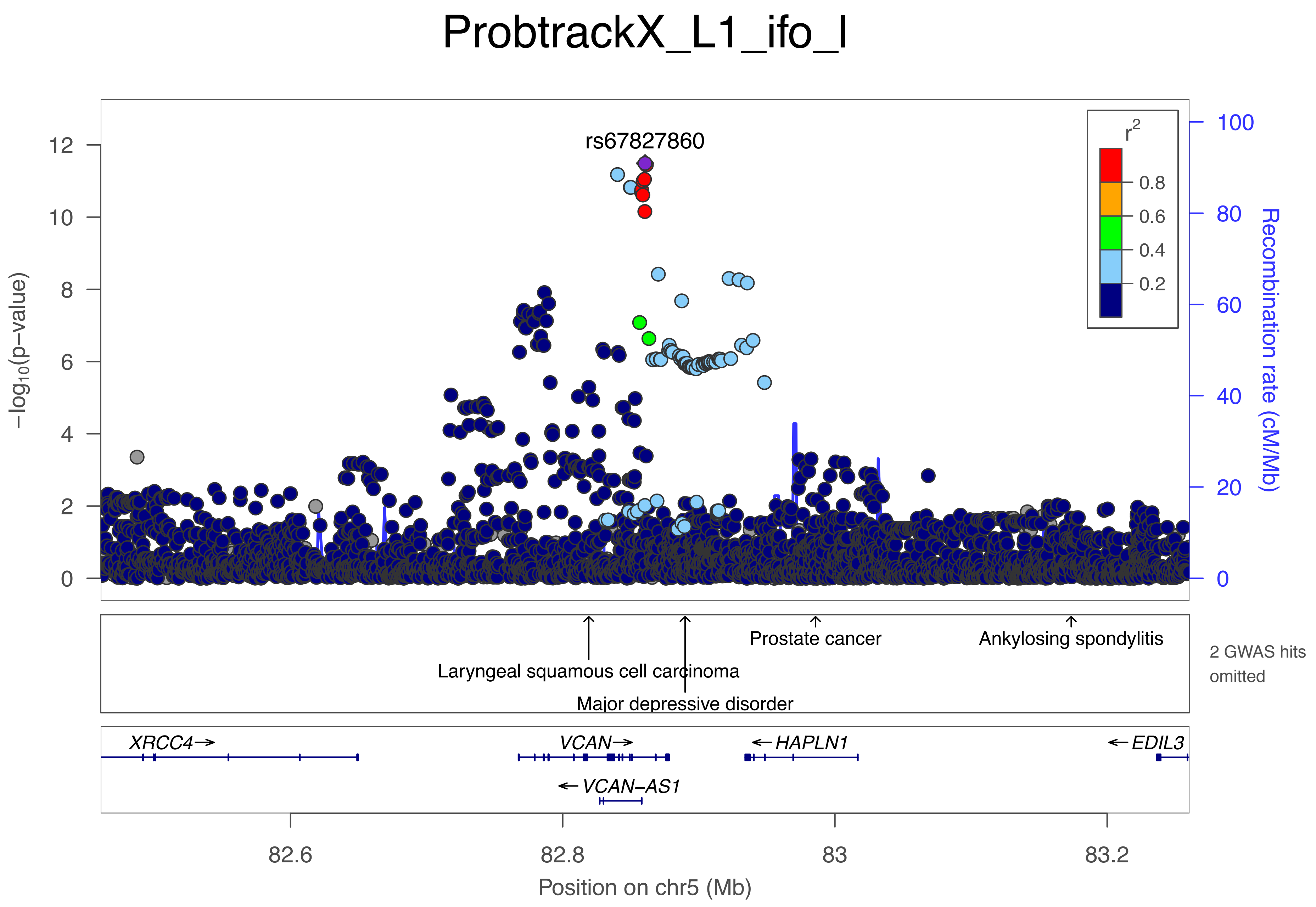

ProbtrackX_L1_ifo_l
rs67827860
−log10(p−value)
Recombination rate (cM/Mb)
r2
0.8
0.6
0.4
0.2
Prostate cancer
Ankylosing spondylitis
Laryngeal squamous cell carcinoma
Major depressive disorder
2 GWAS hits omitted
XRCC4
VCAN
HAPLN1
EDIL3
VCAN−AS1
82.6
82.8
83
83.2
Position on chr5 (Mb)

| | |
|---|---|
| date: | Thu Aug 17 17:52:01 2017 |
| build: | hg19 |
| display range: | chr5:82460485−83260485 [82460485−83260485] |
| hilite range: | 0 – 0 [ 0 – 0 ] |
| reference SNP: | chr5:82860485 |
| number of SNPs plotted: | 3393 |
| min P.value: | 3.25E−12 [chr5:82860485] |
| max P.value: | 10E−1 [chr5:83192371] |
| omitted GWAS Hits: | NA, NA |

Make more plots at http://csg.sph.umich.edu/locuszoom/

GWAS Catalog SNPs in Region

| chr | pos (Mb) | trait | snp |
|---|---|---|---|
| 5 | 82.81912 | Laryngeal squamous cell carcinoma | rs310518 |
| 5 | 82.84549 | Diisocyanate−induced asthma | rs3852186 |
| 5 | 82.88991 | Major depressive disorder | rs310501 |
| 5 | 82.96073 | Visceral fat | rs3846635 |
| 5 | 82.98574 | Prostate cancer | rs4466137 |
| 5 | 83.17359 | Ankylosing spondylitis | rs4552569 |

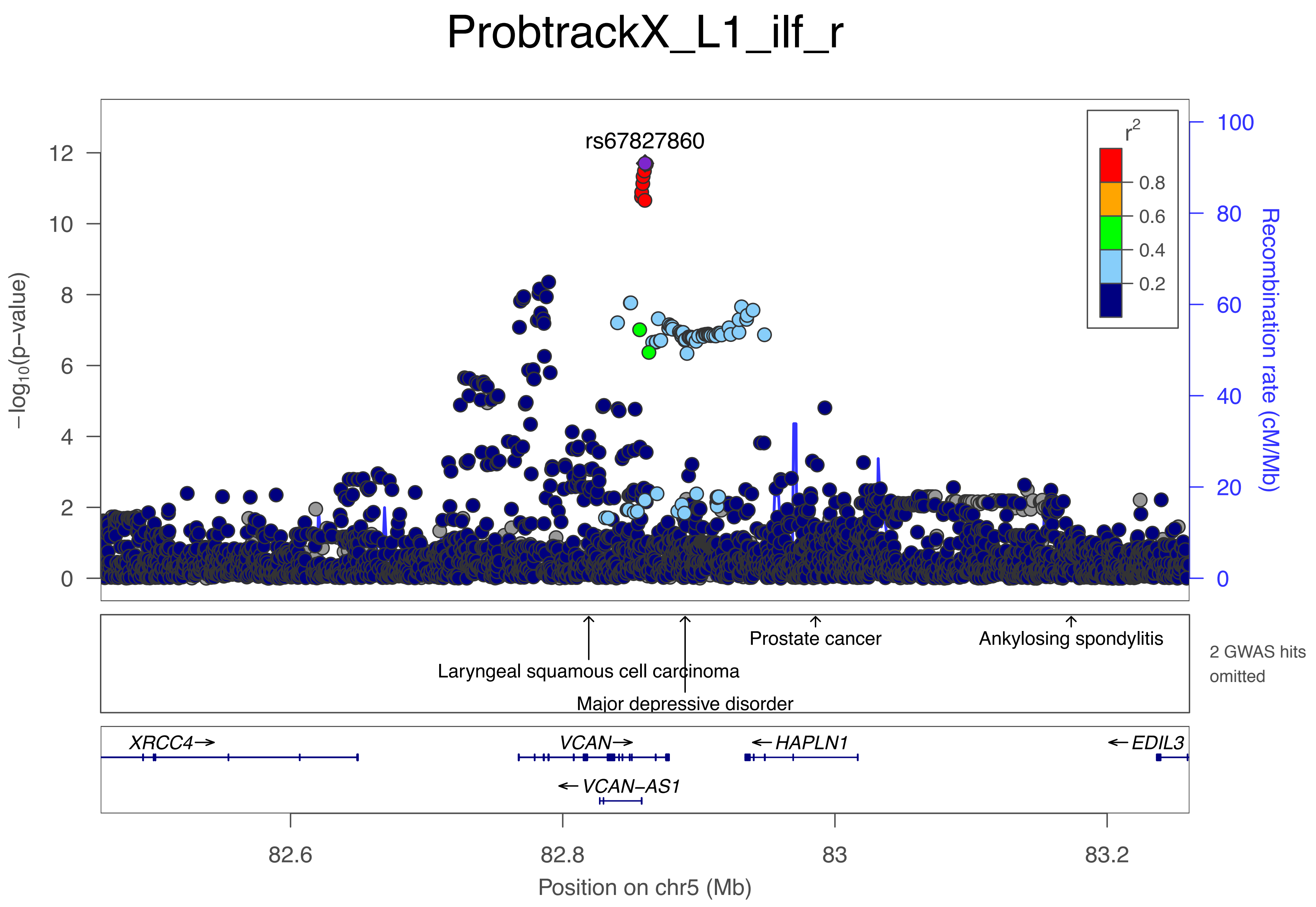

ProbtrackX_L1_ilf_r
rs67827860
−log10(p−value)
Recombination rate (cM/Mb)
r2
0.8
0.6
0.4
0.2
0
2
4
6
8
10
12
20
40
60
80
100
Laryngeal squamous cell carcinoma
Major depressive disorder
Prostate cancer
Ankylosing spondylitis
2 GWAS hits omitted
XRCC4→
VCAN→
←HAPLN1
←EDIL3
←VCAN−AS1
82.6
82.8
83
83.2
Position on chr5 (Mb)

| | |
|---|---|
| date: | Thu Aug 17 17:52:01 2017 |
| build: | hg19 |
| display range: | chr5:82460485−83260485 [82460485−83260485] |
| hilite range: | 0 – 0 [ 0 – 0 ] |
| reference SNP: | chr5:82860485 |
| number of SNPs plotted: | 3393 |
| min P.value: | 1.99E−12 [chr5:82860485] |
| max P.value: | 10E−1 [chr5:83204134] |
| omitted GWAS Hits: | NA, NA |

Make more plots at http://csg.sph.umich.edu/locuszoom/

GWAS Catalog SNPs in Region

| chr | pos (Mb) | trait | snp |
|---|---|---|---|
| 5 | 82.81912 | Laryngeal squamous cell carcinoma | rs310518 |
| 5 | 82.84549 | Diisocyanate–induced asthma | rs3852186 |
| 5 | 82.88991 | Major depressive disorder | rs310501 |
| 5 | 82.96073 | Visceral fat | rs3846635 |
| 5 | 82.98574 | Prostate cancer | rs4466137 |
| 5 | 83.17359 | Ankylosing spondylitis | rs4552569 |

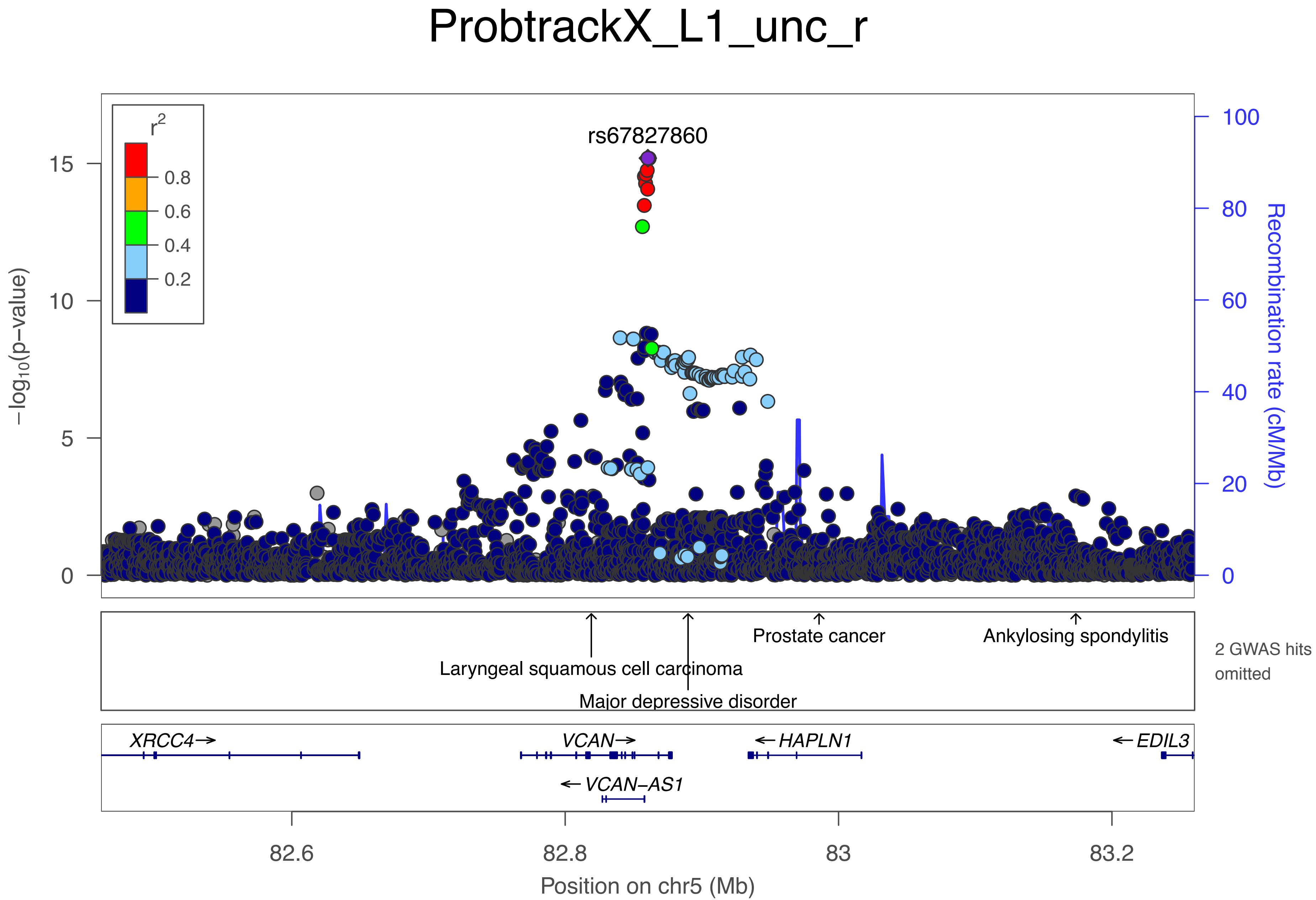
ProbtrackX_L1_unc_r
rs67827860
r2
0.8
0.6
0.4
0.2
−log10(p−value)
Recombination rate (cM/Mb)
Laryngeal squamous cell carcinoma
Major depressive disorder
Prostate cancer
Ankylosing spondylitis
2 GWAS hits omitted
XRCC4
VCAN
VCAN−AS1
HAPLN1
EDIL3
82.6
82.8
83
83.2
Position on chr5 (Mb)

| | |
|---|---|
| date: | Thu Aug 17 17:52:01 2017 |
| build: | hg19 |
| display range: | chr5:82460485−83260485 [82460485−83260485] |
| hilite range: | 0 – 0 [ 0 – 0 ] |
| reference SNP: | chr5:82860485 |
| number of SNPs plotted: | 3393 |
| min P.value: | 6.41E−16 [chr5:82860485] |
| max P.value: | 10E−1 [chr5:83052198] |
| omitted GWAS Hits: | NA, NA |

Make more plots at http://csg.sph.umich.edu/locuszoom/

GWAS Catalog SNPs in Region

| chr | pos (Mb) | trait | snp |
|---|---|---|---|
| 5 | 82.81912 | Laryngeal squamous cell carcinoma | rs310518 |
| 5 | 82.84549 | Diisocyanate–induced asthma | rs3852186 |
| 5 | 82.88991 | Major depressive disorder | rs310501 |
| 5 | 82.96073 | Visceral fat | rs3846635 |
| 5 | 82.98574 | Prostate cancer | rs4466137 |
| 5 | 83.17359 | Ankylosing spondylitis | rs4552569 |

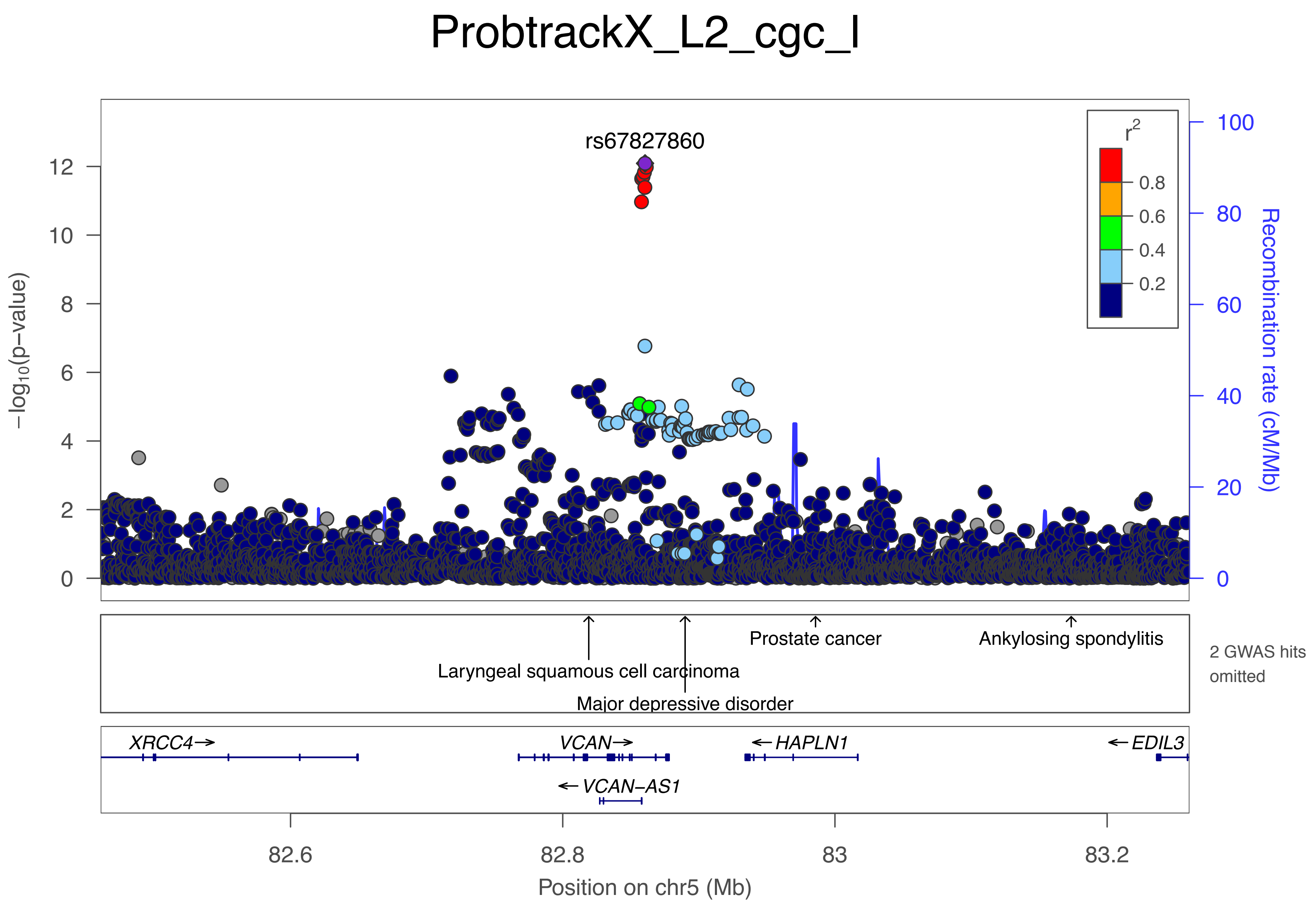

ProbtrackX_L2_cgc_l
rs67827860
r2
0.8
0.6
0.4
0.2
−log10(p−value)
Recombination rate (cM/Mb)
Prostate cancer
Ankylosing spondylitis
Laryngeal squamous cell carcinoma
Major depressive disorder
2 GWAS hits omitted
XRCC4→
VCAN→
←HAPLN1
←EDIL3
←VCAN−AS1
82.6
82.8
83
83.2
Position on chr5 (Mb)

| | |
|---|---|
| date: | Thu Aug 17 17:52:01 2017 |
| build: | hg19 |
| display range: | chr5:82460485−83260485 [82460485−83260485] |
| hilite range: | 0 – 0 [ 0 – 0 ] |
| reference SNP: | chr5:82860485 |
| number of SNPs plotted: | 3393 |
| min P.value: | 8.13E−13 [chr5:82860485] |
| max P.value: | 10E−1 [chr5:82616725] |
| omitted GWAS Hits: | NA, NA |

Make more plots at http://csg.sph.umich.edu/locuszoom/

GWAS Catalog SNPs in Region

| chr | pos (Mb) | trait | snp |
|---|---|---|---|
| 5 | 82.81912 | Laryngeal squamous cell carcinoma | rs310518 |
| 5 | 82.84549 | Diisocyanate–induced asthma | rs3852186 |
| 5 | 82.88991 | Major depressive disorder | rs310501 |
| 5 | 82.96073 | Visceral fat | rs3846635 |
| 5 | 82.98574 | Prostate cancer | rs4466137 |
| 5 | 83.17359 | Ankylosing spondylitis | rs4552569 |

# ProbtrackX_L2_cgc_r

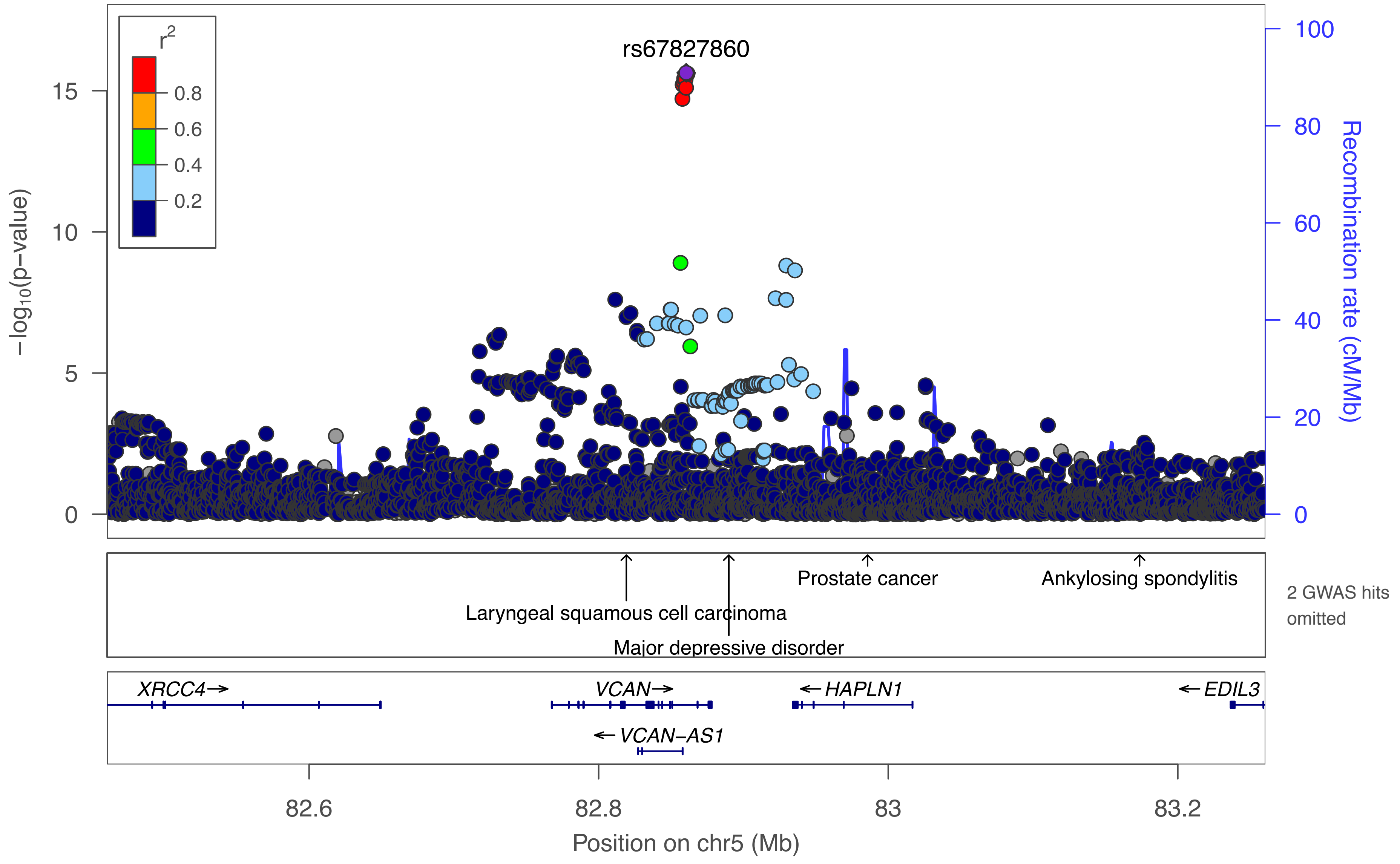

| | |
|---|---|
| date: | Thu Aug 17 17:52:01 2017 |
| build: | hg19 |
| display range: | chr5:82460485−83260485 [82460485−83260485] |
| hilite range: | 0 – 0 [ 0 – 0 ] |
| reference SNP: | chr5:82860485 |
| number of SNPs plotted: | 3393 |
| min P.value: | 2.32E−16 [chr5:82860485] |
| max P.value: | 10E−1 [chr5:82975351] |
| omitted GWAS Hits: | NA, NA |

Make more plots at http://csg.sph.umich.edu/locuszoom/

GWAS Catalog SNPs in Region

| chr | pos (Mb) | trait | snp |
|---|---|---|---|
| 5 | 82.81912 | Laryngeal squamous cell carcinoma | rs310518 |
| 5 | 82.84549 | Diisocyanate–induced asthma | rs3852186 |
| 5 | 82.88991 | Major depressive disorder | rs310501 |
| 5 | 82.96073 | Visceral fat | rs3846635 |
| 5 | 82.98574 | Prostate cancer | rs4466137 |
| 5 | 83.17359 | Ankylosing spondylitis | rs4552569 |

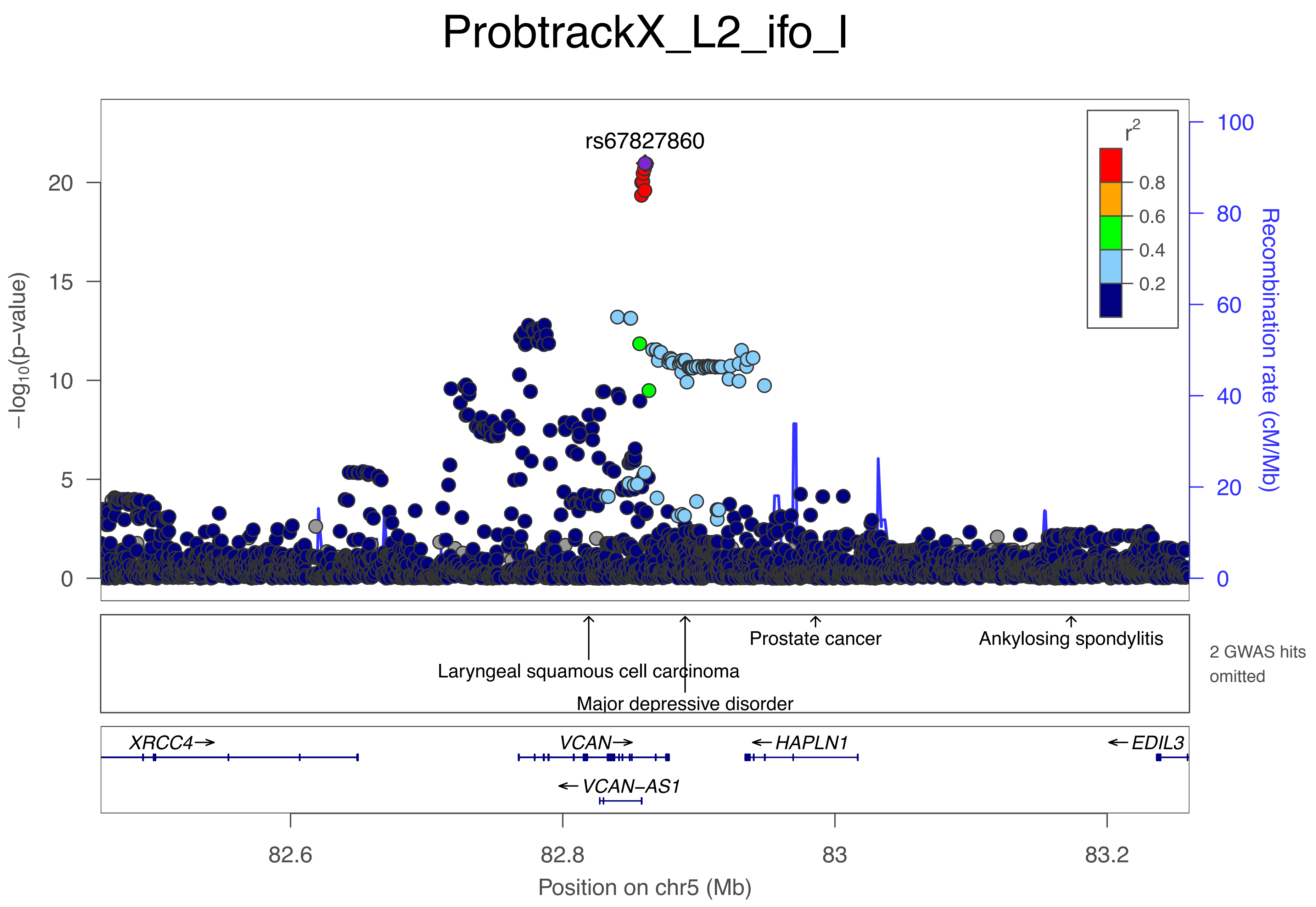
ProbtrackX_L2_ifo_l
rs67827860
r2
0.8
0.6
0.4
0.2
−log10(p−value)
Recombination rate (cM/Mb)
Laryngeal squamous cell carcinoma
Major depressive disorder
Prostate cancer
Ankylosing spondylitis
2 GWAS hits omitted
XRCC4
VCAN
VCAN−AS1
HAPLN1
EDIL3
82.6
82.8
83
83.2
Position on chr5 (Mb)

date: Thu Aug 17 17:52:01 2017

build: hg19

display range: chr5:82460485−83260485 [82460485−83260485]

hilite range: 0 – 0 [ 0 – 0 ]

reference SNP: chr5:82860485

number of SNPs plotted: 3393

min P.value: 1.08E−21 [chr5:82860485]

max P.value: 9.99E−1 [chr5:82901436]

omitted GWAS Hits: NA, NA

Make more plots at http://csg.sph.umich.edu/locuszoom/

GWAS Catalog SNPs in Region

| chr | pos (Mb) | trait | snp |
|---|---|---|---|
| 5 | 82.81912 | Laryngeal squamous cell carcinoma | rs310518 |
| 5 | 82.84549 | Diisocyanate–induced asthma | rs3852186 |
| 5 | 82.88991 | Major depressive disorder | rs310501 |
| 5 | 82.96073 | Visceral fat | rs3846635 |
| 5 | 82.98574 | Prostate cancer | rs4466137 |
| 5 | 83.17359 | Ankylosing spondylitis | rs4552569 |

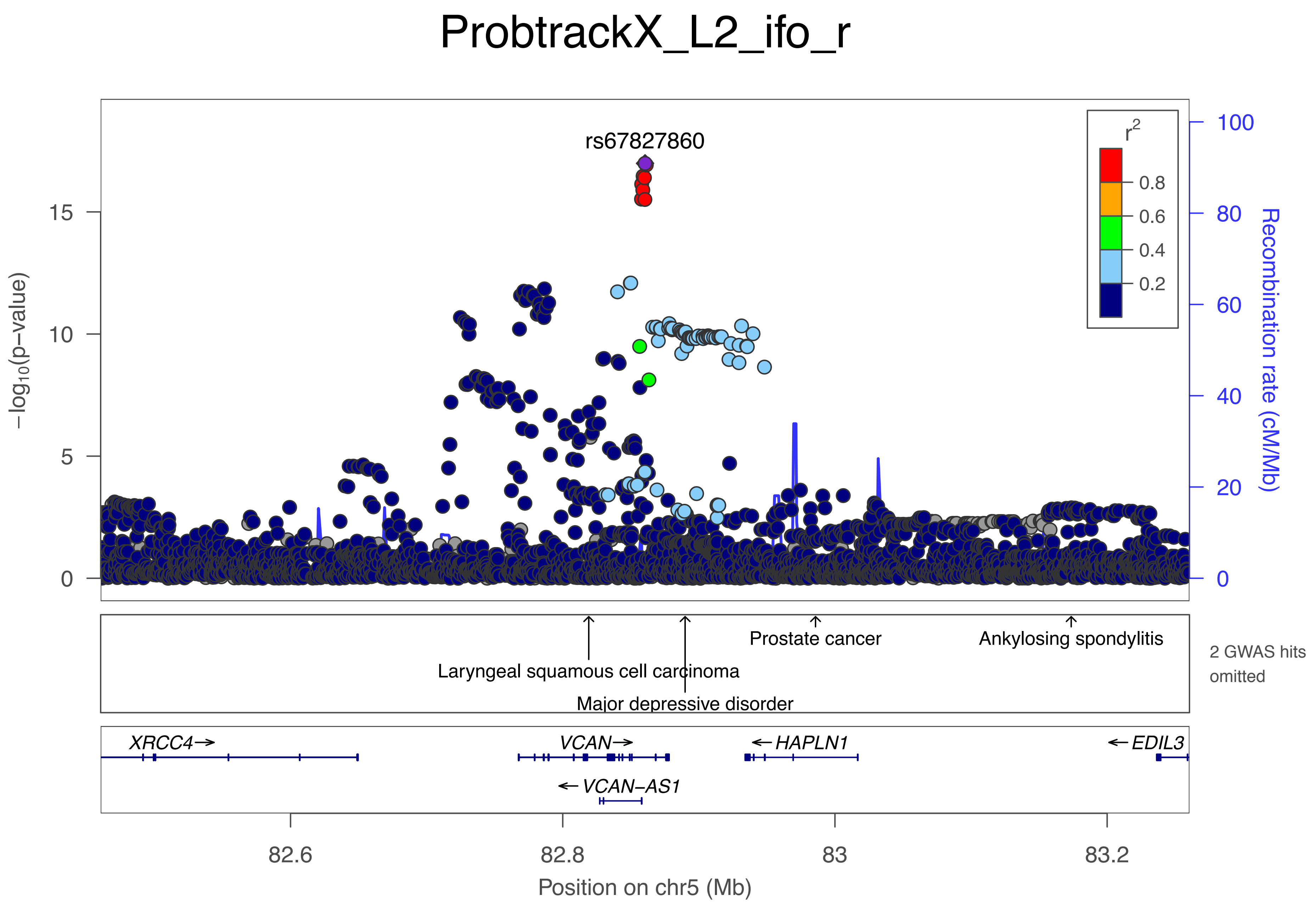

ProbtrackX_L2_ifo_r
rs67827860
−log10(p−value)
Recombination rate (cM/Mb)
r2
0.8
0.6
0.4
0.2
0
5
10
15
0
20
40
60
80
100
Laryngeal squamous cell carcinoma
Major depressive disorder
Prostate cancer
Ankylosing spondylitis
2 GWAS hits omitted
XRCC4→
VCAN→
←HAPLN1
←EDIL3
←VCAN−AS1
82.6
82.8
83
83.2
Position on chr5 (Mb)

| | |
|---|---|
| date: | Thu Aug 17 17:52:01 2017 |
| build: | hg19 |
| display range: | chr5:82460485–83260485 [82460485–83260485] |
| hilite range: | 0 – 0 [ 0 – 0 ] |
| reference SNP: | chr5:82860485 |
| number of SNPs plotted: | 3393 |
| min P.value: | 1.03E–17 [chr5:82860485] |
| max P.value: | 9.99E–1 [chr5:82776020] |
| omitted GWAS Hits: | NA, NA |

Make more plots at http://csg.sph.umich.edu/locuszoom/

GWAS Catalog SNPs in Region

| chr | pos (Mb) | trait | snp |
|---|---|---|---|
| 5 | 82.81912 | Laryngeal squamous cell carcinoma | rs310518 |
| 5 | 82.84549 | Diisocyanate−induced asthma | rs3852186 |
| 5 | 82.88991 | Major depressive disorder | rs310501 |
| 5 | 82.96073 | Visceral fat | rs3846635 |
| 5 | 82.98574 | Prostate cancer | rs4466137 |
| 5 | 83.17359 | Ankylosing spondylitis | rs4552569 |

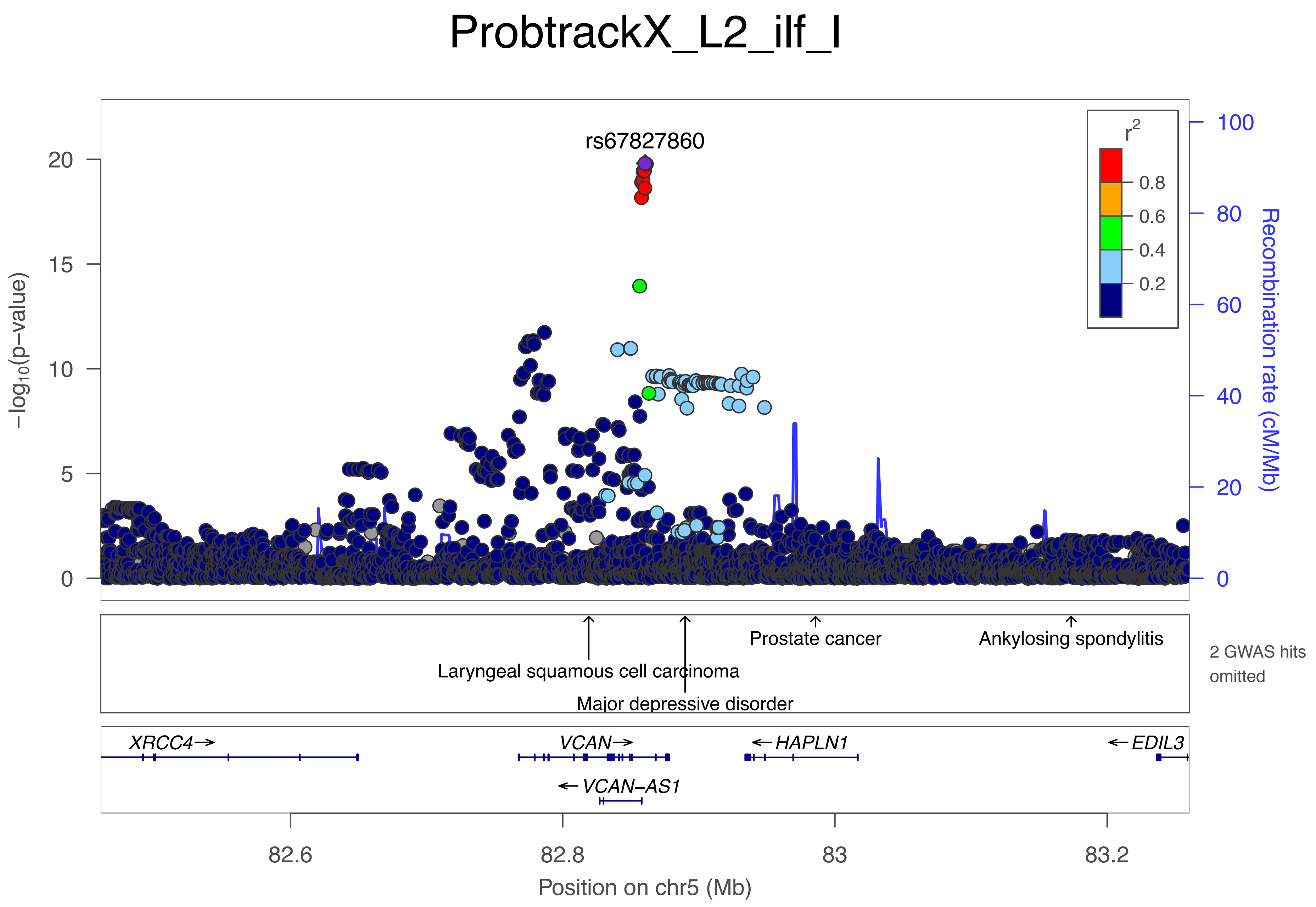
ProbtrackX_L2_ilf_l
rs67827860
r²
0.8
0.6
0.4
0.2
−log10(p−value)
Recombination rate (cM/Mb)
Prostate cancer
Ankylosing spondylitis
Laryngeal squamous cell carcinoma
Major depressive disorder
2 GWAS hits omitted
XRCC4→
VCAN→
←HAPLN1
←EDIL3
←VCAN−AS1
82.6
82.8
83
83.2
Position on chr5 (Mb)

| | |
|---|---|
| date: | Thu Aug 17 17:52:01 2017 |
| build: | hg19 |
| display range: | chr5:82460485–83260485 [82460485–83260485] |
| hilite range: | 0 – 0 [ 0 – 0 ] |
| reference SNP: | chr5:82860485 |
| number of SNPs plotted: | 3393 |
| min P.value: | 1.57E–20 [chr5:82860485] |
| max P.value: | 9.99E–1 [chr5:82649969] |
| omitted GWAS Hits: | NA, NA |

Make more plots at http://csg.sph.umich.edu/locuszoom/

GWAS Catalog SNPs in Region

| chr | pos (Mb) | trait | snp |
|---|---|---|---|
| 5 | 82.81912 | Laryngeal squamous cell carcinoma | rs310518 |
| 5 | 82.84549 | Diisocyanate–induced asthma | rs3852186 |
| 5 | 82.88991 | Major depressive disorder | rs310501 |
| 5 | 82.96073 | Visceral fat | rs3846635 |
| 5 | 82.98574 | Prostate cancer | rs4466137 |
| 5 | 83.17359 | Ankylosing spondylitis | rs4552569 |

# ProbtrackX_L2_ilf_r

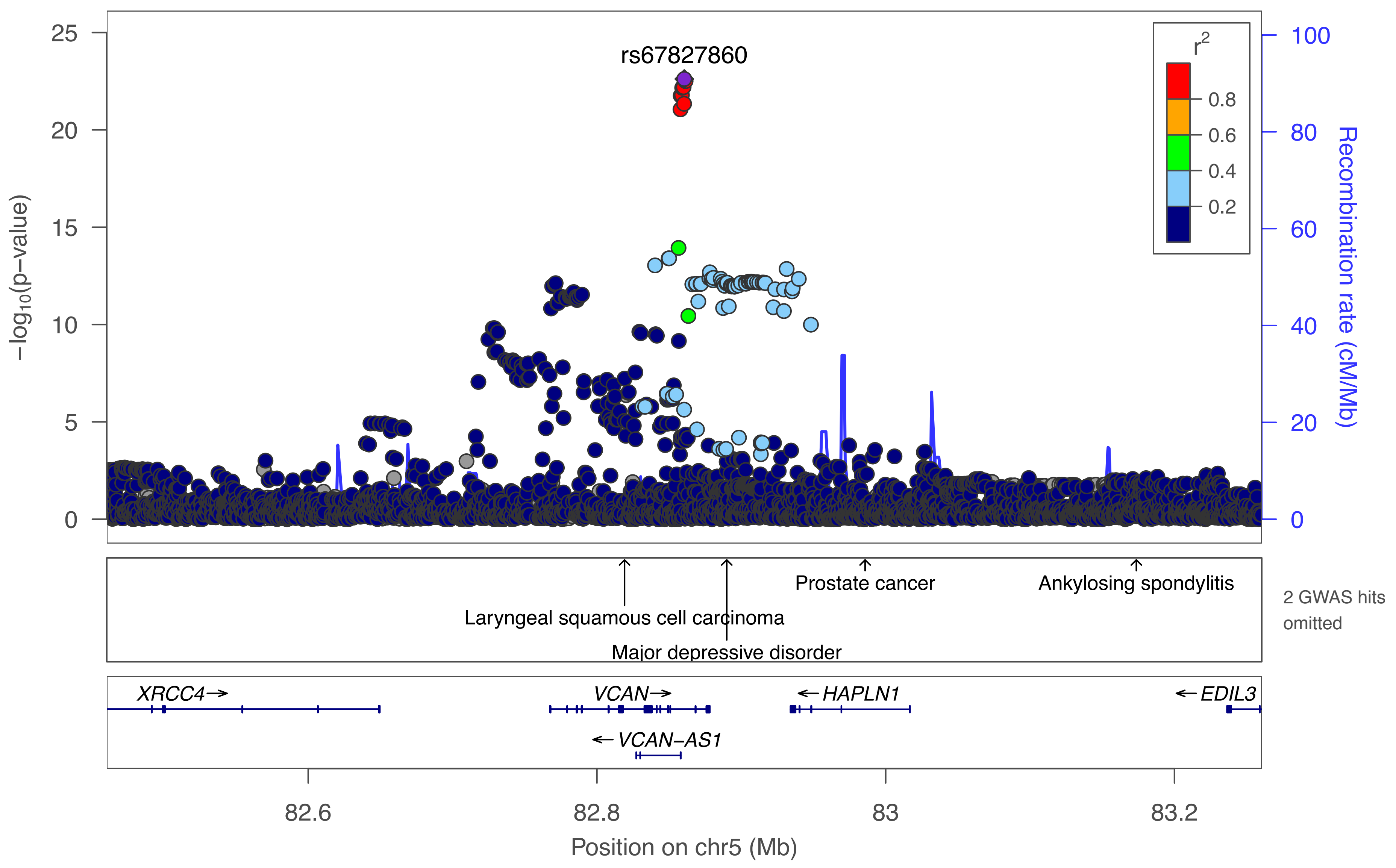

| | |
|---|---|
| date: | Thu Aug 17 17:52:01 2017 |
| build: | hg19 |
| display range: | chr5:82460485−83260485 [82460485−83260485] |
| hilite range: | 0 – 0 [ 0 – 0 ] |
| reference SNP: | chr5:82860485 |
| number of SNPs plotted: | 3393 |
| min P.value: | 2.43E−23 [chr5:82860485] |
| max P.value: | 10E−1 [chr5:82769528] |
| omitted GWAS Hits: | NA, NA |

Make more plots at http://csg.sph.umich.edu/locuszoom/

GWAS Catalog SNPs in Region

| chr | pos (Mb) | trait | snp |
|---|---|---|---|
| 5 | 82.81912 | Laryngeal squamous cell carcinoma | rs310518 |
| 5 | 82.84549 | Diisocyanate−induced asthma | rs3852186 |
| 5 | 82.88991 | Major depressive disorder | rs310501 |
| 5 | 82.96073 | Visceral fat | rs3846635 |
| 5 | 82.98574 | Prostate cancer | rs4466137 |
| 5 | 83.17359 | Ankylosing spondylitis | rs4552569 |

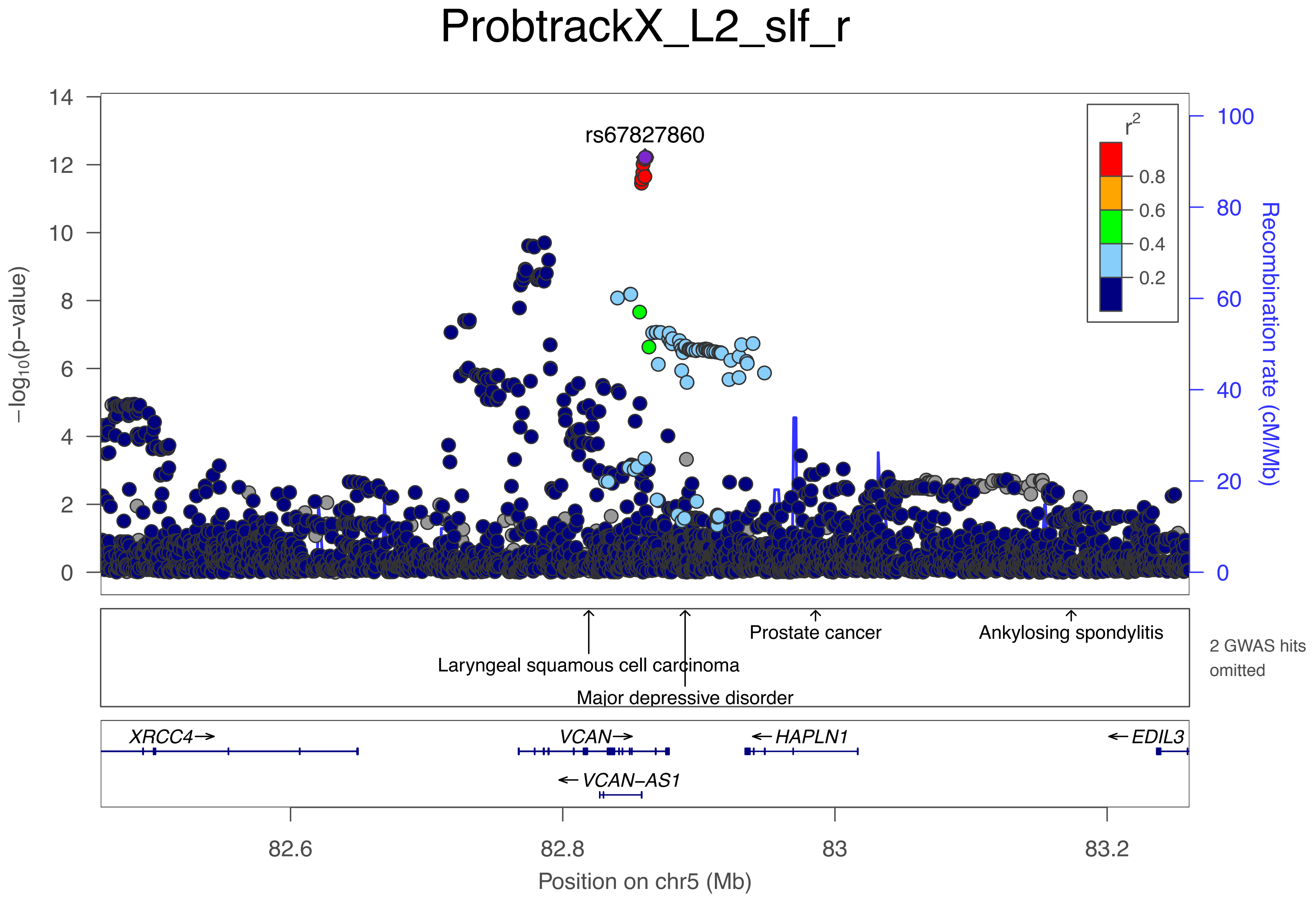
ProbtrackX_L2_slf_r
rs67827860
−log10(p−value)
Recombination rate (cM/Mb)
r2
0.8
0.6
0.4
0.2
Laryngeal squamous cell carcinoma
Major depressive disorder
Prostate cancer
Ankylosing spondylitis
2 GWAS hits omitted
XRCC4→
VCAN→
←VCAN−AS1
←HAPLN1
←EDIL3
82.6
82.8
83
83.2
Position on chr5 (Mb)

| | |
|---|---|
| date: | Thu Aug 17 17:52:01 2017 |
| build: | hg19 |
| display range: | chr5:82460485–83260485 [82460485–83260485] |
| hilite range: | 0 – 0 [ 0 – 0 ] |
| reference SNP: | chr5:82860485 |
| number of SNPs plotted: | 3393 |
| min P.value: | 6.07E–13 [chr5:82860485] |
| max P.value: | 10E–1 [chr5:82768225] |
| omitted GWAS Hits: | NA, NA |

Make more plots at http://csg.sph.umich.edu/locuszoom/

GWAS Catalog SNPs in Region

| chr | pos (Mb) | trait | snp |
|---|---|---|---|
| 5 | 82.81912 | Laryngeal squamous cell carcinoma | rs310518 |
| 5 | 82.84549 | Diisocyanate–induced asthma | rs3852186 |
| 5 | 82.88991 | Major depressive disorder | rs310501 |
| 5 | 82.96073 | Visceral fat | rs3846635 |
| 5 | 82.98574 | Prostate cancer | rs4466137 |
| 5 | 83.17359 | Ankylosing spondylitis | rs4552569 |

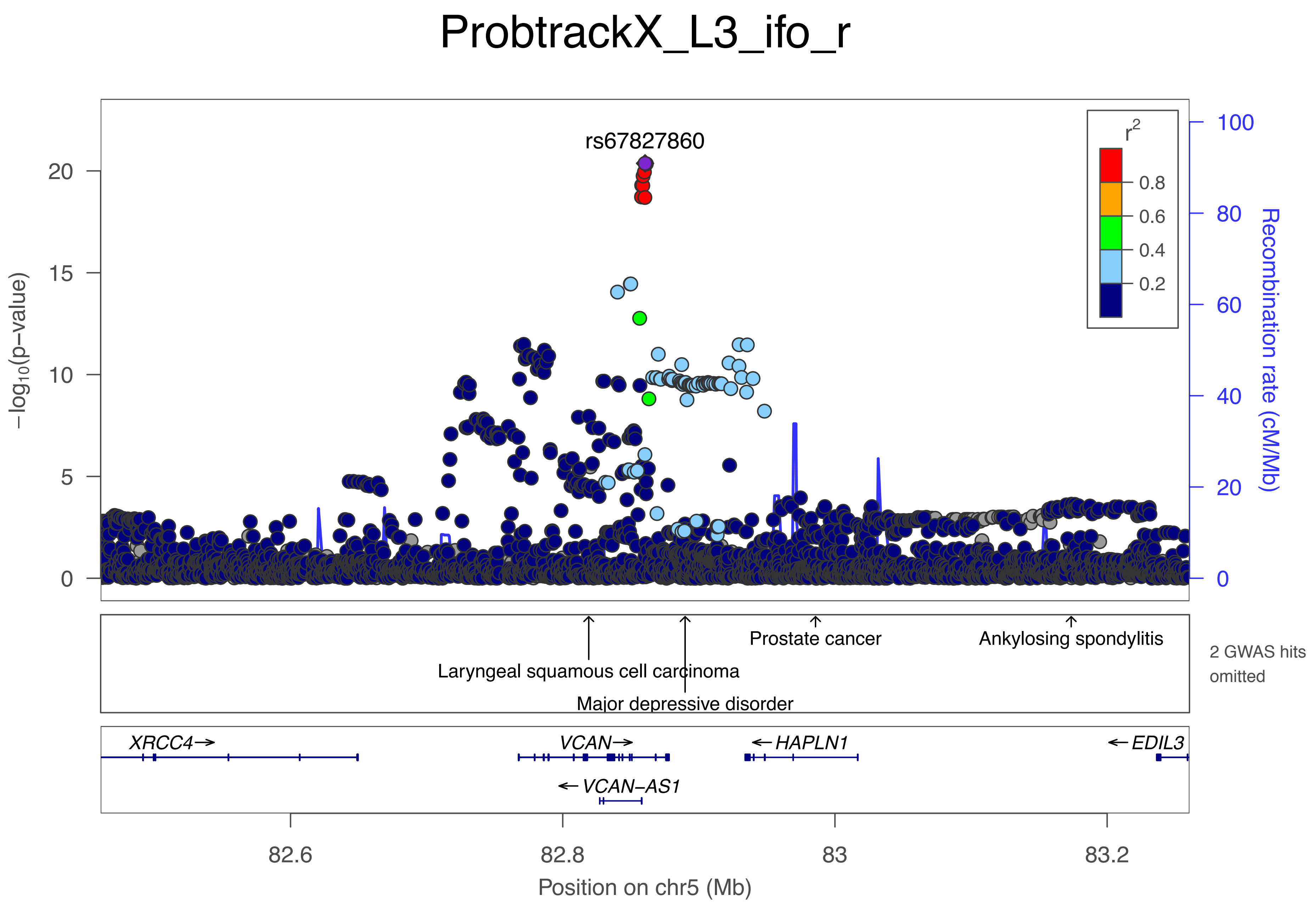
ProbtrackX_L3_ifo_r
rs67827860
−log10(p−value)
Recombination rate (cM/Mb)
r2
0.8
0.6
0.4
0.2
0
5
10
15
20
0
20
40
60
80
100
Laryngeal squamous cell carcinoma
Major depressive disorder
Prostate cancer
Ankylosing spondylitis
2 GWAS hits omitted
XRCC4→
VCAN→
←VCAN−AS1
←HAPLN1
←EDIL3
82.6
82.8
83
83.2
Position on chr5 (Mb)

| | |
|---|---|
| date: | Thu Aug 17 17:52:01 2017 |
| build: | hg19 |
| display range: | chr5:82460485−83260485 [82460485−83260485] |
| hilite range: | 0 – 0 [ 0 – 0 ] |
| reference SNP: | chr5:82860485 |
| number of SNPs plotted: | 3393 |
| min P.value: | 4.27E−21 [chr5:82860485] |
| max P.value: | 10E−1 [chr5:82897384] |
| omitted GWAS Hits: | NA, NA |

Make more plots at http://csg.sph.umich.edu/locuszoom/

GWAS Catalog SNPs in Region

| chr | pos (Mb) | trait | snp |
|---|---|---|---|
| 5 | 82.81912 | Laryngeal squamous cell carcinoma | rs310518 |
| 5 | 82.84549 | Diisocyanate–induced asthma | rs3852186 |
| 5 | 82.88991 | Major depressive disorder | rs310501 |
| 5 | 82.96073 | Visceral fat | rs3846635 |
| 5 | 82.98574 | Prostate cancer | rs4466137 |
| 5 | 83.17359 | Ankylosing spondylitis | rs4552569 |

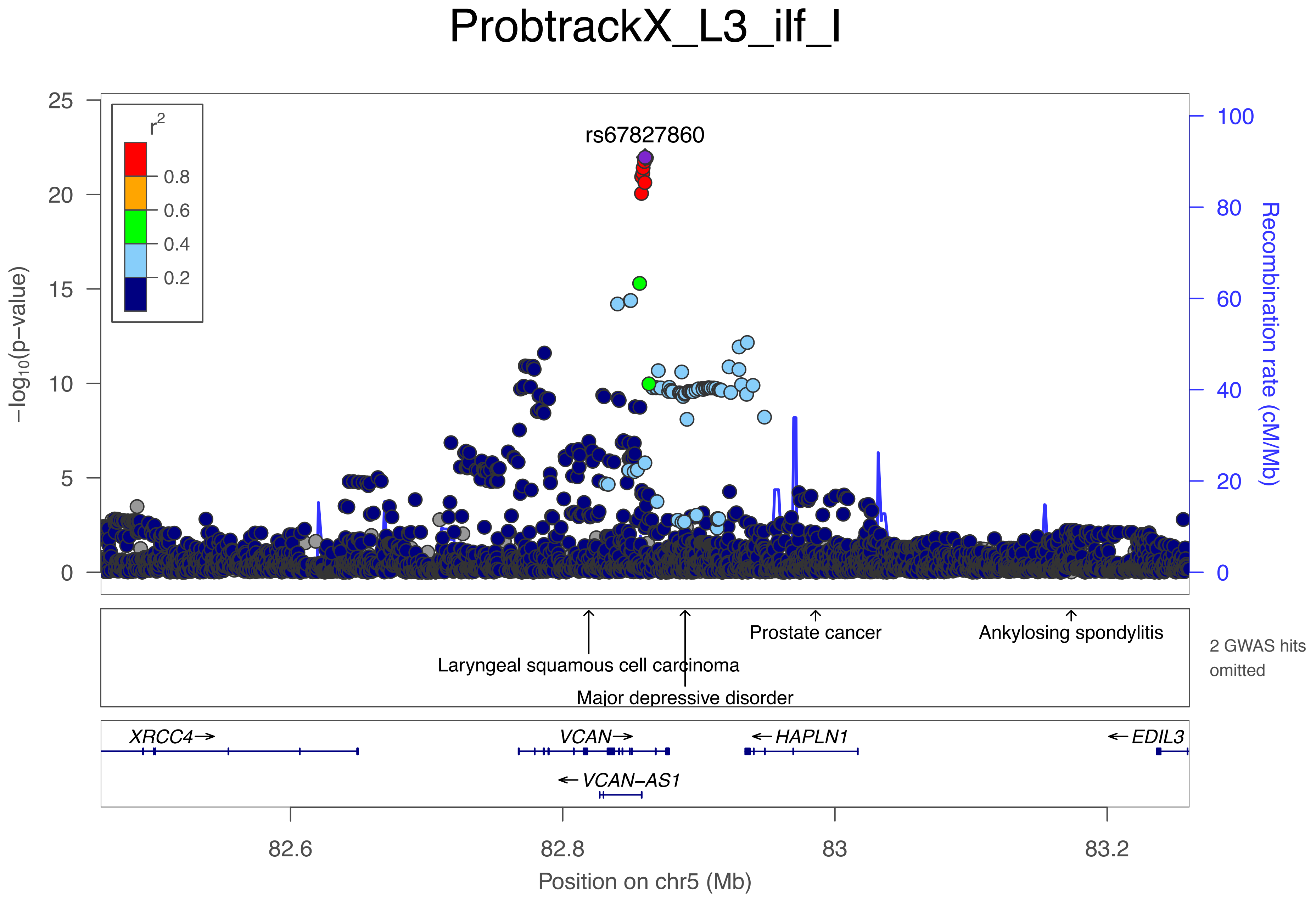

ProbtrackX_L3_ilf_l
r2
0.8
0.6
0.4
0.2
rs67827860
25
20
15
10
5
0
−log10(p−value)
100
80
60
40
20
0
Recombination rate (cM/Mb)
Laryngeal squamous cell carcinoma
Major depressive disorder
Prostate cancer
Ankylosing spondylitis
2 GWAS hits omitted
XRCC4→
VCAN→
←VCAN−AS1
←HAPLN1
←EDIL3
82.6
82.8
83
83.2
Position on chr5 (Mb)

| | |
|---|---|
| date: | Thu Aug 17 17:52:01 2017 |
| build: | hg19 |
| display range: | chr5:82460485−83260485 [82460485−83260485] |
| hilite range: | 0 – 0 [ 0 – 0 ] |
| reference SNP: | chr5:82860485 |
| number of SNPs plotted: | 3393 |
| min P.value: | 1.08E−22 [chr5:82860485] |
| max P.value: | 10E−1 [chr5:83165955] |
| omitted GWAS Hits: | NA, NA |

Make more plots at http://csg.sph.umich.edu/locuszoom/

GWAS Catalog SNPs in Region

| chr | pos (Mb) | trait | snp |
|---|---|---|---|
| 5 | 82.81912 | Laryngeal squamous cell carcinoma | rs310518 |
| 5 | 82.84549 | Diisocyanate–induced asthma | rs3852186 |
| 5 | 82.88991 | Major depressive disorder | rs310501 |
| 5 | 82.96073 | Visceral fat | rs3846635 |
| 5 | 82.98574 | Prostate cancer | rs4466137 |
| 5 | 83.17359 | Ankylosing spondylitis | rs4552569 |

# ProbtrackX_L3_ilf_r

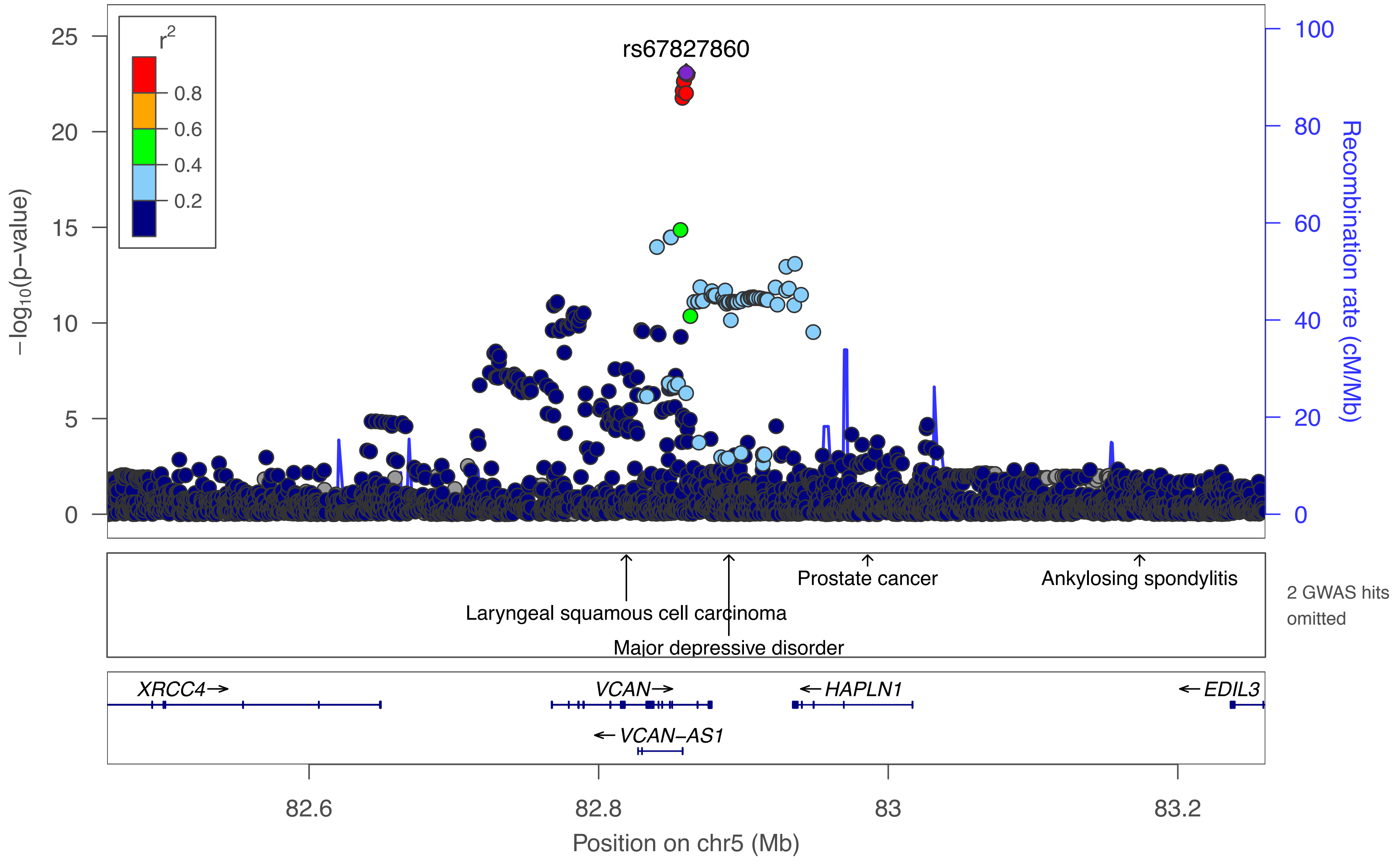

| | |
|---|---|
| date: | Thu Aug 17 17:52:01 2017 |
| build: | hg19 |
| display range: | chr5:82460485–83260485 [82460485–83260485] |
| hilite range: | 0 – 0 [ 0 – 0 ] |
| reference SNP: | chr5:82860485 |
| number of SNPs plotted: | 3393 |
| min P.value: | 8.34E–24 [chr5:82860485] |
| max P.value: | 10E–1 [chr5:82978826] |
| omitted GWAS Hits: | NA, NA |

Make more plots at http://csg.sph.umich.edu/locuszoom/

GWAS Catalog SNPs in Region

| chr | pos (Mb) | trait | snp |
|---|---|---|---|
| 5 | 82.81912 | Laryngeal squamous cell carcinoma | rs310518 |
| 5 | 82.84549 | Diisocyanate–induced asthma | rs3852186 |
| 5 | 82.88991 | Major depressive disorder | rs310501 |
| 5 | 82.96073 | Visceral fat | rs3846635 |
| 5 | 82.98574 | Prostate cancer | rs4466137 |
| 5 | 83.17359 | Ankylosing spondylitis | rs4552569 |

# ProbtrackX_L3_slf_r

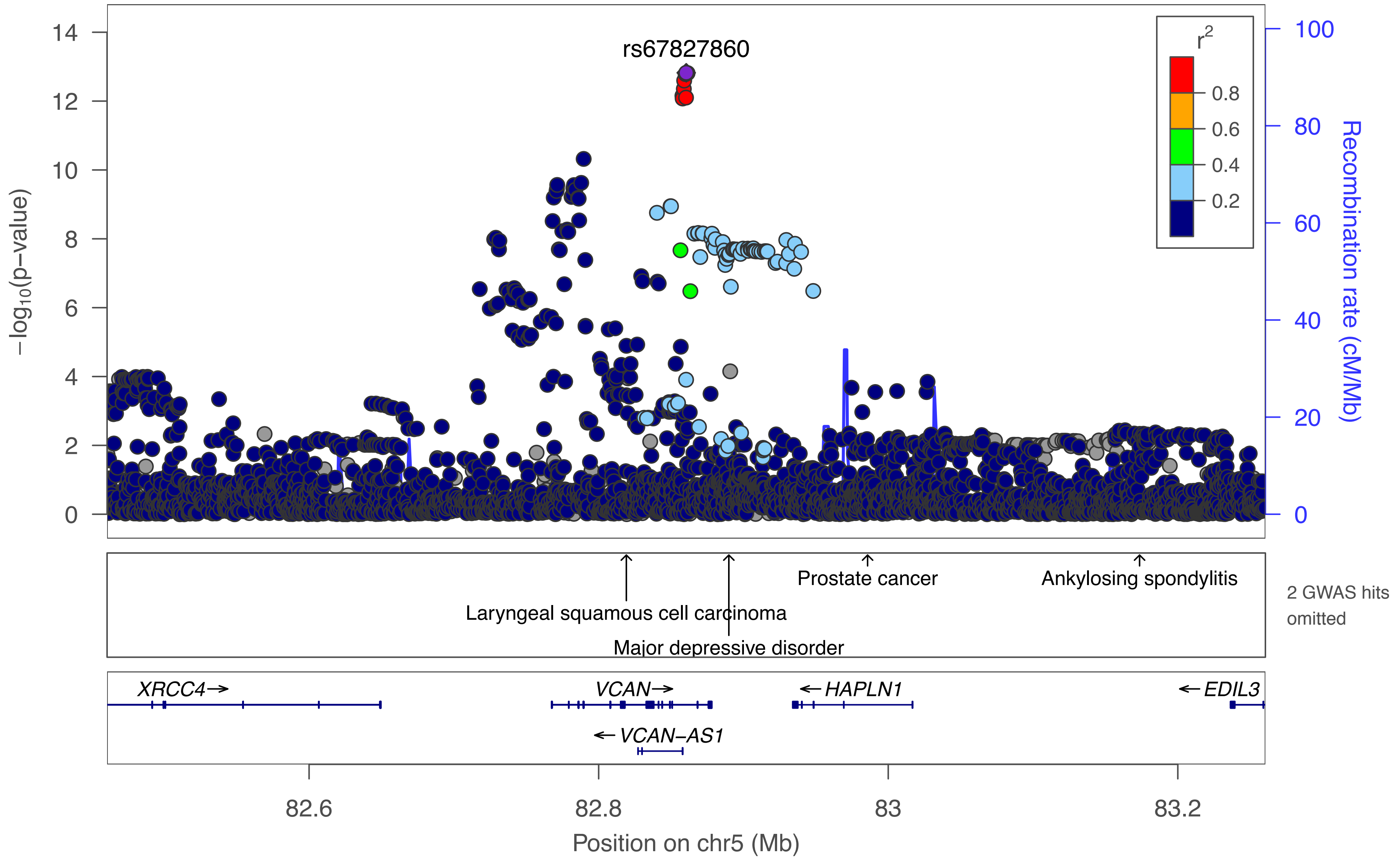

| | |
|---|---|
| date: | Thu Aug 17 17:52:01 2017 |
| build: | hg19 |
| display range: | chr5:82460485−83260485 [82460485−83260485] |
| hilite range: | 0 – 0 [ 0 – 0 ] |
| reference SNP: | chr5:82860485 |
| number of SNPs plotted: | 3393 |
| min P.value: | 1.51E−13 [chr5:82860485] |
| max P.value: | 10E−1 [chr5:82510720] |
| omitted GWAS Hits: | NA, NA |

Make more plots at http://csg.sph.umich.edu/locuszoom/

GWAS Catalog SNPs in Region

| chr | pos (Mb) | trait | snp |
|---|---|---|---|
| 5 | 82.81912 | Laryngeal squamous cell carcinoma | rs310518 |
| 5 | 82.84549 | Diisocyanate–induced asthma | rs3852186 |
| 5 | 82.88991 | Major depressive disorder | rs310501 |
| 5 | 82.96073 | Visceral fat | rs3846635 |
| 5 | 82.98574 | Prostate cancer | rs4466137 |
| 5 | 83.17359 | Ankylosing spondylitis | rs4552569 |

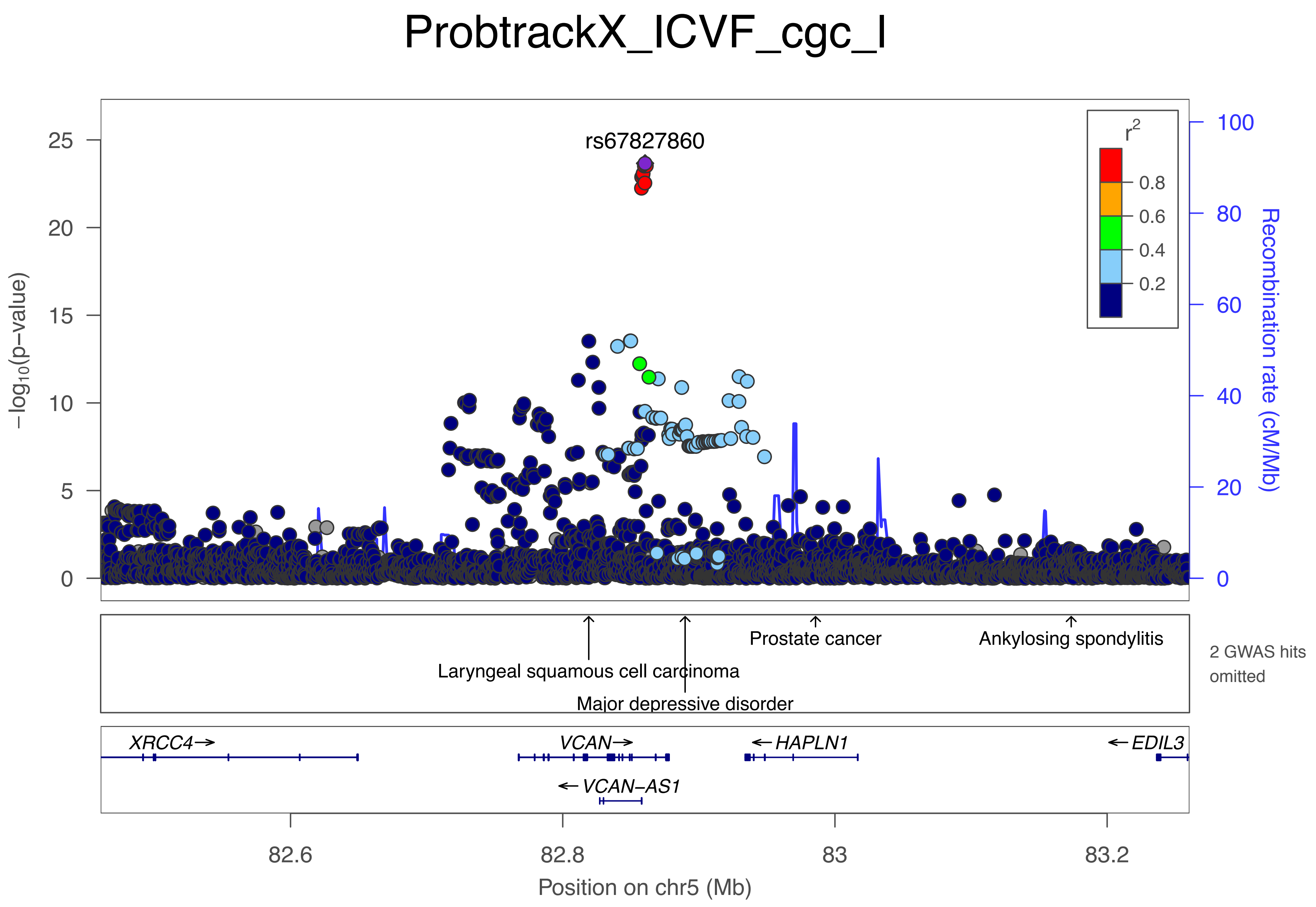

ProbtrackX_ICVF_cgc_l
rs67827860
−log10(p−value)
Recombination rate (cM/Mb)
r2
0.8
0.6
0.4
0.2
25
20
15
10
5
0
100
80
60
40
20
0
Laryngeal squamous cell carcinoma
Major depressive disorder
Prostate cancer
Ankylosing spondylitis
2 GWAS hits omitted
XRCC4→
VCAN→
←VCAN−AS1
←HAPLN1
←EDIL3
82.6
82.8
83
83.2
Position on chr5 (Mb)

| | |
|---|---|
| date: | Thu Aug 17 17:52:01 2017 |
| build: | hg19 |
| display range: | chr5:82460485–83260485 [82460485–83260485] |
| hilite range: | 0 – 0 [ 0 – 0 ] |
| reference SNP: | chr5:82860485 |
| number of SNPs plotted: | 3393 |
| min P.value: | 2.18E–24 [chr5:82860485] |
| max P.value: | 10E–1 [chr5:83139843] |
| omitted GWAS Hits: | NA, NA |

Make more plots at http://csg.sph.umich.edu/locuszoom/

GWAS Catalog SNPs in Region

| chr | pos (Mb) | trait | snp |
|---|---|---|---|
| 5 | 82.81912 | Laryngeal squamous cell carcinoma | rs310518 |
| 5 | 82.84549 | Diisocyanate–induced asthma | rs3852186 |
| 5 | 82.88991 | Major depressive disorder | rs310501 |
| 5 | 82.96073 | Visceral fat | rs3846635 |
| 5 | 82.98574 | Prostate cancer | rs4466137 |
| 5 | 83.17359 | Ankylosing spondylitis | rs4552569 |

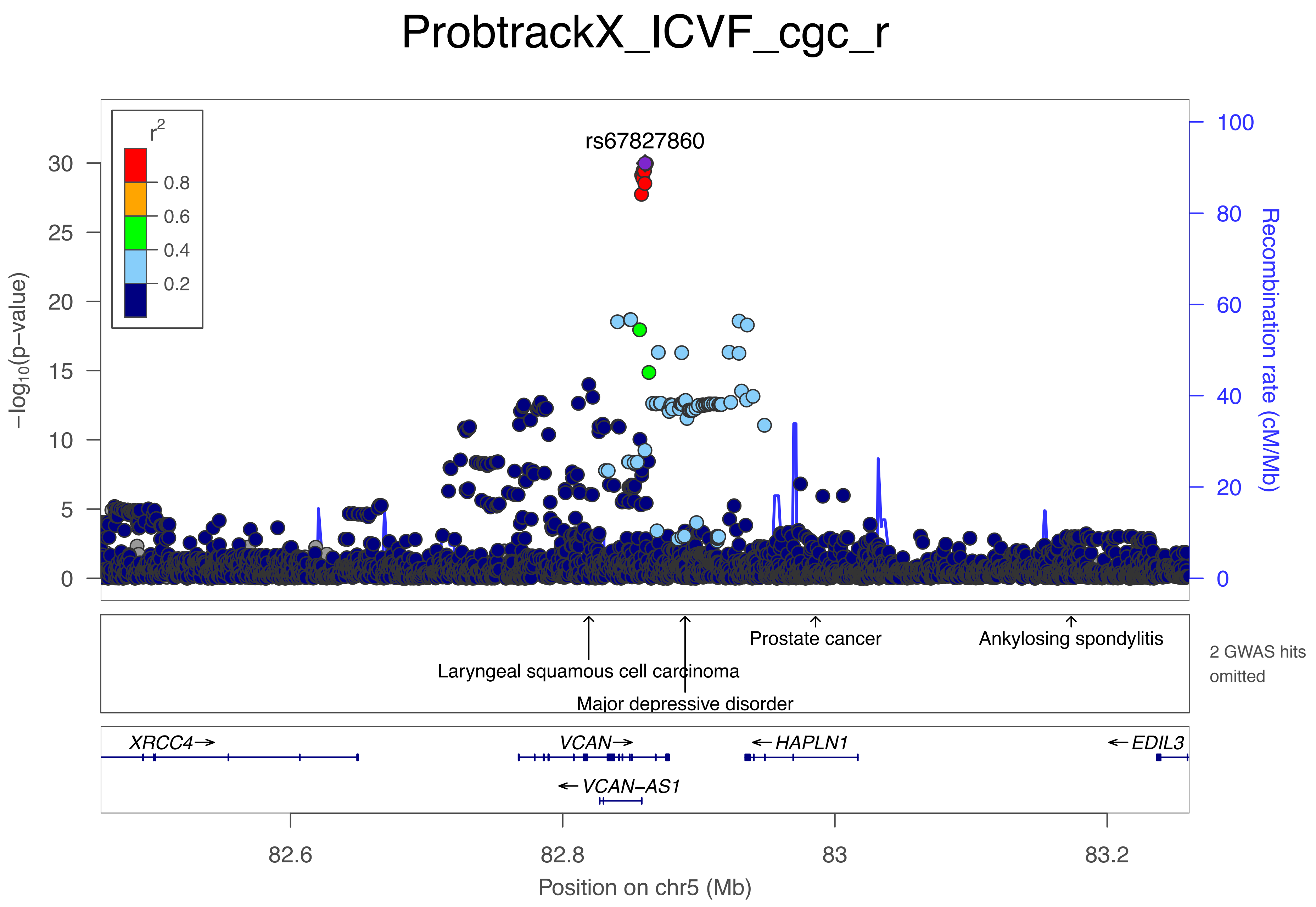
ProbtrackX_ICVF_cgc_r
rs67827860
r2
0.8
0.6
0.4
0.2
−log10(p−value)
Recombination rate (cM/Mb)
Laryngeal squamous cell carcinoma
Major depressive disorder
Prostate cancer
Ankylosing spondylitis
2 GWAS hits omitted
XRCC4
VCAN
VCAN−AS1
HAPLN1
EDIL3
82.6
82.8
83
83.2
Position on chr5 (Mb)

| | |
|---|---|
| date: | Thu Aug 17 17:52:01 2017 |
| build: | hg19 |
| display range: | chr5:82460485−83260485 [82460485−83260485] |
| hilite range: | 0 – 0 [ 0 – 0 ] |
| reference SNP: | chr5:82860485 |
| number of SNPs plotted: | 3393 |
| min P.value: | 1.06E−30 [chr5:82860485] |
| max P.value: | 9.99E−1 [chr5:82581810] |
| omitted GWAS Hits: | NA, NA |

Make more plots at http://csg.sph.umich.edu/locuszoom/

GWAS Catalog SNPs in Region

| chr | pos (Mb) | trait | snp |
|---|---|---|---|
| 5 | 82.81912 | Laryngeal squamous cell carcinoma | rs310518 |
| 5 | 82.84549 | Diisocyanate−induced asthma | rs3852186 |
| 5 | 82.88991 | Major depressive disorder | rs310501 |
| 5 | 82.96073 | Visceral fat | rs3846635 |
| 5 | 82.98574 | Prostate cancer | rs4466137 |
| 5 | 83.17359 | Ankylosing spondylitis | rs4552569 |

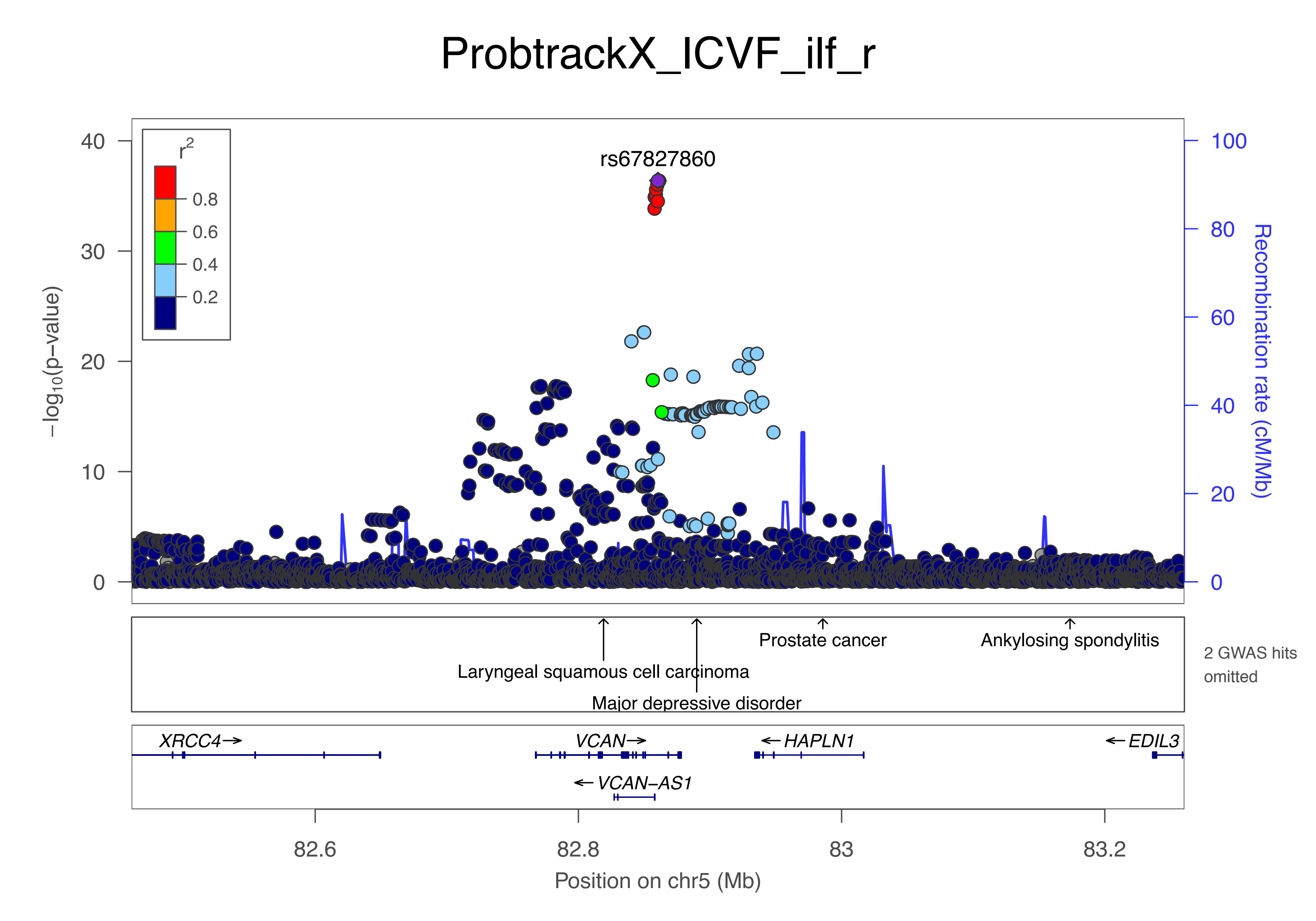
ProbtrackX_ICVF_ilf_r
rs67827860
r²
0.8
0.6
0.4
0.2
40
30
20
10
0
−log10(p−value)
100
80
60
40
20
0
Recombination rate (cM/Mb)
Laryngeal squamous cell carcinoma
Major depressive disorder
Prostate cancer
Ankylosing spondylitis
2 GWAS hits omitted
XRCC4→
VCAN→
←VCAN−AS1
←HAPLN1
←EDIL3
82.6
82.8
83
83.2
Position on chr5 (Mb)

| | |
|---|---|
| date: | Thu Aug 17 17:52:01 2017 |
| build: | hg19 |
| display range: | chr5:82460485–83260485 [82460485–83260485] |
| hilite range: | 0 – 0 [ 0 – 0 ] |
| reference SNP: | chr5:82860485 |
| number of SNPs plotted: | 3393 |
| min P.value: | 4.06E–37 [chr5:82860485] |
| max P.value: | 9.99E–1 [chr5:82522233] |
| omitted GWAS Hits: | NA, NA |

Make more plots at http://csg.sph.umich.edu/locuszoom/

GWAS Catalog SNPs in Region

| chr | pos (Mb) | trait | snp |
|---|---|---|---|
| 5 | 82.81912 | Laryngeal squamous cell carcinoma | rs310518 |
| 5 | 82.84549 | Diisocyanate–induced asthma | rs3852186 |
| 5 | 82.88991 | Major depressive disorder | rs310501 |
| 5 | 82.96073 | Visceral fat | rs3846635 |
| 5 | 82.98574 | Prostate cancer | rs4466137 |
| 5 | 83.17359 | Ankylosing spondylitis | rs4552569 |

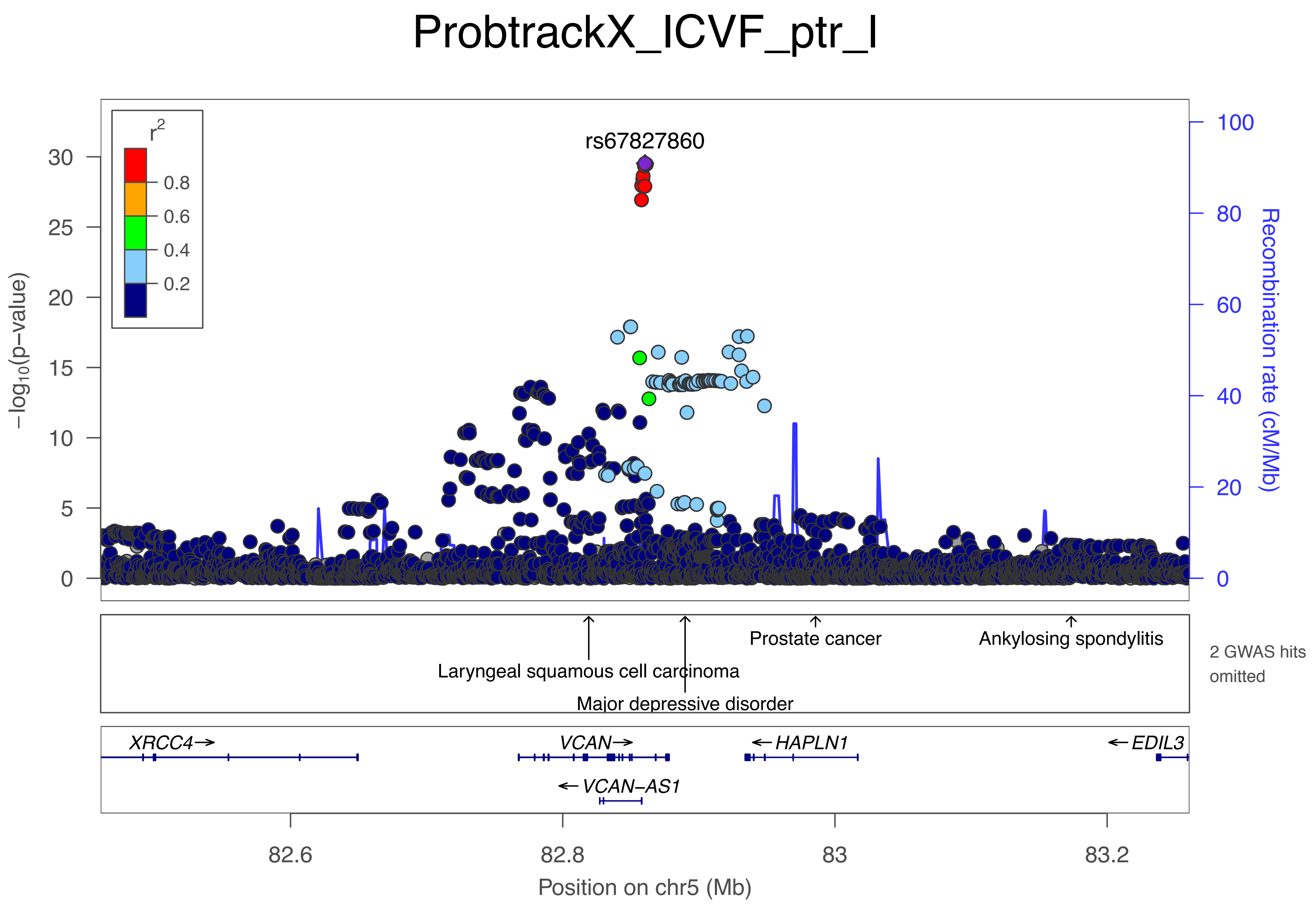

ProbtrackX_ICVF_ptr_l
rs67827860
r²
0.8
0.6
0.4
0.2
−log10(p−value)
Recombination rate (cM/Mb)
Laryngeal squamous cell carcinoma
Major depressive disorder
Prostate cancer
Ankylosing spondylitis
2 GWAS hits omitted
XRCC4→
VCAN→
←VCAN−AS1
←HAPLN1
←EDIL3
82.6
82.8
83
83.2
Position on chr5 (Mb)

| | |
|---|---|
| date: | Thu Aug 17 17:52:01 2017 |
| build: | hg19 |
| display range: | chr5:82460485−83260485 [82460485−83260485] |
| hilite range: | 0 – 0 [ 0 – 0 ] |
| reference SNP: | chr5:82860485 |
| number of SNPs plotted: | 3393 |
| min P.value: | 2.94E−30 [chr5:82860485] |
| max P.value: | 10E−1 [chr5:82633011] |
| omitted GWAS Hits: | NA, NA |

Make more plots at http://csg.sph.umich.edu/locuszoom/

GWAS Catalog SNPs in Region

| chr | pos (Mb) | trait | snp |
|---|---|---|---|
| 5 | 82.81912 | Laryngeal squamous cell carcinoma | rs310518 |
| 5 | 82.84549 | Diisocyanate–induced asthma | rs3852186 |
| 5 | 82.88991 | Major depressive disorder | rs310501 |
| 5 | 82.96073 | Visceral fat | rs3846635 |
| 5 | 82.98574 | Prostate cancer | rs4466137 |
| 5 | 83.17359 | Ankylosing spondylitis | rs4552569 |

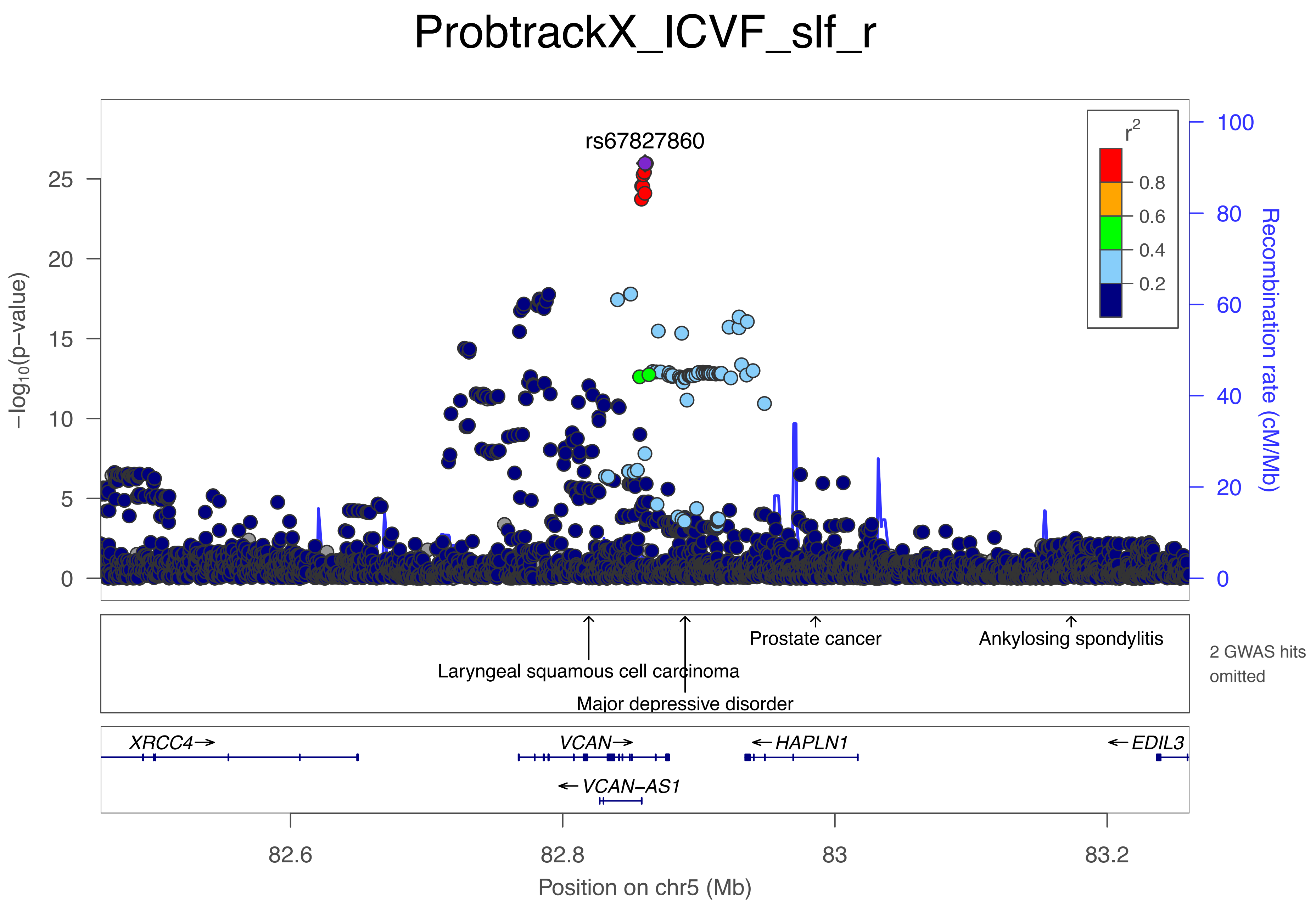
ProbtrackX_ICVF_slf_r
rs67827860
r²
0.8
0.6
0.4
0.2
−log10(p−value)
Recombination rate (cM/Mb)
Prostate cancer
Ankylosing spondylitis
Laryngeal squamous cell carcinoma
Major depressive disorder
2 GWAS hits omitted
XRCC4
VCAN
HAPLN1
EDIL3
VCAN−AS1
82.6
82.8
83
83.2
Position on chr5 (Mb)

| | |
|---|---|
| date: | Thu Aug 17 17:52:01 2017 |
| build: | hg19 |
| display range: | chr5:82460485–83260485 [82460485–83260485] |
| hilite range: | 0 – 0 [ 0 – 0 ] |
| reference SNP: | chr5:82860485 |
| number of SNPs plotted: | 3393 |
| min P.value: | 1.07E–26 [chr5:82860485] |
| max P.value: | 10E–1 [chr5:82735475] |
| omitted GWAS Hits: | NA, NA |

Make more plots at http://csg.sph.umich.edu/locuszoom/

GWAS Catalog SNPs in Region

| chr | pos (Mb) | trait | snp |
|---|---|---|---|
| 5 | 82.81912 | Laryngeal squamous cell carcinoma | rs310518 |
| 5 | 82.84549 | Diisocyanate–induced asthma | rs3852186 |
| 5 | 82.88991 | Major depressive disorder | rs310501 |
| 5 | 82.96073 | Visceral fat | rs3846635 |
| 5 | 82.98574 | Prostate cancer | rs4466137 |
| 5 | 83.17359 | Ankylosing spondylitis | rs4552569 |

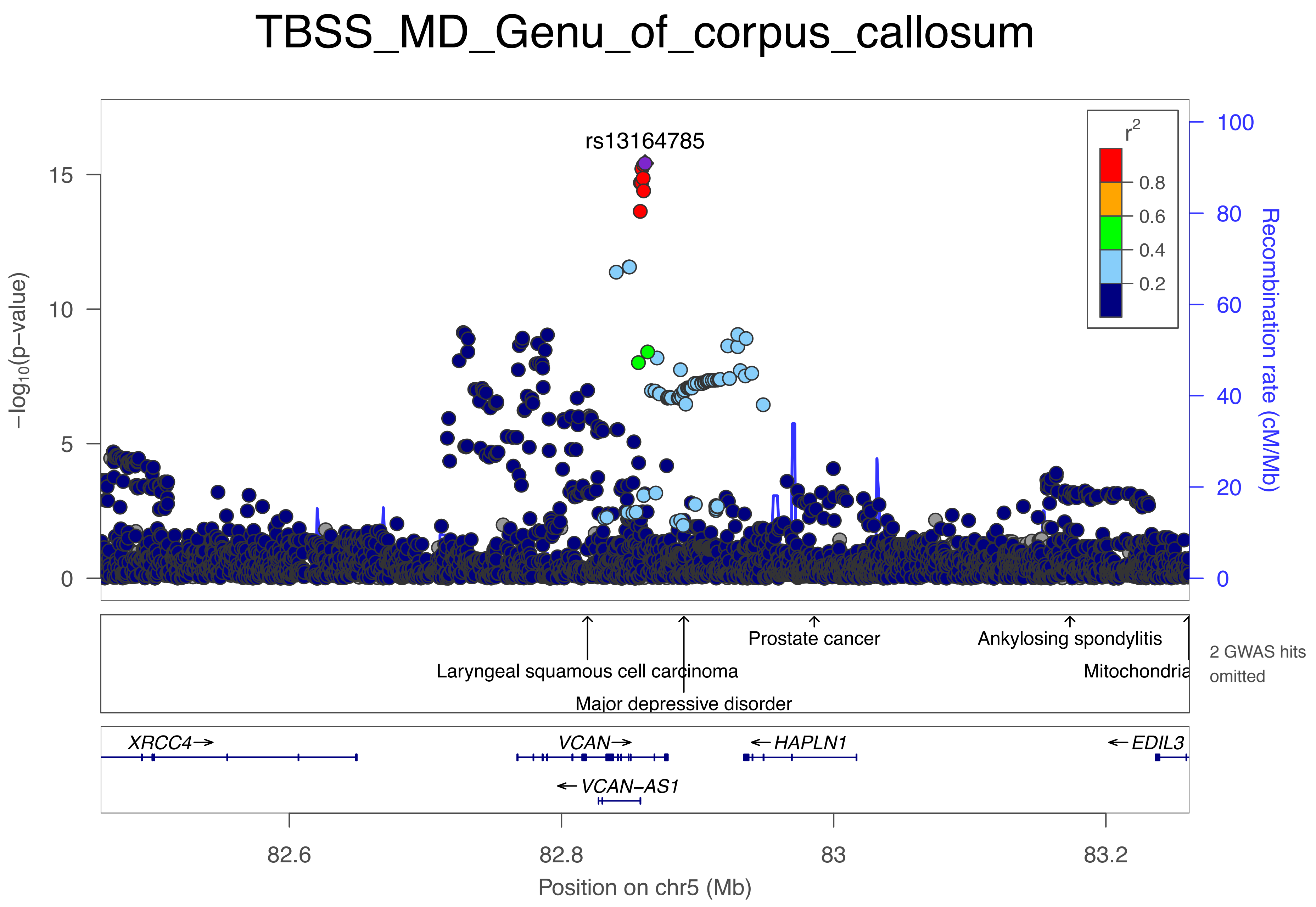
TBSS_MD_Genu_of_corpus_callosum
rs13164785
−log10(p−value)
Recombination rate (cM/Mb)
r2
0.8
0.6
0.4
0.2
Laryngeal squamous cell carcinoma
Major depressive disorder
Prostate cancer
Ankylosing spondylitis
Mitochondria
2 GWAS hits omitted
XRCC4→
VCAN→
← VCAN−AS1
← HAPLN1
← EDIL3
82.6
82.8
83
83.2
Position on chr5 (Mb)

| | |
|---|---|
| date: | Thu Aug 17 17:52:01 2017 |
| build: | hg19 |
| display range: | chr5:82461400−83261400 [82461400−83261400] |
| hilite range: | 0 – 0 [ 0 – 0 ] |
| reference SNP: | chr5:82861400 |
| number of SNPs plotted: | 3396 |
| min P.value: | 3.84E−16 [chr5:82861400] |
| max P.value: | 10E−1 [chr5:82791634] |
| omitted GWAS Hits: | chr5:83.260938−Mitochondrial DNA levels, NA |

Make more plots at http://csg.sph.umich.edu/locuszoom/

GWAS Catalog SNPs in Region

| chr | pos (Mb) | trait | snp |
|---|---|---|---|
| 5 | 82.81912 | Laryngeal squamous cell carcinoma | rs310518 |
| 5 | 82.84549 | Diisocyanate−induced asthma | rs3852186 |
| 5 | 82.88991 | Major depressive disorder | rs310501 |
| 5 | 82.96073 | Visceral fat | rs3846635 |
| 5 | 82.98574 | Prostate cancer | rs4466137 |
| 5 | 83.17359 | Ankylosing spondylitis | rs4552569 |
| 5 | 83.26094 | Mitochondrial DNA levels | rs2301070 |

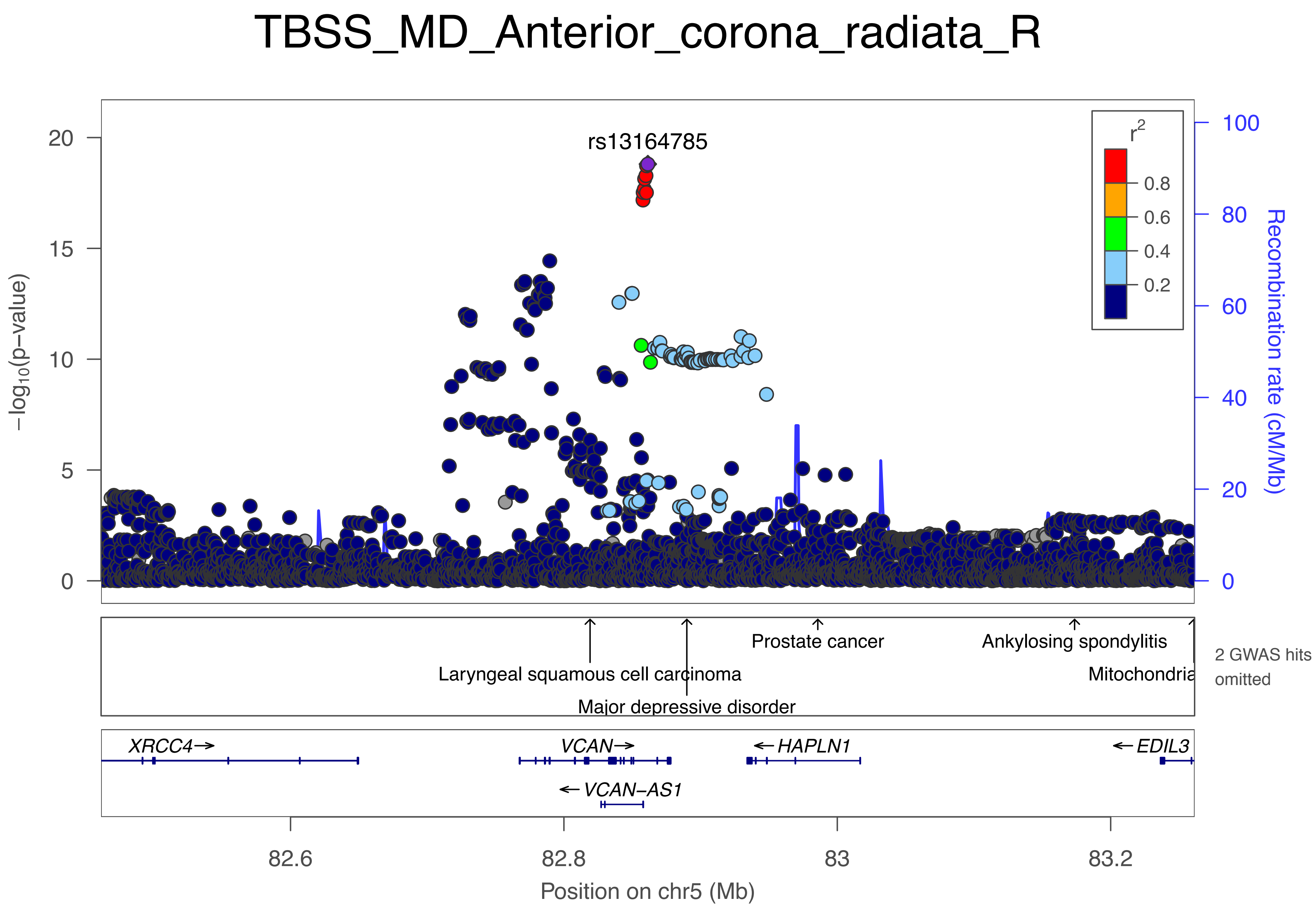

TBSS_MD_Anterior_corona_radiata_R
rs13164785
r²
0.8
0.6
0.4
0.2
20
15
10
5
0
−log10(p−value)
100
80
60
40
20
0
Recombination rate (cM/Mb)
Prostate cancer
Ankylosing spondylitis
Laryngeal squamous cell carcinoma
Mitochondria
Major depressive disorder
2 GWAS hits omitted
XRCC4→
VCAN→
←HAPLN1
←EDIL3
←VCAN−AS1
82.6
82.8
83
83.2
Position on chr5 (Mb)

| | |
|---|---|
| date: | Thu Aug 17 17:52:01 2017 |
| build: | hg19 |
| display range: | chr5:82461400–83261400 [82461400–83261400] |
| hilite range: | 0 – 0 [ 0 – 0 ] |
| reference SNP: | chr5:82861400 |
| number of SNPs plotted: | 3396 |
| min P.value: | 1.57E–19 [chr5:82861400] |
| max P.value: | 9.99E–1 [chr5:82707560] |
| omitted GWAS Hits: | chr5:83.260938–Mitochondrial DNA levels, NA |

Make more plots at http://csg.sph.umich.edu/locuszoom/

GWAS Catalog SNPs in Region

| chr | pos (Mb) | trait | snp |
|---|---|---|---|
| 5 | 82.81912 | Laryngeal squamous cell carcinoma | rs310518 |
| 5 | 82.84549 | Diisocyanate–induced asthma | rs3852186 |
| 5 | 82.88991 | Major depressive disorder | rs310501 |
| 5 | 82.96073 | Visceral fat | rs3846635 |
| 5 | 82.98574 | Prostate cancer | rs4466137 |
| 5 | 83.17359 | Ankylosing spondylitis | rs4552569 |
| 5 | 83.26094 | Mitochondrial DNA levels | rs2301070 |

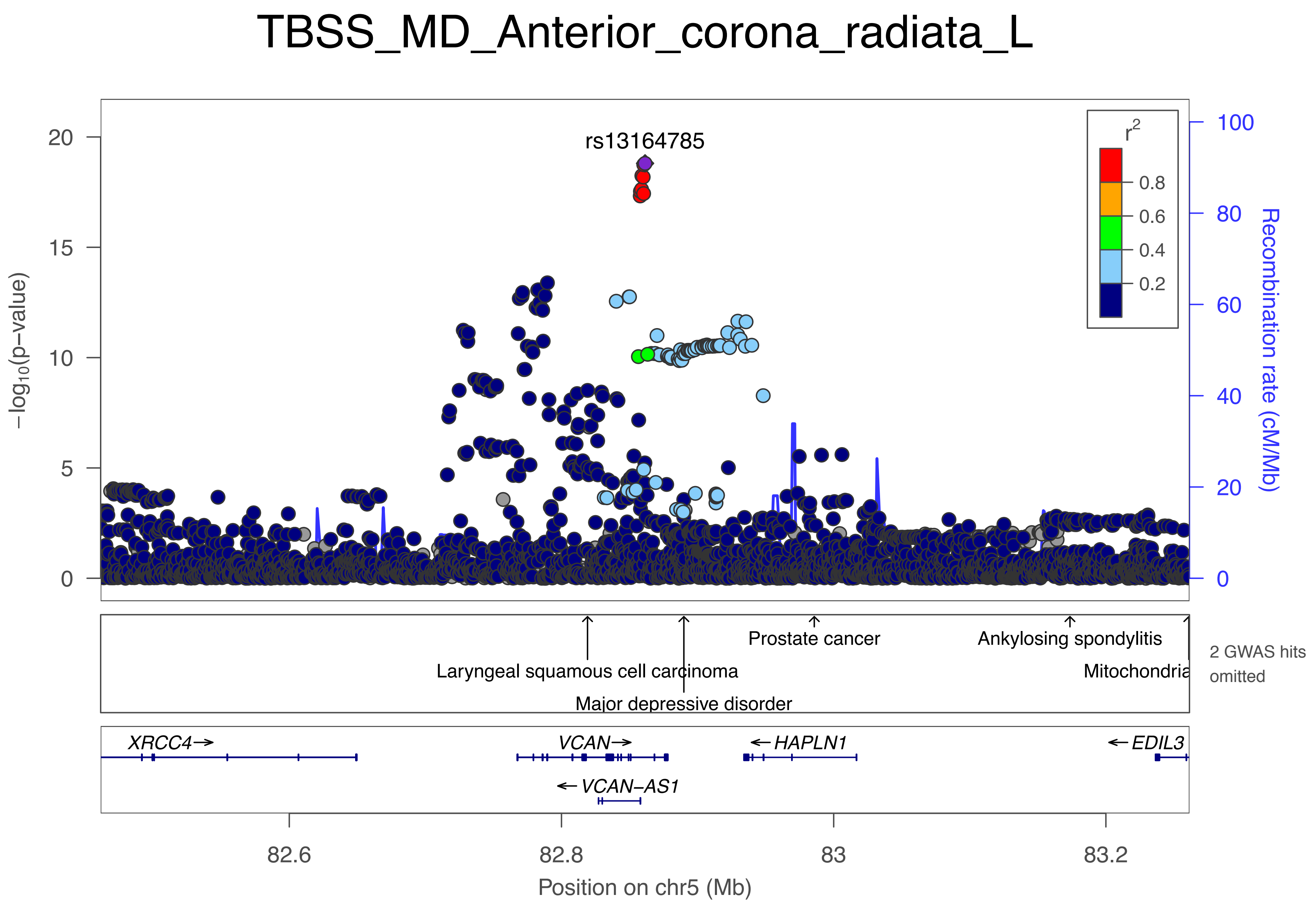
TBSS_MD_Anterior_corona_radiata_L
rs13164785
−log10(p−value)
Recombination rate (cM/Mb)
r2
0.8
0.6
0.4
0.2
Prostate cancer
Ankylosing spondylitis
Laryngeal squamous cell carcinoma
Mitochondria
Major depressive disorder
2 GWAS hits omitted
XRCC4→
VCAN→
←HAPLN1
←EDIL3
←VCAN−AS1
82.6
82.8
83
83.2
Position on chr5 (Mb)

| | |
|---|---|
| date: | Thu Aug 17 17:52:01 2017 |
| build: | hg19 |
| display range: | chr5:82461400−83261400 [82461400−83261400] |
| hilite range: | 0 – 0 [ 0 – 0 ] |
| reference SNP: | chr5:82861400 |
| number of SNPs plotted: | 3396 |
| min P.value: | 1.57E−19 [chr5:82861400] |
| max P.value: | 9.99E−1 [chr5:83077850] |
| omitted GWAS Hits: | chr5:83.260938−Mitochondrial DNA levels, NA |

Make more plots at http://csg.sph.umich.edu/locuszoom/

GWAS Catalog SNPs in Region

| chr | pos (Mb) | trait | snp |
|---|---|---|---|
| 5 | 82.81912 | Laryngeal squamous cell carcinoma | rs310518 |
| 5 | 82.84549 | Diisocyanate–induced asthma | rs3852186 |
| 5 | 82.88991 | Major depressive disorder | rs310501 |
| 5 | 82.96073 | Visceral fat | rs3846635 |
| 5 | 82.98574 | Prostate cancer | rs4466137 |
| 5 | 83.17359 | Ankylosing spondylitis | rs4552569 |
| 5 | 83.26094 | Mitochondrial DNA levels | rs2301070 |

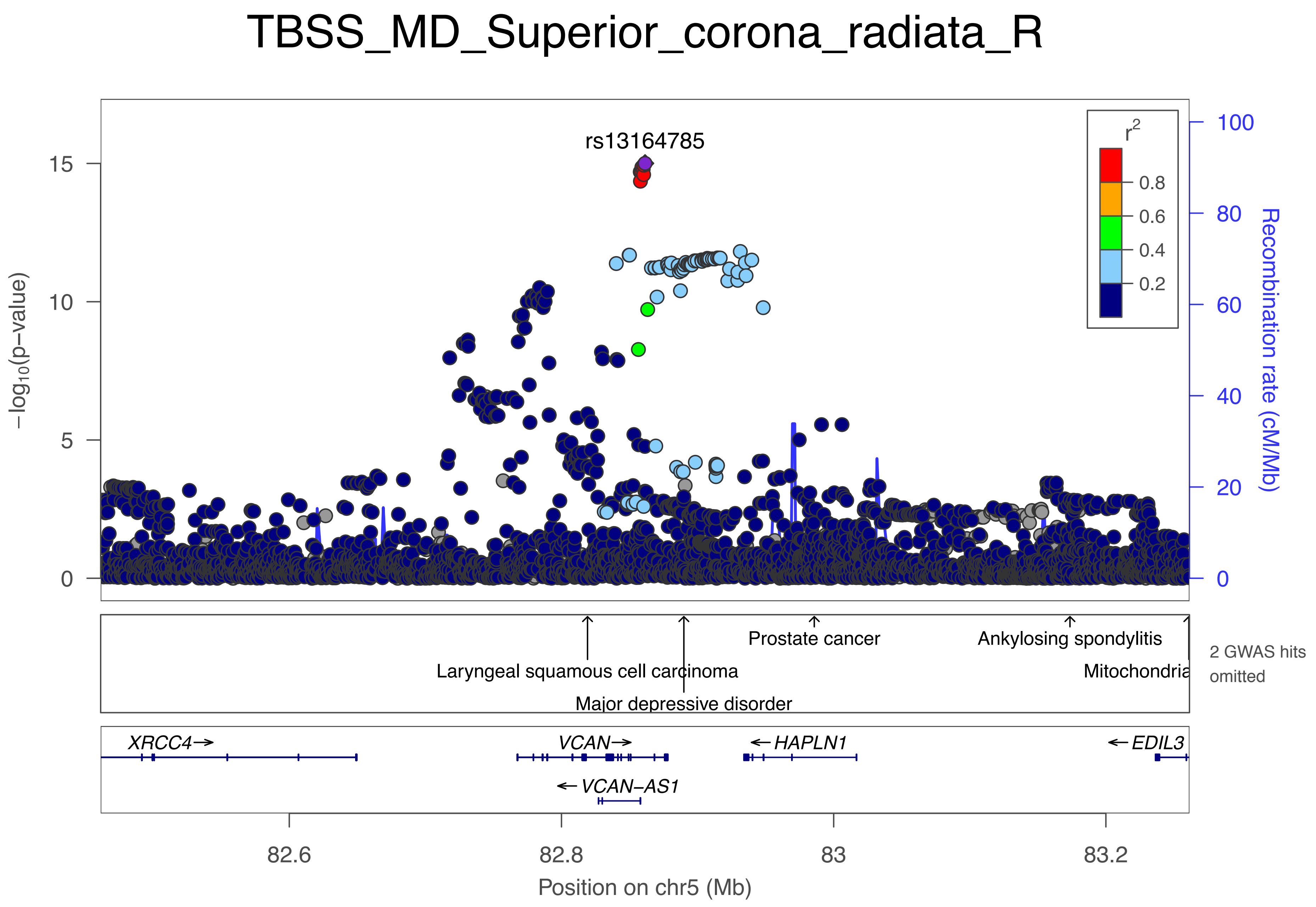
TBSS_MD_Superior_corona_radiata_R
rs13164785
−log10(p−value)
Recombination rate (cM/Mb)
r2
0.8
0.6
0.4
0.2
Prostate cancer
Ankylosing spondylitis
Laryngeal squamous cell carcinoma
Mitochondria
Major depressive disorder
2 GWAS hits omitted
XRCC4
VCAN
HAPLN1
EDIL3
VCAN−AS1
82.6
82.8
83
83.2
Position on chr5 (Mb)

| | |
|---|---|
| date: | Thu Aug 17 17:52:01 2017 |
| build: | hg19 |
| display range: | chr5:82461400−83261400 [82461400−83261400] |
| hilite range: | 0 − 0 [ 0 − 0 ] |
| reference SNP: | chr5:82861400 |
| number of SNPs plotted: | 3396 |
| min P.value: | 9.93E−16 [chr5:82861400] |
| max P.value: | 9.99E−1 [chr5:82693995] |
| omitted GWAS Hits: | chr5:83.260938−Mitochondrial DNA levels, NA |

Make more plots at http://csg.sph.umich.edu/locuszoom/

GWAS Catalog SNPs in Region

| chr | pos (Mb) | trait | snp |
|---|---|---|---|
| 5 | 82.81912 | Laryngeal squamous cell carcinoma | rs310518 |
| 5 | 82.84549 | Diisocyanate–induced asthma | rs3852186 |
| 5 | 82.88991 | Major depressive disorder | rs310501 |
| 5 | 82.96073 | Visceral fat | rs3846635 |
| 5 | 82.98574 | Prostate cancer | rs4466137 |
| 5 | 83.17359 | Ankylosing spondylitis | rs4552569 |
| 5 | 83.26094 | Mitochondrial DNA levels | rs2301070 |

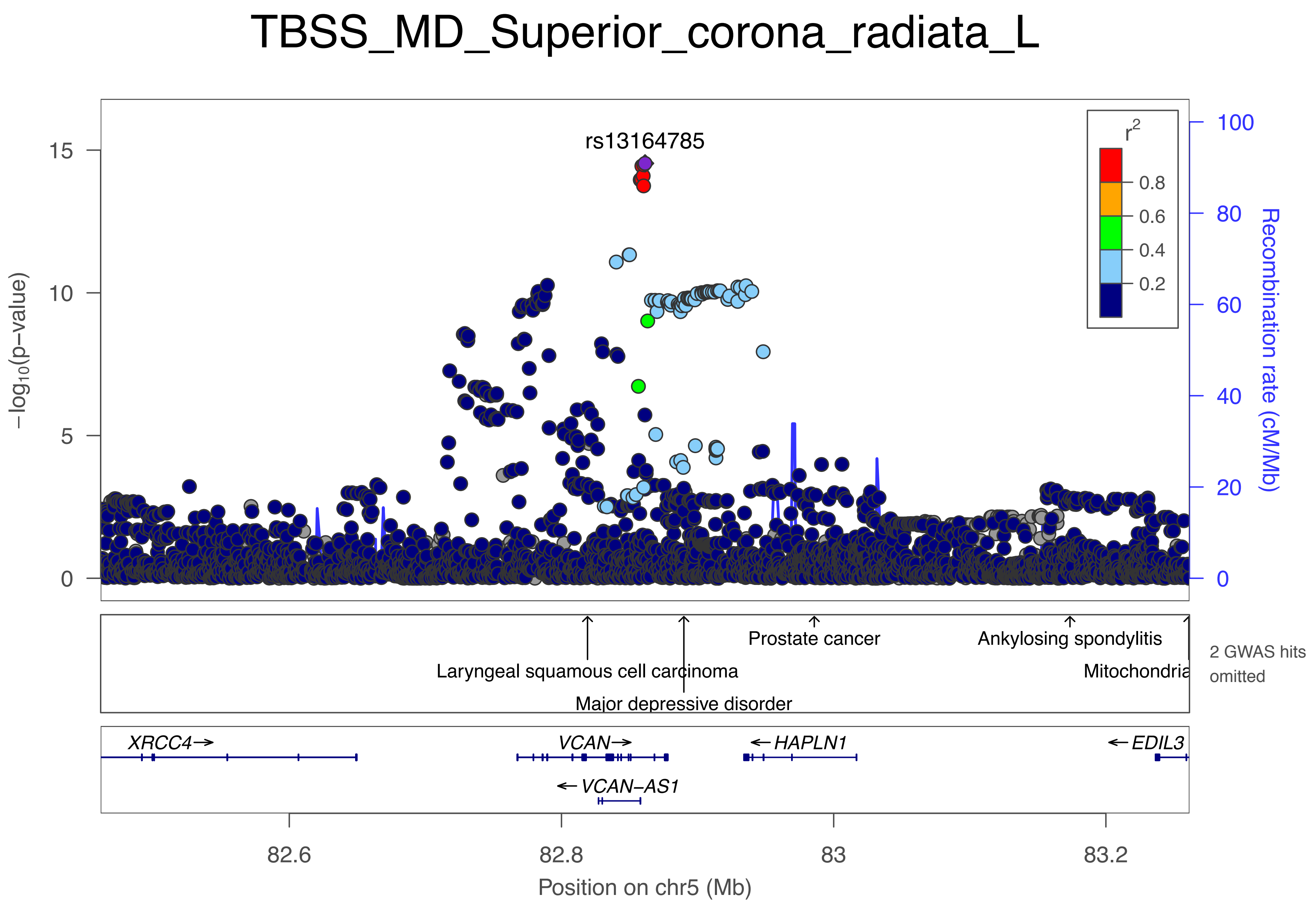
TBSS_MD_Superior_corona_radiata_L
rs13164785
−log10(p−value)
15
10
5
0
r2
0.8
0.6
0.4
0.2
100
80
60
40
20
0
Recombination rate (cM/Mb)
Laryngeal squamous cell carcinoma
Major depressive disorder
Prostate cancer
Ankylosing spondylitis
Mitochondria
2 GWAS hits omitted
XRCC4→
VCAN→
←VCAN−AS1
←HAPLN1
←EDIL3
82.6
82.8
83
83.2
Position on chr5 (Mb)

| | |
|---|---|
| date: | Thu Aug 17 17:52:01 2017 |
| build: | hg19 |
| display range: | chr5:82461400–83261400 [82461400–83261400] |
| hilite range: | 0 – 0 [ 0 – 0 ] |
| reference SNP: | chr5:82861400 |
| number of SNPs plotted: | 3396 |
| min P.value: | 2.91E–15 [chr5:82861400] |
| max P.value: | 10E–1 [chr5:83211071] |
| omitted GWAS Hits: | chr5:83.260938–Mitochondrial DNA levels, NA |

Make more plots at http://csg.sph.umich.edu/locuszoom/

GWAS Catalog SNPs in Region

| chr | pos (Mb) | trait | snp |
|---|---|---|---|
| 5 | 82.81912 | Laryngeal squamous cell carcinoma | rs310518 |
| 5 | 82.84549 | Diisocyanate−induced asthma | rs3852186 |
| 5 | 82.88991 | Major depressive disorder | rs310501 |
| 5 | 82.96073 | Visceral fat | rs3846635 |
| 5 | 82.98574 | Prostate cancer | rs4466137 |
| 5 | 83.17359 | Ankylosing spondylitis | rs4552569 |
| 5 | 83.26094 | Mitochondrial DNA levels | rs2301070 |

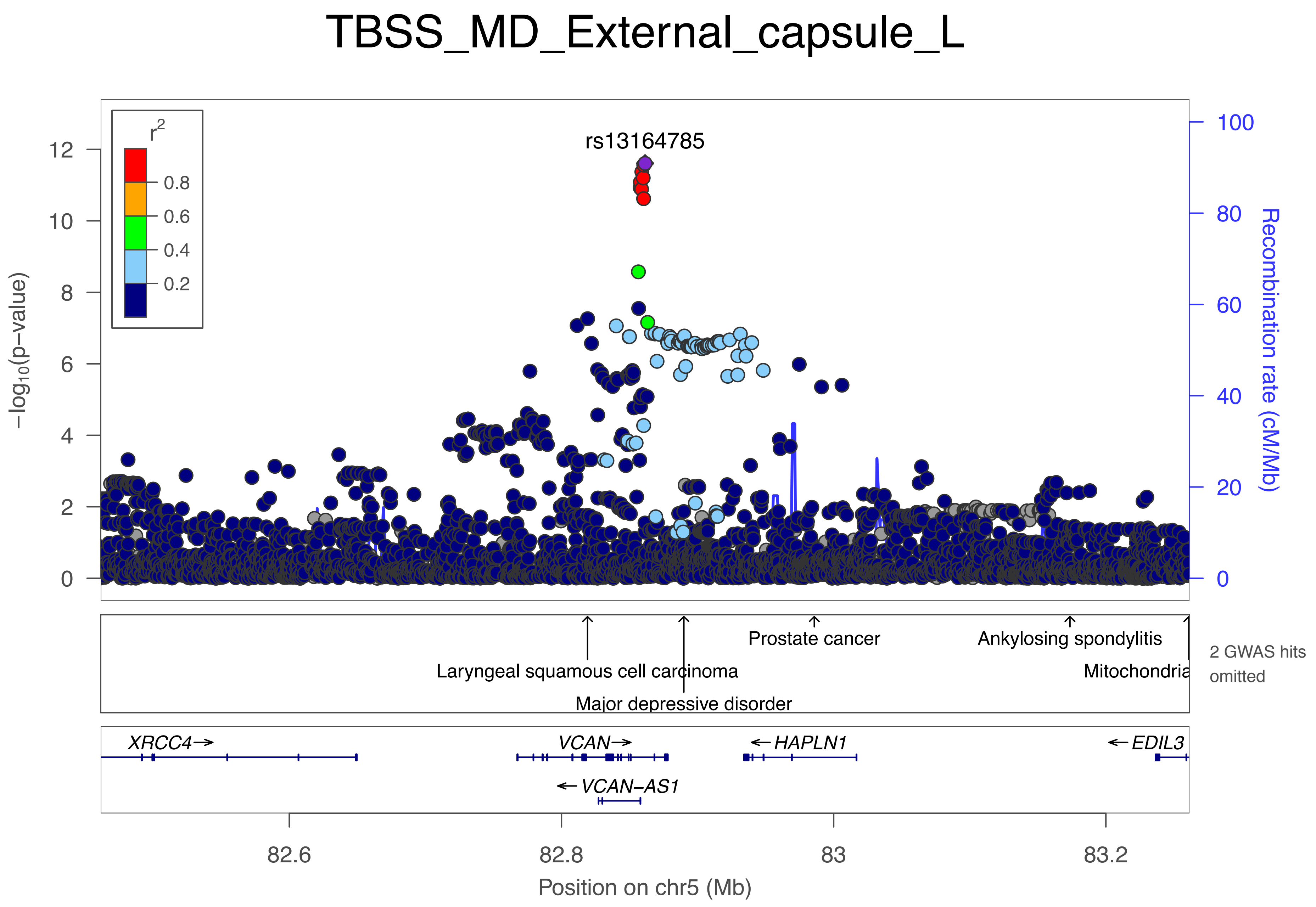

TBSS_MD_External_capsule_L
rs13164785
r2
0.8
0.6
0.4
0.2
−log10(p−value)
Recombination rate (cM/Mb)
Prostate cancer
Ankylosing spondylitis
Laryngeal squamous cell carcinoma
Major depressive disorder
Mitochondria
2 GWAS hits omitted
XRCC4→
VCAN→
←HAPLN1
←EDIL3
←VCAN−AS1
82.6
82.8
83
83.2
Position on chr5 (Mb)

| | |
|---|---|
| date: | Thu Aug 17 17:52:01 2017 |
| build: | hg19 |
| display range: | chr5:82461400–83261400 [82461400–83261400] |
| hilite range: | 0 – 0 [ 0 – 0 ] |
| reference SNP: | chr5:82861400 |
| number of SNPs plotted: | 3396 |
| min P.value: | 2.47E–12 [chr5:82861400] |
| max P.value: | 9.99E–1 [chr5:83227196] |
| omitted GWAS Hits: | chr5:83.260938–Mitochondrial DNA levels, NA |

Make more plots at http://csg.sph.umich.edu/locuszoom/

GWAS Catalog SNPs in Region

| chr | pos (Mb) | trait | snp |
|---|---|---|---|
| 5 | 82.81912 | Laryngeal squamous cell carcinoma | rs310518 |
| 5 | 82.84549 | Diisocyanate–induced asthma | rs3852186 |
| 5 | 82.88991 | Major depressive disorder | rs310501 |
| 5 | 82.96073 | Visceral fat | rs3846635 |
| 5 | 82.98574 | Prostate cancer | rs4466137 |
| 5 | 83.17359 | Ankylosing spondylitis | rs4552569 |
| 5 | 83.26094 | Mitochondrial DNA levels | rs2301070 |

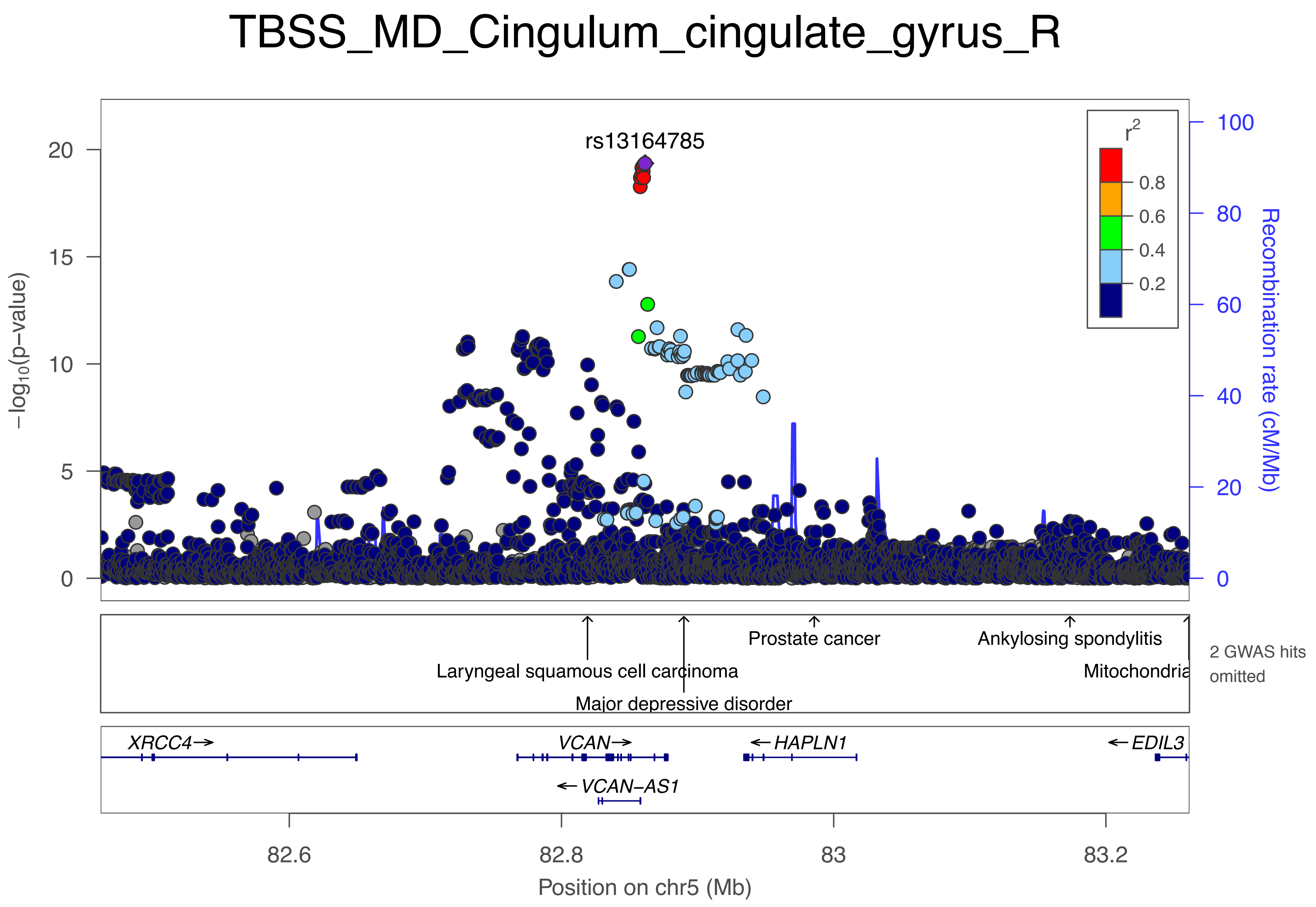
TBSS_MD_Cingulum_cingulate_gyrus_R
rs13164785
−log10(p−value)
Recombination rate (cM/Mb)
r2
0.8
0.6
0.4
0.2
Prostate cancer
Ankylosing spondylitis
Laryngeal squamous cell carcinoma
Mitochondria
Major depressive disorder
2 GWAS hits omitted
XRCC4→
VCAN→
←HAPLN1
←EDIL3
←VCAN−AS1
82.6
82.8
83
83.2
Position on chr5 (Mb)

| | |
|---|---|
| date: | Thu Aug 17 17:52:01 2017 |
| build: | hg19 |
| display range: | chr5:82461400−83261400 [82461400−83261400] |
| hilite range: | 0 – 0 [ 0 – 0 ] |
| reference SNP: | chr5:82861400 |
| number of SNPs plotted: | 3396 |
| min P.value: | 4.4E−20 [chr5:82861400] |
| max P.value: | 10E−1 [chr5:83125888] |
| omitted GWAS Hits: | chr5:83.260938−Mitochondrial DNA levels, NA |

Make more plots at http://csg.sph.umich.edu/locuszoom/

GWAS Catalog SNPs in Region

| chr | pos (Mb) | trait | snp |
|---|---|---|---|
| 5 | 82.81912 | Laryngeal squamous cell carcinoma | rs310518 |
| 5 | 82.84549 | Diisocyanate–induced asthma | rs3852186 |
| 5 | 82.88991 | Major depressive disorder | rs310501 |
| 5 | 82.96073 | Visceral fat | rs3846635 |
| 5 | 82.98574 | Prostate cancer | rs4466137 |
| 5 | 83.17359 | Ankylosing spondylitis | rs4552569 |
| 5 | 83.26094 | Mitochondrial DNA levels | rs2301070 |

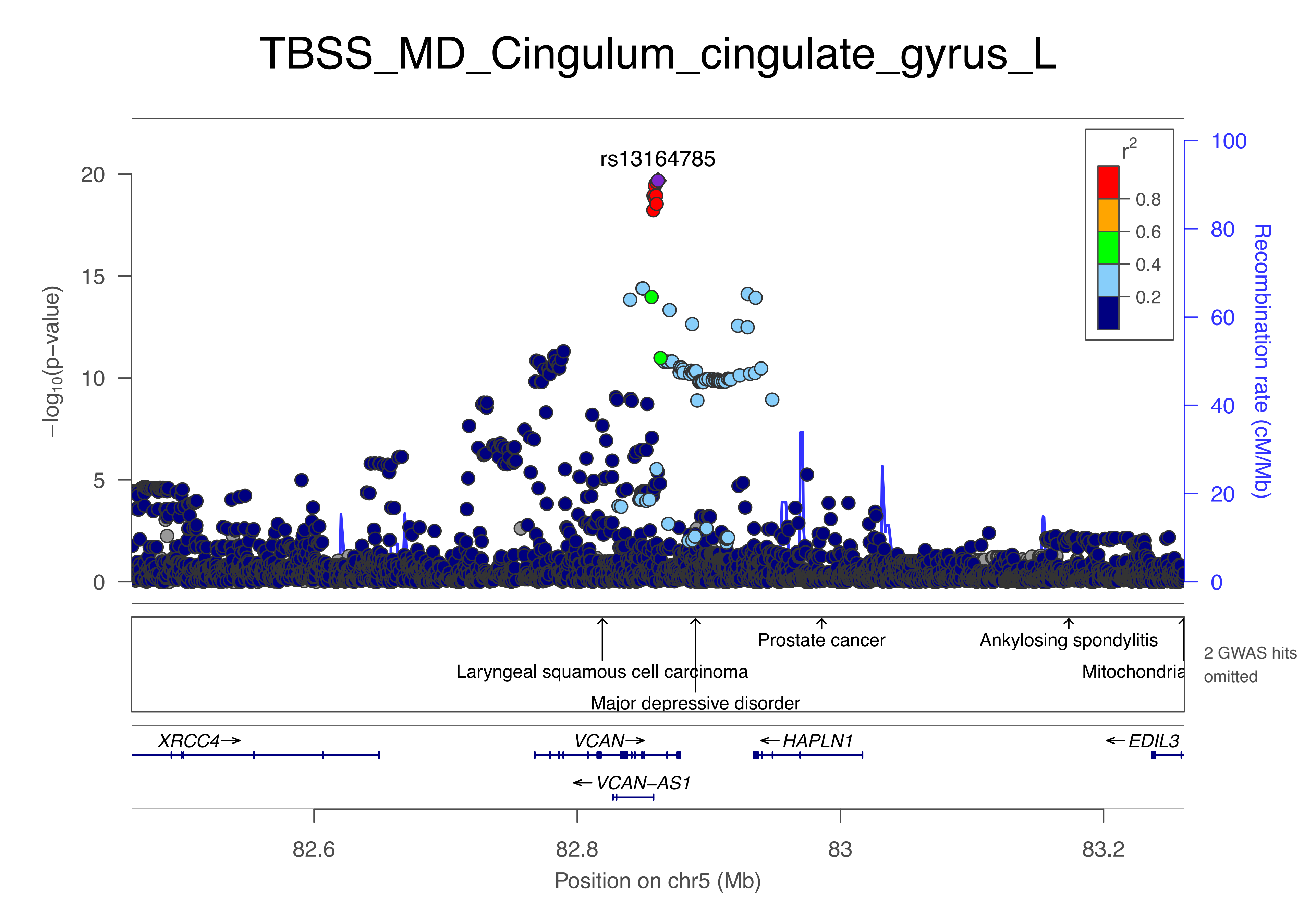
TBSS_MD_Cingulum_cingulate_gyrus_L
rs13164785
−log10(p−value)
Recombination rate (cM/Mb)
r2
0.8
0.6
0.4
0.2
Laryngeal squamous cell carcinoma
Major depressive disorder
Prostate cancer
Ankylosing spondylitis
Mitochondria
2 GWAS hits omitted
XRCC4
VCAN
VCAN−AS1
HAPLN1
EDIL3
82.6
82.8
83
83.2
Position on chr5 (Mb)

| | |
|---|---|
| date: | Thu Aug 17 17:52:01 2017 |
| build: | hg19 |
| display range: | chr5:82461400−83261400 [82461400−83261400] |
| hilite range: | 0 – 0 [ 0 – 0 ] |
| reference SNP: | chr5:82861400 |
| number of SNPs plotted: | 3396 |
| min P.value: | 2.09E−20 [chr5:82861400] |
| max P.value: | 10E−1 [chr5:82557155] |
| omitted GWAS Hits: | chr5:83.260938−Mitochondrial DNA levels, NA |

Make more plots at http://csg.sph.umich.edu/locuszoom/

GWAS Catalog SNPs in Region

| chr | pos (Mb) | trait | snp |
|---|---|---|---|
| 5 | 82.81912 | Laryngeal squamous cell carcinoma | rs310518 |
| 5 | 82.84549 | Diisocyanate–induced asthma | rs3852186 |
| 5 | 82.88991 | Major depressive disorder | rs310501 |
| 5 | 82.96073 | Visceral fat | rs3846635 |
| 5 | 82.98574 | Prostate cancer | rs4466137 |
| 5 | 83.17359 | Ankylosing spondylitis | rs4552569 |
| 5 | 83.26094 | Mitochondrial DNA levels | rs2301070 |

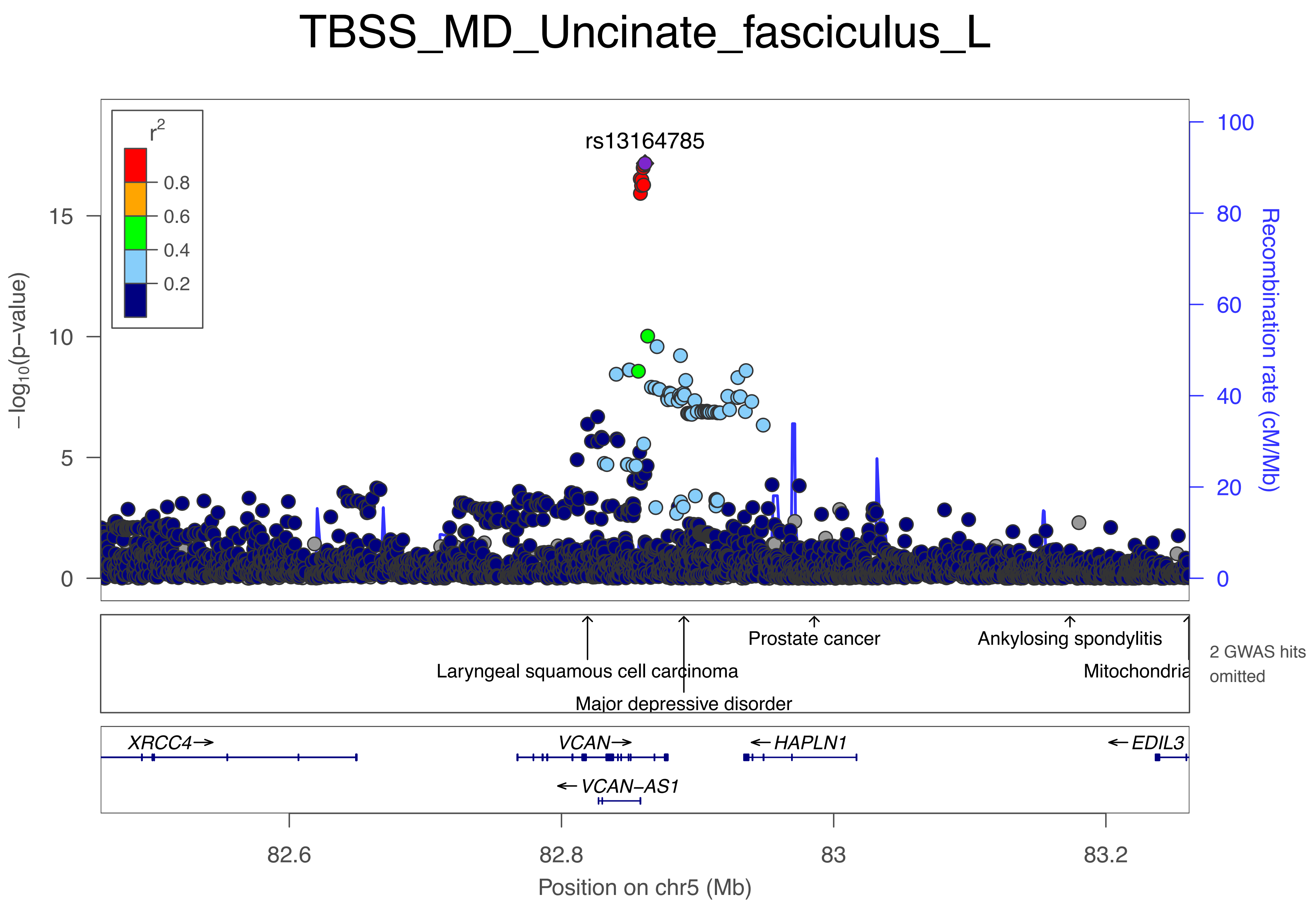
TBSS_MD_Uncinate_fasciculus_L
rs13164785
r2
0.8
0.6
0.4
0.2
−log10(p−value)
Recombination rate (cM/Mb)
Laryngeal squamous cell carcinoma
Major depressive disorder
Prostate cancer
Ankylosing spondylitis
Mitochondria
2 GWAS hits omitted
XRCC4→
VCAN→
← VCAN−AS1
← HAPLN1
← EDIL3
82.6
82.8
83
83.2
Position on chr5 (Mb)

| | |
|---|---|
| date: | Thu Aug 17 17:52:01 2017 |
| build: | hg19 |
| display range: | chr5:82461400–83261400 [82461400–83261400] |
| hilite range: | 0 – 0 [ 0 – 0 ] |
| reference SNP: | chr5:82861400 |
| number of SNPs plotted: | 3396 |
| min P.value: | 6.75E–18 [chr5:82861400] |
| max P.value: | 10E–1 [chr5:83191718] |
| omitted GWAS Hits: | chr5:83.260938–Mitochondrial DNA levels, NA |

Make more plots at http://csg.sph.umich.edu/locuszoom/

GWAS Catalog SNPs in Region

| chr | pos (Mb) | trait | snp |
|---|---|---|---|
| 5 | 82.81912 | Laryngeal squamous cell carcinoma | rs310518 |
| 5 | 82.84549 | Diisocyanate–induced asthma | rs3852186 |
| 5 | 82.88991 | Major depressive disorder | rs310501 |
| 5 | 82.96073 | Visceral fat | rs3846635 |
| 5 | 82.98574 | Prostate cancer | rs4466137 |
| 5 | 83.17359 | Ankylosing spondylitis | rs4552569 |
| 5 | 83.26094 | Mitochondrial DNA levels | rs2301070 |

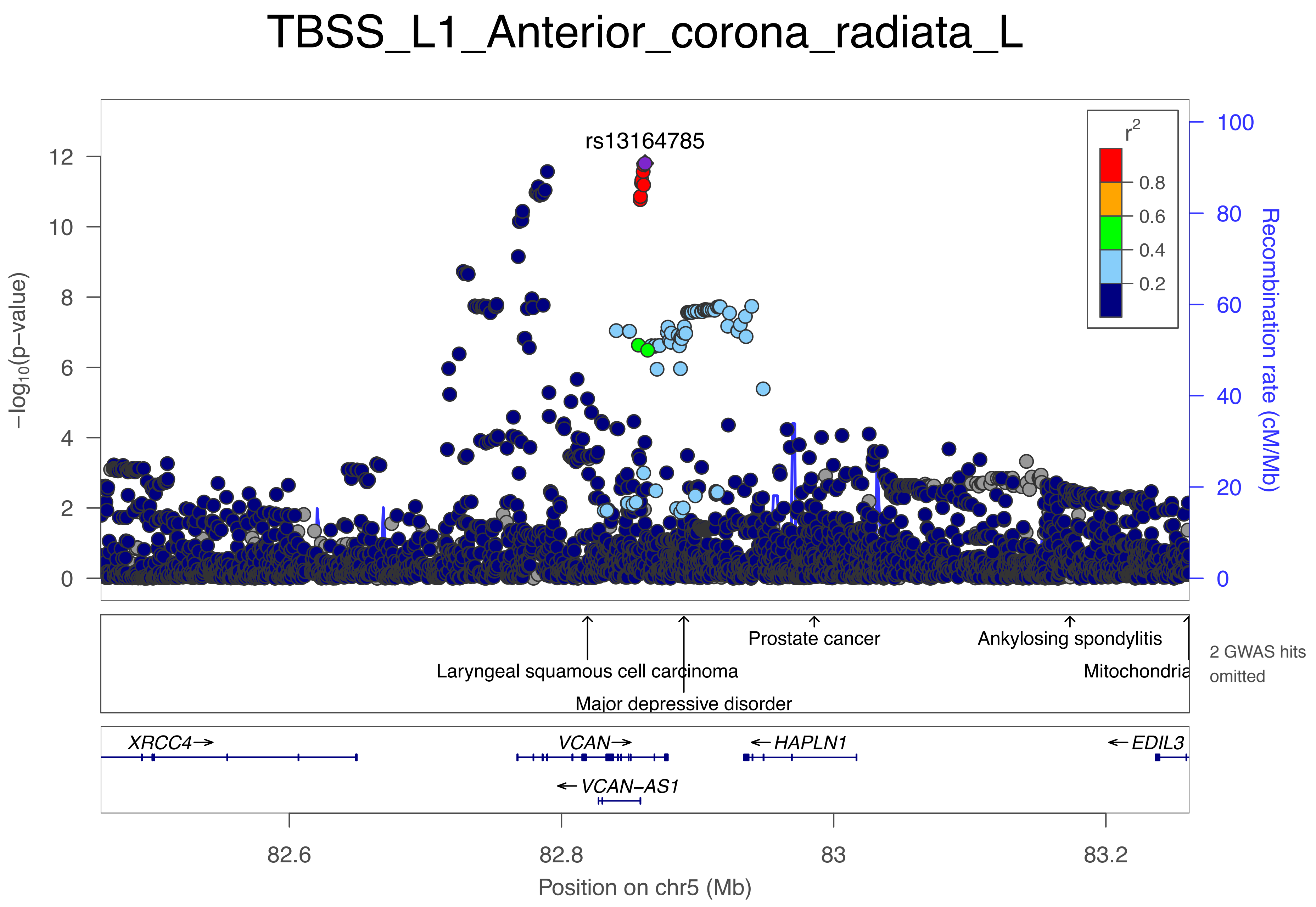
TBSS_L1_Anterior_corona_radiata_L
rs13164785
r2
0.8
0.6
0.4
0.2
−log10(p−value)
Recombination rate (cM/Mb)
Laryngeal squamous cell carcinoma
Major depressive disorder
Prostate cancer
Ankylosing spondylitis
Mitochondria
2 GWAS hits omitted
XRCC4→
VCAN→
←HAPLN1
←EDIL3
←VCAN−AS1
82.6
82.8
83
83.2
Position on chr5 (Mb)

| | |
|---|---|
| date: | Thu Aug 17 17:52:01 2017 |
| build: | hg19 |
| display range: | chr5:82461400−83261400 [82461400−83261400] |
| hilite range: | 0 – 0 [ 0 – 0 ] |
| reference SNP: | chr5:82861400 |
| number of SNPs plotted: | 3396 |
| min P.value: | 1.57E−12 [chr5:82861400] |
| max P.value: | 9.99E−1 [chr5:82939273] |
| omitted GWAS Hits: | chr5:83.260938−Mitochondrial DNA levels, NA |

Make more plots at http://csg.sph.umich.edu/locuszoom/

GWAS Catalog SNPs in Region

| chr | pos (Mb) | trait | snp |
|---|---|---|---|
| 5 | 82.81912 | Laryngeal squamous cell carcinoma | rs310518 |
| 5 | 82.84549 | Diisocyanate−induced asthma | rs3852186 |
| 5 | 82.88991 | Major depressive disorder | rs310501 |
| 5 | 82.96073 | Visceral fat | rs3846635 |
| 5 | 82.98574 | Prostate cancer | rs4466137 |
| 5 | 83.17359 | Ankylosing spondylitis | rs4552569 |
| 5 | 83.26094 | Mitochondrial DNA levels | rs2301070 |

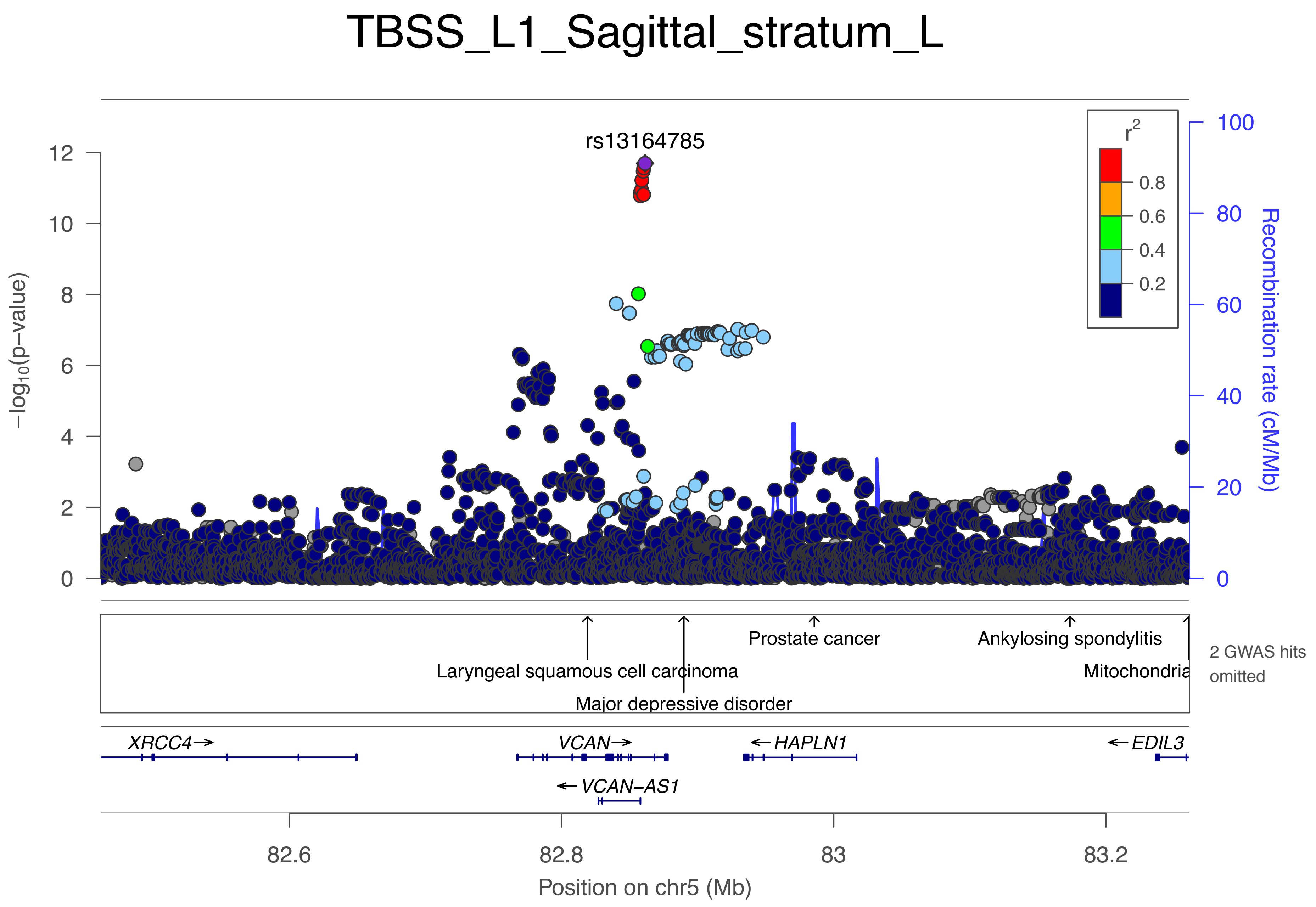

TBSS_L1_Sagittal_stratum_L
rs13164785
r2
0.8
0.6
0.4
0.2
−log10(p−value)
Recombination rate (cM/Mb)
Prostate cancer
Ankylosing spondylitis
Laryngeal squamous cell carcinoma
Mitochondria
Major depressive disorder
2 GWAS hits omitted
XRCC4
VCAN
HAPLN1
EDIL3
VCAN−AS1
82.6
82.8
83
83.2
Position on chr5 (Mb)

| | |
|---|---|
| date: | Thu Aug 17 17:52:01 2017 |
| build: | hg19 |
| display range: | chr5:82461400−83261400 [82461400−83261400] |
| hilite range: | 0 – 0 [ 0 – 0 ] |
| reference SNP: | chr5:82861400 |
| number of SNPs plotted: | 3396 |
| min P.value: | 2.01E−12 [chr5:82861400] |
| max P.value: | 10E−1 [chr5:82659352] |
| omitted GWAS Hits: | chr5:83.260938−Mitochondrial DNA levels, NA |

Make more plots at http://csg.sph.umich.edu/locuszoom/

GWAS Catalog SNPs in Region

| chr | pos (Mb) | trait | snp |
|---|---|---|---|
| 5 | 82.81912 | Laryngeal squamous cell carcinoma | rs310518 |
| 5 | 82.84549 | Diisocyanate–induced asthma | rs3852186 |
| 5 | 82.88991 | Major depressive disorder | rs310501 |
| 5 | 82.96073 | Visceral fat | rs3846635 |
| 5 | 82.98574 | Prostate cancer | rs4466137 |
| 5 | 83.17359 | Ankylosing spondylitis | rs4552569 |
| 5 | 83.26094 | Mitochondrial DNA levels | rs2301070 |

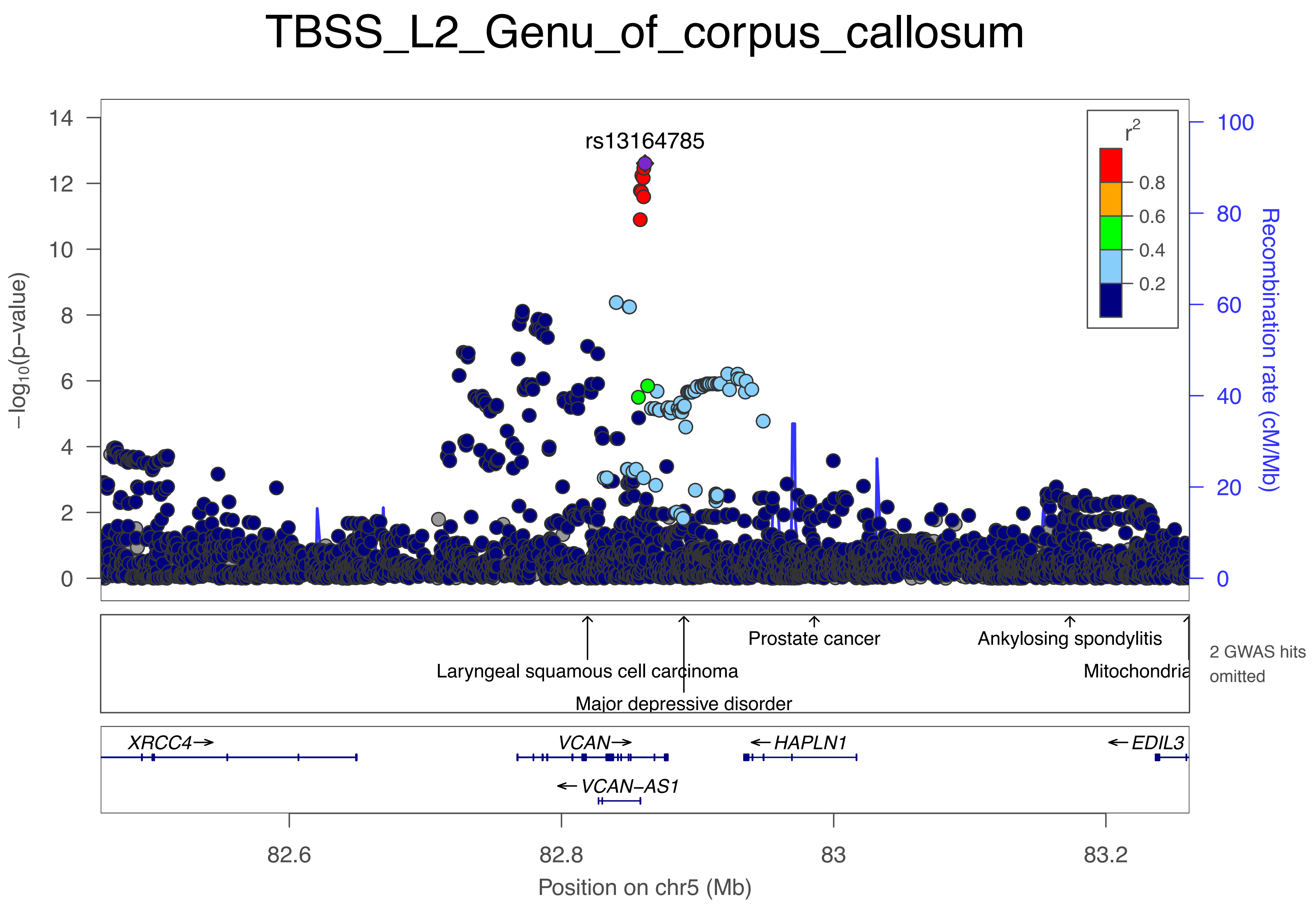
TBSS_L2_Genu_of_corpus_callosum
rs13164785
r²
0.8
0.6
0.4
0.2
−log10(p−value)
Recombination rate (cM/Mb)
Prostate cancer
Ankylosing spondylitis
Laryngeal squamous cell carcinoma
Mitochondria
Major depressive disorder
2 GWAS hits omitted
XRCC4
VCAN
HAPLN1
EDIL3
VCAN−AS1
82.6
82.8
83
83.2
Position on chr5 (Mb)

| | |
|---|---|
| date: | Thu Aug 17 17:52:01 2017 |
| build: | hg19 |
| display range: | chr5:82461400−83261400 [82461400−83261400] |
| hilite range: | 0 – 0 [ 0 – 0 ] |
| reference SNP: | chr5:82861400 |
| number of SNPs plotted: | 3396 |
| min P.value: | 2.47E−13 [chr5:82861400] |
| max P.value: | 10E−1 [chr5:82642540] |
| omitted GWAS Hits: | chr5:83.260938−Mitochondrial DNA levels, NA |

Make more plots at http://csg.sph.umich.edu/locuszoom/

GWAS Catalog SNPs in Region

| chr | pos (Mb) | trait | snp |
|---|---|---|---|
| 5 | 82.81912 | Laryngeal squamous cell carcinoma | rs310518 |
| 5 | 82.84549 | Diisocyanate–induced asthma | rs3852186 |
| 5 | 82.88991 | Major depressive disorder | rs310501 |
| 5 | 82.96073 | Visceral fat | rs3846635 |
| 5 | 82.98574 | Prostate cancer | rs4466137 |
| 5 | 83.17359 | Ankylosing spondylitis | rs4552569 |
| 5 | 83.26094 | Mitochondrial DNA levels | rs2301070 |

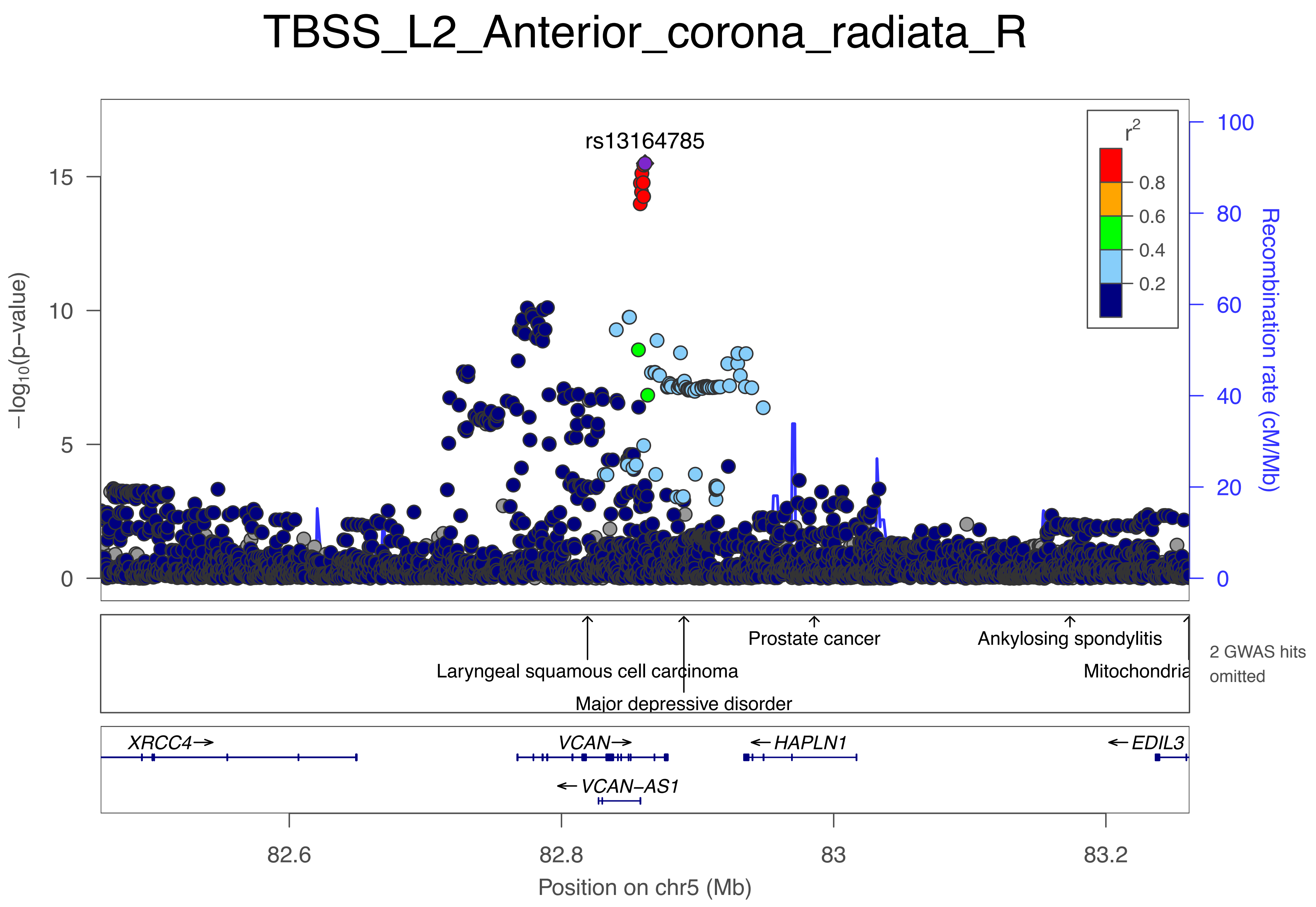
TBSS_L2_Anterior_corona_radiata_R
rs13164785
r²
0.8
0.6
0.4
0.2
15
10
5
0
−log₁₀(p−value)
100
80
60
40
20
0
Recombination rate (cM/Mb)
Prostate cancer
Ankylosing spondylitis
Laryngeal squamous cell carcinoma
Mitochondria
Major depressive disorder
2 GWAS hits omitted
XRCC4→
VCAN→
←HAPLN1
←EDIL3
←VCAN−AS1
82.6
82.8
83
83.2
Position on chr5 (Mb)

| | |
|---|---|
| date: | Thu Aug 17 17:52:01 2017 |
| build: | hg19 |
| display range: | chr5:82461400–83261400 [82461400–83261400] |
| hilite range: | 0 – 0 [ 0 – 0 ] |
| reference SNP: | chr5:82861400 |
| number of SNPs plotted: | 3396 |
| min P.value: | 3.19E–16 [chr5:82861400] |
| max P.value: | 10E–1 [chr5:82492578] |
| omitted GWAS Hits: | chr5:83.260938–Mitochondrial DNA levels, NA |

Make more plots at http://csg.sph.umich.edu/locuszoom/

GWAS Catalog SNPs in Region

| chr | pos (Mb) | trait | snp |
|---|---|---|---|
| 5 | 82.81912 | Laryngeal squamous cell carcinoma | rs310518 |
| 5 | 82.84549 | Diisocyanate–induced asthma | rs3852186 |
| 5 | 82.88991 | Major depressive disorder | rs310501 |
| 5 | 82.96073 | Visceral fat | rs3846635 |
| 5 | 82.98574 | Prostate cancer | rs4466137 |
| 5 | 83.17359 | Ankylosing spondylitis | rs4552569 |
| 5 | 83.26094 | Mitochondrial DNA levels | rs2301070 |

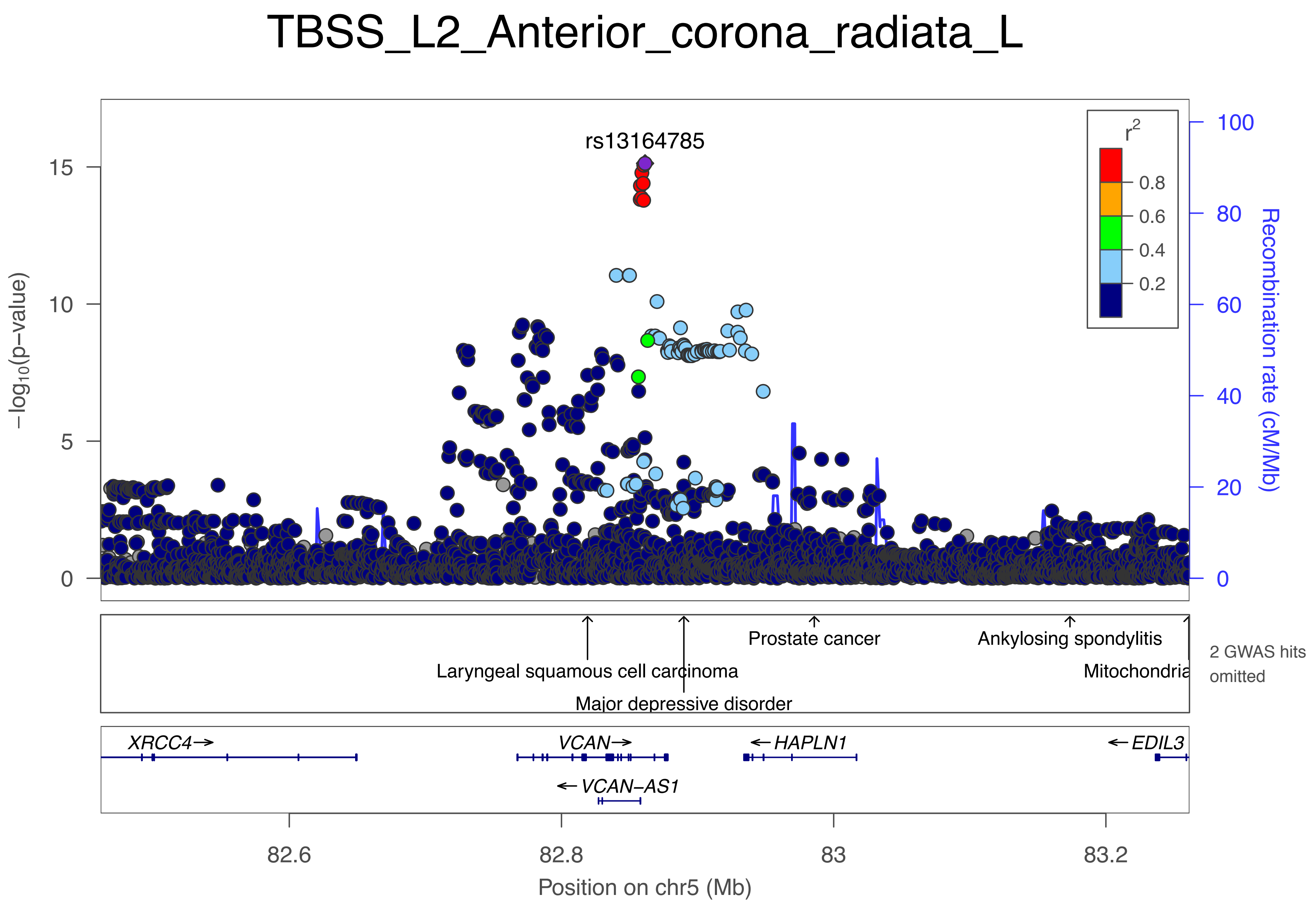

TBSS_L2_Anterior_corona_radiata_L
rs13164785
−log10(p−value)
Recombination rate (cM/Mb)
r2
0.8
0.6
0.4
0.2
Prostate cancer
Ankylosing spondylitis
Laryngeal squamous cell carcinoma
Mitochondria
Major depressive disorder
2 GWAS hits omitted
XRCC4→
VCAN→
←HAPLN1
←EDIL3
←VCAN−AS1
82.6
82.8
83
83.2
Position on chr5 (Mb)

| | |
|---|---|
| date: | Thu Aug 17 17:52:01 2017 |
| build: | hg19 |
| display range: | chr5:82461400−83261400 [82461400−83261400] |
| hilite range: | 0 – 0 [ 0 – 0 ] |
| reference SNP: | chr5:82861400 |
| number of SNPs plotted: | 3396 |
| min P.value: | 7.41E−16 [chr5:82861400] |
| max P.value: | 10E−1 [chr5:82847384] |
| omitted GWAS Hits: | chr5:83.260938−Mitochondrial DNA levels, NA |

Make more plots at http://csg.sph.umich.edu/locuszoom/

GWAS Catalog SNPs in Region

| chr | pos (Mb) | trait | snp |
|---|---|---|---|
| 5 | 82.81912 | Laryngeal squamous cell carcinoma | rs310518 |
| 5 | 82.84549 | Diisocyanate–induced asthma | rs3852186 |
| 5 | 82.88991 | Major depressive disorder | rs310501 |
| 5 | 82.96073 | Visceral fat | rs3846635 |
| 5 | 82.98574 | Prostate cancer | rs4466137 |
| 5 | 83.17359 | Ankylosing spondylitis | rs4552569 |
| 5 | 83.26094 | Mitochondrial DNA levels | rs2301070 |

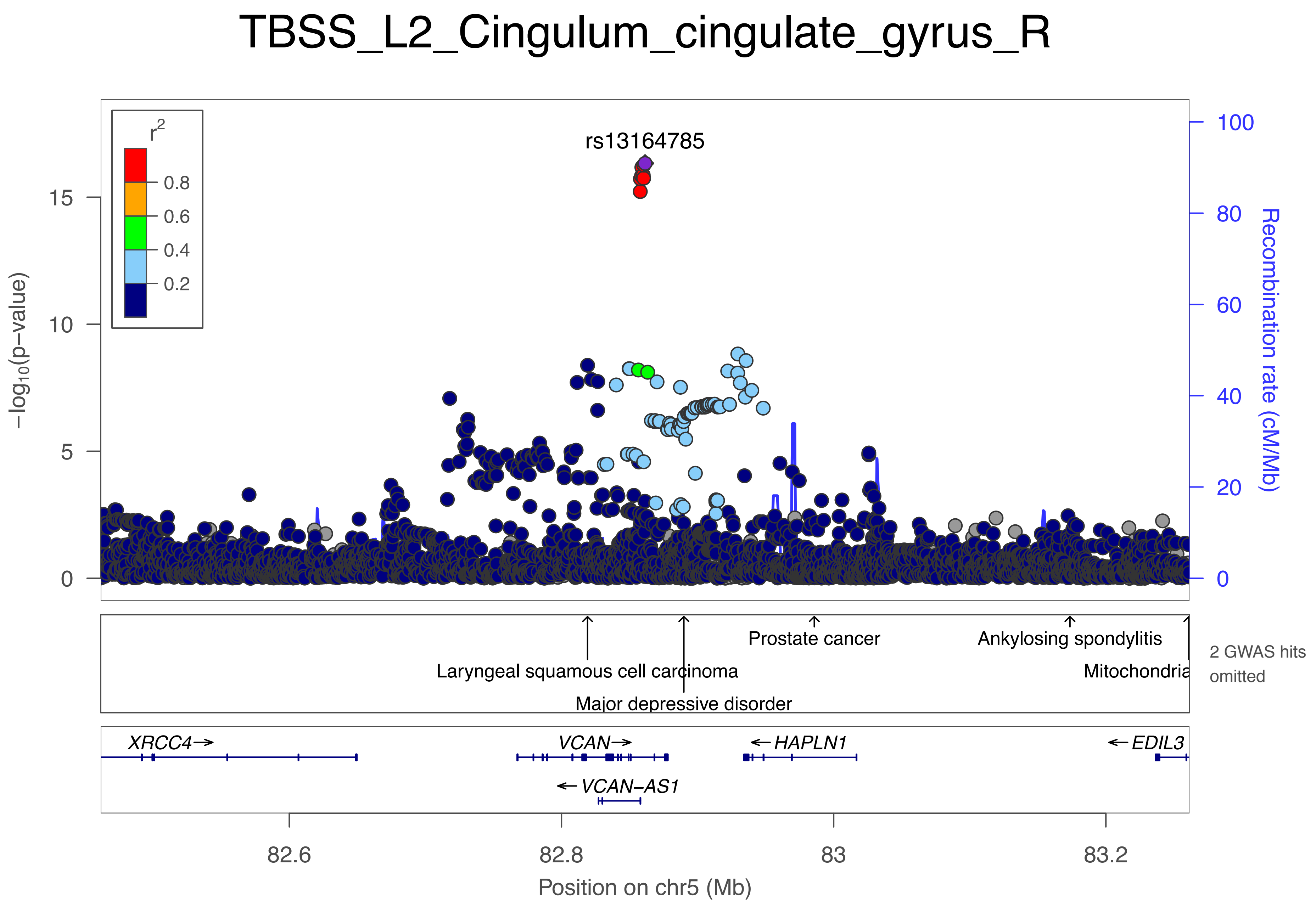
TBSS_L2_Cingulum_cingulate_gyrus_R
rs13164785
r2
0.8
0.6
0.4
0.2
−log10(p−value)
Recombination rate (cM/Mb)
Prostate cancer
Ankylosing spondylitis
Laryngeal squamous cell carcinoma
Mitochondria
Major depressive disorder
2 GWAS hits omitted
XRCC4
VCAN
HAPLN1
EDIL3
VCAN−AS1
82.6
82.8
83
83.2
Position on chr5 (Mb)

| | |
|---|---|
| date: | Thu Aug 17 17:52:01 2017 |
| build: | hg19 |
| display range: | chr5:82461400–83261400 [82461400–83261400] |
| hilite range: | 0 – 0 [ 0 – 0 ] |
| reference SNP: | chr5:82861400 |
| number of SNPs plotted: | 3396 |
| min P.value: | 4.67E–17 [chr5:82861400] |
| max P.value: | 9.99E–1 [chr5:83217296] |
| omitted GWAS Hits: | chr5:83.260938–Mitochondrial DNA levels, NA |

Make more plots at http://csg.sph.umich.edu/locuszoom/

GWAS Catalog SNPs in Region

| chr | pos (Mb) | trait | snp |
|---|---|---|---|
| 5 | 82.81912 | Laryngeal squamous cell carcinoma | rs310518 |
| 5 | 82.84549 | Diisocyanate–induced asthma | rs3852186 |
| 5 | 82.88991 | Major depressive disorder | rs310501 |
| 5 | 82.96073 | Visceral fat | rs3846635 |
| 5 | 82.98574 | Prostate cancer | rs4466137 |
| 5 | 83.17359 | Ankylosing spondylitis | rs4552569 |
| 5 | 83.26094 | Mitochondrial DNA levels | rs2301070 |

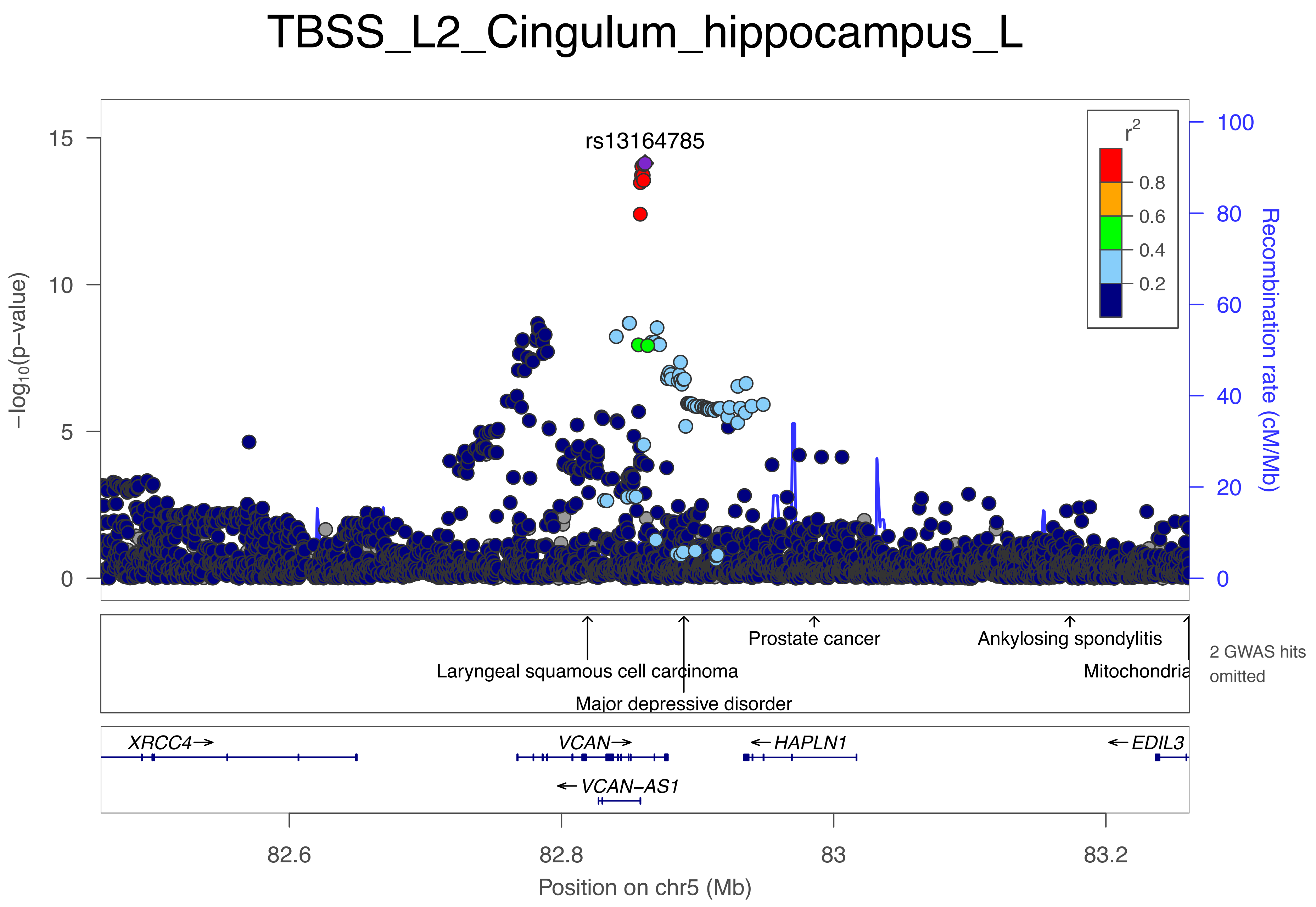
TBSS_L2_Cingulum_hippocampus_L
rs13164785
r²
0.8
0.6
0.4
0.2
−log10(p−value)
Recombination rate (cM/Mb)
Laryngeal squamous cell carcinoma
Major depressive disorder
Prostate cancer
Ankylosing spondylitis
Mitochondria
2 GWAS hits omitted
XRCC4→
VCAN→
←VCAN−AS1
←HAPLN1
←EDIL3
82.6
82.8
83
83.2
Position on chr5 (Mb)

| | |
|---|---|
| date: | Thu Aug 17 17:52:01 2017 |
| build: | hg19 |
| display range: | chr5:82461400−83261400 [82461400−83261400] |
| hilite range: | 0 – 0 [ 0 – 0 ] |
| reference SNP: | chr5:82861400 |
| number of SNPs plotted: | 3396 |
| min P.value: | 7.41E−15 [chr5:82861400] |
| max P.value: | 10E−1 [chr5:83099074] |
| omitted GWAS Hits: | chr5:83.260938−Mitochondrial DNA levels, NA |

Make more plots at http://csg.sph.umich.edu/locuszoom/

GWAS Catalog SNPs in Region

| chr | pos (Mb) | trait | snp |
|---|---|---|---|
| 5 | 82.81912 | Laryngeal squamous cell carcinoma | rs310518 |
| 5 | 82.84549 | Diisocyanate−induced asthma | rs3852186 |
| 5 | 82.88991 | Major depressive disorder | rs310501 |
| 5 | 82.96073 | Visceral fat | rs3846635 |
| 5 | 82.98574 | Prostate cancer | rs4466137 |
| 5 | 83.17359 | Ankylosing spondylitis | rs4552569 |
| 5 | 83.26094 | Mitochondrial DNA levels | rs2301070 |

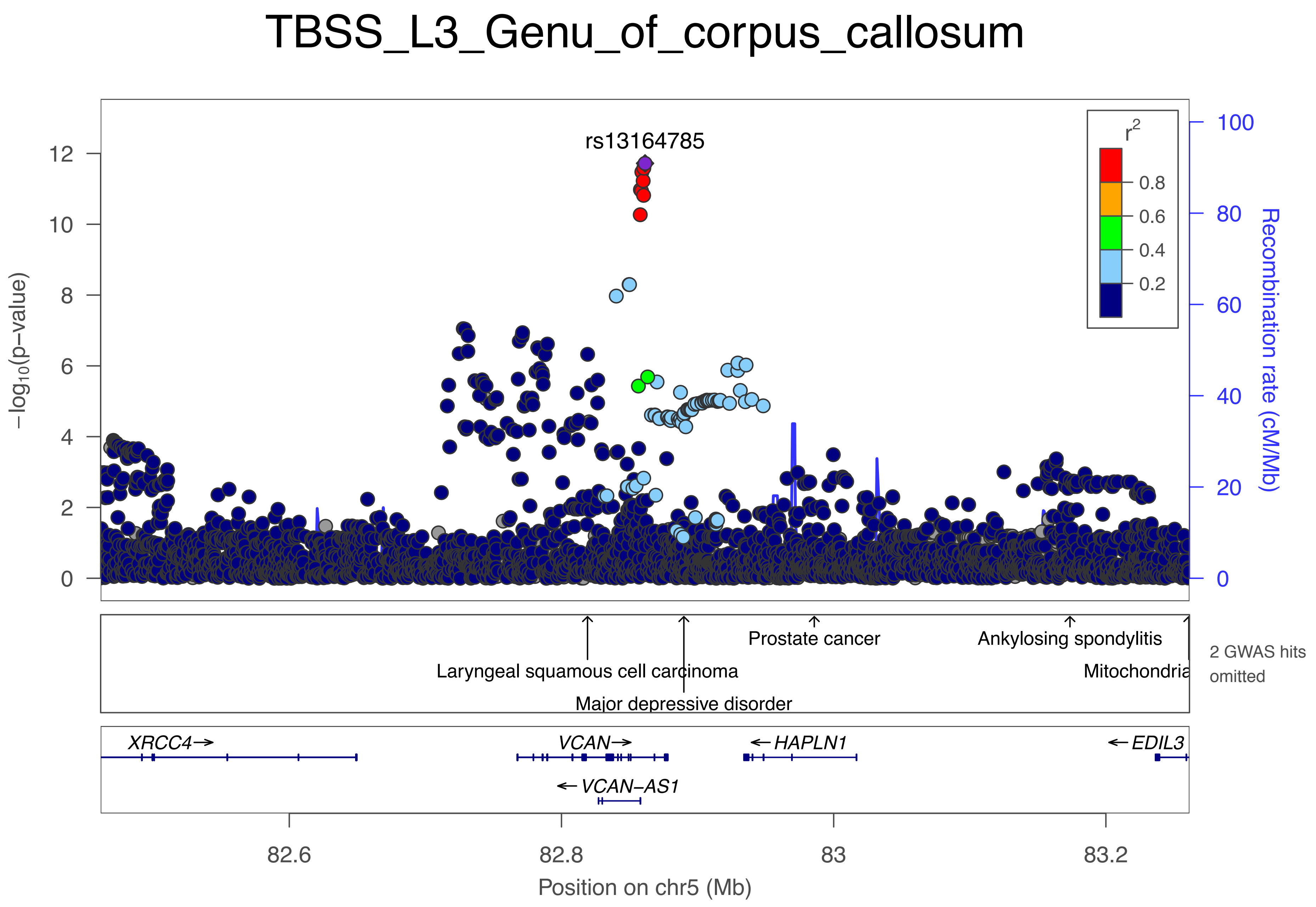
TBSS_L3_Genu_of_corpus_callosum
rs13164785
r2
0.8
0.6
0.4
0.2
−log10(p−value)
Recombination rate (cM/Mb)
12
10
8
6
4
2
0
100
80
60
40
20
0
Prostate cancer
Ankylosing spondylitis
Laryngeal squamous cell carcinoma
Mitochondria
Major depressive disorder
2 GWAS hits
omitted
XRCC4→
VCAN→
←HAPLN1
←EDIL3
←VCAN−AS1
82.6
82.8
83
83.2
Position on chr5 (Mb)

| | |
|---|---|
| date: | Thu Aug 17 17:52:01 2017 |
| build: | hg19 |
| display range: | chr5:82461400−83261400 [82461400−83261400] |
| hilite range: | 0 – 0 [ 0 – 0 ] |
| reference SNP: | chr5:82861400 |
| number of SNPs plotted: | 3396 |
| min P.value: | 1.91E−12 [chr5:82861400] |
| max P.value: | 9.99E−1 [chr5:82588995] |
| omitted GWAS Hits: | chr5:83.260938−Mitochondrial DNA levels, NA |

Make more plots at http://csg.sph.umich.edu/locuszoom/

GWAS Catalog SNPs in Region

| chr | pos (Mb) | trait | snp |
|---|---|---|---|
| 5 | 82.81912 | Laryngeal squamous cell carcinoma | rs310518 |
| 5 | 82.84549 | Diisocyanate–induced asthma | rs3852186 |
| 5 | 82.88991 | Major depressive disorder | rs310501 |
| 5 | 82.96073 | Visceral fat | rs3846635 |
| 5 | 82.98574 | Prostate cancer | rs4466137 |
| 5 | 83.17359 | Ankylosing spondylitis | rs4552569 |
| 5 | 83.26094 | Mitochondrial DNA levels | rs2301070 |

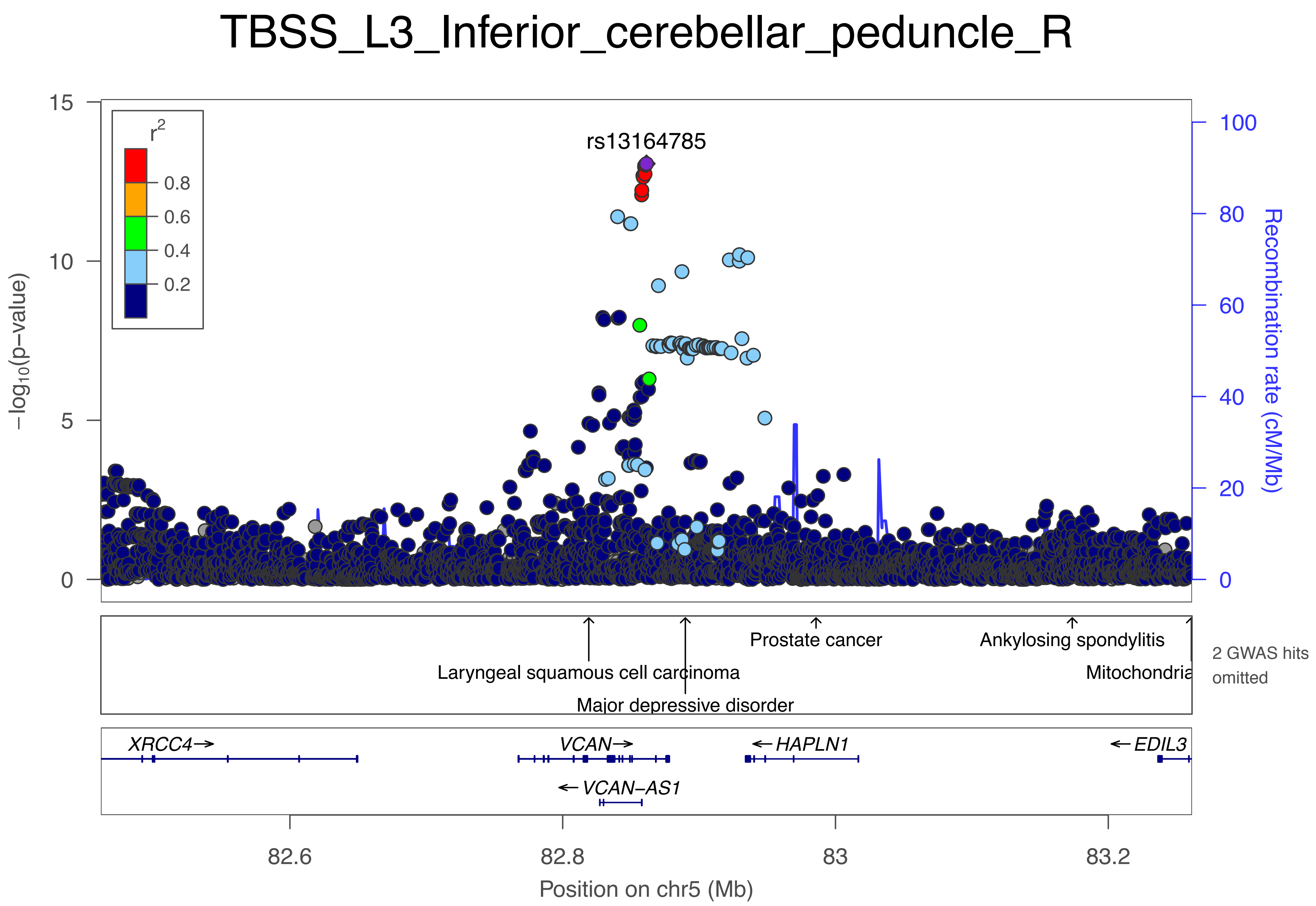
TBSS_L3_Inferior_cerebellar_peduncle_R
rs13164785
r2
0.8
0.6
0.4
0.2
15
10
5
0
−log10(p−value)
100
80
60
40
20
0
Recombination rate (cM/Mb)
Laryngeal squamous cell carcinoma
Major depressive disorder
Prostate cancer
Ankylosing spondylitis
Mitochondria
2 GWAS hits omitted
XRCC4→
VCAN→
←VCAN−AS1
←HAPLN1
←EDIL3
82.6
82.8
83
83.2
Position on chr5 (Mb)

| | |
|---|---|
| date: | Thu Aug 17 17:52:01 2017 |
| build: | hg19 |
| display range: | chr5:82461400−83261400 [82461400−83261400] |
| hilite range: | 0 – 0 [ 0 – 0 ] |
| reference SNP: | chr5:82861400 |
| number of SNPs plotted: | 3396 |
| min P.value: | 8.71E−14 [chr5:82861400] |
| max P.value: | 10E−1 [chr5:82516916] |
| omitted GWAS Hits: | chr5:83.260938−Mitochondrial DNA levels, NA |

Make more plots at http://csg.sph.umich.edu/locuszoom/

GWAS Catalog SNPs in Region

| chr | pos (Mb) | trait | snp |
|---|---|---|---|
| 5 | 82.81912 | Laryngeal squamous cell carcinoma | rs310518 |
| 5 | 82.84549 | Diisocyanate–induced asthma | rs3852186 |
| 5 | 82.88991 | Major depressive disorder | rs310501 |
| 5 | 82.96073 | Visceral fat | rs3846635 |
| 5 | 82.98574 | Prostate cancer | rs4466137 |
| 5 | 83.17359 | Ankylosing spondylitis | rs4552569 |
| 5 | 83.26094 | Mitochondrial DNA levels | rs2301070 |

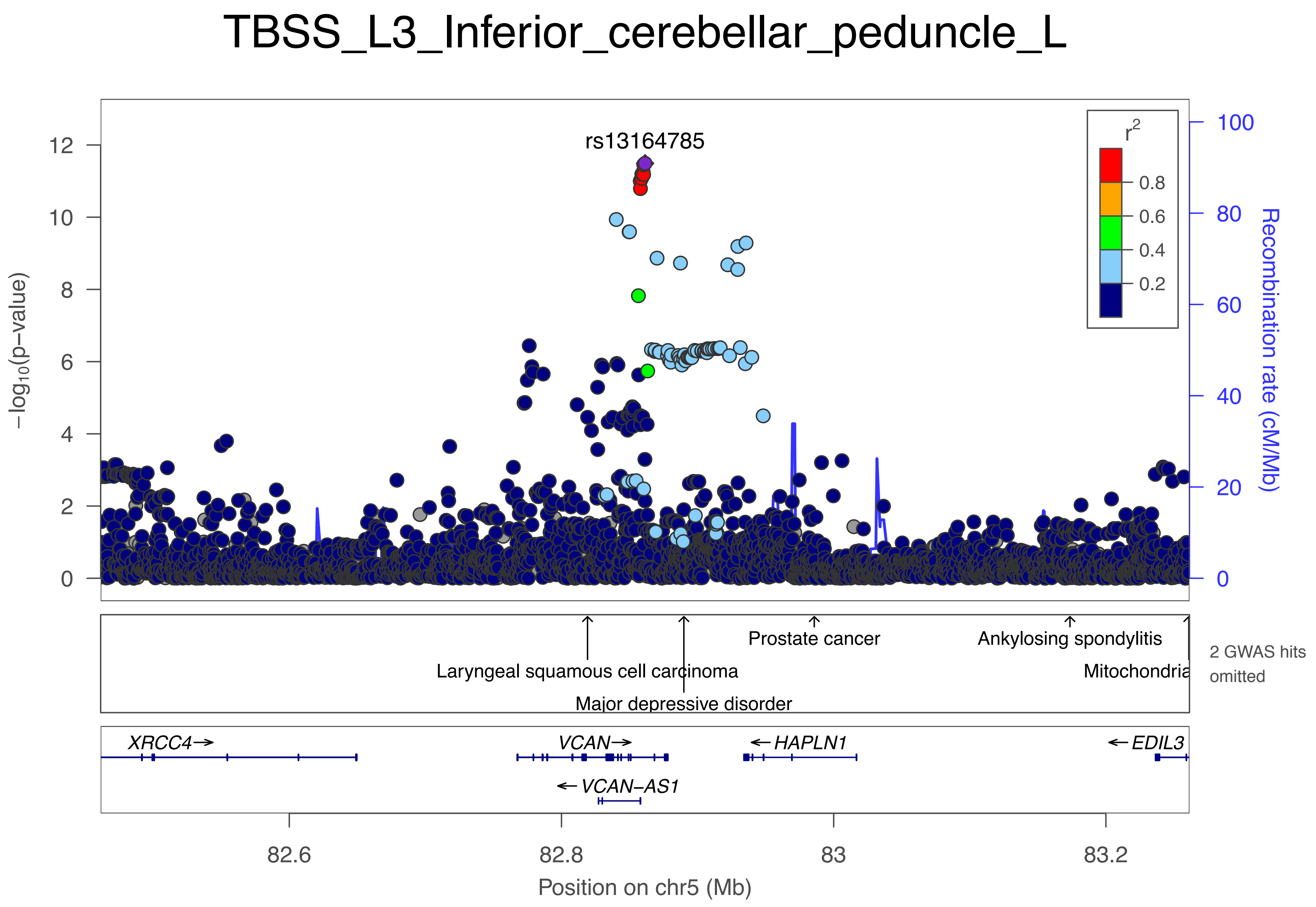
TBSS_L3_Inferior_cerebellar_peduncle_L
rs13164785
−log10(p−value)
Recombination rate (cM/Mb)
r2
0.8
0.6
0.4
0.2
Prostate cancer
Ankylosing spondylitis
Laryngeal squamous cell carcinoma
Mitochondria
Major depressive disorder
2 GWAS hits omitted
XRCC4→
VCAN→
←HAPLN1
←EDIL3
←VCAN−AS1
82.6
82.8
83
83.2
Position on chr5 (Mb)

| | |
|---|---|
| date: | Thu Aug 17 17:52:01 2017 |
| build: | hg19 |
| display range: | chr5:82461400−83261400 [82461400−83261400] |
| hilite range: | 0 – 0 [ 0 – 0 ] |
| reference SNP: | chr5:82861400 |
| number of SNPs plotted: | 3396 |
| min P.value: | 3.23E−12 [chr5:82861400] |
| max P.value: | 10E−1 [chr5:83215237] |
| omitted GWAS Hits: | chr5:83.260938−Mitochondrial DNA levels, NA |

Make more plots at http://csg.sph.umich.edu/locuszoom/

GWAS Catalog SNPs in Region

| chr | pos (Mb) | trait | snp |
|---|---|---|---|
| 5 | 82.81912 | Laryngeal squamous cell carcinoma | rs310518 |
| 5 | 82.84549 | Diisocyanate–induced asthma | rs3852186 |
| 5 | 82.88991 | Major depressive disorder | rs310501 |
| 5 | 82.96073 | Visceral fat | rs3846635 |
| 5 | 82.98574 | Prostate cancer | rs4466137 |
| 5 | 83.17359 | Ankylosing spondylitis | rs4552569 |
| 5 | 83.26094 | Mitochondrial DNA levels | rs2301070 |

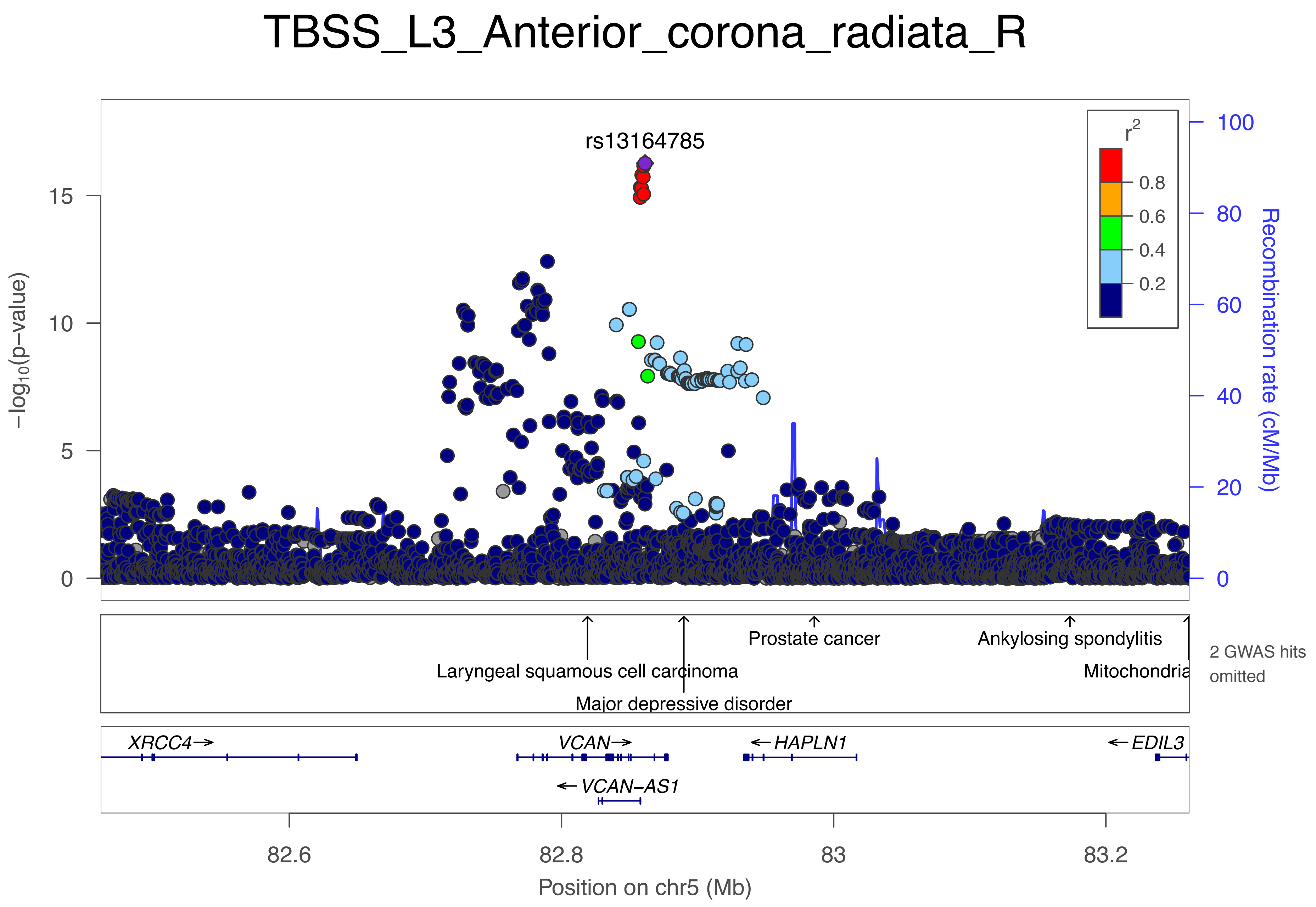

TBSS_L3_Anterior_corona_radiata_R
rs13164785
−log10(p−value)
15
10
5
0
r2
0.8
0.6
0.4
0.2
100
80
60
40
20
0
Recombination rate (cM/Mb)
Prostate cancer
Ankylosing spondylitis
Laryngeal squamous cell carcinoma
Mitochondria
Major depressive disorder
2 GWAS hits omitted
XRCC4→
VCAN→
←HAPLN1
←EDIL3
←VCAN−AS1
82.6
82.8
83
83.2
Position on chr5 (Mb)

| | |
|---|---|
| date: | Thu Aug 17 17:52:01 2017 |
| build: | hg19 |
| display range: | chr5:82461400−83261400 [82461400−83261400] |
| hilite range: | 0 – 0 [ 0 – 0 ] |
| reference SNP: | chr5:82861400 |
| number of SNPs plotted: | 3396 |
| min P.value: | 5.5E−17 [chr5:82861400] |
| max P.value: | 10E−1 [chr5:82723169] |
| omitted GWAS Hits: | chr5:83.260938−Mitochondrial DNA levels, NA |

Make more plots at http://csg.sph.umich.edu/locuszoom/

GWAS Catalog SNPs in Region

| chr | pos (Mb) | trait | snp |
|---|---|---|---|
| 5 | 82.81912 | Laryngeal squamous cell carcinoma | rs310518 |
| 5 | 82.84549 | Diisocyanate–induced asthma | rs3852186 |
| 5 | 82.88991 | Major depressive disorder | rs310501 |
| 5 | 82.96073 | Visceral fat | rs3846635 |
| 5 | 82.98574 | Prostate cancer | rs4466137 |
| 5 | 83.17359 | Ankylosing spondylitis | rs4552569 |
| 5 | 83.26094 | Mitochondrial DNA levels | rs2301070 |

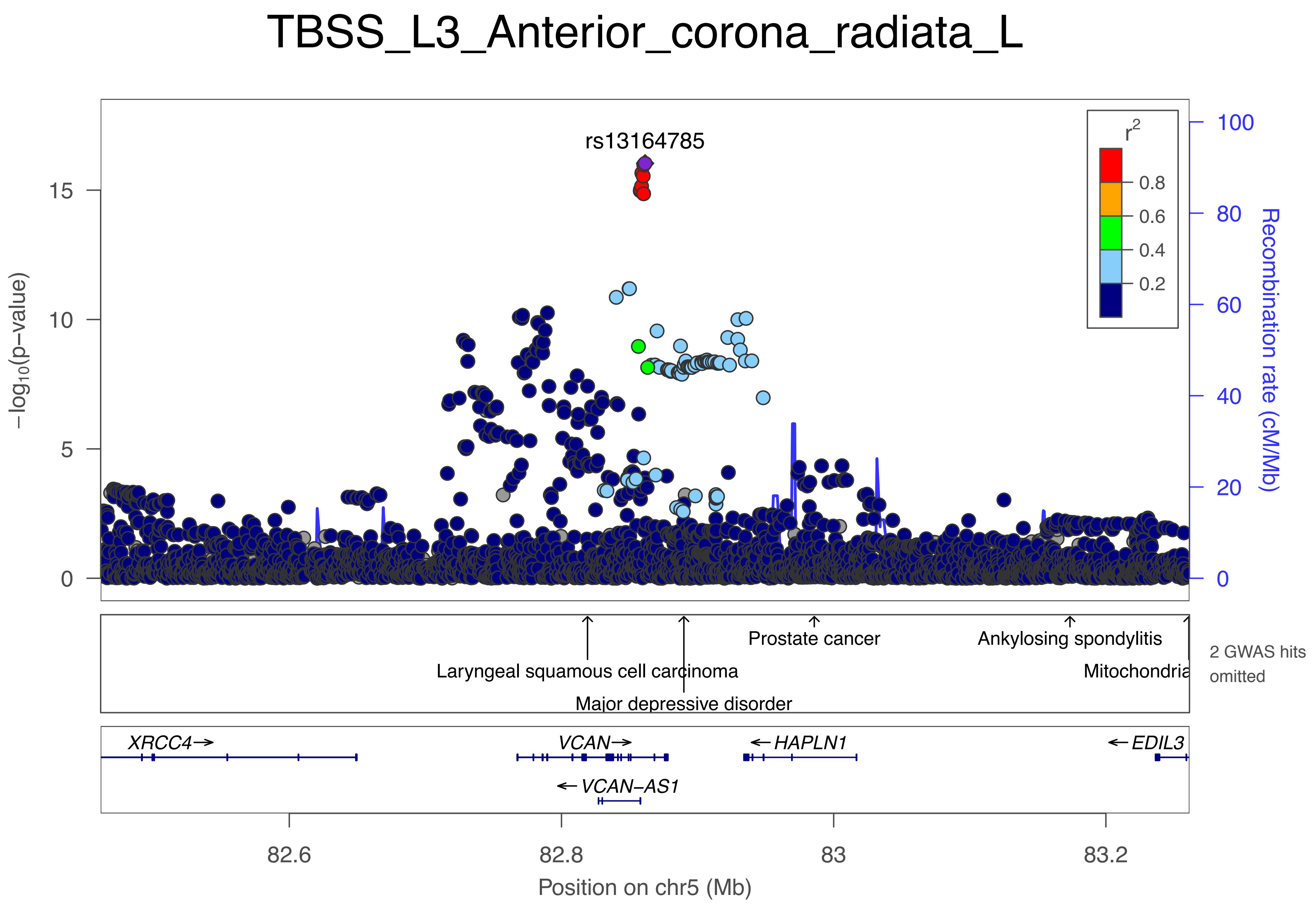

TBSS_L3_Anterior_corona_radiata_L
rs13164785
$-\log_{10}$(p−value)
15
10
5
0
$r^2$
0.8
0.6
0.4
0.2
100
80
60
40
20
0
Recombination rate (cM/Mb)
Prostate cancer
Ankylosing spondylitis
Laryngeal squamous cell carcinoma
Mitochondria
2 GWAS hits omitted
Major depressive disorder
XRCC4→
VCAN→
←HAPLN1
←EDIL3
←VCAN−AS1
82.6
82.8
83
83.2
Position on chr5 (Mb)

| | |
|---|---|
| date: | Thu Aug 17 17:52:01 2017 |
| build: | hg19 |
| display range: | chr5:82461400−83261400 [82461400−83261400] |
| hilite range: | 0 – 0 [ 0 – 0 ] |
| reference SNP: | chr5:82861400 |
| number of SNPs plotted: | 3396 |
| min P.value: | 9.23E−17 [chr5:82861400] |
| max P.value: | 10E−1 [chr5:83097554] |
| omitted GWAS Hits: | chr5:83.260938−Mitochondrial DNA levels, NA |

Make more plots at http://csg.sph.umich.edu/locuszoom/

GWAS Catalog SNPs in Region

| chr | pos (Mb) | trait | snp |
|---|---|---|---|
| 5 | 82.81912 | Laryngeal squamous cell carcinoma | rs310518 |
| 5 | 82.84549 | Diisocyanate−induced asthma | rs3852186 |
| 5 | 82.88991 | Major depressive disorder | rs310501 |
| 5 | 82.96073 | Visceral fat | rs3846635 |
| 5 | 82.98574 | Prostate cancer | rs4466137 |
| 5 | 83.17359 | Ankylosing spondylitis | rs4552569 |
| 5 | 83.26094 | Mitochondrial DNA levels | rs2301070 |

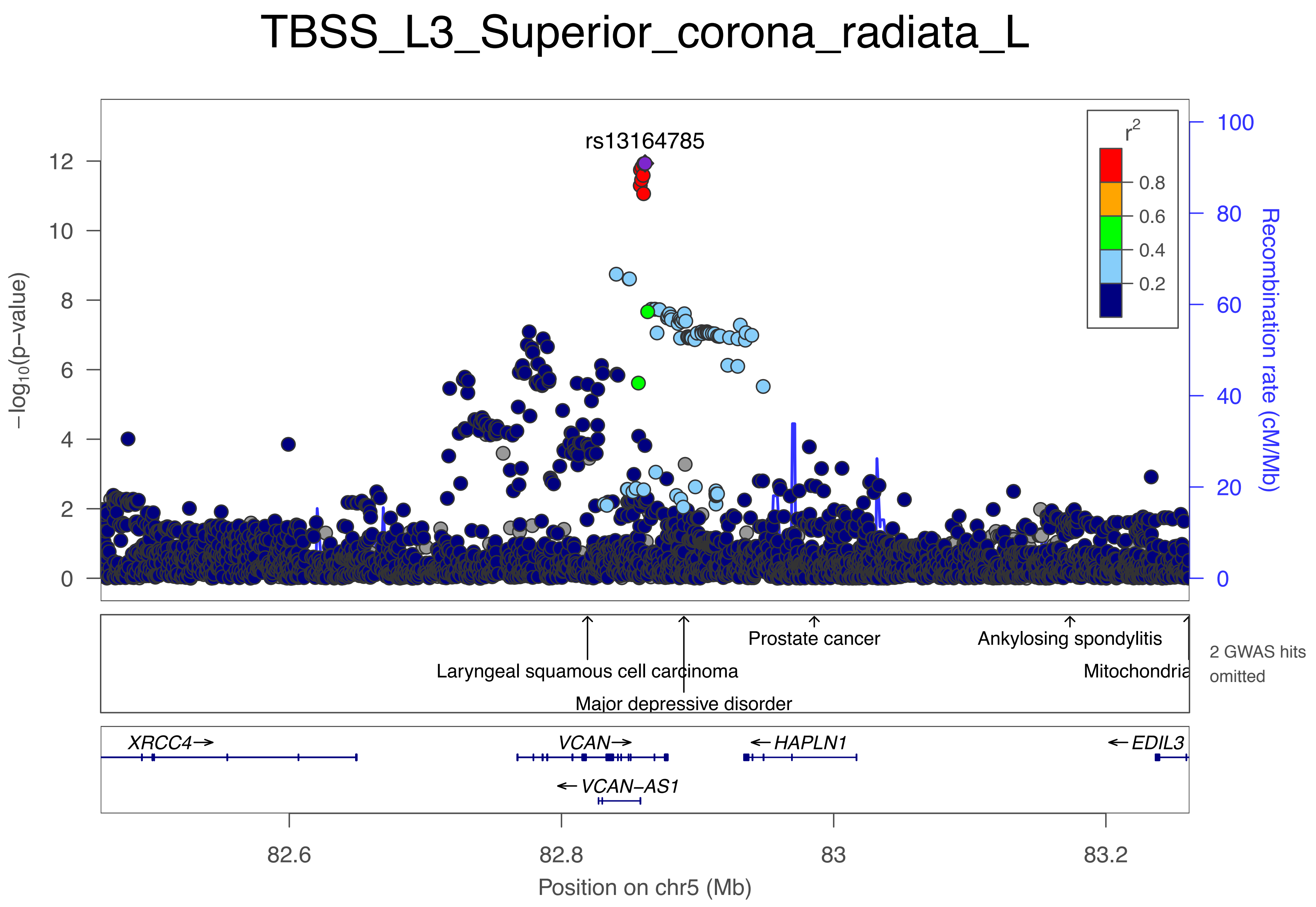
TBSS_L3_Superior_corona_radiata_L
rs13164785
−log10(p−value)
Recombination rate (cM/Mb)
r2
0.8
0.6
0.4
0.2
Prostate cancer
Ankylosing spondylitis
Laryngeal squamous cell carcinoma
Mitochondria
Major depressive disorder
2 GWAS hits omitted
XRCC4→
VCAN→
← HAPLN1
← EDIL3
← VCAN−AS1
82.6
82.8
83
83.2
Position on chr5 (Mb)

| | |
|---|---|
| date: | Thu Aug 17 17:52:01 2017 |
| build: | hg19 |
| display range: | chr5:82461400−83261400 [82461400−83261400] |
| hilite range: | 0 – 0 [ 0 – 0 ] |
| reference SNP: | chr5:82861400 |
| number of SNPs plotted: | 3396 |
| min P.value: | 1.16E−12 [chr5:82861400] |
| max P.value: | 10E−1 [chr5:82966675] |
| omitted GWAS Hits: | chr5:83.260938−Mitochondrial DNA levels, NA |

Make more plots at http://csg.sph.umich.edu/locuszoom/

GWAS Catalog SNPs in Region

| chr | pos (Mb) | trait | snp |
|---|---|---|---|
| 5 | 82.81912 | Laryngeal squamous cell carcinoma | rs310518 |
| 5 | 82.84549 | Diisocyanate–induced asthma | rs3852186 |
| 5 | 82.88991 | Major depressive disorder | rs310501 |
| 5 | 82.96073 | Visceral fat | rs3846635 |
| 5 | 82.98574 | Prostate cancer | rs4466137 |
| 5 | 83.17359 | Ankylosing spondylitis | rs4552569 |
| 5 | 83.26094 | Mitochondrial DNA levels | rs2301070 |

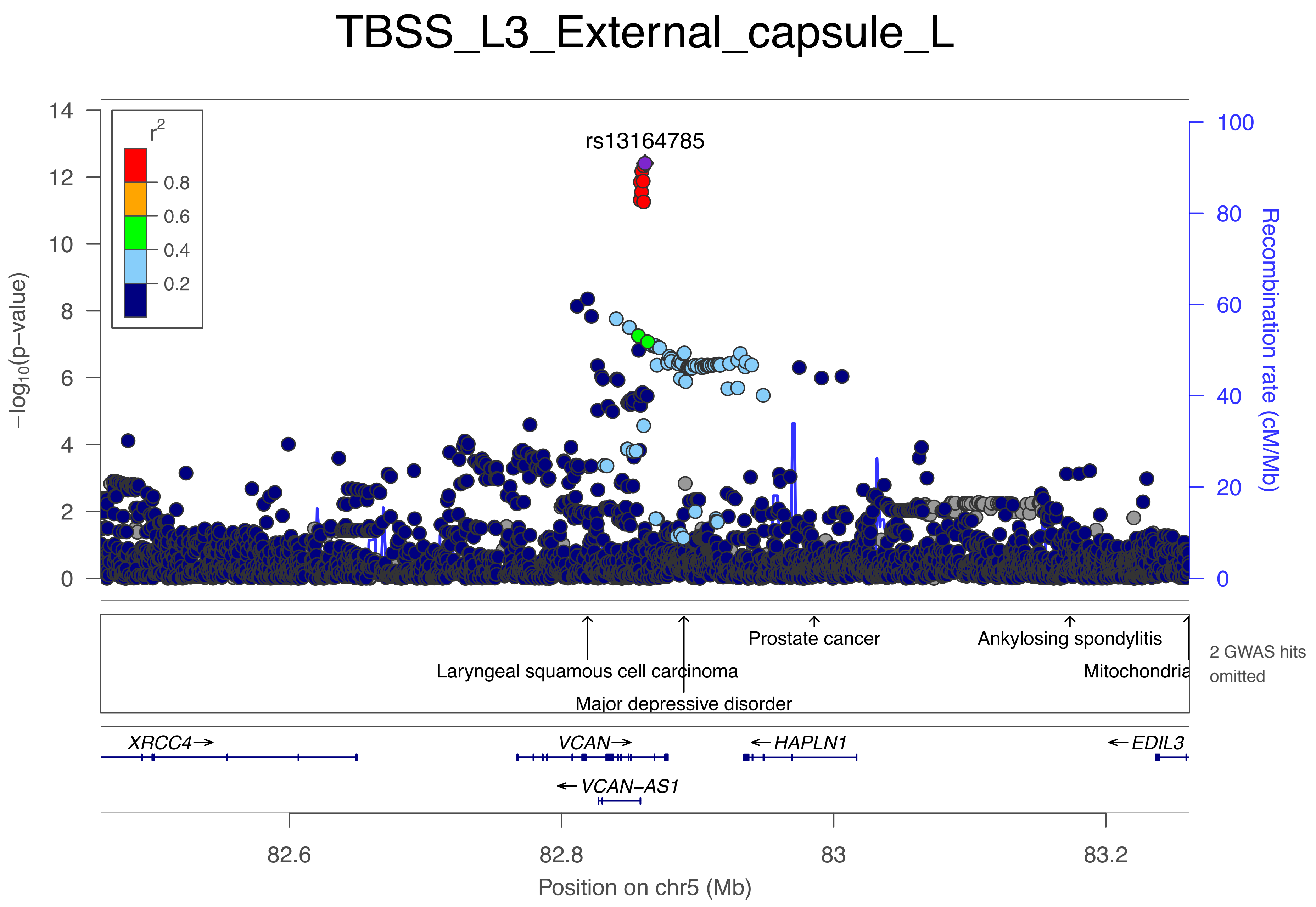
TBSS_L3_External_capsule_L
rs13164785
r2
0.8
0.6
0.4
0.2
−log10(p−value)
Recombination rate (cM/Mb)
Prostate cancer
Ankylosing spondylitis
Laryngeal squamous cell carcinoma
Mitochondria
Major depressive disorder
2 GWAS hits omitted
XRCC4→
VCAN→
←HAPLN1
←EDIL3
←VCAN−AS1
82.6
82.8
83
83.2
Position on chr5 (Mb)

| | |
|---|---|
| date: | Thu Aug 17 17:52:01 2017 |
| build: | hg19 |
| display range: | chr5:82461400–83261400 [82461400–83261400] |
| hilite range: | 0 – 0 [ 0 – 0 ] |
| reference SNP: | chr5:82861400 |
| number of SNPs plotted: | 3396 |
| min P.value: | 3.88E−13 [chr5:82861400] |
| max P.value: | 9.99E−1 [chr5:82864957] |
| omitted GWAS Hits: | chr5:83.260938−Mitochondrial DNA levels, NA |

Make more plots at http://csg.sph.umich.edu/locuszoom/

GWAS Catalog SNPs in Region

| chr | pos (Mb) | trait | snp |
|---|---|---|---|
| 5 | 82.81912 | Laryngeal squamous cell carcinoma | rs310518 |
| 5 | 82.84549 | Diisocyanate−induced asthma | rs3852186 |
| 5 | 82.88991 | Major depressive disorder | rs310501 |
| 5 | 82.96073 | Visceral fat | rs3846635 |
| 5 | 82.98574 | Prostate cancer | rs4466137 |
| 5 | 83.17359 | Ankylosing spondylitis | rs4552569 |
| 5 | 83.26094 | Mitochondrial DNA levels | rs2301070 |

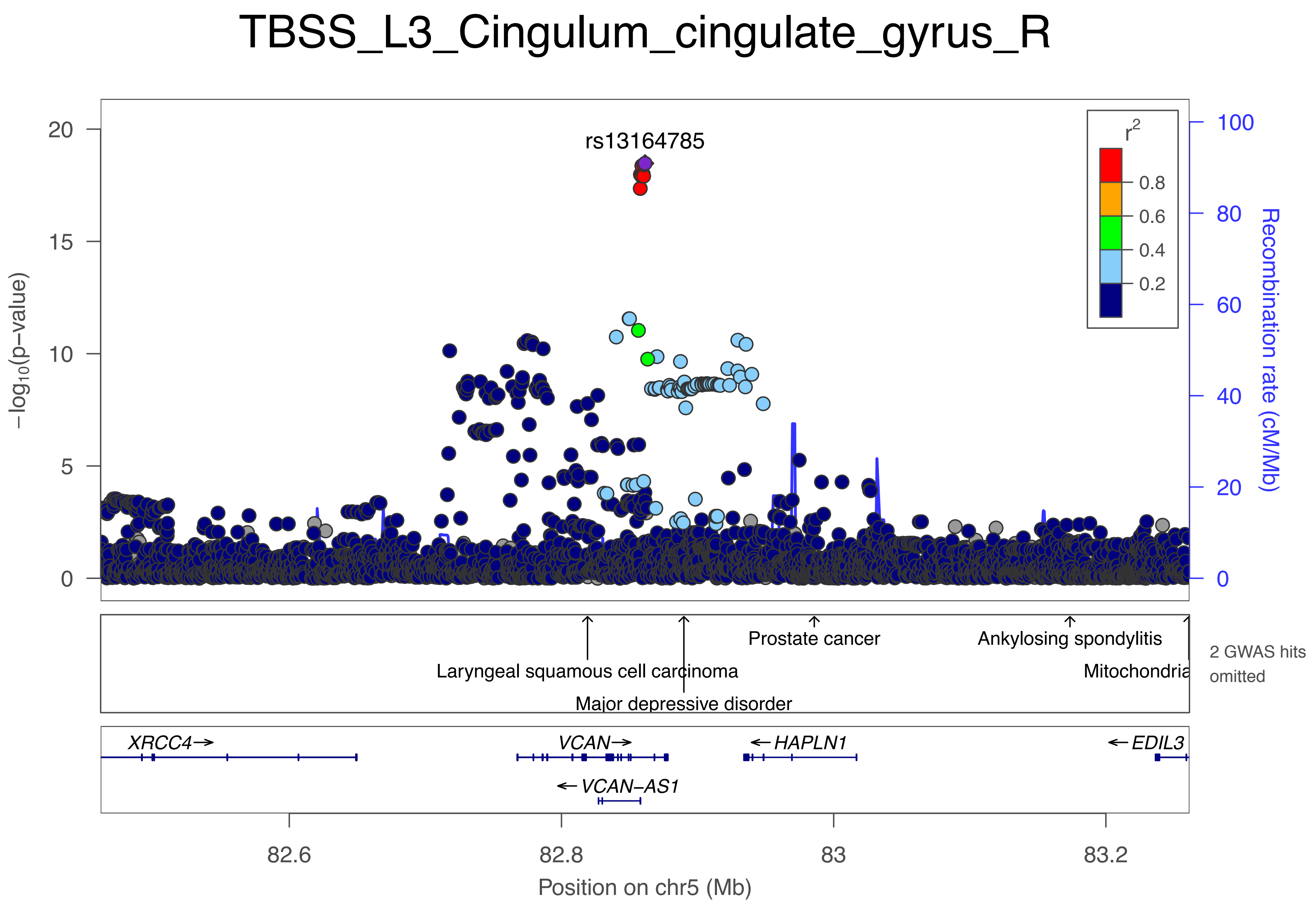
TBSS_L3_Cingulum_cingulate_gyrus_R
rs13164785
r2
0.8
0.6
0.4
0.2
−log10(p−value)
0
5
10
15
20
Recombination rate (cM/Mb)
0
20
40
60
80
100
Prostate cancer
Ankylosing spondylitis
Laryngeal squamous cell carcinoma
Major depressive disorder
Mitochondria
2 GWAS hits omitted
XRCC4→
VCAN→
←HAPLN1
←EDIL3
←VCAN−AS1
82.6
82.8
83
83.2
Position on chr5 (Mb)

| | |
|---|---|
| date: | Thu Aug 17 17:52:01 2017 |
| build: | hg19 |
| display range: | chr5:82461400−83261400 [82461400−83261400] |
| hilite range: | 0 – 0 [ 0 – 0 ] |
| reference SNP: | chr5:82861400 |
| number of SNPs plotted: | 3396 |
| min P.value: | 3.35E−19 [chr5:82861400] |
| max P.value: | 9.99E−1 [chr5:83229426] |
| omitted GWAS Hits: | chr5:83.260938−Mitochondrial DNA levels, NA |

Make more plots at http://csg.sph.umich.edu/locuszoom/

GWAS Catalog SNPs in Region

| chr | pos (Mb) | trait | snp |
|---|---|---|---|
| 5 | 82.81912 | Laryngeal squamous cell carcinoma | rs310518 |
| 5 | 82.84549 | Diisocyanate−induced asthma | rs3852186 |
| 5 | 82.88991 | Major depressive disorder | rs310501 |
| 5 | 82.96073 | Visceral fat | rs3846635 |
| 5 | 82.98574 | Prostate cancer | rs4466137 |
| 5 | 83.17359 | Ankylosing spondylitis | rs4552569 |
| 5 | 83.26094 | Mitochondrial DNA levels | rs2301070 |

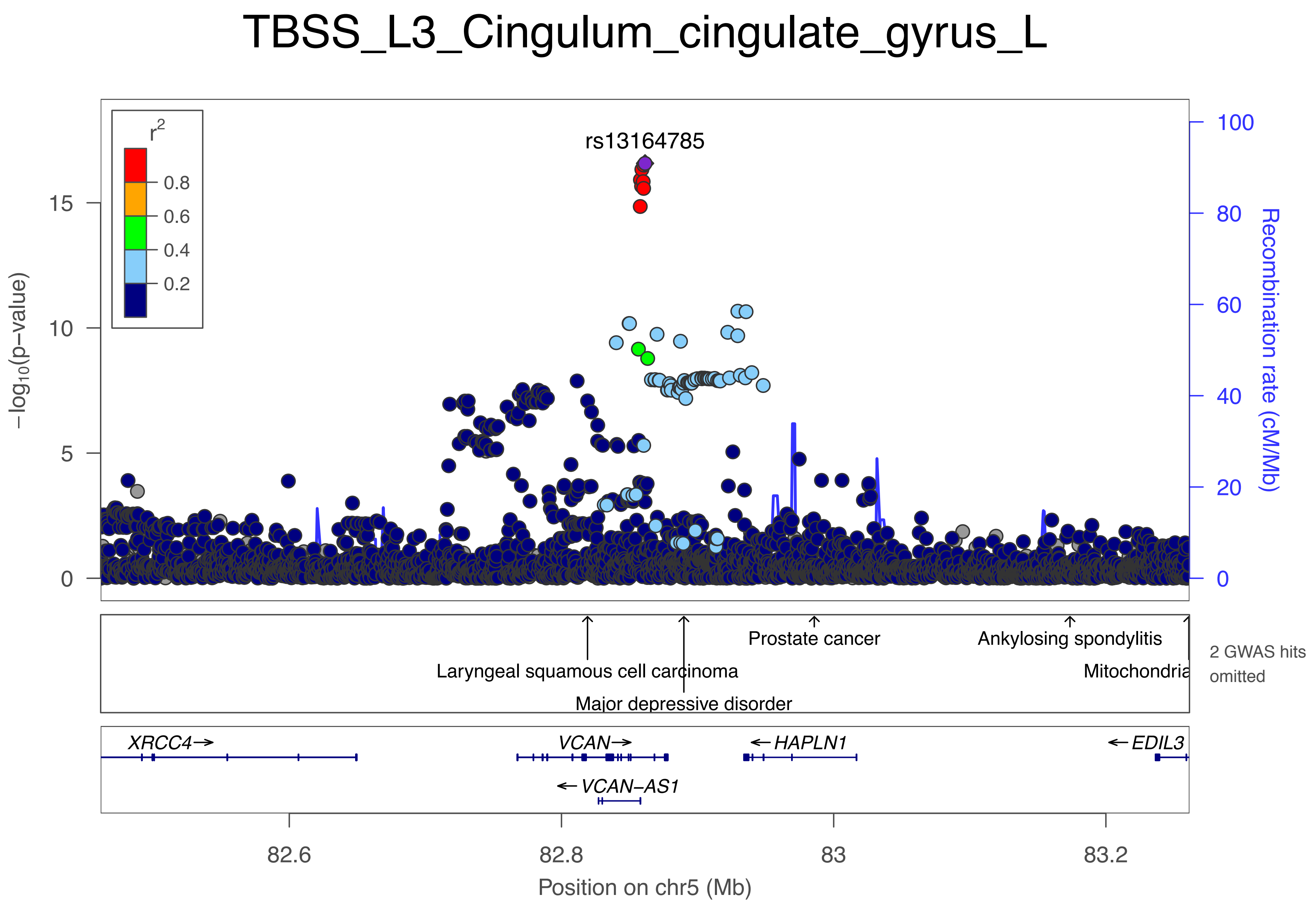
TBSS_L3_Cingulum_cingulate_gyrus_L
r2
0.8
0.6
0.4
0.2
rs13164785
−log10(p−value)
15
10
5
0
100
80
60
40
20
0
Recombination rate (cM/Mb)
Prostate cancer
Ankylosing spondylitis
Laryngeal squamous cell carcinoma
Mitochondria
Major depressive disorder
2 GWAS hits omitted
XRCC4
VCAN
HAPLN1
EDIL3
VCAN−AS1
82.6
82.8
83
83.2
Position on chr5 (Mb)

| | |
|---|---|
| date: | Thu Aug 17 17:52:01 2017 |
| build: | hg19 |
| display range: | chr5:82461400–83261400 [82461400–83261400] |
| hilite range: | 0 – 0 [ 0 – 0 ] |
| reference SNP: | chr5:82861400 |
| number of SNPs plotted: | 3396 |
| min P.value: | 2.67E–17 [chr5:82861400] |
| max P.value: | 10E–1 [chr5:82485682] |
| omitted GWAS Hits: | chr5:83.260938–Mitochondrial DNA levels, NA |

Make more plots at http://csg.sph.umich.edu/locuszoom/

GWAS Catalog SNPs in Region

| chr | pos (Mb) | trait | snp |
|---|---|---|---|
| 5 | 82.81912 | Laryngeal squamous cell carcinoma | rs310518 |
| 5 | 82.84549 | Diisocyanate−induced asthma | rs3852186 |
| 5 | 82.88991 | Major depressive disorder | rs310501 |
| 5 | 82.96073 | Visceral fat | rs3846635 |
| 5 | 82.98574 | Prostate cancer | rs4466137 |
| 5 | 83.17359 | Ankylosing spondylitis | rs4552569 |
| 5 | 83.26094 | Mitochondrial DNA levels | rs2301070 |

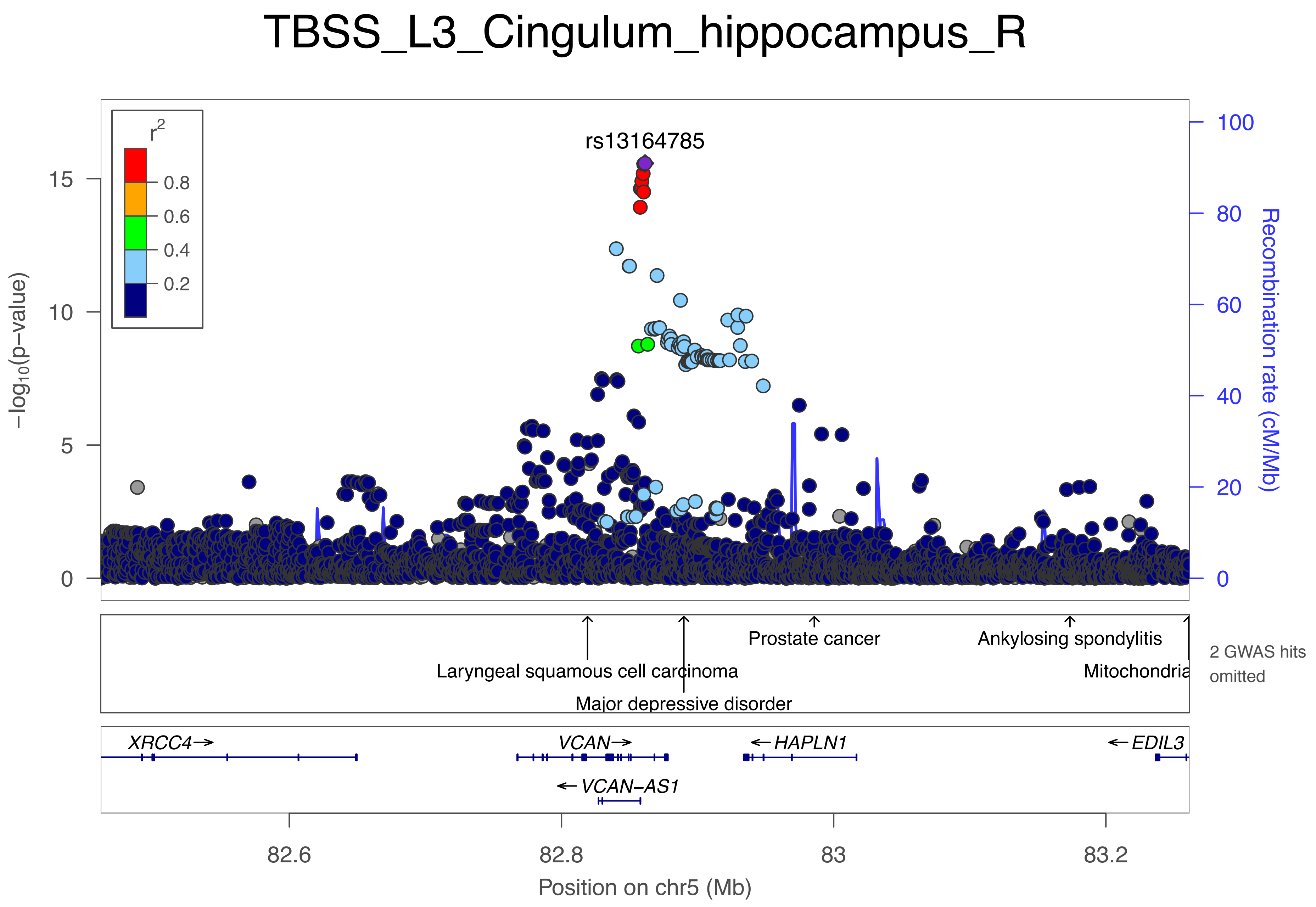

TBSS_L3_Cingulum_hippocampus_R
rs13164785
r2
0.8
0.6
0.4
0.2
−log10(p−value)
Recombination rate (cM/Mb)
Prostate cancer
Ankylosing spondylitis
Laryngeal squamous cell carcinoma
Major depressive disorder
Mitochondria
2 GWAS hits omitted
XRCC4→
VCAN→
←HAPLN1
←EDIL3
←VCAN−AS1
82.6
82.8
83
83.2
Position on chr5 (Mb)

| | |
|---|---|
| date: | Thu Aug 17 17:55:19 2017 |
| build: | hg19 |
| display range: | chr5:82461400–83261400 [82461400–83261400] |
| hilite range: | 0 – 0 [ 0 – 0 ] |
| reference SNP: | chr5:82861400 |
| number of SNPs plotted: | 3396 |
| min P.value: | 2.66E−16 [chr5:82861400] |
| max P.value: | 9.99E−1 [chr5:82597893] |
| omitted GWAS Hits: | chr5:83.260938−Mitochondrial DNA levels, NA |

Make more plots at http://csg.sph.umich.edu/locuszoom/

GWAS Catalog SNPs in Region

| chr | pos (Mb) | trait | snp |
|---|---|---|---|
| 5 | 82.81912 | Laryngeal squamous cell carcinoma | rs310518 |
| 5 | 82.84549 | Diisocyanate–induced asthma | rs3852186 |
| 5 | 82.88991 | Major depressive disorder | rs310501 |
| 5 | 82.96073 | Visceral fat | rs3846635 |
| 5 | 82.98574 | Prostate cancer | rs4466137 |
| 5 | 83.17359 | Ankylosing spondylitis | rs4552569 |
| 5 | 83.26094 | Mitochondrial DNA levels | rs2301070 |

TBSS_L3_Cingulum_hippocampus_L

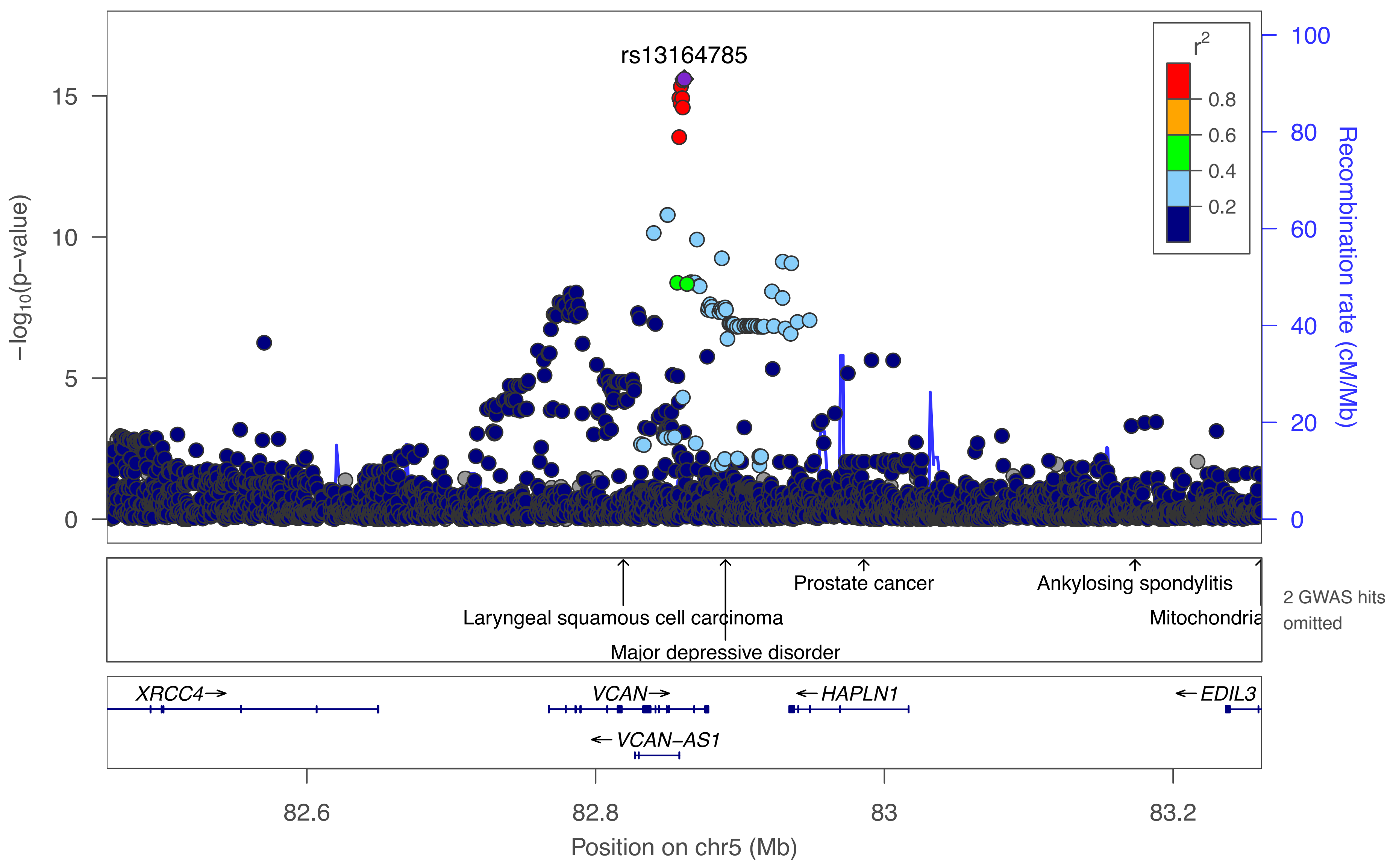

| | |
|---|---|
| date: | Thu Aug 17 17:55:19 2017 |
| build: | hg19 |
| display range: | chr5:82461400−83261400 [82461400−83261400] |
| hilite range: | 0 – 0 [ 0 – 0 ] |
| reference SNP: | chr5:82861400 |
| number of SNPs plotted: | 3396 |
| min P.value: | 2.52E−16 [chr5:82861400] |
| max P.value: | 10E−1 [chr5:82779454] |
| omitted GWAS Hits: | chr5:83.260938−Mitochondrial DNA levels, NA |

Make more plots at http://csg.sph.umich.edu/locuszoom/

GWAS Catalog SNPs in Region

| chr | pos (Mb) | trait | snp |
|---|---|---|---|
| 5 | 82.81912 | Laryngeal squamous cell carcinoma | rs310518 |
| 5 | 82.84549 | Diisocyanate−induced asthma | rs3852186 |
| 5 | 82.88991 | Major depressive disorder | rs310501 |
| 5 | 82.96073 | Visceral fat | rs3846635 |
| 5 | 82.98574 | Prostate cancer | rs4466137 |
| 5 | 83.17359 | Ankylosing spondylitis | rs4552569 |
| 5 | 83.26094 | Mitochondrial DNA levels | rs2301070 |

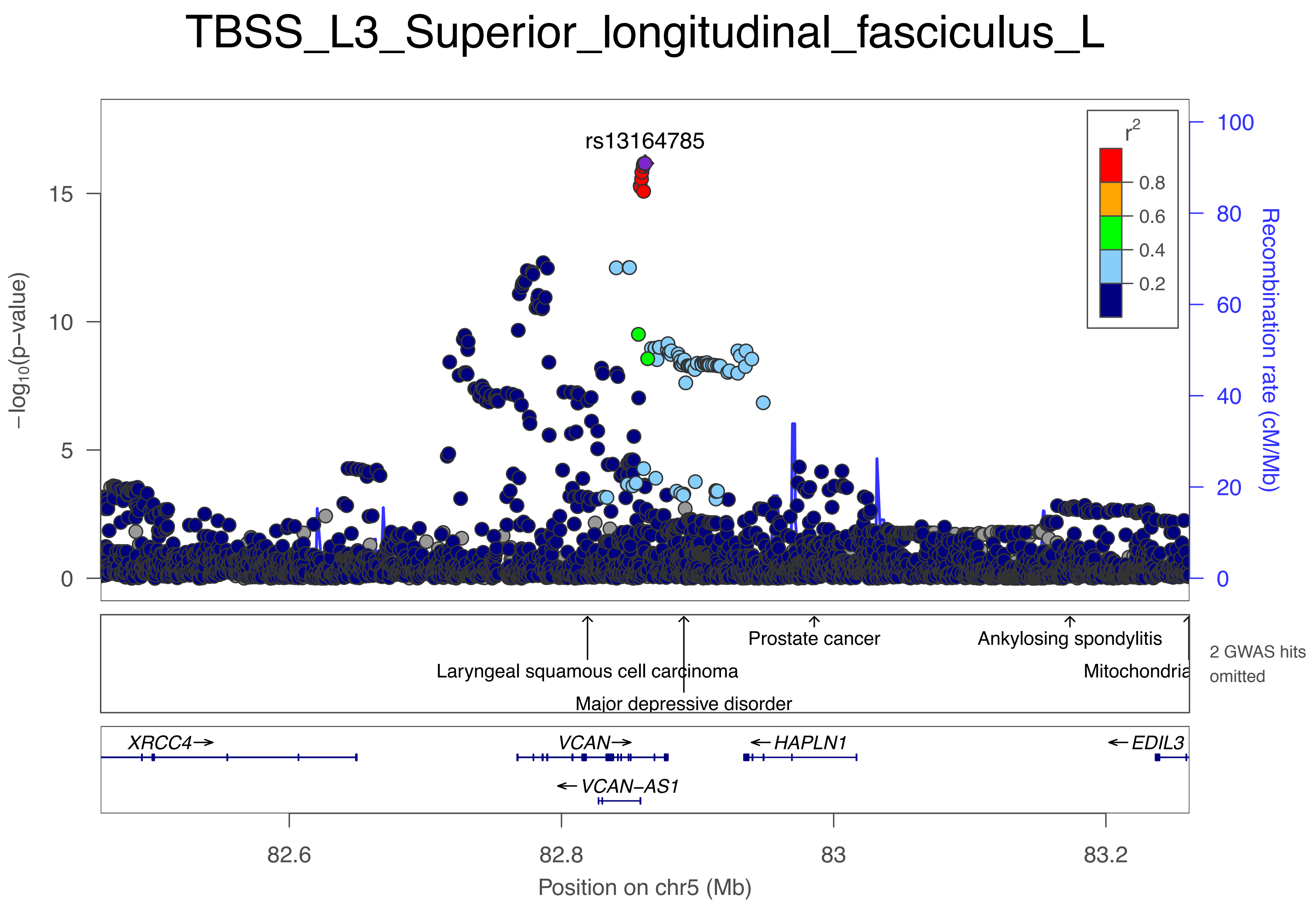

TBSS_L3_Superior_longitudinal_fasciculus_L
rs13164785
r²
0.8
0.6
0.4
0.2
−log10(p−value)
Recombination rate (cM/Mb)
Laryngeal squamous cell carcinoma
Major depressive disorder
Prostate cancer
Ankylosing spondylitis
Mitochondria
2 GWAS hits omitted
XRCC4→
VCAN→
←VCAN−AS1
←HAPLN1
←EDIL3
82.6
82.8
83
83.2
Position on chr5 (Mb)

| | |
|---|---|
| date: | Thu Aug 17 17:55:19 2017 |
| build: | hg19 |
| display range: | chr5:82461400−83261400 [82461400−83261400] |
| hilite range: | 0 – 0 [ 0 – 0 ] |
| reference SNP: | chr5:82861400 |
| number of SNPs plotted: | 3396 |
| min P.value: | 6.71E−17 [chr5:82861400] |
| max P.value: | 10E−1 [chr5:82959109] |
| omitted GWAS Hits: | chr5:83.260938−Mitochondrial DNA levels, NA |

Make more plots at http://csg.sph.umich.edu/locuszoom/

GWAS Catalog SNPs in Region

| chr | pos (Mb) | trait | snp |
|---|---|---|---|
| 5 | 82.81912 | Laryngeal squamous cell carcinoma | rs310518 |
| 5 | 82.84549 | Diisocyanate–induced asthma | rs3852186 |
| 5 | 82.88991 | Major depressive disorder | rs310501 |
| 5 | 82.96073 | Visceral fat | rs3846635 |
| 5 | 82.98574 | Prostate cancer | rs4466137 |
| 5 | 83.17359 | Ankylosing spondylitis | rs4552569 |
| 5 | 83.26094 | Mitochondrial DNA levels | rs2301070 |

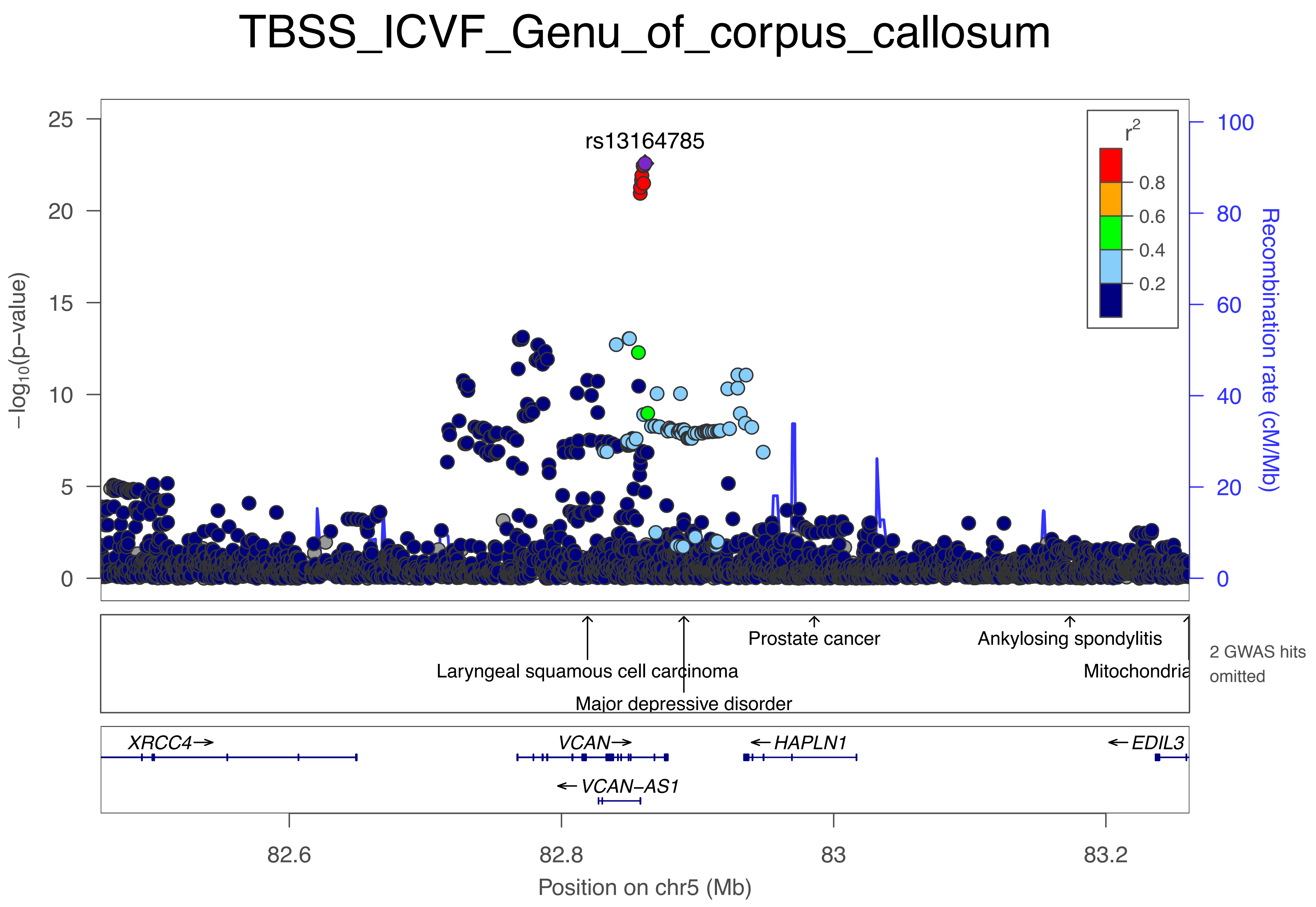

TBSS_ICVF_Genu_of_corpus_callosum
rs13164785
r2
0.8
0.6
0.4
0.2
−log10(p−value)
Recombination rate (cM/Mb)
Prostate cancer
Ankylosing spondylitis
Laryngeal squamous cell carcinoma
Mitochondria
Major depressive disorder
2 GWAS hits omitted
XRCC4→
VCAN→
←HAPLN1
←EDIL3
←VCAN−AS1
82.6
82.8
83
83.2
Position on chr5 (Mb)

| | |
|---|---|
| date: | Thu Aug 17 17:55:19 2017 |
| build: | hg19 |
| display range: | chr5:82461400–83261400 [82461400–83261400] |
| hilite range: | 0 – 0 [ 0 – 0 ] |
| reference SNP: | chr5:82861400 |
| number of SNPs plotted: | 3396 |
| min P.value: | 2.64E–23 [chr5:82861400] |
| max P.value: | 9.99E–1 [chr5:83009083] |
| omitted GWAS Hits: | chr5:83.260938–Mitochondrial DNA levels, NA |

Make more plots at http://csg.sph.umich.edu/locuszoom/

GWAS Catalog SNPs in Region

| chr | pos (Mb) | trait | snp |
|---|---|---|---|
| 5 | 82.81912 | Laryngeal squamous cell carcinoma | rs310518 |
| 5 | 82.84549 | Diisocyanate–induced asthma | rs3852186 |
| 5 | 82.88991 | Major depressive disorder | rs310501 |
| 5 | 82.96073 | Visceral fat | rs3846635 |
| 5 | 82.98574 | Prostate cancer | rs4466137 |
| 5 | 83.17359 | Ankylosing spondylitis | rs4552569 |
| 5 | 83.26094 | Mitochondrial DNA levels | rs2301070 |

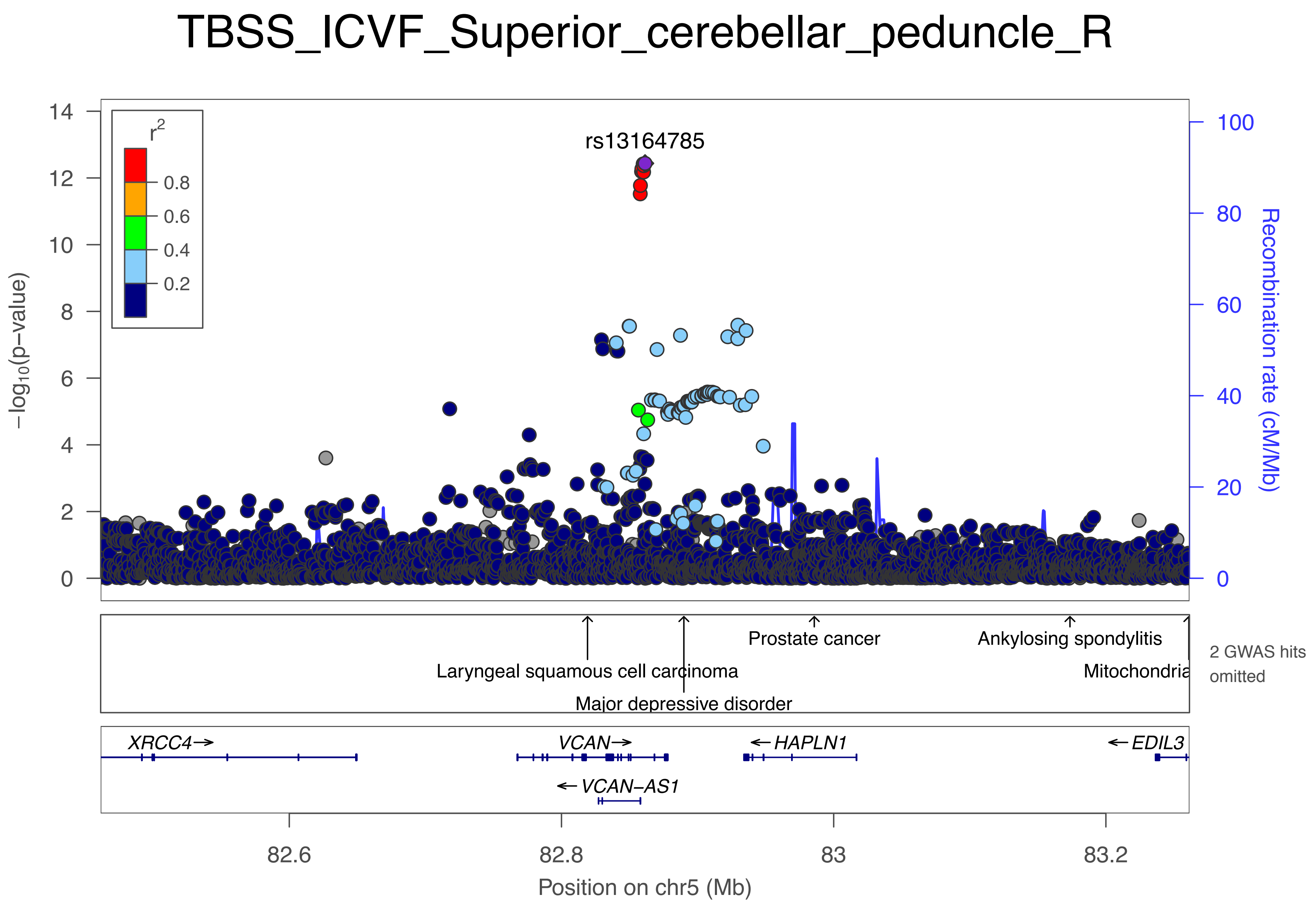

TBSS_ICVF_Superior_cerebellar_peduncle_R
rs13164785
r2
0.8
0.6
0.4
0.2
−log10(p−value)
Recombination rate (cM/Mb)
Prostate cancer
Ankylosing spondylitis
Laryngeal squamous cell carcinoma
Mitochondria
Major depressive disorder
2 GWAS hits omitted
XRCC4
VCAN
HAPLN1
EDIL3
VCAN−AS1
82.6
82.8
83
83.2
Position on chr5 (Mb)

| | |
|---|---|
| date: | Thu Aug 17 17:55:19 2017 |
| build: | hg19 |
| display range: | chr5:82461400–83261400 [82461400–83261400] |
| hilite range: | 0 – 0 [ 0 – 0 ] |
| reference SNP: | chr5:82861400 |
| number of SNPs plotted: | 3396 |
| min P.value: | 3.66E−13 [chr5:82861400] |
| max P.value: | 9.99E−1 [chr5:82489975] |
| omitted GWAS Hits: | chr5:83.260938−Mitochondrial DNA levels, NA |

Make more plots at http://csg.sph.umich.edu/locuszoom/

GWAS Catalog SNPs in Region

| chr | pos (Mb) | trait | snp |
|---|---|---|---|
| 5 | 82.81912 | Laryngeal squamous cell carcinoma | rs310518 |
| 5 | 82.84549 | Diisocyanate–induced asthma | rs3852186 |
| 5 | 82.88991 | Major depressive disorder | rs310501 |
| 5 | 82.96073 | Visceral fat | rs3846635 |
| 5 | 82.98574 | Prostate cancer | rs4466137 |
| 5 | 83.17359 | Ankylosing spondylitis | rs4552569 |
| 5 | 83.26094 | Mitochondrial DNA levels | rs2301070 |

# TBSS_ICVF_Anterior_limb_of_internal_capsule_R

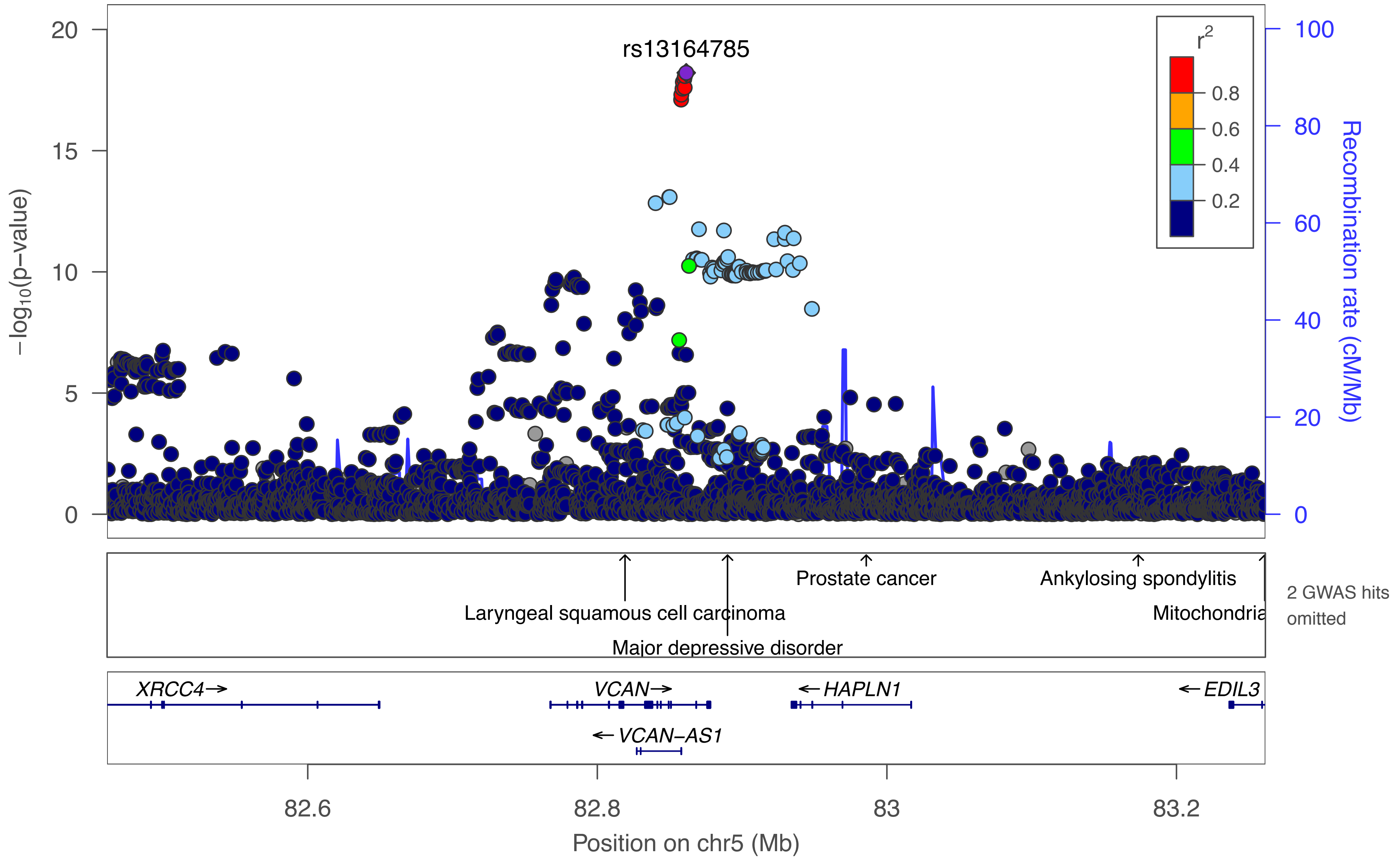

| | |
|---|---|
| date: | Thu Aug 17 17:55:19 2017 |
| build: | hg19 |
| display range: | chr5:82461400–83261400 [82461400–83261400] |
| hilite range: | 0 – 0 [ 0 – 0 ] |
| reference SNP: | chr5:82861400 |
| number of SNPs plotted: | 3396 |
| min P.value: | 6.12E–19 [chr5:82861400] |
| max P.value: | 10E–1 [chr5:83037028] |
| omitted GWAS Hits: | chr5:83.260938–Mitochondrial DNA levels, NA |

Make more plots at http://csg.sph.umich.edu/locuszoom/

GWAS Catalog SNPs in Region

| chr | pos (Mb) | trait | snp |
|---|---|---|---|
| 5 | 82.81912 | Laryngeal squamous cell carcinoma | rs310518 |
| 5 | 82.84549 | Diisocyanate–induced asthma | rs3852186 |
| 5 | 82.88991 | Major depressive disorder | rs310501 |
| 5 | 82.96073 | Visceral fat | rs3846635 |
| 5 | 82.98574 | Prostate cancer | rs4466137 |
| 5 | 83.17359 | Ankylosing spondylitis | rs4552569 |
| 5 | 83.26094 | Mitochondrial DNA levels | rs2301070 |

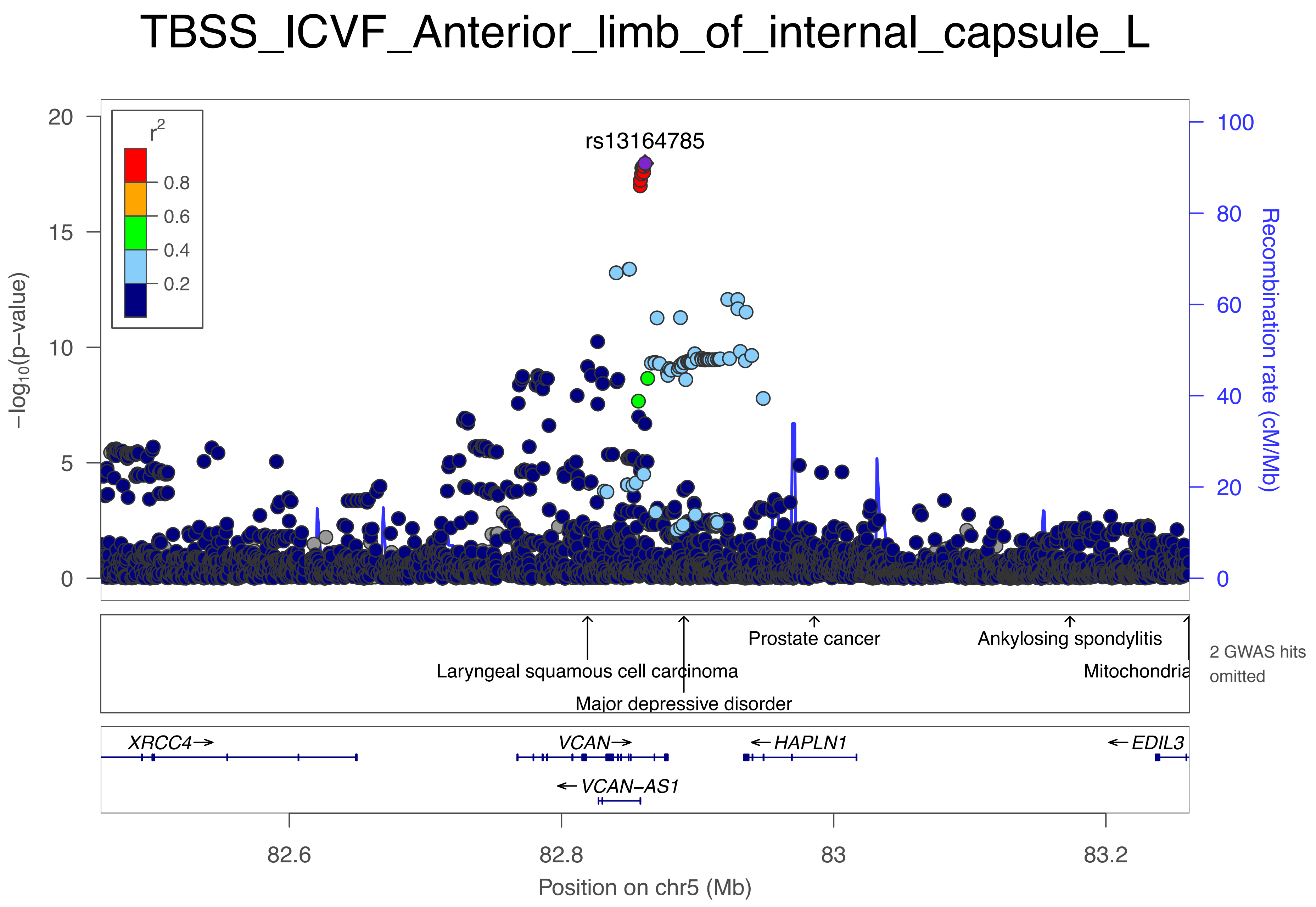

TBSS_ICVF_Anterior_limb_of_internal_capsule_L
rs13164785
r2
0.8
0.6
0.4
0.2
−log10(p−value)
Recombination rate (cM/Mb)
Laryngeal squamous cell carcinoma
Major depressive disorder
Prostate cancer
Ankylosing spondylitis
Mitochondria
2 GWAS hits omitted
XRCC4
VCAN
VCAN−AS1
HAPLN1
EDIL3
82.6
82.8
83
83.2
Position on chr5 (Mb)

| | |
|---|---|
| date: | Thu Aug 17 17:55:19 2017 |
| build: | hg19 |
| display range: | chr5:82461400−83261400 [82461400−83261400] |
| hilite range: | 0 – 0 [ 0 – 0 ] |
| reference SNP: | chr5:82861400 |
| number of SNPs plotted: | 3396 |
| min P.value: | 1.08E−18 [chr5:82861400] |
| max P.value: | 9.99E−1 [chr5:83129334] |
| omitted GWAS Hits: | chr5:83.260938−Mitochondrial DNA levels, NA |

Make more plots at http://csg.sph.umich.edu/locuszoom/

GWAS Catalog SNPs in Region

| chr | pos (Mb) | trait | snp |
|---|---|---|---|
| 5 | 82.81912 | Laryngeal squamous cell carcinoma | rs310518 |
| 5 | 82.84549 | Diisocyanate−induced asthma | rs3852186 |
| 5 | 82.88991 | Major depressive disorder | rs310501 |
| 5 | 82.96073 | Visceral fat | rs3846635 |
| 5 | 82.98574 | Prostate cancer | rs4466137 |
| 5 | 83.17359 | Ankylosing spondylitis | rs4552569 |
| 5 | 83.26094 | Mitochondrial DNA levels | rs2301070 |

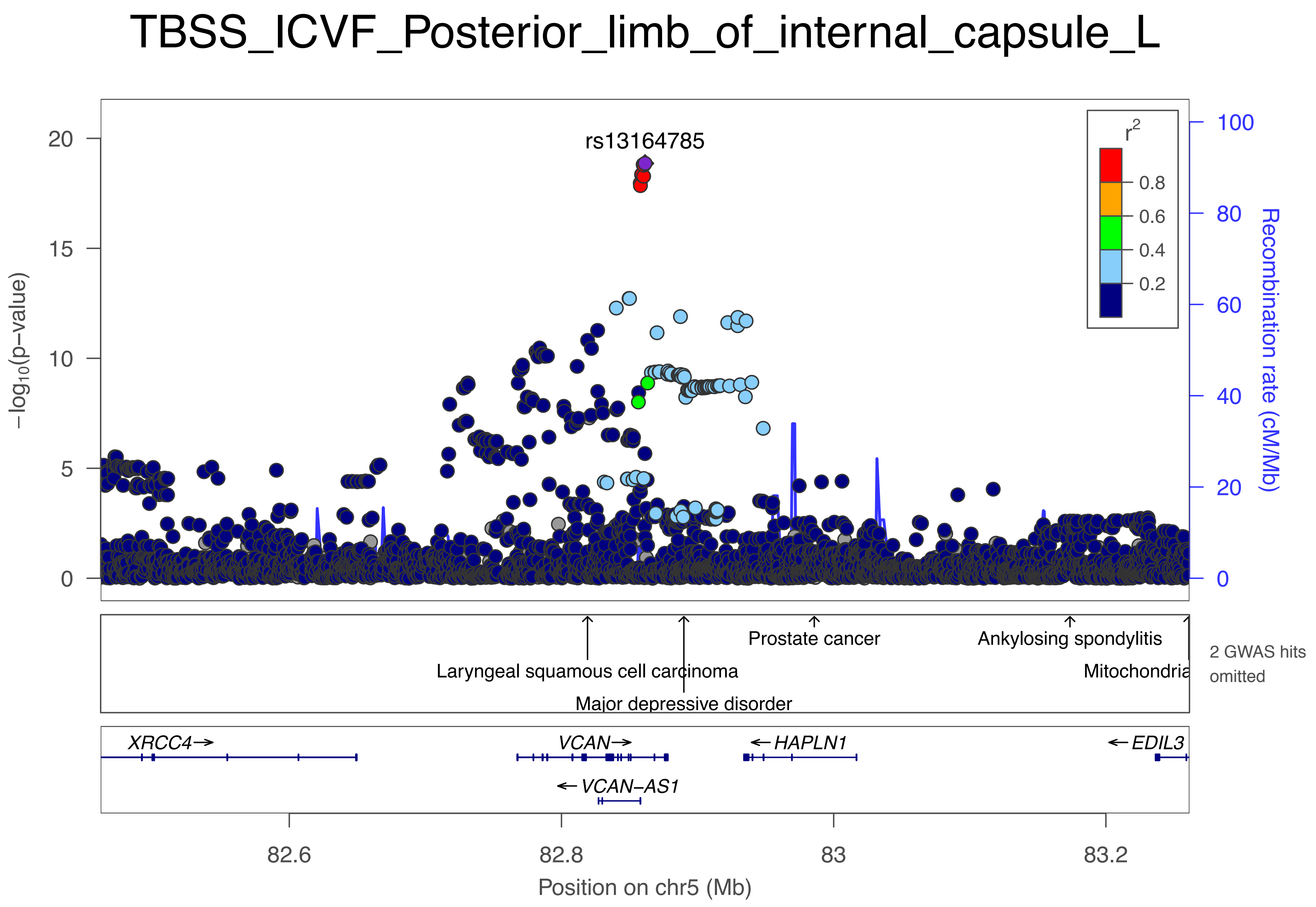

TBSS_ICVF_Posterior_limb_of_internal_capsule_L
rs13164785
r2
0.8
0.6
0.4
0.2
−log10(p−value)
Recombination rate (cM/Mb)
0
5
10
15
20
0
20
40
60
80
100
Prostate cancer
Ankylosing spondylitis
Laryngeal squamous cell carcinoma
Mitochondria
Major depressive disorder
2 GWAS hits omitted
XRCC4→
VCAN→
←HAPLN1
←EDIL3
←VCAN−AS1
82.6
82.8
83
83.2
Position on chr5 (Mb)

| | |
|---|---|
| date: | Thu Aug 17 17:55:19 2017 |
| build: | hg19 |
| display range: | chr5:82461400−83261400 [82461400−83261400] |
| hilite range: | 0 – 0 [ 0 – 0 ] |
| reference SNP: | chr5:82861400 |
| number of SNPs plotted: | 3396 |
| min P.value: | 1.37E−19 [chr5:82861400] |
| max P.value: | 10E−1 [chr5:83098673] |
| omitted GWAS Hits: | chr5:83.260938−Mitochondrial DNA levels, NA |

Make more plots at http://csg.sph.umich.edu/locuszoom/

GWAS Catalog SNPs in Region

| chr | pos (Mb) | trait | snp |
| --- | --- | --- | --- |
| 5 | 82.81912 | Laryngeal squamous cell carcinoma | rs310518 |
| 5 | 82.84549 | Diisocyanate–induced asthma | rs3852186 |
| 5 | 82.88991 | Major depressive disorder | rs310501 |
| 5 | 82.96073 | Visceral fat | rs3846635 |
| 5 | 82.98574 | Prostate cancer | rs4466137 |
| 5 | 83.17359 | Ankylosing spondylitis | rs4552569 |
| 5 | 83.26094 | Mitochondrial DNA levels | rs2301070 |

# TBSS_ICVF_Retrolenticular_part_of_internal_capsule_R

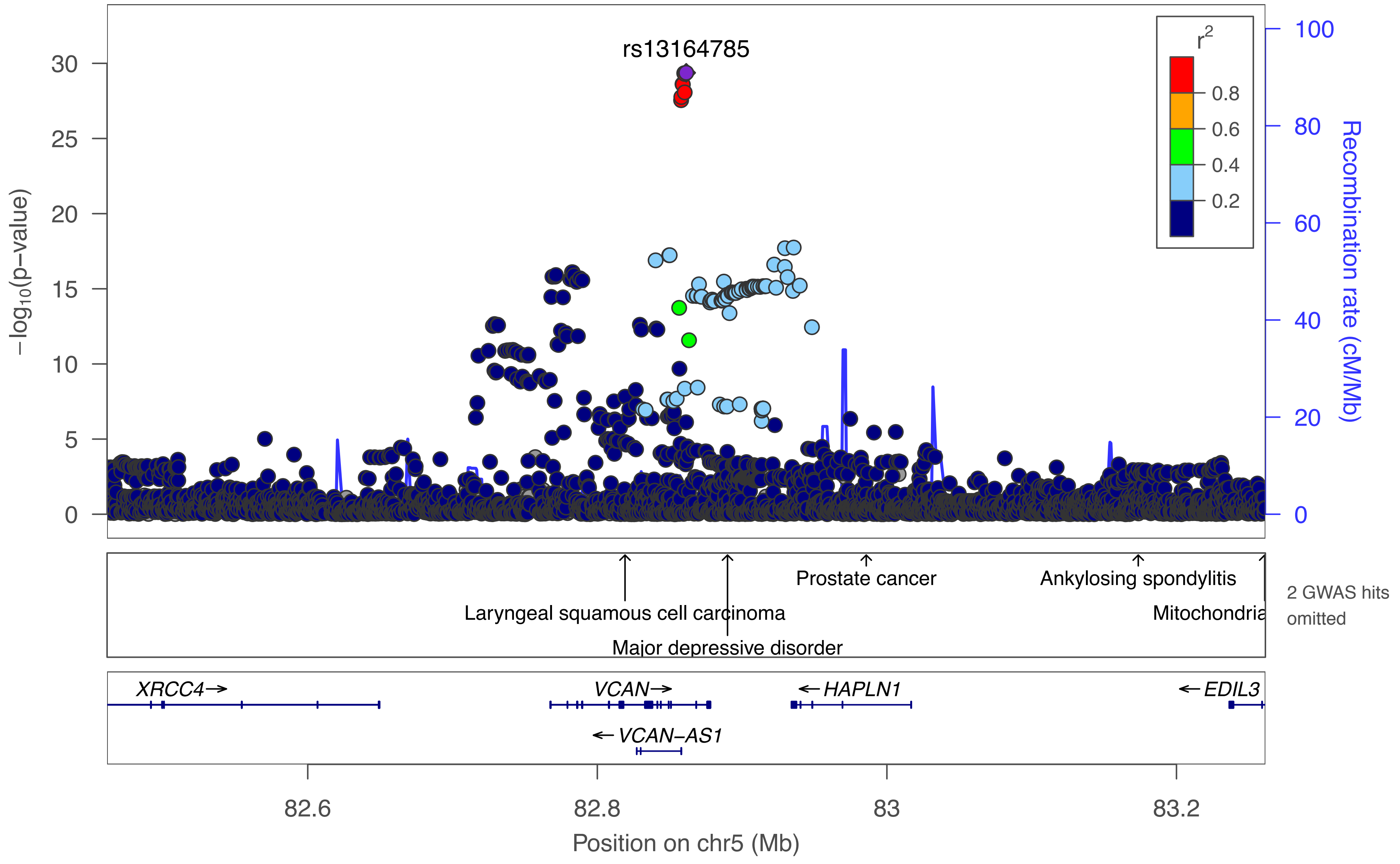

| | |
|---|---|
| date: | Thu Aug 17 17:55:19 2017 |
| build: | hg19 |
| display range: | chr5:82461400−83261400 [82461400−83261400] |
| hilite range: | 0 − 0 [ 0 − 0 ] |
| reference SNP: | chr5:82861400 |
| number of SNPs plotted: | 3396 |
| min P.value: | 4.26E−30 [chr5:82861400] |
| max P.value: | 10E−1 [chr5:83082369] |
| omitted GWAS Hits: | chr5:83.260938−Mitochondrial DNA levels, NA |

Make more plots at http://csg.sph.umich.edu/locuszoom/

GWAS Catalog SNPs in Region

| chr | pos (Mb) | trait | snp |
|---|---|---|---|
| 5 | 82.81912 | Laryngeal squamous cell carcinoma | rs310518 |
| 5 | 82.84549 | Diisocyanate–induced asthma | rs3852186 |
| 5 | 82.88991 | Major depressive disorder | rs310501 |
| 5 | 82.96073 | Visceral fat | rs3846635 |
| 5 | 82.98574 | Prostate cancer | rs4466137 |
| 5 | 83.17359 | Ankylosing spondylitis | rs4552569 |
| 5 | 83.26094 | Mitochondrial DNA levels | rs2301070 |

# TBSS_ICVF_Retrolenticular_part_of_internal_capsule_L

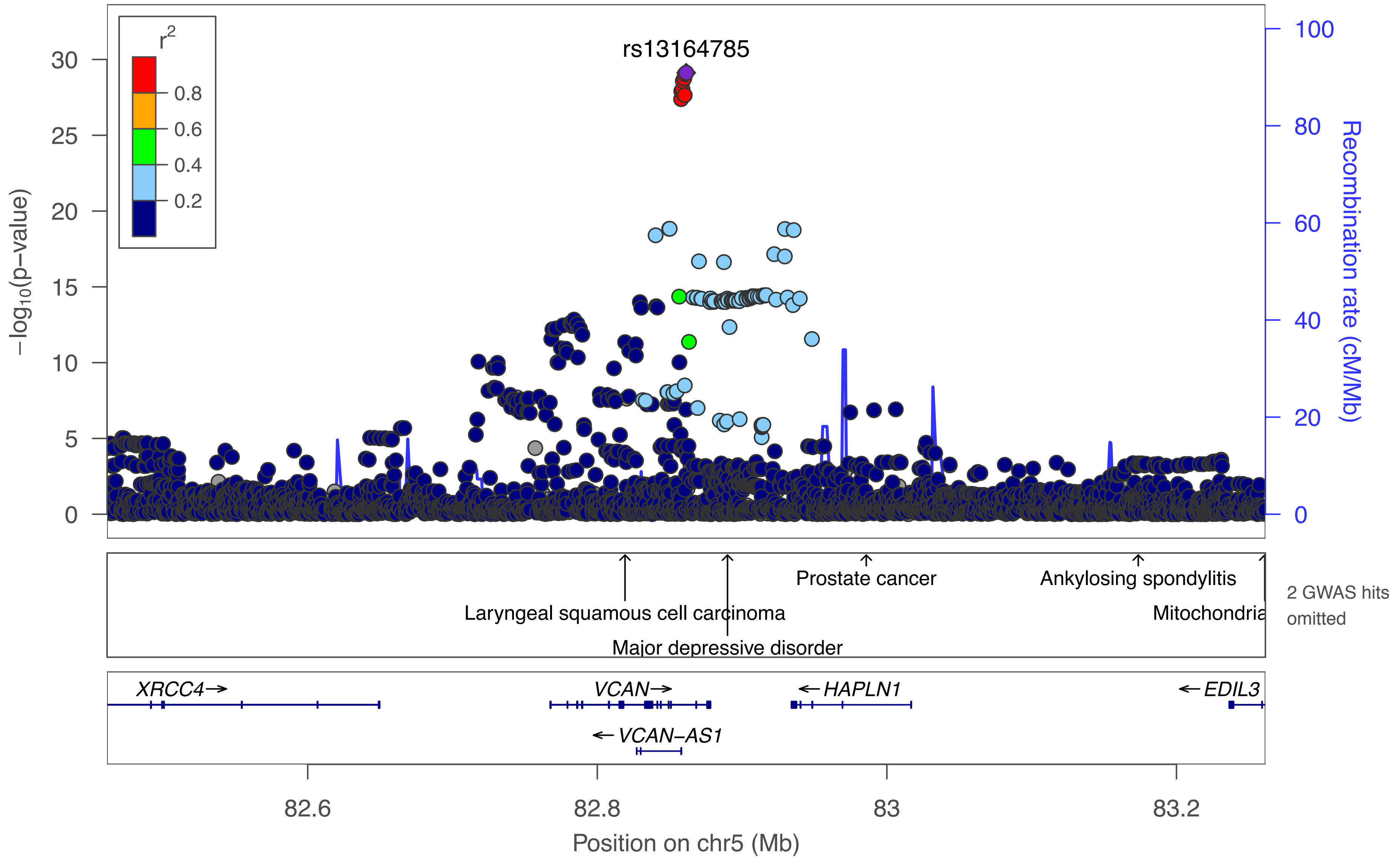

| | |
|---|---|
| date: | Thu Aug 17 17:55:19 2017 |
| build: | hg19 |
| display range: | chr5:82461400–83261400 [82461400–83261400] |
| hilite range: | 0 – 0 [ 0 – 0 ] |
| reference SNP: | chr5:82861400 |
| number of SNPs plotted: | 3396 |
| min P.value: | 7.57E–30 [chr5:82861400] |
| max P.value: | 10E–1 [chr5:83127527] |
| omitted GWAS Hits: | chr5:83.260938–Mitochondrial DNA levels, NA |

Make more plots at http://csg.sph.umich.edu/locuszoom/

GWAS Catalog SNPs in Region

| chr | pos (Mb) | trait | snp |
|---|---|---|---|
| 5 | 82.81912 | Laryngeal squamous cell carcinoma | rs310518 |
| 5 | 82.84549 | Diisocyanate–induced asthma | rs3852186 |
| 5 | 82.88991 | Major depressive disorder | rs310501 |
| 5 | 82.96073 | Visceral fat | rs3846635 |
| 5 | 82.98574 | Prostate cancer | rs4466137 |
| 5 | 83.17359 | Ankylosing spondylitis | rs4552569 |
| 5 | 83.26094 | Mitochondrial DNA levels | rs2301070 |

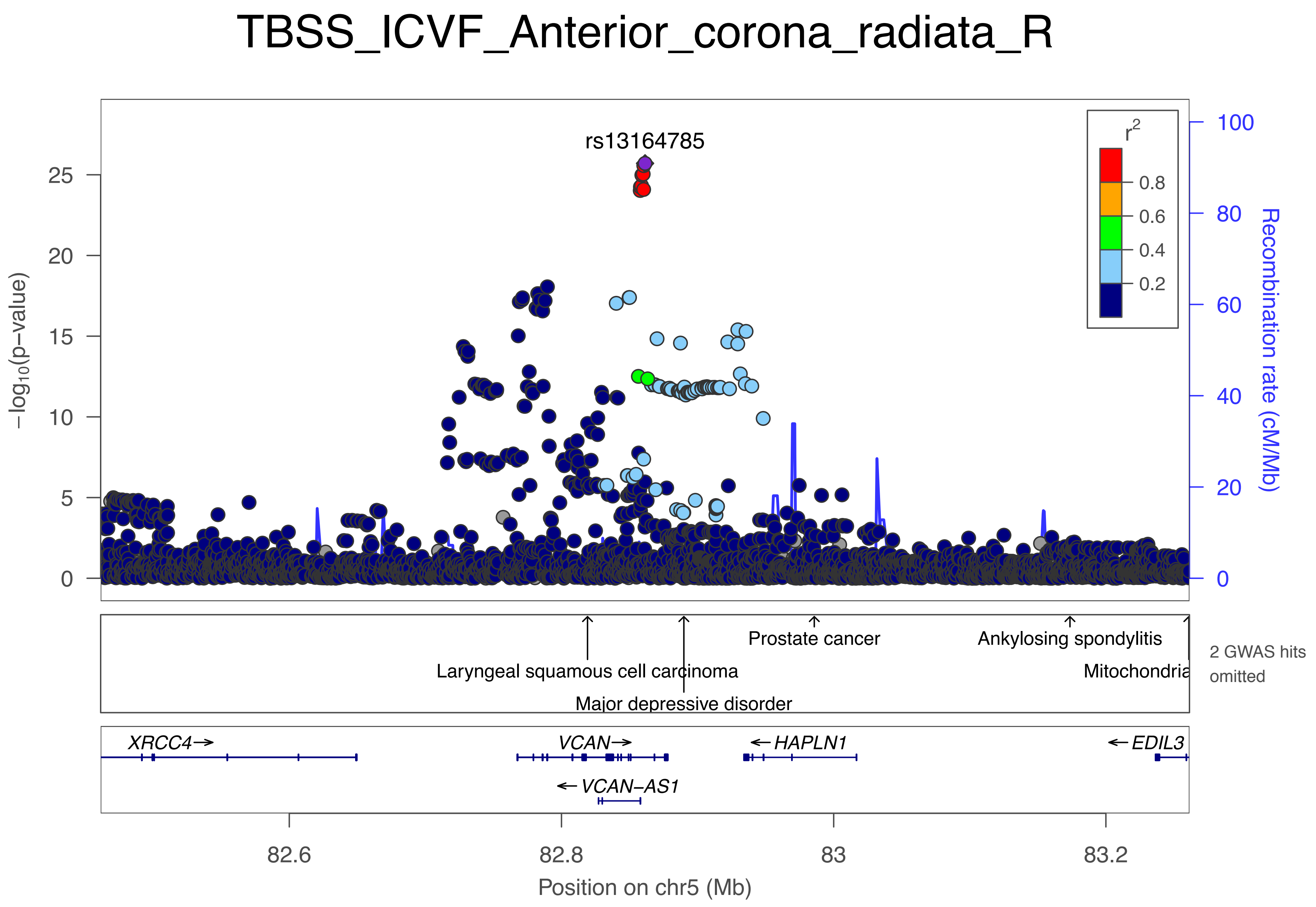

TBSS_ICVF_Anterior_corona_radiata_R
rs13164785
r²
0.8
0.6
0.4
0.2
−log10(p−value)
Recombination rate (cM/Mb)
Prostate cancer
Ankylosing spondylitis
Laryngeal squamous cell carcinoma
Mitochondria
Major depressive disorder
2 GWAS hits omitted
XRCC4→
VCAN→
←HAPLN1
←EDIL3
←VCAN−AS1
82.6
82.8
83
83.2
Position on chr5 (Mb)

| | |
|---|---|
| date: | Thu Aug 17 17:55:19 2017 |
| build: | hg19 |
| display range: | chr5:82461400−83261400 [82461400−83261400] |
| hilite range: | 0 − 0 [ 0 − 0 ] |
| reference SNP: | chr5:82861400 |
| number of SNPs plotted: | 3396 |
| min P.value: | 1.96E−26 [chr5:82861400] |
| max P.value: | 10E−1 [chr5:82586088] |
| omitted GWAS Hits: | chr5:83.260938−Mitochondrial DNA levels, NA |

Make more plots at http://csg.sph.umich.edu/locuszoom/

GWAS Catalog SNPs in Region

| chr | pos (Mb) | trait | snp |
|---|---|---|---|
| 5 | 82.81912 | Laryngeal squamous cell carcinoma | rs310518 |
| 5 | 82.84549 | Diisocyanate–induced asthma | rs3852186 |
| 5 | 82.88991 | Major depressive disorder | rs310501 |
| 5 | 82.96073 | Visceral fat | rs3846635 |
| 5 | 82.98574 | Prostate cancer | rs4466137 |
| 5 | 83.17359 | Ankylosing spondylitis | rs4552569 |
| 5 | 83.26094 | Mitochondrial DNA levels | rs2301070 |

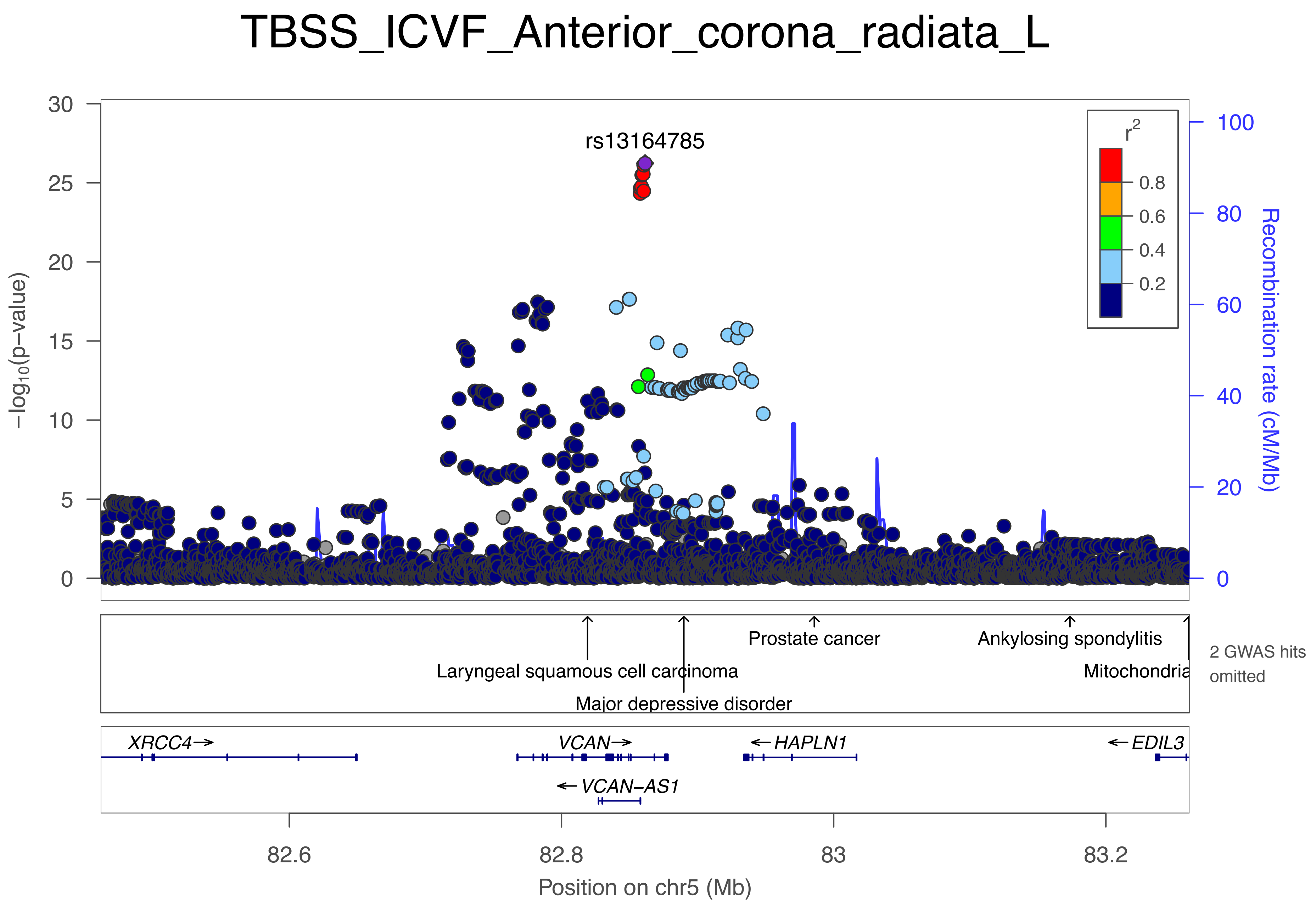
TBSS_ICVF_Anterior_corona_radiata_L
rs13164785
−log10(p−value)
Recombination rate (cM/Mb)
r2
0.8
0.6
0.4
0.2
Laryngeal squamous cell carcinoma
Major depressive disorder
Prostate cancer
Ankylosing spondylitis
Mitochondria
2 GWAS hits omitted
XRCC4→
VCAN→
←HAPLN1
←EDIL3
←VCAN−AS1
82.6
82.8
83
83.2
Position on chr5 (Mb)

| | |
|---|---|
| date: | Thu Aug 17 17:55:19 2017 |
| build: | hg19 |
| display range: | chr5:82461400−83261400 [82461400−83261400] |
| hilite range: | 0 – 0 [ 0 – 0 ] |
| reference SNP: | chr5:82861400 |
| number of SNPs plotted: | 3396 |
| min P.value: | 5.9E−27 [chr5:82861400] |
| max P.value: | 9.99E−1 [chr5:82929723] |
| omitted GWAS Hits: | chr5:83.260938−Mitochondrial DNA levels, NA |

Make more plots at http://csg.sph.umich.edu/locuszoom/

GWAS Catalog SNPs in Region

| chr | pos (Mb) | trait | snp |
|---|---|---|---|
| 5 | 82.81912 | Laryngeal squamous cell carcinoma | rs310518 |
| 5 | 82.84549 | Diisocyanate–induced asthma | rs3852186 |
| 5 | 82.88991 | Major depressive disorder | rs310501 |
| 5 | 82.96073 | Visceral fat | rs3846635 |
| 5 | 82.98574 | Prostate cancer | rs4466137 |
| 5 | 83.17359 | Ankylosing spondylitis | rs4552569 |
| 5 | 83.26094 | Mitochondrial DNA levels | rs2301070 |

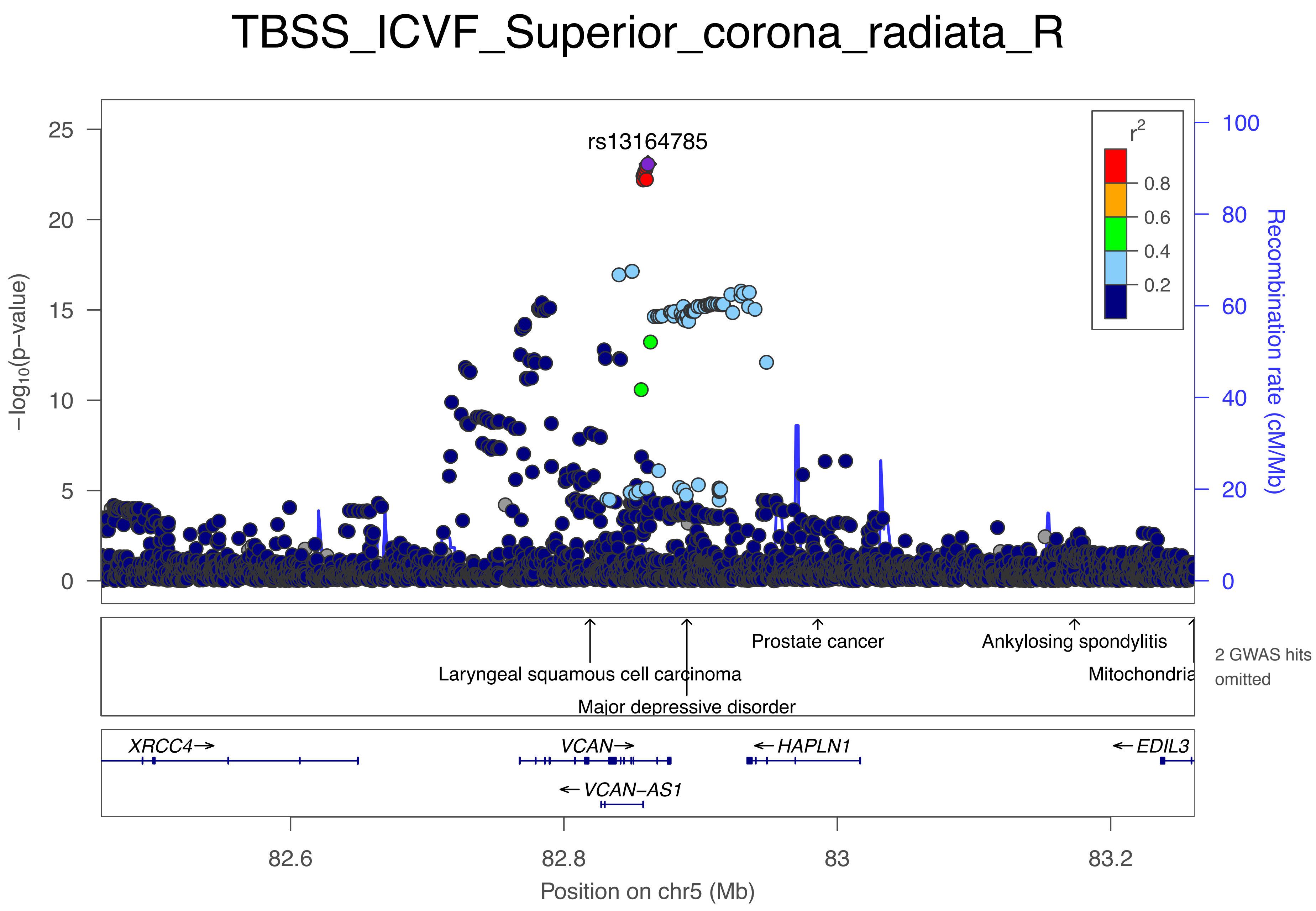
TBSS_ICVF_Superior_corona_radiata_R
rs13164785
r2
0.8
0.6
0.4
0.2
−log10(p−value)
Recombination rate (cM/Mb)
25
20
15
10
5
0
100
80
60
40
20
0
Prostate cancer
Ankylosing spondylitis
Laryngeal squamous cell carcinoma
Mitochondria
Major depressive disorder
2 GWAS hits omitted
XRCC4→
VCAN→
←HAPLN1
←EDIL3
←VCAN−AS1
82.6
82.8
83
83.2
Position on chr5 (Mb)

| | |
|---|---|
| date: | Thu Aug 17 17:55:19 2017 |
| build: | hg19 |
| display range: | chr5:82461400–83261400 [82461400–83261400] |
| hilite range: | 0 – 0 [ 0 – 0 ] |
| reference SNP: | chr5:82861400 |
| number of SNPs plotted: | 3396 |
| min P.value: | 8.41E–24 [chr5:82861400] |
| max P.value: | 9.97E–1 [chr5:82659709] |
| omitted GWAS Hits: | chr5:83.260938–Mitochondrial DNA levels, NA |

Make more plots at http://csg.sph.umich.edu/locuszoom/

GWAS Catalog SNPs in Region

| chr | pos (Mb) | trait | snp |
|---|---|---|---|
| 5 | 82.81912 | Laryngeal squamous cell carcinoma | rs310518 |
| 5 | 82.84549 | Diisocyanate–induced asthma | rs3852186 |
| 5 | 82.88991 | Major depressive disorder | rs310501 |
| 5 | 82.96073 | Visceral fat | rs3846635 |
| 5 | 82.98574 | Prostate cancer | rs4466137 |
| 5 | 83.17359 | Ankylosing spondylitis | rs4552569 |
| 5 | 83.26094 | Mitochondrial DNA levels | rs2301070 |

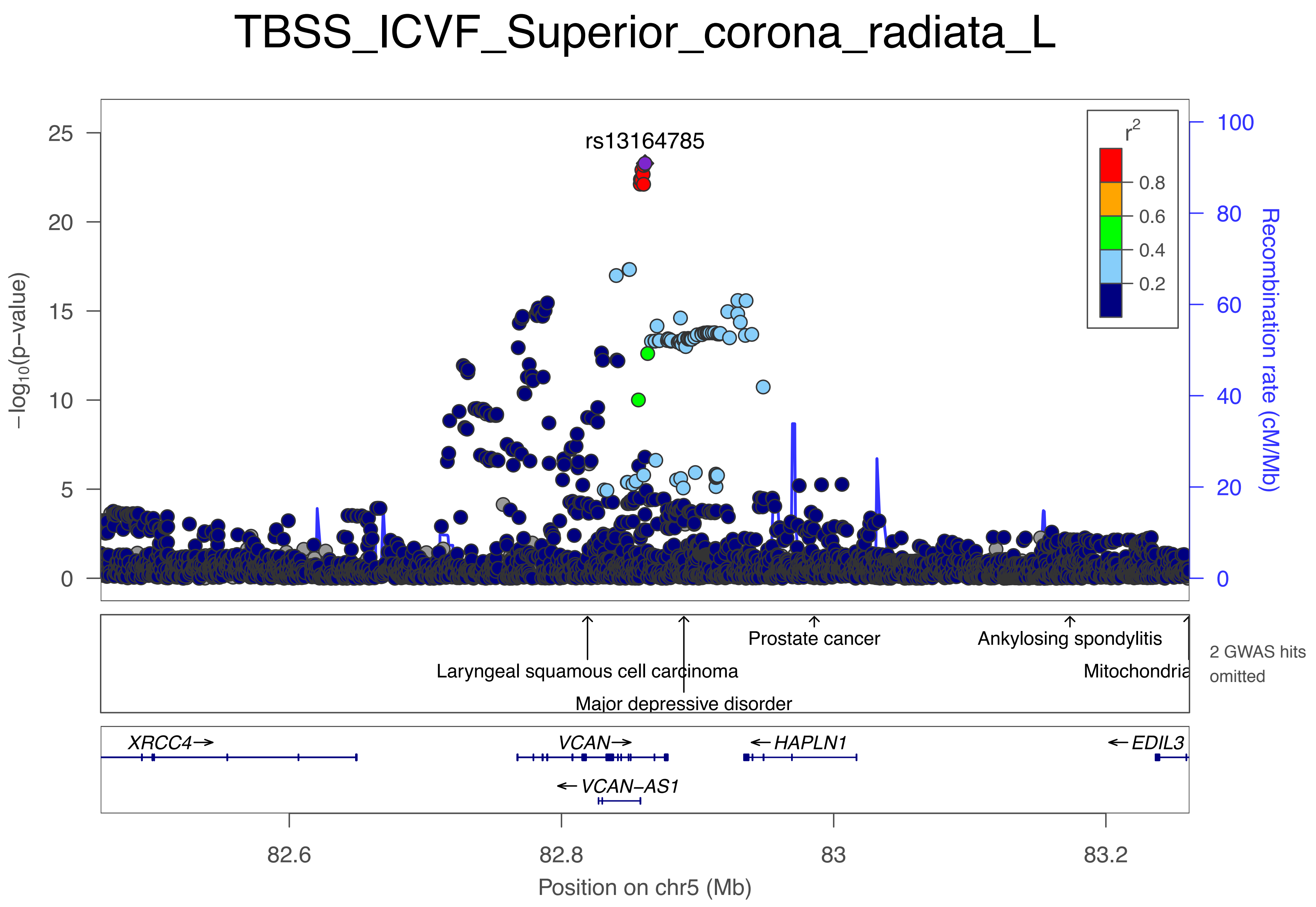

TBSS_ICVF_Superior_corona_radiata_L
rs13164785
−log10(p−value)
25
20
15
10
5
0
r2
0.8
0.6
0.4
0.2
100
80
60
40
20
0
Recombination rate (cM/Mb)
Prostate cancer
Ankylosing spondylitis
Laryngeal squamous cell carcinoma
Mitochondria
Major depressive disorder
2 GWAS hits omitted
XRCC4→
VCAN→
←HAPLN1
←EDIL3
←VCAN−AS1
82.6
82.8
83
83.2
Position on chr5 (Mb)

| | |
|---|---|
| date: | Thu Aug 17 17:55:19 2017 |
| build: | hg19 |
| display range: | chr5:82461400−83261400 [82461400−83261400] |
| hilite range: | 0 – 0 [ 0 – 0 ] |
| reference SNP: | chr5:82861400 |
| number of SNPs plotted: | 3396 |
| min P.value: | 5.2E−24 [chr5:82861400] |
| max P.value: | 10E−1 [chr5:82630687] |
| omitted GWAS Hits: | chr5:83.260938−Mitochondrial DNA levels, NA |

Make more plots at http://csg.sph.umich.edu/locuszoom/

GWAS Catalog SNPs in Region

| chr | pos (Mb) | trait | snp |
|---|---|---|---|
| 5 | 82.81912 | Laryngeal squamous cell carcinoma | rs310518 |
| 5 | 82.84549 | Diisocyanate–induced asthma | rs3852186 |
| 5 | 82.88991 | Major depressive disorder | rs310501 |
| 5 | 82.96073 | Visceral fat | rs3846635 |
| 5 | 82.98574 | Prostate cancer | rs4466137 |
| 5 | 83.17359 | Ankylosing spondylitis | rs4552569 |
| 5 | 83.26094 | Mitochondrial DNA levels | rs2301070 |

# TBSS_ICVF_Posterior_corona_radiata_R

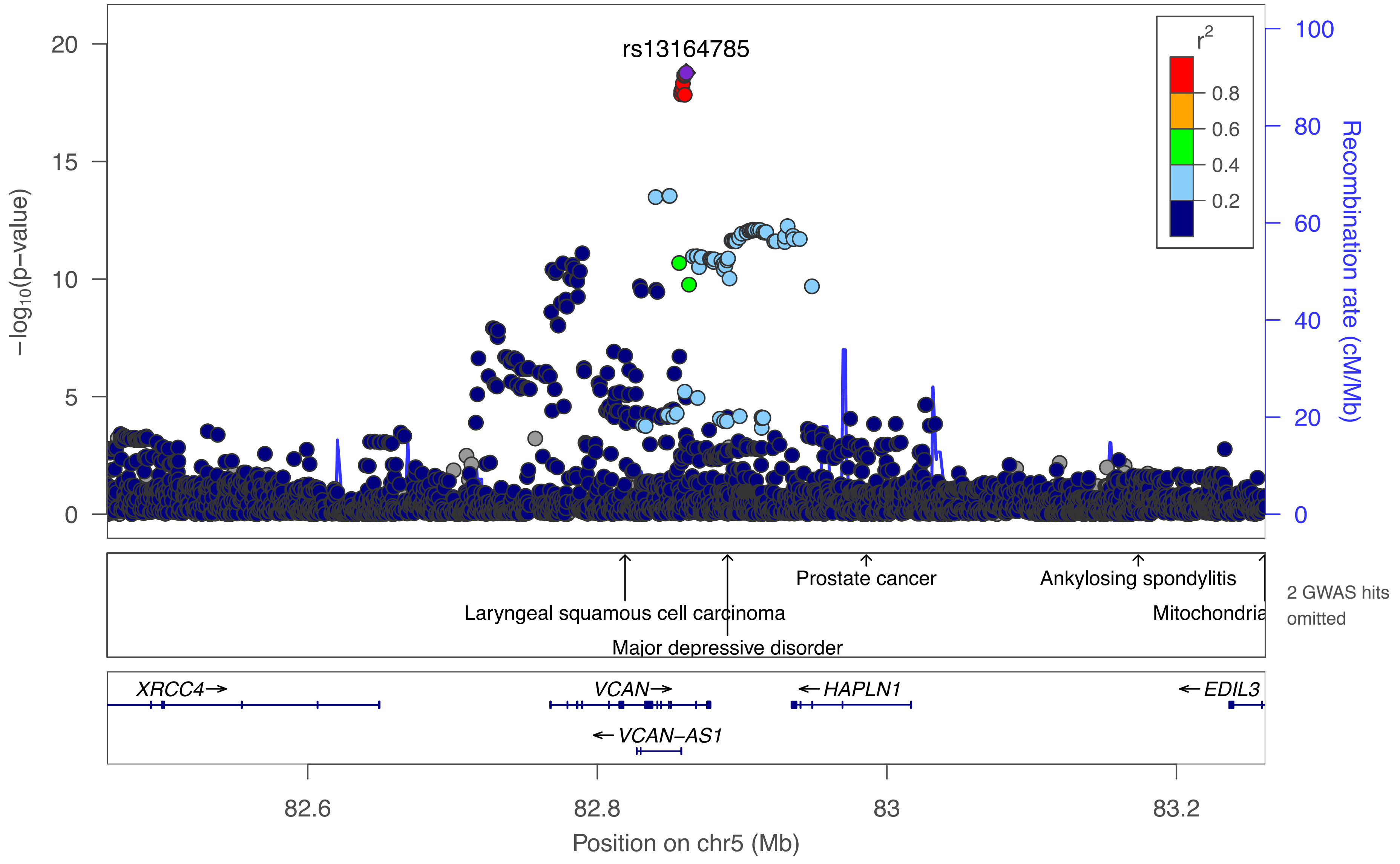

| | |
|---|---|
| date: | Thu Aug 17 17:55:19 2017 |
| build: | hg19 |
| display range: | chr5:82461400−83261400 [82461400−83261400] |
| hilite range: | 0 – 0 [ 0 – 0 ] |
| reference SNP: | chr5:82861400 |
| number of SNPs plotted: | 3396 |
| min P.value: | 1.69E−19 [chr5:82861400] |
| max P.value: | 10E−1 [chr5:82907433] |
| omitted GWAS Hits: | chr5:83.260938−Mitochondrial DNA levels, NA |

Make more plots at http://csg.sph.umich.edu/locuszoom/

GWAS Catalog SNPs in Region

| chr | pos (Mb) | trait | snp |
|---|---|---|---|
| 5 | 82.81912 | Laryngeal squamous cell carcinoma | rs310518 |
| 5 | 82.84549 | Diisocyanate−induced asthma | rs3852186 |
| 5 | 82.88991 | Major depressive disorder | rs310501 |
| 5 | 82.96073 | Visceral fat | rs3846635 |
| 5 | 82.98574 | Prostate cancer | rs4466137 |
| 5 | 83.17359 | Ankylosing spondylitis | rs4552569 |
| 5 | 83.26094 | Mitochondrial DNA levels | rs2301070 |

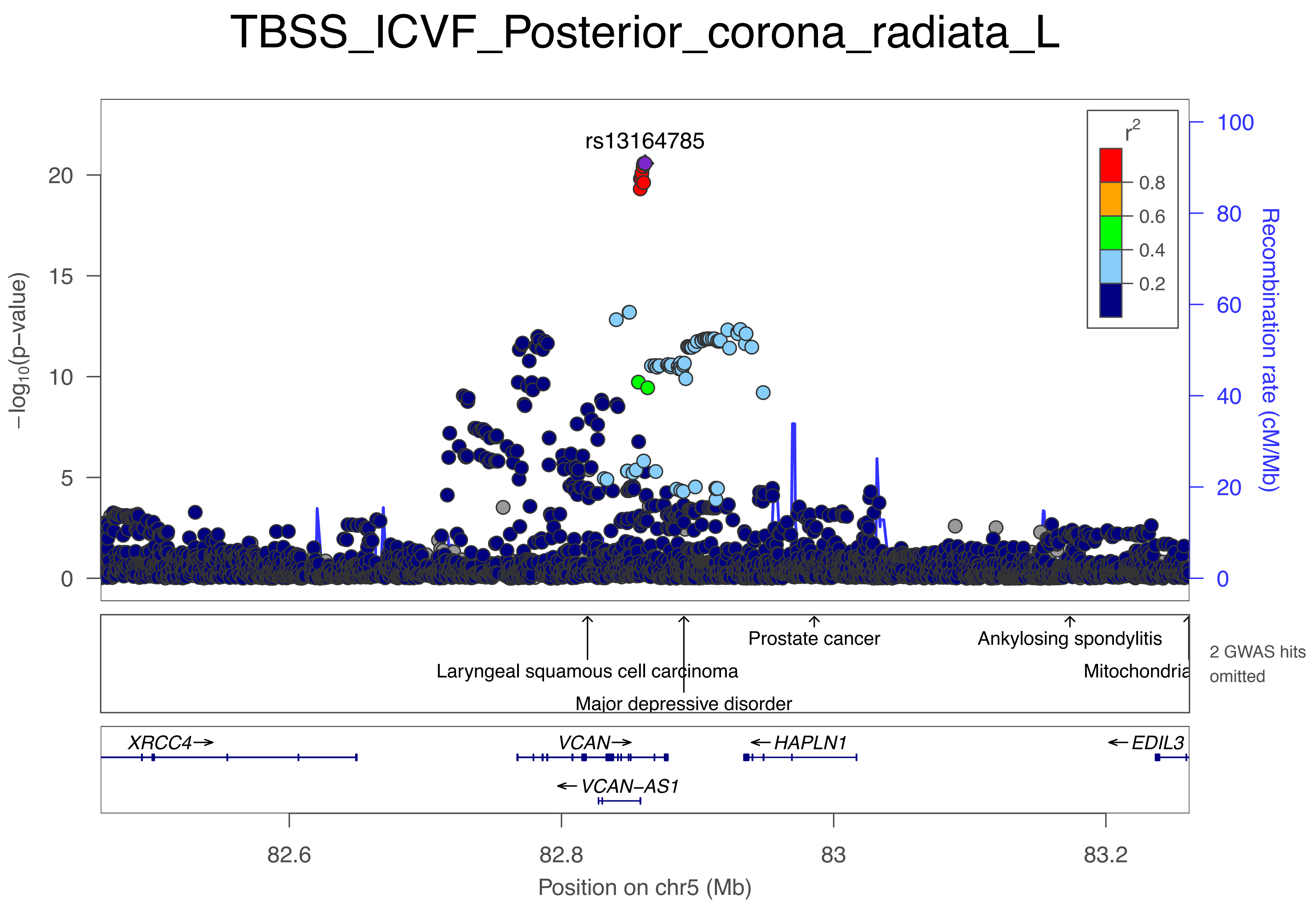
TBSS_ICVF_Posterior_corona_radiata_L
rs13164785
−log10(p−value)
Recombination rate (cM/Mb)
r²
0.8
0.6
0.4
0.2
Prostate cancer
Ankylosing spondylitis
Laryngeal squamous cell carcinoma
Mitochondria
Major depressive disorder
2 GWAS hits omitted
XRCC4→
VCAN→
←HAPLN1
←EDIL3
←VCAN−AS1
82.6
82.8
83
83.2
Position on chr5 (Mb)

| | |
|---|---|
| date: | Thu Aug 17 17:55:19 2017 |
| build: | hg19 |
| display range: | chr5:82461400–83261400 [82461400–83261400] |
| hilite range: | 0 – 0 [ 0 – 0 ] |
| reference SNP: | chr5:82861400 |
| number of SNPs plotted: | 3396 |
| min P.value: | 2.65E–21 [chr5:82861400] |
| max P.value: | 10E–1 [chr5:82587485] |
| omitted GWAS Hits: | chr5:83.260938–Mitochondrial DNA levels, NA |

Make more plots at http://csg.sph.umich.edu/locuszoom/

GWAS Catalog SNPs in Region

| chr | pos (Mb) | trait | snp |
|---|---|---|---|
| 5 | 82.81912 | Laryngeal squamous cell carcinoma | rs310518 |
| 5 | 82.84549 | Diisocyanate–induced asthma | rs3852186 |
| 5 | 82.88991 | Major depressive disorder | rs310501 |
| 5 | 82.96073 | Visceral fat | rs3846635 |
| 5 | 82.98574 | Prostate cancer | rs4466137 |
| 5 | 83.17359 | Ankylosing spondylitis | rs4552569 |
| 5 | 83.26094 | Mitochondrial DNA levels | rs2301070 |

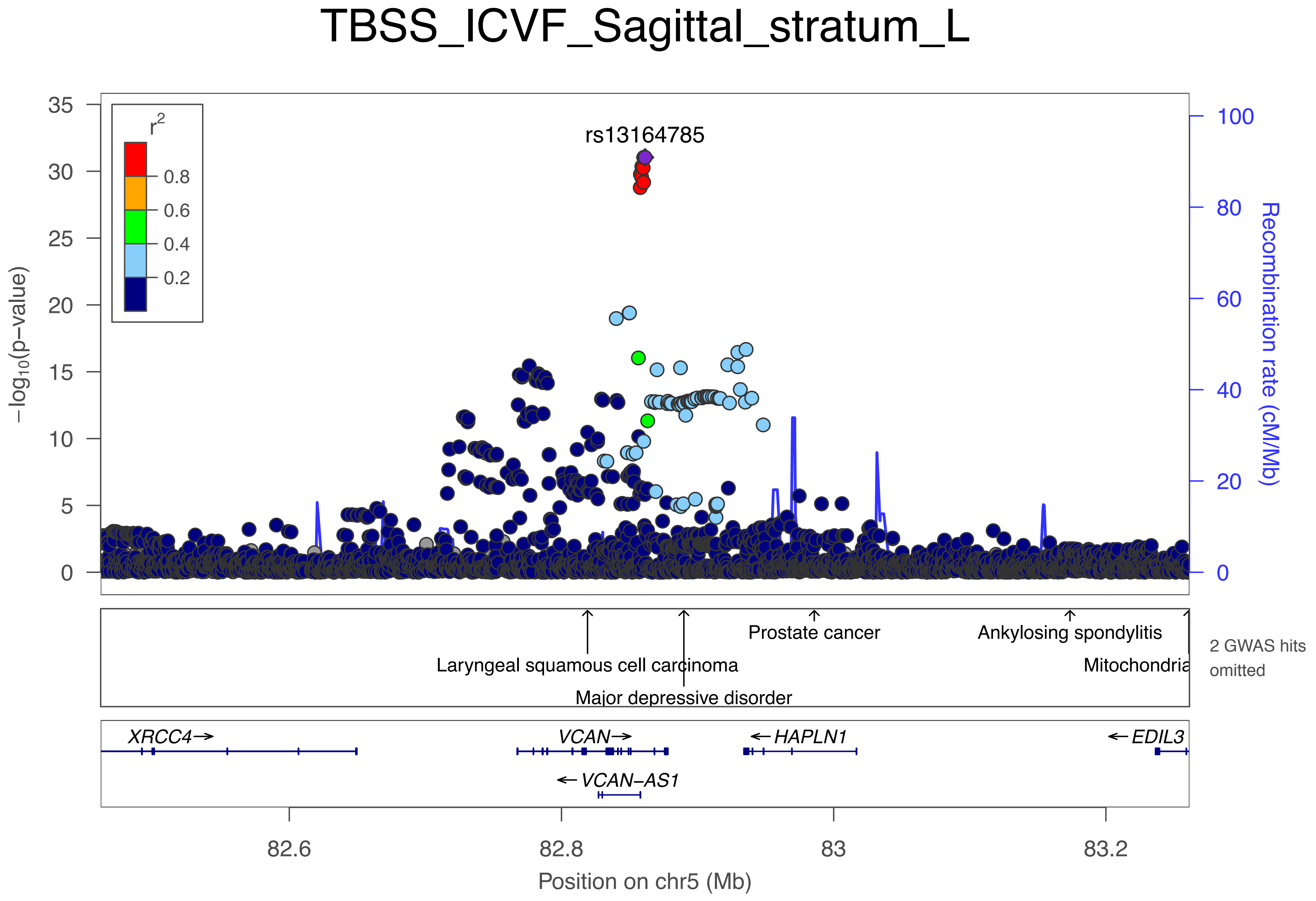

TBSS_ICVF_Sagittal_stratum_L
rs13164785
r2
0.8
0.6
0.4
0.2
35
30
25
20
15
10
5
0
−log10(p−value)
100
80
60
40
20
0
Recombination rate (cM/Mb)
Laryngeal squamous cell carcinoma
Major depressive disorder
Prostate cancer
Ankylosing spondylitis
Mitochondria
2 GWAS hits omitted
XRCC4→
VCAN→
←HAPLN1
←EDIL3
←VCAN−AS1
82.6
82.8
83
83.2
Position on chr5 (Mb)

| | |
|---|---|
| date: | Thu Aug 17 17:55:19 2017 |
| build: | hg19 |
| display range: | chr5:82461400–83261400 [82461400–83261400] |
| hilite range: | 0 – 0 [ 0 – 0 ] |
| reference SNP: | chr5:82861400 |
| number of SNPs plotted: | 3396 |
| min P.value: | 9.12E–32 [chr5:82861400] |
| max P.value: | 10E–1 [chr5:83203721] |
| omitted GWAS Hits: | chr5:83.260938–Mitochondrial DNA levels, NA |

Make more plots at http://csg.sph.umich.edu/locuszoom/

GWAS Catalog SNPs in Region

| chr | pos (Mb) | trait | snp |
|---|---|---|---|
| 5 | 82.81912 | Laryngeal squamous cell carcinoma | rs310518 |
| 5 | 82.84549 | Diisocyanate−induced asthma | rs3852186 |
| 5 | 82.88991 | Major depressive disorder | rs310501 |
| 5 | 82.96073 | Visceral fat | rs3846635 |
| 5 | 82.98574 | Prostate cancer | rs4466137 |
| 5 | 83.17359 | Ankylosing spondylitis | rs4552569 |
| 5 | 83.26094 | Mitochondrial DNA levels | rs2301070 |

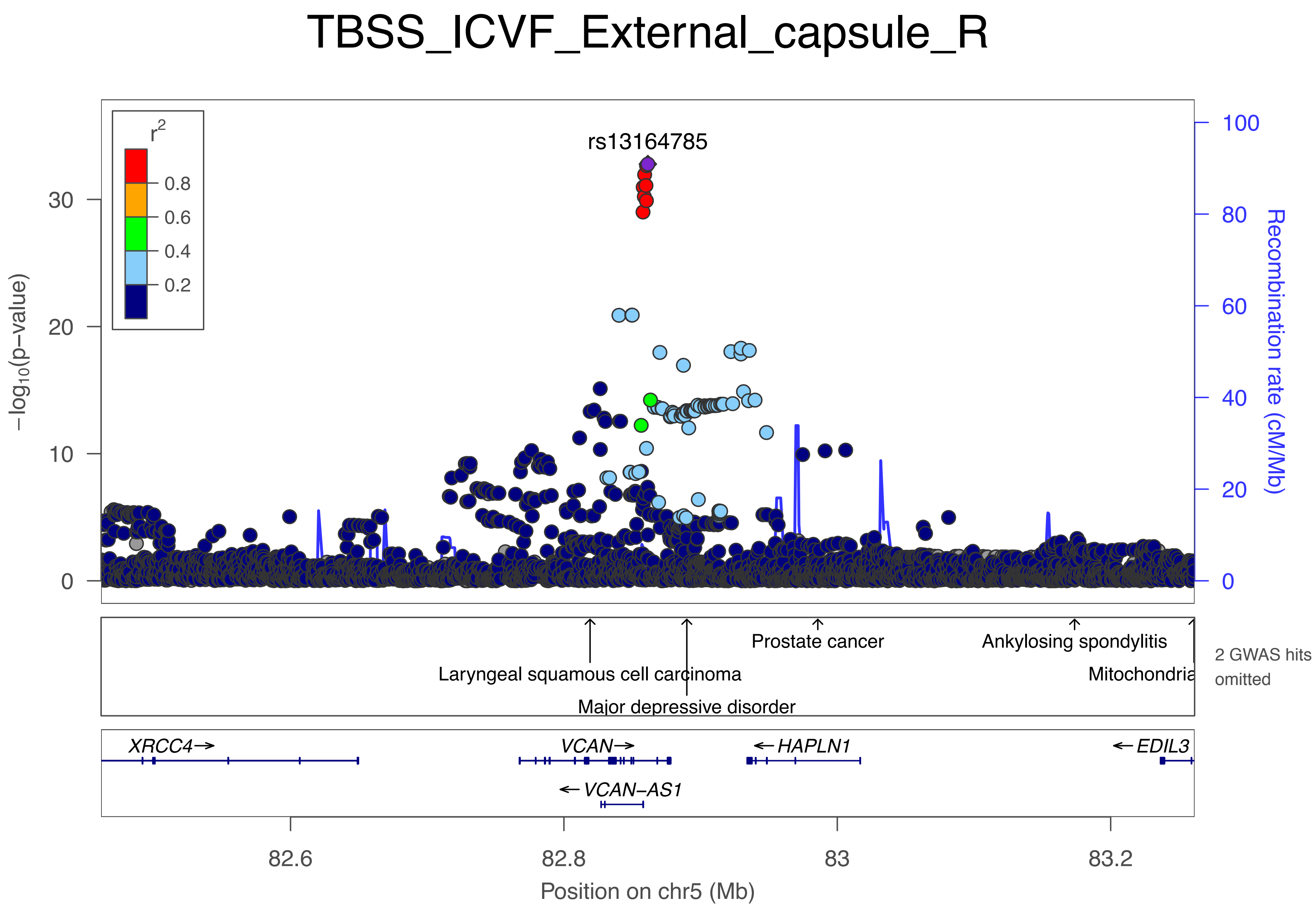
TBSS_ICVF_External_capsule_R
rs13164785
r2
0.8
0.6
0.4
0.2
30
20
10
0
−log10(p−value)
100
80
60
40
20
0
Recombination rate (cM/Mb)
Laryngeal squamous cell carcinoma
Major depressive disorder
Prostate cancer
Ankylosing spondylitis
Mitochondria
2 GWAS hits omitted
XRCC4→
VCAN→
←VCAN−AS1
←HAPLN1
←EDIL3
82.6
82.8
83
83.2
Position on chr5 (Mb)

| | |
|---|---|
| date: | Thu Aug 17 17:55:19 2017 |
| build: | hg19 |
| display range: | chr5:82461400−83261400 [82461400−83261400] |
| hilite range: | 0 – 0 [ 0 – 0 ] |
| reference SNP: | chr5:82861400 |
| number of SNPs plotted: | 3396 |
| min P.value: | 1.66E−33 [chr5:82861400] |
| max P.value: | 10E−1 [chr5:83006443] |
| omitted GWAS Hits: | chr5:83.260938−Mitochondrial DNA levels, NA |

Make more plots at http://csg.sph.umich.edu/locuszoom/

GWAS Catalog SNPs in Region

| chr | pos (Mb) | trait | snp |
|---|---|---|---|
| 5 | 82.81912 | Laryngeal squamous cell carcinoma | rs310518 |
| 5 | 82.84549 | Diisocyanate–induced asthma | rs3852186 |
| 5 | 82.88991 | Major depressive disorder | rs310501 |
| 5 | 82.96073 | Visceral fat | rs3846635 |
| 5 | 82.98574 | Prostate cancer | rs4466137 |
| 5 | 83.17359 | Ankylosing spondylitis | rs4552569 |
| 5 | 83.26094 | Mitochondrial DNA levels | rs2301070 |

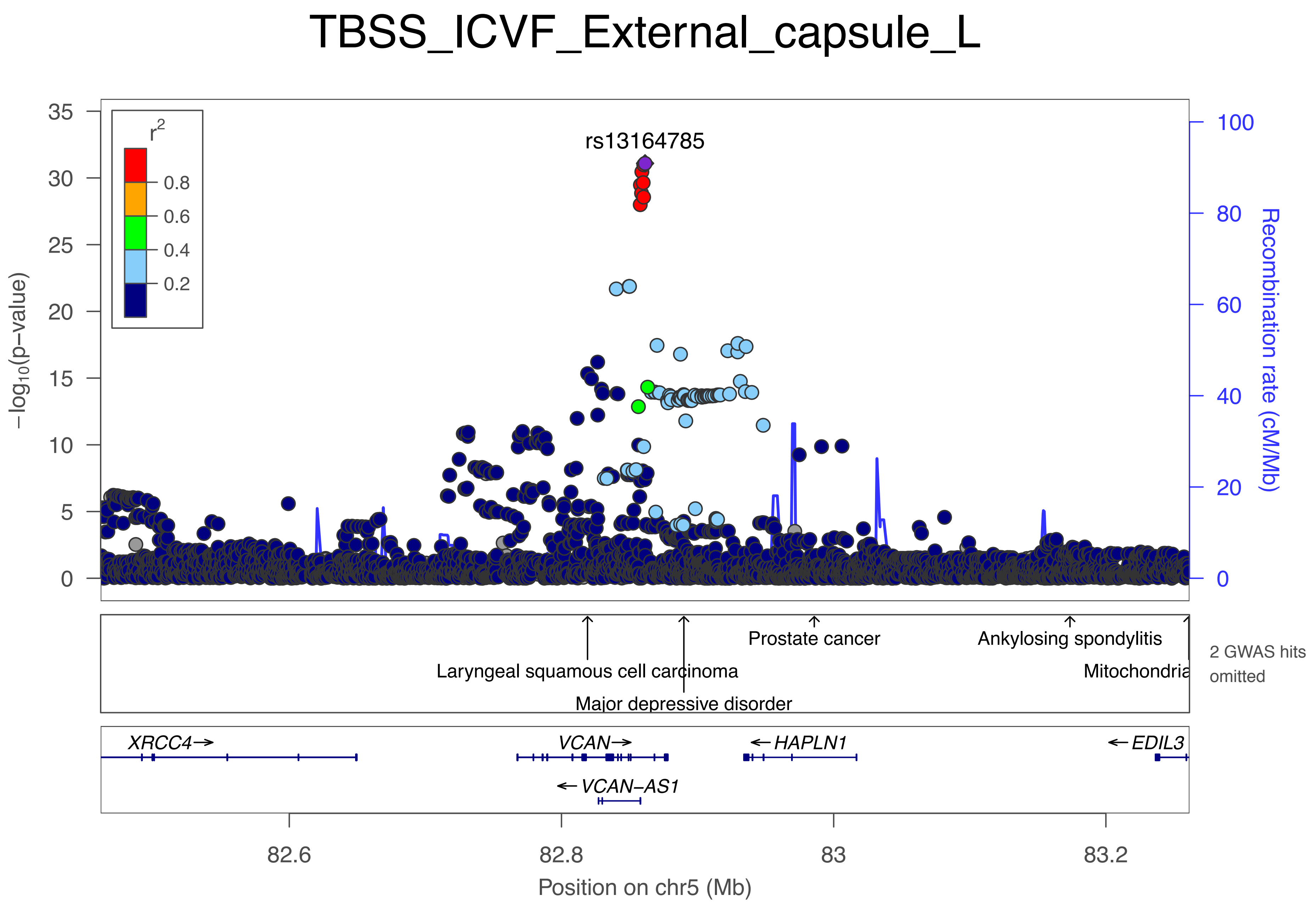
TBSS_ICVF_External_capsule_L
rs13164785
r2
0.8
0.6
0.4
0.2
35
30
25
20
15
10
5
0
−log10(p−value)
100
80
60
40
20
0
Recombination rate (cM/Mb)
Prostate cancer
Ankylosing spondylitis
Laryngeal squamous cell carcinoma
Mitochondria
Major depressive disorder
2 GWAS hits omitted
XRCC4
VCAN
HAPLN1
EDIL3
VCAN−AS1
82.6
82.8
83
83.2
Position on chr5 (Mb)

| | |
|---|---|
| date: | Thu Aug 17 17:55:19 2017 |
| build: | hg19 |
| display range: | chr5:82461400–83261400 [82461400–83261400] |
| hilite range: | 0 – 0 [ 0 – 0 ] |
| reference SNP: | chr5:82861400 |
| number of SNPs plotted: | 3396 |
| min P.value: | 8.11E–32 [chr5:82861400] |
| max P.value: | 10E–1 [chr5:83248345] |
| omitted GWAS Hits: | chr5:83.260938–Mitochondrial DNA levels, NA |

Make more plots at http://csg.sph.umich.edu/locuszoom/

GWAS Catalog SNPs in Region

| chr | pos (Mb) | trait | snp |
|---|---|---|---|
| 5 | 82.81912 | Laryngeal squamous cell carcinoma | rs310518 |
| 5 | 82.84549 | Diisocyanate–induced asthma | rs3852186 |
| 5 | 82.88991 | Major depressive disorder | rs310501 |
| 5 | 82.96073 | Visceral fat | rs3846635 |
| 5 | 82.98574 | Prostate cancer | rs4466137 |
| 5 | 83.17359 | Ankylosing spondylitis | rs4552569 |
| 5 | 83.26094 | Mitochondrial DNA levels | rs2301070 |

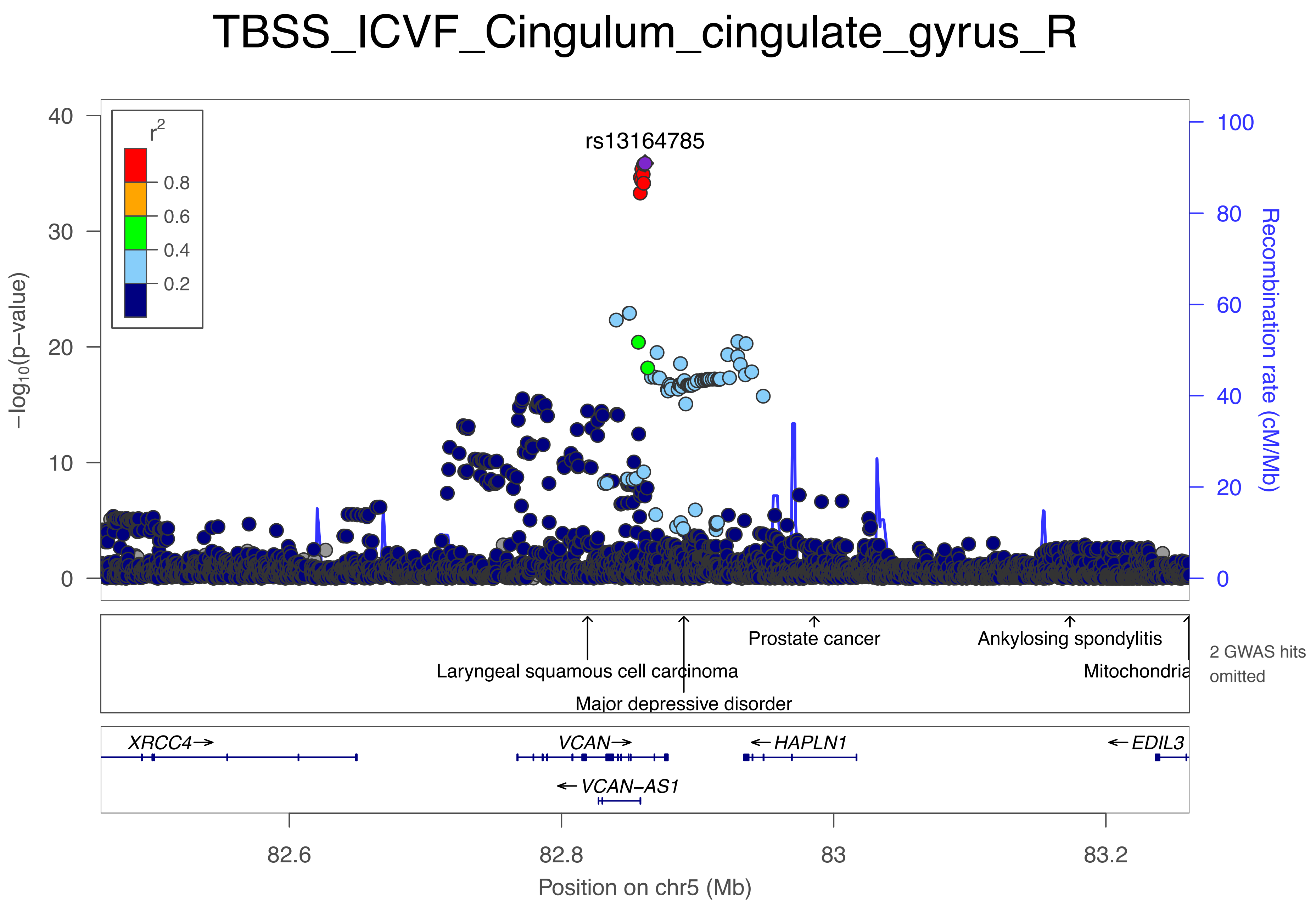

TBSS_ICVF_Cingulum_cingulate_gyrus_R
rs13164785
r2
0.8
0.6
0.4
0.2
−log10(p−value)
Recombination rate (cM/Mb)
Prostate cancer
Ankylosing spondylitis
Laryngeal squamous cell carcinoma
Major depressive disorder
Mitochondria
2 GWAS hits omitted
XRCC4→
VCAN→
←HAPLN1
←EDIL3
←VCAN−AS1
82.6
82.8
83
83.2
Position on chr5 (Mb)

| | |
|---|---|
| date: | Thu Aug 17 17:55:19 2017 |
| build: | hg19 |
| display range: | chr5:82461400−83261400 [82461400−83261400] |
| hilite range: | 0 − 0 [ 0 − 0 ] |
| reference SNP: | chr5:82861400 |
| number of SNPs plotted: | 3396 |
| min P.value: | 1.37E−36 [chr5:82861400] |
| max P.value: | 10E−1 [chr5:83215082] |
| omitted GWAS Hits: | chr5:83.260938−Mitochondrial DNA levels, NA |

Make more plots at http://csg.sph.umich.edu/locuszoom/

GWAS Catalog SNPs in Region

| chr | pos (Mb) | trait | snp |
|---|---|---|---|
| 5 | 82.81912 | Laryngeal squamous cell carcinoma | rs310518 |
| 5 | 82.84549 | Diisocyanate–induced asthma | rs3852186 |
| 5 | 82.88991 | Major depressive disorder | rs310501 |
| 5 | 82.96073 | Visceral fat | rs3846635 |
| 5 | 82.98574 | Prostate cancer | rs4466137 |
| 5 | 83.17359 | Ankylosing spondylitis | rs4552569 |
| 5 | 83.26094 | Mitochondrial DNA levels | rs2301070 |

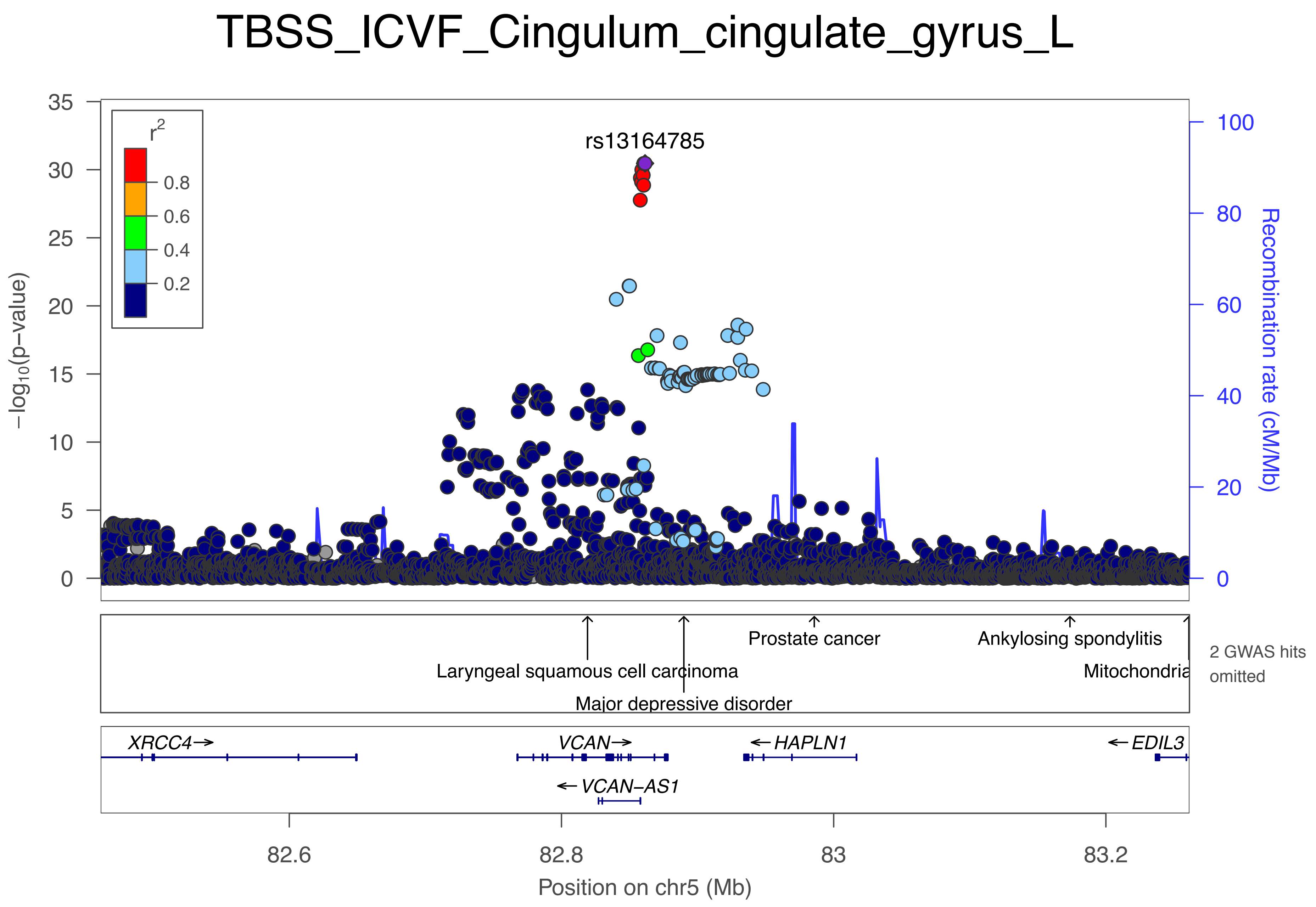
TBSS_ICVF_Cingulum_cingulate_gyrus_L
rs13164785
r2
0.8
0.6
0.4
0.2
−log10(p−value)
Recombination rate (cM/Mb)
Prostate cancer
Ankylosing spondylitis
Laryngeal squamous cell carcinoma
Major depressive disorder
Mitochondria
2 GWAS hits omitted
XRCC4→
VCAN→
←HAPLN1
←EDIL3
←VCAN−AS1
82.6
82.8
83
83.2
Position on chr5 (Mb)

| | |
|---|---|
| date: | Thu Aug 17 17:55:19 2017 |
| build: | hg19 |
| display range: | chr5:82461400−83261400 [82461400−83261400] |
| hilite range: | 0 – 0 [ 0 – 0 ] |
| reference SNP: | chr5:82861400 |
| number of SNPs plotted: | 3396 |
| min P.value: | 3.44E−31 [chr5:82861400] |
| max P.value: | 10E−1 [chr5:82919700] |
| omitted GWAS Hits: | chr5:83.260938−Mitochondrial DNA levels, NA |

Make more plots at http://csg.sph.umich.edu/locuszoom/

GWAS Catalog SNPs in Region

| chr | pos (Mb) | trait | snp |
|---|---|---|---|
| 5 | 82.81912 | Laryngeal squamous cell carcinoma | rs310518 |
| 5 | 82.84549 | Diisocyanate–induced asthma | rs3852186 |
| 5 | 82.88991 | Major depressive disorder | rs310501 |
| 5 | 82.96073 | Visceral fat | rs3846635 |
| 5 | 82.98574 | Prostate cancer | rs4466137 |
| 5 | 83.17359 | Ankylosing spondylitis | rs4552569 |
| 5 | 83.26094 | Mitochondrial DNA levels | rs2301070 |

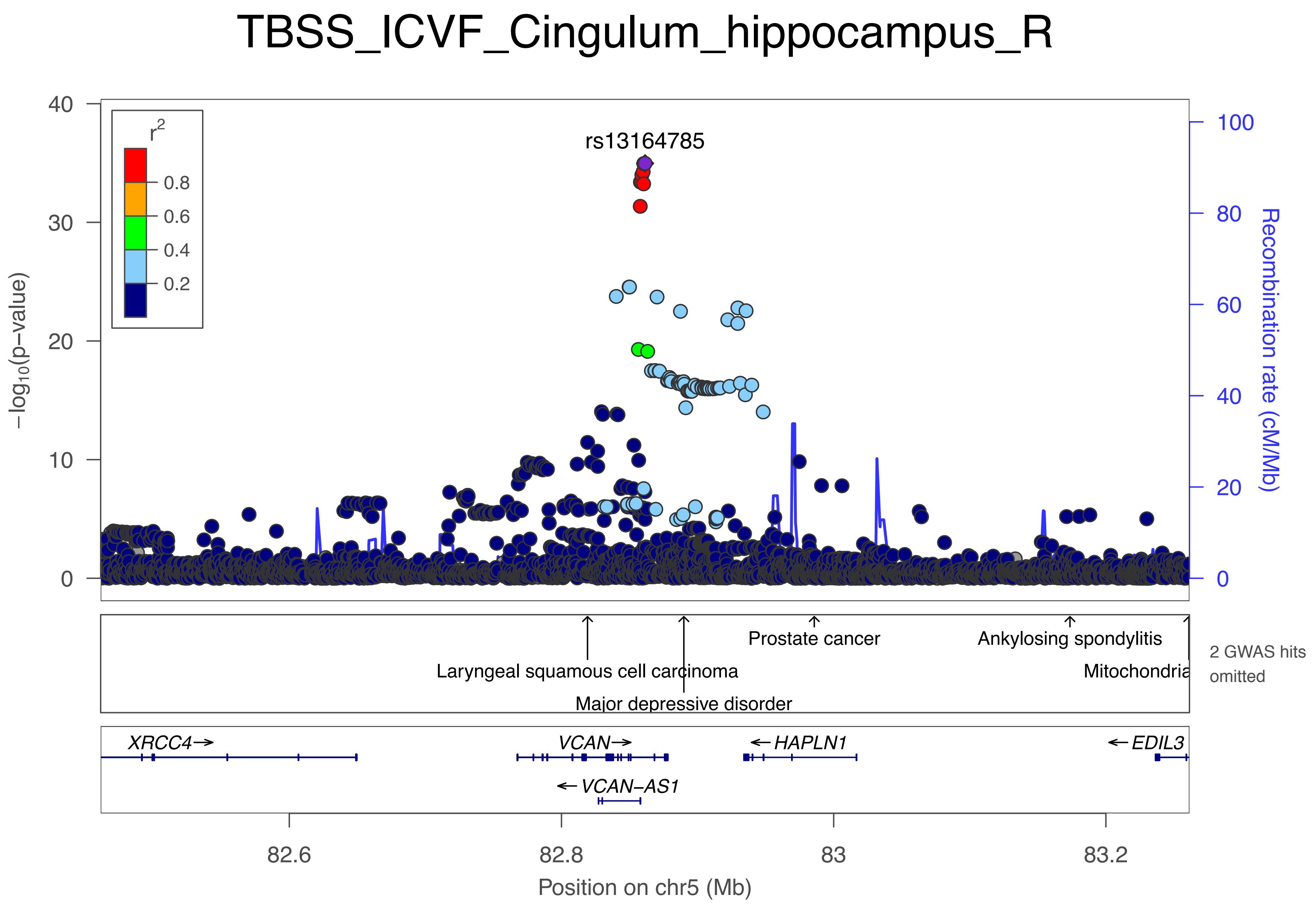
TBSS_ICVF_Cingulum_hippocampus_R
rs13164785
r²
0.8
0.6
0.4
0.2
−log₁₀(p−value)
Recombination rate (cM/Mb)
Prostate cancer
Ankylosing spondylitis
Laryngeal squamous cell carcinoma
Mitochondria
Major depressive disorder
2 GWAS hits omitted
XRCC4
VCAN
HAPLN1
EDIL3
VCAN−AS1
82.6
82.8
83
83.2
Position on chr5 (Mb)

| | |
|---|---|
| date: | Thu Aug 17 17:55:19 2017 |
| build: | hg19 |
| display range: | chr5:82461400–83261400 [82461400–83261400] |
| hilite range: | 0 – 0 [ 0 – 0 ] |
| reference SNP: | chr5:82861400 |
| number of SNPs plotted: | 3396 |
| min P.value: | 1.07E–35 [chr5:82861400] |
| max P.value: | 9.99E–1 [chr5:82510029] |
| omitted GWAS Hits: | chr5:83.260938–Mitochondrial DNA levels, NA |

Make more plots at http://csg.sph.umich.edu/locuszoom/

GWAS Catalog SNPs in Region

| chr | pos (Mb) | trait | snp |
|---|---|---|---|
| 5 | 82.81912 | Laryngeal squamous cell carcinoma | rs310518 |
| 5 | 82.84549 | Diisocyanate–induced asthma | rs3852186 |
| 5 | 82.88991 | Major depressive disorder | rs310501 |
| 5 | 82.96073 | Visceral fat | rs3846635 |
| 5 | 82.98574 | Prostate cancer | rs4466137 |
| 5 | 83.17359 | Ankylosing spondylitis | rs4552569 |
| 5 | 83.26094 | Mitochondrial DNA levels | rs2301070 |

# TBSS_ICVF_Cingulum_hippocampus_L

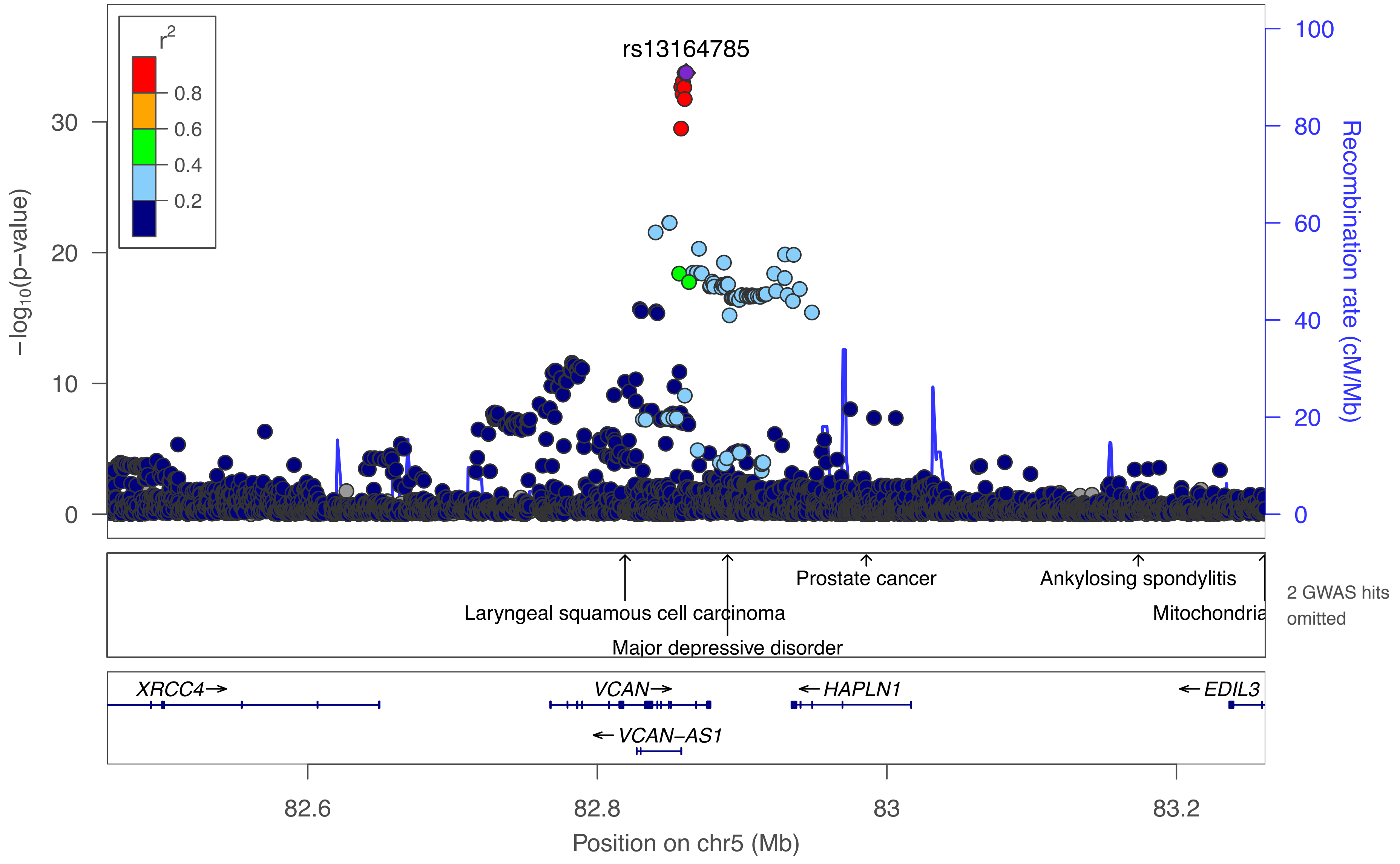

| | |
|---|---|
| date: | Thu Aug 17 17:55:19 2017 |
| build: | hg19 |
| display range: | chr5:82461400–83261400 [82461400–83261400] |
| hilite range: | 0 – 0 [ 0 – 0 ] |
| reference SNP: | chr5:82861400 |
| number of SNPs plotted: | 3396 |
| min P.value: | 1.77E–34 [chr5:82861400] |
| max P.value: | 10E–1 [chr5:83017764] |
| omitted GWAS Hits: | chr5:83.260938–Mitochondrial DNA levels, NA |

Make more plots at http://csg.sph.umich.edu/locuszoom/

GWAS Catalog SNPs in Region

| chr | pos (Mb) | trait | snp |
|---|---|---|---|
| 5 | 82.81912 | Laryngeal squamous cell carcinoma | rs310518 |
| 5 | 82.84549 | Diisocyanate–induced asthma | rs3852186 |
| 5 | 82.88991 | Major depressive disorder | rs310501 |
| 5 | 82.96073 | Visceral fat | rs3846635 |
| 5 | 82.98574 | Prostate cancer | rs4466137 |
| 5 | 83.17359 | Ankylosing spondylitis | rs4552569 |
| 5 | 83.26094 | Mitochondrial DNA levels | rs2301070 |

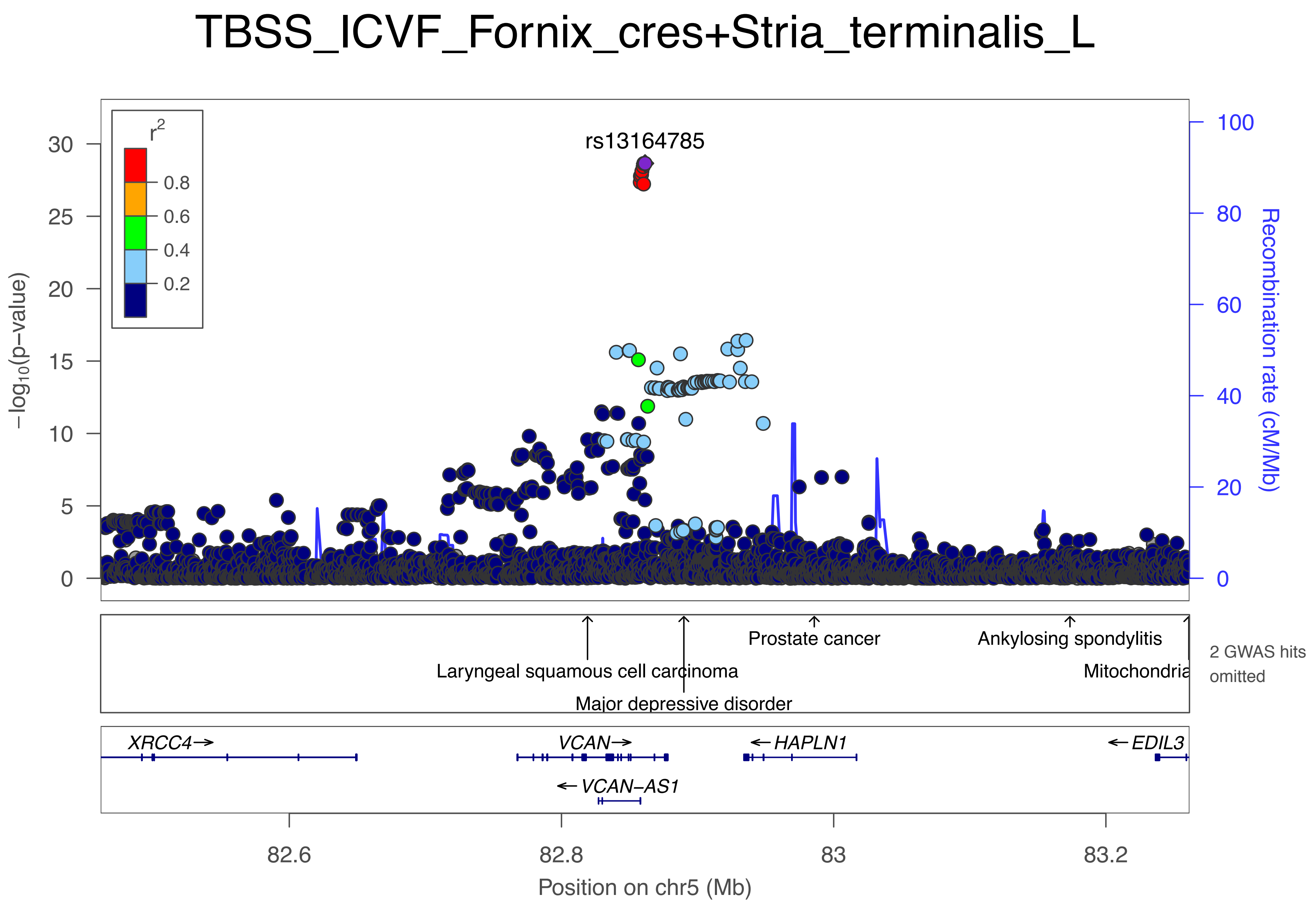

TBSS_ICVF_Fornix_cres+Stria_terminalis_L
rs13164785
$r^2$
0.8
0.6
0.4
0.2
30
25
20
15
10
5
0
$-\log_{10}$(p−value)
100
80
60
40
20
0
Recombination rate (cM/Mb)
Laryngeal squamous cell carcinoma
Major depressive disorder
Prostate cancer
Ankylosing spondylitis
Mitochondria
2 GWAS hits omitted
XRCC4→
VCAN→
←HAPLN1
←EDIL3
←VCAN−AS1
82.6
82.8
83
83.2
Position on chr5 (Mb)

| | |
|---|---|
| date: | Thu Aug 17 17:55:19 2017 |
| build: | hg19 |
| display range: | chr5:82461400−83261400 [82461400−83261400] |
| hilite range: | 0 – 0 [ 0 – 0 ] |
| reference SNP: | chr5:82861400 |
| number of SNPs plotted: | 3396 |
| min P.value: | 2.22E−29 [chr5:82861400] |
| max P.value: | 9.99E−1 [chr5:83092851] |
| omitted GWAS Hits: | chr5:83.260938−Mitochondrial DNA levels, NA |

Make more plots at http://csg.sph.umich.edu/locuszoom/

GWAS Catalog SNPs in Region

| chr | pos (Mb) | trait | snp |
|---|---|---|---|
| 5 | 82.81912 | Laryngeal squamous cell carcinoma | rs310518 |
| 5 | 82.84549 | Diisocyanate−induced asthma | rs3852186 |
| 5 | 82.88991 | Major depressive disorder | rs310501 |
| 5 | 82.96073 | Visceral fat | rs3846635 |
| 5 | 82.98574 | Prostate cancer | rs4466137 |
| 5 | 83.17359 | Ankylosing spondylitis | rs4552569 |
| 5 | 83.26094 | Mitochondrial DNA levels | rs2301070 |

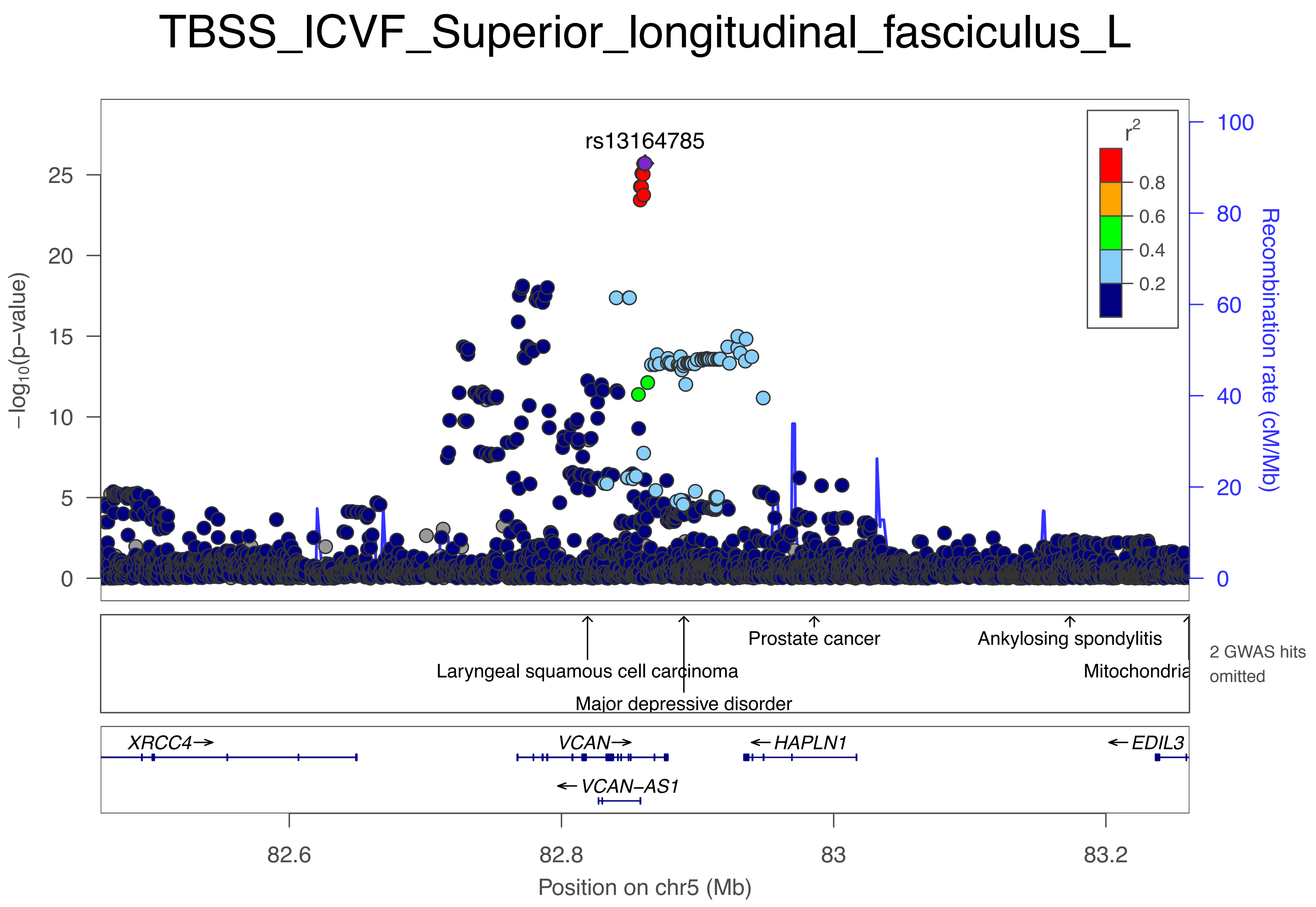
TBSS_ICVF_Superior_longitudinal_fasciculus_L
rs13164785
−log10(p−value)
Recombination rate (cM/Mb)
r2
0.8
0.6
0.4
0.2
Prostate cancer
Ankylosing spondylitis
Laryngeal squamous cell carcinoma
Mitochondria
Major depressive disorder
2 GWAS hits omitted
XRCC4→
VCAN→
←HAPLN1
←EDIL3
←VCAN−AS1
82.6
82.8
83
83.2
Position on chr5 (Mb)

| | |
|---|---|
| date: | Thu Aug 17 18:10:34 2017 |
| build: | hg19 |
| display range: | chr5:82461400–83261400 [82461400–83261400] |
| hilite range: | 0 – 0 [ 0 – 0 ] |
| reference SNP: | chr5:82861400 |
| number of SNPs plotted: | 3396 |
| min P.value: | 1.95E–26 [chr5:82861400] |
| max P.value: | 9.99E–1 [chr5:83169008] |
| omitted GWAS Hits: | chr5:83.260938–Mitochondrial DNA levels, NA |

Make more plots at http://csg.sph.umich.edu/locuszoom/

GWAS Catalog SNPs in Region

| chr | pos (Mb) | trait | snp |
|---|---|---|---|
| 5 | 82.81912 | Laryngeal squamous cell carcinoma | rs310518 |
| 5 | 82.84549 | Diisocyanate−induced asthma | rs3852186 |
| 5 | 82.88991 | Major depressive disorder | rs310501 |
| 5 | 82.96073 | Visceral fat | rs3846635 |
| 5 | 82.98574 | Prostate cancer | rs4466137 |
| 5 | 83.17359 | Ankylosing spondylitis | rs4552569 |
| 5 | 83.26094 | Mitochondrial DNA levels | rs2301070 |

# TBSS_ICVF_Superior_fronto−occipital_fasciculus_R

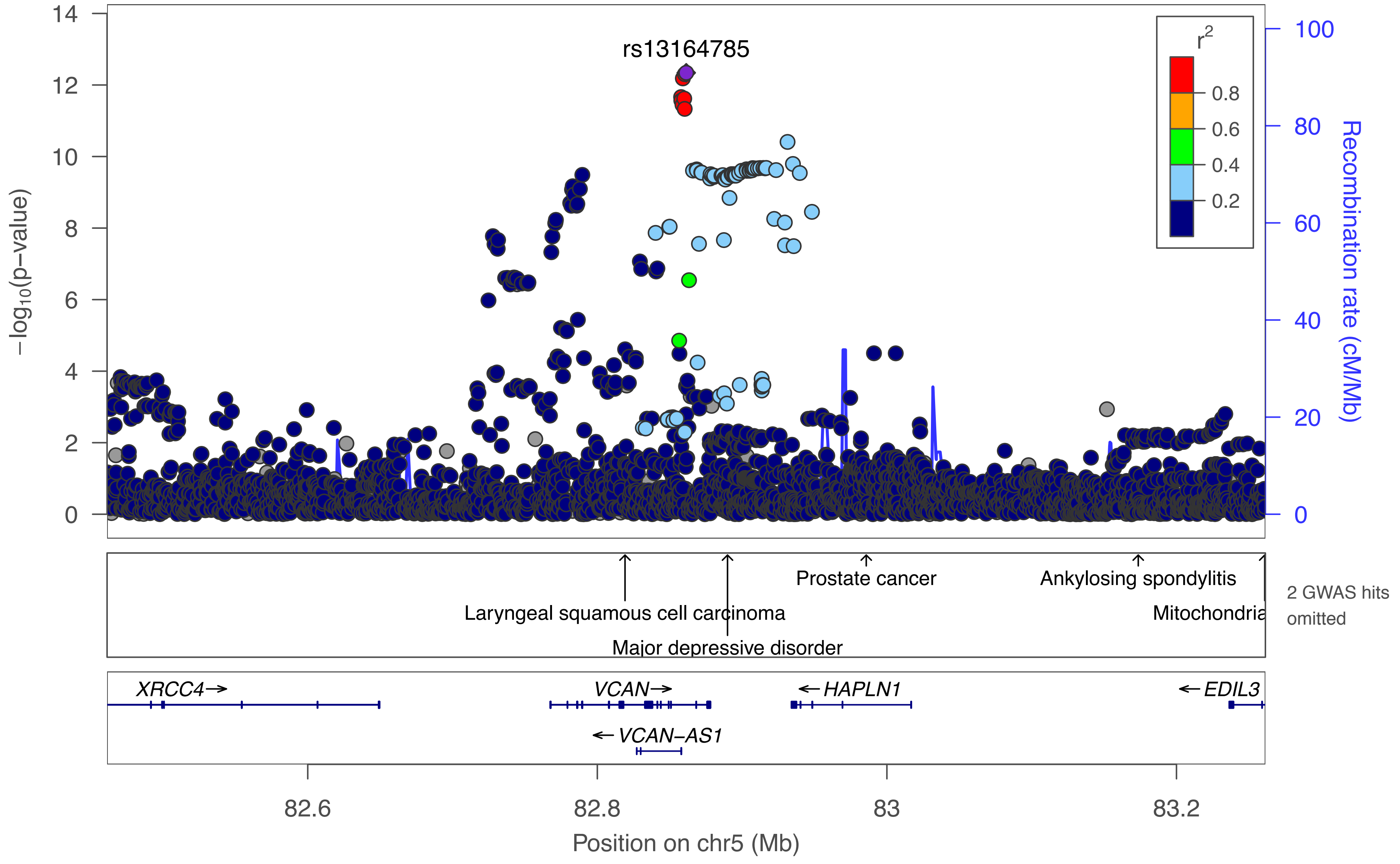

| | |
|---|---|
| date: | Thu Aug 17 17:55:19 2017 |
| build: | hg19 |
| display range: | chr5:82461400−83261400 [82461400−83261400] |
| hilite range: | 0 − 0 [ 0 − 0 ] |
| reference SNP: | chr5:82861400 |
| number of SNPs plotted: | 3396 |
| min P.value: | 4.56E−13 [chr5:82861400] |
| max P.value: | 10E−1 [chr5:82712592] |
| omitted GWAS Hits: | chr5:83.260938−Mitochondrial DNA levels, NA |

Make more plots at http://csg.sph.umich.edu/locuszoom/

GWAS Catalog SNPs in Region

| chr | pos (Mb) | trait | snp |
|---|---|---|---|
| 5 | 82.81912 | Laryngeal squamous cell carcinoma | rs310518 |
| 5 | 82.84549 | Diisocyanate–induced asthma | rs3852186 |
| 5 | 82.88991 | Major depressive disorder | rs310501 |
| 5 | 82.96073 | Visceral fat | rs3846635 |
| 5 | 82.98574 | Prostate cancer | rs4466137 |
| 5 | 83.17359 | Ankylosing spondylitis | rs4552569 |
| 5 | 83.26094 | Mitochondrial DNA levels | rs2301070 |

# TBSS_ICVF_Uncinate_fasciculus_R

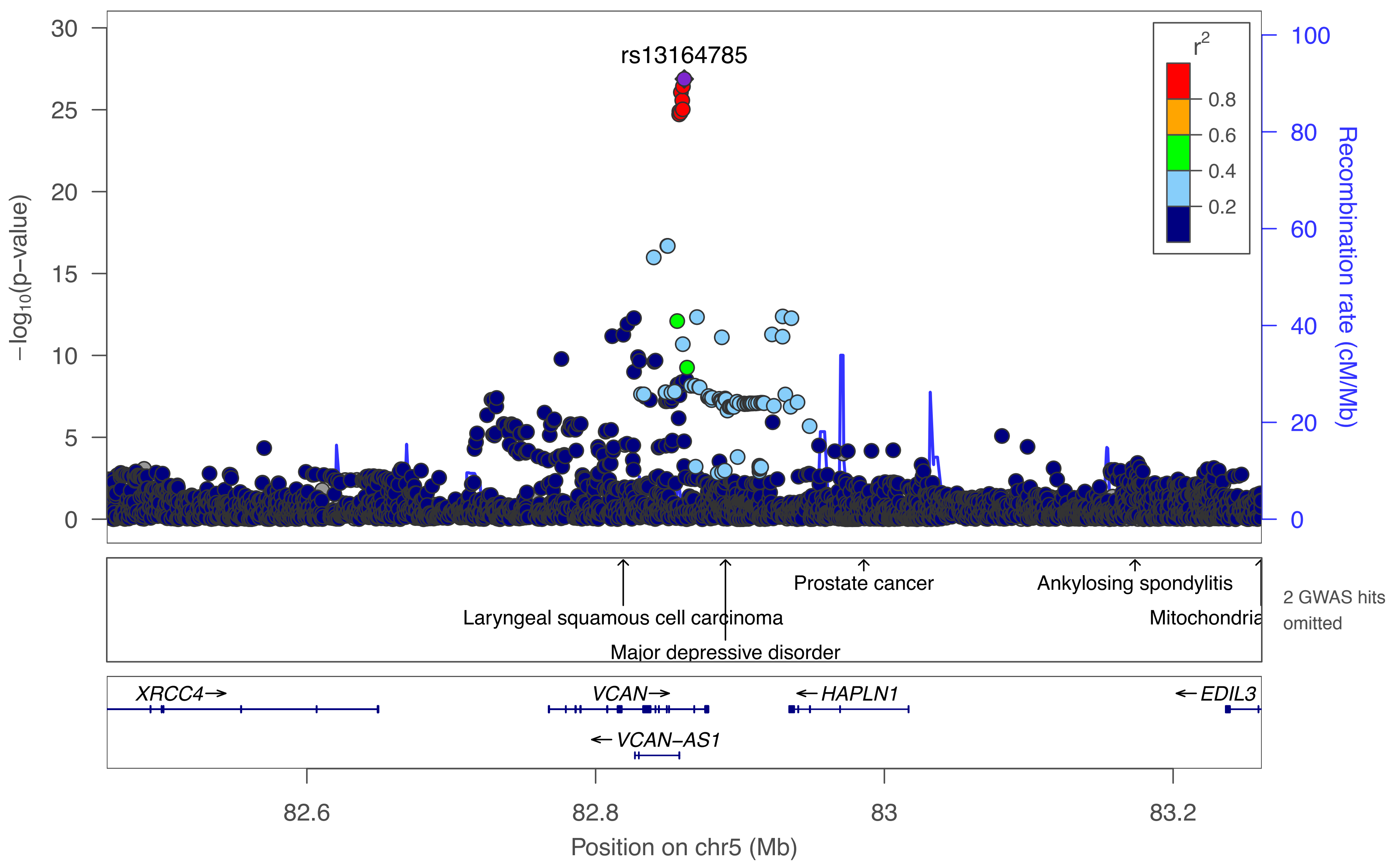

| | |
|---|---|
| date: | Thu Aug 17 17:58:11 2017 |
| build: | hg19 |
| display range: | chr5:82461400–83261400 [82461400–83261400] |
| hilite range: | 0 – 0 [ 0 – 0 ] |
| reference SNP: | chr5:82861400 |
| number of SNPs plotted: | 3396 |
| min P.value: | 1.32E–27 [chr5:82861400] |
| max P.value: | 9.99E–1 [chr5:82973210] |
| omitted GWAS Hits: | chr5:83.260938–Mitochondrial DNA levels, NA |

Make more plots at http://csg.sph.umich.edu/locuszoom/

GWAS Catalog SNPs in Region

| chr | pos (Mb) | trait | snp |
|---|---|---|---|
| 5 | 82.81912 | Laryngeal squamous cell carcinoma | rs310518 |
| 5 | 82.84549 | Diisocyanate−induced asthma | rs3852186 |
| 5 | 82.88991 | Major depressive disorder | rs310501 |
| 5 | 82.96073 | Visceral fat | rs3846635 |
| 5 | 82.98574 | Prostate cancer | rs4466137 |
| 5 | 83.17359 | Ankylosing spondylitis | rs4552569 |
| 5 | 83.26094 | Mitochondrial DNA levels | rs2301070 |

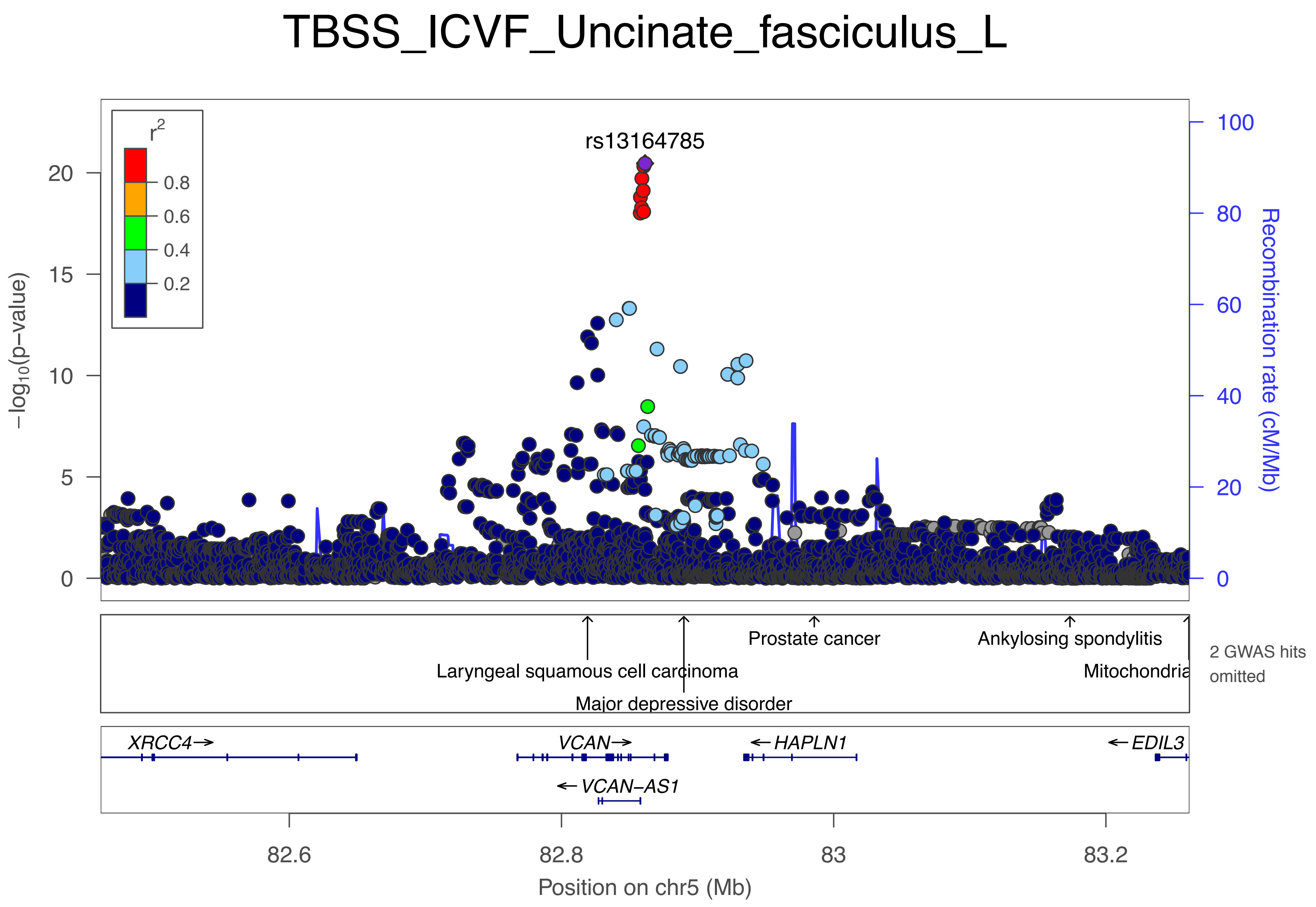
TBSS_ICVF_Uncinate_fasciculus_L
rs13164785
r2
0.8
0.6
0.4
0.2
−log10(p−value)
Recombination rate (cM/Mb)
Laryngeal squamous cell carcinoma
Major depressive disorder
Prostate cancer
Ankylosing spondylitis
Mitochondria
2 GWAS hits omitted
XRCC4→
VCAN→
←HAPLN1
←EDIL3
←VCAN−AS1
82.6
82.8
83
83.2
Position on chr5 (Mb)

| | |
|---|---|
| date: | Thu Aug 17 17:58:11 2017 |
| build: | hg19 |
| display range: | chr5:82461400−83261400 [82461400−83261400] |
| hilite range: | 0 − 0 [ 0 − 0 ] |
| reference SNP: | chr5:82861400 |
| number of SNPs plotted: | 3396 |
| min P.value: | 3.43E−21 [chr5:82861400] |
| max P.value: | 10E−1 [chr5:83117229] |
| omitted GWAS Hits: | chr5:83.260938−Mitochondrial DNA levels, NA |

Make more plots at http://csg.sph.umich.edu/locuszoom/

GWAS Catalog SNPs in Region

| chr | pos (Mb) | trait | snp |
|---|---|---|---|
| 5 | 82.81912 | Laryngeal squamous cell carcinoma | rs310518 |
| 5 | 82.84549 | Diisocyanate–induced asthma | rs3852186 |
| 5 | 82.88991 | Major depressive disorder | rs310501 |
| 5 | 82.96073 | Visceral fat | rs3846635 |
| 5 | 82.98574 | Prostate cancer | rs4466137 |
| 5 | 83.17359 | Ankylosing spondylitis | rs4552569 |
| 5 | 83.26094 | Mitochondrial DNA levels | rs2301070 |

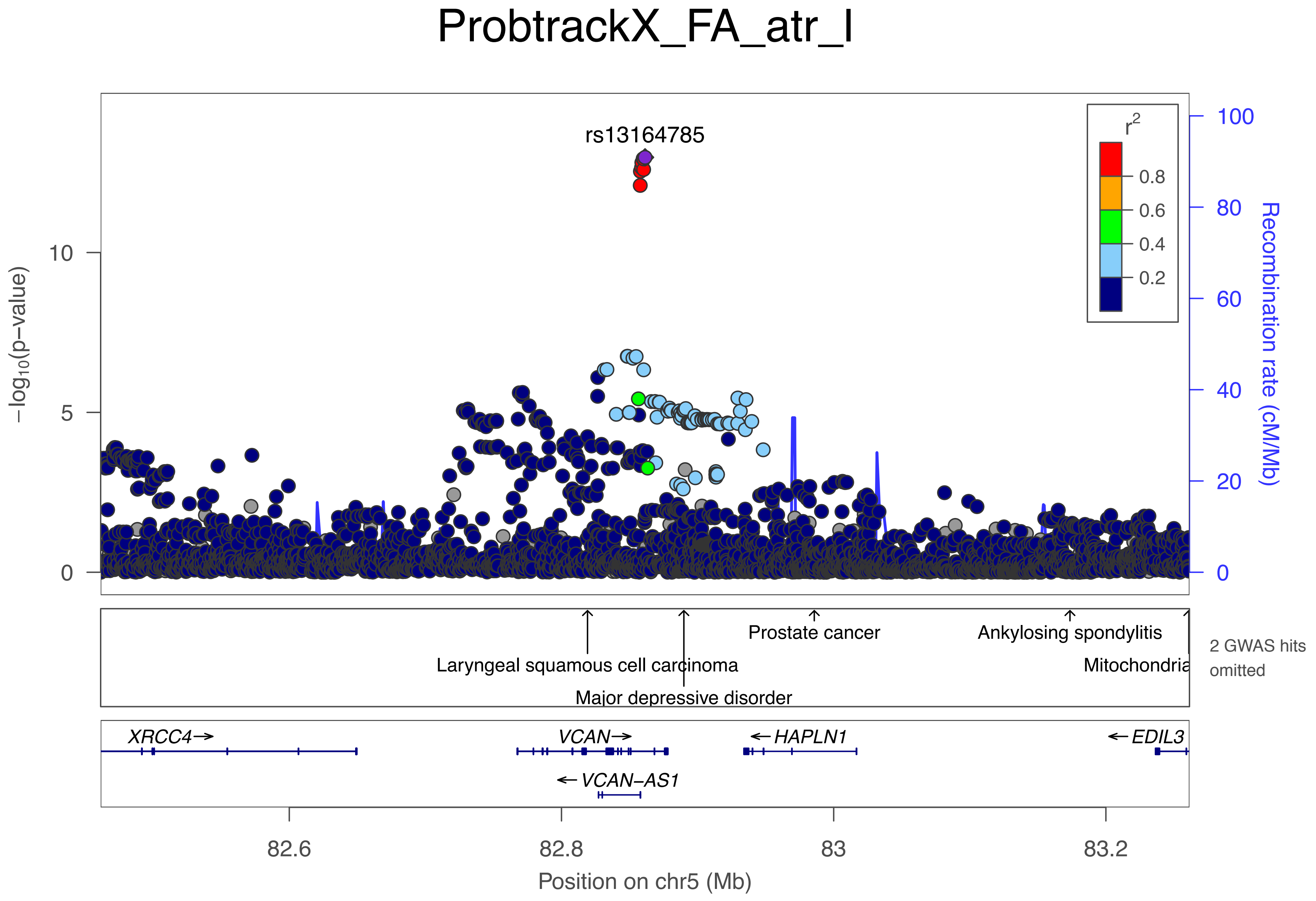

ProbtrackX_FA_atr_l
rs13164785
r2
0.8
0.6
0.4
0.2
−log10(p−value)
Recombination rate (cM/Mb)
10
5
0
100
80
60
40
20
0
Laryngeal squamous cell carcinoma
Major depressive disorder
Prostate cancer
Ankylosing spondylitis
Mitochondria
2 GWAS hits omitted
XRCC4→
VCAN→
←HAPLN1
←EDIL3
←VCAN−AS1
82.6
82.8
83
83.2
Position on chr5 (Mb)

| | |
|---|---|
| date: | Thu Aug 17 17:58:11 2017 |
| build: | hg19 |
| display range: | chr5:82461400–83261400 [82461400–83261400] |
| hilite range: | 0 – 0 [ 0 – 0 ] |
| reference SNP: | chr5:82861400 |
| number of SNPs plotted: | 3396 |
| min P.value: | 1.05E−13 [chr5:82861400] |
| max P.value: | 10E−1 [chr5:82994521] |
| omitted GWAS Hits: | chr5:83.260938–Mitochondrial DNA levels, NA |

Make more plots at http://csg.sph.umich.edu/locuszoom/

GWAS Catalog SNPs in Region

| chr | pos (Mb) | trait | snp |
|---|---|---|---|
| 5 | 82.81912 | Laryngeal squamous cell carcinoma | rs310518 |
| 5 | 82.84549 | Diisocyanate–induced asthma | rs3852186 |
| 5 | 82.88991 | Major depressive disorder | rs310501 |
| 5 | 82.96073 | Visceral fat | rs3846635 |
| 5 | 82.98574 | Prostate cancer | rs4466137 |
| 5 | 83.17359 | Ankylosing spondylitis | rs4552569 |
| 5 | 83.26094 | Mitochondrial DNA levels | rs2301070 |

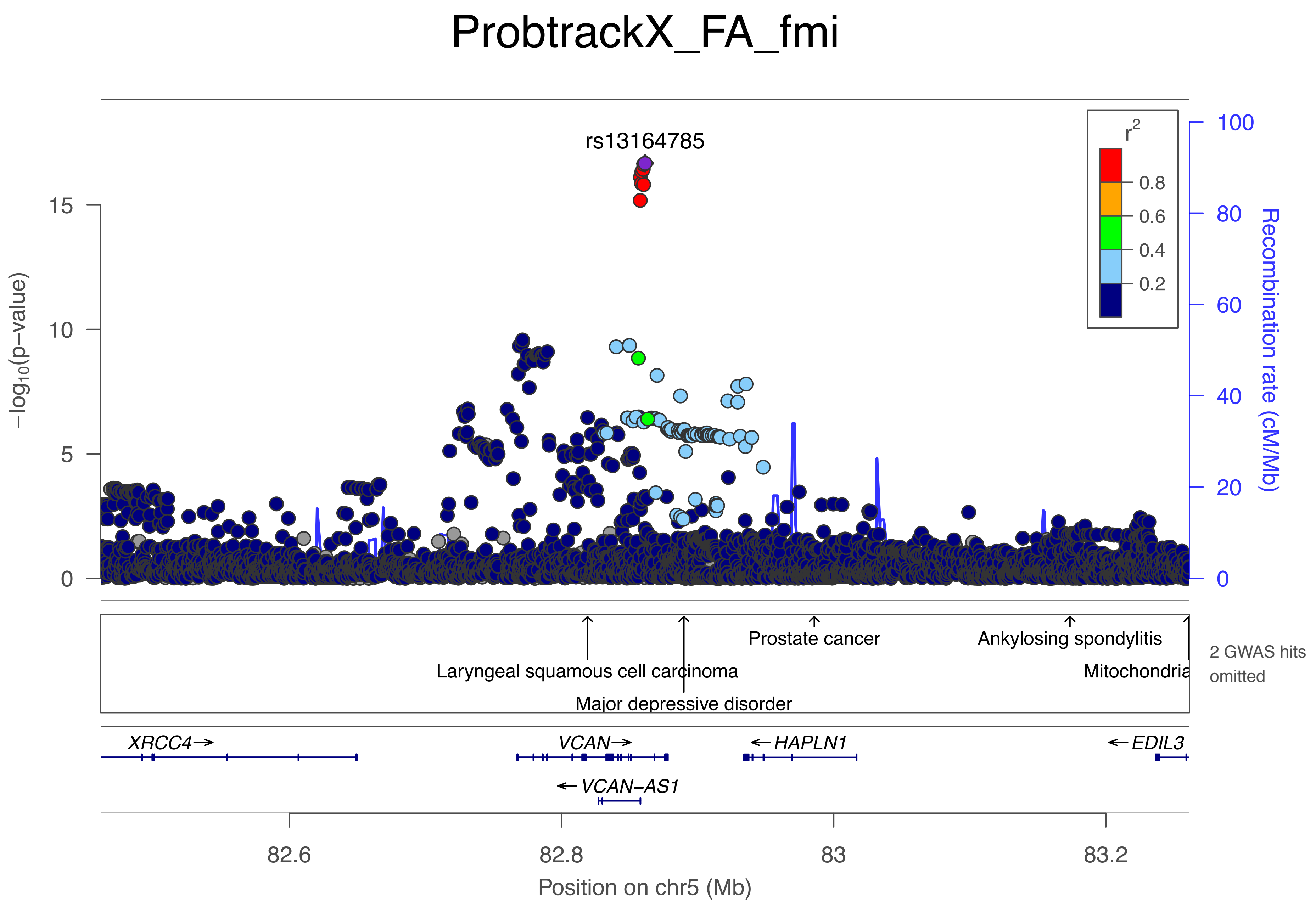

ProbtrackX_FA_fmi
rs13164785
−log10(p−value)
Recombination rate (cM/Mb)
r2
0.8
0.6
0.4
0.2
Prostate cancer
Ankylosing spondylitis
Laryngeal squamous cell carcinoma
Mitochondria
Major depressive disorder
2 GWAS hits omitted
XRCC4
VCAN
HAPLN1
EDIL3
VCAN−AS1
82.6
82.8
83
83.2
Position on chr5 (Mb)

| | |
|---|---|
| date: | Thu Aug 17 17:58:11 2017 |
| build: | hg19 |
| display range: | chr5:82461400−83261400 [82461400−83261400] |
| hilite range: | 0 – 0 [ 0 – 0 ] |
| reference SNP: | chr5:82861400 |
| number of SNPs plotted: | 3396 |
| min P.value: | 2.12E−17 [chr5:82861400] |
| max P.value: | 9.99E−1 [chr5:82642418] |
| omitted GWAS Hits: | chr5:83.260938−Mitochondrial DNA levels, NA |

Make more plots at http://csg.sph.umich.edu/locuszoom/

GWAS Catalog SNPs in Region

| chr | pos (Mb) | trait | snp |
|---|---|---|---|
| 5 | 82.81912 | Laryngeal squamous cell carcinoma | rs310518 |
| 5 | 82.84549 | Diisocyanate–induced asthma | rs3852186 |
| 5 | 82.88991 | Major depressive disorder | rs310501 |
| 5 | 82.96073 | Visceral fat | rs3846635 |
| 5 | 82.98574 | Prostate cancer | rs4466137 |
| 5 | 83.17359 | Ankylosing spondylitis | rs4552569 |
| 5 | 83.26094 | Mitochondrial DNA levels | rs2301070 |

# ProbtrackX_FA_unc_r

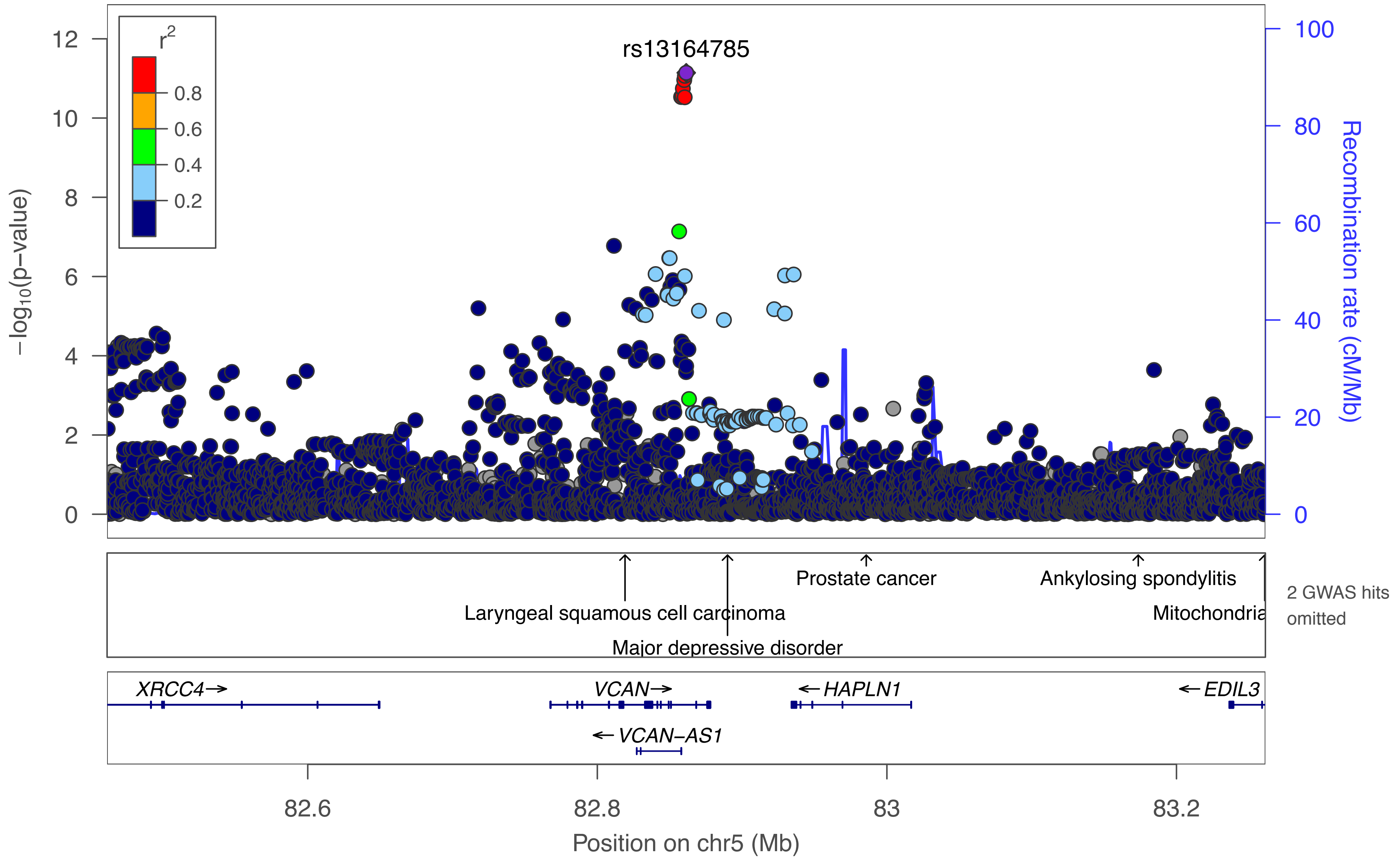

| | |
|---|---|
| date: | Thu Aug 17 17:58:11 2017 |
| build: | hg19 |
| display range: | chr5:82461400–83261400 [82461400–83261400] |
| hilite range: | 0 – 0 [ 0 – 0 ] |
| reference SNP: | chr5:82861400 |
| number of SNPs plotted: | 3396 |
| min P.value: | 7.21E–12 [chr5:82861400] |
| max P.value: | 10E–1 [chr5:83260593] |
| omitted GWAS Hits: | chr5:83.260938–Mitochondrial DNA levels, NA |

Make more plots at http://csg.sph.umich.edu/locuszoom/

GWAS Catalog SNPs in Region

| chr | pos (Mb) | trait | snp |
|---|---|---|---|
| 5 | 82.81912 | Laryngeal squamous cell carcinoma | rs310518 |
| 5 | 82.84549 | Diisocyanate–induced asthma | rs3852186 |
| 5 | 82.88991 | Major depressive disorder | rs310501 |
| 5 | 82.96073 | Visceral fat | rs3846635 |
| 5 | 82.98574 | Prostate cancer | rs4466137 |
| 5 | 83.17359 | Ankylosing spondylitis | rs4552569 |
| 5 | 83.26094 | Mitochondrial DNA levels | rs2301070 |

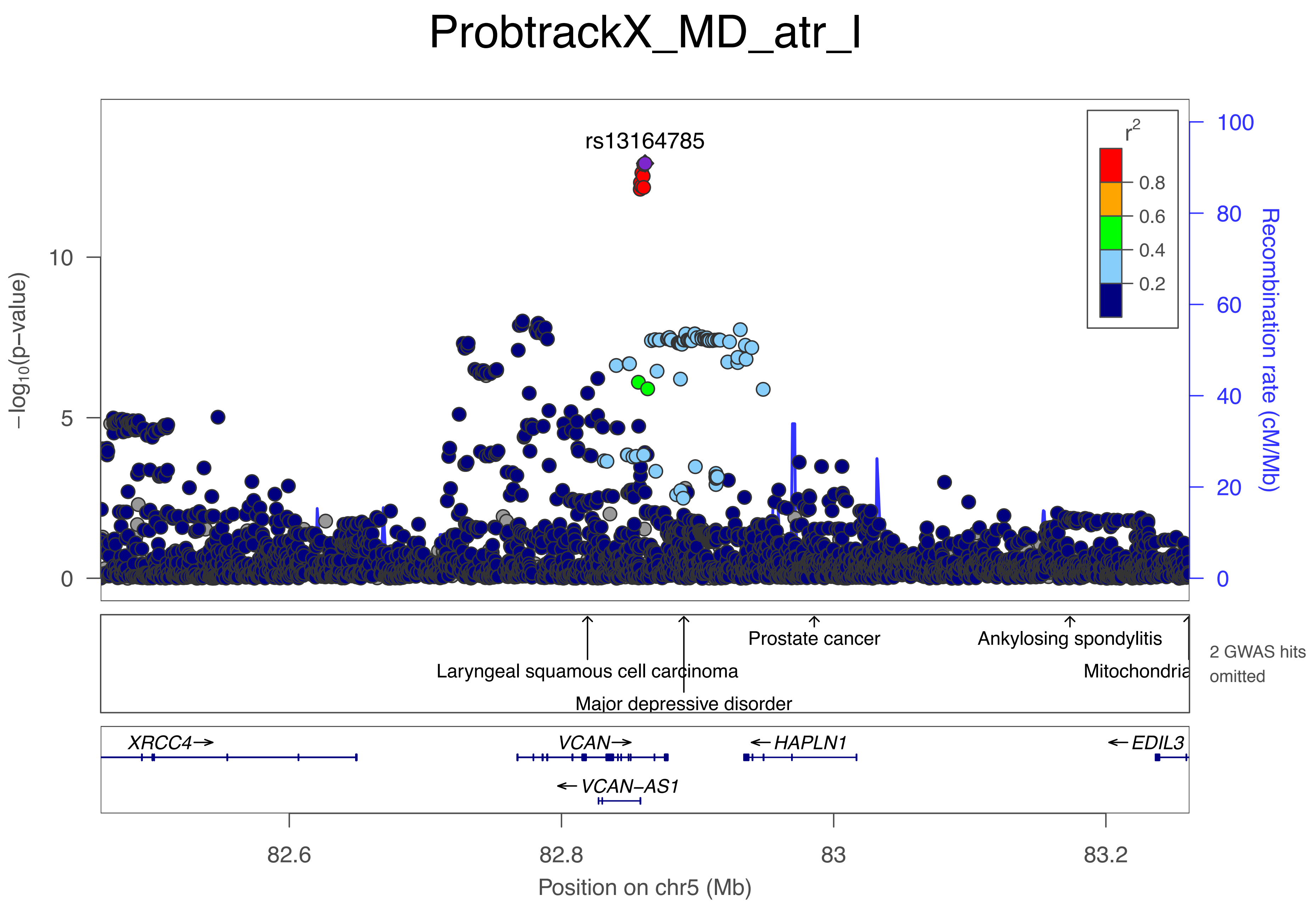

ProbtrackX_MD_atr_l
rs13164785
−log10(p−value)
Recombination rate (cM/Mb)
r2
0.8
0.6
0.4
0.2
Laryngeal squamous cell carcinoma
Major depressive disorder
Prostate cancer
Ankylosing spondylitis
Mitochondria
2 GWAS hits omitted
XRCC4→
VCAN→
←VCAN−AS1
←HAPLN1
←EDIL3
82.6
82.8
83
83.2
Position on chr5 (Mb)

| | |
|---|---|
| date: | Thu Aug 17 17:58:11 2017 |
| build: | hg19 |
| display range: | chr5:82461400−83261400 [82461400−83261400] |
| hilite range: | 0 – 0 [ 0 – 0 ] |
| reference SNP: | chr5:82861400 |
| number of SNPs plotted: | 3396 |
| min P.value: | 1.2E−13 [chr5:82861400] |
| max P.value: | 10E−1 [chr5:83165521] |
| omitted GWAS Hits: | chr5:83.260938−Mitochondrial DNA levels, NA |

Make more plots at http://csg.sph.umich.edu/locuszoom/

GWAS Catalog SNPs in Region

| chr | pos (Mb) | trait | snp |
|---|---|---|---|
| 5 | 82.81912 | Laryngeal squamous cell carcinoma | rs310518 |
| 5 | 82.84549 | Diisocyanate–induced asthma | rs3852186 |
| 5 | 82.88991 | Major depressive disorder | rs310501 |
| 5 | 82.96073 | Visceral fat | rs3846635 |
| 5 | 82.98574 | Prostate cancer | rs4466137 |
| 5 | 83.17359 | Ankylosing spondylitis | rs4552569 |
| 5 | 83.26094 | Mitochondrial DNA levels | rs2301070 |

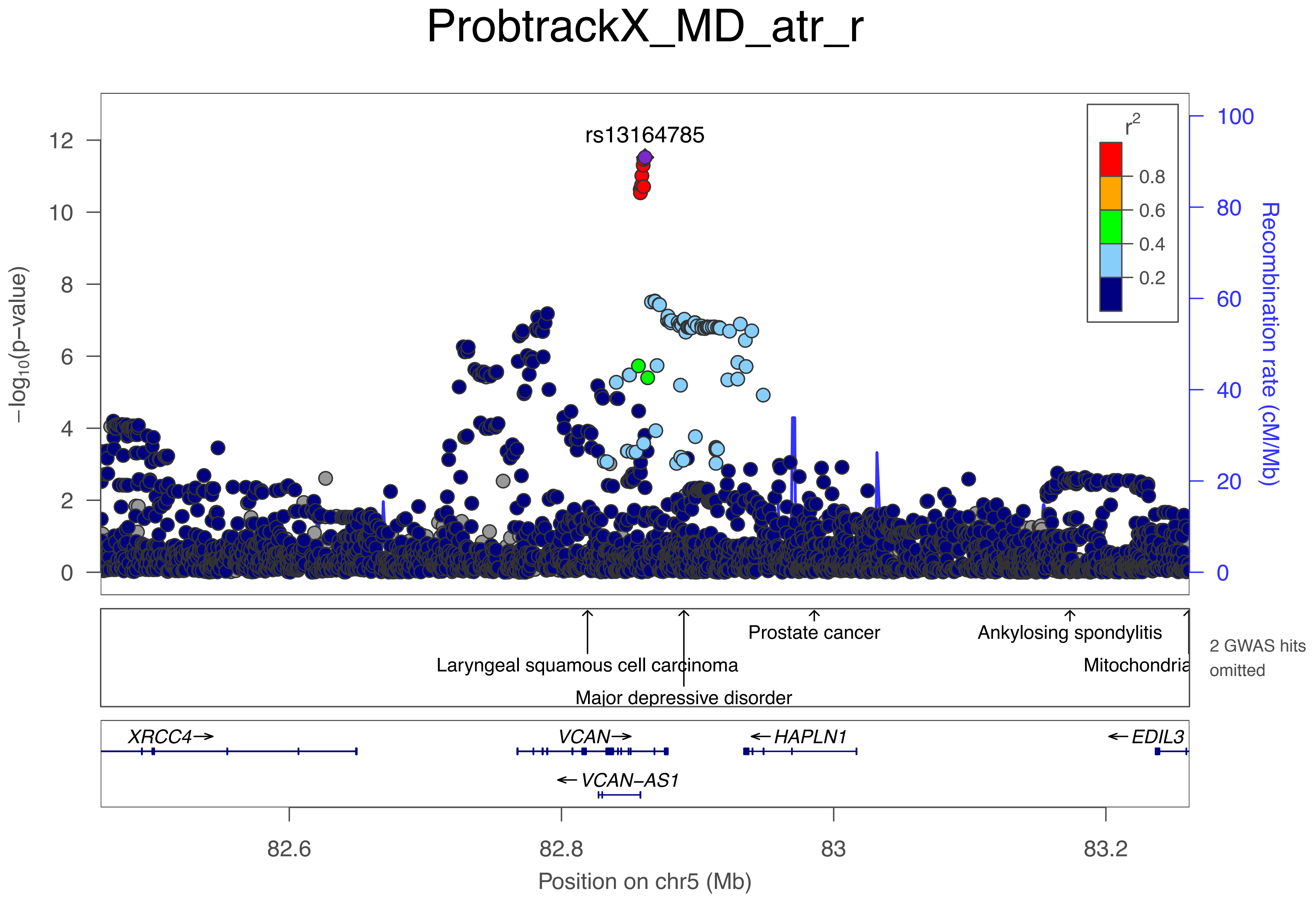
ProbtrackX_MD_atr_r
rs13164785
r²
0.8
0.6
0.4
0.2
−log10(p−value)
Recombination rate (cM/Mb)
Prostate cancer
Ankylosing spondylitis
Laryngeal squamous cell carcinoma
Mitochondria
2 GWAS hits omitted
Major depressive disorder
XRCC4→
VCAN→
←HAPLN1
←EDIL3
←VCAN−AS1
82.6
82.8
83
83.2
Position on chr5 (Mb)

| | |
|---|---|
| date: | Thu Aug 17 17:58:11 2017 |
| build: | hg19 |
| display range: | chr5:82461400−83261400 [82461400−83261400] |
| hilite range: | 0 – 0 [ 0 – 0 ] |
| reference SNP: | chr5:82861400 |
| number of SNPs plotted: | 3396 |
| min P.value: | 3.01E−12 [chr5:82861400] |
| max P.value: | 10E−1 [chr5:82773484] |
| omitted GWAS Hits: | chr5:83.260938−Mitochondrial DNA levels, NA |

Make more plots at http://csg.sph.umich.edu/locuszoom/

GWAS Catalog SNPs in Region

| chr | pos (Mb) | trait | snp |
|---|---|---|---|
| 5 | 82.81912 | Laryngeal squamous cell carcinoma | rs310518 |
| 5 | 82.84549 | Diisocyanate–induced asthma | rs3852186 |
| 5 | 82.88991 | Major depressive disorder | rs310501 |
| 5 | 82.96073 | Visceral fat | rs3846635 |
| 5 | 82.98574 | Prostate cancer | rs4466137 |
| 5 | 83.17359 | Ankylosing spondylitis | rs4552569 |
| 5 | 83.26094 | Mitochondrial DNA levels | rs2301070 |

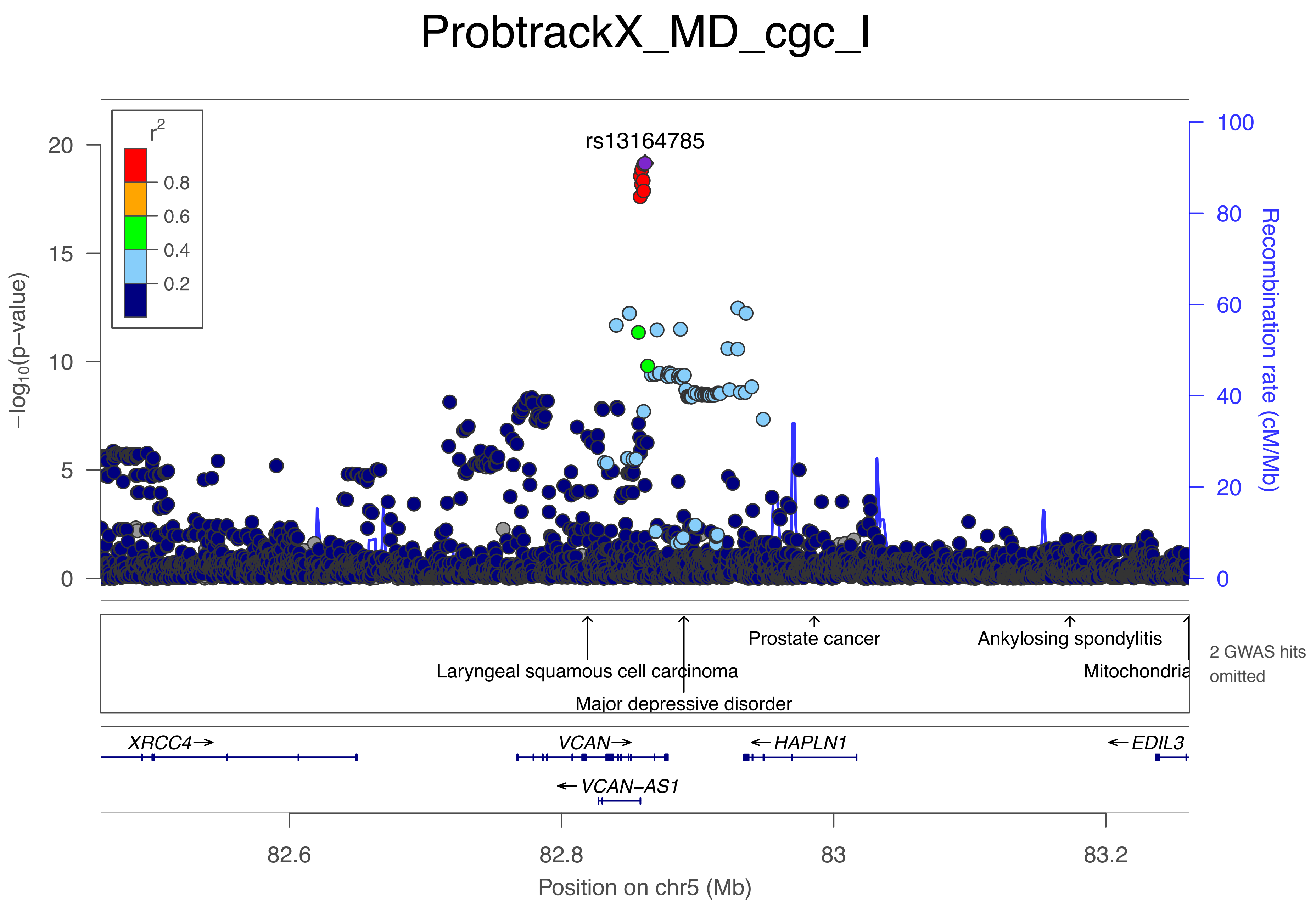

ProbtrackX_MD_cgc_l
rs13164785
r²
0.8
0.6
0.4
0.2
20
15
10
5
0
−log10(p−value)
100
80
60
40
20
0
Recombination rate (cM/Mb)
Laryngeal squamous cell carcinoma
Major depressive disorder
Prostate cancer
Ankylosing spondylitis
Mitochondria
2 GWAS hits omitted
XRCC4→
VCAN→
←VCAN−AS1
←HAPLN1
←EDIL3
82.6
82.8
83
83.2
Position on chr5 (Mb)

| | |
|---|---|
| date: | Thu Aug 17 17:58:11 2017 |
| build: | hg19 |
| display range: | chr5:82461400–83261400 [82461400–83261400] |
| hilite range: | 0 – 0 [ 0 – 0 ] |
| reference SNP: | chr5:82861400 |
| number of SNPs plotted: | 3396 |
| min P.value: | 7.18E–20 [chr5:82861400] |
| max P.value: | 10E–1 [chr5:82958821] |
| omitted GWAS Hits: | chr5:83.260938–Mitochondrial DNA levels, NA |

Make more plots at http://csg.sph.umich.edu/locuszoom/

GWAS Catalog SNPs in Region

| chr | pos (Mb) | trait | snp |
|---|---|---|---|
| 5 | 82.81912 | Laryngeal squamous cell carcinoma | rs310518 |
| 5 | 82.84549 | Diisocyanate–induced asthma | rs3852186 |
| 5 | 82.88991 | Major depressive disorder | rs310501 |
| 5 | 82.96073 | Visceral fat | rs3846635 |
| 5 | 82.98574 | Prostate cancer | rs4466137 |
| 5 | 83.17359 | Ankylosing spondylitis | rs4552569 |
| 5 | 83.26094 | Mitochondrial DNA levels | rs2301070 |

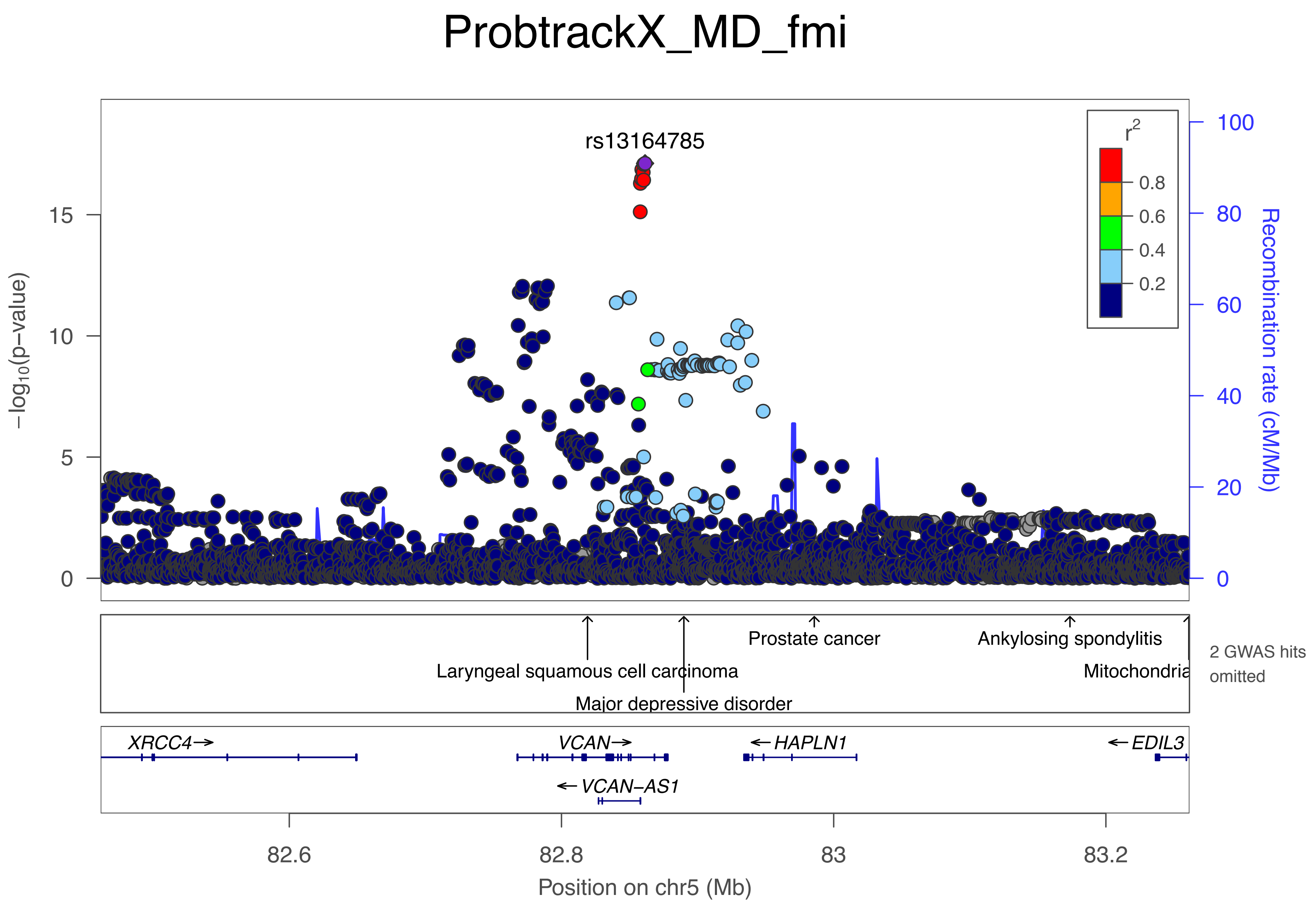

ProbtrackX_MD_fmi
rs13164785
−log10(p−value)
Recombination rate (cM/Mb)
r2
0.8
0.6
0.4
0.2
0
5
10
15
0
20
40
60
80
100
Laryngeal squamous cell carcinoma
Major depressive disorder
Prostate cancer
Ankylosing spondylitis
Mitochondria
2 GWAS hits omitted
XRCC4→
VCAN→
←VCAN−AS1
←HAPLN1
←EDIL3
82.6
82.8
83
83.2
Position on chr5 (Mb)

| | |
|---|---|
| date: | Thu Aug 17 17:58:11 2017 |
| build: | hg19 |
| display range: | chr5:82461400–83261400 [82461400–83261400] |
| hilite range: | 0 – 0 [ 0 – 0 ] |
| reference SNP: | chr5:82861400 |
| number of SNPs plotted: | 3396 |
| min P.value: | 7.62E–18 [chr5:82861400] |
| max P.value: | 10E–1 [chr5:82826483] |
| omitted GWAS Hits: | chr5:83.260938–Mitochondrial DNA levels, NA |

Make more plots at http://csg.sph.umich.edu/locuszoom/

GWAS Catalog SNPs in Region

| chr | pos (Mb) | trait | snp |
|---|---|---|---|
| 5 | 82.81912 | Laryngeal squamous cell carcinoma | rs310518 |
| 5 | 82.84549 | Diisocyanate–induced asthma | rs3852186 |
| 5 | 82.88991 | Major depressive disorder | rs310501 |
| 5 | 82.96073 | Visceral fat | rs3846635 |
| 5 | 82.98574 | Prostate cancer | rs4466137 |
| 5 | 83.17359 | Ankylosing spondylitis | rs4552569 |
| 5 | 83.26094 | Mitochondrial DNA levels | rs2301070 |

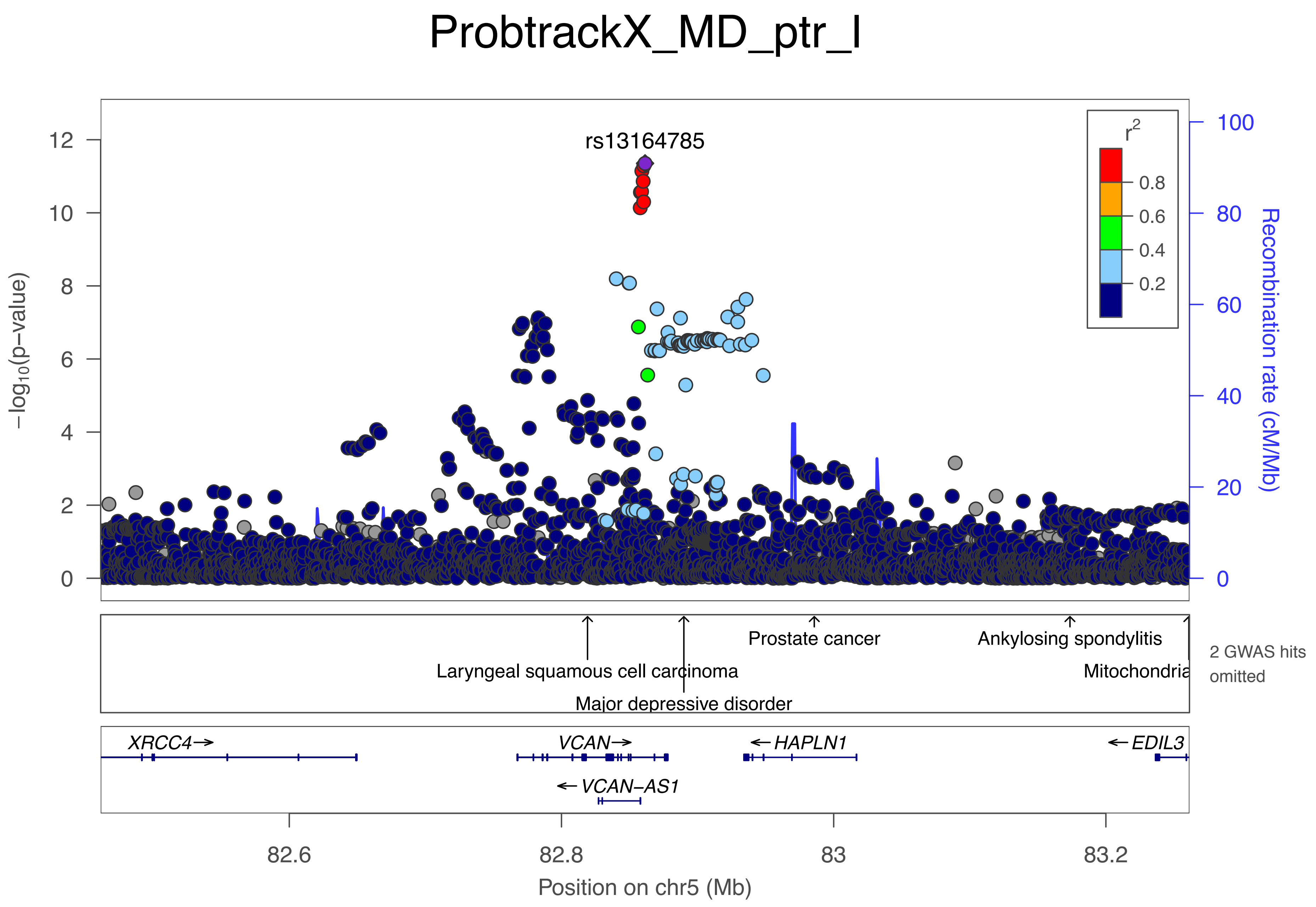
ProbtrackX_MD_ptr_l
rs13164785
−log10(p−value)
Recombination rate (cM/Mb)
r2
0.8
0.6
0.4
0.2
12
10
8
6
4
2
0
100
80
60
40
20
0
Prostate cancer
Ankylosing spondylitis
Laryngeal squamous cell carcinoma
Mitochondria
Major depressive disorder
2 GWAS hits omitted
XRCC4→
VCAN→
←HAPLN1
←EDIL3
←VCAN−AS1
82.6
82.8
83
83.2
Position on chr5 (Mb)

| | |
|---|---|
| date: | Thu Aug 17 17:58:11 2017 |
| build: | hg19 |
| display range: | chr5:82461400−83261400 [82461400−83261400] |
| hilite range: | 0 − 0 [ 0 − 0 ] |
| reference SNP: | chr5:82861400 |
| number of SNPs plotted: | 3396 |
| min P.value: | 4.44E−12 [chr5:82861400] |
| max P.value: | 9.99E−1 [chr5:82874942] |
| omitted GWAS Hits: | chr5:83.260938−Mitochondrial DNA levels, NA |

Make more plots at http://csg.sph.umich.edu/locuszoom/

GWAS Catalog SNPs in Region

| chr | pos (Mb) | trait | snp |
|---|---|---|---|
| 5 | 82.81912 | Laryngeal squamous cell carcinoma | rs310518 |
| 5 | 82.84549 | Diisocyanate−induced asthma | rs3852186 |
| 5 | 82.88991 | Major depressive disorder | rs310501 |
| 5 | 82.96073 | Visceral fat | rs3846635 |
| 5 | 82.98574 | Prostate cancer | rs4466137 |
| 5 | 83.17359 | Ankylosing spondylitis | rs4552569 |
| 5 | 83.26094 | Mitochondrial DNA levels | rs2301070 |

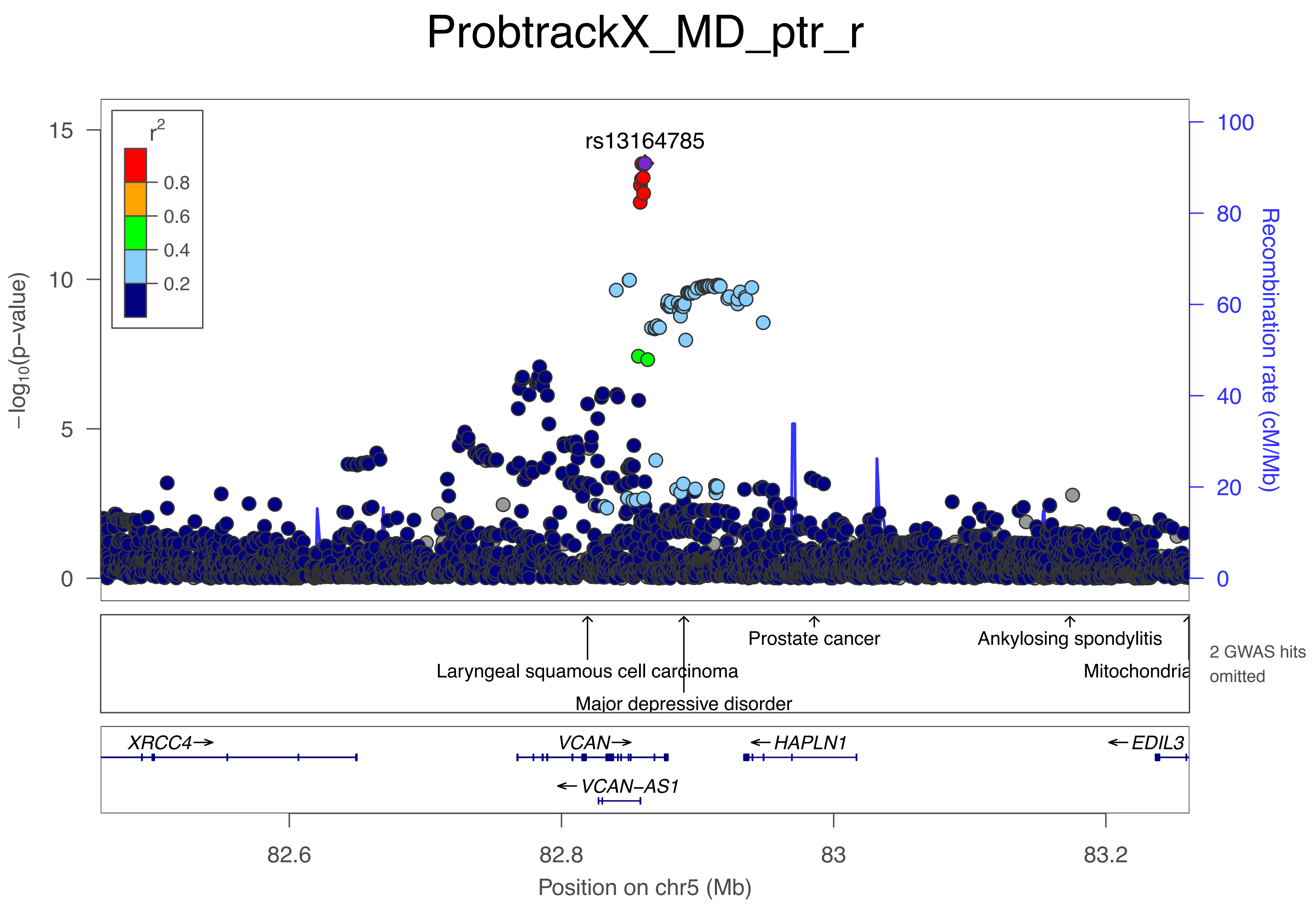

ProbtrackX_MD_ptr_r
rs13164785
r²
0.8
0.6
0.4
0.2
15
10
5
0
−log10(p−value)
100
80
60
40
20
0
Recombination rate (cM/Mb)
Prostate cancer
Ankylosing spondylitis
Laryngeal squamous cell carcinoma
Mitochondria
Major depressive disorder
2 GWAS hits omitted
XRCC4→
VCAN→
←HAPLN1
←EDIL3
←VCAN−AS1
82.6
82.8
83
83.2
Position on chr5 (Mb)

| | |
|---|---|
| date: | Thu Aug 17 17:58:11 2017 |
| build: | hg19 |
| display range: | chr5:82461400−83261400 [82461400−83261400] |
| hilite range: | 0 – 0 [ 0 – 0 ] |
| reference SNP: | chr5:82861400 |
| number of SNPs plotted: | 3396 |
| min P.value: | 1.32E−14 [chr5:82861400] |
| max P.value: | 9.99E−1 [chr5:83184551] |
| omitted GWAS Hits: | chr5:83.260938−Mitochondrial DNA levels, NA |

Make more plots at http://csg.sph.umich.edu/locuszoom/

GWAS Catalog SNPs in Region

| chr | pos (Mb) | trait | snp |
|---|---|---|---|
| 5 | 82.81912 | Laryngeal squamous cell carcinoma | rs310518 |
| 5 | 82.84549 | Diisocyanate−induced asthma | rs3852186 |
| 5 | 82.88991 | Major depressive disorder | rs310501 |
| 5 | 82.96073 | Visceral fat | rs3846635 |
| 5 | 82.98574 | Prostate cancer | rs4466137 |
| 5 | 83.17359 | Ankylosing spondylitis | rs4552569 |
| 5 | 83.26094 | Mitochondrial DNA levels | rs2301070 |

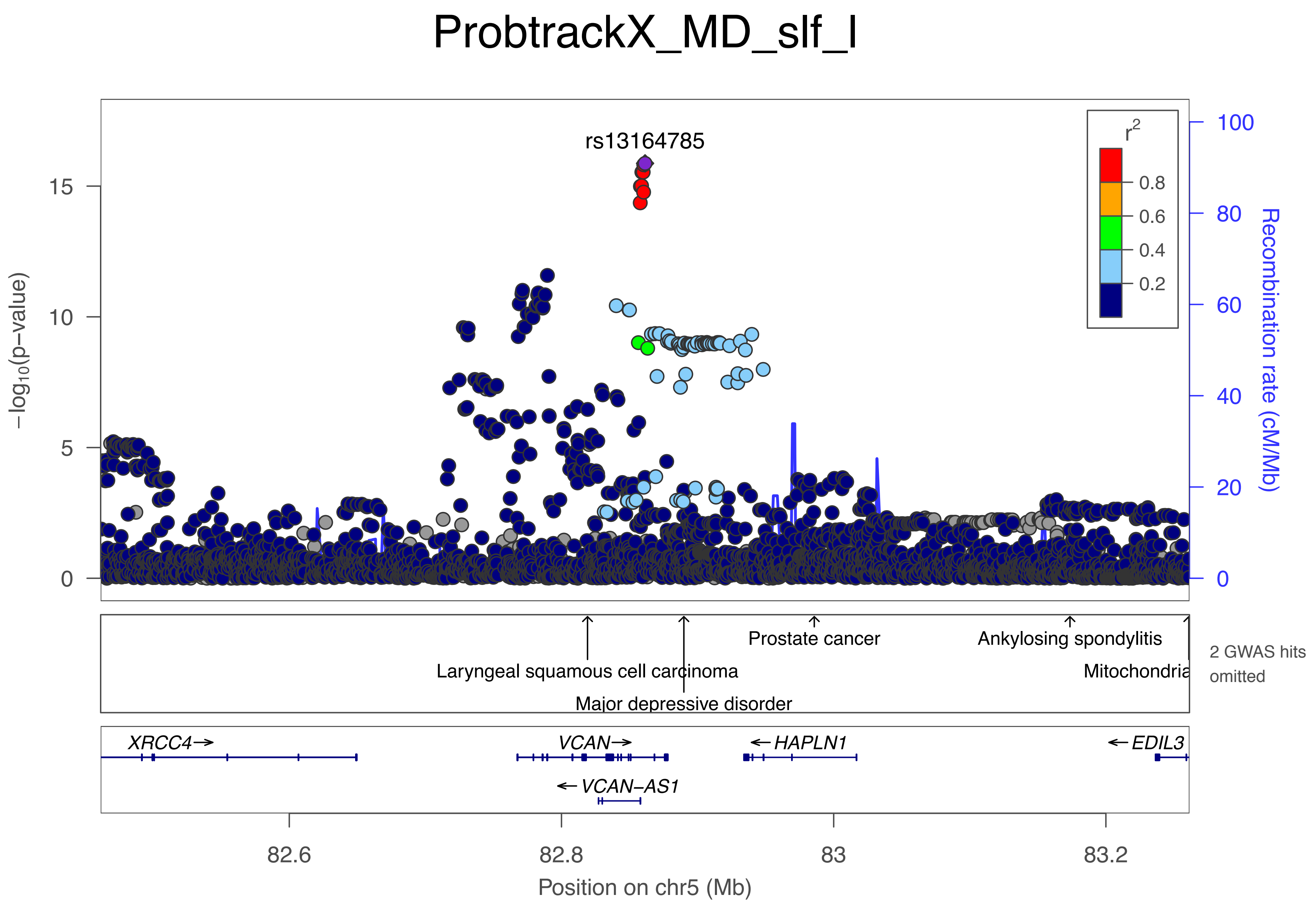
ProbtrackX_MD_slf_I
rs13164785
r²
0.8
0.6
0.4
0.2
15
10
5
0
−log10(p−value)
100
80
60
40
20
0
Recombination rate (cM/Mb)
Prostate cancer
Ankylosing spondylitis
Laryngeal squamous cell carcinoma
Mitochondria
Major depressive disorder
2 GWAS hits omitted
XRCC4→
VCAN→
←HAPLN1
←EDIL3
←VCAN−AS1
82.6
82.8
83
83.2
Position on chr5 (Mb)

| | |
|---|---|
| date: | Thu Aug 17 17:58:11 2017 |
| build: | hg19 |
| display range: | chr5:82461400−83261400 [82461400−83261400] |
| hilite range: | 0 – 0 [ 0 – 0 ] |
| reference SNP: | chr5:82861400 |
| number of SNPs plotted: | 3396 |
| min P.value: | 1.35E−16 [chr5:82861400] |
| max P.value: | 10E−1 [chr5:83080322] |
| omitted GWAS Hits: | chr5:83.260938−Mitochondrial DNA levels, NA |

Make more plots at http://csg.sph.umich.edu/locuszoom/

GWAS Catalog SNPs in Region

| chr | pos (Mb) | trait | snp |
|---|---|---|---|
| 5 | 82.81912 | Laryngeal squamous cell carcinoma | rs310518 |
| 5 | 82.84549 | Diisocyanate–induced asthma | rs3852186 |
| 5 | 82.88991 | Major depressive disorder | rs310501 |
| 5 | 82.96073 | Visceral fat | rs3846635 |
| 5 | 82.98574 | Prostate cancer | rs4466137 |
| 5 | 83.17359 | Ankylosing spondylitis | rs4552569 |
| 5 | 83.26094 | Mitochondrial DNA levels | rs2301070 |

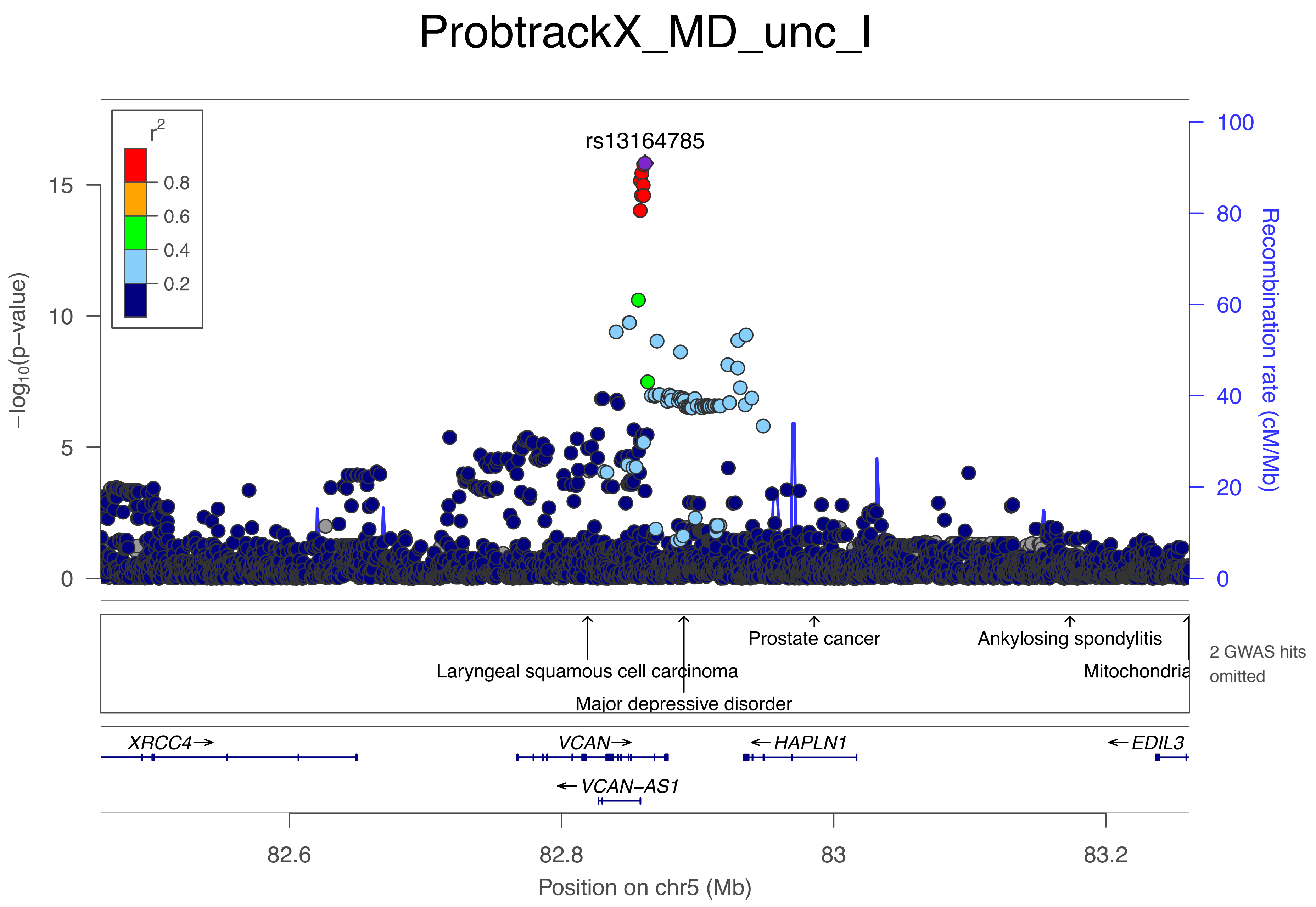
ProbtrackX_MD_unc_l
rs13164785
r2
0.8
0.6
0.4
0.2
−log10(p−value)
Recombination rate (cM/Mb)
Prostate cancer
Ankylosing spondylitis
Laryngeal squamous cell carcinoma
Major depressive disorder
Mitochondria
2 GWAS hits omitted
XRCC4
VCAN
HAPLN1
EDIL3
VCAN−AS1
82.6
82.8
83
83.2
Position on chr5 (Mb)

| | |
|---|---|
| date: | Thu Aug 17 17:58:11 2017 |
| build: | hg19 |
| display range: | chr5:82461400−83261400 [82461400−83261400] |
| hilite range: | 0 – 0 [ 0 – 0 ] |
| reference SNP: | chr5:82861400 |
| number of SNPs plotted: | 3396 |
| min P.value: | 1.51E−16 [chr5:82861400] |
| max P.value: | 10E−1 [chr5:82730532] |
| omitted GWAS Hits: | chr5:83.260938−Mitochondrial DNA levels, NA |

Make more plots at http://csg.sph.umich.edu/locuszoom/

GWAS Catalog SNPs in Region

| chr | pos (Mb) | trait | snp |
|---|---|---|---|
| 5 | 82.81912 | Laryngeal squamous cell carcinoma | rs310518 |
| 5 | 82.84549 | Diisocyanate–induced asthma | rs3852186 |
| 5 | 82.88991 | Major depressive disorder | rs310501 |
| 5 | 82.96073 | Visceral fat | rs3846635 |
| 5 | 82.98574 | Prostate cancer | rs4466137 |
| 5 | 83.17359 | Ankylosing spondylitis | rs4552569 |
| 5 | 83.26094 | Mitochondrial DNA levels | rs2301070 |

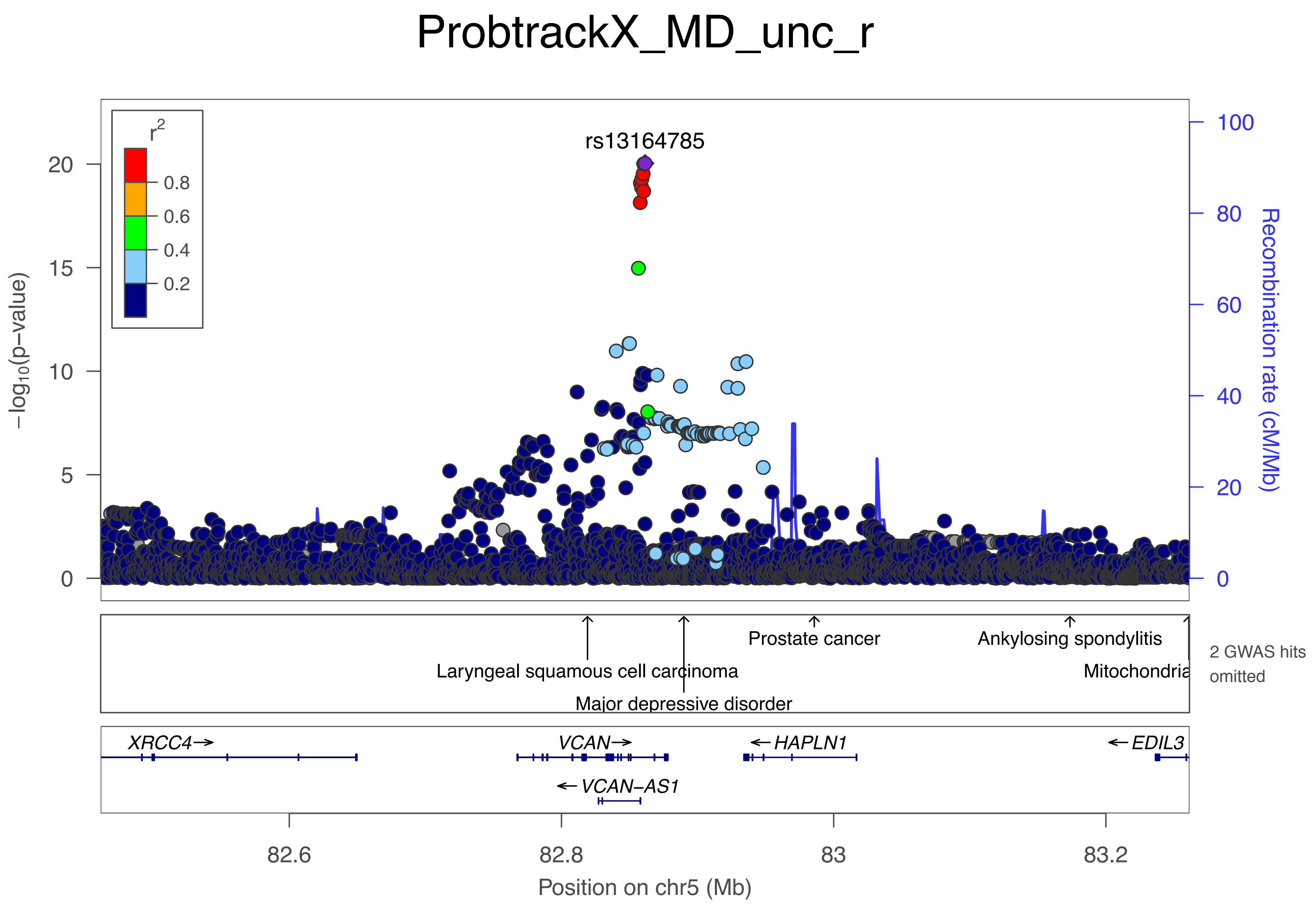

ProbtrackX_MD_unc_r
rs13164785
r2
0.8
0.6
0.4
0.2
−log10(p−value)
Recombination rate (cM/Mb)
0
5
10
15
20
0
20
40
60
80
100
Laryngeal squamous cell carcinoma
Major depressive disorder
Prostate cancer
Ankylosing spondylitis
Mitochondria
2 GWAS hits omitted
XRCC4→
VCAN→
←VCAN−AS1
←HAPLN1
←EDIL3
82.6
82.8
83
83.2
Position on chr5 (Mb)

| | |
|---|---|
| date: | Thu Aug 17 17:58:11 2017 |
| build: | hg19 |
| display range: | chr5:82461400–83261400 [82461400–83261400] |
| hilite range: | 0 – 0 [ 0 – 0 ] |
| reference SNP: | chr5:82861400 |
| number of SNPs plotted: | 3396 |
| min P.value: | 9.18E–21 [chr5:82861400] |
| max P.value: | 10E–1 [chr5:82733693] |
| omitted GWAS Hits: | chr5:83.260938–Mitochondrial DNA levels, NA |

Make more plots at http://csg.sph.umich.edu/locuszoom/

GWAS Catalog SNPs in Region

| chr | pos (Mb) | trait | snp |
|---|---|---|---|
| 5 | 82.81912 | Laryngeal squamous cell carcinoma | rs310518 |
| 5 | 82.84549 | Diisocyanate–induced asthma | rs3852186 |
| 5 | 82.88991 | Major depressive disorder | rs310501 |
| 5 | 82.96073 | Visceral fat | rs3846635 |
| 5 | 82.98574 | Prostate cancer | rs4466137 |
| 5 | 83.17359 | Ankylosing spondylitis | rs4552569 |
| 5 | 83.26094 | Mitochondrial DNA levels | rs2301070 |

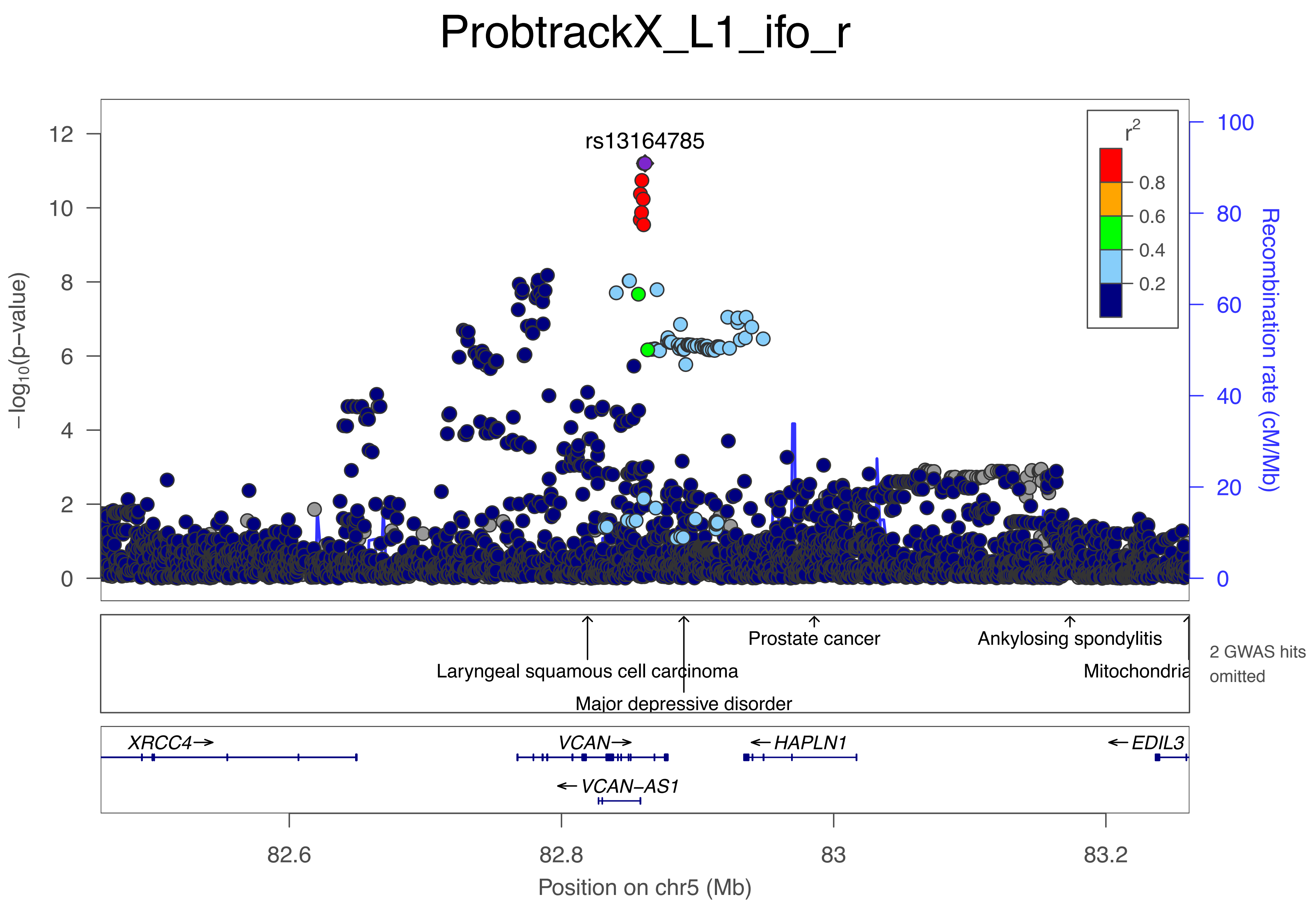

ProbtrackX_L1_ifo_r
rs13164785
−log10(p−value)
Recombination rate (cM/Mb)
r2
0.8
0.6
0.4
0.2
0
2
4
6
8
10
12
20
40
60
80
100
Laryngeal squamous cell carcinoma
Major depressive disorder
Prostate cancer
Ankylosing spondylitis
Mitochondria
2 GWAS hits omitted
XRCC4→
VCAN→
←VCAN−AS1
←HAPLN1
←EDIL3
82.6
82.8
83
83.2
Position on chr5 (Mb)

| | |
|---|---|
| date: | Thu Aug 17 17:58:11 2017 |
| build: | hg19 |
| display range: | chr5:82461400−83261400 [82461400−83261400] |
| hilite range: | 0 − 0 [ 0 − 0 ] |
| reference SNP: | chr5:82861400 |
| number of SNPs plotted: | 3396 |
| min P.value: | 6.31E−12 [chr5:82861400] |
| max P.value: | 10E−1 [chr5:83231254] |
| omitted GWAS Hits: | chr5:83.260938−Mitochondrial DNA levels, NA |

Make more plots at http://csg.sph.umich.edu/locuszoom/

GWAS Catalog SNPs in Region

| chr | pos (Mb) | trait | snp |
| --- | --- | --- | --- |
| 5 | 82.81912 | Laryngeal squamous cell carcinoma | rs310518 |
| 5 | 82.84549 | Diisocyanate–induced asthma | rs3852186 |
| 5 | 82.88991 | Major depressive disorder | rs310501 |
| 5 | 82.96073 | Visceral fat | rs3846635 |
| 5 | 82.98574 | Prostate cancer | rs4466137 |
| 5 | 83.17359 | Ankylosing spondylitis | rs4552569 |
| 5 | 83.26094 | Mitochondrial DNA levels | rs2301070 |

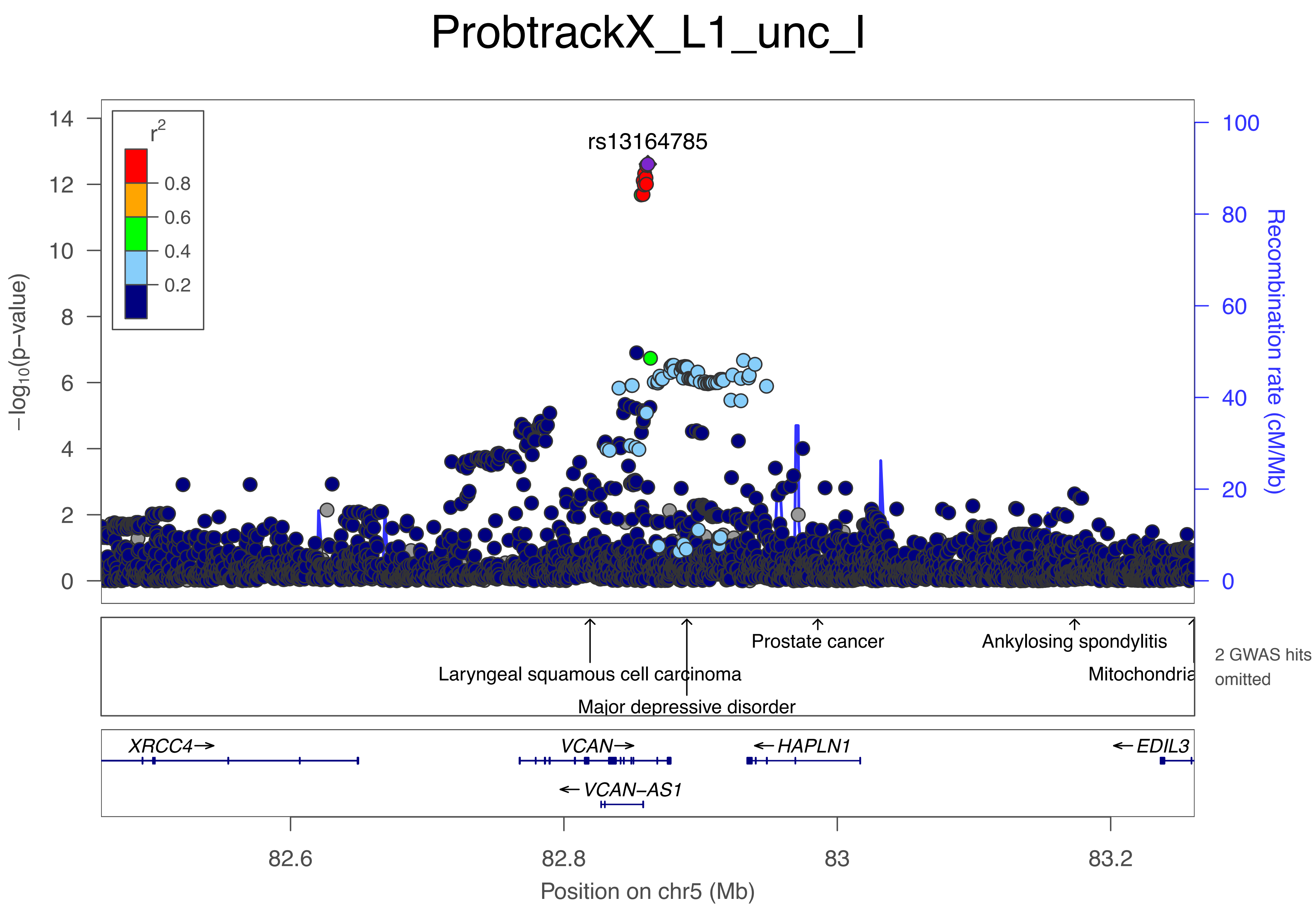

ProbtrackX_L1_unc_l
rs13164785
r²
0.8
0.6
0.4
0.2
−log10(p−value)
Recombination rate (cM/Mb)
14
12
10
8
6
4
2
0
100
80
60
40
20
0
Laryngeal squamous cell carcinoma
Major depressive disorder
Prostate cancer
Ankylosing spondylitis
Mitochondria
2 GWAS hits omitted
XRCC4→
VCAN→
←HAPLN1
←EDIL3
←VCAN−AS1
82.6
82.8
83
83.2
Position on chr5 (Mb)

| | |
|---|---|
| date: | Thu Aug 17 17:58:11 2017 |
| build: | hg19 |
| display range: | chr5:82461400−83261400 [82461400−83261400] |
| hilite range: | 0 – 0 [ 0 – 0 ] |
| reference SNP: | chr5:82861400 |
| number of SNPs plotted: | 3396 |
| min P.value: | 2.44E−13 [chr5:82861400] |
| max P.value: | 10E−1 [chr5:82471279] |
| omitted GWAS Hits: | chr5:83.260938−Mitochondrial DNA levels, NA |

Make more plots at http://csg.sph.umich.edu/locuszoom/

GWAS Catalog SNPs in Region

| chr | pos (Mb) | trait | snp |
|---|---|---|---|
| 5 | 82.81912 | Laryngeal squamous cell carcinoma | rs310518 |
| 5 | 82.84549 | Diisocyanate–induced asthma | rs3852186 |
| 5 | 82.88991 | Major depressive disorder | rs310501 |
| 5 | 82.96073 | Visceral fat | rs3846635 |
| 5 | 82.98574 | Prostate cancer | rs4466137 |
| 5 | 83.17359 | Ankylosing spondylitis | rs4552569 |
| 5 | 83.26094 | Mitochondrial DNA levels | rs2301070 |

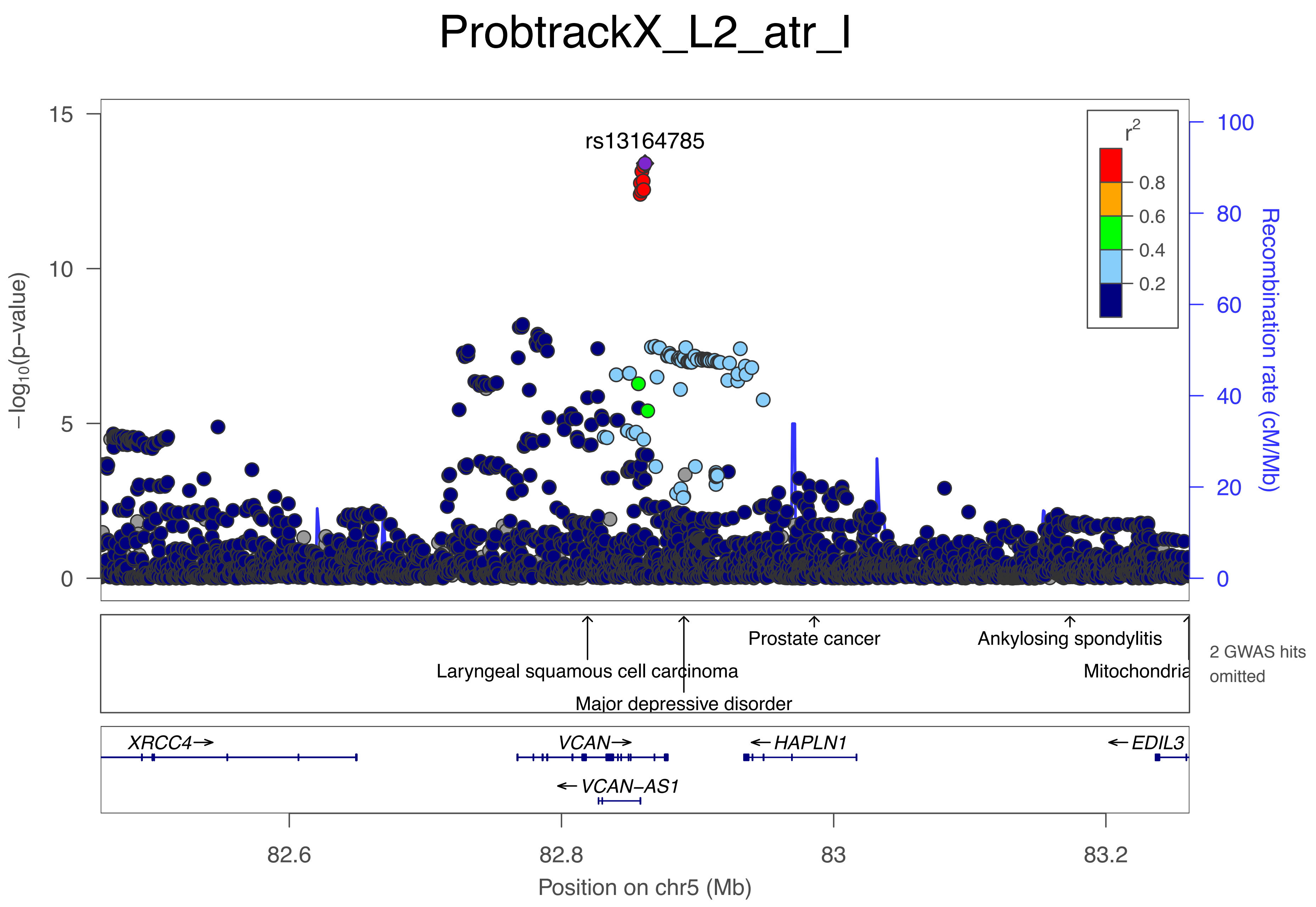

ProbtrackX_L2_atr_l
rs13164785
r²
0.8
0.6
0.4
0.2
15
10
5
0
−log10(p−value)
100
80
60
40
20
0
Recombination rate (cM/Mb)
Prostate cancer
Ankylosing spondylitis
Laryngeal squamous cell carcinoma
Mitochondria
Major depressive disorder
2 GWAS hits omitted
XRCC4→
VCAN→
←HAPLN1
←EDIL3
←VCAN−AS1
82.6
82.8
83
83.2
Position on chr5 (Mb)

| | |
|---|---|
| date: | Thu Aug 17 17:58:11 2017 |
| build: | hg19 |
| display range: | chr5:82461400−83261400 [82461400−83261400] |
| hilite range: | 0 − 0 [ 0 − 0 ] |
| reference SNP: | chr5:82861400 |
| number of SNPs plotted: | 3396 |
| min P.value: | 3.99E−14 [chr5:82861400] |
| max P.value: | 9.99E−1 [chr5:82634569] |
| omitted GWAS Hits: | chr5:83.260938−Mitochondrial DNA levels, NA |

Make more plots at http://csg.sph.umich.edu/locuszoom/

GWAS Catalog SNPs in Region

| chr | pos (Mb) | trait | snp |
|---|---|---|---|
| 5 | 82.81912 | Laryngeal squamous cell carcinoma | rs310518 |
| 5 | 82.84549 | Diisocyanate−induced asthma | rs3852186 |
| 5 | 82.88991 | Major depressive disorder | rs310501 |
| 5 | 82.96073 | Visceral fat | rs3846635 |
| 5 | 82.98574 | Prostate cancer | rs4466137 |
| 5 | 83.17359 | Ankylosing spondylitis | rs4552569 |
| 5 | 83.26094 | Mitochondrial DNA levels | rs2301070 |

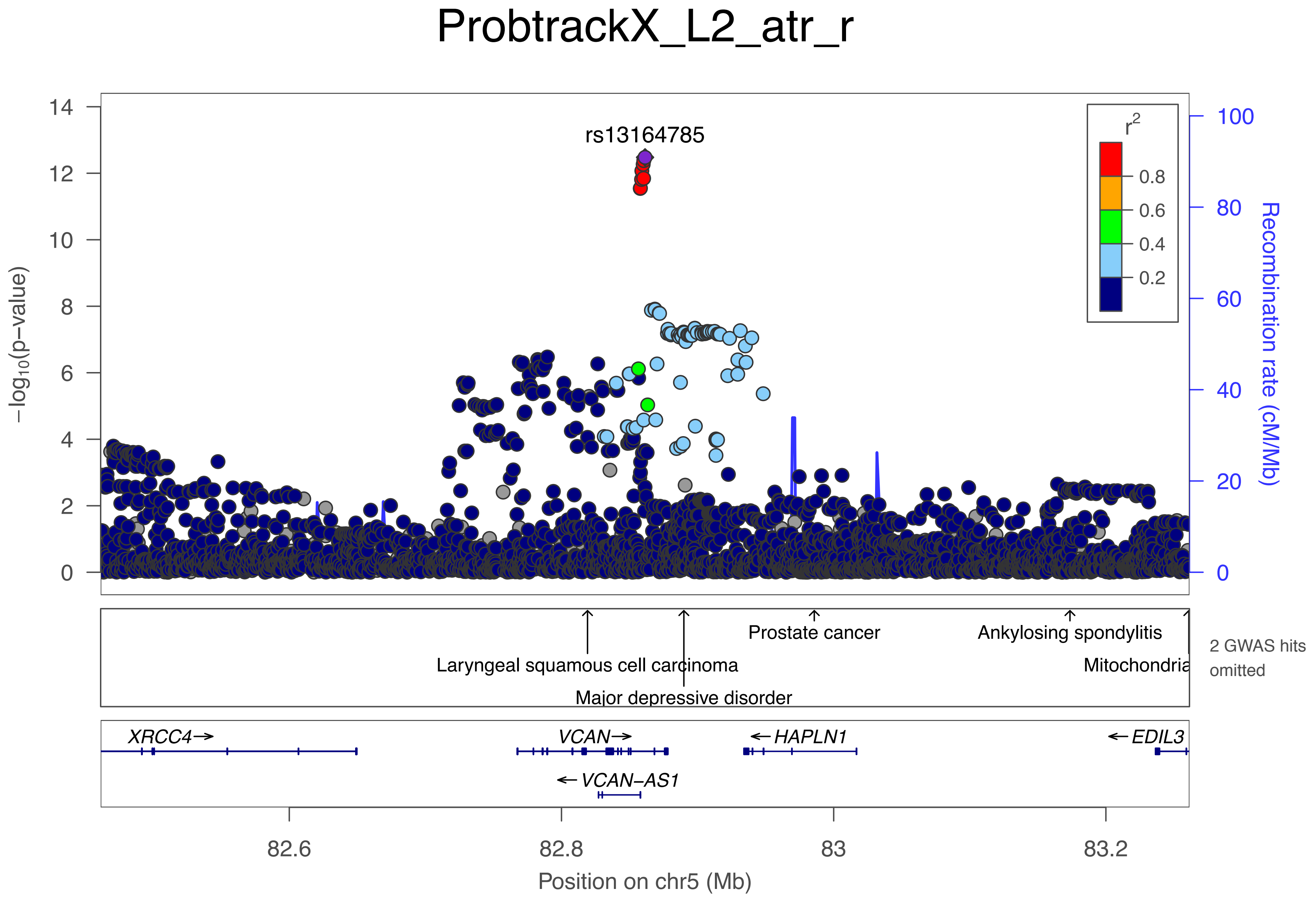
ProbtrackX_L2_atr_r
rs13164785
r2
0.8
0.6
0.4
0.2
14
12
10
8
6
4
2
0
−log10(p−value)
100
80
60
40
20
0
Recombination rate (cM/Mb)
Laryngeal squamous cell carcinoma
Major depressive disorder
Prostate cancer
Ankylosing spondylitis
Mitochondria
2 GWAS hits omitted
XRCC4→
VCAN→
←HAPLN1
←EDIL3
←VCAN−AS1
82.6
82.8
83
83.2
Position on chr5 (Mb)

| | |
|---|---|
| date: | Thu Aug 17 17:58:11 2017 |
| build: | hg19 |
| display range: | chr5:82461400−83261400 [82461400−83261400] |
| hilite range: | 0 – 0 [ 0 – 0 ] |
| reference SNP: | chr5:82861400 |
| number of SNPs plotted: | 3396 |
| min P.value: | 3.33E−13 [chr5:82861400] |
| max P.value: | 10E−1 [chr5:83167859] |
| omitted GWAS Hits: | chr5:83.260938−Mitochondrial DNA levels, NA |

Make more plots at http://csg.sph.umich.edu/locuszoom/

GWAS Catalog SNPs in Region

| chr | pos (Mb) | trait | snp |
|---|---|---|---|
| 5 | 82.81912 | Laryngeal squamous cell carcinoma | rs310518 |
| 5 | 82.84549 | Diisocyanate–induced asthma | rs3852186 |
| 5 | 82.88991 | Major depressive disorder | rs310501 |
| 5 | 82.96073 | Visceral fat | rs3846635 |
| 5 | 82.98574 | Prostate cancer | rs4466137 |
| 5 | 83.17359 | Ankylosing spondylitis | rs4552569 |
| 5 | 83.26094 | Mitochondrial DNA levels | rs2301070 |

# ProbtrackX_L2_fmi

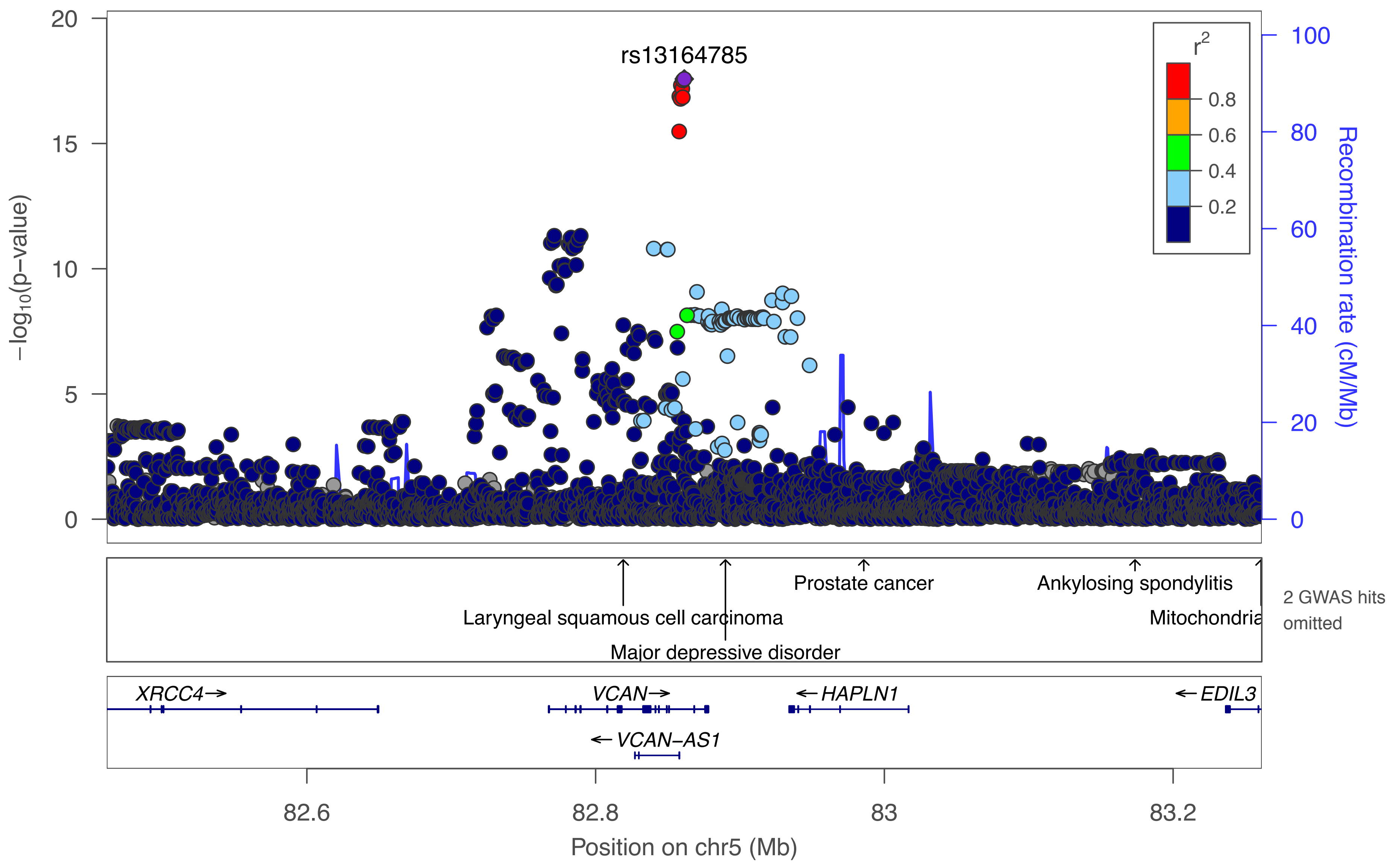

| | |
|---|---|
| date: | Thu Aug 17 17:58:11 2017 |
| build: | hg19 |
| display range: | chr5:82461400−83261400 [82461400−83261400] |
| hilite range: | 0 – 0 [ 0 – 0 ] |
| reference SNP: | chr5:82861400 |
| number of SNPs plotted: | 3396 |
| min P.value: | 2.65E−18 [chr5:82861400] |
| max P.value: | 9.99E−1 [chr5:82676039] |
| omitted GWAS Hits: | chr5:83.260938−Mitochondrial DNA levels, NA |

Make more plots at http://csg.sph.umich.edu/locuszoom/

GWAS Catalog SNPs in Region

| chr | pos (Mb) | trait | snp |
|---|---|---|---|
| 5 | 82.81912 | Laryngeal squamous cell carcinoma | rs310518 |
| 5 | 82.84549 | Diisocyanate−induced asthma | rs3852186 |
| 5 | 82.88991 | Major depressive disorder | rs310501 |
| 5 | 82.96073 | Visceral fat | rs3846635 |
| 5 | 82.98574 | Prostate cancer | rs4466137 |
| 5 | 83.17359 | Ankylosing spondylitis | rs4552569 |
| 5 | 83.26094 | Mitochondrial DNA levels | rs2301070 |

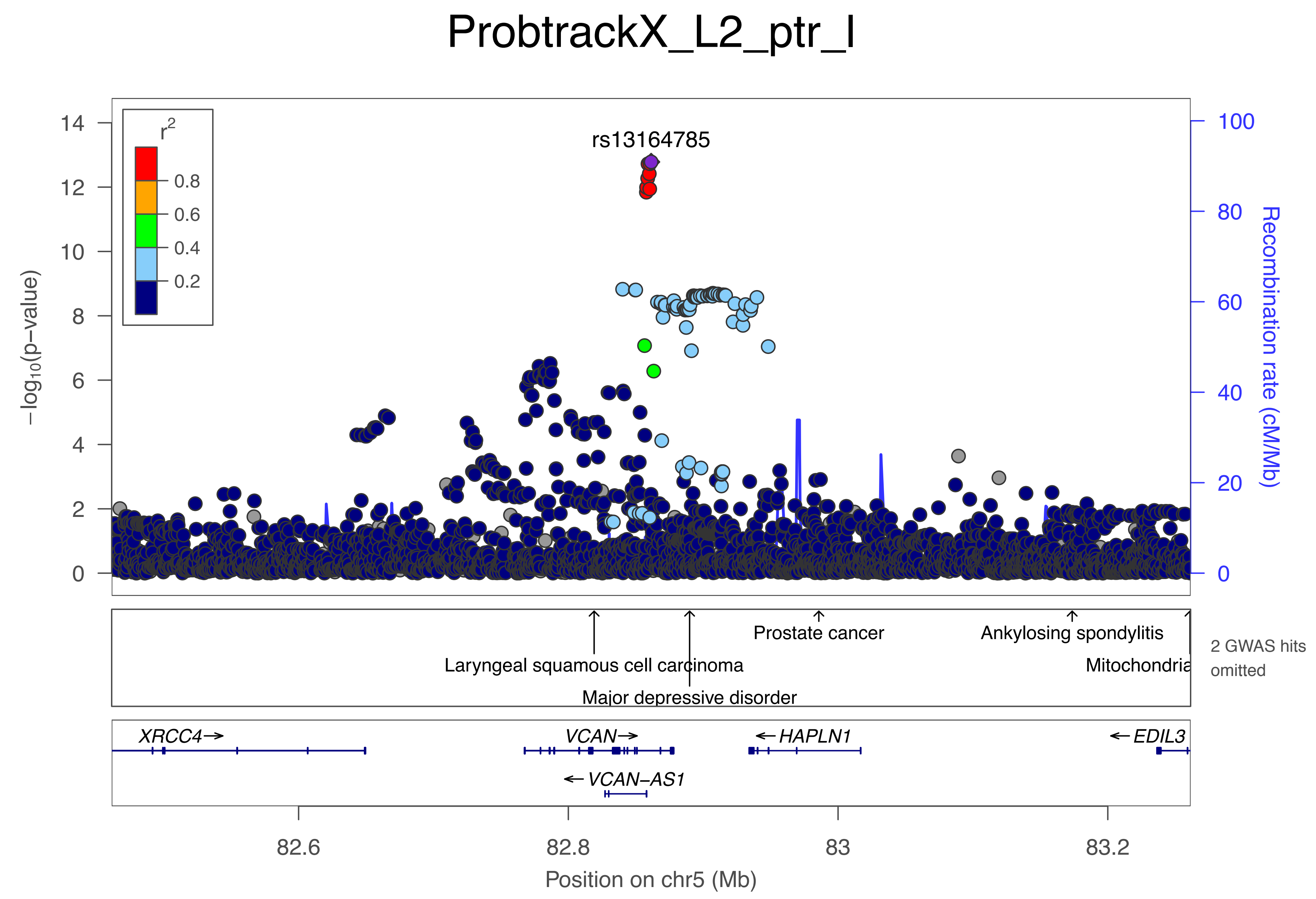
ProbtrackX_L2_ptr_l
rs13164785
r2
0.8
0.6
0.4
0.2
−log10(p−value)
Recombination rate (cM/Mb)
Prostate cancer
Ankylosing spondylitis
Laryngeal squamous cell carcinoma
Mitochondria
Major depressive disorder
2 GWAS hits omitted
XRCC4→
VCAN→
←HAPLN1
←EDIL3
←VCAN−AS1
82.6
82.8
83
83.2
Position on chr5 (Mb)

| | |
|---|---|
| date: | Thu Aug 17 17:58:11 2017 |
| build: | hg19 |
| display range: | chr5:82461400−83261400 [82461400−83261400] |
| hilite range: | 0 – 0 [ 0 – 0 ] |
| reference SNP: | chr5:82861400 |
| number of SNPs plotted: | 3396 |
| min P.value: | 1.65E−13 [chr5:82861400] |
| max P.value: | 10E−1 [chr5:83118236] |
| omitted GWAS Hits: | chr5:83.260938−Mitochondrial DNA levels, NA |

Make more plots at http://csg.sph.umich.edu/locuszoom/

GWAS Catalog SNPs in Region

| chr | pos (Mb) | trait | snp |
|---|---|---|---|
| 5 | 82.81912 | Laryngeal squamous cell carcinoma | rs310518 |
| 5 | 82.84549 | Diisocyanate–induced asthma | rs3852186 |
| 5 | 82.88991 | Major depressive disorder | rs310501 |
| 5 | 82.96073 | Visceral fat | rs3846635 |
| 5 | 82.98574 | Prostate cancer | rs4466137 |
| 5 | 83.17359 | Ankylosing spondylitis | rs4552569 |
| 5 | 83.26094 | Mitochondrial DNA levels | rs2301070 |

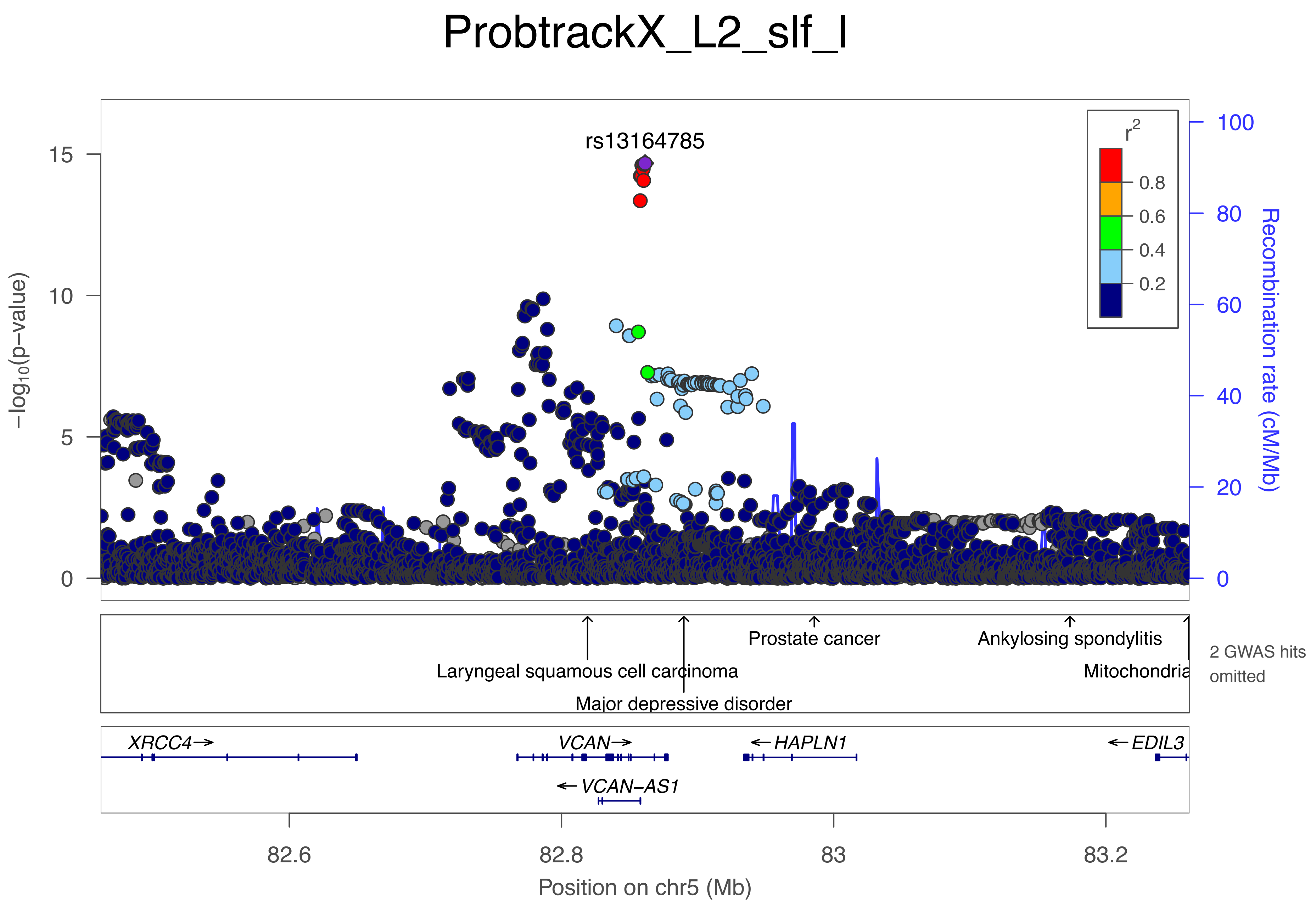

ProbtrackX_L2_slf_l
rs13164785
−log10(p−value)
Recombination rate (cM/Mb)
r2
0.8
0.6
0.4
0.2
Prostate cancer
Ankylosing spondylitis
Laryngeal squamous cell carcinoma
Mitochondria
Major depressive disorder
2 GWAS hits omitted
XRCC4→
VCAN→
←HAPLN1
←EDIL3
←VCAN−AS1
82.6
82.8
83
83.2
Position on chr5 (Mb)

| | |
|---|---|
| date: | Thu Aug 17 17:58:12 2017 |
| build: | hg19 |
| display range: | chr5:82461400−83261400 [82461400−83261400] |
| hilite range: | 0 – 0 [ 0 – 0 ] |
| reference SNP: | chr5:82861400 |
| number of SNPs plotted: | 3396 |
| min P.value: | 2.13E−15 [chr5:82861400] |
| max P.value: | 9.99E−1 [chr5:83022127] |
| omitted GWAS Hits: | chr5:83.260938−Mitochondrial DNA levels, NA |

Make more plots at http://csg.sph.umich.edu/locuszoom/

GWAS Catalog SNPs in Region

| chr | pos (Mb) | trait | snp |
|---|---|---|---|
| 5 | 82.81912 | Laryngeal squamous cell carcinoma | rs310518 |
| 5 | 82.84549 | Diisocyanate−induced asthma | rs3852186 |
| 5 | 82.88991 | Major depressive disorder | rs310501 |
| 5 | 82.96073 | Visceral fat | rs3846635 |
| 5 | 82.98574 | Prostate cancer | rs4466137 |
| 5 | 83.17359 | Ankylosing spondylitis | rs4552569 |
| 5 | 83.26094 | Mitochondrial DNA levels | rs2301070 |

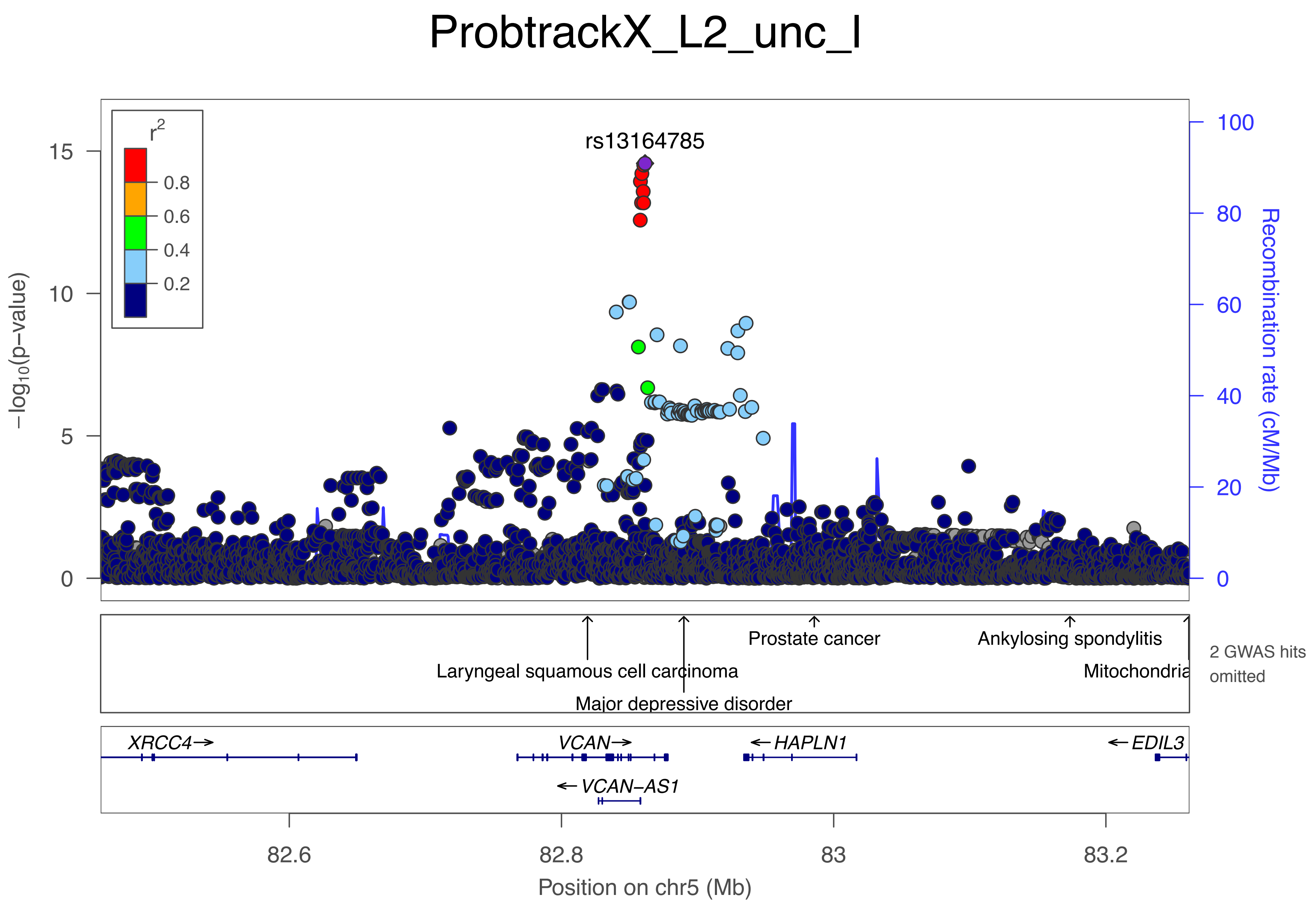
ProbtrackX_L2_unc_l
r²
0.8
0.6
0.4
0.2
rs13164785
15
10
5
0
−log10(p−value)
100
80
60
40
20
0
Recombination rate (cM/Mb)
Prostate cancer
Ankylosing spondylitis
Laryngeal squamous cell carcinoma
Mitochondria
Major depressive disorder
2 GWAS hits omitted
XRCC4→
VCAN→
←HAPLN1
←EDIL3
←VCAN−AS1
82.6
82.8
83
83.2
Position on chr5 (Mb)

| | |
|---|---|
| date: | Thu Aug 17 17:58:12 2017 |
| build: | hg19 |
| display range: | chr5:82461400−83261400 [82461400−83261400] |
| hilite range: | 0 – 0 [ 0 – 0 ] |
| reference SNP: | chr5:82861400 |
| number of SNPs plotted: | 3396 |
| min P.value: | 2.7E−15 [chr5:82861400] |
| max P.value: | 9.99E−1 [chr5:82941836] |
| omitted GWAS Hits: | chr5:83.260938−Mitochondrial DNA levels, NA |

Make more plots at http://csg.sph.umich.edu/locuszoom/

GWAS Catalog SNPs in Region

| chr | pos (Mb) | trait | snp |
|---|---|---|---|
| 5 | 82.81912 | Laryngeal squamous cell carcinoma | rs310518 |
| 5 | 82.84549 | Diisocyanate−induced asthma | rs3852186 |
| 5 | 82.88991 | Major depressive disorder | rs310501 |
| 5 | 82.96073 | Visceral fat | rs3846635 |
| 5 | 82.98574 | Prostate cancer | rs4466137 |
| 5 | 83.17359 | Ankylosing spondylitis | rs4552569 |
| 5 | 83.26094 | Mitochondrial DNA levels | rs2301070 |

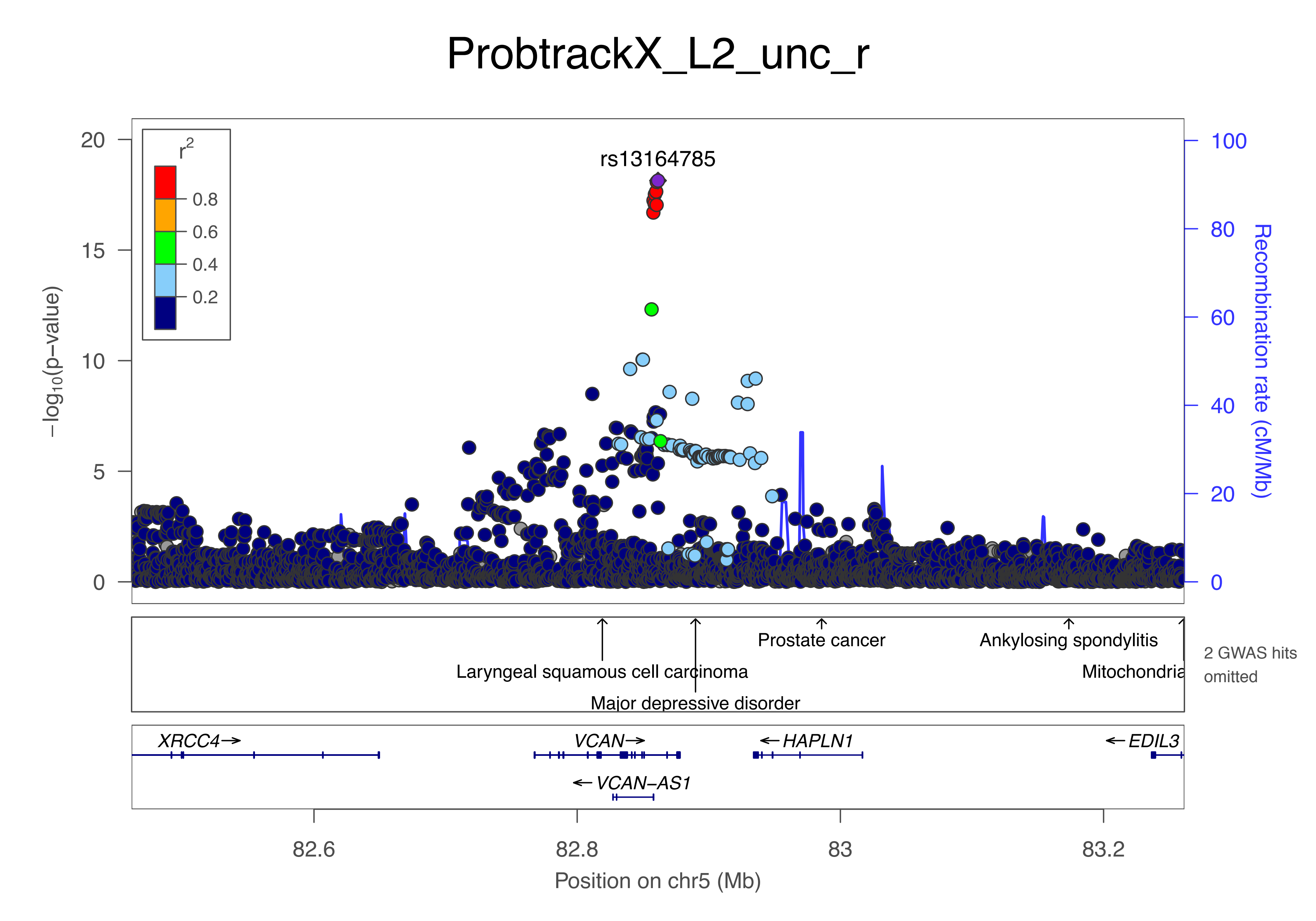
ProbtrackX_L2_unc_r
rs13164785
r2
0.8
0.6
0.4
0.2
−log10(p−value)
20
15
10
5
0
100
80
60
40
20
0
Recombination rate (cM/Mb)
Laryngeal squamous cell carcinoma
Major depressive disorder
Prostate cancer
Ankylosing spondylitis
Mitochondria
2 GWAS hits omitted
XRCC4→
VCAN→
←VCAN−AS1
←HAPLN1
←EDIL3
82.6
82.8
83
83.2
Position on chr5 (Mb)

| | |
|---|---|
| date: | Thu Aug 17 17:58:12 2017 |
| build: | hg19 |
| display range: | chr5:82461400−83261400 [82461400−83261400] |
| hilite range: | 0 – 0 [ 0 – 0 ] |
| reference SNP: | chr5:82861400 |
| number of SNPs plotted: | 3396 |
| min P.value: | 7.24E−19 [chr5:82861400] |
| max P.value: | 10E−1 [chr5:82594107] |
| omitted GWAS Hits: | chr5:83.260938−Mitochondrial DNA levels, NA |

Make more plots at http://csg.sph.umich.edu/locuszoom/

GWAS Catalog SNPs in Region

| chr | pos (Mb) | trait | snp |
|---|---|---|---|
| 5 | 82.81912 | Laryngeal squamous cell carcinoma | rs310518 |
| 5 | 82.84549 | Diisocyanate–induced asthma | rs3852186 |
| 5 | 82.88991 | Major depressive disorder | rs310501 |
| 5 | 82.96073 | Visceral fat | rs3846635 |
| 5 | 82.98574 | Prostate cancer | rs4466137 |
| 5 | 83.17359 | Ankylosing spondylitis | rs4552569 |
| 5 | 83.26094 | Mitochondrial DNA levels | rs2301070 |

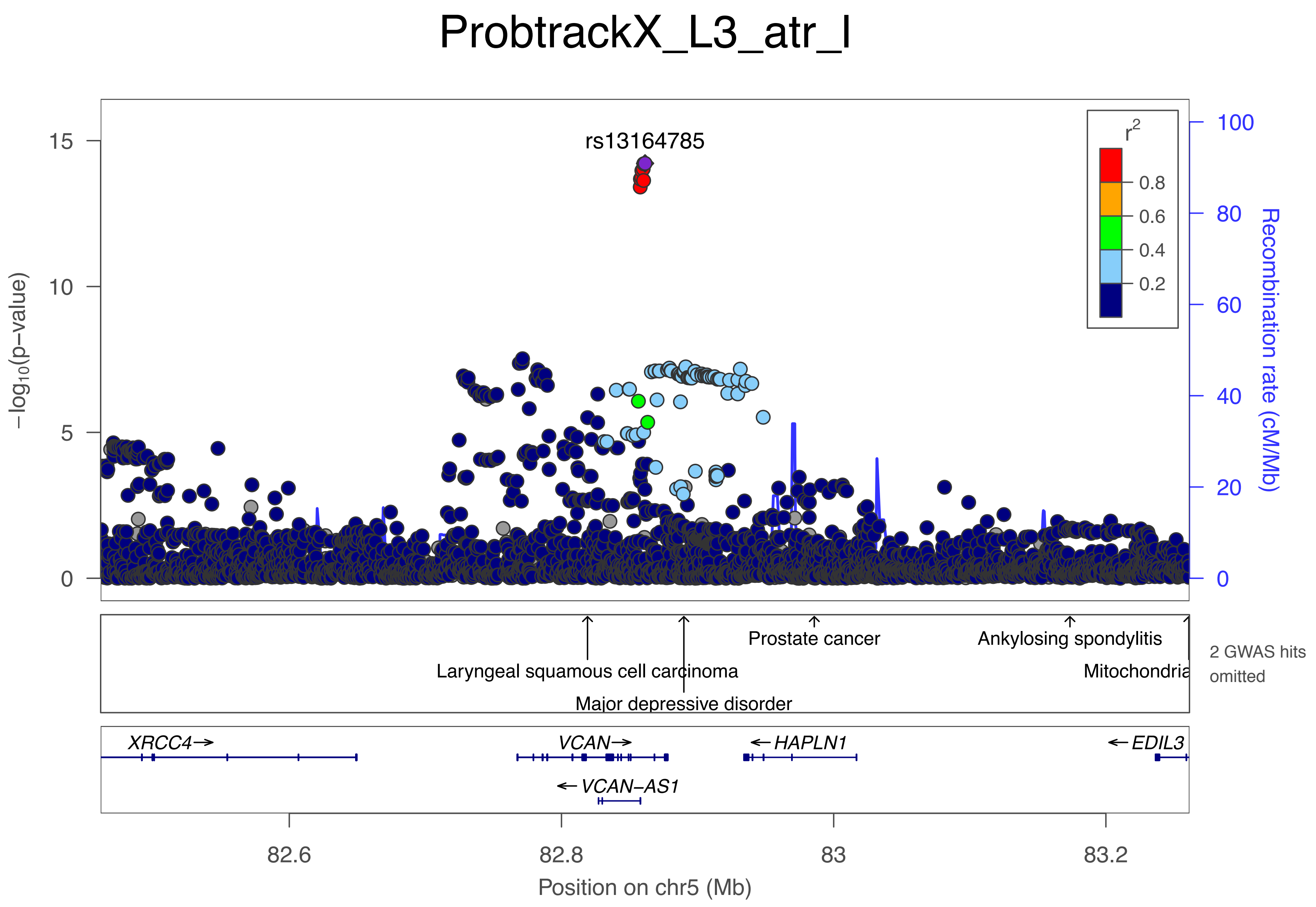
ProbtrackX_L3_atr_l
rs13164785
r2
0.8
0.6
0.4
0.2
15
10
5
0
−log10(p−value)
100
80
60
40
20
0
Recombination rate (cM/Mb)
Prostate cancer
Ankylosing spondylitis
Laryngeal squamous cell carcinoma
Mitochondria
Major depressive disorder
2 GWAS hits omitted
XRCC4→
VCAN→
← HAPLN1
← EDIL3
← VCAN−AS1
82.6
82.8
83
83.2
Position on chr5 (Mb)

| | |
|---|---|
| date: | Thu Aug 17 17:58:12 2017 |
| build: | hg19 |
| display range: | chr5:82461400−83261400 [82461400−83261400] |
| hilite range: | 0 − 0 [ 0 − 0 ] |
| reference SNP: | chr5:82861400 |
| number of SNPs plotted: | 3396 |
| min P.value: | 6.01E−15 [chr5:82861400] |
| max P.value: | 10E−1 [chr5:82974532] |
| omitted GWAS Hits: | chr5:83.260938−Mitochondrial DNA levels, NA |

Make more plots at http://csg.sph.umich.edu/locuszoom/

GWAS Catalog SNPs in Region

| chr | pos (Mb) | trait | snp |
|---|---|---|---|
| 5 | 82.81912 | Laryngeal squamous cell carcinoma | rs310518 |
| 5 | 82.84549 | Diisocyanate–induced asthma | rs3852186 |
| 5 | 82.88991 | Major depressive disorder | rs310501 |
| 5 | 82.96073 | Visceral fat | rs3846635 |
| 5 | 82.98574 | Prostate cancer | rs4466137 |
| 5 | 83.17359 | Ankylosing spondylitis | rs4552569 |
| 5 | 83.26094 | Mitochondrial DNA levels | rs2301070 |

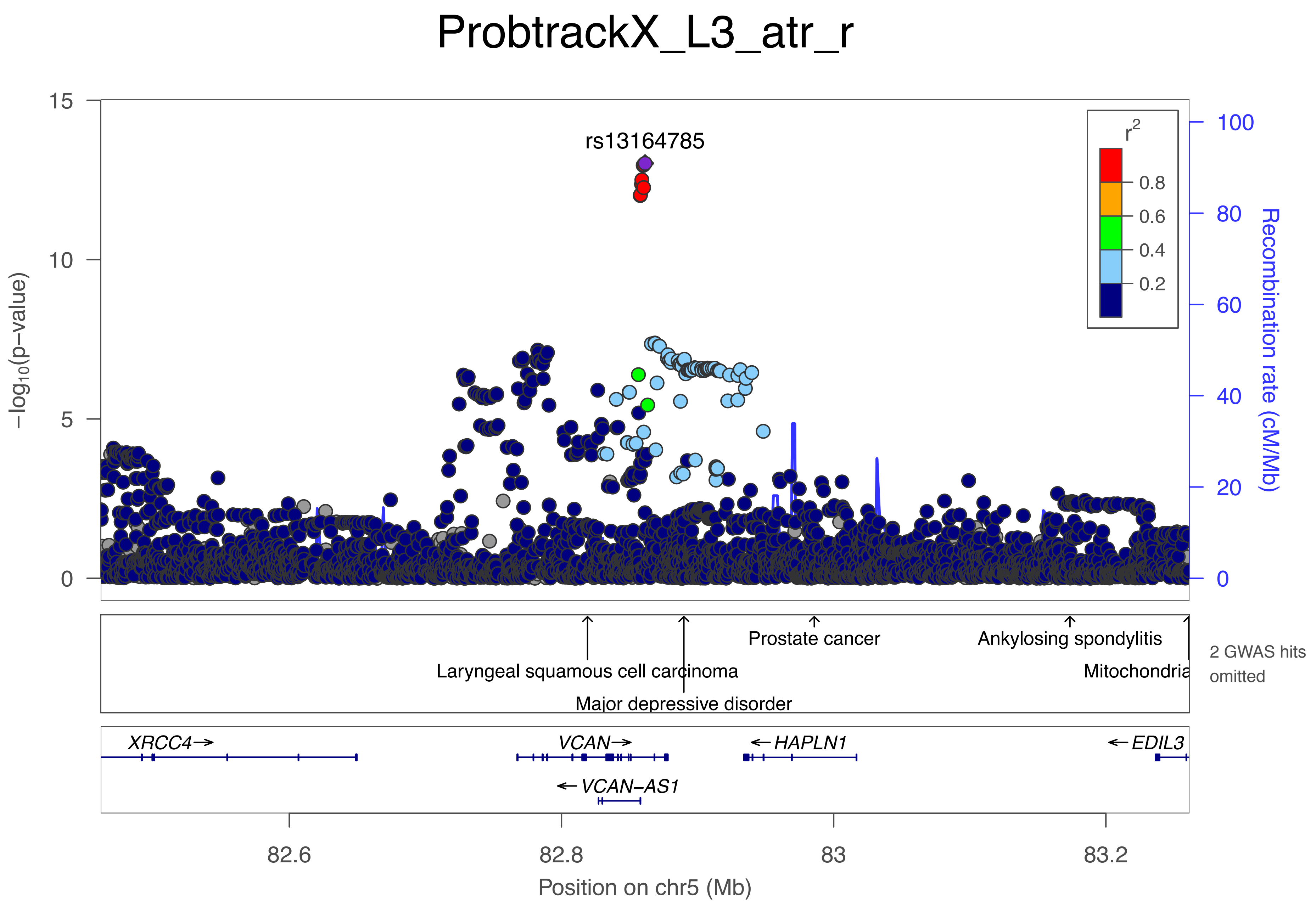
ProbtrackX_L3_atr_r
rs13164785
r2
0.8
0.6
0.4
0.2
−log10(p−value)
15
10
5
0
100
80
60
40
20
0
Recombination rate (cM/Mb)
Prostate cancer
Ankylosing spondylitis
Laryngeal squamous cell carcinoma
Mitochondria
Major depressive disorder
2 GWAS hits omitted
XRCC4→
VCAN→
←HAPLN1
←EDIL3
←VCAN−AS1
82.6
82.8
83
83.2
Position on chr5 (Mb)

| | |
|---|---|
| date: | Thu Aug 17 17:58:12 2017 |
| build: | hg19 |
| display range: | chr5:82461400−83261400 [82461400−83261400] |
| hilite range: | 0 – 0 [ 0 – 0 ] |
| reference SNP: | chr5:82861400 |
| number of SNPs plotted: | 3396 |
| min P.value: | 9.53E−14 [chr5:82861400] |
| max P.value: | 10E−1 [chr5:82706652] |
| omitted GWAS Hits: | chr5:83.260938−Mitochondrial DNA levels, NA |

Make more plots at http://csg.sph.umich.edu/locuszoom/

GWAS Catalog SNPs in Region

| chr | pos (Mb) | trait | snp |
|---|---|---|---|
| 5 | 82.81912 | Laryngeal squamous cell carcinoma | rs310518 |
| 5 | 82.84549 | Diisocyanate–induced asthma | rs3852186 |
| 5 | 82.88991 | Major depressive disorder | rs310501 |
| 5 | 82.96073 | Visceral fat | rs3846635 |
| 5 | 82.98574 | Prostate cancer | rs4466137 |
| 5 | 83.17359 | Ankylosing spondylitis | rs4552569 |
| 5 | 83.26094 | Mitochondrial DNA levels | rs2301070 |

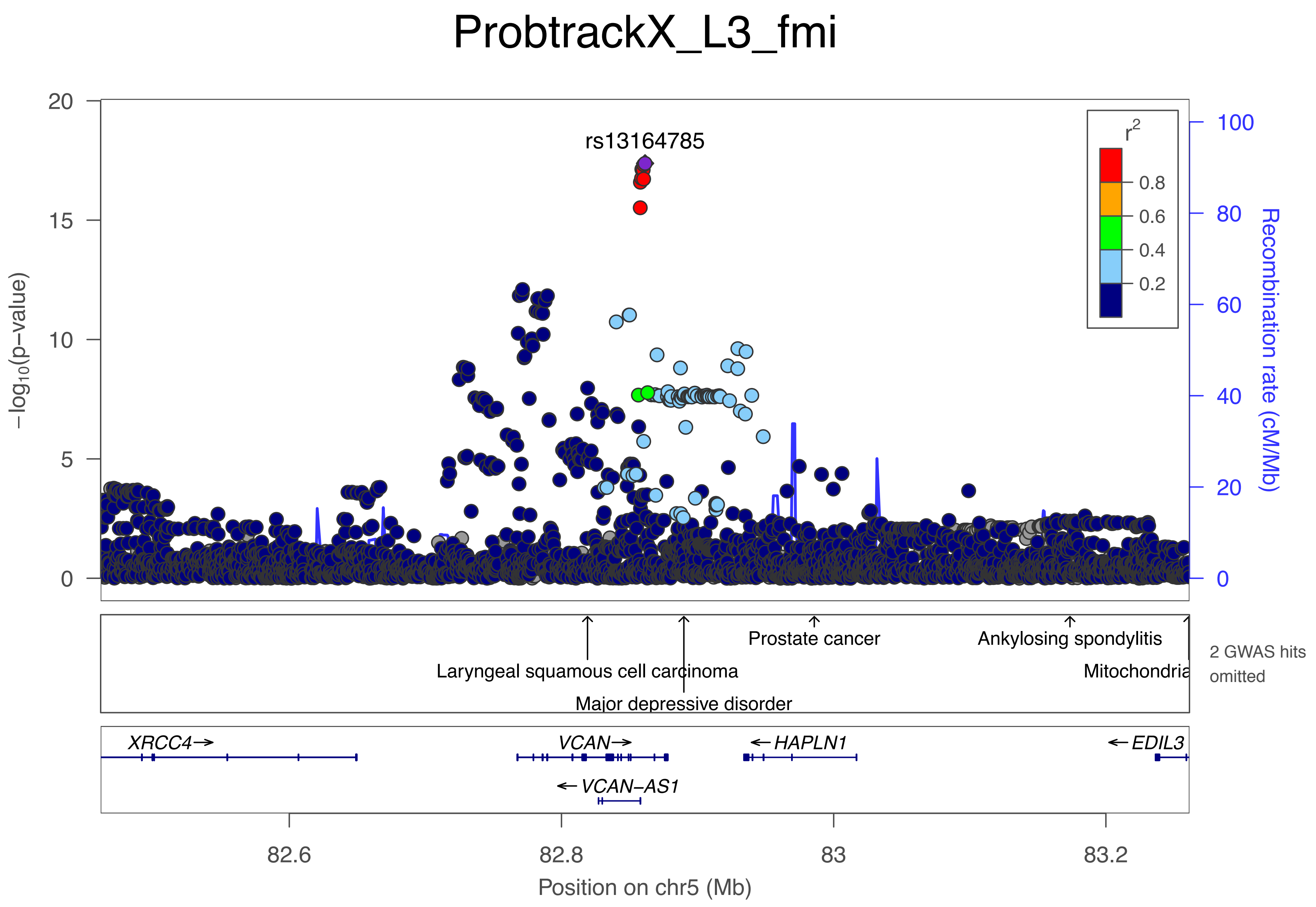

ProbtrackX_L3_fmi
rs13164785
$-\log_{10}$(p−value)
Recombination rate (cM/Mb)
$r^2$
0.8
0.6
0.4
0.2
20
15
10
5
0
100
80
60
40
20
0
Laryngeal squamous cell carcinoma
Major depressive disorder
Prostate cancer
Ankylosing spondylitis
Mitochondria
2 GWAS hits omitted
XRCC4→
VCAN→
←VCAN−AS1
←HAPLN1
←EDIL3
82.6
82.8
83
83.2
Position on chr5 (Mb)

| | |
|---|---|
| date: | Thu Aug 17 17:58:12 2017 |
| build: | hg19 |
| display range: | chr5:82461400−83261400 [82461400−83261400] |
| hilite range: | 0 – 0 [ 0 – 0 ] |
| reference SNP: | chr5:82861400 |
| number of SNPs plotted: | 3396 |
| min P.value: | 4.18E−18 [chr5:82861400] |
| max P.value: | 9.98E−1 [chr5:82601623] |
| omitted GWAS Hits: | chr5:83.260938−Mitochondrial DNA levels, NA |

Make more plots at http://csg.sph.umich.edu/locuszoom/

GWAS Catalog SNPs in Region

| chr | pos (Mb) | trait | snp |
|---|---|---|---|
| 5 | 82.81912 | Laryngeal squamous cell carcinoma | rs310518 |
| 5 | 82.84549 | Diisocyanate–induced asthma | rs3852186 |
| 5 | 82.88991 | Major depressive disorder | rs310501 |
| 5 | 82.96073 | Visceral fat | rs3846635 |
| 5 | 82.98574 | Prostate cancer | rs4466137 |
| 5 | 83.17359 | Ankylosing spondylitis | rs4552569 |
| 5 | 83.26094 | Mitochondrial DNA levels | rs2301070 |

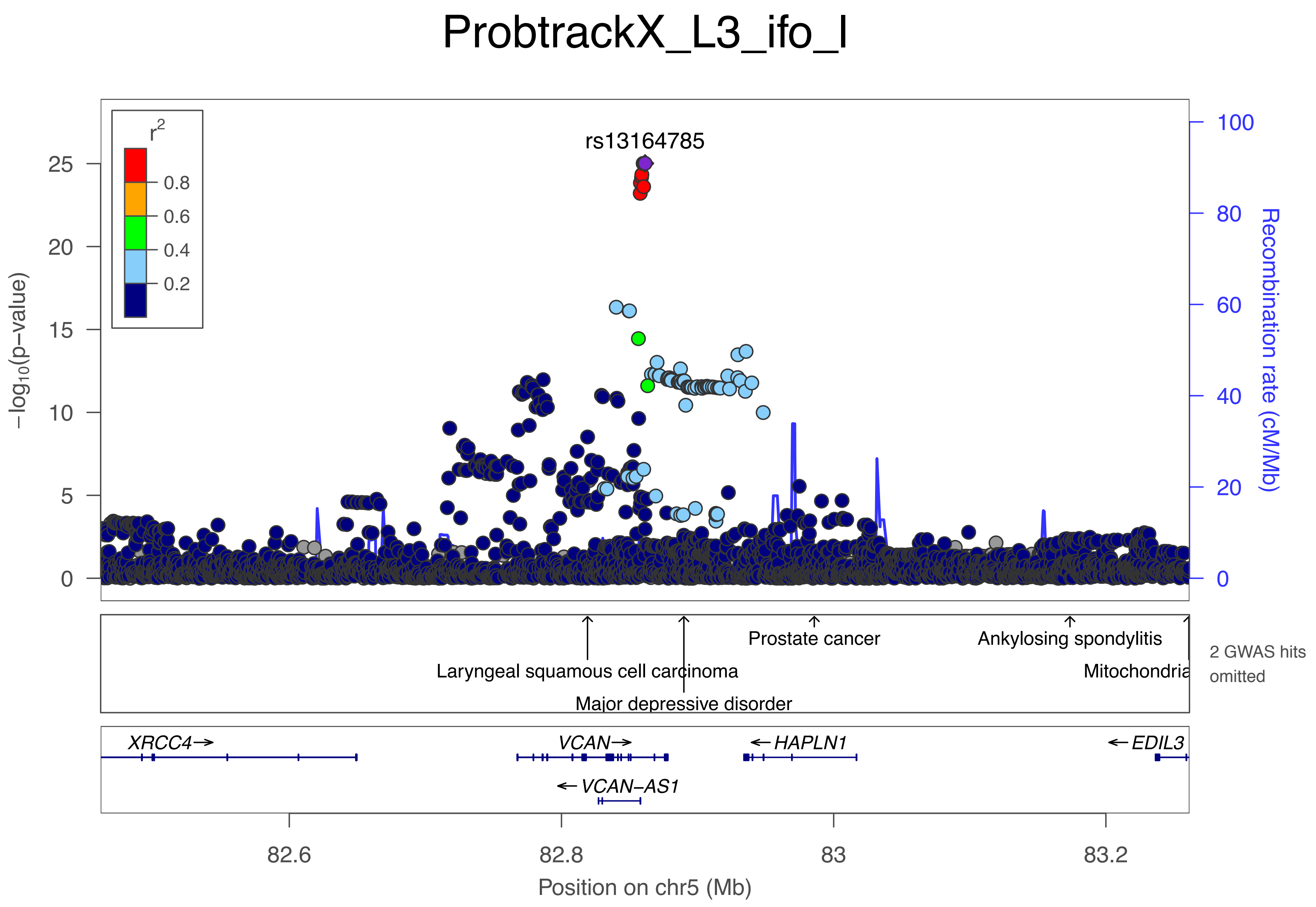

ProbtrackX_L3_ifo_l
rs13164785
r2
0.8
0.6
0.4
0.2
−log10(p−value)
Recombination rate (cM/Mb)
Prostate cancer
Ankylosing spondylitis
Laryngeal squamous cell carcinoma
Mitochondria
Major depressive disorder
2 GWAS hits omitted
XRCC4
VCAN
HAPLN1
EDIL3
VCAN−AS1
82.6
82.8
83
83.2
Position on chr5 (Mb)

| | |
|---|---|
| date: | Thu Aug 17 17:58:12 2017 |
| build: | hg19 |
| display range: | chr5:82461400−83261400 [82461400−83261400] |
| hilite range: | 0 – 0 [ 0 – 0 ] |
| reference SNP: | chr5:82861400 |
| number of SNPs plotted: | 3396 |
| min P.value: | 9.64E−26 [chr5:82861400] |
| max P.value: | 10E−1 [chr5:83215254] |
| omitted GWAS Hits: | chr5:83.260938−Mitochondrial DNA levels, NA |

Make more plots at http://csg.sph.umich.edu/locuszoom/

GWAS Catalog SNPs in Region

| chr | pos (Mb) | trait | snp |
|---|---|---|---|
| 5 | 82.81912 | Laryngeal squamous cell carcinoma | rs310518 |
| 5 | 82.84549 | Diisocyanate–induced asthma | rs3852186 |
| 5 | 82.88991 | Major depressive disorder | rs310501 |
| 5 | 82.96073 | Visceral fat | rs3846635 |
| 5 | 82.98574 | Prostate cancer | rs4466137 |
| 5 | 83.17359 | Ankylosing spondylitis | rs4552569 |
| 5 | 83.26094 | Mitochondrial DNA levels | rs2301070 |

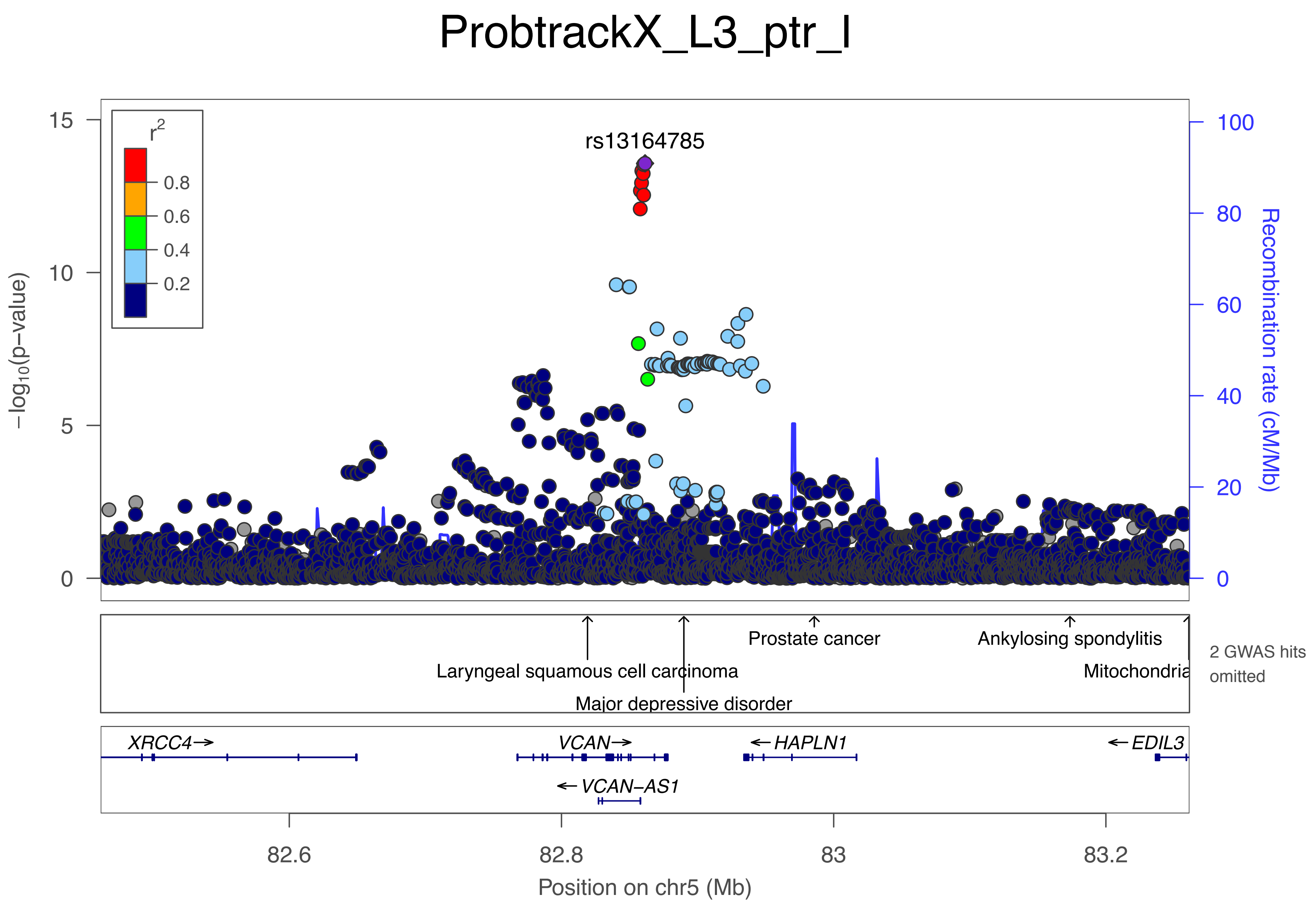
ProbtrackX_L3_ptr_l
rs13164785
r2
0.8
0.6
0.4
0.2
15
10
5
0
−log10(p−value)
100
80
60
40
20
0
Recombination rate (cM/Mb)
Prostate cancer
Ankylosing spondylitis
Laryngeal squamous cell carcinoma
Mitochondria
Major depressive disorder
2 GWAS hits omitted
XRCC4
VCAN
HAPLN1
EDIL3
VCAN−AS1
82.6
82.8
83
83.2
Position on chr5 (Mb)

| | |
|---|---|
| date: | Thu Aug 17 17:58:12 2017 |
| build: | hg19 |
| display range: | chr5:82461400−83261400 [82461400−83261400] |
| hilite range: | 0 – 0  [ 0 – 0 ] |
| reference SNP: | chr5:82861400 |
| number of SNPs plotted: | 3396 |
| min P.value: | 2.69E−14 [chr5:82861400] |
| max P.value: | 10E−1 [chr5:83012450] |
| omitted GWAS Hits: | chr5:83.260938−Mitochondrial DNA levels, NA |

Make more plots at http://csg.sph.umich.edu/locuszoom/

GWAS Catalog SNPs in Region

| chr | pos (Mb) | trait | snp |
|---|---|---|---|
| 5 | 82.81912 | Laryngeal squamous cell carcinoma | rs310518 |
| 5 | 82.84549 | Diisocyanate–induced asthma | rs3852186 |
| 5 | 82.88991 | Major depressive disorder | rs310501 |
| 5 | 82.96073 | Visceral fat | rs3846635 |
| 5 | 82.98574 | Prostate cancer | rs4466137 |
| 5 | 83.17359 | Ankylosing spondylitis | rs4552569 |
| 5 | 83.26094 | Mitochondrial DNA levels | rs2301070 |

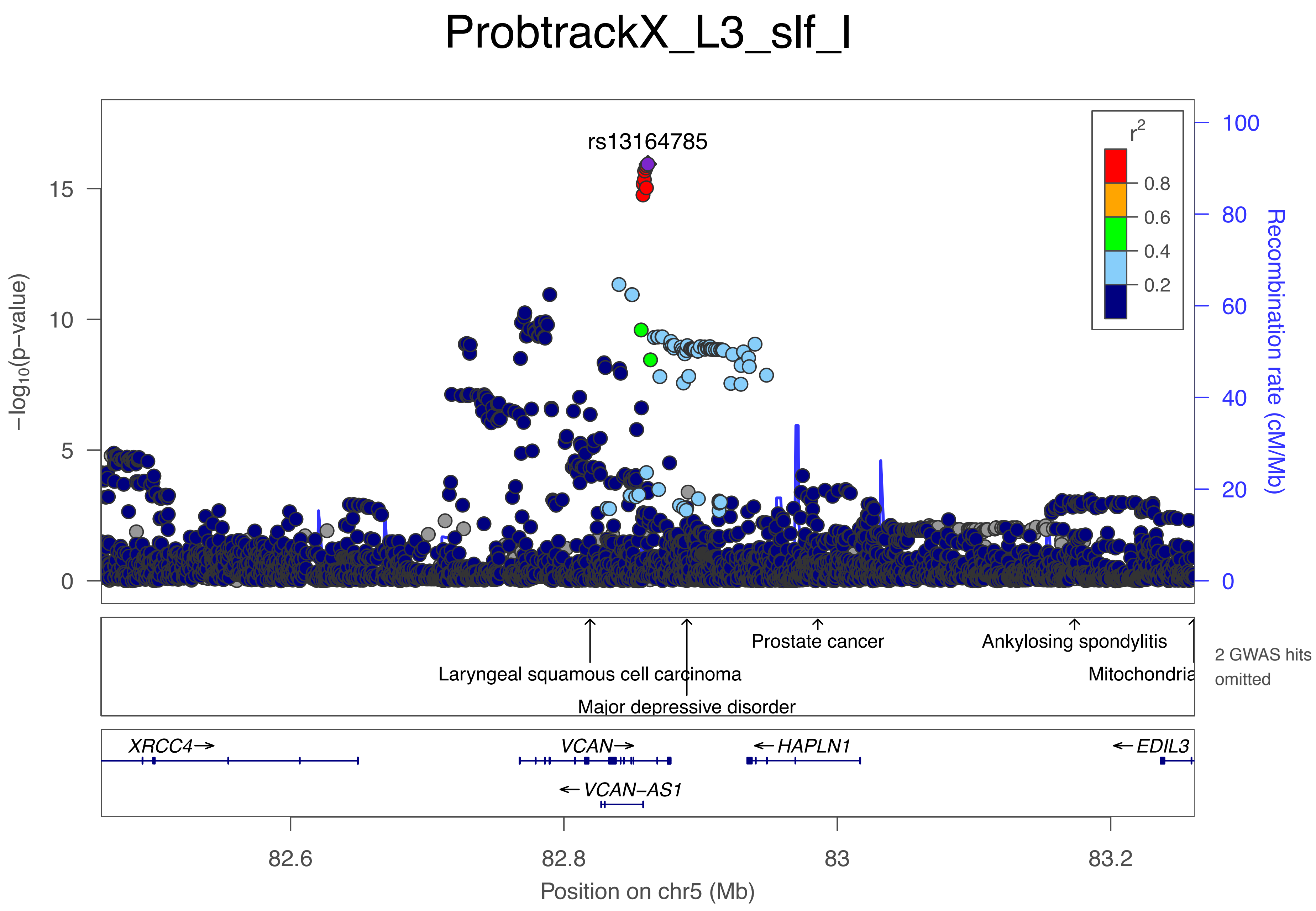
ProbtrackX_L3_slf_l
rs13164785
r²
0.8
0.6
0.4
0.2
−log10(p−value)
0
5
10
15
Recombination rate (cM/Mb)
0
20
40
60
80
100
Laryngeal squamous cell carcinoma
Major depressive disorder
Prostate cancer
Ankylosing spondylitis
Mitochondria
2 GWAS hits omitted
XRCC4→
VCAN→
←HAPLN1
←EDIL3
←VCAN−AS1
82.6
82.8
83
83.2
Position on chr5 (Mb)

| | |
|---|---|
| date: | Thu Aug 17 17:58:12 2017 |
| build: | hg19 |
| display range: | chr5:82461400−83261400 [82461400−83261400] |
| hilite range: | 0 – 0 [ 0 – 0 ] |
| reference SNP: | chr5:82861400 |
| number of SNPs plotted: | 3396 |
| min P.value: | 1.15E−16 [chr5:82861400] |
| max P.value: | 9.99E−1 [chr5:82834009] |
| omitted GWAS Hits: | chr5:83.260938−Mitochondrial DNA levels, NA |

Make more plots at http://csg.sph.umich.edu/locuszoom/

GWAS Catalog SNPs in Region

| chr | pos (Mb) | trait | snp |
|---|---|---|---|
| 5 | 82.81912 | Laryngeal squamous cell carcinoma | rs310518 |
| 5 | 82.84549 | Diisocyanate–induced asthma | rs3852186 |
| 5 | 82.88991 | Major depressive disorder | rs310501 |
| 5 | 82.96073 | Visceral fat | rs3846635 |
| 5 | 82.98574 | Prostate cancer | rs4466137 |
| 5 | 83.17359 | Ankylosing spondylitis | rs4552569 |
| 5 | 83.26094 | Mitochondrial DNA levels | rs2301070 |

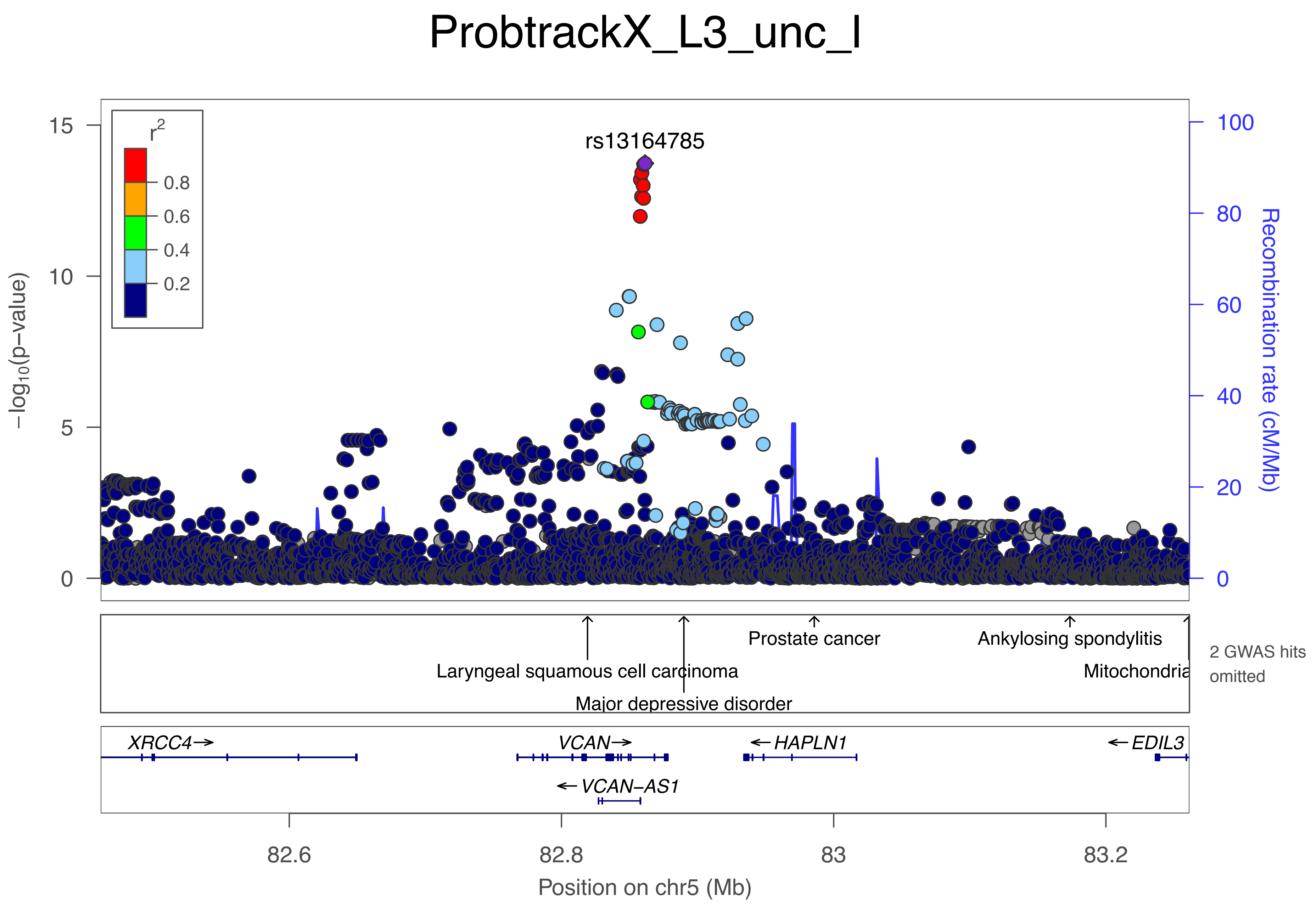

ProbtrackX_L3_unc_l
rs13164785
r²
0.8
0.6
0.4
0.2
15
10
5
0
−log10(p−value)
100
80
60
40
20
0
Recombination rate (cM/Mb)
Laryngeal squamous cell carcinoma
Major depressive disorder
Prostate cancer
Ankylosing spondylitis
Mitochondria
2 GWAS hits omitted
XRCC4→
VCAN→
←VCAN−AS1
←HAPLN1
←EDIL3
82.6
82.8
83
83.2
Position on chr5 (Mb)

| | |
|---|---|
| date: | Thu Aug 17 17:58:12 2017 |
| build: | hg19 |
| display range: | chr5:82461400−83261400 [82461400−83261400] |
| hilite range: | 0 – 0 [ 0 – 0 ] |
| reference SNP: | chr5:82861400 |
| number of SNPs plotted: | 3396 |
| min P.value: | 1.85E−14 [chr5:82861400] |
| max P.value: | 10E−1 [chr5:82526953] |
| omitted GWAS Hits: | chr5:83.260938−Mitochondrial DNA levels, NA |

Make more plots at http://csg.sph.umich.edu/locuszoom/

GWAS Catalog SNPs in Region

| chr | pos (Mb) | trait | snp |
|---|---|---|---|
| 5 | 82.81912 | Laryngeal squamous cell carcinoma | rs310518 |
| 5 | 82.84549 | Diisocyanate–induced asthma | rs3852186 |
| 5 | 82.88991 | Major depressive disorder | rs310501 |
| 5 | 82.96073 | Visceral fat | rs3846635 |
| 5 | 82.98574 | Prostate cancer | rs4466137 |
| 5 | 83.17359 | Ankylosing spondylitis | rs4552569 |
| 5 | 83.26094 | Mitochondrial DNA levels | rs2301070 |

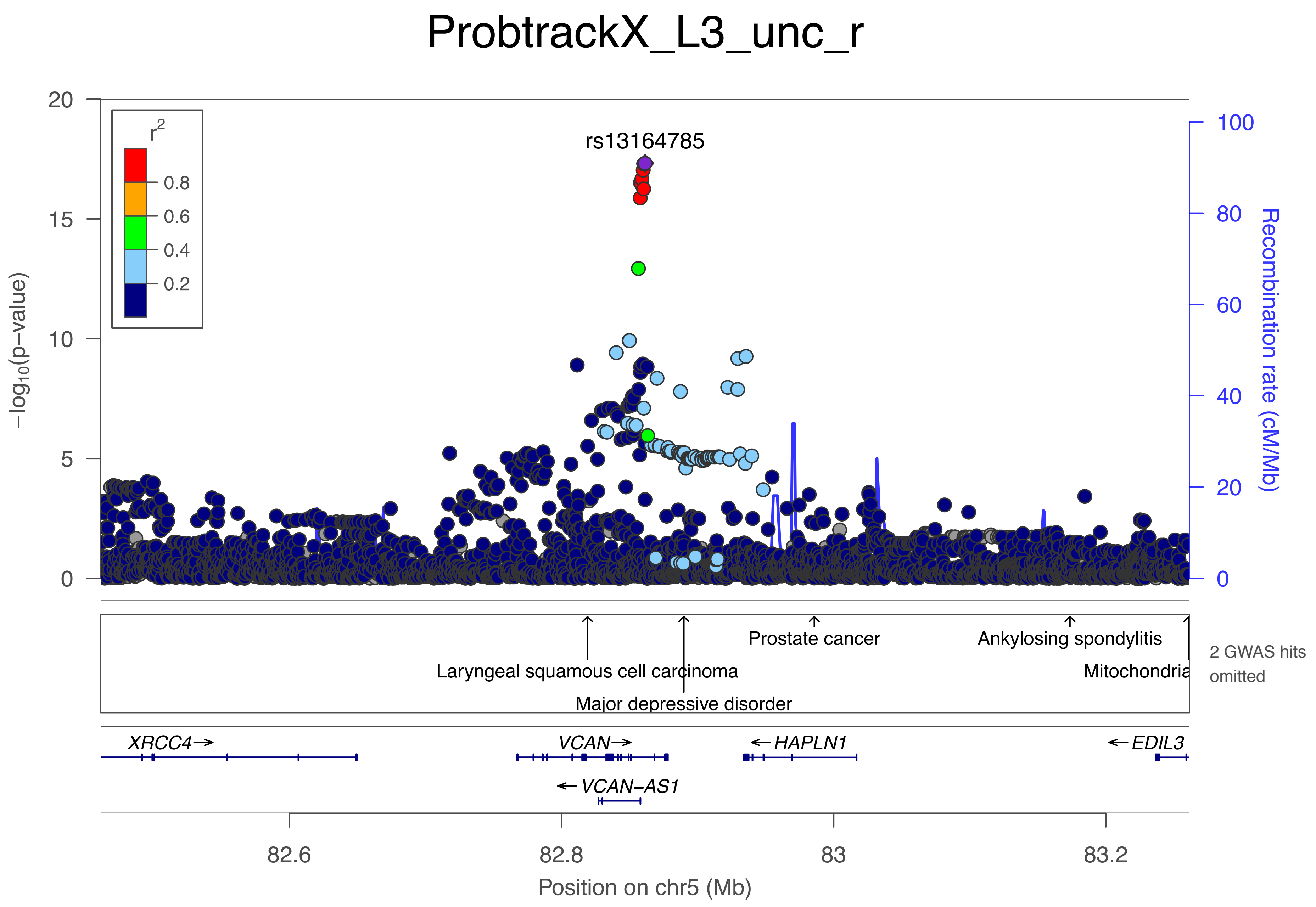

ProbtrackX_L3_unc_r
rs13164785
r2
0.8
0.6
0.4
0.2
20
15
10
5
0
−log10(p−value)
100
80
60
40
20
0
Recombination rate (cM/Mb)
Prostate cancer
Ankylosing spondylitis
Laryngeal squamous cell carcinoma
Mitochondria
Major depressive disorder
2 GWAS hits omitted
XRCC4→
VCAN→
←HAPLN1
←EDIL3
←VCAN−AS1
82.6
82.8
83
83.2
Position on chr5 (Mb)

| | |
|---|---|
| date: | Thu Aug 17 17:58:12 2017 |
| build: | hg19 |
| display range: | chr5:82461400−83261400 [82461400−83261400] |
| hilite range: | 0 – 0 [ 0 – 0 ] |
| reference SNP: | chr5:82861400 |
| number of SNPs plotted: | 3396 |
| min P.value: | 4.79E−18 [chr5:82861400] |
| max P.value: | 9.99E−1 [chr5:82782892] |
| omitted GWAS Hits: | chr5:83.260938−Mitochondrial DNA levels, NA |

Make more plots at http://csg.sph.umich.edu/locuszoom/

GWAS Catalog SNPs in Region

| chr | pos (Mb) | trait | snp |
|---|---|---|---|
| 5 | 82.81912 | Laryngeal squamous cell carcinoma | rs310518 |
| 5 | 82.84549 | Diisocyanate–induced asthma | rs3852186 |
| 5 | 82.88991 | Major depressive disorder | rs310501 |
| 5 | 82.96073 | Visceral fat | rs3846635 |
| 5 | 82.98574 | Prostate cancer | rs4466137 |
| 5 | 83.17359 | Ankylosing spondylitis | rs4552569 |
| 5 | 83.26094 | Mitochondrial DNA levels | rs2301070 |

# ProbtrackX_ICVF_ar_l

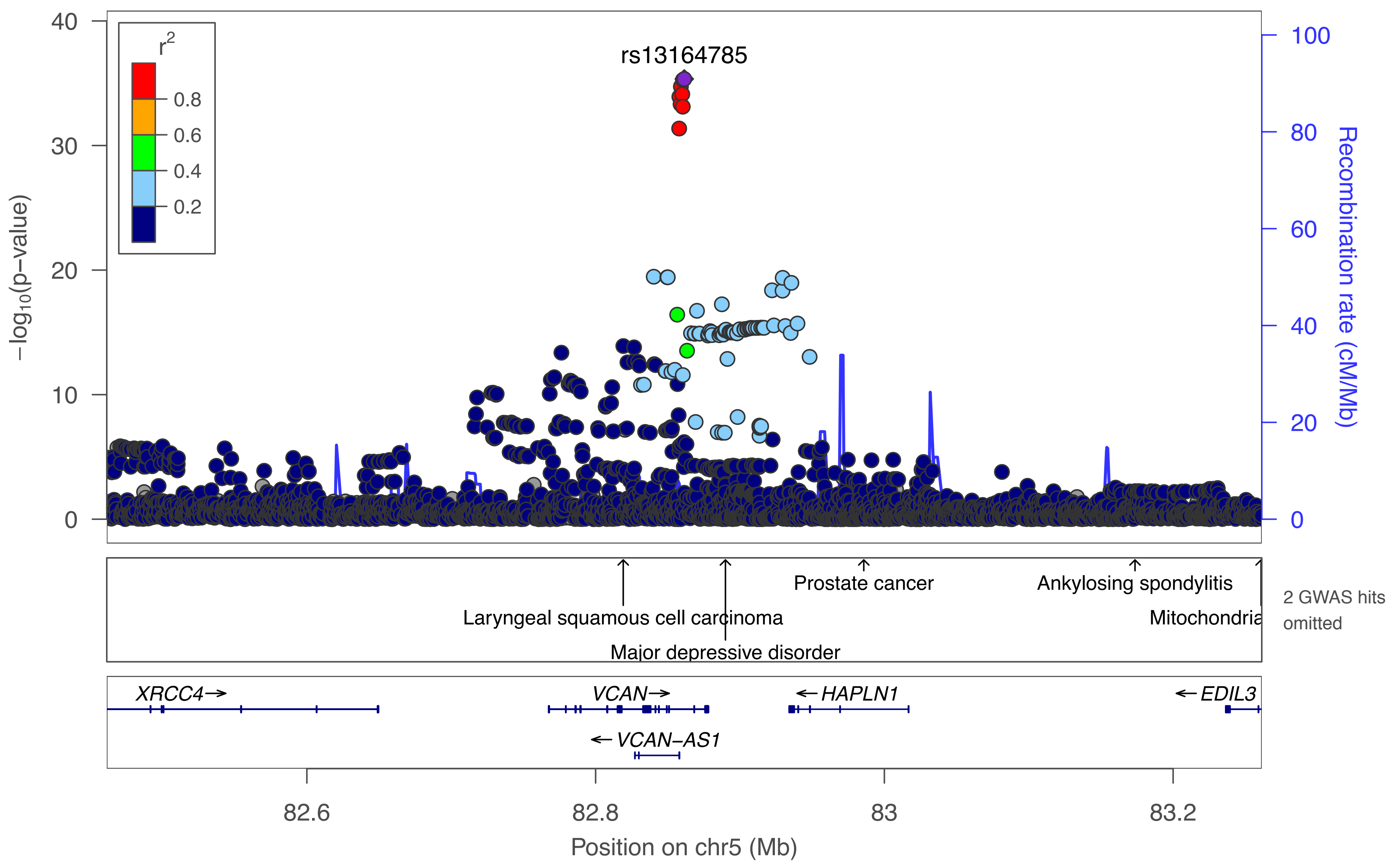

| | |
|---|---|
| date: | Thu Aug 17 17:58:12 2017 |
| build: | hg19 |
| display range: | chr5:82461400–83261400 [82461400–83261400] |
| hilite range: | 0 – 0 [ 0 – 0 ] |
| reference SNP: | chr5:82861400 |
| number of SNPs plotted: | 3396 |
| min P.value: | 4.55E–36 [chr5:82861400] |
| max P.value: | 9.99E–1 [chr5:83165588] |
| omitted GWAS Hits: | chr5:83.260938–Mitochondrial DNA levels, NA |

Make more plots at http://csg.sph.umich.edu/locuszoom/

GWAS Catalog SNPs in Region

| chr | pos (Mb) | trait | snp |
|---|---|---|---|
| 5 | 82.81912 | Laryngeal squamous cell carcinoma | rs310518 |
| 5 | 82.84549 | Diisocyanate–induced asthma | rs3852186 |
| 5 | 82.88991 | Major depressive disorder | rs310501 |
| 5 | 82.96073 | Visceral fat | rs3846635 |
| 5 | 82.98574 | Prostate cancer | rs4466137 |
| 5 | 83.17359 | Ankylosing spondylitis | rs4552569 |
| 5 | 83.26094 | Mitochondrial DNA levels | rs2301070 |

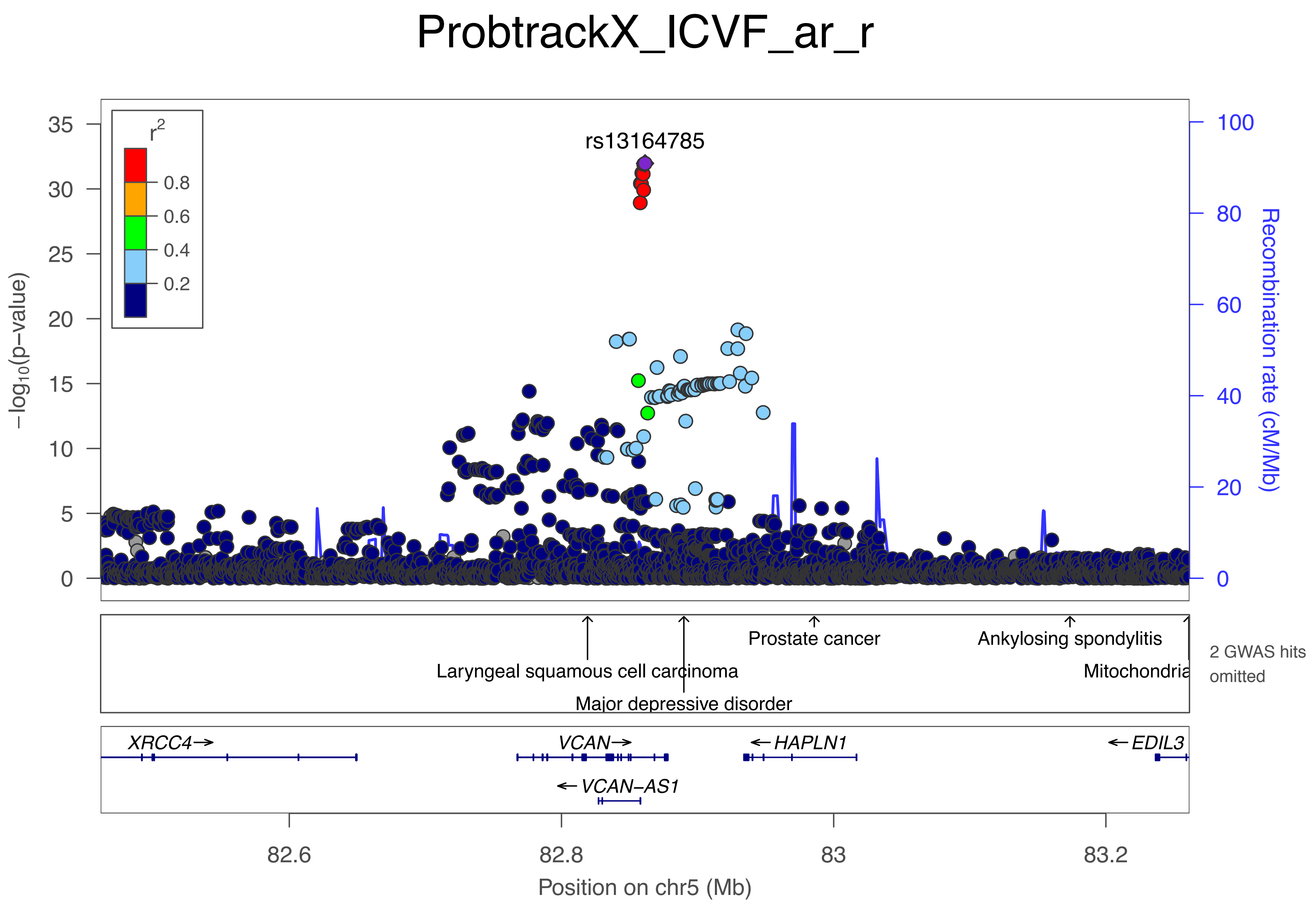
ProbtrackX_ICVF_ar_r
rs13164785
r2
0.8
0.6
0.4
0.2
−log10(p−value)
Recombination rate (cM/Mb)
Prostate cancer
Ankylosing spondylitis
Laryngeal squamous cell carcinoma
Mitochondria
Major depressive disorder
2 GWAS hits omitted
XRCC4→
VCAN→
←HAPLN1
←EDIL3
←VCAN−AS1
82.6
82.8
83
83.2
Position on chr5 (Mb)

| | |
|---|---|
| date: | Thu Aug 17 17:58:12 2017 |
| build: | hg19 |
| display range: | chr5:82461400−83261400 [82461400−83261400] |
| hilite range: | 0 − 0 [ 0 − 0 ] |
| reference SNP: | chr5:82861400 |
| number of SNPs plotted: | 3396 |
| min P.value: | 1.07E−32 [chr5:82861400] |
| max P.value: | 10E−1 [chr5:83227145] |
| omitted GWAS Hits: | chr5:83.260938−Mitochondrial DNA levels, NA |

Make more plots at http://csg.sph.umich.edu/locuszoom/

GWAS Catalog SNPs in Region

| chr | pos (Mb) | trait | snp |
|---|---|---|---|
| 5 | 82.81912 | Laryngeal squamous cell carcinoma | rs310518 |
| 5 | 82.84549 | Diisocyanate−induced asthma | rs3852186 |
| 5 | 82.88991 | Major depressive disorder | rs310501 |
| 5 | 82.96073 | Visceral fat | rs3846635 |
| 5 | 82.98574 | Prostate cancer | rs4466137 |
| 5 | 83.17359 | Ankylosing spondylitis | rs4552569 |
| 5 | 83.26094 | Mitochondrial DNA levels | rs2301070 |

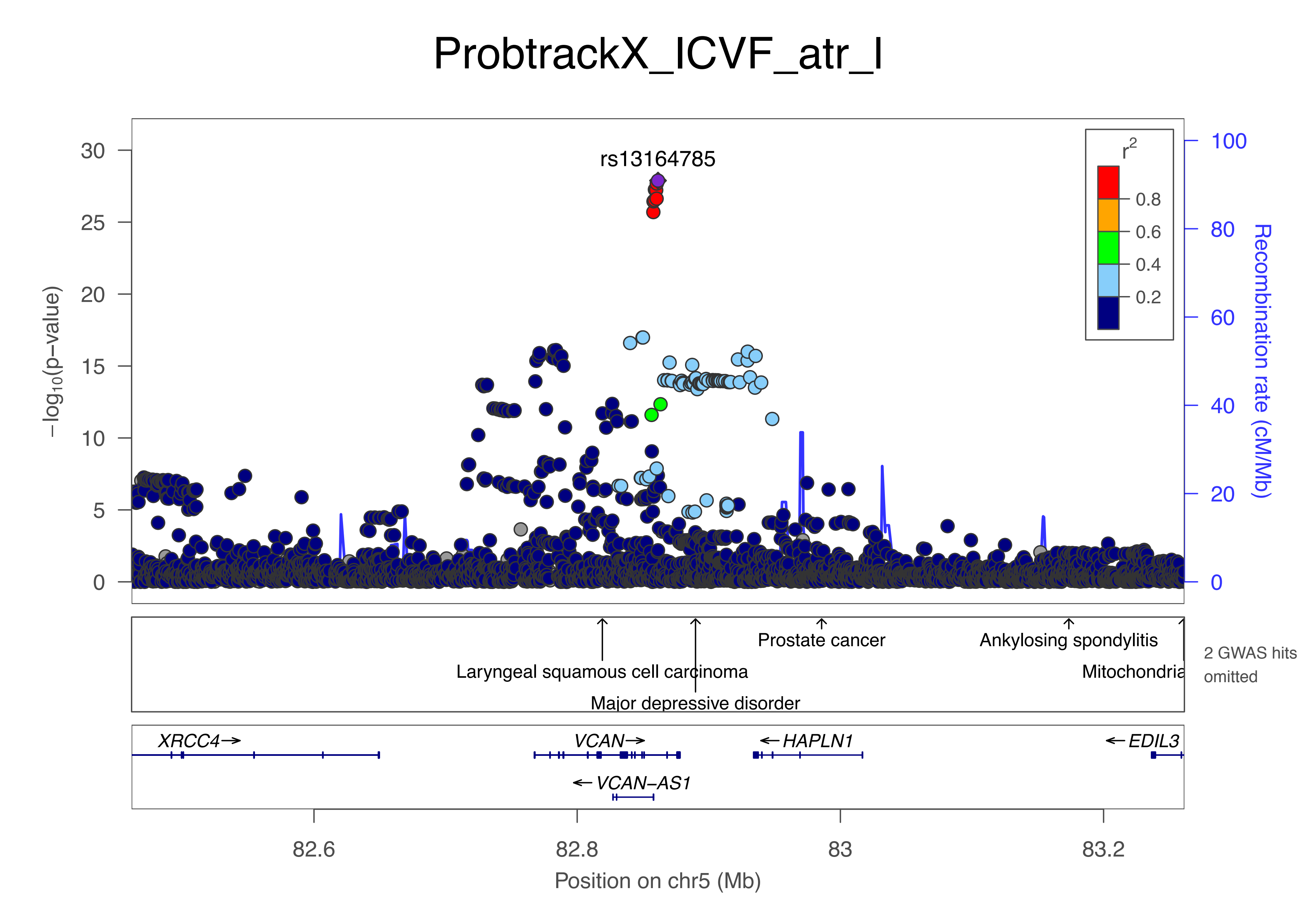
ProbtrackX_ICVF_atr_l
rs13164785
r2
0.8
0.6
0.4
0.2
−log10(p−value)
30
25
20
15
10
5
0
Recombination rate (cM/Mb)
100
80
60
40
20
0
Prostate cancer
Ankylosing spondylitis
Laryngeal squamous cell carcinoma
Mitochondria
Major depressive disorder
2 GWAS hits omitted
XRCC4→
VCAN→
←HAPLN1
←EDIL3
←VCAN−AS1
82.6
82.8
83
83.2
Position on chr5 (Mb)

| | |
|---|---|
| date: | Thu Aug 17 17:58:12 2017 |
| build: | hg19 |
| display range: | chr5:82461400–83261400 [82461400–83261400] |
| hilite range: | 0 – 0 [ 0 – 0 ] |
| reference SNP: | chr5:82861400 |
| number of SNPs plotted: | 3396 |
| min P.value: | 1.3E–28 [chr5:82861400] |
| max P.value: | 9.99E–1 [chr5:82791833] |
| omitted GWAS Hits: | chr5:83.260938–Mitochondrial DNA levels, NA |

Make more plots at http://csg.sph.umich.edu/locuszoom/

GWAS Catalog SNPs in Region

| chr | pos (Mb) | trait | snp |
|---|---|---|---|
| 5 | 82.81912 | Laryngeal squamous cell carcinoma | rs310518 |
| 5 | 82.84549 | Diisocyanate–induced asthma | rs3852186 |
| 5 | 82.88991 | Major depressive disorder | rs310501 |
| 5 | 82.96073 | Visceral fat | rs3846635 |
| 5 | 82.98574 | Prostate cancer | rs4466137 |
| 5 | 83.17359 | Ankylosing spondylitis | rs4552569 |
| 5 | 83.26094 | Mitochondrial DNA levels | rs2301070 |

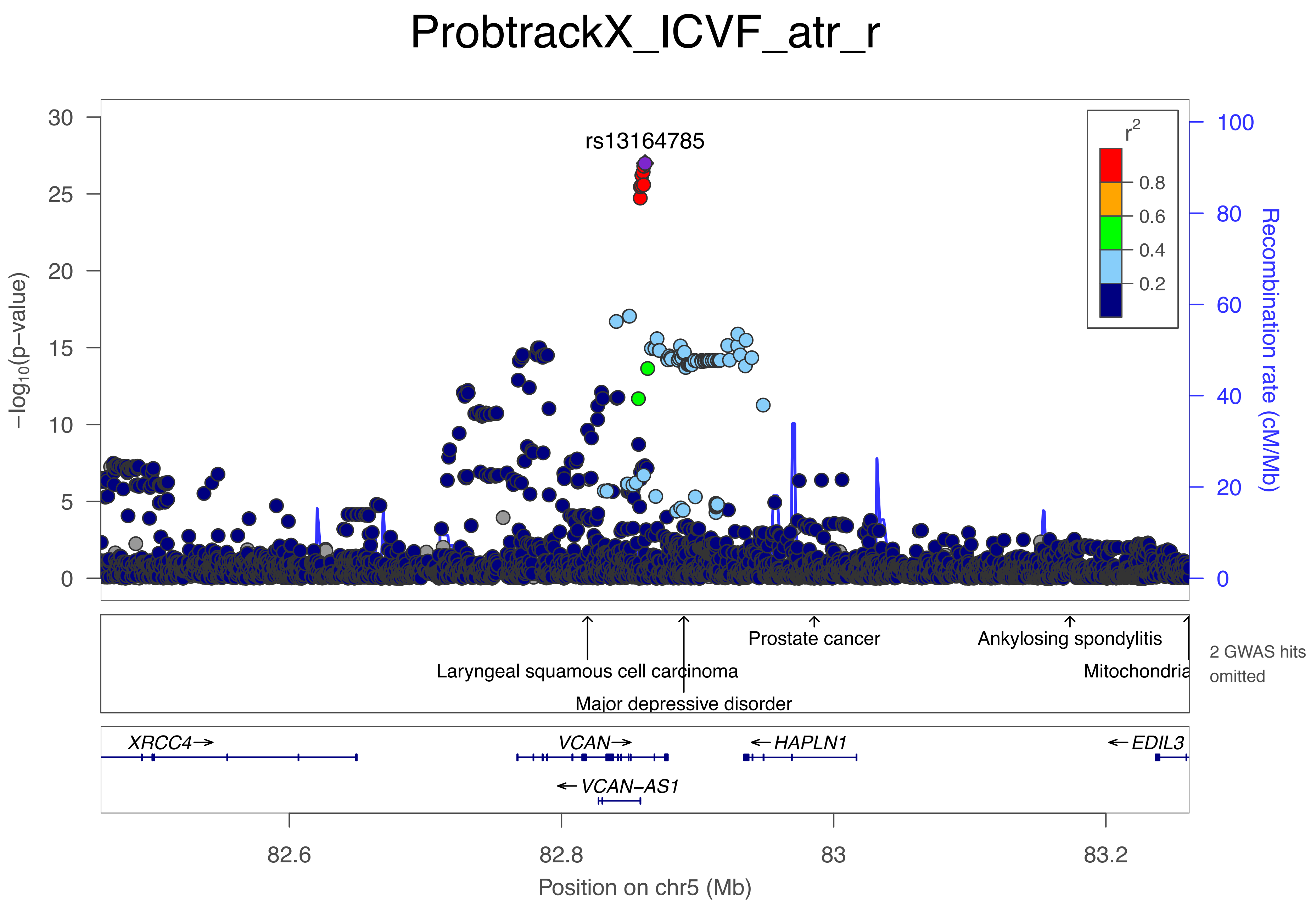

ProbtrackX_ICVF_atr_r
rs13164785
−log10(p−value)
Recombination rate (cM/Mb)
r2
0.8
0.6
0.4
0.2
Prostate cancer
Ankylosing spondylitis
Laryngeal squamous cell carcinoma
Major depressive disorder
Mitochondria
2 GWAS hits omitted
XRCC4
VCAN
HAPLN1
EDIL3
VCAN−AS1
82.6
82.8
83
83.2
Position on chr5 (Mb)

| | |
|---|---|
| date: | Thu Aug 17 17:58:12 2017 |
| build: | hg19 |
| display range: | chr5:82461400–83261400 [82461400–83261400] |
| hilite range: | 0 – 0 [ 0 – 0 ] |
| reference SNP: | chr5:82861400 |
| number of SNPs plotted: | 3396 |
| min P.value: | 1.03E–27 [chr5:82861400] |
| max P.value: | 9.99E–1 [chr5:83159644] |
| omitted GWAS Hits: | chr5:83.260938–Mitochondrial DNA levels, NA |

Make more plots at http://csg.sph.umich.edu/locuszoom/

GWAS Catalog SNPs in Region

| chr | pos (Mb) | trait | snp |
|---|---|---|---|
| 5 | 82.81912 | Laryngeal squamous cell carcinoma | rs310518 |
| 5 | 82.84549 | Diisocyanate–induced asthma | rs3852186 |
| 5 | 82.88991 | Major depressive disorder | rs310501 |
| 5 | 82.96073 | Visceral fat | rs3846635 |
| 5 | 82.98574 | Prostate cancer | rs4466137 |
| 5 | 83.17359 | Ankylosing spondylitis | rs4552569 |
| 5 | 83.26094 | Mitochondrial DNA levels | rs2301070 |

# ProbtrackX_ICVF_cgh_l

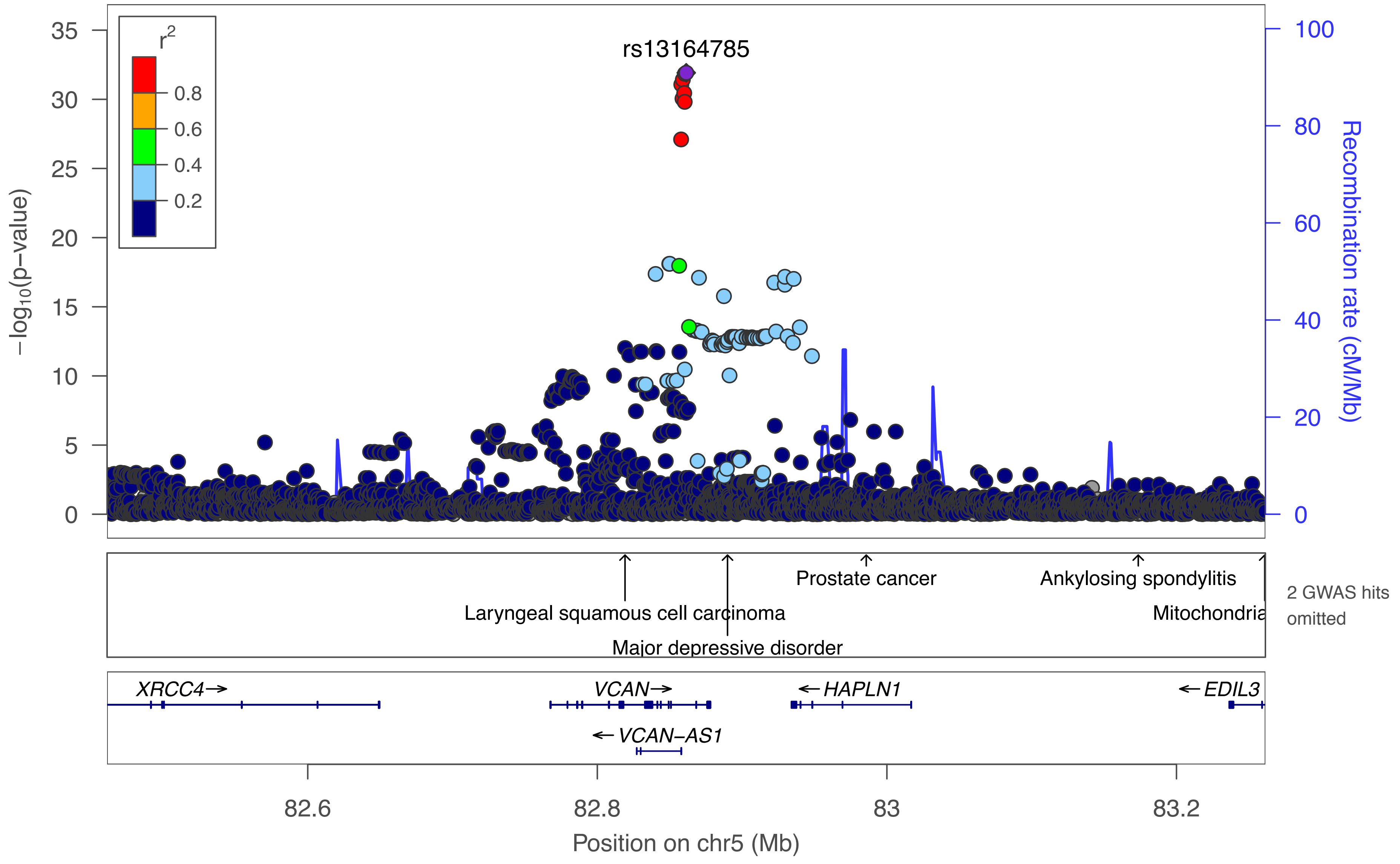

| | |
|---|---|
| date: | Thu Aug 17 17:58:12 2017 |
| build: | hg19 |
| display range: | chr5:82461400–83261400 [82461400–83261400] |
| hilite range: | 0 – 0 [ 0 – 0 ] |
| reference SNP: | chr5:82861400 |
| number of SNPs plotted: | 3396 |
| min P.value: | 1.21E–32 [chr5:82861400] |
| max P.value: | 10E–1 [chr5:82951885] |
| omitted GWAS Hits: | chr5:83.260938–Mitochondrial DNA levels, NA |

Make more plots at http://csg.sph.umich.edu/locuszoom/

GWAS Catalog SNPs in Region

| chr | pos (Mb) | trait | snp |
|---|---|---|---|
| 5 | 82.81912 | Laryngeal squamous cell carcinoma | rs310518 |
| 5 | 82.84549 | Diisocyanate–induced asthma | rs3852186 |
| 5 | 82.88991 | Major depressive disorder | rs310501 |
| 5 | 82.96073 | Visceral fat | rs3846635 |
| 5 | 82.98574 | Prostate cancer | rs4466137 |
| 5 | 83.17359 | Ankylosing spondylitis | rs4552569 |
| 5 | 83.26094 | Mitochondrial DNA levels | rs2301070 |

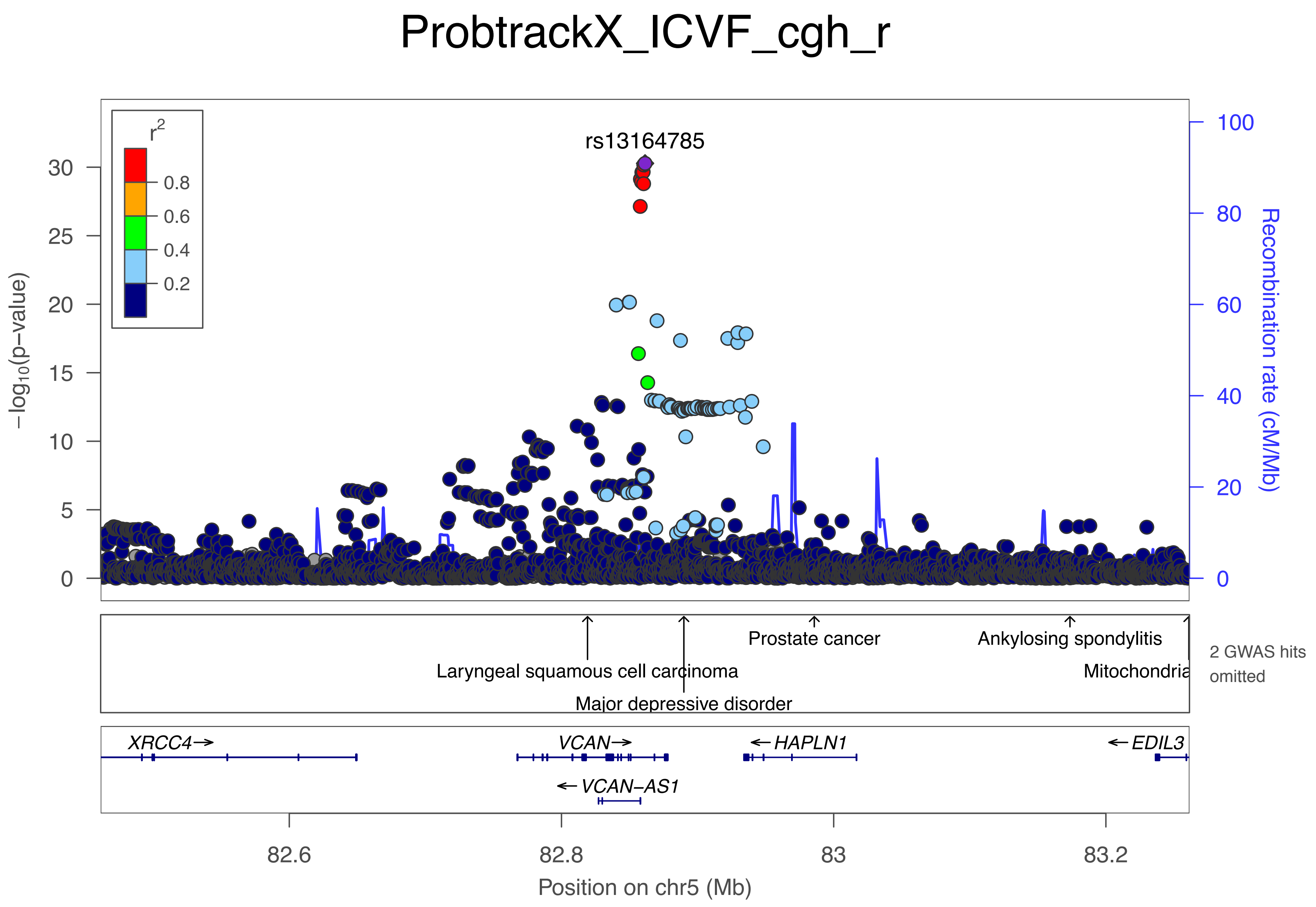
ProbtrackX_ICVF_cgh_r
rs13164785
r2
0.8
0.6
0.4
0.2
30
25
20
15
10
5
0
−log10(p−value)
100
80
60
40
20
0
Recombination rate (cM/Mb)
Laryngeal squamous cell carcinoma
Major depressive disorder
Prostate cancer
Ankylosing spondylitis
Mitochondria
2 GWAS hits omitted
XRCC4→
VCAN→
←VCAN−AS1
←HAPLN1
←EDIL3
82.6
82.8
83
83.2
Position on chr5 (Mb)

| | |
|---|---|
| date: | Thu Aug 17 17:58:12 2017 |
| build: | hg19 |
| display range: | chr5:82461400–83261400 [82461400–83261400] |
| hilite range: | 0 – 0 [ 0 – 0 ] |
| reference SNP: | chr5:82861400 |
| number of SNPs plotted: | 3396 |
| min P.value: | 5.25E–31 [chr5:82861400] |
| max P.value: | 9.98E–1 [chr5:83243278] |
| omitted GWAS Hits: | chr5:83.260938–Mitochondrial DNA levels, NA |

Make more plots at http://csg.sph.umich.edu/locuszoom/

GWAS Catalog SNPs in Region

| chr | pos (Mb) | trait | snp |
|---|---|---|---|
| 5 | 82.81912 | Laryngeal squamous cell carcinoma | rs310518 |
| 5 | 82.84549 | Diisocyanate–induced asthma | rs3852186 |
| 5 | 82.88991 | Major depressive disorder | rs310501 |
| 5 | 82.96073 | Visceral fat | rs3846635 |
| 5 | 82.98574 | Prostate cancer | rs4466137 |
| 5 | 83.17359 | Ankylosing spondylitis | rs4552569 |
| 5 | 83.26094 | Mitochondrial DNA levels | rs2301070 |

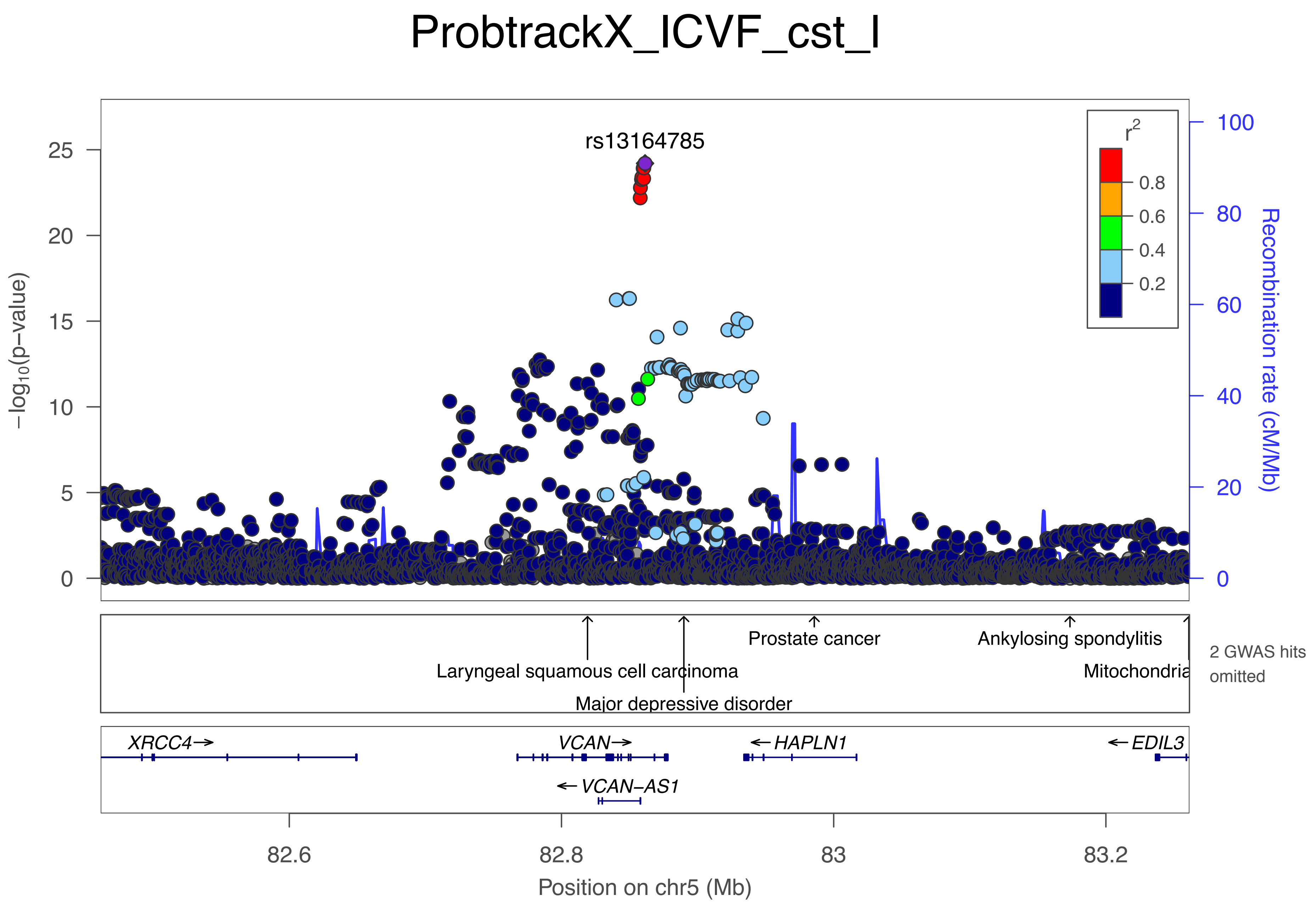
ProbtrackX_ICVF_cst_l
rs13164785
r2
0.8
0.6
0.4
0.2
25
20
15
10
5
0
100
80
60
40
20
0
−log10(p−value)
Recombination rate (cM/Mb)
Prostate cancer
Ankylosing spondylitis
Laryngeal squamous cell carcinoma
Mitochondria
Major depressive disorder
2 GWAS hits omitted
XRCC4→
VCAN→
←HAPLN1
←EDIL3
←VCAN−AS1
82.6
82.8
83
83.2
Position on chr5 (Mb)

| | |
|---|---|
| date: | Thu Aug 17 17:58:12 2017 |
| build: | hg19 |
| display range: | chr5:82461400−83261400 [82461400−83261400] |
| hilite range: | 0 – 0 [ 0 – 0 ] |
| reference SNP: | chr5:82861400 |
| number of SNPs plotted: | 3396 |
| min P.value: | 6.24E−25 [chr5:82861400] |
| max P.value: | 10E−1 [chr5:82605654] |
| omitted GWAS Hits: | chr5:83.260938−Mitochondrial DNA levels, NA |

Make more plots at http://csg.sph.umich.edu/locuszoom/

GWAS Catalog SNPs in Region

| chr | pos (Mb) | trait | snp |
|---|---|---|---|
| 5 | 82.81912 | Laryngeal squamous cell carcinoma | rs310518 |
| 5 | 82.84549 | Diisocyanate–induced asthma | rs3852186 |
| 5 | 82.88991 | Major depressive disorder | rs310501 |
| 5 | 82.96073 | Visceral fat | rs3846635 |
| 5 | 82.98574 | Prostate cancer | rs4466137 |
| 5 | 83.17359 | Ankylosing spondylitis | rs4552569 |
| 5 | 83.26094 | Mitochondrial DNA levels | rs2301070 |

# ProbtrackX_ICVF_cst_r

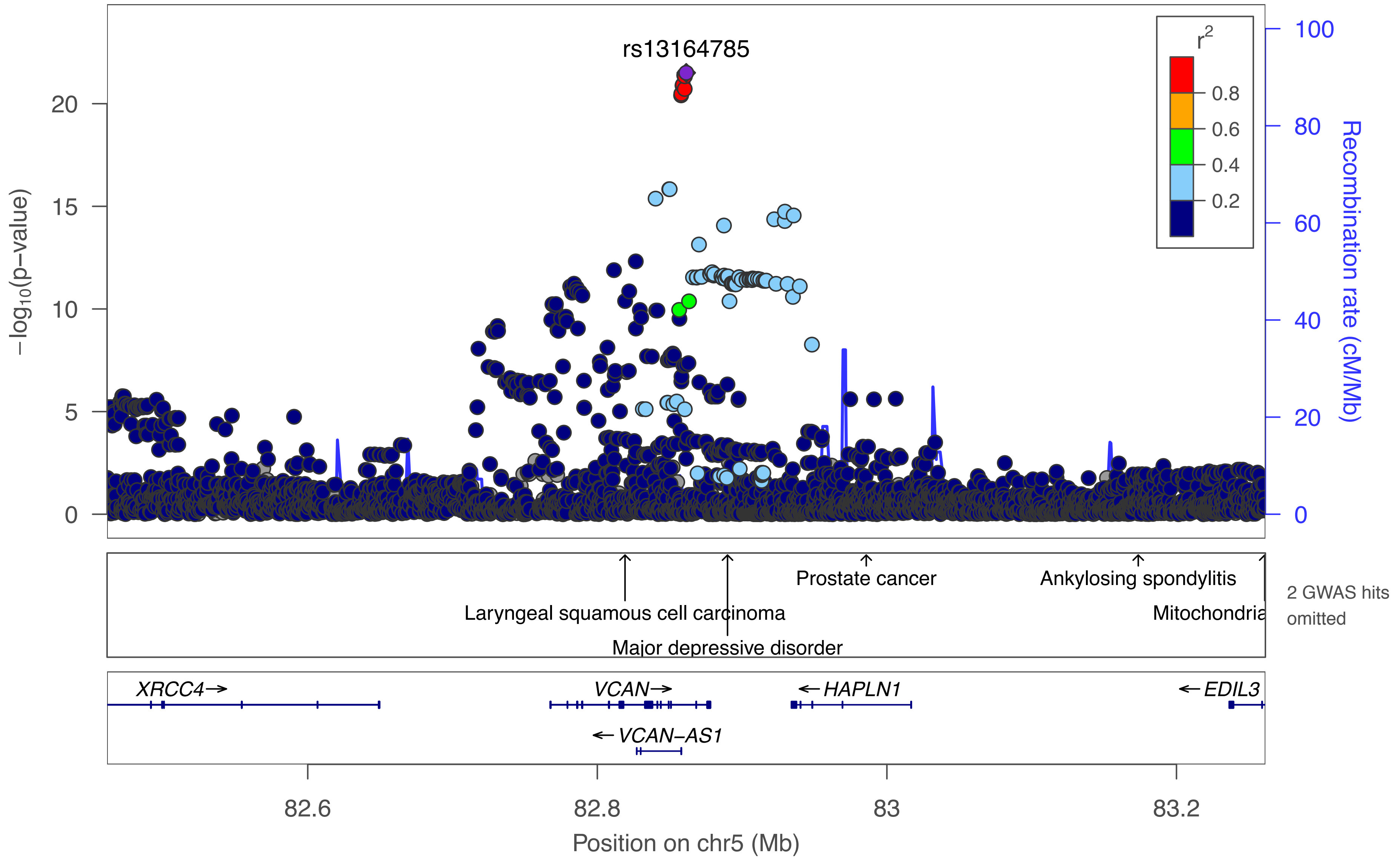

| | |
|---|---|
| date: | Thu Aug 17 17:58:12 2017 |
| build: | hg19 |
| display range: | chr5:82461400–83261400 [82461400–83261400] |
| hilite range: | 0 – 0 [ 0 – 0 ] |
| reference SNP: | chr5:82861400 |
| number of SNPs plotted: | 3396 |
| min P.value: | 3.13E–22 [chr5:82861400] |
| max P.value: | 10E–1 [chr5:82962487] |
| omitted GWAS Hits: | chr5:83.260938–Mitochondrial DNA levels, NA |

Make more plots at http://csg.sph.umich.edu/locuszoom/

GWAS Catalog SNPs in Region

| chr | pos (Mb) | trait | snp |
|---|---|---|---|
| 5 | 82.81912 | Laryngeal squamous cell carcinoma | rs310518 |
| 5 | 82.84549 | Diisocyanate–induced asthma | rs3852186 |
| 5 | 82.88991 | Major depressive disorder | rs310501 |
| 5 | 82.96073 | Visceral fat | rs3846635 |
| 5 | 82.98574 | Prostate cancer | rs4466137 |
| 5 | 83.17359 | Ankylosing spondylitis | rs4552569 |
| 5 | 83.26094 | Mitochondrial DNA levels | rs2301070 |

# ProbtrackX_ICVF_fmi

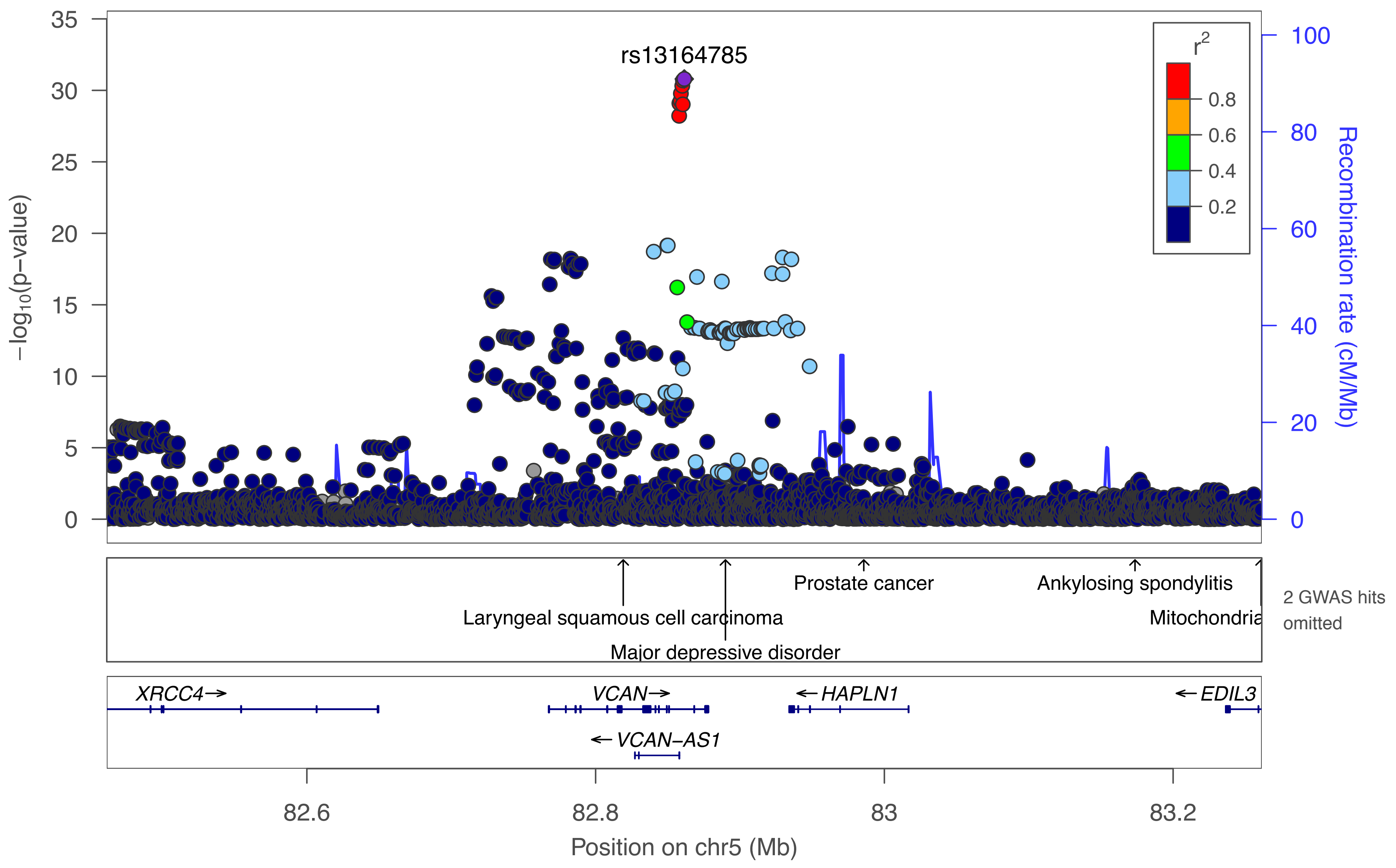

| | |
|---|---|
| date: | Thu Aug 17 17:58:12 2017 |
| build: | hg19 |
| display range: | chr5:82461400–83261400 [82461400–83261400] |
| hilite range: | 0 – 0 [ 0 – 0 ] |
| reference SNP: | chr5:82861400 |
| number of SNPs plotted: | 3396 |
| min P.value: | 1.6E−31 [chr5:82861400] |
| max P.value: | 9.99E−1 [chr5:82847913] |
| omitted GWAS Hits: | chr5:83.260938–Mitochondrial DNA levels, NA |

Make more plots at http://csg.sph.umich.edu/locuszoom/

GWAS Catalog SNPs in Region

| chr | pos (Mb) | trait | snp |
|---|---|---|---|
| 5 | 82.81912 | Laryngeal squamous cell carcinoma | rs310518 |
| 5 | 82.84549 | Diisocyanate−induced asthma | rs3852186 |
| 5 | 82.88991 | Major depressive disorder | rs310501 |
| 5 | 82.96073 | Visceral fat | rs3846635 |
| 5 | 82.98574 | Prostate cancer | rs4466137 |
| 5 | 83.17359 | Ankylosing spondylitis | rs4552569 |
| 5 | 83.26094 | Mitochondrial DNA levels | rs2301070 |

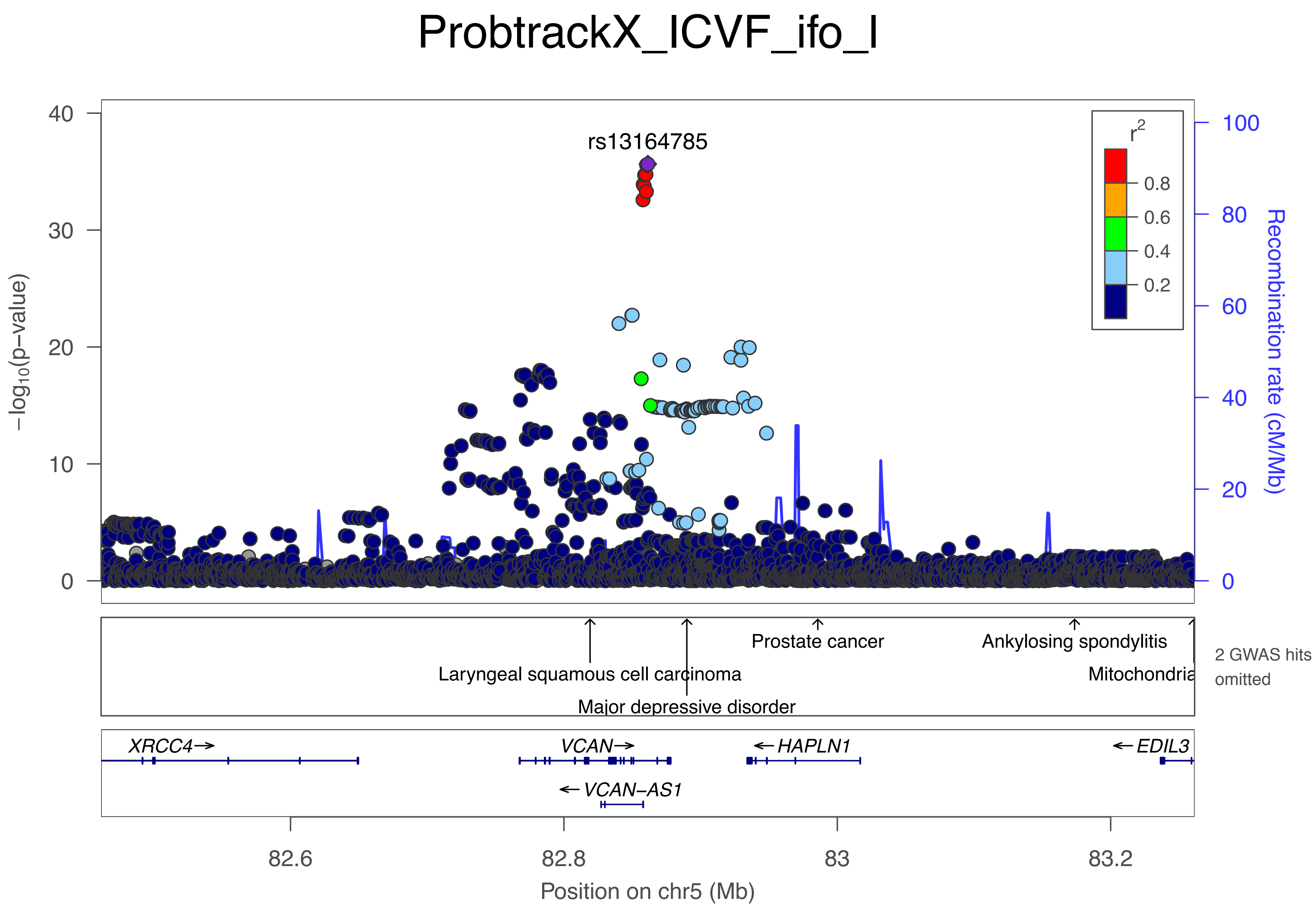

ProbtrackX_ICVF_ifo_l
rs13164785
−log10(p−value)
Recombination rate (cM/Mb)
r2
0.8
0.6
0.4
0.2
40
30
20
10
0
100
80
60
40
20
0
Prostate cancer
Ankylosing spondylitis
Laryngeal squamous cell carcinoma
Mitochondria
Major depressive disorder
2 GWAS hits omitted
XRCC4
VCAN
HAPLN1
EDIL3
VCAN−AS1
82.6
82.8
83
83.2
Position on chr5 (Mb)

| | |
|---|---|
| date: | Thu Aug 17 17:58:12 2017 |
| build: | hg19 |
| display range: | chr5:82461400–83261400 [82461400–83261400] |
| hilite range: | 0 – 0 [ 0 – 0 ] |
| reference SNP: | chr5:82861400 |
| number of SNPs plotted: | 3396 |
| min P.value: | 2.27E–36 [chr5:82861400] |
| max P.value: | 9.98E–1 [chr5:82691419] |
| omitted GWAS Hits: | chr5:83.260938–Mitochondrial DNA levels, NA |

Make more plots at http://csg.sph.umich.edu/locuszoom/

GWAS Catalog SNPs in Region

| chr | pos (Mb) | trait | snp |
|---|---|---|---|
| 5 | 82.81912 | Laryngeal squamous cell carcinoma | rs310518 |
| 5 | 82.84549 | Diisocyanate−induced asthma | rs3852186 |
| 5 | 82.88991 | Major depressive disorder | rs310501 |
| 5 | 82.96073 | Visceral fat | rs3846635 |
| 5 | 82.98574 | Prostate cancer | rs4466137 |
| 5 | 83.17359 | Ankylosing spondylitis | rs4552569 |
| 5 | 83.26094 | Mitochondrial DNA levels | rs2301070 |

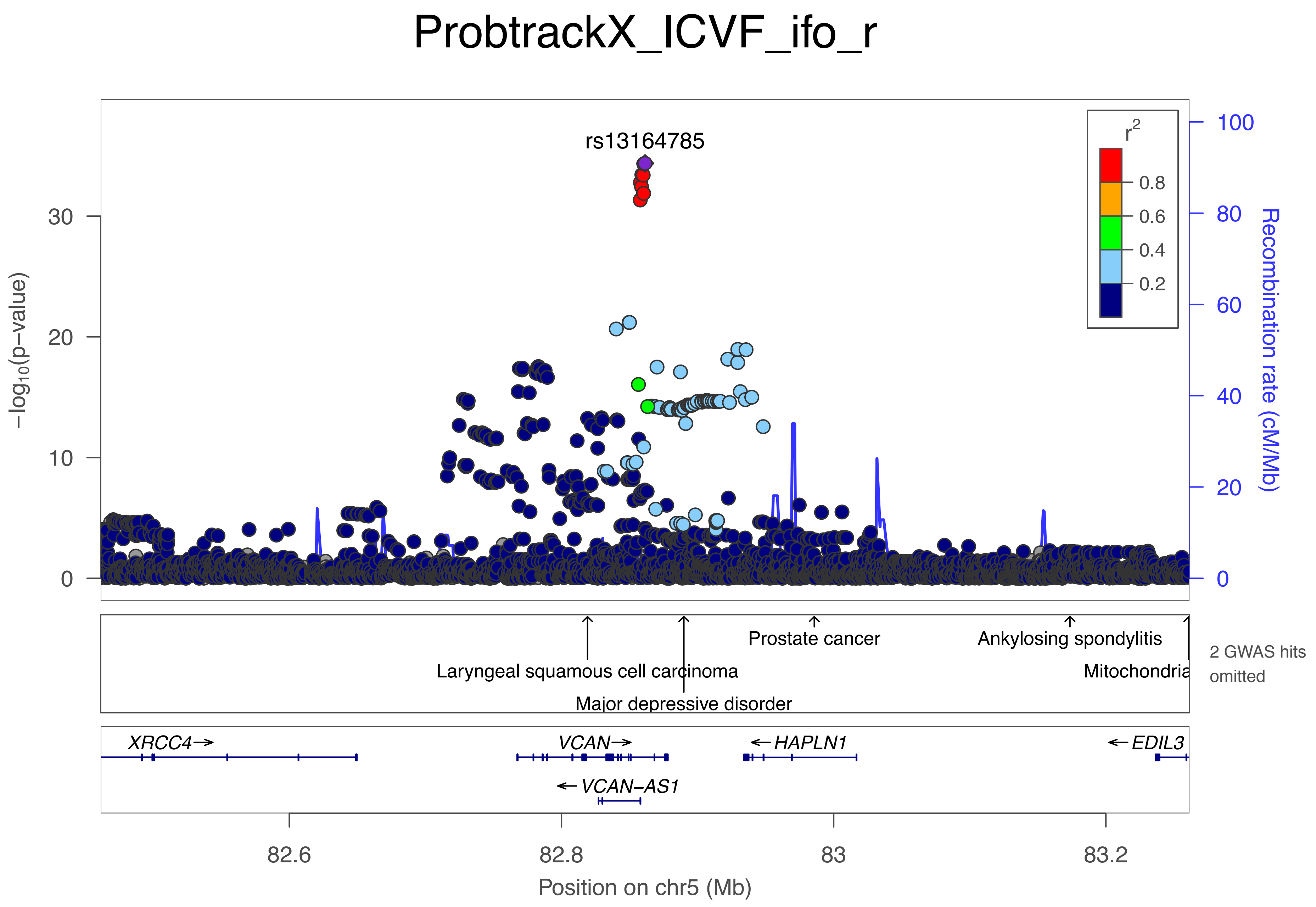
ProbtrackX_ICVF_ifo_r
rs13164785
r2
0.8
0.6
0.4
0.2
−log10(p−value)
30
20
10
0
100
80
60
40
20
0
Recombination rate (cM/Mb)
Prostate cancer
Ankylosing spondylitis
Laryngeal squamous cell carcinoma
Mitochondria
Major depressive disorder
2 GWAS hits omitted
XRCC4→
VCAN→
←HAPLN1
←EDIL3
←VCAN−AS1
82.6
82.8
83
83.2
Position on chr5 (Mb)

| | |
|---|---|
| date: | Thu Aug 17 17:58:12 2017 |
| build: | hg19 |
| display range: | chr5:82461400−83261400 [82461400−83261400] |
| hilite range: | 0 − 0 [ 0 − 0 ] |
| reference SNP: | chr5:82861400 |
| number of SNPs plotted: | 3396 |
| min P.value: | 4.28E−35 [chr5:82861400] |
| max P.value: | 10E−1 [chr5:82653069] |
| omitted GWAS Hits: | chr5:83.260938−Mitochondrial DNA levels, NA |

Make more plots at http://csg.sph.umich.edu/locuszoom/

GWAS Catalog SNPs in Region

| chr | pos (Mb) | trait | snp |
|---|---|---|---|
| 5 | 82.81912 | Laryngeal squamous cell carcinoma | rs310518 |
| 5 | 82.84549 | Diisocyanate–induced asthma | rs3852186 |
| 5 | 82.88991 | Major depressive disorder | rs310501 |
| 5 | 82.96073 | Visceral fat | rs3846635 |
| 5 | 82.98574 | Prostate cancer | rs4466137 |
| 5 | 83.17359 | Ankylosing spondylitis | rs4552569 |
| 5 | 83.26094 | Mitochondrial DNA levels | rs2301070 |

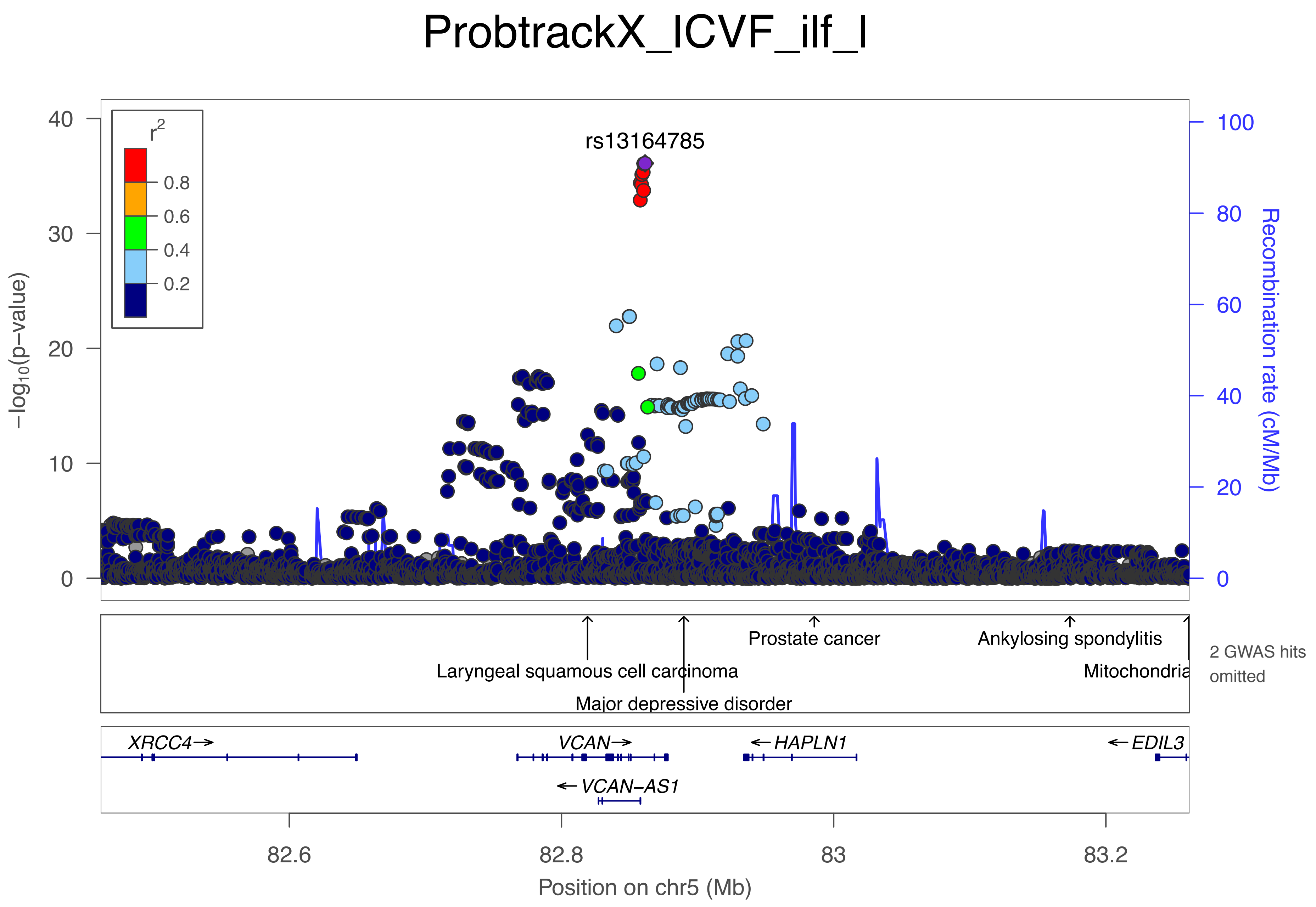

ProbtrackX_ICVF_ilf_l
rs13164785
r2
0.8
0.6
0.4
0.2
−log10(p−value)
Recombination rate (cM/Mb)
Prostate cancer
Ankylosing spondylitis
Laryngeal squamous cell carcinoma
Mitochondria
Major depressive disorder
2 GWAS hits omitted
XRCC4→
VCAN→
←HAPLN1
←EDIL3
←VCAN−AS1
82.6
82.8
83
83.2
Position on chr5 (Mb)

| | |
|---|---|
| date: | Thu Aug 17 17:58:12 2017 |
| build: | hg19 |
| display range: | chr5:82461400–83261400 [82461400–83261400] |
| hilite range: | 0 – 0 [ 0 – 0 ] |
| reference SNP: | chr5:82861400 |
| number of SNPs plotted: | 3396 |
| min P.value: | 8.02E–37 [chr5:82861400] |
| max P.value: | 10E–1 [chr5:83163554] |
| omitted GWAS Hits: | chr5:83.260938–Mitochondrial DNA levels, NA |

Make more plots at http://csg.sph.umich.edu/locuszoom/

GWAS Catalog SNPs in Region

| chr | pos (Mb) | trait | snp |
|---|---|---|---|
| 5 | 82.81912 | Laryngeal squamous cell carcinoma | rs310518 |
| 5 | 82.84549 | Diisocyanate–induced asthma | rs3852186 |
| 5 | 82.88991 | Major depressive disorder | rs310501 |
| 5 | 82.96073 | Visceral fat | rs3846635 |
| 5 | 82.98574 | Prostate cancer | rs4466137 |
| 5 | 83.17359 | Ankylosing spondylitis | rs4552569 |
| 5 | 83.26094 | Mitochondrial DNA levels | rs2301070 |

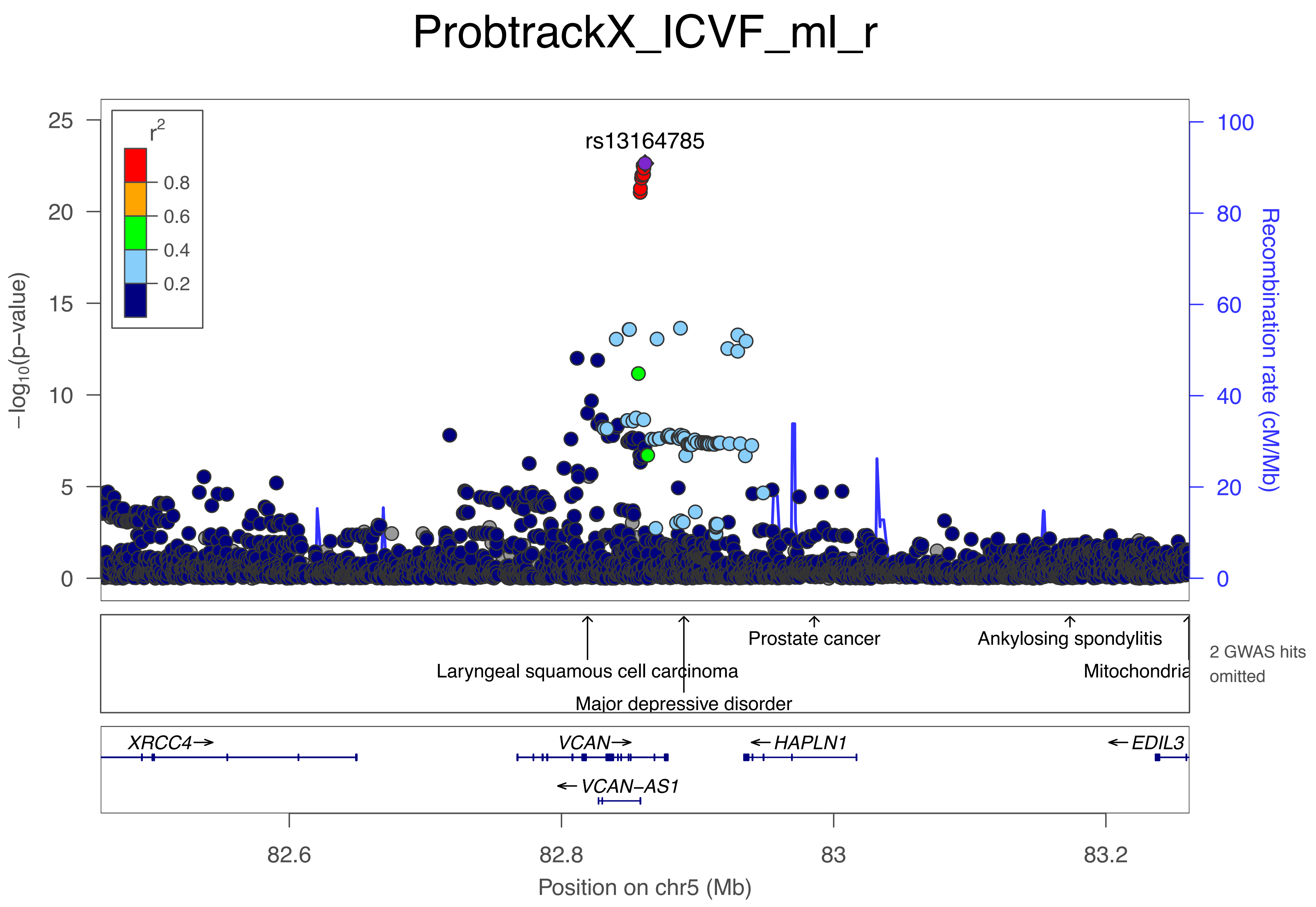
ProbtrackX_ICVF_ml_r
rs13164785
r2
0.8
0.6
0.4
0.2
−log10(p−value)
Recombination rate (cM/Mb)
Prostate cancer
Ankylosing spondylitis
Laryngeal squamous cell carcinoma
Mitochondria
Major depressive disorder
2 GWAS hits omitted
XRCC4
VCAN
HAPLN1
EDIL3
VCAN−AS1
82.6
82.8
83
83.2
Position on chr5 (Mb)

| | |
|---|---|
| date: | Thu Aug 17 17:58:12 2017 |
| build: | hg19 |
| display range: | chr5:82461400–83261400 [82461400–83261400] |
| hilite range: | 0 – 0 [ 0 – 0 ] |
| reference SNP: | chr5:82861400 |
| number of SNPs plotted: | 3396 |
| min P.value: | 2.35E–23 [chr5:82861400] |
| max P.value: | 10E–1 [chr5:82832449] |
| omitted GWAS Hits: | chr5:83.260938–Mitochondrial DNA levels, NA |

Make more plots at http://csg.sph.umich.edu/locuszoom/

GWAS Catalog SNPs in Region

| chr | pos (Mb) | trait | snp |
|---|---|---|---|
| 5 | 82.81912 | Laryngeal squamous cell carcinoma | rs310518 |
| 5 | 82.84549 | Diisocyanate–induced asthma | rs3852186 |
| 5 | 82.88991 | Major depressive disorder | rs310501 |
| 5 | 82.96073 | Visceral fat | rs3846635 |
| 5 | 82.98574 | Prostate cancer | rs4466137 |
| 5 | 83.17359 | Ankylosing spondylitis | rs4552569 |
| 5 | 83.26094 | Mitochondrial DNA levels | rs2301070 |

# ProbtrackX_ICVF_slf_I

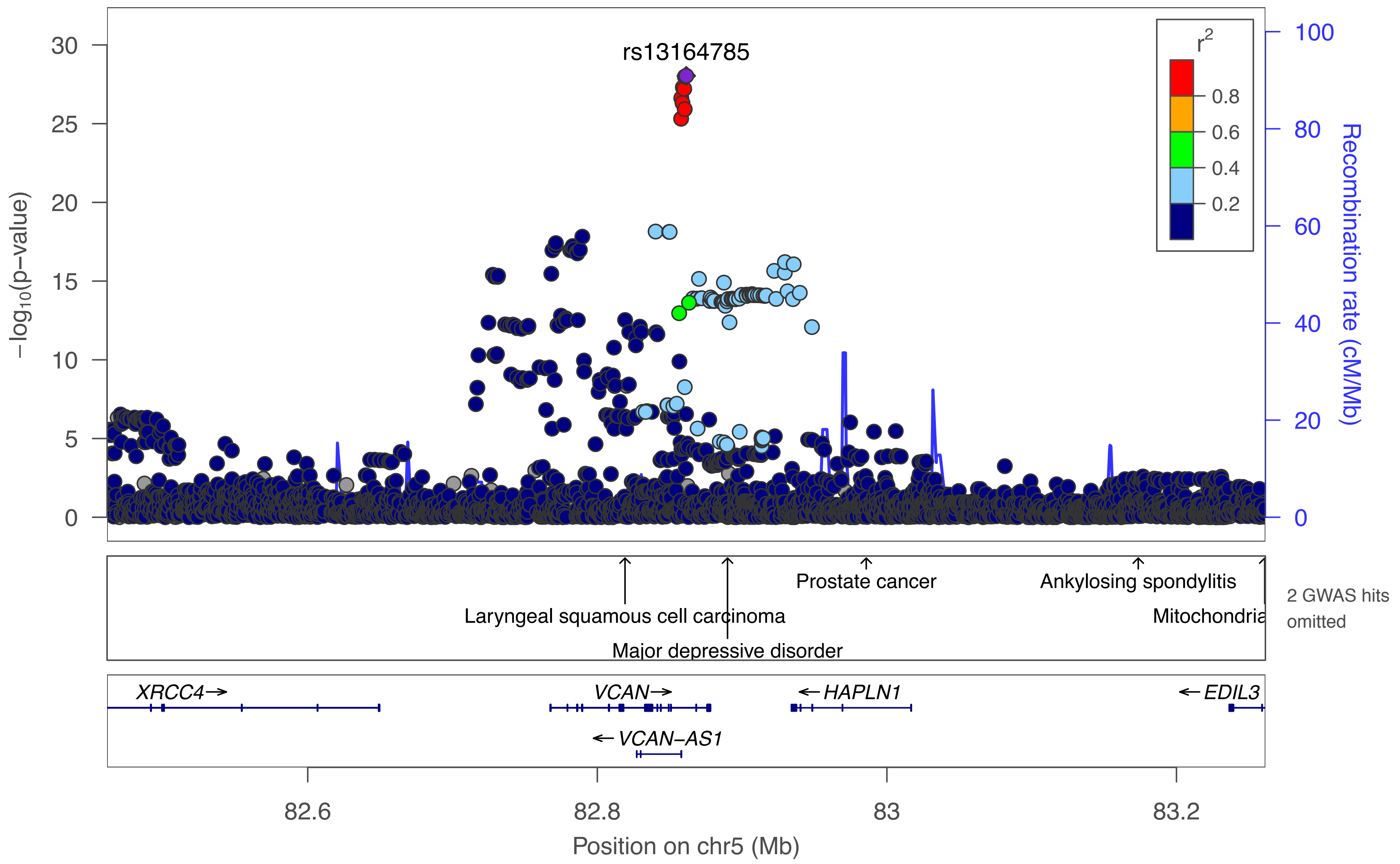

| | |
|---|---|
| date: | Thu Aug 17 18:10:31 2017 |
| build: | hg19 |
| display range: | chr5:82461400−83261400 [82461400−83261400] |
| hilite range: | 0 – 0 [ 0 – 0 ] |
| reference SNP: | chr5:82861400 |
| number of SNPs plotted: | 3396 |
| min P.value: | 9.12E−29 [chr5:82861400] |
| max P.value: | 9.99E−1 [chr5:83228903] |
| omitted GWAS Hits: | chr5:83.260938−Mitochondrial DNA levels, NA |

Make more plots at http://csg.sph.umich.edu/locuszoom/

GWAS Catalog SNPs in Region

| chr | pos (Mb) | trait | snp |
|---|---|---|---|
| 5 | 82.81912 | Laryngeal squamous cell carcinoma | rs310518 |
| 5 | 82.84549 | Diisocyanate–induced asthma | rs3852186 |
| 5 | 82.88991 | Major depressive disorder | rs310501 |
| 5 | 82.96073 | Visceral fat | rs3846635 |
| 5 | 82.98574 | Prostate cancer | rs4466137 |
| 5 | 83.17359 | Ankylosing spondylitis | rs4552569 |
| 5 | 83.26094 | Mitochondrial DNA levels | rs2301070 |

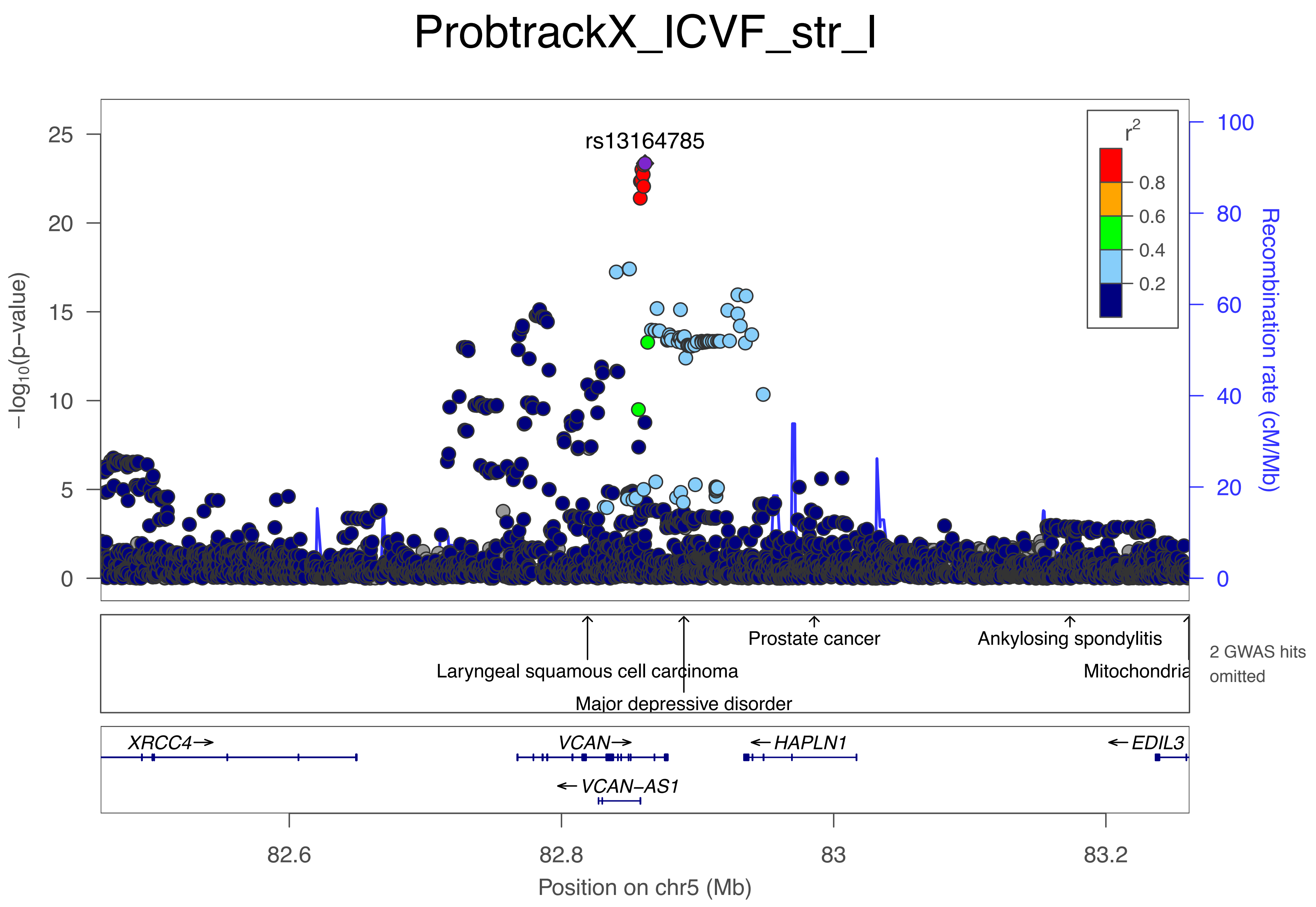
ProbtrackX_ICVF_str_l
rs13164785
−log10(p−value)
Recombination rate (cM/Mb)
r2
0.8
0.6
0.4
0.2
0
5
10
15
20
25
0
20
40
60
80
100
Laryngeal squamous cell carcinoma
Major depressive disorder
Prostate cancer
Ankylosing spondylitis
Mitochondria
2 GWAS hits omitted
XRCC4→
VCAN→
←VCAN−AS1
←HAPLN1
←EDIL3
82.6
82.8
83
83.2
Position on chr5 (Mb)

| | |
|---|---|
| date: | Thu Aug 17 18:23:30 2017 |
| build: | hg19 |
| display range: | chr5:82461400–83261400 [82461400–83261400] |
| hilite range: | 0 – 0 [ 0 – 0 ] |
| reference SNP: | chr5:82861400 |
| number of SNPs plotted: | 3396 |
| min P.value: | 4.42E–24 [chr5:82861400] |
| max P.value: | 10E–1 [chr5:82592509] |
| omitted GWAS Hits: | chr5:83.260938–Mitochondrial DNA levels, NA |

Make more plots at http://csg.sph.umich.edu/locuszoom/

GWAS Catalog SNPs in Region

| chr | pos (Mb) | trait | snp |
|---|---|---|---|
| 5 | 82.81912 | Laryngeal squamous cell carcinoma | rs310518 |
| 5 | 82.84549 | Diisocyanate−induced asthma | rs3852186 |
| 5 | 82.88991 | Major depressive disorder | rs310501 |
| 5 | 82.96073 | Visceral fat | rs3846635 |
| 5 | 82.98574 | Prostate cancer | rs4466137 |
| 5 | 83.17359 | Ankylosing spondylitis | rs4552569 |
| 5 | 83.26094 | Mitochondrial DNA levels | rs2301070 |

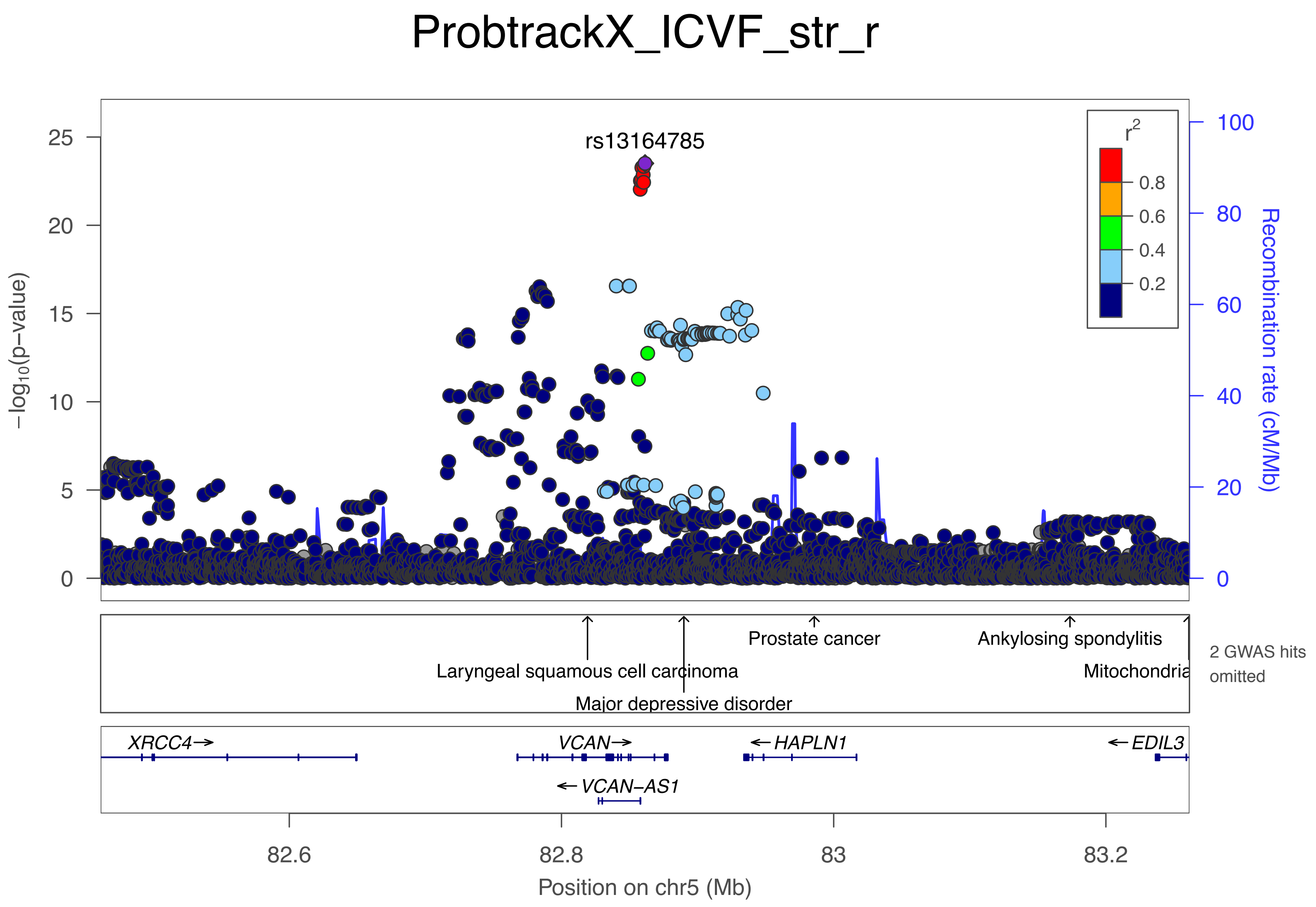
ProbtrackX_ICVF_str_r
rs13164785
−log10(p−value)
Recombination rate (cM/Mb)
r2
0.8
0.6
0.4
0.2
Laryngeal squamous cell carcinoma
Major depressive disorder
Prostate cancer
Ankylosing spondylitis
Mitochondria
2 GWAS hits omitted
XRCC4
VCAN
HAPLN1
EDIL3
VCAN−AS1
82.6
82.8
83
83.2
Position on chr5 (Mb)

| | |
|---|---|
| date: | Thu Aug 17 18:23:30 2017 |
| build: | hg19 |
| display range: | chr5:82461400−83261400 [82461400−83261400] |
| hilite range: | 0 – 0 [ 0 – 0 ] |
| reference SNP: | chr5:82861400 |
| number of SNPs plotted: | 3396 |
| min P.value: | 3.1E−24 [chr5:82861400] |
| max P.value: | 9.98E−1 [chr5:83098673] |
| omitted GWAS Hits: | chr5:83.260938−Mitochondrial DNA levels, NA |

Make more plots at http://csg.sph.umich.edu/locuszoom/

GWAS Catalog SNPs in Region

| chr | pos (Mb) | trait | snp |
|---|---|---|---|
| 5 | 82.81912 | Laryngeal squamous cell carcinoma | rs310518 |
| 5 | 82.84549 | Diisocyanate−induced asthma | rs3852186 |
| 5 | 82.88991 | Major depressive disorder | rs310501 |
| 5 | 82.96073 | Visceral fat | rs3846635 |
| 5 | 82.98574 | Prostate cancer | rs4466137 |
| 5 | 83.17359 | Ankylosing spondylitis | rs4552569 |
| 5 | 83.26094 | Mitochondrial DNA levels | rs2301070 |

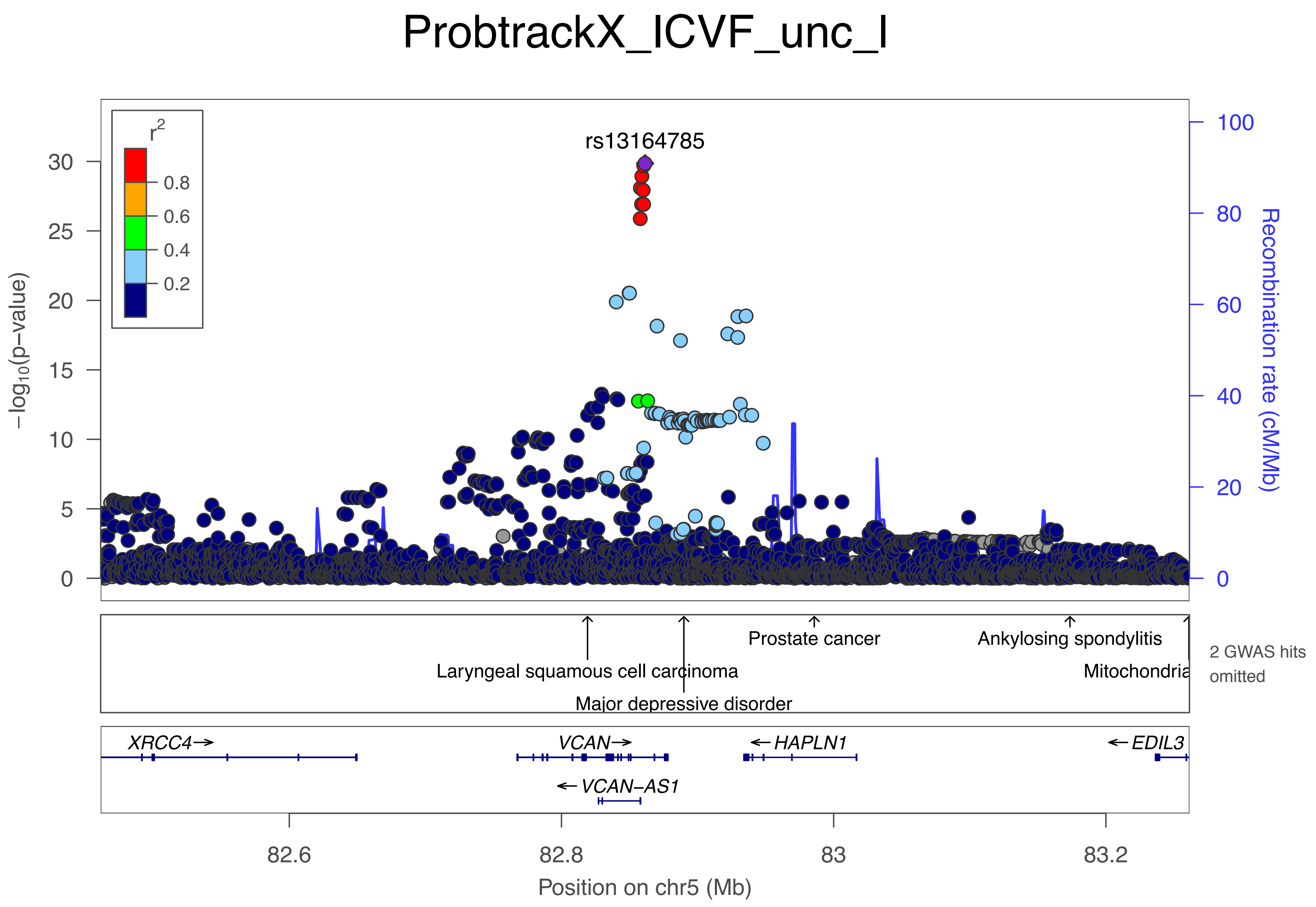
ProbtrackX_ICVF_unc_l
rs13164785
r2
0.8
0.6
0.4
0.2
−log10(p−value)
30
25
20
15
10
5
0
100
80
60
40
20
0
Recombination rate (cM/Mb)
Prostate cancer
Ankylosing spondylitis
Laryngeal squamous cell carcinoma
Mitochondria
Major depressive disorder
2 GWAS hits omitted
XRCC4
VCAN
HAPLN1
EDIL3
VCAN−AS1
82.6
82.8
83
83.2
Position on chr5 (Mb)

| | |
|---|---|
| date: | Thu Aug 17 18:23:31 2017 |
| build: | hg19 |
| display range: | chr5:82461400–83261400 [82461400–83261400] |
| hilite range: | 0 – 0 [ 0 – 0 ] |
| reference SNP: | chr5:82861400 |
| number of SNPs plotted: | 3396 |
| min P.value: | 1.38E–30 [chr5:82861400] |
| max P.value: | 9.99E–1 [chr5:82811969] |
| omitted GWAS Hits: | chr5:83.260938–Mitochondrial DNA levels, NA |

Make more plots at http://csg.sph.umich.edu/locuszoom/

GWAS Catalog SNPs in Region

| chr | pos (Mb) | trait | snp |
|---|---|---|---|
| 5 | 82.81912 | Laryngeal squamous cell carcinoma | rs310518 |
| 5 | 82.84549 | Diisocyanate–induced asthma | rs3852186 |
| 5 | 82.88991 | Major depressive disorder | rs310501 |
| 5 | 82.96073 | Visceral fat | rs3846635 |
| 5 | 82.98574 | Prostate cancer | rs4466137 |
| 5 | 83.17359 | Ankylosing spondylitis | rs4552569 |
| 5 | 83.26094 | Mitochondrial DNA levels | rs2301070 |

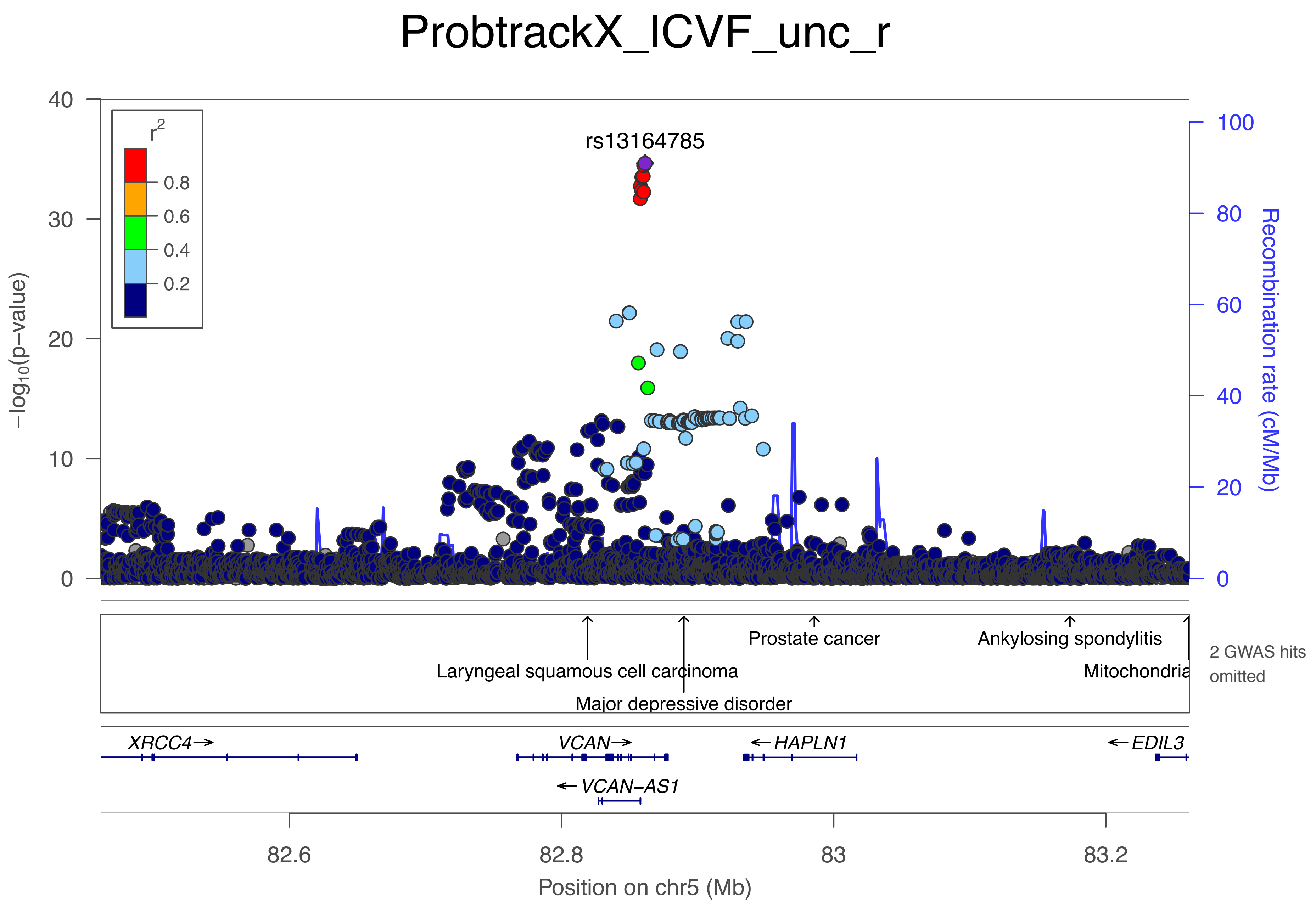
ProbtrackX_ICVF_unc_r
rs13164785
r2
0.8
0.6
0.4
0.2
−log10(p−value)
Recombination rate (cM/Mb)
Prostate cancer
Ankylosing spondylitis
Laryngeal squamous cell carcinoma
Mitochondria
Major depressive disorder
2 GWAS hits omitted
XRCC4→
VCAN→
←HAPLN1
←EDIL3
←VCAN−AS1
82.6
82.8
83
83.2
Position on chr5 (Mb)

| | |
|---|---|
| date: | Thu Aug 17 18:01:49 2017 |
| build: | hg19 |
| display range: | chr5:82461400−83261400 [82461400−83261400] |
| hilite range: | 0 – 0 [ 0 – 0 ] |
| reference SNP: | chr5:82861400 |
| number of SNPs plotted: | 3396 |
| min P.value: | 2.29E−35 [chr5:82861400] |
| max P.value: | 9.97E−1 [chr5:83021163] |
| omitted GWAS Hits: | chr5:83.260938−Mitochondrial DNA levels, NA |

Make more plots at http://csg.sph.umich.edu/locuszoom/

GWAS Catalog SNPs in Region

| chr | pos (Mb) | trait | snp |
|---|---|---|---|
| 5 | 82.81912 | Laryngeal squamous cell carcinoma | rs310518 |
| 5 | 82.84549 | Diisocyanate–induced asthma | rs3852186 |
| 5 | 82.88991 | Major depressive disorder | rs310501 |
| 5 | 82.96073 | Visceral fat | rs3846635 |
| 5 | 82.98574 | Prostate cancer | rs4466137 |
| 5 | 83.17359 | Ankylosing spondylitis | rs4552569 |
| 5 | 83.26094 | Mitochondrial DNA levels | rs2301070 |

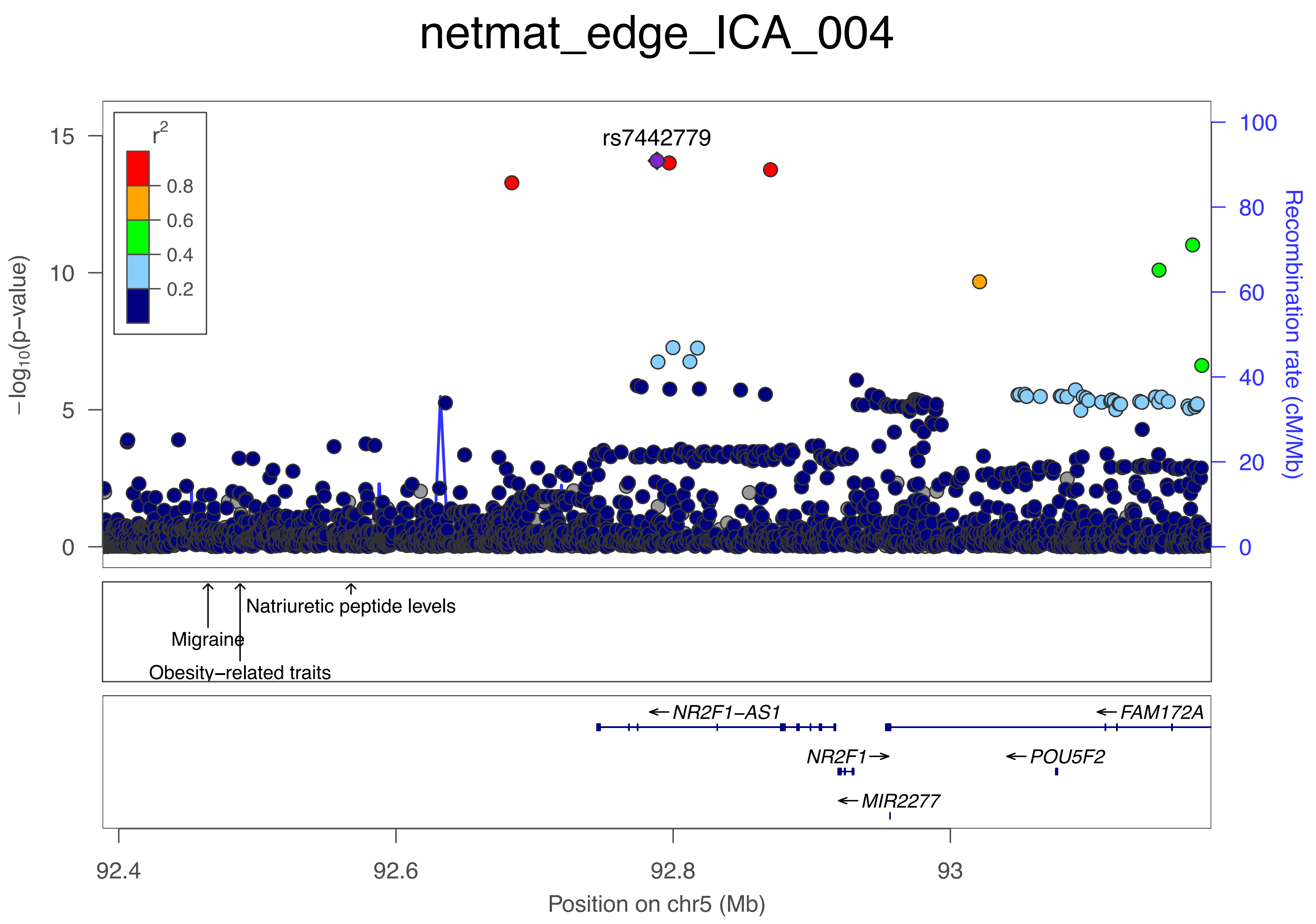
netmat_edge_ICA_004
rs7442779
r2
0.8
0.6
0.4
0.2
15
10
5
0
−log10(p−value)
100
80
60
40
20
0
Recombination rate (cM/Mb)
Natriuretic peptide levels
Migraine
Obesity−related traits
NR2F1−AS1
FAM172A
NR2F1
POU5F2
MIR2277
92.4
92.6
92.8
93
Position on chr5 (Mb)

| | |
|---|---|
| date: | Sat Aug 19 17:26:53 2017 |
| build: | hg19 |
| display range: | chr5:92388278−93188278 [92388278−93188278] |
| hilite range: | 0 – 0 [ 0 – 0 ] |
| reference SNP: | chr5:92788278 |
| number of SNPs plotted: | 2480 |
| min P.value: | 8.18E−15 [chr5:92788278] |
| max P.value: | 10E−1 [chr5:93160560] |

Make more plots at http://csg.sph.umich.edu/locuszoom/

GWAS Catalog SNPs in Region

| chr | pos (Mb) | trait | snp |
|---|---|---|---|
| 5 | 92.46442 | Migraine | rs12519773 |
| 5 | 92.48754 | Obesity–related traits | rs13173682 |
| 5 | 92.56754 | Natriuretic peptide levels | rs4869419 |

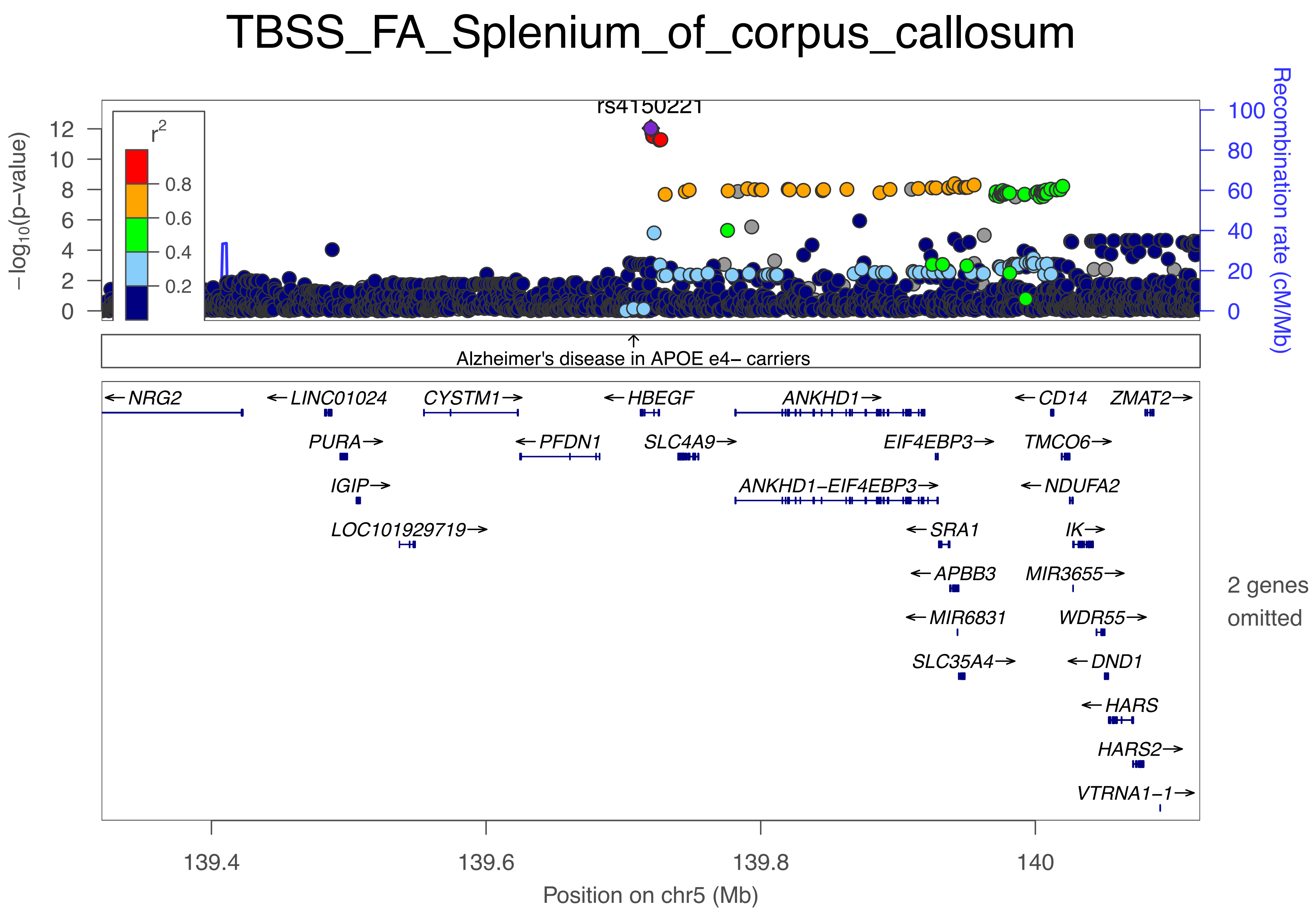
TBSS_FA_Splenium_of_corpus_callosum
rs4150221
r2
0.8
0.6
0.4
0.2
−log10(p−value)
0
2
4
6
8
10
12
Recombination rate (cM/Mb)
0
20
40
60
80
100
Alzheimer's disease in APOE e4− carriers
NRG2
LINC01024
CYSTM1
HBEGF
ANKHD1
CD14
ZMAT2
PURA
PFDN1
SLC4A9
EIF4EBP3
TMCO6
IGIP
ANKHD1−EIF4EBP3
NDUFA2
LOC101929719
SRA1
IK
APBB3
MIR3655
MIR6831
WDR55
SLC35A4
DND1
HARS
HARS2
VTRNA1−1
2 genes omitted
139.4
139.6
139.8
140
Position on chr5 (Mb)

| | |
|---|---|
| date: | Thu Aug 17 17:58:47 2017 |
| build: | hg19 |
| display range: | chr5:139319991−140119991 [139319991−140119991] |
| hilite range: | 0 – 0 [ 0 – 0 ] |
| reference SNP: | chr5:139719991 |
| number of SNPs plotted: | 2368 |
| min P.value: | 9.14E−13 [chr5:139719991] |
| max P.value: | 10E−1 [chr5:139713045] |
| omitted Genes: | VTRNA1−2, VTRNA1−3 |

Make more plots at http://csg.sph.umich.edu/locuszoom/

GWAS Catalog SNPs in Region

| chr | pos (Mb) | trait | snp |
|---|---|---|---|
| 5 | 139.7074 | Alzheimer's disease in APOE e4– carriers | rs11168036 |

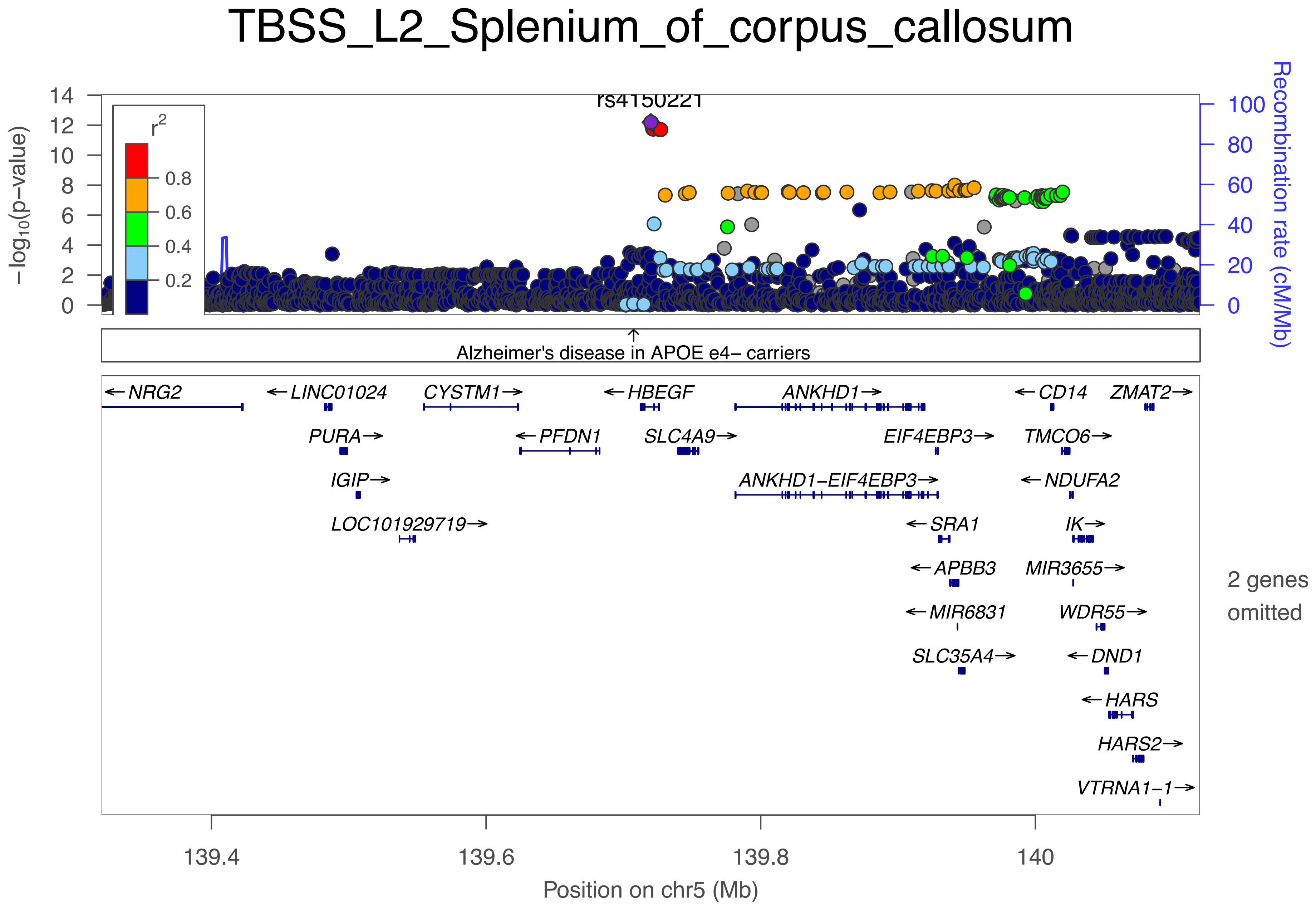

TBSS_L2_Splenium_of_corpus_callosum
rs4150221
r2
0.8
0.6
0.4
0.2
−log10(p−value)
Recombination rate (cM/Mb)
Alzheimer's disease in APOE e4− carriers
NRG2
LINC01024
CYSTM1
HBEGF
ANKHD1
CD14
ZMAT2
PURA
PFDN1
SLC4A9
EIF4EBP3
TMCO6
IGIP
ANKHD1−EIF4EBP3
NDUFA2
LOC101929719
SRA1
IK
APBB3
MIR3655
MIR6831
WDR55
SLC35A4
DND1
HARS
HARS2
VTRNA1−1
2 genes omitted
139.4
139.6
139.8
140
Position on chr5 (Mb)

| | |
|---|---|
| date: | Thu Aug 17 17:58:41 2017 |
| build: | hg19 |
| display range: | chr5:139319991−140119991 [139319991−140119991] |
| hilite range: | 0 – 0 [ 0 – 0 ] |
| reference SNP: | chr5:139719991 |
| number of SNPs plotted: | 2368 |
| min P.value: | 6.43E−13 [chr5:139719991] |
| max P.value: | 10E−1 [chr5:139906014] |
| omitted Genes: | VTRNA1−2, VTRNA1−3 |

Make more plots at http://csg.sph.umich.edu/locuszoom/

GWAS Catalog SNPs in Region

| chr | pos (Mb) | trait | snp |
|---|---|---|---|
| 5 | 139.7074 | Alzheimer's disease in APOE e4– carriers | rs11168036 |

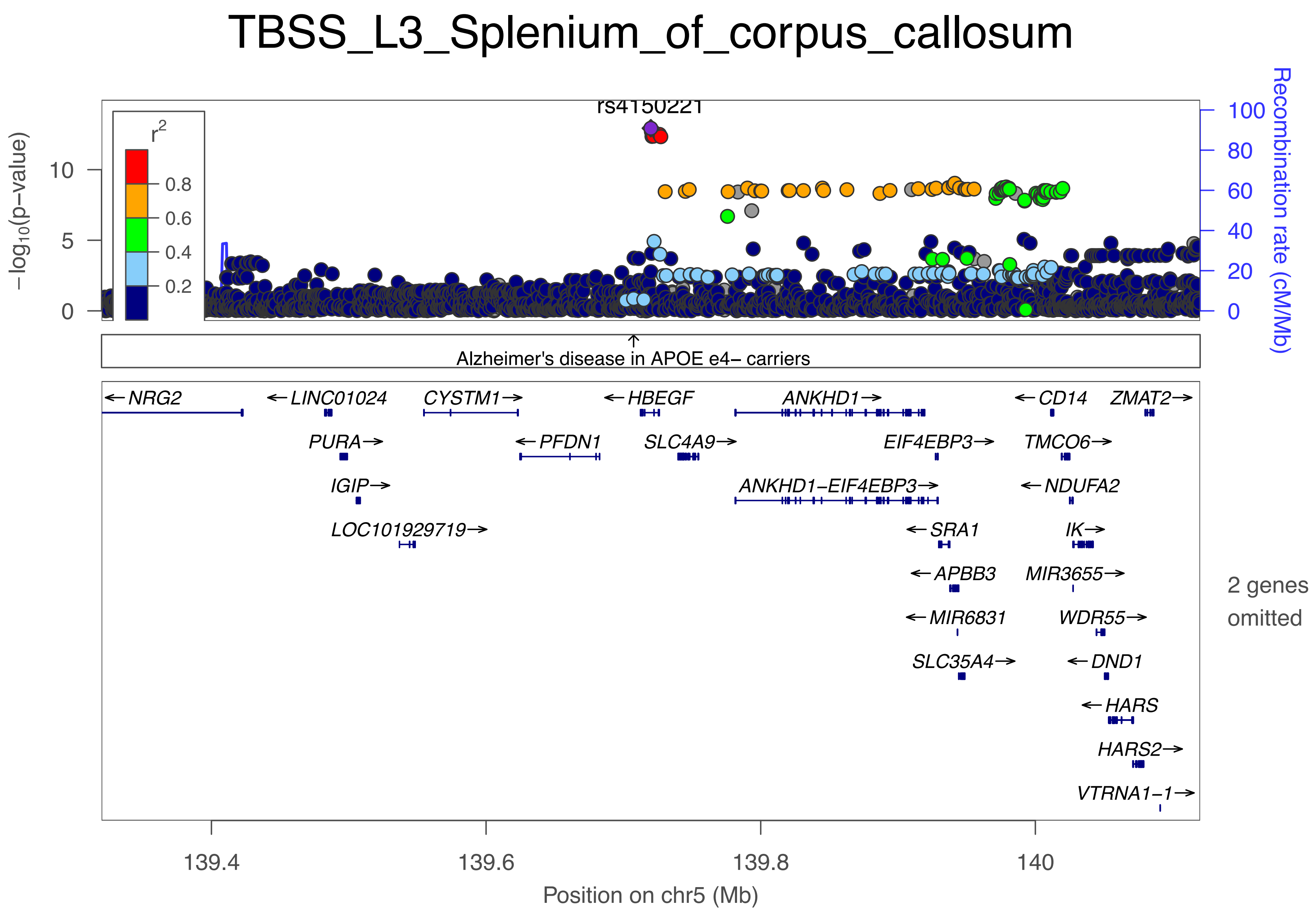

TBSS_L3_Splenium_of_corpus_callosum
rs4150221
r2
0.8
0.6
0.4
0.2
−log10(p−value)
Recombination rate (cM/Mb)
Alzheimer's disease in APOE e4− carriers
NRG2
LINC01024
CYSTM1
HBEGF
ANKHD1
CD14
ZMAT2
PURA
PFDN1
SLC4A9
EIF4EBP3
TMCO6
IGIP
ANKHD1−EIF4EBP3
NDUFA2
LOC101929719
SRA1
IK
APBB3
MIR3655
MIR6831
WDR55
SLC35A4
DND1
HARS
HARS2
VTRNA1−1
2 genes omitted
139.4
139.6
139.8
140
Position on chr5 (Mb)

| | |
|---|---|
| date: | Thu Aug 17 18:05:48 2017 |
| build: | hg19 |
| display range: | chr5:139319991−140119991 [139319991−140119991] |
| hilite range: | 0 – 0 [ 0 – 0 ] |
| reference SNP: | chr5:139719991 |
| number of SNPs plotted: | 2368 |
| min P.value: | 1.17E−13 [chr5:139719991] |
| max P.value: | 9.98E−1 [chr5:139438687] |
| omitted Genes: | VTRNA1−2, VTRNA1−3 |

Make more plots at http://csg.sph.umich.edu/locuszoom/

GWAS Catalog SNPs in Region

| chr | pos (Mb) | trait | snp |
|---|---|---|---|
| 5 | 139.7074 | Alzheimer's disease in APOE e4– carriers | rs11168036 |

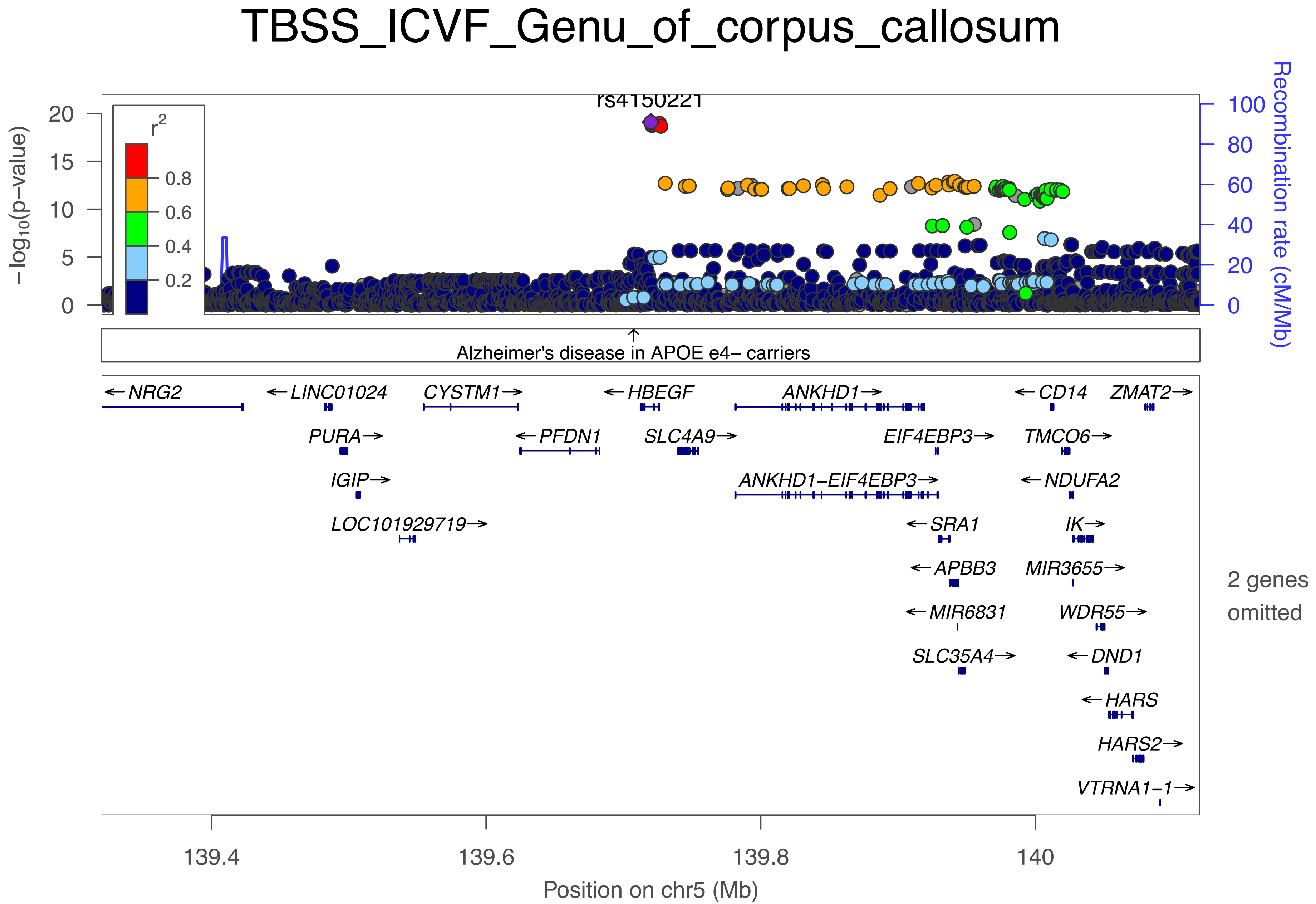
TBSS_ICVF_Genu_of_corpus_callosum
rs4150221
r2
0.8
0.6
0.4
0.2
−log10(p−value)
Recombination rate (cM/Mb)
Alzheimer's disease in APOE e4− carriers
NRG2
LINC01024
CYSTM1
HBEGF
ANKHD1
CD14
ZMAT2
PURA
PFDN1
SLC4A9
EIF4EBP3
TMCO6
IGIP
ANKHD1−EIF4EBP3
NDUFA2
LOC101929719
SRA1
IK
APBB3
MIR3655
MIR6831
WDR55
SLC35A4
DND1
HARS
HARS2
VTRNA1−1
2 genes omitted
139.4
139.6
139.8
140
Position on chr5 (Mb)

| | |
|---|---|
| date: | Thu Aug 17 18:10:46 2017 |
| build: | hg19 |
| display range: | chr5:139319991−140119991 [139319991−140119991] |
| hilite range: | 0 – 0 [ 0 – 0 ] |
| reference SNP: | chr5:139719991 |
| number of SNPs plotted: | 2368 |
| min P.value: | 8.43E−20 [chr5:139719991] |
| max P.value: | 10E−1 [chr5:140008715] |
| omitted Genes: | VTRNA1−2, VTRNA1−3 |

Make more plots at http://csg.sph.umich.edu/locuszoom/

GWAS Catalog SNPs in Region

| chr | pos (Mb) | trait | snp |
|---|---|---|---|
| 5 | 139.7074 | Alzheimer's disease in APOE e4– carriers | rs11168036 |

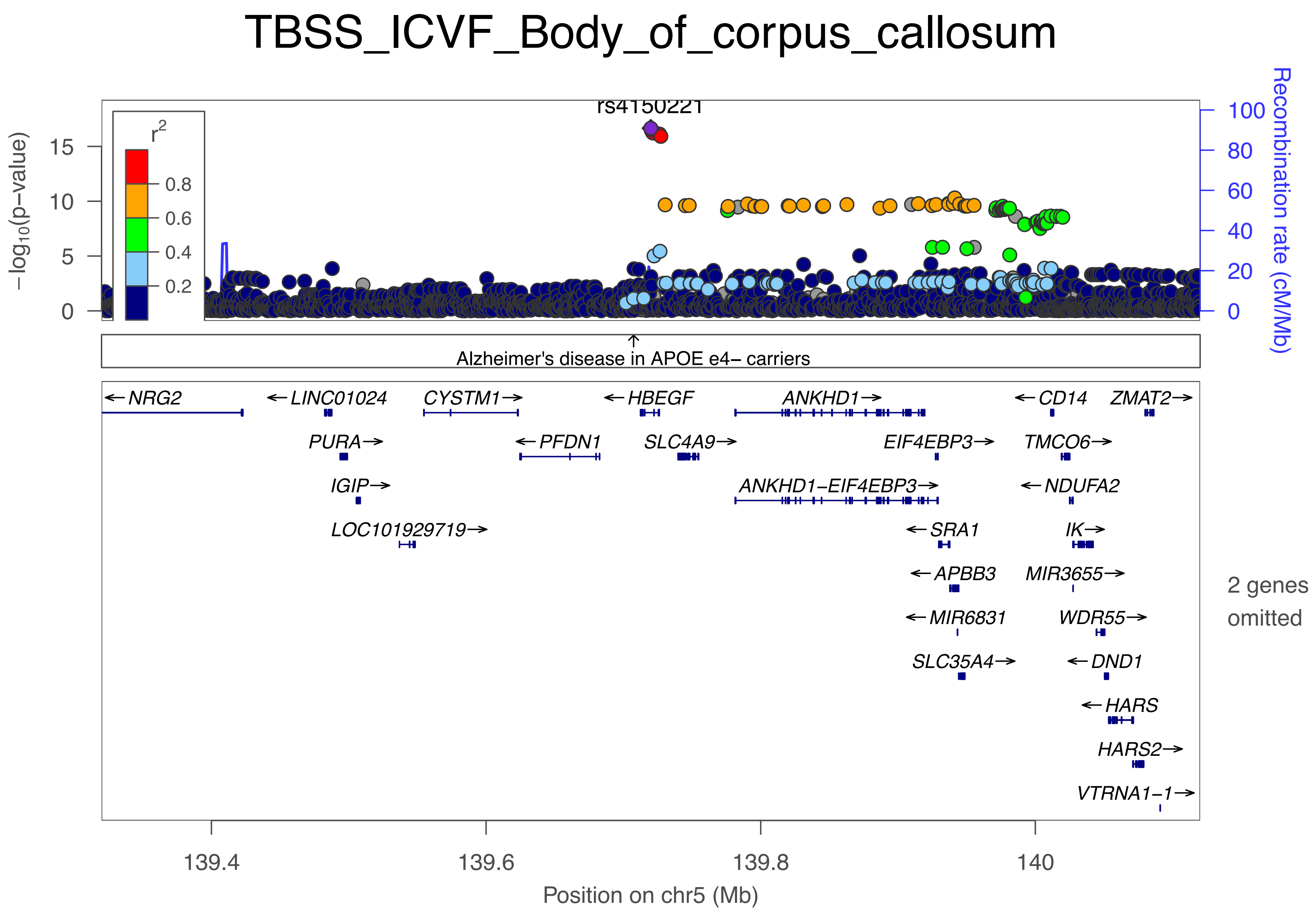

TBSS_ICVF_Body_of_corpus_callosum
rs4150221
r2
0.8
0.6
0.4
0.2
−log10(p−value)
15
10
5
0
Recombination rate (cM/Mb)
100
80
60
40
20
0
Alzheimer's disease in APOE e4− carriers
NRG2
LINC01024
CYSTM1
HBEGF
ANKHD1
CD14
ZMAT2
PURA
PFDN1
SLC4A9
EIF4EBP3
TMCO6
IGIP
ANKHD1−EIF4EBP3
NDUFA2
LOC101929719
SRA1
IK
APBB3
MIR3655
MIR6831
WDR55
SLC35A4
DND1
HARS
HARS2
VTRNA1−1
2 genes omitted
139.4
139.6
139.8
140
Position on chr5 (Mb)

| | |
|---|---|
| date: | Thu Aug 17 18:35:50 2017 |
| build: | hg19 |
| display range: | chr5:139319991−140119991 [139319991−140119991] |
| hilite range: | 0 – 0 [ 0 – 0 ] |
| reference SNP: | chr5:139719991 |
| number of SNPs plotted: | 2368 |
| min P.value: | 2.2E−17 [chr5:139719991] |
| max P.value: | 9.99E−1 [chr5:139656940] |
| omitted Genes: | VTRNA1−2, VTRNA1−3 |

Make more plots at http://csg.sph.umich.edu/locuszoom/

GWAS Catalog SNPs in Region

| chr | pos (Mb) | trait | snp |
|---|---|---|---|
| 5 | 139.7074 | Alzheimer's disease in APOE e4– carriers | rs11168036 |

# TBSS_ICVF_Splenium_of_corpus_callosum

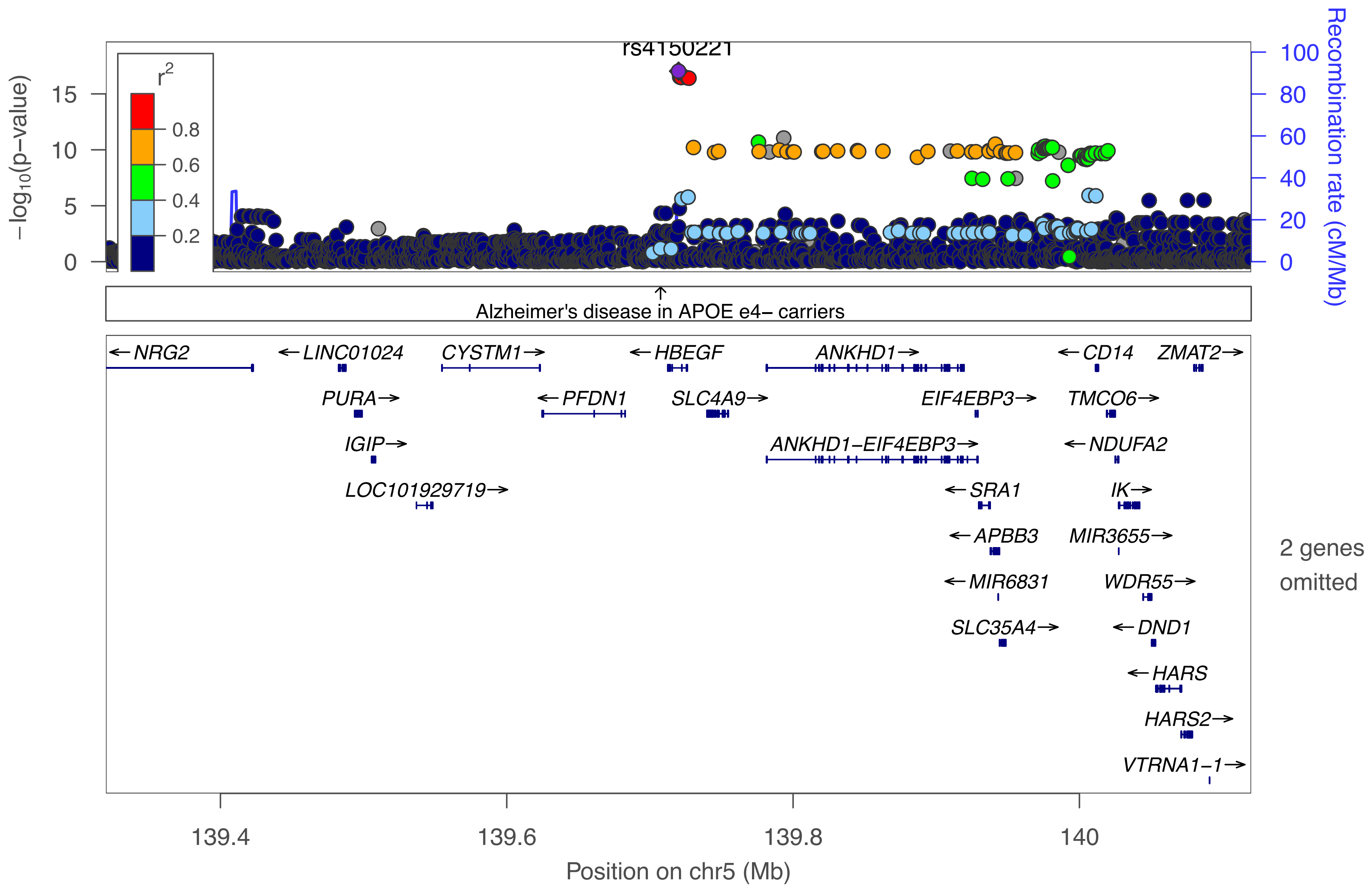

| | |
|---|---|
| date: | Thu Aug 17 18:35:50 2017 |
| build: | hg19 |
| display range: | chr5:139319991−140119991 [139319991−140119991] |
| hilite range: | 0 – 0 [ 0 – 0 ] |
| reference SNP: | chr5:139719991 |
| number of SNPs plotted: | 2368 |
| min P.value: | 9.29E−18 [chr5:139719991] |
| max P.value: | 10E−1 [chr5:139406956] |
| omitted Genes: | VTRNA1−2, VTRNA1−3 |

Make more plots at http://csg.sph.umich.edu/locuszoom/

GWAS Catalog SNPs in Region

| chr | pos (Mb) | trait | snp |
|---|---|---|---|
| 5 | 139.7074 | Alzheimer's disease in APOE e4– carriers | rs11168036 |

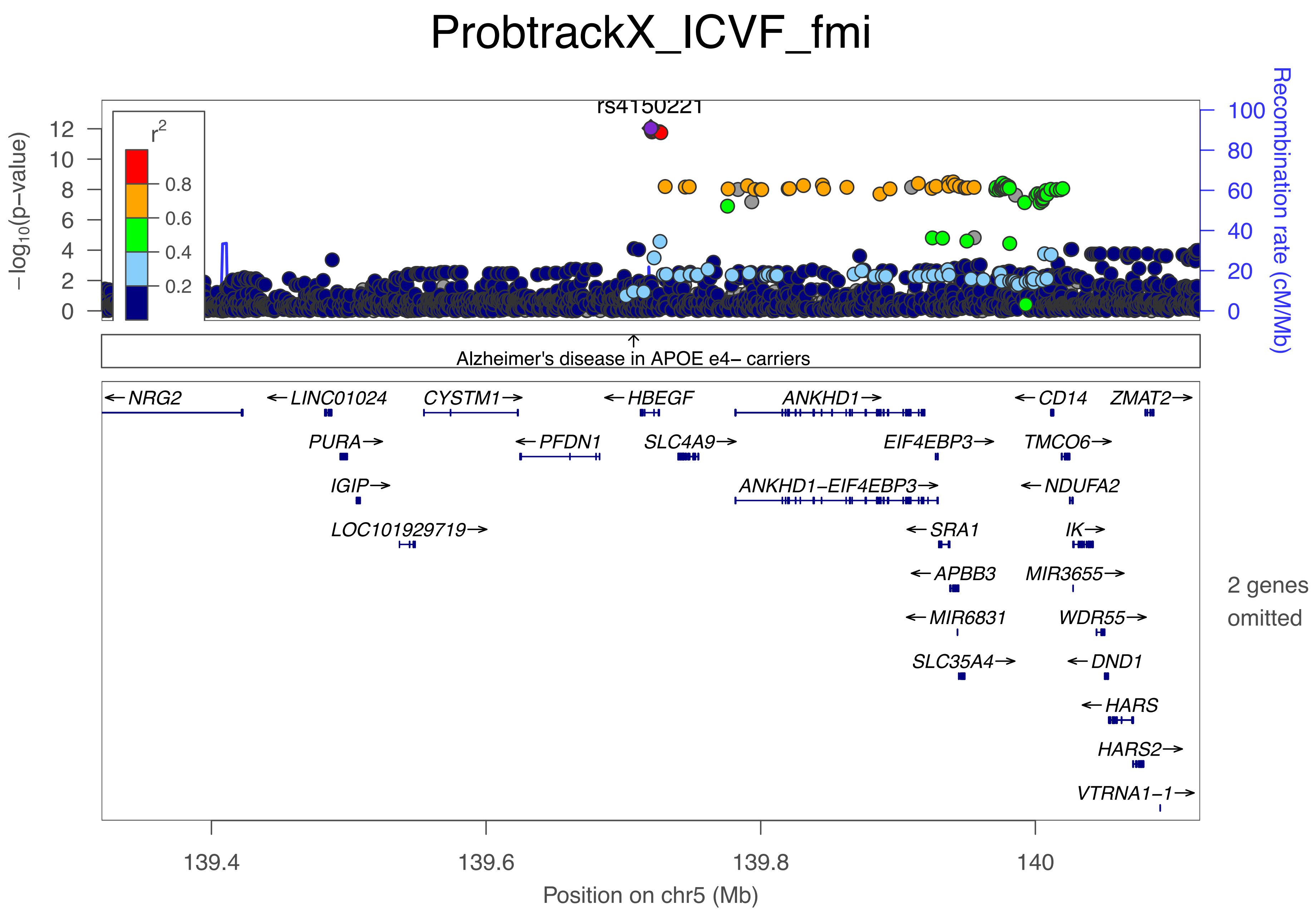
ProbtrackX_ICVF_fmi
rs4150221
r²
0.8
0.6
0.4
0.2
−log10(p−value)
Recombination rate (cM/Mb)
Alzheimer's disease in APOE e4− carriers
←NRG2
←LINC01024
CYSTM1→
←HBEGF
ANKHD1→
←CD14
ZMAT2→
PURA→
←PFDN1
SLC4A9→
EIF4EBP3→
TMCO6→
IGIP→
ANKHD1−EIF4EBP3→
←NDUFA2
LOC101929719→
←SRA1
IK→
←APBB3
MIR3655→
←MIR6831
WDR55→
SLC35A4→
←DND1
←HARS
HARS2→
VTRNA1−1→
2 genes omitted
139.4
139.6
139.8
140
Position on chr5 (Mb)

| | |
|---|---|
| date: | Thu Aug 17 18:35:50 2017 |
| build: | hg19 |
| display range: | chr5:139319991−140119991 [139319991−140119991] |
| hilite range: | 0 – 0 [ 0 – 0 ] |
| reference SNP: | chr5:139719991 |
| number of SNPs plotted: | 2368 |
| min P.value: | 9.23E−13 [chr5:139719991] |
| max P.value: | 9.99E−1 [chr5:139600618] |
| omitted Genes: | VTRNA1−2, VTRNA1−3 |

Make more plots at http://csg.sph.umich.edu/locuszoom/

GWAS Catalog SNPs in Region

| chr | pos (Mb) | trait | snp |
|---|---|---|---|
| 5 | 139.7074 | Alzheimer's disease in APOE e4– carriers | rs11168036 |

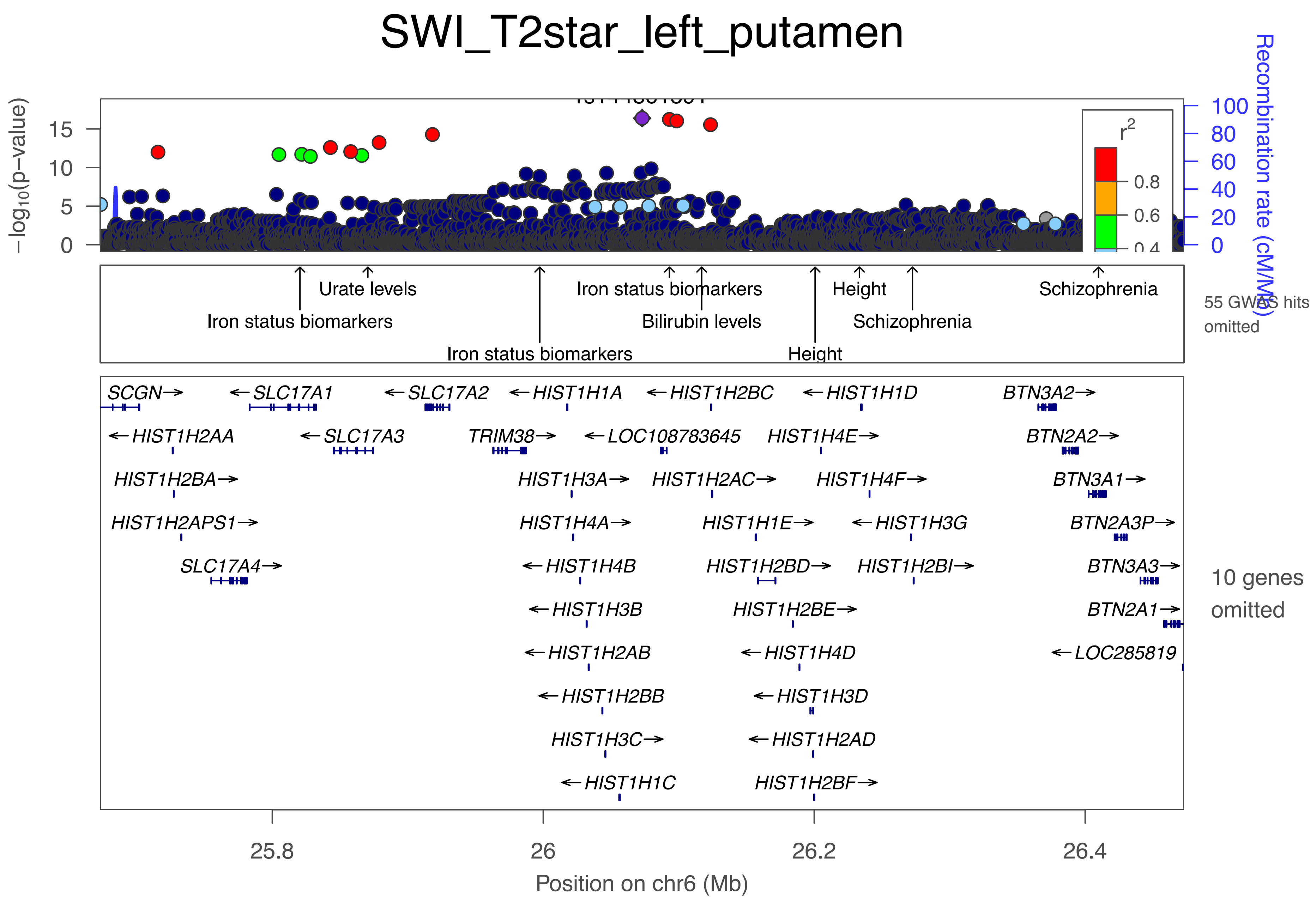
SWI_T2star_left_putamen
−log10(p−value)
Recombination rate (cM/Mb)
r2
0.8
0.6
0.4
55 GWAS hits omitted
Urate levels
Iron status biomarkers
Iron status biomarkers
Iron status biomarkers
Bilirubin levels
Height
Height
Schizophrenia
Schizophrenia
SCGN
SLC17A1
SLC17A2
HIST1H1A
HIST1H2BC
HIST1H1D
BTN3A2
HIST1H2AA
SLC17A3
TRIM38
LOC108783645
HIST1H4E
BTN2A2
HIST1H2BA
HIST1H3A
HIST1H2AC
HIST1H4F
BTN3A1
HIST1H2APS1
HIST1H4A
HIST1H1E
HIST1H3G
BTN2A3P
SLC17A4
HIST1H4B
HIST1H2BD
HIST1H2BI
BTN3A3
HIST1H3B
HIST1H2BE
BTN2A1
HIST1H2AB
HIST1H4D
LOC285819
HIST1H2BB
HIST1H3D
HIST1H3C
HIST1H2AD
HIST1H1C
HIST1H2BF
10 genes omitted
25.8
26
26.2
26.4
Position on chr6 (Mb)

| | |
|---|---|
| date: | Thu Aug 17 18:10:22 2017 |
| build: | hg19 |
| display range: | chr6:25672992−26472992 [25672992−26472992] |
| hilite range: | 0 – 0 [ 0 – 0 ] |
| reference SNP: | chr6:26072992 |
| number of SNPs plotted: | 4565 |
| min P.value: | 4.09E−17 [chr6:26072992] |
| max P.value: | 9.99E−1 [chr6:26074151] |
| omitted Genes: | HFE, HIST1H4C, HIST1H1T |
| omitted Genes: | HIST1H2BG, HIST1H2AE, HIST1H3E |
| omitted Genes: | HIST1H4G, HIST1H3F, HIST1H2BH |
| omitted Genes: | HIST1H4H |
| omitted GWAS Hits: | chr6:26.200677−Height, chr6:26.40989−Schizophrenia |
| omitted GWAS Hits: | NA, NA |
| omitted GWAS Hits: | NA, NA |
| omitted GWAS Hits: | NA, NA |
| omitted GWAS Hits: | NA, NA |
| omitted GWAS Hits: | NA, NA |
| omitted GWAS Hits: | NA, NA |
| omitted GWAS Hits: | NA, NA |
| omitted GWAS Hits: | NA, NA |
| omitted GWAS Hits: | NA, NA |
| omitted GWAS Hits: | NA, NA |

Make more plots at http://csg.sph.umich.edu/locuszoom/

GWAS Catalog SNPs in Region

| chr | pos (Mb) | trait | snp |
|---|---|---|---|
| 6 | 25.77205 | Carotid intima media thickness | rs4712972 |
| 6 | 25.77381 | Urate levels in lean individuals | rs2275906 |
| 6 | 25.77695 | Cardiovascular disease risk factors | rs11754288 |
| 6 | 25.77695 | Blood metabolite levels | rs11754288 |
| 6 | 25.79443 | Blood metabolite levels | rs2762353 |
| 6 | 25.80132 | Urate levels in overweight individuals | rs1165209 |
| 6 | 25.80132 | Urate levels in lean individuals | rs1165209 |
| 6 | 25.81315 | Urate levels | rs1165196 |
| 6 | 25.81315 | Serum uric acid levels | rs1165196 |
| 6 | 25.81315 | Urate levels in overweight individuals | rs1165196 |
| 6 | 25.81859 | Blood metabolite ratios | rs1185567 |
| 6 | 25.81877 | Urate levels in overweight individuals | rs1165152 |
| 6 | 25.82044 | Iron status biomarkers | rs17270561 |
| 6 | 25.82162 | Urate levels | rs1165151 |
| 6 | 25.82177 | Red blood cell traits | rs17342717 |
| 6 | 25.82177 | Iron status biomarkers | rs17342717 |
| 6 | 25.82344 | Uric acid levels | rs1183201 |
| 6 | 25.84295 | Hematology traits | rs1408272 |
| 6 | 25.84295 | Iron status biomarkers | rs1408272 |
| 6 | 25.84295 | Red blood cell traits | rs1408272 |
| 6 | 25.84295 | Mean corpuscular hemoglobin | rs1408272 |

GWAS Catalog SNPs in Region

| chr | pos (Mb) | trait | snp |
|---|---|---|---|
| 6 | 25.86937 | Homocysteine levels | rs548987 |
| 6 | 25.87054 | Urate levels | rs1165205 |
| 6 | 25.87442 | Schizophrenia | rs13198474 |
| 6 | 25.89558 | Blood pressure | rs6910741 |
| 6 | 25.91698 | Height | rs1865760 |
| 6 | 25.99746 | Iron status biomarkers | rs12216125 |
| 6 | 26.03161 | HIV−1 viral setpoint | rs10484434 |
| 6 | 26.09118 | Diastolic blood pressure | rs1799945 |
| 6 | 26.09118 | Hypertension | rs1799945 |
| 6 | 26.09118 | Systolic blood pressure | rs1799945 |
| 6 | 26.09118 | Iron status biomarkers | rs1799945 |
| 6 | 26.09314 | Iron status biomarkers | rs1800562 |
| 6 | 26.09314 | Hematological parameters | rs1800562 |
| 6 | 26.09314 | Hepcidin levels | rs1800562 |
| 6 | 26.09314 | Cardiovascular disease risk factors | rs1800562 |
| 6 | 26.09314 | LDL cholesterol | rs1800562 |
| 6 | 26.09314 | Glycated hemoglobin levels | rs1800562 |
| 6 | 26.09314 | Red blood cell traits | rs1800562 |
| 6 | 26.09314 | Alcohol consumption (transferrin glycosylation) | rs1800562 |
| 6 | 26.09314 | Cholesterol, total | rs1800562 |
| 6 | 26.09314 | Hematology traits | rs1800562 |

GWAS Catalog SNPs in Region

| chr | pos (Mb) | trait | snp |
|---|---|---|---|
| 6 | 26.09314 | Hemoglobin | rs1800562 |
| 6 | 26.09314 | Hematocrit | rs1800562 |
| 6 | 26.09314 | Mean corpuscular volume | rs1800562 |
| 6 | 26.09314 | Iron status biomarkers (ferritin levels) | rs1800562 |
| 6 | 26.09314 | Iron status biomarkers (transferrin saturation) | rs1800562 |
| 6 | 26.09314 | Iron status biomarkers (transferrin levels) | rs1800562 |
| 6 | 26.09314 | Iron status biomarkers (iron levels) | rs1800562 |
| 6 | 26.09314 | Mean corpuscular hemoglobin | rs1800562 |
| 6 | 26.09738 | Schizophrenia | rs6918586 |
| 6 | 26.10746 | Blood pressure | rs198846 |
| 6 | 26.10746 | Hemoglobin | rs198846 |
| 6 | 26.10746 | Mean corpuscular volume | rs198846 |
| 6 | 26.11698 | Bilirubin levels | rs12206204 |
| 6 | 26.12845 | Narcolepsy with cataplexy | rs198811 |
| 6 | 26.15748 | Height | rs4141885 |
| 6 | 26.20068 | Height | rs806794 |
| 6 | 26.23339 | Height | rs10946808 |
| 6 | 26.26007 | Plasma omega−6 polyunsaturated fatty acid levels (linoleic acid) | rs4476815 |
| 6 | 26.27255 | Schizophrenia | rs61747867 |
| 6 | 26.27665 | Educational attainment | rs9393692 |
| 6 | 26.32589 | Educational attainment (years of education) | rs2179152 |

GWAS Catalog SNPs in Region

| chr | pos (Mb) | trait | snp |
|---|---|---|---|
| 6 | 26.40989 | Schizophrenia | rs41266839 |

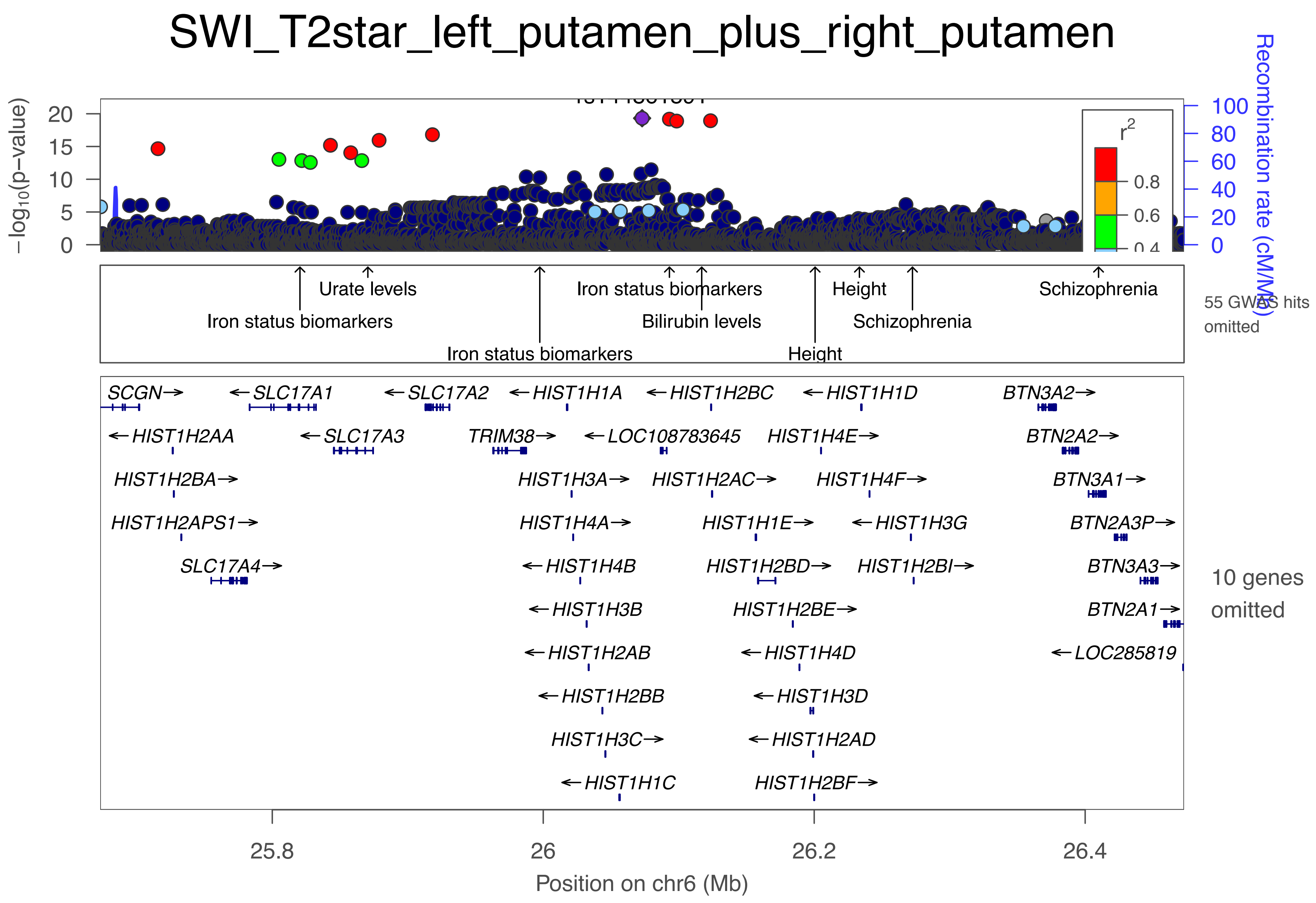

SWI_T2star_left_putamen_plus_right_putamen
−log10(p−value)
Recombination rate (cM/Mb)
r2
0.8
0.6
0.4
Urate levels
Iron status biomarkers
Iron status biomarkers
Iron status biomarkers
Bilirubin levels
Height
Height
Schizophrenia
Schizophrenia
55 GWAS hits omitted
SCGN
HIST1H2AA
HIST1H2BA
HIST1H2APS1
SLC17A4
SLC17A1
SLC17A3
SLC17A2
TRIM38
HIST1H1A
HIST1H3A
HIST1H4A
HIST1H4B
HIST1H3B
HIST1H2AB
HIST1H2BB
HIST1H3C
HIST1H1C
HIST1H2BC
LOC108783645
HIST1H2AC
HIST1H1E
HIST1H2BD
HIST1H2BE
HIST1H4D
HIST1H3D
HIST1H2AD
HIST1H2BF
HIST1H1D
HIST1H4E
HIST1H4F
HIST1H3G
HIST1H2BI
BTN3A2
BTN2A2
BTN3A1
BTN2A3P
BTN3A3
BTN2A1
LOC285819
10 genes omitted
25.8
26
26.2
26.4
Position on chr6 (Mb)

date: Thu Aug 17 18:18:43 2017

build: hg19

display range: chr6:25672992−26472992 [25672992−26472992]

hilite range: 0 – 0 [ 0 – 0 ]

reference SNP: chr6:26072992

number of SNPs plotted: 4565

min P.value: 4.81E−20 [chr6:26072992]

max P.value: 9.99E−1 [chr6:25971279]

omitted Genes: HFE, HIST1H4C, HIST1H1T

omitted Genes: HIST1H2BG, HIST1H2AE, HIST1H3E

omitted Genes: HIST1H4G, HIST1H3F, HIST1H2BH

omitted Genes: HIST1H4H

omitted GWAS Hits: chr6:26.200677−Height, chr6:26.40989−Schizophrenia

omitted GWAS Hits: NA, NA

omitted GWAS Hits: NA, NA

omitted GWAS Hits: NA, NA

omitted GWAS Hits: NA, NA

omitted GWAS Hits: NA, NA

omitted GWAS Hits: NA, NA

omitted GWAS Hits: NA, NA

omitted GWAS Hits: NA, NA

omitted GWAS Hits: NA, NA

Make more plots at http://csg.sph.umich.edu/locuszoom/

omitted GWAS Hits: NA, NA

GWAS Catalog SNPs in Region

| chr | pos (Mb) | trait | snp |
|---|---|---|---|
| 6 | 25.77205 | Carotid intima media thickness | rs4712972 |
| 6 | 25.77381 | Urate levels in lean individuals | rs2275906 |
| 6 | 25.77695 | Cardiovascular disease risk factors | rs11754288 |
| 6 | 25.77695 | Blood metabolite levels | rs11754288 |
| 6 | 25.79443 | Blood metabolite levels | rs2762353 |
| 6 | 25.80132 | Urate levels in overweight individuals | rs1165209 |
| 6 | 25.80132 | Urate levels in lean individuals | rs1165209 |
| 6 | 25.81315 | Urate levels | rs1165196 |
| 6 | 25.81315 | Serum uric acid levels | rs1165196 |
| 6 | 25.81315 | Urate levels in overweight individuals | rs1165196 |
| 6 | 25.81859 | Blood metabolite ratios | rs1185567 |
| 6 | 25.81877 | Urate levels in overweight individuals | rs1165152 |
| 6 | 25.82044 | Iron status biomarkers | rs17270561 |
| 6 | 25.82162 | Urate levels | rs1165151 |
| 6 | 25.82177 | Red blood cell traits | rs17342717 |
| 6 | 25.82177 | Iron status biomarkers | rs17342717 |
| 6 | 25.82344 | Uric acid levels | rs1183201 |
| 6 | 25.84295 | Hematology traits | rs1408272 |
| 6 | 25.84295 | Iron status biomarkers | rs1408272 |
| 6 | 25.84295 | Red blood cell traits | rs1408272 |
| 6 | 25.84295 | Mean corpuscular hemoglobin | rs1408272 |

GWAS Catalog SNPs in Region

| chr | pos (Mb) | trait | snp |
|---|---|---|---|
| 6 | 25.86937 | Homocysteine levels | rs548987 |
| 6 | 25.87054 | Urate levels | rs1165205 |
| 6 | 25.87442 | Schizophrenia | rs13198474 |
| 6 | 25.89558 | Blood pressure | rs6910741 |
| 6 | 25.91698 | Height | rs1865760 |
| 6 | 25.99746 | Iron status biomarkers | rs12216125 |
| 6 | 26.03161 | HIV−1 viral setpoint | rs10484434 |
| 6 | 26.09118 | Diastolic blood pressure | rs1799945 |
| 6 | 26.09118 | Hypertension | rs1799945 |
| 6 | 26.09118 | Systolic blood pressure | rs1799945 |
| 6 | 26.09118 | Iron status biomarkers | rs1799945 |
| 6 | 26.09314 | Iron status biomarkers | rs1800562 |
| 6 | 26.09314 | Hematological parameters | rs1800562 |
| 6 | 26.09314 | Hepcidin levels | rs1800562 |
| 6 | 26.09314 | Cardiovascular disease risk factors | rs1800562 |
| 6 | 26.09314 | LDL cholesterol | rs1800562 |
| 6 | 26.09314 | Glycated hemoglobin levels | rs1800562 |
| 6 | 26.09314 | Red blood cell traits | rs1800562 |
| 6 | 26.09314 | Alcohol consumption (transferrin glycosylation) | rs1800562 |
| 6 | 26.09314 | Cholesterol, total | rs1800562 |
| 6 | 26.09314 | Hematology traits | rs1800562 |

GWAS Catalog SNPs in Region

| chr | pos (Mb) | trait | snp |
|---|---|---|---|
| 6 | 26.09314 | Hemoglobin | rs1800562 |
| 6 | 26.09314 | Hematocrit | rs1800562 |
| 6 | 26.09314 | Mean corpuscular volume | rs1800562 |
| 6 | 26.09314 | Iron status biomarkers (ferritin levels) | rs1800562 |
| 6 | 26.09314 | Iron status biomarkers (transferrin saturation) | rs1800562 |
| 6 | 26.09314 | Iron status biomarkers (transferrin levels) | rs1800562 |
| 6 | 26.09314 | Iron status biomarkers (iron levels) | rs1800562 |
| 6 | 26.09314 | Mean corpuscular hemoglobin | rs1800562 |
| 6 | 26.09738 | Schizophrenia | rs6918586 |
| 6 | 26.10746 | Blood pressure | rs198846 |
| 6 | 26.10746 | Hemoglobin | rs198846 |
| 6 | 26.10746 | Mean corpuscular volume | rs198846 |
| 6 | 26.11698 | Bilirubin levels | rs12206204 |
| 6 | 26.12845 | Narcolepsy with cataplexy | rs198811 |
| 6 | 26.15748 | Height | rs4141885 |
| 6 | 26.20068 | Height | rs806794 |
| 6 | 26.23339 | Height | rs10946808 |
| 6 | 26.26007 | Plasma omega–6 polyunsaturated fatty acid levels (linoleic acid) | rs4476815 |
| 6 | 26.27255 | Schizophrenia | rs61747867 |
| 6 | 26.27665 | Educational attainment | rs9393692 |
| 6 | 26.32589 | Educational attainment (years of education) | rs2179152 |

GWAS Catalog SNPs in Region

| chr | pos (Mb) | trait | snp |
|---|---|---|---|
| 6 | 26.40989 | Schizophrenia | rs41266839 |

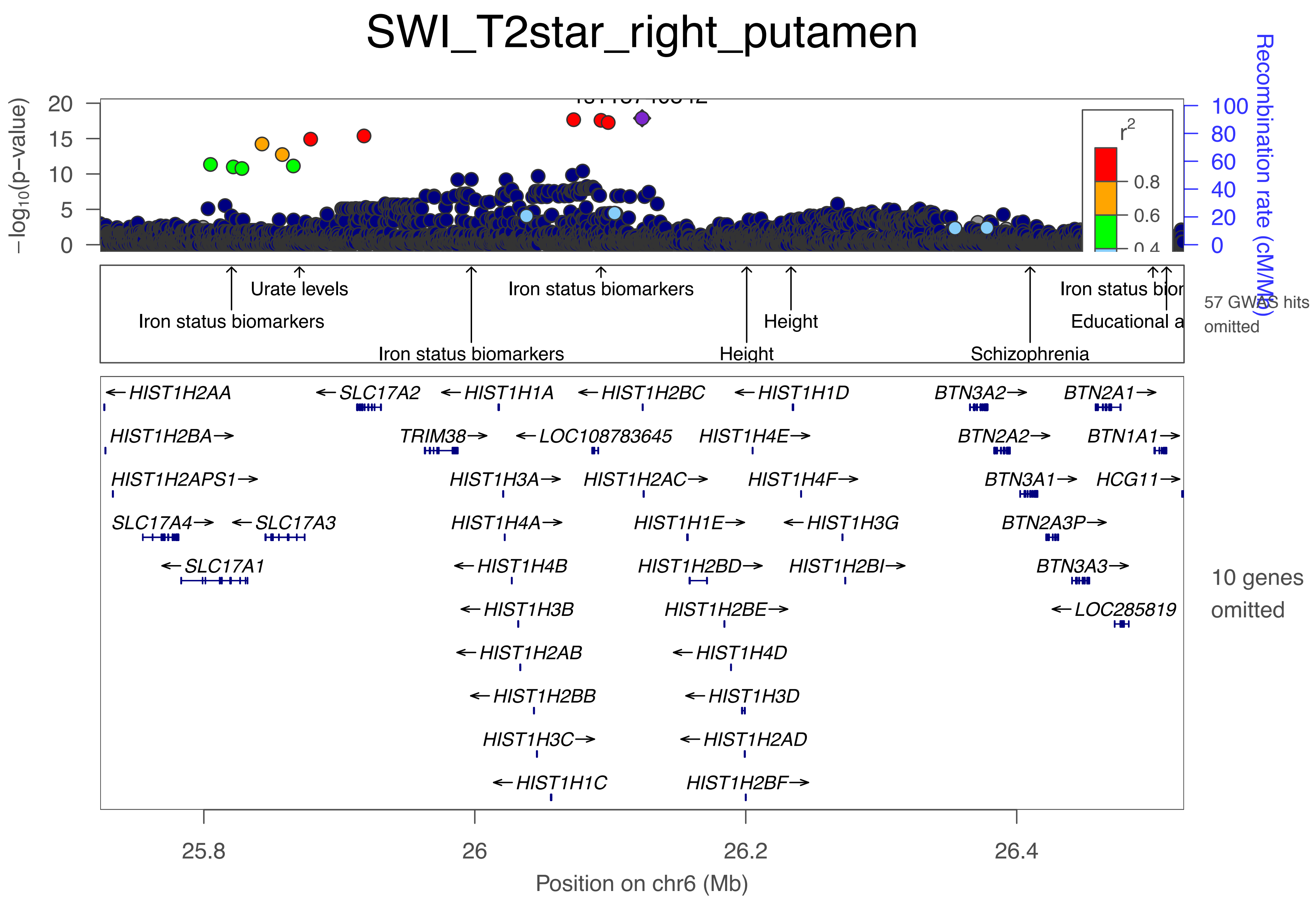

SWI_T2star_right_putamen
−log10(p−value)
Recombination rate (cM/Mb)
r2
0.8
0.6
0.4
Urate levels
Iron status biomarkers
Iron status biomarkers
Iron status biomarkers
Height
Height
Schizophrenia
Iron status bior
Educational a
57 GWAS hits omitted
HIST1H2AA
SLC17A2
HIST1H1A
HIST1H2BC
HIST1H1D
BTN3A2
BTN2A1
HIST1H2BA
TRIM38
LOC108783645
HIST1H4E
BTN2A2
BTN1A1
HIST1H2APS1
HIST1H3A
HIST1H2AC
HIST1H4F
BTN3A1
HCG11
SLC17A4
SLC17A3
HIST1H4A
HIST1H1E
HIST1H3G
BTN2A3P
SLC17A1
HIST1H4B
HIST1H2BD
HIST1H2BI
BTN3A3
HIST1H3B
HIST1H2BE
LOC285819
HIST1H2AB
HIST1H4D
HIST1H2BB
HIST1H3D
HIST1H3C
HIST1H2AD
HIST1H1C
HIST1H2BF
10 genes omitted
25.8
26
26.2
26.4
Position on chr6 (Mb)

date: Thu Aug 17 18:14:04 2017

build: hg19

display range: chr6:25723502−26523502 [25723502−26523502]

hilite range: 0 – 0 [ 0 – 0 ]

reference SNP: chr6:26123502

number of SNPs plotted: 4507

min P.value: 1.33E−18 [chr6:26123502]

max P.value: 9.98E−1 [chr6:26104008]

omitted Genes: HFE, HIST1H4C, HIST1H1T

omitted Genes: HIST1H2BG, HIST1H2AE, HIST1H3E

omitted Genes: HIST1H4G, HIST1H3F, HIST1H2BH

omitted Genes: HIST1H4H

omitted GWAS Hits: chr6:26.510564−Educational attainment, chr6:26.200677−Height

omitted GWAS Hits: NA, NA

omitted GWAS Hits: NA, NA

omitted GWAS Hits: NA, NA

omitted GWAS Hits: NA, NA

omitted GWAS Hits: NA, NA

omitted GWAS Hits: NA, NA

omitted GWAS Hits: NA, NA

omitted GWAS Hits: NA, NA

omitted GWAS Hits: NA, NA

Make more plots at http://csg.sph.umich.edu/locuszoom/

omitted GWAS Hits: NA, NA

GWAS Catalog SNPs in Region

| chr | pos (Mb) | trait | snp |
|---|---|---|---|
| 6 | 25.77205 | Carotid intima media thickness | rs4712972 |
| 6 | 25.77381 | Urate levels in lean individuals | rs2275906 |
| 6 | 25.77695 | Cardiovascular disease risk factors | rs11754288 |
| 6 | 25.77695 | Blood metabolite levels | rs11754288 |
| 6 | 25.79443 | Blood metabolite levels | rs2762353 |
| 6 | 25.80132 | Urate levels in overweight individuals | rs1165209 |
| 6 | 25.80132 | Urate levels in lean individuals | rs1165209 |
| 6 | 25.81315 | Urate levels | rs1165196 |
| 6 | 25.81315 | Serum uric acid levels | rs1165196 |
| 6 | 25.81315 | Urate levels in overweight individuals | rs1165196 |
| 6 | 25.81859 | Blood metabolite ratios | rs1185567 |
| 6 | 25.81877 | Urate levels in overweight individuals | rs1165152 |
| 6 | 25.82044 | Iron status biomarkers | rs17270561 |
| 6 | 25.82162 | Urate levels | rs1165151 |
| 6 | 25.82177 | Red blood cell traits | rs17342717 |
| 6 | 25.82177 | Iron status biomarkers | rs17342717 |
| 6 | 25.82344 | Uric acid levels | rs1183201 |
| 6 | 25.84295 | Hematology traits | rs1408272 |
| 6 | 25.84295 | Iron status biomarkers | rs1408272 |
| 6 | 25.84295 | Red blood cell traits | rs1408272 |
| 6 | 25.84295 | Mean corpuscular hemoglobin | rs1408272 |

GWAS Catalog SNPs in Region

| chr | pos (Mb) | trait | snp |
|---|---|---|---|
| 6 | 25.86937 | Homocysteine levels | rs548987 |
| 6 | 25.87054 | Urate levels | rs1165205 |
| 6 | 25.87442 | Schizophrenia | rs13198474 |
| 6 | 25.89558 | Blood pressure | rs6910741 |
| 6 | 25.91698 | Height | rs1865760 |
| 6 | 25.99746 | Iron status biomarkers | rs12216125 |
| 6 | 26.03161 | HIV−1 viral setpoint | rs10484434 |
| 6 | 26.09118 | Diastolic blood pressure | rs1799945 |
| 6 | 26.09118 | Hypertension | rs1799945 |
| 6 | 26.09118 | Systolic blood pressure | rs1799945 |
| 6 | 26.09118 | Iron status biomarkers | rs1799945 |
| 6 | 26.09314 | Iron status biomarkers | rs1800562 |
| 6 | 26.09314 | Hematological parameters | rs1800562 |
| 6 | 26.09314 | Hepcidin levels | rs1800562 |
| 6 | 26.09314 | Cardiovascular disease risk factors | rs1800562 |
| 6 | 26.09314 | LDL cholesterol | rs1800562 |
| 6 | 26.09314 | Glycated hemoglobin levels | rs1800562 |
| 6 | 26.09314 | Red blood cell traits | rs1800562 |
| 6 | 26.09314 | Alcohol consumption (transferrin glycosylation) | rs1800562 |
| 6 | 26.09314 | Cholesterol, total | rs1800562 |
| 6 | 26.09314 | Hematology traits | rs1800562 |

GWAS Catalog SNPs in Region

| chr | pos (Mb) | trait | snp |
|---|---|---|---|
| 6 | 26.09314 | Hemoglobin | rs1800562 |
| 6 | 26.09314 | Hematocrit | rs1800562 |
| 6 | 26.09314 | Mean corpuscular volume | rs1800562 |
| 6 | 26.09314 | Iron status biomarkers (ferritin levels) | rs1800562 |
| 6 | 26.09314 | Iron status biomarkers (transferrin saturation) | rs1800562 |
| 6 | 26.09314 | Iron status biomarkers (transferrin levels) | rs1800562 |
| 6 | 26.09314 | Iron status biomarkers (iron levels) | rs1800562 |
| 6 | 26.09314 | Mean corpuscular hemoglobin | rs1800562 |
| 6 | 26.09738 | Schizophrenia | rs6918586 |
| 6 | 26.10746 | Blood pressure | rs198846 |
| 6 | 26.10746 | Hemoglobin | rs198846 |
| 6 | 26.10746 | Mean corpuscular volume | rs198846 |
| 6 | 26.11698 | Bilirubin levels | rs12206204 |
| 6 | 26.12845 | Narcolepsy with cataplexy | rs198811 |
| 6 | 26.15748 | Height | rs4141885 |
| 6 | 26.20068 | Height | rs806794 |
| 6 | 26.23339 | Height | rs10946808 |
| 6 | 26.26007 | Plasma omega–6 polyunsaturated fatty acid levels (linoleic acid) | rs4476815 |
| 6 | 26.27255 | Schizophrenia | rs61747867 |
| 6 | 26.27665 | Educational attainment | rs9393692 |
| 6 | 26.32589 | Educational attainment (years of education) | rs2179152 |

GWAS Catalog SNPs in Region

| chr | pos (Mb) | trait | snp |
|---|---|---|---|
| 6 | 26.40989 | Schizophrenia | rs41266839 |
| 6 | 26.50056 | Iron status biomarkers | rs13194984 |
| 6 | 26.51056 | Educational attainment | rs1056667 |

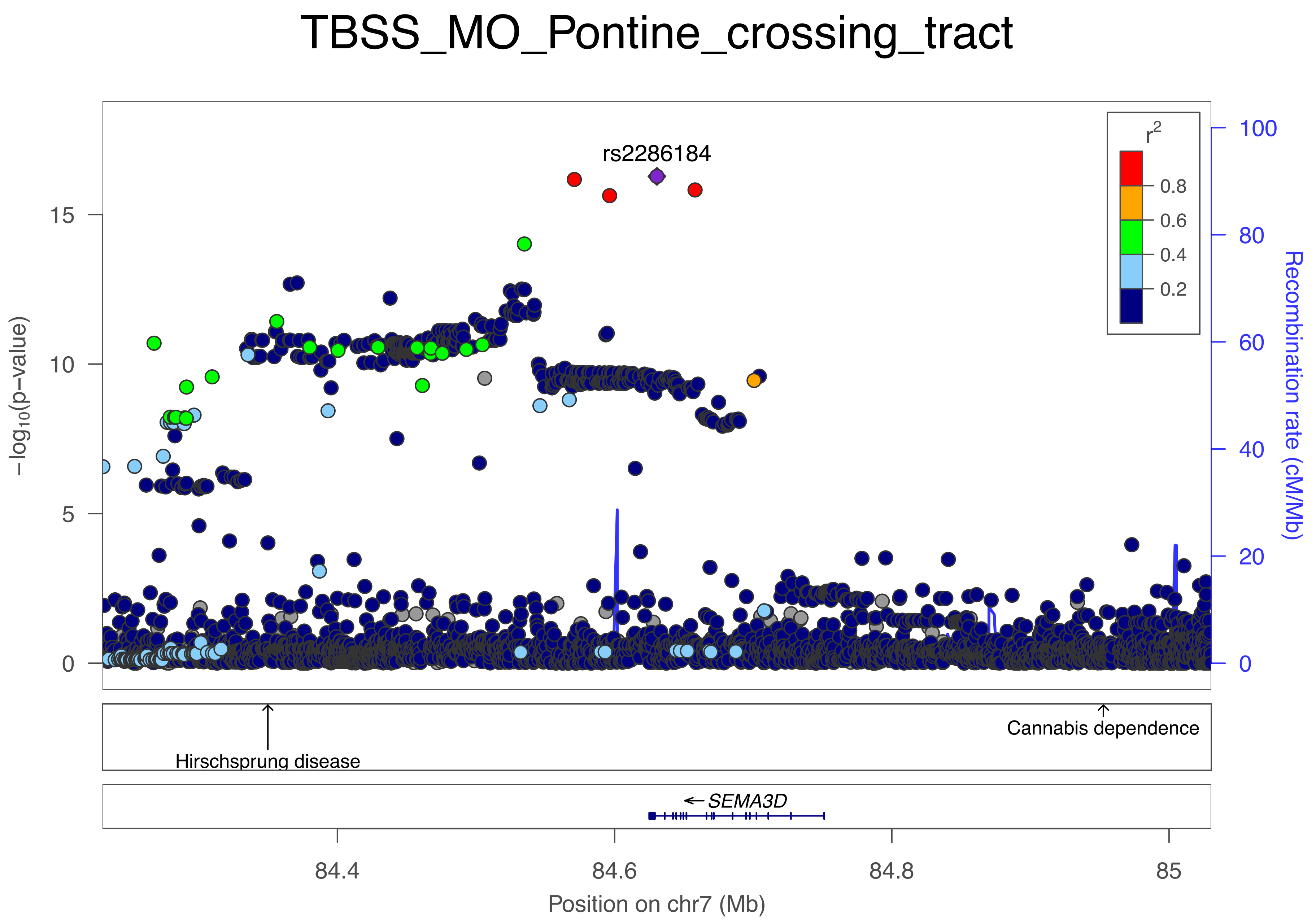

TBSS_MO_Pontine_crossing_tract
rs2286184
r2
0.8
0.6
0.4
0.2
100
80
60
40
20
0
15
10
5
0
−log10(p−value)
Recombination rate (cM/Mb)
Hirschsprung disease
Cannabis dependence
SEMA3D
84.4
84.6
84.8
85
Position on chr7 (Mb)

| | |
|---|---|
| date: | Thu Aug 17 18:06:12 2017 |
| build: | hg19 |
| display range: | chr7:84230516−85030516 [84230516−85030516] |
| hilite range: | 0 – 0 [ 0 – 0 ] |
| reference SNP: | chr7:84630516 |
| number of SNPs plotted: | 3260 |
| min P.value: | 5.31E−17 [chr7:84630516] |
| max P.value: | 10E−1 [chr7:84419665] |

Make more plots at http://csg.sph.umich.edu/locuszoom/

GWAS Catalog SNPs in Region

| chr | pos (Mb) | trait | snp |
|---|---|---|---|
| 7 | 84.34984 | Hirschsprung disease | rs80227144 |
| 7 | 84.95263 | Cannabis dependence | rs12534830 |

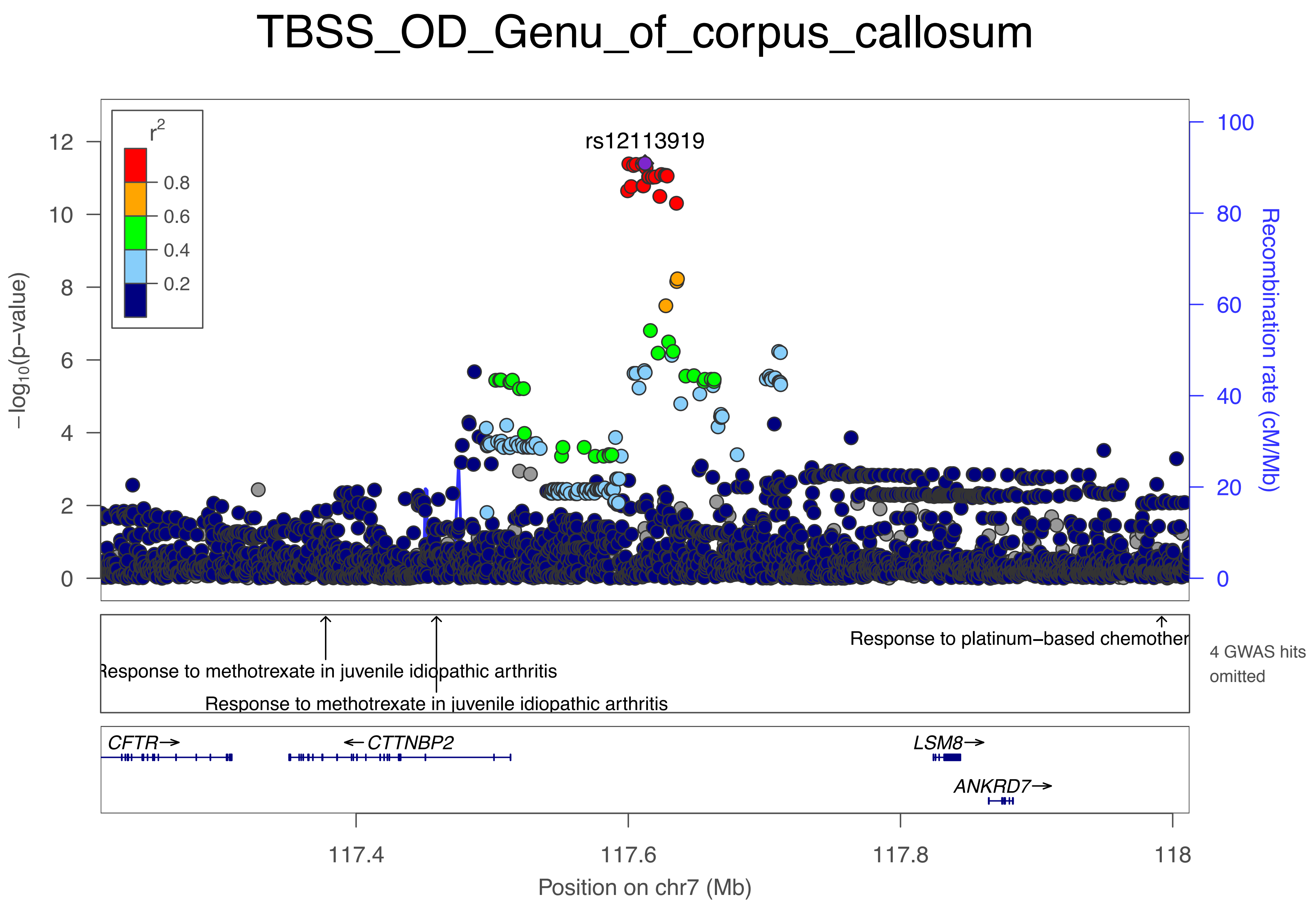

TBSS_OD_Genu_of_corpus_callosum
rs12113919
r2
0.8
0.6
0.4
0.2
−log10(p−value)
Recombination rate (cM/Mb)
Response to platinum−based chemother
Response to methotrexate in juvenile idiopathic arthritis
Response to methotrexate in juvenile idiopathic arthritis
4 GWAS hits omitted
CFTR
CTTNBP2
LSM8
ANKRD7
117.4
117.6
117.8
118
Position on chr7 (Mb)

| | |
|---|---|
| date: | Thu Aug 17 18:07:04 2017 |
| build: | hg19 |
| display range: | chr7:117212315−118012315 [117212315−118012315] |
| hilite range: | 0 – 0 [ 0 – 0 ] |
| reference SNP: | chr7:117612315 |
| number of SNPs plotted: | 3195 |
| min P.value: | 3.96E−12 [chr7:117612315] |
| max P.value: | 10E−1 [chr7:117436438] |
| omitted GWAS Hits: | NA, NA |
| omitted GWAS Hits: | NA |

Make more plots at http://csg.sph.umich.edu/locuszoom/

GWAS Catalog SNPs in Region

| chr | pos (Mb) | trait | snp |
|---|---|---|---|
| 7 | 117.2567 | Barrett's esophagus  or Esophageal adenocarcinoma | rs17451754 |
| 7 | 117.3776 | Response to methotrexate in juvenile idiopathic arthritis | rs757278 |
| 7 | 117.4590 | Response to methotrexate in juvenile idiopathic arthritis | rs7800668 |
| 7 | 117.5237 | Depression | rs10233018 |
| 7 | 117.5296 | Neuroticism | rs10244364 |
| 7 | 117.6748 | Parental longevity (mother's age at death) | rs76108901 |
| 7 | 117.9919 | Response to platinum−based chemotherapy in non−small−cell lung cancer | rs41997 |

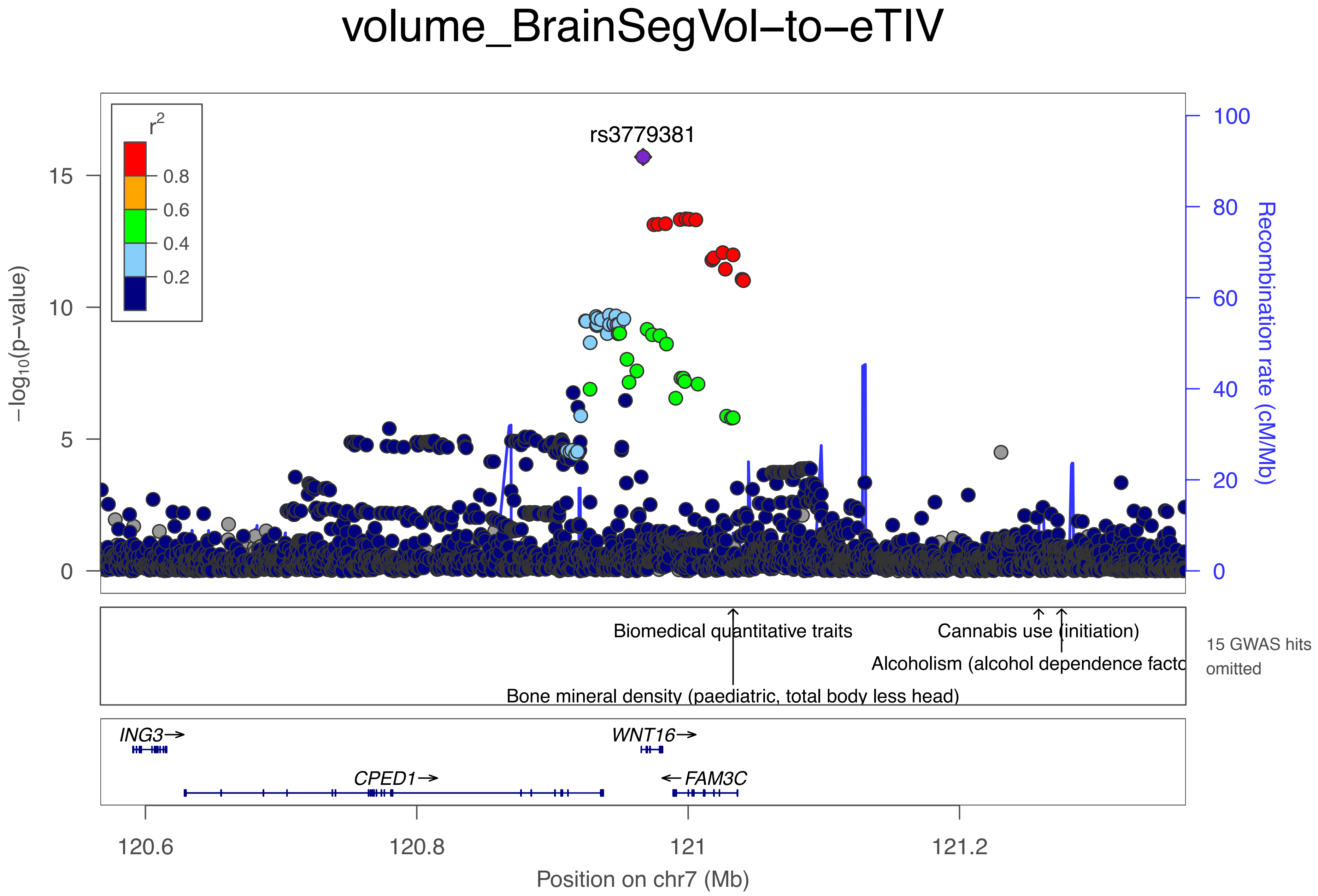

volume_BrainSegVol−to−eTIV
rs3779381
r2
0.8
0.6
0.4
0.2
−log10(p−value)
15
10
5
0
100
80
60
40
20
0
Recombination rate (cM/Mb)
Biomedical quantitative traits
Cannabis use (initiation)
Alcoholism (alcohol dependence facto
Bone mineral density (paediatric, total body less head)
15 GWAS hits omitted
ING3→
WNT16→
CPED1→
←FAM3C
120.6
120.8
121
121.2
Position on chr7 (Mb)

| | |
|---|---|
| date: | Thu Aug 17 18:34:43 2017 |
| build: | hg19 |
| display range: | chr7:120566790−121366790 [120566790−121366790] |
| hilite range: | 0 – 0 [ 0 – 0 ] |
| reference SNP: | chr7:120966790 |
| number of SNPs plotted: | 3489 |
| min P.value: | 2E−16 [chr7:120966790] |
| max P.value: | 10E−1 [chr7:121323676] |
| omitted GWAS Hits: | chr7:121.258371−Cannabis use (initiation), NA |
| omitted GWAS Hits: | NA, NA |
| omitted GWAS Hits: | NA, NA |
| omitted GWAS Hits: | NA, NA |
| omitted GWAS Hits: | NA, NA |

Make more plots at http://csg.sph.umich.edu/locuszoom/

GWAS Catalog SNPs in Region

| chr | pos (Mb) | trait | snp |
|---|---|---|---|
| 7 | 120.7473 | Bone mineral density (paediatric, skull) | rs13223036 |
| 7 | 120.7589 | Bone mineral density (paediatric, upper limb) | rs798943 |
| 7 | 120.7851 | Bone mineral density | rs13245690 |
| 7 | 120.8435 | Pediatric areal bone mineral density (radius) | rs7797976 |
| 7 | 120.8435 | Pediatric bone mineral content (radius) | rs7797976 |
| 7 | 120.9038 | Bone mineral density | rs4609139 |
| 7 | 120.9622 | Bone properties (heel) | rs2908007 |
| 7 | 120.9698 | Bone mineral density (paediatric, lower limb) | rs2908004 |
| 7 | 120.9698 | Bone mineral density | rs2908004 |
| 7 | 120.9748 | Bone mineral density | rs3801387 |
| 7 | 120.9791 | Cortical thickness | rs2707466 |
| 7 | 120.9833 | Bone mineral density | rs10242100 |
| 7 | 121.0186 | Bone mineral density | rs917727 |
| 7 | 121.0331 | Biomedical quantitative traits | rs7776725 |
| 7 | 121.0331 | Bone mineral density (paediatric, total body less head) | rs7776725 |
| 7 | 121.0331 | Bone mineral density (total hip) | rs7776725 |
| 7 | 121.0331 | Bone mineral density (femoral neck) | rs7776725 |
| 7 | 121.2584 | Cannabis use (initiation) | rs28581422 |
| 7 | 121.2752 | Alcoholism (alcohol dependence factor score) | rs10253361 |

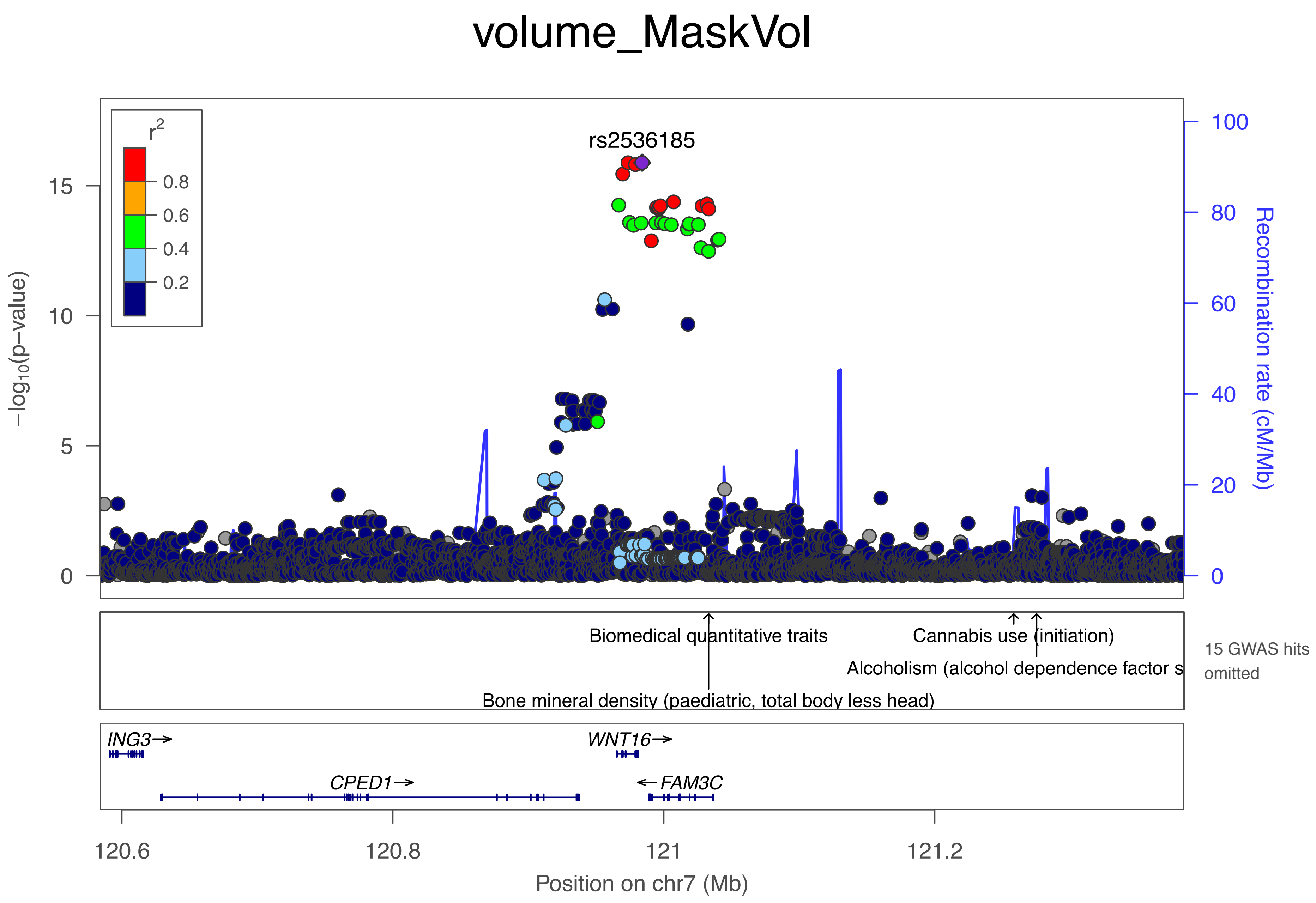
volume_MaskVol
rs2536185
r²
0.8
0.6
0.4
0.2
−log₁₀(p−value)
Recombination rate (cM/Mb)
Biomedical quantitative traits
Cannabis use (initiation)
Alcoholism (alcohol dependence factor s
Bone mineral density (paediatric, total body less head)
15 GWAS hits omitted
ING3
WNT16
CPED1
FAM3C
120.6
120.8
121
121.2
Position on chr7 (Mb)

| | |
|---|---|
| date: | Thu Aug 17 18:35:37 2017 |
| build: | hg19 |
| display range: | chr7:120584041−121384041 [120584041−121384041] |
| hilite range: | 0 – 0 [ 0 – 0 ] |
| reference SNP: | chr7:120984041 |
| number of SNPs plotted: | 3498 |
| min P.value: | 1.3E−16 [chr7:120984041] |
| max P.value: | 10E−1 [chr7:120938077] |
| omitted GWAS Hits: | chr7:121.258371−Cannabis use (initiation), NA |
| omitted GWAS Hits: | NA, NA |
| omitted GWAS Hits: | NA, NA |
| omitted GWAS Hits: | NA, NA |
| omitted GWAS Hits: | NA, NA |

Make more plots at http://csg.sph.umich.edu/locuszoom/

GWAS Catalog SNPs in Region

| chr | pos (Mb) | trait | snp |
|---|---|---|---|
| 7 | 120.7473 | Bone mineral density (paediatric, skull) | rs13223036 |
| 7 | 120.7589 | Bone mineral density (paediatric, upper limb) | rs798943 |
| 7 | 120.7851 | Bone mineral density | rs13245690 |
| 7 | 120.8435 | Pediatric areal bone mineral density (radius) | rs7797976 |
| 7 | 120.8435 | Pediatric bone mineral content (radius) | rs7797976 |
| 7 | 120.9038 | Bone mineral density | rs4609139 |
| 7 | 120.9622 | Bone properties (heel) | rs2908007 |
| 7 | 120.9698 | Bone mineral density (paediatric, lower limb) | rs2908004 |
| 7 | 120.9698 | Bone mineral density | rs2908004 |
| 7 | 120.9748 | Bone mineral density | rs3801387 |
| 7 | 120.9791 | Cortical thickness | rs2707466 |
| 7 | 120.9833 | Bone mineral density | rs10242100 |
| 7 | 121.0186 | Bone mineral density | rs917727 |
| 7 | 121.0331 | Biomedical quantitative traits | rs7776725 |
| 7 | 121.0331 | Bone mineral density (paediatric, total body less head) | rs7776725 |
| 7 | 121.0331 | Bone mineral density (total hip) | rs7776725 |
| 7 | 121.0331 | Bone mineral density (femoral neck) | rs7776725 |
| 7 | 121.2584 | Cannabis use (initiation) | rs28581422 |
| 7 | 121.2752 | Alcoholism (alcohol dependence factor score) | rs10253361 |

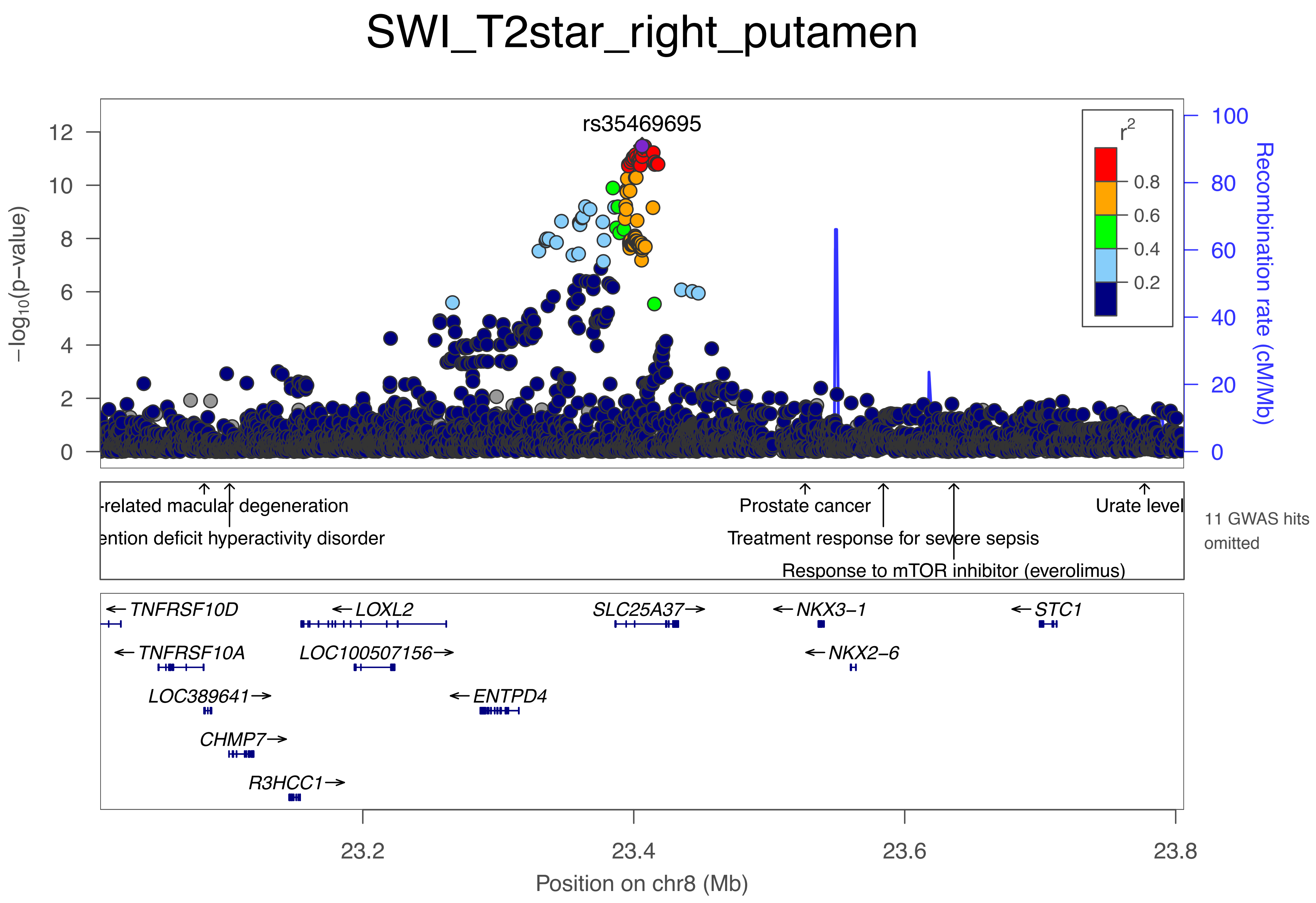
SWI_T2star_right_putamen
rs35469695
−log10(p−value)
Recombination rate (cM/Mb)
r2
0.8
0.6
0.4
0.2
-related macular degeneration
ention deficit hyperactivity disorder
Prostate cancer
Treatment response for severe sepsis
Response to mTOR inhibitor (everolimus)
Urate level
11 GWAS hits omitted
TNFRSF10D
TNFRSF10A
LOC389641
CHMP7
R3HCC1
LOXL2
LOC100507156
ENTPD4
SLC25A37
NKX3−1
NKX2−6
STC1
23.2
23.4
23.6
23.8
Position on chr8 (Mb)

| | |
|---|---|
| date: | Thu Aug 17 18:20:59 2017 |
| build: | hg19 |
| display range: | chr8:23006169−23806169 [23006169−23806169] |
| hilite range: | 0 – 0 [ 0 – 0 ] |
| reference SNP: | chr8:23406169 |
| number of SNPs plotted: | 4695 |
| min P.value: | 3.37E−12 [chr8:23406169] |
| max P.value: | 10E−1 [chr8:23352178] |
| omitted GWAS Hits: | chr8:23.777006−Urate levels, NA |
| omitted GWAS Hits: | NA, NA |
| omitted GWAS Hits: | NA, NA |
| omitted GWAS Hits: | NA, NA |

Make more plots at http://csg.sph.umich.edu/locuszoom/

GWAS Catalog SNPs in Region

| chr | pos (Mb) | trait | snp |
|---|---|---|---|
| 8 | 23.08297 | Age−related macular degeneration | rs13278062 |
| 8 | 23.08297 | Advanced age−related macular degeneration | rs79037040 |
| 8 | 23.10162 | Attention deficit hyperactivity disorder | rs7463256 |
| 8 | 23.50726 | Prostate cancer | rs142463603 |
| 8 | 23.50738 | Post bronchodilator FEV1/FVC ratio in COPD | rs147410223 |
| 8 | 23.50738 | Post bronchodilator FEV1/FVC ratio | rs147410223 |
| 8 | 23.52646 | Prostate cancer | rs1512268 |
| 8 | 23.53402 | Prostate cancer | rs10503733 |
| 8 | 23.58423 | Treatment response for severe sepsis | rs13273073 |
| 8 | 23.60332 | Waist−to−hip ratio adjusted for body mass index | rs7830933 |
| 8 | 23.63628 | Response to mTOR inhibitor (everolimus) | rs218869 |
| 8 | 23.64349 | Preschool internalizing problems | rs310272 |
| 8 | 23.71499 | Glomerular filtration rate (creatinine) | rs3758086 |
| 8 | 23.75115 | Glomerular filtration rate in non diabetics (creatinine) | rs10109414 |
| 8 | 23.75115 | Chronic kidney disease | rs10109414 |
| 8 | 23.75115 | Glomerular filtration rate | rs10109414 |
| 8 | 23.77701 | Urate levels | rs17786744 |

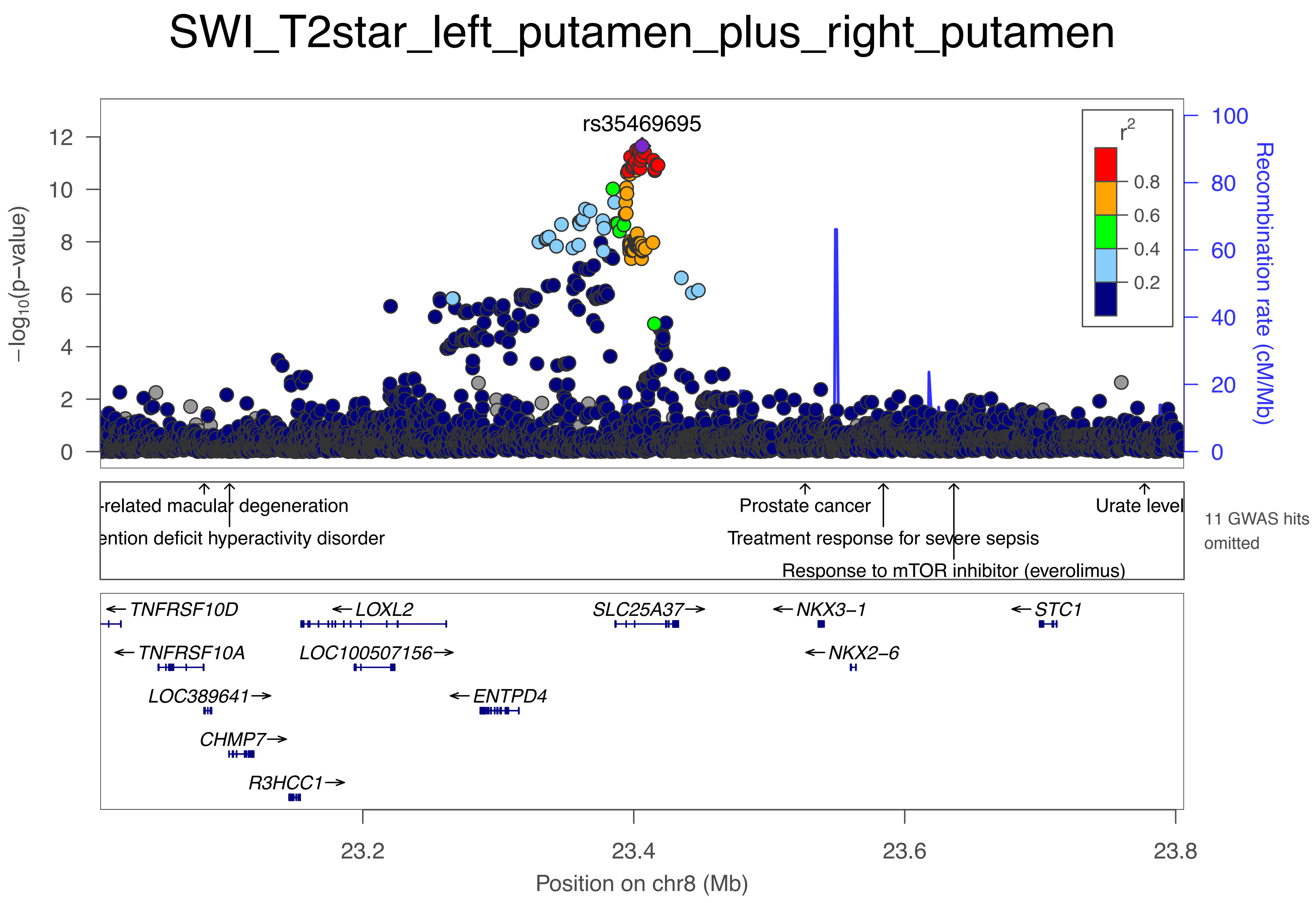

SWI_T2star_left_putamen_plus_right_putamen
rs35469695
−log10(p−value)
Recombination rate (cM/Mb)
r2
0.8
0.6
0.4
0.2
-related macular degeneration
ention deficit hyperactivity disorder
Prostate cancer
Treatment response for severe sepsis
Response to mTOR inhibitor (everolimus)
Urate level
11 GWAS hits omitted
TNFRSF10D
TNFRSF10A
LOC389641
CHMP7
R3HCC1
LOXL2
LOC100507156
ENTPD4
SLC25A37
NKX3−1
NKX2−6
STC1
23.2
23.4
23.6
23.8
Position on chr8 (Mb)

date: Thu Aug 17 18:21:00 2017

build: hg19

display range: chr8:23006169−23806169 [23006169−23806169]

hilite range: 0 – 0 [ 0 – 0 ]

reference SNP: chr8:23406169

number of SNPs plotted: 4695

min P.value: 2.22E−12 [chr8:23406169]

max P.value: 10E−1 [chr8:23155376]

omitted GWAS Hits: chr8:23.777006−Urate levels, NA

omitted GWAS Hits: NA, NA

omitted GWAS Hits: NA, NA

omitted GWAS Hits: NA, NA

Make more plots at http://csg.sph.umich.edu/locuszoom/

GWAS Catalog SNPs in Region

| chr | pos (Mb) | trait | snp |
|---|---|---|---|
| 8 | 23.08297 | Age−related macular degeneration | rs13278062 |
| 8 | 23.08297 | Advanced age−related macular degeneration | rs79037040 |
| 8 | 23.10162 | Attention deficit hyperactivity disorder | rs7463256 |
| 8 | 23.50726 | Prostate cancer | rs142463603 |
| 8 | 23.50738 | Post bronchodilator FEV1/FVC ratio in COPD | rs147410223 |
| 8 | 23.50738 | Post bronchodilator FEV1/FVC ratio | rs147410223 |
| 8 | 23.52646 | Prostate cancer | rs1512268 |
| 8 | 23.53402 | Prostate cancer | rs10503733 |
| 8 | 23.58423 | Treatment response for severe sepsis | rs13273073 |
| 8 | 23.60332 | Waist−to−hip ratio adjusted for body mass index | rs7830933 |
| 8 | 23.63628 | Response to mTOR inhibitor (everolimus) | rs218869 |
| 8 | 23.64349 | Preschool internalizing problems | rs310272 |
| 8 | 23.71499 | Glomerular filtration rate (creatinine) | rs3758086 |
| 8 | 23.75115 | Glomerular filtration rate in non diabetics (creatinine) | rs10109414 |
| 8 | 23.75115 | Chronic kidney disease | rs10109414 |
| 8 | 23.75115 | Glomerular filtration rate | rs10109414 |
| 8 | 23.77701 | Urate levels | rs17786744 |

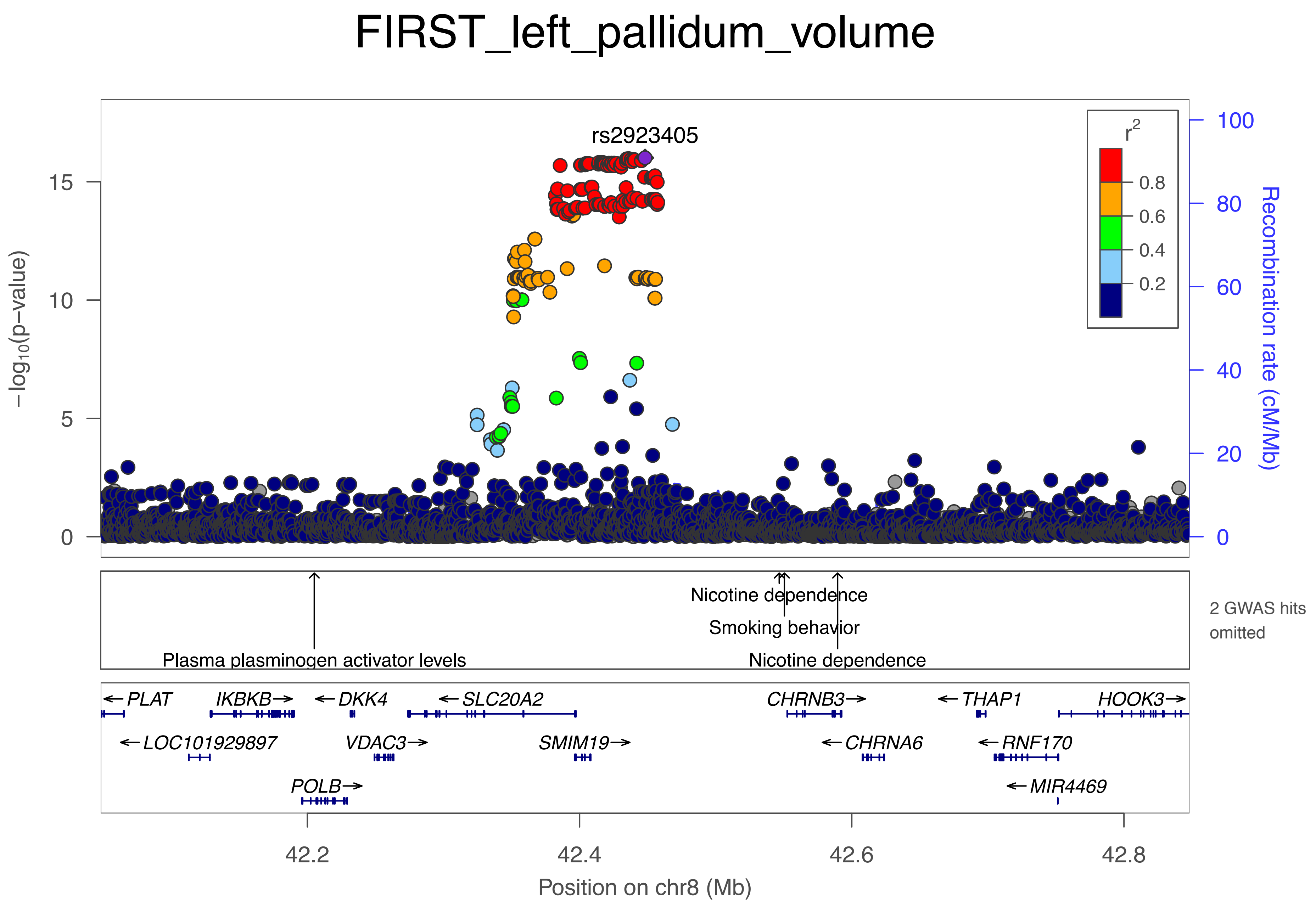
FIRST_left_pallidum_volume
rs2923405
r²
0.8
0.6
0.4
0.2
−log₁₀(p−value)
Recombination rate (cM/Mb)
Plasma plasminogen activator levels
Nicotine dependence
Smoking behavior
Nicotine dependence
2 GWAS hits omitted
PLAT
IKBKB
DKK4
SLC20A2
CHRNB3
THAP1
HOOK3
LOC101929897
VDAC3
SMIM19
CHRNA6
RNF170
POLB
MIR4469
42.2
42.4
42.6
42.8
Position on chr8 (Mb)

| | |
|---|---|
| date: | Thu Aug 17 18:35:28 2017 |
| build: | hg19 |
| display range: | chr8:42048126−42848126 [42048126−42848126] |
| hilite range: | 0 – 0 [ 0 – 0 ] |
| reference SNP: | chr8:42448126 |
| number of SNPs plotted: | 3233 |
| min P.value: | 9.68E−17 [chr8:42448126] |
| max P.value: | 9.99E−1 [chr8:42213126] |
| omitted GWAS Hits: | NA, NA |

Make more plots at http://csg.sph.umich.edu/locuszoom/

GWAS Catalog SNPs in Region

| chr | pos (Mb) | trait | snp |
|---|---|---|---|
| 8 | 42.20508 | Plasma plasminogen activator levels | rs3136739 |
| 8 | 42.47227 | Gut microbiota (functional units) | rs7016086 |
| 8 | 42.54671 | Nicotine dependence | rs1451240 |
| 8 | 42.55050 | Smoking behavior | rs6474412 |
| 8 | 42.58960 | Nicotine dependence | rs55828312 |
| 8 | 42.65818 | Pelvic organ prolapse (moderate/severe) | rs9772809 |

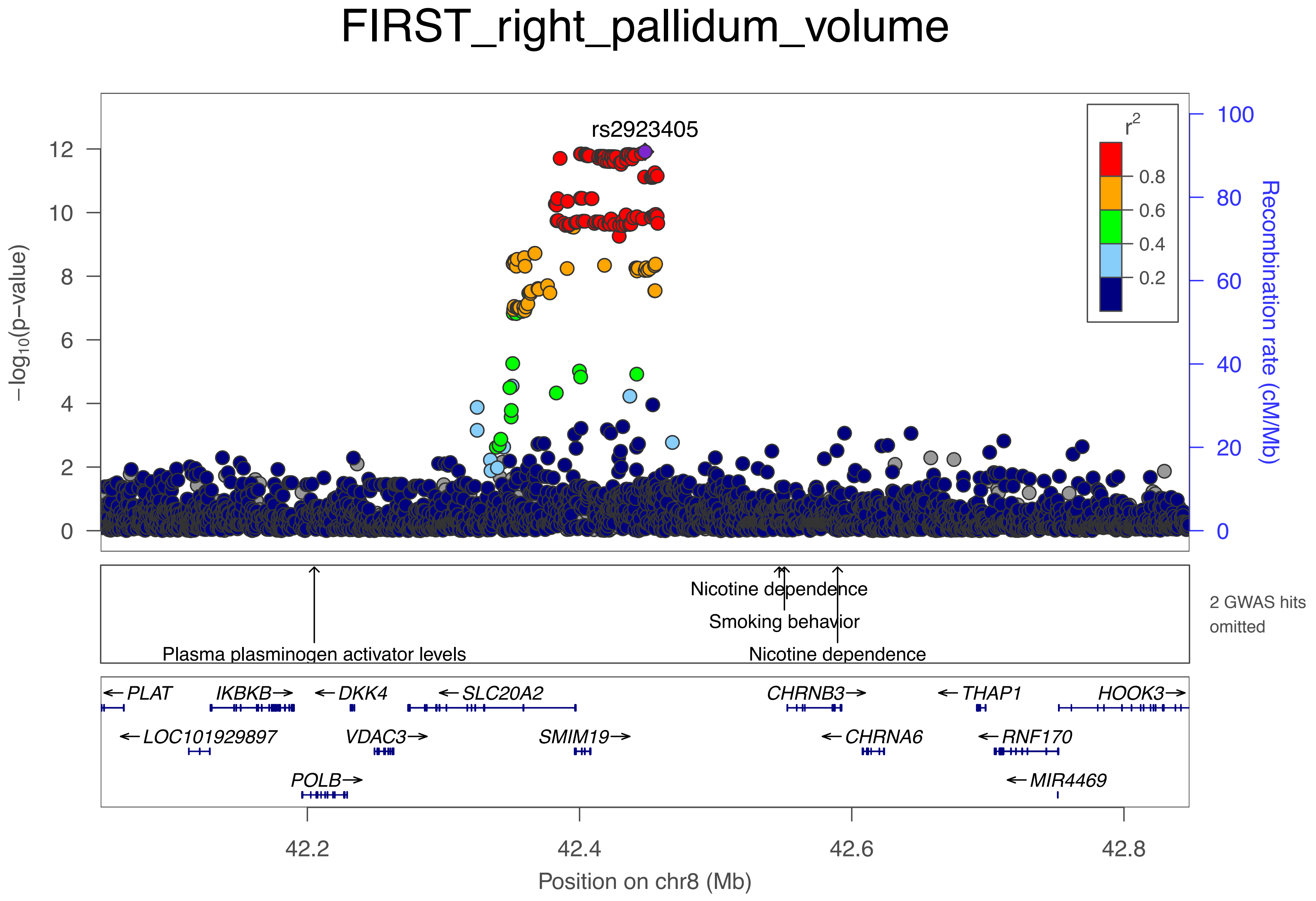

FIRST_right_pallidum_volume
rs2923405
r2
0.8
0.6
0.4
0.2
−log10(p−value)
Recombination rate (cM/Mb)
Nicotine dependence
Smoking behavior
Nicotine dependence
Plasma plasminogen activator levels
2 GWAS hits omitted
PLAT
IKBKB
DKK4
SLC20A2
CHRNB3
THAP1
HOOK3
LOC101929897
VDAC3
SMIM19
CHRNA6
RNF170
POLB
MIR4469
42.2
42.4
42.6
42.8
Position on chr8 (Mb)

| | |
|---|---|
| date: | Thu Aug 17 18:35:27 2017 |
| build: | hg19 |
| display range: | chr8:42048126−42848126 [42048126−42848126] |
| hilite range: | 0 – 0 [ 0 – 0 ] |
| reference SNP: | chr8:42448126 |
| number of SNPs plotted: | 3233 |
| min P.value: | 1.22E−12 [chr8:42448126] |
| max P.value: | 10E−1 [chr8:42443310] |
| omitted GWAS Hits: | NA, NA |

Make more plots at http://csg.sph.umich.edu/locuszoom/

GWAS Catalog SNPs in Region

| chr | pos (Mb) | trait | snp |
|---|---|---|---|
| 8 | 42.20508 | Plasma plasminogen activator levels | rs3136739 |
| 8 | 42.47227 | Gut microbiota (functional units) | rs7016086 |
| 8 | 42.54671 | Nicotine dependence | rs1451240 |
| 8 | 42.55050 | Smoking behavior | rs6474412 |
| 8 | 42.58960 | Nicotine dependence | rs55828312 |
| 8 | 42.65818 | Pelvic organ prolapse (moderate/severe) | rs9772809 |

# FIRST_left_pallidum_volume_plus_FIRST_right_pallidum_volume

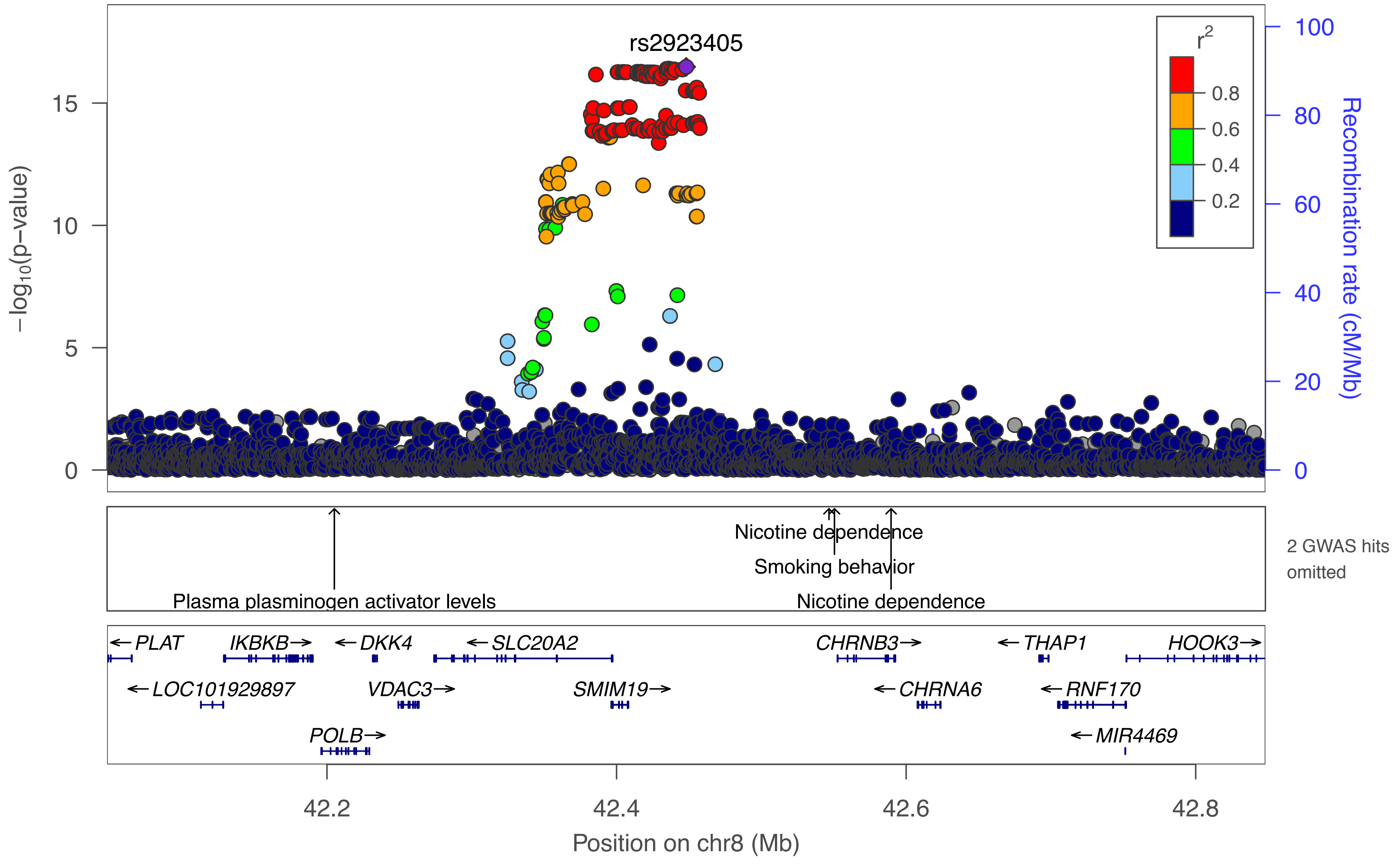

| | |
|---|---|
| date: | Thu Aug 17 18:16:48 2017 |
| build: | hg19 |
| display range: | chr8:42048126−42848126 [42048126−42848126] |
| hilite range: | 0 – 0 [ 0 – 0 ] |
| reference SNP: | chr8:42448126 |
| number of SNPs plotted: | 3233 |
| min P.value: | 3.31E−17 [chr8:42448126] |
| max P.value: | 10E−1 [chr8:42771878] |
| omitted GWAS Hits: | NA, NA |

Make more plots at http://csg.sph.umich.edu/locuszoom/

GWAS Catalog SNPs in Region

| chr | pos (Mb) | trait | snp |
|---|---|---|---|
| 8 | 42.20508 | Plasma plasminogen activator levels | rs3136739 |
| 8 | 42.47227 | Gut microbiota (functional units) | rs7016086 |
| 8 | 42.54671 | Nicotine dependence | rs1451240 |
| 8 | 42.55050 | Smoking behavior | rs6474412 |
| 8 | 42.58960 | Nicotine dependence | rs55828312 |
| 8 | 42.65818 | Pelvic organ prolapse (moderate/severe) | rs9772809 |

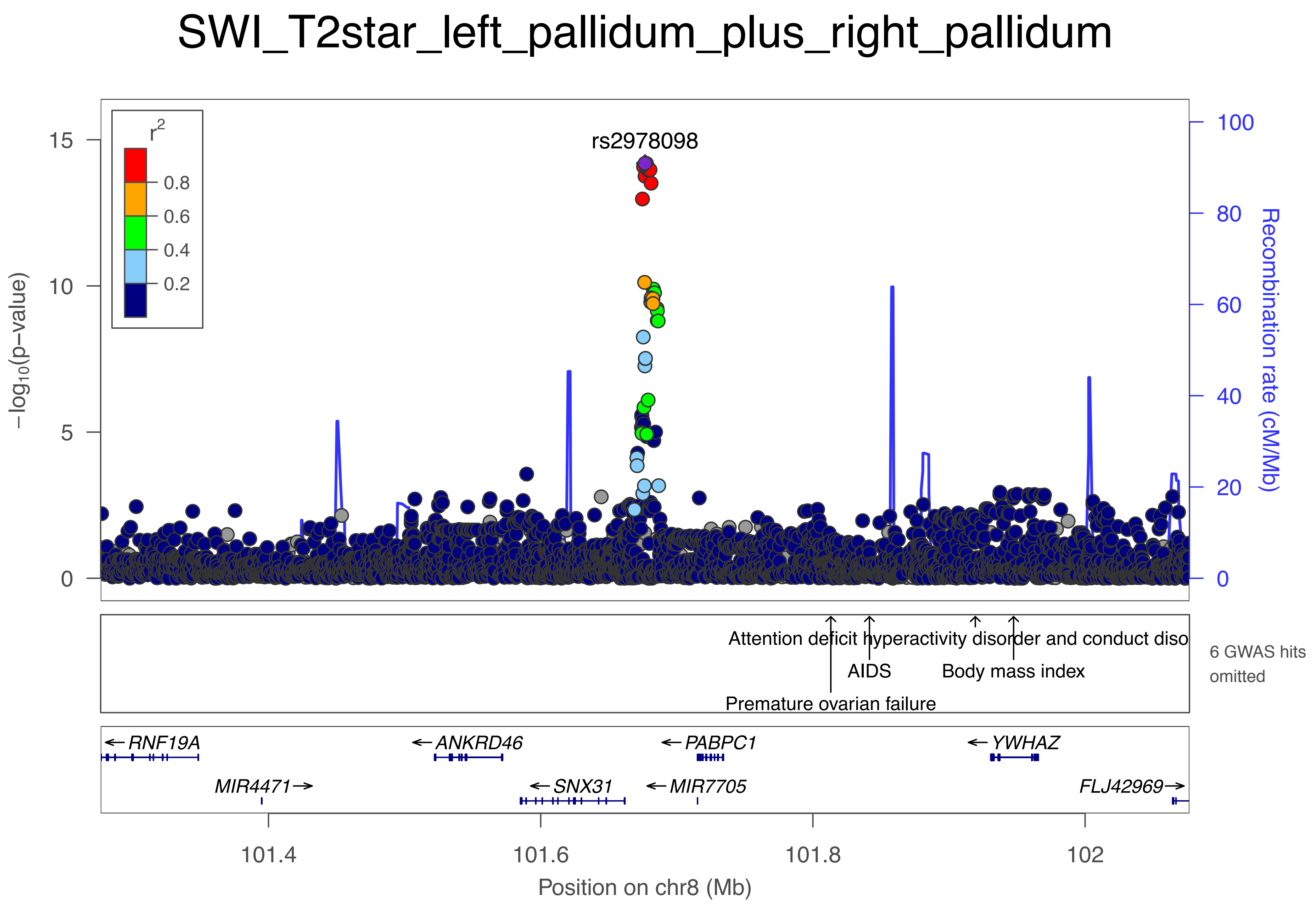

SWI_T2star_left_pallidum_plus_right_pallidum
rs2978098
r2
0.8
0.6
0.4
0.2
15
10
5
0
−log10(p−value)
100
80
60
40
20
0
Recombination rate (cM/Mb)
Attention deficit hyperactivity disorder and conduct diso
AIDS
Body mass index
Premature ovarian failure
6 GWAS hits omitted
RNF19A
ANKRD46
PABPC1
YWHAZ
MIR4471
SNX31
MIR7705
FLJ42969
101.4
101.6
101.8
102
Position on chr8 (Mb)

| | |
|---|---|
| date: | Thu Aug 17 18:38:36 2017 |
| build: | hg19 |
| display range: | chr8:101276675−102076675 [101276675−102076675] |
| hilite range: | 0 – 0 [ 0 – 0 ] |
| reference SNP: | chr8:101676675 |
| number of SNPs plotted: | 3823 |
| min P.value: | 6.43E−15 [chr8:101676675] |
| max P.value: | 10E−1 [chr8:102066162] |
| omitted GWAS Hits: | chr8:101.947453−Body mass index, NA |
| omitted GWAS Hits: | NA, NA |

Make more plots at http://csg.sph.umich.edu/locuszoom/

GWAS Catalog SNPs in Region

| chr | pos (Mb) | trait | snp |
|---|---|---|---|
| 8 | 101.3302 | Atrioventricular conduction | rs1371867 |
| 8 | 101.4318 | Asthma | rs7830057 |
| 8 | 101.5146 | Percentage gas trapping | rs2844036 |
| 8 | 101.6828 | Cerebrospinal fluid clusterin levels | rs1693575 |
| 8 | 101.8072 | Alcohol consumption | rs36061340 |
| 8 | 101.8132 | Premature ovarian failure | rs3847153 |
| 8 | 101.8415 | AIDS | rs3108919 |
| 8 | 101.9193 | Attention deficit hyperactivity disorder and conduct disorder | rs931812 |
| 8 | 101.9475 | Body mass index | rs3134353 |
| 8 | 102.0459 | Cognitive decline rate in late mild cognitive impairment | rs116237496 |

# SWI_T2star_right_pallidum

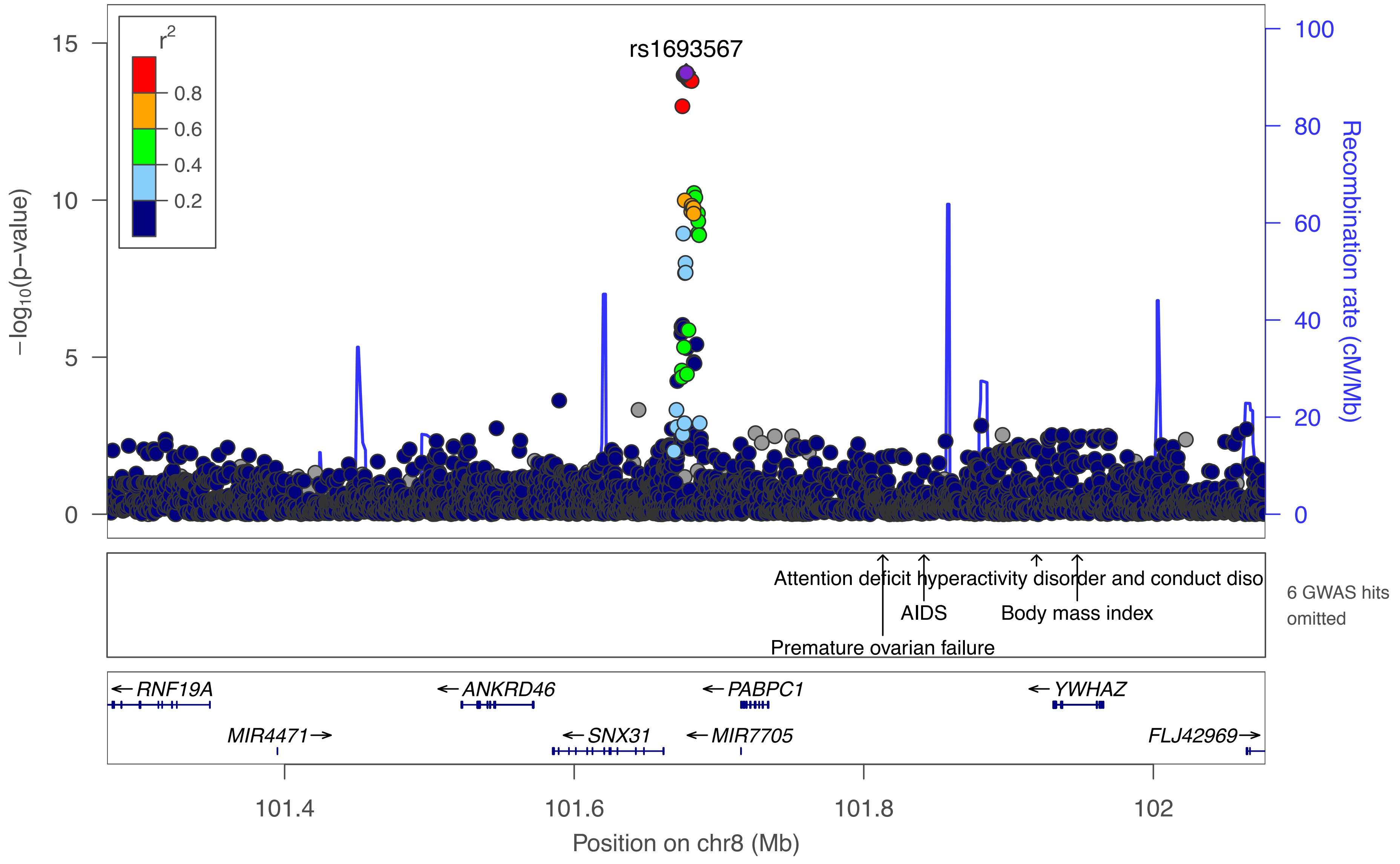

| | |
|---|---|
| date: | Thu Aug 17 18:38:36 2017 |
| build: | hg19 |
| display range: | chr8:101277386−102077386 [101277386−102077386] |
| hilite range: | 0 – 0 [ 0 – 0 ] |
| reference SNP: | chr8:101677386 |
| number of SNPs plotted: | 3822 |
| min P.value: | 8.85E−15 [chr8:101677386] |
| max P.value: | 10E−1 [chr8:101343125] |
| omitted GWAS Hits: | chr8:101.947453−Body mass index, NA |
| omitted GWAS Hits: | NA, NA |

Make more plots at http://csg.sph.umich.edu/locuszoom/

GWAS Catalog SNPs in Region

| chr | pos (Mb) | trait | snp |
|---|---|---|---|
| 8 | 101.3302 | Atrioventricular conduction | rs1371867 |
| 8 | 101.4318 | Asthma | rs7830057 |
| 8 | 101.5146 | Percentage gas trapping | rs2844036 |
| 8 | 101.6828 | Cerebrospinal fluid clusterin levels | rs1693575 |
| 8 | 101.8072 | Alcohol consumption | rs36061340 |
| 8 | 101.8132 | Premature ovarian failure | rs3847153 |
| 8 | 101.8415 | AIDS | rs3108919 |
| 8 | 101.9193 | Attention deficit hyperactivity disorder and conduct disorder | rs931812 |
| 8 | 101.9475 | Body mass index | rs3134353 |
| 8 | 102.0459 | Cognitive decline rate in late mild cognitive impairment | rs116237496 |

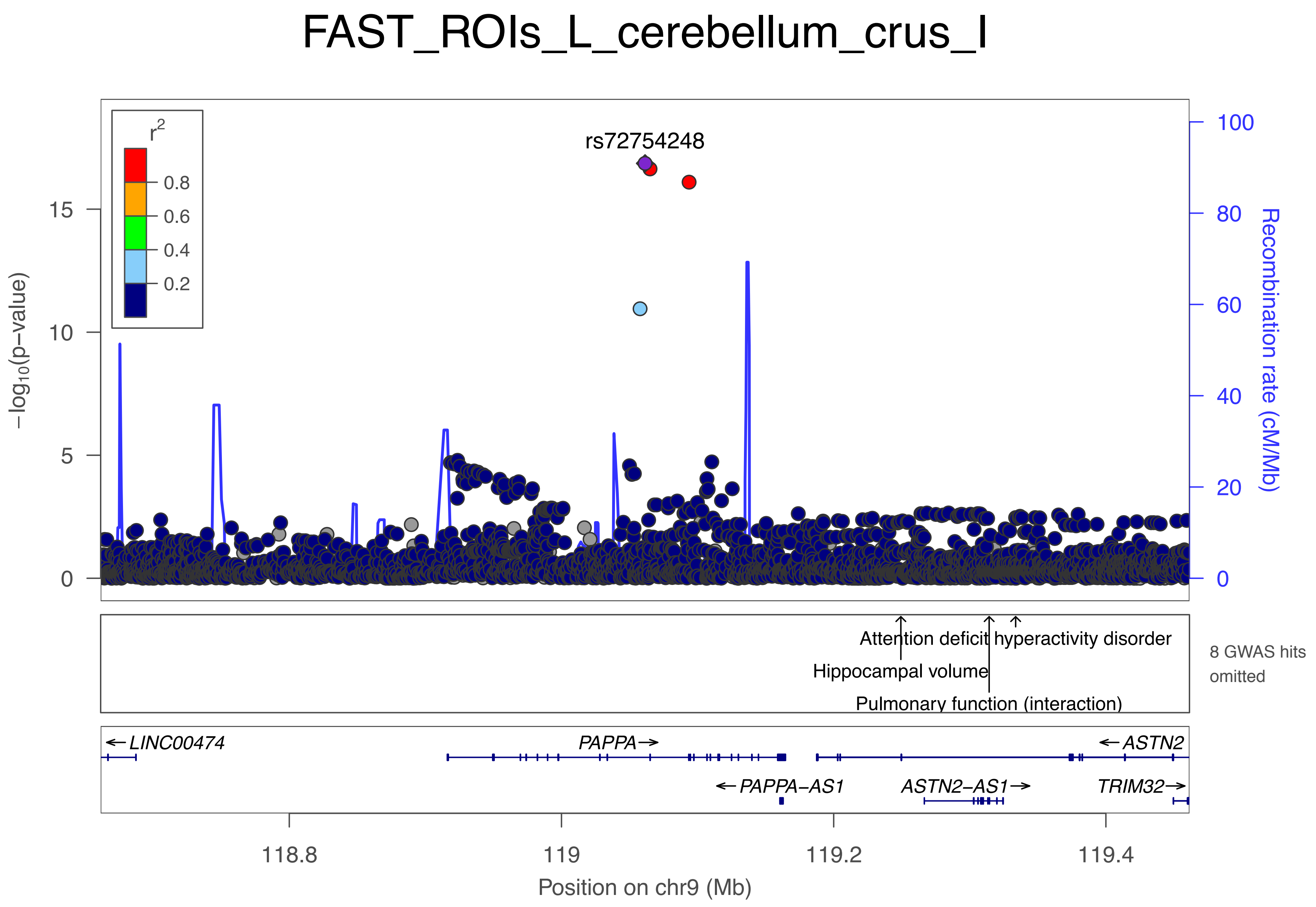

FAST_ROIs_L_cerebellum_crus_I
rs72754248
r2
0.8
0.6
0.4
0.2
15
10
5
0
−log10(p−value)
100
80
60
40
20
0
Recombination rate (cM/Mb)
Attention deficit hyperactivity disorder
Hippocampal volume
Pulmonary function (interaction)
8 GWAS hits omitted
← LINC00474
PAPPA →
← ASTN2
← PAPPA−AS1
ASTN2−AS1 →
TRIM32 →
118.8
119
119.2
119.4
Position on chr9 (Mb)

| | |
|---|---|
| date: | Thu Aug 17 18:28:35 2017 |
| build: | hg19 |
| display range: | chr9:118661396−119461396 [118661396−119461396] |
| hilite range: | 0 – 0 [ 0 – 0 ] |
| reference SNP: | chr9:119061396 |
| number of SNPs plotted: | 3336 |
| min P.value: | 1.38E−17 [chr9:119061396] |
| max P.value: | 10E−1 [chr9:119129825] |
| omitted GWAS Hits: | NA, NA |
| omitted GWAS Hits: | NA, NA |
| omitted GWAS Hits: | NA, NA |

Make more plots at http://csg.sph.umich.edu/locuszoom/

GWAS Catalog SNPs in Region

| chr | pos (Mb) | trait | snp |
|---|---|---|---|
| 9 | 118.9567 | Conotruncal heart defects | rs436582 |
| 9 | 118.9650 | 3−hydroxypropylmercapturic acid levels in smokers | rs1003858 |
| 9 | 119.1223 | Height | rs751543 |
| 9 | 119.1348 | Height | rs7869550 |
| 9 | 119.1818 | Migraine | rs17303101 |
| 9 | 119.2493 | Hippocampal volume | rs7852872 |
| 9 | 119.2526 | Migraine | rs6478241 |
| 9 | 119.2526 | Migraine without aura | rs6478241 |
| 9 | 119.2526 | Migraine − clinic−based | rs6478241 |
| 9 | 119.3142 | Pulmonary function (interaction) | rs13290997 |
| 9 | 119.3337 | Attention deficit hyperactivity disorder | rs10983238 |

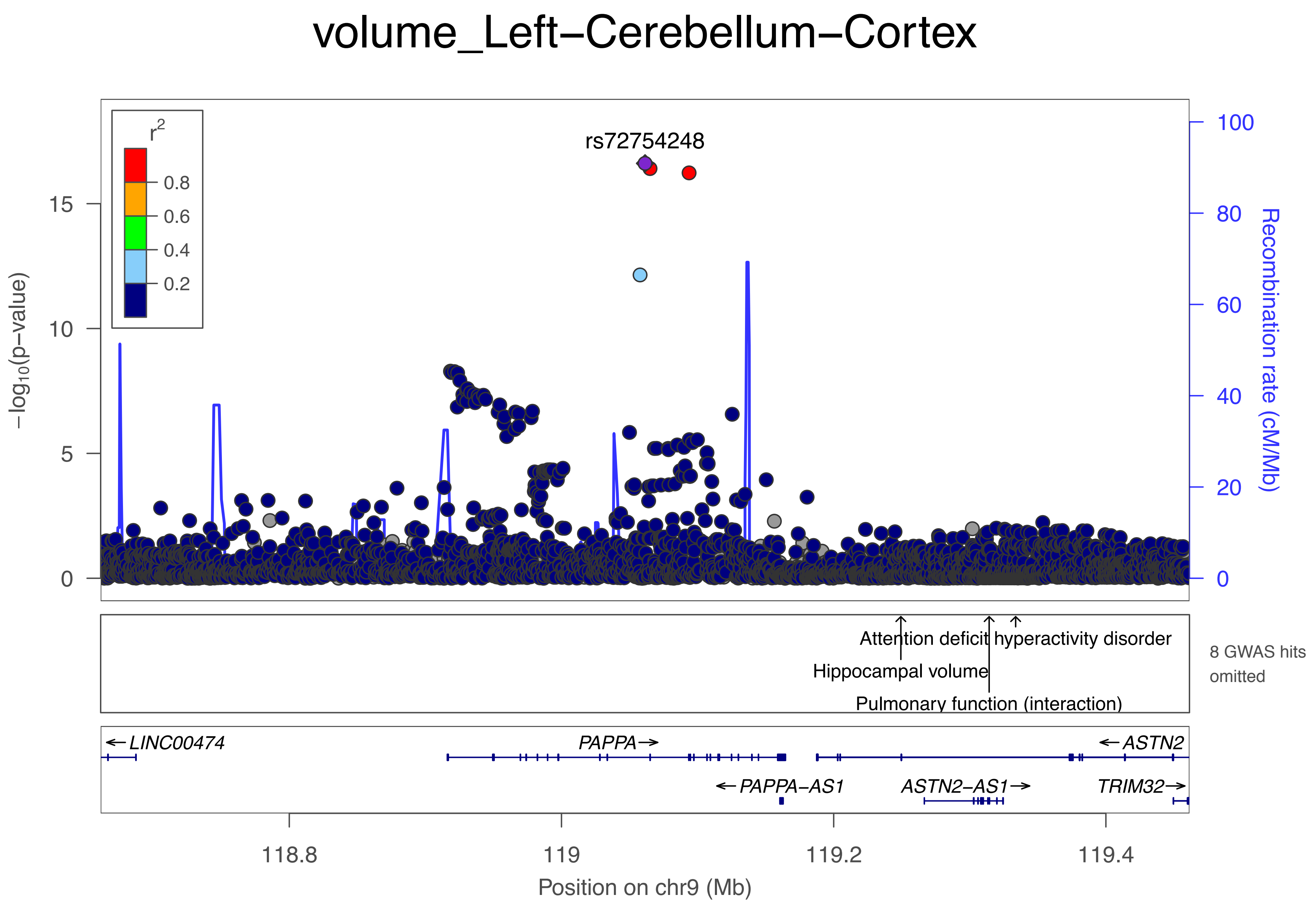

volume_Left−Cerebellum−Cortex
rs72754248
r2
0.8
0.6
0.4
0.2
−log10(p−value)
Recombination rate (cM/Mb)
Attention deficit hyperactivity disorder
Hippocampal volume
Pulmonary function (interaction)
8 GWAS hits omitted
LINC00474
PAPPA
ASTN2
PAPPA−AS1
ASTN2−AS1
TRIM32
118.8
119
119.2
119.4
Position on chr9 (Mb)

| | |
|---|---|
| date: | Thu Aug 17 18:10:37 2017 |
| build: | hg19 |
| display range: | chr9:118661396−119461396 [118661396−119461396] |
| hilite range: | 0 – 0 [ 0 – 0 ] |
| reference SNP: | chr9:119061396 |
| number of SNPs plotted: | 3336 |
| min P.value: | 2.43E−17 [chr9:119061396] |
| max P.value: | 9.99E−1 [chr9:119342366] |
| omitted GWAS Hits: | NA, NA |
| omitted GWAS Hits: | NA, NA |
| omitted GWAS Hits: | NA, NA |

Make more plots at http://csg.sph.umich.edu/locuszoom/

GWAS Catalog SNPs in Region

| chr | pos (Mb) | trait | snp |
|---|---|---|---|
| 9 | 118.9567 | Conotruncal heart defects | rs436582 |
| 9 | 118.9650 | 3–hydroxypropylmercapturic acid levels in smokers | rs1003858 |
| 9 | 119.1223 | Height | rs751543 |
| 9 | 119.1348 | Height | rs7869550 |
| 9 | 119.1818 | Migraine | rs17303101 |
| 9 | 119.2493 | Hippocampal volume | rs7852872 |
| 9 | 119.2526 | Migraine | rs6478241 |
| 9 | 119.2526 | Migraine without aura | rs6478241 |
| 9 | 119.2526 | Migraine – clinic–based | rs6478241 |
| 9 | 119.3142 | Pulmonary function (interaction) | rs13290997 |
| 9 | 119.3337 | Attention deficit hyperactivity disorder | rs10983238 |

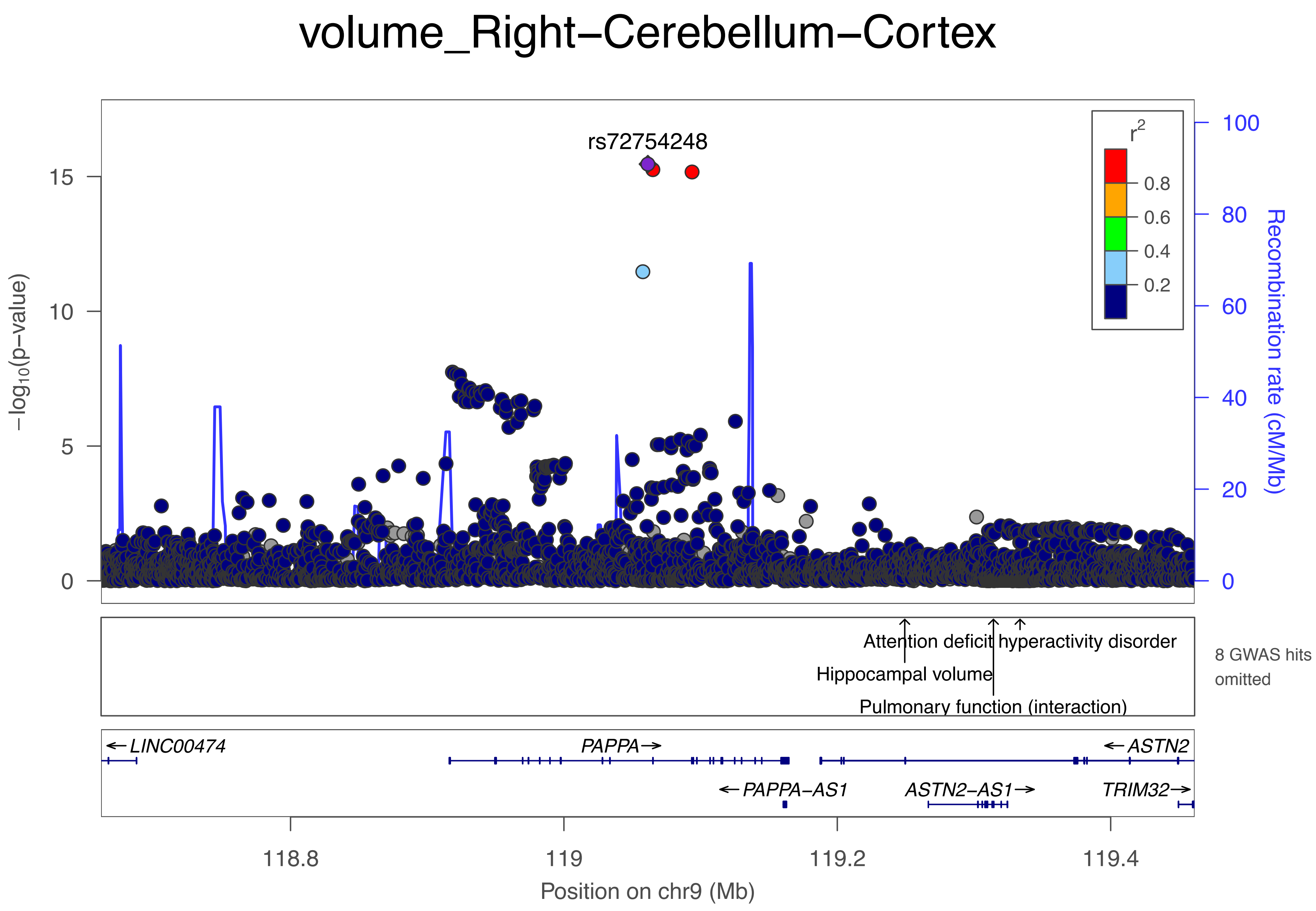

volume_Right−Cerebellum−Cortex
rs72754248
r2
0.8
0.6
0.4
0.2
−log10(p−value)
Recombination rate (cM/Mb)
Attention deficit hyperactivity disorder
Hippocampal volume
Pulmonary function (interaction)
8 GWAS hits omitted
← LINC00474
PAPPA →
← ASTN2
← PAPPA−AS1
ASTN2−AS1 →
TRIM32 →
118.8
119
119.2
119.4
Position on chr9 (Mb)

| | |
|---|---|
| date: | Thu Aug 17 18:22:31 2017 |
| build: | hg19 |
| display range: | chr9:118661396−119461396 [118661396−119461396] |
| hilite range: | 0 – 0 [ 0 – 0 ] |
| reference SNP: | chr9:119061396 |
| number of SNPs plotted: | 3336 |
| min P.value: | 3.45E−16 [chr9:119061396] |
| max P.value: | 10E−1 [chr9:118668675] |
| omitted GWAS Hits: | NA, NA |
| omitted GWAS Hits: | NA, NA |
| omitted GWAS Hits: | NA, NA |

Make more plots at http://csg.sph.umich.edu/locuszoom/

GWAS Catalog SNPs in Region

| chr | pos (Mb) | trait | snp |
|---|---|---|---|
| 9 | 118.9567 | Conotruncal heart defects | rs436582 |
| 9 | 118.9650 | 3–hydroxypropylmercapturic acid levels in smokers | rs1003858 |
| 9 | 119.1223 | Height | rs751543 |
| 9 | 119.1348 | Height | rs7869550 |
| 9 | 119.1818 | Migraine | rs17303101 |
| 9 | 119.2493 | Hippocampal volume | rs7852872 |
| 9 | 119.2526 | Migraine | rs6478241 |
| 9 | 119.2526 | Migraine without aura | rs6478241 |
| 9 | 119.2526 | Migraine – clinic–based | rs6478241 |
| 9 | 119.3142 | Pulmonary function (interaction) | rs13290997 |
| 9 | 119.3337 | Attention deficit hyperactivity disorder | rs10983238 |

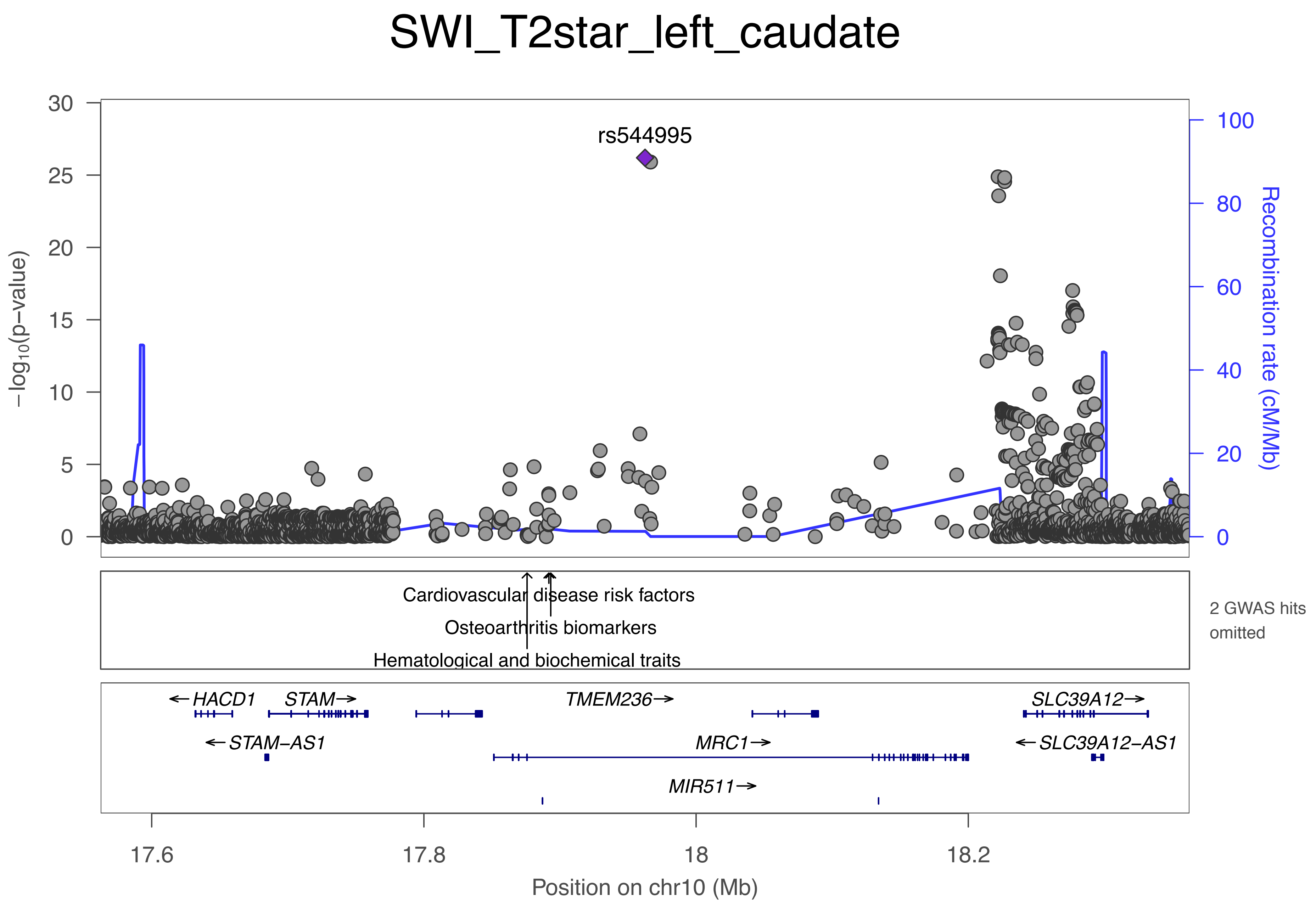

SWI_T2star_left_caudate
rs544995
−log10(p−value)
Recombination rate (cM/Mb)
Cardiovascular disease risk factors
Osteoarthritis biomarkers
Hematological and biochemical traits
2 GWAS hits omitted
←HACD1
STAM→
TMEM236→
SLC39A12→
←STAM−AS1
MRC1→
←SLC39A12−AS1
MIR511→
17.6
17.8
18
18.2
Position on chr10 (Mb)

| | |
|---|---|
| date: | Thu Aug 17 17:52:01 2017 |
| build: | hg19 |
| display range: | chr10:17562506−18362506 [17562506−18362506] |
| hilite range: | 0 – 0 [ 0 – 0 ] |
| reference SNP: | chr10:17962506 |
| number of SNPs plotted: | 2378 |
| min P.value: | 6.37E−27 [chr10:17962506] |
| max P.value: | 9.99E−1 [chr10:17720533] |
| omitted GWAS Hits: | NA, NA |
| Warning: | No usable LD information for reference SNP. |

Make more plots at http://csg.sph.umich.edu/locuszoom/

GWAS Catalog SNPs in Region

| chr | pos (Mb) | trait | snp |
|---|---|---|---|
| 10 | 17.59612 | Glucose homeostasis traits | rs6602203 |
| 10 | 17.87582 | Hematological and biochemical traits | rs2477664 |
| 10 | 17.89182 | Cardiovascular disease risk factors | rs2437258 |
| 10 | 17.89321 | Osteoarthritis biomarkers | rs691461 |
| 10 | 17.96656 | Childhood and early adolescence aggressive behavior | rs10508552 |

# SWI_T2star_right_caudate

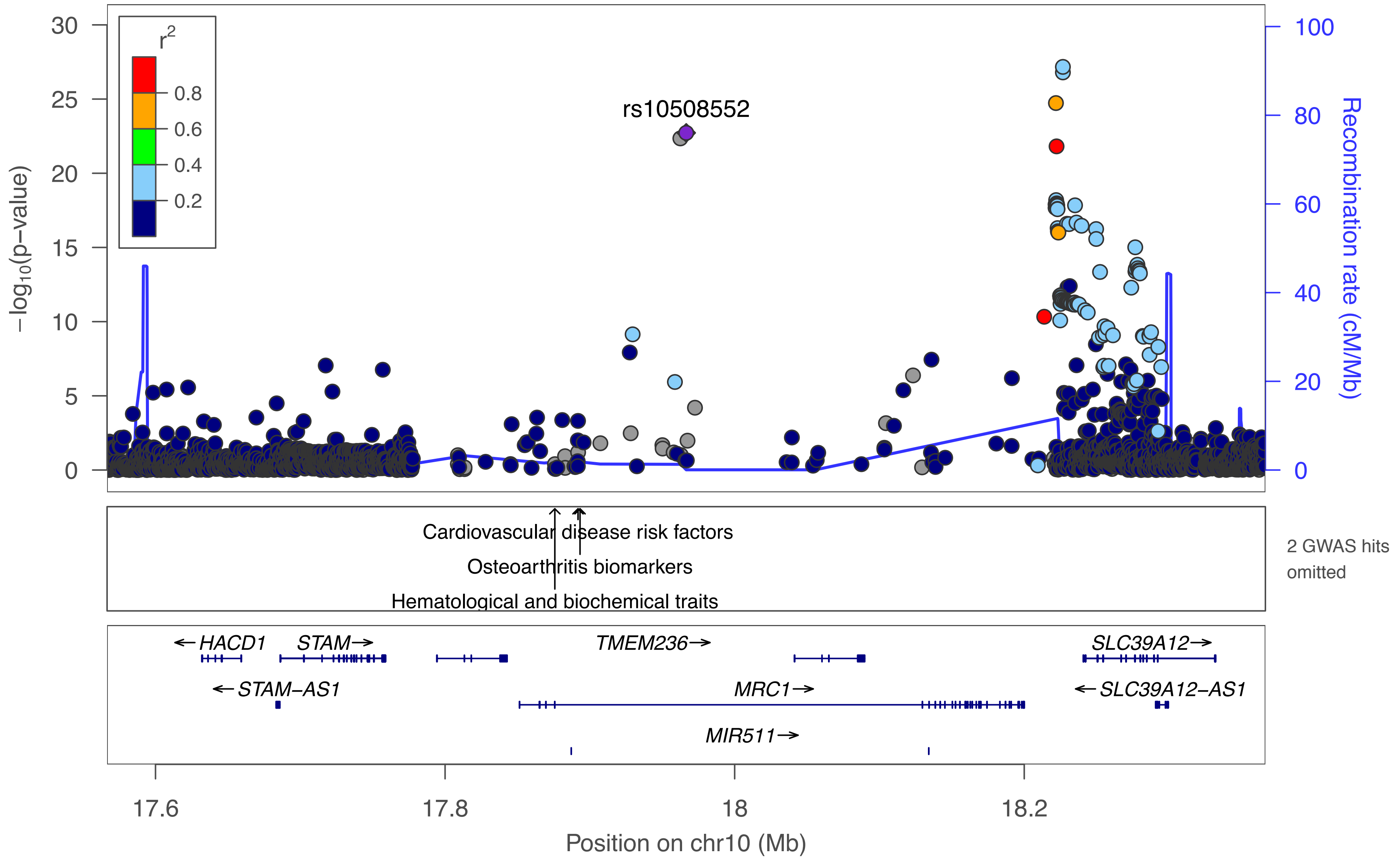

| | |
|---|---|
| date: | Thu Aug 17 18:05:07 2017 |
| build: | hg19 |
| display range: | chr10:17566561−18366561 [17566561−18366561] |
| hilite range: | 0 – 0 [ 0 – 0 ] |
| reference SNP: | chr10:17966561 |
| number of SNPs plotted: | 2390 |
| min P.value: | 6.76E−28 [chr10:18226714] |
| max P.value: | 10E−1 [chr10:17593986] |
| omitted GWAS Hits: | NA, NA |

Make more plots at http://csg.sph.umich.edu/locuszoom/

GWAS Catalog SNPs in Region

| chr | pos (Mb) | trait | snp |
|---|---|---|---|
| 10 | 17.59612 | Glucose homeostasis traits | rs6602203 |
| 10 | 17.87582 | Hematological and biochemical traits | rs2477664 |
| 10 | 17.89182 | Cardiovascular disease risk factors | rs2437258 |
| 10 | 17.89321 | Osteoarthritis biomarkers | rs691461 |
| 10 | 17.96656 | Childhood and early adolescence aggressive behavior | rs10508552 |

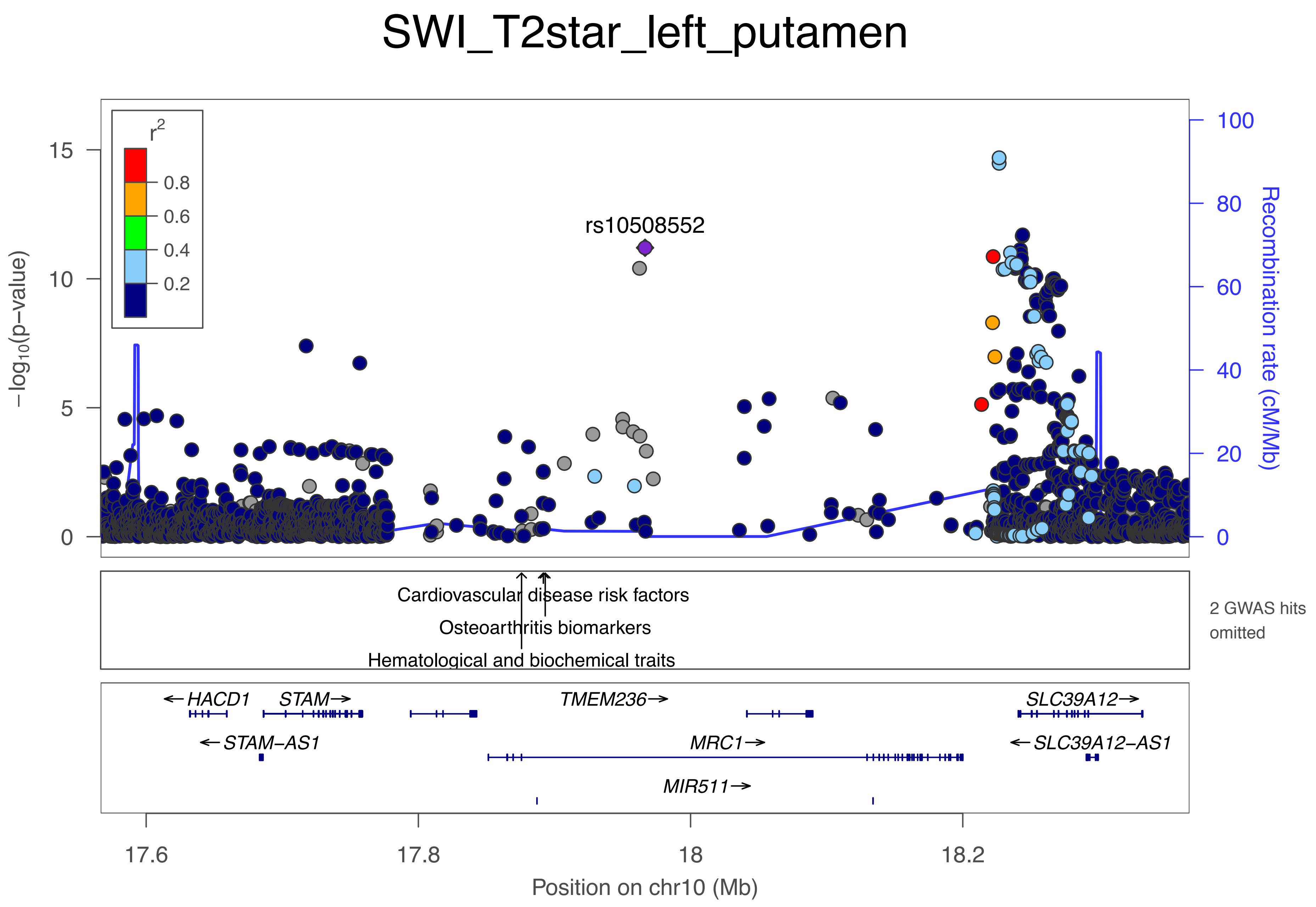

SWI_T2star_left_putamen
r2
0.8
0.6
0.4
0.2
rs10508552
15
10
5
0
−log10(p−value)
100
80
60
40
20
0
Recombination rate (cM/Mb)
Cardiovascular disease risk factors
Osteoarthritis biomarkers
Hematological and biochemical traits
2 GWAS hits omitted
← HACD1
STAM→
TMEM236→
SLC39A12→
← STAM−AS1
MRC1→
← SLC39A12−AS1
MIR511→
17.6
17.8
18
18.2
Position on chr10 (Mb)

| | |
|---|---|
| date: | Thu Aug 17 18:05:08 2017 |
| build: | hg19 |
| display range: | chr10:17566561−18366561 [17566561−18366561] |
| hilite range: | 0 – 0 [ 0 – 0 ] |
| reference SNP: | chr10:17966561 |
| number of SNPs plotted: | 2390 |
| min P.value: | 2.04E−15 [chr10:18226714] |
| max P.value: | 10E−1 [chr10:17685893] |
| omitted GWAS Hits: | NA, NA |

Make more plots at http://csg.sph.umich.edu/locuszoom/

GWAS Catalog SNPs in Region

| chr | pos (Mb) | trait | snp |
|---|---|---|---|
| 10 | 17.59612 | Glucose homeostasis traits | rs6602203 |
| 10 | 17.87582 | Hematological and biochemical traits | rs2477664 |
| 10 | 17.89182 | Cardiovascular disease risk factors | rs2437258 |
| 10 | 17.89321 | Osteoarthritis biomarkers | rs691461 |
| 10 | 17.96656 | Childhood and early adolescence aggressive behavior | rs10508552 |

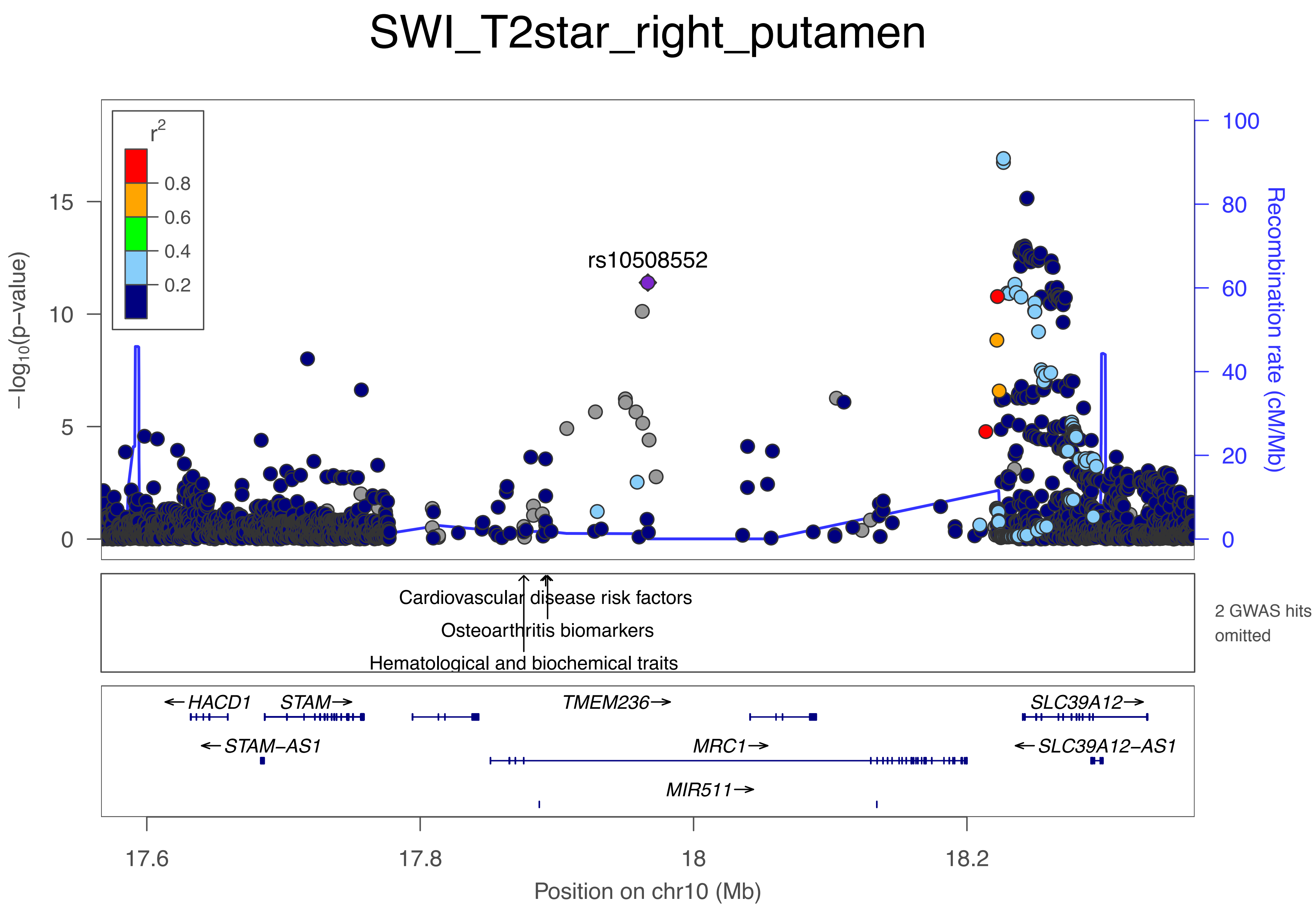

SWI_T2star_right_putamen
r2
0.8
0.6
0.4
0.2
−log10(p−value)
15
10
5
0
100
80
60
40
20
0
Recombination rate (cM/Mb)
rs10508552
Cardiovascular disease risk factors
Osteoarthritis biomarkers
Hematological and biochemical traits
2 GWAS hits omitted
←HACD1
STAM→
TMEM236→
SLC39A12→
←STAM−AS1
MRC1→
←SLC39A12−AS1
MIR511→
17.6
17.8
18
18.2
Position on chr10 (Mb)

| | |
|---|---|
| date: | Thu Aug 17 18:05:07 2017 |
| build: | hg19 |
| display range: | chr10:17566561−18366561 [17566561−18366561] |
| hilite range: | 0 – 0 [ 0 – 0 ] |
| reference SNP: | chr10:17966561 |
| number of SNPs plotted: | 2390 |
| min P.value: | 1.21E−17 [chr10:18226714] |
| max P.value: | 10E−1 [chr10:17582716] |
| omitted GWAS Hits: | NA, NA |

Make more plots at http://csg.sph.umich.edu/locuszoom/

GWAS Catalog SNPs in Region

| chr | pos (Mb) | trait | snp |
| --- | --- | --- | --- |
| 10 | 17.59612 | Glucose homeostasis traits | rs6602203 |
| 10 | 17.87582 | Hematological and biochemical traits | rs2477664 |
| 10 | 17.89182 | Cardiovascular disease risk factors | rs2437258 |
| 10 | 17.89321 | Osteoarthritis biomarkers | rs691461 |
| 10 | 17.96656 | Childhood and early adolescence aggressive behavior | rs10508552 |

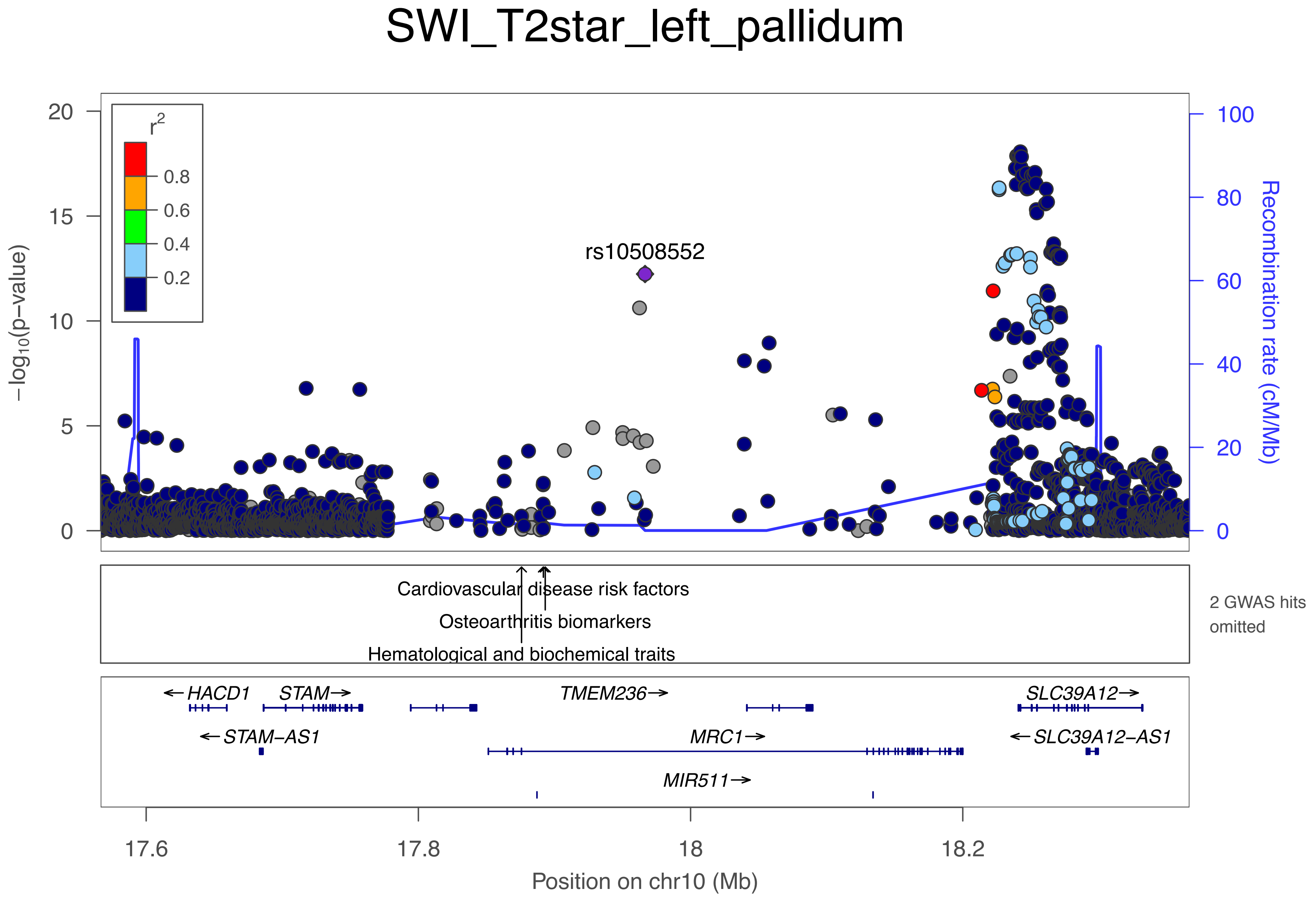

SWI_T2star_left_pallidum
r2
0.8
0.6
0.4
0.2
rs10508552
−log10(p−value)
Recombination rate (cM/Mb)
20
15
10
5
0
100
80
60
40
20
0
Cardiovascular disease risk factors
Osteoarthritis biomarkers
Hematological and biochemical traits
2 GWAS hits omitted
← HACD1
STAM→
TMEM236→
SLC39A12→
← STAM−AS1
MRC1→
← SLC39A12−AS1
MIR511→
17.6
17.8
18
18.2
Position on chr10 (Mb)

| | |
|---|---|
| date: | Thu Aug 17 17:54:38 2017 |
| build: | hg19 |
| display range: | chr10:17566561−18366561 [17566561−18366561] |
| hilite range: | 0 – 0 [ 0 – 0 ] |
| reference SNP: | chr10:17966561 |
| number of SNPs plotted: | 2390 |
| min P.value: | 8.63E−19 [chr10:18242311] |
| max P.value: | 9.99E−1 [chr10:17741046] |
| omitted GWAS Hits: | NA, NA |

Make more plots at http://csg.sph.umich.edu/locuszoom/

GWAS Catalog SNPs in Region

| chr | pos (Mb) | trait | snp |
|---|---|---|---|
| 10 | 17.59612 | Glucose homeostasis traits | rs6602203 |
| 10 | 17.87582 | Hematological and biochemical traits | rs2477664 |
| 10 | 17.89182 | Cardiovascular disease risk factors | rs2437258 |
| 10 | 17.89321 | Osteoarthritis biomarkers | rs691461 |
| 10 | 17.96656 | Childhood and early adolescence aggressive behavior | rs10508552 |

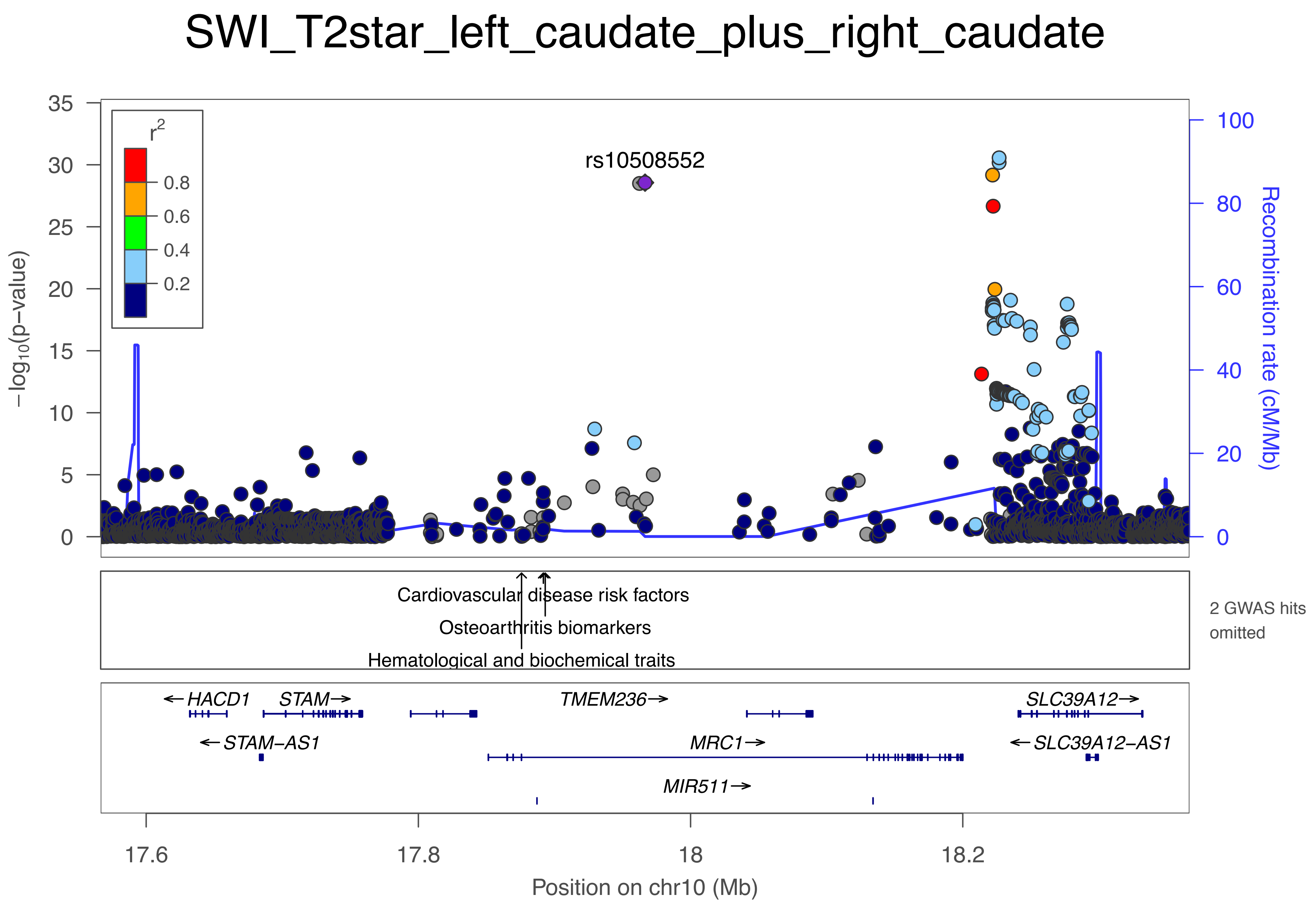
SWI_T2star_left_caudate_plus_right_caudate
rs10508552
r2
0.8
0.6
0.4
0.2
35
30
25
20
15
10
5
0
100
80
60
40
20
0
−log10(p−value)
Recombination rate (cM/Mb)
Cardiovascular disease risk factors
Osteoarthritis biomarkers
Hematological and biochemical traits
2 GWAS hits omitted
←HACD1
STAM→
TMEM236→
SLC39A12→
←STAM−AS1
MRC1→
←SLC39A12−AS1
MIR511→
17.6
17.8
18
18.2
Position on chr10 (Mb)

| | |
|---|---|
| date: | Thu Aug 17 18:00:15 2017 |
| build: | hg19 |
| display range: | chr10:17566561−18366561 [17566561−18366561] |
| hilite range: | 0 – 0 [ 0 – 0 ] |
| reference SNP: | chr10:17966561 |
| number of SNPs plotted: | 2390 |
| min P.value: | 2.73E−31 [chr10:18226714] |
| max P.value: | 9.97E−1 [chr10:18324574] |
| omitted GWAS Hits: | NA, NA |

Make more plots at http://csg.sph.umich.edu/locuszoom/

GWAS Catalog SNPs in Region

| chr | pos (Mb) | trait | snp |
|---|---|---|---|
| 10 | 17.59612 | Glucose homeostasis traits | rs6602203 |
| 10 | 17.87582 | Hematological and biochemical traits | rs2477664 |
| 10 | 17.89182 | Cardiovascular disease risk factors | rs2437258 |
| 10 | 17.89321 | Osteoarthritis biomarkers | rs691461 |
| 10 | 17.96656 | Childhood and early adolescence aggressive behavior | rs10508552 |

# SWI_T2star_left_putamen_plus_right_putamen

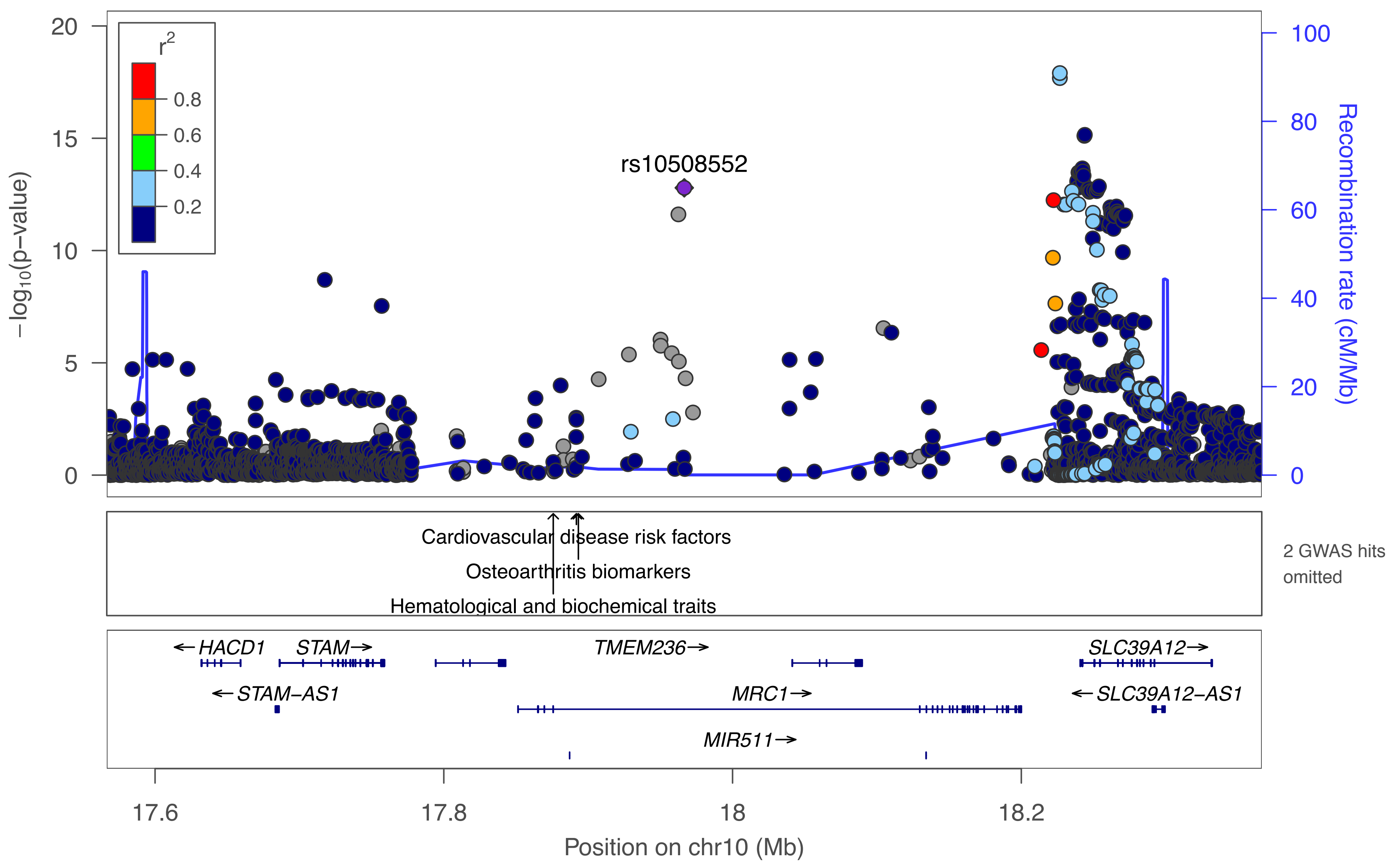

| | |
|---|---|
| date: | Thu Aug 17 17:56:57 2017 |
| build: | hg19 |
| display range: | chr10:17566561−18366561 [17566561−18366561] |
| hilite range: | 0 – 0 [ 0 – 0 ] |
| reference SNP: | chr10:17966561 |
| number of SNPs plotted: | 2390 |
| min P.value: | 1.25E−18 [chr10:18226714] |
| max P.value: | 9.99E−1 [chr10:17650307] |
| omitted GWAS Hits: | NA, NA |

Make more plots at http://csg.sph.umich.edu/locuszoom/

GWAS Catalog SNPs in Region

| chr | pos (Mb) | trait | snp |
|---|---|---|---|
| 10 | 17.59612 | Glucose homeostasis traits | rs6602203 |
| 10 | 17.87582 | Hematological and biochemical traits | rs2477664 |
| 10 | 17.89182 | Cardiovascular disease risk factors | rs2437258 |
| 10 | 17.89321 | Osteoarthritis biomarkers | rs691461 |
| 10 | 17.96656 | Childhood and early adolescence aggressive behavior | rs10508552 |

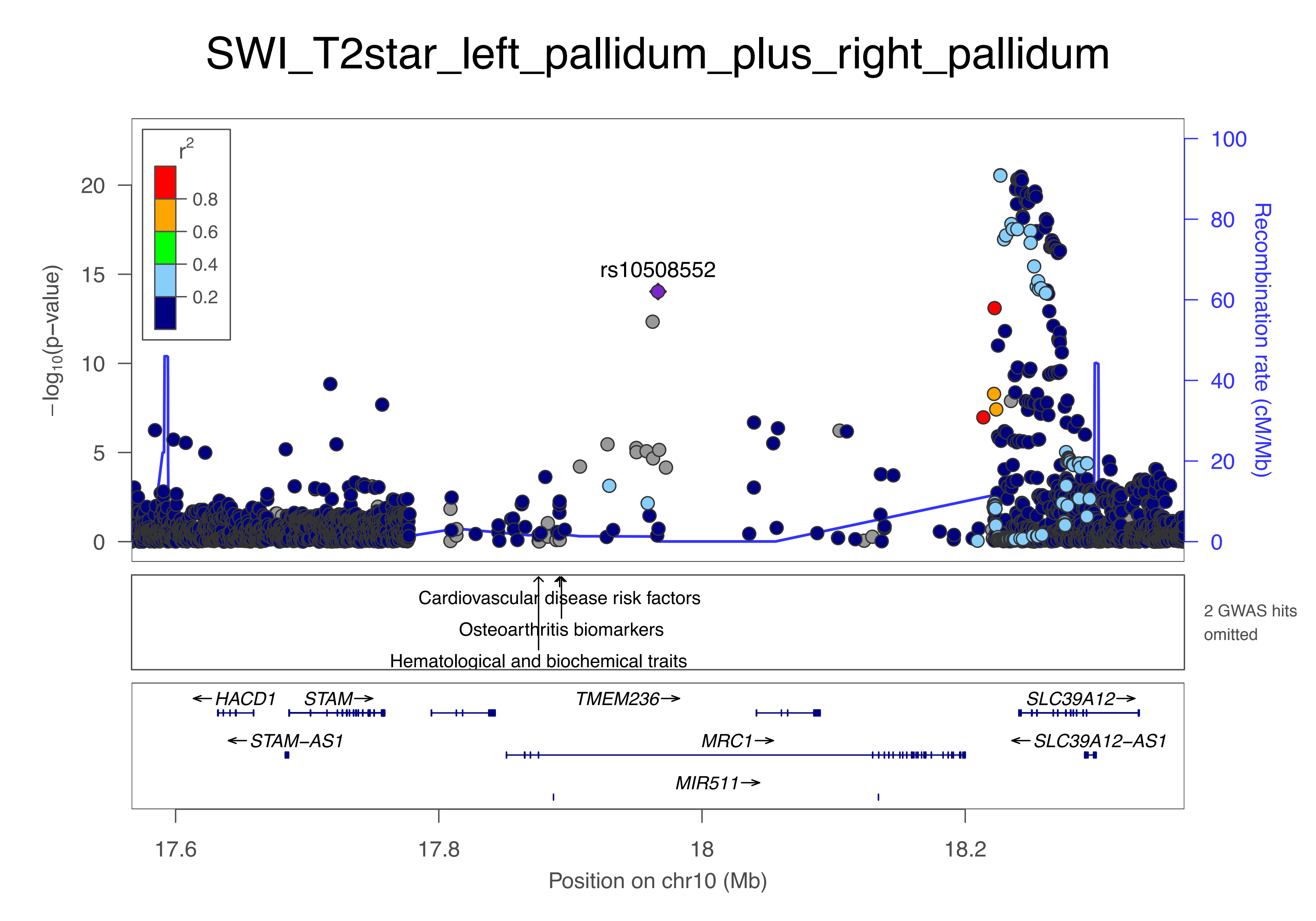
SWI_T2star_left_pallidum_plus_right_pallidum
r2
0.8
0.6
0.4
0.2
rs10508552
−log10(p−value)
Recombination rate (cM/Mb)
Cardiovascular disease risk factors
Osteoarthritis biomarkers
Hematological and biochemical traits
2 GWAS hits omitted
HACD1
STAM
TMEM236
SLC39A12
STAM−AS1
MRC1
SLC39A12−AS1
MIR511
17.6
17.8
18
18.2
Position on chr10 (Mb)

| | |
|---|---|
| date: | Thu Aug 17 18:00:17 2017 |
| build: | hg19 |
| display range: | chr10:17566561−18366561 [17566561−18366561] |
| hilite range: | 0 – 0 [ 0 – 0 ] |
| reference SNP: | chr10:17966561 |
| number of SNPs plotted: | 2390 |
| min P.value: | 2.78E−21 [chr10:18226707] |
| max P.value: | 10E−1 [chr10:17754182] |
| omitted GWAS Hits: | NA, NA |

Make more plots at http://csg.sph.umich.edu/locuszoom/

GWAS Catalog SNPs in Region

| chr | pos (Mb) | trait | snp |
|---|---|---|---|
| 10 | 17.59612 | Glucose homeostasis traits | rs6602203 |
| 10 | 17.87582 | Hematological and biochemical traits | rs2477664 |
| 10 | 17.89182 | Cardiovascular disease risk factors | rs2437258 |
| 10 | 17.89321 | Osteoarthritis biomarkers | rs691461 |
| 10 | 17.96656 | Childhood and early adolescence aggressive behavior | rs10508552 |

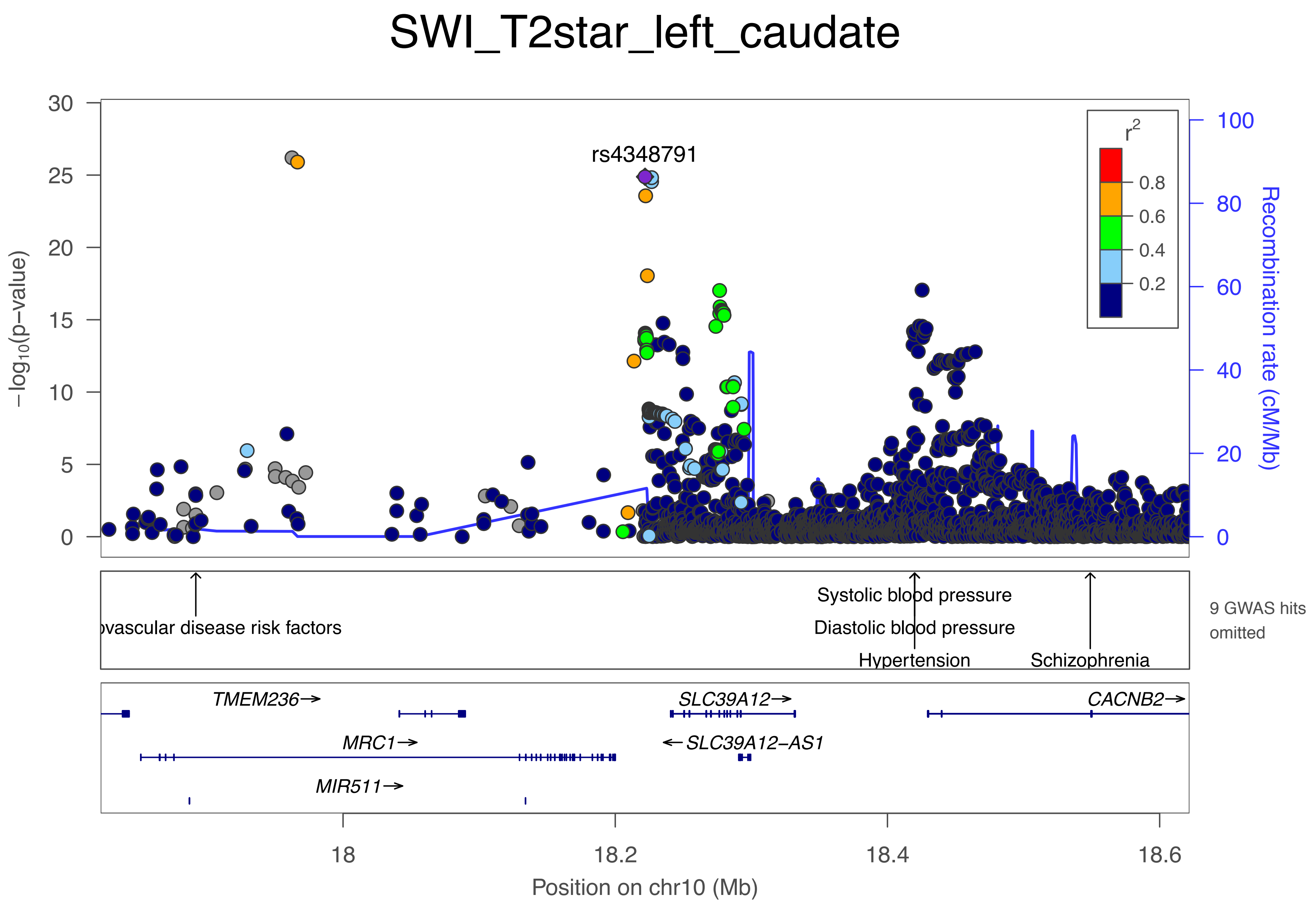
SWI_T2star_left_caudate
rs4348791
r2
0.8
0.6
0.4
0.2
−log10(p−value)
Recombination rate (cM/Mb)
0
5
10
15
20
25
30
0
20
40
60
80
100
ovascular disease risk factors
Systolic blood pressure
Diastolic blood pressure
Hypertension
Schizophrenia
9 GWAS hits omitted
TMEM236→
MRC1→
MIR511→
SLC39A12→
←SLC39A12−AS1
CACNB2→
18
18.2
18.4
18.6
Position on chr10 (Mb)

| | |
|---|---|
| date: | Thu Aug 17 18:16:04 2017 |
| build: | hg19 |
| display range: | chr10:17821908−18621908 [17821908−18621908] |
| hilite range: | 0 – 0 [ 0 – 0 ] |
| reference SNP: | chr10:18221908 |
| number of SNPs plotted: | 2678 |
| min P.value: | 6.37E−27 [chr10:17962506] |
| max P.value: | 9.99E−1 [chr10:18348597] |
| omitted GWAS Hits: | chr10:18.549016−Schizophrenia, NA |
| omitted GWAS Hits: | NA, NA |
| omitted GWAS Hits: | NA, NA |

Make more plots at http://csg.sph.umich.edu/locuszoom/

GWAS Catalog SNPs in Region

| trait |
| --- |
| Hematological and biochemical traits |
| Cardiovascular disease risk factors |
| Osteoarthritis biomarkers |
| Childhood and early adolescence aggressive behavior |
| Bipolar disorder and schizophrenia |
| Response to antipsychotic treatment |
| Systolic blood pressure |
| Diastolic blood pressure |
| Hypertension |
| Primary tooth development (time to first tooth eruption) |
| Primary tooth development (number of teeth) |
| Metabolite levels (HVA/MHPG ratio) |
| Schizophrenia |
| Autism spectrum disorder, attention deficit–hyperactivity disorder, bipolar disorder, major depressive disorder, and schizophrenia (combin |

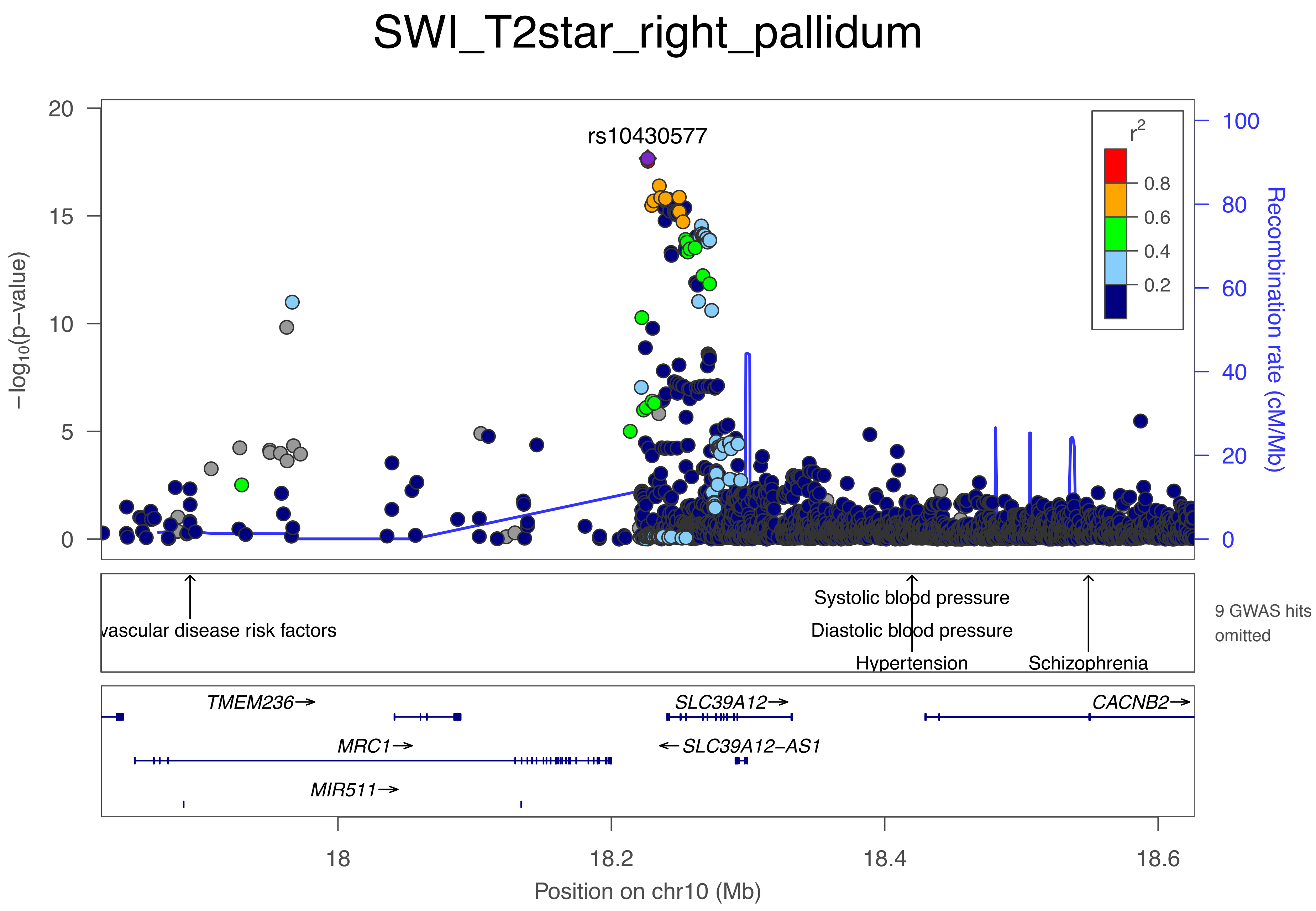

SWI_T2star_right_pallidum
rs10430577
−log10(p−value)
Recombination rate (cM/Mb)
r2
0.8
0.6
0.4
0.2
20
15
10
5
0
100
80
60
40
20
0
vascular disease risk factors
Systolic blood pressure
Diastolic blood pressure
Hypertension
Schizophrenia
9 GWAS hits omitted
TMEM236→
MRC1→
MIR511→
SLC39A12→
←SLC39A12−AS1
CACNB2→
18
18.2
18.4
18.6
Position on chr10 (Mb)

| | |
|---|---|
| date: | Thu Aug 17 18:00:22 2017 |
| build: | hg19 |
| display range: | chr10:17826707−18626707 [17826707−18626707] |
| hilite range: | 0 – 0 [ 0 – 0 ] |
| reference SNP: | chr10:18226707 |
| number of SNPs plotted: | 2697 |
| min P.value: | 2.17E−18 [chr10:18226707] |
| max P.value: | 10E−1 [chr10:18456156] |
| omitted GWAS Hits: | chr10:18.549016−Schizophrenia, NA |
| omitted GWAS Hits: | NA, NA |
| omitted GWAS Hits: | NA, NA |

Make more plots at http://csg.sph.umich.edu/locuszoom/

GWAS Catalog SNPs in Region

| trait |
|---|
| Hematological and biochemical traits |
| Cardiovascular disease risk factors |
| Osteoarthritis biomarkers |
| Childhood and early adolescence aggressive behavior |
| Bipolar disorder and schizophrenia |
| Response to antipsychotic treatment |
| Systolic blood pressure |
| Diastolic blood pressure |
| Hypertension |
| Primary tooth development (time to first tooth eruption) |
| Primary tooth development (number of teeth) |
| Metabolite levels (HVA/MHPG ratio) |
| Schizophrenia |
| Autism spectrum disorder, attention deficit–hyperactivity disorder, bipolar disorder, major depressive disorder, and schizophrenia (combin |

# SWI_T2star_left_pallidum_plus_right_pallidum

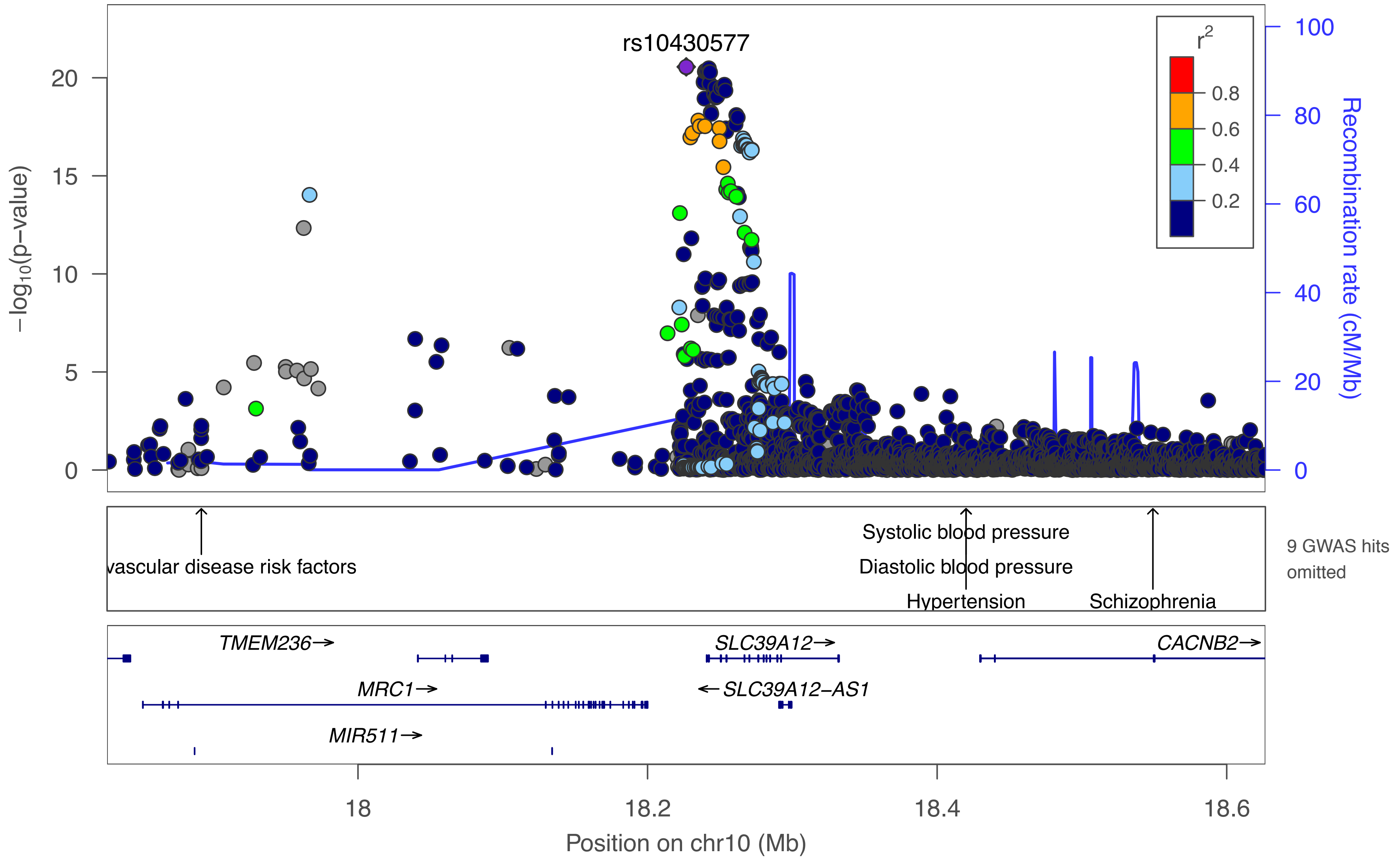

| | |
|---|---|
| date: | Thu Aug 17 18:00:22 2017 |
| build: | hg19 |
| display range: | chr10:17826707−18626707 [17826707−18626707] |
| hilite range: | 0 – 0 [ 0 – 0 ] |
| reference SNP: | chr10:18226707 |
| number of SNPs plotted: | 2697 |
| min P.value: | 2.78E−21 [chr10:18226707] |
| max P.value: | 9.99E−1 [chr10:18613497] |
| omitted GWAS Hits: | chr10:18.549016−Schizophrenia, NA |
| omitted GWAS Hits: | NA, NA |
| omitted GWAS Hits: | NA, NA |

Make more plots at http://csg.sph.umich.edu/locuszoom/

GWAS Catalog SNPs in Region

| trait |
|---|
| Hematological and biochemical traits |
| Cardiovascular disease risk factors |
| Osteoarthritis biomarkers |
| Childhood and early adolescence aggressive behavior |
| Bipolar disorder and schizophrenia |
| Response to antipsychotic treatment |
| Systolic blood pressure |
| Diastolic blood pressure |
| Hypertension |
| Primary tooth development (time to first tooth eruption) |
| Primary tooth development (number of teeth) |
| Metabolite levels (HVA/MHPG ratio) |
| Schizophrenia |
| Autism spectrum disorder, attention deficit–hyperactivity disorder, bipolar disorder, major depressive disorder, and schizophrenia (combin |

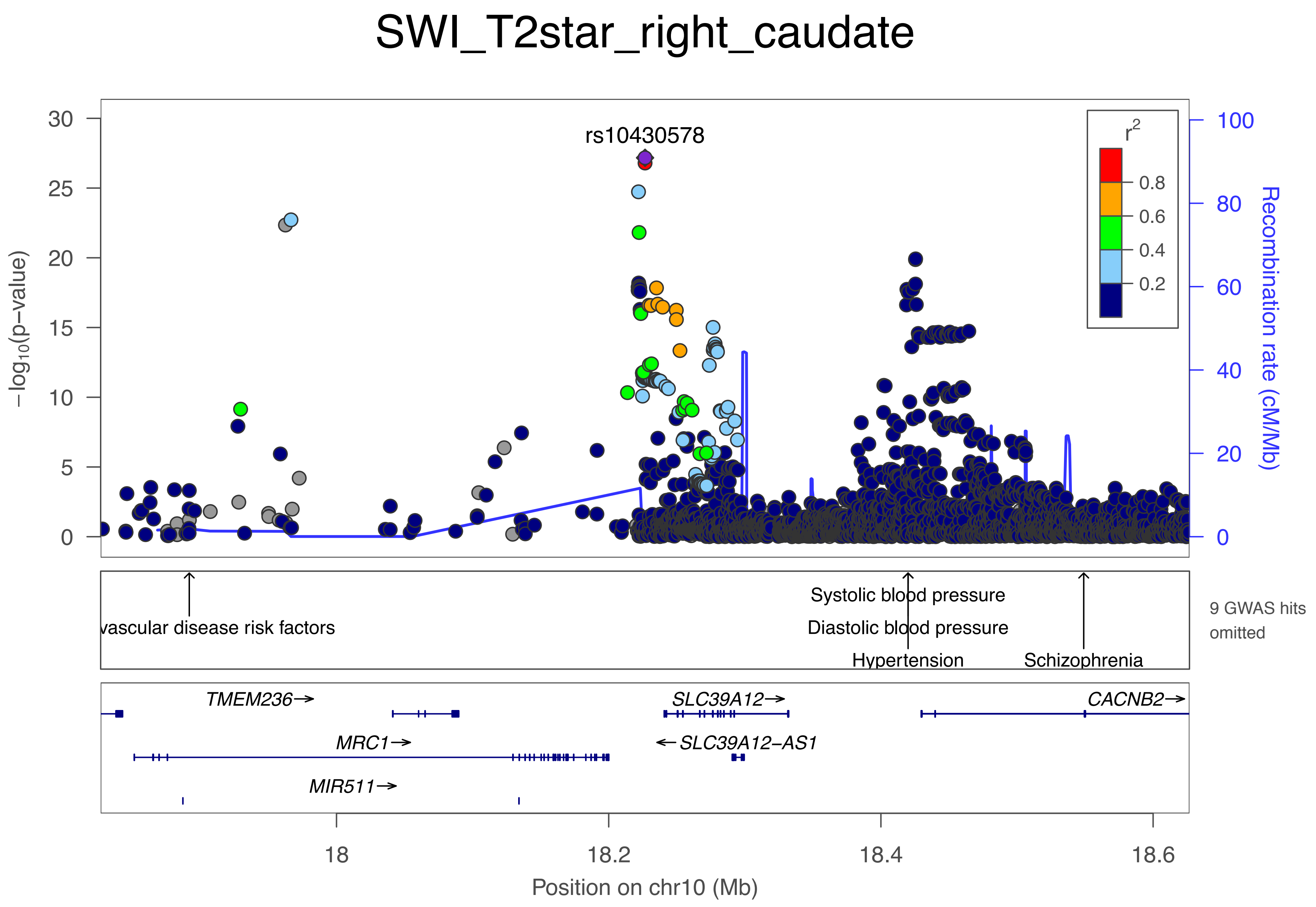
SWI_T2star_right_caudate
rs10430578
−log10(p−value)
Recombination rate (cM/Mb)
r2
0.8
0.6
0.4
0.2
vascular disease risk factors
Systolic blood pressure
Diastolic blood pressure
Hypertension
Schizophrenia
9 GWAS hits omitted
TMEM236→
SLC39A12→
CACNB2→
MRC1→
←SLC39A12−AS1
MIR511→
18
18.2
18.4
18.6
Position on chr10 (Mb)

| | |
|---|---|
| date: | Thu Aug 17 18:26:51 2017 |
| build: | hg19 |
| display range: | chr10:17826714−18626714 [17826714−18626714] |
| hilite range: | 0 – 0 [ 0 – 0 ] |
| reference SNP: | chr10:18226714 |
| number of SNPs plotted: | 2697 |
| min P.value: | 6.76E−28 [chr10:18226714] |
| max P.value: | 10E−1 [chr10:18624476] |
| omitted GWAS Hits: | chr10:18.549016−Schizophrenia, NA |
| omitted GWAS Hits: | NA, NA |
| omitted GWAS Hits: | NA, NA |

Make more plots at http://csg.sph.umich.edu/locuszoom/

GWAS Catalog SNPs in Region

| trait |
|---|
| Hematological and biochemical traits |
| Cardiovascular disease risk factors |
| Osteoarthritis biomarkers |
| Childhood and early adolescence aggressive behavior |
| Bipolar disorder and schizophrenia |
| Response to antipsychotic treatment |
| Systolic blood pressure |
| Diastolic blood pressure |
| Hypertension |
| Primary tooth development (time to first tooth eruption) |
| Primary tooth development (number of teeth) |
| Metabolite levels (HVA/MHPG ratio) |
| Schizophrenia |
| Autism spectrum disorder, attention deficit–hyperactivity disorder, bipolar disorder, major depressive disorder, and schizophrenia (combin |

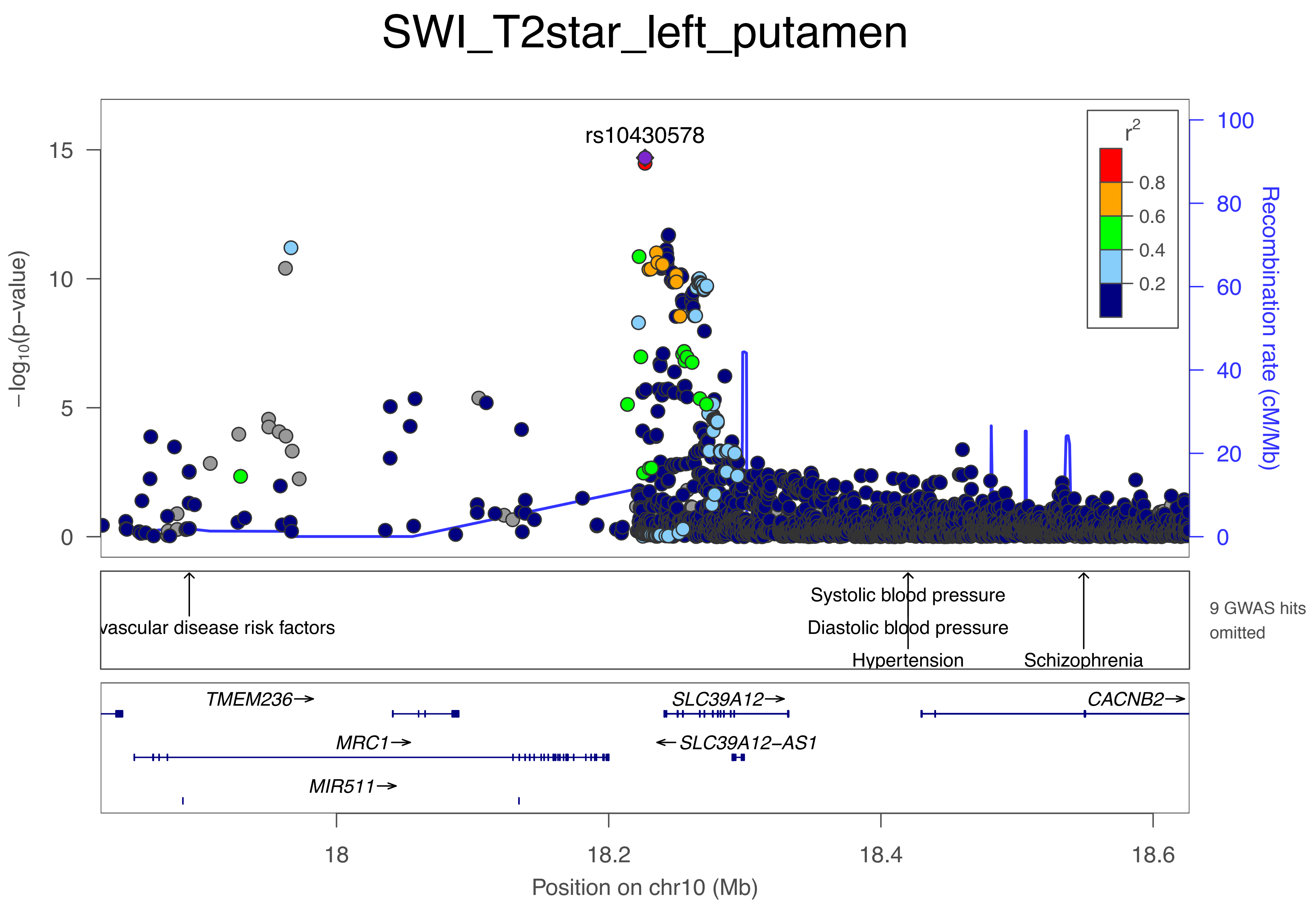

SWI_T2star_left_putamen
rs10430578
r2
0.8
0.6
0.4
0.2
15
10
5
0
−log10(p−value)
100
80
60
40
20
0
Recombination rate (cM/Mb)
vascular disease risk factors
Systolic blood pressure
Diastolic blood pressure
Hypertension
Schizophrenia
9 GWAS hits omitted
TMEM236→
MRC1→
MIR511→
SLC39A12→
←SLC39A12−AS1
CACNB2→
18
18.2
18.4
18.6
Position on chr10 (Mb)

| | |
|---|---|
| date: | Thu Aug 17 18:26:51 2017 |
| build: | hg19 |
| display range: | chr10:17826714−18626714 [17826714−18626714] |
| hilite range: | 0 – 0 [ 0 – 0 ] |
| reference SNP: | chr10:18226714 |
| number of SNPs plotted: | 2697 |
| min P.value: | 2.04E−15 [chr10:18226714] |
| max P.value: | 10E−1 [chr10:18571506] |
| omitted GWAS Hits: | chr10:18.549016−Schizophrenia, NA |
| omitted GWAS Hits: | NA, NA |
| omitted GWAS Hits: | NA, NA |

Make more plots at http://csg.sph.umich.edu/locuszoom/

GWAS Catalog SNPs in Region

| trait |
| --- |
| Hematological and biochemical traits |
| Cardiovascular disease risk factors |
| Osteoarthritis biomarkers |
| Childhood and early adolescence aggressive behavior |
| Bipolar disorder and schizophrenia |
| Response to antipsychotic treatment |
| Systolic blood pressure |
| Diastolic blood pressure |
| Hypertension |
| Primary tooth development (time to first tooth eruption) |
| Primary tooth development (number of teeth) |
| Metabolite levels (HVA/MHPG ratio) |
| Schizophrenia |
| Autism spectrum disorder, attention deficit–hyperactivity disorder, bipolar disorder, major depressive disorder, and schizophrenia (combin |

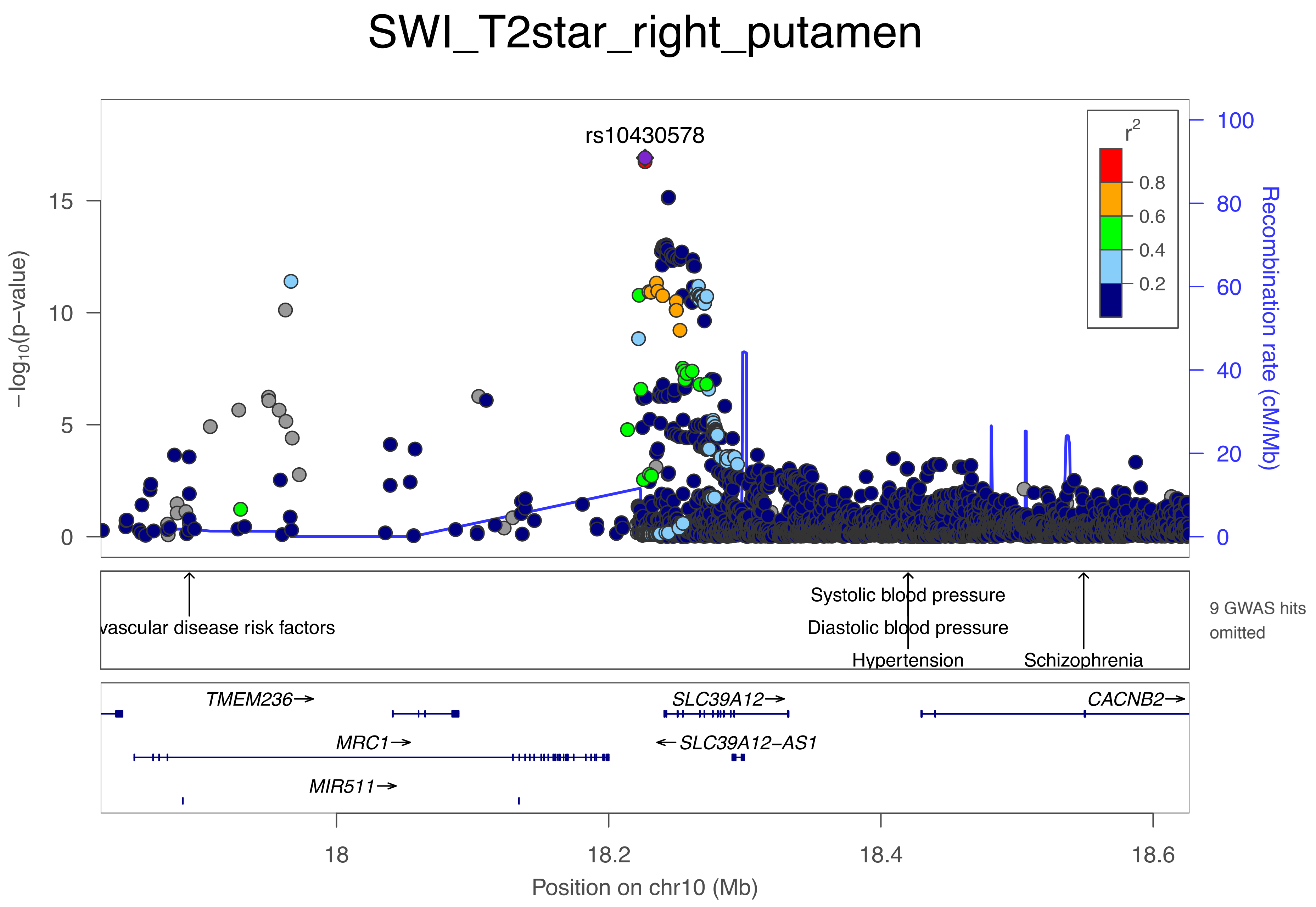
SWI_T2star_right_putamen
rs10430578
−log10(p−value)
Recombination rate (cM/Mb)
r2
0.8
0.6
0.4
0.2
0
5
10
15
0
20
40
60
80
100
vascular disease risk factors
Systolic blood pressure
Diastolic blood pressure
Hypertension
Schizophrenia
9 GWAS hits omitted
TMEM236→
MRC1→
MIR511→
SLC39A12→
←SLC39A12−AS1
CACNB2→
18
18.2
18.4
18.6
Position on chr10 (Mb)

| | |
|---|---|
| date: | Thu Aug 17 18:35:05 2017 |
| build: | hg19 |
| display range: | chr10:17826714−18626714 [17826714−18626714] |
| hilite range: | 0 − 0 [ 0 − 0 ] |
| reference SNP: | chr10:18226714 |
| number of SNPs plotted: | 2697 |
| min P.value: | 1.21E−17 [chr10:18226714] |
| max P.value: | 10E−1 [chr10:18577835] |
| omitted GWAS Hits: | chr10:18.549016−Schizophrenia, NA |
| omitted GWAS Hits: | NA, NA |
| omitted GWAS Hits: | NA, NA |

Make more plots at http://csg.sph.umich.edu/locuszoom/

GWAS Catalog SNPs in Region

| trait |
|---|
| Hematological and biochemical traits |
| Cardiovascular disease risk factors |
| Osteoarthritis biomarkers |
| Childhood and early adolescence aggressive behavior |
| Bipolar disorder and schizophrenia |
| Response to antipsychotic treatment |
| Systolic blood pressure |
| Diastolic blood pressure |
| Hypertension |
| Primary tooth development (time to first tooth eruption) |
| Primary tooth development (number of teeth) |
| Metabolite levels (HVA/MHPG ratio) |
| Schizophrenia |
| Autism spectrum disorder, attention deficit–hyperactivity disorder, bipolar disorder, major depressive disorder, and schizophrenia (combin |

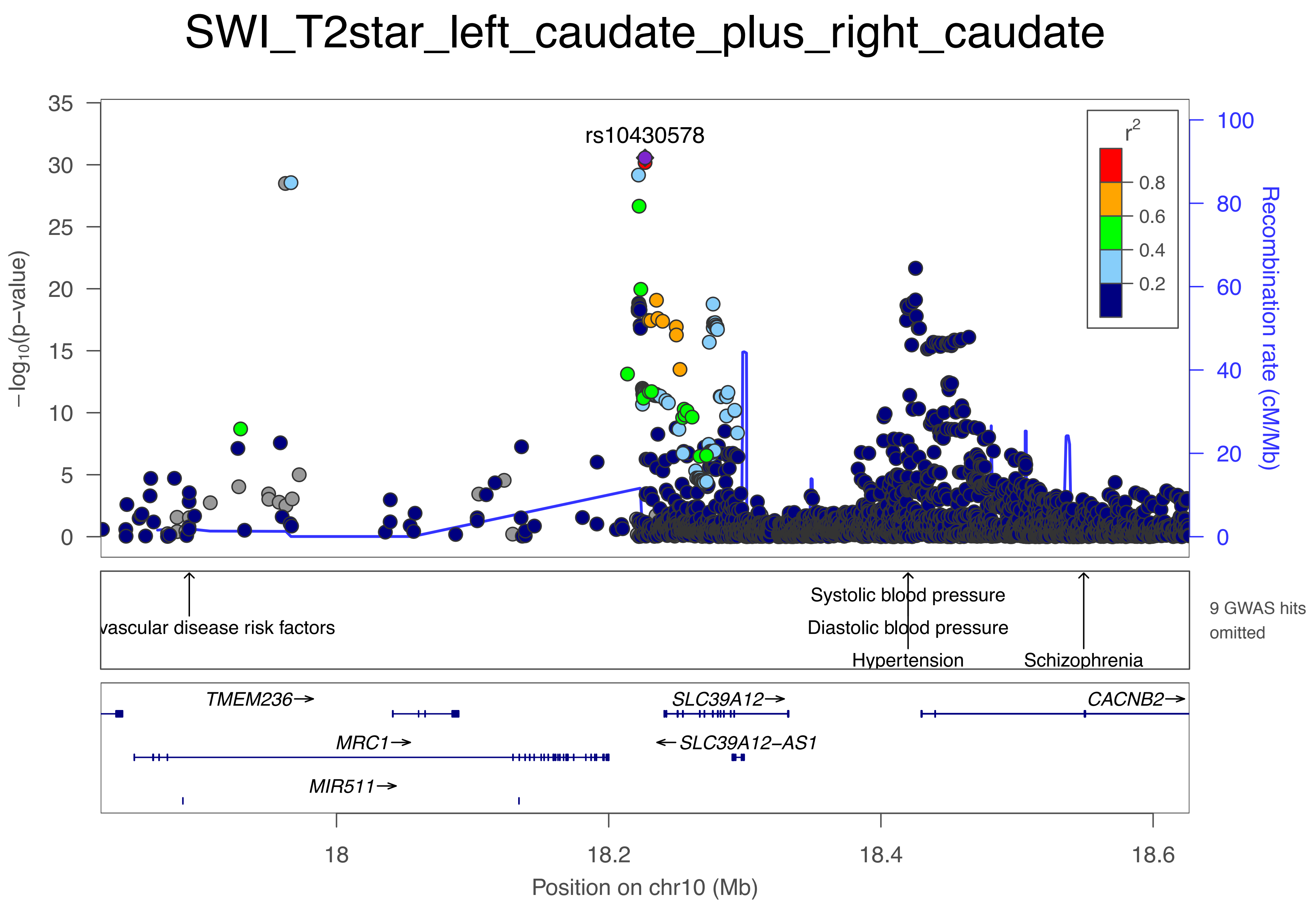
SWI_T2star_left_caudate_plus_right_caudate
rs10430578
−log10(p−value)
Recombination rate (cM/Mb)
r2
0.8
0.6
0.4
0.2
vascular disease risk factors
Systolic blood pressure
Diastolic blood pressure
Hypertension
Schizophrenia
9 GWAS hits omitted
TMEM236→
MRC1→
MIR511→
SLC39A12→
←SLC39A12−AS1
CACNB2→
18
18.2
18.4
18.6
Position on chr10 (Mb)

| | |
|---|---|
| date: | Thu Aug 17 18:35:05 2017 |
| build: | hg19 |
| display range: | chr10:17826714−18626714 [17826714−18626714] |
| hilite range: | 0 – 0 [ 0 – 0 ] |
| reference SNP: | chr10:18226714 |
| number of SNPs plotted: | 2697 |
| min P.value: | 2.73E−31 [chr10:18226714] |
| max P.value: | 9.98E−1 [chr10:18541427] |
| omitted GWAS Hits: | chr10:18.549016−Schizophrenia, NA |
| omitted GWAS Hits: | NA, NA |
| omitted GWAS Hits: | NA, NA |

Make more plots at http://csg.sph.umich.edu/locuszoom/

GWAS Catalog SNPs in Region

| trait |
| --- |
| Hematological and biochemical traits |
| Cardiovascular disease risk factors |
| Osteoarthritis biomarkers |
| Childhood and early adolescence aggressive behavior |
| Bipolar disorder and schizophrenia |
| Response to antipsychotic treatment |
| Systolic blood pressure |
| Diastolic blood pressure |
| Hypertension |
| Primary tooth development (time to first tooth eruption) |
| Primary tooth development (number of teeth) |
| Metabolite levels (HVA/MHPG ratio) |
| Schizophrenia |
| Autism spectrum disorder, attention deficit–hyperactivity disorder, bipolar disorder, major depressive disorder, and schizophrenia (combin |

# SWI_T2star_left_putamen_plus_right_putamen

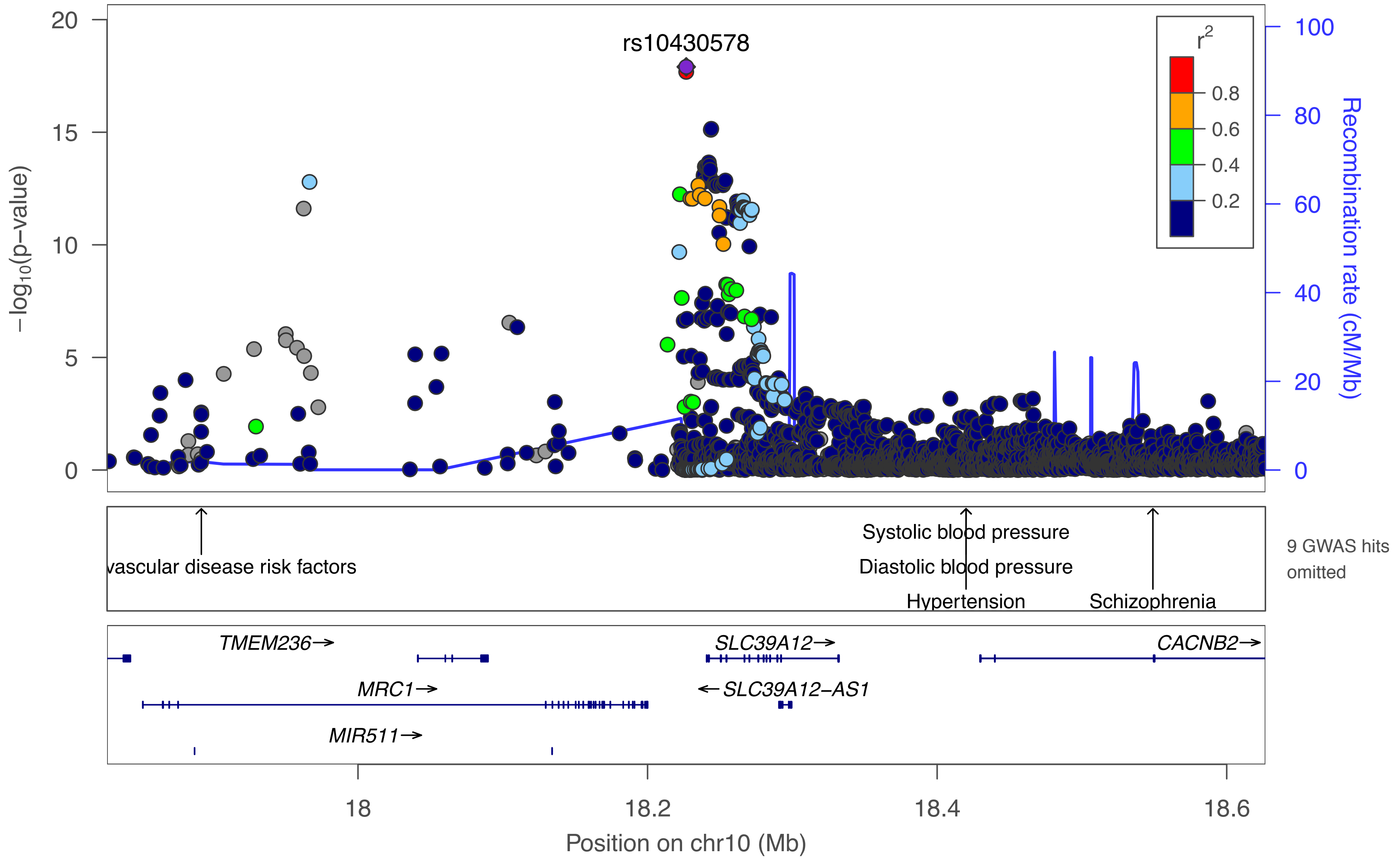

| | |
|---|---|
| date: | Thu Aug 17 18:35:05 2017 |
| build: | hg19 |
| display range: | chr10:17826714−18626714 [17826714−18626714] |
| hilite range: | 0 – 0 [ 0 – 0 ] |
| reference SNP: | chr10:18226714 |
| number of SNPs plotted: | 2697 |
| min P.value: | 1.25E−18 [chr10:18226714] |
| max P.value: | 9.99E−1 [chr10:18339376] |
| omitted GWAS Hits: | chr10:18.549016−Schizophrenia, NA |
| omitted GWAS Hits: | NA, NA |
| omitted GWAS Hits: | NA, NA |

Make more plots at http://csg.sph.umich.edu/locuszoom/

GWAS Catalog SNPs in Region

| trait |
|---|
| Hematological and biochemical traits |
| Cardiovascular disease risk factors |
| Osteoarthritis biomarkers |
| Childhood and early adolescence aggressive behavior |
| Bipolar disorder and schizophrenia |
| Response to antipsychotic treatment |
| Systolic blood pressure |
| Diastolic blood pressure |
| Hypertension |
| Primary tooth development (time to first tooth eruption) |
| Primary tooth development (number of teeth) |
| Metabolite levels (HVA/MHPG ratio) |
| Schizophrenia |
| Autism spectrum disorder, attention deficit–hyperactivity disorder, bipolar disorder, major depressive disorder, and schizophrenia (combin |

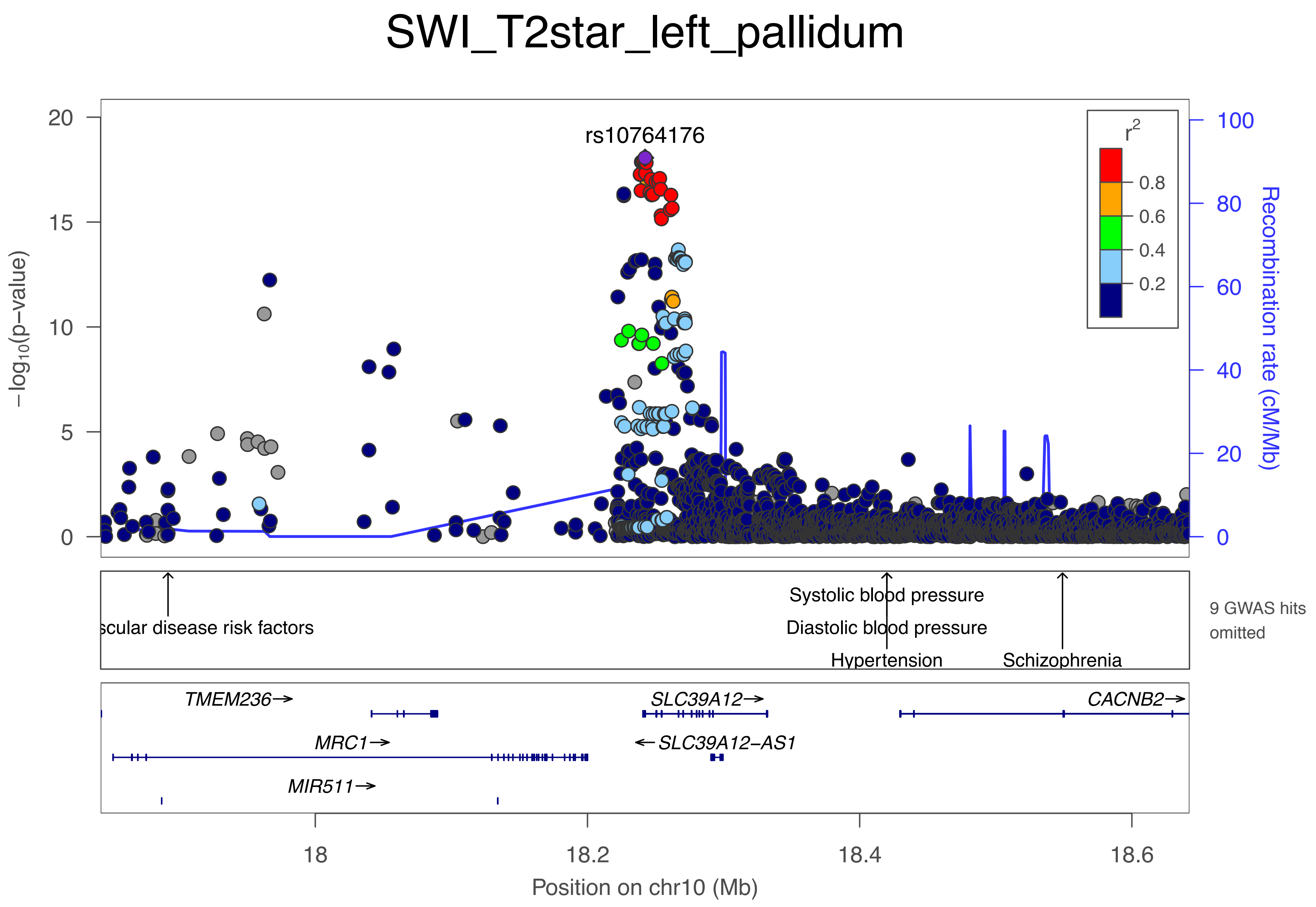
SWI_T2star_left_pallidum
rs10764176
r²
0.8
0.6
0.4
0.2
−log10(p−value)
Recombination rate (cM/Mb)
scular disease risk factors
Systolic blood pressure
Diastolic blood pressure
Hypertension
Schizophrenia
9 GWAS hits omitted
TMEM236→
MRC1→
MIR511→
SLC39A12→
←SLC39A12−AS1
CACNB2→
18
18.2
18.4
18.6
Position on chr10 (Mb)

| | |
|---|---|
| date: | Thu Aug 17 18:32:35 2017 |
| build: | hg19 |
| display range: | chr10:17842311−18642311 [17842311−18642311] |
| hilite range: | 0 – 0 [ 0 – 0 ] |
| reference SNP: | chr10:18242311 |
| number of SNPs plotted: | 2775 |
| min P.value: | 8.63E−19 [chr10:18242311] |
| max P.value: | 9.99E−1 [chr10:18559835] |
| omitted GWAS Hits: | chr10:18.549016−Schizophrenia, NA |
| omitted GWAS Hits: | NA, NA |
| omitted GWAS Hits: | NA, NA |

Make more plots at http://csg.sph.umich.edu/locuszoom/

GWAS Catalog SNPs in Region

| trait |
| --- |
| Hematological and biochemical traits |
| Cardiovascular disease risk factors |
| Osteoarthritis biomarkers |
| Childhood and early adolescence aggressive behavior |
| Bipolar disorder and schizophrenia |
| Response to antipsychotic treatment |
| Systolic blood pressure |
| Diastolic blood pressure |
| Hypertension |
| Primary tooth development (time to first tooth eruption) |
| Primary tooth development (number of teeth) |
| Metabolite levels (HVA/MHPG ratio) |
| Schizophrenia |
| Autism spectrum disorder, attention deficit–hyperactivity disorder, bipolar disorder, major depressive disorder, and schizophrenia (combin |

# SWI_T2star_left_caudate

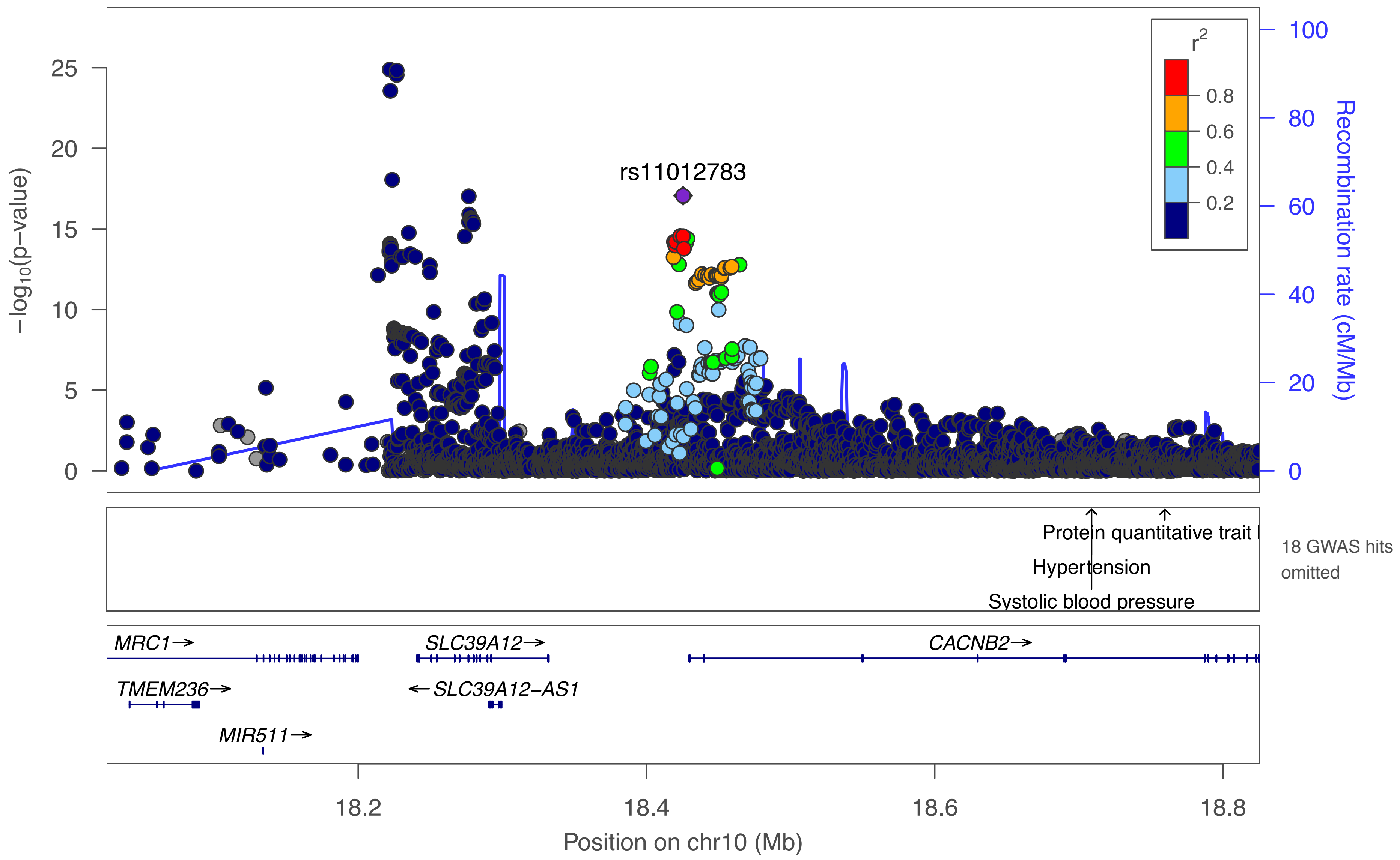

| | |
|---|---|
| date: | Thu Aug 17 18:46:01 2017 |
| build: | hg19 |
| display range: | chr10:18025418−18825418 [18025418−18825418] |
| hilite range: | 0 – 0 [ 0 – 0 ] |
| reference SNP: | chr10:18425418 |
| number of SNPs plotted: | 3878 |
| min P.value: | 1.31E−25 [chr10:18221908] |
| max P.value: | 10E−1 [chr10:18761474] |
| omitted GWAS Hits: | NA, NA |
| omitted GWAS Hits: | NA, NA |
| omitted GWAS Hits: | NA, NA |
| omitted GWAS Hits: | NA, NA |
| omitted GWAS Hits: | NA, NA |
| omitted GWAS Hits: | NA, NA |

Make more plots at http://csg.sph.umich.edu/locuszoom/

GWAS Catalog SNPs in Region

| trait |
| --- |
| Bipolar disorder and schizophrenia |
| Response to antipsychotic treatment |
| Systolic blood pressure |
| Diastolic blood pressure |
| Hypertension |
| Primary tooth development (time to first tooth eruption) |
| Primary tooth development (number of teeth) |
| Metabolite levels (HVA/MHPG ratio) |
| Schizophrenia |
| Autism spectrum disorder, attention deficit−hyperactivity disorder, bipolar disorder, major depressive disorder, and schizophrenia (combin |
| QRS complex (Sokolow−Lyon) |
| Hypertension |
| Systolic blood pressure |
| Diastolic blood pressure |
| Blood pressure |
| Small vessel stroke |
| Quantitative traits |
| Schizophrenia |
| Schizophrenia |
| Protein quantitative trait loci |
| Obesity−related traits |

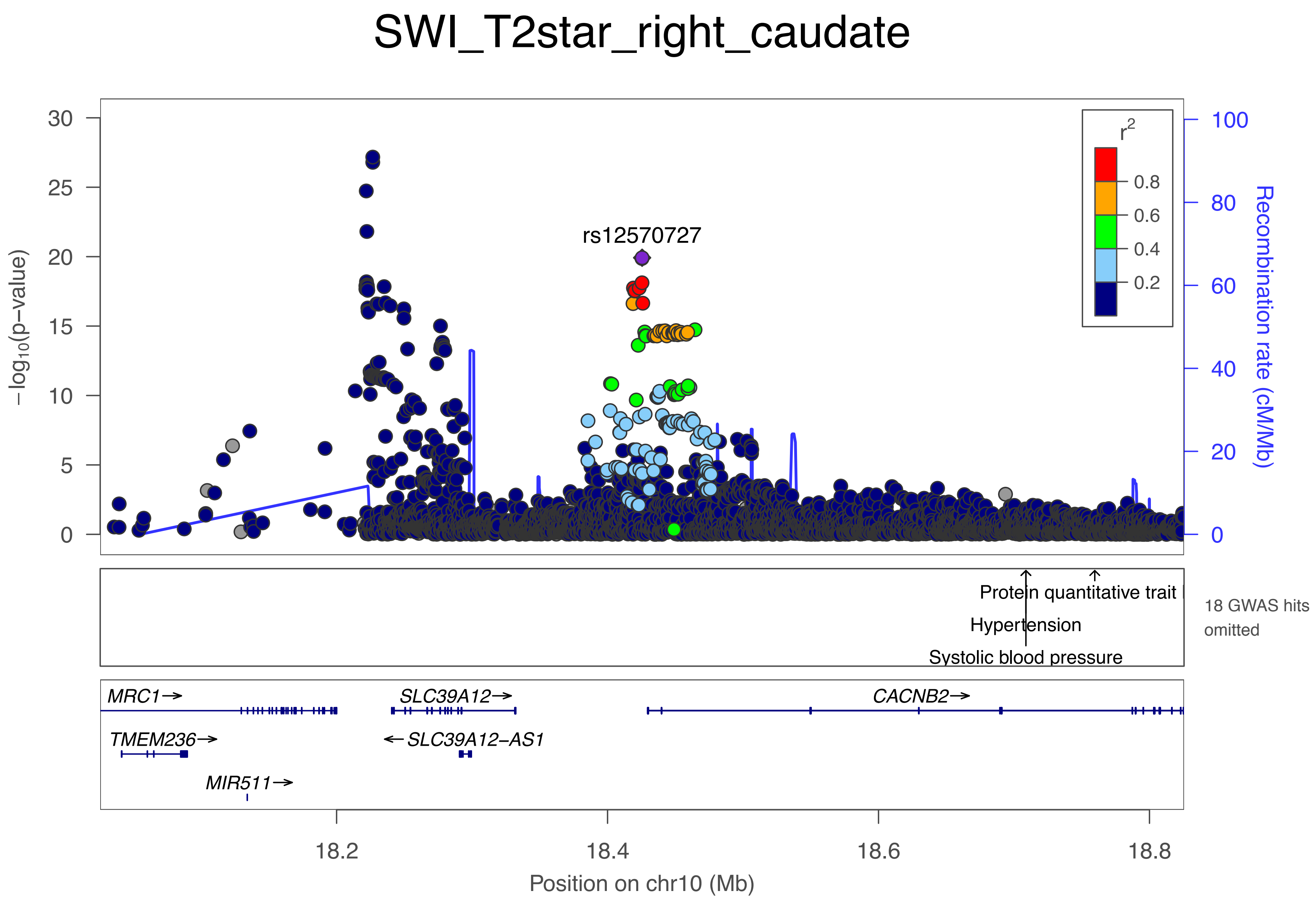
SWI_T2star_right_caudate
rs12570727
r2
0.8
0.6
0.4
0.2
−log10(p−value)
Recombination rate (cM/Mb)
Protein quantitative trait
Hypertension
Systolic blood pressure
18 GWAS hits omitted
MRC1→
SLC39A12→
CACNB2→
TMEM236→
←SLC39A12−AS1
MIR511→
18.2
18.4
18.6
18.8
Position on chr10 (Mb)

| | |
|---|---|
| date: | Thu Aug 17 18:46:01 2017 |
| build: | hg19 |
| display range: | chr10:18025519−18825519 [18025519−18825519] |
| hilite range: | 0 – 0 [ 0 – 0 ] |
| reference SNP: | chr10:18425519 |
| number of SNPs plotted: | 3878 |
| min P.value: | 6.76E−28 [chr10:18226714] |
| max P.value: | 10E−1 [chr10:18624476] |
| omitted GWAS Hits: | NA, NA |
| omitted GWAS Hits: | NA, NA |
| omitted GWAS Hits: | NA, NA |
| omitted GWAS Hits: | NA, NA |
| omitted GWAS Hits: | NA, NA |
| omitted GWAS Hits: | NA, NA |

Make more plots at http://csg.sph.umich.edu/locuszoom/

GWAS Catalog SNPs in Region

| trait |
| --- |
| Bipolar disorder and schizophrenia |
| Response to antipsychotic treatment |
| Systolic blood pressure |
| Diastolic blood pressure |
| Hypertension |
| Primary tooth development (time to first tooth eruption) |
| Primary tooth development (number of teeth) |
| Metabolite levels (HVA/MHPG ratio) |
| Schizophrenia |
| Autism spectrum disorder, attention deficit–hyperactivity disorder, bipolar disorder, major depressive disorder, and schizophrenia (combin |
| QRS complex (Sokolow–Lyon) |
| Hypertension |
| Systolic blood pressure |
| Diastolic blood pressure |
| Blood pressure |
| Small vessel stroke |
| Quantitative traits |
| Schizophrenia |
| Schizophrenia |
| Protein quantitative trait loci |
| Obesity–related traits |

# SWI_T2star_left_caudate_plus_right_caudate

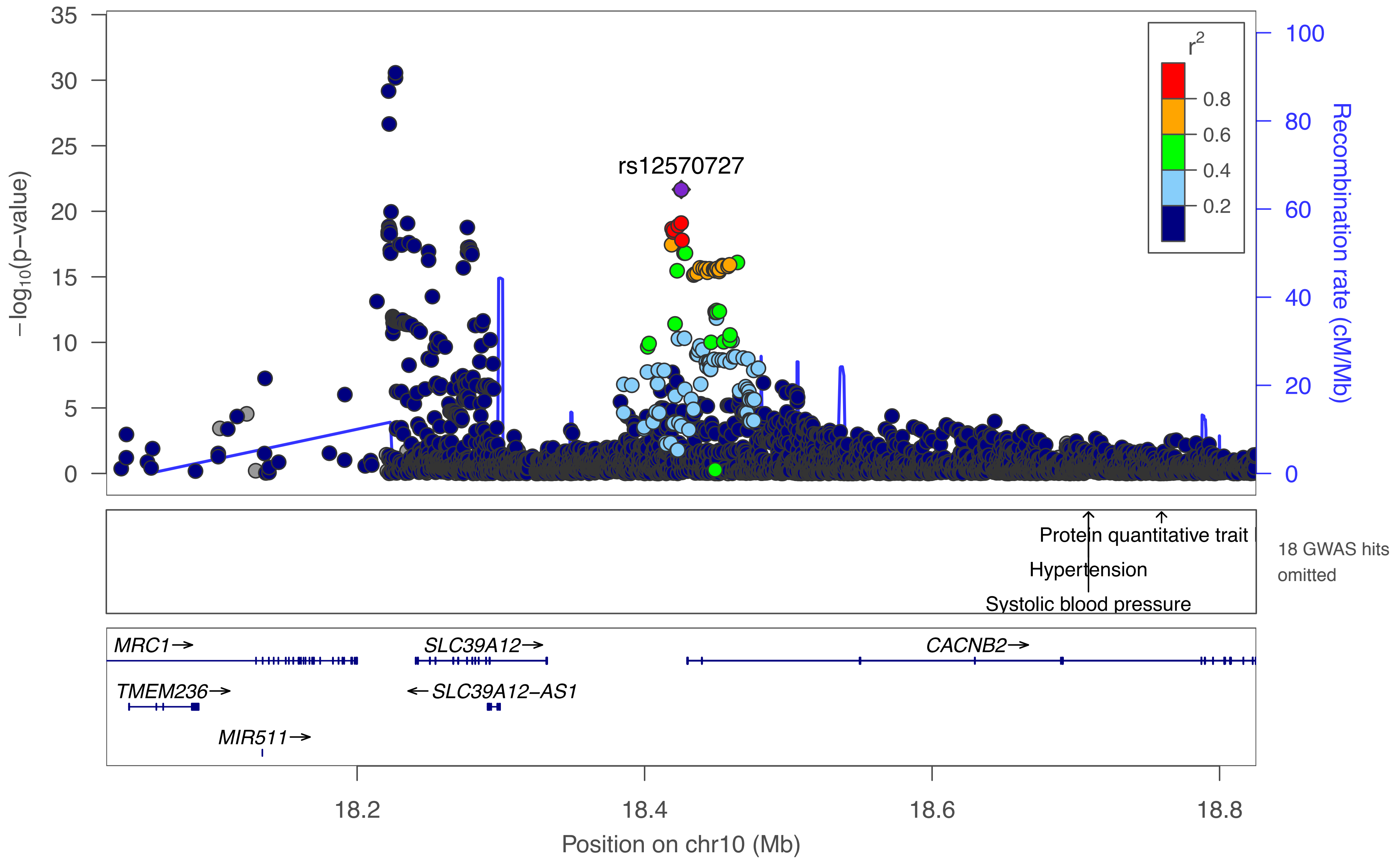

| | |
|---|---|
| date: | Thu Aug 17 18:44:47 2017 |
| build: | hg19 |
| display range: | chr10:18025519−18825519 [18025519−18825519] |
| hilite range: | 0 – 0 [ 0 – 0 ] |
| reference SNP: | chr10:18425519 |
| number of SNPs plotted: | 3878 |
| min P.value: | 2.73E−31 [chr10:18226714] |
| max P.value: | 10E−1 [chr10:18781803] |
| omitted GWAS Hits: | NA, NA |
| omitted GWAS Hits: | NA, NA |
| omitted GWAS Hits: | NA, NA |
| omitted GWAS Hits: | NA, NA |
| omitted GWAS Hits: | NA, NA |
| omitted GWAS Hits: | NA, NA |

Make more plots at http://csg.sph.umich.edu/locuszoom/

GWAS Catalog SNPs in Region

| trait |
|---|
| Bipolar disorder and schizophrenia |
| Response to antipsychotic treatment |
| Systolic blood pressure |
| Diastolic blood pressure |
| Hypertension |
| Primary tooth development (time to first tooth eruption) |
| Primary tooth development (number of teeth) |
| Metabolite levels (HVA/MHPG ratio) |
| Schizophrenia |
| Autism spectrum disorder, attention deficit−hyperactivity disorder, bipolar disorder, major depressive disorder, and schizophrenia (combin |
| QRS complex (Sokolow−Lyon) |
| Hypertension |
| Systolic blood pressure |
| Diastolic blood pressure |
| Blood pressure |
| Small vessel stroke |
| Quantitative traits |
| Schizophrenia |
| Schizophrenia |
| Protein quantitative trait loci |
| Obesity−related traits |

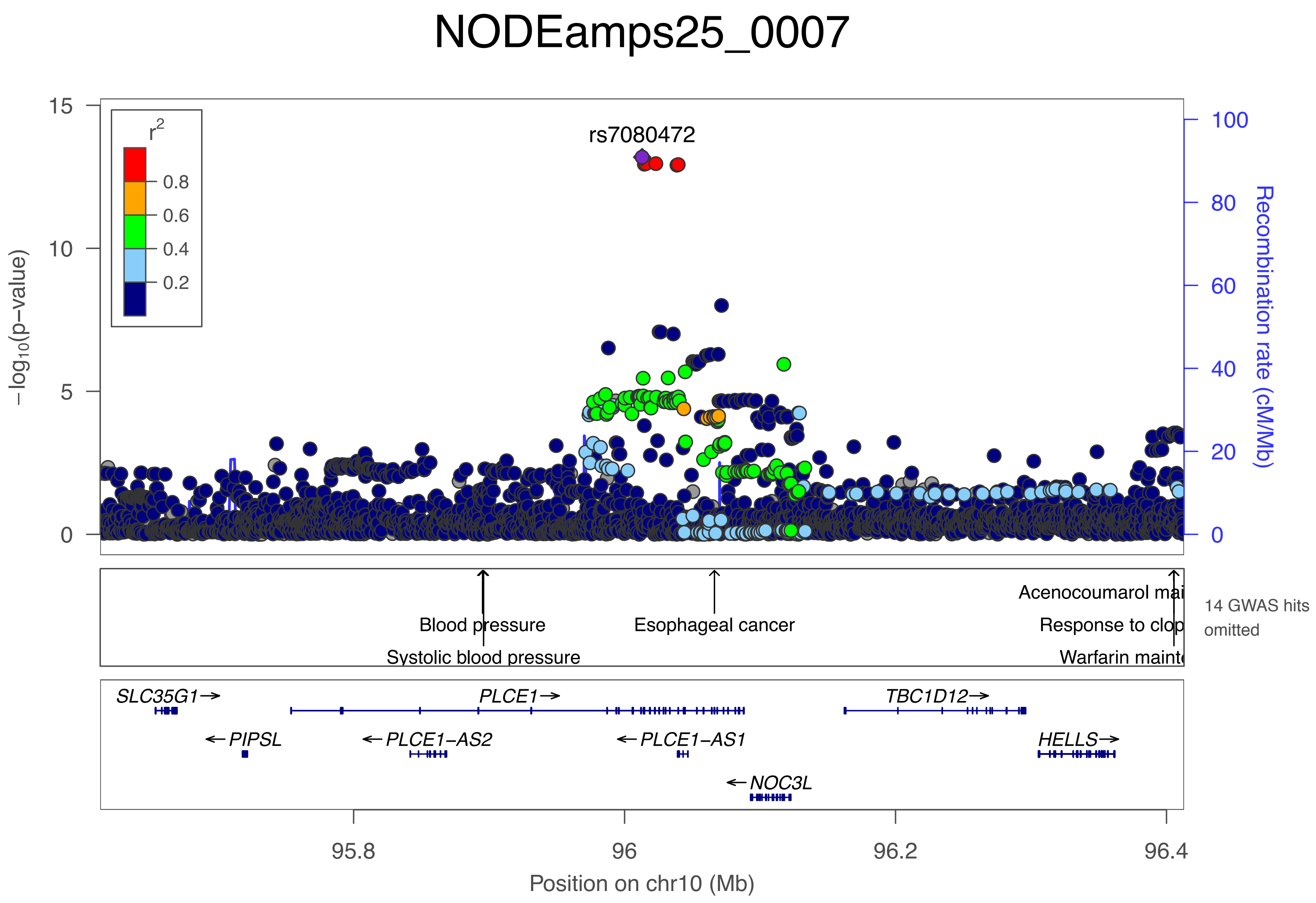
NODEamps25_0007
rs7080472
r2
0.8
0.6
0.4
0.2
15
10
5
0
−log10(p−value)
100
80
60
40
20
0
Recombination rate (cM/Mb)
Blood pressure
Systolic blood pressure
Esophageal cancer
Acenocoumarol mai
Response to clop
Warfarin maint
14 GWAS hits omitted
SLC35G1→
PLCE1→
TBC1D12→
←PIPSL
←PLCE1−AS2
←PLCE1−AS1
HELLS→
←NOC3L
95.8
96
96.2
96.4
Position on chr10 (Mb)

| | |
|---|---|
| date: | Thu Aug 17 18:48:50 2017 |
| build: | hg19 |
| display range: | chr10:95612950−96412950 [95612950−96412950] |
| hilite range: | 0 – 0 [ 0 – 0 ] |
| reference SNP: | chr10:96012950 |
| number of SNPs plotted: | 3177 |
| min P.value: | 6.47E−14 [chr10:96012950] |
| max P.value: | 10E−1 [chr10:95669893] |
| omitted GWAS Hits: | chr10:96.066341−Esophageal cancer, chr10:96.405502−Response to clopidogrel therapy |
| omitted GWAS Hits: | NA, NA |
| omitted GWAS Hits: | NA, NA |
| omitted GWAS Hits: | NA, NA |
| omitted GWAS Hits: | NA, NA |

Make more plots at http://csg.sph.umich.edu/locuszoom/

GWAS Catalog SNPs in Region

| chr | pos (Mb) | trait | snp |
|---|---|---|---|
| 10 | 95.65996 | Cannabis dependence | rs146091982 |
| 10 | 95.89518 | Blood pressure | rs9663362 |
| 10 | 95.89594 | Systolic blood pressure | rs932764 |
| 10 | 95.89594 | Diastolic blood pressure | rs932764 |
| 10 | 95.89594 | Hypertension | rs932764 |
| 10 | 95.90632 | Glucose homeostasis traits | rs1223629 |
| 10 | 96.01371 | Personality traits in bipolar disorder | rs9419788 |
| 10 | 96.01462 | Migraine | rs10786156 |
| 10 | 96.01903 | Migraine | rs75473620 |
| 10 | 96.03598 | Sudden cardiac arrest | rs11187837 |
| 10 | 96.03631 | Vertical cup–disc ratio | rs7072574 |
| 10 | 96.05830 | Dengue shock syndrome | rs3765524 |
| 10 | 96.05830 | Esophageal cancer and gastric cancer | rs3765524 |
| 10 | 96.05864 | Lifetime average cigarettes per day in chronic obstructive pulmonary disease | rs117607728 |
| 10 | 96.06634 | Esophageal cancer | rs2274223 |
| 10 | 96.06634 | Esophageal squamous cell carcinoma | rs2274223 |
| 10 | 96.07037 | Esophageal cancer and gastric cancer | rs3781264 |
| 10 | 96.40533 | Acenocoumarol maintenance dosage | rs12772169 |
| 10 | 96.40550 | Response to clopidogrel therapy | rs12777823 |
| 10 | 96.40550 | Warfarin maintenance dose | rs12777823 |

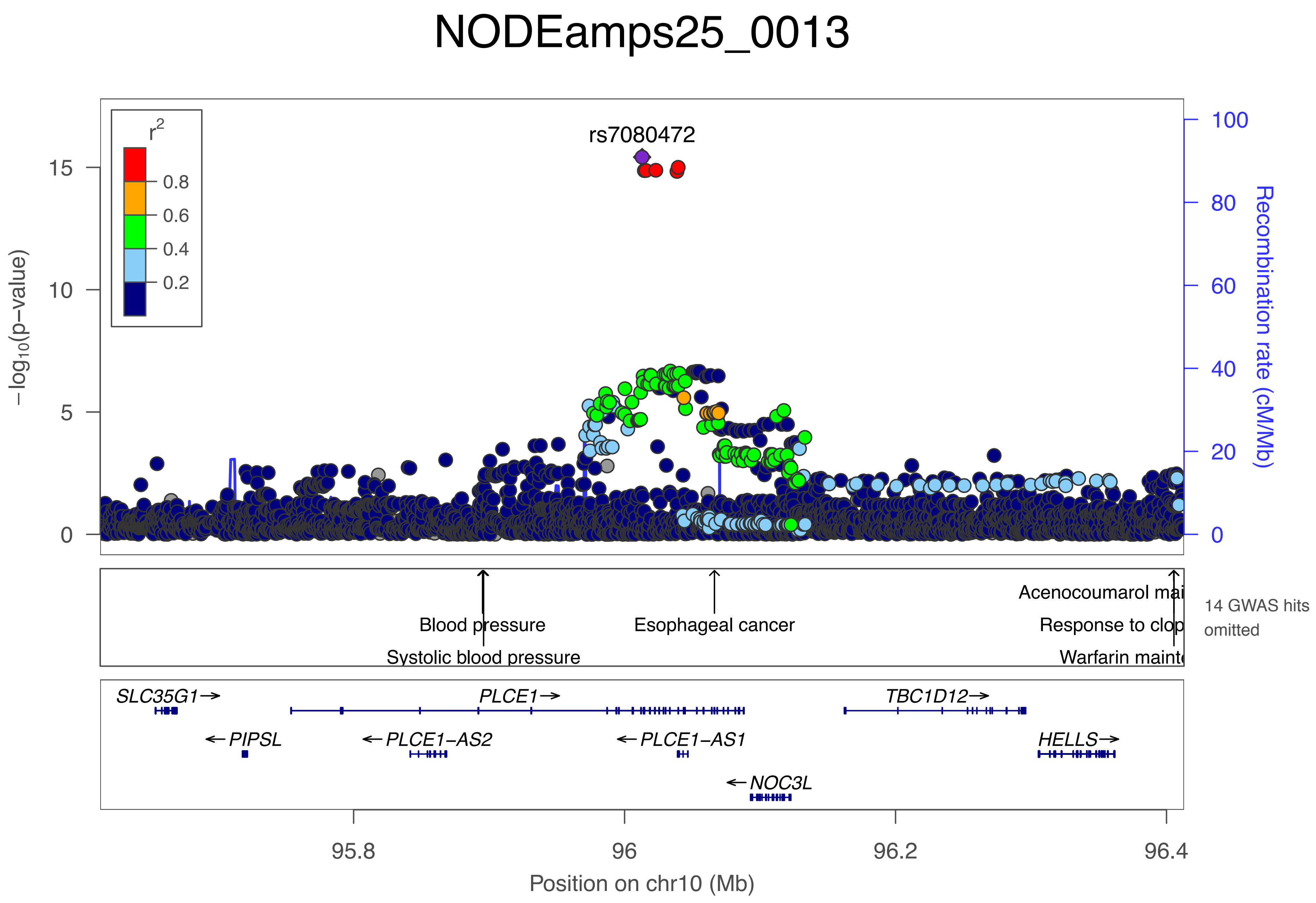

NODEamps25_0013
rs7080472
r2
0.8
0.6
0.4
0.2
−log10(p−value)
Recombination rate (cM/Mb)
Blood pressure
Systolic blood pressure
Esophageal cancer
Acenocoumarol mai
Response to clop
Warfarin mainte
14 GWAS hits omitted
SLC35G1→
PLCE1→
TBC1D12→
←PIPSL
←PLCE1−AS2
←PLCE1−AS1
HELLS→
←NOC3L
95.8
96
96.2
96.4
Position on chr10 (Mb)

| | |
|---|---|
| date: | Thu Aug 17 18:48:50 2017 |
| build: | hg19 |
| display range: | chr10:95612950−96412950 [95612950−96412950] |
| hilite range: | 0 – 0 [ 0 – 0 ] |
| reference SNP: | chr10:96012950 |
| number of SNPs plotted: | 3177 |
| min P.value: | 3.8E−16 [chr10:96012950] |
| max P.value: | 10E−1 [chr10:95975547] |
| omitted GWAS Hits: | chr10:96.066341−Esophageal cancer, chr10:96.405502−Response to clopidogrel therapy |
| omitted GWAS Hits: | NA, NA |
| omitted GWAS Hits: | NA, NA |
| omitted GWAS Hits: | NA, NA |
| omitted GWAS Hits: | NA, NA |

Make more plots at http://csg.sph.umich.edu/locuszoom/

GWAS Catalog SNPs in Region

| chr | pos (Mb) | trait | snp |
|---|---|---|---|
| 10 | 95.65996 | Cannabis dependence | rs146091982 |
| 10 | 95.89518 | Blood pressure | rs9663362 |
| 10 | 95.89594 | Systolic blood pressure | rs932764 |
| 10 | 95.89594 | Diastolic blood pressure | rs932764 |
| 10 | 95.89594 | Hypertension | rs932764 |
| 10 | 95.90632 | Glucose homeostasis traits | rs1223629 |
| 10 | 96.01371 | Personality traits in bipolar disorder | rs9419788 |
| 10 | 96.01462 | Migraine | rs10786156 |
| 10 | 96.01903 | Migraine | rs75473620 |
| 10 | 96.03598 | Sudden cardiac arrest | rs11187837 |
| 10 | 96.03631 | Vertical cup–disc ratio | rs7072574 |
| 10 | 96.05830 | Dengue shock syndrome | rs3765524 |
| 10 | 96.05830 | Esophageal cancer and gastric cancer | rs3765524 |
| 10 | 96.05864 | Lifetime average cigarettes per day in chronic obstructive pulmonary disease | rs117607728 |
| 10 | 96.06634 | Esophageal cancer | rs2274223 |
| 10 | 96.06634 | Esophageal squamous cell carcinoma | rs2274223 |
| 10 | 96.07037 | Esophageal cancer and gastric cancer | rs3781264 |
| 10 | 96.40533 | Acenocoumarol maintenance dosage | rs12772169 |
| 10 | 96.40550 | Response to clopidogrel therapy | rs12777823 |
| 10 | 96.40550 | Warfarin maintenance dose | rs12777823 |

# NODEamps100_0010

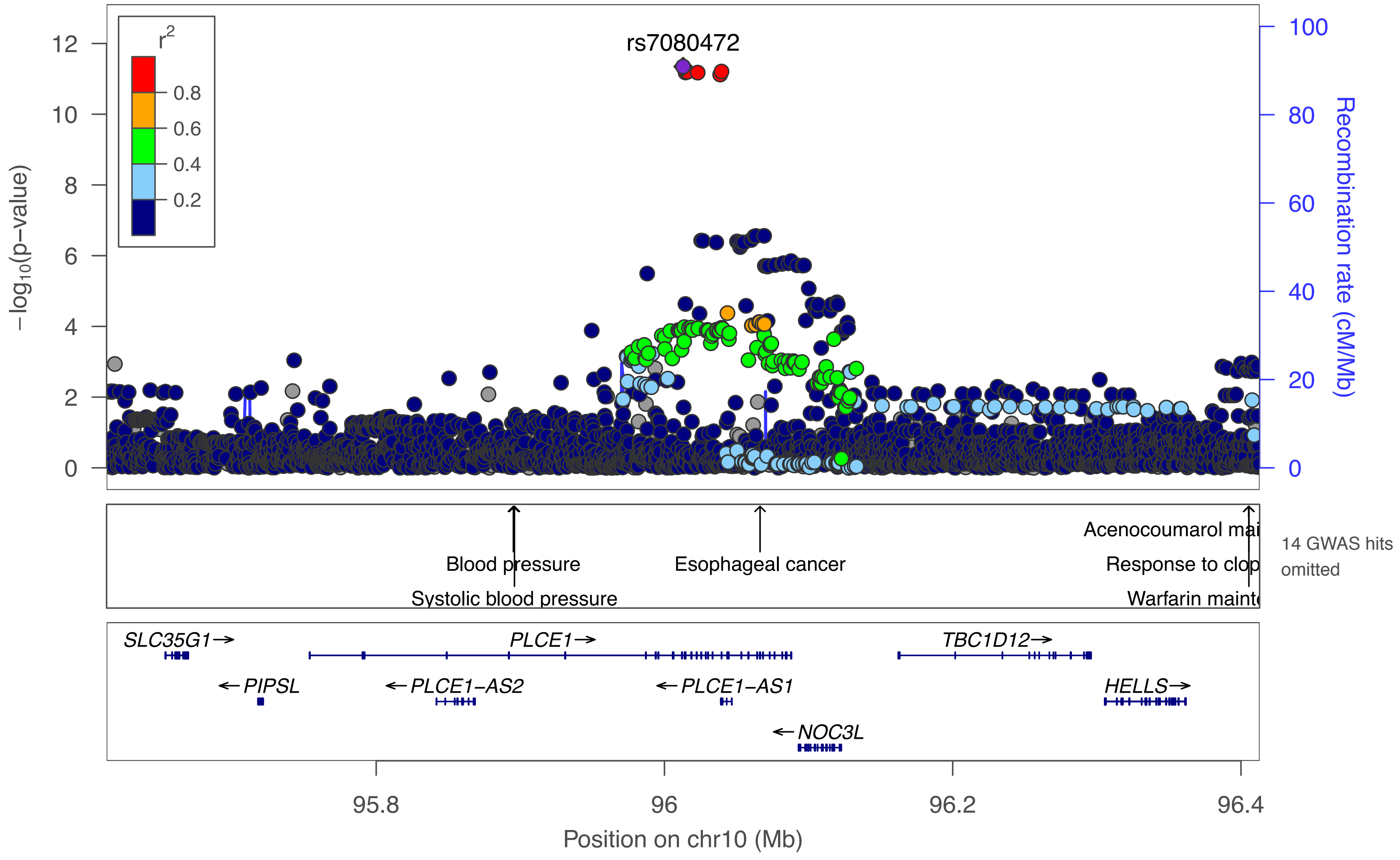

| | |
|---|---|
| date: | Thu Aug 17 18:48:08 2017 |
| build: | hg19 |
| display range: | chr10:95612950−96412950 [95612950−96412950] |
| hilite range: | 0 – 0 [ 0 – 0 ] |
| reference SNP: | chr10:96012950 |
| number of SNPs plotted: | 3177 |
| min P.value: | 4.49E−12 [chr10:96012950] |
| max P.value: | 10E−1 [chr10:96062523] |
| omitted GWAS Hits: | chr10:96.066341−Esophageal cancer, chr10:96.405502−Response to clopidogrel therapy |
| omitted GWAS Hits: | NA, NA |
| omitted GWAS Hits: | NA, NA |
| omitted GWAS Hits: | NA, NA |
| omitted GWAS Hits: | NA, NA |

Make more plots at http://csg.sph.umich.edu/locuszoom/

GWAS Catalog SNPs in Region

| chr | pos (Mb) | trait | snp |
|---|---|---|---|
| 10 | 95.65996 | Cannabis dependence | rs146091982 |
| 10 | 95.89518 | Blood pressure | rs9663362 |
| 10 | 95.89594 | Systolic blood pressure | rs932764 |
| 10 | 95.89594 | Diastolic blood pressure | rs932764 |
| 10 | 95.89594 | Hypertension | rs932764 |
| 10 | 95.90632 | Glucose homeostasis traits | rs1223629 |
| 10 | 96.01371 | Personality traits in bipolar disorder | rs9419788 |
| 10 | 96.01462 | Migraine | rs10786156 |
| 10 | 96.01903 | Migraine | rs75473620 |
| 10 | 96.03598 | Sudden cardiac arrest | rs11187837 |
| 10 | 96.03631 | Vertical cup–disc ratio | rs7072574 |
| 10 | 96.05830 | Dengue shock syndrome | rs3765524 |
| 10 | 96.05830 | Esophageal cancer and gastric cancer | rs3765524 |
| 10 | 96.05864 | Lifetime average cigarettes per day in chronic obstructive pulmonary disease | rs117607728 |
| 10 | 96.06634 | Esophageal cancer | rs2274223 |
| 10 | 96.06634 | Esophageal squamous cell carcinoma | rs2274223 |
| 10 | 96.07037 | Esophageal cancer and gastric cancer | rs3781264 |
| 10 | 96.40533 | Acenocoumarol maintenance dosage | rs12772169 |
| 10 | 96.40550 | Response to clopidogrel therapy | rs12777823 |
| 10 | 96.40550 | Warfarin maintenance dose | rs12777823 |

# NODEamps100_0011

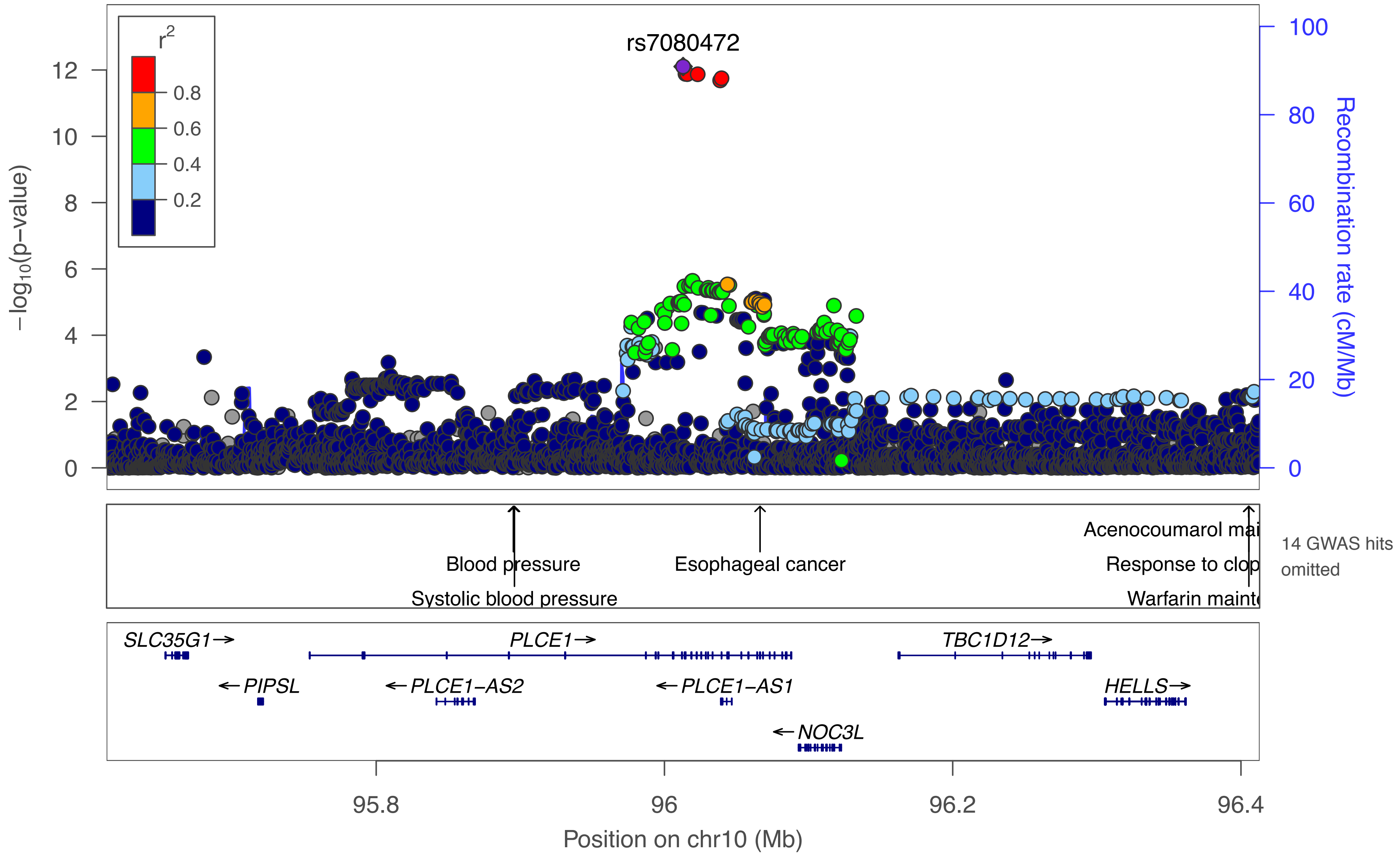

| | |
|---|---|
| date: | Thu Aug 17 18:48:08 2017 |
| build: | hg19 |
| display range: | chr10:95612950−96412950 [95612950−96412950] |
| hilite range: | 0 – 0 [ 0 – 0 ] |
| reference SNP: | chr10:96012950 |
| number of SNPs plotted: | 3177 |
| min P.value: | 7.87E−13 [chr10:96012950] |
| max P.value: | 10E−1 [chr10:95615108] |
| omitted GWAS Hits: | chr10:96.066341−Esophageal cancer, chr10:96.405502−Response to clopidogrel therapy |
| omitted GWAS Hits: | NA, NA |
| omitted GWAS Hits: | NA, NA |
| omitted GWAS Hits: | NA, NA |
| omitted GWAS Hits: | NA, NA |

Make more plots at http://csg.sph.umich.edu/locuszoom/

GWAS Catalog SNPs in Region

| chr | pos (Mb) | trait | snp |
|---|---|---|---|
| 10 | 95.65996 | Cannabis dependence | rs146091982 |
| 10 | 95.89518 | Blood pressure | rs9663362 |
| 10 | 95.89594 | Systolic blood pressure | rs932764 |
| 10 | 95.89594 | Diastolic blood pressure | rs932764 |
| 10 | 95.89594 | Hypertension | rs932764 |
| 10 | 95.90632 | Glucose homeostasis traits | rs1223629 |
| 10 | 96.01371 | Personality traits in bipolar disorder | rs9419788 |
| 10 | 96.01462 | Migraine | rs10786156 |
| 10 | 96.01903 | Migraine | rs75473620 |
| 10 | 96.03598 | Sudden cardiac arrest | rs11187837 |
| 10 | 96.03631 | Vertical cup–disc ratio | rs7072574 |
| 10 | 96.05830 | Dengue shock syndrome | rs3765524 |
| 10 | 96.05830 | Esophageal cancer and gastric cancer | rs3765524 |
| 10 | 96.05864 | Lifetime average cigarettes per day in chronic obstructive pulmonary disease | rs117607728 |
| 10 | 96.06634 | Esophageal cancer | rs2274223 |
| 10 | 96.06634 | Esophageal squamous cell carcinoma | rs2274223 |
| 10 | 96.07037 | Esophageal cancer and gastric cancer | rs3781264 |
| 10 | 96.40533 | Acenocoumarol maintenance dosage | rs12772169 |
| 10 | 96.40550 | Response to clopidogrel therapy | rs12777823 |
| 10 | 96.40550 | Warfarin maintenance dose | rs12777823 |

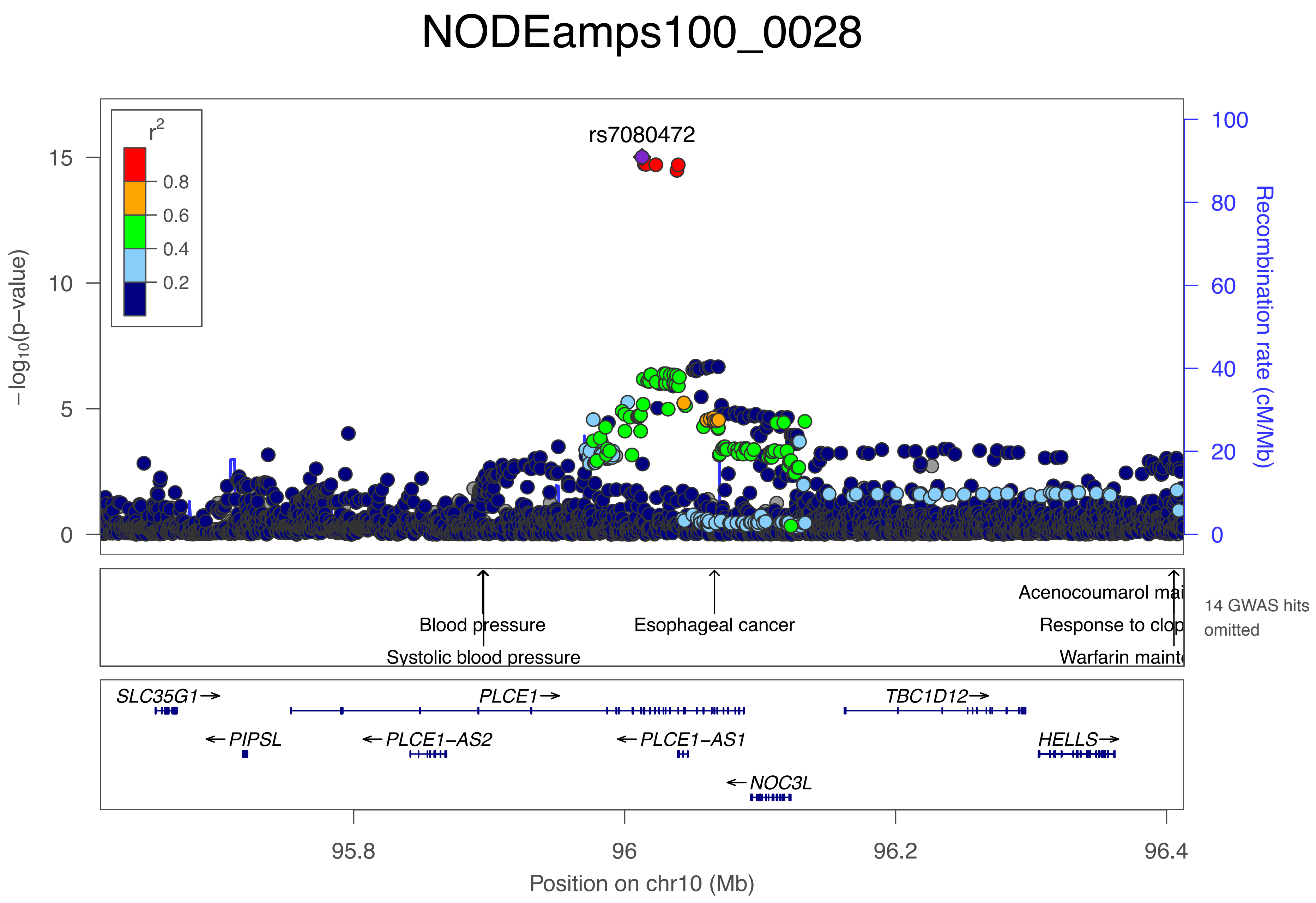
NODEamps100_0028
rs7080472
r2
0.8
0.6
0.4
0.2
15
10
5
0
−log10(p−value)
100
80
60
40
20
0
Recombination rate (cM/Mb)
Acenocoumarol mai
Blood pressure
Esophageal cancer
Response to clop
Systolic blood pressure
Warfarin mainte
14 GWAS hits omitted
SLC35G1→
PLCE1→
TBC1D12→
←PIPSL
←PLCE1−AS2
←PLCE1−AS1
HELLS→
←NOC3L
95.8
96
96.2
96.4
Position on chr10 (Mb)

| | |
|---|---|
| date: | Thu Aug 17 18:48:08 2017 |
| build: | hg19 |
| display range: | chr10:95612950−96412950 [95612950−96412950] |
| hilite range: | 0 – 0 [ 0 – 0 ] |
| reference SNP: | chr10:96012950 |
| number of SNPs plotted: | 3177 |
| min P.value: | 9.73E−16 [chr10:96012950] |
| max P.value: | 10E−1 [chr10:96015298] |
| omitted GWAS Hits: | chr10:96.066341−Esophageal cancer, chr10:96.405502−Response to clopidogrel therapy |
| omitted GWAS Hits: | NA, NA |
| omitted GWAS Hits: | NA, NA |
| omitted GWAS Hits: | NA, NA |
| omitted GWAS Hits: | NA, NA |

Make more plots at http://csg.sph.umich.edu/locuszoom/

GWAS Catalog SNPs in Region

| chr | pos (Mb) | trait | snp |
|---|---|---|---|
| 10 | 95.65996 | Cannabis dependence | rs146091982 |
| 10 | 95.89518 | Blood pressure | rs9663362 |
| 10 | 95.89594 | Systolic blood pressure | rs932764 |
| 10 | 95.89594 | Diastolic blood pressure | rs932764 |
| 10 | 95.89594 | Hypertension | rs932764 |
| 10 | 95.90632 | Glucose homeostasis traits | rs1223629 |
| 10 | 96.01371 | Personality traits in bipolar disorder | rs9419788 |
| 10 | 96.01462 | Migraine | rs10786156 |
| 10 | 96.01903 | Migraine | rs75473620 |
| 10 | 96.03598 | Sudden cardiac arrest | rs11187837 |
| 10 | 96.03631 | Vertical cup–disc ratio | rs7072574 |
| 10 | 96.05830 | Dengue shock syndrome | rs3765524 |
| 10 | 96.05830 | Esophageal cancer and gastric cancer | rs3765524 |
| 10 | 96.05864 | Lifetime average cigarettes per day in chronic obstructive pulmonary disease | rs117607728 |
| 10 | 96.06634 | Esophageal cancer | rs2274223 |
| 10 | 96.06634 | Esophageal squamous cell carcinoma | rs2274223 |
| 10 | 96.07037 | Esophageal cancer and gastric cancer | rs3781264 |
| 10 | 96.40533 | Acenocoumarol maintenance dosage | rs12772169 |
| 10 | 96.40550 | Response to clopidogrel therapy | rs12777823 |
| 10 | 96.40550 | Warfarin maintenance dose | rs12777823 |

# NODEamps100_0029

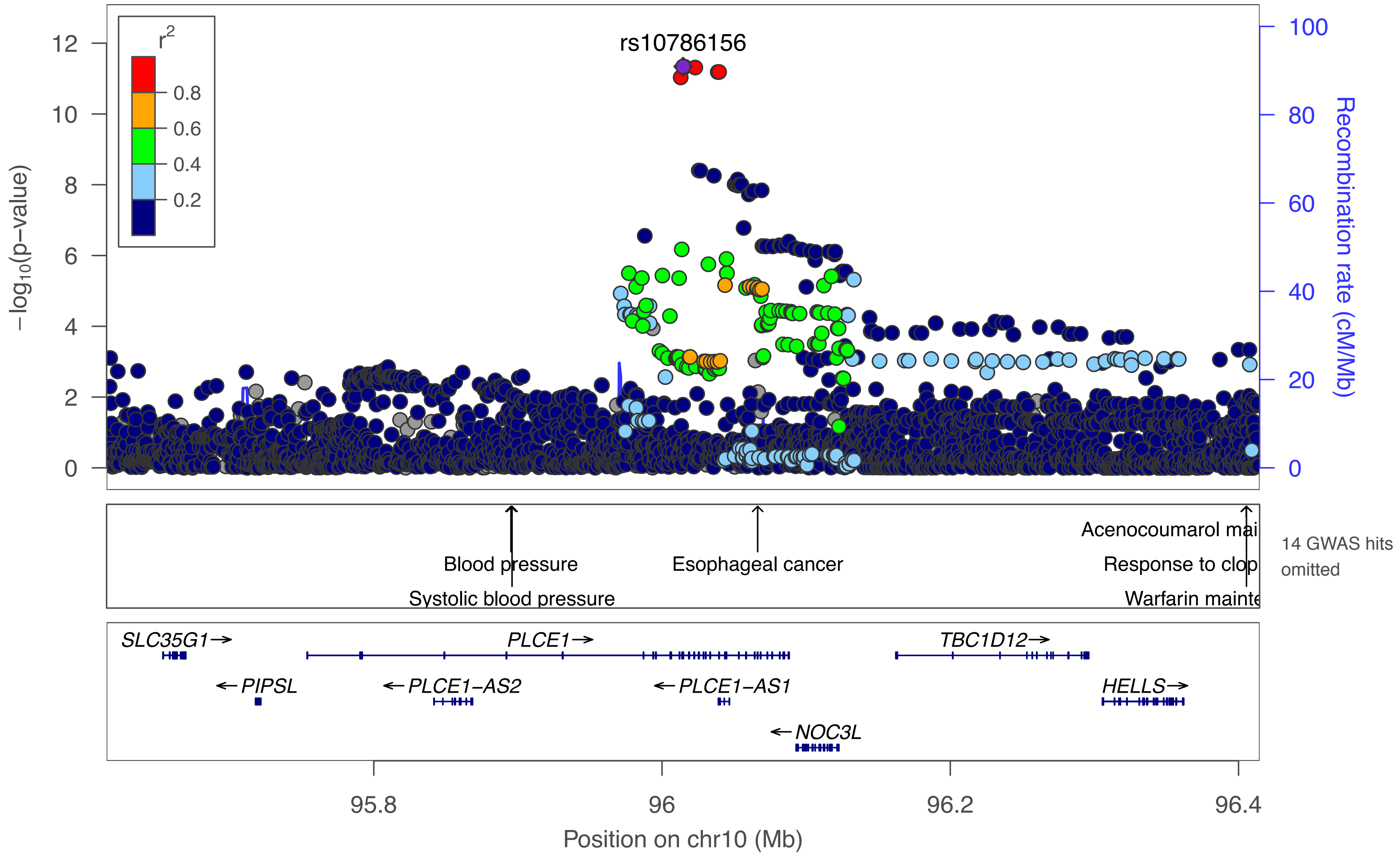

| | |
|---|---|
| date: | Thu Aug 17 18:48:50 2017 |
| build: | hg19 |
| display range: | chr10:95614622−96414622 [95614622−96414622] |
| hilite range: | 0 – 0 [ 0 – 0 ] |
| reference SNP: | chr10:96014622 |
| number of SNPs plotted: | 3178 |
| min P.value: | 4.57E−12 [chr10:96014622] |
| max P.value: | 9.99E−1 [chr10:96278592] |
| omitted GWAS Hits: | chr10:96.066341−Esophageal cancer, chr10:96.405502−Response to clopidogrel therapy |
| omitted GWAS Hits: | NA, NA |
| omitted GWAS Hits: | NA, NA |
| omitted GWAS Hits: | NA, NA |
| omitted GWAS Hits: | NA, NA |

Make more plots at http://csg.sph.umich.edu/locuszoom/

GWAS Catalog SNPs in Region

| chr | pos (Mb) | trait | snp |
|---|---|---|---|
| 10 | 95.65996 | Cannabis dependence | rs146091982 |
| 10 | 95.89518 | Blood pressure | rs9663362 |
| 10 | 95.89594 | Systolic blood pressure | rs932764 |
| 10 | 95.89594 | Diastolic blood pressure | rs932764 |
| 10 | 95.89594 | Hypertension | rs932764 |
| 10 | 95.90632 | Glucose homeostasis traits | rs1223629 |
| 10 | 96.01371 | Personality traits in bipolar disorder | rs9419788 |
| 10 | 96.01462 | Migraine | rs10786156 |
| 10 | 96.01903 | Migraine | rs75473620 |
| 10 | 96.03598 | Sudden cardiac arrest | rs11187837 |
| 10 | 96.03631 | Vertical cup–disc ratio | rs7072574 |
| 10 | 96.05830 | Dengue shock syndrome | rs3765524 |
| 10 | 96.05830 | Esophageal cancer and gastric cancer | rs3765524 |
| 10 | 96.05864 | Lifetime average cigarettes per day in chronic obstructive pulmonary disease | rs117607728 |
| 10 | 96.06634 | Esophageal cancer | rs2274223 |
| 10 | 96.06634 | Esophageal squamous cell carcinoma | rs2274223 |
| 10 | 96.07037 | Esophageal cancer and gastric cancer | rs3781264 |
| 10 | 96.40533 | Acenocoumarol maintenance dosage | rs12772169 |
| 10 | 96.40550 | Response to clopidogrel therapy | rs12777823 |
| 10 | 96.40550 | Warfarin maintenance dose | rs12777823 |

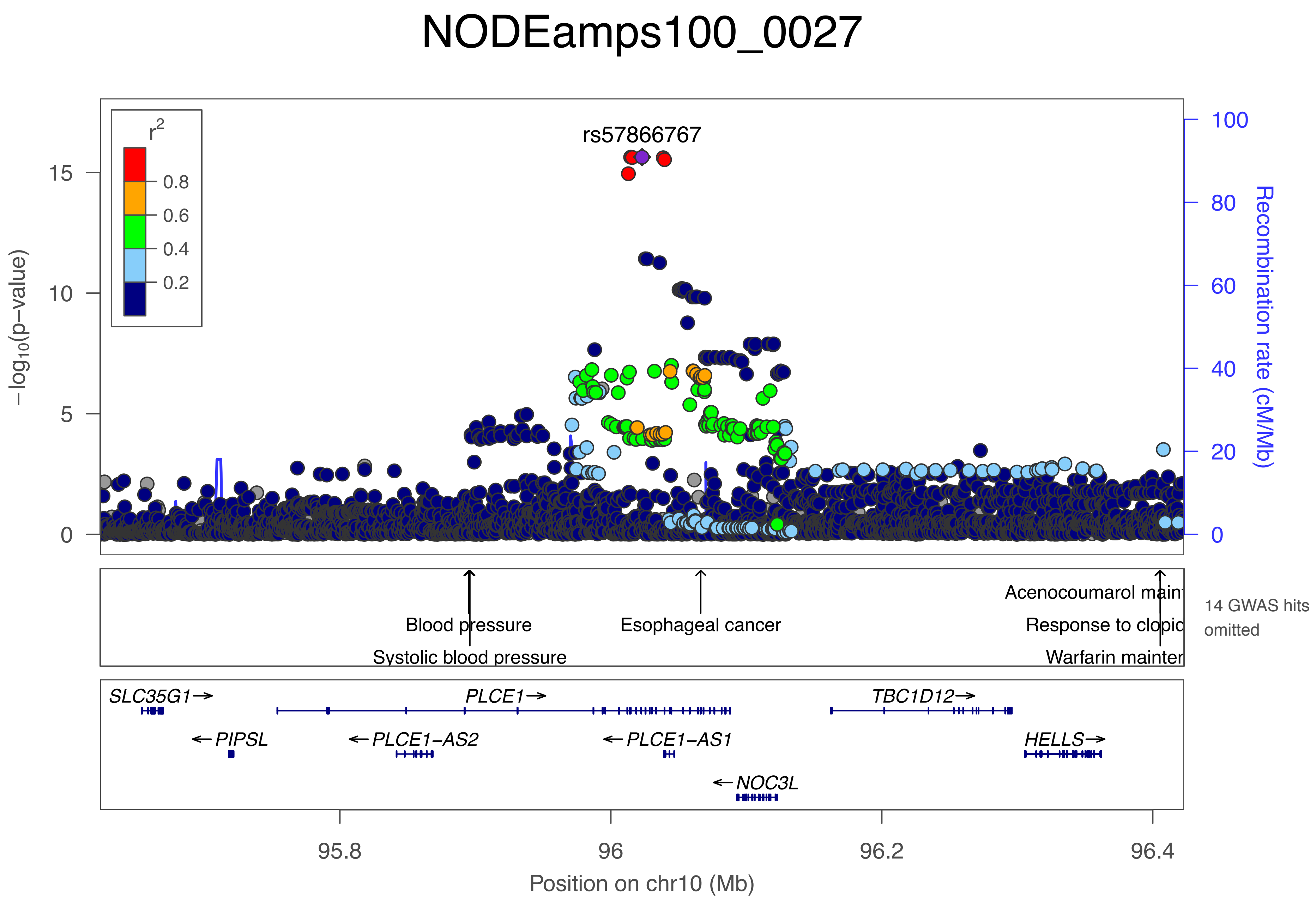
NODEamps100_0027
rs57866767
r2
0.8
0.6
0.4
0.2
15
10
5
0
−log10(p−value)
100
80
60
40
20
0
Recombination rate (cM/Mb)
Blood pressure
Systolic blood pressure
Esophageal cancer
Acenocoumarol maint
Response to clopid
Warfarin mainter
14 GWAS hits omitted
SLC35G1→
PLCE1→
TBC1D12→
←PIPSL
←PLCE1−AS2
←PLCE1−AS1
HELLS→
←NOC3L
95.8
96
96.2
96.4
Position on chr10 (Mb)

| | |
|---|---|
| date: | Thu Aug 17 18:49:10 2017 |
| build: | hg19 |
| display range: | chr10:95623077−96423077 [95623077−96423077] |
| hilite range: | 0 – 0 [ 0 – 0 ] |
| reference SNP: | chr10:96023077 |
| number of SNPs plotted: | 3184 |
| min P.value: | 2.31E−16 [chr10:96023077] |
| max P.value: | 9.99E−1 [chr10:96119218] |
| omitted GWAS Hits: | chr10:96.066341−Esophageal cancer, chr10:96.405502−Response to clopidogrel therapy |
| omitted GWAS Hits: | NA, NA |
| omitted GWAS Hits: | NA, NA |
| omitted GWAS Hits: | NA, NA |
| omitted GWAS Hits: | NA, NA |

Make more plots at http://csg.sph.umich.edu/locuszoom/

GWAS Catalog SNPs in Region

| chr | pos (Mb) | trait | snp |
|---|---|---|---|
| 10 | 95.65996 | Cannabis dependence | rs146091982 |
| 10 | 95.89518 | Blood pressure | rs9663362 |
| 10 | 95.89594 | Systolic blood pressure | rs932764 |
| 10 | 95.89594 | Diastolic blood pressure | rs932764 |
| 10 | 95.89594 | Hypertension | rs932764 |
| 10 | 95.90632 | Glucose homeostasis traits | rs1223629 |
| 10 | 96.01371 | Personality traits in bipolar disorder | rs9419788 |
| 10 | 96.01462 | Migraine | rs10786156 |
| 10 | 96.01903 | Migraine | rs75473620 |
| 10 | 96.03598 | Sudden cardiac arrest | rs11187837 |
| 10 | 96.03631 | Vertical cup–disc ratio | rs7072574 |
| 10 | 96.05830 | Dengue shock syndrome | rs3765524 |
| 10 | 96.05830 | Esophageal cancer and gastric cancer | rs3765524 |
| 10 | 96.05864 | Lifetime average cigarettes per day in chronic obstructive pulmonary disease | rs117607728 |
| 10 | 96.06634 | Esophageal cancer | rs2274223 |
| 10 | 96.06634 | Esophageal squamous cell carcinoma | rs2274223 |
| 10 | 96.07037 | Esophageal cancer and gastric cancer | rs3781264 |
| 10 | 96.40533 | Acenocoumarol maintenance dosage | rs12772169 |
| 10 | 96.40550 | Response to clopidogrel therapy | rs12777823 |
| 10 | 96.40550 | Warfarin maintenance dose | rs12777823 |

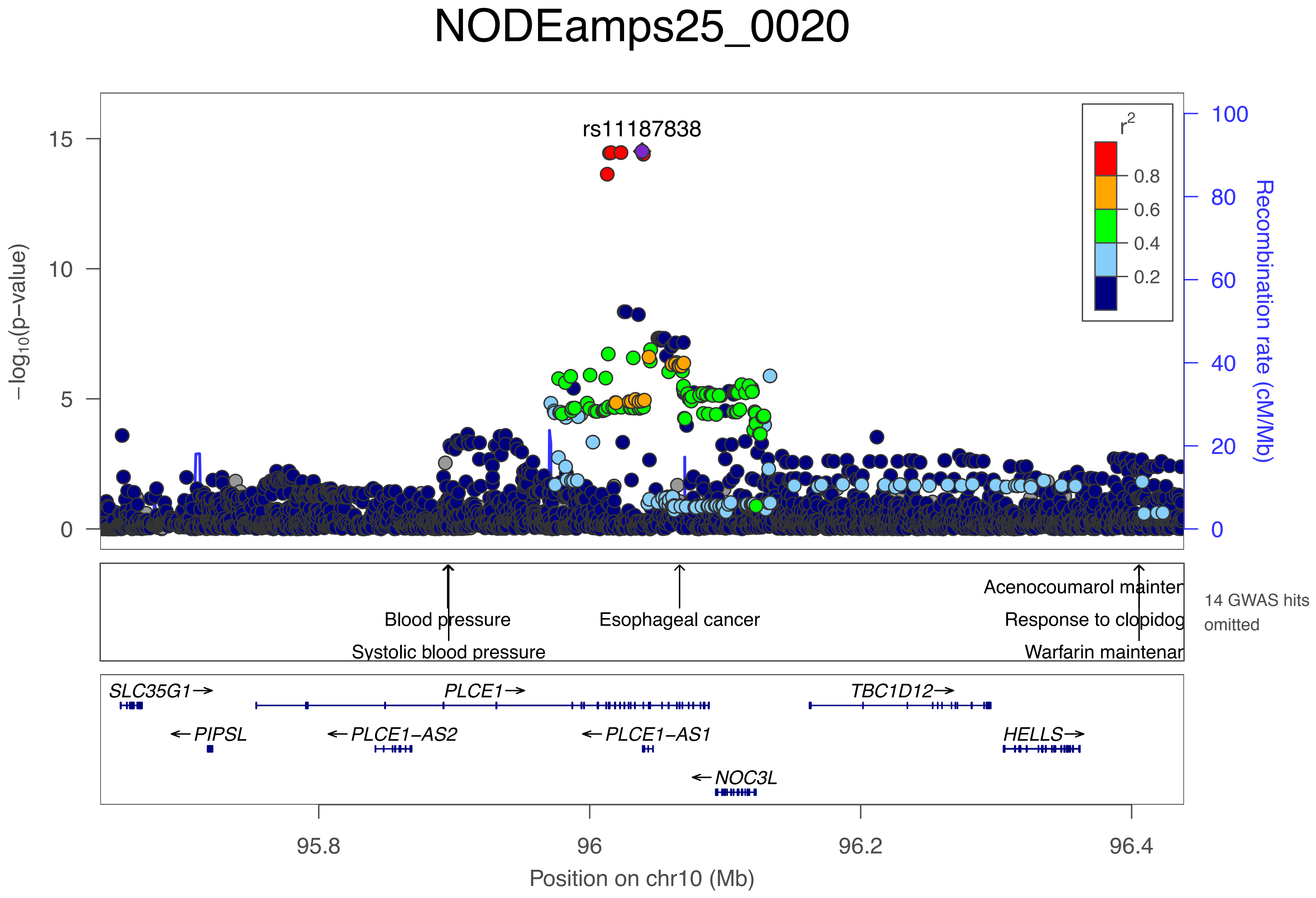
NODEamps25_0020
rs11187838
−log10(p−value)
15
10
5
0
r2
0.8
0.6
0.4
0.2
100
80
60
40
20
0
Recombination rate (cM/Mb)
Blood pressure
Systolic blood pressure
Esophageal cancer
Acenocoumarol mainten
Response to clopidog
Warfarin maintenar
14 GWAS hits omitted
SLC35G1→
PLCE1→
TBC1D12→
←PIPSL
←PLCE1−AS2
←PLCE1−AS1
HELLS→
←NOC3L
95.8
96
96.2
96.4
Position on chr10 (Mb)

| | |
|---|---|
| date: | Thu Aug 17 18:47:34 2017 |
| build: | hg19 |
| display range: | chr10:95638686−96438686 [95638686−96438686] |
| hilite range: | 0 – 0 [ 0 – 0 ] |
| reference SNP: | chr10:96038686 |
| number of SNPs plotted: | 3190 |
| min P.value: | 3.05E−15 [chr10:96038686] |
| max P.value: | 9.99E−1 [chr10:96260800] |
| omitted GWAS Hits: | chr10:96.066341−Esophageal cancer, chr10:96.405502−Response to clopidogrel therapy |
| omitted GWAS Hits: | NA, NA |
| omitted GWAS Hits: | NA, NA |
| omitted GWAS Hits: | NA, NA |
| omitted GWAS Hits: | NA, NA |

Make more plots at http://csg.sph.umich.edu/locuszoom/

GWAS Catalog SNPs in Region

| chr | pos (Mb) | trait | snp |
|---|---|---|---|
| 10 | 95.65996 | Cannabis dependence | rs146091982 |
| 10 | 95.89518 | Blood pressure | rs9663362 |
| 10 | 95.89594 | Systolic blood pressure | rs932764 |
| 10 | 95.89594 | Diastolic blood pressure | rs932764 |
| 10 | 95.89594 | Hypertension | rs932764 |
| 10 | 95.90632 | Glucose homeostasis traits | rs1223629 |
| 10 | 96.01371 | Personality traits in bipolar disorder | rs9419788 |
| 10 | 96.01462 | Migraine | rs10786156 |
| 10 | 96.01903 | Migraine | rs75473620 |
| 10 | 96.03598 | Sudden cardiac arrest | rs11187837 |
| 10 | 96.03631 | Vertical cup–disc ratio | rs7072574 |
| 10 | 96.05830 | Dengue shock syndrome | rs3765524 |
| 10 | 96.05830 | Esophageal cancer and gastric cancer | rs3765524 |
| 10 | 96.05864 | Lifetime average cigarettes per day in chronic obstructive pulmonary disease | rs117607728 |
| 10 | 96.06634 | Esophageal cancer | rs2274223 |
| 10 | 96.06634 | Esophageal squamous cell carcinoma | rs2274223 |
| 10 | 96.07037 | Esophageal cancer and gastric cancer | rs3781264 |
| 10 | 96.40533 | Acenocoumarol maintenance dosage | rs12772169 |
| 10 | 96.40550 | Response to clopidogrel therapy | rs12777823 |
| 10 | 96.40550 | Warfarin maintenance dose | rs12777823 |

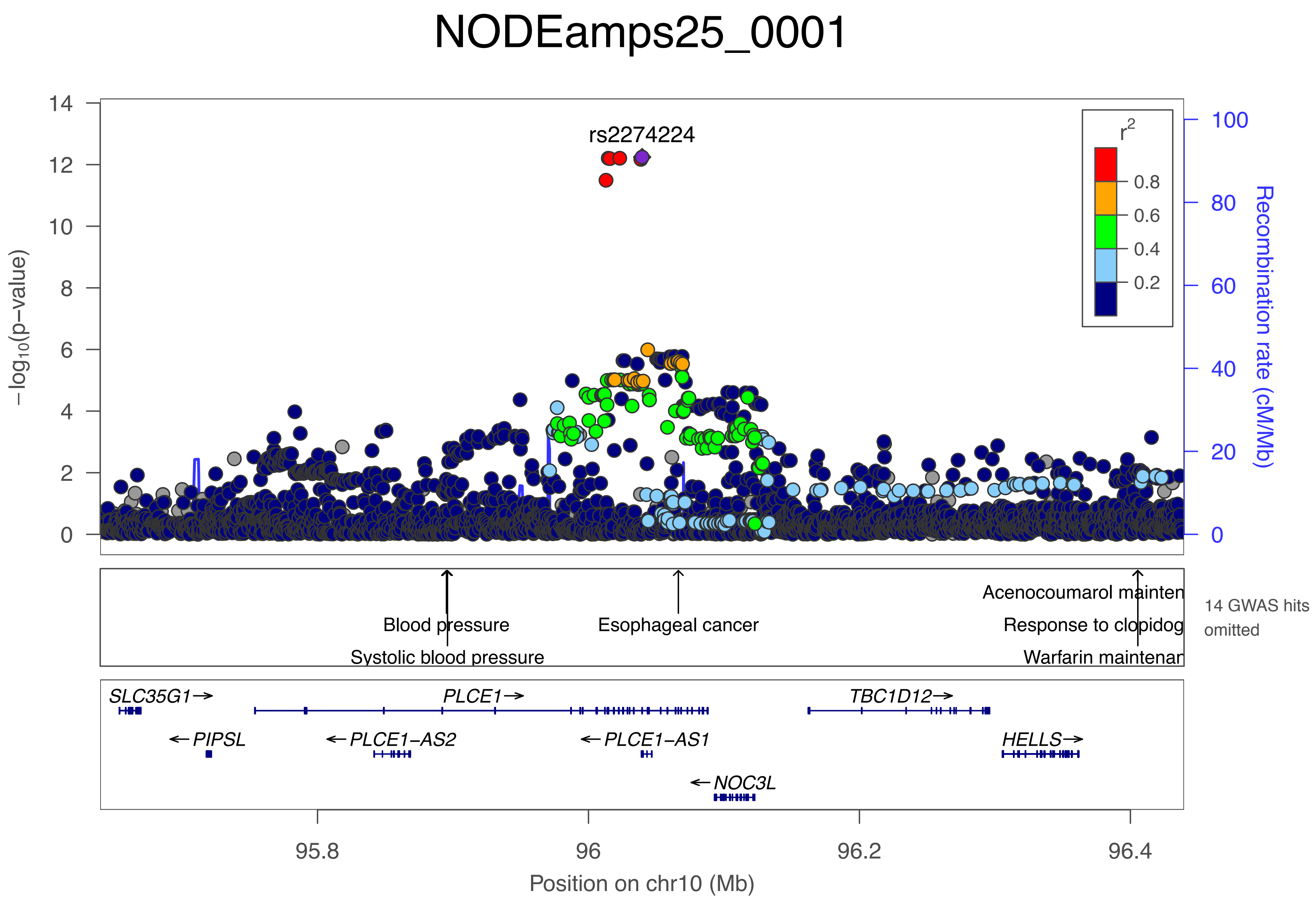

NODEamps25_0001
rs2274224
−log10(p−value)
Recombination rate (cM/Mb)
r2
0.8
0.6
0.4
0.2
Blood pressure
Systolic blood pressure
Esophageal cancer
Acenocoumarol mainten
Response to clopidog
Warfarin maintenan
14 GWAS hits omitted
SLC35G1→
PLCE1→
TBC1D12→
←PIPSL
←PLCE1−AS2
←PLCE1−AS1
HELLS→
←NOC3L
95.8
96
96.2
96.4
Position on chr10 (Mb)

| | |
|---|---|
| date: | Thu Aug 17 18:49:43 2017 |
| build: | hg19 |
| display range: | chr10:95639597−96439597 [95639597−96439597] |
| hilite range: | 0 – 0 [ 0 – 0 ] |
| reference SNP: | chr10:96039597 |
| number of SNPs plotted: | 3187 |
| min P.value: | 5.7E−13 [chr10:96039597] |
| max P.value: | 9.99E−1 [chr10:96176921] |
| omitted GWAS Hits: | chr10:96.066341−Esophageal cancer, chr10:96.405502−Response to clopidogrel therapy |
| omitted GWAS Hits: | NA, NA |
| omitted GWAS Hits: | NA, NA |
| omitted GWAS Hits: | NA, NA |
| omitted GWAS Hits: | NA, NA |

Make more plots at http://csg.sph.umich.edu/locuszoom/

GWAS Catalog SNPs in Region

| chr | pos (Mb) | trait | snp |
|---|---|---|---|
| 10 | 95.65996 | Cannabis dependence | rs146091982 |
| 10 | 95.89518 | Blood pressure | rs9663362 |
| 10 | 95.89594 | Systolic blood pressure | rs932764 |
| 10 | 95.89594 | Diastolic blood pressure | rs932764 |
| 10 | 95.89594 | Hypertension | rs932764 |
| 10 | 95.90632 | Glucose homeostasis traits | rs1223629 |
| 10 | 96.01371 | Personality traits in bipolar disorder | rs9419788 |
| 10 | 96.01462 | Migraine | rs10786156 |
| 10 | 96.01903 | Migraine | rs75473620 |
| 10 | 96.03598 | Sudden cardiac arrest | rs11187837 |
| 10 | 96.03631 | Vertical cup–disc ratio | rs7072574 |
| 10 | 96.05830 | Dengue shock syndrome | rs3765524 |
| 10 | 96.05830 | Esophageal cancer and gastric cancer | rs3765524 |
| 10 | 96.05864 | Lifetime average cigarettes per day in chronic obstructive pulmonary disease | rs117607728 |
| 10 | 96.06634 | Esophageal cancer | rs2274223 |
| 10 | 96.06634 | Esophageal squamous cell carcinoma | rs2274223 |
| 10 | 96.07037 | Esophageal cancer and gastric cancer | rs3781264 |
| 10 | 96.40533 | Acenocoumarol maintenance dosage | rs12772169 |
| 10 | 96.40550 | Response to clopidogrel therapy | rs12777823 |
| 10 | 96.40550 | Warfarin maintenance dose | rs12777823 |

# NODEamps25_0005

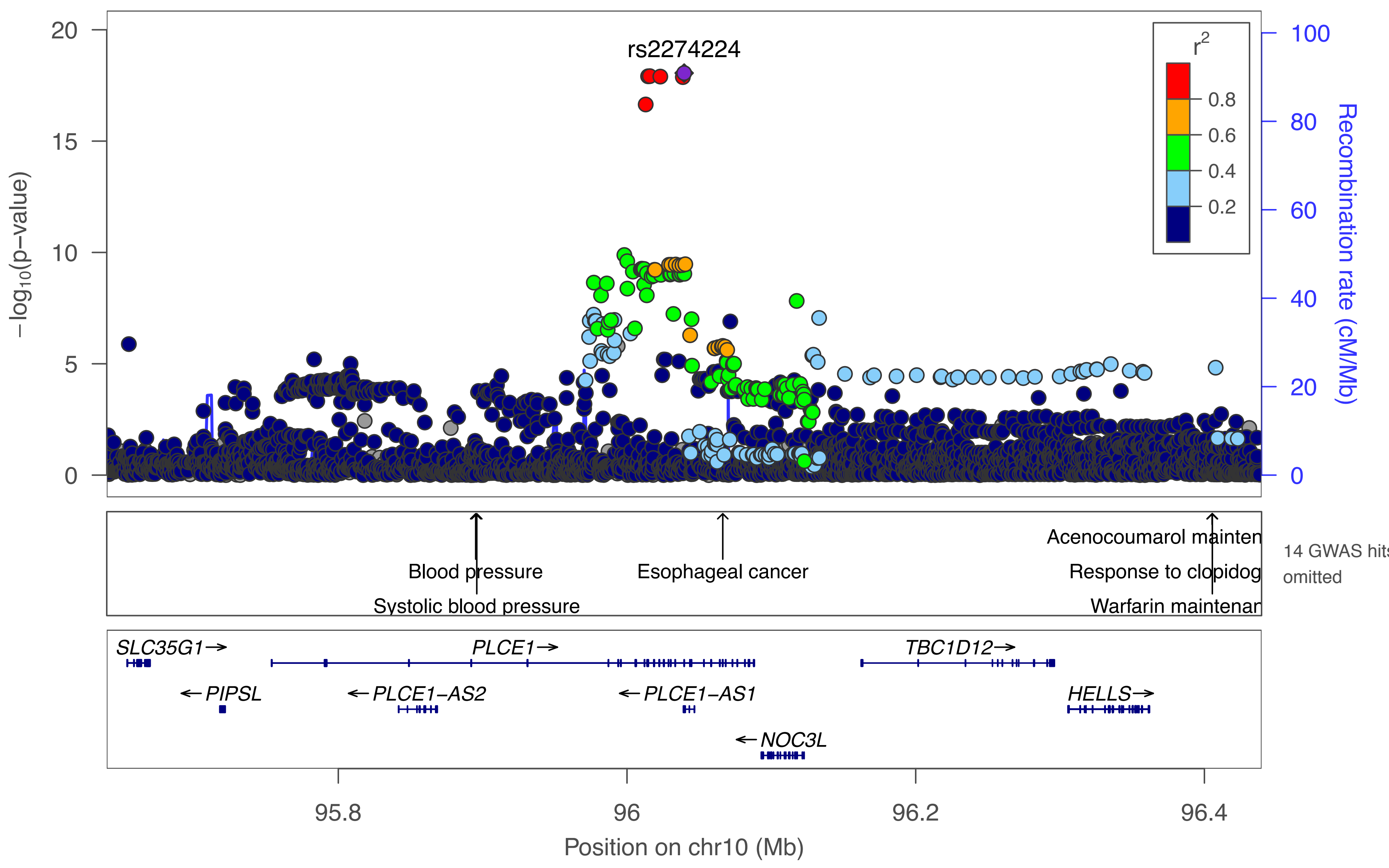

| | |
|---|---|
| date: | Thu Aug 17 18:49:43 2017 |
| build: | hg19 |
| display range: | chr10:95639597−96439597 [95639597−96439597] |
| hilite range: | 0 – 0 [ 0 – 0 ] |
| reference SNP: | chr10:96039597 |
| number of SNPs plotted: | 3187 |
| min P.value: | 8.79E−19 [chr10:96039597] |
| max P.value: | 10E−1 [chr10:96056694] |
| omitted GWAS Hits: | chr10:96.066341−Esophageal cancer, chr10:96.405502−Response to clopidogrel therapy |
| omitted GWAS Hits: | NA, NA |
| omitted GWAS Hits: | NA, NA |
| omitted GWAS Hits: | NA, NA |
| omitted GWAS Hits: | NA, NA |

Make more plots at http://csg.sph.umich.edu/locuszoom/

GWAS Catalog SNPs in Region

| chr | pos (Mb) | trait | snp |
|---|---|---|---|
| 10 | 95.65996 | Cannabis dependence | rs146091982 |
| 10 | 95.89518 | Blood pressure | rs9663362 |
| 10 | 95.89594 | Systolic blood pressure | rs932764 |
| 10 | 95.89594 | Diastolic blood pressure | rs932764 |
| 10 | 95.89594 | Hypertension | rs932764 |
| 10 | 95.90632 | Glucose homeostasis traits | rs1223629 |
| 10 | 96.01371 | Personality traits in bipolar disorder | rs9419788 |
| 10 | 96.01462 | Migraine | rs10786156 |
| 10 | 96.01903 | Migraine | rs75473620 |
| 10 | 96.03598 | Sudden cardiac arrest | rs11187837 |
| 10 | 96.03631 | Vertical cup–disc ratio | rs7072574 |
| 10 | 96.05830 | Dengue shock syndrome | rs3765524 |
| 10 | 96.05830 | Esophageal cancer and gastric cancer | rs3765524 |
| 10 | 96.05864 | Lifetime average cigarettes per day in chronic obstructive pulmonary disease | rs117607728 |
| 10 | 96.06634 | Esophageal cancer | rs2274223 |
| 10 | 96.06634 | Esophageal squamous cell carcinoma | rs2274223 |
| 10 | 96.07037 | Esophageal cancer and gastric cancer | rs3781264 |
| 10 | 96.40533 | Acenocoumarol maintenance dosage | rs12772169 |
| 10 | 96.40550 | Response to clopidogrel therapy | rs12777823 |
| 10 | 96.40550 | Warfarin maintenance dose | rs12777823 |

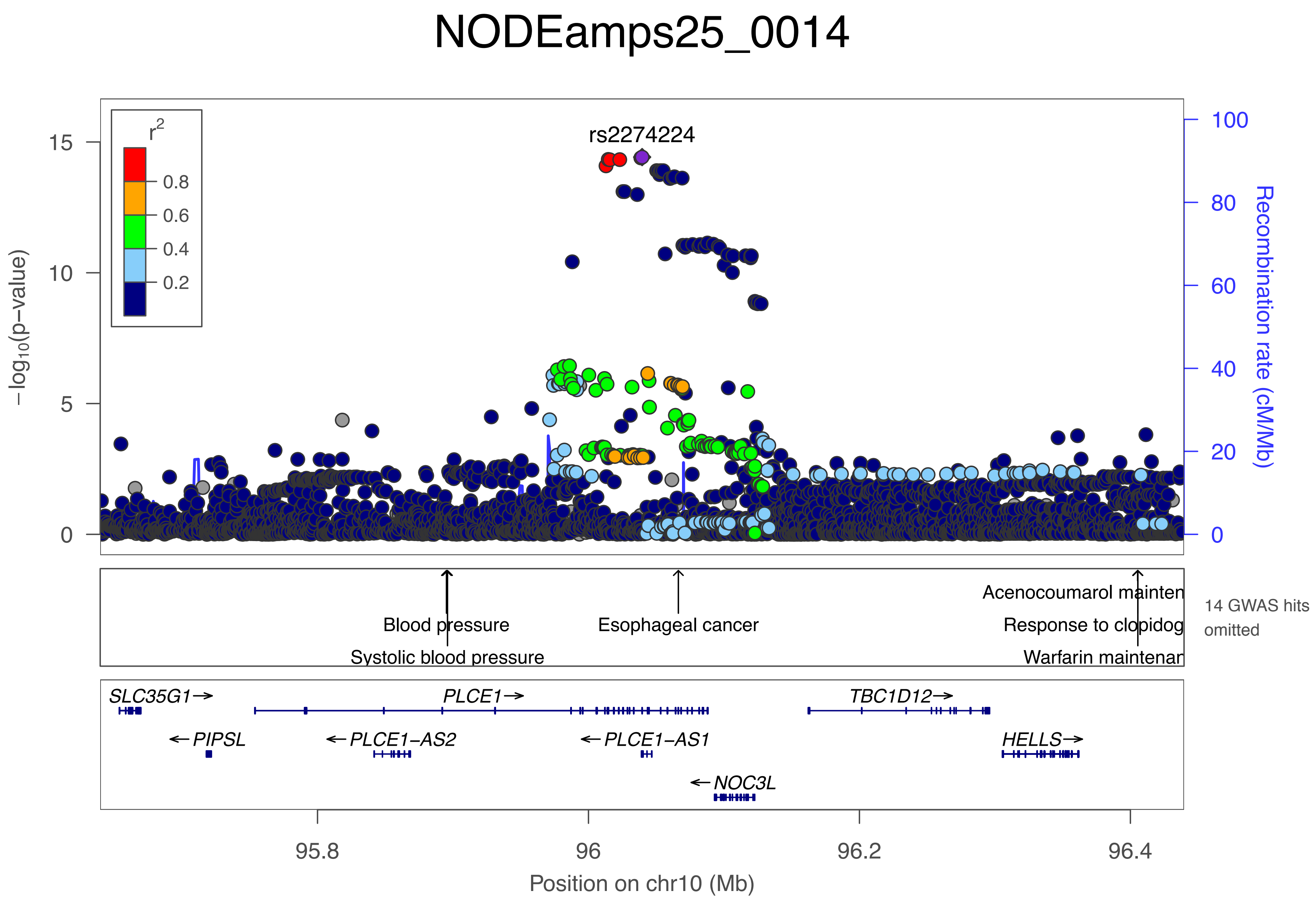
NODEamps25_0014
rs2274224
r2
0.8
0.6
0.4
0.2
15
10
5
0
−log10(p−value)
100
80
60
40
20
0
Recombination rate (cM/Mb)
Blood pressure
Systolic blood pressure
Esophageal cancer
Acenocoumarol mainten
Response to clopidog
Warfarin maintenar
14 GWAS hits
omitted
SLC35G1→
PLCE1→
TBC1D12→
←PIPSL
←PLCE1−AS2
←PLCE1−AS1
HELLS→
←NOC3L
95.8
96
96.2
96.4
Position on chr10 (Mb)

| | |
|---|---|
| date: | Thu Aug 17 18:47:34 2017 |
| build: | hg19 |
| display range: | chr10:95639597−96439597 [95639597−96439597] |
| hilite range: | 0 – 0 [ 0 – 0 ] |
| reference SNP: | chr10:96039597 |
| number of SNPs plotted: | 3187 |
| min P.value: | 3.78E−15 [chr10:96039597] |
| max P.value: | 9.99E−1 [chr10:95718356] |
| omitted GWAS Hits: | chr10:96.066341−Esophageal cancer, chr10:96.405502−Response to clopidogrel therapy |
| omitted GWAS Hits: | NA, NA |
| omitted GWAS Hits: | NA, NA |
| omitted GWAS Hits: | NA, NA |
| omitted GWAS Hits: | NA, NA |

Make more plots at http://csg.sph.umich.edu/locuszoom/

GWAS Catalog SNPs in Region

| chr | pos (Mb) | trait | snp |
|---|---|---|---|
| 10 | 95.65996 | Cannabis dependence | rs146091982 |
| 10 | 95.89518 | Blood pressure | rs9663362 |
| 10 | 95.89594 | Systolic blood pressure | rs932764 |
| 10 | 95.89594 | Diastolic blood pressure | rs932764 |
| 10 | 95.89594 | Hypertension | rs932764 |
| 10 | 95.90632 | Glucose homeostasis traits | rs1223629 |
| 10 | 96.01371 | Personality traits in bipolar disorder | rs9419788 |
| 10 | 96.01462 | Migraine | rs10786156 |
| 10 | 96.01903 | Migraine | rs75473620 |
| 10 | 96.03598 | Sudden cardiac arrest | rs11187837 |
| 10 | 96.03631 | Vertical cup–disc ratio | rs7072574 |
| 10 | 96.05830 | Dengue shock syndrome | rs3765524 |
| 10 | 96.05830 | Esophageal cancer and gastric cancer | rs3765524 |
| 10 | 96.05864 | Lifetime average cigarettes per day in chronic obstructive pulmonary disease | rs117607728 |
| 10 | 96.06634 | Esophageal cancer | rs2274223 |
| 10 | 96.06634 | Esophageal squamous cell carcinoma | rs2274223 |
| 10 | 96.07037 | Esophageal cancer and gastric cancer | rs3781264 |
| 10 | 96.40533 | Acenocoumarol maintenance dosage | rs12772169 |
| 10 | 96.40550 | Response to clopidogrel therapy | rs12777823 |
| 10 | 96.40550 | Warfarin maintenance dose | rs12777823 |

# NODEamps25_0021

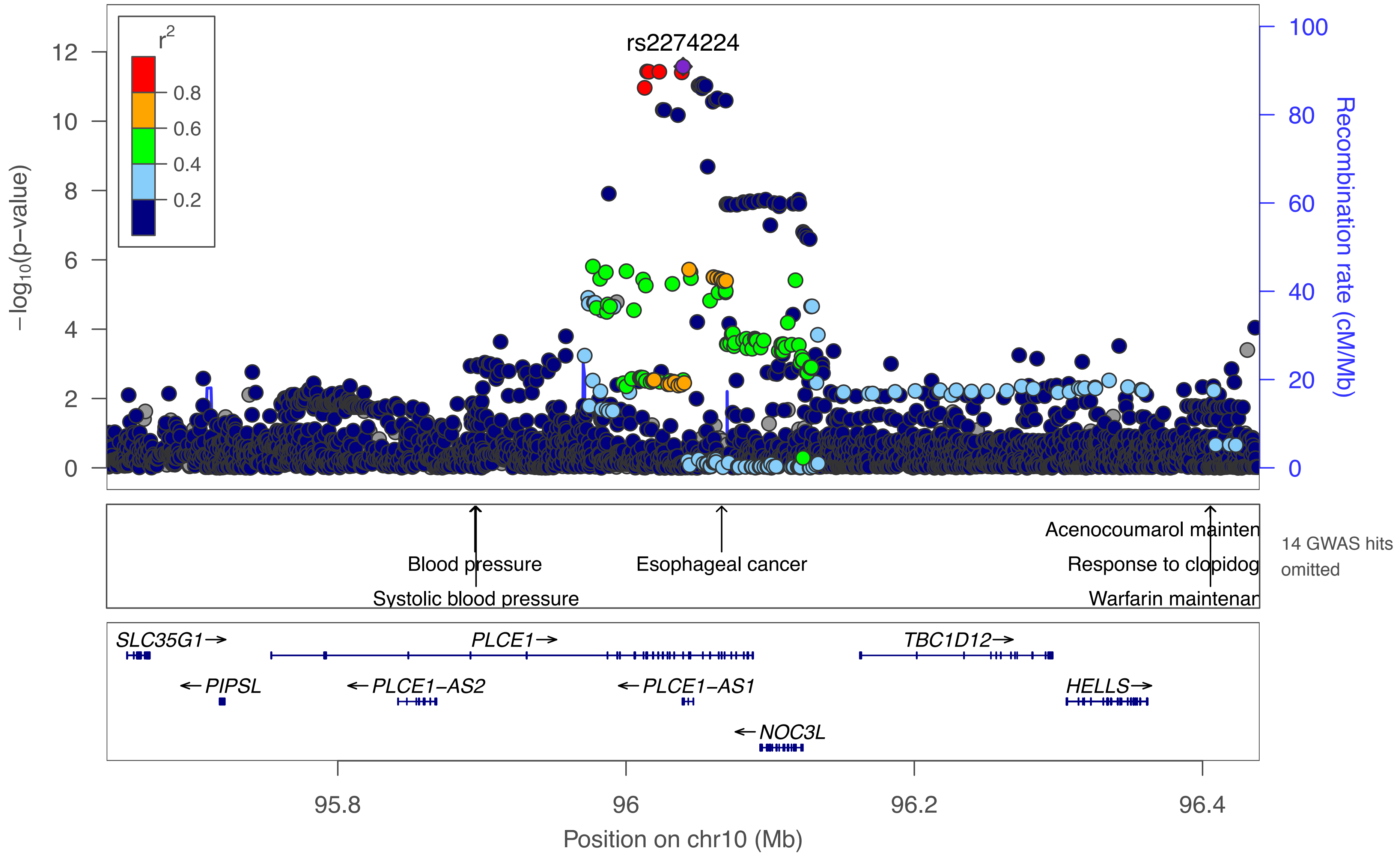

| | |
|---|---|
| date: | Thu Aug 17 18:49:43 2017 |
| build: | hg19 |
| display range: | chr10:95639597−96439597 [95639597−96439597] |
| hilite range: | 0 – 0 [ 0 – 0 ] |
| reference SNP: | chr10:96039597 |
| number of SNPs plotted: | 3187 |
| min P.value: | 2.65E−12 [chr10:96039597] |
| max P.value: | 10E−1 [chr10:96036405] |
| omitted GWAS Hits: | chr10:96.066341−Esophageal cancer, chr10:96.405502−Response to clopidogrel therapy |
| omitted GWAS Hits: | NA, NA |
| omitted GWAS Hits: | NA, NA |
| omitted GWAS Hits: | NA, NA |
| omitted GWAS Hits: | NA, NA |

Make more plots at http://csg.sph.umich.edu/locuszoom/

GWAS Catalog SNPs in Region

| chr | pos (Mb) | trait | snp |
|---|---|---|---|
| 10 | 95.65996 | Cannabis dependence | rs146091982 |
| 10 | 95.89518 | Blood pressure | rs9663362 |
| 10 | 95.89594 | Systolic blood pressure | rs932764 |
| 10 | 95.89594 | Diastolic blood pressure | rs932764 |
| 10 | 95.89594 | Hypertension | rs932764 |
| 10 | 95.90632 | Glucose homeostasis traits | rs1223629 |
| 10 | 96.01371 | Personality traits in bipolar disorder | rs9419788 |
| 10 | 96.01462 | Migraine | rs10786156 |
| 10 | 96.01903 | Migraine | rs75473620 |
| 10 | 96.03598 | Sudden cardiac arrest | rs11187837 |
| 10 | 96.03631 | Vertical cup−disc ratio | rs7072574 |
| 10 | 96.05830 | Dengue shock syndrome | rs3765524 |
| 10 | 96.05830 | Esophageal cancer and gastric cancer | rs3765524 |
| 10 | 96.05864 | Lifetime average cigarettes per day in chronic obstructive pulmonary disease | rs117607728 |
| 10 | 96.06634 | Esophageal cancer | rs2274223 |
| 10 | 96.06634 | Esophageal squamous cell carcinoma | rs2274223 |
| 10 | 96.07037 | Esophageal cancer and gastric cancer | rs3781264 |
| 10 | 96.40533 | Acenocoumarol maintenance dosage | rs12772169 |
| 10 | 96.40550 | Response to clopidogrel therapy | rs12777823 |
| 10 | 96.40550 | Warfarin maintenance dose | rs12777823 |

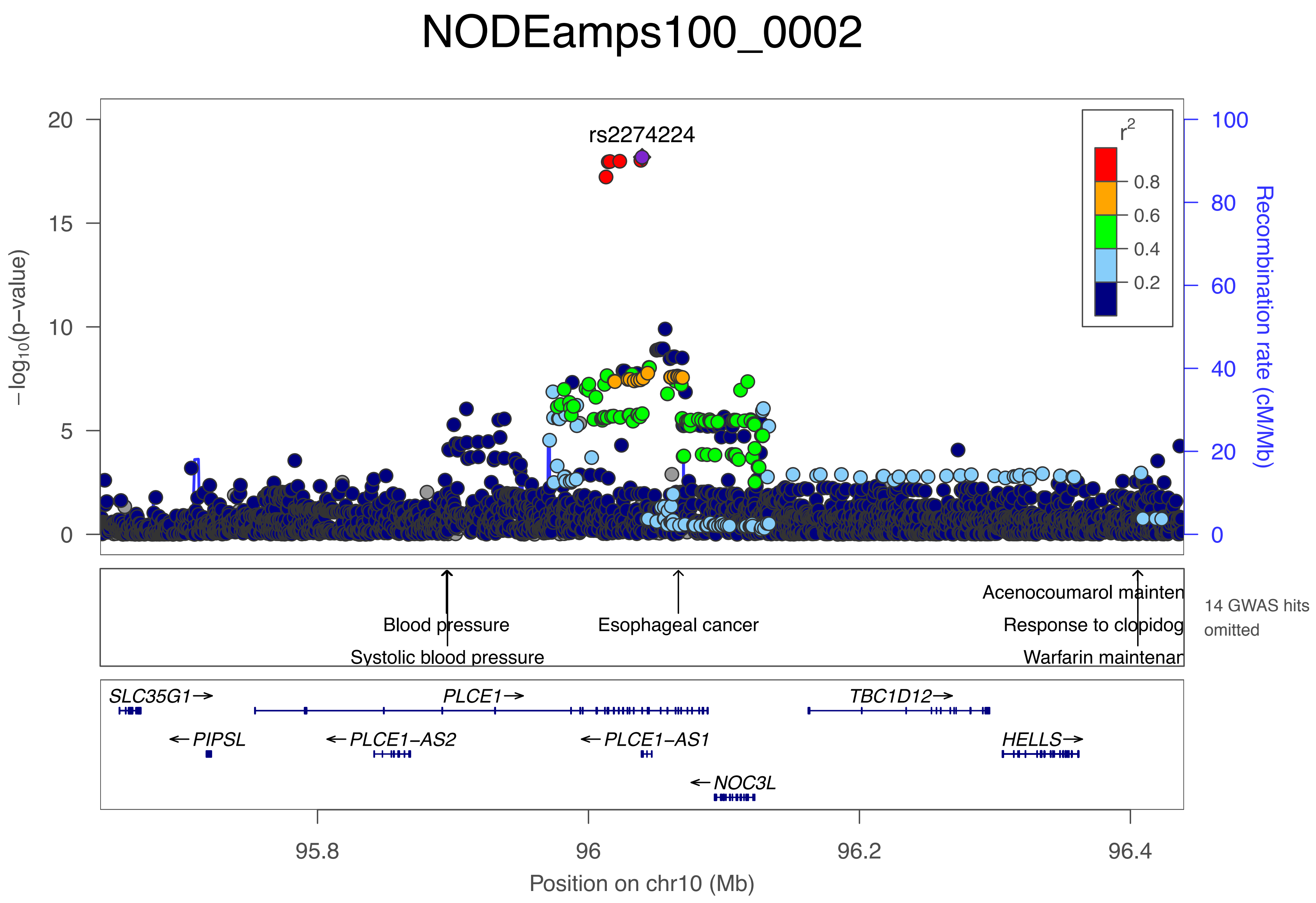

NODEamps100_0002
rs2274224
−log10(p−value)
Recombination rate (cM/Mb)
r2
0.8
0.6
0.4
0.2
20
15
10
5
0
100
80
60
40
20
0
Blood pressure
Systolic blood pressure
Esophageal cancer
Acenocoumarol mainten
Response to clopidog
Warfarin maintenar
14 GWAS hits omitted
SLC35G1→
PLCE1→
TBC1D12→
←PIPSL
←PLCE1−AS2
←PLCE1−AS1
HELLS→
←NOC3L
95.8
96
96.2
96.4
Position on chr10 (Mb)

| | |
|---|---|
| date: | Thu Aug 17 18:49:43 2017 |
| build: | hg19 |
| display range: | chr10:95639597−96439597 [95639597−96439597] |
| hilite range: | 0 – 0 [ 0 – 0 ] |
| reference SNP: | chr10:96039597 |
| number of SNPs plotted: | 3187 |
| min P.value: | 6.55E−19 [chr10:96039597] |
| max P.value: | 9.99E−1 [chr10:95693377] |
| omitted GWAS Hits: | chr10:96.066341−Esophageal cancer, chr10:96.405502−Response to clopidogrel therapy |
| omitted GWAS Hits: | NA, NA |
| omitted GWAS Hits: | NA, NA |
| omitted GWAS Hits: | NA, NA |
| omitted GWAS Hits: | NA, NA |

Make more plots at http://csg.sph.umich.edu/locuszoom/

## GWAS Catalog SNPs in Region

| chr | pos (Mb) | trait | snp |
|---|---|---|---|
| 10 | 95.65996 | Cannabis dependence | rs146091982 |
| 10 | 95.89518 | Blood pressure | rs9663362 |
| 10 | 95.89594 | Systolic blood pressure | rs932764 |
| 10 | 95.89594 | Diastolic blood pressure | rs932764 |
| 10 | 95.89594 | Hypertension | rs932764 |
| 10 | 95.90632 | Glucose homeostasis traits | rs1223629 |
| 10 | 96.01371 | Personality traits in bipolar disorder | rs9419788 |
| 10 | 96.01462 | Migraine | rs10786156 |
| 10 | 96.01903 | Migraine | rs75473620 |
| 10 | 96.03598 | Sudden cardiac arrest | rs11187837 |
| 10 | 96.03631 | Vertical cup–disc ratio | rs7072574 |
| 10 | 96.05830 | Dengue shock syndrome | rs3765524 |
| 10 | 96.05830 | Esophageal cancer and gastric cancer | rs3765524 |
| 10 | 96.05864 | Lifetime average cigarettes per day in chronic obstructive pulmonary disease | rs117607728 |
| 10 | 96.06634 | Esophageal cancer | rs2274223 |
| 10 | 96.06634 | Esophageal squamous cell carcinoma | rs2274223 |
| 10 | 96.07037 | Esophageal cancer and gastric cancer | rs3781264 |
| 10 | 96.40533 | Acenocoumarol maintenance dosage | rs12772169 |
| 10 | 96.40550 | Response to clopidogrel therapy | rs12777823 |
| 10 | 96.40550 | Warfarin maintenance dose | rs12777823 |

# NODEamps100_0012

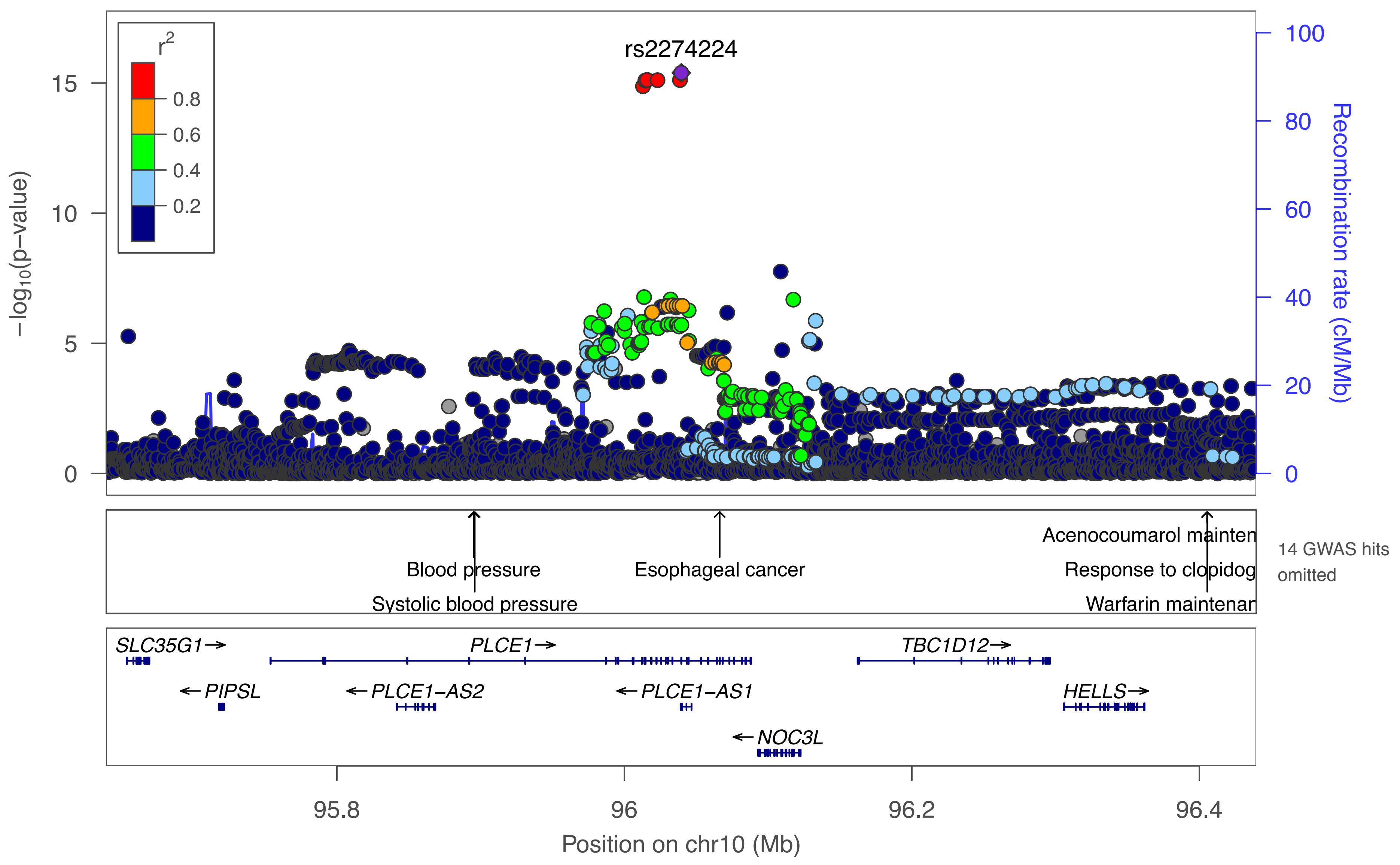

| | |
|---|---|
| date: | Thu Aug 17 18:49:43 2017 |
| build: | hg19 |
| display range: | chr10:95639597−96439597 [95639597−96439597] |
| hilite range: | 0 – 0 [ 0 – 0 ] |
| reference SNP: | chr10:96039597 |
| number of SNPs plotted: | 3187 |
| min P.value: | 4.04E−16 [chr10:96039597] |
| max P.value: | 10E−1 [chr10:95800687] |
| omitted GWAS Hits: | chr10:96.066341−Esophageal cancer, chr10:96.405502−Response to clopidogrel therapy |
| omitted GWAS Hits: | NA, NA |
| omitted GWAS Hits: | NA, NA |
| omitted GWAS Hits: | NA, NA |
| omitted GWAS Hits: | NA, NA |

Make more plots at http://csg.sph.umich.edu/locuszoom/

GWAS Catalog SNPs in Region

| chr | pos (Mb) | trait | snp |
|---|---|---|---|
| 10 | 95.65996 | Cannabis dependence | rs146091982 |
| 10 | 95.89518 | Blood pressure | rs9663362 |
| 10 | 95.89594 | Systolic blood pressure | rs932764 |
| 10 | 95.89594 | Diastolic blood pressure | rs932764 |
| 10 | 95.89594 | Hypertension | rs932764 |
| 10 | 95.90632 | Glucose homeostasis traits | rs1223629 |
| 10 | 96.01371 | Personality traits in bipolar disorder | rs9419788 |
| 10 | 96.01462 | Migraine | rs10786156 |
| 10 | 96.01903 | Migraine | rs75473620 |
| 10 | 96.03598 | Sudden cardiac arrest | rs11187837 |
| 10 | 96.03631 | Vertical cup−disc ratio | rs7072574 |
| 10 | 96.05830 | Dengue shock syndrome | rs3765524 |
| 10 | 96.05830 | Esophageal cancer and gastric cancer | rs3765524 |
| 10 | 96.05864 | Lifetime average cigarettes per day in chronic obstructive pulmonary disease | rs117607728 |
| 10 | 96.06634 | Esophageal cancer | rs2274223 |
| 10 | 96.06634 | Esophageal squamous cell carcinoma | rs2274223 |
| 10 | 96.07037 | Esophageal cancer and gastric cancer | rs3781264 |
| 10 | 96.40533 | Acenocoumarol maintenance dosage | rs12772169 |
| 10 | 96.40550 | Response to clopidogrel therapy | rs12777823 |
| 10 | 96.40550 | Warfarin maintenance dose | rs12777823 |

# NODEamps100_0015

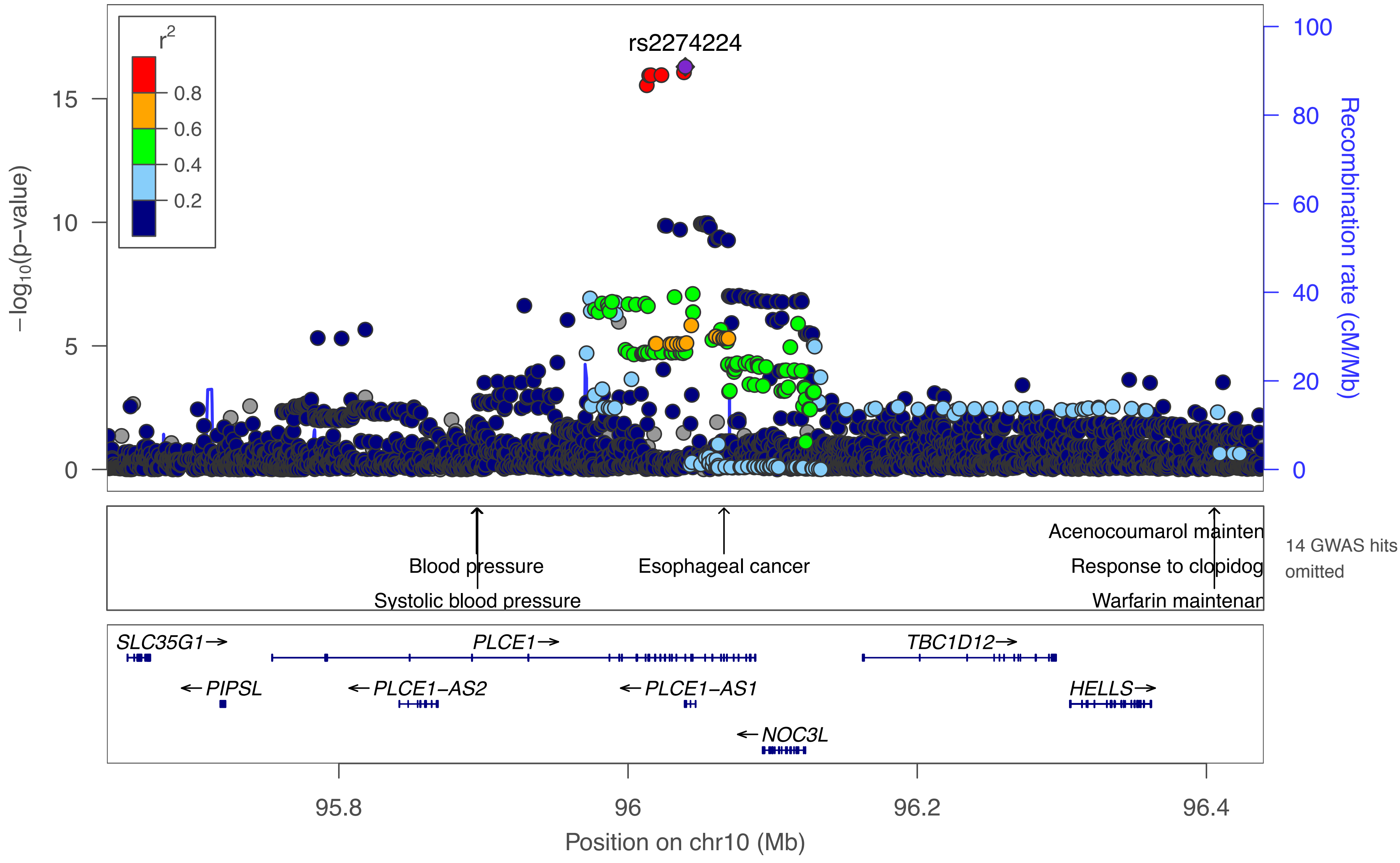

| | |
|---|---|
| date: | Thu Aug 17 18:43:21 2017 |
| build: | hg19 |
| display range: | chr10:95639597−96439597 [95639597−96439597] |
| hilite range: | 0 – 0 [ 0 – 0 ] |
| reference SNP: | chr10:96039597 |
| number of SNPs plotted: | 3187 |
| min P.value: | 5.11E−17 [chr10:96039597] |
| max P.value: | 10E−1 [chr10:95769538] |
| omitted GWAS Hits: | chr10:96.066341−Esophageal cancer, chr10:96.405502−Response to clopidogrel therapy |
| omitted GWAS Hits: | NA, NA |
| omitted GWAS Hits: | NA, NA |
| omitted GWAS Hits: | NA, NA |
| omitted GWAS Hits: | NA, NA |

Make more plots at http://csg.sph.umich.edu/locuszoom/

GWAS Catalog SNPs in Region

| chr | pos (Mb) | trait | snp |
|---|---|---|---|
| 10 | 95.65996 | Cannabis dependence | rs146091982 |
| 10 | 95.89518 | Blood pressure | rs9663362 |
| 10 | 95.89594 | Systolic blood pressure | rs932764 |
| 10 | 95.89594 | Diastolic blood pressure | rs932764 |
| 10 | 95.89594 | Hypertension | rs932764 |
| 10 | 95.90632 | Glucose homeostasis traits | rs1223629 |
| 10 | 96.01371 | Personality traits in bipolar disorder | rs9419788 |
| 10 | 96.01462 | Migraine | rs10786156 |
| 10 | 96.01903 | Migraine | rs75473620 |
| 10 | 96.03598 | Sudden cardiac arrest | rs11187837 |
| 10 | 96.03631 | Vertical cup−disc ratio | rs7072574 |
| 10 | 96.05830 | Dengue shock syndrome | rs3765524 |
| 10 | 96.05830 | Esophageal cancer and gastric cancer | rs3765524 |
| 10 | 96.05864 | Lifetime average cigarettes per day in chronic obstructive pulmonary disease | rs117607728 |
| 10 | 96.06634 | Esophageal cancer | rs2274223 |
| 10 | 96.06634 | Esophageal squamous cell carcinoma | rs2274223 |
| 10 | 96.07037 | Esophageal cancer and gastric cancer | rs3781264 |
| 10 | 96.40533 | Acenocoumarol maintenance dosage | rs12772169 |
| 10 | 96.40550 | Response to clopidogrel therapy | rs12777823 |
| 10 | 96.40550 | Warfarin maintenance dose | rs12777823 |

# NODEamps100_0019

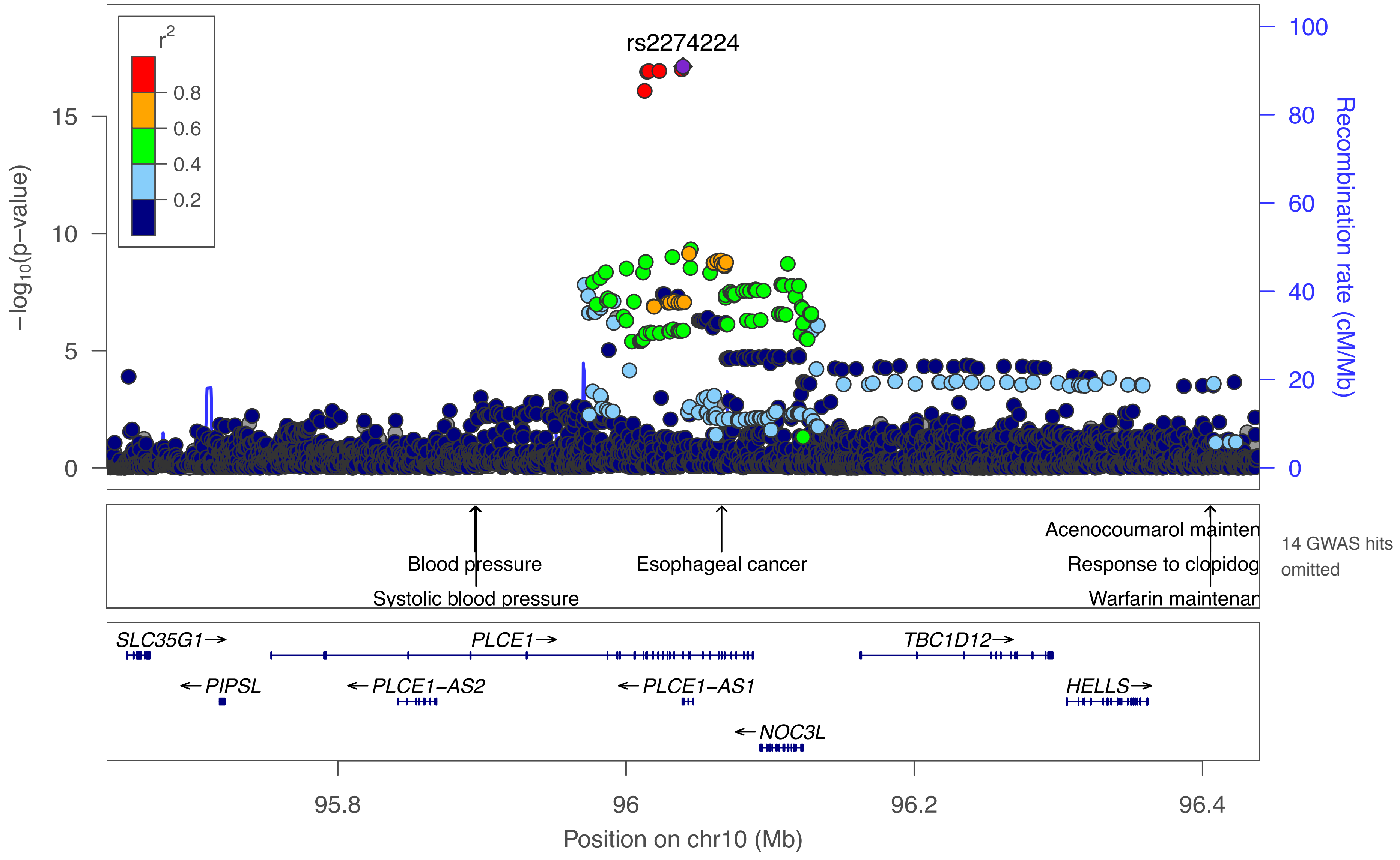

| | |
|---|---|
| date: | Thu Aug 17 18:47:36 2017 |
| build: | hg19 |
| display range: | chr10:95639597−96439597 [95639597−96439597] |
| hilite range: | 0 – 0 [ 0 – 0 ] |
| reference SNP: | chr10:96039597 |
| number of SNPs plotted: | 3187 |
| min P.value: | 7.59E−18 [chr10:96039597] |
| max P.value: | 9.99E−1 [chr10:96193476] |
| omitted GWAS Hits: | chr10:96.066341−Esophageal cancer, chr10:96.405502−Response to clopidogrel therapy |
| omitted GWAS Hits: | NA, NA |
| omitted GWAS Hits: | NA, NA |
| omitted GWAS Hits: | NA, NA |
| omitted GWAS Hits: | NA, NA |

Make more plots at http://csg.sph.umich.edu/locuszoom/

GWAS Catalog SNPs in Region

| chr | pos (Mb) | trait | snp |
|---|---|---|---|
| 10 | 95.65996 | Cannabis dependence | rs146091982 |
| 10 | 95.89518 | Blood pressure | rs9663362 |
| 10 | 95.89594 | Systolic blood pressure | rs932764 |
| 10 | 95.89594 | Diastolic blood pressure | rs932764 |
| 10 | 95.89594 | Hypertension | rs932764 |
| 10 | 95.90632 | Glucose homeostasis traits | rs1223629 |
| 10 | 96.01371 | Personality traits in bipolar disorder | rs9419788 |
| 10 | 96.01462 | Migraine | rs10786156 |
| 10 | 96.01903 | Migraine | rs75473620 |
| 10 | 96.03598 | Sudden cardiac arrest | rs11187837 |
| 10 | 96.03631 | Vertical cup–disc ratio | rs7072574 |
| 10 | 96.05830 | Dengue shock syndrome | rs3765524 |
| 10 | 96.05830 | Esophageal cancer and gastric cancer | rs3765524 |
| 10 | 96.05864 | Lifetime average cigarettes per day in chronic obstructive pulmonary disease | rs117607728 |
| 10 | 96.06634 | Esophageal cancer | rs2274223 |
| 10 | 96.06634 | Esophageal squamous cell carcinoma | rs2274223 |
| 10 | 96.07037 | Esophageal cancer and gastric cancer | rs3781264 |
| 10 | 96.40533 | Acenocoumarol maintenance dosage | rs12772169 |
| 10 | 96.40550 | Response to clopidogrel therapy | rs12777823 |
| 10 | 96.40550 | Warfarin maintenance dose | rs12777823 |

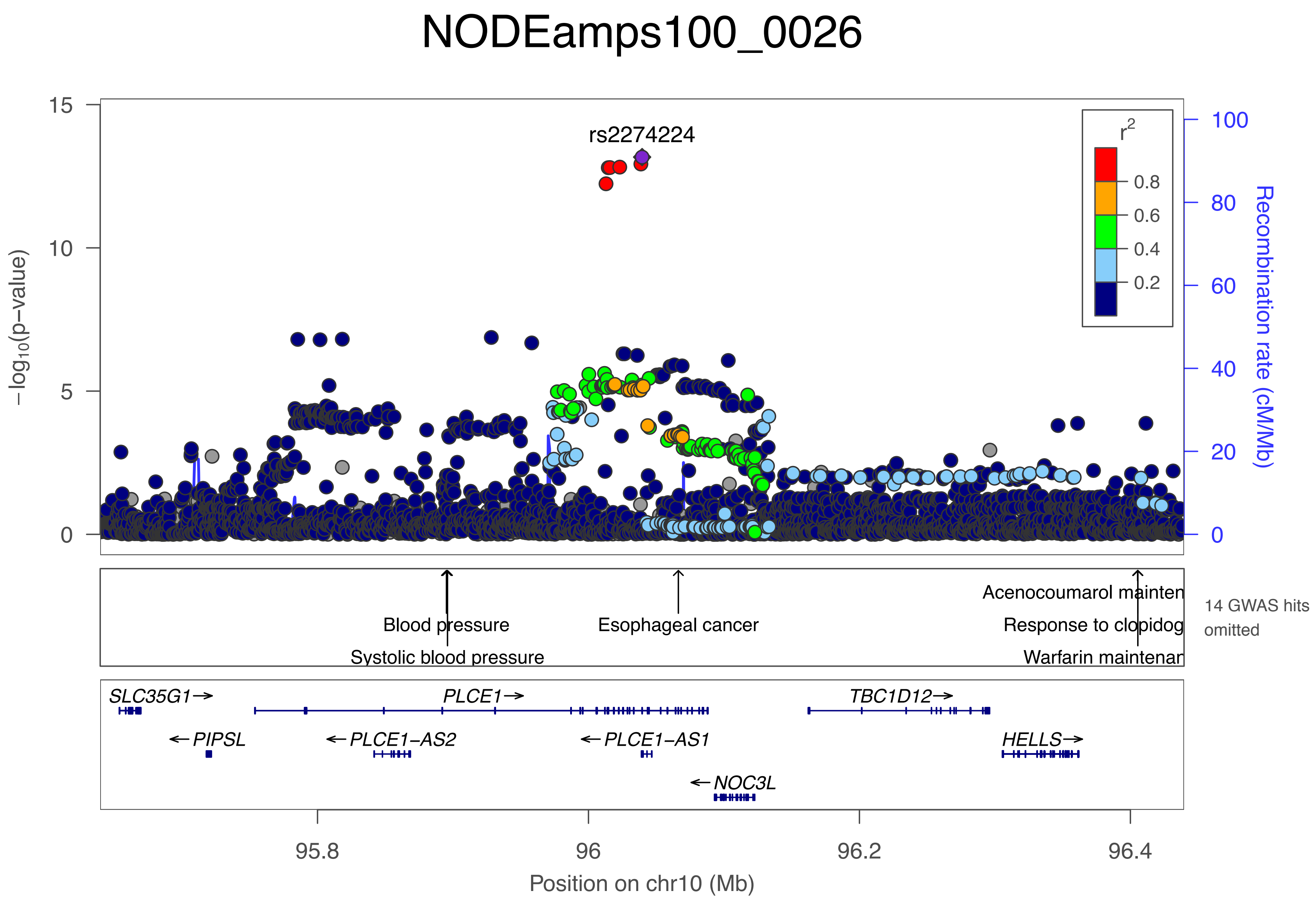

NODEamps100_0026
rs2274224
−log10(p−value)
Recombination rate (cM/Mb)
r2
0.8
0.6
0.4
0.2
Blood pressure
Systolic blood pressure
Esophageal cancer
Acenocoumarol mainten
Response to clopidog
Warfarin maintenar
14 GWAS hits omitted
SLC35G1→
PLCE1→
TBC1D12→
←PIPSL
←PLCE1−AS2
←PLCE1−AS1
HELLS→
←NOC3L
95.8
96
96.2
96.4
Position on chr10 (Mb)

| | |
|---|---|
| date: | Thu Aug 17 18:43:22 2017 |
| build: | hg19 |
| display range: | chr10:95639597−96439597 [95639597−96439597] |
| hilite range: | 0 – 0 [ 0 – 0 ] |
| reference SNP: | chr10:96039597 |
| number of SNPs plotted: | 3187 |
| min P.value: | 6.79E−14 [chr10:96039597] |
| max P.value: | 10E−1 [chr10:96106408] |
| omitted GWAS Hits: | chr10:96.066341−Esophageal cancer, chr10:96.405502−Response to clopidogrel therapy |
| omitted GWAS Hits: | NA, NA |
| omitted GWAS Hits: | NA, NA |
| omitted GWAS Hits: | NA, NA |
| omitted GWAS Hits: | NA, NA |

Make more plots at http://csg.sph.umich.edu/locuszoom/

GWAS Catalog SNPs in Region

| chr | pos (Mb) | trait | snp |
|---|---|---|---|
| 10 | 95.65996 | Cannabis dependence | rs146091982 |
| 10 | 95.89518 | Blood pressure | rs9663362 |
| 10 | 95.89594 | Systolic blood pressure | rs932764 |
| 10 | 95.89594 | Diastolic blood pressure | rs932764 |
| 10 | 95.89594 | Hypertension | rs932764 |
| 10 | 95.90632 | Glucose homeostasis traits | rs1223629 |
| 10 | 96.01371 | Personality traits in bipolar disorder | rs9419788 |
| 10 | 96.01462 | Migraine | rs10786156 |
| 10 | 96.01903 | Migraine | rs75473620 |
| 10 | 96.03598 | Sudden cardiac arrest | rs11187837 |
| 10 | 96.03631 | Vertical cup–disc ratio | rs7072574 |
| 10 | 96.05830 | Dengue shock syndrome | rs3765524 |
| 10 | 96.05830 | Esophageal cancer and gastric cancer | rs3765524 |
| 10 | 96.05864 | Lifetime average cigarettes per day in chronic obstructive pulmonary disease | rs117607728 |
| 10 | 96.06634 | Esophageal cancer | rs2274223 |
| 10 | 96.06634 | Esophageal squamous cell carcinoma | rs2274223 |
| 10 | 96.07037 | Esophageal cancer and gastric cancer | rs3781264 |
| 10 | 96.40533 | Acenocoumarol maintenance dosage | rs12772169 |
| 10 | 96.40550 | Response to clopidogrel therapy | rs12777823 |
| 10 | 96.40550 | Warfarin maintenance dose | rs12777823 |

# NODEamps100_0035

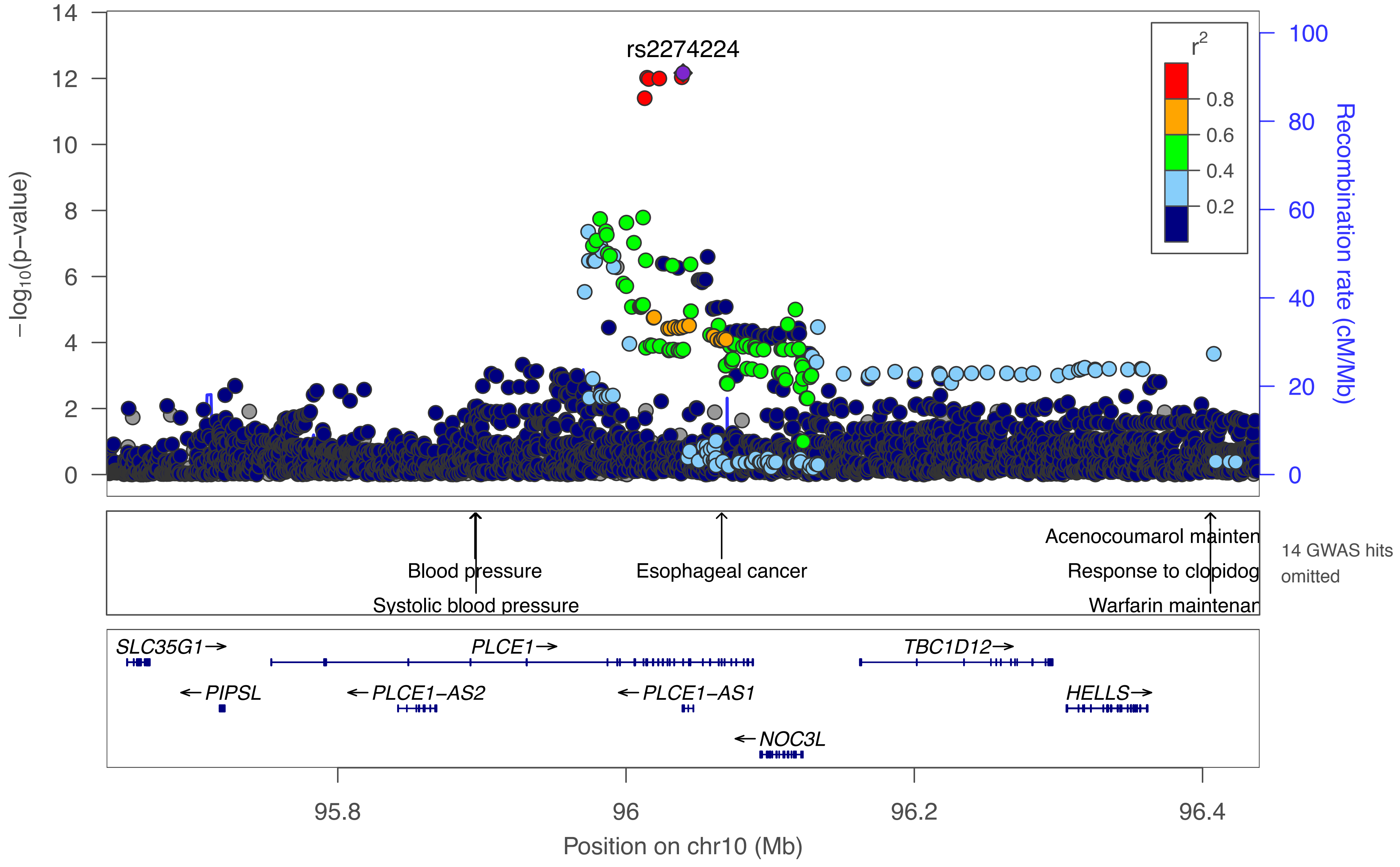

| | |
|---|---|
| date: | Thu Aug 17 18:47:34 2017 |
| build: | hg19 |
| display range: | chr10:95639597−96439597 [95639597−96439597] |
| hilite range: | 0 – 0 [ 0 – 0 ] |
| reference SNP: | chr10:96039597 |
| number of SNPs plotted: | 3187 |
| min P.value: | 6.84E−13 [chr10:96039597] |
| max P.value: | 9.99E−1 [chr10:96380933] |
| omitted GWAS Hits: | chr10:96.066341−Esophageal cancer, chr10:96.405502−Response to clopidogrel therapy |
| omitted GWAS Hits: | NA, NA |
| omitted GWAS Hits: | NA, NA |
| omitted GWAS Hits: | NA, NA |
| omitted GWAS Hits: | NA, NA |

Make more plots at http://csg.sph.umich.edu/locuszoom/

GWAS Catalog SNPs in Region

| chr | pos (Mb) | trait | snp |
|---|---|---|---|
| 10 | 95.65996 | Cannabis dependence | rs146091982 |
| 10 | 95.89518 | Blood pressure | rs9663362 |
| 10 | 95.89594 | Systolic blood pressure | rs932764 |
| 10 | 95.89594 | Diastolic blood pressure | rs932764 |
| 10 | 95.89594 | Hypertension | rs932764 |
| 10 | 95.90632 | Glucose homeostasis traits | rs1223629 |
| 10 | 96.01371 | Personality traits in bipolar disorder | rs9419788 |
| 10 | 96.01462 | Migraine | rs10786156 |
| 10 | 96.01903 | Migraine | rs75473620 |
| 10 | 96.03598 | Sudden cardiac arrest | rs11187837 |
| 10 | 96.03631 | Vertical cup–disc ratio | rs7072574 |
| 10 | 96.05830 | Dengue shock syndrome | rs3765524 |
| 10 | 96.05830 | Esophageal cancer and gastric cancer | rs3765524 |
| 10 | 96.05864 | Lifetime average cigarettes per day in chronic obstructive pulmonary disease | rs117607728 |
| 10 | 96.06634 | Esophageal cancer | rs2274223 |
| 10 | 96.06634 | Esophageal squamous cell carcinoma | rs2274223 |
| 10 | 96.07037 | Esophageal cancer and gastric cancer | rs3781264 |
| 10 | 96.40533 | Acenocoumarol maintenance dosage | rs12772169 |
| 10 | 96.40550 | Response to clopidogrel therapy | rs12777823 |
| 10 | 96.40550 | Warfarin maintenance dose | rs12777823 |

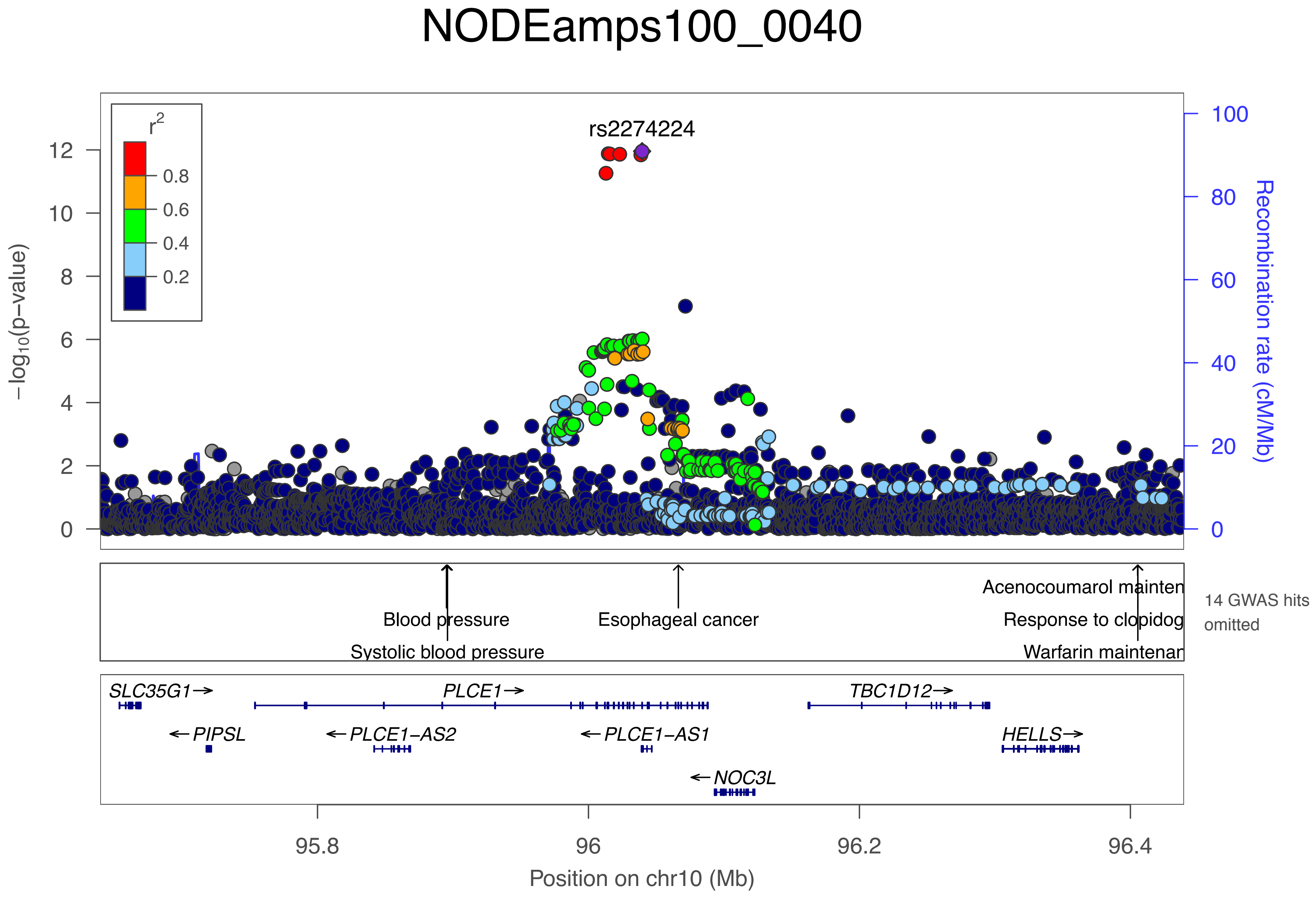
NODEamps100_0040
rs2274224
r2
0.8
0.6
0.4
0.2
12
10
8
6
4
2
0
−log10(p−value)
100
80
60
40
20
0
Recombination rate (cM/Mb)
Blood pressure
Systolic blood pressure
Esophageal cancer
Acenocoumarol mainten
Response to clopidog
Warfarin maintenar
14 GWAS hits omitted
SLC35G1→
PLCE1→
TBC1D12→
←PIPSL
←PLCE1−AS2
←PLCE1−AS1
HELLS→
←NOC3L
95.8
96
96.2
96.4
Position on chr10 (Mb)

| | |
|---|---|
| date: | Thu Aug 17 18:50:05 2017 |
| build: | hg19 |
| display range: | chr10:95639597−96439597 [95639597−96439597] |
| hilite range: | 0 – 0 [ 0 – 0 ] |
| reference SNP: | chr10:96039597 |
| number of SNPs plotted: | 3187 |
| min P.value: | 1.1E−12 [chr10:96039597] |
| max P.value: | 10E−1 [chr10:95829334] |
| omitted GWAS Hits: | chr10:96.066341−Esophageal cancer, chr10:96.405502−Response to clopidogrel therapy |
| omitted GWAS Hits: | NA, NA |
| omitted GWAS Hits: | NA, NA |
| omitted GWAS Hits: | NA, NA |
| omitted GWAS Hits: | NA, NA |

Make more plots at http://csg.sph.umich.edu/locuszoom/

GWAS Catalog SNPs in Region

| chr | pos (Mb) | trait | snp |
|---|---|---|---|
| 10 | 95.65996 | Cannabis dependence | rs146091982 |
| 10 | 95.89518 | Blood pressure | rs9663362 |
| 10 | 95.89594 | Systolic blood pressure | rs932764 |
| 10 | 95.89594 | Diastolic blood pressure | rs932764 |
| 10 | 95.89594 | Hypertension | rs932764 |
| 10 | 95.90632 | Glucose homeostasis traits | rs1223629 |
| 10 | 96.01371 | Personality traits in bipolar disorder | rs9419788 |
| 10 | 96.01462 | Migraine | rs10786156 |
| 10 | 96.01903 | Migraine | rs75473620 |
| 10 | 96.03598 | Sudden cardiac arrest | rs11187837 |
| 10 | 96.03631 | Vertical cup–disc ratio | rs7072574 |
| 10 | 96.05830 | Dengue shock syndrome | rs3765524 |
| 10 | 96.05830 | Esophageal cancer and gastric cancer | rs3765524 |
| 10 | 96.05864 | Lifetime average cigarettes per day in chronic obstructive pulmonary disease | rs117607728 |
| 10 | 96.06634 | Esophageal cancer | rs2274223 |
| 10 | 96.06634 | Esophageal squamous cell carcinoma | rs2274223 |
| 10 | 96.07037 | Esophageal cancer and gastric cancer | rs3781264 |
| 10 | 96.40533 | Acenocoumarol maintenance dosage | rs12772169 |
| 10 | 96.40550 | Response to clopidogrel therapy | rs12777823 |
| 10 | 96.40550 | Warfarin maintenance dose | rs12777823 |

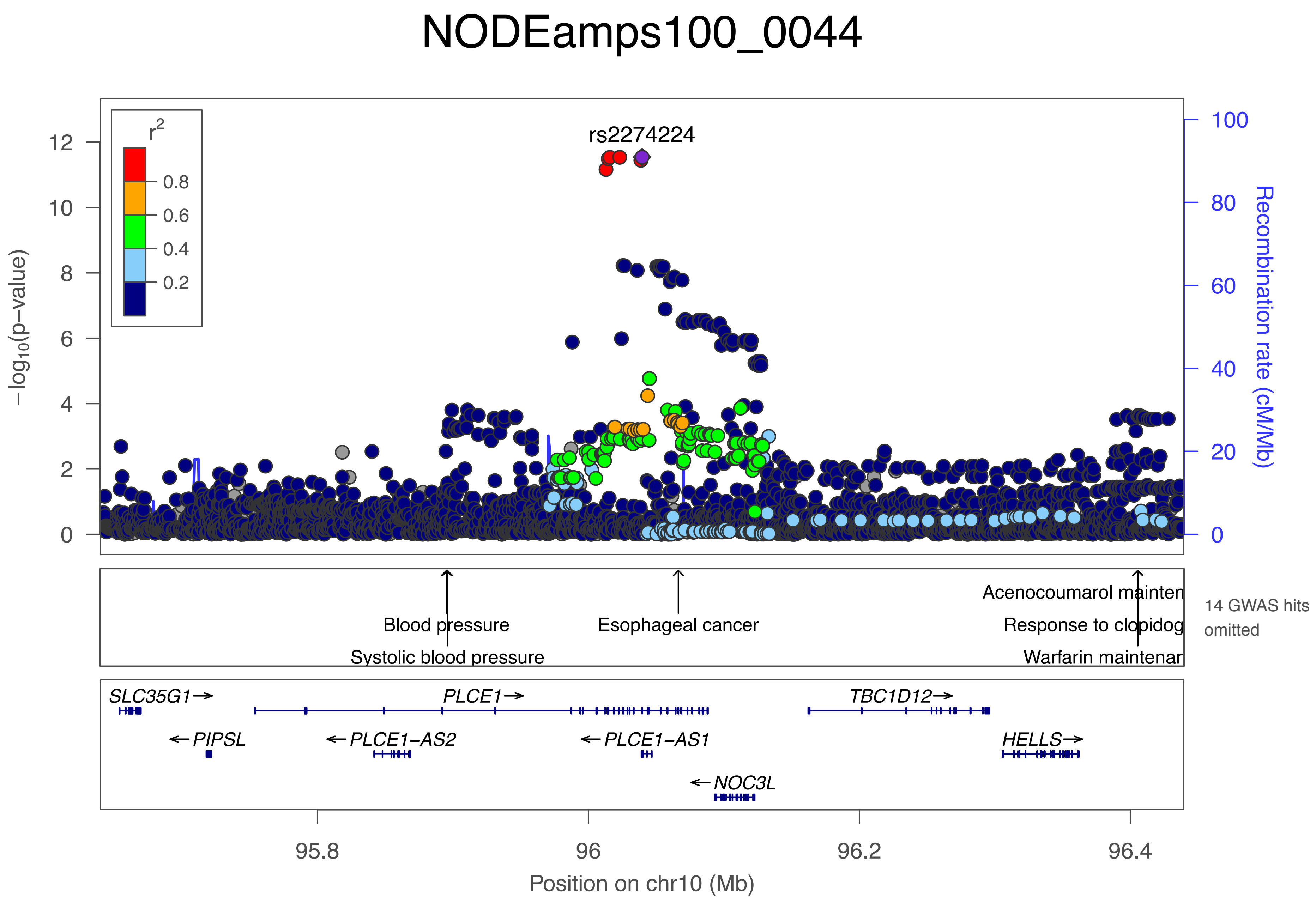

NODEamps100_0044
rs2274224
r2
0.8
0.6
0.4
0.2
−log10(p−value)
Recombination rate (cM/Mb)
12
10
8
6
4
2
0
100
80
60
40
20
0
Blood pressure
Systolic blood pressure
Esophageal cancer
Acenocoumarol mainten
Response to clopidog
Warfarin maintenar
14 GWAS hits omitted
SLC35G1→
PLCE1→
TBC1D12→
←PIPSL
←PLCE1−AS2
←PLCE1−AS1
HELLS→
←NOC3L
95.8
96
96.2
96.4
Position on chr10 (Mb)

| | |
|---|---|
| date: | Thu Aug 17 18:57:13 2017 |
| build: | hg19 |
| display range: | chr10:95639597−96439597 [95639597−96439597] |
| hilite range: | 0 – 0 [ 0 – 0 ] |
| reference SNP: | chr10:96039597 |
| number of SNPs plotted: | 3187 |
| min P.value: | 2.87E−12 [chr10:96039597] |
| max P.value: | 10E−1 [chr10:96178337] |
| omitted GWAS Hits: | chr10:96.066341−Esophageal cancer, chr10:96.405502−Response to clopidogrel therapy |
| omitted GWAS Hits: | NA, NA |
| omitted GWAS Hits: | NA, NA |
| omitted GWAS Hits: | NA, NA |
| omitted GWAS Hits: | NA, NA |

Make more plots at http://csg.sph.umich.edu/locuszoom/

GWAS Catalog SNPs in Region

| chr | pos (Mb) | trait | snp |
|---|---|---|---|
| 10 | 95.65996 | Cannabis dependence | rs146091982 |
| 10 | 95.89518 | Blood pressure | rs9663362 |
| 10 | 95.89594 | Systolic blood pressure | rs932764 |
| 10 | 95.89594 | Diastolic blood pressure | rs932764 |
| 10 | 95.89594 | Hypertension | rs932764 |
| 10 | 95.90632 | Glucose homeostasis traits | rs1223629 |
| 10 | 96.01371 | Personality traits in bipolar disorder | rs9419788 |
| 10 | 96.01462 | Migraine | rs10786156 |
| 10 | 96.01903 | Migraine | rs75473620 |
| 10 | 96.03598 | Sudden cardiac arrest | rs11187837 |
| 10 | 96.03631 | Vertical cup–disc ratio | rs7072574 |
| 10 | 96.05830 | Dengue shock syndrome | rs3765524 |
| 10 | 96.05830 | Esophageal cancer and gastric cancer | rs3765524 |
| 10 | 96.05864 | Lifetime average cigarettes per day in chronic obstructive pulmonary disease | rs117607728 |
| 10 | 96.06634 | Esophageal cancer | rs2274223 |
| 10 | 96.06634 | Esophageal squamous cell carcinoma | rs2274223 |
| 10 | 96.07037 | Esophageal cancer and gastric cancer | rs3781264 |
| 10 | 96.40533 | Acenocoumarol maintenance dosage | rs12772169 |
| 10 | 96.40550 | Response to clopidogrel therapy | rs12777823 |
| 10 | 96.40550 | Warfarin maintenance dose | rs12777823 |

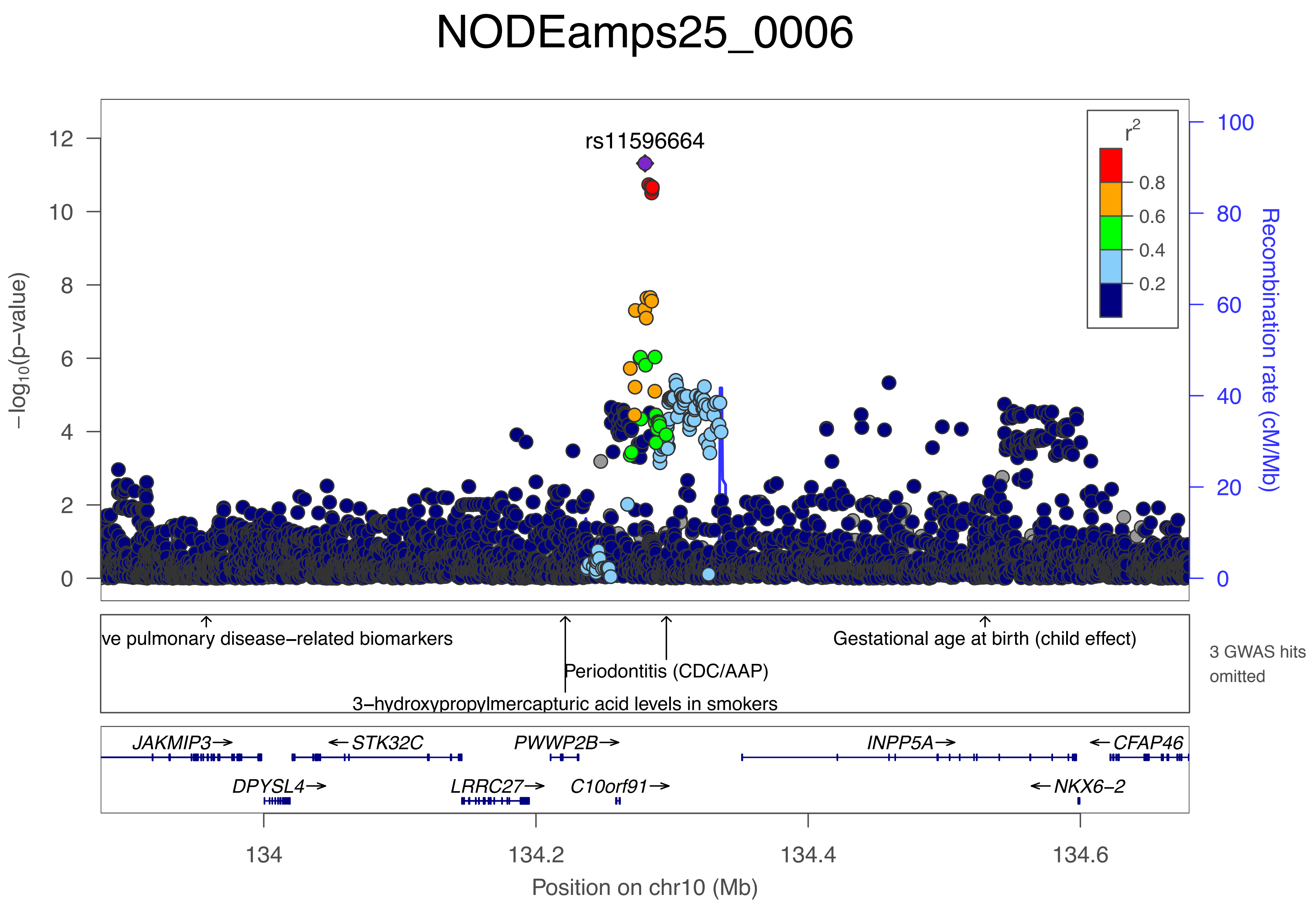

NODEamps25_0006
rs11596664
−log10(p−value)
Recombination rate (cM/Mb)
r2
0.8
0.6
0.4
0.2
ve pulmonary disease−related biomarkers
Periodontitis (CDC/AAP)
3−hydroxypropylmercapturic acid levels in smokers
Gestational age at birth (child effect)
3 GWAS hits omitted
JAKMIP3→
←STK32C
PWWP2B→
INPP5A→
←CFAP46
DPYSL4→
LRRC27→
C10orf91→
←NKX6−2
134
134.2
134.4
134.6
Position on chr10 (Mb)

| | |
|---|---|
| date: | Thu Aug 17 19:17:52 2017 |
| build: | hg19 |
| display range: | chr10:133880157−134680157 [133880157−134680157] |
| hilite range: | 0 – 0 [ 0 – 0 ] |
| reference SNP: | chr10:134280157 |
| number of SNPs plotted: | 5001 |
| min P.value: | 4.84E−12 [chr10:134280157] |
| max P.value: | 10E−1 [chr10:134191867] |
| omitted GWAS Hits: | chr10:134.529972−Gestational age at birth (child effect), NA |

Make more plots at http://csg.sph.umich.edu/locuszoom/

GWAS Catalog SNPs in Region

| chr | pos (Mb) | trait | snp |
|---|---|---|---|
| 10 | 133.9578 | Chronic obstructive pulmonary disease−related biomarkers | rs954820 |
| 10 | 134.2054 | Neuroticism | rs3923857 |
| 10 | 134.2215 | 3−hydroxypropylmercapturic acid levels in smokers | rs74383458 |
| 10 | 134.2215 | 3−hydroxy−1−methylpropylmercapturic acid levels in smokers | rs74383458 |
| 10 | 134.2958 | Periodontitis (CDC/AAP) | rs73389468 |
| 10 | 134.5300 | Gestational age at birth (child effect) | rs873946 |
| 10 | 134.5300 | Gestational age at birth in labor−initiated deliveries (child effect) | rs873946 |

# NODEamps25_0013

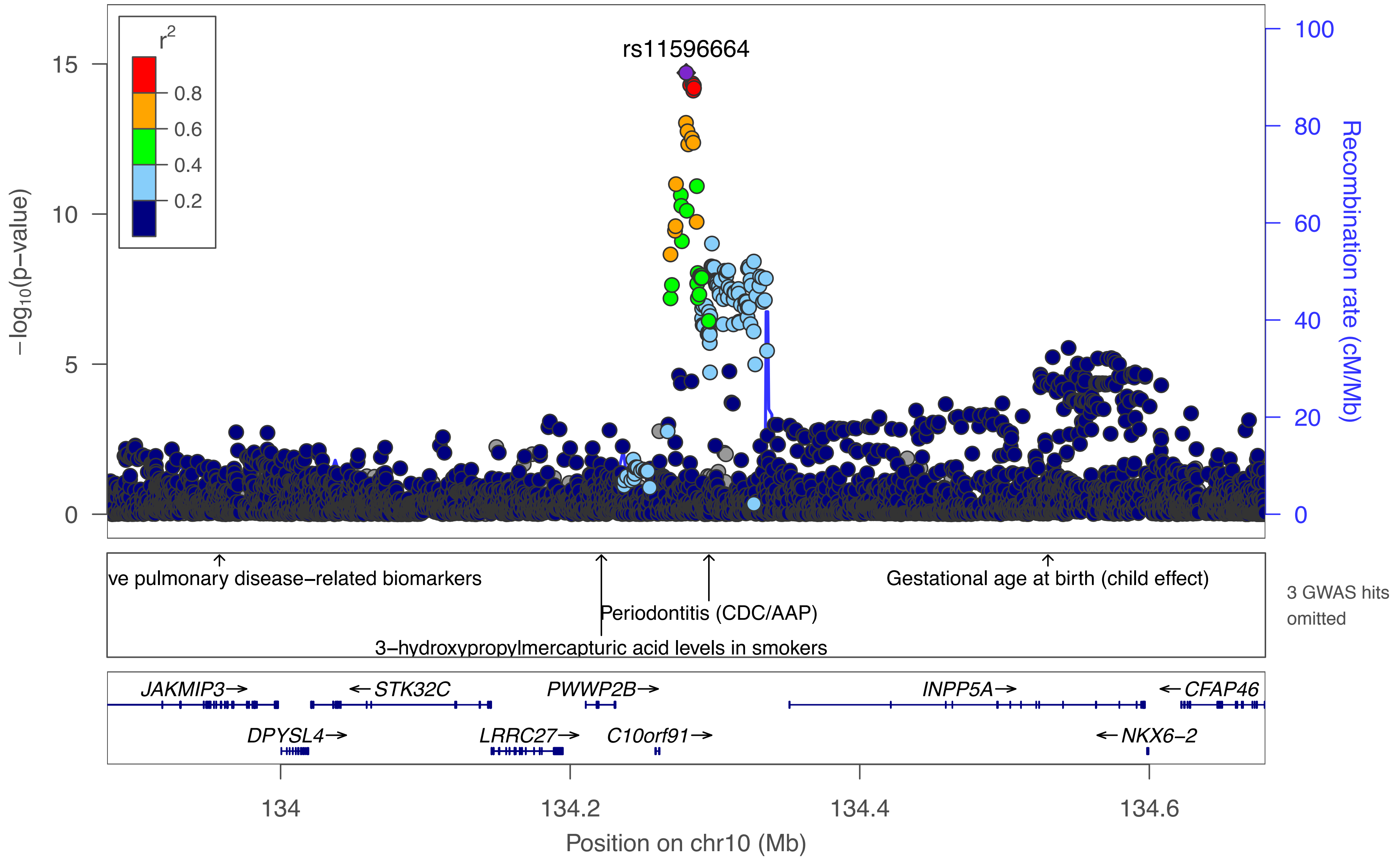

| | |
|---|---|
| date: | Thu Aug 17 19:17:52 2017 |
| build: | hg19 |
| display range: | chr10:133880157−134680157 [133880157−134680157] |
| hilite range: | 0 – 0 [ 0 – 0 ] |
| reference SNP: | chr10:134280157 |
| number of SNPs plotted: | 5001 |
| min P.value: | 1.97E−15 [chr10:134280157] |
| max P.value: | 10E−1 [chr10:134201077] |
| omitted GWAS Hits: | chr10:134.529972−Gestational age at birth (child effect), NA |

Make more plots at http://csg.sph.umich.edu/locuszoom/

GWAS Catalog SNPs in Region

| chr | pos (Mb) | trait | snp |
|---|---|---|---|
| 10 | 133.9578 | Chronic obstructive pulmonary disease−related biomarkers | rs954820 |
| 10 | 134.2054 | Neuroticism | rs3923857 |
| 10 | 134.2215 | 3−hydroxypropylmercapturic acid levels in smokers | rs74383458 |
| 10 | 134.2215 | 3−hydroxy−1−methylpropylmercapturic acid levels in smokers | rs74383458 |
| 10 | 134.2958 | Periodontitis (CDC/AAP) | rs73389468 |
| 10 | 134.5300 | Gestational age at birth (child effect) | rs873946 |
| 10 | 134.5300 | Gestational age at birth in labor−initiated deliveries (child effect) | rs873946 |

# NODEamps25_0016

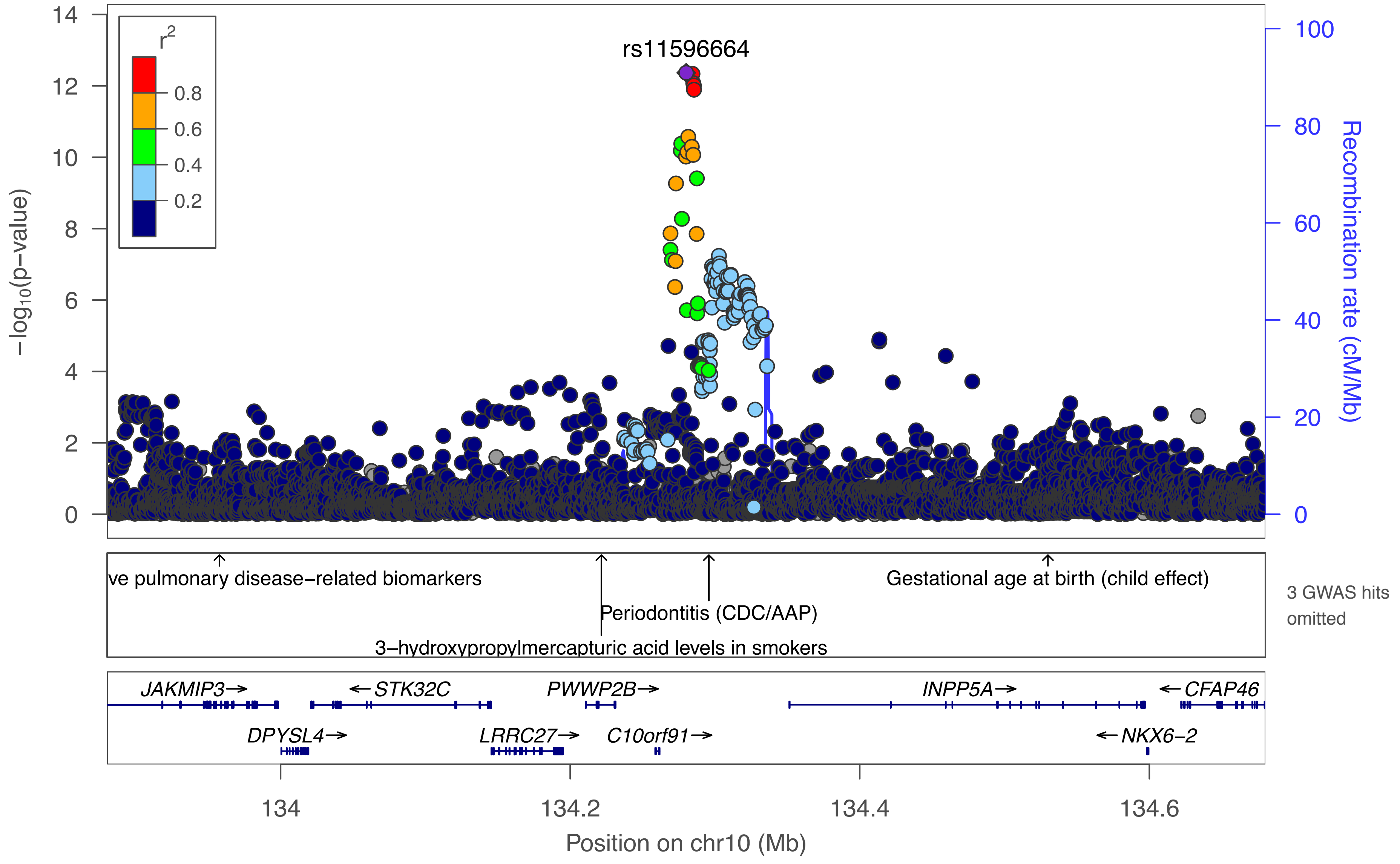

| | |
|---|---|
| date: | Thu Aug 17 19:15:57 2017 |
| build: | hg19 |
| display range: | chr10:133880157−134680157 [133880157−134680157] |
| hilite range: | 0 – 0  [ 0 – 0 ] |
| reference SNP: | chr10:134280157 |
| number of SNPs plotted: | 5001 |
| min P.value: | 4.31E−13 [chr10:134280157] |
| max P.value: | 10E−1 [chr10:134664537] |
| omitted GWAS Hits: | chr10:134.529972−Gestational age at birth (child effect), NA |

Make more plots at http://csg.sph.umich.edu/locuszoom/

GWAS Catalog SNPs in Region

| chr | pos (Mb) | trait | snp |
|---|---|---|---|
| 10 | 133.9578 | Chronic obstructive pulmonary disease−related biomarkers | rs954820 |
| 10 | 134.2054 | Neuroticism | rs3923857 |
| 10 | 134.2215 | 3−hydroxypropylmercapturic acid levels in smokers | rs74383458 |
| 10 | 134.2215 | 3−hydroxy−1−methylpropylmercapturic acid levels in smokers | rs74383458 |
| 10 | 134.2958 | Periodontitis (CDC/AAP) | rs73389468 |
| 10 | 134.5300 | Gestational age at birth (child effect) | rs873946 |
| 10 | 134.5300 | Gestational age at birth in labor−initiated deliveries (child effect) | rs873946 |

# NODEamps25_0021

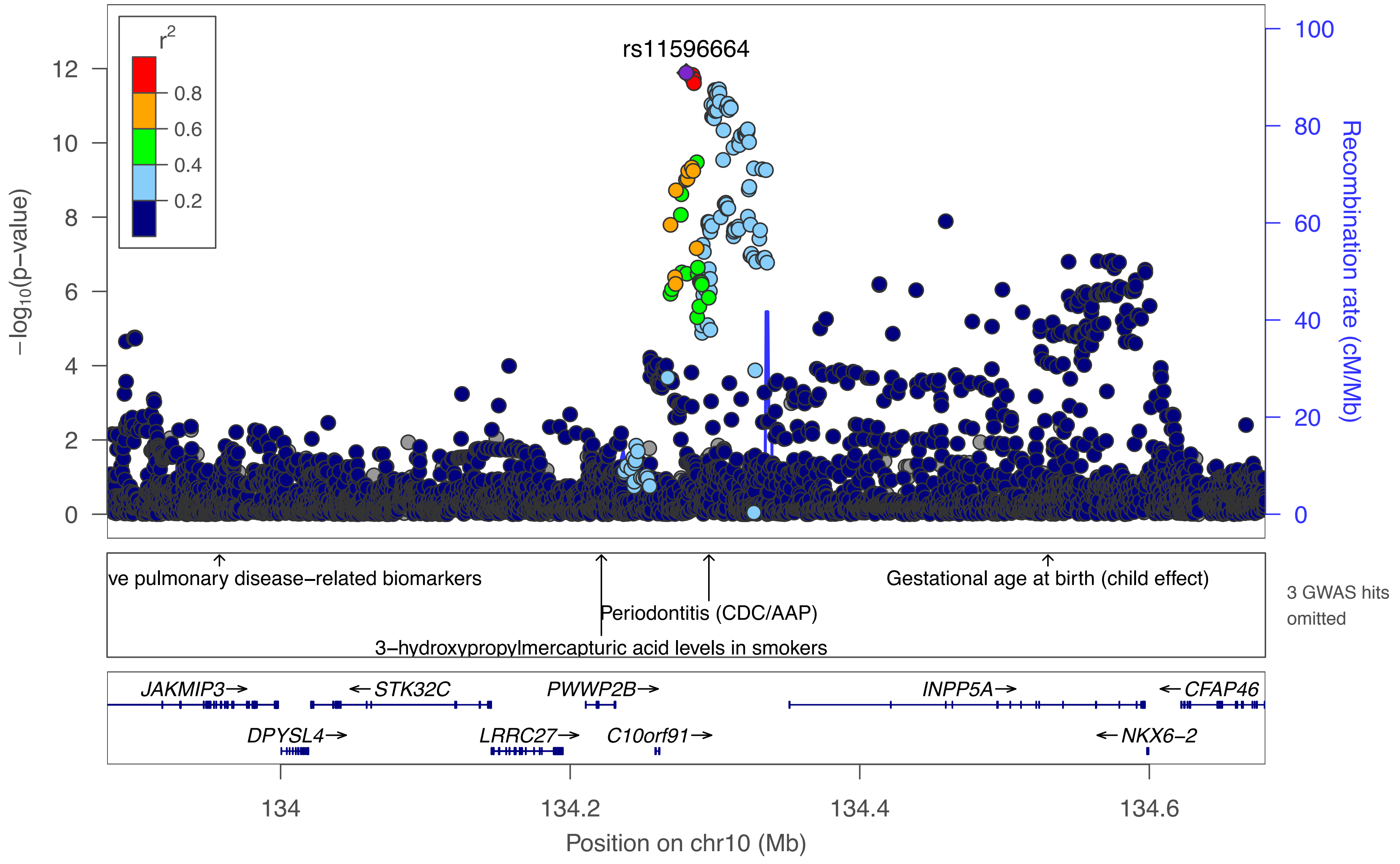

| | |
|---|---|
| date: | Thu Aug 17 19:15:57 2017 |
| build: | hg19 |
| display range: | chr10:133880157−134680157 [133880157−134680157] |
| hilite range: | 0 – 0 [ 0 – 0 ] |
| reference SNP: | chr10:134280157 |
| number of SNPs plotted: | 5001 |
| min P.value: | 1.3E−12 [chr10:134280157] |
| max P.value: | 9.99E−1 [chr10:133935352] |
| omitted GWAS Hits: | chr10:134.529972−Gestational age at birth (child effect), NA |

Make more plots at http://csg.sph.umich.edu/locuszoom/

GWAS Catalog SNPs in Region

| chr | pos (Mb) | trait | snp |
|---|---|---|---|
| 10 | 133.9578 | Chronic obstructive pulmonary disease–related biomarkers | rs954820 |
| 10 | 134.2054 | Neuroticism | rs3923857 |
| 10 | 134.2215 | 3–hydroxypropylmercapturic acid levels in smokers | rs74383458 |
| 10 | 134.2215 | 3–hydroxy–1–methylpropylmercapturic acid levels in smokers | rs74383458 |
| 10 | 134.2958 | Periodontitis (CDC/AAP) | rs73389468 |
| 10 | 134.5300 | Gestational age at birth (child effect) | rs873946 |
| 10 | 134.5300 | Gestational age at birth in labor–initiated deliveries (child effect) | rs873946 |

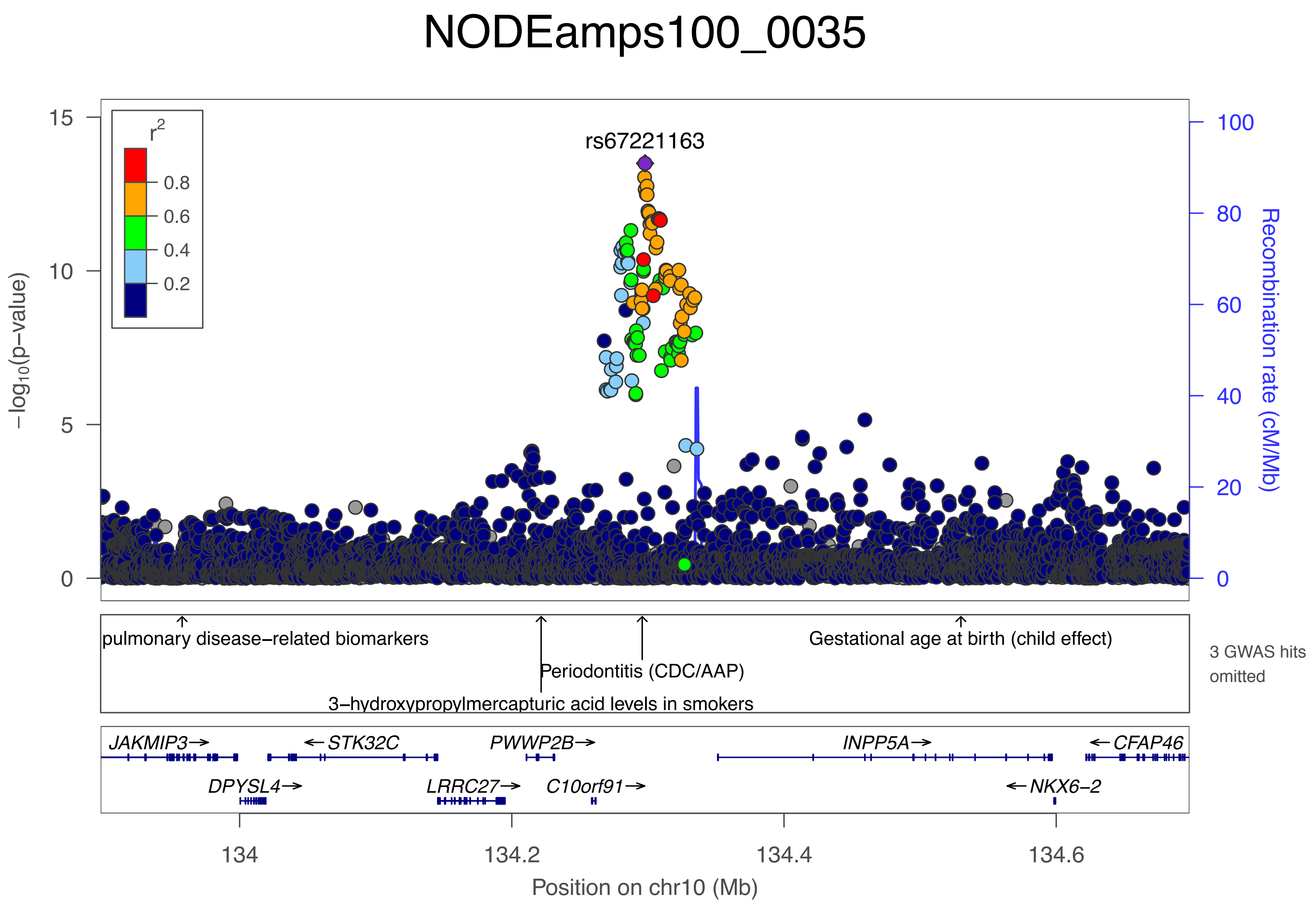

NODEamps100_0035
rs67221163
r2
0.8
0.6
0.4
0.2
−log10(p−value)
Recombination rate (cM/Mb)
pulmonary disease−related biomarkers
Periodontitis (CDC/AAP)
3−hydroxypropylmercapturic acid levels in smokers
Gestational age at birth (child effect)
3 GWAS hits omitted
JAKMIP3→
←STK32C
PWWP2B→
INPP5A→
←CFAP46
DPYSL4→
LRRC27→
C10orf91→
←NKX6−2
134
134.2
134.4
134.6
Position on chr10 (Mb)

| | |
|---|---|
| date: | Thu Aug 17 18:58:29 2017 |
| build: | hg19 |
| display range: | chr10:133897909−134697909 [133897909−134697909] |
| hilite range: | 0 – 0 [ 0 – 0 ] |
| reference SNP: | chr10:134297909 |
| number of SNPs plotted: | 5069 |
| min P.value: | 3.18E−14 [chr10:134297909] |
| max P.value: | 10E−1 [chr10:134697143] |
| omitted GWAS Hits: | chr10:134.529972−Gestational age at birth (child effect), NA |

Make more plots at http://csg.sph.umich.edu/locuszoom/

GWAS Catalog SNPs in Region

| chr | pos (Mb) | trait | snp |
|---|---|---|---|
| 10 | 133.9578 | Chronic obstructive pulmonary disease−related biomarkers | rs954820 |
| 10 | 134.2054 | Neuroticism | rs3923857 |
| 10 | 134.2215 | 3−hydroxypropylmercapturic acid levels in smokers | rs74383458 |
| 10 | 134.2215 | 3−hydroxy−1−methylpropylmercapturic acid levels in smokers | rs74383458 |
| 10 | 134.2958 | Periodontitis (CDC/AAP) | rs73389468 |
| 10 | 134.5300 | Gestational age at birth (child effect) | rs873946 |
| 10 | 134.5300 | Gestational age at birth in labor−initiated deliveries (child effect) | rs873946 |

# NODEamps100_0027

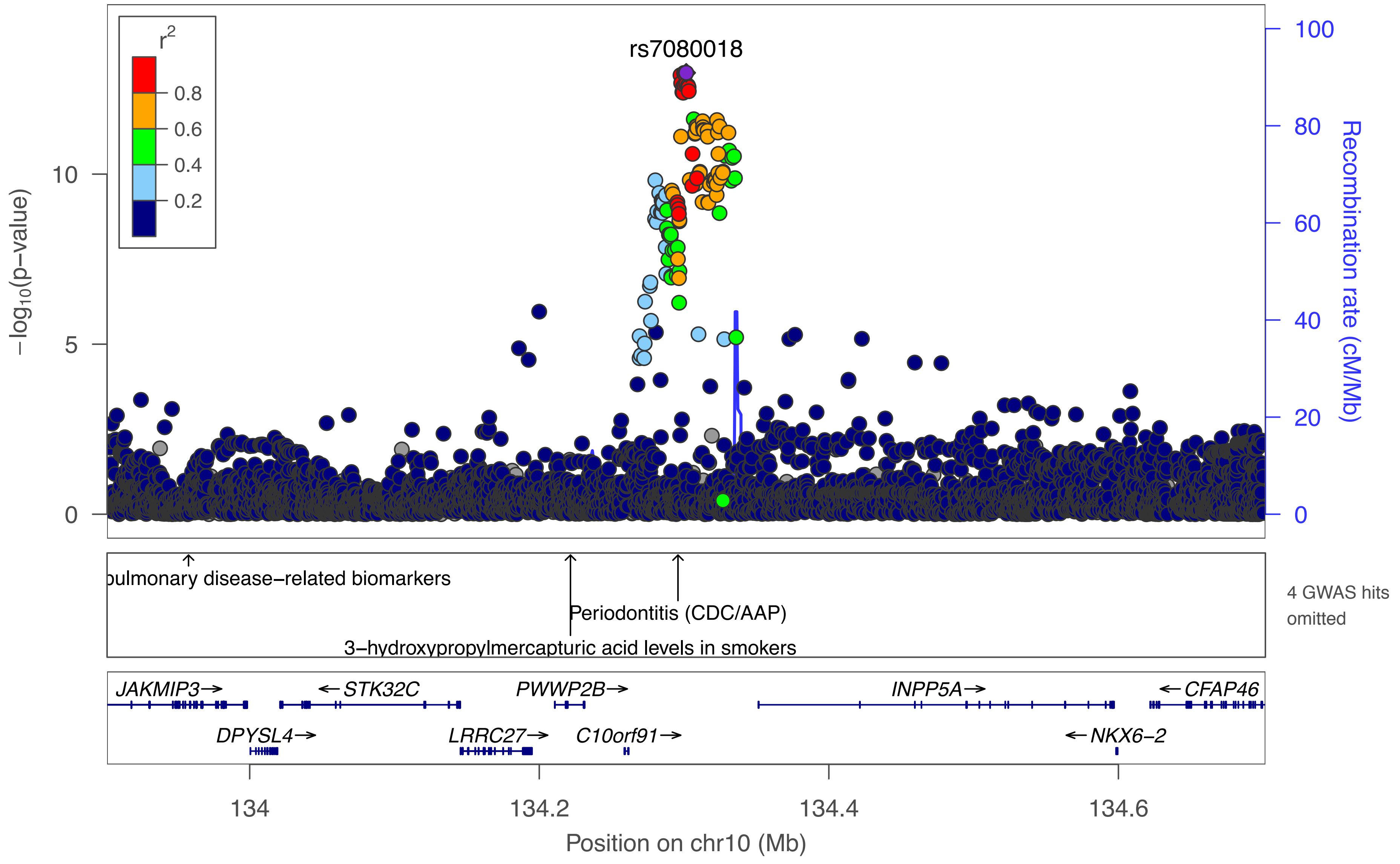

| | |
|---|---|
| date: | Thu Aug 17 18:59:23 2017 |
| build: | hg19 |
| display range: | chr10:133901505–134701505 [133901505–134701505] |
| hilite range: | 0 – 0 [ 0 – 0 ] |
| reference SNP: | chr10:134301505 |
| number of SNPs plotted: | 5069 |
| min P.value: | 1.05E–13 [chr10:134301505] |
| max P.value: | 10E–1 [chr10:134033082] |
| omitted GWAS Hits: | NA, NA |
| omitted GWAS Hits: | NA |

Make more plots at http://csg.sph.umich.edu/locuszoom/

GWAS Catalog SNPs in Region

| chr | pos (Mb) | trait | snp |
|---|---|---|---|
| 10 | 133.9578 | Chronic obstructive pulmonary disease−related biomarkers | rs954820 |
| 10 | 134.2054 | Neuroticism | rs3923857 |
| 10 | 134.2215 | 3−hydroxypropylmercapturic acid levels in smokers | rs74383458 |
| 10 | 134.2215 | 3−hydroxy−1−methylpropylmercapturic acid levels in smokers | rs74383458 |
| 10 | 134.2958 | Periodontitis (CDC/AAP) | rs73389468 |
| 10 | 134.5300 | Gestational age at birth (child effect) | rs873946 |
| 10 | 134.5300 | Gestational age at birth in labor−initiated deliveries (child effect) | rs873946 |

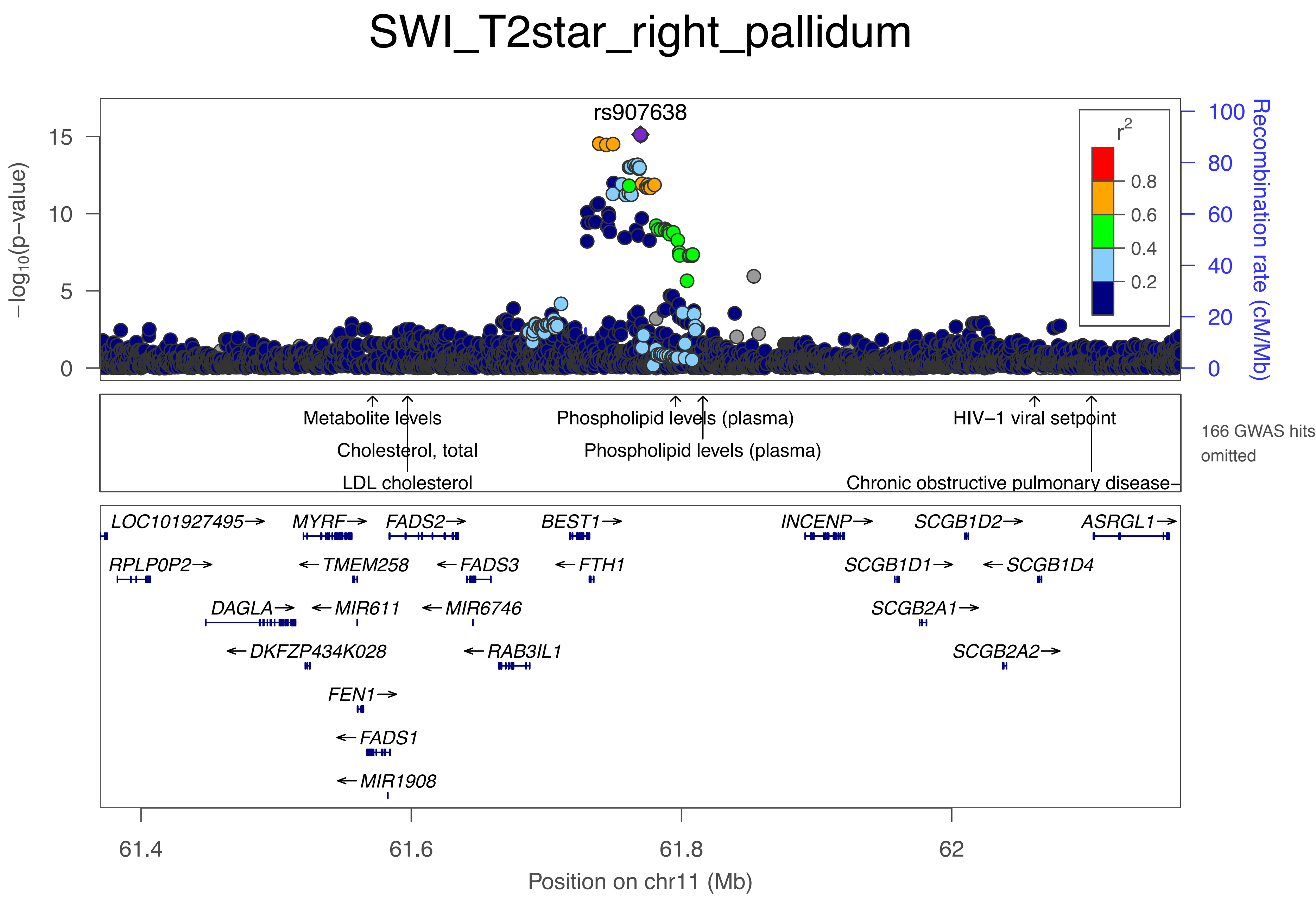
SWI_T2star_right_pallidum
rs907638
−log10(p−value)
Recombination rate (cM/Mb)
r2
0.8
0.6
0.4
0.2
0
5
10
15
20
40
60
80
100
Metabolite levels
Cholesterol, total
LDL cholesterol
Phospholipid levels (plasma)
Phospholipid levels (plasma)
HIV−1 viral setpoint
Chronic obstructive pulmonary disease
166 GWAS hits omitted
LOC101927495→
MYRF→
FADS2→
BEST1→
INCENP→
SCGB1D2→
ASRGL1→
RPLP0P2→
←TMEM258
←FADS3
←FTH1
SCGB1D1→
←SCGB1D4
DAGLA→
←MIR611
←MIR6746
SCGB2A1→
←DKFZP434K028
←RAB3IL1
SCGB2A2→
FEN1→
←FADS1
←MIR1908
61.4
61.6
61.8
62
Position on chr11 (Mb)

date: Thu Aug 17 18:50:05 2017

build: hg19

display range: chr11:61369588−62169588 [61369588−62169588]

hilite range: 0 – 0 [ 0 – 0 ]

reference SNP: chr11:61769588

number of SNPs plotted: 3658

min P.value: 7.46E−16 [chr11:61769588]

max P.value: 10E−1 [chr11:61861500]

omitted GWAS Hits: chr11:61.795586−Phospholipid levels (plasma), chr11:61.815803−Phospholipid levels (plasma)

omitted GWAS Hits: chr11:62.061349−HIV−1 viral setpoint, NA

omitted GWAS Hits: NA, NA

omitted GWAS Hits: NA, NA

omitted GWAS Hits: NA, NA

omitted GWAS Hits: NA, NA

omitted GWAS Hits: NA, NA

omitted GWAS Hits: NA, NA

omitted GWAS Hits: NA, NA

omitted GWAS Hits: NA, NA

omitted GWAS Hits: NA, NA

omitted GWAS Hits: NA, NA

omitted GWAS Hits: NA, NA

omitted GWAS Hits: NA, NA

Make more plots at http://csg.sph.umich.edu/locuszoom/

omitted GWAS Hits: NA, NA

GWAS Catalog SNPs in Region

| chr | pos (Mb) | trait | snp |
|---|---|---|---|
| 11 | 61.41747 | Phospholipid levels (plasma) | rs1692120 |
| 11 | 61.44249 | Plasma omega−6 polyunsaturated fatty acid levels (gamma−linolenic acid) | rs7925523 |
| 11 | 61.45860 | Plasma omega−6 polyunsaturated fatty acid levels (linoleic acid) | rs11827215 |
| 11 | 61.48516 | 3−hydroxypropylmercapturic acid levels in smokers | rs10488693 |
| 11 | 61.49049 | Phospholipid levels (plasma) | rs198426 |
| 11 | 61.49433 | Immune reponse to smallpox (secreted IL−2) | rs4963243 |
| 11 | 61.54350 | Plasma omega−6 polyunsaturated fatty acid levels (arachidonic acid) | rs174528 |
| 11 | 61.54350 | Trans fatty acid levels | rs174528 |
| 11 | 61.54396 | HDL cholesterol | rs174529 |
| 11 | 61.54396 | Triglycerides | rs174529 |
| 11 | 61.54724 | Trans fatty acid levels | rs108499 |
| 11 | 61.54856 | Plasma omega−3 polyunsaturated fatty acid levels (alphalinolenic acid) | rs509360 |
| 11 | 61.54856 | Trans fatty acid levels | rs509360 |
| 11 | 61.54946 | Plasma omega−6 polyunsaturated fatty acid levels (dihomo−gamma−linolenic acid) | rs174534 |
| 11 | 61.54946 | Trans fatty acid levels | rs174534 |
| 11 | 61.55136 | Phospholipid levels (plasma) | rs174535 |
| 11 | 61.55136 | Blood metabolite levels | rs174535 |
| 11 | 61.55136 | Red blood cell fatty acid levels | rs174535 |
| 11 | 61.55136 | Trans fatty acid levels | rs174535 |
| 11 | 61.55193 | Phospholipid levels (plasma) | rs174536 |
| 11 | 61.55193 | Glycerophospholipid levels | rs174536 |

GWAS Catalog SNPs in Region

| chr | pos (Mb) | trait | snp |
|---|---|---|---|
| 11 | 61.55193 | Plasma omega−3 polyunsaturated fatty acid levels (alphalinolenic acid) | rs174536 |
| 11 | 61.55193 | Trans fatty acid levels | rs174536 |
| 11 | 61.55193 | Resting heart rate | rs174536 |
| 11 | 61.55268 | Crohn's disease | rs174537 |
| 11 | 61.55268 | Glycerophospholipid levels | rs174537 |
| 11 | 61.55268 | Plasma omega−6 polyunsaturated fatty acid levels (gamma−linolenic acid) | rs174537 |
| 11 | 61.55268 | Colorectal cancer | rs174537 |
| 11 | 61.55268 | Trans fatty acid levels | rs174537 |
| 11 | 61.55780 | Phospholipid levels (plasma) | rs102275 |
| 11 | 61.55780 | Metabolic syndrome | rs102275 |
| 11 | 61.55780 | Plasma omega−6 polyunsaturated fatty acid levels (arachidonic acid) | rs102275 |
| 11 | 61.55780 | Metabolite levels | rs102275 |
| 11 | 61.55780 | Oleic acid (18:1n−9) plasma levels | rs102275 |
| 11 | 61.55780 | Crohn's disease | rs102275 |
| 11 | 61.55780 | Glycerophospholipid levels | rs102275 |
| 11 | 61.55780 | Trans fatty acid levels | rs102275 |
| 11 | 61.55780 | Palmitoleic acid (16:1n−7) plasma levels | rs102275 |
| 11 | 61.55780 | Stearic acid (18:0) plasma levels | rs102275 |
| 11 | 61.56008 | Phospholipid levels (plasma) | rs174538 |
| 11 | 61.56008 | Blood metabolite levels | rs174538 |
| 11 | 61.56008 | Trans fatty acid levels | rs174538 |

GWAS Catalog SNPs in Region

| chr | pos (Mb) | trait | snp |
|---|---|---|---|
| 11 | 61.56430 | Phospholipid levels (plasma) | rs4246215 |
| 11 | 61.56430 | Platelet count | rs4246215 |
| 11 | 61.56430 | Inflammatory bowel disease | rs4246215 |
| 11 | 61.56430 | Red blood cell fatty acid levels | rs4246215 |
| 11 | 61.56430 | Trans fatty acid levels | rs4246215 |
| 11 | 61.56591 | Metabolite levels | rs174541 |
| 11 | 61.56591 | Red blood cell fatty acid levels | rs174541 |
| 11 | 61.56591 | Trans fatty acid levels | rs174541 |
| 11 | 61.56931 | Delta−6 desaturase activity | rs174545 |
| 11 | 61.56931 | Red blood cell fatty acid levels | rs174545 |
| 11 | 61.56931 | Trans fatty acid levels | rs174545 |
| 11 | 61.56983 | LDL cholesterol | rs174546 |
| 11 | 61.56983 | HDL cholesterol | rs174546 |
| 11 | 61.56983 | Triglycerides | rs174546 |
| 11 | 61.56983 | Cholesterol, total | rs174546 |
| 11 | 61.56983 | Metabolic syndrome | rs174546 |
| 11 | 61.56983 | Glycerophospholipid levels | rs174546 |
| 11 | 61.56983 | Plasma omega−6 polyunsaturated fatty acid levels (gamma−linolenic acid) | rs174546 |
| 11 | 61.56983 | Trans fatty acid levels | rs174546 |
| 11 | 61.56983 | C−reactive protein levels or triglyceride levels (pleiotropy) | rs174546 |
| 11 | 61.56983 | C−reactive protein levels or HDL−cholesterol levels (pleiotropy) | rs174546 |

GWAS Catalog SNPs in Region

| chr | pos (Mb) | trait | snp |
|---|---|---|---|
| 11 | 61.57078 | HDL cholesterol | rs174547 |
| 11 | 61.57078 | Triglycerides | rs174547 |
| 11 | 61.57078 | Phospholipid levels (plasma) | rs174547 |
| 11 | 61.57078 | Resting heart rate | rs174547 |
| 11 | 61.57078 | Metabolic traits | rs174547 |
| 11 | 61.57078 | Lipid metabolism phenotypes | rs174547 |
| 11 | 61.57078 | Comprehensive strength and appendicular lean mass | rs174547 |
| 11 | 61.57078 | Glycerophospholipid levels | rs174547 |
| 11 | 61.57078 | Sphingolipid levels | rs174547 |
| 11 | 61.57078 | Metabolite levels (lipid measures) | rs174547 |
| 11 | 61.57078 | Plasma omega−6 polyunsaturated fatty acid levels (linoleic acid) | rs174547 |
| 11 | 61.57078 | Plasma omega−6 polyunsaturated fatty acid levels (gamma−linolenic acid) | rs174547 |
| 11 | 61.57078 | Plasma omega−6 polyunsaturated fatty acid levels (arachidonic acid) | rs174547 |
| 11 | 61.57078 | Height | rs174547 |
| 11 | 61.57078 | Trans fatty acid levels | rs174547 |
| 11 | 61.57135 | Metabolite levels | rs174548 |
| 11 | 61.57135 | HDL cholesterol | rs174548 |
| 11 | 61.57135 | Triglycerides | rs174548 |
| 11 | 61.57135 | Blood metabolite ratios | rs174548 |
| 11 | 61.57135 | Plasma omega−6 polyunsaturated fatty acid levels (dihomo−gamma−linolenic acid) | rs174548 |
| 11 | 61.57135 | Delta−6 desaturase activity | rs174548 |

GWAS Catalog SNPs in Region

| chr | pos (Mb) | trait | snp |
|---|---|---|---|
| 11 | 61.57135 | Blood metabolite levels | rs174548 |
| 11 | 61.57135 | Hematology traits | rs174548 |
| 11 | 61.57135 | Trans fatty acid levels | rs174548 |
| 11 | 61.57138 | Laryngeal squamous cell carcinoma | rs174549 |
| 11 | 61.57138 | Comprehensive strength and appendicular lean mass | rs174549 |
| 11 | 61.57138 | Heart rate | rs174549 |
| 11 | 61.57138 | Metabolite levels | rs174549 |
| 11 | 61.57138 | Red blood cell fatty acid levels | rs174549 |
| 11 | 61.57138 | Trans fatty acid levels | rs174549 |
| 11 | 61.57148 | Phospholipid levels (plasma) | rs174550 |
| 11 | 61.57148 | Fasting glucose−related traits | rs174550 |
| 11 | 61.57148 | Fasting glucose−related traits (interaction with BMI) | rs174550 |
| 11 | 61.57148 | Glycerophospholipid levels | rs174550 |
| 11 | 61.57148 | Plasma omega−6 polyunsaturated fatty acid levels (linoleic acid) | rs174550 |
| 11 | 61.57148 | Plasma omega−6 polyunsaturated fatty acid levels (adrenic acid) | rs174550 |
| 11 | 61.57148 | Blood metabolite levels | rs174550 |
| 11 | 61.57148 | Red blood cell fatty acid levels | rs174550 |
| 11 | 61.57148 | Trans fatty acid levels | rs174550 |
| 11 | 61.57368 | LDL cholesterol | rs174551 |
| 11 | 61.57946 | Cholesterol, total | rs174554 |
| 11 | 61.57976 | Glycerophospholipid levels | rs174555 |

GWAS Catalog SNPs in Region

| chr | pos (Mb) | trait | snp |
|---|---|---|---|
| 11 | 61.57976 | Plasma omega−6 polyunsaturated fatty acid levels (dihomo−gamma−linolenic acid) | rs174555 |
| 11 | 61.57976 | Trans fatty acid levels | rs174555 |
| 11 | 61.58064 | Blood metabolite levels | rs174556 |
| 11 | 61.58064 | Trans fatty acid levels | rs174556 |
| 11 | 61.59556 | Glycerophospholipid levels | rs968567 |
| 11 | 61.59556 | Rheumatoid arthritis | rs968567 |
| 11 | 61.59556 | Blood metabolite levels | rs968567 |
| 11 | 61.59721 | Cholesterol, total | rs174570 |
| 11 | 61.59721 | LDL cholesterol | rs174570 |
| 11 | 61.59721 | HDL cholesterol | rs174570 |
| 11 | 61.59721 | Glycated hemoglobin levels | rs174570 |
| 11 | 61.59721 | Trans fatty acid levels | rs174570 |
| 11 | 61.59797 | Phospholipid levels (plasma) | rs1535 |
| 11 | 61.59797 | Metabolic syndrome | rs1535 |
| 11 | 61.59797 | Response to statin therapy | rs1535 |
| 11 | 61.59797 | Glycerophospholipid levels | rs1535 |
| 11 | 61.59797 | Inflammatory bowel disease | rs1535 |
| 11 | 61.59797 | Trans fatty acid levels | rs1535 |
| 11 | 61.60034 | Phospholipid levels (plasma) | rs174574 |
| 11 | 61.60034 | Trans fatty acid levels | rs174574 |
| 11 | 61.60034 | C−reactive protein levels or LDL−cholesterol levels (pleiotropy) | rs174574 |

GWAS Catalog SNPs in Region

| chr | pos (Mb) | trait | snp |
|---|---|---|---|
| 11 | 61.60191 | Trans fatty acid levels | rs2845573 |
| 11 | 61.60200 | Trans fatty acid levels | rs174575 |
| 11 | 61.60324 | Plasma omega–6 polyunsaturated fatty acid levels (linoleic acid) | rs2727270 |
| 11 | 61.60324 | Trans fatty acid levels | rs2727270 |
| 11 | 61.60336 | Blood metabolite levels | rs2727271 |
| 11 | 61.60336 | Trans fatty acid levels | rs2727271 |
| 11 | 61.60351 | Glycerophospholipid levels | rs174576 |
| 11 | 61.60351 | Trans fatty acid levels | rs174576 |
| 11 | 61.60478 | Trans fatty acid levels | rs2524299 |
| 11 | 61.60481 | Iron status biomarkers (transferrin levels) | rs174577 |
| 11 | 61.60481 | Plasma omega–6 polyunsaturated fatty acid levels (arachidonic acid) | rs174577 |
| 11 | 61.60481 | P wave duration | rs174577 |
| 11 | 61.60481 | Trans fatty acid levels | rs174577 |
| 11 | 61.60481 | QRS duration | rs174577 |
| 11 | 61.60522 | Trans fatty acid levels | rs2072114 |
| 11 | 61.60550 | Glycerophospholipid levels | rs174578 |
| 11 | 61.60550 | Plasma omega–6 polyunsaturated fatty acid levels (linoleic acid) | rs174578 |
| 11 | 61.60550 | Blood metabolite levels | rs174578 |
| 11 | 61.60550 | Trans fatty acid levels | rs174578 |
| 11 | 61.60975 | Response to statin therapy | rs174583 |
| 11 | 61.60975 | QT interval | rs174583 |

GWAS Catalog SNPs in Region

| chr | pos (Mb) | trait | snp |
|---|---|---|---|
| 11 | 61.60975 | Trans fatty acid levels | rs174583 |
| 11 | 61.61601 | Trans fatty acid levels | rs2851682 |
| 11 | 61.61601 | Gestational age at birth (child effect) | rs2851682 |
| 11 | 61.62314 | Liver enzyme levels (alkaline phosphatase) | rs174601 |
| 11 | 61.62314 | Blood metabolite levels | rs174601 |
| 11 | 61.62314 | Red blood cell fatty acid levels | rs174601 |
| 11 | 61.62314 | Trans fatty acid levels | rs174601 |
| 11 | 61.62379 | Trans fatty acid levels | rs2526678 |
| 11 | 61.63949 | Trans fatty acid levels | rs422249 |
| 11 | 61.63957 | Phospholipid levels (plasma) | rs174448 |
| 11 | 61.63957 | Trans fatty acid levels | rs174448 |
| 11 | 61.64038 | Trans fatty acid levels | rs174449 |
| 11 | 61.65530 | Sphingolipid levels | rs1000778 |
| 11 | 61.66369 | Phospholipid levels (plasma) | rs174468 |
| 11 | 61.67875 | Sphingolipid levels | rs174479 |
| 11 | 61.71148 | Phospholipid levels (plasma) | rs2521572 |
| 11 | 61.71213 | Estradiol levels | rs2727261 |
| 11 | 61.72264 | Phospholipid levels (plasma) | rs1109748 |
| 11 | 61.74629 | Phospholipid levels (plasma) | rs10792320 |
| 11 | 61.79559 | Phospholipid levels (plasma) | rs11230874 |
| 11 | 61.81580 | Phospholipid levels (plasma) | rs4963452 |

GWAS Catalog SNPs in Region

| chr | pos (Mb) | trait | snp |
|---|---|---|---|
| 11 | 61.96393 | 3−hydroxypropylmercapturic acid levels in smokers | rs189478926 |
| 11 | 62.00141 | Plasma omega−6 polyunsaturated fatty acid levels (gamma−linolenic acid) | rs12806663 |
| 11 | 62.06135 | HIV−1 viral setpoint | rs11231017 |
| 11 | 62.10342 | Chronic obstructive pulmonary disease−related biomarkers | rs2463822 |
| 11 | 62.14169 | 3−hydroxypropylmercapturic acid levels in smokers | rs184485201 |

# SWI_T2star_left_pallidum

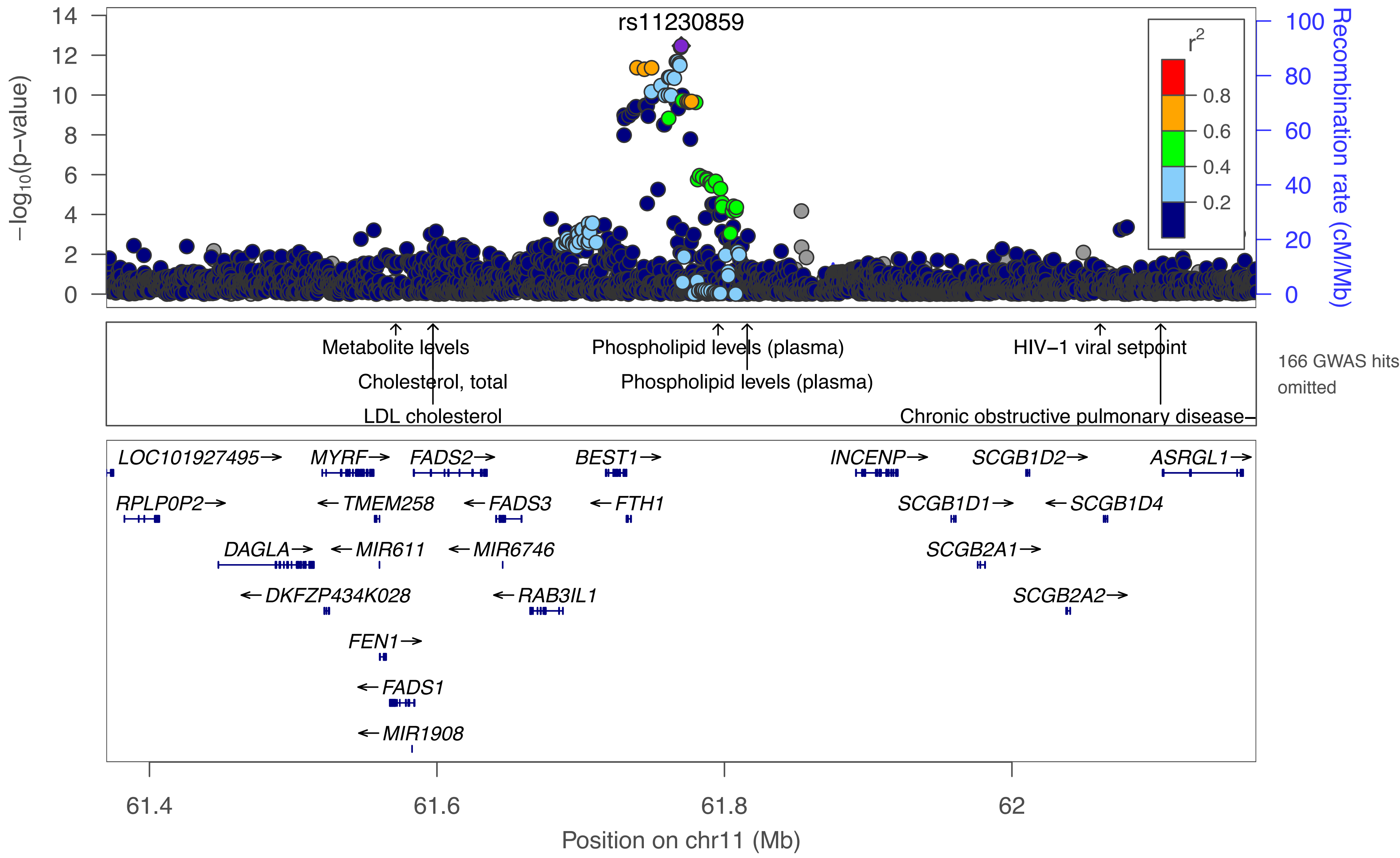

| | |
|---|---|
| date: | Thu Aug 17 19:04:48 2017 |
| build: | hg19 |
| display range: | chr11:61369972−62169972 [61369972−62169972] |
| hilite range: | 0 – 0 [ 0 – 0 ] |
| reference SNP: | chr11:61769972 |
| number of SNPs plotted: | 3667 |
| min P.value: | 3.37E−13 [chr11:61769972] |
| max P.value: | 10E−1 [chr11:61923657] |
| omitted GWAS Hits: | chr11:61.795586−Phospholipid levels (plasma), chr11:61.815803−Phospholipid levels (plasma) |
| omitted GWAS Hits: | chr11:62.061349−HIV−1 viral setpoint, NA |
| omitted GWAS Hits: | NA, NA |
| omitted GWAS Hits: | NA, NA |
| omitted GWAS Hits: | NA, NA |
| omitted GWAS Hits: | NA, NA |
| omitted GWAS Hits: | NA, NA |
| omitted GWAS Hits: | NA, NA |
| omitted GWAS Hits: | NA, NA |
| omitted GWAS Hits: | NA, NA |
| omitted GWAS Hits: | NA, NA |
| omitted GWAS Hits: | NA, NA |
| omitted GWAS Hits: | NA, NA |
| omitted GWAS Hits: | NA, NA |
| omitted GWAS Hits: | NA, NA |

Make more plots at http://csg.sph.umich.edu/locuszoom/

GWAS Catalog SNPs in Region

| chr | pos (Mb) | trait | snp |
|---|---|---|---|
| 11 | 61.41747 | Phospholipid levels (plasma) | rs1692120 |
| 11 | 61.44249 | Plasma omega−6 polyunsaturated fatty acid levels (gamma−linolenic acid) | rs7925523 |
| 11 | 61.45860 | Plasma omega−6 polyunsaturated fatty acid levels (linoleic acid) | rs11827215 |
| 11 | 61.48516 | 3−hydroxypropylmercapturic acid levels in smokers | rs10488693 |
| 11 | 61.49049 | Phospholipid levels (plasma) | rs198426 |
| 11 | 61.49433 | Immune reponse to smallpox (secreted IL−2) | rs4963243 |
| 11 | 61.54350 | Plasma omega−6 polyunsaturated fatty acid levels (arachidonic acid) | rs174528 |
| 11 | 61.54350 | Trans fatty acid levels | rs174528 |
| 11 | 61.54396 | HDL cholesterol | rs174529 |
| 11 | 61.54396 | Triglycerides | rs174529 |
| 11 | 61.54724 | Trans fatty acid levels | rs108499 |
| 11 | 61.54856 | Plasma omega−3 polyunsaturated fatty acid levels (alphalinolenic acid) | rs509360 |
| 11 | 61.54856 | Trans fatty acid levels | rs509360 |
| 11 | 61.54946 | Plasma omega−6 polyunsaturated fatty acid levels (dihomo−gamma−linolenic acid) | rs174534 |
| 11 | 61.54946 | Trans fatty acid levels | rs174534 |
| 11 | 61.55136 | Phospholipid levels (plasma) | rs174535 |
| 11 | 61.55136 | Blood metabolite levels | rs174535 |
| 11 | 61.55136 | Red blood cell fatty acid levels | rs174535 |
| 11 | 61.55136 | Trans fatty acid levels | rs174535 |
| 11 | 61.55193 | Phospholipid levels (plasma) | rs174536 |
| 11 | 61.55193 | Glycerophospholipid levels | rs174536 |

GWAS Catalog SNPs in Region

| chr | pos (Mb) | trait | snp |
|---|---|---|---|
| 11 | 61.55193 | Plasma omega−3 polyunsaturated fatty acid levels (alphalinolenic acid) | rs174536 |
| 11 | 61.55193 | Trans fatty acid levels | rs174536 |
| 11 | 61.55193 | Resting heart rate | rs174536 |
| 11 | 61.55268 | Crohn's disease | rs174537 |
| 11 | 61.55268 | Glycerophospholipid levels | rs174537 |
| 11 | 61.55268 | Plasma omega−6 polyunsaturated fatty acid levels (gamma−linolenic acid) | rs174537 |
| 11 | 61.55268 | Colorectal cancer | rs174537 |
| 11 | 61.55268 | Trans fatty acid levels | rs174537 |
| 11 | 61.55780 | Phospholipid levels (plasma) | rs102275 |
| 11 | 61.55780 | Metabolic syndrome | rs102275 |
| 11 | 61.55780 | Plasma omega−6 polyunsaturated fatty acid levels (arachidonic acid) | rs102275 |
| 11 | 61.55780 | Metabolite levels | rs102275 |
| 11 | 61.55780 | Oleic acid (18:1n−9) plasma levels | rs102275 |
| 11 | 61.55780 | Crohn's disease | rs102275 |
| 11 | 61.55780 | Glycerophospholipid levels | rs102275 |
| 11 | 61.55780 | Trans fatty acid levels | rs102275 |
| 11 | 61.55780 | Palmitoleic acid (16:1n−7) plasma levels | rs102275 |
| 11 | 61.55780 | Stearic acid (18:0) plasma levels | rs102275 |
| 11 | 61.56008 | Phospholipid levels (plasma) | rs174538 |
| 11 | 61.56008 | Blood metabolite levels | rs174538 |
| 11 | 61.56008 | Trans fatty acid levels | rs174538 |

GWAS Catalog SNPs in Region

| chr | pos (Mb) | trait | snp |
|---|---|---|---|
| 11 | 61.56430 | Phospholipid levels (plasma) | rs4246215 |
| 11 | 61.56430 | Platelet count | rs4246215 |
| 11 | 61.56430 | Inflammatory bowel disease | rs4246215 |
| 11 | 61.56430 | Red blood cell fatty acid levels | rs4246215 |
| 11 | 61.56430 | Trans fatty acid levels | rs4246215 |
| 11 | 61.56591 | Metabolite levels | rs174541 |
| 11 | 61.56591 | Red blood cell fatty acid levels | rs174541 |
| 11 | 61.56591 | Trans fatty acid levels | rs174541 |
| 11 | 61.56931 | Delta−6 desaturase activity | rs174545 |
| 11 | 61.56931 | Red blood cell fatty acid levels | rs174545 |
| 11 | 61.56931 | Trans fatty acid levels | rs174545 |
| 11 | 61.56983 | LDL cholesterol | rs174546 |
| 11 | 61.56983 | HDL cholesterol | rs174546 |
| 11 | 61.56983 | Triglycerides | rs174546 |
| 11 | 61.56983 | Cholesterol, total | rs174546 |
| 11 | 61.56983 | Metabolic syndrome | rs174546 |
| 11 | 61.56983 | Glycerophospholipid levels | rs174546 |
| 11 | 61.56983 | Plasma omega−6 polyunsaturated fatty acid levels (gamma−linolenic acid) | rs174546 |
| 11 | 61.56983 | Trans fatty acid levels | rs174546 |
| 11 | 61.56983 | C−reactive protein levels or triglyceride levels (pleiotropy) | rs174546 |
| 11 | 61.56983 | C−reactive protein levels or HDL−cholesterol levels (pleiotropy) | rs174546 |

GWAS Catalog SNPs in Region

| chr | pos (Mb) | trait | snp |
|---|---|---|---|
| 11 | 61.57078 | HDL cholesterol | rs174547 |
| 11 | 61.57078 | Triglycerides | rs174547 |
| 11 | 61.57078 | Phospholipid levels (plasma) | rs174547 |
| 11 | 61.57078 | Resting heart rate | rs174547 |
| 11 | 61.57078 | Metabolic traits | rs174547 |
| 11 | 61.57078 | Lipid metabolism phenotypes | rs174547 |
| 11 | 61.57078 | Comprehensive strength and appendicular lean mass | rs174547 |
| 11 | 61.57078 | Glycerophospholipid levels | rs174547 |
| 11 | 61.57078 | Sphingolipid levels | rs174547 |
| 11 | 61.57078 | Metabolite levels (lipid measures) | rs174547 |
| 11 | 61.57078 | Plasma omega−6 polyunsaturated fatty acid levels (linoleic acid) | rs174547 |
| 11 | 61.57078 | Plasma omega−6 polyunsaturated fatty acid levels (gamma−linolenic acid) | rs174547 |
| 11 | 61.57078 | Plasma omega−6 polyunsaturated fatty acid levels (arachidonic acid) | rs174547 |
| 11 | 61.57078 | Height | rs174547 |
| 11 | 61.57078 | Trans fatty acid levels | rs174547 |
| 11 | 61.57135 | Metabolite levels | rs174548 |
| 11 | 61.57135 | HDL cholesterol | rs174548 |
| 11 | 61.57135 | Triglycerides | rs174548 |
| 11 | 61.57135 | Blood metabolite ratios | rs174548 |
| 11 | 61.57135 | Plasma omega−6 polyunsaturated fatty acid levels (dihomo−gamma−linolenic acid) | rs174548 |
| 11 | 61.57135 | Delta−6 desaturase activity | rs174548 |

GWAS Catalog SNPs in Region

| chr | pos (Mb) | trait | snp |
|---|---|---|---|
| 11 | 61.57135 | Blood metabolite levels | rs174548 |
| 11 | 61.57135 | Hematology traits | rs174548 |
| 11 | 61.57135 | Trans fatty acid levels | rs174548 |
| 11 | 61.57138 | Laryngeal squamous cell carcinoma | rs174549 |
| 11 | 61.57138 | Comprehensive strength and appendicular lean mass | rs174549 |
| 11 | 61.57138 | Heart rate | rs174549 |
| 11 | 61.57138 | Metabolite levels | rs174549 |
| 11 | 61.57138 | Red blood cell fatty acid levels | rs174549 |
| 11 | 61.57138 | Trans fatty acid levels | rs174549 |
| 11 | 61.57148 | Phospholipid levels (plasma) | rs174550 |
| 11 | 61.57148 | Fasting glucose−related traits | rs174550 |
| 11 | 61.57148 | Fasting glucose−related traits (interaction with BMI) | rs174550 |
| 11 | 61.57148 | Glycerophospholipid levels | rs174550 |
| 11 | 61.57148 | Plasma omega−6 polyunsaturated fatty acid levels (linoleic acid) | rs174550 |
| 11 | 61.57148 | Plasma omega−6 polyunsaturated fatty acid levels (adrenic acid) | rs174550 |
| 11 | 61.57148 | Blood metabolite levels | rs174550 |
| 11 | 61.57148 | Red blood cell fatty acid levels | rs174550 |
| 11 | 61.57148 | Trans fatty acid levels | rs174550 |
| 11 | 61.57368 | LDL cholesterol | rs174551 |
| 11 | 61.57946 | Cholesterol, total | rs174554 |
| 11 | 61.57976 | Glycerophospholipid levels | rs174555 |

GWAS Catalog SNPs in Region

| chr | pos (Mb) | trait | snp |
|---|---|---|---|
| 11 | 61.57976 | Plasma omega−6 polyunsaturated fatty acid levels (dihomo−gamma−linolenic acid) | rs174555 |
| 11 | 61.57976 | Trans fatty acid levels | rs174555 |
| 11 | 61.58064 | Blood metabolite levels | rs174556 |
| 11 | 61.58064 | Trans fatty acid levels | rs174556 |
| 11 | 61.59556 | Glycerophospholipid levels | rs968567 |
| 11 | 61.59556 | Rheumatoid arthritis | rs968567 |
| 11 | 61.59556 | Blood metabolite levels | rs968567 |
| 11 | 61.59721 | Cholesterol, total | rs174570 |
| 11 | 61.59721 | LDL cholesterol | rs174570 |
| 11 | 61.59721 | HDL cholesterol | rs174570 |
| 11 | 61.59721 | Glycated hemoglobin levels | rs174570 |
| 11 | 61.59721 | Trans fatty acid levels | rs174570 |
| 11 | 61.59797 | Phospholipid levels (plasma) | rs1535 |
| 11 | 61.59797 | Metabolic syndrome | rs1535 |
| 11 | 61.59797 | Response to statin therapy | rs1535 |
| 11 | 61.59797 | Glycerophospholipid levels | rs1535 |
| 11 | 61.59797 | Inflammatory bowel disease | rs1535 |
| 11 | 61.59797 | Trans fatty acid levels | rs1535 |
| 11 | 61.60034 | Phospholipid levels (plasma) | rs174574 |
| 11 | 61.60034 | Trans fatty acid levels | rs174574 |
| 11 | 61.60034 | C−reactive protein levels or LDL−cholesterol levels (pleiotropy) | rs174574 |

GWAS Catalog SNPs in Region

| chr | pos (Mb) | trait | snp |
|---|---|---|---|
| 11 | 61.60191 | Trans fatty acid levels | rs2845573 |
| 11 | 61.60200 | Trans fatty acid levels | rs174575 |
| 11 | 61.60324 | Plasma omega–6 polyunsaturated fatty acid levels (linoleic acid) | rs2727270 |
| 11 | 61.60324 | Trans fatty acid levels | rs2727270 |
| 11 | 61.60336 | Blood metabolite levels | rs2727271 |
| 11 | 61.60336 | Trans fatty acid levels | rs2727271 |
| 11 | 61.60351 | Glycerophospholipid levels | rs174576 |
| 11 | 61.60351 | Trans fatty acid levels | rs174576 |
| 11 | 61.60478 | Trans fatty acid levels | rs2524299 |
| 11 | 61.60481 | Iron status biomarkers (transferrin levels) | rs174577 |
| 11 | 61.60481 | Plasma omega–6 polyunsaturated fatty acid levels (arachidonic acid) | rs174577 |
| 11 | 61.60481 | P wave duration | rs174577 |
| 11 | 61.60481 | Trans fatty acid levels | rs174577 |
| 11 | 61.60481 | QRS duration | rs174577 |
| 11 | 61.60522 | Trans fatty acid levels | rs2072114 |
| 11 | 61.60550 | Glycerophospholipid levels | rs174578 |
| 11 | 61.60550 | Plasma omega–6 polyunsaturated fatty acid levels (linoleic acid) | rs174578 |
| 11 | 61.60550 | Blood metabolite levels | rs174578 |
| 11 | 61.60550 | Trans fatty acid levels | rs174578 |
| 11 | 61.60975 | Response to statin therapy | rs174583 |
| 11 | 61.60975 | QT interval | rs174583 |

GWAS Catalog SNPs in Region

| chr | pos (Mb) | trait | snp |
|---|---|---|---|
| 11 | 61.60975 | Trans fatty acid levels | rs174583 |
| 11 | 61.61601 | Trans fatty acid levels | rs2851682 |
| 11 | 61.61601 | Gestational age at birth (child effect) | rs2851682 |
| 11 | 61.62314 | Liver enzyme levels (alkaline phosphatase) | rs174601 |
| 11 | 61.62314 | Blood metabolite levels | rs174601 |
| 11 | 61.62314 | Red blood cell fatty acid levels | rs174601 |
| 11 | 61.62314 | Trans fatty acid levels | rs174601 |
| 11 | 61.62379 | Trans fatty acid levels | rs2526678 |
| 11 | 61.63949 | Trans fatty acid levels | rs422249 |
| 11 | 61.63957 | Phospholipid levels (plasma) | rs174448 |
| 11 | 61.63957 | Trans fatty acid levels | rs174448 |
| 11 | 61.64038 | Trans fatty acid levels | rs174449 |
| 11 | 61.65530 | Sphingolipid levels | rs1000778 |
| 11 | 61.66369 | Phospholipid levels (plasma) | rs174468 |
| 11 | 61.67875 | Sphingolipid levels | rs174479 |
| 11 | 61.71148 | Phospholipid levels (plasma) | rs2521572 |
| 11 | 61.71213 | Estradiol levels | rs2727261 |
| 11 | 61.72264 | Phospholipid levels (plasma) | rs1109748 |
| 11 | 61.74629 | Phospholipid levels (plasma) | rs10792320 |
| 11 | 61.79559 | Phospholipid levels (plasma) | rs11230874 |
| 11 | 61.81580 | Phospholipid levels (plasma) | rs4963452 |

GWAS Catalog SNPs in Region

| chr | pos (Mb) | trait | snp |
|---|---|---|---|
| 11 | 61.96393 | 3−hydroxypropylmercapturic acid levels in smokers | rs189478926 |
| 11 | 62.00141 | Plasma omega−6 polyunsaturated fatty acid levels (gamma−linolenic acid) | rs12806663 |
| 11 | 62.06135 | HIV−1 viral setpoint | rs11231017 |
| 11 | 62.10342 | Chronic obstructive pulmonary disease−related biomarkers | rs2463822 |
| 11 | 62.14169 | 3−hydroxypropylmercapturic acid levels in smokers | rs184485201 |

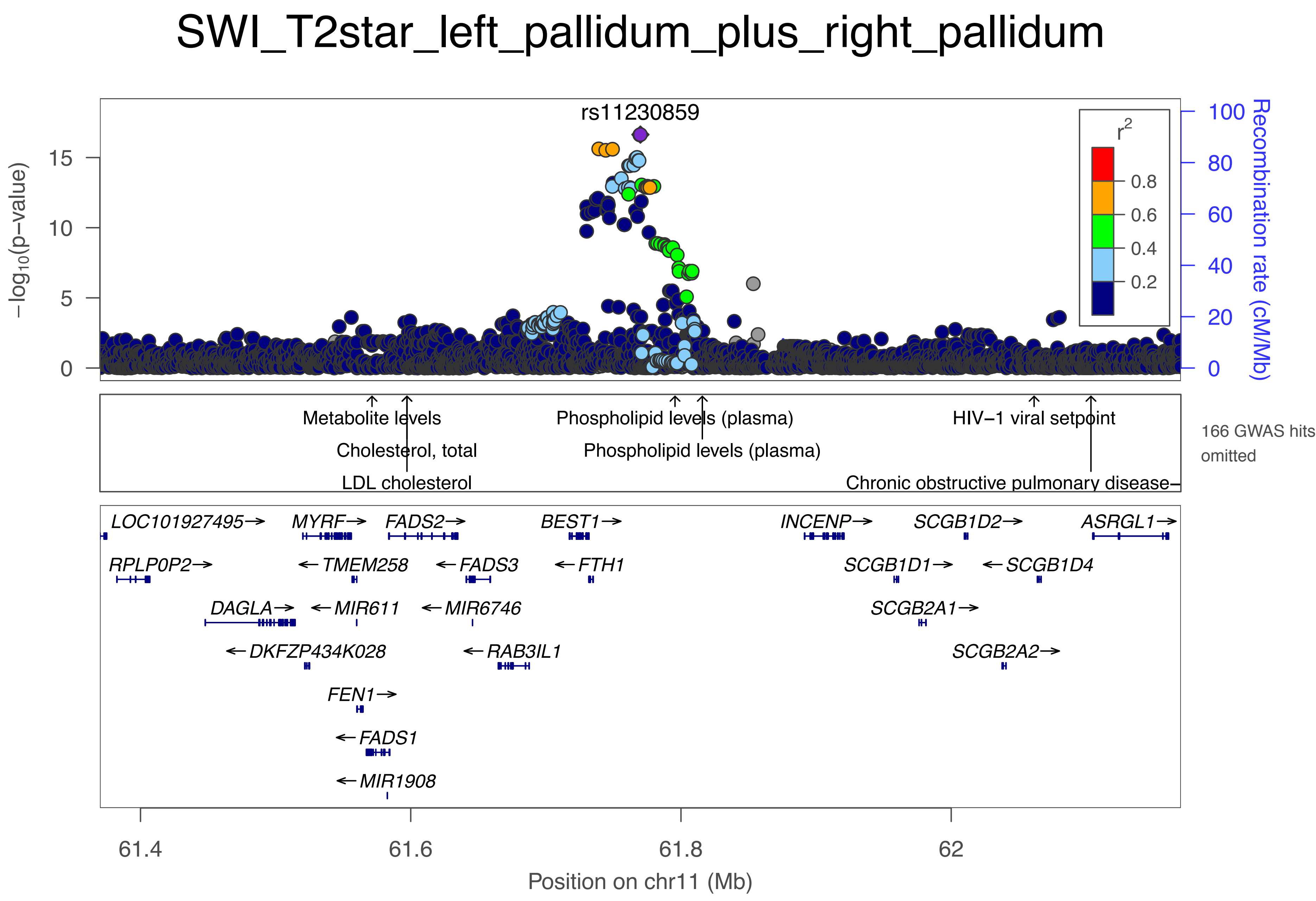

SWI_T2star_left_pallidum_plus_right_pallidum
rs11230859
−log10(p−value)
Recombination rate (cM/Mb)
r2
0.8
0.6
0.4
0.2
Metabolite levels
Cholesterol, total
LDL cholesterol
Phospholipid levels (plasma)
Phospholipid levels (plasma)
HIV−1 viral setpoint
Chronic obstructive pulmonary disease
166 GWAS hits omitted
LOC101927495→
RPLP0P2→
DAGLA→
←DKFZP434K028
MYRF→
←TMEM258
←MIR611
FEN1→
←FADS1
←MIR1908
FADS2→
←FADS3
←MIR6746
←RAB3IL1
BEST1→
←FTH1
INCENP→
SCGB1D1→
SCGB2A1→
SCGB1D2→
←SCGB1D4
SCGB2A2→
ASRGL1→
61.4
61.6
61.8
62
Position on chr11 (Mb)

| | |
|---|---|
| date: | Thu Aug 17 19:04:48 2017 |
| build: | hg19 |
| display range: | chr11:61369972−62169972 [61369972−62169972] |
| hilite range: | 0 – 0 [ 0 – 0 ] |
| reference SNP: | chr11:61769972 |
| number of SNPs plotted: | 3667 |
| min P.value: | 2.31E−17 [chr11:61769972] |
| max P.value: | 9.98E−1 [chr11:61529267] |
| omitted GWAS Hits: | chr11:61.795586−Phospholipid levels (plasma), chr11:61.815803−Phospholipid levels (plasma) |
| omitted GWAS Hits: | chr11:62.061349−HIV−1 viral setpoint, NA |
| omitted GWAS Hits: | NA, NA |
| omitted GWAS Hits: | NA, NA |
| omitted GWAS Hits: | NA, NA |
| omitted GWAS Hits: | NA, NA |
| omitted GWAS Hits: | NA, NA |
| omitted GWAS Hits: | NA, NA |
| omitted GWAS Hits: | NA, NA |
| omitted GWAS Hits: | NA, NA |
| omitted GWAS Hits: | NA, NA |
| omitted GWAS Hits: | NA, NA |
| omitted GWAS Hits: | NA, NA |
| omitted GWAS Hits: | NA, NA |
| omitted GWAS Hits: | NA, NA |

Make more plots at http://csg.sph.umich.edu/locuszoom/

GWAS Catalog SNPs in Region

| chr | pos (Mb) | trait | snp |
|---|---|---|---|
| 11 | 61.41747 | Phospholipid levels (plasma) | rs1692120 |
| 11 | 61.44249 | Plasma omega−6 polyunsaturated fatty acid levels (gamma−linolenic acid) | rs7925523 |
| 11 | 61.45860 | Plasma omega−6 polyunsaturated fatty acid levels (linoleic acid) | rs11827215 |
| 11 | 61.48516 | 3−hydroxypropylmercapturic acid levels in smokers | rs10488693 |
| 11 | 61.49049 | Phospholipid levels (plasma) | rs198426 |
| 11 | 61.49433 | Immune reponse to smallpox (secreted IL−2) | rs4963243 |
| 11 | 61.54350 | Plasma omega−6 polyunsaturated fatty acid levels (arachidonic acid) | rs174528 |
| 11 | 61.54350 | Trans fatty acid levels | rs174528 |
| 11 | 61.54396 | HDL cholesterol | rs174529 |
| 11 | 61.54396 | Triglycerides | rs174529 |
| 11 | 61.54724 | Trans fatty acid levels | rs108499 |
| 11 | 61.54856 | Plasma omega−3 polyunsaturated fatty acid levels (alphalinolenic acid) | rs509360 |
| 11 | 61.54856 | Trans fatty acid levels | rs509360 |
| 11 | 61.54946 | Plasma omega−6 polyunsaturated fatty acid levels (dihomo−gamma−linolenic acid) | rs174534 |
| 11 | 61.54946 | Trans fatty acid levels | rs174534 |
| 11 | 61.55136 | Phospholipid levels (plasma) | rs174535 |
| 11 | 61.55136 | Blood metabolite levels | rs174535 |
| 11 | 61.55136 | Red blood cell fatty acid levels | rs174535 |
| 11 | 61.55136 | Trans fatty acid levels | rs174535 |
| 11 | 61.55193 | Phospholipid levels (plasma) | rs174536 |
| 11 | 61.55193 | Glycerophospholipid levels | rs174536 |

GWAS Catalog SNPs in Region

| chr | pos (Mb) | trait | snp |
|---|---|---|---|
| 11 | 61.55193 | Plasma omega−3 polyunsaturated fatty acid levels (alphalinolenic acid) | rs174536 |
| 11 | 61.55193 | Trans fatty acid levels | rs174536 |
| 11 | 61.55193 | Resting heart rate | rs174536 |
| 11 | 61.55268 | Crohn's disease | rs174537 |
| 11 | 61.55268 | Glycerophospholipid levels | rs174537 |
| 11 | 61.55268 | Plasma omega−6 polyunsaturated fatty acid levels (gamma−linolenic acid) | rs174537 |
| 11 | 61.55268 | Colorectal cancer | rs174537 |
| 11 | 61.55268 | Trans fatty acid levels | rs174537 |
| 11 | 61.55780 | Phospholipid levels (plasma) | rs102275 |
| 11 | 61.55780 | Metabolic syndrome | rs102275 |
| 11 | 61.55780 | Plasma omega−6 polyunsaturated fatty acid levels (arachidonic acid) | rs102275 |
| 11 | 61.55780 | Metabolite levels | rs102275 |
| 11 | 61.55780 | Oleic acid (18:1n−9) plasma levels | rs102275 |
| 11 | 61.55780 | Crohn's disease | rs102275 |
| 11 | 61.55780 | Glycerophospholipid levels | rs102275 |
| 11 | 61.55780 | Trans fatty acid levels | rs102275 |
| 11 | 61.55780 | Palmitoleic acid (16:1n−7) plasma levels | rs102275 |
| 11 | 61.55780 | Stearic acid (18:0) plasma levels | rs102275 |
| 11 | 61.56008 | Phospholipid levels (plasma) | rs174538 |
| 11 | 61.56008 | Blood metabolite levels | rs174538 |
| 11 | 61.56008 | Trans fatty acid levels | rs174538 |

GWAS Catalog SNPs in Region

| chr | pos (Mb) | trait | snp |
|---|---|---|---|
| 11 | 61.56430 | Phospholipid levels (plasma) | rs4246215 |
| 11 | 61.56430 | Platelet count | rs4246215 |
| 11 | 61.56430 | Inflammatory bowel disease | rs4246215 |
| 11 | 61.56430 | Red blood cell fatty acid levels | rs4246215 |
| 11 | 61.56430 | Trans fatty acid levels | rs4246215 |
| 11 | 61.56591 | Metabolite levels | rs174541 |
| 11 | 61.56591 | Red blood cell fatty acid levels | rs174541 |
| 11 | 61.56591 | Trans fatty acid levels | rs174541 |
| 11 | 61.56931 | Delta−6 desaturase activity | rs174545 |
| 11 | 61.56931 | Red blood cell fatty acid levels | rs174545 |
| 11 | 61.56931 | Trans fatty acid levels | rs174545 |
| 11 | 61.56983 | LDL cholesterol | rs174546 |
| 11 | 61.56983 | HDL cholesterol | rs174546 |
| 11 | 61.56983 | Triglycerides | rs174546 |
| 11 | 61.56983 | Cholesterol, total | rs174546 |
| 11 | 61.56983 | Metabolic syndrome | rs174546 |
| 11 | 61.56983 | Glycerophospholipid levels | rs174546 |
| 11 | 61.56983 | Plasma omega−6 polyunsaturated fatty acid levels (gamma−linolenic acid) | rs174546 |
| 11 | 61.56983 | Trans fatty acid levels | rs174546 |
| 11 | 61.56983 | C−reactive protein levels or triglyceride levels (pleiotropy) | rs174546 |
| 11 | 61.56983 | C−reactive protein levels or HDL−cholesterol levels (pleiotropy) | rs174546 |

GWAS Catalog SNPs in Region

| chr | pos (Mb) | trait | snp |
|---|---|---|---|
| 11 | 61.57078 | HDL cholesterol | rs174547 |
| 11 | 61.57078 | Triglycerides | rs174547 |
| 11 | 61.57078 | Phospholipid levels (plasma) | rs174547 |
| 11 | 61.57078 | Resting heart rate | rs174547 |
| 11 | 61.57078 | Metabolic traits | rs174547 |
| 11 | 61.57078 | Lipid metabolism phenotypes | rs174547 |
| 11 | 61.57078 | Comprehensive strength and appendicular lean mass | rs174547 |
| 11 | 61.57078 | Glycerophospholipid levels | rs174547 |
| 11 | 61.57078 | Sphingolipid levels | rs174547 |
| 11 | 61.57078 | Metabolite levels (lipid measures) | rs174547 |
| 11 | 61.57078 | Plasma omega−6 polyunsaturated fatty acid levels (linoleic acid) | rs174547 |
| 11 | 61.57078 | Plasma omega−6 polyunsaturated fatty acid levels (gamma−linolenic acid) | rs174547 |
| 11 | 61.57078 | Plasma omega−6 polyunsaturated fatty acid levels (arachidonic acid) | rs174547 |
| 11 | 61.57078 | Height | rs174547 |
| 11 | 61.57078 | Trans fatty acid levels | rs174547 |
| 11 | 61.57135 | Metabolite levels | rs174548 |
| 11 | 61.57135 | HDL cholesterol | rs174548 |
| 11 | 61.57135 | Triglycerides | rs174548 |
| 11 | 61.57135 | Blood metabolite ratios | rs174548 |
| 11 | 61.57135 | Plasma omega−6 polyunsaturated fatty acid levels (dihomo−gamma−linolenic acid) | rs174548 |
| 11 | 61.57135 | Delta−6 desaturase activity | rs174548 |

GWAS Catalog SNPs in Region

| chr | pos (Mb) | trait | snp |
|---|---|---|---|
| 11 | 61.57135 | Blood metabolite levels | rs174548 |
| 11 | 61.57135 | Hematology traits | rs174548 |
| 11 | 61.57135 | Trans fatty acid levels | rs174548 |
| 11 | 61.57138 | Laryngeal squamous cell carcinoma | rs174549 |
| 11 | 61.57138 | Comprehensive strength and appendicular lean mass | rs174549 |
| 11 | 61.57138 | Heart rate | rs174549 |
| 11 | 61.57138 | Metabolite levels | rs174549 |
| 11 | 61.57138 | Red blood cell fatty acid levels | rs174549 |
| 11 | 61.57138 | Trans fatty acid levels | rs174549 |
| 11 | 61.57148 | Phospholipid levels (plasma) | rs174550 |
| 11 | 61.57148 | Fasting glucose−related traits | rs174550 |
| 11 | 61.57148 | Fasting glucose−related traits (interaction with BMI) | rs174550 |
| 11 | 61.57148 | Glycerophospholipid levels | rs174550 |
| 11 | 61.57148 | Plasma omega−6 polyunsaturated fatty acid levels (linoleic acid) | rs174550 |
| 11 | 61.57148 | Plasma omega−6 polyunsaturated fatty acid levels (adrenic acid) | rs174550 |
| 11 | 61.57148 | Blood metabolite levels | rs174550 |
| 11 | 61.57148 | Red blood cell fatty acid levels | rs174550 |
| 11 | 61.57148 | Trans fatty acid levels | rs174550 |
| 11 | 61.57368 | LDL cholesterol | rs174551 |
| 11 | 61.57946 | Cholesterol, total | rs174554 |
| 11 | 61.57976 | Glycerophospholipid levels | rs174555 |

GWAS Catalog SNPs in Region

| chr | pos (Mb) | trait | snp |
|---|---|---|---|
| 11 | 61.57976 | Plasma omega−6 polyunsaturated fatty acid levels (dihomo−gamma−linolenic acid) | rs174555 |
| 11 | 61.57976 | Trans fatty acid levels | rs174555 |
| 11 | 61.58064 | Blood metabolite levels | rs174556 |
| 11 | 61.58064 | Trans fatty acid levels | rs174556 |
| 11 | 61.59556 | Glycerophospholipid levels | rs968567 |
| 11 | 61.59556 | Rheumatoid arthritis | rs968567 |
| 11 | 61.59556 | Blood metabolite levels | rs968567 |
| 11 | 61.59721 | Cholesterol, total | rs174570 |
| 11 | 61.59721 | LDL cholesterol | rs174570 |
| 11 | 61.59721 | HDL cholesterol | rs174570 |
| 11 | 61.59721 | Glycated hemoglobin levels | rs174570 |
| 11 | 61.59721 | Trans fatty acid levels | rs174570 |
| 11 | 61.59797 | Phospholipid levels (plasma) | rs1535 |
| 11 | 61.59797 | Metabolic syndrome | rs1535 |
| 11 | 61.59797 | Response to statin therapy | rs1535 |
| 11 | 61.59797 | Glycerophospholipid levels | rs1535 |
| 11 | 61.59797 | Inflammatory bowel disease | rs1535 |
| 11 | 61.59797 | Trans fatty acid levels | rs1535 |
| 11 | 61.60034 | Phospholipid levels (plasma) | rs174574 |
| 11 | 61.60034 | Trans fatty acid levels | rs174574 |
| 11 | 61.60034 | C−reactive protein levels or LDL−cholesterol levels (pleiotropy) | rs174574 |

GWAS Catalog SNPs in Region

| chr | pos (Mb) | trait | snp |
|---|---|---|---|
| 11 | 61.60191 | Trans fatty acid levels | rs2845573 |
| 11 | 61.60200 | Trans fatty acid levels | rs174575 |
| 11 | 61.60324 | Plasma omega−6 polyunsaturated fatty acid levels (linoleic acid) | rs2727270 |
| 11 | 61.60324 | Trans fatty acid levels | rs2727270 |
| 11 | 61.60336 | Blood metabolite levels | rs2727271 |
| 11 | 61.60336 | Trans fatty acid levels | rs2727271 |
| 11 | 61.60351 | Glycerophospholipid levels | rs174576 |
| 11 | 61.60351 | Trans fatty acid levels | rs174576 |
| 11 | 61.60478 | Trans fatty acid levels | rs2524299 |
| 11 | 61.60481 | Iron status biomarkers (transferrin levels) | rs174577 |
| 11 | 61.60481 | Plasma omega−6 polyunsaturated fatty acid levels (arachidonic acid) | rs174577 |
| 11 | 61.60481 | P wave duration | rs174577 |
| 11 | 61.60481 | Trans fatty acid levels | rs174577 |
| 11 | 61.60481 | QRS duration | rs174577 |
| 11 | 61.60522 | Trans fatty acid levels | rs2072114 |
| 11 | 61.60550 | Glycerophospholipid levels | rs174578 |
| 11 | 61.60550 | Plasma omega−6 polyunsaturated fatty acid levels (linoleic acid) | rs174578 |
| 11 | 61.60550 | Blood metabolite levels | rs174578 |
| 11 | 61.60550 | Trans fatty acid levels | rs174578 |
| 11 | 61.60975 | Response to statin therapy | rs174583 |
| 11 | 61.60975 | QT interval | rs174583 |

GWAS Catalog SNPs in Region

| chr | pos (Mb) | trait | snp |
|---|---|---|---|
| 11 | 61.60975 | Trans fatty acid levels | rs174583 |
| 11 | 61.61601 | Trans fatty acid levels | rs2851682 |
| 11 | 61.61601 | Gestational age at birth (child effect) | rs2851682 |
| 11 | 61.62314 | Liver enzyme levels (alkaline phosphatase) | rs174601 |
| 11 | 61.62314 | Blood metabolite levels | rs174601 |
| 11 | 61.62314 | Red blood cell fatty acid levels | rs174601 |
| 11 | 61.62314 | Trans fatty acid levels | rs174601 |
| 11 | 61.62379 | Trans fatty acid levels | rs2526678 |
| 11 | 61.63949 | Trans fatty acid levels | rs422249 |
| 11 | 61.63957 | Phospholipid levels (plasma) | rs174448 |
| 11 | 61.63957 | Trans fatty acid levels | rs174448 |
| 11 | 61.64038 | Trans fatty acid levels | rs174449 |
| 11 | 61.65530 | Sphingolipid levels | rs1000778 |
| 11 | 61.66369 | Phospholipid levels (plasma) | rs174468 |
| 11 | 61.67875 | Sphingolipid levels | rs174479 |
| 11 | 61.71148 | Phospholipid levels (plasma) | rs2521572 |
| 11 | 61.71213 | Estradiol levels | rs2727261 |
| 11 | 61.72264 | Phospholipid levels (plasma) | rs1109748 |
| 11 | 61.74629 | Phospholipid levels (plasma) | rs10792320 |
| 11 | 61.79559 | Phospholipid levels (plasma) | rs11230874 |
| 11 | 61.81580 | Phospholipid levels (plasma) | rs4963452 |

GWAS Catalog SNPs in Region

| chr | pos (Mb) | trait | snp |
|---|---|---|---|
| 11 | 61.96393 | 3−hydroxypropylmercapturic acid levels in smokers | rs189478926 |
| 11 | 62.00141 | Plasma omega−6 polyunsaturated fatty acid levels (gamma−linolenic acid) | rs12806663 |
| 11 | 62.06135 | HIV−1 viral setpoint | rs11231017 |
| 11 | 62.10342 | Chronic obstructive pulmonary disease−related biomarkers | rs2463822 |
| 11 | 62.14169 | 3−hydroxypropylmercapturic acid levels in smokers | rs184485201 |

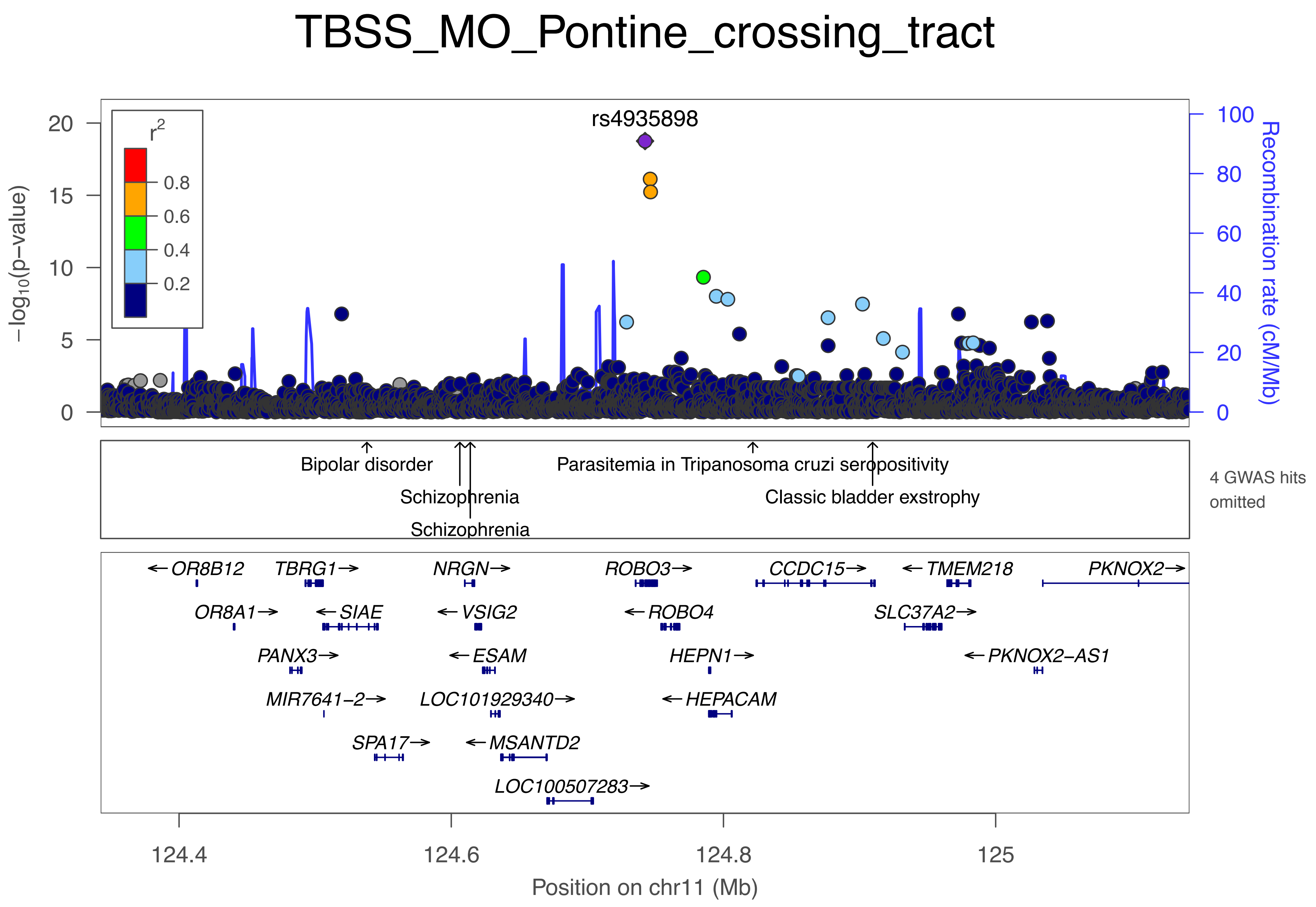

TBSS_MO_Pontine_crossing_tract
rs4935898
r2
0.8
0.6
0.4
0.2
−log10(p−value)
Recombination rate (cM/Mb)
Bipolar disorder
Schizophrenia
Schizophrenia
Parasitemia in Tripanosoma cruzi seropositivity
Classic bladder exstrophy
4 GWAS hits omitted
OR8B12
TBRG1
NRGN
ROBO3
CCDC15
TMEM218
PKNOX2
OR8A1
SIAE
VSIG2
ROBO4
SLC37A2
PANX3
ESAM
HEPN1
PKNOX2−AS1
MIR7641−2
LOC101929340
HEPACAM
SPA17
MSANTD2
LOC100507283
124.4
124.6
124.8
125
Position on chr11 (Mb)

| | |
|---|---|
| date: | Thu Aug 17 18:53:13 2017 |
| build: | hg19 |
| display range: | chr11:124342385−125142385 [124342385−125142385] |
| hilite range: | 0 – 0 [ 0 – 0 ] |
| reference SNP: | chr11:124742385 |
| number of SNPs plotted: | 3762 |
| min P.value: | 1.76E−19 [chr11:124742385] |
| max P.value: | 9.98E−1 [chr11:124882546] |
| omitted GWAS Hits: | chr11:124.909582−Classic bladder exstrophy, NA |
| omitted GWAS Hits: | NA |

Make more plots at http://csg.sph.umich.edu/locuszoom/

GWAS Catalog SNPs in Region

| chr | pos (Mb) | trait | snp |
|---|---|---|---|
| 11 | 124.4386 | Anxiety disorder | rs10893268 |
| 11 | 124.4613 | Schizophrenia | rs7127399 |
| 11 | 124.5380 | Bipolar disorder | rs544368 |
| 11 | 124.5393 | Serum metabolite levels | rs12282107 |
| 11 | 124.6063 | Schizophrenia | rs12807809 |
| 11 | 124.6140 | Schizophrenia | rs55661361 |
| 11 | 124.8216 | Parasitemia in Tripanosoma cruzi seropositivity | rs4408325 |
| 11 | 124.9096 | Classic bladder exstrophy | rs76239813 |
| 11 | 124.9849 | Exploratory eye movement dysfunction in schizophrenia (cognitive search score) | rs663734 |

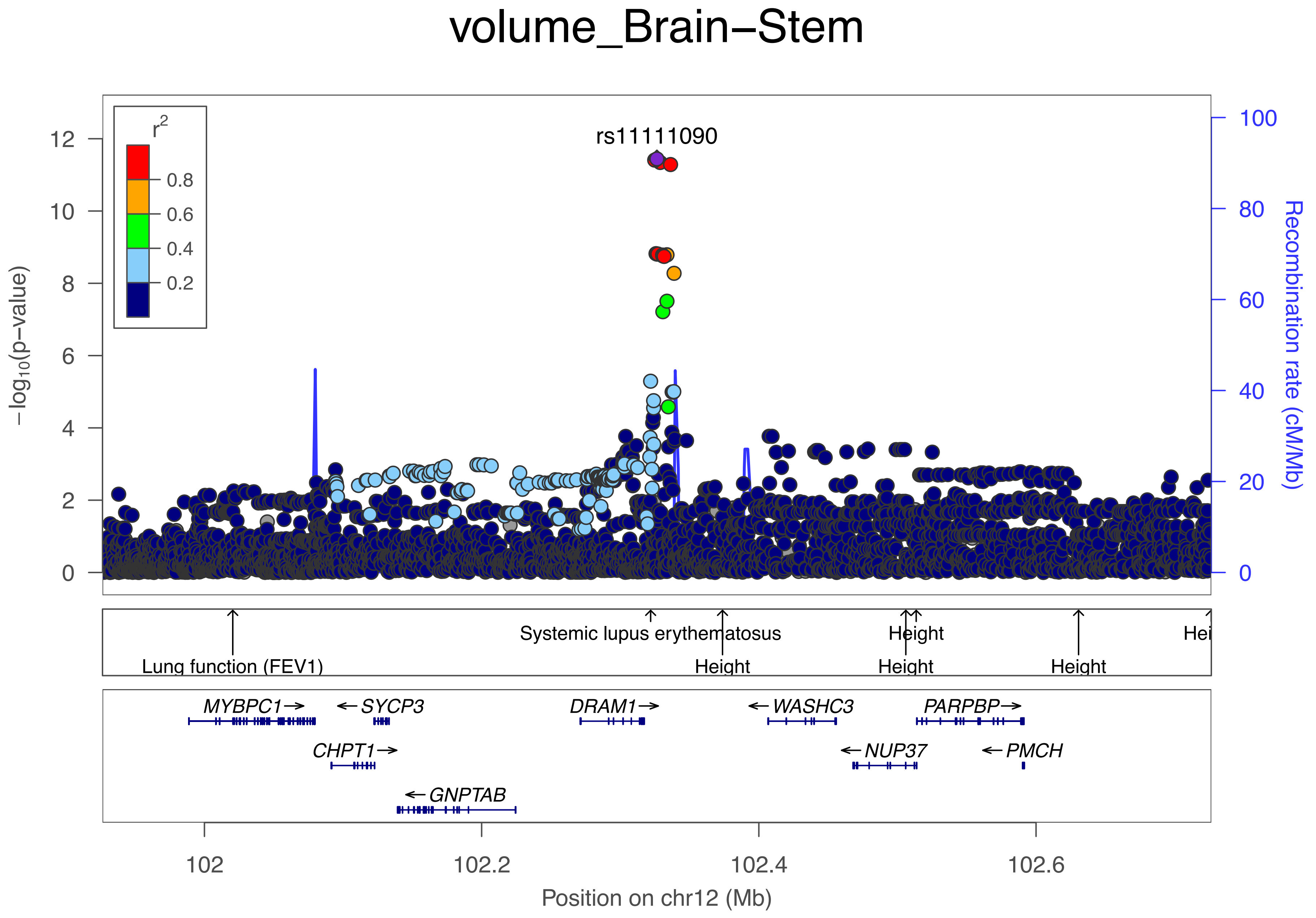
volume_Brain−Stem
rs11111090
r2
0.8
0.6
0.4
0.2
−log10(p−value)
Recombination rate (cM/Mb)
Lung function (FEV1)
Systemic lupus erythematosus
Height
Height
Height
Height
Height
Hei
MYBPC1→
←SYCP3
DRAM1→
←WASHC3
PARPBP→
CHPT1→
←NUP37
←PMCH
←GNPTAB
102
102.2
102.4
102.6
Position on chr12 (Mb)

| | |
|---|---|
| date: | Thu Aug 17 19:07:51 2017 |
| build: | hg19 |
| display range: | chr12:101926461−102726461 [101926461−102726461] |
| hilite range: | 0 – 0 [ 0 – 0 ] |
| reference SNP: | chr12:102326461 |
| number of SNPs plotted: | 3709 |
| min P.value: | 3.61E−12 [chr12:102326461] |
| max P.value: | 10E−1 [chr12:101931920] |

Make more plots at http://csg.sph.umich.edu/locuszoom/

GWAS Catalog SNPs in Region

| chr | pos (Mb) | trait | snp |
|---|---|---|---|
| 12 | 102.0204 | Lung function (FEV1) | rs10860757 |
| 12 | 102.3219 | Systemic lupus erythematosus | rs4622329 |
| 12 | 102.3738 | Height | rs7971536 |
| 12 | 102.5060 | Height | rs2271266 |
| 12 | 102.5135 | Height | rs2292303 |
| 12 | 102.6307 | Height | rs7313075 |
| 12 | 102.7263 | Height | rs1520223 |

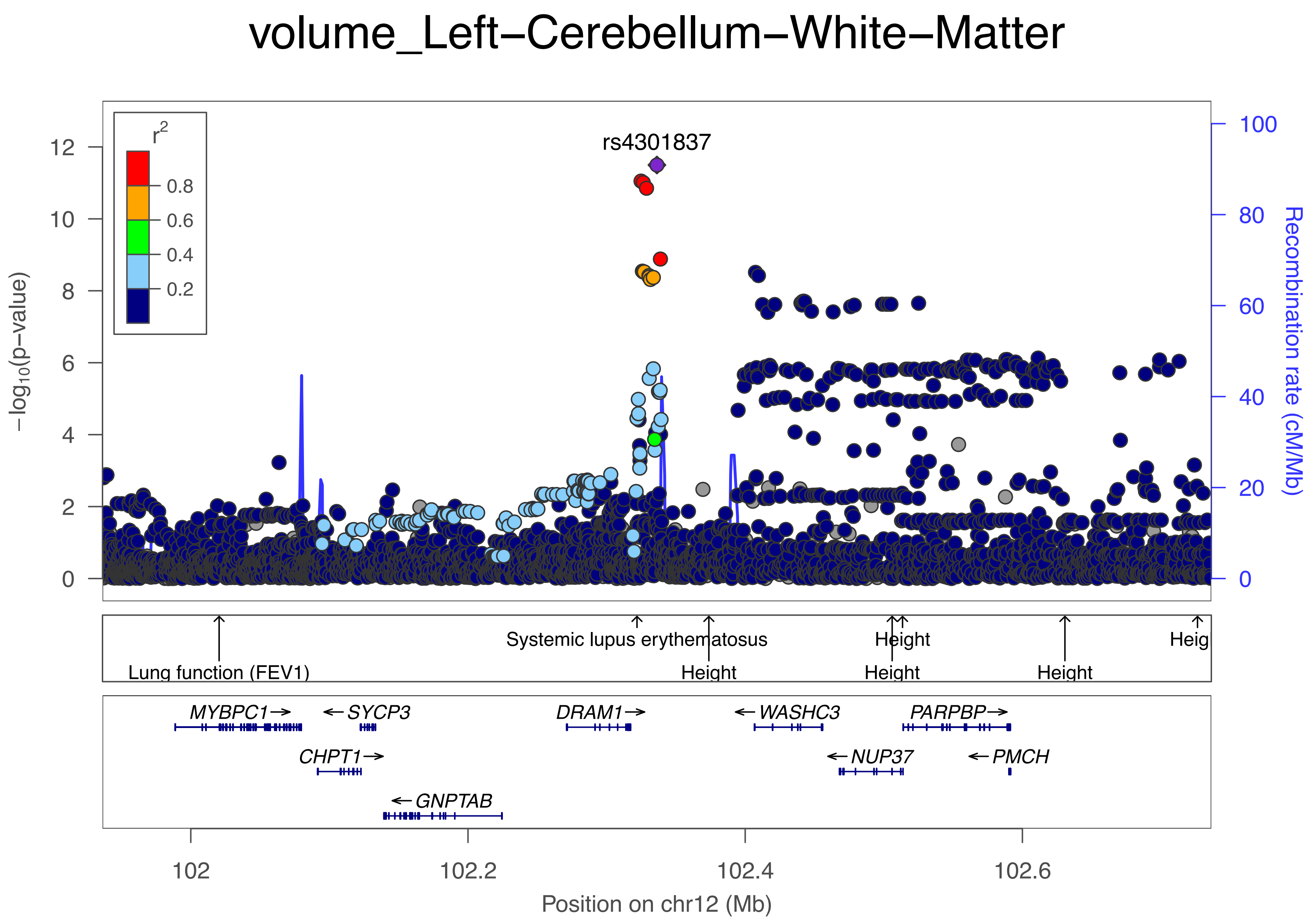

volume_Left−Cerebellum−White−Matter
rs4301837
r2
0.8
0.6
0.4
0.2
−log10(p−value)
Recombination rate (cM/Mb)
Lung function (FEV1)
Systemic lupus erythematosus
Height
Height
Height
Height
Height
Heig
MYBPC1
SYCP3
DRAM1
WASHC3
PARPBP
CHPT1
NUP37
PMCH
GNPTAB
102
102.2
102.4
102.6
Position on chr12 (Mb)

| | |
|---|---|
| date: | Thu Aug 17 19:08:20 2017 |
| build: | hg19 |
| display range: | chr12:101936310−102736310 [101936310−102736310] |
| hilite range: | 0 – 0 [ 0 – 0 ] |
| reference SNP: | chr12:102336310 |
| number of SNPs plotted: | 3703 |
| min P.value: | 3.18E−12 [chr12:102336310] |
| max P.value: | 10E−1 [chr12:102529636] |

Make more plots at http://csg.sph.umich.edu/locuszoom/

GWAS Catalog SNPs in Region

| chr | pos (Mb) | trait | snp |
|---|---|---|---|
| 12 | 102.0204 | Lung function (FEV1) | rs10860757 |
| 12 | 102.3219 | Systemic lupus erythematosus | rs4622329 |
| 12 | 102.3738 | Height | rs7971536 |
| 12 | 102.5060 | Height | rs2271266 |
| 12 | 102.5135 | Height | rs2292303 |
| 12 | 102.6307 | Height | rs7313075 |
| 12 | 102.7263 | Height | rs1520223 |

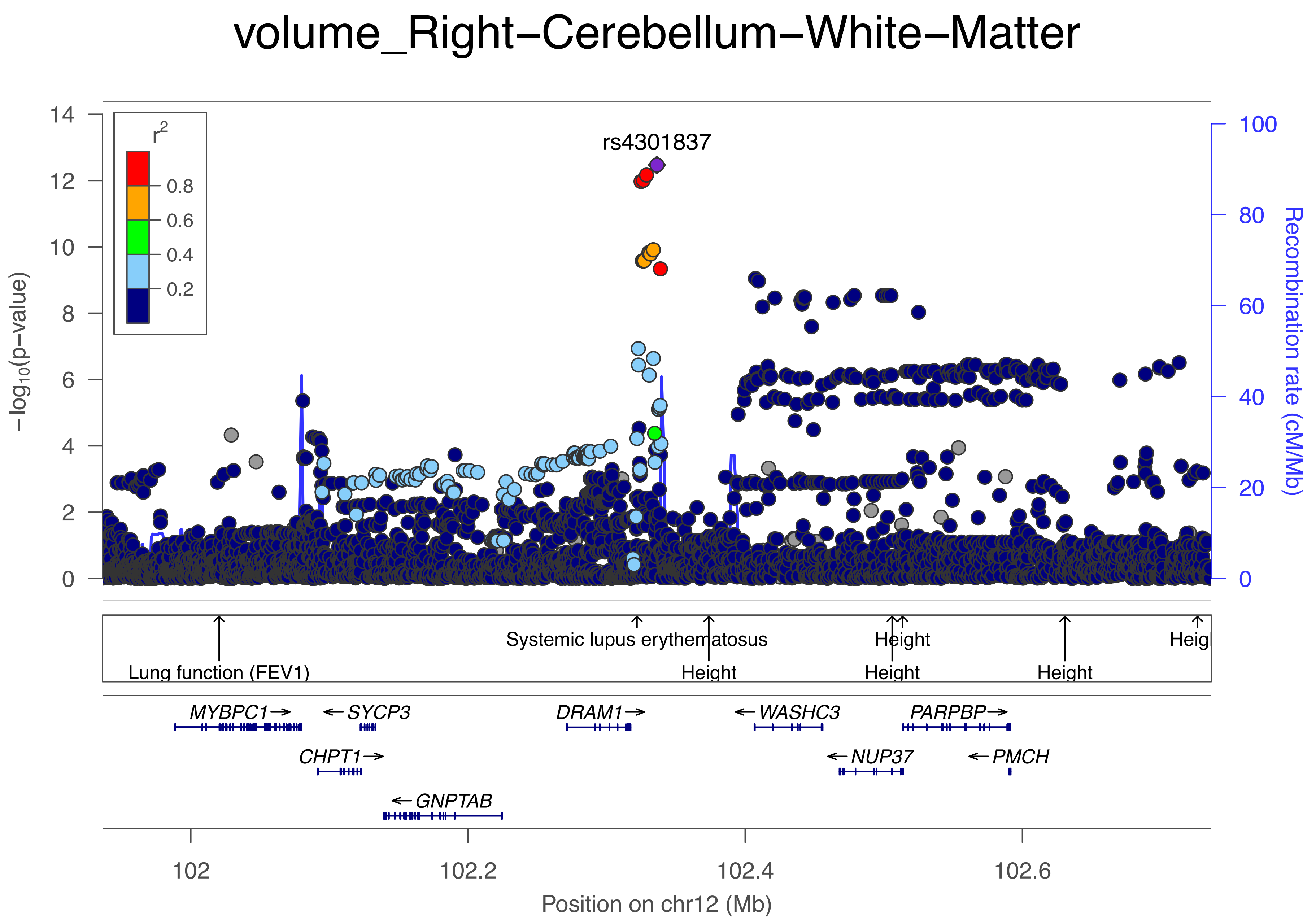
volume_Right−Cerebellum−White−Matter
rs4301837
r²
0.8
0.6
0.4
0.2
−log10(p−value)
Recombination rate (cM/Mb)
Lung function (FEV1)
Systemic lupus erythematosus
Height
Height
Height
Height
Heig
MYBPC1
SYCP3
DRAM1
WASHC3
PARPBP
CHPT1
NUP37
PMCH
GNPTAB
102
102.2
102.4
102.6
Position on chr12 (Mb)

| | |
|---|---|
| date: | Thu Aug 17 18:53:18 2017 |
| build: | hg19 |
| display range: | chr12:101936310−102736310 [101936310−102736310] |
| hilite range: | 0 – 0 [ 0 – 0 ] |
| reference SNP: | chr12:102336310 |
| number of SNPs plotted: | 3703 |
| min P.value: | 3.4E−13 [chr12:102336310] |
| max P.value: | 10E−1 [chr12:102689357] |

Make more plots at http://csg.sph.umich.edu/locuszoom/

GWAS Catalog SNPs in Region

| chr | pos (Mb) | trait | snp |
|---|---|---|---|
| 12 | 102.0204 | Lung function (FEV1) | rs10860757 |
| 12 | 102.3219 | Systemic lupus erythematosus | rs4622329 |
| 12 | 102.3738 | Height | rs7971536 |
| 12 | 102.5060 | Height | rs2271266 |
| 12 | 102.5135 | Height | rs2292303 |
| 12 | 102.6307 | Height | rs7313075 |
| 12 | 102.7263 | Height | rs1520223 |

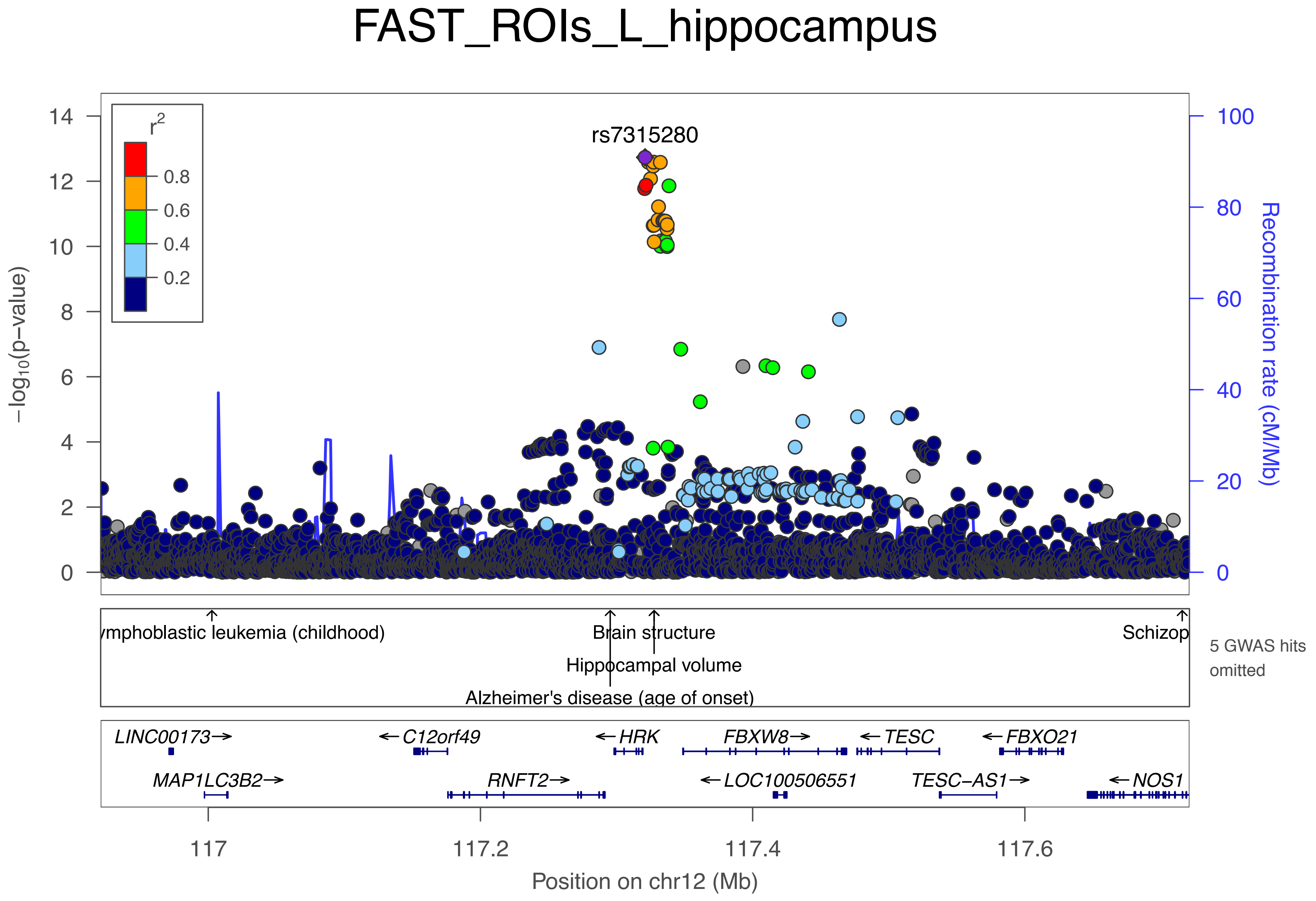
FAST_ROIs_L_hippocampus
rs7315280
r2
0.8
0.6
0.4
0.2
−log10(p−value)
Recombination rate (cM/Mb)
14
12
10
8
6
4
2
0
100
80
60
40
20
0
ymphoblastic leukemia (childhood)
Brain structure
Hippocampal volume
Alzheimer's disease (age of onset)
Schizop
5 GWAS hits
omitted
LINC00173→
←C12orf49
←HRK
FBXW8→
←TESC
←FBXO21
MAP1LC3B2→
RNFT2→
←LOC100506551
TESC−AS1→
←NOS1
117
117.2
117.4
117.6
Position on chr12 (Mb)

| | |
|---|---|
| date: | Thu Aug 17 18:58:48 2017 |
| build: | hg19 |
| display range: | chr12:116920938−117720938 [116920938−117720938] |
| hilite range: | 0 – 0 [ 0 – 0 ] |
| reference SNP: | chr12:117320938 |
| number of SNPs plotted: | 3873 |
| min P.value: | 1.85E−13 [chr12:117320938] |
| max P.value: | 10E−1 [chr12:117036380] |
| omitted GWAS Hits: | NA, NA |
| omitted GWAS Hits: | NA, NA |

Make more plots at http://csg.sph.umich.edu/locuszoom/

GWAS Catalog SNPs in Region

| chr | pos (Mb) | trait | snp |
|---|---|---|---|
| 12 | 117.0027 | Acute lymphoblastic leukemia (childhood) | rs2089222 |
| 12 | 117.1543 | Diisocyanate−induced asthma | rs2279695 |
| 12 | 117.1994 | Obesity−related traits | rs12322695 |
| 12 | 117.2953 | Alzheimer's disease (age of onset) | rs17429217 |
| 12 | 117.3234 | Subcortical brain region volumes | rs77956314 |
| 12 | 117.3276 | Brain structure | rs7294919 |
| 12 | 117.3276 | Hippocampal volume | rs7294919 |
| 12 | 117.3276 | Subcortical brain region volumes | rs7294919 |
| 12 | 117.5106 | Pre−treatment pain in head and neck squamous cell carcinoma | rs11068315 |
| 12 | 117.7156 | Schizophrenia | rs2293052 |

# FAST_ROIs_R_hippocampus

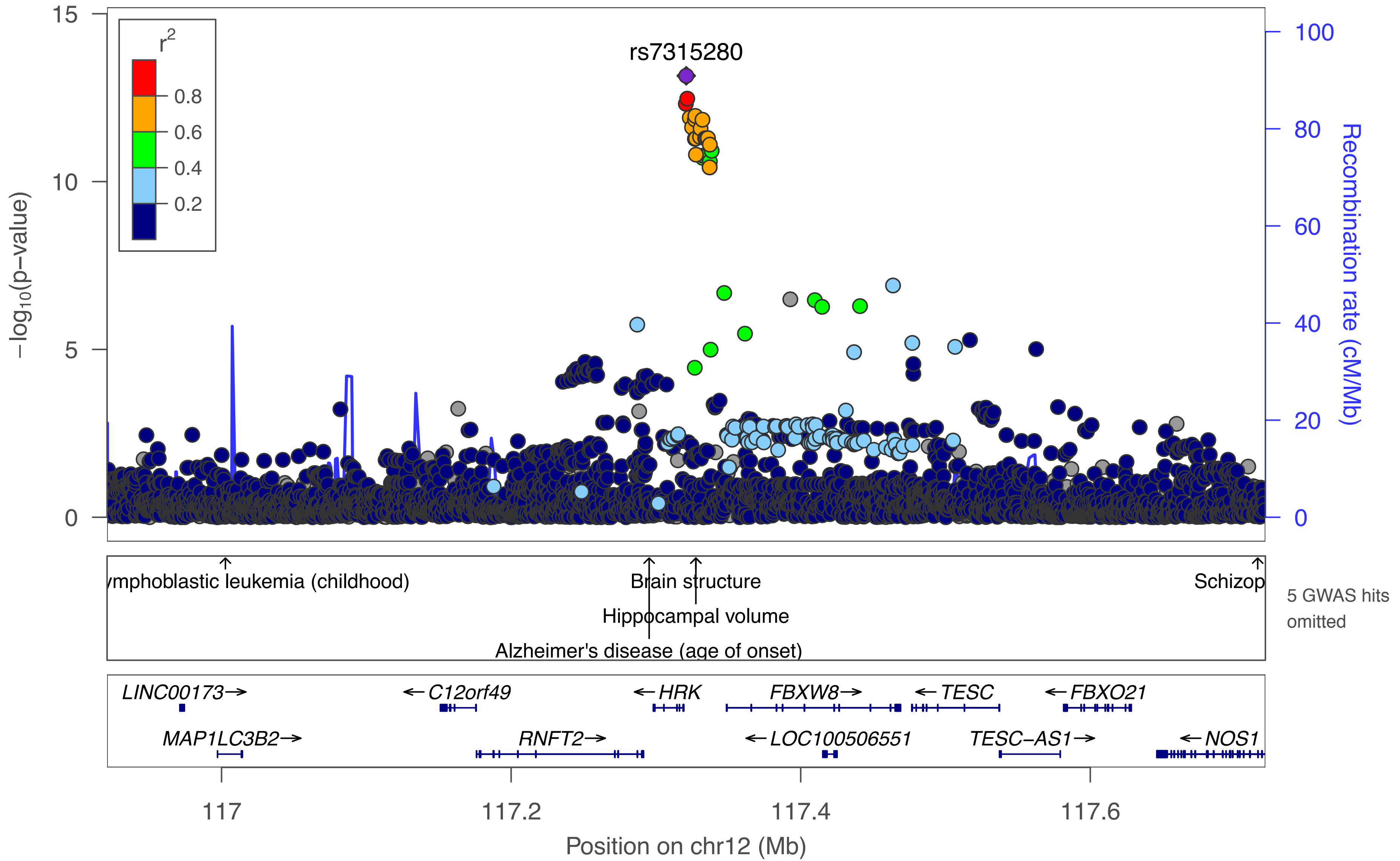

| | |
|---|---|
| date: | Thu Aug 17 18:52:17 2017 |
| build: | hg19 |
| display range: | chr12:116920938−117720938 [116920938−117720938] |
| hilite range: | 0 – 0 [ 0 – 0 ] |
| reference SNP: | chr12:117320938 |
| number of SNPs plotted: | 3873 |
| min P.value: | 7.06E−14 [chr12:117320938] |
| max P.value: | 9.99E−1 [chr12:117185788] |
| omitted GWAS Hits: | NA, NA |
| omitted GWAS Hits: | NA, NA |

Make more plots at http://csg.sph.umich.edu/locuszoom/

GWAS Catalog SNPs in Region

| chr | pos (Mb) | trait | snp |
|---|---|---|---|
| 12 | 117.0027 | Acute lymphoblastic leukemia (childhood) | rs2089222 |
| 12 | 117.1543 | Diisocyanate−induced asthma | rs2279695 |
| 12 | 117.1994 | Obesity−related traits | rs12322695 |
| 12 | 117.2953 | Alzheimer's disease (age of onset) | rs17429217 |
| 12 | 117.3234 | Subcortical brain region volumes | rs77956314 |
| 12 | 117.3276 | Brain structure | rs7294919 |
| 12 | 117.3276 | Hippocampal volume | rs7294919 |
| 12 | 117.3276 | Subcortical brain region volumes | rs7294919 |
| 12 | 117.5106 | Pre−treatment pain in head and neck squamous cell carcinoma | rs11068315 |
| 12 | 117.7156 | Schizophrenia | rs2293052 |

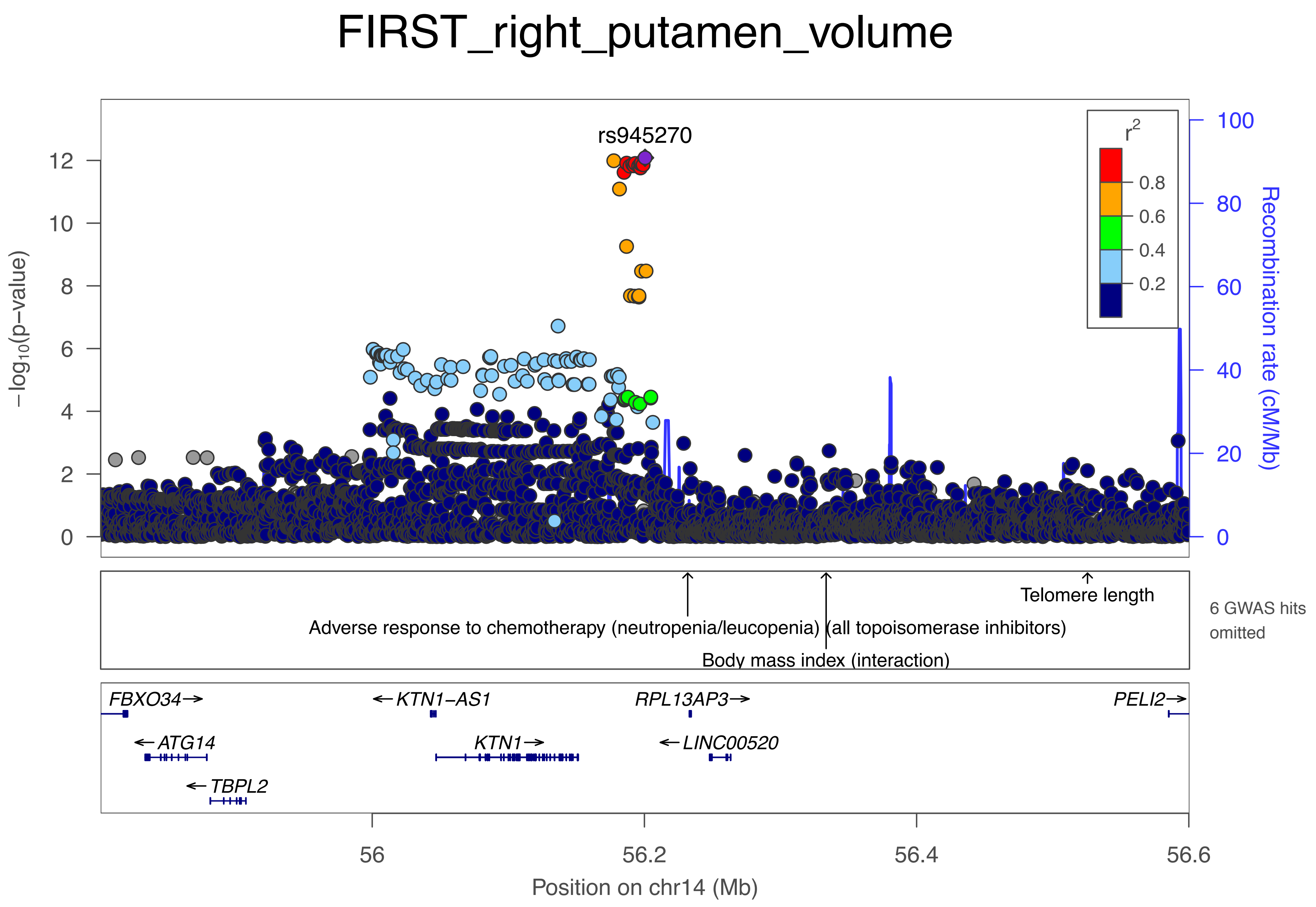
FIRST_right_putamen_volume
rs945270
−log10(p−value)
Recombination rate (cM/Mb)
r2
0.8
0.6
0.4
0.2
Telomere length
Adverse response to chemotherapy (neutropenia/leucopenia) (all topoisomerase inhibitors)
Body mass index (interaction)
6 GWAS hits omitted
FBXO34→
←KTN1−AS1
RPL13AP3→
PELI2→
←ATG14
KTN1→
←LINC00520
←TBPL2
56
56.2
56.4
56.6
Position on chr14 (Mb)

| | |
|---|---|
| date: | Thu Aug 17 19:08:40 2017 |
| build: | hg19 |
| display range: | chr14:55800473–56600473 [55800473–56600473] |
| hilite range: | 0 – 0 [ 0 – 0 ] |
| reference SNP: | chr14:56200473 |
| number of SNPs plotted: | 4468 |
| min P.value: | 8.24E–13 [chr14:56200473] |
| max P.value: | 10E–1 [chr14:55902967] |
| omitted GWAS Hits: | NA, NA |
| omitted GWAS Hits: | NA, NA |

Make more plots at http://csg.sph.umich.edu/locuszoom/

GWAS Catalog SNPs in Region

| chr | pos (Mb) | trait | snp |
|---|---|---|---|
| 14 | 56.20047 | Subcortical brain region volumes | rs945270 |
| 14 | 56.23180 | Adverse response to chemotherapy (neutropenia/leucopenia) (all topoisomerase inhibitors) | rs7494275 |
| 14 | 56.25340 | Parental extreme longevity (95 years and older) | rs12898084 |
| 14 | 56.33351 | Body mass index (interaction) | rs7350721 |
| 14 | 56.35692 | Post bronchodilator FEV1/FVC ratio | rs73276024 |
| 14 | 56.36174 | Dementia and core Alzheimer's disease neuropathologic changes | rs35862341 |
| 14 | 56.39311 | Height | rs4898878 |
| 14 | 56.41812 | Obesity−related traits | rs10151037 |
| 14 | 56.52557 | Telomere length | rs398652 |

# FIRST_left_putamen_volume_plus_FIRST_right_putamen_volume

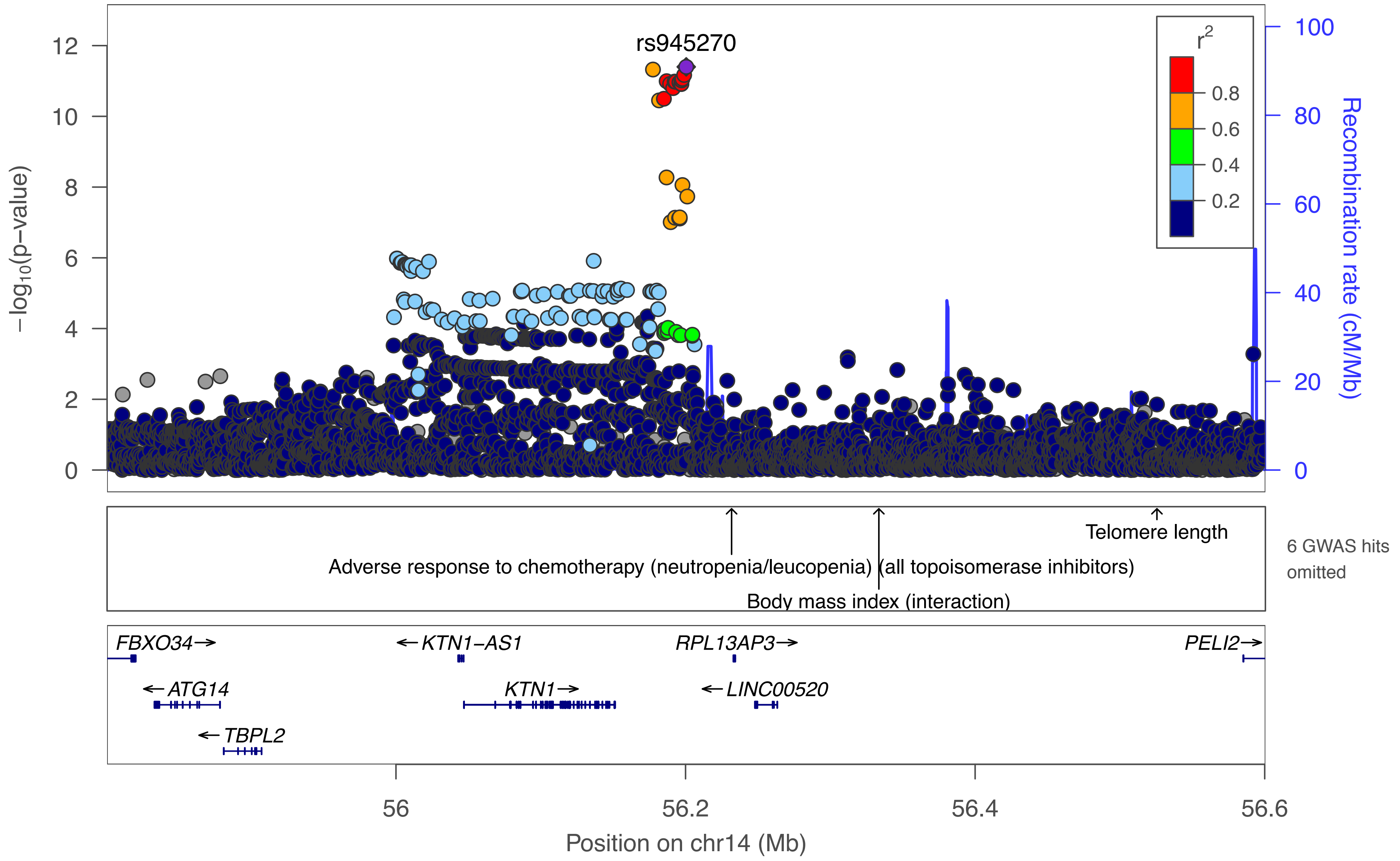

| | |
|---|---|
| date: | Thu Aug 17 19:08:40 2017 |
| build: | hg19 |
| display range: | chr14:55800473–56600473 [55800473–56600473] |
| hilite range: | 0 – 0 [ 0 – 0 ] |
| reference SNP: | chr14:56200473 |
| number of SNPs plotted: | 4468 |
| min P.value: | 3.99E−12 [chr14:56200473] |
| max P.value: | 9.99E−1 [chr14:56286466] |
| omitted GWAS Hits: | NA, NA |
| omitted GWAS Hits: | NA, NA |

Make more plots at http://csg.sph.umich.edu/locuszoom/

GWAS Catalog SNPs in Region

| chr | pos (Mb) | trait | snp |
|---|---|---|---|
| 14 | 56.20047 | Subcortical brain region volumes | rs945270 |
| 14 | 56.23180 | Adverse response to chemotherapy (neutropenia/leucopenia) (all topoisomerase inhibitors) | rs7494275 |
| 14 | 56.25340 | Parental extreme longevity (95 years and older) | rs12898084 |
| 14 | 56.33351 | Body mass index (interaction) | rs7350721 |
| 14 | 56.35692 | Post bronchodilator FEV1/FVC ratio | rs73276024 |
| 14 | 56.36174 | Dementia and core Alzheimer's disease neuropathologic changes | rs35862341 |
| 14 | 56.39311 | Height | rs4898878 |
| 14 | 56.41812 | Obesity−related traits | rs10151037 |
| 14 | 56.52557 | Telomere length | rs398652 |

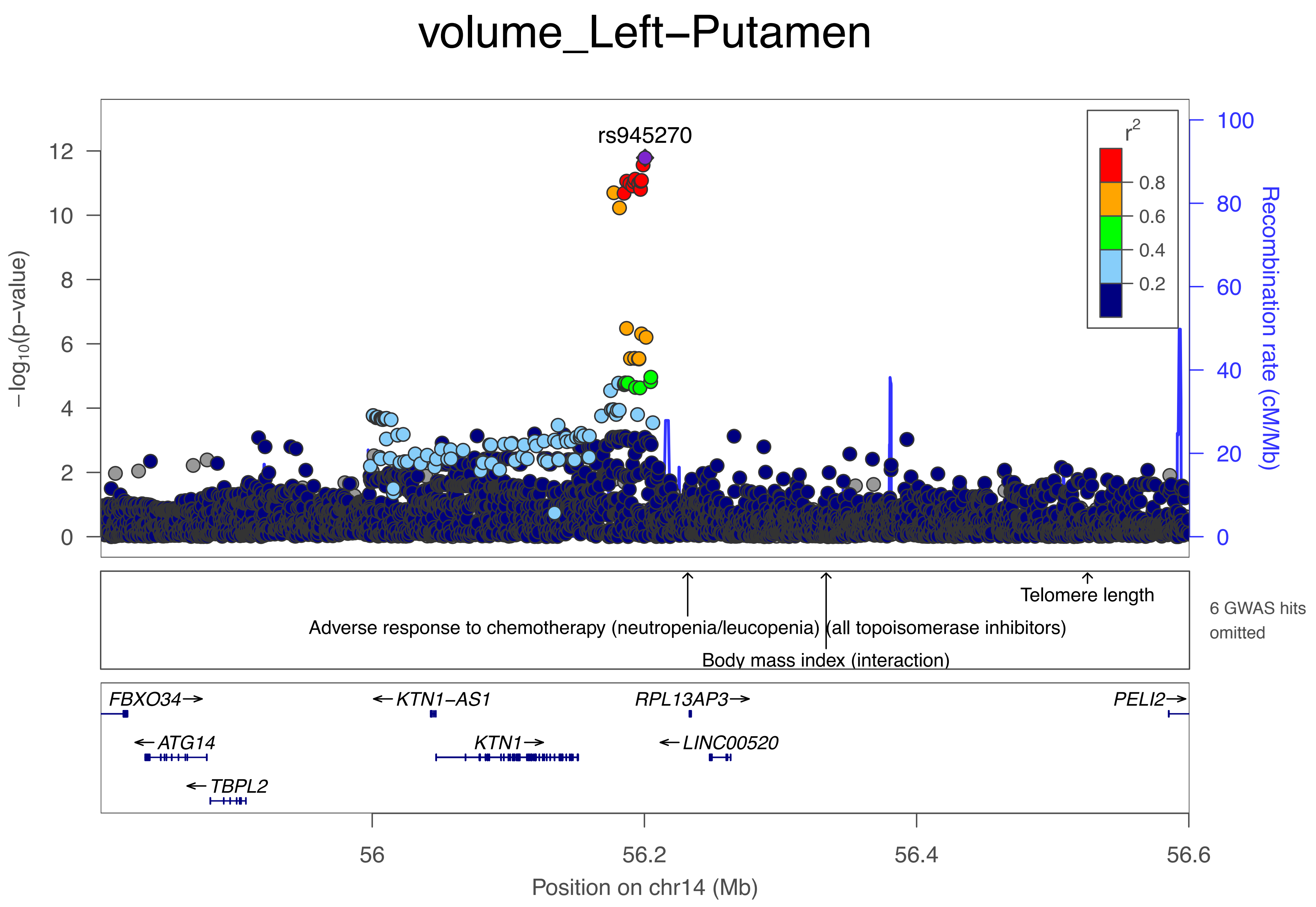

volume_Left−Putamen
rs945270
−log10(p−value)
Recombination rate (cM/Mb)
r2
0.8
0.6
0.4
0.2
Telomere length
Adverse response to chemotherapy (neutropenia/leucopenia) (all topoisomerase inhibitors)
Body mass index (interaction)
6 GWAS hits omitted
FBXO34→
←KTN1−AS1
RPL13AP3→
PELI2→
←ATG14
KTN1→
←LINC00520
←TBPL2
56
56.2
56.4
56.6
Position on chr14 (Mb)

| | |
|---|---|
| date: | Thu Aug 17 19:09:55 2017 |
| build: | hg19 |
| display range: | chr14:55800473−56600473 [55800473−56600473] |
| hilite range: | 0 – 0 [ 0 – 0 ] |
| reference SNP: | chr14:56200473 |
| number of SNPs plotted: | 4468 |
| min P.value: | 1.63E−12 [chr14:56200473] |
| max P.value: | 10E−1 [chr14:55808282] |
| omitted GWAS Hits: | NA, NA |
| omitted GWAS Hits: | NA, NA |

Make more plots at http://csg.sph.umich.edu/locuszoom/

GWAS Catalog SNPs in Region

| chr | pos (Mb) | trait | snp |
|---|---|---|---|
| 14 | 56.20047 | Subcortical brain region volumes | rs945270 |
| 14 | 56.23180 | Adverse response to chemotherapy (neutropenia/leucopenia) (all topoisomerase inhibitors) | rs7494275 |
| 14 | 56.25340 | Parental extreme longevity (95 years and older) | rs12898084 |
| 14 | 56.33351 | Body mass index (interaction) | rs7350721 |
| 14 | 56.35692 | Post bronchodilator FEV1/FVC ratio | rs73276024 |
| 14 | 56.36174 | Dementia and core Alzheimer's disease neuropathologic changes | rs35862341 |
| 14 | 56.39311 | Height | rs4898878 |
| 14 | 56.41812 | Obesity−related traits | rs10151037 |
| 14 | 56.52557 | Telomere length | rs398652 |

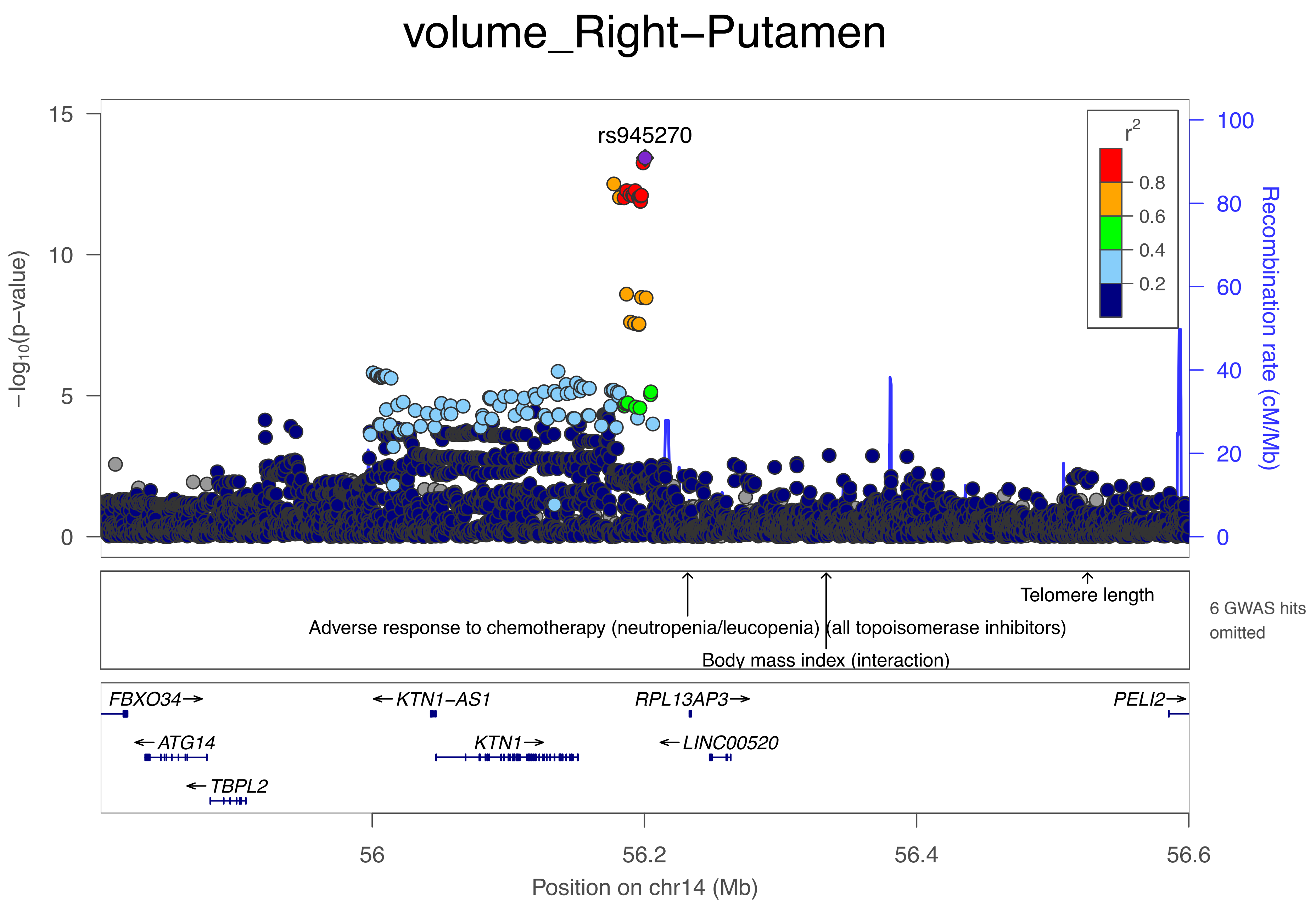
volume_Right–Putamen
rs945270
−log10(p−value)
Recombination rate (cM/Mb)
r2
0.8
0.6
0.4
0.2
15
10
5
0
100
80
60
40
20
0
Telomere length
Adverse response to chemotherapy (neutropenia/leucopenia) (all topoisomerase inhibitors)
Body mass index (interaction)
6 GWAS hits omitted
FBXO34→
←KTN1−AS1
RPL13AP3→
PELI2→
←ATG14
KTN1→
←LINC00520
←TBPL2
56
56.2
56.4
56.6
Position on chr14 (Mb)

| | |
|---|---|
| date: | Thu Aug 17 19:14:48 2017 |
| build: | hg19 |
| display range: | chr14:55800473−56600473 [55800473−56600473] |
| hilite range: | 0 – 0 [ 0 – 0 ] |
| reference SNP: | chr14:56200473 |
| number of SNPs plotted: | 4468 |
| min P.value: | 3.67E−14 [chr14:56200473] |
| max P.value: | 10E−1 [chr14:56527105] |
| omitted GWAS Hits: | NA, NA |
| omitted GWAS Hits: | NA, NA |

Make more plots at http://csg.sph.umich.edu/locuszoom/

GWAS Catalog SNPs in Region

| chr | pos (Mb) | trait | snp |
|---|---|---|---|
| 14 | 56.20047 | Subcortical brain region volumes | rs945270 |
| 14 | 56.23180 | Adverse response to chemotherapy (neutropenia/leucopenia) (all topoisomerase inhibitors) | rs7494275 |
| 14 | 56.25340 | Parental extreme longevity (95 years and older) | rs12898084 |
| 14 | 56.33351 | Body mass index (interaction) | rs7350721 |
| 14 | 56.35692 | Post bronchodilator FEV1/FVC ratio | rs73276024 |
| 14 | 56.36174 | Dementia and core Alzheimer's disease neuropathologic changes | rs35862341 |
| 14 | 56.39311 | Height | rs4898878 |
| 14 | 56.41812 | Obesity−related traits | rs10151037 |
| 14 | 56.52557 | Telomere length | rs398652 |

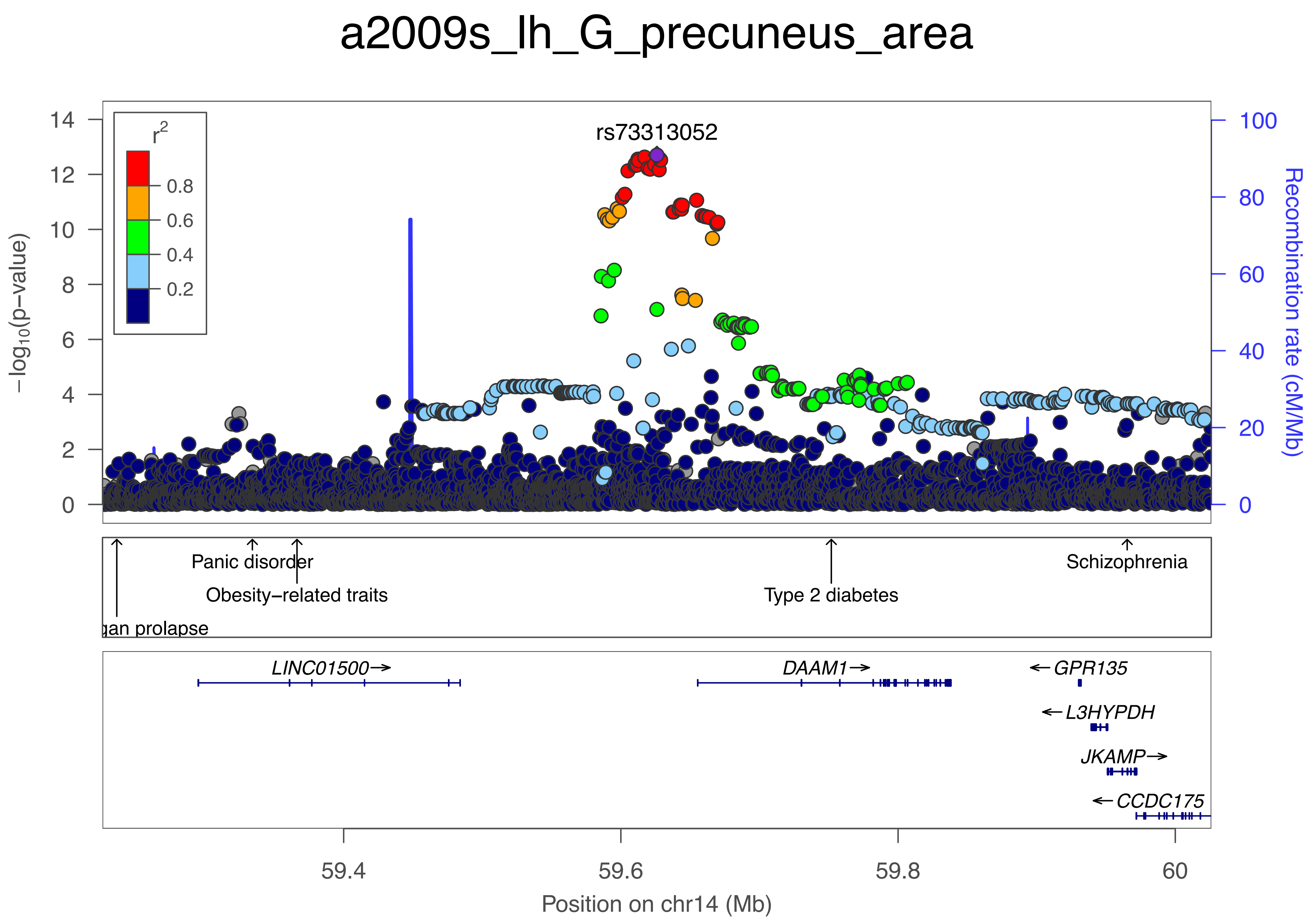
a2009s_lh_G_precuneus_area
rs73313052
r²
0.8
0.6
0.4
0.2
−log10(p−value)
Recombination rate (cM/Mb)
Panic disorder
Obesity−related traits
gan prolapse
Type 2 diabetes
Schizophrenia
LINC01500→
DAAM1→
←GPR135
←L3HYPDH
JKAMP→
←CCDC175
59.4
59.6
59.8
60
Position on chr14 (Mb)

| | |
|---|---|
| date: | Thu Aug 17 19:03:55 2017 |
| build: | hg19 |
| display range: | chr14:59225997−60025997 [59225997−60025997] |
| hilite range: | 0 – 0 [ 0 – 0 ] |
| reference SNP: | chr14:59625997 |
| number of SNPs plotted: | 4164 |
| min P.value: | 1.98E−13 [chr14:59625997] |
| max P.value: | 10E−1 [chr14:59512546] |

Make more plots at http://csg.sph.umich.edu/locuszoom/

GWAS Catalog SNPs in Region

| chr | pos (Mb) | trait | snp |
|---|---|---|---|
| 14 | 59.23631 | Pelvic organ prolapse | rs7147087 |
| 14 | 59.33413 | Panic disorder | rs4901869 |
| 14 | 59.36632 | Obesity–related traits | rs405460 |
| 14 | 59.75183 | Type 2 diabetes | rs35209784 |
| 14 | 59.96529 | Schizophrenia | rs111803315 |

# DKTatlas_lh_cuneus_area

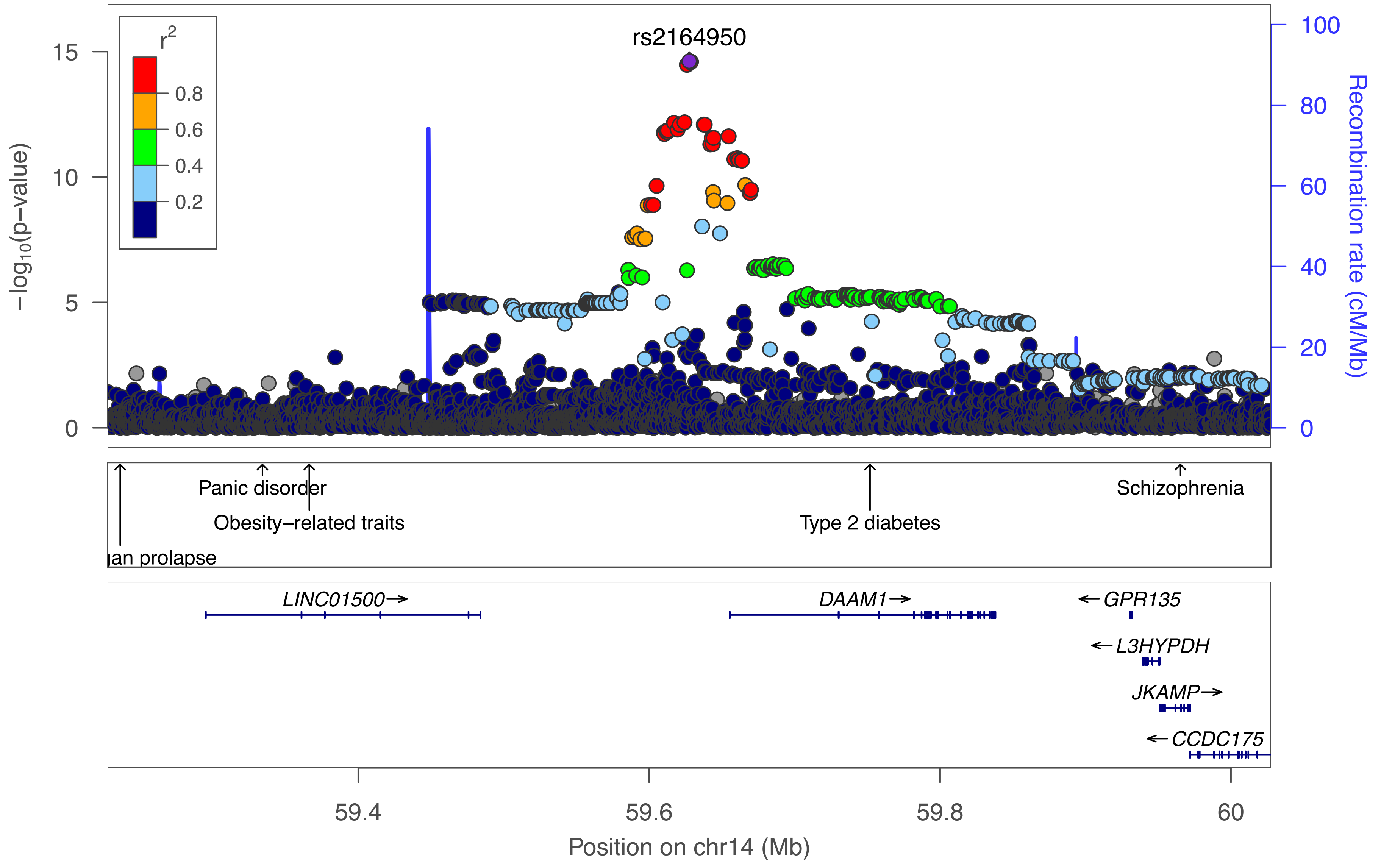

| | |
|---|---|
| date: | Thu Aug 17 19:08:58 2017 |
| build: | hg19 |
| display range: | chr14:59227631−60027631 [59227631−60027631] |
| hilite range: | 0 – 0 [ 0 – 0 ] |
| reference SNP: | chr14:59627631 |
| number of SNPs plotted: | 4163 |
| min P.value: | 2.42E−15 [chr14:59627631] |
| max P.value: | 10E−1 [chr14:59700041] |

Make more plots at http://csg.sph.umich.edu/locuszoom/

GWAS Catalog SNPs in Region

| chr | pos (Mb) | trait | snp |
|---|---|---|---|
| 14 | 59.23631 | Pelvic organ prolapse | rs7147087 |
| 14 | 59.33413 | Panic disorder | rs4901869 |
| 14 | 59.36632 | Obesity–related traits | rs405460 |
| 14 | 59.75183 | Type 2 diabetes | rs35209784 |
| 14 | 59.96529 | Schizophrenia | rs111803315 |

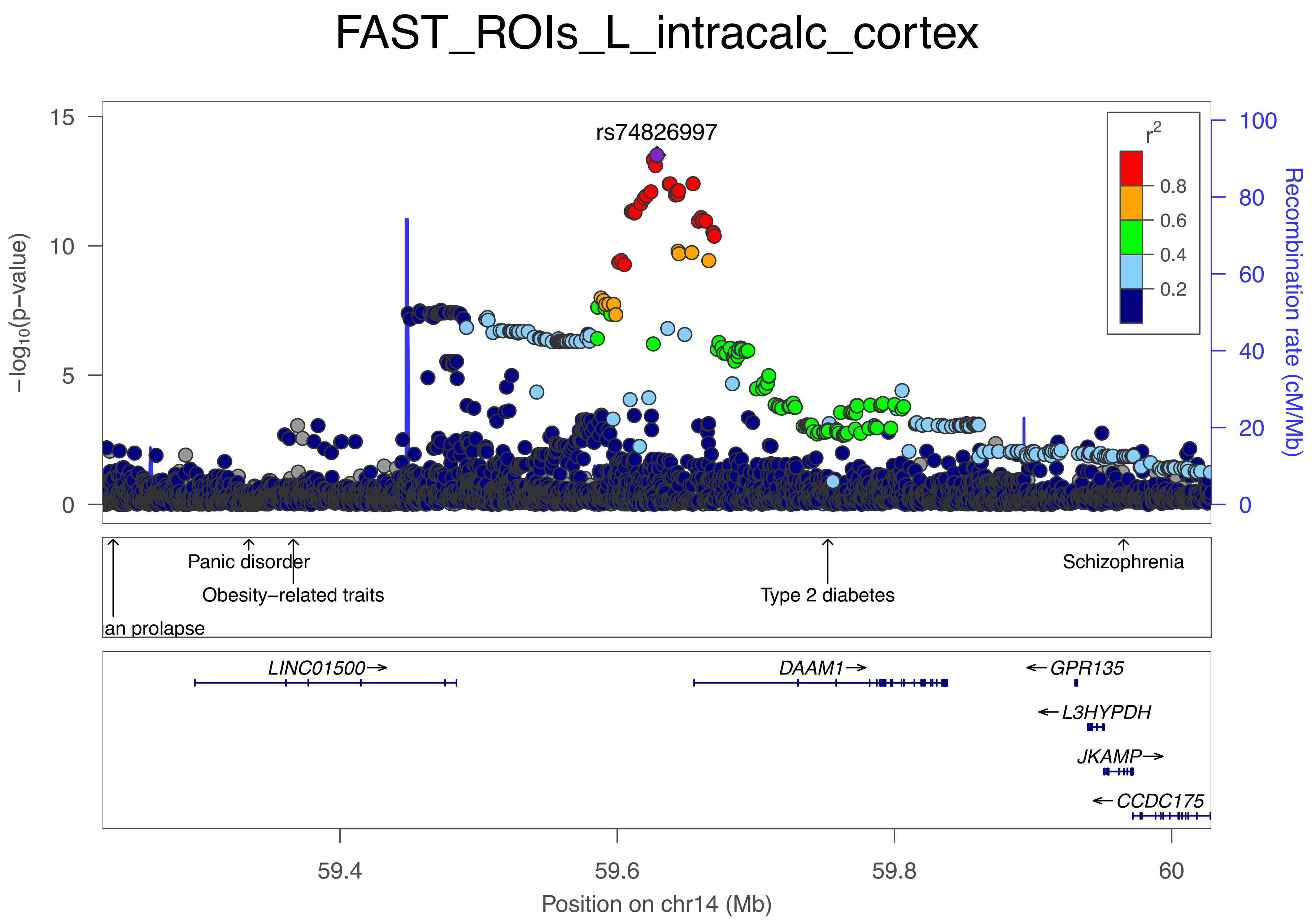

FAST_ROIs_L_intracalc_cortex
rs74826997
−log10(p−value)
Recombination rate (cM/Mb)
r2
0.8
0.6
0.4
0.2
15
10
5
0
100
80
60
40
20
0
Panic disorder
Obesity−related traits
an prolapse
Type 2 diabetes
Schizophrenia
LINC01500→
DAAM1→
←GPR135
←L3HYPDH
JKAMP→
←CCDC175
59.4
59.6
59.8
60
Position on chr14 (Mb)

| | |
|---|---|
| date: | Thu Aug 17 18:46:28 2017 |
| build: | hg19 |
| display range: | chr14:59228609−60028609 [59228609−60028609] |
| hilite range: | 0 – 0 [ 0 – 0 ] |
| reference SNP: | chr14:59628609 |
| number of SNPs plotted: | 4162 |
| min P.value: | 3.08E−14 [chr14:59628609] |
| max P.value: | 10E−1 [chr14:59334863] |

Make more plots at http://csg.sph.umich.edu/locuszoom/

# GWAS Catalog SNPs in Region

| chr | pos (Mb) | trait | snp |
|---|---|---|---|
| 14 | 59.23631 | Pelvic organ prolapse | rs7147087 |
| 14 | 59.33413 | Panic disorder | rs4901869 |
| 14 | 59.36632 | Obesity–related traits | rs405460 |
| 14 | 59.75183 | Type 2 diabetes | rs35209784 |
| 14 | 59.96529 | Schizophrenia | rs111803315 |

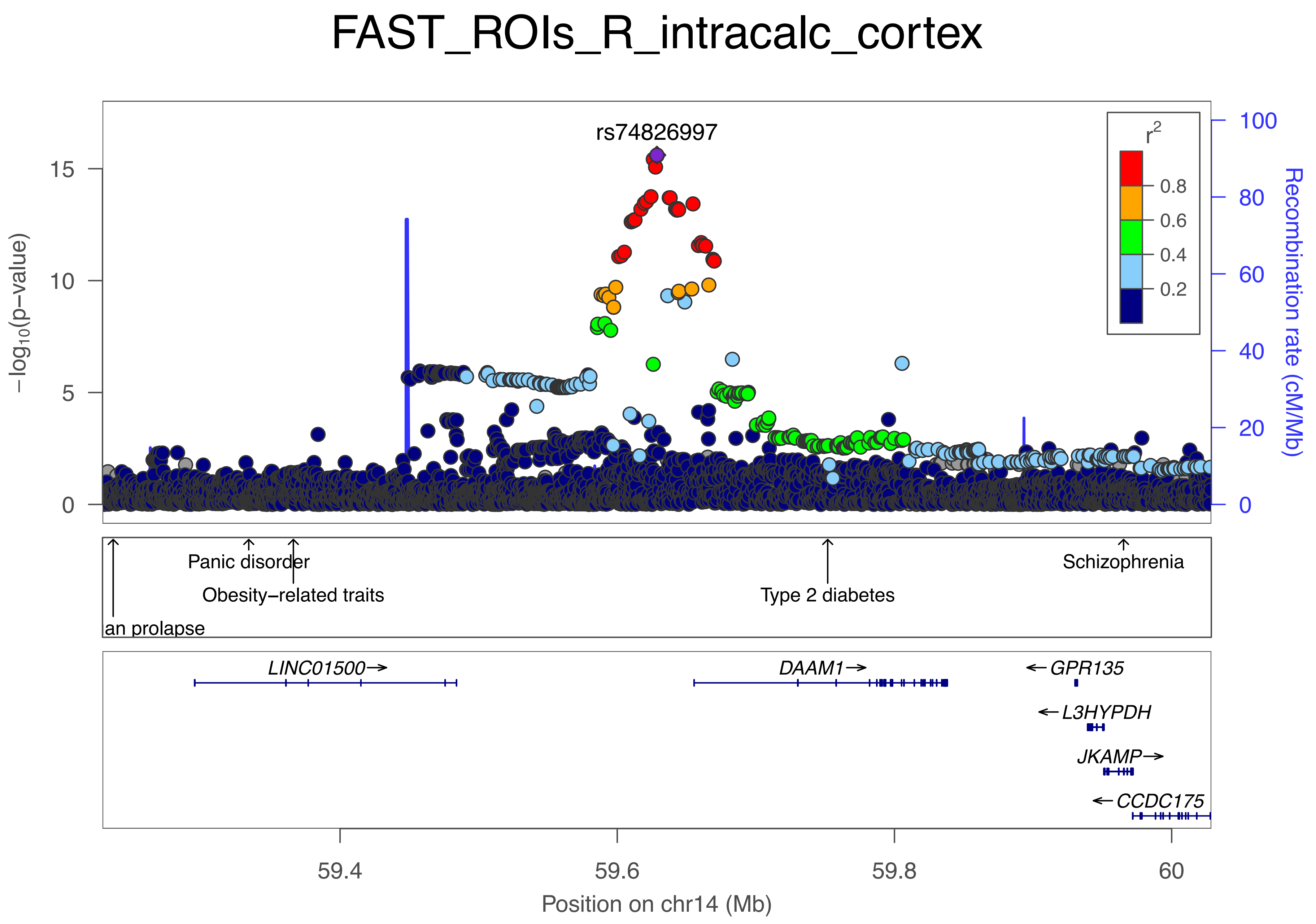
FAST_ROIs_R_intracalc_cortex
rs74826997
−log10(p−value)
Recombination rate (cM/Mb)
r2
0.8
0.6
0.4
0.2
15
10
5
0
100
80
60
40
20
0
Panic disorder
Obesity−related traits
an prolapse
Type 2 diabetes
Schizophrenia
LINC01500→
DAAM1→
←GPR135
←L3HYPDH
JKAMP→
←CCDC175
59.4
59.6
59.8
60
Position on chr14 (Mb)

| | |
|---|---|
| date: | Thu Aug 17 18:46:27 2017 |
| build: | hg19 |
| display range: | chr14:59228609−60028609 [59228609−60028609] |
| hilite range: | 0 – 0 [ 0 – 0 ] |
| reference SNP: | chr14:59628609 |
| number of SNPs plotted: | 4162 |
| min P.value: | 2.46E−16 [chr14:59628609] |
| max P.value: | 10E−1 [chr14:59746864] |

Make more plots at http://csg.sph.umich.edu/locuszoom/

GWAS Catalog SNPs in Region

| chr | pos (Mb) | trait | snp |
|---|---|---|---|
| 14 | 59.23631 | Pelvic organ prolapse | rs7147087 |
| 14 | 59.33413 | Panic disorder | rs4901869 |
| 14 | 59.36632 | Obesity–related traits | rs405460 |
| 14 | 59.75183 | Type 2 diabetes | rs35209784 |
| 14 | 59.96529 | Schizophrenia | rs111803315 |

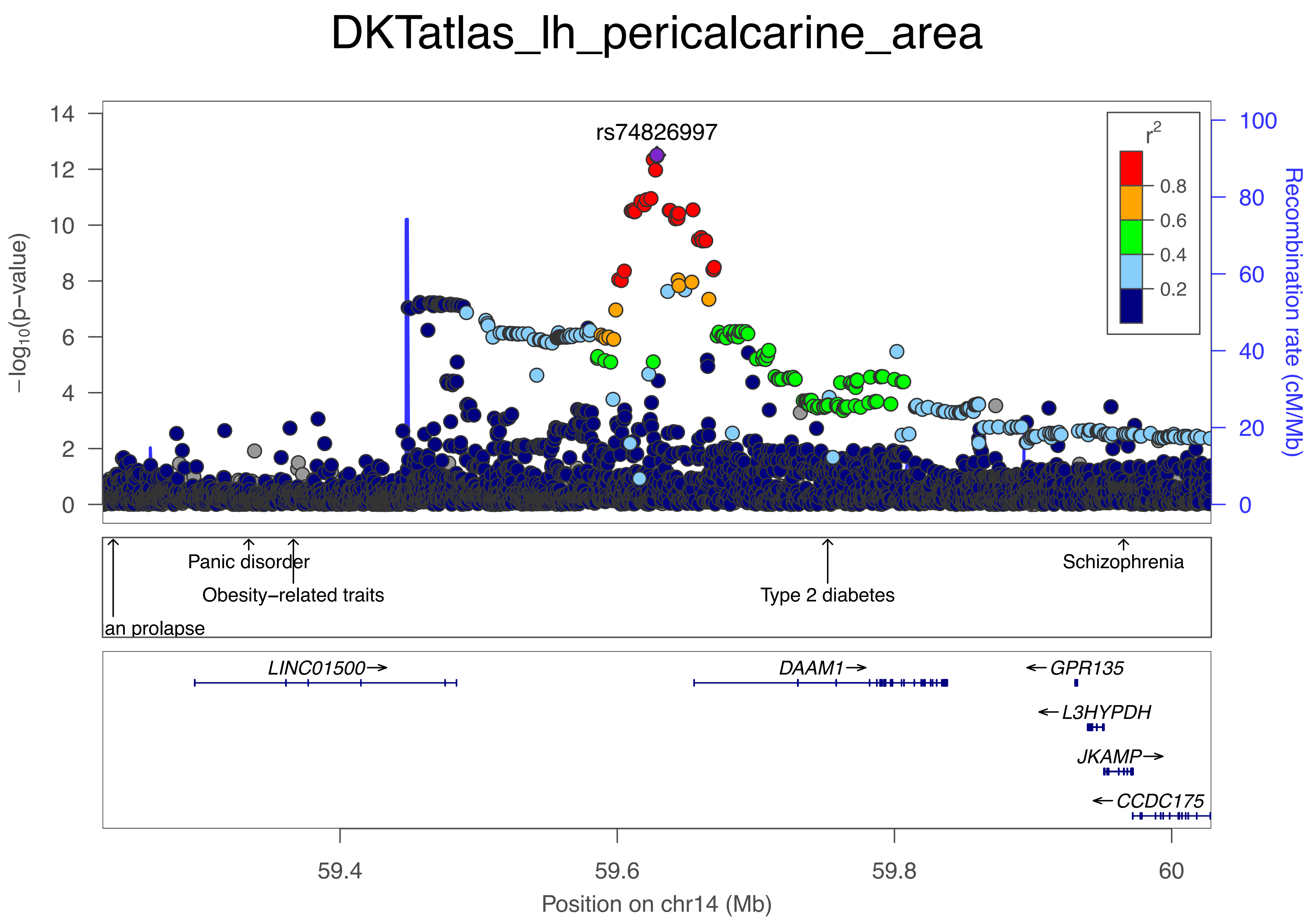

DKTatlas_lh_pericalcarine_area
rs74826997
−log10(p−value)
0
2
4
6
8
10
12
14
r2
0.8
0.6
0.4
0.2
100
80
60
40
20
0
Recombination rate (cM/Mb)
Panic disorder
Obesity−related traits
an prolapse
Type 2 diabetes
Schizophrenia
LINC01500→
DAAM1→
←GPR135
←L3HYPDH
JKAMP→
←CCDC175
59.4
59.6
59.8
60
Position on chr14 (Mb)

| | |
|---|---|
| date: | Thu Aug 17 19:09:22 2017 |
| build: | hg19 |
| display range: | chr14:59228609−60028609 [59228609−60028609] |
| hilite range: | 0 – 0  [ 0 – 0 ] |
| reference SNP: | chr14:59628609 |
| number of SNPs plotted: | 4162 |
| min P.value: | 3.1E−13 [chr14:59628609] |
| max P.value: | 10E−1 [chr14:59452591] |

Make more plots at http://csg.sph.umich.edu/locuszoom/

GWAS Catalog SNPs in Region

| chr | pos (Mb) | trait | snp |
|---|---|---|---|
| 14 | 59.23631 | Pelvic organ prolapse | rs7147087 |
| 14 | 59.33413 | Panic disorder | rs4901869 |
| 14 | 59.36632 | Obesity–related traits | rs405460 |
| 14 | 59.75183 | Type 2 diabetes | rs35209784 |
| 14 | 59.96529 | Schizophrenia | rs111803315 |

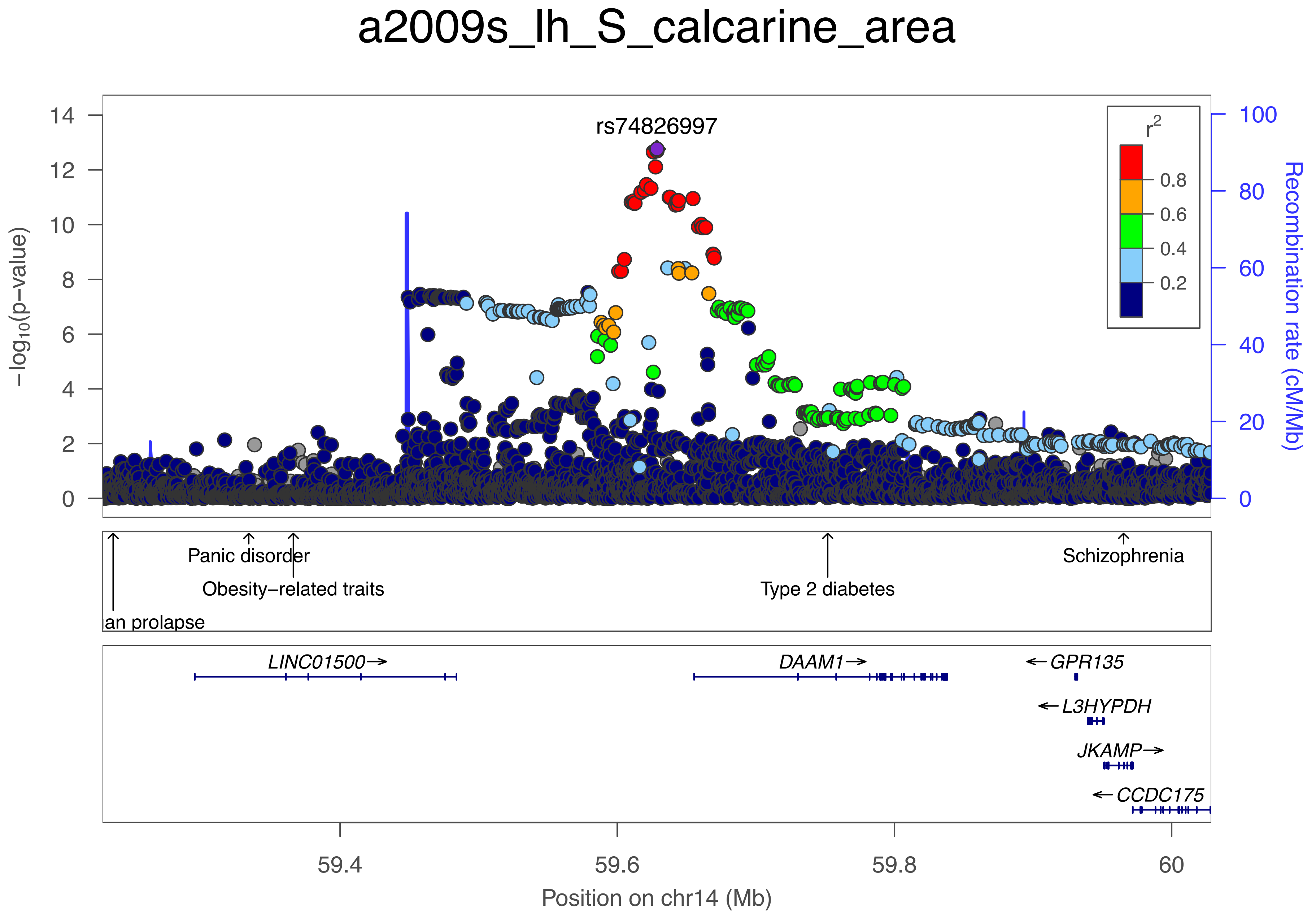
a2009s_lh_S_calcarine_area
rs74826997
−log10(p−value)
Recombination rate (cM/Mb)
r2
0.8
0.6
0.4
0.2
Panic disorder
Obesity−related traits
an prolapse
Type 2 diabetes
Schizophrenia
LINC01500→
DAAM1→
←GPR135
←L3HYPDH
JKAMP→
←CCDC175
59.4
59.6
59.8
60
Position on chr14 (Mb)

| | |
|---|---|
| date: | Thu Aug 17 19:08:59 2017 |
| build: | hg19 |
| display range: | chr14:59228609−60028609 [59228609−60028609] |
| hilite range: | 0 – 0  [ 0 – 0 ] |
| reference SNP: | chr14:59628609 |
| number of SNPs plotted: | 4162 |
| min P.value: | 1.72E−13 [chr14:59628609] |
| max P.value: | 10E−1 [chr14:59263090] |

Make more plots at http://csg.sph.umich.edu/locuszoom/

GWAS Catalog SNPs in Region

| chr | pos (Mb) | trait | snp |
|---|---|---|---|
| 14 | 59.23631 | Pelvic organ prolapse | rs7147087 |
| 14 | 59.33413 | Panic disorder | rs4901869 |
| 14 | 59.36632 | Obesity–related traits | rs405460 |
| 14 | 59.75183 | Type 2 diabetes | rs35209784 |
| 14 | 59.96529 | Schizophrenia | rs111803315 |

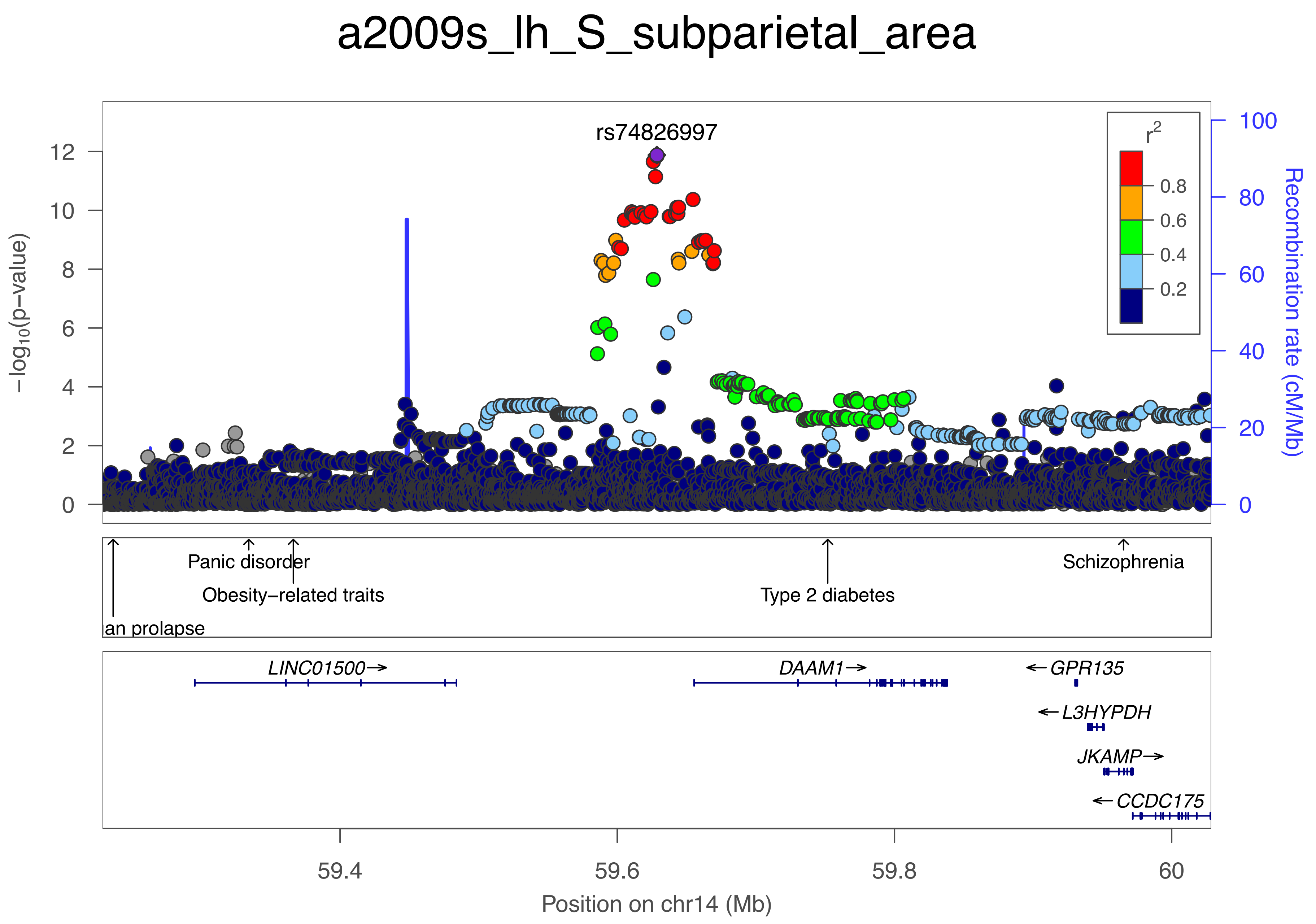
a2009s_lh_S_subparietal_area
rs74826997
r2
0.8
0.6
0.4
0.2
−log10(p−value)
Recombination rate (cM/Mb)
Panic disorder
Obesity−related traits
an prolapse
Type 2 diabetes
Schizophrenia
LINC01500
DAAM1
GPR135
L3HYPDH
JKAMP
CCDC175
59.4
59.6
59.8
60
Position on chr14 (Mb)

| | |
|---|---|
| date: | Thu Aug 17 18:55:02 2017 |
| build: | hg19 |
| display range: | chr14:59228609−60028609 [59228609−60028609] |
| hilite range: | 0 – 0 [ 0 – 0 ] |
| reference SNP: | chr14:59628609 |
| number of SNPs plotted: | 4162 |
| min P.value: | 1.32E−12 [chr14:59628609] |
| max P.value: | 10E−1 [chr14:59768278] |

Make more plots at http://csg.sph.umich.edu/locuszoom/

GWAS Catalog SNPs in Region

| chr | pos (Mb) | trait | snp |
|---|---|---|---|
| 14 | 59.23631 | Pelvic organ prolapse | rs7147087 |
| 14 | 59.33413 | Panic disorder | rs4901869 |
| 14 | 59.36632 | Obesity–related traits | rs405460 |
| 14 | 59.75183 | Type 2 diabetes | rs35209784 |
| 14 | 59.96529 | Schizophrenia | rs111803315 |

# DKTatlas_rh_pericalcarine_area

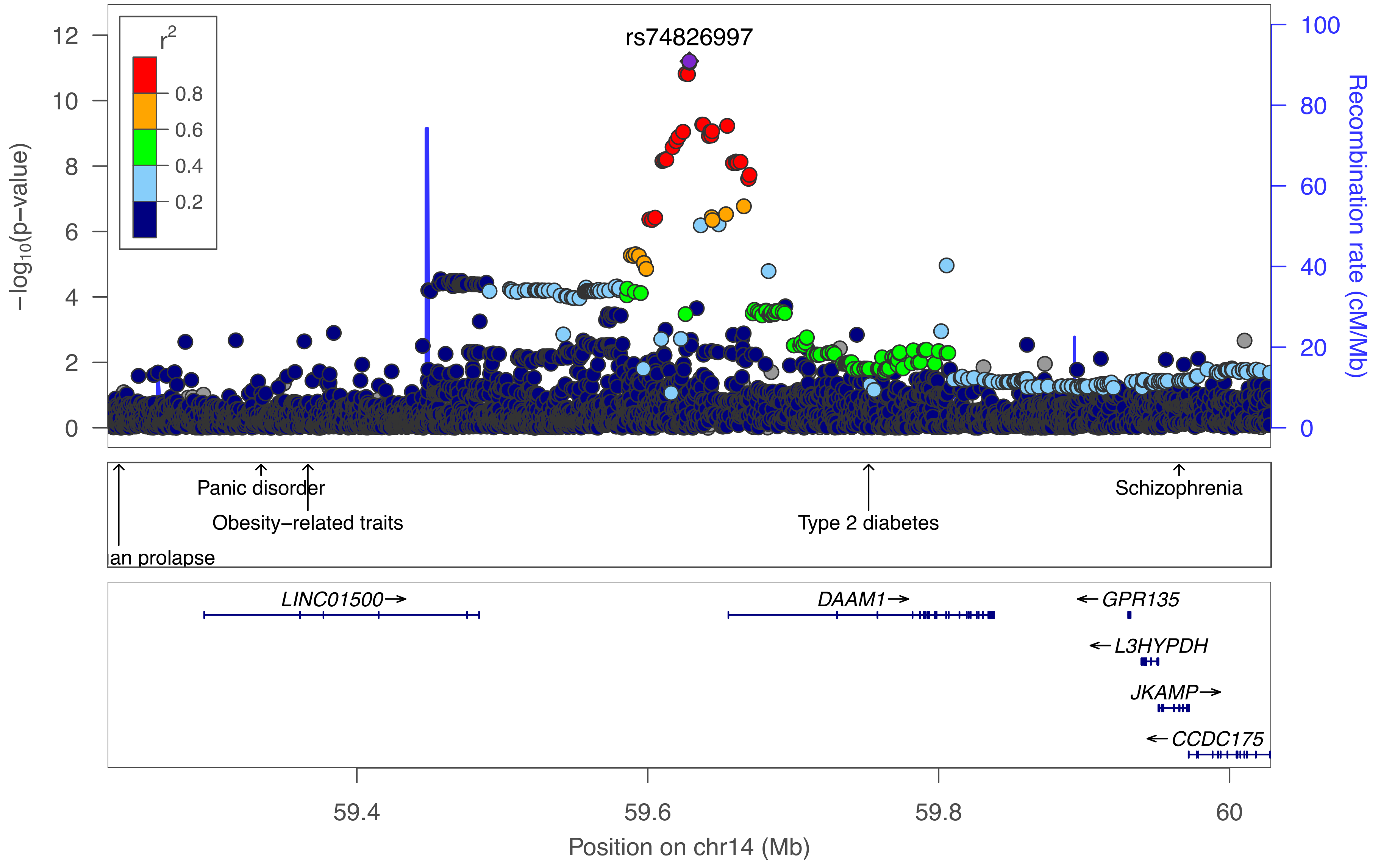

| | |
|---|---|
| date: | Thu Aug 17 18:58:26 2017 |
| build: | hg19 |
| display range: | chr14:59228609−60028609 [59228609−60028609] |
| hilite range: | 0 – 0 [ 0 – 0 ] |
| reference SNP: | chr14:59628609 |
| number of SNPs plotted: | 4162 |
| min P.value: | 6.25E−12 [chr14:59628609] |
| max P.value: | 10E−1 [chr14:59347121] |

Make more plots at http://csg.sph.umich.edu/locuszoom/

GWAS Catalog SNPs in Region

| chr | pos (Mb) | trait | snp |
|---|---|---|---|
| 14 | 59.23631 | Pelvic organ prolapse | rs7147087 |
| 14 | 59.33413 | Panic disorder | rs4901869 |
| 14 | 59.36632 | Obesity–related traits | rs405460 |
| 14 | 59.75183 | Type 2 diabetes | rs35209784 |
| 14 | 59.96529 | Schizophrenia | rs111803315 |

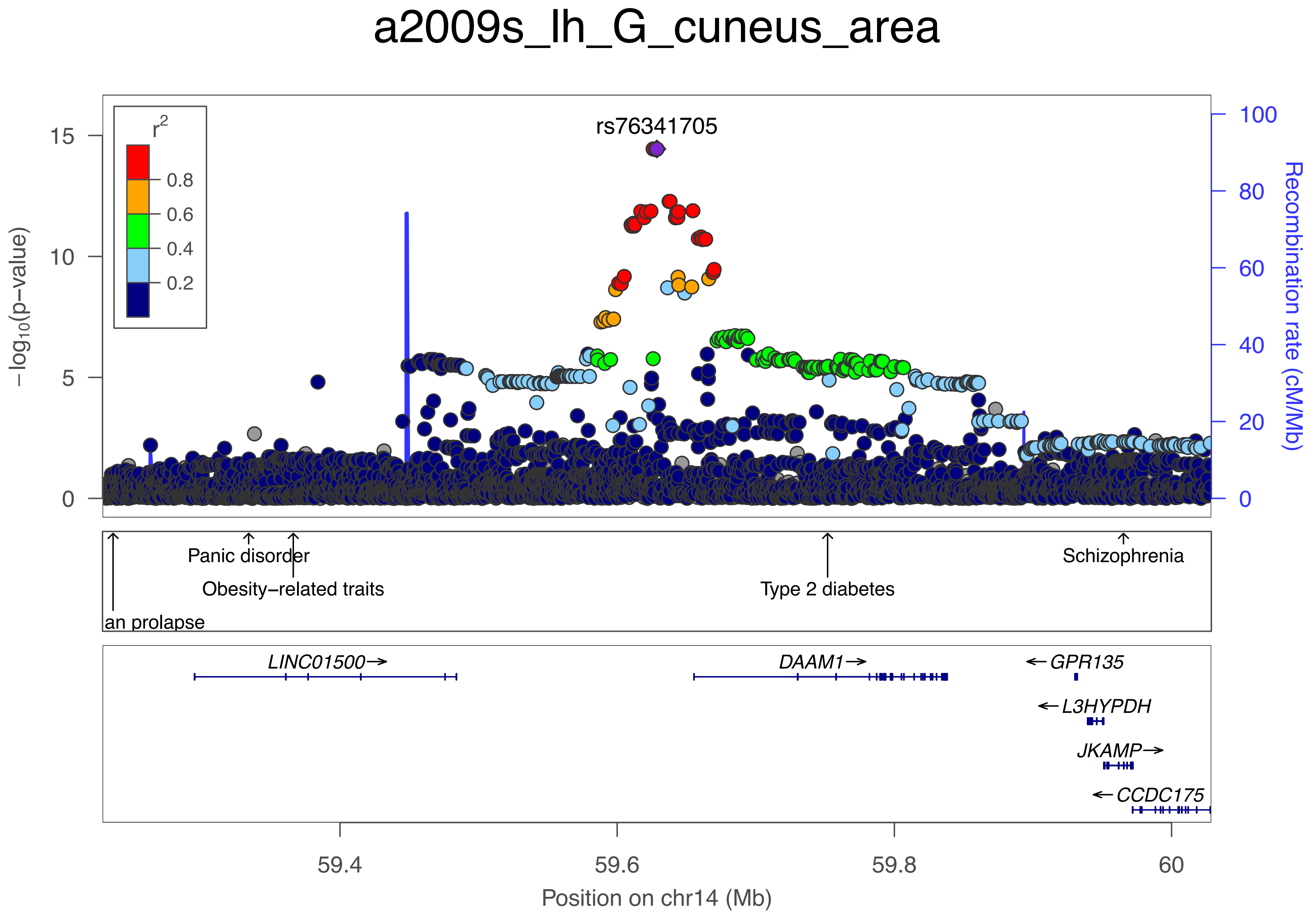
a2009s_lh_G_cuneus_area
rs76341705
r2
0.8
0.6
0.4
0.2
15
10
5
0
−log10(p−value)
100
80
60
40
20
0
Recombination rate (cM/Mb)
Panic disorder
Obesity−related traits
an prolapse
Type 2 diabetes
Schizophrenia
LINC01500→
DAAM1→
←GPR135
←L3HYPDH
JKAMP→
←CCDC175
59.4
59.6
59.8
60
Position on chr14 (Mb)

| | |
|---|---|
| date: | Thu Aug 17 18:58:26 2017 |
| build: | hg19 |
| display range: | chr14:59228679−60028679 [59228679−60028679] |
| hilite range: | 0 – 0 [ 0 – 0 ] |
| reference SNP: | chr14:59628679 |
| number of SNPs plotted: | 4162 |
| min P.value: | 3.61E−15 [chr14:59625997] |
| max P.value: | 9.99E−1 [chr14:59439189] |

Make more plots at http://csg.sph.umich.edu/locuszoom/

GWAS Catalog SNPs in Region

| chr | pos (Mb) | trait | snp |
|---|---|---|---|
| 14 | 59.23631 | Pelvic organ prolapse | rs7147087 |
| 14 | 59.33413 | Panic disorder | rs4901869 |
| 14 | 59.36632 | Obesity–related traits | rs405460 |
| 14 | 59.75183 | Type 2 diabetes | rs35209784 |
| 14 | 59.96529 | Schizophrenia | rs111803315 |

# DKTatlas_rh_lingual_area

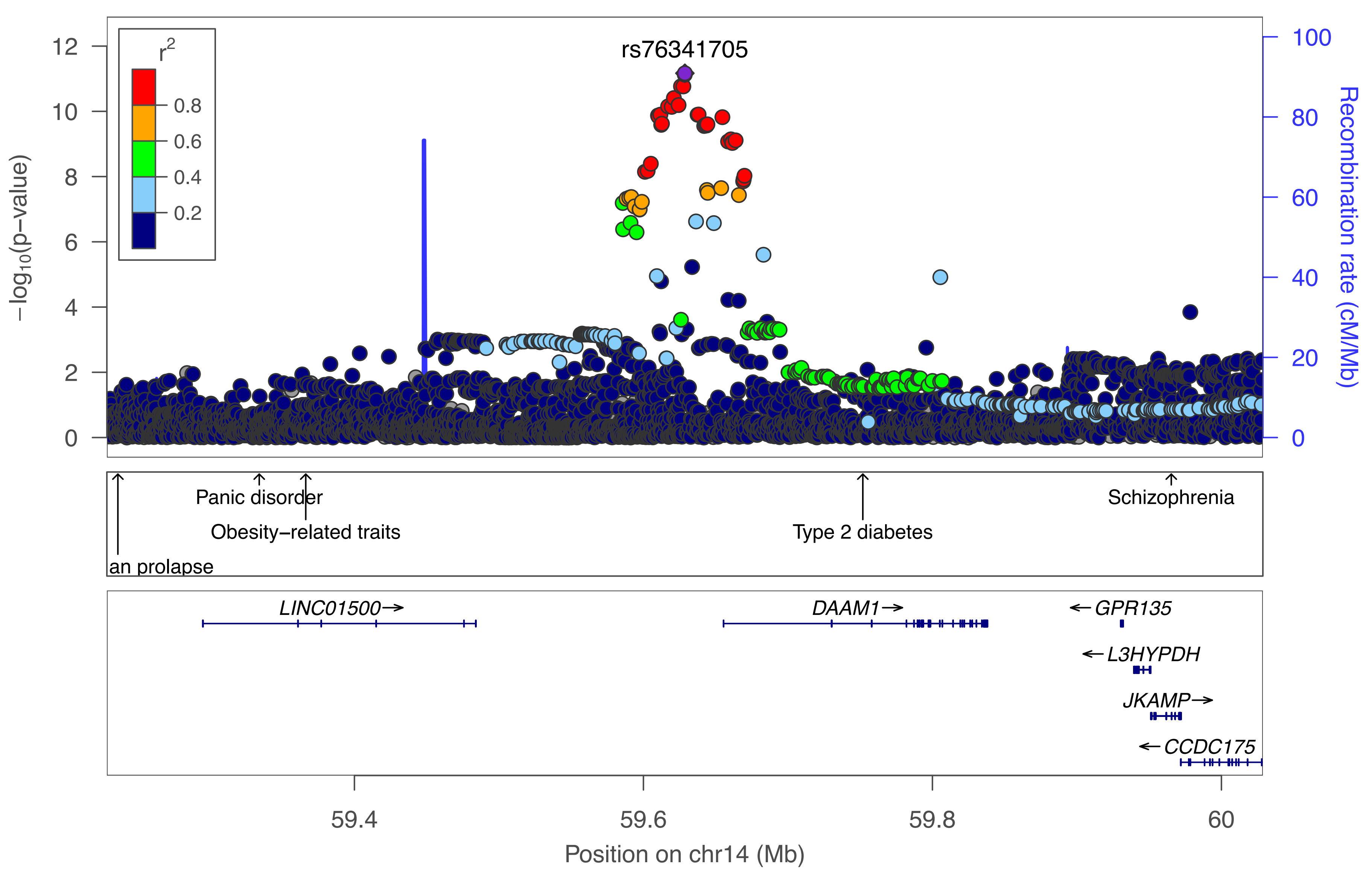

| | |
|---|---|
| date: | Thu Aug 17 18:58:37 2017 |
| build: | hg19 |
| display range: | chr14:59228679−60028679 [59228679−60028679] |
| hilite range: | 0 – 0 [ 0 – 0 ] |
| reference SNP: | chr14:59628679 |
| number of SNPs plotted: | 4162 |
| min P.value: | 6.79E−12 [chr14:59628679] |
| max P.value: | 10E−1 [chr14:59824861] |

Make more plots at http://csg.sph.umich.edu/locuszoom/

GWAS Catalog SNPs in Region

| chr | pos (Mb) | trait | snp |
|---|---|---|---|
| 14 | 59.23631 | Pelvic organ prolapse | rs7147087 |
| 14 | 59.33413 | Panic disorder | rs4901869 |
| 14 | 59.36632 | Obesity–related traits | rs405460 |
| 14 | 59.75183 | Type 2 diabetes | rs35209784 |
| 14 | 59.96529 | Schizophrenia | rs111803315 |

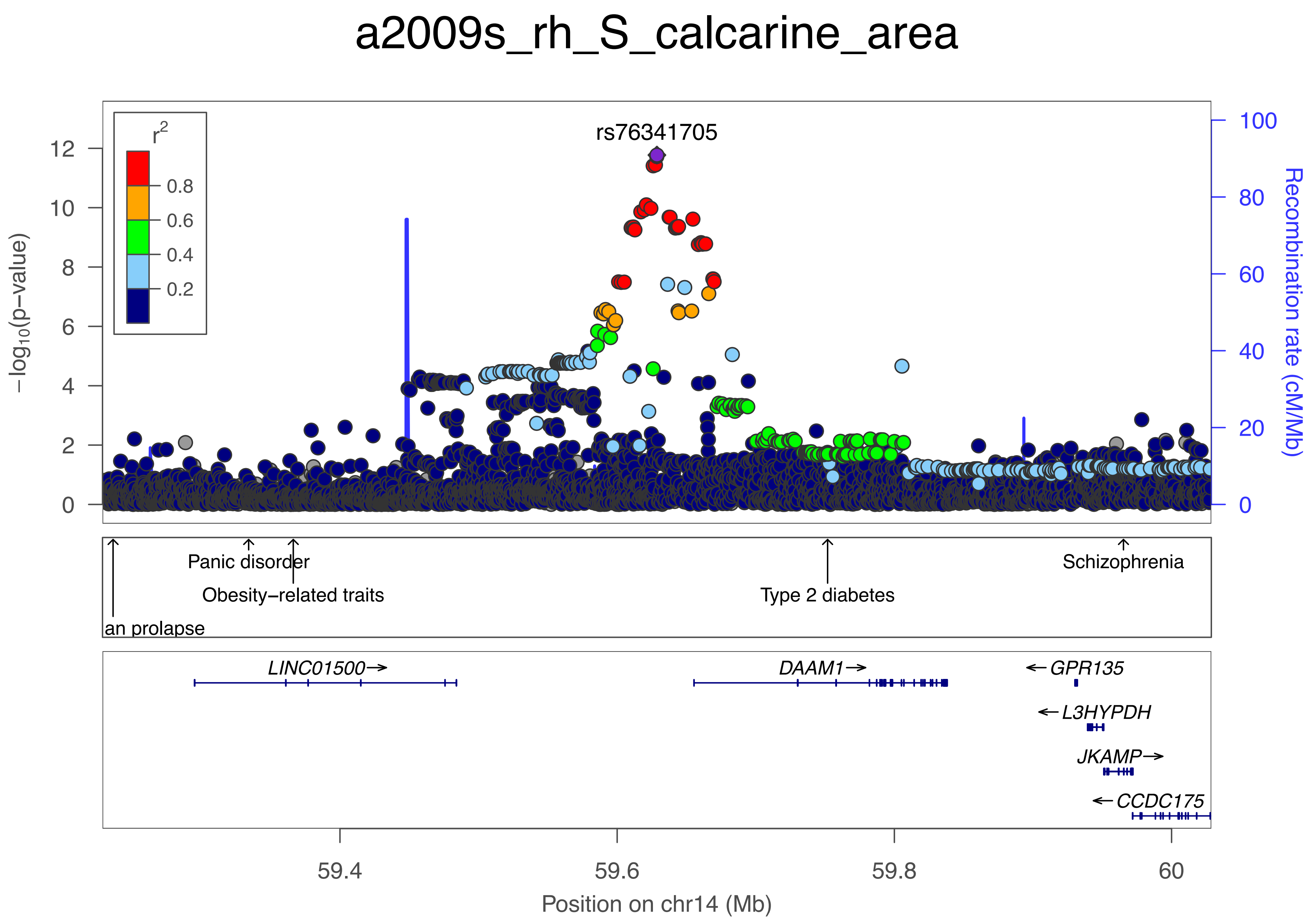
a2009s_rh_S_calcarine_area
rs76341705
r²
0.8
0.6
0.4
0.2
−log10(p−value)
Recombination rate (cM/Mb)
12
10
8
6
4
2
0
100
80
60
40
20
0
Panic disorder
Obesity−related traits
an prolapse
Type 2 diabetes
Schizophrenia
LINC01500→
DAAM1→
←GPR135
←L3HYPDH
JKAMP→
←CCDC175
59.4
59.6
59.8
60
Position on chr14 (Mb)

| | |
|---|---|
| date: | Thu Aug 17 18:58:36 2017 |
| build: | hg19 |
| display range: | chr14:59228679−60028679 [59228679−60028679] |
| hilite range: | 0 – 0  [ 0 – 0 ] |
| reference SNP: | chr14:59628679 |
| number of SNPs plotted: | 4162 |
| min P.value: | 1.69E−12 [chr14:59628679] |
| max P.value: | 10E−1 [chr14:59429871] |

Make more plots at http://csg.sph.umich.edu/locuszoom/

GWAS Catalog SNPs in Region

| chr | pos (Mb) | trait | snp |
|---|---|---|---|
| 14 | 59.23631 | Pelvic organ prolapse | rs7147087 |
| 14 | 59.33413 | Panic disorder | rs4901869 |
| 14 | 59.36632 | Obesity–related traits | rs405460 |
| 14 | 59.75183 | Type 2 diabetes | rs35209784 |
| 14 | 59.96529 | Schizophrenia | rs111803315 |

# FAST_ROIs_L_precentral_gyrus

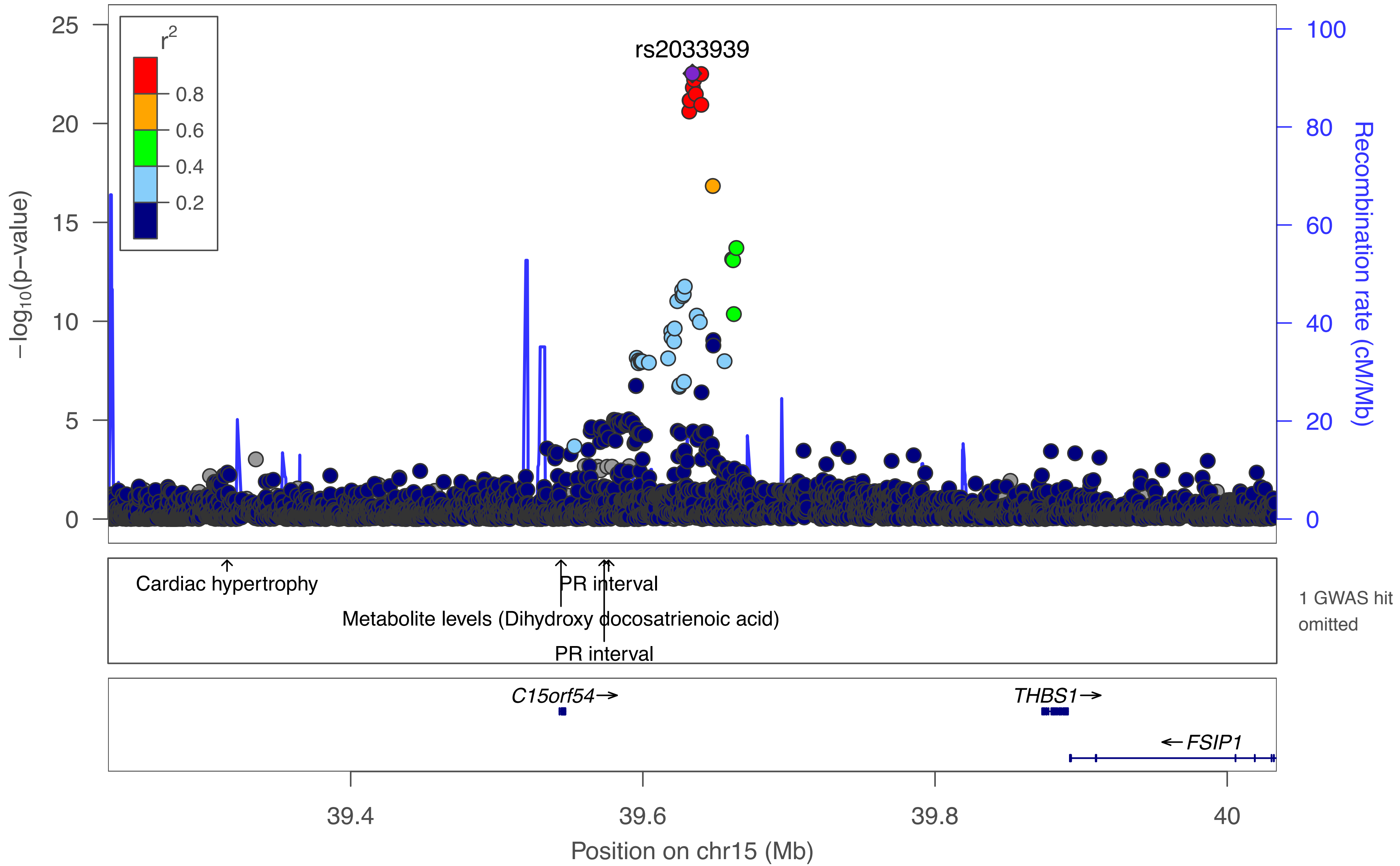

| | |
|---|---|
| date: | Thu Aug 17 19:08:08 2017 |
| build: | hg19 |
| display range: | chr15:39233904−40033904 [39233904−40033904] |
| hilite range: | 0 − 0  [ 0 − 0 ] |
| reference SNP: | chr15:39633904 |
| number of SNPs plotted: | 4352 |
| min P.value: | 2.96E−23 [chr15:39633904] |
| max P.value: | 10E−1 [chr15:39368012] |
| omitted GWAS Hits: | NA |

Make more plots at http://csg.sph.umich.edu/locuszoom/

GWAS Catalog SNPs in Region

| chr | pos (Mb) | trait | snp |
|---|---|---|---|
| 15 | 39.31536 | Cardiac hypertrophy | rs12907914 |
| 15 | 39.34705 | Parental extreme longevity (95 years and older) | rs78539969 |
| 15 | 39.54395 | Metabolite levels (Dihydroxy docosatrienoic acid) | rs17691453 |
| 15 | 39.57353 | PR interval | rs12595668 |
| 15 | 39.57656 | PR interval | rs746265 |

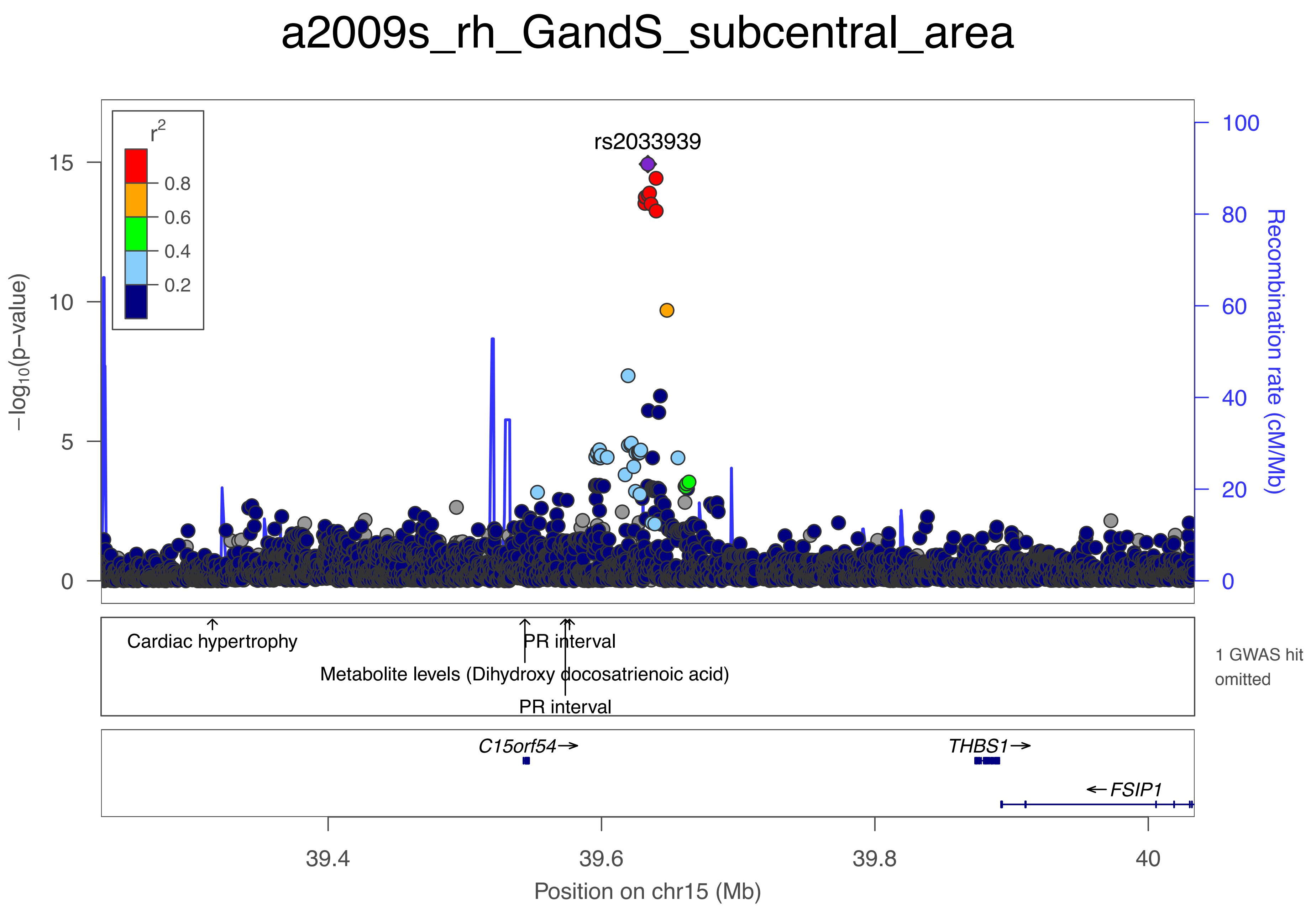
a2009s_rh_GandS_subcentral_area
rs2033939
r2
0.8
0.6
0.4
0.2
15
10
5
0
−log10(p−value)
100
80
60
40
20
0
Recombination rate (cM/Mb)
Cardiac hypertrophy
PR interval
Metabolite levels (Dihydroxy docosatrienoic acid)
PR interval
1 GWAS hit omitted
C15orf54
THBS1
FSIP1
39.4
39.6
39.8
40
Position on chr15 (Mb)

| | |
|---|---|
| date: | Thu Aug 17 18:47:49 2017 |
| build: | hg19 |
| display range: | chr15:39233904−40033904 [39233904−40033904] |
| hilite range: | 0 – 0 [ 0 – 0 ] |
| reference SNP: | chr15:39633904 |
| number of SNPs plotted: | 4352 |
| min P.value: | 1.17E−15 [chr15:39633904] |
| max P.value: | 10E−1 [chr15:39874973] |
| omitted GWAS Hits: | NA |

Make more plots at http://csg.sph.umich.edu/locuszoom/

GWAS Catalog SNPs in Region

| chr | pos (Mb) | trait | snp |
|---|---|---|---|
| 15 | 39.31536 | Cardiac hypertrophy | rs12907914 |
| 15 | 39.34705 | Parental extreme longevity (95 years and older) | rs78539969 |
| 15 | 39.54395 | Metabolite levels (Dihydroxy docosatrienoic acid) | rs17691453 |
| 15 | 39.57353 | PR interval | rs12595668 |
| 15 | 39.57656 | PR interval | rs746265 |

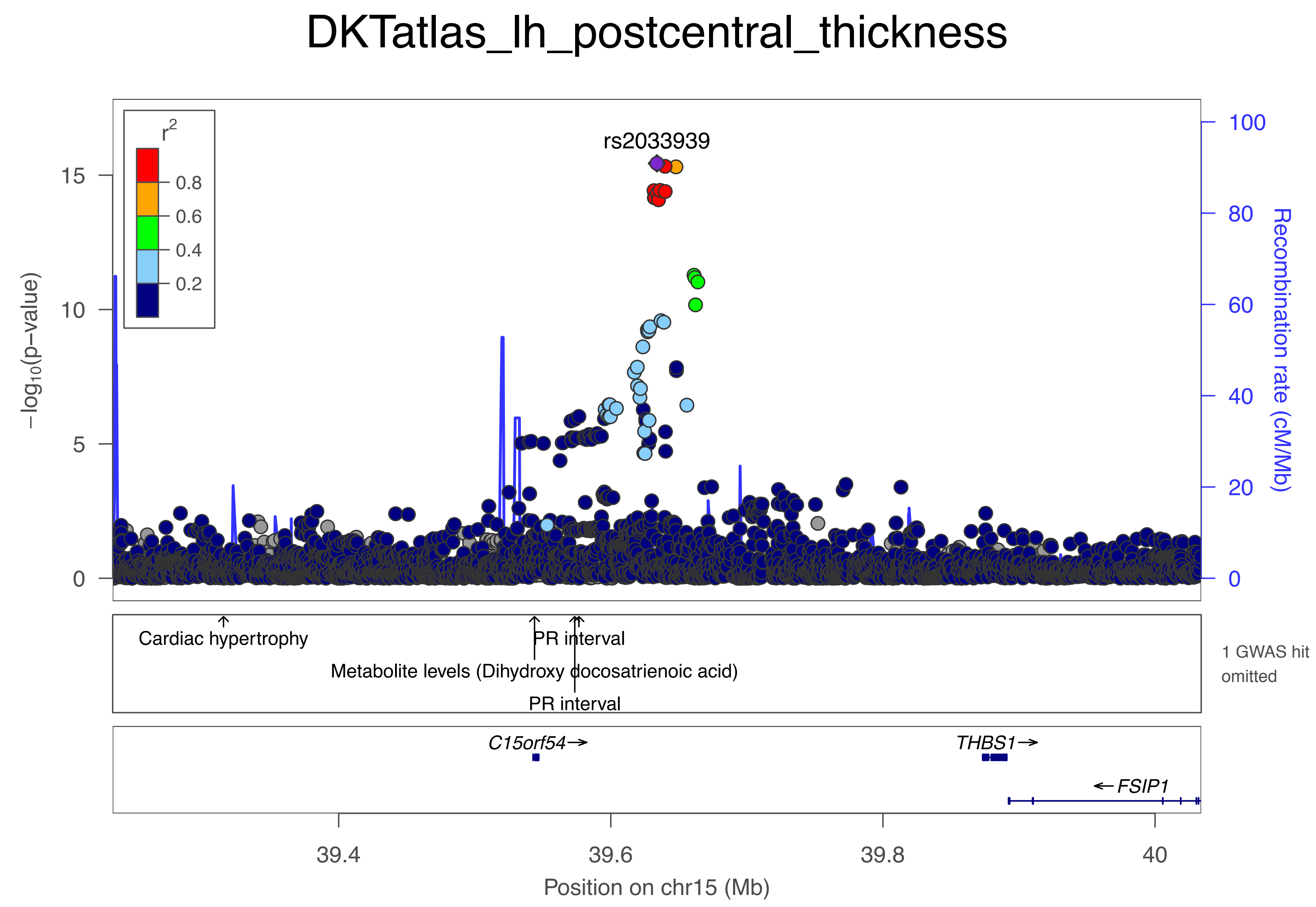
DKTatlas_lh_postcentral_thickness
r2
0.8
0.6
0.4
0.2
rs2033939
15
10
5
0
−log10(p−value)
100
80
60
40
20
0
Recombination rate (cM/Mb)
Cardiac hypertrophy
PR interval
Metabolite levels (Dihydroxy docosatrienoic acid)
PR interval
1 GWAS hit omitted
C15orf54→
THBS1→
←FSIP1
39.4
39.6
39.8
40
Position on chr15 (Mb)

| | |
|---|---|
| date: | Thu Aug 17 18:55:24 2017 |
| build: | hg19 |
| display range: | chr15:39233904−40033904 [39233904−40033904] |
| hilite range: | 0 – 0 [ 0 – 0 ] |
| reference SNP: | chr15:39633904 |
| number of SNPs plotted: | 4352 |
| min P.value: | 3.64E−16 [chr15:39633904] |
| max P.value: | 10E−1 [chr15:39807423] |
| omitted GWAS Hits: | NA |

Make more plots at http://csg.sph.umich.edu/locuszoom/

GWAS Catalog SNPs in Region

| chr | pos (Mb) | trait | snp |
|---|---|---|---|
| 15 | 39.31536 | Cardiac hypertrophy | rs12907914 |
| 15 | 39.34705 | Parental extreme longevity (95 years and older) | rs78539969 |
| 15 | 39.54395 | Metabolite levels (Dihydroxy docosatrienoic acid) | rs17691453 |
| 15 | 39.57353 | PR interval | rs12595668 |
| 15 | 39.57656 | PR interval | rs746265 |

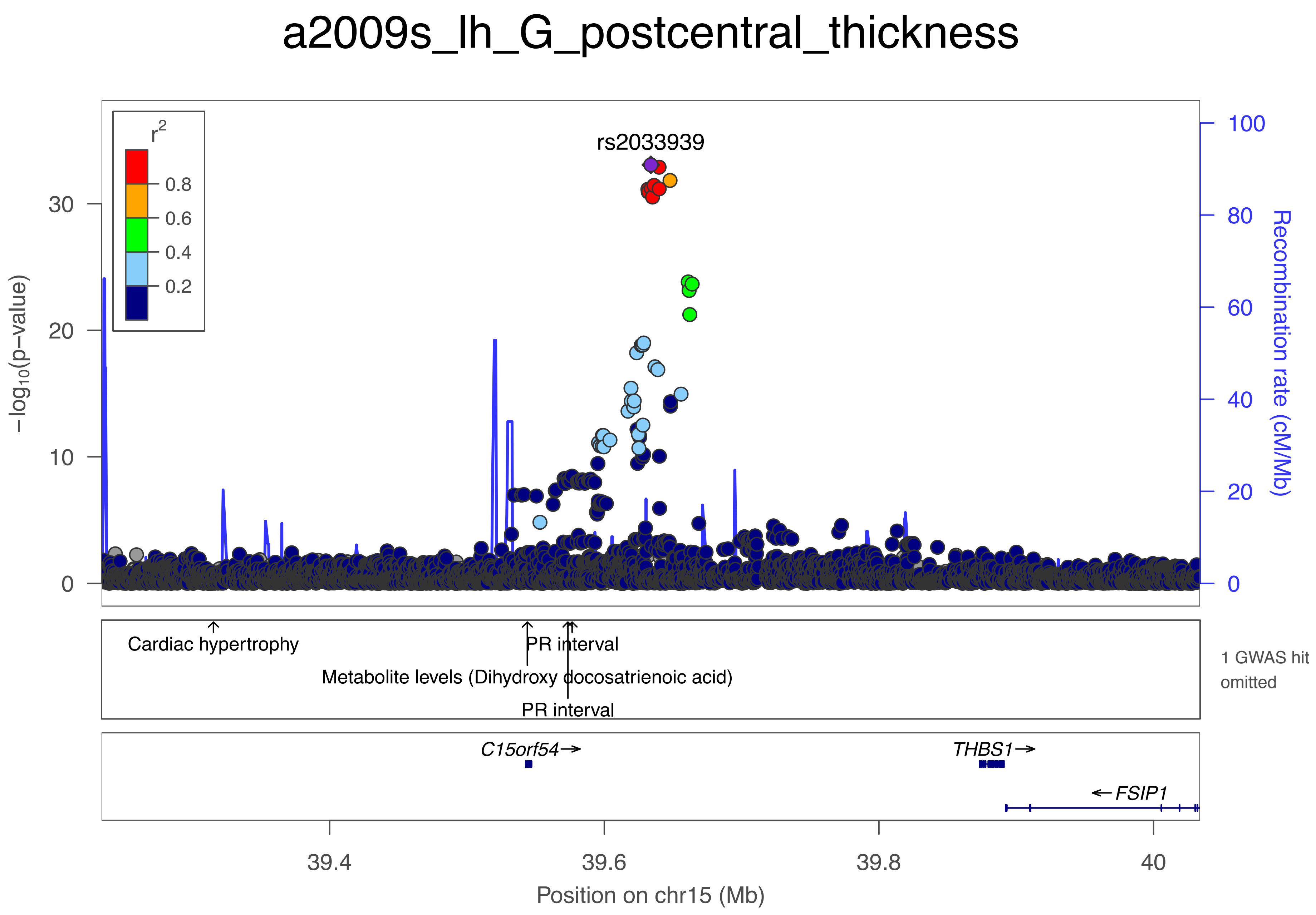
a2009s_lh_G_postcentral_thickness
rs2033939
r2
0.8
0.6
0.4
0.2
−log10(p−value)
Recombination rate (cM/Mb)
Cardiac hypertrophy
PR interval
Metabolite levels (Dihydroxy docosatrienoic acid)
PR interval
1 GWAS hit omitted
C15orf54
THBS1
FSIP1
39.4
39.6
39.8
40
Position on chr15 (Mb)

| | |
|---|---|
| date: | Thu Aug 17 19:07:32 2017 |
| build: | hg19 |
| display range: | chr15:39233904−40033904 [39233904−40033904] |
| hilite range: | 0 – 0 [ 0 – 0 ] |
| reference SNP: | chr15:39633904 |
| number of SNPs plotted: | 4352 |
| min P.value: | 8.36E−34 [chr15:39633904] |
| max P.value: | 10E−1 [chr15:39982927] |
| omitted GWAS Hits: | NA |

Make more plots at http://csg.sph.umich.edu/locuszoom/

GWAS Catalog SNPs in Region

| chr | pos (Mb) | trait | snp |
|---|---|---|---|
| 15 | 39.31536 | Cardiac hypertrophy | rs12907914 |
| 15 | 39.34705 | Parental extreme longevity (95 years and older) | rs78539969 |
| 15 | 39.54395 | Metabolite levels (Dihydroxy docosatrienoic acid) | rs17691453 |
| 15 | 39.57353 | PR interval | rs12595668 |
| 15 | 39.57656 | PR interval | rs746265 |

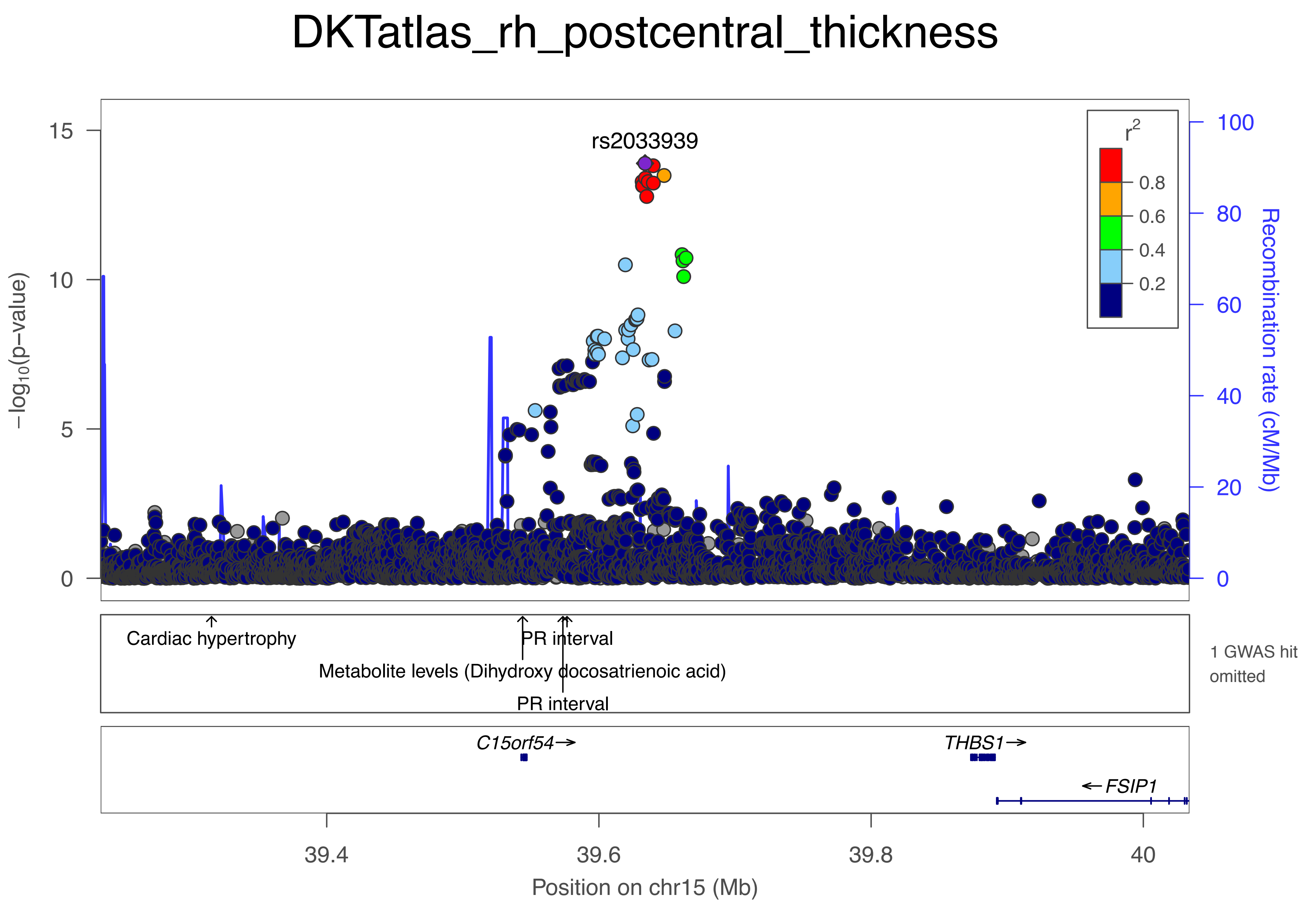
DKTatlas_rh_postcentral_thickness
rs2033939
−log10(p−value)
Recombination rate (cM/Mb)
r2
0.8
0.6
0.4
0.2
15
10
5
0
100
80
60
40
20
Cardiac hypertrophy
PR interval
Metabolite levels (Dihydroxy docosatrienoic acid)
PR interval
1 GWAS hit omitted
C15orf54→
THBS1→
←FSIP1
39.4
39.6
39.8
40
Position on chr15 (Mb)

| | |
|---|---|
| date: | Thu Aug 17 18:47:48 2017 |
| build: | hg19 |
| display range: | chr15:39233904–40033904 [39233904–40033904] |
| hilite range: | 0 – 0 [ 0 – 0 ] |
| reference SNP: | chr15:39633904 |
| number of SNPs plotted: | 4352 |
| min P.value: | 1.28E–14 [chr15:39633904] |
| max P.value: | 9.99E–1 [chr15:39265911] |
| omitted GWAS Hits: | NA |

Make more plots at http://csg.sph.umich.edu/locuszoom/

GWAS Catalog SNPs in Region

| chr | pos (Mb) | trait | snp |
| --- | --- | --- | --- |
| 15 | 39.31536 | Cardiac hypertrophy | rs12907914 |
| 15 | 39.34705 | Parental extreme longevity (95 years and older) | rs78539969 |
| 15 | 39.54395 | Metabolite levels (Dihydroxy docosatrienoic acid) | rs17691453 |
| 15 | 39.57353 | PR interval | rs12595668 |
| 15 | 39.57656 | PR interval | rs746265 |

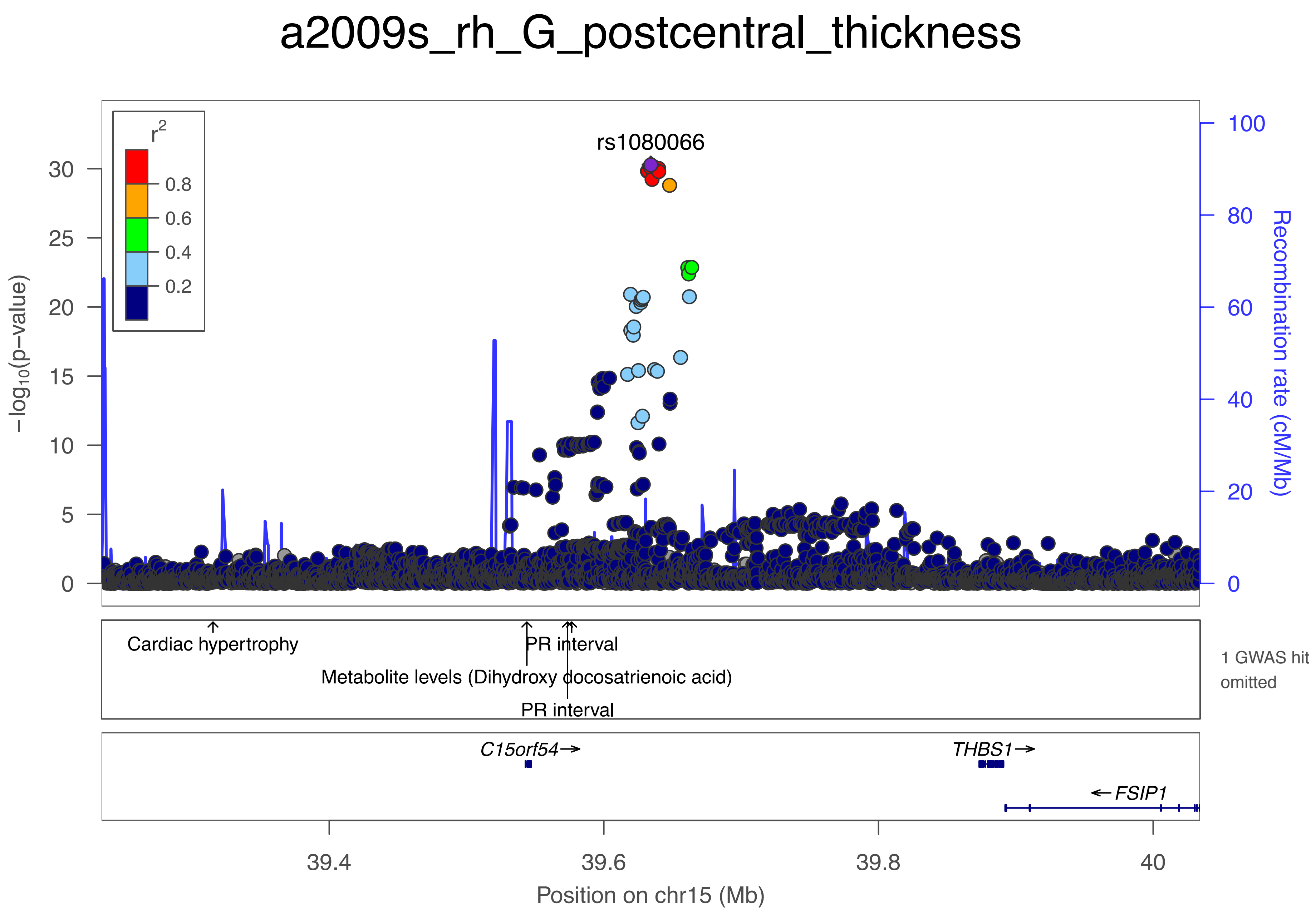
a2009s_rh_G_postcentral_thickness
rs1080066
r2
0.8
0.6
0.4
0.2
−log10(p−value)
30
25
20
15
10
5
0
100
80
60
40
20
0
Recombination rate (cM/Mb)
Cardiac hypertrophy
PR interval
Metabolite levels (Dihydroxy docosatrienoic acid)
PR interval
1 GWAS hit omitted
C15orf54→
THBS1→
←FSIP1
39.4
39.6
39.8
40
Position on chr15 (Mb)

| | |
|---|---|
| date: | Thu Aug 17 19:07:32 2017 |
| build: | hg19 |
| display range: | chr15:39234222−40034222 [39234222−40034222] |
| hilite range: | 0 – 0 [ 0 – 0 ] |
| reference SNP: | chr15:39634222 |
| number of SNPs plotted: | 4350 |
| min P.value: | 5.15E−31 [chr15:39634222] |
| max P.value: | 10E−1 [chr15:39396921] |
| omitted GWAS Hits: | NA |

Make more plots at http://csg.sph.umich.edu/locuszoom/

GWAS Catalog SNPs in Region

| chr | pos (Mb) | trait | snp |
|---|---|---|---|
| 15 | 39.31536 | Cardiac hypertrophy | rs12907914 |
| 15 | 39.34705 | Parental extreme longevity (95 years and older) | rs78539969 |
| 15 | 39.54395 | Metabolite levels (Dihydroxy docosatrienoic acid) | rs17691453 |
| 15 | 39.57353 | PR interval | rs12595668 |
| 15 | 39.57656 | PR interval | rs746265 |

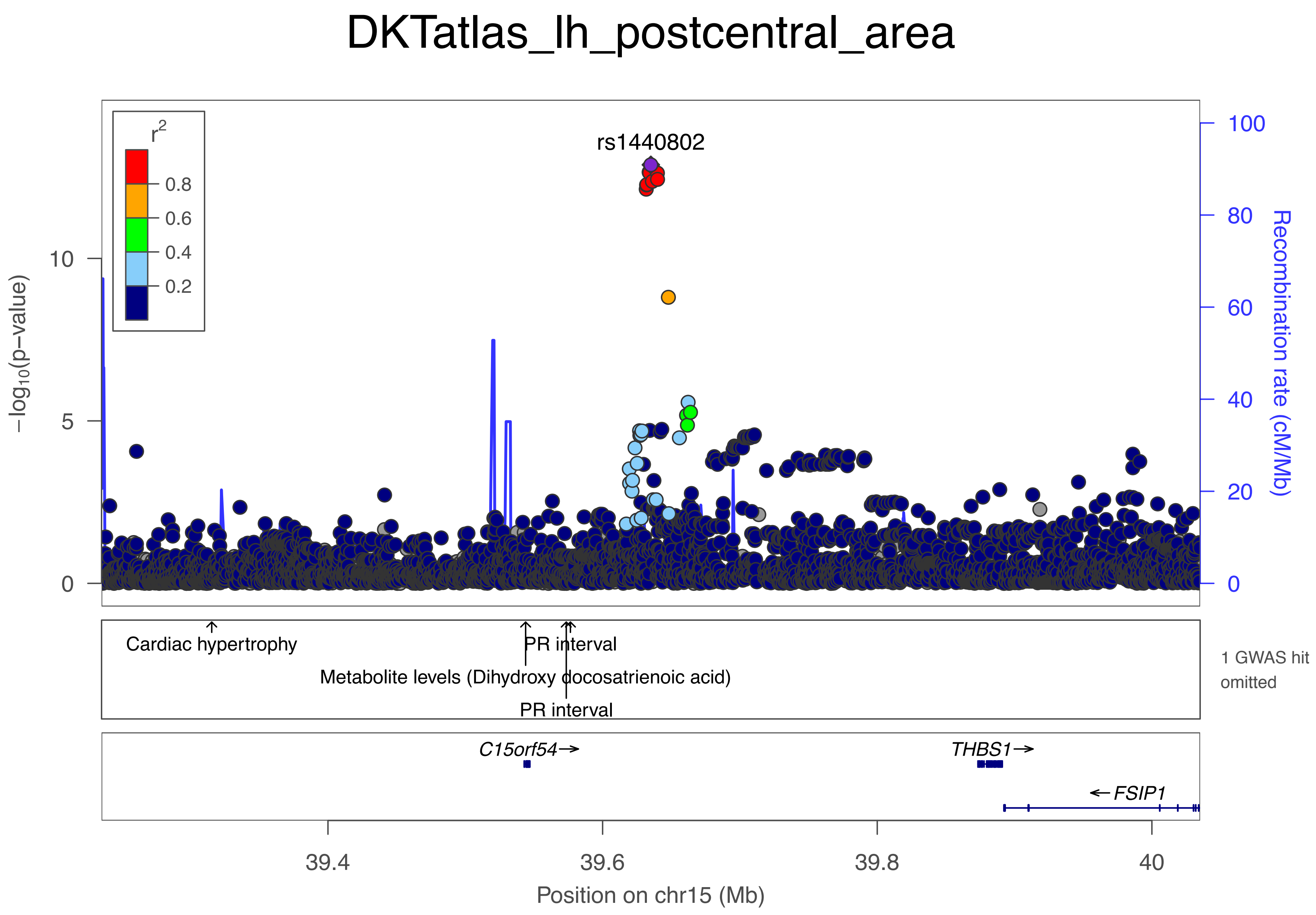

DKTatlas_lh_postcentral_area
r2
0.8
0.6
0.4
0.2
rs1440802
10
5
0
−log10(p−value)
100
80
60
40
20
0
Recombination rate (cM/Mb)
Cardiac hypertrophy
PR interval
Metabolite levels (Dihydroxy docosatrienoic acid)
PR interval
1 GWAS hit
omitted
C15orf54→
THBS1→
←FSIP1
39.4
39.6
39.8
40
Position on chr15 (Mb)

| | |
|---|---|
| date: | Thu Aug 17 18:55:29 2017 |
| build: | hg19 |
| display range: | chr15:39235124−40035124 [39235124−40035124] |
| hilite range: | 0 – 0 [ 0 – 0 ] |
| reference SNP: | chr15:39635124 |
| number of SNPs plotted: | 4346 |
| min P.value: | 1.32E−13 [chr15:39635124] |
| max P.value: | 10E−1 [chr15:39573686] |
| omitted GWAS Hits: | NA |

Make more plots at http://csg.sph.umich.edu/locuszoom/

GWAS Catalog SNPs in Region

| chr | pos (Mb) | trait | snp |
|---|---|---|---|
| 15 | 39.31536 | Cardiac hypertrophy | rs12907914 |
| 15 | 39.34705 | Parental extreme longevity (95 years and older) | rs78539969 |
| 15 | 39.54395 | Metabolite levels (Dihydroxy docosatrienoic acid) | rs17691453 |
| 15 | 39.57353 | PR interval | rs12595668 |
| 15 | 39.57656 | PR interval | rs746265 |

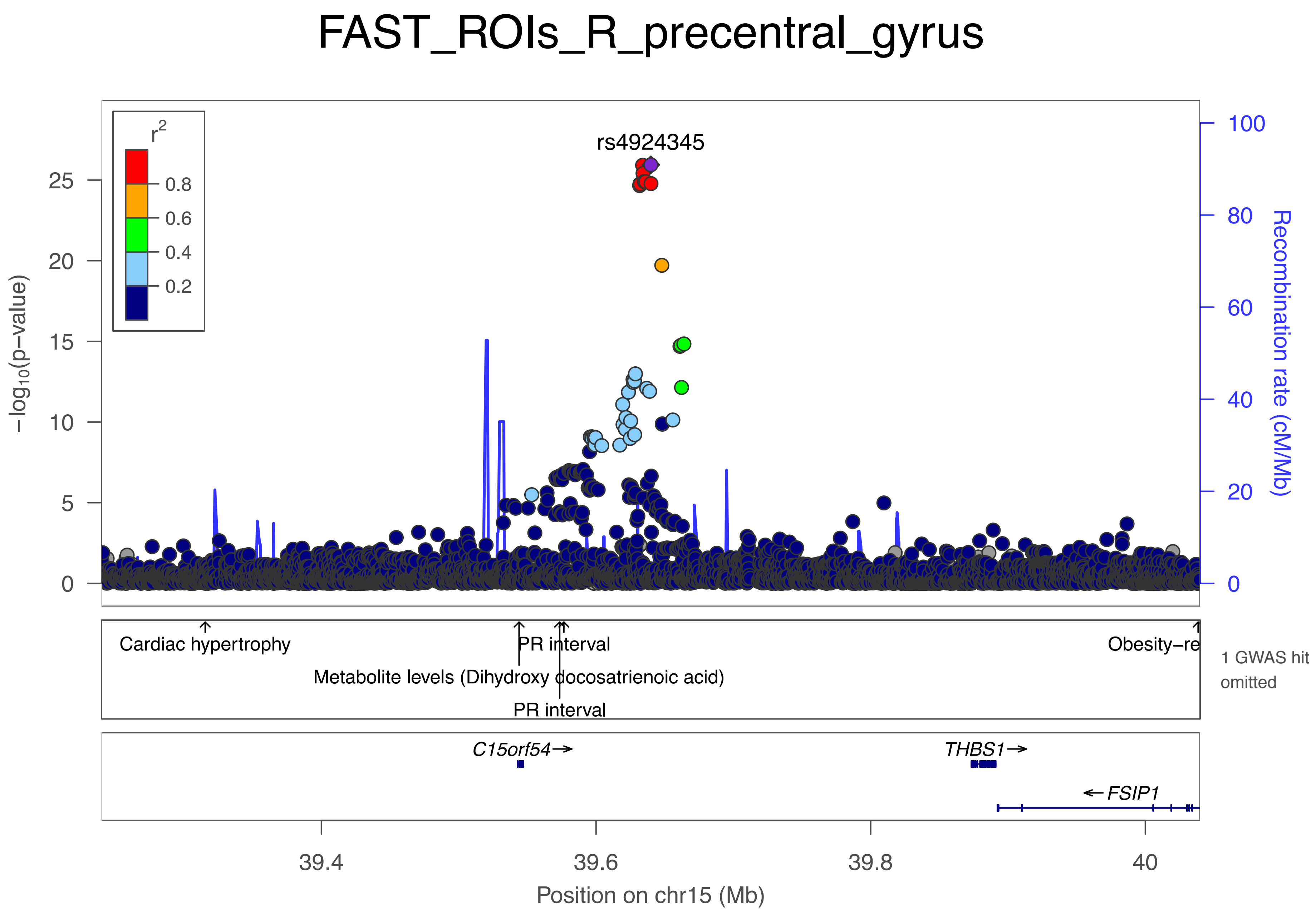

FAST_ROIs_R_precentral_gyrus
r2
0.8
0.6
0.4
0.2
rs4924345
−log10(p−value)
Recombination rate (cM/Mb)
25
20
15
10
5
0
100
80
60
40
20
0
Cardiac hypertrophy
PR interval
Metabolite levels (Dihydroxy docosatrienoic acid)
PR interval
Obesity−re
1 GWAS hit omitted
C15orf54→
THBS1→
←FSIP1
39.4
39.6
39.8
40
Position on chr15 (Mb)

| | |
|---|---|
| date: | Thu Aug 17 18:55:45 2017 |
| build: | hg19 |
| display range: | chr15:39239898−40039898 [39239898−40039898] |
| hilite range: | 0 – 0 [ 0 – 0 ] |
| reference SNP: | chr15:39639898 |
| number of SNPs plotted: | 4344 |
| min P.value: | 1.13E−26 [chr15:39639898] |
| max P.value: | 10E−1 [chr15:39865883] |
| omitted GWAS Hits: | NA |

Make more plots at http://csg.sph.umich.edu/locuszoom/

GWAS Catalog SNPs in Region

| chr | pos (Mb) | trait | snp |
|---|---|---|---|
| 15 | 39.31536 | Cardiac hypertrophy | rs12907914 |
| 15 | 39.34705 | Parental extreme longevity (95 years and older) | rs78539969 |
| 15 | 39.54395 | Metabolite levels (Dihydroxy docosatrienoic acid) | rs17691453 |
| 15 | 39.57353 | PR interval | rs12595668 |
| 15 | 39.57656 | PR interval | rs746265 |
| 15 | 40.03843 | Obesity−related traits | rs8033957 |

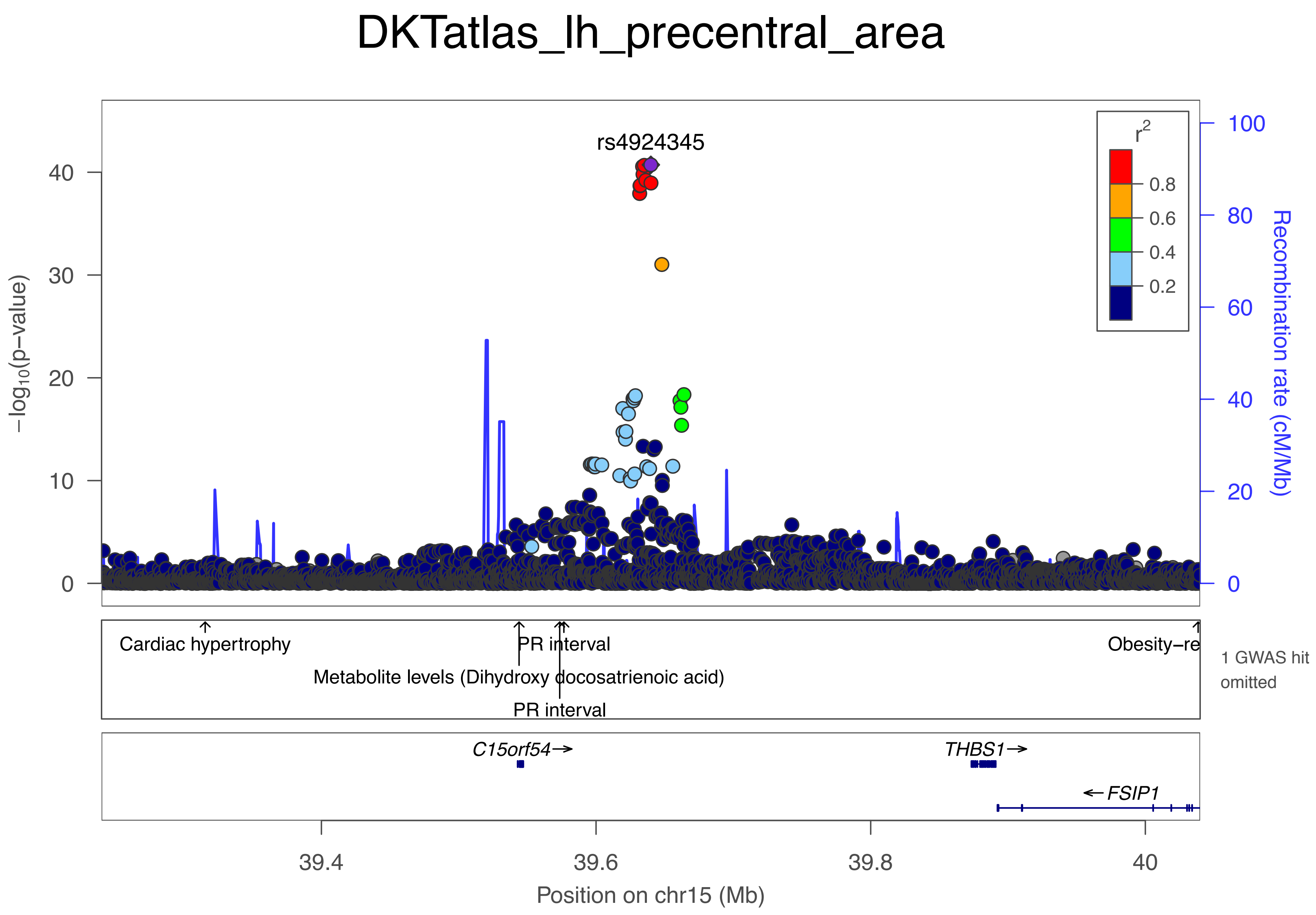

DKTatlas_lh_precentral_area
rs4924345
−log10(p−value)
40
30
20
10
0
r2
0.8
0.6
0.4
0.2
100
80
60
40
20
0
Recombination rate (cM/Mb)
Cardiac hypertrophy
PR interval
Metabolite levels (Dihydroxy docosatrienoic acid)
PR interval
Obesity−re
1 GWAS hit omitted
C15orf54→
THBS1→
←FSIP1
39.4
39.6
39.8
40
Position on chr15 (Mb)

| | |
|---|---|
| date: | Thu Aug 17 18:53:41 2017 |
| build: | hg19 |
| display range: | chr15:39239898−40039898 [39239898−40039898] |
| hilite range: | 0 – 0 [ 0 – 0 ] |
| reference SNP: | chr15:39639898 |
| number of SNPs plotted: | 4344 |
| min P.value: | 1.89E−41 [chr15:39639898] |
| max P.value: | 10E−1 [chr15:39844150] |
| omitted GWAS Hits: | NA |

Make more plots at http://csg.sph.umich.edu/locuszoom/

GWAS Catalog SNPs in Region

| chr | pos (Mb) | trait | snp |
|---|---|---|---|
| 15 | 39.31536 | Cardiac hypertrophy | rs12907914 |
| 15 | 39.34705 | Parental extreme longevity (95 years and older) | rs78539969 |
| 15 | 39.54395 | Metabolite levels (Dihydroxy docosatrienoic acid) | rs17691453 |
| 15 | 39.57353 | PR interval | rs12595668 |
| 15 | 39.57656 | PR interval | rs746265 |
| 15 | 40.03843 | Obesity–related traits | rs8033957 |

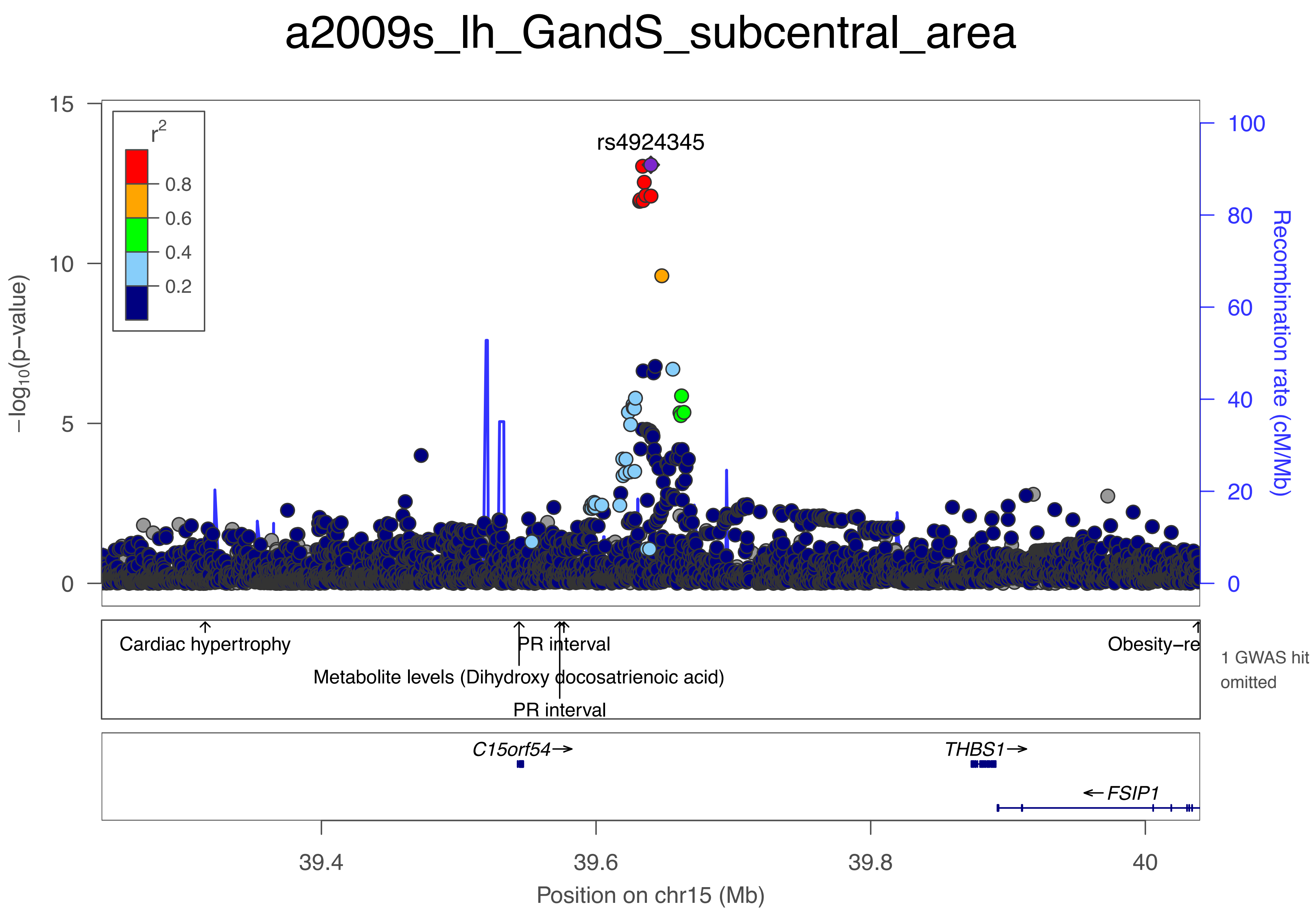

a2009s_lh_GandS_subcentral_area
r2
0.8
0.6
0.4
0.2
rs4924345
15
10
5
0
−log10(p−value)
100
80
60
40
20
0
Recombination rate (cM/Mb)
Cardiac hypertrophy
PR interval
Metabolite levels (Dihydroxy docosatrienoic acid)
PR interval
Obesity−re
1 GWAS hit omitted
C15orf54→
THBS1→
←FSIP1
39.4
39.6
39.8
40
Position on chr15 (Mb)

| | |
|---|---|
| date: | Thu Aug 17 18:48:08 2017 |
| build: | hg19 |
| display range: | chr15:39239898−40039898 [39239898−40039898] |
| hilite range: | 0 – 0 [ 0 – 0 ] |
| reference SNP: | chr15:39639898 |
| number of SNPs plotted: | 4344 |
| min P.value: | 8.26E−14 [chr15:39639898] |
| max P.value: | 10E−1 [chr15:39555303] |
| omitted GWAS Hits: | NA |

Make more plots at http://csg.sph.umich.edu/locuszoom/

GWAS Catalog SNPs in Region

| chr | pos (Mb) | trait | snp |
|---|---|---|---|
| 15 | 39.31536 | Cardiac hypertrophy | rs12907914 |
| 15 | 39.34705 | Parental extreme longevity (95 years and older) | rs78539969 |
| 15 | 39.54395 | Metabolite levels (Dihydroxy docosatrienoic acid) | rs17691453 |
| 15 | 39.57353 | PR interval | rs12595668 |
| 15 | 39.57656 | PR interval | rs746265 |
| 15 | 40.03843 | Obesity−related traits | rs8033957 |

# a2009s_lh_G_precentral_area

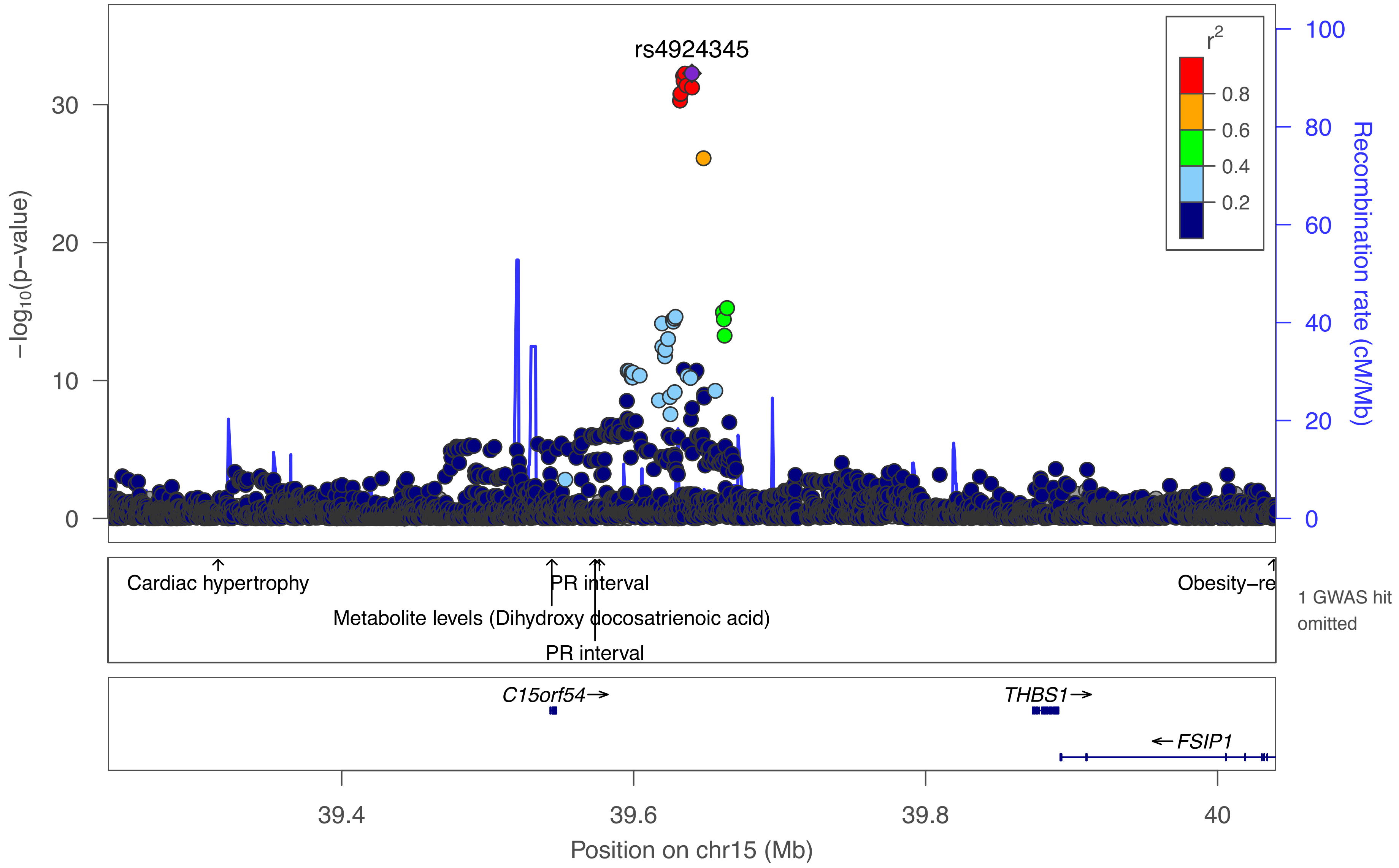

| | |
|---|---|
| date: | Thu Aug 17 18:57:11 2017 |
| build: | hg19 |
| display range: | chr15:39239898−40039898 [39239898−40039898] |
| hilite range: | 0 – 0 [ 0 – 0 ] |
| reference SNP: | chr15:39639898 |
| number of SNPs plotted: | 4344 |
| min P.value: | 5.47E−33 [chr15:39639898] |
| max P.value: | 10E−1 [chr15:39703982] |
| omitted GWAS Hits: | NA |

Make more plots at http://csg.sph.umich.edu/locuszoom/

GWAS Catalog SNPs in Region

| chr | pos (Mb) | trait | snp |
|---|---|---|---|
| 15 | 39.31536 | Cardiac hypertrophy | rs12907914 |
| 15 | 39.34705 | Parental extreme longevity (95 years and older) | rs78539969 |
| 15 | 39.54395 | Metabolite levels (Dihydroxy docosatrienoic acid) | rs17691453 |
| 15 | 39.57353 | PR interval | rs12595668 |
| 15 | 39.57656 | PR interval | rs746265 |
| 15 | 40.03843 | Obesity−related traits | rs8033957 |

# a2009s_lh_S_central_area

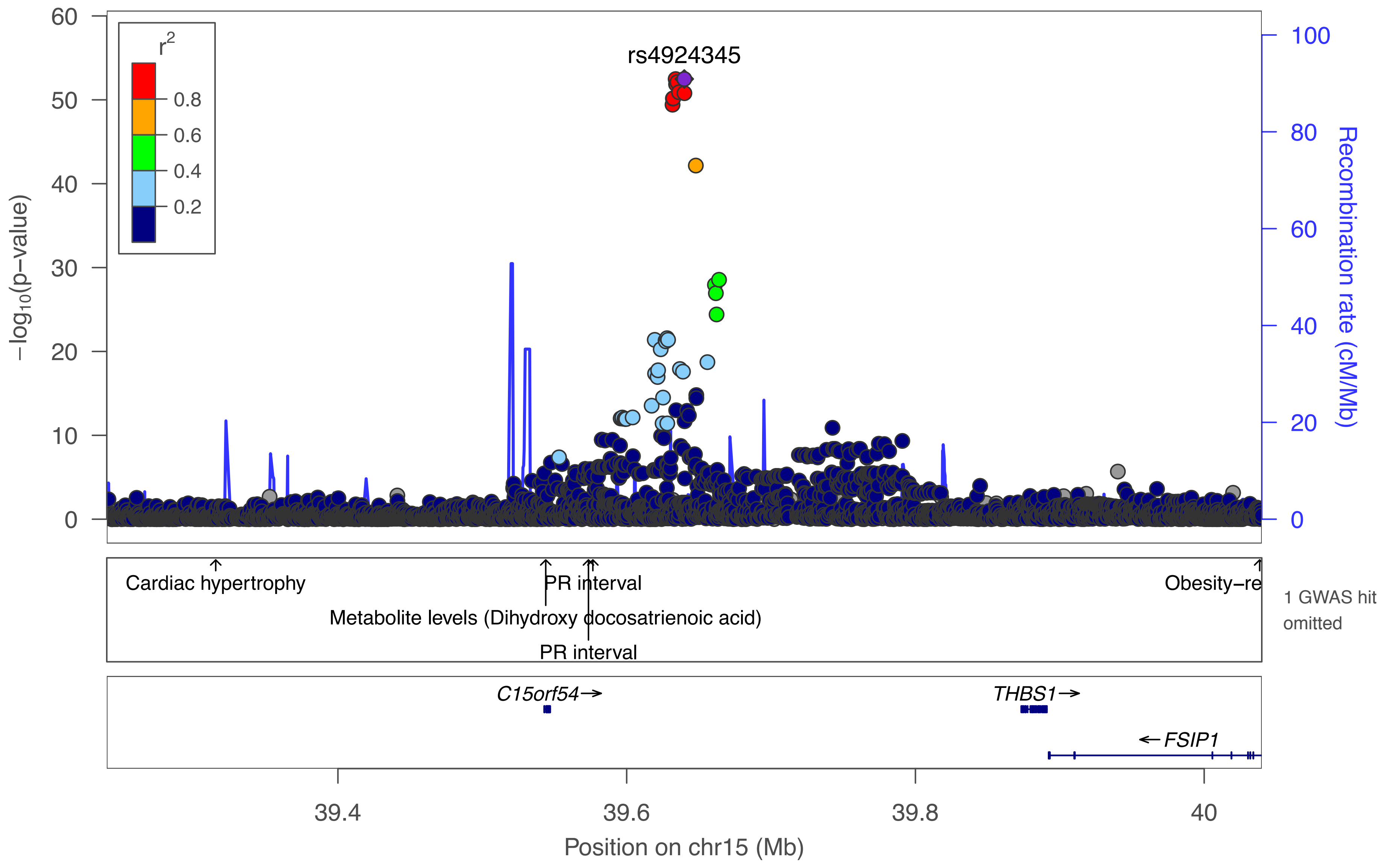

| | |
|---|---|
| date: | Thu Aug 17 18:53:27 2017 |
| build: | hg19 |
| display range: | chr15:39239898−40039898 [39239898−40039898] |
| hilite range: | 0 – 0 [ 0 – 0 ] |
| reference SNP: | chr15:39639898 |
| number of SNPs plotted: | 4344 |
| min P.value: | 3.27E−53 [chr15:39639898] |
| max P.value: | 10E−1 [chr15:39282979] |
| omitted GWAS Hits: | NA |

Make more plots at http://csg.sph.umich.edu/locuszoom/

GWAS Catalog SNPs in Region

| chr | pos (Mb) | trait | snp |
|---|---|---|---|
| 15 | 39.31536 | Cardiac hypertrophy | rs12907914 |
| 15 | 39.34705 | Parental extreme longevity (95 years and older) | rs78539969 |
| 15 | 39.54395 | Metabolite levels (Dihydroxy docosatrienoic acid) | rs17691453 |
| 15 | 39.57353 | PR interval | rs12595668 |
| 15 | 39.57656 | PR interval | rs746265 |
| 15 | 40.03843 | Obesity–related traits | rs8033957 |

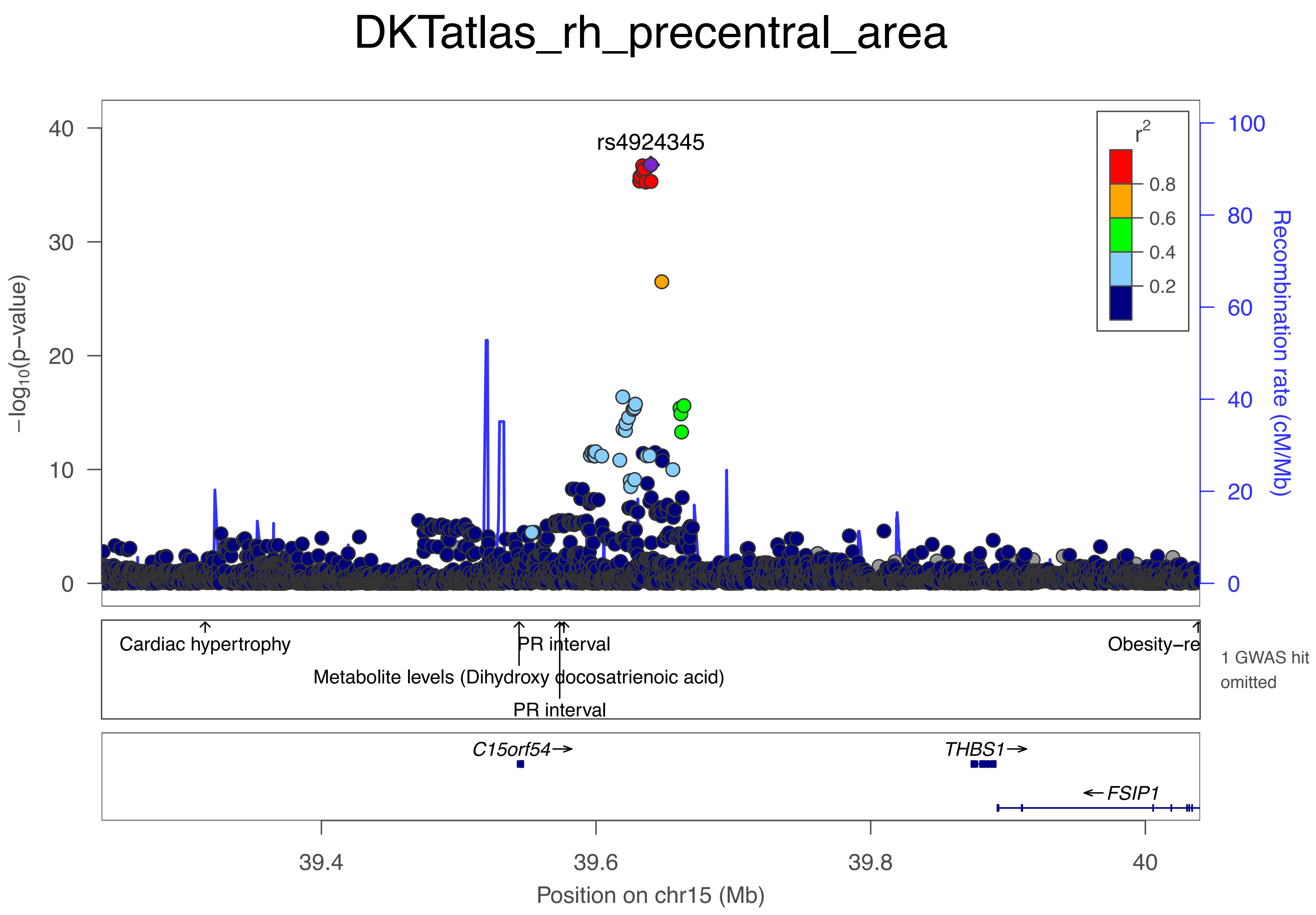
DKTatlas_rh_precentral_area
rs4924345
−log10(p−value)
Recombination rate (cM/Mb)
r2
0.8
0.6
0.4
0.2
Cardiac hypertrophy
PR interval
Metabolite levels (Dihydroxy docosatrienoic acid)
PR interval
Obesity−re
1 GWAS hit omitted
C15orf54→
THBS1→
←FSIP1
39.4
39.6
39.8
40
Position on chr15 (Mb)

| | |
|---|---|
| date: | Thu Aug 17 18:57:12 2017 |
| build: | hg19 |
| display range: | chr15:39239898–40039898 [39239898–40039898] |
| hilite range: | 0 – 0 [ 0 – 0 ] |
| reference SNP: | chr15:39639898 |
| number of SNPs plotted: | 4344 |
| min P.value: | 1.71E–37 [chr15:39639898] |
| max P.value: | 10E–1 [chr15:39334288] |
| omitted GWAS Hits: | NA |

Make more plots at http://csg.sph.umich.edu/locuszoom/

GWAS Catalog SNPs in Region

| chr | pos (Mb) | trait | snp |
|---|---|---|---|
| 15 | 39.31536 | Cardiac hypertrophy | rs12907914 |
| 15 | 39.34705 | Parental extreme longevity (95 years and older) | rs78539969 |
| 15 | 39.54395 | Metabolite levels (Dihydroxy docosatrienoic acid) | rs17691453 |
| 15 | 39.57353 | PR interval | rs12595668 |
| 15 | 39.57656 | PR interval | rs746265 |
| 15 | 40.03843 | Obesity–related traits | rs8033957 |

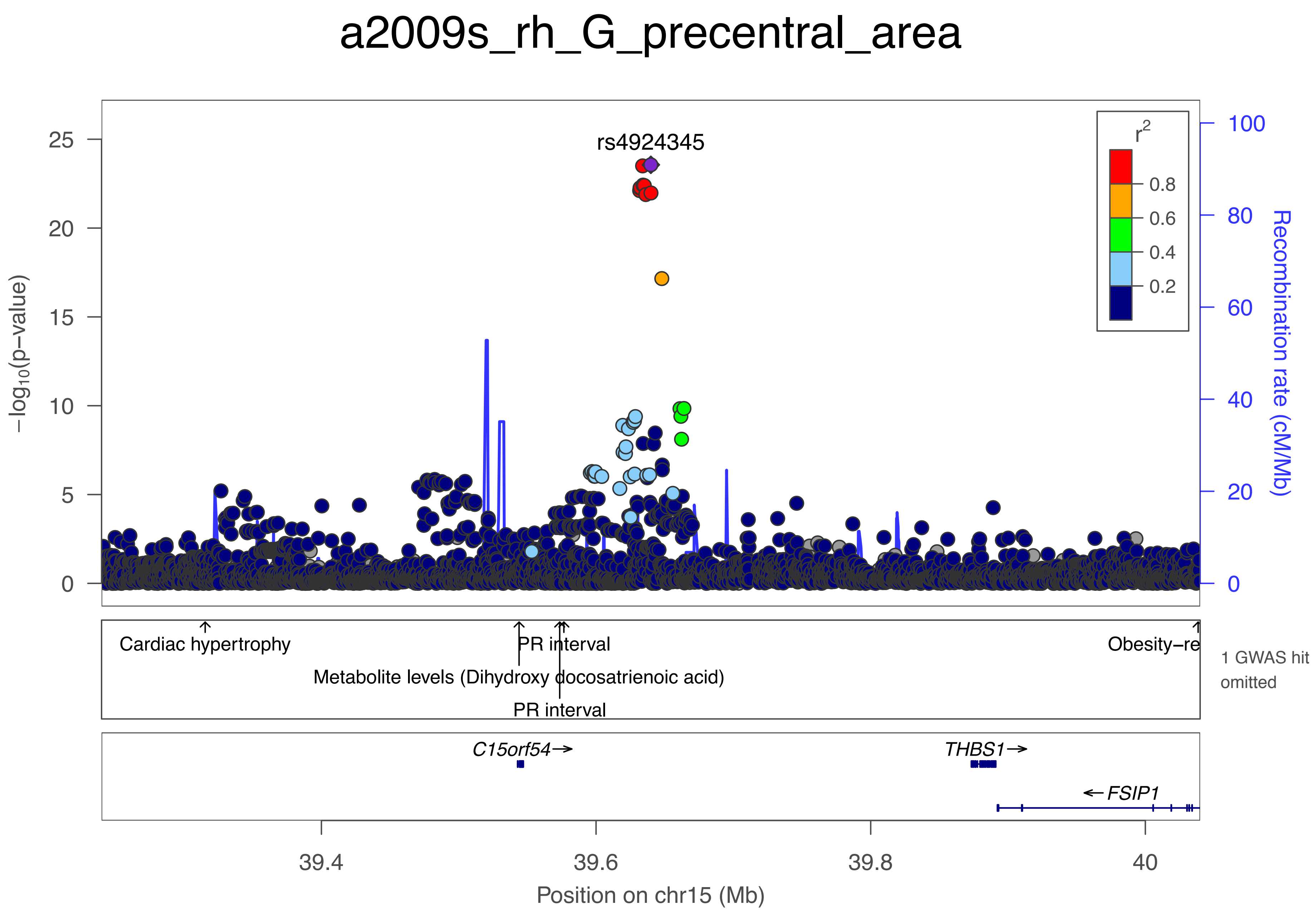

a2009s_rh_G_precentral_area
rs4924345
−log10(p−value)
Recombination rate (cM/Mb)
r2
0.8
0.6
0.4
0.2
0
5
10
15
20
25
0
20
40
60
80
100
Cardiac hypertrophy
PR interval
Metabolite levels (Dihydroxy docosatrienoic acid)
PR interval
Obesity−re
1 GWAS hit omitted
C15orf54→
THBS1→
←FSIP1
39.4
39.6
39.8
40
Position on chr15 (Mb)

| | |
|---|---|
| date: | Thu Aug 17 18:53:34 2017 |
| build: | hg19 |
| display range: | chr15:39239898−40039898 [39239898−40039898] |
| hilite range: | 0 – 0 [ 0 – 0 ] |
| reference SNP: | chr15:39639898 |
| number of SNPs plotted: | 4344 |
| min P.value: | 2.75E−24 [chr15:39639898] |
| max P.value: | 9.99E−1 [chr15:39600386] |
| omitted GWAS Hits: | NA |

Make more plots at http://csg.sph.umich.edu/locuszoom/

GWAS Catalog SNPs in Region

| chr | pos (Mb) | trait | snp |
|---|---|---|---|
| 15 | 39.31536 | Cardiac hypertrophy | rs12907914 |
| 15 | 39.34705 | Parental extreme longevity (95 years and older) | rs78539969 |
| 15 | 39.54395 | Metabolite levels (Dihydroxy docosatrienoic acid) | rs17691453 |
| 15 | 39.57353 | PR interval | rs12595668 |
| 15 | 39.57656 | PR interval | rs746265 |
| 15 | 40.03843 | Obesity−related traits | rs8033957 |

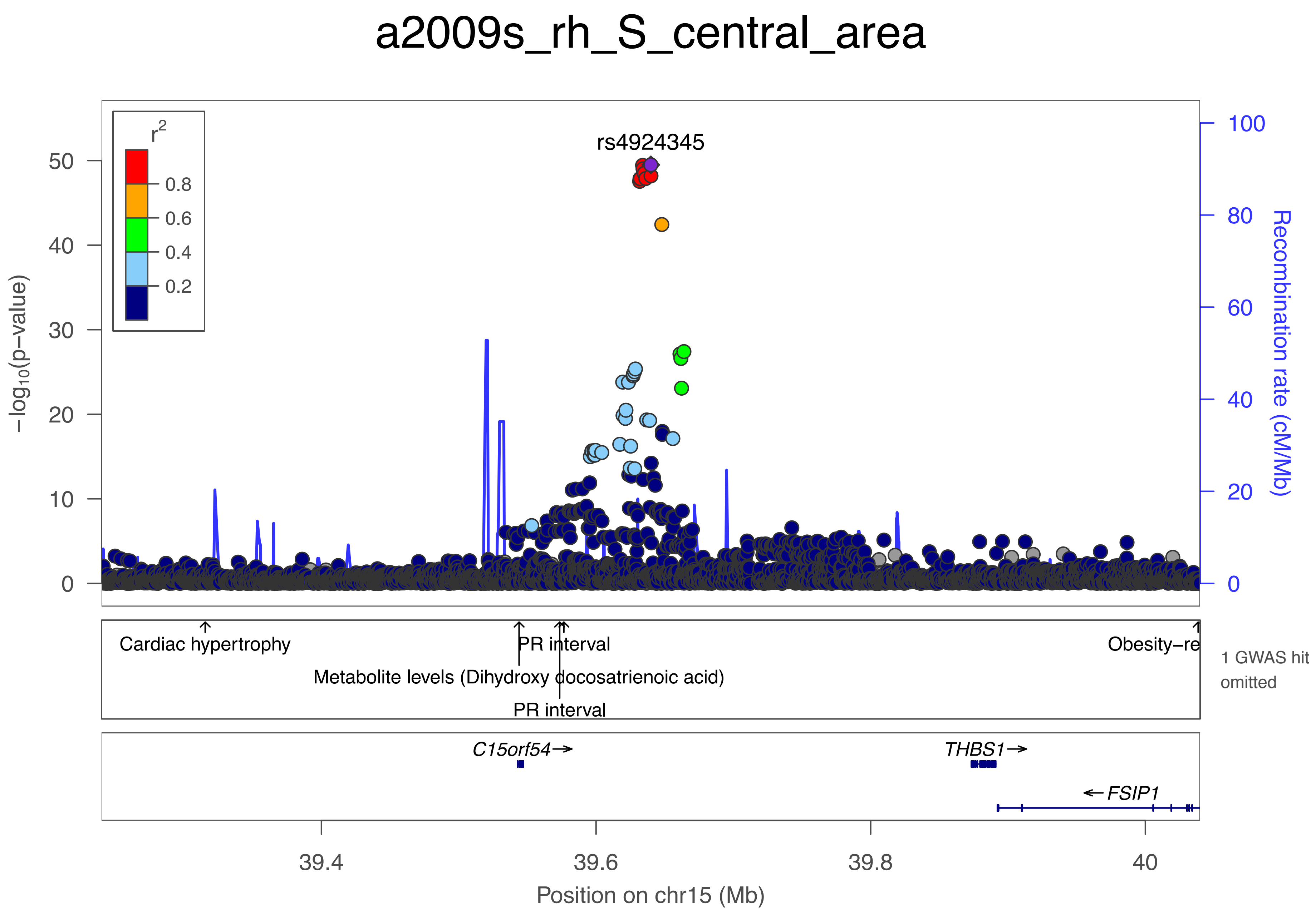

a2009s_rh_S_central_area
rs4924345
r2
0.8
0.6
0.4
0.2
−log10(p−value)
Recombination rate (cM/Mb)
Cardiac hypertrophy
PR interval
Metabolite levels (Dihydroxy docosatrienoic acid)
PR interval
Obesity−re
1 GWAS hit omitted
C15orf54→
THBS1→
←FSIP1
Position on chr15 (Mb)

| | |
|---|---|
| date: | Thu Aug 17 18:53:26 2017 |
| build: | hg19 |
| display range: | chr15:39239898−40039898 [39239898−40039898] |
| hilite range: | 0 – 0 [ 0 – 0 ] |
| reference SNP: | chr15:39639898 |
| number of SNPs plotted: | 4344 |
| min P.value: | 3.15E−50 [chr15:39639898] |
| max P.value: | 10E−1 [chr15:39507763] |
| omitted GWAS Hits: | NA |

Make more plots at http://csg.sph.umich.edu/locuszoom/

GWAS Catalog SNPs in Region

| chr | pos (Mb) | trait | snp |
|---|---|---|---|
| 15 | 39.31536 | Cardiac hypertrophy | rs12907914 |
| 15 | 39.34705 | Parental extreme longevity (95 years and older) | rs78539969 |
| 15 | 39.54395 | Metabolite levels (Dihydroxy docosatrienoic acid) | rs17691453 |
| 15 | 39.57353 | PR interval | rs12595668 |
| 15 | 39.57656 | PR interval | rs746265 |
| 15 | 40.03843 | Obesity–related traits | rs8033957 |

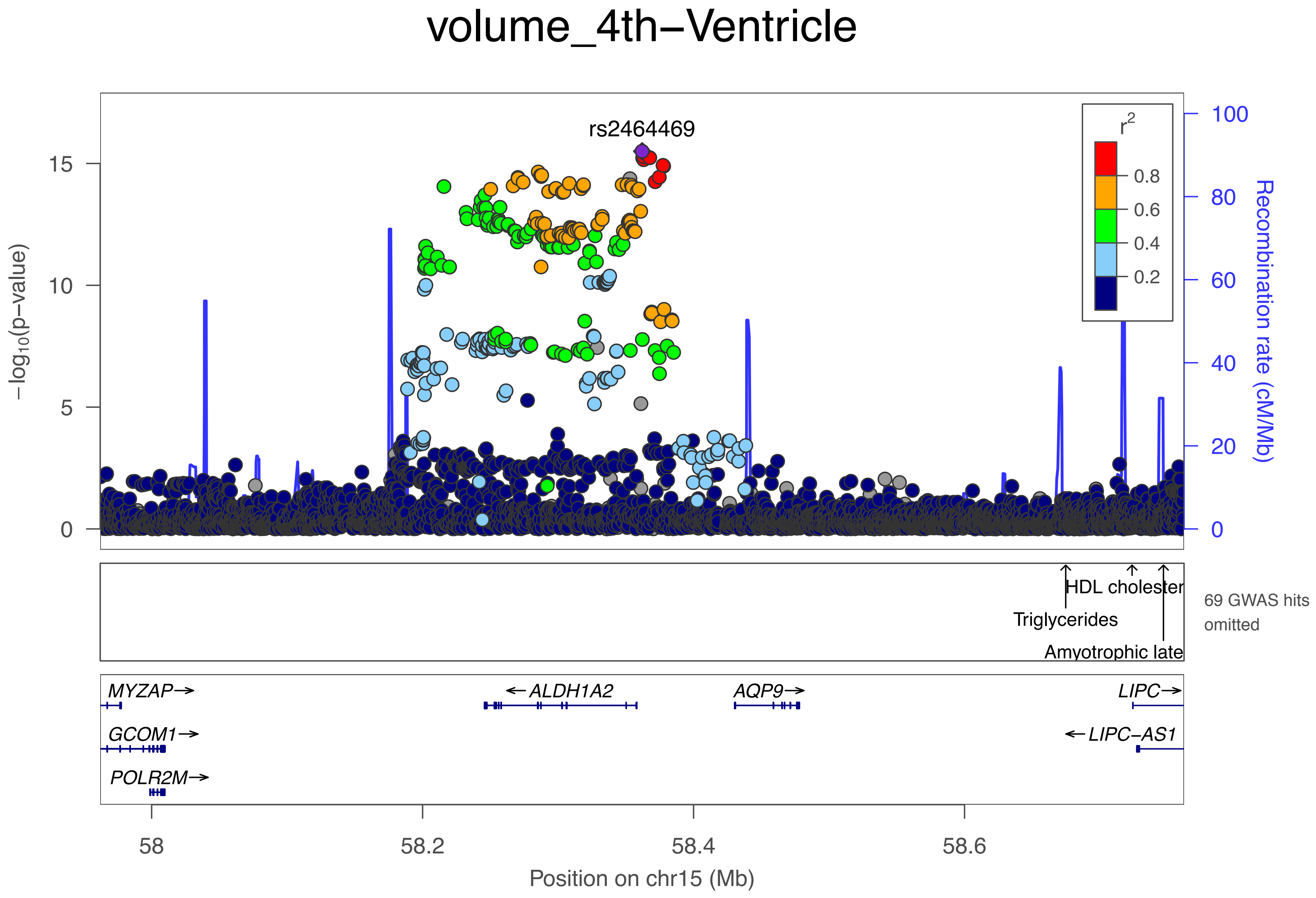
volume_4th−Ventricle
rs2464469
r2
0.8
0.6
0.4
0.2
−log10(p−value)
15
10
5
0
Recombination rate (cM/Mb)
100
80
60
40
20
0
HDL cholester
Triglycerides
Amyotrophic late
69 GWAS hits omitted
MYZAP→
GCOM1→
POLR2M→
←ALDH1A2
AQP9→
LIPC→
←LIPC−AS1
58
58.2
58.4
58.6
Position on chr15 (Mb)

date: Thu Aug 17 19:01:30 2017

build: hg19

display range: chr15:57962025−58762025 [57962025−58762025]

hilite range: 0 – 0 [ 0 – 0 ]

reference SNP: chr15:58362025

number of SNPs plotted: 4625

min P.value: 3.16E−16 [chr15:58362025]

max P.value: 10E−1 [chr15:58464691]

omitted GWAS Hits: NA, NA

omitted GWAS Hits: NA, NA

omitted GWAS Hits: NA, NA

omitted GWAS Hits: NA, NA

omitted GWAS Hits: NA, NA

omitted GWAS Hits: NA, NA

omitted GWAS Hits: NA, NA

omitted GWAS Hits: NA, NA

omitted GWAS Hits: NA, NA

omitted GWAS Hits: NA, NA

omitted GWAS Hits: NA, NA

omitted GWAS Hits: NA, NA

omitted GWAS Hits: NA, NA

omitted GWAS Hits: NA, NA

Make more plots at http://csg.sph.umich.edu/locuszoom/

omitted GWAS Hits: NA, NA

GWAS Catalog SNPs in Region

| chr | pos (Mb) | trait | snp |
|---|---|---|---|
| 15 | 58.00976 | Seasonality | rs1808478 |
| 15 | 58.03386 | Diisocyanate−induced asthma | rs487836 |
| 15 | 58.08062 | Myopia (pathological) | rs1559777 |
| 15 | 58.08237 | Diisocyanate−induced asthma | rs566767 |
| 15 | 58.08537 | Post bronchodilator FEV1/FVC ratio | rs114831632 |
| 15 | 58.08889 | Post bronchodilator FEV1/FVC ratio | rs149074315 |
| 15 | 58.08933 | Post bronchodilator FEV1/FVC ratio | rs140526800 |
| 15 | 58.10059 | Post bronchodilator FEV1/FVC ratio | rs145330511 |
| 15 | 58.10186 | Post bronchodilator FEV1/FVC ratio | rs140949321 |
| 15 | 58.10198 | Post bronchodilator FEV1/FVC ratio | rs148246786 |
| 15 | 58.10224 | Post bronchodilator FEV1/FVC ratio | rs7164035 |
| 15 | 58.10423 | Post bronchodilator FEV1/FVC ratio | rs146071159 |
| 15 | 58.10480 | Post bronchodilator FEV1/FVC ratio | rs113870173 |
| 15 | 58.10503 | Post bronchodilator FEV1/FVC ratio | rs115942227 |
| 15 | 58.21341 | Hypertension | rs1550576 |
| 15 | 58.24680 | Osteoarthritis (hand, severe) | rs3204689 |
| 15 | 58.25311 | Barrett's esophagus | rs3784262 |
| 15 | 58.25311 | Digestive system disease (Barrett's esophagus and esophageal adenocarcinoma combined) | rs3784262 |
| 15 | 58.26742 | Barrett's esophagus | rs66725070 |
| 15 | 58.36203 | Barrett's esophagus  or Esophageal adenocarcinoma | rs2464469 |
| 15 | 58.47198 | Metabolite levels | rs16939881 |

GWAS Catalog SNPs in Region

| chr | pos (Mb) | trait | snp |
|---|---|---|---|
| 15 | 58.55169 | Stroke | rs4471613 |
| 15 | 58.55169 | Ischemic stroke | rs4471613 |
| 15 | 58.57497 | Metabolite levels (lipoprotein measures) | rs56050415 |
| 15 | 58.63558 | Cognitive performance | rs4775031 |
| 15 | 58.67177 | Cholesterol, total | rs1601935 |
| 15 | 58.67467 | Non−small cell lung cancer | rs539901 |
| 15 | 58.67469 | Triglycerides | rs4775041 |
| 15 | 58.67469 | Metabolite levels | rs4775041 |
| 15 | 58.67469 | HDL cholesterol | rs4775041 |
| 15 | 58.67789 | Gout | rs187775 |
| 15 | 58.67851 | HDL cholesterol | rs10468017 |
| 15 | 58.67851 | Cardiovascular disease risk factors | rs10468017 |
| 15 | 58.67851 | Metabolic syndrome (bivariate traits) | rs10468017 |
| 15 | 58.67851 | Age−related macular degeneration | rs10468017 |
| 15 | 58.67851 | Phospholipid levels (plasma) | rs10468017 |
| 15 | 58.67851 | Blood metabolite levels | rs10468017 |
| 15 | 58.68018 | HDL cholesterol | rs261291 |
| 15 | 58.68064 | Lipid metabolism phenotypes | rs35853021 |
| 15 | 58.68095 | Metabolic syndrome (bivariate traits) | rs2043085 |
| 15 | 58.68095 | Lipid traits | rs2043085 |
| 15 | 58.68095 | Advanced age−related macular degeneration | rs2043085 |

GWAS Catalog SNPs in Region

| chr | pos (Mb) | trait | snp |
|---|---|---|---|
| 15 | 58.68337 | HDL cholesterol | rs1532085 |
| 15 | 58.68337 | Cholesterol, total | rs1532085 |
| 15 | 58.68337 | Triglycerides | rs1532085 |
| 15 | 58.68337 | Metabolic syndrome | rs1532085 |
| 15 | 58.68337 | Lipid metabolism phenotypes | rs1532085 |
| 15 | 58.68337 | Red blood cell traits | rs1532085 |
| 15 | 58.68337 | Metabolite levels | rs1532085 |
| 15 | 58.68337 | Metabolite levels (lipoprotein measures) | rs1532085 |
| 15 | 58.68788 | Age−related macular degeneration | rs493258 |
| 15 | 58.68847 | Age−related macular degeneration | rs920915 |
| 15 | 58.69402 | HDL cholesterol | rs16940212 |
| 15 | 58.71220 | Obesity−related traits | rs11857380 |
| 15 | 58.72343 | HDL cholesterol | rs1077835 |
| 15 | 58.72343 | Triglycerides | rs1077835 |
| 15 | 58.72348 | Hematological and biochemical traits | rs1077834 |
| 15 | 58.72348 | C−reactive protein levels or HDL−cholesterol levels (pleiotropy) | rs1077834 |
| 15 | 58.72368 | HDL cholesterol | rs1800588 |
| 15 | 58.72368 | Triglycerides | rs1800588 |
| 15 | 58.72368 | Cholesterol, total | rs1800588 |
| 15 | 58.72394 | Blood metabolite levels | rs2070895 |
| 15 | 58.72394 | Advanced age−related macular degeneration | rs2070895 |

GWAS Catalog SNPs in Region

| chr | pos (Mb) | trait | snp |
|---|---|---|---|
| 15 | 58.72479 | HDL cholesterol | rs8034802 |
| 15 | 58.72674 | HDL cholesterol | rs261334 |
| 15 | 58.72674 | Metabolite levels (lipoprotein measures) | rs261334 |
| 15 | 58.73050 | Lipid traits | rs588136 |
| 15 | 58.73050 | Blood metabolite levels | rs588136 |
| 15 | 58.73064 | Alzheimer's disease (cognitive decline) | rs17301739 |
| 15 | 58.73115 | Metabolite levels | rs261342 |
| 15 | 58.73842 | Schizophrenia | rs12914626 |
| 15 | 58.74683 | Amyotrophic lateral sclerosis | rs3825776 |

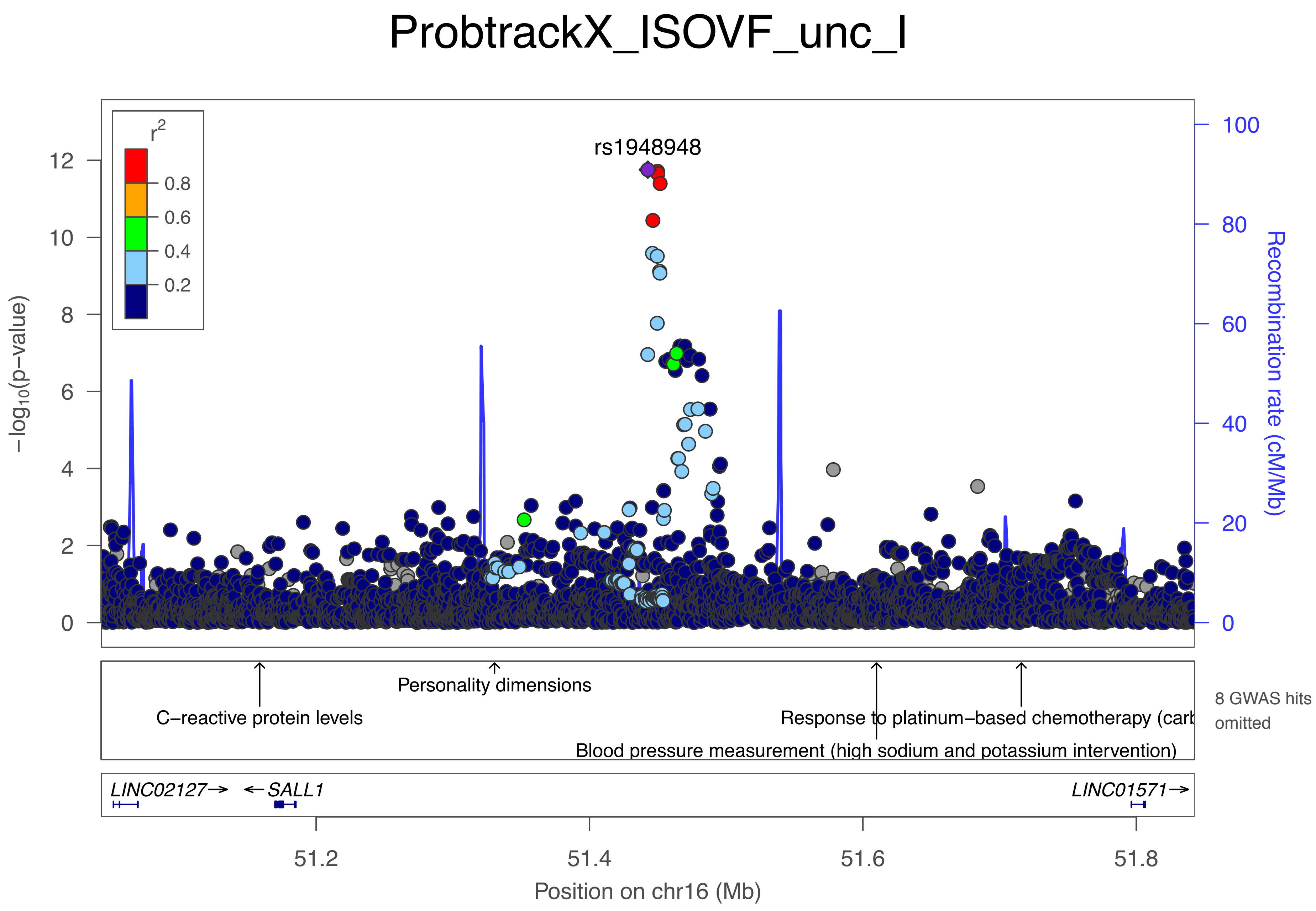

ProbtrackX_ISOVF_unc_l
rs1948948
r2
0.8
0.6
0.4
0.2
−log10(p−value)
Recombination rate (cM/Mb)
Personality dimensions
C−reactive protein levels
Response to platinum−based chemotherapy (carb
Blood pressure measurement (high sodium and potassium intervention)
8 GWAS hits omitted
LINC02127
SALL1
LINC01571
51.2
51.4
51.6
51.8
Position on chr16 (Mb)

| | |
|---|---|
| date: | Thu Aug 17 19:11:22 2017 |
| build: | hg19 |
| display range: | chr16:51042679–51842679 [51042679–51842679] |
| hilite range: | 0 – 0 [ 0 – 0 ] |
| reference SNP: | chr16:51442679 |
| number of SNPs plotted: | 3808 |
| min P.value: | 1.75E–12 [chr16:51442679] |
| max P.value: | 10E–1 [chr16:51602902] |
| omitted GWAS Hits: | NA, NA |
| omitted GWAS Hits: | NA, NA |
| omitted GWAS Hits: | NA, NA |

Make more plots at http://csg.sph.umich.edu/locuszoom/

GWAS Catalog SNPs in Region

| chr | pos (Mb) | trait | snp |
|---|---|---|---|
| 16 | 51.06153 | Post bronchodilator FEV1/FVC ratio | rs147153117 |
| 16 | 51.15871 | C−reactive protein levels | rs10521222 |
| 16 | 51.18373 | Educational attainment (years of education) | rs11643654 |
| 16 | 51.24549 | Weight loss (gastric bypass surgery) | rs7185923 |
| 16 | 51.33053 | Personality dimensions | rs4131099 |
| 16 | 51.35748 | Left superior temporal gyrus thickness (schizophrenia interaction) | rs17338034 |
| 16 | 51.42891 | Optic cup area | rs11646917 |
| 16 | 51.45829 | Optic disc parameters | rs1362756 |
| 16 | 51.45829 | Optic disc area | rs1362756 |
| 16 | 51.48232 | Vertical cup−disc ratio | rs1345467 |
| 16 | 51.60995 | Blood pressure measurement (high sodium and potassium intervention) | rs2030114 |
| 16 | 51.71587 | Response to platinum−based chemotherapy (carboplatin) | rs1381451 |

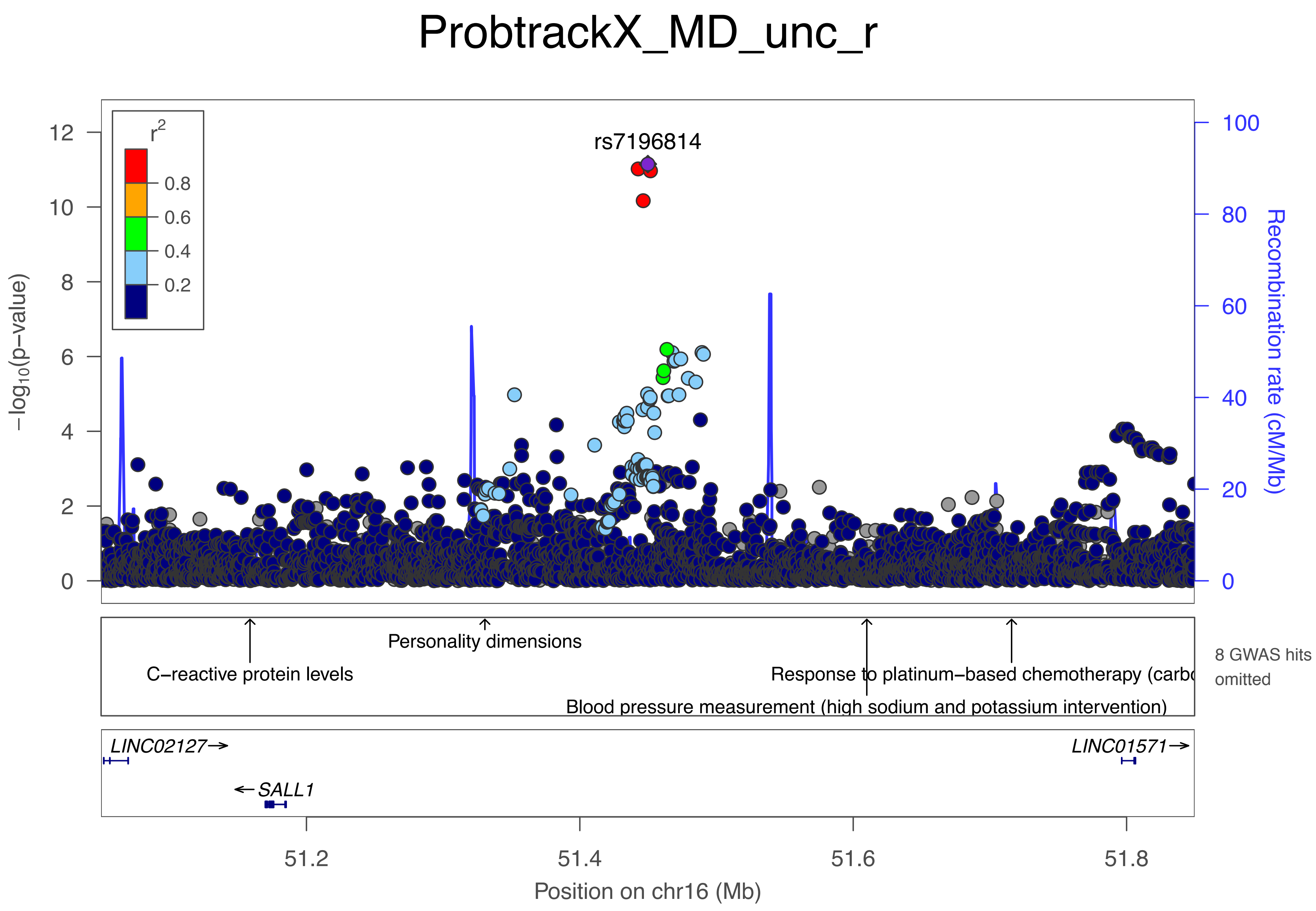
ProbtrackX_MD_unc_r
rs7196814
r2
0.8
0.6
0.4
0.2
−log10(p−value)
Recombination rate (cM/Mb)
Personality dimensions
C−reactive protein levels
Response to platinum−based chemotherapy (carbo
Blood pressure measurement (high sodium and potassium intervention)
8 GWAS hits omitted
LINC02127→
←SALL1
LINC01571→
51.2
51.4
51.6
51.8
Position on chr16 (Mb)

| | |
|---|---|
| date: | Thu Aug 17 19:11:44 2017 |
| build: | hg19 |
| display range: | chr16:51049760−51849760 [51049760−51849760] |
| hilite range: | 0 – 0 [ 0 – 0 ] |
| reference SNP: | chr16:51449760 |
| number of SNPs plotted: | 3808 |
| min P.value: | 7.11E−12 [chr16:51449760] |
| max P.value: | 10E−1 [chr16:51818989] |
| omitted GWAS Hits: | NA, NA |
| omitted GWAS Hits: | NA, NA |
| omitted GWAS Hits: | NA, NA |

Make more plots at http://csg.sph.umich.edu/locuszoom/

GWAS Catalog SNPs in Region

| chr | pos (Mb) | trait | snp |
|---|---|---|---|
| 16 | 51.06153 | Post bronchodilator FEV1/FVC ratio | rs147153117 |
| 16 | 51.15871 | C–reactive protein levels | rs10521222 |
| 16 | 51.18373 | Educational attainment (years of education) | rs11643654 |
| 16 | 51.24549 | Weight loss (gastric bypass surgery) | rs7185923 |
| 16 | 51.33053 | Personality dimensions | rs4131099 |
| 16 | 51.35748 | Left superior temporal gyrus thickness (schizophrenia interaction) | rs17338034 |
| 16 | 51.42891 | Optic cup area | rs11646917 |
| 16 | 51.45829 | Optic disc parameters | rs1362756 |
| 16 | 51.45829 | Optic disc area | rs1362756 |
| 16 | 51.48232 | Vertical cup–disc ratio | rs1345467 |
| 16 | 51.60995 | Blood pressure measurement (high sodium and potassium intervention) | rs2030114 |
| 16 | 51.71587 | Response to platinum–based chemotherapy (carboplatin) | rs1381451 |

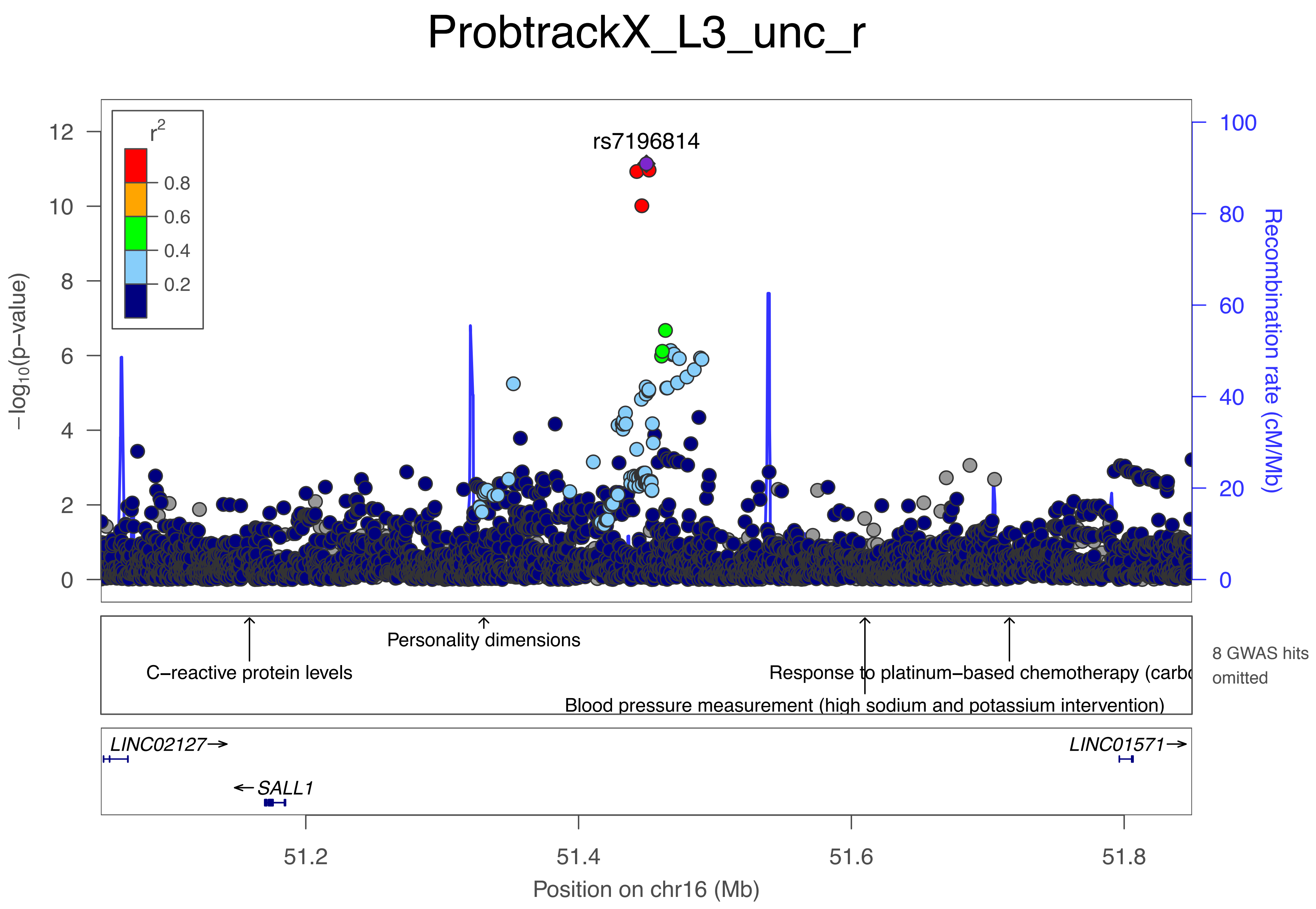

ProbtrackX_L3_unc_r
rs7196814
r2
0.8
0.6
0.4
0.2
−log10(p−value)
12
10
8
6
4
2
0
Recombination rate (cM/Mb)
100
80
60
40
20
0
Personality dimensions
C−reactive protein levels
Response to platinum−based chemotherapy (carbo
Blood pressure measurement (high sodium and potassium intervention)
8 GWAS hits omitted
LINC02127→
←SALL1
LINC01571→
51.2
51.4
51.6
51.8
Position on chr16 (Mb)

| | |
|---|---|
| date: | Thu Aug 17 18:52:05 2017 |
| build: | hg19 |
| display range: | chr16:51049760–51849760 [51049760–51849760] |
| hilite range: | 0 – 0 [ 0 – 0 ] |
| reference SNP: | chr16:51449760 |
| number of SNPs plotted: | 3808 |
| min P.value: | 7.28E−12 [chr16:51449760] |
| max P.value: | 10E−1 [chr16:51142360] |
| omitted GWAS Hits: | NA, NA |
| omitted GWAS Hits: | NA, NA |
| omitted GWAS Hits: | NA, NA |

Make more plots at http://csg.sph.umich.edu/locuszoom/

GWAS Catalog SNPs in Region

| chr | pos (Mb) | trait | snp |
|---|---|---|---|
| 16 | 51.06153 | Post bronchodilator FEV1/FVC ratio | rs147153117 |
| 16 | 51.15871 | C−reactive protein levels | rs10521222 |
| 16 | 51.18373 | Educational attainment (years of education) | rs11643654 |
| 16 | 51.24549 | Weight loss (gastric bypass surgery) | rs7185923 |
| 16 | 51.33053 | Personality dimensions | rs4131099 |
| 16 | 51.35748 | Left superior temporal gyrus thickness (schizophrenia interaction) | rs17338034 |
| 16 | 51.42891 | Optic cup area | rs11646917 |
| 16 | 51.45829 | Optic disc parameters | rs1362756 |
| 16 | 51.45829 | Optic disc area | rs1362756 |
| 16 | 51.48232 | Vertical cup−disc ratio | rs1345467 |
| 16 | 51.60995 | Blood pressure measurement (high sodium and potassium intervention) | rs2030114 |
| 16 | 51.71587 | Response to platinum−based chemotherapy (carboplatin) | rs1381451 |

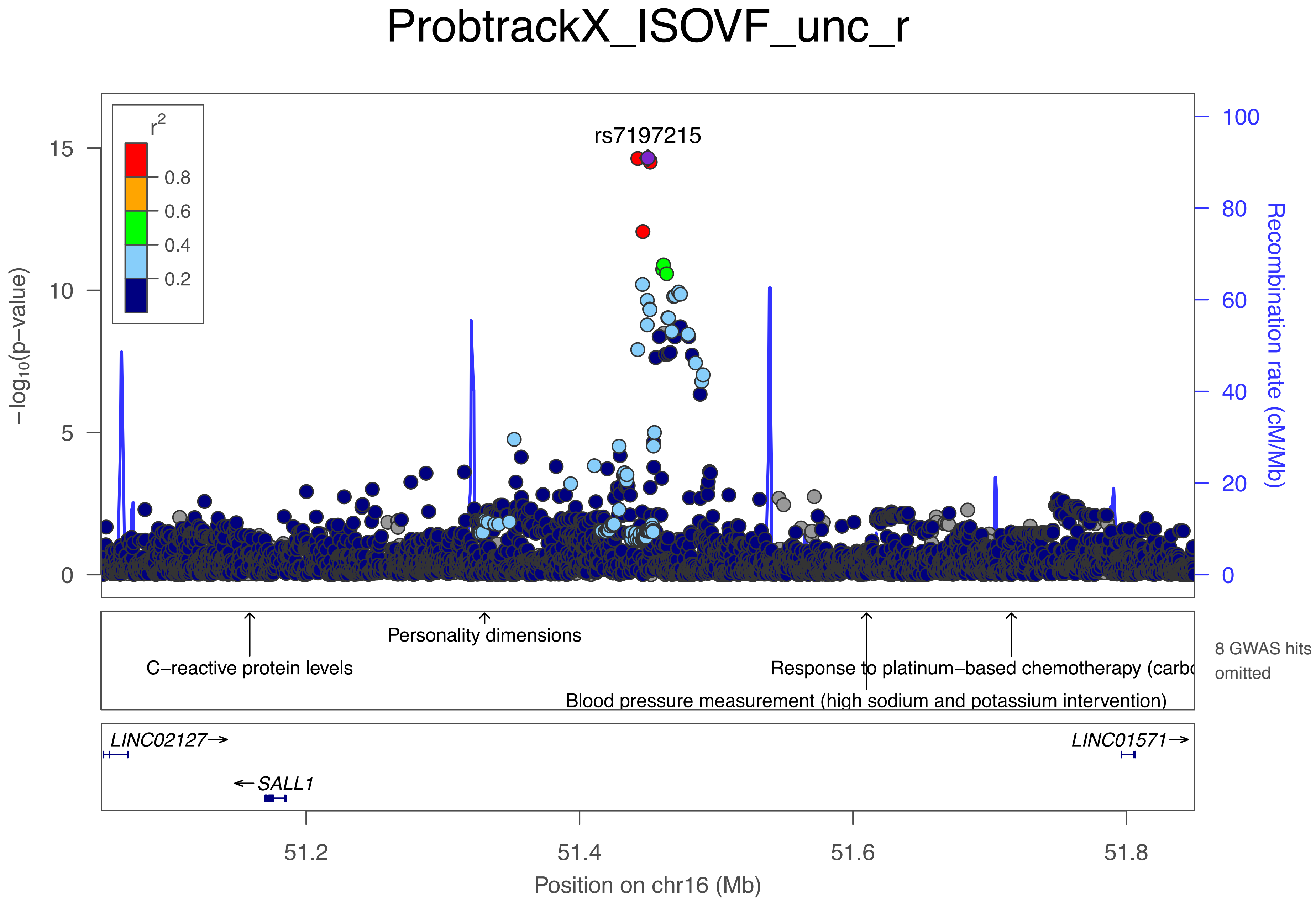
ProbtrackX_ISOVF_unc_r
rs7197215
r2
0.8
0.6
0.4
0.2
15
10
5
0
−log10(p−value)
100
80
60
40
20
0
Recombination rate (cM/Mb)
Personality dimensions
C−reactive protein levels
Response to platinum−based chemotherapy (carbo
Blood pressure measurement (high sodium and potassium intervention)
8 GWAS hits omitted
LINC02127
SALL1
LINC01571
51.2
51.4
51.6
51.8
Position on chr16 (Mb)

| | |
|---|---|
| date: | Thu Aug 17 18:52:16 2017 |
| build: | hg19 |
| display range: | chr16:51049978–51849978 [51049978–51849978] |
| hilite range: | 0 – 0 [ 0 – 0 ] |
| reference SNP: | chr16:51449978 |
| number of SNPs plotted: | 3805 |
| min P.value: | 2.24E–15 [chr16:51449978] |
| max P.value: | 10E–1 [chr16:51130463] |
| omitted GWAS Hits: | NA, NA |
| omitted GWAS Hits: | NA, NA |
| omitted GWAS Hits: | NA, NA |

Make more plots at http://csg.sph.umich.edu/locuszoom/

GWAS Catalog SNPs in Region

| chr | pos (Mb) | trait | snp |
|---|---|---|---|
| 16 | 51.06153 | Post bronchodilator FEV1/FVC ratio | rs147153117 |
| 16 | 51.15871 | C−reactive protein levels | rs10521222 |
| 16 | 51.18373 | Educational attainment (years of education) | rs11643654 |
| 16 | 51.24549 | Weight loss (gastric bypass surgery) | rs7185923 |
| 16 | 51.33053 | Personality dimensions | rs4131099 |
| 16 | 51.35748 | Left superior temporal gyrus thickness (schizophrenia interaction) | rs17338034 |
| 16 | 51.42891 | Optic cup area | rs11646917 |
| 16 | 51.45829 | Optic disc parameters | rs1362756 |
| 16 | 51.45829 | Optic disc area | rs1362756 |
| 16 | 51.48232 | Vertical cup−disc ratio | rs1345467 |
| 16 | 51.60995 | Blood pressure measurement (high sodium and potassium intervention) | rs2030114 |
| 16 | 51.71587 | Response to platinum−based chemotherapy (carboplatin) | rs1381451 |

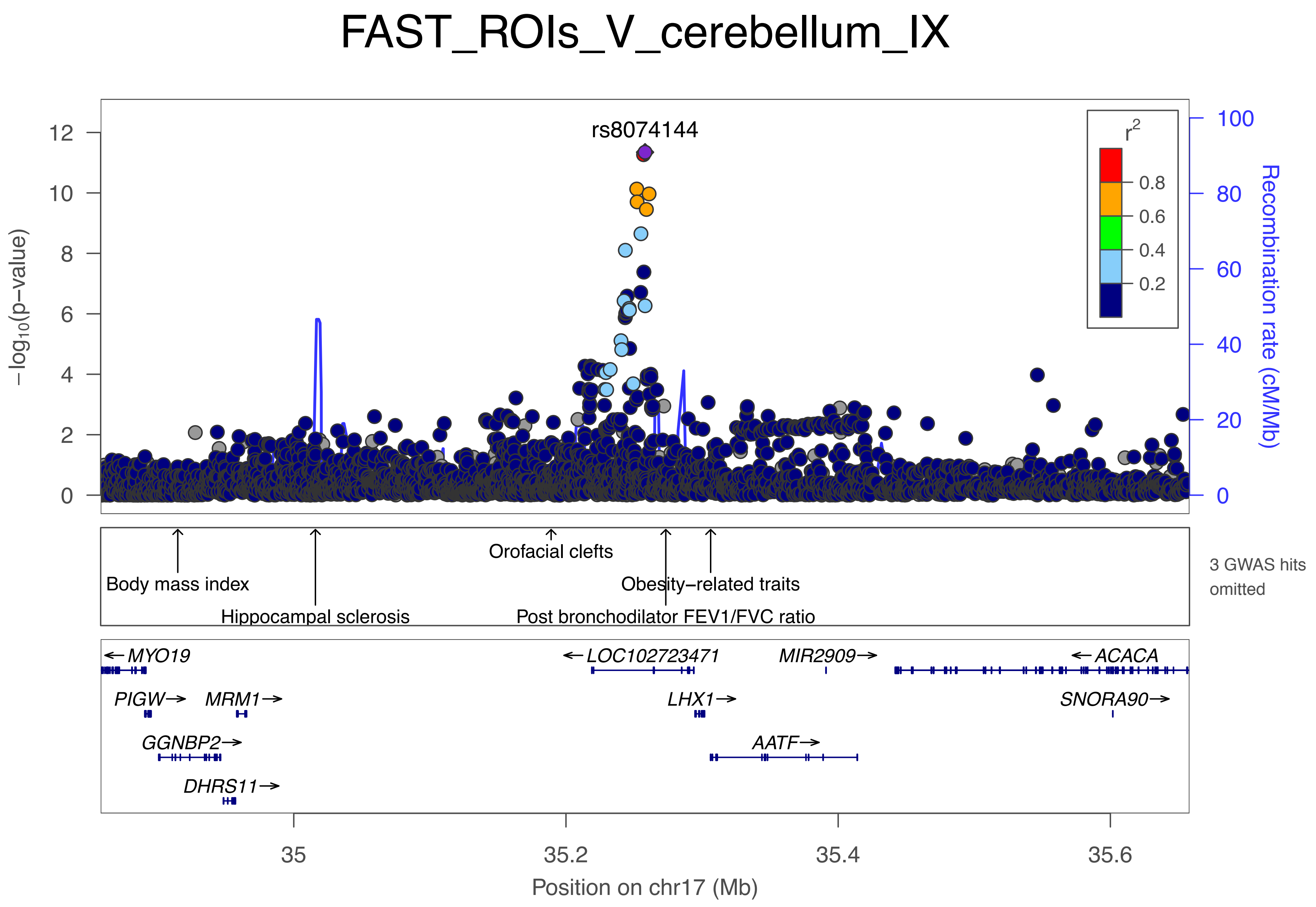
FAST_ROIs_V_cerebellum_IX
rs8074144
−log10(p−value)
Recombination rate (cM/Mb)
r2
0.8
0.6
0.4
0.2
Body mass index
Hippocampal sclerosis
Orofacial clefts
Obesity−related traits
Post bronchodilator FEV1/FVC ratio
3 GWAS hits omitted
MYO19
PIGW
MRM1
GGNBP2
DHRS11
LOC102723471
LHX1
MIR2909
AATF
ACACA
SNORA90
35
35.2
35.4
35.6
Position on chr17 (Mb)

| | |
|---|---|
| date: | Thu Aug 17 19:10:54 2017 |
| build: | hg19 |
| display range: | chr17:34858114–35658114 [34858114–35658114] |
| hilite range: | 0 – 0 [ 0 – 0 ] |
| reference SNP: | chr17:35258114 |
| number of SNPs plotted: | 3369 |
| min P.value: | 4.5E−12 [chr17:35258114] |
| max P.value: | 10E−1 [chr17:35195623] |
| omitted GWAS Hits: | NA, NA |

Make more plots at http://csg.sph.umich.edu/locuszoom/

GWAS Catalog SNPs in Region

| chr | pos (Mb) | trait | snp |
|---|---|---|---|
| 17 | 34.91479 | Body mass index | rs12150665 |
| 17 | 35.01590 | Hippocampal sclerosis | rs12950363 |
| 17 | 35.18200 | Obstructive sleep apnea trait (apnea hipopnea index) | rs4796285 |
| 17 | 35.18909 | Orofacial clefts | rs17138064 |
| 17 | 35.27092 | Post bronchodilator FEV1/FVC ratio | rs55955238 |
| 17 | 35.27334 | Post bronchodilator FEV1/FVC ratio | rs2003347 |
| 17 | 35.27428 | Post bronchodilator FEV1/FVC ratio | rs12185265 |
| 17 | 35.30631 | Obesity−related traits | rs2306658 |

# FAST_ROIs_L_cerebellum_IX

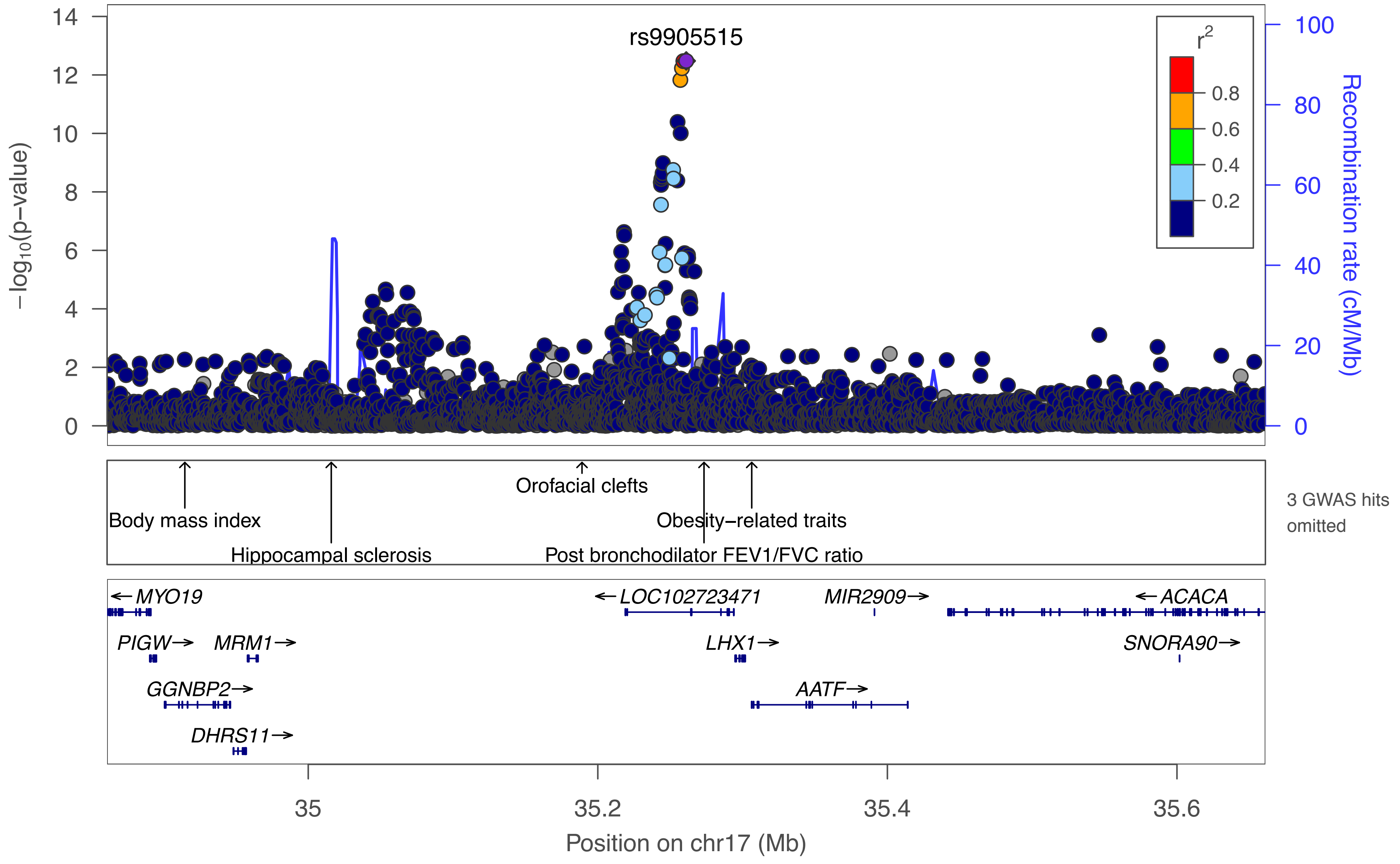

| | |
|---|---|
| date: | Thu Aug 17 19:10:55 2017 |
| build: | hg19 |
| display range: | chr17:34861073–35661073 [34861073–35661073] |
| hilite range: | 0 – 0 [ 0 – 0 ] |
| reference SNP: | chr17:35261073 |
| number of SNPs plotted: | 3367 |
| min P.value: | 3.32E−13 [chr17:35261073] |
| max P.value: | 9.99E−1 [chr17:35070327] |
| omitted GWAS Hits: | NA, NA |

Make more plots at http://csg.sph.umich.edu/locuszoom/

GWAS Catalog SNPs in Region

| chr | pos (Mb) | trait | snp |
|---|---|---|---|
| 17 | 34.91479 | Body mass index | rs12150665 |
| 17 | 35.01590 | Hippocampal sclerosis | rs12950363 |
| 17 | 35.18200 | Obstructive sleep apnea trait (apnea hipopnea index) | rs4796285 |
| 17 | 35.18909 | Orofacial clefts | rs17138064 |
| 17 | 35.27092 | Post bronchodilator FEV1/FVC ratio | rs55955238 |
| 17 | 35.27334 | Post bronchodilator FEV1/FVC ratio | rs2003347 |
| 17 | 35.27428 | Post bronchodilator FEV1/FVC ratio | rs12185265 |
| 17 | 35.30631 | Obesity–related traits | rs2306658 |

# SWI_T2star_left_caudate

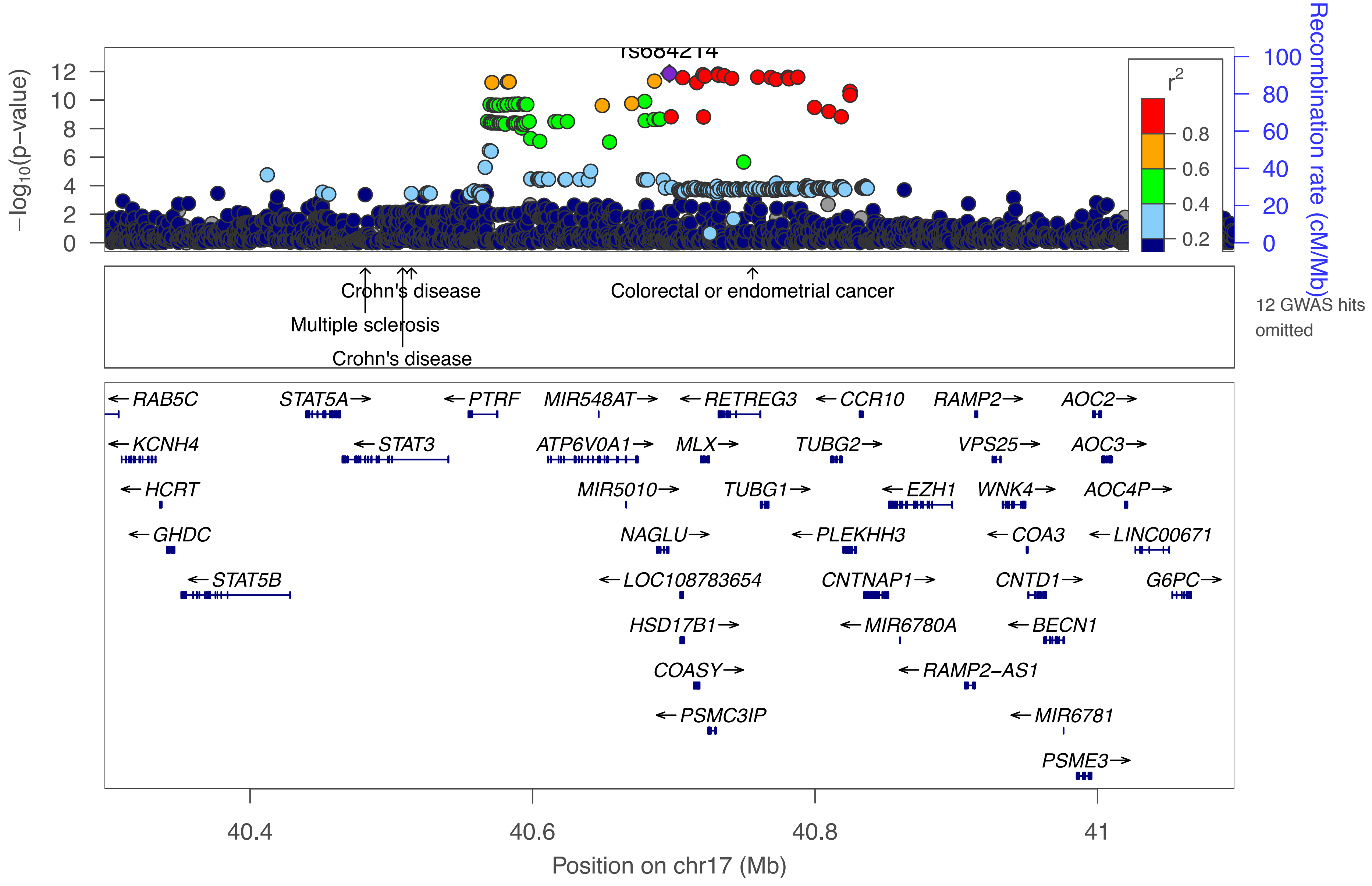

| | |
|---|---|
| date: | Thu Aug 17 19:02:56 2017 |
| build: | hg19 |
| display range: | chr17:40296915−41096915 [40296915−41096915] |
| hilite range: | 0 – 0 [ 0 – 0 ] |
| reference SNP: | chr17:40696915 |
| number of SNPs plotted: | 2522 |
| min P.value: | 1.38E−12 [chr17:40696915] |
| max P.value: | 10E−1 [chr17:40698530] |
| omitted GWAS Hits: | chr17:40.755811−Colorectal or endometrial cancer, NA |
| omitted GWAS Hits: | NA, NA |
| omitted GWAS Hits: | NA, NA |
| omitted GWAS Hits: | NA, NA |

Make more plots at http://csg.sph.umich.edu/locuszoom/

GWAS Catalog SNPs in Region

| chr | pos (Mb) | trait | snp |
|---|---|---|---|
| 17 | 40.48153 | Multiple sclerosis | rs2293152 |
| 17 | 40.48524 | Atopic dermatitis | rs17881320 |
| 17 | 40.49953 | Crohn's disease | rs6503695 |
| 17 | 40.50798 | Crohn's disease | rs9891119 |
| 17 | 40.50798 | Multiple sclerosis | rs9891119 |
| 17 | 40.51420 | Crohn's disease | rs744166 |
| 17 | 40.51420 | Multiple sclerosis | rs744166 |
| 17 | 40.51420 | Inflammatory bowel disease | rs744166 |
| 17 | 40.52754 | Ulcerative colitis | rs12942547 |
| 17 | 40.52754 | Crohn's disease | rs12942547 |
| 17 | 40.52754 | Inflammatory bowel disease | rs12942547 |
| 17 | 40.54221 | Atopic dermatitis | rs4796793 |
| 17 | 40.56504 | Psoriasis | rs56364076 |
| 17 | 40.57077 | Crohn's disease | rs11871801 |
| 17 | 40.69334 | Systolic blood pressure change trajectories | rs630539 |
| 17 | 40.75581 | Colorectal or endometrial cancer | rs9901225 |

# SWI_T2star_left_caudate_plus_right_caudate

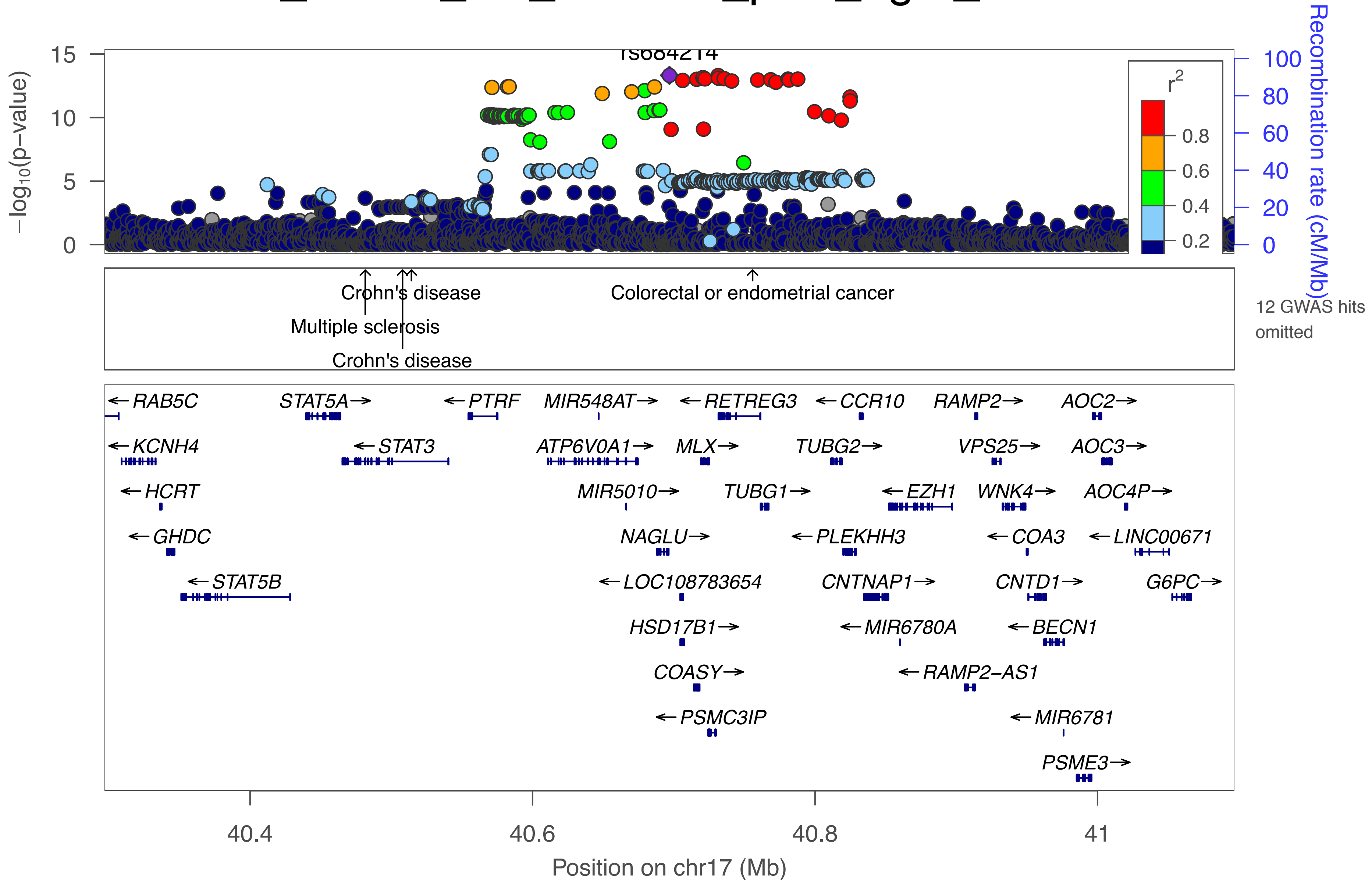

| | |
|---|---|
| date: | Thu Aug 17 19:03:05 2017 |
| build: | hg19 |
| display range: | chr17:40296915−41096915 [40296915−41096915] |
| hilite range: | 0 – 0 [ 0 – 0 ] |
| reference SNP: | chr17:40696915 |
| number of SNPs plotted: | 2522 |
| min P.value: | 4.78E−14 [chr17:40696915] |
| max P.value: | 10E−1 [chr17:40880002] |
| omitted GWAS Hits: | chr17:40.755811−Colorectal or endometrial cancer, NA |
| omitted GWAS Hits: | NA, NA |
| omitted GWAS Hits: | NA, NA |
| omitted GWAS Hits: | NA, NA |

Make more plots at http://csg.sph.umich.edu/locuszoom/

GWAS Catalog SNPs in Region

| chr | pos (Mb) | trait | snp |
|---|---|---|---|
| 17 | 40.48153 | Multiple sclerosis | rs2293152 |
| 17 | 40.48524 | Atopic dermatitis | rs17881320 |
| 17 | 40.49953 | Crohn's disease | rs6503695 |
| 17 | 40.50798 | Crohn's disease | rs9891119 |
| 17 | 40.50798 | Multiple sclerosis | rs9891119 |
| 17 | 40.51420 | Crohn's disease | rs744166 |
| 17 | 40.51420 | Multiple sclerosis | rs744166 |
| 17 | 40.51420 | Inflammatory bowel disease | rs744166 |
| 17 | 40.52754 | Ulcerative colitis | rs12942547 |
| 17 | 40.52754 | Crohn's disease | rs12942547 |
| 17 | 40.52754 | Inflammatory bowel disease | rs12942547 |
| 17 | 40.54221 | Atopic dermatitis | rs4796793 |
| 17 | 40.56504 | Psoriasis | rs56364076 |
| 17 | 40.57077 | Crohn's disease | rs11871801 |
| 17 | 40.69334 | Systolic blood pressure change trajectories | rs630539 |
| 17 | 40.75581 | Colorectal or endometrial cancer | rs9901225 |

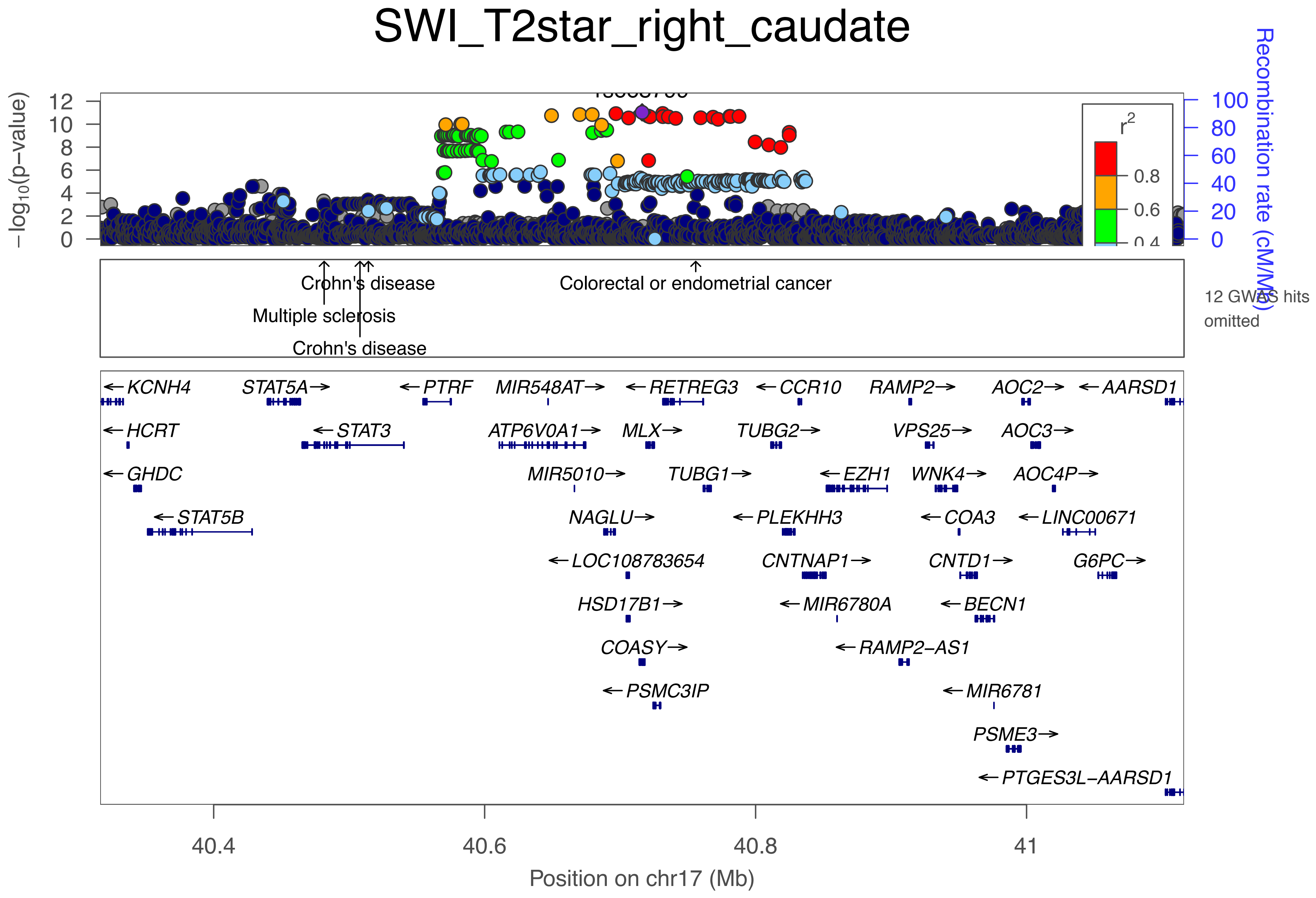

SWI_T2star_right_caudate
−log10(p−value)
Recombination rate (cM/Mb)
r2
0.8
0.6
0.4
Crohn's disease
Multiple sclerosis
Crohn's disease
Colorectal or endometrial cancer
12 GWAS hits omitted
KCNH4
HCRT
GHDC
STAT5B
STAT5A
STAT3
PTRF
MIR548AT
ATP6V0A1
MIR5010
NAGLU
LOC108783654
HSD17B1
COASY
PSMC3IP
RETREG3
MLX
TUBG1
TUBG2
PLEKHH3
CNTNAP1
MIR6780A
RAMP2−AS1
CCR10
EZH1
RAMP2
VPS25
WNK4
COA3
CNTD1
BECN1
MIR6781
PSME3
PTGES3L−AARSD1
AOC2
AOC3
AOC4P
LINC00671
G6PC
AARSD1
40.4
40.6
40.8
41
Position on chr17 (Mb)

| | |
|---|---|
| date: | Thu Aug 17 19:17:10 2017 |
| build: | hg19 |
| display range: | chr17:40316235−41116235 [40316235−41116235] |
| hilite range: | 0 – 0 [ 0 – 0 ] |
| reference SNP: | chr17:40716235 |
| number of SNPs plotted: | 2501 |
| min P.value: | 9.33E−12 [chr17:40716235] |
| max P.value: | 10E−1 [chr17:40892181] |
| omitted GWAS Hits: | chr17:40.755811−Colorectal or endometrial cancer, NA |
| omitted GWAS Hits: | NA, NA |
| omitted GWAS Hits: | NA, NA |
| omitted GWAS Hits: | NA, NA |

Make more plots at http://csg.sph.umich.edu/locuszoom/

GWAS Catalog SNPs in Region

| chr | pos (Mb) | trait | snp |
|---|---|---|---|
| 17 | 40.48153 | Multiple sclerosis | rs2293152 |
| 17 | 40.48524 | Atopic dermatitis | rs17881320 |
| 17 | 40.49953 | Crohn's disease | rs6503695 |
| 17 | 40.50798 | Crohn's disease | rs9891119 |
| 17 | 40.50798 | Multiple sclerosis | rs9891119 |
| 17 | 40.51420 | Crohn's disease | rs744166 |
| 17 | 40.51420 | Multiple sclerosis | rs744166 |
| 17 | 40.51420 | Inflammatory bowel disease | rs744166 |
| 17 | 40.52754 | Ulcerative colitis | rs12942547 |
| 17 | 40.52754 | Crohn's disease | rs12942547 |
| 17 | 40.52754 | Inflammatory bowel disease | rs12942547 |
| 17 | 40.54221 | Atopic dermatitis | rs4796793 |
| 17 | 40.56504 | Psoriasis | rs56364076 |
| 17 | 40.57077 | Crohn's disease | rs11871801 |
| 17 | 40.69334 | Systolic blood pressure change trajectories | rs630539 |
| 17 | 40.75581 | Colorectal or endometrial cancer | rs9901225 |

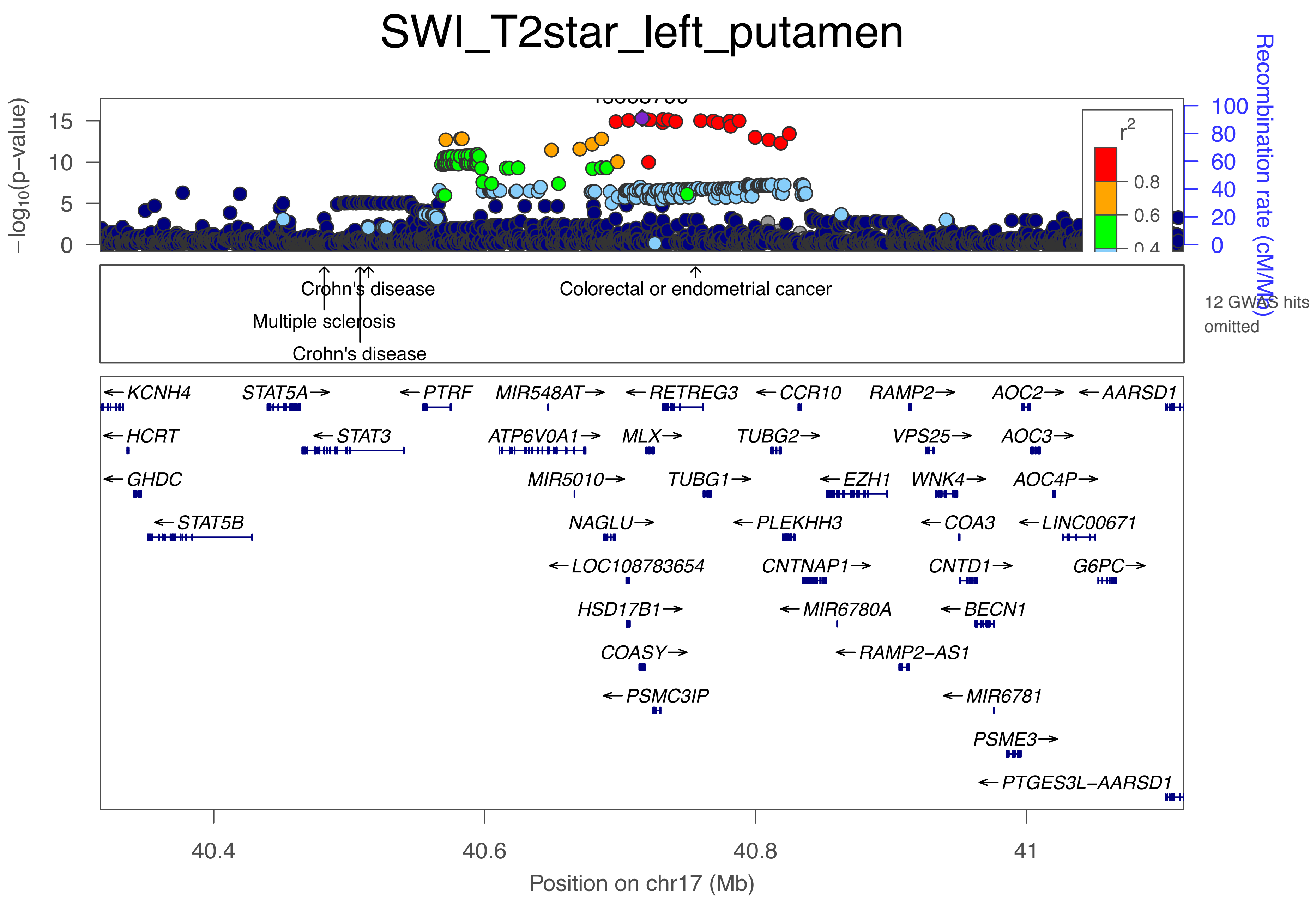

SWI_T2star_left_putamen
−log10(p−value)
Recombination rate (cM/Mb)
r2
0.8
0.6
0.4
Crohn's disease
Multiple sclerosis
Crohn's disease
Colorectal or endometrial cancer
12 GWAS hits omitted
KCNH4
HCRT
GHDC
STAT5B
STAT5A
STAT3
PTRF
MIR548AT
ATP6V0A1
MIR5010
NAGLU
LOC108783654
HSD17B1
COASY
PSMC3IP
RETREG3
MLX
TUBG1
PLEKHH3
CNTNAP1
MIR6780A
RAMP2−AS1
CCR10
TUBG2
EZH1
RAMP2
VPS25
WNK4
COA3
CNTD1
BECN1
MIR6781
PSME3
PTGES3L−AARSD1
AOC2
AOC3
AOC4P
LINC00671
G6PC
AARSD1
Position on chr17 (Mb)

| | |
|---|---|
| date: | Thu Aug 17 19:17:10 2017 |
| build: | hg19 |
| display range: | chr17:40316235−41116235 [40316235−41116235] |
| hilite range: | 0 – 0 [ 0 – 0 ] |
| reference SNP: | chr17:40716235 |
| number of SNPs plotted: | 2501 |
| min P.value: | 4.91E−16 [chr17:40716235] |
| max P.value: | 9.99E−1 [chr17:40910657] |
| omitted GWAS Hits: | chr17:40.755811−Colorectal or endometrial cancer, NA |
| omitted GWAS Hits: | NA, NA |
| omitted GWAS Hits: | NA, NA |
| omitted GWAS Hits: | NA, NA |

Make more plots at http://csg.sph.umich.edu/locuszoom/

GWAS Catalog SNPs in Region

| chr | pos (Mb) | trait | snp |
|---|---|---|---|
| 17 | 40.48153 | Multiple sclerosis | rs2293152 |
| 17 | 40.48524 | Atopic dermatitis | rs17881320 |
| 17 | 40.49953 | Crohn's disease | rs6503695 |
| 17 | 40.50798 | Crohn's disease | rs9891119 |
| 17 | 40.50798 | Multiple sclerosis | rs9891119 |
| 17 | 40.51420 | Crohn's disease | rs744166 |
| 17 | 40.51420 | Multiple sclerosis | rs744166 |
| 17 | 40.51420 | Inflammatory bowel disease | rs744166 |
| 17 | 40.52754 | Ulcerative colitis | rs12942547 |
| 17 | 40.52754 | Crohn's disease | rs12942547 |
| 17 | 40.52754 | Inflammatory bowel disease | rs12942547 |
| 17 | 40.54221 | Atopic dermatitis | rs4796793 |
| 17 | 40.56504 | Psoriasis | rs56364076 |
| 17 | 40.57077 | Crohn's disease | rs11871801 |
| 17 | 40.69334 | Systolic blood pressure change trajectories | rs630539 |
| 17 | 40.75581 | Colorectal or endometrial cancer | rs9901225 |

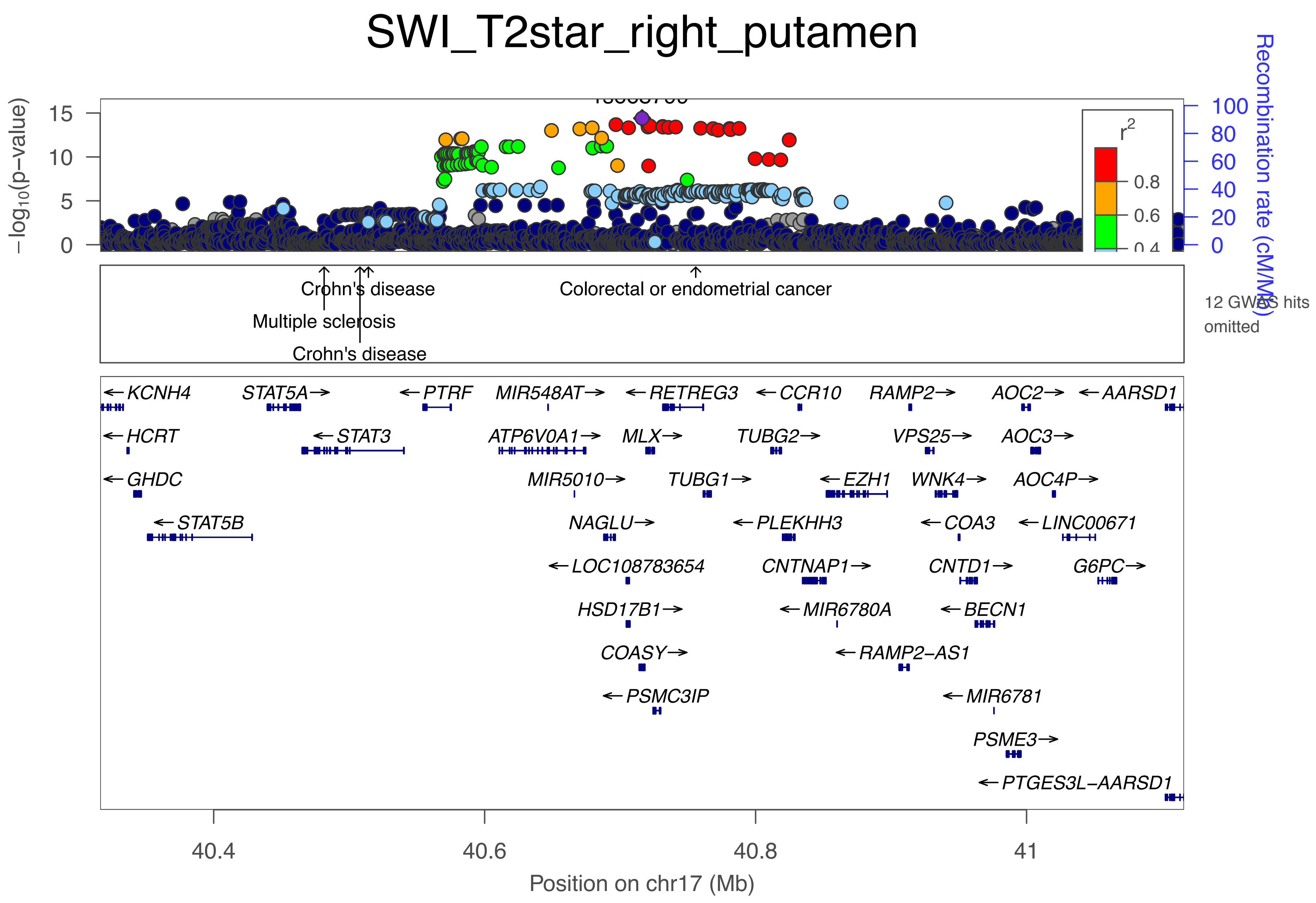
SWI_T2star_right_putamen
−log10(p−value)
Recombination rate (cM/Mb)
r2
0.8
0.6
0.4
Crohn's disease
Multiple sclerosis
Crohn's disease
Colorectal or endometrial cancer
12 GWAS hits omitted
KCNH4
HCRT
GHDC
STAT5B
STAT5A
STAT3
PTRF
MIR548AT
ATP6V0A1
MIR5010
NAGLU
LOC108783654
HSD17B1
COASY
PSMC3IP
RETREG3
MLX
TUBG2
TUBG1
PLEKHH3
CNTNAP1
MIR6780A
RAMP2-AS1
CCR10
EZH1
RAMP2
VPS25
WNK4
COA3
CNTD1
BECN1
MIR6781
PSME3
AOC2
AOC3
AOC4P
LINC00671
G6PC
AARSD1
PTGES3L-AARSD1
Position on chr17 (Mb)

| | |
|---|---|
| date: | Thu Aug 17 19:04:35 2017 |
| build: | hg19 |
| display range: | chr17:40316235−41116235 [40316235−41116235] |
| hilite range: | 0 – 0 [ 0 – 0 ] |
| reference SNP: | chr17:40716235 |
| number of SNPs plotted: | 2501 |
| min P.value: | 3.9E−15 [chr17:40716235] |
| max P.value: | 10E−1 [chr17:40916235] |
| omitted GWAS Hits: | chr17:40.755811−Colorectal or endometrial cancer, NA |
| omitted GWAS Hits: | NA, NA |
| omitted GWAS Hits: | NA, NA |
| omitted GWAS Hits: | NA, NA |

Make more plots at http://csg.sph.umich.edu/locuszoom/

GWAS Catalog SNPs in Region

| chr | pos (Mb) | trait | snp |
|---|---|---|---|
| 17 | 40.48153 | Multiple sclerosis | rs2293152 |
| 17 | 40.48524 | Atopic dermatitis | rs17881320 |
| 17 | 40.49953 | Crohn's disease | rs6503695 |
| 17 | 40.50798 | Crohn's disease | rs9891119 |
| 17 | 40.50798 | Multiple sclerosis | rs9891119 |
| 17 | 40.51420 | Crohn's disease | rs744166 |
| 17 | 40.51420 | Multiple sclerosis | rs744166 |
| 17 | 40.51420 | Inflammatory bowel disease | rs744166 |
| 17 | 40.52754 | Ulcerative colitis | rs12942547 |
| 17 | 40.52754 | Crohn's disease | rs12942547 |
| 17 | 40.52754 | Inflammatory bowel disease | rs12942547 |
| 17 | 40.54221 | Atopic dermatitis | rs4796793 |
| 17 | 40.56504 | Psoriasis | rs56364076 |
| 17 | 40.57077 | Crohn's disease | rs11871801 |
| 17 | 40.69334 | Systolic blood pressure change trajectories | rs630539 |
| 17 | 40.75581 | Colorectal or endometrial cancer | rs9901225 |

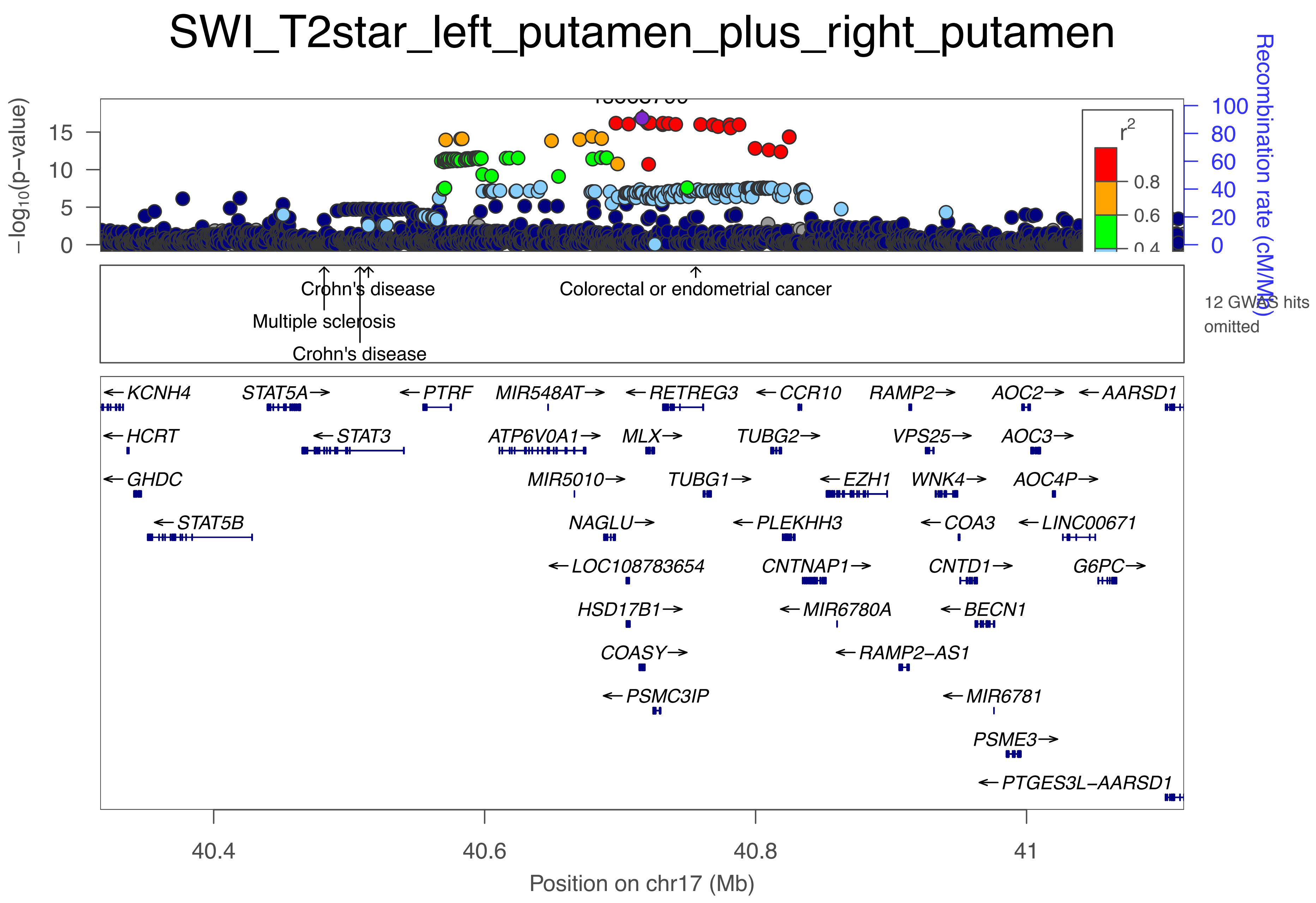
SWI_T2star_left_putamen_plus_right_putamen
−log10(p−value)
Recombination rate (cM/Mb)
r2
0.8
0.6
0.4
Crohn's disease
Multiple sclerosis
Crohn's disease
Colorectal or endometrial cancer
12 GWAS hits omitted
KCNH4
HCRT
GHDC
STAT5B
STAT5A
STAT3
PTRF
MIR548AT
ATP6V0A1
MIR5010
NAGLU
LOC108783654
HSD17B1
COASY
PSMC3IP
RETREG3
MLX
TUBG1
TUBG2
CCR10
PLEKHH3
CNTNAP1
MIR6780A
EZH1
RAMP2-AS1
RAMP2
VPS25
WNK4
COA3
CNTD1
BECN1
MIR6781
PSME3
PTGES3L-AARSD1
AOC2
AOC3
AOC4P
LINC00671
G6PC
AARSD1
40.4
40.6
40.8
41
Position on chr17 (Mb)

date: Thu Aug 17 19:04:35 2017

build: hg19

display range: chr17:40316235−41116235 [40316235−41116235]

hilite range: 0 – 0 [ 0 – 0 ]

reference SNP: chr17:40716235

number of SNPs plotted: 2501

min P.value: 1.43E−17 [chr17:40716235]

max P.value: 10E−1 [chr17:40366396]

omitted GWAS Hits: chr17:40.755811−Colorectal or endometrial cancer, NA

omitted GWAS Hits: NA, NA

omitted GWAS Hits: NA, NA

omitted GWAS Hits: NA, NA

Make more plots at http://csg.sph.umich.edu/locuszoom/

GWAS Catalog SNPs in Region

| chr | pos (Mb) | trait | snp |
|---|---|---|---|
| 17 | 40.48153 | Multiple sclerosis | rs2293152 |
| 17 | 40.48524 | Atopic dermatitis | rs17881320 |
| 17 | 40.49953 | Crohn's disease | rs6503695 |
| 17 | 40.50798 | Crohn's disease | rs9891119 |
| 17 | 40.50798 | Multiple sclerosis | rs9891119 |
| 17 | 40.51420 | Crohn's disease | rs744166 |
| 17 | 40.51420 | Multiple sclerosis | rs744166 |
| 17 | 40.51420 | Inflammatory bowel disease | rs744166 |
| 17 | 40.52754 | Ulcerative colitis | rs12942547 |
| 17 | 40.52754 | Crohn's disease | rs12942547 |
| 17 | 40.52754 | Inflammatory bowel disease | rs12942547 |
| 17 | 40.54221 | Atopic dermatitis | rs4796793 |
| 17 | 40.56504 | Psoriasis | rs56364076 |
| 17 | 40.57077 | Crohn's disease | rs11871801 |
| 17 | 40.69334 | Systolic blood pressure change trajectories | rs630539 |
| 17 | 40.75581 | Colorectal or endometrial cancer | rs9901225 |

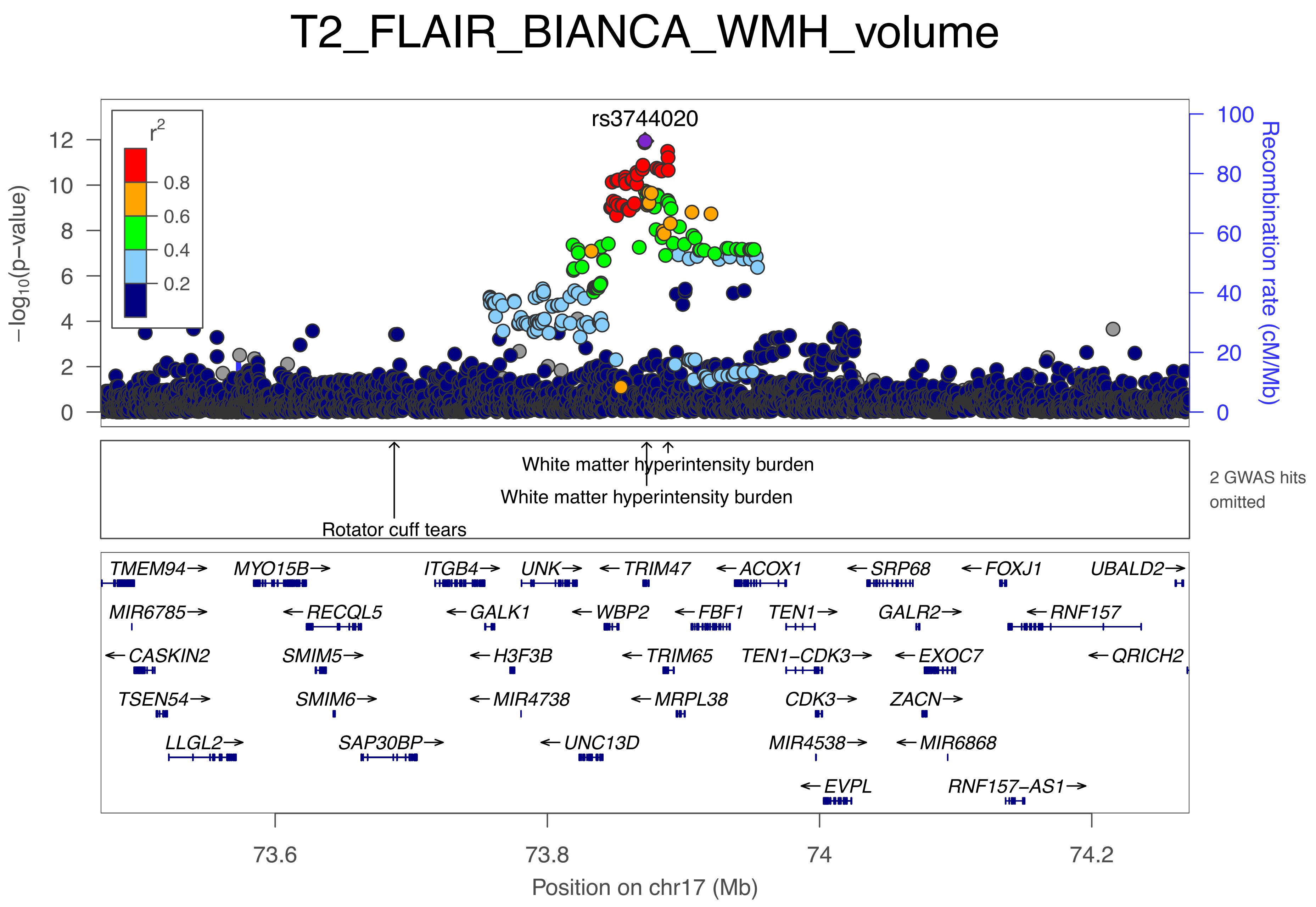
T2_FLAIR_BIANCA_WMH_volume
rs3744020
r2
0.8
0.6
0.4
0.2
−log10(p−value)
Recombination rate (cM/Mb)
White matter hyperintensity burden
White matter hyperintensity burden
Rotator cuff tears
2 GWAS hits omitted
TMEM94→
MYO15B→
ITGB4→
UNK→
←TRIM47
←ACOX1
←SRP68
←FOXJ1
UBALD2→
MIR6785→
←RECQL5
←GALK1
←WBP2
←FBF1
TEN1→
GALR2→
←RNF157
←CASKIN2
SMIM5→
←H3F3B
←TRIM65
TEN1−CDK3→
←EXOC7
←QRICH2
TSEN54→
SMIM6→
←MIR4738
←MRPL38
CDK3→
ZACN→
LLGL2→
SAP30BP→
←UNC13D
MIR4538→
←MIR6868
←EVPL
RNF157−AS1→
73.6
73.8
74
74.2
Position on chr17 (Mb)

| | |
|---|---|
| date: | Thu Aug 17 19:11:55 2017 |
| build: | hg19 |
| display range: | chr17:73471773−74271773 [73471773−74271773] |
| hilite range: | 0 – 0 [ 0 – 0 ] |
| reference SNP: | chr17:73871773 |
| number of SNPs plotted: | 3811 |
| min P.value: | 1.15E−12 [chr17:73871773] |
| max P.value: | 10E−1 [chr17:73572178] |
| omitted GWAS Hits: | NA, NA |

Make more plots at http://csg.sph.umich.edu/locuszoom/

GWAS Catalog SNPs in Region

| chr | pos (Mb) | trait | snp |
|---|---|---|---|
| 17 | 73.68749 | Rotator cuff tears | rs820218 |
| 17 | 73.68829 | Lip morphology | rs820217 |
| 17 | 73.87295 | White matter hyperintensity burden | rs1055129 |
| 17 | 73.88215 | White matter hyperintensity burden | rs7214628 |
| 17 | 73.88867 | White matter hyperintensity burden | rs3744028 |

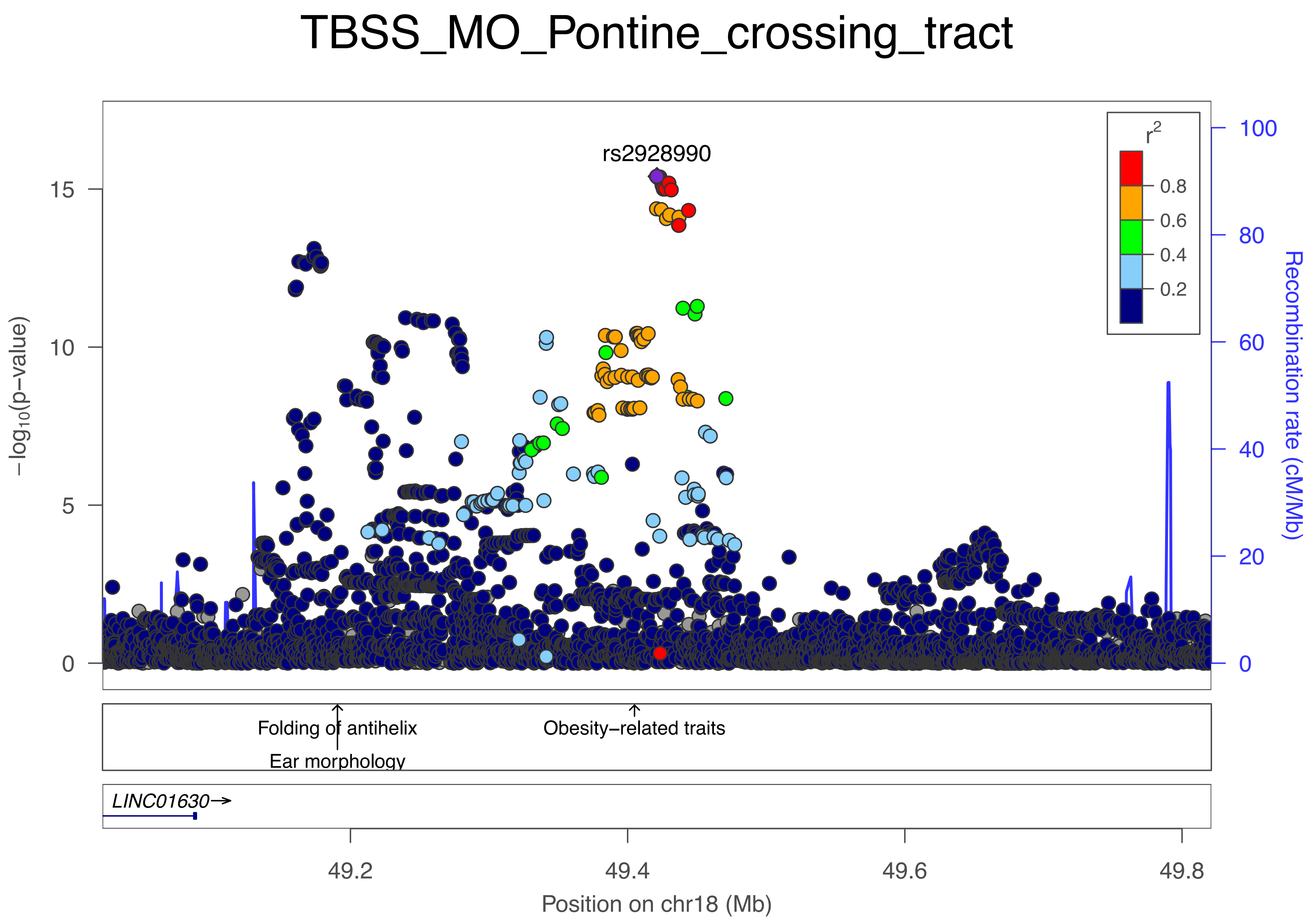
TBSS_MO_Pontine_crossing_tract
rs2928990
r2
0.8
0.6
0.4
0.2
−log10(p−value)
15
10
5
0
100
80
60
40
20
0
Recombination rate (cM/Mb)
Folding of antihelix
Ear morphology
Obesity−related traits
LINC01630→
49.2
49.4
49.6
49.8
Position on chr18 (Mb)

| | |
|---|---|
| date: | Thu Aug 17 19:01:51 2017 |
| build: | hg19 |
| display range: | chr18:49021125−49821125 [49021125−49821125] |
| hilite range: | 0 – 0 [ 0 – 0 ] |
| reference SNP: | chr18:49421125 |
| number of SNPs plotted: | 4230 |
| min P.value: | 3.97E−16 [chr18:49421125] |
| max P.value: | 10E−1 [chr18:49667076] |

Make more plots at http://csg.sph.umich.edu/locuszoom/

GWAS Catalog SNPs in Region

| chr | pos (Mb) | trait | snp |
|---|---|---|---|
| 18 | 49.19064 | Folding of antihelix | rs1619249 |
| 18 | 49.19064 | Ear morphology | rs1619249 |
| 18 | 49.40496 | Obesity–related traits | rs1351435 |
