# Supplementary material for: Genome-wide association studies of brain imaging phenotypes in UK Biobank
Source: Nature. 2018 Oct 10;562(7726):210–6. doi: 10.1038/s41586-018-0571-7 (PMC6786974; doi:10.1038/s41586-018-0571-7)
Supplement: Supplementary file 3 — This file contains Supplementary Figures S1-S22. [file 41586_2018_571_MOESM3_ESM.zip › Figure-S5.pdf]

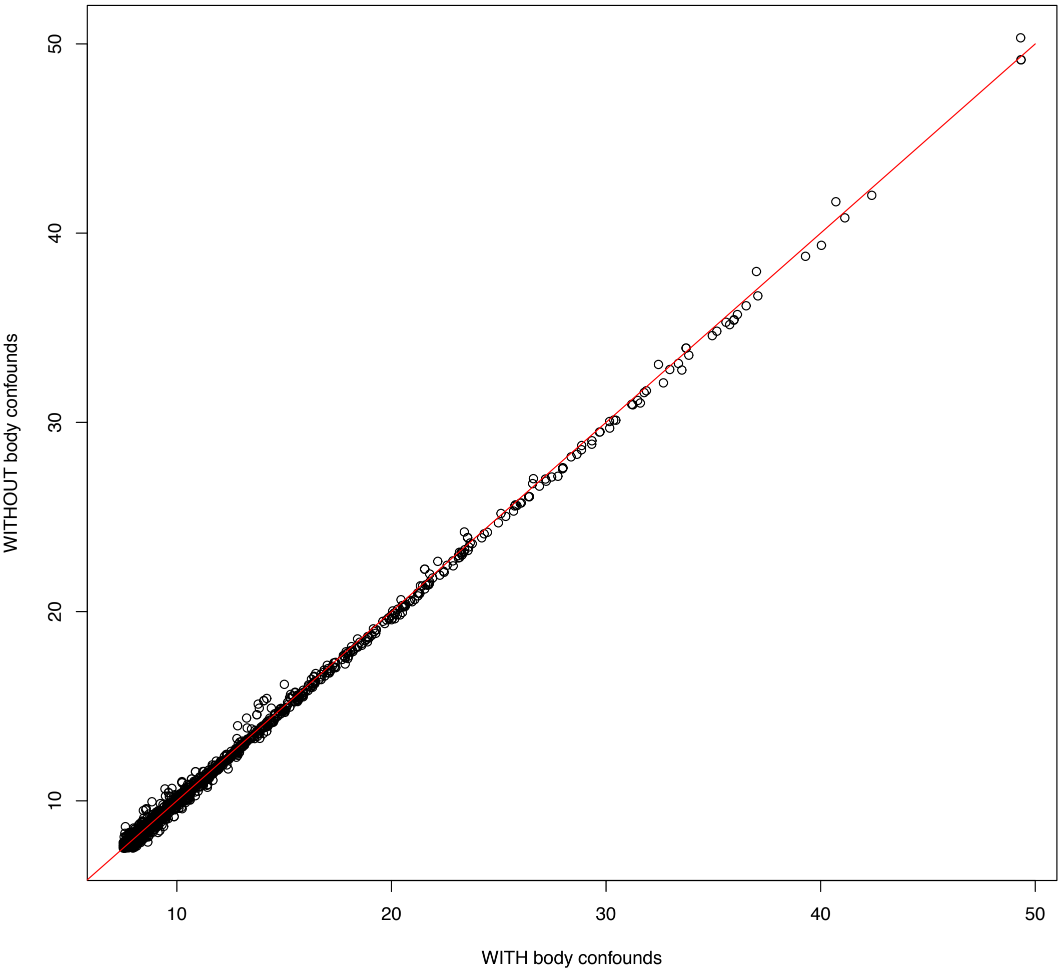

Supplementary Figure 5. Association of IDPs with and without Body Confounds. The GWAS for all 3,144 IDPs was run with and without correcting for the 4 classes of body confounds (blood pressure, height, weight and bone density). The top associations across all the GWAS (-log10 p-value > 7.5) with and without body confound correction are plotted against each other in the figure.
