# Supplementary material for: Genome-wide association studies of brain imaging phenotypes in UK Biobank
Source: Nature. 2018 Oct 10;562(7726):210–6. doi: 10.1038/s41586-018-0571-7 (PMC6786974; doi:10.1038/s41586-018-0571-7)
Supplement: Supplementary file 4 — This file contains Supplementary Tables S1-S13. [file 41586_2018_571_MOESM4_ESM.zip › SuppTable_12.pdf]

IDP Group name	Description	Number of IDPs	Number of complete samples (subjects)
**T1-SIENAX**	White, grey and cerebrospinal fluid (CSF) volumes	10	8,428
**T1-FIRST**	7 Sub-cortical volumes x3 (left, right and left+right); brain-stem volume	22	8,428
**T1-FAST_ROIs**	Grey matter partial volume summed in 139 regions of interest (ROIs)	139	8,427
**T2-FLAIR-BIANCA**	Total white matter hyperintensity volume	1	7,705
**SWI-T2***	T2* signal intensity in 7 distinct subcortical structures x 3 (left, right and left+right)	21	7,778
**FreeSurfer**	Cortical areas and thicknesses based on 2 different cortical atlases; subcortical volumes	483	8,411
**dMRI**	6 Diffusion tensor and 3 microstructure modelling measures, on each of 75 white matter tract regions	675	7,532
**tfMRI**	Signal strength in task activated regions	16	7,612
**rfMRI**	Resting state fluctuation amplitudes in regions from two functional parcellations, and functional network connectivity between all pairs of regions + ICA dimension reduced connectivity	1,777	7,916

Supplementary Table 12: Imaging derived phenotype (IDP) grouping. This table shows the 3,144 IDPs grouped according to modality and missing data patterns.
