# Supplementary material for: Genome-wide association studies of brain imaging phenotypes in UK Biobank
Source: Nature. 2018 Oct 10;562(7726):210–6. doi: 10.1038/s41586-018-0571-7 (PMC6786974; doi:10.1038/s41586-018-0571-7)
Supplement: Supplementary file 4 — This file contains Supplementary Tables S1-S13. [file 41586_2018_571_MOESM4_ESM.zip › SuppTable_13.pdf]

Supplementary Table 13*:* Sample sizes and DOIs for studies of the 9 traits considered in the coheritability analysis.

Trait	Sample Size	DOI
Attention deficit hyperactivity disorder	55,374	DOI:10.1101/145581. Discovery of the first genome-wide significant risk loci for ADHD. 2017. Biorxiv preprint 2017/06/03/145581.
Alzheimer's disease	54,162	DOI:10.1038/ng.2802. Meta-analysis of 74,046 {Jones:2016do}individuals identifies 11 new susceptibility loci for Alzheimer's disease. 2013. Nature Genetics. 45(12) p1452-8.
Autism spectrum	13,574	DOI:10.1186/s13229-017-0137-9. Meta-analysis of GWAS of over 16,000 individuals with autism spectrum disorder highlights a novel locus at 10q24.32 and a significant overlap with schizophrenia. 2017. Brain, Cognition and Behavior. 8(21).
Major depressive disorder	18,759	DOI:10.1038/mp.2012.21. A mega-analysis of genome-wide association studies for major depressive disorder. 2013. Molecular Psychiatry. 18(4) p497-511.
Schizophrenia	70,100	DOI:10.1038/nature13595. Biological insights from 108 schizophrenia-associated genetic loci. 2014. 511(7510) p421-7.
Stroke	29,633	DOI:10.1212/WNL.0000000000002528. Low-frequency and common genetic variation in ischemic stroke: The METASTROKE collaboration. 2016. Neurology 86(13) p1217-26.
Amyotrophic lateral sclerosis	36,052	DOI: 10.1038/ng.3622. Genome-wide association analyses identify new risk variants and the genetic architecture of amyotrophic lateral sclerosis. 2016. Nature Genetics. 48(9) 1043-8.
Neuroticism	170,911	DOI:10.1038/ng.3552. Genetic variants associated with subjective well-being, depressive symptoms, and neuroticism identified through genome-wide analyses. 2016. Nature. 48(6) p624-33.
Sleep duration	127,573	DOI:10.1371/journal.pgen.1006125. Genome-wide association analyses in 128,266 individuals identifies new morningness and sleep duration loci. 2016. PLOS Genetics. 12(8).
Bipolar disorder	16,731	DOI: 10.1038/ng.943. Large-scale genome-wide association analysis of bipolar disorder identifies a new susceptibility locus near ODZ4. 2011. Nature Genetics 43(977).
