# Supplementary material for: Genome-wide association studies of brain imaging phenotypes in UK Biobank
Source: Nature. 2018 Oct 10;562(7726):210–6. doi: 10.1038/s41586-018-0571-7 (PMC6786974; doi:10.1038/s41586-018-0571-7)
Supplement: Supplementary file 4 — This file contains Supplementary Tables S1-S13. [file 41586_2018_571_MOESM4_ESM.zip › SuppTable_1.pdf]

Modality name	Type	Description	Number of complete samples (subjects)
T1-weighted image (**T1**)	Structural	Measures anatomical features based on contrast between grey and white matter and other tissues.	8,411- 8,428
T2-weighted FLAIR image (**T2 FLAIR**)	Structural	Provides a different contrast between tissues (compared with T1), and is sensitive to some pathologies such as white matter lesions.	7.705
Susceptibility-weighted imaging (**swMRI** or **SWI**)	Structural	Can be processed in multiple ways to reflect venous vasculature, microbleeds, aspects of micro-structure (local cellular structure) and biochemical processes such as iron deposition.	7,778
Diffusion-weighted imaging (**dMRI**)	Structural connectivity	Measures movement of water molecules within their local tissue environment, allowing for the estimation of long-range structural connectivity and local microstructure.	7,532
Resting-state functional MRI (**rfMRI**)	Functional connectivity	Measures dynamic changes in blood oxygenation associated with intrinsic brain activity, to assess functional connectivity via temporal similarities between brain regions.	7,916
Task functional MRI (**tfMRI**)	Functional activation	Functional imaging while subject performs a particular task or experiences a sensory stimulus.	7,612

Supplementary Table 1: **Brain Imaging modalities.** An overview of the 6 different brain imaging types used in the UK Biobank study.
