# Supplementary material for: Genome-wide association studies of brain imaging phenotypes in UK Biobank
Source: Nature. 2018 Oct 10;562(7726):210–6. doi: 10.1038/s41586-018-0571-7 (PMC6786974; doi:10.1038/s41586-018-0571-7)
Supplement: Supplementary file 4 — This file contains Supplementary Tables S1-S13. [file 41586_2018_571_MOESM4_ESM.zip › SuppTable_3.pdf]

Hibar et al. Volume	UK Biobank IDP	r_g	Standard error	p-value
Caudate	T1_FIRST.Caudate.Left	0.9007	0.111	4.84E-16
	T1_FIRST.Caudate.Right	0.971	0.1141	1.73E-17
	T1_FIRST.Caudate.L_plus_R	0.9368	0.1083	5.14E-18
	Freesurfer.Caudate.Left	0.8973	0.1162	1.17E-14
	Freesurfer.Caudate.Right	0.9169	0.1183	9.20E-15
Putamen	T1_FIRST.Putamen.Left	0.9981	0.1549	1.18E-10
	T1_FIRST.Putamen.Right	0.9275	0.126	1.82E-13
	T1_FIRST.Putamen.L_plus_R	0.9699	0.1274	2.66E-14
	Freesurfer.Putamen.Left	0.9896	0.1376	6.44E-13
	Freesurfer.Putamen.Right	0.9425	0.1352	3.15E-12
Accumbens	T1_FIRST.Accumbens.Left	1.1475	0.3899	3.25E-03
	T1_FIRST.Accumbens.Right	0.9778	0.3445	4.54E-03
	T1_FIRST.Accumbens.L_plus_R	1.0816	0.3382	1.38E-03
	Freesurfer.Accumbens.Left	1.1835	0.2999	7.95E-05
	Freesurfer.Accumbens.Right	1.1172	0.276	5.17E-05
Pallidum	T1_FIRST.Pallidum.Left	0.9259	0.2039	5.61E-06
	T1_FIRST.Pallidum.Right	0.8372	0.1707	9.39E-07
	T1_FIRST.Pallidum.L_plus_R	0.8872	0.1724	2.65E-07
	Freesurfer.Pallidum.Left	0.7965	0.162	8.74E-07
	Freesurfer.Pallidum.Right	0.7036	0.1632	1.61E-05
Hippocampus	T1_FIRST.Hippocampus.Left	0.6951	0.2015	5.60E-04
	T1_FIRST.Hippocampus.Right	0.6945	0.2463	4.81E-03
	T1_FIRST.Hippocampus.L_plus_R	0.6998	0.2004	4.80E-04
	Freesurfer.Hippocampus.Left	0.7325	0.1659	1.01E-05
	Freesurfer.Hippocampus.Right	0.7329	0.1667	1.11E-05
Thalamus	T1_FIRST.Thalamus.Left	0.6903	0.1916	0.0003
	T1_FIRST.Thalamus.Right	0.6688	0.2054	0.0011
	T1_FIRST.Thalamus.L_plus_R	0.6827	0.1929	0.0004
	Freesurfer.Thalamus.Left	0.8172	0.2663	0.0022
	Freesurfer.Thalamus.Right	0.7992	0.2323	0.0006

Supplementary Table 3 : Comparison of genetic architectures. LDSCORE regression was used to estimate the correlation between the heritable components that we inferred for the volumes of subcortical structures with those inferred by a previous study (Hibar et al. 2015). The first column lists the structures for which Hibar et al. provide summary statistics for tests of association with aggregate measurements of volume. The second column lists comparable IDPs from our study. Amygdala is absent from the table as LDSCORE regression was unable to compute a genetic correlation for this structure. The last three columns list the corresponding estimates for genetic correlations (r_g), the standard errors of those estimates and p-values for a test of non-zero correlation. For example, the second row compares the heritable component of aggregate caudate volume inferred by Hibar et al., with the heritable component of caudate volume in the right hemisphere as inferred by our study using FIRST segmentation. The genetic correlation for these two phenotypes is 0.971 with SE 0.1141. In all cases, the 95% confidence intervals cover 1. Consequently, there is no evidence of any difference in genetic architecture inferred by the two studies.
