# Supplementary material for: Genome-wide association studies of brain imaging phenotypes in UK Biobank
Source: Nature. 2018 Oct 10;562(7726):210–6. doi: 10.1038/s41586-018-0571-7 (PMC6786974; doi:10.1038/s41586-018-0571-7)
Supplement: Supplementary file 4 — This file contains Supplementary Tables S1-S13. [file 41586_2018_571_MOESM4_ESM.zip › SuppTable_4.pdf]

IDP Group name	Number of IDPs	Number of associated loci with –log10 p > 11	Number of associated loci with –log10 p > 7.5	Average heritability per IDP group
**T1-SIENAX**	10	0	14	0.413
**T1-FIRST**	22	5	18	0.337
**T1-FAST_ROIs**	139	24	88	0.288
**T2-FLAIR-BIANCA**	1	2	3	0.441
**SWI-T2***	21	47	89	0.320
**FreeSurfer**	483	33	185	0.250
**dMRI**	675	225	599	0.363
**tfMRI**	16	0	0	0.071
**rfMRI-nodes (25)**	21	32	266	0.186
**rfMRI-edges (25)**	210	0	22	0.054
**rfMRI-nodes (100)**	55	15	83	0.196
**rfMRI-edges (100)**	1485	1	121	0.054
**rfMRI-ICA features**	6	4	11	0.349

Supplementary Table 4: **Summary of associations by IDP group**. The table shows the number of associated genetic loci stratified by IDP group. Column 2: the number of IDPs in each group. Column 3: the number of associated genetic loci (from **Supplementary Table 6**) for each group. Column 4: the number of associated genetic loci (from **Supplementary Table 5**) for each group. Column 5: the average heritability per IDP group (from **Figure 1**). Columns 3 and 4 also include the number of associated loci per IDP for each group. The rfMRI IDP groups are split according to the size of the netmat parcellation (25 or 100 parcels).
