# Supplementary material for: Genome-wide association studies of brain imaging phenotypes in UK Biobank
Source: Nature. 2018 Oct 10;562(7726):210–6. doi: 10.1038/s41586-018-0571-7 (PMC6786974; doi:10.1038/s41586-018-0571-7)
Supplement: Supplementary file 4 — This file contains Supplementary Tables S1-S13. [file 41586_2018_571_MOESM4_ESM.zip › SuppTable_7.pdf]

Supplementary Table 7: This table shows the 23 groups of IDPs that were for the multi-phenotype tests.

IDP group	Number of IDPs per group
T1_brain_vol_all	10
T1_Subcortical_all	15
T1_Subcortical_L_plus_R	7
T1_FAST_ROIs	139
Freesurfer_volume	59
Freesurfer_area	212
Freesurfer_thickness	212
T2_star	14
T2_star_L_plus_R	7
dMRI_Probtrackx	243
dMRI_FA	75
dMRI_MD	75
dMRI_MO	75
dMRI_L1	75
dMRI_L2	75
dMRI_L3	75
dMRI_ICVF	75
dMRI_OD	75
dMRI_ISOVF	75
rfMRI_ICA_Features	6
tfMRI	16
rfMRI_25	21
rfMRI_100	55
