# Supplementary material for: Genome-wide association studies of brain imaging phenotypes in UK Biobank
Source: Nature. 2018 Oct 10;562(7726):210–6. doi: 10.1038/s41586-018-0571-7 (PMC6786974; doi:10.1038/s41586-018-0571-7)
Supplement: Supplementary file 3 — This file contains Supplementary Figures S1-S22. [file Figure-S17.pdf › Figure-S17.pdf]

Supplementary Figure 17: Partitioning of heritability by functional category – Enrichment/depletion for each individual IDP. The figures that follow show the estimated enrichment of 24 functional annotations for heritability for individual IDPs (N = 8,428 subjects, see Methods for calculation details). The figure on each page corresponds to a single IDP. The vertical axis denotes the annotations. Each annotation corresponds to its extended version as defined in Finucane et al. (2015). The horizontal axis corresponds to the enrichment (proportion of heritability attributed to an annotation normalised by the proportion of SNPs with that annotation). Each dot represents the estimated enrichment and error bars represent 95% confidence intervals. Dots and error bars are colour-coded consistently to enable easier identification of the same annotation when scanning multiple pages. The grey vertical line denotes an enrichment of 1. If an annotation has an enrichment of 1, this means that SNPs with this annotation explain no more (or less) heritability than an equivalent number of SNPs selected at random without regard to annotation. Annotations exhibiting nominally significant (p value < 0.05) departures from an enrichment of 1 have ** appearing before their name on the vertical axis. These p values are produced by a block jackknife procedure as described in Finucane et al. (2015) and not using the standard errors of the estimated enrichments. Consequently, significance of enrichment and coverage of 1 by the 95% CI are not always consistent.

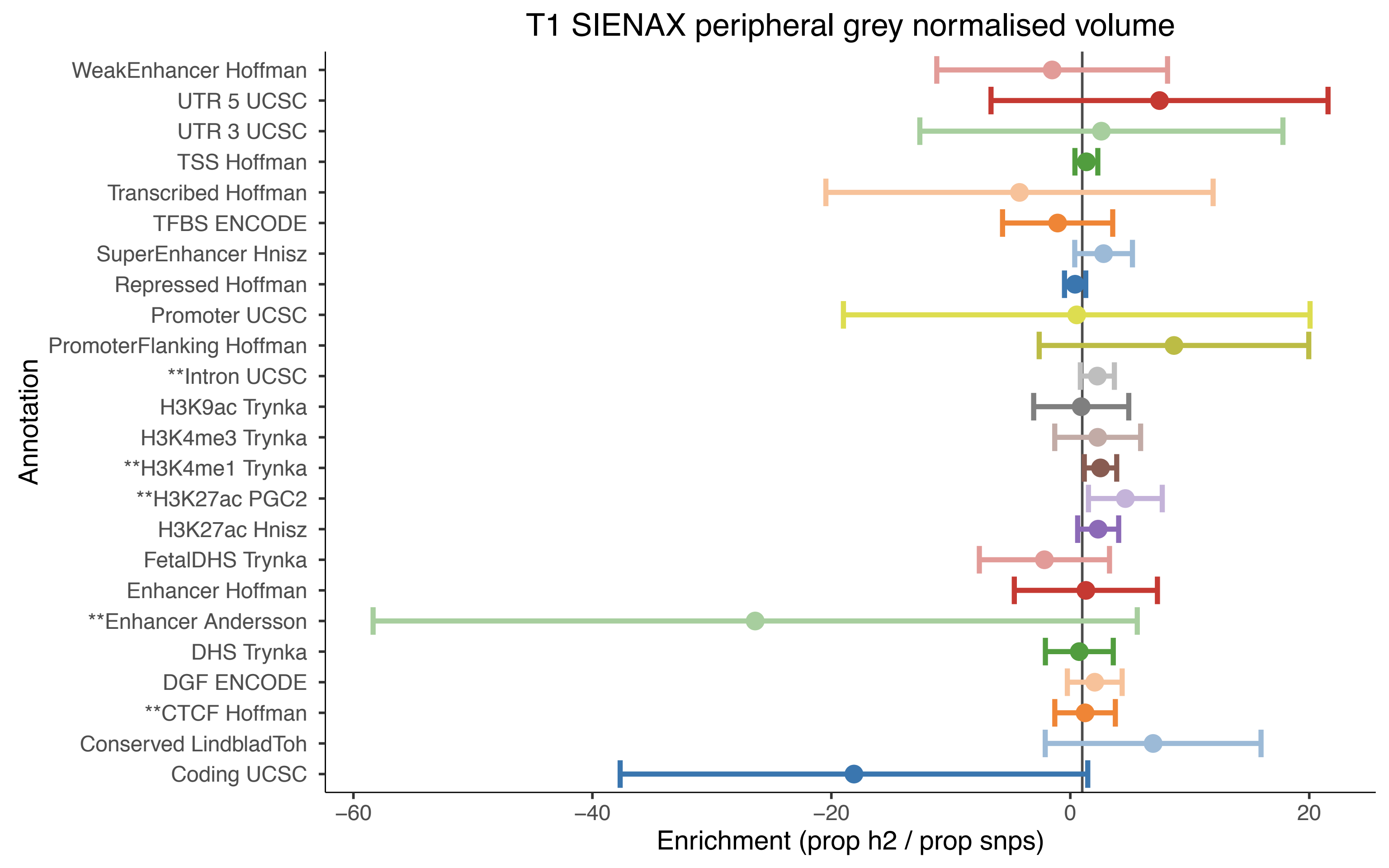
T1 SIENAX peripheral grey normalised volume
WeakEnhancer Hoffman
UTR 5 UCSC
UTR 3 UCSC
TSS Hoffman
Transcribed Hoffman
TFBS ENCODE
SuperEnhancer Hnisz
Repressed Hoffman
Promoter UCSC
PromoterFlanking Hoffman
**Intron UCSC
H3K9ac Trynka
H3K4me3 Trynka
**H3K4me1 Trynka
**H3K27ac PGC2
H3K27ac Hnisz
FetalDHS Trynka
Enhancer Hoffman
**Enhancer Andersson
DHS Trynka
DGF ENCODE
**CTCF Hoffman
Conserved LindbladToh
Coding UCSC
Annotation
−60
−40
−20
0
20
Enrichment (prop h2 / prop snps)

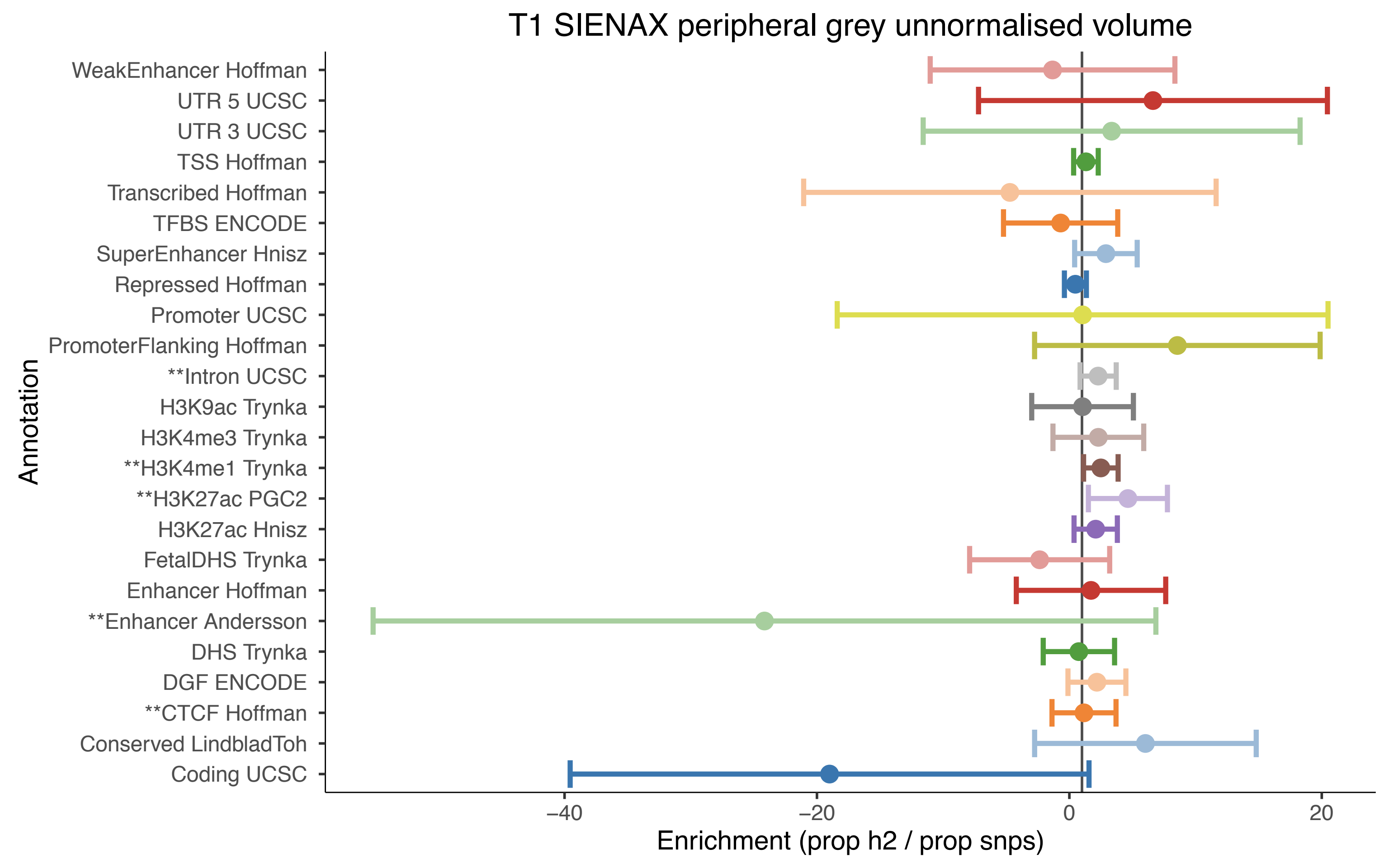
T1 SIENAX peripheral grey unnormalised volume
WeakEnhancer Hoffman
UTR 5 UCSC
UTR 3 UCSC
TSS Hoffman
Transcribed Hoffman
TFBS ENCODE
SuperEnhancer Hnisz
Repressed Hoffman
Promoter UCSC
PromoterFlanking Hoffman
**Intron UCSC
H3K9ac Trynka
H3K4me3 Trynka
**H3K4me1 Trynka
**H3K27ac PGC2
H3K27ac Hnisz
FetalDHS Trynka
Enhancer Hoffman
**Enhancer Andersson
DHS Trynka
DGF ENCODE
**CTCF Hoffman
Conserved LindbladToh
Coding UCSC
Annotation
−40
−20
0
20
Enrichment (prop h2 / prop snps)

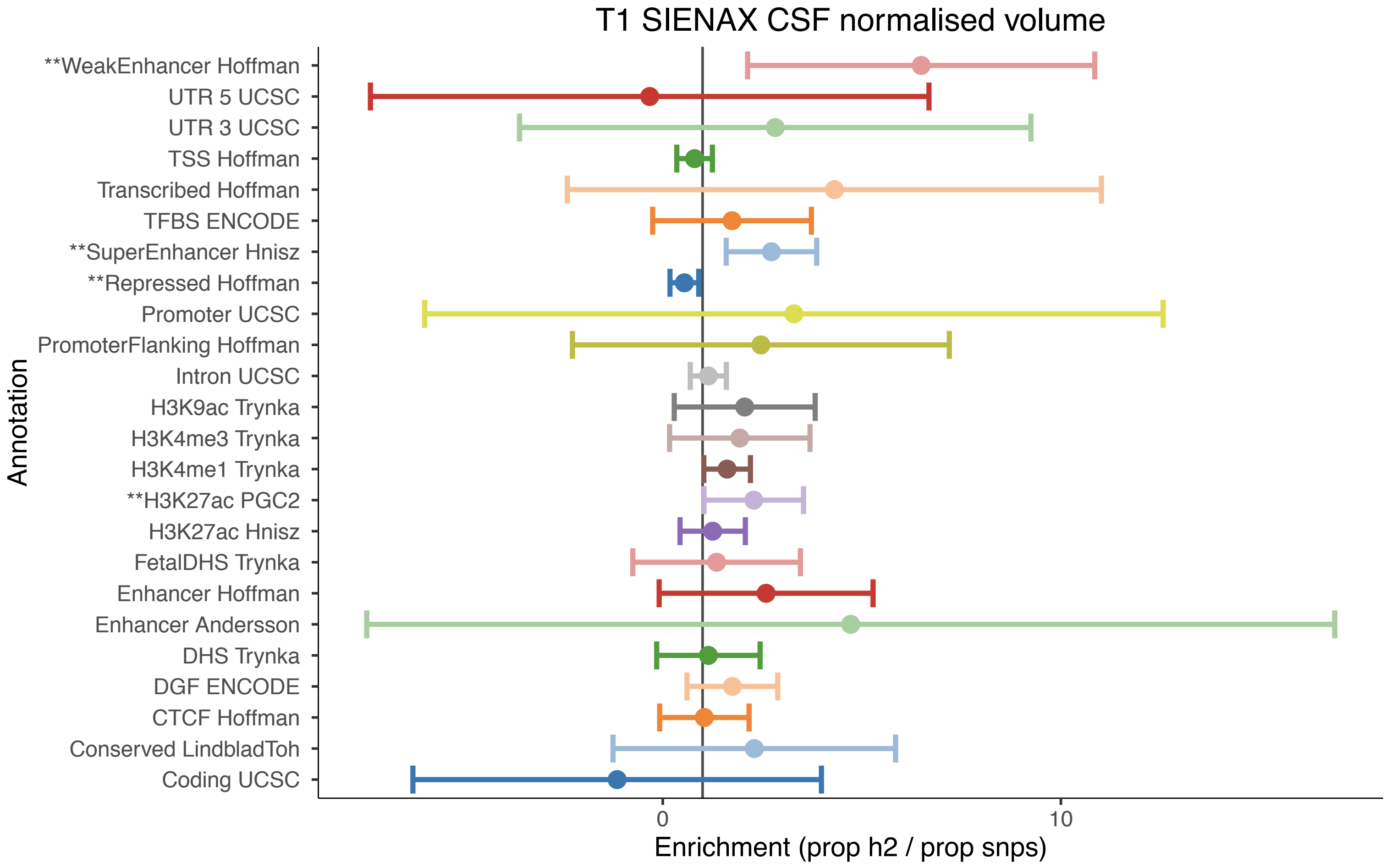

T1 SIENAX CSF normalised volume
Annotation
**WeakEnhancer Hoffman
UTR 5 UCSC
UTR 3 UCSC
TSS Hoffman
Transcribed Hoffman
TFBS ENCODE
**SuperEnhancer Hnisz
**Repressed Hoffman
Promoter UCSC
PromoterFlanking Hoffman
Intron UCSC
H3K9ac Trynka
H3K4me3 Trynka
H3K4me1 Trynka
**H3K27ac PGC2
H3K27ac Hnisz
FetalDHS Trynka
Enhancer Hoffman
Enhancer Andersson
DHS Trynka
DGF ENCODE
CTCF Hoffman
Conserved LindbladToh
Coding UCSC
0
10
Enrichment (prop h2 / prop snps)

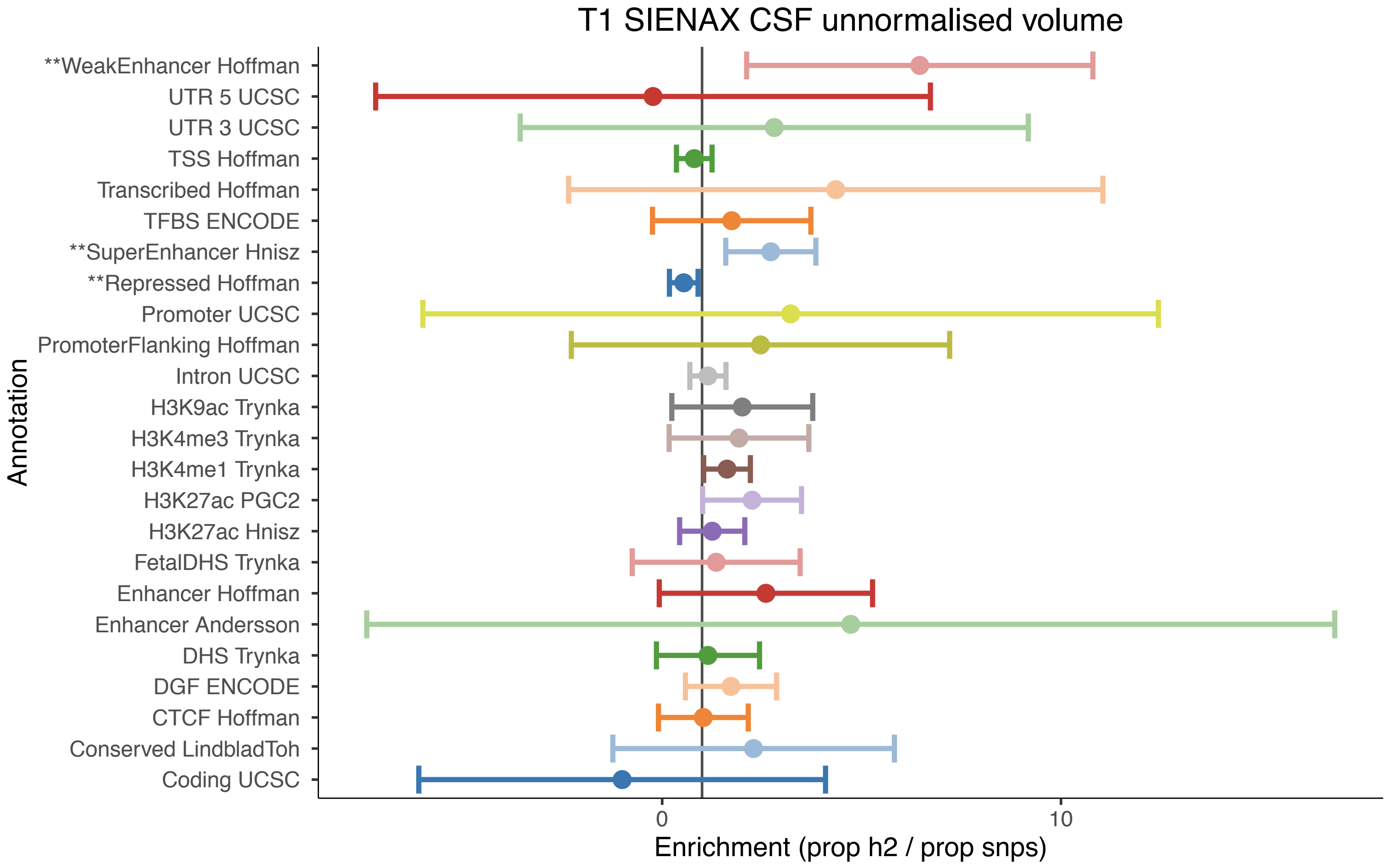
T1 SIENAX CSF unnormalised volume
**WeakEnhancer Hoffman
UTR 5 UCSC
UTR 3 UCSC
TSS Hoffman
Transcribed Hoffman
TFBS ENCODE
**SuperEnhancer Hnisz
**Repressed Hoffman
Promoter UCSC
PromoterFlanking Hoffman
Intron UCSC
H3K9ac Trynka
H3K4me3 Trynka
H3K4me1 Trynka
H3K27ac PGC2
H3K27ac Hnisz
FetalDHS Trynka
Enhancer Hoffman
Enhancer Andersson
DHS Trynka
DGF ENCODE
CTCF Hoffman
Conserved LindbladToh
Coding UCSC
Annotation
0
10
Enrichment (prop h2 / prop snps)

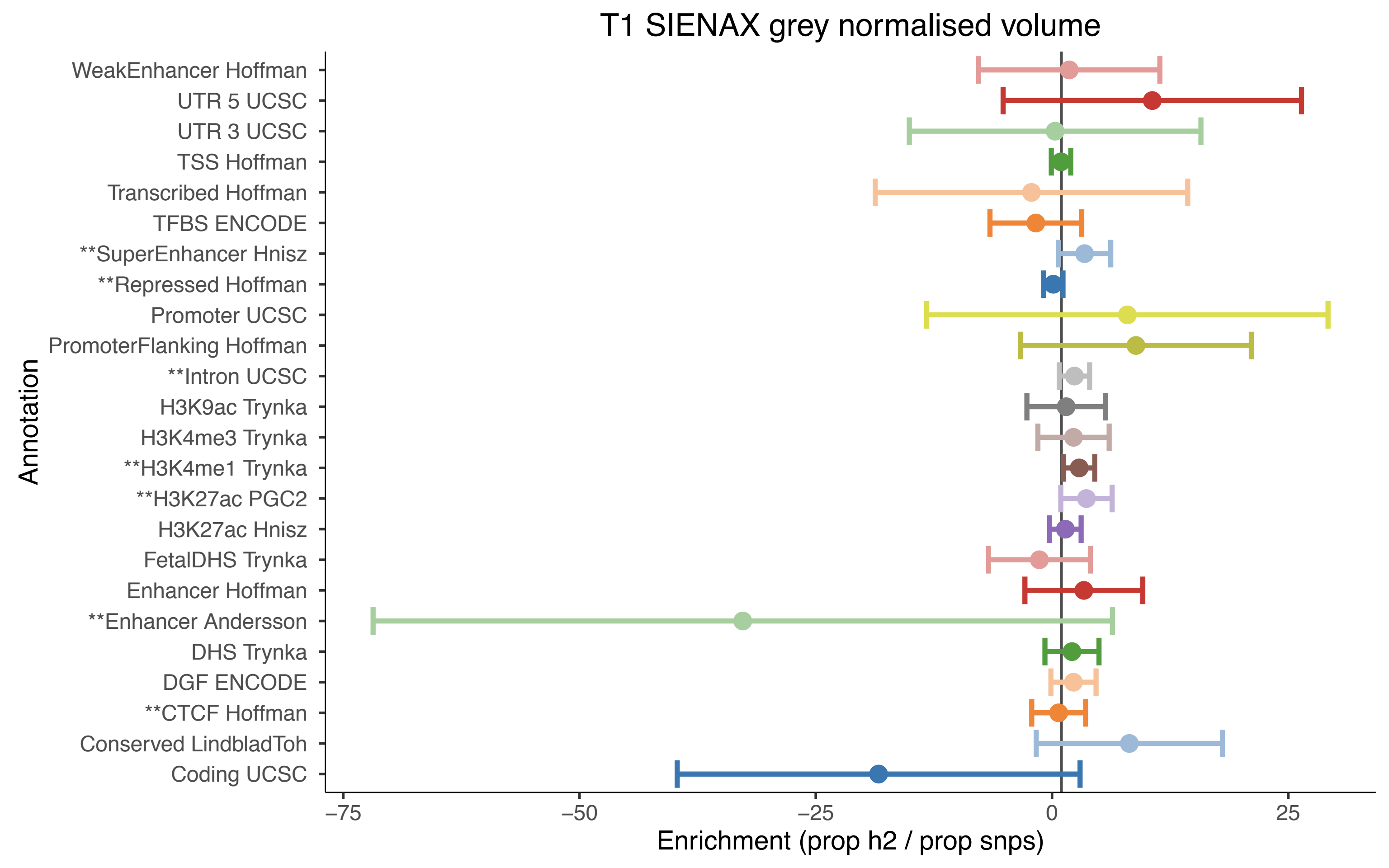
T1 SIENAX grey normalised volume
WeakEnhancer Hoffman
UTR 5 UCSC
UTR 3 UCSC
TSS Hoffman
Transcribed Hoffman
TFBS ENCODE
**SuperEnhancer Hnisz
**Repressed Hoffman
Promoter UCSC
PromoterFlanking Hoffman
**Intron UCSC
H3K9ac Trynka
H3K4me3 Trynka
**H3K4me1 Trynka
**H3K27ac PGC2
H3K27ac Hnisz
FetalDHS Trynka
Enhancer Hoffman
**Enhancer Andersson
DHS Trynka
DGF ENCODE
**CTCF Hoffman
Conserved LindbladToh
Coding UCSC
Annotation
−75
−50
−25
0
25
Enrichment (prop h2 / prop snps)

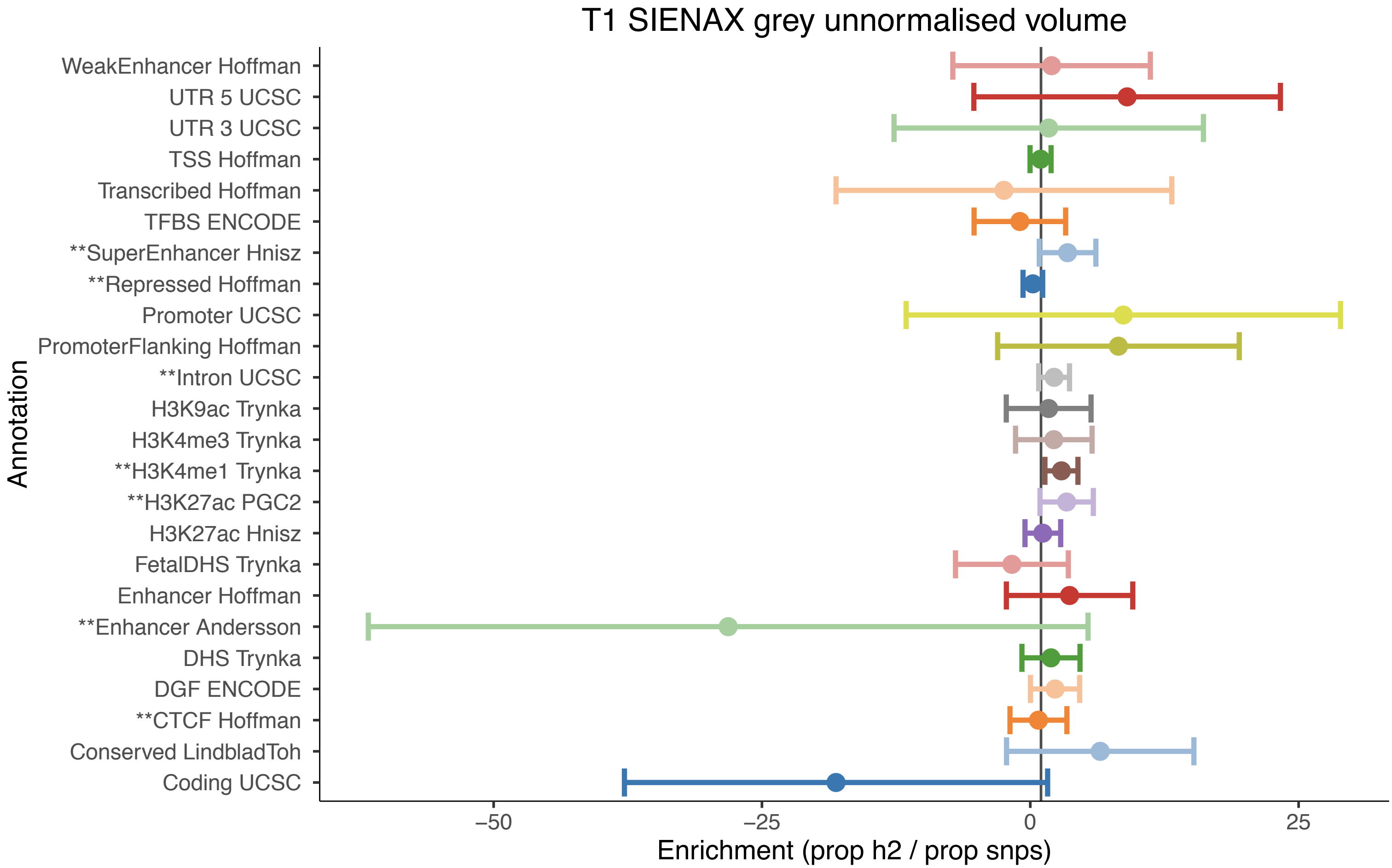

T1 SIENAX grey unnormalised volume
WeakEnhancer Hoffman
UTR 5 UCSC
UTR 3 UCSC
TSS Hoffman
Transcribed Hoffman
TFBS ENCODE
**SuperEnhancer Hnisz
**Repressed Hoffman
Promoter UCSC
PromoterFlanking Hoffman
**Intron UCSC
H3K9ac Trynka
H3K4me3 Trynka
**H3K4me1 Trynka
**H3K27ac PGC2
H3K27ac Hnisz
FetalDHS Trynka
Enhancer Hoffman
**Enhancer Andersson
DHS Trynka
DGF ENCODE
**CTCF Hoffman
Conserved LindbladToh
Coding UCSC
Annotation
−50
−25
0
25
Enrichment (prop h2 / prop snps)

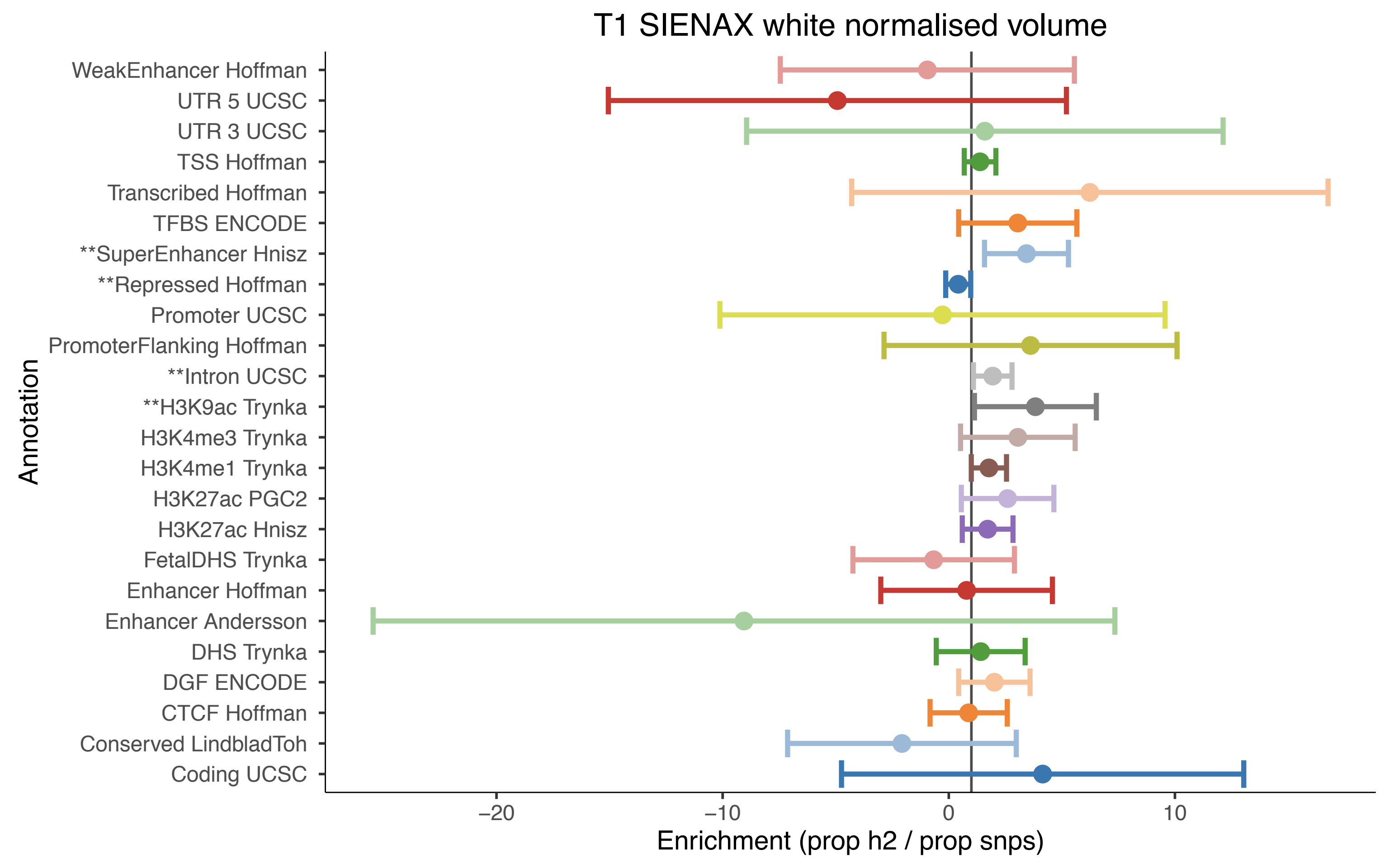
T1 SIENAX white normalised volume
WeakEnhancer Hoffman
UTR 5 UCSC
UTR 3 UCSC
TSS Hoffman
Transcribed Hoffman
TFBS ENCODE
**SuperEnhancer Hnisz
**Repressed Hoffman
Promoter UCSC
PromoterFlanking Hoffman
**Intron UCSC
**H3K9ac Trynka
H3K4me3 Trynka
H3K4me1 Trynka
H3K27ac PGC2
H3K27ac Hnisz
FetalDHS Trynka
Enhancer Hoffman
Enhancer Andersson
DHS Trynka
DGF ENCODE
CTCF Hoffman
Conserved LindbladToh
Coding UCSC
Annotation
−20
−10
0
10
Enrichment (prop h2 / prop snps)

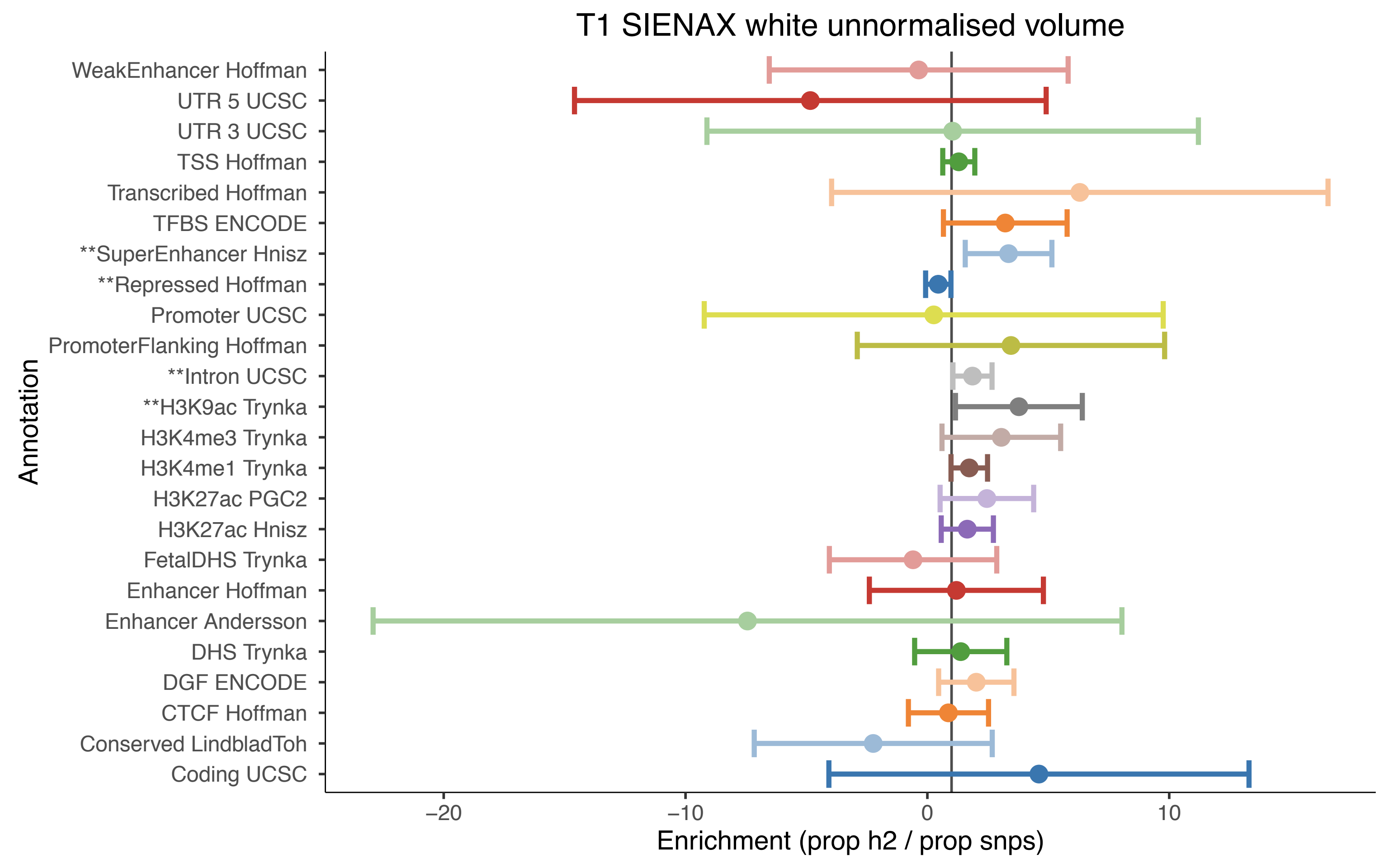

T1 SIENAX white unnormalised volume
WeakEnhancer Hoffman
UTR 5 UCSC
UTR 3 UCSC
TSS Hoffman
Transcribed Hoffman
TFBS ENCODE
**SuperEnhancer Hnisz
**Repressed Hoffman
Promoter UCSC
PromoterFlanking Hoffman
**Intron UCSC
**H3K9ac Trynka
H3K4me3 Trynka
H3K4me1 Trynka
H3K27ac PGC2
H3K27ac Hnisz
FetalDHS Trynka
Enhancer Hoffman
Enhancer Andersson
DHS Trynka
DGF ENCODE
CTCF Hoffman
Conserved LindbladToh
Coding UCSC
Annotation
−20
−10
0
10
Enrichment (prop h2 / prop snps)

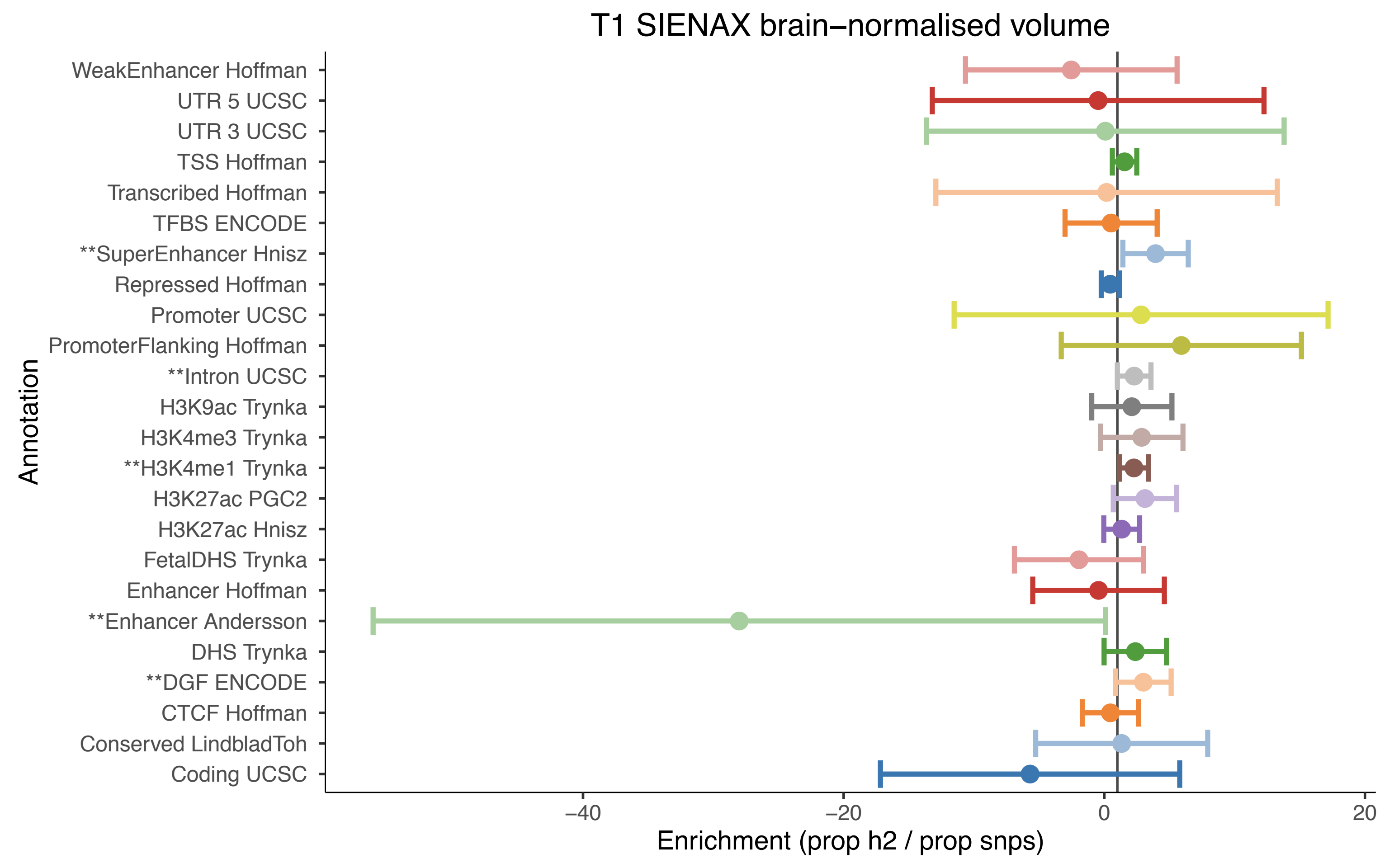

T1 SIENAX brain−normalised volume
WeakEnhancer Hoffman
UTR 5 UCSC
UTR 3 UCSC
TSS Hoffman
Transcribed Hoffman
TFBS ENCODE
**SuperEnhancer Hnisz
Repressed Hoffman
Promoter UCSC
PromoterFlanking Hoffman
**Intron UCSC
H3K9ac Trynka
H3K4me3 Trynka
**H3K4me1 Trynka
H3K27ac PGC2
H3K27ac Hnisz
FetalDHS Trynka
Enhancer Hoffman
**Enhancer Andersson
DHS Trynka
**DGF ENCODE
CTCF Hoffman
Conserved LindbladToh
Coding UCSC
Annotation
−40
−20
0
20
Enrichment (prop h2 / prop snps)

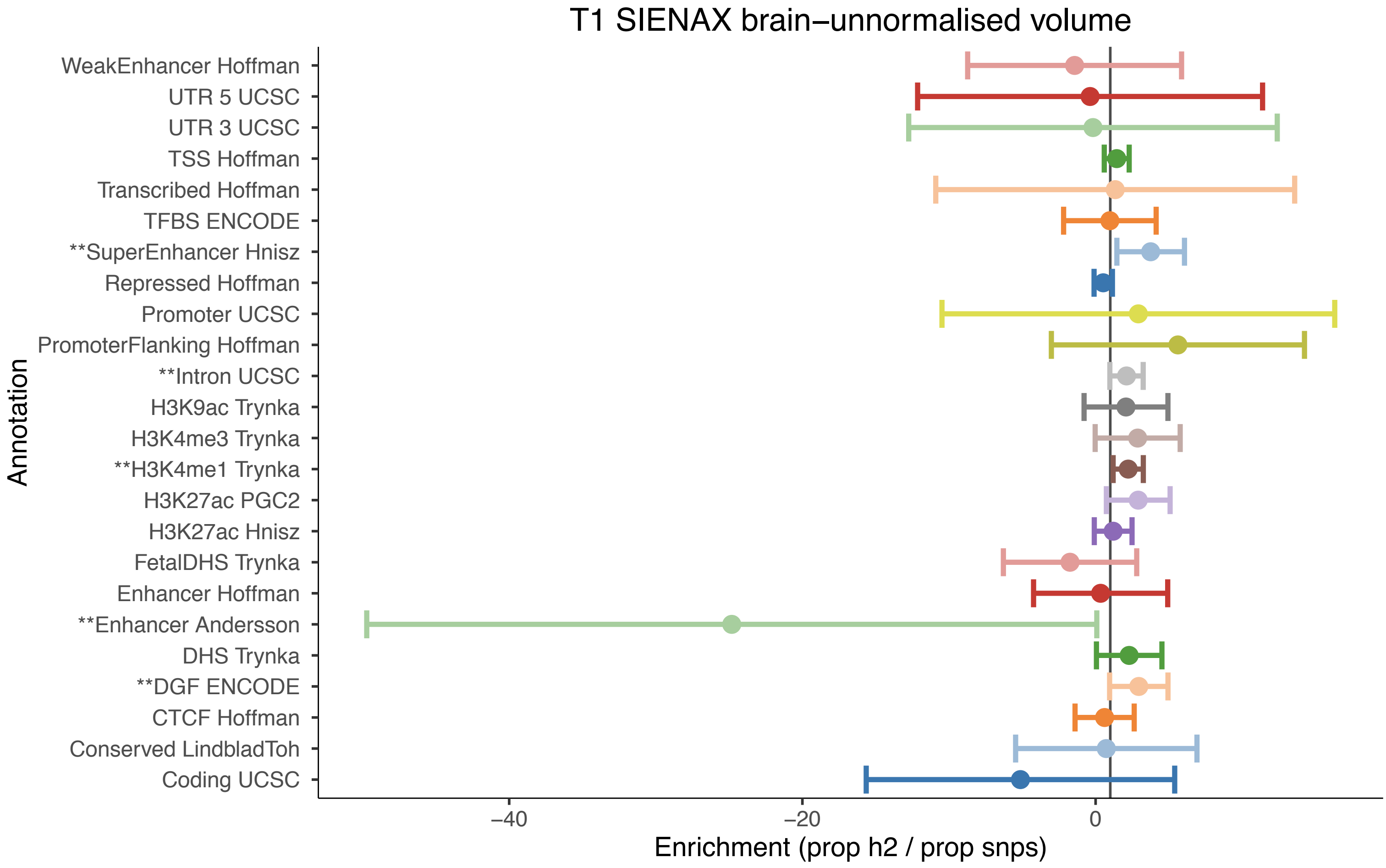

T1 SIENAX brain−unnormalised volume
WeakEnhancer Hoffman
UTR 5 UCSC
UTR 3 UCSC
TSS Hoffman
Transcribed Hoffman
TFBS ENCODE
**SuperEnhancer Hnisz
Repressed Hoffman
Promoter UCSC
PromoterFlanking Hoffman
**Intron UCSC
H3K9ac Trynka
H3K4me3 Trynka
**H3K4me1 Trynka
H3K27ac PGC2
H3K27ac Hnisz
FetalDHS Trynka
Enhancer Hoffman
**Enhancer Andersson
DHS Trynka
**DGF ENCODE
CTCF Hoffman
Conserved LindbladToh
Coding UCSC
Annotation
−40
−20
0
Enrichment (prop h2 / prop snps)

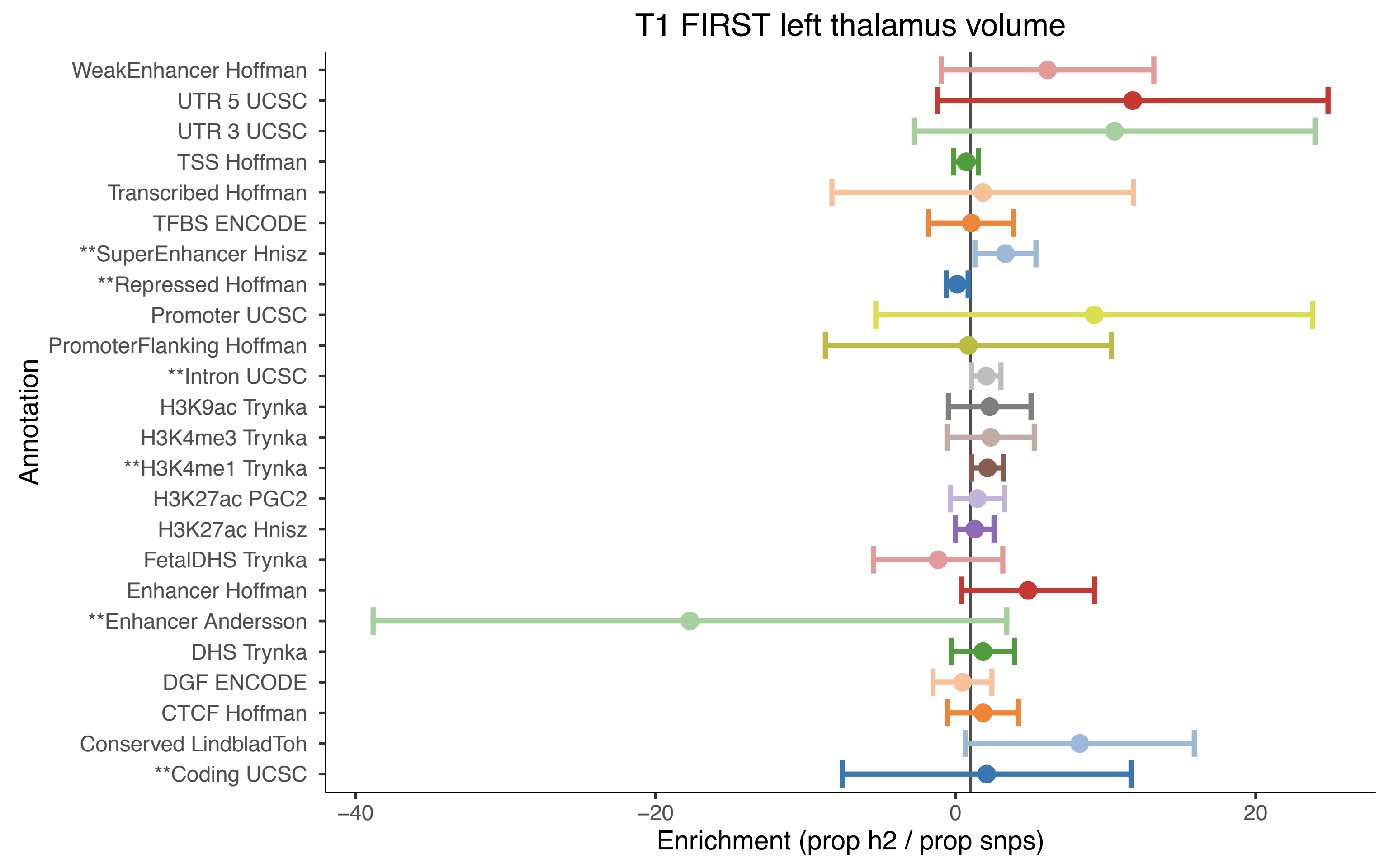

T1 FIRST left thalamus volume
WeakEnhancer Hoffman
UTR 5 UCSC
UTR 3 UCSC
TSS Hoffman
Transcribed Hoffman
TFBS ENCODE
**SuperEnhancer Hnisz
**Repressed Hoffman
Promoter UCSC
PromoterFlanking Hoffman
**Intron UCSC
H3K9ac Trynka
H3K4me3 Trynka
**H3K4me1 Trynka
H3K27ac PGC2
H3K27ac Hnisz
FetalDHS Trynka
Enhancer Hoffman
**Enhancer Andersson
DHS Trynka
DGF ENCODE
CTCF Hoffman
Conserved LindbladToh
**Coding UCSC
Annotation
−40
−20
0
20
Enrichment (prop h2 / prop snps)

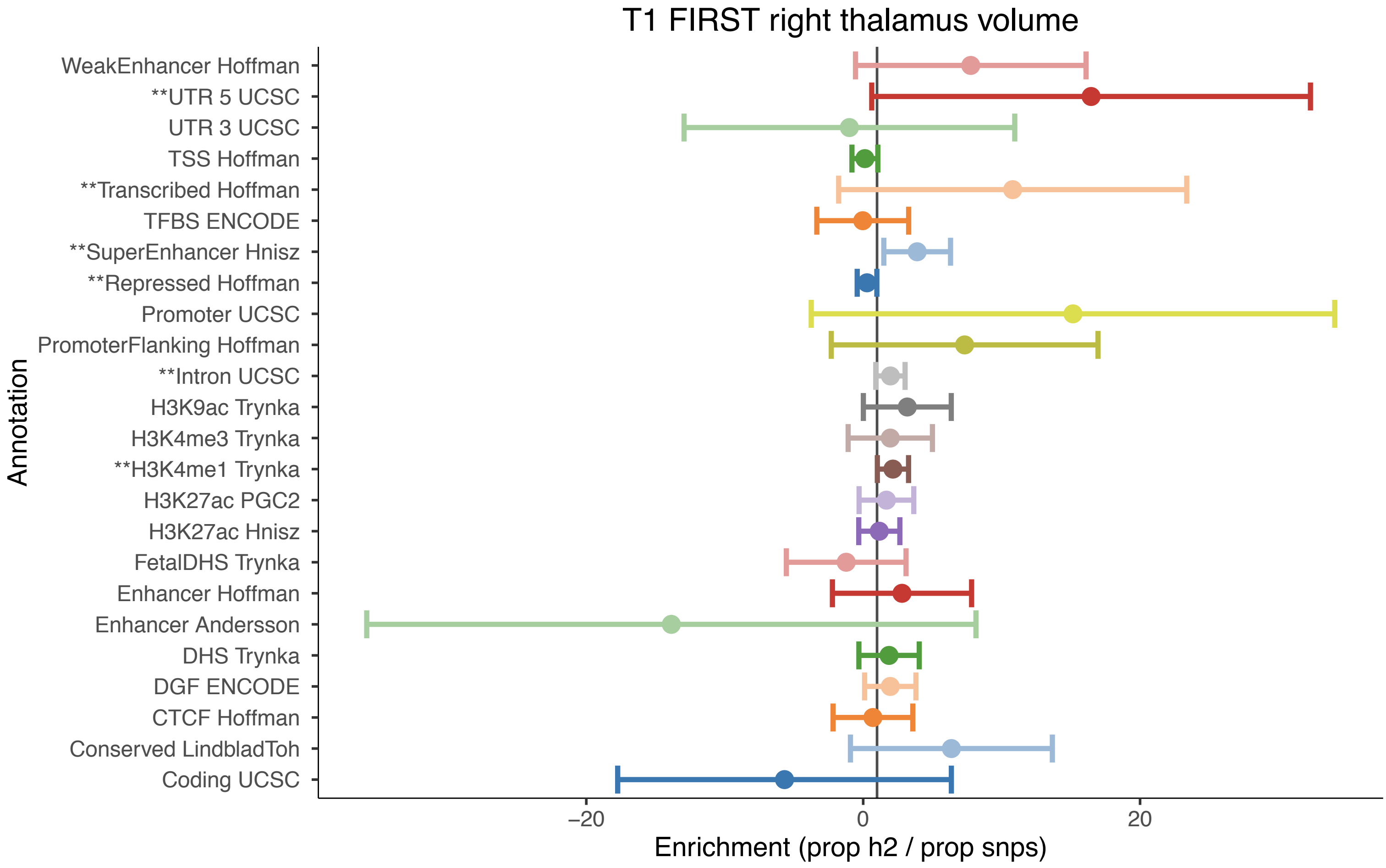
T1 FIRST right thalamus volume
WeakEnhancer Hoffman
**UTR 5 UCSC
UTR 3 UCSC
TSS Hoffman
**Transcribed Hoffman
TFBS ENCODE
**SuperEnhancer Hnisz
**Repressed Hoffman
Promoter UCSC
PromoterFlanking Hoffman
**Intron UCSC
H3K9ac Trynka
H3K4me3 Trynka
**H3K4me1 Trynka
H3K27ac PGC2
H3K27ac Hnisz
FetalDHS Trynka
Enhancer Hoffman
Enhancer Andersson
DHS Trynka
DGF ENCODE
CTCF Hoffman
Conserved LindbladToh
Coding UCSC
Annotation
−20
0
20
Enrichment (prop h2 / prop snps)

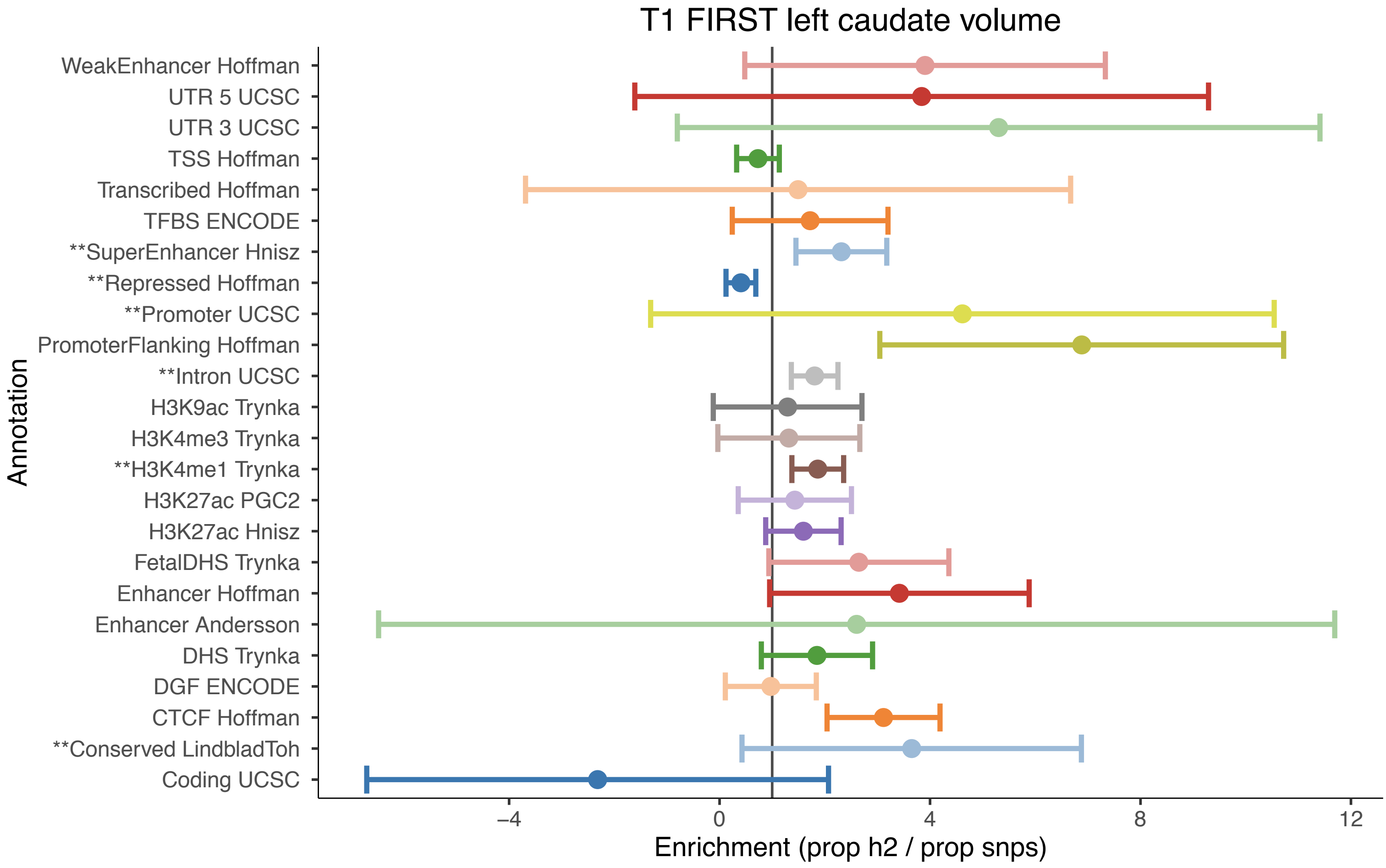

T1 FIRST left caudate volume
WeakEnhancer Hoffman
UTR 5 UCSC
UTR 3 UCSC
TSS Hoffman
Transcribed Hoffman
TFBS ENCODE
**SuperEnhancer Hnisz
**Repressed Hoffman
**Promoter UCSC
PromoterFlanking Hoffman
**Intron UCSC
H3K9ac Trynka
H3K4me3 Trynka
**H3K4me1 Trynka
H3K27ac PGC2
H3K27ac Hnisz
FetalDHS Trynka
Enhancer Hoffman
Enhancer Andersson
DHS Trynka
DGF ENCODE
CTCF Hoffman
**Conserved LindbladToh
Coding UCSC
Annotation
−4
0
4
8
12
Enrichment (prop h2 / prop snps)

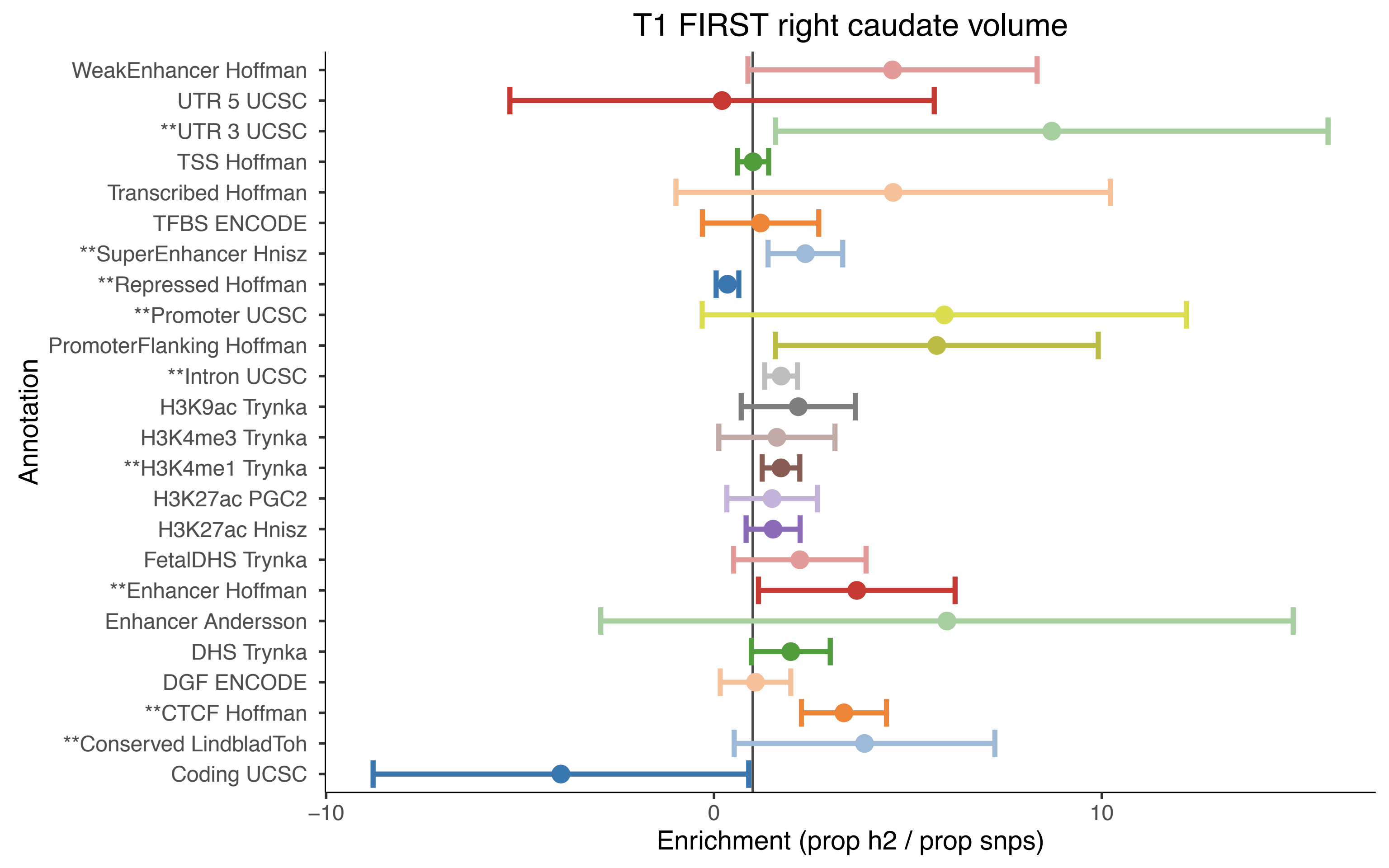

T1 FIRST right caudate volume
WeakEnhancer Hoffman
UTR 5 UCSC
**UTR 3 UCSC
TSS Hoffman
Transcribed Hoffman
TFBS ENCODE
**SuperEnhancer Hnisz
**Repressed Hoffman
**Promoter UCSC
PromoterFlanking Hoffman
**Intron UCSC
H3K9ac Trynka
H3K4me3 Trynka
**H3K4me1 Trynka
H3K27ac PGC2
H3K27ac Hnisz
FetalDHS Trynka
**Enhancer Hoffman
Enhancer Andersson
DHS Trynka
DGF ENCODE
**CTCF Hoffman
**Conserved LindbladToh
Coding UCSC
Annotation
−10
0
10
Enrichment (prop h2 / prop snps)

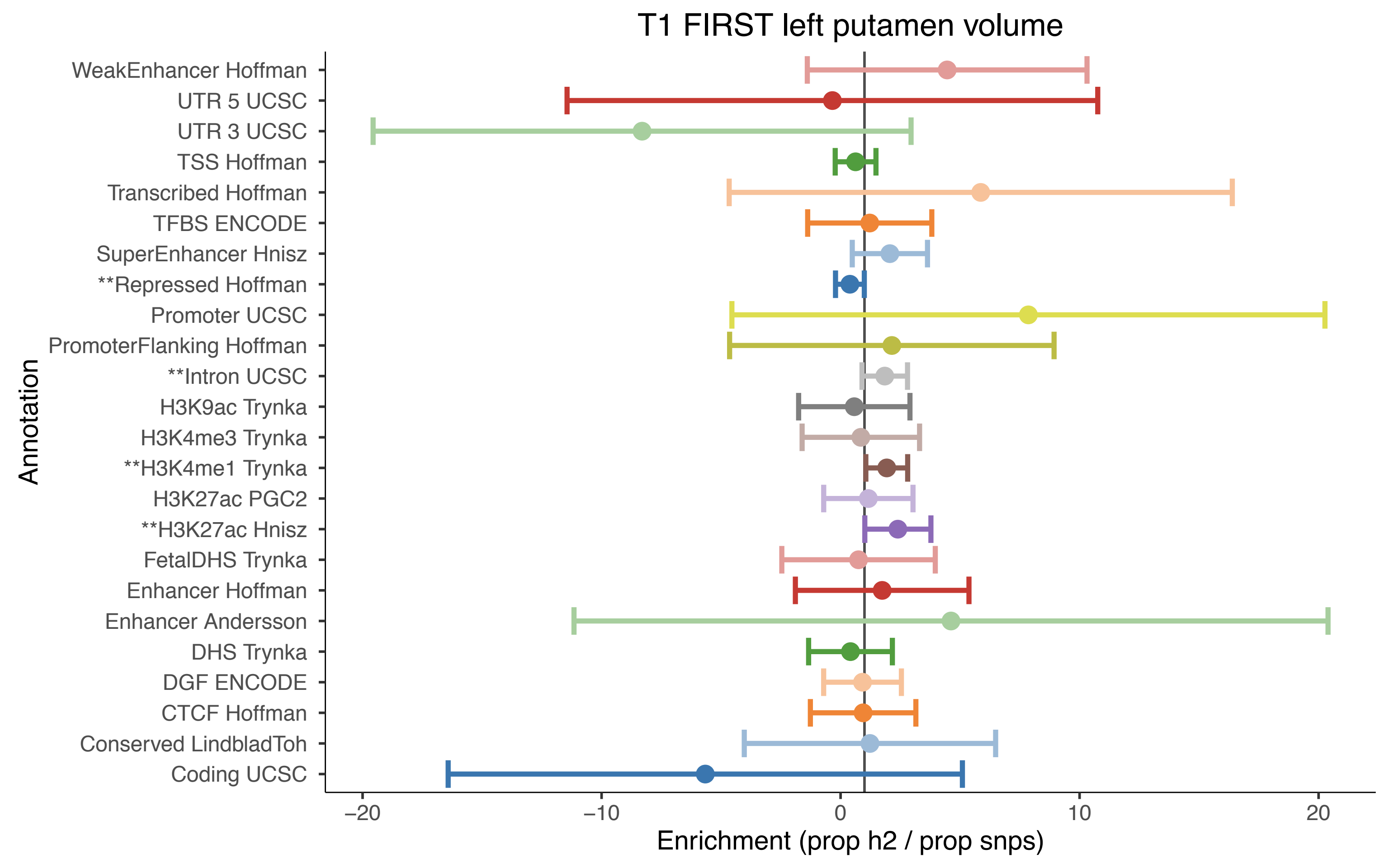

T1 FIRST left putamen volume
WeakEnhancer Hoffman
UTR 5 UCSC
UTR 3 UCSC
TSS Hoffman
Transcribed Hoffman
TFBS ENCODE
SuperEnhancer Hnisz
**Repressed Hoffman
Promoter UCSC
PromoterFlanking Hoffman
**Intron UCSC
H3K9ac Trynka
H3K4me3 Trynka
**H3K4me1 Trynka
H3K27ac PGC2
**H3K27ac Hnisz
FetalDHS Trynka
Enhancer Hoffman
Enhancer Andersson
DHS Trynka
DGF ENCODE
CTCF Hoffman
Conserved LindbladToh
Coding UCSC
Annotation
−20
−10
0
10
20
Enrichment (prop h2 / prop snps)

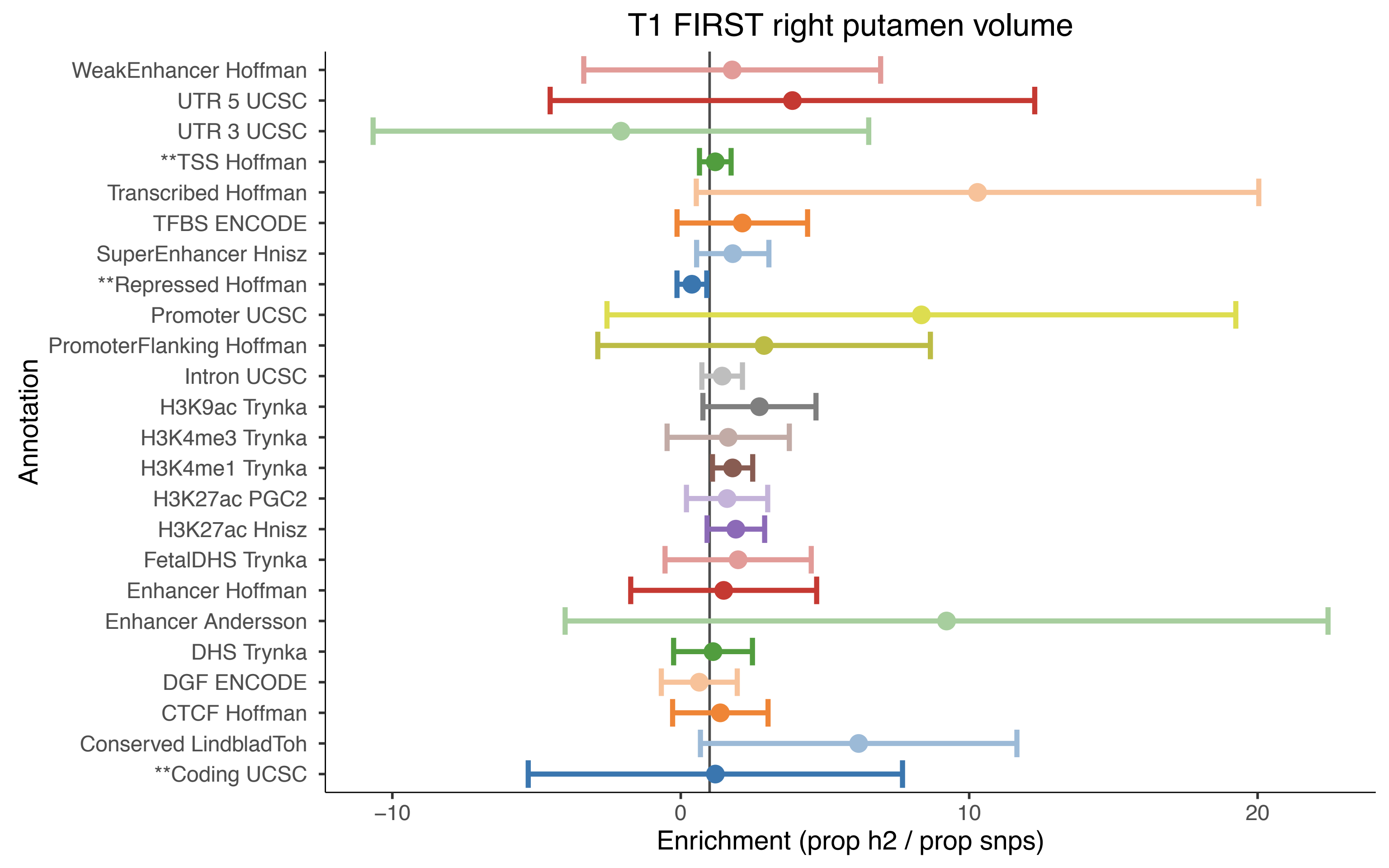
T1 FIRST right putamen volume
WeakEnhancer Hoffman
UTR 5 UCSC
UTR 3 UCSC
**TSS Hoffman
Transcribed Hoffman
TFBS ENCODE
SuperEnhancer Hnisz
**Repressed Hoffman
Promoter UCSC
PromoterFlanking Hoffman
Intron UCSC
H3K9ac Trynka
H3K4me3 Trynka
H3K4me1 Trynka
H3K27ac PGC2
H3K27ac Hnisz
FetalDHS Trynka
Enhancer Hoffman
Enhancer Andersson
DHS Trynka
DGF ENCODE
CTCF Hoffman
Conserved LindbladToh
**Coding UCSC
Annotation
−10
0
10
20
Enrichment (prop h2 / prop snps)

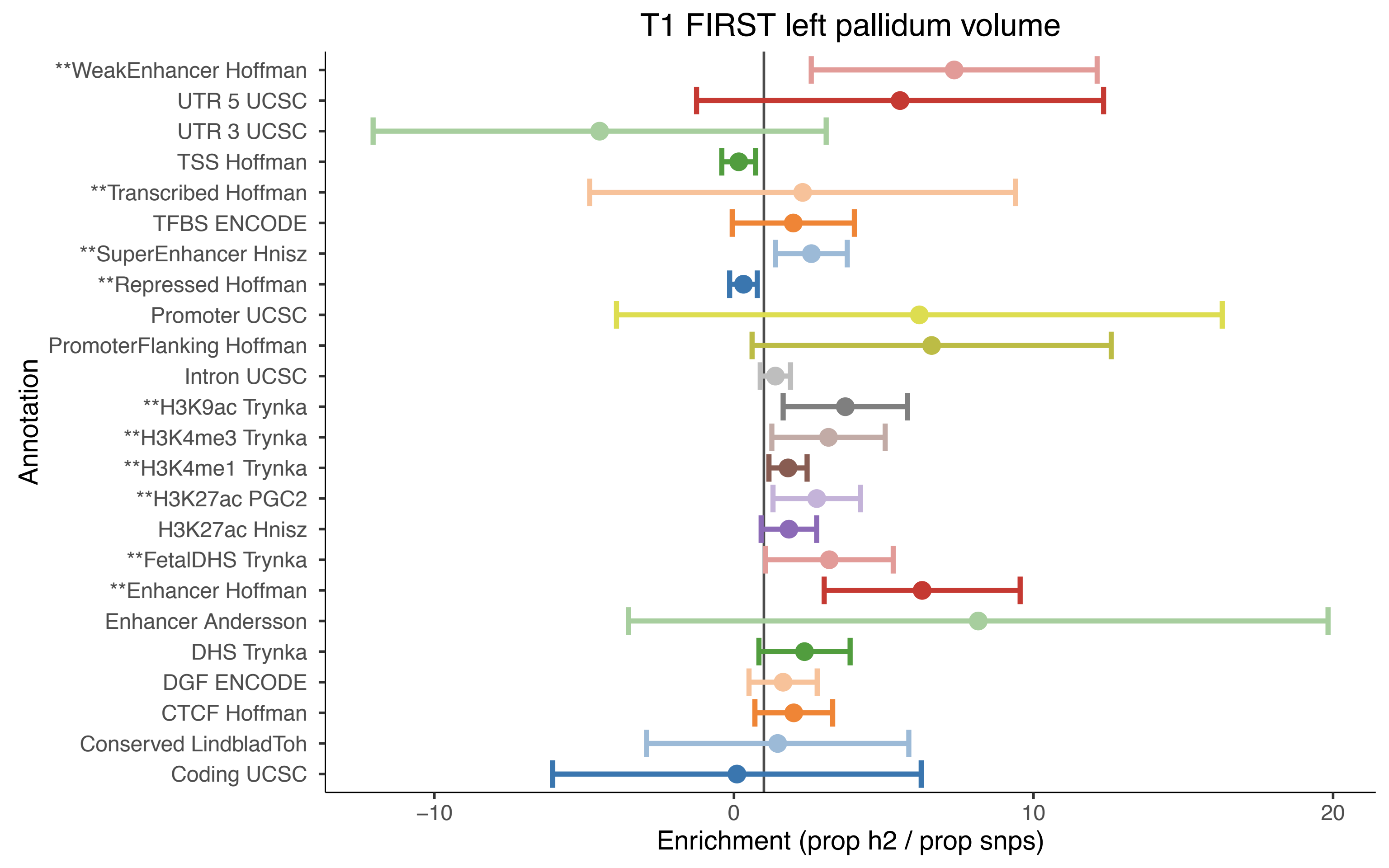
T1 FIRST left pallidum volume
**WeakEnhancer Hoffman
UTR 5 UCSC
UTR 3 UCSC
TSS Hoffman
**Transcribed Hoffman
TFBS ENCODE
**SuperEnhancer Hnisz
**Repressed Hoffman
Promoter UCSC
PromoterFlanking Hoffman
Intron UCSC
**H3K9ac Trynka
**H3K4me3 Trynka
**H3K4me1 Trynka
**H3K27ac PGC2
H3K27ac Hnisz
**FetalDHS Trynka
**Enhancer Hoffman
Enhancer Andersson
DHS Trynka
DGF ENCODE
CTCF Hoffman
Conserved LindbladToh
Coding UCSC
Annotation
−10
0
10
20
Enrichment (prop h2 / prop snps)

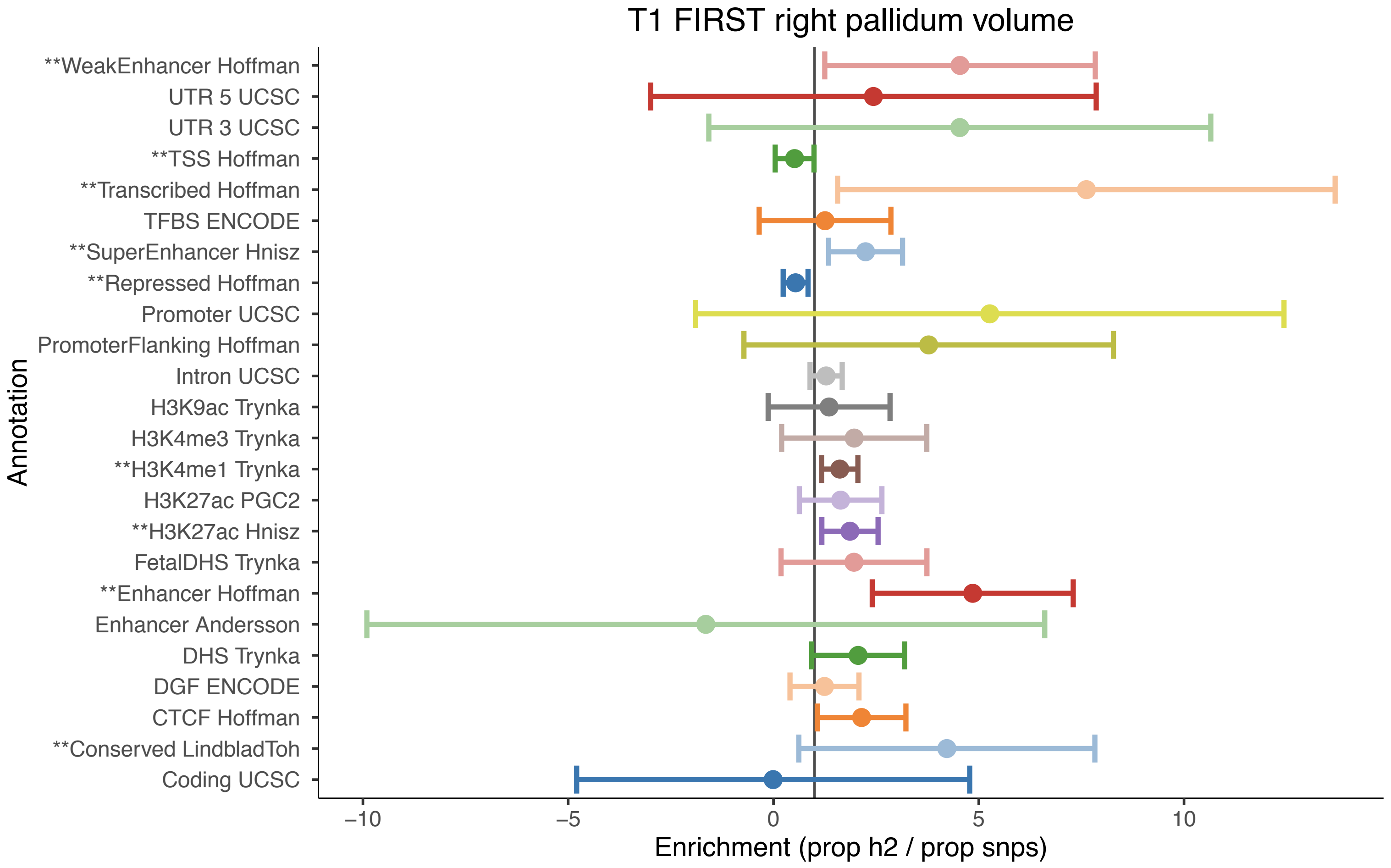
T1 FIRST right pallidum volume
**WeakEnhancer Hoffman
UTR 5 UCSC
UTR 3 UCSC
**TSS Hoffman
**Transcribed Hoffman
TFBS ENCODE
**SuperEnhancer Hnisz
**Repressed Hoffman
Promoter UCSC
PromoterFlanking Hoffman
Intron UCSC
H3K9ac Trynka
H3K4me3 Trynka
**H3K4me1 Trynka
H3K27ac PGC2
**H3K27ac Hnisz
FetalDHS Trynka
**Enhancer Hoffman
Enhancer Andersson
DHS Trynka
DGF ENCODE
CTCF Hoffman
**Conserved LindbladToh
Coding UCSC
Annotation
−10
−5
0
5
10
Enrichment (prop h2 / prop snps)

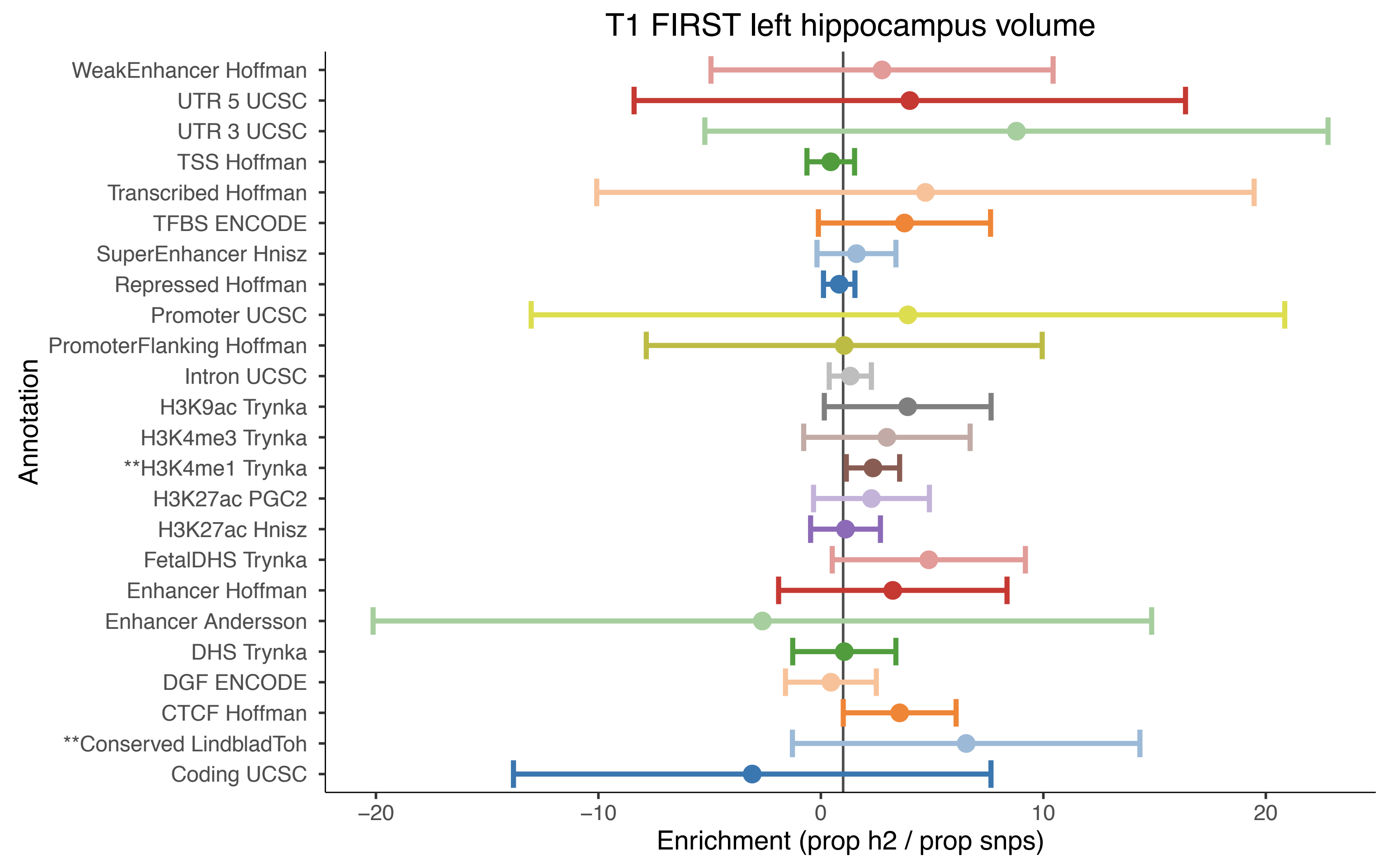

T1 FIRST left hippocampus volume
WeakEnhancer Hoffman
UTR 5 UCSC
UTR 3 UCSC
TSS Hoffman
Transcribed Hoffman
TFBS ENCODE
SuperEnhancer Hnisz
Repressed Hoffman
Promoter UCSC
PromoterFlanking Hoffman
Intron UCSC
H3K9ac Trynka
H3K4me3 Trynka
**H3K4me1 Trynka
H3K27ac PGC2
H3K27ac Hnisz
FetalDHS Trynka
Enhancer Hoffman
Enhancer Andersson
DHS Trynka
DGF ENCODE
CTCF Hoffman
**Conserved LindbladToh
Coding UCSC
Annotation
−20
−10
0
10
20
Enrichment (prop h2 / prop snps)

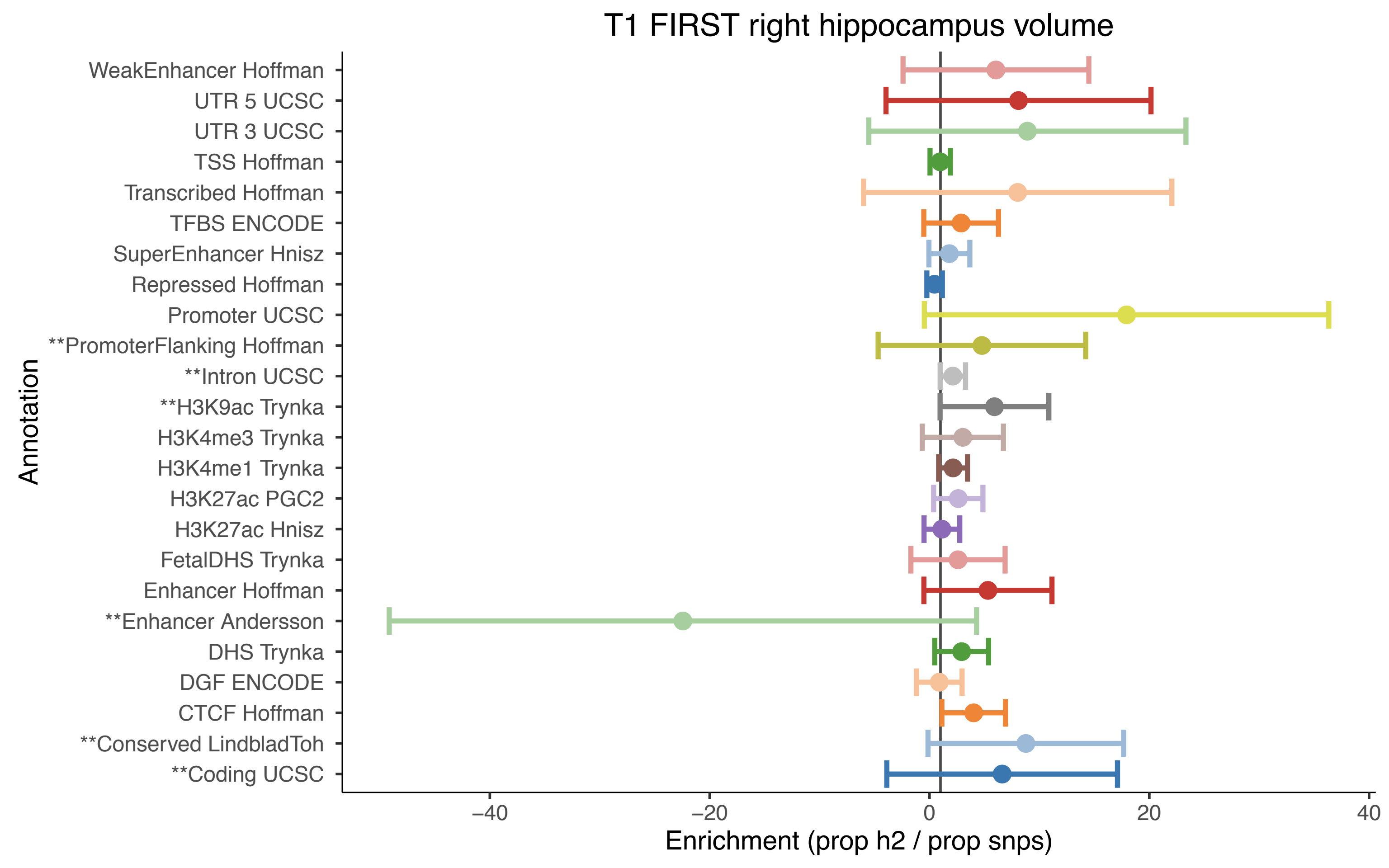

T1 FIRST right hippocampus volume
WeakEnhancer Hoffman
UTR 5 UCSC
UTR 3 UCSC
TSS Hoffman
Transcribed Hoffman
TFBS ENCODE
SuperEnhancer Hnisz
Repressed Hoffman
Promoter UCSC
**PromoterFlanking Hoffman
**Intron UCSC
**H3K9ac Trynka
H3K4me3 Trynka
H3K4me1 Trynka
H3K27ac PGC2
H3K27ac Hnisz
FetalDHS Trynka
Enhancer Hoffman
**Enhancer Andersson
DHS Trynka
DGF ENCODE
CTCF Hoffman
**Conserved LindbladToh
**Coding UCSC
Annotation
−40
−20
0
20
40
Enrichment (prop h2 / prop snps)

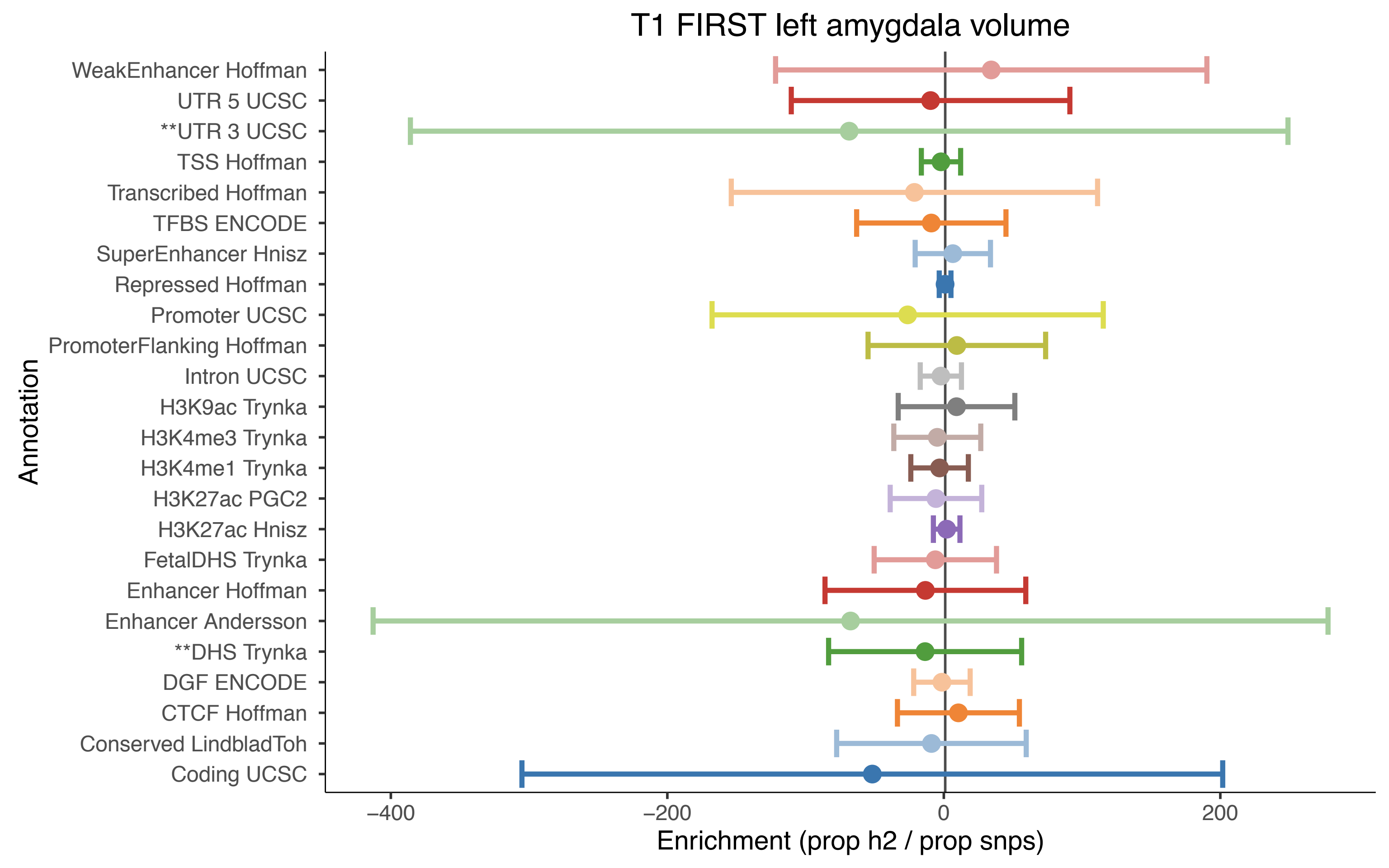

T1 FIRST left amygdala volume
WeakEnhancer Hoffman
UTR 5 UCSC
**UTR 3 UCSC
TSS Hoffman
Transcribed Hoffman
TFBS ENCODE
SuperEnhancer Hnisz
Repressed Hoffman
Promoter UCSC
PromoterFlanking Hoffman
Intron UCSC
H3K9ac Trynka
H3K4me3 Trynka
H3K4me1 Trynka
H3K27ac PGC2
H3K27ac Hnisz
FetalDHS Trynka
Enhancer Hoffman
Enhancer Andersson
**DHS Trynka
DGF ENCODE
CTCF Hoffman
Conserved LindbladToh
Coding UCSC
Annotation
−400
−200
0
200
Enrichment (prop h2 / prop snps)

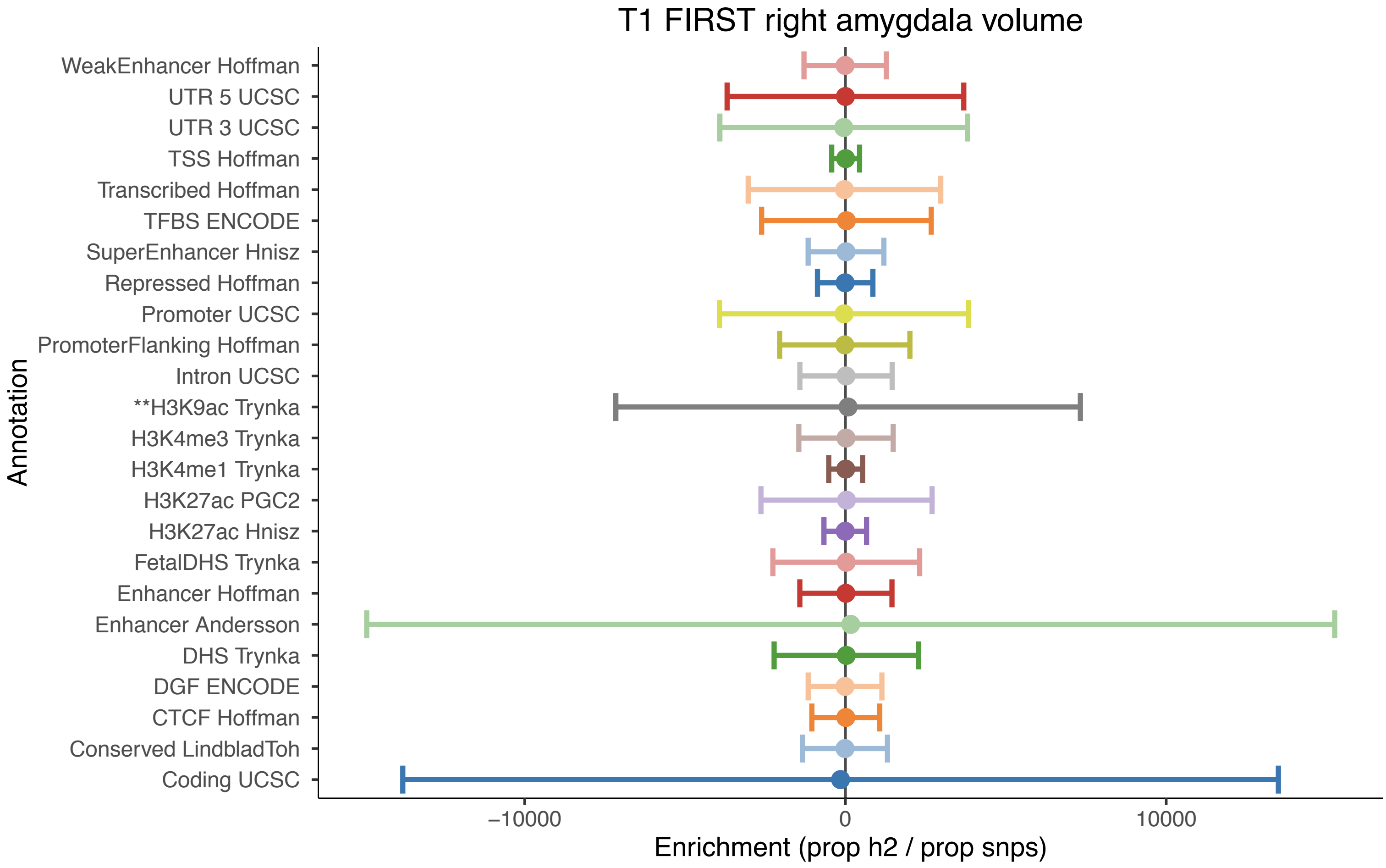

T1 FIRST right amygdala volume
WeakEnhancer Hoffman
UTR 5 UCSC
UTR 3 UCSC
TSS Hoffman
Transcribed Hoffman
TFBS ENCODE
SuperEnhancer Hnisz
Repressed Hoffman
Promoter UCSC
PromoterFlanking Hoffman
Intron UCSC
**H3K9ac Trynka
H3K4me3 Trynka
H3K4me1 Trynka
H3K27ac PGC2
H3K27ac Hnisz
FetalDHS Trynka
Enhancer Hoffman
Enhancer Andersson
DHS Trynka
DGF ENCODE
CTCF Hoffman
Conserved LindbladToh
Coding UCSC
Annotation
−10000
0
10000
Enrichment (prop h2 / prop snps)

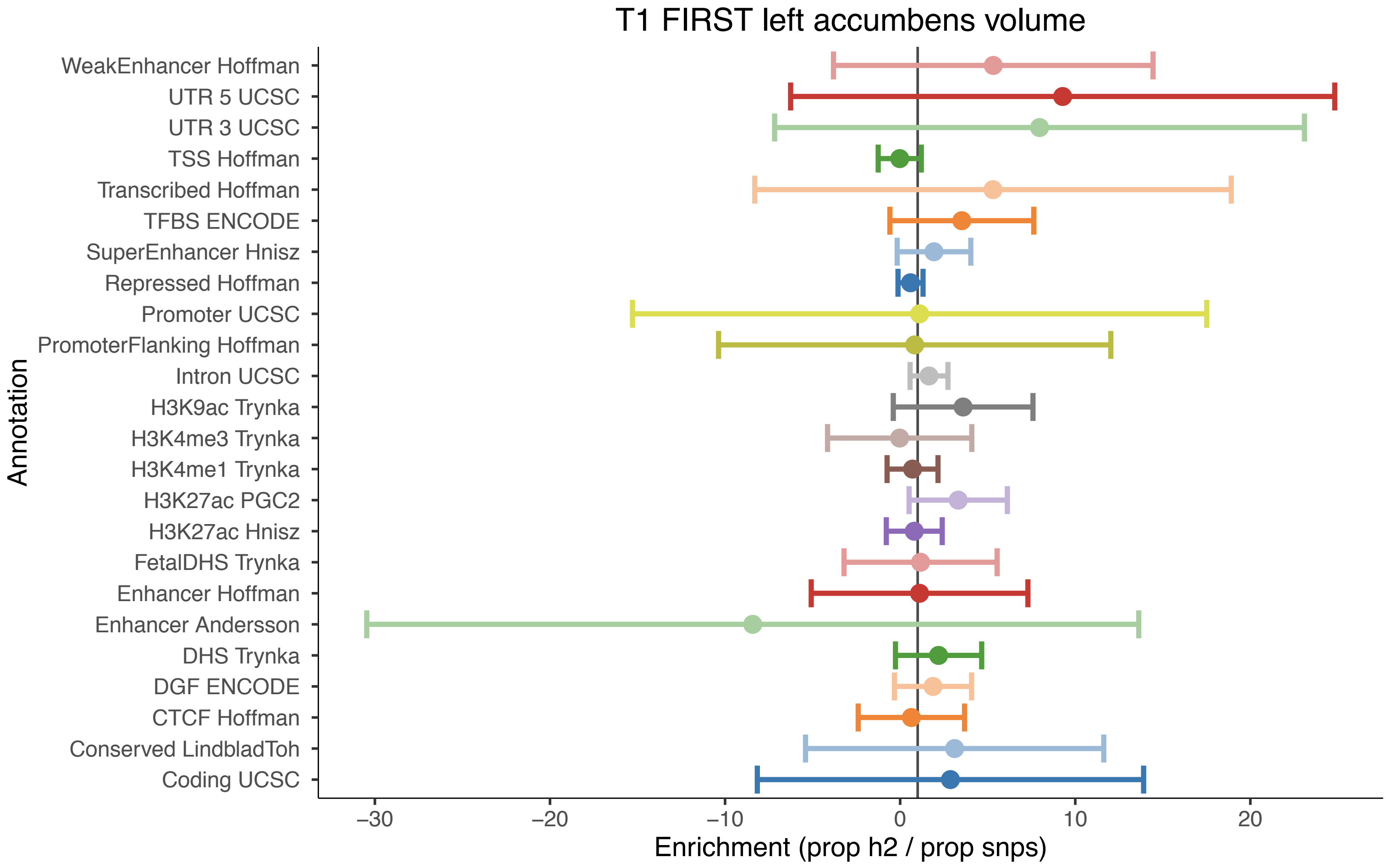

T1 FIRST left accumbens volume
WeakEnhancer Hoffman
UTR 5 UCSC
UTR 3 UCSC
TSS Hoffman
Transcribed Hoffman
TFBS ENCODE
SuperEnhancer Hnisz
Repressed Hoffman
Promoter UCSC
PromoterFlanking Hoffman
Intron UCSC
H3K9ac Trynka
H3K4me3 Trynka
H3K4me1 Trynka
H3K27ac PGC2
H3K27ac Hnisz
FetalDHS Trynka
Enhancer Hoffman
Enhancer Andersson
DHS Trynka
DGF ENCODE
CTCF Hoffman
Conserved LindbladToh
Coding UCSC
Annotation
−30
−20
−10
0
10
20
Enrichment (prop h2 / prop snps)

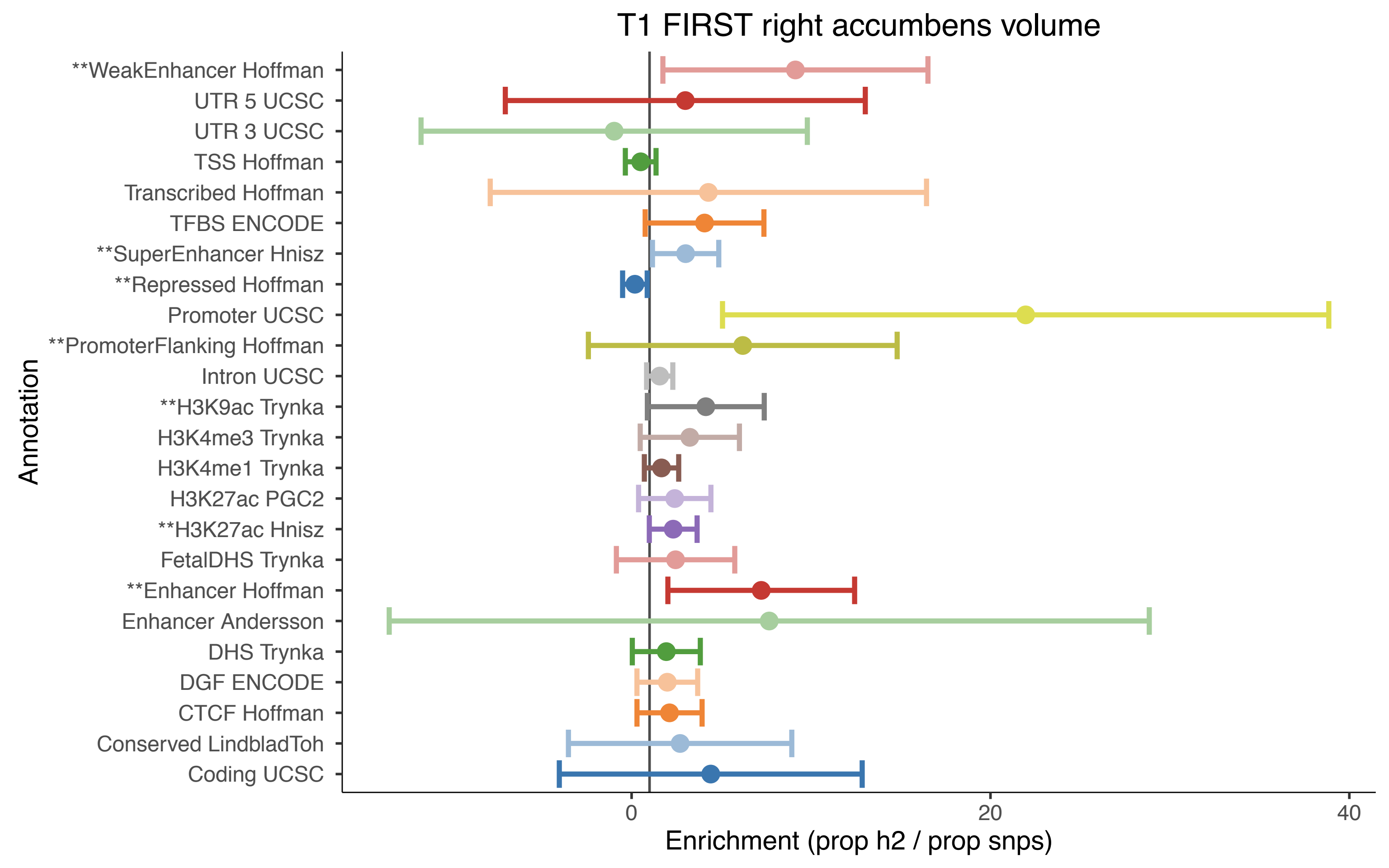
T1 FIRST right accumbens volume
**WeakEnhancer Hoffman
UTR 5 UCSC
UTR 3 UCSC
TSS Hoffman
Transcribed Hoffman
TFBS ENCODE
**SuperEnhancer Hnisz
**Repressed Hoffman
Promoter UCSC
**PromoterFlanking Hoffman
Intron UCSC
**H3K9ac Trynka
H3K4me3 Trynka
H3K4me1 Trynka
H3K27ac PGC2
**H3K27ac Hnisz
FetalDHS Trynka
**Enhancer Hoffman
Enhancer Andersson
DHS Trynka
DGF ENCODE
CTCF Hoffman
Conserved LindbladToh
Coding UCSC
Annotation
0
20
40
Enrichment (prop h2 / prop snps)

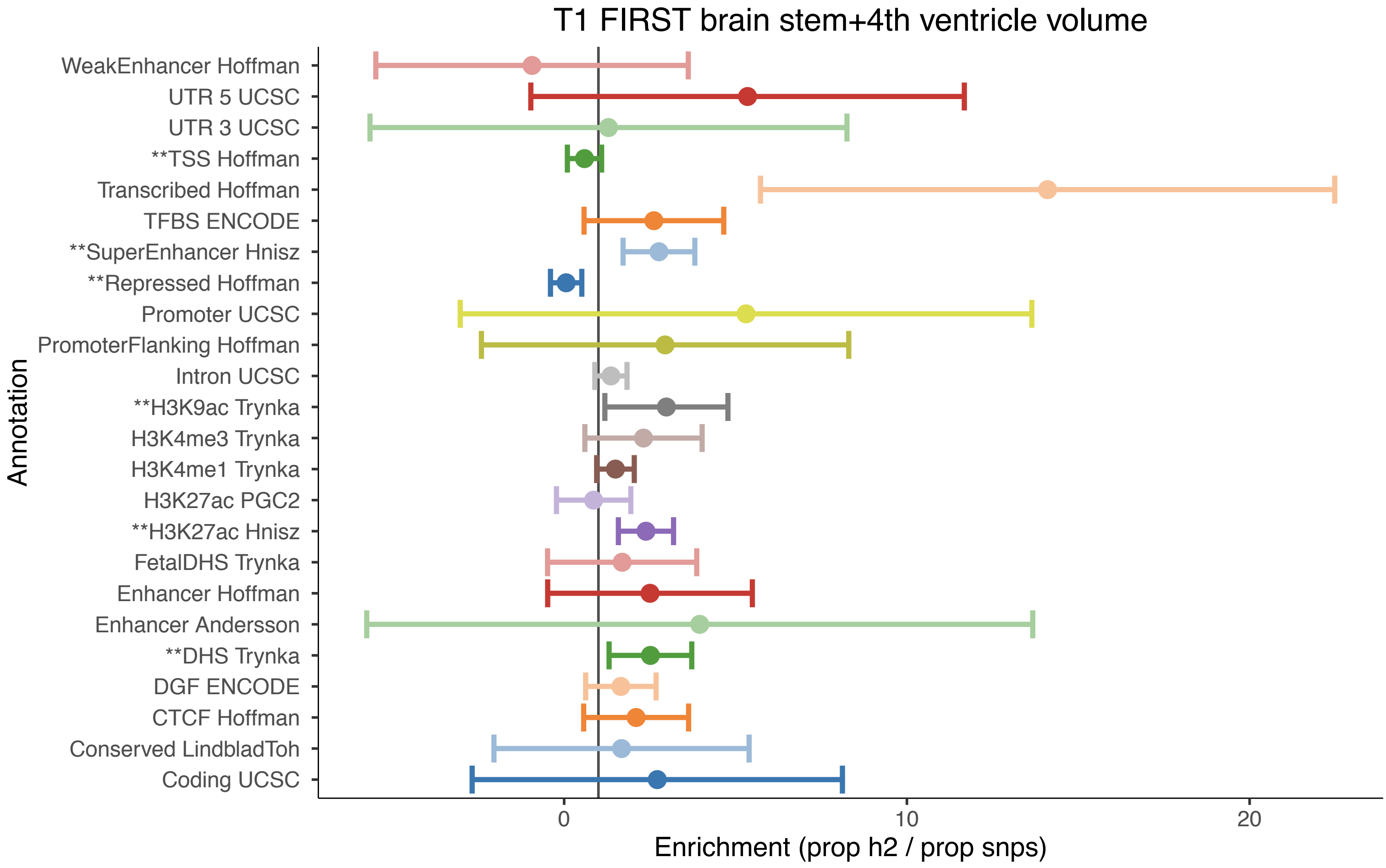
T1 FIRST brain stem+4th ventricle volume
WeakEnhancer Hoffman
UTR 5 UCSC
UTR 3 UCSC
**TSS Hoffman
Transcribed Hoffman
TFBS ENCODE
**SuperEnhancer Hnisz
**Repressed Hoffman
Promoter UCSC
PromoterFlanking Hoffman
Intron UCSC
**H3K9ac Trynka
H3K4me3 Trynka
H3K4me1 Trynka
H3K27ac PGC2
**H3K27ac Hnisz
FetalDHS Trynka
Enhancer Hoffman
Enhancer Andersson
**DHS Trynka
DGF ENCODE
CTCF Hoffman
Conserved LindbladToh
Coding UCSC
Annotation
0
10
20
Enrichment (prop h2 / prop snps)

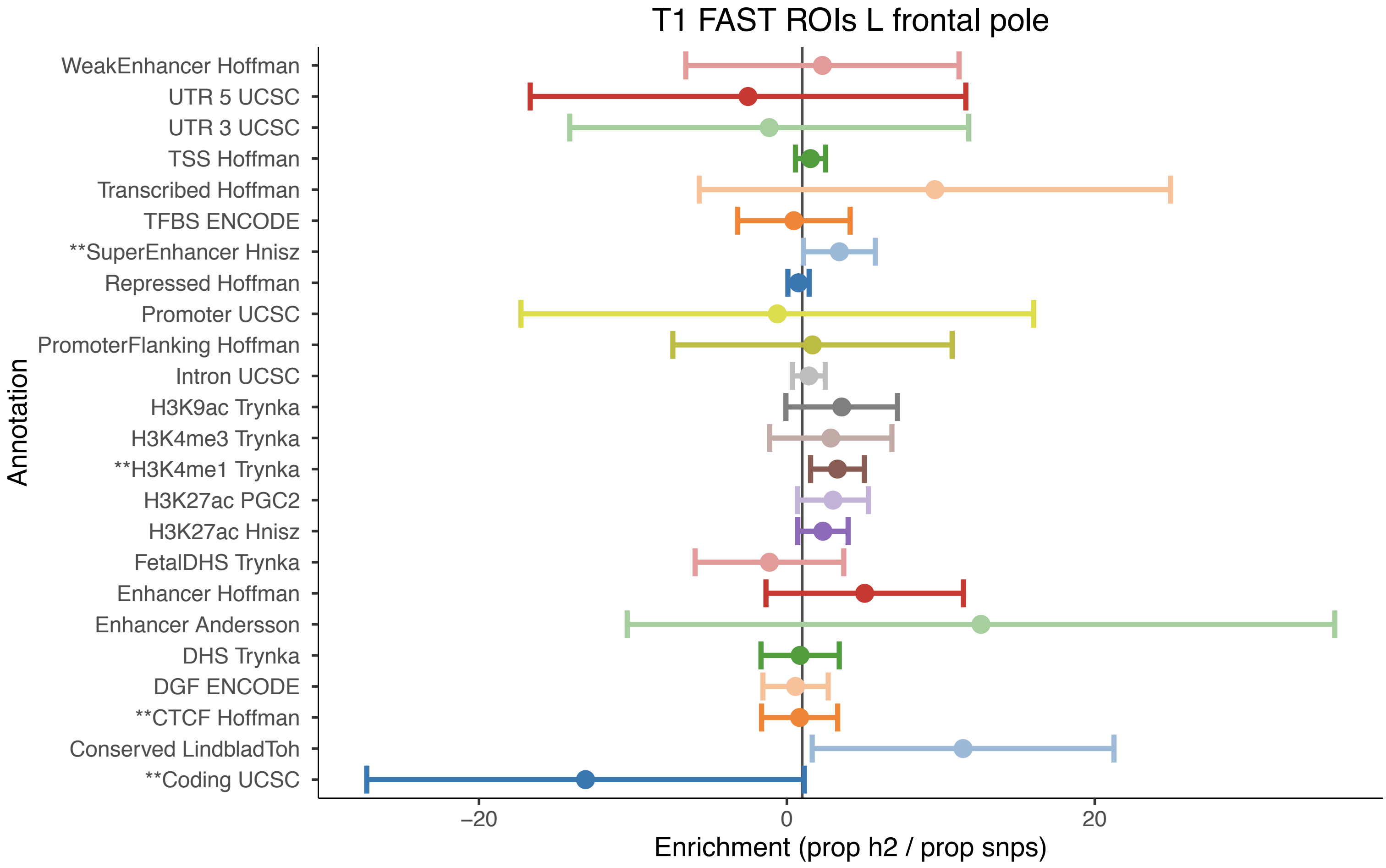

T1 FAST ROIs L frontal pole
WeakEnhancer Hoffman
UTR 5 UCSC
UTR 3 UCSC
TSS Hoffman
Transcribed Hoffman
TFBS ENCODE
**SuperEnhancer Hnisz
Repressed Hoffman
Promoter UCSC
PromoterFlanking Hoffman
Intron UCSC
H3K9ac Trynka
H3K4me3 Trynka
**H3K4me1 Trynka
H3K27ac PGC2
H3K27ac Hnisz
FetalDHS Trynka
Enhancer Hoffman
Enhancer Andersson
DHS Trynka
DGF ENCODE
**CTCF Hoffman
Conserved LindbladToh
**Coding UCSC
Annotation
−20
0
20
Enrichment (prop h2 / prop snps)

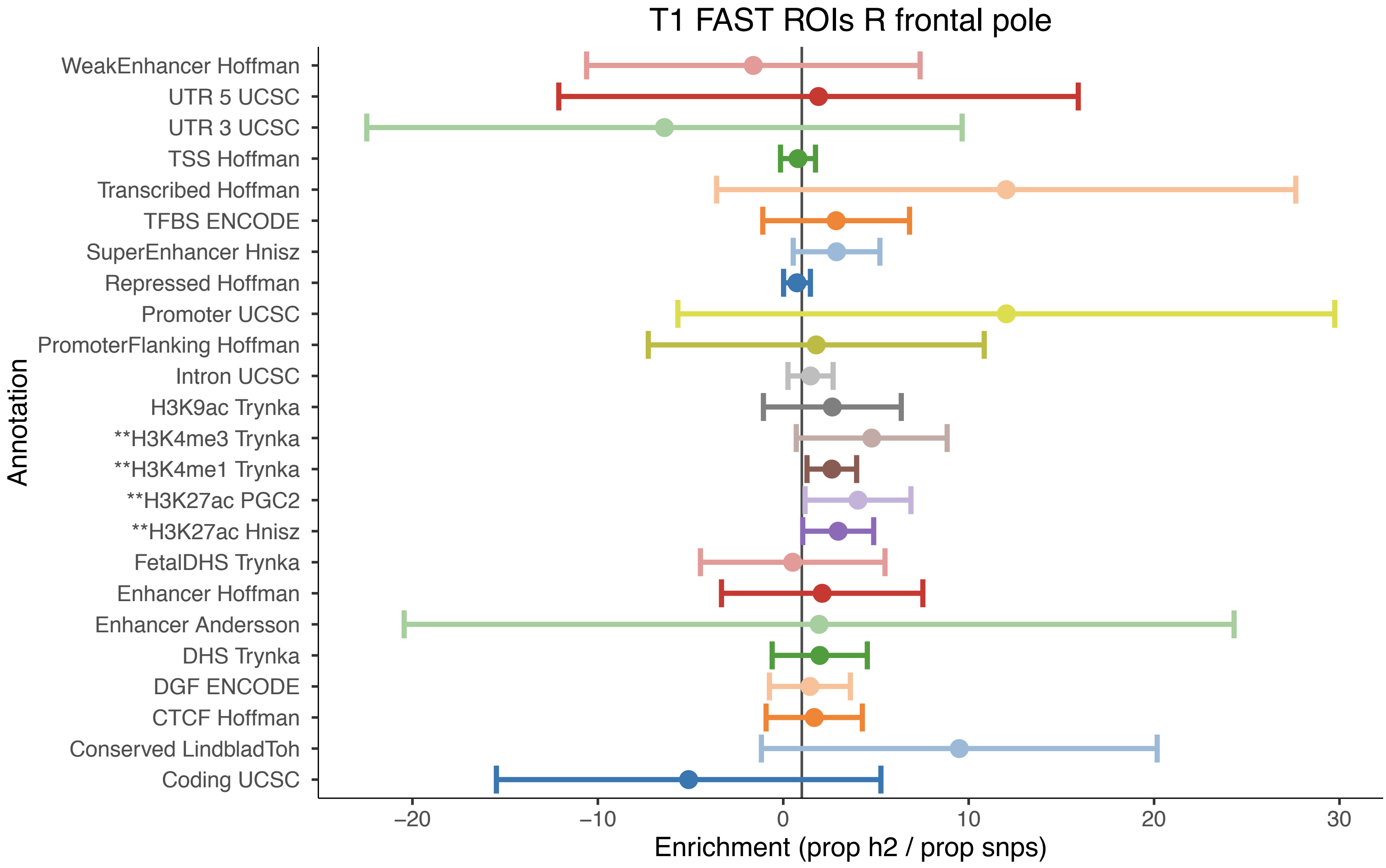

T1 FAST ROIs R frontal pole
WeakEnhancer Hoffman
UTR 5 UCSC
UTR 3 UCSC
TSS Hoffman
Transcribed Hoffman
TFBS ENCODE
SuperEnhancer Hnisz
Repressed Hoffman
Promoter UCSC
PromoterFlanking Hoffman
Intron UCSC
H3K9ac Trynka
**H3K4me3 Trynka
**H3K4me1 Trynka
**H3K27ac PGC2
**H3K27ac Hnisz
FetalDHS Trynka
Enhancer Hoffman
Enhancer Andersson
DHS Trynka
DGF ENCODE
CTCF Hoffman
Conserved LindbladToh
Coding UCSC
Annotation
−20
−10
0
10
20
30
Enrichment (prop h2 / prop snps)

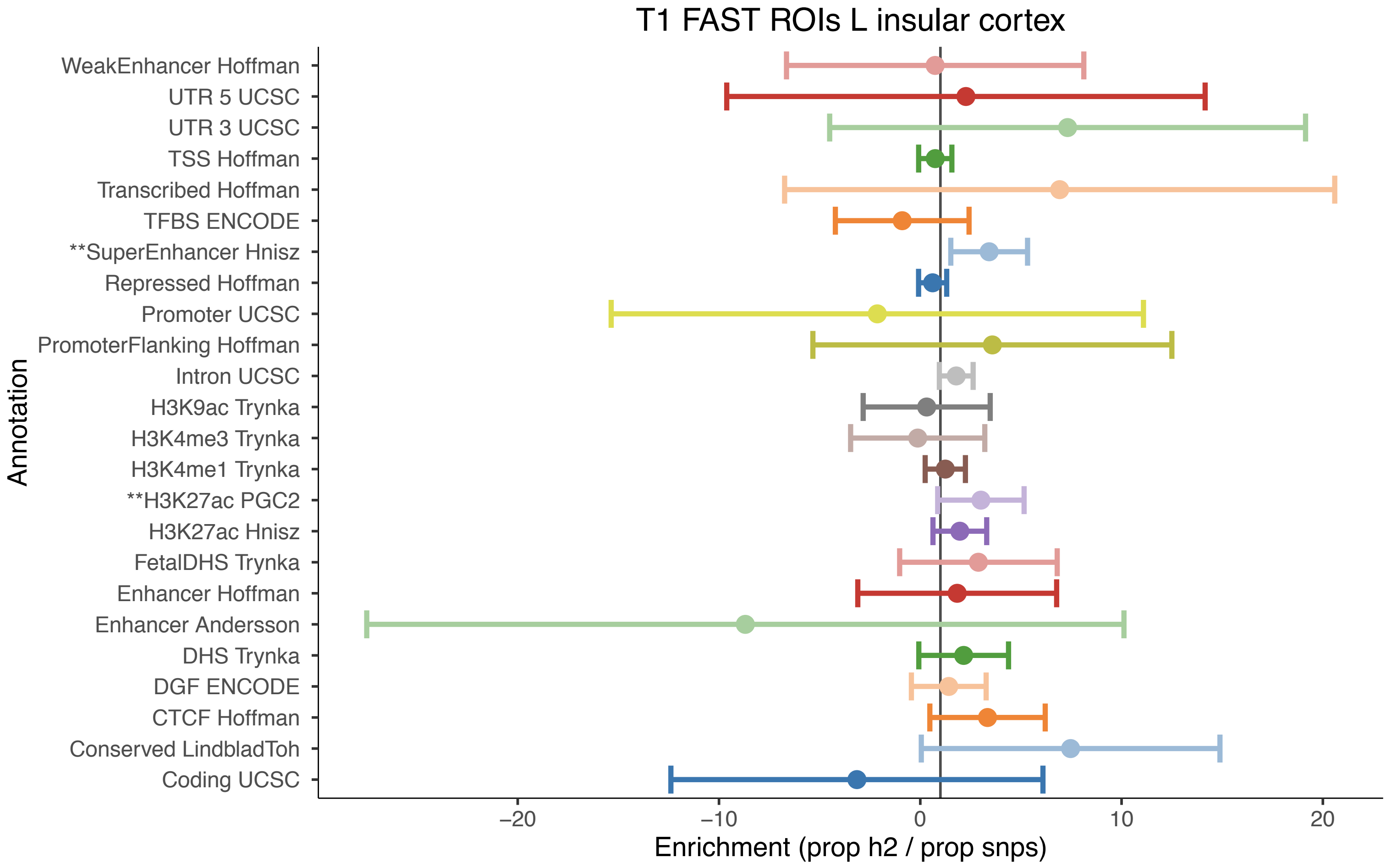
T1 FAST ROIs L insular cortex
WeakEnhancer Hoffman
UTR 5 UCSC
UTR 3 UCSC
TSS Hoffman
Transcribed Hoffman
TFBS ENCODE
**SuperEnhancer Hnisz
Repressed Hoffman
Promoter UCSC
PromoterFlanking Hoffman
Intron UCSC
H3K9ac Trynka
H3K4me3 Trynka
H3K4me1 Trynka
**H3K27ac PGC2
H3K27ac Hnisz
FetalDHS Trynka
Enhancer Hoffman
Enhancer Andersson
DHS Trynka
DGF ENCODE
CTCF Hoffman
Conserved LindbladToh
Coding UCSC
Annotation
−20
−10
0
10
20
Enrichment (prop h2 / prop snps)

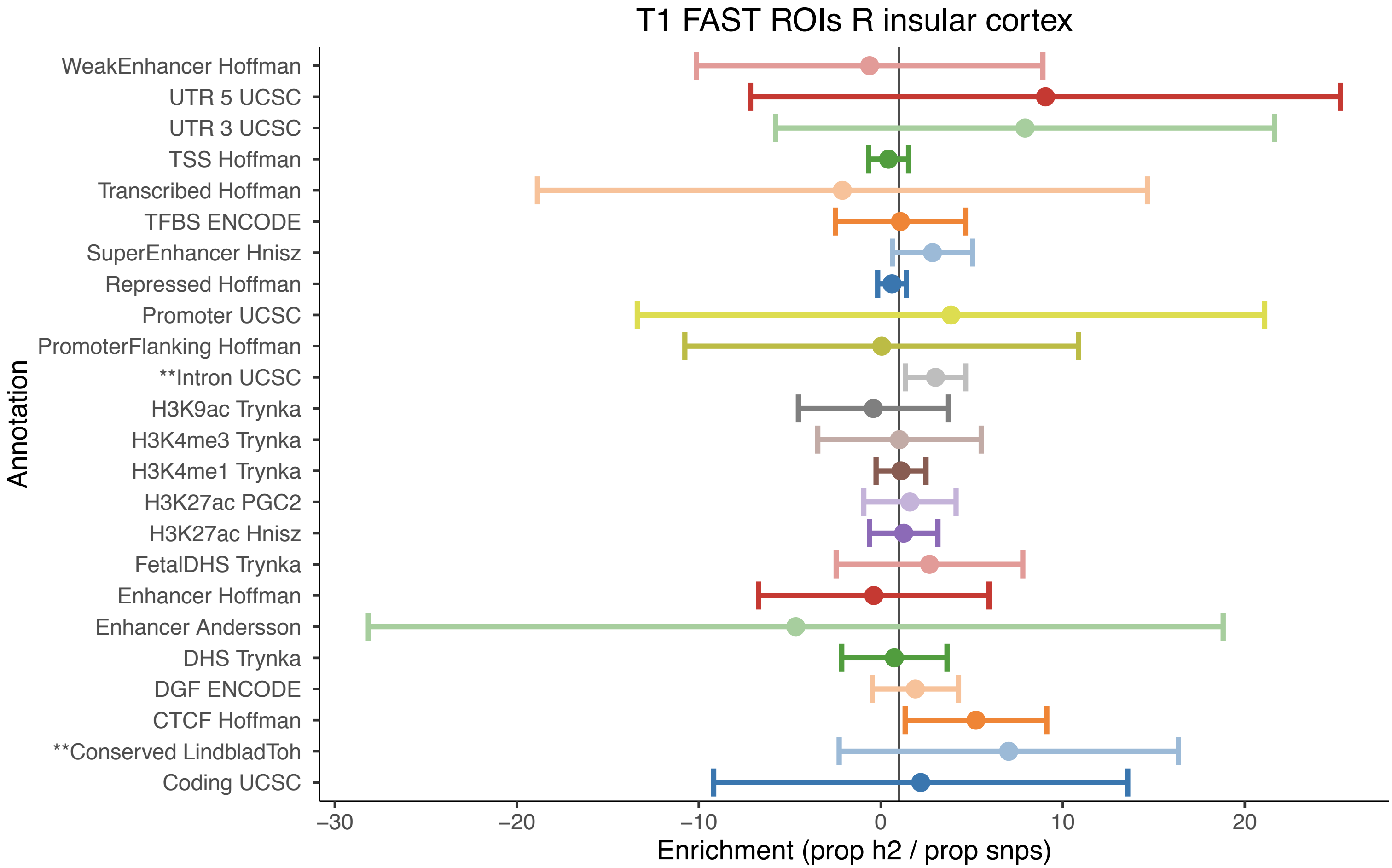

T1 FAST ROIs R insular cortex
WeakEnhancer Hoffman
UTR 5 UCSC
UTR 3 UCSC
TSS Hoffman
Transcribed Hoffman
TFBS ENCODE
SuperEnhancer Hnisz
Repressed Hoffman
Promoter UCSC
PromoterFlanking Hoffman
**Intron UCSC
H3K9ac Trynka
H3K4me3 Trynka
H3K4me1 Trynka
H3K27ac PGC2
H3K27ac Hnisz
FetalDHS Trynka
Enhancer Hoffman
Enhancer Andersson
DHS Trynka
DGF ENCODE
CTCF Hoffman
**Conserved LindbladToh
Coding UCSC
Annotation
−30
−20
−10
0
10
20
Enrichment (prop h2 / prop snps)

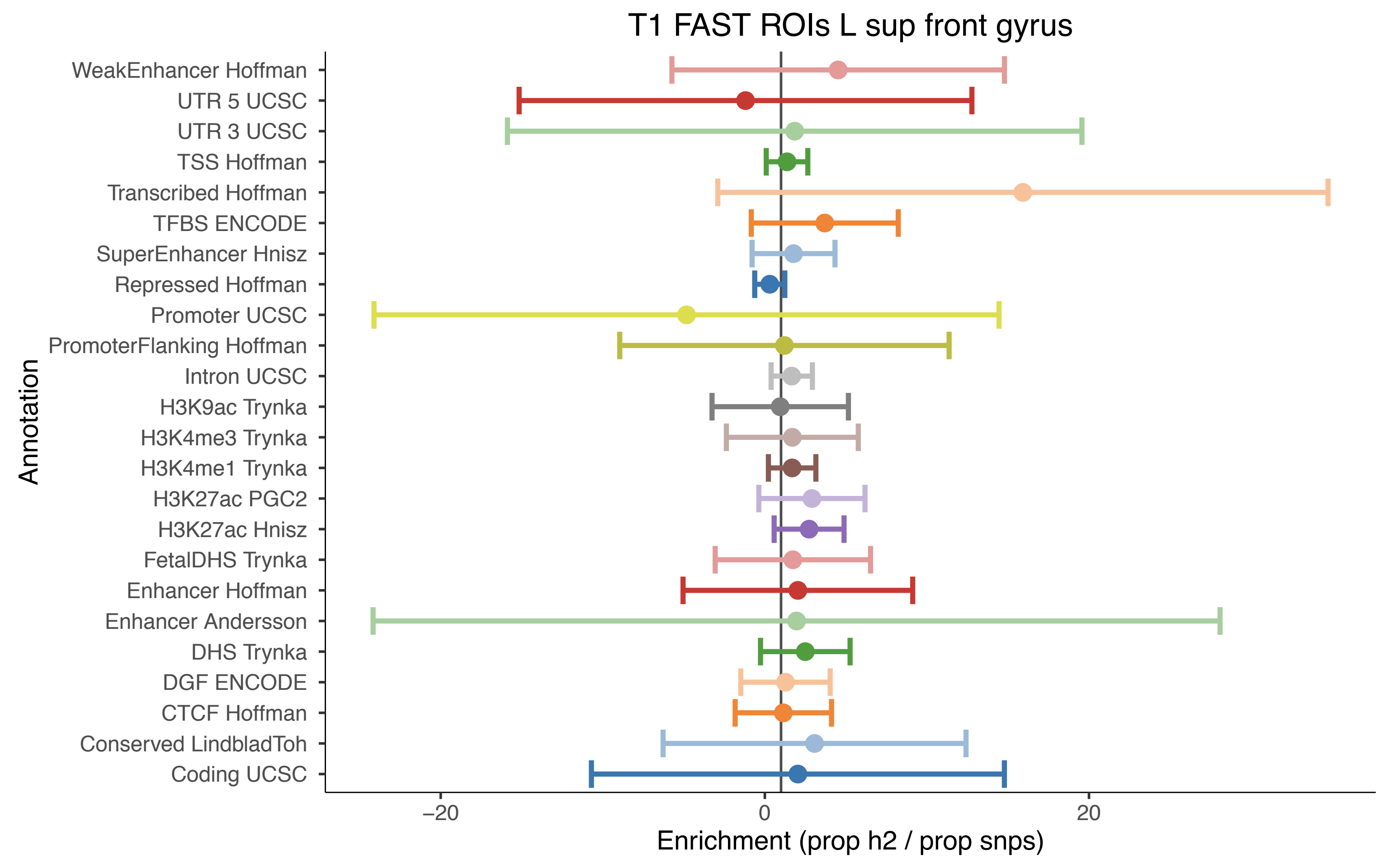
T1 FAST ROIs L sup front gyrus
WeakEnhancer Hoffman
UTR 5 UCSC
UTR 3 UCSC
TSS Hoffman
Transcribed Hoffman
TFBS ENCODE
SuperEnhancer Hnisz
Repressed Hoffman
Promoter UCSC
PromoterFlanking Hoffman
Intron UCSC
H3K9ac Trynka
H3K4me3 Trynka
H3K4me1 Trynka
H3K27ac PGC2
H3K27ac Hnisz
FetalDHS Trynka
Enhancer Hoffman
Enhancer Andersson
DHS Trynka
DGF ENCODE
CTCF Hoffman
Conserved LindbladToh
Coding UCSC
Annotation
−20
0
20
Enrichment (prop h2 / prop snps)

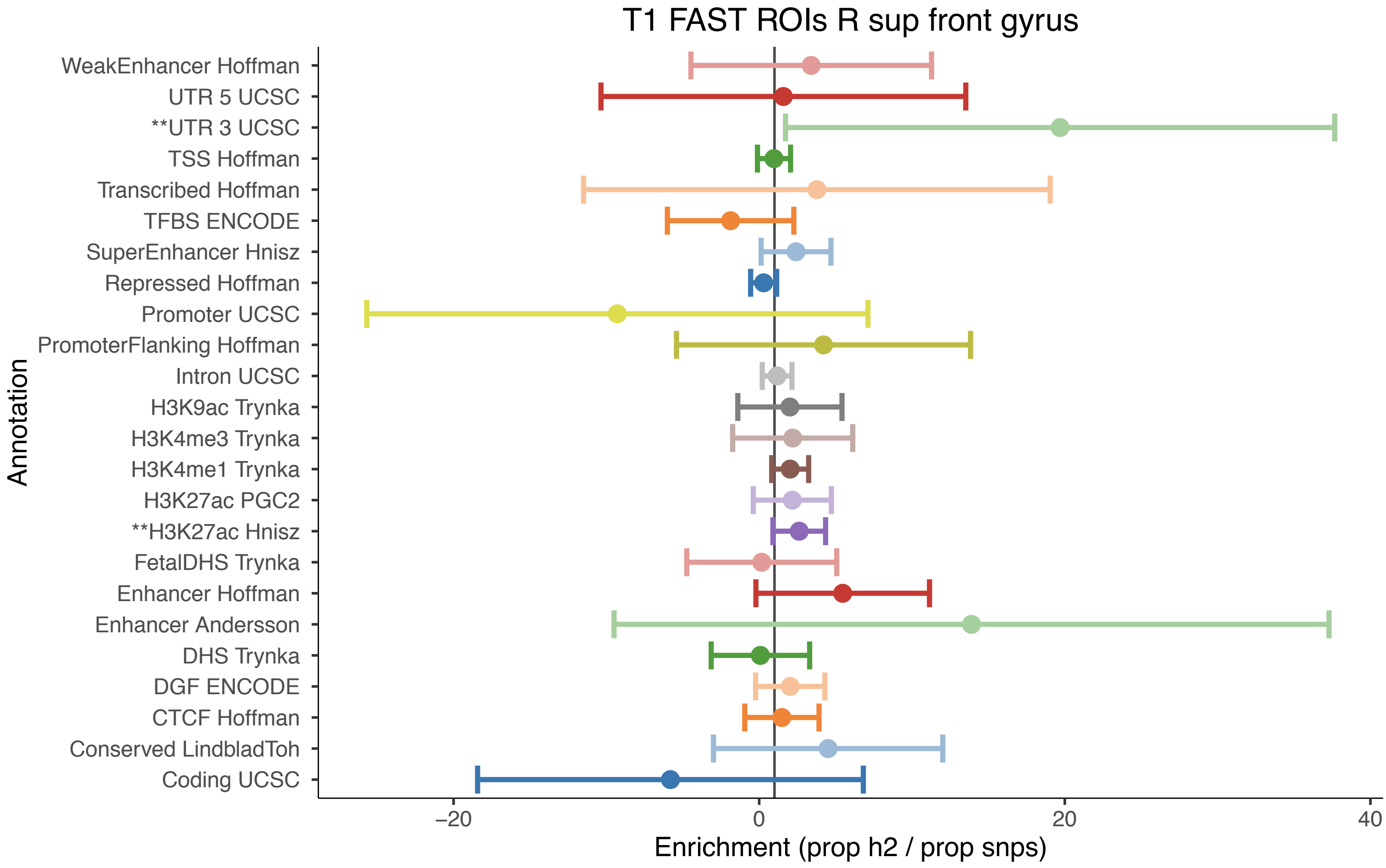
T1 FAST ROIs R sup front gyrus
WeakEnhancer Hoffman
UTR 5 UCSC
**UTR 3 UCSC
TSS Hoffman
Transcribed Hoffman
TFBS ENCODE
SuperEnhancer Hnisz
Repressed Hoffman
Promoter UCSC
PromoterFlanking Hoffman
Intron UCSC
H3K9ac Trynka
H3K4me3 Trynka
H3K4me1 Trynka
H3K27ac PGC2
**H3K27ac Hnisz
FetalDHS Trynka
Enhancer Hoffman
Enhancer Andersson
DHS Trynka
DGF ENCODE
CTCF Hoffman
Conserved LindbladToh
Coding UCSC
Annotation
−20
0
20
40
Enrichment (prop h2 / prop snps)

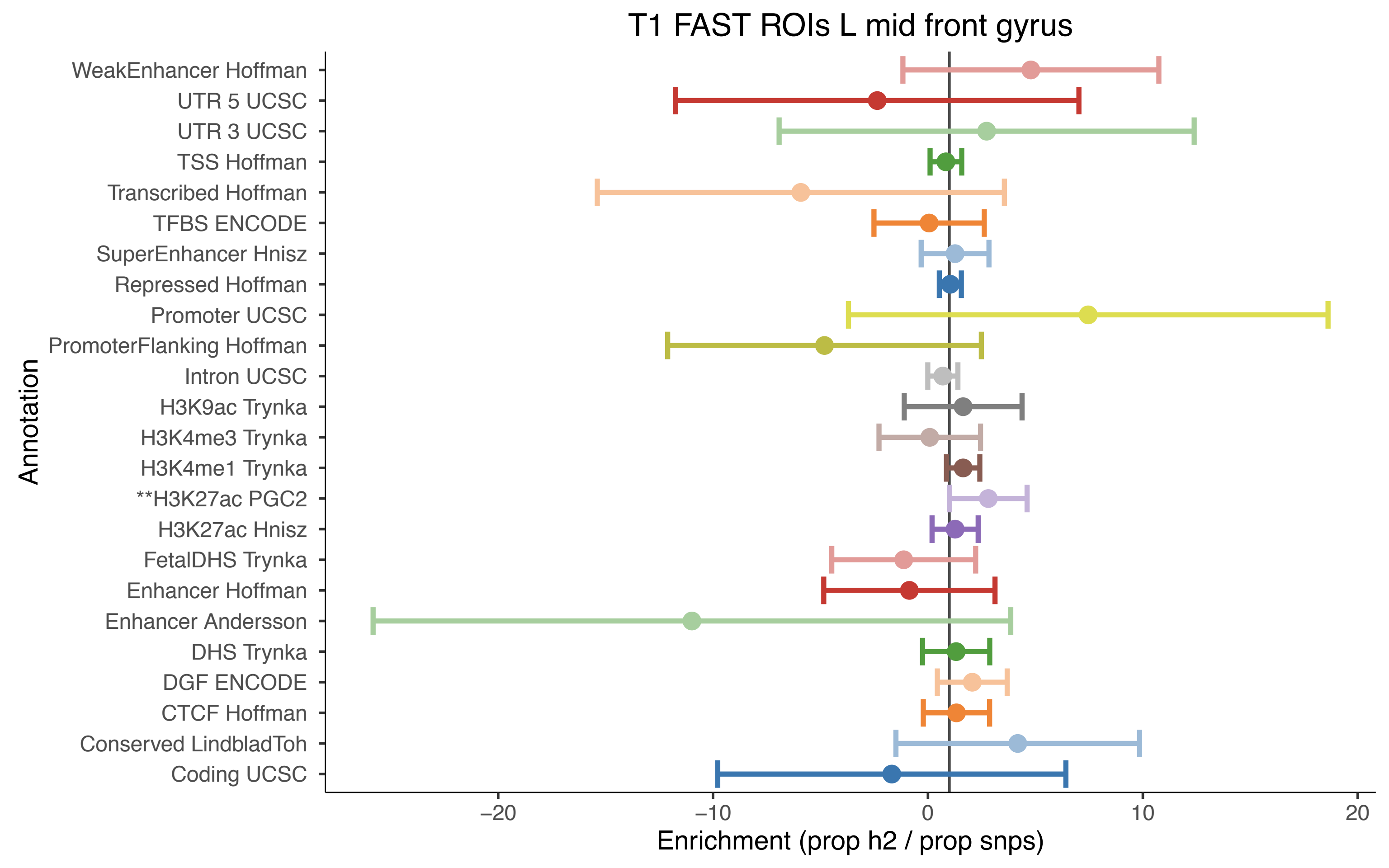
T1 FAST ROIs L mid front gyrus
WeakEnhancer Hoffman
UTR 5 UCSC
UTR 3 UCSC
TSS Hoffman
Transcribed Hoffman
TFBS ENCODE
SuperEnhancer Hnisz
Repressed Hoffman
Promoter UCSC
PromoterFlanking Hoffman
Intron UCSC
H3K9ac Trynka
H3K4me3 Trynka
H3K4me1 Trynka
**H3K27ac PGC2
H3K27ac Hnisz
FetalDHS Trynka
Enhancer Hoffman
Enhancer Andersson
DHS Trynka
DGF ENCODE
CTCF Hoffman
Conserved LindbladToh
Coding UCSC
Annotation
−20
−10
0
10
20
Enrichment (prop h2 / prop snps)

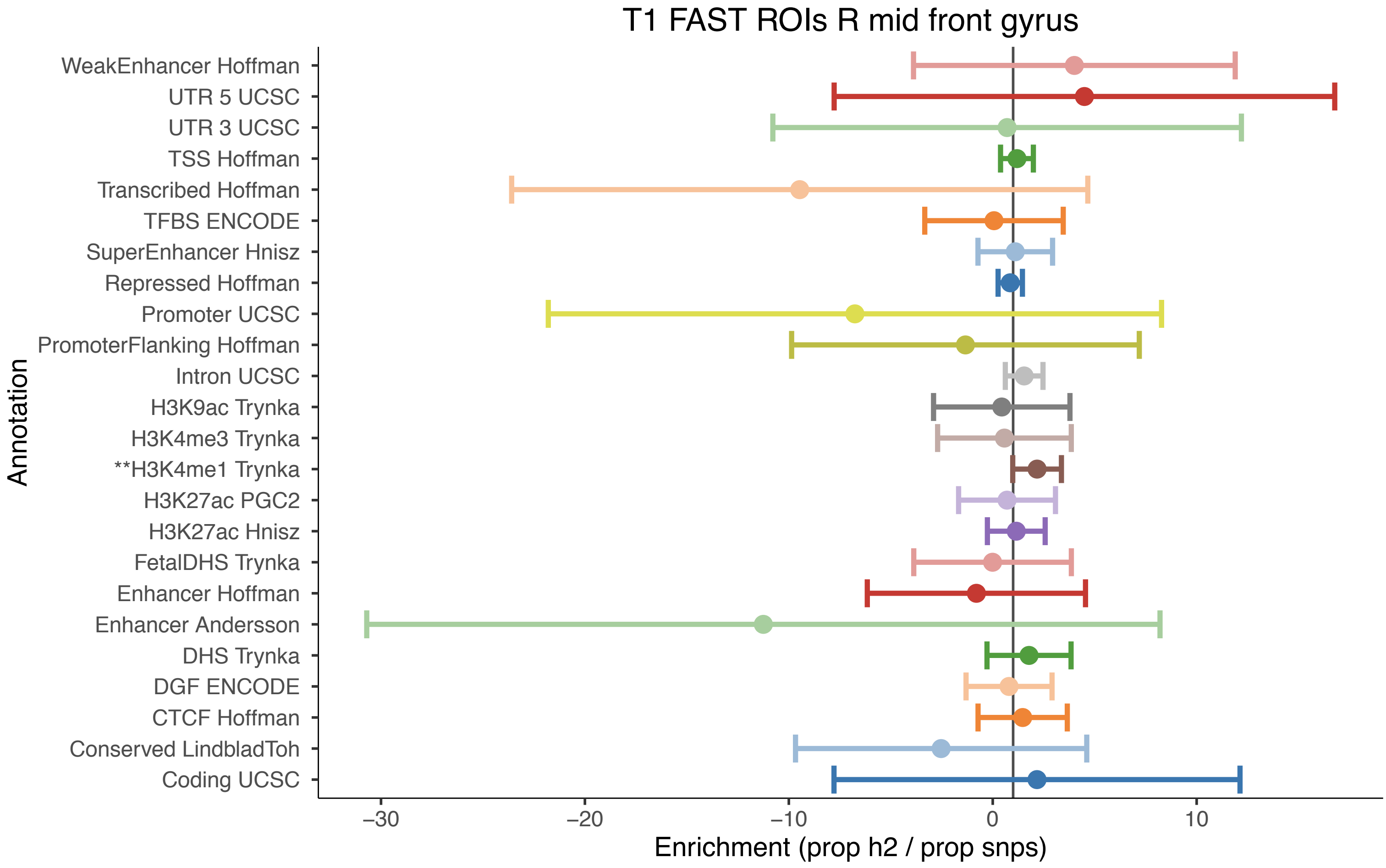

T1 FAST ROIs R mid front gyrus
WeakEnhancer Hoffman
UTR 5 UCSC
UTR 3 UCSC
TSS Hoffman
Transcribed Hoffman
TFBS ENCODE
SuperEnhancer Hnisz
Repressed Hoffman
Promoter UCSC
PromoterFlanking Hoffman
Intron UCSC
H3K9ac Trynka
H3K4me3 Trynka
**H3K4me1 Trynka
H3K27ac PGC2
H3K27ac Hnisz
FetalDHS Trynka
Enhancer Hoffman
Enhancer Andersson
DHS Trynka
DGF ENCODE
CTCF Hoffman
Conserved LindbladToh
Coding UCSC
Annotation
−30
−20
−10
0
10
Enrichment (prop h2 / prop snps)

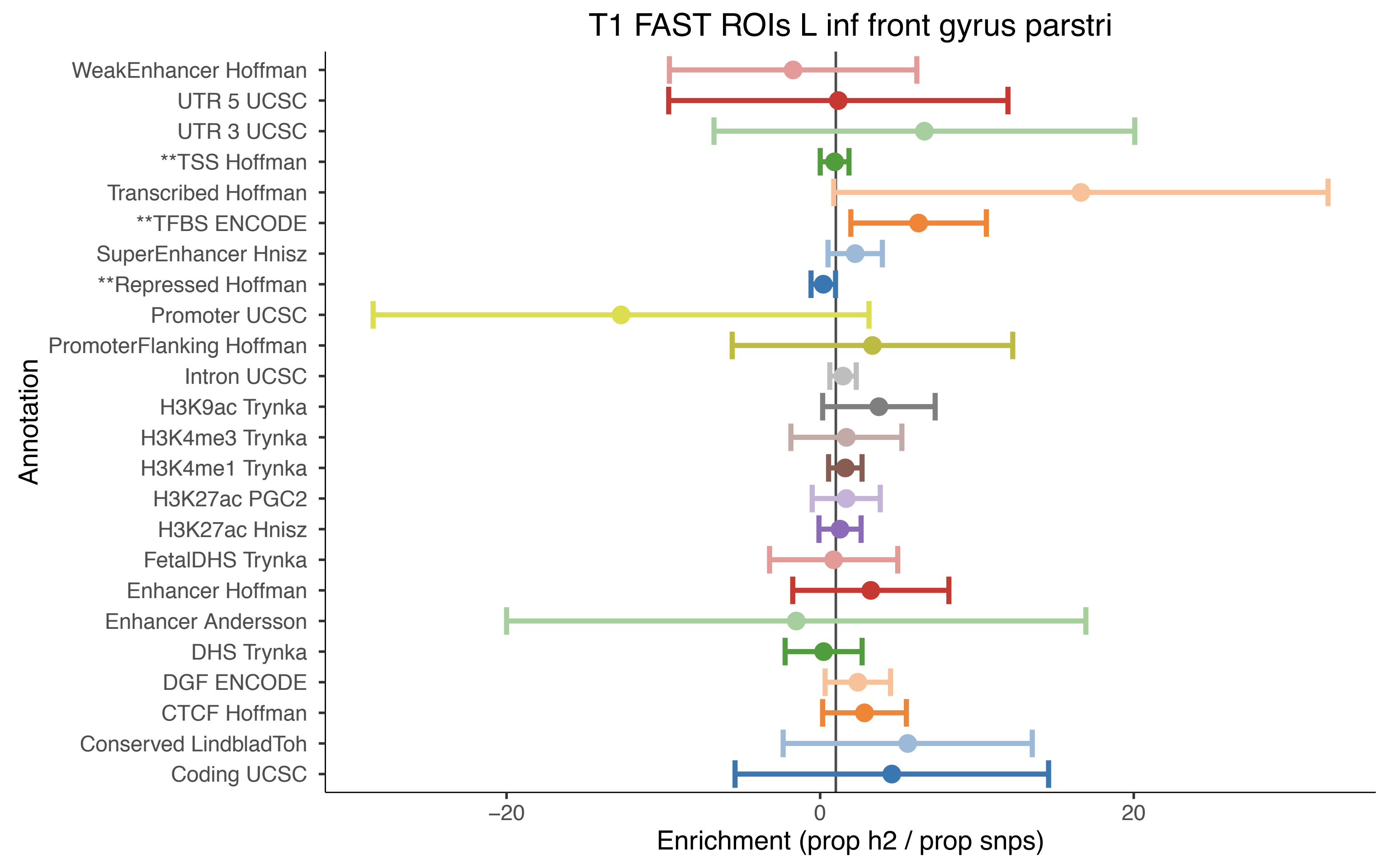
T1 FAST ROIs L inf front gyrus parstri
WeakEnhancer Hoffman
UTR 5 UCSC
UTR 3 UCSC
**TSS Hoffman
Transcribed Hoffman
**TFBS ENCODE
SuperEnhancer Hnisz
**Repressed Hoffman
Promoter UCSC
PromoterFlanking Hoffman
Intron UCSC
H3K9ac Trynka
H3K4me3 Trynka
H3K4me1 Trynka
H3K27ac PGC2
H3K27ac Hnisz
FetalDHS Trynka
Enhancer Hoffman
Enhancer Andersson
DHS Trynka
DGF ENCODE
CTCF Hoffman
Conserved LindbladToh
Coding UCSC
Annotation
−20
0
20
Enrichment (prop h2 / prop snps)

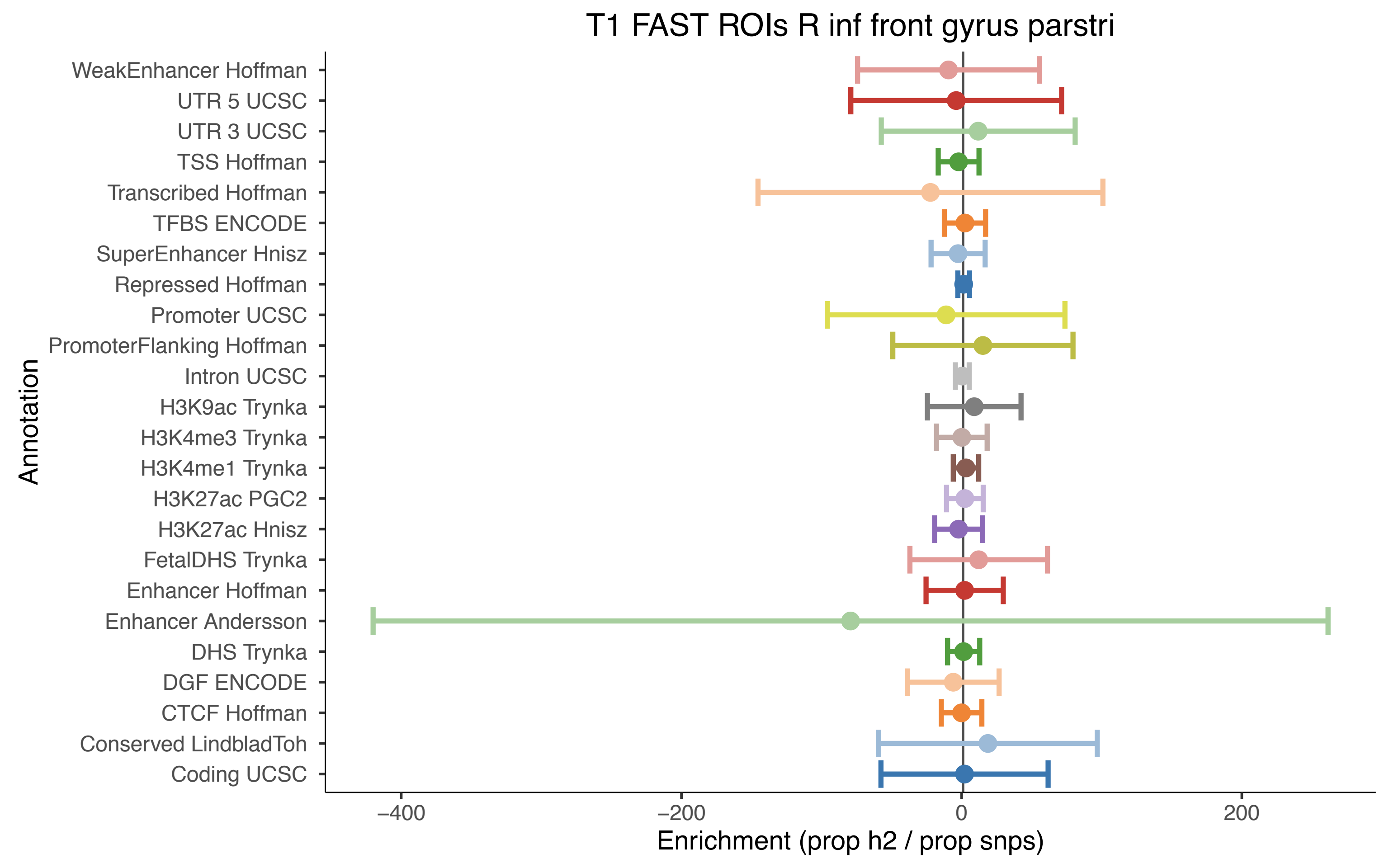
T1 FAST ROIs R inf front gyrus parstri
WeakEnhancer Hoffman
UTR 5 UCSC
UTR 3 UCSC
TSS Hoffman
Transcribed Hoffman
TFBS ENCODE
SuperEnhancer Hnisz
Repressed Hoffman
Promoter UCSC
PromoterFlanking Hoffman
Intron UCSC
H3K9ac Trynka
H3K4me3 Trynka
H3K4me1 Trynka
H3K27ac PGC2
H3K27ac Hnisz
FetalDHS Trynka
Enhancer Hoffman
Enhancer Andersson
DHS Trynka
DGF ENCODE
CTCF Hoffman
Conserved LindbladToh
Coding UCSC
Annotation
−400
−200
0
200
Enrichment (prop h2 / prop snps)

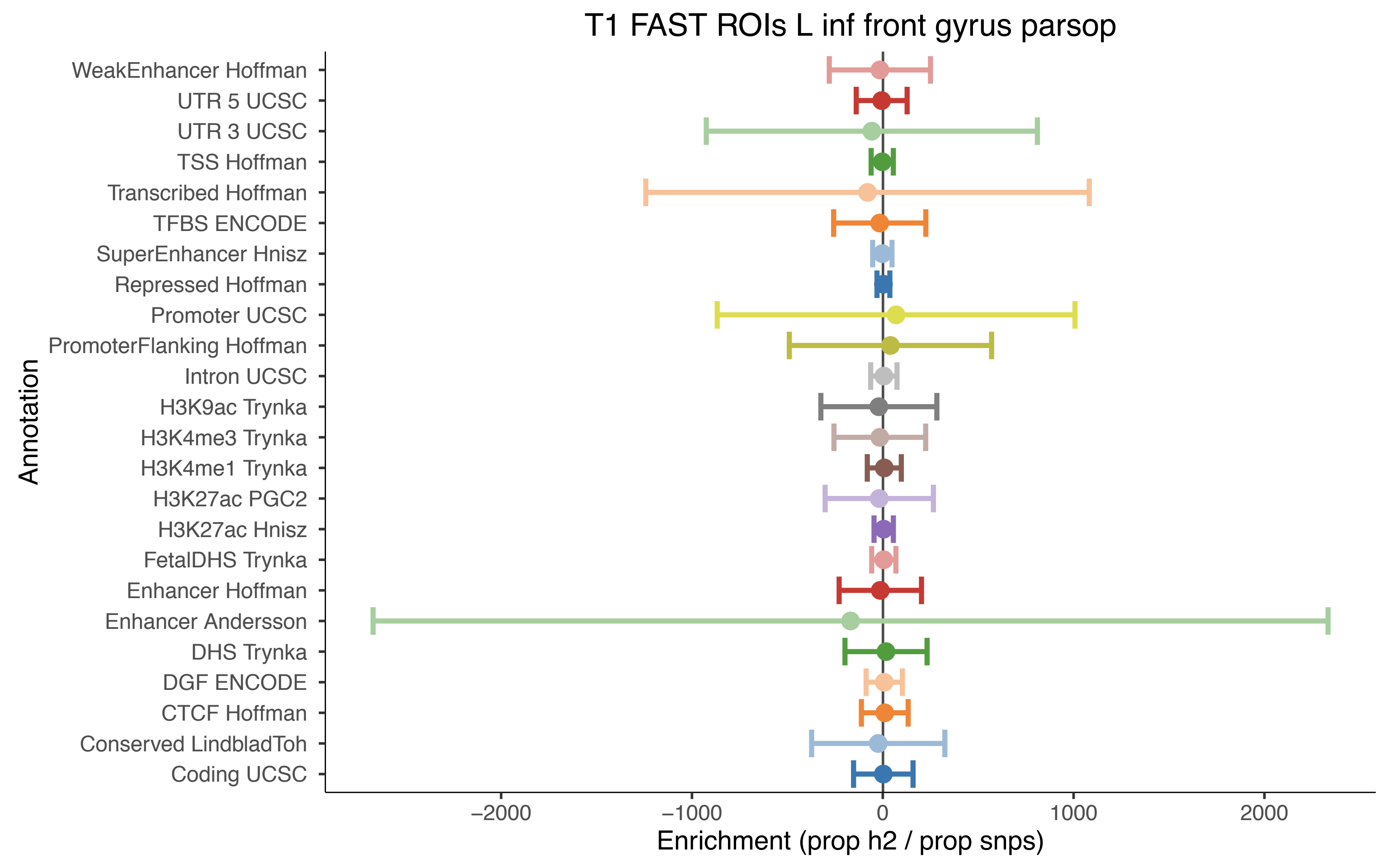
T1 FAST ROIs L inf front gyrus parsop
WeakEnhancer Hoffman
UTR 5 UCSC
UTR 3 UCSC
TSS Hoffman
Transcribed Hoffman
TFBS ENCODE
SuperEnhancer Hnisz
Repressed Hoffman
Promoter UCSC
PromoterFlanking Hoffman
Intron UCSC
H3K9ac Trynka
H3K4me3 Trynka
H3K4me1 Trynka
H3K27ac PGC2
H3K27ac Hnisz
FetalDHS Trynka
Enhancer Hoffman
Enhancer Andersson
DHS Trynka
DGF ENCODE
CTCF Hoffman
Conserved LindbladToh
Coding UCSC
Annotation
−2000
−1000
0
1000
2000
Enrichment (prop h2 / prop snps)

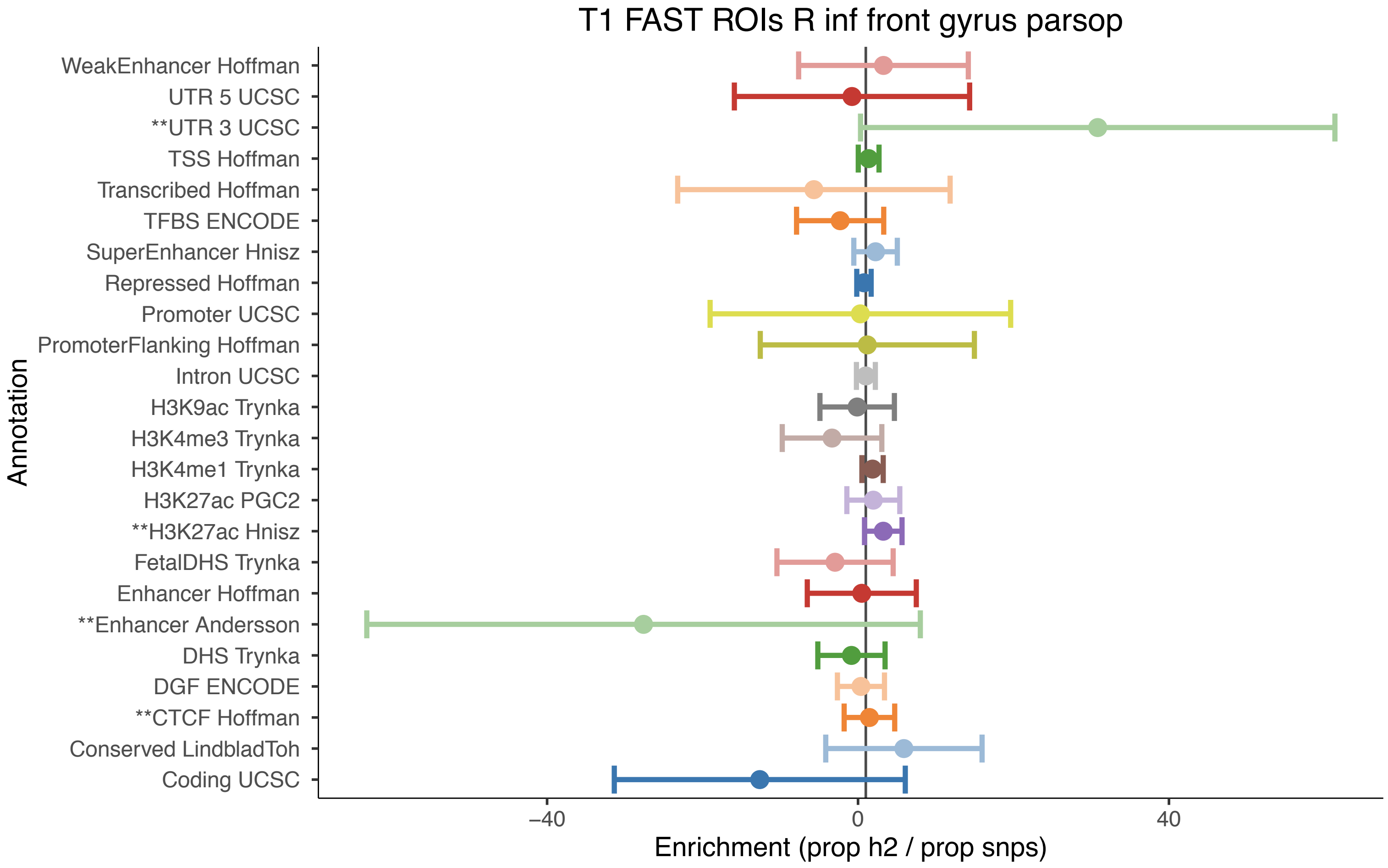

T1 FAST ROIs R inf front gyrus parsop
WeakEnhancer Hoffman
UTR 5 UCSC
**UTR 3 UCSC
TSS Hoffman
Transcribed Hoffman
TFBS ENCODE
SuperEnhancer Hnisz
Repressed Hoffman
Promoter UCSC
PromoterFlanking Hoffman
Intron UCSC
H3K9ac Trynka
H3K4me3 Trynka
H3K4me1 Trynka
H3K27ac PGC2
**H3K27ac Hnisz
FetalDHS Trynka
Enhancer Hoffman
**Enhancer Andersson
DHS Trynka
DGF ENCODE
**CTCF Hoffman
Conserved LindbladToh
Coding UCSC
Annotation
−40
0
40
Enrichment (prop h2 / prop snps)

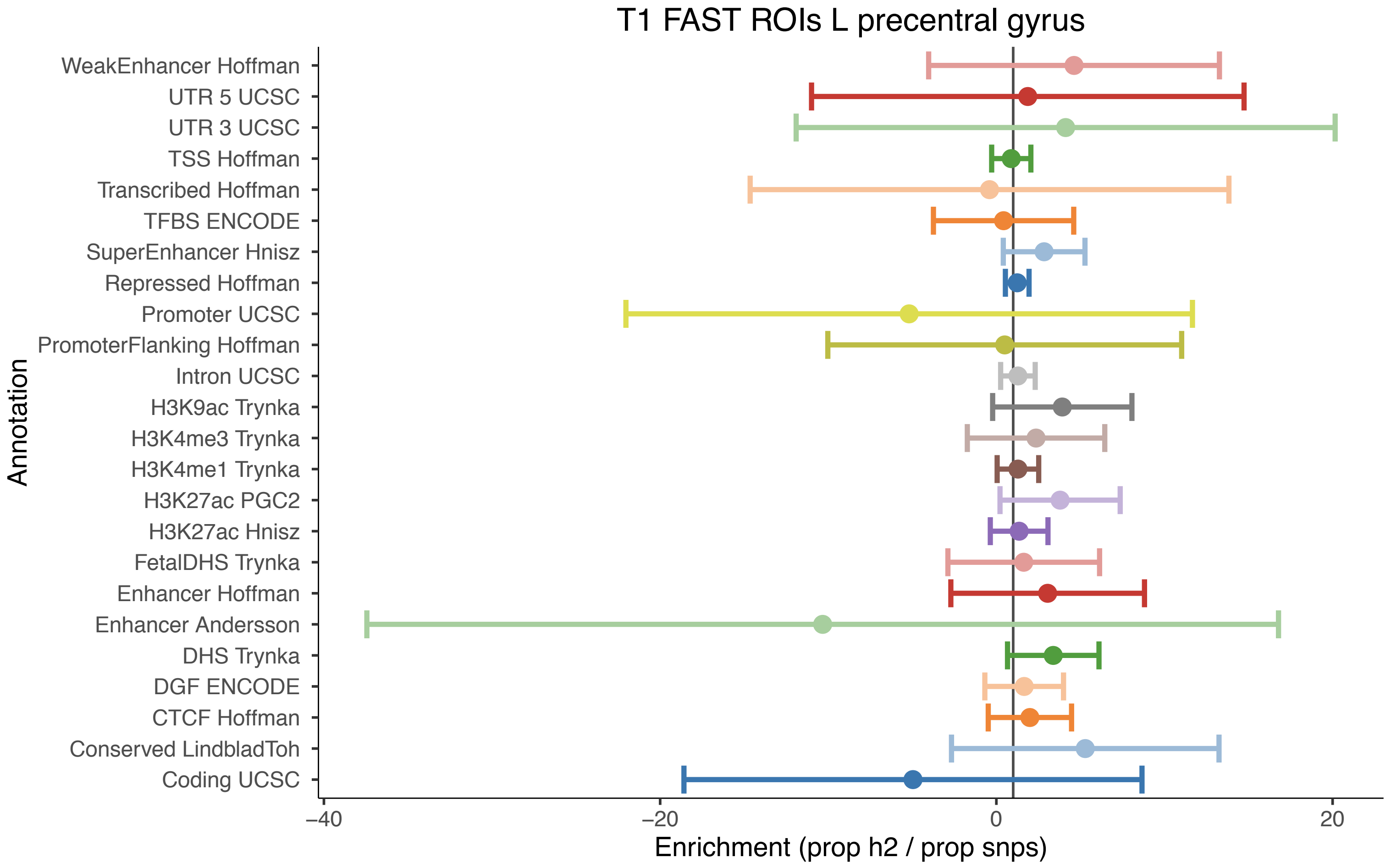

T1 FAST ROIs L precentral gyrus
WeakEnhancer Hoffman
UTR 5 UCSC
UTR 3 UCSC
TSS Hoffman
Transcribed Hoffman
TFBS ENCODE
SuperEnhancer Hnisz
Repressed Hoffman
Promoter UCSC
PromoterFlanking Hoffman
Intron UCSC
H3K9ac Trynka
H3K4me3 Trynka
H3K4me1 Trynka
H3K27ac PGC2
H3K27ac Hnisz
FetalDHS Trynka
Enhancer Hoffman
Enhancer Andersson
DHS Trynka
DGF ENCODE
CTCF Hoffman
Conserved LindbladToh
Coding UCSC
Annotation
−40
−20
0
20
Enrichment (prop h2 / prop snps)

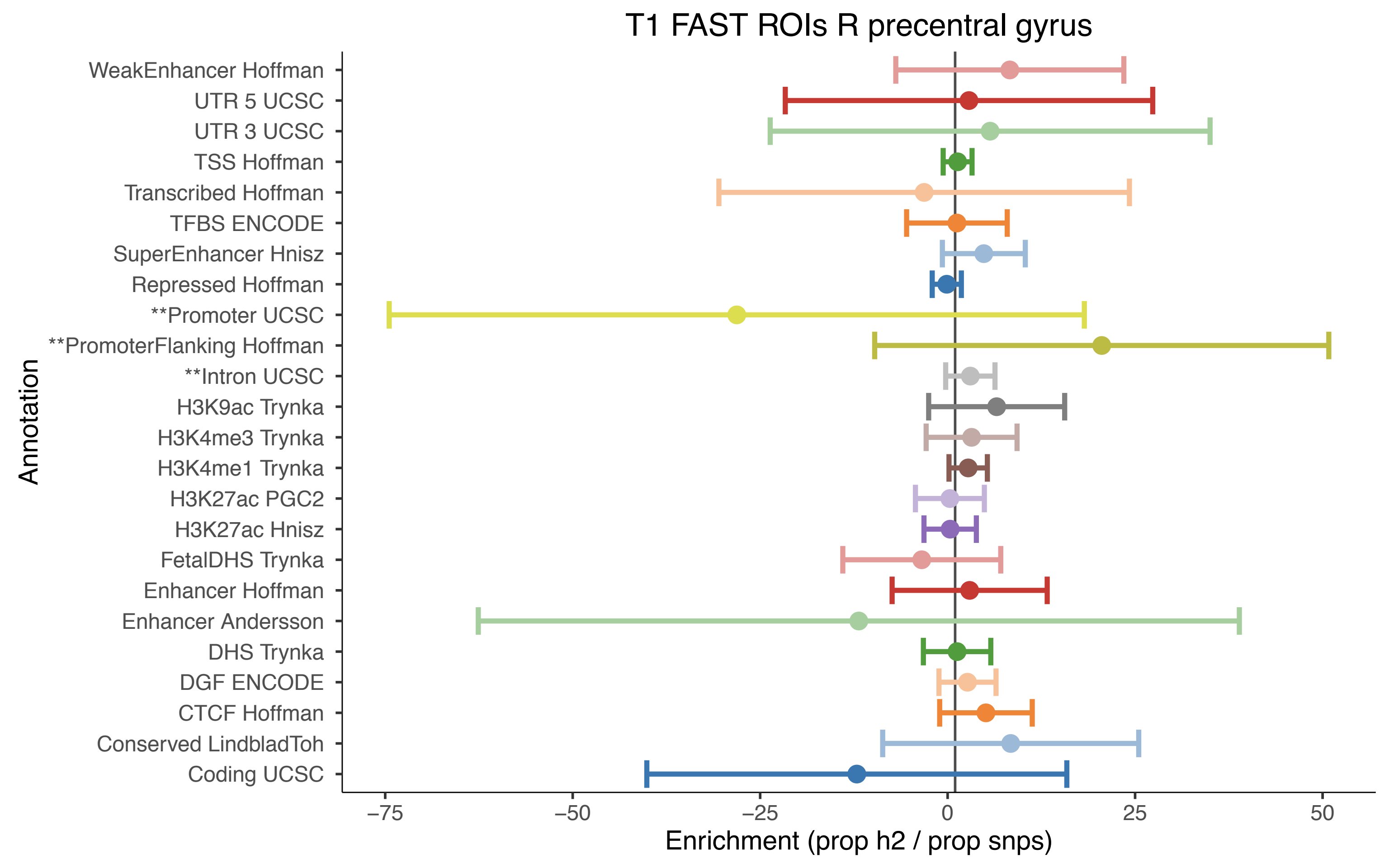
T1 FAST ROIs R precentral gyrus
WeakEnhancer Hoffman
UTR 5 UCSC
UTR 3 UCSC
TSS Hoffman
Transcribed Hoffman
TFBS ENCODE
SuperEnhancer Hnisz
Repressed Hoffman
**Promoter UCSC
**PromoterFlanking Hoffman
**Intron UCSC
H3K9ac Trynka
H3K4me3 Trynka
H3K4me1 Trynka
H3K27ac PGC2
H3K27ac Hnisz
FetalDHS Trynka
Enhancer Hoffman
Enhancer Andersson
DHS Trynka
DGF ENCODE
CTCF Hoffman
Conserved LindbladToh
Coding UCSC
Annotation
−75
−50
−25
0
25
50
Enrichment (prop h2 / prop snps)

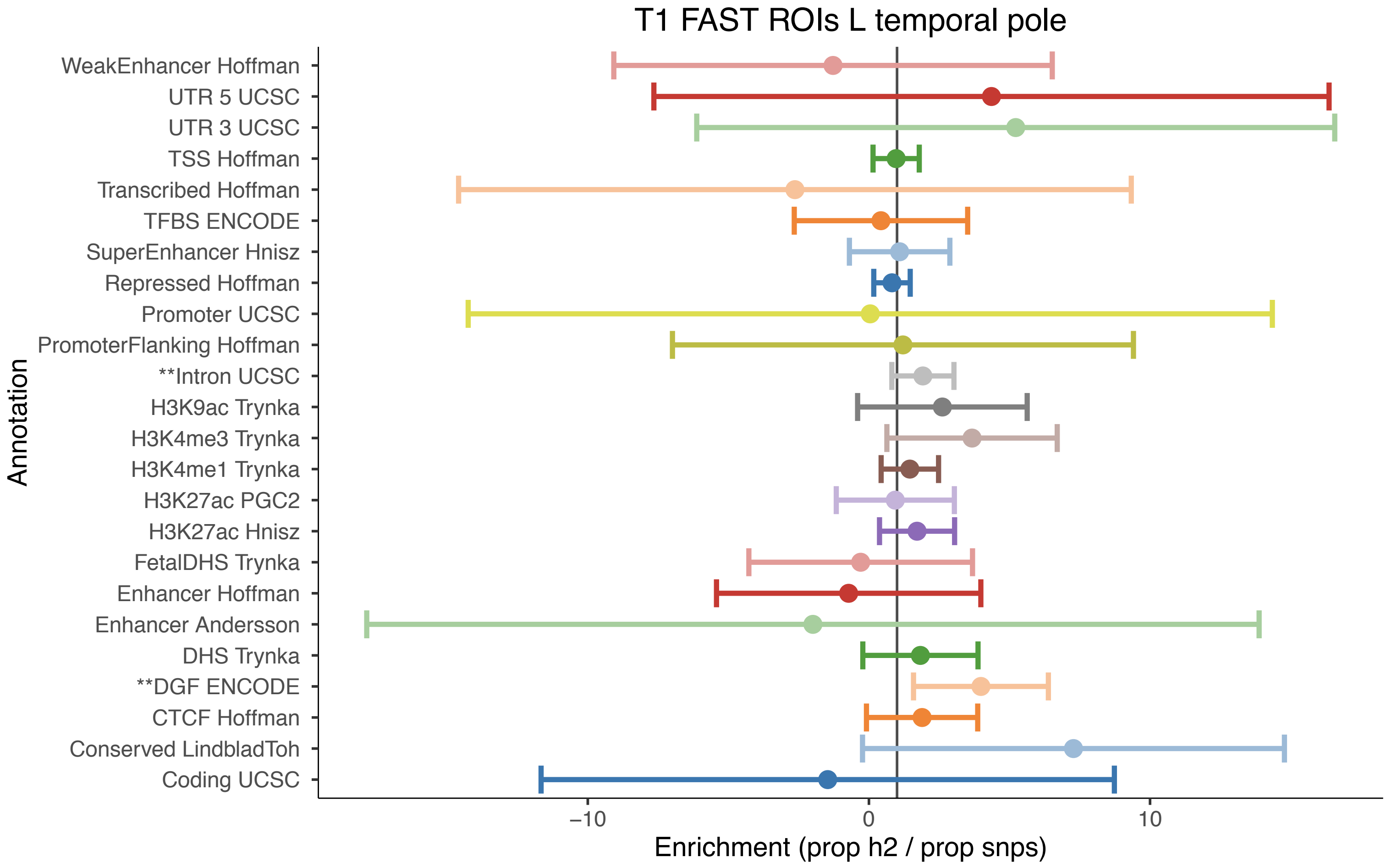

T1 FAST ROIs L temporal pole
WeakEnhancer Hoffman
UTR 5 UCSC
UTR 3 UCSC
TSS Hoffman
Transcribed Hoffman
TFBS ENCODE
SuperEnhancer Hnisz
Repressed Hoffman
Promoter UCSC
PromoterFlanking Hoffman
**Intron UCSC
H3K9ac Trynka
H3K4me3 Trynka
H3K4me1 Trynka
H3K27ac PGC2
H3K27ac Hnisz
FetalDHS Trynka
Enhancer Hoffman
Enhancer Andersson
DHS Trynka
**DGF ENCODE
CTCF Hoffman
Conserved LindbladToh
Coding UCSC
Annotation
−10
0
10
Enrichment (prop h2 / prop snps)

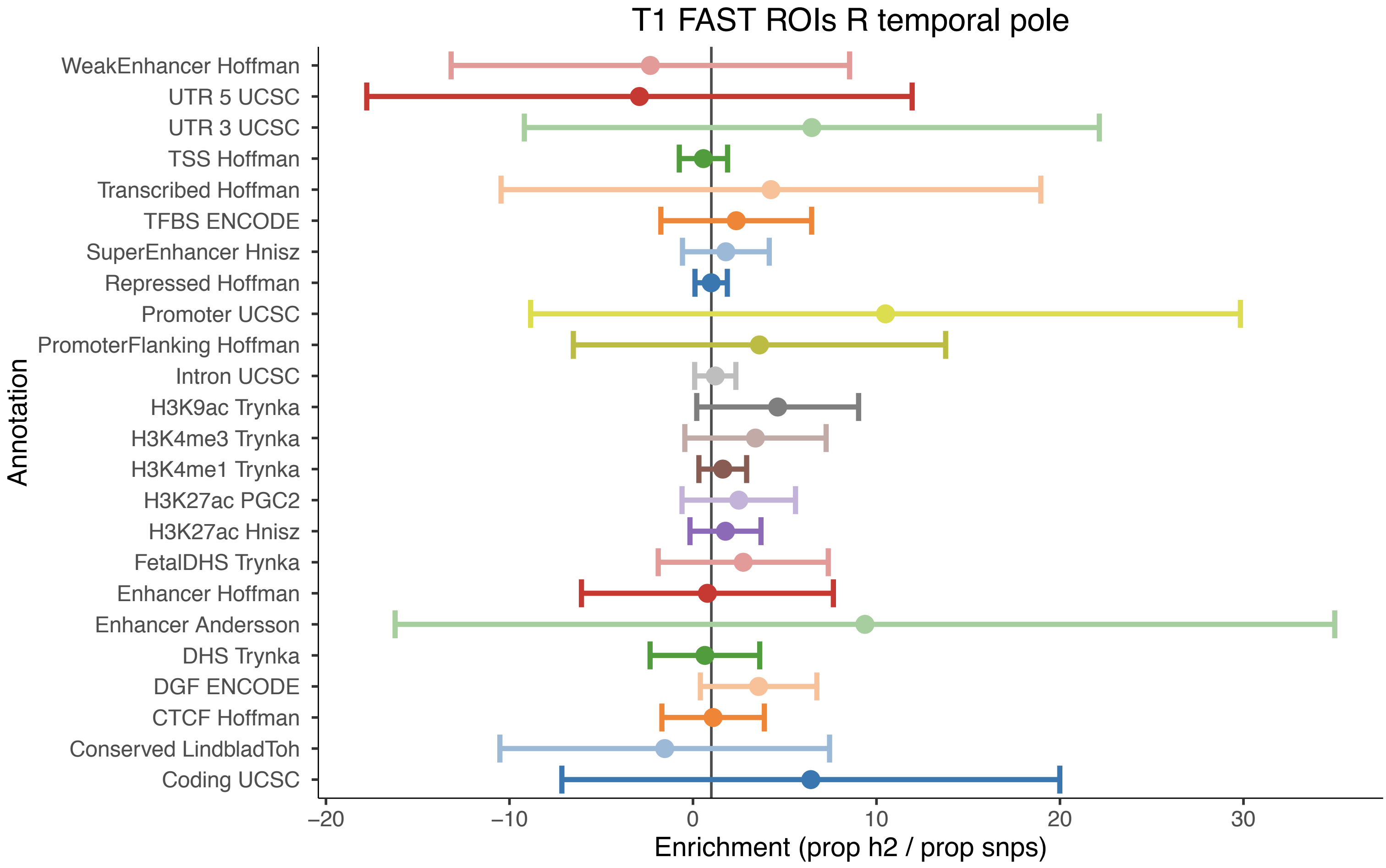
T1 FAST ROIs R temporal pole
WeakEnhancer Hoffman
UTR 5 UCSC
UTR 3 UCSC
TSS Hoffman
Transcribed Hoffman
TFBS ENCODE
SuperEnhancer Hnisz
Repressed Hoffman
Promoter UCSC
PromoterFlanking Hoffman
Intron UCSC
H3K9ac Trynka
H3K4me3 Trynka
H3K4me1 Trynka
H3K27ac PGC2
H3K27ac Hnisz
FetalDHS Trynka
Enhancer Hoffman
Enhancer Andersson
DHS Trynka
DGF ENCODE
CTCF Hoffman
Conserved LindbladToh
Coding UCSC
Annotation
−20
−10
0
10
20
30
Enrichment (prop h2 / prop snps)

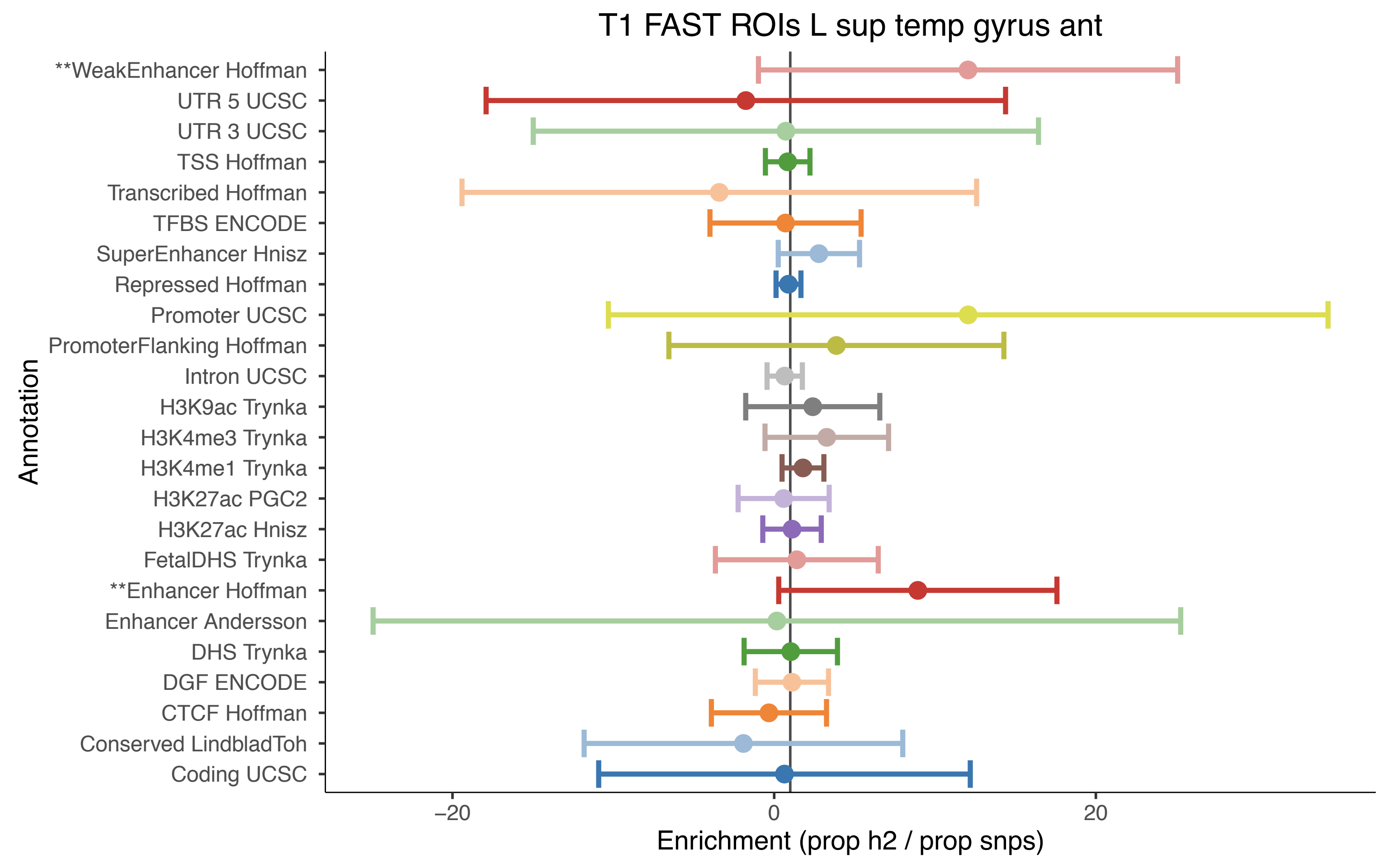
T1 FAST ROIs L sup temp gyrus ant
**WeakEnhancer Hoffman
UTR 5 UCSC
UTR 3 UCSC
TSS Hoffman
Transcribed Hoffman
TFBS ENCODE
SuperEnhancer Hnisz
Repressed Hoffman
Promoter UCSC
PromoterFlanking Hoffman
Intron UCSC
H3K9ac Trynka
H3K4me3 Trynka
H3K4me1 Trynka
H3K27ac PGC2
H3K27ac Hnisz
FetalDHS Trynka
**Enhancer Hoffman
Enhancer Andersson
DHS Trynka
DGF ENCODE
CTCF Hoffman
Conserved LindbladToh
Coding UCSC
Annotation
−20
0
20
Enrichment (prop h2 / prop snps)

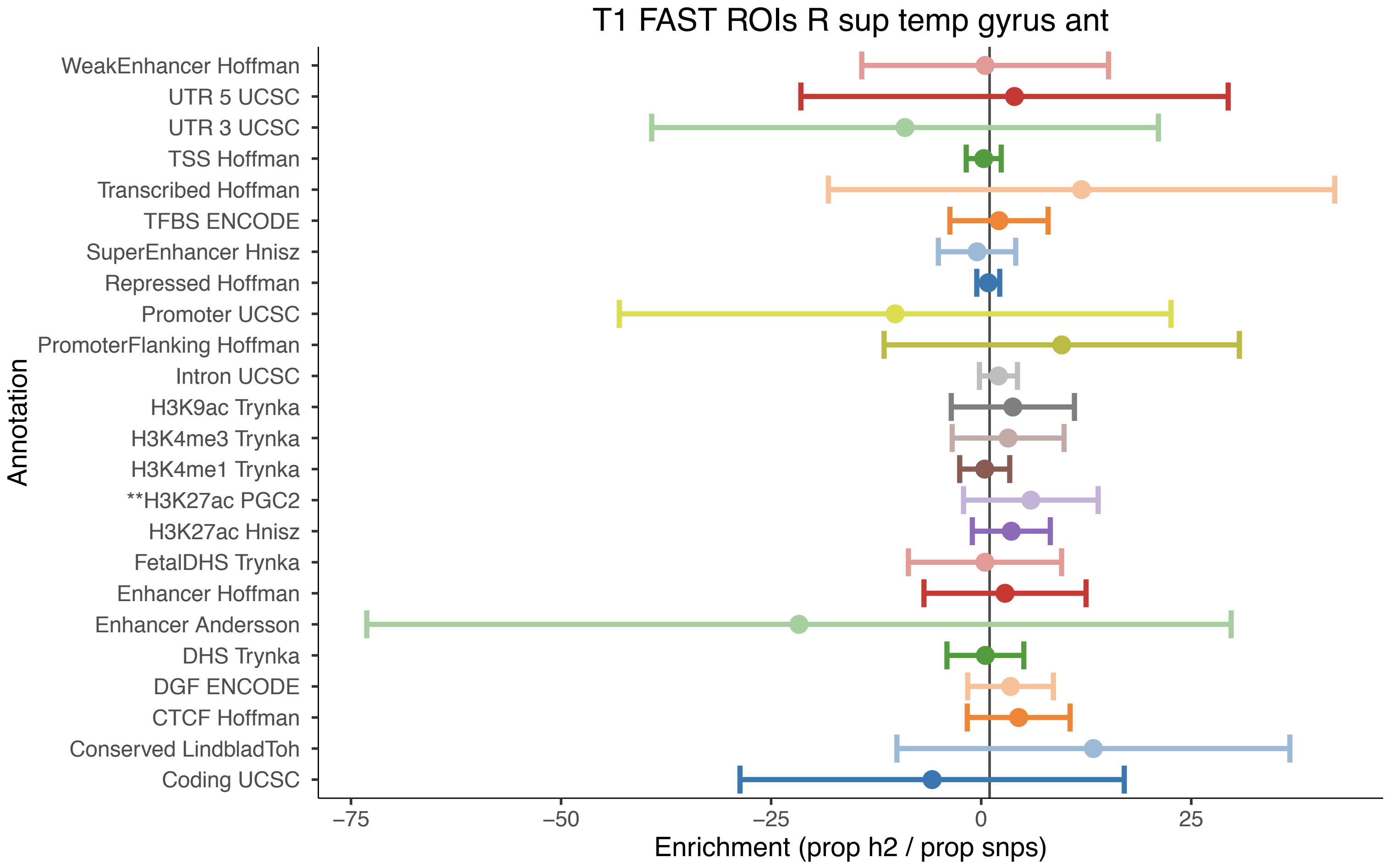

T1 FAST ROIs R sup temp gyrus ant
WeakEnhancer Hoffman
UTR 5 UCSC
UTR 3 UCSC
TSS Hoffman
Transcribed Hoffman
TFBS ENCODE
SuperEnhancer Hnisz
Repressed Hoffman
Promoter UCSC
PromoterFlanking Hoffman
Intron UCSC
H3K9ac Trynka
H3K4me3 Trynka
H3K4me1 Trynka
**H3K27ac PGC2
H3K27ac Hnisz
FetalDHS Trynka
Enhancer Hoffman
Enhancer Andersson
DHS Trynka
DGF ENCODE
CTCF Hoffman
Conserved LindbladToh
Coding UCSC
Annotation
−75
−50
−25
0
25
Enrichment (prop h2 / prop snps)

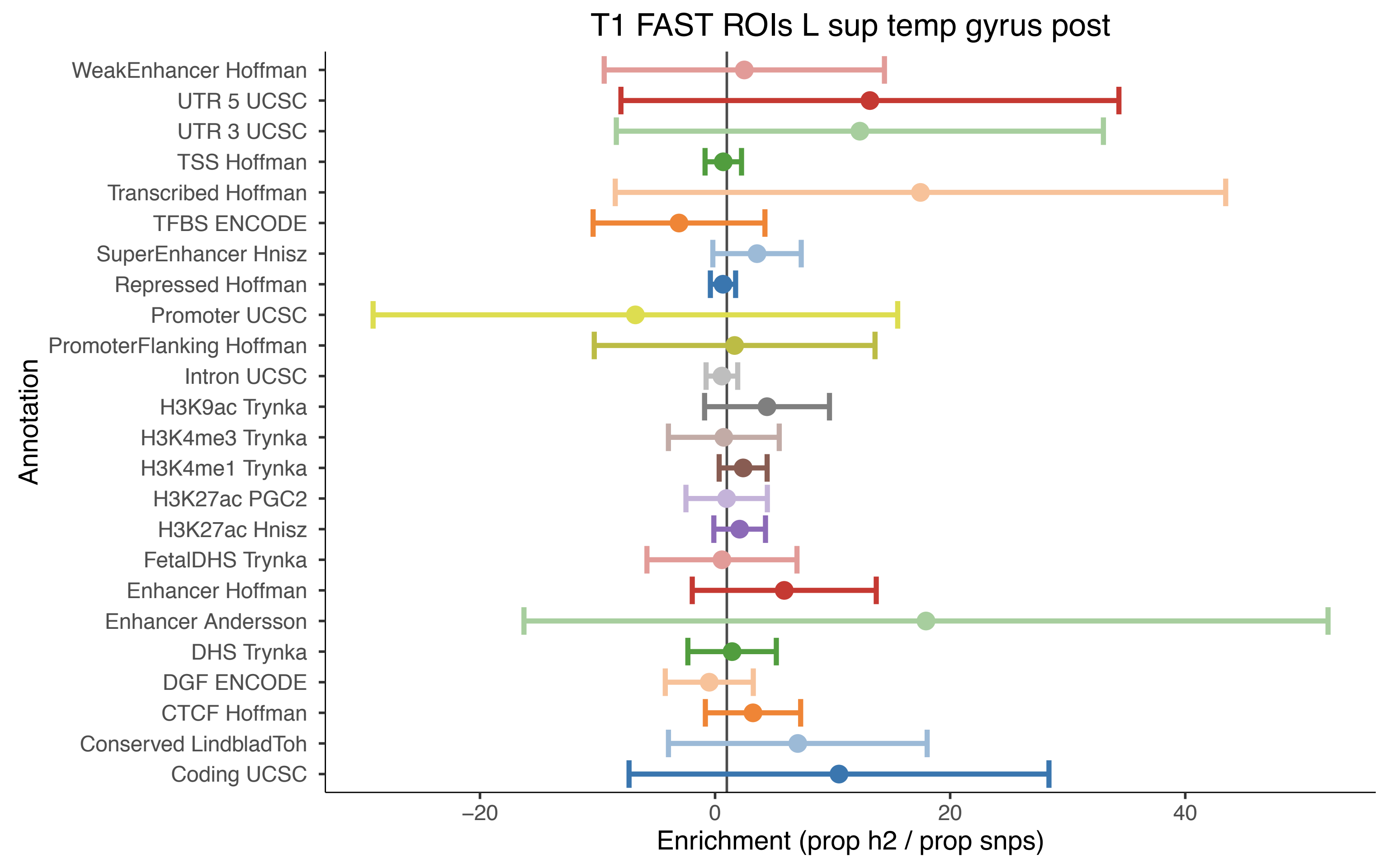
T1 FAST ROIs L sup temp gyrus post
WeakEnhancer Hoffman
UTR 5 UCSC
UTR 3 UCSC
TSS Hoffman
Transcribed Hoffman
TFBS ENCODE
SuperEnhancer Hnisz
Repressed Hoffman
Promoter UCSC
PromoterFlanking Hoffman
Intron UCSC
H3K9ac Trynka
H3K4me3 Trynka
H3K4me1 Trynka
H3K27ac PGC2
H3K27ac Hnisz
FetalDHS Trynka
Enhancer Hoffman
Enhancer Andersson
DHS Trynka
DGF ENCODE
CTCF Hoffman
Conserved LindbladToh
Coding UCSC
Annotation
−20
0
20
40
Enrichment (prop h2 / prop snps)

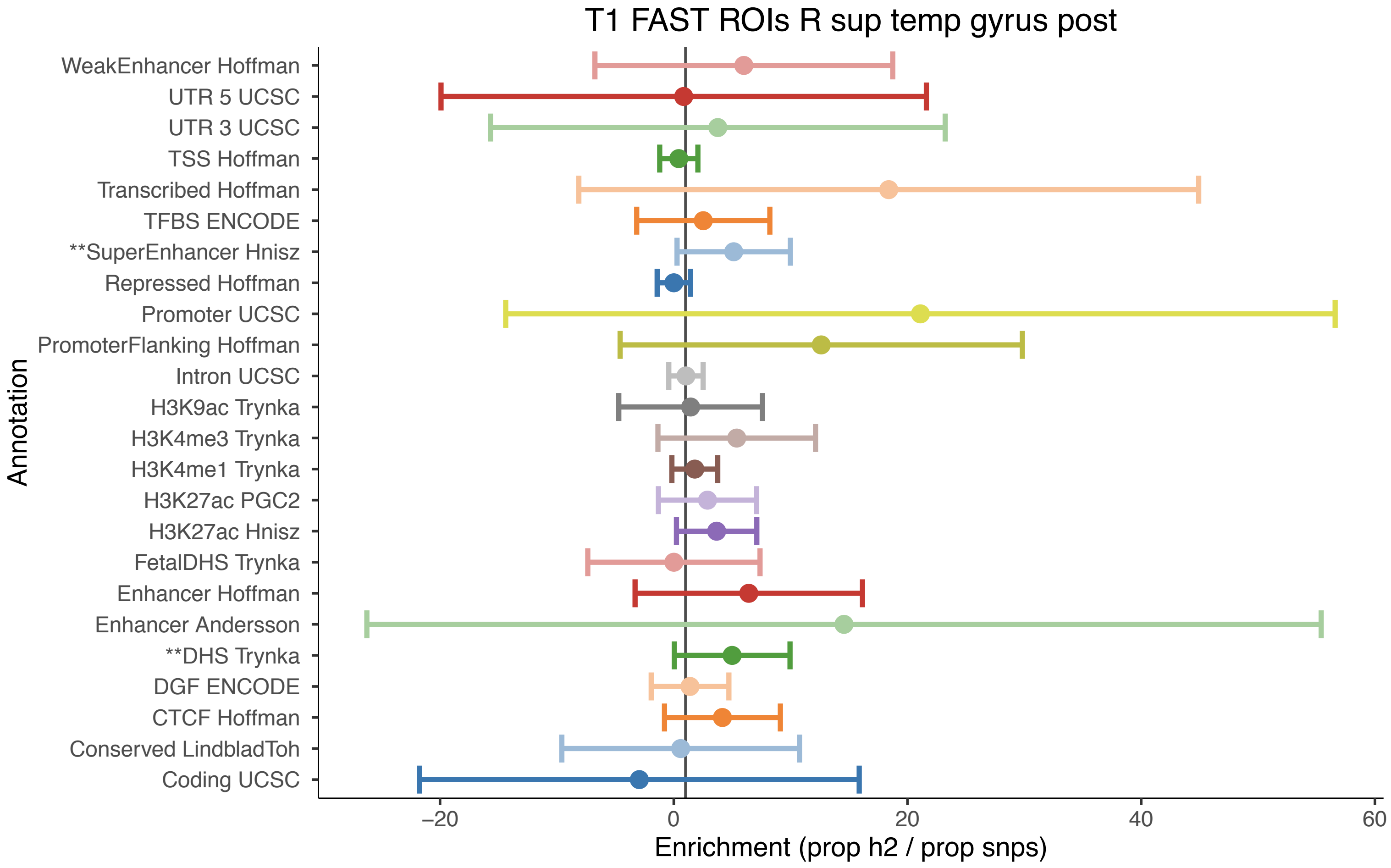

T1 FAST ROIs R sup temp gyrus post
WeakEnhancer Hoffman
UTR 5 UCSC
UTR 3 UCSC
TSS Hoffman
Transcribed Hoffman
TFBS ENCODE
**SuperEnhancer Hnisz
Repressed Hoffman
Promoter UCSC
PromoterFlanking Hoffman
Intron UCSC
H3K9ac Trynka
H3K4me3 Trynka
H3K4me1 Trynka
H3K27ac PGC2
H3K27ac Hnisz
FetalDHS Trynka
Enhancer Hoffman
Enhancer Andersson
**DHS Trynka
DGF ENCODE
CTCF Hoffman
Conserved LindbladToh
Coding UCSC
Annotation
−20
0
20
40
60
Enrichment (prop h2 / prop snps)

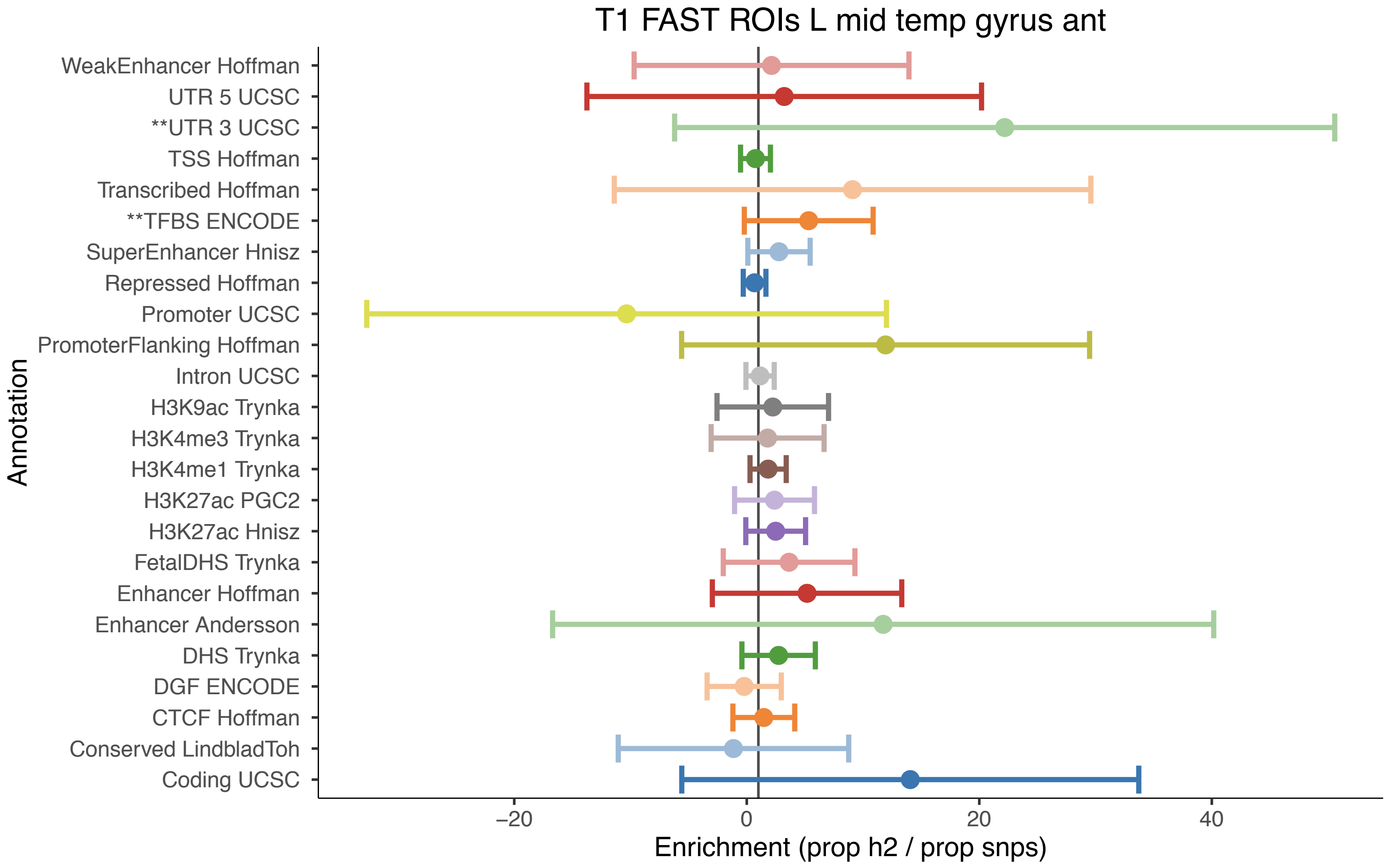

T1 FAST ROIs L mid temp gyrus ant
WeakEnhancer Hoffman
UTR 5 UCSC
**UTR 3 UCSC
TSS Hoffman
Transcribed Hoffman
**TFBS ENCODE
SuperEnhancer Hnisz
Repressed Hoffman
Promoter UCSC
PromoterFlanking Hoffman
Intron UCSC
H3K9ac Trynka
H3K4me3 Trynka
H3K4me1 Trynka
H3K27ac PGC2
H3K27ac Hnisz
FetalDHS Trynka
Enhancer Hoffman
Enhancer Andersson
DHS Trynka
DGF ENCODE
CTCF Hoffman
Conserved LindbladToh
Coding UCSC
Annotation
−20
0
20
40
Enrichment (prop h2 / prop snps)

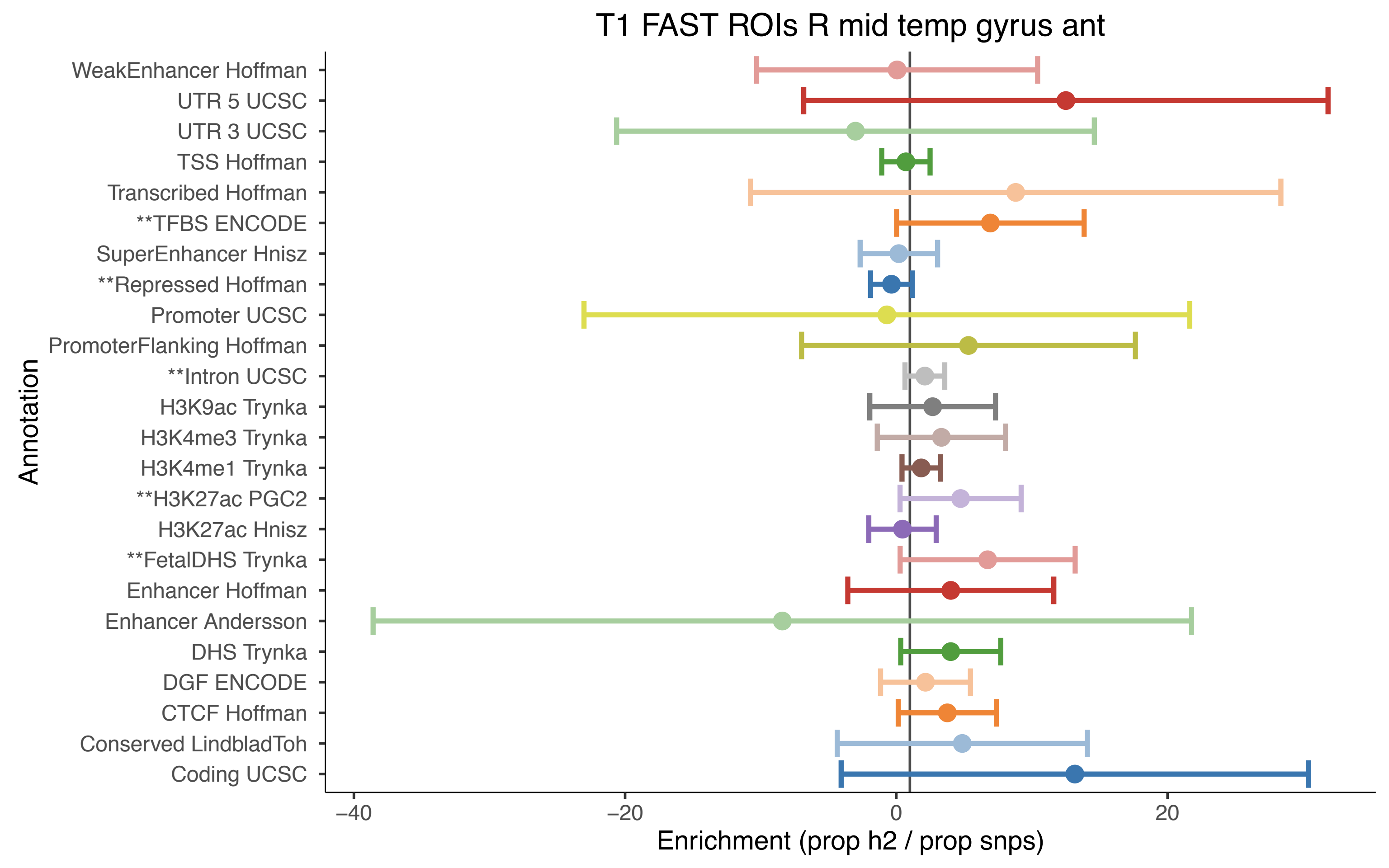
T1 FAST ROIs R mid temp gyrus ant
WeakEnhancer Hoffman
UTR 5 UCSC
UTR 3 UCSC
TSS Hoffman
Transcribed Hoffman
**TFBS ENCODE
SuperEnhancer Hnisz
**Repressed Hoffman
Promoter UCSC
PromoterFlanking Hoffman
**Intron UCSC
H3K9ac Trynka
H3K4me3 Trynka
H3K4me1 Trynka
**H3K27ac PGC2
H3K27ac Hnisz
**FetalDHS Trynka
Enhancer Hoffman
Enhancer Andersson
DHS Trynka
DGF ENCODE
CTCF Hoffman
Conserved LindbladToh
Coding UCSC
Annotation
−40
−20
0
20
Enrichment (prop h2 / prop snps)

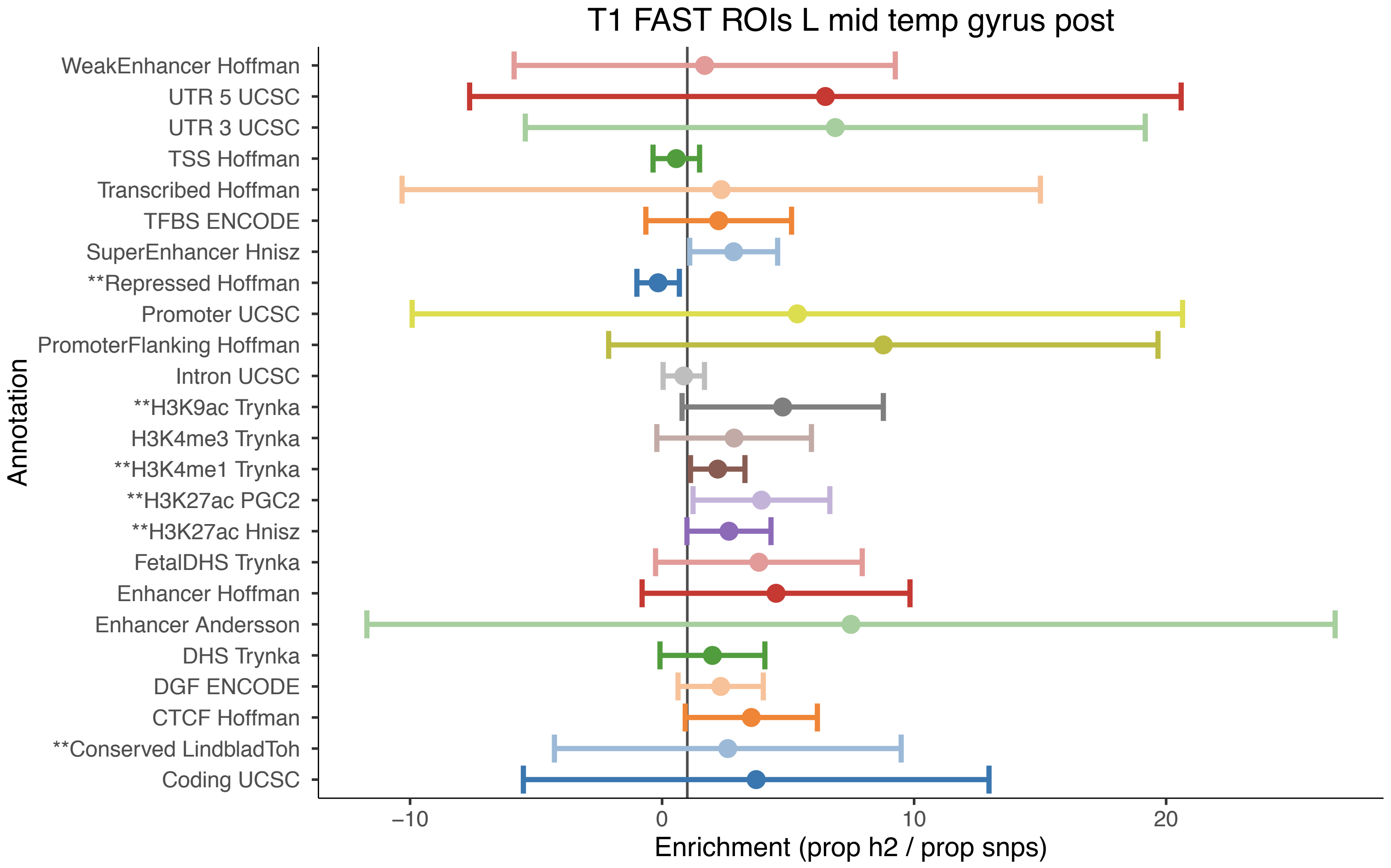

T1 FAST ROIs L mid temp gyrus post
WeakEnhancer Hoffman
UTR 5 UCSC
UTR 3 UCSC
TSS Hoffman
Transcribed Hoffman
TFBS ENCODE
SuperEnhancer Hnisz
**Repressed Hoffman
Promoter UCSC
PromoterFlanking Hoffman
Intron UCSC
**H3K9ac Trynka
H3K4me3 Trynka
**H3K4me1 Trynka
**H3K27ac PGC2
**H3K27ac Hnisz
FetalDHS Trynka
Enhancer Hoffman
Enhancer Andersson
DHS Trynka
DGF ENCODE
CTCF Hoffman
**Conserved LindbladToh
Coding UCSC
Annotation
−10
0
10
20
Enrichment (prop h2 / prop snps)

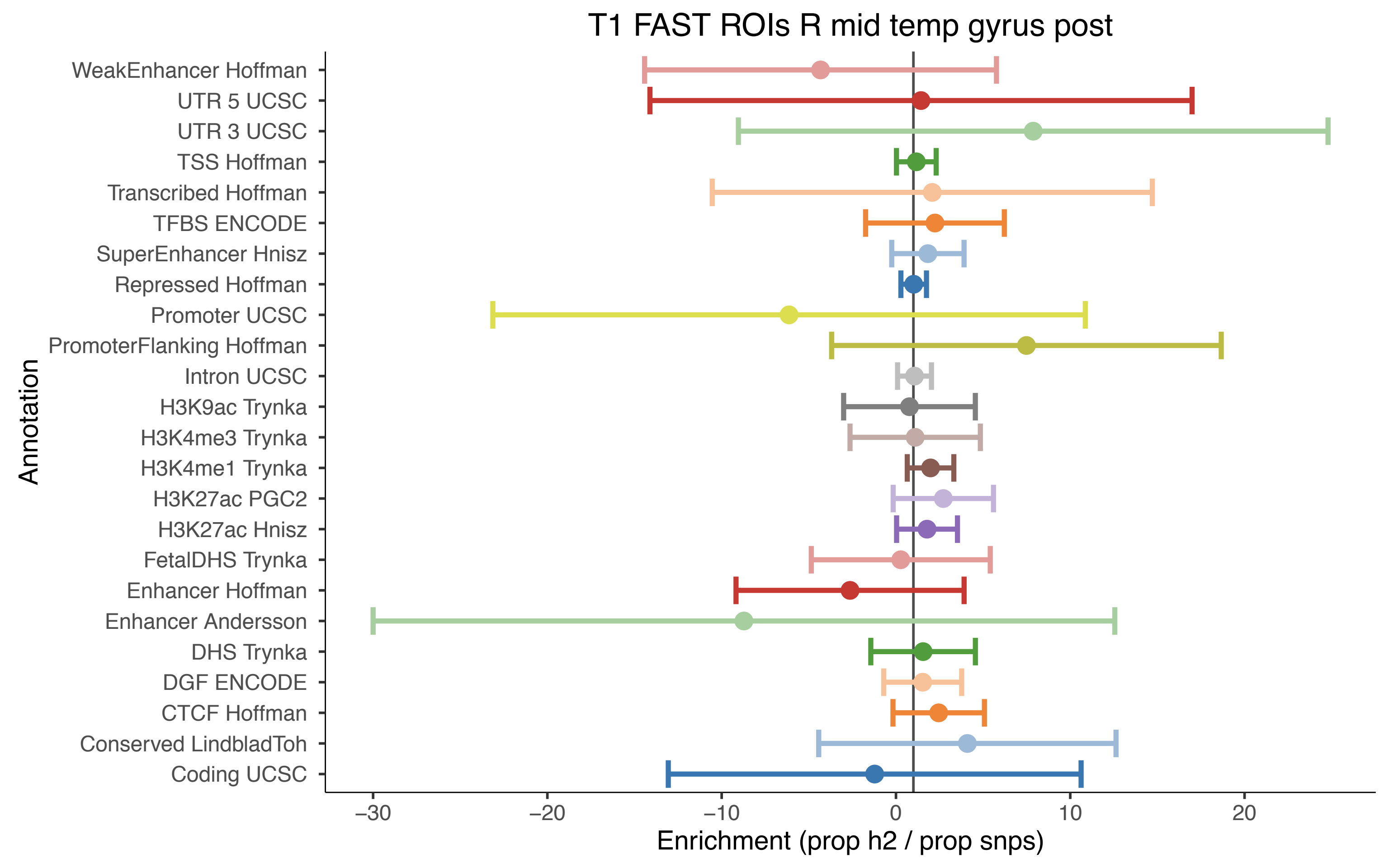
T1 FAST ROIs R mid temp gyrus post
Annotation
WeakEnhancer Hoffman
UTR 5 UCSC
UTR 3 UCSC
TSS Hoffman
Transcribed Hoffman
TFBS ENCODE
SuperEnhancer Hnisz
Repressed Hoffman
Promoter UCSC
PromoterFlanking Hoffman
Intron UCSC
H3K9ac Trynka
H3K4me3 Trynka
H3K4me1 Trynka
H3K27ac PGC2
H3K27ac Hnisz
FetalDHS Trynka
Enhancer Hoffman
Enhancer Andersson
DHS Trynka
DGF ENCODE
CTCF Hoffman
Conserved LindbladToh
Coding UCSC
−30
−20
−10
0
10
20
Enrichment (prop h2 / prop snps)

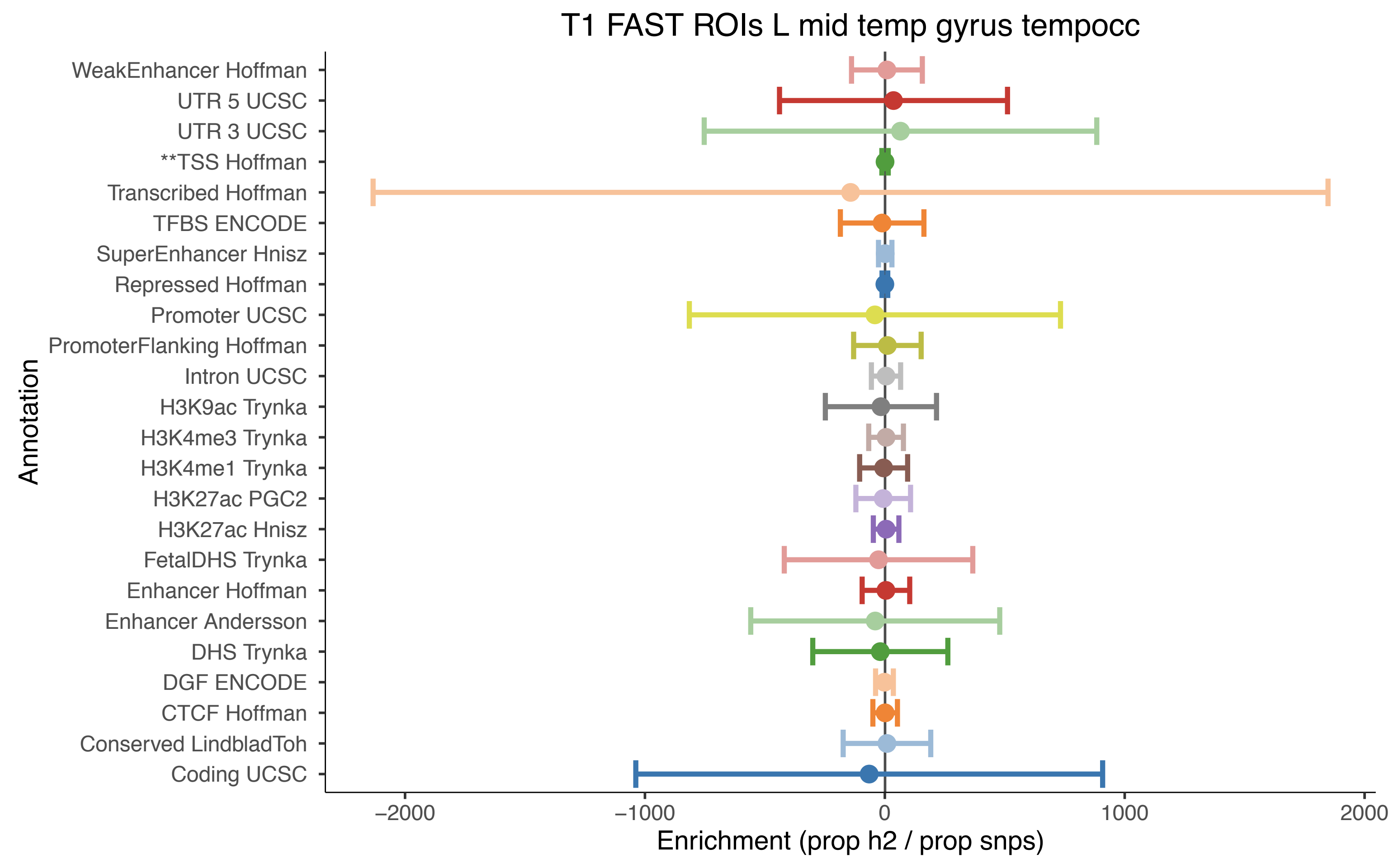
T1 FAST ROIs L mid temp gyrus tempocc
WeakEnhancer Hoffman
UTR 5 UCSC
UTR 3 UCSC
**TSS Hoffman
Transcribed Hoffman
TFBS ENCODE
SuperEnhancer Hnisz
Repressed Hoffman
Promoter UCSC
PromoterFlanking Hoffman
Intron UCSC
H3K9ac Trynka
H3K4me3 Trynka
H3K4me1 Trynka
H3K27ac PGC2
H3K27ac Hnisz
FetalDHS Trynka
Enhancer Hoffman
Enhancer Andersson
DHS Trynka
DGF ENCODE
CTCF Hoffman
Conserved LindbladToh
Coding UCSC
Annotation
−2000
−1000
0
1000
2000
Enrichment (prop h2 / prop snps)

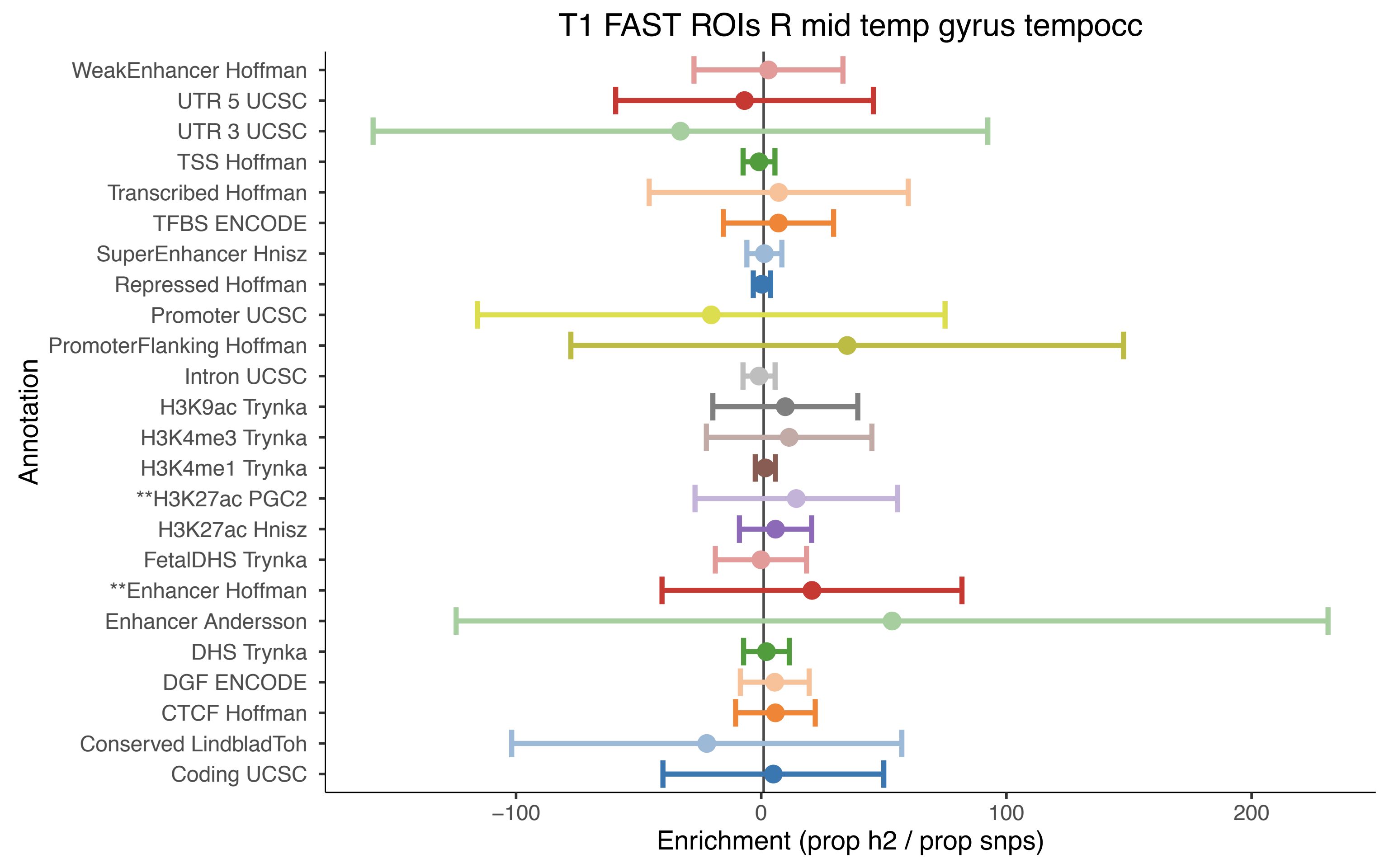
T1 FAST ROIs R mid temp gyrus tempocc
WeakEnhancer Hoffman
UTR 5 UCSC
UTR 3 UCSC
TSS Hoffman
Transcribed Hoffman
TFBS ENCODE
SuperEnhancer Hnisz
Repressed Hoffman
Promoter UCSC
PromoterFlanking Hoffman
Intron UCSC
H3K9ac Trynka
H3K4me3 Trynka
H3K4me1 Trynka
**H3K27ac PGC2
H3K27ac Hnisz
FetalDHS Trynka
**Enhancer Hoffman
Enhancer Andersson
DHS Trynka
DGF ENCODE
CTCF Hoffman
Conserved LindbladToh
Coding UCSC
Annotation
−100
0
100
200
Enrichment (prop h2 / prop snps)

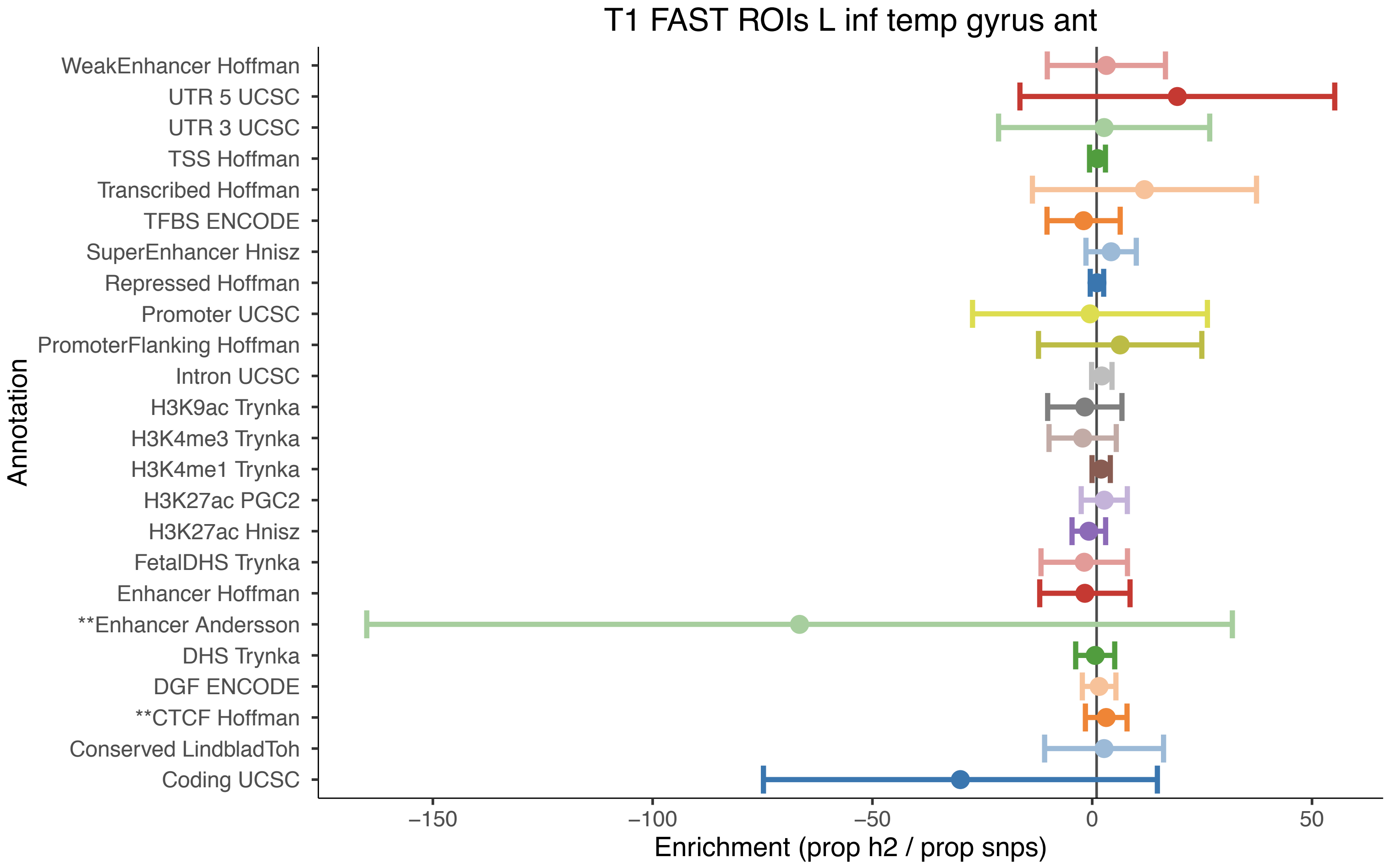

T1 FAST ROIs L inf temp gyrus ant
WeakEnhancer Hoffman
UTR 5 UCSC
UTR 3 UCSC
TSS Hoffman
Transcribed Hoffman
TFBS ENCODE
SuperEnhancer Hnisz
Repressed Hoffman
Promoter UCSC
PromoterFlanking Hoffman
Intron UCSC
H3K9ac Trynka
H3K4me3 Trynka
H3K4me1 Trynka
H3K27ac PGC2
H3K27ac Hnisz
FetalDHS Trynka
Enhancer Hoffman
**Enhancer Andersson
DHS Trynka
DGF ENCODE
**CTCF Hoffman
Conserved LindbladToh
Coding UCSC
Annotation
−150
−100
−50
0
50
Enrichment (prop h2 / prop snps)

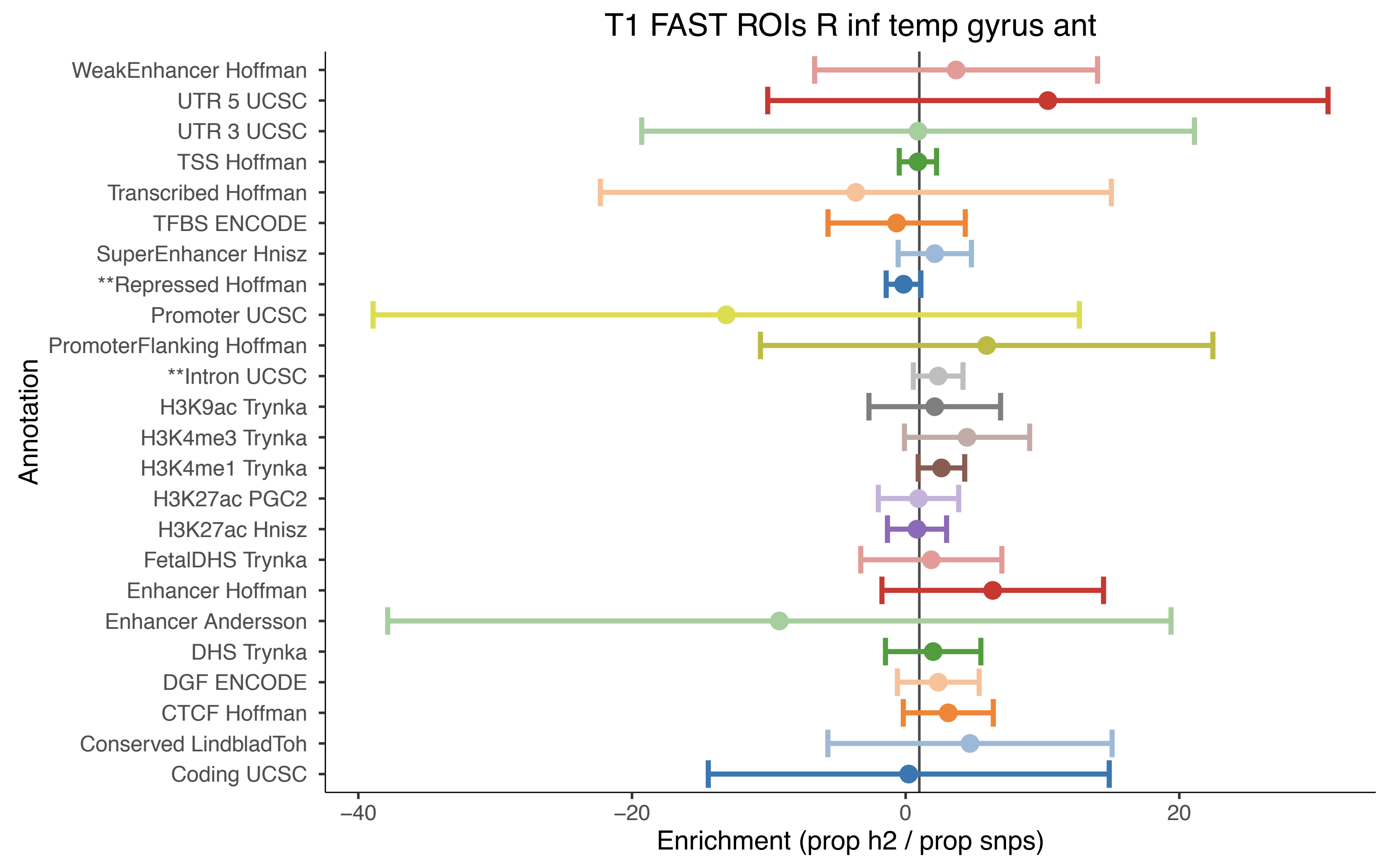
T1 FAST ROIs R inf temp gyrus ant
WeakEnhancer Hoffman
UTR 5 UCSC
UTR 3 UCSC
TSS Hoffman
Transcribed Hoffman
TFBS ENCODE
SuperEnhancer Hnisz
**Repressed Hoffman
Promoter UCSC
PromoterFlanking Hoffman
**Intron UCSC
H3K9ac Trynka
H3K4me3 Trynka
H3K4me1 Trynka
H3K27ac PGC2
H3K27ac Hnisz
FetalDHS Trynka
Enhancer Hoffman
Enhancer Andersson
DHS Trynka
DGF ENCODE
CTCF Hoffman
Conserved LindbladToh
Coding UCSC
Annotation
−40
−20
0
20
Enrichment (prop h2 / prop snps)

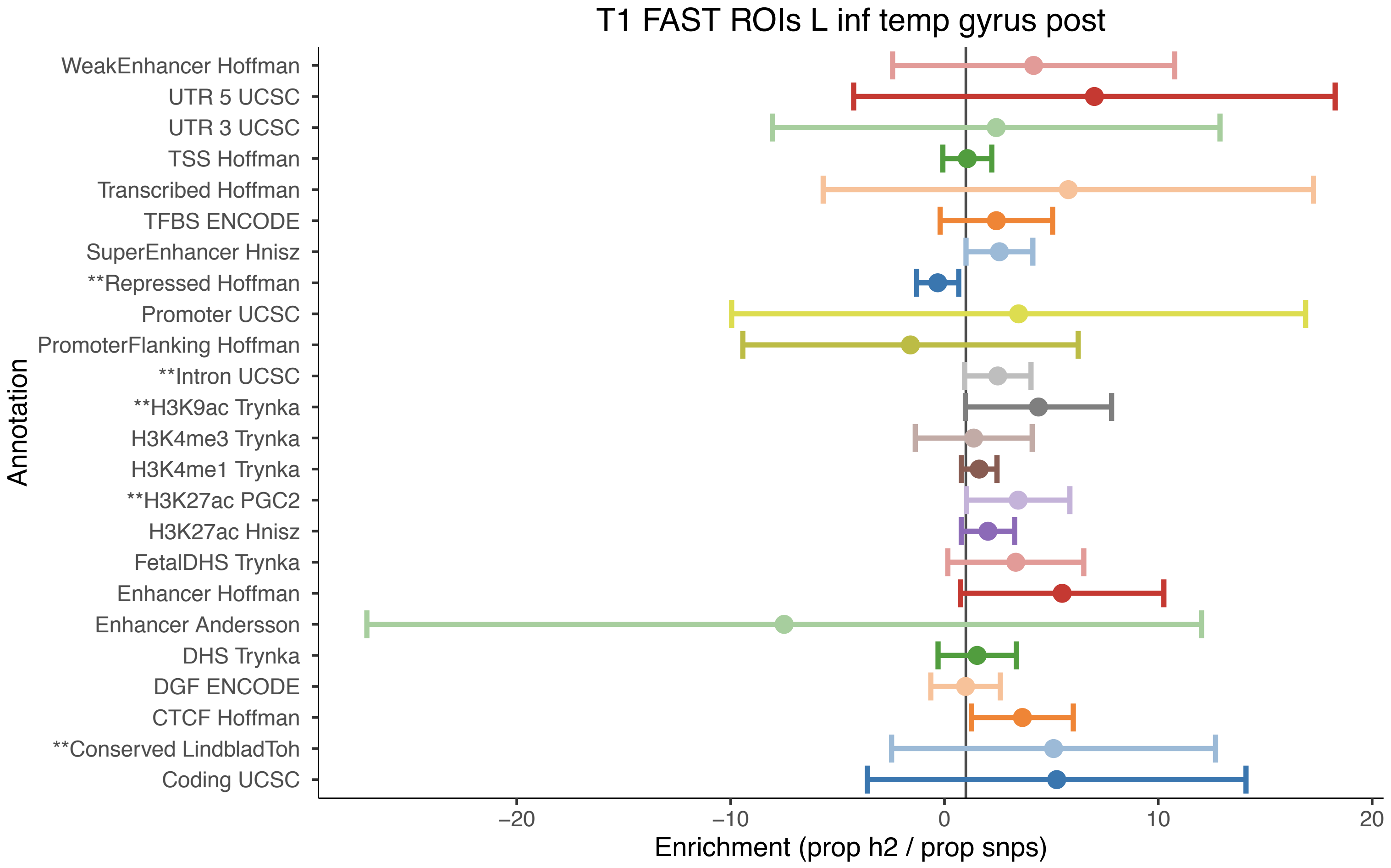
T1 FAST ROIs L inf temp gyrus post
WeakEnhancer Hoffman
UTR 5 UCSC
UTR 3 UCSC
TSS Hoffman
Transcribed Hoffman
TFBS ENCODE
SuperEnhancer Hnisz
**Repressed Hoffman
Promoter UCSC
PromoterFlanking Hoffman
**Intron UCSC
**H3K9ac Trynka
H3K4me3 Trynka
H3K4me1 Trynka
**H3K27ac PGC2
H3K27ac Hnisz
FetalDHS Trynka
Enhancer Hoffman
Enhancer Andersson
DHS Trynka
DGF ENCODE
CTCF Hoffman
**Conserved LindbladToh
Coding UCSC
Annotation
−20
−10
0
10
20
Enrichment (prop h2 / prop snps)

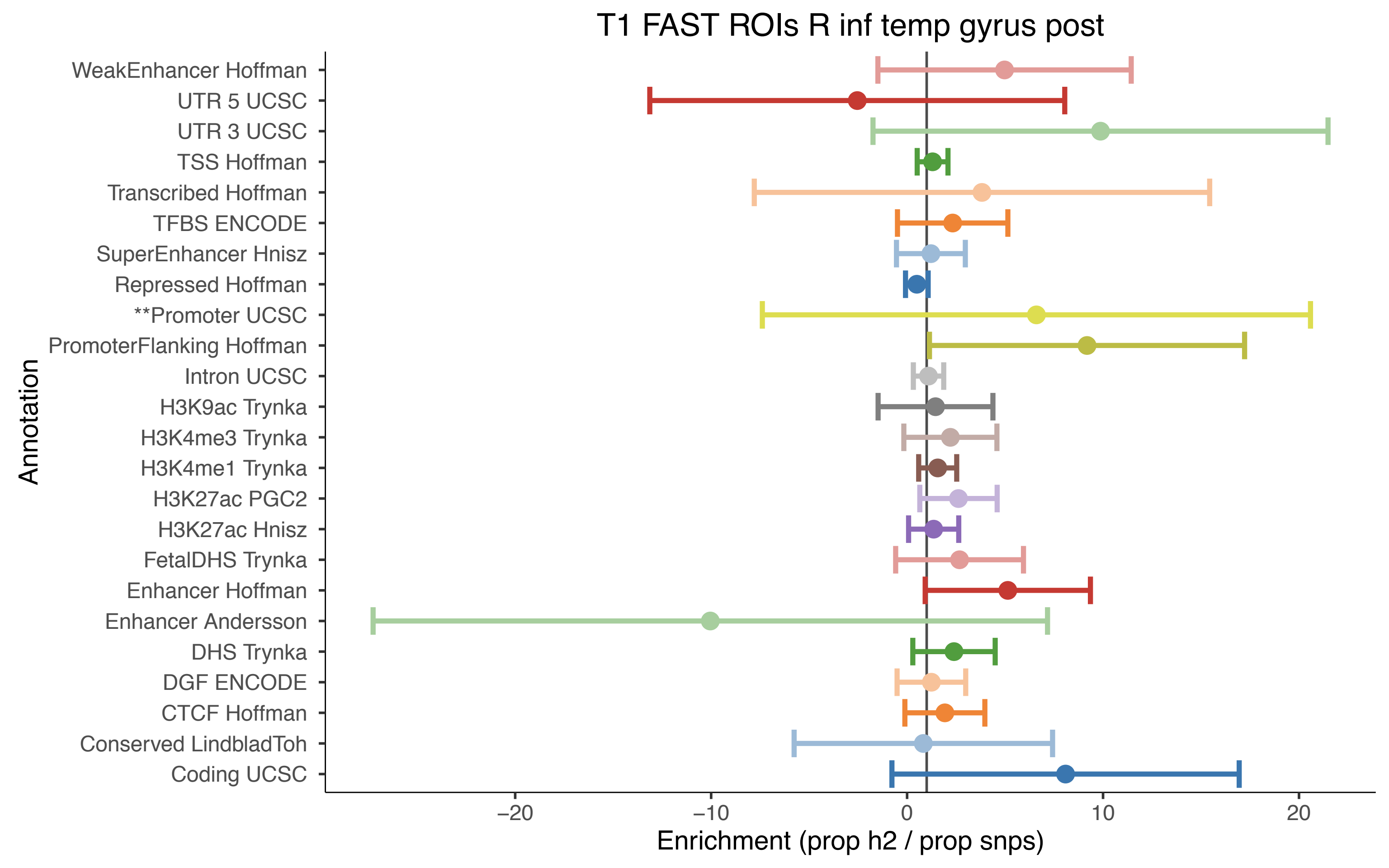

T1 FAST ROIs R inf temp gyrus post
WeakEnhancer Hoffman
UTR 5 UCSC
UTR 3 UCSC
TSS Hoffman
Transcribed Hoffman
TFBS ENCODE
SuperEnhancer Hnisz
Repressed Hoffman
**Promoter UCSC
PromoterFlanking Hoffman
Intron UCSC
H3K9ac Trynka
H3K4me3 Trynka
H3K4me1 Trynka
H3K27ac PGC2
H3K27ac Hnisz
FetalDHS Trynka
Enhancer Hoffman
Enhancer Andersson
DHS Trynka
DGF ENCODE
CTCF Hoffman
Conserved LindbladToh
Coding UCSC
Annotation
−20
−10
0
10
20
Enrichment (prop h2 / prop snps)

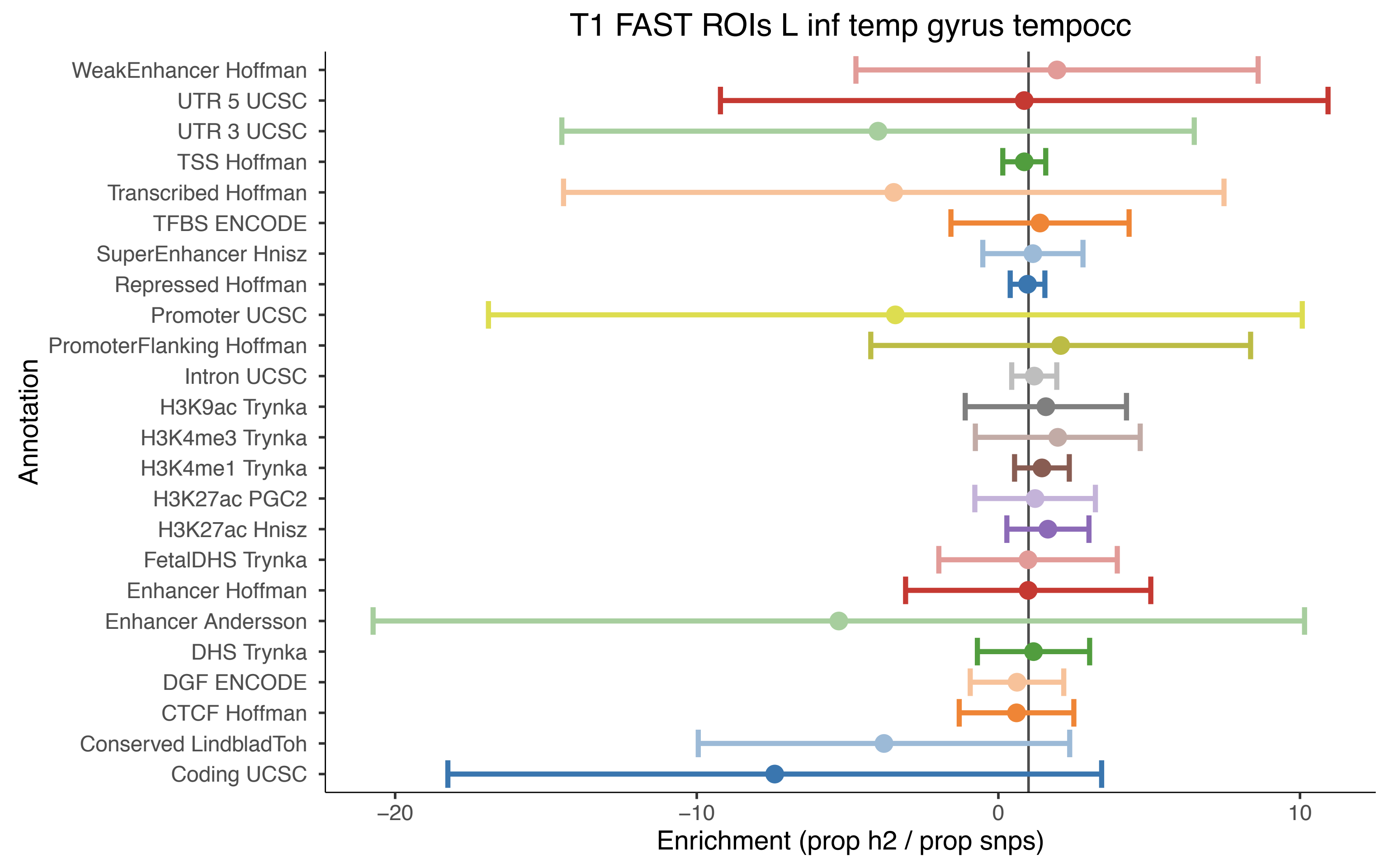
T1 FAST ROIs L inf temp gyrus tempocc
WeakEnhancer Hoffman
UTR 5 UCSC
UTR 3 UCSC
TSS Hoffman
Transcribed Hoffman
TFBS ENCODE
SuperEnhancer Hnisz
Repressed Hoffman
Promoter UCSC
PromoterFlanking Hoffman
Intron UCSC
H3K9ac Trynka
H3K4me3 Trynka
H3K4me1 Trynka
H3K27ac PGC2
H3K27ac Hnisz
FetalDHS Trynka
Enhancer Hoffman
Enhancer Andersson
DHS Trynka
DGF ENCODE
CTCF Hoffman
Conserved LindbladToh
Coding UCSC
Annotation
−20
−10
0
10
Enrichment (prop h2 / prop snps)

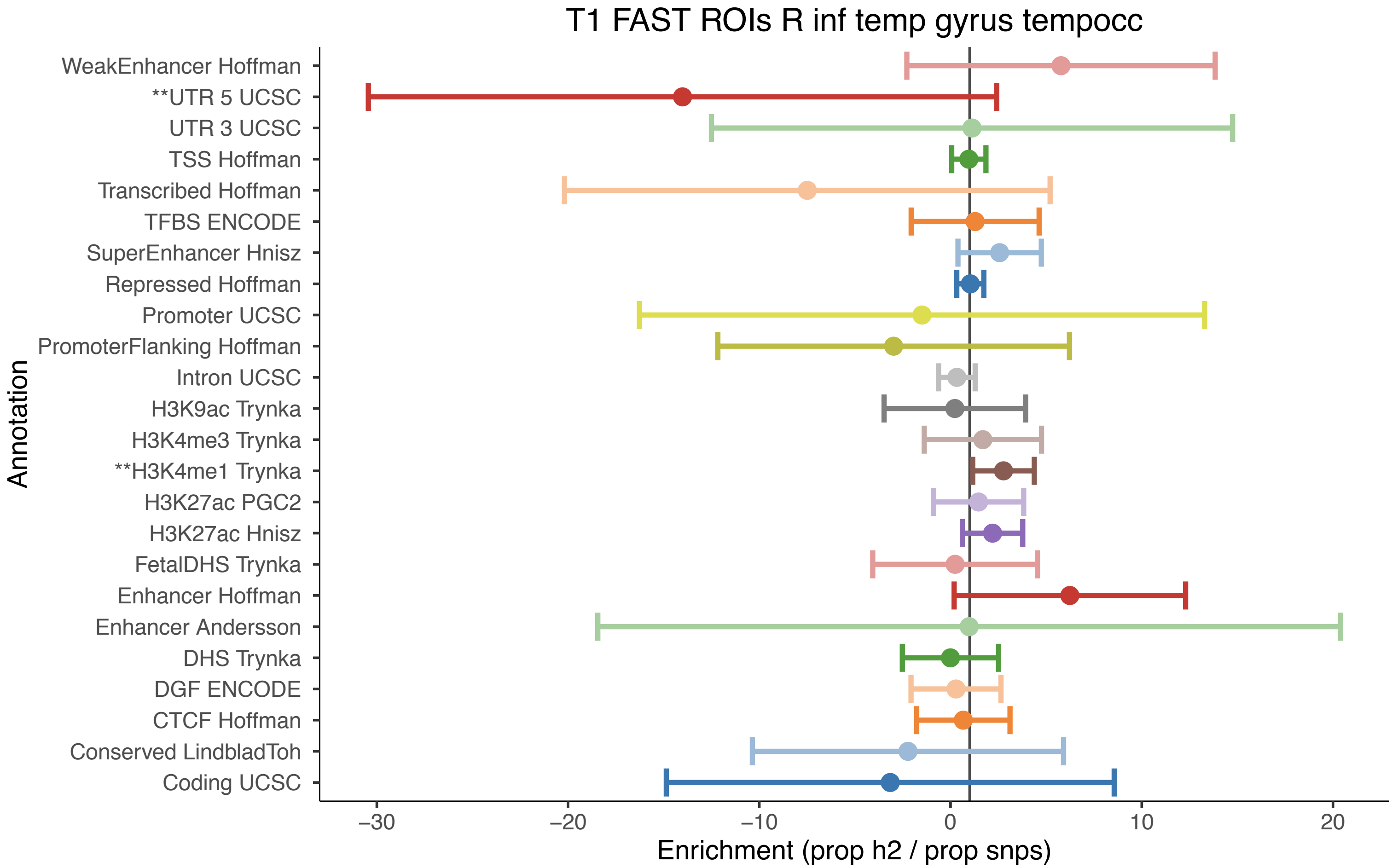

T1 FAST ROIs R inf temp gyrus tempocc
WeakEnhancer Hoffman
**UTR 5 UCSC
UTR 3 UCSC
TSS Hoffman
Transcribed Hoffman
TFBS ENCODE
SuperEnhancer Hnisz
Repressed Hoffman
Promoter UCSC
PromoterFlanking Hoffman
Intron UCSC
H3K9ac Trynka
H3K4me3 Trynka
**H3K4me1 Trynka
H3K27ac PGC2
H3K27ac Hnisz
FetalDHS Trynka
Enhancer Hoffman
Enhancer Andersson
DHS Trynka
DGF ENCODE
CTCF Hoffman
Conserved LindbladToh
Coding UCSC
Annotation
−30
−20
−10
0
10
20
Enrichment (prop h2 / prop snps)

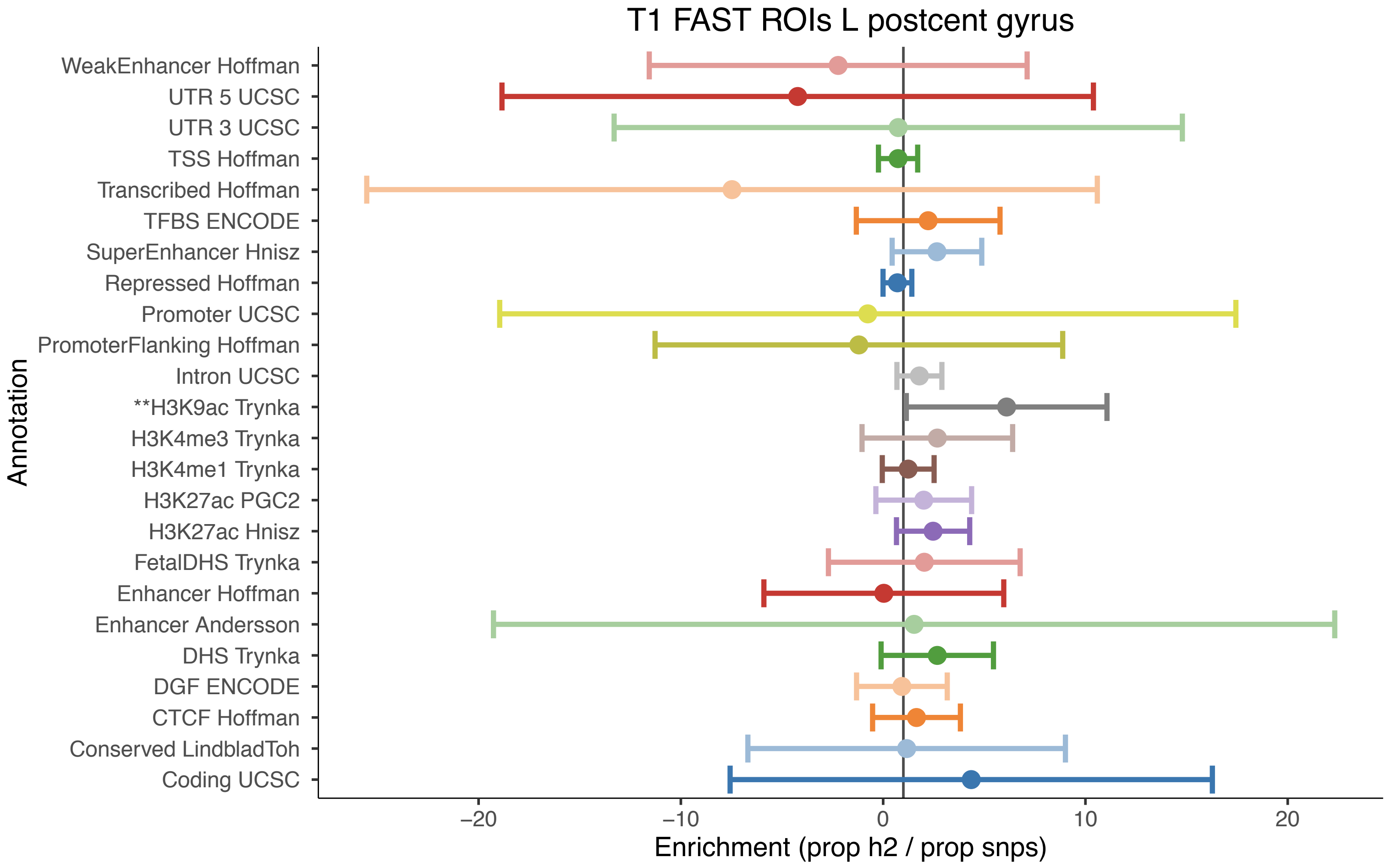
T1 FAST ROIs L postcent gyrus
WeakEnhancer Hoffman
UTR 5 UCSC
UTR 3 UCSC
TSS Hoffman
Transcribed Hoffman
TFBS ENCODE
SuperEnhancer Hnisz
Repressed Hoffman
Promoter UCSC
PromoterFlanking Hoffman
Intron UCSC
**H3K9ac Trynka
H3K4me3 Trynka
H3K4me1 Trynka
H3K27ac PGC2
H3K27ac Hnisz
FetalDHS Trynka
Enhancer Hoffman
Enhancer Andersson
DHS Trynka
DGF ENCODE
CTCF Hoffman
Conserved LindbladToh
Coding UCSC
Annotation
−20
−10
0
10
20
Enrichment (prop h2 / prop snps)

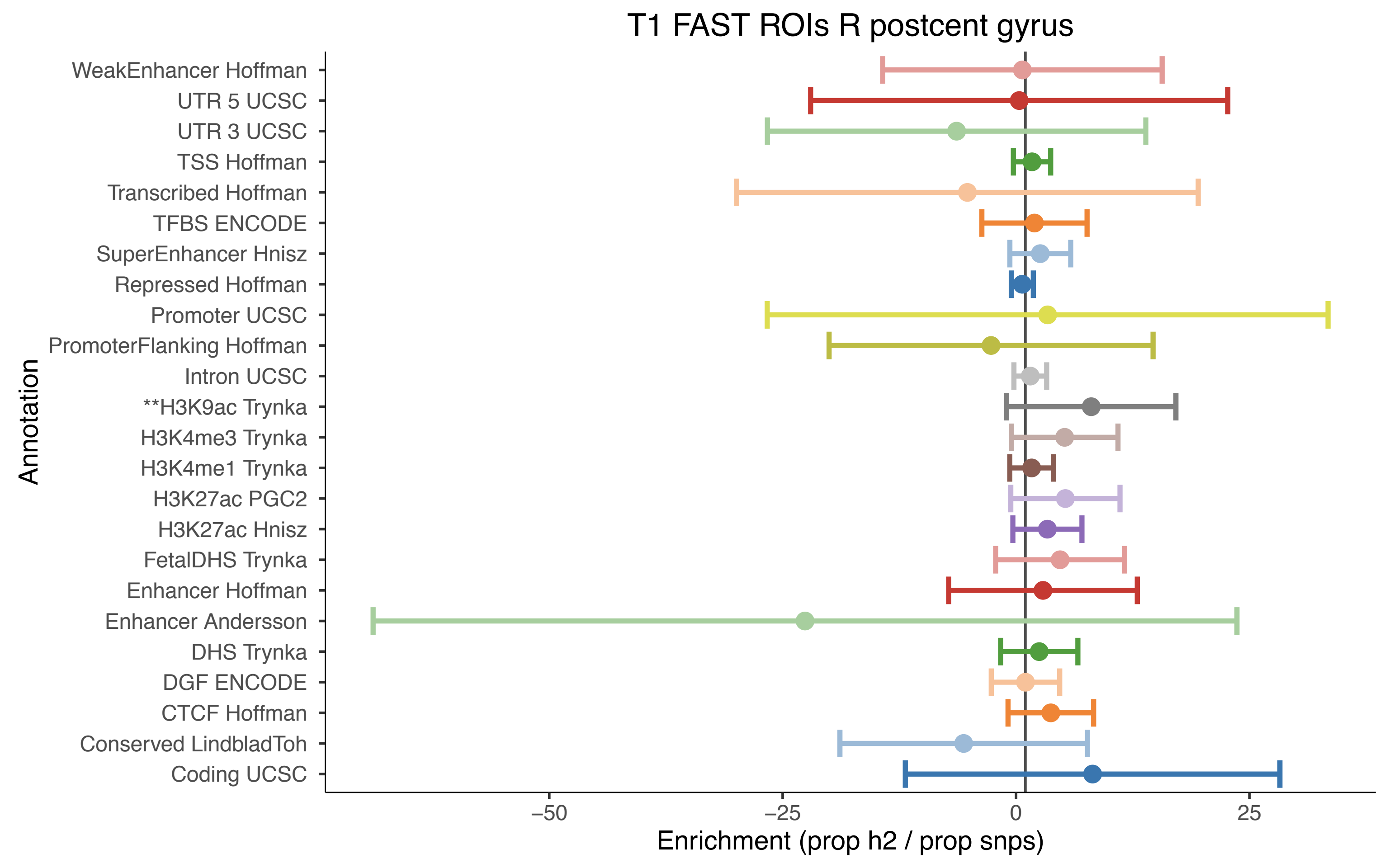
T1 FAST ROIs R postcent gyrus
WeakEnhancer Hoffman
UTR 5 UCSC
UTR 3 UCSC
TSS Hoffman
Transcribed Hoffman
TFBS ENCODE
SuperEnhancer Hnisz
Repressed Hoffman
Promoter UCSC
PromoterFlanking Hoffman
Intron UCSC
**H3K9ac Trynka
H3K4me3 Trynka
H3K4me1 Trynka
H3K27ac PGC2
H3K27ac Hnisz
FetalDHS Trynka
Enhancer Hoffman
Enhancer Andersson
DHS Trynka
DGF ENCODE
CTCF Hoffman
Conserved LindbladToh
Coding UCSC
Annotation
−50
−25
0
25
Enrichment (prop h2 / prop snps)

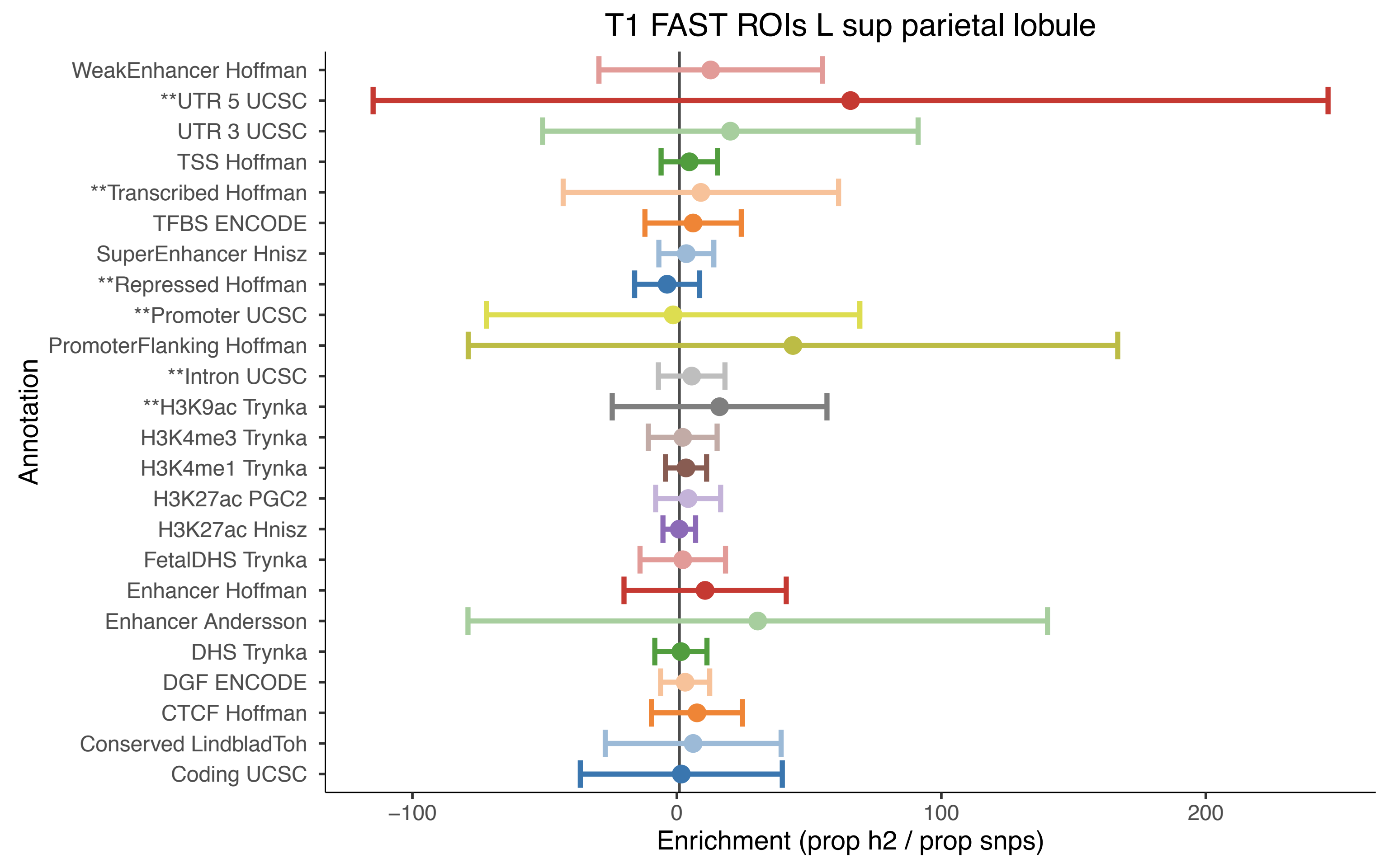

T1 FAST ROIs L sup parietal lobule
WeakEnhancer Hoffman
**UTR 5 UCSC
UTR 3 UCSC
TSS Hoffman
**Transcribed Hoffman
TFBS ENCODE
SuperEnhancer Hnisz
**Repressed Hoffman
**Promoter UCSC
PromoterFlanking Hoffman
**Intron UCSC
**H3K9ac Trynka
H3K4me3 Trynka
H3K4me1 Trynka
H3K27ac PGC2
H3K27ac Hnisz
FetalDHS Trynka
Enhancer Hoffman
Enhancer Andersson
DHS Trynka
DGF ENCODE
CTCF Hoffman
Conserved LindbladToh
Coding UCSC
Annotation
−100
0
100
200
Enrichment (prop h2 / prop snps)

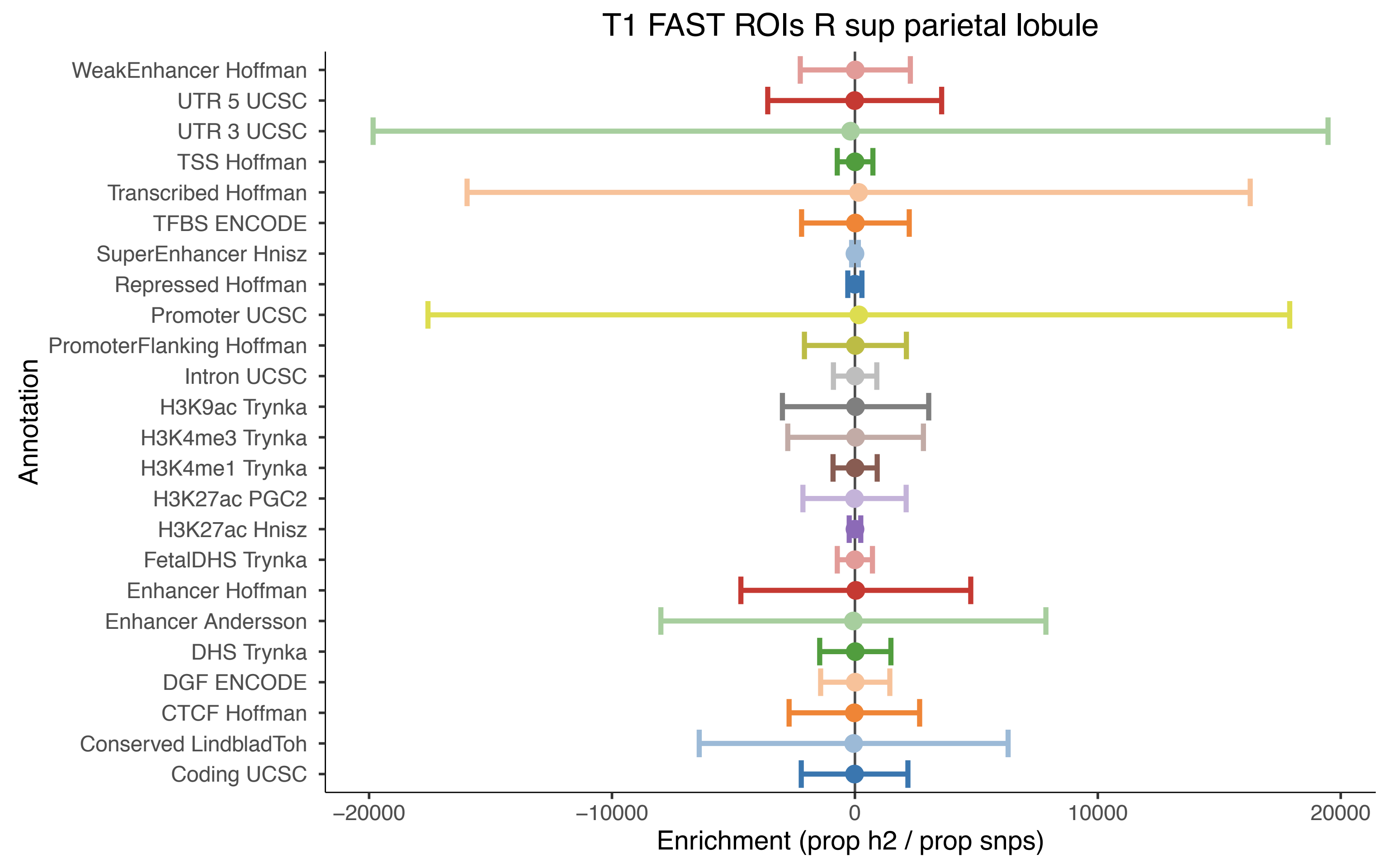

T1 FAST ROIs R sup parietal lobule
WeakEnhancer Hoffman
UTR 5 UCSC
UTR 3 UCSC
TSS Hoffman
Transcribed Hoffman
TFBS ENCODE
SuperEnhancer Hnisz
Repressed Hoffman
Promoter UCSC
PromoterFlanking Hoffman
Intron UCSC
H3K9ac Trynka
H3K4me3 Trynka
H3K4me1 Trynka
H3K27ac PGC2
H3K27ac Hnisz
FetalDHS Trynka
Enhancer Hoffman
Enhancer Andersson
DHS Trynka
DGF ENCODE
CTCF Hoffman
Conserved LindbladToh
Coding UCSC
Annotation
−20000
−10000
0
10000
20000
Enrichment (prop h2 / prop snps)

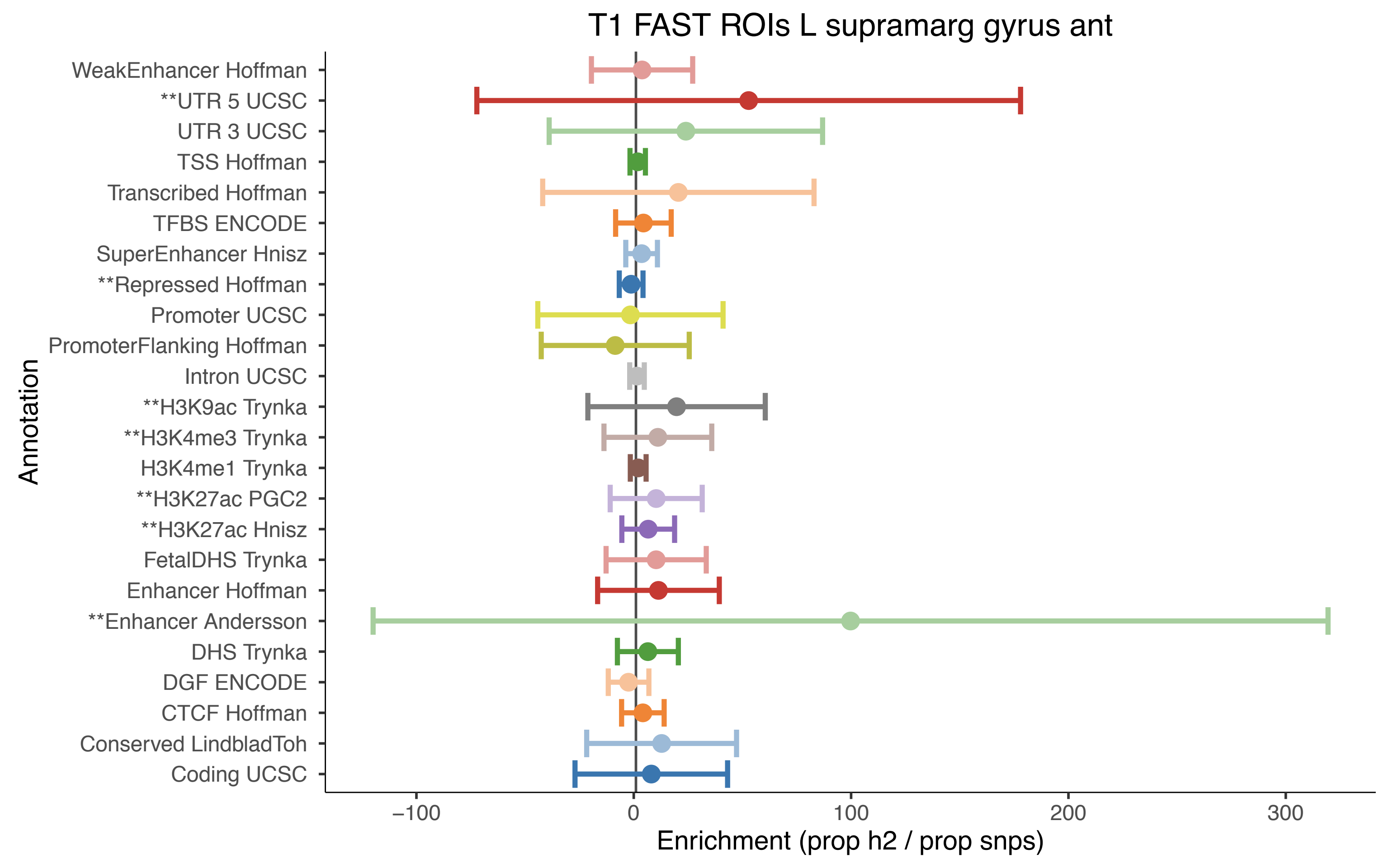

T1 FAST ROIs L supramarg gyrus ant
Annotation
WeakEnhancer Hoffman
**UTR 5 UCSC
UTR 3 UCSC
TSS Hoffman
Transcribed Hoffman
TFBS ENCODE
SuperEnhancer Hnisz
**Repressed Hoffman
Promoter UCSC
PromoterFlanking Hoffman
Intron UCSC
**H3K9ac Trynka
**H3K4me3 Trynka
H3K4me1 Trynka
**H3K27ac PGC2
**H3K27ac Hnisz
FetalDHS Trynka
Enhancer Hoffman
**Enhancer Andersson
DHS Trynka
DGF ENCODE
CTCF Hoffman
Conserved LindbladToh
Coding UCSC
−100
0
100
200
300
Enrichment (prop h2 / prop snps)

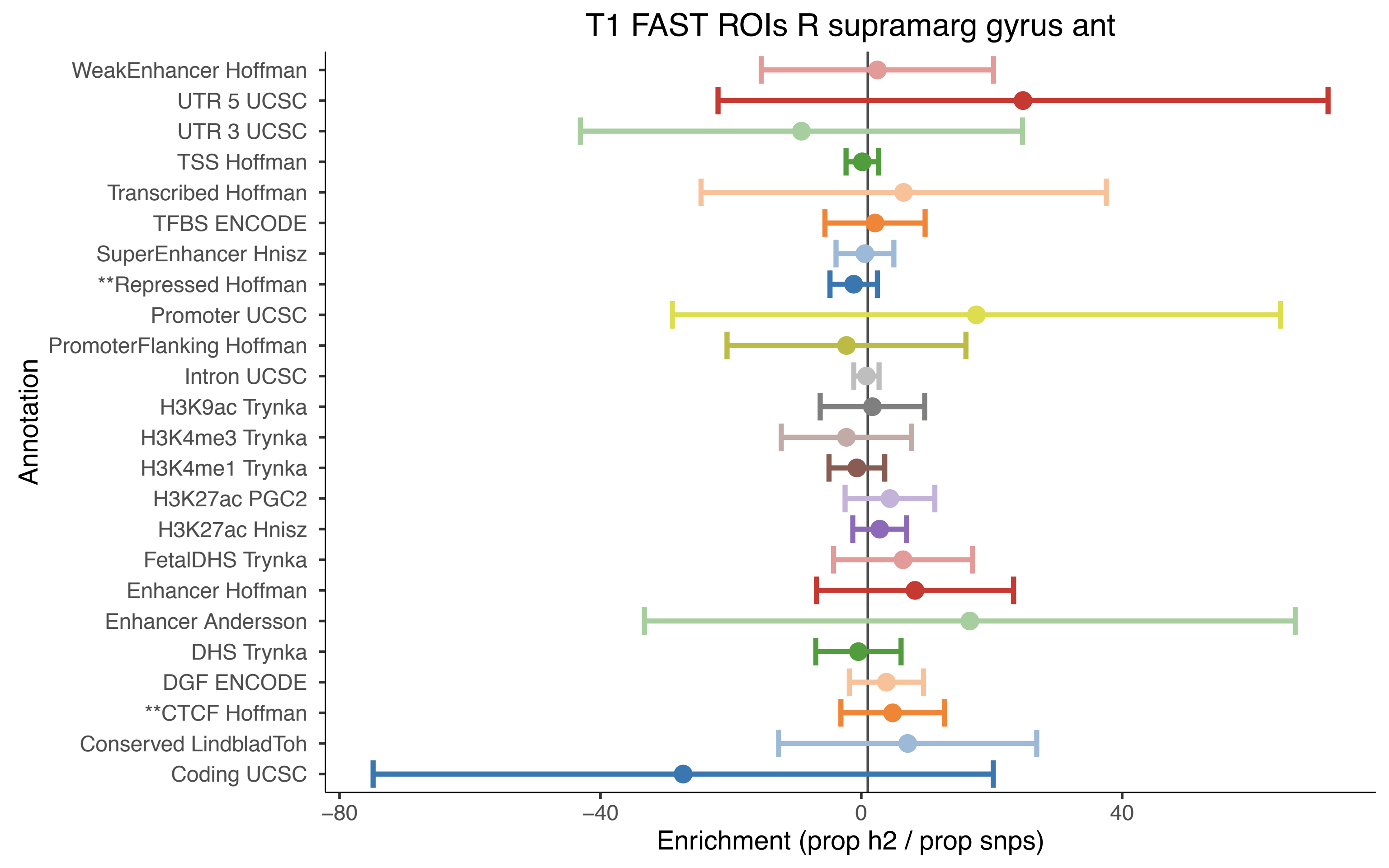

T1 FAST ROIs R supramarg gyrus ant
WeakEnhancer Hoffman
UTR 5 UCSC
UTR 3 UCSC
TSS Hoffman
Transcribed Hoffman
TFBS ENCODE
SuperEnhancer Hnisz
**Repressed Hoffman
Promoter UCSC
PromoterFlanking Hoffman
Intron UCSC
H3K9ac Trynka
H3K4me3 Trynka
H3K4me1 Trynka
H3K27ac PGC2
H3K27ac Hnisz
FetalDHS Trynka
Enhancer Hoffman
Enhancer Andersson
DHS Trynka
DGF ENCODE
**CTCF Hoffman
Conserved LindbladToh
Coding UCSC
Annotation
−80
−40
0
40
Enrichment (prop h2 / prop snps)

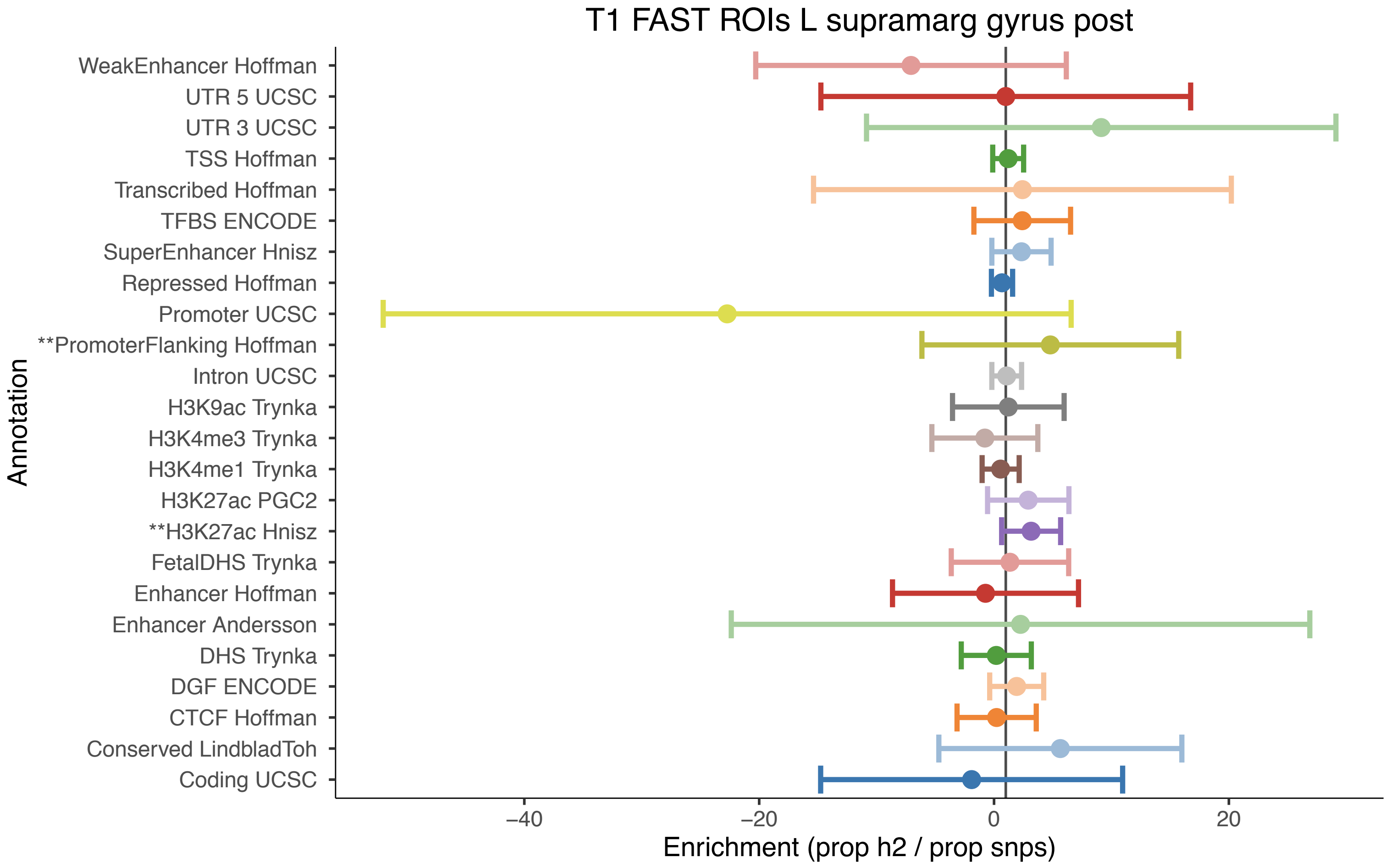

T1 FAST ROIs L supramarg gyrus post
WeakEnhancer Hoffman
UTR 5 UCSC
UTR 3 UCSC
TSS Hoffman
Transcribed Hoffman
TFBS ENCODE
SuperEnhancer Hnisz
Repressed Hoffman
Promoter UCSC
**PromoterFlanking Hoffman
Intron UCSC
H3K9ac Trynka
H3K4me3 Trynka
H3K4me1 Trynka
H3K27ac PGC2
**H3K27ac Hnisz
FetalDHS Trynka
Enhancer Hoffman
Enhancer Andersson
DHS Trynka
DGF ENCODE
CTCF Hoffman
Conserved LindbladToh
Coding UCSC
Annotation
−40
−20
0
20
Enrichment (prop h2 / prop snps)

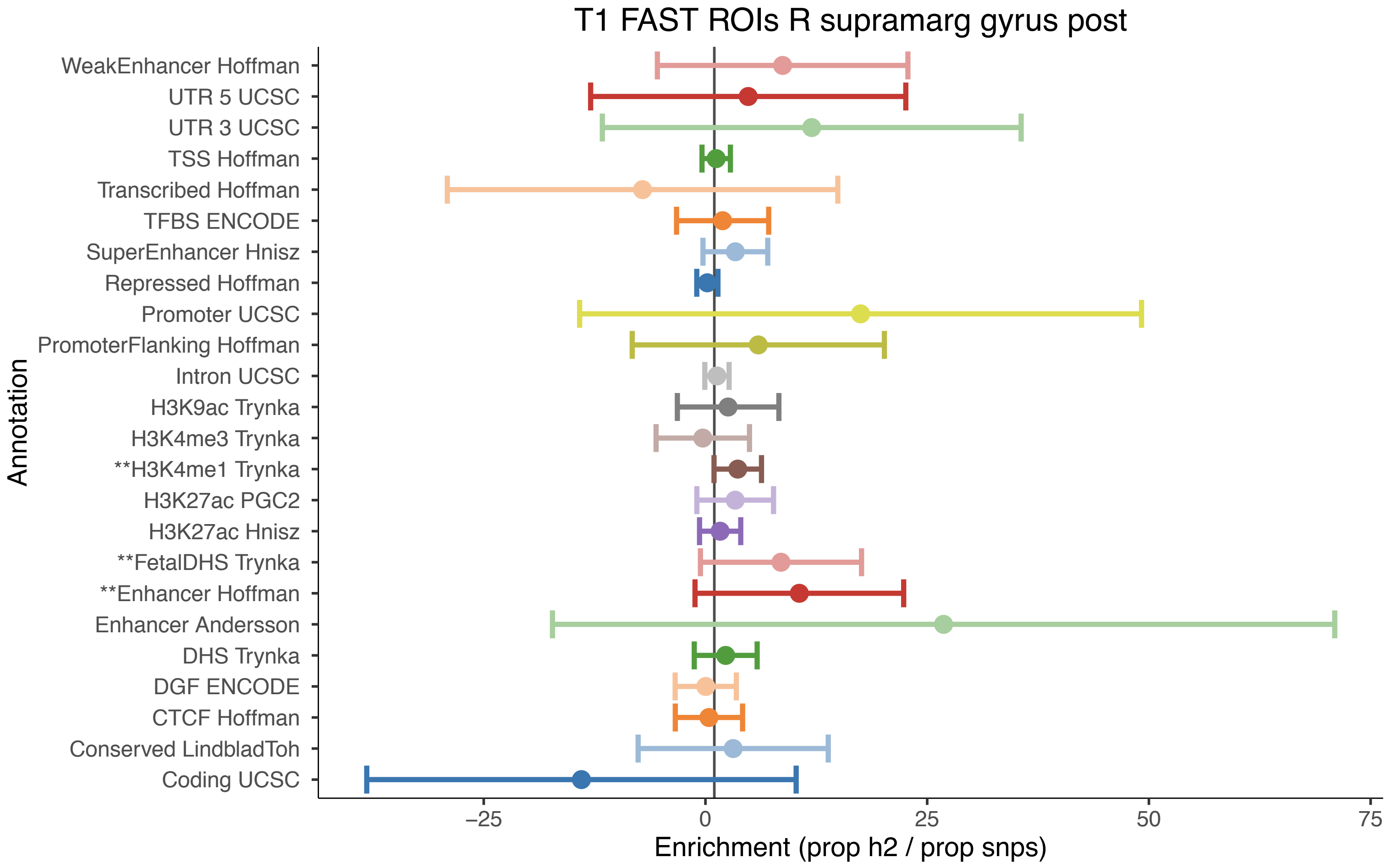
T1 FAST ROIs R supramarg gyrus post
WeakEnhancer Hoffman
UTR 5 UCSC
UTR 3 UCSC
TSS Hoffman
Transcribed Hoffman
TFBS ENCODE
SuperEnhancer Hnisz
Repressed Hoffman
Promoter UCSC
PromoterFlanking Hoffman
Intron UCSC
H3K9ac Trynka
H3K4me3 Trynka
**H3K4me1 Trynka
H3K27ac PGC2
H3K27ac Hnisz
**FetalDHS Trynka
**Enhancer Hoffman
Enhancer Andersson
DHS Trynka
DGF ENCODE
CTCF Hoffman
Conserved LindbladToh
Coding UCSC
Annotation
−25
0
25
50
75
Enrichment (prop h2 / prop snps)

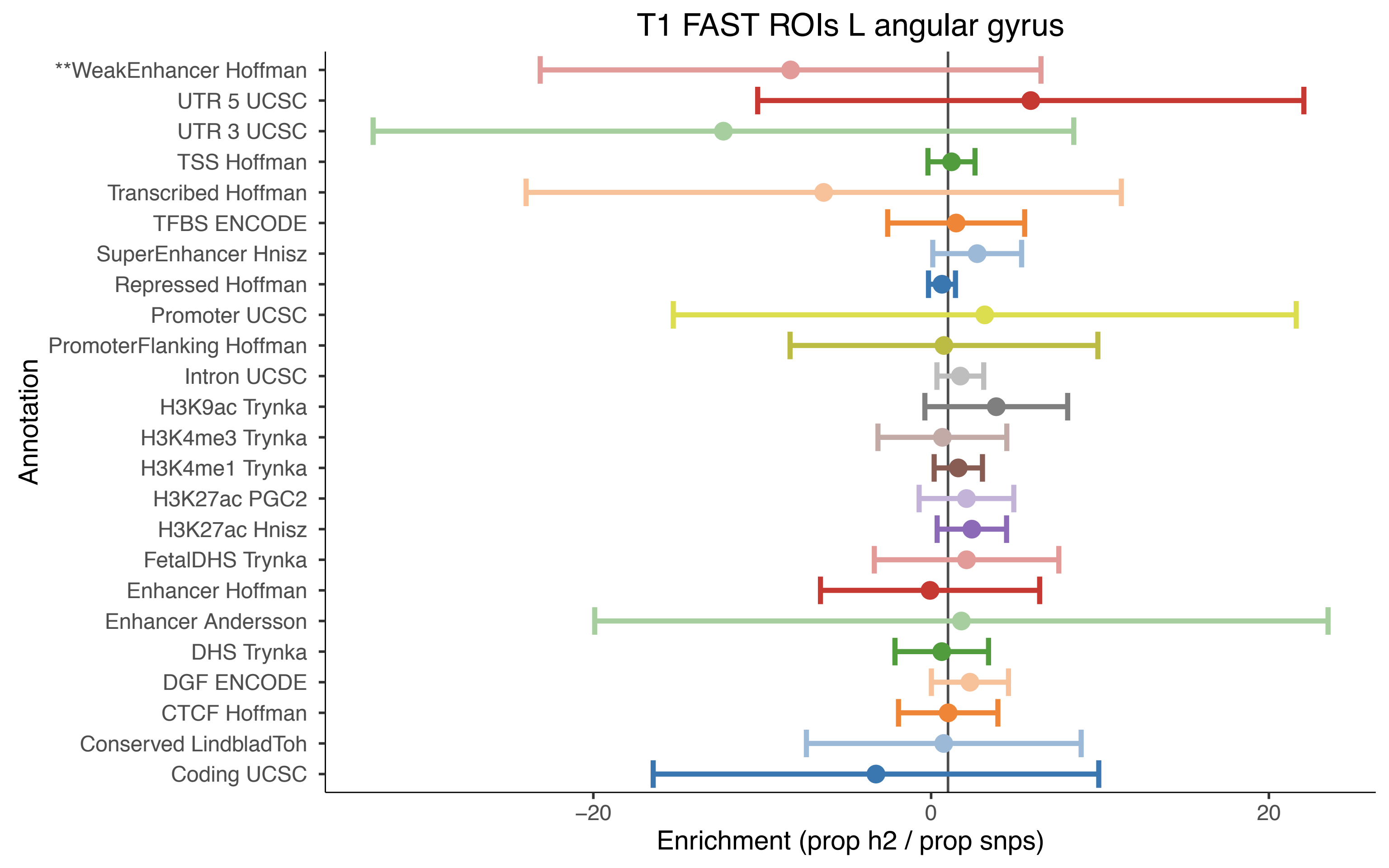

T1 FAST ROIs L angular gyrus
**WeakEnhancer Hoffman
UTR 5 UCSC
UTR 3 UCSC
TSS Hoffman
Transcribed Hoffman
TFBS ENCODE
SuperEnhancer Hnisz
Repressed Hoffman
Promoter UCSC
PromoterFlanking Hoffman
Intron UCSC
H3K9ac Trynka
H3K4me3 Trynka
H3K4me1 Trynka
H3K27ac PGC2
H3K27ac Hnisz
FetalDHS Trynka
Enhancer Hoffman
Enhancer Andersson
DHS Trynka
DGF ENCODE
CTCF Hoffman
Conserved LindbladToh
Coding UCSC
Annotation
−20
0
20
Enrichment (prop h2 / prop snps)

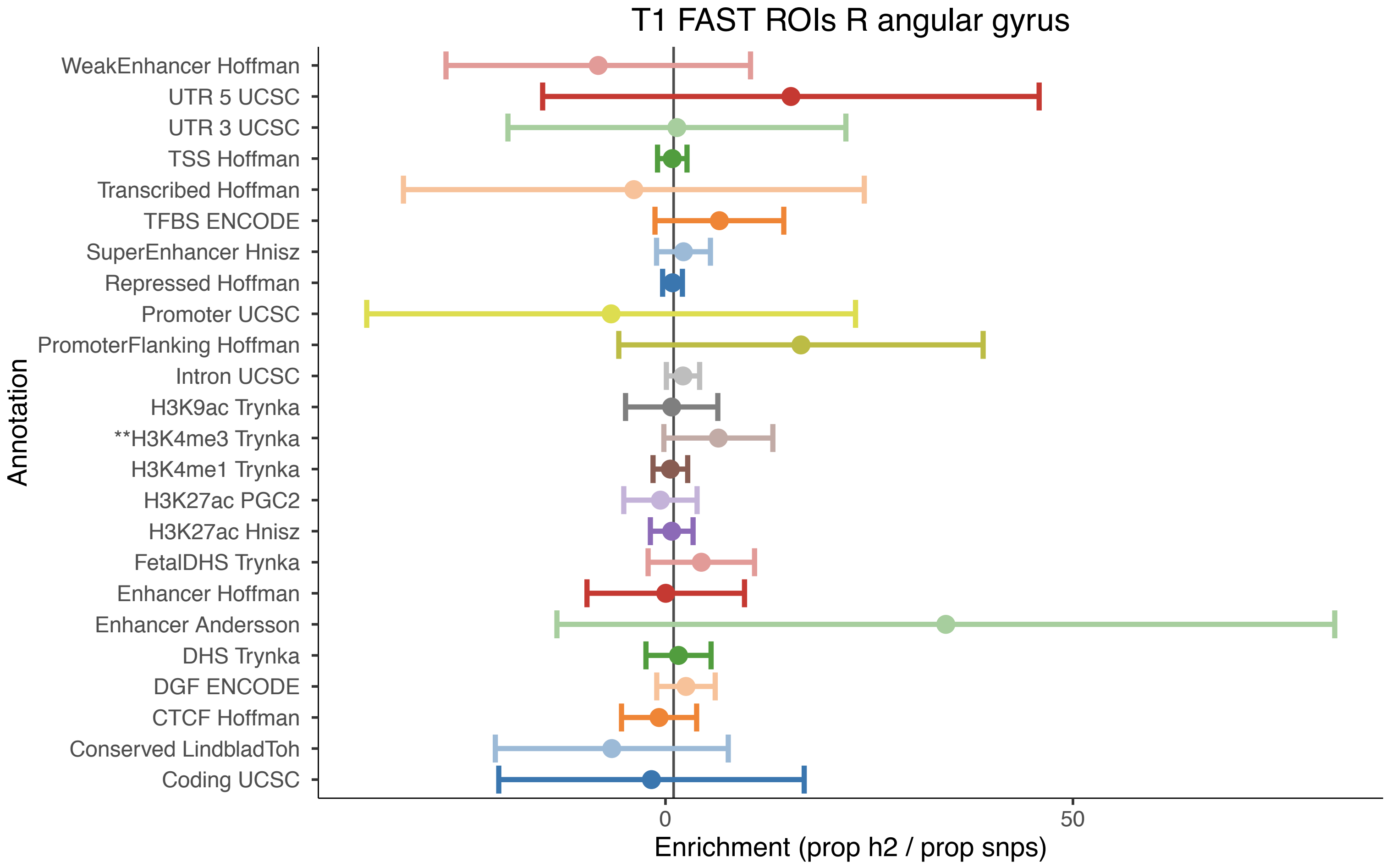

T1 FAST ROIs R angular gyrus
WeakEnhancer Hoffman
UTR 5 UCSC
UTR 3 UCSC
TSS Hoffman
Transcribed Hoffman
TFBS ENCODE
SuperEnhancer Hnisz
Repressed Hoffman
Promoter UCSC
PromoterFlanking Hoffman
Intron UCSC
H3K9ac Trynka
**H3K4me3 Trynka
H3K4me1 Trynka
H3K27ac PGC2
H3K27ac Hnisz
FetalDHS Trynka
Enhancer Hoffman
Enhancer Andersson
DHS Trynka
DGF ENCODE
CTCF Hoffman
Conserved LindbladToh
Coding UCSC
Annotation
0
50
Enrichment (prop h2 / prop snps)

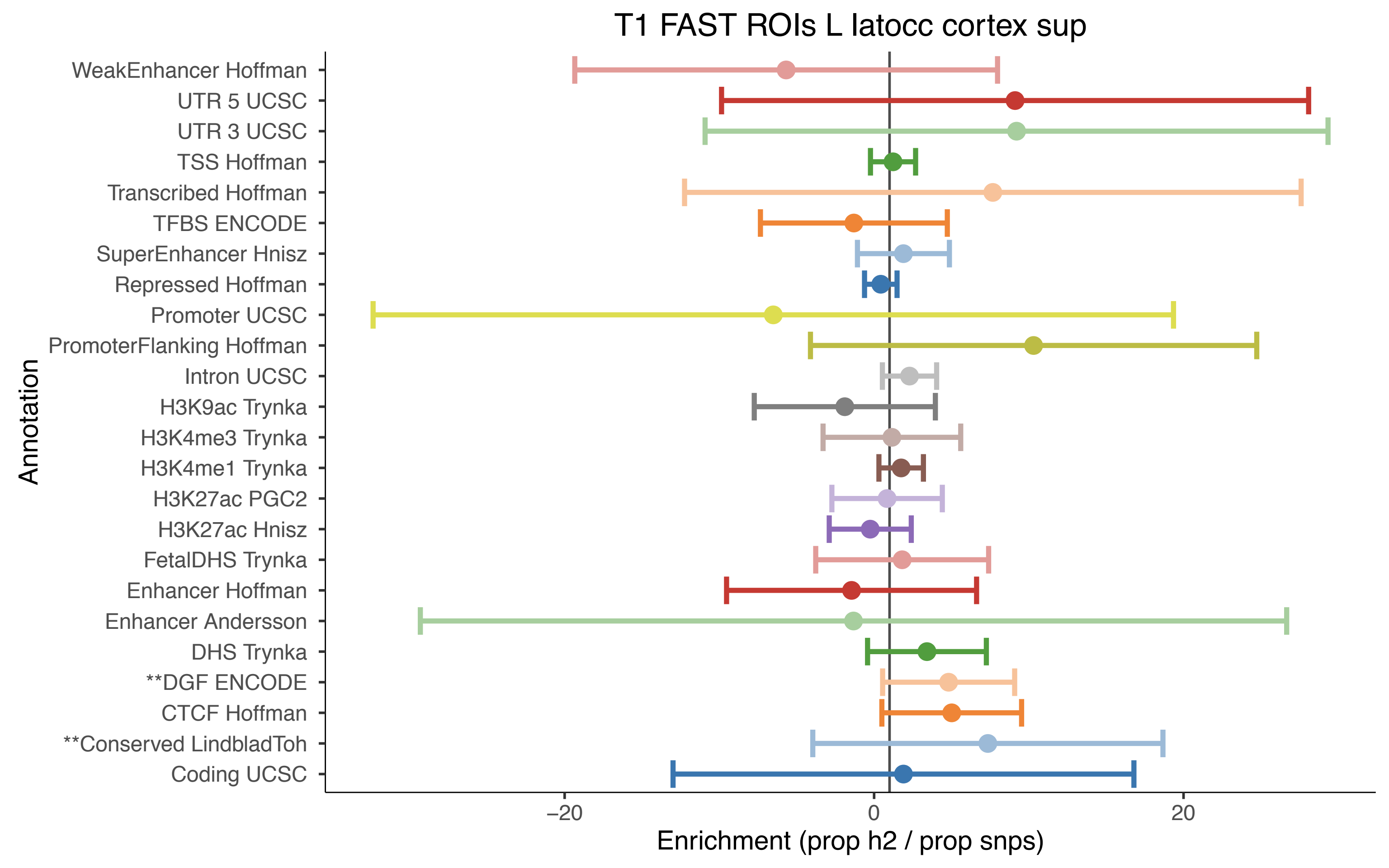
T1 FAST ROIs L latocc cortex sup
WeakEnhancer Hoffman
UTR 5 UCSC
UTR 3 UCSC
TSS Hoffman
Transcribed Hoffman
TFBS ENCODE
SuperEnhancer Hnisz
Repressed Hoffman
Promoter UCSC
PromoterFlanking Hoffman
Intron UCSC
H3K9ac Trynka
H3K4me3 Trynka
H3K4me1 Trynka
H3K27ac PGC2
H3K27ac Hnisz
FetalDHS Trynka
Enhancer Hoffman
Enhancer Andersson
DHS Trynka
**DGF ENCODE
CTCF Hoffman
**Conserved LindbladToh
Coding UCSC
Annotation
−20
0
20
Enrichment (prop h2 / prop snps)

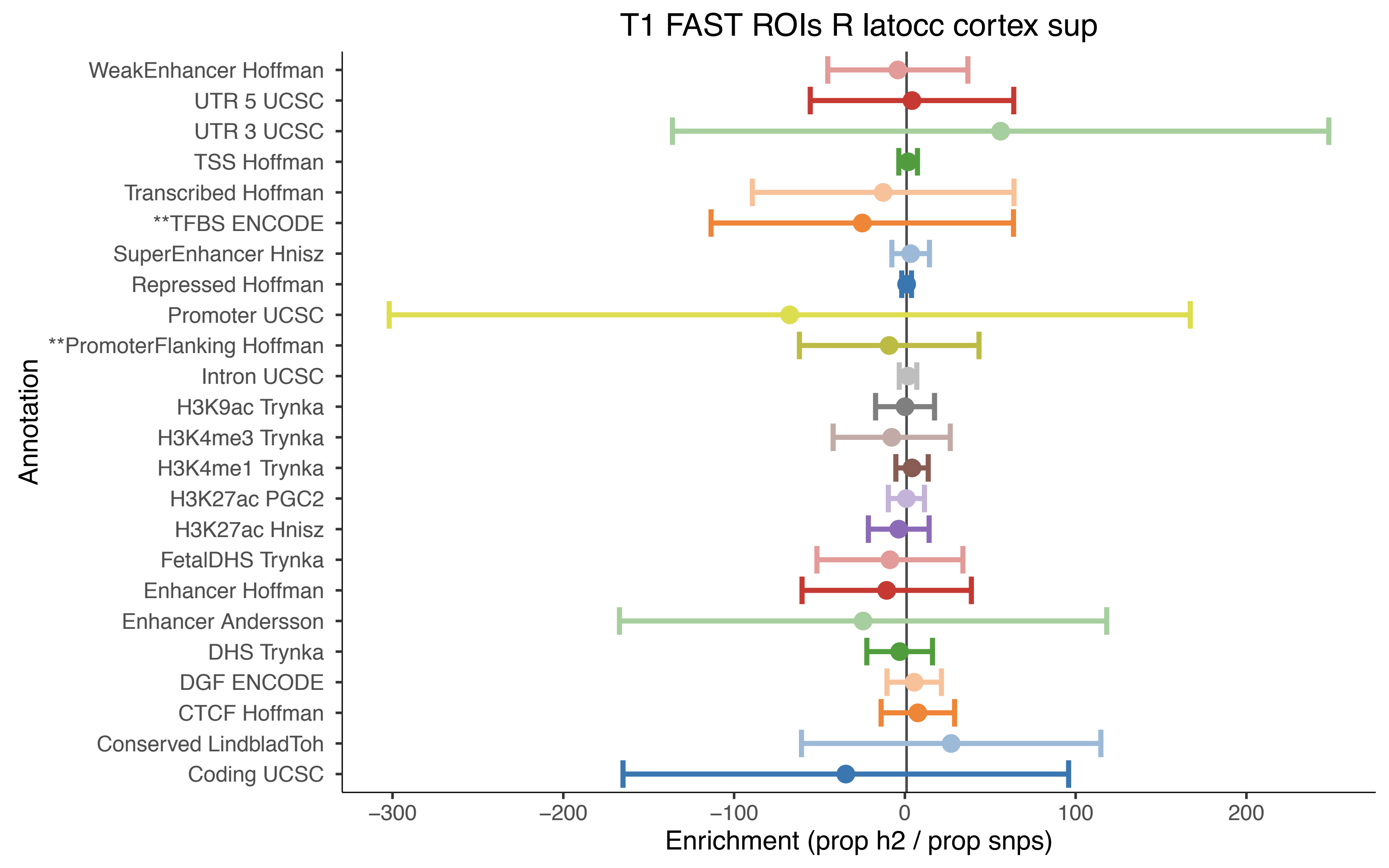

T1 FAST ROIs R latocc cortex sup
WeakEnhancer Hoffman
UTR 5 UCSC
UTR 3 UCSC
TSS Hoffman
Transcribed Hoffman
**TFBS ENCODE
SuperEnhancer Hnisz
Repressed Hoffman
Promoter UCSC
**PromoterFlanking Hoffman
Intron UCSC
H3K9ac Trynka
H3K4me3 Trynka
H3K4me1 Trynka
H3K27ac PGC2
H3K27ac Hnisz
FetalDHS Trynka
Enhancer Hoffman
Enhancer Andersson
DHS Trynka
DGF ENCODE
CTCF Hoffman
Conserved LindbladToh
Coding UCSC
Annotation
−300
−200
−100
0
100
200
Enrichment (prop h2 / prop snps)

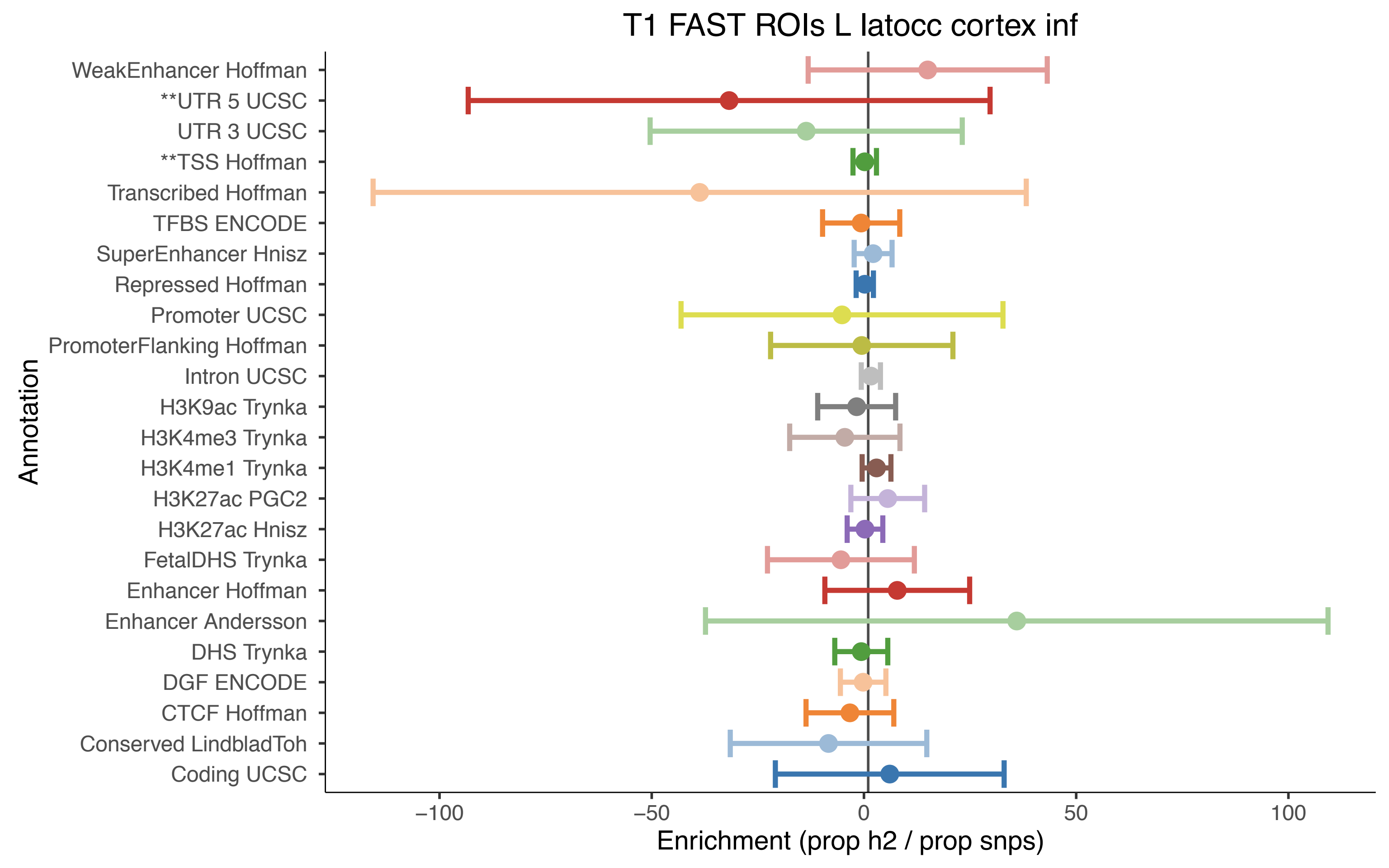
T1 FAST ROIs L latocc cortex inf
WeakEnhancer Hoffman
**UTR 5 UCSC
UTR 3 UCSC
**TSS Hoffman
Transcribed Hoffman
TFBS ENCODE
SuperEnhancer Hnisz
Repressed Hoffman
Promoter UCSC
PromoterFlanking Hoffman
Intron UCSC
H3K9ac Trynka
H3K4me3 Trynka
H3K4me1 Trynka
H3K27ac PGC2
H3K27ac Hnisz
FetalDHS Trynka
Enhancer Hoffman
Enhancer Andersson
DHS Trynka
DGF ENCODE
CTCF Hoffman
Conserved LindbladToh
Coding UCSC
Annotation
−100
−50
0
50
100
Enrichment (prop h2 / prop snps)

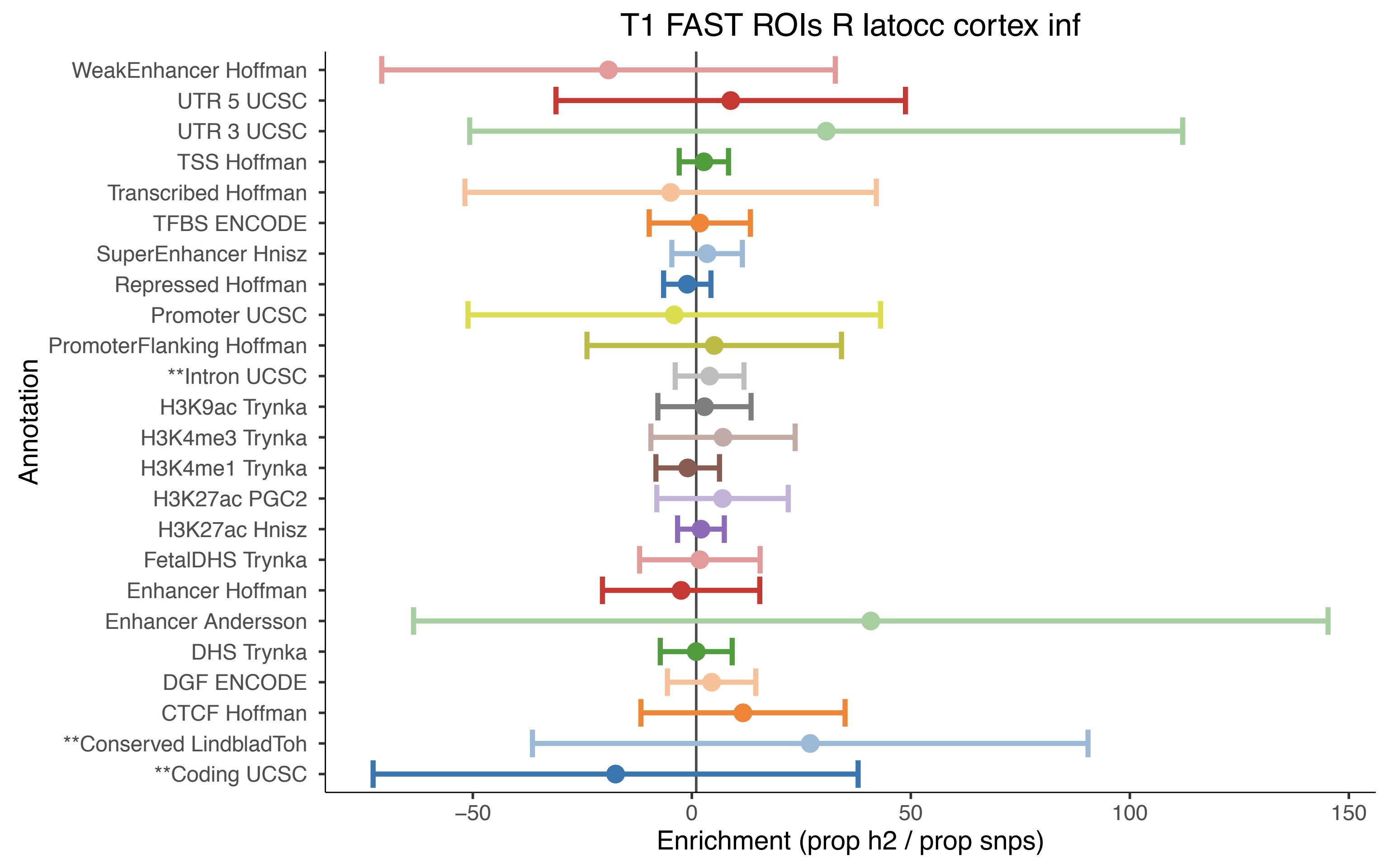
T1 FAST ROIs R latocc cortex inf
WeakEnhancer Hoffman
UTR 5 UCSC
UTR 3 UCSC
TSS Hoffman
Transcribed Hoffman
TFBS ENCODE
SuperEnhancer Hnisz
Repressed Hoffman
Promoter UCSC
PromoterFlanking Hoffman
**Intron UCSC
H3K9ac Trynka
H3K4me3 Trynka
H3K4me1 Trynka
H3K27ac PGC2
H3K27ac Hnisz
FetalDHS Trynka
Enhancer Hoffman
Enhancer Andersson
DHS Trynka
DGF ENCODE
CTCF Hoffman
**Conserved LindbladToh
**Coding UCSC
Annotation
−50
0
50
100
150
Enrichment (prop h2 / prop snps)

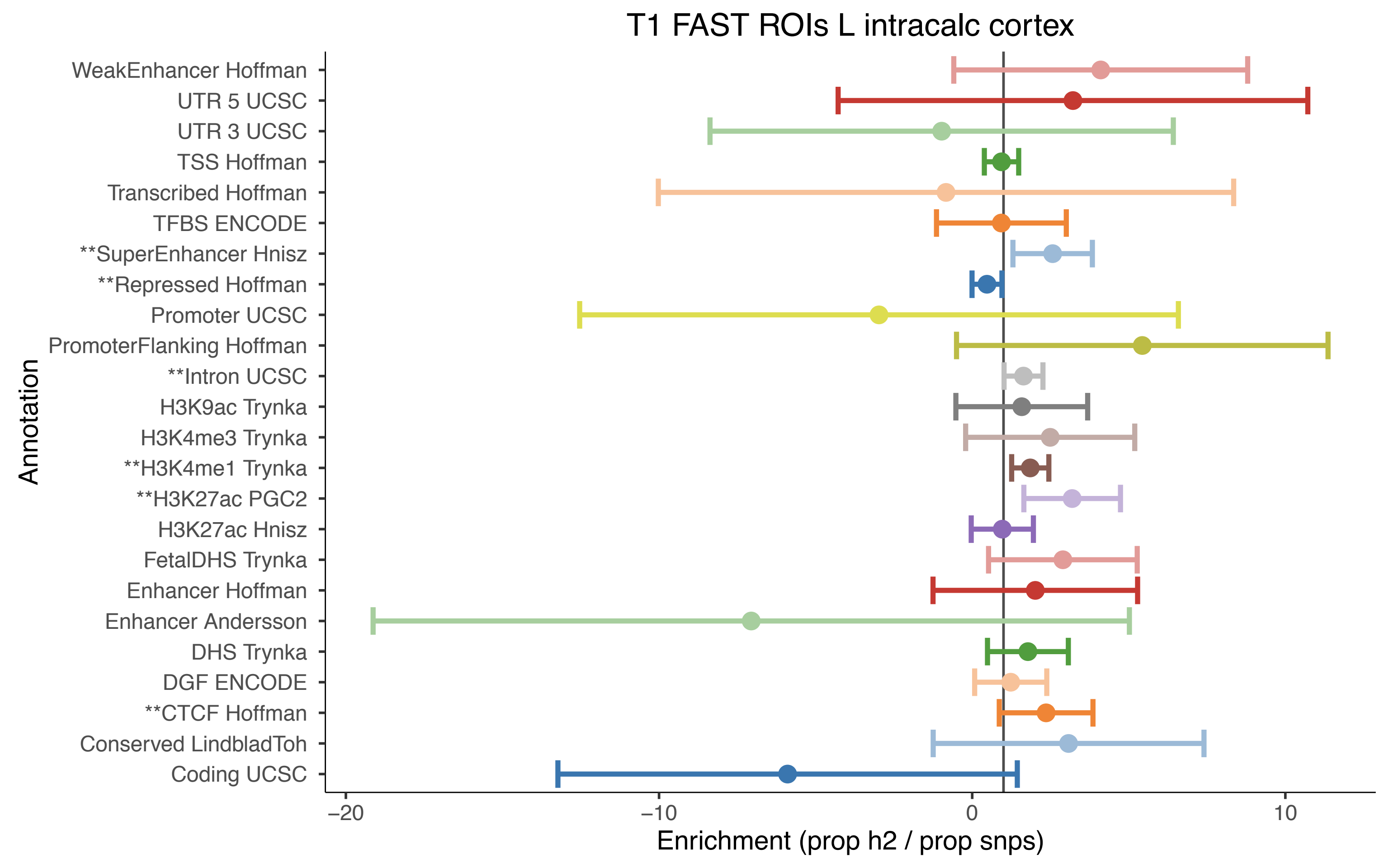

T1 FAST ROIs L intracalc cortex
WeakEnhancer Hoffman
UTR 5 UCSC
UTR 3 UCSC
TSS Hoffman
Transcribed Hoffman
TFBS ENCODE
**SuperEnhancer Hnisz
**Repressed Hoffman
Promoter UCSC
PromoterFlanking Hoffman
**Intron UCSC
H3K9ac Trynka
H3K4me3 Trynka
**H3K4me1 Trynka
**H3K27ac PGC2
H3K27ac Hnisz
FetalDHS Trynka
Enhancer Hoffman
Enhancer Andersson
DHS Trynka
DGF ENCODE
**CTCF Hoffman
Conserved LindbladToh
Coding UCSC
Annotation
−20
−10
0
10
Enrichment (prop h2 / prop snps)

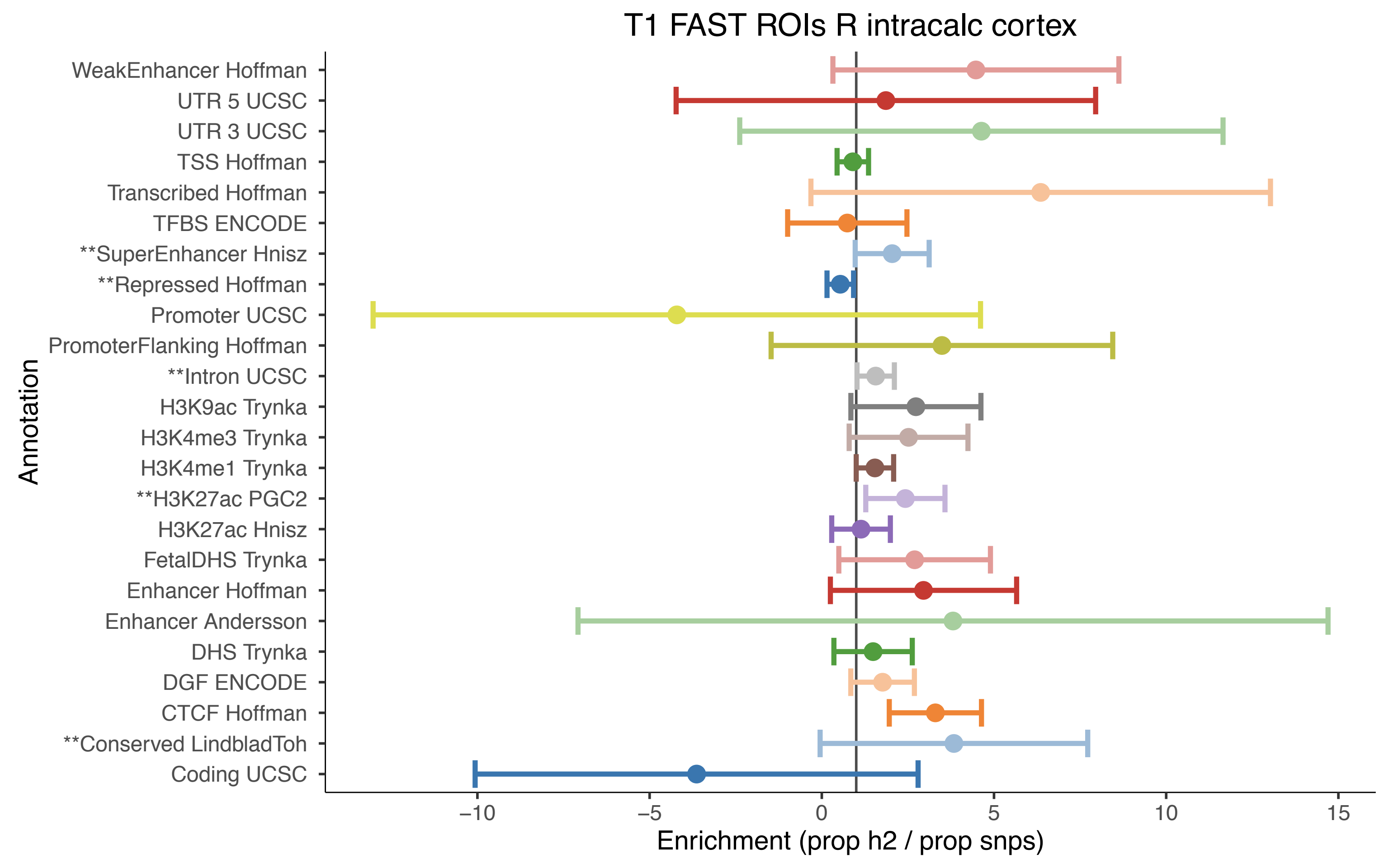
T1 FAST ROIs R intracalc cortex
WeakEnhancer Hoffman
UTR 5 UCSC
UTR 3 UCSC
TSS Hoffman
Transcribed Hoffman
TFBS ENCODE
**SuperEnhancer Hnisz
**Repressed Hoffman
Promoter UCSC
PromoterFlanking Hoffman
**Intron UCSC
H3K9ac Trynka
H3K4me3 Trynka
H3K4me1 Trynka
**H3K27ac PGC2
H3K27ac Hnisz
FetalDHS Trynka
Enhancer Hoffman
Enhancer Andersson
DHS Trynka
DGF ENCODE
CTCF Hoffman
**Conserved LindbladToh
Coding UCSC
Annotation
−10
−5
0
5
10
15
Enrichment (prop h2 / prop snps)

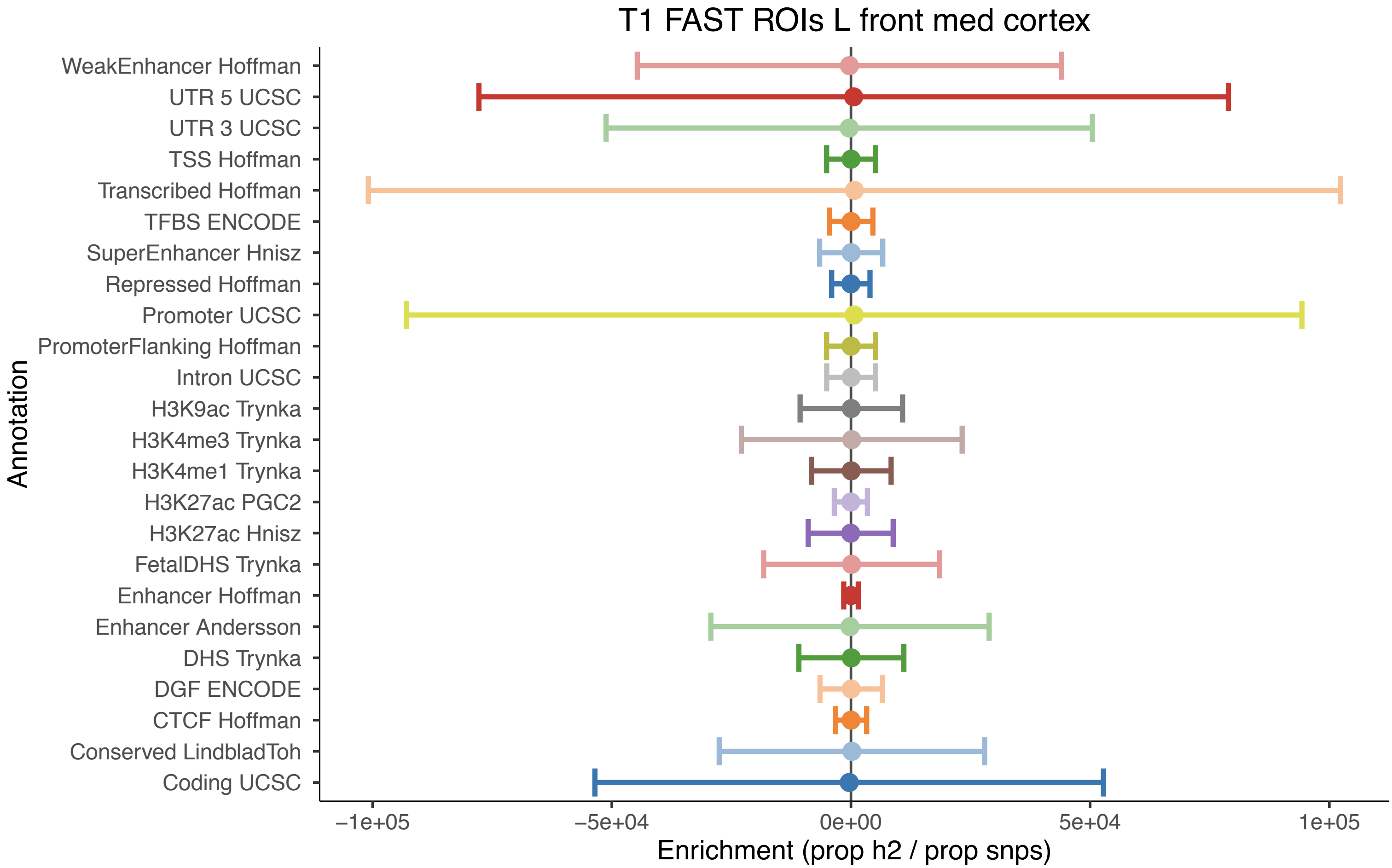
T1 FAST ROIs L front med cortex
WeakEnhancer Hoffman
UTR 5 UCSC
UTR 3 UCSC
TSS Hoffman
Transcribed Hoffman
TFBS ENCODE
SuperEnhancer Hnisz
Repressed Hoffman
Promoter UCSC
PromoterFlanking Hoffman
Intron UCSC
H3K9ac Trynka
H3K4me3 Trynka
H3K4me1 Trynka
H3K27ac PGC2
H3K27ac Hnisz
FetalDHS Trynka
Enhancer Hoffman
Enhancer Andersson
DHS Trynka
DGF ENCODE
CTCF Hoffman
Conserved LindbladToh
Coding UCSC
Annotation
−1e+05
−5e+04
0e+00
5e+04
1e+05
Enrichment (prop h2 / prop snps)

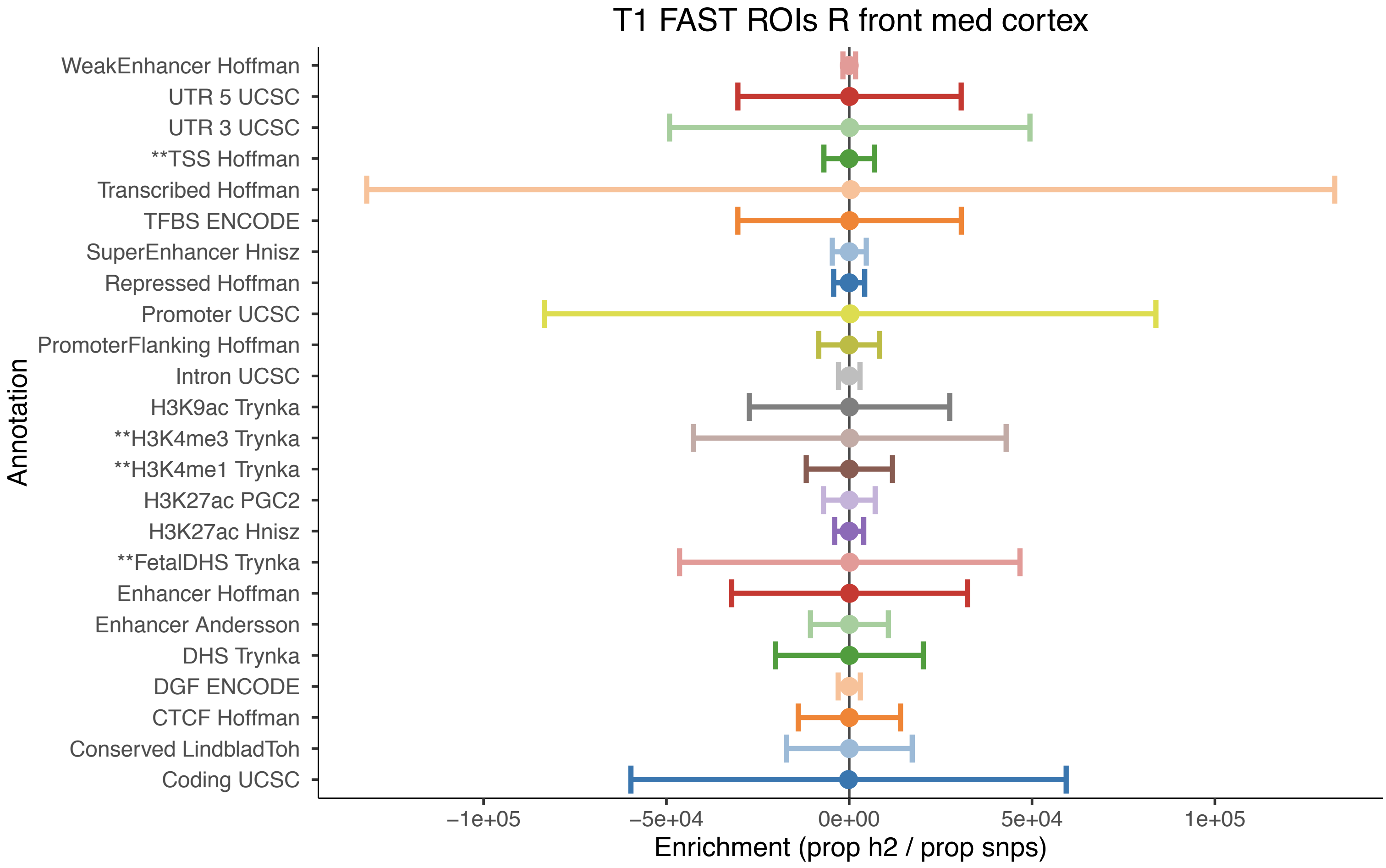
T1 FAST ROIs R front med cortex
WeakEnhancer Hoffman
UTR 5 UCSC
UTR 3 UCSC
**TSS Hoffman
Transcribed Hoffman
TFBS ENCODE
SuperEnhancer Hnisz
Repressed Hoffman
Promoter UCSC
PromoterFlanking Hoffman
Intron UCSC
H3K9ac Trynka
**H3K4me3 Trynka
**H3K4me1 Trynka
H3K27ac PGC2
H3K27ac Hnisz
**FetalDHS Trynka
Enhancer Hoffman
Enhancer Andersson
DHS Trynka
DGF ENCODE
CTCF Hoffman
Conserved LindbladToh
Coding UCSC
Annotation
−1e+05
−5e+04
0e+00
5e+04
1e+05
Enrichment (prop h2 / prop snps)

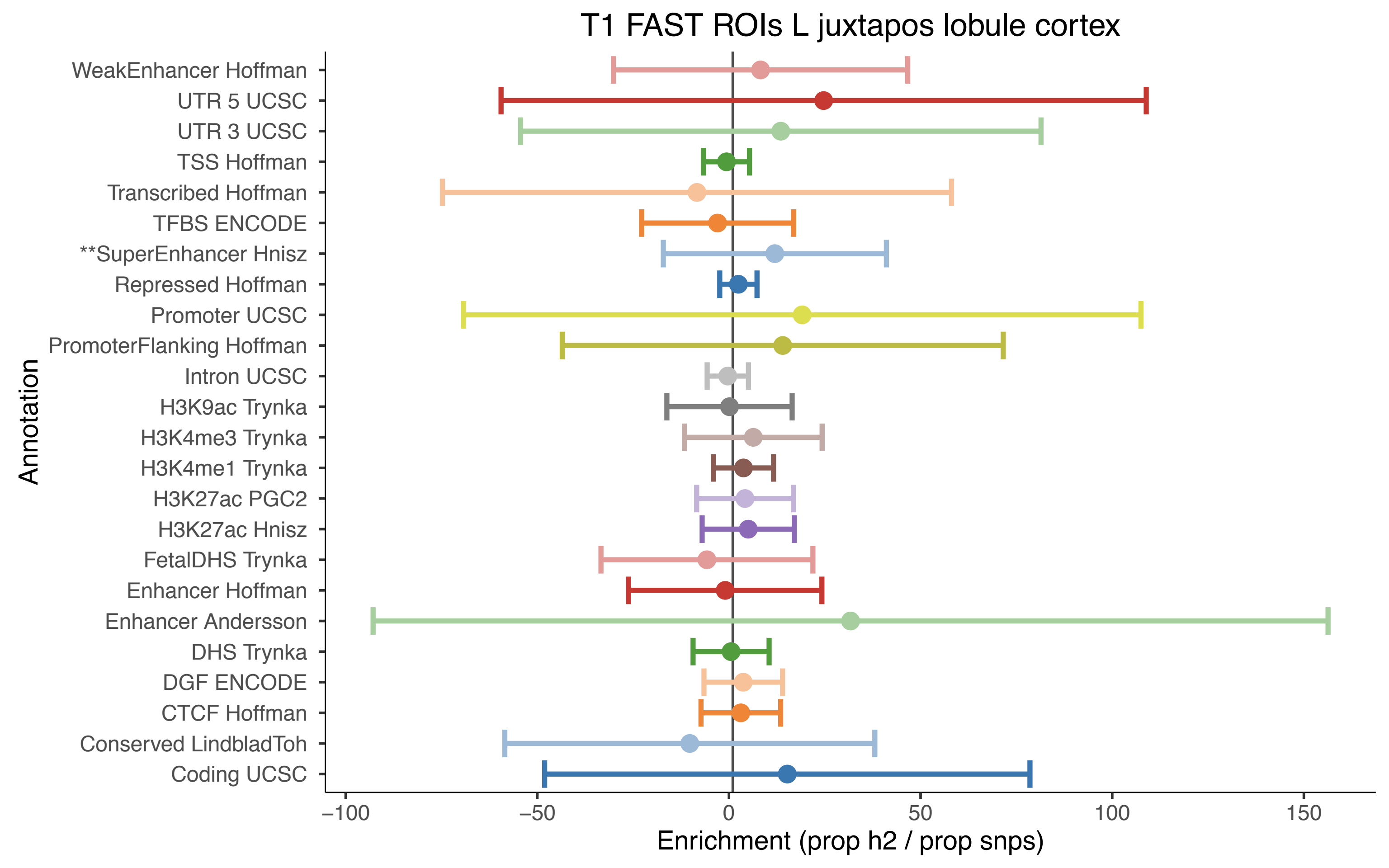
T1 FAST ROIs L juxtapos lobule cortex
WeakEnhancer Hoffman
UTR 5 UCSC
UTR 3 UCSC
TSS Hoffman
Transcribed Hoffman
TFBS ENCODE
**SuperEnhancer Hnisz
Repressed Hoffman
Promoter UCSC
PromoterFlanking Hoffman
Intron UCSC
H3K9ac Trynka
H3K4me3 Trynka
H3K4me1 Trynka
H3K27ac PGC2
H3K27ac Hnisz
FetalDHS Trynka
Enhancer Hoffman
Enhancer Andersson
DHS Trynka
DGF ENCODE
CTCF Hoffman
Conserved LindbladToh
Coding UCSC
Annotation
−100
−50
0
50
100
150
Enrichment (prop h2 / prop snps)

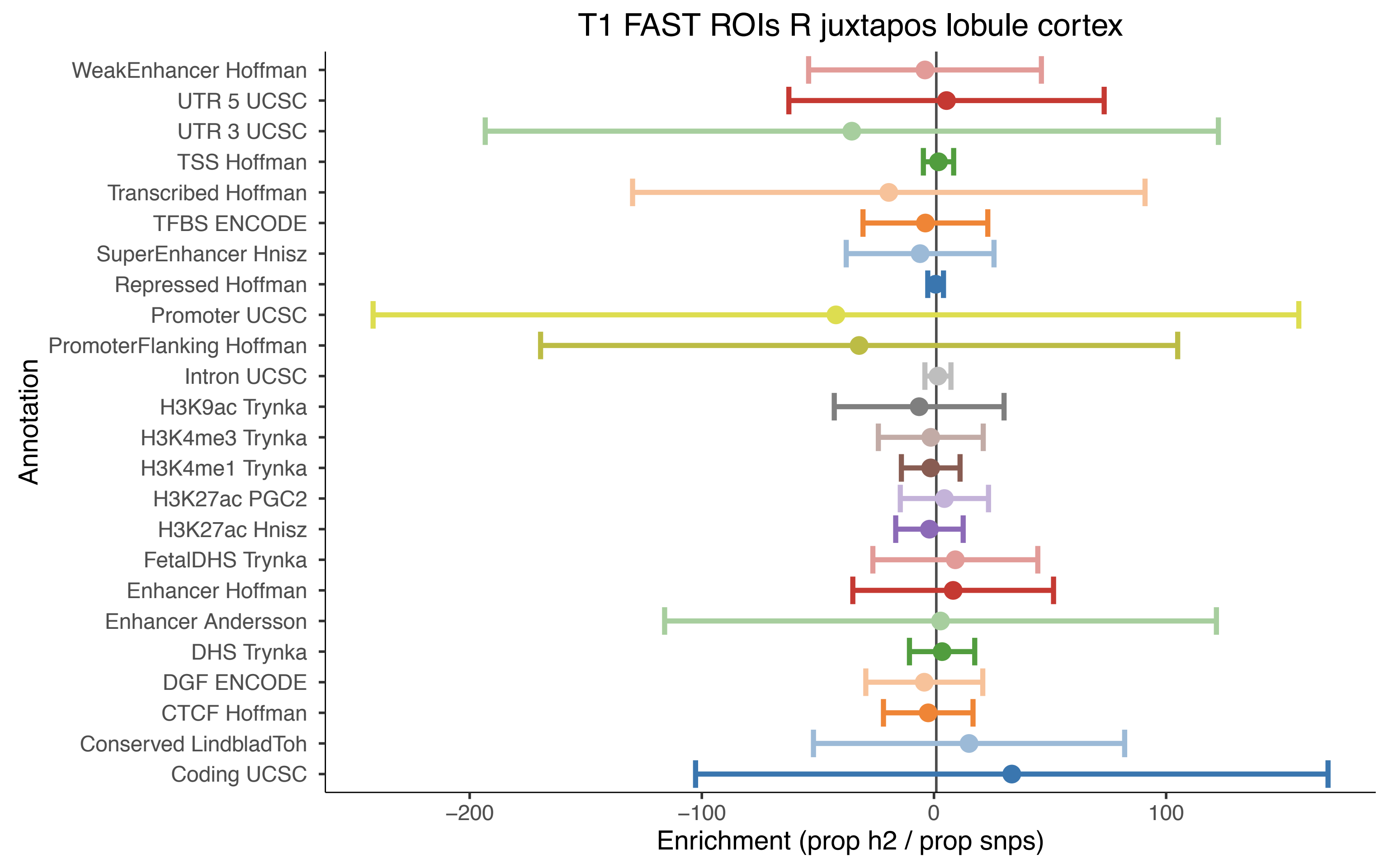
T1 FAST ROIs R juxtapos lobule cortex
WeakEnhancer Hoffman
UTR 5 UCSC
UTR 3 UCSC
TSS Hoffman
Transcribed Hoffman
TFBS ENCODE
SuperEnhancer Hnisz
Repressed Hoffman
Promoter UCSC
PromoterFlanking Hoffman
Intron UCSC
H3K9ac Trynka
H3K4me3 Trynka
H3K4me1 Trynka
H3K27ac PGC2
H3K27ac Hnisz
FetalDHS Trynka
Enhancer Hoffman
Enhancer Andersson
DHS Trynka
DGF ENCODE
CTCF Hoffman
Conserved LindbladToh
Coding UCSC
Annotation
−200
−100
0
100
Enrichment (prop h2 / prop snps)

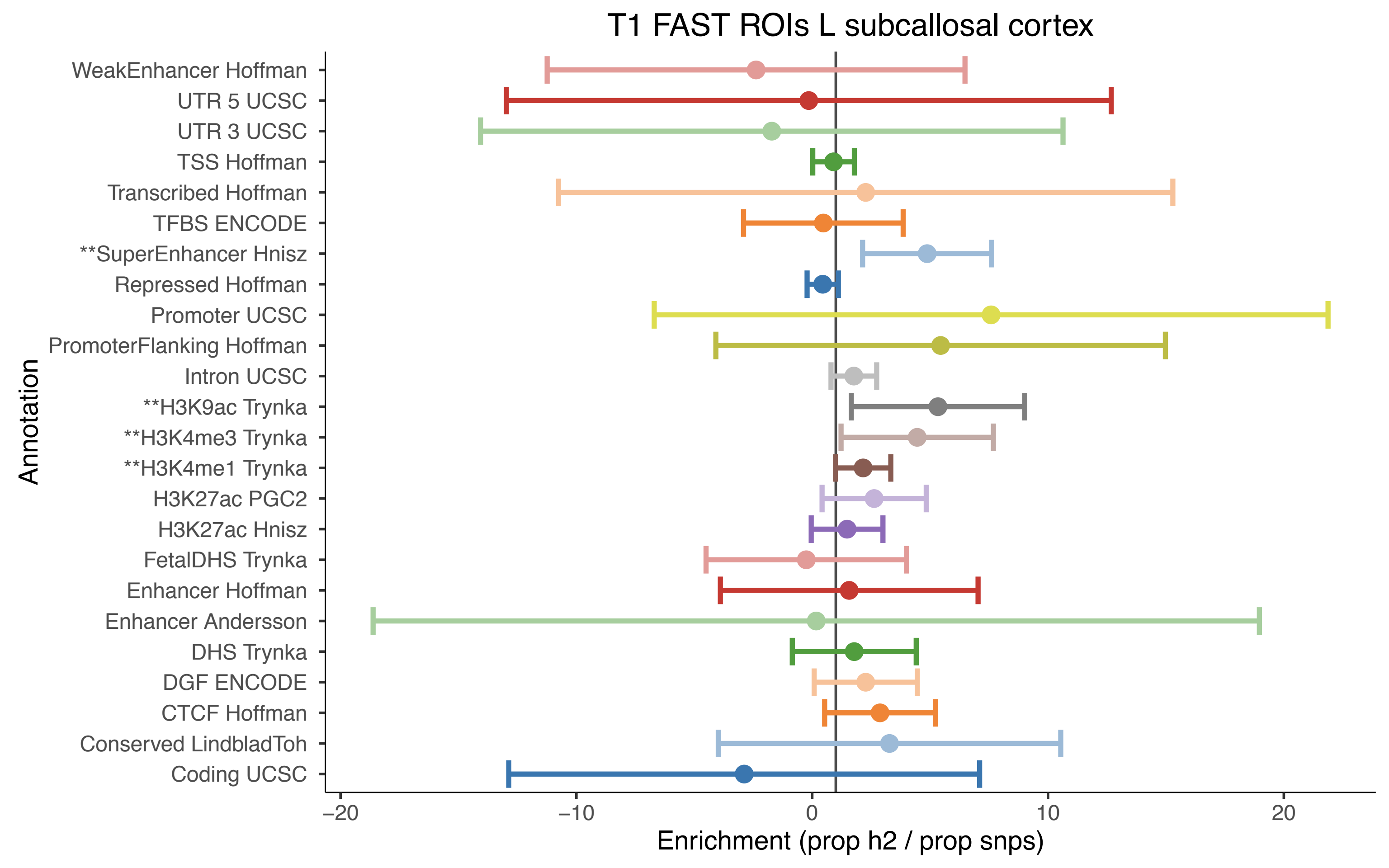

T1 FAST ROIs L subcallosal cortex
WeakEnhancer Hoffman
UTR 5 UCSC
UTR 3 UCSC
TSS Hoffman
Transcribed Hoffman
TFBS ENCODE
**SuperEnhancer Hnisz
Repressed Hoffman
Promoter UCSC
PromoterFlanking Hoffman
Intron UCSC
**H3K9ac Trynka
**H3K4me3 Trynka
**H3K4me1 Trynka
H3K27ac PGC2
H3K27ac Hnisz
FetalDHS Trynka
Enhancer Hoffman
Enhancer Andersson
DHS Trynka
DGF ENCODE
CTCF Hoffman
Conserved LindbladToh
Coding UCSC
Annotation
−20
−10
0
10
20
Enrichment (prop h2 / prop snps)

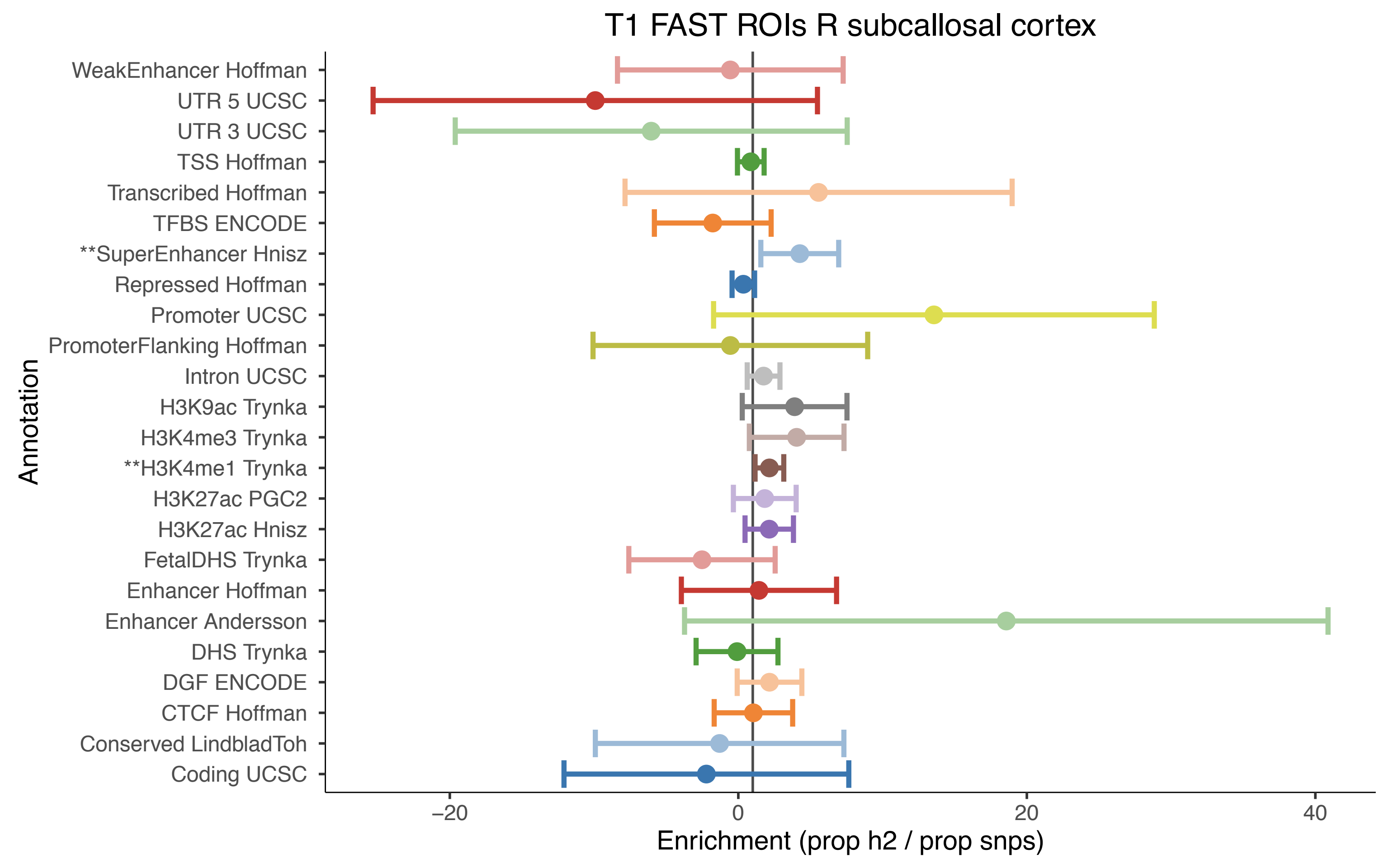

T1 FAST ROIs R subcallosal cortex
WeakEnhancer Hoffman
UTR 5 UCSC
UTR 3 UCSC
TSS Hoffman
Transcribed Hoffman
TFBS ENCODE
**SuperEnhancer Hnisz
Repressed Hoffman
Promoter UCSC
PromoterFlanking Hoffman
Intron UCSC
H3K9ac Trynka
H3K4me3 Trynka
**H3K4me1 Trynka
H3K27ac PGC2
H3K27ac Hnisz
FetalDHS Trynka
Enhancer Hoffman
Enhancer Andersson
DHS Trynka
DGF ENCODE
CTCF Hoffman
Conserved LindbladToh
Coding UCSC
Annotation
−20
0
20
40
Enrichment (prop h2 / prop snps)

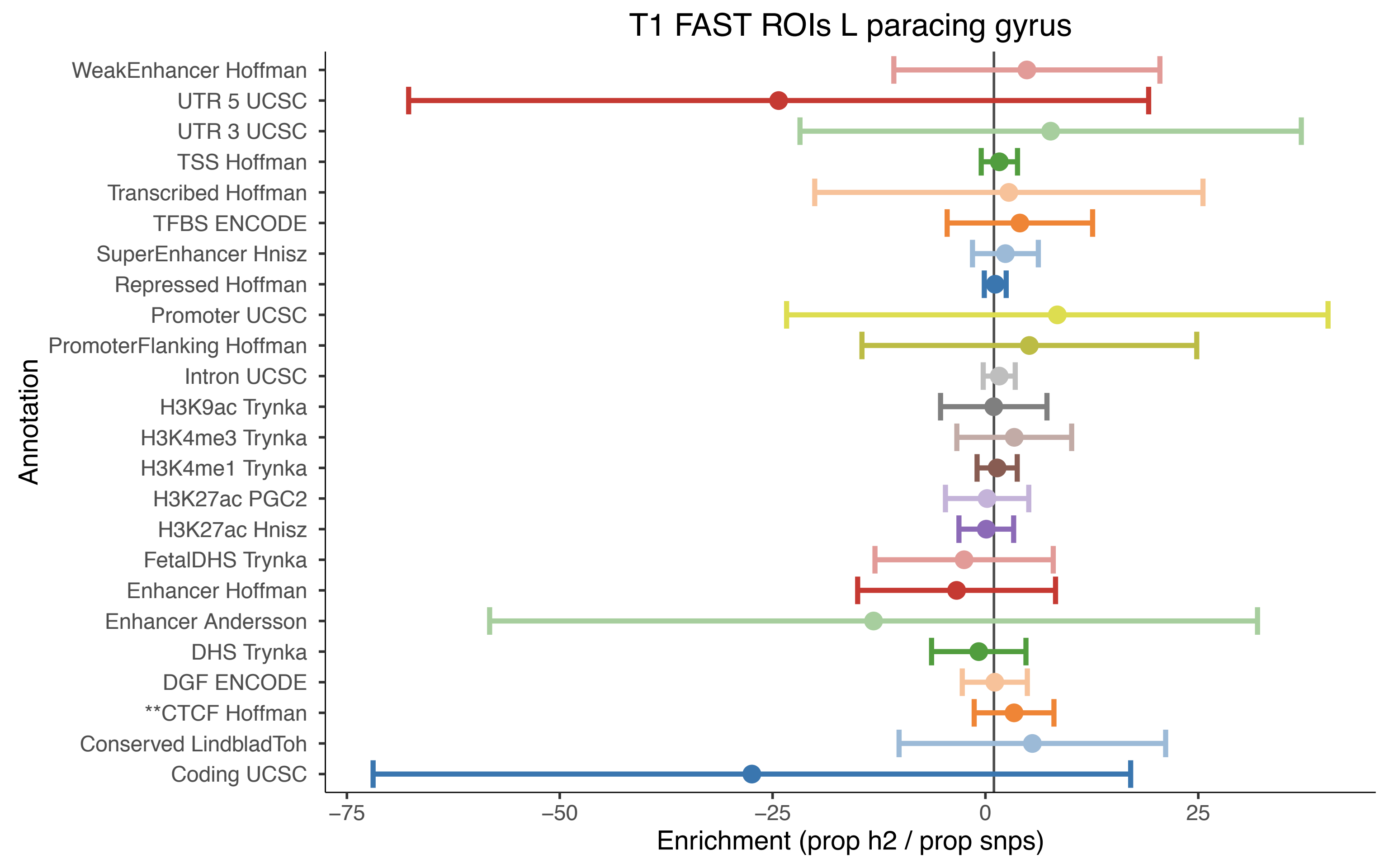

T1 FAST ROIs L paracing gyrus
Annotation
WeakEnhancer Hoffman
UTR 5 UCSC
UTR 3 UCSC
TSS Hoffman
Transcribed Hoffman
TFBS ENCODE
SuperEnhancer Hnisz
Repressed Hoffman
Promoter UCSC
PromoterFlanking Hoffman
Intron UCSC
H3K9ac Trynka
H3K4me3 Trynka
H3K4me1 Trynka
H3K27ac PGC2
H3K27ac Hnisz
FetalDHS Trynka
Enhancer Hoffman
Enhancer Andersson
DHS Trynka
DGF ENCODE
**CTCF Hoffman
Conserved LindbladToh
Coding UCSC
−75
−50
−25
0
25
Enrichment (prop h2 / prop snps)

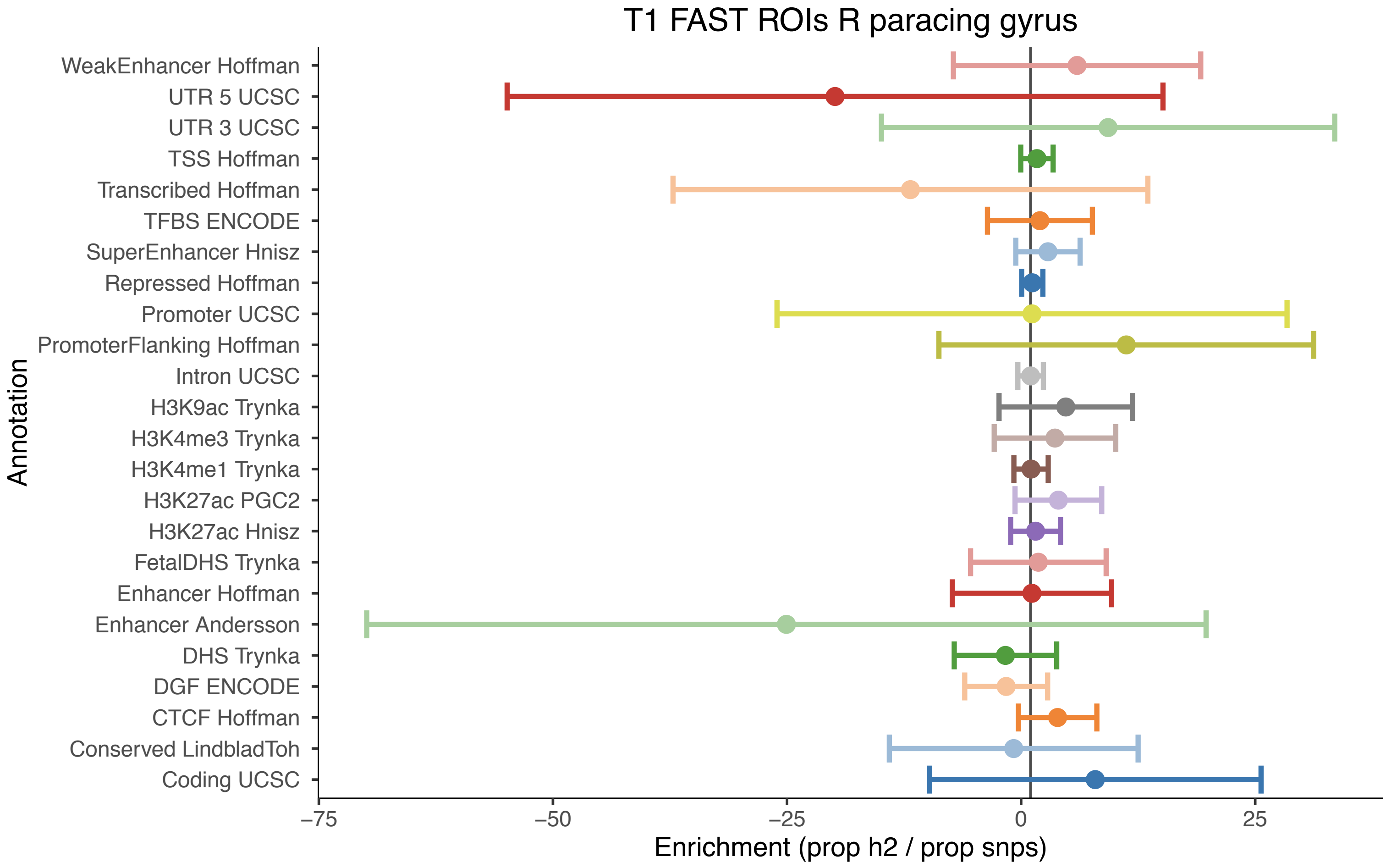

T1 FAST ROIs R paracing gyrus
WeakEnhancer Hoffman
UTR 5 UCSC
UTR 3 UCSC
TSS Hoffman
Transcribed Hoffman
TFBS ENCODE
SuperEnhancer Hnisz
Repressed Hoffman
Promoter UCSC
PromoterFlanking Hoffman
Intron UCSC
H3K9ac Trynka
H3K4me3 Trynka
H3K4me1 Trynka
H3K27ac PGC2
H3K27ac Hnisz
FetalDHS Trynka
Enhancer Hoffman
Enhancer Andersson
DHS Trynka
DGF ENCODE
CTCF Hoffman
Conserved LindbladToh
Coding UCSC
Annotation
−75
−50
−25
0
25
Enrichment (prop h2 / prop snps)

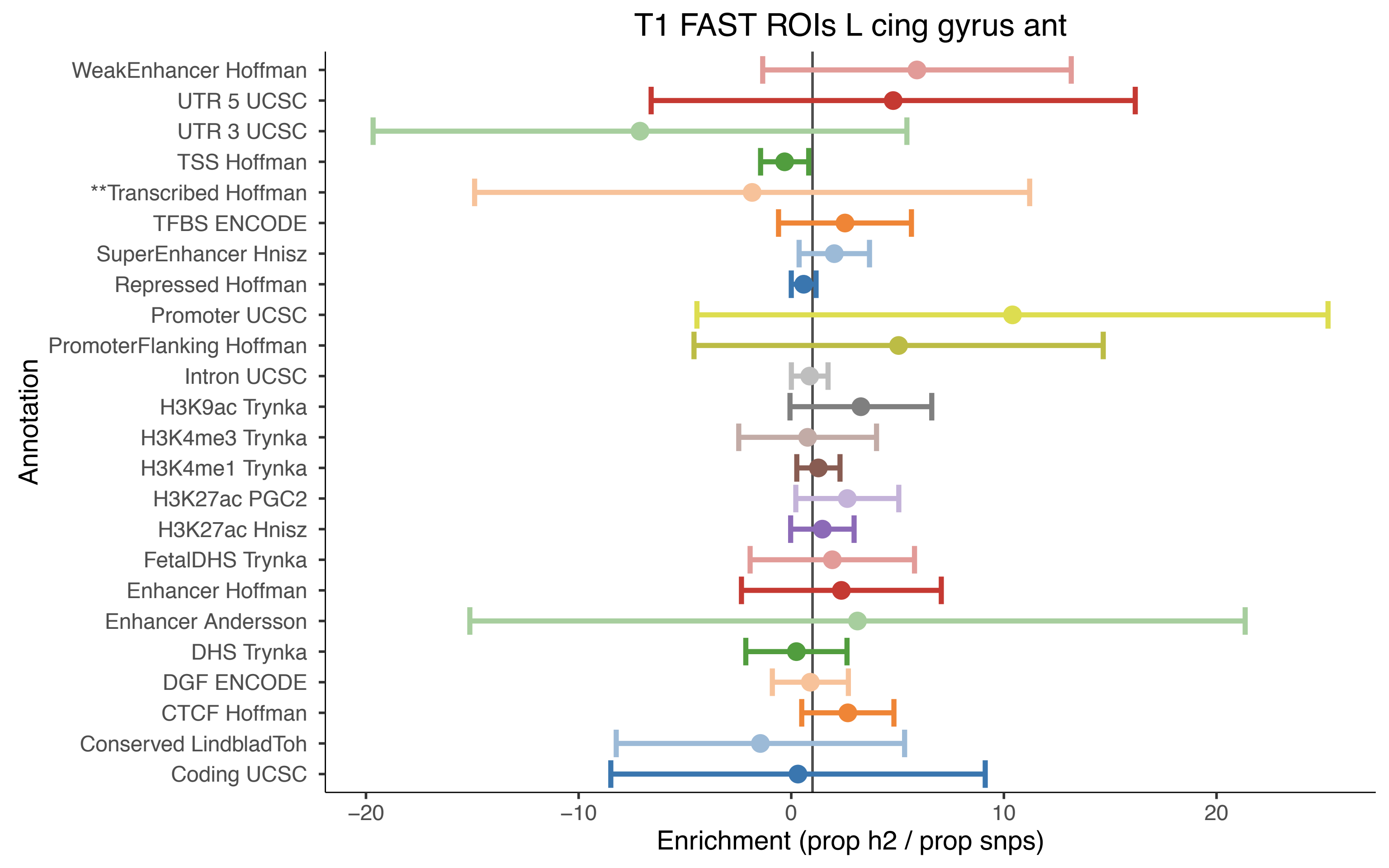
T1 FAST ROIs L cing gyrus ant
WeakEnhancer Hoffman
UTR 5 UCSC
UTR 3 UCSC
TSS Hoffman
**Transcribed Hoffman
TFBS ENCODE
SuperEnhancer Hnisz
Repressed Hoffman
Promoter UCSC
PromoterFlanking Hoffman
Intron UCSC
H3K9ac Trynka
H3K4me3 Trynka
H3K4me1 Trynka
H3K27ac PGC2
H3K27ac Hnisz
FetalDHS Trynka
Enhancer Hoffman
Enhancer Andersson
DHS Trynka
DGF ENCODE
CTCF Hoffman
Conserved LindbladToh
Coding UCSC
Annotation
−20
−10
0
10
20
Enrichment (prop h2 / prop snps)

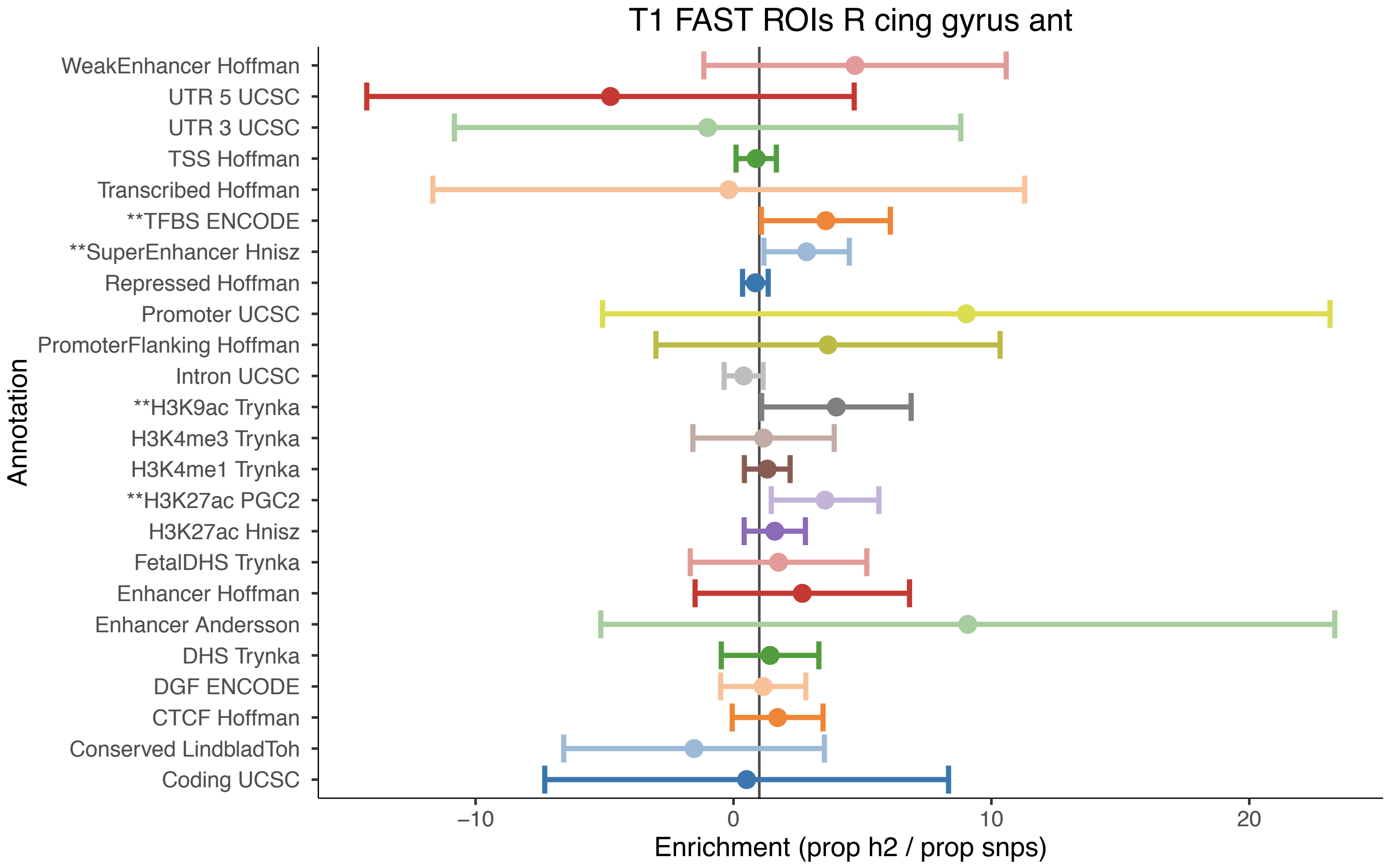
T1 FAST ROIs R cing gyrus ant
WeakEnhancer Hoffman
UTR 5 UCSC
UTR 3 UCSC
TSS Hoffman
Transcribed Hoffman
**TFBS ENCODE
**SuperEnhancer Hnisz
Repressed Hoffman
Promoter UCSC
PromoterFlanking Hoffman
Intron UCSC
**H3K9ac Trynka
H3K4me3 Trynka
H3K4me1 Trynka
**H3K27ac PGC2
H3K27ac Hnisz
FetalDHS Trynka
Enhancer Hoffman
Enhancer Andersson
DHS Trynka
DGF ENCODE
CTCF Hoffman
Conserved LindbladToh
Coding UCSC
Annotation
−10
0
10
20
Enrichment (prop h2 / prop snps)

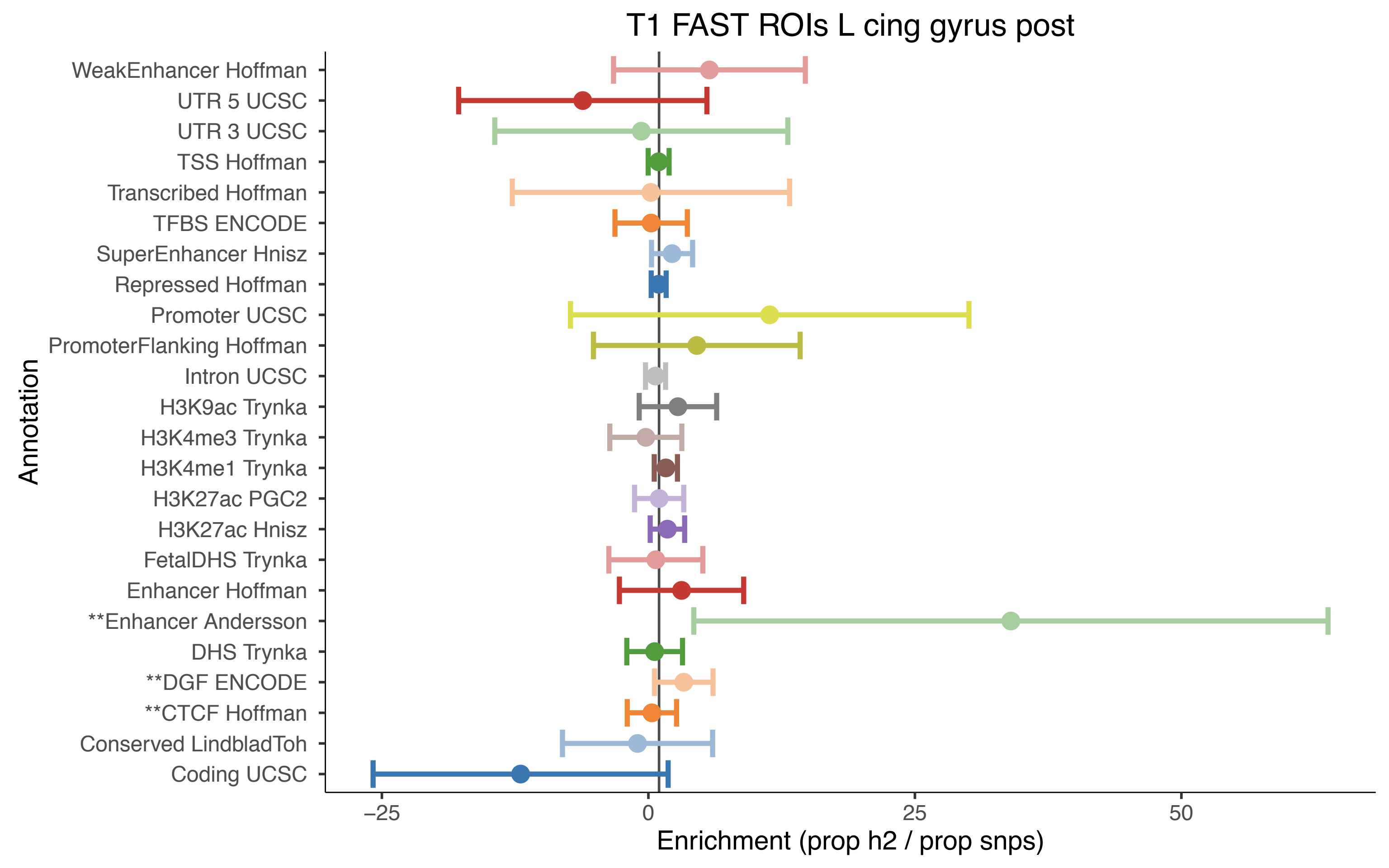

T1 FAST ROIs L cing gyrus post
WeakEnhancer Hoffman
UTR 5 UCSC
UTR 3 UCSC
TSS Hoffman
Transcribed Hoffman
TFBS ENCODE
SuperEnhancer Hnisz
Repressed Hoffman
Promoter UCSC
PromoterFlanking Hoffman
Intron UCSC
H3K9ac Trynka
H3K4me3 Trynka
H3K4me1 Trynka
H3K27ac PGC2
H3K27ac Hnisz
FetalDHS Trynka
Enhancer Hoffman
**Enhancer Andersson
DHS Trynka
**DGF ENCODE
**CTCF Hoffman
Conserved LindbladToh
Coding UCSC
Annotation
−25
0
25
50
Enrichment (prop h2 / prop snps)

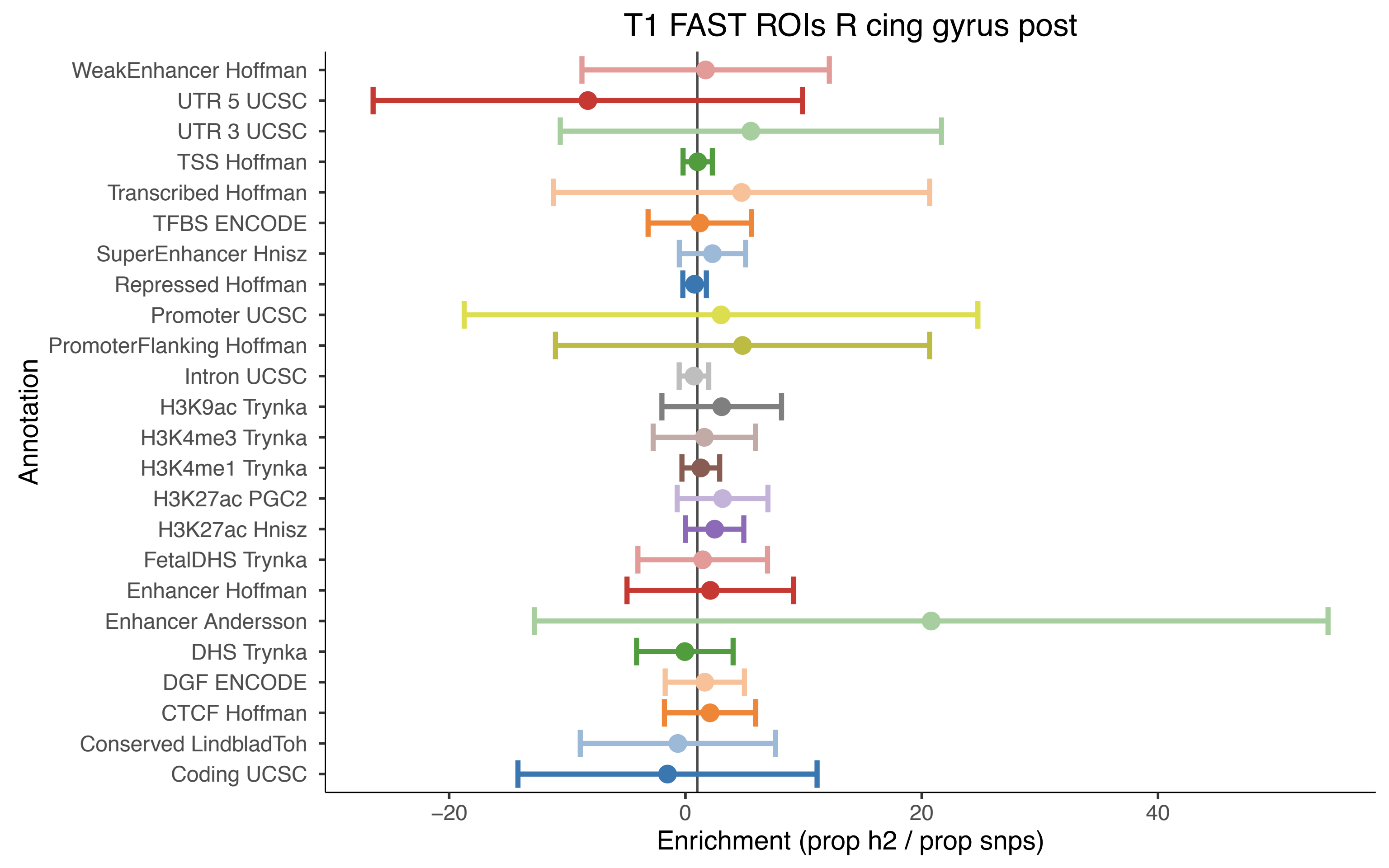
T1 FAST ROIs R cing gyrus post
WeakEnhancer Hoffman
UTR 5 UCSC
UTR 3 UCSC
TSS Hoffman
Transcribed Hoffman
TFBS ENCODE
SuperEnhancer Hnisz
Repressed Hoffman
Promoter UCSC
PromoterFlanking Hoffman
Intron UCSC
H3K9ac Trynka
H3K4me3 Trynka
H3K4me1 Trynka
H3K27ac PGC2
H3K27ac Hnisz
FetalDHS Trynka
Enhancer Hoffman
Enhancer Andersson
DHS Trynka
DGF ENCODE
CTCF Hoffman
Conserved LindbladToh
Coding UCSC
Annotation
−20
0
20
40
Enrichment (prop h2 / prop snps)

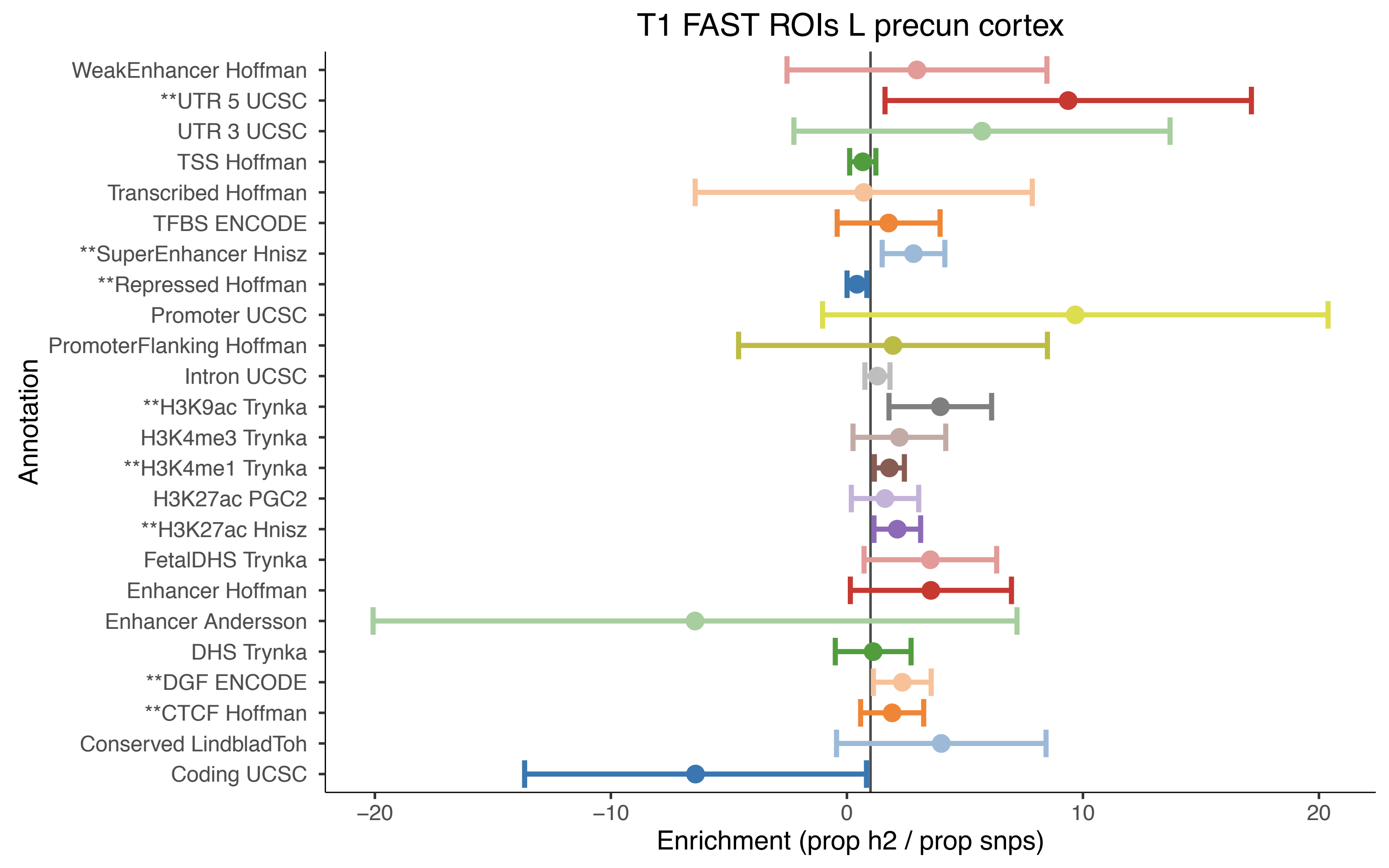
T1 FAST ROIs L precun cortex
WeakEnhancer Hoffman
**UTR 5 UCSC
UTR 3 UCSC
TSS Hoffman
Transcribed Hoffman
TFBS ENCODE
**SuperEnhancer Hnisz
**Repressed Hoffman
Promoter UCSC
PromoterFlanking Hoffman
Intron UCSC
**H3K9ac Trynka
H3K4me3 Trynka
**H3K4me1 Trynka
H3K27ac PGC2
**H3K27ac Hnisz
FetalDHS Trynka
Enhancer Hoffman
Enhancer Andersson
DHS Trynka
**DGF ENCODE
**CTCF Hoffman
Conserved LindbladToh
Coding UCSC
Annotation
−20
−10
0
10
20
Enrichment (prop h2 / prop snps)

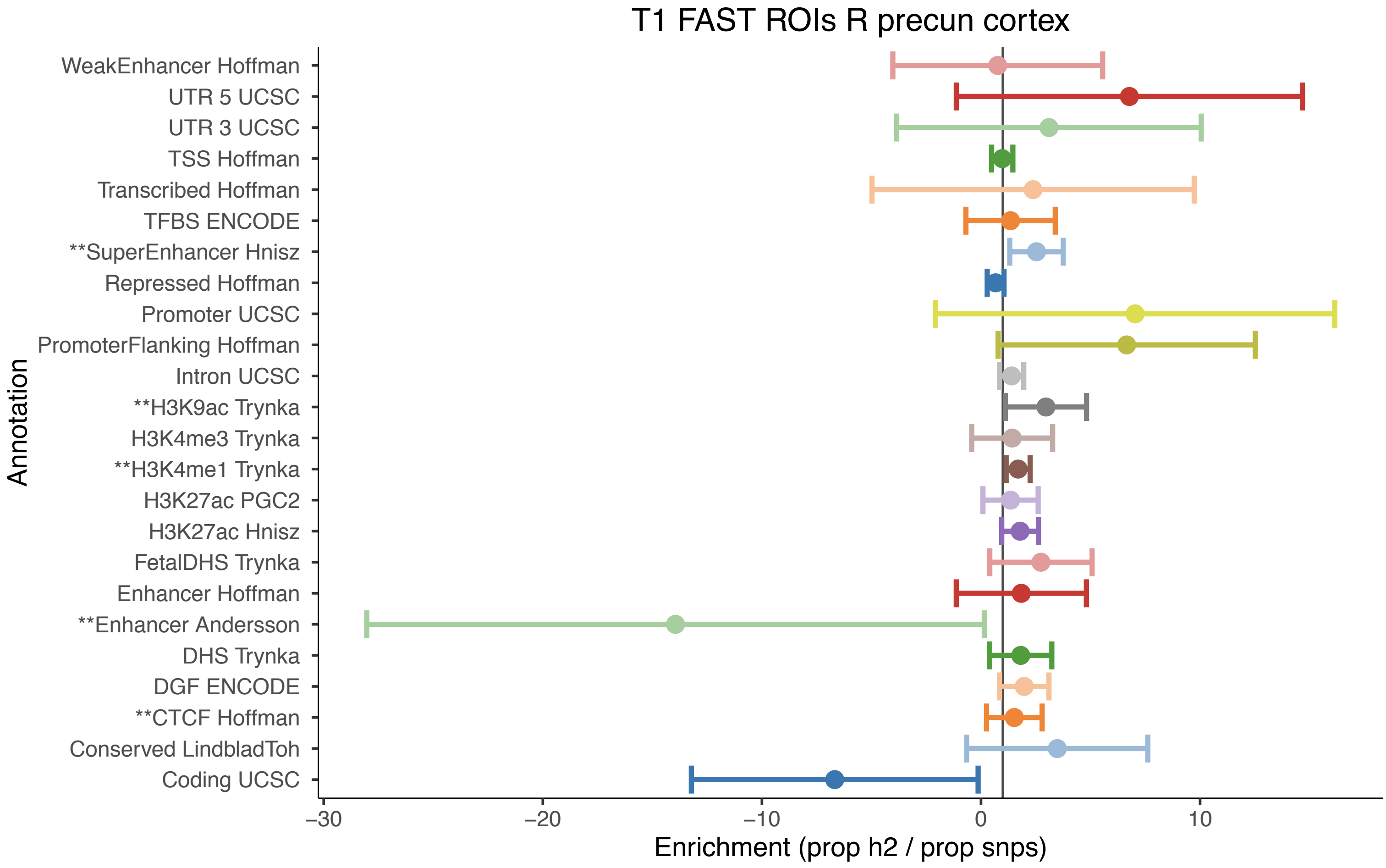
T1 FAST ROIs R precun cortex
WeakEnhancer Hoffman
UTR 5 UCSC
UTR 3 UCSC
TSS Hoffman
Transcribed Hoffman
TFBS ENCODE
**SuperEnhancer Hnisz
Repressed Hoffman
Promoter UCSC
PromoterFlanking Hoffman
Intron UCSC
**H3K9ac Trynka
H3K4me3 Trynka
**H3K4me1 Trynka
H3K27ac PGC2
H3K27ac Hnisz
FetalDHS Trynka
Enhancer Hoffman
**Enhancer Andersson
DHS Trynka
DGF ENCODE
**CTCF Hoffman
Conserved LindbladToh
Coding UCSC
Annotation
−30
−20
−10
0
10
Enrichment (prop h2 / prop snps)

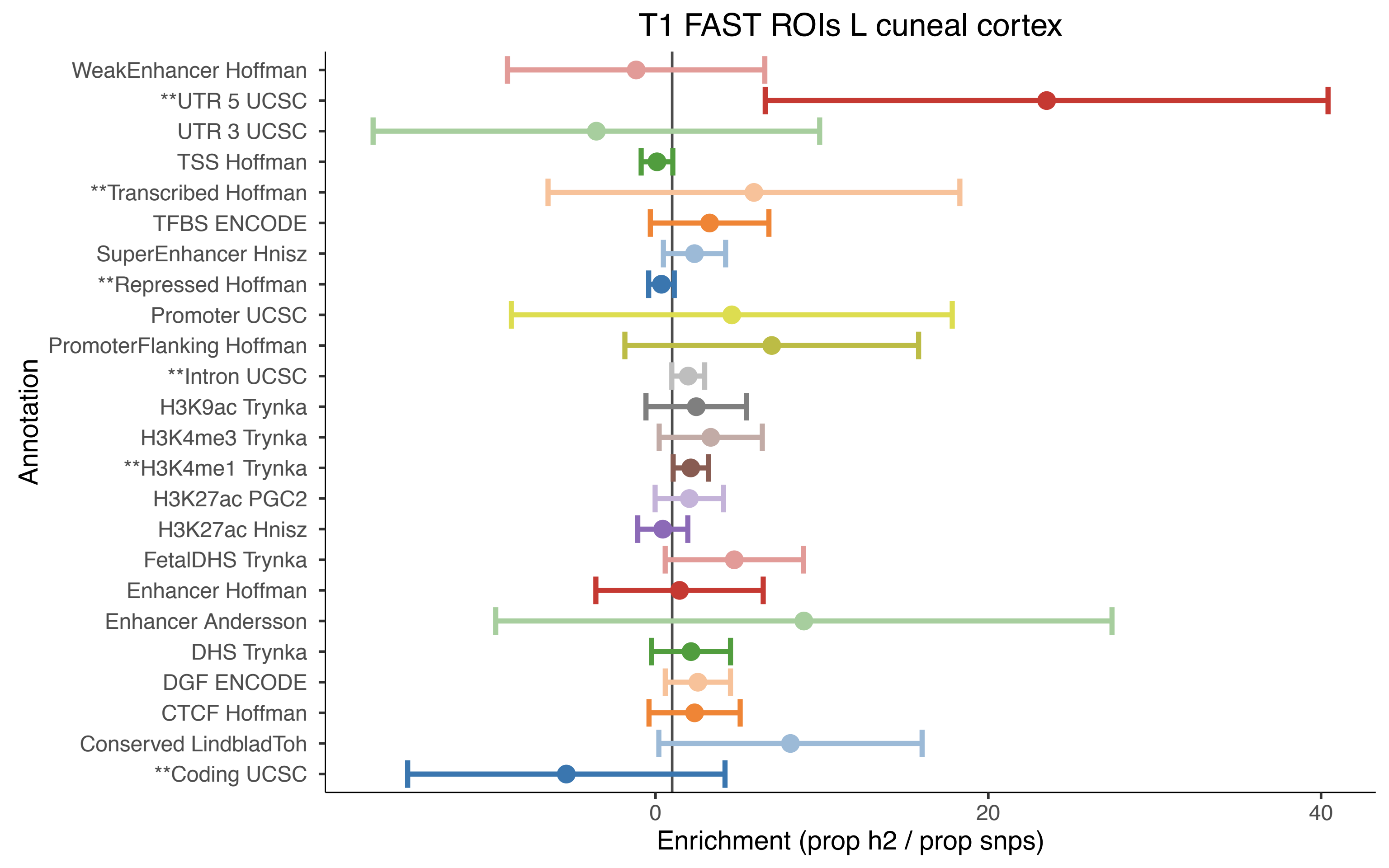

T1 FAST ROIs L cuneal cortex
WeakEnhancer Hoffman
**UTR 5 UCSC
UTR 3 UCSC
TSS Hoffman
**Transcribed Hoffman
TFBS ENCODE
SuperEnhancer Hnisz
**Repressed Hoffman
Promoter UCSC
PromoterFlanking Hoffman
**Intron UCSC
H3K9ac Trynka
H3K4me3 Trynka
**H3K4me1 Trynka
H3K27ac PGC2
H3K27ac Hnisz
FetalDHS Trynka
Enhancer Hoffman
Enhancer Andersson
DHS Trynka
DGF ENCODE
CTCF Hoffman
Conserved LindbladToh
**Coding UCSC
Annotation
0
20
40
Enrichment (prop h2 / prop snps)

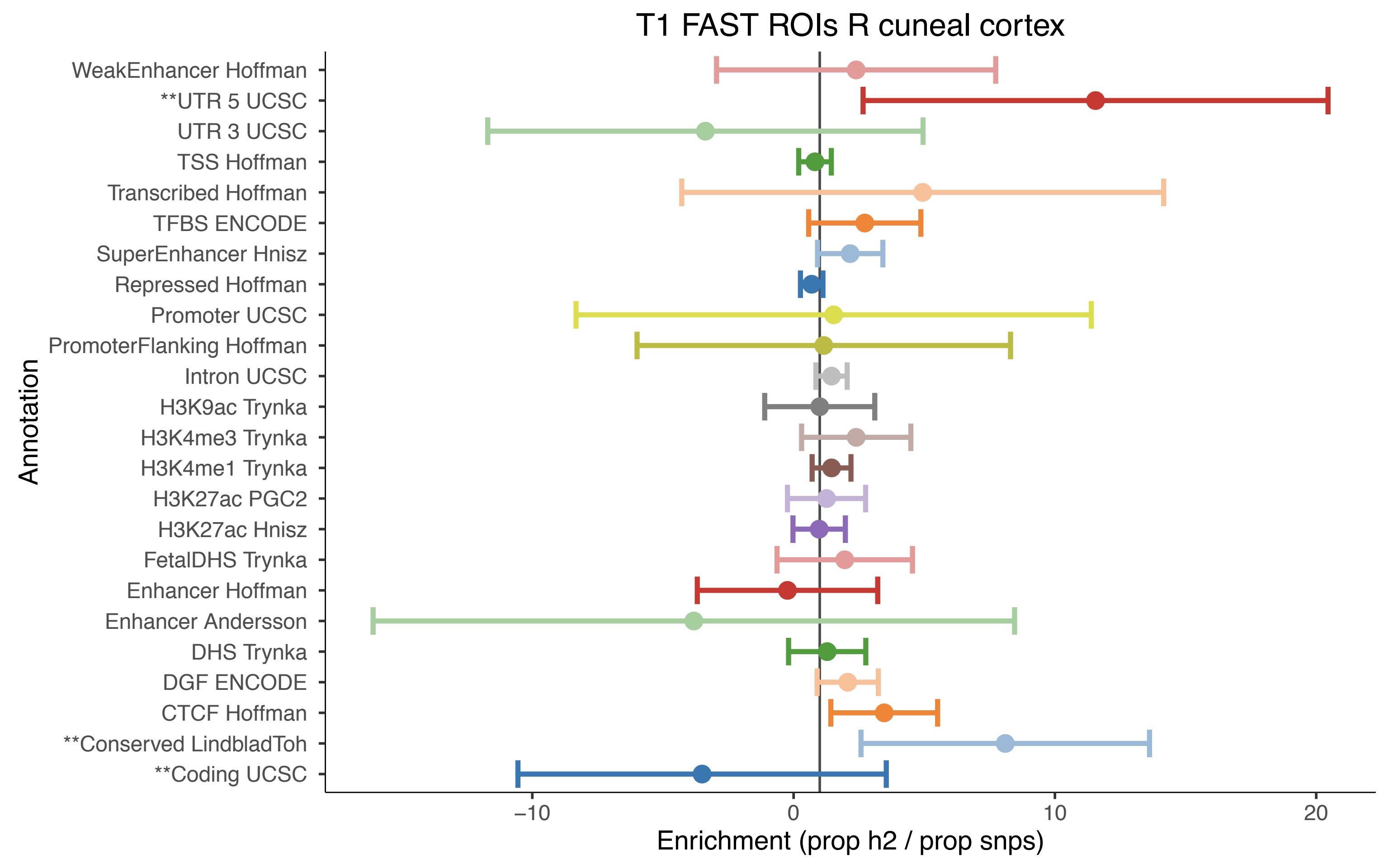
T1 FAST ROIs R cuneal cortex
WeakEnhancer Hoffman
**UTR 5 UCSC
UTR 3 UCSC
TSS Hoffman
Transcribed Hoffman
TFBS ENCODE
SuperEnhancer Hnisz
Repressed Hoffman
Promoter UCSC
PromoterFlanking Hoffman
Intron UCSC
H3K9ac Trynka
H3K4me3 Trynka
H3K4me1 Trynka
H3K27ac PGC2
H3K27ac Hnisz
FetalDHS Trynka
Enhancer Hoffman
Enhancer Andersson
DHS Trynka
DGF ENCODE
CTCF Hoffman
**Conserved LindbladToh
**Coding UCSC
Annotation
−10
0
10
20
Enrichment (prop h2 / prop snps)

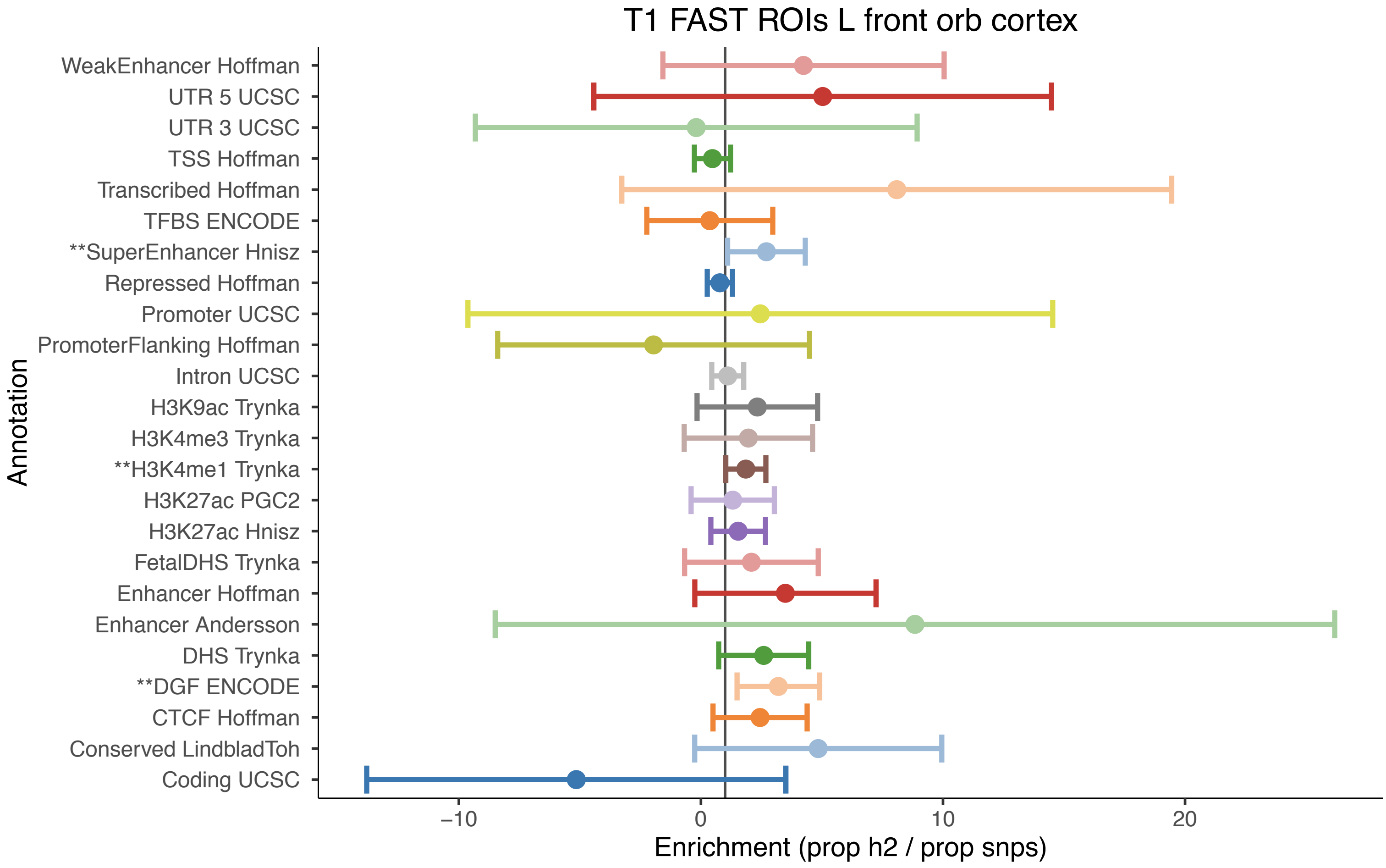
T1 FAST ROIs L front orb cortex
WeakEnhancer Hoffman
UTR 5 UCSC
UTR 3 UCSC
TSS Hoffman
Transcribed Hoffman
TFBS ENCODE
**SuperEnhancer Hnisz
Repressed Hoffman
Promoter UCSC
PromoterFlanking Hoffman
Intron UCSC
H3K9ac Trynka
H3K4me3 Trynka
**H3K4me1 Trynka
H3K27ac PGC2
H3K27ac Hnisz
FetalDHS Trynka
Enhancer Hoffman
Enhancer Andersson
DHS Trynka
**DGF ENCODE
CTCF Hoffman
Conserved LindbladToh
Coding UCSC
Annotation
−10
0
10
20
Enrichment (prop h2 / prop snps)

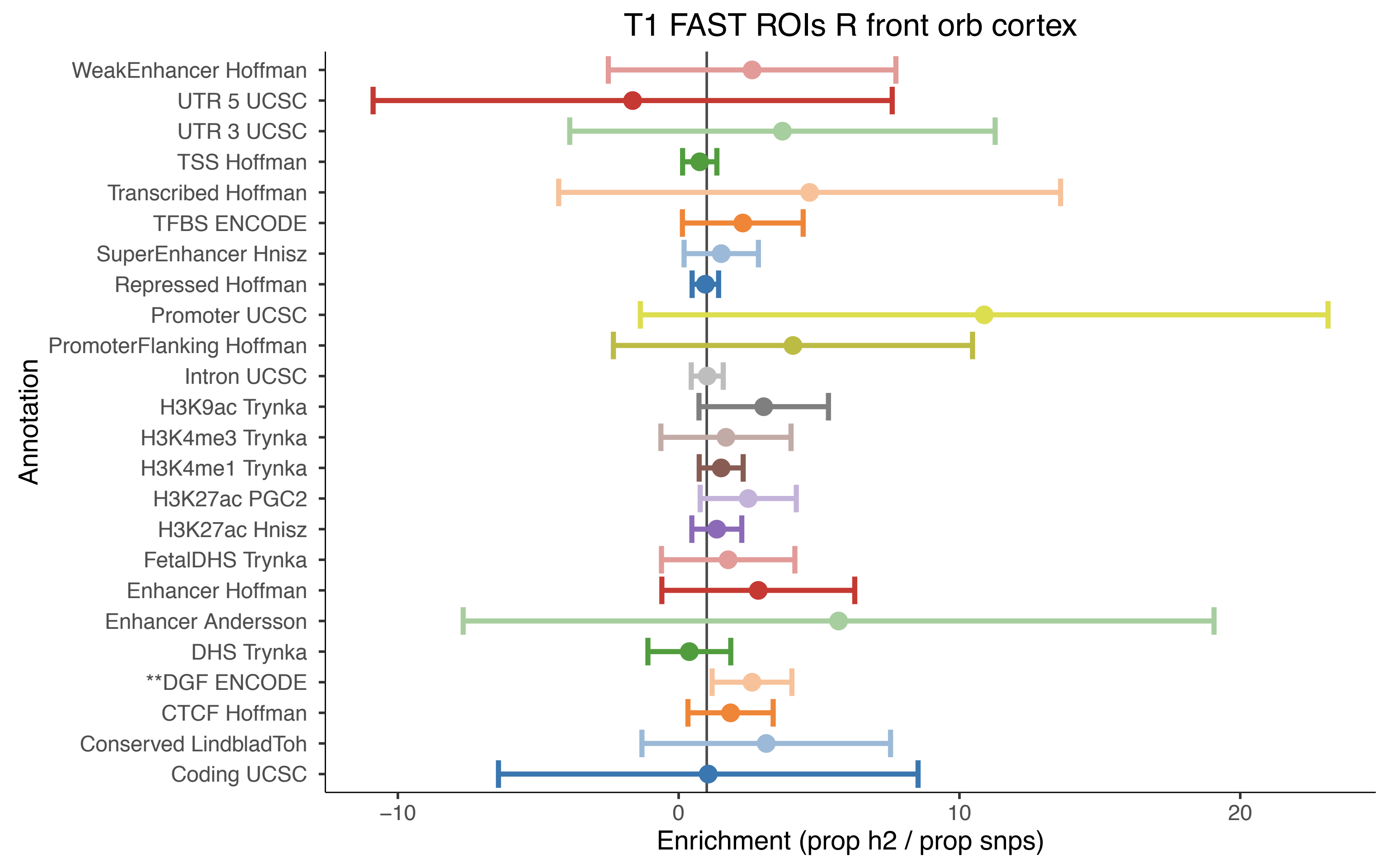
T1 FAST ROIs R front orb cortex
WeakEnhancer Hoffman
UTR 5 UCSC
UTR 3 UCSC
TSS Hoffman
Transcribed Hoffman
TFBS ENCODE
SuperEnhancer Hnisz
Repressed Hoffman
Promoter UCSC
PromoterFlanking Hoffman
Intron UCSC
H3K9ac Trynka
H3K4me3 Trynka
H3K4me1 Trynka
H3K27ac PGC2
H3K27ac Hnisz
FetalDHS Trynka
Enhancer Hoffman
Enhancer Andersson
DHS Trynka
**DGF ENCODE
CTCF Hoffman
Conserved LindbladToh
Coding UCSC
Annotation
−10
0
10
20
Enrichment (prop h2 / prop snps)

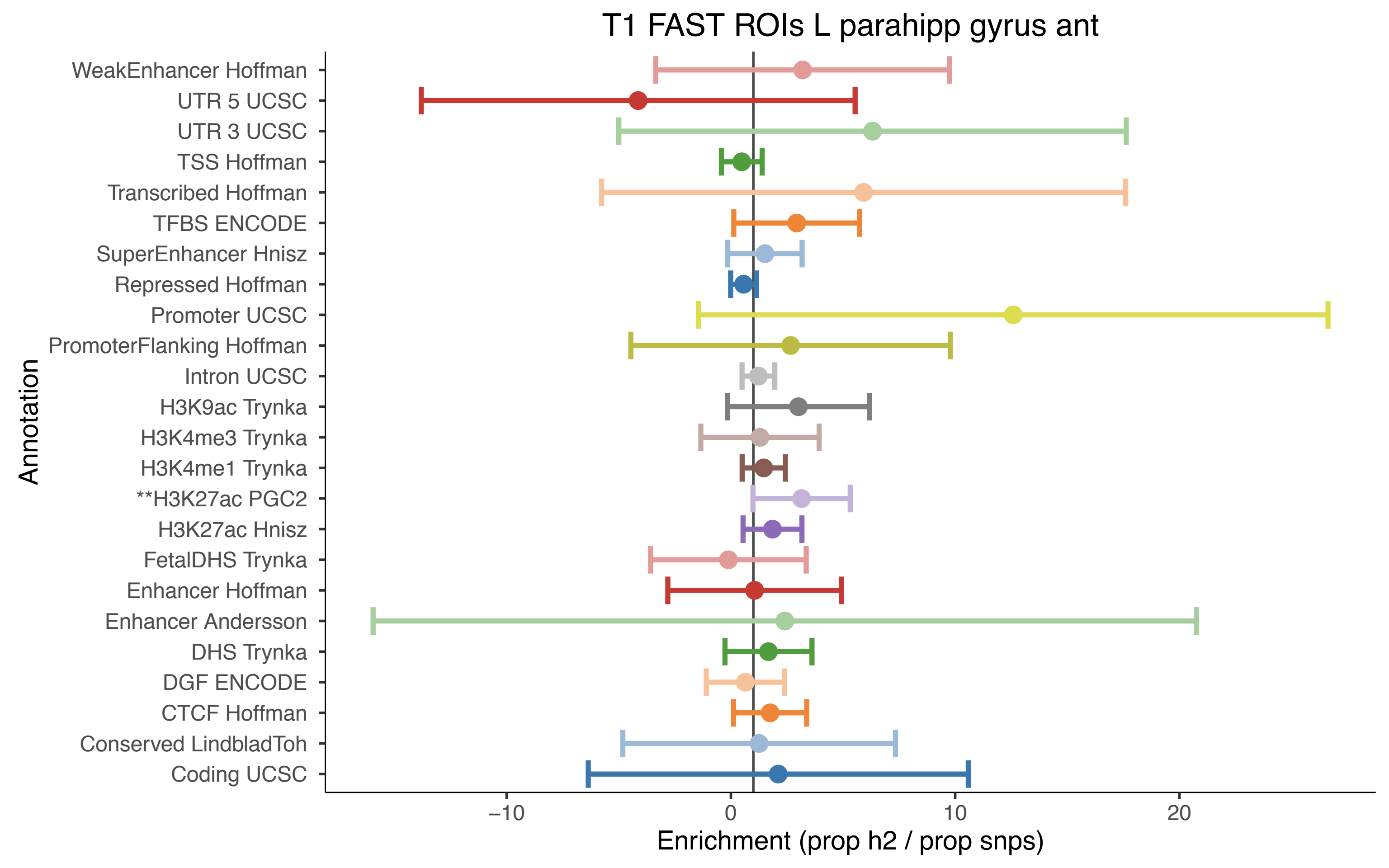
T1 FAST ROIs L parahipp gyrus ant
WeakEnhancer Hoffman
UTR 5 UCSC
UTR 3 UCSC
TSS Hoffman
Transcribed Hoffman
TFBS ENCODE
SuperEnhancer Hnisz
Repressed Hoffman
Promoter UCSC
PromoterFlanking Hoffman
Intron UCSC
H3K9ac Trynka
H3K4me3 Trynka
H3K4me1 Trynka
**H3K27ac PGC2
H3K27ac Hnisz
FetalDHS Trynka
Enhancer Hoffman
Enhancer Andersson
DHS Trynka
DGF ENCODE
CTCF Hoffman
Conserved LindbladToh
Coding UCSC
Annotation
−10
0
10
20
Enrichment (prop h2 / prop snps)

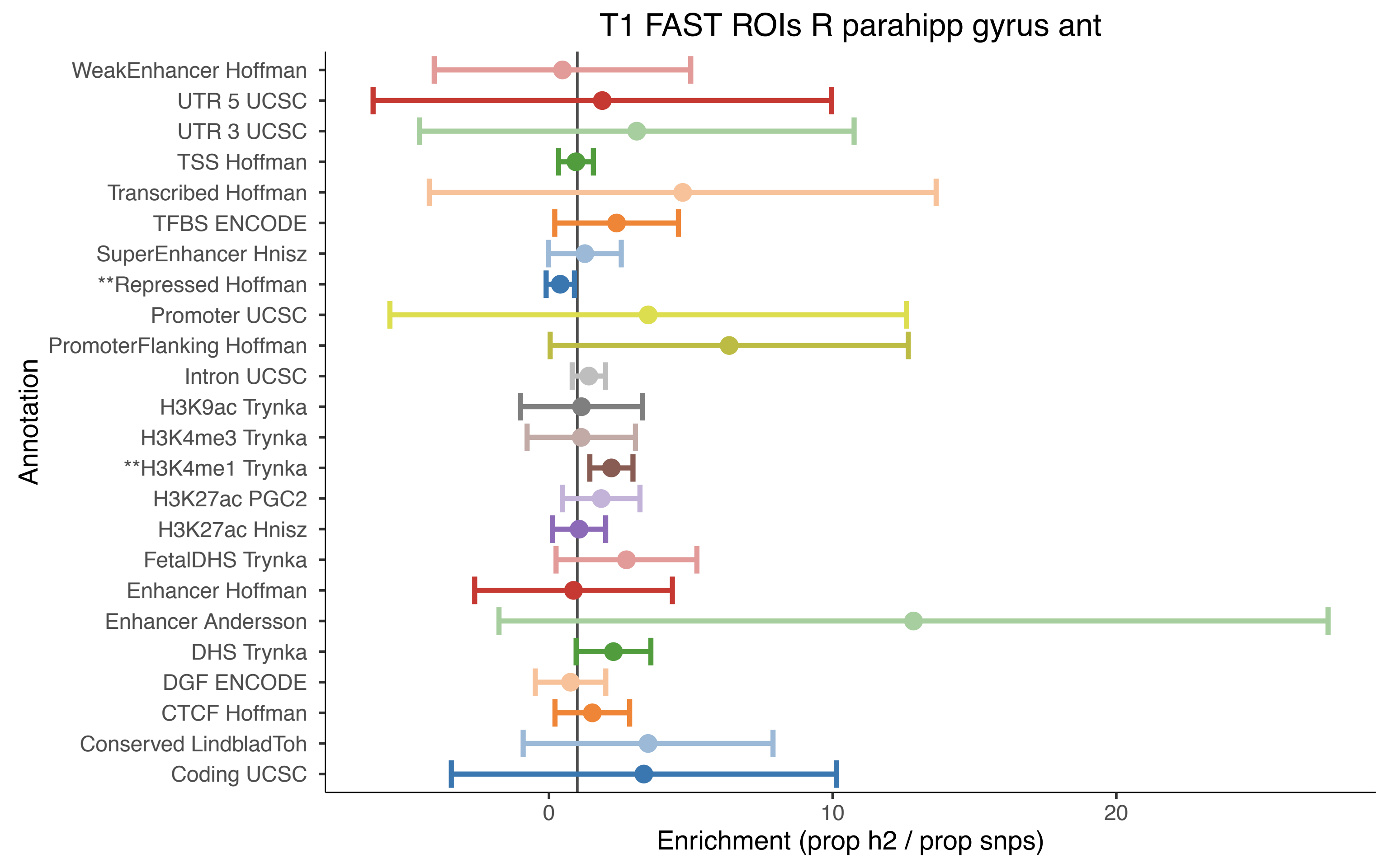
T1 FAST ROIs R parahipp gyrus ant
WeakEnhancer Hoffman
UTR 5 UCSC
UTR 3 UCSC
TSS Hoffman
Transcribed Hoffman
TFBS ENCODE
SuperEnhancer Hnisz
**Repressed Hoffman
Promoter UCSC
PromoterFlanking Hoffman
Intron UCSC
H3K9ac Trynka
H3K4me3 Trynka
**H3K4me1 Trynka
H3K27ac PGC2
H3K27ac Hnisz
FetalDHS Trynka
Enhancer Hoffman
Enhancer Andersson
DHS Trynka
DGF ENCODE
CTCF Hoffman
Conserved LindbladToh
Coding UCSC
Annotation
0
10
20
Enrichment (prop h2 / prop snps)

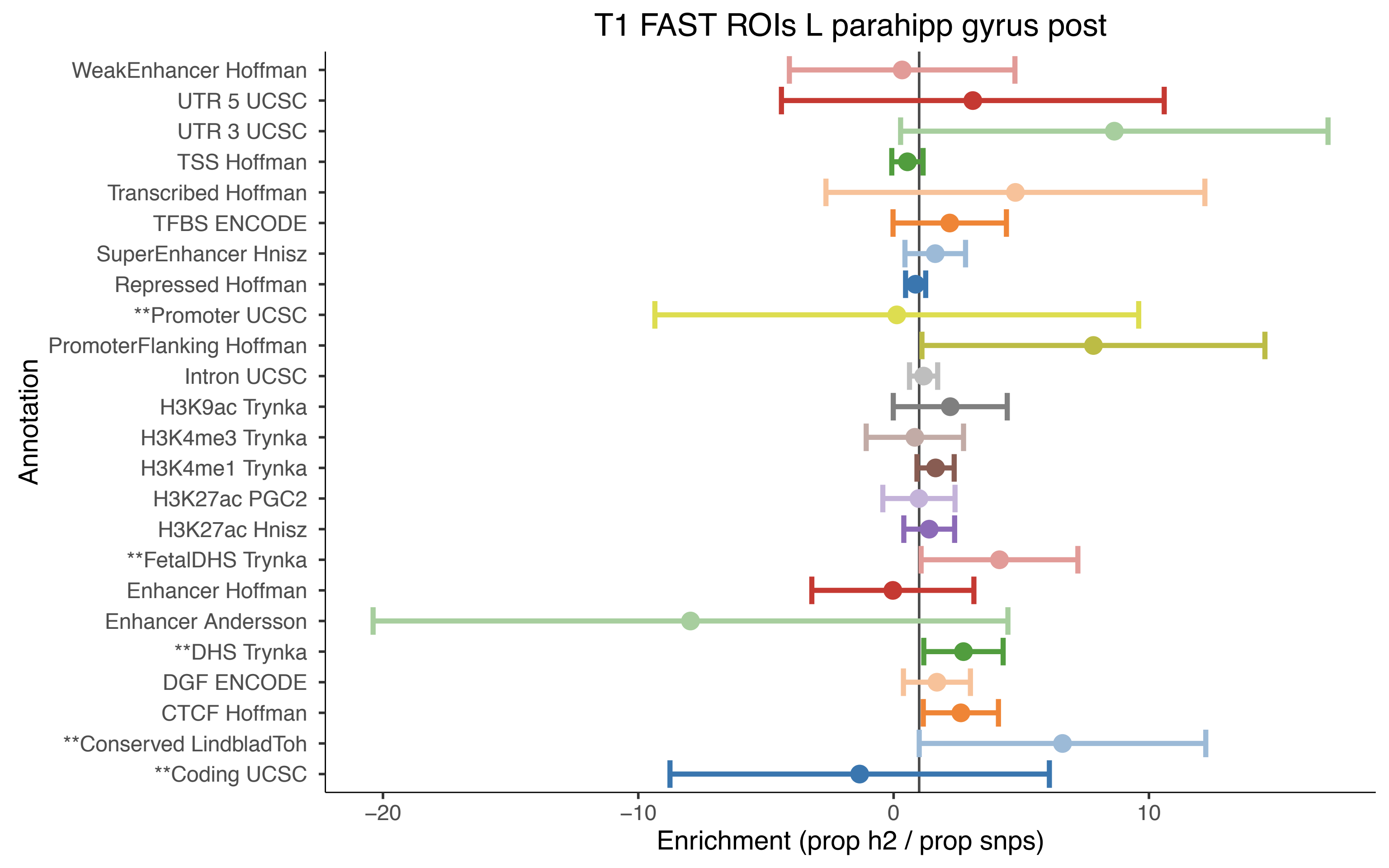
T1 FAST ROIs L parahipp gyrus post
WeakEnhancer Hoffman
UTR 5 UCSC
UTR 3 UCSC
TSS Hoffman
Transcribed Hoffman
TFBS ENCODE
SuperEnhancer Hnisz
Repressed Hoffman
**Promoter UCSC
PromoterFlanking Hoffman
Intron UCSC
H3K9ac Trynka
H3K4me3 Trynka
H3K4me1 Trynka
H3K27ac PGC2
H3K27ac Hnisz
**FetalDHS Trynka
Enhancer Hoffman
Enhancer Andersson
**DHS Trynka
DGF ENCODE
CTCF Hoffman
**Conserved LindbladToh
**Coding UCSC
Annotation
−20
−10
0
10
Enrichment (prop h2 / prop snps)

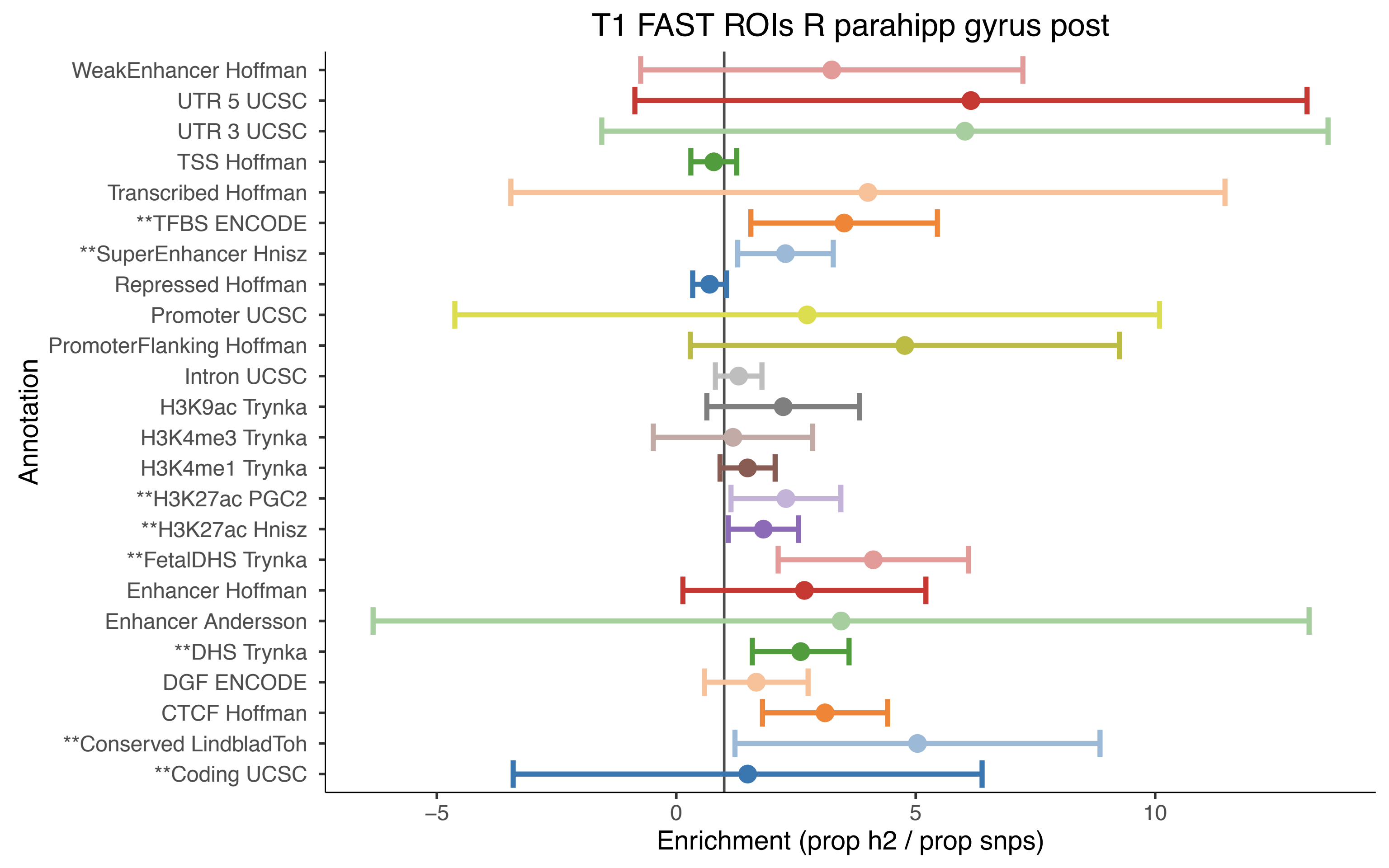
T1 FAST ROIs R parahipp gyrus post
WeakEnhancer Hoffman
UTR 5 UCSC
UTR 3 UCSC
TSS Hoffman
Transcribed Hoffman
**TFBS ENCODE
**SuperEnhancer Hnisz
Repressed Hoffman
Promoter UCSC
PromoterFlanking Hoffman
Intron UCSC
H3K9ac Trynka
H3K4me3 Trynka
H3K4me1 Trynka
**H3K27ac PGC2
**H3K27ac Hnisz
**FetalDHS Trynka
Enhancer Hoffman
Enhancer Andersson
**DHS Trynka
DGF ENCODE
CTCF Hoffman
**Conserved LindbladToh
**Coding UCSC
Annotation
−5
0
5
10
Enrichment (prop h2 / prop snps)

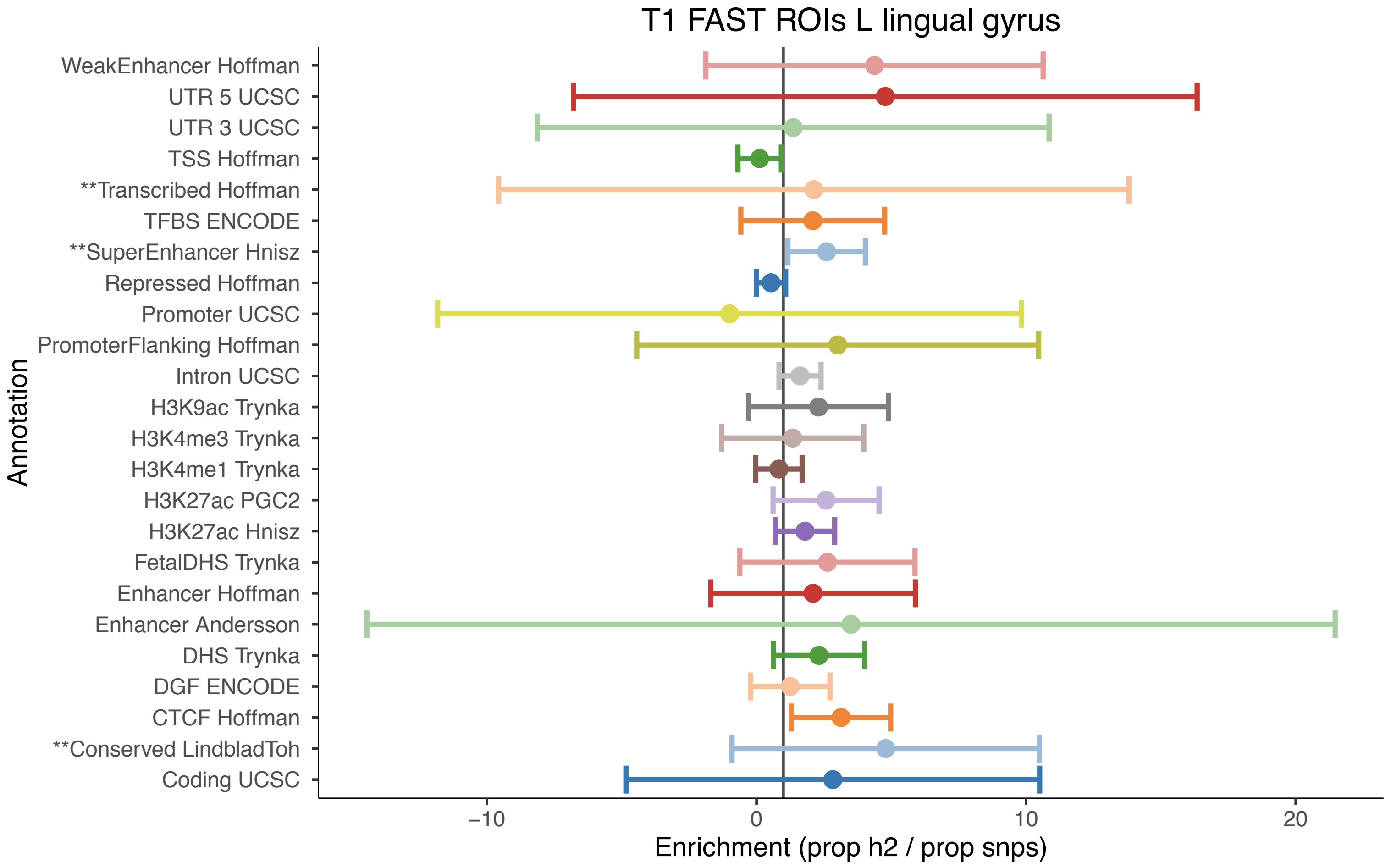

T1 FAST ROIs L lingual gyrus
WeakEnhancer Hoffman
UTR 5 UCSC
UTR 3 UCSC
TSS Hoffman
**Transcribed Hoffman
TFBS ENCODE
**SuperEnhancer Hnisz
Repressed Hoffman
Promoter UCSC
PromoterFlanking Hoffman
Intron UCSC
H3K9ac Trynka
H3K4me3 Trynka
H3K4me1 Trynka
H3K27ac PGC2
H3K27ac Hnisz
FetalDHS Trynka
Enhancer Hoffman
Enhancer Andersson
DHS Trynka
DGF ENCODE
CTCF Hoffman
**Conserved LindbladToh
Coding UCSC
Annotation
−10
0
10
20
Enrichment (prop h2 / prop snps)

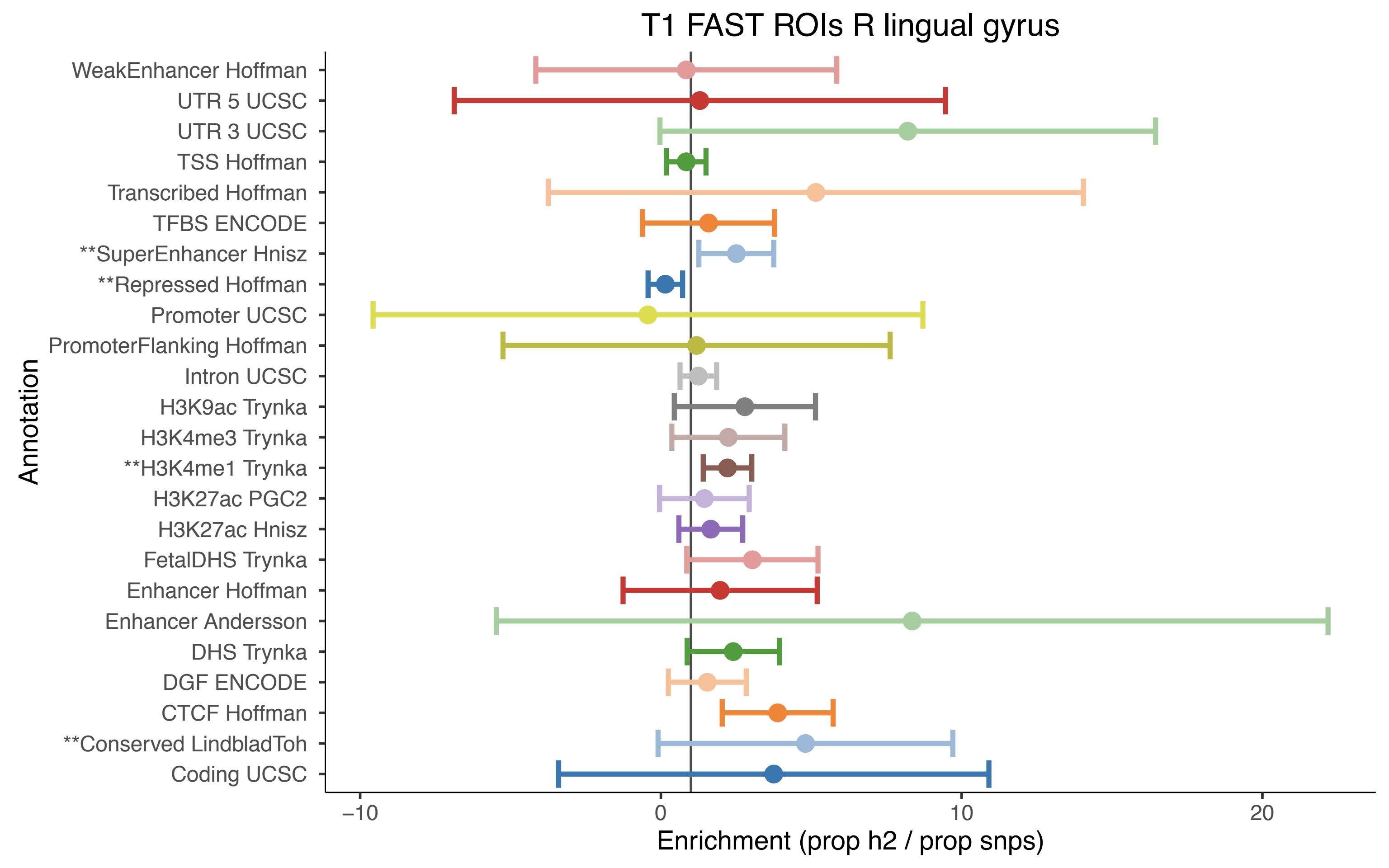
T1 FAST ROIs R lingual gyrus
WeakEnhancer Hoffman
UTR 5 UCSC
UTR 3 UCSC
TSS Hoffman
Transcribed Hoffman
TFBS ENCODE
**SuperEnhancer Hnisz
**Repressed Hoffman
Promoter UCSC
PromoterFlanking Hoffman
Intron UCSC
H3K9ac Trynka
H3K4me3 Trynka
**H3K4me1 Trynka
H3K27ac PGC2
H3K27ac Hnisz
FetalDHS Trynka
Enhancer Hoffman
Enhancer Andersson
DHS Trynka
DGF ENCODE
CTCF Hoffman
**Conserved LindbladToh
Coding UCSC
Annotation
−10
0
10
20
Enrichment (prop h2 / prop snps)

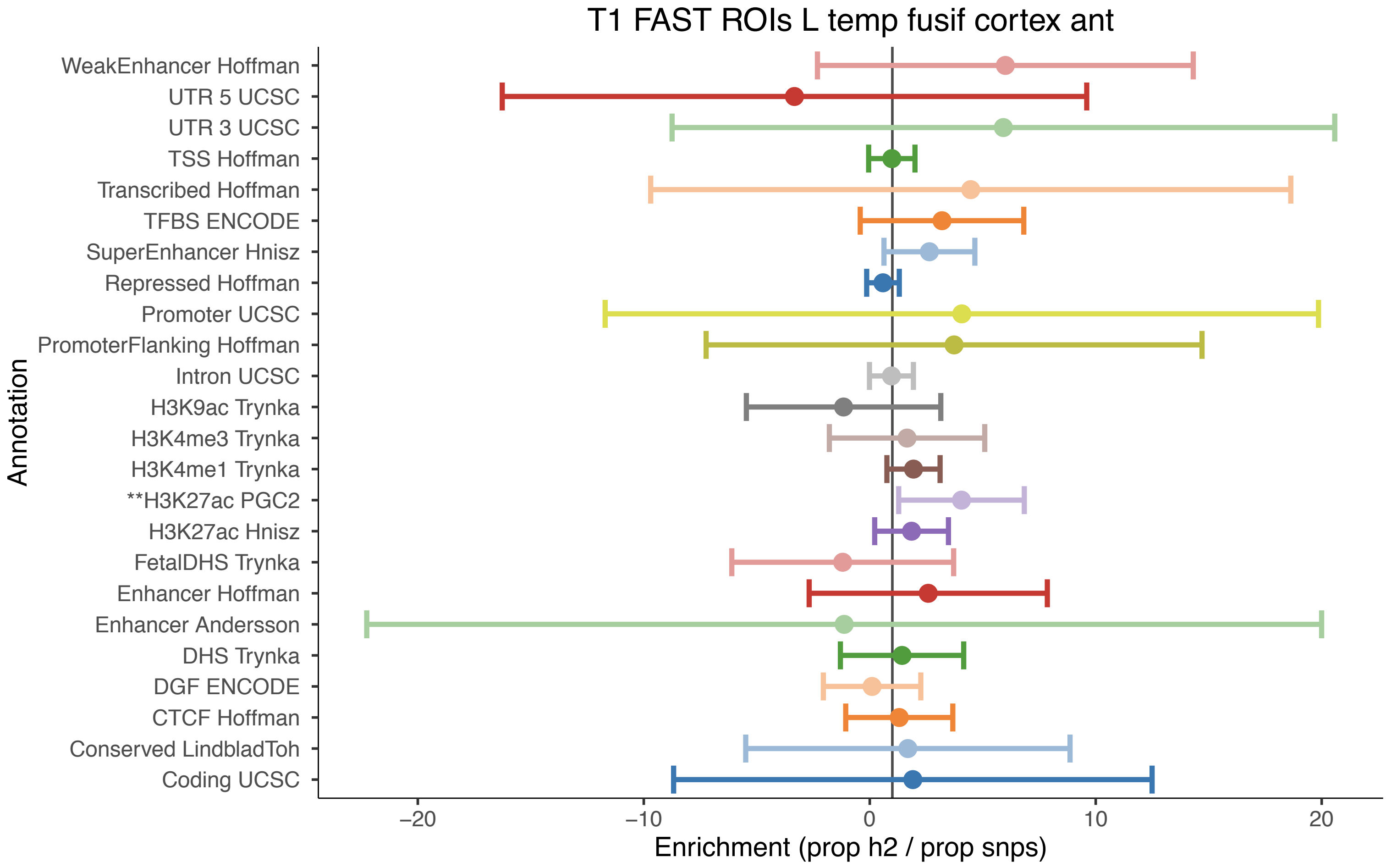

T1 FAST ROIs L temp fusif cortex ant
WeakEnhancer Hoffman
UTR 5 UCSC
UTR 3 UCSC
TSS Hoffman
Transcribed Hoffman
TFBS ENCODE
SuperEnhancer Hnisz
Repressed Hoffman
Promoter UCSC
PromoterFlanking Hoffman
Intron UCSC
H3K9ac Trynka
H3K4me3 Trynka
H3K4me1 Trynka
**H3K27ac PGC2
H3K27ac Hnisz
FetalDHS Trynka
Enhancer Hoffman
Enhancer Andersson
DHS Trynka
DGF ENCODE
CTCF Hoffman
Conserved LindbladToh
Coding UCSC
Annotation
−20
−10
0
10
20
Enrichment (prop h2 / prop snps)

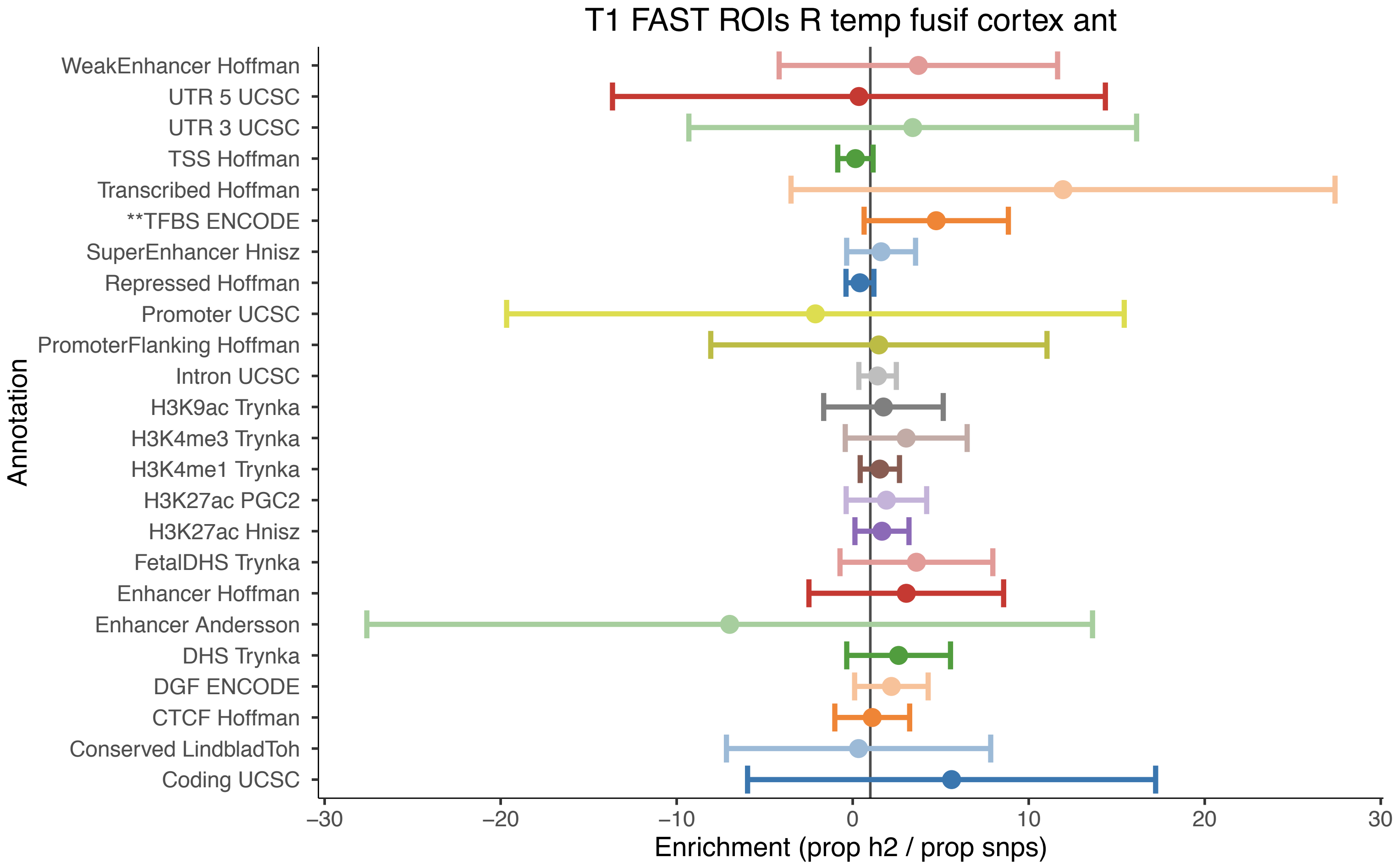

T1 FAST ROIs R temp fusif cortex ant
WeakEnhancer Hoffman
UTR 5 UCSC
UTR 3 UCSC
TSS Hoffman
Transcribed Hoffman
**TFBS ENCODE
SuperEnhancer Hnisz
Repressed Hoffman
Promoter UCSC
PromoterFlanking Hoffman
Intron UCSC
H3K9ac Trynka
H3K4me3 Trynka
H3K4me1 Trynka
H3K27ac PGC2
H3K27ac Hnisz
FetalDHS Trynka
Enhancer Hoffman
Enhancer Andersson
DHS Trynka
DGF ENCODE
CTCF Hoffman
Conserved LindbladToh
Coding UCSC
Annotation
−30
−20
−10
0
10
20
30
Enrichment (prop h2 / prop snps)

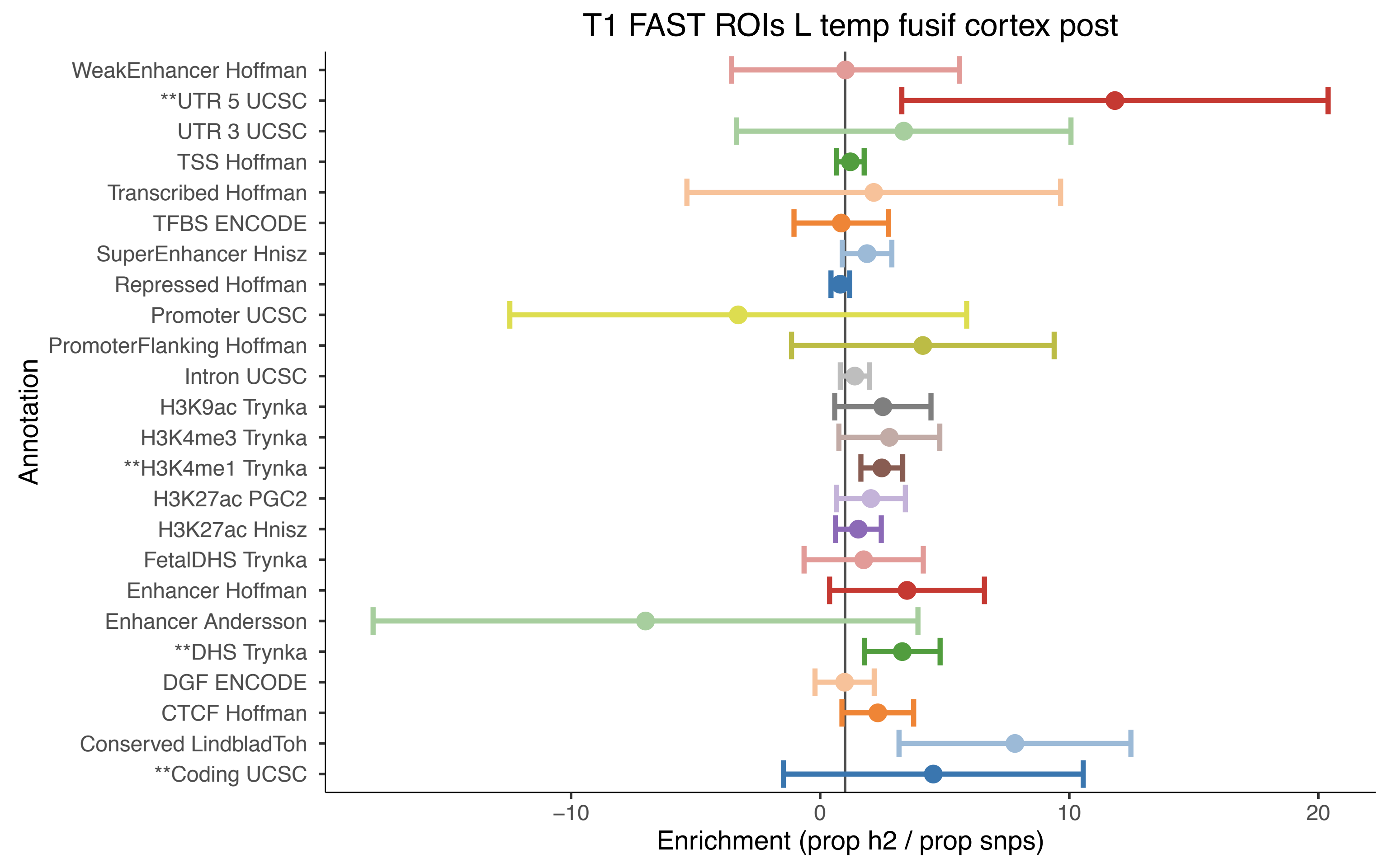

T1 FAST ROIs L temp fusif cortex post
WeakEnhancer Hoffman
**UTR 5 UCSC
UTR 3 UCSC
TSS Hoffman
Transcribed Hoffman
TFBS ENCODE
SuperEnhancer Hnisz
Repressed Hoffman
Promoter UCSC
PromoterFlanking Hoffman
Intron UCSC
H3K9ac Trynka
H3K4me3 Trynka
**H3K4me1 Trynka
H3K27ac PGC2
H3K27ac Hnisz
FetalDHS Trynka
Enhancer Hoffman
Enhancer Andersson
**DHS Trynka
DGF ENCODE
CTCF Hoffman
Conserved LindbladToh
**Coding UCSC
Annotation
−10
0
10
20
Enrichment (prop h2 / prop snps)

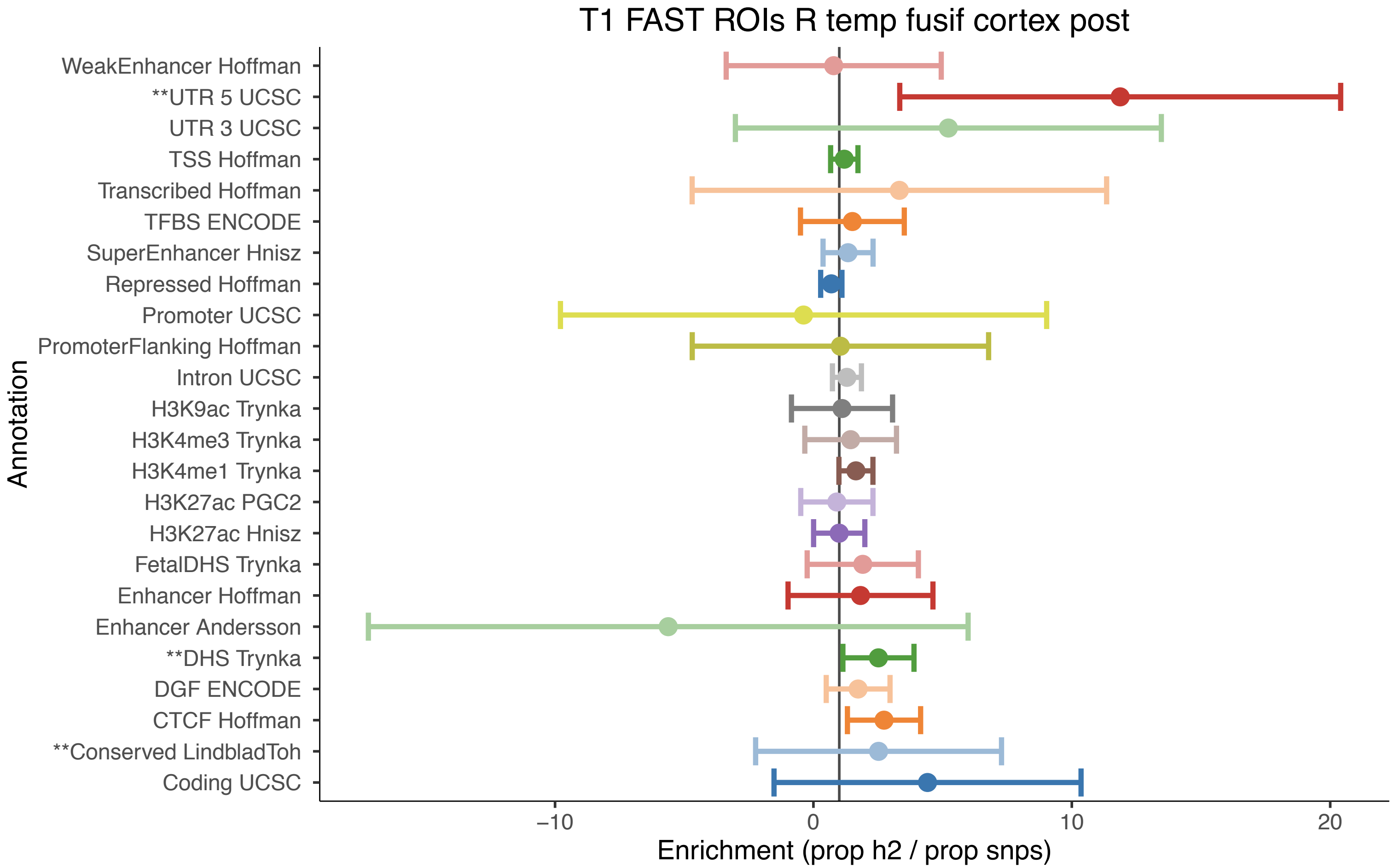

T1 FAST ROIs R temp fusif cortex post
WeakEnhancer Hoffman
**UTR 5 UCSC
UTR 3 UCSC
TSS Hoffman
Transcribed Hoffman
TFBS ENCODE
SuperEnhancer Hnisz
Repressed Hoffman
Promoter UCSC
PromoterFlanking Hoffman
Intron UCSC
H3K9ac Trynka
H3K4me3 Trynka
H3K4me1 Trynka
H3K27ac PGC2
H3K27ac Hnisz
FetalDHS Trynka
Enhancer Hoffman
Enhancer Andersson
**DHS Trynka
DGF ENCODE
CTCF Hoffman
**Conserved LindbladToh
Coding UCSC
Annotation
−10
0
10
20
Enrichment (prop h2 / prop snps)

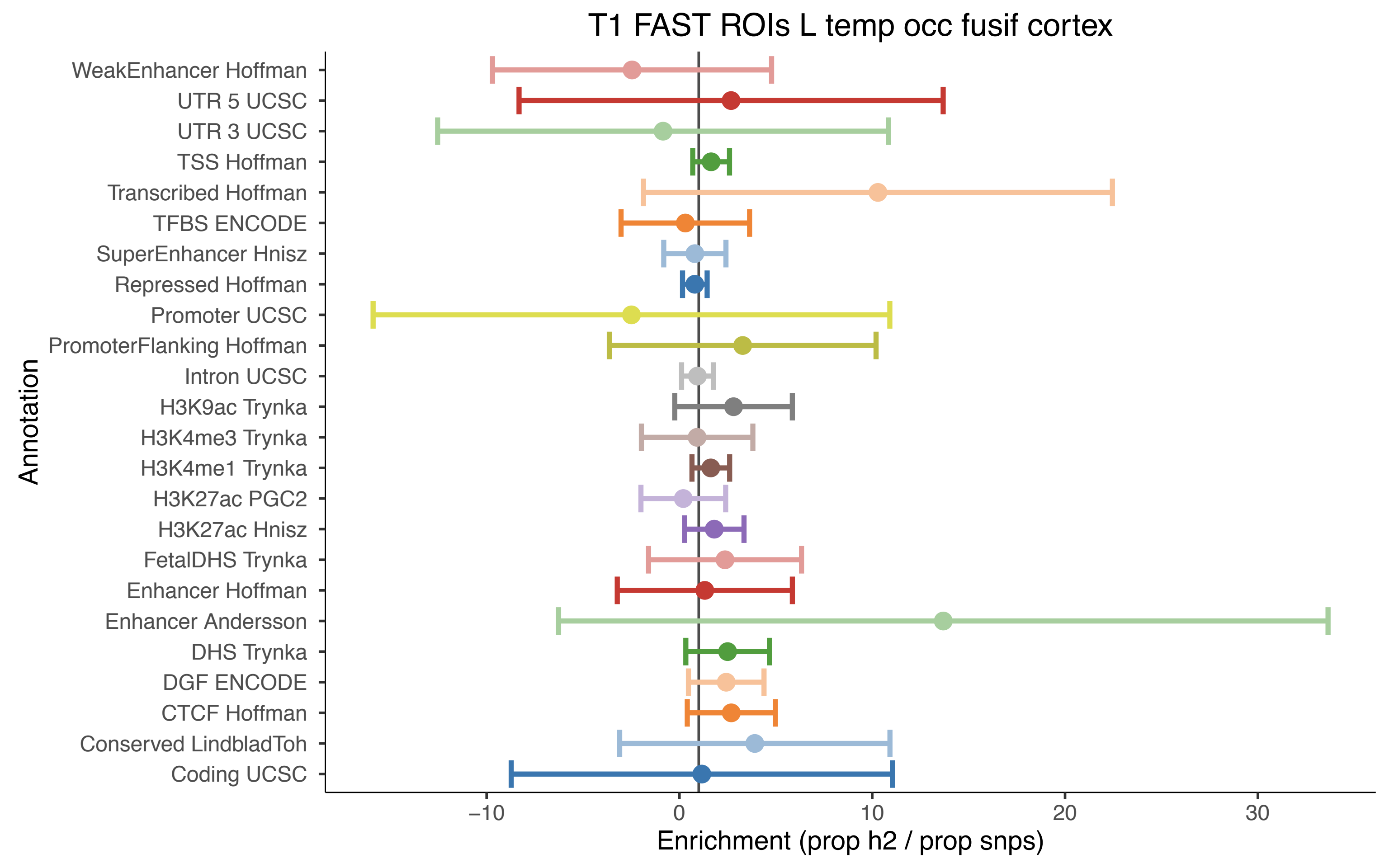
T1 FAST ROIs L temp occ fusif cortex
WeakEnhancer Hoffman
UTR 5 UCSC
UTR 3 UCSC
TSS Hoffman
Transcribed Hoffman
TFBS ENCODE
SuperEnhancer Hnisz
Repressed Hoffman
Promoter UCSC
PromoterFlanking Hoffman
Intron UCSC
H3K9ac Trynka
H3K4me3 Trynka
H3K4me1 Trynka
H3K27ac PGC2
H3K27ac Hnisz
FetalDHS Trynka
Enhancer Hoffman
Enhancer Andersson
DHS Trynka
DGF ENCODE
CTCF Hoffman
Conserved LindbladToh
Coding UCSC
Annotation
−10
0
10
20
30
Enrichment (prop h2 / prop snps)

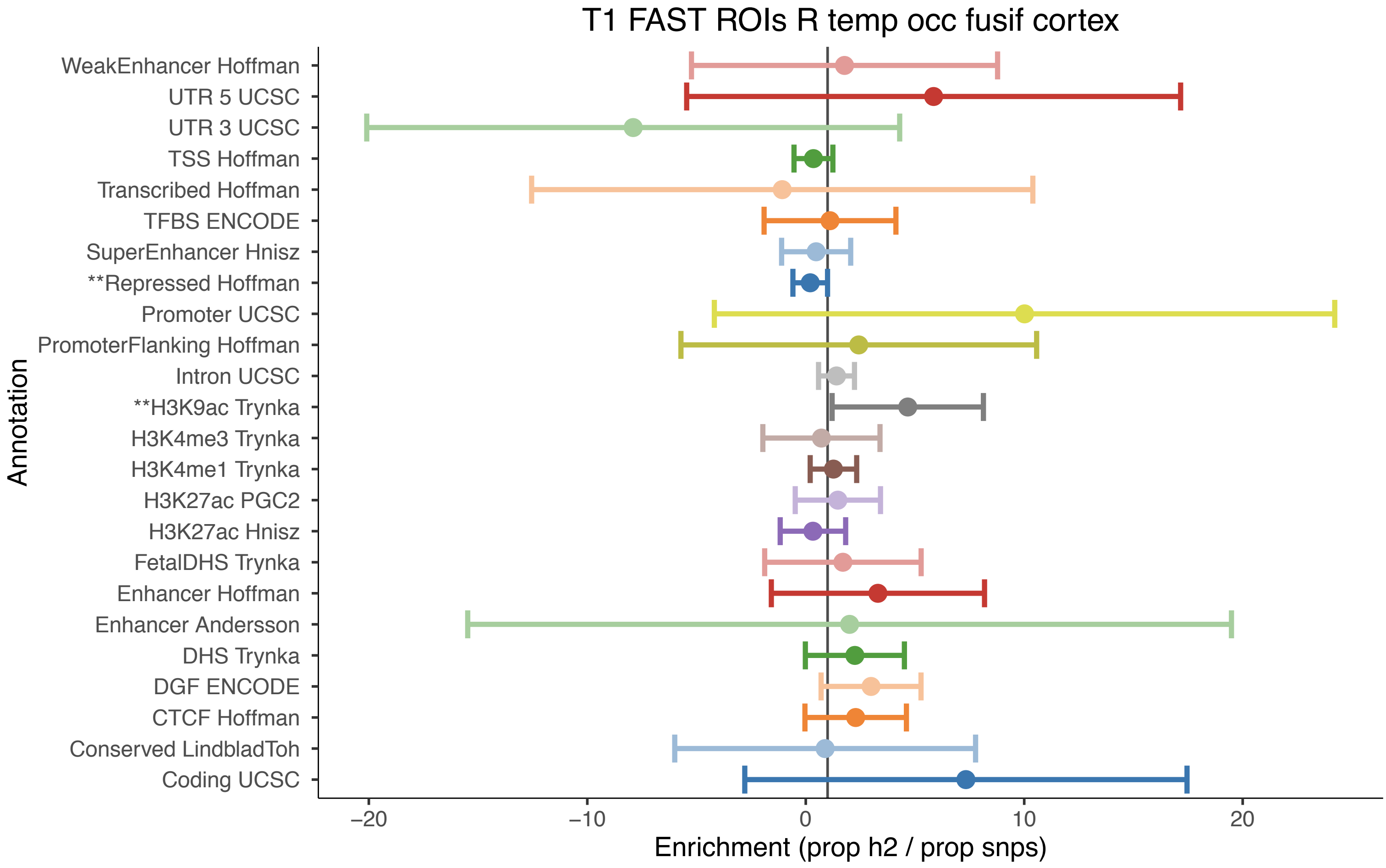

T1 FAST ROIs R temp occ fusif cortex
WeakEnhancer Hoffman
UTR 5 UCSC
UTR 3 UCSC
TSS Hoffman
Transcribed Hoffman
TFBS ENCODE
SuperEnhancer Hnisz
**Repressed Hoffman
Promoter UCSC
PromoterFlanking Hoffman
Intron UCSC
**H3K9ac Trynka
H3K4me3 Trynka
H3K4me1 Trynka
H3K27ac PGC2
H3K27ac Hnisz
FetalDHS Trynka
Enhancer Hoffman
Enhancer Andersson
DHS Trynka
DGF ENCODE
CTCF Hoffman
Conserved LindbladToh
Coding UCSC
Annotation
−20
−10
0
10
20
Enrichment (prop h2 / prop snps)

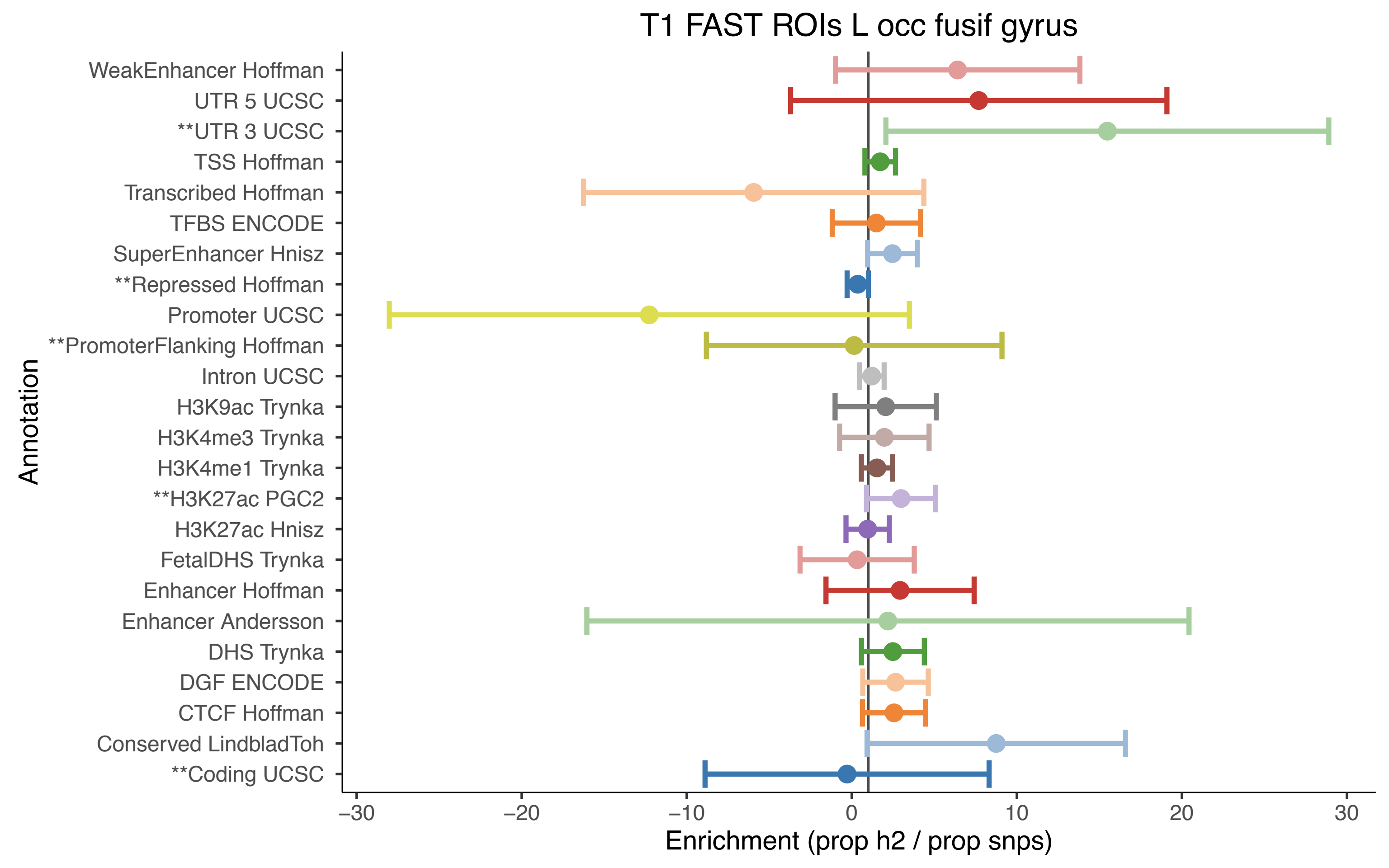
T1 FAST ROIs L occ fusif gyrus
WeakEnhancer Hoffman
UTR 5 UCSC
**UTR 3 UCSC
TSS Hoffman
Transcribed Hoffman
TFBS ENCODE
SuperEnhancer Hnisz
**Repressed Hoffman
Promoter UCSC
**PromoterFlanking Hoffman
Intron UCSC
H3K9ac Trynka
H3K4me3 Trynka
H3K4me1 Trynka
**H3K27ac PGC2
H3K27ac Hnisz
FetalDHS Trynka
Enhancer Hoffman
Enhancer Andersson
DHS Trynka
DGF ENCODE
CTCF Hoffman
Conserved LindbladToh
**Coding UCSC
Annotation
−30
−20
−10
0
10
20
30
Enrichment (prop h2 / prop snps)

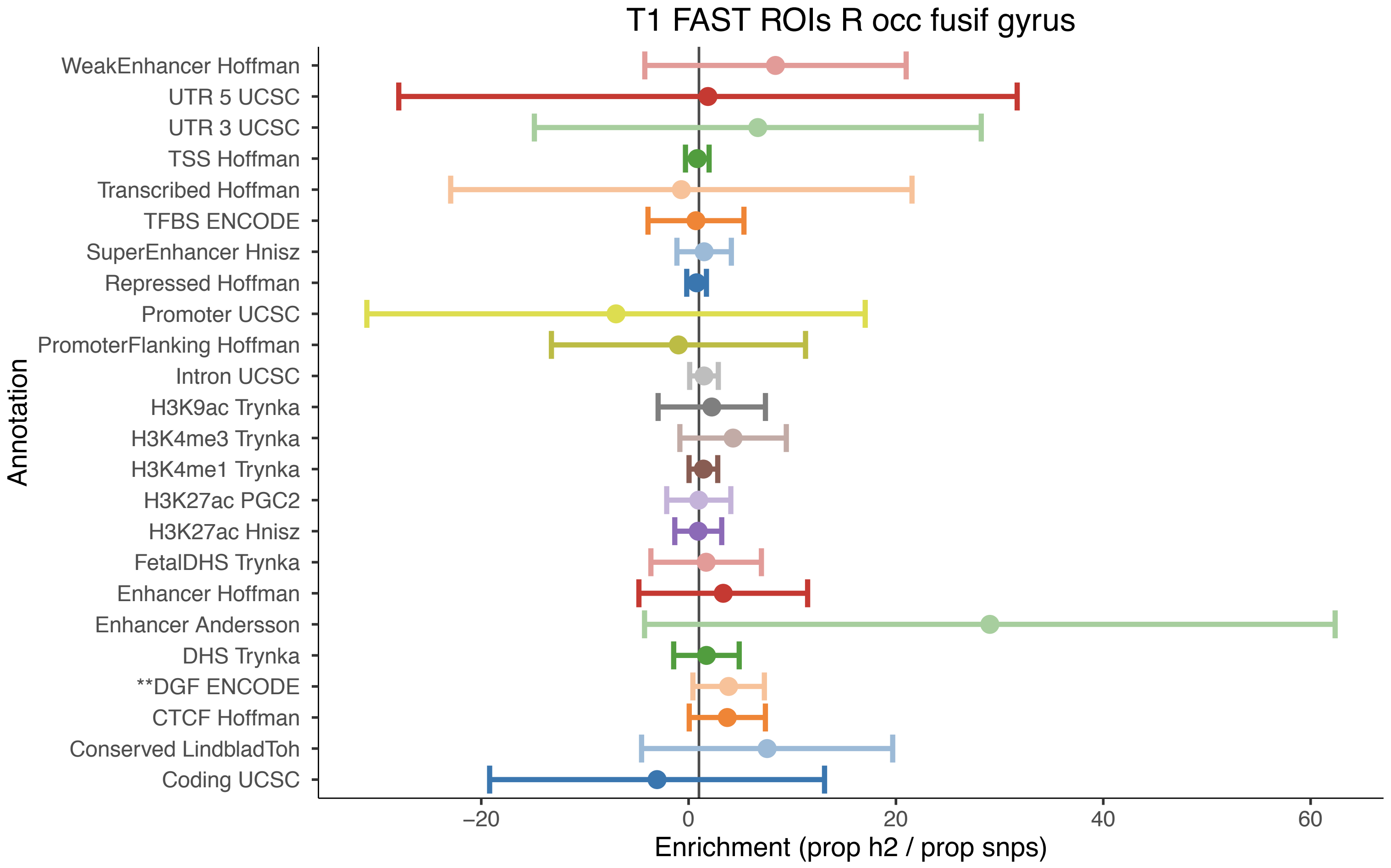
T1 FAST ROIs R occ fusif gyrus
WeakEnhancer Hoffman
UTR 5 UCSC
UTR 3 UCSC
TSS Hoffman
Transcribed Hoffman
TFBS ENCODE
SuperEnhancer Hnisz
Repressed Hoffman
Promoter UCSC
PromoterFlanking Hoffman
Intron UCSC
H3K9ac Trynka
H3K4me3 Trynka
H3K4me1 Trynka
H3K27ac PGC2
H3K27ac Hnisz
FetalDHS Trynka
Enhancer Hoffman
Enhancer Andersson
DHS Trynka
**DGF ENCODE
CTCF Hoffman
Conserved LindbladToh
Coding UCSC
Annotation
−20
0
20
40
60
Enrichment (prop h2 / prop snps)

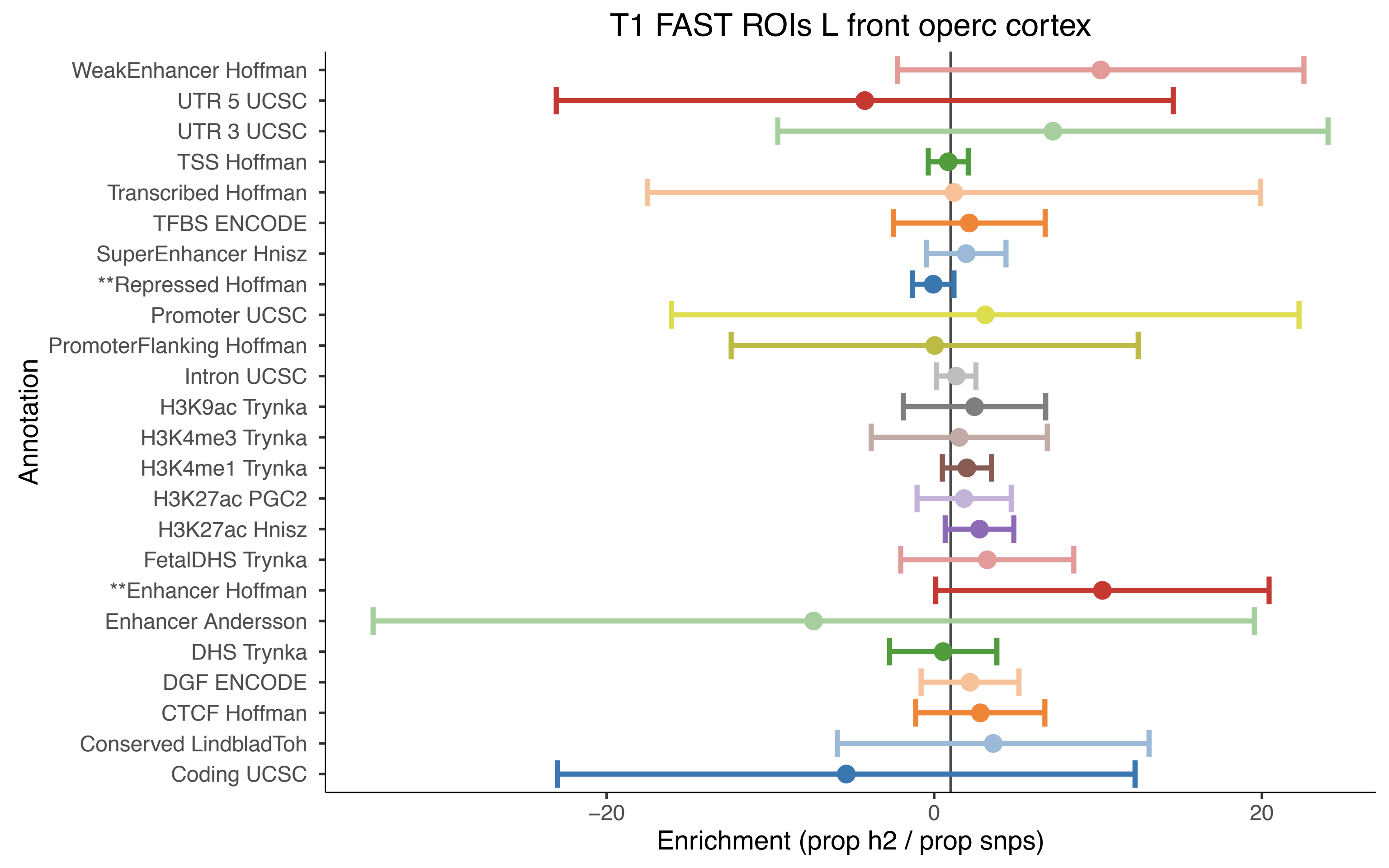

T1 FAST ROIs L front operc cortex
WeakEnhancer Hoffman
UTR 5 UCSC
UTR 3 UCSC
TSS Hoffman
Transcribed Hoffman
TFBS ENCODE
SuperEnhancer Hnisz
**Repressed Hoffman
Promoter UCSC
PromoterFlanking Hoffman
Intron UCSC
H3K9ac Trynka
H3K4me3 Trynka
H3K4me1 Trynka
H3K27ac PGC2
H3K27ac Hnisz
FetalDHS Trynka
**Enhancer Hoffman
Enhancer Andersson
DHS Trynka
DGF ENCODE
CTCF Hoffman
Conserved LindbladToh
Coding UCSC
Annotation
−20
0
20
Enrichment (prop h2 / prop snps)

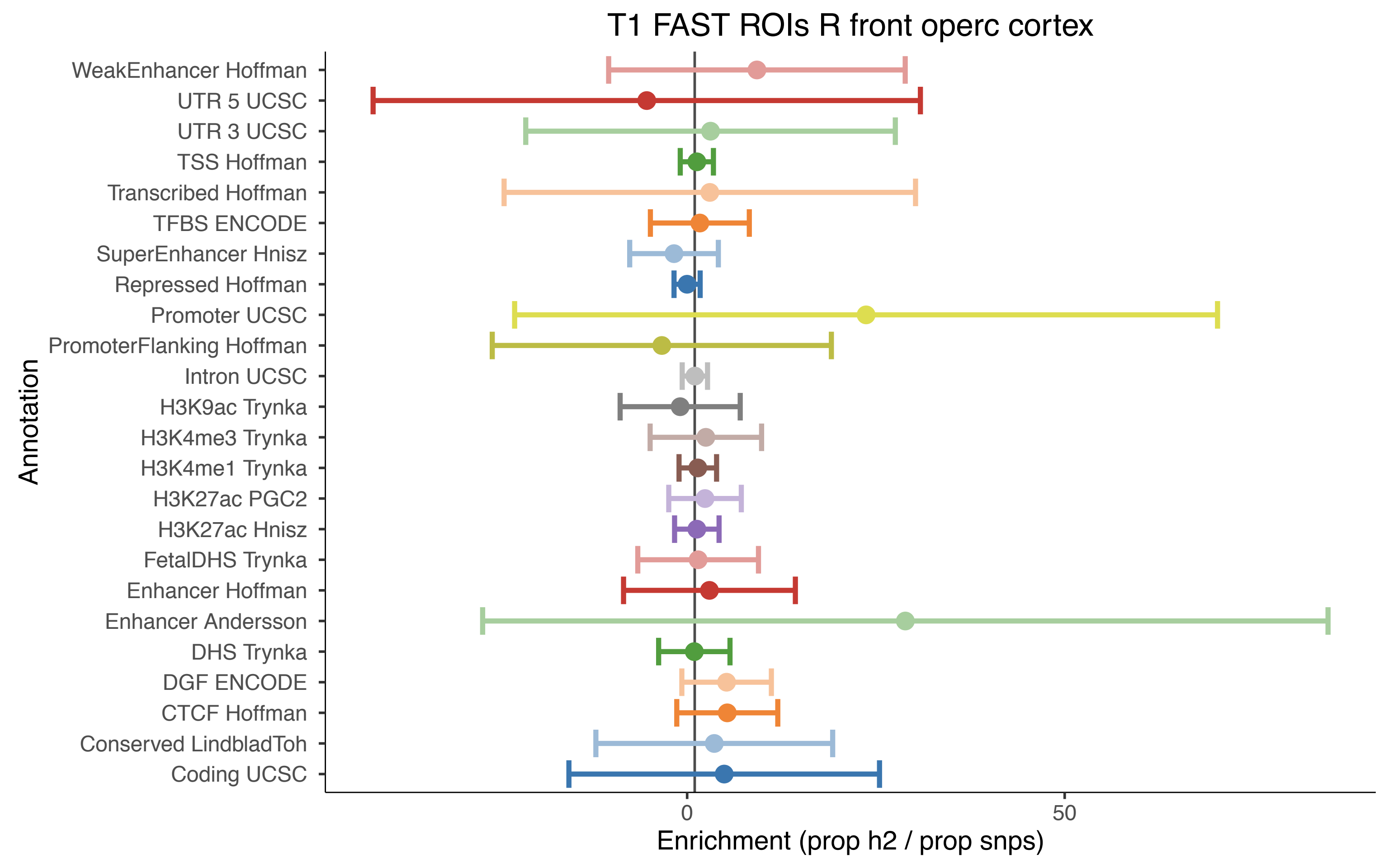

T1 FAST ROIs R front operc cortex
Annotation
WeakEnhancer Hoffman
UTR 5 UCSC
UTR 3 UCSC
TSS Hoffman
Transcribed Hoffman
TFBS ENCODE
SuperEnhancer Hnisz
Repressed Hoffman
Promoter UCSC
PromoterFlanking Hoffman
Intron UCSC
H3K9ac Trynka
H3K4me3 Trynka
H3K4me1 Trynka
H3K27ac PGC2
H3K27ac Hnisz
FetalDHS Trynka
Enhancer Hoffman
Enhancer Andersson
DHS Trynka
DGF ENCODE
CTCF Hoffman
Conserved LindbladToh
Coding UCSC
0
50
Enrichment (prop h2 / prop snps)

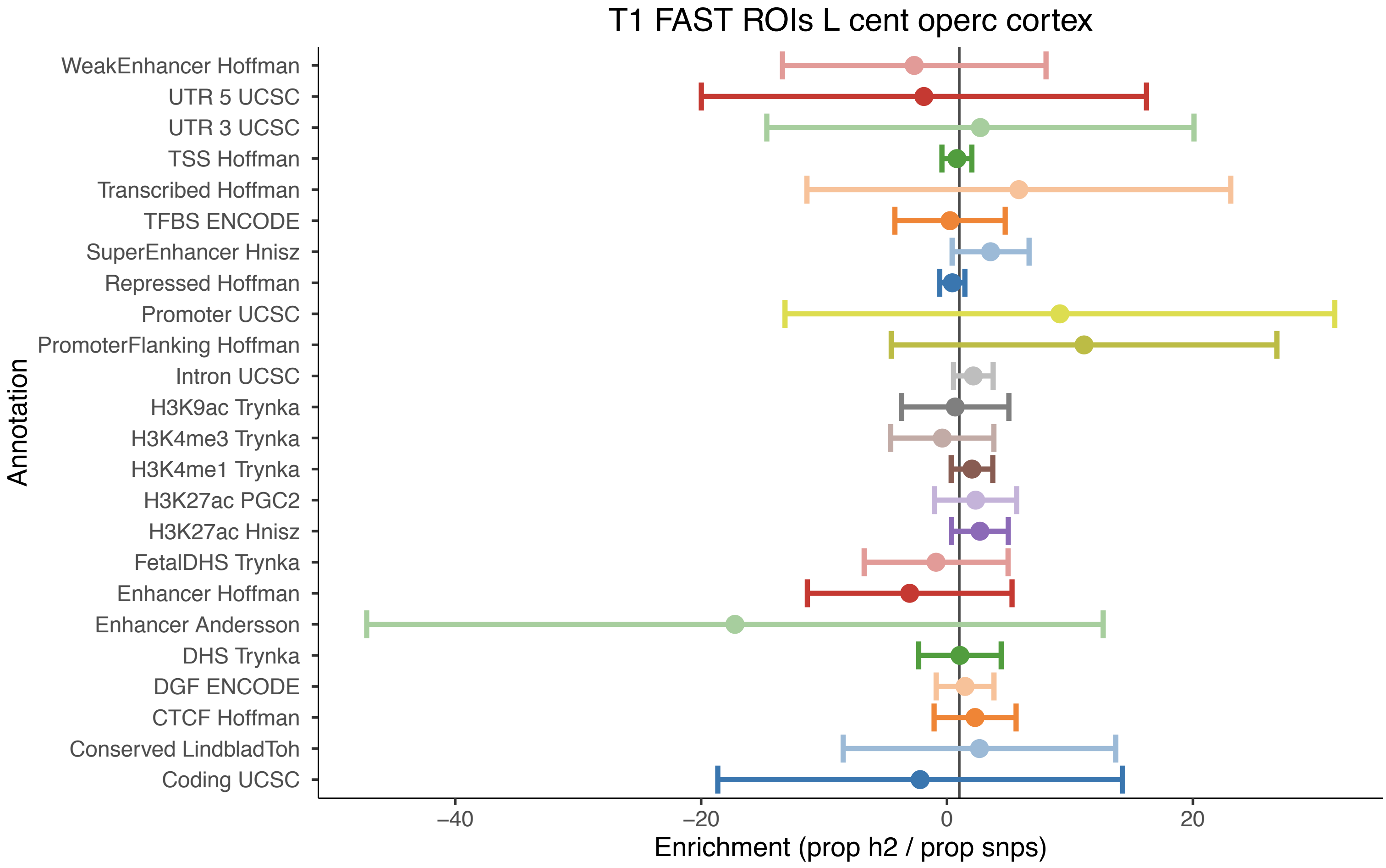

T1 FAST ROIs L cent operc cortex
WeakEnhancer Hoffman
UTR 5 UCSC
UTR 3 UCSC
TSS Hoffman
Transcribed Hoffman
TFBS ENCODE
SuperEnhancer Hnisz
Repressed Hoffman
Promoter UCSC
PromoterFlanking Hoffman
Intron UCSC
H3K9ac Trynka
H3K4me3 Trynka
H3K4me1 Trynka
H3K27ac PGC2
H3K27ac Hnisz
FetalDHS Trynka
Enhancer Hoffman
Enhancer Andersson
DHS Trynka
DGF ENCODE
CTCF Hoffman
Conserved LindbladToh
Coding UCSC
Annotation
−40
−20
0
20
Enrichment (prop h2 / prop snps)

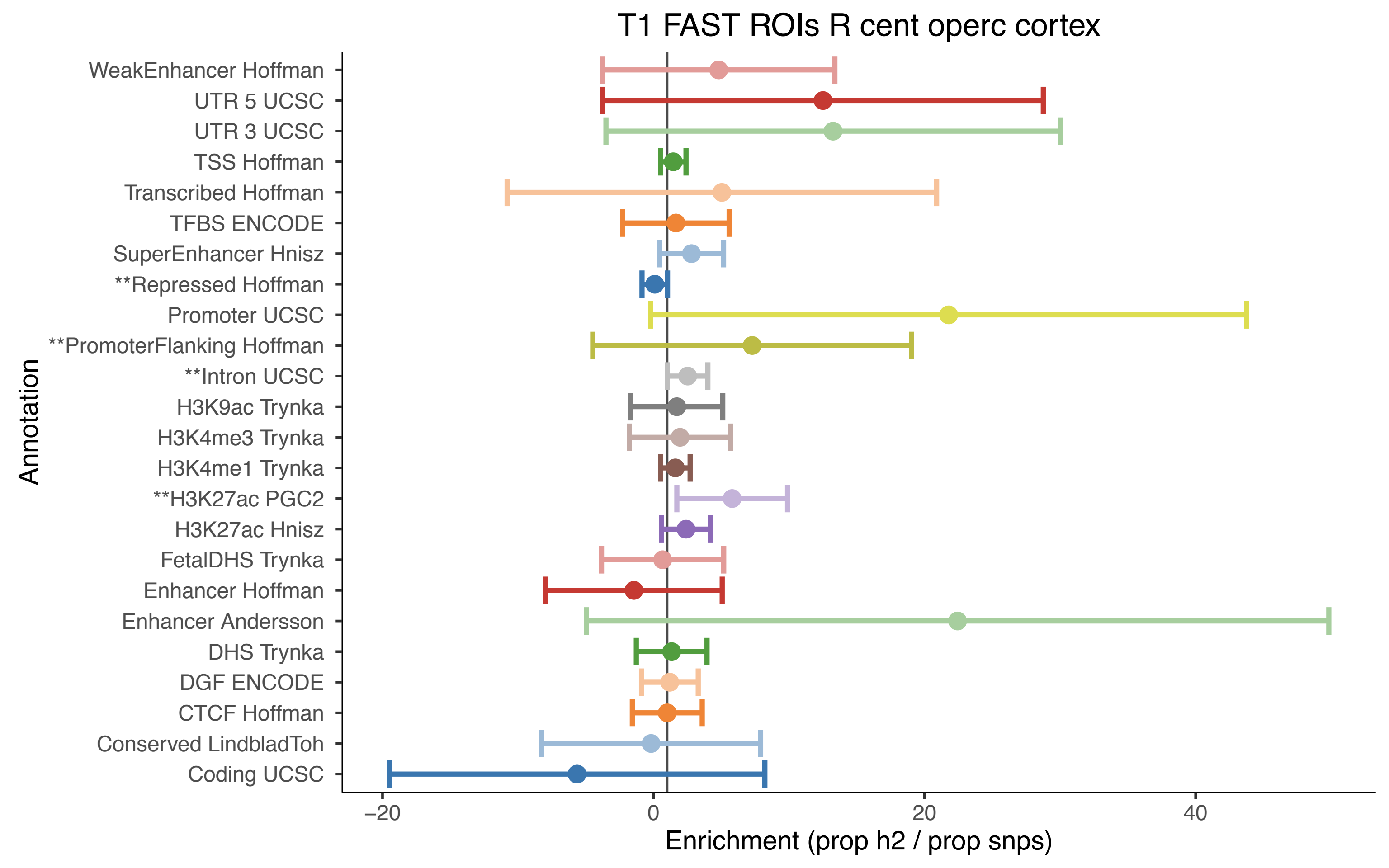
T1 FAST ROIs R cent operc cortex
Annotation
WeakEnhancer Hoffman
UTR 5 UCSC
UTR 3 UCSC
TSS Hoffman
Transcribed Hoffman
TFBS ENCODE
SuperEnhancer Hnisz
**Repressed Hoffman
Promoter UCSC
**PromoterFlanking Hoffman
**Intron UCSC
H3K9ac Trynka
H3K4me3 Trynka
H3K4me1 Trynka
**H3K27ac PGC2
H3K27ac Hnisz
FetalDHS Trynka
Enhancer Hoffman
Enhancer Andersson
DHS Trynka
DGF ENCODE
CTCF Hoffman
Conserved LindbladToh
Coding UCSC
−20
0
20
40
Enrichment (prop h2 / prop snps)

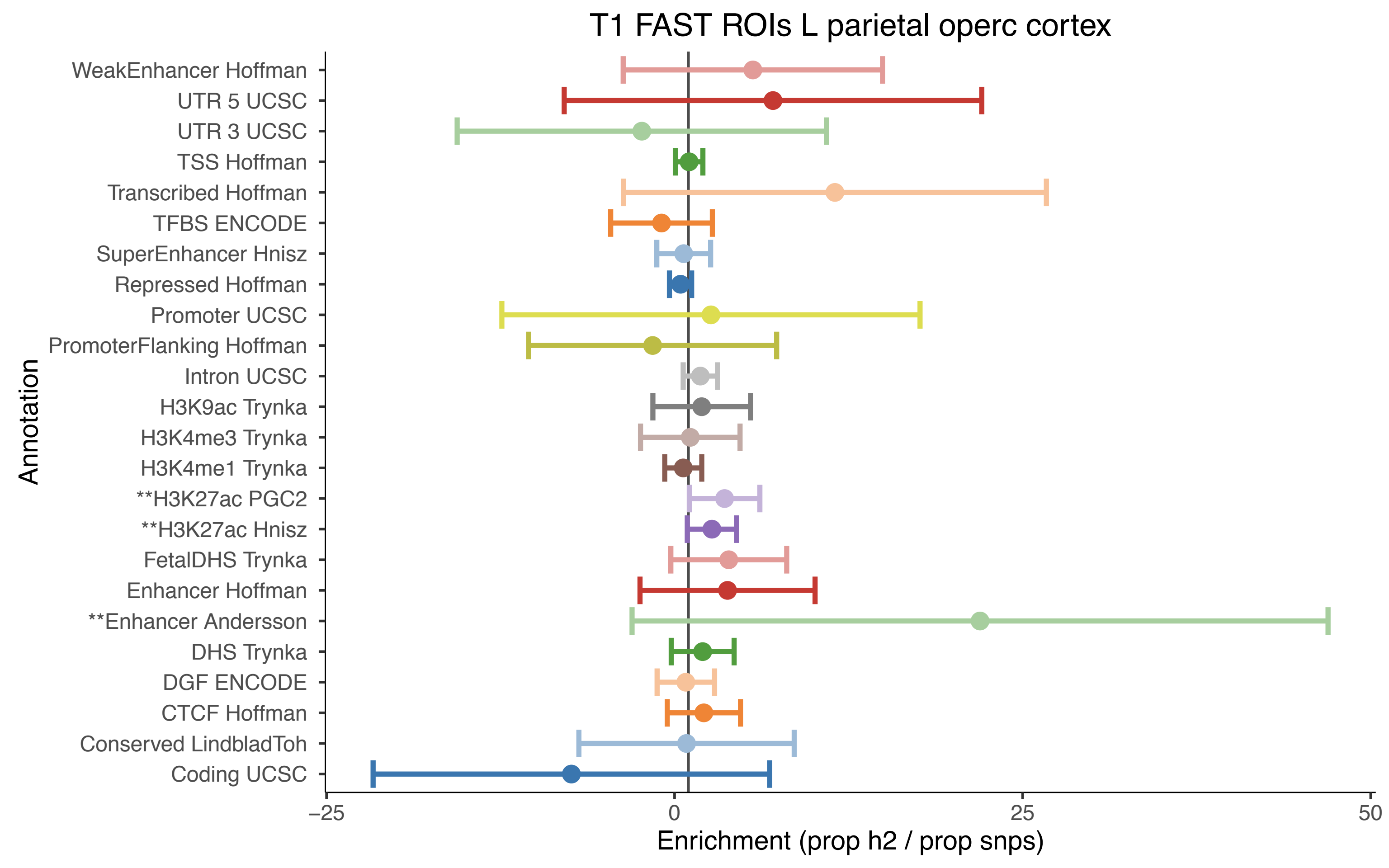
T1 FAST ROIs L parietal operc cortex
WeakEnhancer Hoffman
UTR 5 UCSC
UTR 3 UCSC
TSS Hoffman
Transcribed Hoffman
TFBS ENCODE
SuperEnhancer Hnisz
Repressed Hoffman
Promoter UCSC
PromoterFlanking Hoffman
Intron UCSC
H3K9ac Trynka
H3K4me3 Trynka
H3K4me1 Trynka
**H3K27ac PGC2
**H3K27ac Hnisz
FetalDHS Trynka
Enhancer Hoffman
**Enhancer Andersson
DHS Trynka
DGF ENCODE
CTCF Hoffman
Conserved LindbladToh
Coding UCSC
Annotation
−25
0
25
50
Enrichment (prop h2 / prop snps)

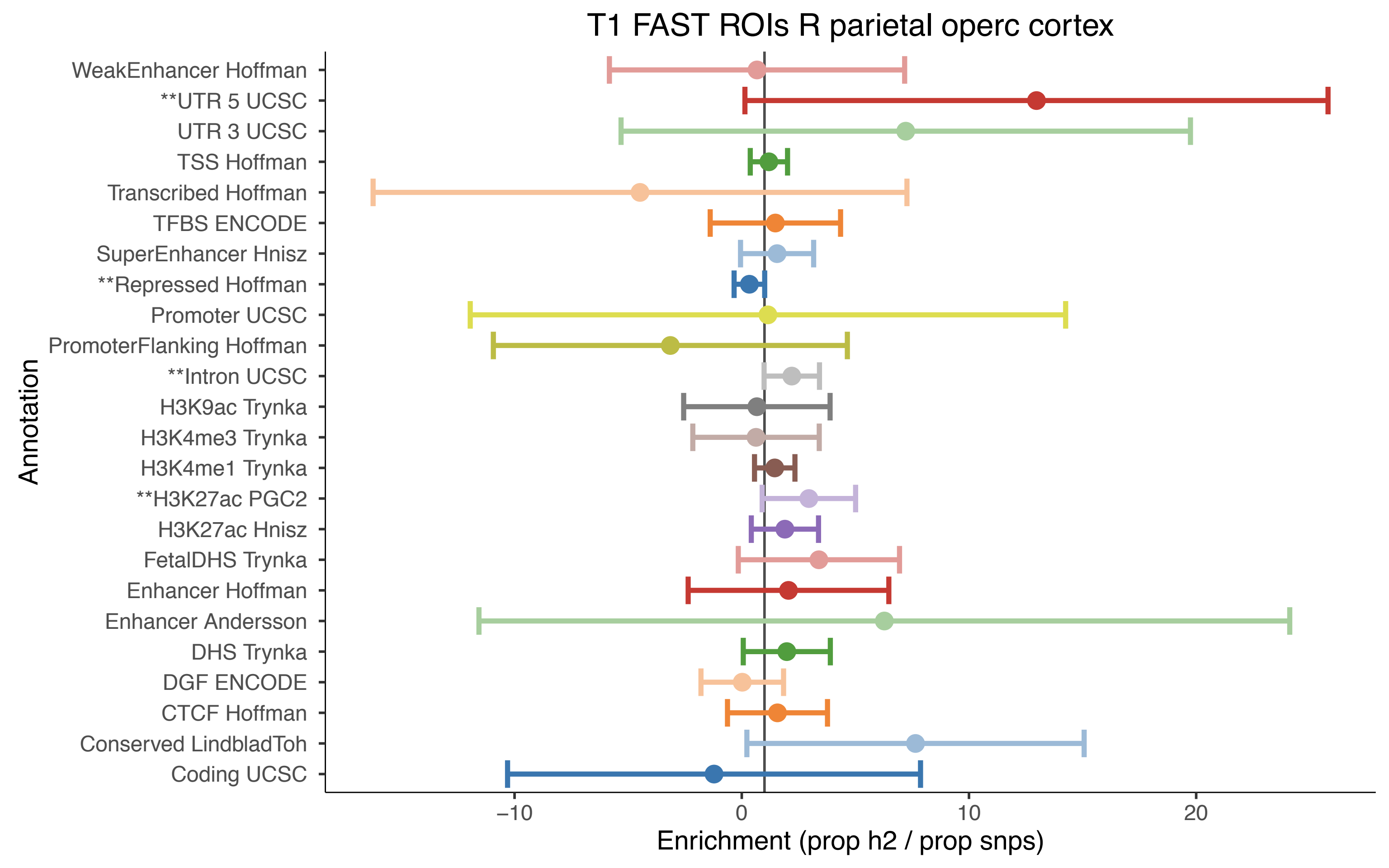
T1 FAST ROIs R parietal operc cortex
WeakEnhancer Hoffman
**UTR 5 UCSC
UTR 3 UCSC
TSS Hoffman
Transcribed Hoffman
TFBS ENCODE
SuperEnhancer Hnisz
**Repressed Hoffman
Promoter UCSC
PromoterFlanking Hoffman
**Intron UCSC
H3K9ac Trynka
H3K4me3 Trynka
H3K4me1 Trynka
**H3K27ac PGC2
H3K27ac Hnisz
FetalDHS Trynka
Enhancer Hoffman
Enhancer Andersson
DHS Trynka
DGF ENCODE
CTCF Hoffman
Conserved LindbladToh
Coding UCSC
Annotation
−10
0
10
20
Enrichment (prop h2 / prop snps)

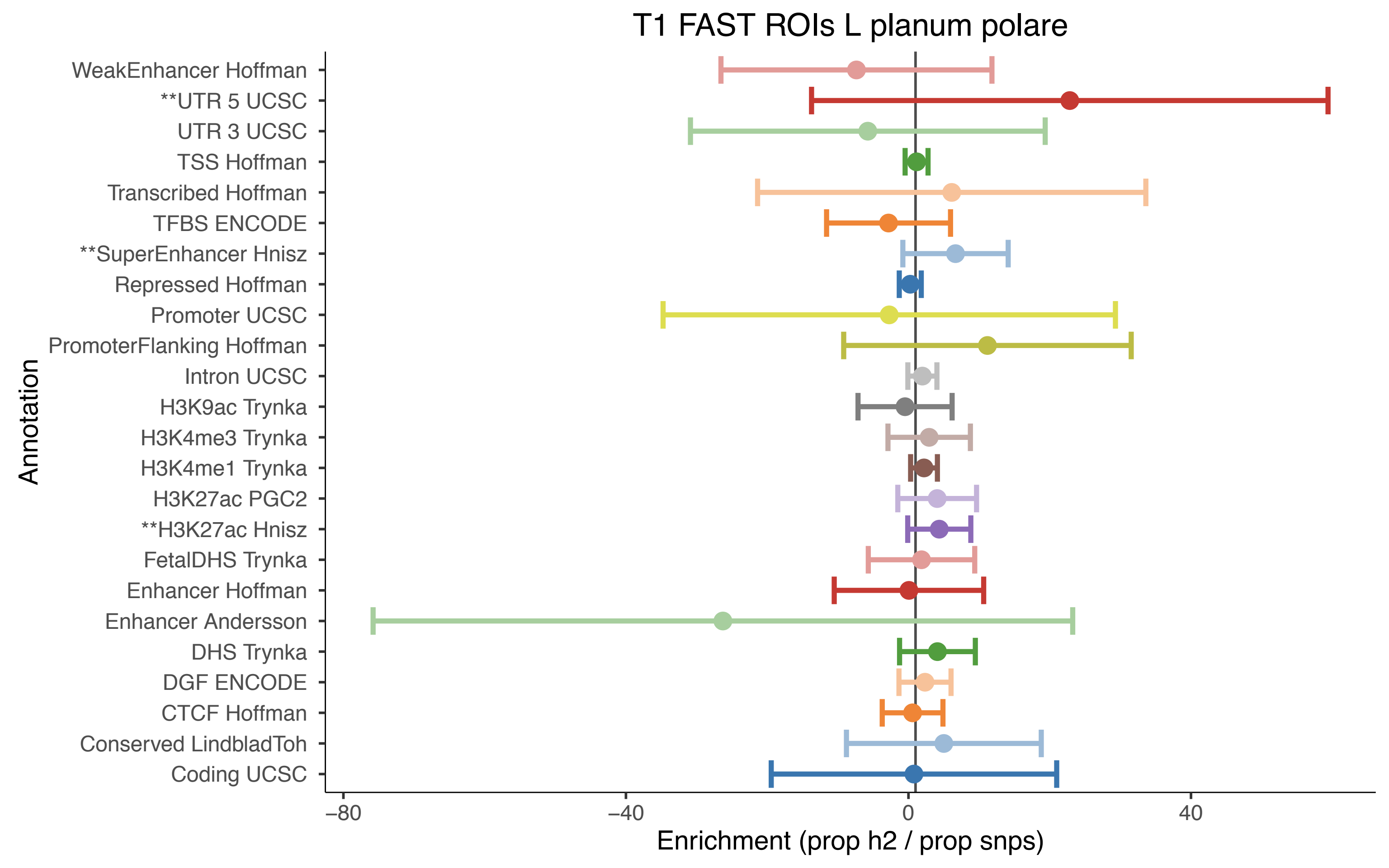

T1 FAST ROIs L planum polare
WeakEnhancer Hoffman
**UTR 5 UCSC
UTR 3 UCSC
TSS Hoffman
Transcribed Hoffman
TFBS ENCODE
**SuperEnhancer Hnisz
Repressed Hoffman
Promoter UCSC
PromoterFlanking Hoffman
Intron UCSC
H3K9ac Trynka
H3K4me3 Trynka
H3K4me1 Trynka
H3K27ac PGC2
**H3K27ac Hnisz
FetalDHS Trynka
Enhancer Hoffman
Enhancer Andersson
DHS Trynka
DGF ENCODE
CTCF Hoffman
Conserved LindbladToh
Coding UCSC
Annotation
−80
−40
0
40
Enrichment (prop h2 / prop snps)

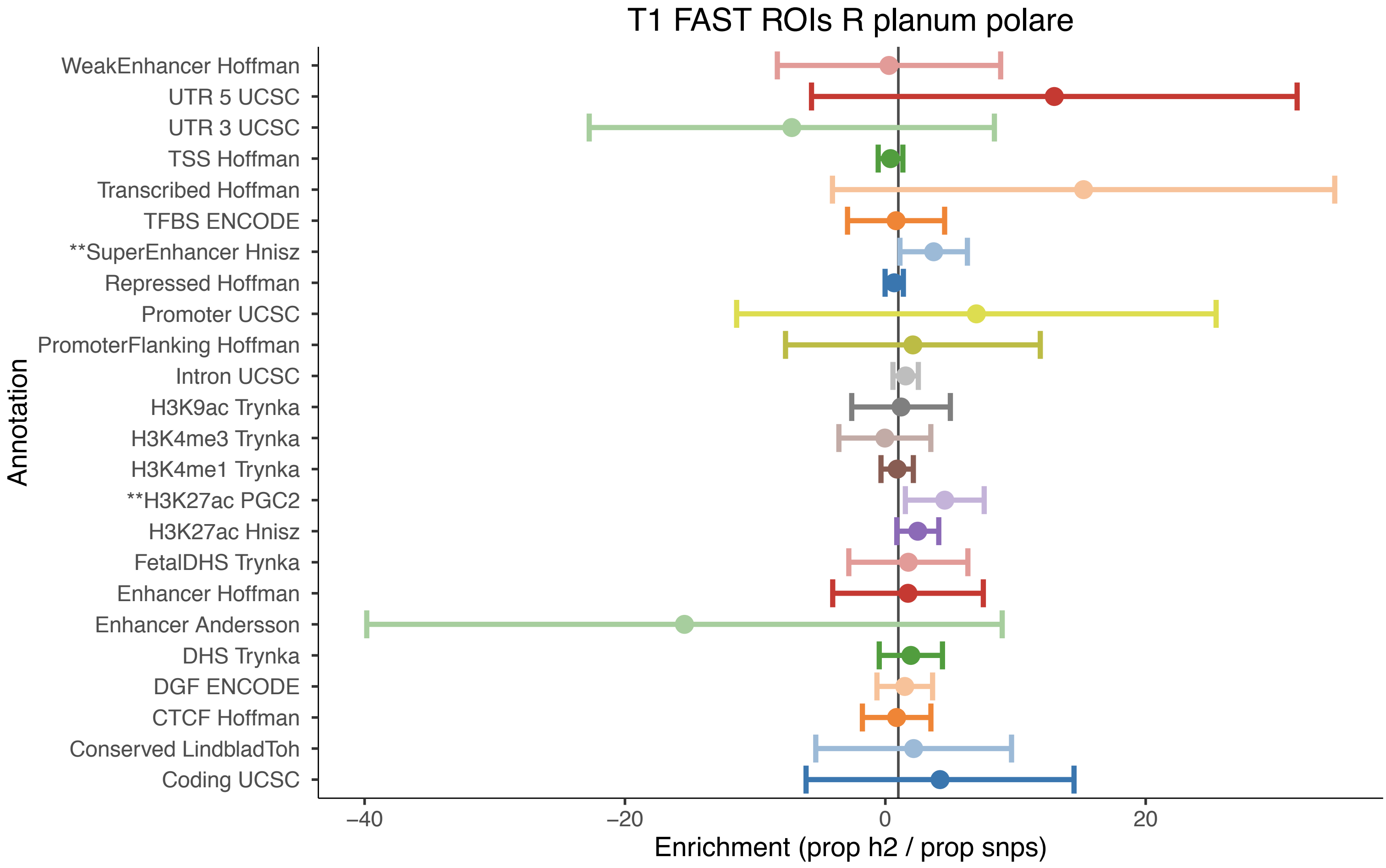

T1 FAST ROIs R planum polare
WeakEnhancer Hoffman
UTR 5 UCSC
UTR 3 UCSC
TSS Hoffman
Transcribed Hoffman
TFBS ENCODE
**SuperEnhancer Hnisz
Repressed Hoffman
Promoter UCSC
PromoterFlanking Hoffman
Intron UCSC
H3K9ac Trynka
H3K4me3 Trynka
H3K4me1 Trynka
**H3K27ac PGC2
H3K27ac Hnisz
FetalDHS Trynka
Enhancer Hoffman
Enhancer Andersson
DHS Trynka
DGF ENCODE
CTCF Hoffman
Conserved LindbladToh
Coding UCSC
Annotation
−40
−20
0
20
Enrichment (prop h2 / prop snps)

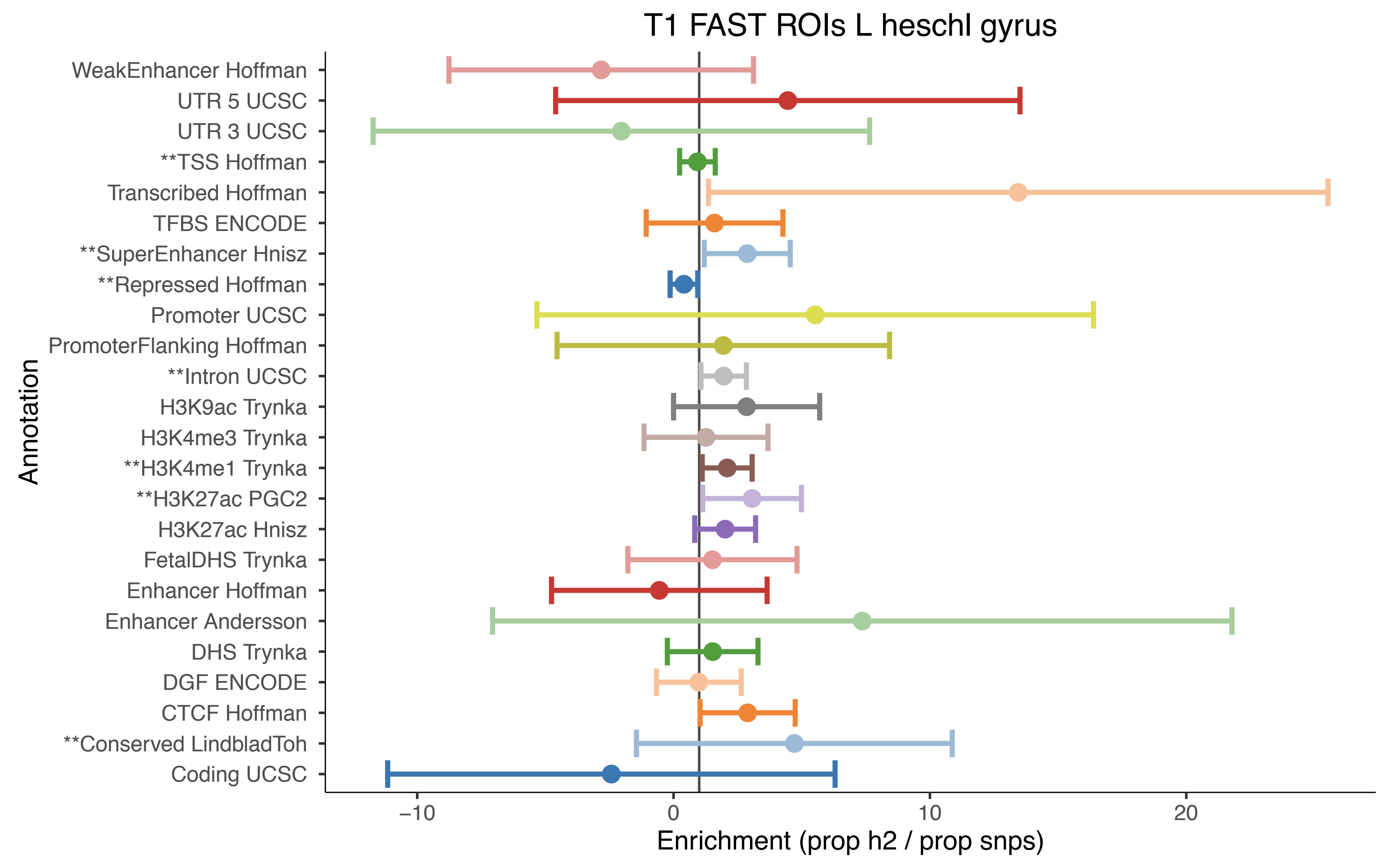
T1 FAST ROIs L heschl gyrus
WeakEnhancer Hoffman
UTR 5 UCSC
UTR 3 UCSC
**TSS Hoffman
Transcribed Hoffman
TFBS ENCODE
**SuperEnhancer Hnisz
**Repressed Hoffman
Promoter UCSC
PromoterFlanking Hoffman
**Intron UCSC
H3K9ac Trynka
H3K4me3 Trynka
**H3K4me1 Trynka
**H3K27ac PGC2
H3K27ac Hnisz
FetalDHS Trynka
Enhancer Hoffman
Enhancer Andersson
DHS Trynka
DGF ENCODE
CTCF Hoffman
**Conserved LindbladToh
Coding UCSC
Annotation
−10
0
10
20
Enrichment (prop h2 / prop snps)

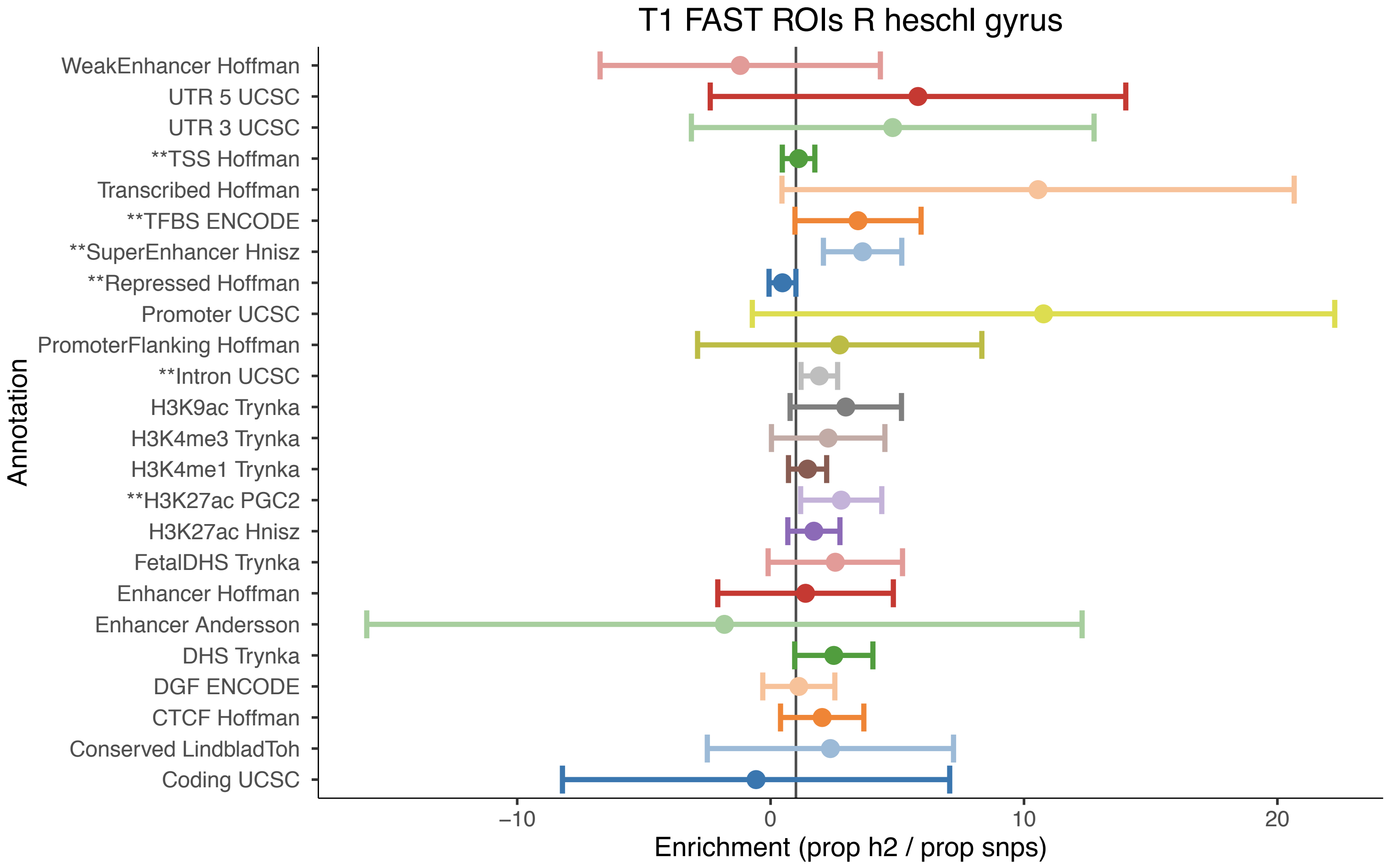

T1 FAST ROIs R heschl gyrus
WeakEnhancer Hoffman
UTR 5 UCSC
UTR 3 UCSC
**TSS Hoffman
Transcribed Hoffman
**TFBS ENCODE
**SuperEnhancer Hnisz
**Repressed Hoffman
Promoter UCSC
PromoterFlanking Hoffman
**Intron UCSC
H3K9ac Trynka
H3K4me3 Trynka
H3K4me1 Trynka
**H3K27ac PGC2
H3K27ac Hnisz
FetalDHS Trynka
Enhancer Hoffman
Enhancer Andersson
DHS Trynka
DGF ENCODE
CTCF Hoffman
Conserved LindbladToh
Coding UCSC
Annotation
−10
0
10
20
Enrichment (prop h2 / prop snps)

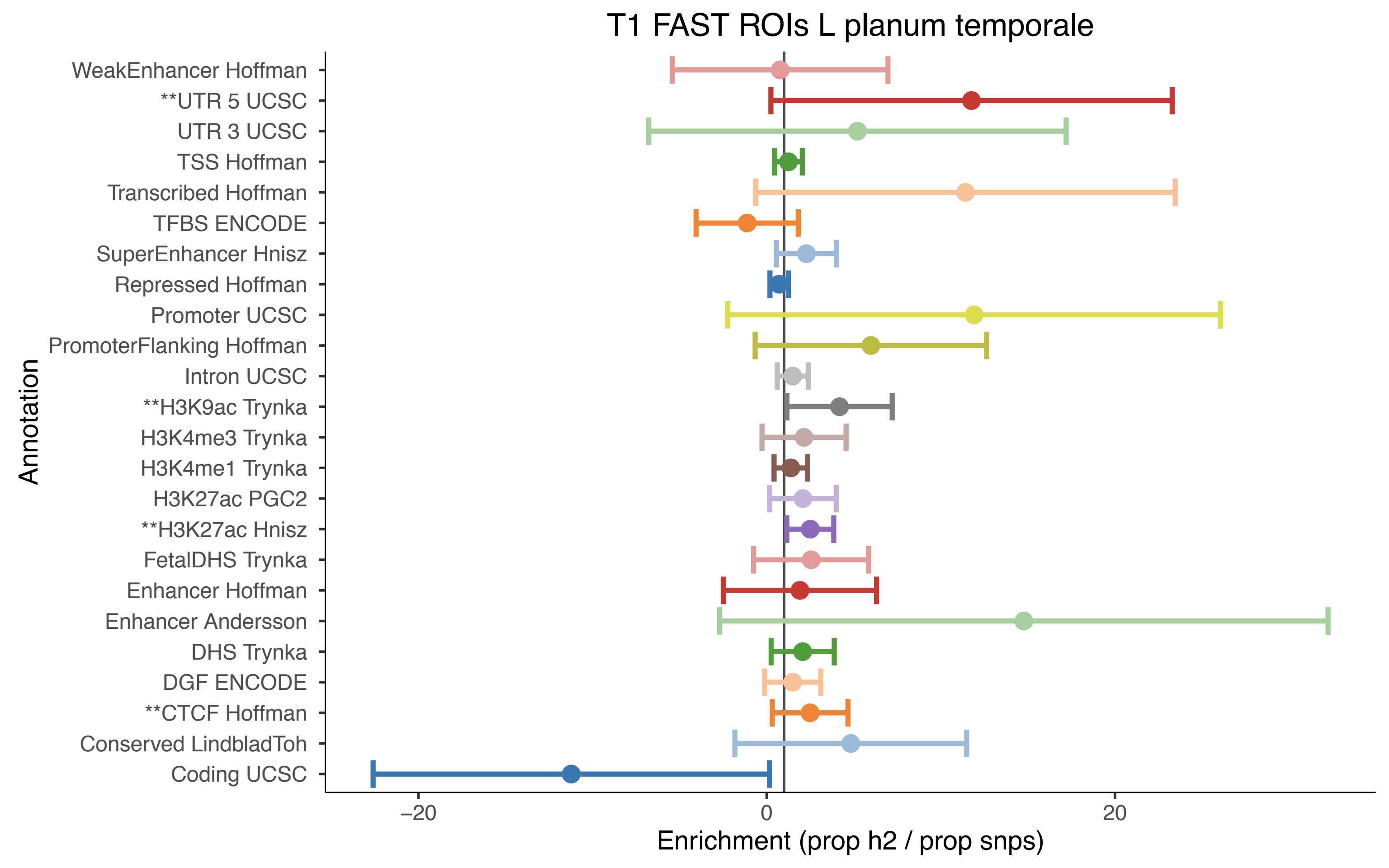

T1 FAST ROIs L planum temporale
Annotation
WeakEnhancer Hoffman
**UTR 5 UCSC
UTR 3 UCSC
TSS Hoffman
Transcribed Hoffman
TFBS ENCODE
SuperEnhancer Hnisz
Repressed Hoffman
Promoter UCSC
PromoterFlanking Hoffman
Intron UCSC
**H3K9ac Trynka
H3K4me3 Trynka
H3K4me1 Trynka
H3K27ac PGC2
**H3K27ac Hnisz
FetalDHS Trynka
Enhancer Hoffman
Enhancer Andersson
DHS Trynka
DGF ENCODE
**CTCF Hoffman
Conserved LindbladToh
Coding UCSC
−20
0
20
Enrichment (prop h2 / prop snps)

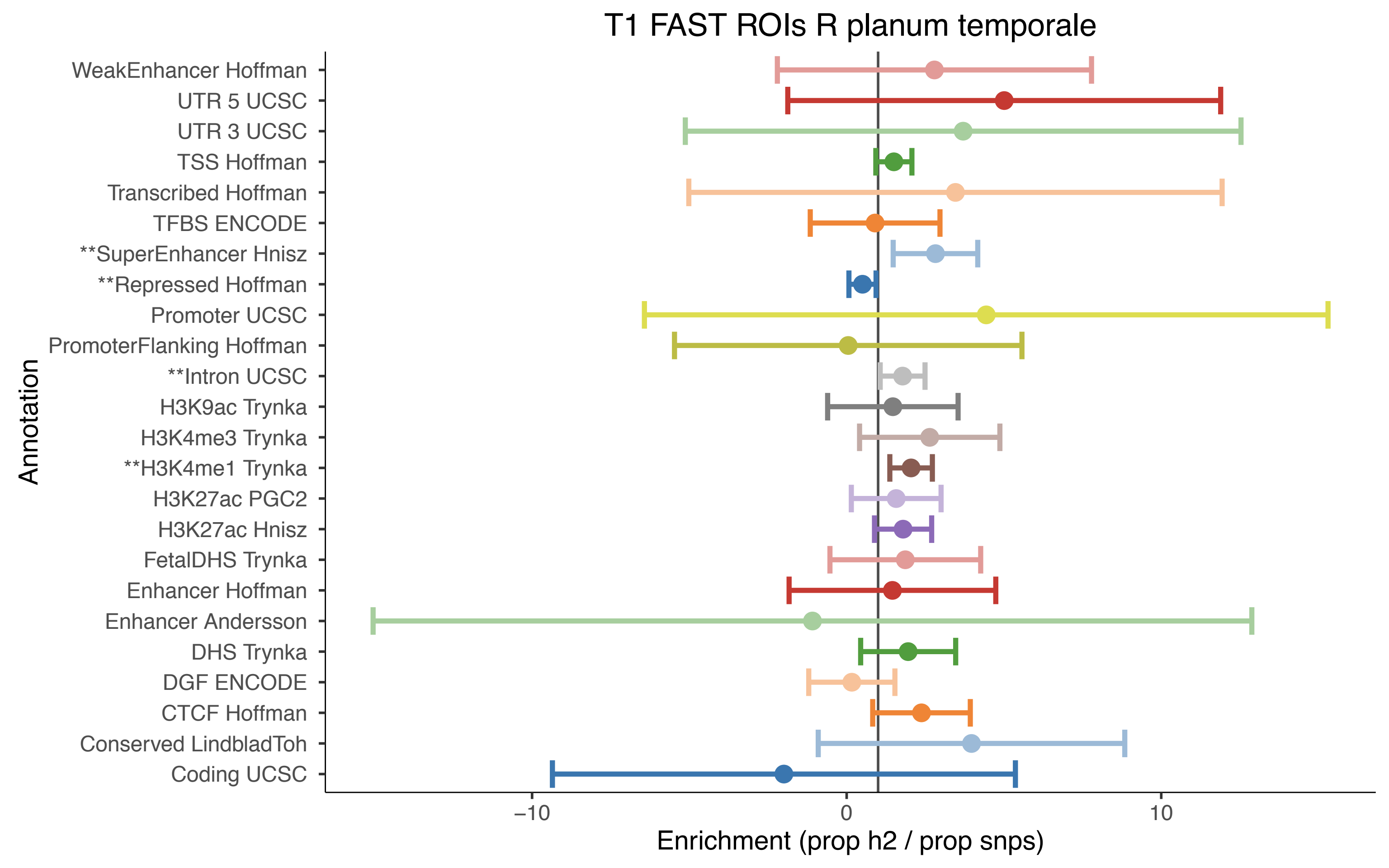

T1 FAST ROIs R planum temporale
WeakEnhancer Hoffman
UTR 5 UCSC
UTR 3 UCSC
TSS Hoffman
Transcribed Hoffman
TFBS ENCODE
**SuperEnhancer Hnisz
**Repressed Hoffman
Promoter UCSC
PromoterFlanking Hoffman
**Intron UCSC
H3K9ac Trynka
H3K4me3 Trynka
**H3K4me1 Trynka
H3K27ac PGC2
H3K27ac Hnisz
FetalDHS Trynka
Enhancer Hoffman
Enhancer Andersson
DHS Trynka
DGF ENCODE
CTCF Hoffman
Conserved LindbladToh
Coding UCSC
Annotation
−10
0
10
Enrichment (prop h2 / prop snps)

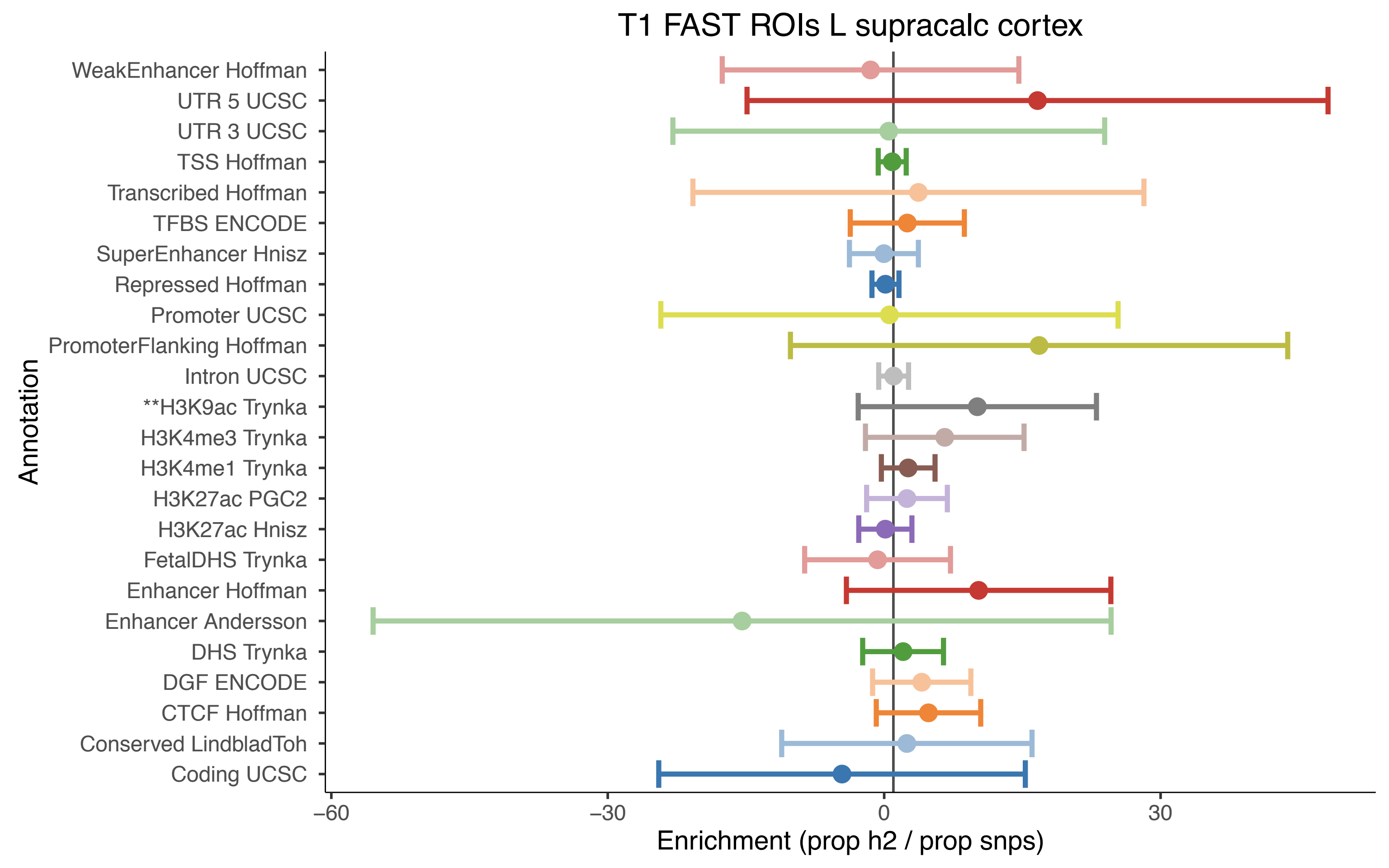

T1 FAST ROIs L supracalc cortex
WeakEnhancer Hoffman
UTR 5 UCSC
UTR 3 UCSC
TSS Hoffman
Transcribed Hoffman
TFBS ENCODE
SuperEnhancer Hnisz
Repressed Hoffman
Promoter UCSC
PromoterFlanking Hoffman
Intron UCSC
**H3K9ac Trynka
H3K4me3 Trynka
H3K4me1 Trynka
H3K27ac PGC2
H3K27ac Hnisz
FetalDHS Trynka
Enhancer Hoffman
Enhancer Andersson
DHS Trynka
DGF ENCODE
CTCF Hoffman
Conserved LindbladToh
Coding UCSC
Annotation
−60
−30
0
30
Enrichment (prop h2 / prop snps)

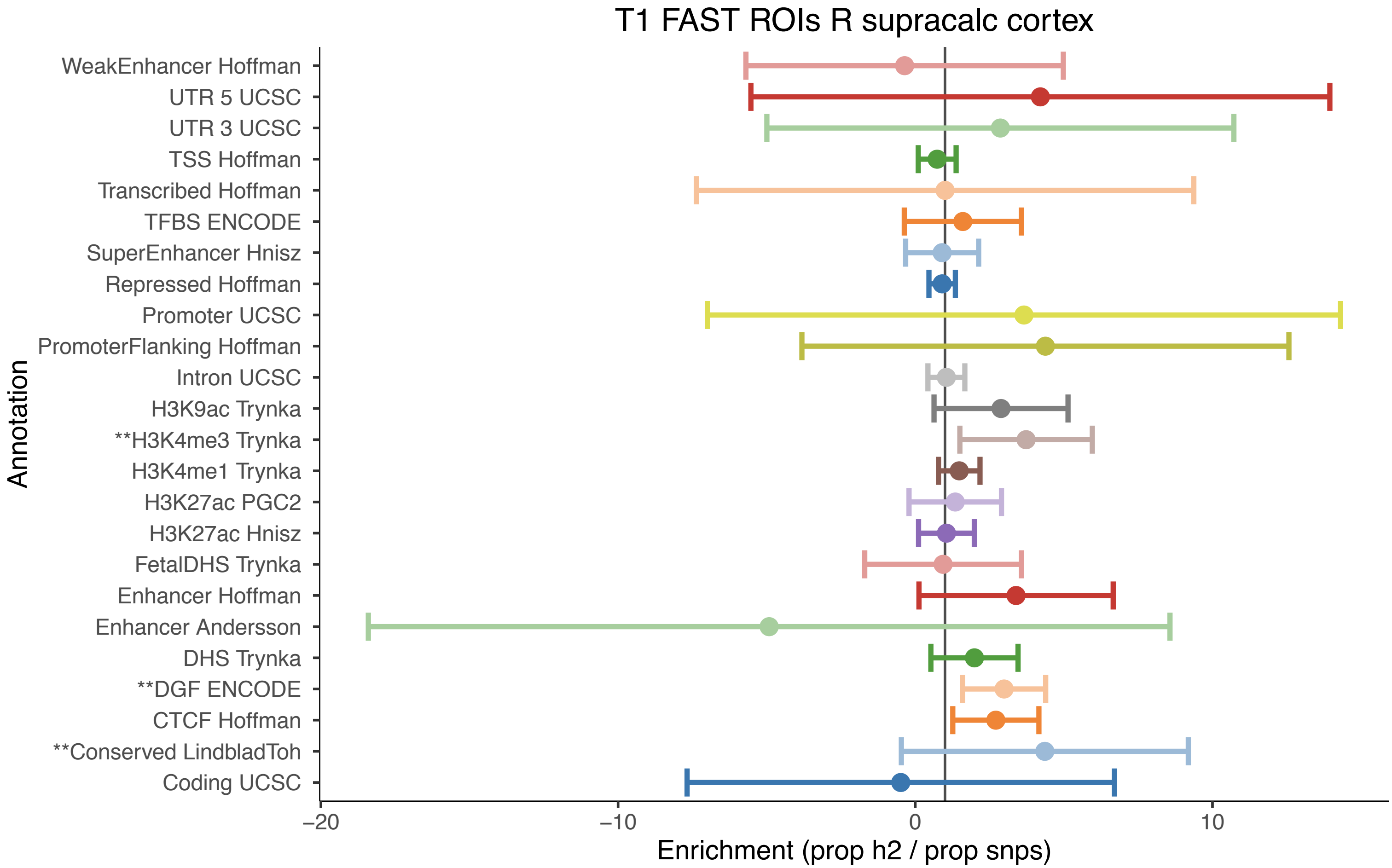
T1 FAST ROIs R supracalc cortex
WeakEnhancer Hoffman
UTR 5 UCSC
UTR 3 UCSC
TSS Hoffman
Transcribed Hoffman
TFBS ENCODE
SuperEnhancer Hnisz
Repressed Hoffman
Promoter UCSC
PromoterFlanking Hoffman
Intron UCSC
H3K9ac Trynka
**H3K4me3 Trynka
H3K4me1 Trynka
H3K27ac PGC2
H3K27ac Hnisz
FetalDHS Trynka
Enhancer Hoffman
Enhancer Andersson
DHS Trynka
**DGF ENCODE
CTCF Hoffman
**Conserved LindbladToh
Coding UCSC
Annotation
−20
−10
0
10
Enrichment (prop h2 / prop snps)

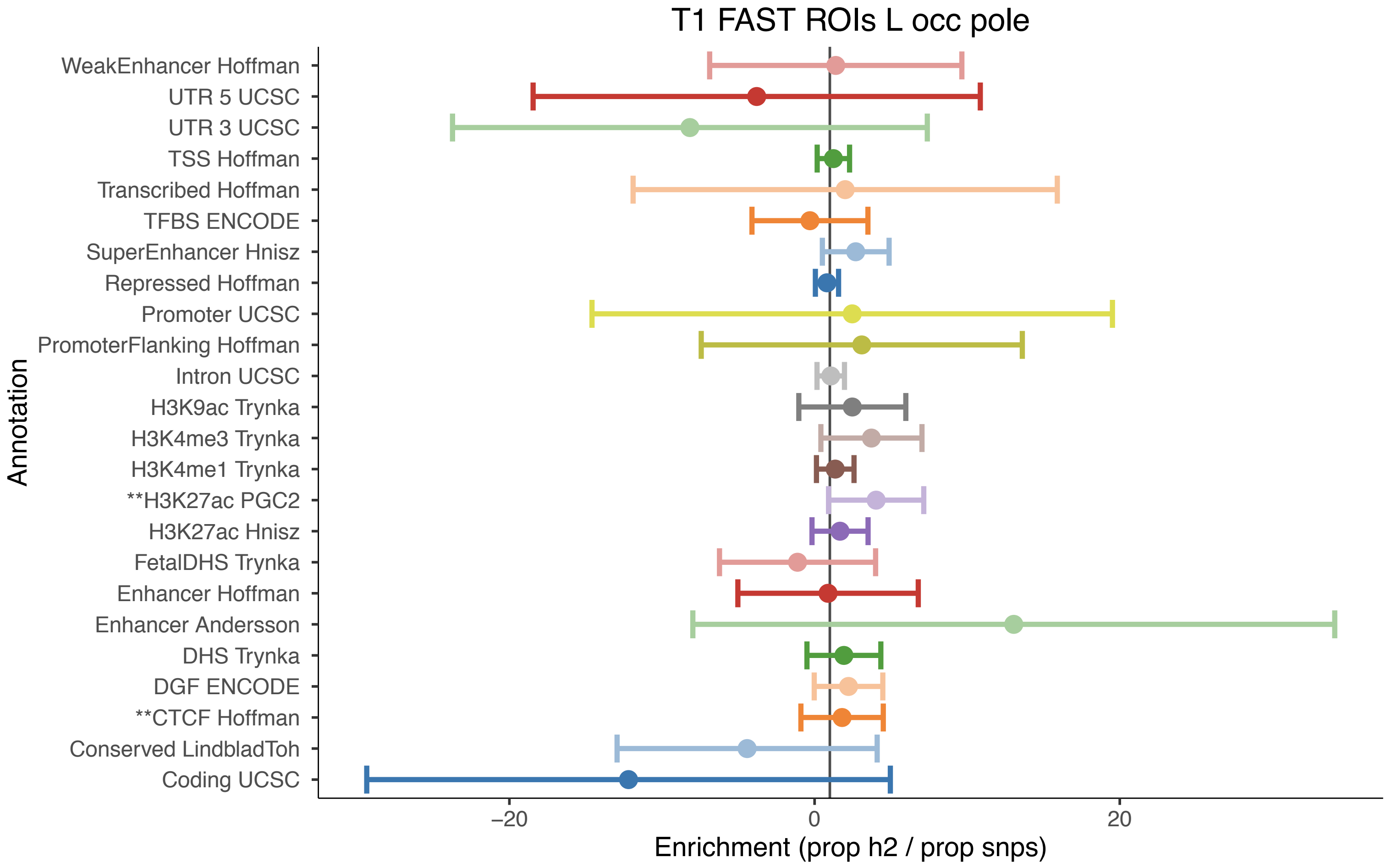
T1 FAST ROIs L occ pole
WeakEnhancer Hoffman
UTR 5 UCSC
UTR 3 UCSC
TSS Hoffman
Transcribed Hoffman
TFBS ENCODE
SuperEnhancer Hnisz
Repressed Hoffman
Promoter UCSC
PromoterFlanking Hoffman
Intron UCSC
H3K9ac Trynka
H3K4me3 Trynka
H3K4me1 Trynka
**H3K27ac PGC2
H3K27ac Hnisz
FetalDHS Trynka
Enhancer Hoffman
Enhancer Andersson
DHS Trynka
DGF ENCODE
**CTCF Hoffman
Conserved LindbladToh
Coding UCSC
Annotation
−20
0
20
Enrichment (prop h2 / prop snps)

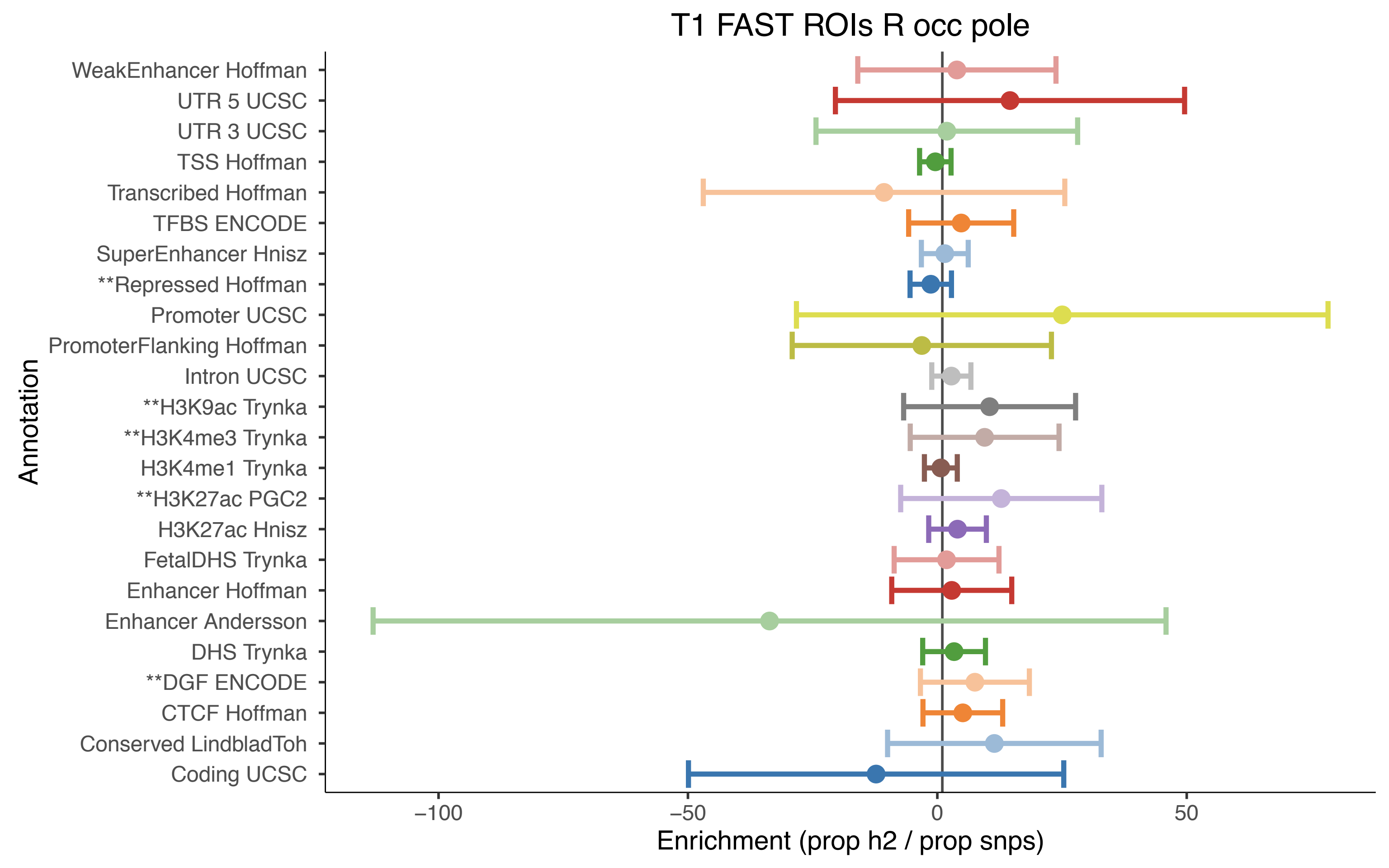

T1 FAST ROIs R occ pole
WeakEnhancer Hoffman
UTR 5 UCSC
UTR 3 UCSC
TSS Hoffman
Transcribed Hoffman
TFBS ENCODE
SuperEnhancer Hnisz
**Repressed Hoffman
Promoter UCSC
PromoterFlanking Hoffman
Intron UCSC
**H3K9ac Trynka
**H3K4me3 Trynka
H3K4me1 Trynka
**H3K27ac PGC2
H3K27ac Hnisz
FetalDHS Trynka
Enhancer Hoffman
Enhancer Andersson
DHS Trynka
**DGF ENCODE
CTCF Hoffman
Conserved LindbladToh
Coding UCSC
Annotation
−100
−50
0
50
Enrichment (prop h2 / prop snps)

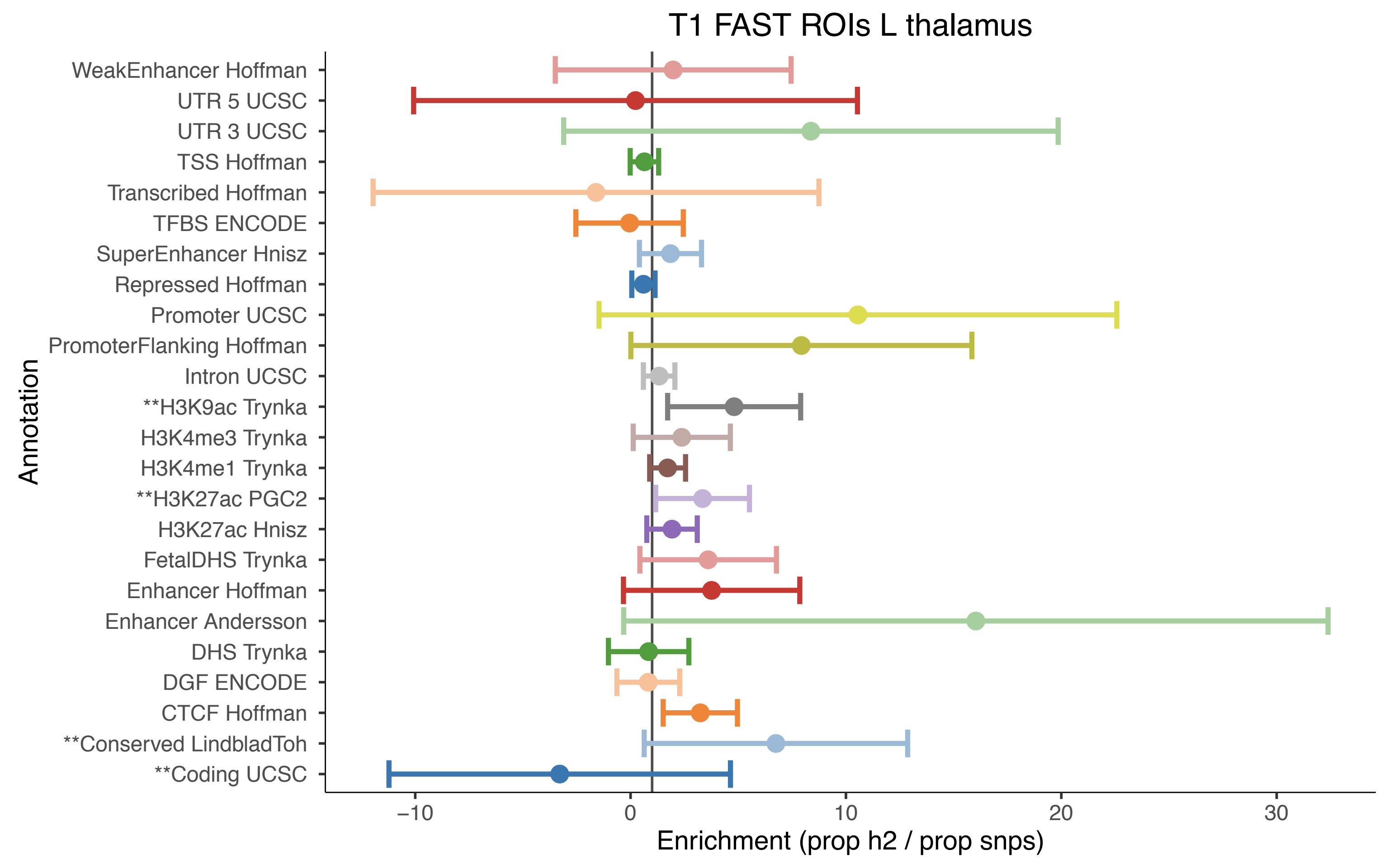

T1 FAST ROIs L thalamus
WeakEnhancer Hoffman
UTR 5 UCSC
UTR 3 UCSC
TSS Hoffman
Transcribed Hoffman
TFBS ENCODE
SuperEnhancer Hnisz
Repressed Hoffman
Promoter UCSC
PromoterFlanking Hoffman
Intron UCSC
**H3K9ac Trynka
H3K4me3 Trynka
H3K4me1 Trynka
**H3K27ac PGC2
H3K27ac Hnisz
FetalDHS Trynka
Enhancer Hoffman
Enhancer Andersson
DHS Trynka
DGF ENCODE
CTCF Hoffman
**Conserved LindbladToh
**Coding UCSC
Annotation
−10
0
10
20
30
Enrichment (prop h2 / prop snps)

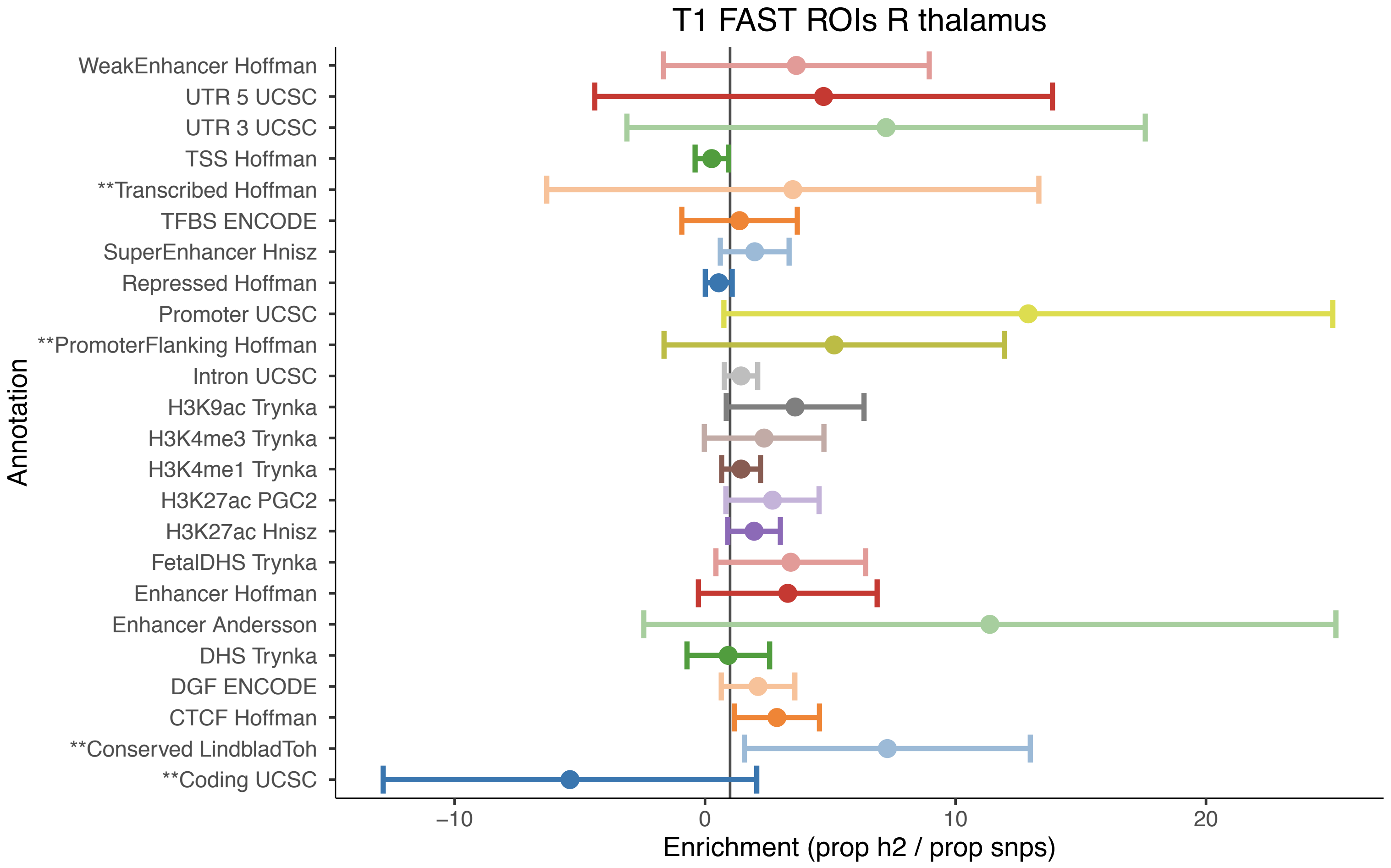

T1 FAST ROIs R thalamus
WeakEnhancer Hoffman
UTR 5 UCSC
UTR 3 UCSC
TSS Hoffman
**Transcribed Hoffman
TFBS ENCODE
SuperEnhancer Hnisz
Repressed Hoffman
Promoter UCSC
**PromoterFlanking Hoffman
Intron UCSC
H3K9ac Trynka
H3K4me3 Trynka
H3K4me1 Trynka
H3K27ac PGC2
H3K27ac Hnisz
FetalDHS Trynka
Enhancer Hoffman
Enhancer Andersson
DHS Trynka
DGF ENCODE
CTCF Hoffman
**Conserved LindbladToh
**Coding UCSC
Annotation
−10
0
10
20
Enrichment (prop h2 / prop snps)

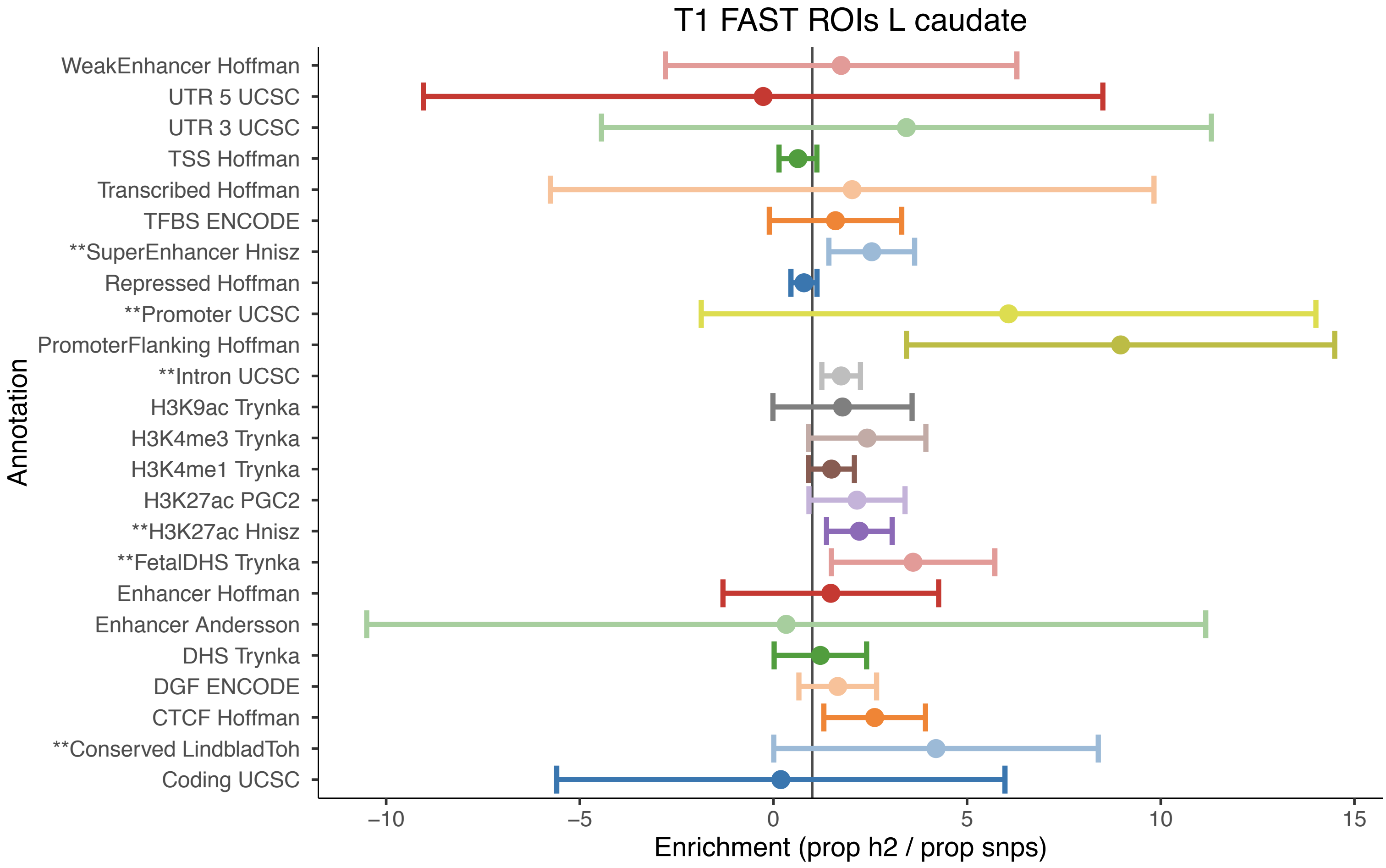
T1 FAST ROIs L caudate
Annotation
WeakEnhancer Hoffman
UTR 5 UCSC
UTR 3 UCSC
TSS Hoffman
Transcribed Hoffman
TFBS ENCODE
**SuperEnhancer Hnisz
Repressed Hoffman
**Promoter UCSC
PromoterFlanking Hoffman
**Intron UCSC
H3K9ac Trynka
H3K4me3 Trynka
H3K4me1 Trynka
H3K27ac PGC2
**H3K27ac Hnisz
**FetalDHS Trynka
Enhancer Hoffman
Enhancer Andersson
DHS Trynka
DGF ENCODE
CTCF Hoffman
**Conserved LindbladToh
Coding UCSC
−10
−5
0
5
10
15
Enrichment (prop h2 / prop snps)

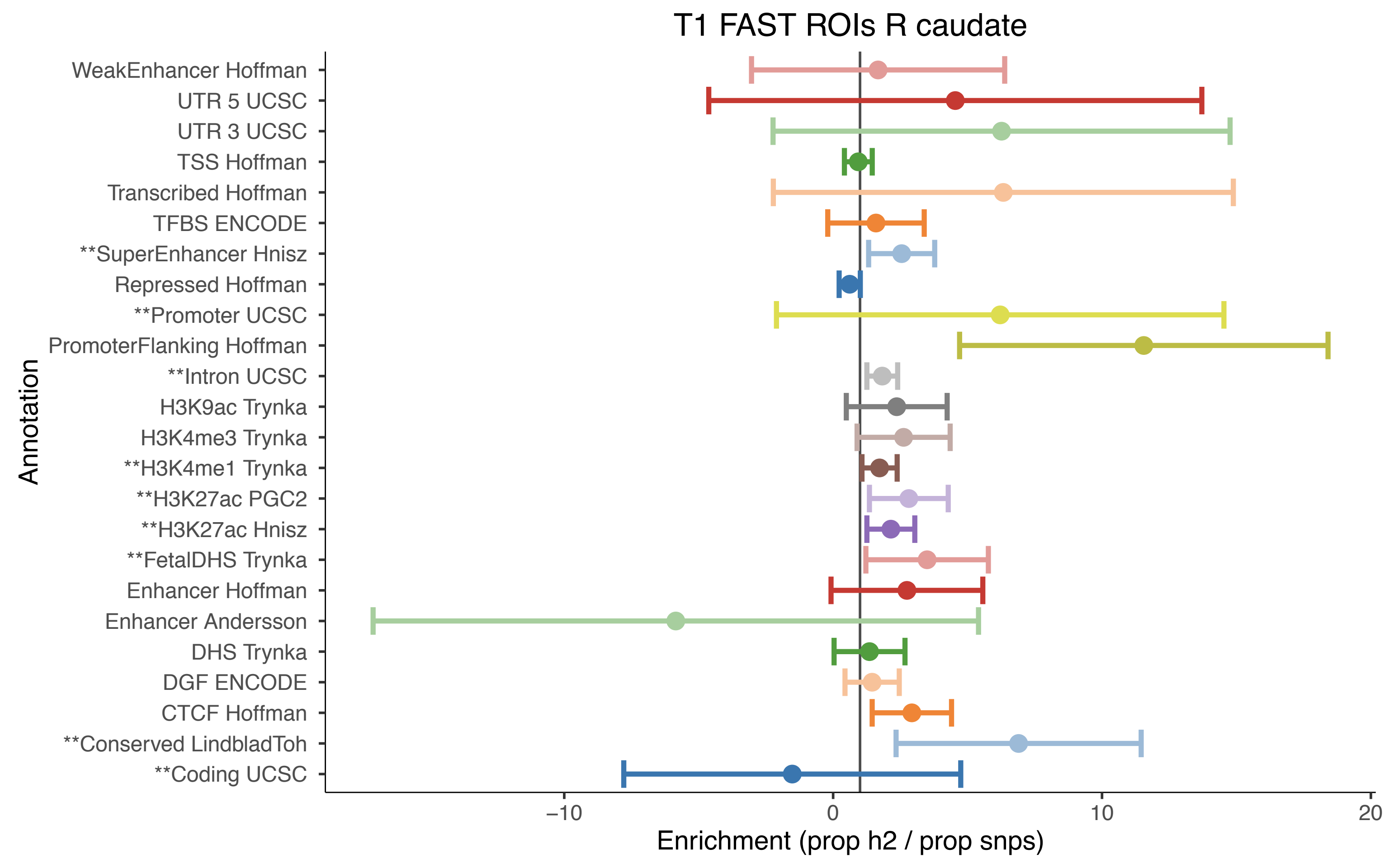
T1 FAST ROIs R caudate
WeakEnhancer Hoffman
UTR 5 UCSC
UTR 3 UCSC
TSS Hoffman
Transcribed Hoffman
TFBS ENCODE
**SuperEnhancer Hnisz
Repressed Hoffman
**Promoter UCSC
PromoterFlanking Hoffman
**Intron UCSC
H3K9ac Trynka
H3K4me3 Trynka
**H3K4me1 Trynka
**H3K27ac PGC2
**H3K27ac Hnisz
**FetalDHS Trynka
Enhancer Hoffman
Enhancer Andersson
DHS Trynka
DGF ENCODE
CTCF Hoffman
**Conserved LindbladToh
**Coding UCSC
Annotation
−10
0
10
20
Enrichment (prop h2 / prop snps)

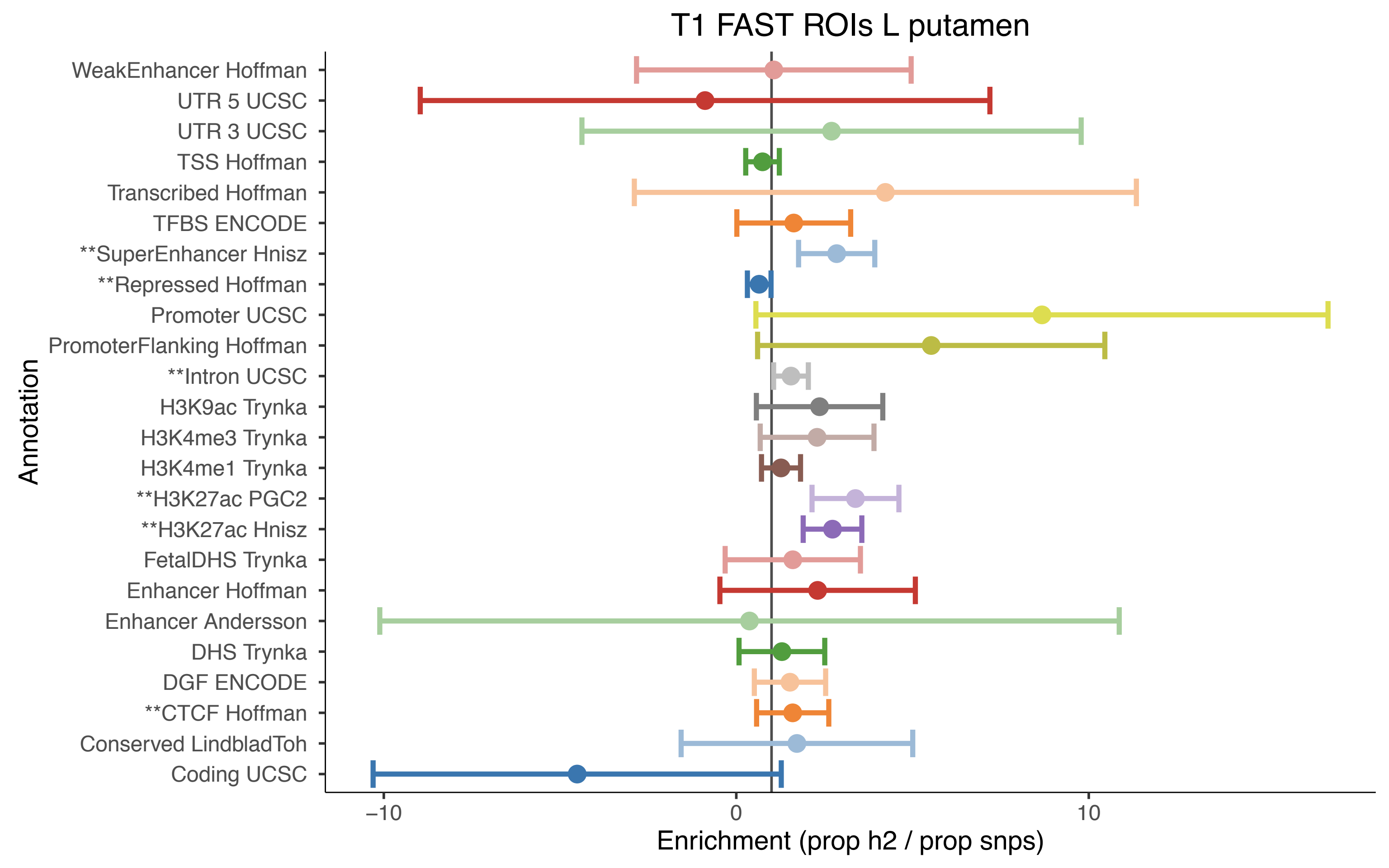
T1 FAST ROIs L putamen
WeakEnhancer Hoffman
UTR 5 UCSC
UTR 3 UCSC
TSS Hoffman
Transcribed Hoffman
TFBS ENCODE
**SuperEnhancer Hnisz
**Repressed Hoffman
Promoter UCSC
PromoterFlanking Hoffman
**Intron UCSC
H3K9ac Trynka
H3K4me3 Trynka
H3K4me1 Trynka
**H3K27ac PGC2
**H3K27ac Hnisz
FetalDHS Trynka
Enhancer Hoffman
Enhancer Andersson
DHS Trynka
DGF ENCODE
**CTCF Hoffman
Conserved LindbladToh
Coding UCSC
Annotation
−10
0
10
Enrichment (prop h2 / prop snps)

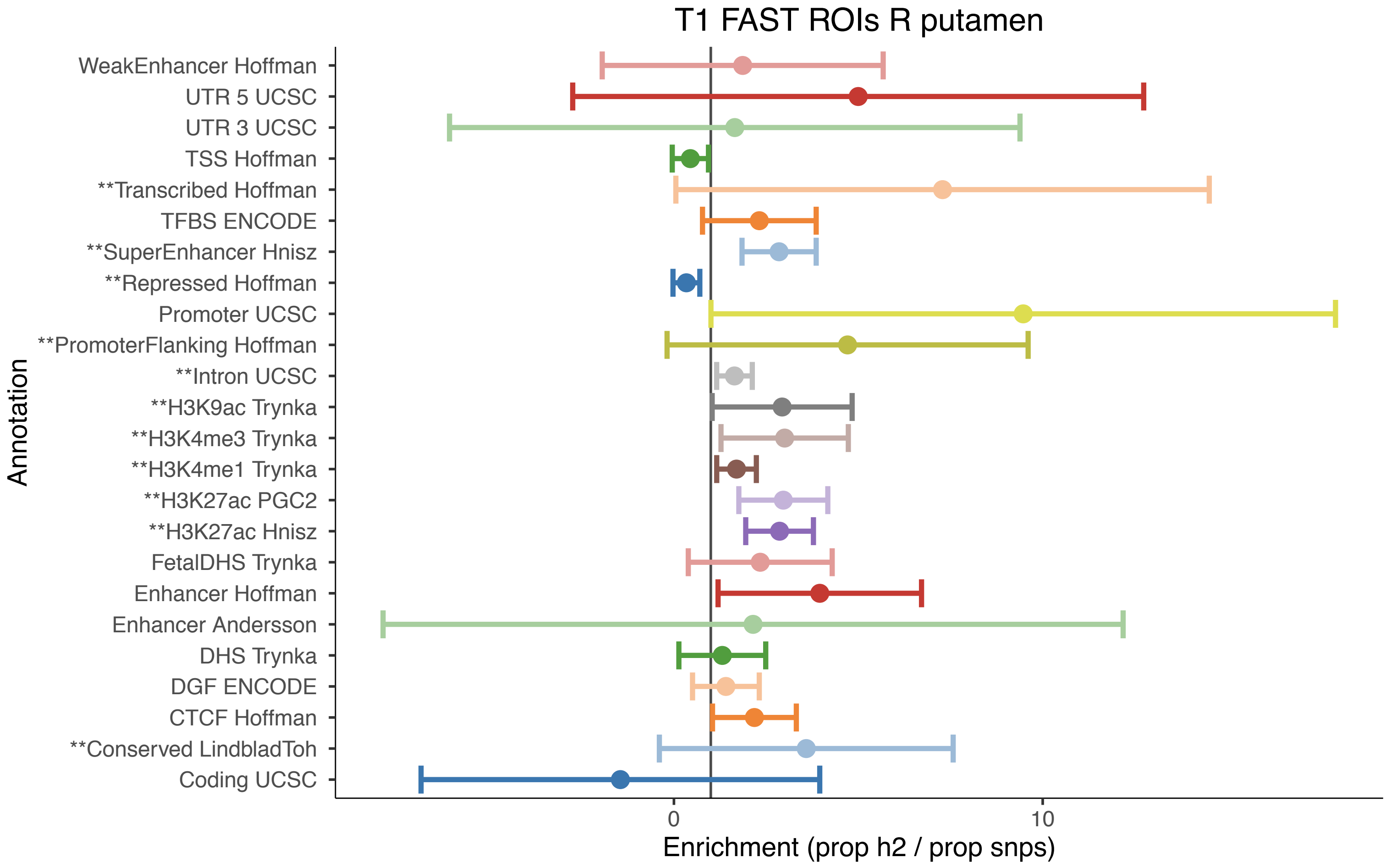
T1 FAST ROIs R putamen
WeakEnhancer Hoffman
UTR 5 UCSC
UTR 3 UCSC
TSS Hoffman
**Transcribed Hoffman
TFBS ENCODE
**SuperEnhancer Hnisz
**Repressed Hoffman
Promoter UCSC
**PromoterFlanking Hoffman
**Intron UCSC
**H3K9ac Trynka
**H3K4me3 Trynka
**H3K4me1 Trynka
**H3K27ac PGC2
**H3K27ac Hnisz
FetalDHS Trynka
Enhancer Hoffman
Enhancer Andersson
DHS Trynka
DGF ENCODE
CTCF Hoffman
**Conserved LindbladToh
Coding UCSC
Annotation
0
10
Enrichment (prop h2 / prop snps)

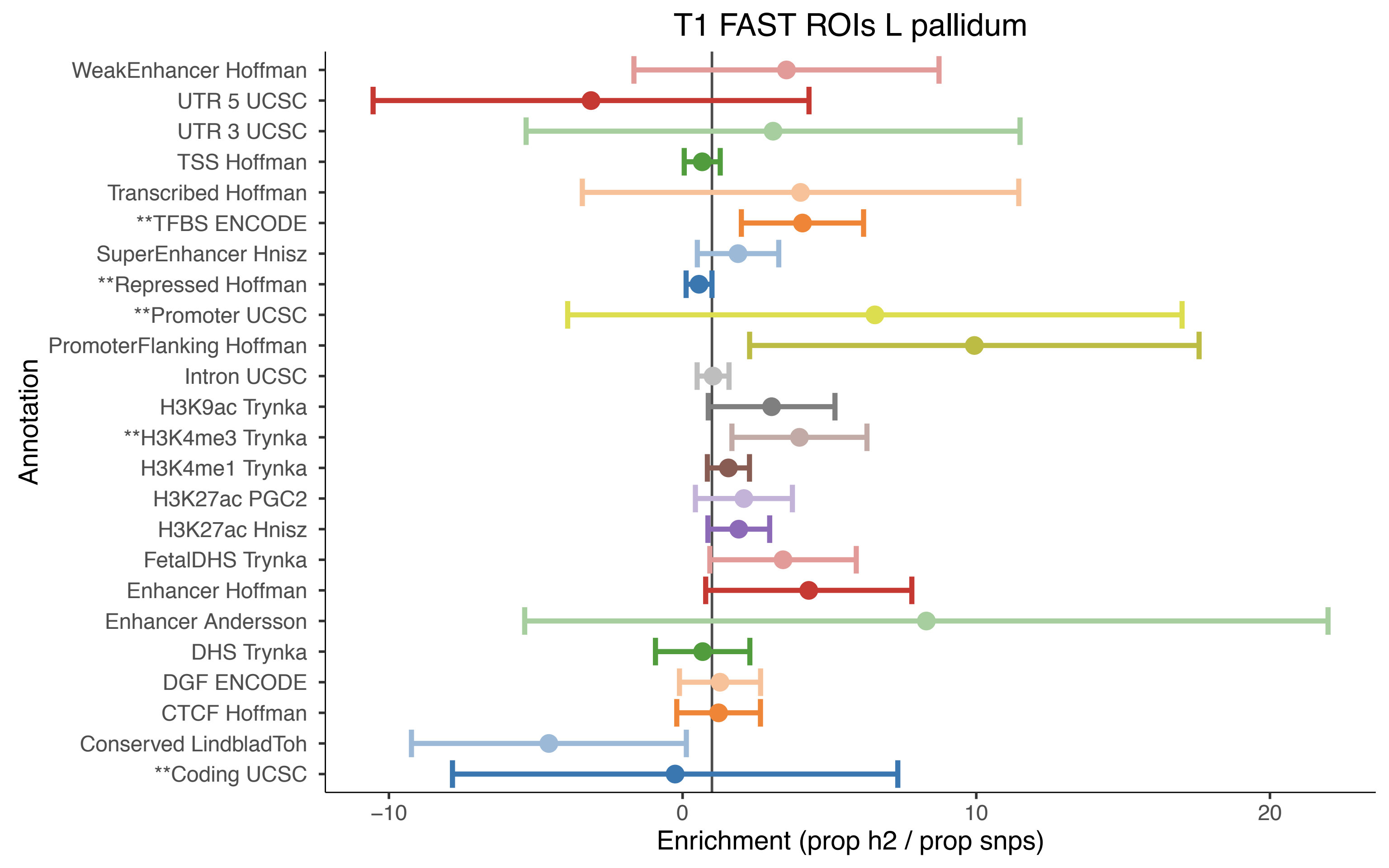
T1 FAST ROIs L pallidum
WeakEnhancer Hoffman
UTR 5 UCSC
UTR 3 UCSC
TSS Hoffman
Transcribed Hoffman
**TFBS ENCODE
SuperEnhancer Hnisz
**Repressed Hoffman
**Promoter UCSC
PromoterFlanking Hoffman
Intron UCSC
H3K9ac Trynka
**H3K4me3 Trynka
H3K4me1 Trynka
H3K27ac PGC2
H3K27ac Hnisz
FetalDHS Trynka
Enhancer Hoffman
Enhancer Andersson
DHS Trynka
DGF ENCODE
CTCF Hoffman
Conserved LindbladToh
**Coding UCSC
Annotation
−10
0
10
20
Enrichment (prop h2 / prop snps)

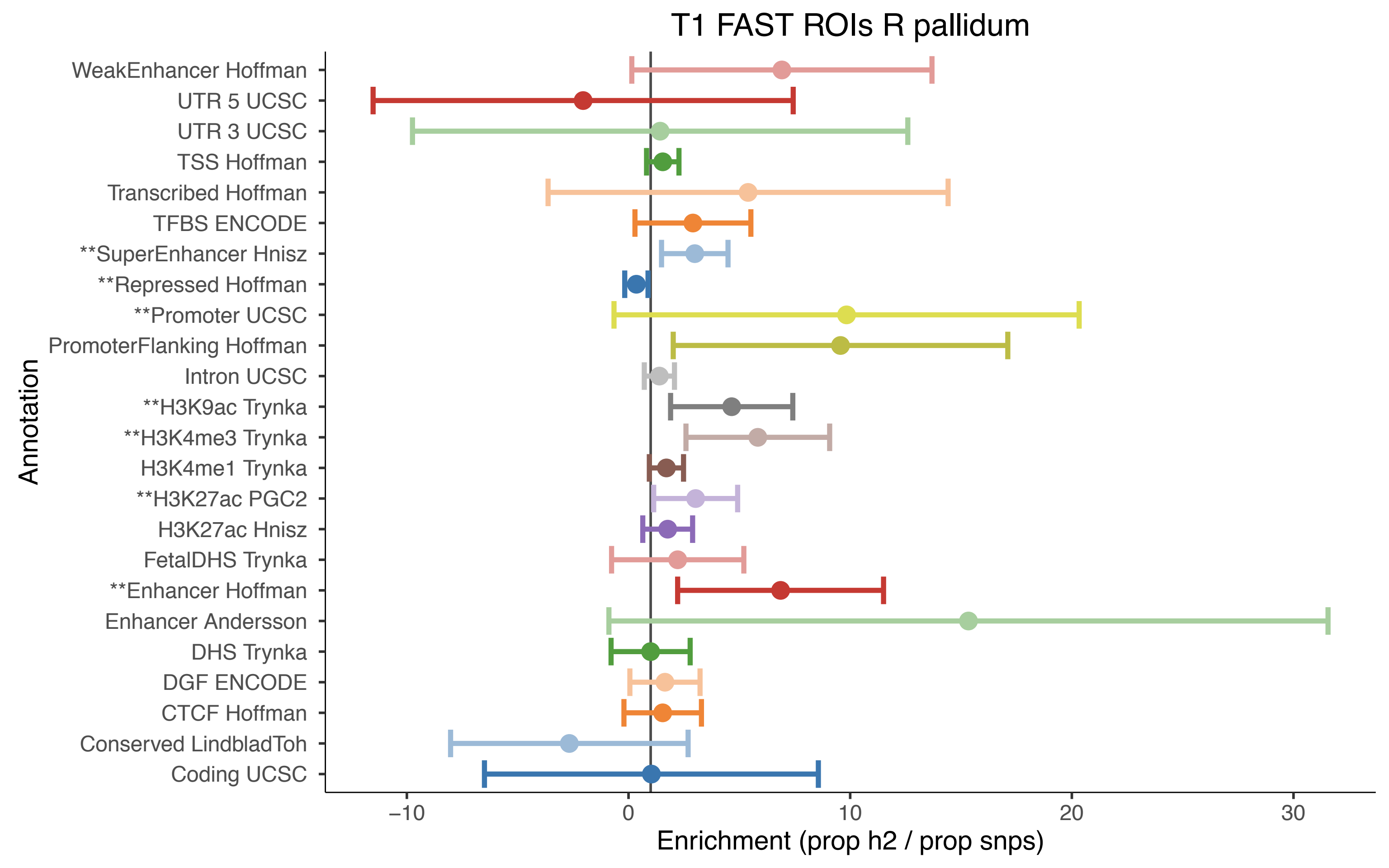
T1 FAST ROIs R pallidum
Annotation
WeakEnhancer Hoffman
UTR 5 UCSC
UTR 3 UCSC
TSS Hoffman
Transcribed Hoffman
TFBS ENCODE
**SuperEnhancer Hnisz
**Repressed Hoffman
**Promoter UCSC
PromoterFlanking Hoffman
Intron UCSC
**H3K9ac Trynka
**H3K4me3 Trynka
H3K4me1 Trynka
**H3K27ac PGC2
H3K27ac Hnisz
FetalDHS Trynka
**Enhancer Hoffman
Enhancer Andersson
DHS Trynka
DGF ENCODE
CTCF Hoffman
Conserved LindbladToh
Coding UCSC
−10
0
10
20
30
Enrichment (prop h2 / prop snps)

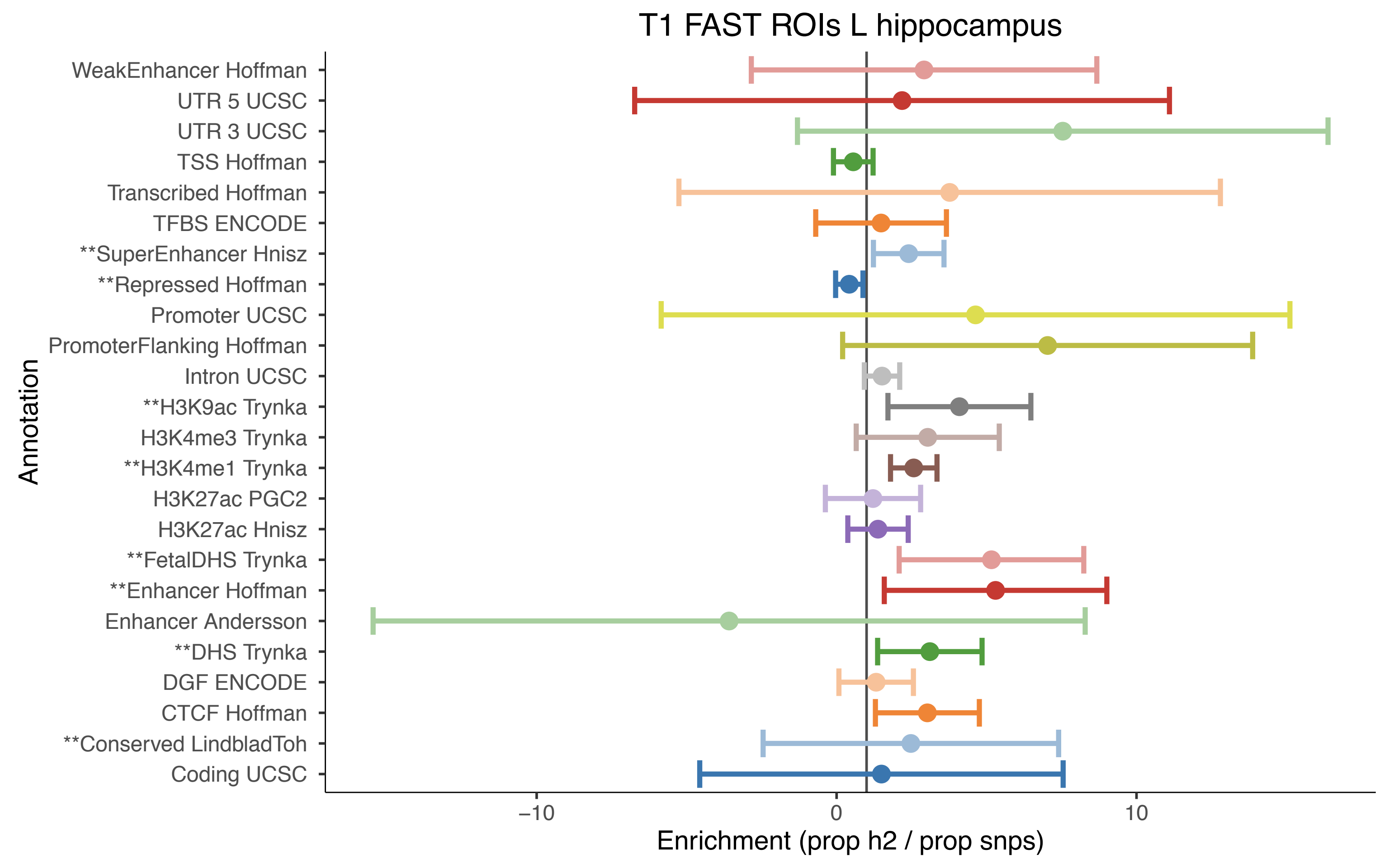

T1 FAST ROIs L hippocampus
WeakEnhancer Hoffman
UTR 5 UCSC
UTR 3 UCSC
TSS Hoffman
Transcribed Hoffman
TFBS ENCODE
**SuperEnhancer Hnisz
**Repressed Hoffman
Promoter UCSC
PromoterFlanking Hoffman
Intron UCSC
**H3K9ac Trynka
H3K4me3 Trynka
**H3K4me1 Trynka
H3K27ac PGC2
H3K27ac Hnisz
**FetalDHS Trynka
**Enhancer Hoffman
Enhancer Andersson
**DHS Trynka
DGF ENCODE
CTCF Hoffman
**Conserved LindbladToh
Coding UCSC
Annotation
−10
0
10
Enrichment (prop h2 / prop snps)

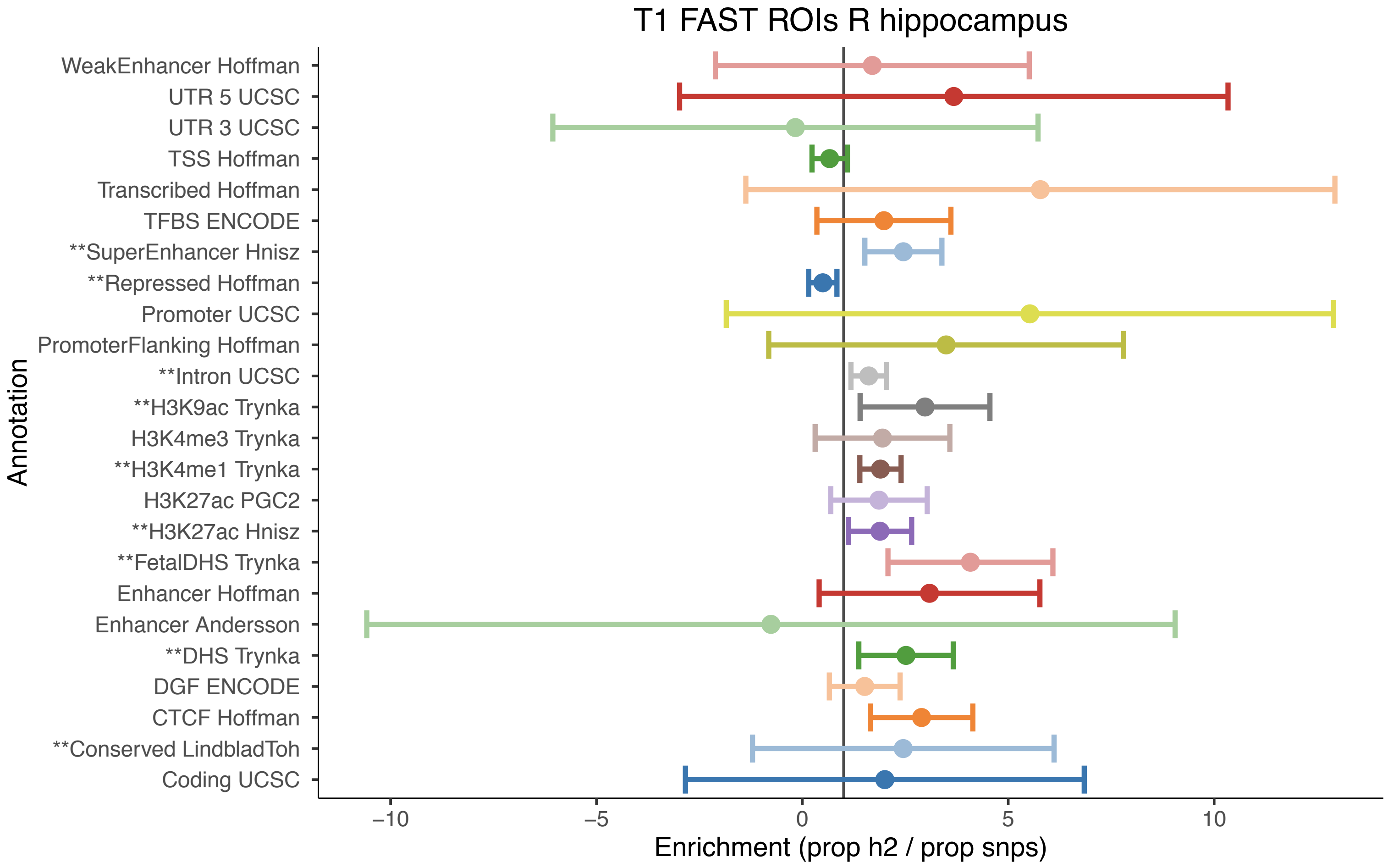

T1 FAST ROIs R hippocampus
WeakEnhancer Hoffman
UTR 5 UCSC
UTR 3 UCSC
TSS Hoffman
Transcribed Hoffman
TFBS ENCODE
**SuperEnhancer Hnisz
**Repressed Hoffman
Promoter UCSC
PromoterFlanking Hoffman
**Intron UCSC
**H3K9ac Trynka
H3K4me3 Trynka
**H3K4me1 Trynka
H3K27ac PGC2
**H3K27ac Hnisz
**FetalDHS Trynka
Enhancer Hoffman
Enhancer Andersson
**DHS Trynka
DGF ENCODE
CTCF Hoffman
**Conserved LindbladToh
Coding UCSC
Annotation
−10
−5
0
5
10
Enrichment (prop h2 / prop snps)

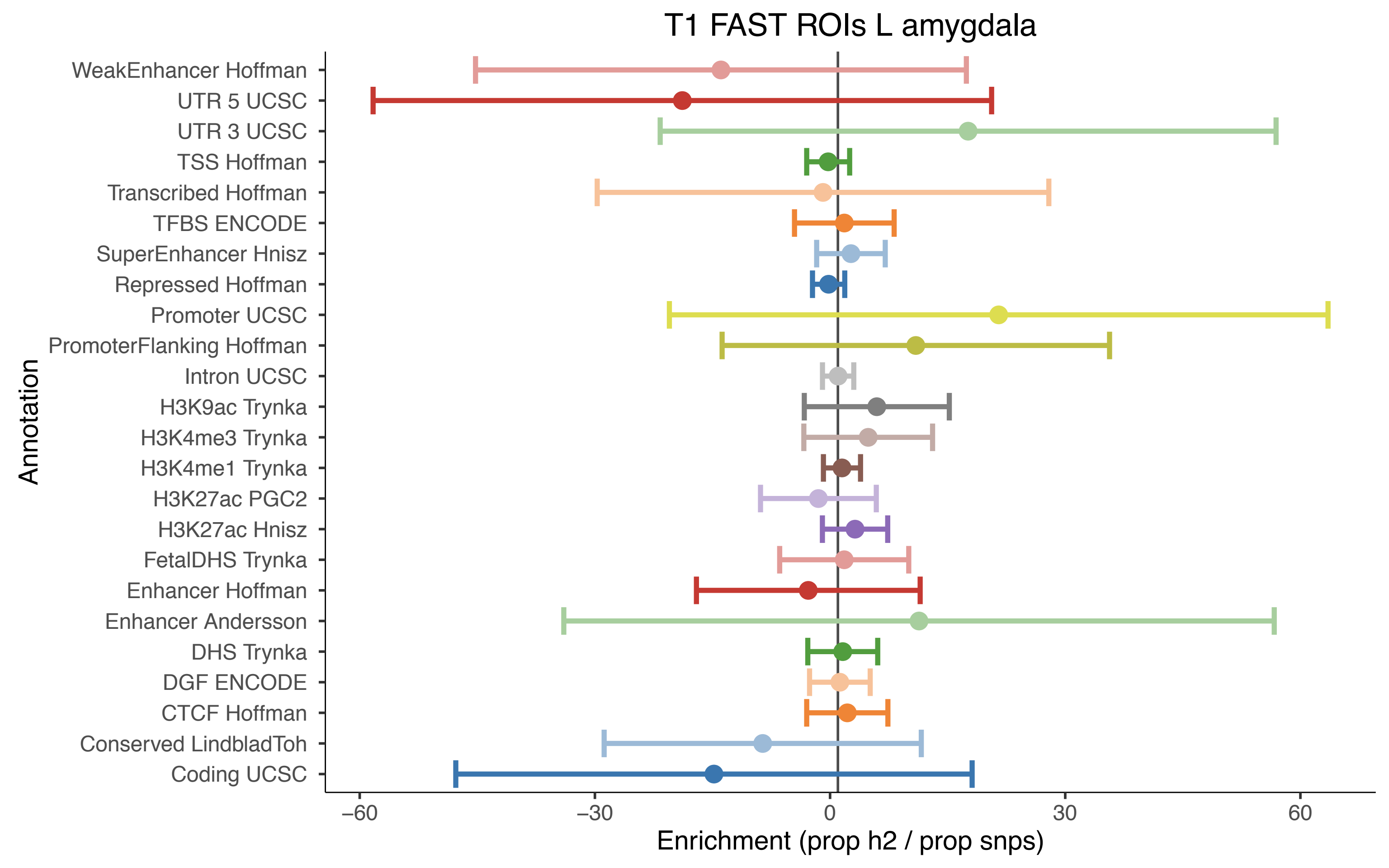
T1 FAST ROIs L amygdala
WeakEnhancer Hoffman
UTR 5 UCSC
UTR 3 UCSC
TSS Hoffman
Transcribed Hoffman
TFBS ENCODE
SuperEnhancer Hnisz
Repressed Hoffman
Promoter UCSC
PromoterFlanking Hoffman
Intron UCSC
H3K9ac Trynka
H3K4me3 Trynka
H3K4me1 Trynka
H3K27ac PGC2
H3K27ac Hnisz
FetalDHS Trynka
Enhancer Hoffman
Enhancer Andersson
DHS Trynka
DGF ENCODE
CTCF Hoffman
Conserved LindbladToh
Coding UCSC
Annotation
−60
−30
0
30
60
Enrichment (prop h2 / prop snps)

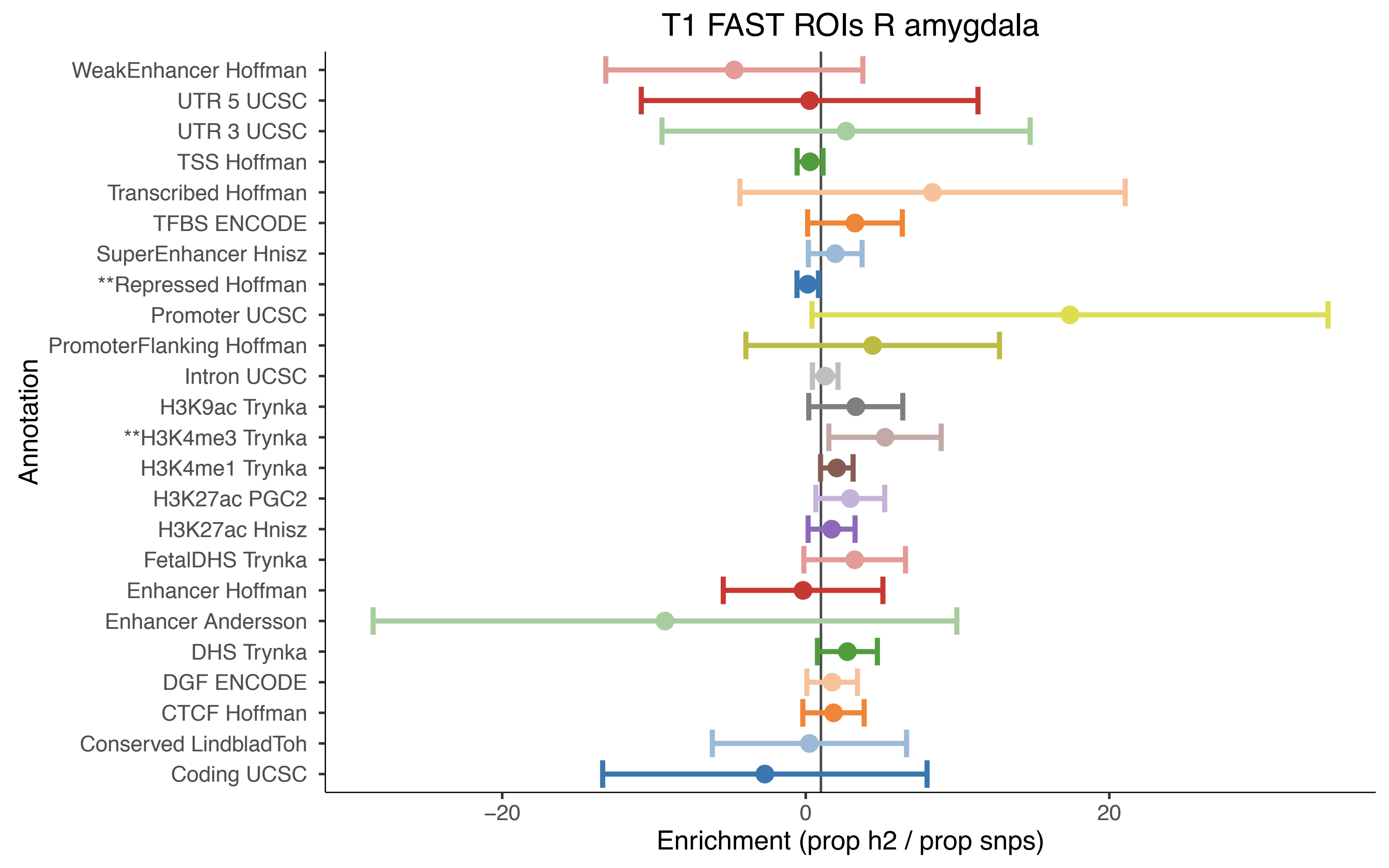
T1 FAST ROIs R amygdala
WeakEnhancer Hoffman
UTR 5 UCSC
UTR 3 UCSC
TSS Hoffman
Transcribed Hoffman
TFBS ENCODE
SuperEnhancer Hnisz
**Repressed Hoffman
Promoter UCSC
PromoterFlanking Hoffman
Intron UCSC
H3K9ac Trynka
**H3K4me3 Trynka
H3K4me1 Trynka
H3K27ac PGC2
H3K27ac Hnisz
FetalDHS Trynka
Enhancer Hoffman
Enhancer Andersson
DHS Trynka
DGF ENCODE
CTCF Hoffman
Conserved LindbladToh
Coding UCSC
Annotation
−20
0
20
Enrichment (prop h2 / prop snps)

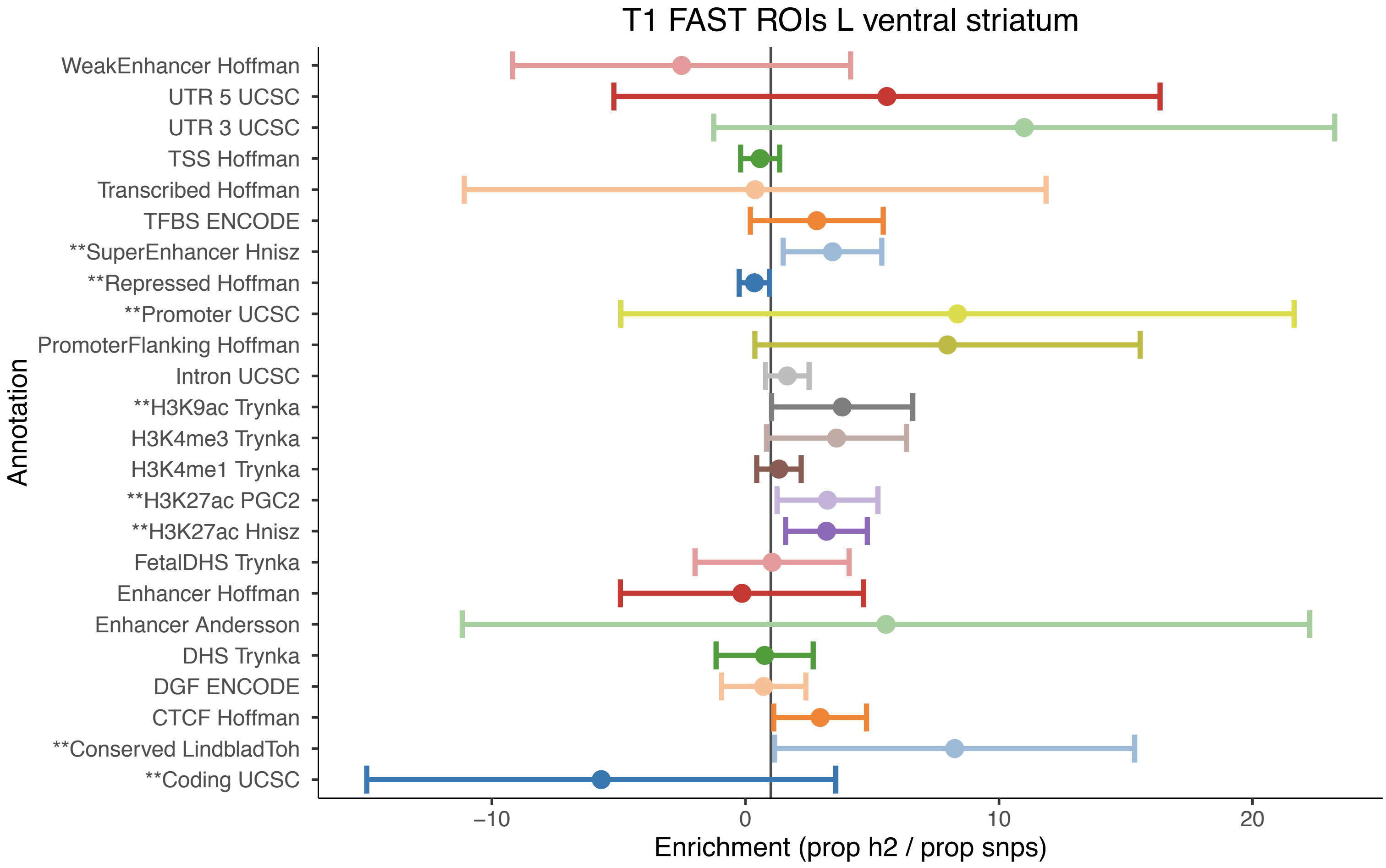
T1 FAST ROIs L ventral striatum
WeakEnhancer Hoffman
UTR 5 UCSC
UTR 3 UCSC
TSS Hoffman
Transcribed Hoffman
TFBS ENCODE
**SuperEnhancer Hnisz
**Repressed Hoffman
**Promoter UCSC
PromoterFlanking Hoffman
Intron UCSC
**H3K9ac Trynka
H3K4me3 Trynka
H3K4me1 Trynka
**H3K27ac PGC2
**H3K27ac Hnisz
FetalDHS Trynka
Enhancer Hoffman
Enhancer Andersson
DHS Trynka
DGF ENCODE
CTCF Hoffman
**Conserved LindbladToh
**Coding UCSC
Annotation
−10
0
10
20
Enrichment (prop h2 / prop snps)

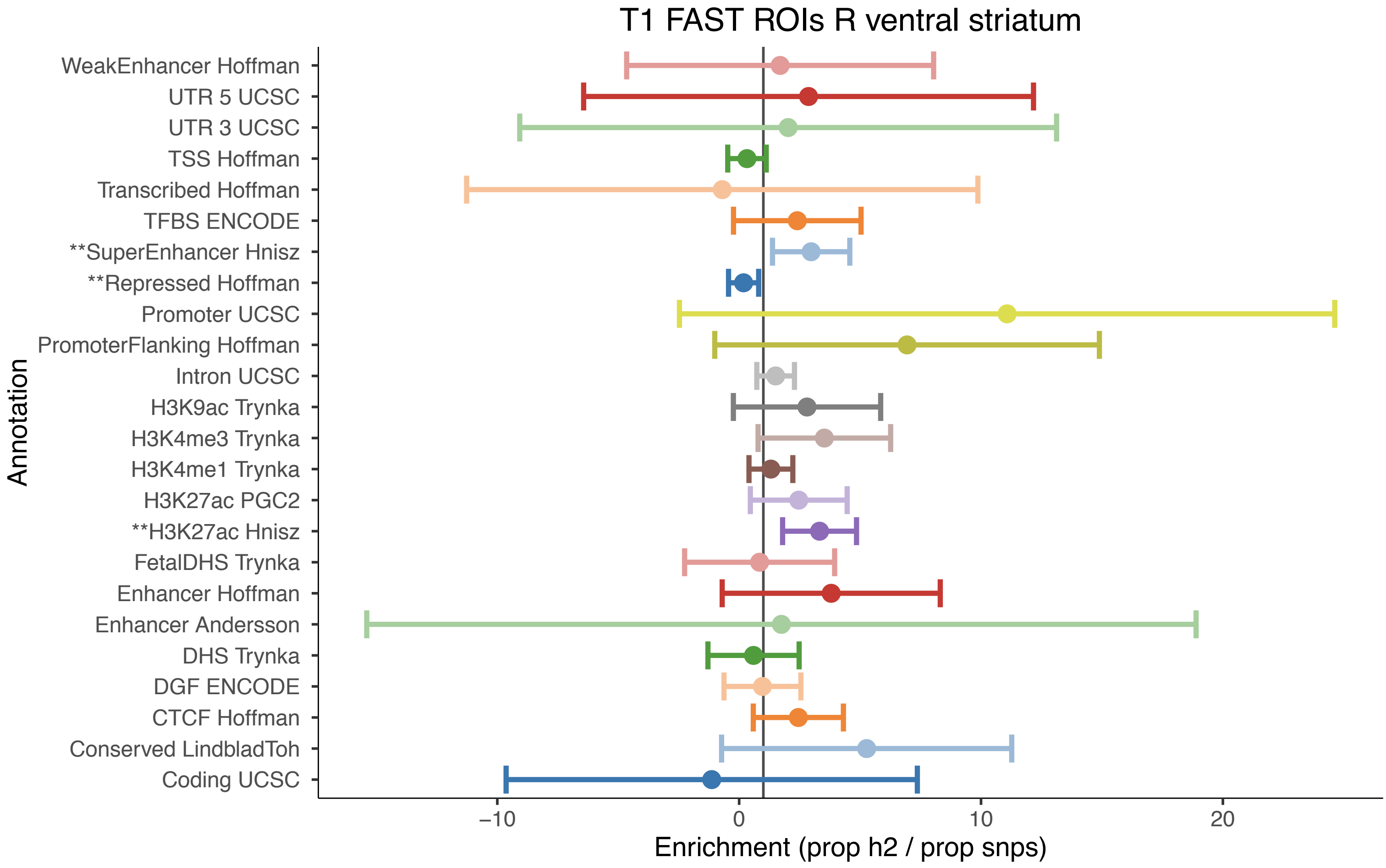
T1 FAST ROIs R ventral striatum
WeakEnhancer Hoffman
UTR 5 UCSC
UTR 3 UCSC
TSS Hoffman
Transcribed Hoffman
TFBS ENCODE
**SuperEnhancer Hnisz
**Repressed Hoffman
Promoter UCSC
PromoterFlanking Hoffman
Intron UCSC
H3K9ac Trynka
H3K4me3 Trynka
H3K4me1 Trynka
H3K27ac PGC2
**H3K27ac Hnisz
FetalDHS Trynka
Enhancer Hoffman
Enhancer Andersson
DHS Trynka
DGF ENCODE
CTCF Hoffman
Conserved LindbladToh
Coding UCSC
Annotation
−10
0
10
20
Enrichment (prop h2 / prop snps)

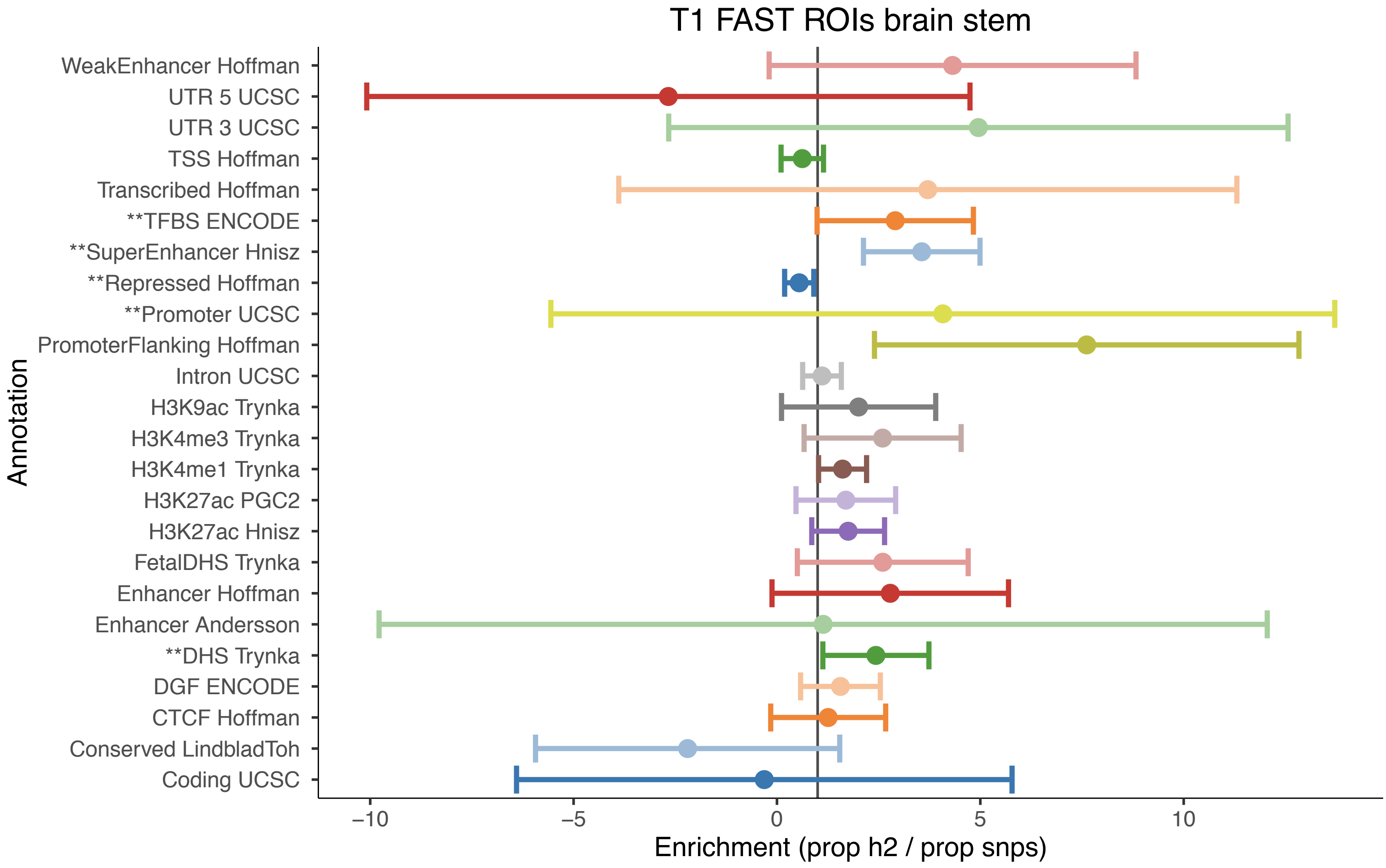

T1 FAST ROIs brain stem
WeakEnhancer Hoffman
UTR 5 UCSC
UTR 3 UCSC
TSS Hoffman
Transcribed Hoffman
**TFBS ENCODE
**SuperEnhancer Hnisz
**Repressed Hoffman
**Promoter UCSC
PromoterFlanking Hoffman
Intron UCSC
H3K9ac Trynka
H3K4me3 Trynka
H3K4me1 Trynka
H3K27ac PGC2
H3K27ac Hnisz
FetalDHS Trynka
Enhancer Hoffman
Enhancer Andersson
**DHS Trynka
DGF ENCODE
CTCF Hoffman
Conserved LindbladToh
Coding UCSC
Annotation
−10
−5
0
5
10
Enrichment (prop h2 / prop snps)

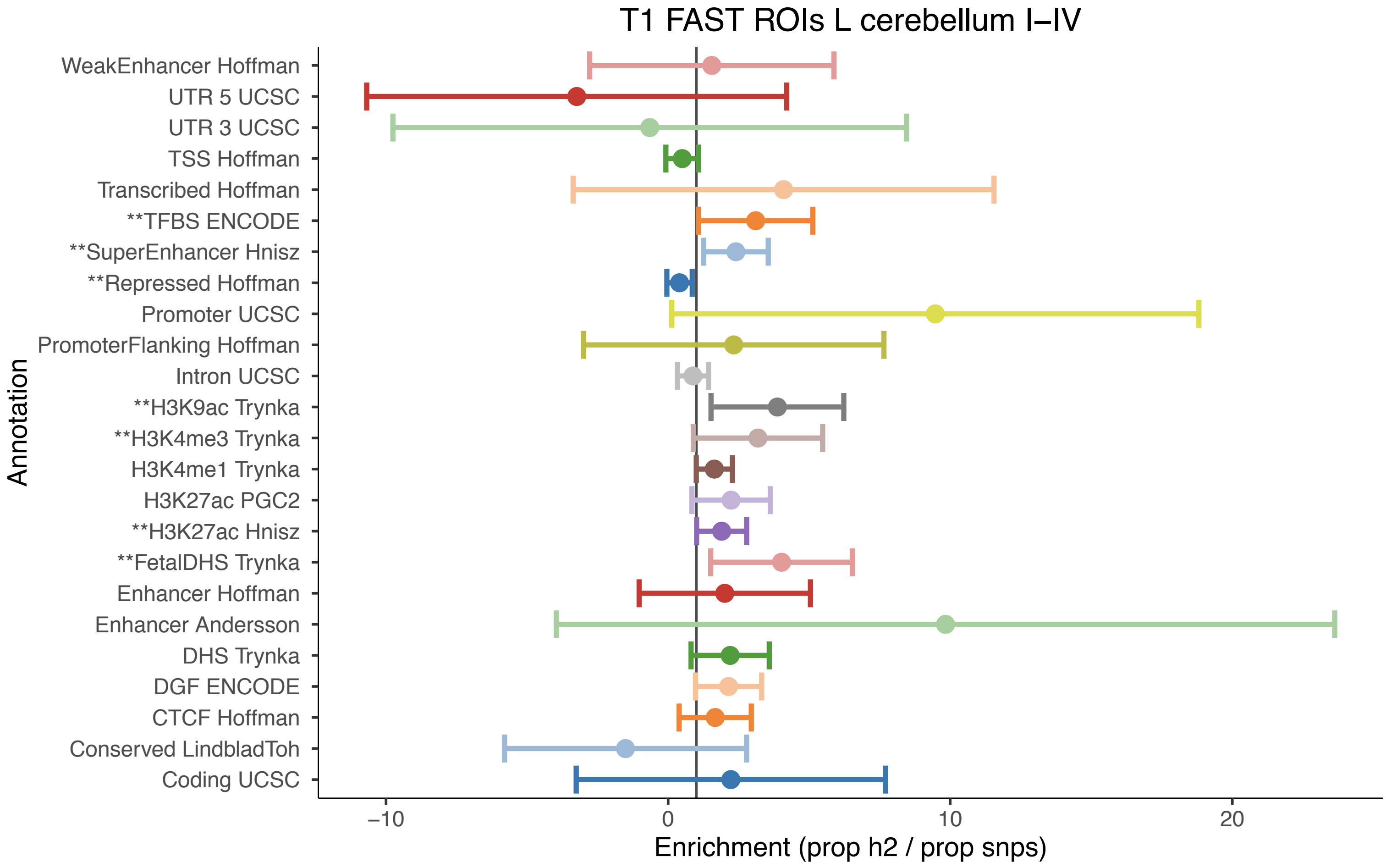

T1 FAST ROIs L cerebellum I–IV
WeakEnhancer Hoffman
UTR 5 UCSC
UTR 3 UCSC
TSS Hoffman
Transcribed Hoffman
**TFBS ENCODE
**SuperEnhancer Hnisz
**Repressed Hoffman
Promoter UCSC
PromoterFlanking Hoffman
Intron UCSC
**H3K9ac Trynka
**H3K4me3 Trynka
H3K4me1 Trynka
H3K27ac PGC2
**H3K27ac Hnisz
**FetalDHS Trynka
Enhancer Hoffman
Enhancer Andersson
DHS Trynka
DGF ENCODE
CTCF Hoffman
Conserved LindbladToh
Coding UCSC
Annotation
−10
0
10
20
Enrichment (prop h2 / prop snps)

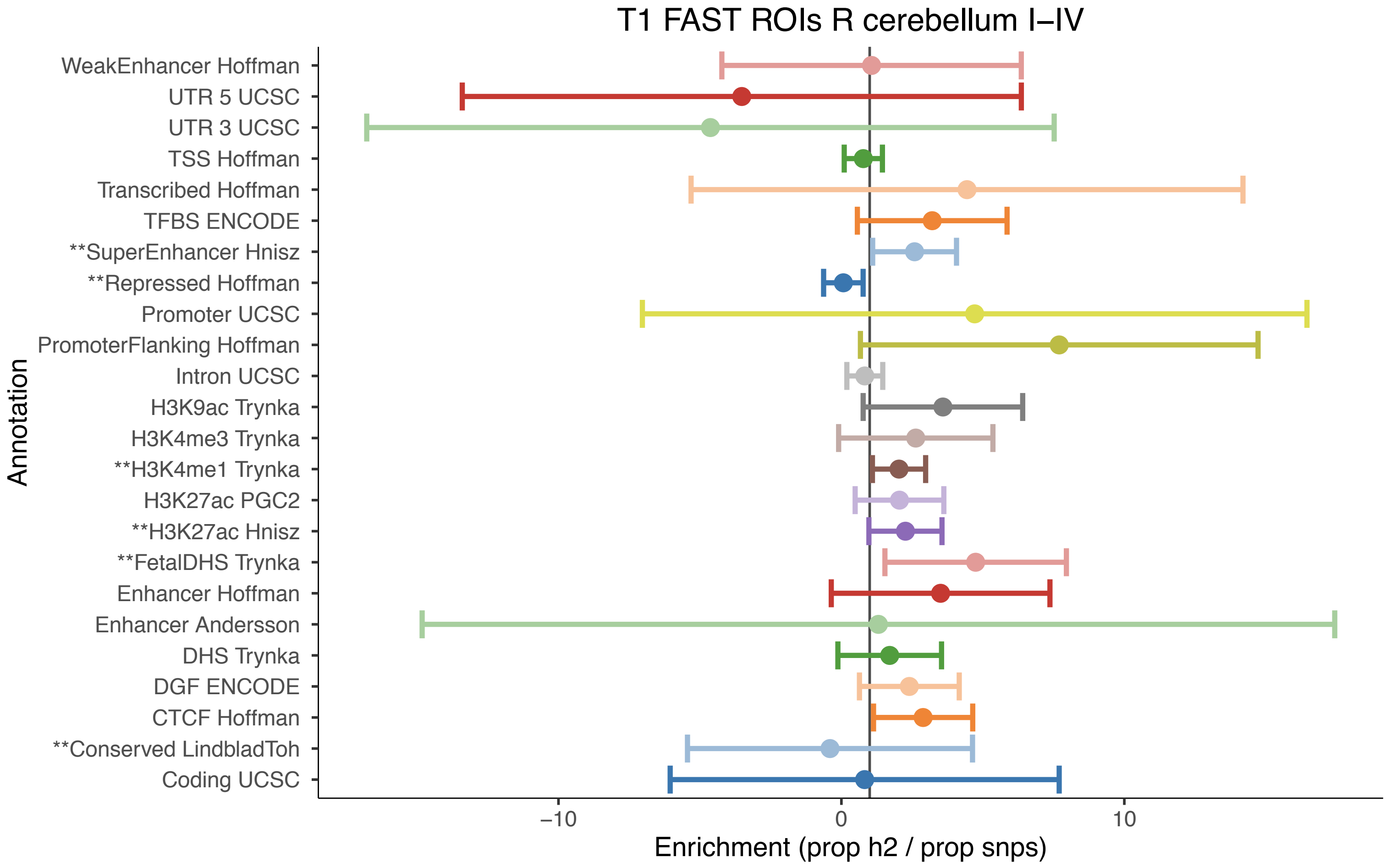
T1 FAST ROIs R cerebellum I–IV
WeakEnhancer Hoffman
UTR 5 UCSC
UTR 3 UCSC
TSS Hoffman
Transcribed Hoffman
TFBS ENCODE
**SuperEnhancer Hnisz
**Repressed Hoffman
Promoter UCSC
PromoterFlanking Hoffman
Intron UCSC
H3K9ac Trynka
H3K4me3 Trynka
**H3K4me1 Trynka
H3K27ac PGC2
**H3K27ac Hnisz
**FetalDHS Trynka
Enhancer Hoffman
Enhancer Andersson
DHS Trynka
DGF ENCODE
CTCF Hoffman
**Conserved LindbladToh
Coding UCSC
Annotation
–10
0
10
Enrichment (prop h2 / prop snps)

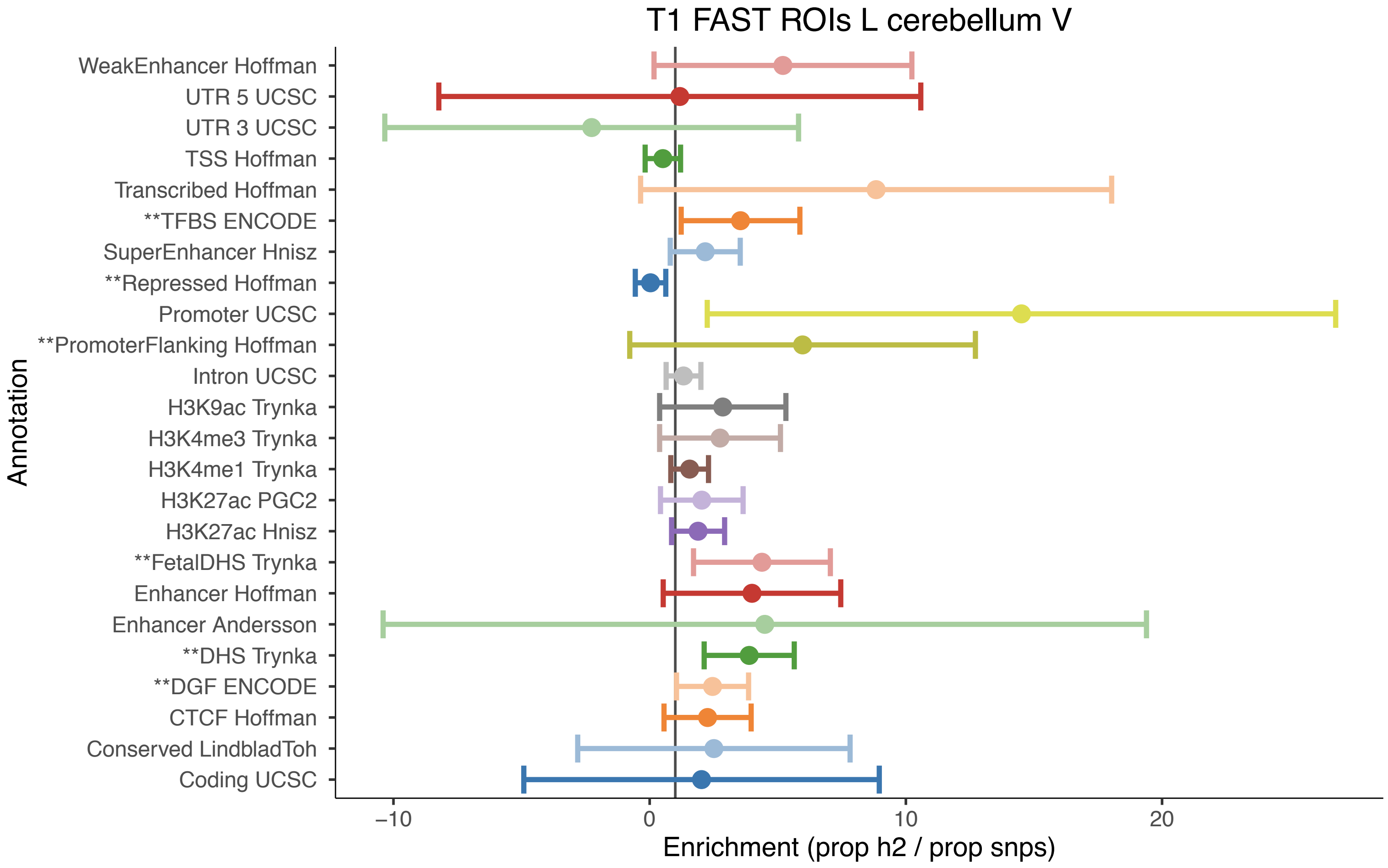

T1 FAST ROIs L cerebellum V
WeakEnhancer Hoffman
UTR 5 UCSC
UTR 3 UCSC
TSS Hoffman
Transcribed Hoffman
**TFBS ENCODE
SuperEnhancer Hnisz
**Repressed Hoffman
Promoter UCSC
**PromoterFlanking Hoffman
Intron UCSC
H3K9ac Trynka
H3K4me3 Trynka
H3K4me1 Trynka
H3K27ac PGC2
H3K27ac Hnisz
**FetalDHS Trynka
Enhancer Hoffman
Enhancer Andersson
**DHS Trynka
**DGF ENCODE
CTCF Hoffman
Conserved LindbladToh
Coding UCSC
Annotation
−10
0
10
20
Enrichment (prop h2 / prop snps)

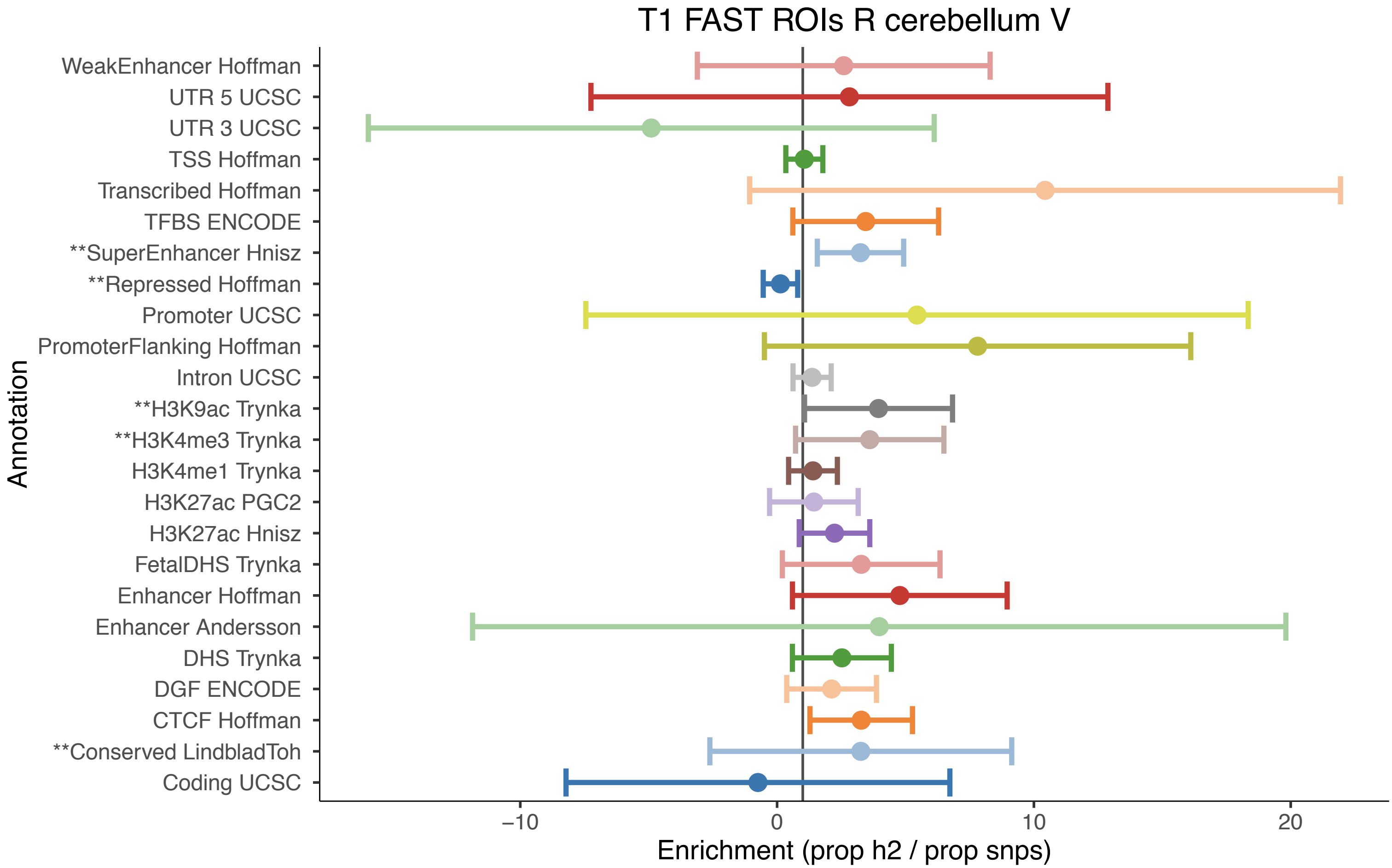
T1 FAST ROIs R cerebellum V
WeakEnhancer Hoffman
UTR 5 UCSC
UTR 3 UCSC
TSS Hoffman
Transcribed Hoffman
TFBS ENCODE
**SuperEnhancer Hnisz
**Repressed Hoffman
Promoter UCSC
PromoterFlanking Hoffman
Intron UCSC
**H3K9ac Trynka
**H3K4me3 Trynka
H3K4me1 Trynka
H3K27ac PGC2
H3K27ac Hnisz
FetalDHS Trynka
Enhancer Hoffman
Enhancer Andersson
DHS Trynka
DGF ENCODE
CTCF Hoffman
**Conserved LindbladToh
Coding UCSC
Annotation
−10
0
10
20
Enrichment (prop h2 / prop snps)

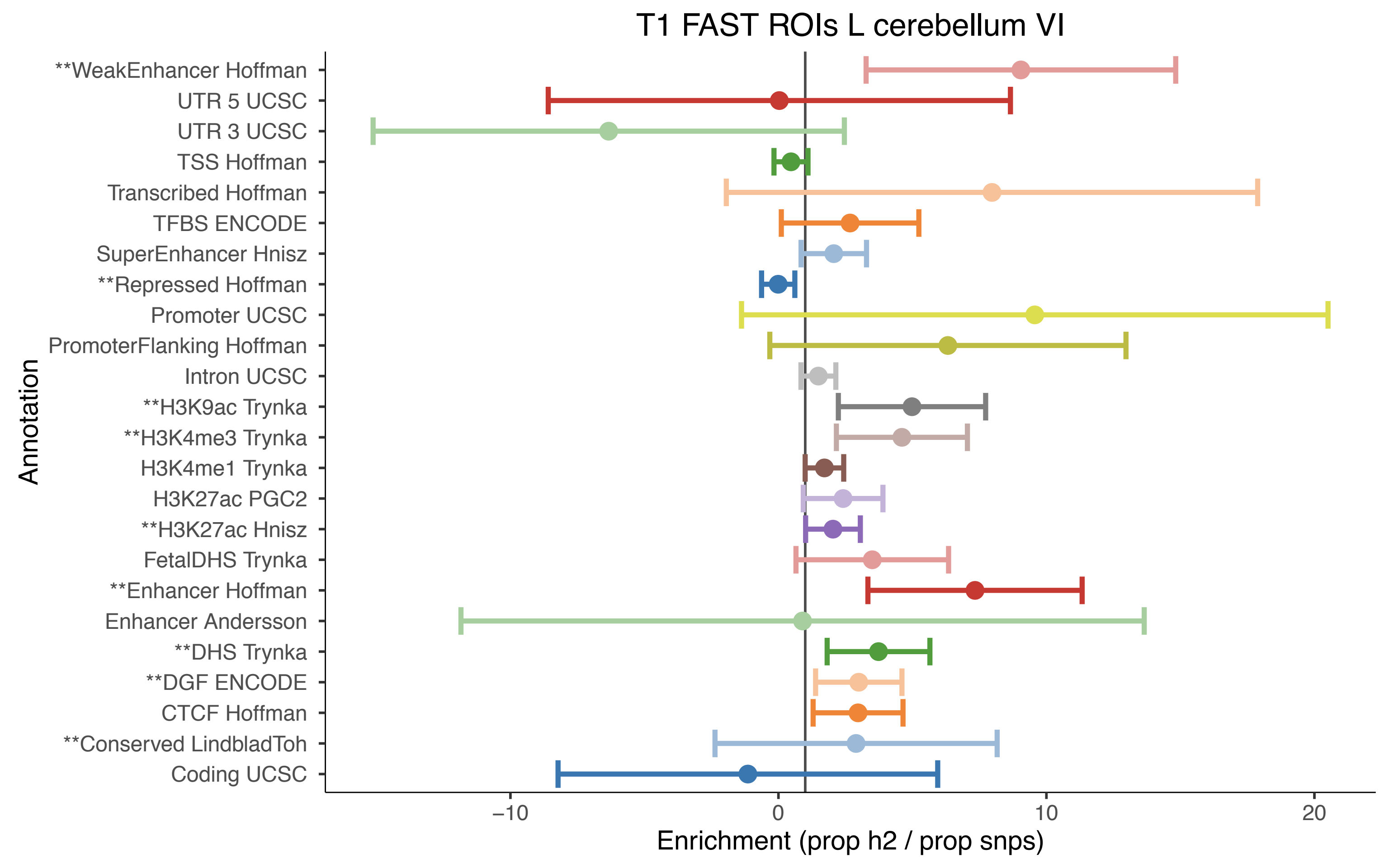
T1 FAST ROIs L cerebellum VI
**WeakEnhancer Hoffman
UTR 5 UCSC
UTR 3 UCSC
TSS Hoffman
Transcribed Hoffman
TFBS ENCODE
SuperEnhancer Hnisz
**Repressed Hoffman
Promoter UCSC
PromoterFlanking Hoffman
Intron UCSC
**H3K9ac Trynka
**H3K4me3 Trynka
H3K4me1 Trynka
H3K27ac PGC2
**H3K27ac Hnisz
FetalDHS Trynka
**Enhancer Hoffman
Enhancer Andersson
**DHS Trynka
**DGF ENCODE
CTCF Hoffman
**Conserved LindbladToh
Coding UCSC
Annotation
−10
0
10
20
Enrichment (prop h2 / prop snps)

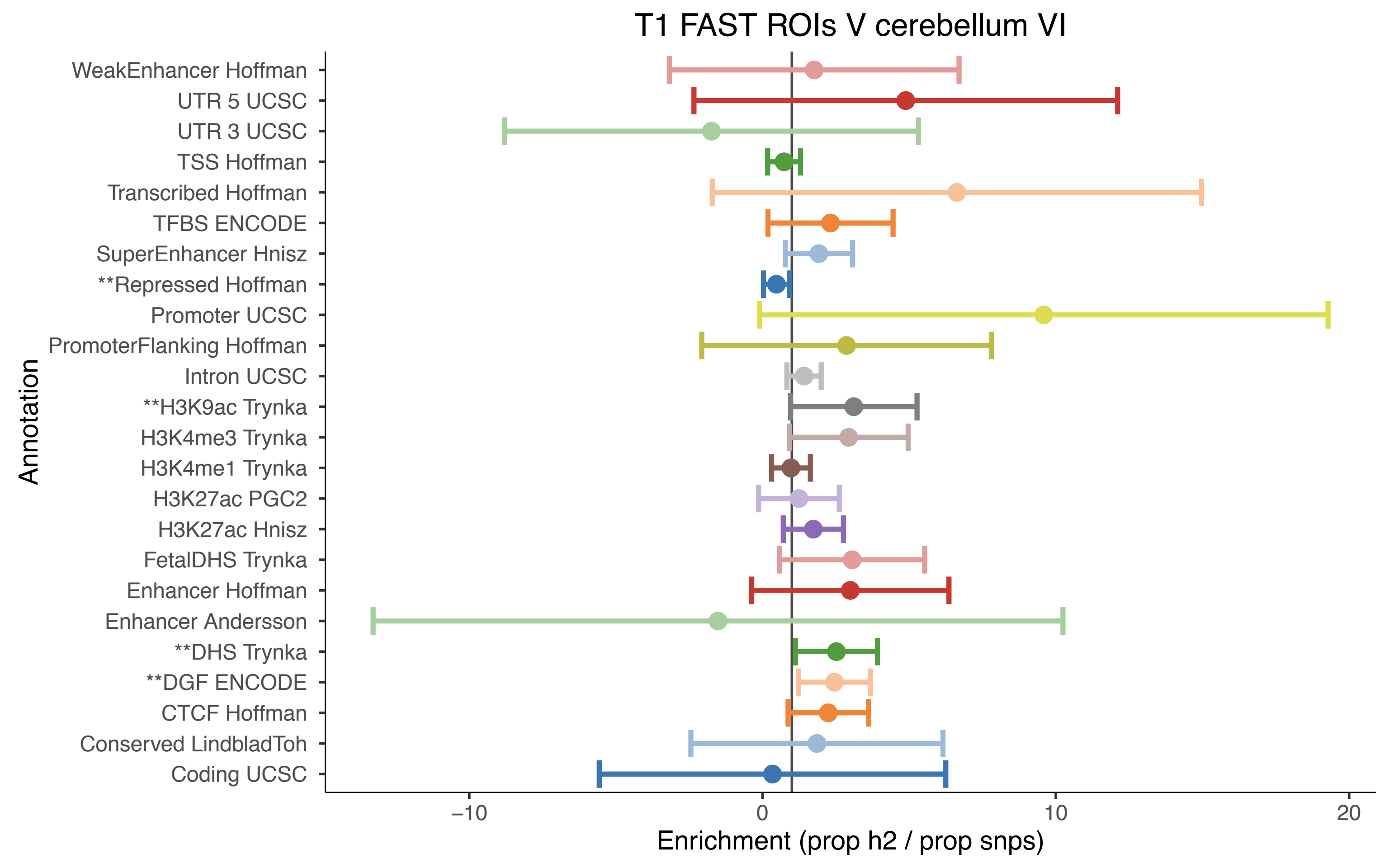
T1 FAST ROIs V cerebellum VI
WeakEnhancer Hoffman
UTR 5 UCSC
UTR 3 UCSC
TSS Hoffman
Transcribed Hoffman
TFBS ENCODE
SuperEnhancer Hnisz
**Repressed Hoffman
Promoter UCSC
PromoterFlanking Hoffman
Intron UCSC
**H3K9ac Trynka
H3K4me3 Trynka
H3K4me1 Trynka
H3K27ac PGC2
H3K27ac Hnisz
FetalDHS Trynka
Enhancer Hoffman
Enhancer Andersson
**DHS Trynka
**DGF ENCODE
CTCF Hoffman
Conserved LindbladToh
Coding UCSC
Annotation
−10
0
10
20
Enrichment (prop h2 / prop snps)

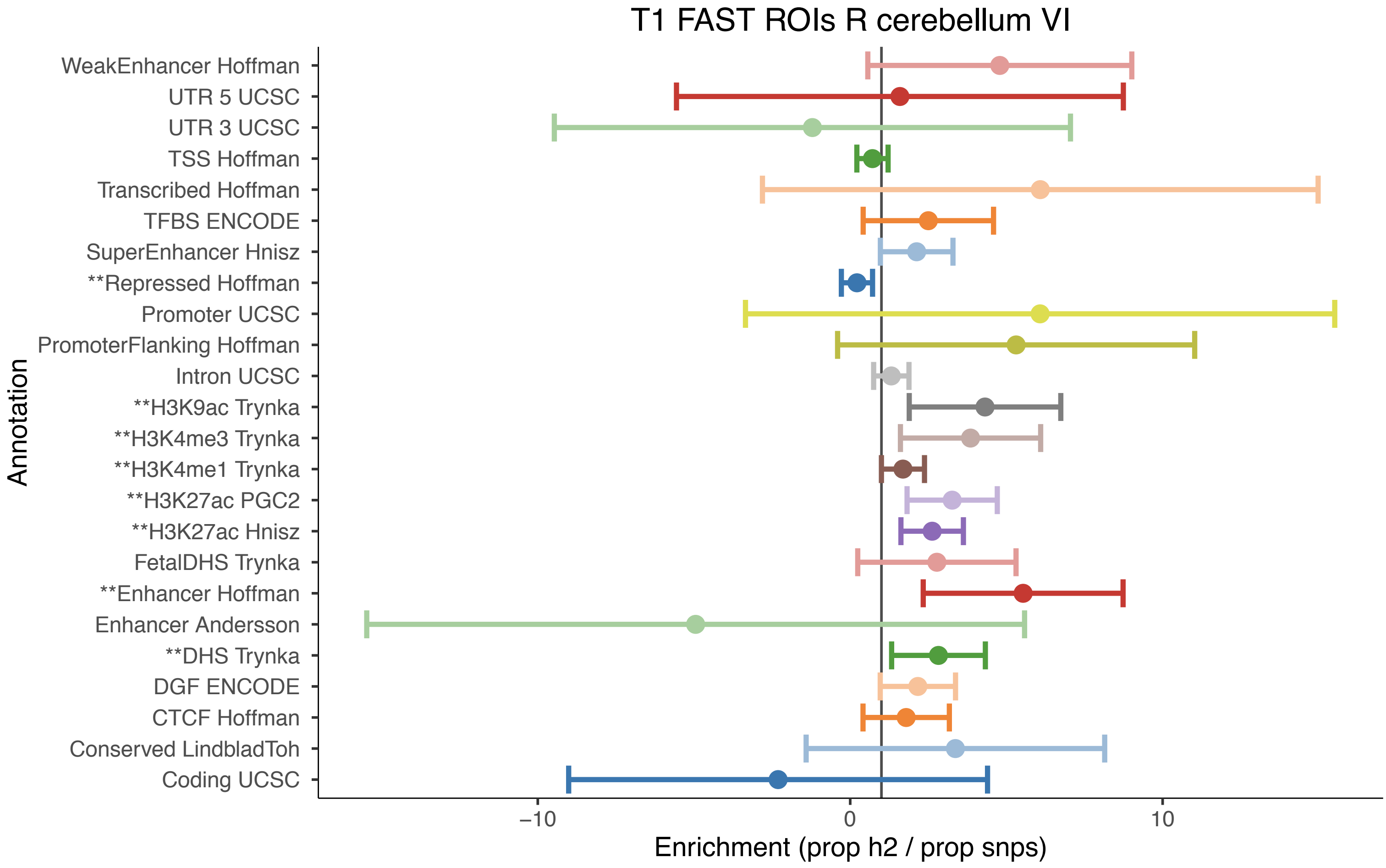
T1 FAST ROIs R cerebellum VI
WeakEnhancer Hoffman
UTR 5 UCSC
UTR 3 UCSC
TSS Hoffman
Transcribed Hoffman
TFBS ENCODE
SuperEnhancer Hnisz
**Repressed Hoffman
Promoter UCSC
PromoterFlanking Hoffman
Intron UCSC
**H3K9ac Trynka
**H3K4me3 Trynka
**H3K4me1 Trynka
**H3K27ac PGC2
**H3K27ac Hnisz
FetalDHS Trynka
**Enhancer Hoffman
Enhancer Andersson
**DHS Trynka
DGF ENCODE
CTCF Hoffman
Conserved LindbladToh
Coding UCSC
Annotation
−10
0
10
Enrichment (prop h2 / prop snps)

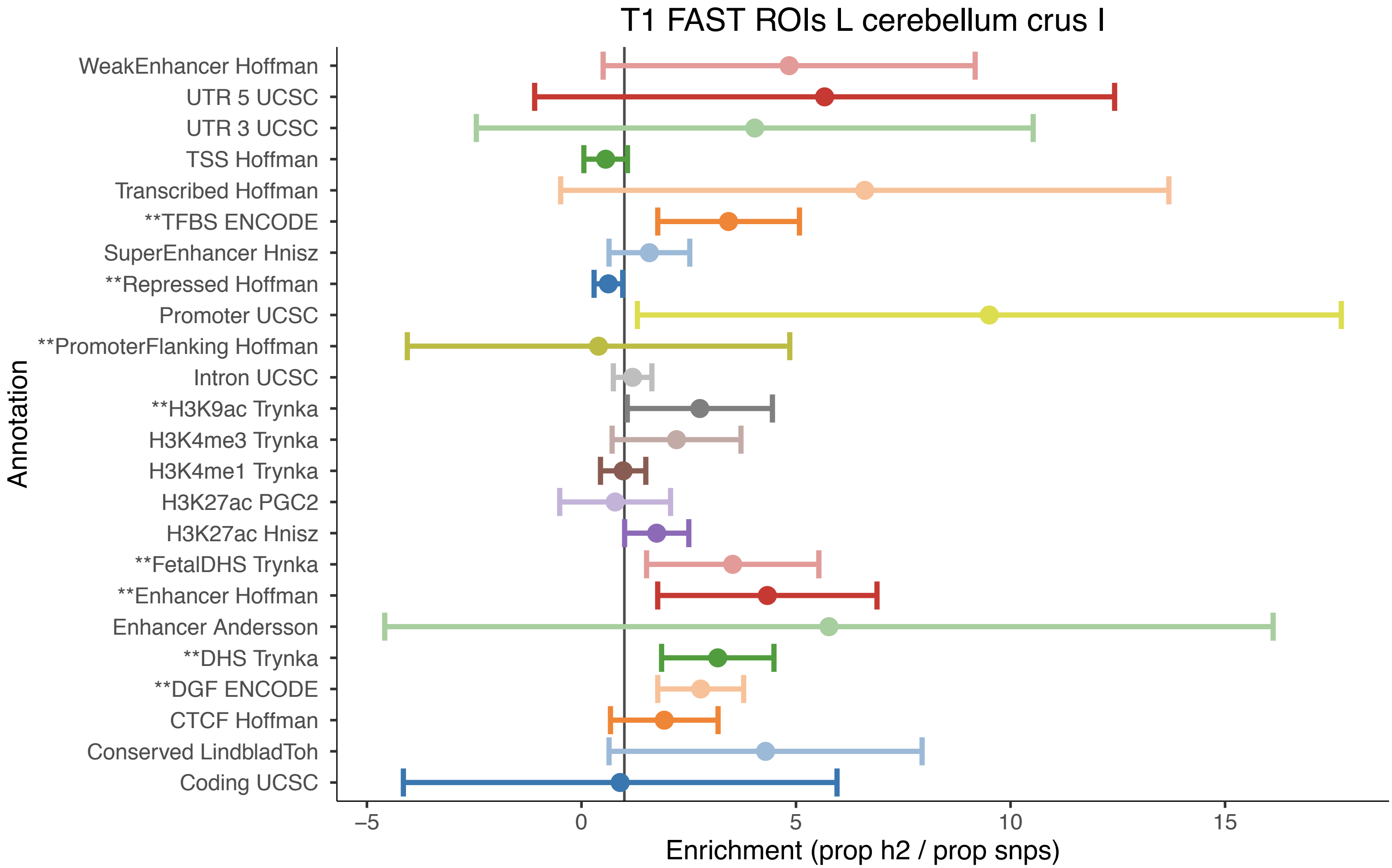

T1 FAST ROIs L cerebellum crus I
WeakEnhancer Hoffman
UTR 5 UCSC
UTR 3 UCSC
TSS Hoffman
Transcribed Hoffman
**TFBS ENCODE
SuperEnhancer Hnisz
**Repressed Hoffman
Promoter UCSC
**PromoterFlanking Hoffman
Intron UCSC
**H3K9ac Trynka
H3K4me3 Trynka
H3K4me1 Trynka
H3K27ac PGC2
H3K27ac Hnisz
**FetalDHS Trynka
**Enhancer Hoffman
Enhancer Andersson
**DHS Trynka
**DGF ENCODE
CTCF Hoffman
Conserved LindbladToh
Coding UCSC
Annotation
−5
0
5
10
15
Enrichment (prop h2 / prop snps)

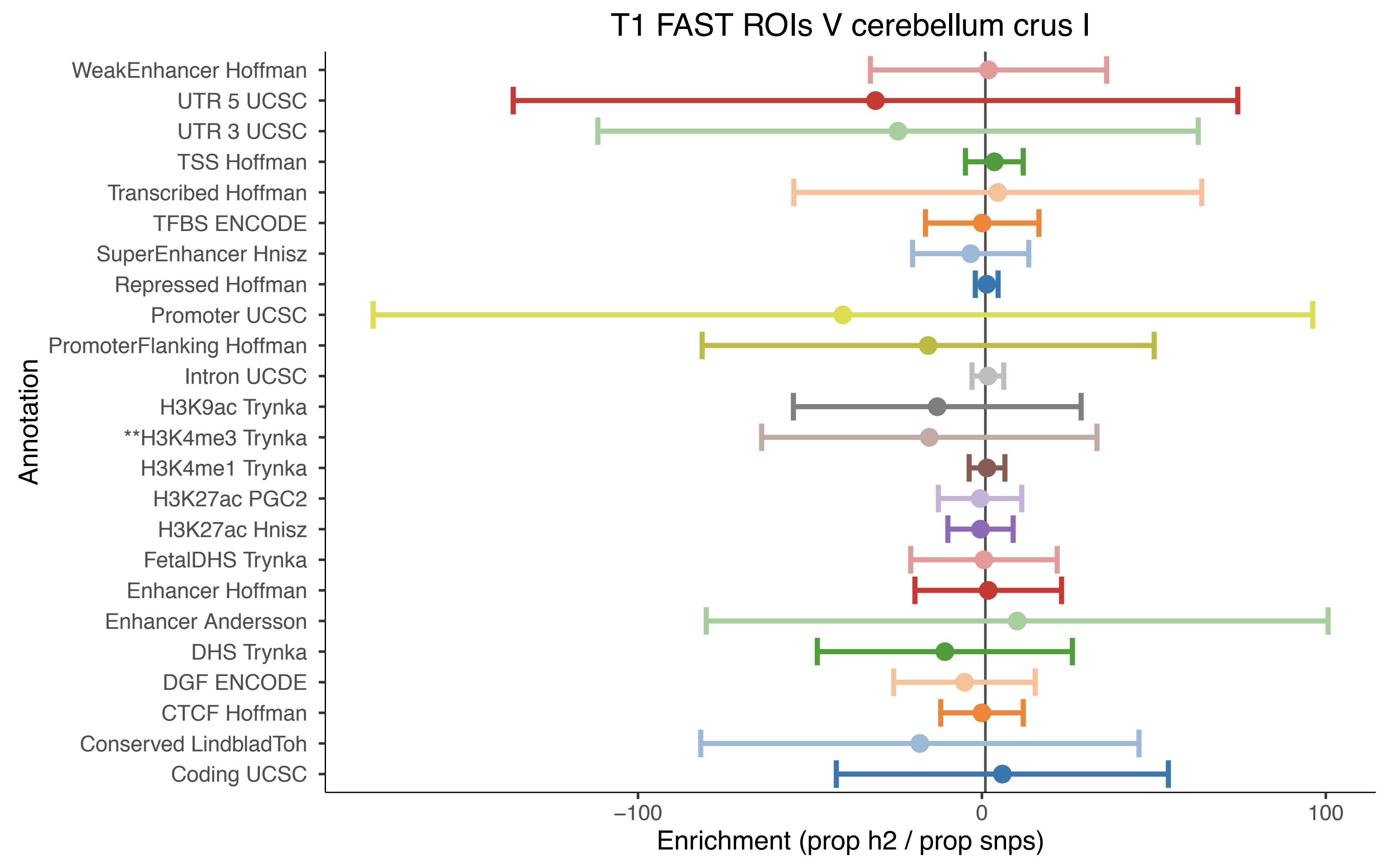
T1 FAST ROIs V cerebellum crus I
WeakEnhancer Hoffman
UTR 5 UCSC
UTR 3 UCSC
TSS Hoffman
Transcribed Hoffman
TFBS ENCODE
SuperEnhancer Hnisz
Repressed Hoffman
Promoter UCSC
PromoterFlanking Hoffman
Intron UCSC
H3K9ac Trynka
**H3K4me3 Trynka
H3K4me1 Trynka
H3K27ac PGC2
H3K27ac Hnisz
FetalDHS Trynka
Enhancer Hoffman
Enhancer Andersson
DHS Trynka
DGF ENCODE
CTCF Hoffman
Conserved LindbladToh
Coding UCSC
Annotation
−100
0
100
Enrichment (prop h2 / prop snps)

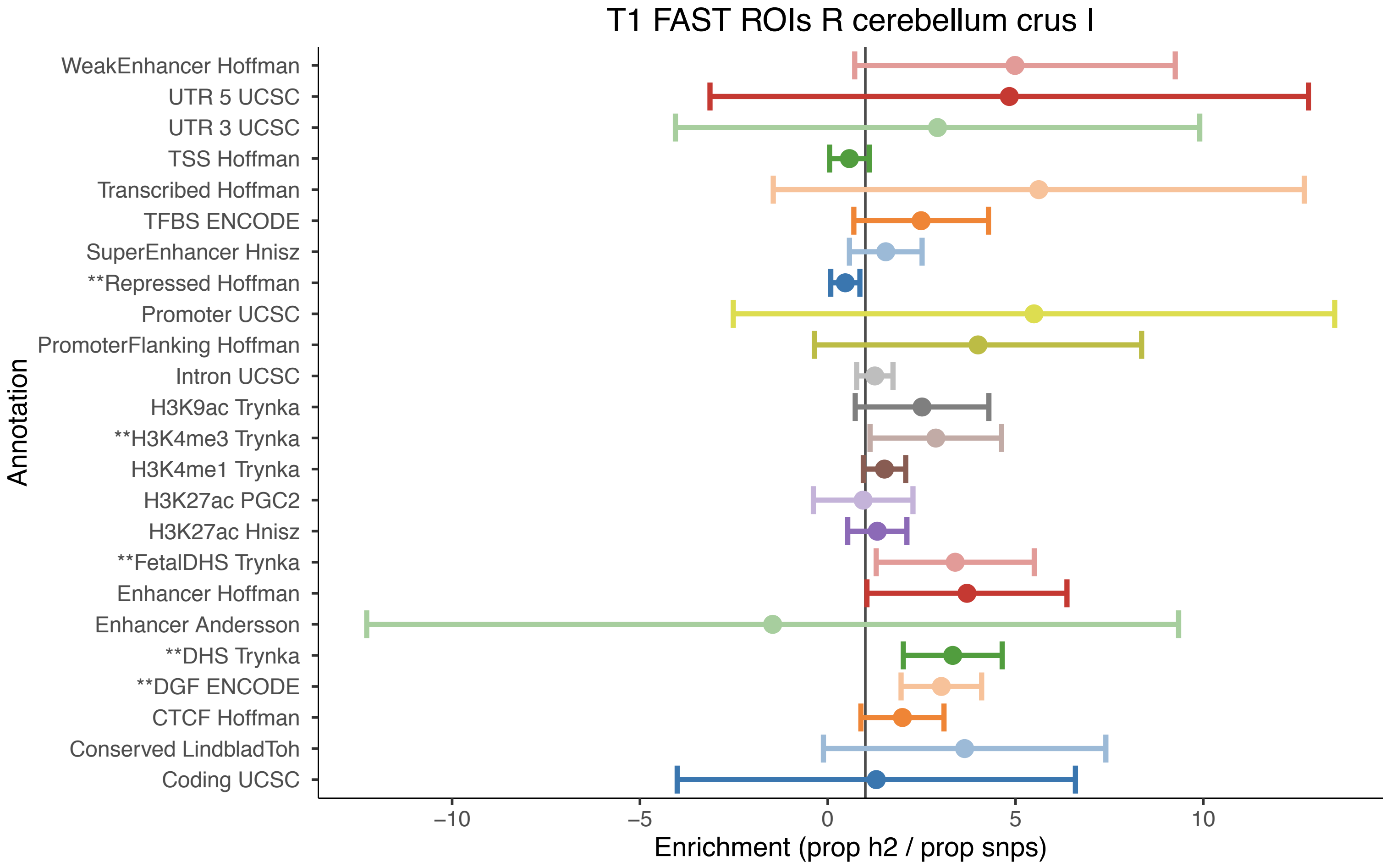

T1 FAST ROIs R cerebellum crus I
WeakEnhancer Hoffman
UTR 5 UCSC
UTR 3 UCSC
TSS Hoffman
Transcribed Hoffman
TFBS ENCODE
SuperEnhancer Hnisz
**Repressed Hoffman
Promoter UCSC
PromoterFlanking Hoffman
Intron UCSC
H3K9ac Trynka
**H3K4me3 Trynka
H3K4me1 Trynka
H3K27ac PGC2
H3K27ac Hnisz
**FetalDHS Trynka
Enhancer Hoffman
Enhancer Andersson
**DHS Trynka
**DGF ENCODE
CTCF Hoffman
Conserved LindbladToh
Coding UCSC
Annotation
−10
−5
0
5
10
Enrichment (prop h2 / prop snps)

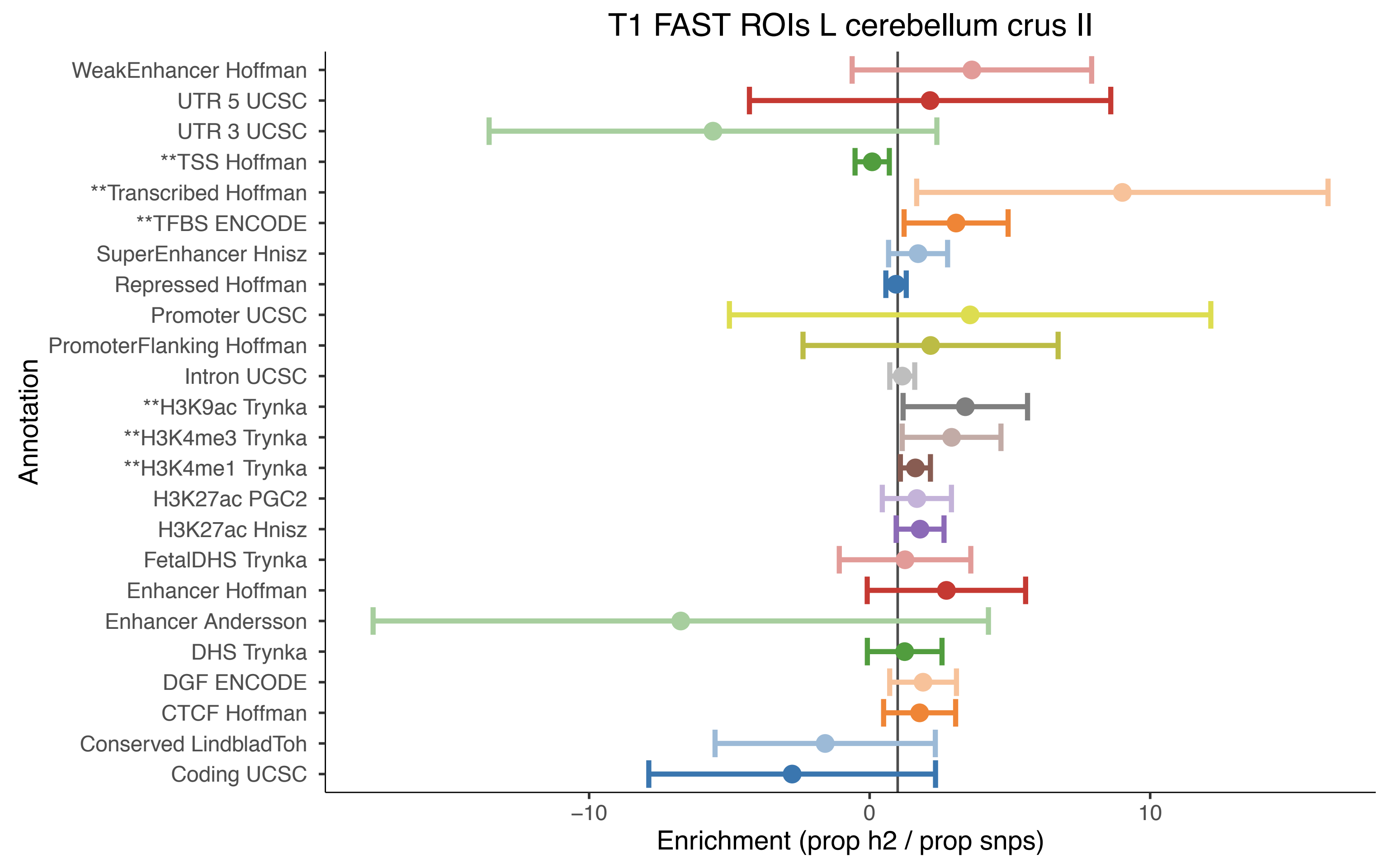

T1 FAST ROIs L cerebellum crus II
WeakEnhancer Hoffman
UTR 5 UCSC
UTR 3 UCSC
**TSS Hoffman
**Transcribed Hoffman
**TFBS ENCODE
SuperEnhancer Hnisz
Repressed Hoffman
Promoter UCSC
PromoterFlanking Hoffman
Intron UCSC
**H3K9ac Trynka
**H3K4me3 Trynka
**H3K4me1 Trynka
H3K27ac PGC2
H3K27ac Hnisz
FetalDHS Trynka
Enhancer Hoffman
Enhancer Andersson
DHS Trynka
DGF ENCODE
CTCF Hoffman
Conserved LindbladToh
Coding UCSC
Annotation
−10
0
10
Enrichment (prop h2 / prop snps)

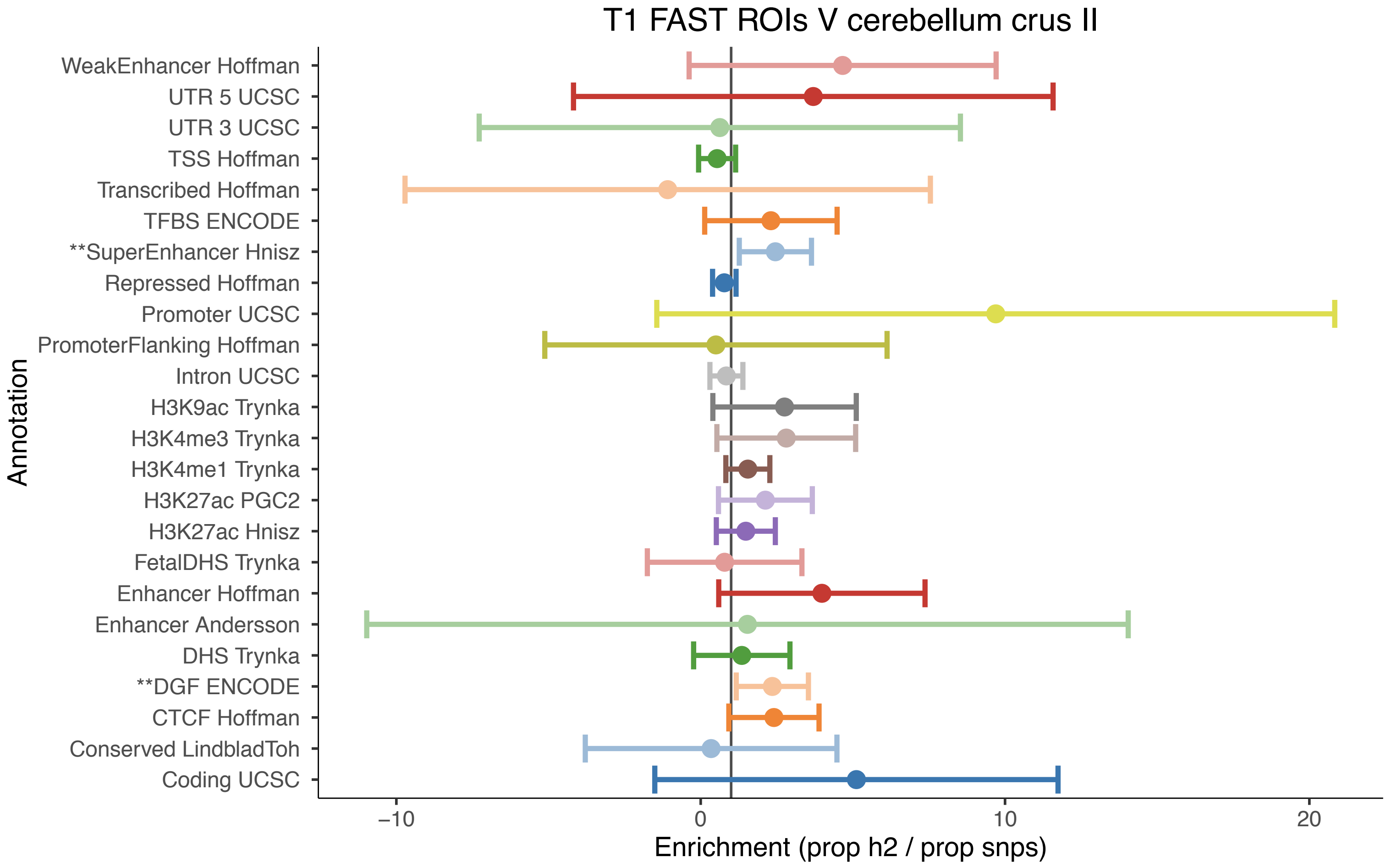

T1 FAST ROIs V cerebellum crus II
WeakEnhancer Hoffman
UTR 5 UCSC
UTR 3 UCSC
TSS Hoffman
Transcribed Hoffman
TFBS ENCODE
**SuperEnhancer Hnisz
Repressed Hoffman
Promoter UCSC
PromoterFlanking Hoffman
Intron UCSC
H3K9ac Trynka
H3K4me3 Trynka
H3K4me1 Trynka
H3K27ac PGC2
H3K27ac Hnisz
FetalDHS Trynka
Enhancer Hoffman
Enhancer Andersson
DHS Trynka
**DGF ENCODE
CTCF Hoffman
Conserved LindbladToh
Coding UCSC
Annotation
−10
0
10
20
Enrichment (prop h2 / prop snps)

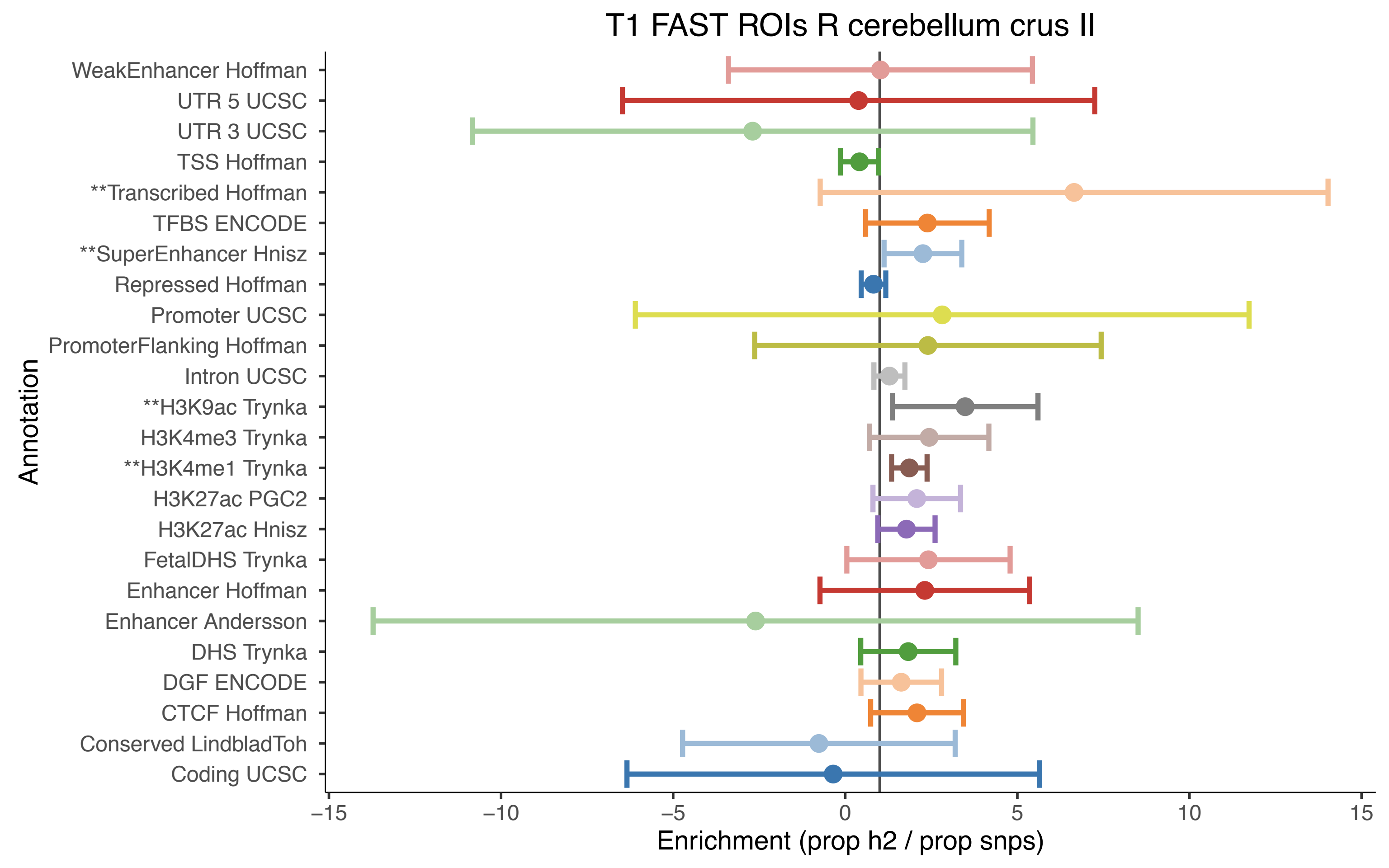

T1 FAST ROIs R cerebellum crus II
WeakEnhancer Hoffman
UTR 5 UCSC
UTR 3 UCSC
TSS Hoffman
**Transcribed Hoffman
TFBS ENCODE
**SuperEnhancer Hnisz
Repressed Hoffman
Promoter UCSC
PromoterFlanking Hoffman
Intron UCSC
**H3K9ac Trynka
H3K4me3 Trynka
**H3K4me1 Trynka
H3K27ac PGC2
H3K27ac Hnisz
FetalDHS Trynka
Enhancer Hoffman
Enhancer Andersson
DHS Trynka
DGF ENCODE
CTCF Hoffman
Conserved LindbladToh
Coding UCSC
Annotation
−15
−10
−5
0
5
10
15
Enrichment (prop h2 / prop snps)

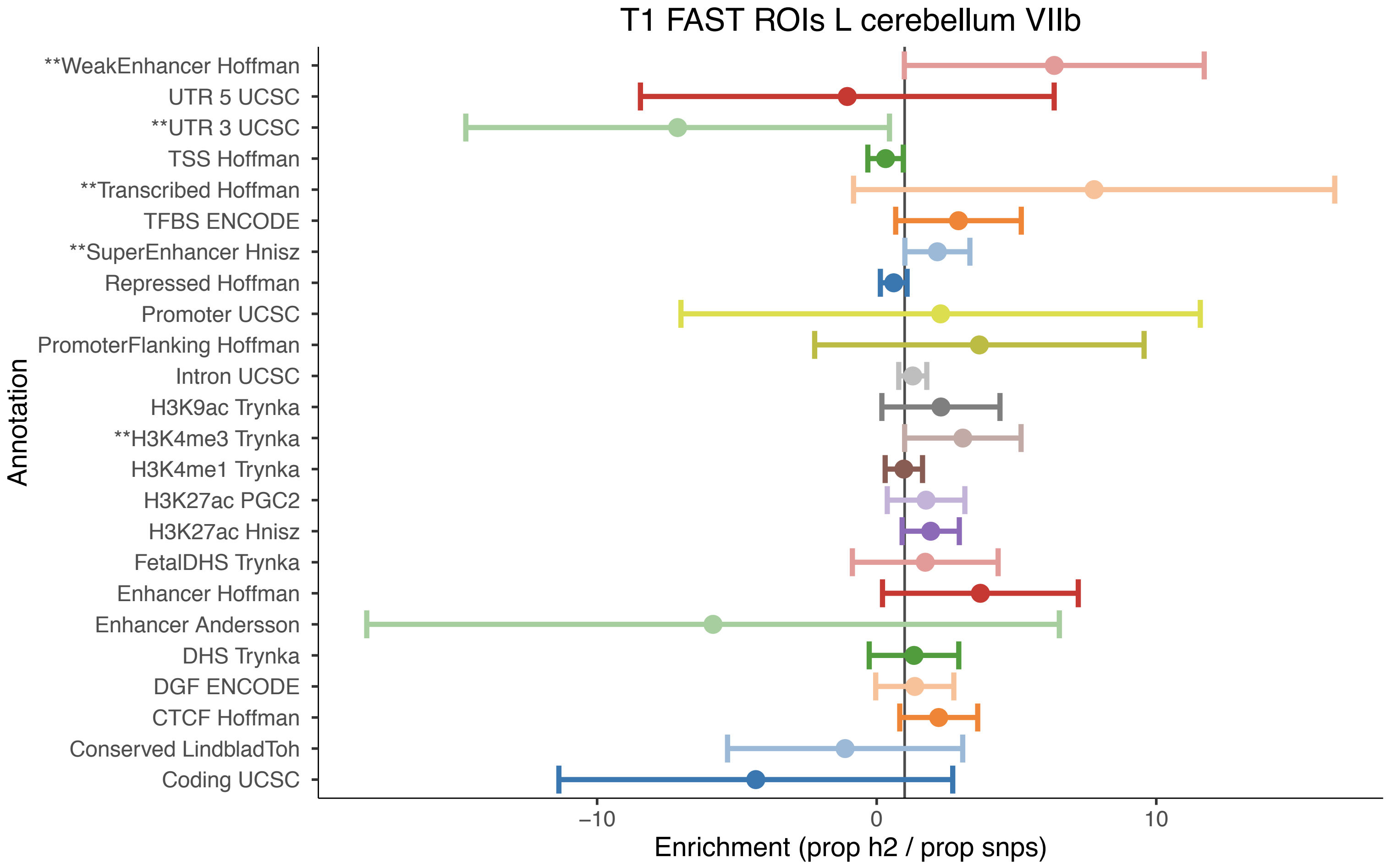
T1 FAST ROIs L cerebellum VIIb
**WeakEnhancer Hoffman
UTR 5 UCSC
**UTR 3 UCSC
TSS Hoffman
**Transcribed Hoffman
TFBS ENCODE
**SuperEnhancer Hnisz
Repressed Hoffman
Promoter UCSC
PromoterFlanking Hoffman
Intron UCSC
H3K9ac Trynka
**H3K4me3 Trynka
H3K4me1 Trynka
H3K27ac PGC2
H3K27ac Hnisz
FetalDHS Trynka
Enhancer Hoffman
Enhancer Andersson
DHS Trynka
DGF ENCODE
CTCF Hoffman
Conserved LindbladToh
Coding UCSC
Annotation
−10
0
10
Enrichment (prop h2 / prop snps)

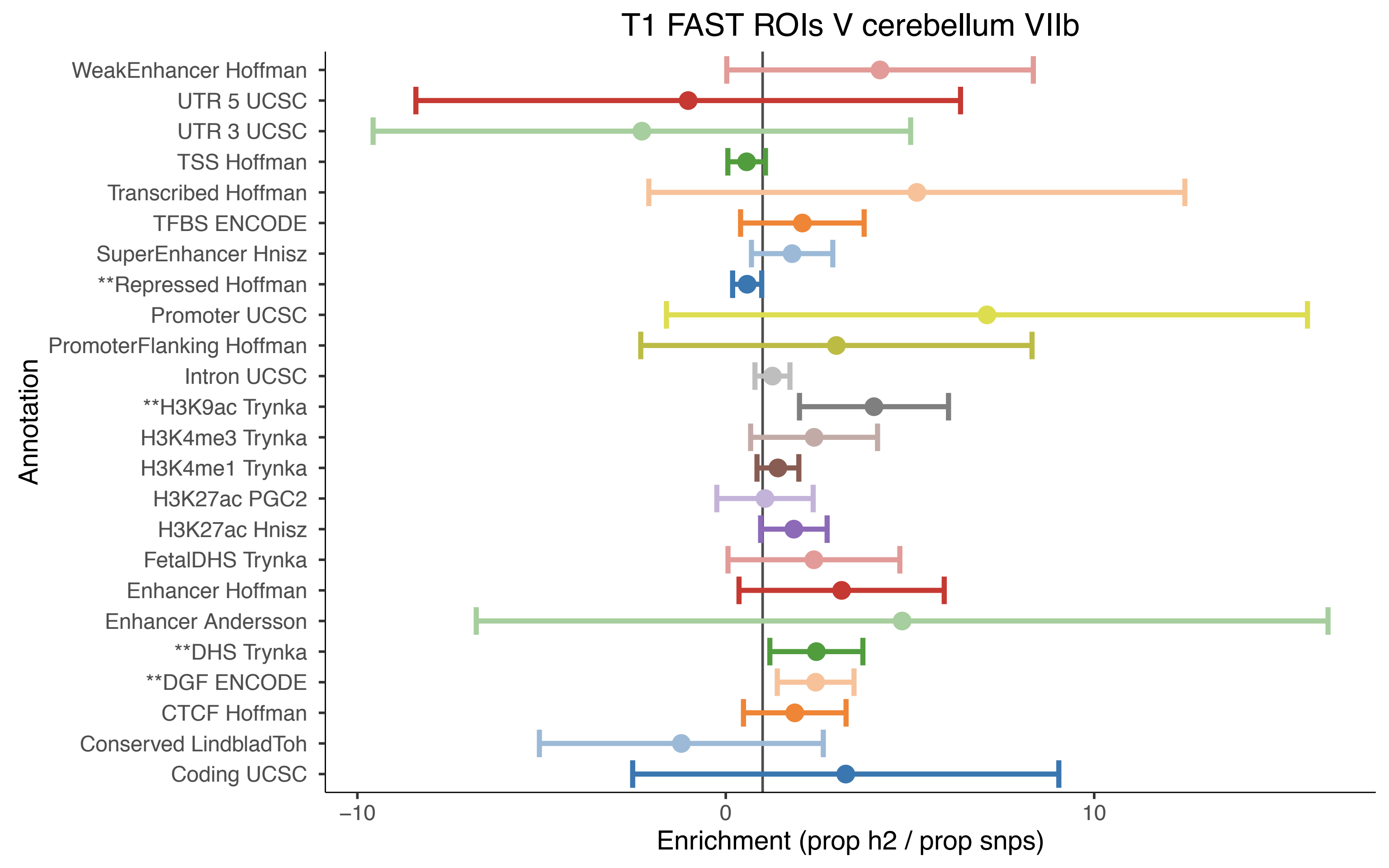
T1 FAST ROIs V cerebellum VIIb
WeakEnhancer Hoffman
UTR 5 UCSC
UTR 3 UCSC
TSS Hoffman
Transcribed Hoffman
TFBS ENCODE
SuperEnhancer Hnisz
**Repressed Hoffman
Promoter UCSC
PromoterFlanking Hoffman
Intron UCSC
**H3K9ac Trynka
H3K4me3 Trynka
H3K4me1 Trynka
H3K27ac PGC2
H3K27ac Hnisz
FetalDHS Trynka
Enhancer Hoffman
Enhancer Andersson
**DHS Trynka
**DGF ENCODE
CTCF Hoffman
Conserved LindbladToh
Coding UCSC
Annotation
−10
0
10
Enrichment (prop h2 / prop snps)

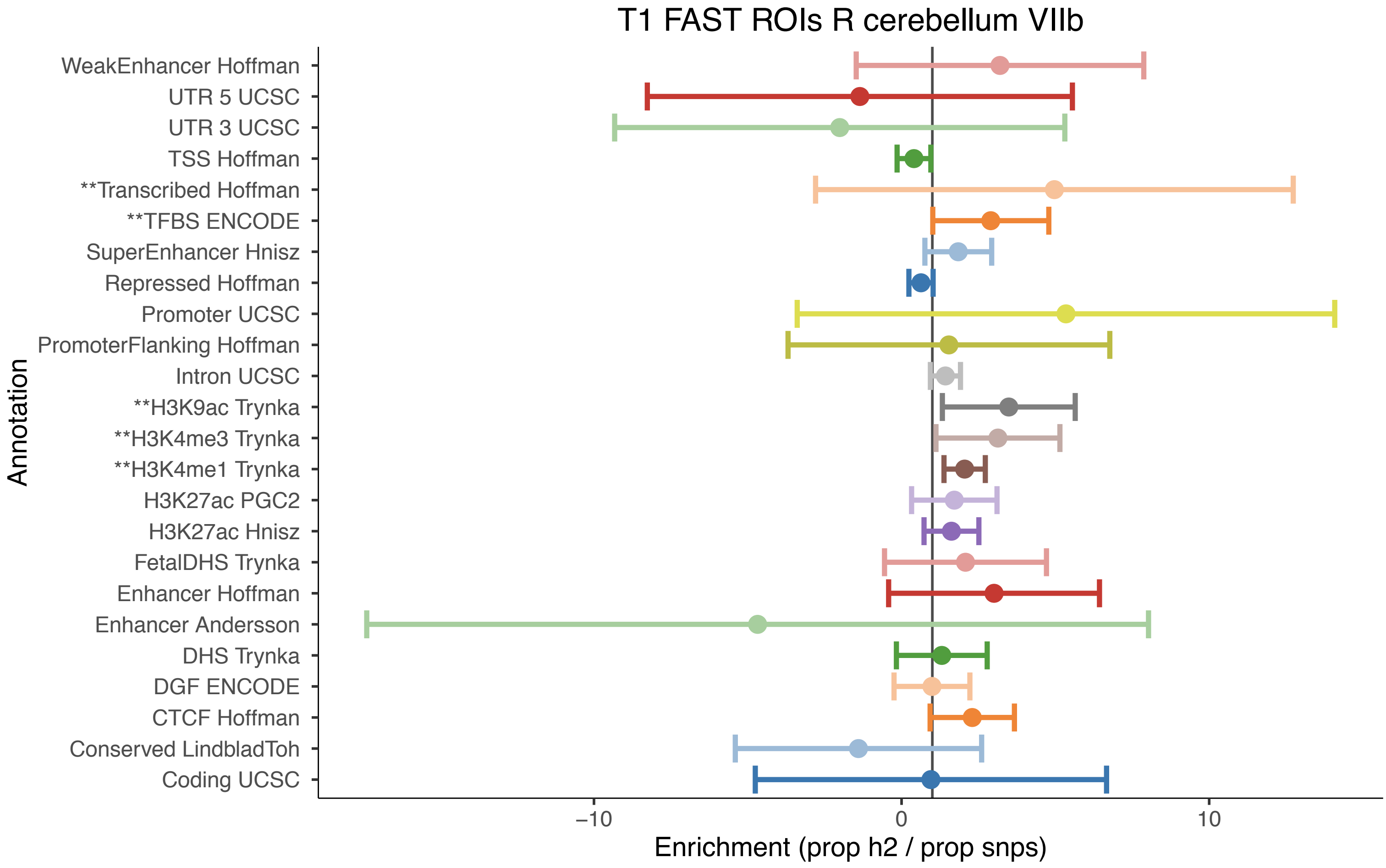
T1 FAST ROIs R cerebellum VIIb
WeakEnhancer Hoffman
UTR 5 UCSC
UTR 3 UCSC
TSS Hoffman
**Transcribed Hoffman
**TFBS ENCODE
SuperEnhancer Hnisz
Repressed Hoffman
Promoter UCSC
PromoterFlanking Hoffman
Intron UCSC
**H3K9ac Trynka
**H3K4me3 Trynka
**H3K4me1 Trynka
H3K27ac PGC2
H3K27ac Hnisz
FetalDHS Trynka
Enhancer Hoffman
Enhancer Andersson
DHS Trynka
DGF ENCODE
CTCF Hoffman
Conserved LindbladToh
Coding UCSC
Annotation
−10
0
10
Enrichment (prop h2 / prop snps)

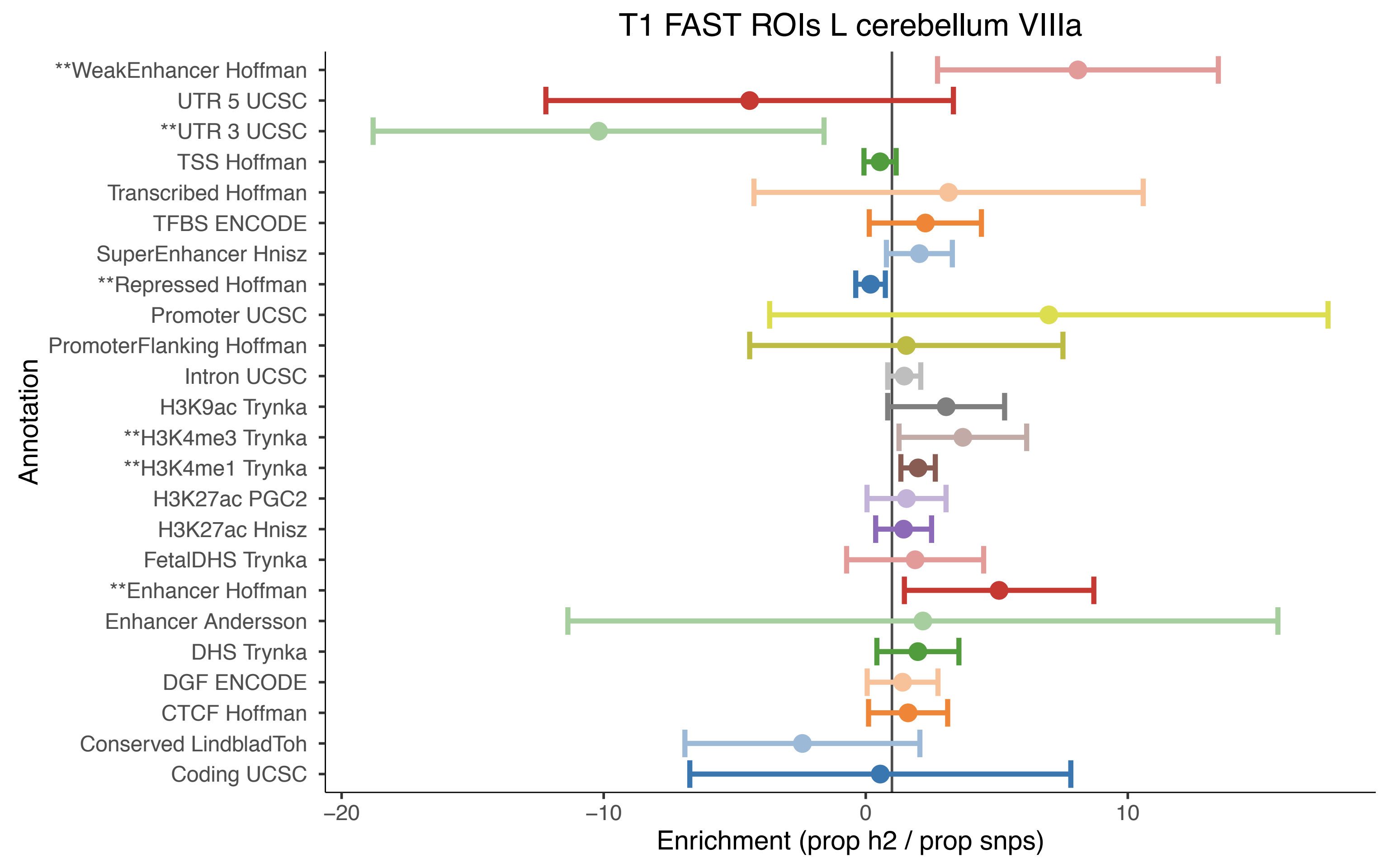
T1 FAST ROIs L cerebellum VIIIa
**WeakEnhancer Hoffman
UTR 5 UCSC
**UTR 3 UCSC
TSS Hoffman
Transcribed Hoffman
TFBS ENCODE
SuperEnhancer Hnisz
**Repressed Hoffman
Promoter UCSC
PromoterFlanking Hoffman
Intron UCSC
H3K9ac Trynka
**H3K4me3 Trynka
**H3K4me1 Trynka
H3K27ac PGC2
H3K27ac Hnisz
FetalDHS Trynka
**Enhancer Hoffman
Enhancer Andersson
DHS Trynka
DGF ENCODE
CTCF Hoffman
Conserved LindbladToh
Coding UCSC
Annotation
−20
−10
0
10
Enrichment (prop h2 / prop snps)

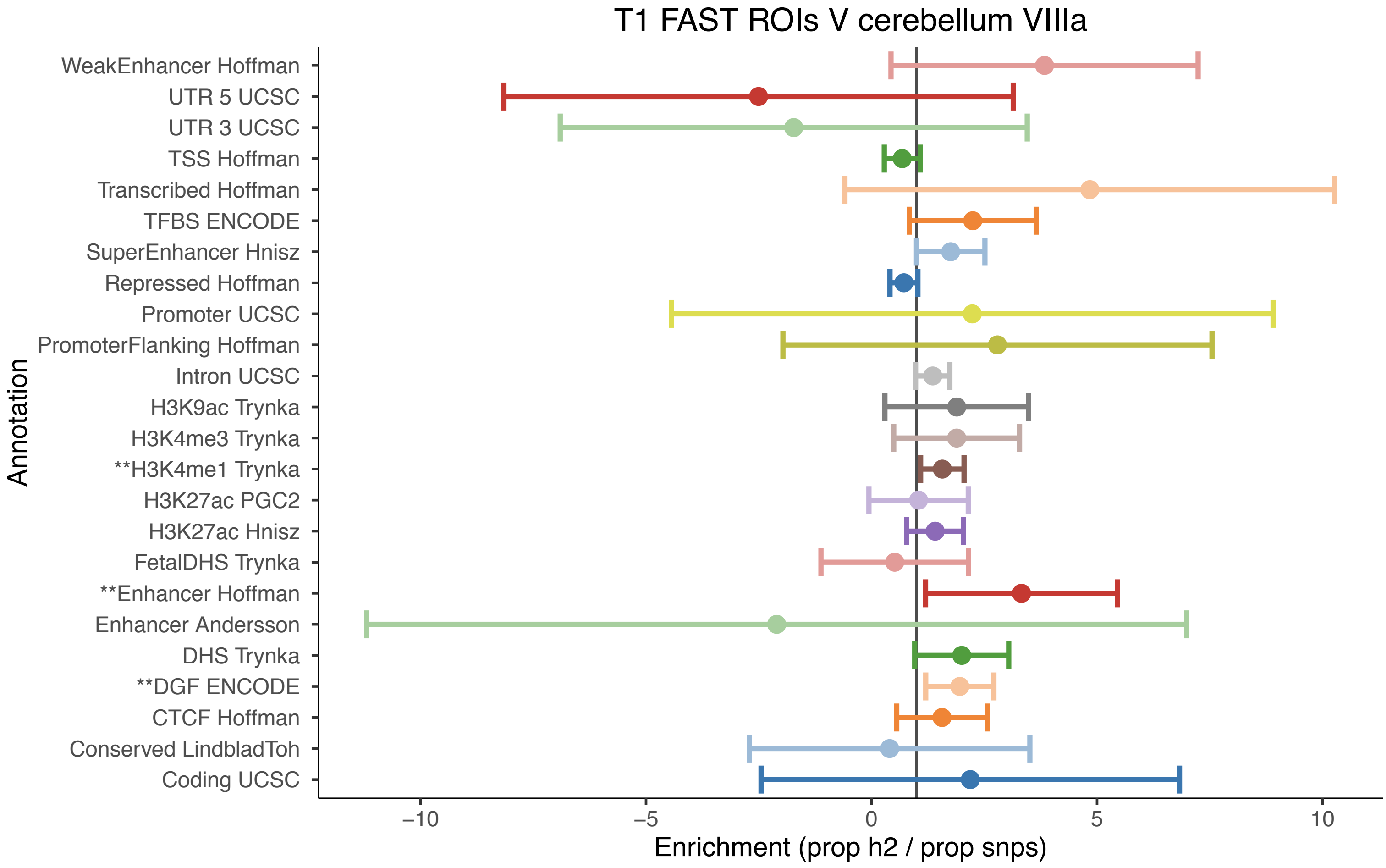

T1 FAST ROIs V cerebellum VIIIa
WeakEnhancer Hoffman
UTR 5 UCSC
UTR 3 UCSC
TSS Hoffman
Transcribed Hoffman
TFBS ENCODE
SuperEnhancer Hnisz
Repressed Hoffman
Promoter UCSC
PromoterFlanking Hoffman
Intron UCSC
H3K9ac Trynka
H3K4me3 Trynka
**H3K4me1 Trynka
H3K27ac PGC2
H3K27ac Hnisz
FetalDHS Trynka
**Enhancer Hoffman
Enhancer Andersson
DHS Trynka
**DGF ENCODE
CTCF Hoffman
Conserved LindbladToh
Coding UCSC
Annotation
−10
−5
0
5
10
Enrichment (prop h2 / prop snps)

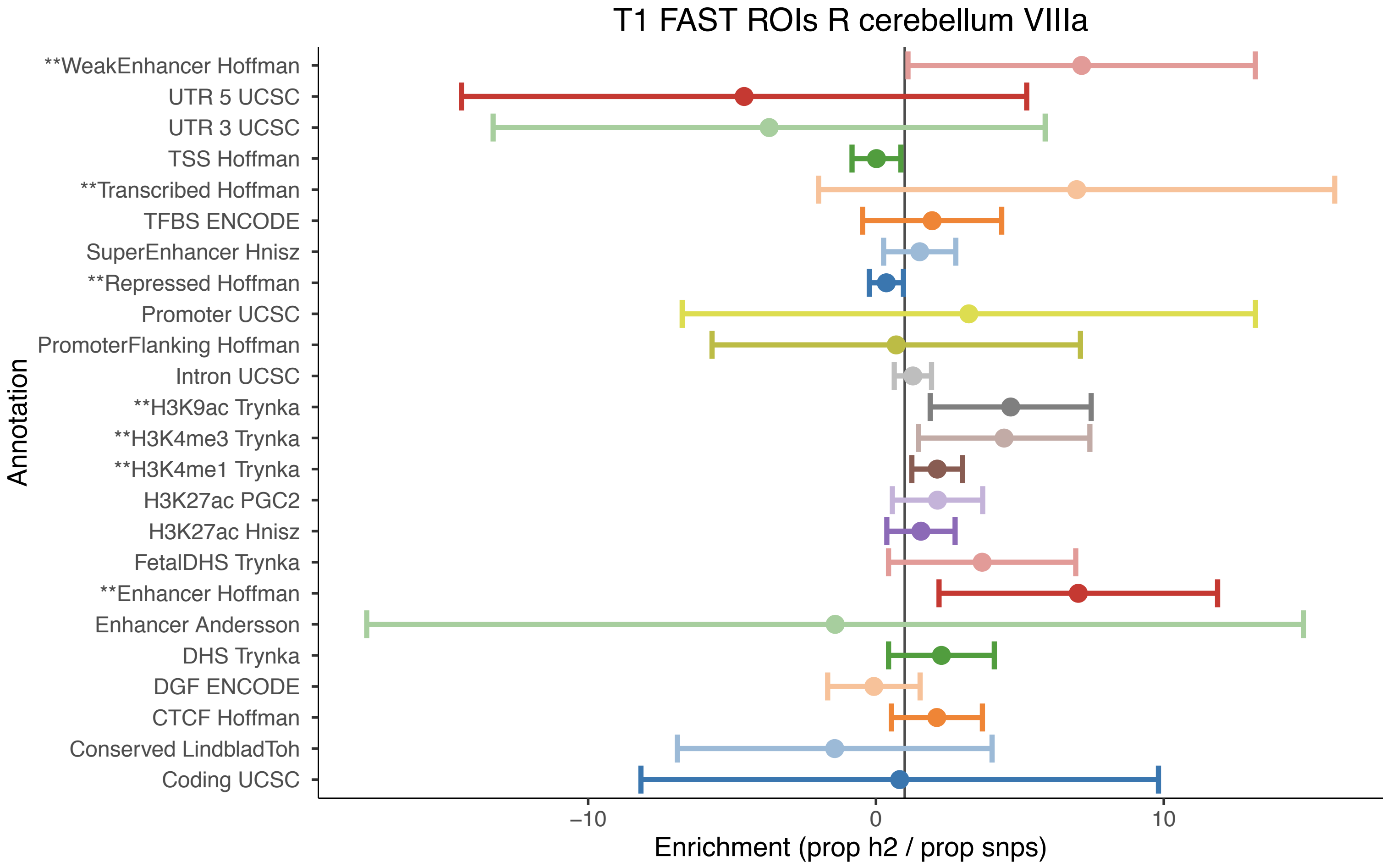
T1 FAST ROIs R cerebellum VIIIa
**WeakEnhancer Hoffman
UTR 5 UCSC
UTR 3 UCSC
TSS Hoffman
**Transcribed Hoffman
TFBS ENCODE
SuperEnhancer Hnisz
**Repressed Hoffman
Promoter UCSC
PromoterFlanking Hoffman
Intron UCSC
**H3K9ac Trynka
**H3K4me3 Trynka
**H3K4me1 Trynka
H3K27ac PGC2
H3K27ac Hnisz
FetalDHS Trynka
**Enhancer Hoffman
Enhancer Andersson
DHS Trynka
DGF ENCODE
CTCF Hoffman
Conserved LindbladToh
Coding UCSC
Annotation
−10
0
10
Enrichment (prop h2 / prop snps)

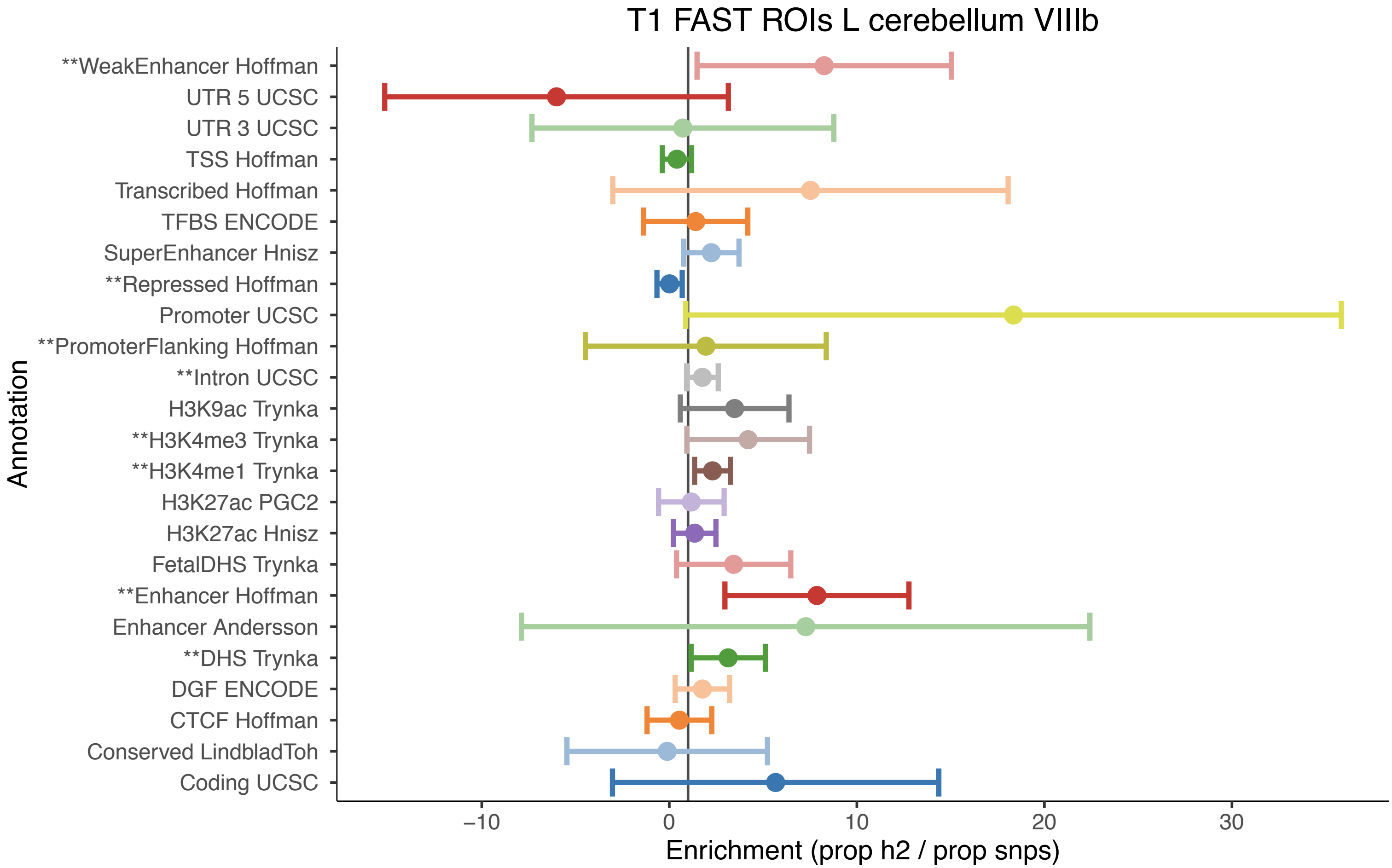

T1 FAST ROIs L cerebellum VIIIb
**WeakEnhancer Hoffman
UTR 5 UCSC
UTR 3 UCSC
TSS Hoffman
Transcribed Hoffman
TFBS ENCODE
SuperEnhancer Hnisz
**Repressed Hoffman
Promoter UCSC
**PromoterFlanking Hoffman
**Intron UCSC
H3K9ac Trynka
**H3K4me3 Trynka
**H3K4me1 Trynka
H3K27ac PGC2
H3K27ac Hnisz
FetalDHS Trynka
**Enhancer Hoffman
Enhancer Andersson
**DHS Trynka
DGF ENCODE
CTCF Hoffman
Conserved LindbladToh
Coding UCSC
Annotation
–10
0
10
20
30
Enrichment (prop h2 / prop snps)

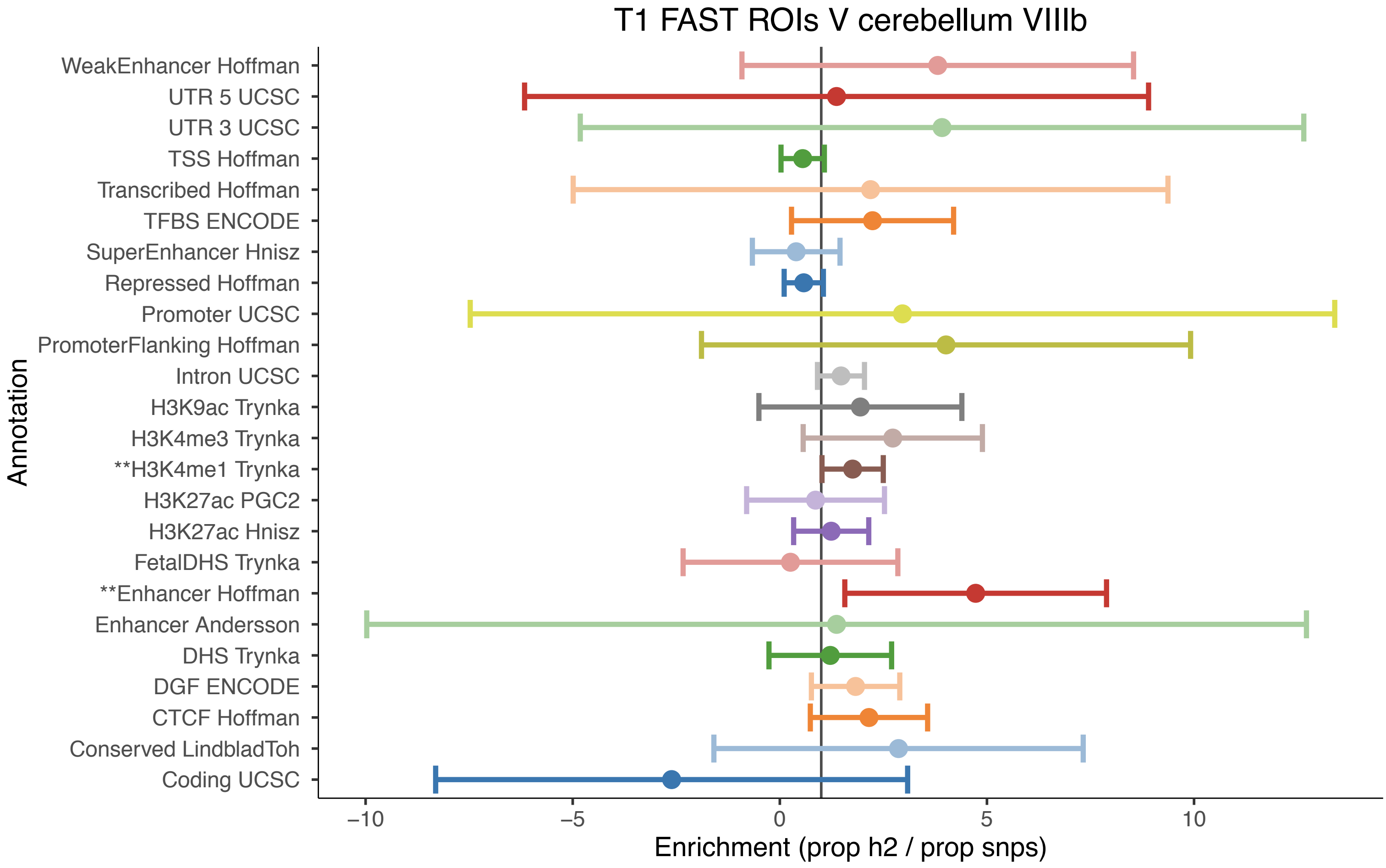

T1 FAST ROIs V cerebellum VIIIb
Annotation
WeakEnhancer Hoffman
UTR 5 UCSC
UTR 3 UCSC
TSS Hoffman
Transcribed Hoffman
TFBS ENCODE
SuperEnhancer Hnisz
Repressed Hoffman
Promoter UCSC
PromoterFlanking Hoffman
Intron UCSC
H3K9ac Trynka
H3K4me3 Trynka
**H3K4me1 Trynka
H3K27ac PGC2
H3K27ac Hnisz
FetalDHS Trynka
**Enhancer Hoffman
Enhancer Andersson
DHS Trynka
DGF ENCODE
CTCF Hoffman
Conserved LindbladToh
Coding UCSC
−10
−5
0
5
10
Enrichment (prop h2 / prop snps)

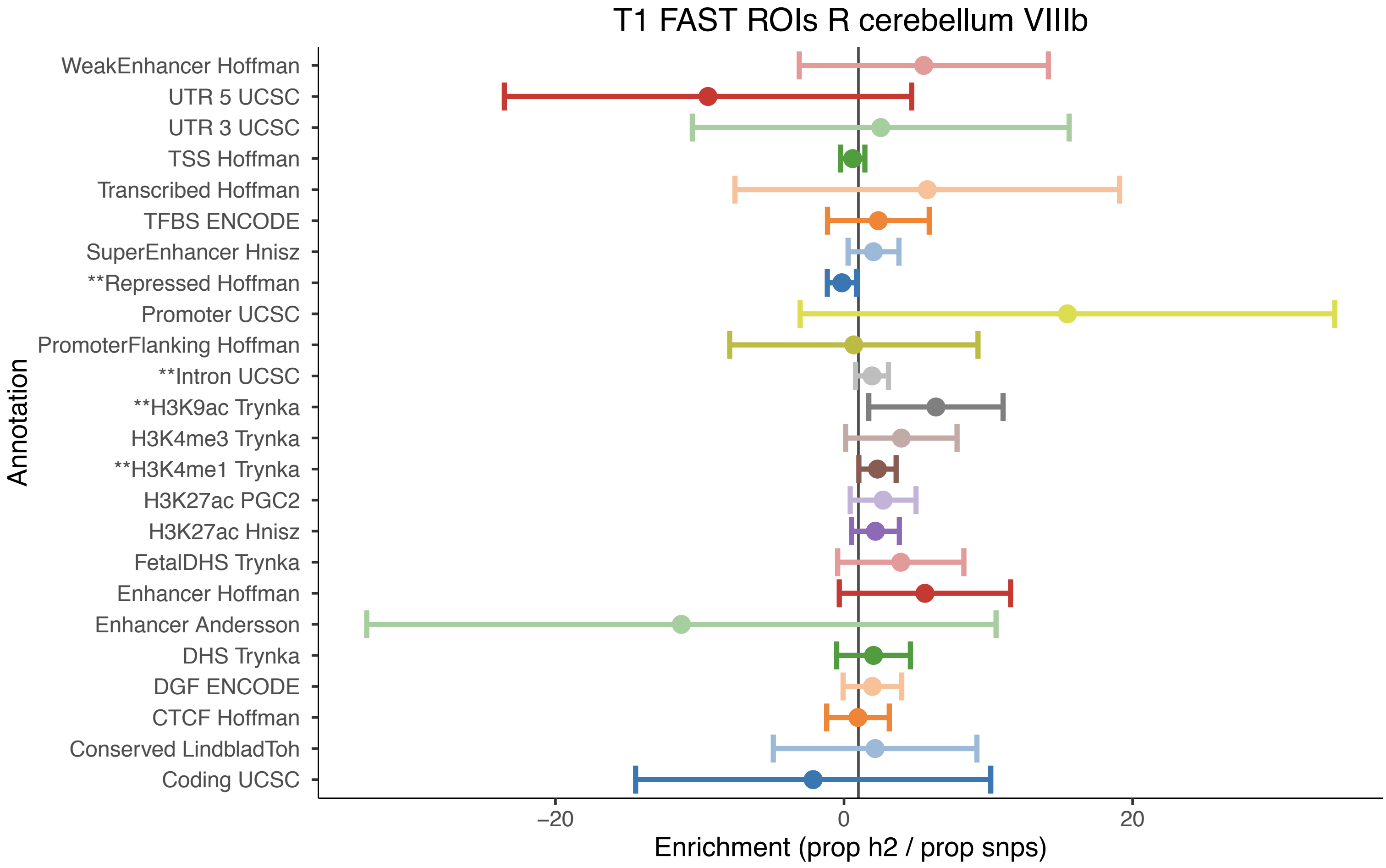
T1 FAST ROIs R cerebellum VIIIb
WeakEnhancer Hoffman
UTR 5 UCSC
UTR 3 UCSC
TSS Hoffman
Transcribed Hoffman
TFBS ENCODE
SuperEnhancer Hnisz
**Repressed Hoffman
Promoter UCSC
PromoterFlanking Hoffman
**Intron UCSC
**H3K9ac Trynka
H3K4me3 Trynka
**H3K4me1 Trynka
H3K27ac PGC2
H3K27ac Hnisz
FetalDHS Trynka
Enhancer Hoffman
Enhancer Andersson
DHS Trynka
DGF ENCODE
CTCF Hoffman
Conserved LindbladToh
Coding UCSC
Annotation
−20
0
20
Enrichment (prop h2 / prop snps)

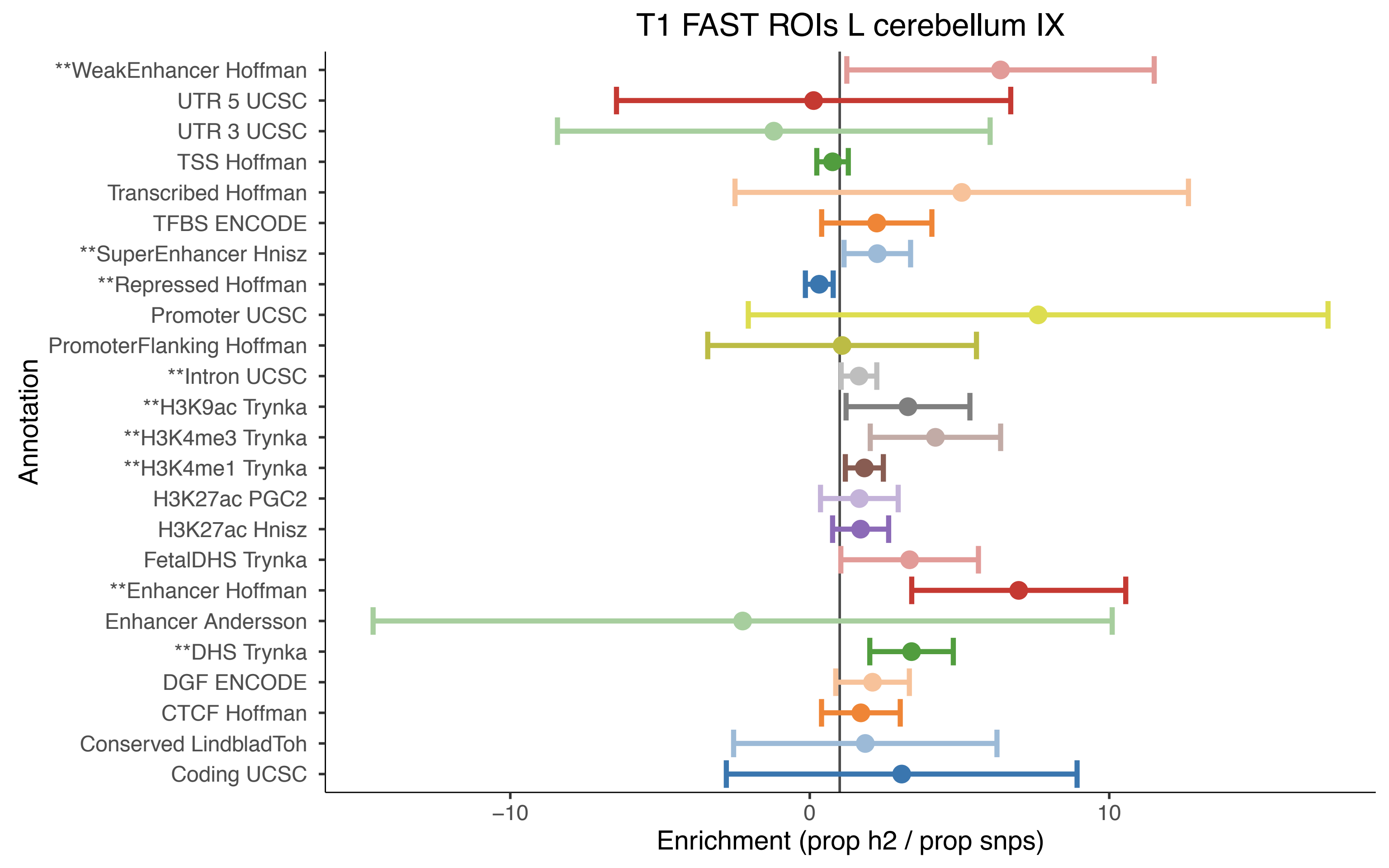
T1 FAST ROIs L cerebellum IX
**WeakEnhancer Hoffman
UTR 5 UCSC
UTR 3 UCSC
TSS Hoffman
Transcribed Hoffman
TFBS ENCODE
**SuperEnhancer Hnisz
**Repressed Hoffman
Promoter UCSC
PromoterFlanking Hoffman
**Intron UCSC
**H3K9ac Trynka
**H3K4me3 Trynka
**H3K4me1 Trynka
H3K27ac PGC2
H3K27ac Hnisz
FetalDHS Trynka
**Enhancer Hoffman
Enhancer Andersson
**DHS Trynka
DGF ENCODE
CTCF Hoffman
Conserved LindbladToh
Coding UCSC
Annotation
−10
0
10
Enrichment (prop h2 / prop snps)

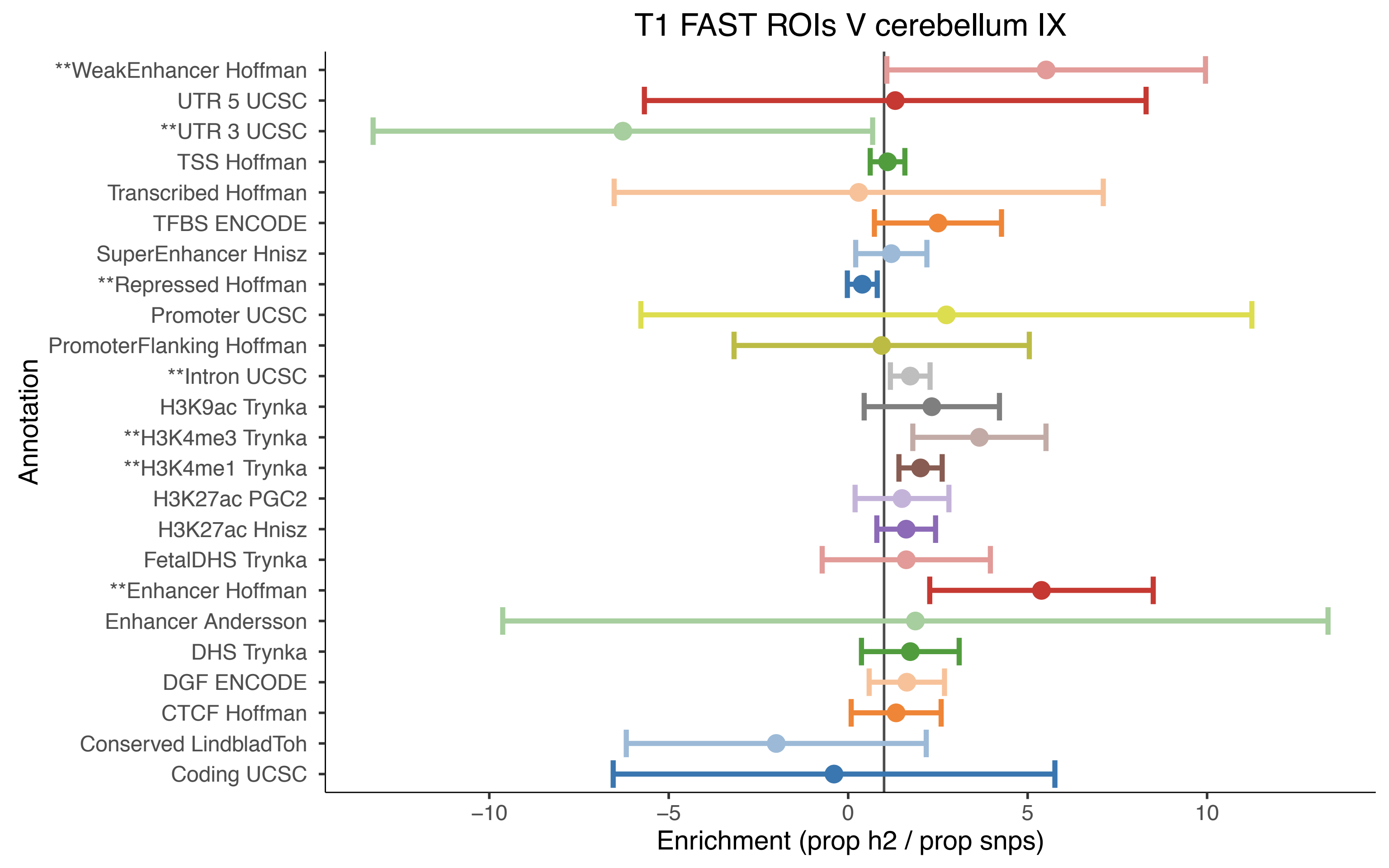

T1 FAST ROIs V cerebellum IX
**WeakEnhancer Hoffman
UTR 5 UCSC
**UTR 3 UCSC
TSS Hoffman
Transcribed Hoffman
TFBS ENCODE
SuperEnhancer Hnisz
**Repressed Hoffman
Promoter UCSC
PromoterFlanking Hoffman
**Intron UCSC
H3K9ac Trynka
**H3K4me3 Trynka
**H3K4me1 Trynka
H3K27ac PGC2
H3K27ac Hnisz
FetalDHS Trynka
**Enhancer Hoffman
Enhancer Andersson
DHS Trynka
DGF ENCODE
CTCF Hoffman
Conserved LindbladToh
Coding UCSC
Annotation
−10
−5
0
5
10
Enrichment (prop h2 / prop snps)

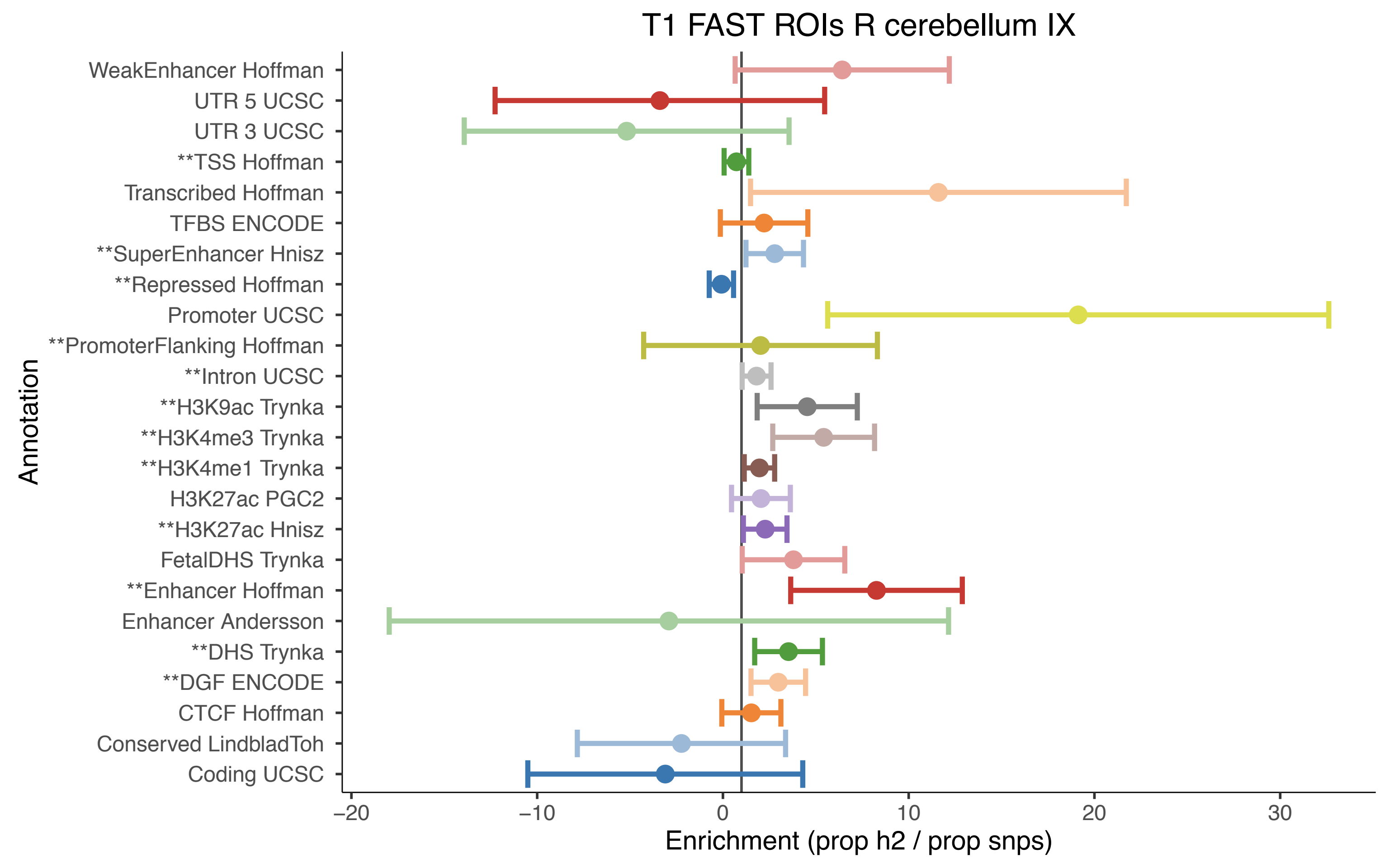

T1 FAST ROIs R cerebellum IX
WeakEnhancer Hoffman
UTR 5 UCSC
UTR 3 UCSC
**TSS Hoffman
Transcribed Hoffman
TFBS ENCODE
**SuperEnhancer Hnisz
**Repressed Hoffman
Promoter UCSC
**PromoterFlanking Hoffman
**Intron UCSC
**H3K9ac Trynka
**H3K4me3 Trynka
**H3K4me1 Trynka
H3K27ac PGC2
**H3K27ac Hnisz
FetalDHS Trynka
**Enhancer Hoffman
Enhancer Andersson
**DHS Trynka
**DGF ENCODE
CTCF Hoffman
Conserved LindbladToh
Coding UCSC
Annotation
−20
−10
0
10
20
30
Enrichment (prop h2 / prop snps)

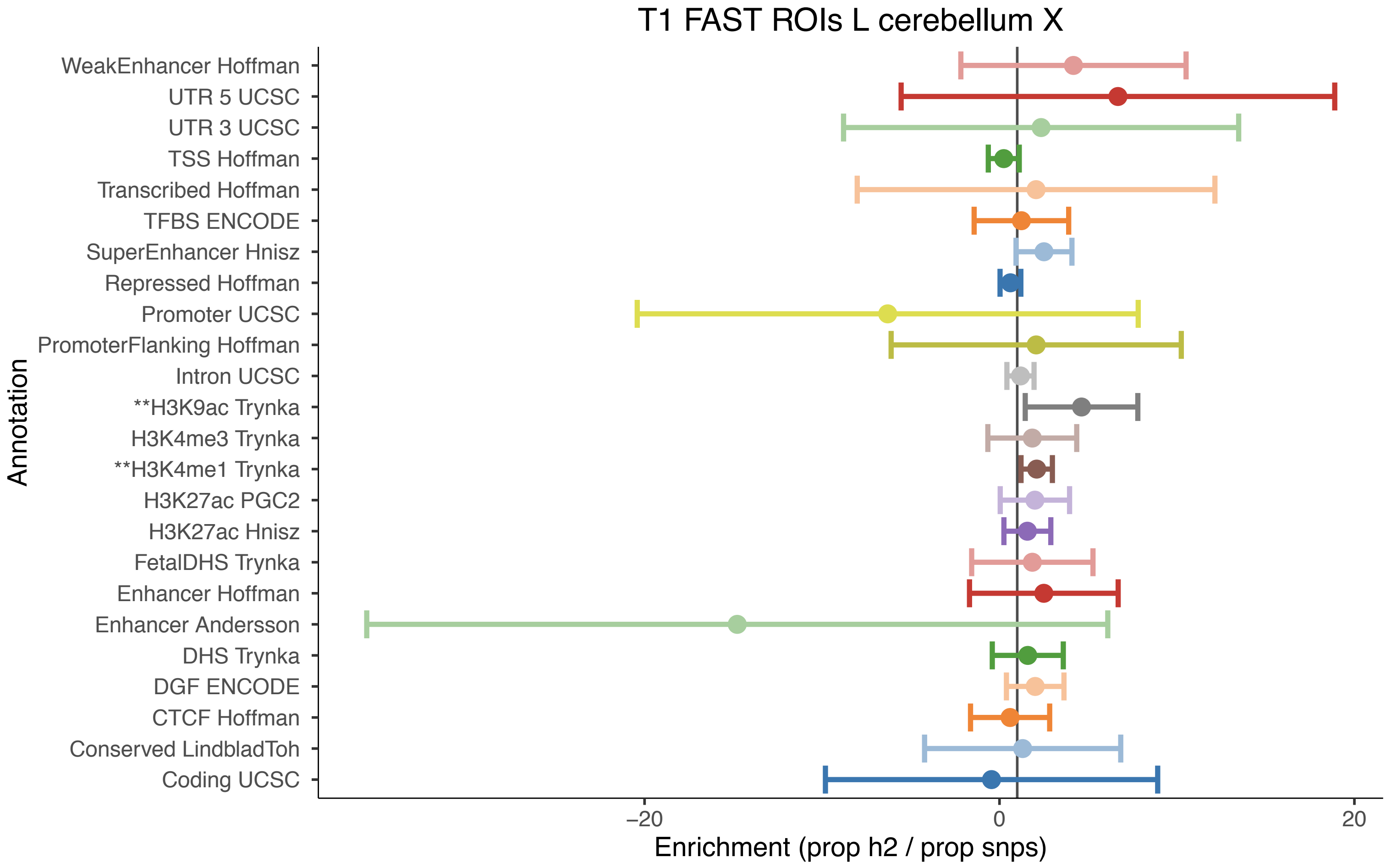

T1 FAST ROIs L cerebellum X
Annotation
WeakEnhancer Hoffman
UTR 5 UCSC
UTR 3 UCSC
TSS Hoffman
Transcribed Hoffman
TFBS ENCODE
SuperEnhancer Hnisz
Repressed Hoffman
Promoter UCSC
PromoterFlanking Hoffman
Intron UCSC
**H3K9ac Trynka
H3K4me3 Trynka
**H3K4me1 Trynka
H3K27ac PGC2
H3K27ac Hnisz
FetalDHS Trynka
Enhancer Hoffman
Enhancer Andersson
DHS Trynka
DGF ENCODE
CTCF Hoffman
Conserved LindbladToh
Coding UCSC
−20
0
20
Enrichment (prop h2 / prop snps)

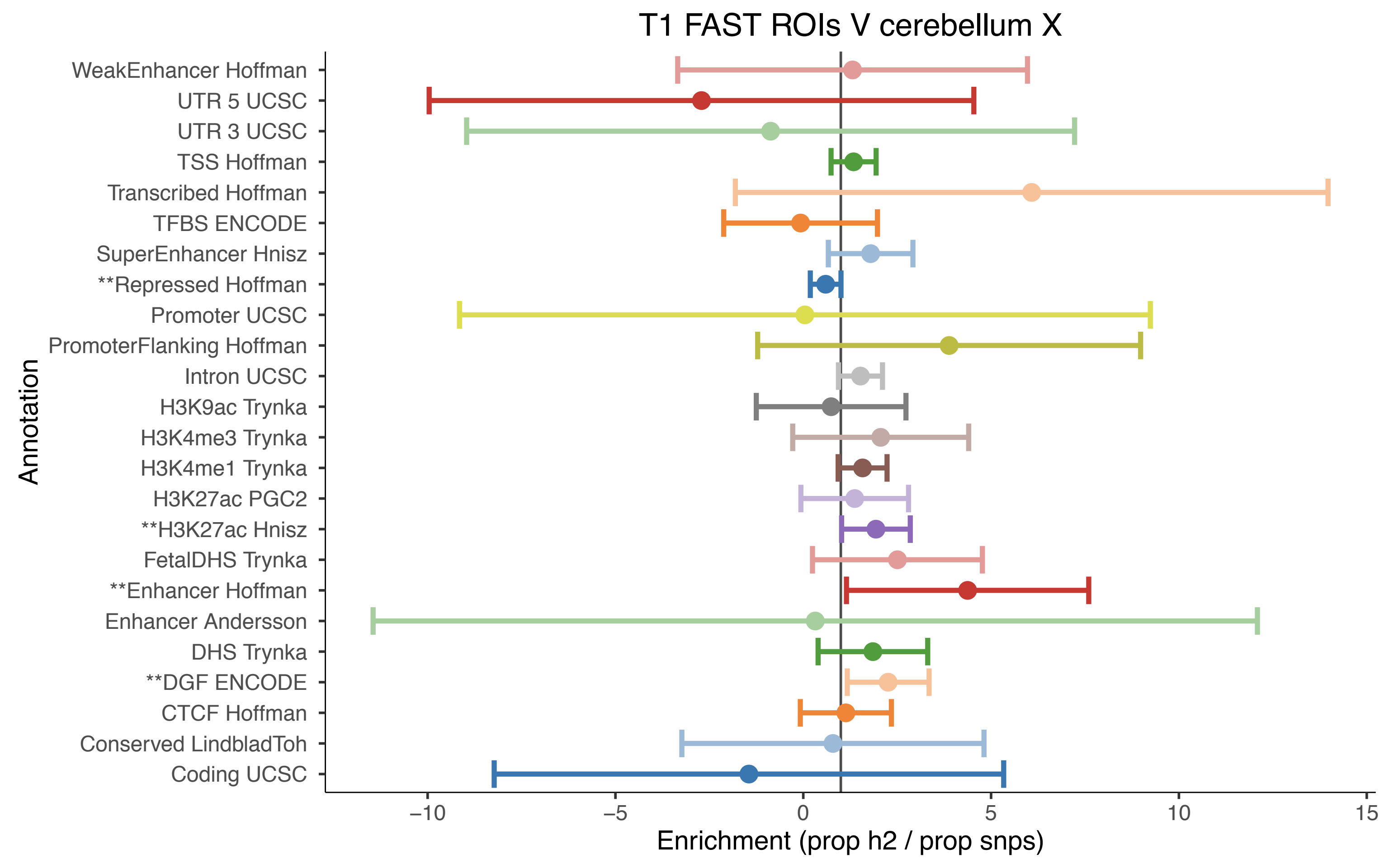

T1 FAST ROIs V cerebellum X
WeakEnhancer Hoffman
UTR 5 UCSC
UTR 3 UCSC
TSS Hoffman
Transcribed Hoffman
TFBS ENCODE
SuperEnhancer Hnisz
**Repressed Hoffman
Promoter UCSC
PromoterFlanking Hoffman
Intron UCSC
H3K9ac Trynka
H3K4me3 Trynka
H3K4me1 Trynka
H3K27ac PGC2
**H3K27ac Hnisz
FetalDHS Trynka
**Enhancer Hoffman
Enhancer Andersson
DHS Trynka
**DGF ENCODE
CTCF Hoffman
Conserved LindbladToh
Coding UCSC
Annotation
−10
−5
0
5
10
15
Enrichment (prop h2 / prop snps)

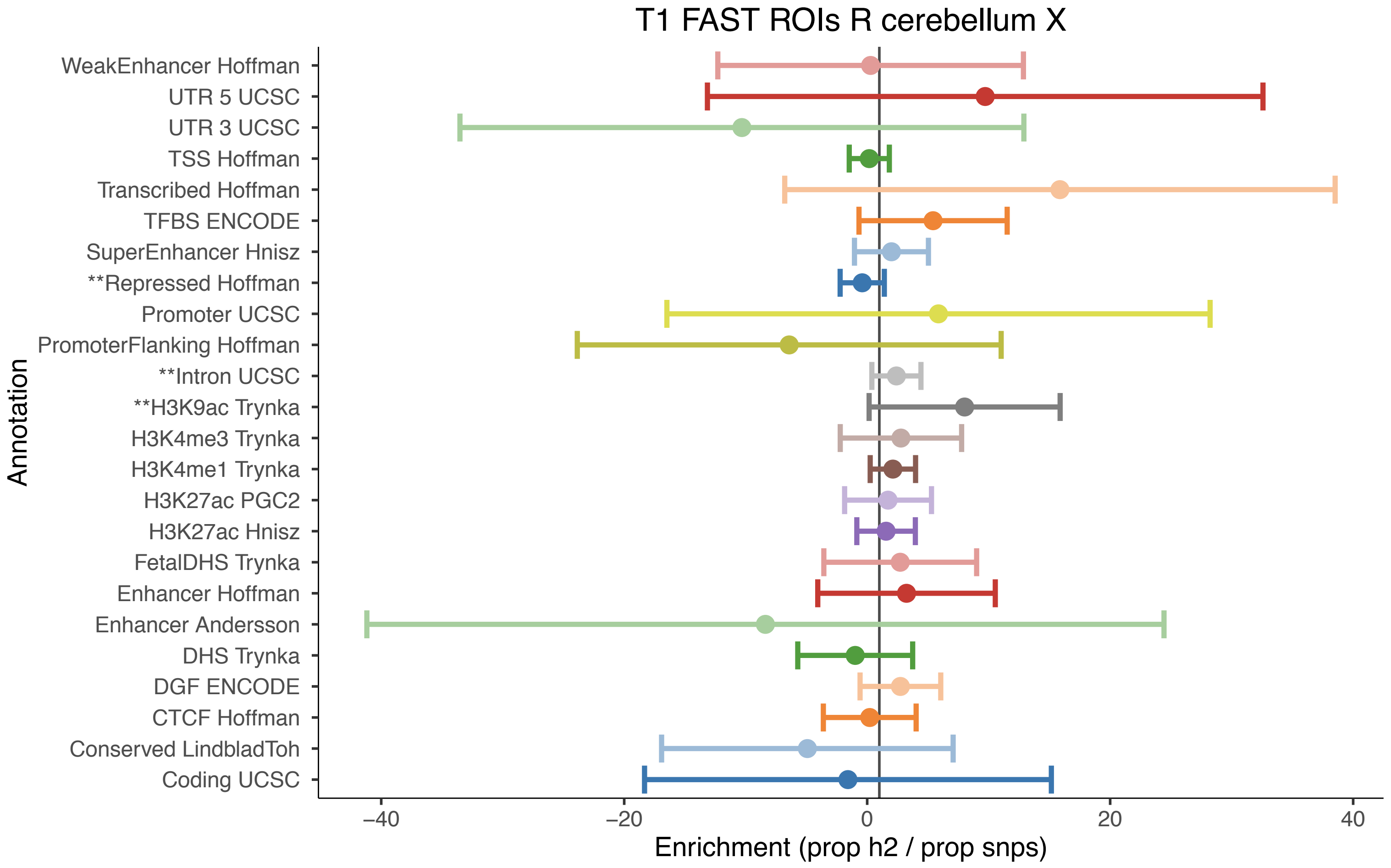
T1 FAST ROIs R cerebellum X
WeakEnhancer Hoffman
UTR 5 UCSC
UTR 3 UCSC
TSS Hoffman
Transcribed Hoffman
TFBS ENCODE
SuperEnhancer Hnisz
**Repressed Hoffman
Promoter UCSC
PromoterFlanking Hoffman
**Intron UCSC
**H3K9ac Trynka
H3K4me3 Trynka
H3K4me1 Trynka
H3K27ac PGC2
H3K27ac Hnisz
FetalDHS Trynka
Enhancer Hoffman
Enhancer Andersson
DHS Trynka
DGF ENCODE
CTCF Hoffman
Conserved LindbladToh
Coding UCSC
Annotation
−40
−20
0
20
40
Enrichment (prop h2 / prop snps)

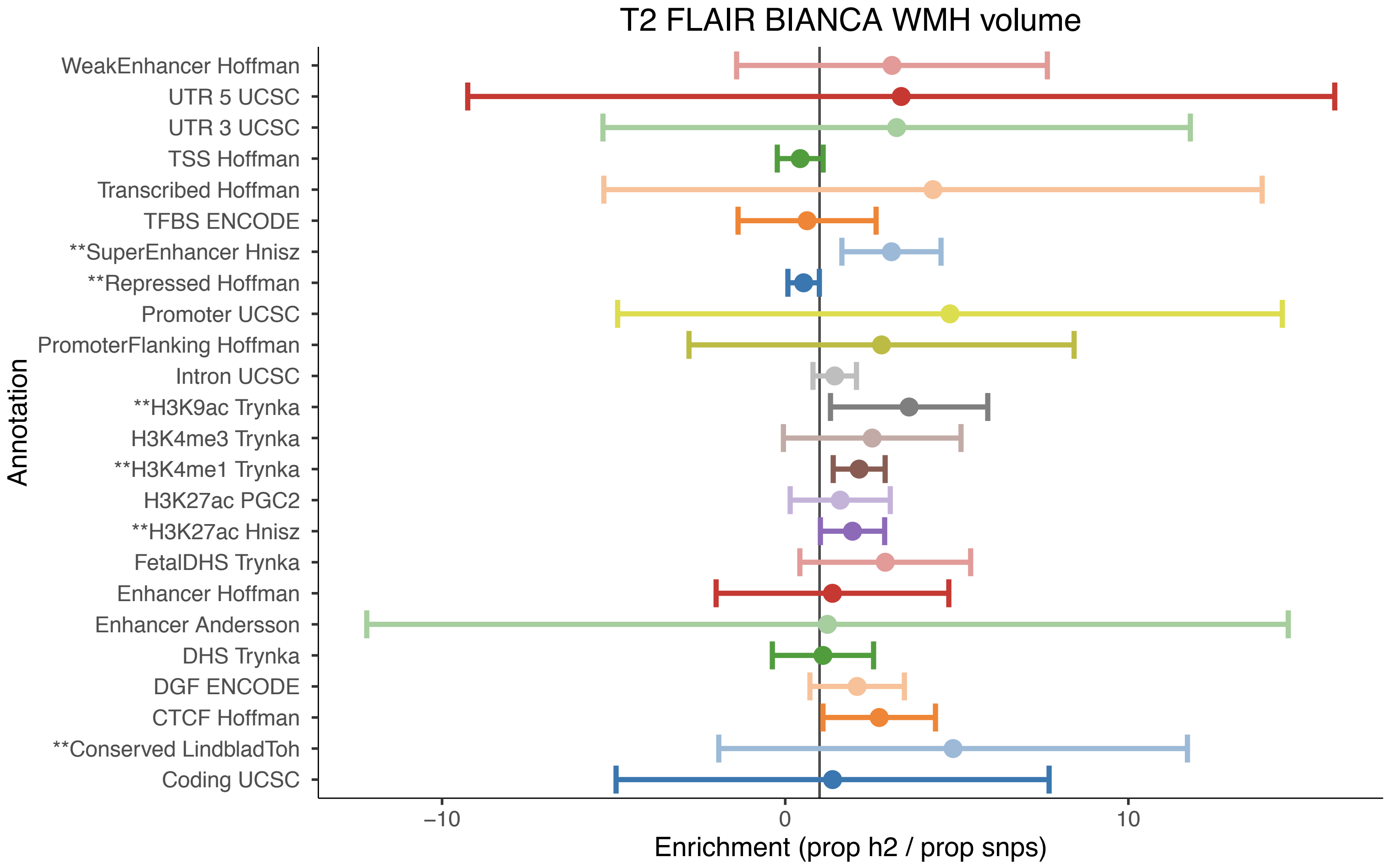

T2 FLAIR BIANCA WMH volume
WeakEnhancer Hoffman
UTR 5 UCSC
UTR 3 UCSC
TSS Hoffman
Transcribed Hoffman
TFBS ENCODE
**SuperEnhancer Hnisz
**Repressed Hoffman
Promoter UCSC
PromoterFlanking Hoffman
Intron UCSC
**H3K9ac Trynka
H3K4me3 Trynka
**H3K4me1 Trynka
H3K27ac PGC2
**H3K27ac Hnisz
FetalDHS Trynka
Enhancer Hoffman
Enhancer Andersson
DHS Trynka
DGF ENCODE
CTCF Hoffman
**Conserved LindbladToh
Coding UCSC
Annotation
−10
0
10
Enrichment (prop h2 / prop snps)

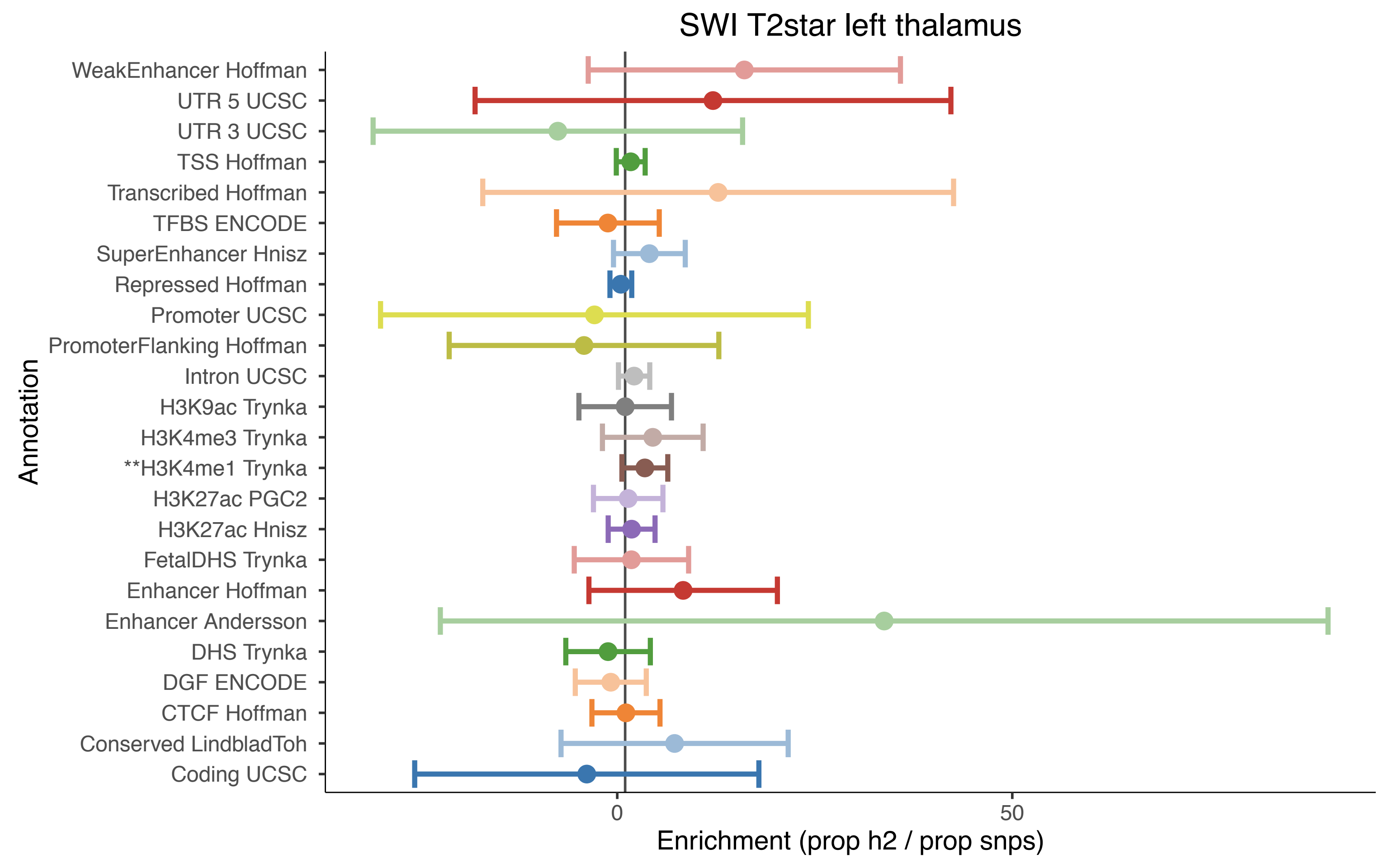

SWI T2star left thalamus
WeakEnhancer Hoffman
UTR 5 UCSC
UTR 3 UCSC
TSS Hoffman
Transcribed Hoffman
TFBS ENCODE
SuperEnhancer Hnisz
Repressed Hoffman
Promoter UCSC
PromoterFlanking Hoffman
Intron UCSC
H3K9ac Trynka
H3K4me3 Trynka
**H3K4me1 Trynka
H3K27ac PGC2
H3K27ac Hnisz
FetalDHS Trynka
Enhancer Hoffman
Enhancer Andersson
DHS Trynka
DGF ENCODE
CTCF Hoffman
Conserved LindbladToh
Coding UCSC
Annotation
0
50
Enrichment (prop h2 / prop snps)

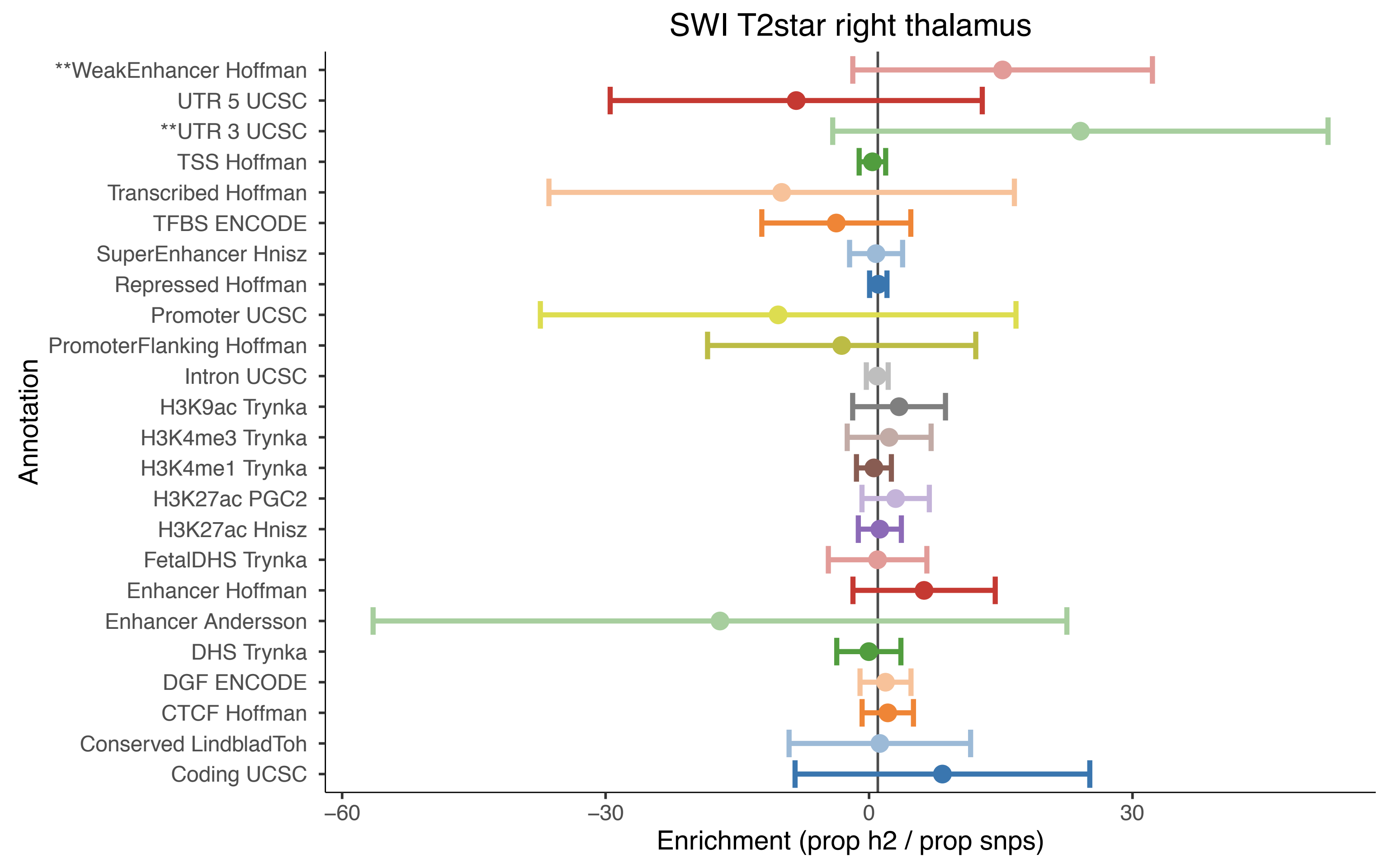
SWI T2star right thalamus
**WeakEnhancer Hoffman
UTR 5 UCSC
**UTR 3 UCSC
TSS Hoffman
Transcribed Hoffman
TFBS ENCODE
SuperEnhancer Hnisz
Repressed Hoffman
Promoter UCSC
PromoterFlanking Hoffman
Intron UCSC
H3K9ac Trynka
H3K4me3 Trynka
H3K4me1 Trynka
H3K27ac PGC2
H3K27ac Hnisz
FetalDHS Trynka
Enhancer Hoffman
Enhancer Andersson
DHS Trynka
DGF ENCODE
CTCF Hoffman
Conserved LindbladToh
Coding UCSC
Annotation
−60
−30
0
30
Enrichment (prop h2 / prop snps)

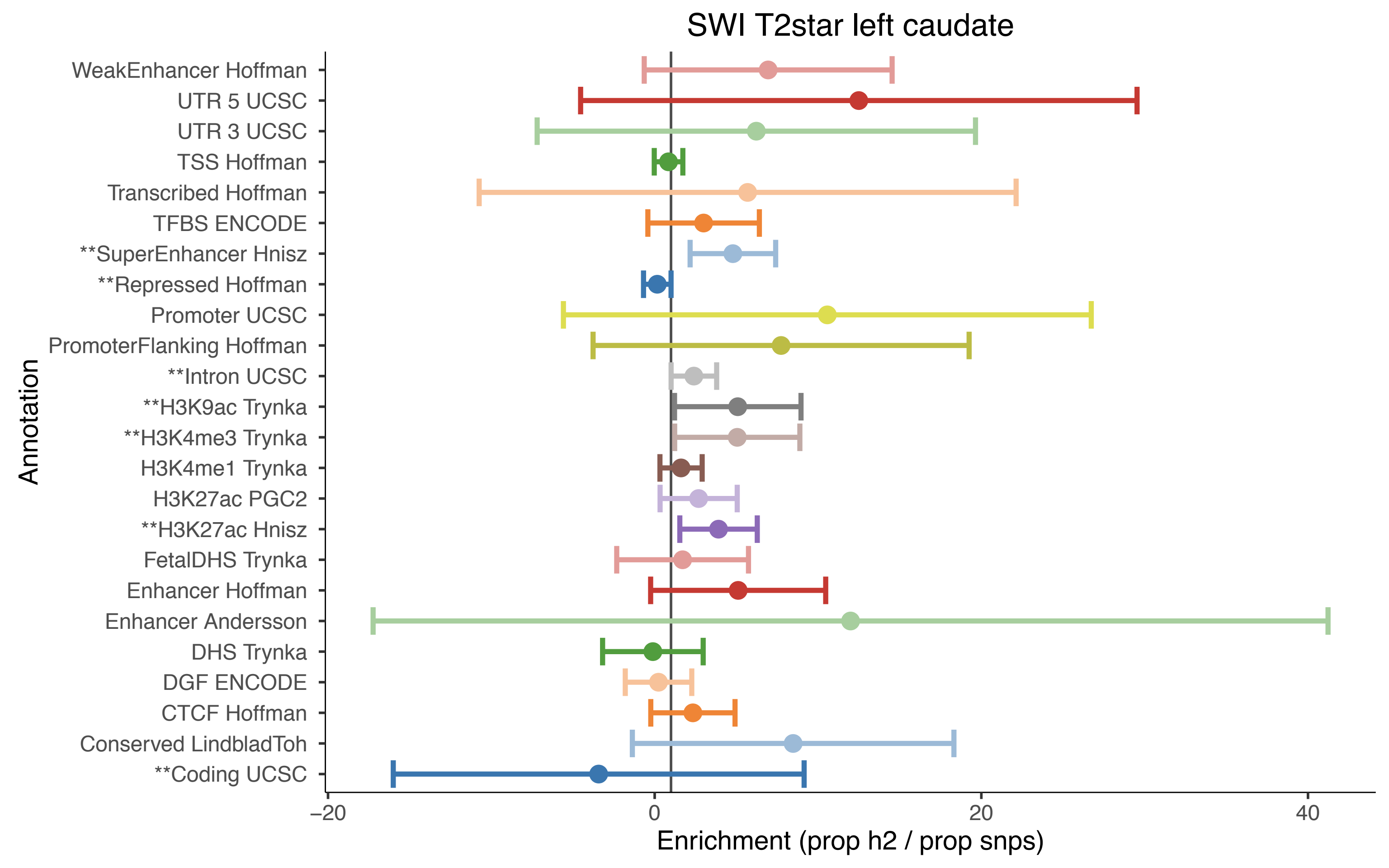
SWI T2star left caudate
WeakEnhancer Hoffman
UTR 5 UCSC
UTR 3 UCSC
TSS Hoffman
Transcribed Hoffman
TFBS ENCODE
**SuperEnhancer Hnisz
**Repressed Hoffman
Promoter UCSC
PromoterFlanking Hoffman
**Intron UCSC
**H3K9ac Trynka
**H3K4me3 Trynka
H3K4me1 Trynka
H3K27ac PGC2
**H3K27ac Hnisz
FetalDHS Trynka
Enhancer Hoffman
Enhancer Andersson
DHS Trynka
DGF ENCODE
CTCF Hoffman
Conserved LindbladToh
**Coding UCSC
Annotation
−20
0
20
40
Enrichment (prop h2 / prop snps)

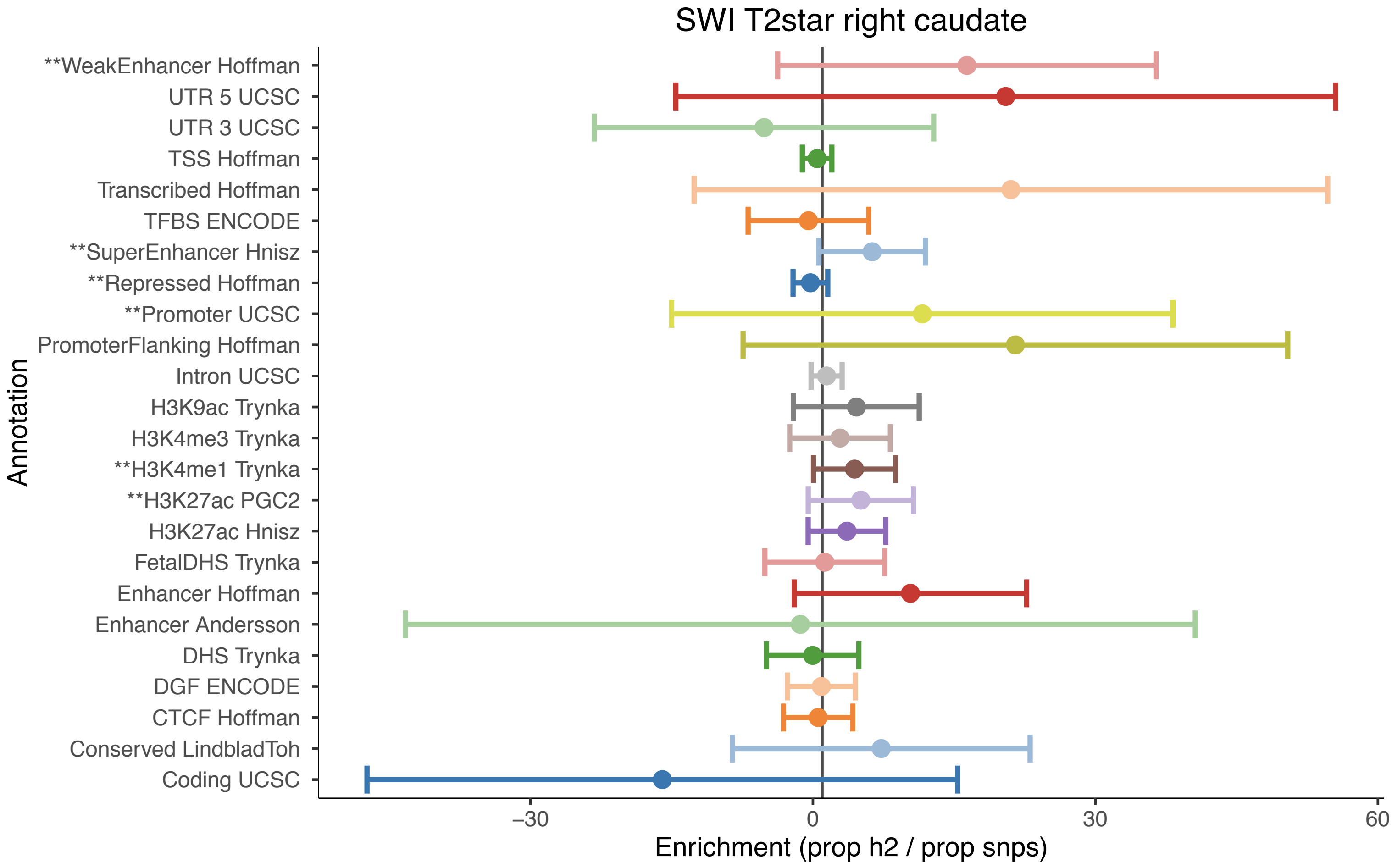
SWI T2star right caudate
**WeakEnhancer Hoffman
UTR 5 UCSC
UTR 3 UCSC
TSS Hoffman
Transcribed Hoffman
TFBS ENCODE
**SuperEnhancer Hnisz
**Repressed Hoffman
**Promoter UCSC
PromoterFlanking Hoffman
Intron UCSC
H3K9ac Trynka
H3K4me3 Trynka
**H3K4me1 Trynka
**H3K27ac PGC2
H3K27ac Hnisz
FetalDHS Trynka
Enhancer Hoffman
Enhancer Andersson
DHS Trynka
DGF ENCODE
CTCF Hoffman
Conserved LindbladToh
Coding UCSC
Annotation
−30
0
30
60
Enrichment (prop h2 / prop snps)

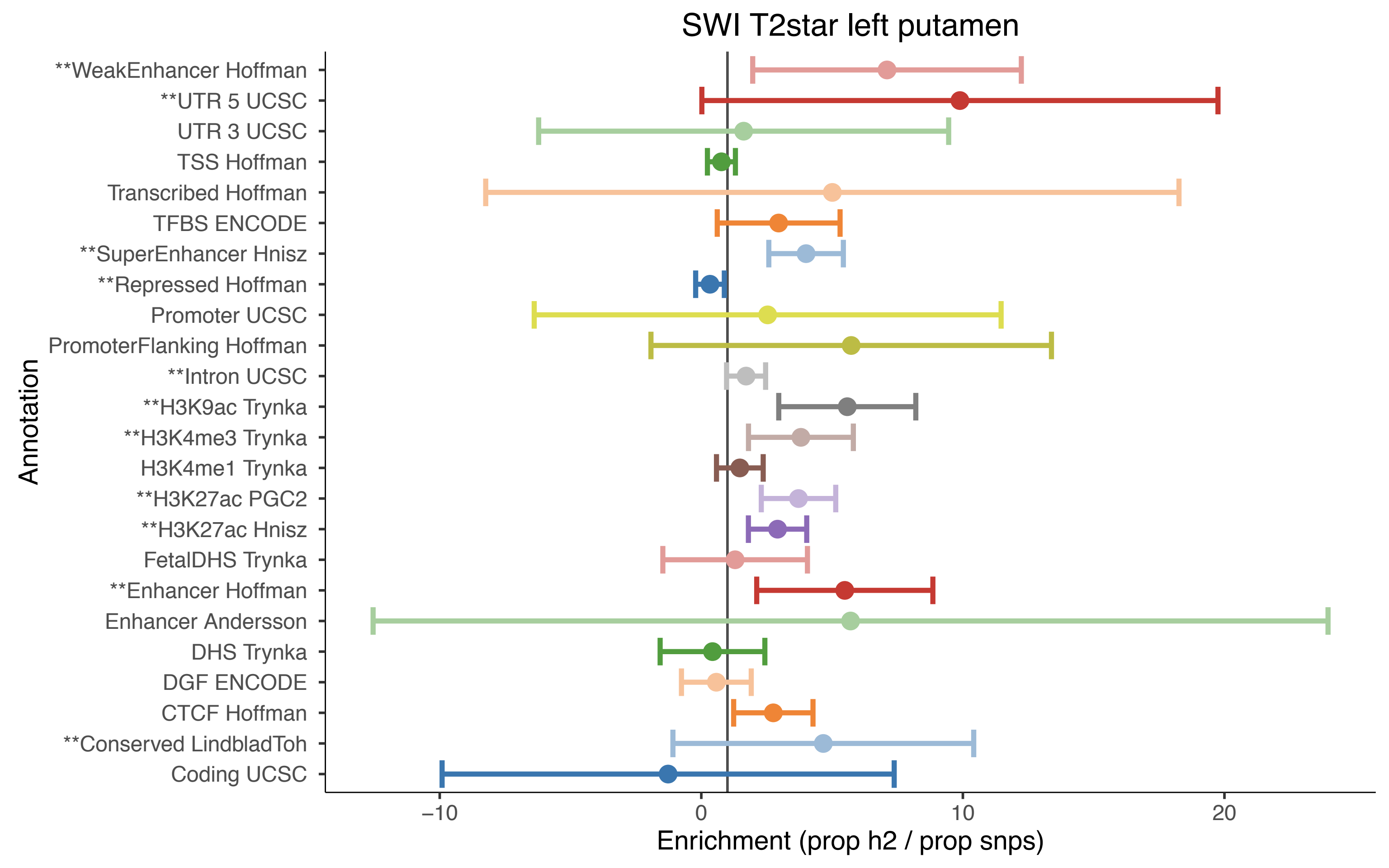

SWI T2star left putamen
**WeakEnhancer Hoffman
**UTR 5 UCSC
UTR 3 UCSC
TSS Hoffman
Transcribed Hoffman
TFBS ENCODE
**SuperEnhancer Hnisz
**Repressed Hoffman
Promoter UCSC
PromoterFlanking Hoffman
**Intron UCSC
**H3K9ac Trynka
**H3K4me3 Trynka
H3K4me1 Trynka
**H3K27ac PGC2
**H3K27ac Hnisz
FetalDHS Trynka
**Enhancer Hoffman
Enhancer Andersson
DHS Trynka
DGF ENCODE
CTCF Hoffman
**Conserved LindbladToh
Coding UCSC
Annotation
−10
0
10
20
Enrichment (prop h2 / prop snps)

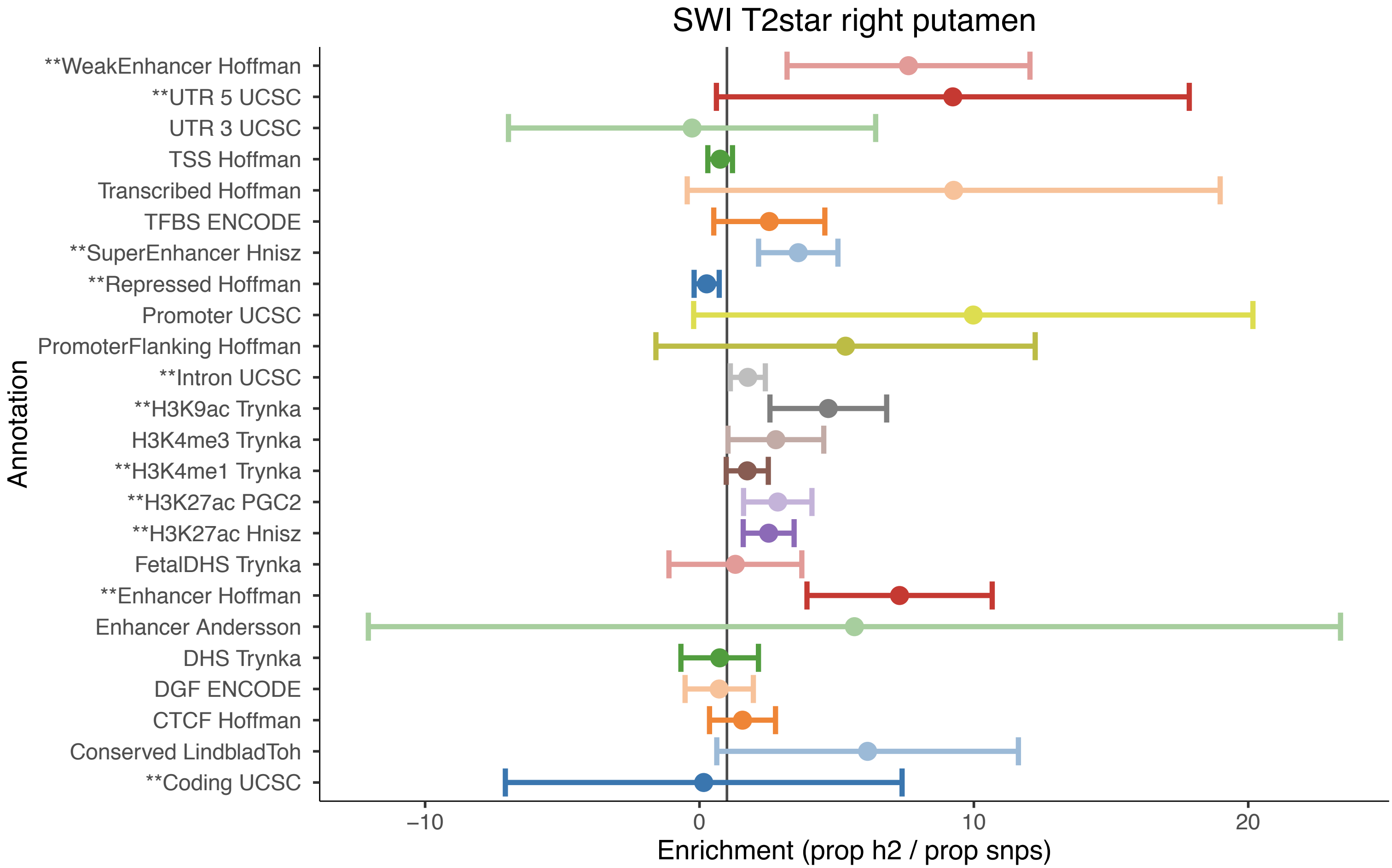
SWI T2star right putamen
**WeakEnhancer Hoffman
**UTR 5 UCSC
UTR 3 UCSC
TSS Hoffman
Transcribed Hoffman
TFBS ENCODE
**SuperEnhancer Hnisz
**Repressed Hoffman
Promoter UCSC
PromoterFlanking Hoffman
**Intron UCSC
**H3K9ac Trynka
H3K4me3 Trynka
**H3K4me1 Trynka
**H3K27ac PGC2
**H3K27ac Hnisz
FetalDHS Trynka
**Enhancer Hoffman
Enhancer Andersson
DHS Trynka
DGF ENCODE
CTCF Hoffman
Conserved LindbladToh
**Coding UCSC
Annotation
−10
0
10
20
Enrichment (prop h2 / prop snps)

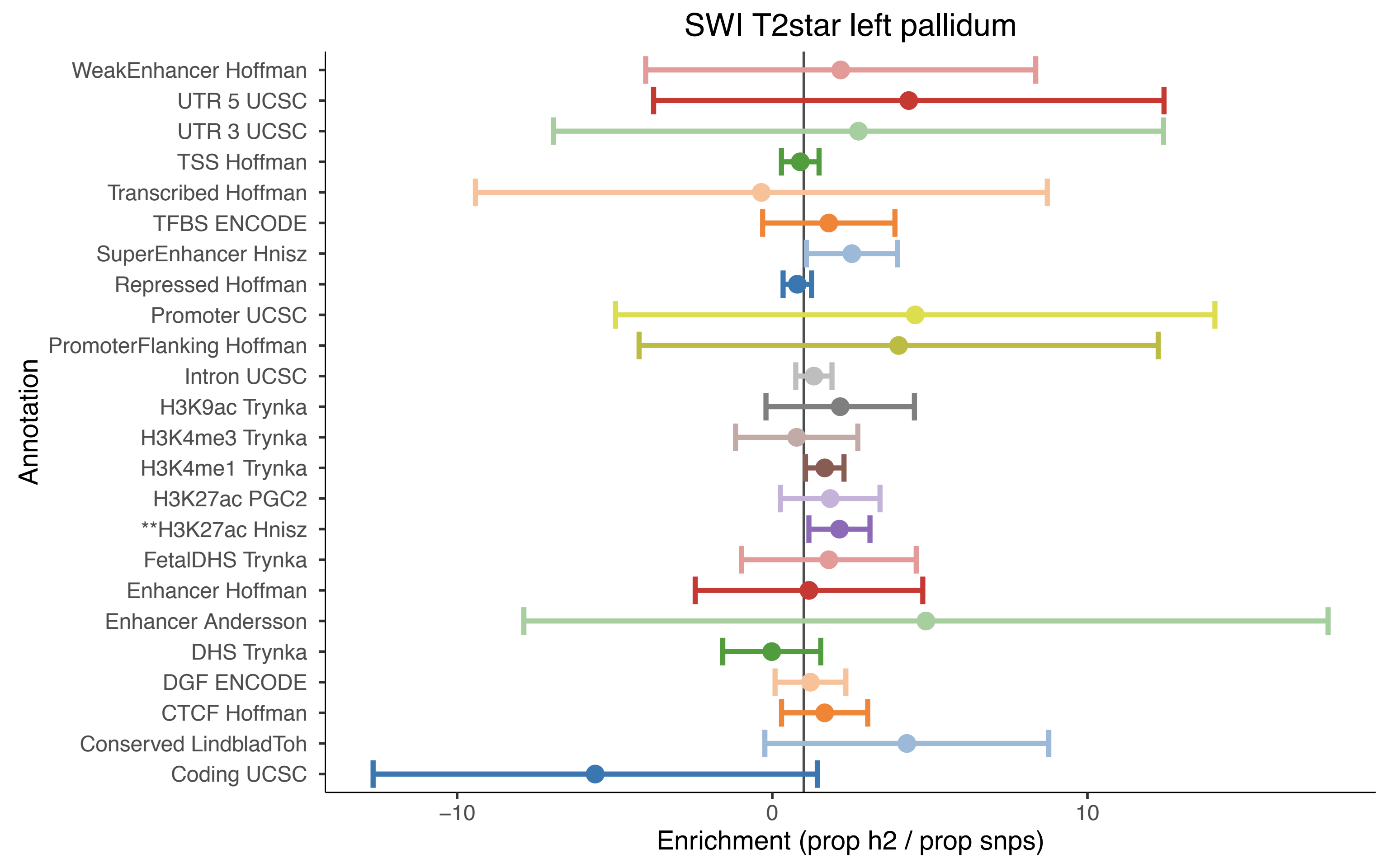

SWI T2star left pallidum
WeakEnhancer Hoffman
UTR 5 UCSC
UTR 3 UCSC
TSS Hoffman
Transcribed Hoffman
TFBS ENCODE
SuperEnhancer Hnisz
Repressed Hoffman
Promoter UCSC
PromoterFlanking Hoffman
Intron UCSC
H3K9ac Trynka
H3K4me3 Trynka
H3K4me1 Trynka
H3K27ac PGC2
**H3K27ac Hnisz
FetalDHS Trynka
Enhancer Hoffman
Enhancer Andersson
DHS Trynka
DGF ENCODE
CTCF Hoffman
Conserved LindbladToh
Coding UCSC
Annotation
−10
0
10
Enrichment (prop h2 / prop snps)

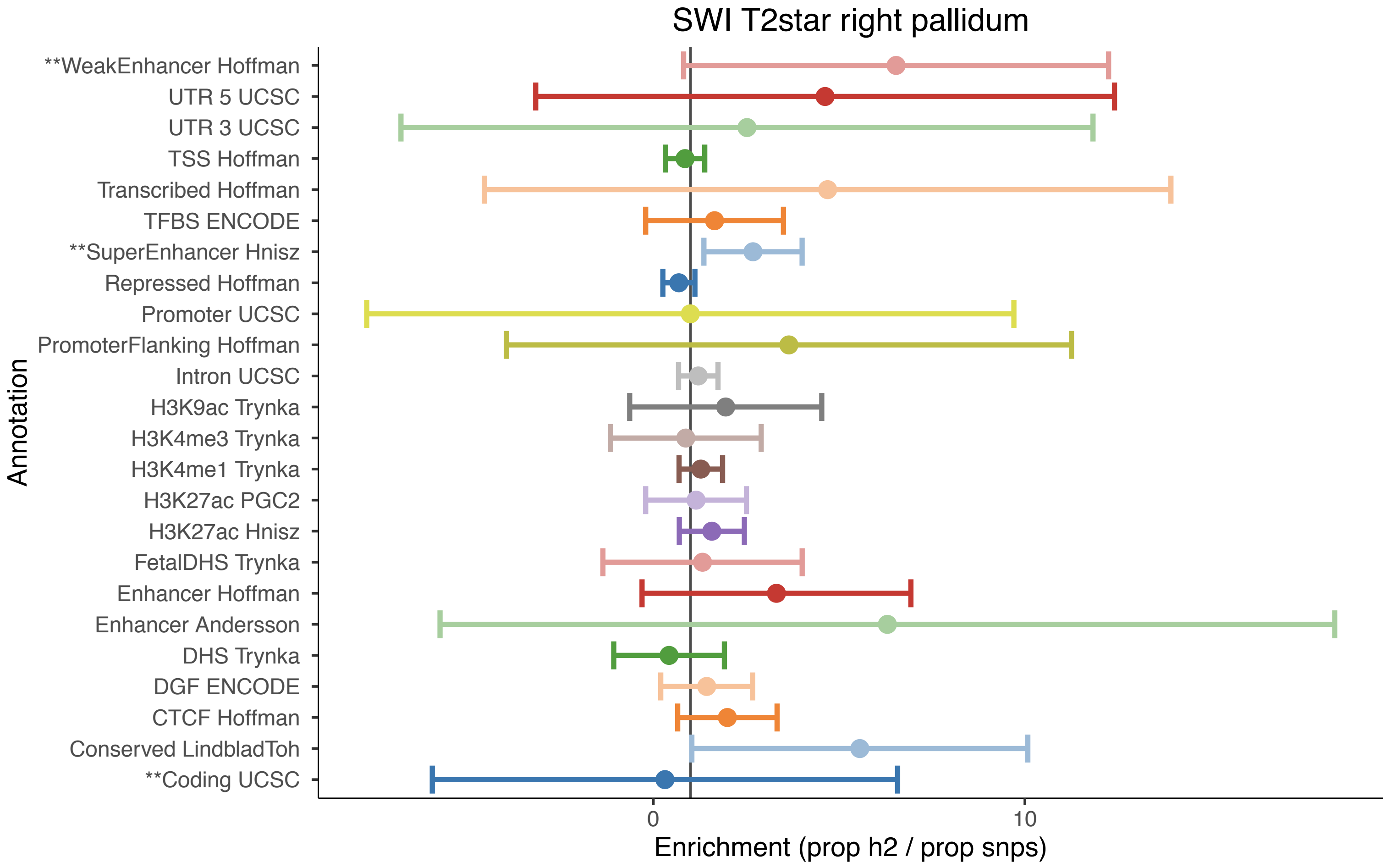

SWI T2star right pallidum
**WeakEnhancer Hoffman
UTR 5 UCSC
UTR 3 UCSC
TSS Hoffman
Transcribed Hoffman
TFBS ENCODE
**SuperEnhancer Hnisz
Repressed Hoffman
Promoter UCSC
PromoterFlanking Hoffman
Intron UCSC
H3K9ac Trynka
H3K4me3 Trynka
H3K4me1 Trynka
H3K27ac PGC2
H3K27ac Hnisz
FetalDHS Trynka
Enhancer Hoffman
Enhancer Andersson
DHS Trynka
DGF ENCODE
CTCF Hoffman
Conserved LindbladToh
**Coding UCSC
Annotation
0
10
Enrichment (prop h2 / prop snps)

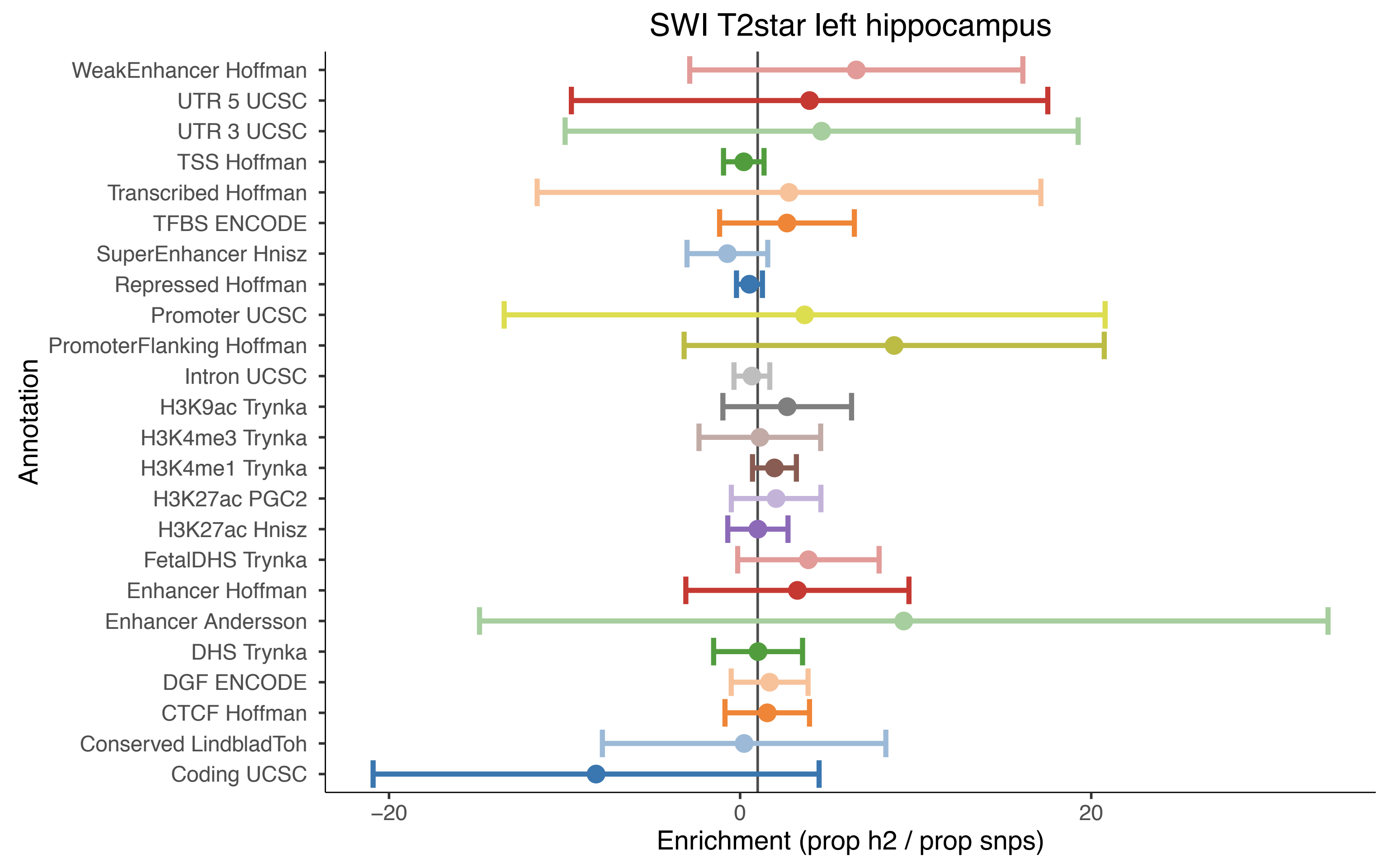
SWI T2star left hippocampus
WeakEnhancer Hoffman
UTR 5 UCSC
UTR 3 UCSC
TSS Hoffman
Transcribed Hoffman
TFBS ENCODE
SuperEnhancer Hnisz
Repressed Hoffman
Promoter UCSC
PromoterFlanking Hoffman
Intron UCSC
H3K9ac Trynka
H3K4me3 Trynka
H3K4me1 Trynka
H3K27ac PGC2
H3K27ac Hnisz
FetalDHS Trynka
Enhancer Hoffman
Enhancer Andersson
DHS Trynka
DGF ENCODE
CTCF Hoffman
Conserved LindbladToh
Coding UCSC
Annotation
−20
0
20
Enrichment (prop h2 / prop snps)

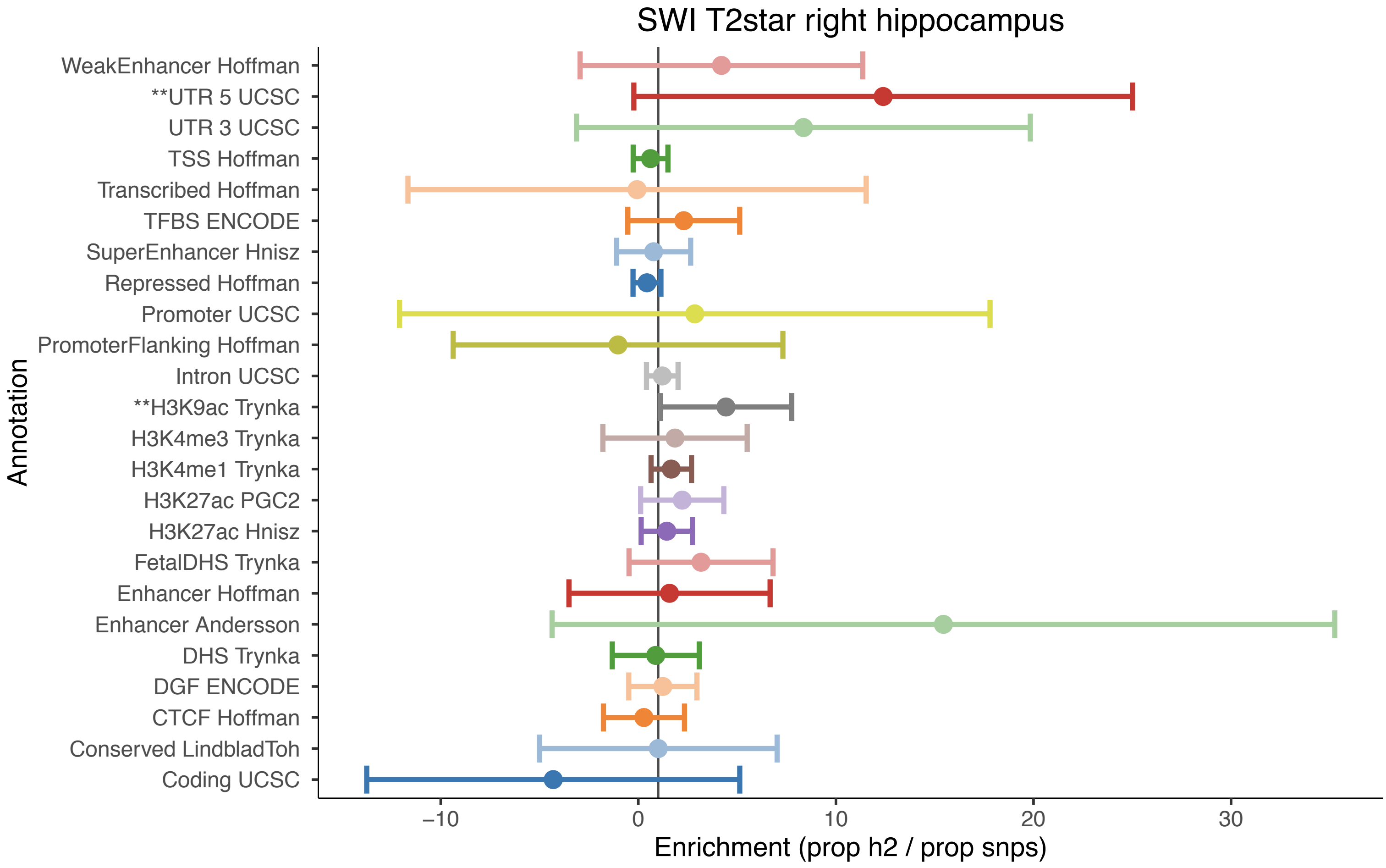

SWI T2star right hippocampus
WeakEnhancer Hoffman
**UTR 5 UCSC
UTR 3 UCSC
TSS Hoffman
Transcribed Hoffman
TFBS ENCODE
SuperEnhancer Hnisz
Repressed Hoffman
Promoter UCSC
PromoterFlanking Hoffman
Intron UCSC
**H3K9ac Trynka
H3K4me3 Trynka
H3K4me1 Trynka
H3K27ac PGC2
H3K27ac Hnisz
FetalDHS Trynka
Enhancer Hoffman
Enhancer Andersson
DHS Trynka
DGF ENCODE
CTCF Hoffman
Conserved LindbladToh
Coding UCSC
Annotation
−10
0
10
20
30
Enrichment (prop h2 / prop snps)

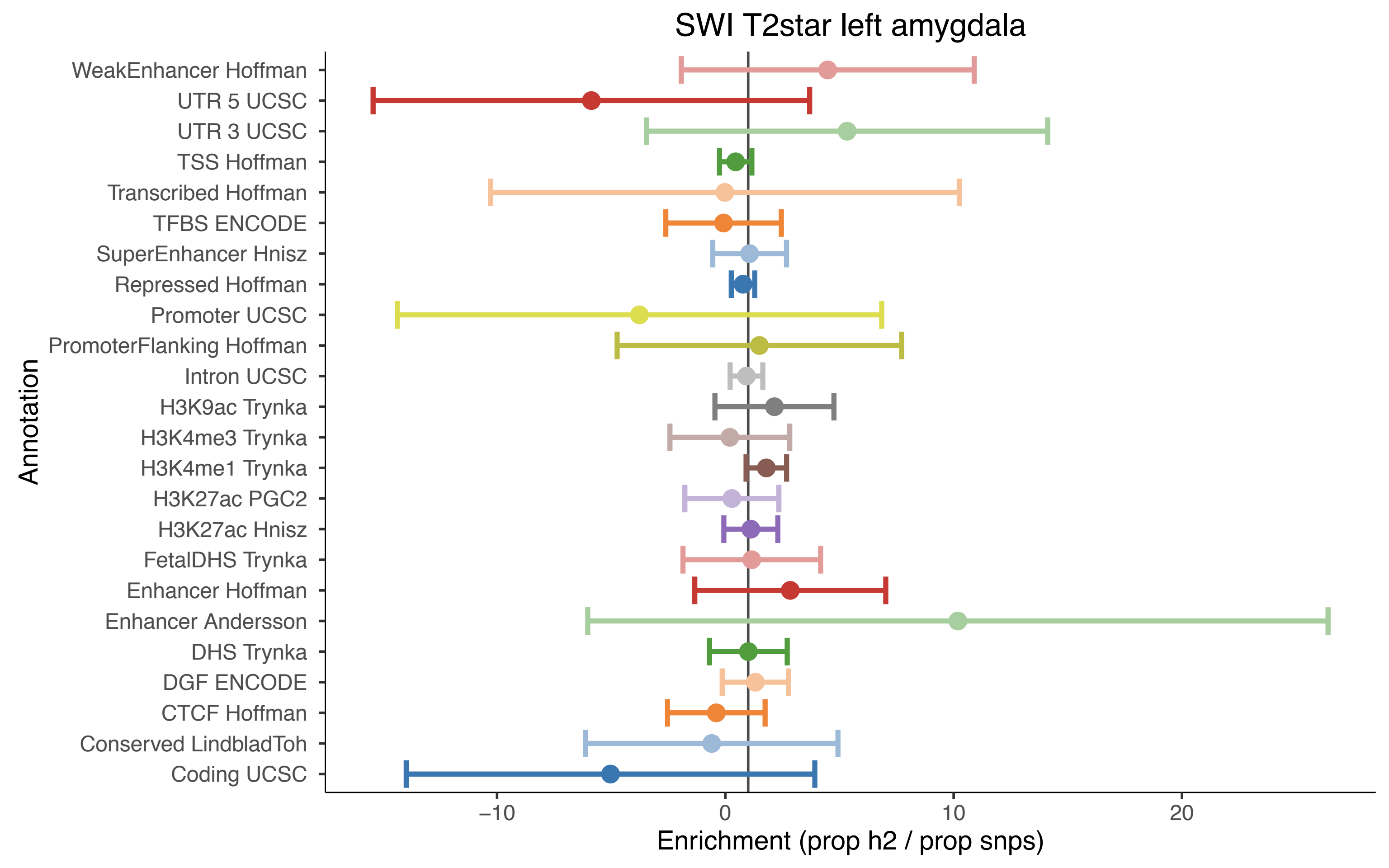
SWI T2star left amygdala
WeakEnhancer Hoffman
UTR 5 UCSC
UTR 3 UCSC
TSS Hoffman
Transcribed Hoffman
TFBS ENCODE
SuperEnhancer Hnisz
Repressed Hoffman
Promoter UCSC
PromoterFlanking Hoffman
Intron UCSC
H3K9ac Trynka
H3K4me3 Trynka
H3K4me1 Trynka
H3K27ac PGC2
H3K27ac Hnisz
FetalDHS Trynka
Enhancer Hoffman
Enhancer Andersson
DHS Trynka
DGF ENCODE
CTCF Hoffman
Conserved LindbladToh
Coding UCSC
Annotation
−10
0
10
20
Enrichment (prop h2 / prop snps)

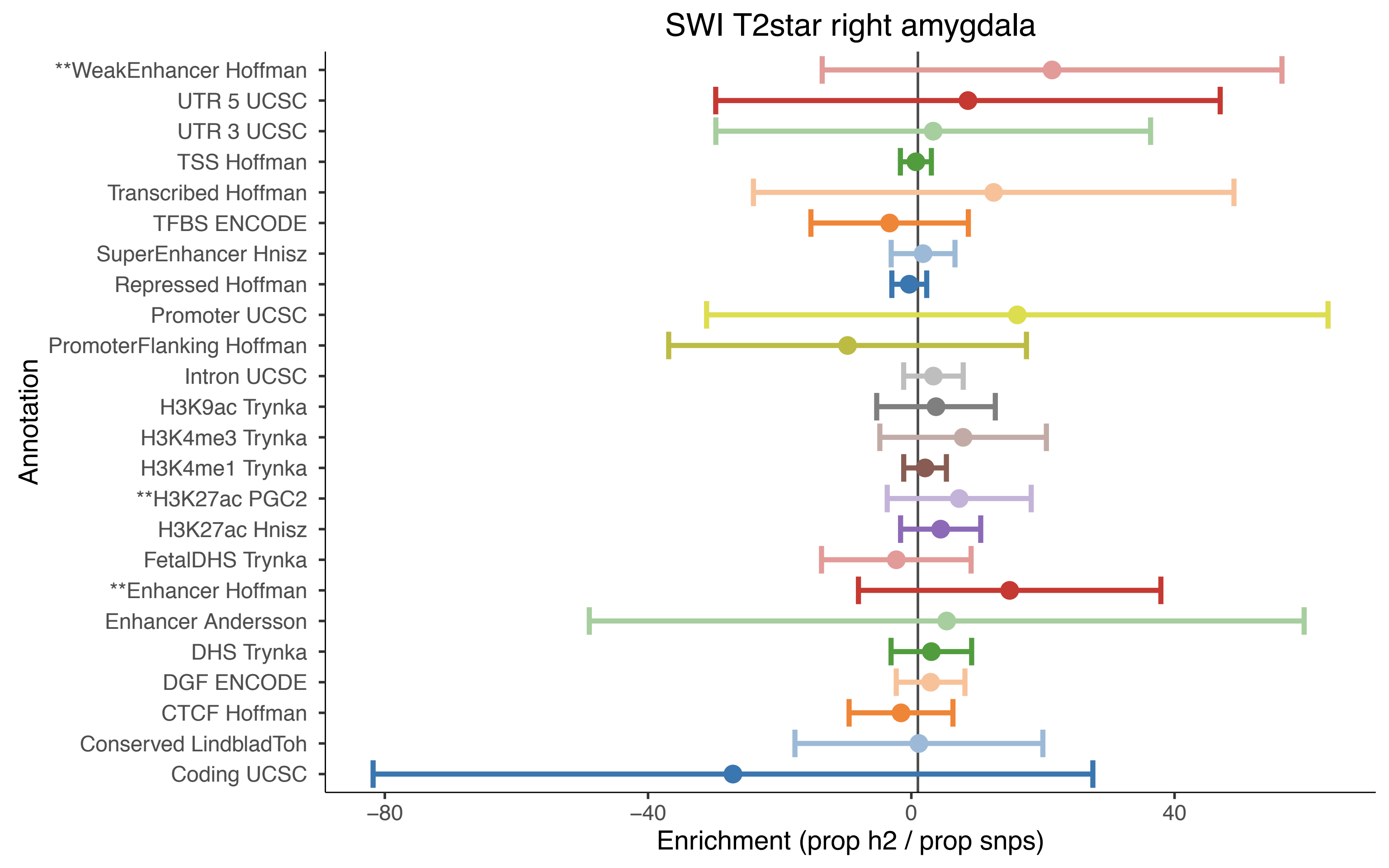
SWI T2star right amygdala
**WeakEnhancer Hoffman
UTR 5 UCSC
UTR 3 UCSC
TSS Hoffman
Transcribed Hoffman
TFBS ENCODE
SuperEnhancer Hnisz
Repressed Hoffman
Promoter UCSC
PromoterFlanking Hoffman
Intron UCSC
H3K9ac Trynka
H3K4me3 Trynka
H3K4me1 Trynka
**H3K27ac PGC2
H3K27ac Hnisz
FetalDHS Trynka
**Enhancer Hoffman
Enhancer Andersson
DHS Trynka
DGF ENCODE
CTCF Hoffman
Conserved LindbladToh
Coding UCSC
Annotation
−80
−40
0
40
Enrichment (prop h2 / prop snps)

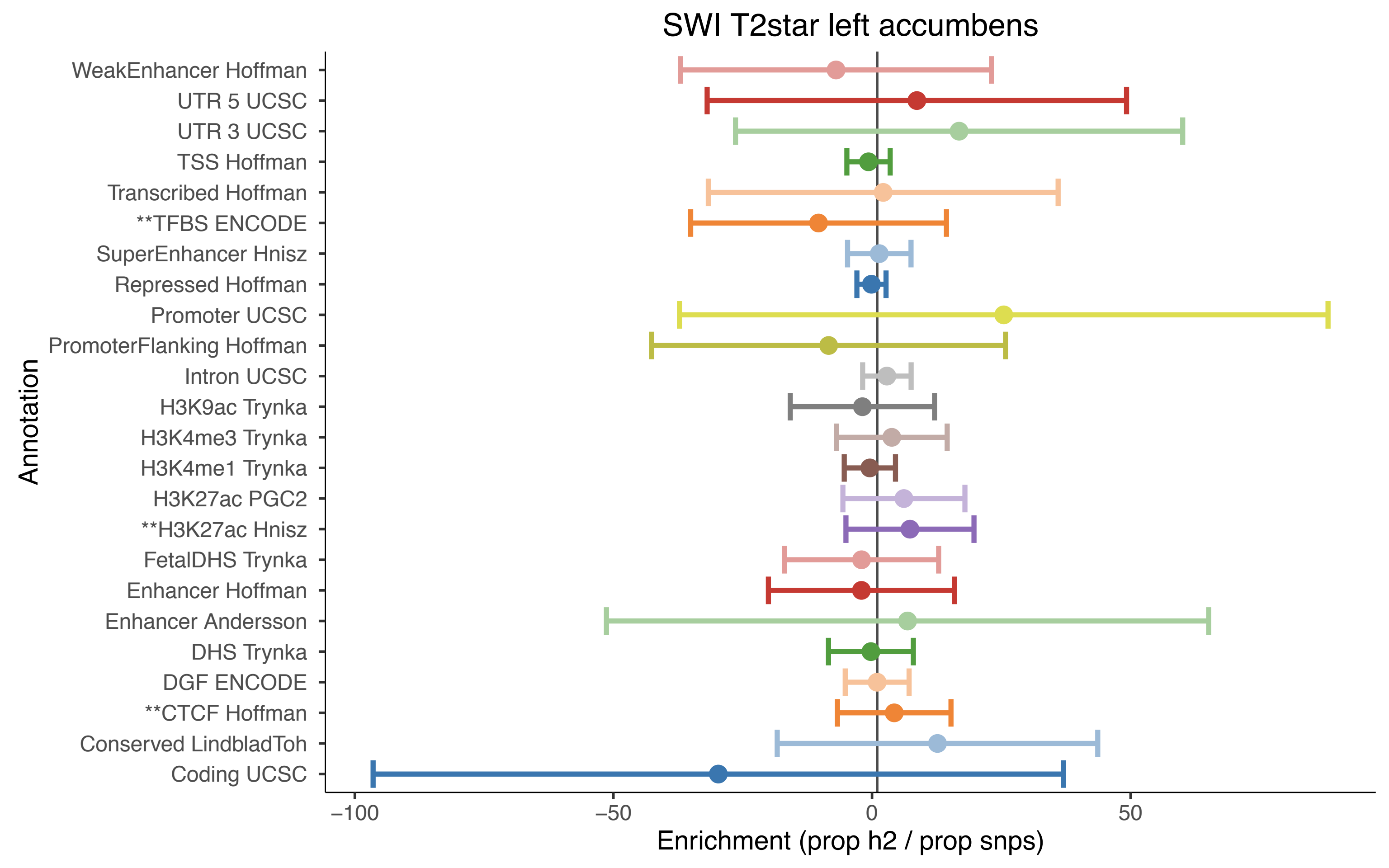

SWI T2star left accumbens
WeakEnhancer Hoffman
UTR 5 UCSC
UTR 3 UCSC
TSS Hoffman
Transcribed Hoffman
**TFBS ENCODE
SuperEnhancer Hnisz
Repressed Hoffman
Promoter UCSC
PromoterFlanking Hoffman
Intron UCSC
H3K9ac Trynka
H3K4me3 Trynka
H3K4me1 Trynka
H3K27ac PGC2
**H3K27ac Hnisz
FetalDHS Trynka
Enhancer Hoffman
Enhancer Andersson
DHS Trynka
DGF ENCODE
**CTCF Hoffman
Conserved LindbladToh
Coding UCSC
Annotation
−100
−50
0
50
Enrichment (prop h2 / prop snps)

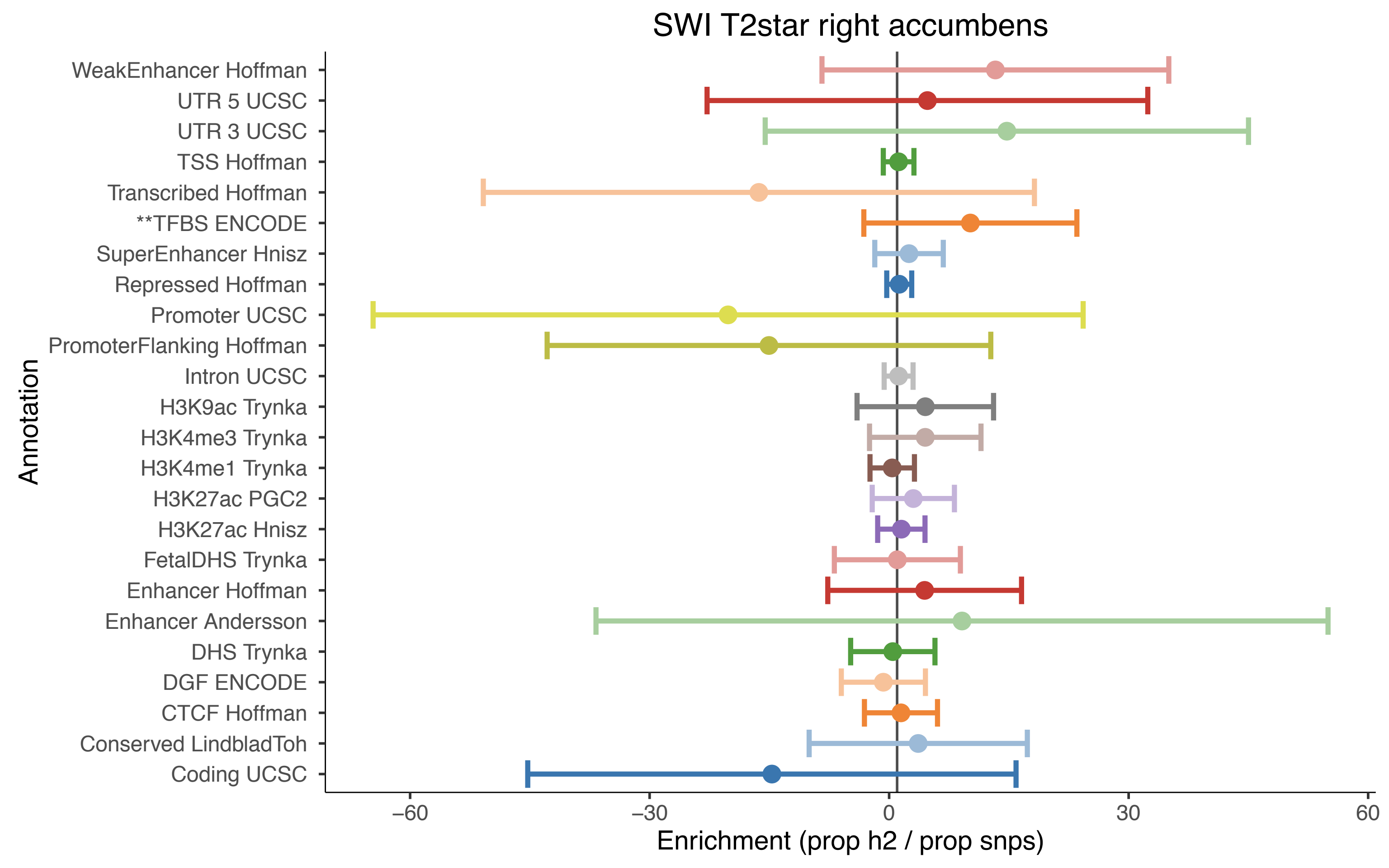
SWI T2star right accumbens
WeakEnhancer Hoffman
UTR 5 UCSC
UTR 3 UCSC
TSS Hoffman
Transcribed Hoffman
**TFBS ENCODE
SuperEnhancer Hnisz
Repressed Hoffman
Promoter UCSC
PromoterFlanking Hoffman
Intron UCSC
H3K9ac Trynka
H3K4me3 Trynka
H3K4me1 Trynka
H3K27ac PGC2
H3K27ac Hnisz
FetalDHS Trynka
Enhancer Hoffman
Enhancer Andersson
DHS Trynka
DGF ENCODE
CTCF Hoffman
Conserved LindbladToh
Coding UCSC
Annotation
−60
−30
0
30
60
Enrichment (prop h2 / prop snps)

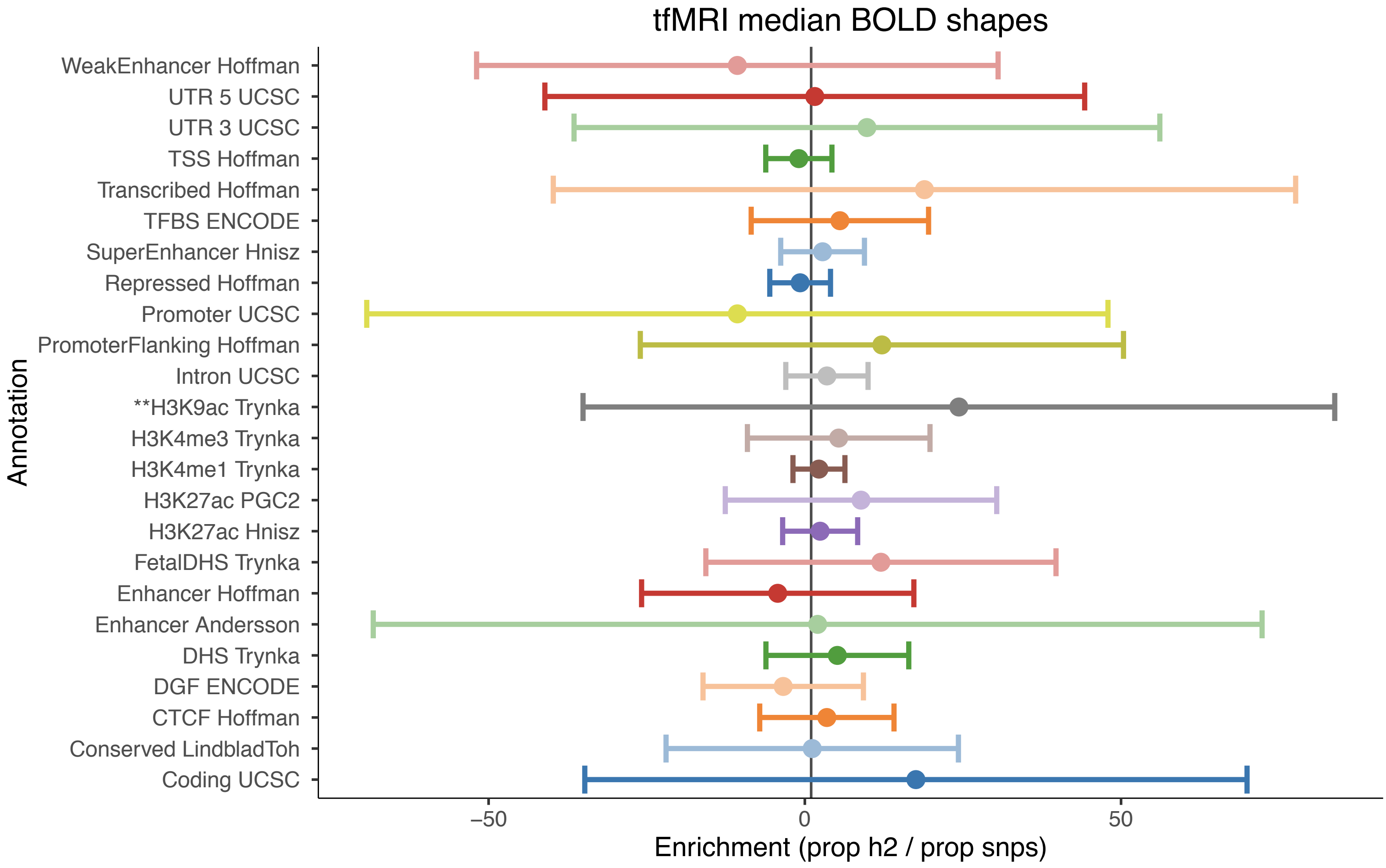
tfMRI median BOLD shapes
Annotation
WeakEnhancer Hoffman
UTR 5 UCSC
UTR 3 UCSC
TSS Hoffman
Transcribed Hoffman
TFBS ENCODE
SuperEnhancer Hnisz
Repressed Hoffman
Promoter UCSC
PromoterFlanking Hoffman
Intron UCSC
**H3K9ac Trynka
H3K4me3 Trynka
H3K4me1 Trynka
H3K27ac PGC2
H3K27ac Hnisz
FetalDHS Trynka
Enhancer Hoffman
Enhancer Andersson
DHS Trynka
DGF ENCODE
CTCF Hoffman
Conserved LindbladToh
Coding UCSC
−50
0
50
Enrichment (prop h2 / prop snps)

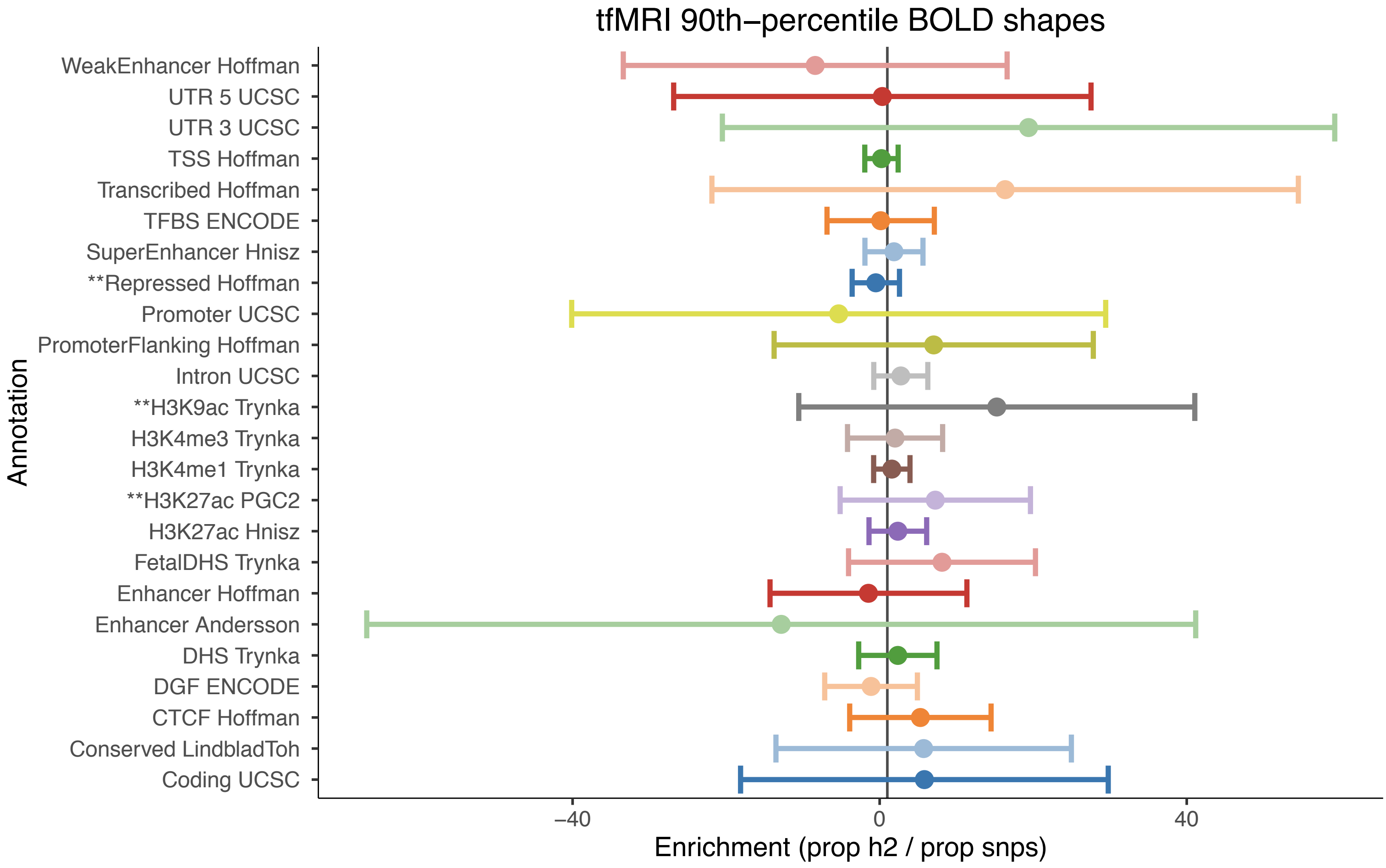
tfMRI 90th−percentile BOLD shapes
WeakEnhancer Hoffman
UTR 5 UCSC
UTR 3 UCSC
TSS Hoffman
Transcribed Hoffman
TFBS ENCODE
SuperEnhancer Hnisz
**Repressed Hoffman
Promoter UCSC
PromoterFlanking Hoffman
Intron UCSC
**H3K9ac Trynka
H3K4me3 Trynka
H3K4me1 Trynka
**H3K27ac PGC2
H3K27ac Hnisz
FetalDHS Trynka
Enhancer Hoffman
Enhancer Andersson
DHS Trynka
DGF ENCODE
CTCF Hoffman
Conserved LindbladToh
Coding UCSC
Annotation
−40
0
40
Enrichment (prop h2 / prop snps)

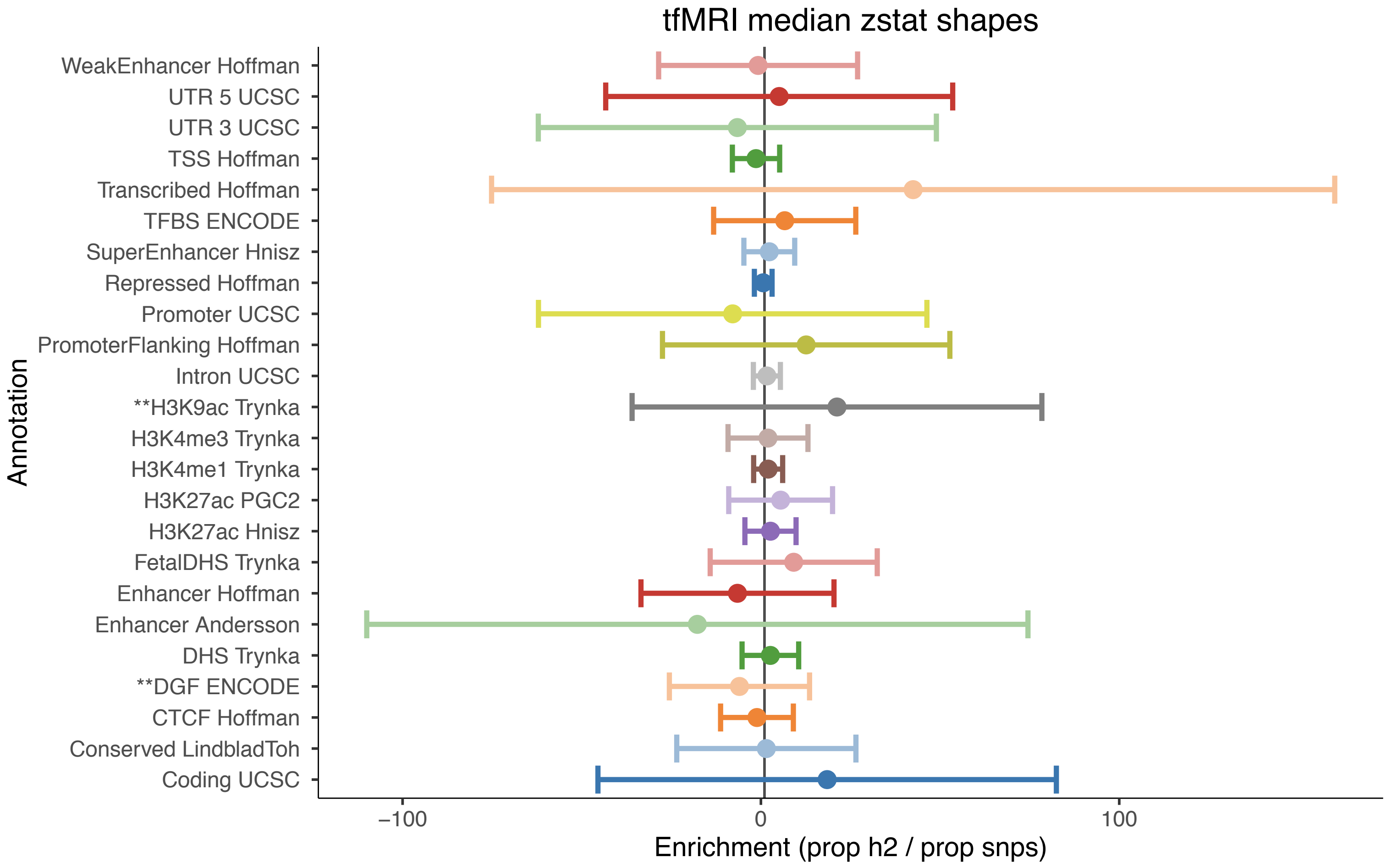
tfMRI median zstat shapes
WeakEnhancer Hoffman
UTR 5 UCSC
UTR 3 UCSC
TSS Hoffman
Transcribed Hoffman
TFBS ENCODE
SuperEnhancer Hnisz
Repressed Hoffman
Promoter UCSC
PromoterFlanking Hoffman
Intron UCSC
**H3K9ac Trynka
H3K4me3 Trynka
H3K4me1 Trynka
H3K27ac PGC2
H3K27ac Hnisz
FetalDHS Trynka
Enhancer Hoffman
Enhancer Andersson
DHS Trynka
**DGF ENCODE
CTCF Hoffman
Conserved LindbladToh
Coding UCSC
Annotation
−100
0
100
Enrichment (prop h2 / prop snps)

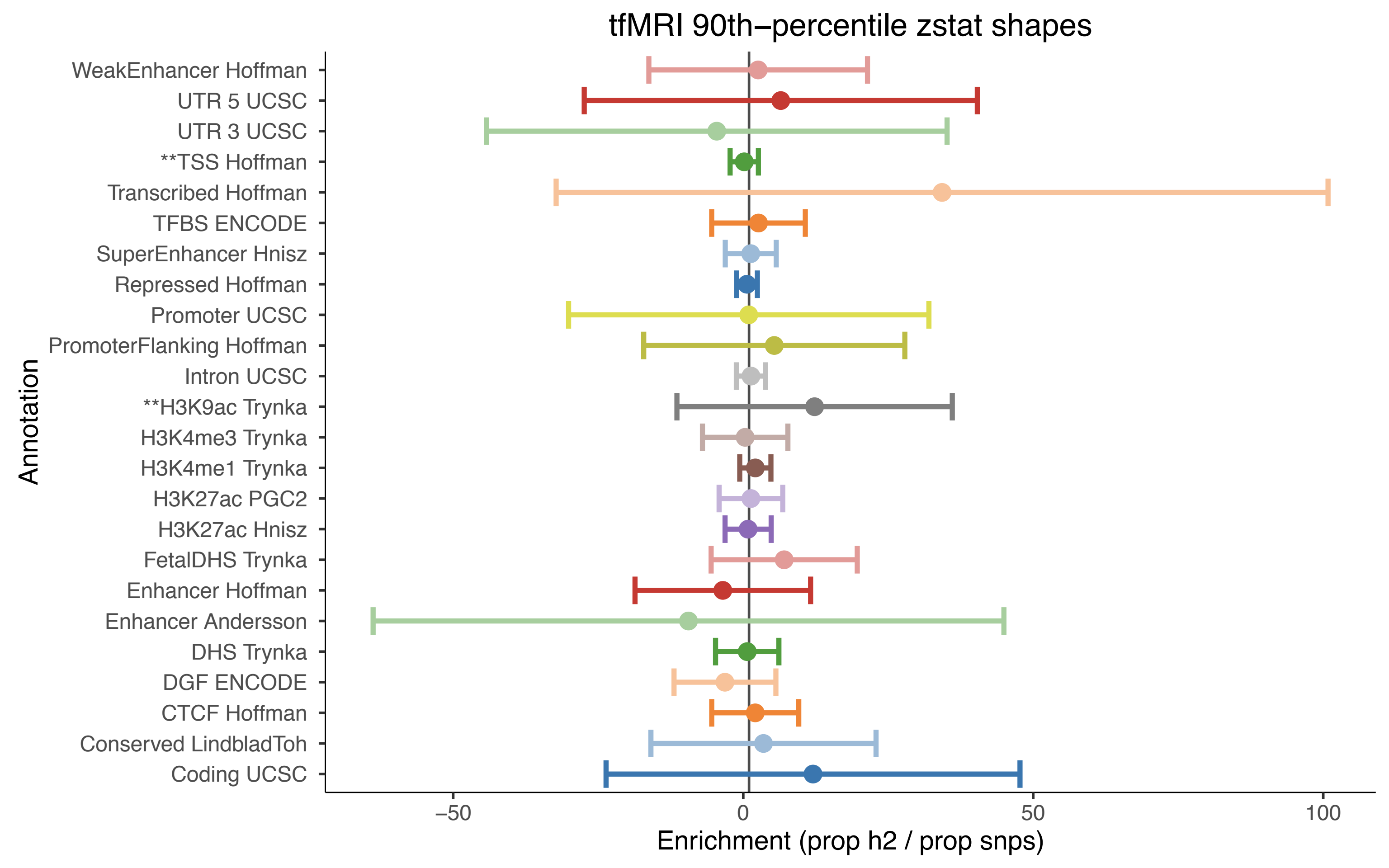
tfMRI 90th−percentile zstat shapes
WeakEnhancer Hoffman
UTR 5 UCSC
UTR 3 UCSC
**TSS Hoffman
Transcribed Hoffman
TFBS ENCODE
SuperEnhancer Hnisz
Repressed Hoffman
Promoter UCSC
PromoterFlanking Hoffman
Intron UCSC
**H3K9ac Trynka
H3K4me3 Trynka
H3K4me1 Trynka
H3K27ac PGC2
H3K27ac Hnisz
FetalDHS Trynka
Enhancer Hoffman
Enhancer Andersson
DHS Trynka
DGF ENCODE
CTCF Hoffman
Conserved LindbladToh
Coding UCSC
Annotation
−50
0
50
100
Enrichment (prop h2 / prop snps)

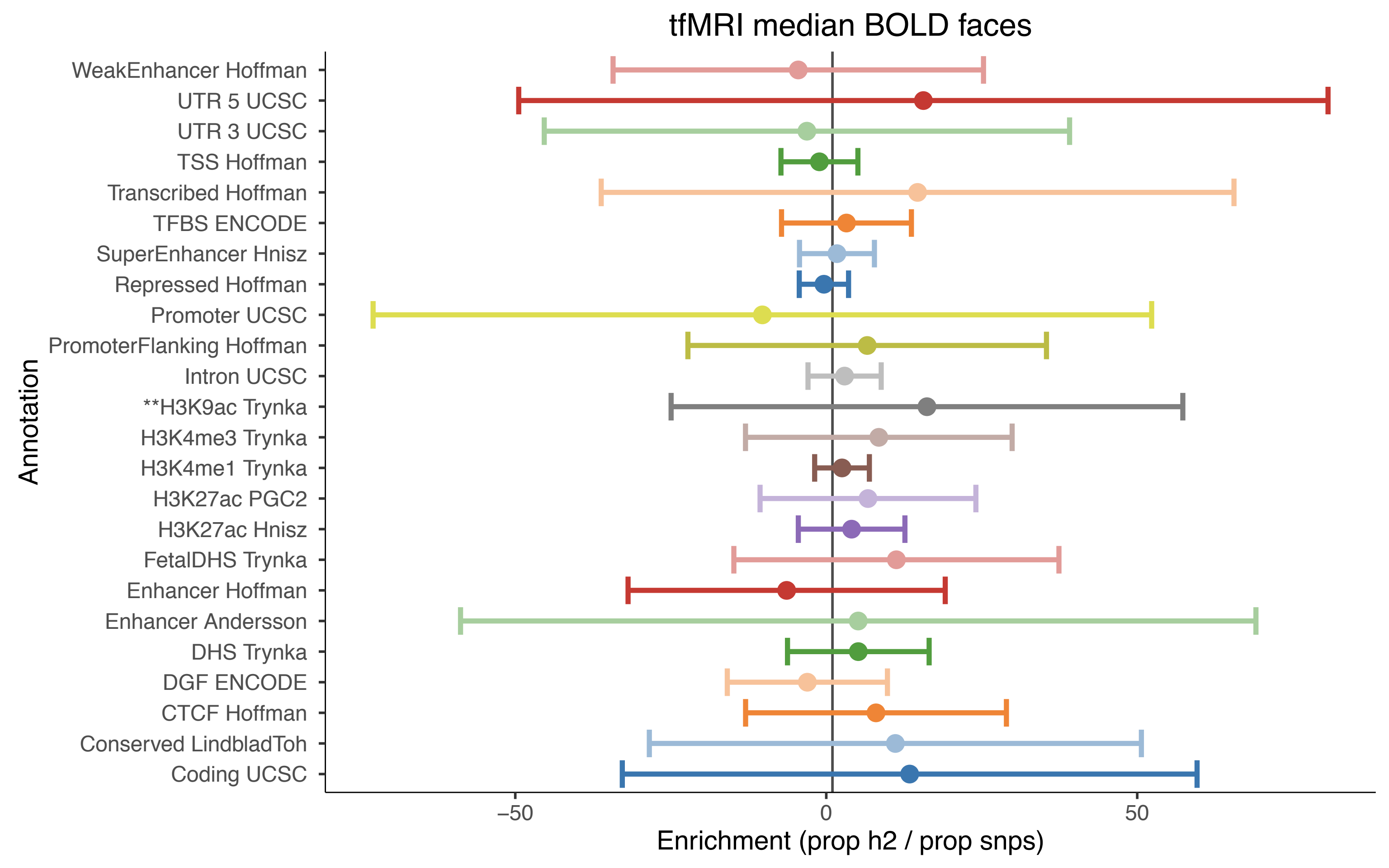
tfMRI median BOLD faces
WeakEnhancer Hoffman
UTR 5 UCSC
UTR 3 UCSC
TSS Hoffman
Transcribed Hoffman
TFBS ENCODE
SuperEnhancer Hnisz
Repressed Hoffman
Promoter UCSC
PromoterFlanking Hoffman
Intron UCSC
**H3K9ac Trynka
H3K4me3 Trynka
H3K4me1 Trynka
H3K27ac PGC2
H3K27ac Hnisz
FetalDHS Trynka
Enhancer Hoffman
Enhancer Andersson
DHS Trynka
DGF ENCODE
CTCF Hoffman
Conserved LindbladToh
Coding UCSC
Annotation
−50
0
50
Enrichment (prop h2 / prop snps)

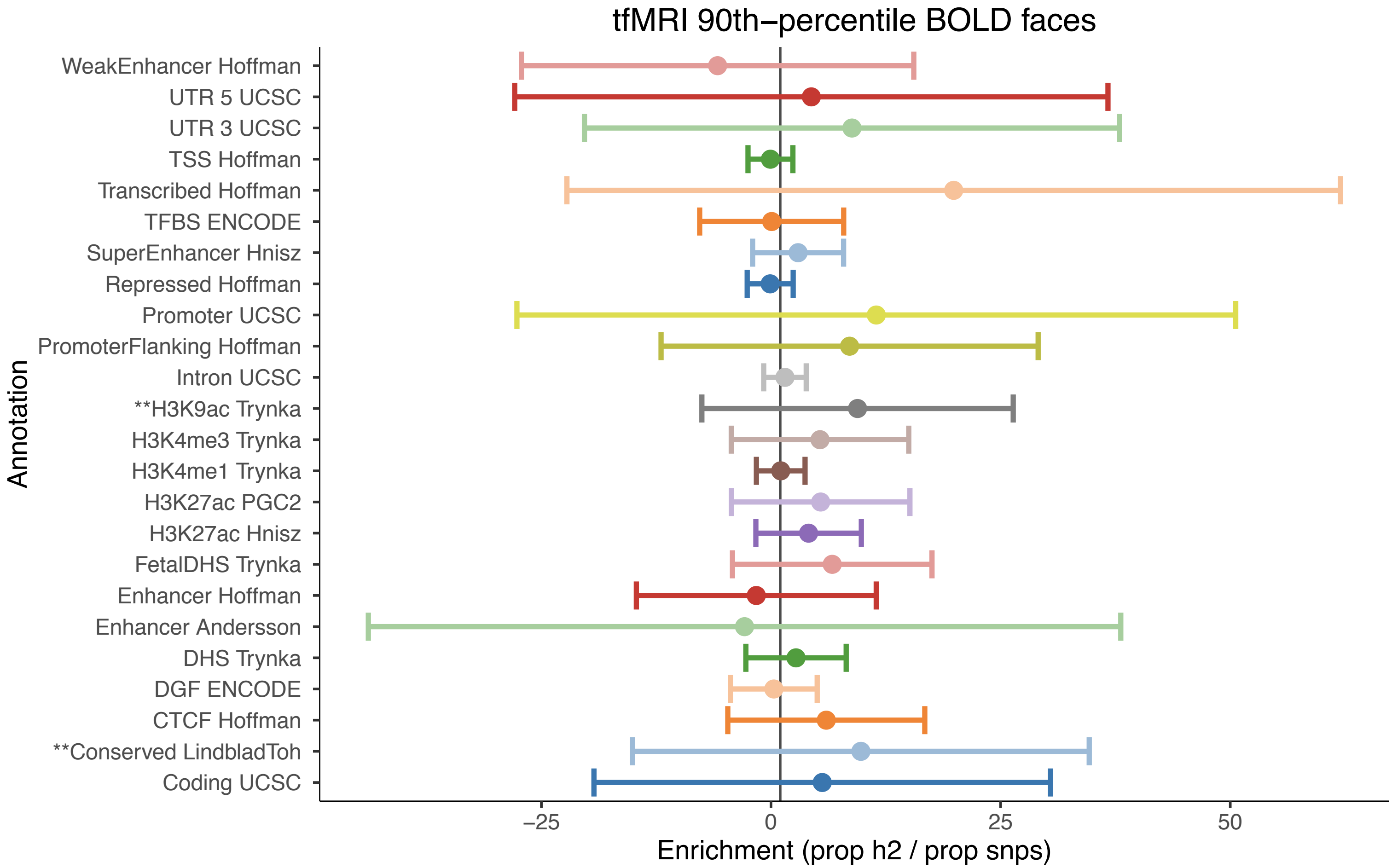
tfMRI 90th−percentile BOLD faces
WeakEnhancer Hoffman
UTR 5 UCSC
UTR 3 UCSC
TSS Hoffman
Transcribed Hoffman
TFBS ENCODE
SuperEnhancer Hnisz
Repressed Hoffman
Promoter UCSC
PromoterFlanking Hoffman
Intron UCSC
**H3K9ac Trynka
H3K4me3 Trynka
H3K4me1 Trynka
H3K27ac PGC2
H3K27ac Hnisz
FetalDHS Trynka
Enhancer Hoffman
Enhancer Andersson
DHS Trynka
DGF ENCODE
CTCF Hoffman
**Conserved LindbladToh
Coding UCSC
Annotation
−25
0
25
50
Enrichment (prop h2 / prop snps)

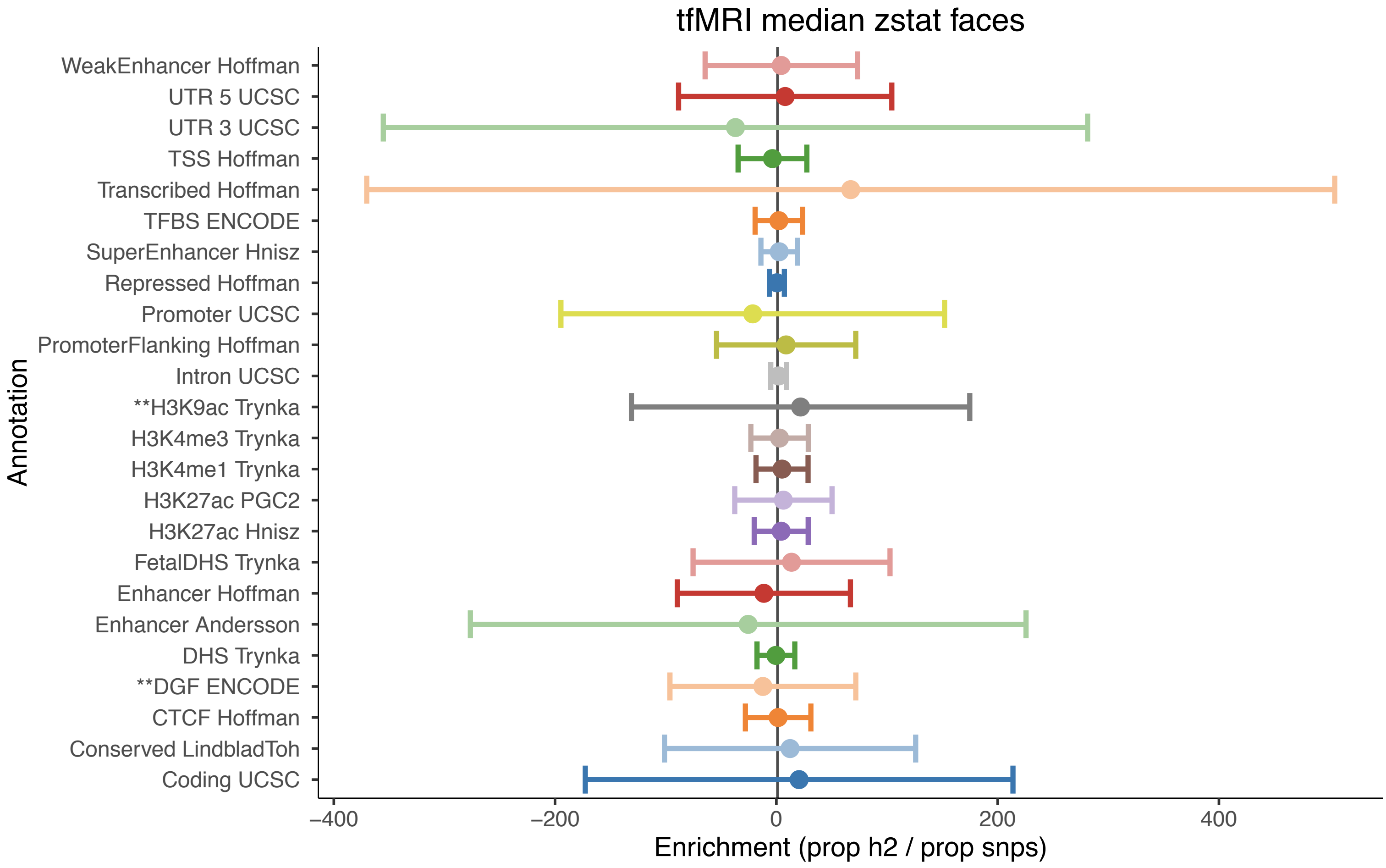

tfMRI median zstat faces
WeakEnhancer Hoffman
UTR 5 UCSC
UTR 3 UCSC
TSS Hoffman
Transcribed Hoffman
TFBS ENCODE
SuperEnhancer Hnisz
Repressed Hoffman
Promoter UCSC
PromoterFlanking Hoffman
Intron UCSC
**H3K9ac Trynka
H3K4me3 Trynka
H3K4me1 Trynka
H3K27ac PGC2
H3K27ac Hnisz
FetalDHS Trynka
Enhancer Hoffman
Enhancer Andersson
DHS Trynka
**DGF ENCODE
CTCF Hoffman
Conserved LindbladToh
Coding UCSC
Annotation
−400
−200
0
200
400
Enrichment (prop h2 / prop snps)

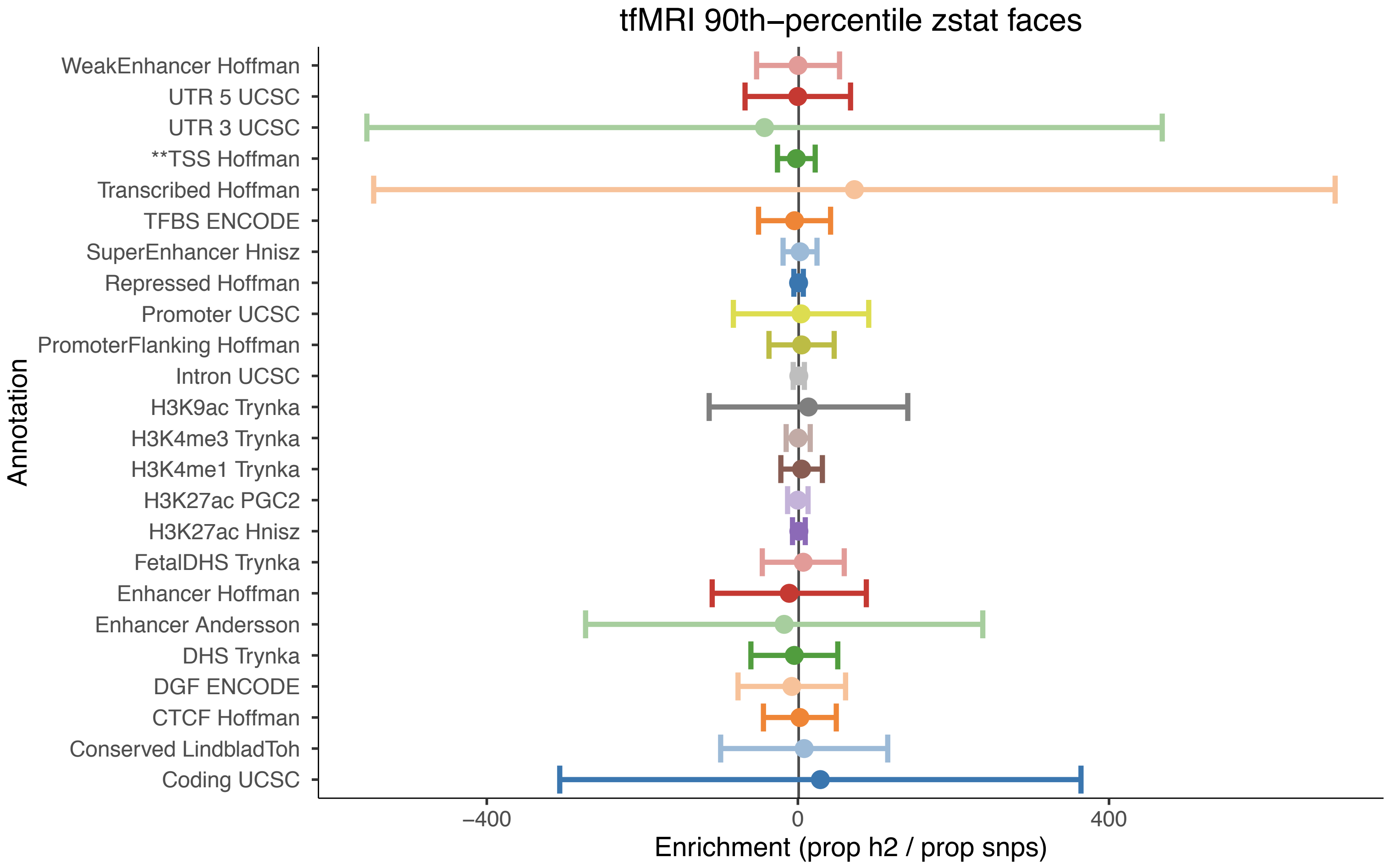

tfMRI 90th−percentile zstat faces
WeakEnhancer Hoffman
UTR 5 UCSC
UTR 3 UCSC
**TSS Hoffman
Transcribed Hoffman
TFBS ENCODE
SuperEnhancer Hnisz
Repressed Hoffman
Promoter UCSC
PromoterFlanking Hoffman
Intron UCSC
H3K9ac Trynka
H3K4me3 Trynka
H3K4me1 Trynka
H3K27ac PGC2
H3K27ac Hnisz
FetalDHS Trynka
Enhancer Hoffman
Enhancer Andersson
DHS Trynka
DGF ENCODE
CTCF Hoffman
Conserved LindbladToh
Coding UCSC
Annotation
−400
0
400
Enrichment (prop h2 / prop snps)

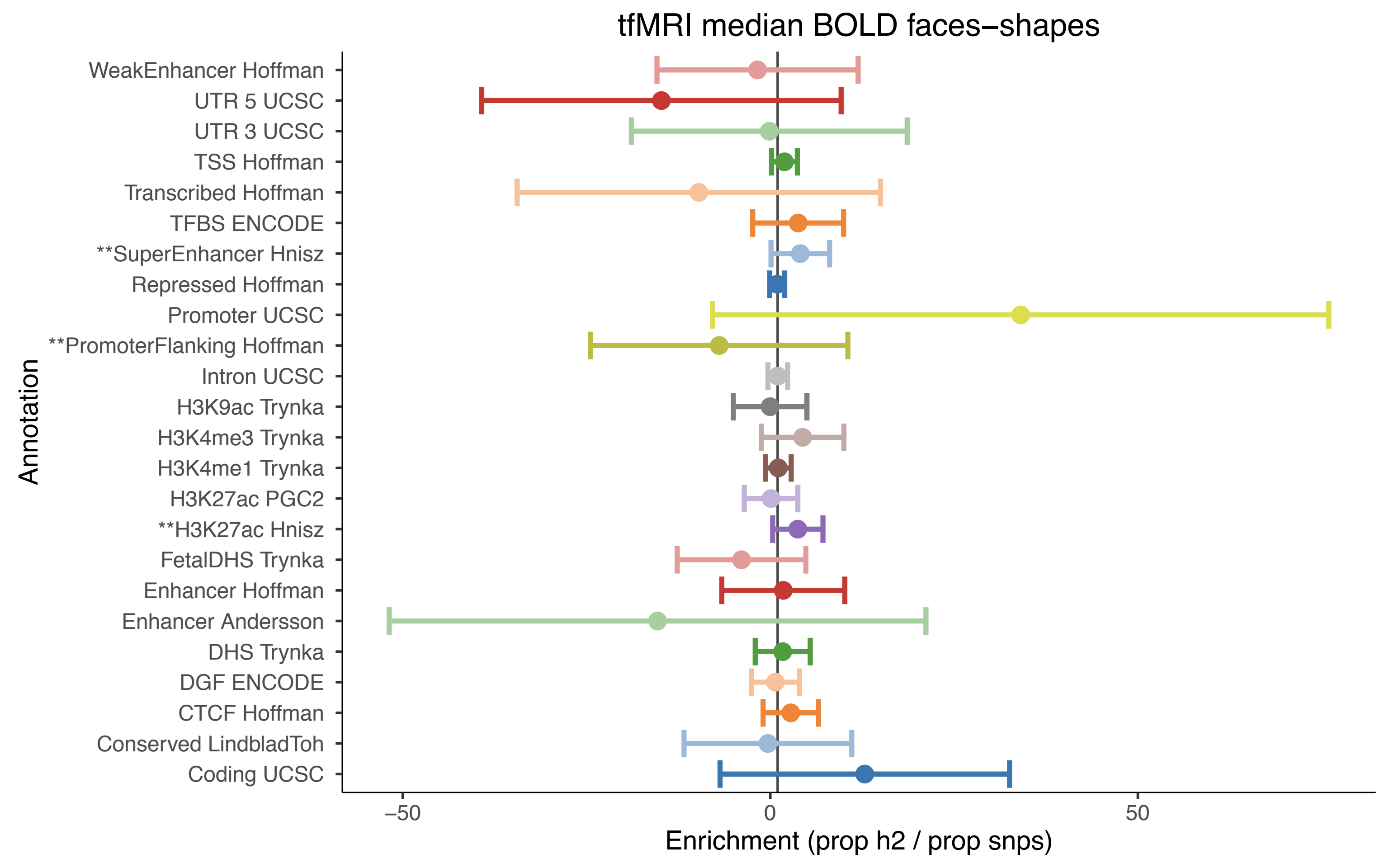

tfMRI median BOLD faces−shapes
WeakEnhancer Hoffman
UTR 5 UCSC
UTR 3 UCSC
TSS Hoffman
Transcribed Hoffman
TFBS ENCODE
**SuperEnhancer Hnisz
Repressed Hoffman
Promoter UCSC
**PromoterFlanking Hoffman
Intron UCSC
H3K9ac Trynka
H3K4me3 Trynka
H3K4me1 Trynka
H3K27ac PGC2
**H3K27ac Hnisz
FetalDHS Trynka
Enhancer Hoffman
Enhancer Andersson
DHS Trynka
DGF ENCODE
CTCF Hoffman
Conserved LindbladToh
Coding UCSC
Annotation
−50
0
50
Enrichment (prop h2 / prop snps)

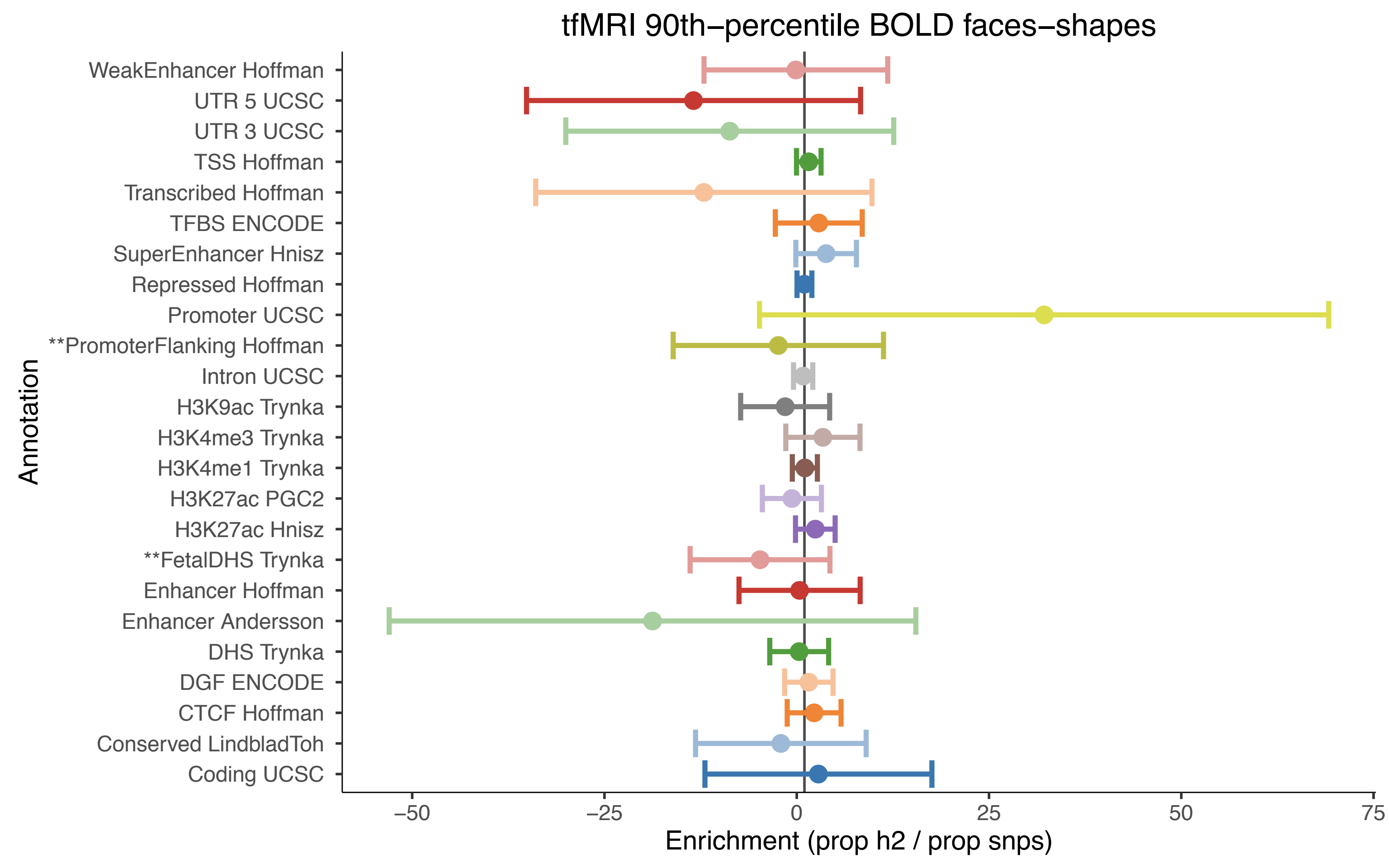
tfMRI 90th−percentile BOLD faces−shapes
WeakEnhancer Hoffman
UTR 5 UCSC
UTR 3 UCSC
TSS Hoffman
Transcribed Hoffman
TFBS ENCODE
SuperEnhancer Hnisz
Repressed Hoffman
Promoter UCSC
**PromoterFlanking Hoffman
Intron UCSC
H3K9ac Trynka
H3K4me3 Trynka
H3K4me1 Trynka
H3K27ac PGC2
H3K27ac Hnisz
**FetalDHS Trynka
Enhancer Hoffman
Enhancer Andersson
DHS Trynka
DGF ENCODE
CTCF Hoffman
Conserved LindbladToh
Coding UCSC
Annotation
−50
−25
0
25
50
75
Enrichment (prop h2 / prop snps)

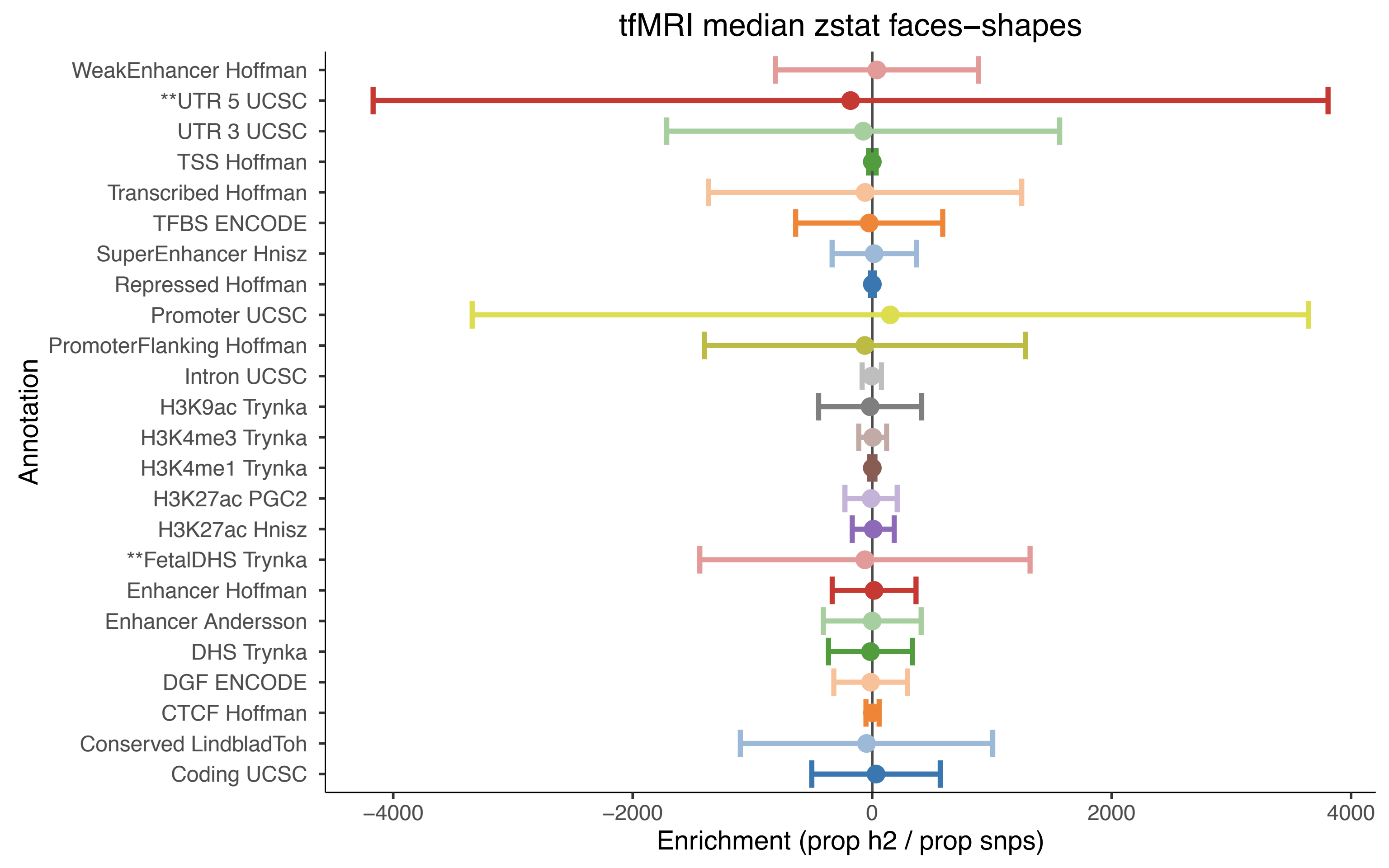

tfMRI median zstat faces–shapes
WeakEnhancer Hoffman
**UTR 5 UCSC
UTR 3 UCSC
TSS Hoffman
Transcribed Hoffman
TFBS ENCODE
SuperEnhancer Hnisz
Repressed Hoffman
Promoter UCSC
PromoterFlanking Hoffman
Intron UCSC
H3K9ac Trynka
H3K4me3 Trynka
H3K4me1 Trynka
H3K27ac PGC2
H3K27ac Hnisz
**FetalDHS Trynka
Enhancer Hoffman
Enhancer Andersson
DHS Trynka
DGF ENCODE
CTCF Hoffman
Conserved LindbladToh
Coding UCSC
Annotation
−4000
−2000
0
2000
4000
Enrichment (prop h2 / prop snps)

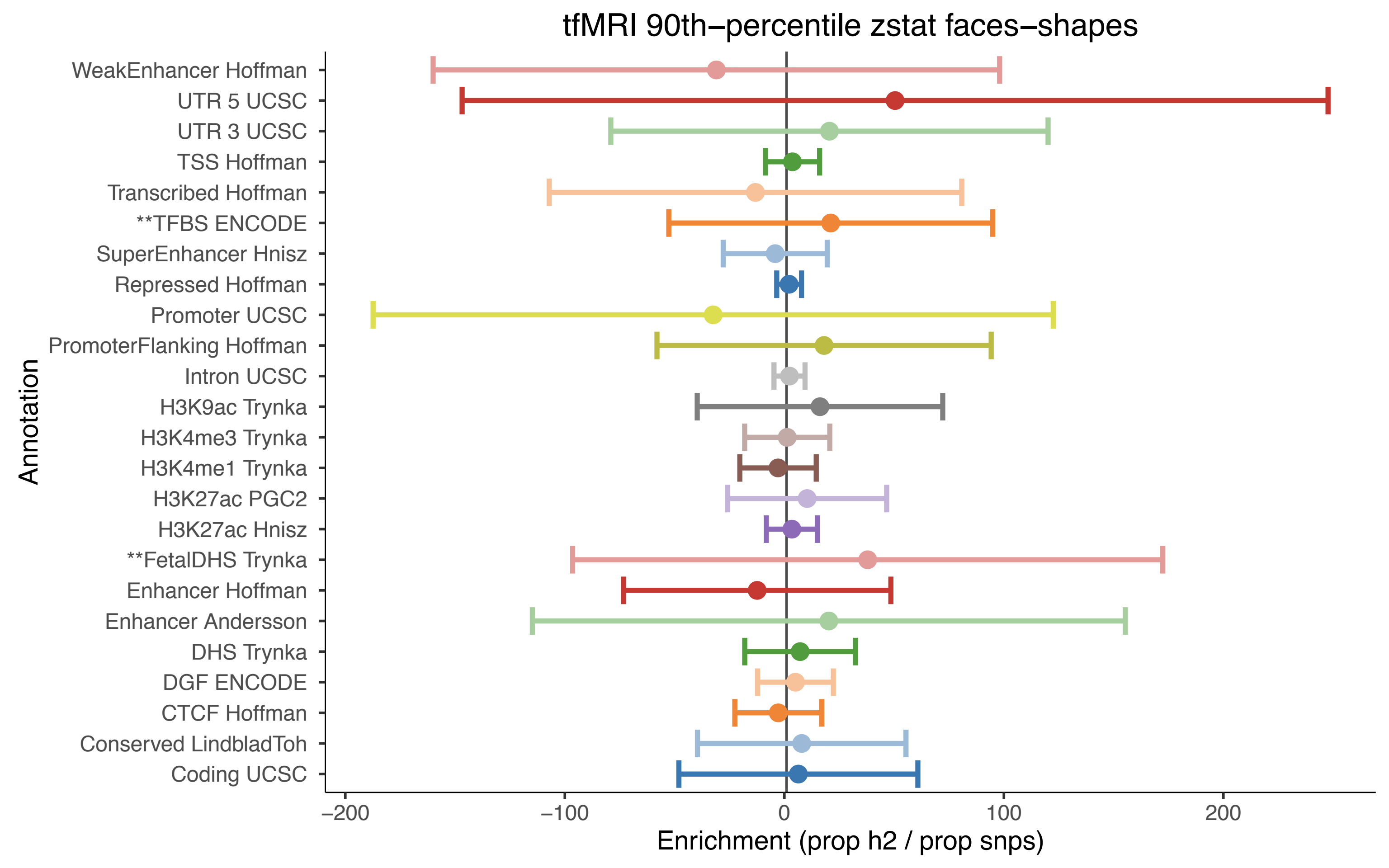

tfMRI 90th−percentile zstat faces−shapes
WeakEnhancer Hoffman
UTR 5 UCSC
UTR 3 UCSC
TSS Hoffman
Transcribed Hoffman
**TFBS ENCODE
SuperEnhancer Hnisz
Repressed Hoffman
Promoter UCSC
PromoterFlanking Hoffman
Intron UCSC
H3K9ac Trynka
H3K4me3 Trynka
H3K4me1 Trynka
H3K27ac PGC2
H3K27ac Hnisz
**FetalDHS Trynka
Enhancer Hoffman
Enhancer Andersson
DHS Trynka
DGF ENCODE
CTCF Hoffman
Conserved LindbladToh
Coding UCSC
Annotation
−200
−100
0
100
200
Enrichment (prop h2 / prop snps)

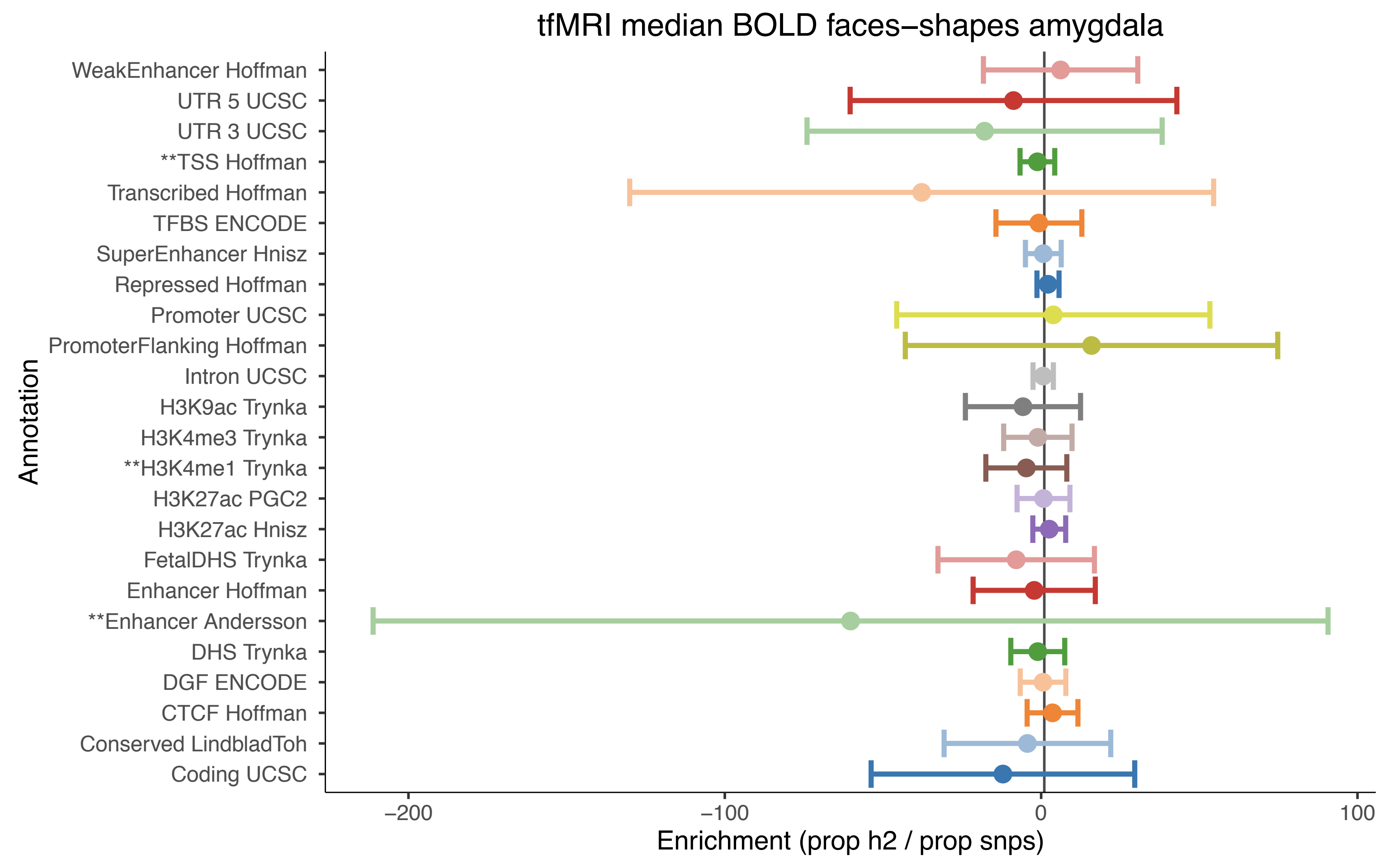

tfMRI median BOLD faces−shapes amygdala
WeakEnhancer Hoffman
UTR 5 UCSC
UTR 3 UCSC
**TSS Hoffman
Transcribed Hoffman
TFBS ENCODE
SuperEnhancer Hnisz
Repressed Hoffman
Promoter UCSC
PromoterFlanking Hoffman
Intron UCSC
H3K9ac Trynka
H3K4me3 Trynka
**H3K4me1 Trynka
H3K27ac PGC2
H3K27ac Hnisz
FetalDHS Trynka
Enhancer Hoffman
**Enhancer Andersson
DHS Trynka
DGF ENCODE
CTCF Hoffman
Conserved LindbladToh
Coding UCSC
Annotation
−200
−100
0
100
Enrichment (prop h2 / prop snps)

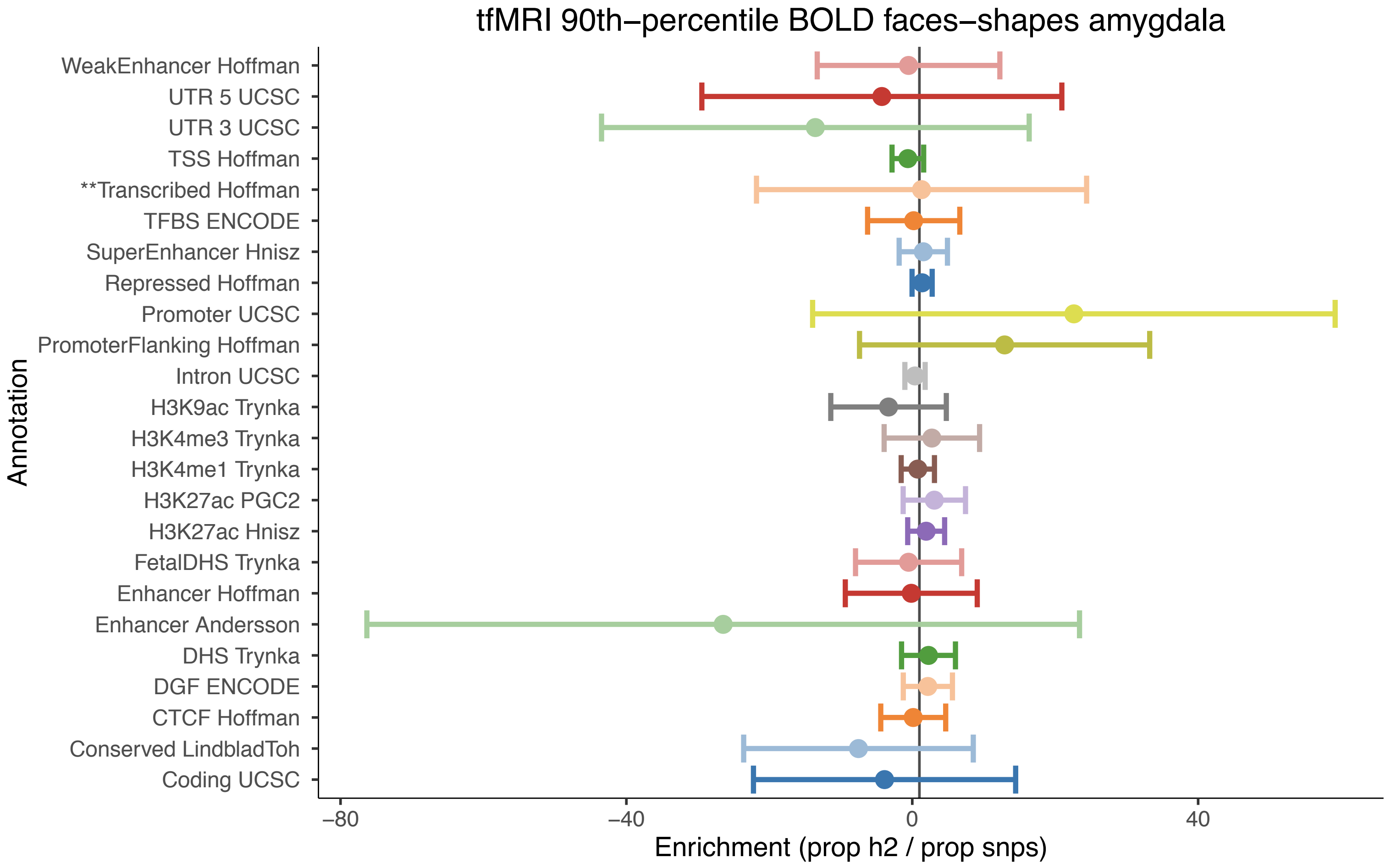

tfMRI 90th−percentile BOLD faces−shapes amygdala
WeakEnhancer Hoffman
UTR 5 UCSC
UTR 3 UCSC
TSS Hoffman
**Transcribed Hoffman
TFBS ENCODE
SuperEnhancer Hnisz
Repressed Hoffman
Promoter UCSC
PromoterFlanking Hoffman
Intron UCSC
H3K9ac Trynka
H3K4me3 Trynka
H3K4me1 Trynka
H3K27ac PGC2
H3K27ac Hnisz
FetalDHS Trynka
Enhancer Hoffman
Enhancer Andersson
DHS Trynka
DGF ENCODE
CTCF Hoffman
Conserved LindbladToh
Coding UCSC
Annotation
−80
−40
0
40
Enrichment (prop h2 / prop snps)

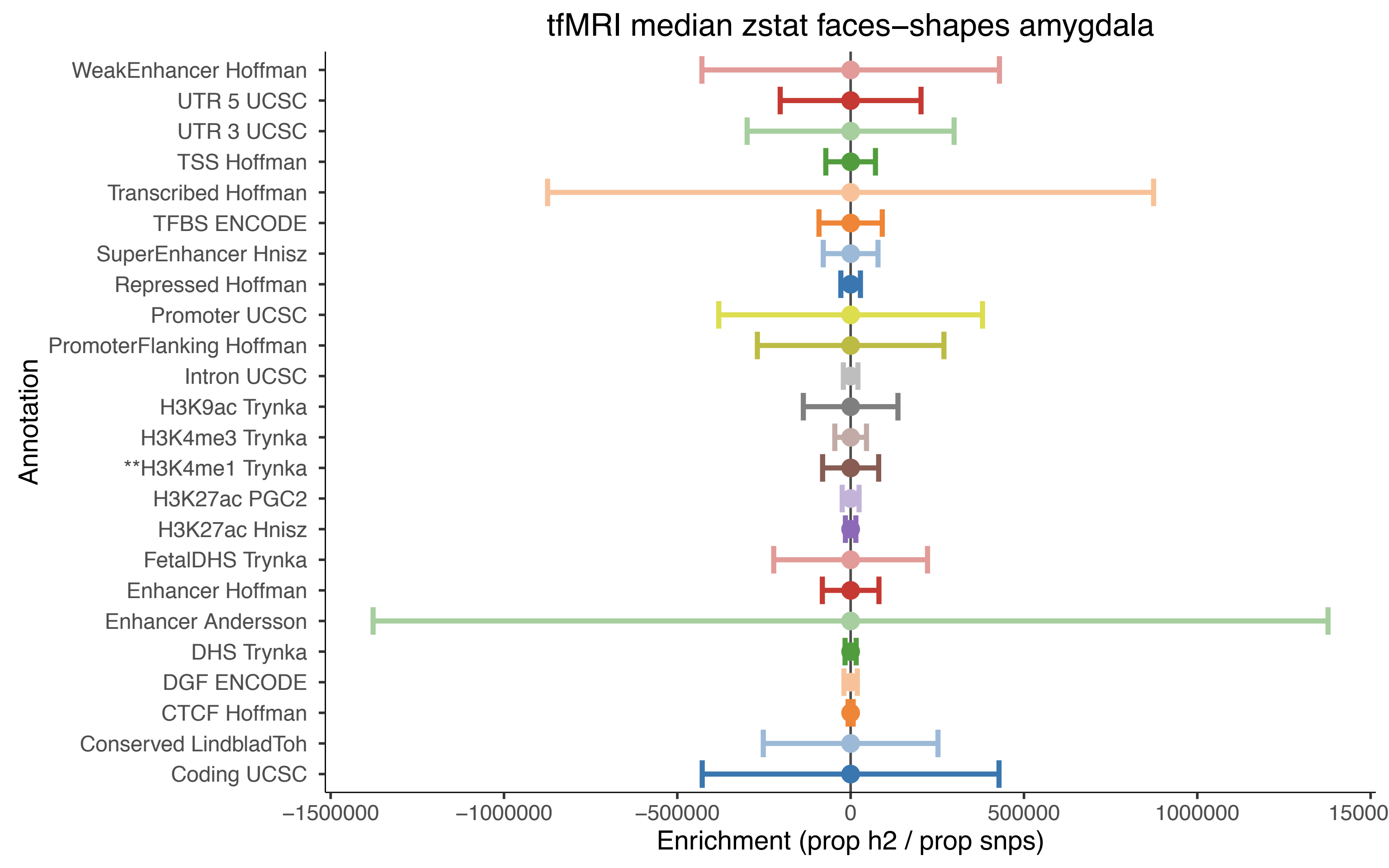

tfMRI median zstat faces−shapes amygdala
WeakEnhancer Hoffman
UTR 5 UCSC
UTR 3 UCSC
TSS Hoffman
Transcribed Hoffman
TFBS ENCODE
SuperEnhancer Hnisz
Repressed Hoffman
Promoter UCSC
PromoterFlanking Hoffman
Intron UCSC
H3K9ac Trynka
H3K4me3 Trynka
**H3K4me1 Trynka
H3K27ac PGC2
H3K27ac Hnisz
FetalDHS Trynka
Enhancer Hoffman
Enhancer Andersson
DHS Trynka
DGF ENCODE
CTCF Hoffman
Conserved LindbladToh
Coding UCSC
Annotation
−1500000
−1000000
−500000
0
500000
1000000
15000
Enrichment (prop h2 / prop snps)

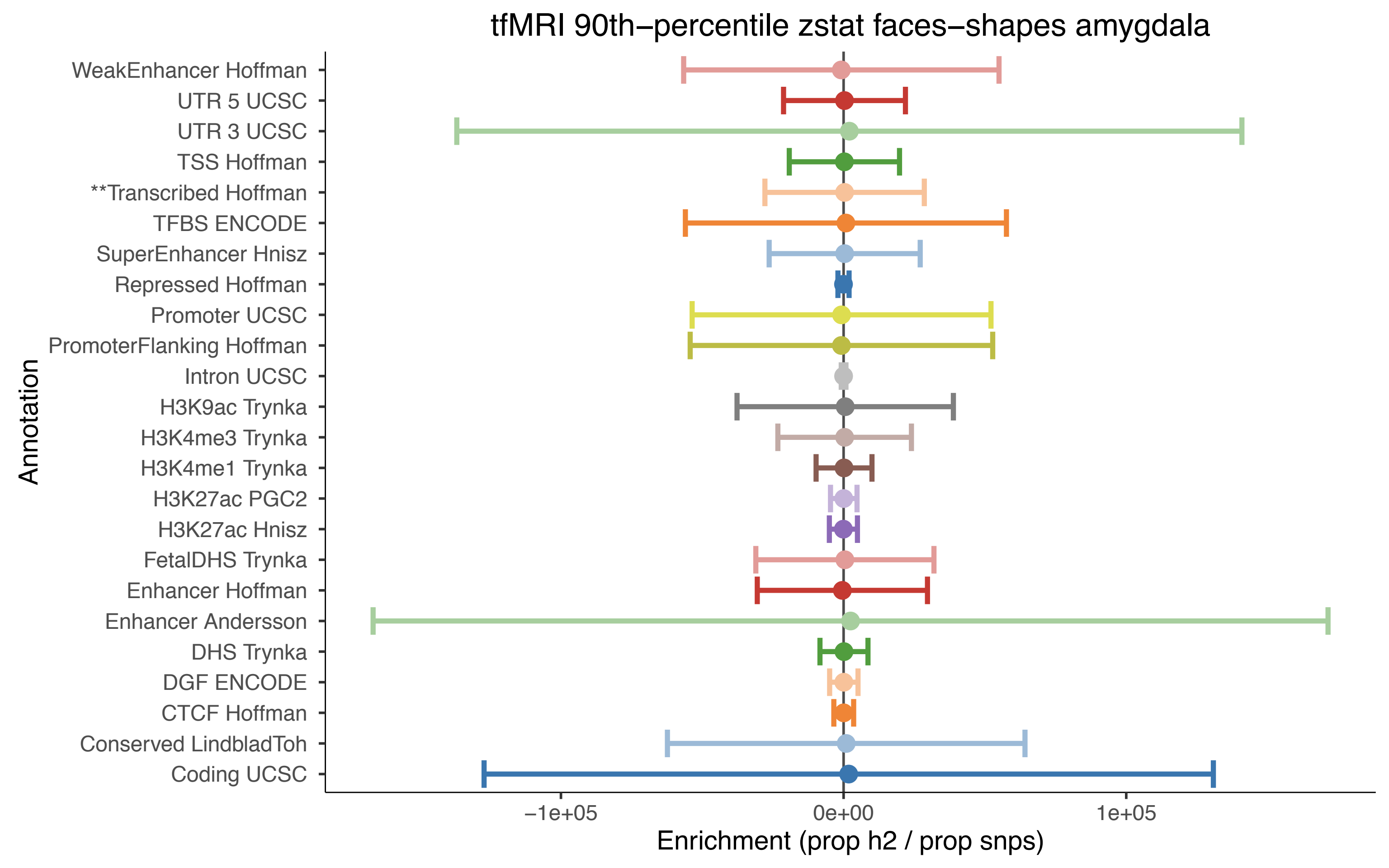

tfMRI 90th−percentile zstat faces−shapes amygdala
WeakEnhancer Hoffman
UTR 5 UCSC
UTR 3 UCSC
TSS Hoffman
**Transcribed Hoffman
TFBS ENCODE
SuperEnhancer Hnisz
Repressed Hoffman
Promoter UCSC
PromoterFlanking Hoffman
Intron UCSC
H3K9ac Trynka
H3K4me3 Trynka
H3K4me1 Trynka
H3K27ac PGC2
H3K27ac Hnisz
FetalDHS Trynka
Enhancer Hoffman
Enhancer Andersson
DHS Trynka
DGF ENCODE
CTCF Hoffman
Conserved LindbladToh
Coding UCSC
Annotation
−1e+05
0e+00
1e+05
Enrichment (prop h2 / prop snps)

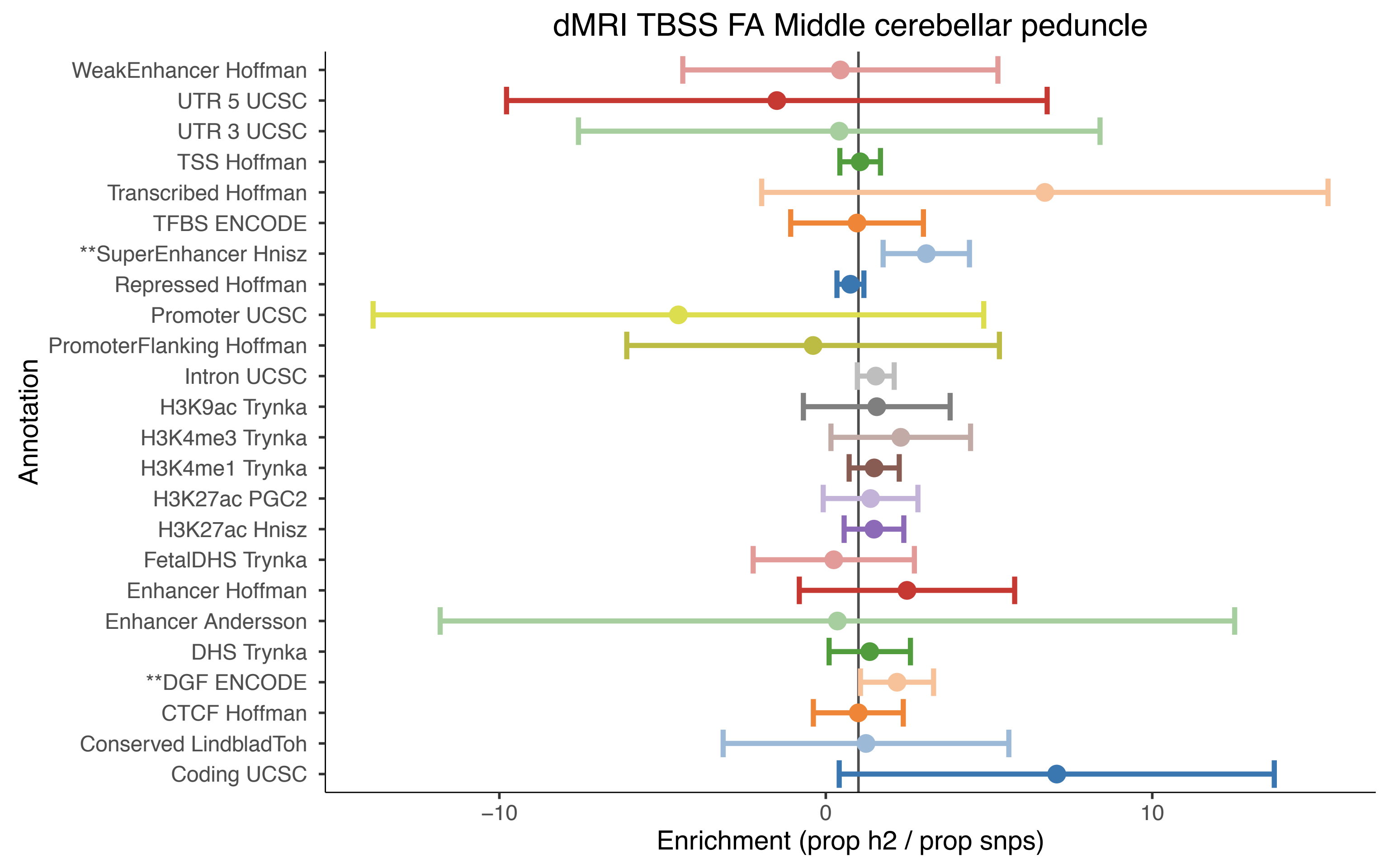

dMRI TBSS FA Middle cerebellar peduncle
WeakEnhancer Hoffman
UTR 5 UCSC
UTR 3 UCSC
TSS Hoffman
Transcribed Hoffman
TFBS ENCODE
**SuperEnhancer Hnisz
Repressed Hoffman
Promoter UCSC
PromoterFlanking Hoffman
Intron UCSC
H3K9ac Trynka
H3K4me3 Trynka
H3K4me1 Trynka
H3K27ac PGC2
H3K27ac Hnisz
FetalDHS Trynka
Enhancer Hoffman
Enhancer Andersson
DHS Trynka
**DGF ENCODE
CTCF Hoffman
Conserved LindbladToh
Coding UCSC
Annotation
−10
0
10
Enrichment (prop h2 / prop snps)

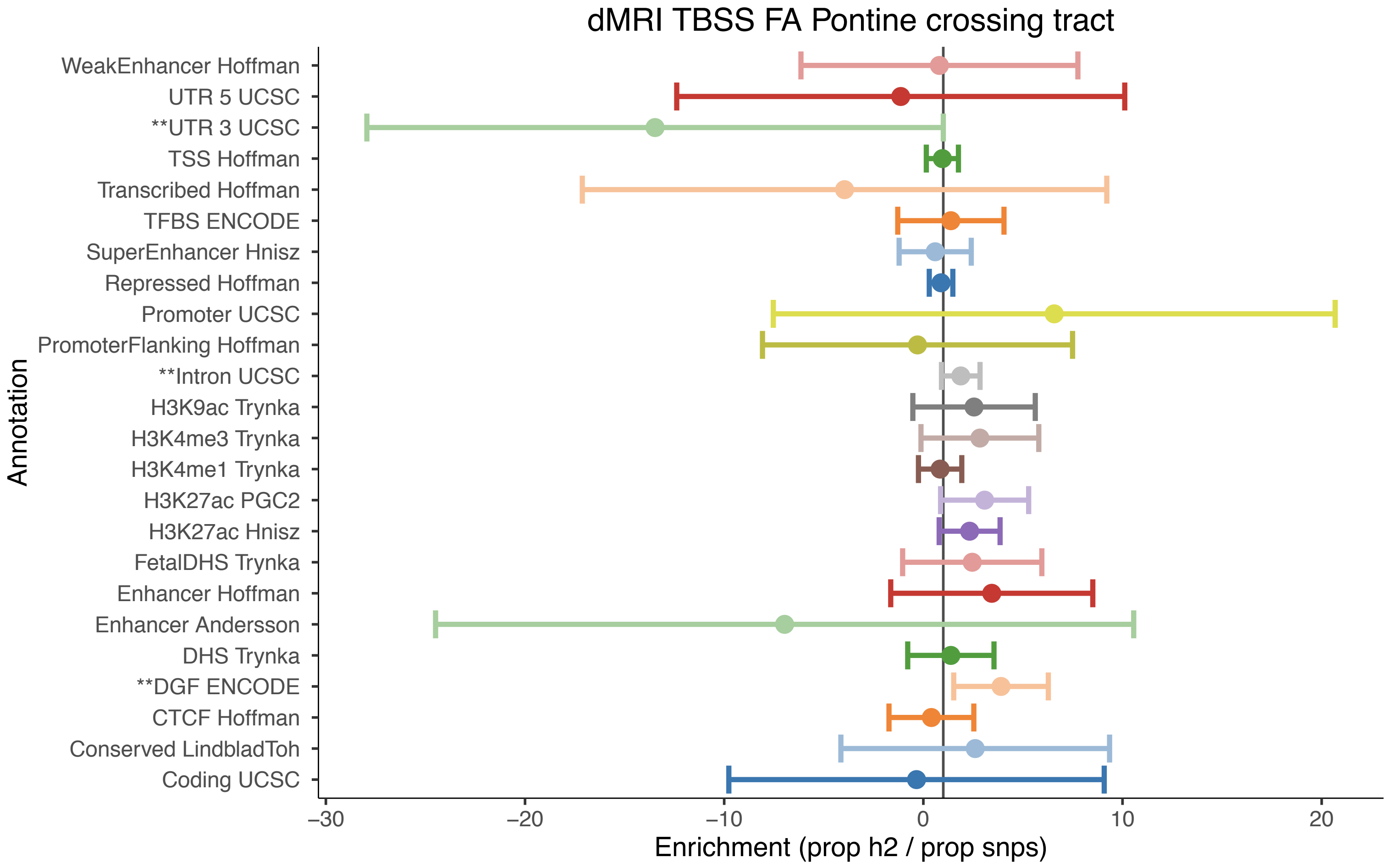

dMRI TBSS FA Pontine crossing tract
WeakEnhancer Hoffman
UTR 5 UCSC
**UTR 3 UCSC
TSS Hoffman
Transcribed Hoffman
TFBS ENCODE
SuperEnhancer Hnisz
Repressed Hoffman
Promoter UCSC
PromoterFlanking Hoffman
**Intron UCSC
H3K9ac Trynka
H3K4me3 Trynka
H3K4me1 Trynka
H3K27ac PGC2
H3K27ac Hnisz
FetalDHS Trynka
Enhancer Hoffman
Enhancer Andersson
DHS Trynka
**DGF ENCODE
CTCF Hoffman
Conserved LindbladToh
Coding UCSC
Annotation
−30
−20
−10
0
10
20
Enrichment (prop h2 / prop snps)

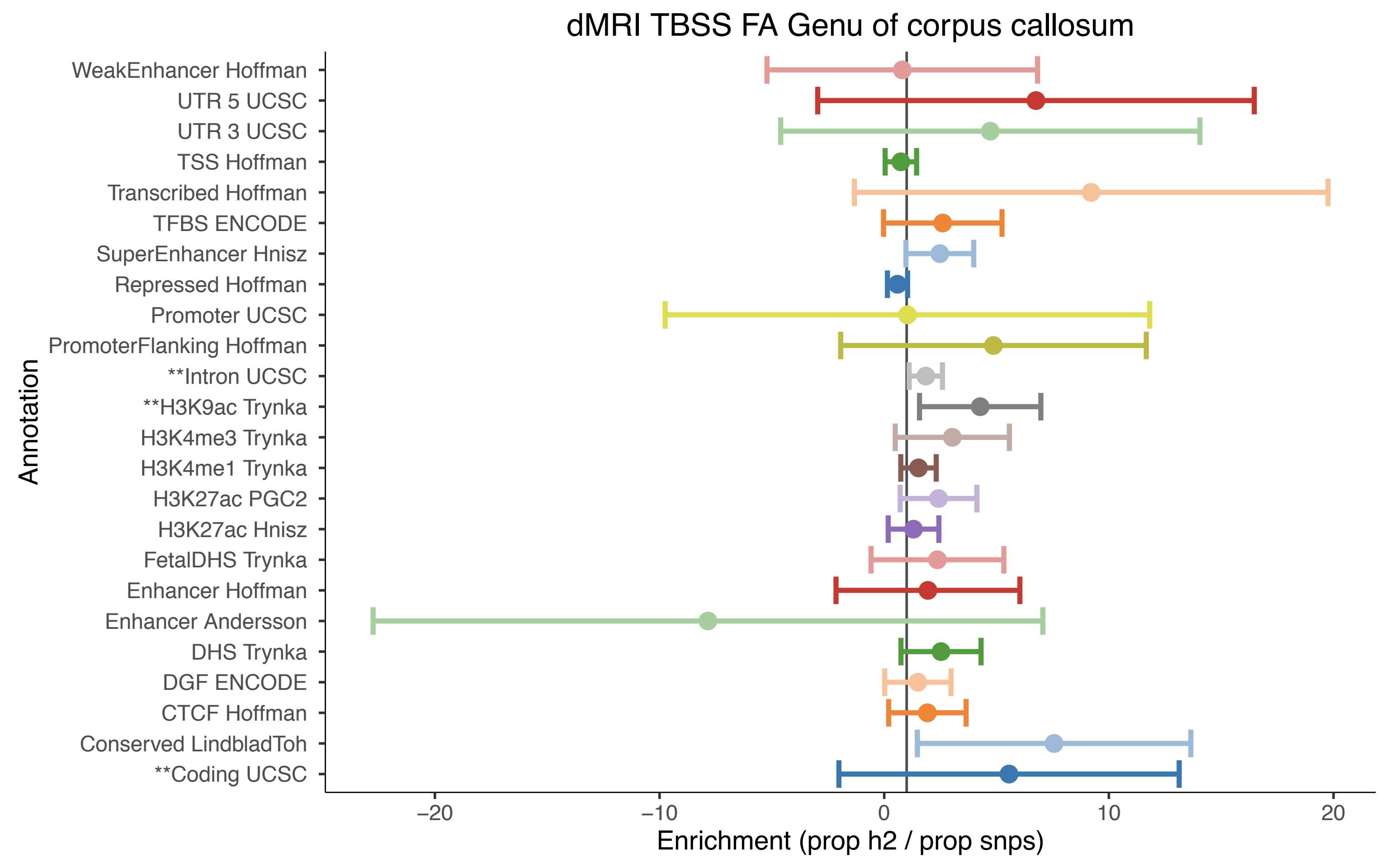
dMRI TBSS FA Genu of corpus callosum
WeakEnhancer Hoffman
UTR 5 UCSC
UTR 3 UCSC
TSS Hoffman
Transcribed Hoffman
TFBS ENCODE
SuperEnhancer Hnisz
Repressed Hoffman
Promoter UCSC
PromoterFlanking Hoffman
**Intron UCSC
**H3K9ac Trynka
H3K4me3 Trynka
H3K4me1 Trynka
H3K27ac PGC2
H3K27ac Hnisz
FetalDHS Trynka
Enhancer Hoffman
Enhancer Andersson
DHS Trynka
DGF ENCODE
CTCF Hoffman
Conserved LindbladToh
**Coding UCSC
Annotation
−20
−10
0
10
20
Enrichment (prop h2 / prop snps)

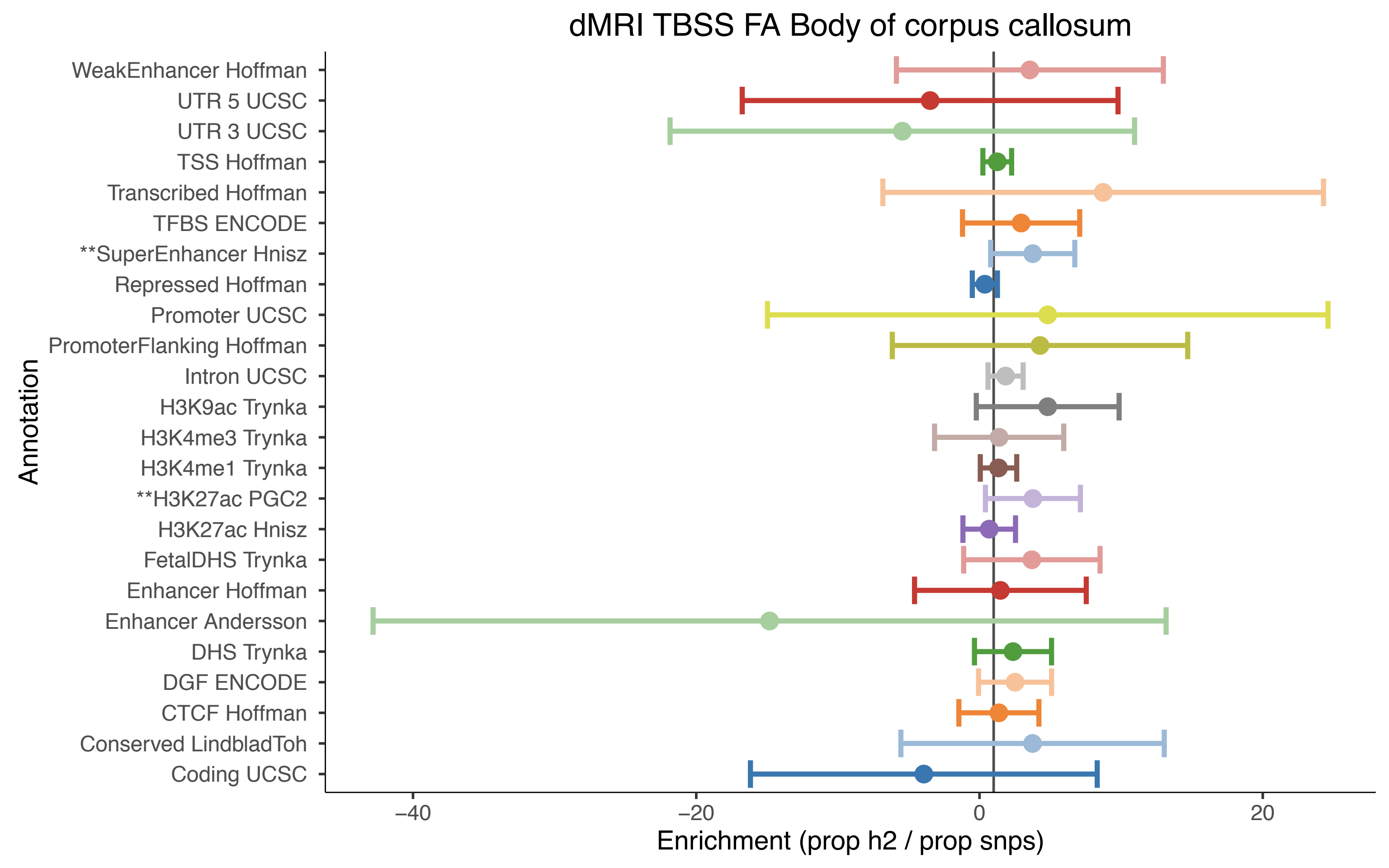
dMRI TBSS FA Body of corpus callosum
WeakEnhancer Hoffman
UTR 5 UCSC
UTR 3 UCSC
TSS Hoffman
Transcribed Hoffman
TFBS ENCODE
**SuperEnhancer Hnisz
Repressed Hoffman
Promoter UCSC
PromoterFlanking Hoffman
Intron UCSC
H3K9ac Trynka
H3K4me3 Trynka
H3K4me1 Trynka
**H3K27ac PGC2
H3K27ac Hnisz
FetalDHS Trynka
Enhancer Hoffman
Enhancer Andersson
DHS Trynka
DGF ENCODE
CTCF Hoffman
Conserved LindbladToh
Coding UCSC
Annotation
−40
−20
0
20
Enrichment (prop h2 / prop snps)

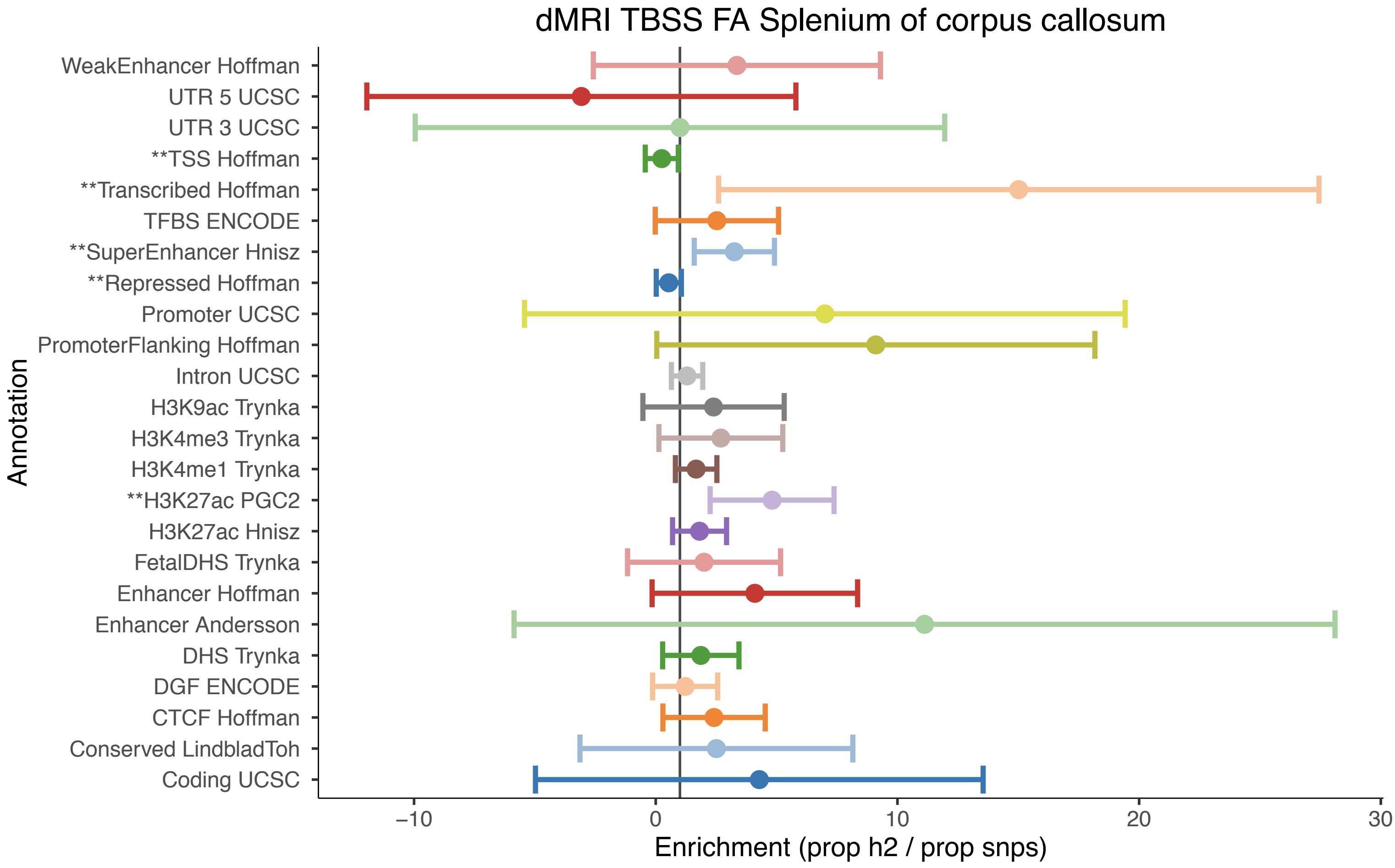

dMRI TBSS FA Splenium of corpus callosum
WeakEnhancer Hoffman
UTR 5 UCSC
UTR 3 UCSC
**TSS Hoffman
**Transcribed Hoffman
TFBS ENCODE
**SuperEnhancer Hnisz
**Repressed Hoffman
Promoter UCSC
PromoterFlanking Hoffman
Intron UCSC
H3K9ac Trynka
H3K4me3 Trynka
H3K4me1 Trynka
**H3K27ac PGC2
H3K27ac Hnisz
FetalDHS Trynka
Enhancer Hoffman
Enhancer Andersson
DHS Trynka
DGF ENCODE
CTCF Hoffman
Conserved LindbladToh
Coding UCSC
Annotation
−10
0
10
20
30
Enrichment (prop h2 / prop snps)

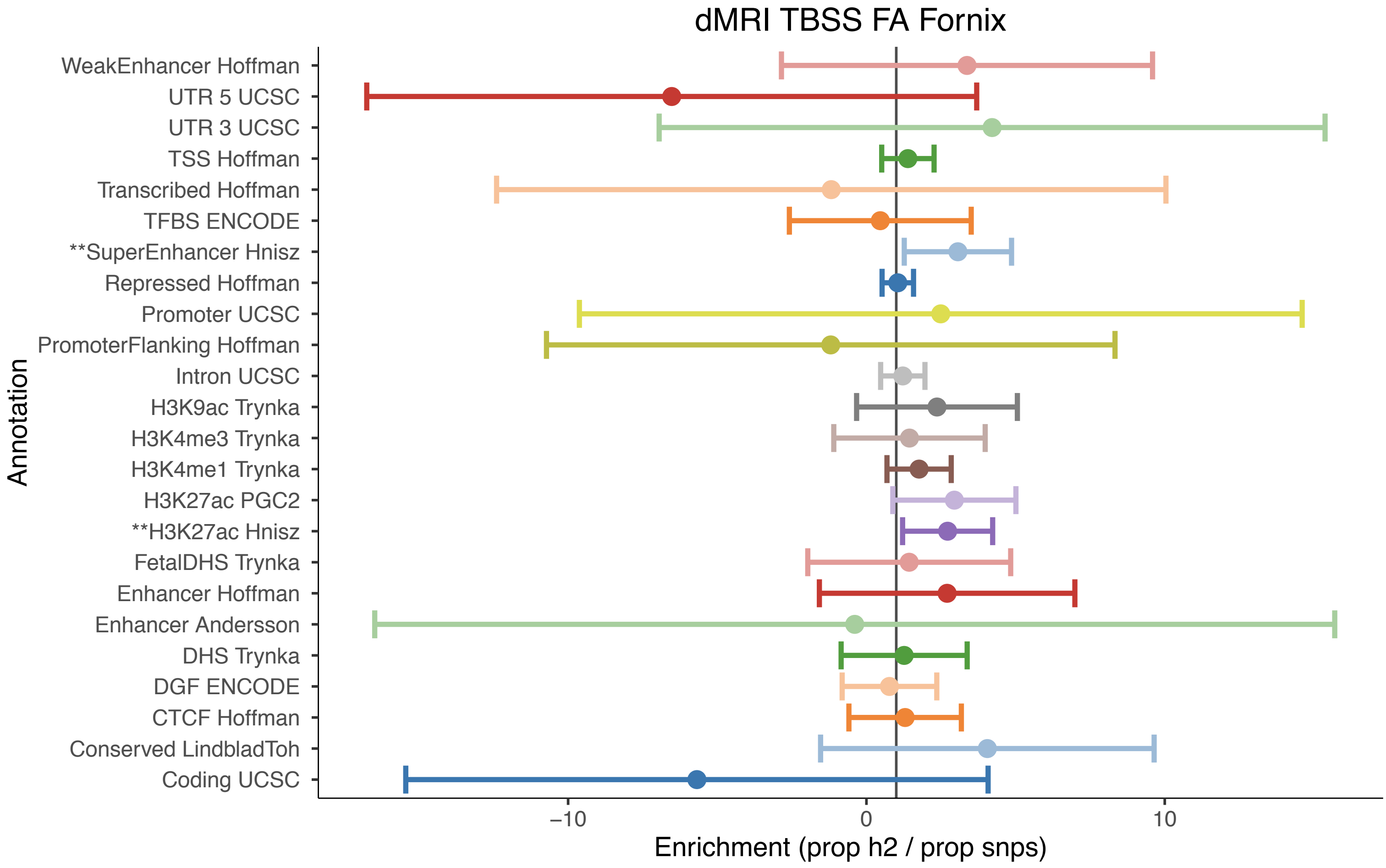
dMRI TBSS FA Fornix
WeakEnhancer Hoffman
UTR 5 UCSC
UTR 3 UCSC
TSS Hoffman
Transcribed Hoffman
TFBS ENCODE
**SuperEnhancer Hnisz
Repressed Hoffman
Promoter UCSC
PromoterFlanking Hoffman
Intron UCSC
H3K9ac Trynka
H3K4me3 Trynka
H3K4me1 Trynka
H3K27ac PGC2
**H3K27ac Hnisz
FetalDHS Trynka
Enhancer Hoffman
Enhancer Andersson
DHS Trynka
DGF ENCODE
CTCF Hoffman
Conserved LindbladToh
Coding UCSC
Annotation
−10
0
10
Enrichment (prop h2 / prop snps)

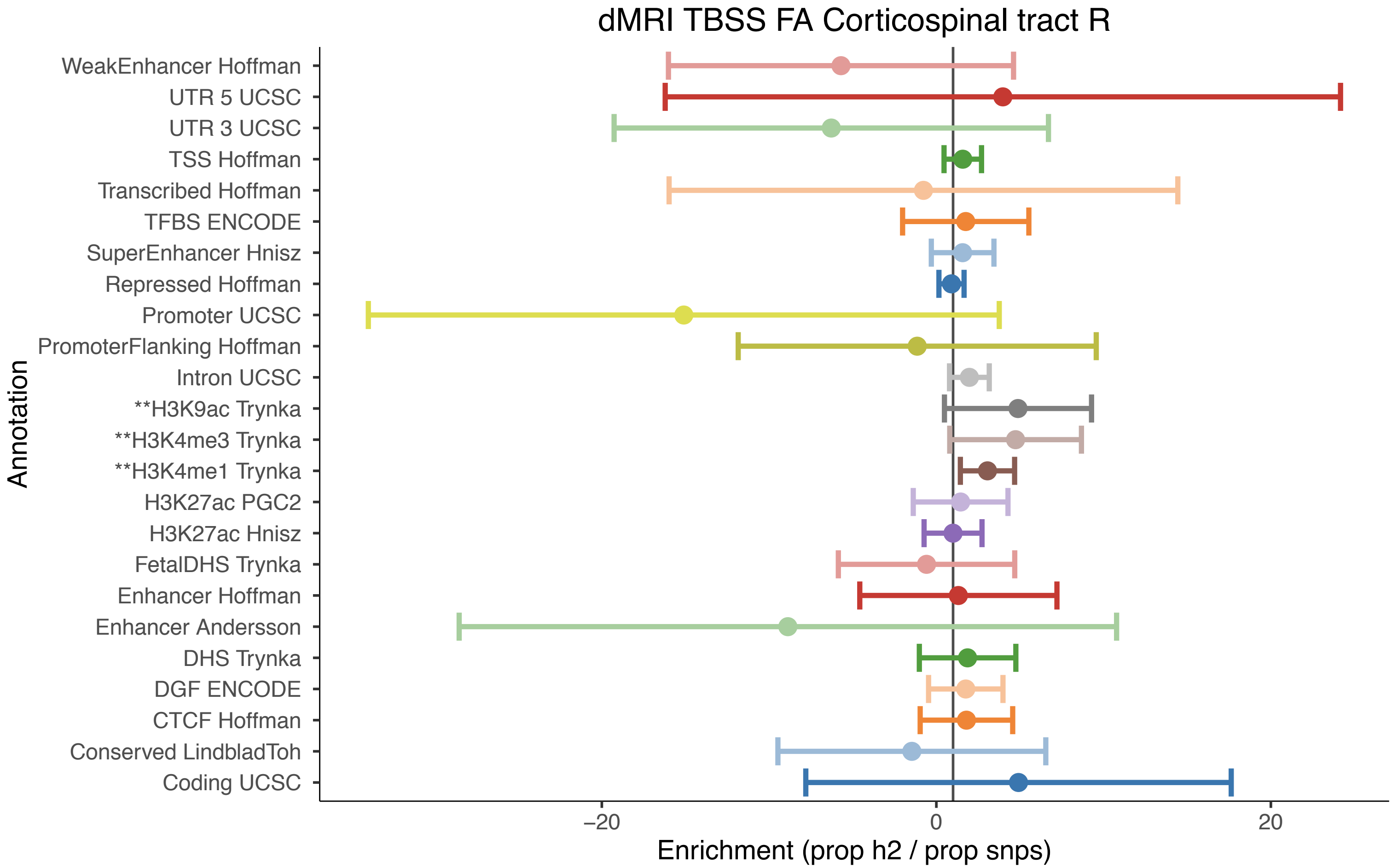

dMRI TBSS FA Corticospinal tract R
WeakEnhancer Hoffman
UTR 5 UCSC
UTR 3 UCSC
TSS Hoffman
Transcribed Hoffman
TFBS ENCODE
SuperEnhancer Hnisz
Repressed Hoffman
Promoter UCSC
PromoterFlanking Hoffman
Intron UCSC
**H3K9ac Trynka
**H3K4me3 Trynka
**H3K4me1 Trynka
H3K27ac PGC2
H3K27ac Hnisz
FetalDHS Trynka
Enhancer Hoffman
Enhancer Andersson
DHS Trynka
DGF ENCODE
CTCF Hoffman
Conserved LindbladToh
Coding UCSC
Annotation
−20
0
20
Enrichment (prop h2 / prop snps)

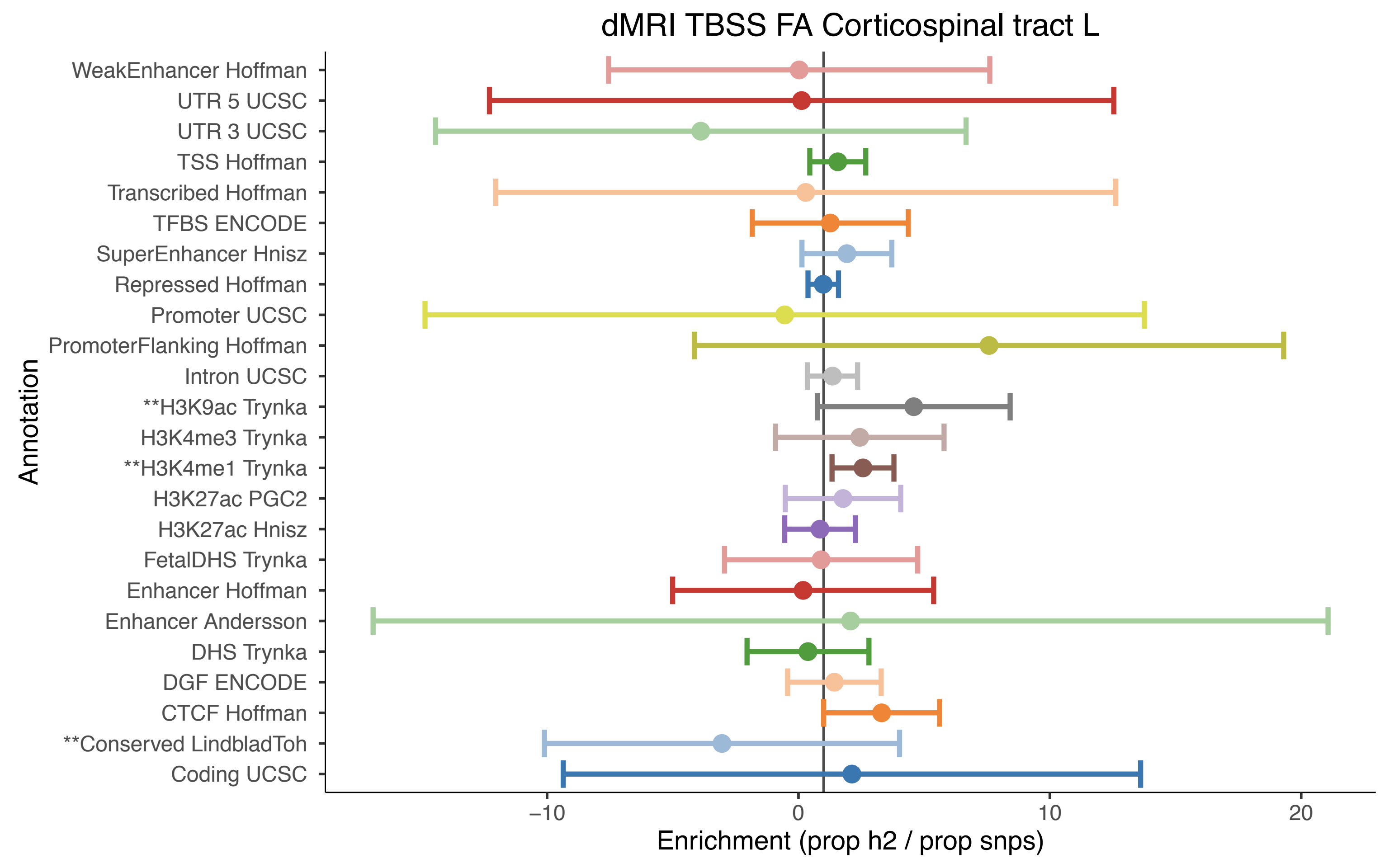
dMRI TBSS FA Corticospinal tract L
WeakEnhancer Hoffman
UTR 5 UCSC
UTR 3 UCSC
TSS Hoffman
Transcribed Hoffman
TFBS ENCODE
SuperEnhancer Hnisz
Repressed Hoffman
Promoter UCSC
PromoterFlanking Hoffman
Intron UCSC
**H3K9ac Trynka
H3K4me3 Trynka
**H3K4me1 Trynka
H3K27ac PGC2
H3K27ac Hnisz
FetalDHS Trynka
Enhancer Hoffman
Enhancer Andersson
DHS Trynka
DGF ENCODE
CTCF Hoffman
**Conserved LindbladToh
Coding UCSC
Annotation
−10
0
10
20
Enrichment (prop h2 / prop snps)

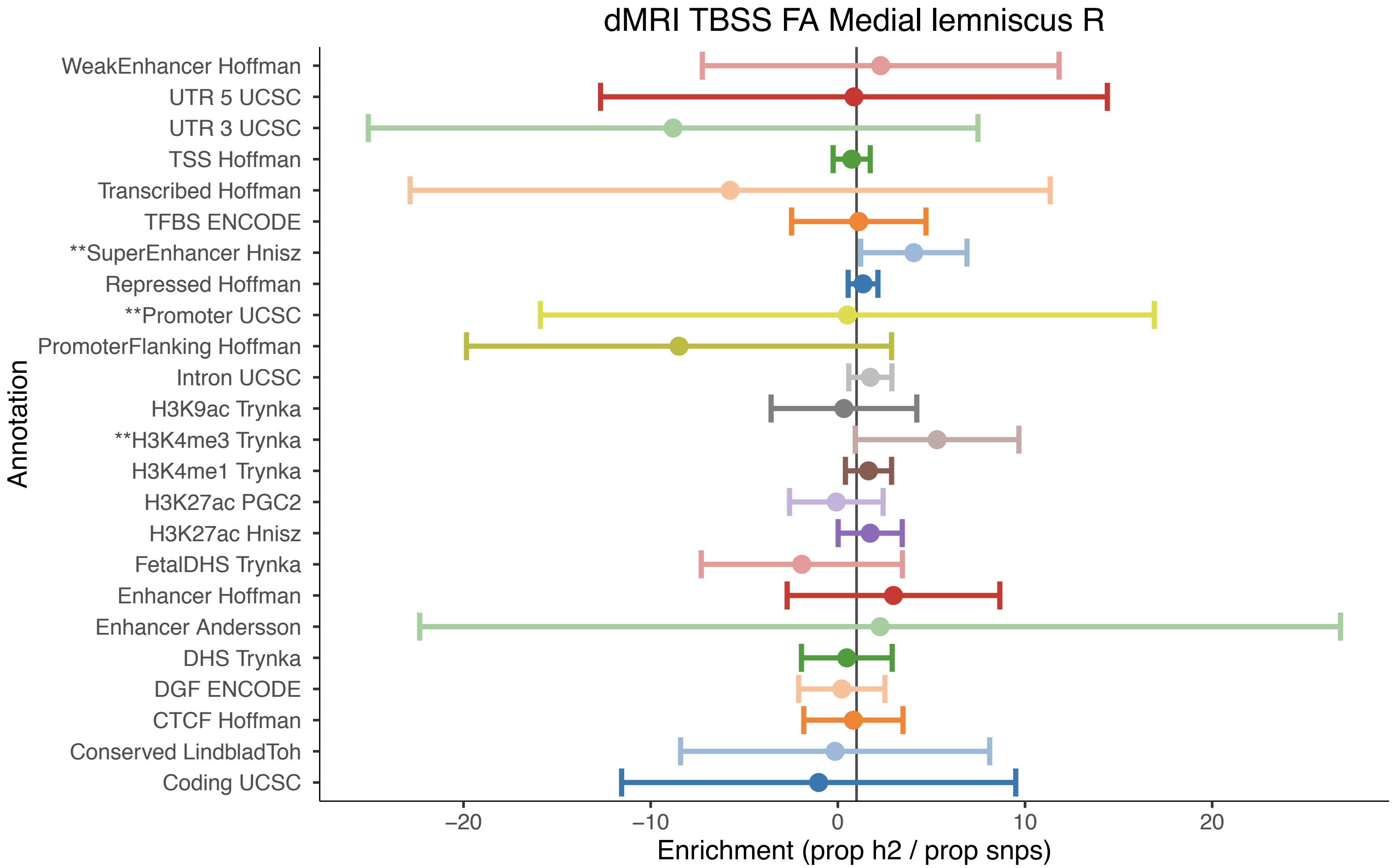
dMRI TBSS FA Medial lemniscus R
WeakEnhancer Hoffman
UTR 5 UCSC
UTR 3 UCSC
TSS Hoffman
Transcribed Hoffman
TFBS ENCODE
**SuperEnhancer Hnisz
Repressed Hoffman
**Promoter UCSC
PromoterFlanking Hoffman
Intron UCSC
H3K9ac Trynka
**H3K4me3 Trynka
H3K4me1 Trynka
H3K27ac PGC2
H3K27ac Hnisz
FetalDHS Trynka
Enhancer Hoffman
Enhancer Andersson
DHS Trynka
DGF ENCODE
CTCF Hoffman
Conserved LindbladToh
Coding UCSC
Annotation
−20
−10
0
10
20
Enrichment (prop h2 / prop snps)

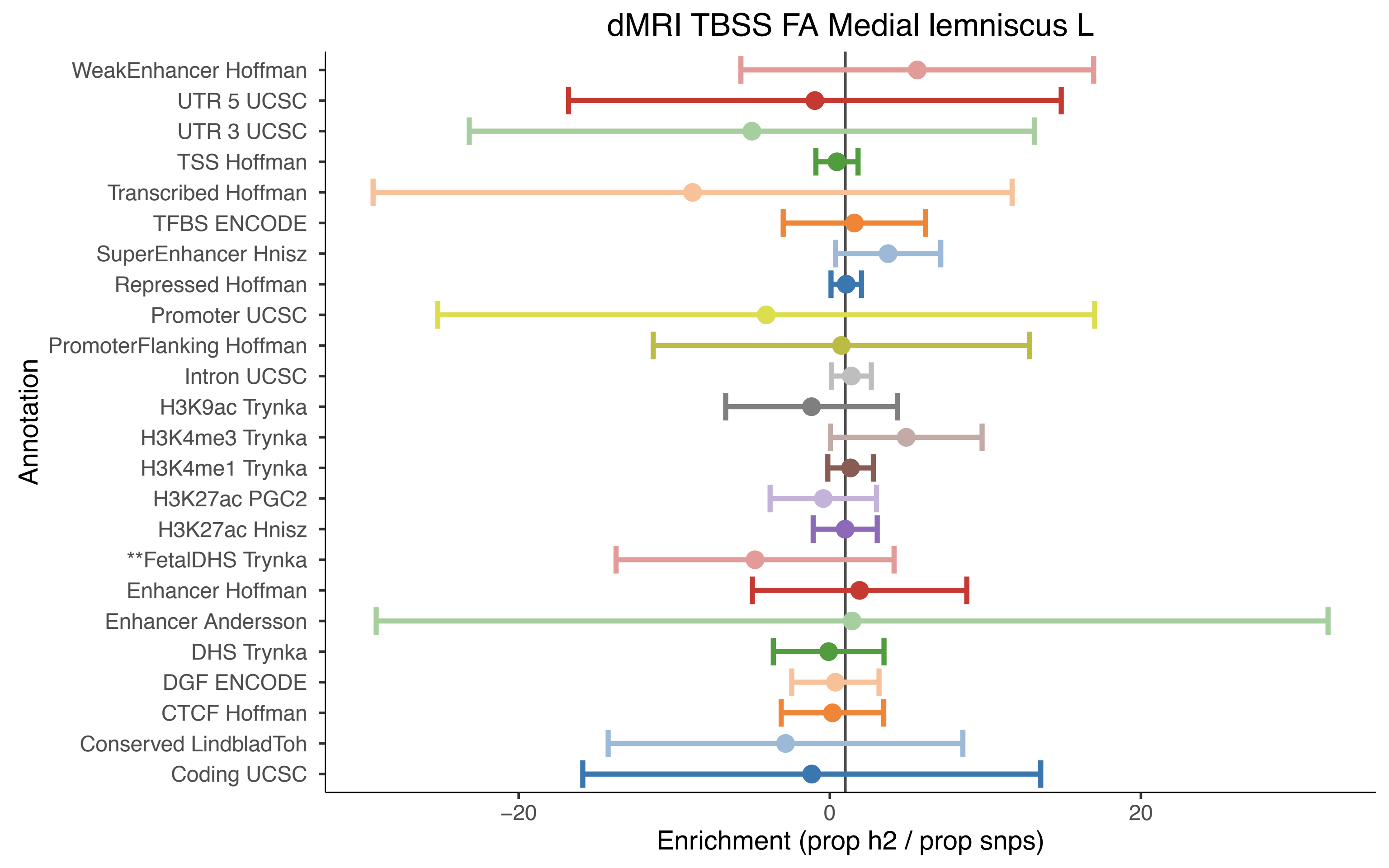

dMRI TBSS FA Medial lemniscus L
WeakEnhancer Hoffman
UTR 5 UCSC
UTR 3 UCSC
TSS Hoffman
Transcribed Hoffman
TFBS ENCODE
SuperEnhancer Hnisz
Repressed Hoffman
Promoter UCSC
PromoterFlanking Hoffman
Intron UCSC
H3K9ac Trynka
H3K4me3 Trynka
H3K4me1 Trynka
H3K27ac PGC2
H3K27ac Hnisz
**FetalDHS Trynka
Enhancer Hoffman
Enhancer Andersson
DHS Trynka
DGF ENCODE
CTCF Hoffman
Conserved LindbladToh
Coding UCSC
Annotation
−20
0
20
Enrichment (prop h2 / prop snps)

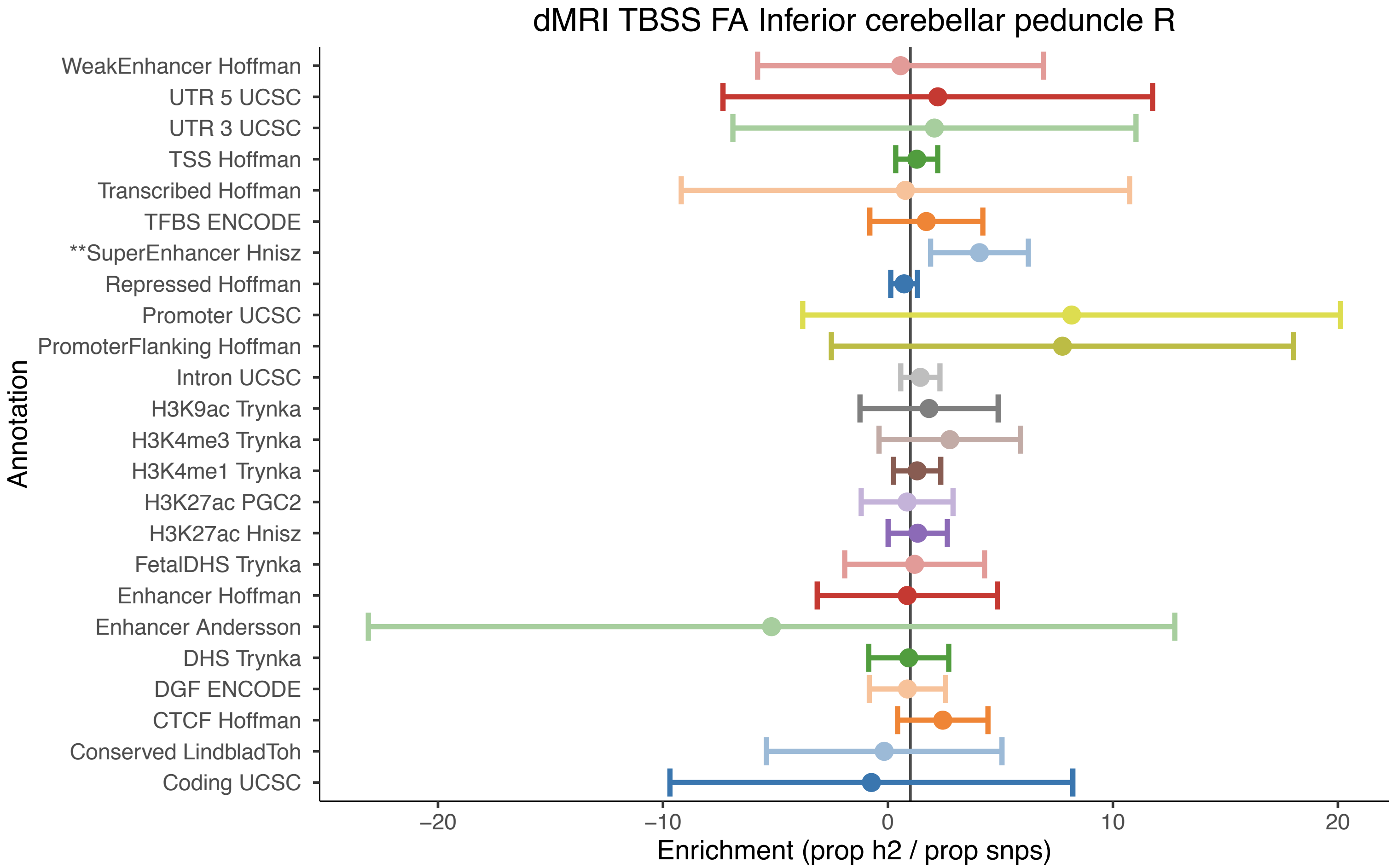

dMRI TBSS FA Inferior cerebellar peduncle R
WeakEnhancer Hoffman
UTR 5 UCSC
UTR 3 UCSC
TSS Hoffman
Transcribed Hoffman
TFBS ENCODE
**SuperEnhancer Hnisz
Repressed Hoffman
Promoter UCSC
PromoterFlanking Hoffman
Intron UCSC
H3K9ac Trynka
H3K4me3 Trynka
H3K4me1 Trynka
H3K27ac PGC2
H3K27ac Hnisz
FetalDHS Trynka
Enhancer Hoffman
Enhancer Andersson
DHS Trynka
DGF ENCODE
CTCF Hoffman
Conserved LindbladToh
Coding UCSC
Annotation
−20
−10
0
10
20
Enrichment (prop h2 / prop snps)

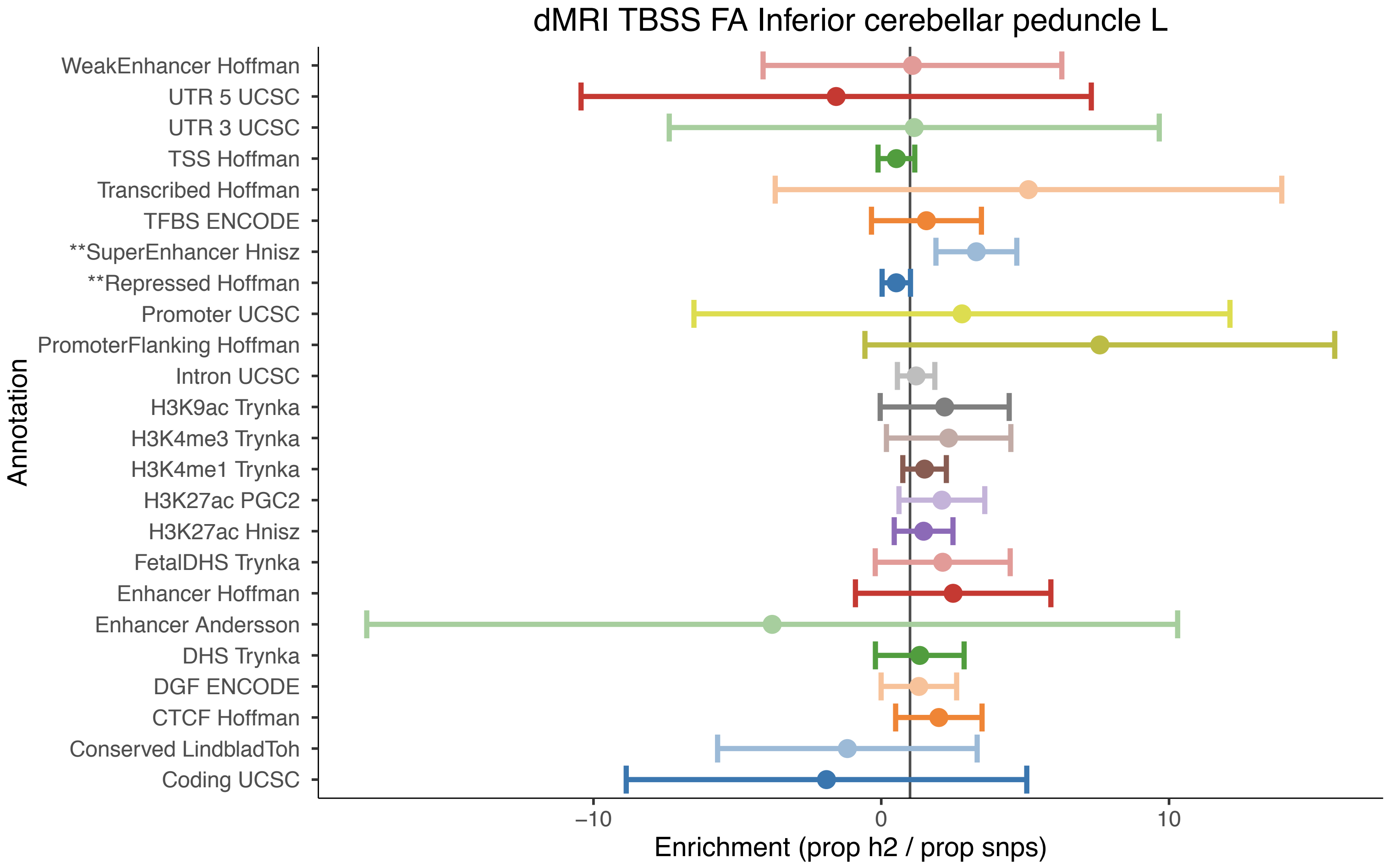

dMRI TBSS FA Inferior cerebellar peduncle L
Annotation
WeakEnhancer Hoffman
UTR 5 UCSC
UTR 3 UCSC
TSS Hoffman
Transcribed Hoffman
TFBS ENCODE
**SuperEnhancer Hnisz
**Repressed Hoffman
Promoter UCSC
PromoterFlanking Hoffman
Intron UCSC
H3K9ac Trynka
H3K4me3 Trynka
H3K4me1 Trynka
H3K27ac PGC2
H3K27ac Hnisz
FetalDHS Trynka
Enhancer Hoffman
Enhancer Andersson
DHS Trynka
DGF ENCODE
CTCF Hoffman
Conserved LindbladToh
Coding UCSC
−10
0
10
Enrichment (prop h2 / prop snps)

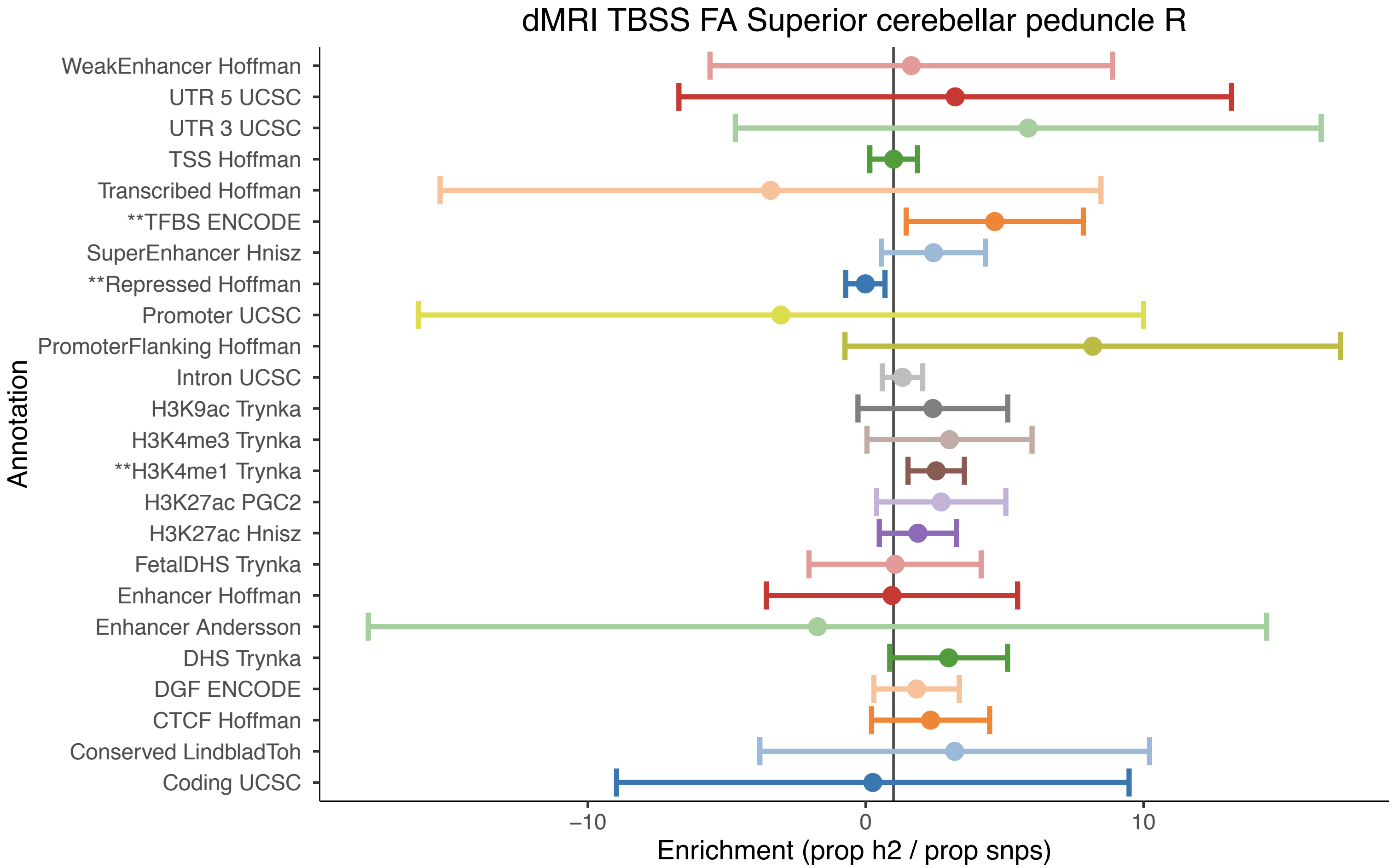

dMRI TBSS FA Superior cerebellar peduncle R
WeakEnhancer Hoffman
UTR 5 UCSC
UTR 3 UCSC
TSS Hoffman
Transcribed Hoffman
**TFBS ENCODE
SuperEnhancer Hnisz
**Repressed Hoffman
Promoter UCSC
PromoterFlanking Hoffman
Intron UCSC
H3K9ac Trynka
H3K4me3 Trynka
**H3K4me1 Trynka
H3K27ac PGC2
H3K27ac Hnisz
FetalDHS Trynka
Enhancer Hoffman
Enhancer Andersson
DHS Trynka
DGF ENCODE
CTCF Hoffman
Conserved LindbladToh
Coding UCSC
Annotation
−10
0
10
Enrichment (prop h2 / prop snps)

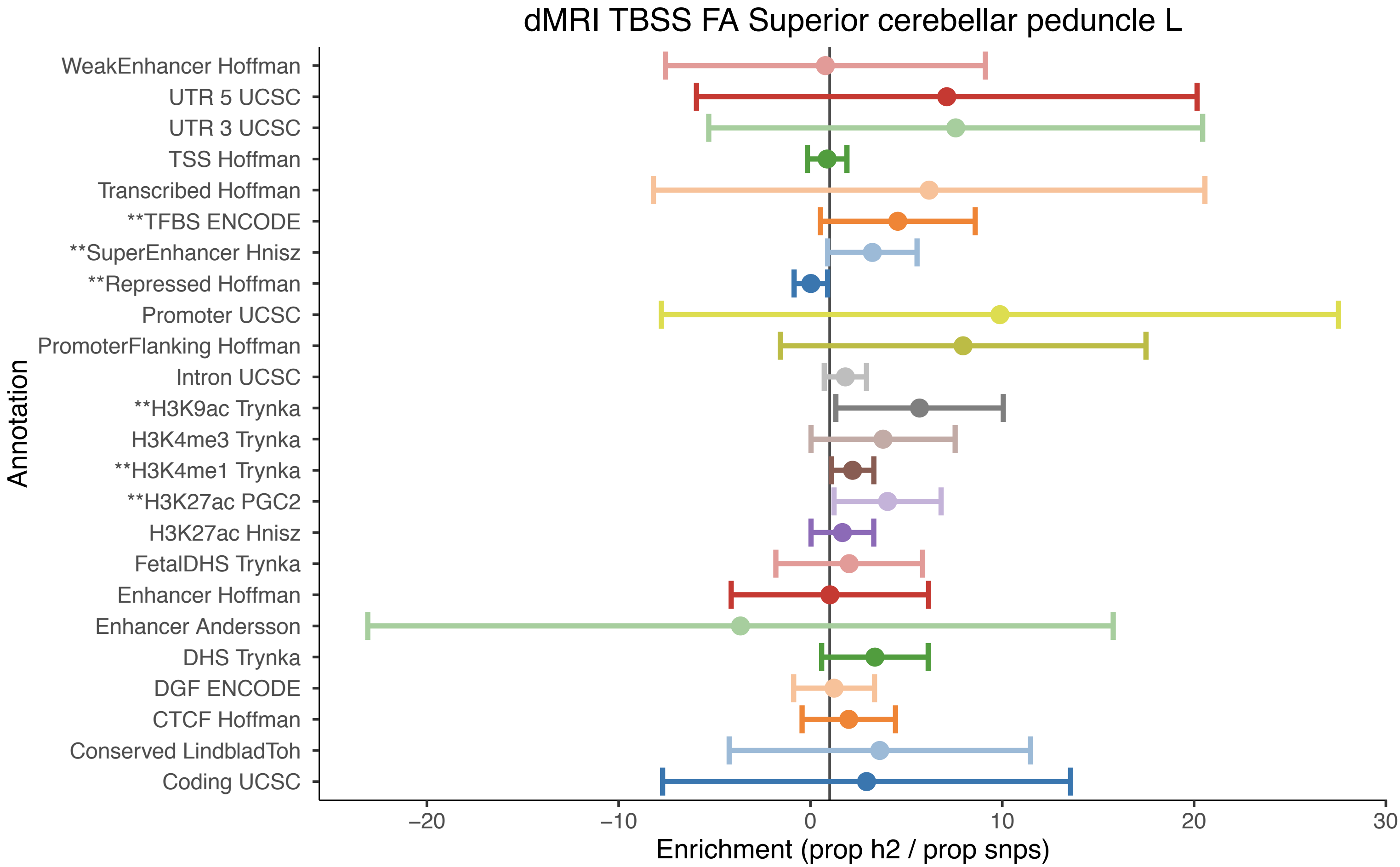
dMRI TBSS FA Superior cerebellar peduncle L
WeakEnhancer Hoffman
UTR 5 UCSC
UTR 3 UCSC
TSS Hoffman
Transcribed Hoffman
**TFBS ENCODE
**SuperEnhancer Hnisz
**Repressed Hoffman
Promoter UCSC
PromoterFlanking Hoffman
Intron UCSC
**H3K9ac Trynka
H3K4me3 Trynka
**H3K4me1 Trynka
**H3K27ac PGC2
H3K27ac Hnisz
FetalDHS Trynka
Enhancer Hoffman
Enhancer Andersson
DHS Trynka
DGF ENCODE
CTCF Hoffman
Conserved LindbladToh
Coding UCSC
Annotation
−20
−10
0
10
20
30
Enrichment (prop h2 / prop snps)

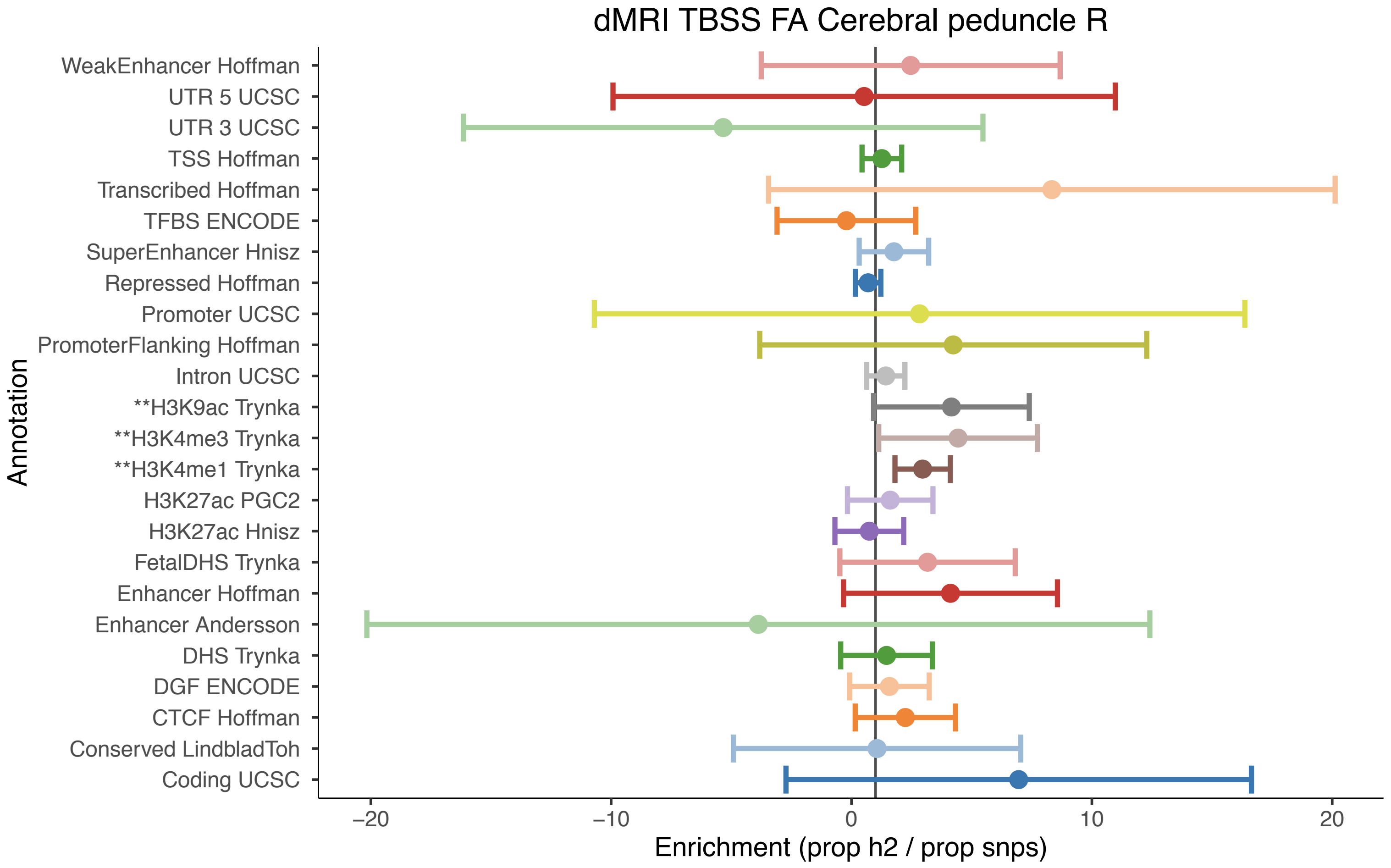
dMRI TBSS FA Cerebral peduncle R
WeakEnhancer Hoffman
UTR 5 UCSC
UTR 3 UCSC
TSS Hoffman
Transcribed Hoffman
TFBS ENCODE
SuperEnhancer Hnisz
Repressed Hoffman
Promoter UCSC
PromoterFlanking Hoffman
Intron UCSC
**H3K9ac Trynka
**H3K4me3 Trynka
**H3K4me1 Trynka
H3K27ac PGC2
H3K27ac Hnisz
FetalDHS Trynka
Enhancer Hoffman
Enhancer Andersson
DHS Trynka
DGF ENCODE
CTCF Hoffman
Conserved LindbladToh
Coding UCSC
Annotation
−20
−10
0
10
20
Enrichment (prop h2 / prop snps)

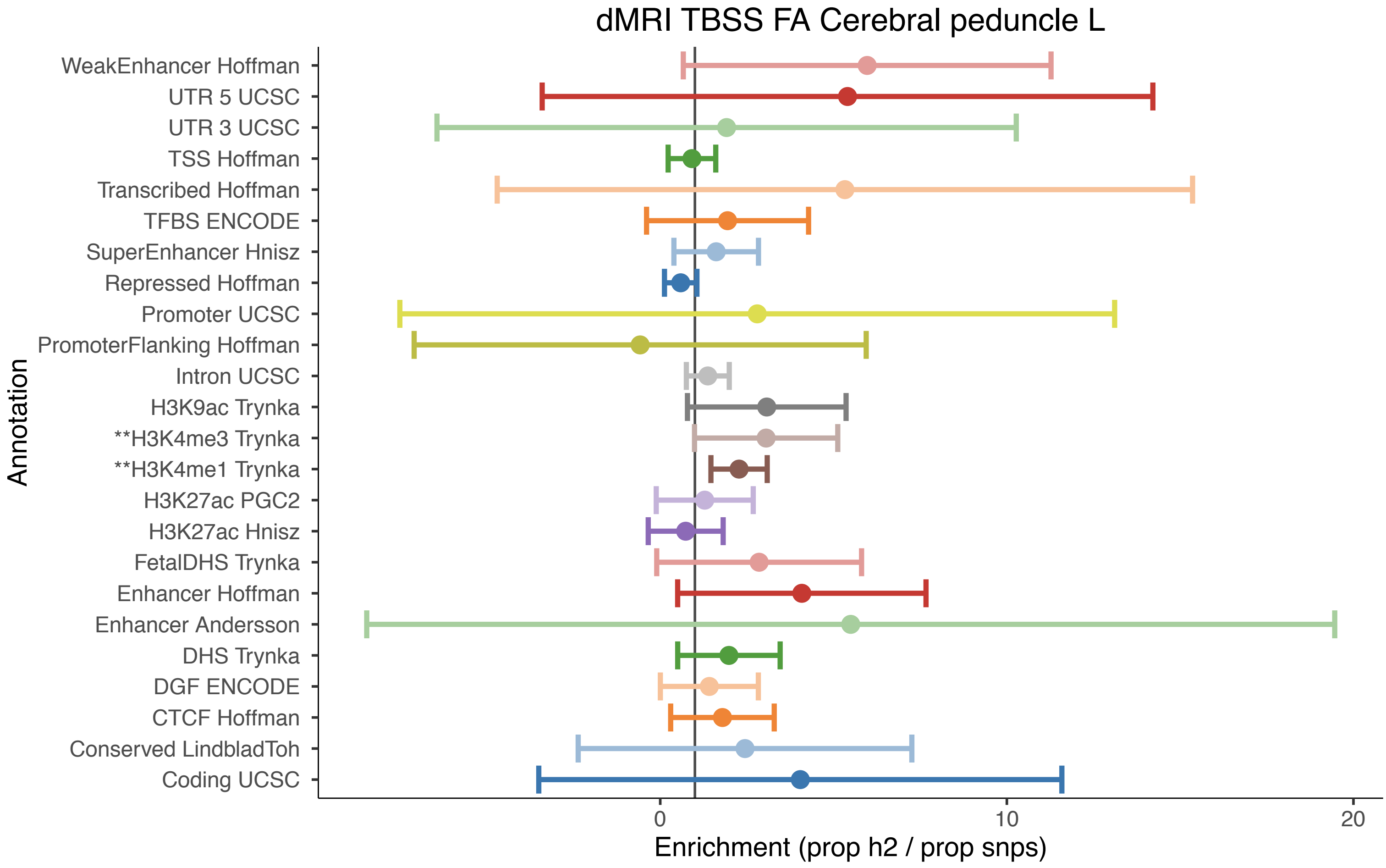

dMRI TBSS FA Cerebral peduncle L
WeakEnhancer Hoffman
UTR 5 UCSC
UTR 3 UCSC
TSS Hoffman
Transcribed Hoffman
TFBS ENCODE
SuperEnhancer Hnisz
Repressed Hoffman
Promoter UCSC
PromoterFlanking Hoffman
Intron UCSC
H3K9ac Trynka
**H3K4me3 Trynka
**H3K4me1 Trynka
H3K27ac PGC2
H3K27ac Hnisz
FetalDHS Trynka
Enhancer Hoffman
Enhancer Andersson
DHS Trynka
DGF ENCODE
CTCF Hoffman
Conserved LindbladToh
Coding UCSC
Annotation
0
10
20
Enrichment (prop h2 / prop snps)

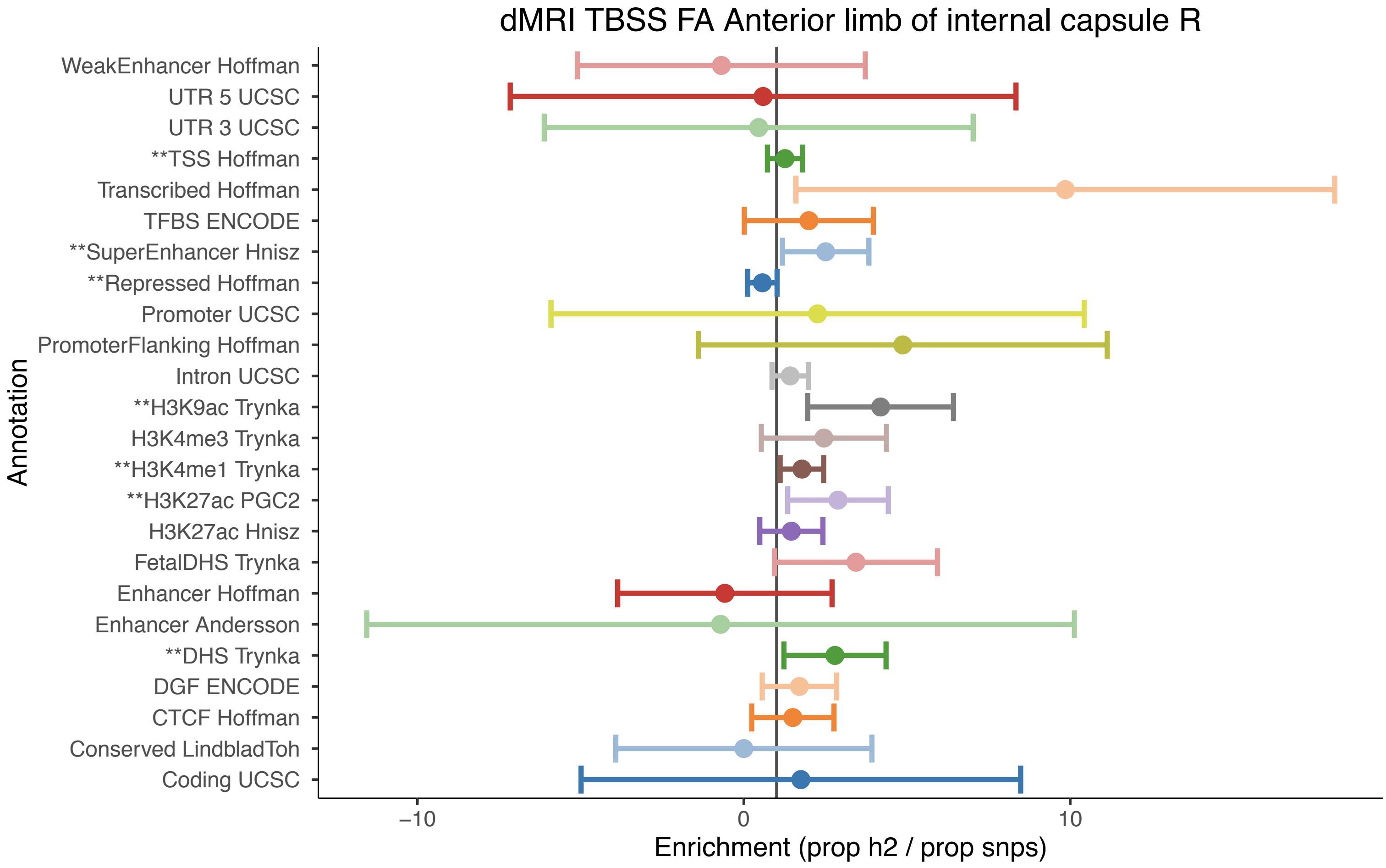

dMRI TBSS FA Anterior limb of internal capsule R
WeakEnhancer Hoffman
UTR 5 UCSC
UTR 3 UCSC
**TSS Hoffman
Transcribed Hoffman
TFBS ENCODE
**SuperEnhancer Hnisz
**Repressed Hoffman
Promoter UCSC
PromoterFlanking Hoffman
Intron UCSC
**H3K9ac Trynka
H3K4me3 Trynka
**H3K4me1 Trynka
**H3K27ac PGC2
H3K27ac Hnisz
FetalDHS Trynka
Enhancer Hoffman
Enhancer Andersson
**DHS Trynka
DGF ENCODE
CTCF Hoffman
Conserved LindbladToh
Coding UCSC
Annotation
−10
0
10
Enrichment (prop h2 / prop snps)

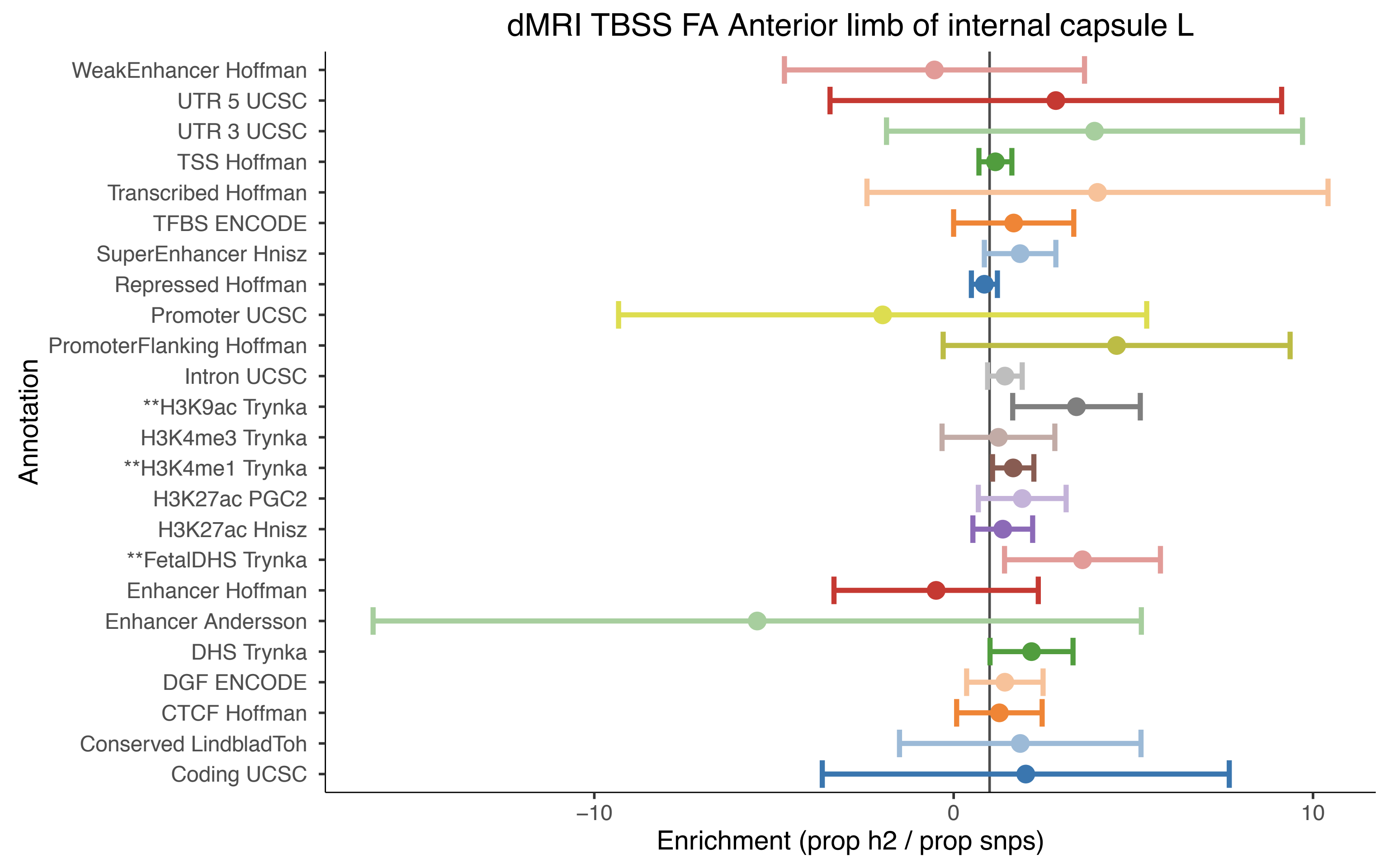

dMRI TBSS FA Anterior limb of internal capsule L
WeakEnhancer Hoffman
UTR 5 UCSC
UTR 3 UCSC
TSS Hoffman
Transcribed Hoffman
TFBS ENCODE
SuperEnhancer Hnisz
Repressed Hoffman
Promoter UCSC
PromoterFlanking Hoffman
Intron UCSC
**H3K9ac Trynka
H3K4me3 Trynka
**H3K4me1 Trynka
H3K27ac PGC2
H3K27ac Hnisz
**FetalDHS Trynka
Enhancer Hoffman
Enhancer Andersson
DHS Trynka
DGF ENCODE
CTCF Hoffman
Conserved LindbladToh
Coding UCSC
Annotation
−10
0
10
Enrichment (prop h2 / prop snps)

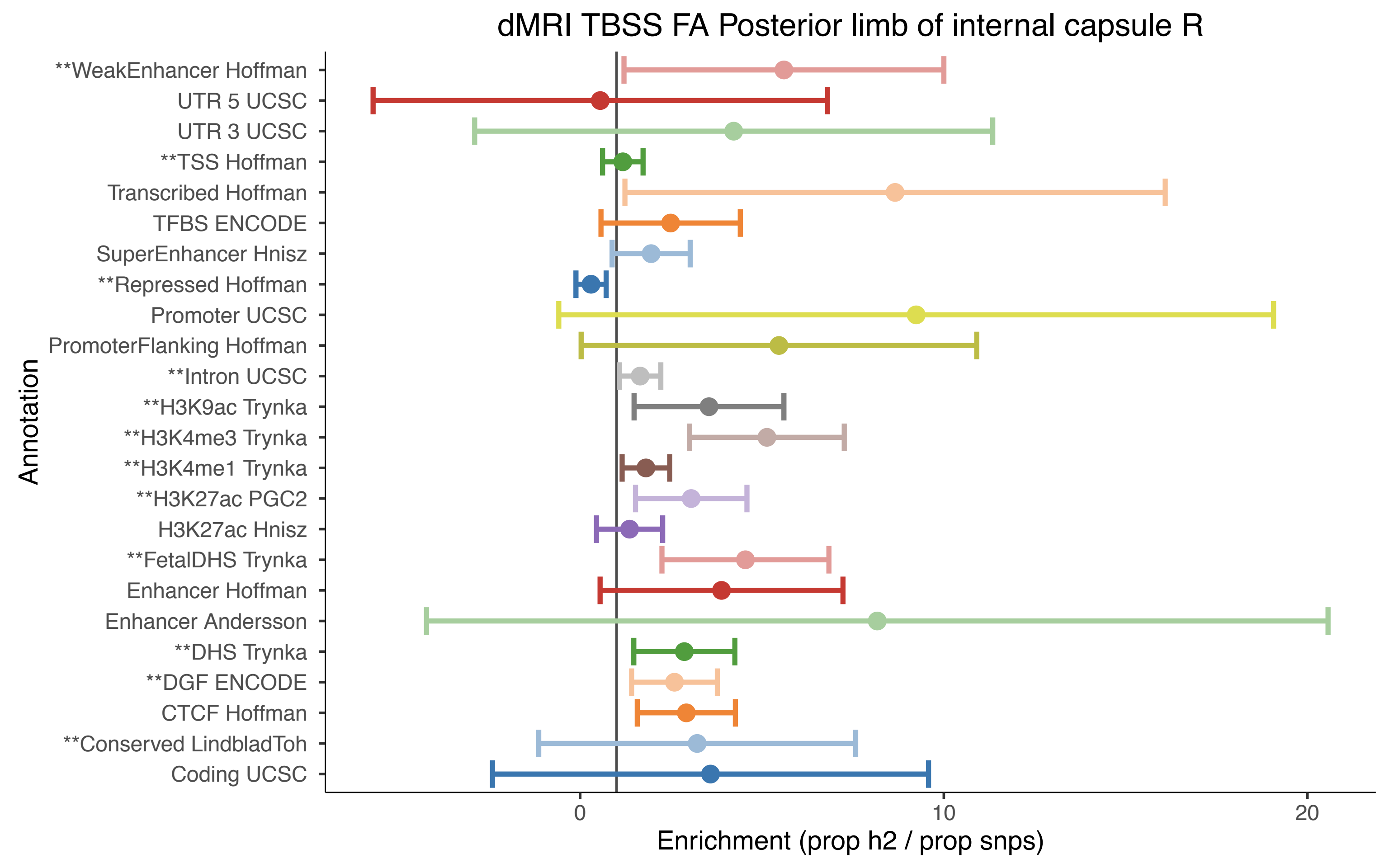

dMRI TBSS FA Posterior limb of internal capsule R
Annotation
**WeakEnhancer Hoffman
UTR 5 UCSC
UTR 3 UCSC
**TSS Hoffman
Transcribed Hoffman
TFBS ENCODE
SuperEnhancer Hnisz
**Repressed Hoffman
Promoter UCSC
PromoterFlanking Hoffman
**Intron UCSC
**H3K9ac Trynka
**H3K4me3 Trynka
**H3K4me1 Trynka
**H3K27ac PGC2
H3K27ac Hnisz
**FetalDHS Trynka
Enhancer Hoffman
Enhancer Andersson
**DHS Trynka
**DGF ENCODE
CTCF Hoffman
**Conserved LindbladToh
Coding UCSC
0
10
20
Enrichment (prop h2 / prop snps)

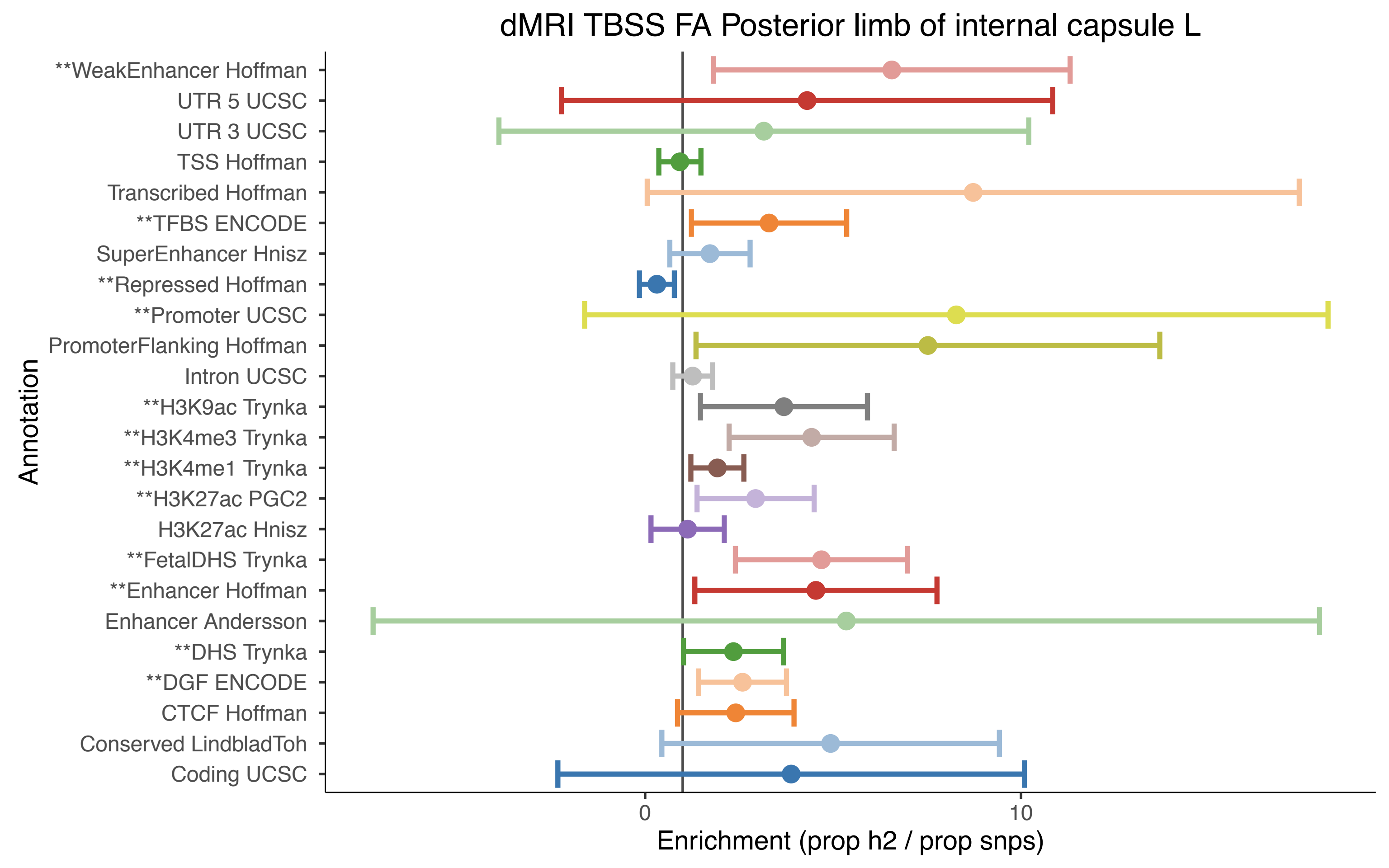
dMRI TBSS FA Posterior limb of internal capsule L
**WeakEnhancer Hoffman
UTR 5 UCSC
UTR 3 UCSC
TSS Hoffman
Transcribed Hoffman
**TFBS ENCODE
SuperEnhancer Hnisz
**Repressed Hoffman
**Promoter UCSC
PromoterFlanking Hoffman
Intron UCSC
**H3K9ac Trynka
**H3K4me3 Trynka
**H3K4me1 Trynka
**H3K27ac PGC2
H3K27ac Hnisz
**FetalDHS Trynka
**Enhancer Hoffman
Enhancer Andersson
**DHS Trynka
**DGF ENCODE
CTCF Hoffman
Conserved LindbladToh
Coding UCSC
Annotation
0
10
Enrichment (prop h2 / prop snps)

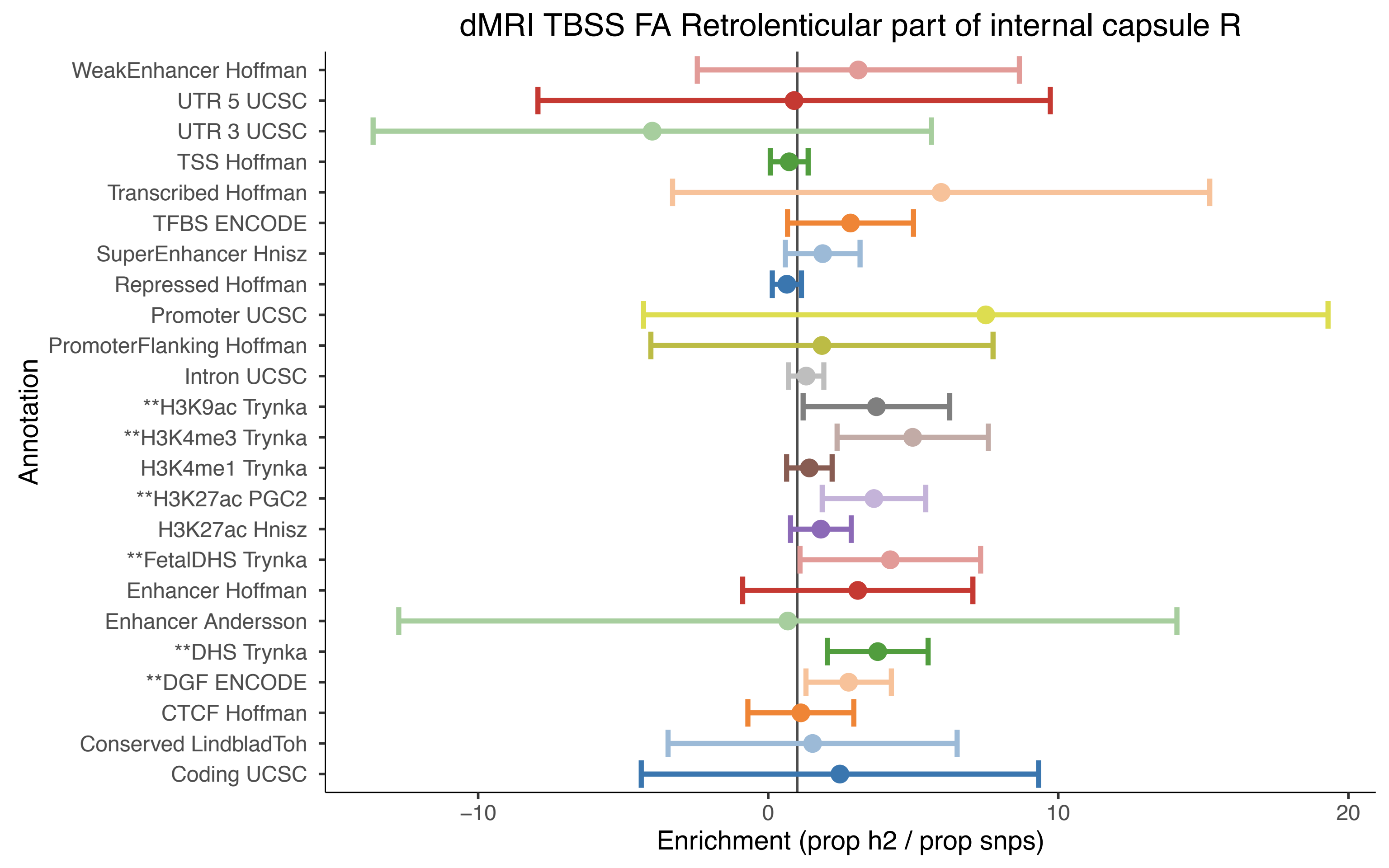
dMRI TBSS FA Retrolenticular part of internal capsule R
WeakEnhancer Hoffman
UTR 5 UCSC
UTR 3 UCSC
TSS Hoffman
Transcribed Hoffman
TFBS ENCODE
SuperEnhancer Hnisz
Repressed Hoffman
Promoter UCSC
PromoterFlanking Hoffman
Intron UCSC
**H3K9ac Trynka
**H3K4me3 Trynka
H3K4me1 Trynka
**H3K27ac PGC2
H3K27ac Hnisz
**FetalDHS Trynka
Enhancer Hoffman
Enhancer Andersson
**DHS Trynka
**DGF ENCODE
CTCF Hoffman
Conserved LindbladToh
Coding UCSC
Annotation
−10
0
10
20
Enrichment (prop h2 / prop snps)

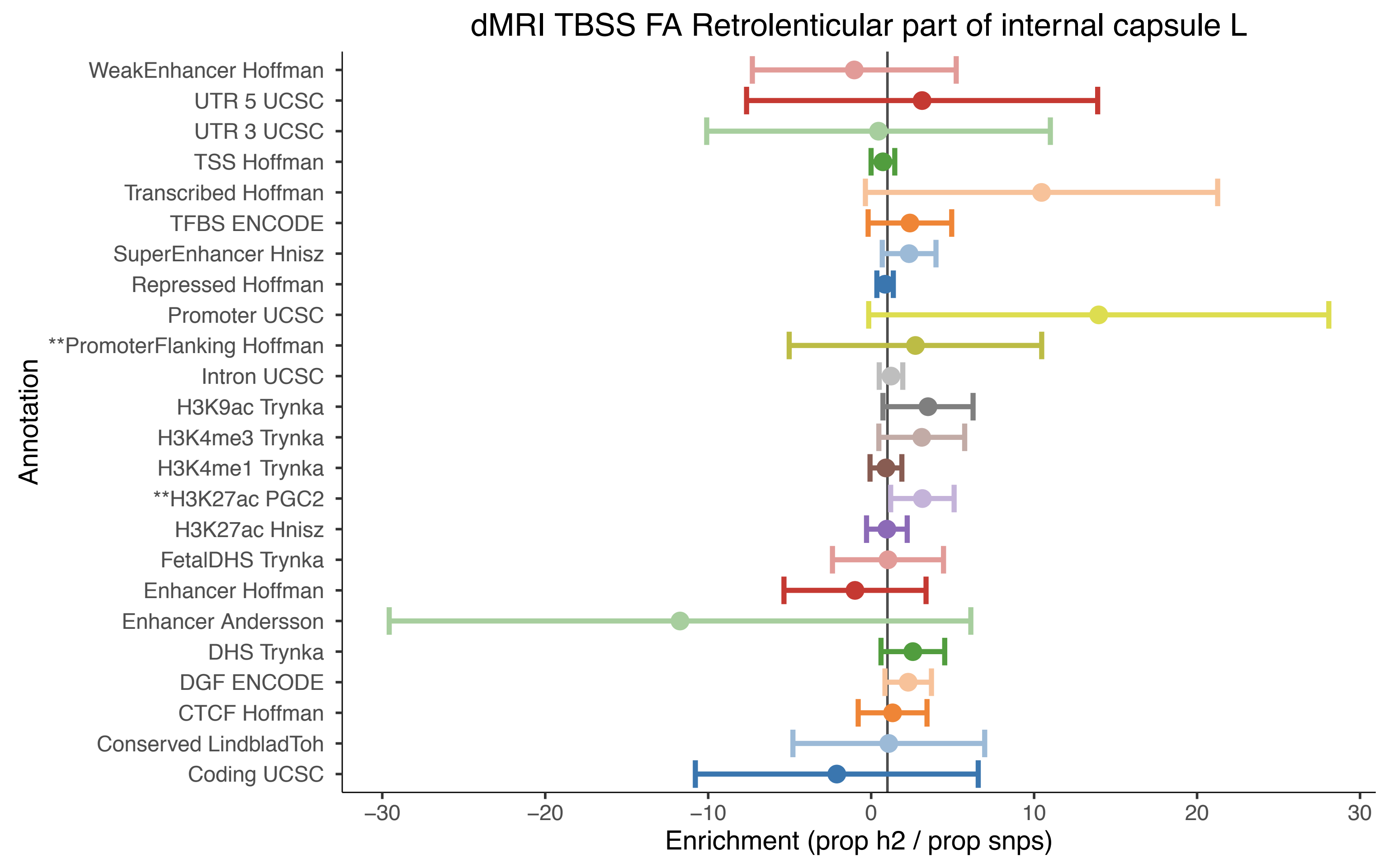

dMRI TBSS FA Retrolenticular part of internal capsule L
WeakEnhancer Hoffman
UTR 5 UCSC
UTR 3 UCSC
TSS Hoffman
Transcribed Hoffman
TFBS ENCODE
SuperEnhancer Hnisz
Repressed Hoffman
Promoter UCSC
**PromoterFlanking Hoffman
Intron UCSC
H3K9ac Trynka
H3K4me3 Trynka
H3K4me1 Trynka
**H3K27ac PGC2
H3K27ac Hnisz
FetalDHS Trynka
Enhancer Hoffman
Enhancer Andersson
DHS Trynka
DGF ENCODE
CTCF Hoffman
Conserved LindbladToh
Coding UCSC
Annotation
−30
−20
−10
0
10
20
30
Enrichment (prop h2 / prop snps)

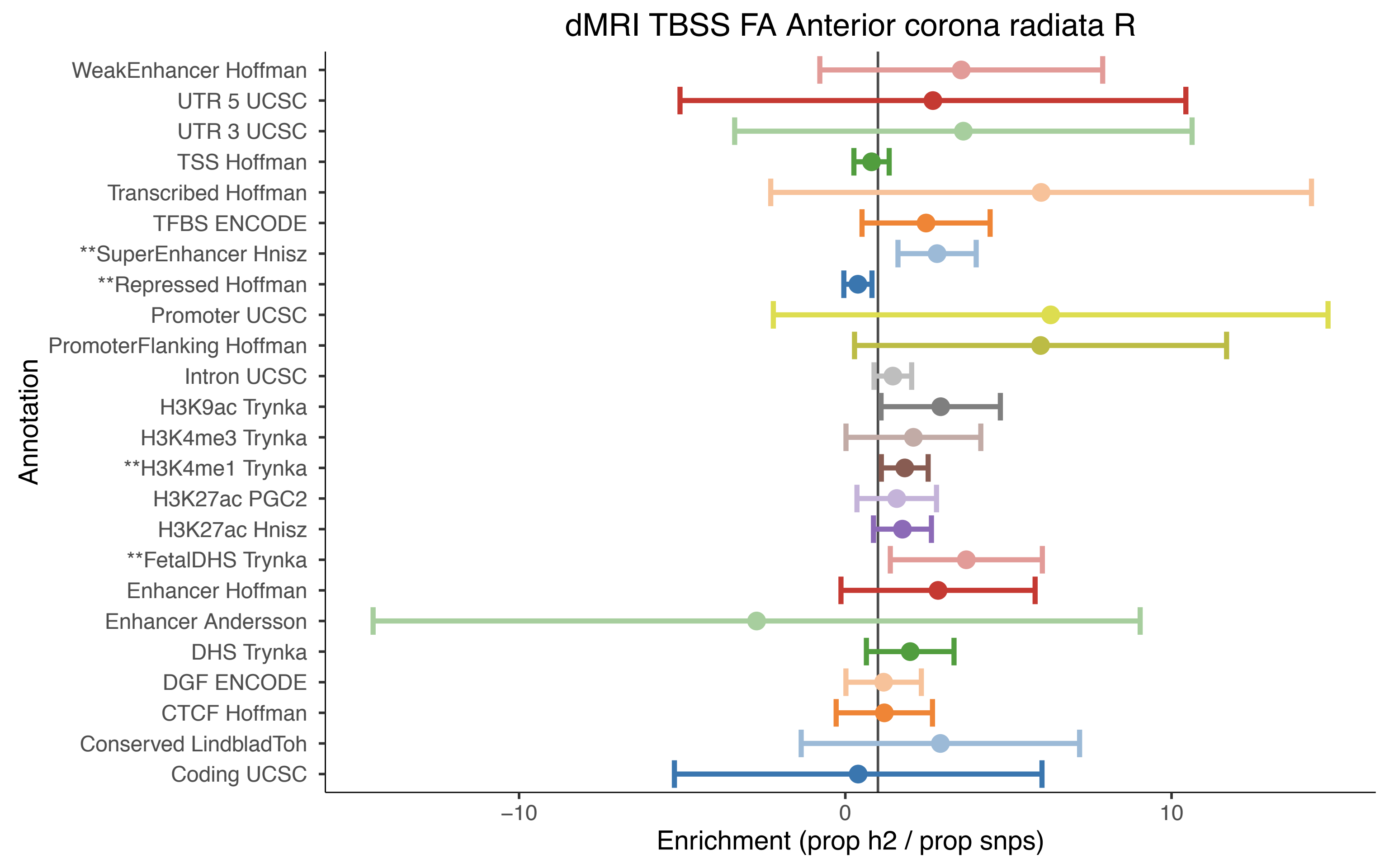
dMRI TBSS FA Anterior corona radiata R
Annotation
WeakEnhancer Hoffman
UTR 5 UCSC
UTR 3 UCSC
TSS Hoffman
Transcribed Hoffman
TFBS ENCODE
**SuperEnhancer Hnisz
**Repressed Hoffman
Promoter UCSC
PromoterFlanking Hoffman
Intron UCSC
H3K9ac Trynka
H3K4me3 Trynka
**H3K4me1 Trynka
H3K27ac PGC2
H3K27ac Hnisz
**FetalDHS Trynka
Enhancer Hoffman
Enhancer Andersson
DHS Trynka
DGF ENCODE
CTCF Hoffman
Conserved LindbladToh
Coding UCSC
−10
0
10
Enrichment (prop h2 / prop snps)

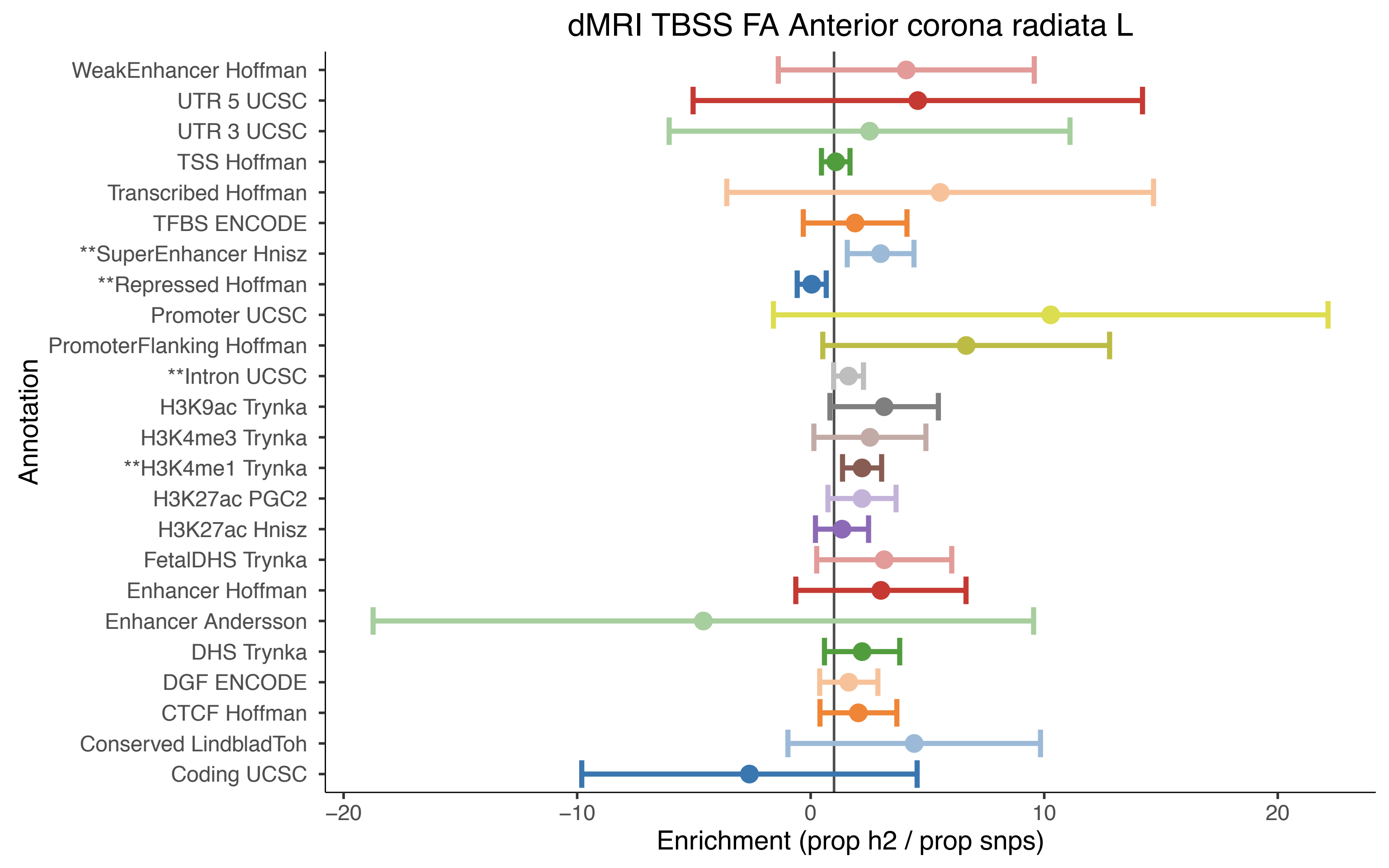

dMRI TBSS FA Anterior corona radiata L
WeakEnhancer Hoffman
UTR 5 UCSC
UTR 3 UCSC
TSS Hoffman
Transcribed Hoffman
TFBS ENCODE
**SuperEnhancer Hnisz
**Repressed Hoffman
Promoter UCSC
PromoterFlanking Hoffman
**Intron UCSC
H3K9ac Trynka
H3K4me3 Trynka
**H3K4me1 Trynka
H3K27ac PGC2
H3K27ac Hnisz
FetalDHS Trynka
Enhancer Hoffman
Enhancer Andersson
DHS Trynka
DGF ENCODE
CTCF Hoffman
Conserved LindbladToh
Coding UCSC
Annotation
−20
−10
0
10
20
Enrichment (prop h2 / prop snps)

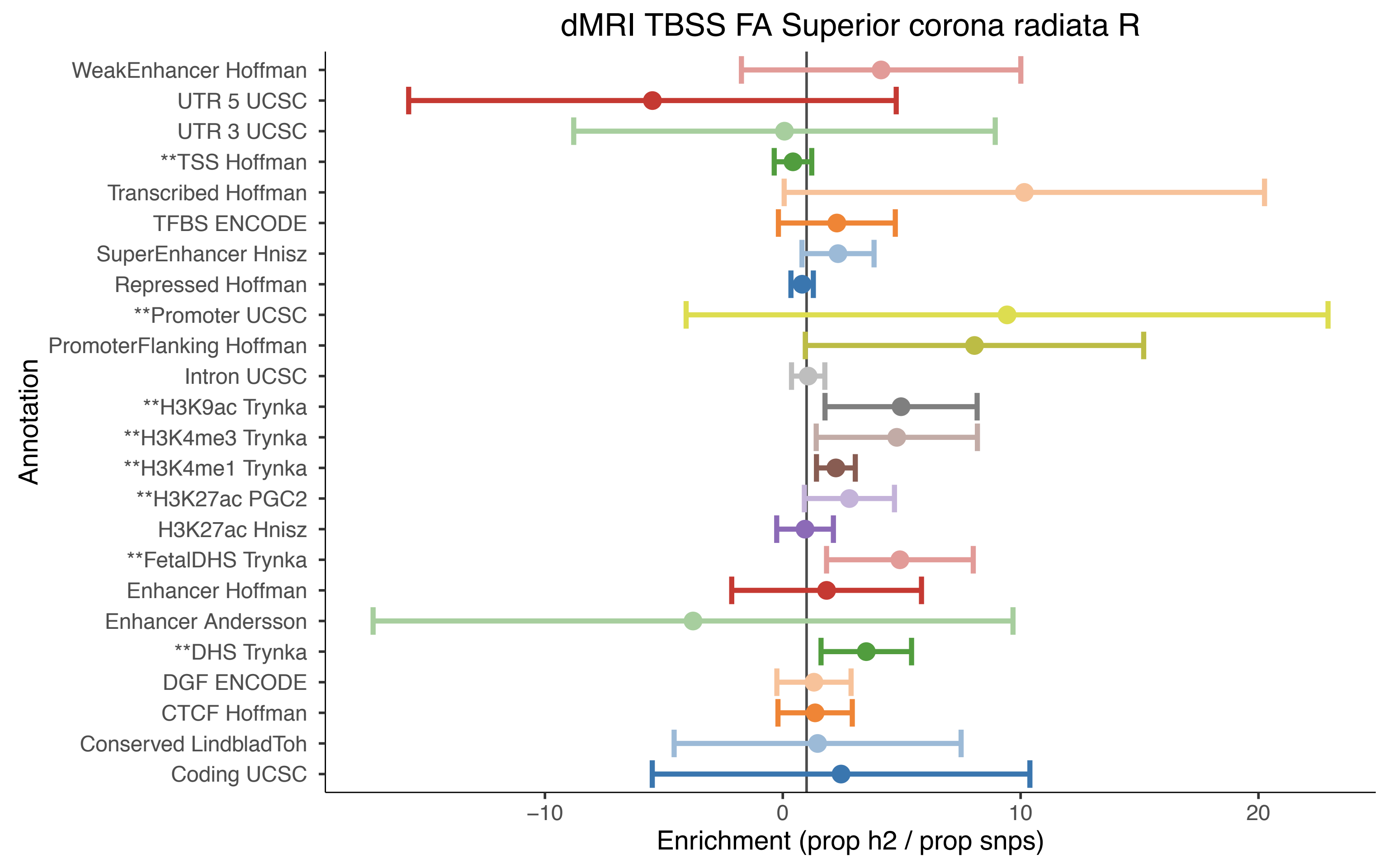

dMRI TBSS FA Superior corona radiata R
WeakEnhancer Hoffman
UTR 5 UCSC
UTR 3 UCSC
**TSS Hoffman
Transcribed Hoffman
TFBS ENCODE
SuperEnhancer Hnisz
Repressed Hoffman
**Promoter UCSC
PromoterFlanking Hoffman
Intron UCSC
**H3K9ac Trynka
**H3K4me3 Trynka
**H3K4me1 Trynka
**H3K27ac PGC2
H3K27ac Hnisz
**FetalDHS Trynka
Enhancer Hoffman
Enhancer Andersson
**DHS Trynka
DGF ENCODE
CTCF Hoffman
Conserved LindbladToh
Coding UCSC
Annotation
−10
0
10
20
Enrichment (prop h2 / prop snps)

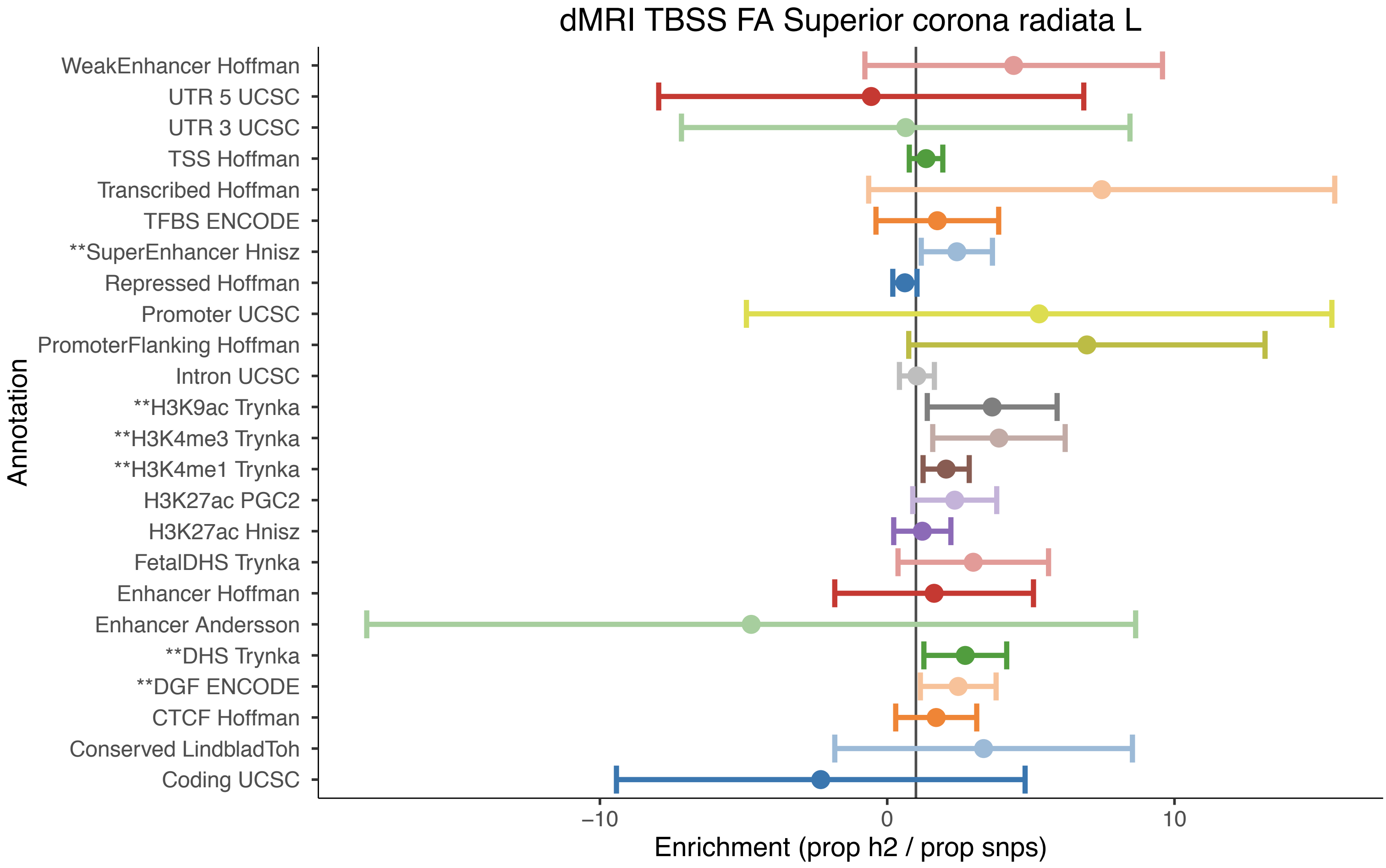
dMRI TBSS FA Superior corona radiata L
WeakEnhancer Hoffman
UTR 5 UCSC
UTR 3 UCSC
TSS Hoffman
Transcribed Hoffman
TFBS ENCODE
**SuperEnhancer Hnisz
Repressed Hoffman
Promoter UCSC
PromoterFlanking Hoffman
Intron UCSC
**H3K9ac Trynka
**H3K4me3 Trynka
**H3K4me1 Trynka
H3K27ac PGC2
H3K27ac Hnisz
FetalDHS Trynka
Enhancer Hoffman
Enhancer Andersson
**DHS Trynka
**DGF ENCODE
CTCF Hoffman
Conserved LindbladToh
Coding UCSC
Annotation
−10
0
10
Enrichment (prop h2 / prop snps)

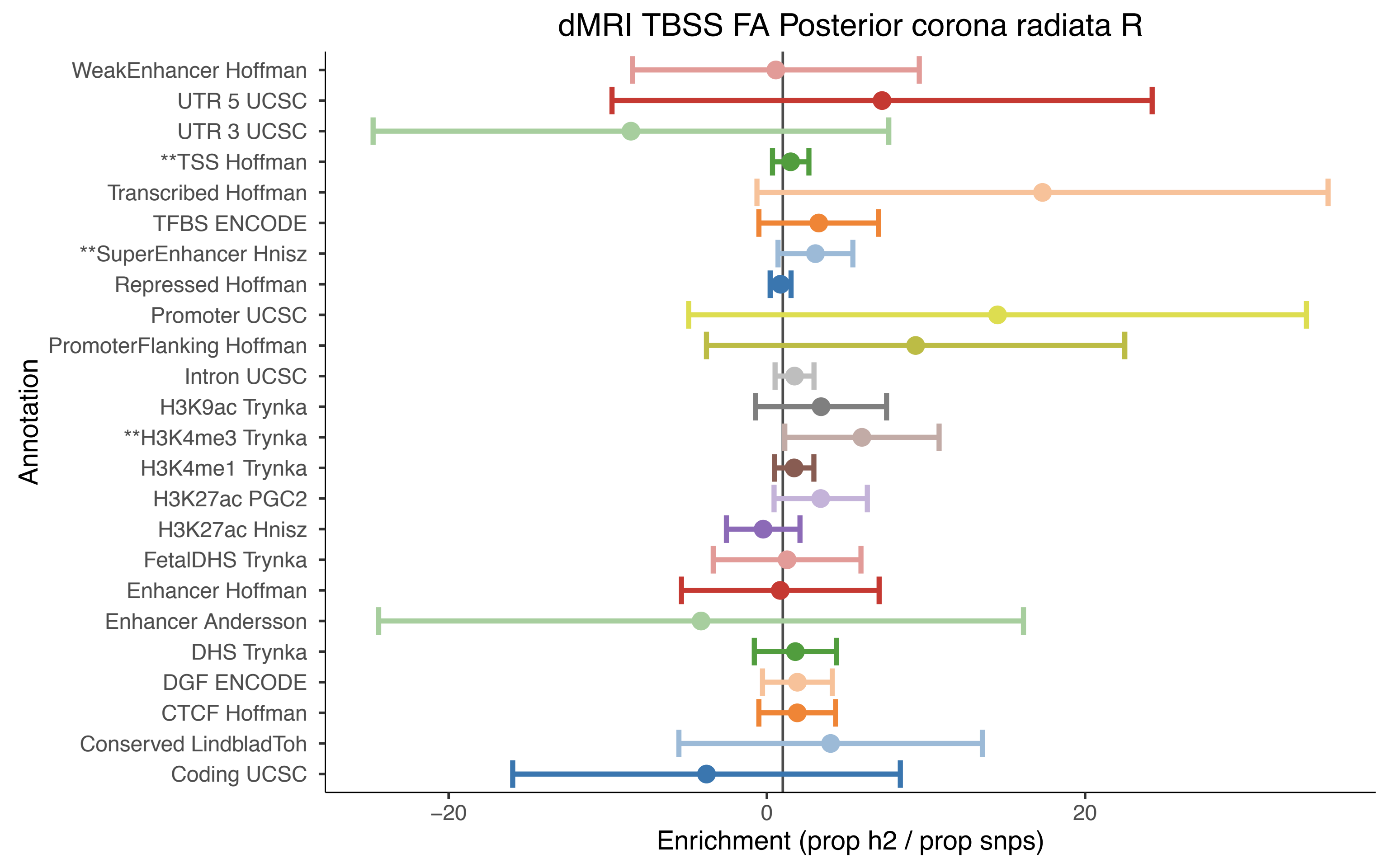
dMRI TBSS FA Posterior corona radiata R
WeakEnhancer Hoffman
UTR 5 UCSC
UTR 3 UCSC
**TSS Hoffman
Transcribed Hoffman
TFBS ENCODE
**SuperEnhancer Hnisz
Repressed Hoffman
Promoter UCSC
PromoterFlanking Hoffman
Intron UCSC
H3K9ac Trynka
**H3K4me3 Trynka
H3K4me1 Trynka
H3K27ac PGC2
H3K27ac Hnisz
FetalDHS Trynka
Enhancer Hoffman
Enhancer Andersson
DHS Trynka
DGF ENCODE
CTCF Hoffman
Conserved LindbladToh
Coding UCSC
Annotation
−20
0
20
Enrichment (prop h2 / prop snps)

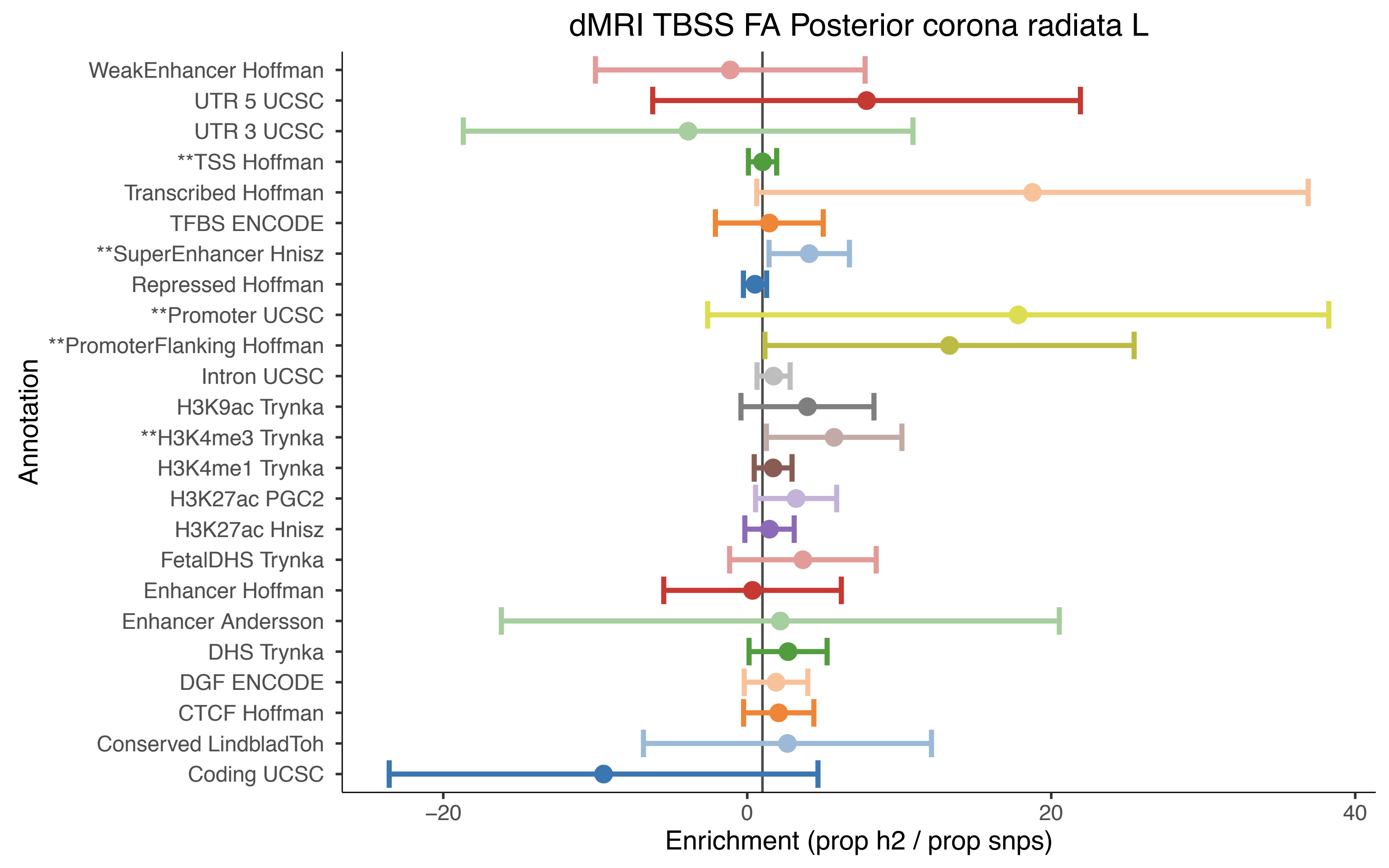

dMRI TBSS FA Posterior corona radiata L
WeakEnhancer Hoffman
UTR 5 UCSC
UTR 3 UCSC
**TSS Hoffman
Transcribed Hoffman
TFBS ENCODE
**SuperEnhancer Hnisz
Repressed Hoffman
**Promoter UCSC
**PromoterFlanking Hoffman
Intron UCSC
H3K9ac Trynka
**H3K4me3 Trynka
H3K4me1 Trynka
H3K27ac PGC2
H3K27ac Hnisz
FetalDHS Trynka
Enhancer Hoffman
Enhancer Andersson
DHS Trynka
DGF ENCODE
CTCF Hoffman
Conserved LindbladToh
Coding UCSC
Annotation
−20
0
20
40
Enrichment (prop h2 / prop snps)

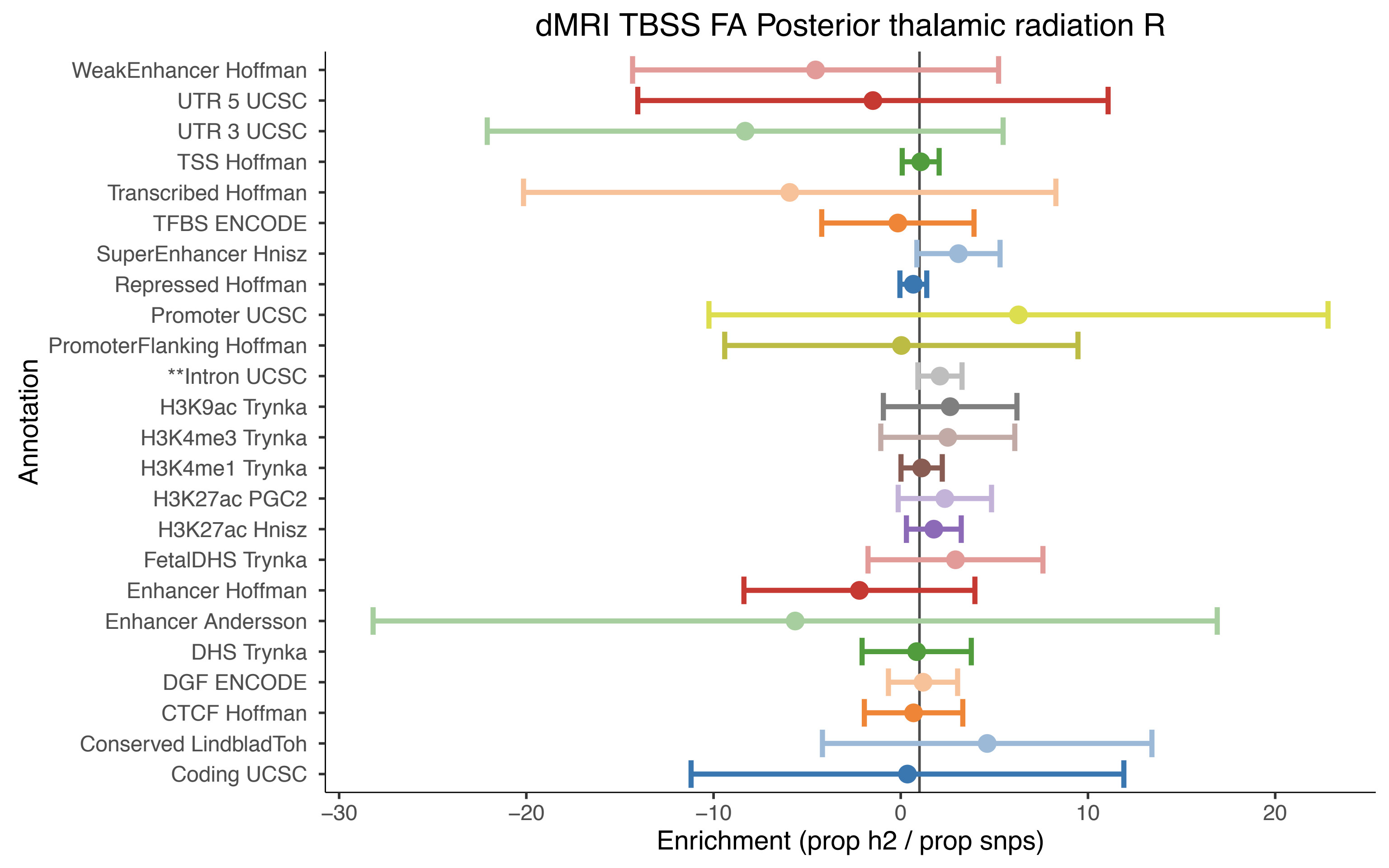

dMRI TBSS FA Posterior thalamic radiation R
WeakEnhancer Hoffman
UTR 5 UCSC
UTR 3 UCSC
TSS Hoffman
Transcribed Hoffman
TFBS ENCODE
SuperEnhancer Hnisz
Repressed Hoffman
Promoter UCSC
PromoterFlanking Hoffman
**Intron UCSC
H3K9ac Trynka
H3K4me3 Trynka
H3K4me1 Trynka
H3K27ac PGC2
H3K27ac Hnisz
FetalDHS Trynka
Enhancer Hoffman
Enhancer Andersson
DHS Trynka
DGF ENCODE
CTCF Hoffman
Conserved LindbladToh
Coding UCSC
Annotation
−30
−20
−10
0
10
20
Enrichment (prop h2 / prop snps)

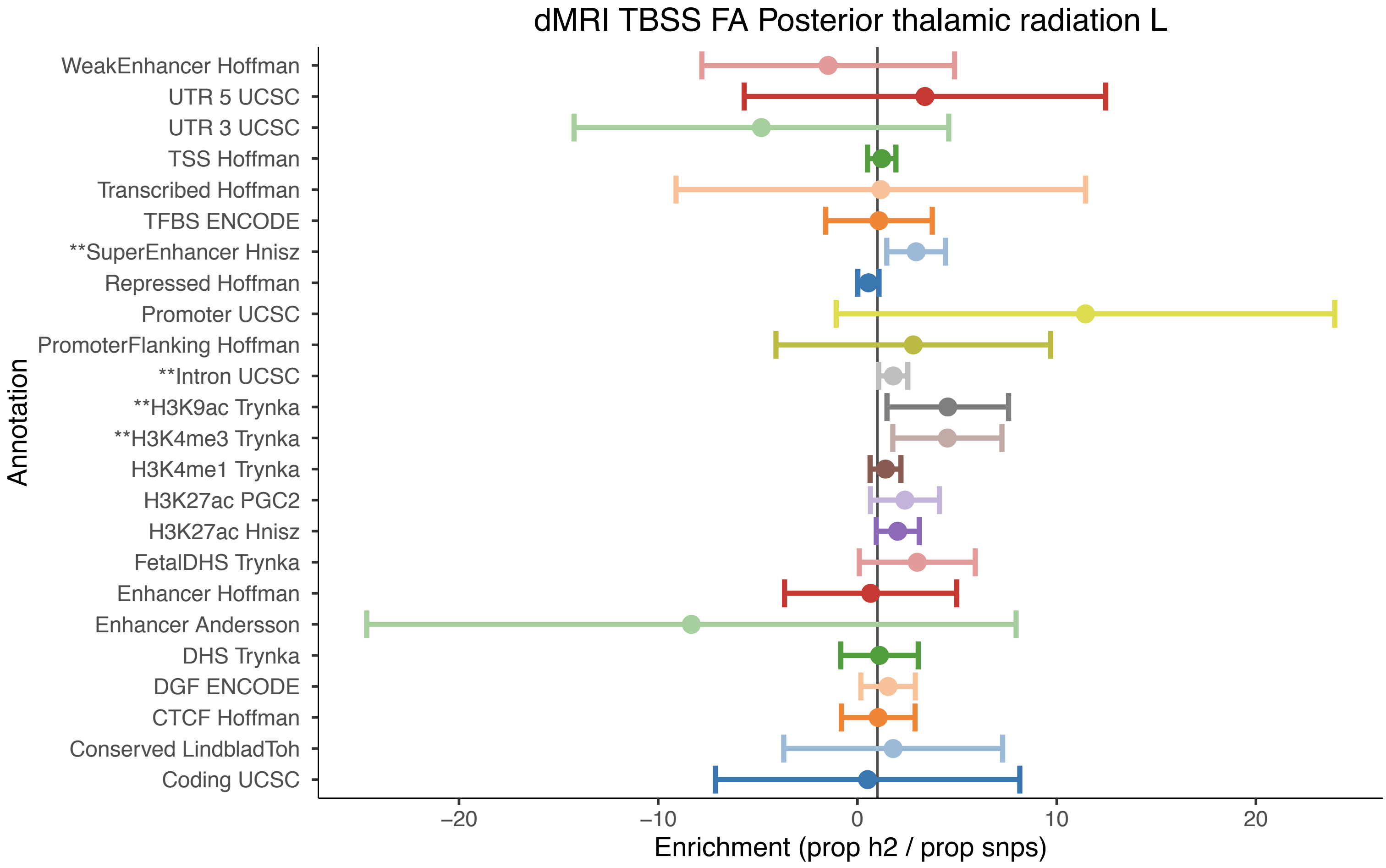

dMRI TBSS FA Posterior thalamic radiation L
WeakEnhancer Hoffman
UTR 5 UCSC
UTR 3 UCSC
TSS Hoffman
Transcribed Hoffman
TFBS ENCODE
**SuperEnhancer Hnisz
Repressed Hoffman
Promoter UCSC
PromoterFlanking Hoffman
**Intron UCSC
**H3K9ac Trynka
**H3K4me3 Trynka
H3K4me1 Trynka
H3K27ac PGC2
H3K27ac Hnisz
FetalDHS Trynka
Enhancer Hoffman
Enhancer Andersson
DHS Trynka
DGF ENCODE
CTCF Hoffman
Conserved LindbladToh
Coding UCSC
Annotation
−20
−10
0
10
20
Enrichment (prop h2 / prop snps)

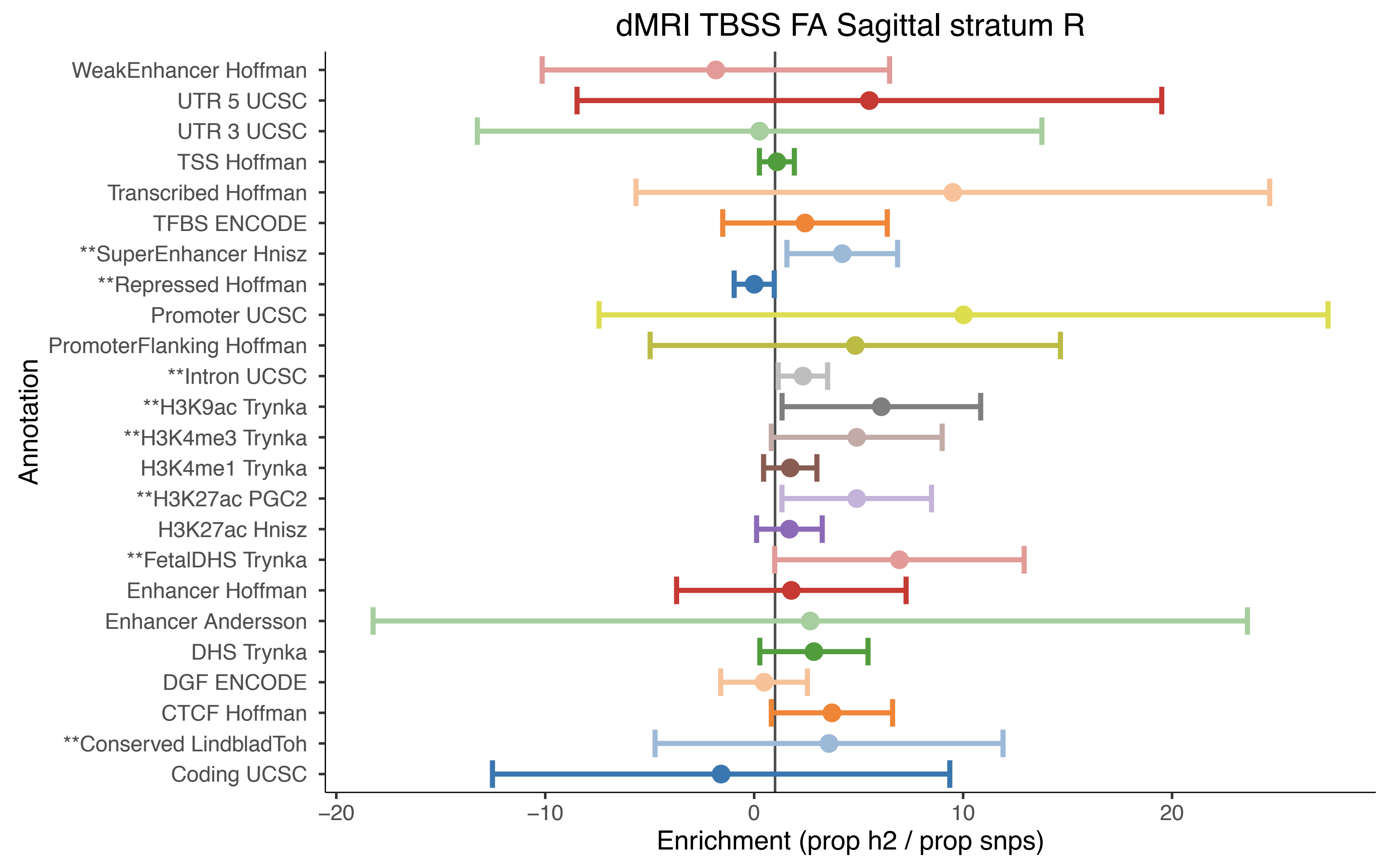

dMRI TBSS FA Sagittal stratum R
WeakEnhancer Hoffman
UTR 5 UCSC
UTR 3 UCSC
TSS Hoffman
Transcribed Hoffman
TFBS ENCODE
**SuperEnhancer Hnisz
**Repressed Hoffman
Promoter UCSC
PromoterFlanking Hoffman
**Intron UCSC
**H3K9ac Trynka
**H3K4me3 Trynka
H3K4me1 Trynka
**H3K27ac PGC2
H3K27ac Hnisz
**FetalDHS Trynka
Enhancer Hoffman
Enhancer Andersson
DHS Trynka
DGF ENCODE
CTCF Hoffman
**Conserved LindbladToh
Coding UCSC
Annotation
−20
−10
0
10
20
Enrichment (prop h2 / prop snps)

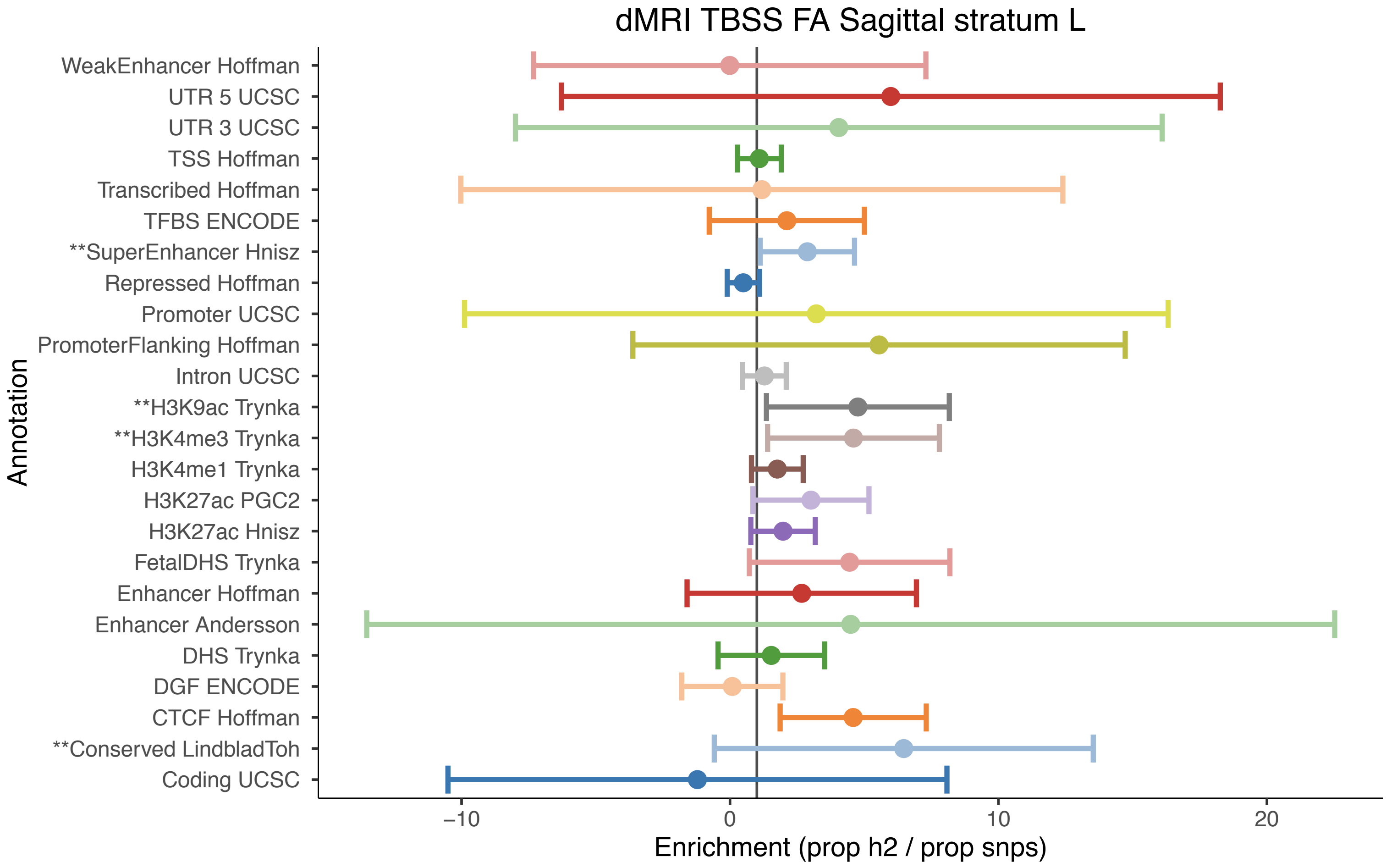

dMRI TBSS FA Sagittal stratum L
WeakEnhancer Hoffman
UTR 5 UCSC
UTR 3 UCSC
TSS Hoffman
Transcribed Hoffman
TFBS ENCODE
**SuperEnhancer Hnisz
Repressed Hoffman
Promoter UCSC
PromoterFlanking Hoffman
Intron UCSC
**H3K9ac Trynka
**H3K4me3 Trynka
H3K4me1 Trynka
H3K27ac PGC2
H3K27ac Hnisz
FetalDHS Trynka
Enhancer Hoffman
Enhancer Andersson
DHS Trynka
DGF ENCODE
CTCF Hoffman
**Conserved LindbladToh
Coding UCSC
Annotation
−10
0
10
20
Enrichment (prop h2 / prop snps)

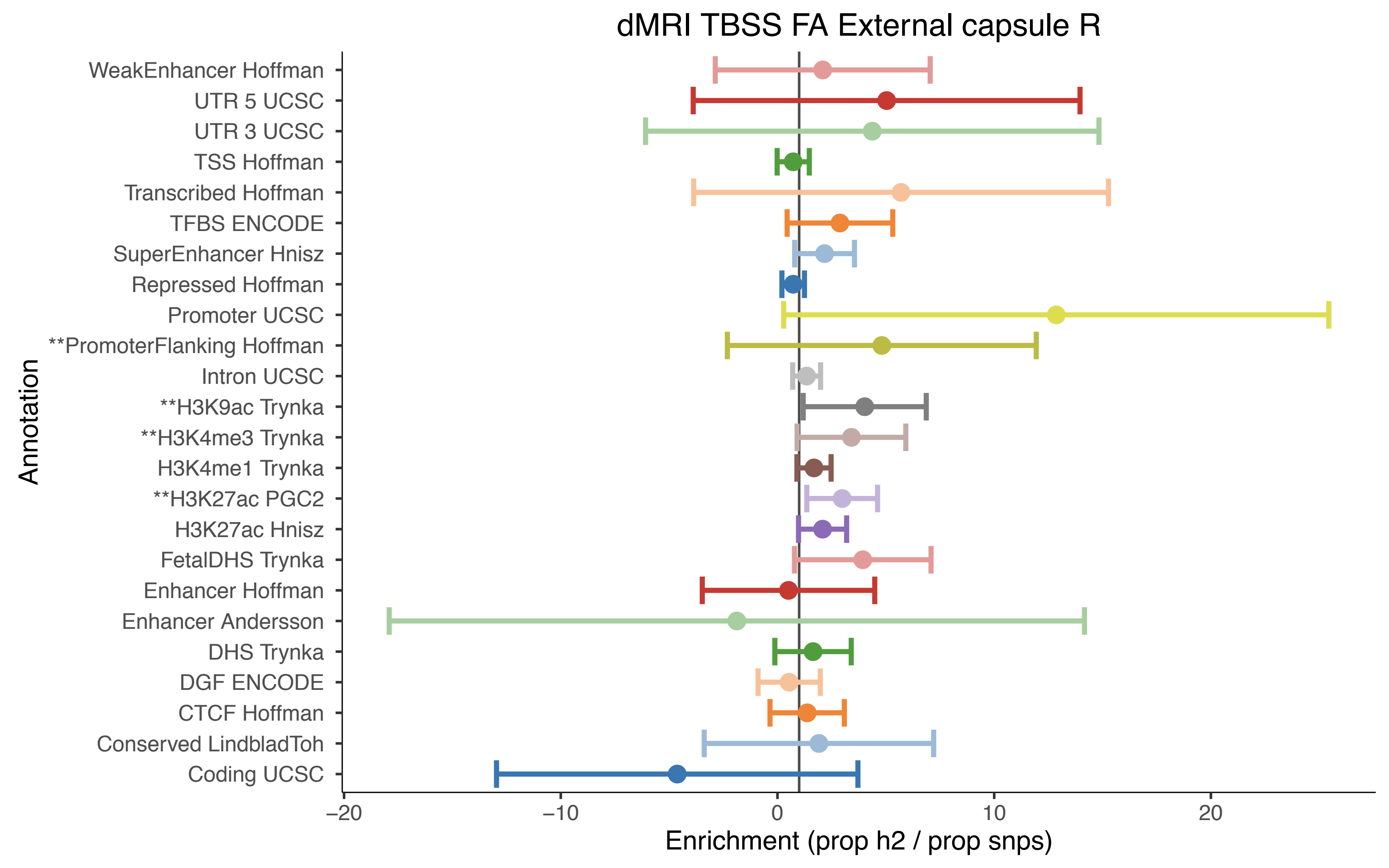
dMRI TBSS FA External capsule R
WeakEnhancer Hoffman
UTR 5 UCSC
UTR 3 UCSC
TSS Hoffman
Transcribed Hoffman
TFBS ENCODE
SuperEnhancer Hnisz
Repressed Hoffman
Promoter UCSC
**PromoterFlanking Hoffman
Intron UCSC
**H3K9ac Trynka
**H3K4me3 Trynka
H3K4me1 Trynka
**H3K27ac PGC2
H3K27ac Hnisz
FetalDHS Trynka
Enhancer Hoffman
Enhancer Andersson
DHS Trynka
DGF ENCODE
CTCF Hoffman
Conserved LindbladToh
Coding UCSC
Annotation
−20
−10
0
10
20
Enrichment (prop h2 / prop snps)

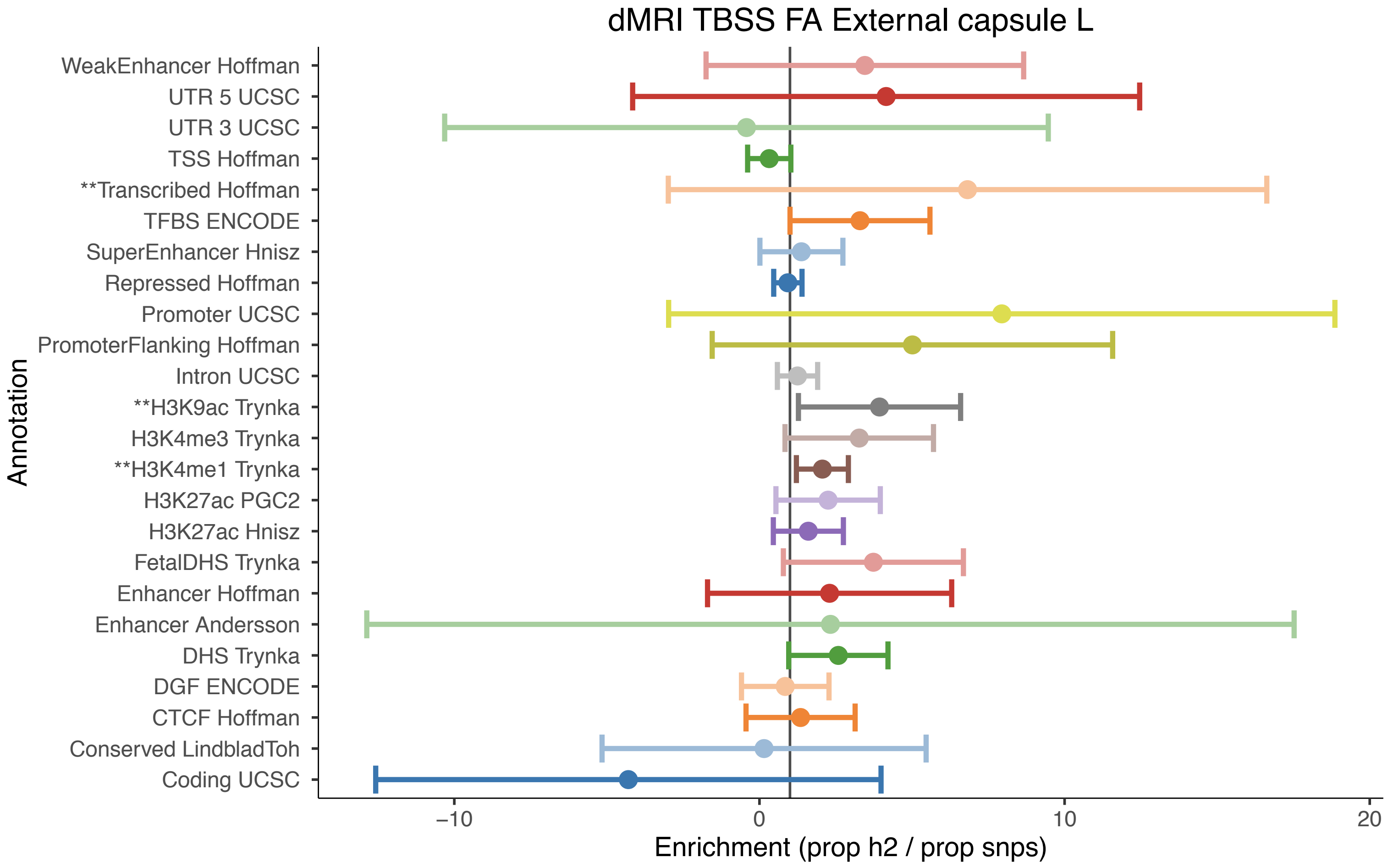

dMRI TBSS FA External capsule L
WeakEnhancer Hoffman
UTR 5 UCSC
UTR 3 UCSC
TSS Hoffman
**Transcribed Hoffman
TFBS ENCODE
SuperEnhancer Hnisz
Repressed Hoffman
Promoter UCSC
PromoterFlanking Hoffman
Intron UCSC
**H3K9ac Trynka
H3K4me3 Trynka
**H3K4me1 Trynka
H3K27ac PGC2
H3K27ac Hnisz
FetalDHS Trynka
Enhancer Hoffman
Enhancer Andersson
DHS Trynka
DGF ENCODE
CTCF Hoffman
Conserved LindbladToh
Coding UCSC
Annotation
−10
0
10
20
Enrichment (prop h2 / prop snps)

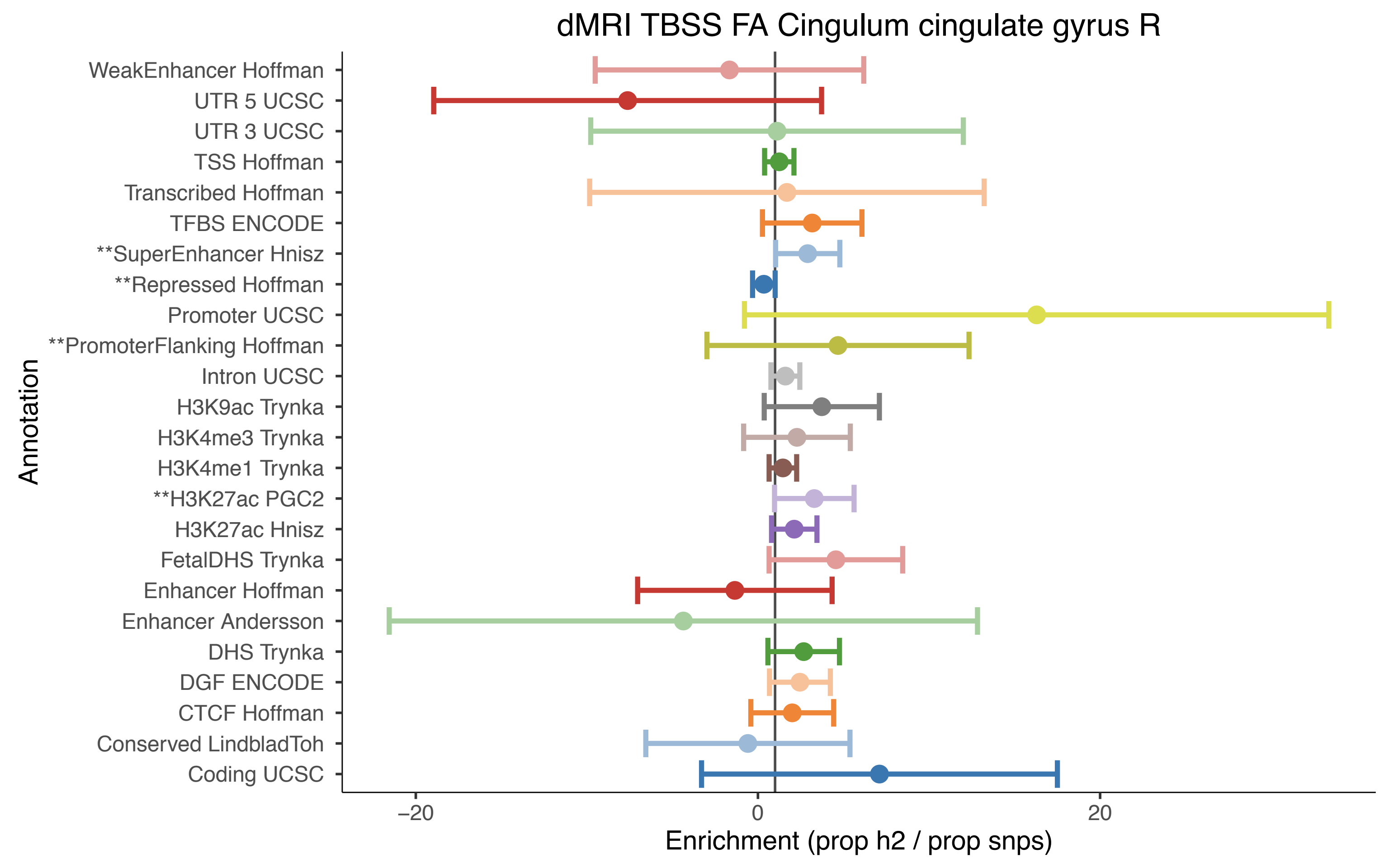

dMRI TBSS FA Cingulum cingulate gyrus R
WeakEnhancer Hoffman
UTR 5 UCSC
UTR 3 UCSC
TSS Hoffman
Transcribed Hoffman
TFBS ENCODE
**SuperEnhancer Hnisz
**Repressed Hoffman
Promoter UCSC
**PromoterFlanking Hoffman
Intron UCSC
H3K9ac Trynka
H3K4me3 Trynka
H3K4me1 Trynka
**H3K27ac PGC2
H3K27ac Hnisz
FetalDHS Trynka
Enhancer Hoffman
Enhancer Andersson
DHS Trynka
DGF ENCODE
CTCF Hoffman
Conserved LindbladToh
Coding UCSC
Annotation
−20
0
20
Enrichment (prop h2 / prop snps)

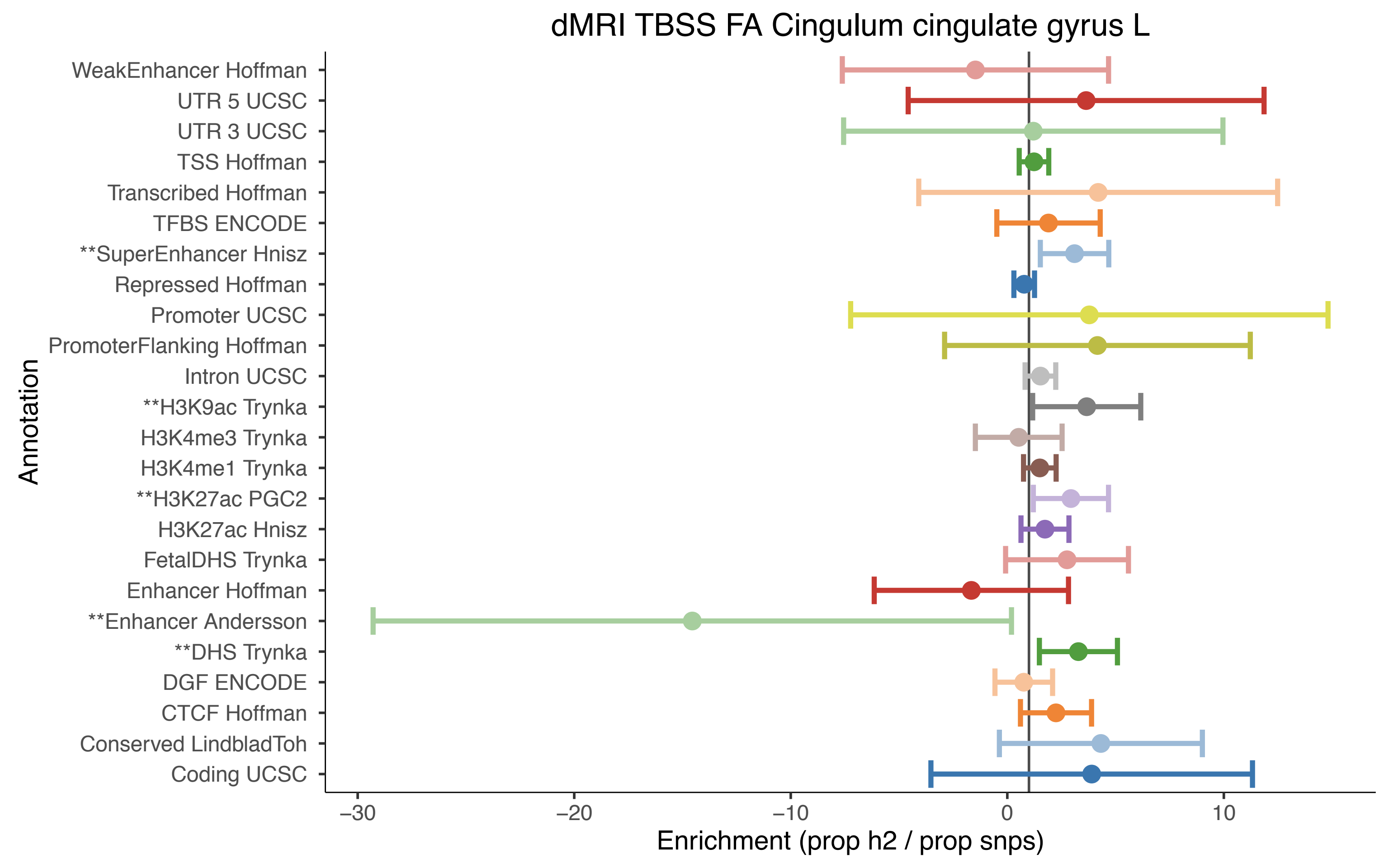
dMRI TBSS FA Cingulum cingulate gyrus L
WeakEnhancer Hoffman
UTR 5 UCSC
UTR 3 UCSC
TSS Hoffman
Transcribed Hoffman
TFBS ENCODE
**SuperEnhancer Hnisz
Repressed Hoffman
Promoter UCSC
PromoterFlanking Hoffman
Intron UCSC
**H3K9ac Trynka
H3K4me3 Trynka
H3K4me1 Trynka
**H3K27ac PGC2
H3K27ac Hnisz
FetalDHS Trynka
Enhancer Hoffman
**Enhancer Andersson
**DHS Trynka
DGF ENCODE
CTCF Hoffman
Conserved LindbladToh
Coding UCSC
Annotation
−30
−20
−10
0
10
Enrichment (prop h2 / prop snps)

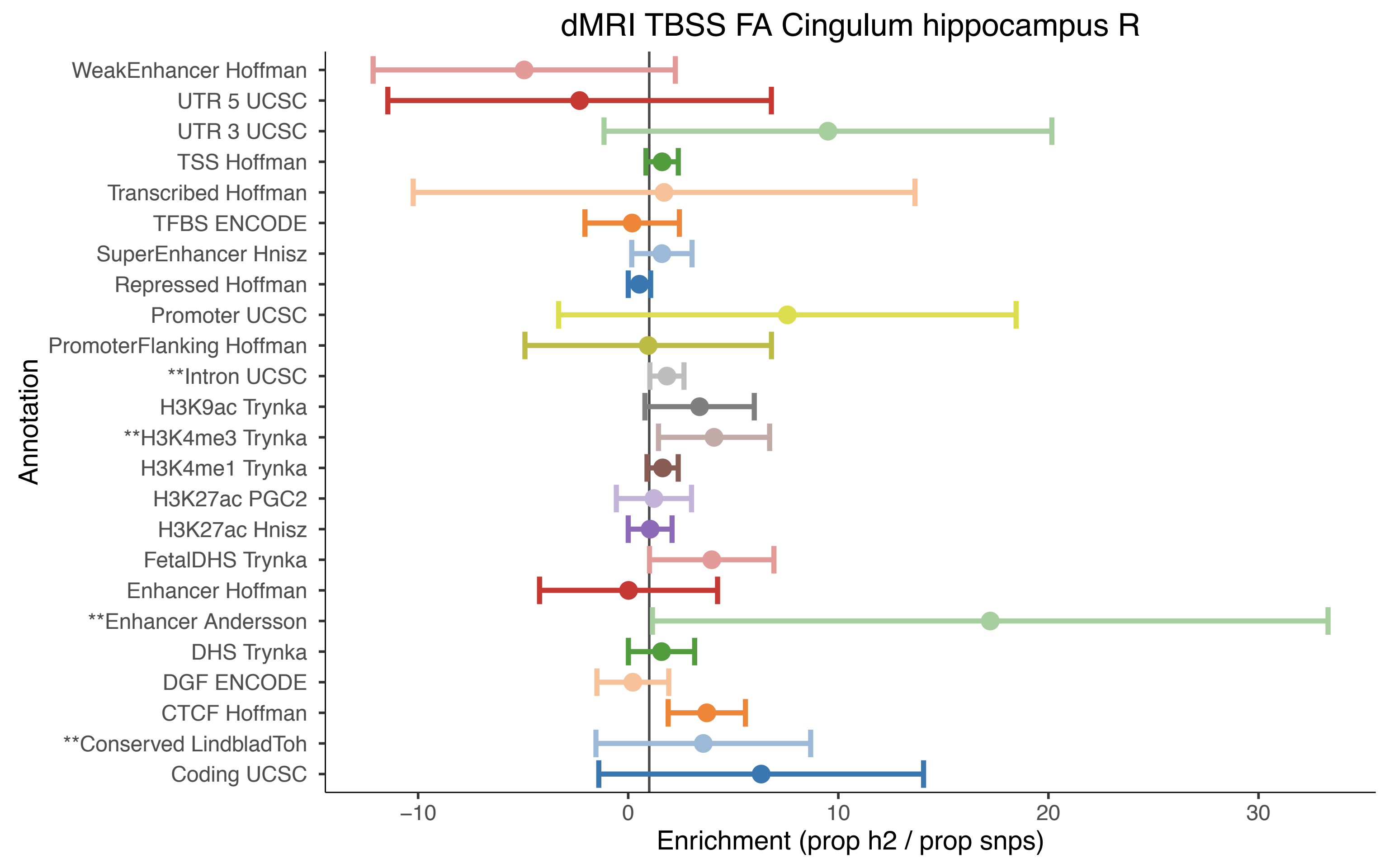

dMRI TBSS FA Cingulum hippocampus R
WeakEnhancer Hoffman
UTR 5 UCSC
UTR 3 UCSC
TSS Hoffman
Transcribed Hoffman
TFBS ENCODE
SuperEnhancer Hnisz
Repressed Hoffman
Promoter UCSC
PromoterFlanking Hoffman
**Intron UCSC
H3K9ac Trynka
**H3K4me3 Trynka
H3K4me1 Trynka
H3K27ac PGC2
H3K27ac Hnisz
FetalDHS Trynka
Enhancer Hoffman
**Enhancer Andersson
DHS Trynka
DGF ENCODE
CTCF Hoffman
**Conserved LindbladToh
Coding UCSC
Annotation
−10
0
10
20
30
Enrichment (prop h2 / prop snps)

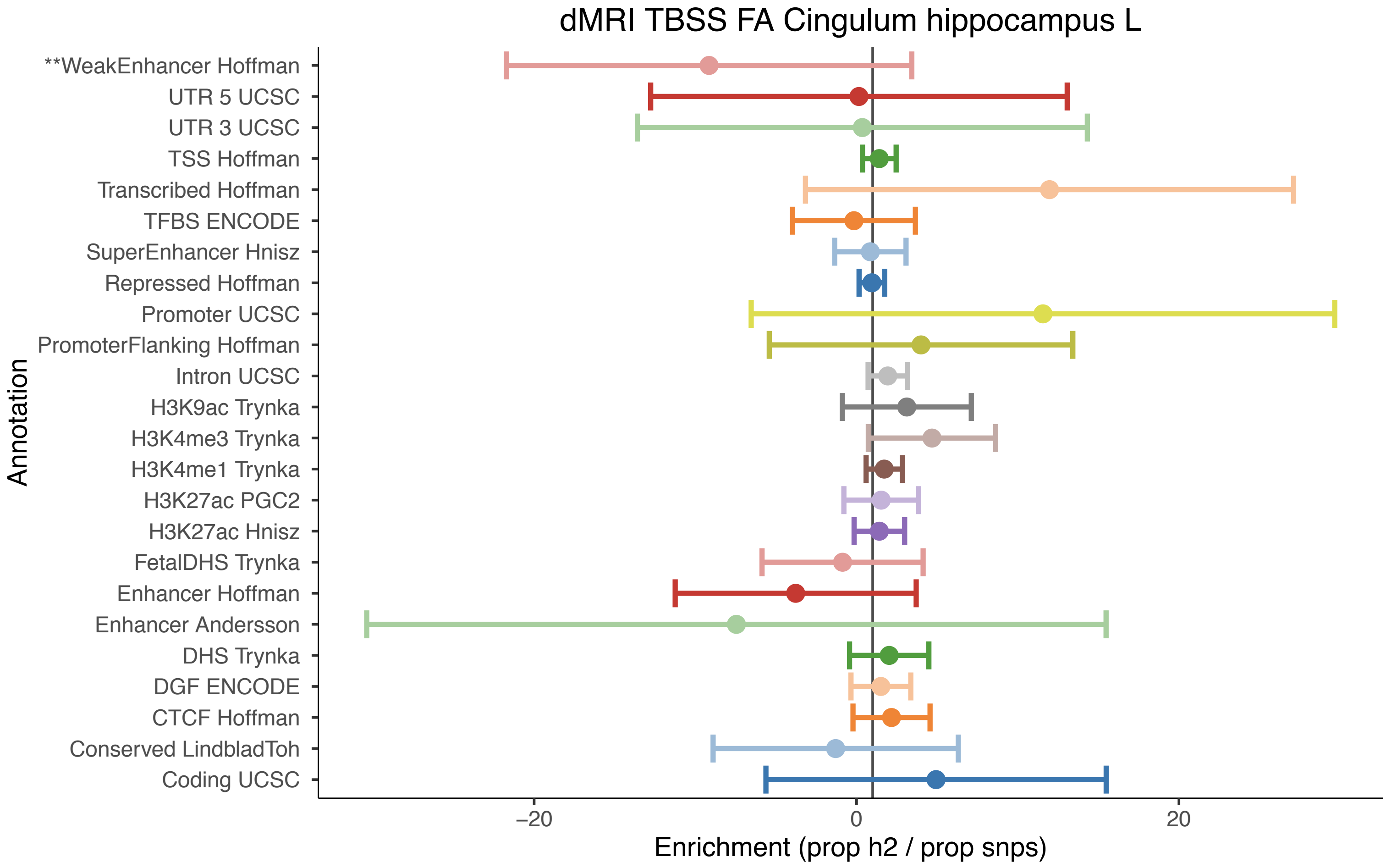

dMRI TBSS FA Cingulum hippocampus L
**WeakEnhancer Hoffman
UTR 5 UCSC
UTR 3 UCSC
TSS Hoffman
Transcribed Hoffman
TFBS ENCODE
SuperEnhancer Hnisz
Repressed Hoffman
Promoter UCSC
PromoterFlanking Hoffman
Intron UCSC
H3K9ac Trynka
H3K4me3 Trynka
H3K4me1 Trynka
H3K27ac PGC2
H3K27ac Hnisz
FetalDHS Trynka
Enhancer Hoffman
Enhancer Andersson
DHS Trynka
DGF ENCODE
CTCF Hoffman
Conserved LindbladToh
Coding UCSC
Annotation
−20
0
20
Enrichment (prop h2 / prop snps)

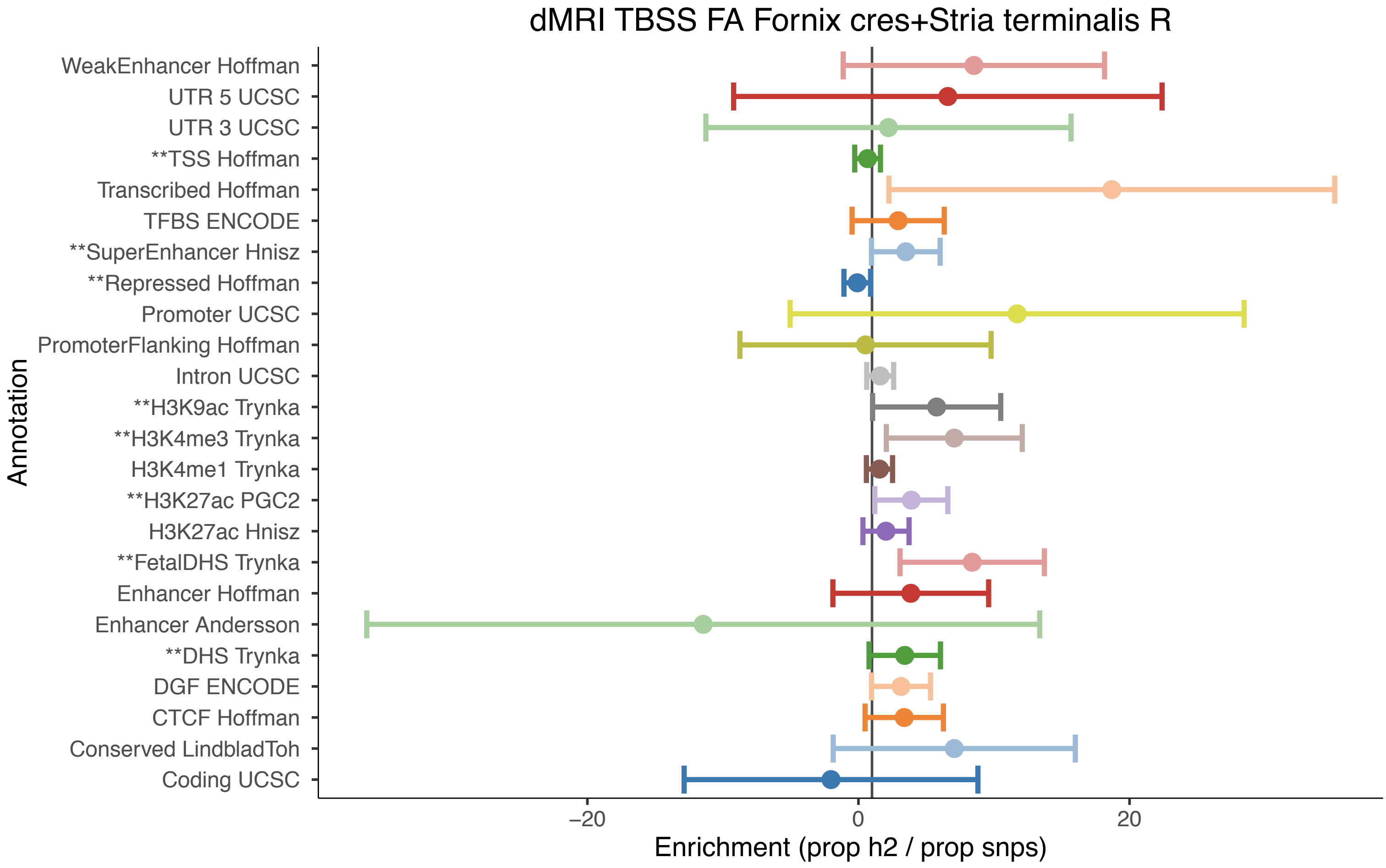
dMRI TBSS FA Fornix cres+Stria terminalis R
WeakEnhancer Hoffman
UTR 5 UCSC
UTR 3 UCSC
**TSS Hoffman
Transcribed Hoffman
TFBS ENCODE
**SuperEnhancer Hnisz
**Repressed Hoffman
Promoter UCSC
PromoterFlanking Hoffman
Intron UCSC
**H3K9ac Trynka
**H3K4me3 Trynka
H3K4me1 Trynka
**H3K27ac PGC2
H3K27ac Hnisz
**FetalDHS Trynka
Enhancer Hoffman
Enhancer Andersson
**DHS Trynka
DGF ENCODE
CTCF Hoffman
Conserved LindbladToh
Coding UCSC
Annotation
−20
0
20
Enrichment (prop h2 / prop snps)

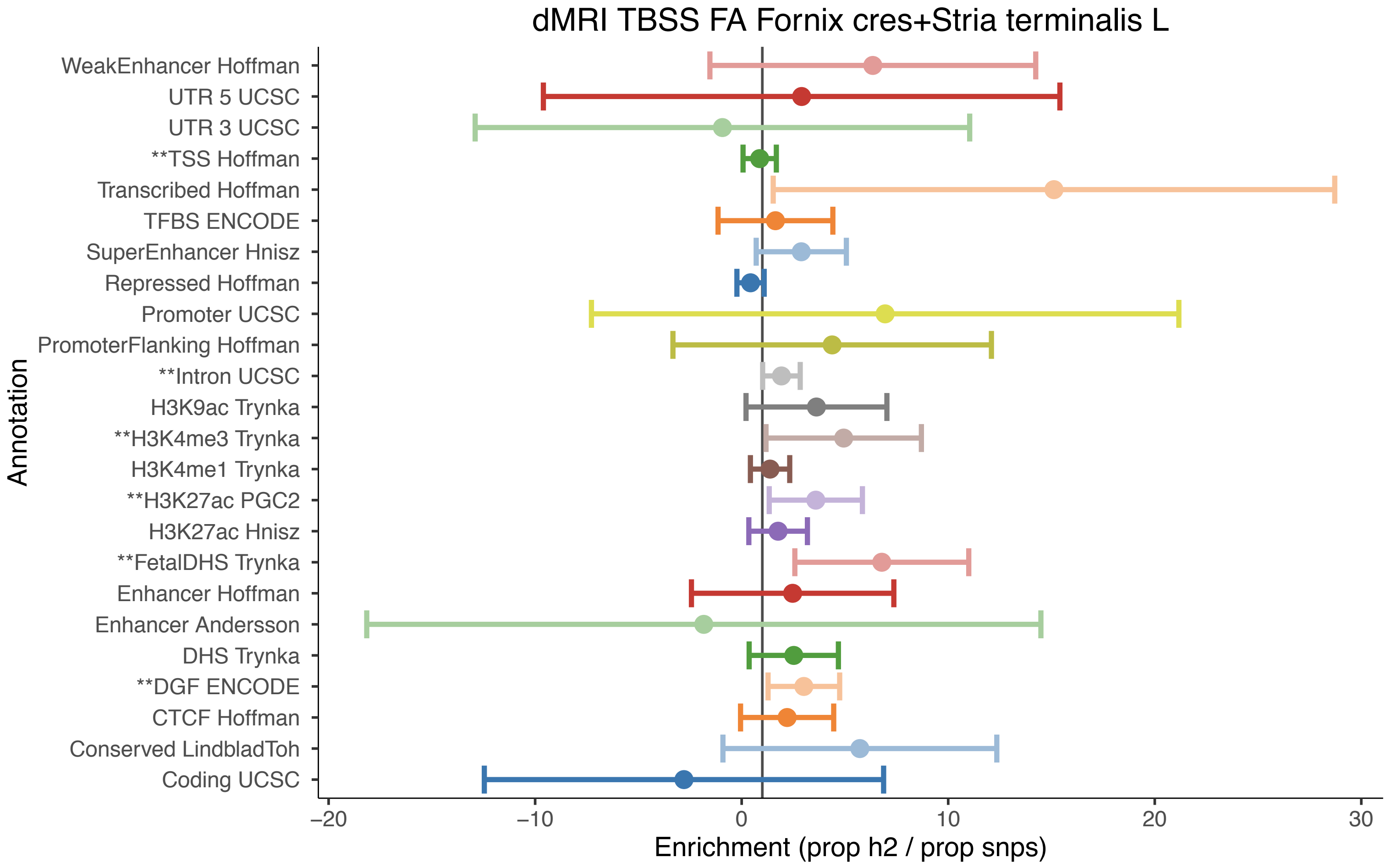
dMRI TBSS FA Fornix cres+Stria terminalis L
WeakEnhancer Hoffman
UTR 5 UCSC
UTR 3 UCSC
**TSS Hoffman
Transcribed Hoffman
TFBS ENCODE
SuperEnhancer Hnisz
Repressed Hoffman
Promoter UCSC
PromoterFlanking Hoffman
**Intron UCSC
H3K9ac Trynka
**H3K4me3 Trynka
H3K4me1 Trynka
**H3K27ac PGC2
H3K27ac Hnisz
**FetalDHS Trynka
Enhancer Hoffman
Enhancer Andersson
DHS Trynka
**DGF ENCODE
CTCF Hoffman
Conserved LindbladToh
Coding UCSC
Annotation
−20
−10
0
10
20
30
Enrichment (prop h2 / prop snps)

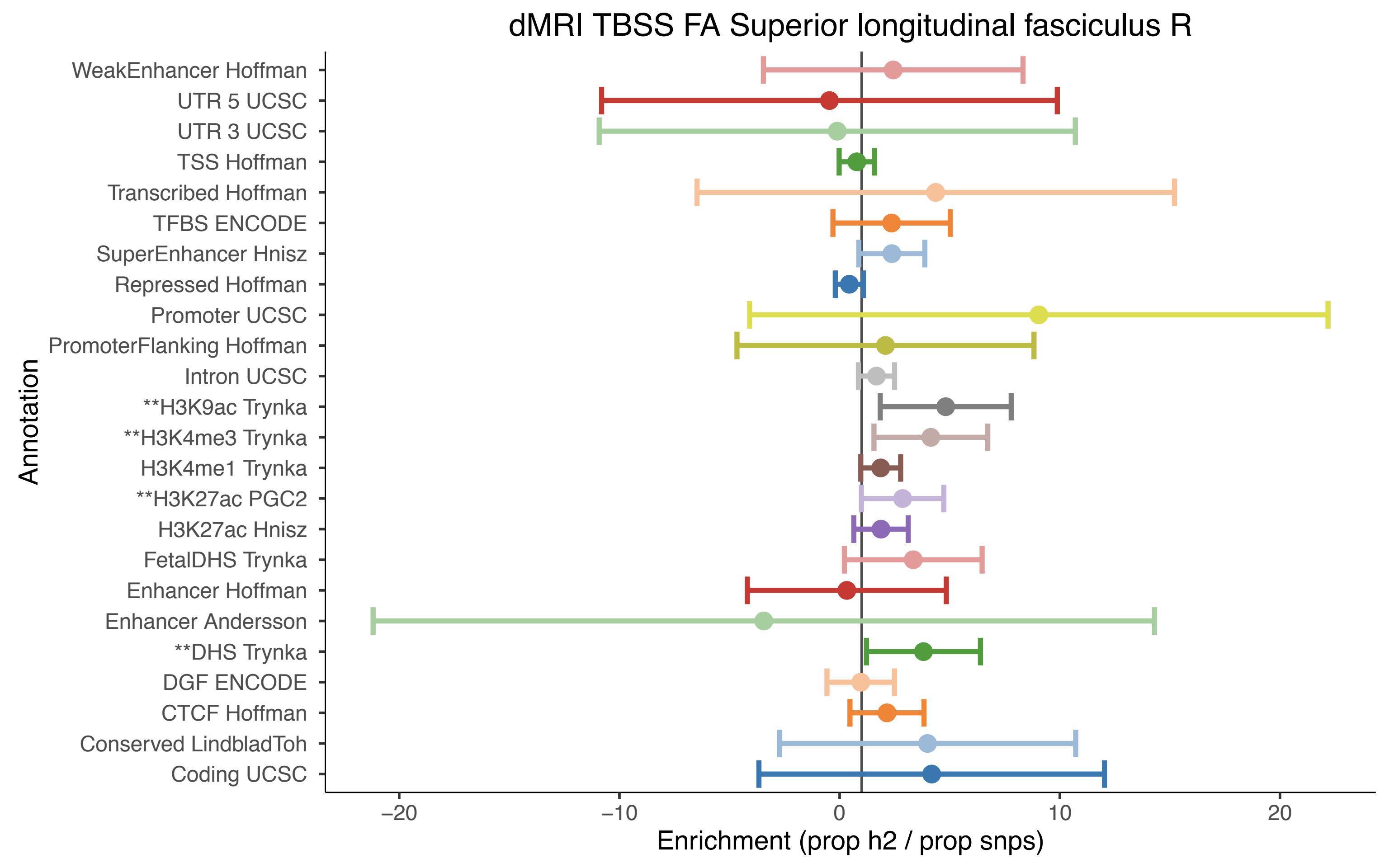

dMRI TBSS FA Superior longitudinal fasciculus R
WeakEnhancer Hoffman
UTR 5 UCSC
UTR 3 UCSC
TSS Hoffman
Transcribed Hoffman
TFBS ENCODE
SuperEnhancer Hnisz
Repressed Hoffman
Promoter UCSC
PromoterFlanking Hoffman
Intron UCSC
**H3K9ac Trynka
**H3K4me3 Trynka
H3K4me1 Trynka
**H3K27ac PGC2
H3K27ac Hnisz
FetalDHS Trynka
Enhancer Hoffman
Enhancer Andersson
**DHS Trynka
DGF ENCODE
CTCF Hoffman
Conserved LindbladToh
Coding UCSC
Annotation
−20
−10
0
10
20
Enrichment (prop h2 / prop snps)

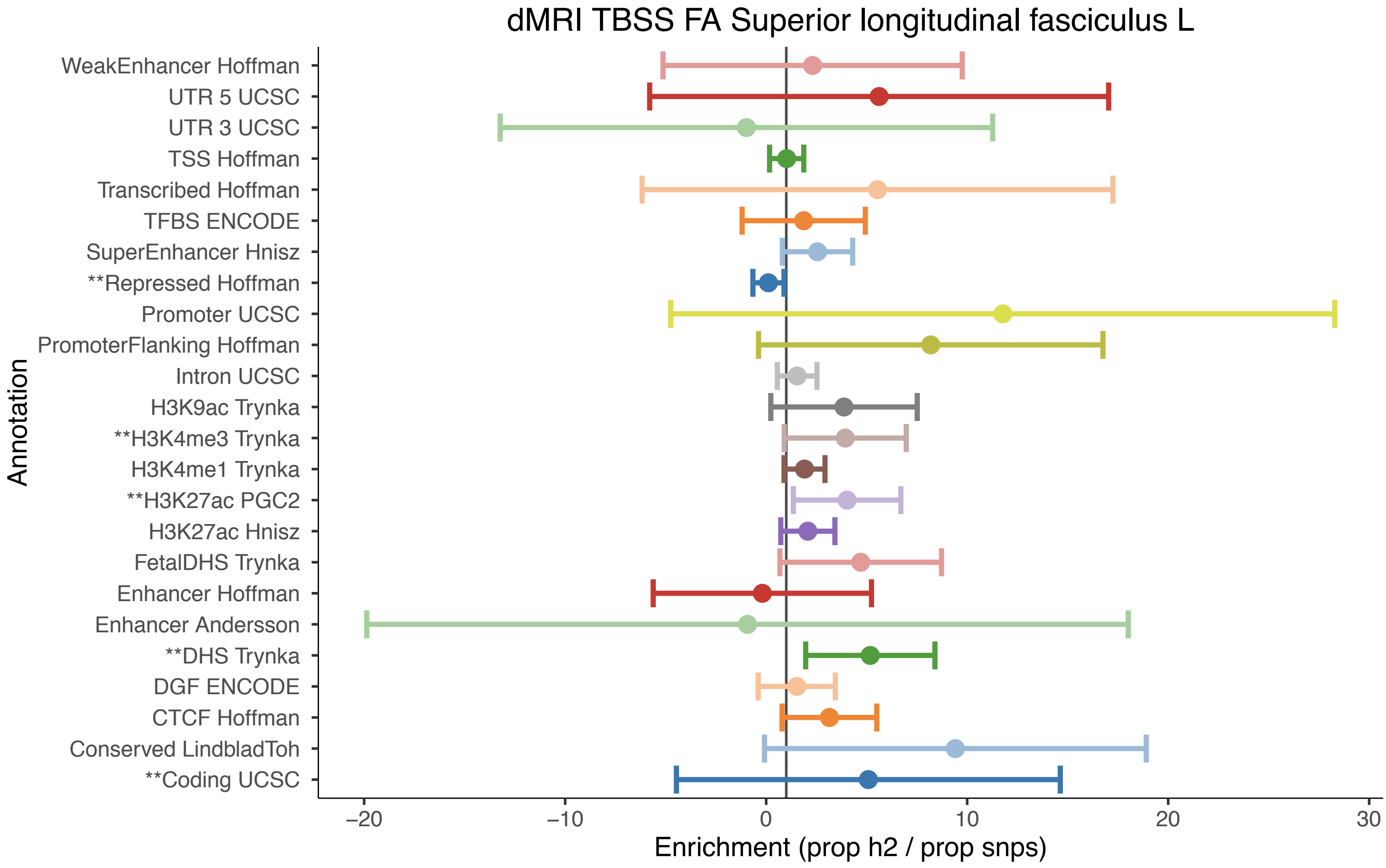
dMRI TBSS FA Superior longitudinal fasciculus L
Annotation
WeakEnhancer Hoffman
UTR 5 UCSC
UTR 3 UCSC
TSS Hoffman
Transcribed Hoffman
TFBS ENCODE
SuperEnhancer Hnisz
**Repressed Hoffman
Promoter UCSC
PromoterFlanking Hoffman
Intron UCSC
H3K9ac Trynka
**H3K4me3 Trynka
H3K4me1 Trynka
**H3K27ac PGC2
H3K27ac Hnisz
FetalDHS Trynka
Enhancer Hoffman
Enhancer Andersson
**DHS Trynka
DGF ENCODE
CTCF Hoffman
Conserved LindbladToh
**Coding UCSC
−20
−10
0
10
20
30
Enrichment (prop h2 / prop snps)

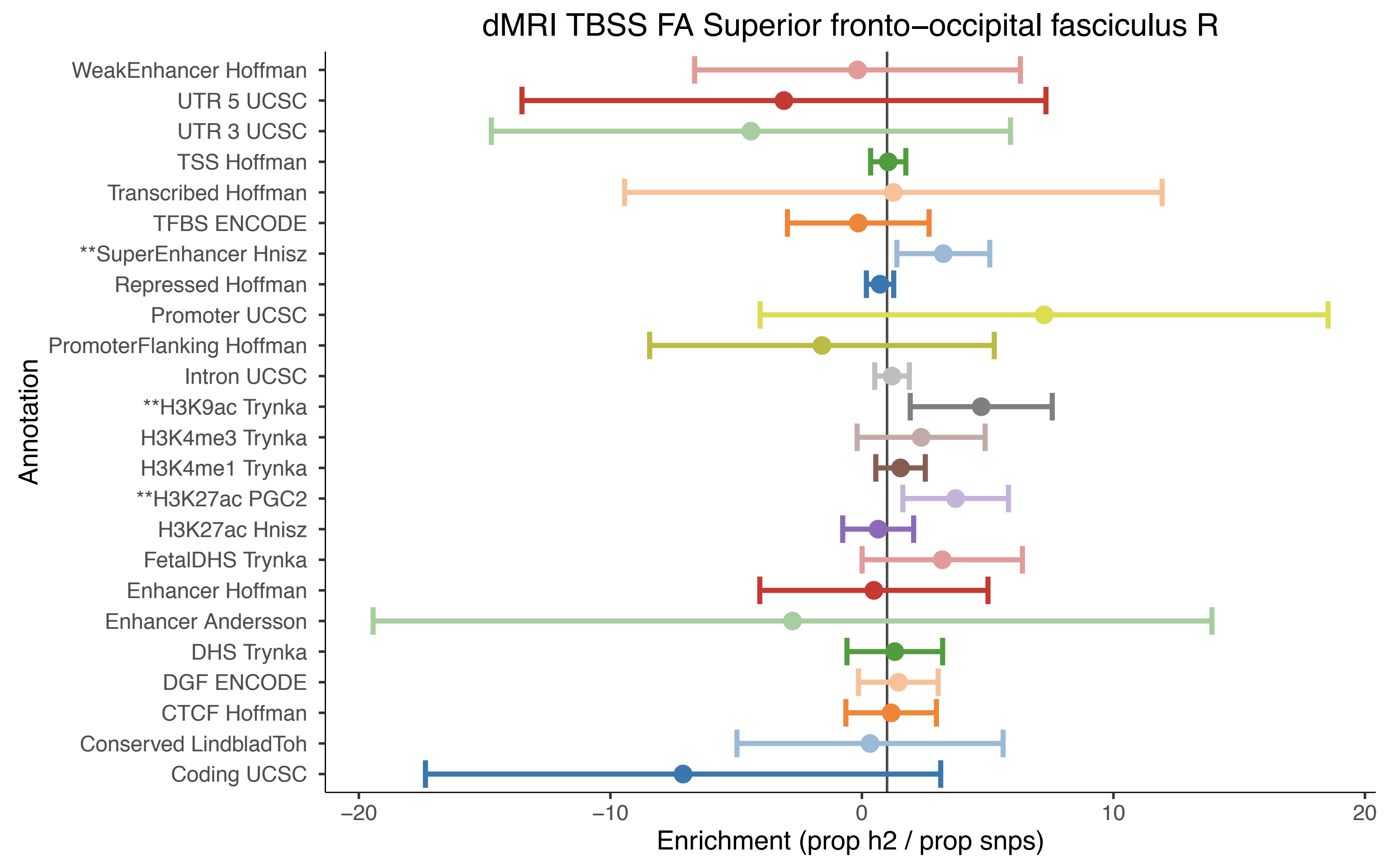
dMRI TBSS FA Superior fronto−occipital fasciculus R
WeakEnhancer Hoffman
UTR 5 UCSC
UTR 3 UCSC
TSS Hoffman
Transcribed Hoffman
TFBS ENCODE
**SuperEnhancer Hnisz
Repressed Hoffman
Promoter UCSC
PromoterFlanking Hoffman
Intron UCSC
**H3K9ac Trynka
H3K4me3 Trynka
H3K4me1 Trynka
**H3K27ac PGC2
H3K27ac Hnisz
FetalDHS Trynka
Enhancer Hoffman
Enhancer Andersson
DHS Trynka
DGF ENCODE
CTCF Hoffman
Conserved LindbladToh
Coding UCSC
Annotation
−20
−10
0
10
20
Enrichment (prop h2 / prop snps)

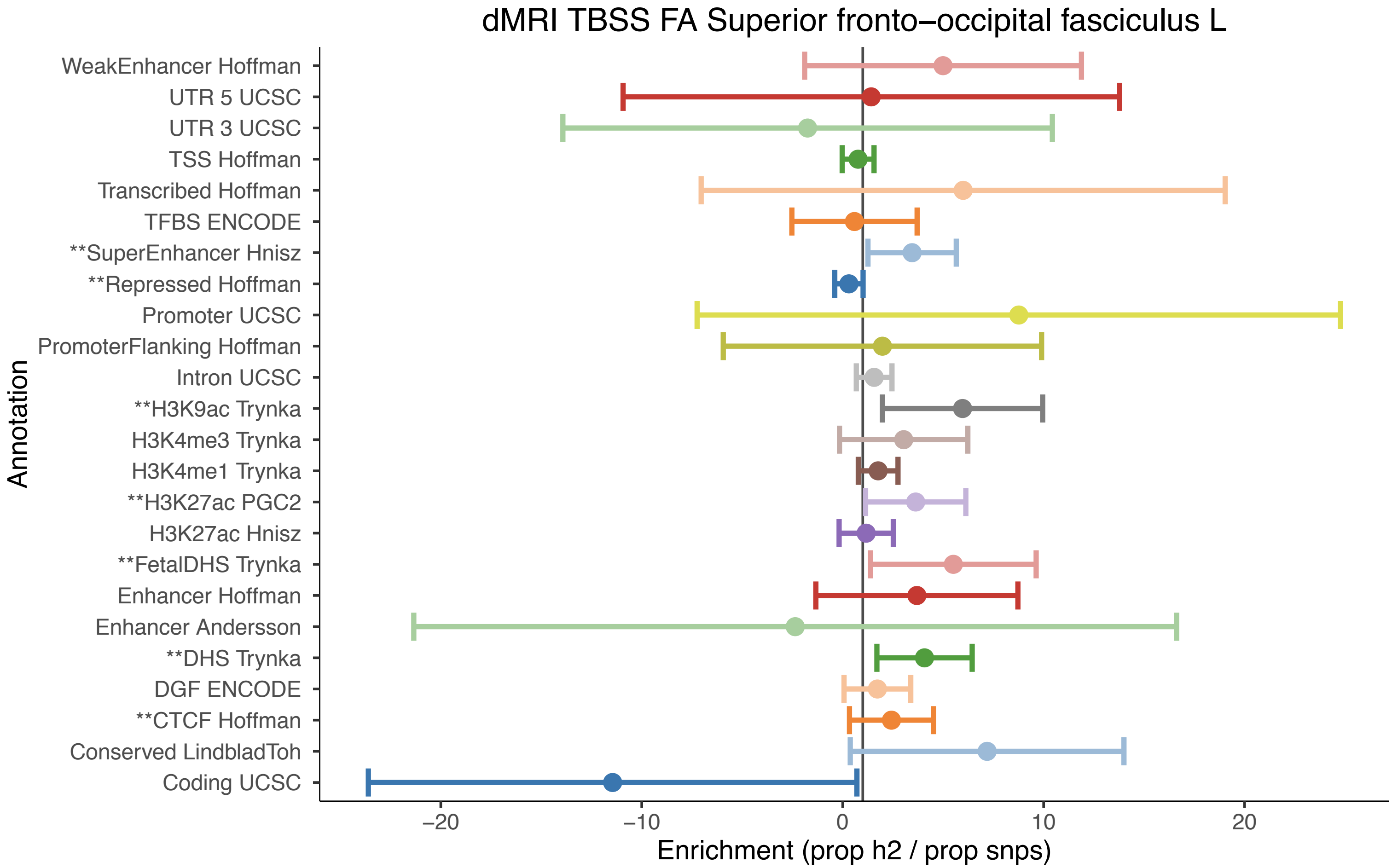

dMRI TBSS FA Superior fronto−occipital fasciculus L
WeakEnhancer Hoffman
UTR 5 UCSC
UTR 3 UCSC
TSS Hoffman
Transcribed Hoffman
TFBS ENCODE
**SuperEnhancer Hnisz
**Repressed Hoffman
Promoter UCSC
PromoterFlanking Hoffman
Intron UCSC
**H3K9ac Trynka
H3K4me3 Trynka
H3K4me1 Trynka
**H3K27ac PGC2
H3K27ac Hnisz
**FetalDHS Trynka
Enhancer Hoffman
Enhancer Andersson
**DHS Trynka
DGF ENCODE
**CTCF Hoffman
Conserved LindbladToh
Coding UCSC
Annotation
−20
−10
0
10
20
Enrichment (prop h2 / prop snps)

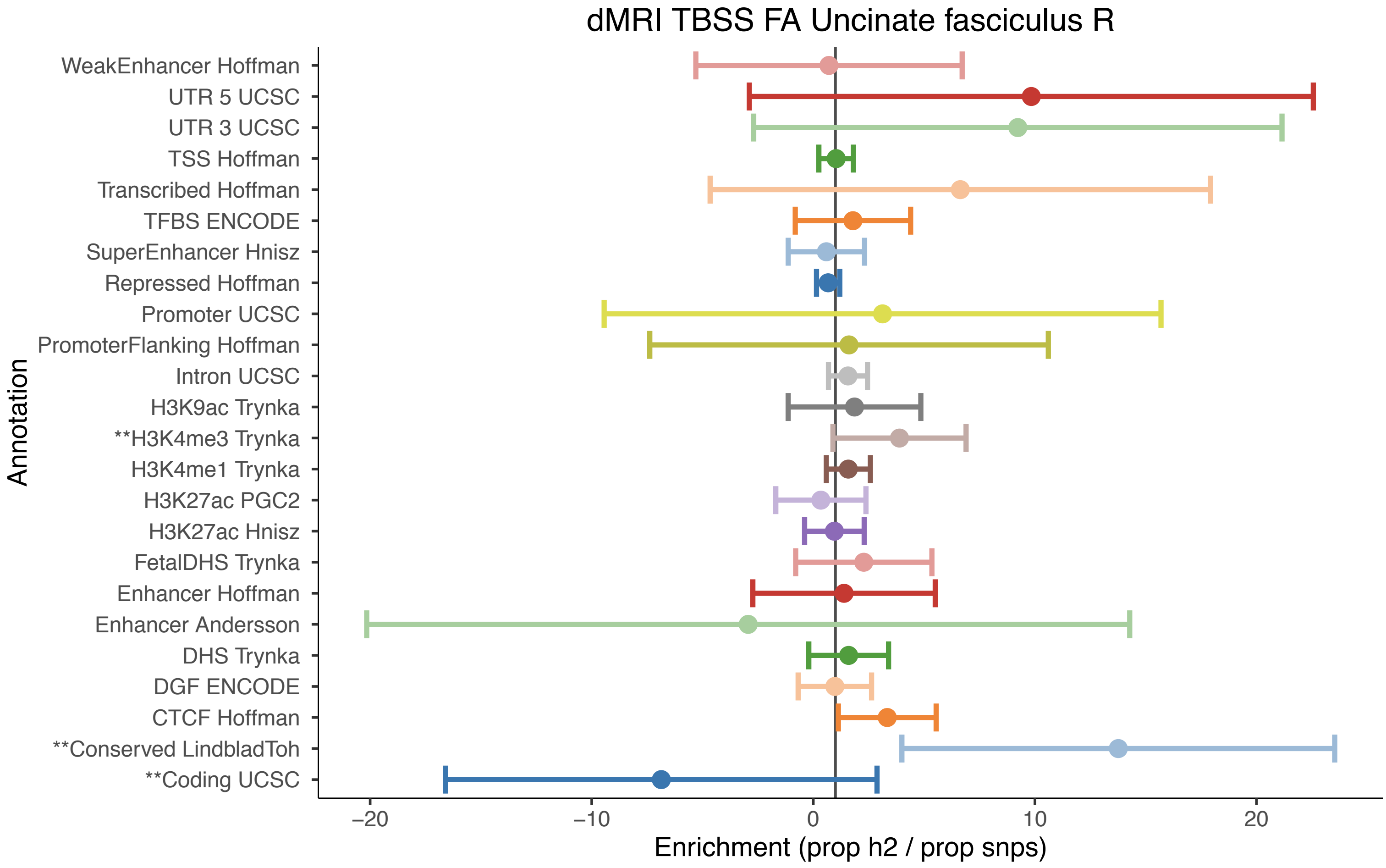

dMRI TBSS FA Uncinate fasciculus R
WeakEnhancer Hoffman
UTR 5 UCSC
UTR 3 UCSC
TSS Hoffman
Transcribed Hoffman
TFBS ENCODE
SuperEnhancer Hnisz
Repressed Hoffman
Promoter UCSC
PromoterFlanking Hoffman
Intron UCSC
H3K9ac Trynka
**H3K4me3 Trynka
H3K4me1 Trynka
H3K27ac PGC2
H3K27ac Hnisz
FetalDHS Trynka
Enhancer Hoffman
Enhancer Andersson
DHS Trynka
DGF ENCODE
CTCF Hoffman
**Conserved LindbladToh
**Coding UCSC
Annotation
−20
−10
0
10
20
Enrichment (prop h2 / prop snps)

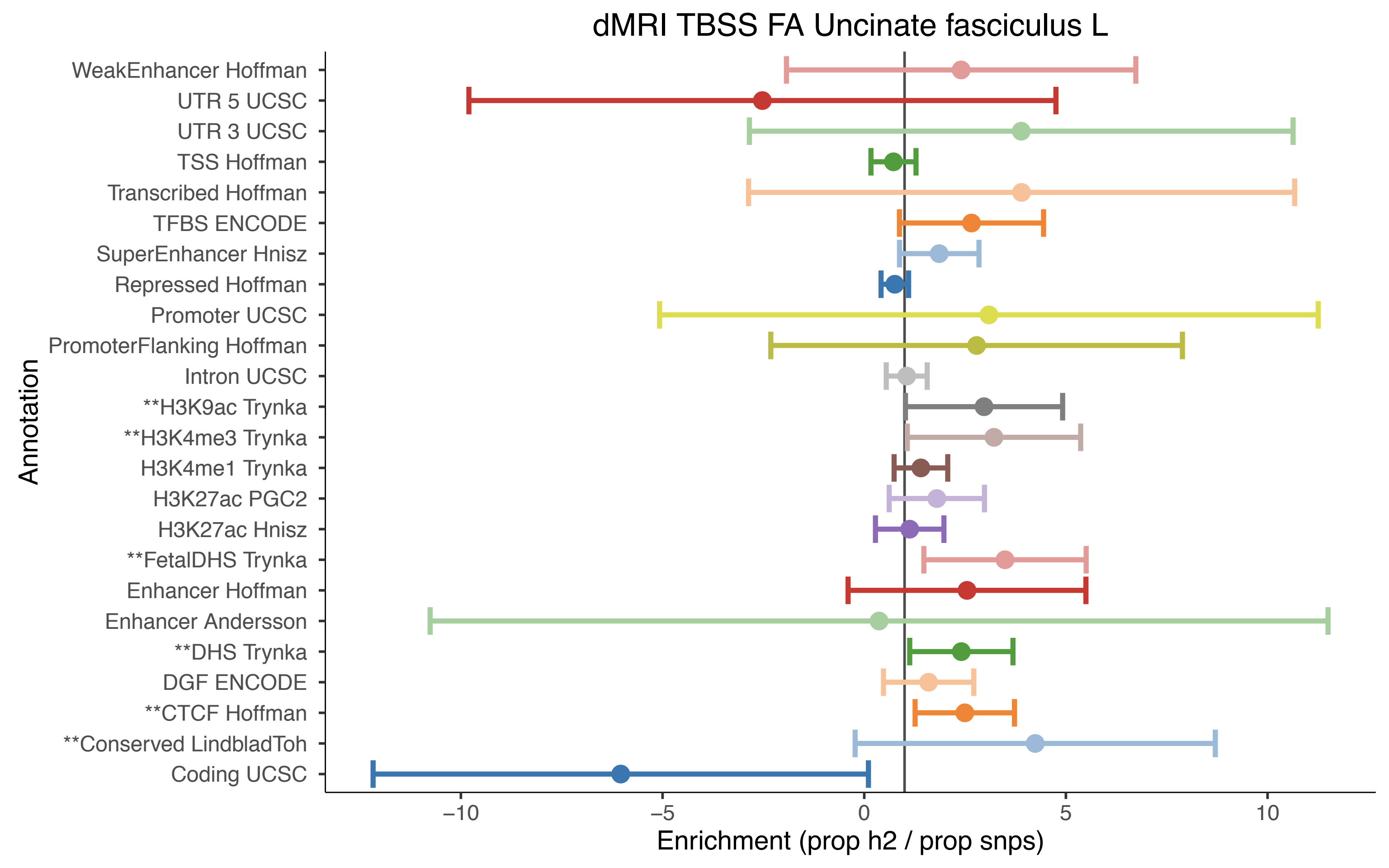

dMRI TBSS FA Uncinate fasciculus L
WeakEnhancer Hoffman
UTR 5 UCSC
UTR 3 UCSC
TSS Hoffman
Transcribed Hoffman
TFBS ENCODE
SuperEnhancer Hnisz
Repressed Hoffman
Promoter UCSC
PromoterFlanking Hoffman
Intron UCSC
**H3K9ac Trynka
**H3K4me3 Trynka
H3K4me1 Trynka
H3K27ac PGC2
H3K27ac Hnisz
**FetalDHS Trynka
Enhancer Hoffman
Enhancer Andersson
**DHS Trynka
DGF ENCODE
**CTCF Hoffman
**Conserved LindbladToh
Coding UCSC
Annotation
−10
−5
0
5
10
Enrichment (prop h2 / prop snps)

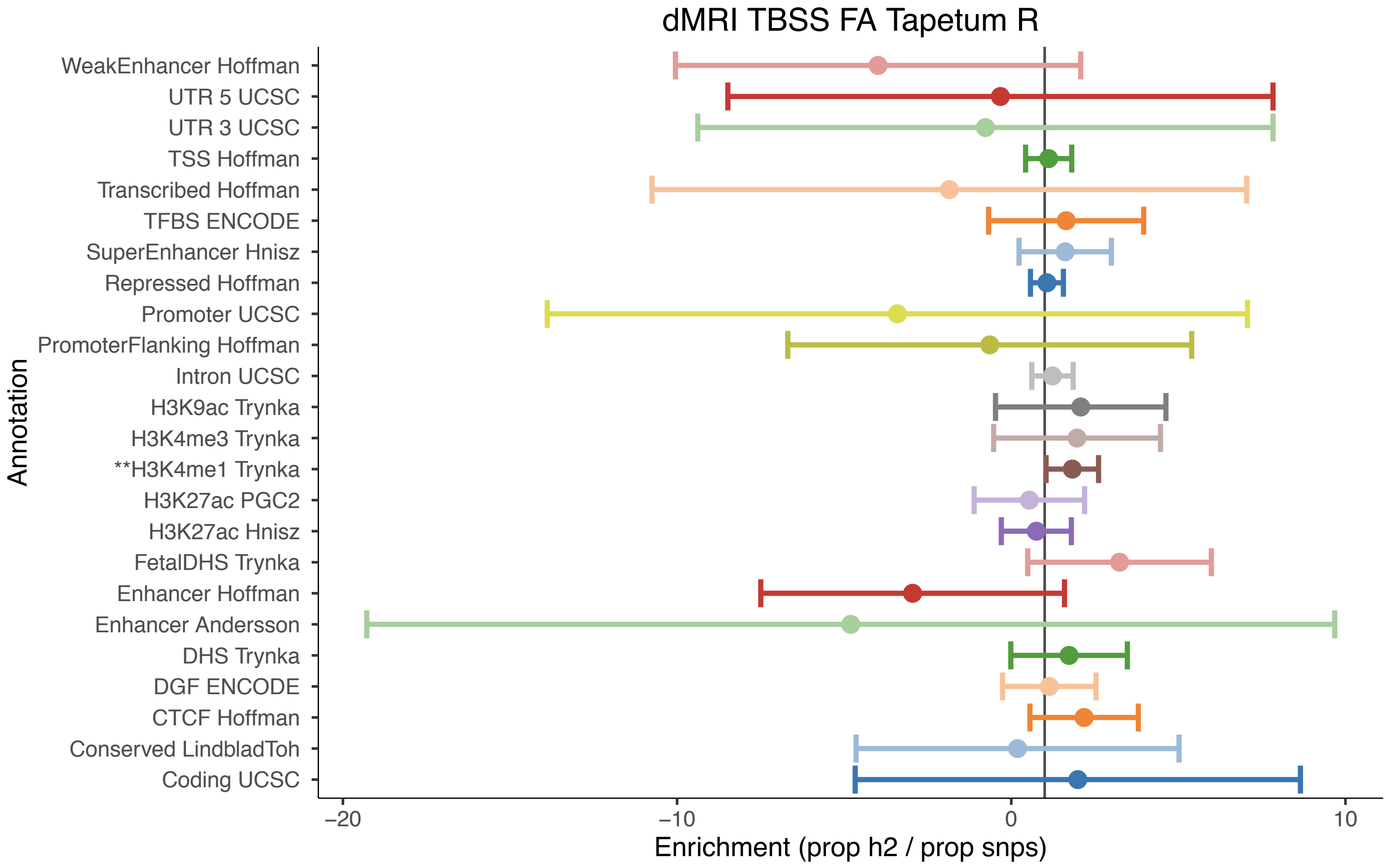
dMRI TBSS FA Tapetum R
WeakEnhancer Hoffman
UTR 5 UCSC
UTR 3 UCSC
TSS Hoffman
Transcribed Hoffman
TFBS ENCODE
SuperEnhancer Hnisz
Repressed Hoffman
Promoter UCSC
PromoterFlanking Hoffman
Intron UCSC
H3K9ac Trynka
H3K4me3 Trynka
**H3K4me1 Trynka
H3K27ac PGC2
H3K27ac Hnisz
FetalDHS Trynka
Enhancer Hoffman
Enhancer Andersson
DHS Trynka
DGF ENCODE
CTCF Hoffman
Conserved LindbladToh
Coding UCSC
Annotation
−20
−10
0
10
Enrichment (prop h2 / prop snps)

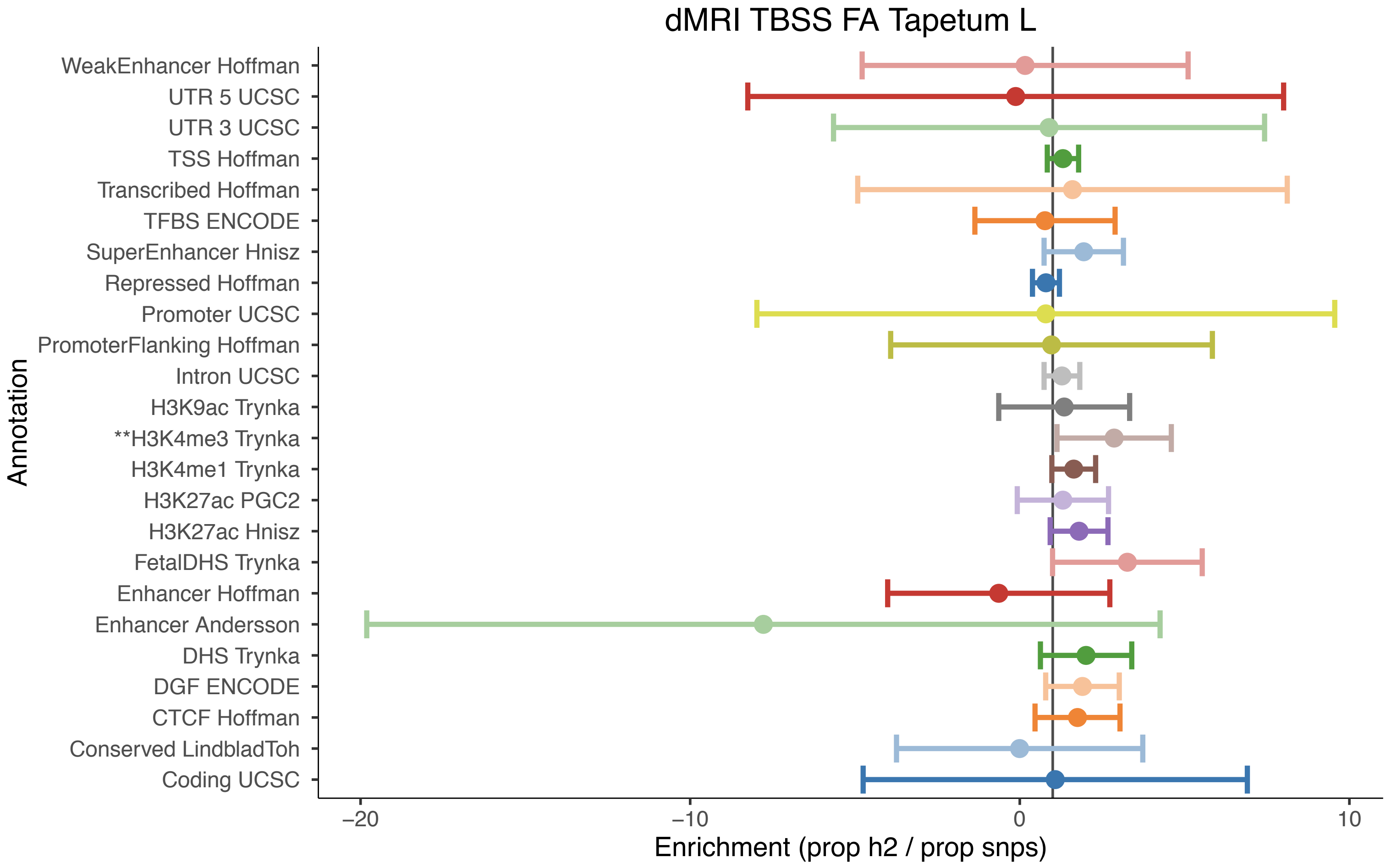

dMRI TBSS FA Tapetum L
WeakEnhancer Hoffman
UTR 5 UCSC
UTR 3 UCSC
TSS Hoffman
Transcribed Hoffman
TFBS ENCODE
SuperEnhancer Hnisz
Repressed Hoffman
Promoter UCSC
PromoterFlanking Hoffman
Intron UCSC
H3K9ac Trynka
**H3K4me3 Trynka
H3K4me1 Trynka
H3K27ac PGC2
H3K27ac Hnisz
FetalDHS Trynka
Enhancer Hoffman
Enhancer Andersson
DHS Trynka
DGF ENCODE
CTCF Hoffman
Conserved LindbladToh
Coding UCSC
Annotation
−20
−10
0
10
Enrichment (prop h2 / prop snps)

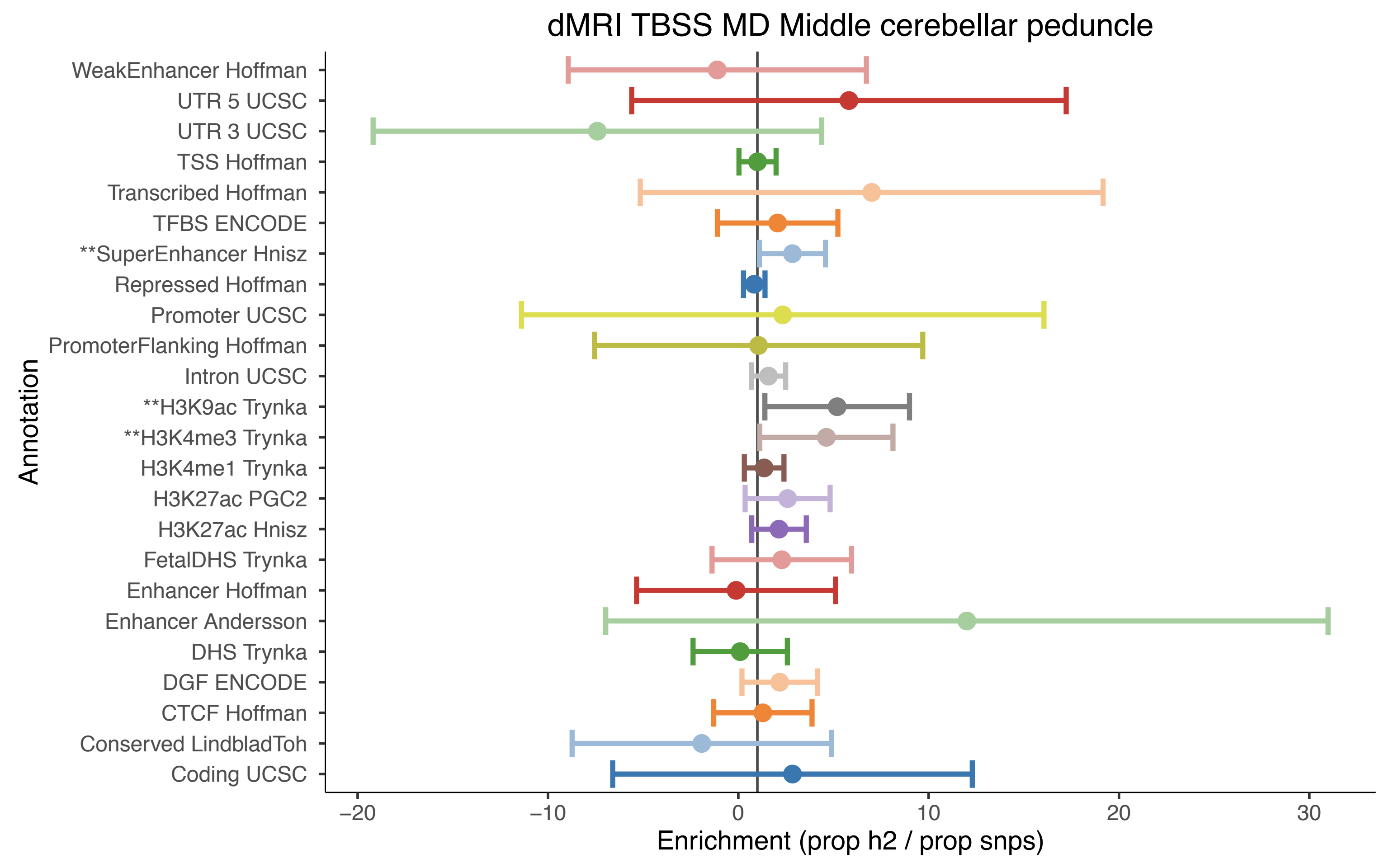

dMRI TBSS MD Middle cerebellar peduncle
WeakEnhancer Hoffman
UTR 5 UCSC
UTR 3 UCSC
TSS Hoffman
Transcribed Hoffman
TFBS ENCODE
**SuperEnhancer Hnisz
Repressed Hoffman
Promoter UCSC
PromoterFlanking Hoffman
Intron UCSC
**H3K9ac Trynka
**H3K4me3 Trynka
H3K4me1 Trynka
H3K27ac PGC2
H3K27ac Hnisz
FetalDHS Trynka
Enhancer Hoffman
Enhancer Andersson
DHS Trynka
DGF ENCODE
CTCF Hoffman
Conserved LindbladToh
Coding UCSC
Annotation
−20
−10
0
10
20
30
Enrichment (prop h2 / prop snps)

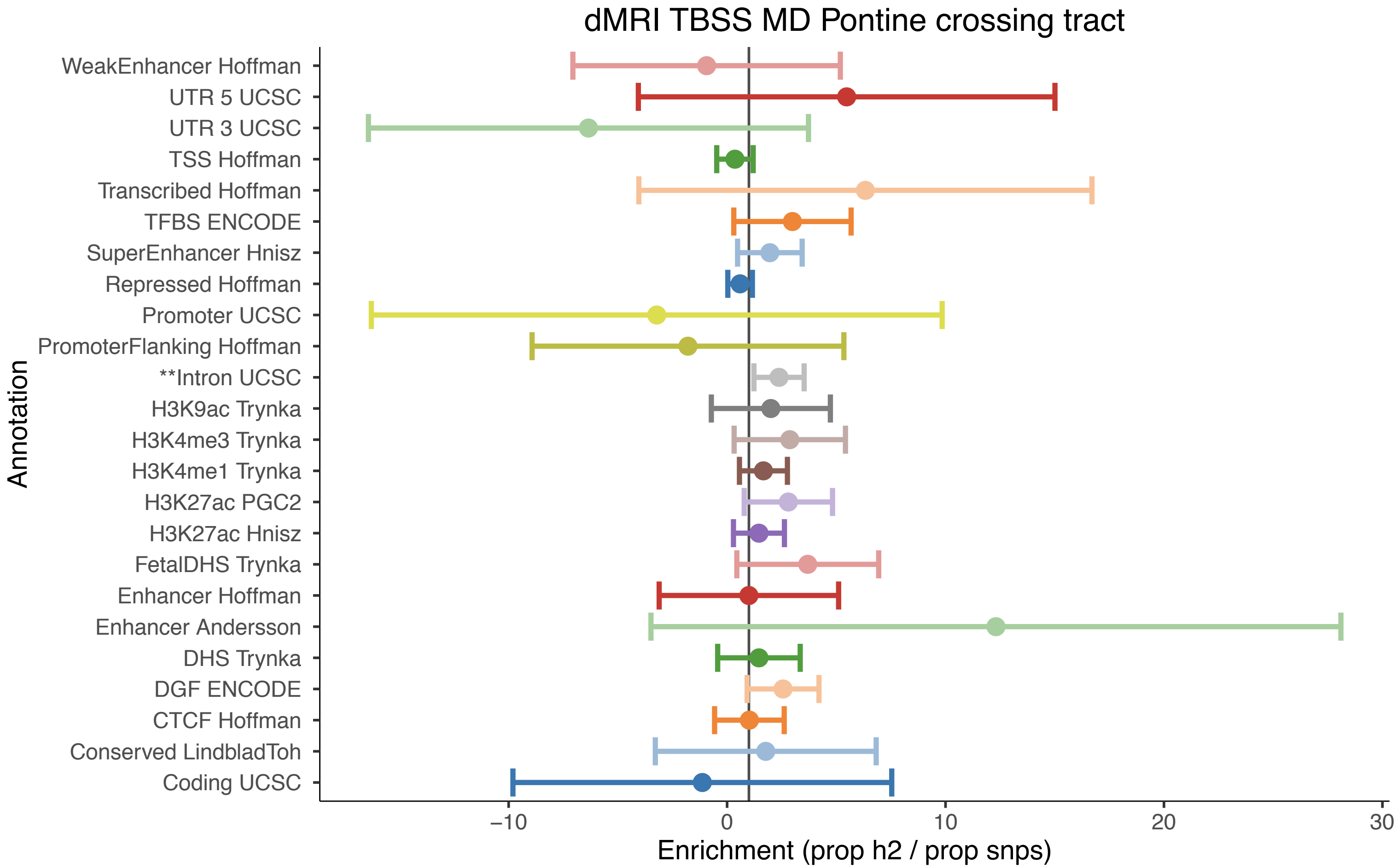

dMRI TBSS MD Pontine crossing tract
WeakEnhancer Hoffman
UTR 5 UCSC
UTR 3 UCSC
TSS Hoffman
Transcribed Hoffman
TFBS ENCODE
SuperEnhancer Hnisz
Repressed Hoffman
Promoter UCSC
PromoterFlanking Hoffman
**Intron UCSC
H3K9ac Trynka
H3K4me3 Trynka
H3K4me1 Trynka
H3K27ac PGC2
H3K27ac Hnisz
FetalDHS Trynka
Enhancer Hoffman
Enhancer Andersson
DHS Trynka
DGF ENCODE
CTCF Hoffman
Conserved LindbladToh
Coding UCSC
Annotation
−10
0
10
20
30
Enrichment (prop h2 / prop snps)

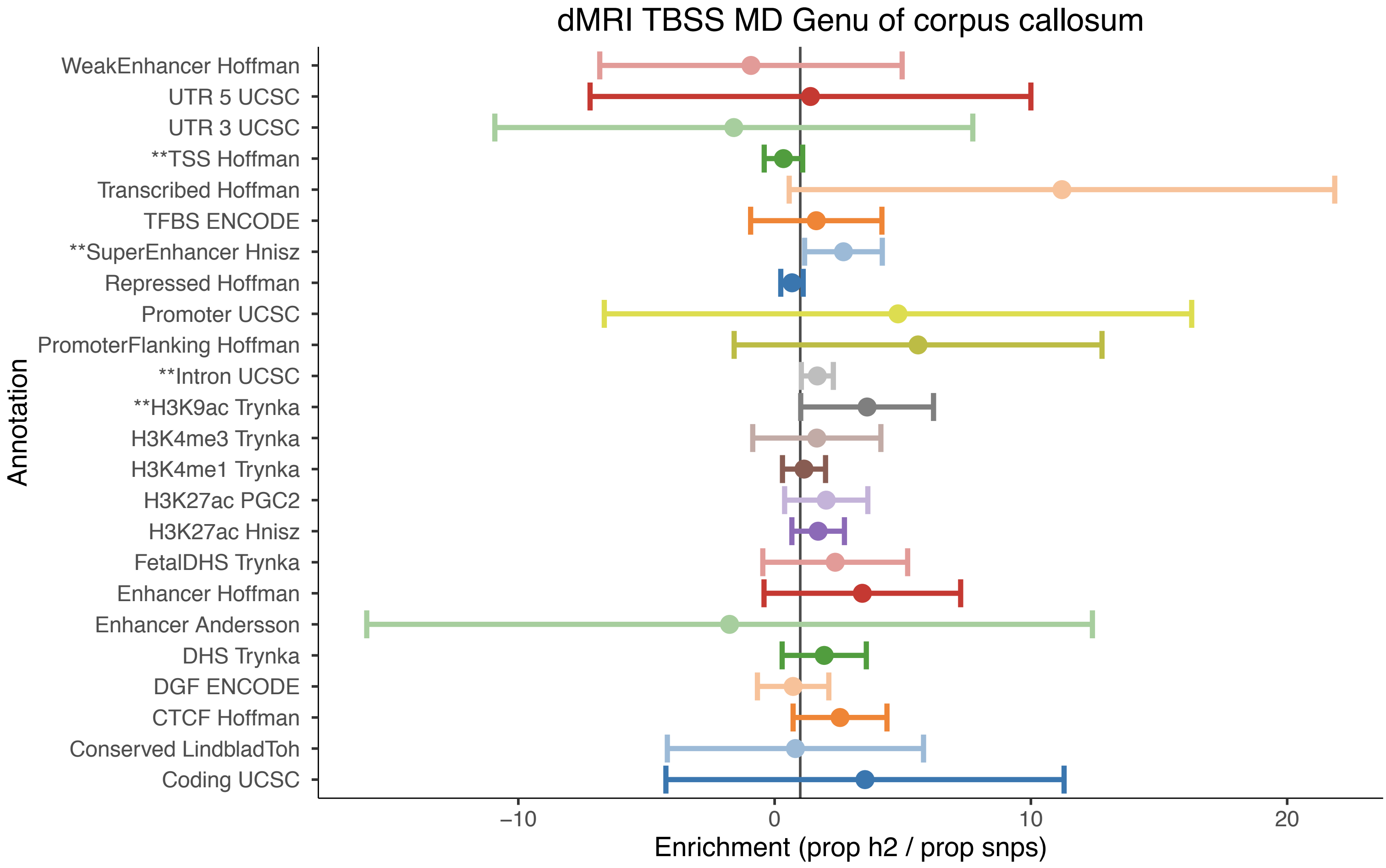

dMRI TBSS MD Genu of corpus callosum
WeakEnhancer Hoffman
UTR 5 UCSC
UTR 3 UCSC
**TSS Hoffman
Transcribed Hoffman
TFBS ENCODE
**SuperEnhancer Hnisz
Repressed Hoffman
Promoter UCSC
PromoterFlanking Hoffman
**Intron UCSC
**H3K9ac Trynka
H3K4me3 Trynka
H3K4me1 Trynka
H3K27ac PGC2
H3K27ac Hnisz
FetalDHS Trynka
Enhancer Hoffman
Enhancer Andersson
DHS Trynka
DGF ENCODE
CTCF Hoffman
Conserved LindbladToh
Coding UCSC
Annotation
−10
0
10
20
Enrichment (prop h2 / prop snps)

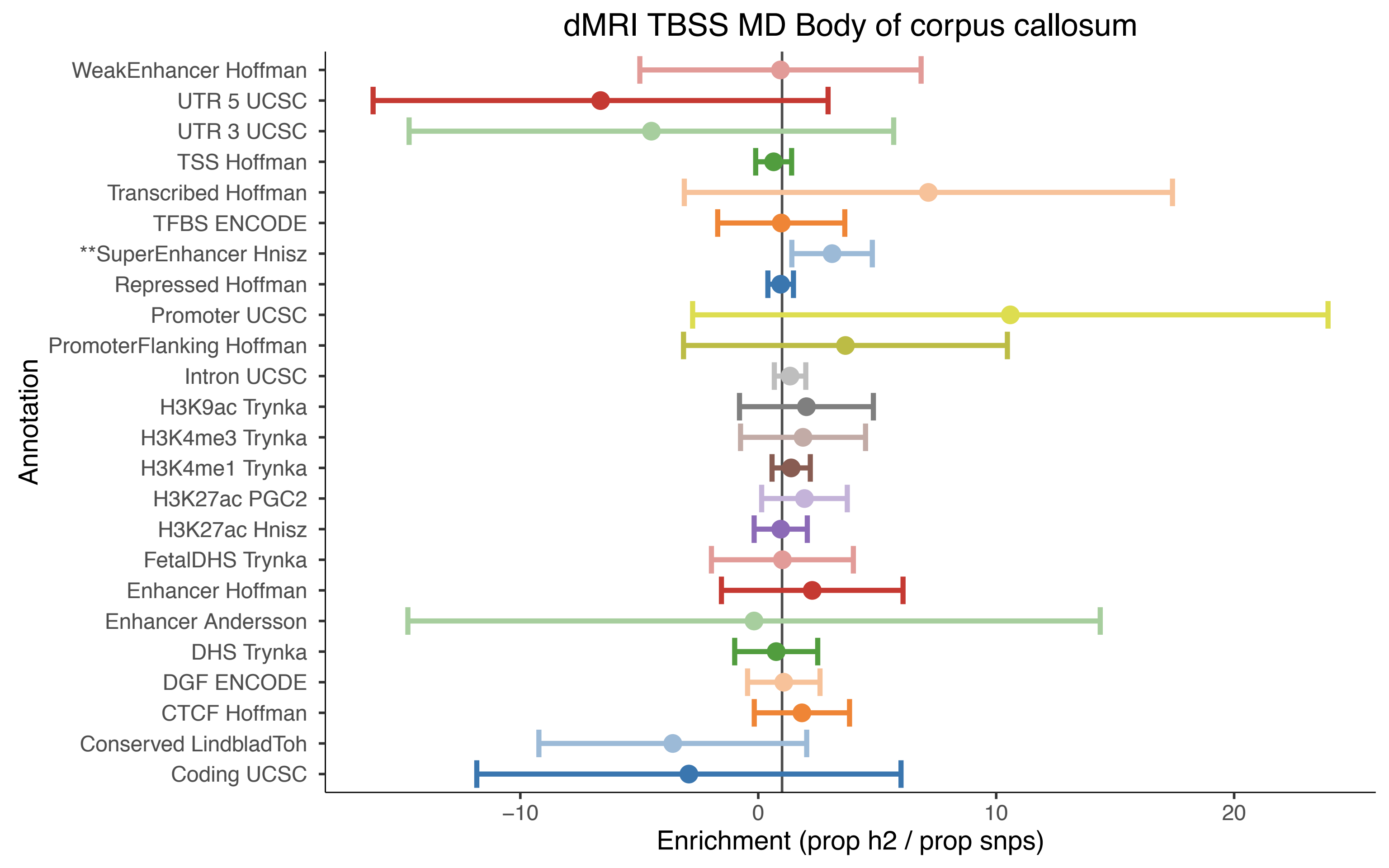
dMRI TBSS MD Body of corpus callosum
WeakEnhancer Hoffman
UTR 5 UCSC
UTR 3 UCSC
TSS Hoffman
Transcribed Hoffman
TFBS ENCODE
**SuperEnhancer Hnisz
Repressed Hoffman
Promoter UCSC
PromoterFlanking Hoffman
Intron UCSC
H3K9ac Trynka
H3K4me3 Trynka
H3K4me1 Trynka
H3K27ac PGC2
H3K27ac Hnisz
FetalDHS Trynka
Enhancer Hoffman
Enhancer Andersson
DHS Trynka
DGF ENCODE
CTCF Hoffman
Conserved LindbladToh
Coding UCSC
Annotation
−10
0
10
20
Enrichment (prop h2 / prop snps)

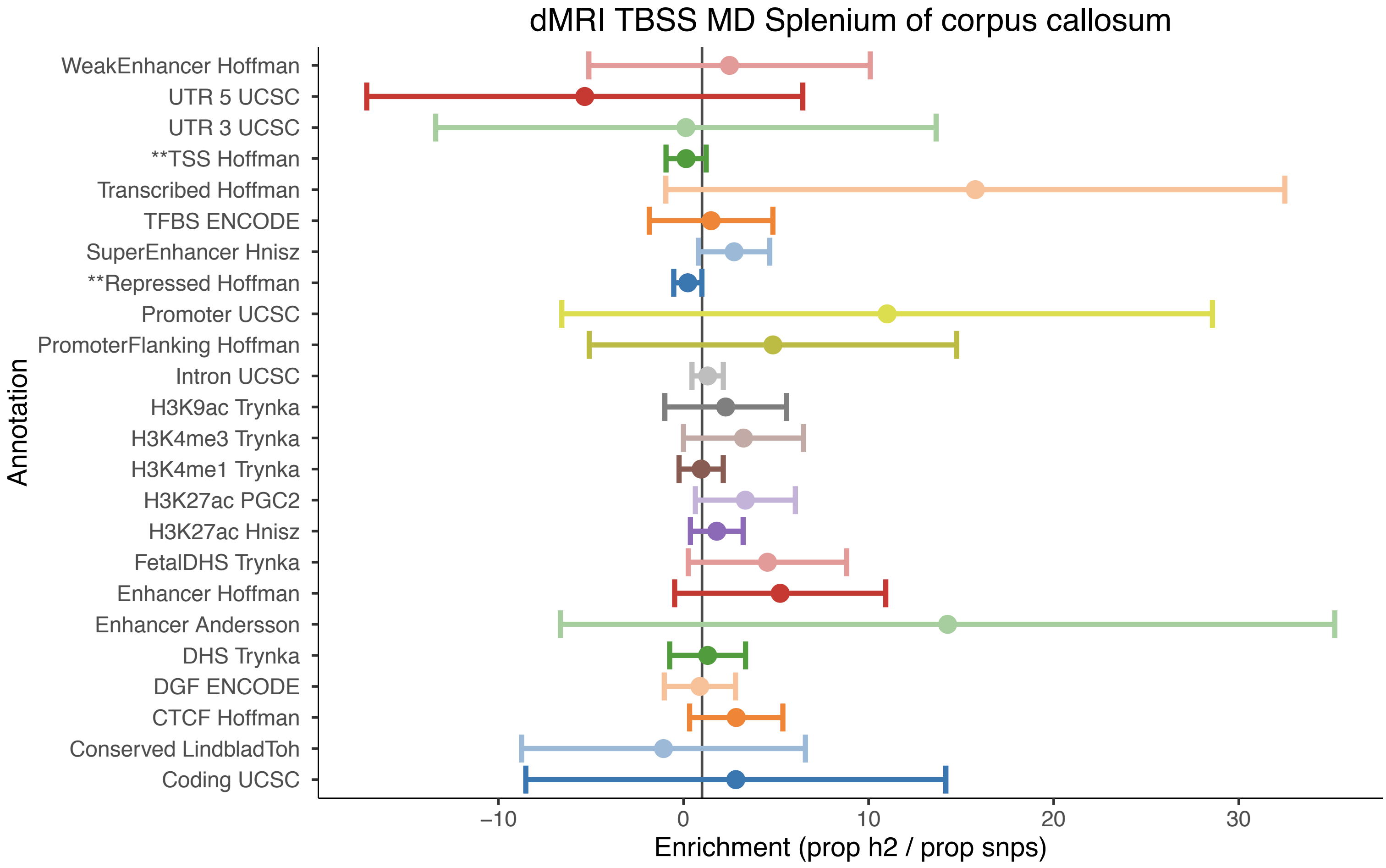
dMRI TBSS MD Splenium of corpus callosum
WeakEnhancer Hoffman
UTR 5 UCSC
UTR 3 UCSC
**TSS Hoffman
Transcribed Hoffman
TFBS ENCODE
SuperEnhancer Hnisz
**Repressed Hoffman
Promoter UCSC
PromoterFlanking Hoffman
Intron UCSC
H3K9ac Trynka
H3K4me3 Trynka
H3K4me1 Trynka
H3K27ac PGC2
H3K27ac Hnisz
FetalDHS Trynka
Enhancer Hoffman
Enhancer Andersson
DHS Trynka
DGF ENCODE
CTCF Hoffman
Conserved LindbladToh
Coding UCSC
Annotation
−10
0
10
20
30
Enrichment (prop h2 / prop snps)

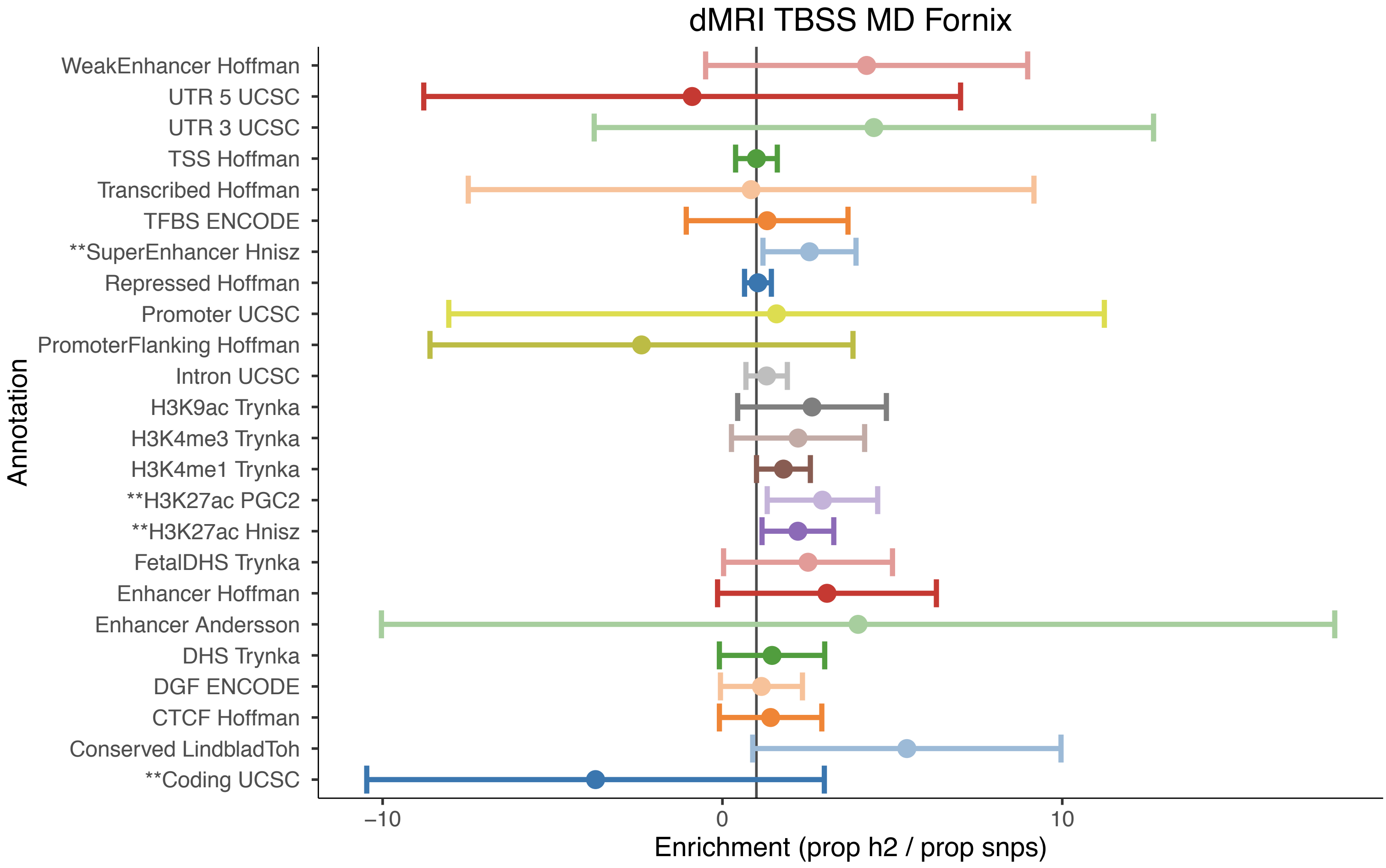

dMRI TBSS MD Fornix
WeakEnhancer Hoffman
UTR 5 UCSC
UTR 3 UCSC
TSS Hoffman
Transcribed Hoffman
TFBS ENCODE
**SuperEnhancer Hnisz
Repressed Hoffman
Promoter UCSC
PromoterFlanking Hoffman
Intron UCSC
H3K9ac Trynka
H3K4me3 Trynka
H3K4me1 Trynka
**H3K27ac PGC2
**H3K27ac Hnisz
FetalDHS Trynka
Enhancer Hoffman
Enhancer Andersson
DHS Trynka
DGF ENCODE
CTCF Hoffman
Conserved LindbladToh
**Coding UCSC
Annotation
−10
0
10
Enrichment (prop h2 / prop snps)

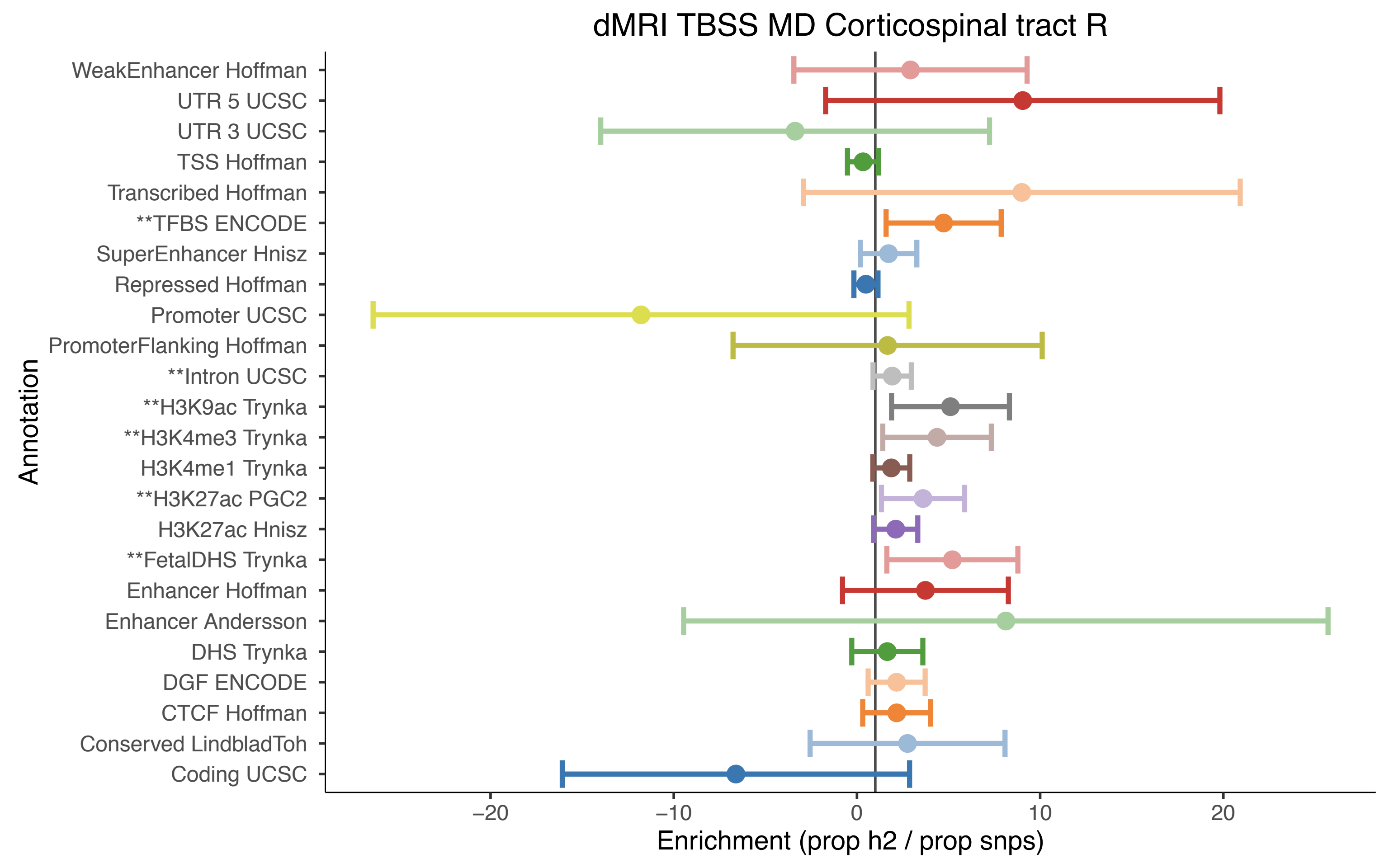
dMRI TBSS MD Corticospinal tract R
WeakEnhancer Hoffman
UTR 5 UCSC
UTR 3 UCSC
TSS Hoffman
Transcribed Hoffman
**TFBS ENCODE
SuperEnhancer Hnisz
Repressed Hoffman
Promoter UCSC
PromoterFlanking Hoffman
**Intron UCSC
**H3K9ac Trynka
**H3K4me3 Trynka
H3K4me1 Trynka
**H3K27ac PGC2
H3K27ac Hnisz
**FetalDHS Trynka
Enhancer Hoffman
Enhancer Andersson
DHS Trynka
DGF ENCODE
CTCF Hoffman
Conserved LindbladToh
Coding UCSC
Annotation
−20
−10
0
10
20
Enrichment (prop h2 / prop snps)

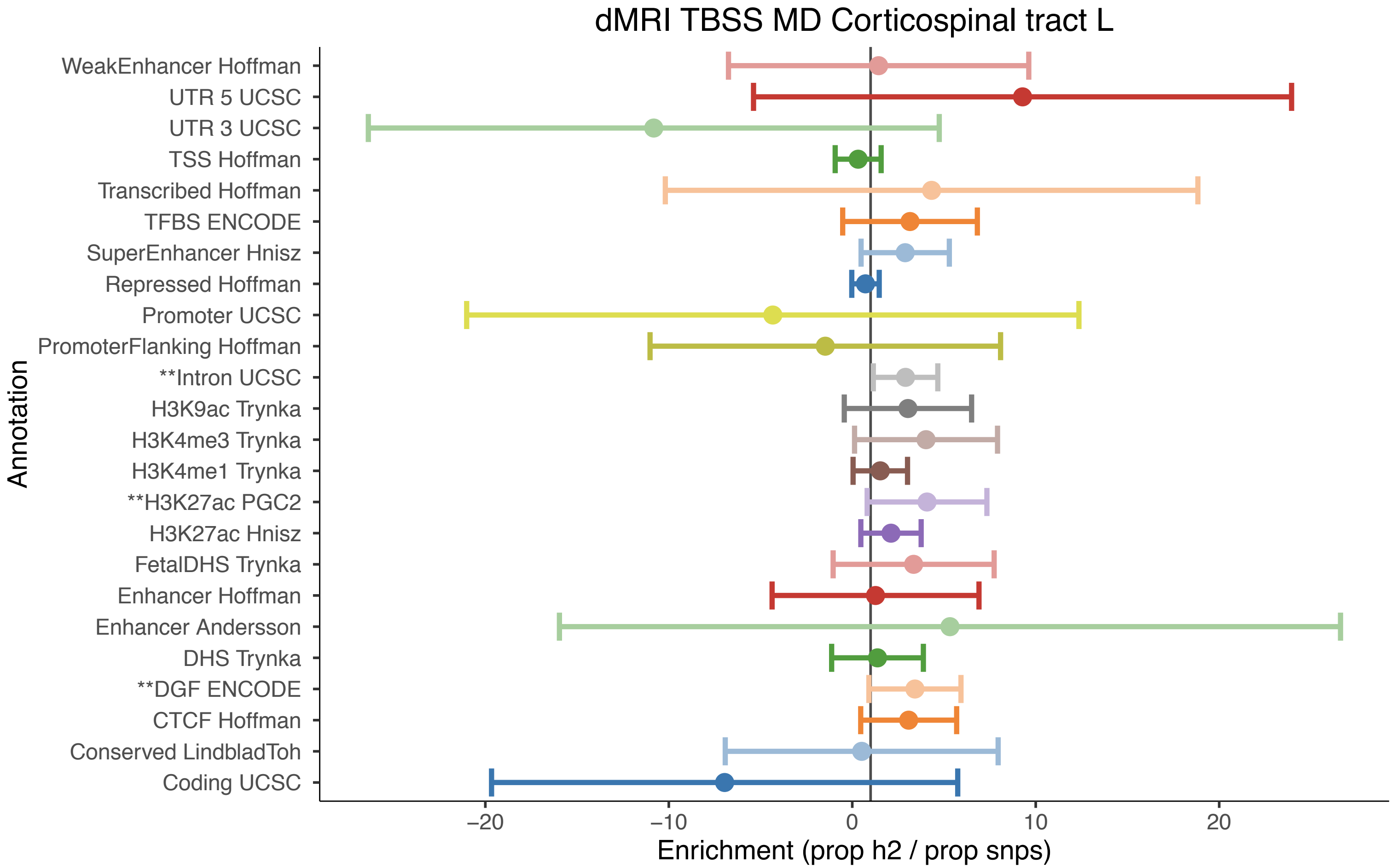

dMRI TBSS MD Corticospinal tract L
WeakEnhancer Hoffman
UTR 5 UCSC
UTR 3 UCSC
TSS Hoffman
Transcribed Hoffman
TFBS ENCODE
SuperEnhancer Hnisz
Repressed Hoffman
Promoter UCSC
PromoterFlanking Hoffman
**Intron UCSC
H3K9ac Trynka
H3K4me3 Trynka
H3K4me1 Trynka
**H3K27ac PGC2
H3K27ac Hnisz
FetalDHS Trynka
Enhancer Hoffman
Enhancer Andersson
DHS Trynka
**DGF ENCODE
CTCF Hoffman
Conserved LindbladToh
Coding UCSC
Annotation
−20
−10
0
10
20
Enrichment (prop h2 / prop snps)

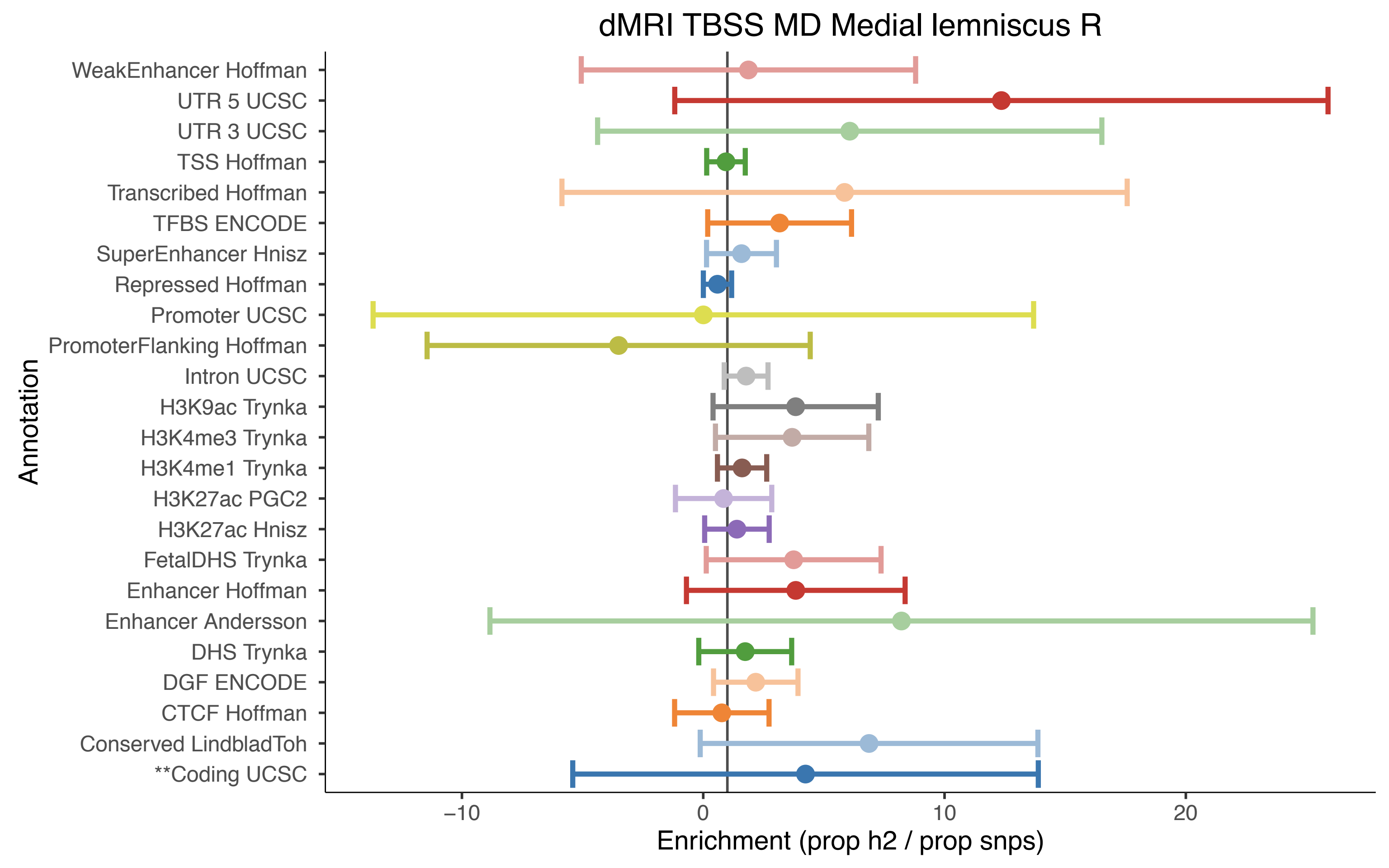
dMRI TBSS MD Medial lemniscus R
WeakEnhancer Hoffman
UTR 5 UCSC
UTR 3 UCSC
TSS Hoffman
Transcribed Hoffman
TFBS ENCODE
SuperEnhancer Hnisz
Repressed Hoffman
Promoter UCSC
PromoterFlanking Hoffman
Intron UCSC
H3K9ac Trynka
H3K4me3 Trynka
H3K4me1 Trynka
H3K27ac PGC2
H3K27ac Hnisz
FetalDHS Trynka
Enhancer Hoffman
Enhancer Andersson
DHS Trynka
DGF ENCODE
CTCF Hoffman
Conserved LindbladToh
**Coding UCSC
Annotation
−10
0
10
20
Enrichment (prop h2 / prop snps)

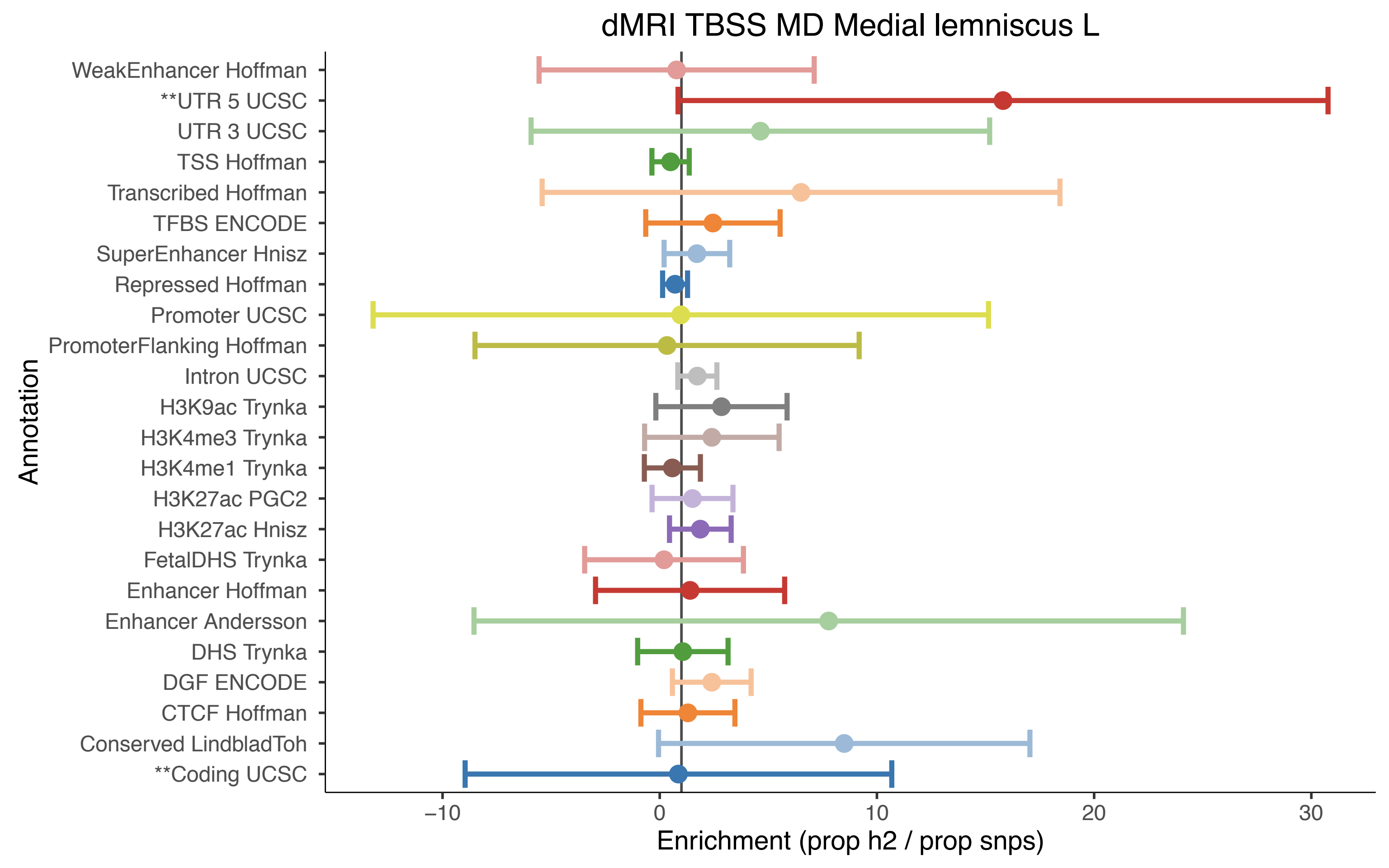
dMRI TBSS MD Medial lemniscus L
WeakEnhancer Hoffman
**UTR 5 UCSC
UTR 3 UCSC
TSS Hoffman
Transcribed Hoffman
TFBS ENCODE
SuperEnhancer Hnisz
Repressed Hoffman
Promoter UCSC
PromoterFlanking Hoffman
Intron UCSC
H3K9ac Trynka
H3K4me3 Trynka
H3K4me1 Trynka
H3K27ac PGC2
H3K27ac Hnisz
FetalDHS Trynka
Enhancer Hoffman
Enhancer Andersson
DHS Trynka
DGF ENCODE
CTCF Hoffman
Conserved LindbladToh
**Coding UCSC
Annotation
−10
0
10
20
30
Enrichment (prop h2 / prop snps)

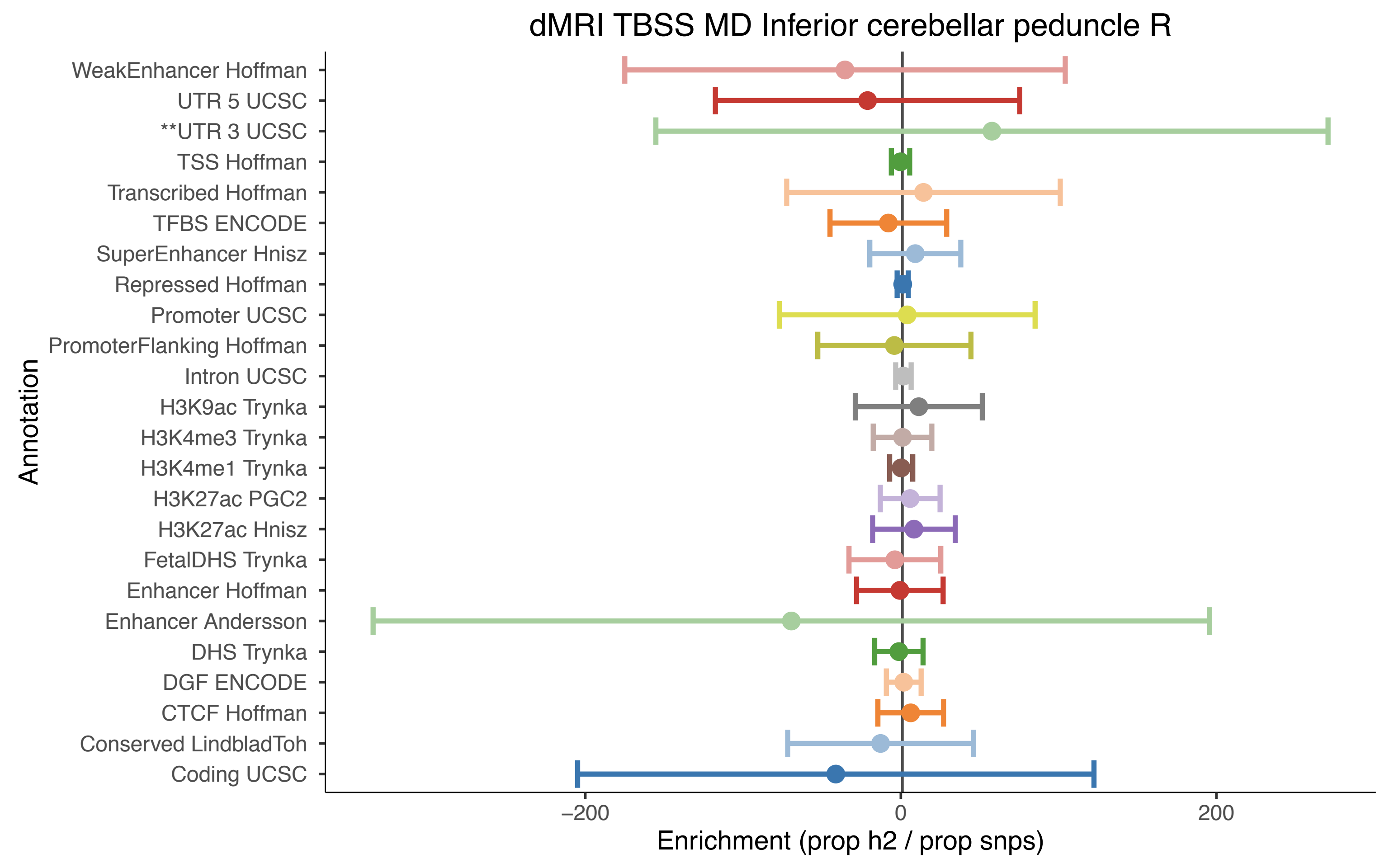
dMRI TBSS MD Inferior cerebellar peduncle R
WeakEnhancer Hoffman
UTR 5 UCSC
**UTR 3 UCSC
TSS Hoffman
Transcribed Hoffman
TFBS ENCODE
SuperEnhancer Hnisz
Repressed Hoffman
Promoter UCSC
PromoterFlanking Hoffman
Intron UCSC
H3K9ac Trynka
H3K4me3 Trynka
H3K4me1 Trynka
H3K27ac PGC2
H3K27ac Hnisz
FetalDHS Trynka
Enhancer Hoffman
Enhancer Andersson
DHS Trynka
DGF ENCODE
CTCF Hoffman
Conserved LindbladToh
Coding UCSC
Annotation
−200
0
200
Enrichment (prop h2 / prop snps)

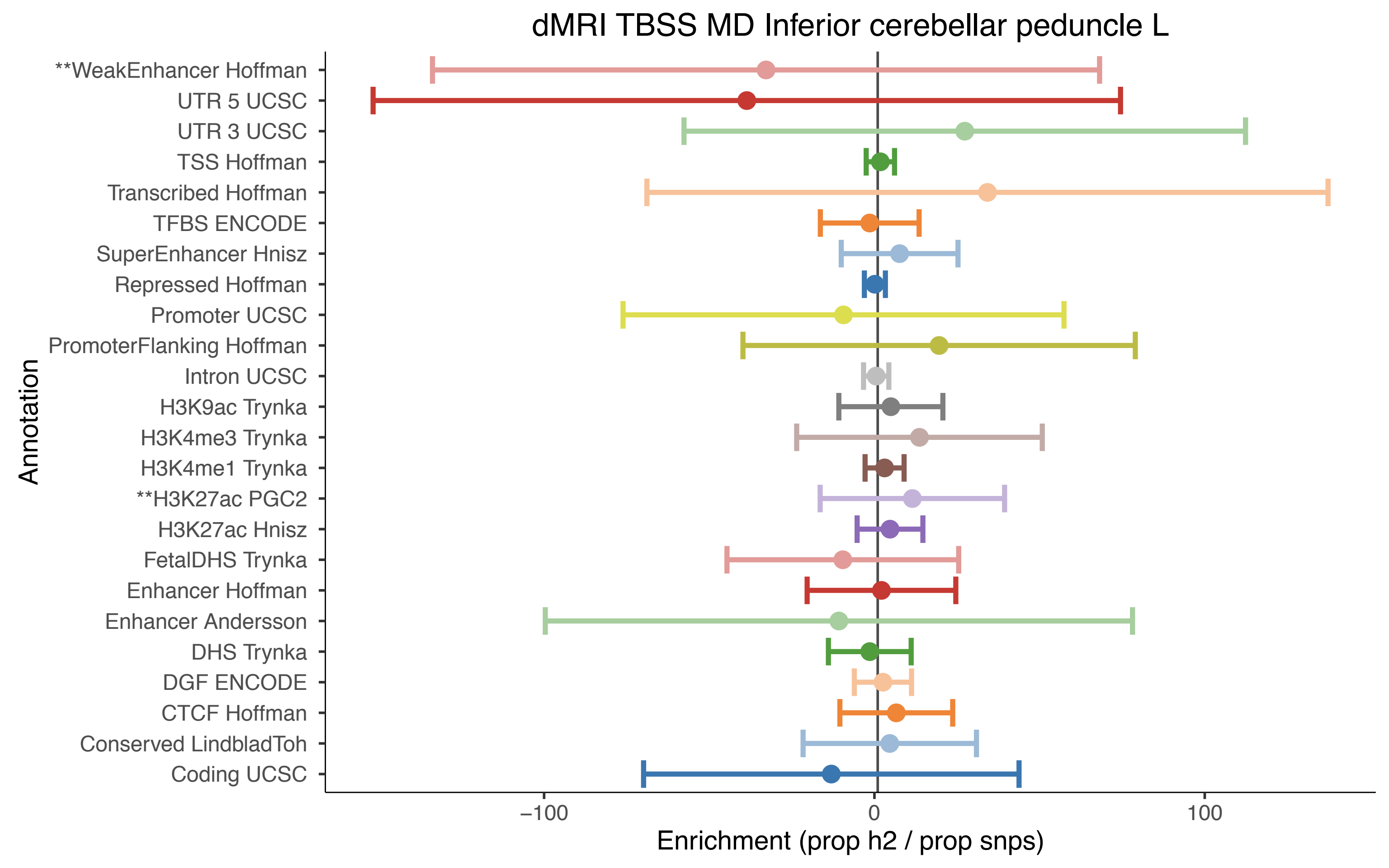
dMRI TBSS MD Inferior cerebellar peduncle L
**WeakEnhancer Hoffman
UTR 5 UCSC
UTR 3 UCSC
TSS Hoffman
Transcribed Hoffman
TFBS ENCODE
SuperEnhancer Hnisz
Repressed Hoffman
Promoter UCSC
PromoterFlanking Hoffman
Intron UCSC
H3K9ac Trynka
H3K4me3 Trynka
H3K4me1 Trynka
**H3K27ac PGC2
H3K27ac Hnisz
FetalDHS Trynka
Enhancer Hoffman
Enhancer Andersson
DHS Trynka
DGF ENCODE
CTCF Hoffman
Conserved LindbladToh
Coding UCSC
Annotation
−100
0
100
Enrichment (prop h2 / prop snps)

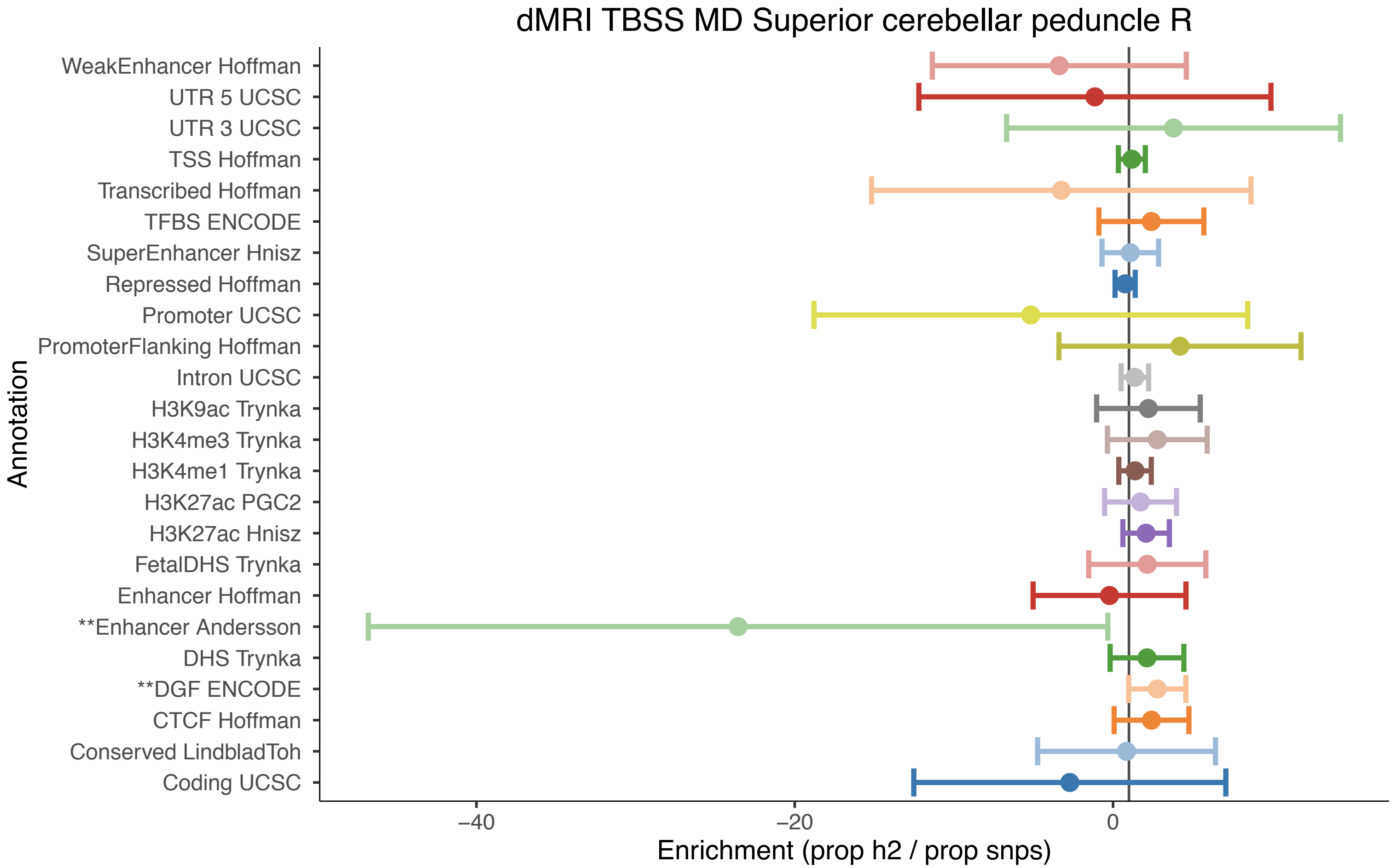

dMRI TBSS MD Superior cerebellar peduncle R
Annotation
WeakEnhancer Hoffman
UTR 5 UCSC
UTR 3 UCSC
TSS Hoffman
Transcribed Hoffman
TFBS ENCODE
SuperEnhancer Hnisz
Repressed Hoffman
Promoter UCSC
PromoterFlanking Hoffman
Intron UCSC
H3K9ac Trynka
H3K4me3 Trynka
H3K4me1 Trynka
H3K27ac PGC2
H3K27ac Hnisz
FetalDHS Trynka
Enhancer Hoffman
**Enhancer Andersson
DHS Trynka
**DGF ENCODE
CTCF Hoffman
Conserved LindbladToh
Coding UCSC
−40
−20
0
Enrichment (prop h2 / prop snps)

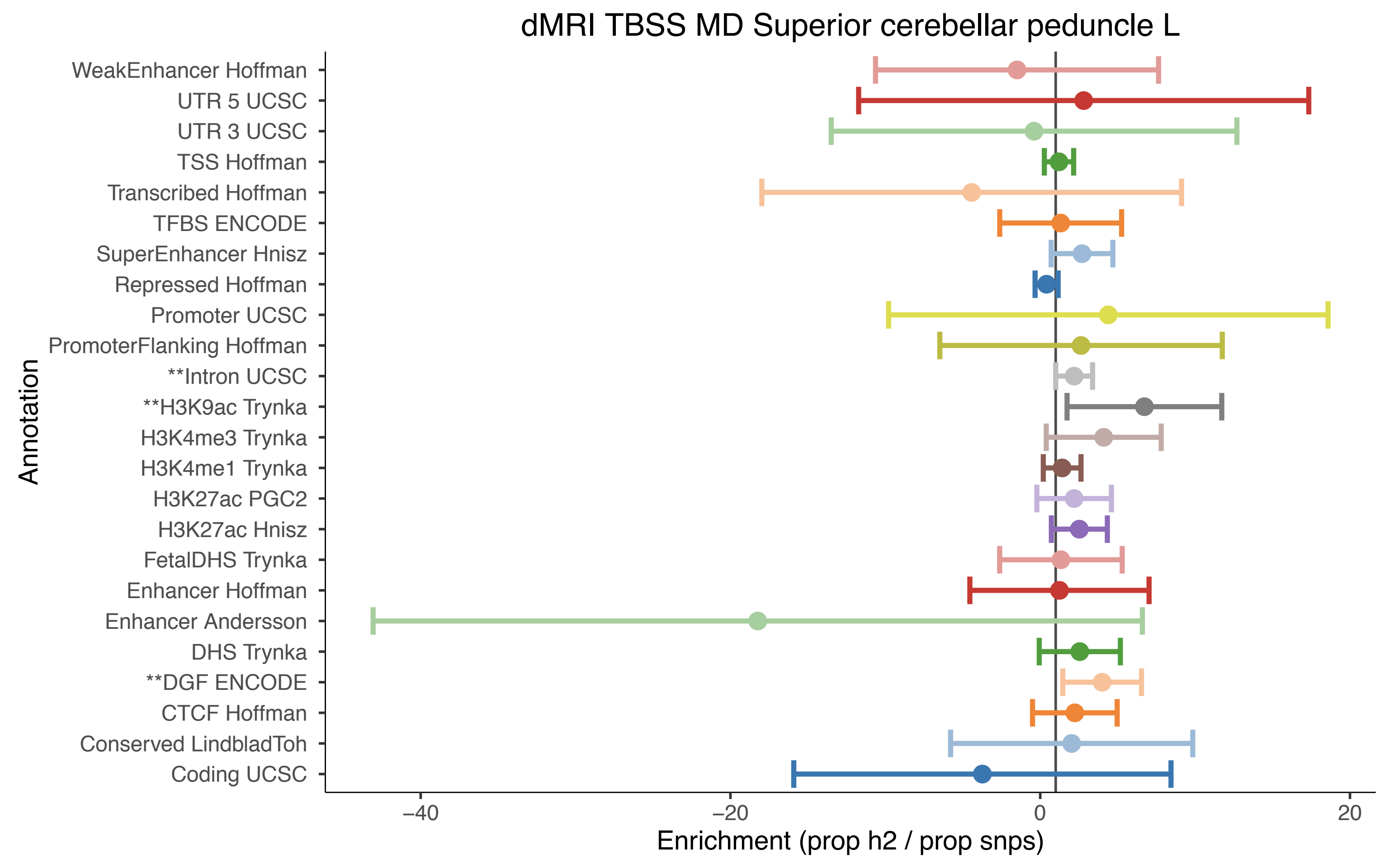

dMRI TBSS MD Superior cerebellar peduncle L
WeakEnhancer Hoffman
UTR 5 UCSC
UTR 3 UCSC
TSS Hoffman
Transcribed Hoffman
TFBS ENCODE
SuperEnhancer Hnisz
Repressed Hoffman
Promoter UCSC
PromoterFlanking Hoffman
**Intron UCSC
**H3K9ac Trynka
H3K4me3 Trynka
H3K4me1 Trynka
H3K27ac PGC2
H3K27ac Hnisz
FetalDHS Trynka
Enhancer Hoffman
Enhancer Andersson
DHS Trynka
**DGF ENCODE
CTCF Hoffman
Conserved LindbladToh
Coding UCSC
Annotation
−40
−20
0
20
Enrichment (prop h2 / prop snps)

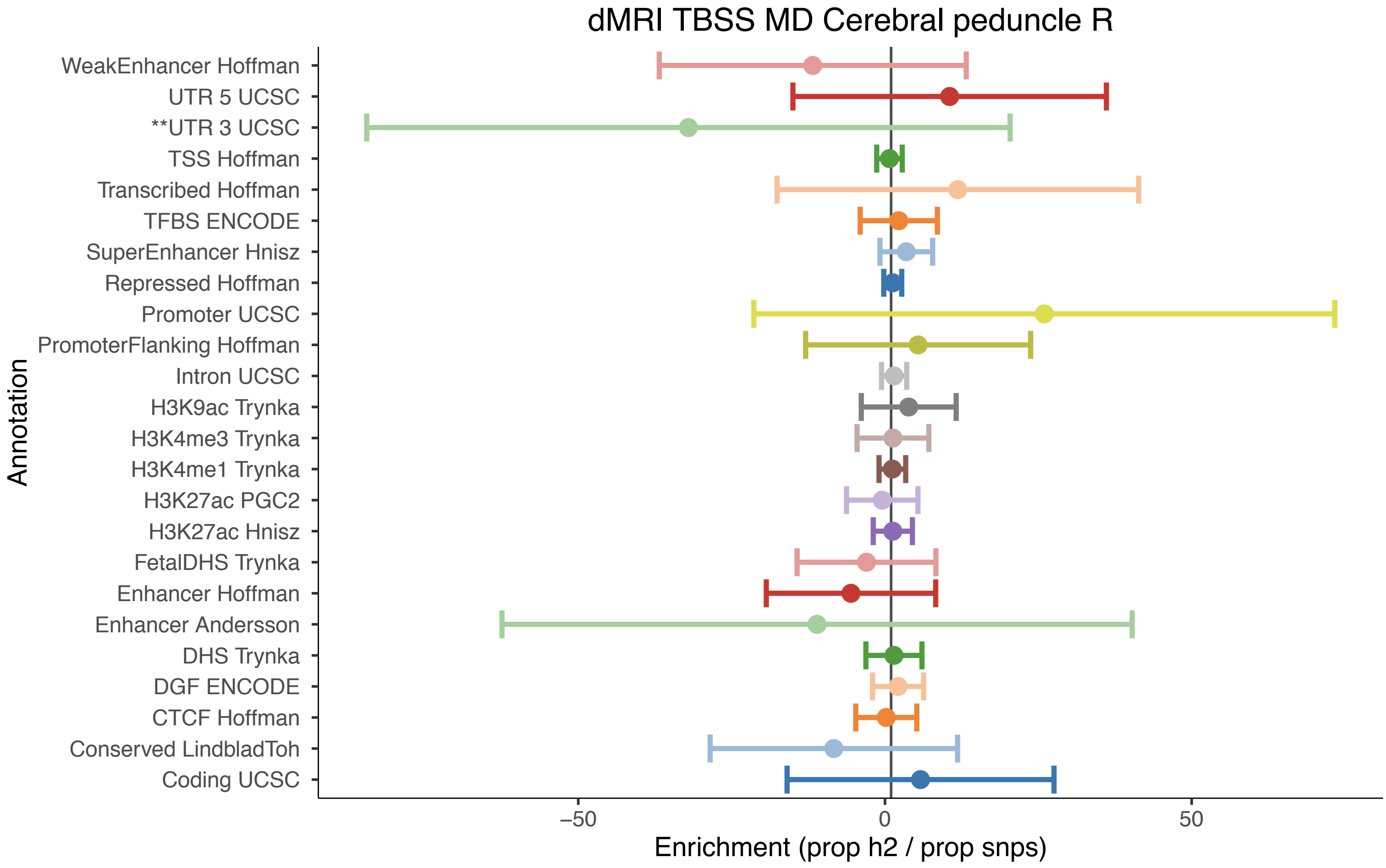

dMRI TBSS MD Cerebral peduncle R
WeakEnhancer Hoffman
UTR 5 UCSC
**UTR 3 UCSC
TSS Hoffman
Transcribed Hoffman
TFBS ENCODE
SuperEnhancer Hnisz
Repressed Hoffman
Promoter UCSC
PromoterFlanking Hoffman
Intron UCSC
H3K9ac Trynka
H3K4me3 Trynka
H3K4me1 Trynka
H3K27ac PGC2
H3K27ac Hnisz
FetalDHS Trynka
Enhancer Hoffman
Enhancer Andersson
DHS Trynka
DGF ENCODE
CTCF Hoffman
Conserved LindbladToh
Coding UCSC
Annotation
−50
0
50
Enrichment (prop h2 / prop snps)

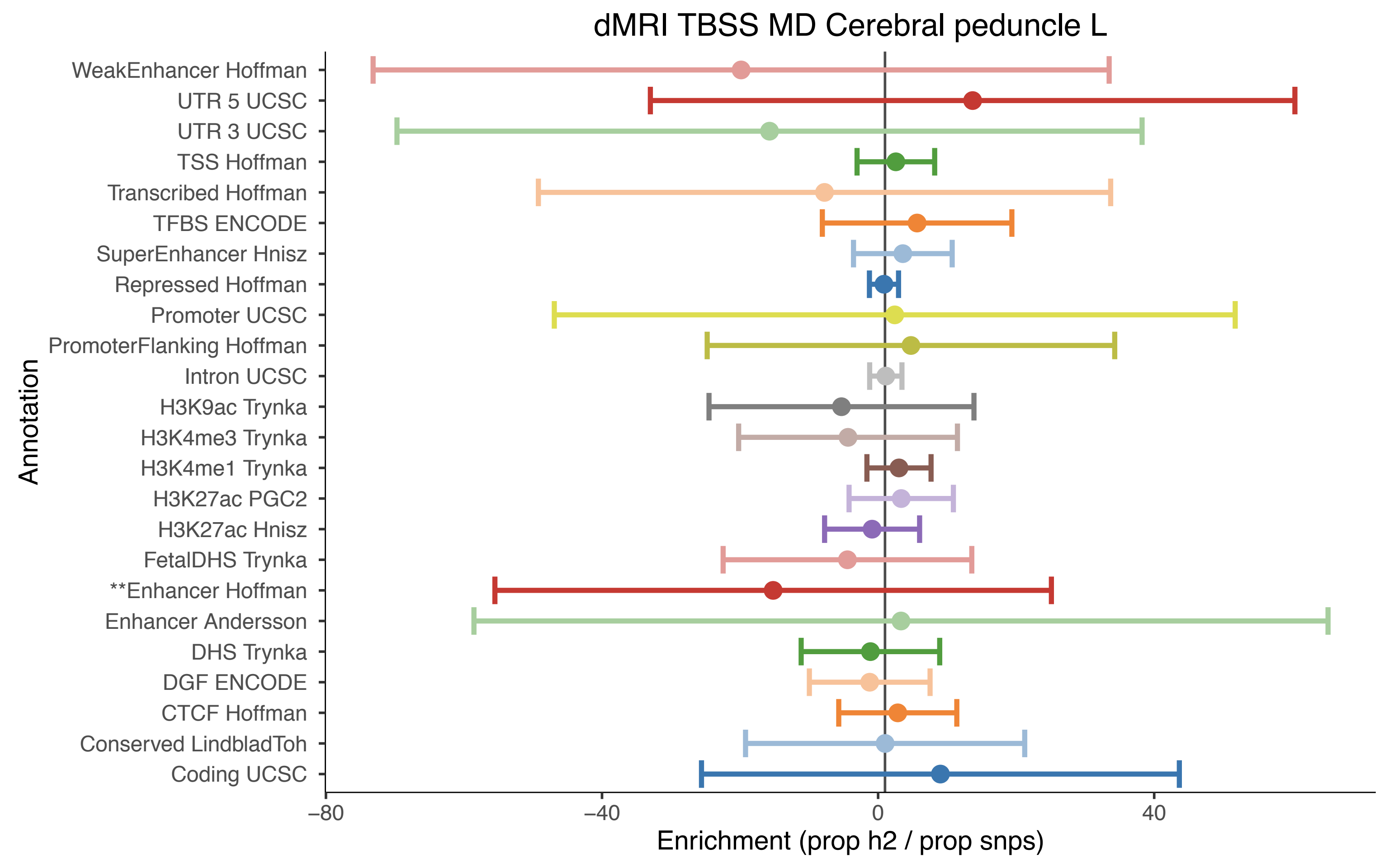
dMRI TBSS MD Cerebral peduncle L
WeakEnhancer Hoffman
UTR 5 UCSC
UTR 3 UCSC
TSS Hoffman
Transcribed Hoffman
TFBS ENCODE
SuperEnhancer Hnisz
Repressed Hoffman
Promoter UCSC
PromoterFlanking Hoffman
Intron UCSC
H3K9ac Trynka
H3K4me3 Trynka
H3K4me1 Trynka
H3K27ac PGC2
H3K27ac Hnisz
FetalDHS Trynka
**Enhancer Hoffman
Enhancer Andersson
DHS Trynka
DGF ENCODE
CTCF Hoffman
Conserved LindbladToh
Coding UCSC
Annotation
−80
−40
0
40
Enrichment (prop h2 / prop snps)

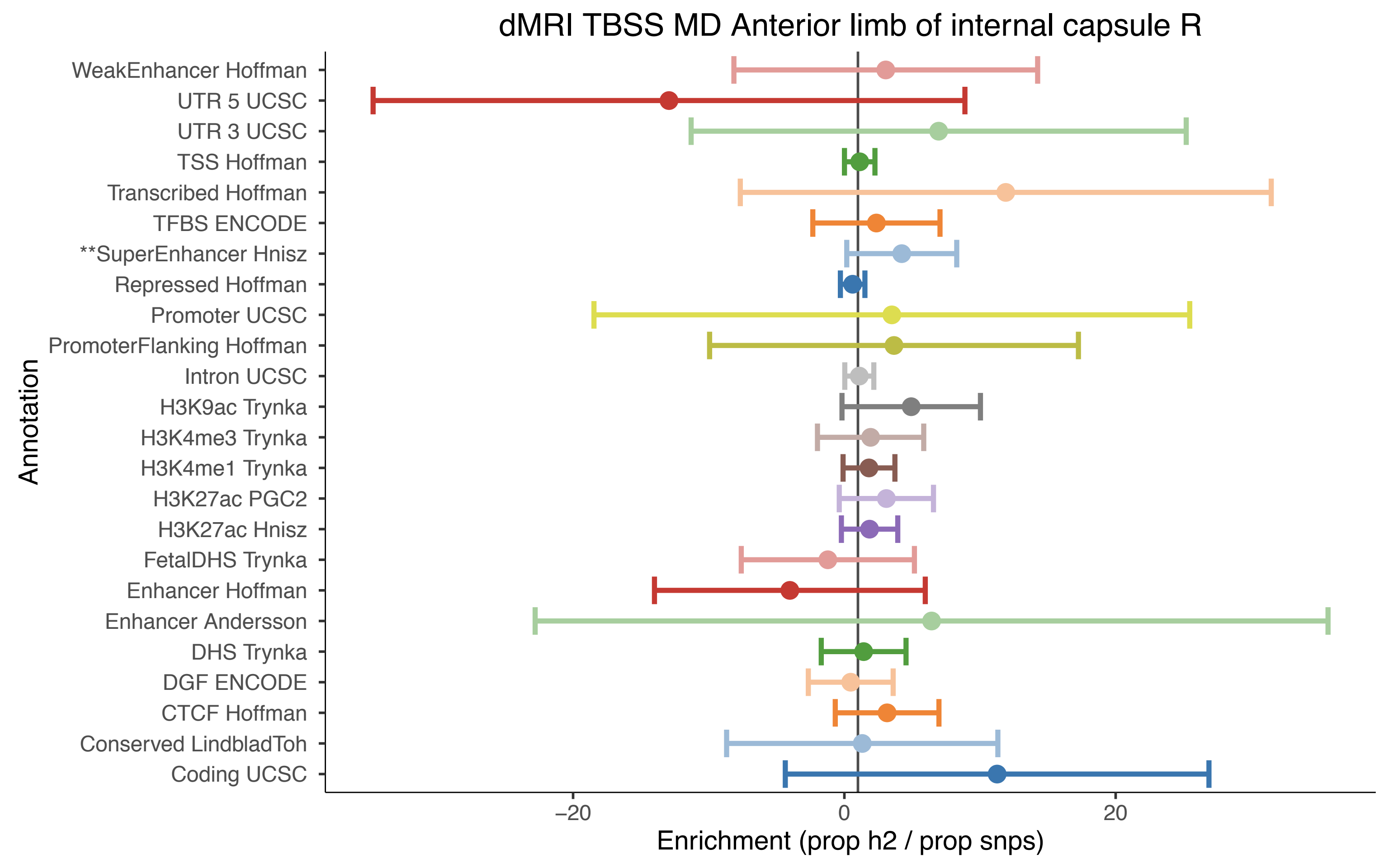
dMRI TBSS MD Anterior limb of internal capsule R
WeakEnhancer Hoffman
UTR 5 UCSC
UTR 3 UCSC
TSS Hoffman
Transcribed Hoffman
TFBS ENCODE
**SuperEnhancer Hnisz
Repressed Hoffman
Promoter UCSC
PromoterFlanking Hoffman
Intron UCSC
H3K9ac Trynka
H3K4me3 Trynka
H3K4me1 Trynka
H3K27ac PGC2
H3K27ac Hnisz
FetalDHS Trynka
Enhancer Hoffman
Enhancer Andersson
DHS Trynka
DGF ENCODE
CTCF Hoffman
Conserved LindbladToh
Coding UCSC
Annotation
−20
0
20
Enrichment (prop h2 / prop snps)

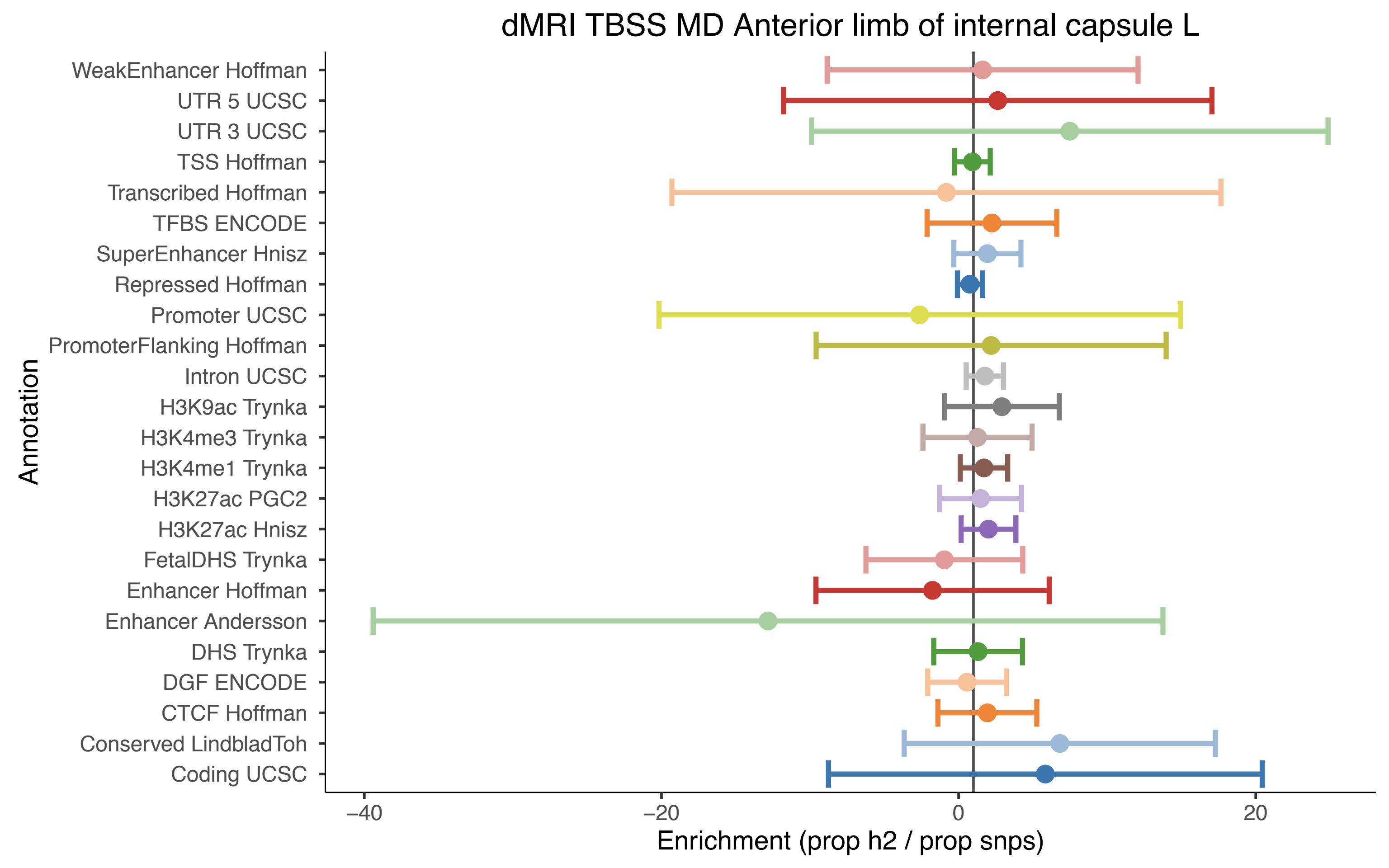
dMRI TBSS MD Anterior limb of internal capsule L
Annotation
WeakEnhancer Hoffman
UTR 5 UCSC
UTR 3 UCSC
TSS Hoffman
Transcribed Hoffman
TFBS ENCODE
SuperEnhancer Hnisz
Repressed Hoffman
Promoter UCSC
PromoterFlanking Hoffman
Intron UCSC
H3K9ac Trynka
H3K4me3 Trynka
H3K4me1 Trynka
H3K27ac PGC2
H3K27ac Hnisz
FetalDHS Trynka
Enhancer Hoffman
Enhancer Andersson
DHS Trynka
DGF ENCODE
CTCF Hoffman
Conserved LindbladToh
Coding UCSC
−40
−20
0
20
Enrichment (prop h2 / prop snps)

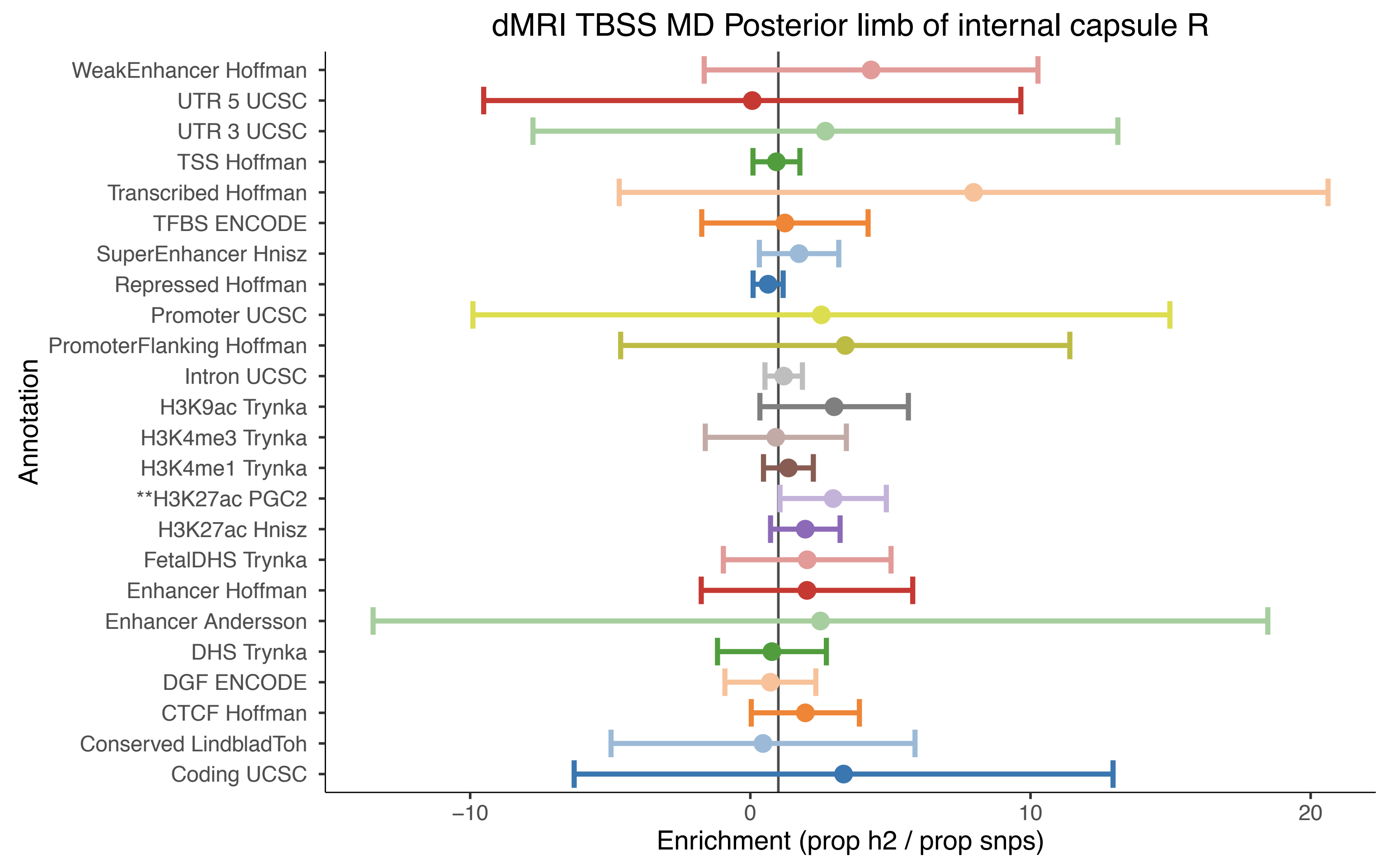
dMRI TBSS MD Posterior limb of internal capsule R
Annotation
WeakEnhancer Hoffman
UTR 5 UCSC
UTR 3 UCSC
TSS Hoffman
Transcribed Hoffman
TFBS ENCODE
SuperEnhancer Hnisz
Repressed Hoffman
Promoter UCSC
PromoterFlanking Hoffman
Intron UCSC
H3K9ac Trynka
H3K4me3 Trynka
H3K4me1 Trynka
**H3K27ac PGC2
H3K27ac Hnisz
FetalDHS Trynka
Enhancer Hoffman
Enhancer Andersson
DHS Trynka
DGF ENCODE
CTCF Hoffman
Conserved LindbladToh
Coding UCSC
−10
0
10
20
Enrichment (prop h2 / prop snps)

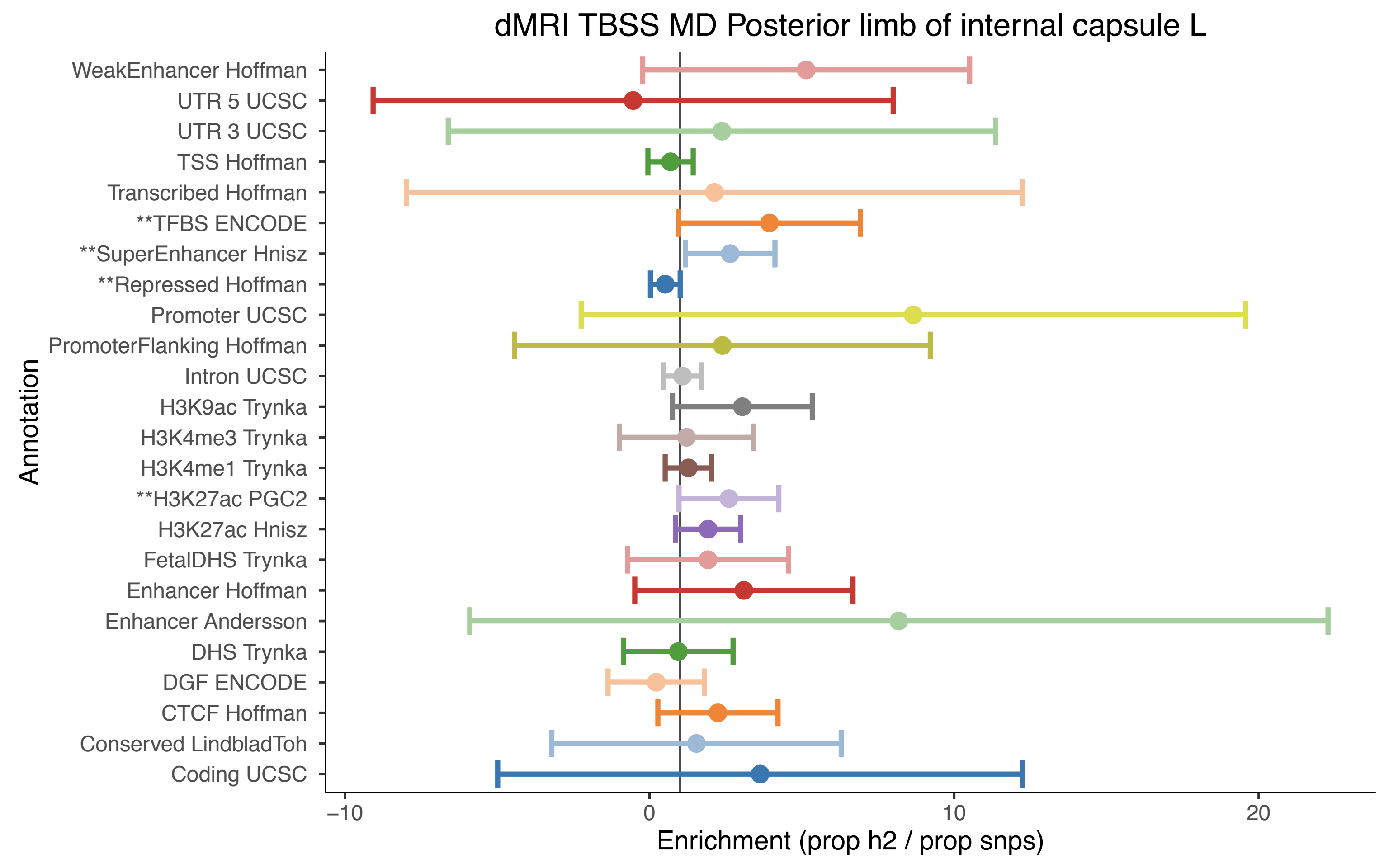

dMRI TBSS MD Posterior limb of internal capsule L
WeakEnhancer Hoffman
UTR 5 UCSC
UTR 3 UCSC
TSS Hoffman
Transcribed Hoffman
**TFBS ENCODE
**SuperEnhancer Hnisz
**Repressed Hoffman
Promoter UCSC
PromoterFlanking Hoffman
Intron UCSC
H3K9ac Trynka
H3K4me3 Trynka
H3K4me1 Trynka
**H3K27ac PGC2
H3K27ac Hnisz
FetalDHS Trynka
Enhancer Hoffman
Enhancer Andersson
DHS Trynka
DGF ENCODE
CTCF Hoffman
Conserved LindbladToh
Coding UCSC
Annotation
−10
0
10
20
Enrichment (prop h2 / prop snps)

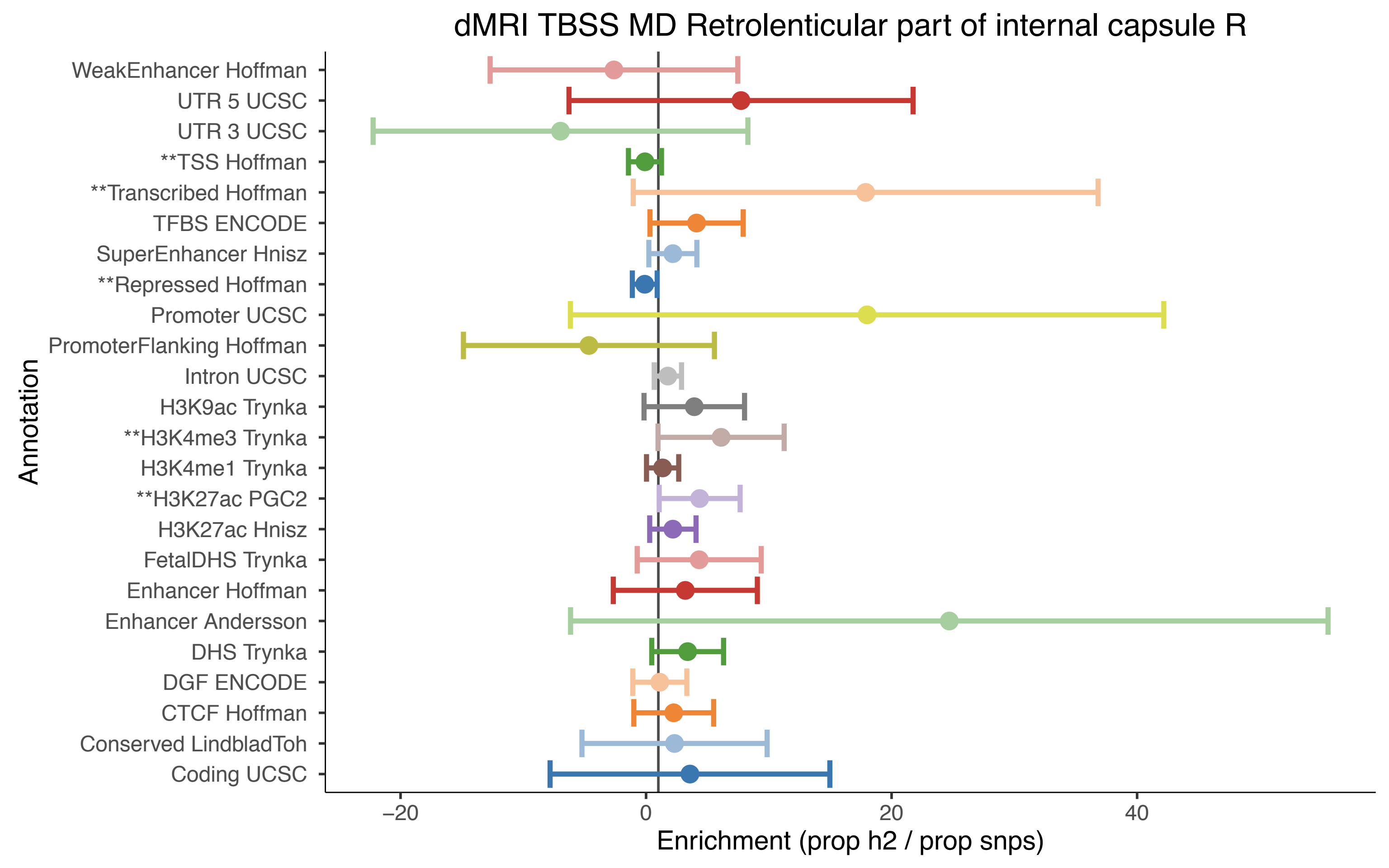

dMRI TBSS MD Retrolenticular part of internal capsule R
WeakEnhancer Hoffman
UTR 5 UCSC
UTR 3 UCSC
**TSS Hoffman
**Transcribed Hoffman
TFBS ENCODE
SuperEnhancer Hnisz
**Repressed Hoffman
Promoter UCSC
PromoterFlanking Hoffman
Intron UCSC
H3K9ac Trynka
**H3K4me3 Trynka
H3K4me1 Trynka
**H3K27ac PGC2
H3K27ac Hnisz
FetalDHS Trynka
Enhancer Hoffman
Enhancer Andersson
DHS Trynka
DGF ENCODE
CTCF Hoffman
Conserved LindbladToh
Coding UCSC
Annotation
−20
0
20
40
Enrichment (prop h2 / prop snps)

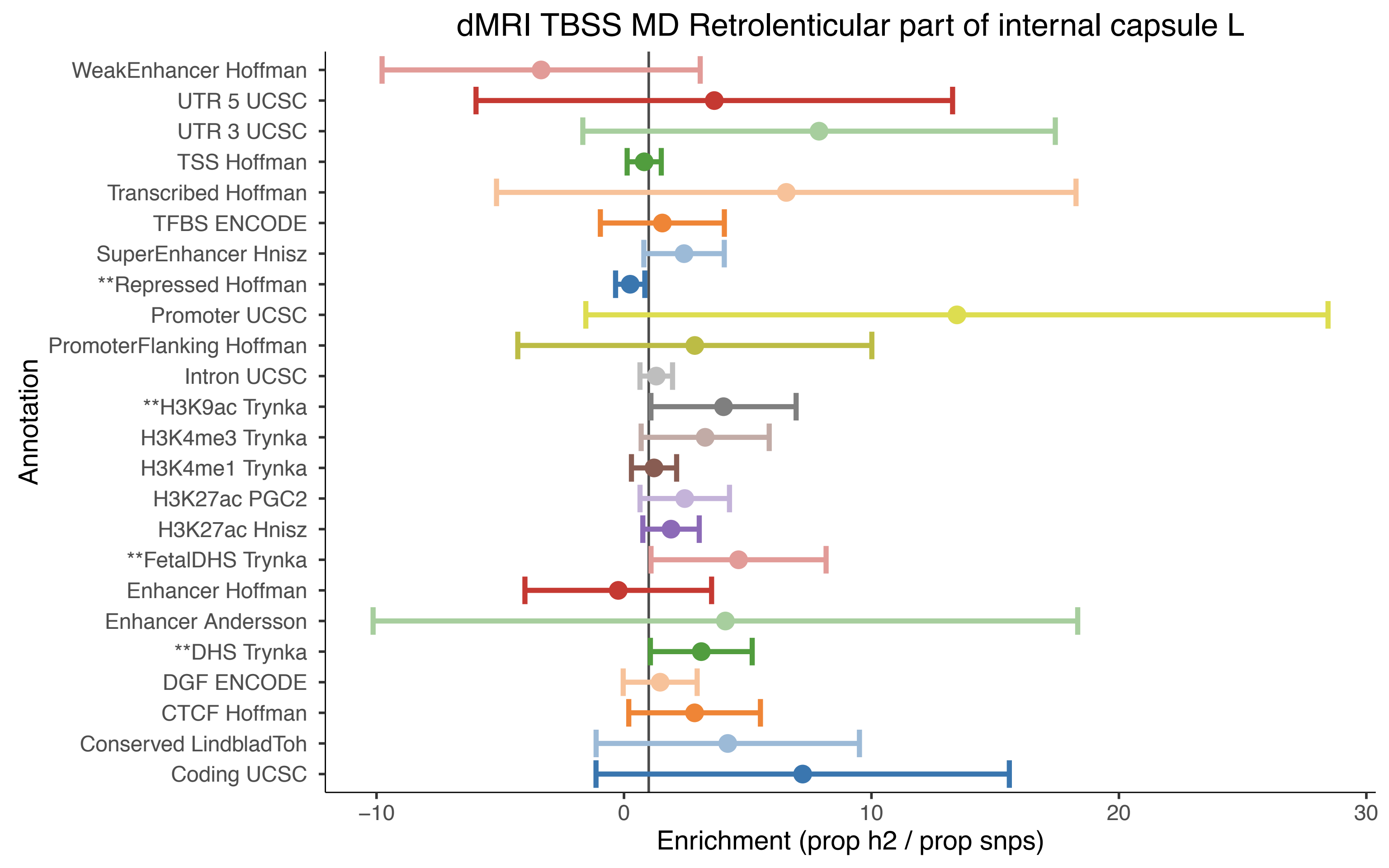

dMRI TBSS MD Retrolenticular part of internal capsule L
WeakEnhancer Hoffman
UTR 5 UCSC
UTR 3 UCSC
TSS Hoffman
Transcribed Hoffman
TFBS ENCODE
SuperEnhancer Hnisz
**Repressed Hoffman
Promoter UCSC
PromoterFlanking Hoffman
Intron UCSC
**H3K9ac Trynka
H3K4me3 Trynka
H3K4me1 Trynka
H3K27ac PGC2
H3K27ac Hnisz
**FetalDHS Trynka
Enhancer Hoffman
Enhancer Andersson
**DHS Trynka
DGF ENCODE
CTCF Hoffman
Conserved LindbladToh
Coding UCSC
Annotation
−10
0
10
20
30
Enrichment (prop h2 / prop snps)

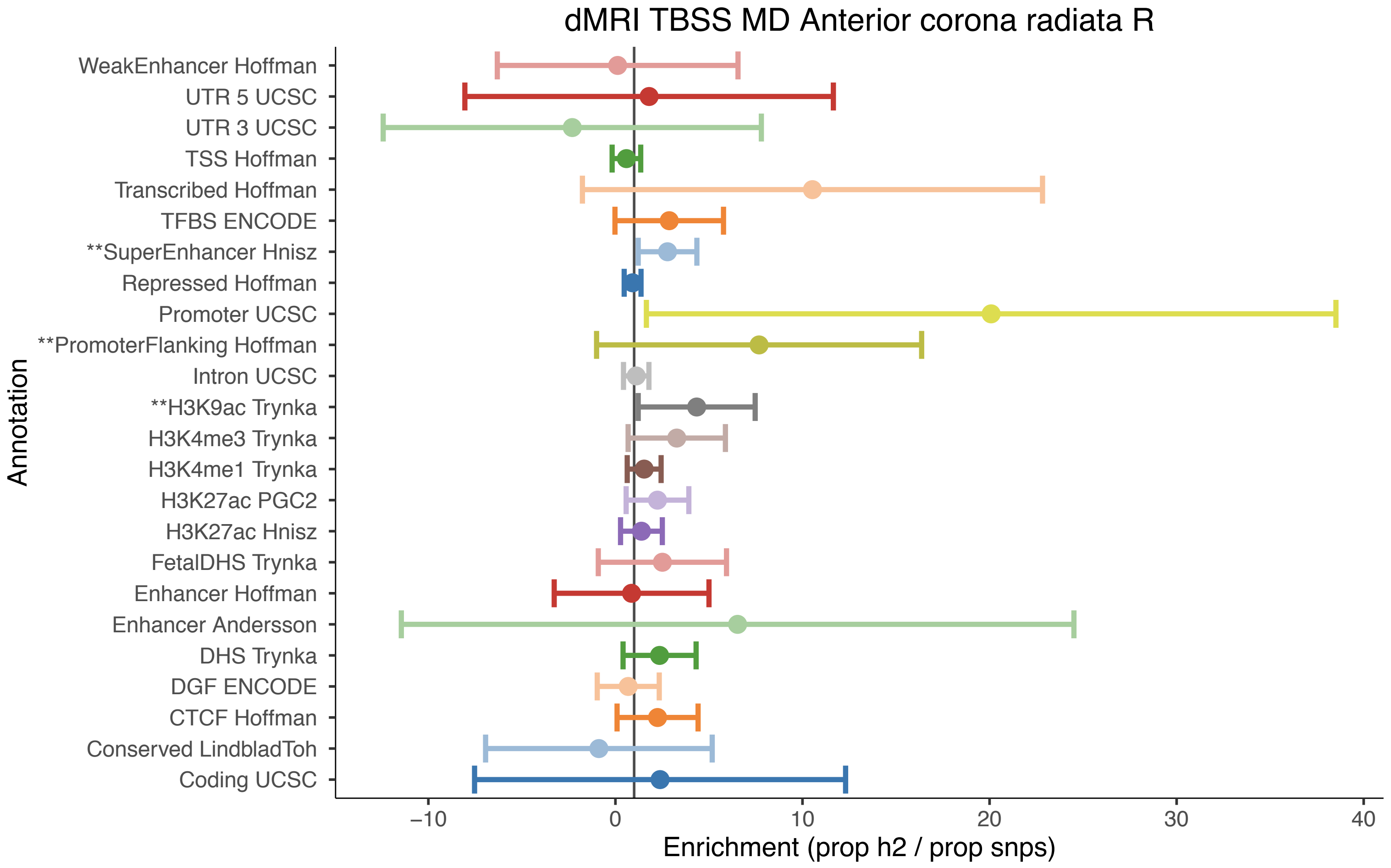
dMRI TBSS MD Anterior corona radiata R
WeakEnhancer Hoffman
UTR 5 UCSC
UTR 3 UCSC
TSS Hoffman
Transcribed Hoffman
TFBS ENCODE
**SuperEnhancer Hnisz
Repressed Hoffman
Promoter UCSC
**PromoterFlanking Hoffman
Intron UCSC
**H3K9ac Trynka
H3K4me3 Trynka
H3K4me1 Trynka
H3K27ac PGC2
H3K27ac Hnisz
FetalDHS Trynka
Enhancer Hoffman
Enhancer Andersson
DHS Trynka
DGF ENCODE
CTCF Hoffman
Conserved LindbladToh
Coding UCSC
Annotation
−10
0
10
20
30
40
Enrichment (prop h2 / prop snps)

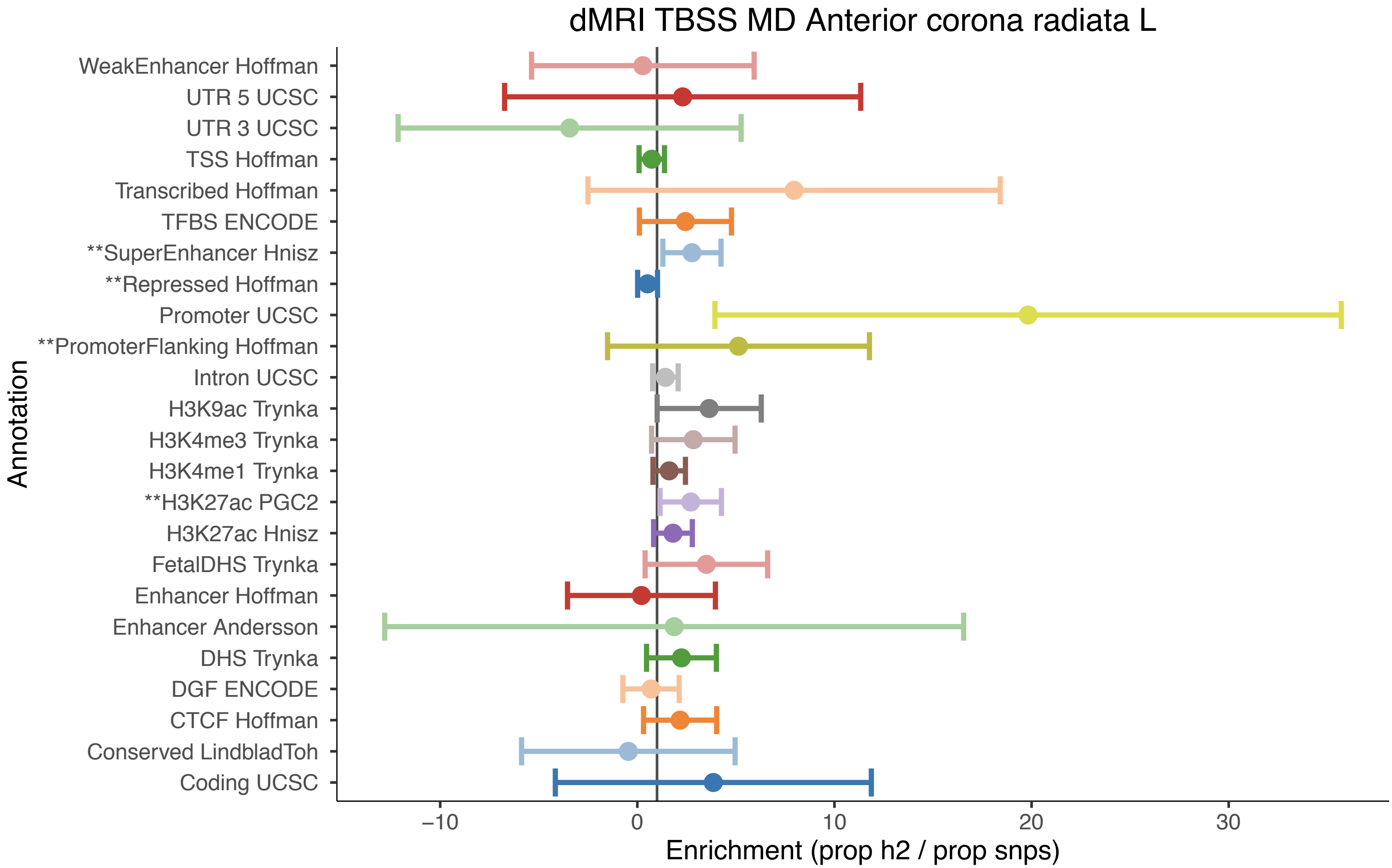
dMRI TBSS MD Anterior corona radiata L
Annotation
WeakEnhancer Hoffman
UTR 5 UCSC
UTR 3 UCSC
TSS Hoffman
Transcribed Hoffman
TFBS ENCODE
**SuperEnhancer Hnisz
**Repressed Hoffman
Promoter UCSC
**PromoterFlanking Hoffman
Intron UCSC
H3K9ac Trynka
H3K4me3 Trynka
H3K4me1 Trynka
**H3K27ac PGC2
H3K27ac Hnisz
FetalDHS Trynka
Enhancer Hoffman
Enhancer Andersson
DHS Trynka
DGF ENCODE
CTCF Hoffman
Conserved LindbladToh
Coding UCSC
−10
0
10
20
30
Enrichment (prop h2 / prop snps)

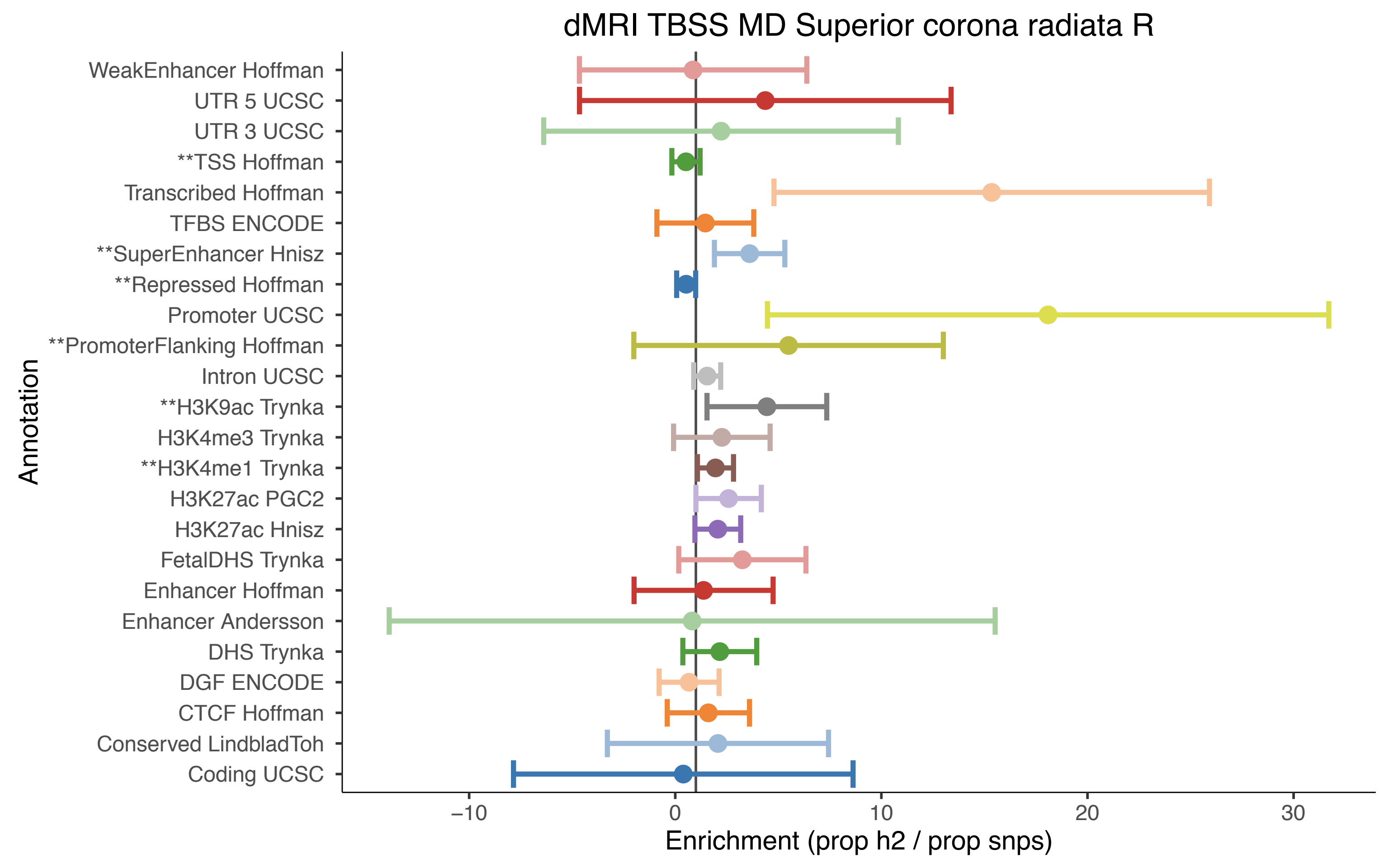

dMRI TBSS MD Superior corona radiata R
WeakEnhancer Hoffman
UTR 5 UCSC
UTR 3 UCSC
**TSS Hoffman
Transcribed Hoffman
TFBS ENCODE
**SuperEnhancer Hnisz
**Repressed Hoffman
Promoter UCSC
**PromoterFlanking Hoffman
Intron UCSC
**H3K9ac Trynka
H3K4me3 Trynka
**H3K4me1 Trynka
H3K27ac PGC2
H3K27ac Hnisz
FetalDHS Trynka
Enhancer Hoffman
Enhancer Andersson
DHS Trynka
DGF ENCODE
CTCF Hoffman
Conserved LindbladToh
Coding UCSC
Annotation
−10
0
10
20
30
Enrichment (prop h2 / prop snps)

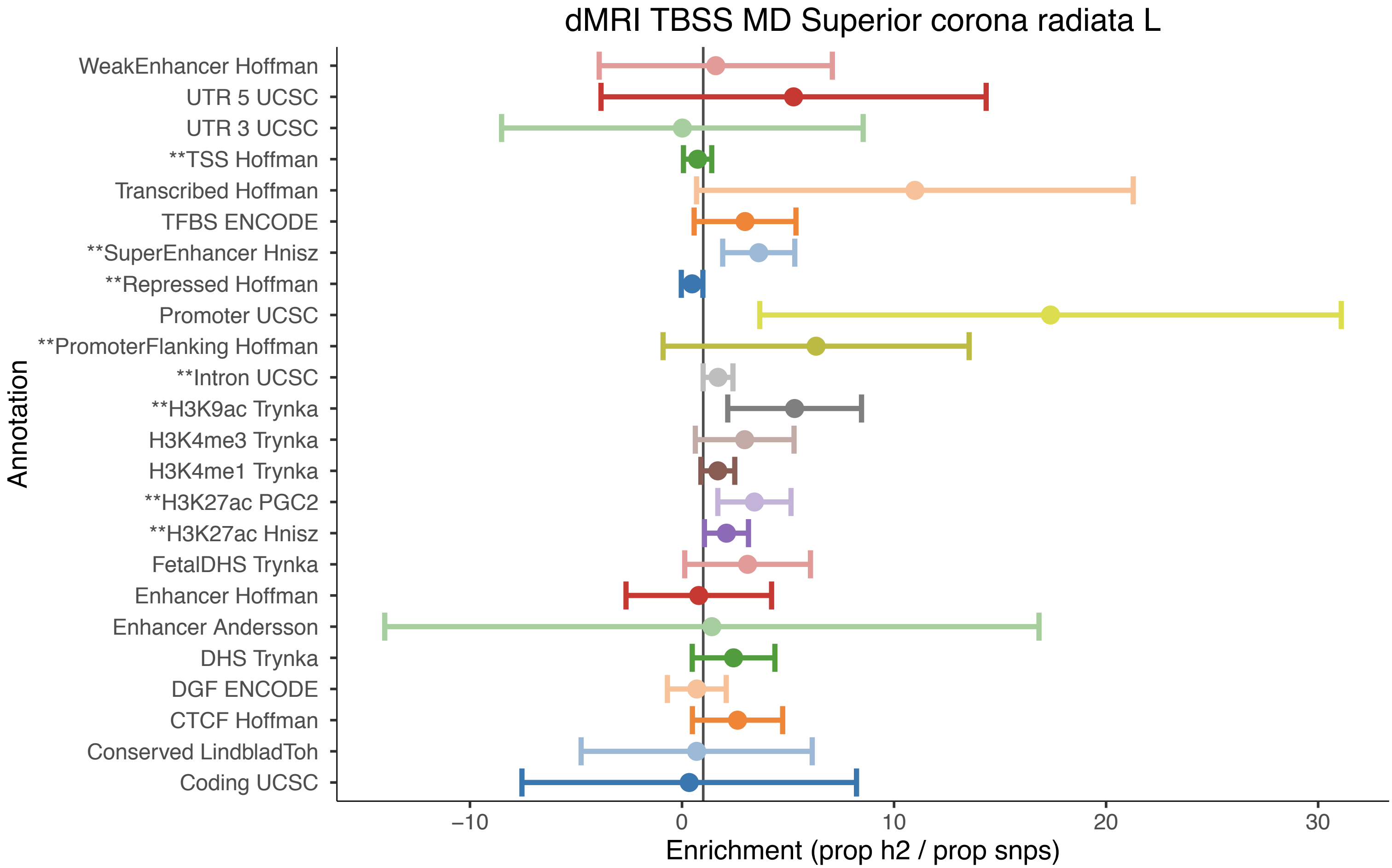
dMRI TBSS MD Superior corona radiata L
WeakEnhancer Hoffman
UTR 5 UCSC
UTR 3 UCSC
**TSS Hoffman
Transcribed Hoffman
TFBS ENCODE
**SuperEnhancer Hnisz
**Repressed Hoffman
Promoter UCSC
**PromoterFlanking Hoffman
**Intron UCSC
**H3K9ac Trynka
H3K4me3 Trynka
H3K4me1 Trynka
**H3K27ac PGC2
**H3K27ac Hnisz
FetalDHS Trynka
Enhancer Hoffman
Enhancer Andersson
DHS Trynka
DGF ENCODE
CTCF Hoffman
Conserved LindbladToh
Coding UCSC
Annotation
−10
0
10
20
30
Enrichment (prop h2 / prop snps)

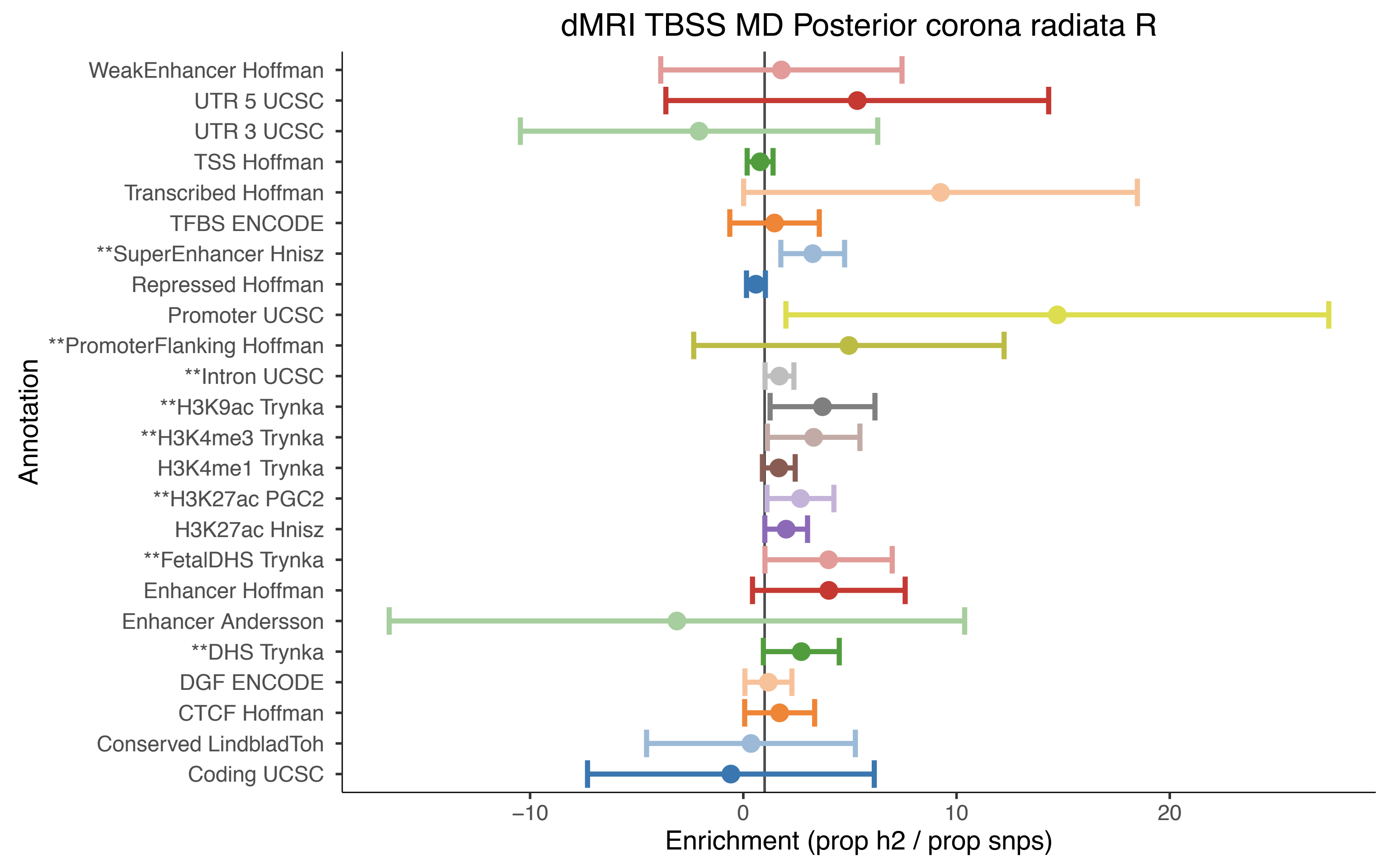

dMRI TBSS MD Posterior corona radiata R
Annotation
WeakEnhancer Hoffman
UTR 5 UCSC
UTR 3 UCSC
TSS Hoffman
Transcribed Hoffman
TFBS ENCODE
**SuperEnhancer Hnisz
Repressed Hoffman
Promoter UCSC
**PromoterFlanking Hoffman
**Intron UCSC
**H3K9ac Trynka
**H3K4me3 Trynka
H3K4me1 Trynka
**H3K27ac PGC2
H3K27ac Hnisz
**FetalDHS Trynka
Enhancer Hoffman
Enhancer Andersson
**DHS Trynka
DGF ENCODE
CTCF Hoffman
Conserved LindbladToh
Coding UCSC
−10
0
10
20
Enrichment (prop h2 / prop snps)

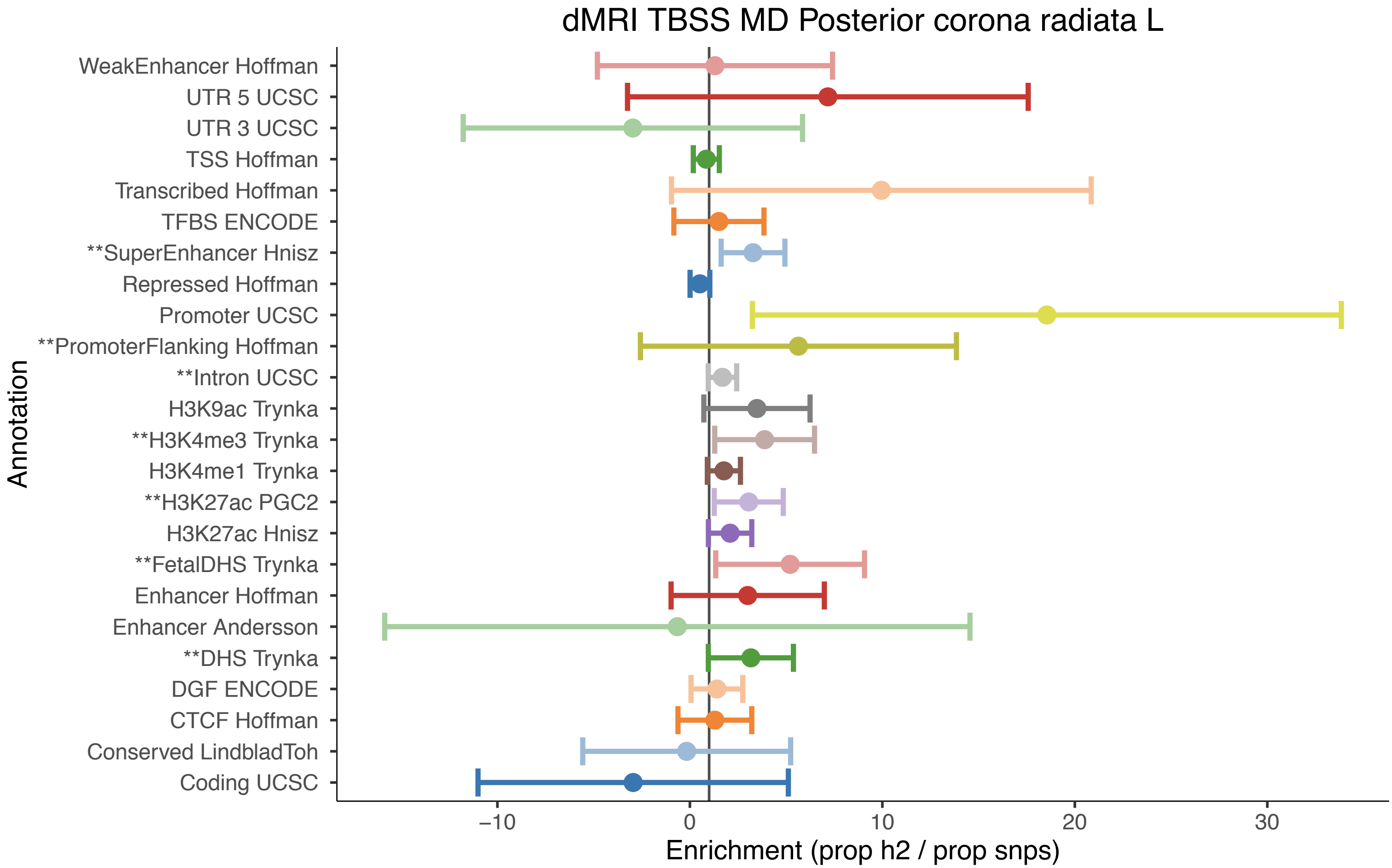

dMRI TBSS MD Posterior corona radiata L
WeakEnhancer Hoffman
UTR 5 UCSC
UTR 3 UCSC
TSS Hoffman
Transcribed Hoffman
TFBS ENCODE
**SuperEnhancer Hnisz
Repressed Hoffman
Promoter UCSC
**PromoterFlanking Hoffman
**Intron UCSC
H3K9ac Trynka
**H3K4me3 Trynka
H3K4me1 Trynka
**H3K27ac PGC2
H3K27ac Hnisz
**FetalDHS Trynka
Enhancer Hoffman
Enhancer Andersson
**DHS Trynka
DGF ENCODE
CTCF Hoffman
Conserved LindbladToh
Coding UCSC
Annotation
−10
0
10
20
30
Enrichment (prop h2 / prop snps)

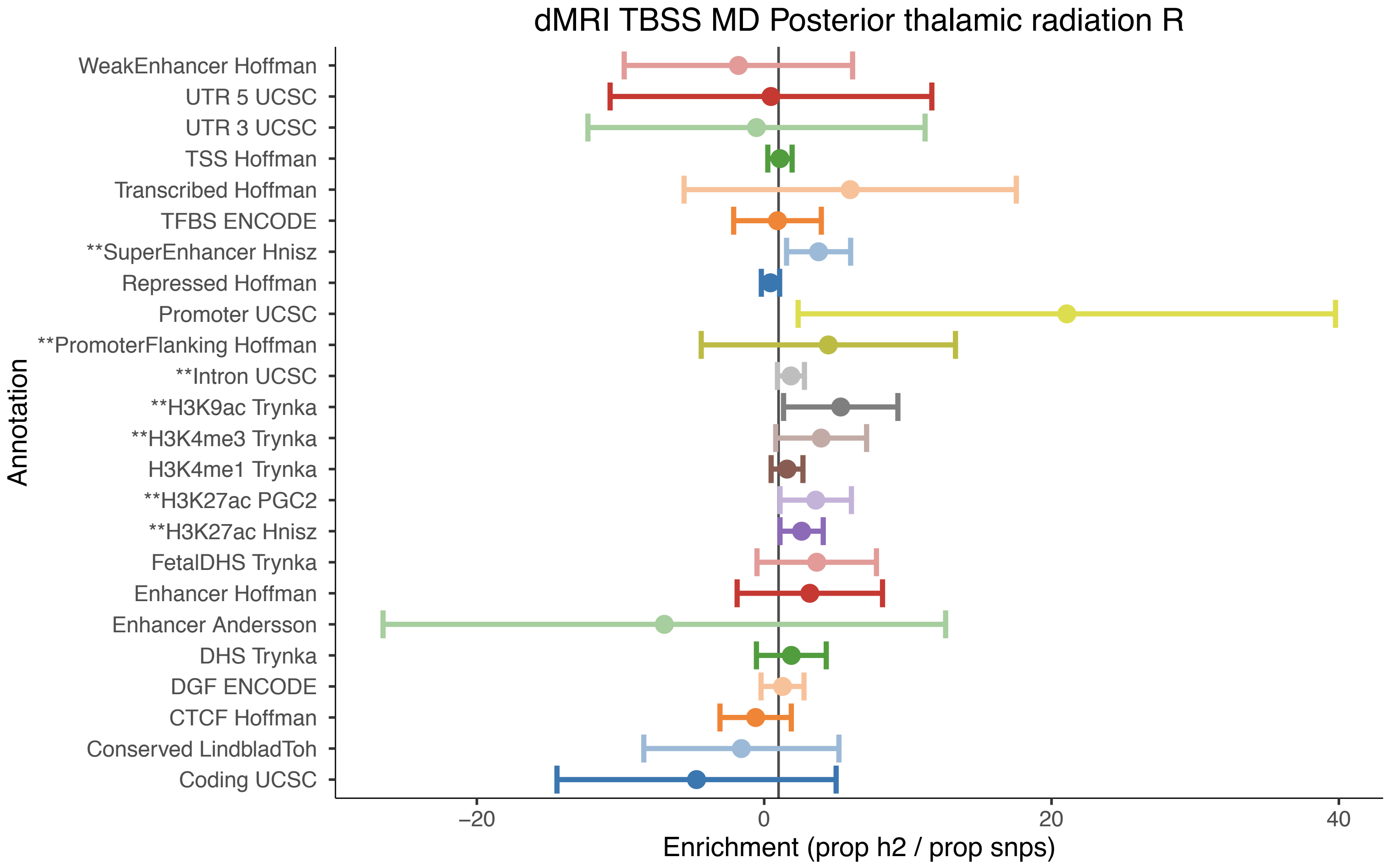

dMRI TBSS MD Posterior thalamic radiation R
WeakEnhancer Hoffman
UTR 5 UCSC
UTR 3 UCSC
TSS Hoffman
Transcribed Hoffman
TFBS ENCODE
**SuperEnhancer Hnisz
Repressed Hoffman
Promoter UCSC
**PromoterFlanking Hoffman
**Intron UCSC
**H3K9ac Trynka
**H3K4me3 Trynka
H3K4me1 Trynka
**H3K27ac PGC2
**H3K27ac Hnisz
FetalDHS Trynka
Enhancer Hoffman
Enhancer Andersson
DHS Trynka
DGF ENCODE
CTCF Hoffman
Conserved LindbladToh
Coding UCSC
Annotation
−20
0
20
40
Enrichment (prop h2 / prop snps)

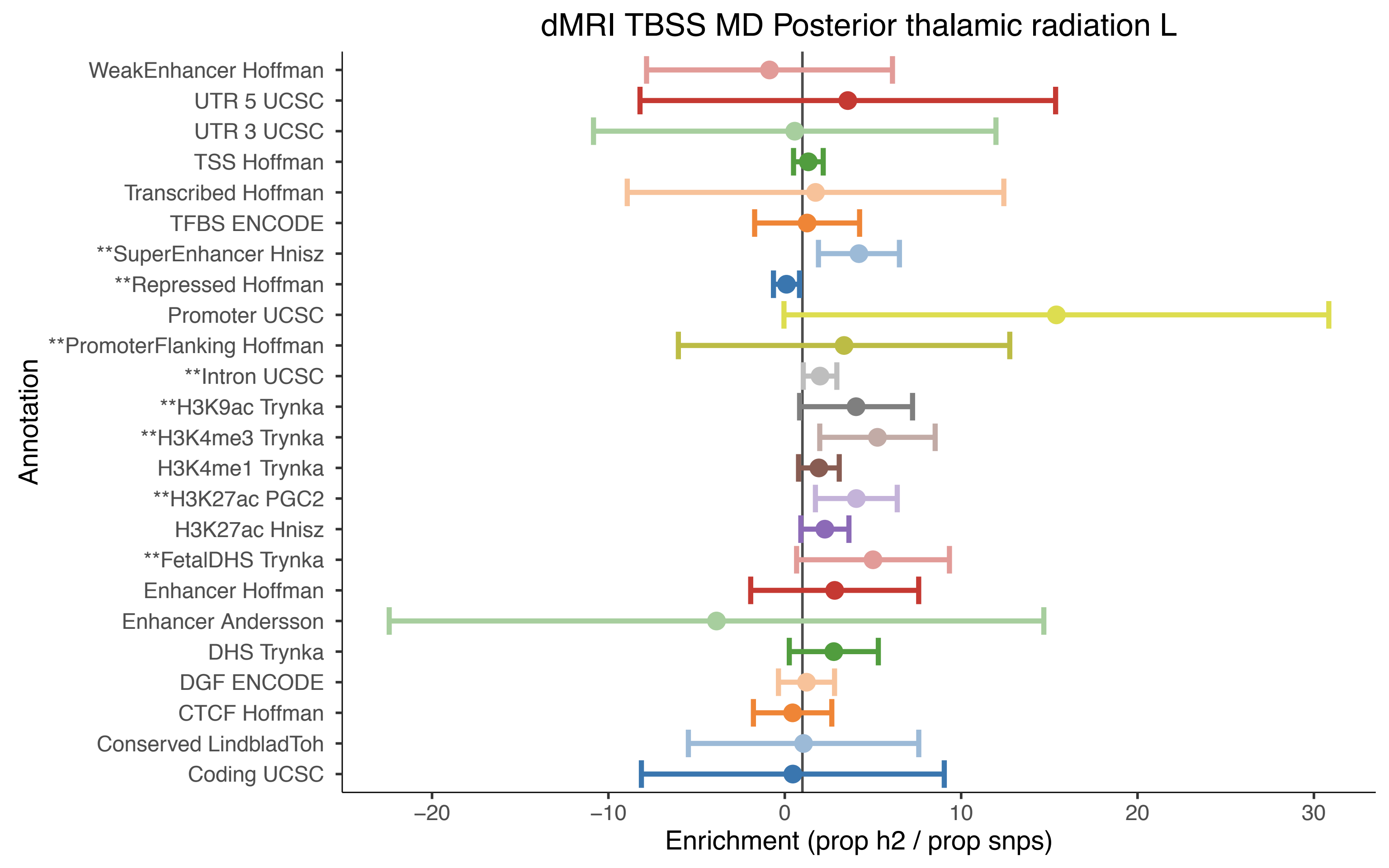

dMRI TBSS MD Posterior thalamic radiation L
WeakEnhancer Hoffman
UTR 5 UCSC
UTR 3 UCSC
TSS Hoffman
Transcribed Hoffman
TFBS ENCODE
**SuperEnhancer Hnisz
**Repressed Hoffman
Promoter UCSC
**PromoterFlanking Hoffman
**Intron UCSC
**H3K9ac Trynka
**H3K4me3 Trynka
H3K4me1 Trynka
**H3K27ac PGC2
H3K27ac Hnisz
**FetalDHS Trynka
Enhancer Hoffman
Enhancer Andersson
DHS Trynka
DGF ENCODE
CTCF Hoffman
Conserved LindbladToh
Coding UCSC
Annotation
−20
−10
0
10
20
30
Enrichment (prop h2 / prop snps)

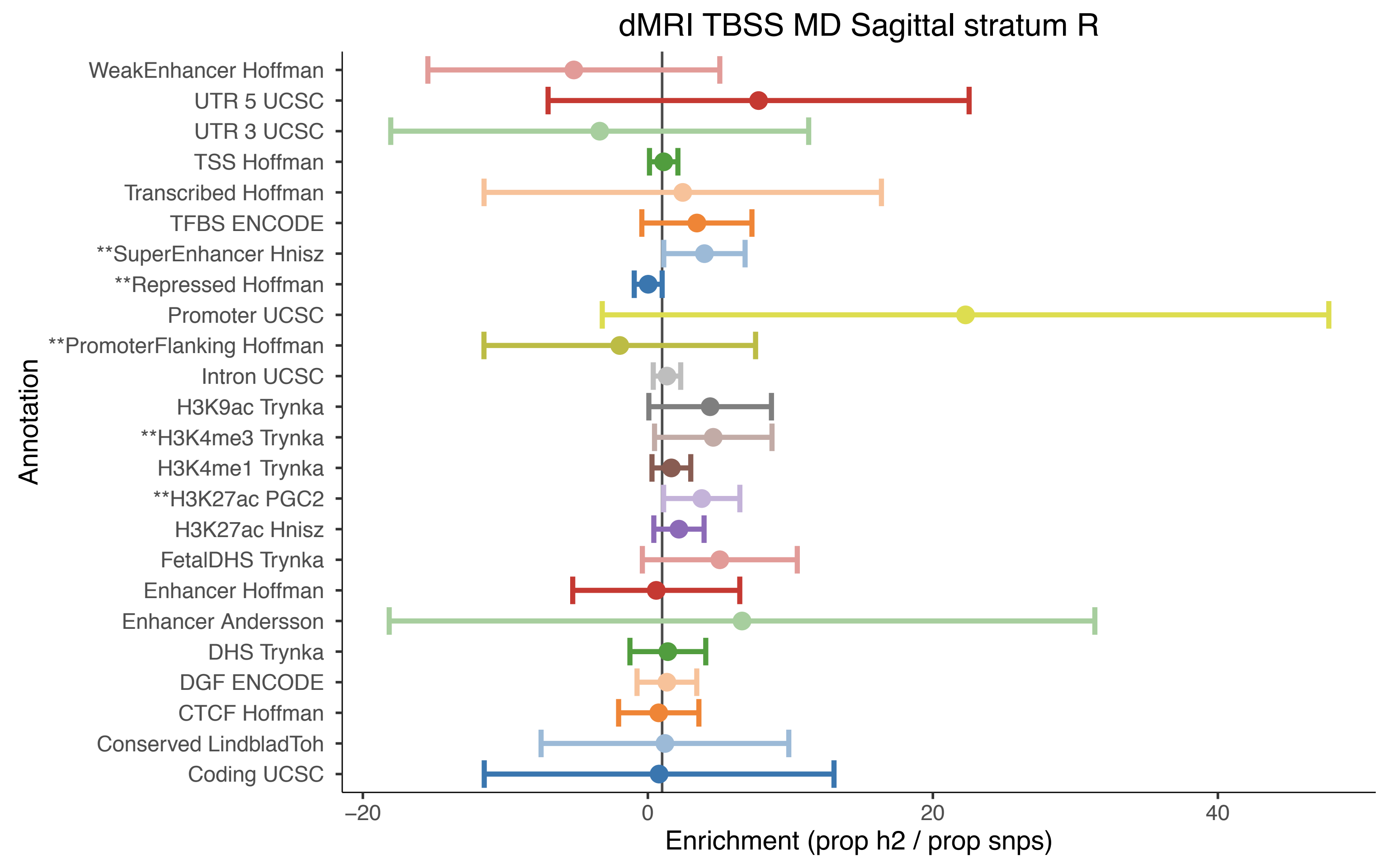
dMRI TBSS MD Sagittal stratum R
WeakEnhancer Hoffman
UTR 5 UCSC
UTR 3 UCSC
TSS Hoffman
Transcribed Hoffman
TFBS ENCODE
**SuperEnhancer Hnisz
**Repressed Hoffman
Promoter UCSC
**PromoterFlanking Hoffman
Intron UCSC
H3K9ac Trynka
**H3K4me3 Trynka
H3K4me1 Trynka
**H3K27ac PGC2
H3K27ac Hnisz
FetalDHS Trynka
Enhancer Hoffman
Enhancer Andersson
DHS Trynka
DGF ENCODE
CTCF Hoffman
Conserved LindbladToh
Coding UCSC
Annotation
−20
0
20
40
Enrichment (prop h2 / prop snps)

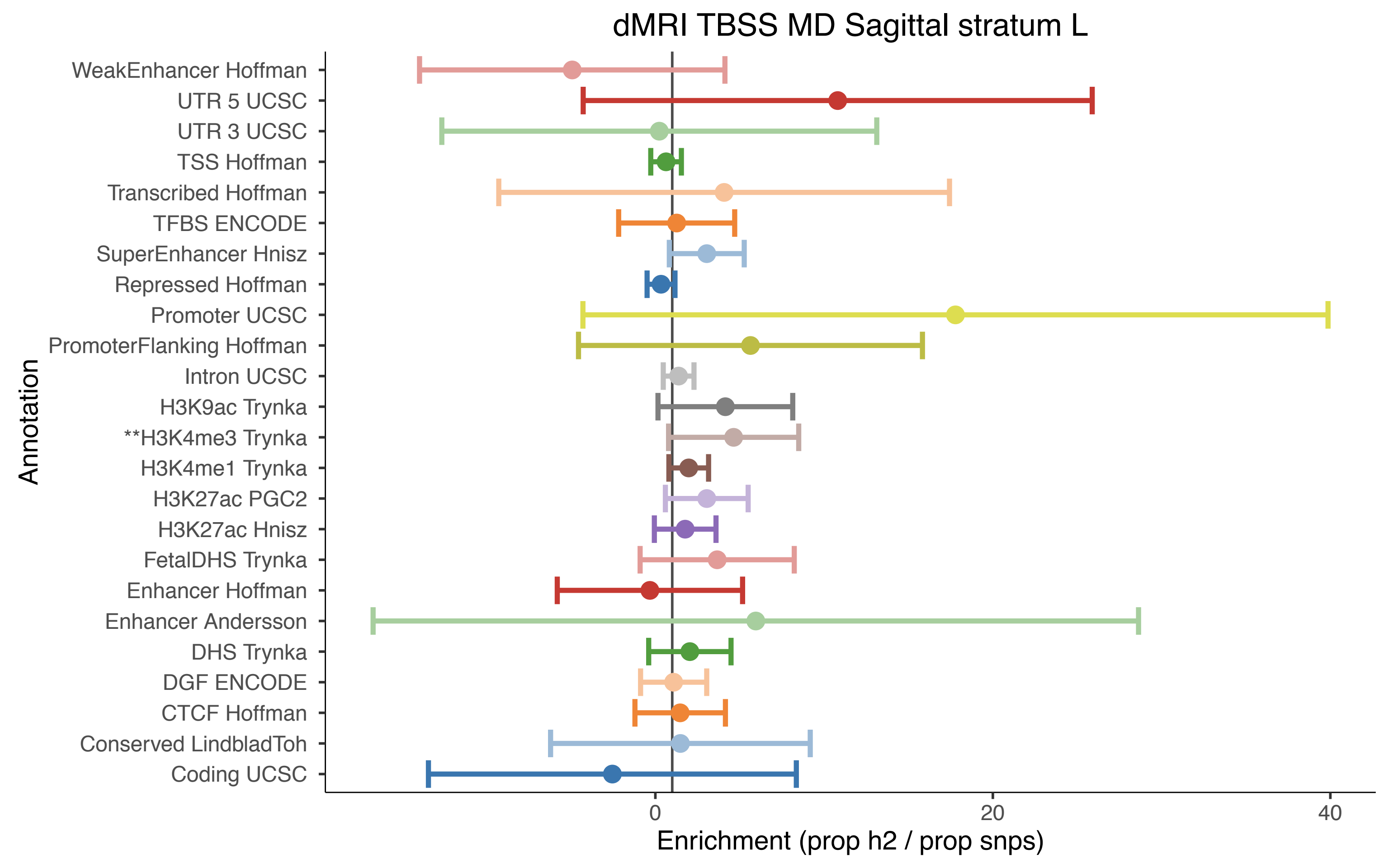
dMRI TBSS MD Sagittal stratum L
WeakEnhancer Hoffman
UTR 5 UCSC
UTR 3 UCSC
TSS Hoffman
Transcribed Hoffman
TFBS ENCODE
SuperEnhancer Hnisz
Repressed Hoffman
Promoter UCSC
PromoterFlanking Hoffman
Intron UCSC
H3K9ac Trynka
**H3K4me3 Trynka
H3K4me1 Trynka
H3K27ac PGC2
H3K27ac Hnisz
FetalDHS Trynka
Enhancer Hoffman
Enhancer Andersson
DHS Trynka
DGF ENCODE
CTCF Hoffman
Conserved LindbladToh
Coding UCSC
Annotation
0
20
40
Enrichment (prop h2 / prop snps)

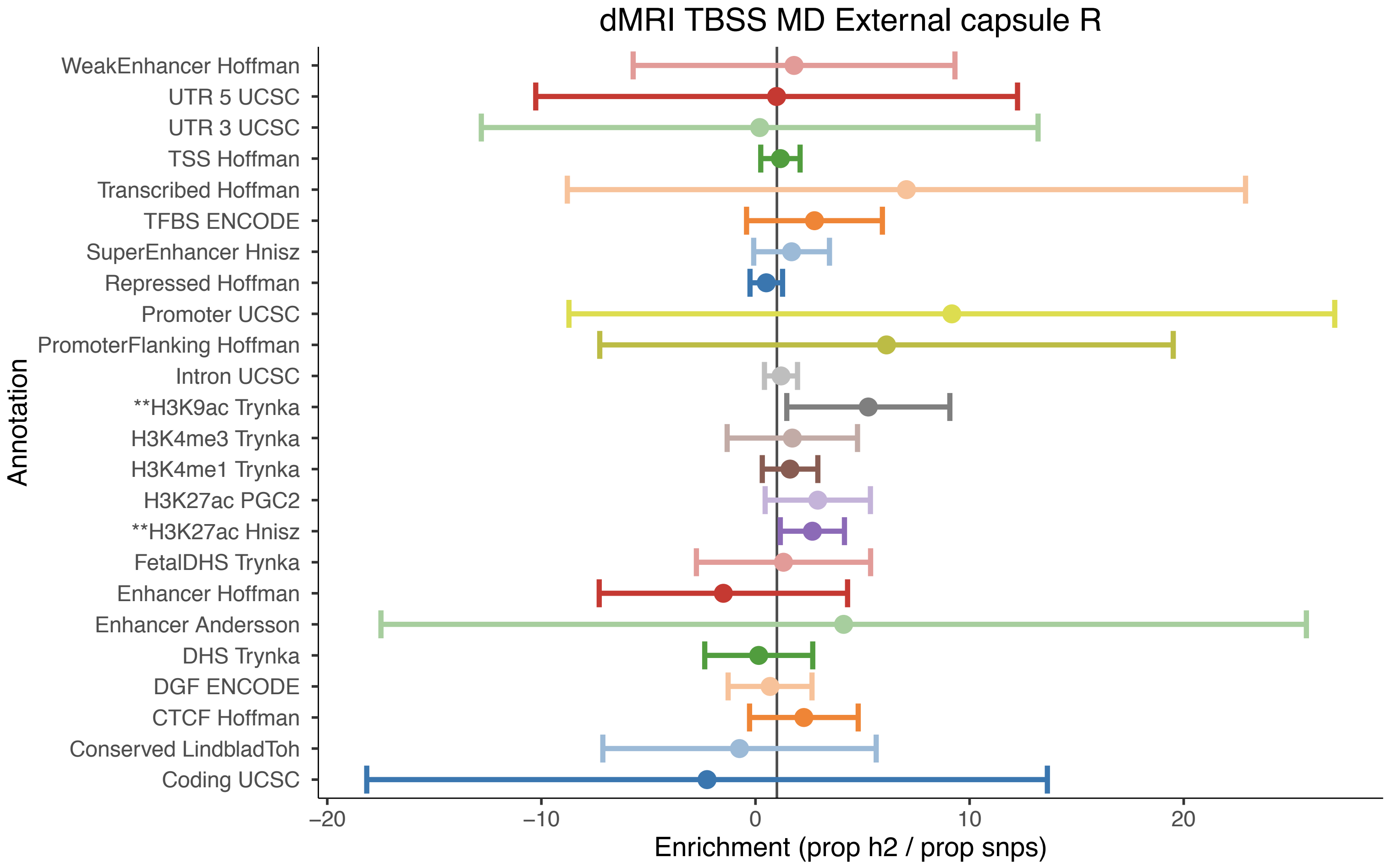

dMRI TBSS MD External capsule R
WeakEnhancer Hoffman
UTR 5 UCSC
UTR 3 UCSC
TSS Hoffman
Transcribed Hoffman
TFBS ENCODE
SuperEnhancer Hnisz
Repressed Hoffman
Promoter UCSC
PromoterFlanking Hoffman
Intron UCSC
**H3K9ac Trynka
H3K4me3 Trynka
H3K4me1 Trynka
H3K27ac PGC2
**H3K27ac Hnisz
FetalDHS Trynka
Enhancer Hoffman
Enhancer Andersson
DHS Trynka
DGF ENCODE
CTCF Hoffman
Conserved LindbladToh
Coding UCSC
Annotation
−20
−10
0
10
20
Enrichment (prop h2 / prop snps)

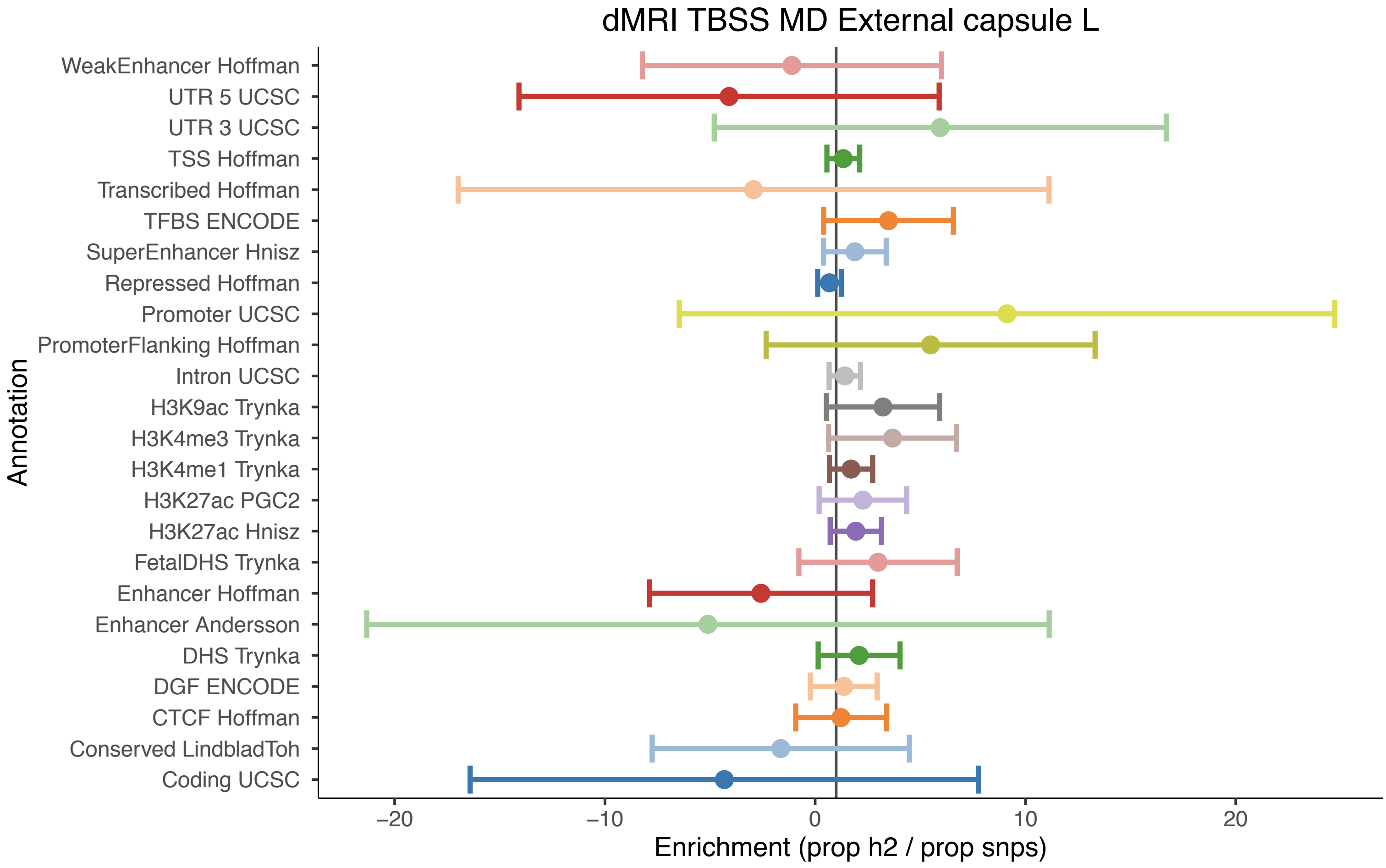
dMRI TBSS MD External capsule L
WeakEnhancer Hoffman
UTR 5 UCSC
UTR 3 UCSC
TSS Hoffman
Transcribed Hoffman
TFBS ENCODE
SuperEnhancer Hnisz
Repressed Hoffman
Promoter UCSC
PromoterFlanking Hoffman
Intron UCSC
H3K9ac Trynka
H3K4me3 Trynka
H3K4me1 Trynka
H3K27ac PGC2
H3K27ac Hnisz
FetalDHS Trynka
Enhancer Hoffman
Enhancer Andersson
DHS Trynka
DGF ENCODE
CTCF Hoffman
Conserved LindbladToh
Coding UCSC
Annotation
−20
−10
0
10
20
Enrichment (prop h2 / prop snps)

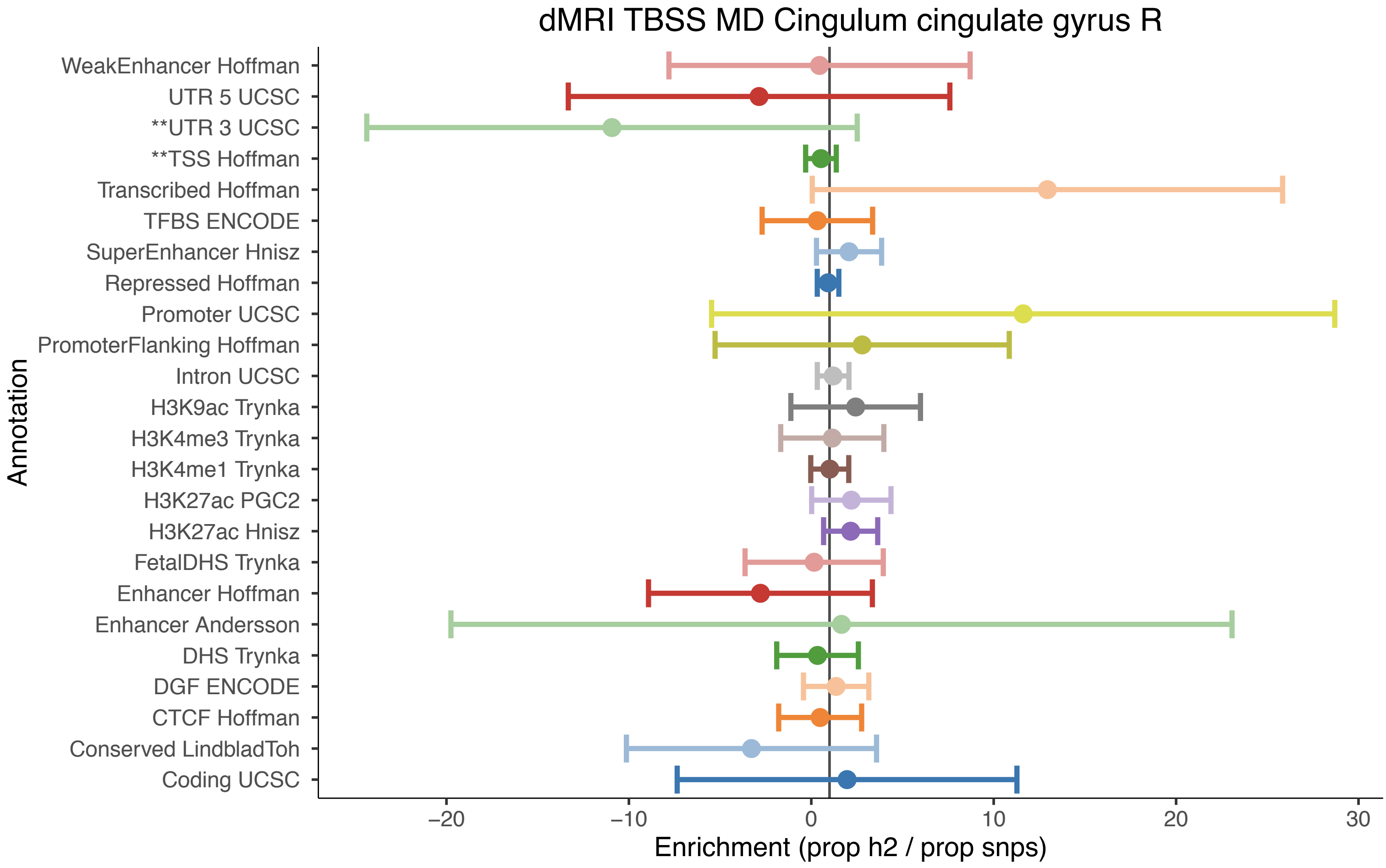

dMRI TBSS MD Cingulum cingulate gyrus R
WeakEnhancer Hoffman
UTR 5 UCSC
**UTR 3 UCSC
**TSS Hoffman
Transcribed Hoffman
TFBS ENCODE
SuperEnhancer Hnisz
Repressed Hoffman
Promoter UCSC
PromoterFlanking Hoffman
Intron UCSC
H3K9ac Trynka
H3K4me3 Trynka
H3K4me1 Trynka
H3K27ac PGC2
H3K27ac Hnisz
FetalDHS Trynka
Enhancer Hoffman
Enhancer Andersson
DHS Trynka
DGF ENCODE
CTCF Hoffman
Conserved LindbladToh
Coding UCSC
Annotation
−20
−10
0
10
20
30
Enrichment (prop h2 / prop snps)

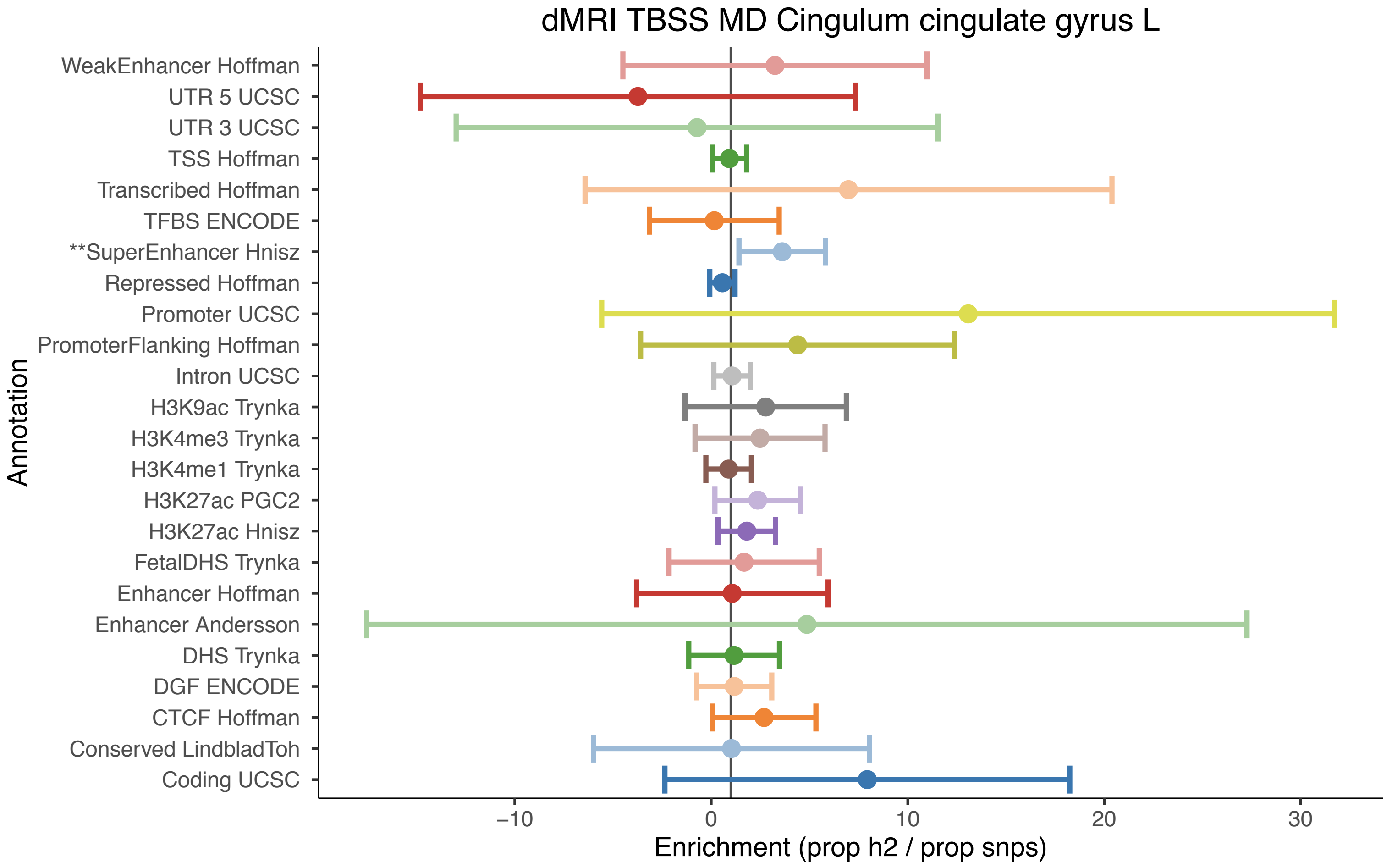
dMRI TBSS MD Cingulum cingulate gyrus L
WeakEnhancer Hoffman
UTR 5 UCSC
UTR 3 UCSC
TSS Hoffman
Transcribed Hoffman
TFBS ENCODE
**SuperEnhancer Hnisz
Repressed Hoffman
Promoter UCSC
PromoterFlanking Hoffman
Intron UCSC
H3K9ac Trynka
H3K4me3 Trynka
H3K4me1 Trynka
H3K27ac PGC2
H3K27ac Hnisz
FetalDHS Trynka
Enhancer Hoffman
Enhancer Andersson
DHS Trynka
DGF ENCODE
CTCF Hoffman
Conserved LindbladToh
Coding UCSC
Annotation
−10
0
10
20
30
Enrichment (prop h2 / prop snps)

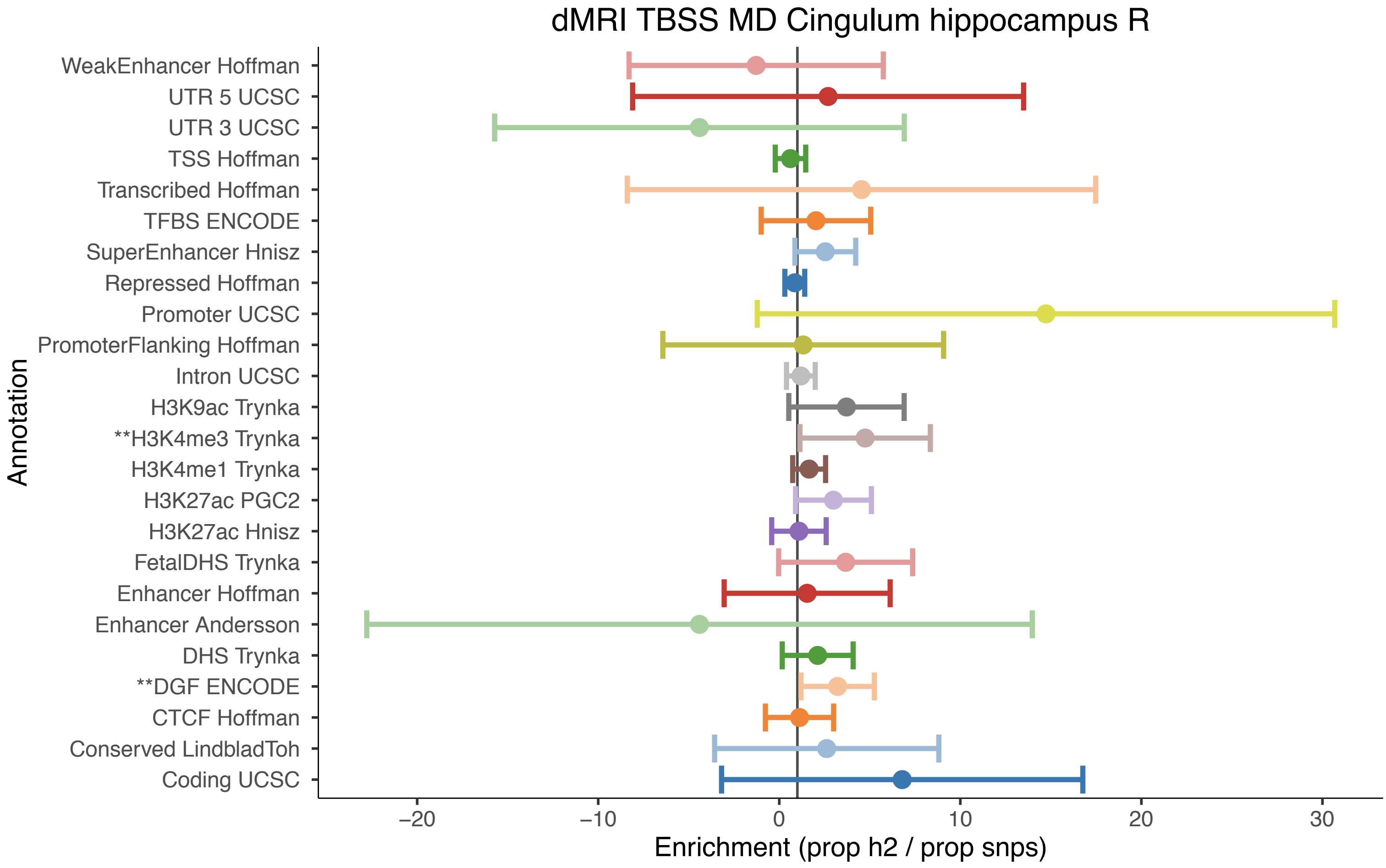

dMRI TBSS MD Cingulum hippocampus R
WeakEnhancer Hoffman
UTR 5 UCSC
UTR 3 UCSC
TSS Hoffman
Transcribed Hoffman
TFBS ENCODE
SuperEnhancer Hnisz
Repressed Hoffman
Promoter UCSC
PromoterFlanking Hoffman
Intron UCSC
H3K9ac Trynka
**H3K4me3 Trynka
H3K4me1 Trynka
H3K27ac PGC2
H3K27ac Hnisz
FetalDHS Trynka
Enhancer Hoffman
Enhancer Andersson
DHS Trynka
**DGF ENCODE
CTCF Hoffman
Conserved LindbladToh
Coding UCSC
Annotation
−20
−10
0
10
20
30
Enrichment (prop h2 / prop snps)

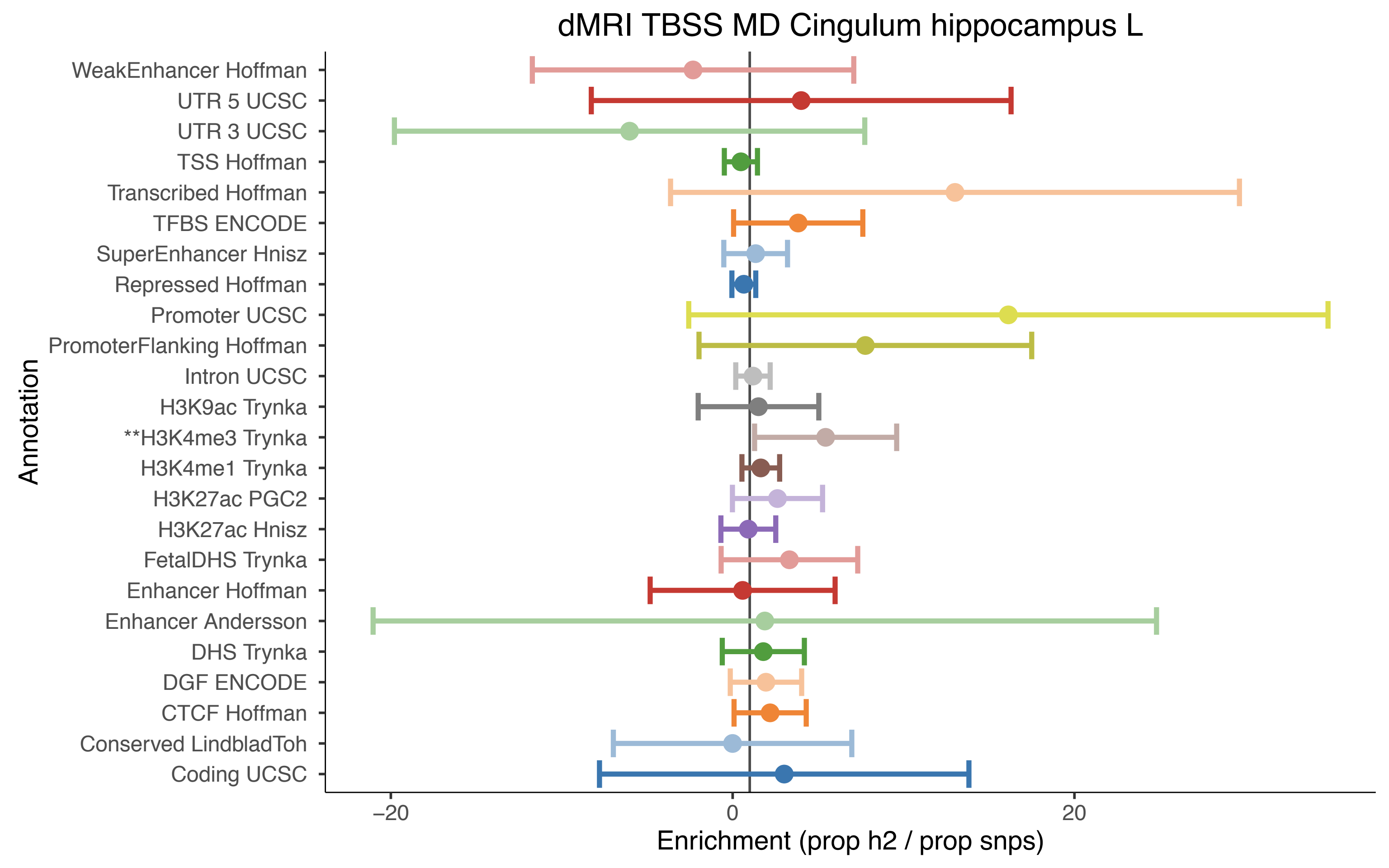
dMRI TBSS MD Cingulum hippocampus L
WeakEnhancer Hoffman
UTR 5 UCSC
UTR 3 UCSC
TSS Hoffman
Transcribed Hoffman
TFBS ENCODE
SuperEnhancer Hnisz
Repressed Hoffman
Promoter UCSC
PromoterFlanking Hoffman
Intron UCSC
H3K9ac Trynka
**H3K4me3 Trynka
H3K4me1 Trynka
H3K27ac PGC2
H3K27ac Hnisz
FetalDHS Trynka
Enhancer Hoffman
Enhancer Andersson
DHS Trynka
DGF ENCODE
CTCF Hoffman
Conserved LindbladToh
Coding UCSC
Annotation
−20
0
20
Enrichment (prop h2 / prop snps)

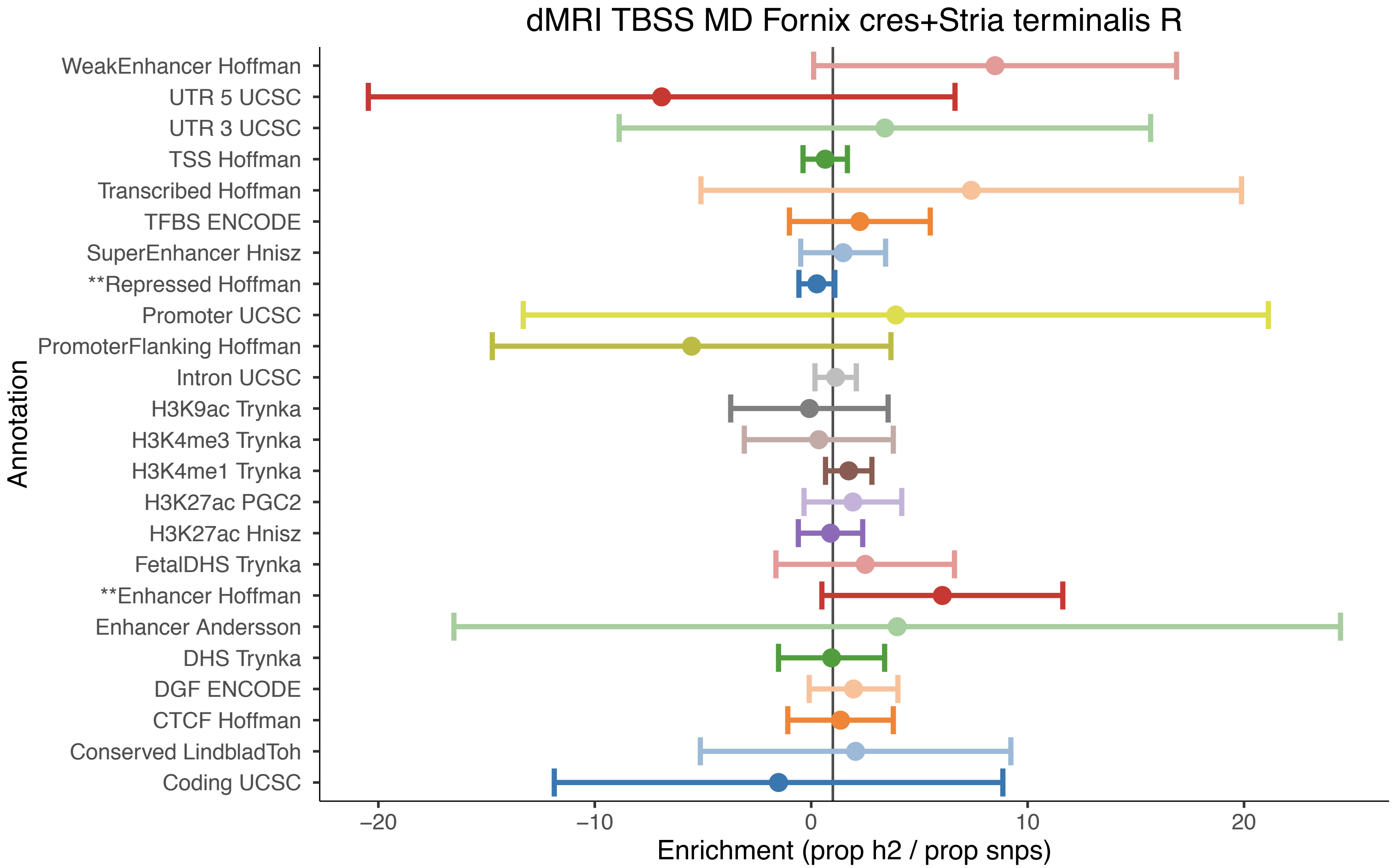
dMRI TBSS MD Fornix cres+Stria terminalis R
WeakEnhancer Hoffman
UTR 5 UCSC
UTR 3 UCSC
TSS Hoffman
Transcribed Hoffman
TFBS ENCODE
SuperEnhancer Hnisz
**Repressed Hoffman
Promoter UCSC
PromoterFlanking Hoffman
Intron UCSC
H3K9ac Trynka
H3K4me3 Trynka
H3K4me1 Trynka
H3K27ac PGC2
H3K27ac Hnisz
FetalDHS Trynka
**Enhancer Hoffman
Enhancer Andersson
DHS Trynka
DGF ENCODE
CTCF Hoffman
Conserved LindbladToh
Coding UCSC
Annotation
−20
−10
0
10
20
Enrichment (prop h2 / prop snps)

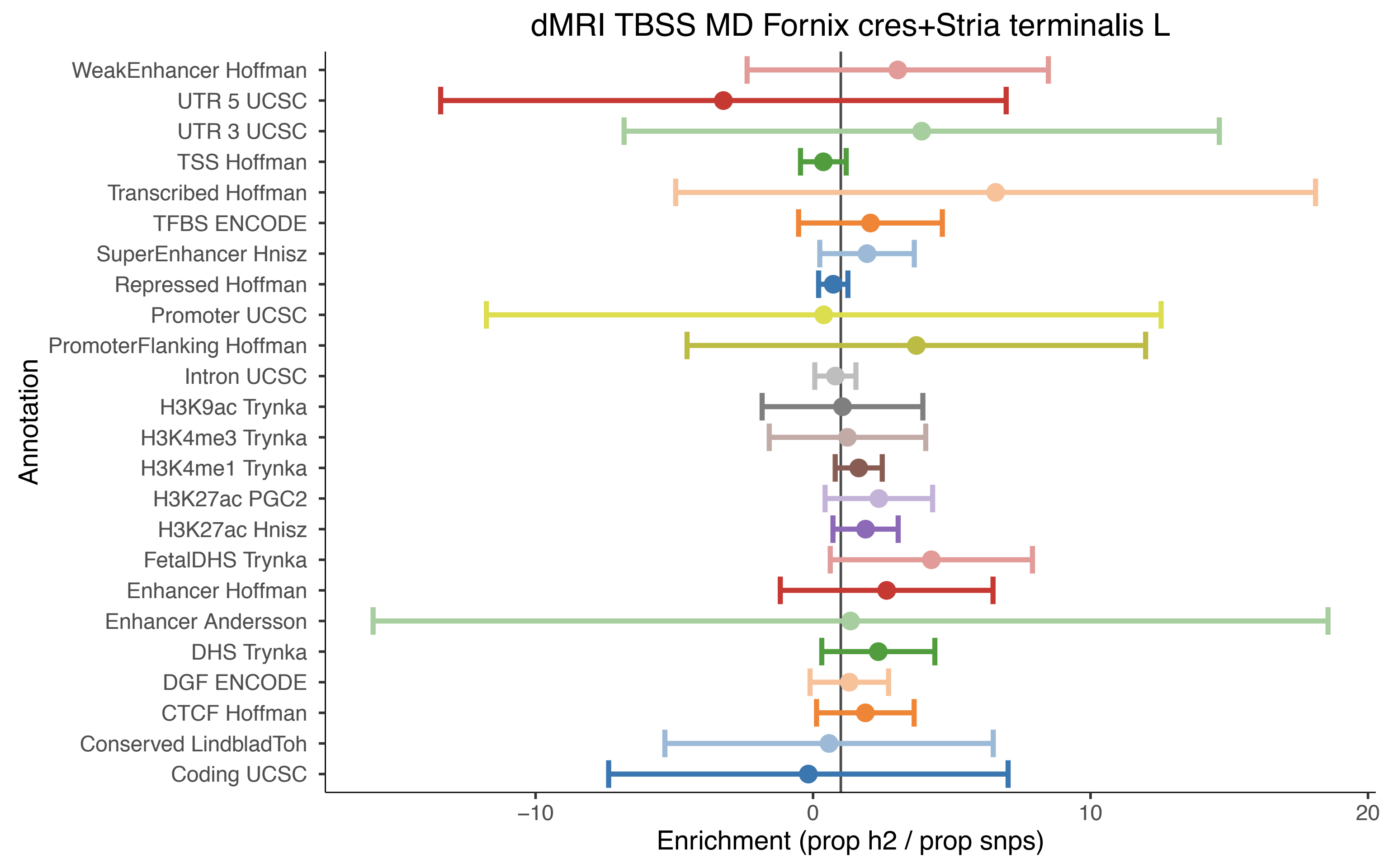
dMRI TBSS MD Fornix cres+Stria terminalis L
WeakEnhancer Hoffman
UTR 5 UCSC
UTR 3 UCSC
TSS Hoffman
Transcribed Hoffman
TFBS ENCODE
SuperEnhancer Hnisz
Repressed Hoffman
Promoter UCSC
PromoterFlanking Hoffman
Intron UCSC
H3K9ac Trynka
H3K4me3 Trynka
H3K4me1 Trynka
H3K27ac PGC2
H3K27ac Hnisz
FetalDHS Trynka
Enhancer Hoffman
Enhancer Andersson
DHS Trynka
DGF ENCODE
CTCF Hoffman
Conserved LindbladToh
Coding UCSC
Annotation
−10
0
10
20
Enrichment (prop h2 / prop snps)

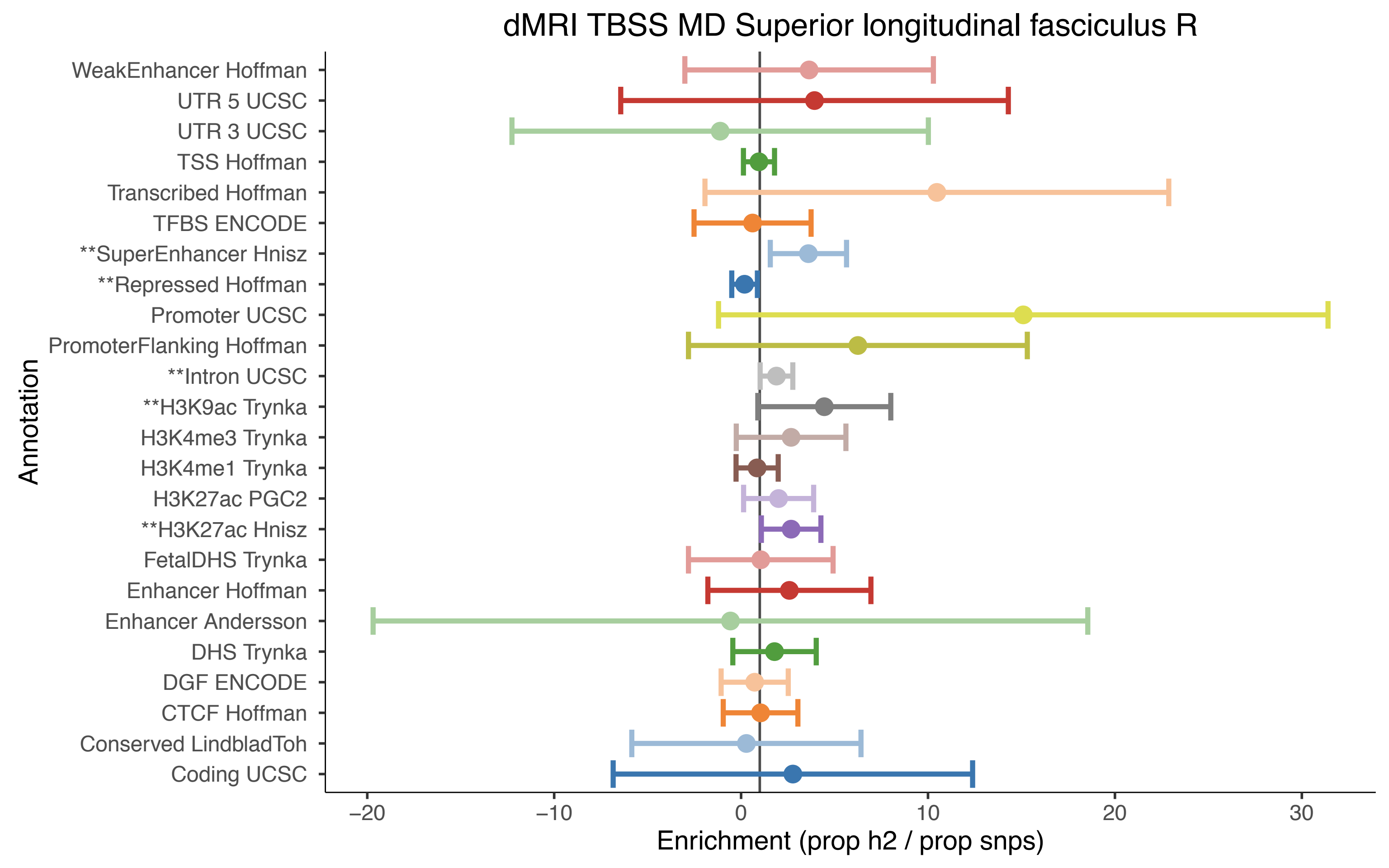
dMRI TBSS MD Superior longitudinal fasciculus R
WeakEnhancer Hoffman
UTR 5 UCSC
UTR 3 UCSC
TSS Hoffman
Transcribed Hoffman
TFBS ENCODE
**SuperEnhancer Hnisz
**Repressed Hoffman
Promoter UCSC
PromoterFlanking Hoffman
**Intron UCSC
**H3K9ac Trynka
H3K4me3 Trynka
H3K4me1 Trynka
H3K27ac PGC2
**H3K27ac Hnisz
FetalDHS Trynka
Enhancer Hoffman
Enhancer Andersson
DHS Trynka
DGF ENCODE
CTCF Hoffman
Conserved LindbladToh
Coding UCSC
Annotation
−20
−10
0
10
20
30
Enrichment (prop h2 / prop snps)

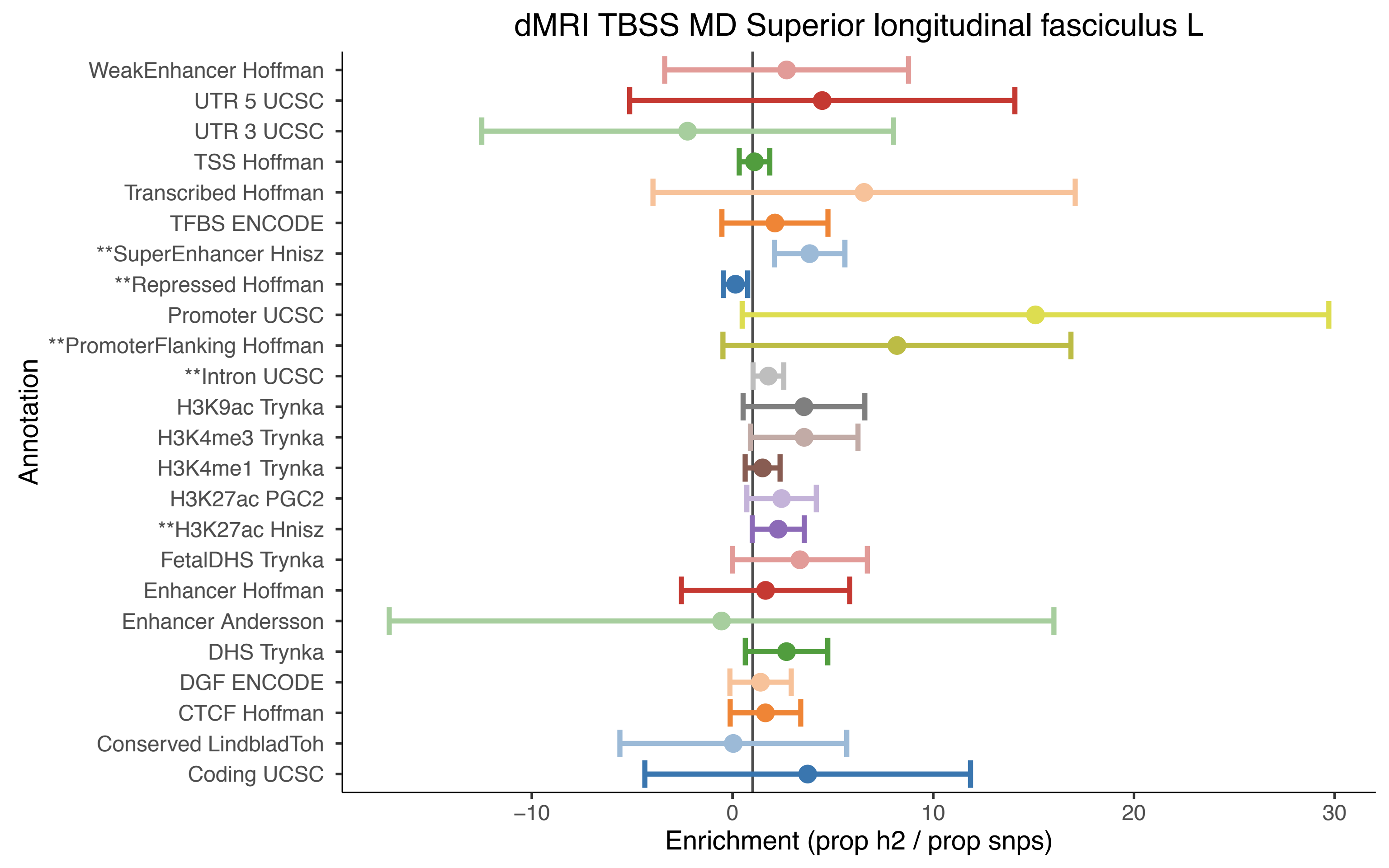

dMRI TBSS MD Superior longitudinal fasciculus L
WeakEnhancer Hoffman
UTR 5 UCSC
UTR 3 UCSC
TSS Hoffman
Transcribed Hoffman
TFBS ENCODE
**SuperEnhancer Hnisz
**Repressed Hoffman
Promoter UCSC
**PromoterFlanking Hoffman
**Intron UCSC
H3K9ac Trynka
H3K4me3 Trynka
H3K4me1 Trynka
H3K27ac PGC2
**H3K27ac Hnisz
FetalDHS Trynka
Enhancer Hoffman
Enhancer Andersson
DHS Trynka
DGF ENCODE
CTCF Hoffman
Conserved LindbladToh
Coding UCSC
Annotation
−10
0
10
20
30
Enrichment (prop h2 / prop snps)

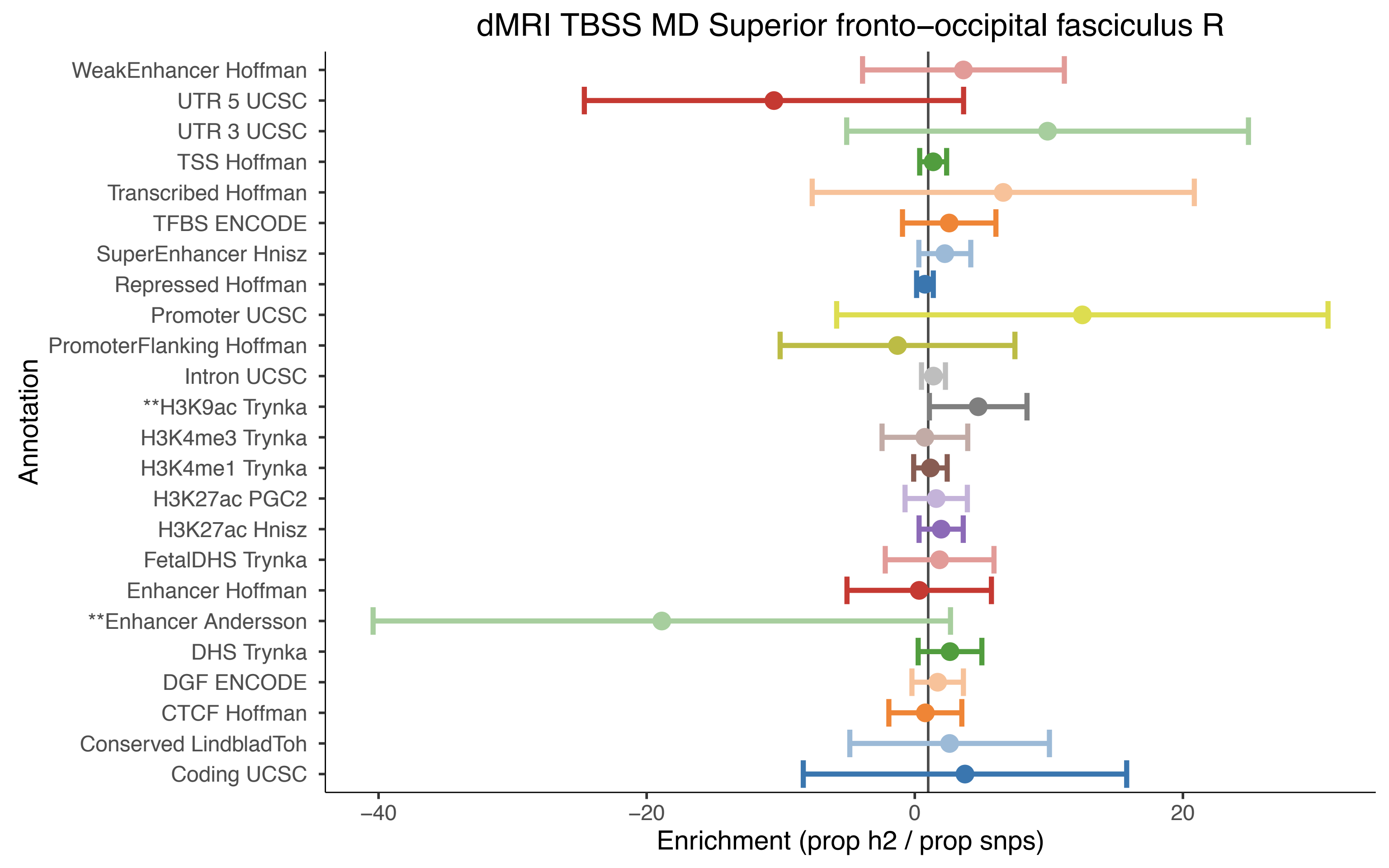

dMRI TBSS MD Superior fronto−occipital fasciculus R
WeakEnhancer Hoffman
UTR 5 UCSC
UTR 3 UCSC
TSS Hoffman
Transcribed Hoffman
TFBS ENCODE
SuperEnhancer Hnisz
Repressed Hoffman
Promoter UCSC
PromoterFlanking Hoffman
Intron UCSC
**H3K9ac Trynka
H3K4me3 Trynka
H3K4me1 Trynka
H3K27ac PGC2
H3K27ac Hnisz
FetalDHS Trynka
Enhancer Hoffman
**Enhancer Andersson
DHS Trynka
DGF ENCODE
CTCF Hoffman
Conserved LindbladToh
Coding UCSC
Annotation
−40
−20
0
20
Enrichment (prop h2 / prop snps)

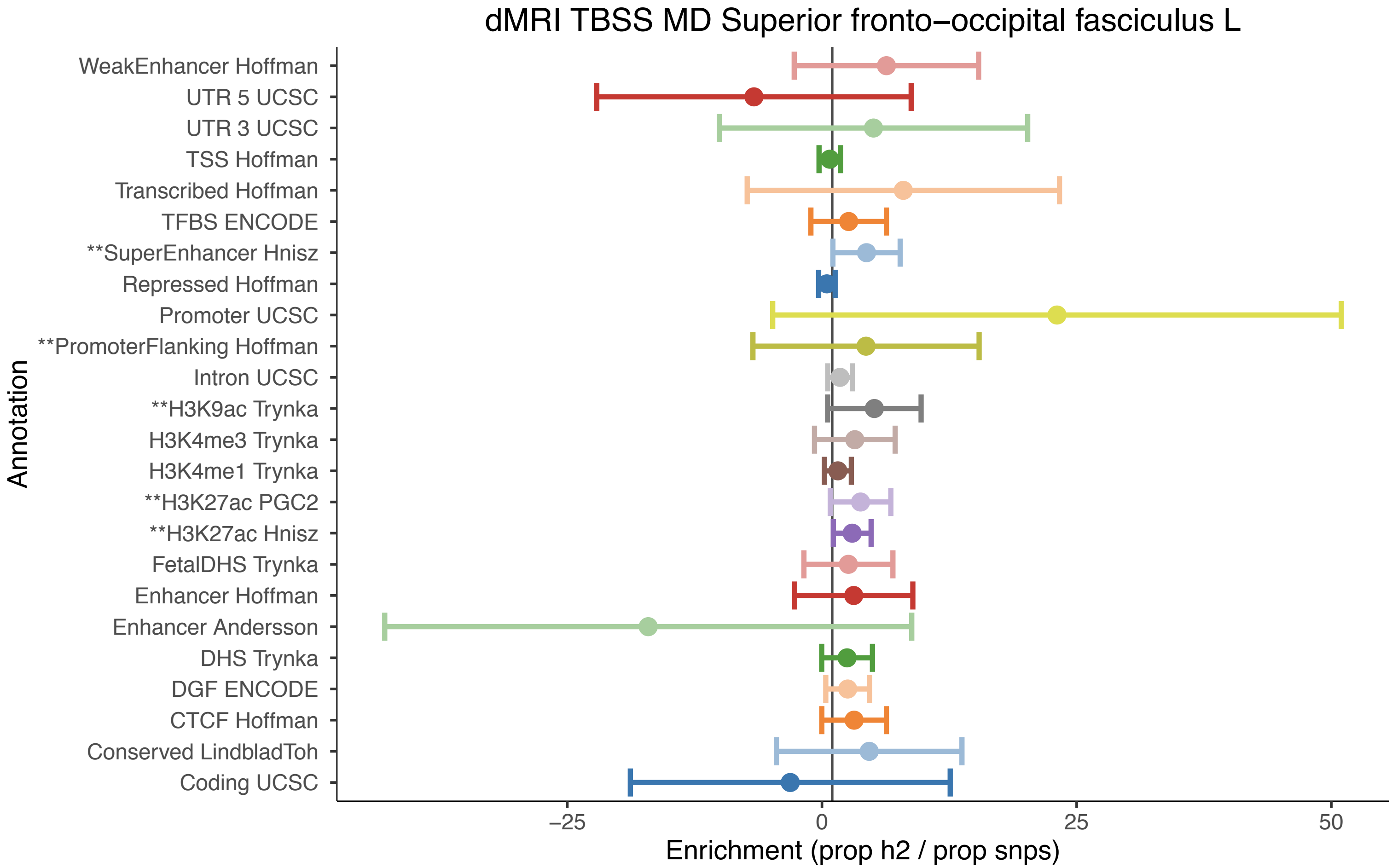

dMRI TBSS MD Superior fronto−occipital fasciculus L
WeakEnhancer Hoffman
UTR 5 UCSC
UTR 3 UCSC
TSS Hoffman
Transcribed Hoffman
TFBS ENCODE
**SuperEnhancer Hnisz
Repressed Hoffman
Promoter UCSC
**PromoterFlanking Hoffman
Intron UCSC
**H3K9ac Trynka
H3K4me3 Trynka
H3K4me1 Trynka
**H3K27ac PGC2
**H3K27ac Hnisz
FetalDHS Trynka
Enhancer Hoffman
Enhancer Andersson
DHS Trynka
DGF ENCODE
CTCF Hoffman
Conserved LindbladToh
Coding UCSC
Annotation
−25
0
25
50
Enrichment (prop h2 / prop snps)

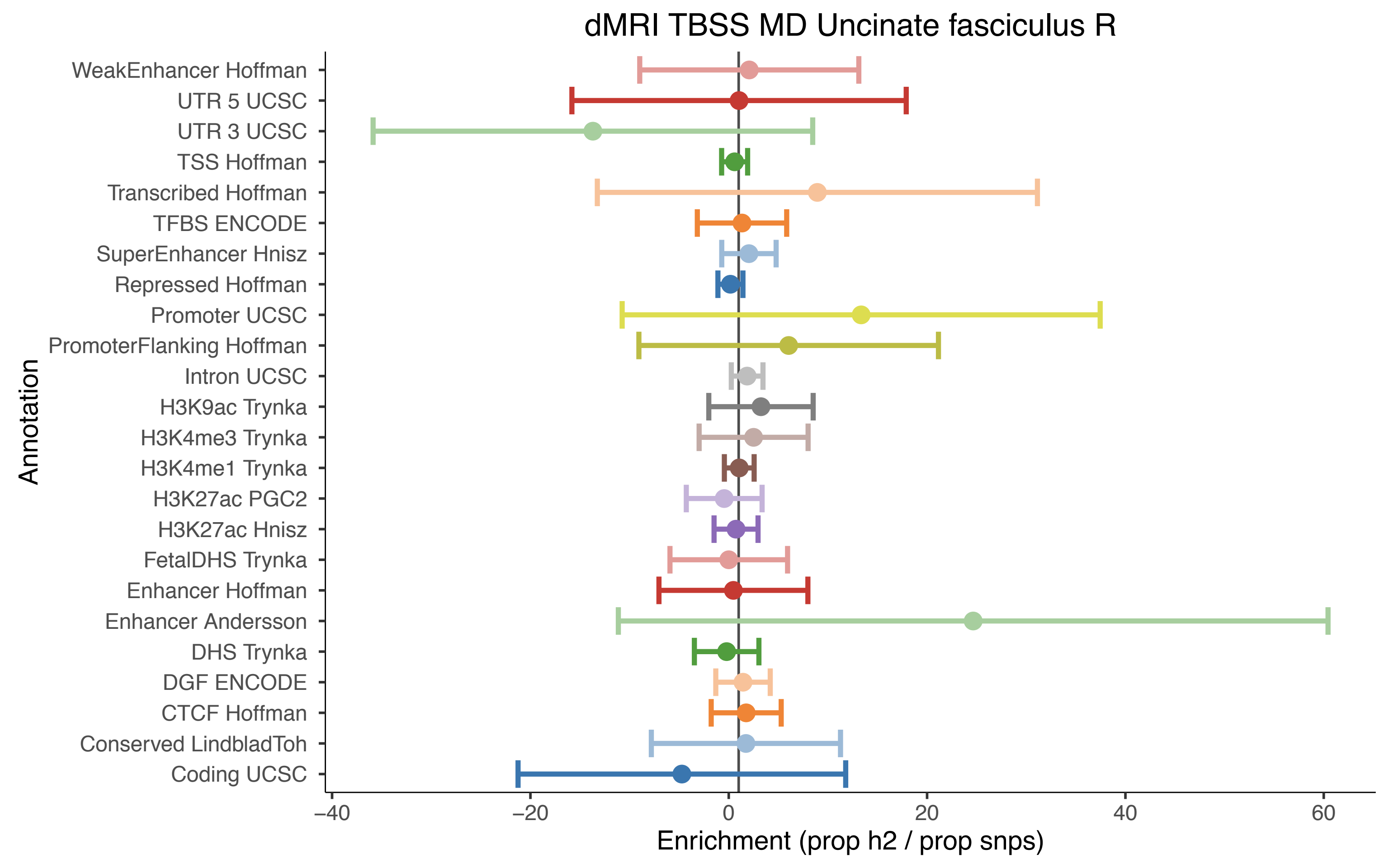
dMRI TBSS MD Uncinate fasciculus R
WeakEnhancer Hoffman
UTR 5 UCSC
UTR 3 UCSC
TSS Hoffman
Transcribed Hoffman
TFBS ENCODE
SuperEnhancer Hnisz
Repressed Hoffman
Promoter UCSC
PromoterFlanking Hoffman
Intron UCSC
H3K9ac Trynka
H3K4me3 Trynka
H3K4me1 Trynka
H3K27ac PGC2
H3K27ac Hnisz
FetalDHS Trynka
Enhancer Hoffman
Enhancer Andersson
DHS Trynka
DGF ENCODE
CTCF Hoffman
Conserved LindbladToh
Coding UCSC
Annotation
−40
−20
0
20
40
60
Enrichment (prop h2 / prop snps)

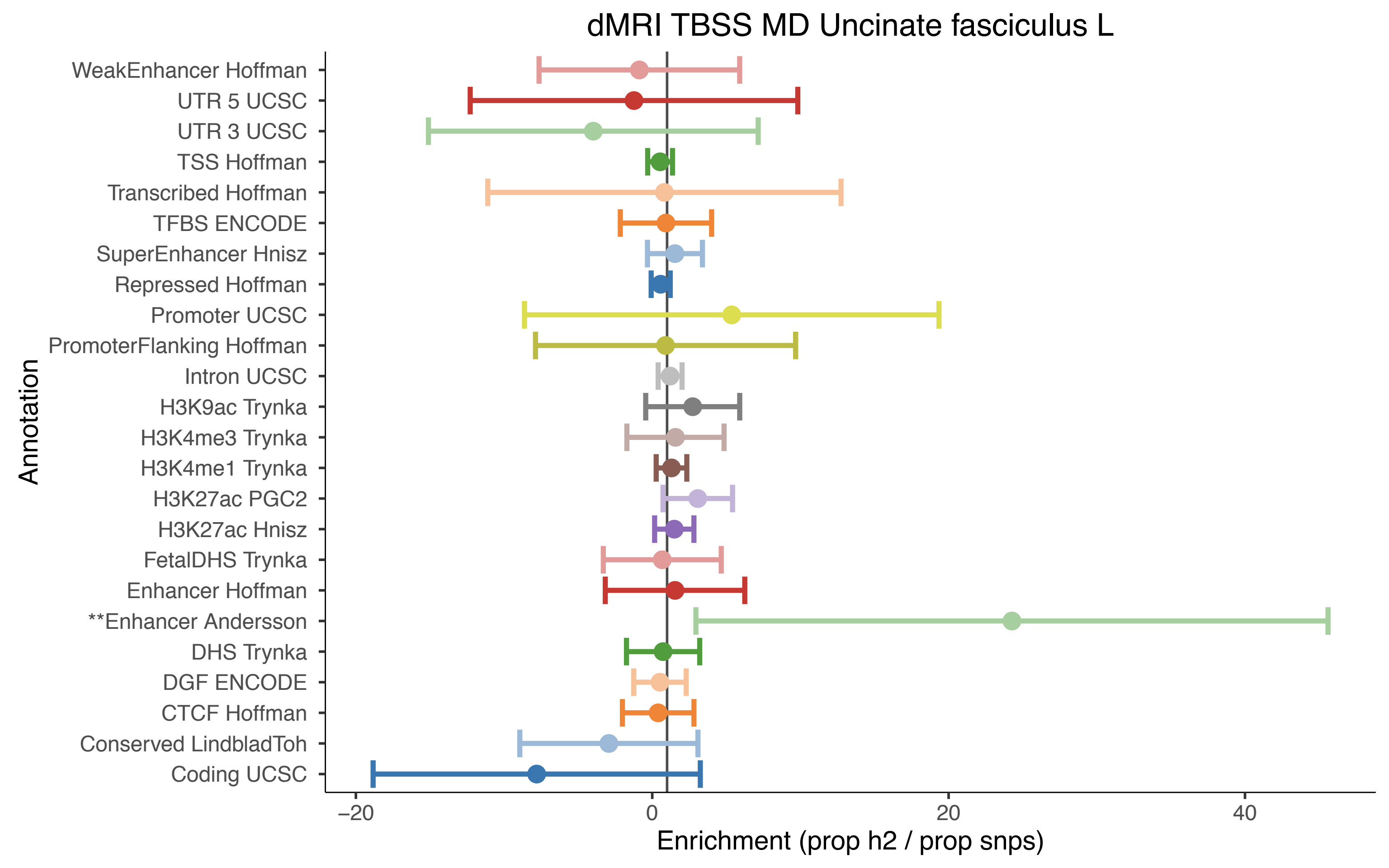

dMRI TBSS MD Uncinate fasciculus L
WeakEnhancer Hoffman
UTR 5 UCSC
UTR 3 UCSC
TSS Hoffman
Transcribed Hoffman
TFBS ENCODE
SuperEnhancer Hnisz
Repressed Hoffman
Promoter UCSC
PromoterFlanking Hoffman
Intron UCSC
H3K9ac Trynka
H3K4me3 Trynka
H3K4me1 Trynka
H3K27ac PGC2
H3K27ac Hnisz
FetalDHS Trynka
Enhancer Hoffman
**Enhancer Andersson
DHS Trynka
DGF ENCODE
CTCF Hoffman
Conserved LindbladToh
Coding UCSC
Annotation
−20
0
20
40
Enrichment (prop h2 / prop snps)

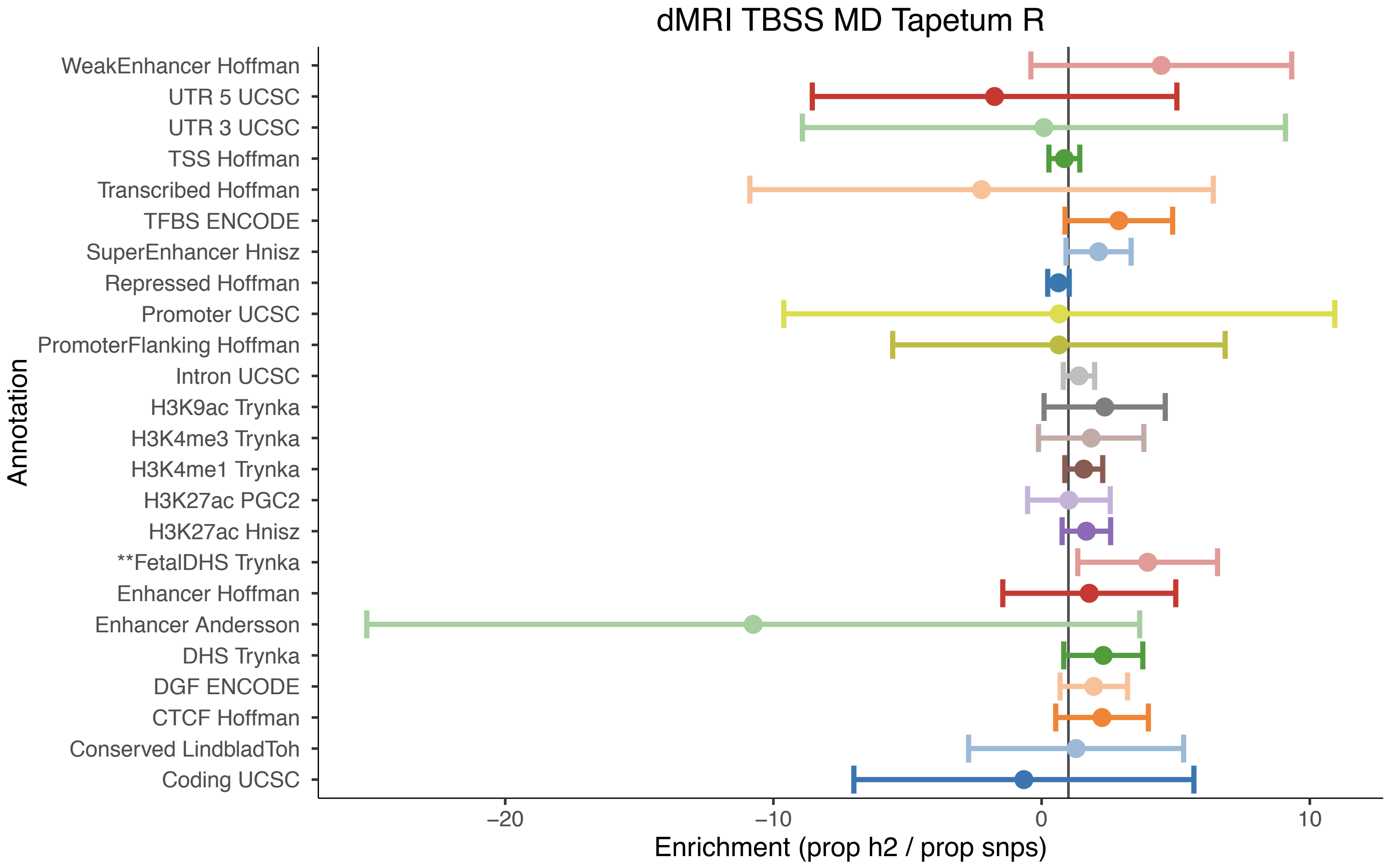
dMRI TBSS MD Tapetum R
WeakEnhancer Hoffman
UTR 5 UCSC
UTR 3 UCSC
TSS Hoffman
Transcribed Hoffman
TFBS ENCODE
SuperEnhancer Hnisz
Repressed Hoffman
Promoter UCSC
PromoterFlanking Hoffman
Intron UCSC
H3K9ac Trynka
H3K4me3 Trynka
H3K4me1 Trynka
H3K27ac PGC2
H3K27ac Hnisz
**FetalDHS Trynka
Enhancer Hoffman
Enhancer Andersson
DHS Trynka
DGF ENCODE
CTCF Hoffman
Conserved LindbladToh
Coding UCSC
Annotation
−20
−10
0
10
Enrichment (prop h2 / prop snps)

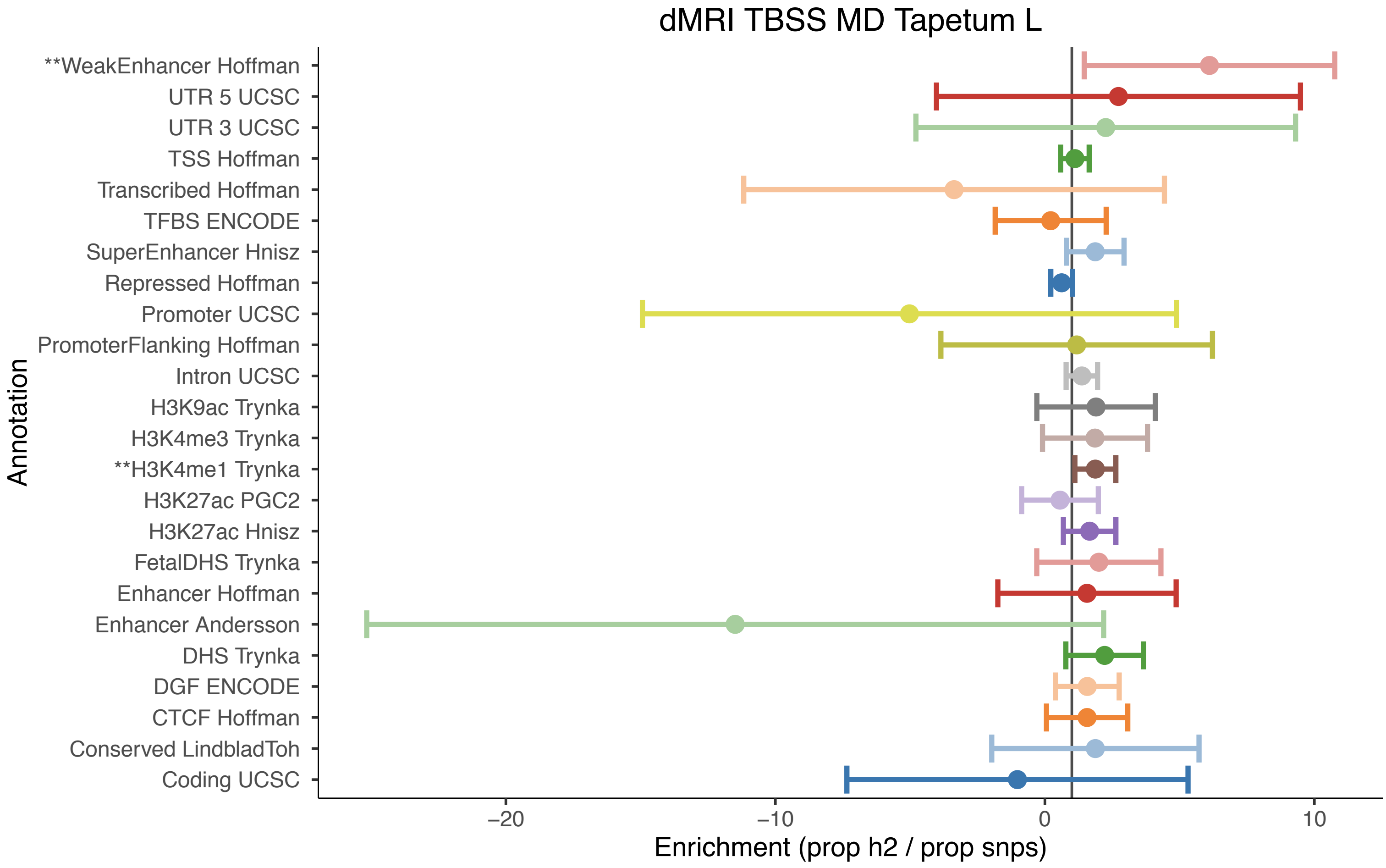

dMRI TBSS MD Tapetum L
**WeakEnhancer Hoffman
UTR 5 UCSC
UTR 3 UCSC
TSS Hoffman
Transcribed Hoffman
TFBS ENCODE
SuperEnhancer Hnisz
Repressed Hoffman
Promoter UCSC
PromoterFlanking Hoffman
Intron UCSC
H3K9ac Trynka
H3K4me3 Trynka
**H3K4me1 Trynka
H3K27ac PGC2
H3K27ac Hnisz
FetalDHS Trynka
Enhancer Hoffman
Enhancer Andersson
DHS Trynka
DGF ENCODE
CTCF Hoffman
Conserved LindbladToh
Coding UCSC
Annotation
−20
−10
0
10
Enrichment (prop h2 / prop snps)

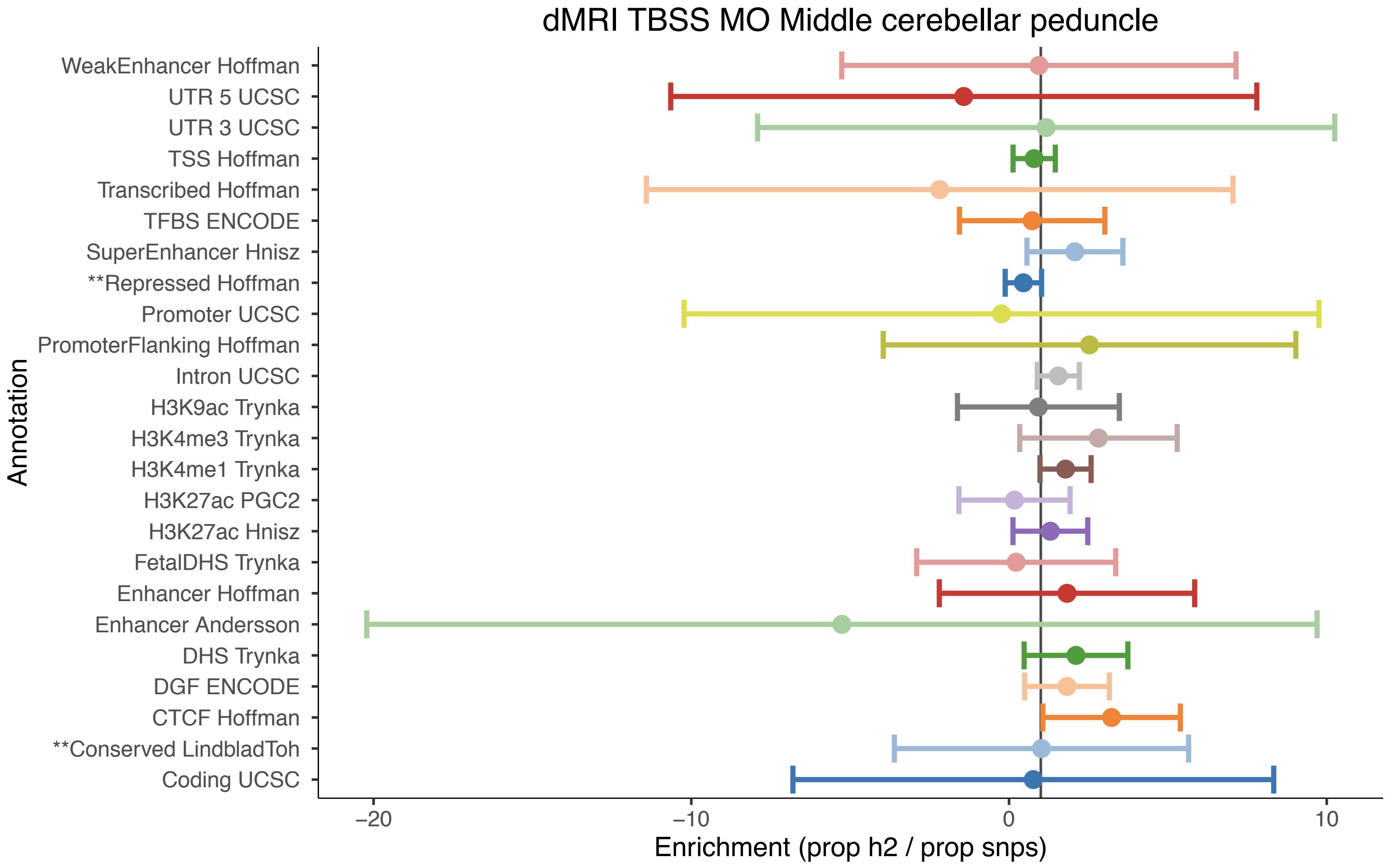
dMRI TBSS MO Middle cerebellar peduncle
WeakEnhancer Hoffman
UTR 5 UCSC
UTR 3 UCSC
TSS Hoffman
Transcribed Hoffman
TFBS ENCODE
SuperEnhancer Hnisz
**Repressed Hoffman
Promoter UCSC
PromoterFlanking Hoffman
Intron UCSC
H3K9ac Trynka
H3K4me3 Trynka
H3K4me1 Trynka
H3K27ac PGC2
H3K27ac Hnisz
FetalDHS Trynka
Enhancer Hoffman
Enhancer Andersson
DHS Trynka
DGF ENCODE
CTCF Hoffman
**Conserved LindbladToh
Coding UCSC
Annotation
−20
−10
0
10
Enrichment (prop h2 / prop snps)

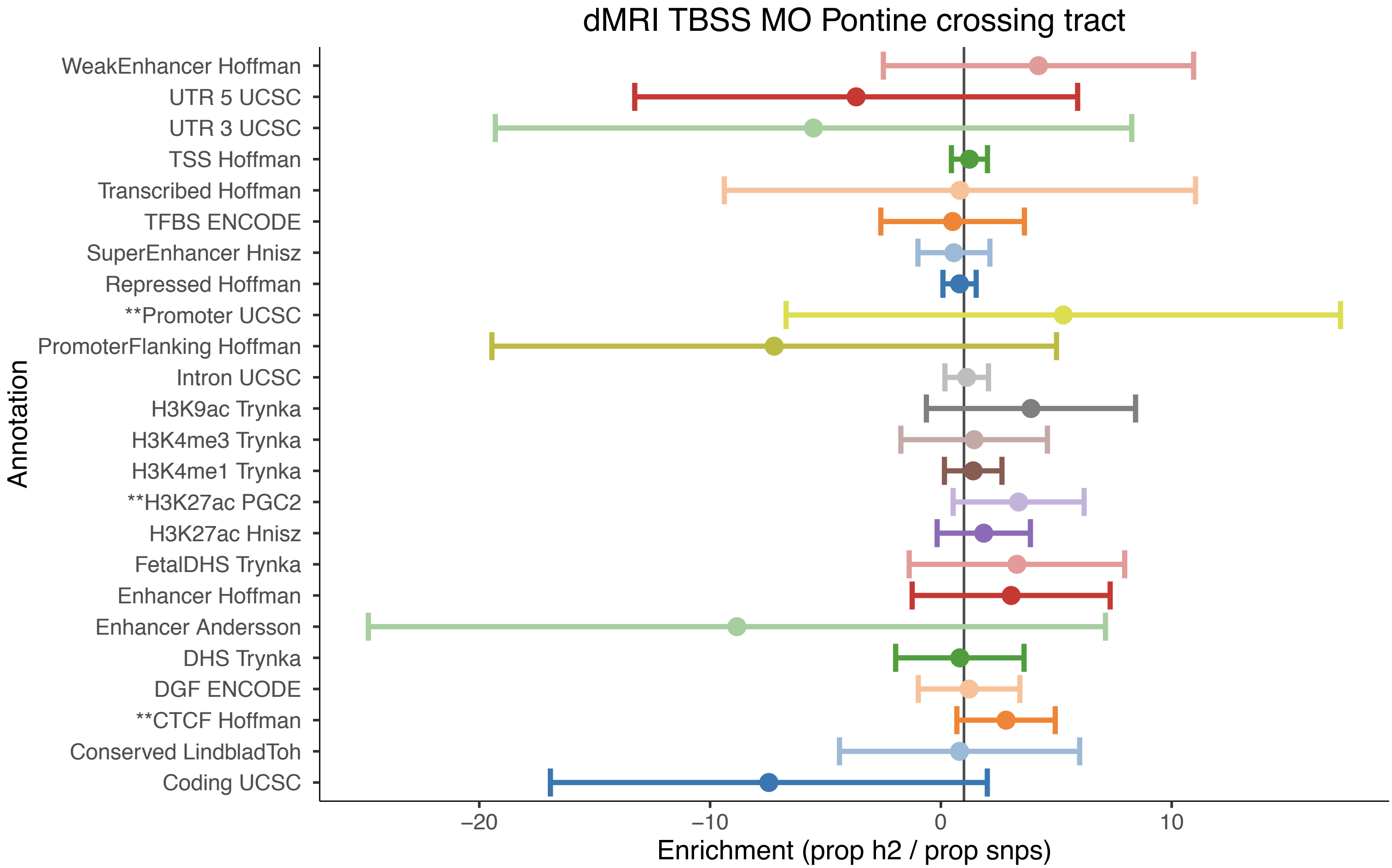
dMRI TBSS MO Pontine crossing tract
WeakEnhancer Hoffman
UTR 5 UCSC
UTR 3 UCSC
TSS Hoffman
Transcribed Hoffman
TFBS ENCODE
SuperEnhancer Hnisz
Repressed Hoffman
**Promoter UCSC
PromoterFlanking Hoffman
Intron UCSC
H3K9ac Trynka
H3K4me3 Trynka
H3K4me1 Trynka
**H3K27ac PGC2
H3K27ac Hnisz
FetalDHS Trynka
Enhancer Hoffman
Enhancer Andersson
DHS Trynka
DGF ENCODE
**CTCF Hoffman
Conserved LindbladToh
Coding UCSC
Annotation
−20
−10
0
10
Enrichment (prop h2 / prop snps)

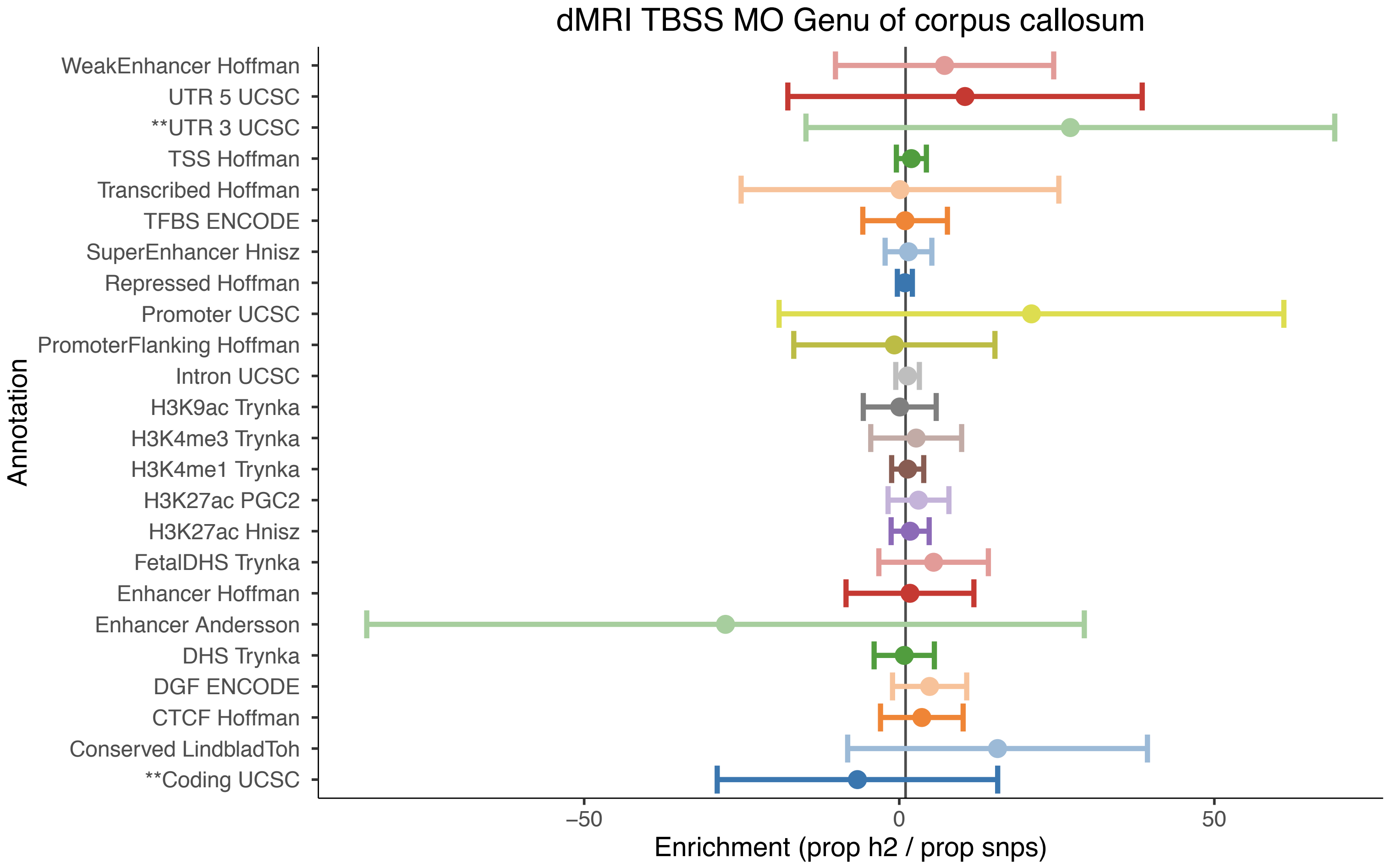

dMRI TBSS MO Genu of corpus callosum
WeakEnhancer Hoffman
UTR 5 UCSC
**UTR 3 UCSC
TSS Hoffman
Transcribed Hoffman
TFBS ENCODE
SuperEnhancer Hnisz
Repressed Hoffman
Promoter UCSC
PromoterFlanking Hoffman
Intron UCSC
H3K9ac Trynka
H3K4me3 Trynka
H3K4me1 Trynka
H3K27ac PGC2
H3K27ac Hnisz
FetalDHS Trynka
Enhancer Hoffman
Enhancer Andersson
DHS Trynka
DGF ENCODE
CTCF Hoffman
Conserved LindbladToh
**Coding UCSC
Annotation
−50
0
50
Enrichment (prop h2 / prop snps)

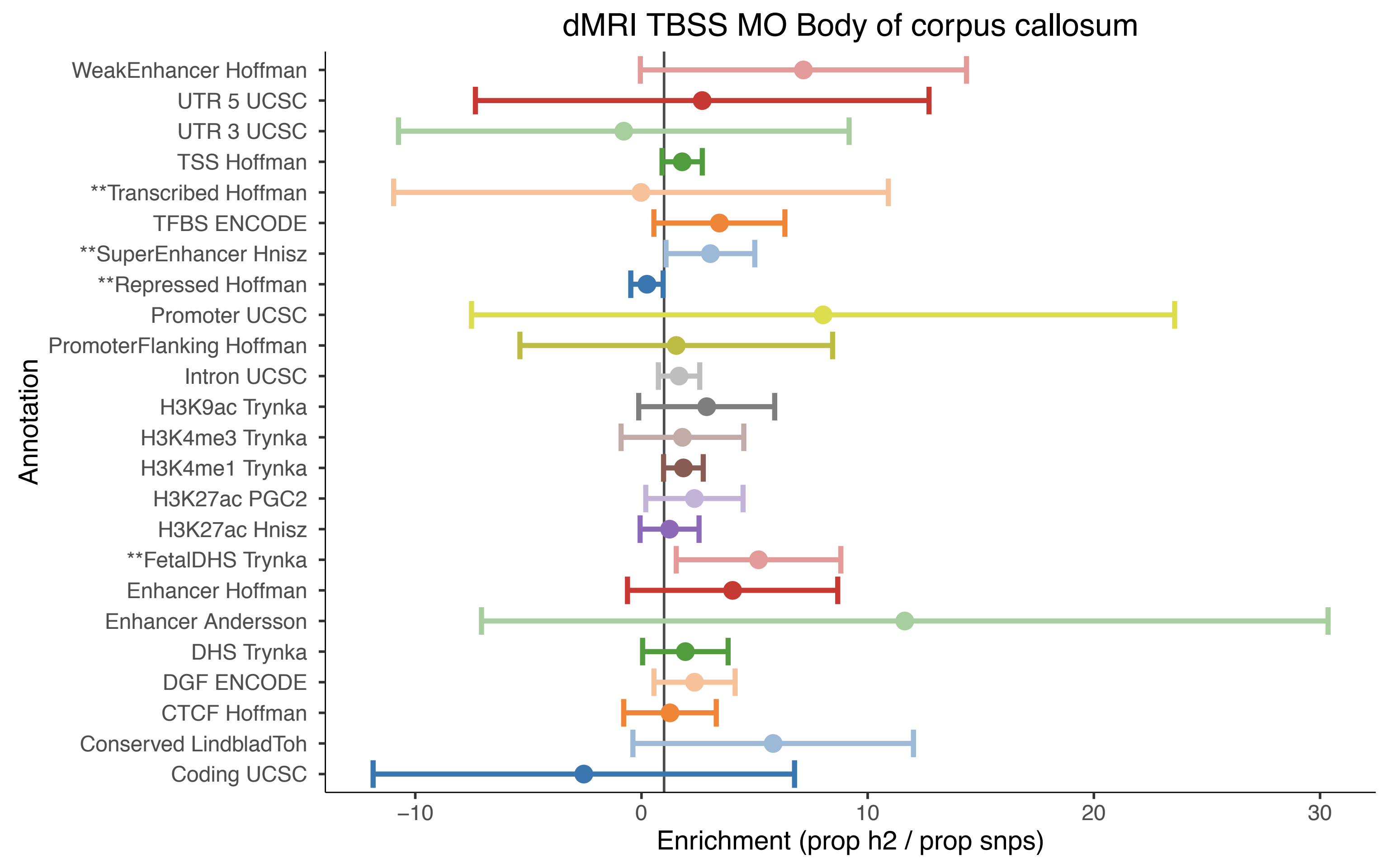
dMRI TBSS MO Body of corpus callosum
WeakEnhancer Hoffman
UTR 5 UCSC
UTR 3 UCSC
TSS Hoffman
**Transcribed Hoffman
TFBS ENCODE
**SuperEnhancer Hnisz
**Repressed Hoffman
Promoter UCSC
PromoterFlanking Hoffman
Intron UCSC
H3K9ac Trynka
H3K4me3 Trynka
H3K4me1 Trynka
H3K27ac PGC2
H3K27ac Hnisz
**FetalDHS Trynka
Enhancer Hoffman
Enhancer Andersson
DHS Trynka
DGF ENCODE
CTCF Hoffman
Conserved LindbladToh
Coding UCSC
Annotation
−10
0
10
20
30
Enrichment (prop h2 / prop snps)

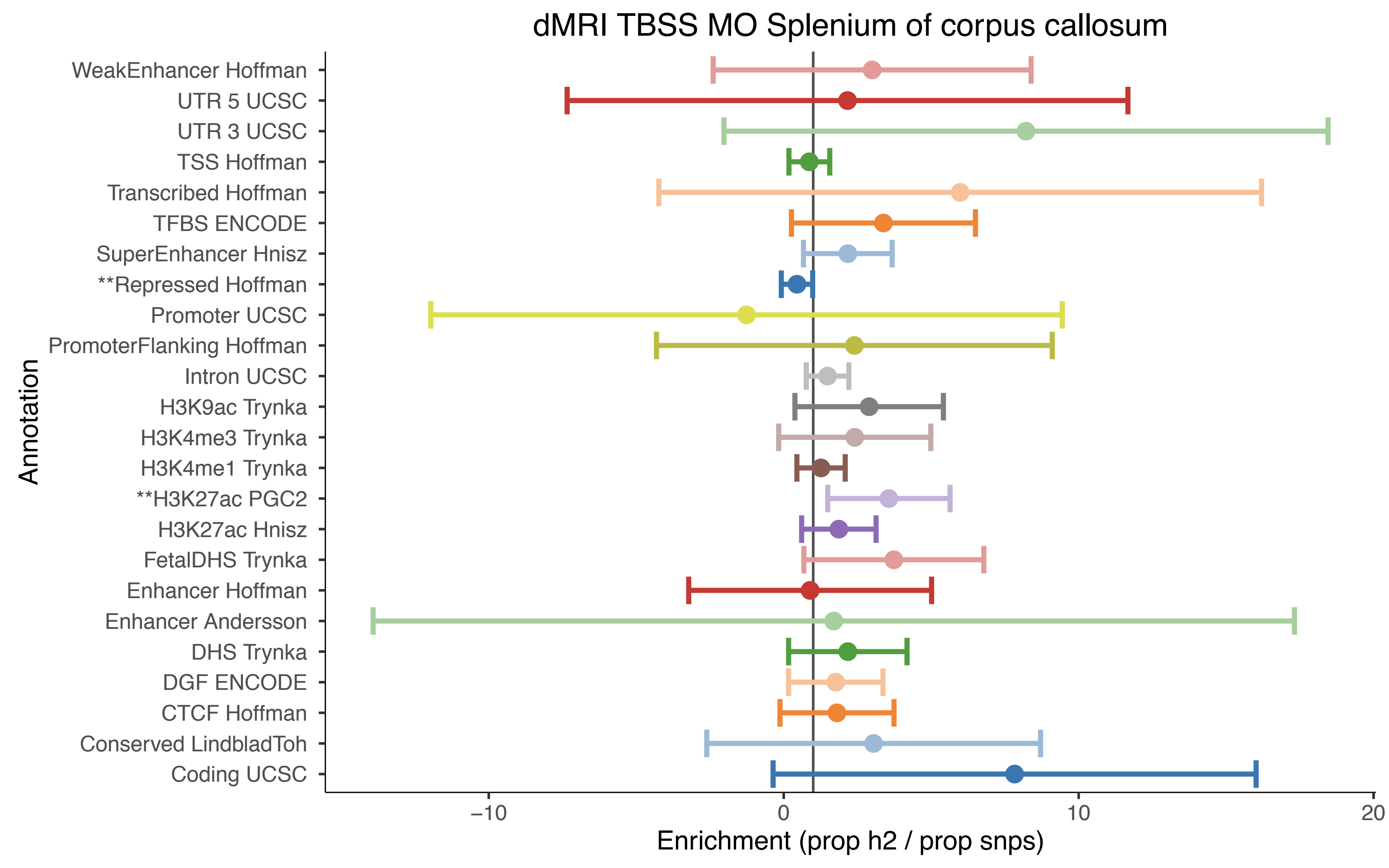

dMRI TBSS MO Splenium of corpus callosum
WeakEnhancer Hoffman
UTR 5 UCSC
UTR 3 UCSC
TSS Hoffman
Transcribed Hoffman
TFBS ENCODE
SuperEnhancer Hnisz
**Repressed Hoffman
Promoter UCSC
PromoterFlanking Hoffman
Intron UCSC
H3K9ac Trynka
H3K4me3 Trynka
H3K4me1 Trynka
**H3K27ac PGC2
H3K27ac Hnisz
FetalDHS Trynka
Enhancer Hoffman
Enhancer Andersson
DHS Trynka
DGF ENCODE
CTCF Hoffman
Conserved LindbladToh
Coding UCSC
Annotation
−10
0
10
20
Enrichment (prop h2 / prop snps)

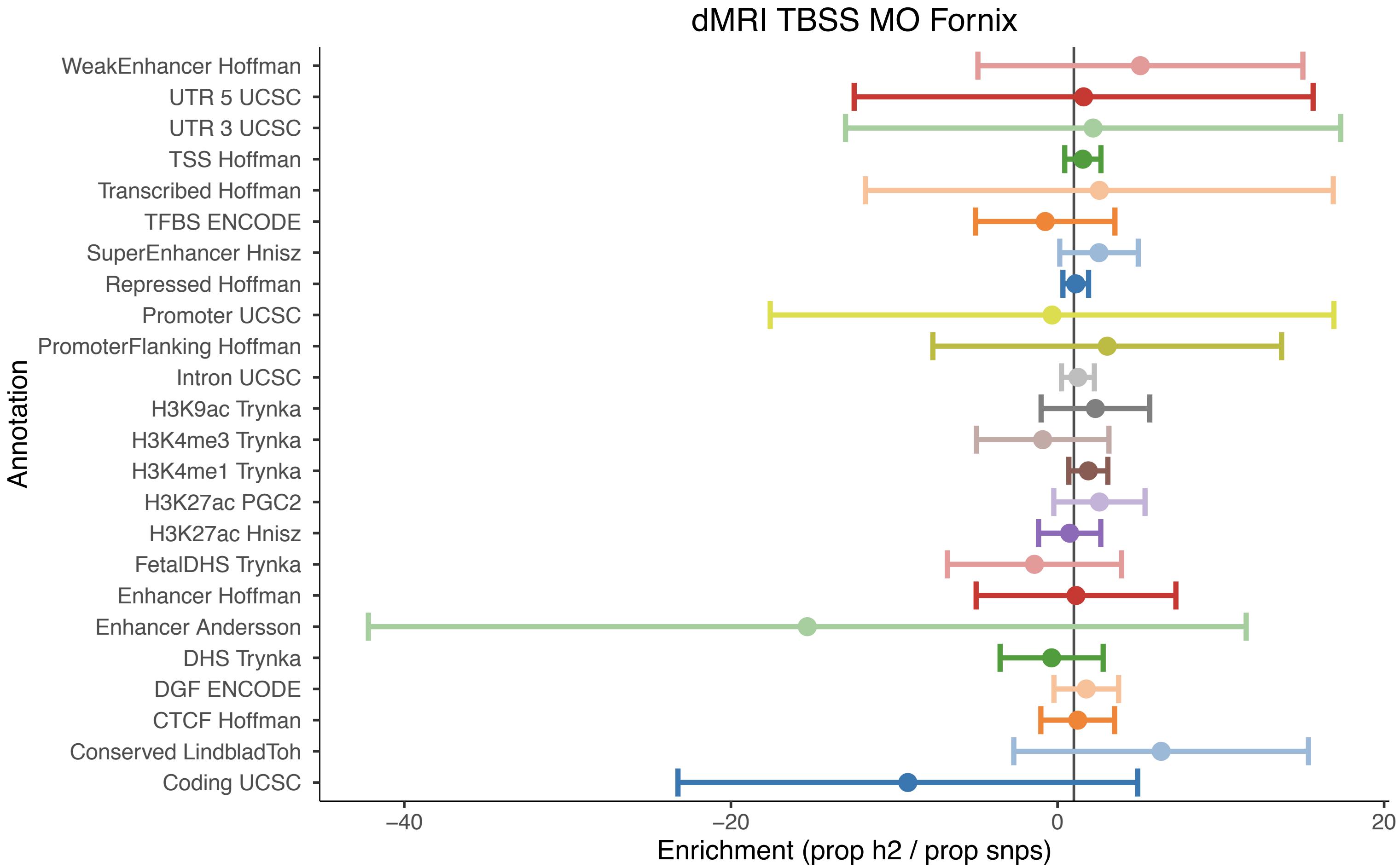
dMRI TBSS MO Fornix
WeakEnhancer Hoffman
UTR 5 UCSC
UTR 3 UCSC
TSS Hoffman
Transcribed Hoffman
TFBS ENCODE
SuperEnhancer Hnisz
Repressed Hoffman
Promoter UCSC
PromoterFlanking Hoffman
Intron UCSC
H3K9ac Trynka
H3K4me3 Trynka
H3K4me1 Trynka
H3K27ac PGC2
H3K27ac Hnisz
FetalDHS Trynka
Enhancer Hoffman
Enhancer Andersson
DHS Trynka
DGF ENCODE
CTCF Hoffman
Conserved LindbladToh
Coding UCSC
Annotation
−40
−20
0
20
Enrichment (prop h2 / prop snps)

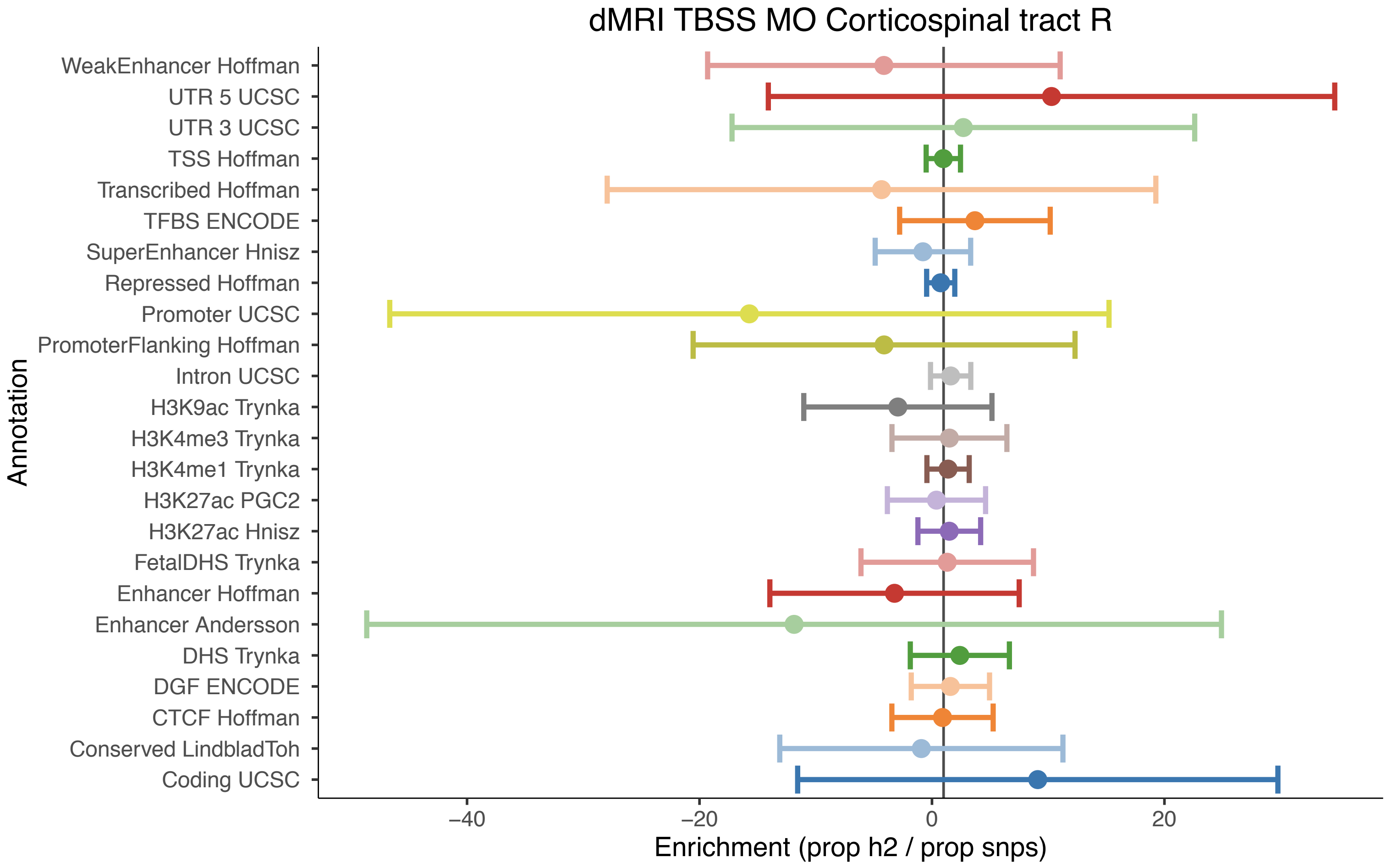

dMRI TBSS MO Corticospinal tract R
WeakEnhancer Hoffman
UTR 5 UCSC
UTR 3 UCSC
TSS Hoffman
Transcribed Hoffman
TFBS ENCODE
SuperEnhancer Hnisz
Repressed Hoffman
Promoter UCSC
PromoterFlanking Hoffman
Intron UCSC
H3K9ac Trynka
H3K4me3 Trynka
H3K4me1 Trynka
H3K27ac PGC2
H3K27ac Hnisz
FetalDHS Trynka
Enhancer Hoffman
Enhancer Andersson
DHS Trynka
DGF ENCODE
CTCF Hoffman
Conserved LindbladToh
Coding UCSC
Annotation
−40
−20
0
20
Enrichment (prop h2 / prop snps)

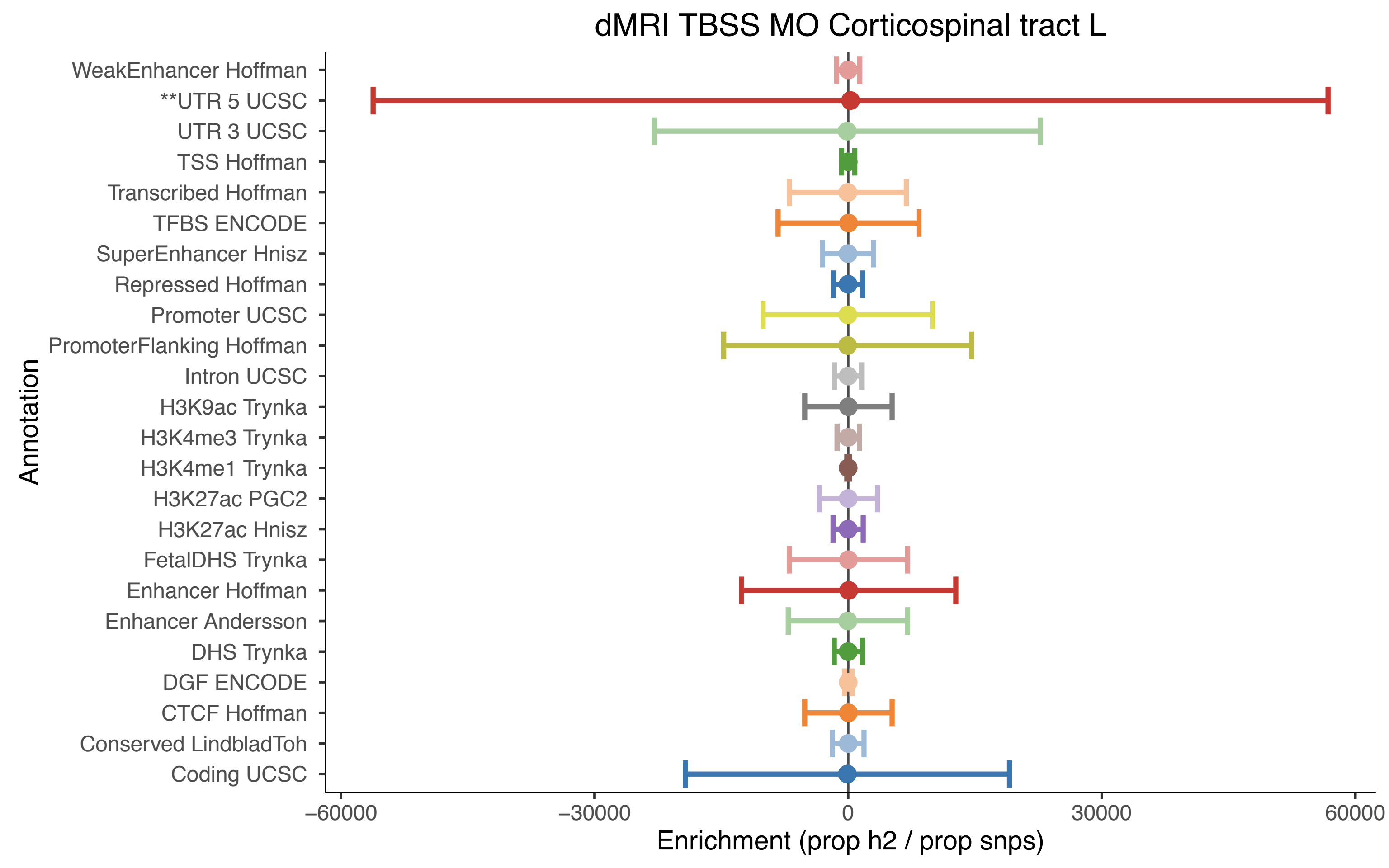

dMRI TBSS MO Corticospinal tract L
WeakEnhancer Hoffman
**UTR 5 UCSC
UTR 3 UCSC
TSS Hoffman
Transcribed Hoffman
TFBS ENCODE
SuperEnhancer Hnisz
Repressed Hoffman
Promoter UCSC
PromoterFlanking Hoffman
Intron UCSC
H3K9ac Trynka
H3K4me3 Trynka
H3K4me1 Trynka
H3K27ac PGC2
H3K27ac Hnisz
FetalDHS Trynka
Enhancer Hoffman
Enhancer Andersson
DHS Trynka
DGF ENCODE
CTCF Hoffman
Conserved LindbladToh
Coding UCSC
Annotation
−60000
−30000
0
30000
60000
Enrichment (prop h2 / prop snps)

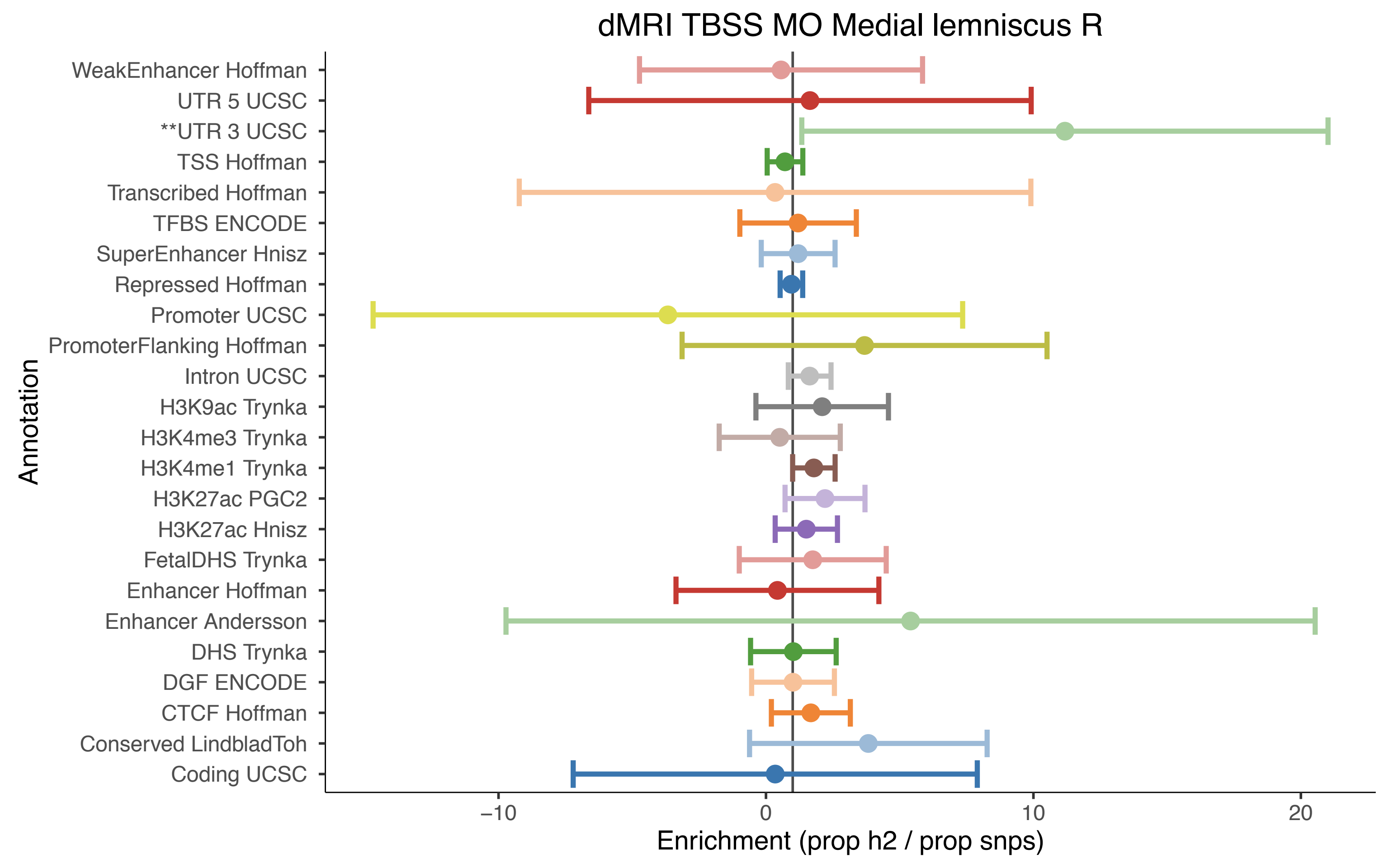

dMRI TBSS MO Medial lemniscus R
WeakEnhancer Hoffman
UTR 5 UCSC
**UTR 3 UCSC
TSS Hoffman
Transcribed Hoffman
TFBS ENCODE
SuperEnhancer Hnisz
Repressed Hoffman
Promoter UCSC
PromoterFlanking Hoffman
Intron UCSC
H3K9ac Trynka
H3K4me3 Trynka
H3K4me1 Trynka
H3K27ac PGC2
H3K27ac Hnisz
FetalDHS Trynka
Enhancer Hoffman
Enhancer Andersson
DHS Trynka
DGF ENCODE
CTCF Hoffman
Conserved LindbladToh
Coding UCSC
Annotation
−10
0
10
20
Enrichment (prop h2 / prop snps)

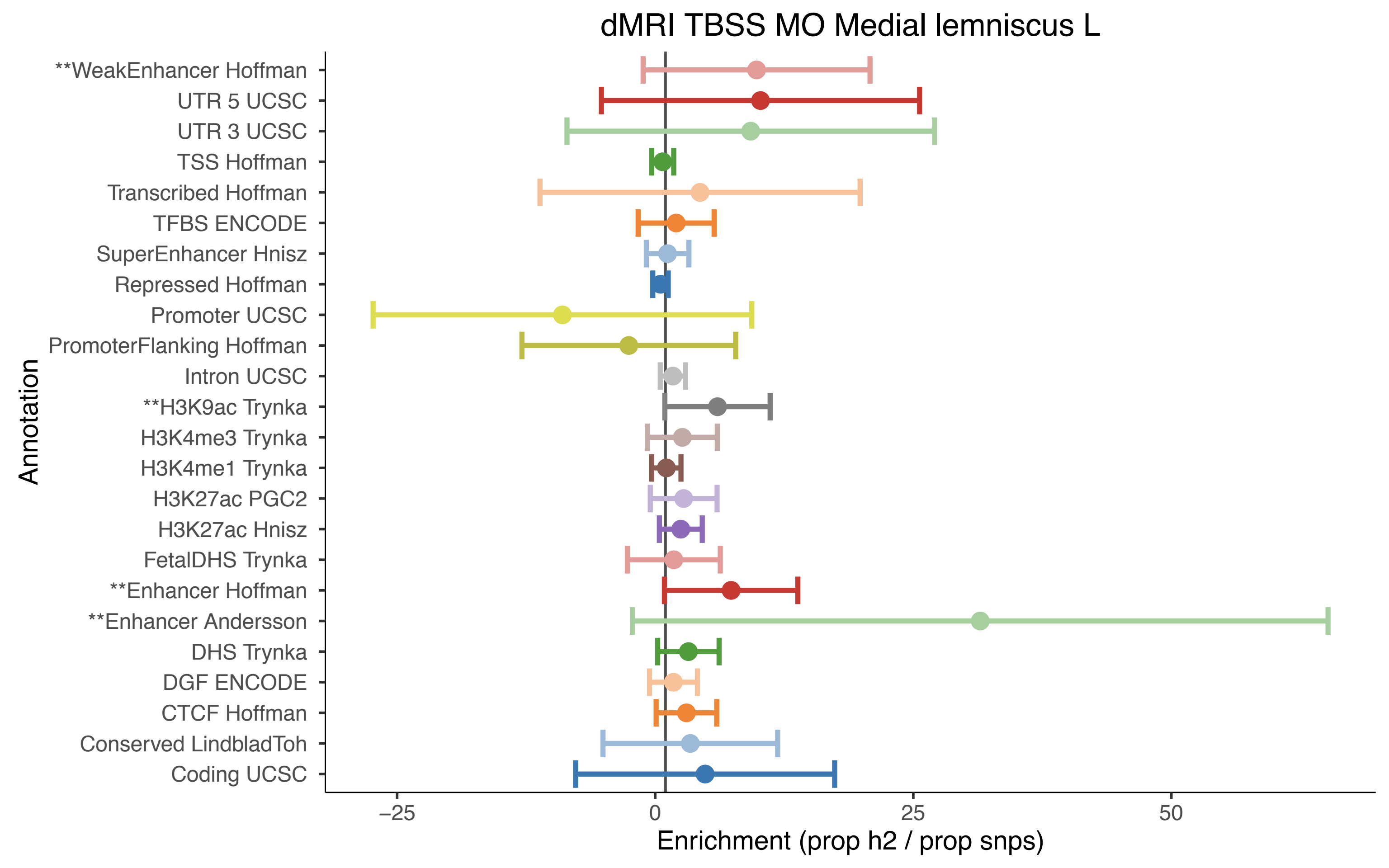

dMRI TBSS MO Medial lemniscus L
**WeakEnhancer Hoffman
UTR 5 UCSC
UTR 3 UCSC
TSS Hoffman
Transcribed Hoffman
TFBS ENCODE
SuperEnhancer Hnisz
Repressed Hoffman
Promoter UCSC
PromoterFlanking Hoffman
Intron UCSC
**H3K9ac Trynka
H3K4me3 Trynka
H3K4me1 Trynka
H3K27ac PGC2
H3K27ac Hnisz
FetalDHS Trynka
**Enhancer Hoffman
**Enhancer Andersson
DHS Trynka
DGF ENCODE
CTCF Hoffman
Conserved LindbladToh
Coding UCSC
Annotation
−25
0
25
50
Enrichment (prop h2 / prop snps)

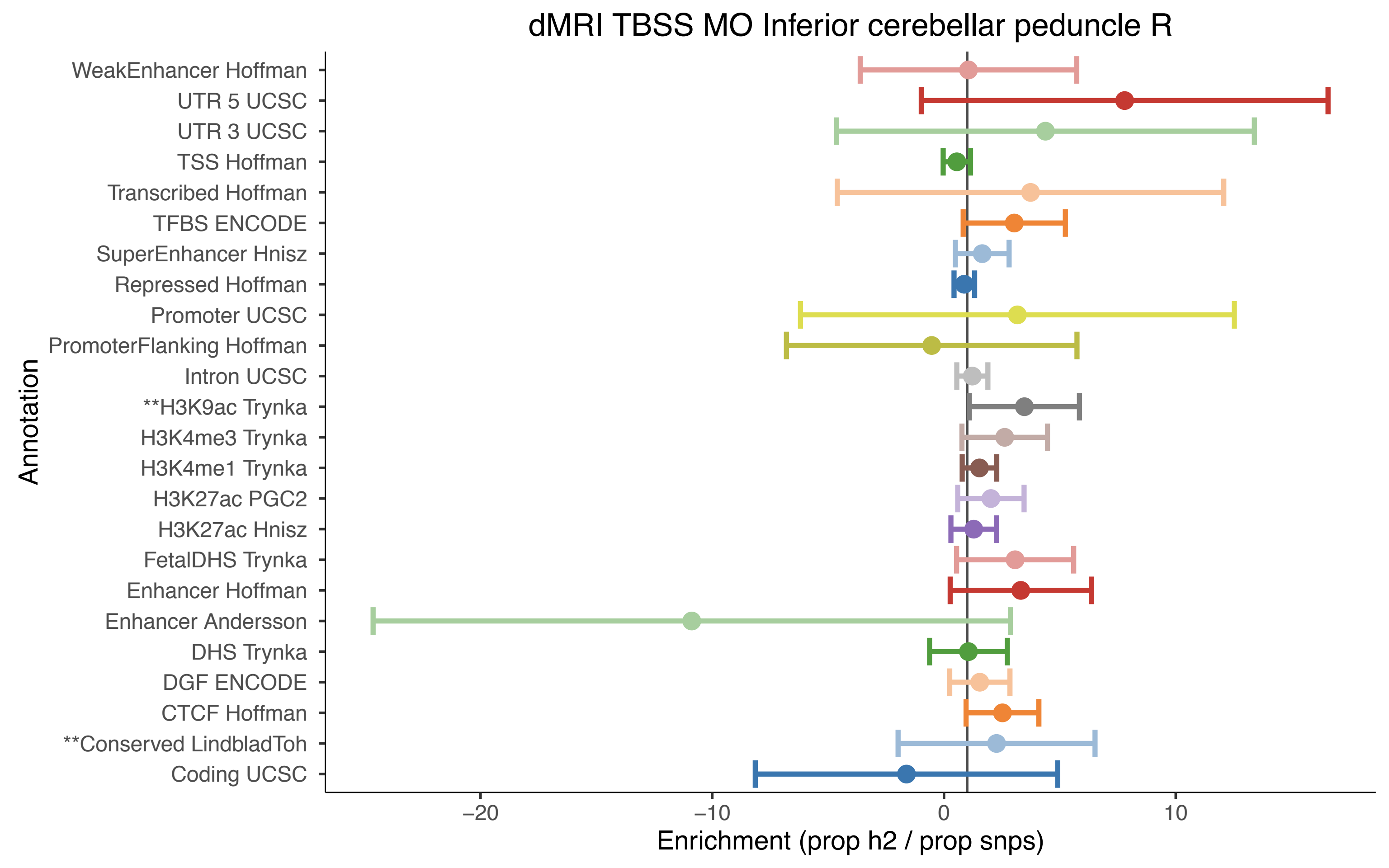

dMRI TBSS MO Inferior cerebellar peduncle R
WeakEnhancer Hoffman
UTR 5 UCSC
UTR 3 UCSC
TSS Hoffman
Transcribed Hoffman
TFBS ENCODE
SuperEnhancer Hnisz
Repressed Hoffman
Promoter UCSC
PromoterFlanking Hoffman
Intron UCSC
**H3K9ac Trynka
H3K4me3 Trynka
H3K4me1 Trynka
H3K27ac PGC2
H3K27ac Hnisz
FetalDHS Trynka
Enhancer Hoffman
Enhancer Andersson
DHS Trynka
DGF ENCODE
CTCF Hoffman
**Conserved LindbladToh
Coding UCSC
Annotation
−20
−10
0
10
Enrichment (prop h2 / prop snps)

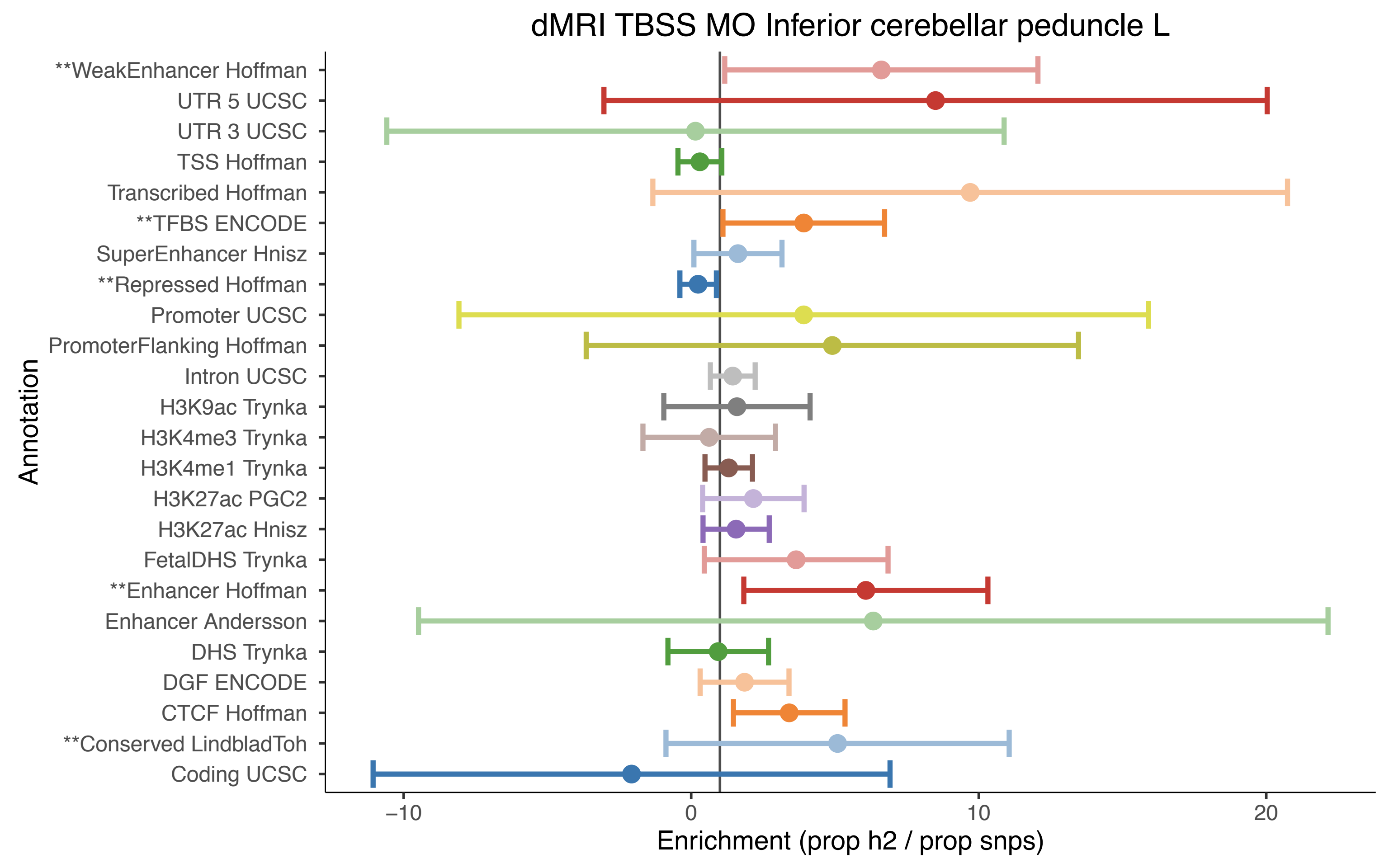
dMRI TBSS MO Inferior cerebellar peduncle L
**WeakEnhancer Hoffman
UTR 5 UCSC
UTR 3 UCSC
TSS Hoffman
Transcribed Hoffman
**TFBS ENCODE
SuperEnhancer Hnisz
**Repressed Hoffman
Promoter UCSC
PromoterFlanking Hoffman
Intron UCSC
H3K9ac Trynka
H3K4me3 Trynka
H3K4me1 Trynka
H3K27ac PGC2
H3K27ac Hnisz
FetalDHS Trynka
**Enhancer Hoffman
Enhancer Andersson
DHS Trynka
DGF ENCODE
CTCF Hoffman
**Conserved LindbladToh
Coding UCSC
Annotation
−10
0
10
20
Enrichment (prop h2 / prop snps)

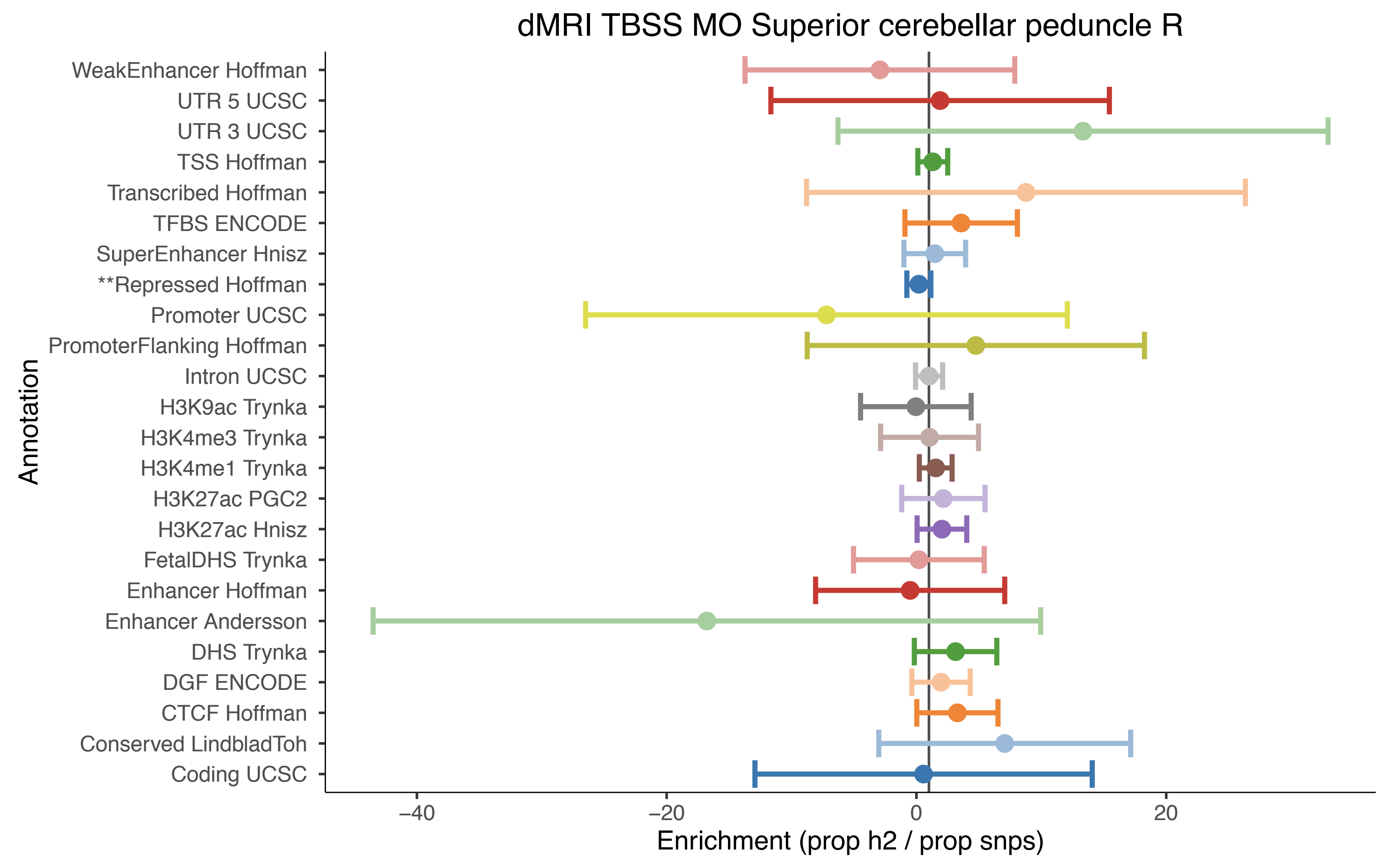
dMRI TBSS MO Superior cerebellar peduncle R
WeakEnhancer Hoffman
UTR 5 UCSC
UTR 3 UCSC
TSS Hoffman
Transcribed Hoffman
TFBS ENCODE
SuperEnhancer Hnisz
**Repressed Hoffman
Promoter UCSC
PromoterFlanking Hoffman
Intron UCSC
H3K9ac Trynka
H3K4me3 Trynka
H3K4me1 Trynka
H3K27ac PGC2
H3K27ac Hnisz
FetalDHS Trynka
Enhancer Hoffman
Enhancer Andersson
DHS Trynka
DGF ENCODE
CTCF Hoffman
Conserved LindbladToh
Coding UCSC
Annotation
−40
−20
0
20
Enrichment (prop h2 / prop snps)

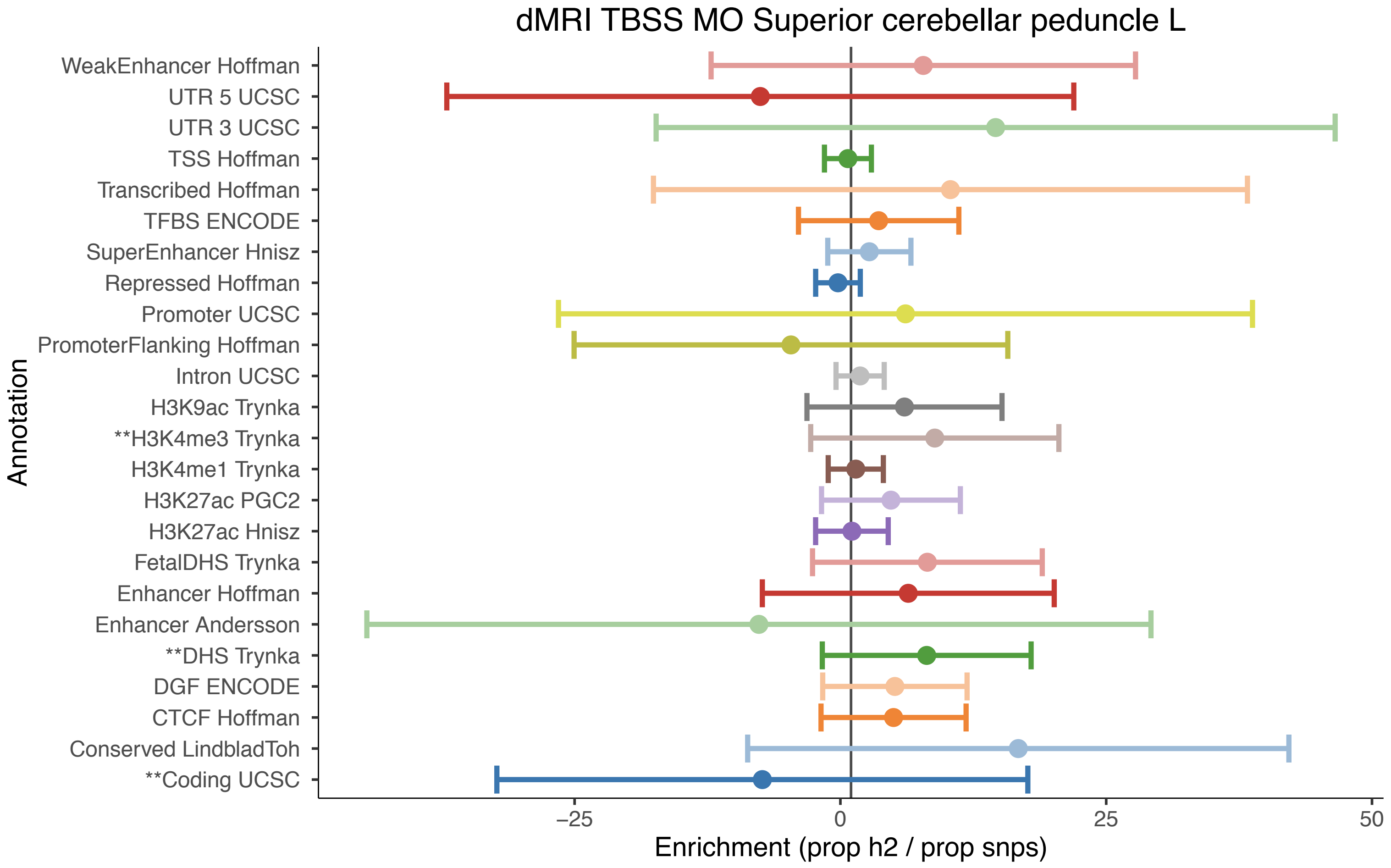

dMRI TBSS MO Superior cerebellar peduncle L
WeakEnhancer Hoffman
UTR 5 UCSC
UTR 3 UCSC
TSS Hoffman
Transcribed Hoffman
TFBS ENCODE
SuperEnhancer Hnisz
Repressed Hoffman
Promoter UCSC
PromoterFlanking Hoffman
Intron UCSC
H3K9ac Trynka
**H3K4me3 Trynka
H3K4me1 Trynka
H3K27ac PGC2
H3K27ac Hnisz
FetalDHS Trynka
Enhancer Hoffman
Enhancer Andersson
**DHS Trynka
DGF ENCODE
CTCF Hoffman
Conserved LindbladToh
**Coding UCSC
Annotation
−25
0
25
50
Enrichment (prop h2 / prop snps)

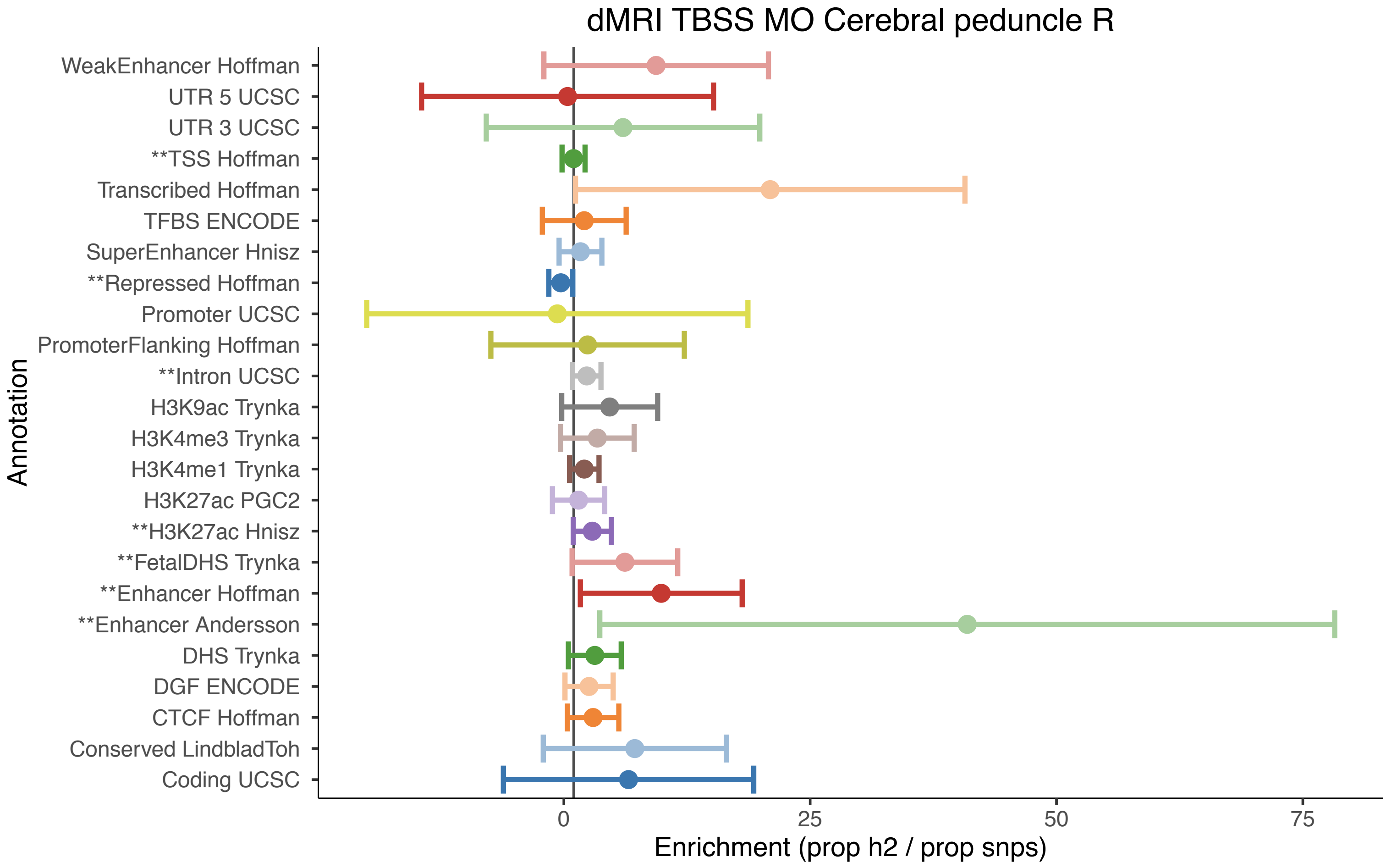
dMRI TBSS MO Cerebral peduncle R
WeakEnhancer Hoffman
UTR 5 UCSC
UTR 3 UCSC
**TSS Hoffman
Transcribed Hoffman
TFBS ENCODE
SuperEnhancer Hnisz
**Repressed Hoffman
Promoter UCSC
PromoterFlanking Hoffman
**Intron UCSC
H3K9ac Trynka
H3K4me3 Trynka
H3K4me1 Trynka
H3K27ac PGC2
**H3K27ac Hnisz
**FetalDHS Trynka
**Enhancer Hoffman
**Enhancer Andersson
DHS Trynka
DGF ENCODE
CTCF Hoffman
Conserved LindbladToh
Coding UCSC
Annotation
0
25
50
75
Enrichment (prop h2 / prop snps)

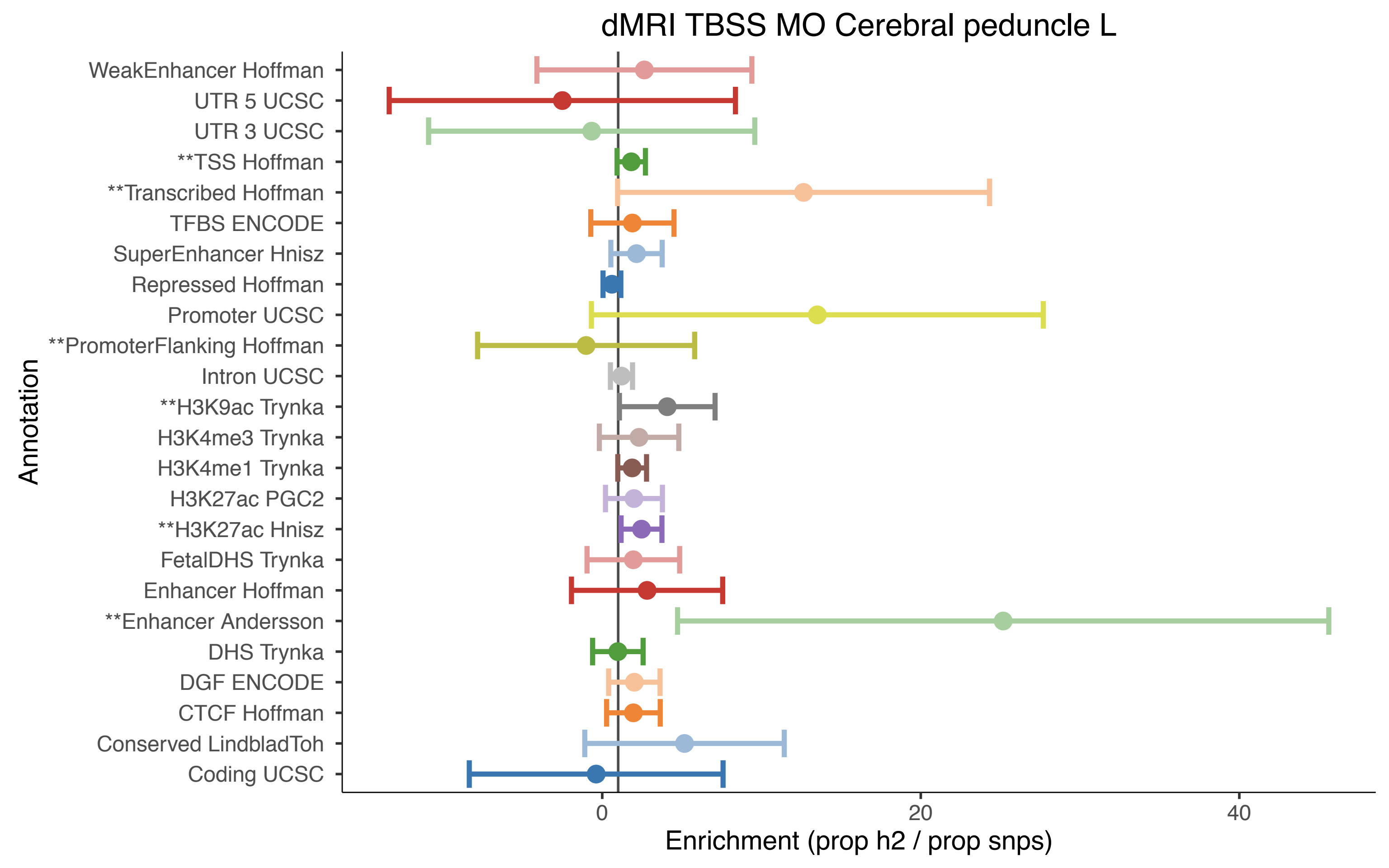
dMRI TBSS MO Cerebral peduncle L
WeakEnhancer Hoffman
UTR 5 UCSC
UTR 3 UCSC
**TSS Hoffman
**Transcribed Hoffman
TFBS ENCODE
SuperEnhancer Hnisz
Repressed Hoffman
Promoter UCSC
**PromoterFlanking Hoffman
Intron UCSC
**H3K9ac Trynka
H3K4me3 Trynka
H3K4me1 Trynka
H3K27ac PGC2
**H3K27ac Hnisz
FetalDHS Trynka
Enhancer Hoffman
**Enhancer Andersson
DHS Trynka
DGF ENCODE
CTCF Hoffman
Conserved LindbladToh
Coding UCSC
Annotation
0
20
40
Enrichment (prop h2 / prop snps)

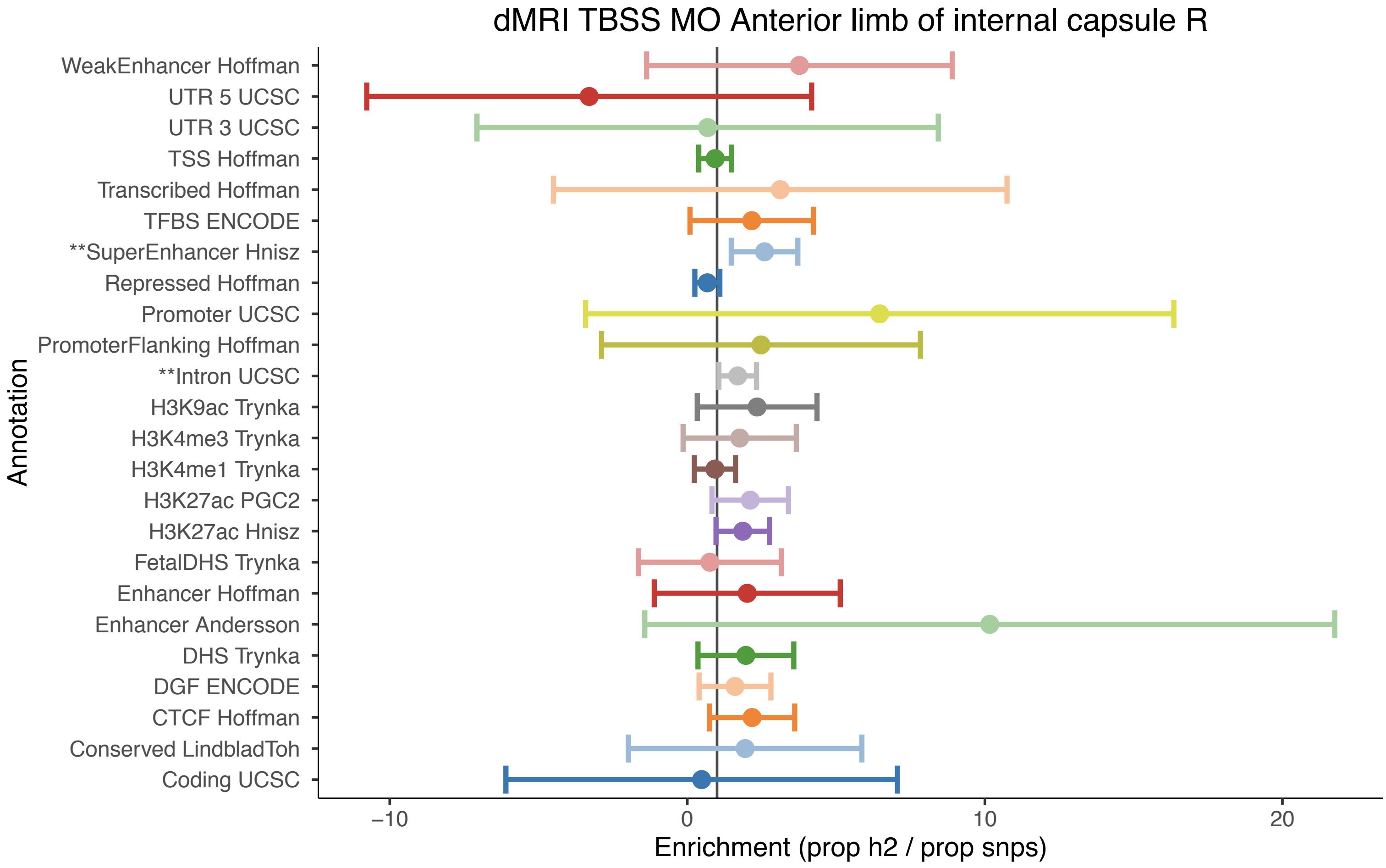
dMRI TBSS MO Anterior limb of internal capsule R
WeakEnhancer Hoffman
UTR 5 UCSC
UTR 3 UCSC
TSS Hoffman
Transcribed Hoffman
TFBS ENCODE
**SuperEnhancer Hnisz
Repressed Hoffman
Promoter UCSC
PromoterFlanking Hoffman
**Intron UCSC
H3K9ac Trynka
H3K4me3 Trynka
H3K4me1 Trynka
H3K27ac PGC2
H3K27ac Hnisz
FetalDHS Trynka
Enhancer Hoffman
Enhancer Andersson
DHS Trynka
DGF ENCODE
CTCF Hoffman
Conserved LindbladToh
Coding UCSC
Annotation
−10
0
10
20
Enrichment (prop h2 / prop snps)

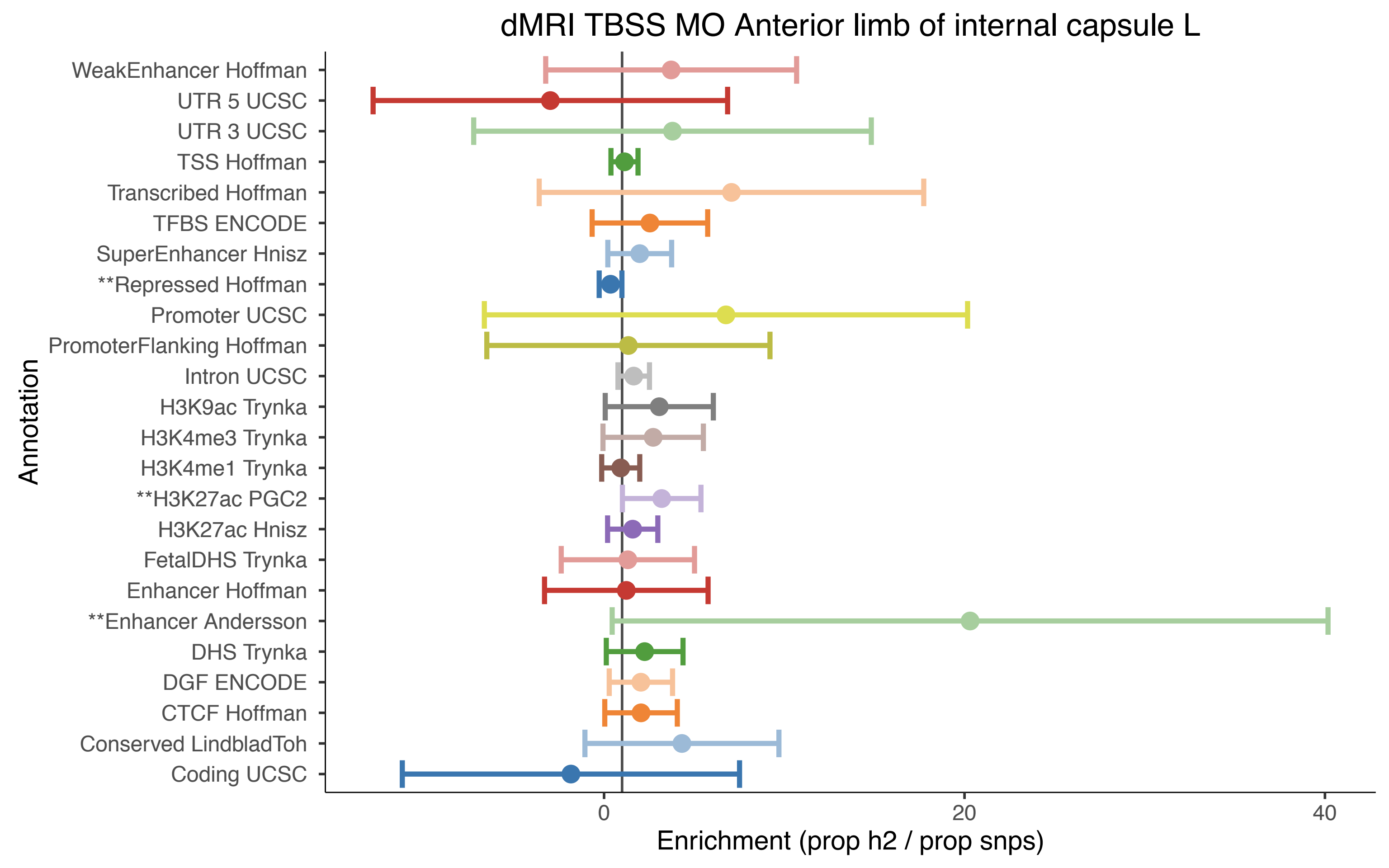
dMRI TBSS MO Anterior limb of internal capsule L
WeakEnhancer Hoffman
UTR 5 UCSC
UTR 3 UCSC
TSS Hoffman
Transcribed Hoffman
TFBS ENCODE
SuperEnhancer Hnisz
**Repressed Hoffman
Promoter UCSC
PromoterFlanking Hoffman
Intron UCSC
H3K9ac Trynka
H3K4me3 Trynka
H3K4me1 Trynka
**H3K27ac PGC2
H3K27ac Hnisz
FetalDHS Trynka
Enhancer Hoffman
**Enhancer Andersson
DHS Trynka
DGF ENCODE
CTCF Hoffman
Conserved LindbladToh
Coding UCSC
Annotation
0
20
40
Enrichment (prop h2 / prop snps)

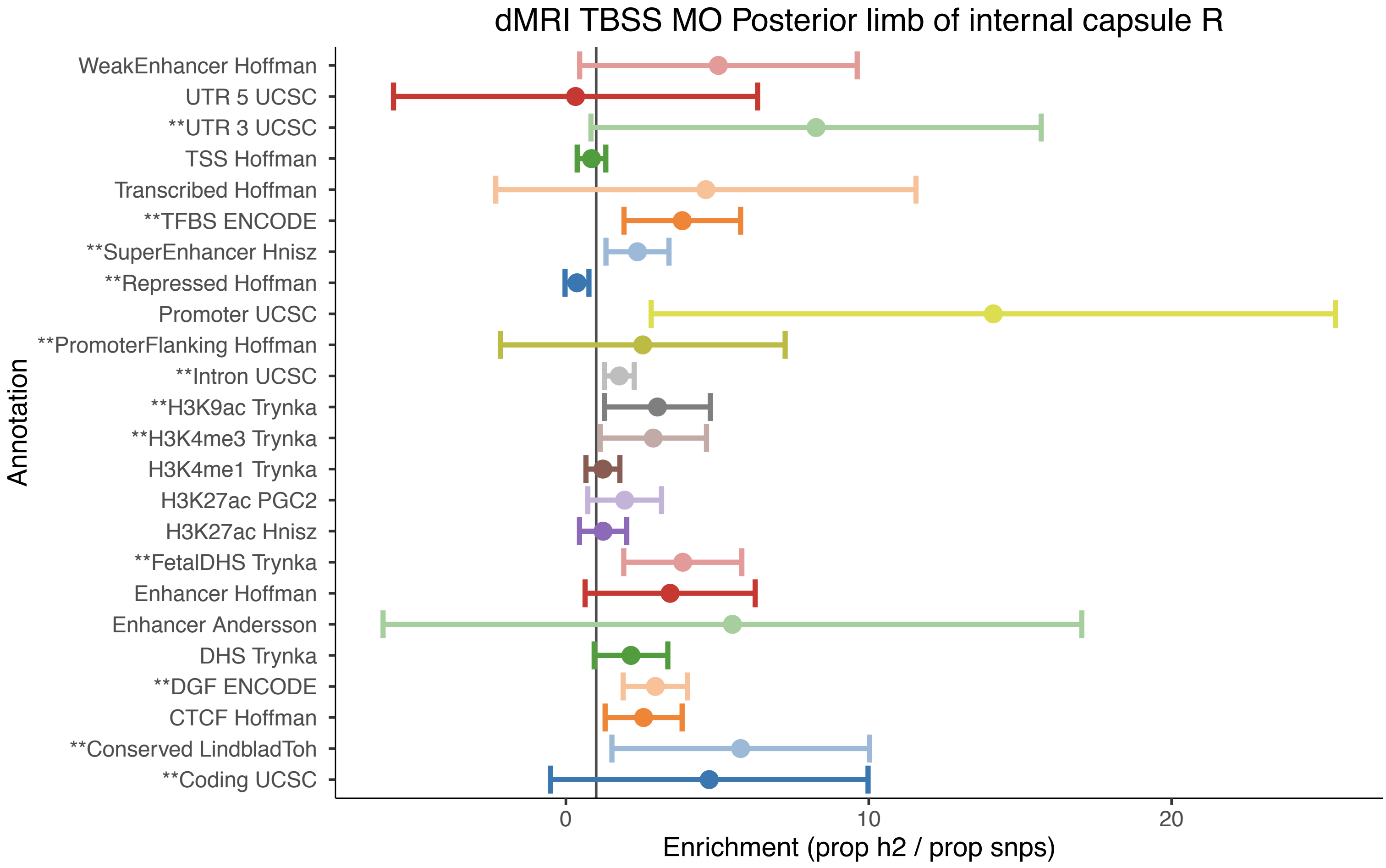

dMRI TBSS MO Posterior limb of internal capsule R
WeakEnhancer Hoffman
UTR 5 UCSC
**UTR 3 UCSC
TSS Hoffman
Transcribed Hoffman
**TFBS ENCODE
**SuperEnhancer Hnisz
**Repressed Hoffman
Promoter UCSC
**PromoterFlanking Hoffman
**Intron UCSC
**H3K9ac Trynka
**H3K4me3 Trynka
H3K4me1 Trynka
H3K27ac PGC2
H3K27ac Hnisz
**FetalDHS Trynka
Enhancer Hoffman
Enhancer Andersson
DHS Trynka
**DGF ENCODE
CTCF Hoffman
**Conserved LindbladToh
**Coding UCSC
Annotation
0
10
20
Enrichment (prop h2 / prop snps)

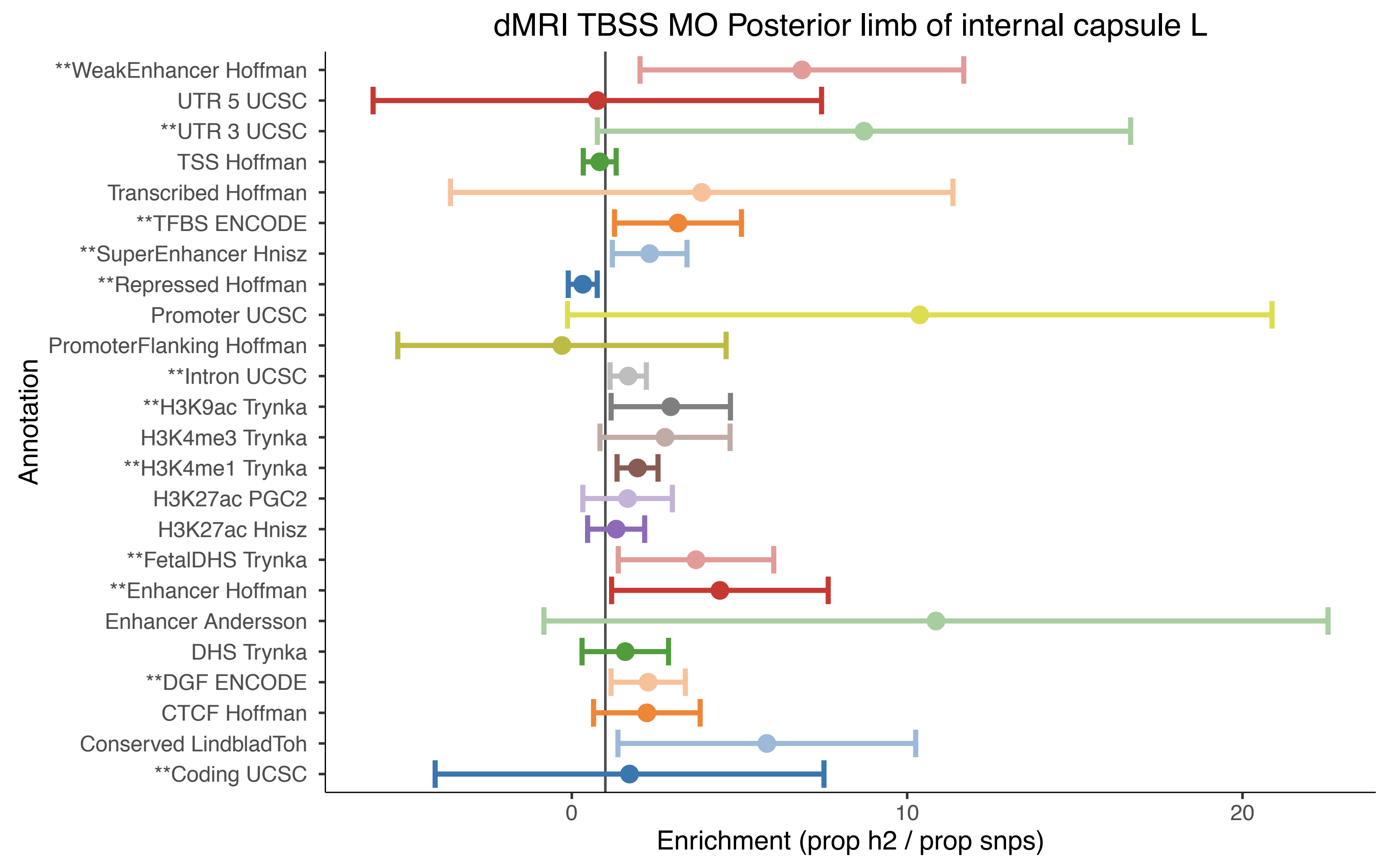
dMRI TBSS MO Posterior limb of internal capsule L
**WeakEnhancer Hoffman
UTR 5 UCSC
**UTR 3 UCSC
TSS Hoffman
Transcribed Hoffman
**TFBS ENCODE
**SuperEnhancer Hnisz
**Repressed Hoffman
Promoter UCSC
PromoterFlanking Hoffman
**Intron UCSC
**H3K9ac Trynka
H3K4me3 Trynka
**H3K4me1 Trynka
H3K27ac PGC2
H3K27ac Hnisz
**FetalDHS Trynka
**Enhancer Hoffman
Enhancer Andersson
DHS Trynka
**DGF ENCODE
CTCF Hoffman
Conserved LindbladToh
**Coding UCSC
Annotation
0
10
20
Enrichment (prop h2 / prop snps)

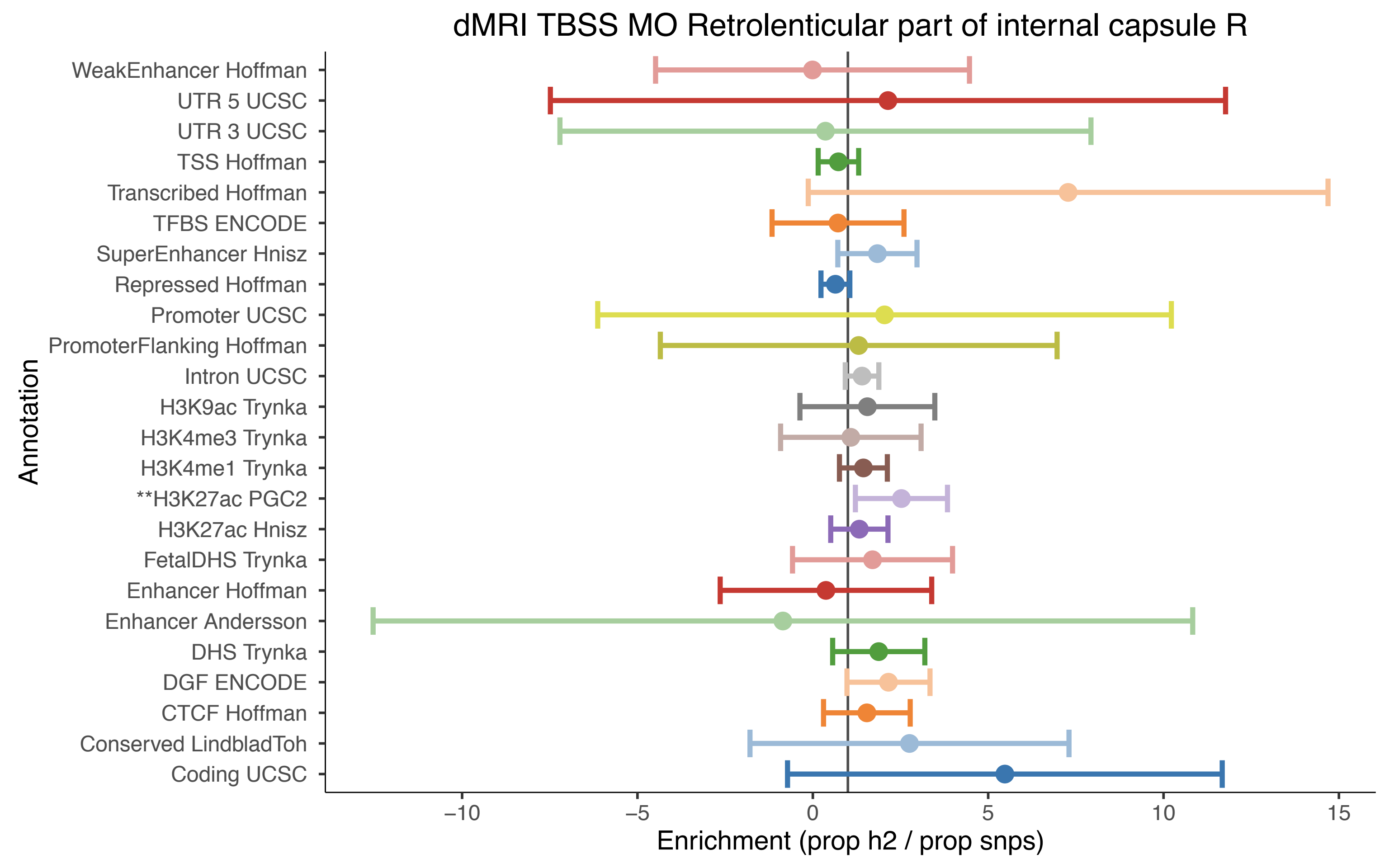
dMRI TBSS MO Retrolenticular part of internal capsule R
Annotation
WeakEnhancer Hoffman
UTR 5 UCSC
UTR 3 UCSC
TSS Hoffman
Transcribed Hoffman
TFBS ENCODE
SuperEnhancer Hnisz
Repressed Hoffman
Promoter UCSC
PromoterFlanking Hoffman
Intron UCSC
H3K9ac Trynka
H3K4me3 Trynka
H3K4me1 Trynka
**H3K27ac PGC2
H3K27ac Hnisz
FetalDHS Trynka
Enhancer Hoffman
Enhancer Andersson
DHS Trynka
DGF ENCODE
CTCF Hoffman
Conserved LindbladToh
Coding UCSC
−10
−5
0
5
10
15
Enrichment (prop h2 / prop snps)

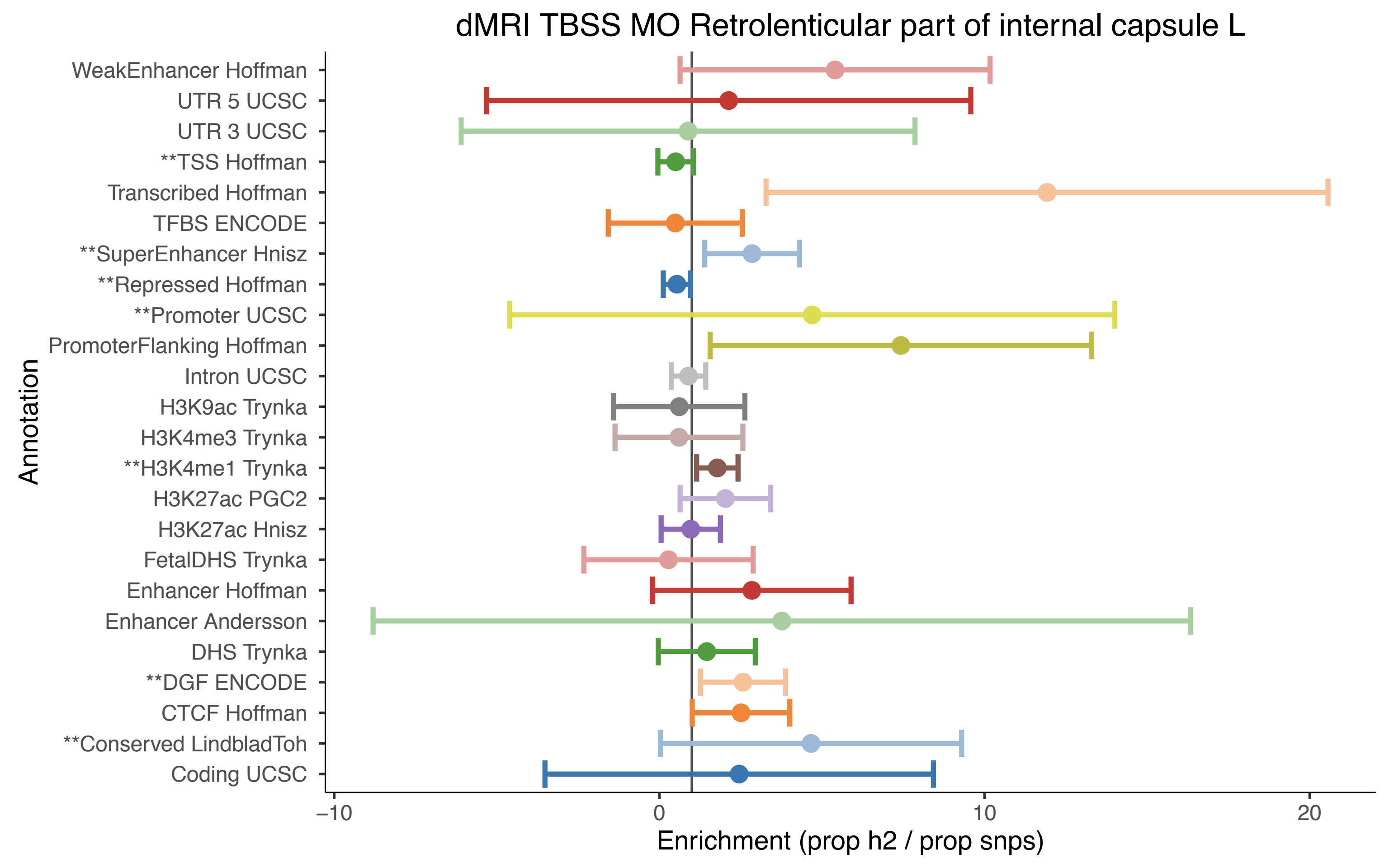
dMRI TBSS MO Retrolenticular part of internal capsule L
WeakEnhancer Hoffman
UTR 5 UCSC
UTR 3 UCSC
**TSS Hoffman
Transcribed Hoffman
TFBS ENCODE
**SuperEnhancer Hnisz
**Repressed Hoffman
**Promoter UCSC
PromoterFlanking Hoffman
Intron UCSC
H3K9ac Trynka
H3K4me3 Trynka
**H3K4me1 Trynka
H3K27ac PGC2
H3K27ac Hnisz
FetalDHS Trynka
Enhancer Hoffman
Enhancer Andersson
DHS Trynka
**DGF ENCODE
CTCF Hoffman
**Conserved LindbladToh
Coding UCSC
Annotation
−10
0
10
20
Enrichment (prop h2 / prop snps)

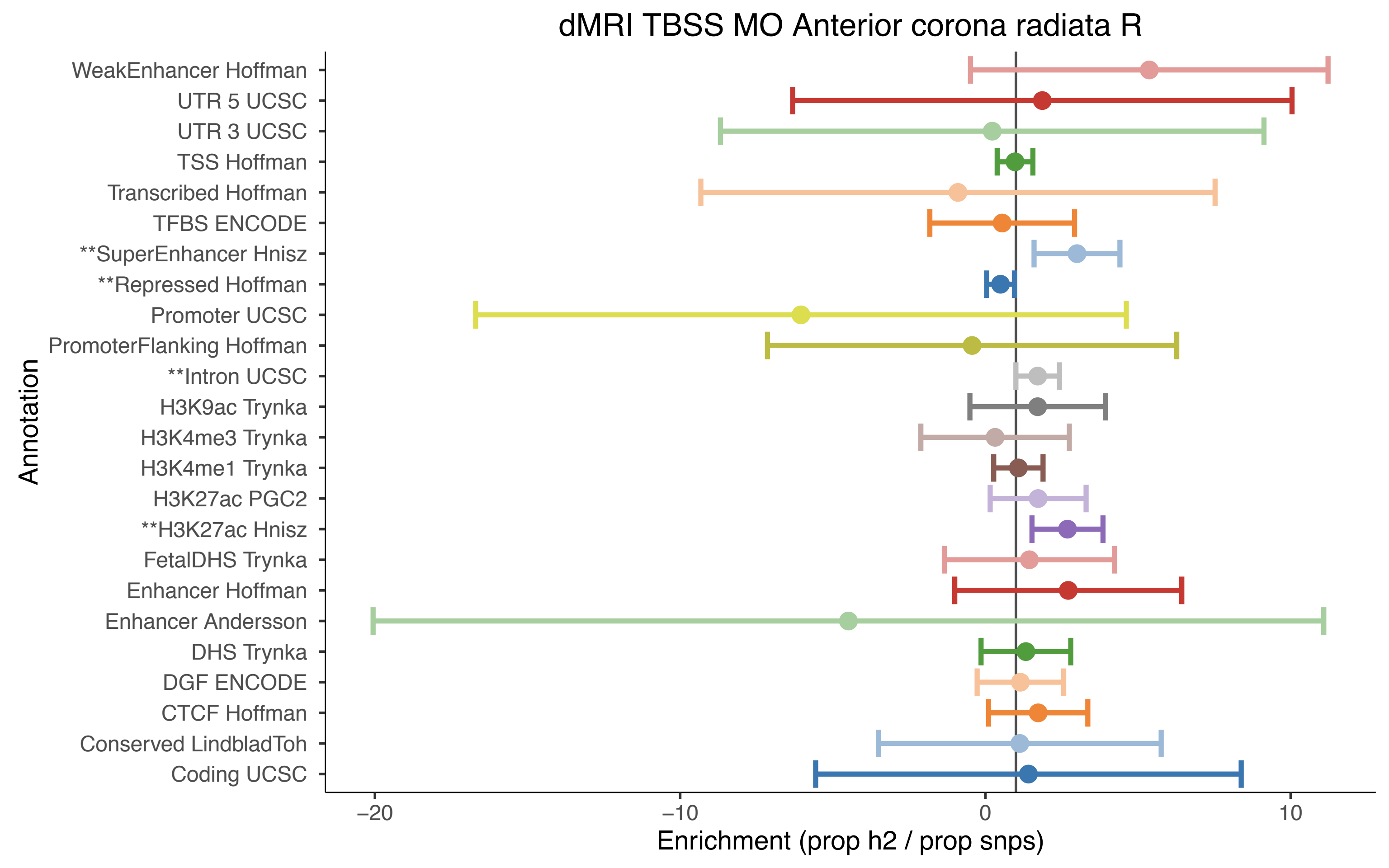
dMRI TBSS MO Anterior corona radiata R
WeakEnhancer Hoffman
UTR 5 UCSC
UTR 3 UCSC
TSS Hoffman
Transcribed Hoffman
TFBS ENCODE
**SuperEnhancer Hnisz
**Repressed Hoffman
Promoter UCSC
PromoterFlanking Hoffman
**Intron UCSC
H3K9ac Trynka
H3K4me3 Trynka
H3K4me1 Trynka
H3K27ac PGC2
**H3K27ac Hnisz
FetalDHS Trynka
Enhancer Hoffman
Enhancer Andersson
DHS Trynka
DGF ENCODE
CTCF Hoffman
Conserved LindbladToh
Coding UCSC
Annotation
−20
−10
0
10
Enrichment (prop h2 / prop snps)

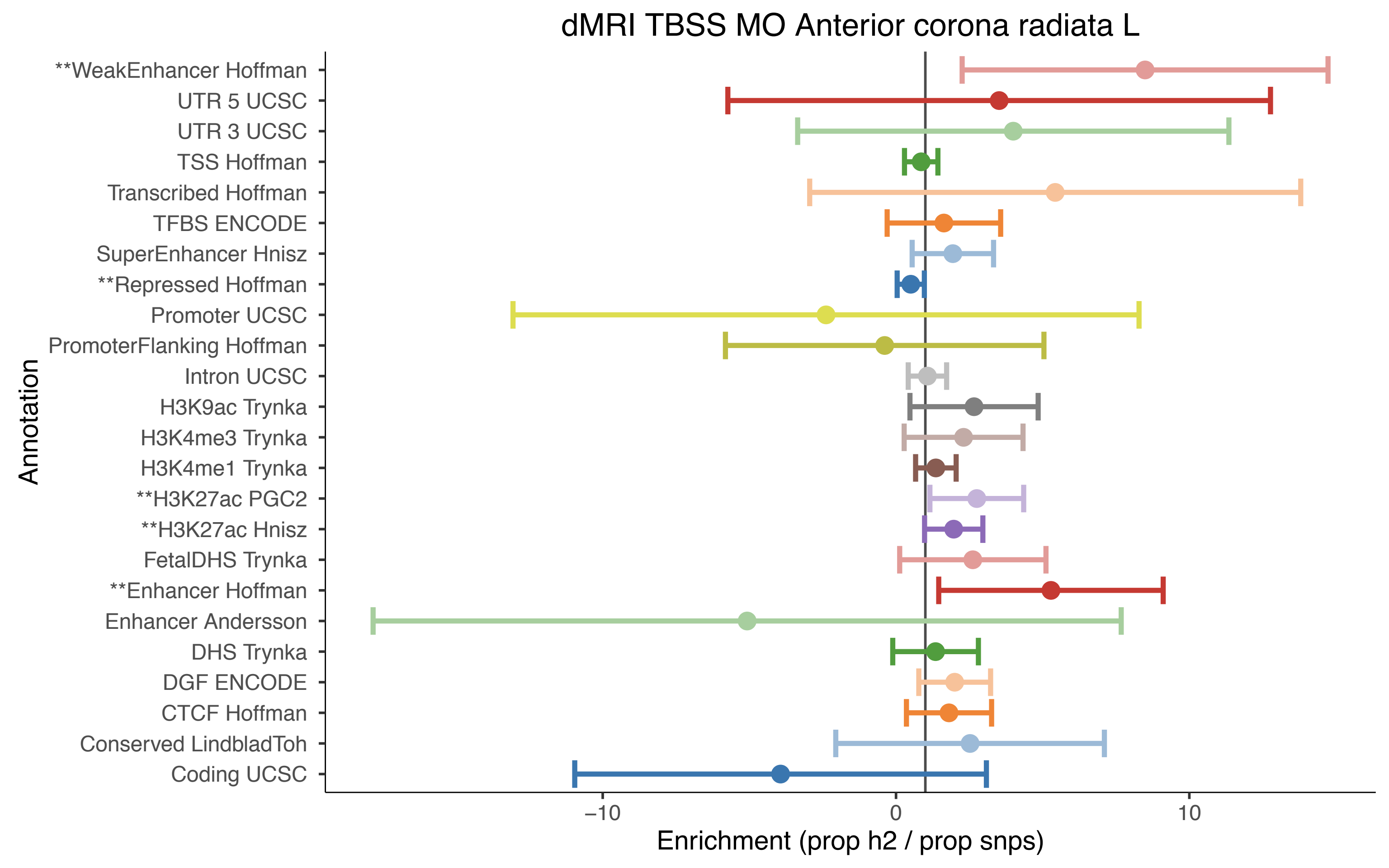

dMRI TBSS MO Anterior corona radiata L
**WeakEnhancer Hoffman
UTR 5 UCSC
UTR 3 UCSC
TSS Hoffman
Transcribed Hoffman
TFBS ENCODE
SuperEnhancer Hnisz
**Repressed Hoffman
Promoter UCSC
PromoterFlanking Hoffman
Intron UCSC
H3K9ac Trynka
H3K4me3 Trynka
H3K4me1 Trynka
**H3K27ac PGC2
**H3K27ac Hnisz
FetalDHS Trynka
**Enhancer Hoffman
Enhancer Andersson
DHS Trynka
DGF ENCODE
CTCF Hoffman
Conserved LindbladToh
Coding UCSC
Annotation
−10
0
10
Enrichment (prop h2 / prop snps)

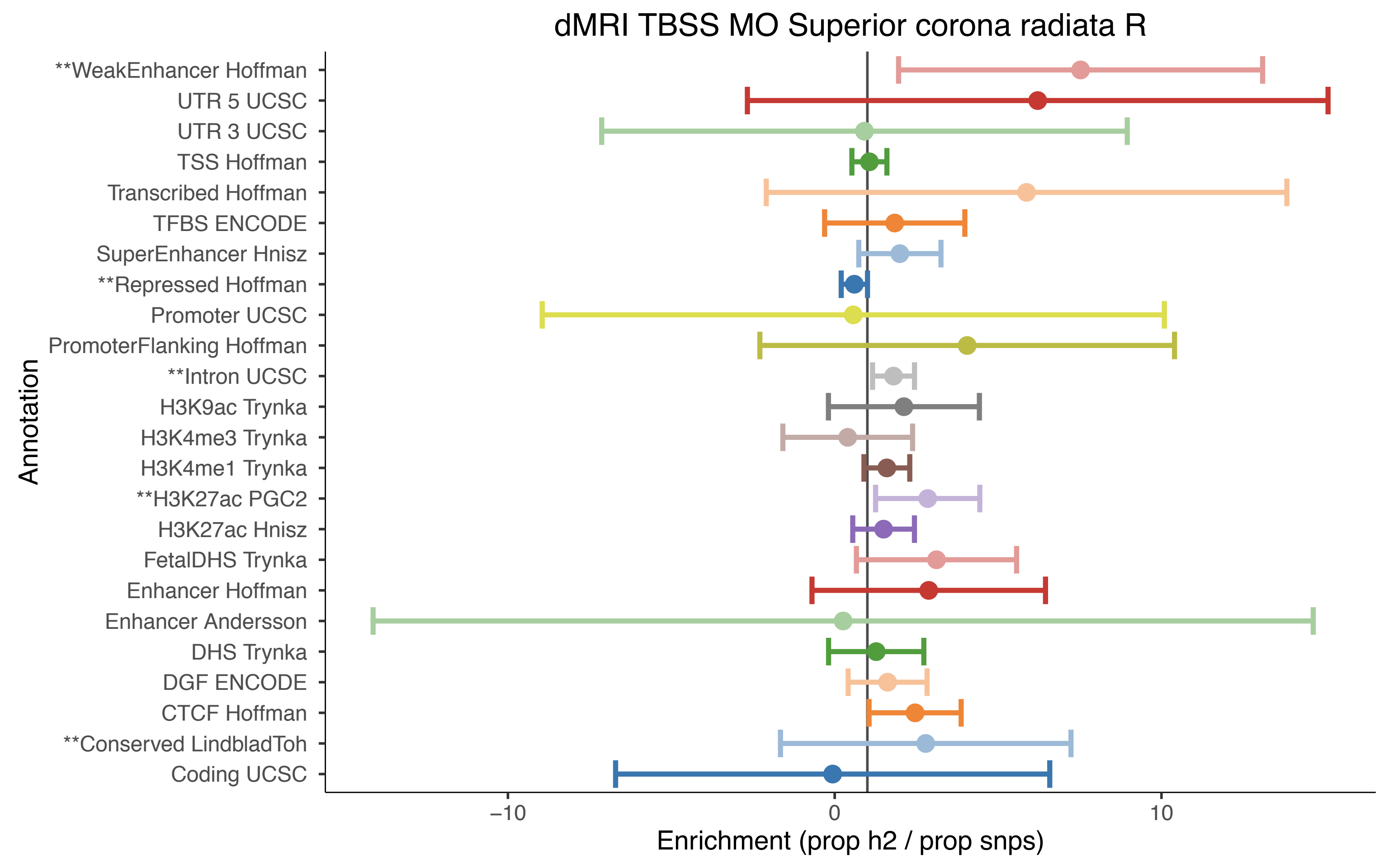
dMRI TBSS MO Superior corona radiata R
**WeakEnhancer Hoffman
UTR 5 UCSC
UTR 3 UCSC
TSS Hoffman
Transcribed Hoffman
TFBS ENCODE
SuperEnhancer Hnisz
**Repressed Hoffman
Promoter UCSC
PromoterFlanking Hoffman
**Intron UCSC
H3K9ac Trynka
H3K4me3 Trynka
H3K4me1 Trynka
**H3K27ac PGC2
H3K27ac Hnisz
FetalDHS Trynka
Enhancer Hoffman
Enhancer Andersson
DHS Trynka
DGF ENCODE
CTCF Hoffman
**Conserved LindbladToh
Coding UCSC
Annotation
−10
0
10
Enrichment (prop h2 / prop snps)

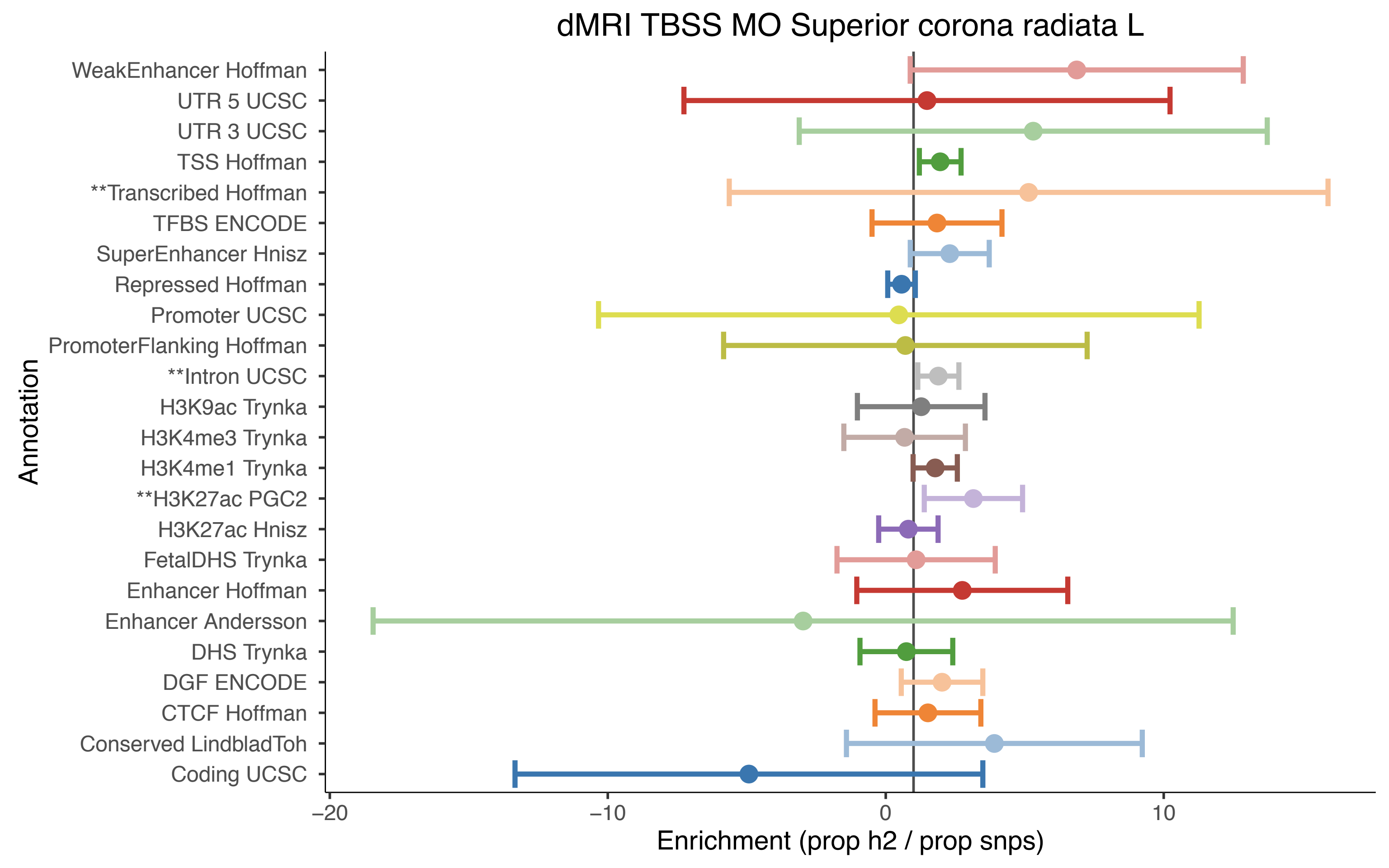

dMRI TBSS MO Superior corona radiata L
WeakEnhancer Hoffman
UTR 5 UCSC
UTR 3 UCSC
TSS Hoffman
**Transcribed Hoffman
TFBS ENCODE
SuperEnhancer Hnisz
Repressed Hoffman
Promoter UCSC
PromoterFlanking Hoffman
**Intron UCSC
H3K9ac Trynka
H3K4me3 Trynka
H3K4me1 Trynka
**H3K27ac PGC2
H3K27ac Hnisz
FetalDHS Trynka
Enhancer Hoffman
Enhancer Andersson
DHS Trynka
DGF ENCODE
CTCF Hoffman
Conserved LindbladToh
Coding UCSC
Annotation
−20
−10
0
10
Enrichment (prop h2 / prop snps)

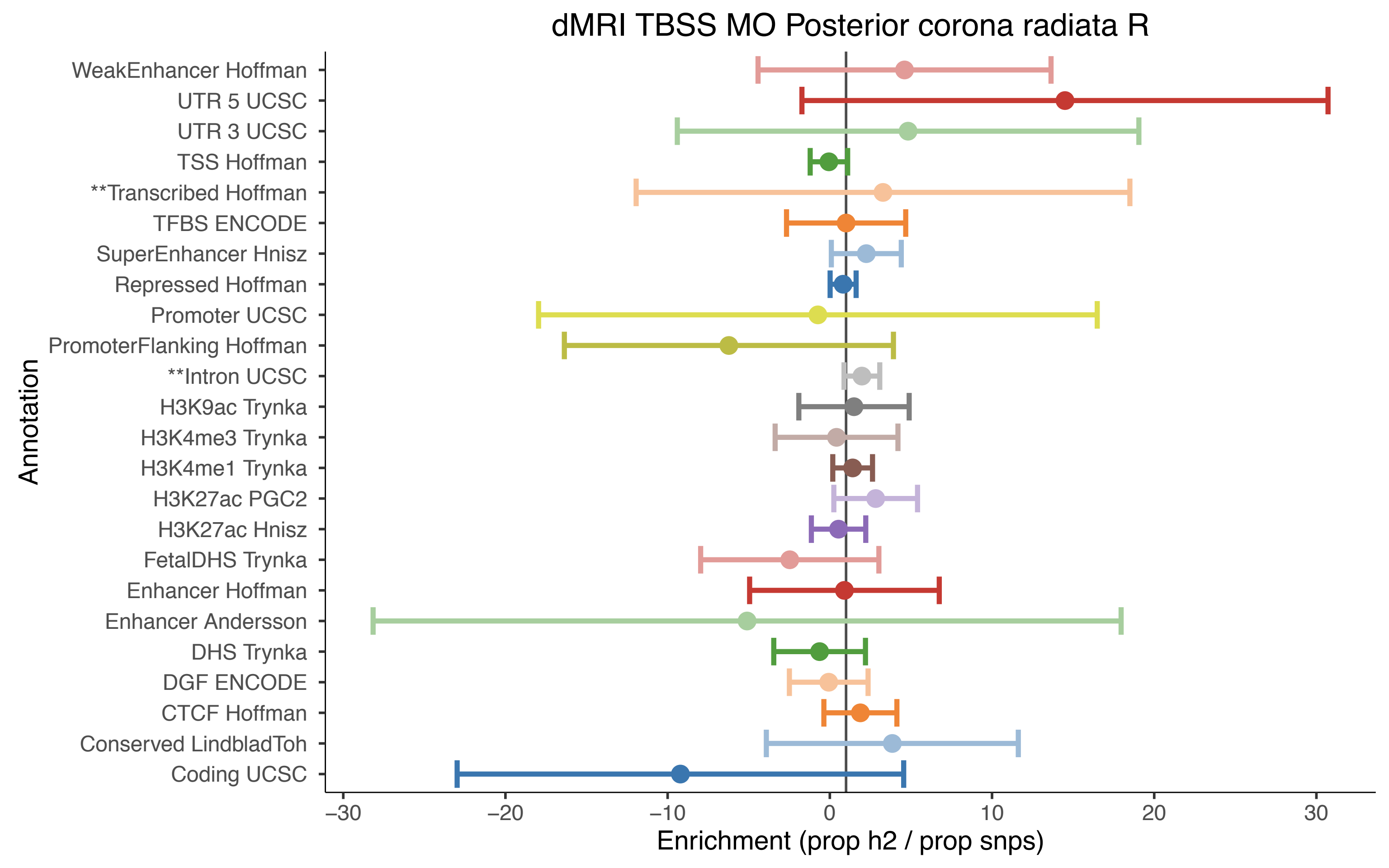
dMRI TBSS MO Posterior corona radiata R
WeakEnhancer Hoffman
UTR 5 UCSC
UTR 3 UCSC
TSS Hoffman
**Transcribed Hoffman
TFBS ENCODE
SuperEnhancer Hnisz
Repressed Hoffman
Promoter UCSC
PromoterFlanking Hoffman
**Intron UCSC
H3K9ac Trynka
H3K4me3 Trynka
H3K4me1 Trynka
H3K27ac PGC2
H3K27ac Hnisz
FetalDHS Trynka
Enhancer Hoffman
Enhancer Andersson
DHS Trynka
DGF ENCODE
CTCF Hoffman
Conserved LindbladToh
Coding UCSC
Annotation
−30
−20
−10
0
10
20
30
Enrichment (prop h2 / prop snps)

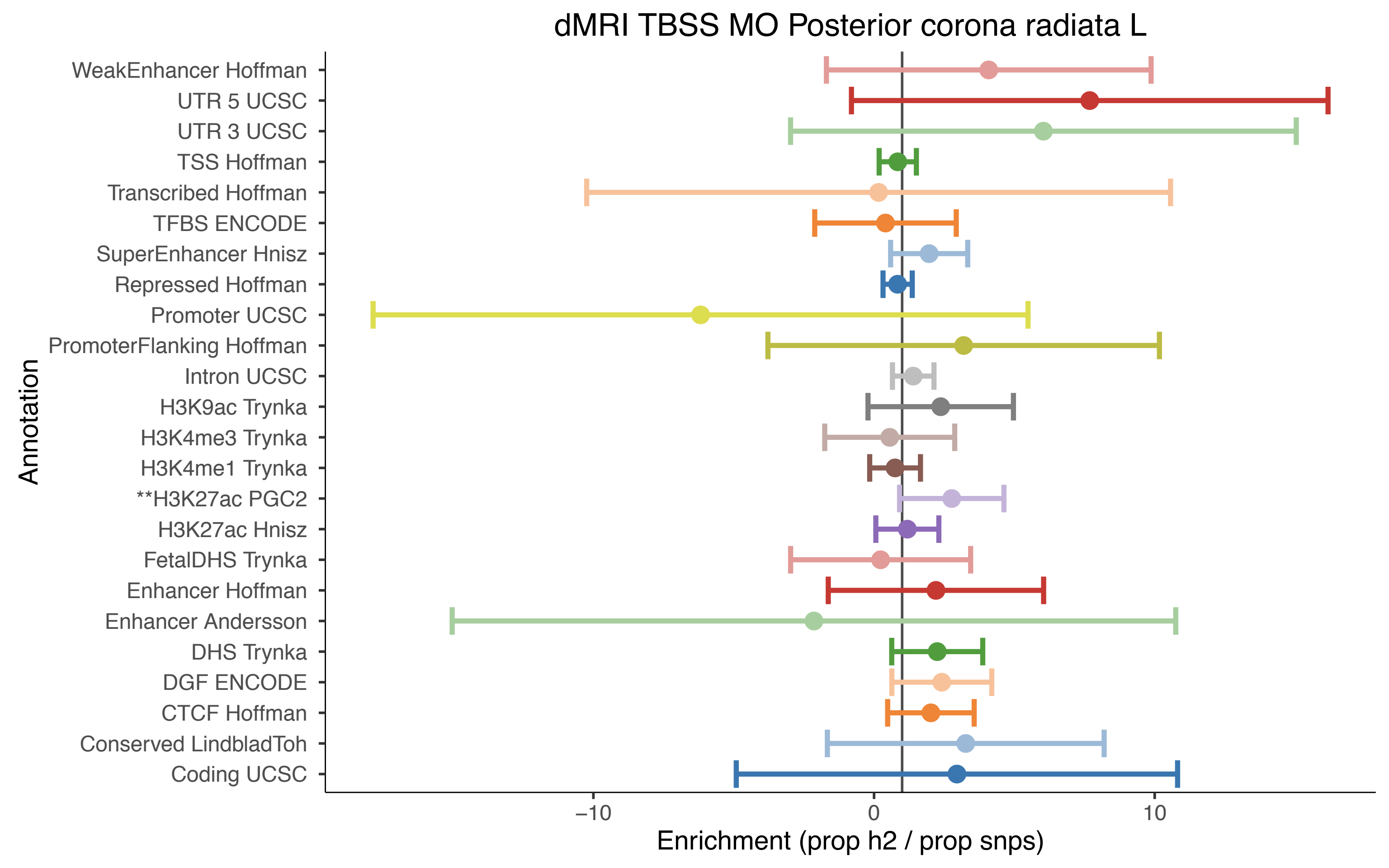

dMRI TBSS MO Posterior corona radiata L
WeakEnhancer Hoffman
UTR 5 UCSC
UTR 3 UCSC
TSS Hoffman
Transcribed Hoffman
TFBS ENCODE
SuperEnhancer Hnisz
Repressed Hoffman
Promoter UCSC
PromoterFlanking Hoffman
Intron UCSC
H3K9ac Trynka
H3K4me3 Trynka
H3K4me1 Trynka
**H3K27ac PGC2
H3K27ac Hnisz
FetalDHS Trynka
Enhancer Hoffman
Enhancer Andersson
DHS Trynka
DGF ENCODE
CTCF Hoffman
Conserved LindbladToh
Coding UCSC
Annotation
−10
0
10
Enrichment (prop h2 / prop snps)

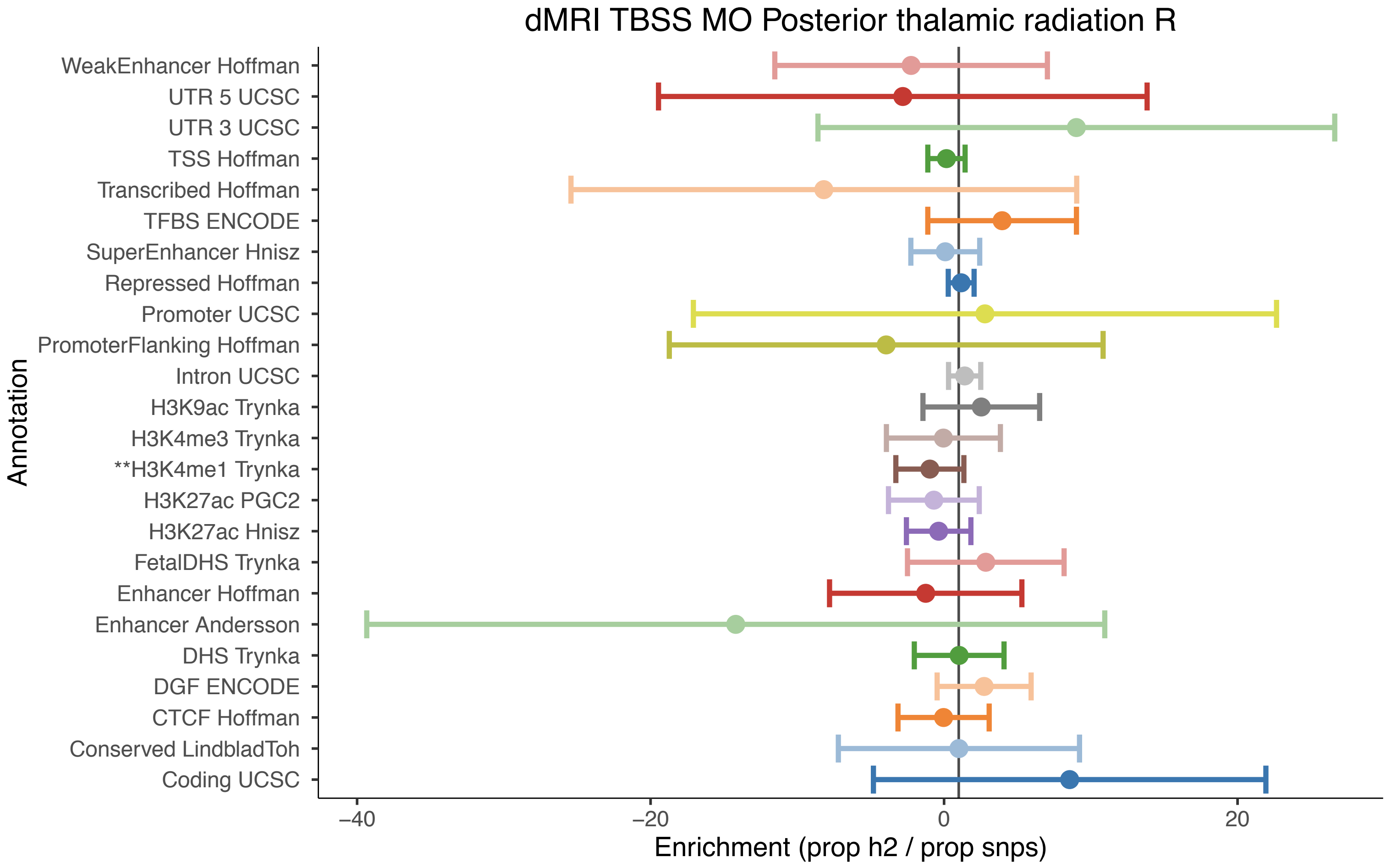
dMRI TBSS MO Posterior thalamic radiation R
WeakEnhancer Hoffman
UTR 5 UCSC
UTR 3 UCSC
TSS Hoffman
Transcribed Hoffman
TFBS ENCODE
SuperEnhancer Hnisz
Repressed Hoffman
Promoter UCSC
PromoterFlanking Hoffman
Intron UCSC
H3K9ac Trynka
H3K4me3 Trynka
**H3K4me1 Trynka
H3K27ac PGC2
H3K27ac Hnisz
FetalDHS Trynka
Enhancer Hoffman
Enhancer Andersson
DHS Trynka
DGF ENCODE
CTCF Hoffman
Conserved LindbladToh
Coding UCSC
Annotation
−40
−20
0
20
Enrichment (prop h2 / prop snps)

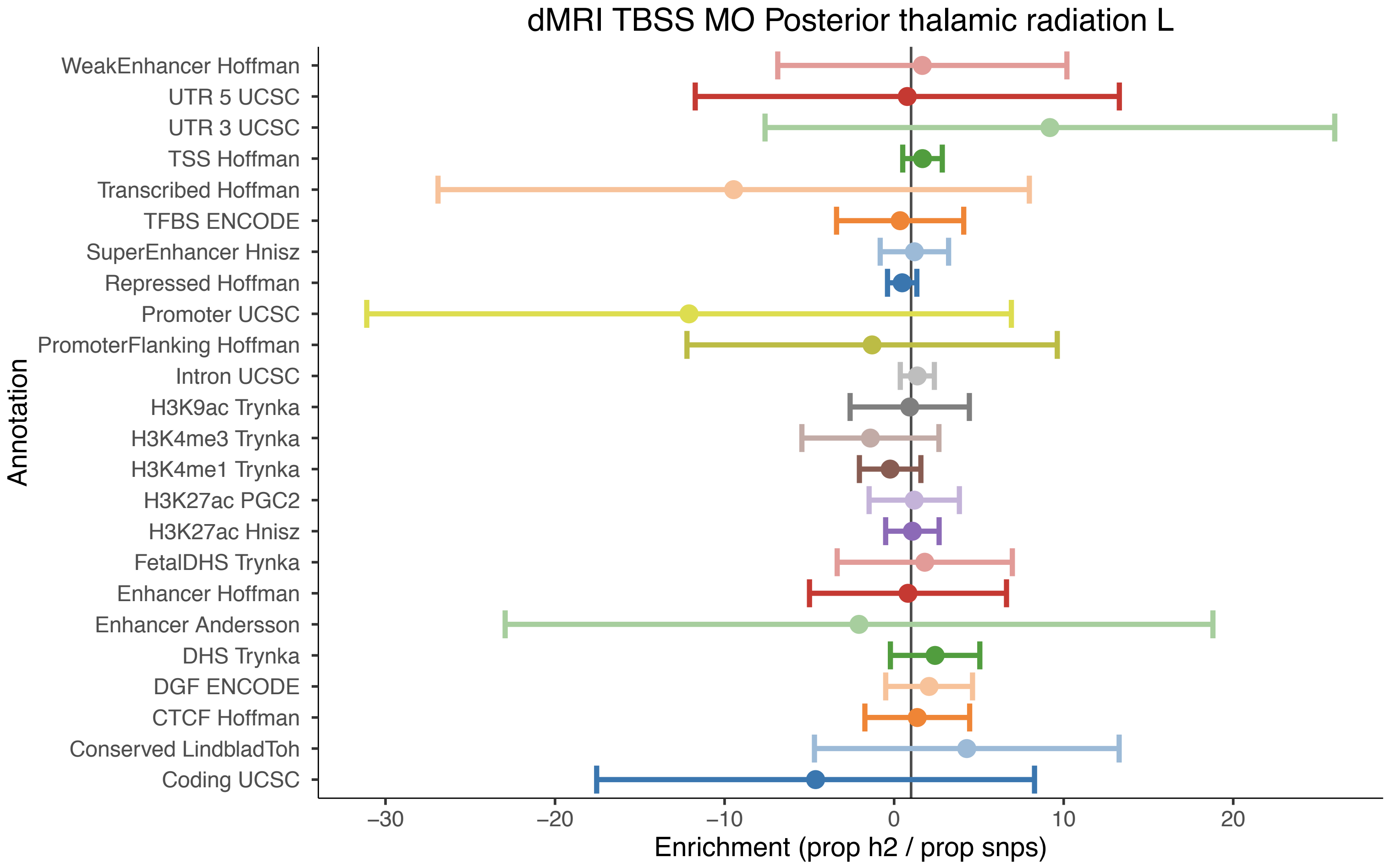
dMRI TBSS MO Posterior thalamic radiation L
Annotation
WeakEnhancer Hoffman
UTR 5 UCSC
UTR 3 UCSC
TSS Hoffman
Transcribed Hoffman
TFBS ENCODE
SuperEnhancer Hnisz
Repressed Hoffman
Promoter UCSC
PromoterFlanking Hoffman
Intron UCSC
H3K9ac Trynka
H3K4me3 Trynka
H3K4me1 Trynka
H3K27ac PGC2
H3K27ac Hnisz
FetalDHS Trynka
Enhancer Hoffman
Enhancer Andersson
DHS Trynka
DGF ENCODE
CTCF Hoffman
Conserved LindbladToh
Coding UCSC
−30
−20
−10
0
10
20
Enrichment (prop h2 / prop snps)

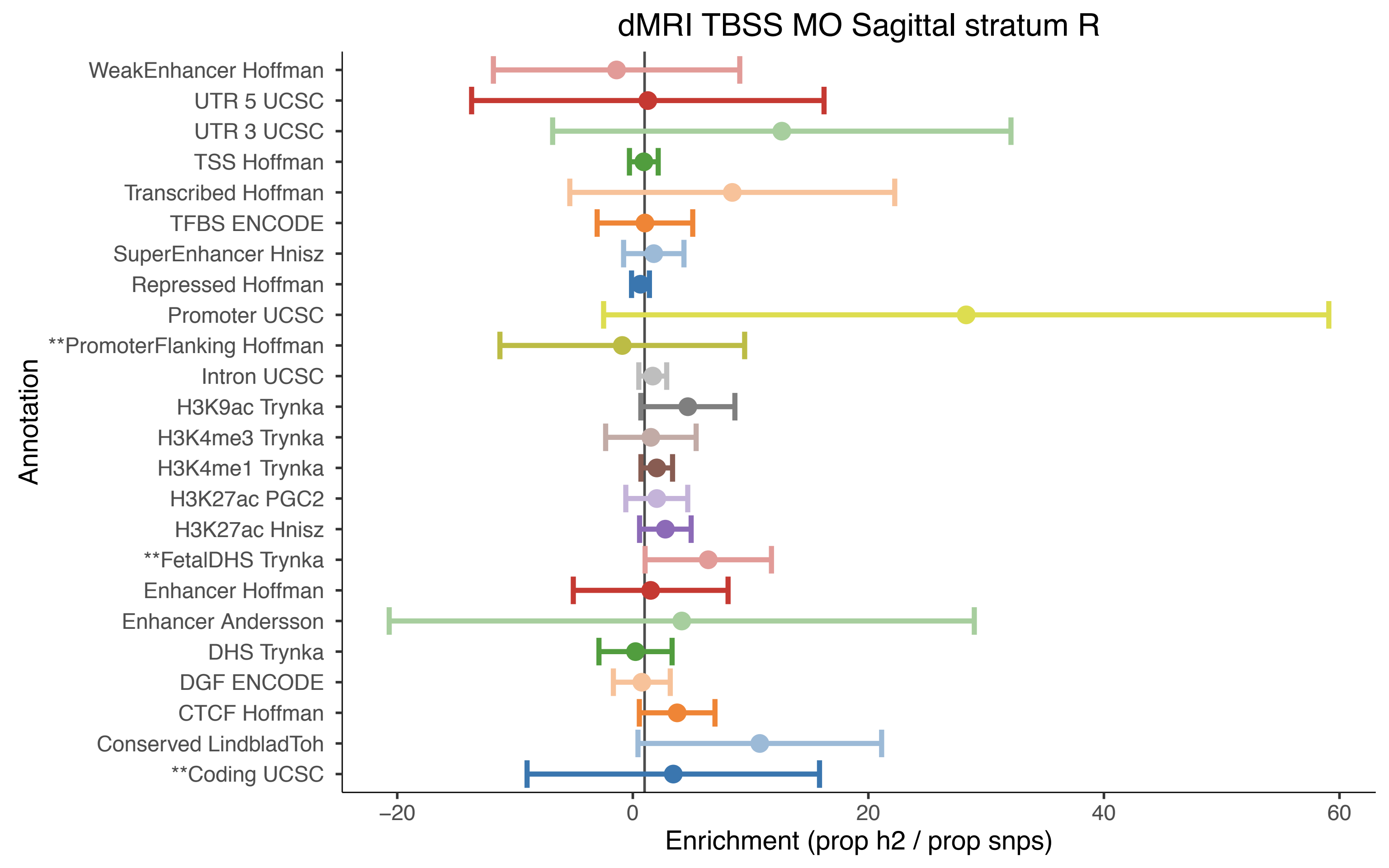
dMRI TBSS MO Sagittal stratum R
WeakEnhancer Hoffman
UTR 5 UCSC
UTR 3 UCSC
TSS Hoffman
Transcribed Hoffman
TFBS ENCODE
SuperEnhancer Hnisz
Repressed Hoffman
Promoter UCSC
**PromoterFlanking Hoffman
Intron UCSC
H3K9ac Trynka
H3K4me3 Trynka
H3K4me1 Trynka
H3K27ac PGC2
H3K27ac Hnisz
**FetalDHS Trynka
Enhancer Hoffman
Enhancer Andersson
DHS Trynka
DGF ENCODE
CTCF Hoffman
Conserved LindbladToh
**Coding UCSC
Annotation
−20
0
20
40
60
Enrichment (prop h2 / prop snps)

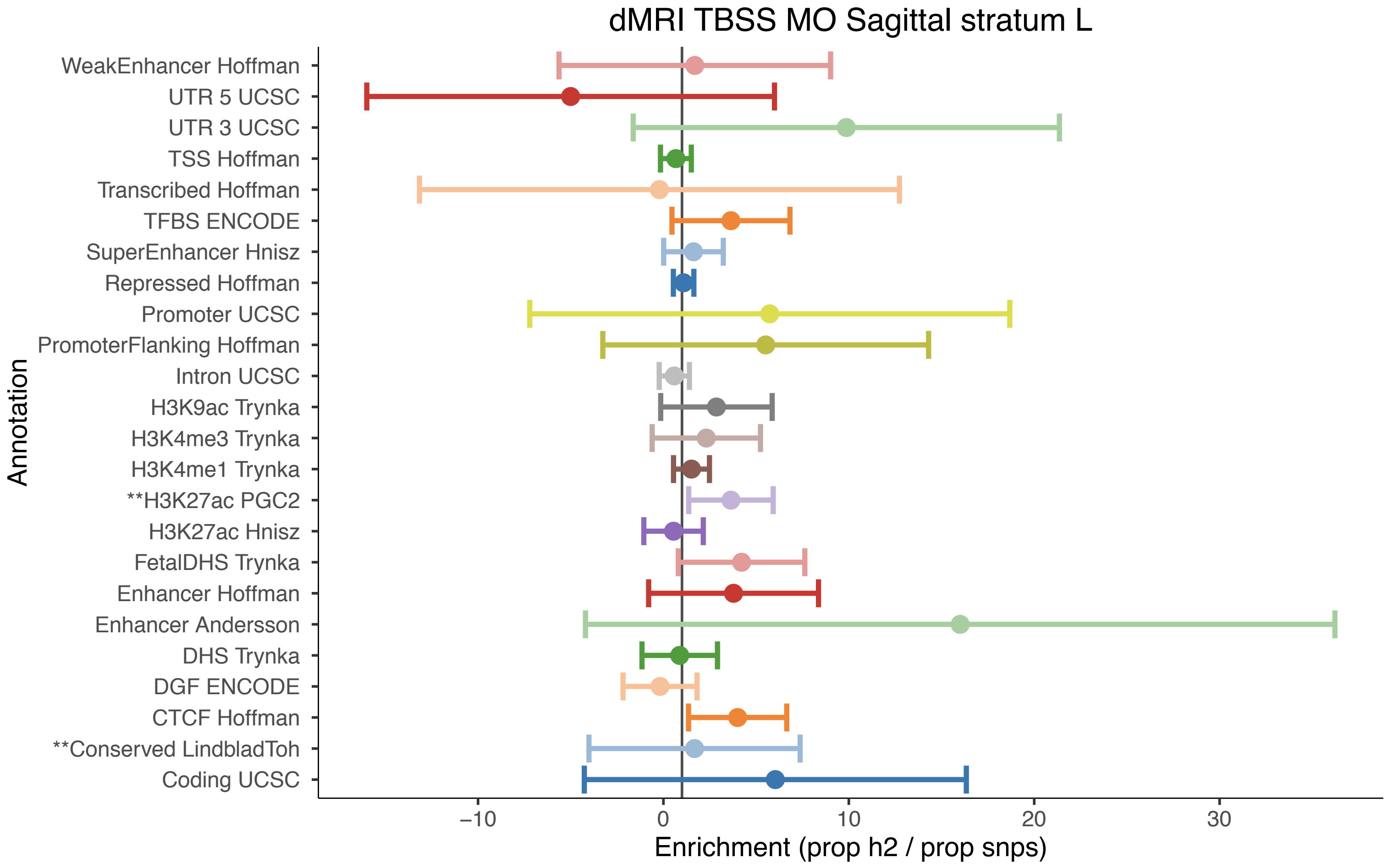
dMRI TBSS MO Sagittal stratum L
WeakEnhancer Hoffman
UTR 5 UCSC
UTR 3 UCSC
TSS Hoffman
Transcribed Hoffman
TFBS ENCODE
SuperEnhancer Hnisz
Repressed Hoffman
Promoter UCSC
PromoterFlanking Hoffman
Intron UCSC
H3K9ac Trynka
H3K4me3 Trynka
H3K4me1 Trynka
**H3K27ac PGC2
H3K27ac Hnisz
FetalDHS Trynka
Enhancer Hoffman
Enhancer Andersson
DHS Trynka
DGF ENCODE
CTCF Hoffman
**Conserved LindbladToh
Coding UCSC
Annotation
−10
0
10
20
30
Enrichment (prop h2 / prop snps)

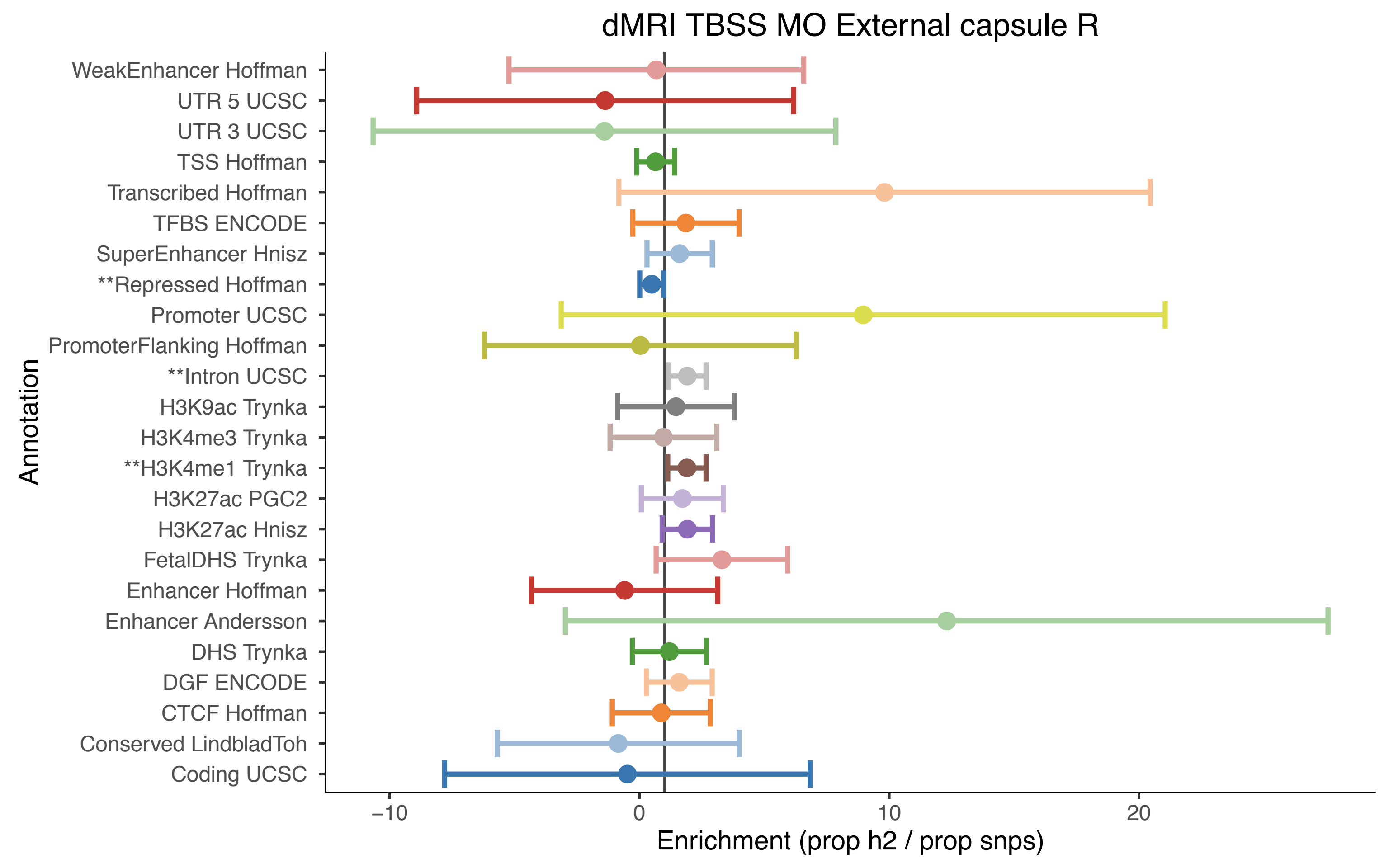

dMRI TBSS MO External capsule R
WeakEnhancer Hoffman
UTR 5 UCSC
UTR 3 UCSC
TSS Hoffman
Transcribed Hoffman
TFBS ENCODE
SuperEnhancer Hnisz
**Repressed Hoffman
Promoter UCSC
PromoterFlanking Hoffman
**Intron UCSC
H3K9ac Trynka
H3K4me3 Trynka
**H3K4me1 Trynka
H3K27ac PGC2
H3K27ac Hnisz
FetalDHS Trynka
Enhancer Hoffman
Enhancer Andersson
DHS Trynka
DGF ENCODE
CTCF Hoffman
Conserved LindbladToh
Coding UCSC
Annotation
−10
0
10
20
Enrichment (prop h2 / prop snps)

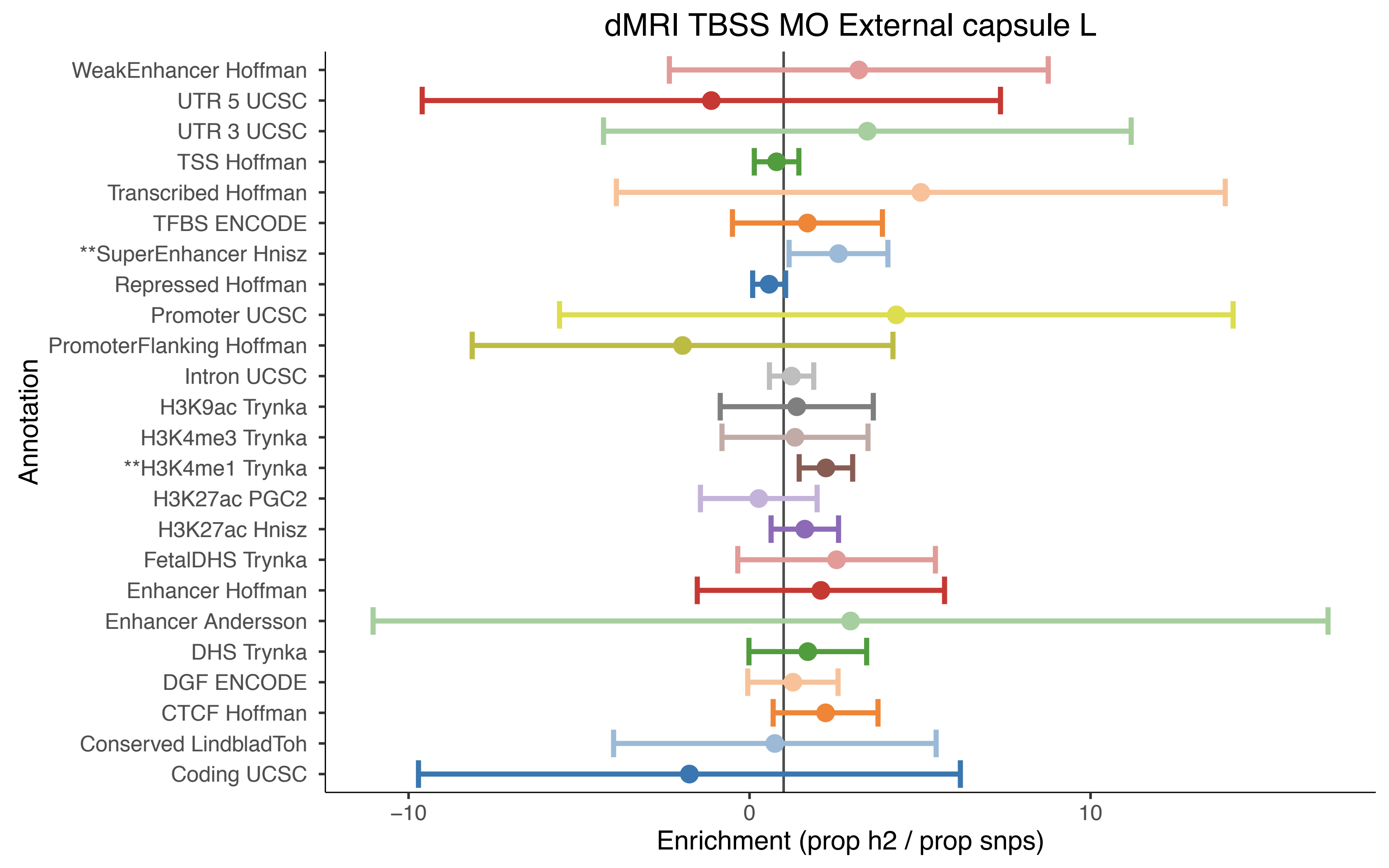
dMRI TBSS MO External capsule L
WeakEnhancer Hoffman
UTR 5 UCSC
UTR 3 UCSC
TSS Hoffman
Transcribed Hoffman
TFBS ENCODE
**SuperEnhancer Hnisz
Repressed Hoffman
Promoter UCSC
PromoterFlanking Hoffman
Intron UCSC
H3K9ac Trynka
H3K4me3 Trynka
**H3K4me1 Trynka
H3K27ac PGC2
H3K27ac Hnisz
FetalDHS Trynka
Enhancer Hoffman
Enhancer Andersson
DHS Trynka
DGF ENCODE
CTCF Hoffman
Conserved LindbladToh
Coding UCSC
Annotation
−10
0
10
Enrichment (prop h2 / prop snps)

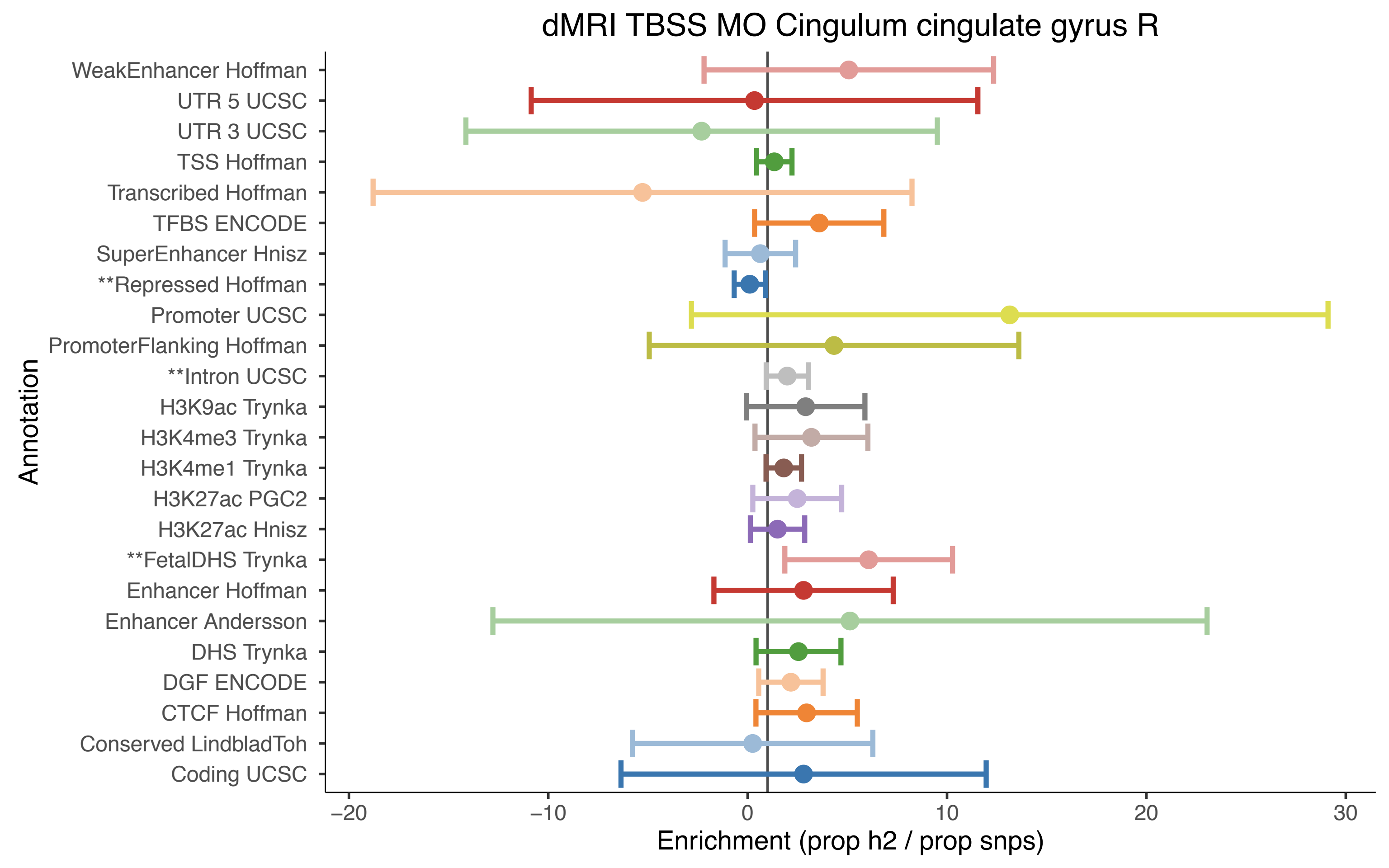

dMRI TBSS MO Cingulum cingulate gyrus R
WeakEnhancer Hoffman
UTR 5 UCSC
UTR 3 UCSC
TSS Hoffman
Transcribed Hoffman
TFBS ENCODE
SuperEnhancer Hnisz
**Repressed Hoffman
Promoter UCSC
PromoterFlanking Hoffman
**Intron UCSC
H3K9ac Trynka
H3K4me3 Trynka
H3K4me1 Trynka
H3K27ac PGC2
H3K27ac Hnisz
**FetalDHS Trynka
Enhancer Hoffman
Enhancer Andersson
DHS Trynka
DGF ENCODE
CTCF Hoffman
Conserved LindbladToh
Coding UCSC
Annotation
−20
−10
0
10
20
30
Enrichment (prop h2 / prop snps)

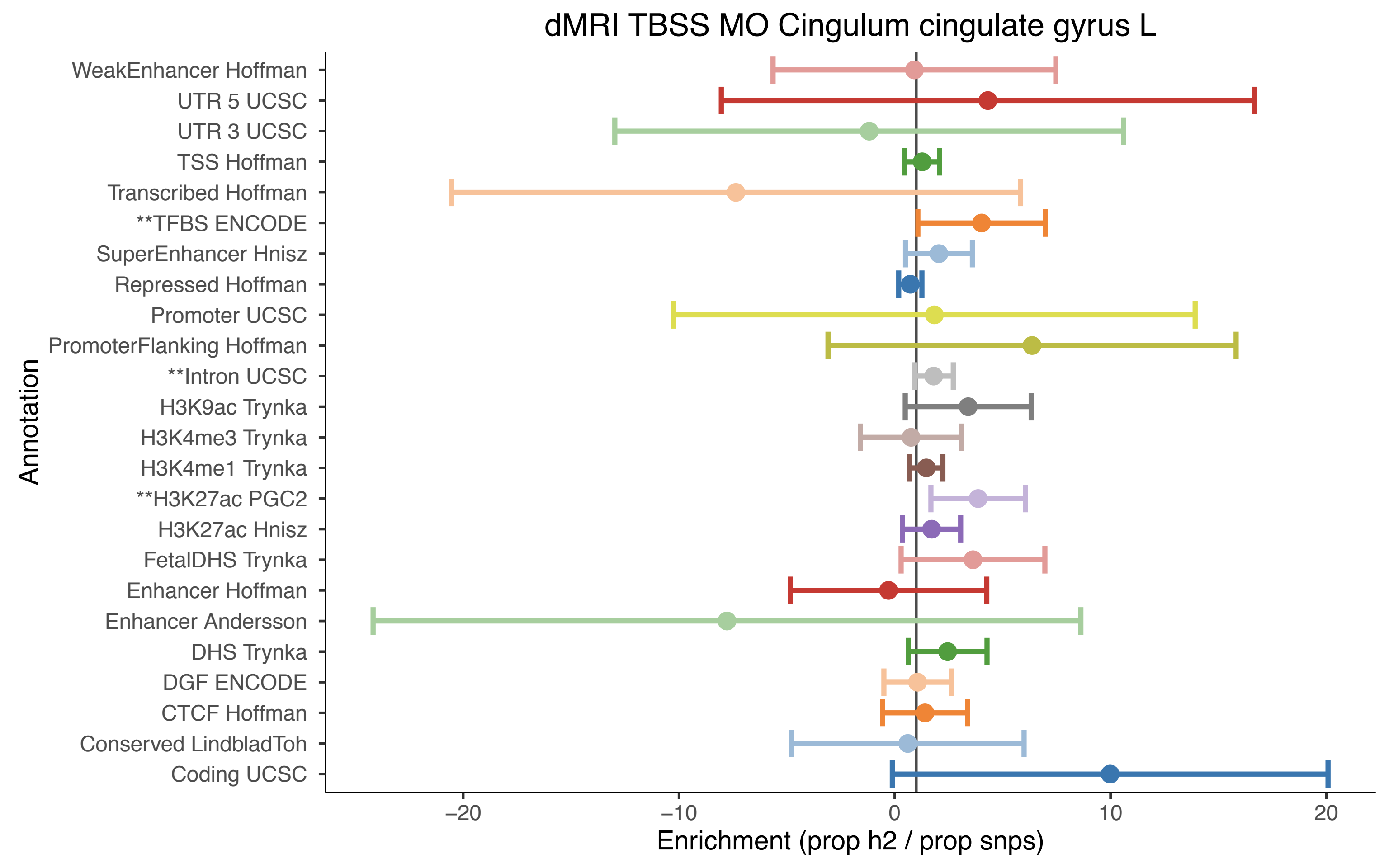
dMRI TBSS MO Cingulum cingulate gyrus L
WeakEnhancer Hoffman
UTR 5 UCSC
UTR 3 UCSC
TSS Hoffman
Transcribed Hoffman
**TFBS ENCODE
SuperEnhancer Hnisz
Repressed Hoffman
Promoter UCSC
PromoterFlanking Hoffman
**Intron UCSC
H3K9ac Trynka
H3K4me3 Trynka
H3K4me1 Trynka
**H3K27ac PGC2
H3K27ac Hnisz
FetalDHS Trynka
Enhancer Hoffman
Enhancer Andersson
DHS Trynka
DGF ENCODE
CTCF Hoffman
Conserved LindbladToh
Coding UCSC
Annotation
−20
−10
0
10
20
Enrichment (prop h2 / prop snps)

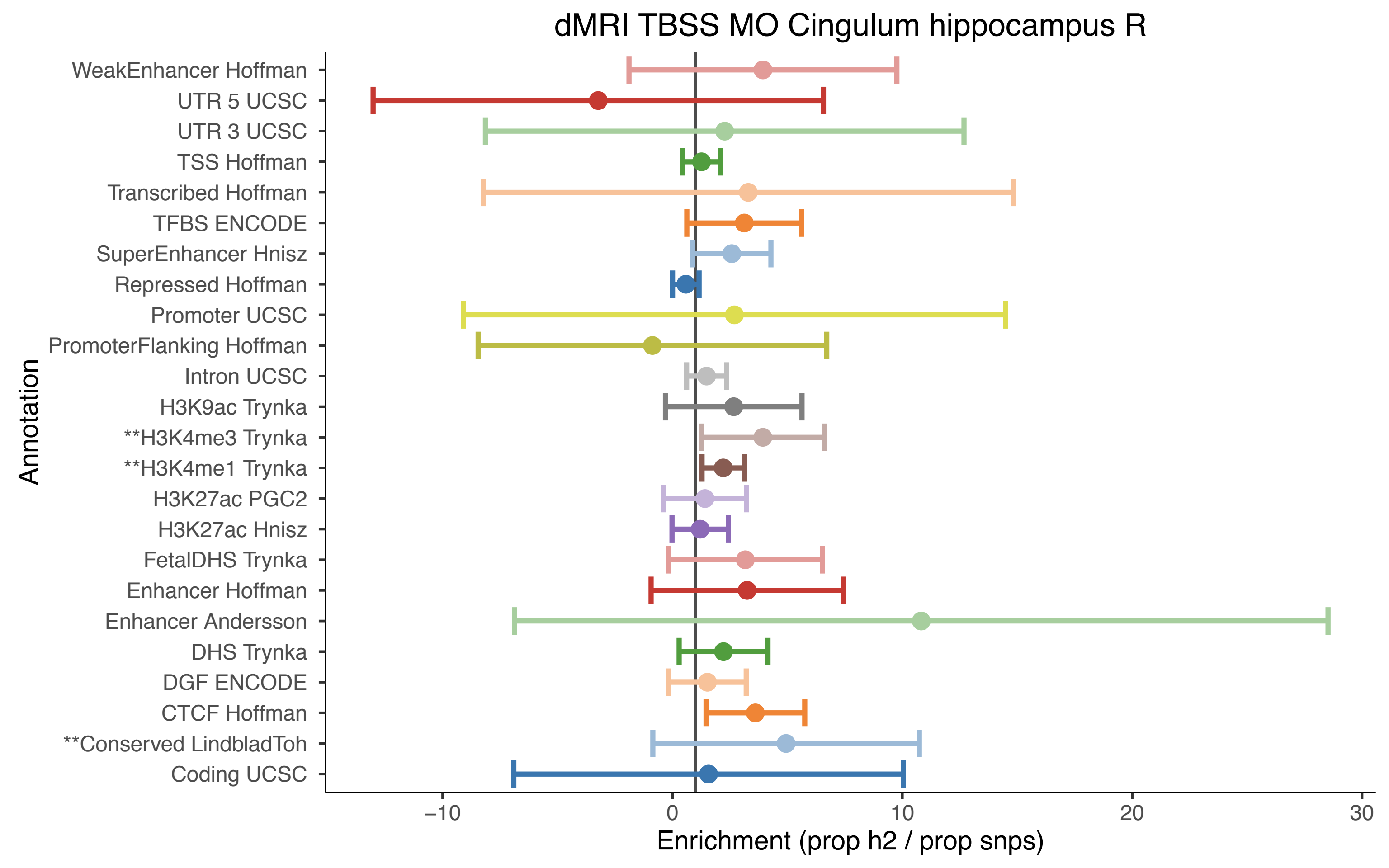
dMRI TBSS MO Cingulum hippocampus R
WeakEnhancer Hoffman
UTR 5 UCSC
UTR 3 UCSC
TSS Hoffman
Transcribed Hoffman
TFBS ENCODE
SuperEnhancer Hnisz
Repressed Hoffman
Promoter UCSC
PromoterFlanking Hoffman
Intron UCSC
H3K9ac Trynka
**H3K4me3 Trynka
**H3K4me1 Trynka
H3K27ac PGC2
H3K27ac Hnisz
FetalDHS Trynka
Enhancer Hoffman
Enhancer Andersson
DHS Trynka
DGF ENCODE
CTCF Hoffman
**Conserved LindbladToh
Coding UCSC
Annotation
−10
0
10
20
30
Enrichment (prop h2 / prop snps)

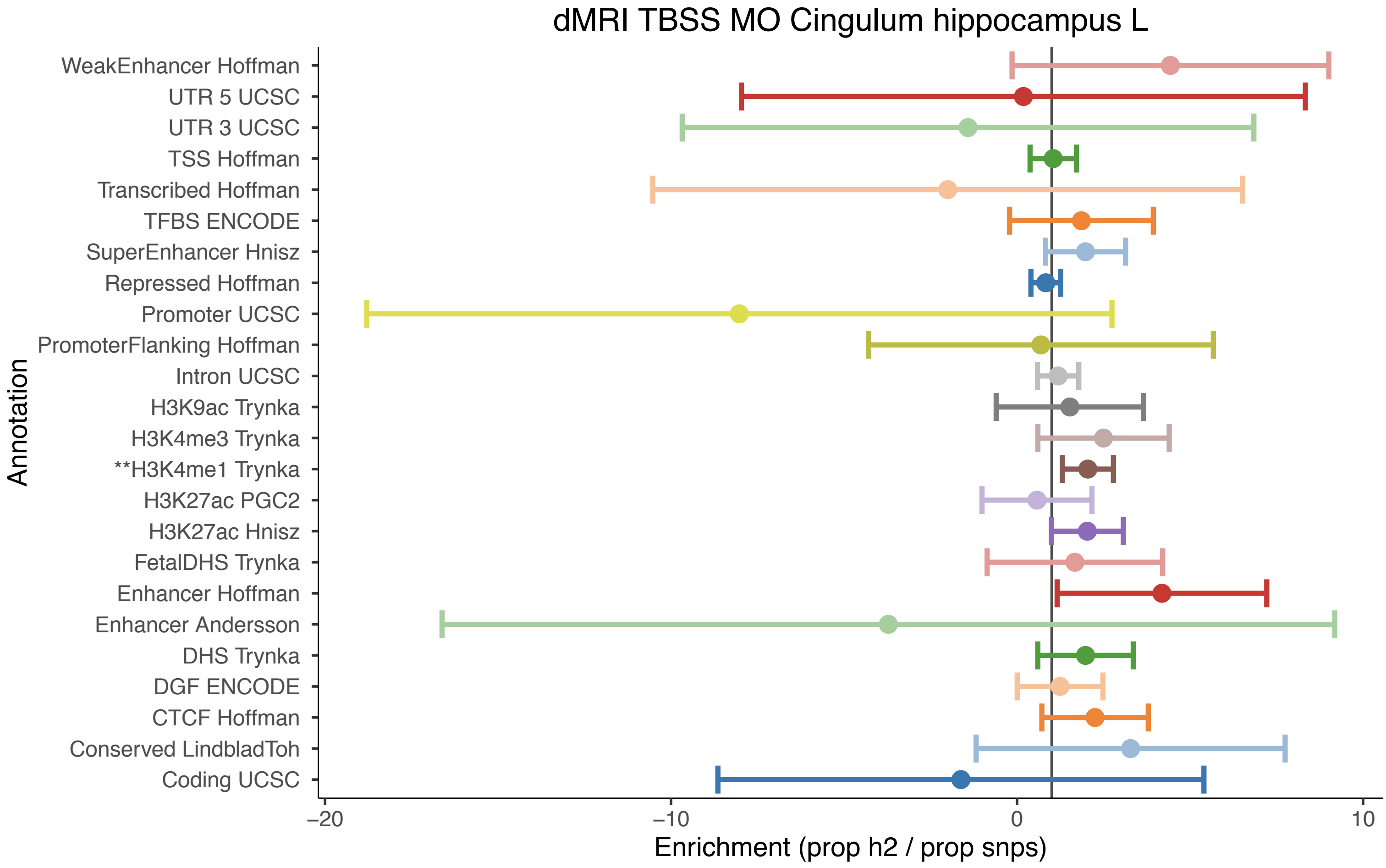

dMRI TBSS MO Cingulum hippocampus L
WeakEnhancer Hoffman
UTR 5 UCSC
UTR 3 UCSC
TSS Hoffman
Transcribed Hoffman
TFBS ENCODE
SuperEnhancer Hnisz
Repressed Hoffman
Promoter UCSC
PromoterFlanking Hoffman
Intron UCSC
H3K9ac Trynka
H3K4me3 Trynka
**H3K4me1 Trynka
H3K27ac PGC2
H3K27ac Hnisz
FetalDHS Trynka
Enhancer Hoffman
Enhancer Andersson
DHS Trynka
DGF ENCODE
CTCF Hoffman
Conserved LindbladToh
Coding UCSC
Annotation
−20
−10
0
10
Enrichment (prop h2 / prop snps)

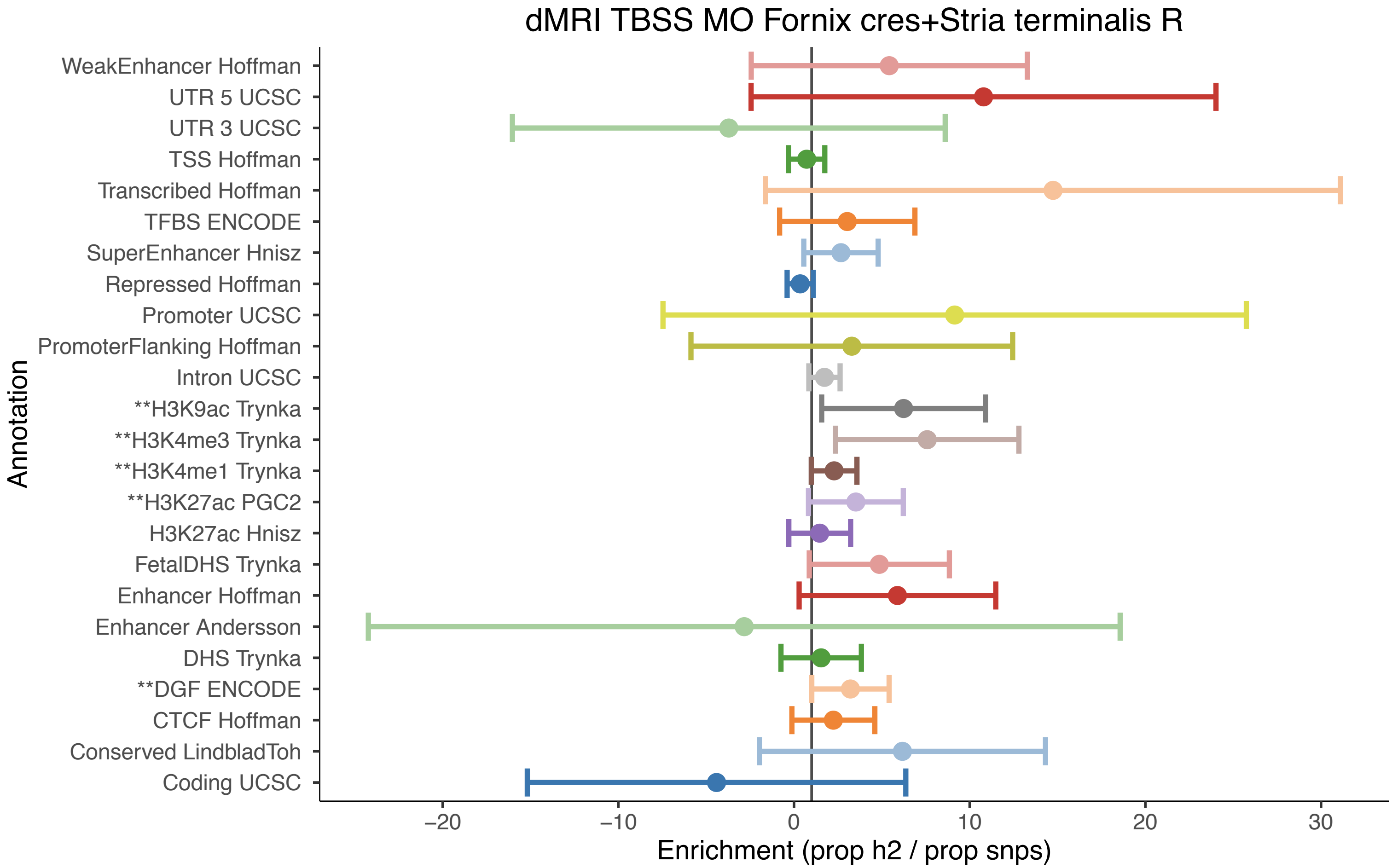
dMRI TBSS MO Fornix cres+Stria terminalis R
WeakEnhancer Hoffman
UTR 5 UCSC
UTR 3 UCSC
TSS Hoffman
Transcribed Hoffman
TFBS ENCODE
SuperEnhancer Hnisz
Repressed Hoffman
Promoter UCSC
PromoterFlanking Hoffman
Intron UCSC
**H3K9ac Trynka
**H3K4me3 Trynka
**H3K4me1 Trynka
**H3K27ac PGC2
H3K27ac Hnisz
FetalDHS Trynka
Enhancer Hoffman
Enhancer Andersson
DHS Trynka
**DGF ENCODE
CTCF Hoffman
Conserved LindbladToh
Coding UCSC
Annotation
−20
−10
0
10
20
30
Enrichment (prop h2 / prop snps)

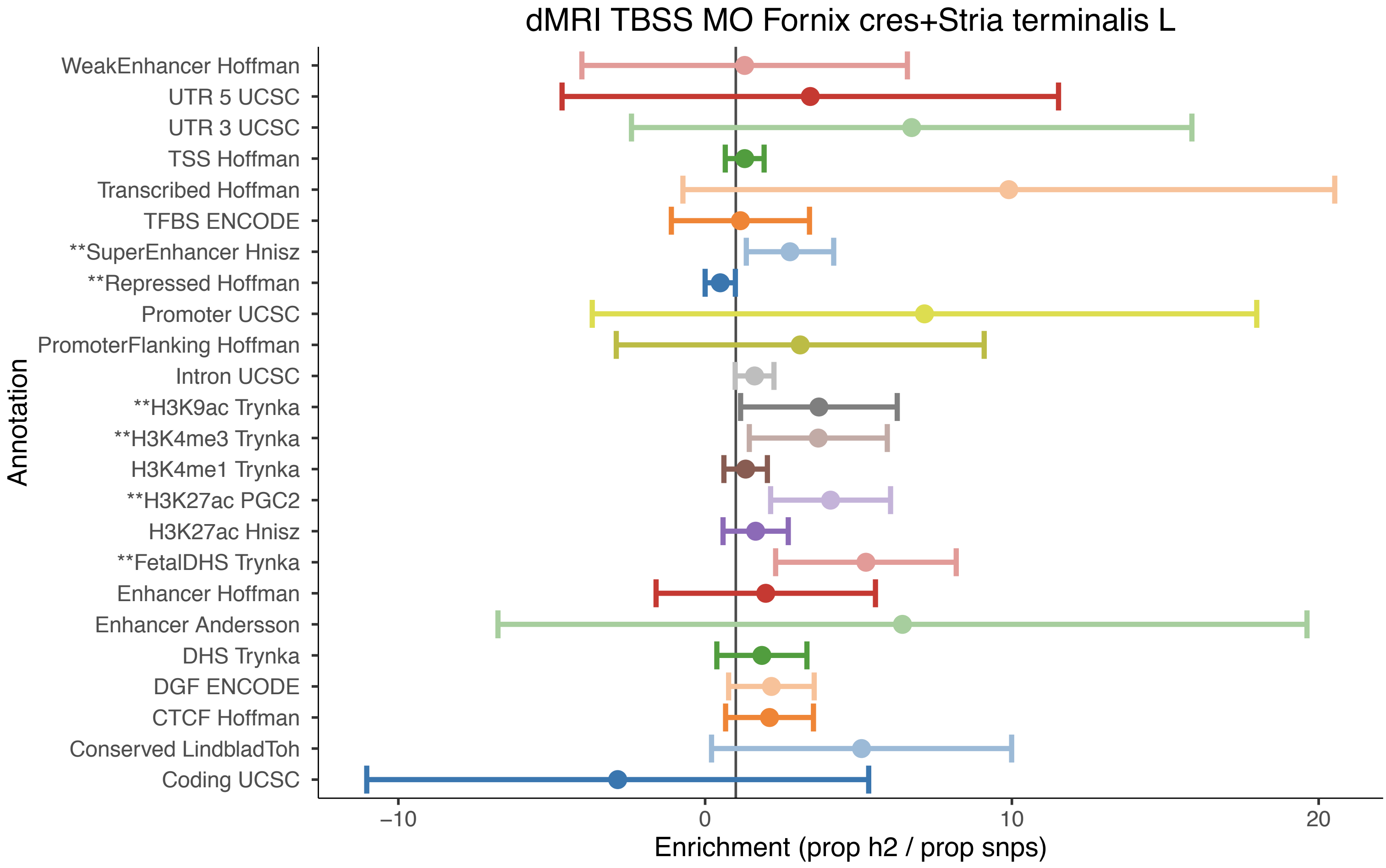
dMRI TBSS MO Fornix cres+Stria terminalis L
WeakEnhancer Hoffman
UTR 5 UCSC
UTR 3 UCSC
TSS Hoffman
Transcribed Hoffman
TFBS ENCODE
**SuperEnhancer Hnisz
**Repressed Hoffman
Promoter UCSC
PromoterFlanking Hoffman
Intron UCSC
**H3K9ac Trynka
**H3K4me3 Trynka
H3K4me1 Trynka
**H3K27ac PGC2
H3K27ac Hnisz
**FetalDHS Trynka
Enhancer Hoffman
Enhancer Andersson
DHS Trynka
DGF ENCODE
CTCF Hoffman
Conserved LindbladToh
Coding UCSC
Annotation
−10
0
10
20
Enrichment (prop h2 / prop snps)

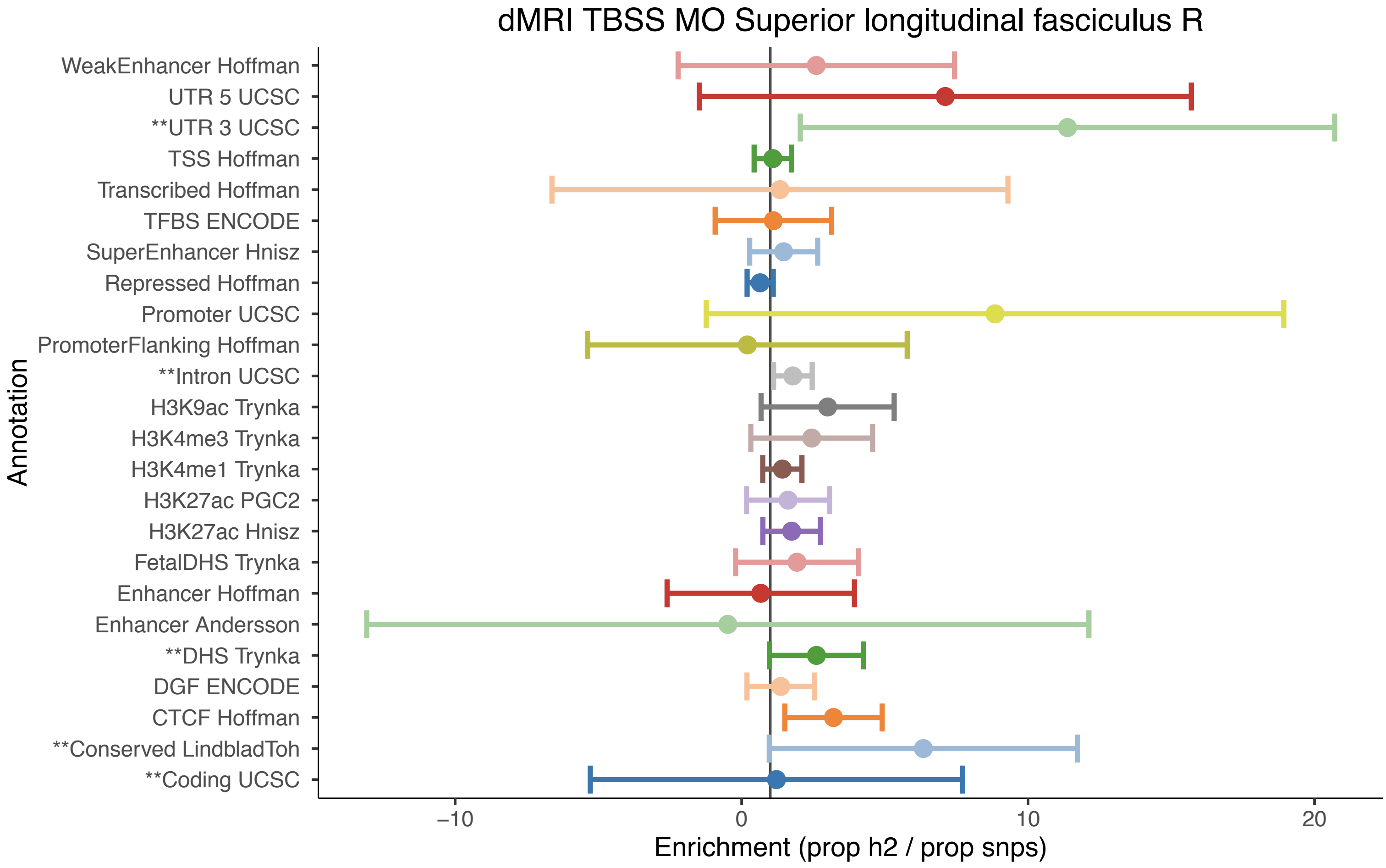

dMRI TBSS MO Superior longitudinal fasciculus R
WeakEnhancer Hoffman
UTR 5 UCSC
**UTR 3 UCSC
TSS Hoffman
Transcribed Hoffman
TFBS ENCODE
SuperEnhancer Hnisz
Repressed Hoffman
Promoter UCSC
PromoterFlanking Hoffman
**Intron UCSC
H3K9ac Trynka
H3K4me3 Trynka
H3K4me1 Trynka
H3K27ac PGC2
H3K27ac Hnisz
FetalDHS Trynka
Enhancer Hoffman
Enhancer Andersson
**DHS Trynka
DGF ENCODE
CTCF Hoffman
**Conserved LindbladToh
**Coding UCSC
Annotation
−10
0
10
20
Enrichment (prop h2 / prop snps)

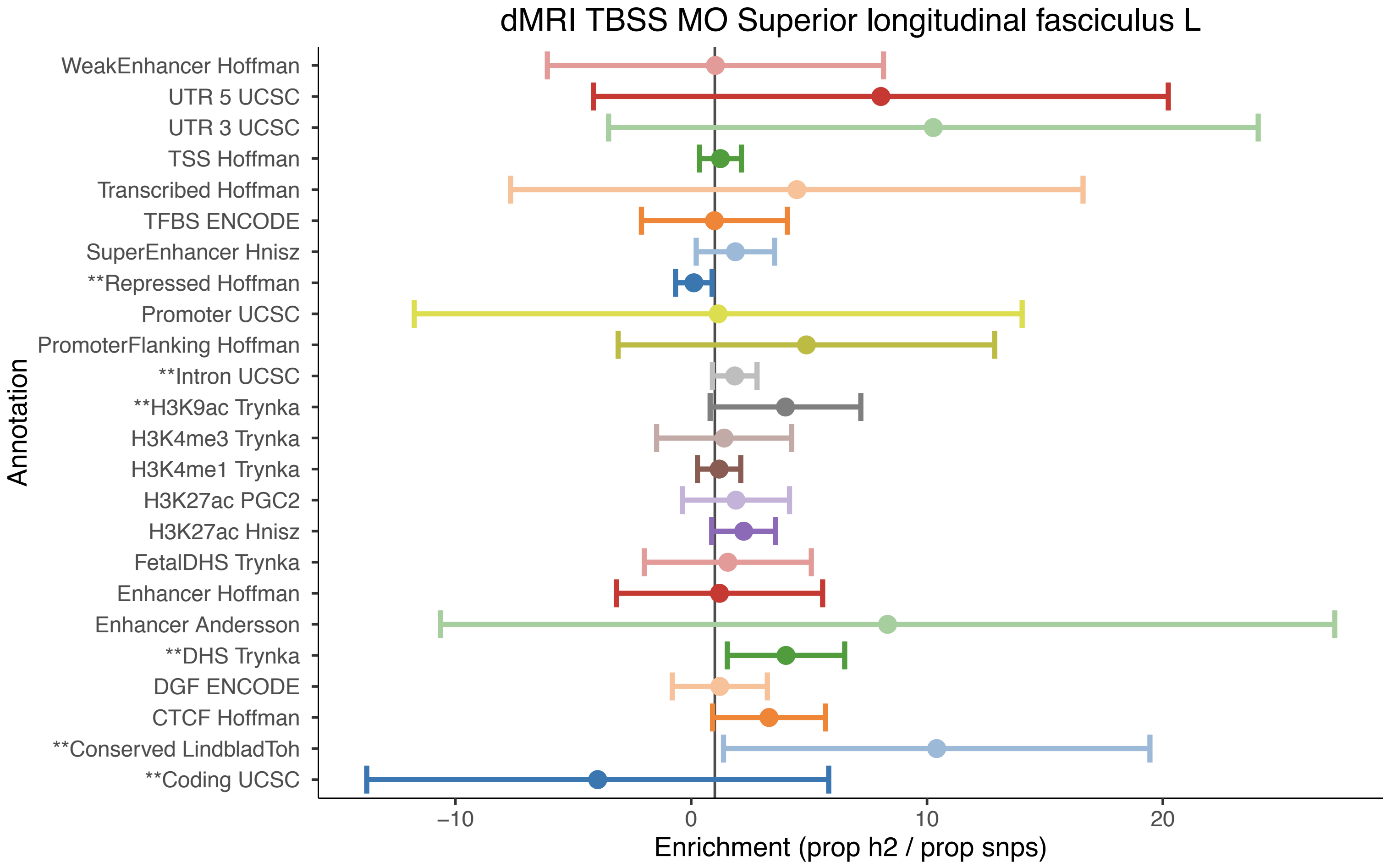
dMRI TBSS MO Superior longitudinal fasciculus L
WeakEnhancer Hoffman
UTR 5 UCSC
UTR 3 UCSC
TSS Hoffman
Transcribed Hoffman
TFBS ENCODE
SuperEnhancer Hnisz
**Repressed Hoffman
Promoter UCSC
PromoterFlanking Hoffman
**Intron UCSC
**H3K9ac Trynka
H3K4me3 Trynka
H3K4me1 Trynka
H3K27ac PGC2
H3K27ac Hnisz
FetalDHS Trynka
Enhancer Hoffman
Enhancer Andersson
**DHS Trynka
DGF ENCODE
CTCF Hoffman
**Conserved LindbladToh
**Coding UCSC
Annotation
−10
0
10
20
Enrichment (prop h2 / prop snps)

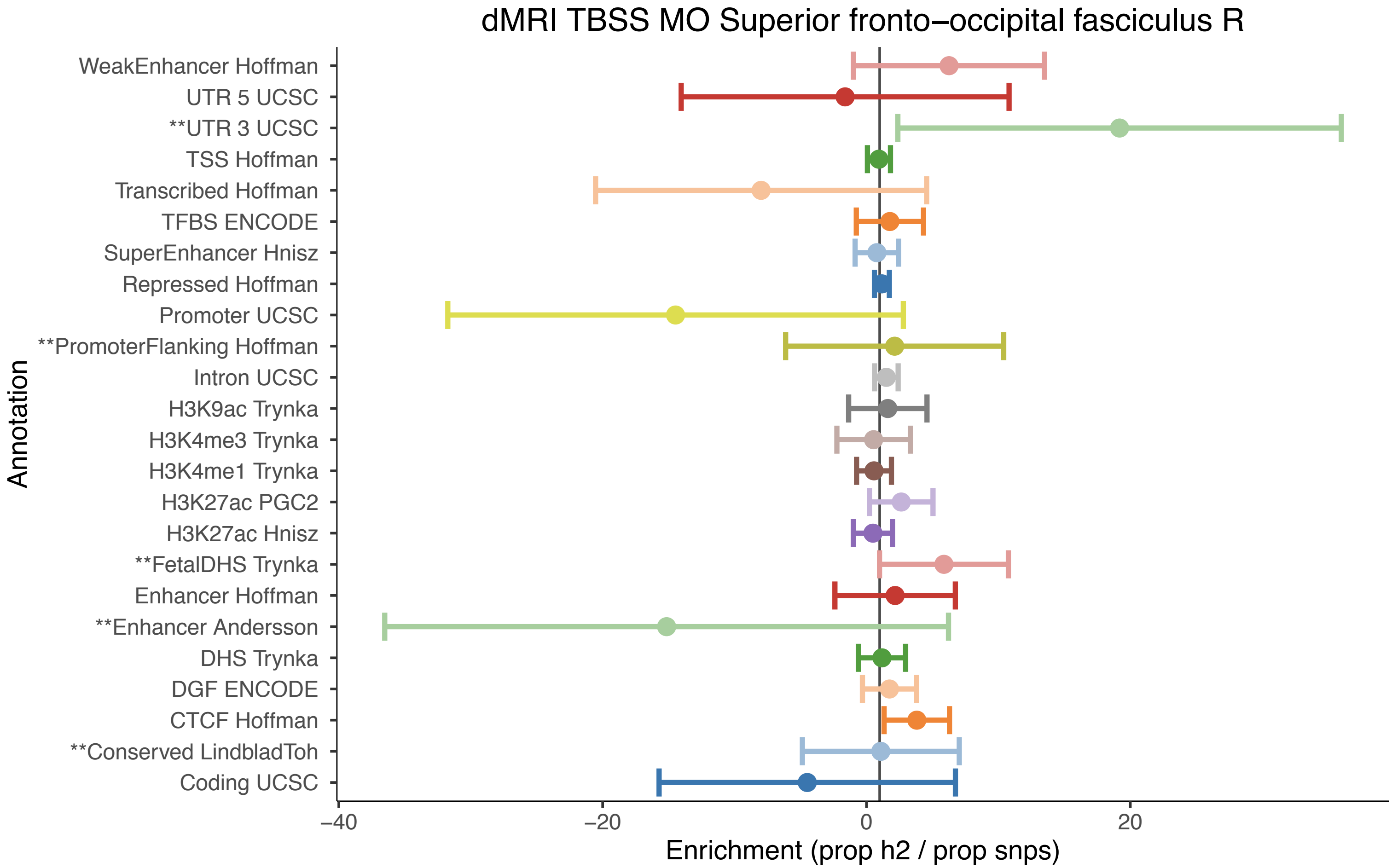

dMRI TBSS MO Superior fronto−occipital fasciculus R
WeakEnhancer Hoffman
UTR 5 UCSC
**UTR 3 UCSC
TSS Hoffman
Transcribed Hoffman
TFBS ENCODE
SuperEnhancer Hnisz
Repressed Hoffman
Promoter UCSC
**PromoterFlanking Hoffman
Intron UCSC
H3K9ac Trynka
H3K4me3 Trynka
H3K4me1 Trynka
H3K27ac PGC2
H3K27ac Hnisz
**FetalDHS Trynka
Enhancer Hoffman
**Enhancer Andersson
DHS Trynka
DGF ENCODE
CTCF Hoffman
**Conserved LindbladToh
Coding UCSC
Annotation
−40
−20
0
20
Enrichment (prop h2 / prop snps)

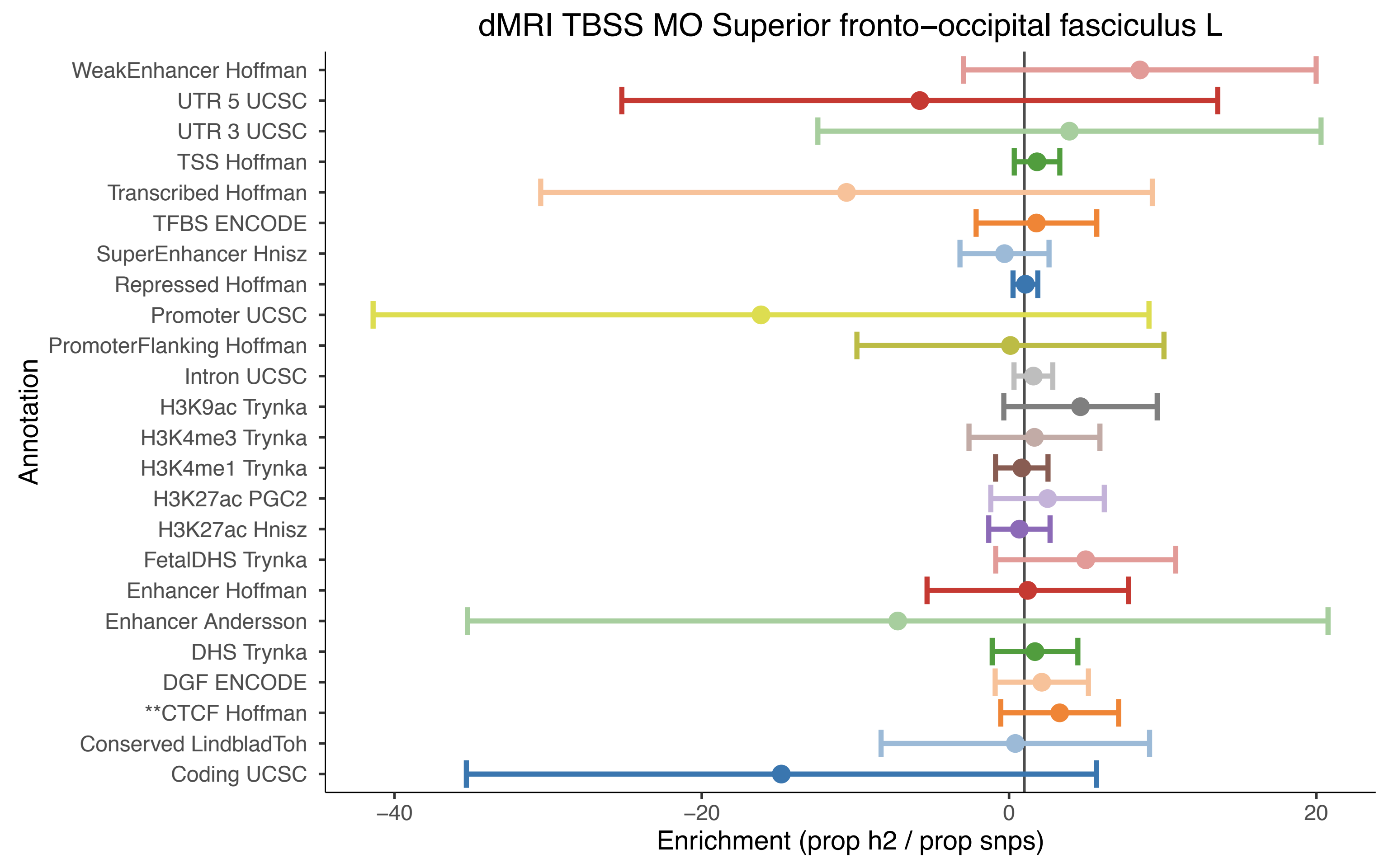

dMRI TBSS MO Superior fronto−occipital fasciculus L
Annotation
WeakEnhancer Hoffman
UTR 5 UCSC
UTR 3 UCSC
TSS Hoffman
Transcribed Hoffman
TFBS ENCODE
SuperEnhancer Hnisz
Repressed Hoffman
Promoter UCSC
PromoterFlanking Hoffman
Intron UCSC
H3K9ac Trynka
H3K4me3 Trynka
H3K4me1 Trynka
H3K27ac PGC2
H3K27ac Hnisz
FetalDHS Trynka
Enhancer Hoffman
Enhancer Andersson
DHS Trynka
DGF ENCODE
**CTCF Hoffman
Conserved LindbladToh
Coding UCSC
−40
−20
0
20
Enrichment (prop h2 / prop snps)

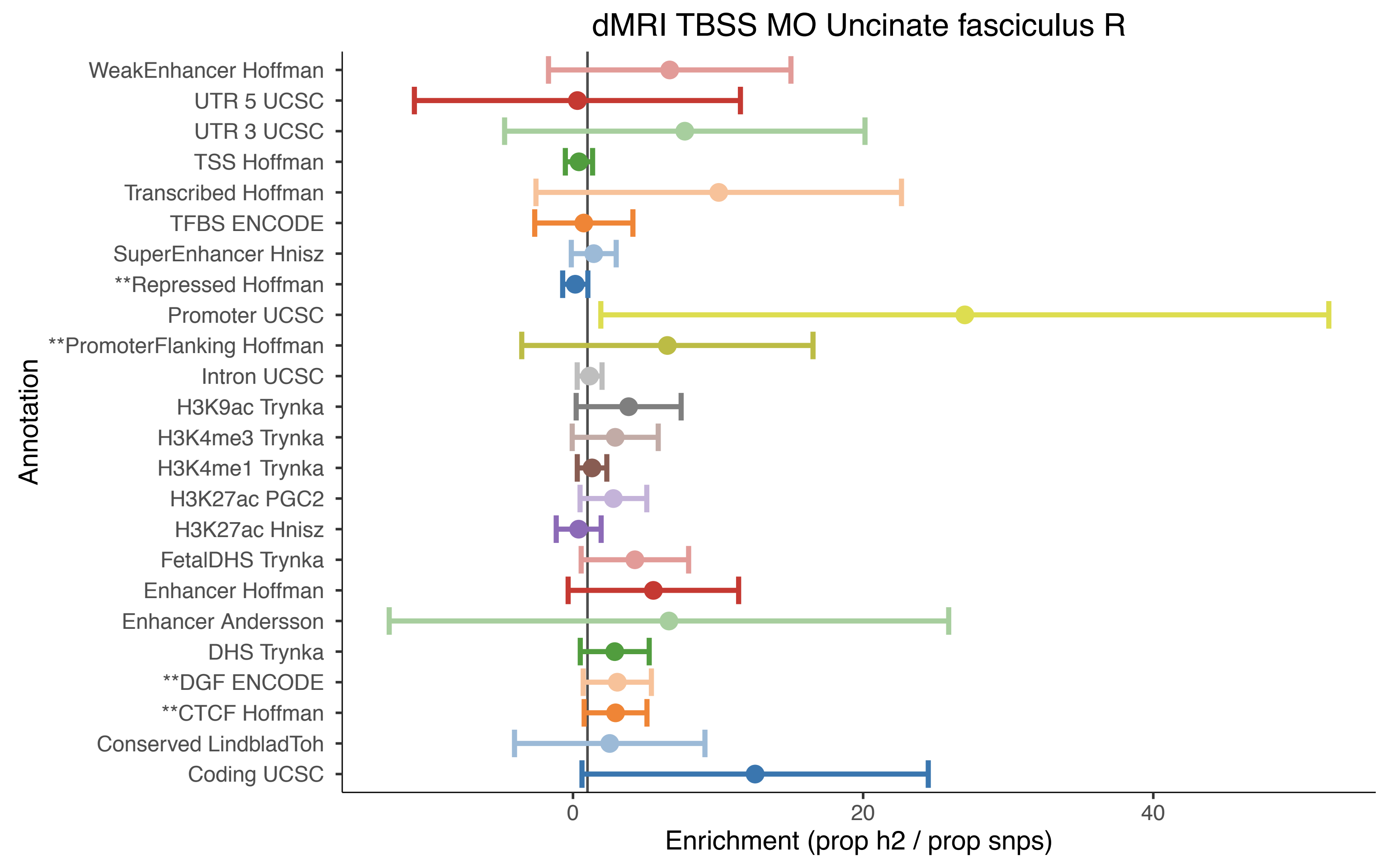

dMRI TBSS MO Uncinate fasciculus R
Annotation
WeakEnhancer Hoffman
UTR 5 UCSC
UTR 3 UCSC
TSS Hoffman
Transcribed Hoffman
TFBS ENCODE
SuperEnhancer Hnisz
**Repressed Hoffman
Promoter UCSC
**PromoterFlanking Hoffman
Intron UCSC
H3K9ac Trynka
H3K4me3 Trynka
H3K4me1 Trynka
H3K27ac PGC2
H3K27ac Hnisz
FetalDHS Trynka
Enhancer Hoffman
Enhancer Andersson
DHS Trynka
**DGF ENCODE
**CTCF Hoffman
Conserved LindbladToh
Coding UCSC
0
20
40
Enrichment (prop h2 / prop snps)

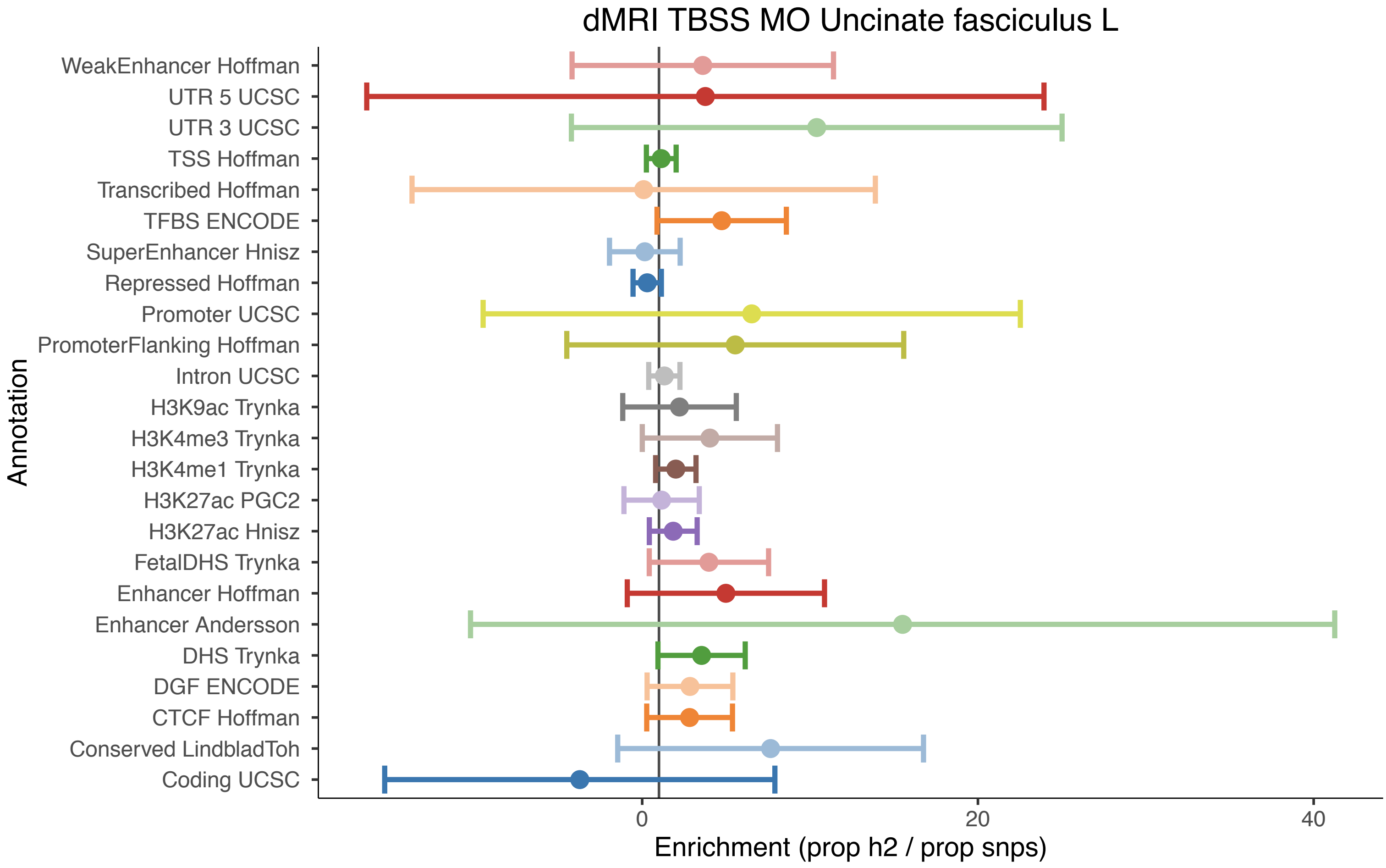

dMRI TBSS MO Uncinate fasciculus L
Annotation
WeakEnhancer Hoffman
UTR 5 UCSC
UTR 3 UCSC
TSS Hoffman
Transcribed Hoffman
TFBS ENCODE
SuperEnhancer Hnisz
Repressed Hoffman
Promoter UCSC
PromoterFlanking Hoffman
Intron UCSC
H3K9ac Trynka
H3K4me3 Trynka
H3K4me1 Trynka
H3K27ac PGC2
H3K27ac Hnisz
FetalDHS Trynka
Enhancer Hoffman
Enhancer Andersson
DHS Trynka
DGF ENCODE
CTCF Hoffman
Conserved LindbladToh
Coding UCSC
0
20
40
Enrichment (prop h2 / prop snps)

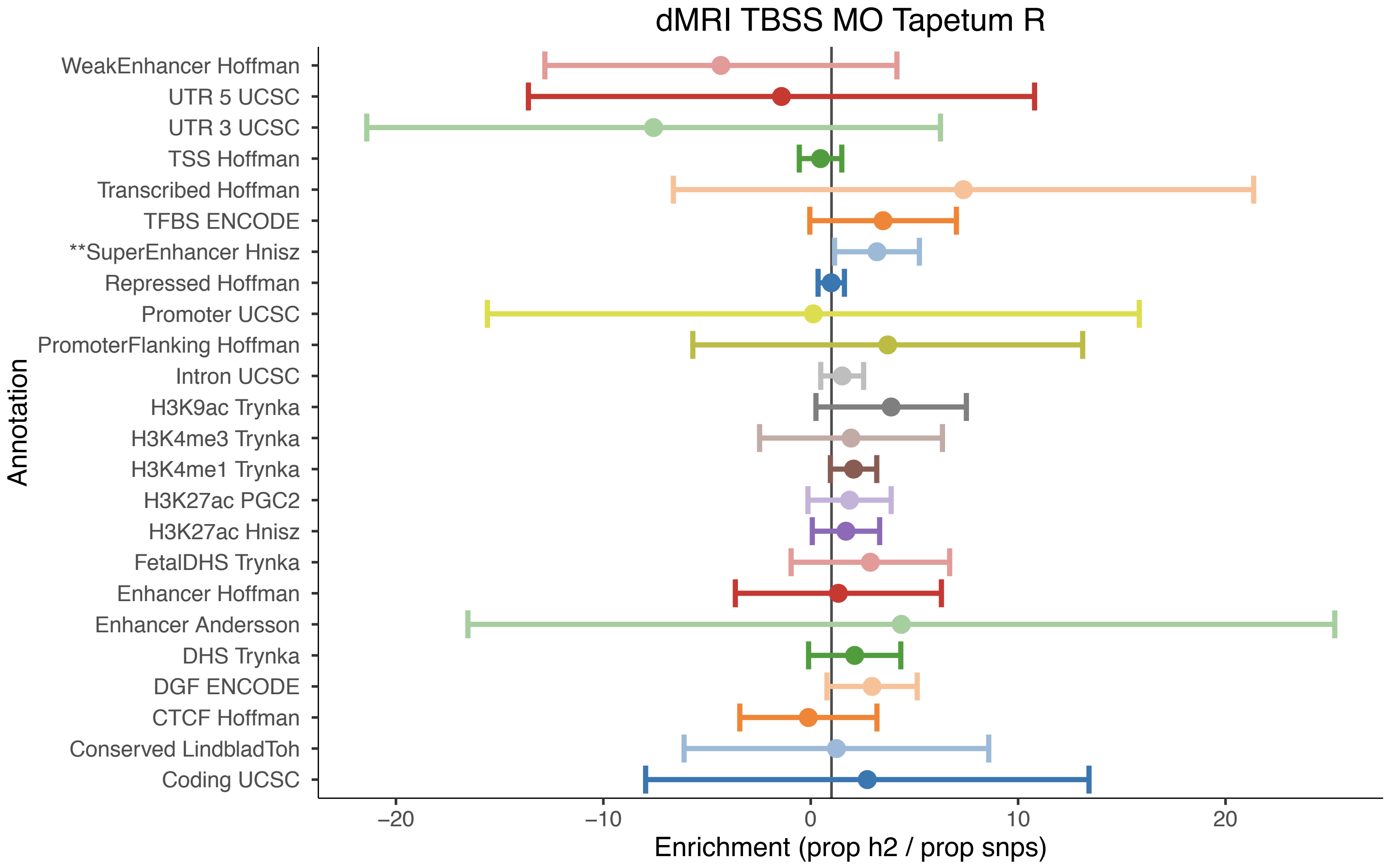

dMRI TBSS MO Tapetum R
WeakEnhancer Hoffman
UTR 5 UCSC
UTR 3 UCSC
TSS Hoffman
Transcribed Hoffman
TFBS ENCODE
**SuperEnhancer Hnisz
Repressed Hoffman
Promoter UCSC
PromoterFlanking Hoffman
Intron UCSC
H3K9ac Trynka
H3K4me3 Trynka
H3K4me1 Trynka
H3K27ac PGC2
H3K27ac Hnisz
FetalDHS Trynka
Enhancer Hoffman
Enhancer Andersson
DHS Trynka
DGF ENCODE
CTCF Hoffman
Conserved LindbladToh
Coding UCSC
Annotation
−20
−10
0
10
20
Enrichment (prop h2 / prop snps)

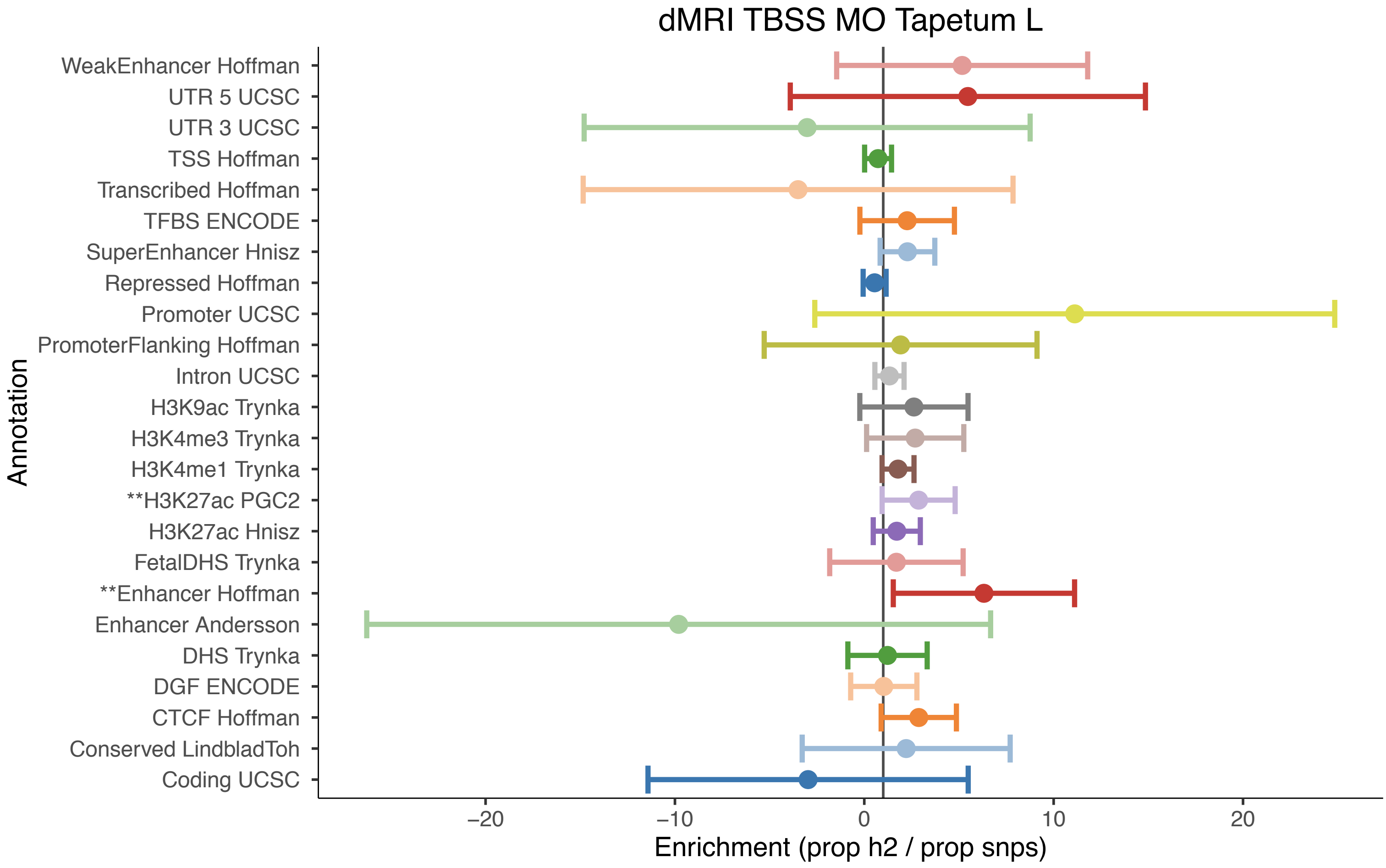

dMRI TBSS MO Tapetum L
WeakEnhancer Hoffman
UTR 5 UCSC
UTR 3 UCSC
TSS Hoffman
Transcribed Hoffman
TFBS ENCODE
SuperEnhancer Hnisz
Repressed Hoffman
Promoter UCSC
PromoterFlanking Hoffman
Intron UCSC
H3K9ac Trynka
H3K4me3 Trynka
H3K4me1 Trynka
**H3K27ac PGC2
H3K27ac Hnisz
FetalDHS Trynka
**Enhancer Hoffman
Enhancer Andersson
DHS Trynka
DGF ENCODE
CTCF Hoffman
Conserved LindbladToh
Coding UCSC
Annotation
−20
−10
0
10
20
Enrichment (prop h2 / prop snps)

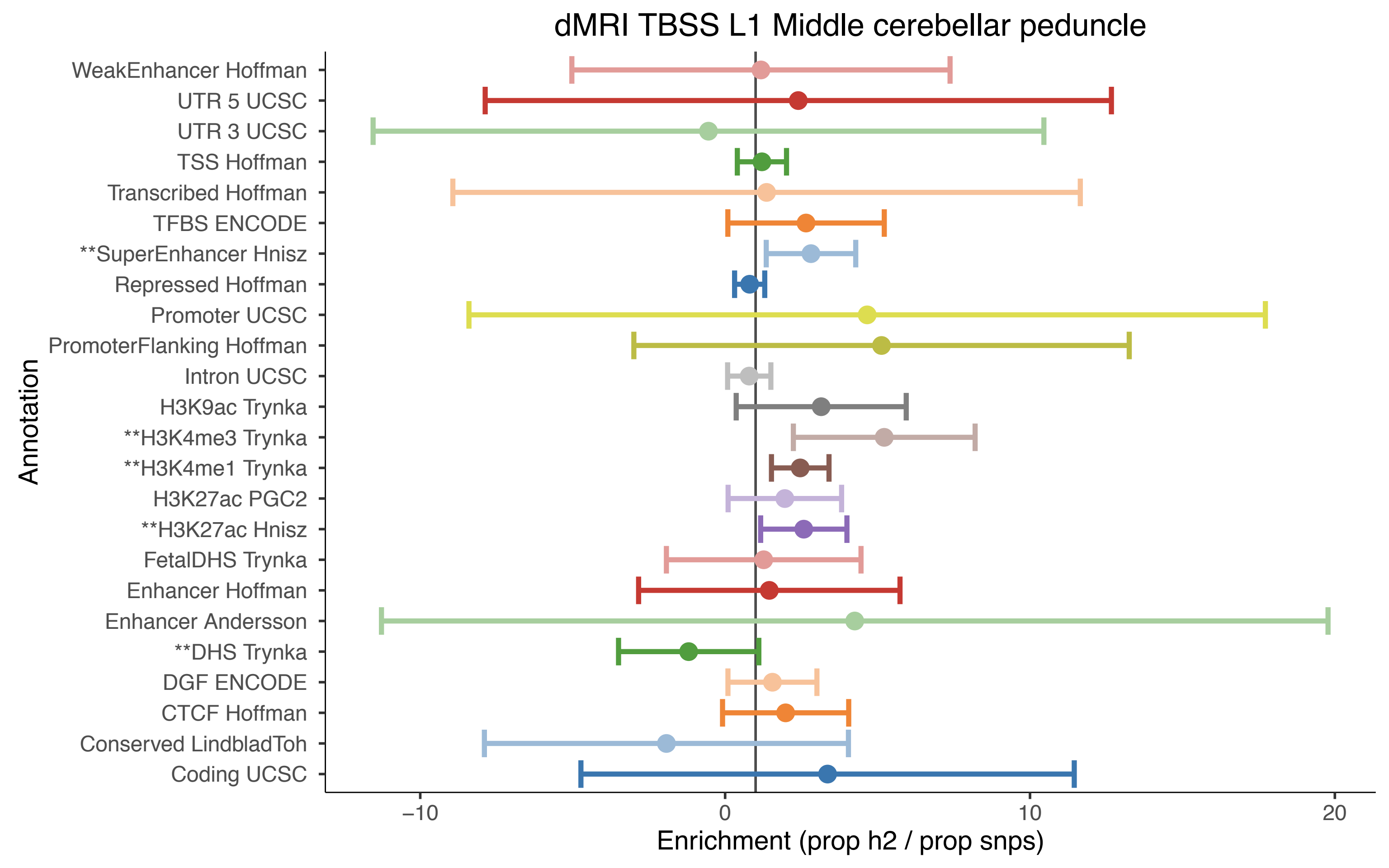

dMRI TBSS L1 Middle cerebellar peduncle
WeakEnhancer Hoffman
UTR 5 UCSC
UTR 3 UCSC
TSS Hoffman
Transcribed Hoffman
TFBS ENCODE
**SuperEnhancer Hnisz
Repressed Hoffman
Promoter UCSC
PromoterFlanking Hoffman
Intron UCSC
H3K9ac Trynka
**H3K4me3 Trynka
**H3K4me1 Trynka
H3K27ac PGC2
**H3K27ac Hnisz
FetalDHS Trynka
Enhancer Hoffman
Enhancer Andersson
**DHS Trynka
DGF ENCODE
CTCF Hoffman
Conserved LindbladToh
Coding UCSC
Annotation
−10
0
10
20
Enrichment (prop h2 / prop snps)

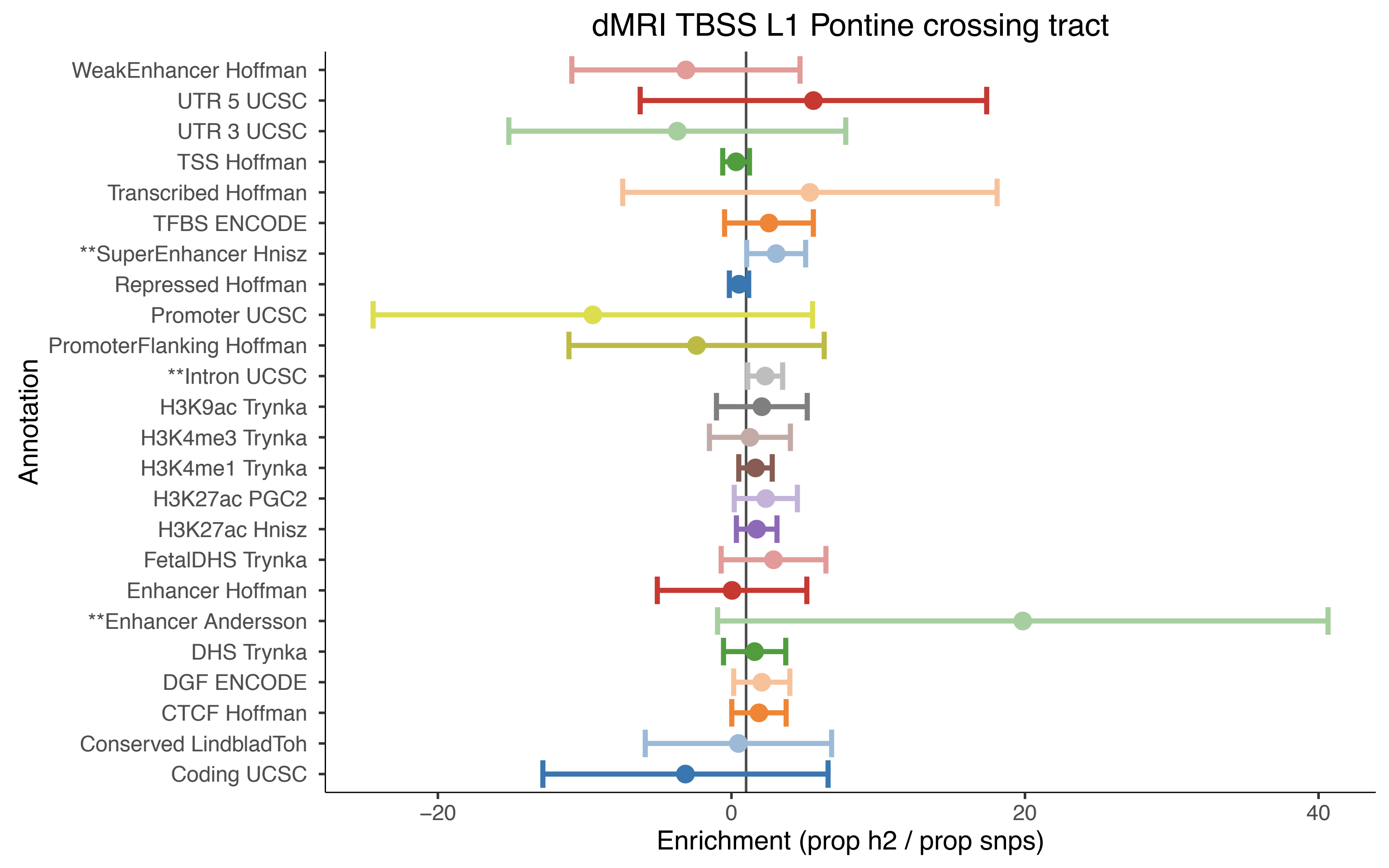

dMRI TBSS L1 Pontine crossing tract
WeakEnhancer Hoffman
UTR 5 UCSC
UTR 3 UCSC
TSS Hoffman
Transcribed Hoffman
TFBS ENCODE
**SuperEnhancer Hnisz
Repressed Hoffman
Promoter UCSC
PromoterFlanking Hoffman
**Intron UCSC
H3K9ac Trynka
H3K4me3 Trynka
H3K4me1 Trynka
H3K27ac PGC2
H3K27ac Hnisz
FetalDHS Trynka
Enhancer Hoffman
**Enhancer Andersson
DHS Trynka
DGF ENCODE
CTCF Hoffman
Conserved LindbladToh
Coding UCSC
Annotation
−20
0
20
40
Enrichment (prop h2 / prop snps)

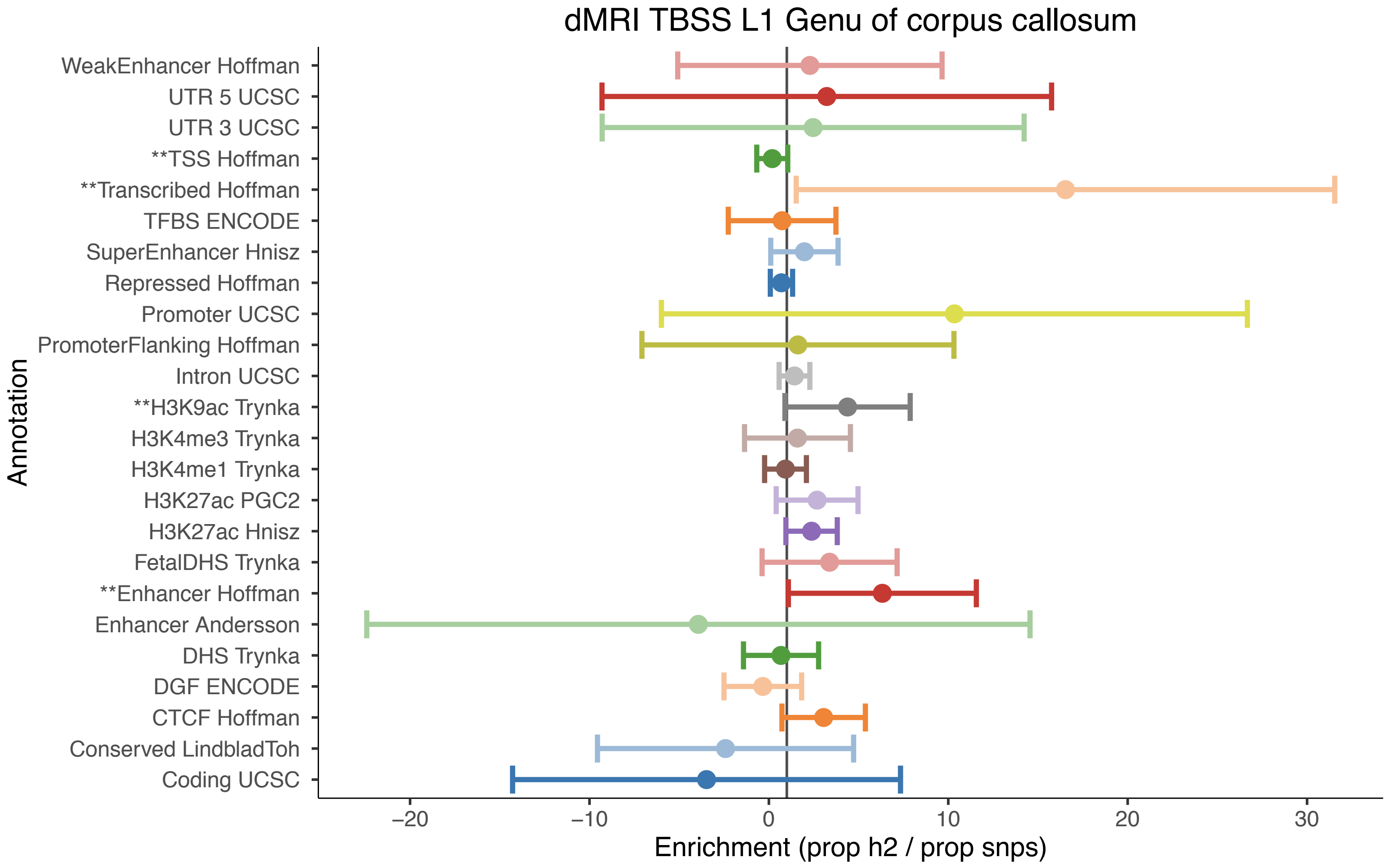
dMRI TBSS L1 Genu of corpus callosum
WeakEnhancer Hoffman
UTR 5 UCSC
UTR 3 UCSC
**TSS Hoffman
**Transcribed Hoffman
TFBS ENCODE
SuperEnhancer Hnisz
Repressed Hoffman
Promoter UCSC
PromoterFlanking Hoffman
Intron UCSC
**H3K9ac Trynka
H3K4me3 Trynka
H3K4me1 Trynka
H3K27ac PGC2
H3K27ac Hnisz
FetalDHS Trynka
**Enhancer Hoffman
Enhancer Andersson
DHS Trynka
DGF ENCODE
CTCF Hoffman
Conserved LindbladToh
Coding UCSC
Annotation
−20
−10
0
10
20
30
Enrichment (prop h2 / prop snps)

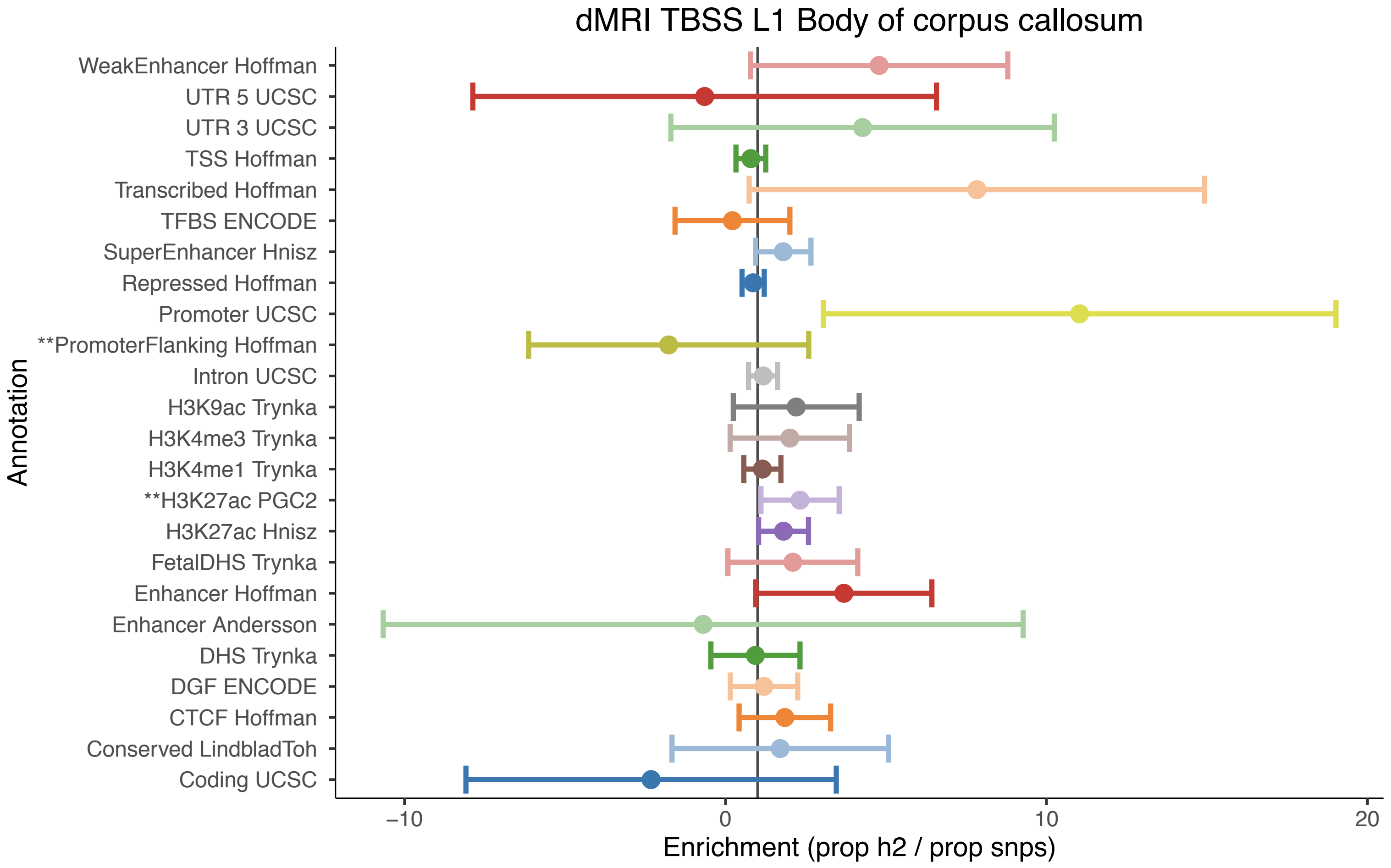

dMRI TBSS L1 Body of corpus callosum
WeakEnhancer Hoffman
UTR 5 UCSC
UTR 3 UCSC
TSS Hoffman
Transcribed Hoffman
TFBS ENCODE
SuperEnhancer Hnisz
Repressed Hoffman
Promoter UCSC
**PromoterFlanking Hoffman
Intron UCSC
H3K9ac Trynka
H3K4me3 Trynka
H3K4me1 Trynka
**H3K27ac PGC2
H3K27ac Hnisz
FetalDHS Trynka
Enhancer Hoffman
Enhancer Andersson
DHS Trynka
DGF ENCODE
CTCF Hoffman
Conserved LindbladToh
Coding UCSC
Annotation
−10
0
10
20
Enrichment (prop h2 / prop snps)

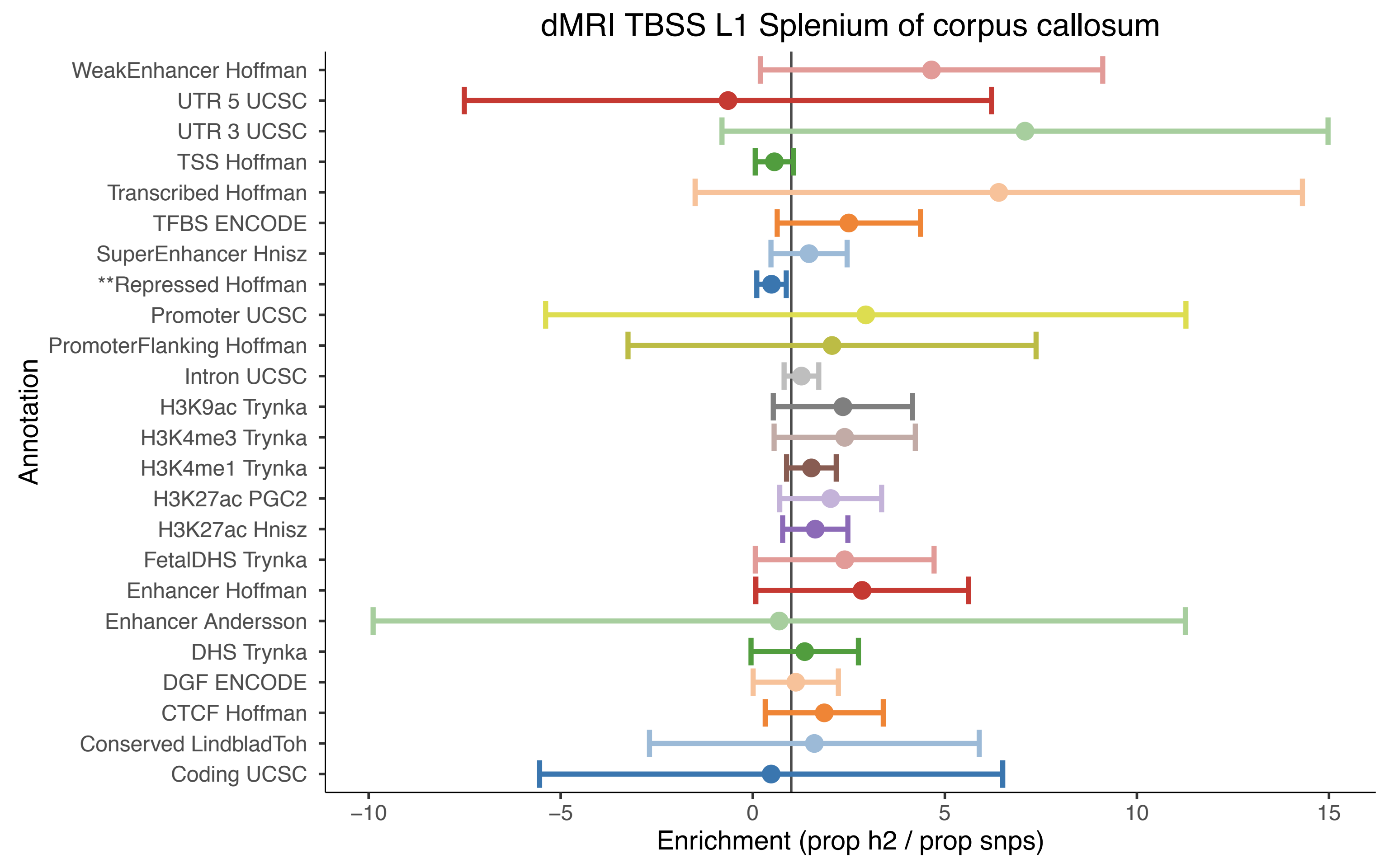

dMRI TBSS L1 Splenium of corpus callosum
WeakEnhancer Hoffman
UTR 5 UCSC
UTR 3 UCSC
TSS Hoffman
Transcribed Hoffman
TFBS ENCODE
SuperEnhancer Hnisz
**Repressed Hoffman
Promoter UCSC
PromoterFlanking Hoffman
Intron UCSC
H3K9ac Trynka
H3K4me3 Trynka
H3K4me1 Trynka
H3K27ac PGC2
H3K27ac Hnisz
FetalDHS Trynka
Enhancer Hoffman
Enhancer Andersson
DHS Trynka
DGF ENCODE
CTCF Hoffman
Conserved LindbladToh
Coding UCSC
Annotation
−10
−5
0
5
10
15
Enrichment (prop h2 / prop snps)

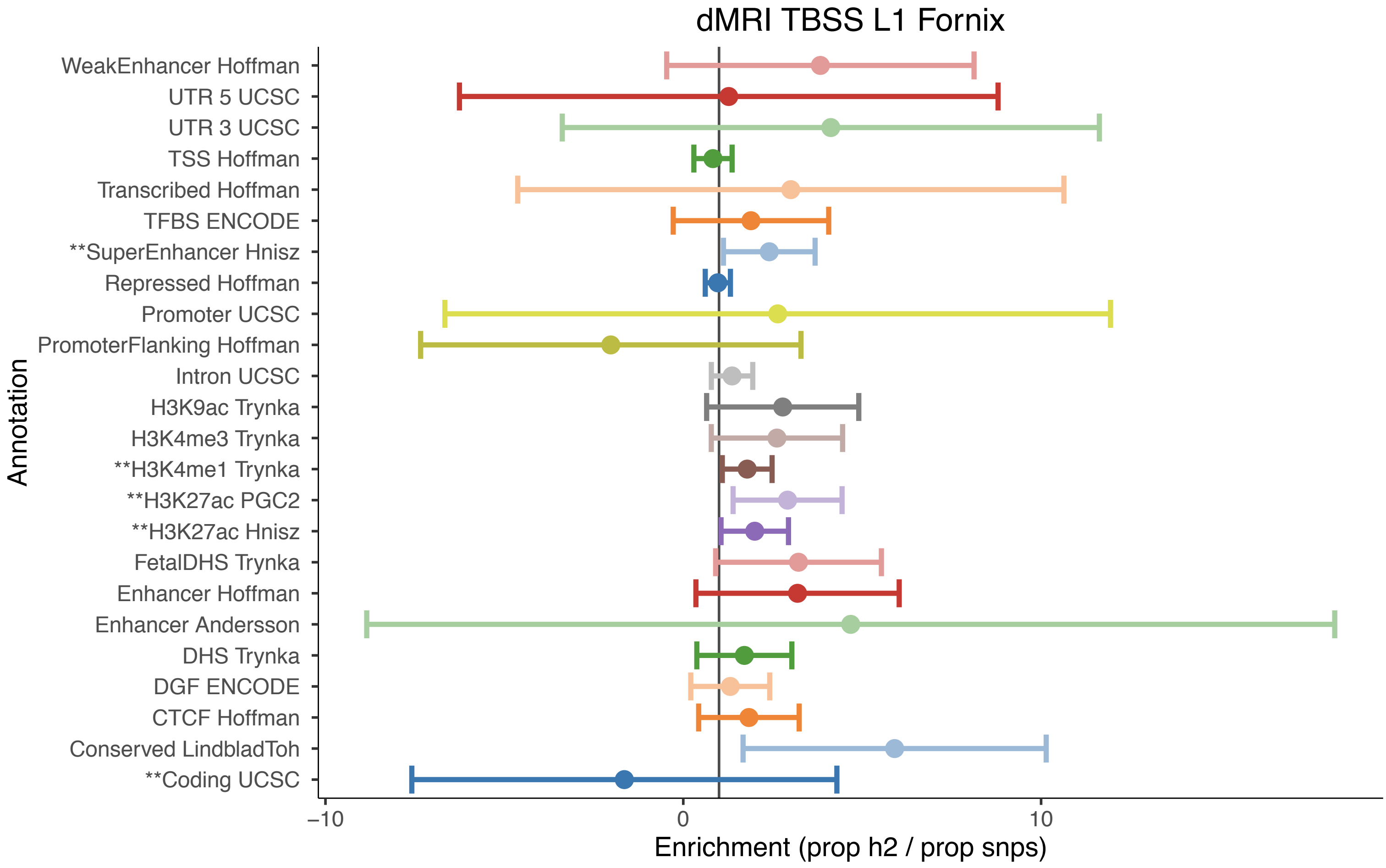

dMRI TBSS L1 Fornix
Annotation
WeakEnhancer Hoffman
UTR 5 UCSC
UTR 3 UCSC
TSS Hoffman
Transcribed Hoffman
TFBS ENCODE
**SuperEnhancer Hnisz
Repressed Hoffman
Promoter UCSC
PromoterFlanking Hoffman
Intron UCSC
H3K9ac Trynka
H3K4me3 Trynka
**H3K4me1 Trynka
**H3K27ac PGC2
**H3K27ac Hnisz
FetalDHS Trynka
Enhancer Hoffman
Enhancer Andersson
DHS Trynka
DGF ENCODE
CTCF Hoffman
Conserved LindbladToh
**Coding UCSC
−10
0
10
Enrichment (prop h2 / prop snps)

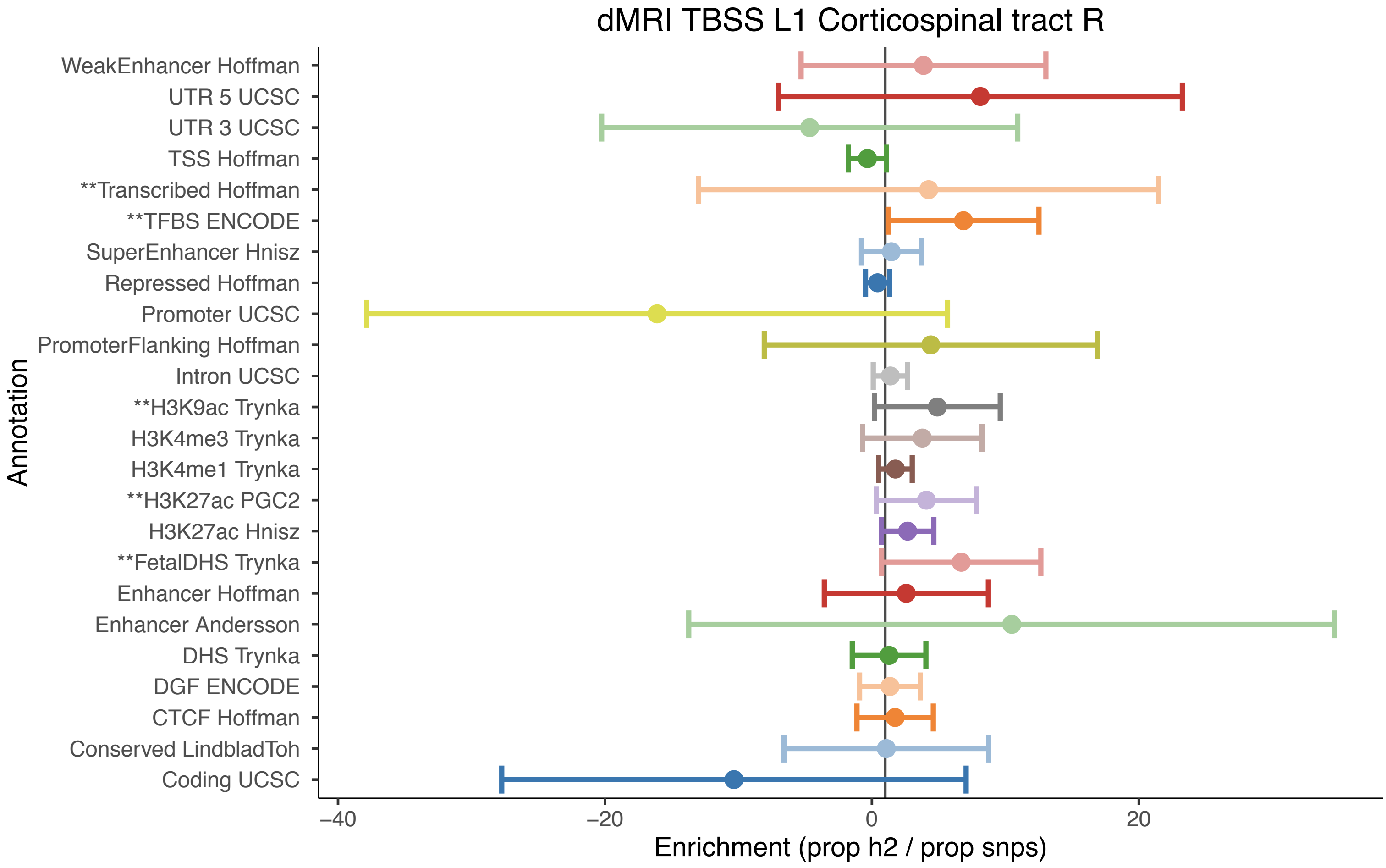

dMRI TBSS L1 Corticospinal tract R
WeakEnhancer Hoffman
UTR 5 UCSC
UTR 3 UCSC
TSS Hoffman
**Transcribed Hoffman
**TFBS ENCODE
SuperEnhancer Hnisz
Repressed Hoffman
Promoter UCSC
PromoterFlanking Hoffman
Intron UCSC
**H3K9ac Trynka
H3K4me3 Trynka
H3K4me1 Trynka
**H3K27ac PGC2
H3K27ac Hnisz
**FetalDHS Trynka
Enhancer Hoffman
Enhancer Andersson
DHS Trynka
DGF ENCODE
CTCF Hoffman
Conserved LindbladToh
Coding UCSC
Annotation
−40
−20
0
20
Enrichment (prop h2 / prop snps)

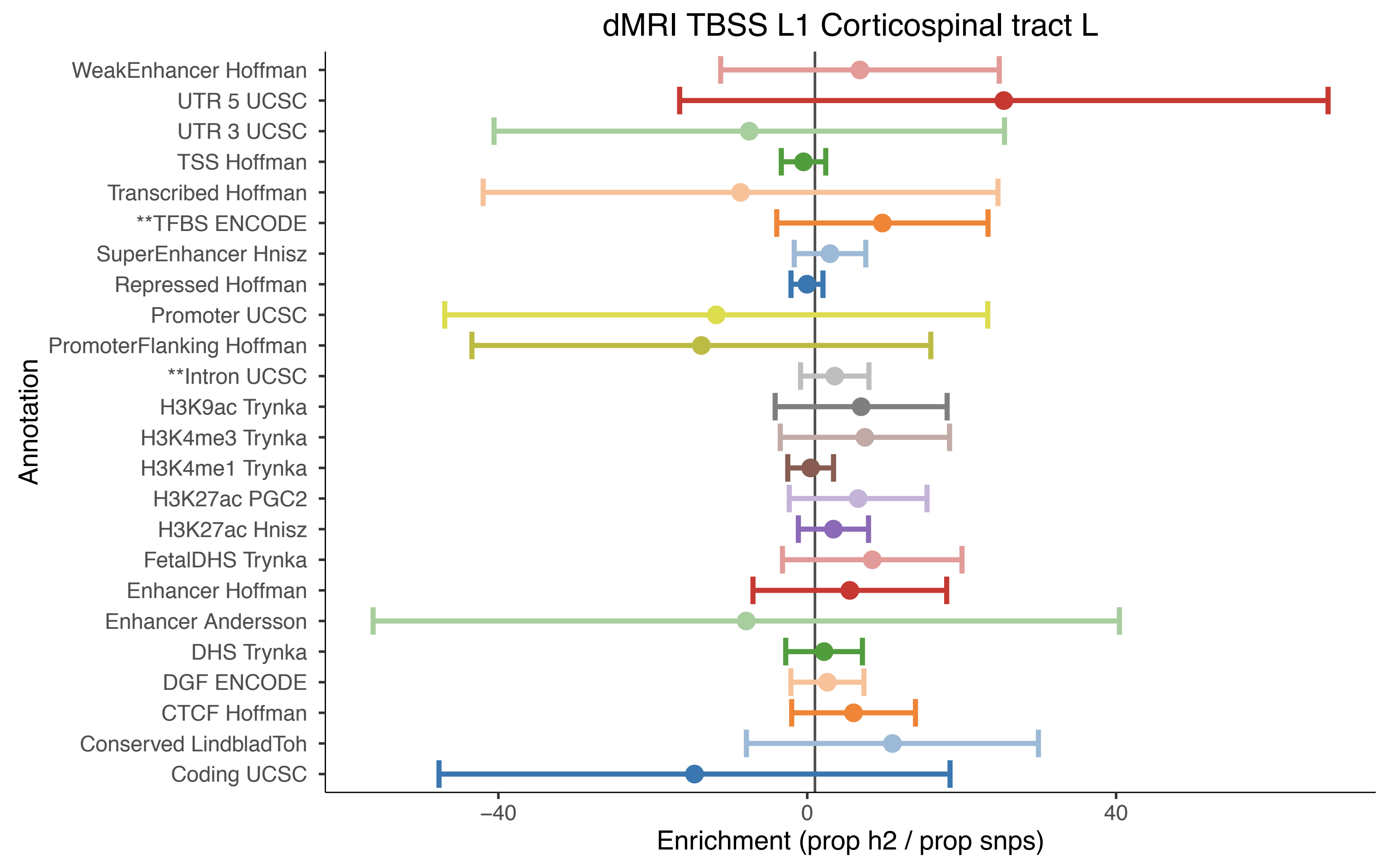
dMRI TBSS L1 Corticospinal tract L
WeakEnhancer Hoffman
UTR 5 UCSC
UTR 3 UCSC
TSS Hoffman
Transcribed Hoffman
**TFBS ENCODE
SuperEnhancer Hnisz
Repressed Hoffman
Promoter UCSC
PromoterFlanking Hoffman
**Intron UCSC
H3K9ac Trynka
H3K4me3 Trynka
H3K4me1 Trynka
H3K27ac PGC2
H3K27ac Hnisz
FetalDHS Trynka
Enhancer Hoffman
Enhancer Andersson
DHS Trynka
DGF ENCODE
CTCF Hoffman
Conserved LindbladToh
Coding UCSC
Annotation
−40
0
40
Enrichment (prop h2 / prop snps)

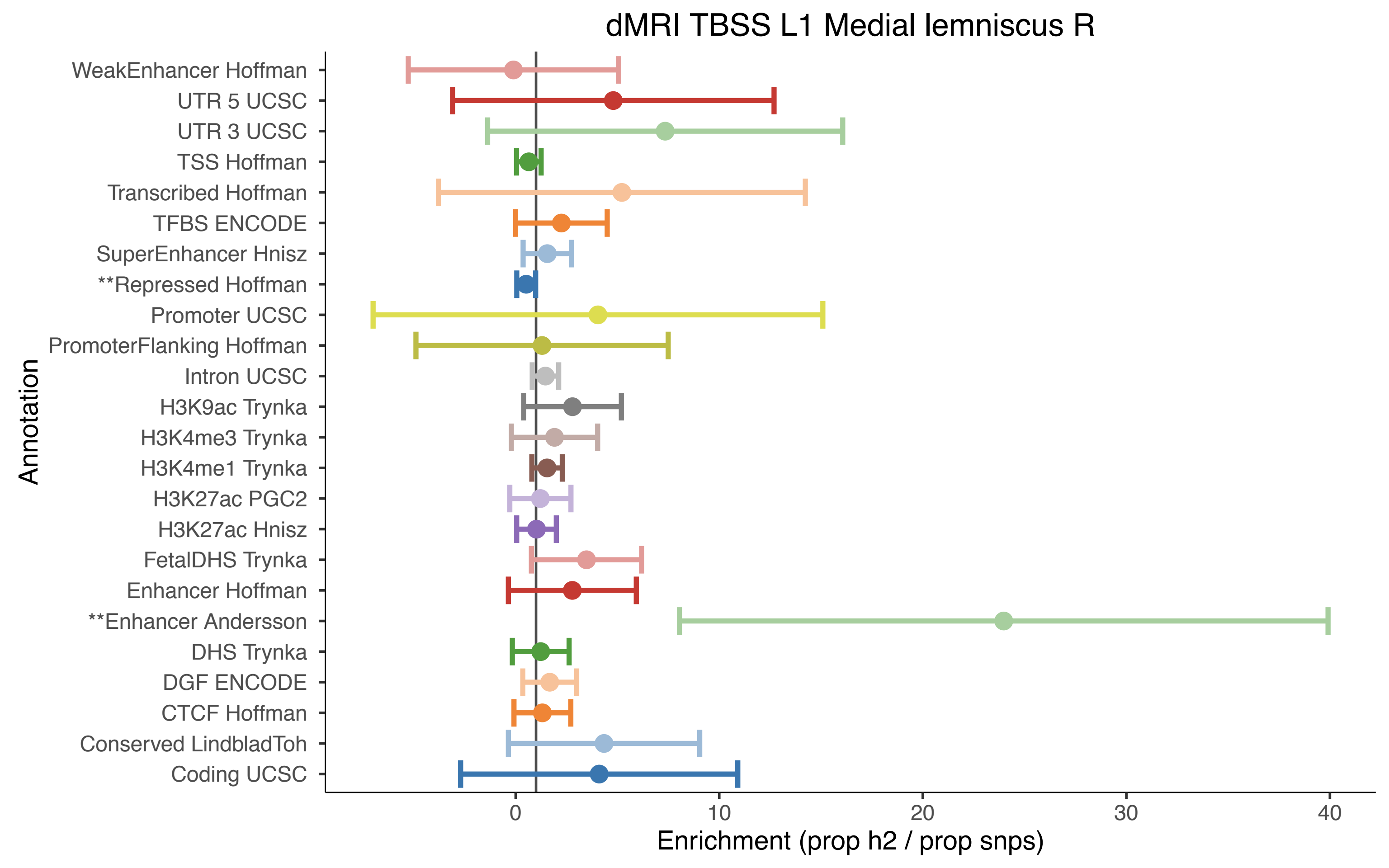

dMRI TBSS L1 Medial lemniscus R
WeakEnhancer Hoffman
UTR 5 UCSC
UTR 3 UCSC
TSS Hoffman
Transcribed Hoffman
TFBS ENCODE
SuperEnhancer Hnisz
**Repressed Hoffman
Promoter UCSC
PromoterFlanking Hoffman
Intron UCSC
H3K9ac Trynka
H3K4me3 Trynka
H3K4me1 Trynka
H3K27ac PGC2
H3K27ac Hnisz
FetalDHS Trynka
Enhancer Hoffman
**Enhancer Andersson
DHS Trynka
DGF ENCODE
CTCF Hoffman
Conserved LindbladToh
Coding UCSC
Annotation
0
10
20
30
40
Enrichment (prop h2 / prop snps)

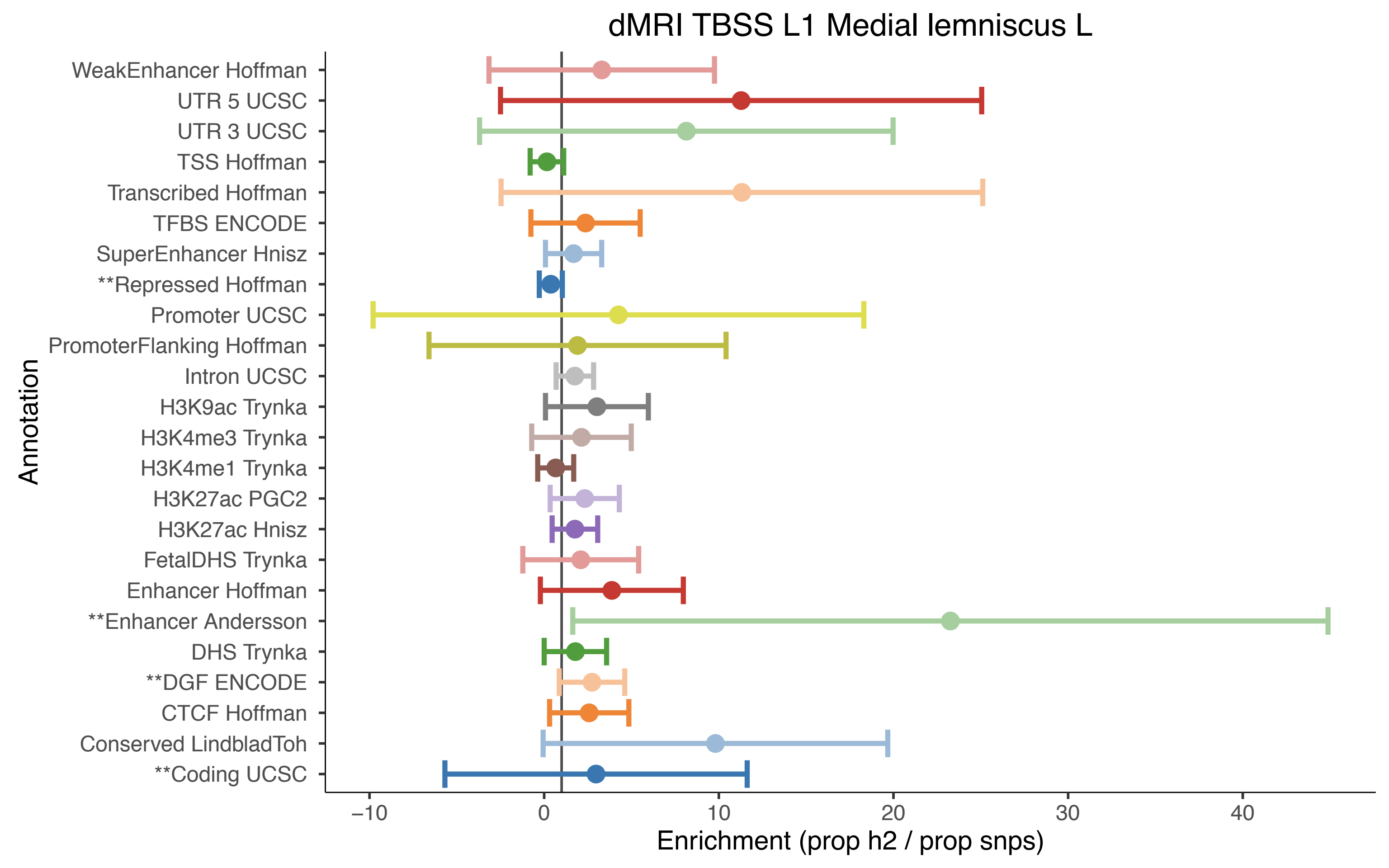
dMRI TBSS L1 Medial lemniscus L
WeakEnhancer Hoffman
UTR 5 UCSC
UTR 3 UCSC
TSS Hoffman
Transcribed Hoffman
TFBS ENCODE
SuperEnhancer Hnisz
**Repressed Hoffman
Promoter UCSC
PromoterFlanking Hoffman
Intron UCSC
H3K9ac Trynka
H3K4me3 Trynka
H3K4me1 Trynka
H3K27ac PGC2
H3K27ac Hnisz
FetalDHS Trynka
Enhancer Hoffman
**Enhancer Andersson
DHS Trynka
**DGF ENCODE
CTCF Hoffman
Conserved LindbladToh
**Coding UCSC
Annotation
−10
0
10
20
30
40
Enrichment (prop h2 / prop snps)

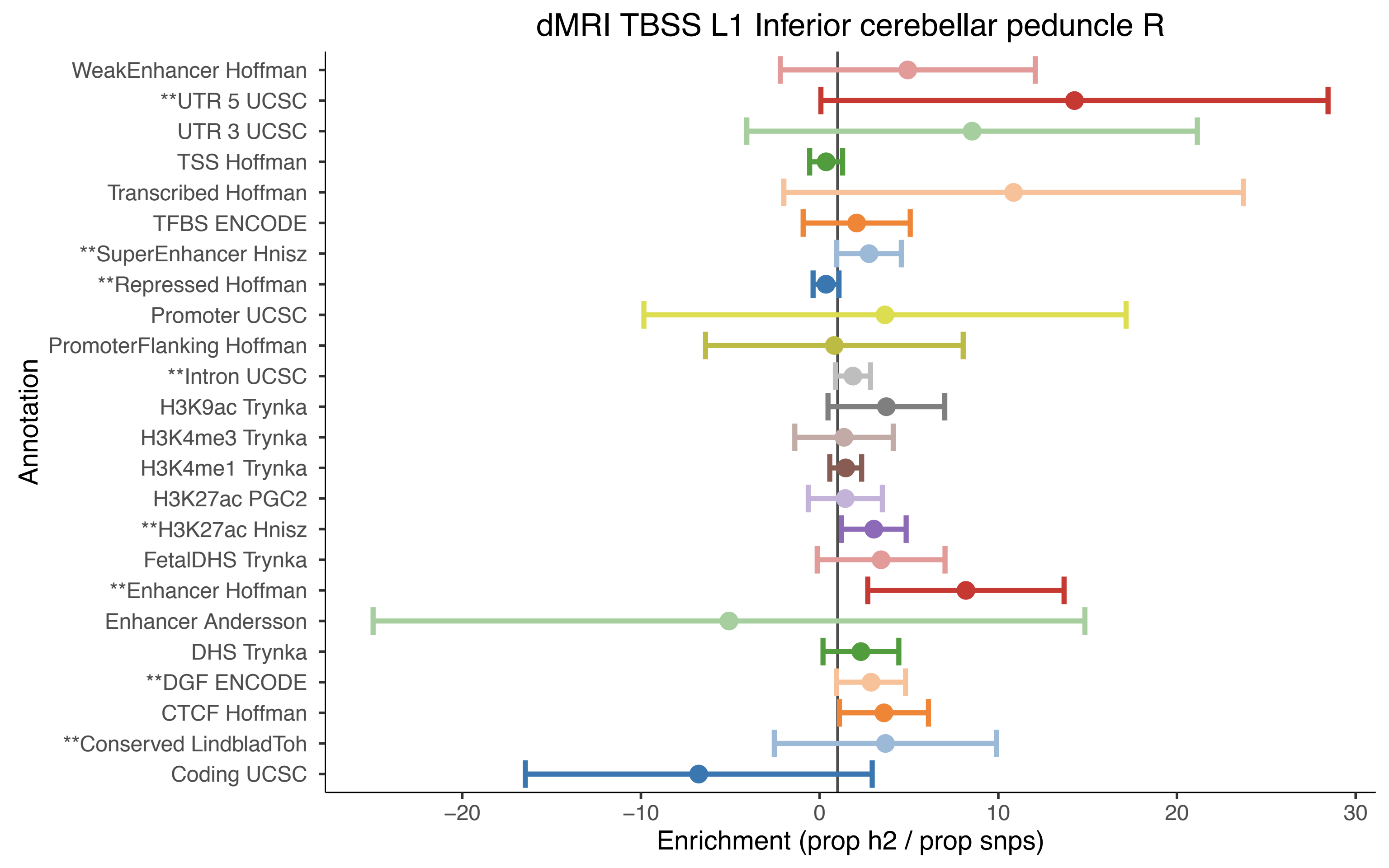

dMRI TBSS L1 Inferior cerebellar peduncle R
WeakEnhancer Hoffman
**UTR 5 UCSC
UTR 3 UCSC
TSS Hoffman
Transcribed Hoffman
TFBS ENCODE
**SuperEnhancer Hnisz
**Repressed Hoffman
Promoter UCSC
PromoterFlanking Hoffman
**Intron UCSC
H3K9ac Trynka
H3K4me3 Trynka
H3K4me1 Trynka
H3K27ac PGC2
**H3K27ac Hnisz
FetalDHS Trynka
**Enhancer Hoffman
Enhancer Andersson
DHS Trynka
**DGF ENCODE
CTCF Hoffman
**Conserved LindbladToh
Coding UCSC
Annotation
−20
−10
0
10
20
30
Enrichment (prop h2 / prop snps)

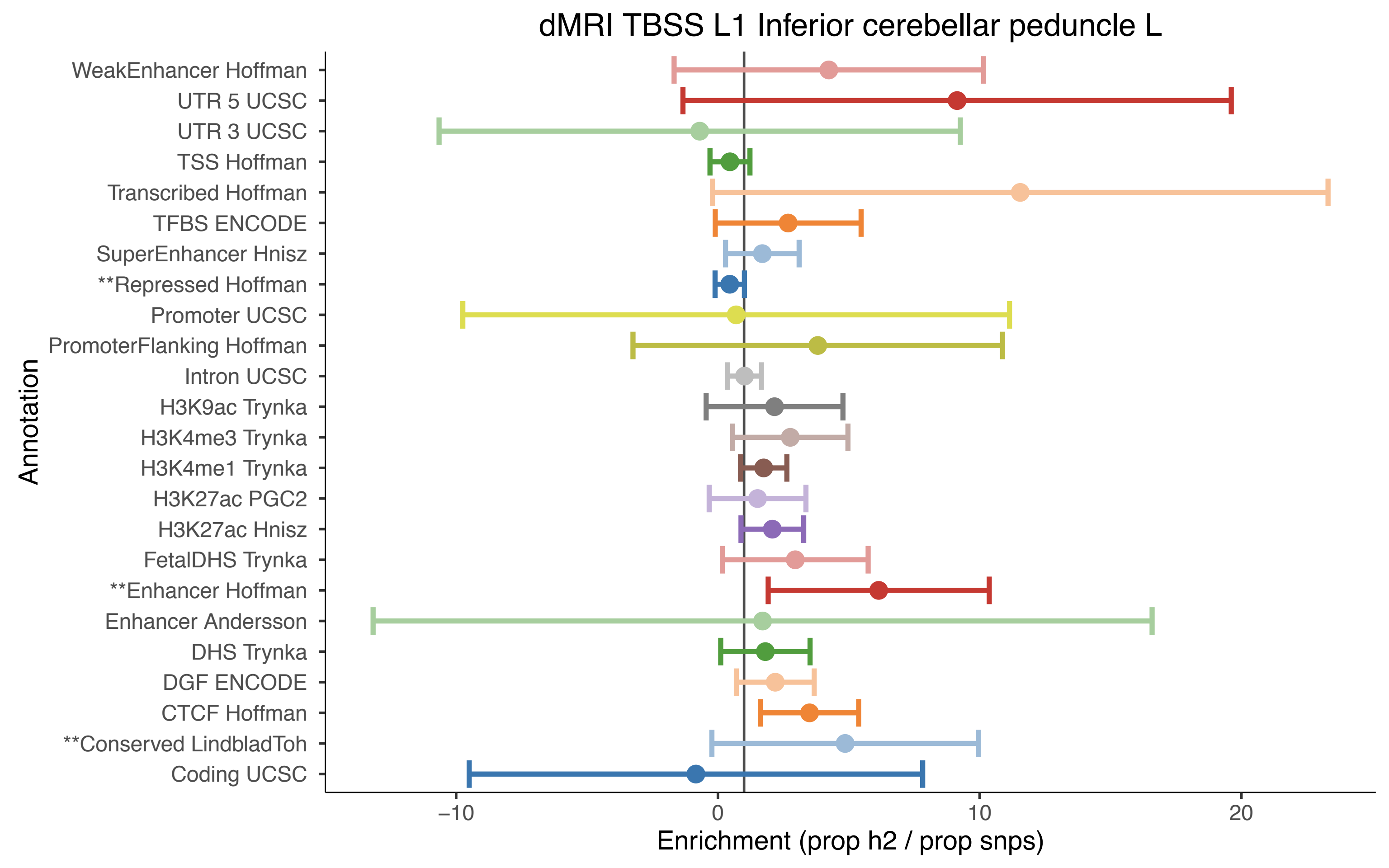

dMRI TBSS L1 Inferior cerebellar peduncle L
Annotation
WeakEnhancer Hoffman
UTR 5 UCSC
UTR 3 UCSC
TSS Hoffman
Transcribed Hoffman
TFBS ENCODE
SuperEnhancer Hnisz
**Repressed Hoffman
Promoter UCSC
PromoterFlanking Hoffman
Intron UCSC
H3K9ac Trynka
H3K4me3 Trynka
H3K4me1 Trynka
H3K27ac PGC2
H3K27ac Hnisz
FetalDHS Trynka
**Enhancer Hoffman
Enhancer Andersson
DHS Trynka
DGF ENCODE
CTCF Hoffman
**Conserved LindbladToh
Coding UCSC
−10
0
10
20
Enrichment (prop h2 / prop snps)

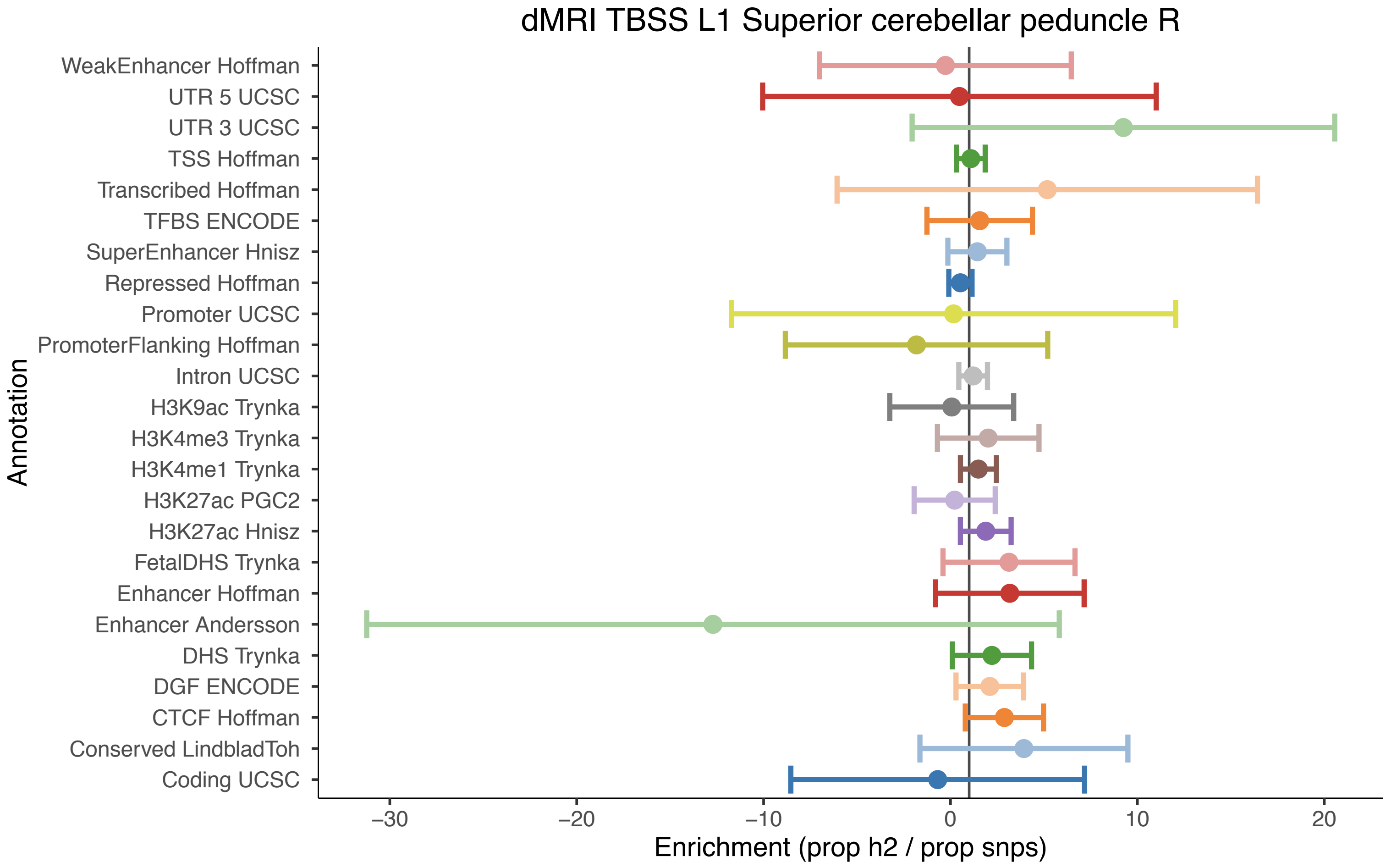

dMRI TBSS L1 Superior cerebellar peduncle R
WeakEnhancer Hoffman
UTR 5 UCSC
UTR 3 UCSC
TSS Hoffman
Transcribed Hoffman
TFBS ENCODE
SuperEnhancer Hnisz
Repressed Hoffman
Promoter UCSC
PromoterFlanking Hoffman
Intron UCSC
H3K9ac Trynka
H3K4me3 Trynka
H3K4me1 Trynka
H3K27ac PGC2
H3K27ac Hnisz
FetalDHS Trynka
Enhancer Hoffman
Enhancer Andersson
DHS Trynka
DGF ENCODE
CTCF Hoffman
Conserved LindbladToh
Coding UCSC
Annotation
−30
−20
−10
0
10
20
Enrichment (prop h2 / prop snps)

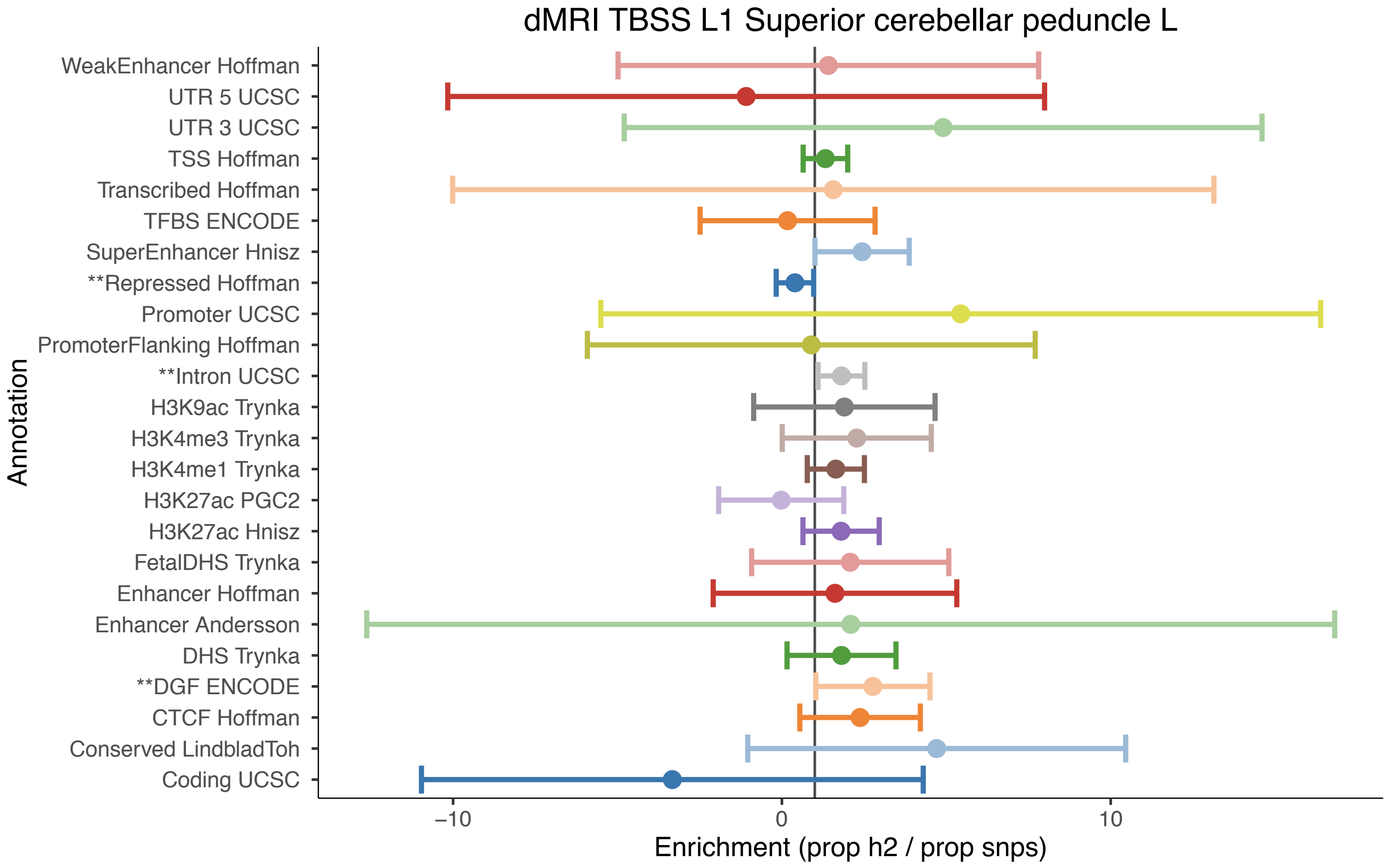

dMRI TBSS L1 Superior cerebellar peduncle L
WeakEnhancer Hoffman
UTR 5 UCSC
UTR 3 UCSC
TSS Hoffman
Transcribed Hoffman
TFBS ENCODE
SuperEnhancer Hnisz
**Repressed Hoffman
Promoter UCSC
PromoterFlanking Hoffman
**Intron UCSC
H3K9ac Trynka
H3K4me3 Trynka
H3K4me1 Trynka
H3K27ac PGC2
H3K27ac Hnisz
FetalDHS Trynka
Enhancer Hoffman
Enhancer Andersson
DHS Trynka
**DGF ENCODE
CTCF Hoffman
Conserved LindbladToh
Coding UCSC
Annotation
−10
0
10
Enrichment (prop h2 / prop snps)

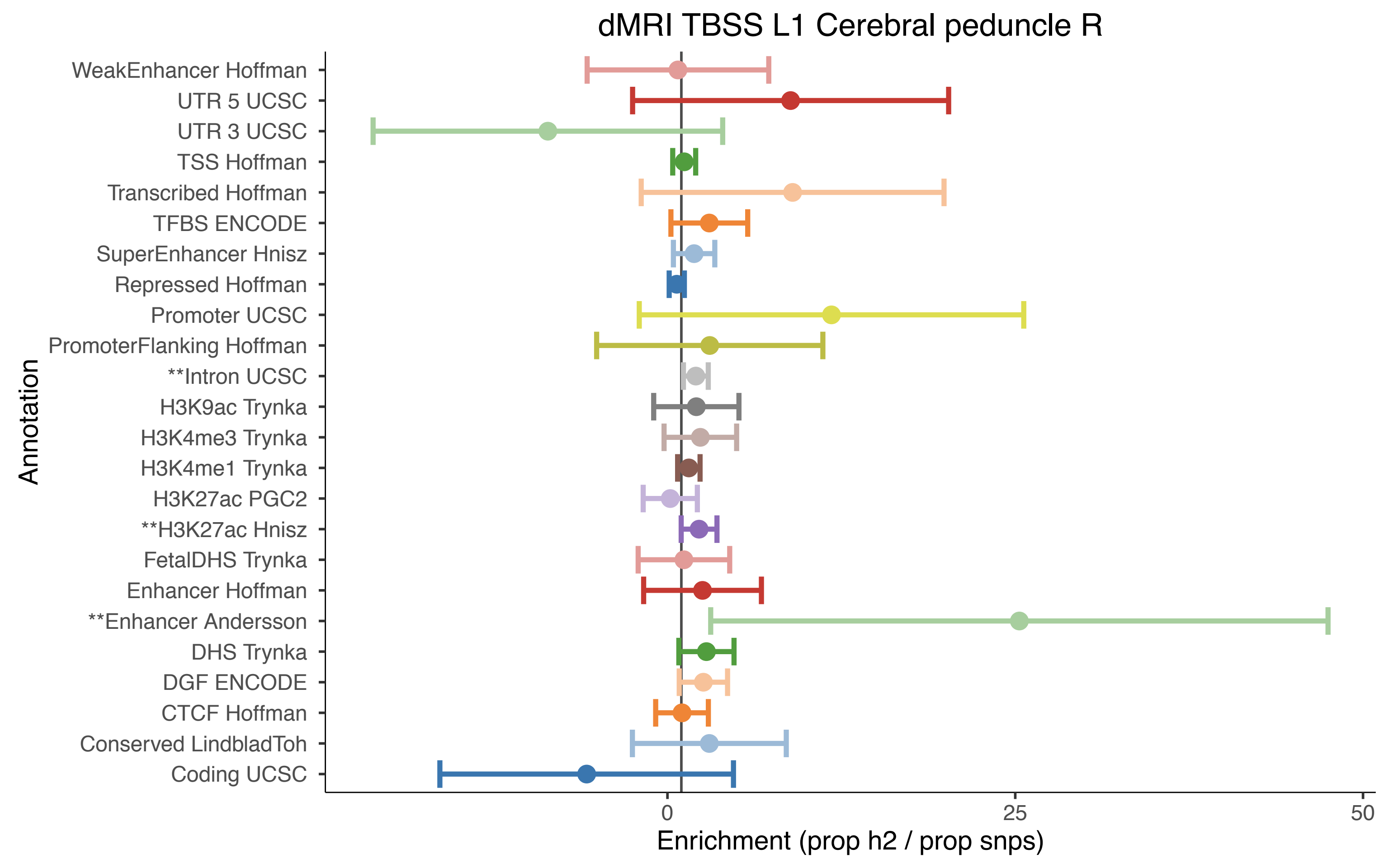
dMRI TBSS L1 Cerebral peduncle R
WeakEnhancer Hoffman
UTR 5 UCSC
UTR 3 UCSC
TSS Hoffman
Transcribed Hoffman
TFBS ENCODE
SuperEnhancer Hnisz
Repressed Hoffman
Promoter UCSC
PromoterFlanking Hoffman
**Intron UCSC
H3K9ac Trynka
H3K4me3 Trynka
H3K4me1 Trynka
H3K27ac PGC2
**H3K27ac Hnisz
FetalDHS Trynka
Enhancer Hoffman
**Enhancer Andersson
DHS Trynka
DGF ENCODE
CTCF Hoffman
Conserved LindbladToh
Coding UCSC
Annotation
0
25
50
Enrichment (prop h2 / prop snps)

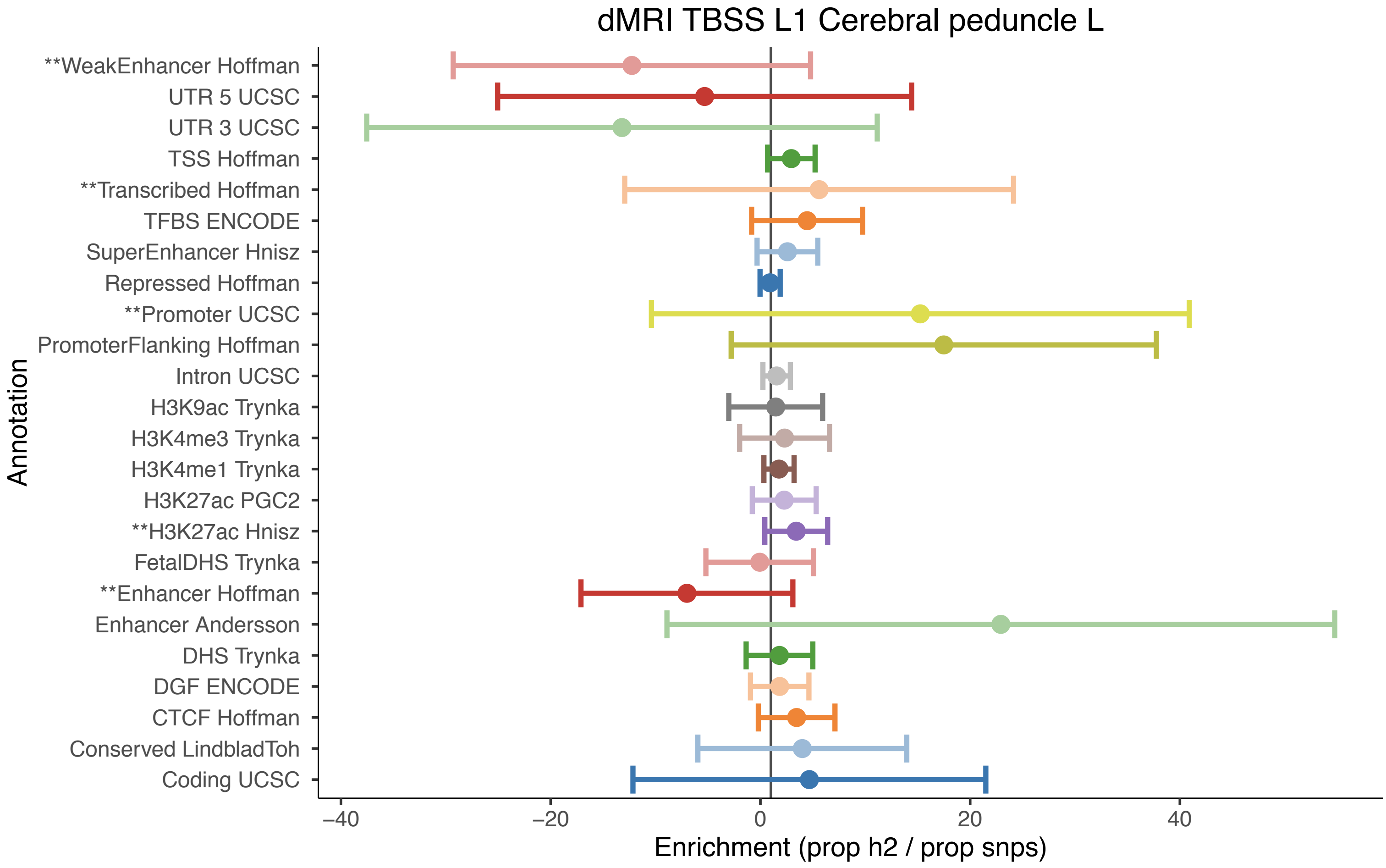
dMRI TBSS L1 Cerebral peduncle L
**WeakEnhancer Hoffman
UTR 5 UCSC
UTR 3 UCSC
TSS Hoffman
**Transcribed Hoffman
TFBS ENCODE
SuperEnhancer Hnisz
Repressed Hoffman
**Promoter UCSC
PromoterFlanking Hoffman
Intron UCSC
H3K9ac Trynka
H3K4me3 Trynka
H3K4me1 Trynka
H3K27ac PGC2
**H3K27ac Hnisz
FetalDHS Trynka
**Enhancer Hoffman
Enhancer Andersson
DHS Trynka
DGF ENCODE
CTCF Hoffman
Conserved LindbladToh
Coding UCSC
Annotation
−40
−20
0
20
40
Enrichment (prop h2 / prop snps)

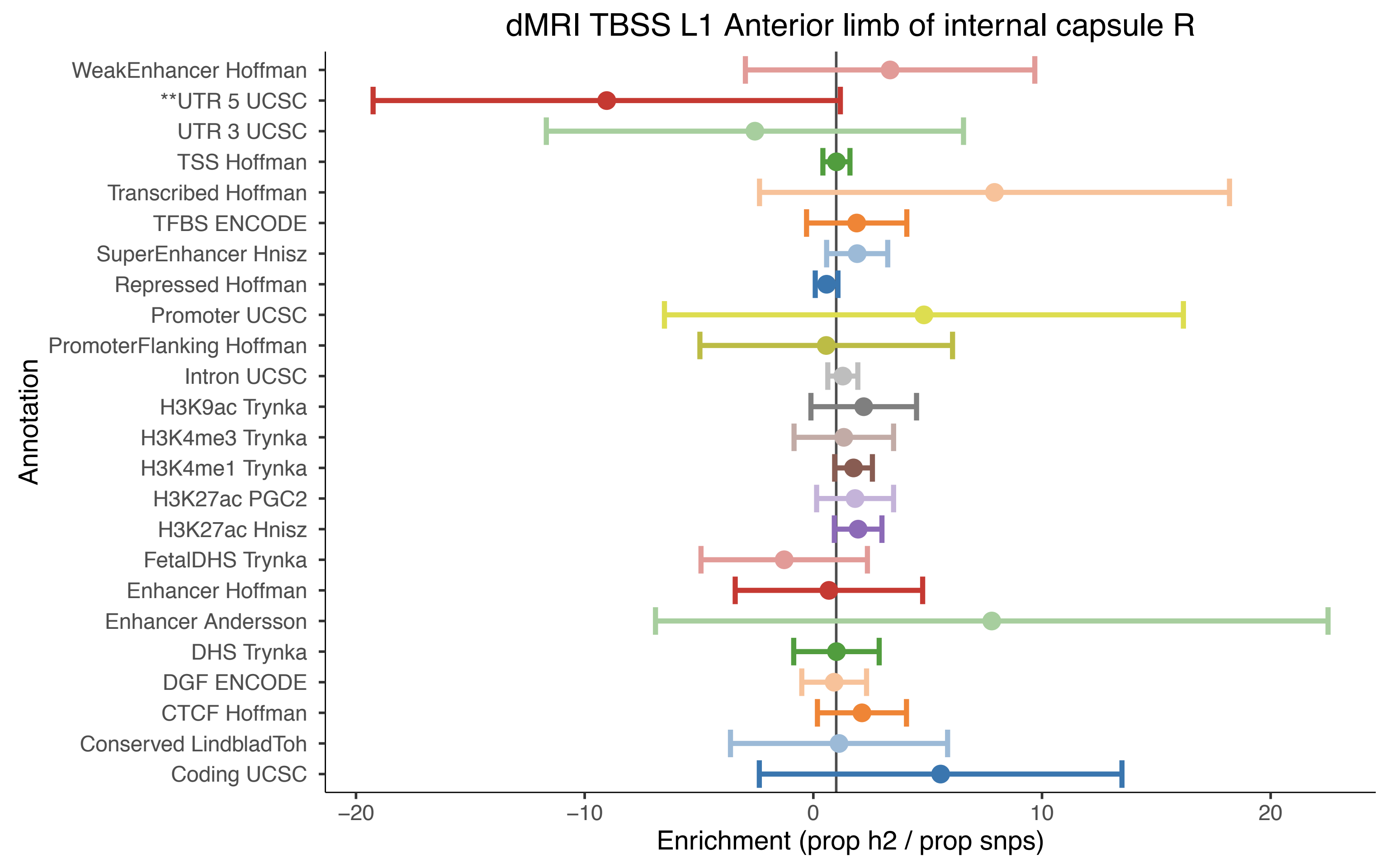

dMRI TBSS L1 Anterior limb of internal capsule R
WeakEnhancer Hoffman
**UTR 5 UCSC
UTR 3 UCSC
TSS Hoffman
Transcribed Hoffman
TFBS ENCODE
SuperEnhancer Hnisz
Repressed Hoffman
Promoter UCSC
PromoterFlanking Hoffman
Intron UCSC
H3K9ac Trynka
H3K4me3 Trynka
H3K4me1 Trynka
H3K27ac PGC2
H3K27ac Hnisz
FetalDHS Trynka
Enhancer Hoffman
Enhancer Andersson
DHS Trynka
DGF ENCODE
CTCF Hoffman
Conserved LindbladToh
Coding UCSC
Annotation
−20
−10
0
10
20
Enrichment (prop h2 / prop snps)

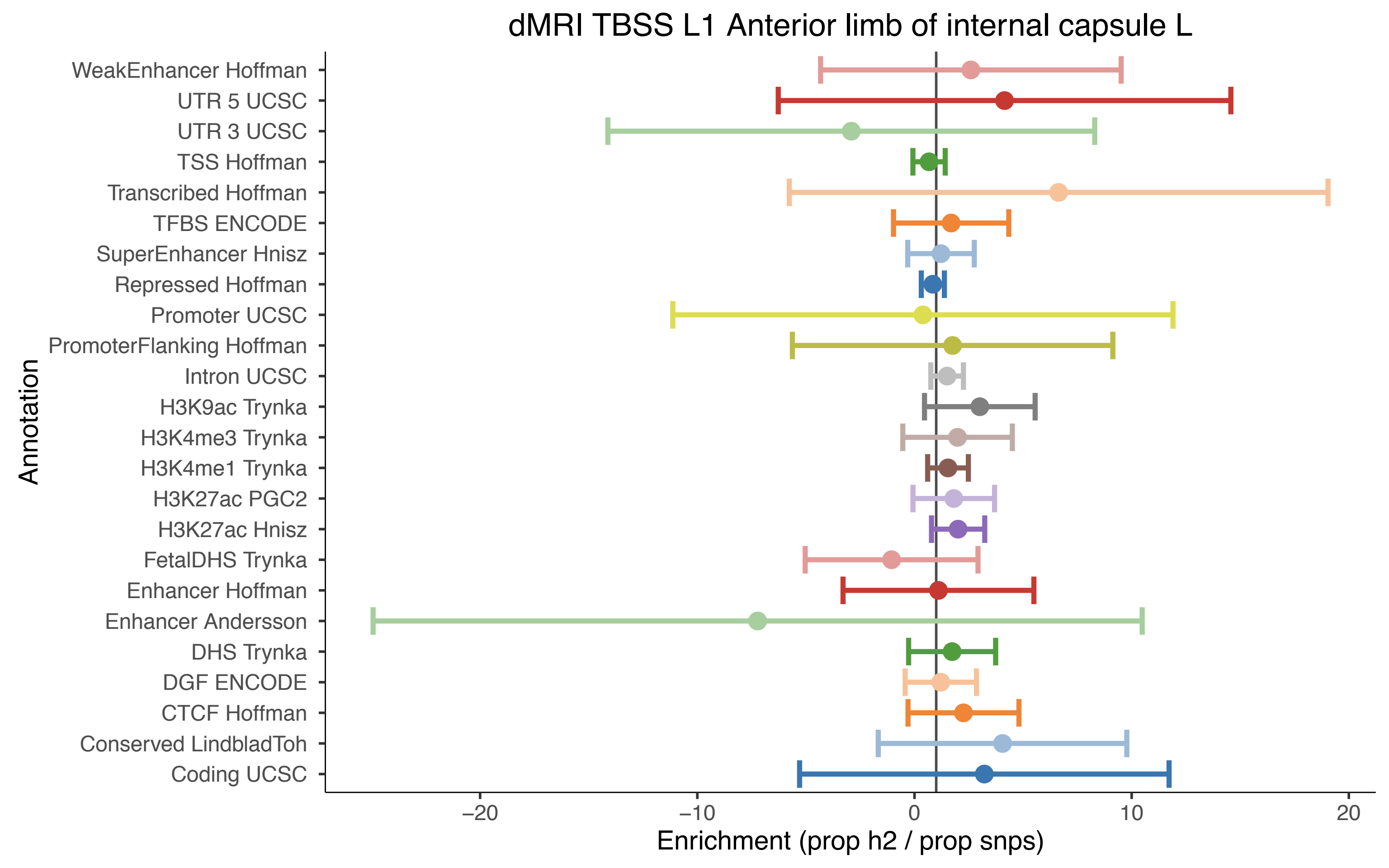
dMRI TBSS L1 Anterior limb of internal capsule L
Annotation
WeakEnhancer Hoffman
UTR 5 UCSC
UTR 3 UCSC
TSS Hoffman
Transcribed Hoffman
TFBS ENCODE
SuperEnhancer Hnisz
Repressed Hoffman
Promoter UCSC
PromoterFlanking Hoffman
Intron UCSC
H3K9ac Trynka
H3K4me3 Trynka
H3K4me1 Trynka
H3K27ac PGC2
H3K27ac Hnisz
FetalDHS Trynka
Enhancer Hoffman
Enhancer Andersson
DHS Trynka
DGF ENCODE
CTCF Hoffman
Conserved LindbladToh
Coding UCSC
−20
−10
0
10
20
Enrichment (prop h2 / prop snps)

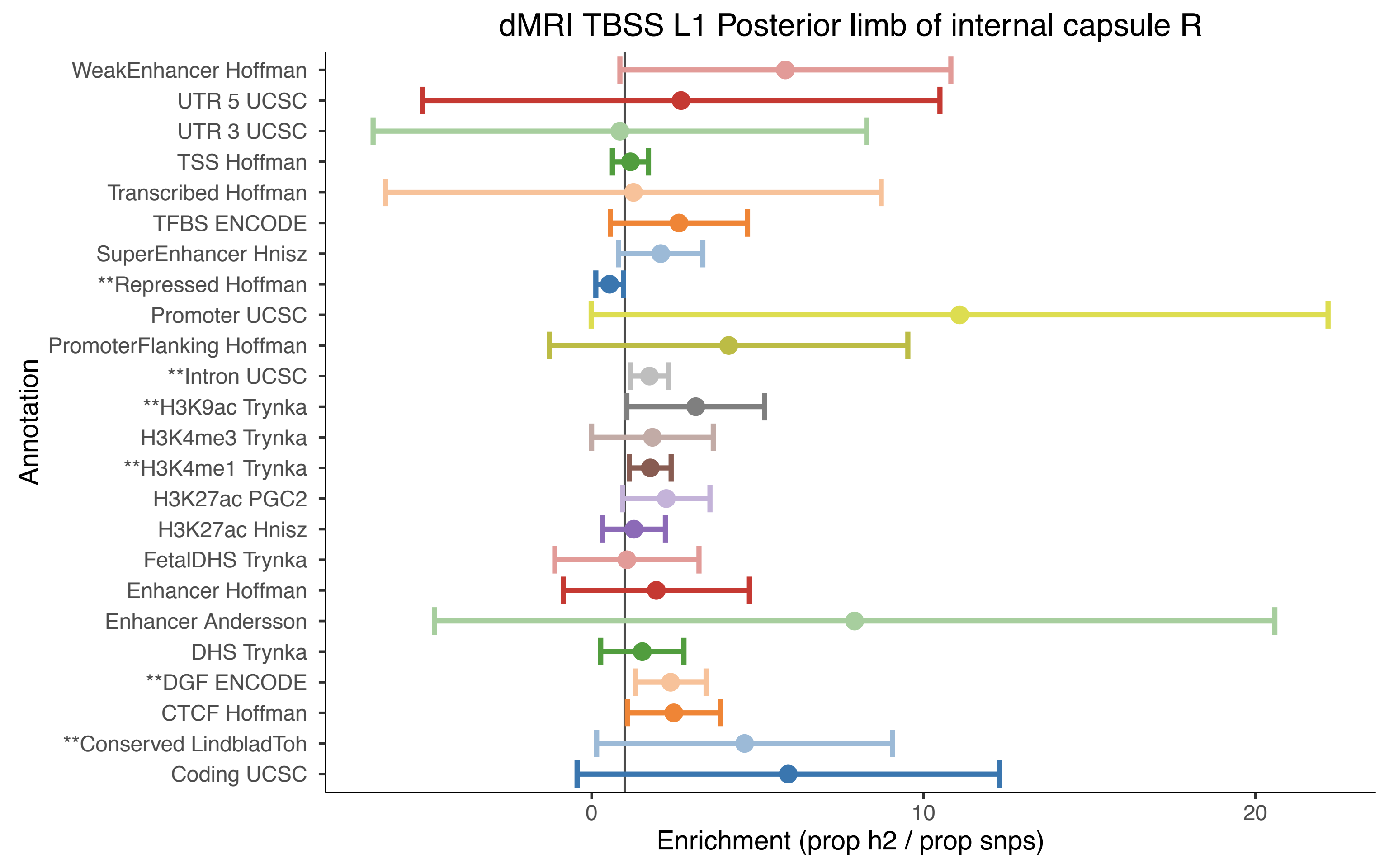
dMRI TBSS L1 Posterior limb of internal capsule R
WeakEnhancer Hoffman
UTR 5 UCSC
UTR 3 UCSC
TSS Hoffman
Transcribed Hoffman
TFBS ENCODE
SuperEnhancer Hnisz
**Repressed Hoffman
Promoter UCSC
PromoterFlanking Hoffman
**Intron UCSC
**H3K9ac Trynka
H3K4me3 Trynka
**H3K4me1 Trynka
H3K27ac PGC2
H3K27ac Hnisz
FetalDHS Trynka
Enhancer Hoffman
Enhancer Andersson
DHS Trynka
**DGF ENCODE
CTCF Hoffman
**Conserved LindbladToh
Coding UCSC
Annotation
0
10
20
Enrichment (prop h2 / prop snps)

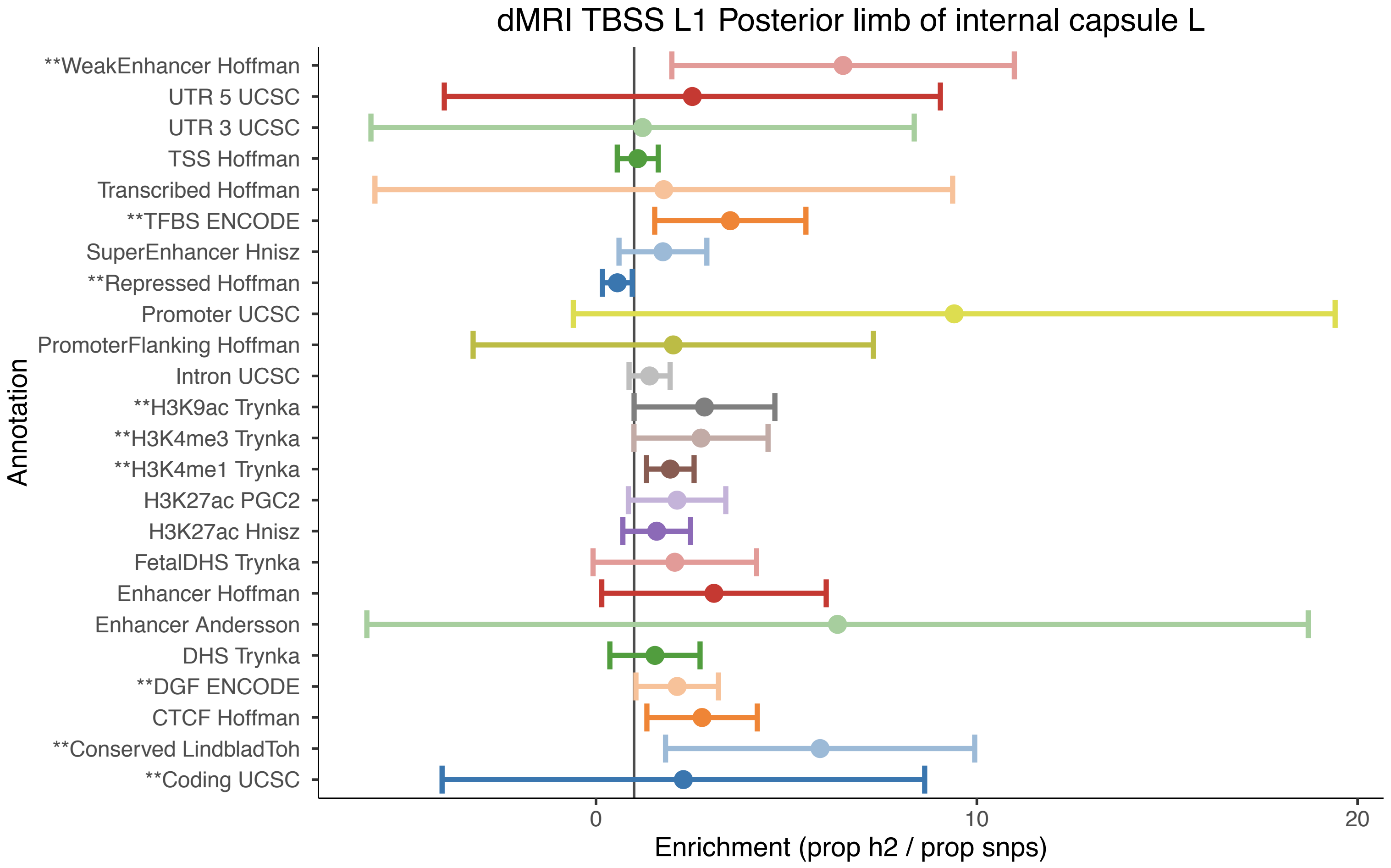
dMRI TBSS L1 Posterior limb of internal capsule L
**WeakEnhancer Hoffman
UTR 5 UCSC
UTR 3 UCSC
TSS Hoffman
Transcribed Hoffman
**TFBS ENCODE
SuperEnhancer Hnisz
**Repressed Hoffman
Promoter UCSC
PromoterFlanking Hoffman
Intron UCSC
**H3K9ac Trynka
**H3K4me3 Trynka
**H3K4me1 Trynka
H3K27ac PGC2
H3K27ac Hnisz
FetalDHS Trynka
Enhancer Hoffman
Enhancer Andersson
DHS Trynka
**DGF ENCODE
CTCF Hoffman
**Conserved LindbladToh
**Coding UCSC
Annotation
0
10
20
Enrichment (prop h2 / prop snps)

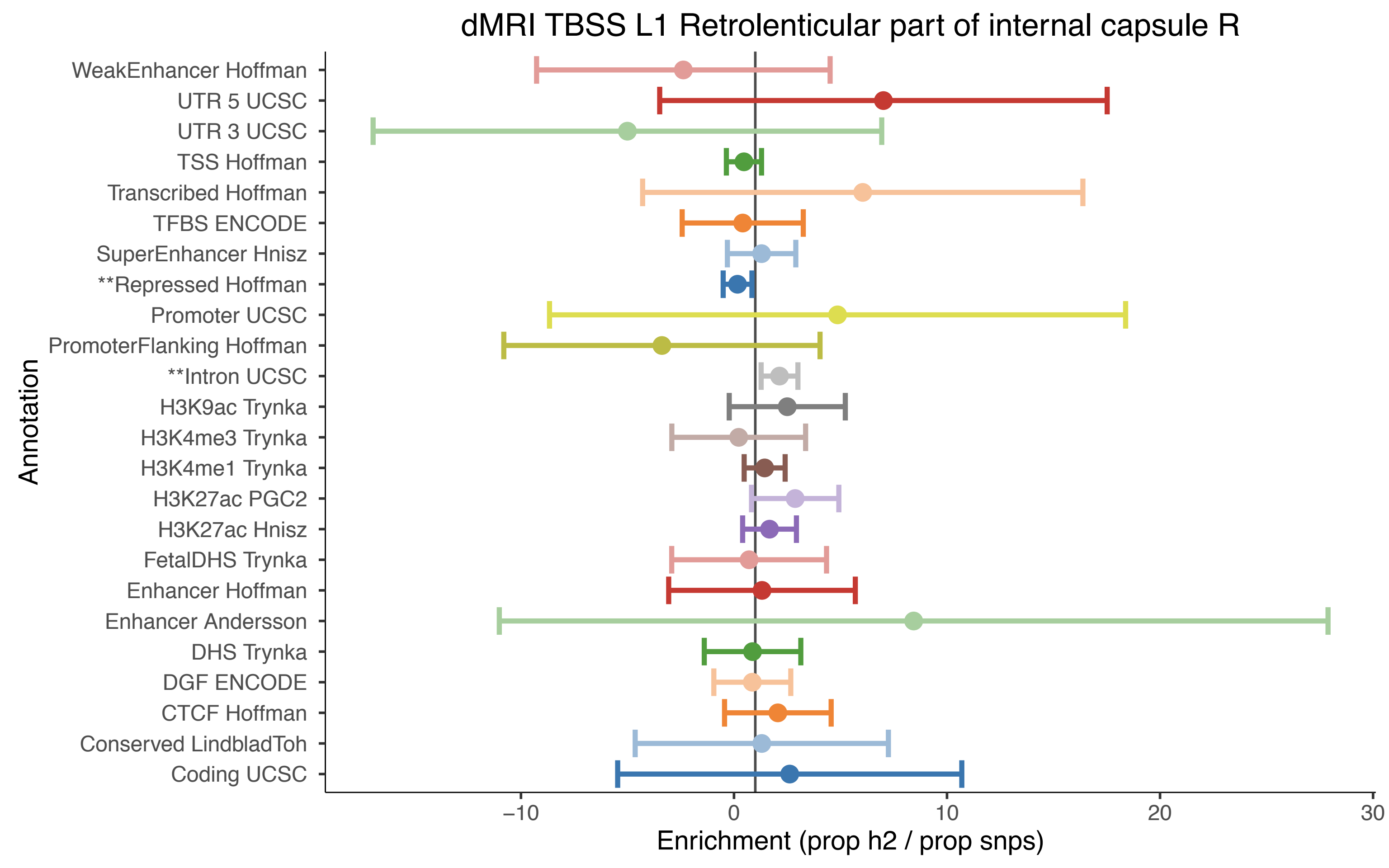
dMRI TBSS L1 Retrolenticular part of internal capsule R
WeakEnhancer Hoffman
UTR 5 UCSC
UTR 3 UCSC
TSS Hoffman
Transcribed Hoffman
TFBS ENCODE
SuperEnhancer Hnisz
**Repressed Hoffman
Promoter UCSC
PromoterFlanking Hoffman
**Intron UCSC
H3K9ac Trynka
H3K4me3 Trynka
H3K4me1 Trynka
H3K27ac PGC2
H3K27ac Hnisz
FetalDHS Trynka
Enhancer Hoffman
Enhancer Andersson
DHS Trynka
DGF ENCODE
CTCF Hoffman
Conserved LindbladToh
Coding UCSC
Annotation
−10
0
10
20
30
Enrichment (prop h2 / prop snps)

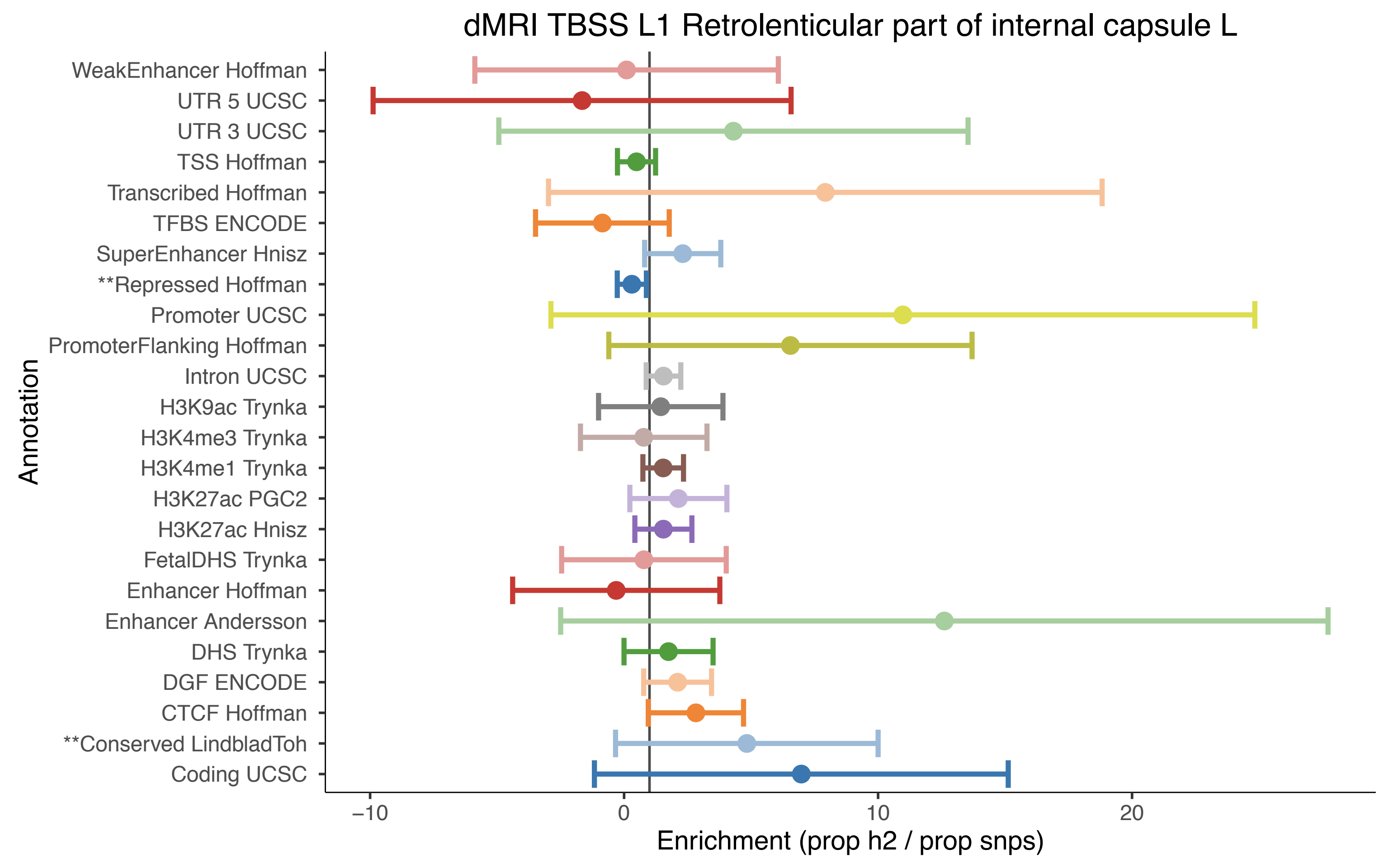

dMRI TBSS L1 Retrolenticular part of internal capsule L
WeakEnhancer Hoffman
UTR 5 UCSC
UTR 3 UCSC
TSS Hoffman
Transcribed Hoffman
TFBS ENCODE
SuperEnhancer Hnisz
**Repressed Hoffman
Promoter UCSC
PromoterFlanking Hoffman
Intron UCSC
H3K9ac Trynka
H3K4me3 Trynka
H3K4me1 Trynka
H3K27ac PGC2
H3K27ac Hnisz
FetalDHS Trynka
Enhancer Hoffman
Enhancer Andersson
DHS Trynka
DGF ENCODE
CTCF Hoffman
**Conserved LindbladToh
Coding UCSC
Annotation
−10
0
10
20
Enrichment (prop h2 / prop snps)

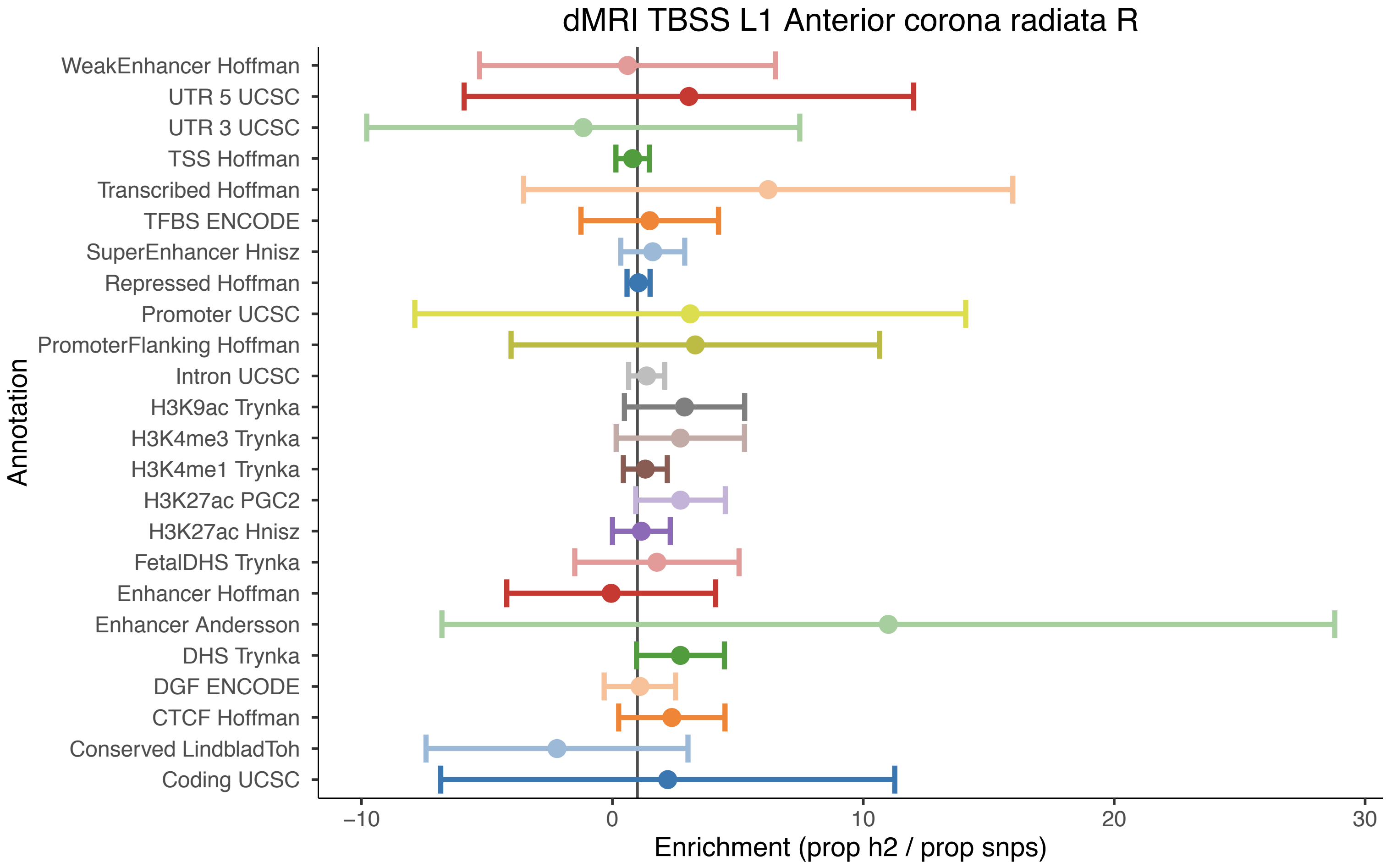

dMRI TBSS L1 Anterior corona radiata R
WeakEnhancer Hoffman
UTR 5 UCSC
UTR 3 UCSC
TSS Hoffman
Transcribed Hoffman
TFBS ENCODE
SuperEnhancer Hnisz
Repressed Hoffman
Promoter UCSC
PromoterFlanking Hoffman
Intron UCSC
H3K9ac Trynka
H3K4me3 Trynka
H3K4me1 Trynka
H3K27ac PGC2
H3K27ac Hnisz
FetalDHS Trynka
Enhancer Hoffman
Enhancer Andersson
DHS Trynka
DGF ENCODE
CTCF Hoffman
Conserved LindbladToh
Coding UCSC
Annotation
−10
0
10
20
30
Enrichment (prop h2 / prop snps)

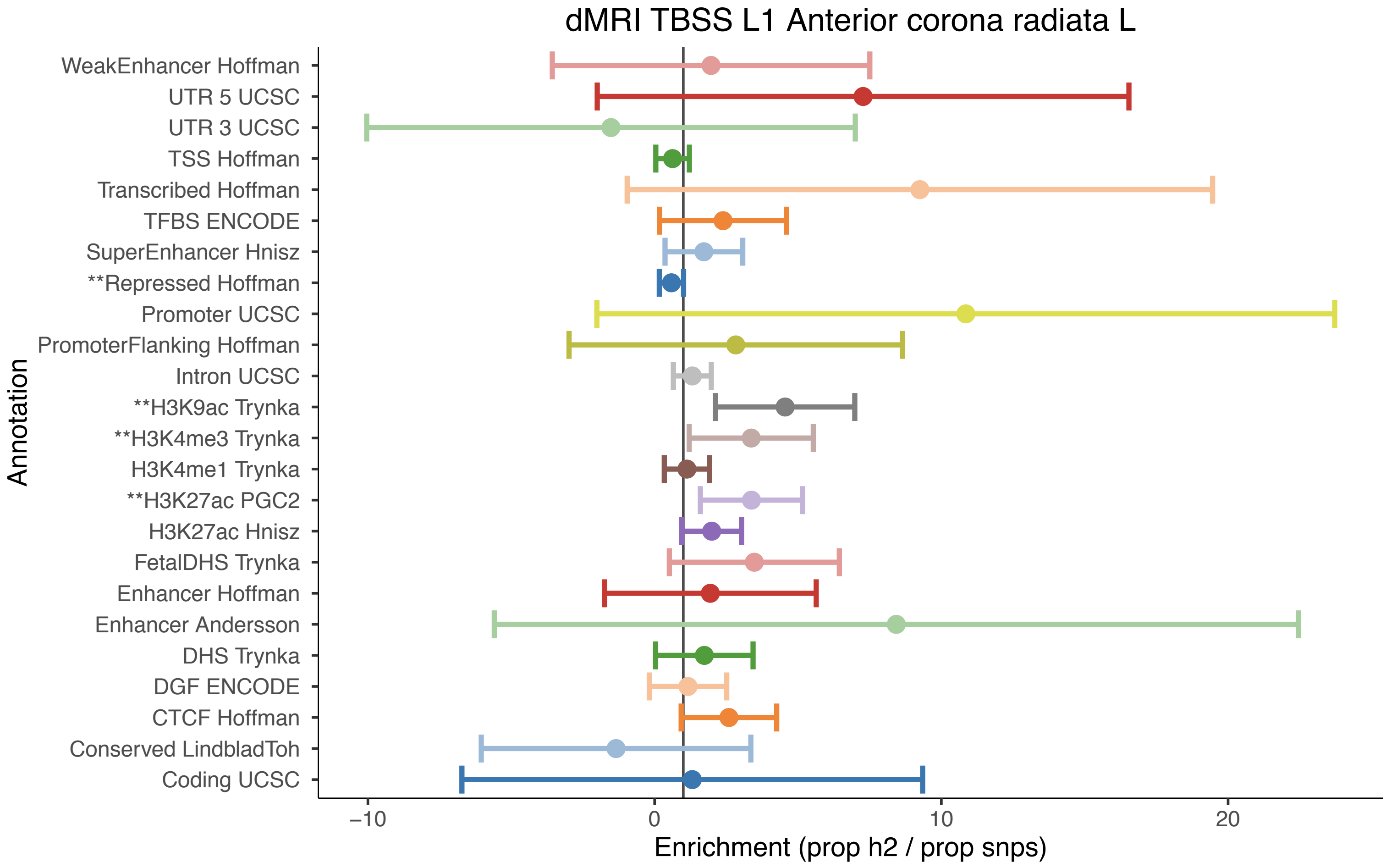

dMRI TBSS L1 Anterior corona radiata L
WeakEnhancer Hoffman
UTR 5 UCSC
UTR 3 UCSC
TSS Hoffman
Transcribed Hoffman
TFBS ENCODE
SuperEnhancer Hnisz
**Repressed Hoffman
Promoter UCSC
PromoterFlanking Hoffman
Intron UCSC
**H3K9ac Trynka
**H3K4me3 Trynka
H3K4me1 Trynka
**H3K27ac PGC2
H3K27ac Hnisz
FetalDHS Trynka
Enhancer Hoffman
Enhancer Andersson
DHS Trynka
DGF ENCODE
CTCF Hoffman
Conserved LindbladToh
Coding UCSC
Annotation
−10
0
10
20
Enrichment (prop h2 / prop snps)

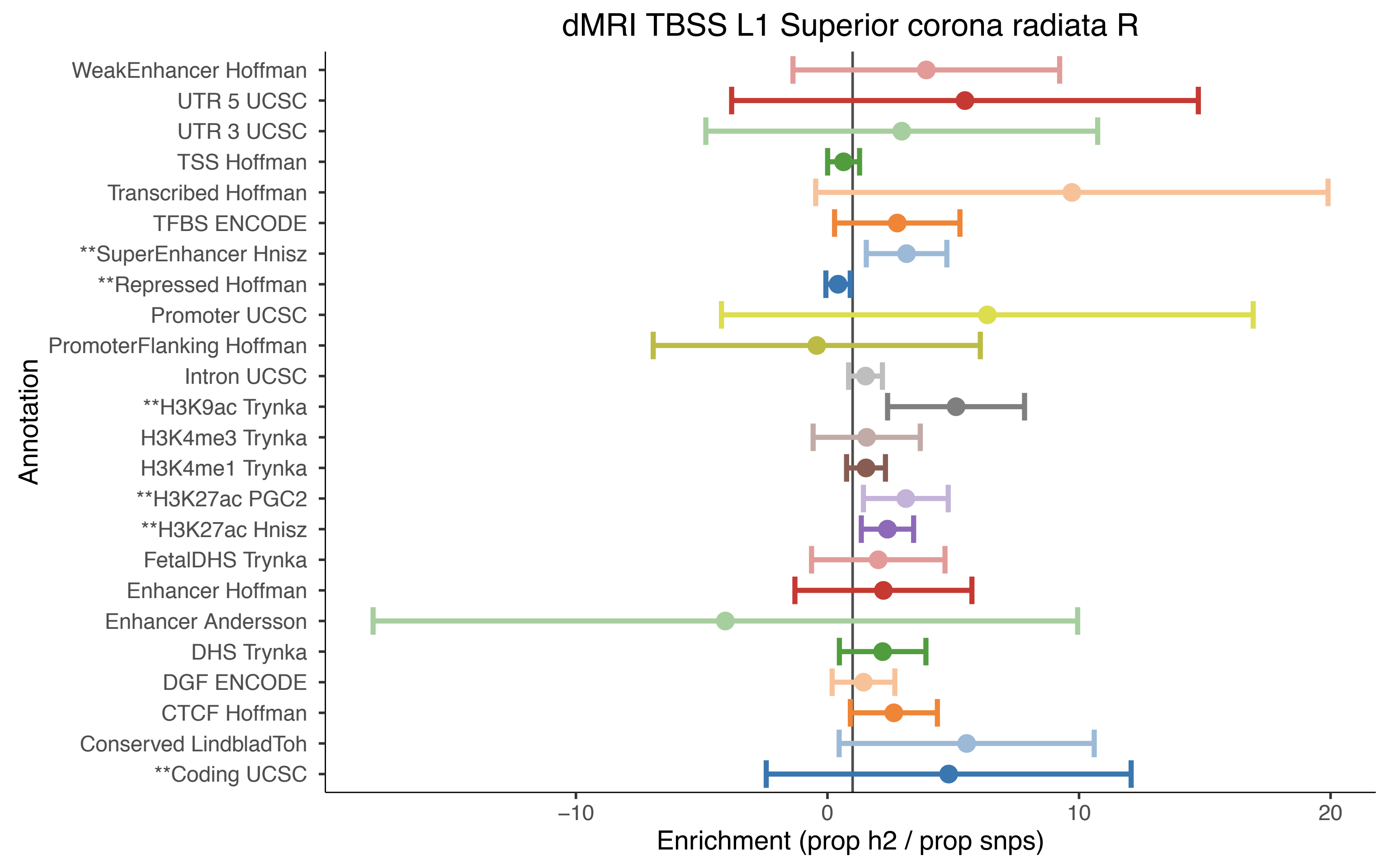
dMRI TBSS L1 Superior corona radiata R
WeakEnhancer Hoffman
UTR 5 UCSC
UTR 3 UCSC
TSS Hoffman
Transcribed Hoffman
TFBS ENCODE
**SuperEnhancer Hnisz
**Repressed Hoffman
Promoter UCSC
PromoterFlanking Hoffman
Intron UCSC
**H3K9ac Trynka
H3K4me3 Trynka
H3K4me1 Trynka
**H3K27ac PGC2
**H3K27ac Hnisz
FetalDHS Trynka
Enhancer Hoffman
Enhancer Andersson
DHS Trynka
DGF ENCODE
CTCF Hoffman
Conserved LindbladToh
**Coding UCSC
Annotation
−10
0
10
20
Enrichment (prop h2 / prop snps)

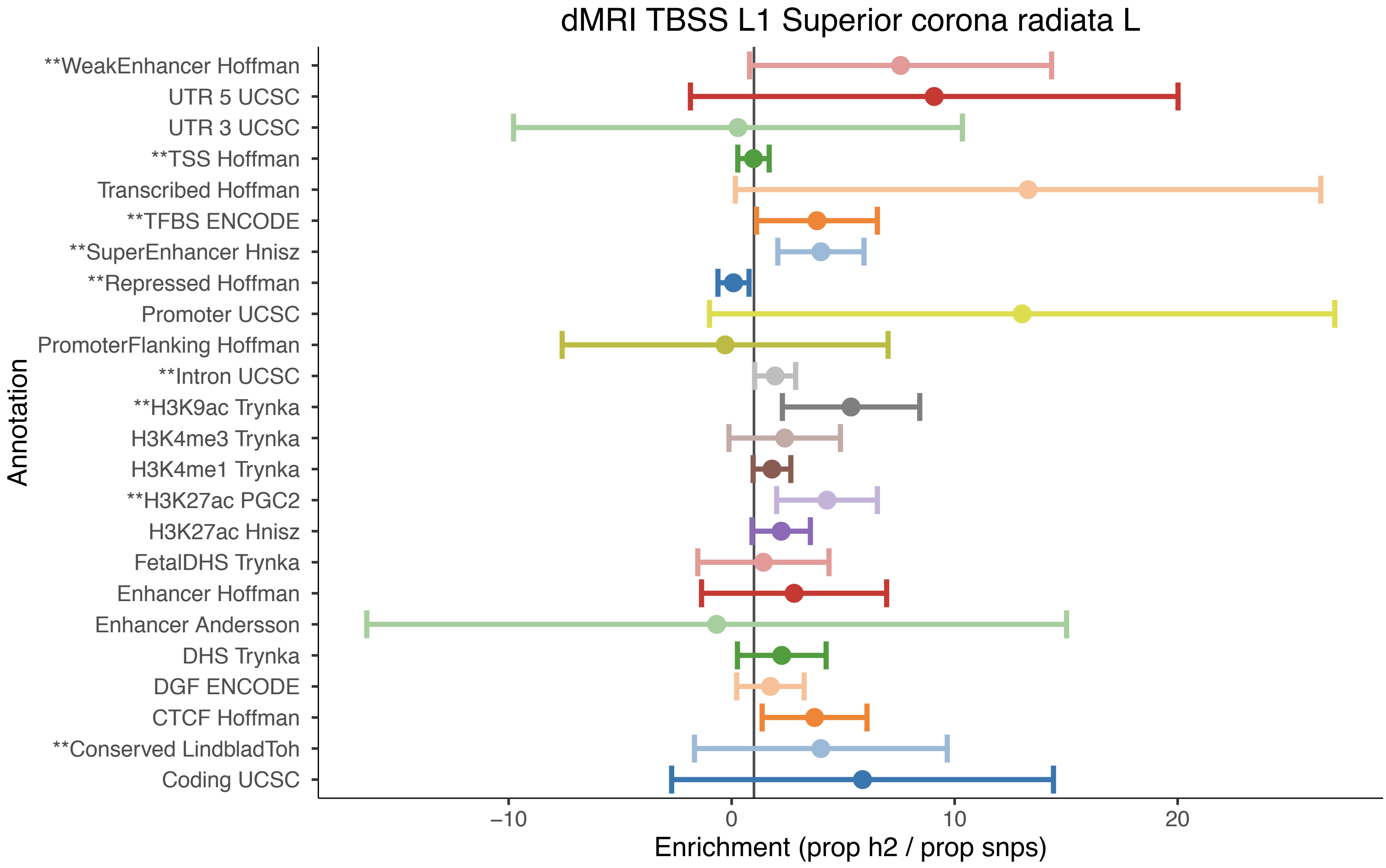

dMRI TBSS L1 Superior corona radiata L
Annotation
**WeakEnhancer Hoffman
UTR 5 UCSC
UTR 3 UCSC
**TSS Hoffman
Transcribed Hoffman
**TFBS ENCODE
**SuperEnhancer Hnisz
**Repressed Hoffman
Promoter UCSC
PromoterFlanking Hoffman
**Intron UCSC
**H3K9ac Trynka
H3K4me3 Trynka
H3K4me1 Trynka
**H3K27ac PGC2
H3K27ac Hnisz
FetalDHS Trynka
Enhancer Hoffman
Enhancer Andersson
DHS Trynka
DGF ENCODE
CTCF Hoffman
**Conserved LindbladToh
Coding UCSC
−10
0
10
20
Enrichment (prop h2 / prop snps)

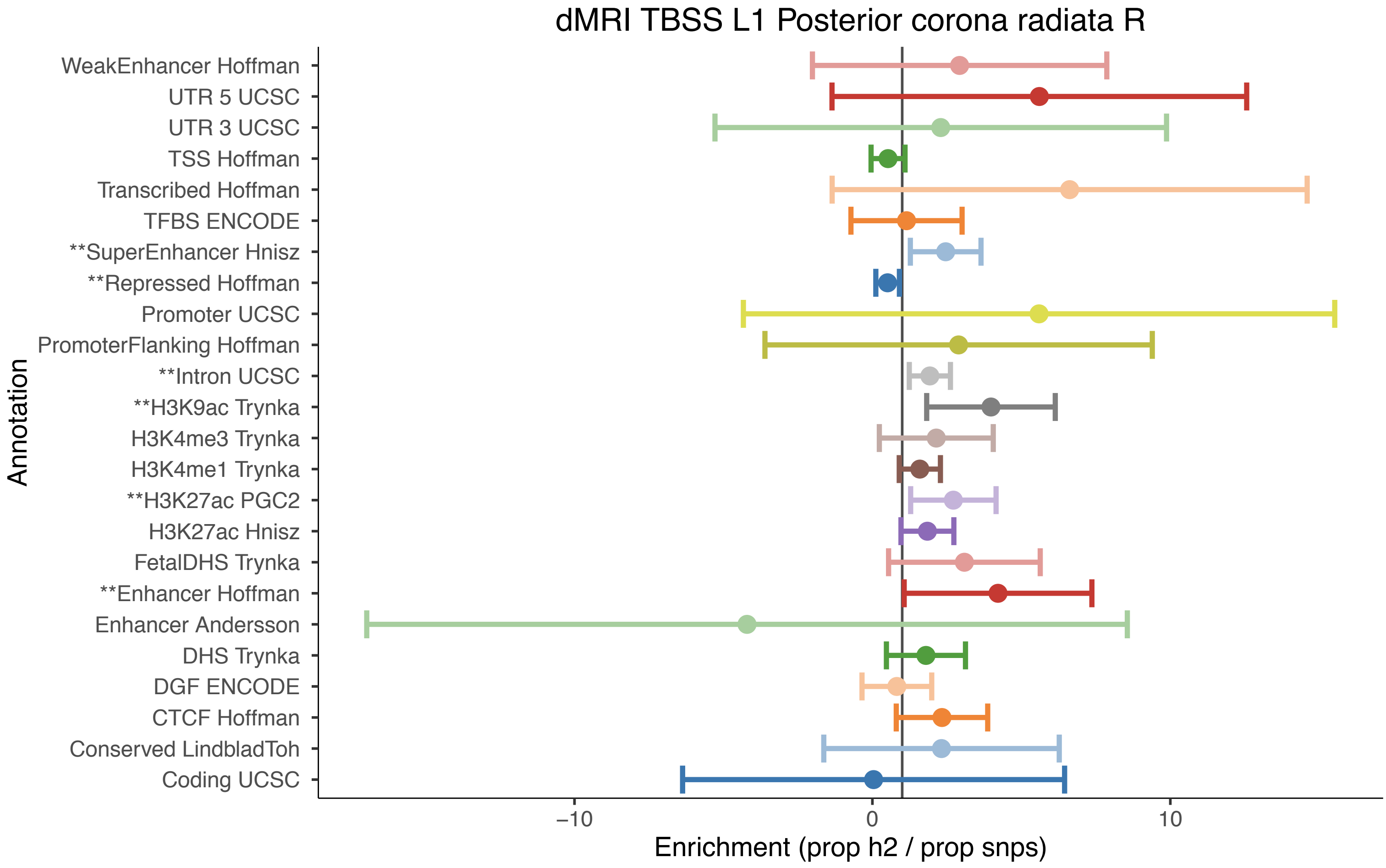

dMRI TBSS L1 Posterior corona radiata R
Annotation
WeakEnhancer Hoffman
UTR 5 UCSC
UTR 3 UCSC
TSS Hoffman
Transcribed Hoffman
TFBS ENCODE
**SuperEnhancer Hnisz
**Repressed Hoffman
Promoter UCSC
PromoterFlanking Hoffman
**Intron UCSC
**H3K9ac Trynka
H3K4me3 Trynka
H3K4me1 Trynka
**H3K27ac PGC2
H3K27ac Hnisz
FetalDHS Trynka
**Enhancer Hoffman
Enhancer Andersson
DHS Trynka
DGF ENCODE
CTCF Hoffman
Conserved LindbladToh
Coding UCSC
−10
0
10
Enrichment (prop h2 / prop snps)

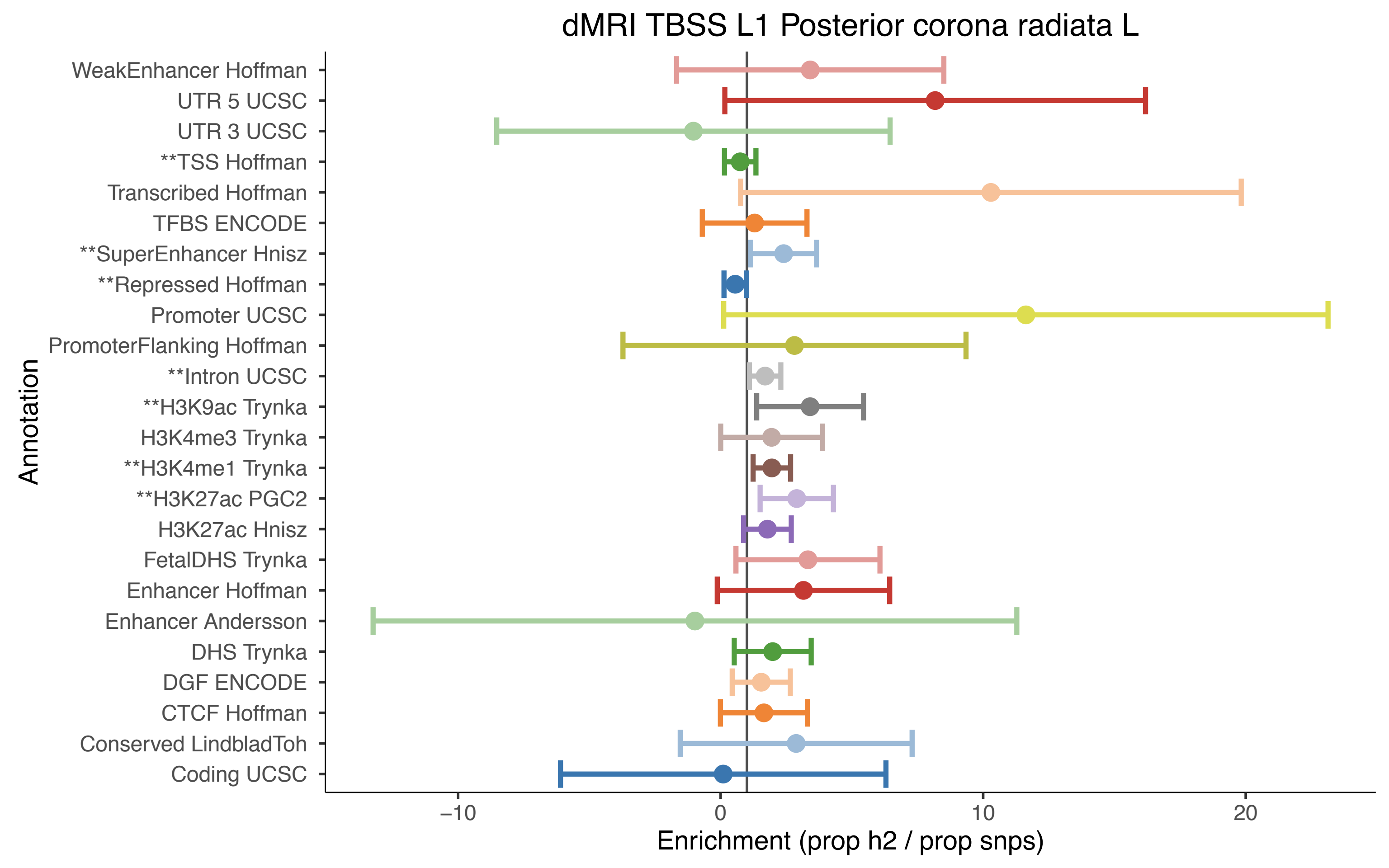
dMRI TBSS L1 Posterior corona radiata L
WeakEnhancer Hoffman
UTR 5 UCSC
UTR 3 UCSC
**TSS Hoffman
Transcribed Hoffman
TFBS ENCODE
**SuperEnhancer Hnisz
**Repressed Hoffman
Promoter UCSC
PromoterFlanking Hoffman
**Intron UCSC
**H3K9ac Trynka
H3K4me3 Trynka
**H3K4me1 Trynka
**H3K27ac PGC2
H3K27ac Hnisz
FetalDHS Trynka
Enhancer Hoffman
Enhancer Andersson
DHS Trynka
DGF ENCODE
CTCF Hoffman
Conserved LindbladToh
Coding UCSC
Annotation
−10
0
10
20
Enrichment (prop h2 / prop snps)

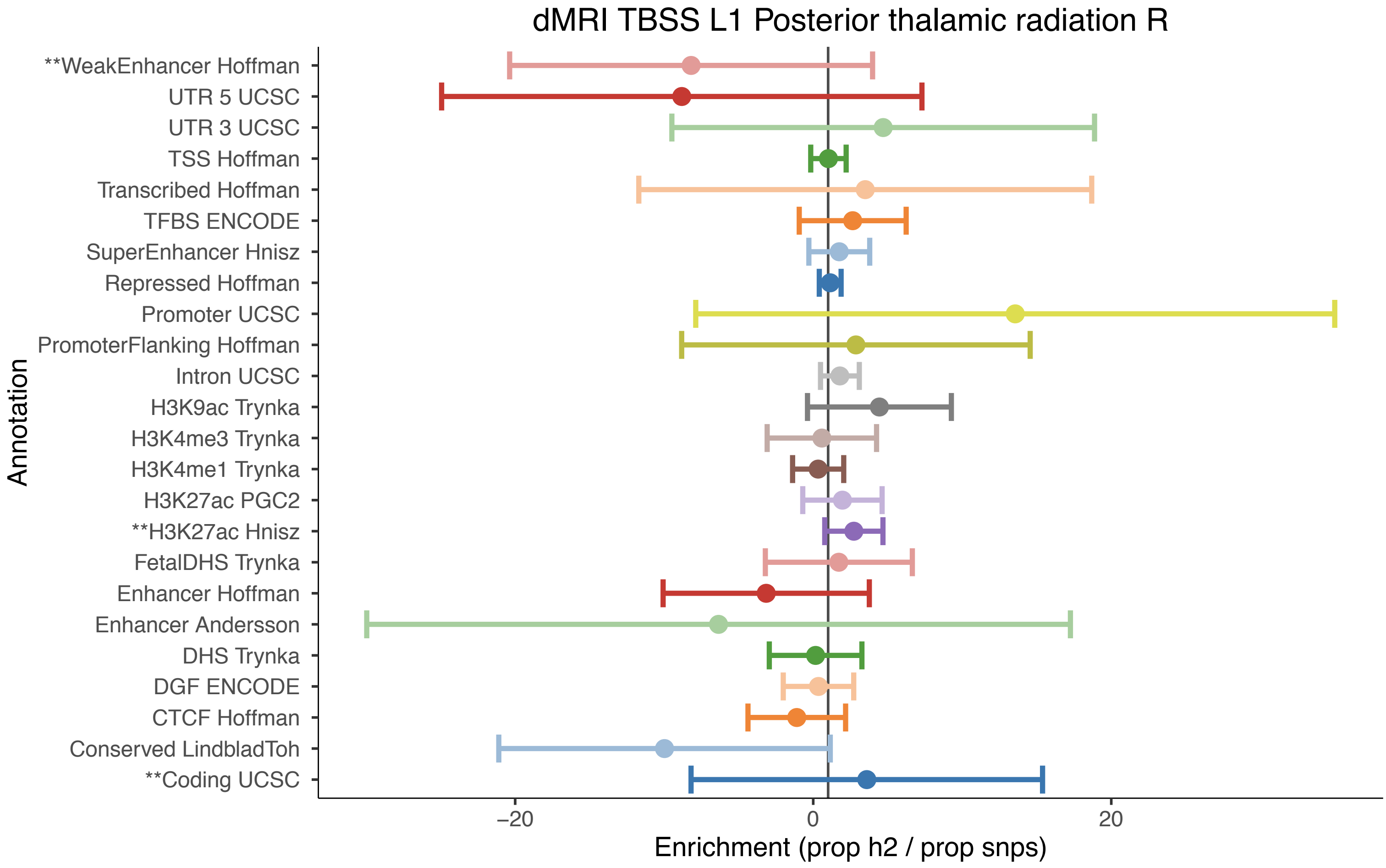
dMRI TBSS L1 Posterior thalamic radiation R
**WeakEnhancer Hoffman
UTR 5 UCSC
UTR 3 UCSC
TSS Hoffman
Transcribed Hoffman
TFBS ENCODE
SuperEnhancer Hnisz
Repressed Hoffman
Promoter UCSC
PromoterFlanking Hoffman
Intron UCSC
H3K9ac Trynka
H3K4me3 Trynka
H3K4me1 Trynka
H3K27ac PGC2
**H3K27ac Hnisz
FetalDHS Trynka
Enhancer Hoffman
Enhancer Andersson
DHS Trynka
DGF ENCODE
CTCF Hoffman
Conserved LindbladToh
**Coding UCSC
Annotation
−20
0
20
Enrichment (prop h2 / prop snps)

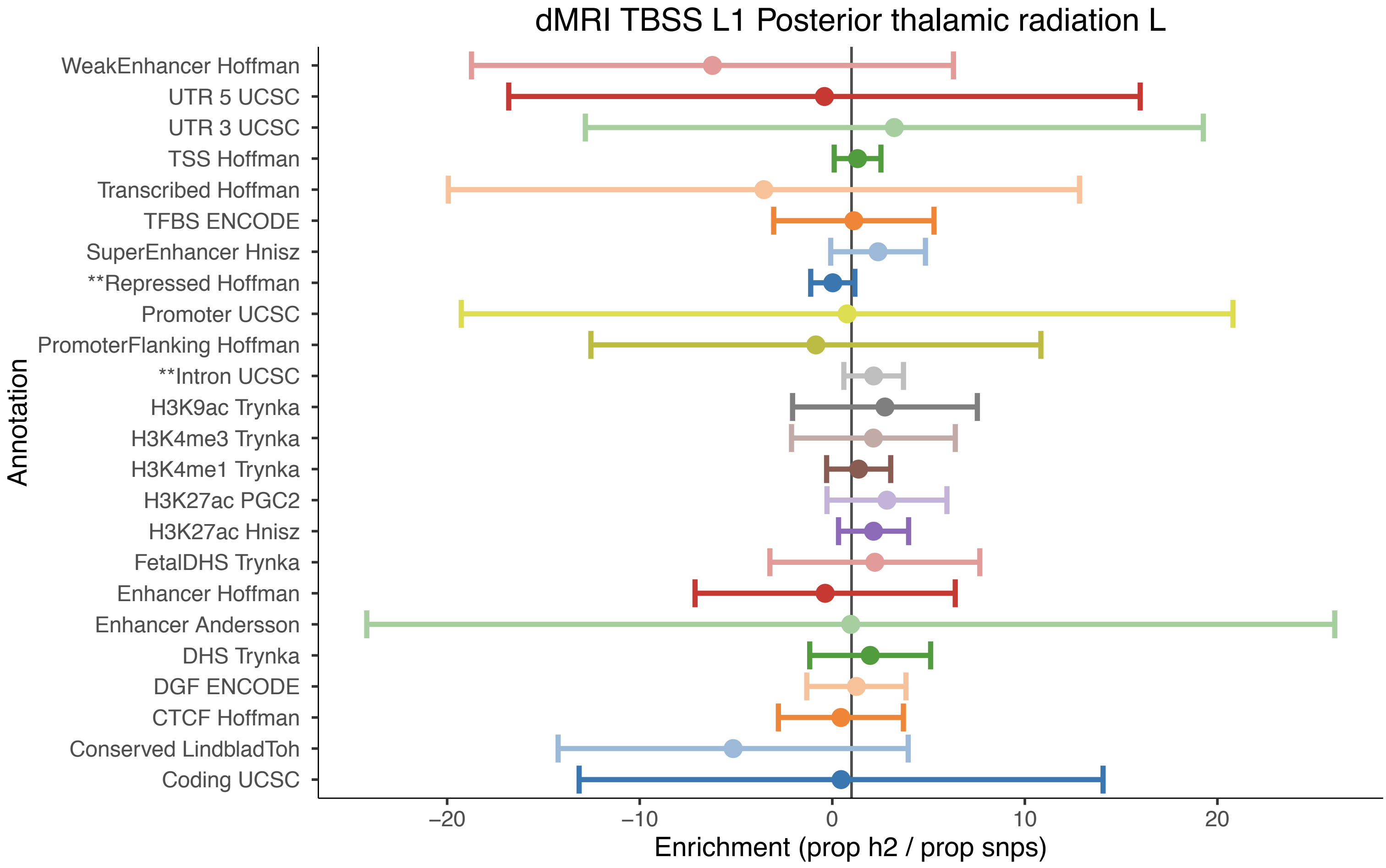
dMRI TBSS L1 Posterior thalamic radiation L
WeakEnhancer Hoffman
UTR 5 UCSC
UTR 3 UCSC
TSS Hoffman
Transcribed Hoffman
TFBS ENCODE
SuperEnhancer Hnisz
**Repressed Hoffman
Promoter UCSC
PromoterFlanking Hoffman
**Intron UCSC
H3K9ac Trynka
H3K4me3 Trynka
H3K4me1 Trynka
H3K27ac PGC2
H3K27ac Hnisz
FetalDHS Trynka
Enhancer Hoffman
Enhancer Andersson
DHS Trynka
DGF ENCODE
CTCF Hoffman
Conserved LindbladToh
Coding UCSC
Annotation
−20
−10
0
10
20
Enrichment (prop h2 / prop snps)

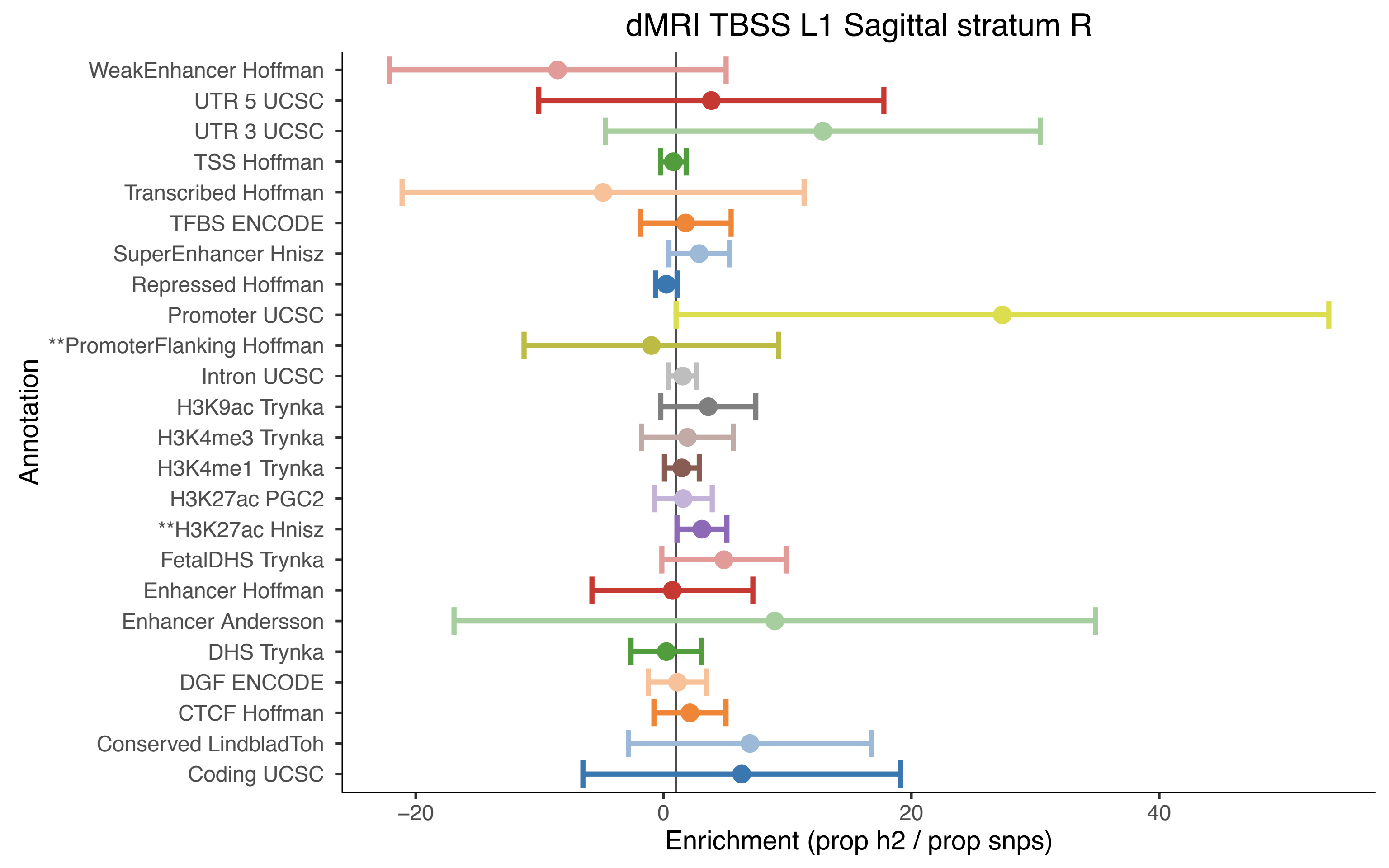

dMRI TBSS L1 Sagittal stratum R
WeakEnhancer Hoffman
UTR 5 UCSC
UTR 3 UCSC
TSS Hoffman
Transcribed Hoffman
TFBS ENCODE
SuperEnhancer Hnisz
Repressed Hoffman
Promoter UCSC
**PromoterFlanking Hoffman
Intron UCSC
H3K9ac Trynka
H3K4me3 Trynka
H3K4me1 Trynka
H3K27ac PGC2
**H3K27ac Hnisz
FetalDHS Trynka
Enhancer Hoffman
Enhancer Andersson
DHS Trynka
DGF ENCODE
CTCF Hoffman
Conserved LindbladToh
Coding UCSC
Annotation
−20
0
20
40
Enrichment (prop h2 / prop snps)

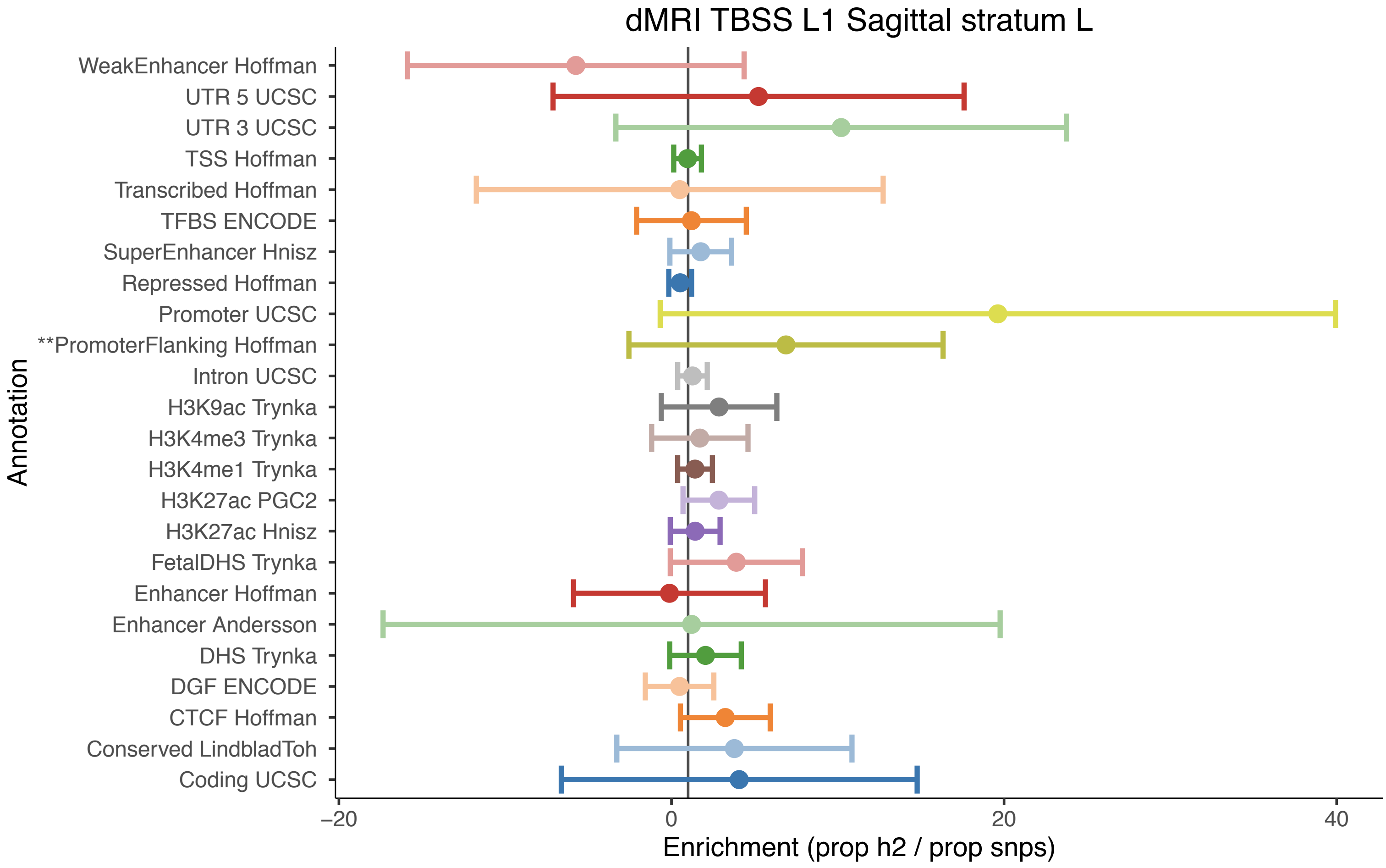

dMRI TBSS L1 Sagittal stratum L
WeakEnhancer Hoffman
UTR 5 UCSC
UTR 3 UCSC
TSS Hoffman
Transcribed Hoffman
TFBS ENCODE
SuperEnhancer Hnisz
Repressed Hoffman
Promoter UCSC
**PromoterFlanking Hoffman
Intron UCSC
H3K9ac Trynka
H3K4me3 Trynka
H3K4me1 Trynka
H3K27ac PGC2
H3K27ac Hnisz
FetalDHS Trynka
Enhancer Hoffman
Enhancer Andersson
DHS Trynka
DGF ENCODE
CTCF Hoffman
Conserved LindbladToh
Coding UCSC
Annotation
−20
0
20
40
Enrichment (prop h2 / prop snps)

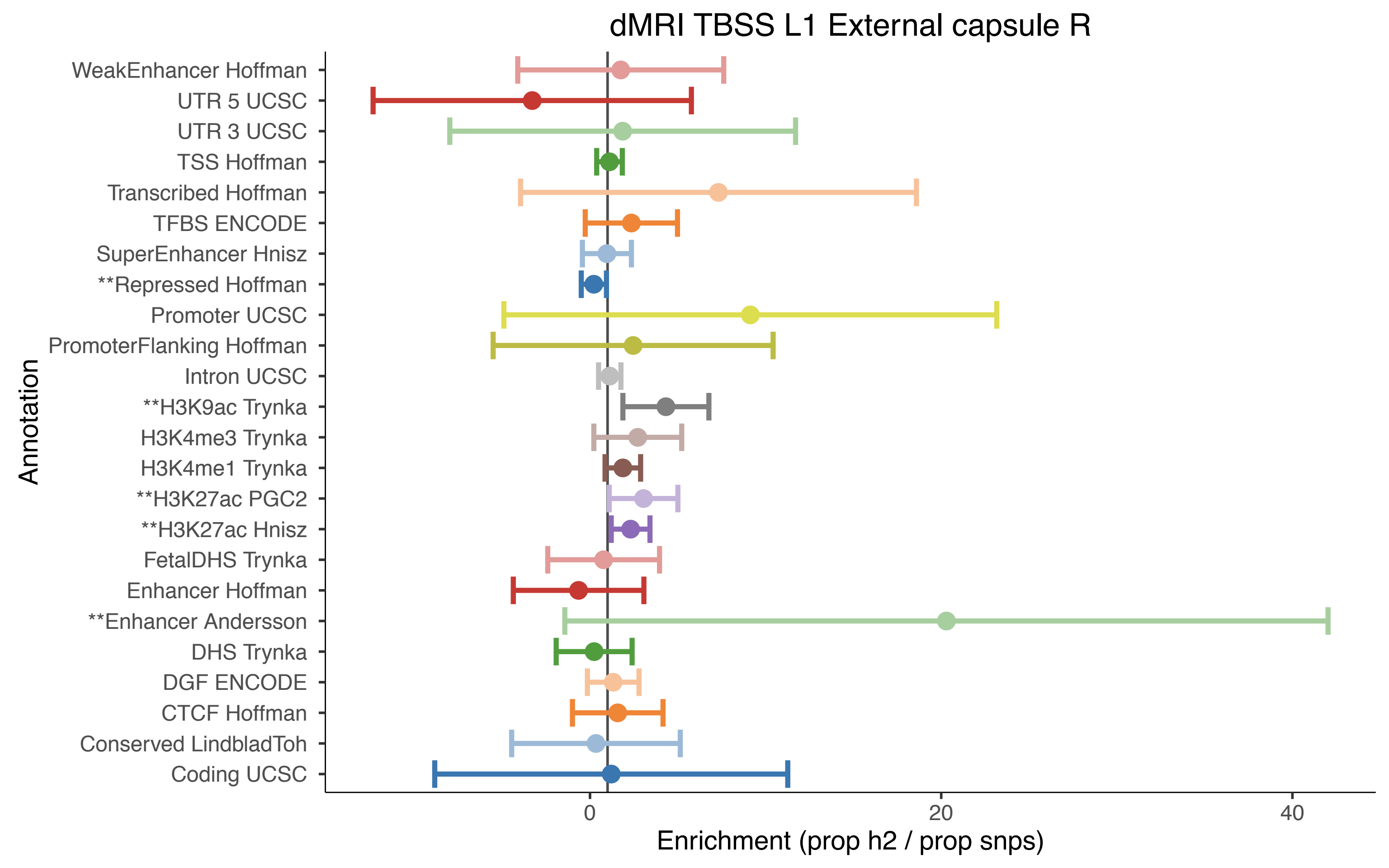

dMRI TBSS L1 External capsule R
WeakEnhancer Hoffman
UTR 5 UCSC
UTR 3 UCSC
TSS Hoffman
Transcribed Hoffman
TFBS ENCODE
SuperEnhancer Hnisz
**Repressed Hoffman
Promoter UCSC
PromoterFlanking Hoffman
Intron UCSC
**H3K9ac Trynka
H3K4me3 Trynka
H3K4me1 Trynka
**H3K27ac PGC2
**H3K27ac Hnisz
FetalDHS Trynka
Enhancer Hoffman
**Enhancer Andersson
DHS Trynka
DGF ENCODE
CTCF Hoffman
Conserved LindbladToh
Coding UCSC
Annotation
0
20
40
Enrichment (prop h2 / prop snps)

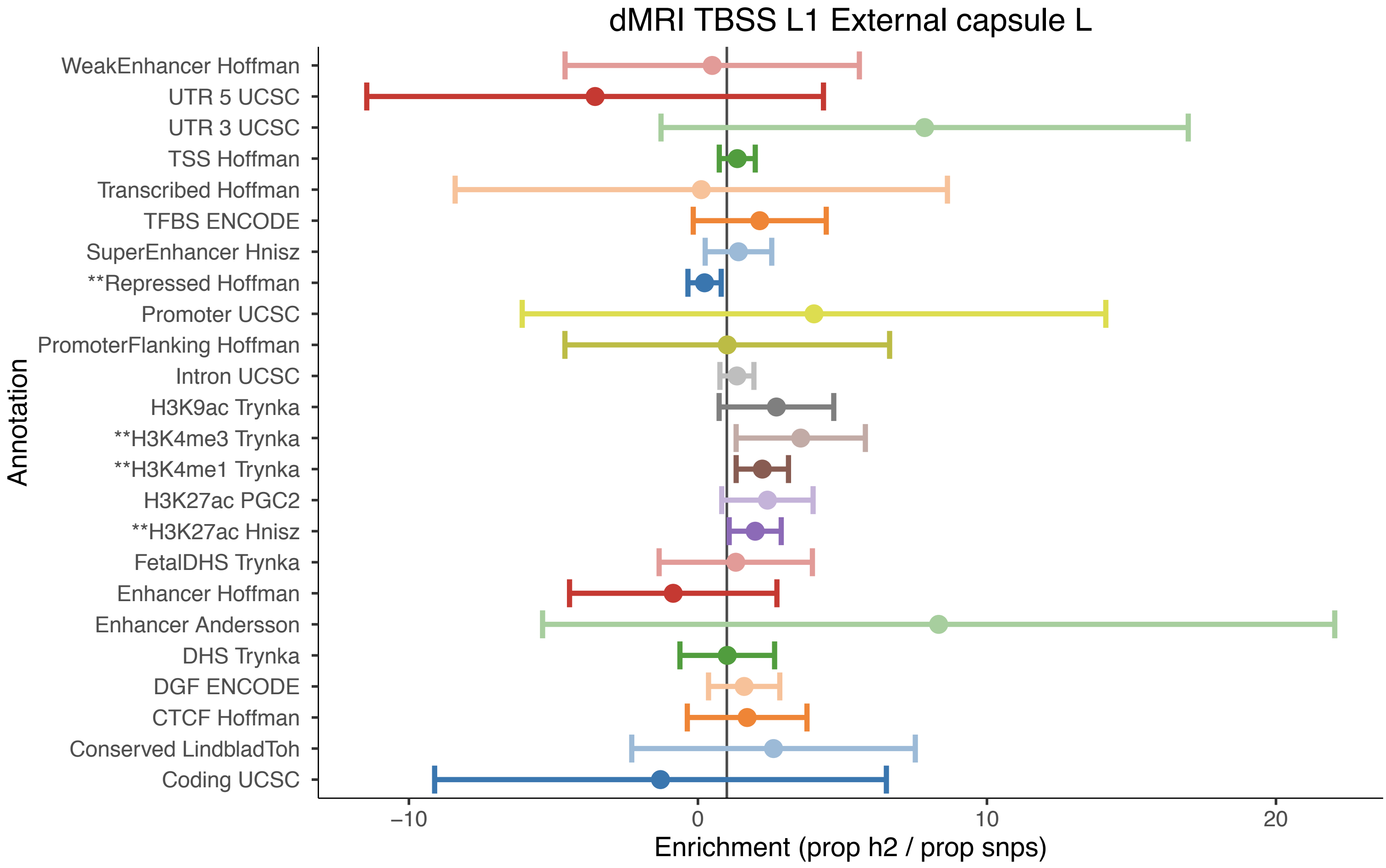

dMRI TBSS L1 External capsule L
Annotation
WeakEnhancer Hoffman
UTR 5 UCSC
UTR 3 UCSC
TSS Hoffman
Transcribed Hoffman
TFBS ENCODE
SuperEnhancer Hnisz
**Repressed Hoffman
Promoter UCSC
PromoterFlanking Hoffman
Intron UCSC
H3K9ac Trynka
**H3K4me3 Trynka
**H3K4me1 Trynka
H3K27ac PGC2
**H3K27ac Hnisz
FetalDHS Trynka
Enhancer Hoffman
Enhancer Andersson
DHS Trynka
DGF ENCODE
CTCF Hoffman
Conserved LindbladToh
Coding UCSC
−10
0
10
20
Enrichment (prop h2 / prop snps)

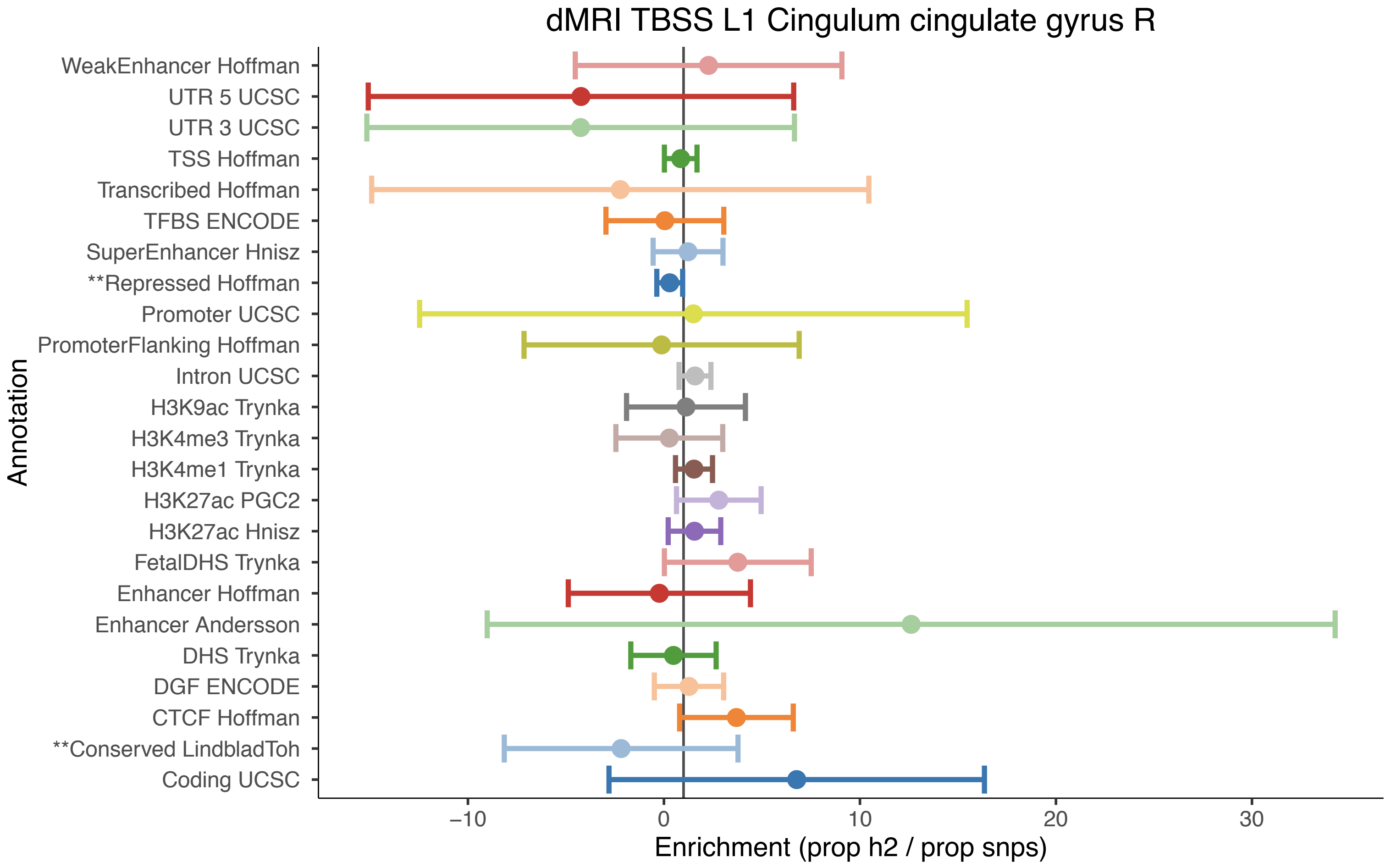

dMRI TBSS L1 Cingulum cingulate gyrus R
WeakEnhancer Hoffman
UTR 5 UCSC
UTR 3 UCSC
TSS Hoffman
Transcribed Hoffman
TFBS ENCODE
SuperEnhancer Hnisz
**Repressed Hoffman
Promoter UCSC
PromoterFlanking Hoffman
Intron UCSC
H3K9ac Trynka
H3K4me3 Trynka
H3K4me1 Trynka
H3K27ac PGC2
H3K27ac Hnisz
FetalDHS Trynka
Enhancer Hoffman
Enhancer Andersson
DHS Trynka
DGF ENCODE
CTCF Hoffman
**Conserved LindbladToh
Coding UCSC
Annotation
−10
0
10
20
30
Enrichment (prop h2 / prop snps)

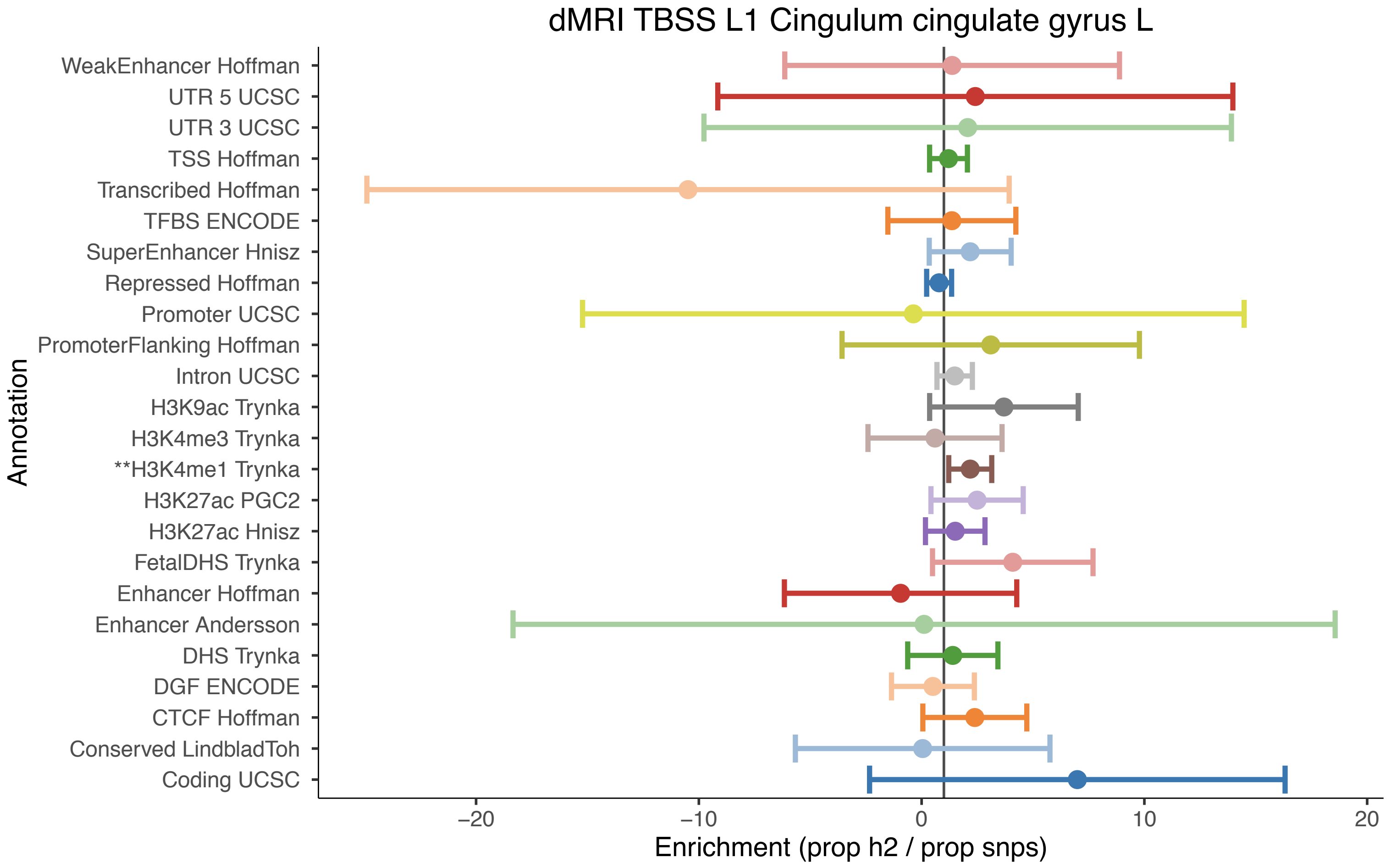

dMRI TBSS L1 Cingulum cingulate gyrus L
WeakEnhancer Hoffman
UTR 5 UCSC
UTR 3 UCSC
TSS Hoffman
Transcribed Hoffman
TFBS ENCODE
SuperEnhancer Hnisz
Repressed Hoffman
Promoter UCSC
PromoterFlanking Hoffman
Intron UCSC
H3K9ac Trynka
H3K4me3 Trynka
**H3K4me1 Trynka
H3K27ac PGC2
H3K27ac Hnisz
FetalDHS Trynka
Enhancer Hoffman
Enhancer Andersson
DHS Trynka
DGF ENCODE
CTCF Hoffman
Conserved LindbladToh
Coding UCSC
Annotation
−20
−10
0
10
20
Enrichment (prop h2 / prop snps)

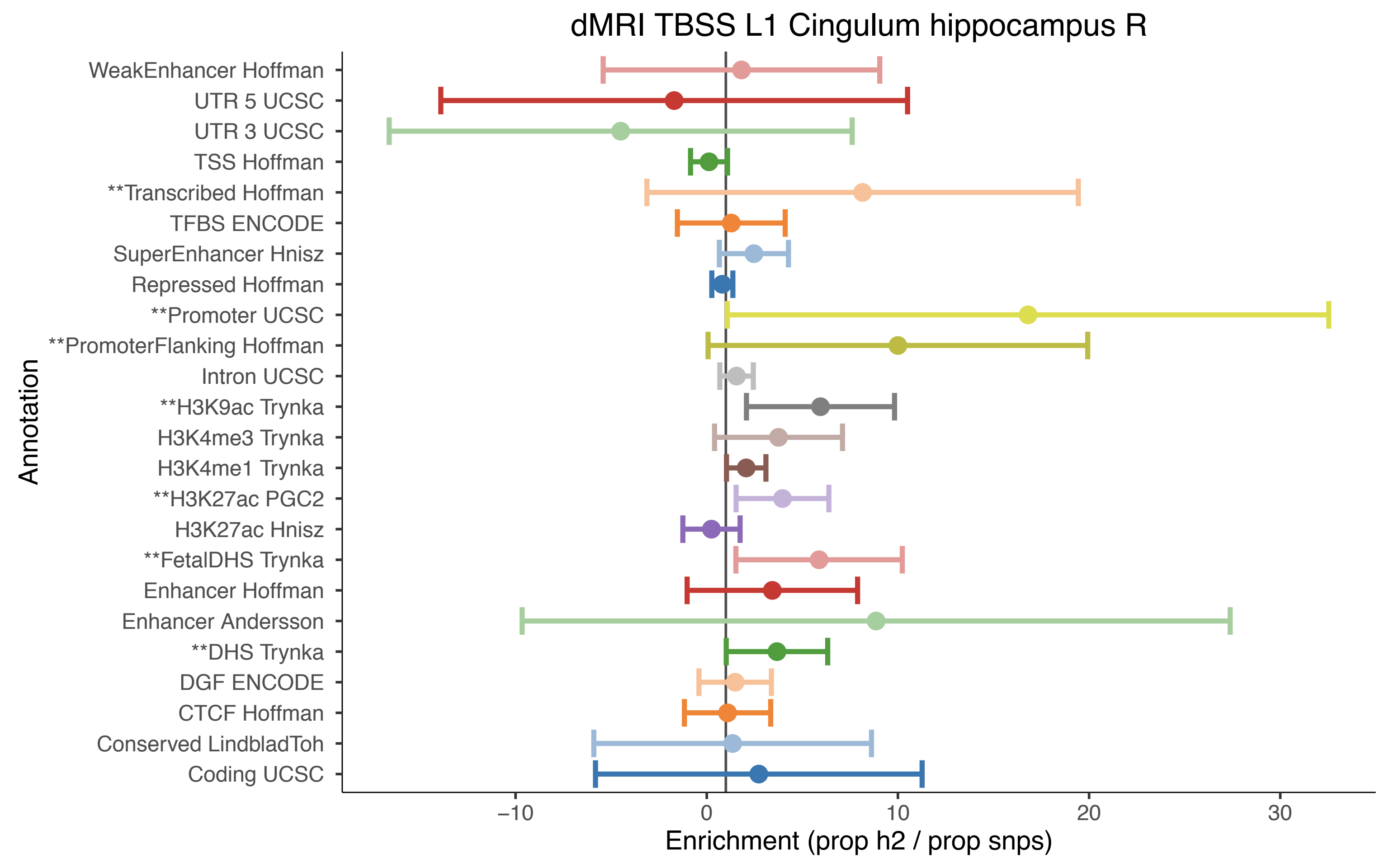

dMRI TBSS L1 Cingulum hippocampus R
WeakEnhancer Hoffman
UTR 5 UCSC
UTR 3 UCSC
TSS Hoffman
**Transcribed Hoffman
TFBS ENCODE
SuperEnhancer Hnisz
Repressed Hoffman
**Promoter UCSC
**PromoterFlanking Hoffman
Intron UCSC
**H3K9ac Trynka
H3K4me3 Trynka
H3K4me1 Trynka
**H3K27ac PGC2
H3K27ac Hnisz
**FetalDHS Trynka
Enhancer Hoffman
Enhancer Andersson
**DHS Trynka
DGF ENCODE
CTCF Hoffman
Conserved LindbladToh
Coding UCSC
Annotation
−10
0
10
20
30
Enrichment (prop h2 / prop snps)

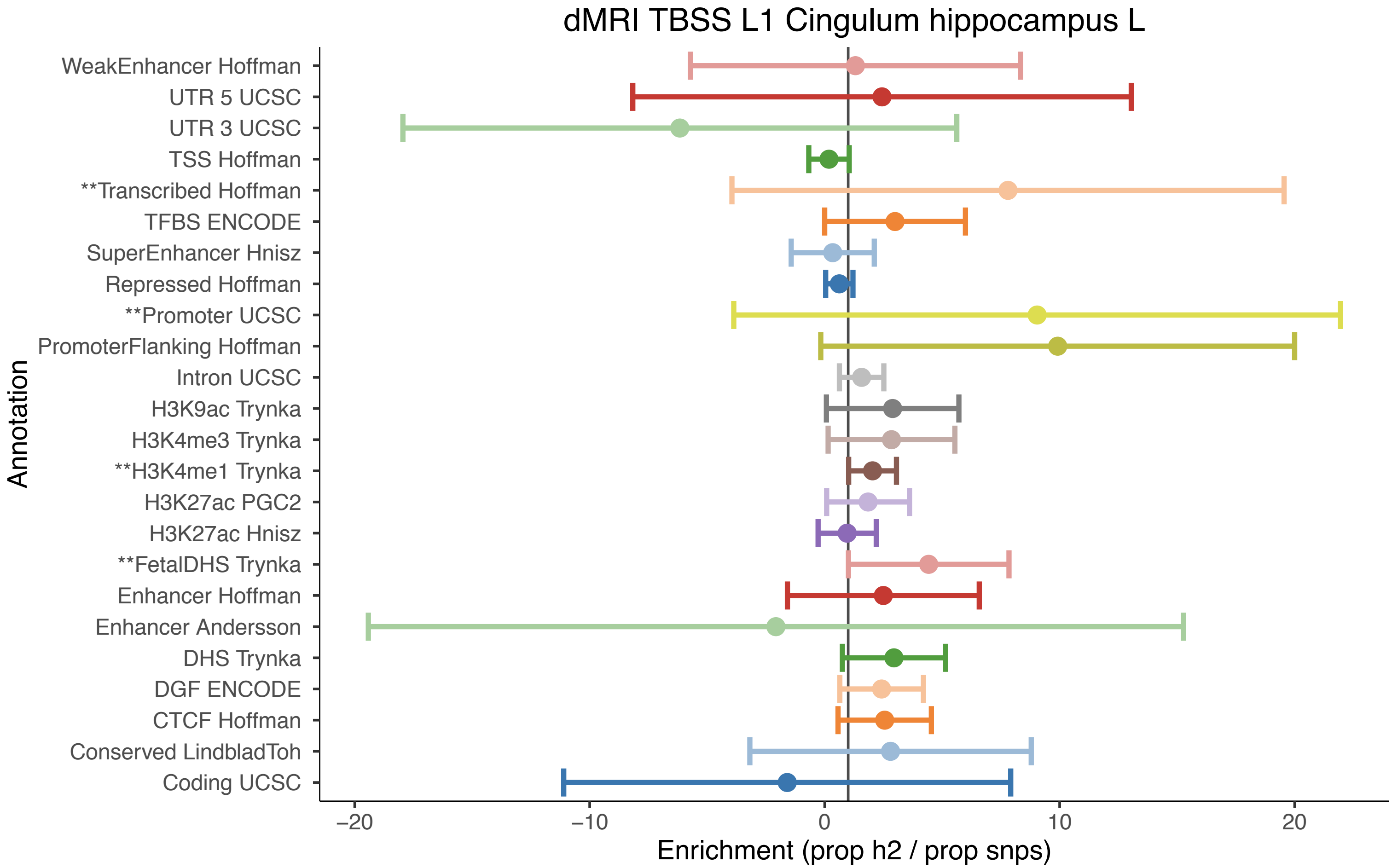

dMRI TBSS L1 Cingulum hippocampus L
WeakEnhancer Hoffman
UTR 5 UCSC
UTR 3 UCSC
TSS Hoffman
**Transcribed Hoffman
TFBS ENCODE
SuperEnhancer Hnisz
Repressed Hoffman
**Promoter UCSC
PromoterFlanking Hoffman
Intron UCSC
H3K9ac Trynka
H3K4me3 Trynka
**H3K4me1 Trynka
H3K27ac PGC2
H3K27ac Hnisz
**FetalDHS Trynka
Enhancer Hoffman
Enhancer Andersson
DHS Trynka
DGF ENCODE
CTCF Hoffman
Conserved LindbladToh
Coding UCSC
Annotation
−20
−10
0
10
20
Enrichment (prop h2 / prop snps)

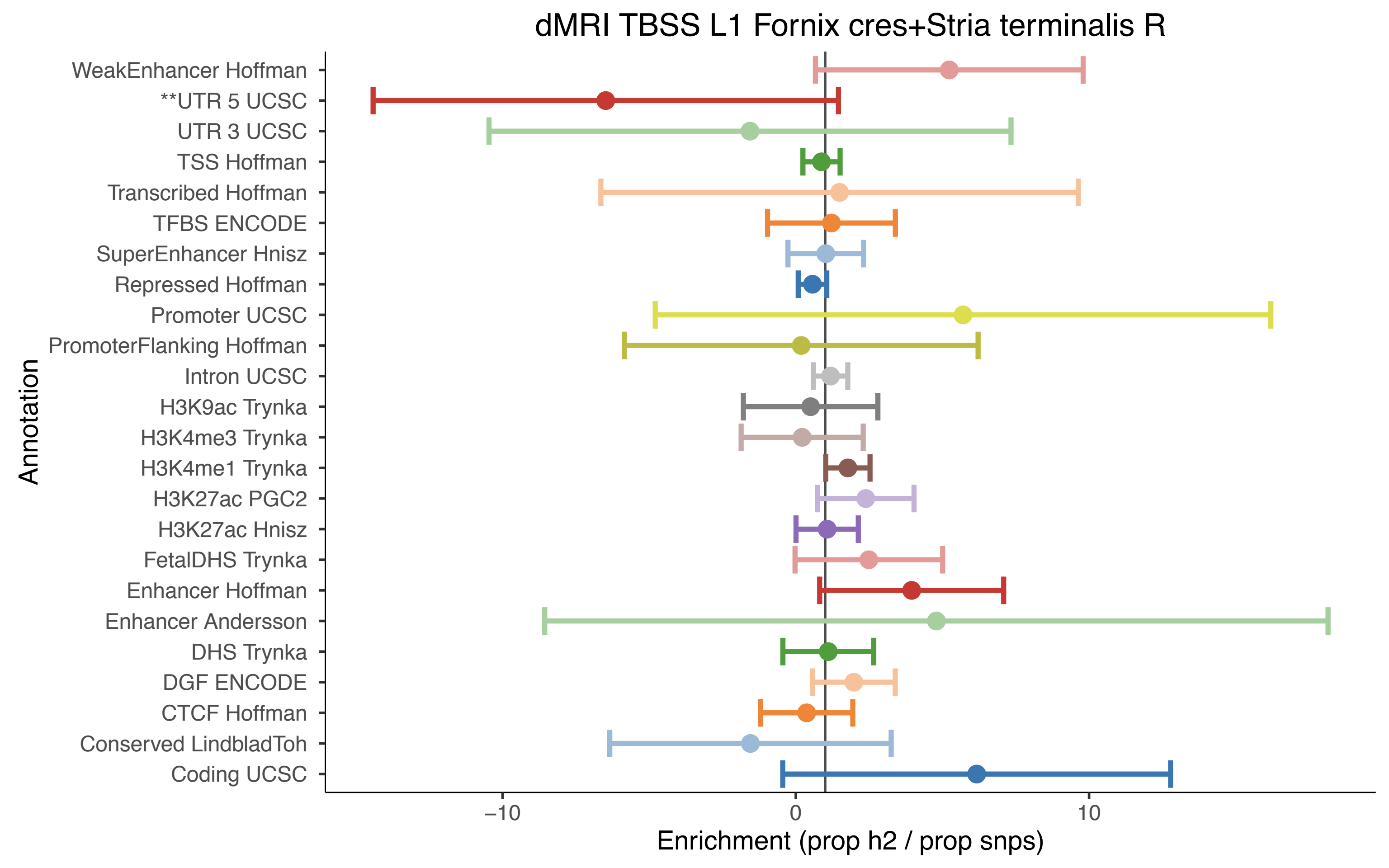

dMRI TBSS L1 Fornix cres+Stria terminalis R
WeakEnhancer Hoffman
**UTR 5 UCSC
UTR 3 UCSC
TSS Hoffman
Transcribed Hoffman
TFBS ENCODE
SuperEnhancer Hnisz
Repressed Hoffman
Promoter UCSC
PromoterFlanking Hoffman
Intron UCSC
H3K9ac Trynka
H3K4me3 Trynka
H3K4me1 Trynka
H3K27ac PGC2
H3K27ac Hnisz
FetalDHS Trynka
Enhancer Hoffman
Enhancer Andersson
DHS Trynka
DGF ENCODE
CTCF Hoffman
Conserved LindbladToh
Coding UCSC
Annotation
−10
0
10
Enrichment (prop h2 / prop snps)

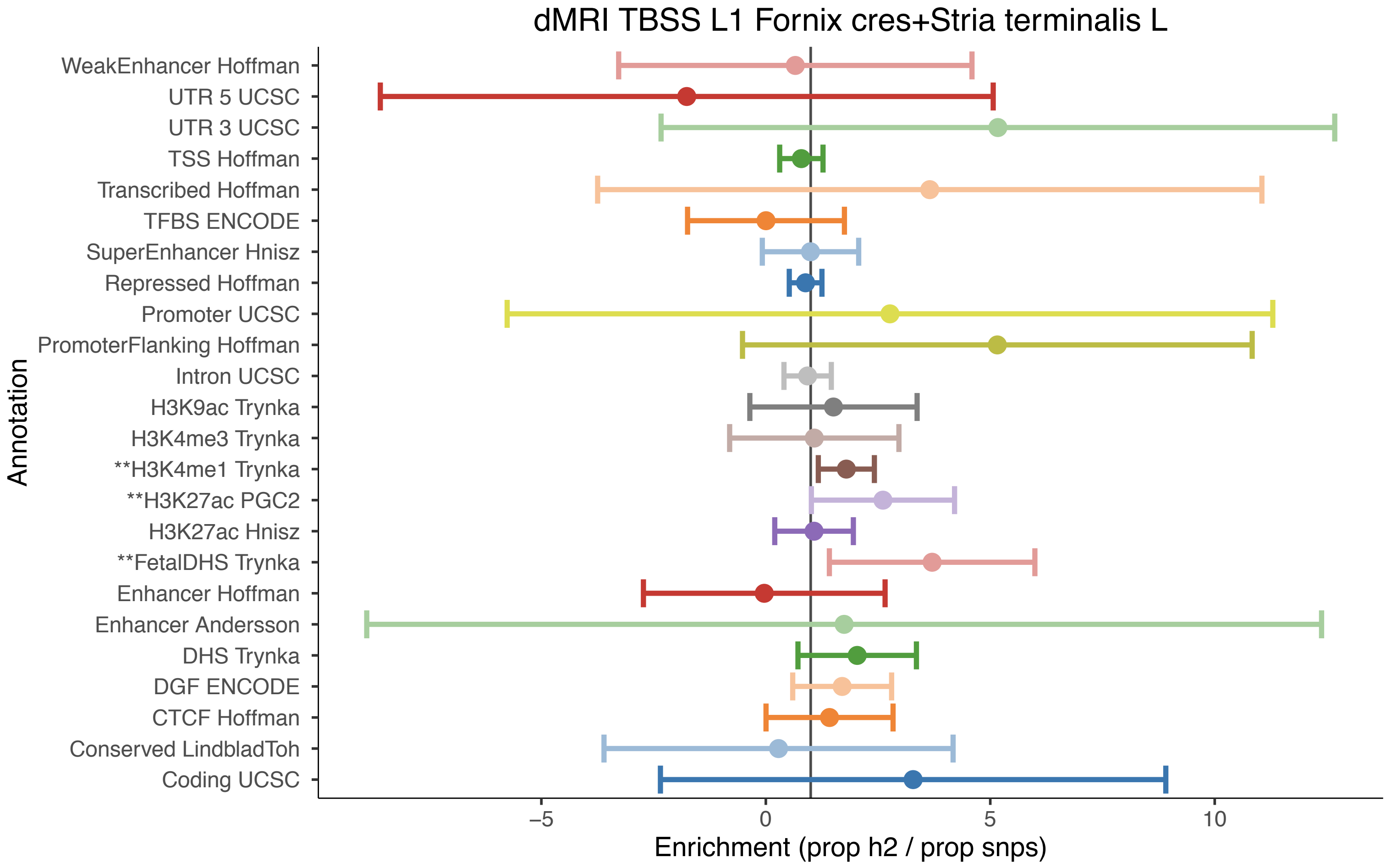

dMRI TBSS L1 Fornix cres+Stria terminalis L
WeakEnhancer Hoffman
UTR 5 UCSC
UTR 3 UCSC
TSS Hoffman
Transcribed Hoffman
TFBS ENCODE
SuperEnhancer Hnisz
Repressed Hoffman
Promoter UCSC
PromoterFlanking Hoffman
Intron UCSC
H3K9ac Trynka
H3K4me3 Trynka
**H3K4me1 Trynka
**H3K27ac PGC2
H3K27ac Hnisz
**FetalDHS Trynka
Enhancer Hoffman
Enhancer Andersson
DHS Trynka
DGF ENCODE
CTCF Hoffman
Conserved LindbladToh
Coding UCSC
Annotation
−5
0
5
10
Enrichment (prop h2 / prop snps)

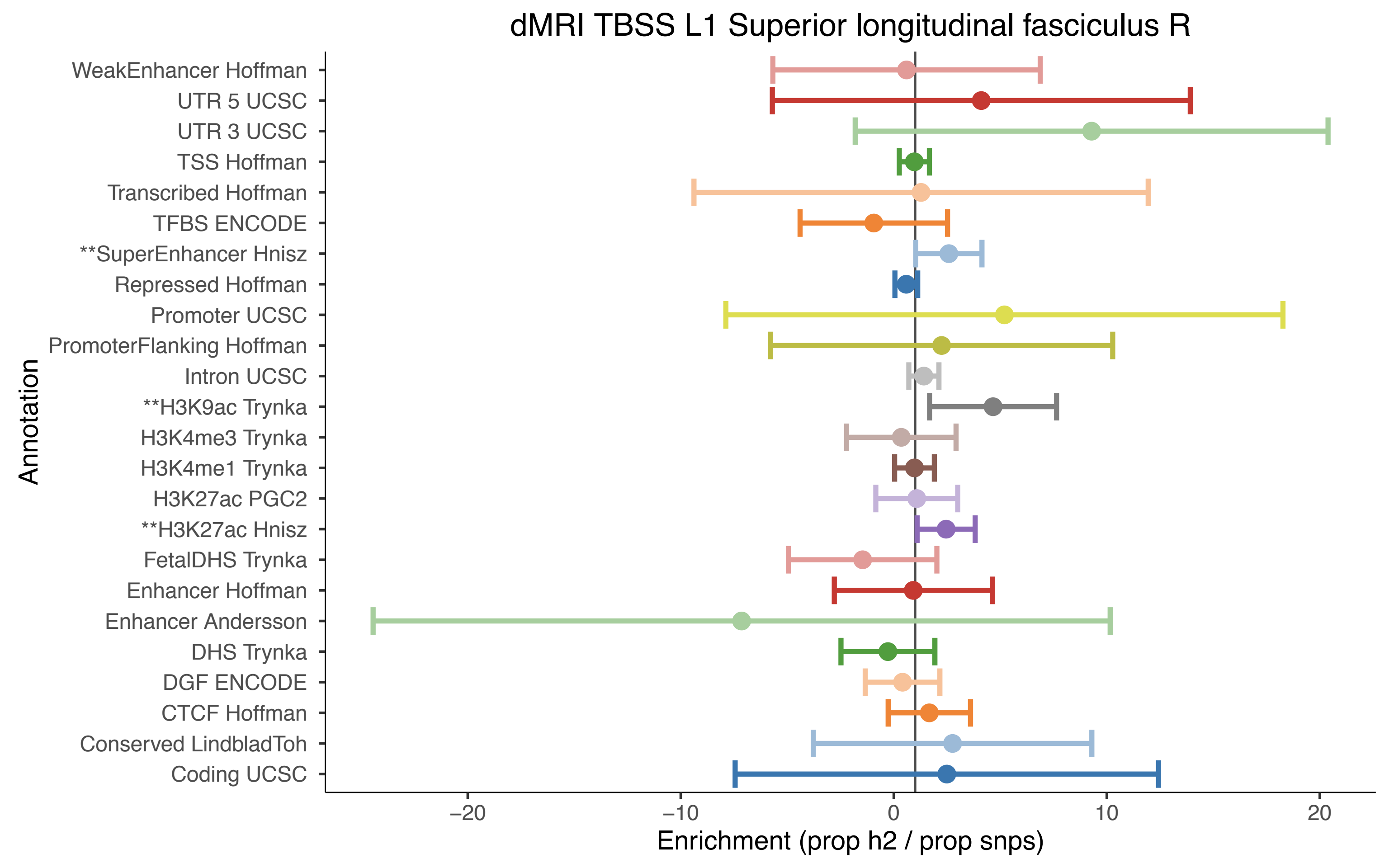
dMRI TBSS L1 Superior longitudinal fasciculus R
WeakEnhancer Hoffman
UTR 5 UCSC
UTR 3 UCSC
TSS Hoffman
Transcribed Hoffman
TFBS ENCODE
**SuperEnhancer Hnisz
Repressed Hoffman
Promoter UCSC
PromoterFlanking Hoffman
Intron UCSC
**H3K9ac Trynka
H3K4me3 Trynka
H3K4me1 Trynka
H3K27ac PGC2
**H3K27ac Hnisz
FetalDHS Trynka
Enhancer Hoffman
Enhancer Andersson
DHS Trynka
DGF ENCODE
CTCF Hoffman
Conserved LindbladToh
Coding UCSC
Annotation
−20
−10
0
10
20
Enrichment (prop h2 / prop snps)

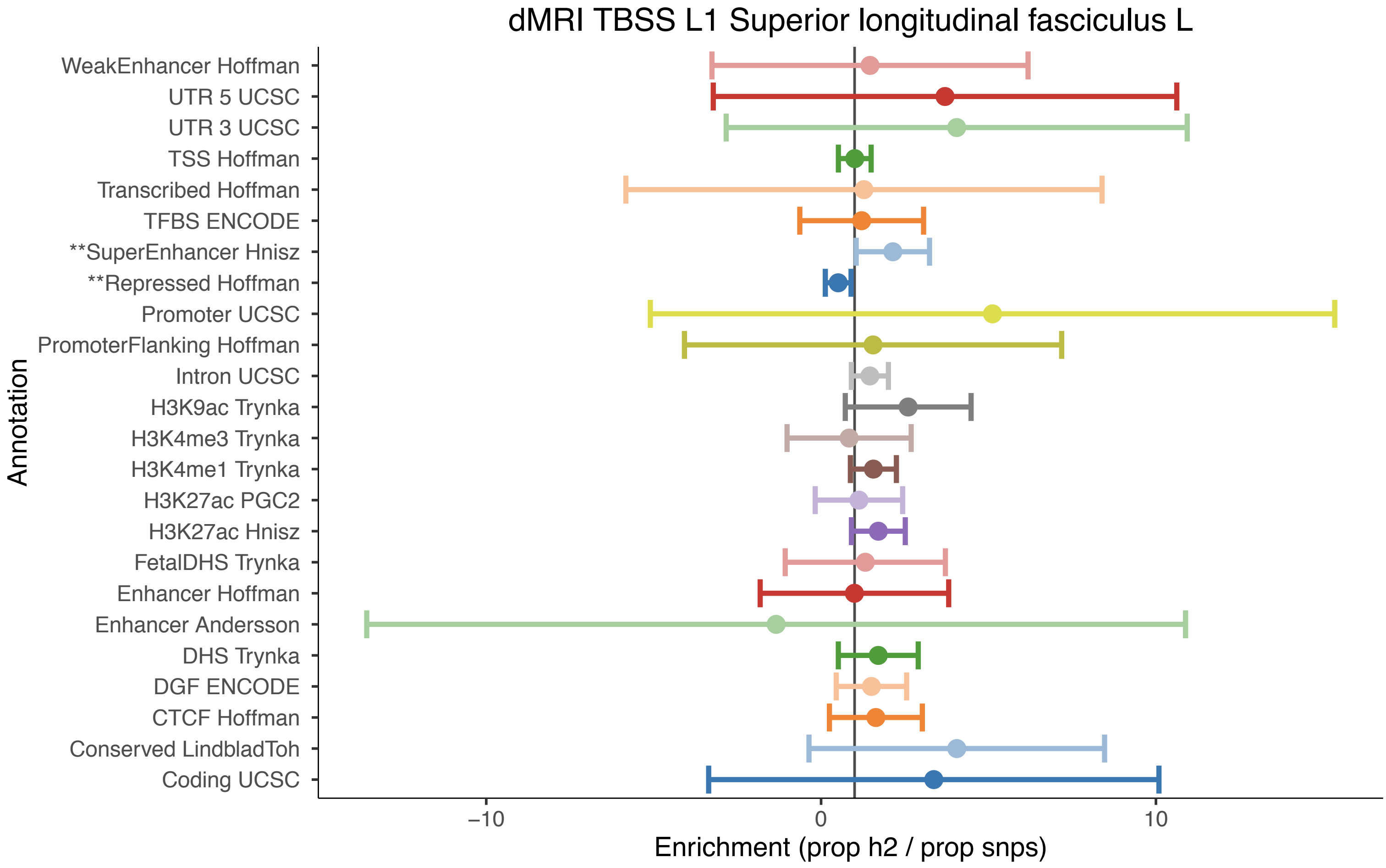
dMRI TBSS L1 Superior longitudinal fasciculus L
WeakEnhancer Hoffman
UTR 5 UCSC
UTR 3 UCSC
TSS Hoffman
Transcribed Hoffman
TFBS ENCODE
**SuperEnhancer Hnisz
**Repressed Hoffman
Promoter UCSC
PromoterFlanking Hoffman
Intron UCSC
H3K9ac Trynka
H3K4me3 Trynka
H3K4me1 Trynka
H3K27ac PGC2
H3K27ac Hnisz
FetalDHS Trynka
Enhancer Hoffman
Enhancer Andersson
DHS Trynka
DGF ENCODE
CTCF Hoffman
Conserved LindbladToh
Coding UCSC
Annotation
−10
0
10
Enrichment (prop h2 / prop snps)

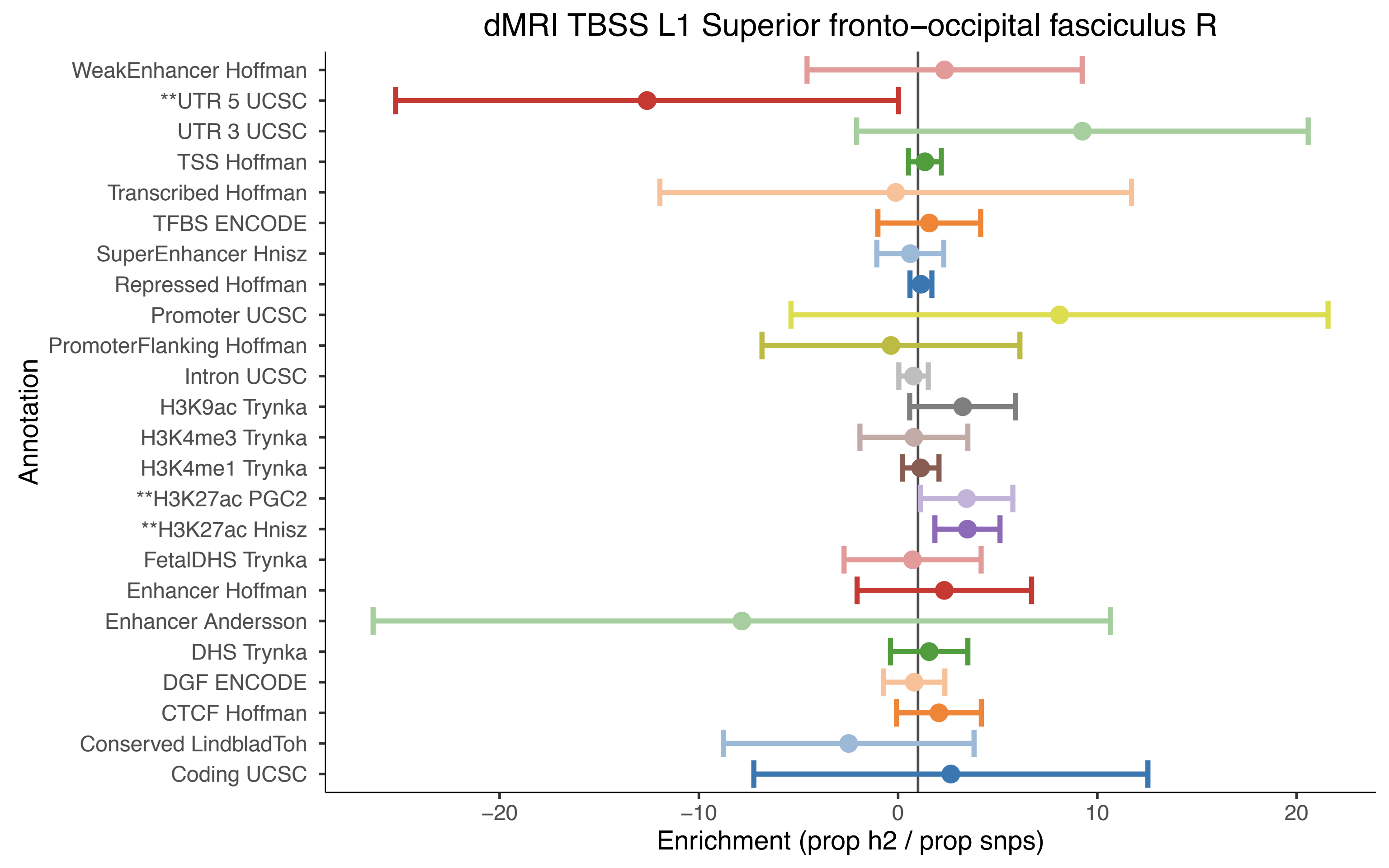
dMRI TBSS L1 Superior fronto–occipital fasciculus R
WeakEnhancer Hoffman
**UTR 5 UCSC
UTR 3 UCSC
TSS Hoffman
Transcribed Hoffman
TFBS ENCODE
SuperEnhancer Hnisz
Repressed Hoffman
Promoter UCSC
PromoterFlanking Hoffman
Intron UCSC
H3K9ac Trynka
H3K4me3 Trynka
H3K4me1 Trynka
**H3K27ac PGC2
**H3K27ac Hnisz
FetalDHS Trynka
Enhancer Hoffman
Enhancer Andersson
DHS Trynka
DGF ENCODE
CTCF Hoffman
Conserved LindbladToh
Coding UCSC
Annotation
−20
−10
0
10
20
Enrichment (prop h2 / prop snps)

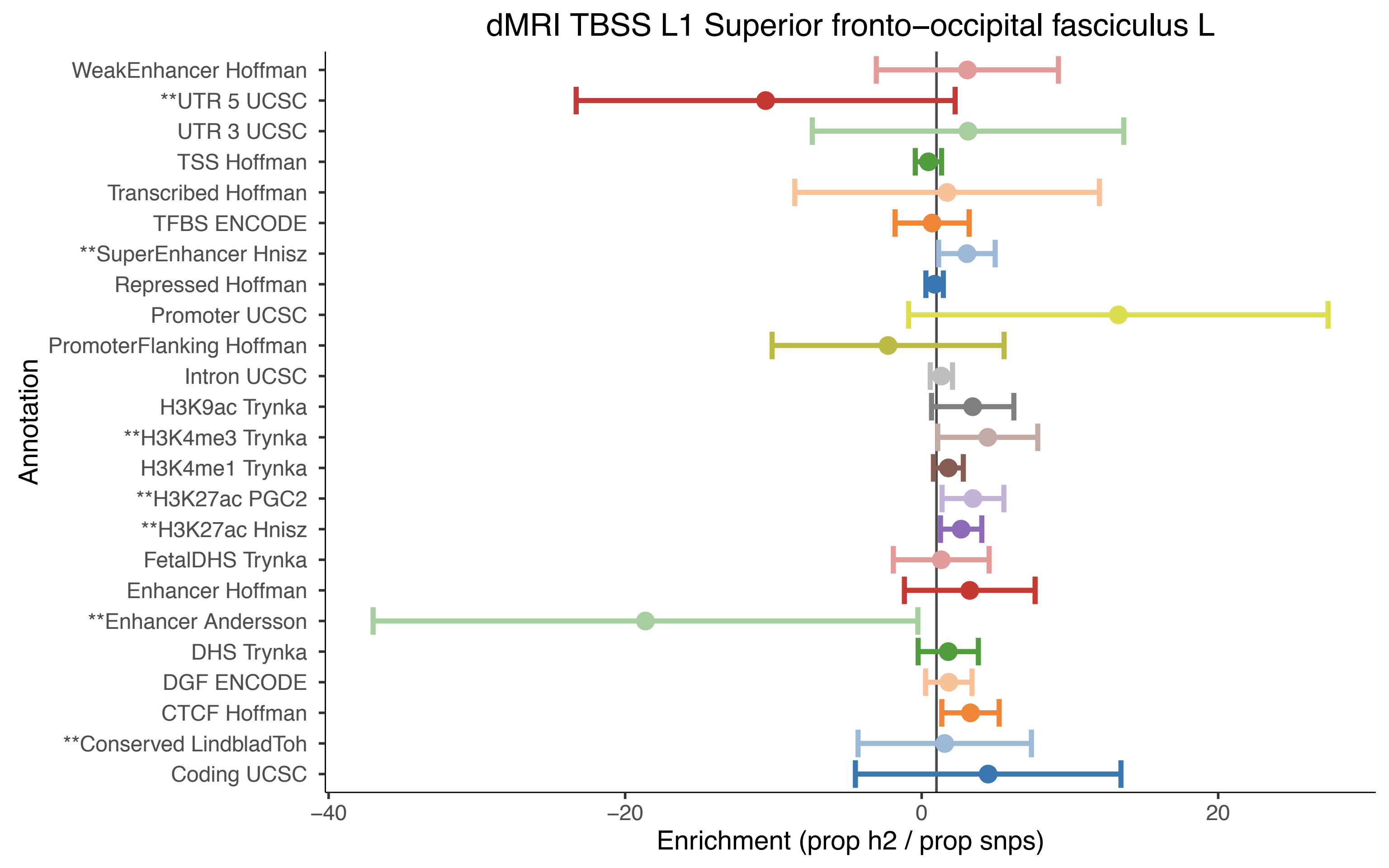

dMRI TBSS L1 Superior fronto−occipital fasciculus L
Annotation
WeakEnhancer Hoffman
**UTR 5 UCSC
UTR 3 UCSC
TSS Hoffman
Transcribed Hoffman
TFBS ENCODE
**SuperEnhancer Hnisz
Repressed Hoffman
Promoter UCSC
PromoterFlanking Hoffman
Intron UCSC
H3K9ac Trynka
**H3K4me3 Trynka
H3K4me1 Trynka
**H3K27ac PGC2
**H3K27ac Hnisz
FetalDHS Trynka
Enhancer Hoffman
**Enhancer Andersson
DHS Trynka
DGF ENCODE
CTCF Hoffman
**Conserved LindbladToh
Coding UCSC
−40
−20
0
20
Enrichment (prop h2 / prop snps)

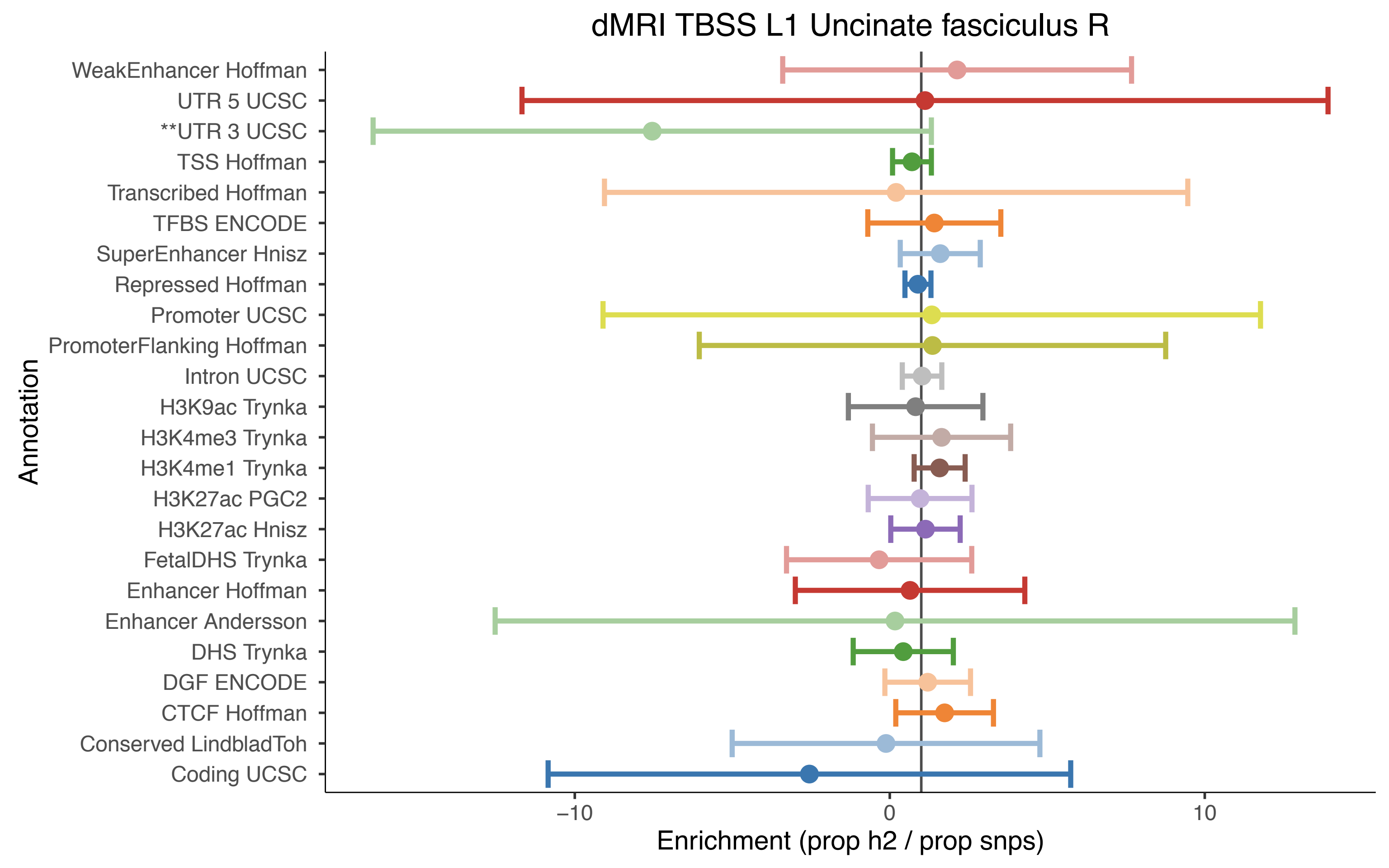
dMRI TBSS L1 Uncinate fasciculus R
WeakEnhancer Hoffman
UTR 5 UCSC
**UTR 3 UCSC
TSS Hoffman
Transcribed Hoffman
TFBS ENCODE
SuperEnhancer Hnisz
Repressed Hoffman
Promoter UCSC
PromoterFlanking Hoffman
Intron UCSC
H3K9ac Trynka
H3K4me3 Trynka
H3K4me1 Trynka
H3K27ac PGC2
H3K27ac Hnisz
FetalDHS Trynka
Enhancer Hoffman
Enhancer Andersson
DHS Trynka
DGF ENCODE
CTCF Hoffman
Conserved LindbladToh
Coding UCSC
Annotation
−10
0
10
Enrichment (prop h2 / prop snps)

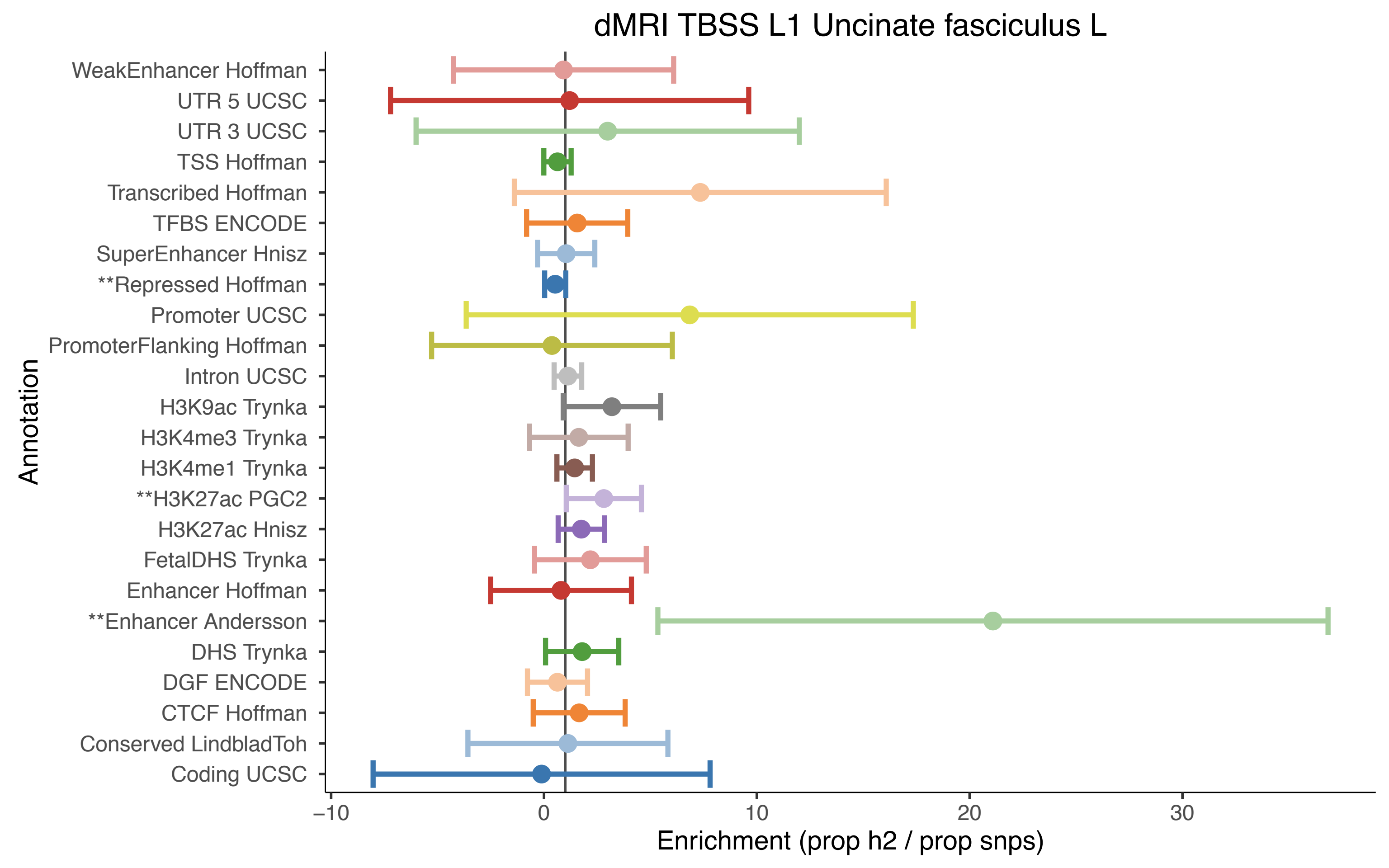

dMRI TBSS L1 Uncinate fasciculus L
WeakEnhancer Hoffman
UTR 5 UCSC
UTR 3 UCSC
TSS Hoffman
Transcribed Hoffman
TFBS ENCODE
SuperEnhancer Hnisz
**Repressed Hoffman
Promoter UCSC
PromoterFlanking Hoffman
Intron UCSC
H3K9ac Trynka
H3K4me3 Trynka
H3K4me1 Trynka
**H3K27ac PGC2
H3K27ac Hnisz
FetalDHS Trynka
Enhancer Hoffman
**Enhancer Andersson
DHS Trynka
DGF ENCODE
CTCF Hoffman
Conserved LindbladToh
Coding UCSC
Annotation
−10
0
10
20
30
Enrichment (prop h2 / prop snps)

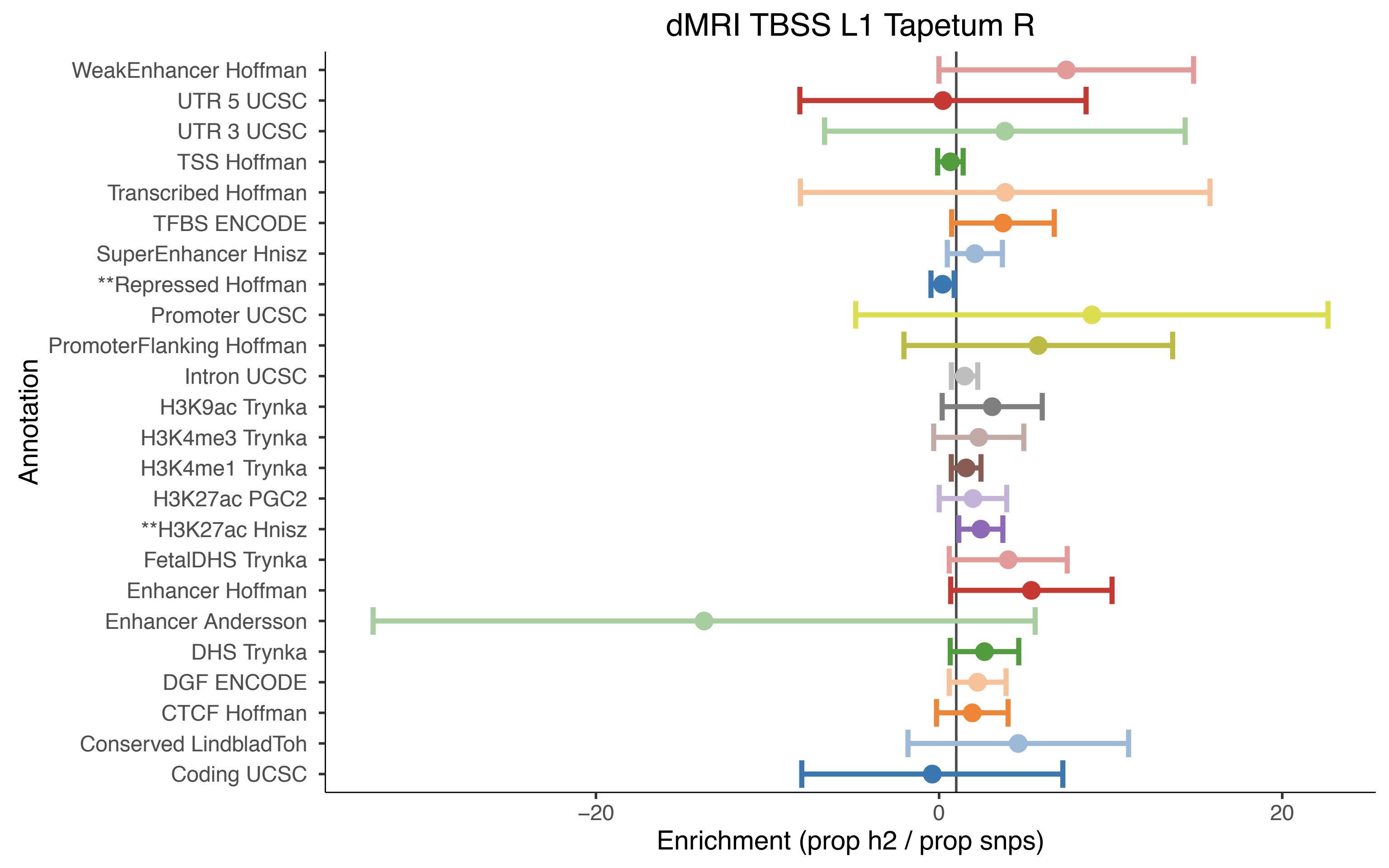
dMRI TBSS L1 Tapetum R
WeakEnhancer Hoffman
UTR 5 UCSC
UTR 3 UCSC
TSS Hoffman
Transcribed Hoffman
TFBS ENCODE
SuperEnhancer Hnisz
**Repressed Hoffman
Promoter UCSC
PromoterFlanking Hoffman
Intron UCSC
H3K9ac Trynka
H3K4me3 Trynka
H3K4me1 Trynka
H3K27ac PGC2
**H3K27ac Hnisz
FetalDHS Trynka
Enhancer Hoffman
Enhancer Andersson
DHS Trynka
DGF ENCODE
CTCF Hoffman
Conserved LindbladToh
Coding UCSC
Annotation
−20
0
20
Enrichment (prop h2 / prop snps)

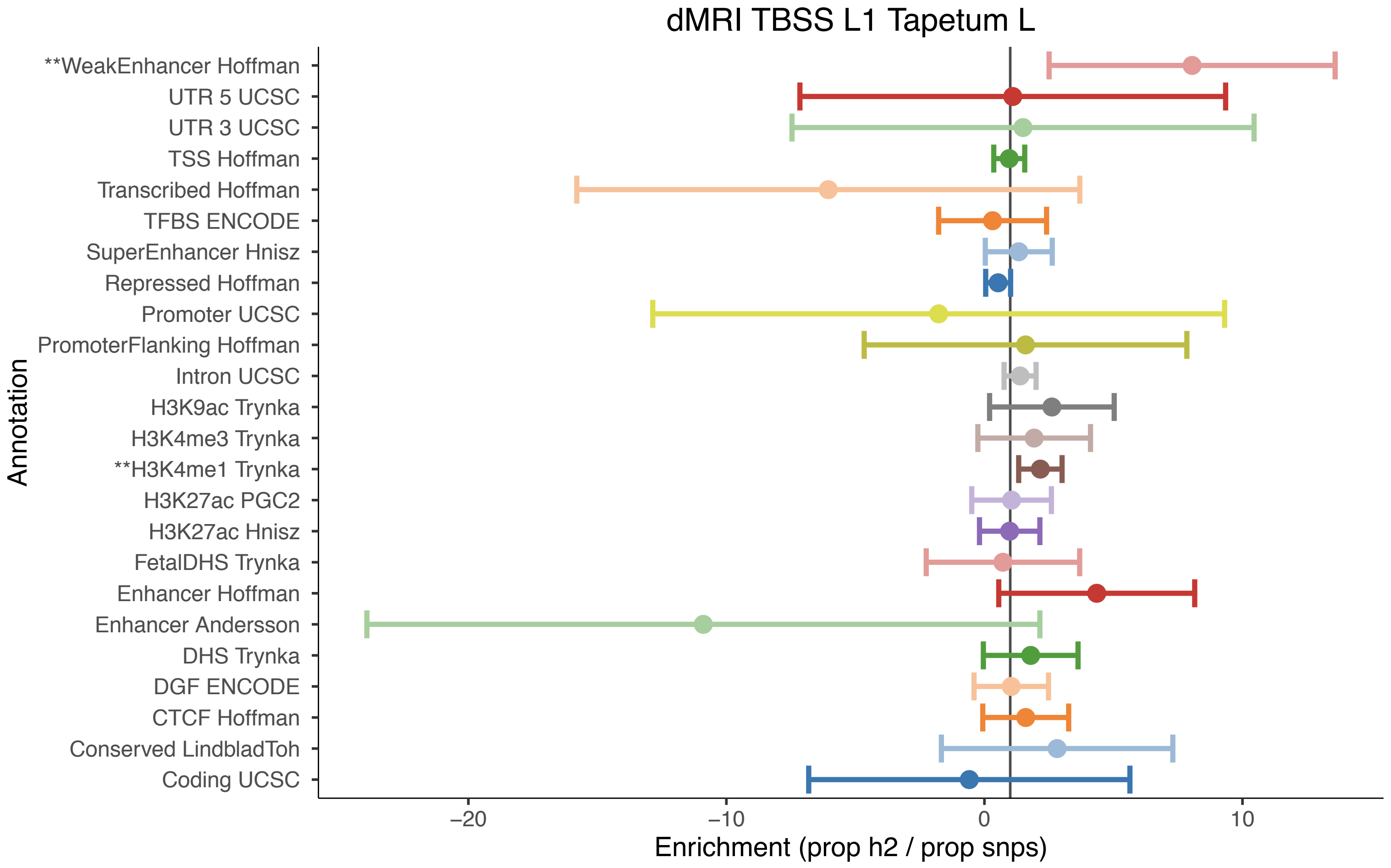

dMRI TBSS L1 Tapetum L
Annotation
**WeakEnhancer Hoffman
UTR 5 UCSC
UTR 3 UCSC
TSS Hoffman
Transcribed Hoffman
TFBS ENCODE
SuperEnhancer Hnisz
Repressed Hoffman
Promoter UCSC
PromoterFlanking Hoffman
Intron UCSC
H3K9ac Trynka
H3K4me3 Trynka
**H3K4me1 Trynka
H3K27ac PGC2
H3K27ac Hnisz
FetalDHS Trynka
Enhancer Hoffman
Enhancer Andersson
DHS Trynka
DGF ENCODE
CTCF Hoffman
Conserved LindbladToh
Coding UCSC
−20
−10
0
10
Enrichment (prop h2 / prop snps)

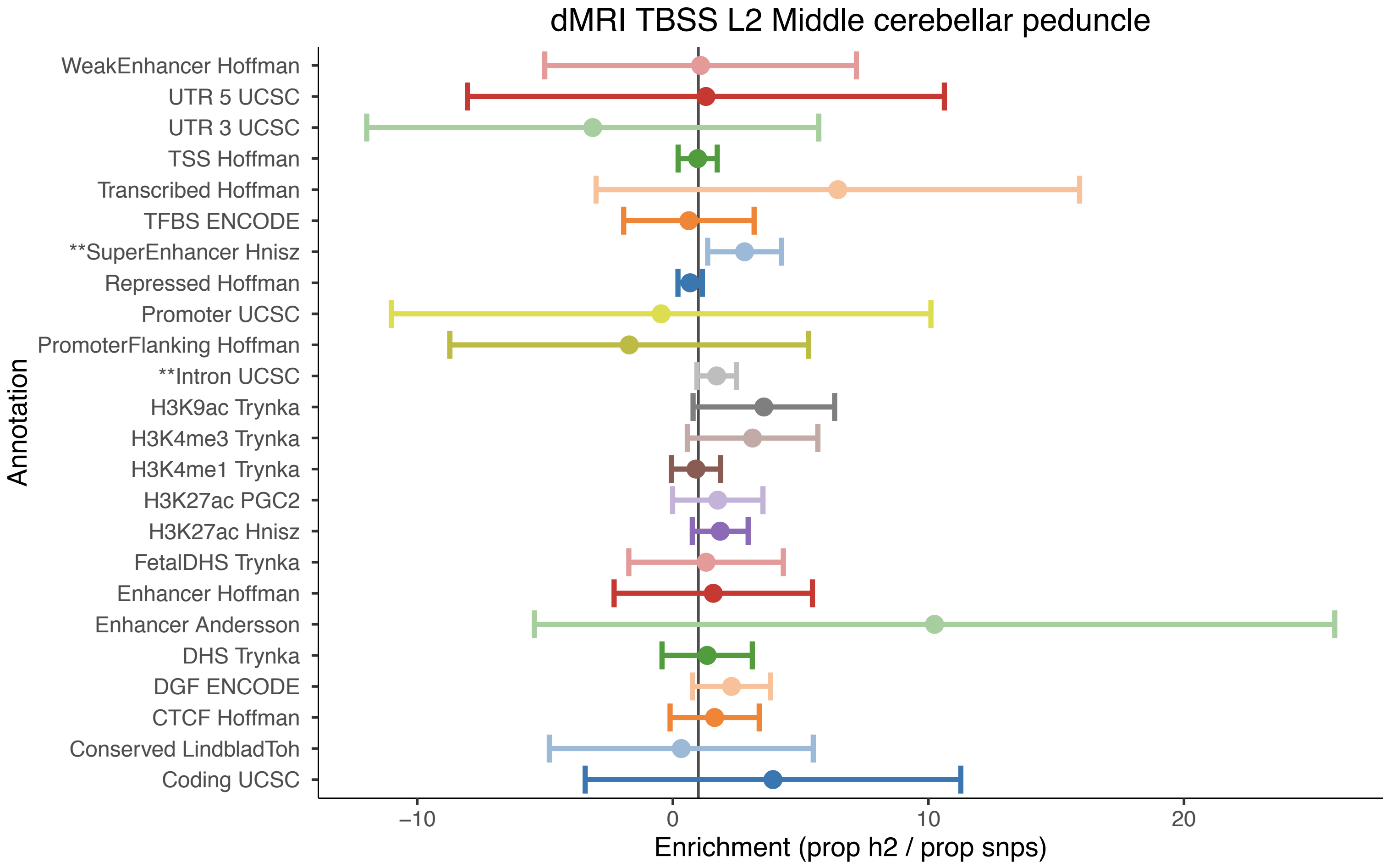
dMRI TBSS L2 Middle cerebellar peduncle
WeakEnhancer Hoffman
UTR 5 UCSC
UTR 3 UCSC
TSS Hoffman
Transcribed Hoffman
TFBS ENCODE
**SuperEnhancer Hnisz
Repressed Hoffman
Promoter UCSC
PromoterFlanking Hoffman
**Intron UCSC
H3K9ac Trynka
H3K4me3 Trynka
H3K4me1 Trynka
H3K27ac PGC2
H3K27ac Hnisz
FetalDHS Trynka
Enhancer Hoffman
Enhancer Andersson
DHS Trynka
DGF ENCODE
CTCF Hoffman
Conserved LindbladToh
Coding UCSC
Annotation
−10
0
10
20
Enrichment (prop h2 / prop snps)

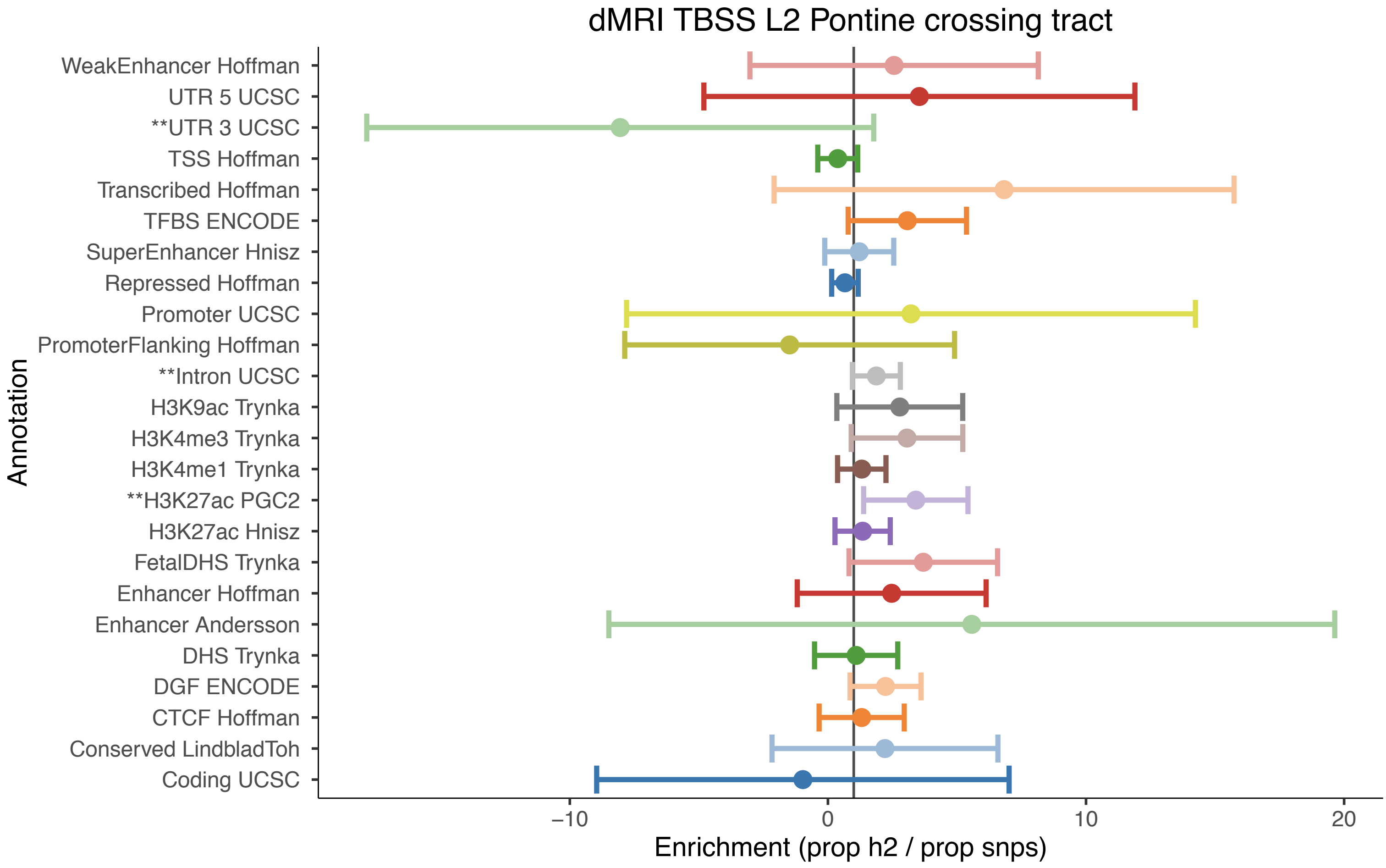
dMRI TBSS L2 Pontine crossing tract
WeakEnhancer Hoffman
UTR 5 UCSC
**UTR 3 UCSC
TSS Hoffman
Transcribed Hoffman
TFBS ENCODE
SuperEnhancer Hnisz
Repressed Hoffman
Promoter UCSC
PromoterFlanking Hoffman
**Intron UCSC
H3K9ac Trynka
H3K4me3 Trynka
H3K4me1 Trynka
**H3K27ac PGC2
H3K27ac Hnisz
FetalDHS Trynka
Enhancer Hoffman
Enhancer Andersson
DHS Trynka
DGF ENCODE
CTCF Hoffman
Conserved LindbladToh
Coding UCSC
Annotation
−10
0
10
20
Enrichment (prop h2 / prop snps)

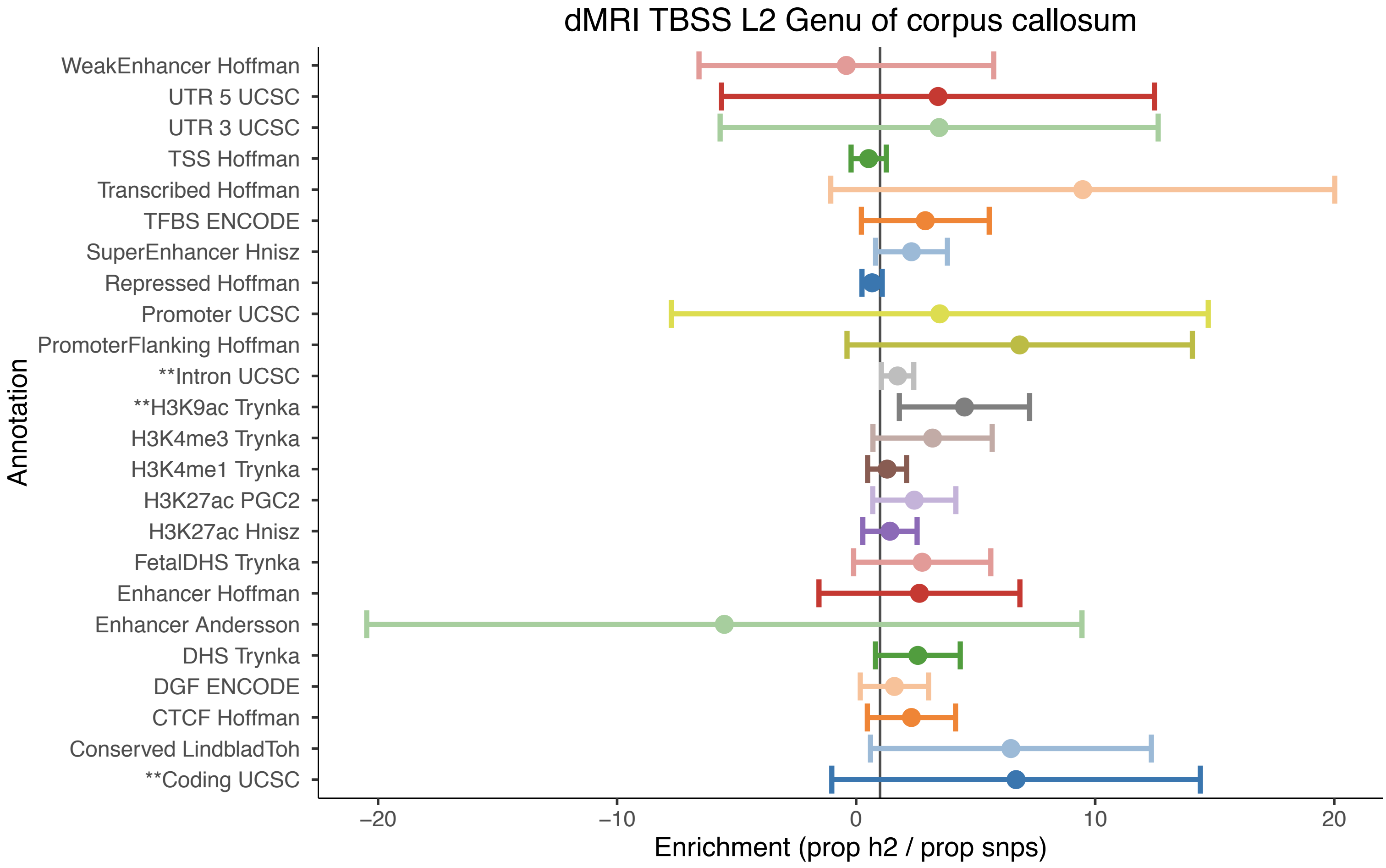
dMRI TBSS L2 Genu of corpus callosum
WeakEnhancer Hoffman
UTR 5 UCSC
UTR 3 UCSC
TSS Hoffman
Transcribed Hoffman
TFBS ENCODE
SuperEnhancer Hnisz
Repressed Hoffman
Promoter UCSC
PromoterFlanking Hoffman
**Intron UCSC
**H3K9ac Trynka
H3K4me3 Trynka
H3K4me1 Trynka
H3K27ac PGC2
H3K27ac Hnisz
FetalDHS Trynka
Enhancer Hoffman
Enhancer Andersson
DHS Trynka
DGF ENCODE
CTCF Hoffman
Conserved LindbladToh
**Coding UCSC
Annotation
−20
−10
0
10
20
Enrichment (prop h2 / prop snps)

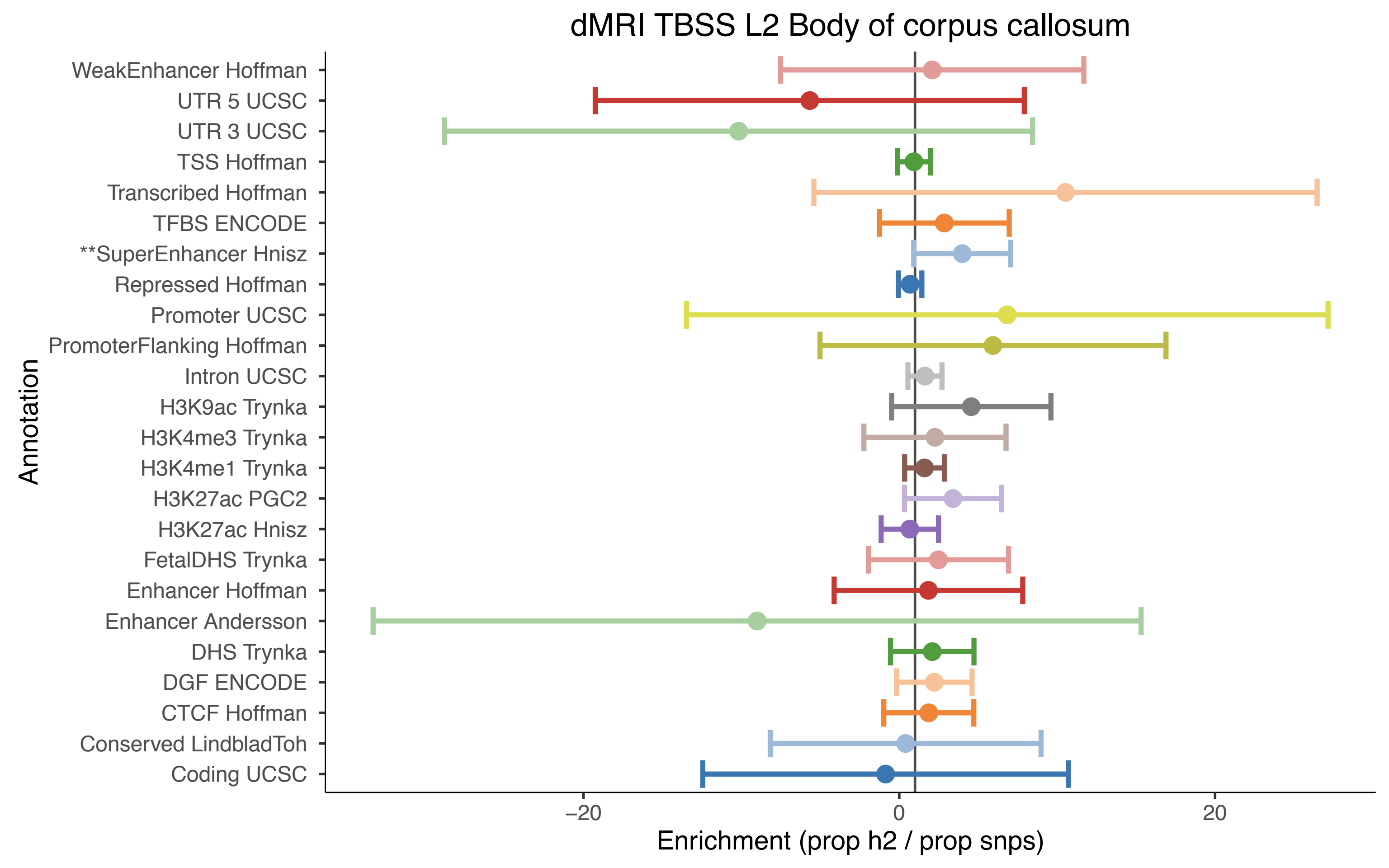
dMRI TBSS L2 Body of corpus callosum
WeakEnhancer Hoffman
UTR 5 UCSC
UTR 3 UCSC
TSS Hoffman
Transcribed Hoffman
TFBS ENCODE
**SuperEnhancer Hnisz
Repressed Hoffman
Promoter UCSC
PromoterFlanking Hoffman
Intron UCSC
H3K9ac Trynka
H3K4me3 Trynka
H3K4me1 Trynka
H3K27ac PGC2
H3K27ac Hnisz
FetalDHS Trynka
Enhancer Hoffman
Enhancer Andersson
DHS Trynka
DGF ENCODE
CTCF Hoffman
Conserved LindbladToh
Coding UCSC
Annotation
−20
0
20
Enrichment (prop h2 / prop snps)

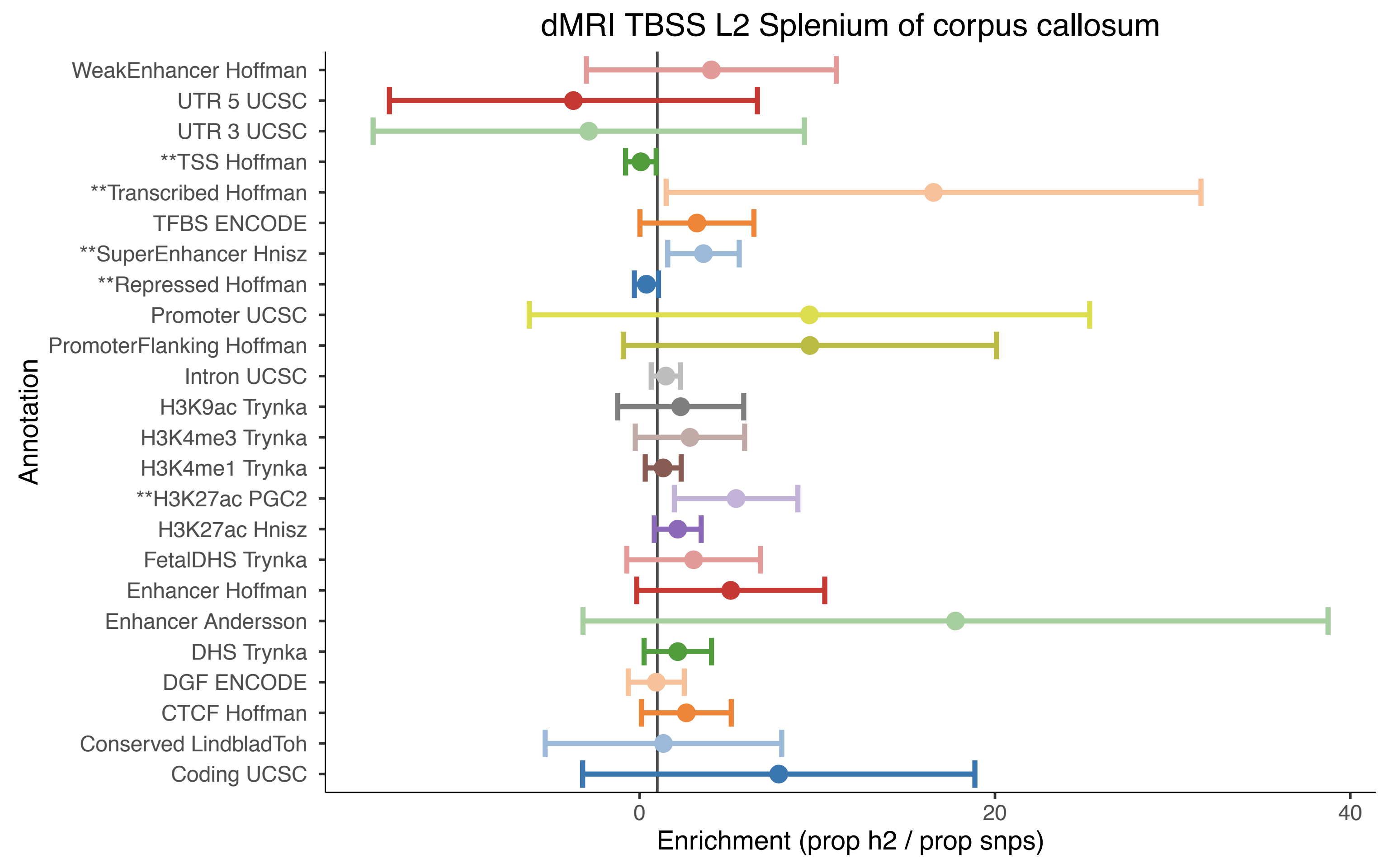

dMRI TBSS L2 Splenium of corpus callosum
WeakEnhancer Hoffman
UTR 5 UCSC
UTR 3 UCSC
**TSS Hoffman
**Transcribed Hoffman
TFBS ENCODE
**SuperEnhancer Hnisz
**Repressed Hoffman
Promoter UCSC
PromoterFlanking Hoffman
Intron UCSC
H3K9ac Trynka
H3K4me3 Trynka
H3K4me1 Trynka
**H3K27ac PGC2
H3K27ac Hnisz
FetalDHS Trynka
Enhancer Hoffman
Enhancer Andersson
DHS Trynka
DGF ENCODE
CTCF Hoffman
Conserved LindbladToh
Coding UCSC
Annotation
0
20
40
Enrichment (prop h2 / prop snps)

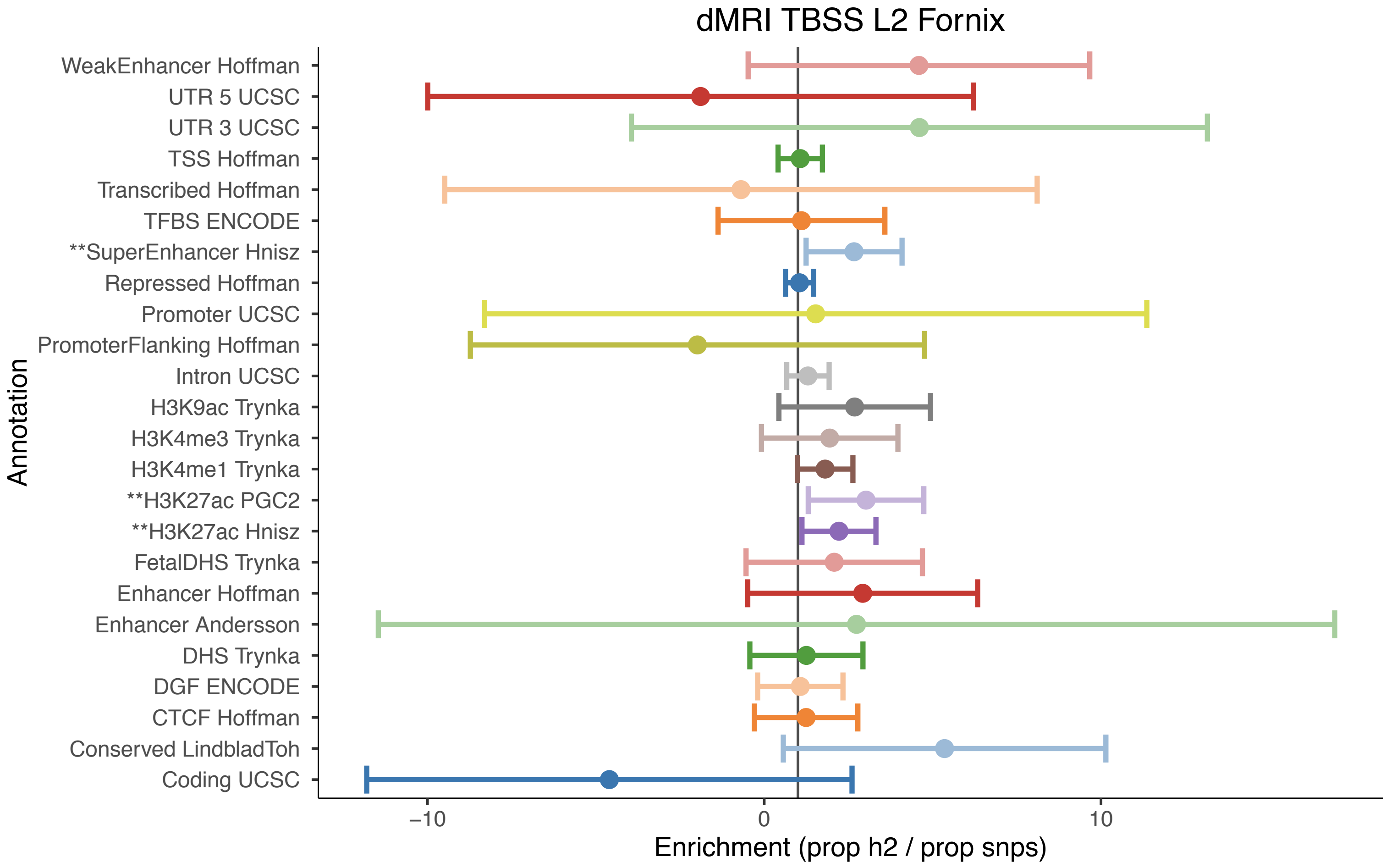
dMRI TBSS L2 Fornix
WeakEnhancer Hoffman
UTR 5 UCSC
UTR 3 UCSC
TSS Hoffman
Transcribed Hoffman
TFBS ENCODE
**SuperEnhancer Hnisz
Repressed Hoffman
Promoter UCSC
PromoterFlanking Hoffman
Intron UCSC
H3K9ac Trynka
H3K4me3 Trynka
H3K4me1 Trynka
**H3K27ac PGC2
**H3K27ac Hnisz
FetalDHS Trynka
Enhancer Hoffman
Enhancer Andersson
DHS Trynka
DGF ENCODE
CTCF Hoffman
Conserved LindbladToh
Coding UCSC
Annotation
−10
0
10
Enrichment (prop h2 / prop snps)

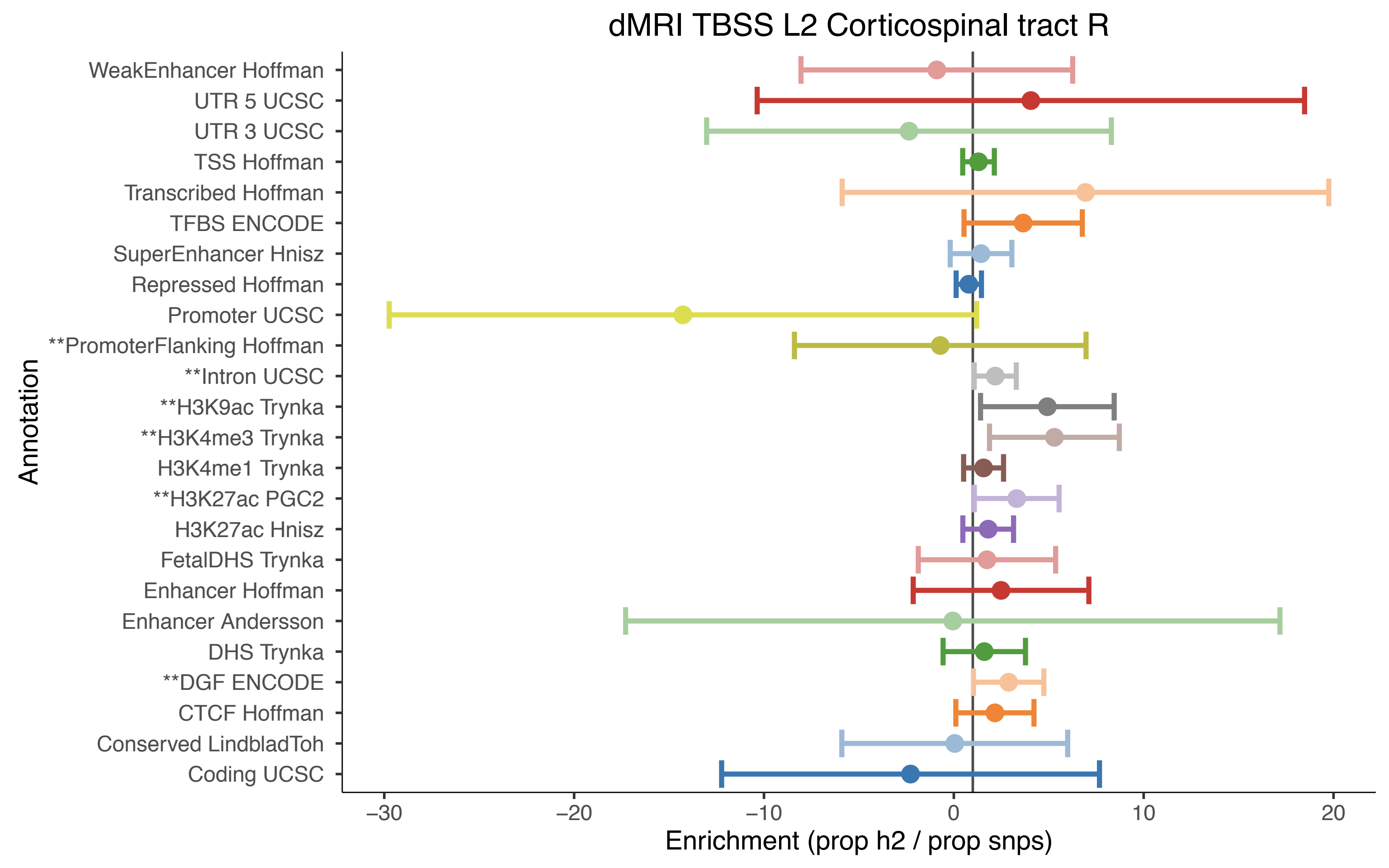
dMRI TBSS L2 Corticospinal tract R
WeakEnhancer Hoffman
UTR 5 UCSC
UTR 3 UCSC
TSS Hoffman
Transcribed Hoffman
TFBS ENCODE
SuperEnhancer Hnisz
Repressed Hoffman
Promoter UCSC
**PromoterFlanking Hoffman
**Intron UCSC
**H3K9ac Trynka
**H3K4me3 Trynka
H3K4me1 Trynka
**H3K27ac PGC2
H3K27ac Hnisz
FetalDHS Trynka
Enhancer Hoffman
Enhancer Andersson
DHS Trynka
**DGF ENCODE
CTCF Hoffman
Conserved LindbladToh
Coding UCSC
Annotation
−30
−20
−10
0
10
20
Enrichment (prop h2 / prop snps)

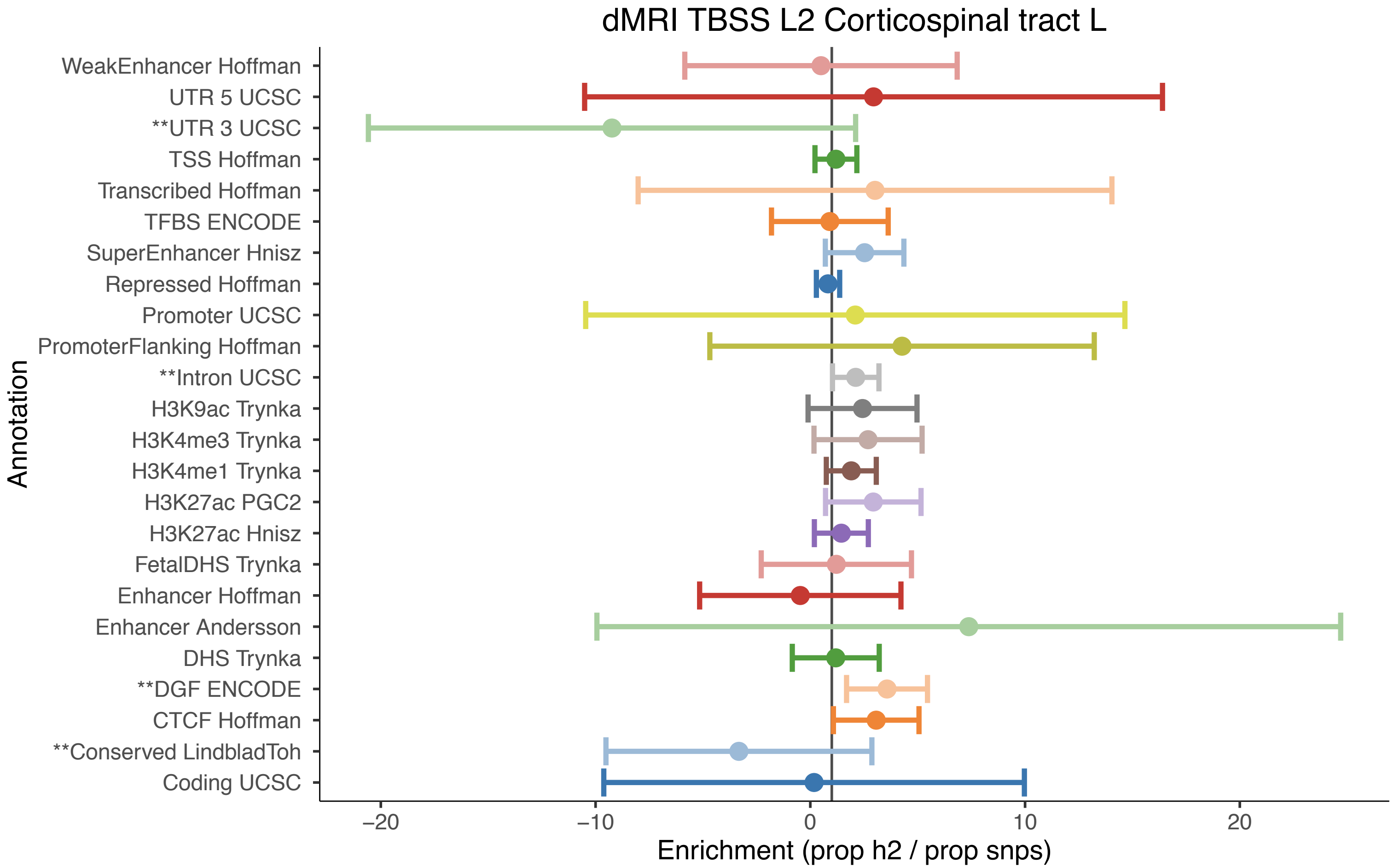

dMRI TBSS L2 Corticospinal tract L
WeakEnhancer Hoffman
UTR 5 UCSC
**UTR 3 UCSC
TSS Hoffman
Transcribed Hoffman
TFBS ENCODE
SuperEnhancer Hnisz
Repressed Hoffman
Promoter UCSC
PromoterFlanking Hoffman
**Intron UCSC
H3K9ac Trynka
H3K4me3 Trynka
H3K4me1 Trynka
H3K27ac PGC2
H3K27ac Hnisz
FetalDHS Trynka
Enhancer Hoffman
Enhancer Andersson
DHS Trynka
**DGF ENCODE
CTCF Hoffman
**Conserved LindbladToh
Coding UCSC
Annotation
−20
−10
0
10
20
Enrichment (prop h2 / prop snps)

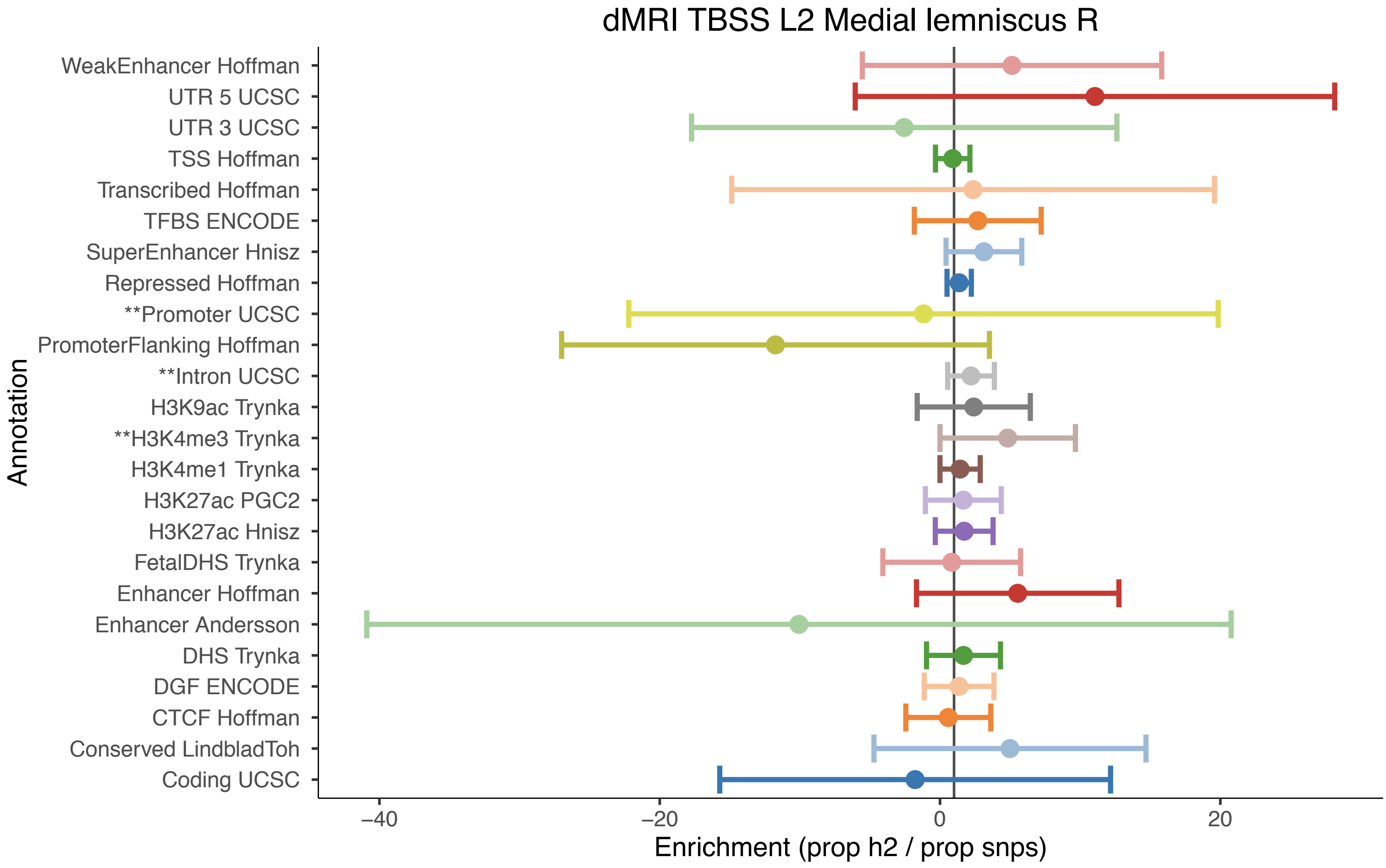
dMRI TBSS L2 Medial lemniscus R
Annotation
WeakEnhancer Hoffman
UTR 5 UCSC
UTR 3 UCSC
TSS Hoffman
Transcribed Hoffman
TFBS ENCODE
SuperEnhancer Hnisz
Repressed Hoffman
**Promoter UCSC
PromoterFlanking Hoffman
**Intron UCSC
H3K9ac Trynka
**H3K4me3 Trynka
H3K4me1 Trynka
H3K27ac PGC2
H3K27ac Hnisz
FetalDHS Trynka
Enhancer Hoffman
Enhancer Andersson
DHS Trynka
DGF ENCODE
CTCF Hoffman
Conserved LindbladToh
Coding UCSC
−40
−20
0
20
Enrichment (prop h2 / prop snps)

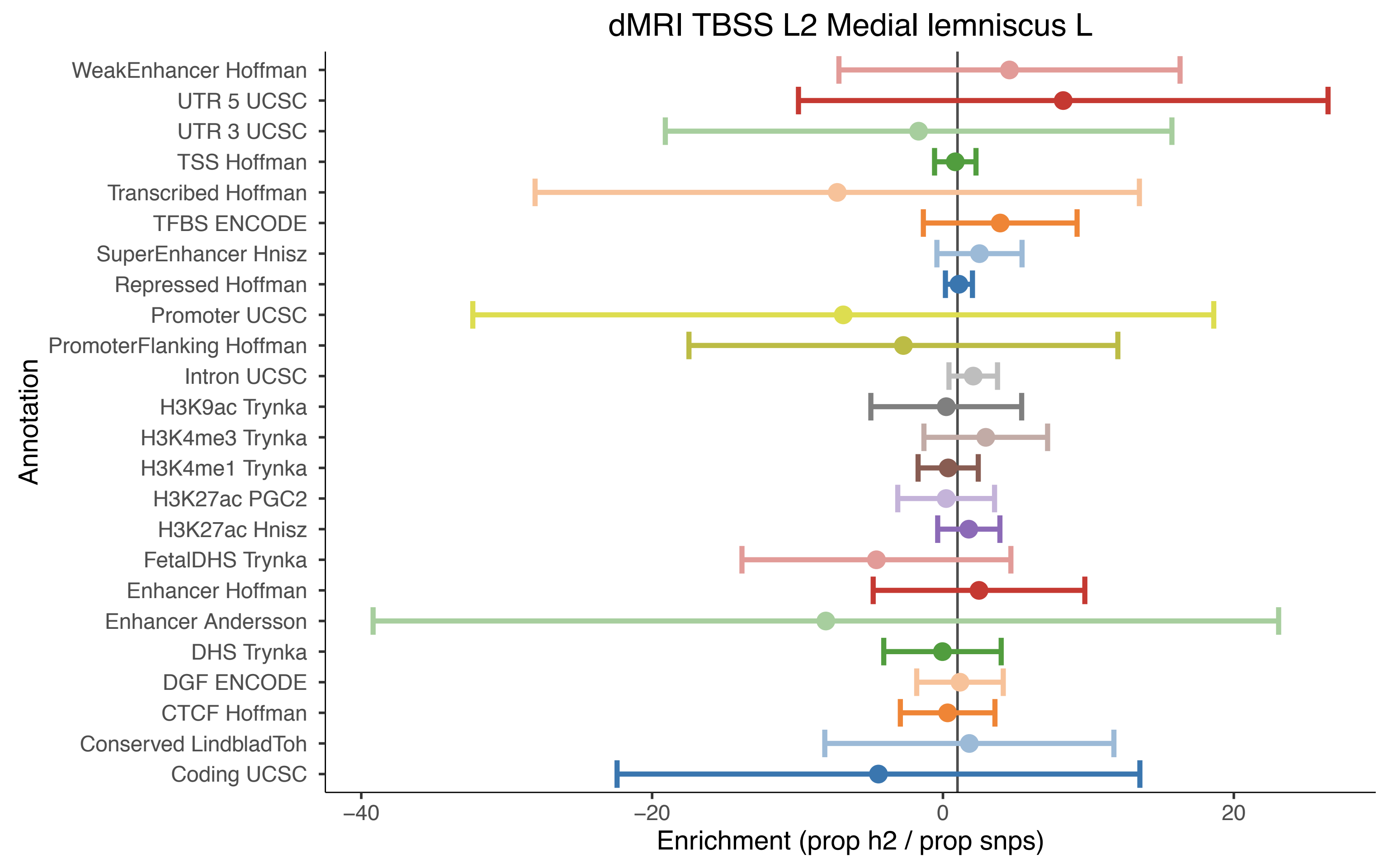

dMRI TBSS L2 Medial lemniscus L
Annotation
WeakEnhancer Hoffman
UTR 5 UCSC
UTR 3 UCSC
TSS Hoffman
Transcribed Hoffman
TFBS ENCODE
SuperEnhancer Hnisz
Repressed Hoffman
Promoter UCSC
PromoterFlanking Hoffman
Intron UCSC
H3K9ac Trynka
H3K4me3 Trynka
H3K4me1 Trynka
H3K27ac PGC2
H3K27ac Hnisz
FetalDHS Trynka
Enhancer Hoffman
Enhancer Andersson
DHS Trynka
DGF ENCODE
CTCF Hoffman
Conserved LindbladToh
Coding UCSC
−40
−20
0
20
Enrichment (prop h2 / prop snps)

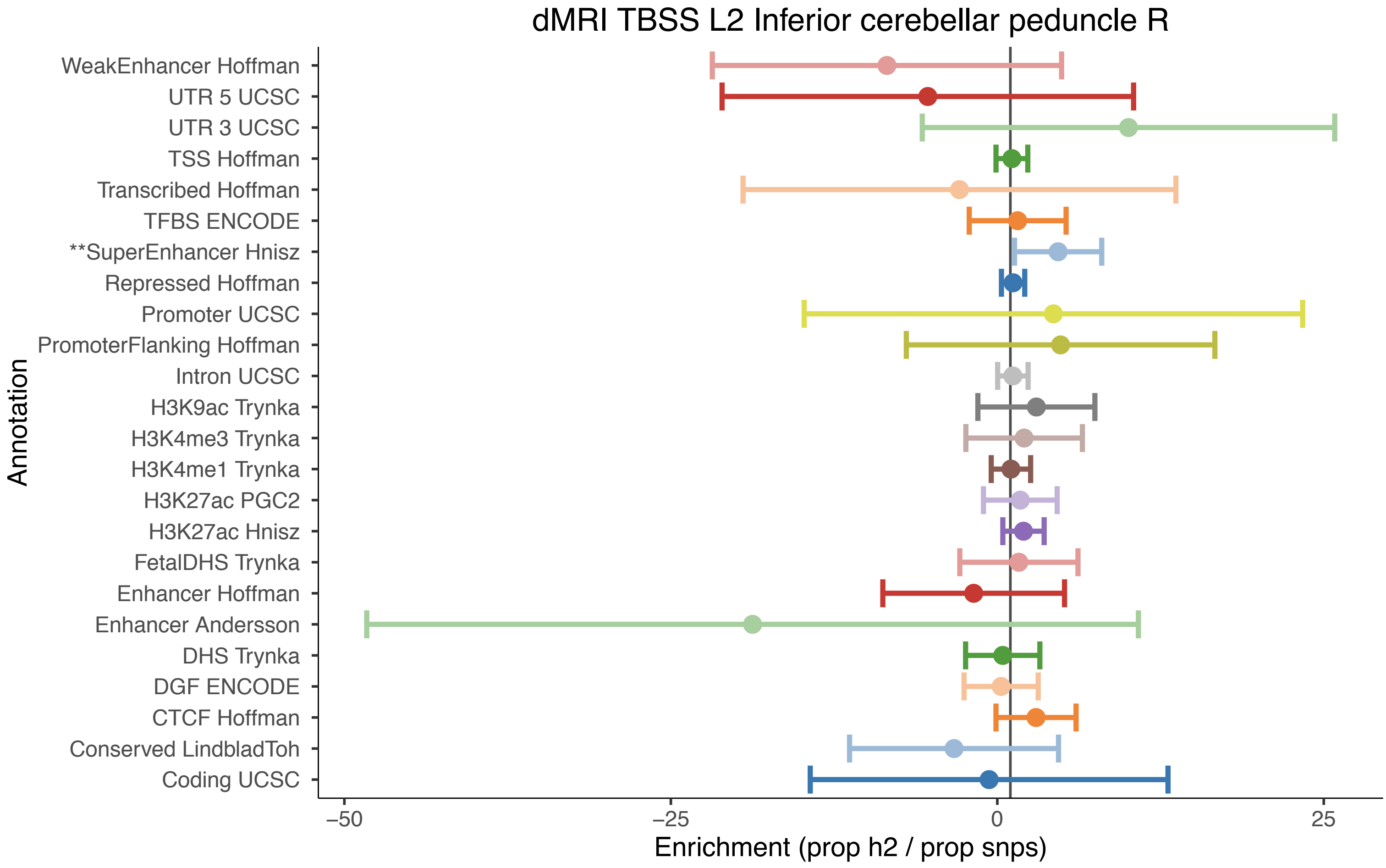

dMRI TBSS L2 Inferior cerebellar peduncle R
WeakEnhancer Hoffman
UTR 5 UCSC
UTR 3 UCSC
TSS Hoffman
Transcribed Hoffman
TFBS ENCODE
**SuperEnhancer Hnisz
Repressed Hoffman
Promoter UCSC
PromoterFlanking Hoffman
Intron UCSC
H3K9ac Trynka
H3K4me3 Trynka
H3K4me1 Trynka
H3K27ac PGC2
H3K27ac Hnisz
FetalDHS Trynka
Enhancer Hoffman
Enhancer Andersson
DHS Trynka
DGF ENCODE
CTCF Hoffman
Conserved LindbladToh
Coding UCSC
Annotation
−50
−25
0
25
Enrichment (prop h2 / prop snps)

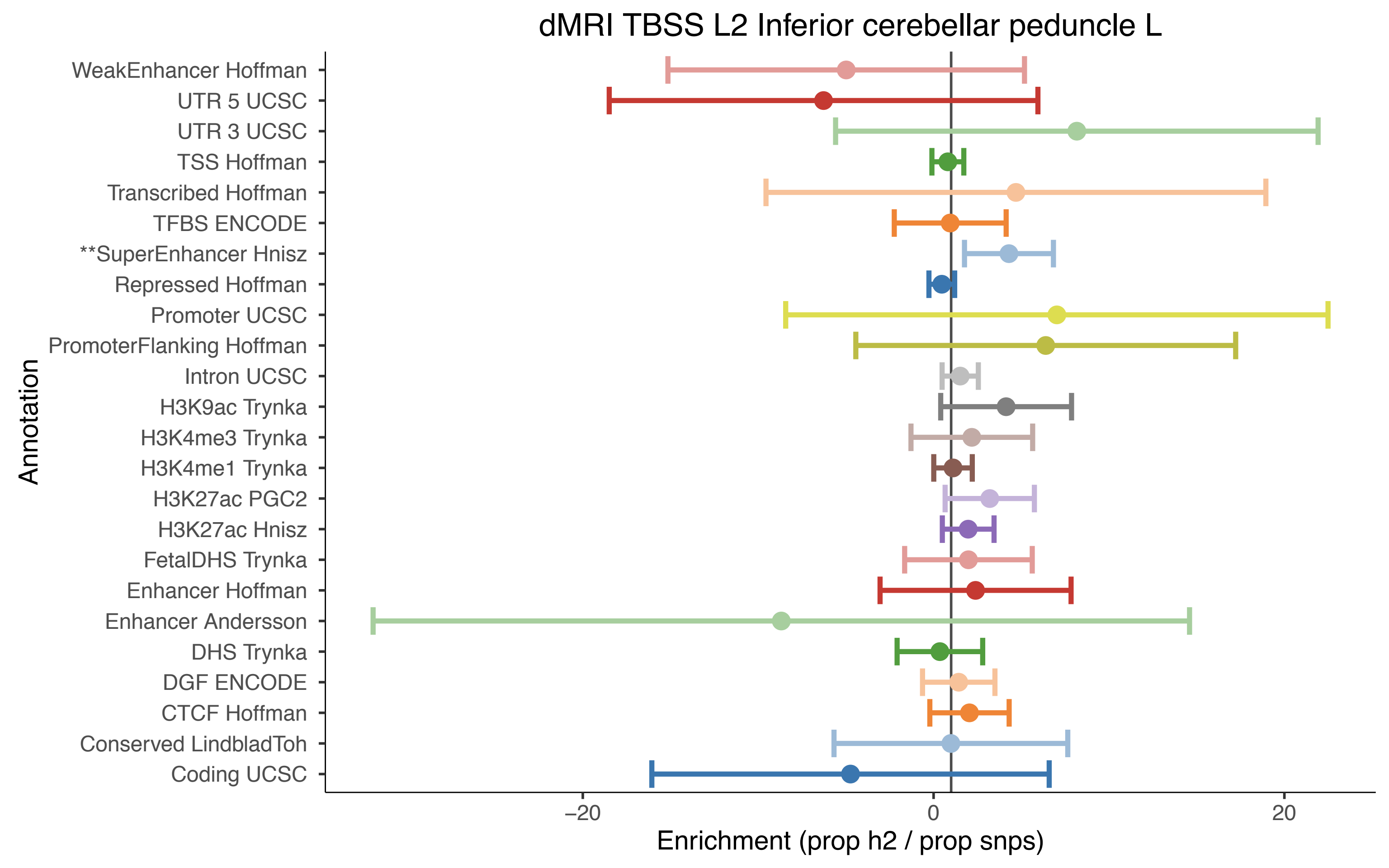
dMRI TBSS L2 Inferior cerebellar peduncle L
Annotation
WeakEnhancer Hoffman
UTR 5 UCSC
UTR 3 UCSC
TSS Hoffman
Transcribed Hoffman
TFBS ENCODE
**SuperEnhancer Hnisz
Repressed Hoffman
Promoter UCSC
PromoterFlanking Hoffman
Intron UCSC
H3K9ac Trynka
H3K4me3 Trynka
H3K4me1 Trynka
H3K27ac PGC2
H3K27ac Hnisz
FetalDHS Trynka
Enhancer Hoffman
Enhancer Andersson
DHS Trynka
DGF ENCODE
CTCF Hoffman
Conserved LindbladToh
Coding UCSC
−20
0
20
Enrichment (prop h2 / prop snps)

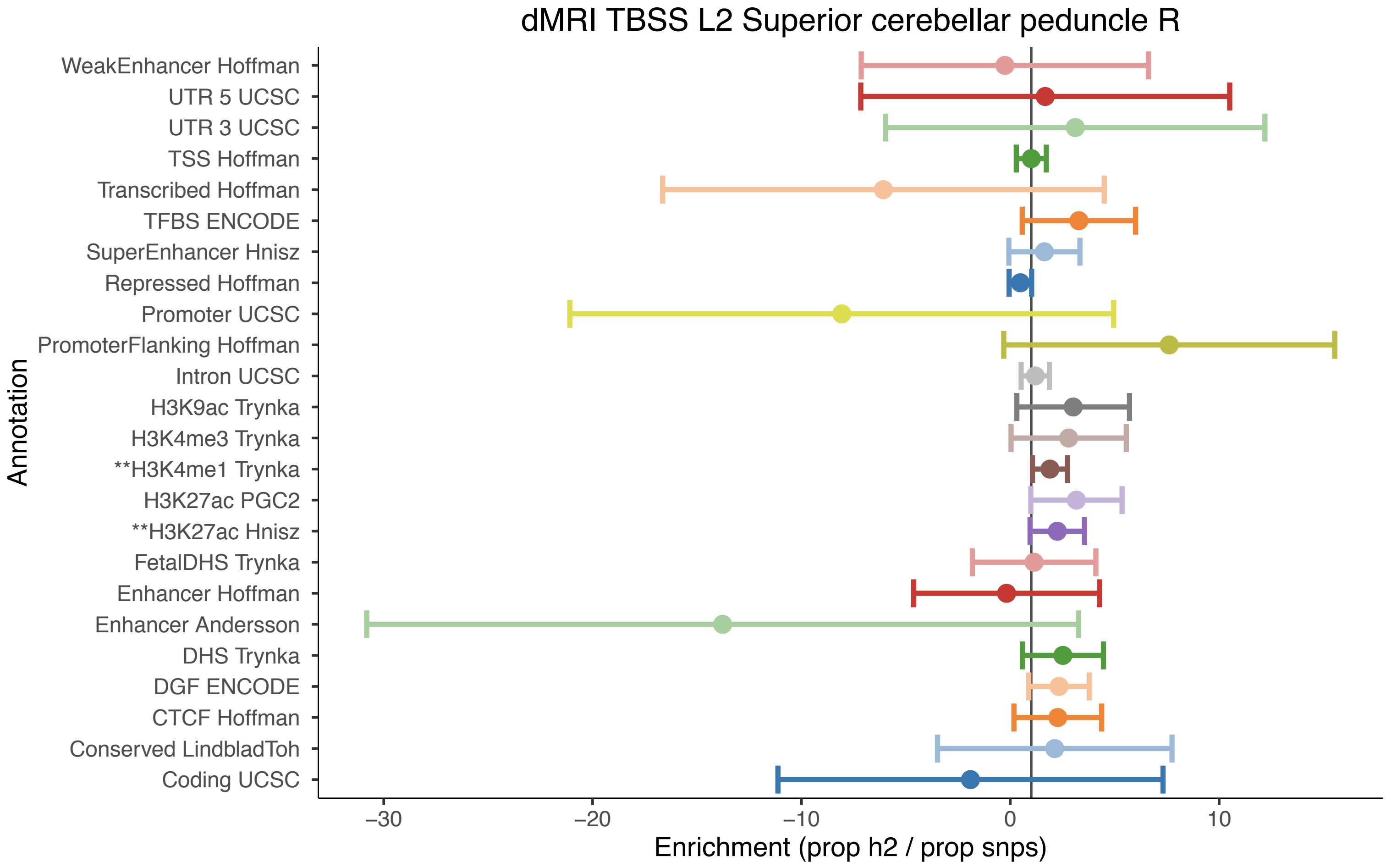

dMRI TBSS L2 Superior cerebellar peduncle R
WeakEnhancer Hoffman
UTR 5 UCSC
UTR 3 UCSC
TSS Hoffman
Transcribed Hoffman
TFBS ENCODE
SuperEnhancer Hnisz
Repressed Hoffman
Promoter UCSC
PromoterFlanking Hoffman
Intron UCSC
H3K9ac Trynka
H3K4me3 Trynka
**H3K4me1 Trynka
H3K27ac PGC2
**H3K27ac Hnisz
FetalDHS Trynka
Enhancer Hoffman
Enhancer Andersson
DHS Trynka
DGF ENCODE
CTCF Hoffman
Conserved LindbladToh
Coding UCSC
Annotation
−30
−20
−10
0
10
Enrichment (prop h2 / prop snps)

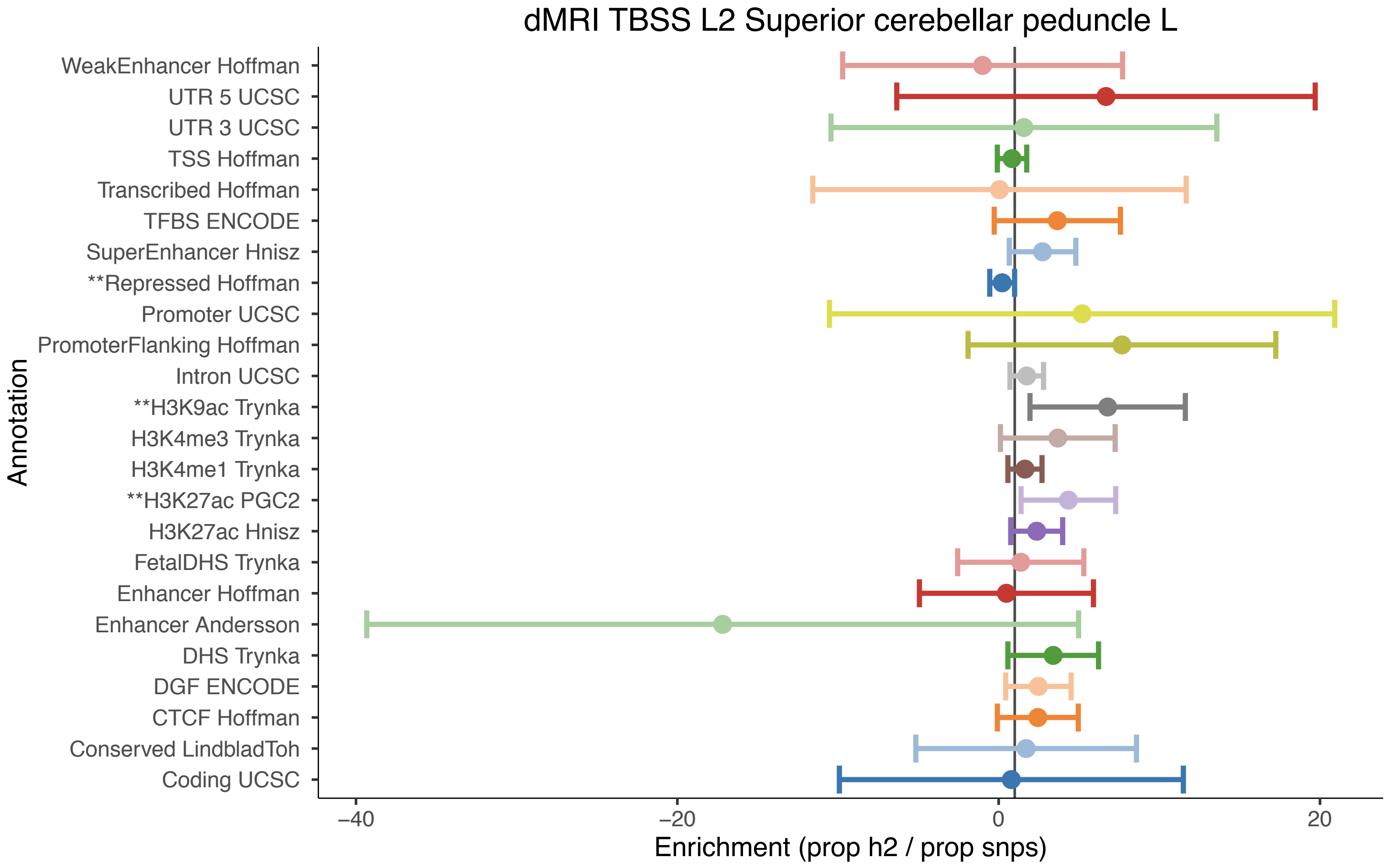
dMRI TBSS L2 Superior cerebellar peduncle L
Annotation
WeakEnhancer Hoffman
UTR 5 UCSC
UTR 3 UCSC
TSS Hoffman
Transcribed Hoffman
TFBS ENCODE
SuperEnhancer Hnisz
**Repressed Hoffman
Promoter UCSC
PromoterFlanking Hoffman
Intron UCSC
**H3K9ac Trynka
H3K4me3 Trynka
H3K4me1 Trynka
**H3K27ac PGC2
H3K27ac Hnisz
FetalDHS Trynka
Enhancer Hoffman
Enhancer Andersson
DHS Trynka
DGF ENCODE
CTCF Hoffman
Conserved LindbladToh
Coding UCSC
−40
−20
0
20
Enrichment (prop h2 / prop snps)

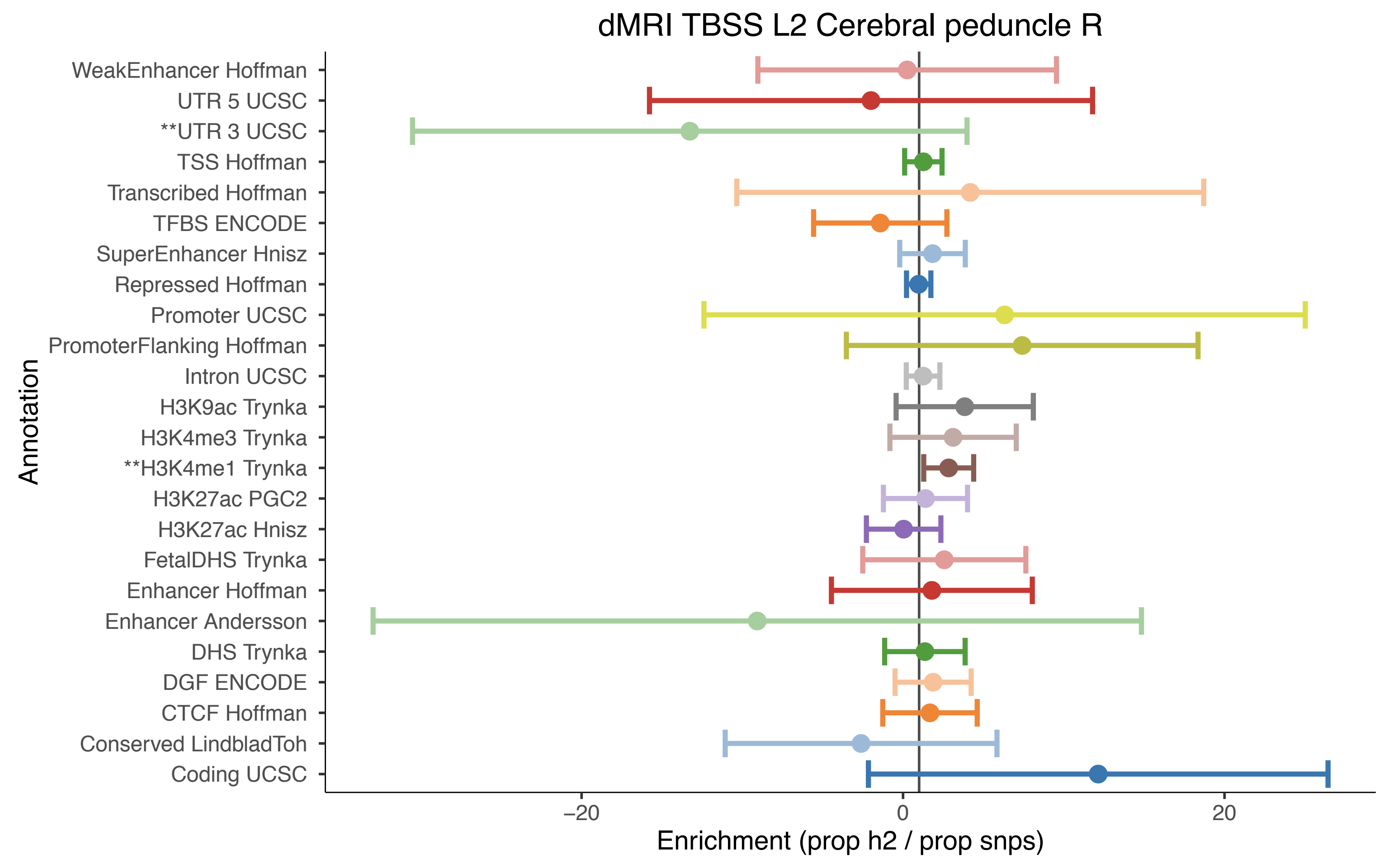

dMRI TBSS L2 Cerebral peduncle R
WeakEnhancer Hoffman
UTR 5 UCSC
**UTR 3 UCSC
TSS Hoffman
Transcribed Hoffman
TFBS ENCODE
SuperEnhancer Hnisz
Repressed Hoffman
Promoter UCSC
PromoterFlanking Hoffman
Intron UCSC
H3K9ac Trynka
H3K4me3 Trynka
**H3K4me1 Trynka
H3K27ac PGC2
H3K27ac Hnisz
FetalDHS Trynka
Enhancer Hoffman
Enhancer Andersson
DHS Trynka
DGF ENCODE
CTCF Hoffman
Conserved LindbladToh
Coding UCSC
Annotation
−20
0
20
Enrichment (prop h2 / prop snps)

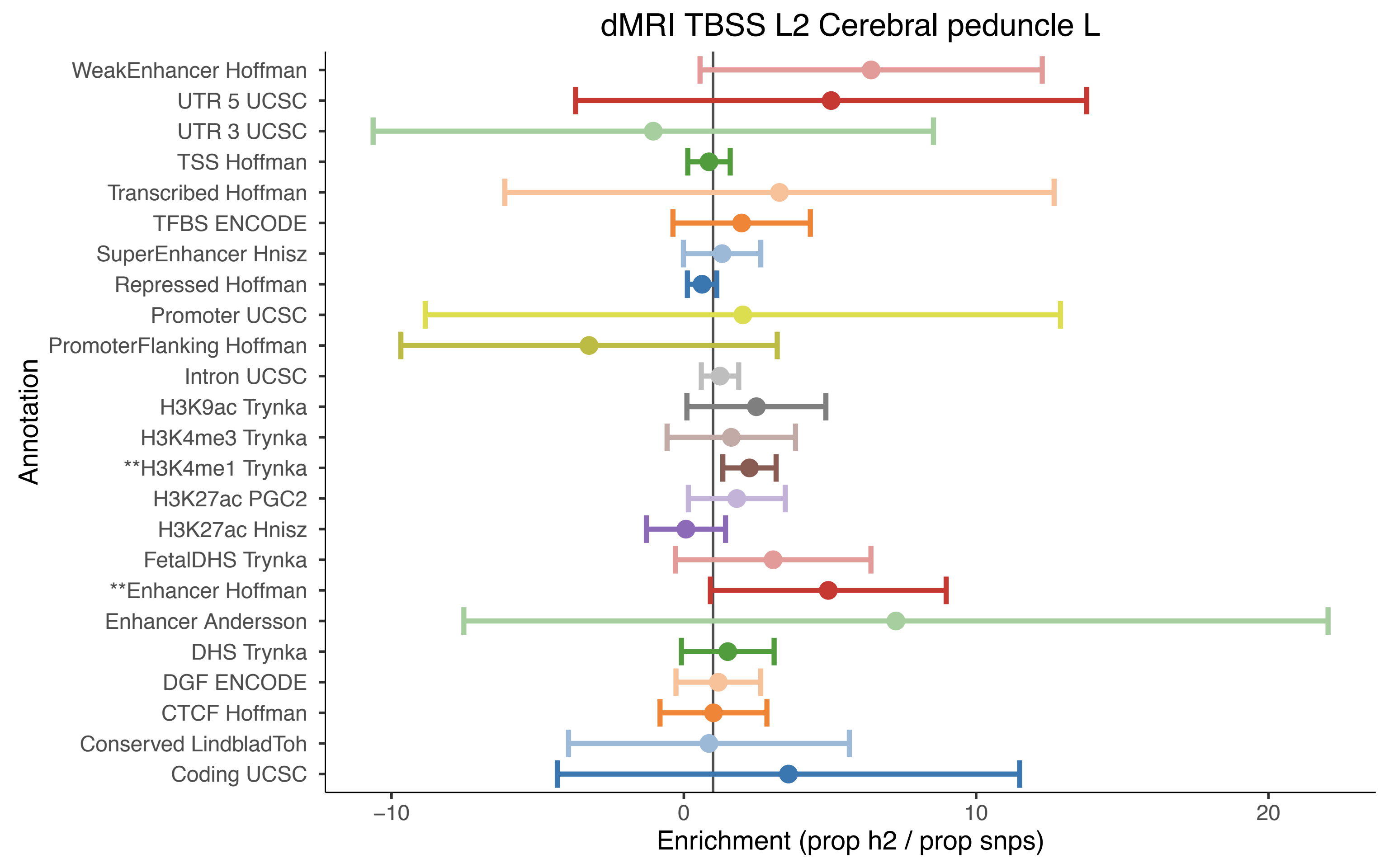

dMRI TBSS L2 Cerebral peduncle L
Annotation
WeakEnhancer Hoffman
UTR 5 UCSC
UTR 3 UCSC
TSS Hoffman
Transcribed Hoffman
TFBS ENCODE
SuperEnhancer Hnisz
Repressed Hoffman
Promoter UCSC
PromoterFlanking Hoffman
Intron UCSC
H3K9ac Trynka
H3K4me3 Trynka
**H3K4me1 Trynka
H3K27ac PGC2
H3K27ac Hnisz
FetalDHS Trynka
**Enhancer Hoffman
Enhancer Andersson
DHS Trynka
DGF ENCODE
CTCF Hoffman
Conserved LindbladToh
Coding UCSC
−10
0
10
20
Enrichment (prop h2 / prop snps)

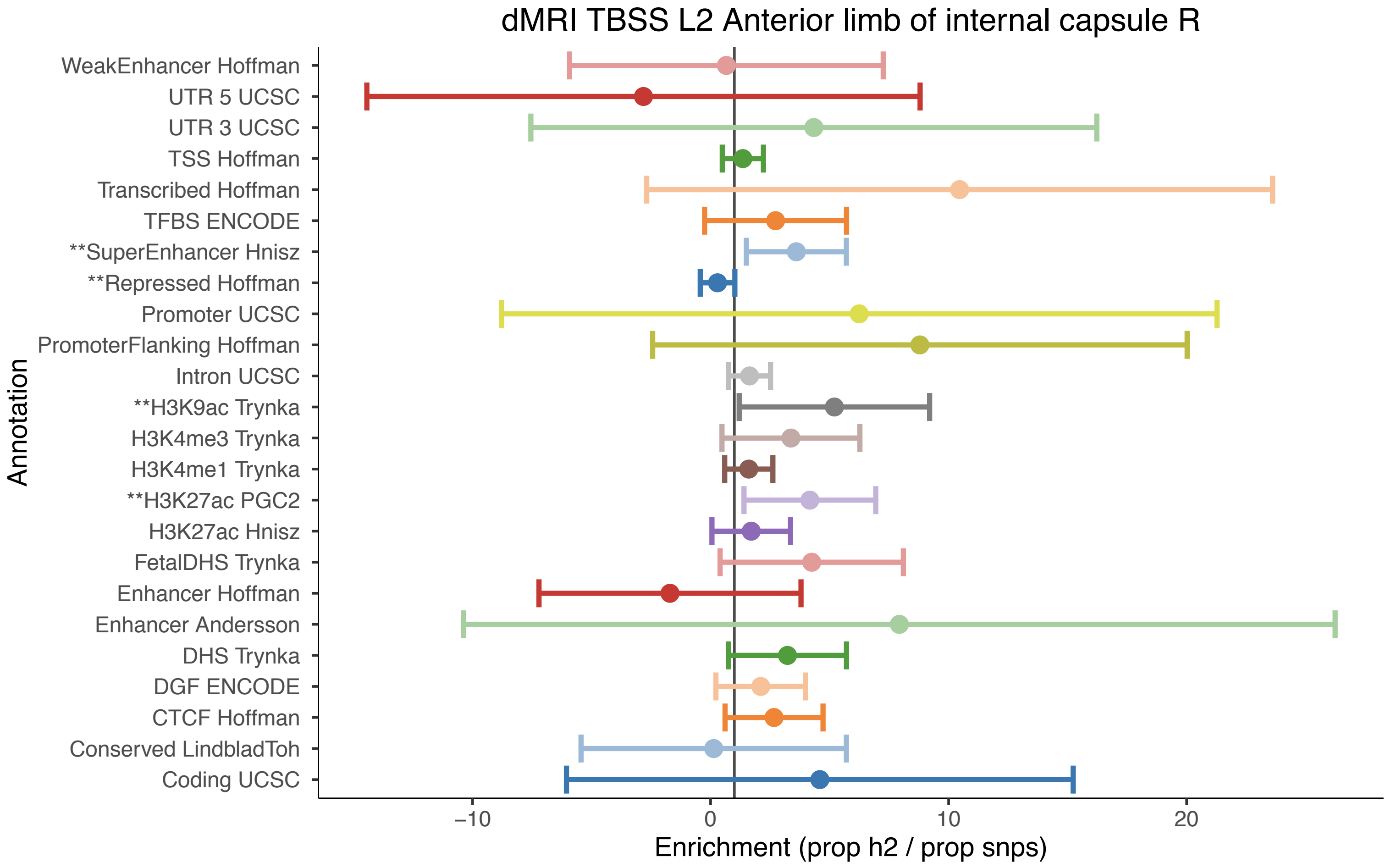

dMRI TBSS L2 Anterior limb of internal capsule R
Annotation
WeakEnhancer Hoffman
UTR 5 UCSC
UTR 3 UCSC
TSS Hoffman
Transcribed Hoffman
TFBS ENCODE
**SuperEnhancer Hnisz
**Repressed Hoffman
Promoter UCSC
PromoterFlanking Hoffman
Intron UCSC
**H3K9ac Trynka
H3K4me3 Trynka
H3K4me1 Trynka
**H3K27ac PGC2
H3K27ac Hnisz
FetalDHS Trynka
Enhancer Hoffman
Enhancer Andersson
DHS Trynka
DGF ENCODE
CTCF Hoffman
Conserved LindbladToh
Coding UCSC
−10
0
10
20
Enrichment (prop h2 / prop snps)

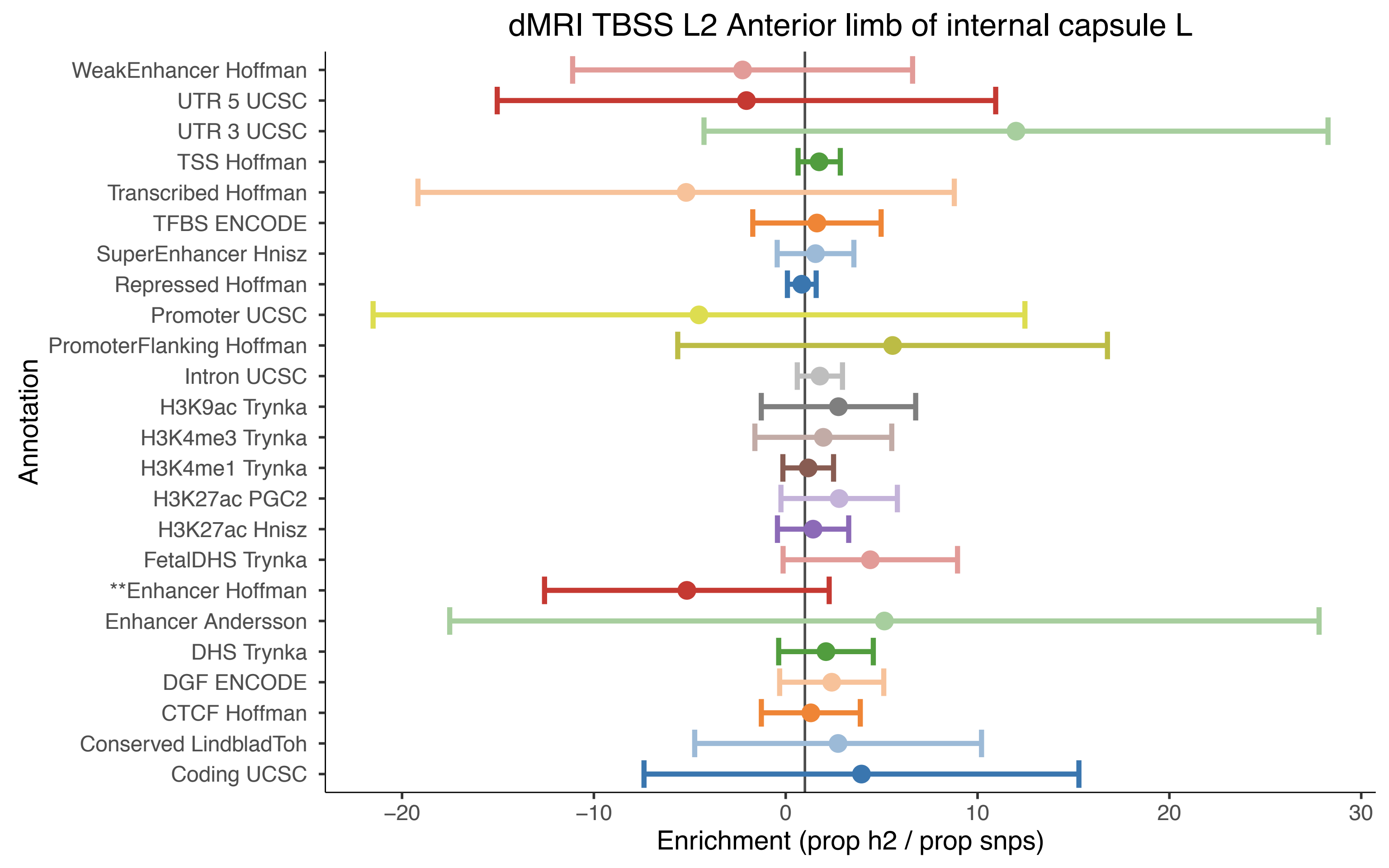

dMRI TBSS L2 Anterior limb of internal capsule L
WeakEnhancer Hoffman
UTR 5 UCSC
UTR 3 UCSC
TSS Hoffman
Transcribed Hoffman
TFBS ENCODE
SuperEnhancer Hnisz
Repressed Hoffman
Promoter UCSC
PromoterFlanking Hoffman
Intron UCSC
H3K9ac Trynka
H3K4me3 Trynka
H3K4me1 Trynka
H3K27ac PGC2
H3K27ac Hnisz
FetalDHS Trynka
**Enhancer Hoffman
Enhancer Andersson
DHS Trynka
DGF ENCODE
CTCF Hoffman
Conserved LindbladToh
Coding UCSC
Annotation
−20
−10
0
10
20
30
Enrichment (prop h2 / prop snps)

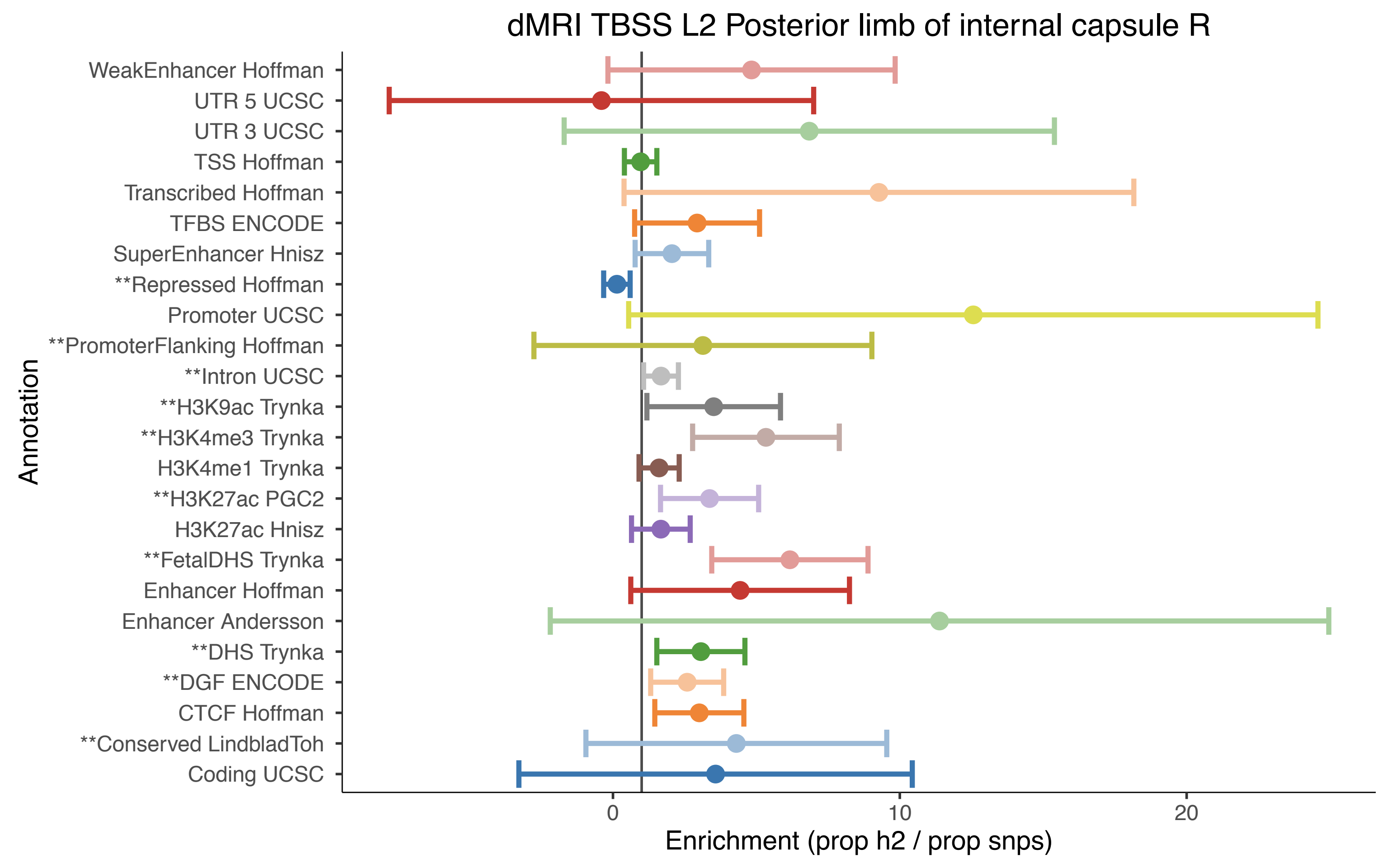

dMRI TBSS L2 Posterior limb of internal capsule R
WeakEnhancer Hoffman
UTR 5 UCSC
UTR 3 UCSC
TSS Hoffman
Transcribed Hoffman
TFBS ENCODE
SuperEnhancer Hnisz
**Repressed Hoffman
Promoter UCSC
**PromoterFlanking Hoffman
**Intron UCSC
**H3K9ac Trynka
**H3K4me3 Trynka
H3K4me1 Trynka
**H3K27ac PGC2
H3K27ac Hnisz
**FetalDHS Trynka
Enhancer Hoffman
Enhancer Andersson
**DHS Trynka
**DGF ENCODE
CTCF Hoffman
**Conserved LindbladToh
Coding UCSC
Annotation
0
10
20
Enrichment (prop h2 / prop snps)

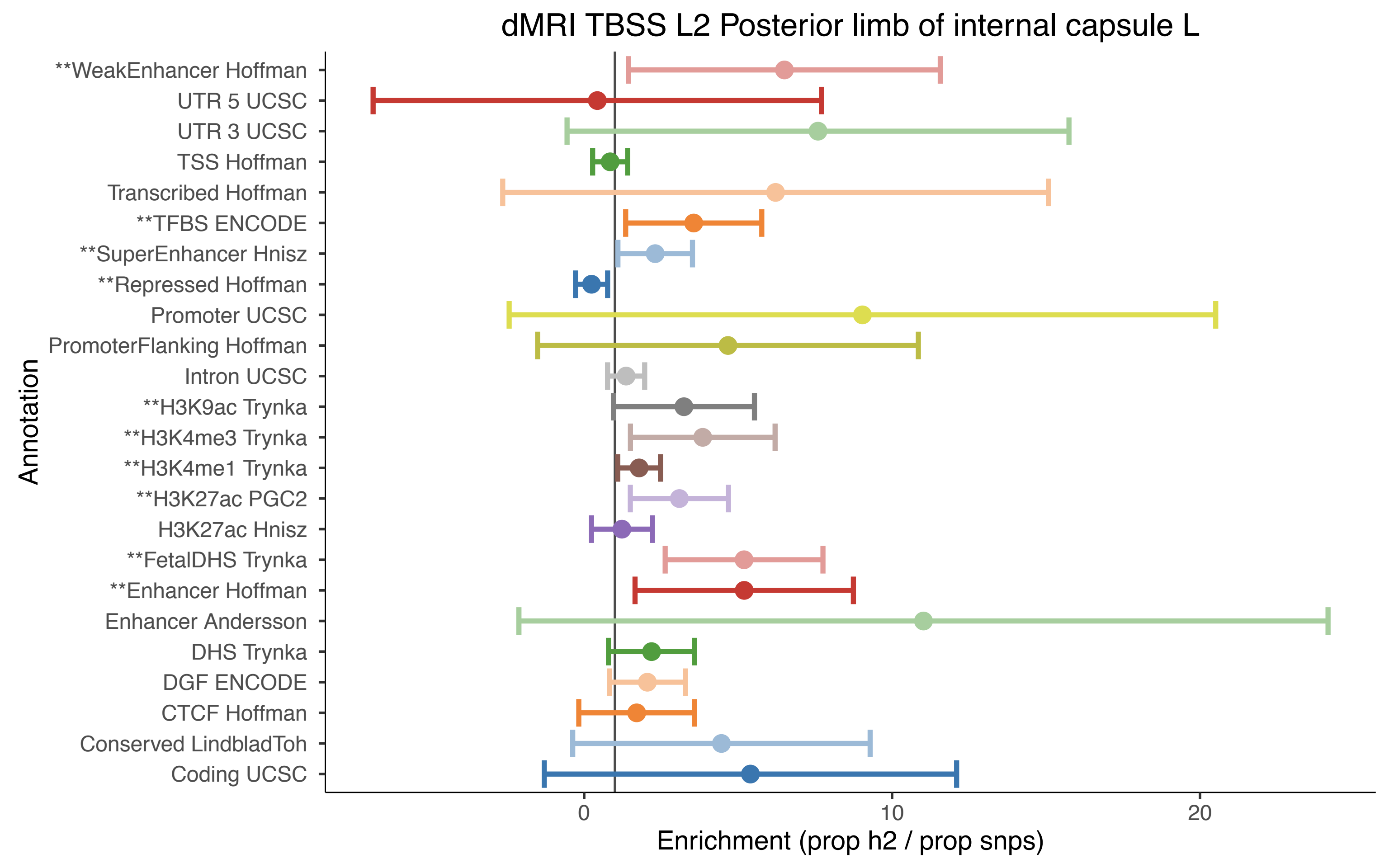

dMRI TBSS L2 Posterior limb of internal capsule L
**WeakEnhancer Hoffman
UTR 5 UCSC
UTR 3 UCSC
TSS Hoffman
Transcribed Hoffman
**TFBS ENCODE
**SuperEnhancer Hnisz
**Repressed Hoffman
Promoter UCSC
PromoterFlanking Hoffman
Intron UCSC
**H3K9ac Trynka
**H3K4me3 Trynka
**H3K4me1 Trynka
**H3K27ac PGC2
H3K27ac Hnisz
**FetalDHS Trynka
**Enhancer Hoffman
Enhancer Andersson
DHS Trynka
DGF ENCODE
CTCF Hoffman
Conserved LindbladToh
Coding UCSC
Annotation
0
10
20
Enrichment (prop h2 / prop snps)

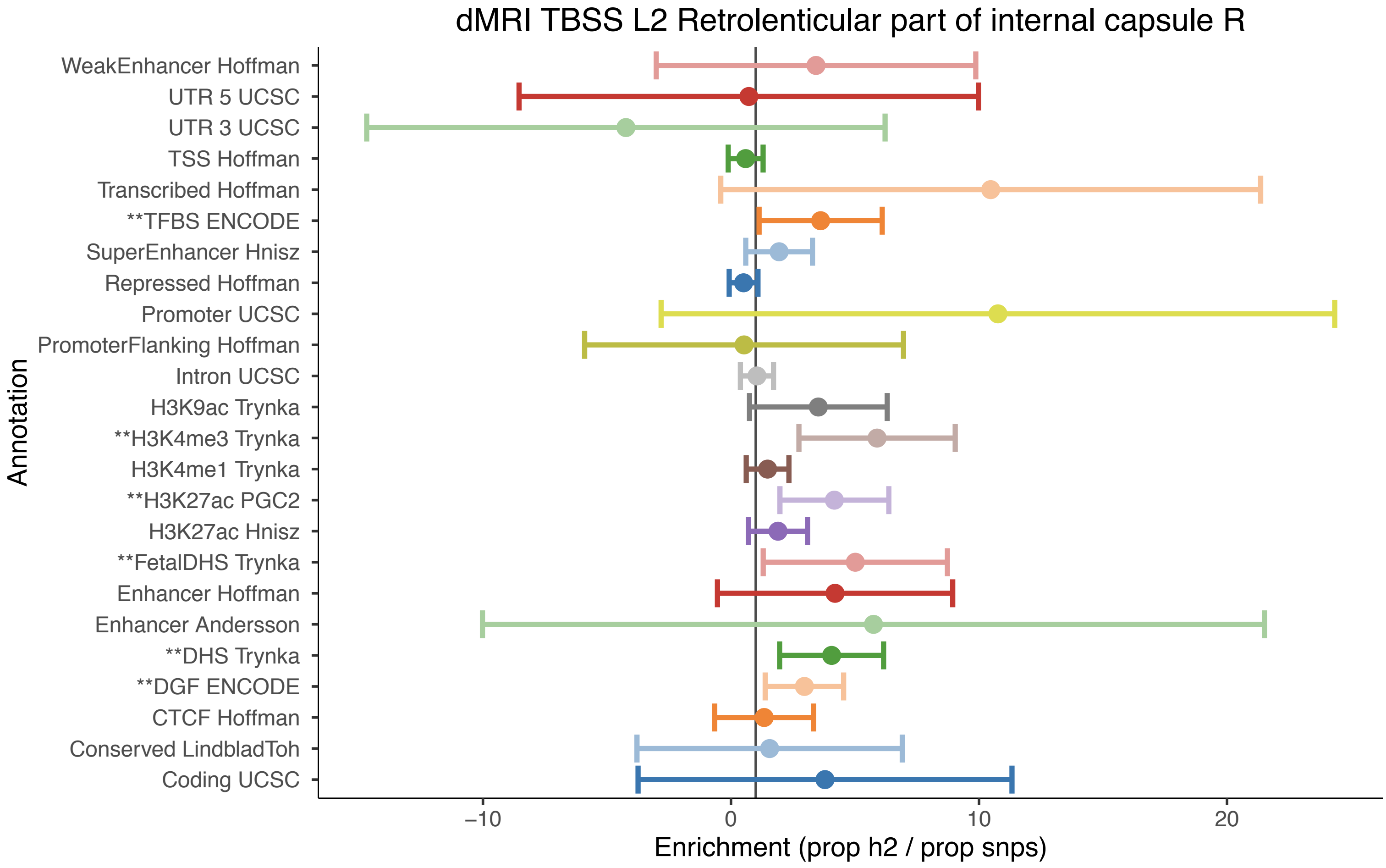

dMRI TBSS L2 Retrolenticular part of internal capsule R
WeakEnhancer Hoffman
UTR 5 UCSC
UTR 3 UCSC
TSS Hoffman
Transcribed Hoffman
**TFBS ENCODE
SuperEnhancer Hnisz
Repressed Hoffman
Promoter UCSC
PromoterFlanking Hoffman
Intron UCSC
H3K9ac Trynka
**H3K4me3 Trynka
H3K4me1 Trynka
**H3K27ac PGC2
H3K27ac Hnisz
**FetalDHS Trynka
Enhancer Hoffman
Enhancer Andersson
**DHS Trynka
**DGF ENCODE
CTCF Hoffman
Conserved LindbladToh
Coding UCSC
Annotation
−10
0
10
20
Enrichment (prop h2 / prop snps)

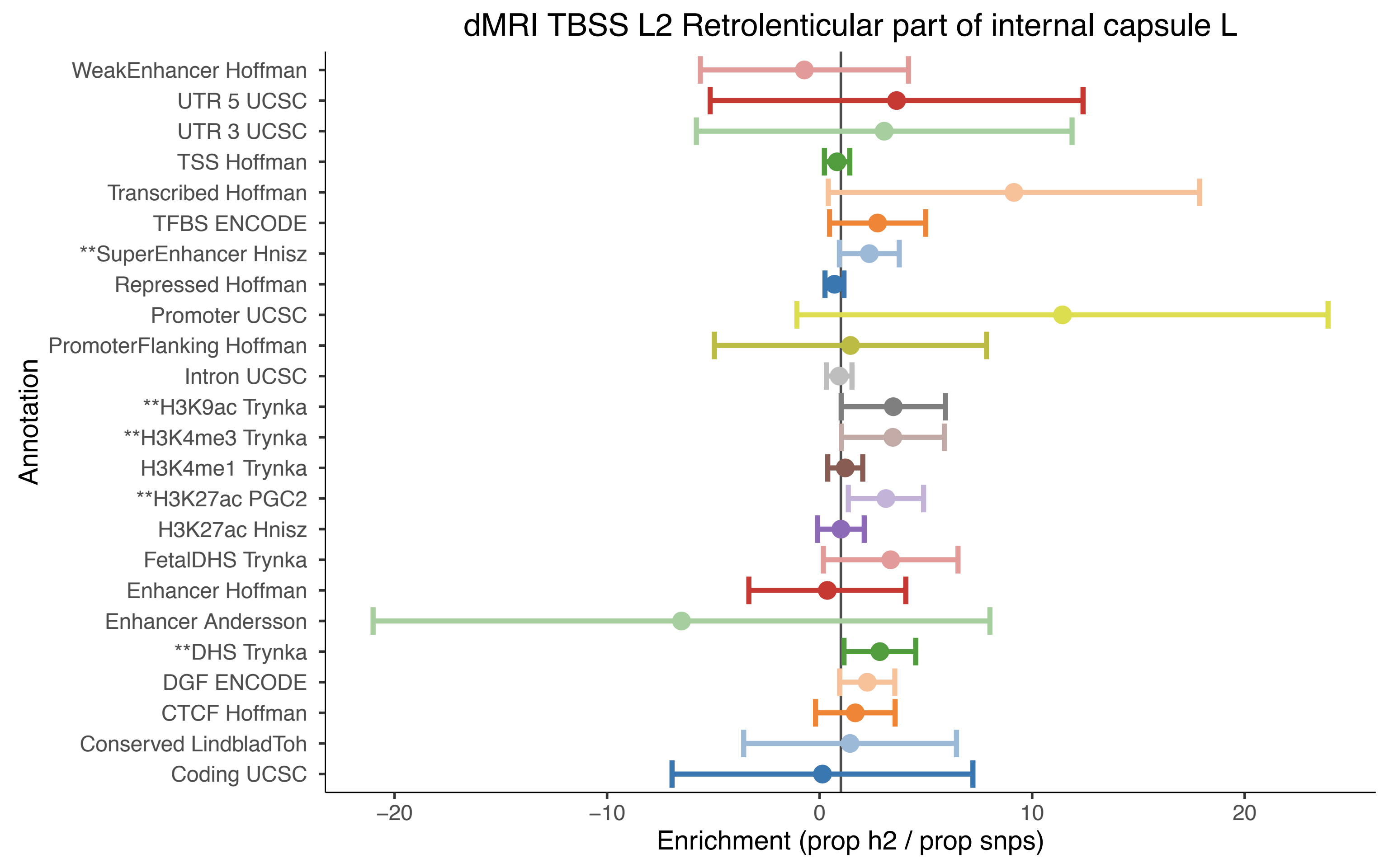
dMRI TBSS L2 Retrolenticular part of internal capsule L
WeakEnhancer Hoffman
UTR 5 UCSC
UTR 3 UCSC
TSS Hoffman
Transcribed Hoffman
TFBS ENCODE
**SuperEnhancer Hnisz
Repressed Hoffman
Promoter UCSC
PromoterFlanking Hoffman
Intron UCSC
**H3K9ac Trynka
**H3K4me3 Trynka
H3K4me1 Trynka
**H3K27ac PGC2
H3K27ac Hnisz
FetalDHS Trynka
Enhancer Hoffman
Enhancer Andersson
**DHS Trynka
DGF ENCODE
CTCF Hoffman
Conserved LindbladToh
Coding UCSC
Annotation
−20
−10
0
10
20
Enrichment (prop h2 / prop snps)

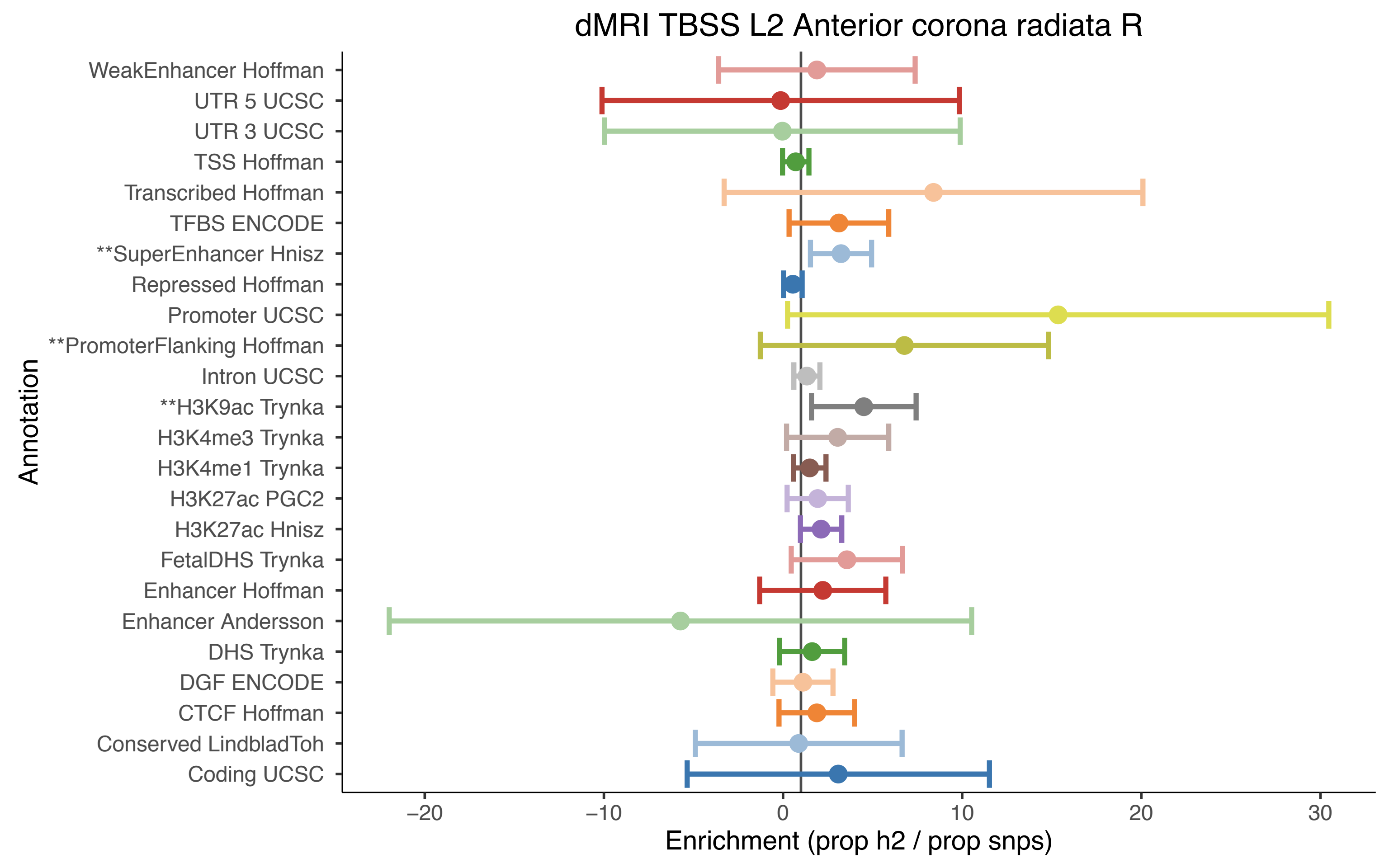

dMRI TBSS L2 Anterior corona radiata R
WeakEnhancer Hoffman
UTR 5 UCSC
UTR 3 UCSC
TSS Hoffman
Transcribed Hoffman
TFBS ENCODE
**SuperEnhancer Hnisz
Repressed Hoffman
Promoter UCSC
**PromoterFlanking Hoffman
Intron UCSC
**H3K9ac Trynka
H3K4me3 Trynka
H3K4me1 Trynka
H3K27ac PGC2
H3K27ac Hnisz
FetalDHS Trynka
Enhancer Hoffman
Enhancer Andersson
DHS Trynka
DGF ENCODE
CTCF Hoffman
Conserved LindbladToh
Coding UCSC
Annotation
−20
−10
0
10
20
30
Enrichment (prop h2 / prop snps)

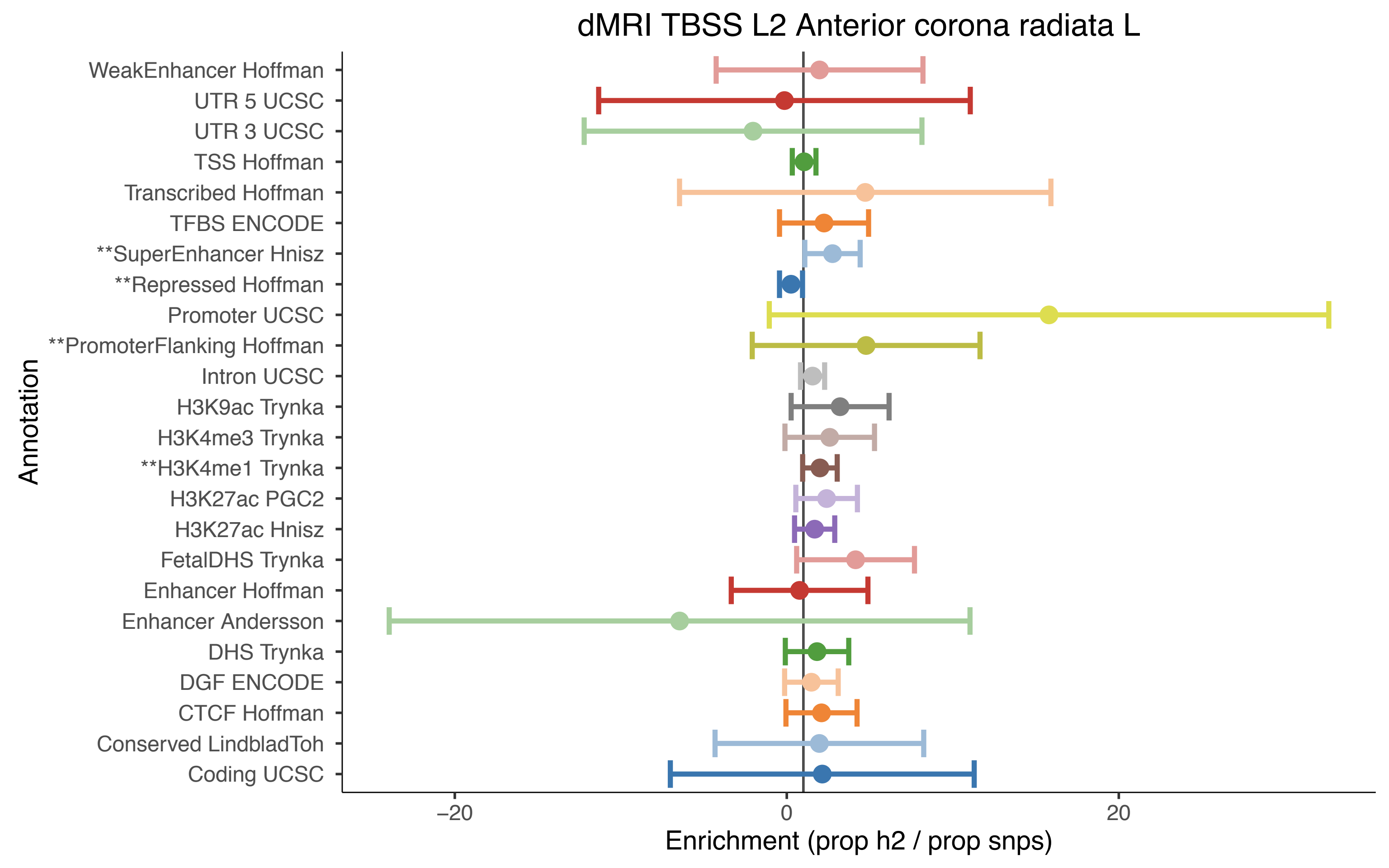

dMRI TBSS L2 Anterior corona radiata L
WeakEnhancer Hoffman
UTR 5 UCSC
UTR 3 UCSC
TSS Hoffman
Transcribed Hoffman
TFBS ENCODE
**SuperEnhancer Hnisz
**Repressed Hoffman
Promoter UCSC
**PromoterFlanking Hoffman
Intron UCSC
H3K9ac Trynka
H3K4me3 Trynka
**H3K4me1 Trynka
H3K27ac PGC2
H3K27ac Hnisz
FetalDHS Trynka
Enhancer Hoffman
Enhancer Andersson
DHS Trynka
DGF ENCODE
CTCF Hoffman
Conserved LindbladToh
Coding UCSC
Annotation
−20
0
20
Enrichment (prop h2 / prop snps)

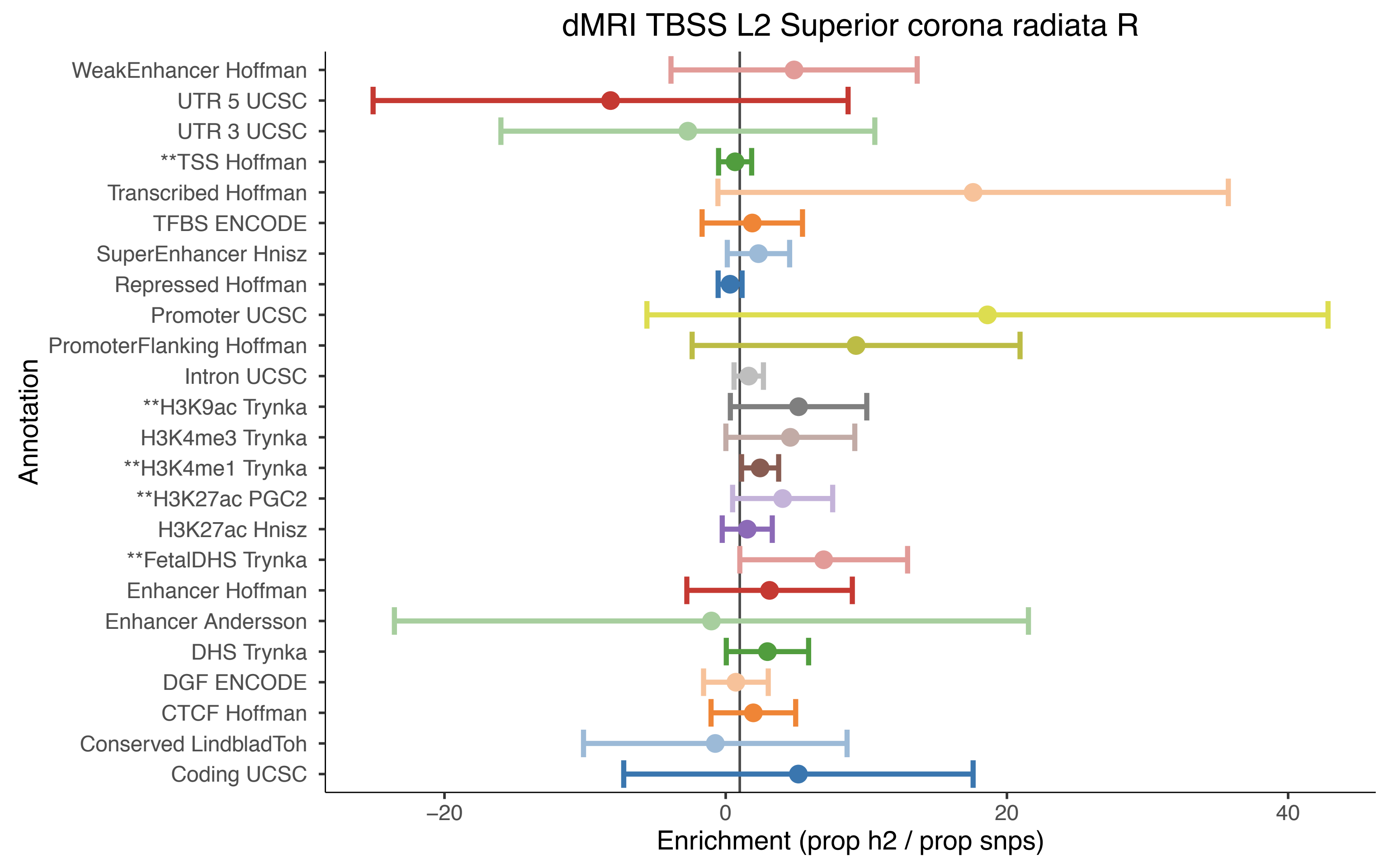

dMRI TBSS L2 Superior corona radiata R
WeakEnhancer Hoffman
UTR 5 UCSC
UTR 3 UCSC
**TSS Hoffman
Transcribed Hoffman
TFBS ENCODE
SuperEnhancer Hnisz
Repressed Hoffman
Promoter UCSC
PromoterFlanking Hoffman
Intron UCSC
**H3K9ac Trynka
H3K4me3 Trynka
**H3K4me1 Trynka
**H3K27ac PGC2
H3K27ac Hnisz
**FetalDHS Trynka
Enhancer Hoffman
Enhancer Andersson
DHS Trynka
DGF ENCODE
CTCF Hoffman
Conserved LindbladToh
Coding UCSC
Annotation
−20
0
20
40
Enrichment (prop h2 / prop snps)

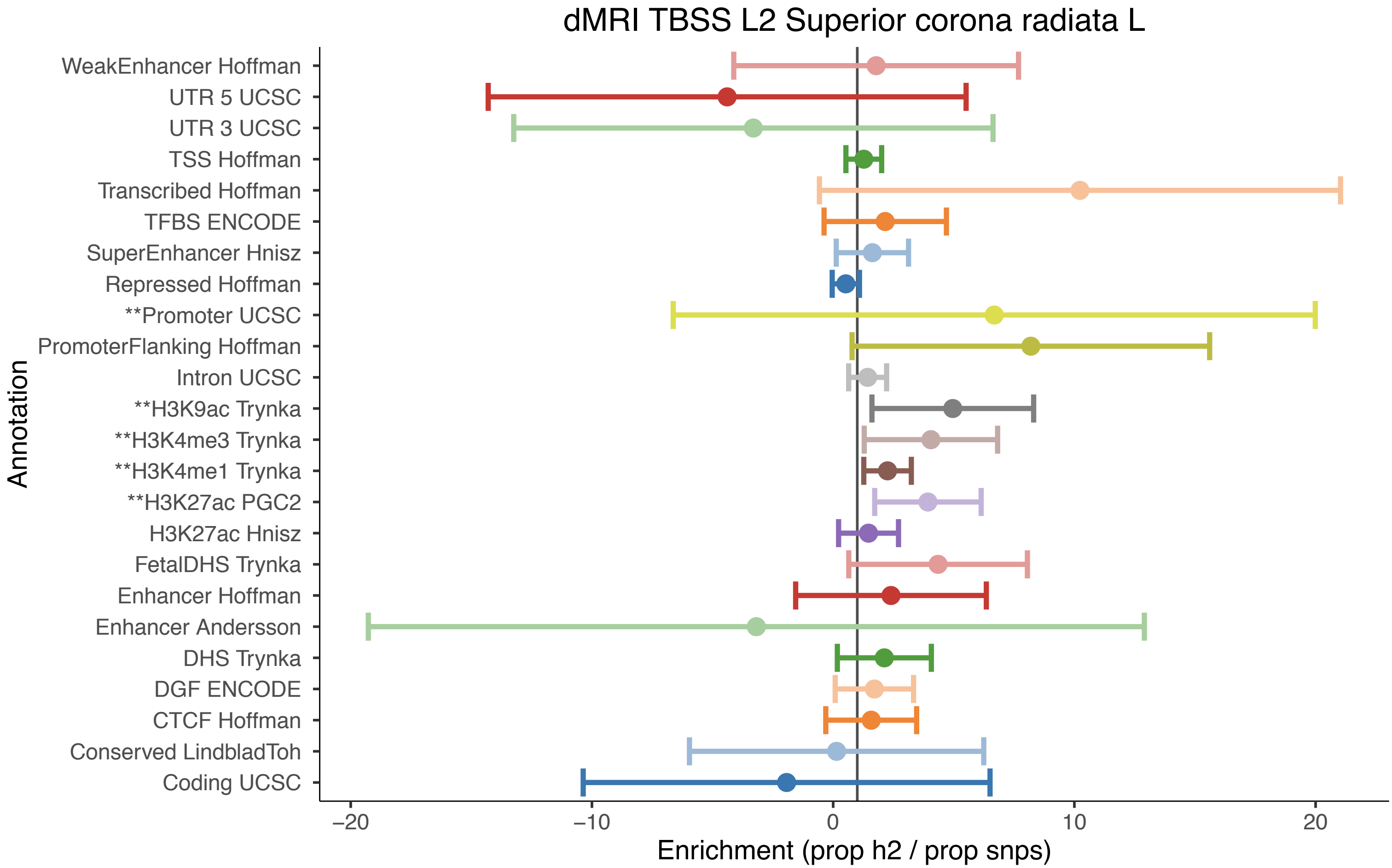

dMRI TBSS L2 Superior corona radiata L
WeakEnhancer Hoffman
UTR 5 UCSC
UTR 3 UCSC
TSS Hoffman
Transcribed Hoffman
TFBS ENCODE
SuperEnhancer Hnisz
Repressed Hoffman
**Promoter UCSC
PromoterFlanking Hoffman
Intron UCSC
**H3K9ac Trynka
**H3K4me3 Trynka
**H3K4me1 Trynka
**H3K27ac PGC2
H3K27ac Hnisz
FetalDHS Trynka
Enhancer Hoffman
Enhancer Andersson
DHS Trynka
DGF ENCODE
CTCF Hoffman
Conserved LindbladToh
Coding UCSC
Annotation
−20
−10
0
10
20
Enrichment (prop h2 / prop snps)

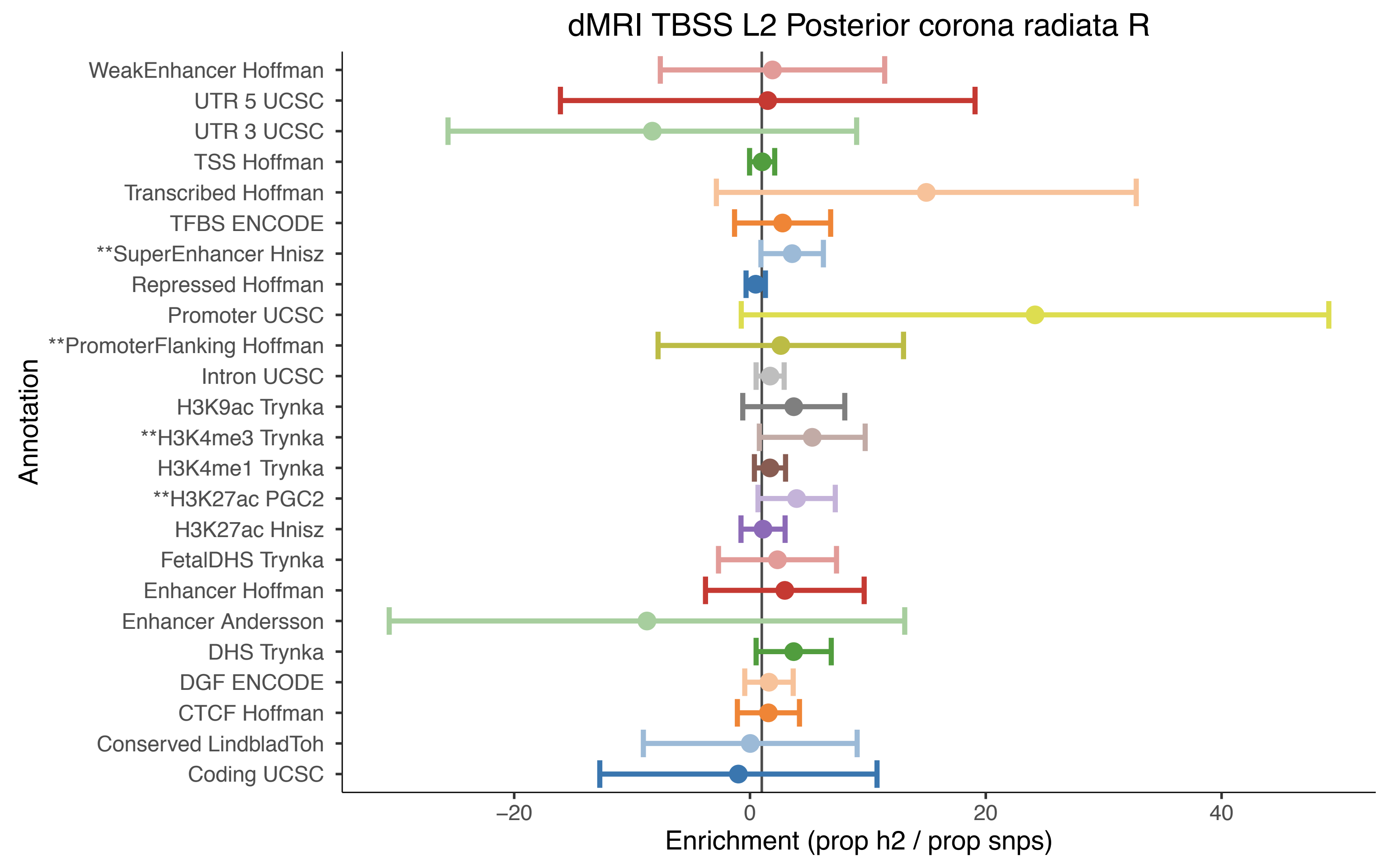
dMRI TBSS L2 Posterior corona radiata R
WeakEnhancer Hoffman
UTR 5 UCSC
UTR 3 UCSC
TSS Hoffman
Transcribed Hoffman
TFBS ENCODE
**SuperEnhancer Hnisz
Repressed Hoffman
Promoter UCSC
**PromoterFlanking Hoffman
Intron UCSC
H3K9ac Trynka
**H3K4me3 Trynka
H3K4me1 Trynka
**H3K27ac PGC2
H3K27ac Hnisz
FetalDHS Trynka
Enhancer Hoffman
Enhancer Andersson
DHS Trynka
DGF ENCODE
CTCF Hoffman
Conserved LindbladToh
Coding UCSC
Annotation
−20
0
20
40
Enrichment (prop h2 / prop snps)

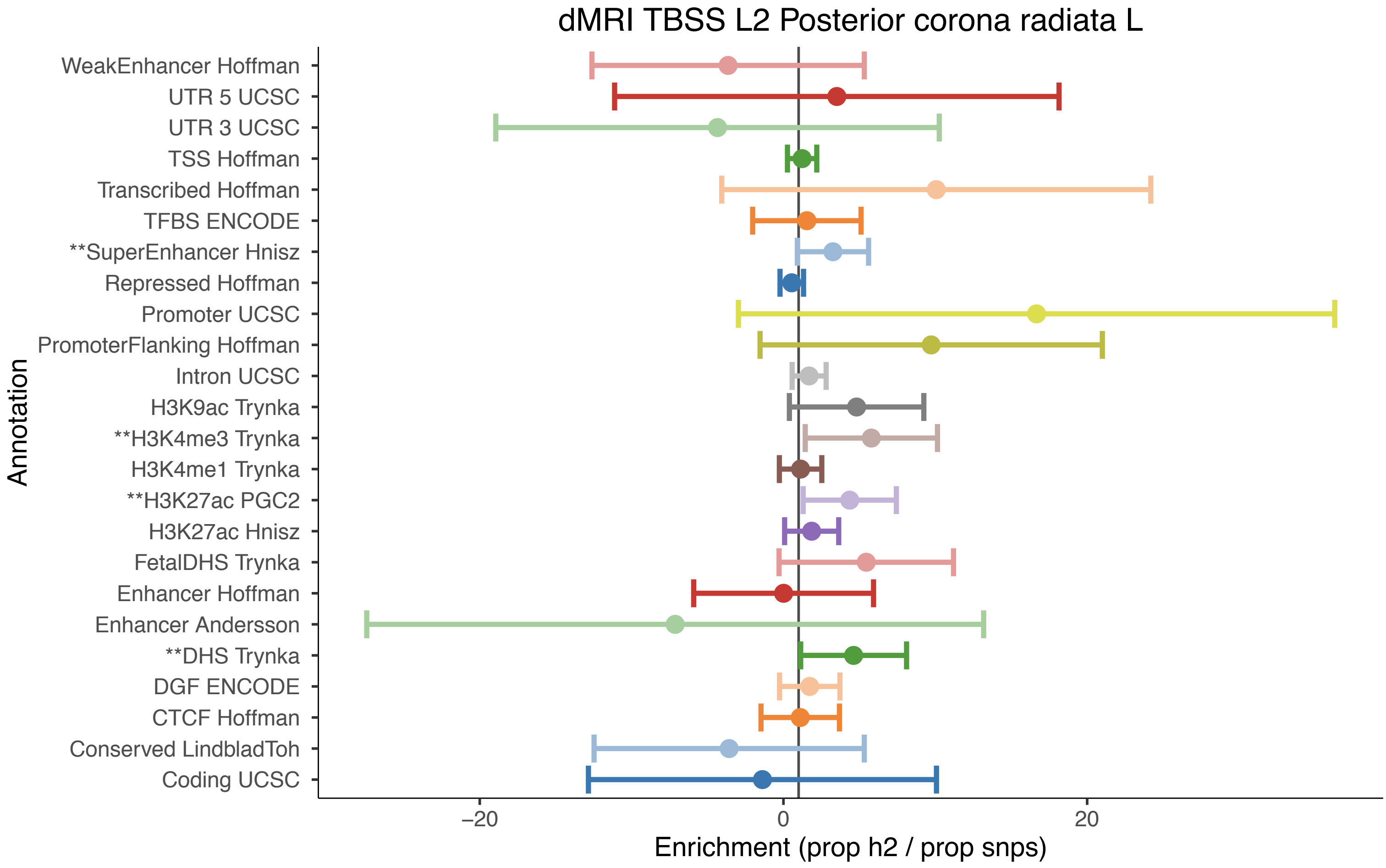

dMRI TBSS L2 Posterior corona radiata L
WeakEnhancer Hoffman
UTR 5 UCSC
UTR 3 UCSC
TSS Hoffman
Transcribed Hoffman
TFBS ENCODE
**SuperEnhancer Hnisz
Repressed Hoffman
Promoter UCSC
PromoterFlanking Hoffman
Intron UCSC
H3K9ac Trynka
**H3K4me3 Trynka
H3K4me1 Trynka
**H3K27ac PGC2
H3K27ac Hnisz
FetalDHS Trynka
Enhancer Hoffman
Enhancer Andersson
**DHS Trynka
DGF ENCODE
CTCF Hoffman
Conserved LindbladToh
Coding UCSC
Annotation
−20
0
20
Enrichment (prop h2 / prop snps)

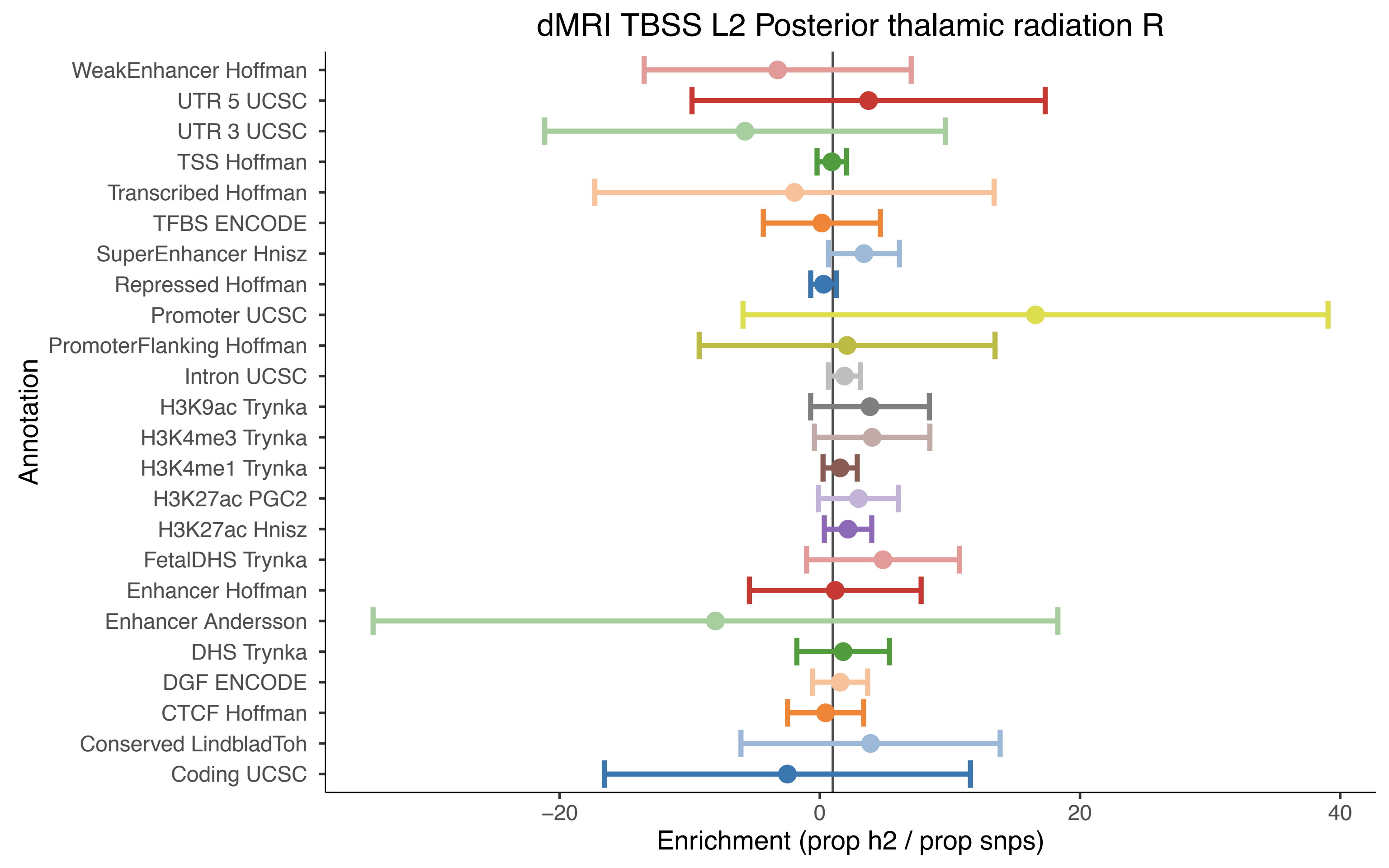
dMRI TBSS L2 Posterior thalamic radiation R
WeakEnhancer Hoffman
UTR 5 UCSC
UTR 3 UCSC
TSS Hoffman
Transcribed Hoffman
TFBS ENCODE
SuperEnhancer Hnisz
Repressed Hoffman
Promoter UCSC
PromoterFlanking Hoffman
Intron UCSC
H3K9ac Trynka
H3K4me3 Trynka
H3K4me1 Trynka
H3K27ac PGC2
H3K27ac Hnisz
FetalDHS Trynka
Enhancer Hoffman
Enhancer Andersson
DHS Trynka
DGF ENCODE
CTCF Hoffman
Conserved LindbladToh
Coding UCSC
Annotation
−20
0
20
40
Enrichment (prop h2 / prop snps)

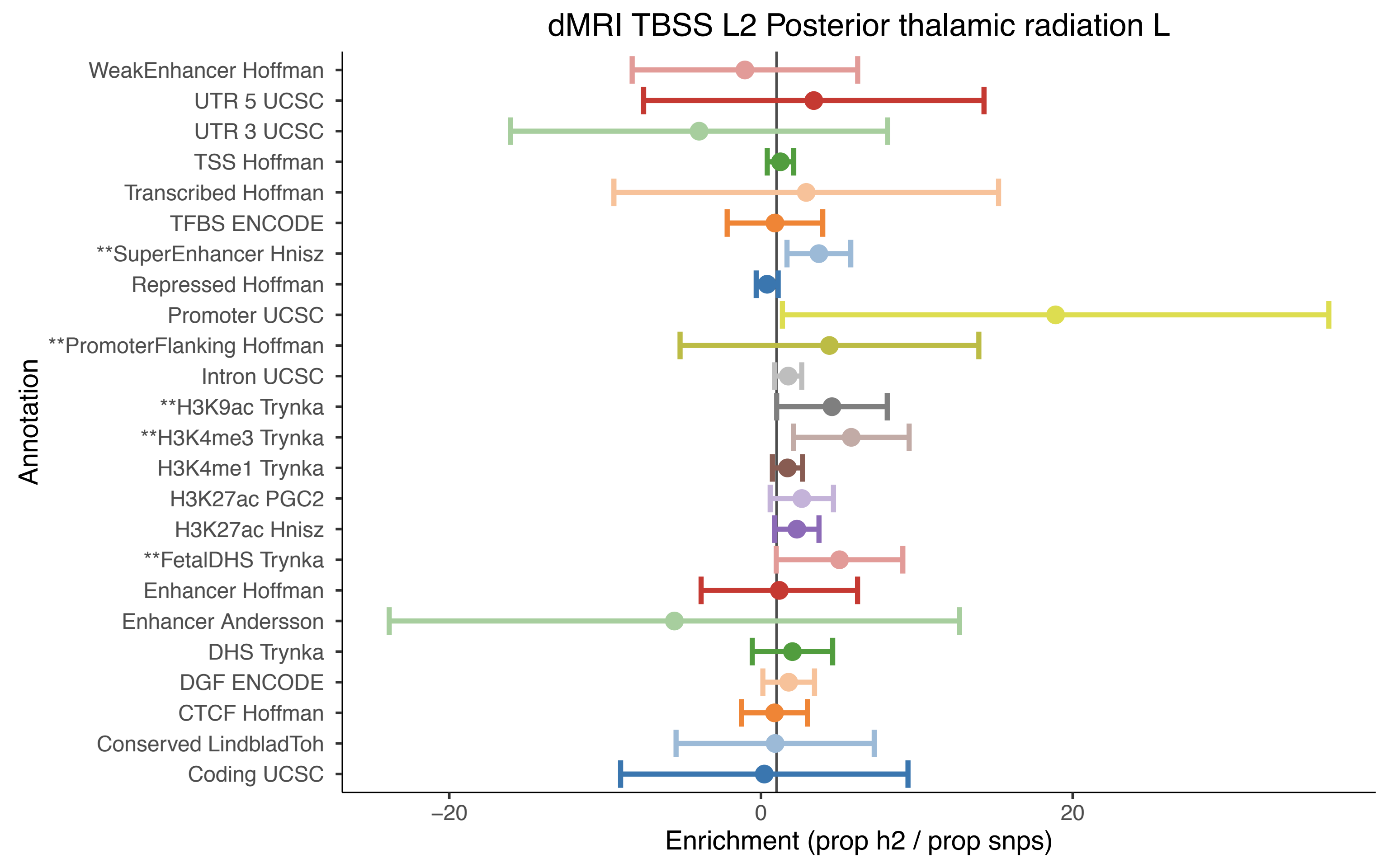

dMRI TBSS L2 Posterior thalamic radiation L
WeakEnhancer Hoffman
UTR 5 UCSC
UTR 3 UCSC
TSS Hoffman
Transcribed Hoffman
TFBS ENCODE
**SuperEnhancer Hnisz
Repressed Hoffman
Promoter UCSC
**PromoterFlanking Hoffman
Intron UCSC
**H3K9ac Trynka
**H3K4me3 Trynka
H3K4me1 Trynka
H3K27ac PGC2
H3K27ac Hnisz
**FetalDHS Trynka
Enhancer Hoffman
Enhancer Andersson
DHS Trynka
DGF ENCODE
CTCF Hoffman
Conserved LindbladToh
Coding UCSC
Annotation
−20
0
20
Enrichment (prop h2 / prop snps)

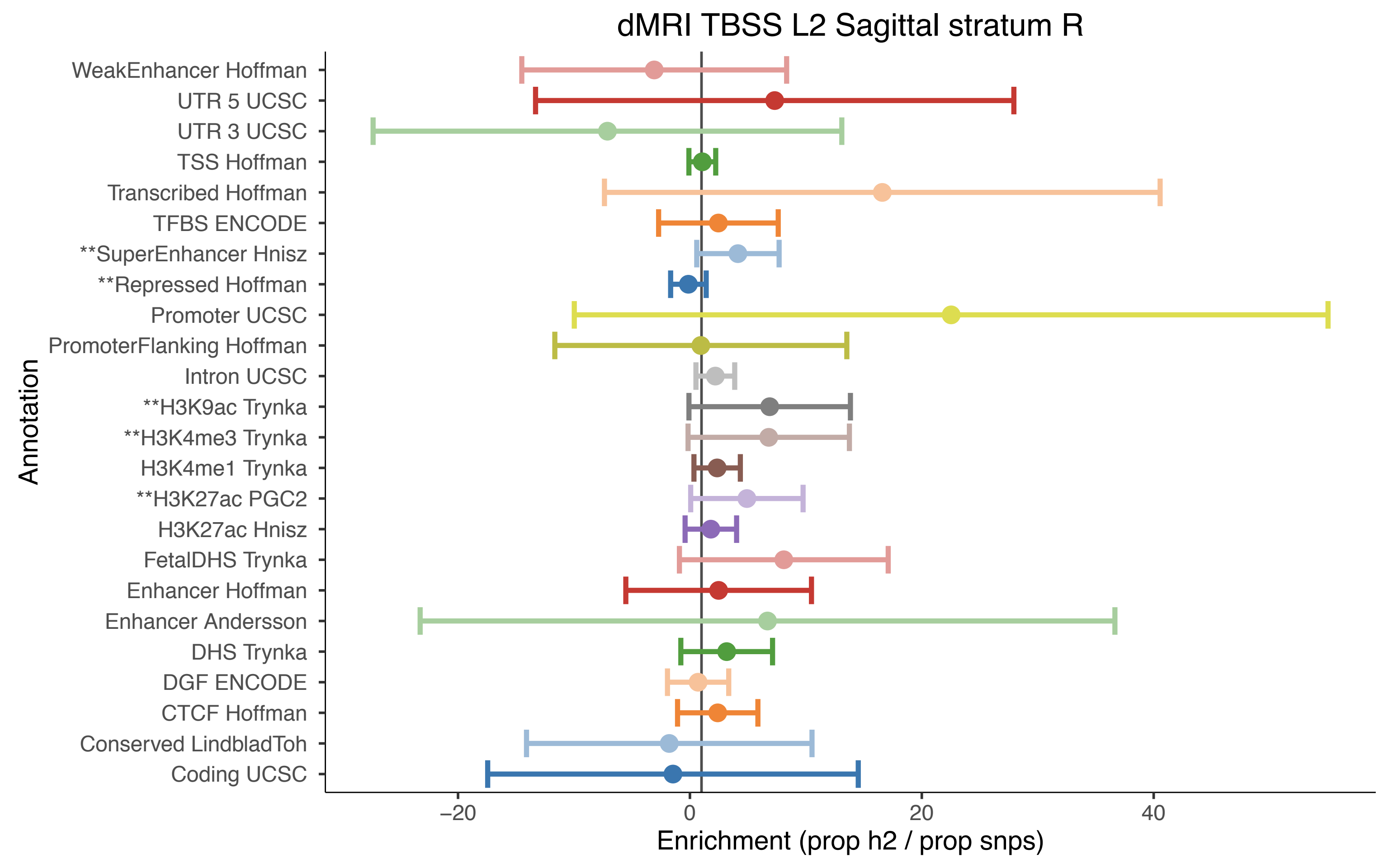
dMRI TBSS L2 Sagittal stratum R
Annotation
WeakEnhancer Hoffman
UTR 5 UCSC
UTR 3 UCSC
TSS Hoffman
Transcribed Hoffman
TFBS ENCODE
**SuperEnhancer Hnisz
**Repressed Hoffman
Promoter UCSC
PromoterFlanking Hoffman
Intron UCSC
**H3K9ac Trynka
**H3K4me3 Trynka
H3K4me1 Trynka
**H3K27ac PGC2
H3K27ac Hnisz
FetalDHS Trynka
Enhancer Hoffman
Enhancer Andersson
DHS Trynka
DGF ENCODE
CTCF Hoffman
Conserved LindbladToh
Coding UCSC
−20
0
20
40
Enrichment (prop h2 / prop snps)

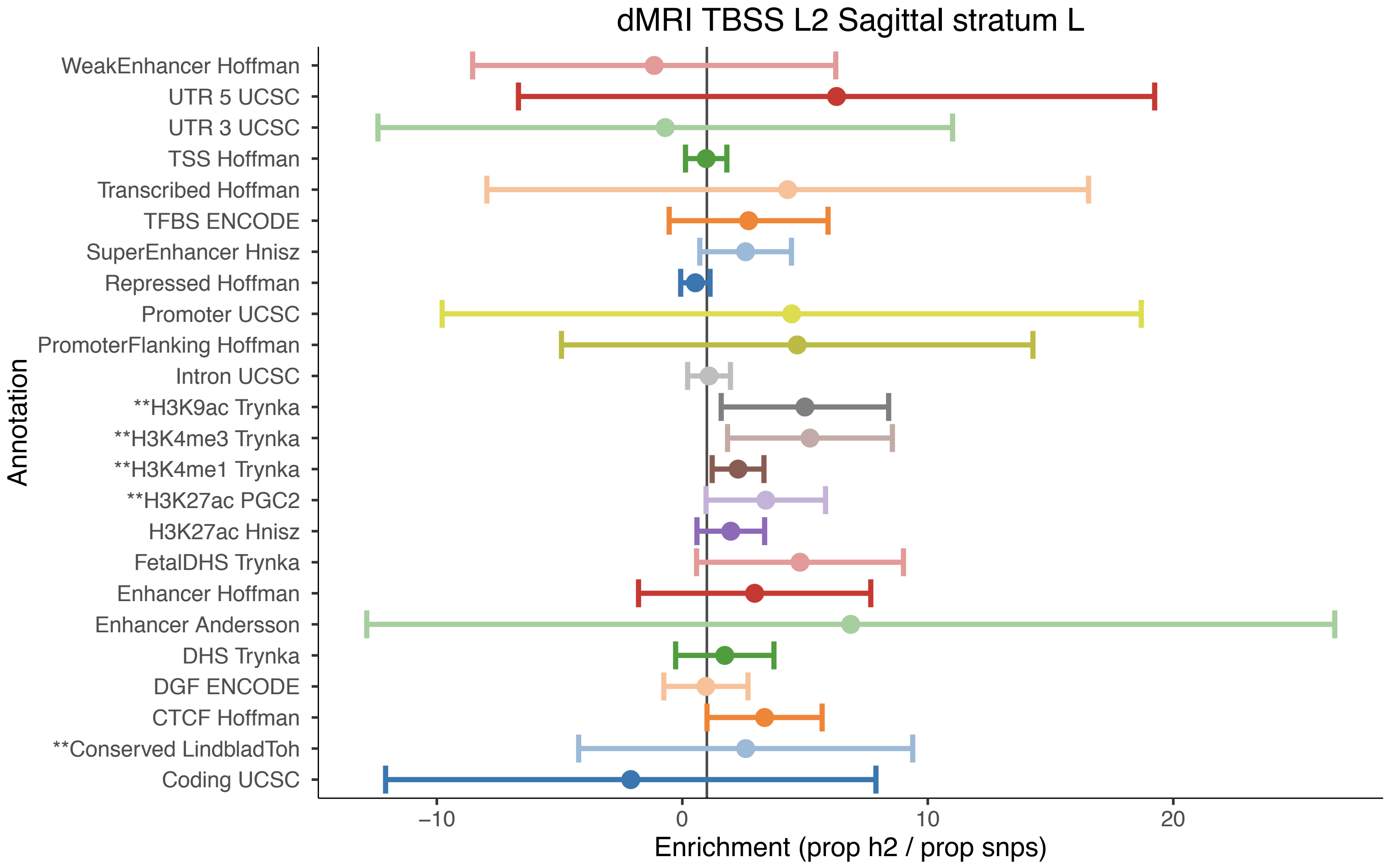

dMRI TBSS L2 Sagittal stratum L
WeakEnhancer Hoffman
UTR 5 UCSC
UTR 3 UCSC
TSS Hoffman
Transcribed Hoffman
TFBS ENCODE
SuperEnhancer Hnisz
Repressed Hoffman
Promoter UCSC
PromoterFlanking Hoffman
Intron UCSC
**H3K9ac Trynka
**H3K4me3 Trynka
**H3K4me1 Trynka
**H3K27ac PGC2
H3K27ac Hnisz
FetalDHS Trynka
Enhancer Hoffman
Enhancer Andersson
DHS Trynka
DGF ENCODE
CTCF Hoffman
**Conserved LindbladToh
Coding UCSC
Annotation
−10
0
10
20
Enrichment (prop h2 / prop snps)

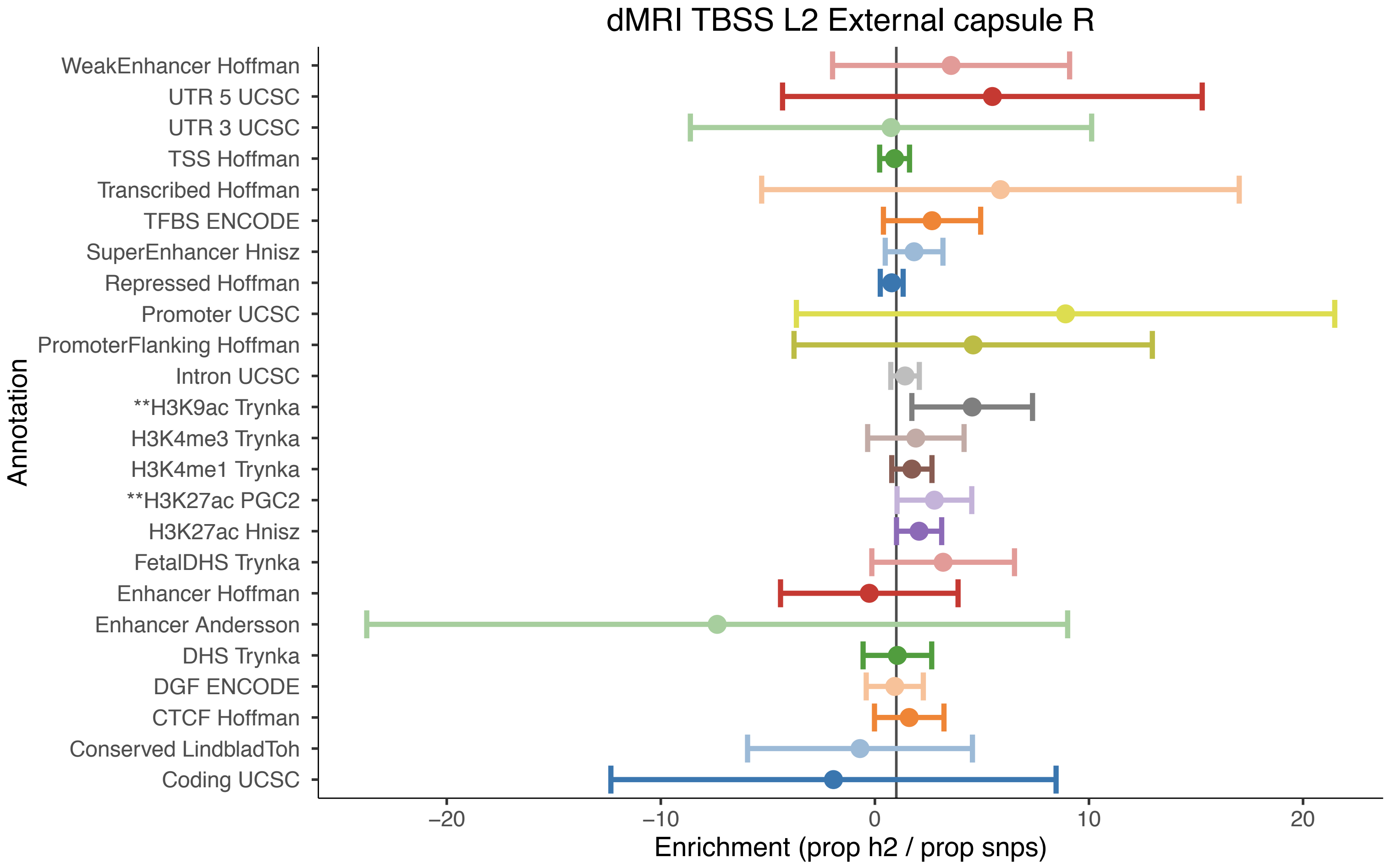
dMRI TBSS L2 External capsule R
WeakEnhancer Hoffman
UTR 5 UCSC
UTR 3 UCSC
TSS Hoffman
Transcribed Hoffman
TFBS ENCODE
SuperEnhancer Hnisz
Repressed Hoffman
Promoter UCSC
PromoterFlanking Hoffman
Intron UCSC
**H3K9ac Trynka
H3K4me3 Trynka
H3K4me1 Trynka
**H3K27ac PGC2
H3K27ac Hnisz
FetalDHS Trynka
Enhancer Hoffman
Enhancer Andersson
DHS Trynka
DGF ENCODE
CTCF Hoffman
Conserved LindbladToh
Coding UCSC
Annotation
−20
−10
0
10
20
Enrichment (prop h2 / prop snps)

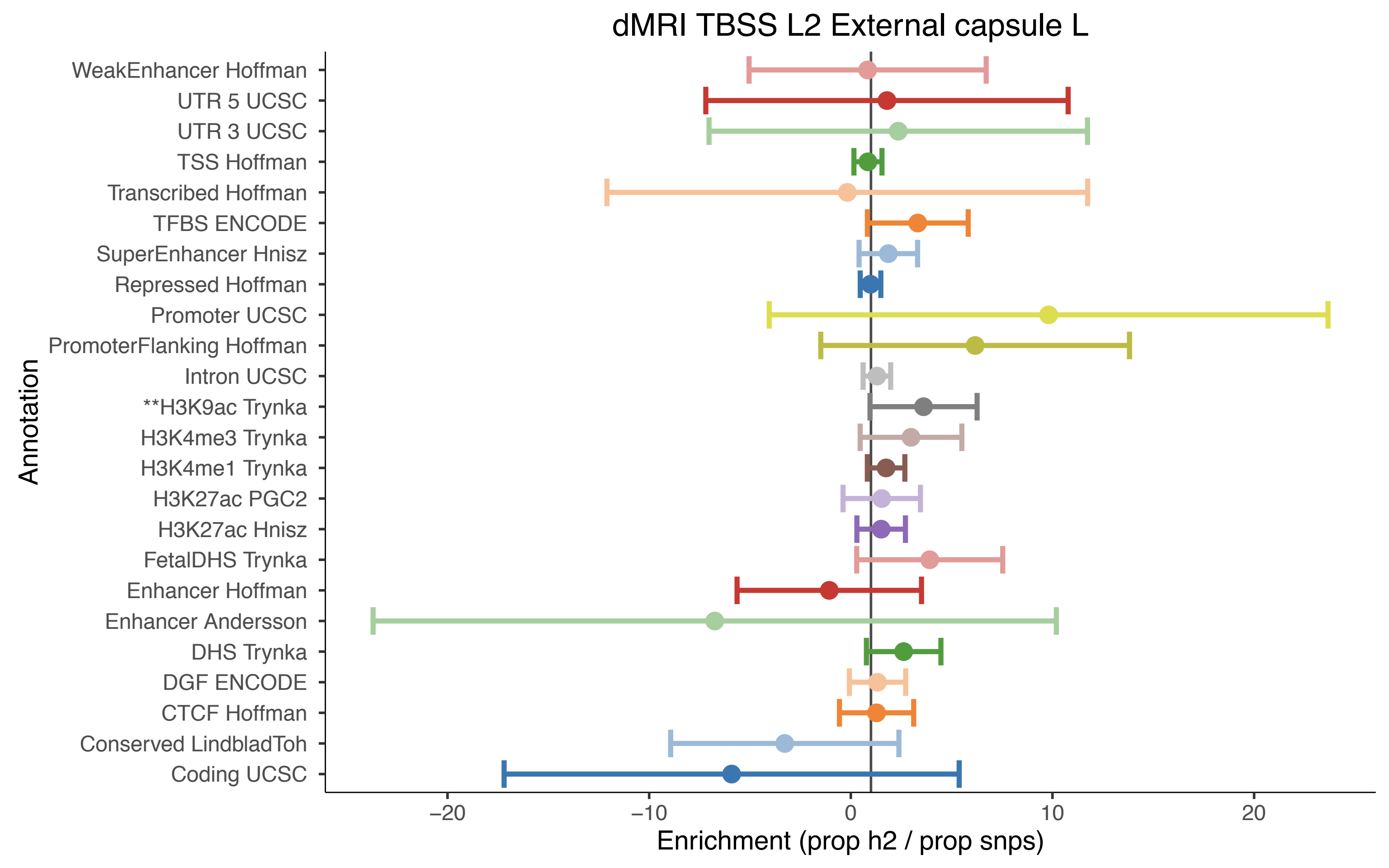

dMRI TBSS L2 External capsule L
WeakEnhancer Hoffman
UTR 5 UCSC
UTR 3 UCSC
TSS Hoffman
Transcribed Hoffman
TFBS ENCODE
SuperEnhancer Hnisz
Repressed Hoffman
Promoter UCSC
PromoterFlanking Hoffman
Intron UCSC
**H3K9ac Trynka
H3K4me3 Trynka
H3K4me1 Trynka
H3K27ac PGC2
H3K27ac Hnisz
FetalDHS Trynka
Enhancer Hoffman
Enhancer Andersson
DHS Trynka
DGF ENCODE
CTCF Hoffman
Conserved LindbladToh
Coding UCSC
Annotation
−20
−10
0
10
20
Enrichment (prop h2 / prop snps)

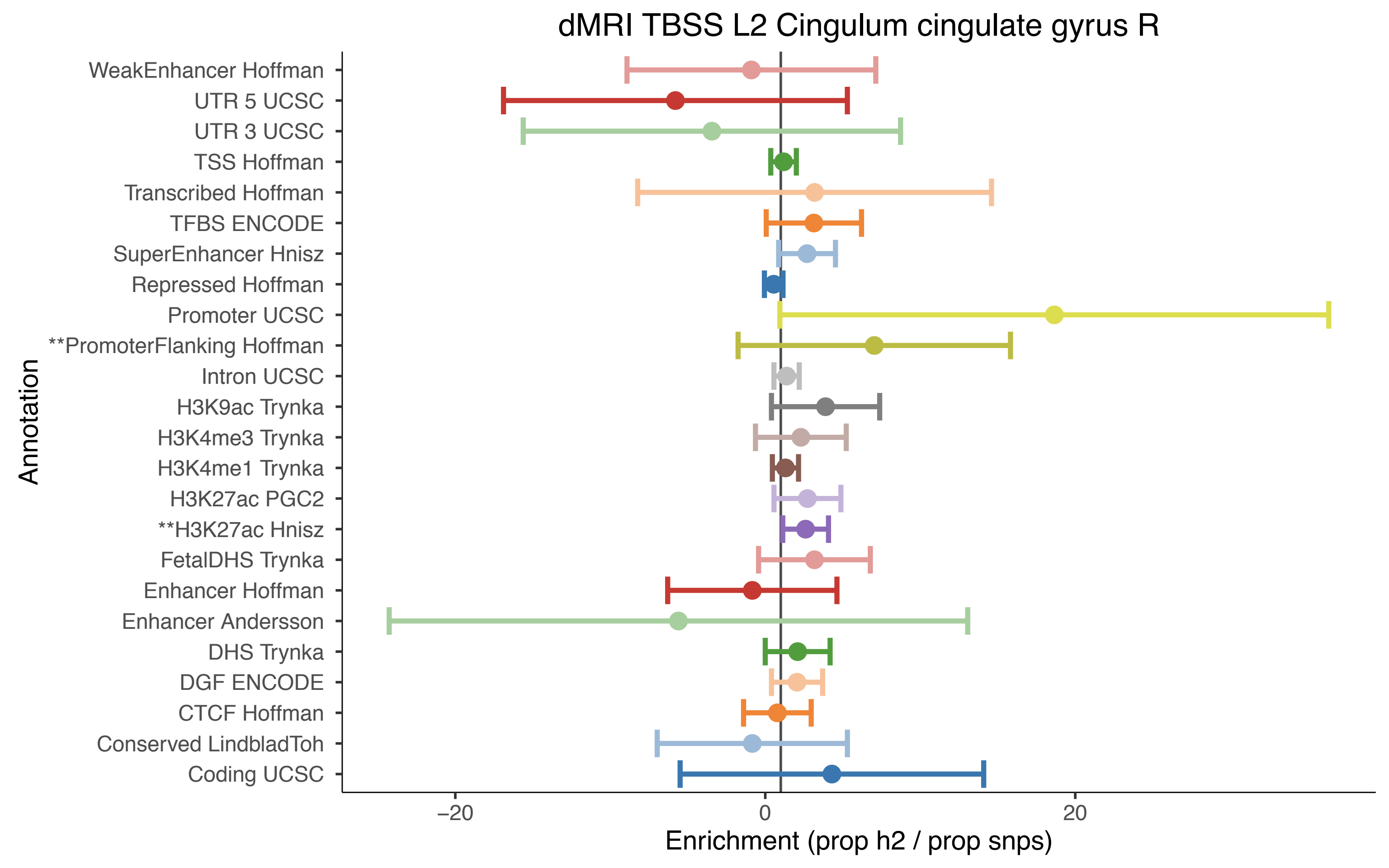

dMRI TBSS L2 Cingulum cingulate gyrus R
WeakEnhancer Hoffman
UTR 5 UCSC
UTR 3 UCSC
TSS Hoffman
Transcribed Hoffman
TFBS ENCODE
SuperEnhancer Hnisz
Repressed Hoffman
Promoter UCSC
**PromoterFlanking Hoffman
Intron UCSC
H3K9ac Trynka
H3K4me3 Trynka
H3K4me1 Trynka
H3K27ac PGC2
**H3K27ac Hnisz
FetalDHS Trynka
Enhancer Hoffman
Enhancer Andersson
DHS Trynka
DGF ENCODE
CTCF Hoffman
Conserved LindbladToh
Coding UCSC
Annotation
−20
0
20
Enrichment (prop h2 / prop snps)

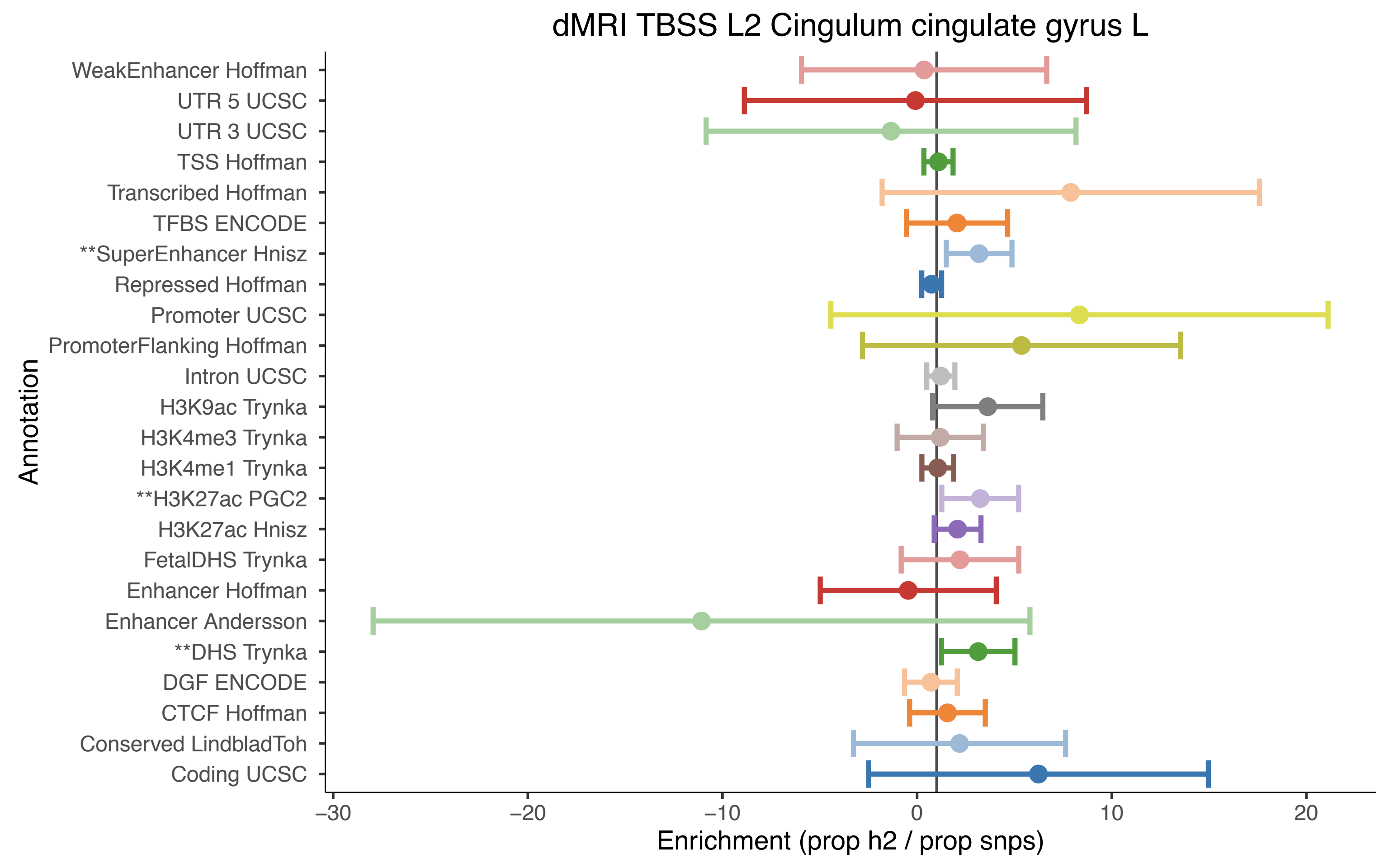
dMRI TBSS L2 Cingulum cingulate gyrus L
WeakEnhancer Hoffman
UTR 5 UCSC
UTR 3 UCSC
TSS Hoffman
Transcribed Hoffman
TFBS ENCODE
**SuperEnhancer Hnisz
Repressed Hoffman
Promoter UCSC
PromoterFlanking Hoffman
Intron UCSC
H3K9ac Trynka
H3K4me3 Trynka
H3K4me1 Trynka
**H3K27ac PGC2
H3K27ac Hnisz
FetalDHS Trynka
Enhancer Hoffman
Enhancer Andersson
**DHS Trynka
DGF ENCODE
CTCF Hoffman
Conserved LindbladToh
Coding UCSC
Annotation
−30
−20
−10
0
10
20
Enrichment (prop h2 / prop snps)

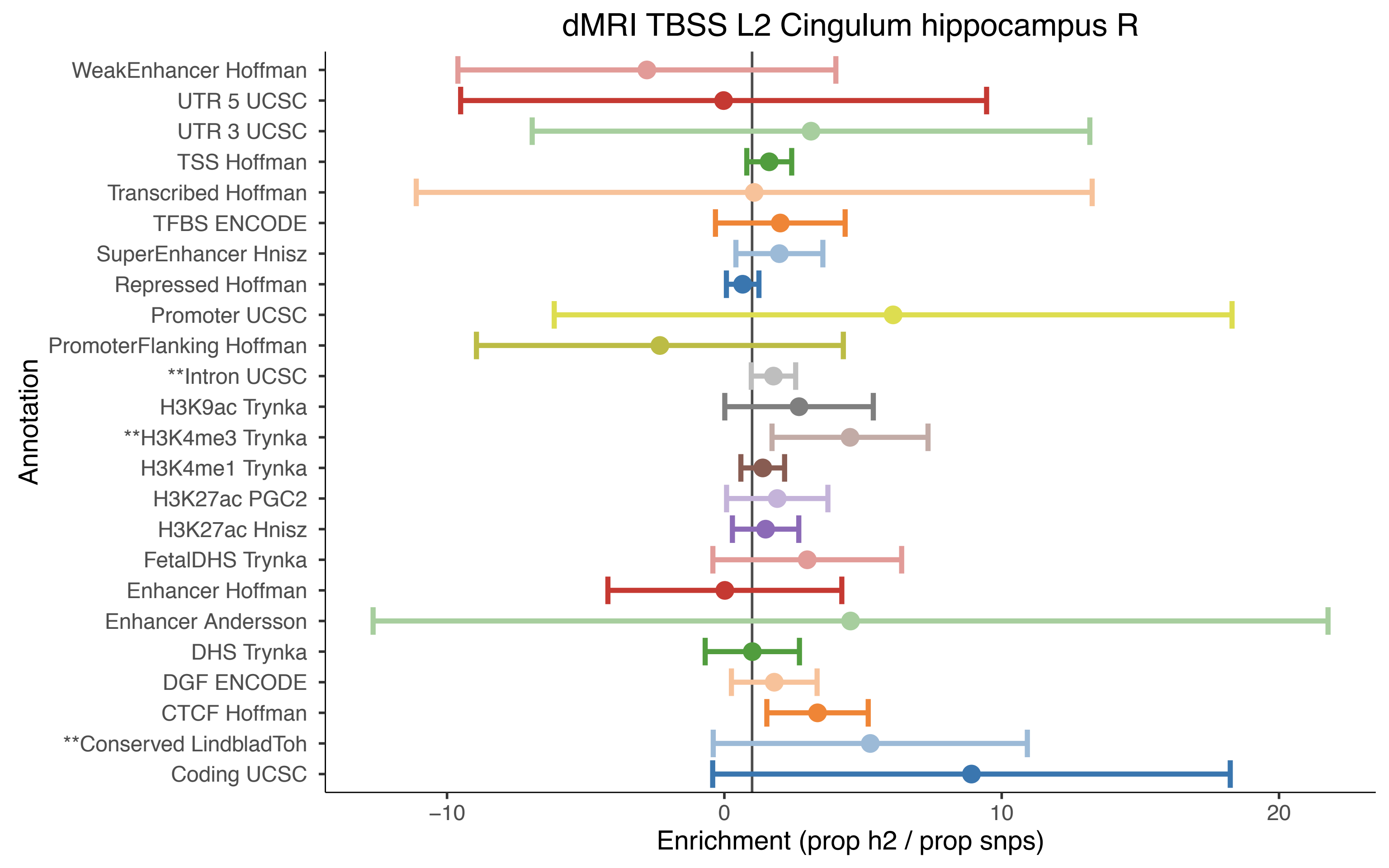
dMRI TBSS L2 Cingulum hippocampus R
WeakEnhancer Hoffman
UTR 5 UCSC
UTR 3 UCSC
TSS Hoffman
Transcribed Hoffman
TFBS ENCODE
SuperEnhancer Hnisz
Repressed Hoffman
Promoter UCSC
PromoterFlanking Hoffman
**Intron UCSC
H3K9ac Trynka
**H3K4me3 Trynka
H3K4me1 Trynka
H3K27ac PGC2
H3K27ac Hnisz
FetalDHS Trynka
Enhancer Hoffman
Enhancer Andersson
DHS Trynka
DGF ENCODE
CTCF Hoffman
**Conserved LindbladToh
Coding UCSC
Annotation
−10
0
10
20
Enrichment (prop h2 / prop snps)

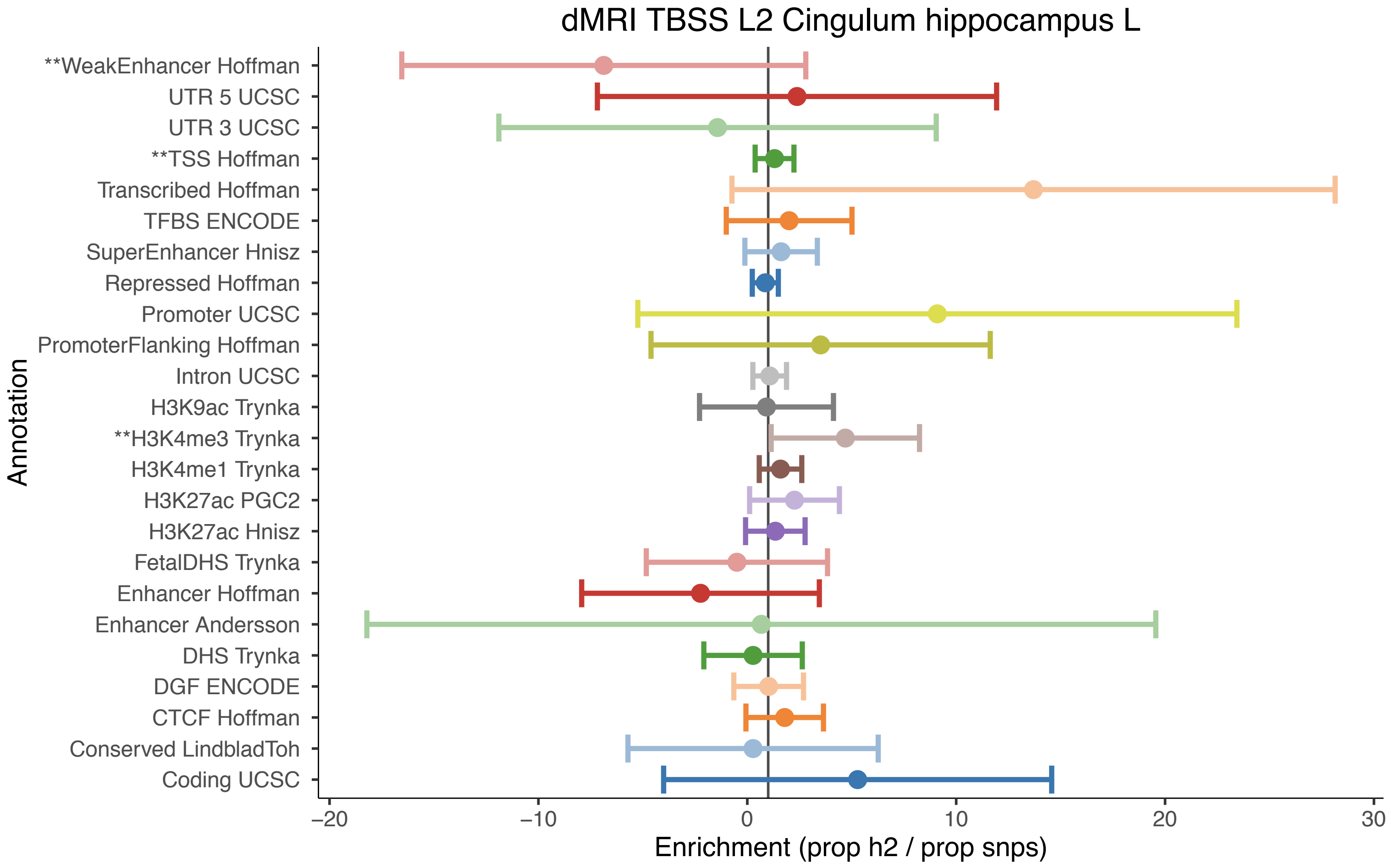
dMRI TBSS L2 Cingulum hippocampus L
**WeakEnhancer Hoffman
UTR 5 UCSC
UTR 3 UCSC
**TSS Hoffman
Transcribed Hoffman
TFBS ENCODE
SuperEnhancer Hnisz
Repressed Hoffman
Promoter UCSC
PromoterFlanking Hoffman
Intron UCSC
H3K9ac Trynka
**H3K4me3 Trynka
H3K4me1 Trynka
H3K27ac PGC2
H3K27ac Hnisz
FetalDHS Trynka
Enhancer Hoffman
Enhancer Andersson
DHS Trynka
DGF ENCODE
CTCF Hoffman
Conserved LindbladToh
Coding UCSC
Annotation
−20
−10
0
10
20
30
Enrichment (prop h2 / prop snps)

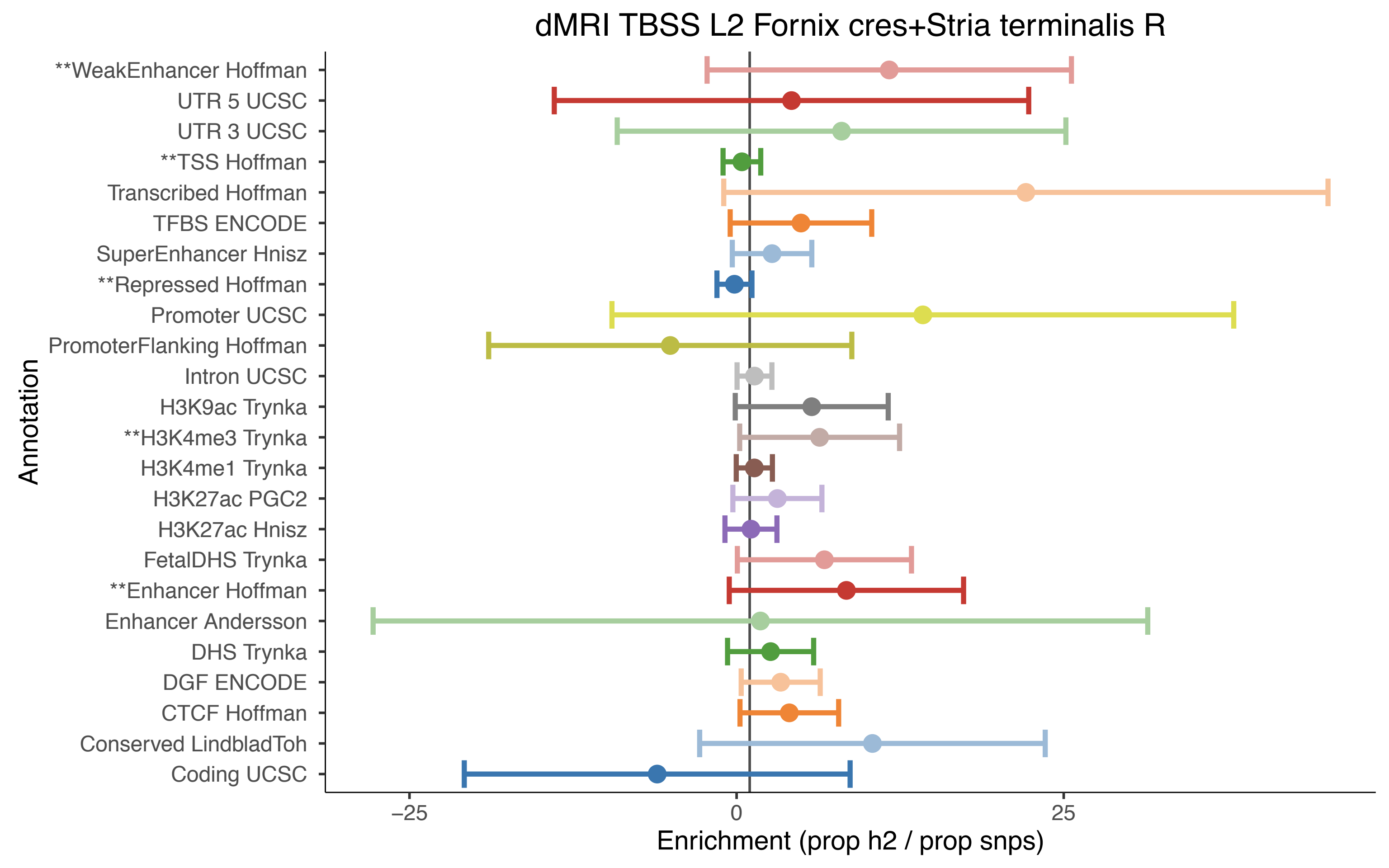

dMRI TBSS L2 Fornix cres+Stria terminalis R
**WeakEnhancer Hoffman
UTR 5 UCSC
UTR 3 UCSC
**TSS Hoffman
Transcribed Hoffman
TFBS ENCODE
SuperEnhancer Hnisz
**Repressed Hoffman
Promoter UCSC
PromoterFlanking Hoffman
Intron UCSC
H3K9ac Trynka
**H3K4me3 Trynka
H3K4me1 Trynka
H3K27ac PGC2
H3K27ac Hnisz
FetalDHS Trynka
**Enhancer Hoffman
Enhancer Andersson
DHS Trynka
DGF ENCODE
CTCF Hoffman
Conserved LindbladToh
Coding UCSC
Annotation
−25
0
25
Enrichment (prop h2 / prop snps)

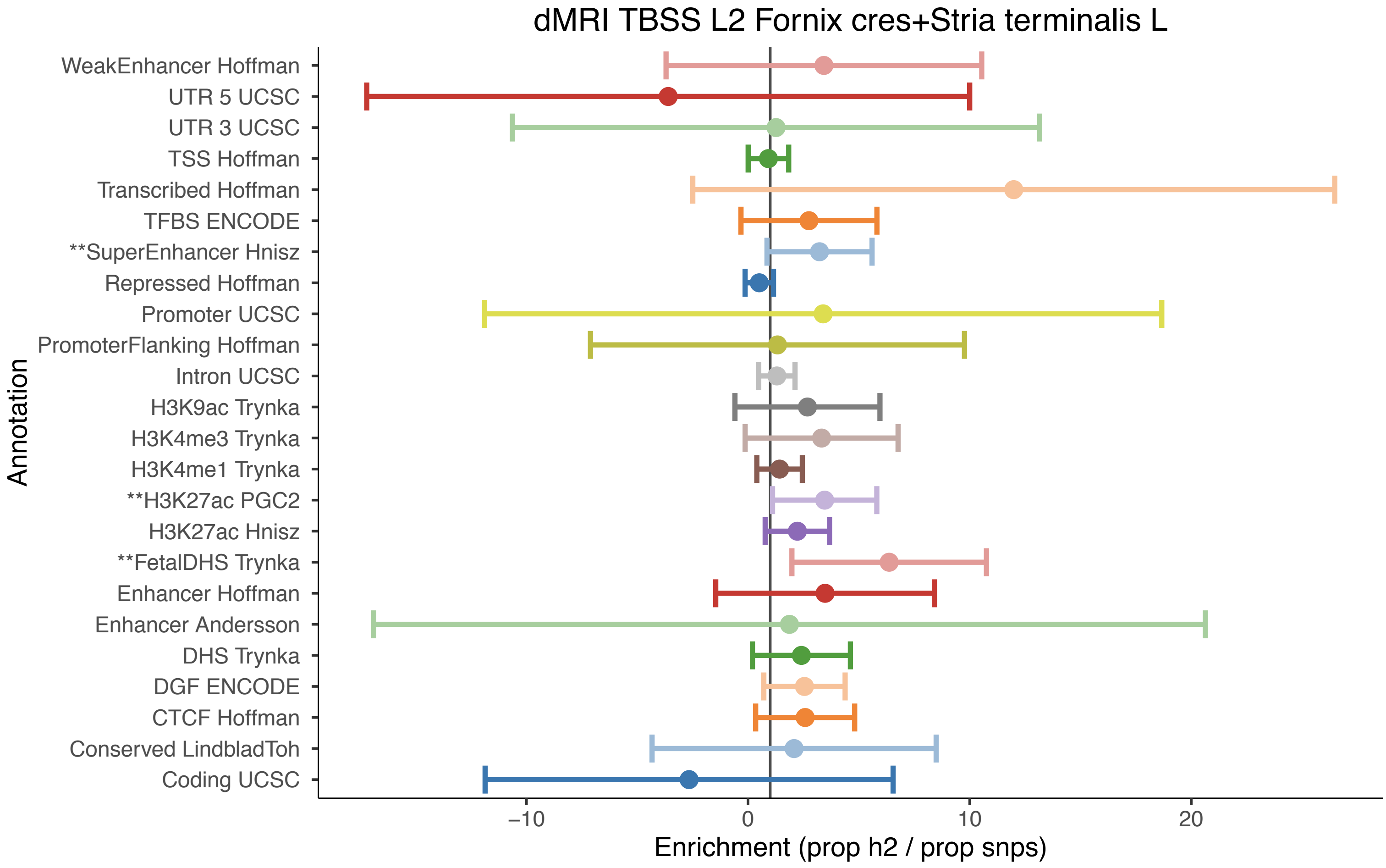

dMRI TBSS L2 Fornix cres+Stria terminalis L
WeakEnhancer Hoffman
UTR 5 UCSC
UTR 3 UCSC
TSS Hoffman
Transcribed Hoffman
TFBS ENCODE
**SuperEnhancer Hnisz
Repressed Hoffman
Promoter UCSC
PromoterFlanking Hoffman
Intron UCSC
H3K9ac Trynka
H3K4me3 Trynka
H3K4me1 Trynka
**H3K27ac PGC2
H3K27ac Hnisz
**FetalDHS Trynka
Enhancer Hoffman
Enhancer Andersson
DHS Trynka
DGF ENCODE
CTCF Hoffman
Conserved LindbladToh
Coding UCSC
Annotation
−10
0
10
20
Enrichment (prop h2 / prop snps)

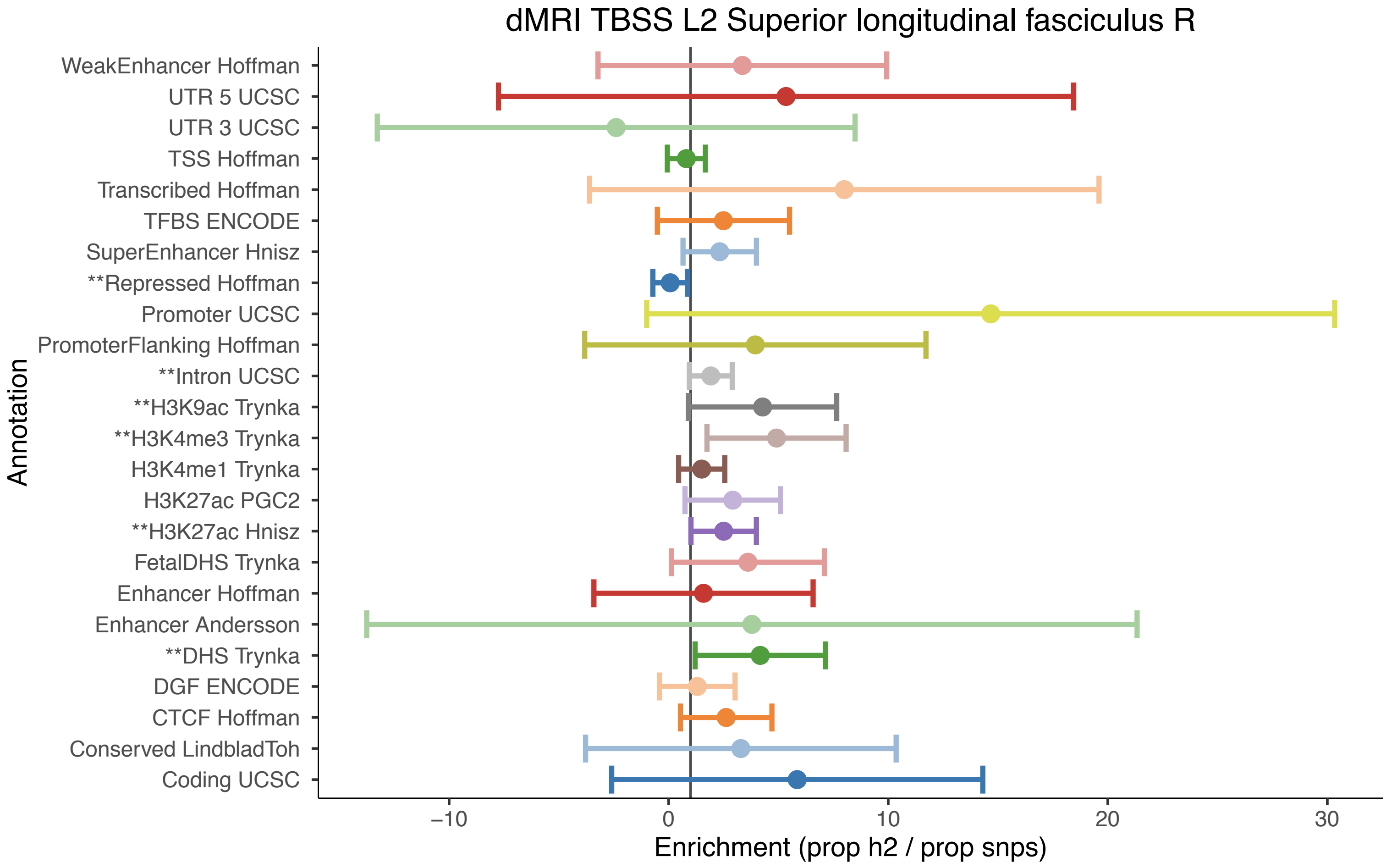

dMRI TBSS L2 Superior longitudinal fasciculus R
WeakEnhancer Hoffman
UTR 5 UCSC
UTR 3 UCSC
TSS Hoffman
Transcribed Hoffman
TFBS ENCODE
SuperEnhancer Hnisz
**Repressed Hoffman
Promoter UCSC
PromoterFlanking Hoffman
**Intron UCSC
**H3K9ac Trynka
**H3K4me3 Trynka
H3K4me1 Trynka
H3K27ac PGC2
**H3K27ac Hnisz
FetalDHS Trynka
Enhancer Hoffman
Enhancer Andersson
**DHS Trynka
DGF ENCODE
CTCF Hoffman
Conserved LindbladToh
Coding UCSC
Annotation
−10
0
10
20
30
Enrichment (prop h2 / prop snps)

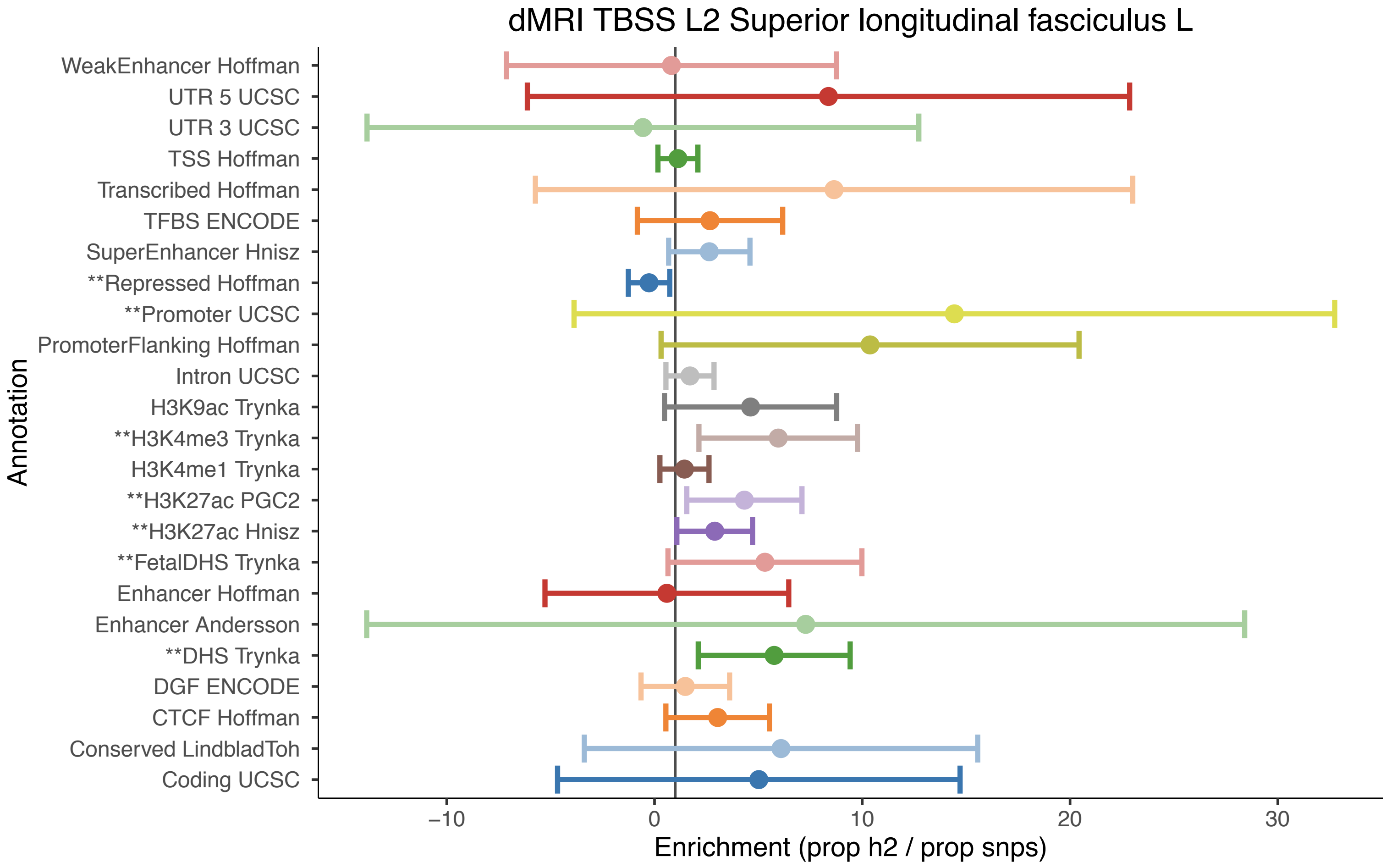

dMRI TBSS L2 Superior longitudinal fasciculus L
WeakEnhancer Hoffman
UTR 5 UCSC
UTR 3 UCSC
TSS Hoffman
Transcribed Hoffman
TFBS ENCODE
SuperEnhancer Hnisz
**Repressed Hoffman
**Promoter UCSC
PromoterFlanking Hoffman
Intron UCSC
H3K9ac Trynka
**H3K4me3 Trynka
H3K4me1 Trynka
**H3K27ac PGC2
**H3K27ac Hnisz
**FetalDHS Trynka
Enhancer Hoffman
Enhancer Andersson
**DHS Trynka
DGF ENCODE
CTCF Hoffman
Conserved LindbladToh
Coding UCSC
Annotation
−10
0
10
20
30
Enrichment (prop h2 / prop snps)

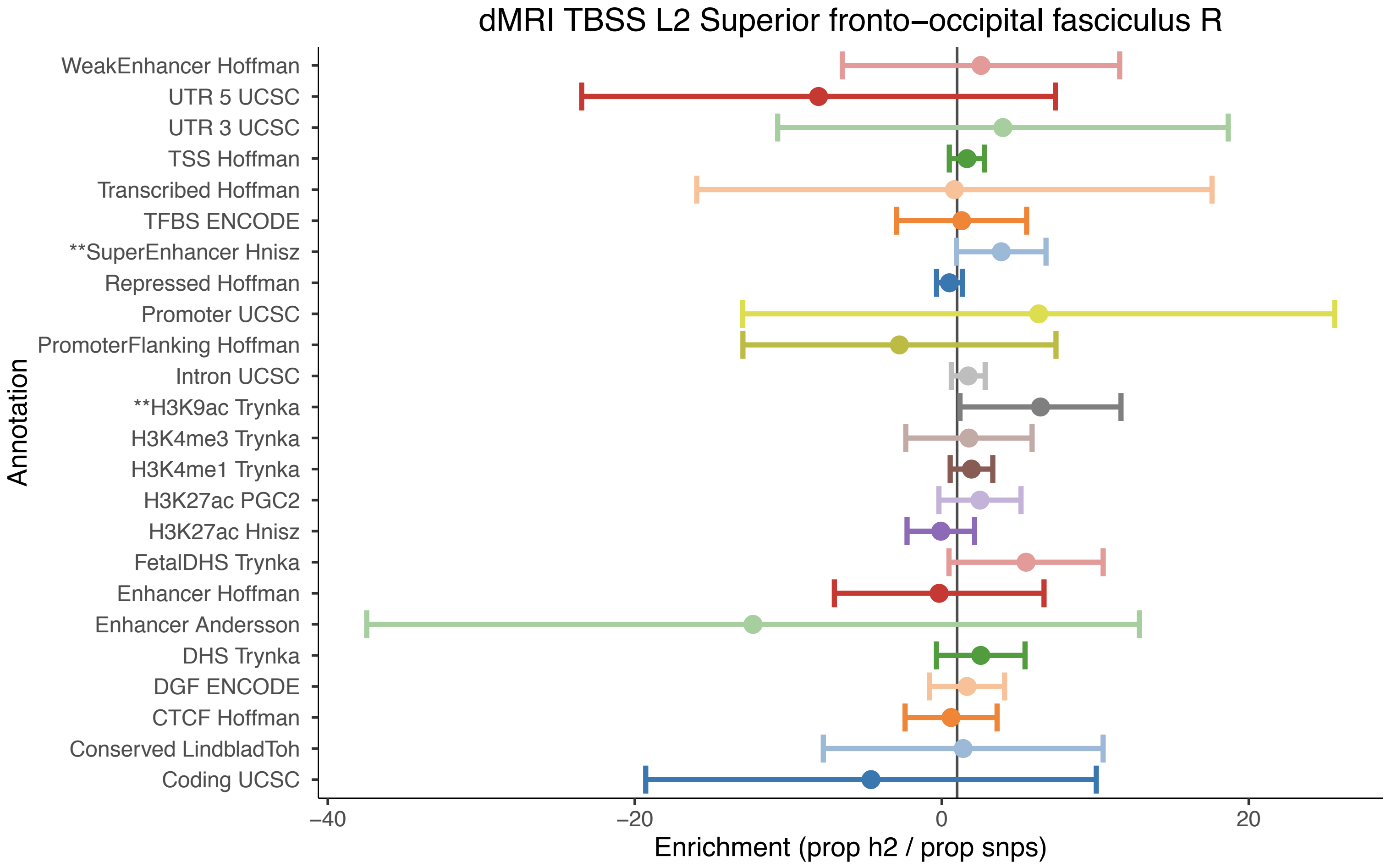

dMRI TBSS L2 Superior fronto−occipital fasciculus R
WeakEnhancer Hoffman
UTR 5 UCSC
UTR 3 UCSC
TSS Hoffman
Transcribed Hoffman
TFBS ENCODE
**SuperEnhancer Hnisz
Repressed Hoffman
Promoter UCSC
PromoterFlanking Hoffman
Intron UCSC
**H3K9ac Trynka
H3K4me3 Trynka
H3K4me1 Trynka
H3K27ac PGC2
H3K27ac Hnisz
FetalDHS Trynka
Enhancer Hoffman
Enhancer Andersson
DHS Trynka
DGF ENCODE
CTCF Hoffman
Conserved LindbladToh
Coding UCSC
Annotation
−40
−20
0
20
Enrichment (prop h2 / prop snps)

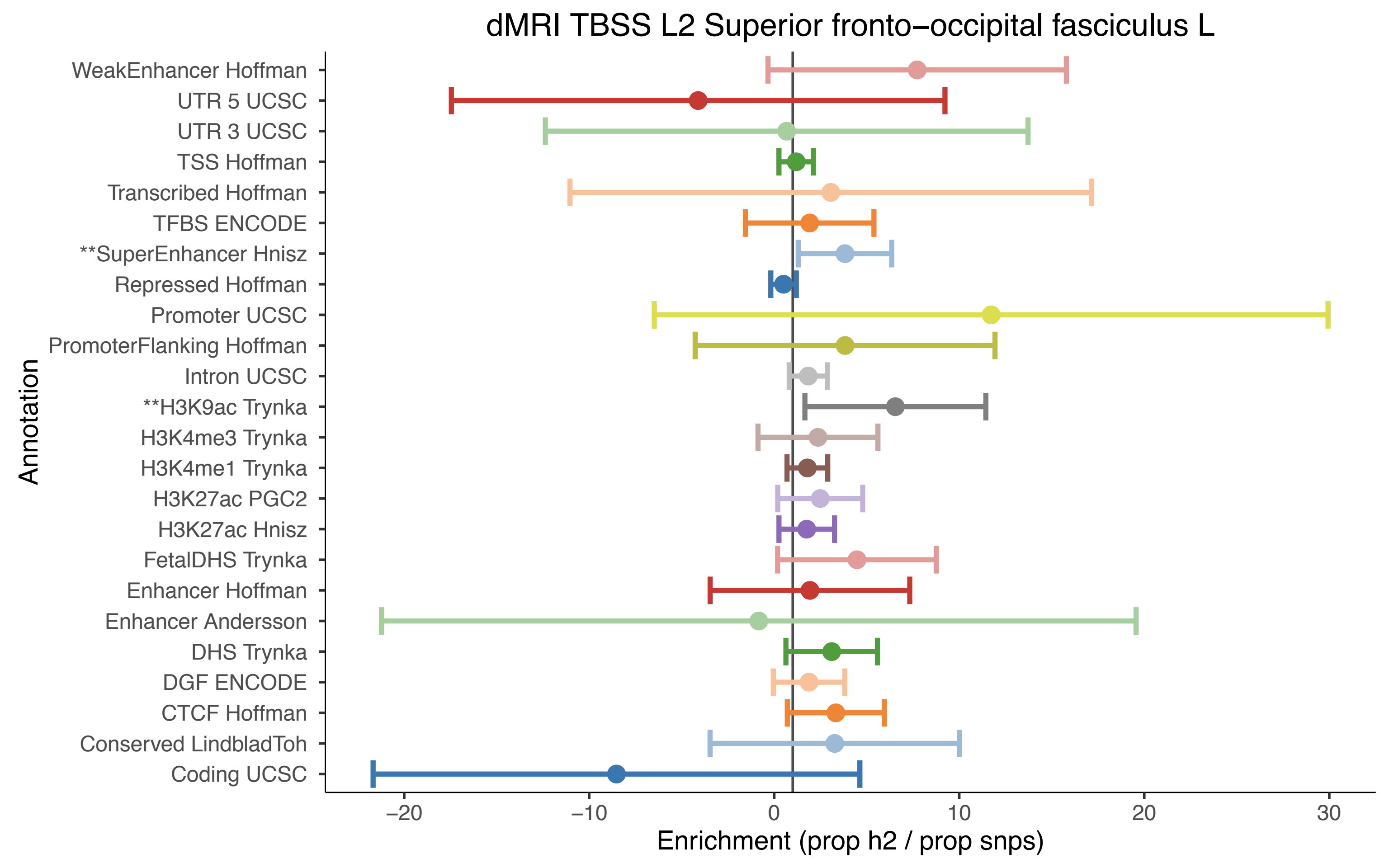
dMRI TBSS L2 Superior fronto−occipital fasciculus L
WeakEnhancer Hoffman
UTR 5 UCSC
UTR 3 UCSC
TSS Hoffman
Transcribed Hoffman
TFBS ENCODE
**SuperEnhancer Hnisz
Repressed Hoffman
Promoter UCSC
PromoterFlanking Hoffman
Intron UCSC
**H3K9ac Trynka
H3K4me3 Trynka
H3K4me1 Trynka
H3K27ac PGC2
H3K27ac Hnisz
FetalDHS Trynka
Enhancer Hoffman
Enhancer Andersson
DHS Trynka
DGF ENCODE
CTCF Hoffman
Conserved LindbladToh
Coding UCSC
Annotation
−20
−10
0
10
20
30
Enrichment (prop h2 / prop snps)

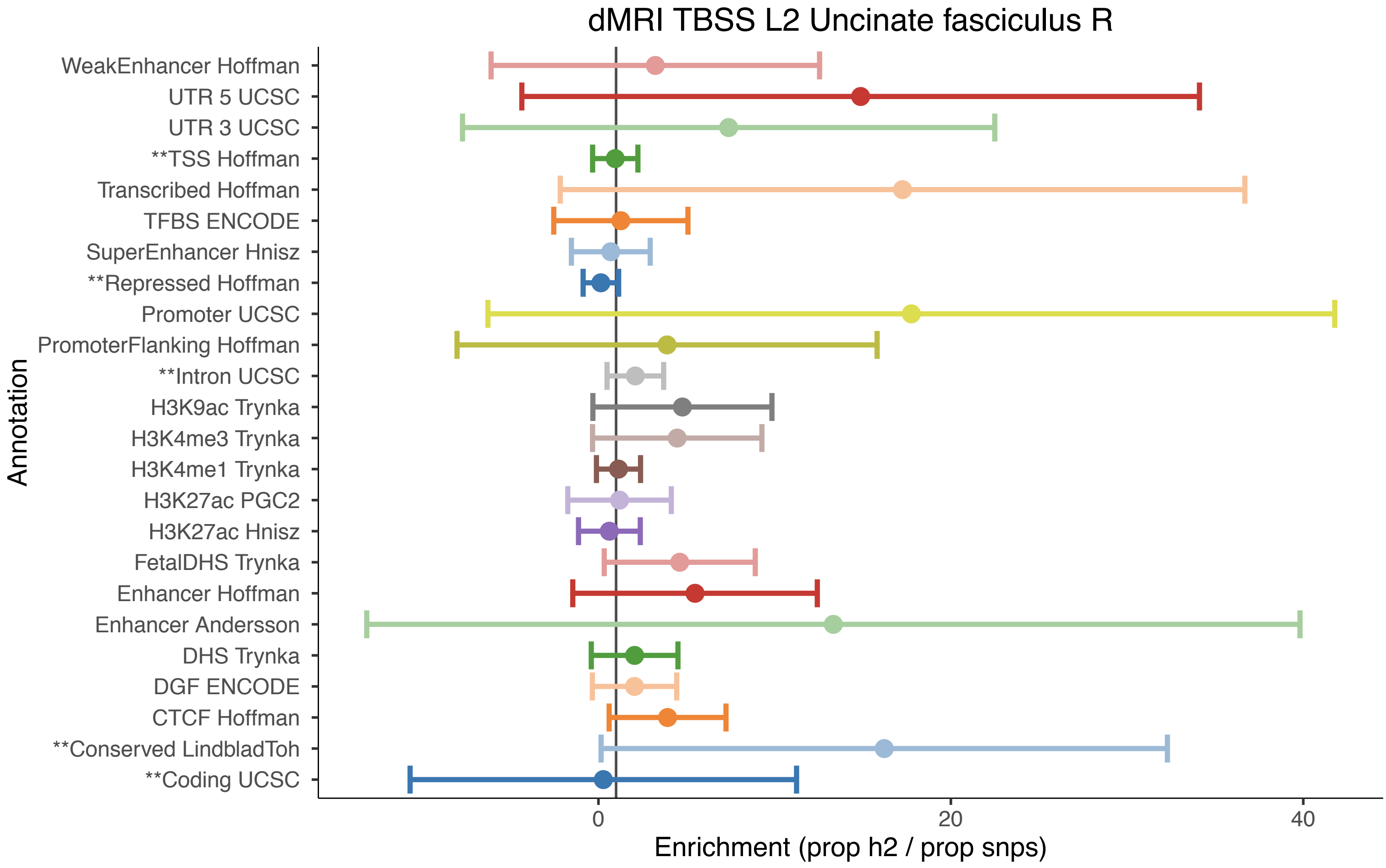
dMRI TBSS L2 Uncinate fasciculus R
WeakEnhancer Hoffman
UTR 5 UCSC
UTR 3 UCSC
**TSS Hoffman
Transcribed Hoffman
TFBS ENCODE
SuperEnhancer Hnisz
**Repressed Hoffman
Promoter UCSC
PromoterFlanking Hoffman
**Intron UCSC
H3K9ac Trynka
H3K4me3 Trynka
H3K4me1 Trynka
H3K27ac PGC2
H3K27ac Hnisz
FetalDHS Trynka
Enhancer Hoffman
Enhancer Andersson
DHS Trynka
DGF ENCODE
CTCF Hoffman
**Conserved LindbladToh
**Coding UCSC
Annotation
0
20
40
Enrichment (prop h2 / prop snps)

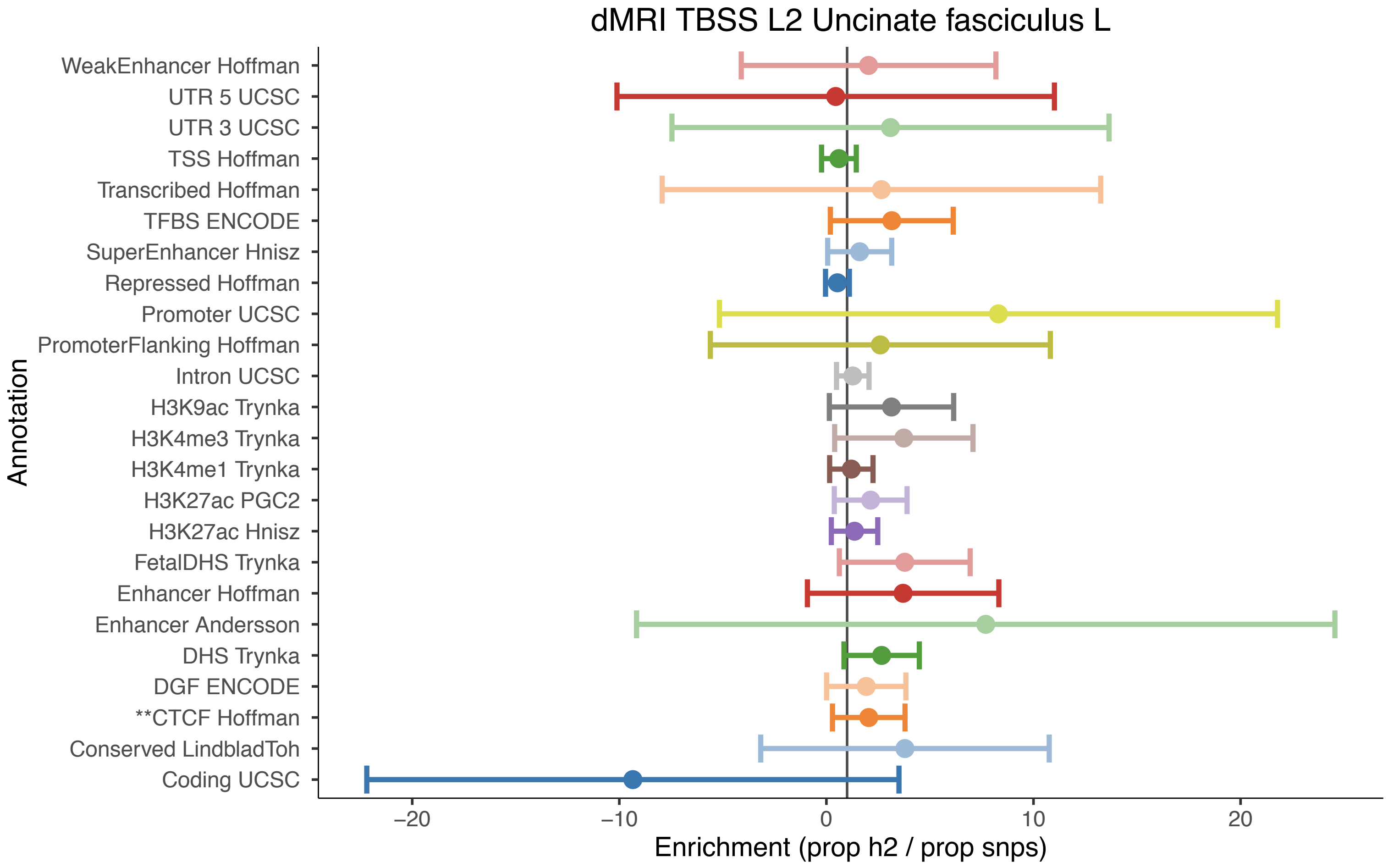

dMRI TBSS L2 Uncinate fasciculus L
WeakEnhancer Hoffman
UTR 5 UCSC
UTR 3 UCSC
TSS Hoffman
Transcribed Hoffman
TFBS ENCODE
SuperEnhancer Hnisz
Repressed Hoffman
Promoter UCSC
PromoterFlanking Hoffman
Intron UCSC
H3K9ac Trynka
H3K4me3 Trynka
H3K4me1 Trynka
H3K27ac PGC2
H3K27ac Hnisz
FetalDHS Trynka
Enhancer Hoffman
Enhancer Andersson
DHS Trynka
DGF ENCODE
**CTCF Hoffman
Conserved LindbladToh
Coding UCSC
Annotation
−20
−10
0
10
20
Enrichment (prop h2 / prop snps)

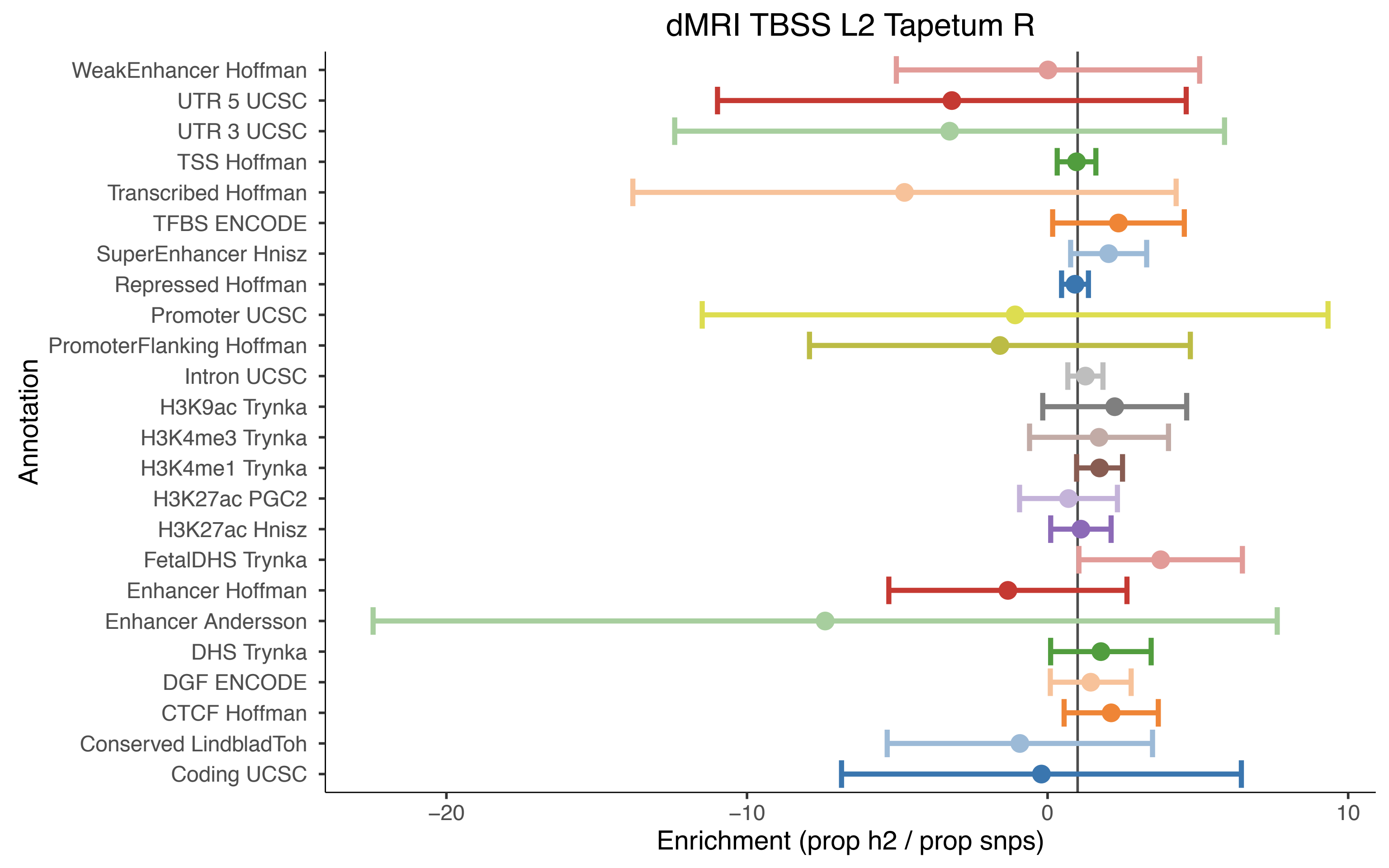

dMRI TBSS L2 Tapetum R
Annotation
WeakEnhancer Hoffman
UTR 5 UCSC
UTR 3 UCSC
TSS Hoffman
Transcribed Hoffman
TFBS ENCODE
SuperEnhancer Hnisz
Repressed Hoffman
Promoter UCSC
PromoterFlanking Hoffman
Intron UCSC
H3K9ac Trynka
H3K4me3 Trynka
H3K4me1 Trynka
H3K27ac PGC2
H3K27ac Hnisz
FetalDHS Trynka
Enhancer Hoffman
Enhancer Andersson
DHS Trynka
DGF ENCODE
CTCF Hoffman
Conserved LindbladToh
Coding UCSC
−20
−10
0
10
Enrichment (prop h2 / prop snps)

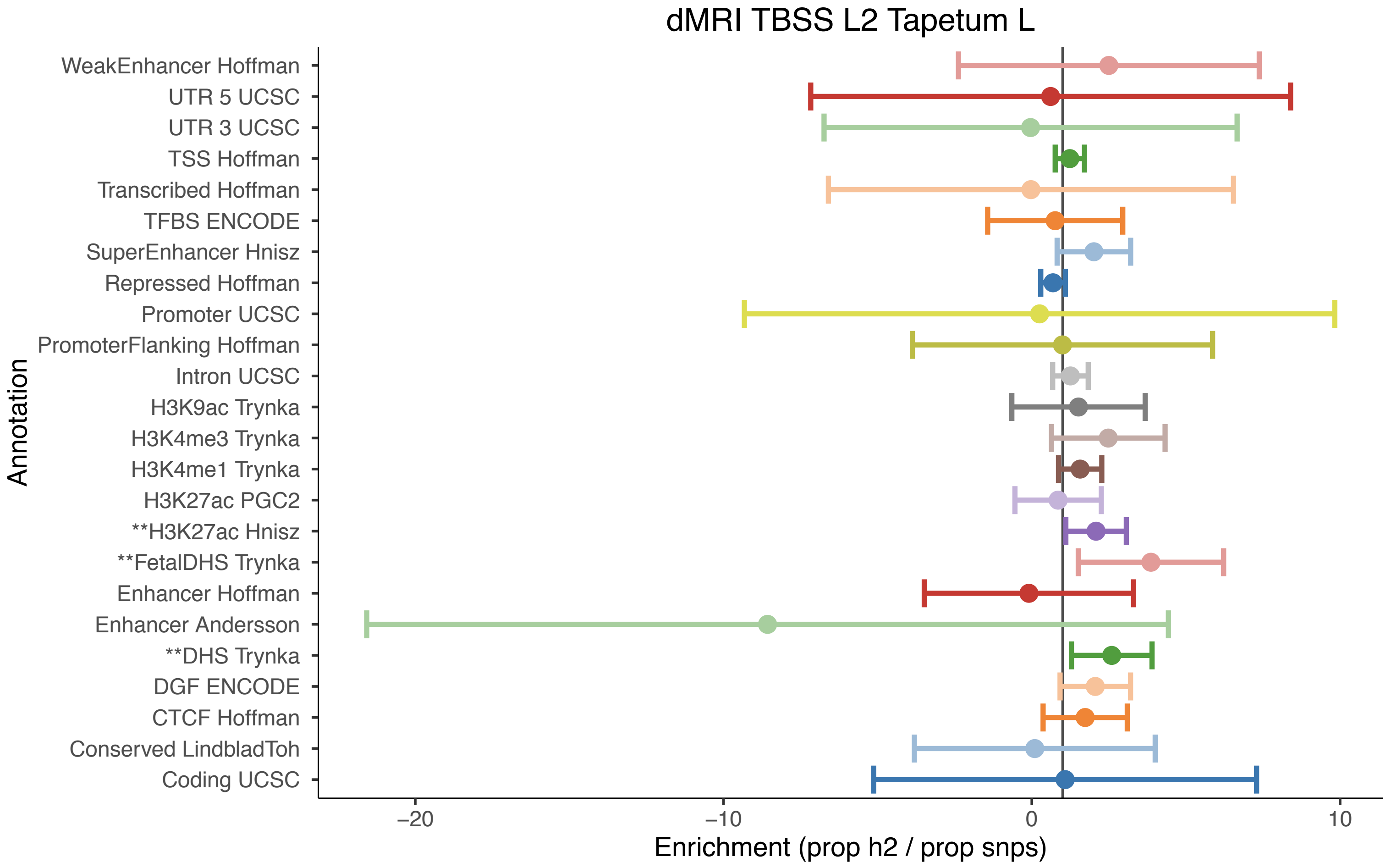

dMRI TBSS L2 Tapetum L
Annotation
WeakEnhancer Hoffman
UTR 5 UCSC
UTR 3 UCSC
TSS Hoffman
Transcribed Hoffman
TFBS ENCODE
SuperEnhancer Hnisz
Repressed Hoffman
Promoter UCSC
PromoterFlanking Hoffman
Intron UCSC
H3K9ac Trynka
H3K4me3 Trynka
H3K4me1 Trynka
H3K27ac PGC2
**H3K27ac Hnisz
**FetalDHS Trynka
Enhancer Hoffman
Enhancer Andersson
**DHS Trynka
DGF ENCODE
CTCF Hoffman
Conserved LindbladToh
Coding UCSC
−20
−10
0
10
Enrichment (prop h2 / prop snps)

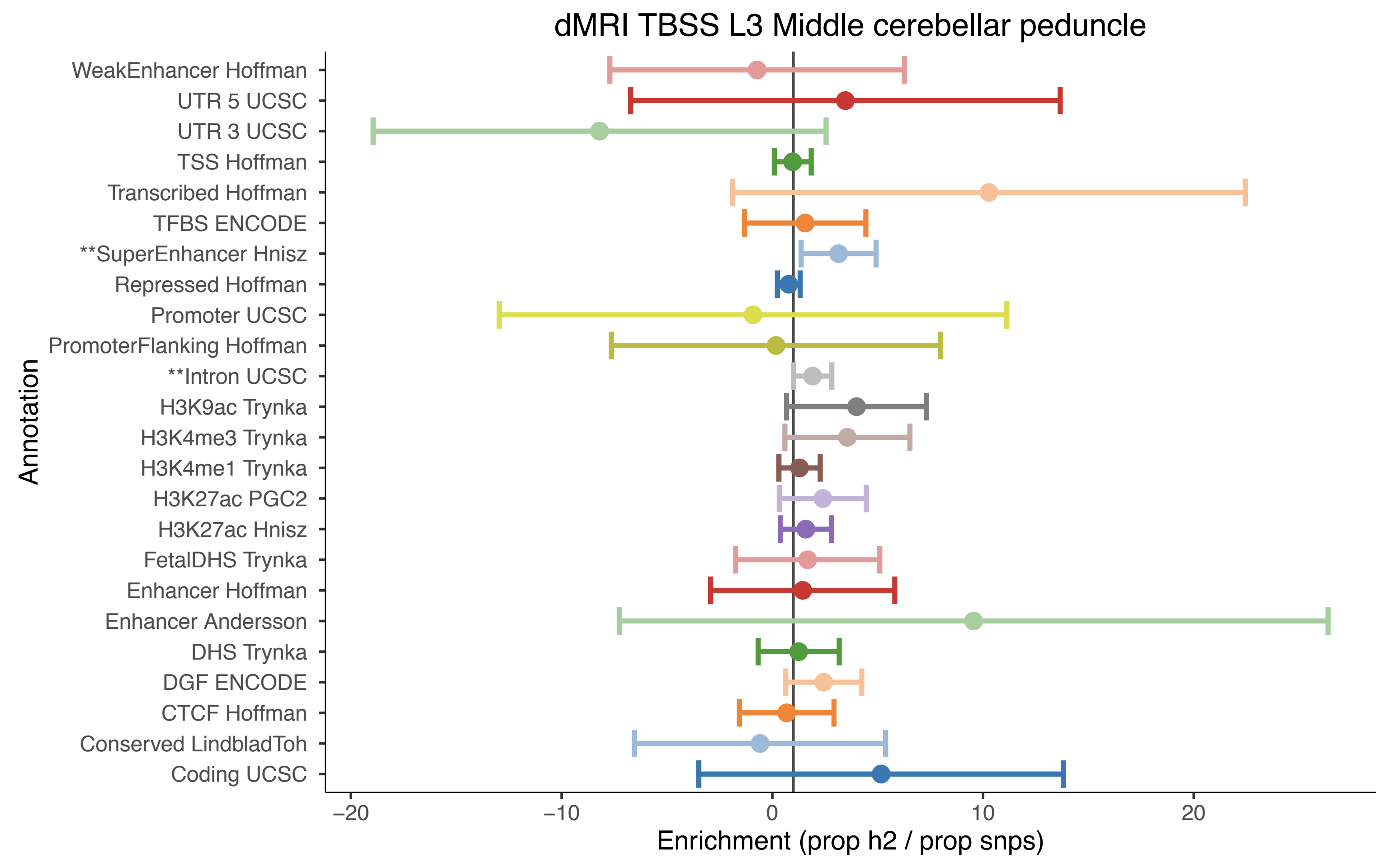
dMRI TBSS L3 Middle cerebellar peduncle
Annotation
WeakEnhancer Hoffman
UTR 5 UCSC
UTR 3 UCSC
TSS Hoffman
Transcribed Hoffman
TFBS ENCODE
**SuperEnhancer Hnisz
Repressed Hoffman
Promoter UCSC
PromoterFlanking Hoffman
**Intron UCSC
H3K9ac Trynka
H3K4me3 Trynka
H3K4me1 Trynka
H3K27ac PGC2
H3K27ac Hnisz
FetalDHS Trynka
Enhancer Hoffman
Enhancer Andersson
DHS Trynka
DGF ENCODE
CTCF Hoffman
Conserved LindbladToh
Coding UCSC
−20
−10
0
10
20
Enrichment (prop h2 / prop snps)

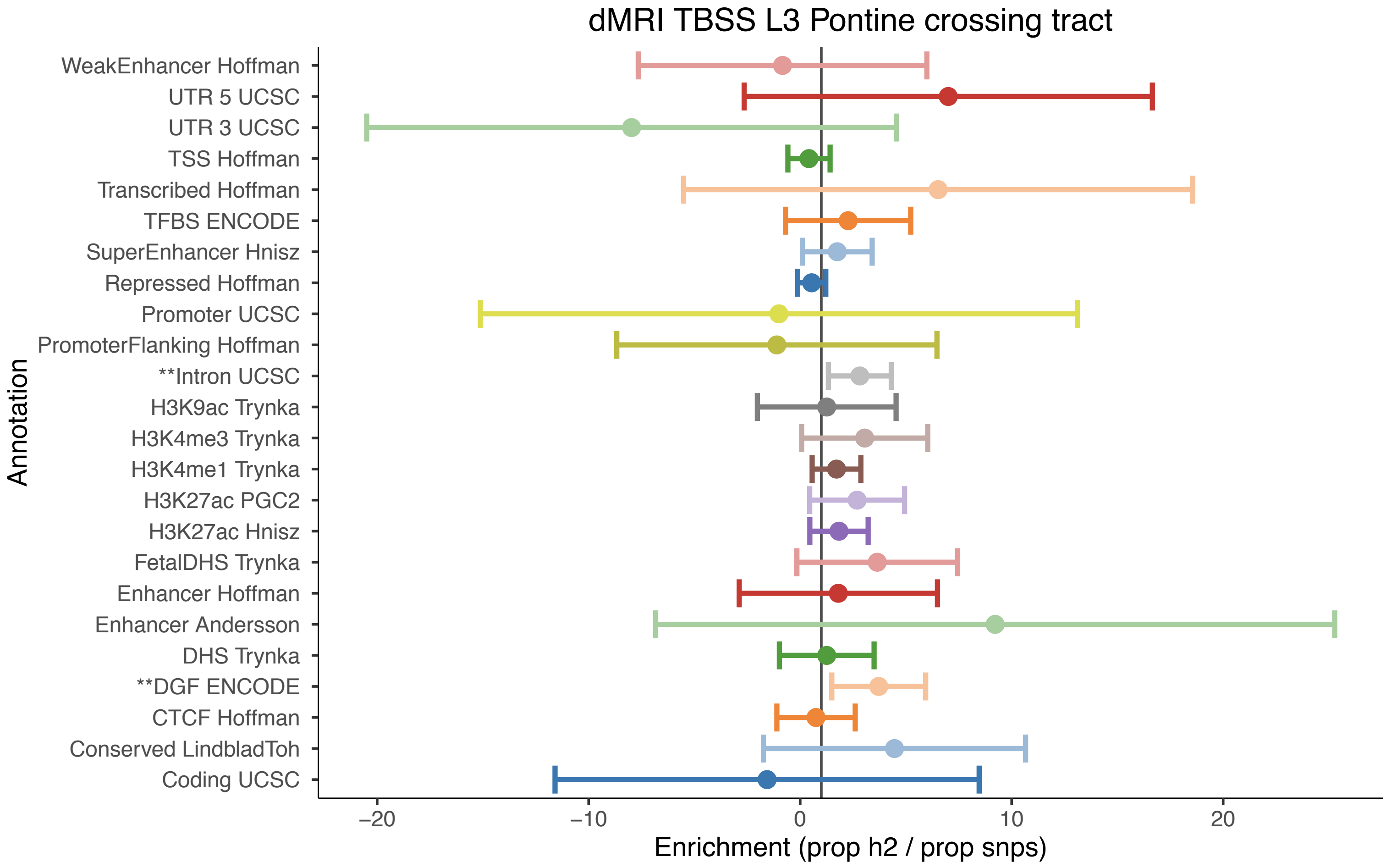

dMRI TBSS L3 Pontine crossing tract
WeakEnhancer Hoffman
UTR 5 UCSC
UTR 3 UCSC
TSS Hoffman
Transcribed Hoffman
TFBS ENCODE
SuperEnhancer Hnisz
Repressed Hoffman
Promoter UCSC
PromoterFlanking Hoffman
**Intron UCSC
H3K9ac Trynka
H3K4me3 Trynka
H3K4me1 Trynka
H3K27ac PGC2
H3K27ac Hnisz
FetalDHS Trynka
Enhancer Hoffman
Enhancer Andersson
DHS Trynka
**DGF ENCODE
CTCF Hoffman
Conserved LindbladToh
Coding UCSC
Annotation
−20
−10
0
10
20
Enrichment (prop h2 / prop snps)

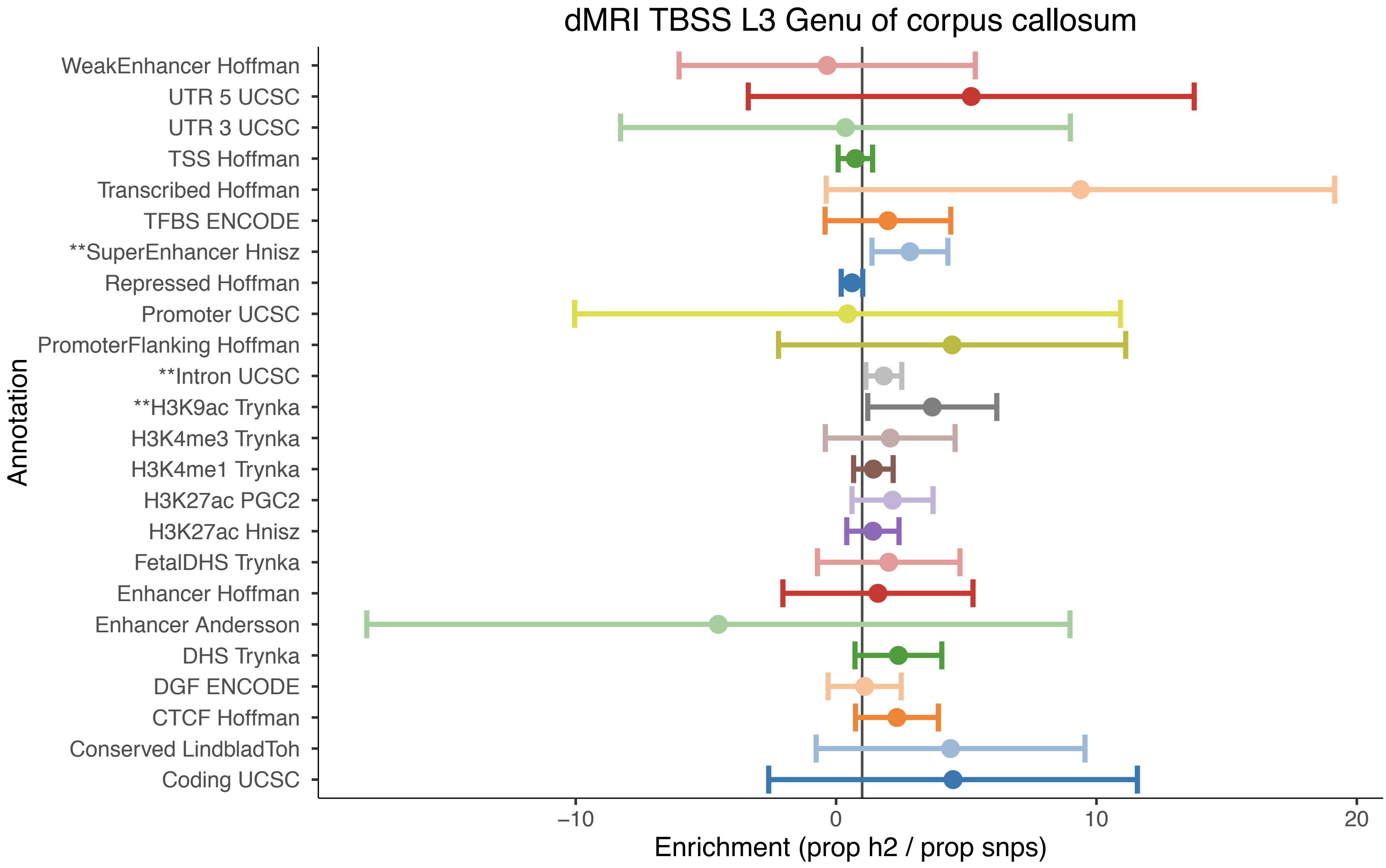

dMRI TBSS L3 Genu of corpus callosum
WeakEnhancer Hoffman
UTR 5 UCSC
UTR 3 UCSC
TSS Hoffman
Transcribed Hoffman
TFBS ENCODE
**SuperEnhancer Hnisz
Repressed Hoffman
Promoter UCSC
PromoterFlanking Hoffman
**Intron UCSC
**H3K9ac Trynka
H3K4me3 Trynka
H3K4me1 Trynka
H3K27ac PGC2
H3K27ac Hnisz
FetalDHS Trynka
Enhancer Hoffman
Enhancer Andersson
DHS Trynka
DGF ENCODE
CTCF Hoffman
Conserved LindbladToh
Coding UCSC
Annotation
−10
0
10
20
Enrichment (prop h2 / prop snps)

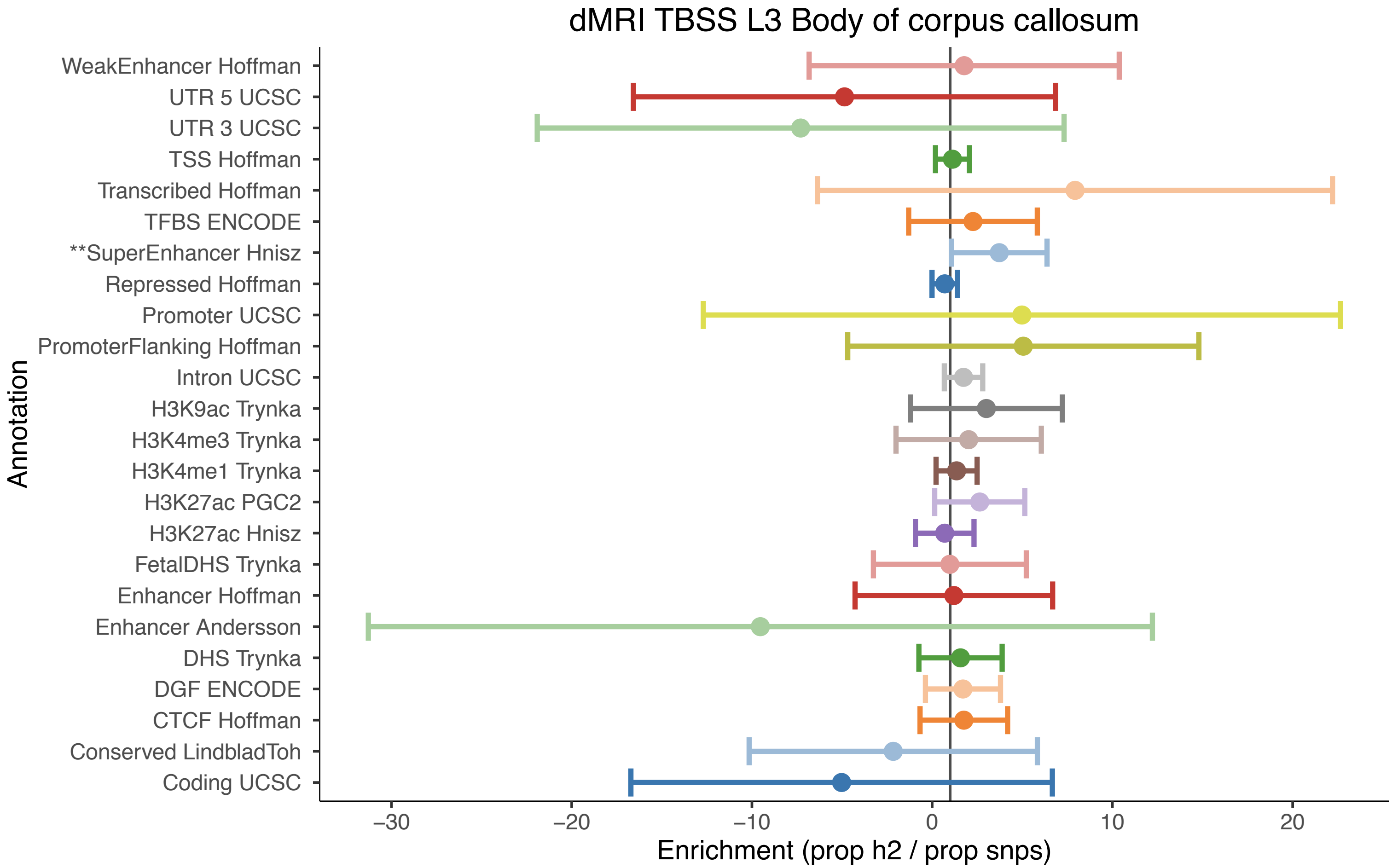

dMRI TBSS L3 Body of corpus callosum
WeakEnhancer Hoffman
UTR 5 UCSC
UTR 3 UCSC
TSS Hoffman
Transcribed Hoffman
TFBS ENCODE
**SuperEnhancer Hnisz
Repressed Hoffman
Promoter UCSC
PromoterFlanking Hoffman
Intron UCSC
H3K9ac Trynka
H3K4me3 Trynka
H3K4me1 Trynka
H3K27ac PGC2
H3K27ac Hnisz
FetalDHS Trynka
Enhancer Hoffman
Enhancer Andersson
DHS Trynka
DGF ENCODE
CTCF Hoffman
Conserved LindbladToh
Coding UCSC
Annotation
−30
−20
−10
0
10
20
Enrichment (prop h2 / prop snps)

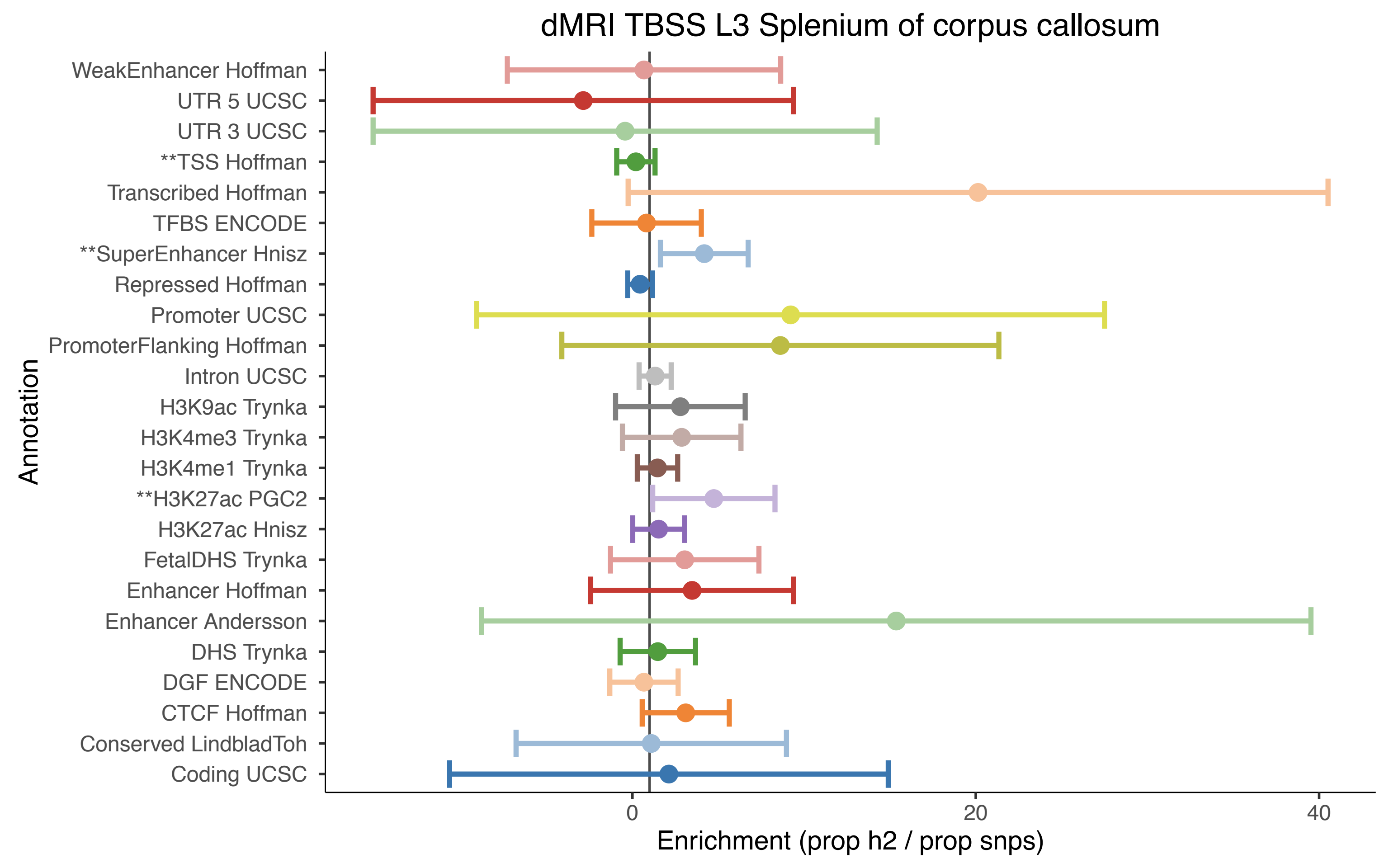

dMRI TBSS L3 Splenium of corpus callosum
Annotation
WeakEnhancer Hoffman
UTR 5 UCSC
UTR 3 UCSC
**TSS Hoffman
Transcribed Hoffman
TFBS ENCODE
**SuperEnhancer Hnisz
Repressed Hoffman
Promoter UCSC
PromoterFlanking Hoffman
Intron UCSC
H3K9ac Trynka
H3K4me3 Trynka
H3K4me1 Trynka
**H3K27ac PGC2
H3K27ac Hnisz
FetalDHS Trynka
Enhancer Hoffman
Enhancer Andersson
DHS Trynka
DGF ENCODE
CTCF Hoffman
Conserved LindbladToh
Coding UCSC
0
20
40
Enrichment (prop h2 / prop snps)

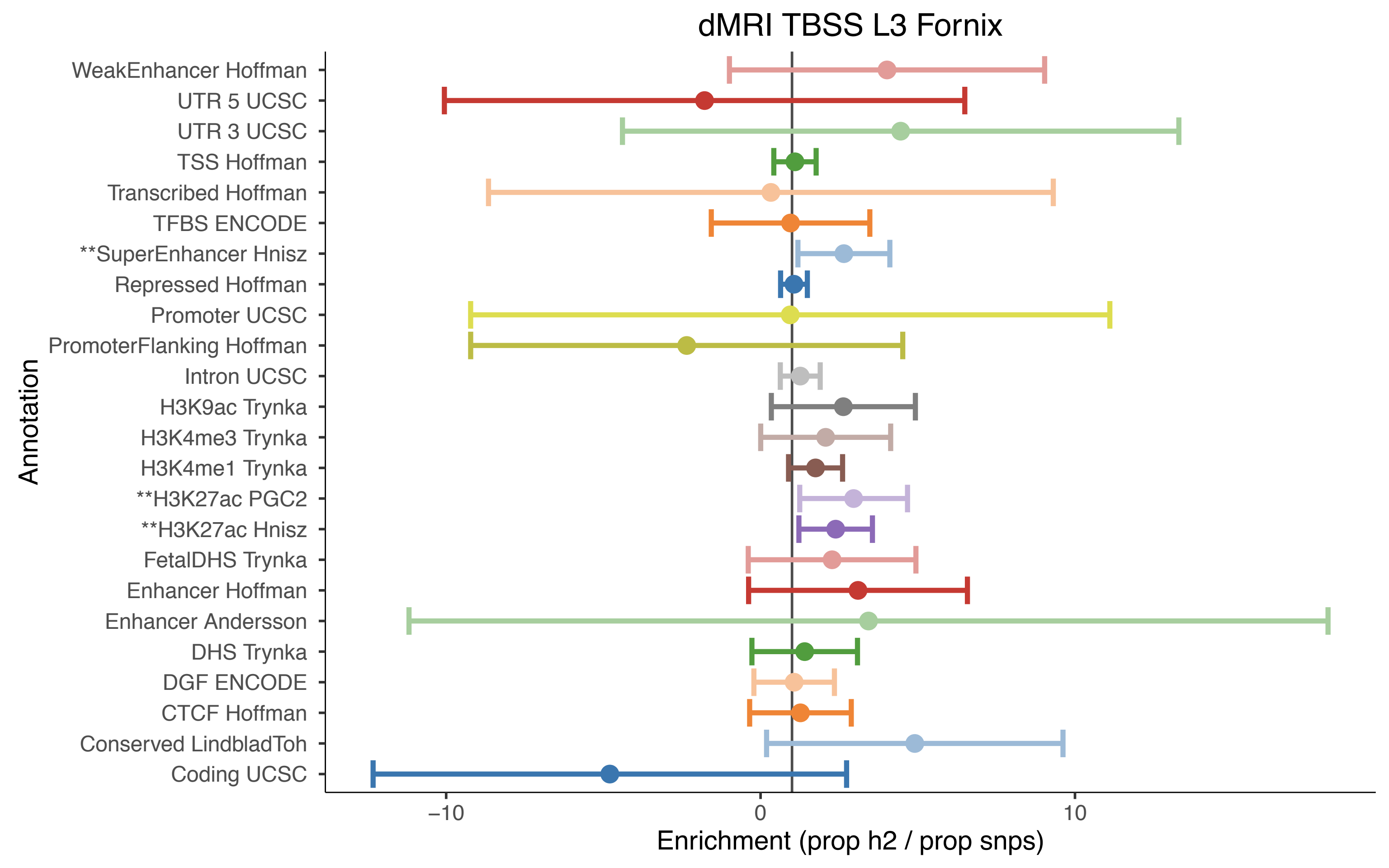

dMRI TBSS L3 Fornix
WeakEnhancer Hoffman
UTR 5 UCSC
UTR 3 UCSC
TSS Hoffman
Transcribed Hoffman
TFBS ENCODE
**SuperEnhancer Hnisz
Repressed Hoffman
Promoter UCSC
PromoterFlanking Hoffman
Intron UCSC
H3K9ac Trynka
H3K4me3 Trynka
H3K4me1 Trynka
**H3K27ac PGC2
**H3K27ac Hnisz
FetalDHS Trynka
Enhancer Hoffman
Enhancer Andersson
DHS Trynka
DGF ENCODE
CTCF Hoffman
Conserved LindbladToh
Coding UCSC
Annotation
−10
0
10
Enrichment (prop h2 / prop snps)

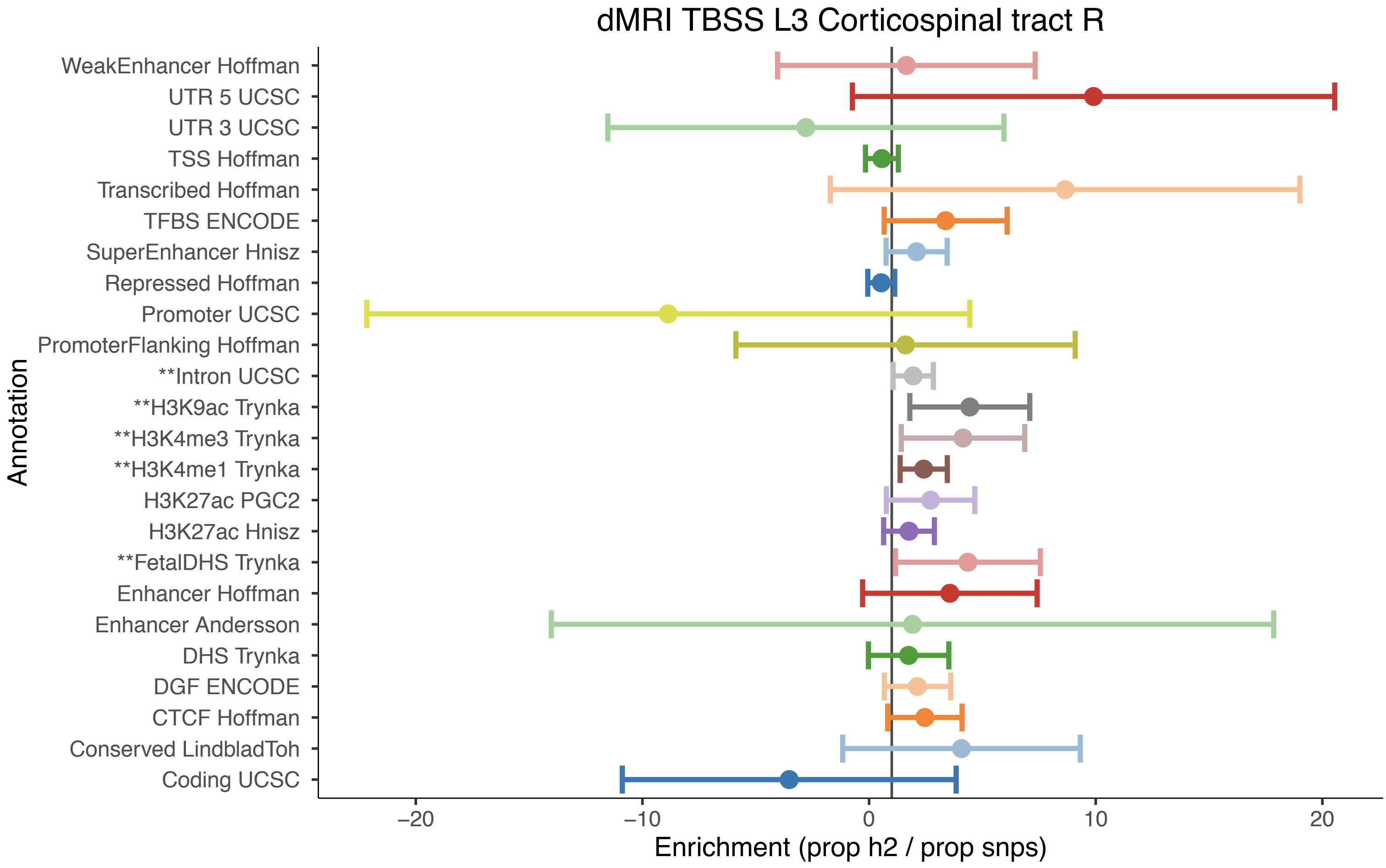

dMRI TBSS L3 Corticospinal tract R
WeakEnhancer Hoffman
UTR 5 UCSC
UTR 3 UCSC
TSS Hoffman
Transcribed Hoffman
TFBS ENCODE
SuperEnhancer Hnisz
Repressed Hoffman
Promoter UCSC
PromoterFlanking Hoffman
**Intron UCSC
**H3K9ac Trynka
**H3K4me3 Trynka
**H3K4me1 Trynka
H3K27ac PGC2
H3K27ac Hnisz
**FetalDHS Trynka
Enhancer Hoffman
Enhancer Andersson
DHS Trynka
DGF ENCODE
CTCF Hoffman
Conserved LindbladToh
Coding UCSC
Annotation
−20
−10
0
10
20
Enrichment (prop h2 / prop snps)

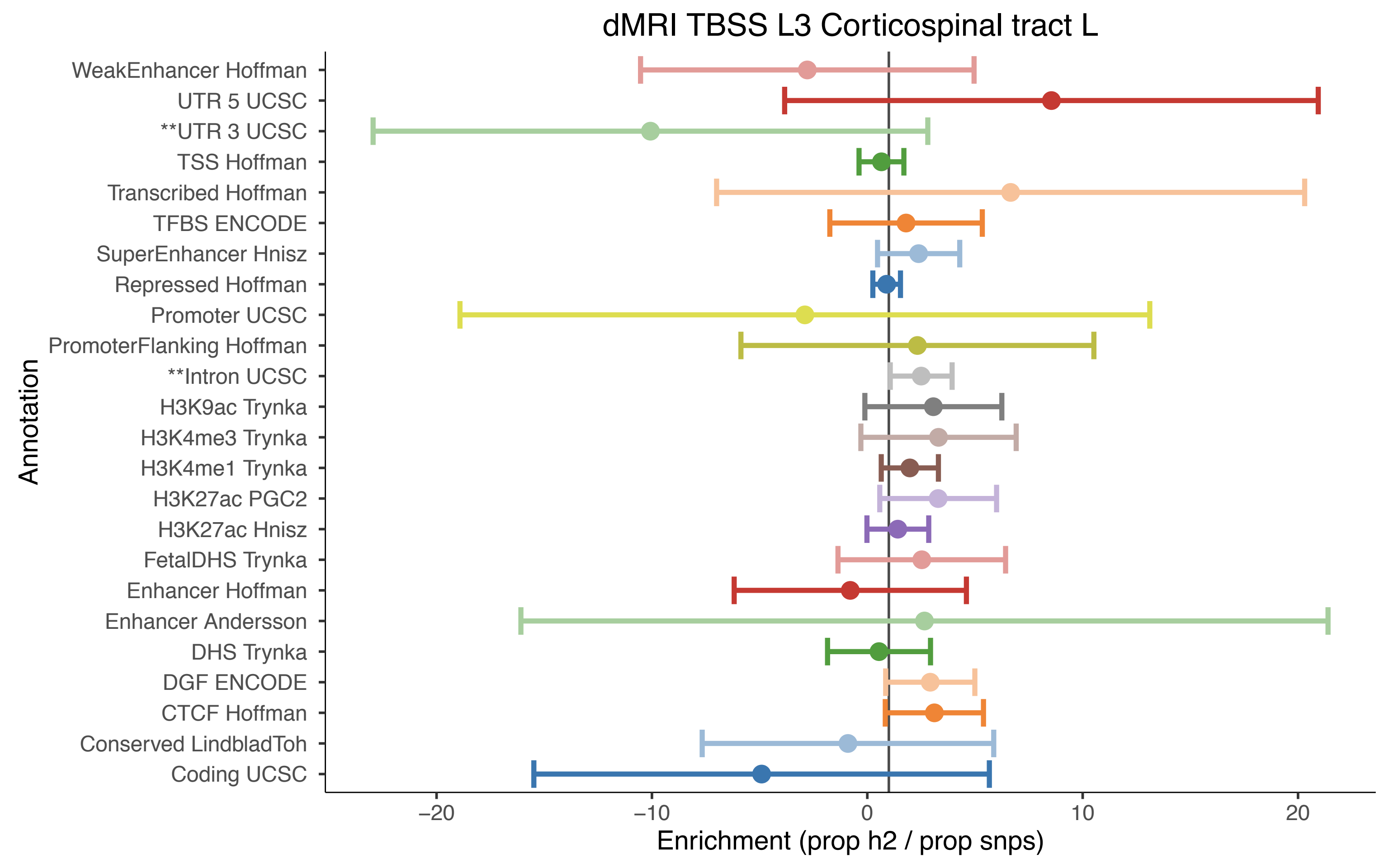

dMRI TBSS L3 Corticospinal tract L
Annotation
WeakEnhancer Hoffman
UTR 5 UCSC
**UTR 3 UCSC
TSS Hoffman
Transcribed Hoffman
TFBS ENCODE
SuperEnhancer Hnisz
Repressed Hoffman
Promoter UCSC
PromoterFlanking Hoffman
**Intron UCSC
H3K9ac Trynka
H3K4me3 Trynka
H3K4me1 Trynka
H3K27ac PGC2
H3K27ac Hnisz
FetalDHS Trynka
Enhancer Hoffman
Enhancer Andersson
DHS Trynka
DGF ENCODE
CTCF Hoffman
Conserved LindbladToh
Coding UCSC
−20
−10
0
10
20
Enrichment (prop h2 / prop snps)

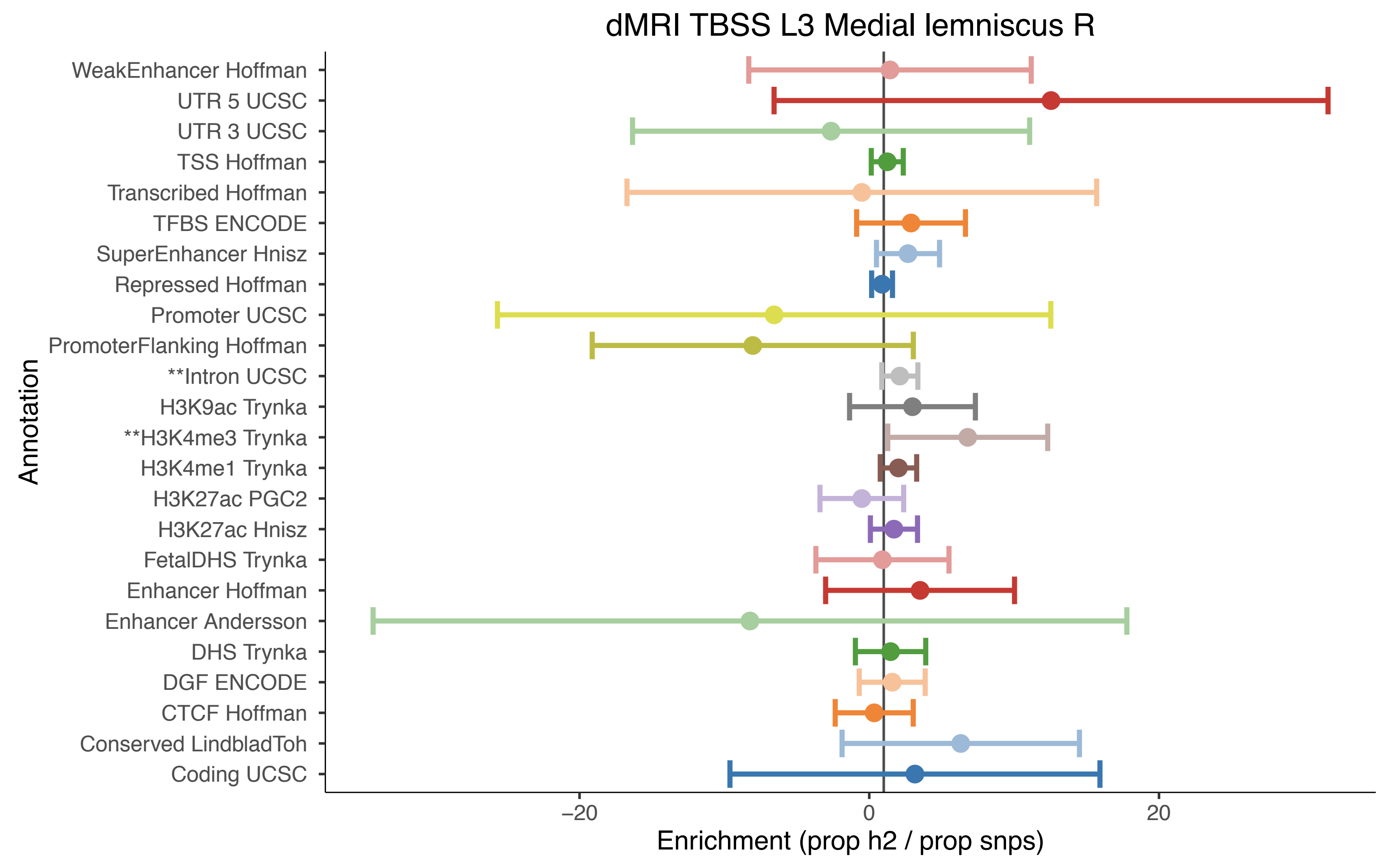
dMRI TBSS L3 Medial lemniscus R
WeakEnhancer Hoffman
UTR 5 UCSC
UTR 3 UCSC
TSS Hoffman
Transcribed Hoffman
TFBS ENCODE
SuperEnhancer Hnisz
Repressed Hoffman
Promoter UCSC
PromoterFlanking Hoffman
**Intron UCSC
H3K9ac Trynka
**H3K4me3 Trynka
H3K4me1 Trynka
H3K27ac PGC2
H3K27ac Hnisz
FetalDHS Trynka
Enhancer Hoffman
Enhancer Andersson
DHS Trynka
DGF ENCODE
CTCF Hoffman
Conserved LindbladToh
Coding UCSC
Annotation
−20
0
20
Enrichment (prop h2 / prop snps)

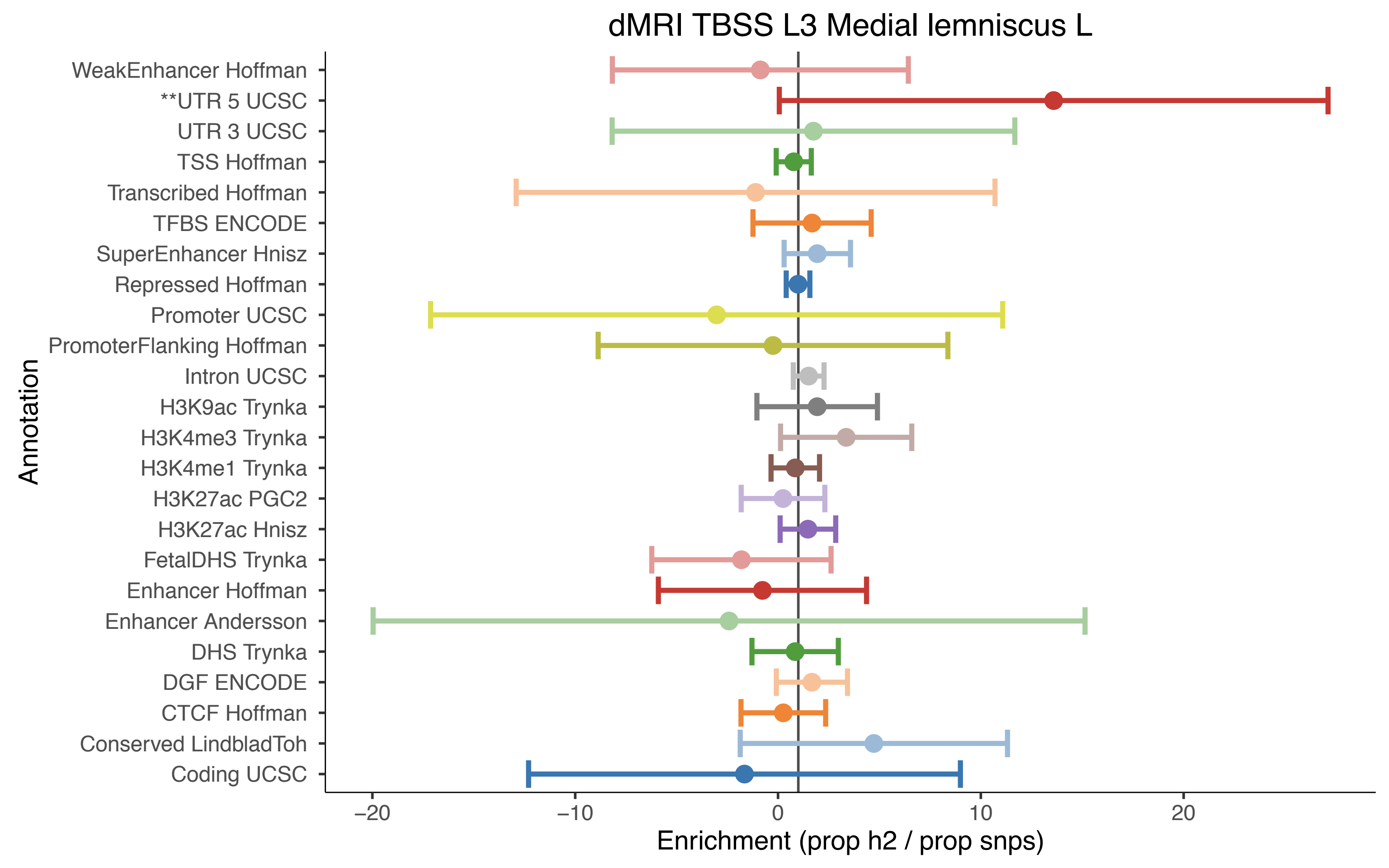
dMRI TBSS L3 Medial lemniscus L
WeakEnhancer Hoffman
**UTR 5 UCSC
UTR 3 UCSC
TSS Hoffman
Transcribed Hoffman
TFBS ENCODE
SuperEnhancer Hnisz
Repressed Hoffman
Promoter UCSC
PromoterFlanking Hoffman
Intron UCSC
H3K9ac Trynka
H3K4me3 Trynka
H3K4me1 Trynka
H3K27ac PGC2
H3K27ac Hnisz
FetalDHS Trynka
Enhancer Hoffman
Enhancer Andersson
DHS Trynka
DGF ENCODE
CTCF Hoffman
Conserved LindbladToh
Coding UCSC
Annotation
−20
−10
0
10
20
Enrichment (prop h2 / prop snps)

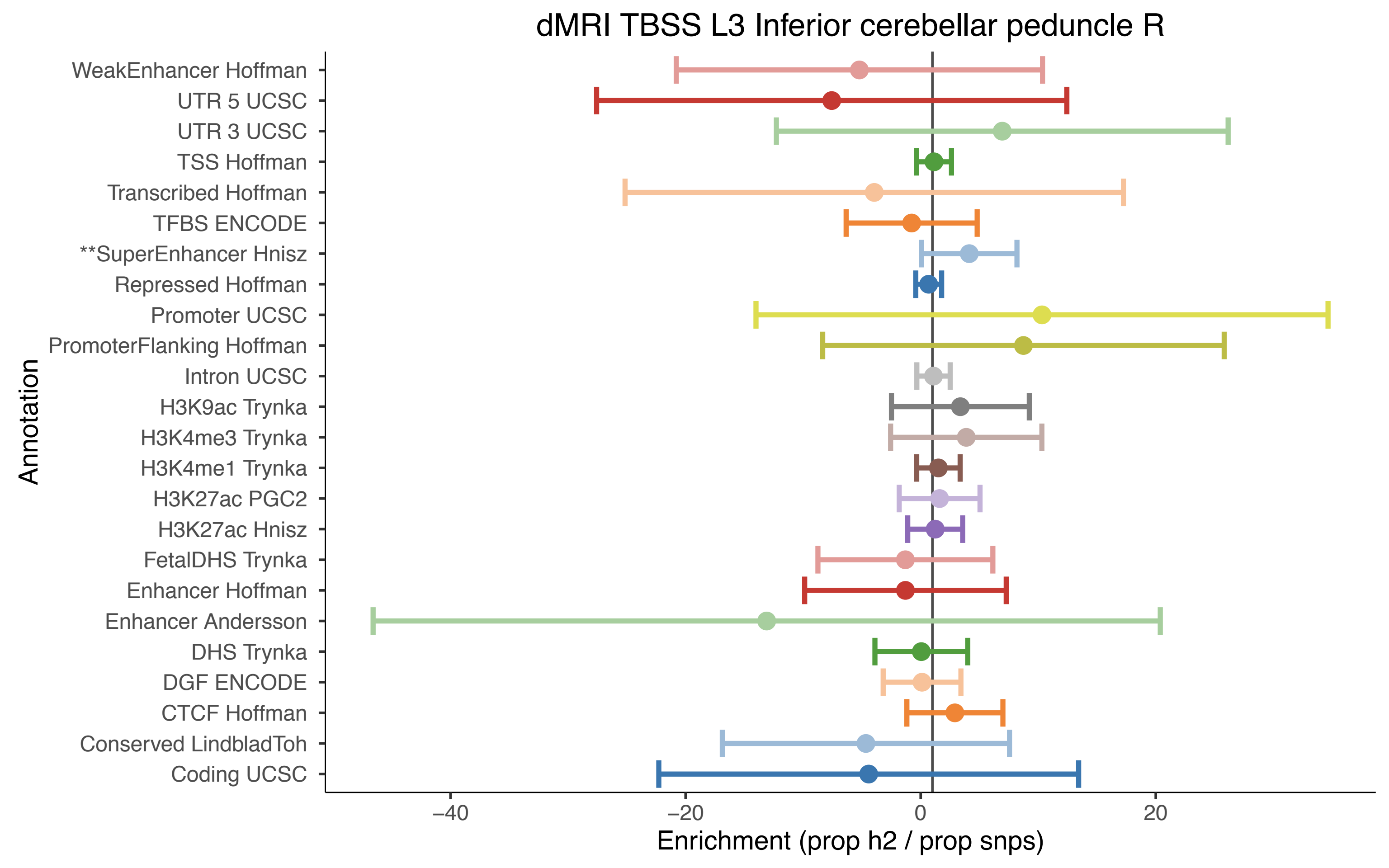

dMRI TBSS L3 Inferior cerebellar peduncle R
WeakEnhancer Hoffman
UTR 5 UCSC
UTR 3 UCSC
TSS Hoffman
Transcribed Hoffman
TFBS ENCODE
**SuperEnhancer Hnisz
Repressed Hoffman
Promoter UCSC
PromoterFlanking Hoffman
Intron UCSC
H3K9ac Trynka
H3K4me3 Trynka
H3K4me1 Trynka
H3K27ac PGC2
H3K27ac Hnisz
FetalDHS Trynka
Enhancer Hoffman
Enhancer Andersson
DHS Trynka
DGF ENCODE
CTCF Hoffman
Conserved LindbladToh
Coding UCSC
Annotation
−40
−20
0
20
Enrichment (prop h2 / prop snps)

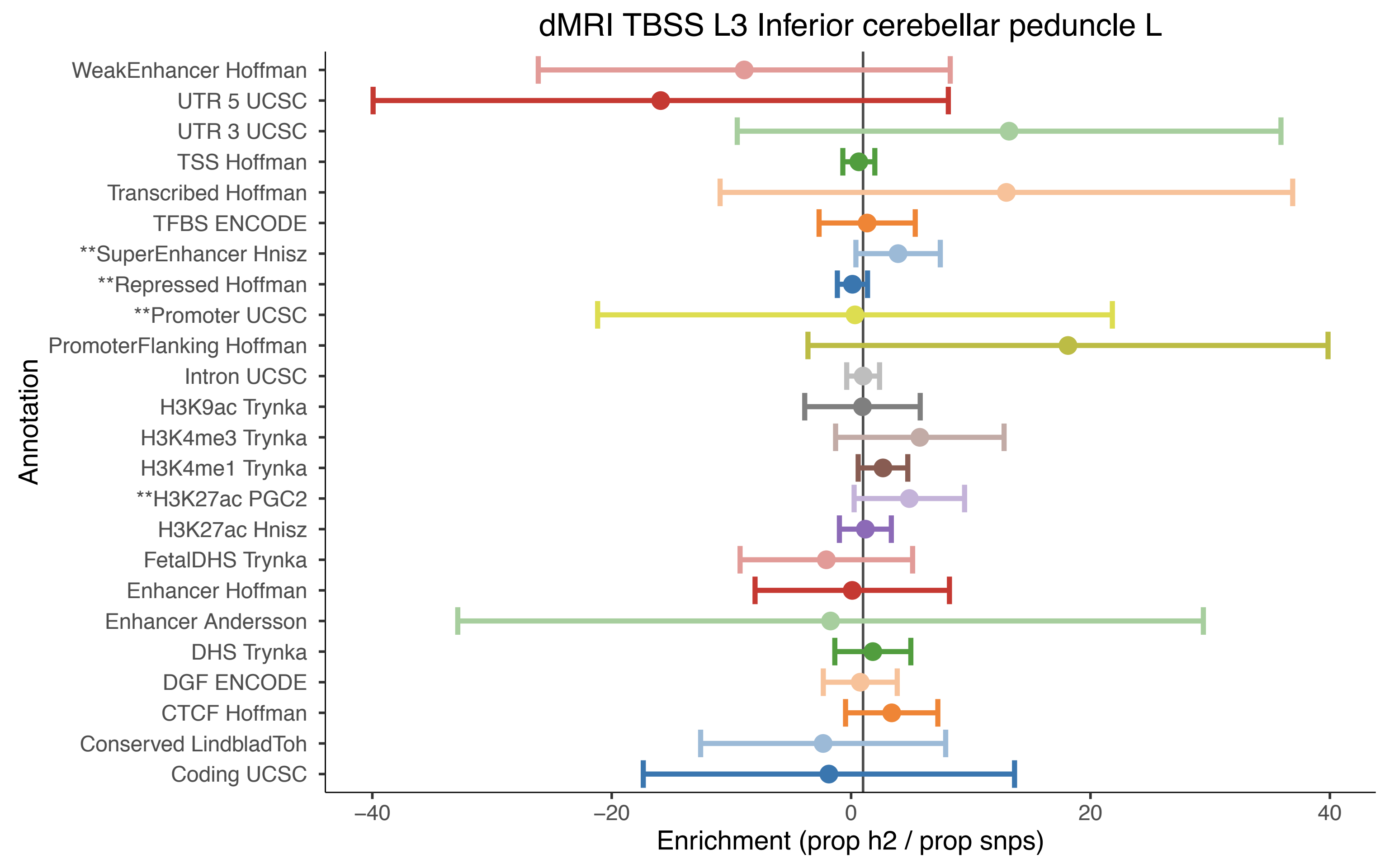

dMRI TBSS L3 Inferior cerebellar peduncle L
WeakEnhancer Hoffman
UTR 5 UCSC
UTR 3 UCSC
TSS Hoffman
Transcribed Hoffman
TFBS ENCODE
**SuperEnhancer Hnisz
**Repressed Hoffman
**Promoter UCSC
PromoterFlanking Hoffman
Intron UCSC
H3K9ac Trynka
H3K4me3 Trynka
H3K4me1 Trynka
**H3K27ac PGC2
H3K27ac Hnisz
FetalDHS Trynka
Enhancer Hoffman
Enhancer Andersson
DHS Trynka
DGF ENCODE
CTCF Hoffman
Conserved LindbladToh
Coding UCSC
Annotation
−40
−20
0
20
40
Enrichment (prop h2 / prop snps)

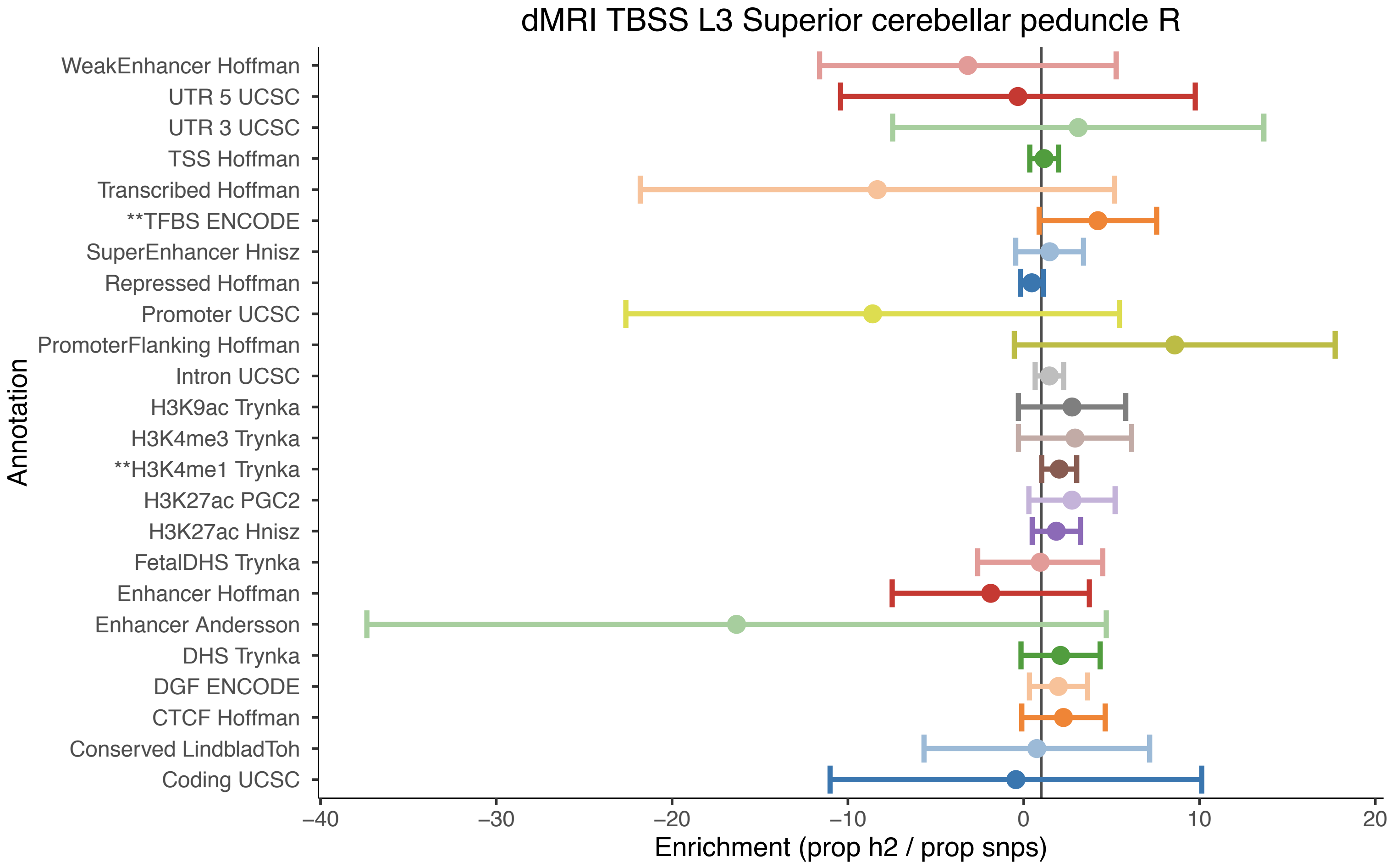
dMRI TBSS L3 Superior cerebellar peduncle R
WeakEnhancer Hoffman
UTR 5 UCSC
UTR 3 UCSC
TSS Hoffman
Transcribed Hoffman
**TFBS ENCODE
SuperEnhancer Hnisz
Repressed Hoffman
Promoter UCSC
PromoterFlanking Hoffman
Intron UCSC
H3K9ac Trynka
H3K4me3 Trynka
**H3K4me1 Trynka
H3K27ac PGC2
H3K27ac Hnisz
FetalDHS Trynka
Enhancer Hoffman
Enhancer Andersson
DHS Trynka
DGF ENCODE
CTCF Hoffman
Conserved LindbladToh
Coding UCSC
Annotation
−40
−30
−20
−10
0
10
20
Enrichment (prop h2 / prop snps)

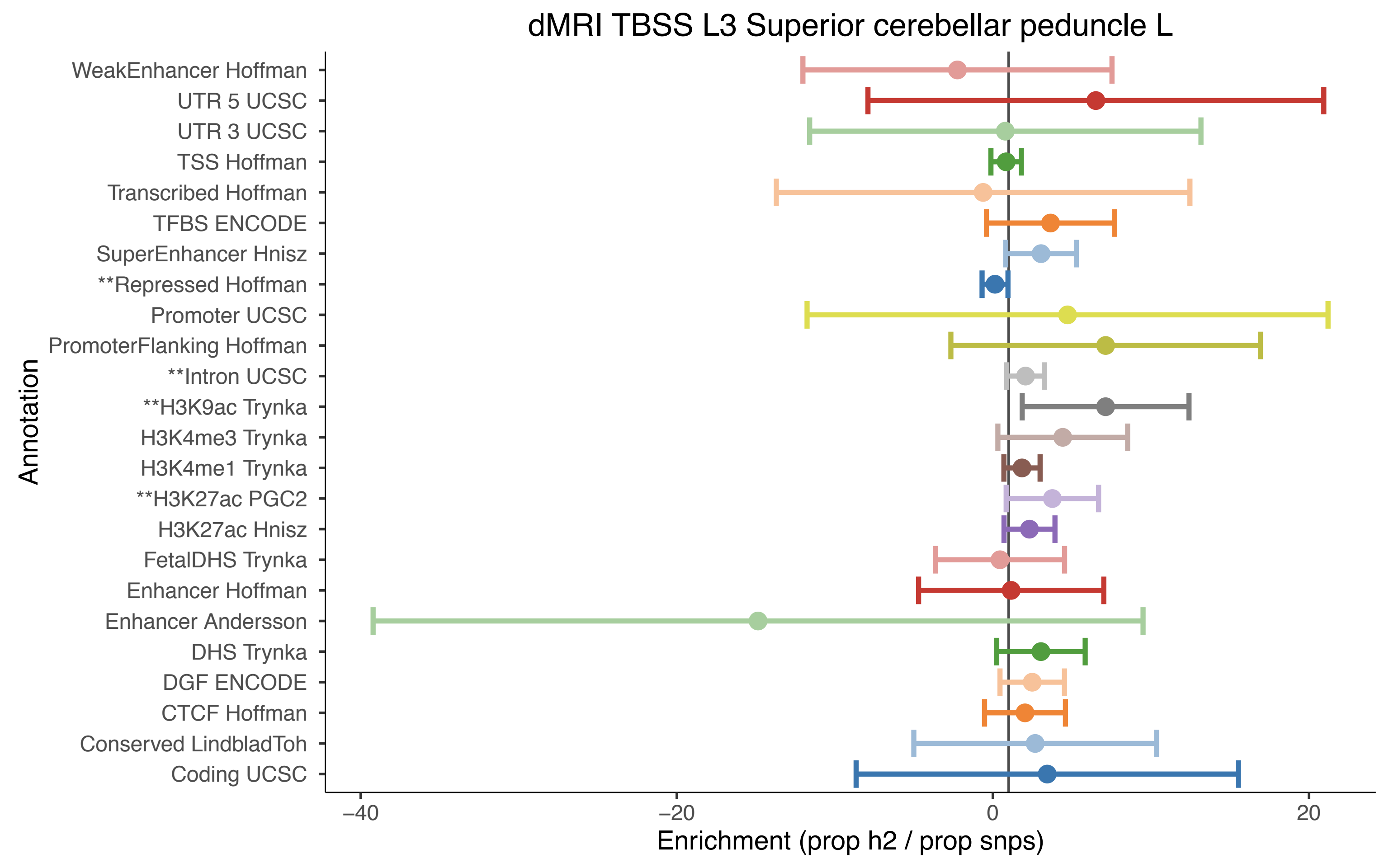

dMRI TBSS L3 Superior cerebellar peduncle L
WeakEnhancer Hoffman
UTR 5 UCSC
UTR 3 UCSC
TSS Hoffman
Transcribed Hoffman
TFBS ENCODE
SuperEnhancer Hnisz
**Repressed Hoffman
Promoter UCSC
PromoterFlanking Hoffman
**Intron UCSC
**H3K9ac Trynka
H3K4me3 Trynka
H3K4me1 Trynka
**H3K27ac PGC2
H3K27ac Hnisz
FetalDHS Trynka
Enhancer Hoffman
Enhancer Andersson
DHS Trynka
DGF ENCODE
CTCF Hoffman
Conserved LindbladToh
Coding UCSC
Annotation
−40
−20
0
20
Enrichment (prop h2 / prop snps)

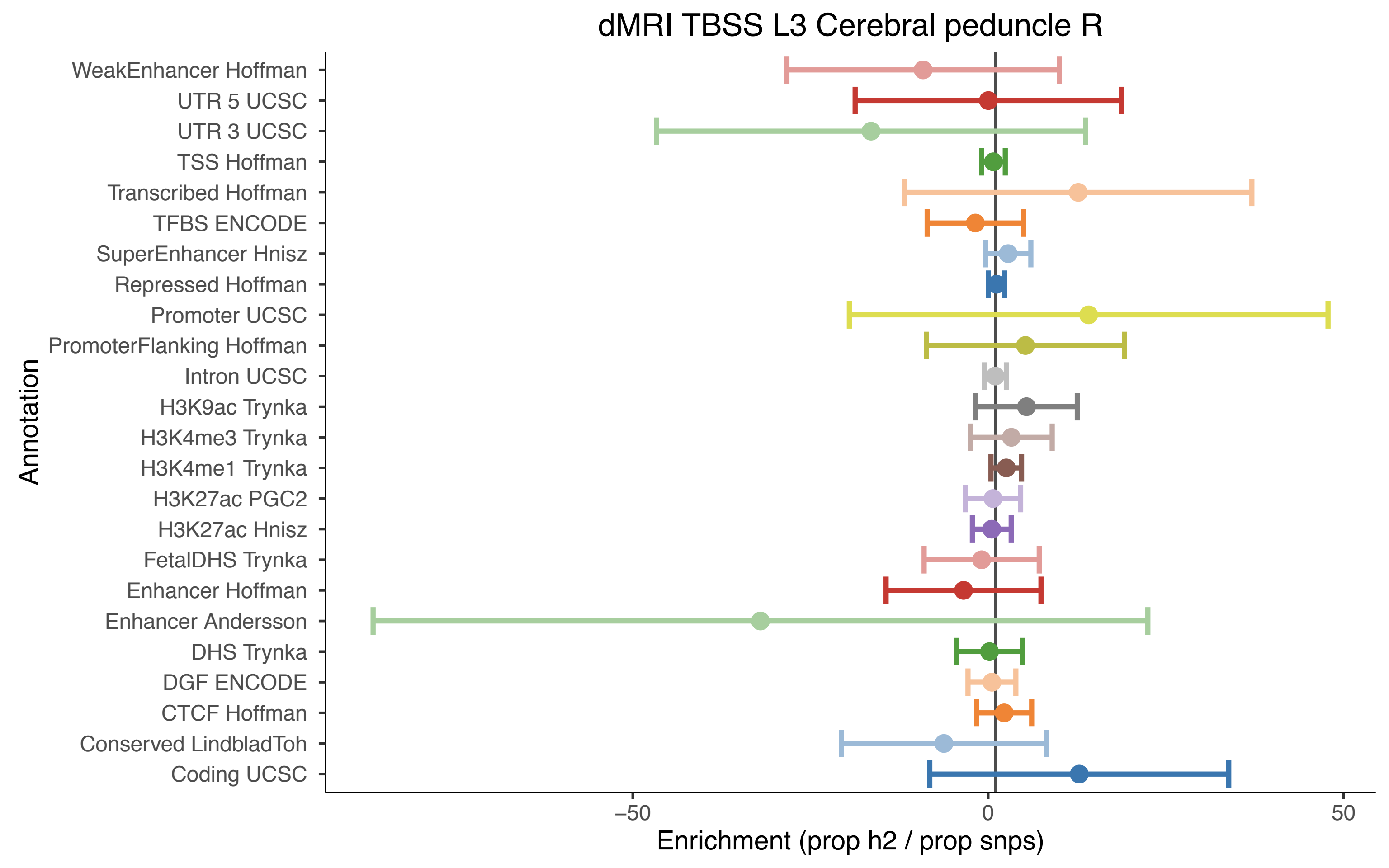
dMRI TBSS L3 Cerebral peduncle R
Annotation
WeakEnhancer Hoffman
UTR 5 UCSC
UTR 3 UCSC
TSS Hoffman
Transcribed Hoffman
TFBS ENCODE
SuperEnhancer Hnisz
Repressed Hoffman
Promoter UCSC
PromoterFlanking Hoffman
Intron UCSC
H3K9ac Trynka
H3K4me3 Trynka
H3K4me1 Trynka
H3K27ac PGC2
H3K27ac Hnisz
FetalDHS Trynka
Enhancer Hoffman
Enhancer Andersson
DHS Trynka
DGF ENCODE
CTCF Hoffman
Conserved LindbladToh
Coding UCSC
−50
0
50
Enrichment (prop h2 / prop snps)

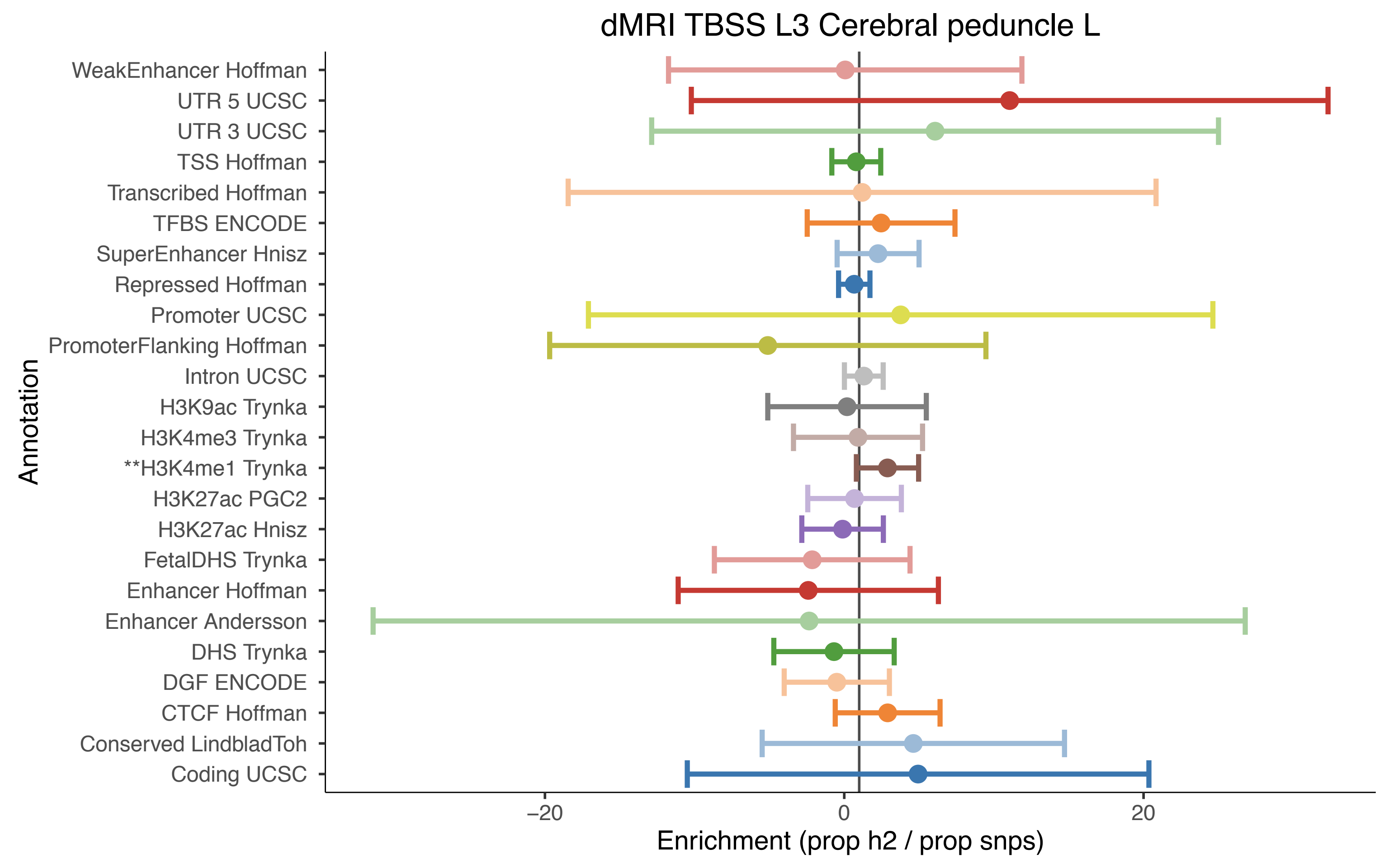
dMRI TBSS L3 Cerebral peduncle L
WeakEnhancer Hoffman
UTR 5 UCSC
UTR 3 UCSC
TSS Hoffman
Transcribed Hoffman
TFBS ENCODE
SuperEnhancer Hnisz
Repressed Hoffman
Promoter UCSC
PromoterFlanking Hoffman
Intron UCSC
H3K9ac Trynka
H3K4me3 Trynka
**H3K4me1 Trynka
H3K27ac PGC2
H3K27ac Hnisz
FetalDHS Trynka
Enhancer Hoffman
Enhancer Andersson
DHS Trynka
DGF ENCODE
CTCF Hoffman
Conserved LindbladToh
Coding UCSC
Annotation
−20
0
20
Enrichment (prop h2 / prop snps)

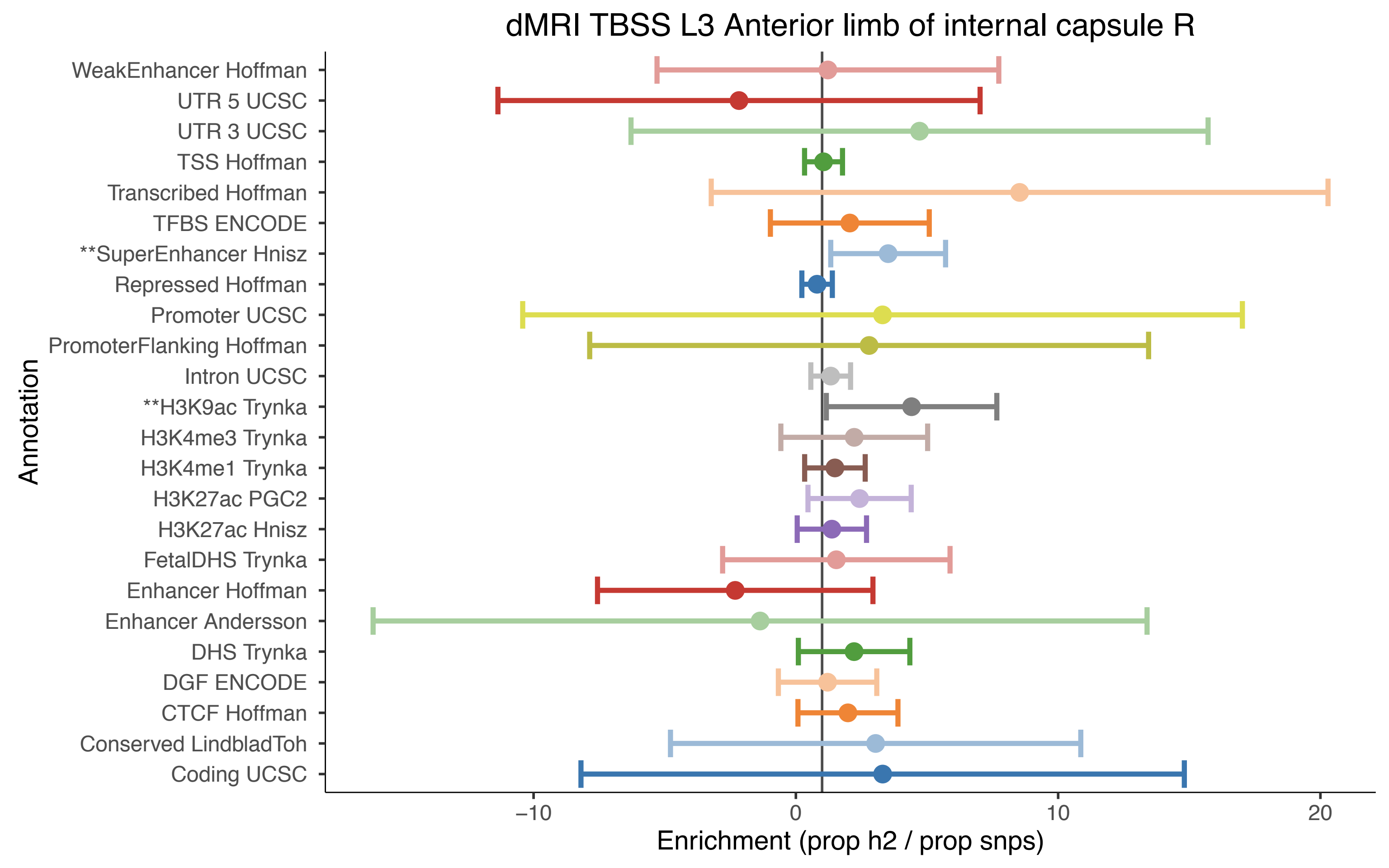
dMRI TBSS L3 Anterior limb of internal capsule R
WeakEnhancer Hoffman
UTR 5 UCSC
UTR 3 UCSC
TSS Hoffman
Transcribed Hoffman
TFBS ENCODE
**SuperEnhancer Hnisz
Repressed Hoffman
Promoter UCSC
PromoterFlanking Hoffman
Intron UCSC
**H3K9ac Trynka
H3K4me3 Trynka
H3K4me1 Trynka
H3K27ac PGC2
H3K27ac Hnisz
FetalDHS Trynka
Enhancer Hoffman
Enhancer Andersson
DHS Trynka
DGF ENCODE
CTCF Hoffman
Conserved LindbladToh
Coding UCSC
Annotation
−10
0
10
20
Enrichment (prop h2 / prop snps)

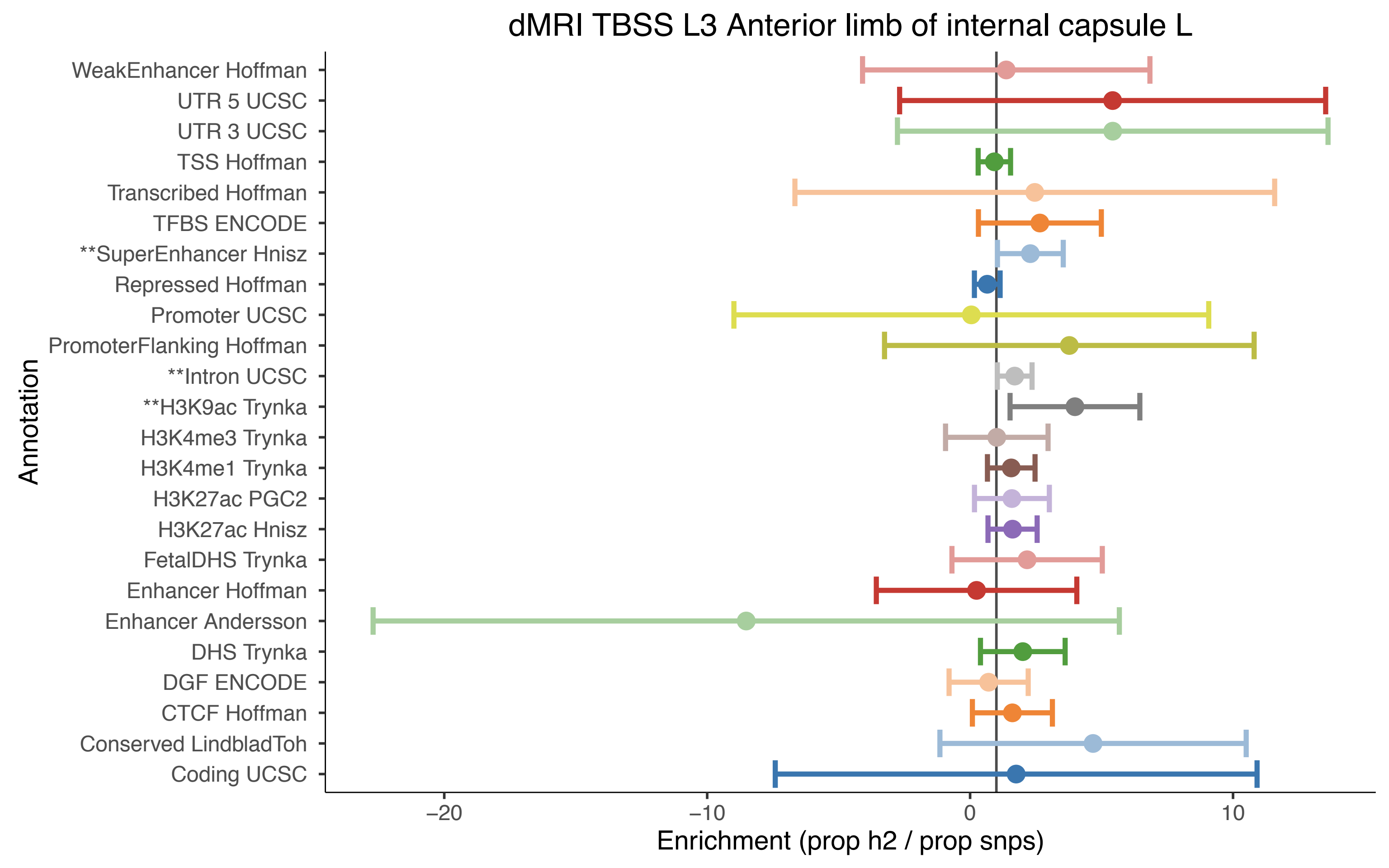

dMRI TBSS L3 Anterior limb of internal capsule L
WeakEnhancer Hoffman
UTR 5 UCSC
UTR 3 UCSC
TSS Hoffman
Transcribed Hoffman
TFBS ENCODE
**SuperEnhancer Hnisz
Repressed Hoffman
Promoter UCSC
PromoterFlanking Hoffman
**Intron UCSC
**H3K9ac Trynka
H3K4me3 Trynka
H3K4me1 Trynka
H3K27ac PGC2
H3K27ac Hnisz
FetalDHS Trynka
Enhancer Hoffman
Enhancer Andersson
DHS Trynka
DGF ENCODE
CTCF Hoffman
Conserved LindbladToh
Coding UCSC
Annotation
−20
−10
0
10
Enrichment (prop h2 / prop snps)

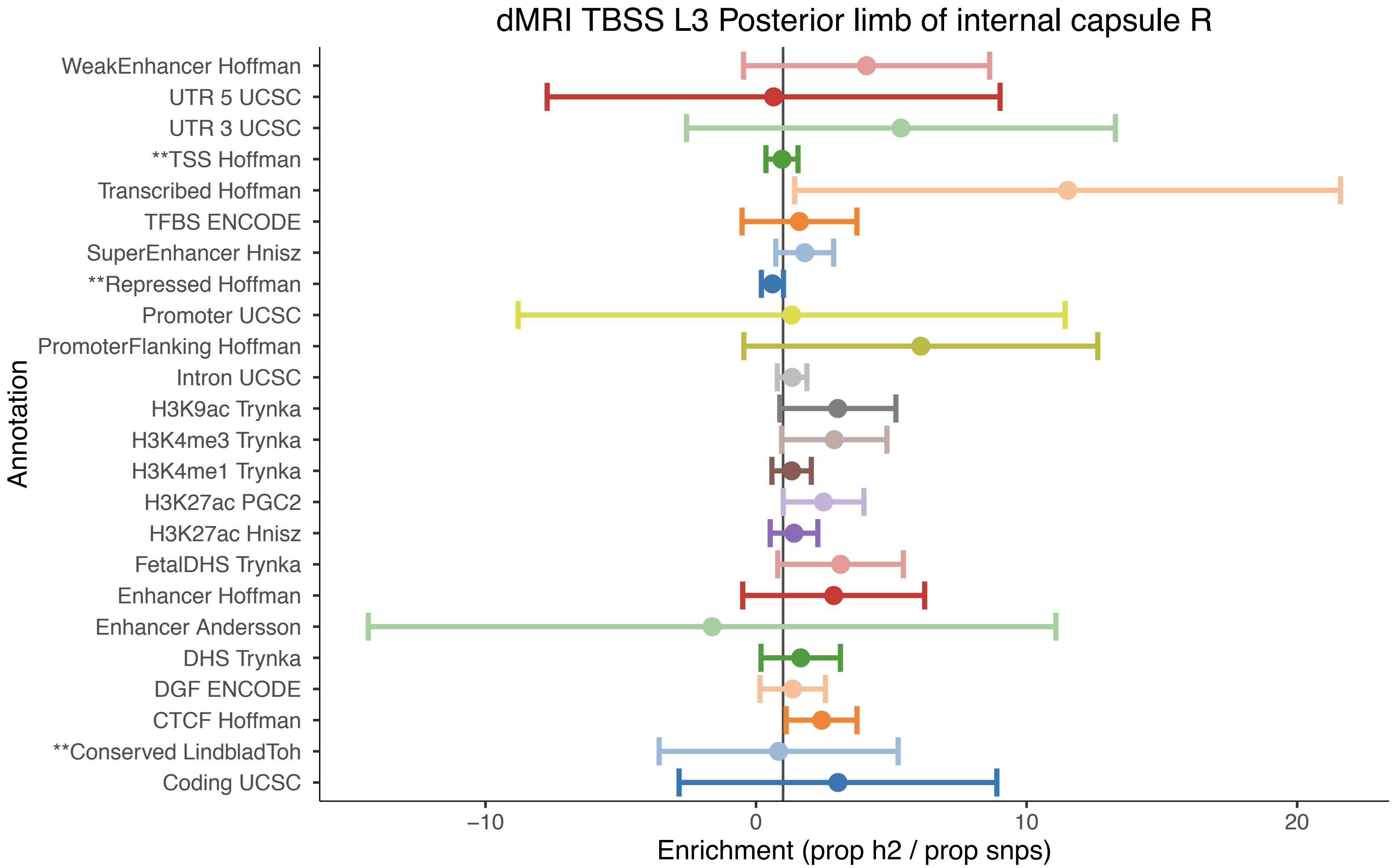

dMRI TBSS L3 Posterior limb of internal capsule R
WeakEnhancer Hoffman
UTR 5 UCSC
UTR 3 UCSC
**TSS Hoffman
Transcribed Hoffman
TFBS ENCODE
SuperEnhancer Hnisz
**Repressed Hoffman
Promoter UCSC
PromoterFlanking Hoffman
Intron UCSC
H3K9ac Trynka
H3K4me3 Trynka
H3K4me1 Trynka
H3K27ac PGC2
H3K27ac Hnisz
FetalDHS Trynka
Enhancer Hoffman
Enhancer Andersson
DHS Trynka
DGF ENCODE
CTCF Hoffman
**Conserved LindbladToh
Coding UCSC
Annotation
−10
0
10
20
Enrichment (prop h2 / prop snps)

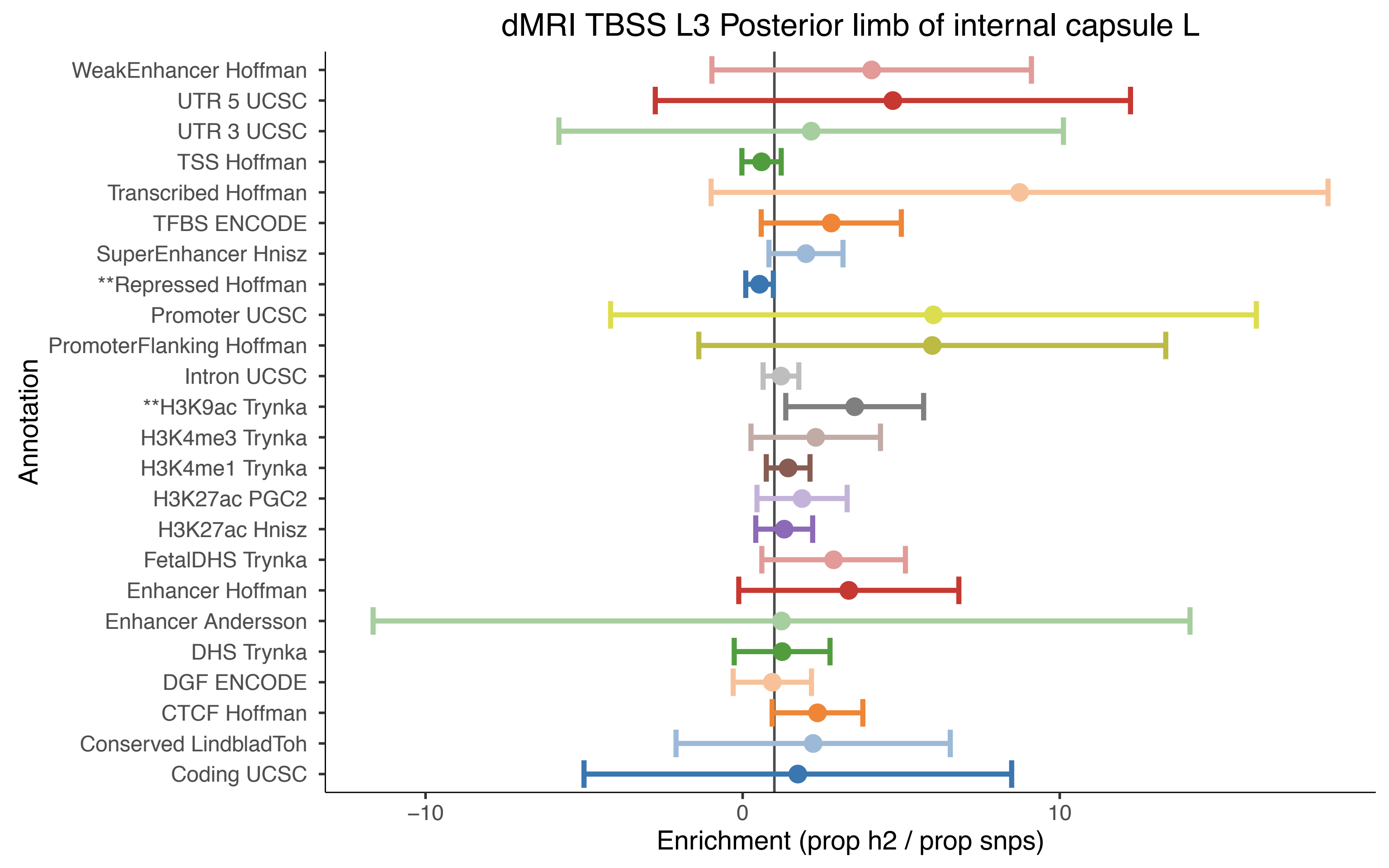

dMRI TBSS L3 Posterior limb of internal capsule L
Annotation
WeakEnhancer Hoffman
UTR 5 UCSC
UTR 3 UCSC
TSS Hoffman
Transcribed Hoffman
TFBS ENCODE
SuperEnhancer Hnisz
**Repressed Hoffman
Promoter UCSC
PromoterFlanking Hoffman
Intron UCSC
**H3K9ac Trynka
H3K4me3 Trynka
H3K4me1 Trynka
H3K27ac PGC2
H3K27ac Hnisz
FetalDHS Trynka
Enhancer Hoffman
Enhancer Andersson
DHS Trynka
DGF ENCODE
CTCF Hoffman
Conserved LindbladToh
Coding UCSC
−10
0
10
Enrichment (prop h2 / prop snps)

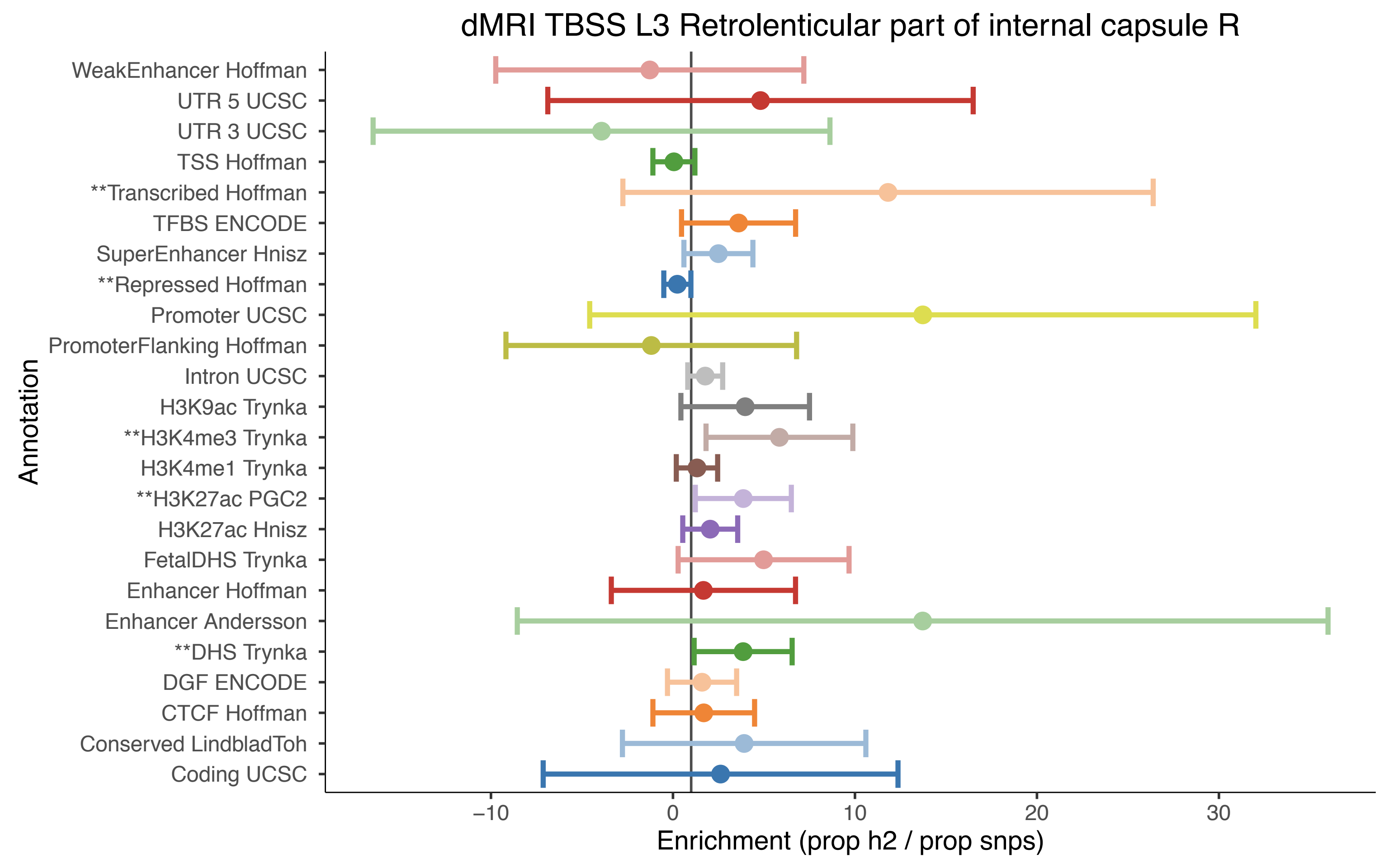
dMRI TBSS L3 Retrolenticular part of internal capsule R
WeakEnhancer Hoffman
UTR 5 UCSC
UTR 3 UCSC
TSS Hoffman
**Transcribed Hoffman
TFBS ENCODE
SuperEnhancer Hnisz
**Repressed Hoffman
Promoter UCSC
PromoterFlanking Hoffman
Intron UCSC
H3K9ac Trynka
**H3K4me3 Trynka
H3K4me1 Trynka
**H3K27ac PGC2
H3K27ac Hnisz
FetalDHS Trynka
Enhancer Hoffman
Enhancer Andersson
**DHS Trynka
DGF ENCODE
CTCF Hoffman
Conserved LindbladToh
Coding UCSC
Annotation
−10
0
10
20
30
Enrichment (prop h2 / prop snps)

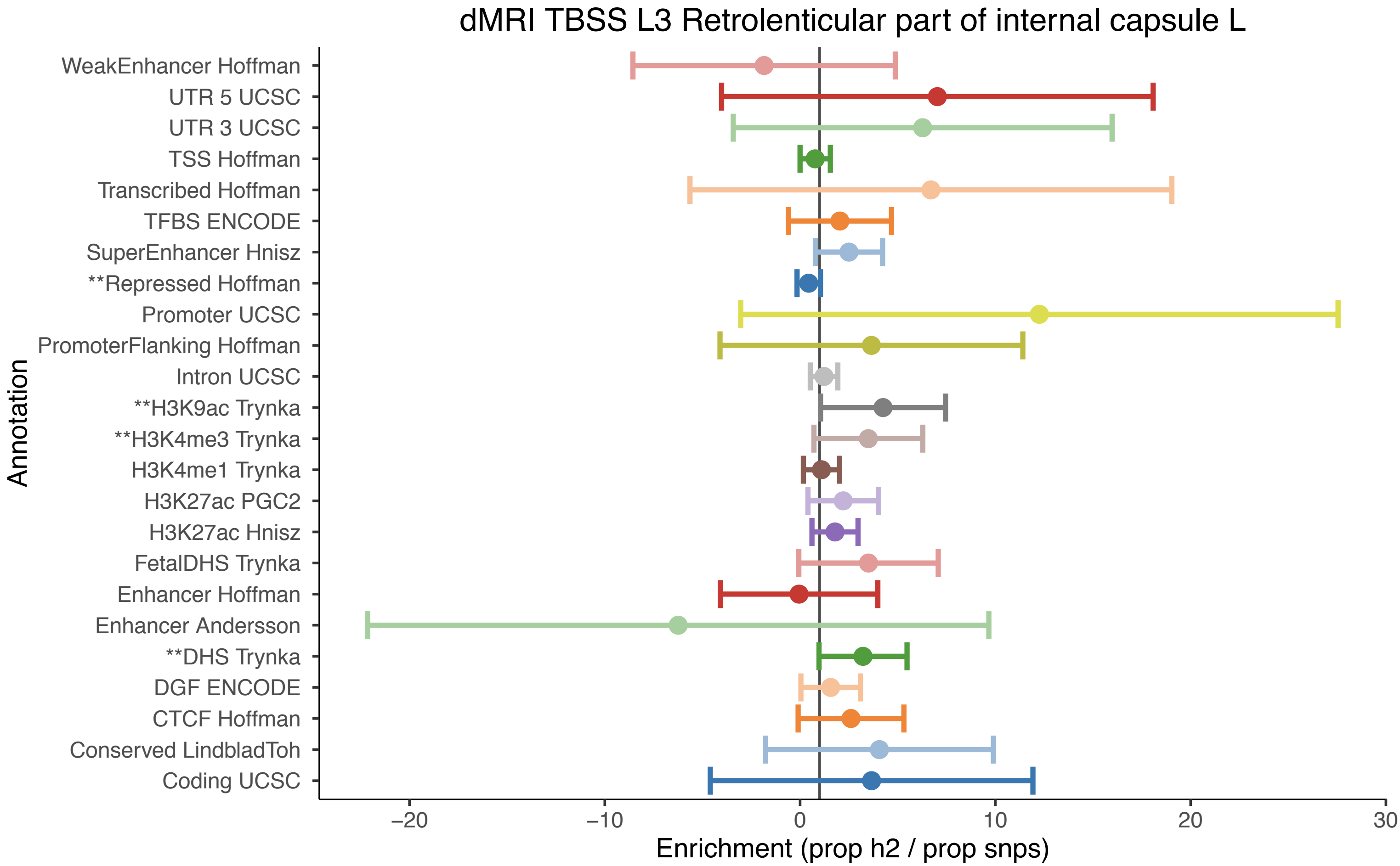
dMRI TBSS L3 Retrolenticular part of internal capsule L
WeakEnhancer Hoffman
UTR 5 UCSC
UTR 3 UCSC
TSS Hoffman
Transcribed Hoffman
TFBS ENCODE
SuperEnhancer Hnisz
**Repressed Hoffman
Promoter UCSC
PromoterFlanking Hoffman
Intron UCSC
**H3K9ac Trynka
**H3K4me3 Trynka
H3K4me1 Trynka
H3K27ac PGC2
H3K27ac Hnisz
FetalDHS Trynka
Enhancer Hoffman
Enhancer Andersson
**DHS Trynka
DGF ENCODE
CTCF Hoffman
Conserved LindbladToh
Coding UCSC
Annotation
−20
−10
0
10
20
30
Enrichment (prop h2 / prop snps)

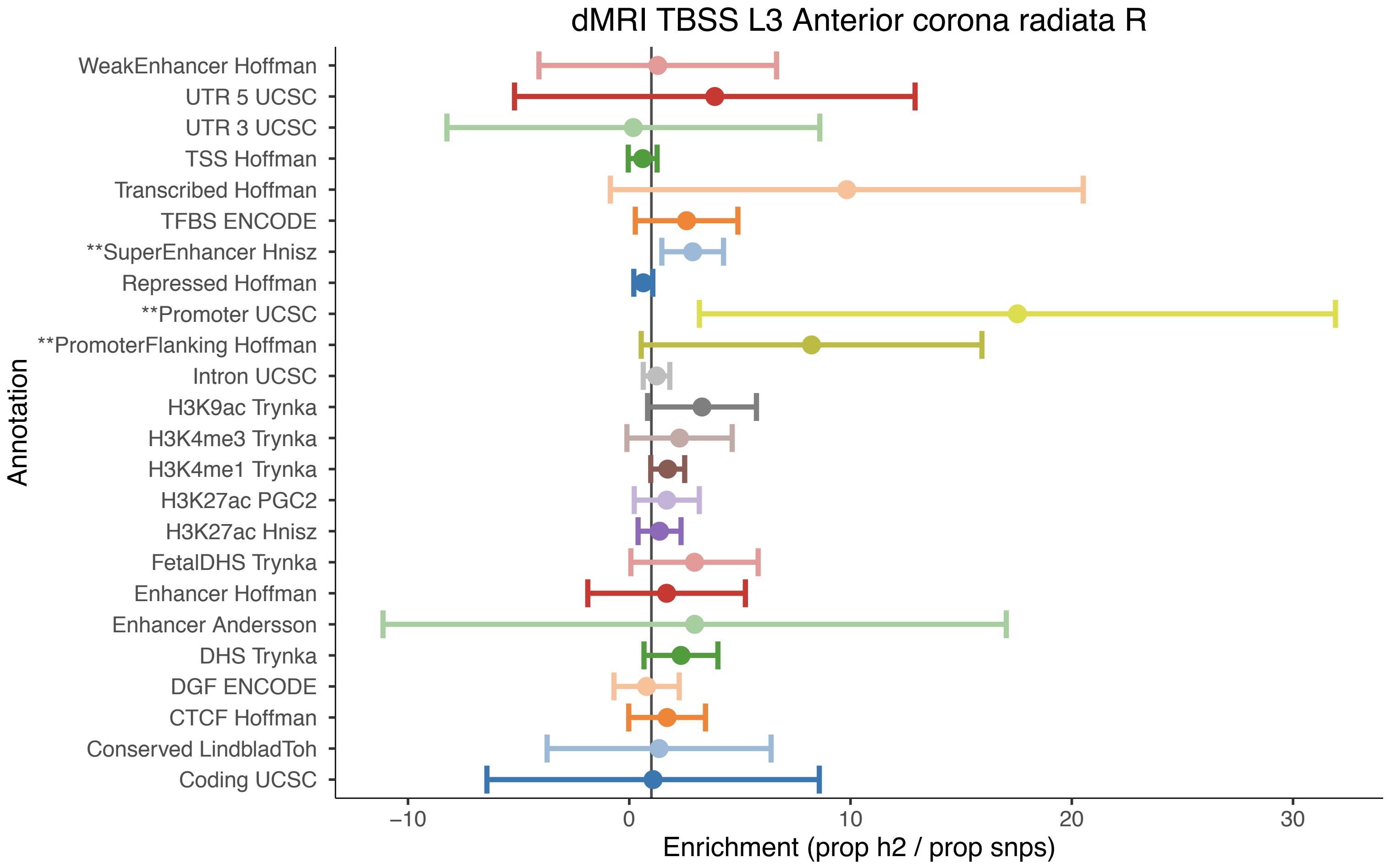
dMRI TBSS L3 Anterior corona radiata R
WeakEnhancer Hoffman
UTR 5 UCSC
UTR 3 UCSC
TSS Hoffman
Transcribed Hoffman
TFBS ENCODE
**SuperEnhancer Hnisz
Repressed Hoffman
**Promoter UCSC
**PromoterFlanking Hoffman
Intron UCSC
H3K9ac Trynka
H3K4me3 Trynka
H3K4me1 Trynka
H3K27ac PGC2
H3K27ac Hnisz
FetalDHS Trynka
Enhancer Hoffman
Enhancer Andersson
DHS Trynka
DGF ENCODE
CTCF Hoffman
Conserved LindbladToh
Coding UCSC
Annotation
−10
0
10
20
30
Enrichment (prop h2 / prop snps)

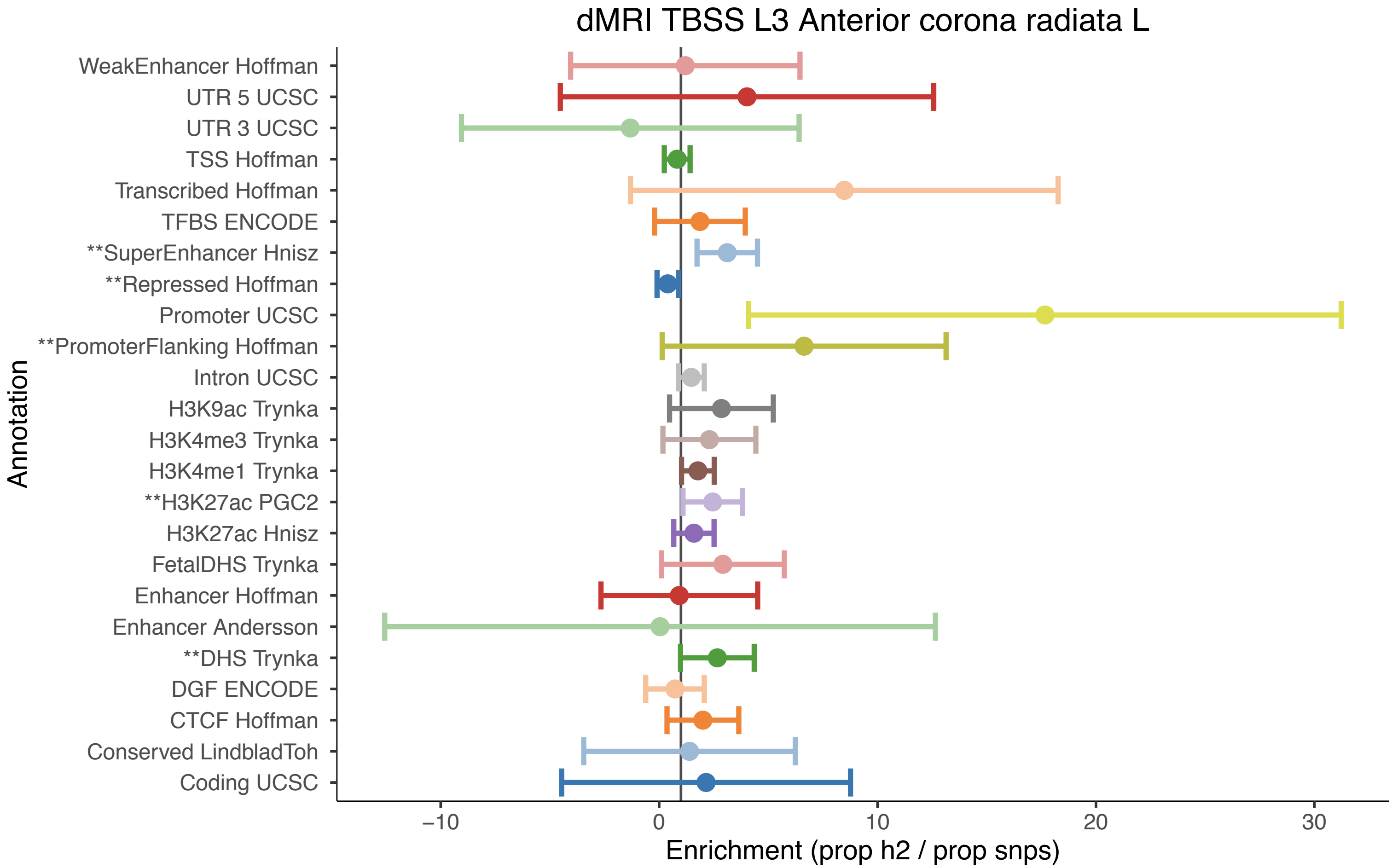

dMRI TBSS L3 Anterior corona radiata L
Annotation
WeakEnhancer Hoffman
UTR 5 UCSC
UTR 3 UCSC
TSS Hoffman
Transcribed Hoffman
TFBS ENCODE
**SuperEnhancer Hnisz
**Repressed Hoffman
Promoter UCSC
**PromoterFlanking Hoffman
Intron UCSC
H3K9ac Trynka
H3K4me3 Trynka
H3K4me1 Trynka
**H3K27ac PGC2
H3K27ac Hnisz
FetalDHS Trynka
Enhancer Hoffman
Enhancer Andersson
**DHS Trynka
DGF ENCODE
CTCF Hoffman
Conserved LindbladToh
Coding UCSC
−10
0
10
20
30
Enrichment (prop h2 / prop snps)

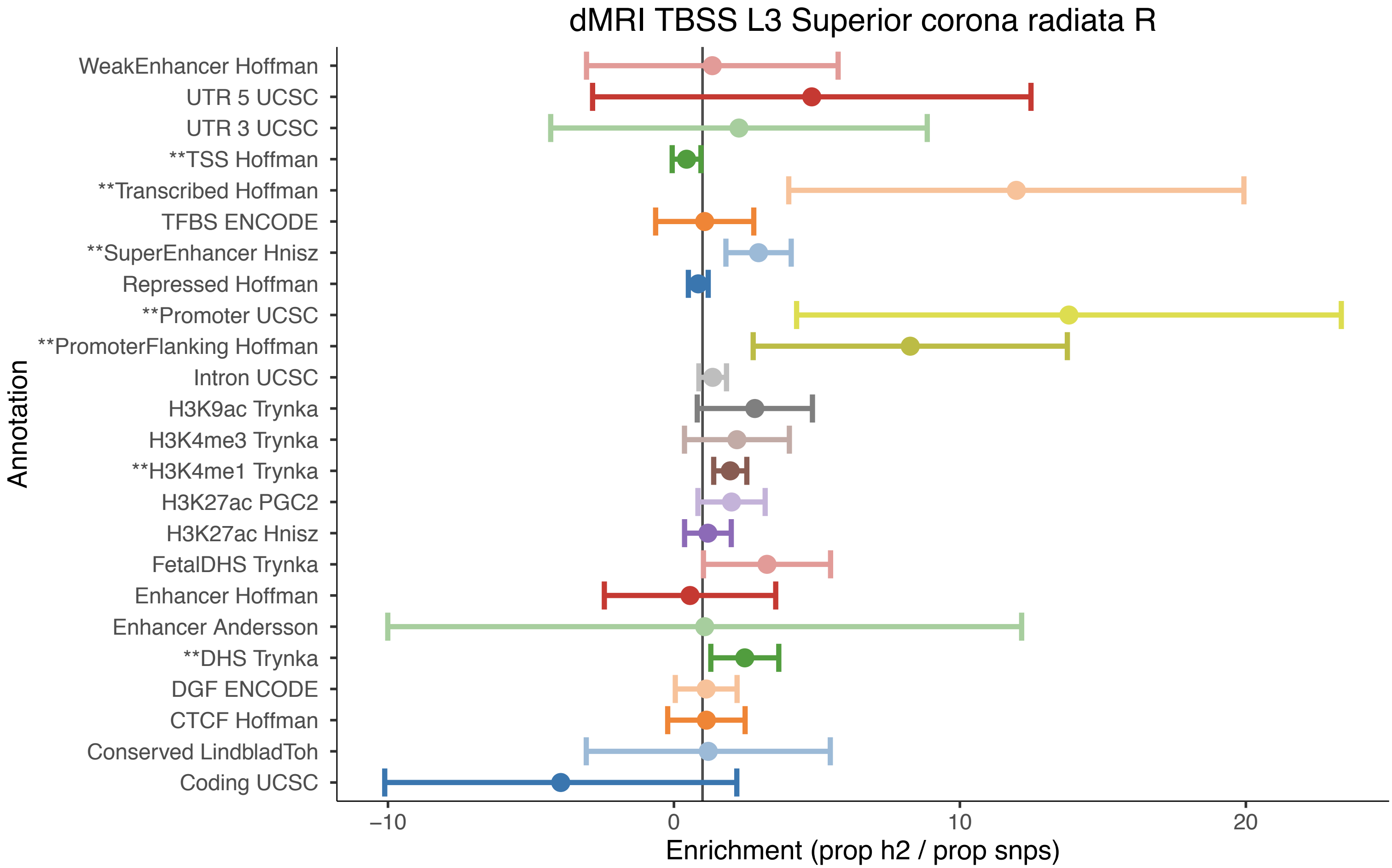

dMRI TBSS L3 Superior corona radiata R
Annotation
WeakEnhancer Hoffman
UTR 5 UCSC
UTR 3 UCSC
**TSS Hoffman
**Transcribed Hoffman
TFBS ENCODE
**SuperEnhancer Hnisz
Repressed Hoffman
**Promoter UCSC
**PromoterFlanking Hoffman
Intron UCSC
H3K9ac Trynka
H3K4me3 Trynka
**H3K4me1 Trynka
H3K27ac PGC2
H3K27ac Hnisz
FetalDHS Trynka
Enhancer Hoffman
Enhancer Andersson
**DHS Trynka
DGF ENCODE
CTCF Hoffman
Conserved LindbladToh
Coding UCSC
−10
0
10
20
Enrichment (prop h2 / prop snps)

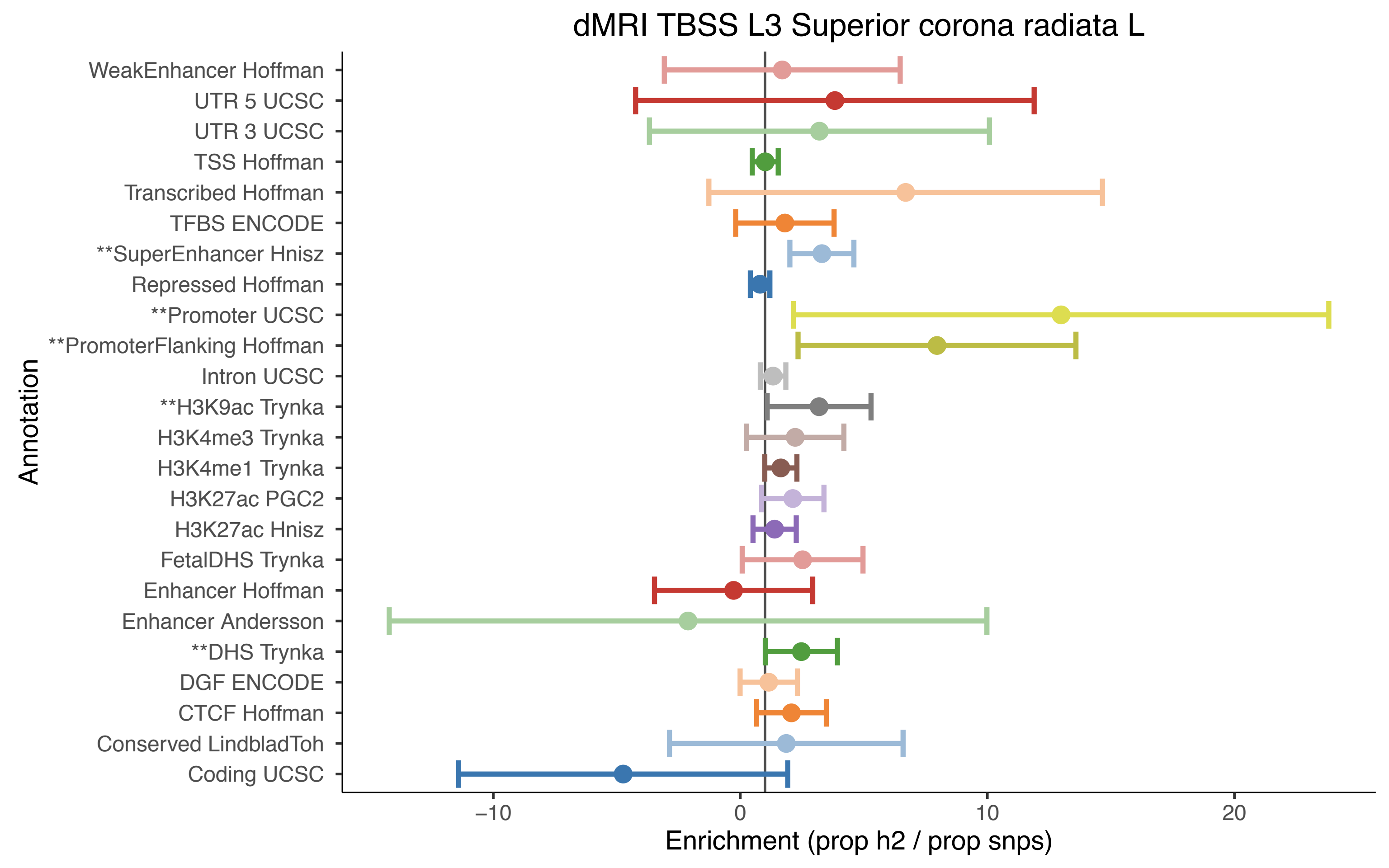

dMRI TBSS L3 Superior corona radiata L
WeakEnhancer Hoffman
UTR 5 UCSC
UTR 3 UCSC
TSS Hoffman
Transcribed Hoffman
TFBS ENCODE
**SuperEnhancer Hnisz
Repressed Hoffman
**Promoter UCSC
**PromoterFlanking Hoffman
Intron UCSC
**H3K9ac Trynka
H3K4me3 Trynka
H3K4me1 Trynka
H3K27ac PGC2
H3K27ac Hnisz
FetalDHS Trynka
Enhancer Hoffman
Enhancer Andersson
**DHS Trynka
DGF ENCODE
CTCF Hoffman
Conserved LindbladToh
Coding UCSC
Annotation
−10
0
10
20
Enrichment (prop h2 / prop snps)

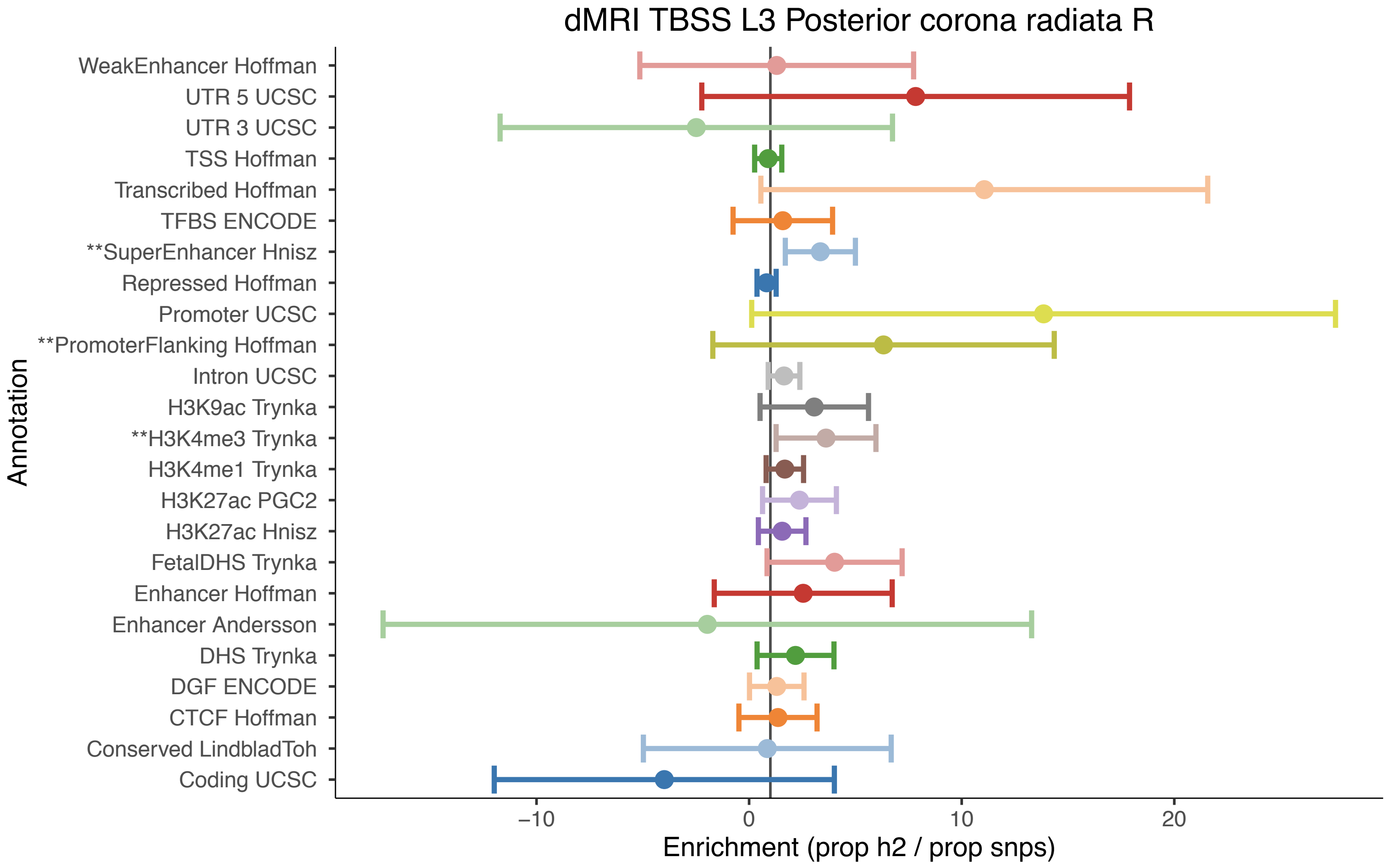
dMRI TBSS L3 Posterior corona radiata R
Annotation
WeakEnhancer Hoffman
UTR 5 UCSC
UTR 3 UCSC
TSS Hoffman
Transcribed Hoffman
TFBS ENCODE
**SuperEnhancer Hnisz
Repressed Hoffman
Promoter UCSC
**PromoterFlanking Hoffman
Intron UCSC
H3K9ac Trynka
**H3K4me3 Trynka
H3K4me1 Trynka
H3K27ac PGC2
H3K27ac Hnisz
FetalDHS Trynka
Enhancer Hoffman
Enhancer Andersson
DHS Trynka
DGF ENCODE
CTCF Hoffman
Conserved LindbladToh
Coding UCSC
−10
0
10
20
Enrichment (prop h2 / prop snps)

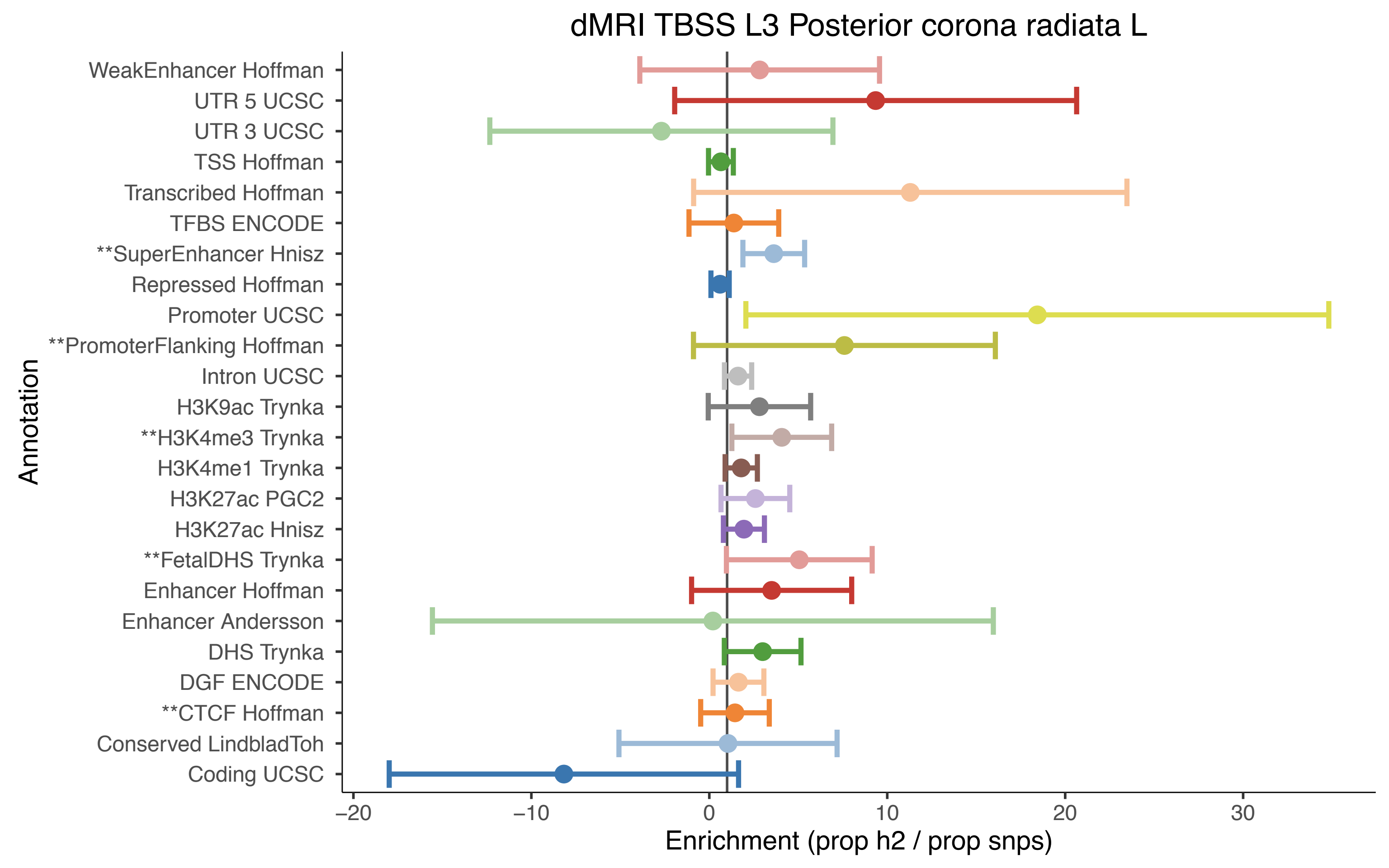

dMRI TBSS L3 Posterior corona radiata L
Annotation
WeakEnhancer Hoffman
UTR 5 UCSC
UTR 3 UCSC
TSS Hoffman
Transcribed Hoffman
TFBS ENCODE
**SuperEnhancer Hnisz
Repressed Hoffman
Promoter UCSC
**PromoterFlanking Hoffman
Intron UCSC
H3K9ac Trynka
**H3K4me3 Trynka
H3K4me1 Trynka
H3K27ac PGC2
H3K27ac Hnisz
**FetalDHS Trynka
Enhancer Hoffman
Enhancer Andersson
DHS Trynka
DGF ENCODE
**CTCF Hoffman
Conserved LindbladToh
Coding UCSC
−20
−10
0
10
20
30
Enrichment (prop h2 / prop snps)

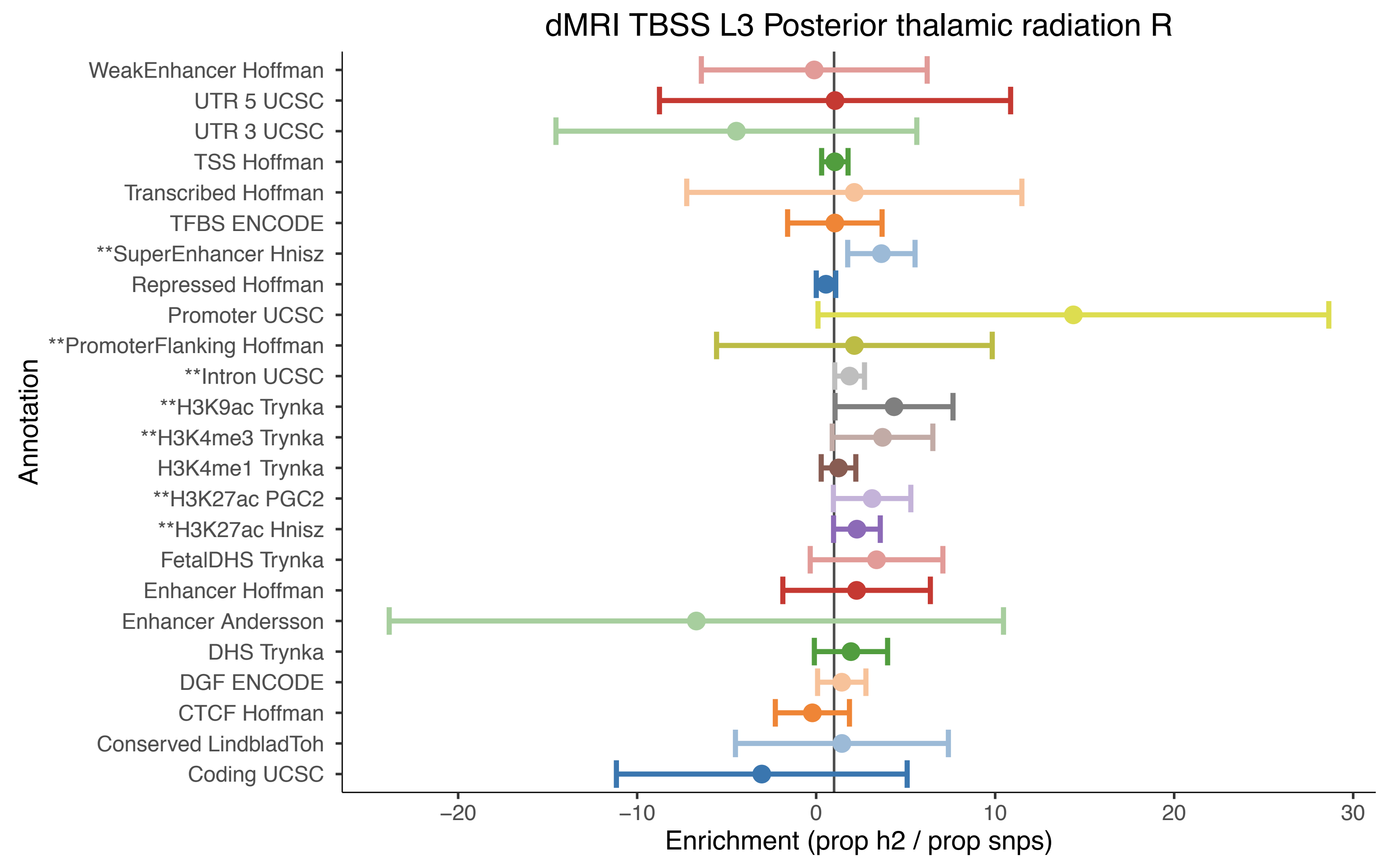
dMRI TBSS L3 Posterior thalamic radiation R
Annotation
WeakEnhancer Hoffman
UTR 5 UCSC
UTR 3 UCSC
TSS Hoffman
Transcribed Hoffman
TFBS ENCODE
**SuperEnhancer Hnisz
Repressed Hoffman
Promoter UCSC
**PromoterFlanking Hoffman
**Intron UCSC
**H3K9ac Trynka
**H3K4me3 Trynka
H3K4me1 Trynka
**H3K27ac PGC2
**H3K27ac Hnisz
FetalDHS Trynka
Enhancer Hoffman
Enhancer Andersson
DHS Trynka
DGF ENCODE
CTCF Hoffman
Conserved LindbladToh
Coding UCSC
−20
−10
0
10
20
30
Enrichment (prop h2 / prop snps)

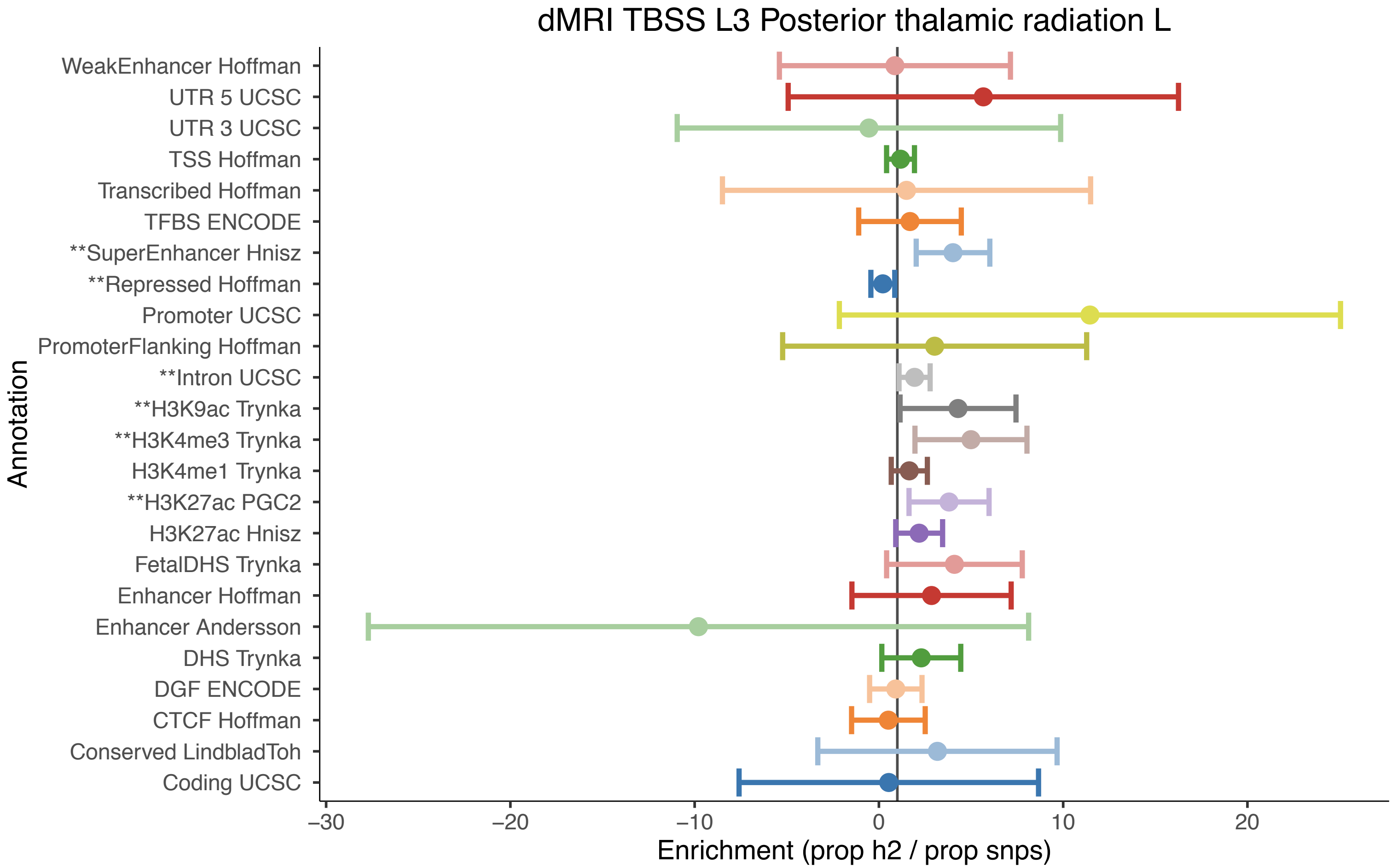

dMRI TBSS L3 Posterior thalamic radiation L
WeakEnhancer Hoffman
UTR 5 UCSC
UTR 3 UCSC
TSS Hoffman
Transcribed Hoffman
TFBS ENCODE
**SuperEnhancer Hnisz
**Repressed Hoffman
Promoter UCSC
PromoterFlanking Hoffman
**Intron UCSC
**H3K9ac Trynka
**H3K4me3 Trynka
H3K4me1 Trynka
**H3K27ac PGC2
H3K27ac Hnisz
FetalDHS Trynka
Enhancer Hoffman
Enhancer Andersson
DHS Trynka
DGF ENCODE
CTCF Hoffman
Conserved LindbladToh
Coding UCSC
Annotation
−30
−20
−10
0
10
20
Enrichment (prop h2 / prop snps)

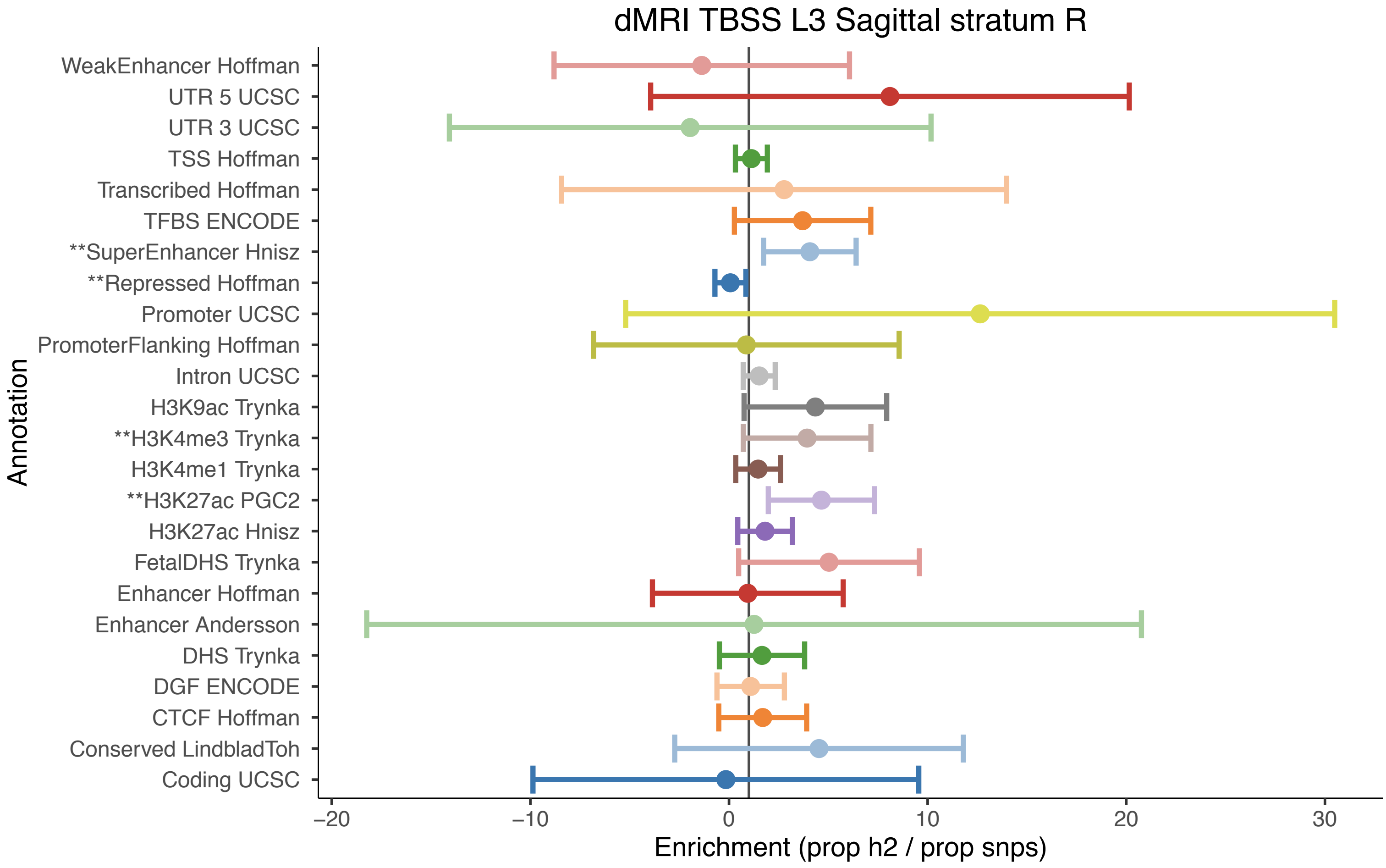

dMRI TBSS L3 Sagittal stratum R
WeakEnhancer Hoffman
UTR 5 UCSC
UTR 3 UCSC
TSS Hoffman
Transcribed Hoffman
TFBS ENCODE
**SuperEnhancer Hnisz
**Repressed Hoffman
Promoter UCSC
PromoterFlanking Hoffman
Intron UCSC
H3K9ac Trynka
**H3K4me3 Trynka
H3K4me1 Trynka
**H3K27ac PGC2
H3K27ac Hnisz
FetalDHS Trynka
Enhancer Hoffman
Enhancer Andersson
DHS Trynka
DGF ENCODE
CTCF Hoffman
Conserved LindbladToh
Coding UCSC
Annotation
−20
−10
0
10
20
30
Enrichment (prop h2 / prop snps)

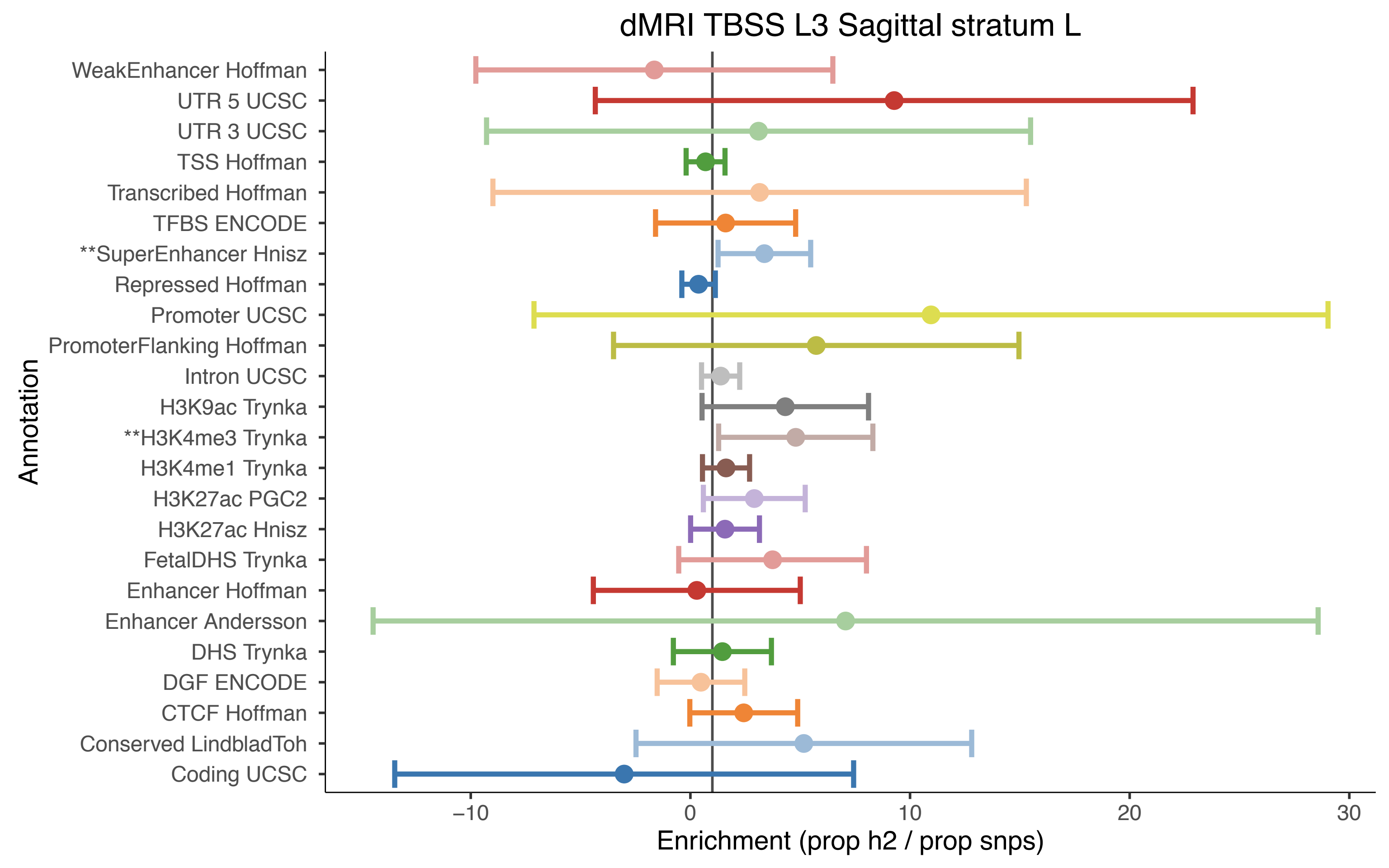
dMRI TBSS L3 Sagittal stratum L
WeakEnhancer Hoffman
UTR 5 UCSC
UTR 3 UCSC
TSS Hoffman
Transcribed Hoffman
TFBS ENCODE
**SuperEnhancer Hnisz
Repressed Hoffman
Promoter UCSC
PromoterFlanking Hoffman
Intron UCSC
H3K9ac Trynka
**H3K4me3 Trynka
H3K4me1 Trynka
H3K27ac PGC2
H3K27ac Hnisz
FetalDHS Trynka
Enhancer Hoffman
Enhancer Andersson
DHS Trynka
DGF ENCODE
CTCF Hoffman
Conserved LindbladToh
Coding UCSC
Annotation
−10
0
10
20
30
Enrichment (prop h2 / prop snps)

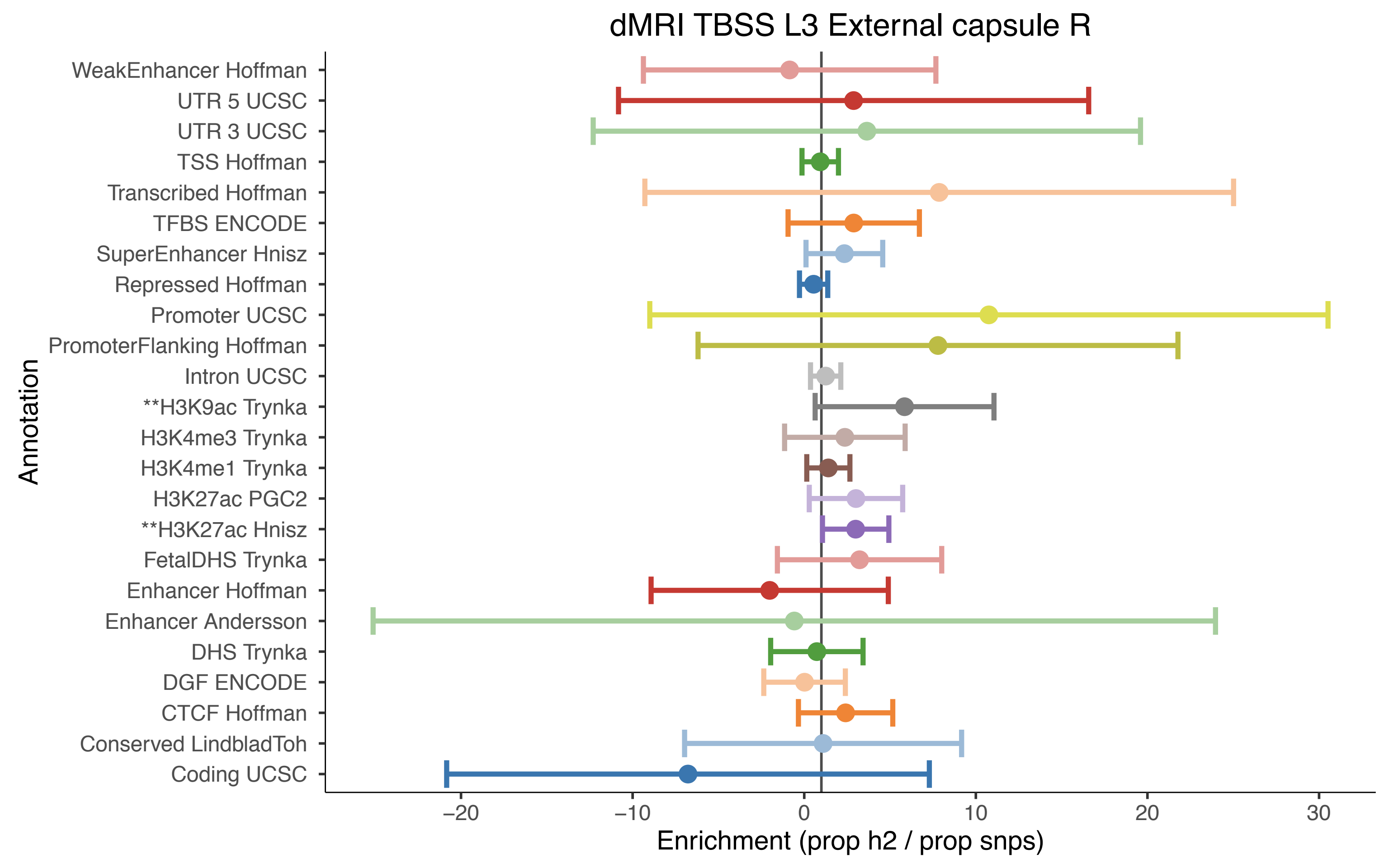

dMRI TBSS L3 External capsule R
WeakEnhancer Hoffman
UTR 5 UCSC
UTR 3 UCSC
TSS Hoffman
Transcribed Hoffman
TFBS ENCODE
SuperEnhancer Hnisz
Repressed Hoffman
Promoter UCSC
PromoterFlanking Hoffman
Intron UCSC
**H3K9ac Trynka
H3K4me3 Trynka
H3K4me1 Trynka
H3K27ac PGC2
**H3K27ac Hnisz
FetalDHS Trynka
Enhancer Hoffman
Enhancer Andersson
DHS Trynka
DGF ENCODE
CTCF Hoffman
Conserved LindbladToh
Coding UCSC
Annotation
−20
−10
0
10
20
30
Enrichment (prop h2 / prop snps)

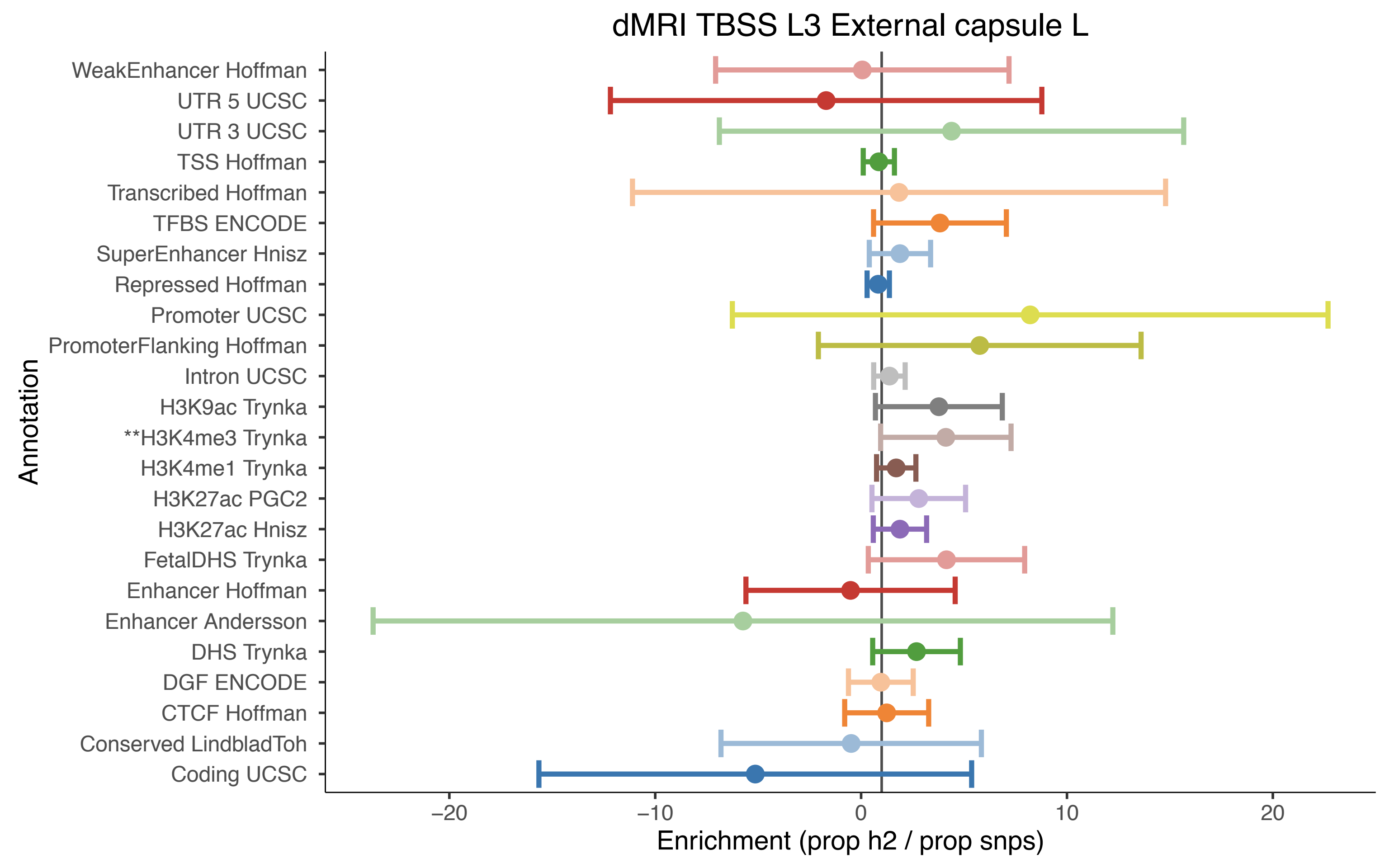

dMRI TBSS L3 External capsule L
WeakEnhancer Hoffman
UTR 5 UCSC
UTR 3 UCSC
TSS Hoffman
Transcribed Hoffman
TFBS ENCODE
SuperEnhancer Hnisz
Repressed Hoffman
Promoter UCSC
PromoterFlanking Hoffman
Intron UCSC
H3K9ac Trynka
**H3K4me3 Trynka
H3K4me1 Trynka
H3K27ac PGC2
H3K27ac Hnisz
FetalDHS Trynka
Enhancer Hoffman
Enhancer Andersson
DHS Trynka
DGF ENCODE
CTCF Hoffman
Conserved LindbladToh
Coding UCSC
Annotation
−20
−10
0
10
20
Enrichment (prop h2 / prop snps)

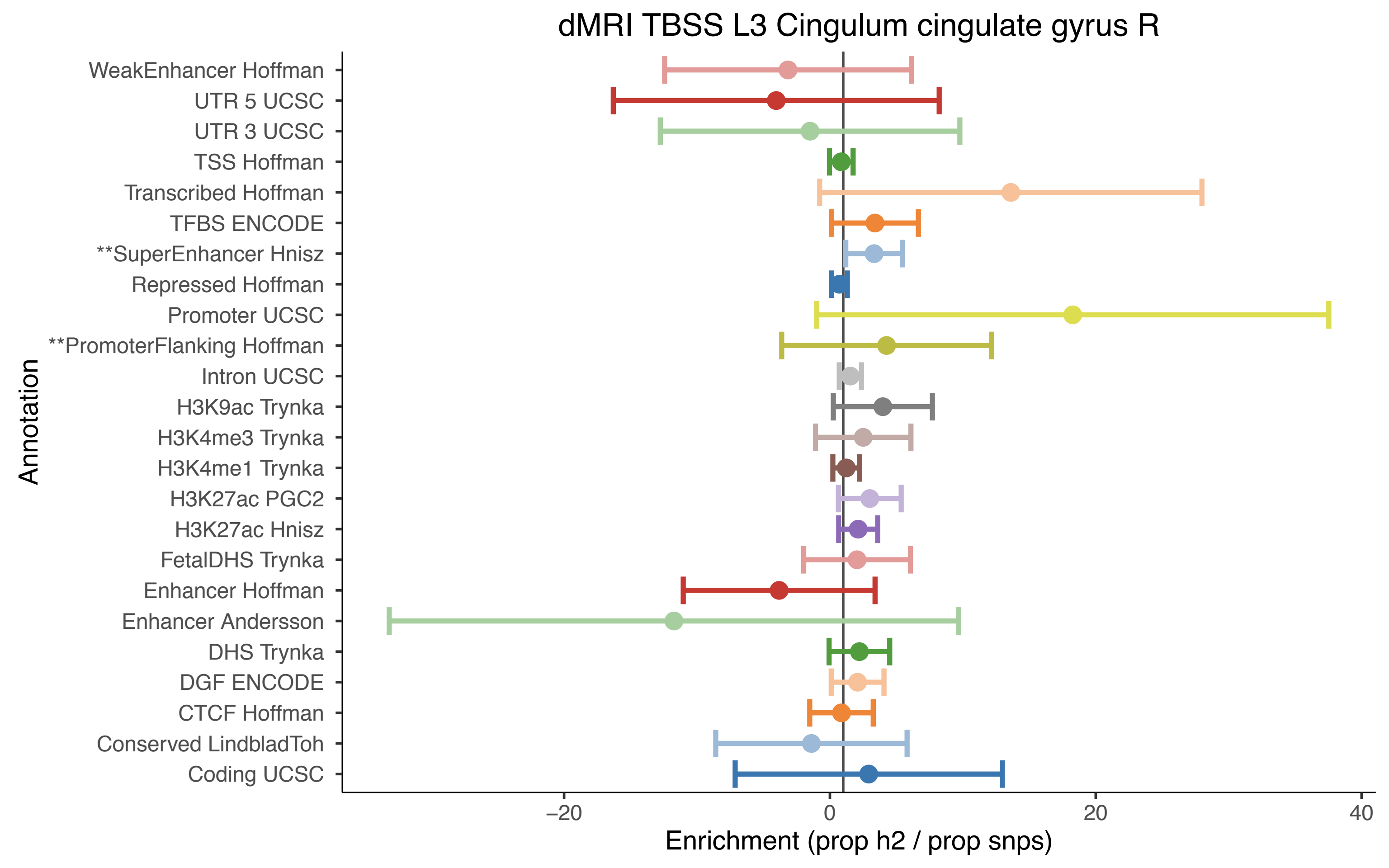
dMRI TBSS L3 Cingulum cingulate gyrus R
WeakEnhancer Hoffman
UTR 5 UCSC
UTR 3 UCSC
TSS Hoffman
Transcribed Hoffman
TFBS ENCODE
**SuperEnhancer Hnisz
Repressed Hoffman
Promoter UCSC
**PromoterFlanking Hoffman
Intron UCSC
H3K9ac Trynka
H3K4me3 Trynka
H3K4me1 Trynka
H3K27ac PGC2
H3K27ac Hnisz
FetalDHS Trynka
Enhancer Hoffman
Enhancer Andersson
DHS Trynka
DGF ENCODE
CTCF Hoffman
Conserved LindbladToh
Coding UCSC
Annotation
−20
0
20
40
Enrichment (prop h2 / prop snps)

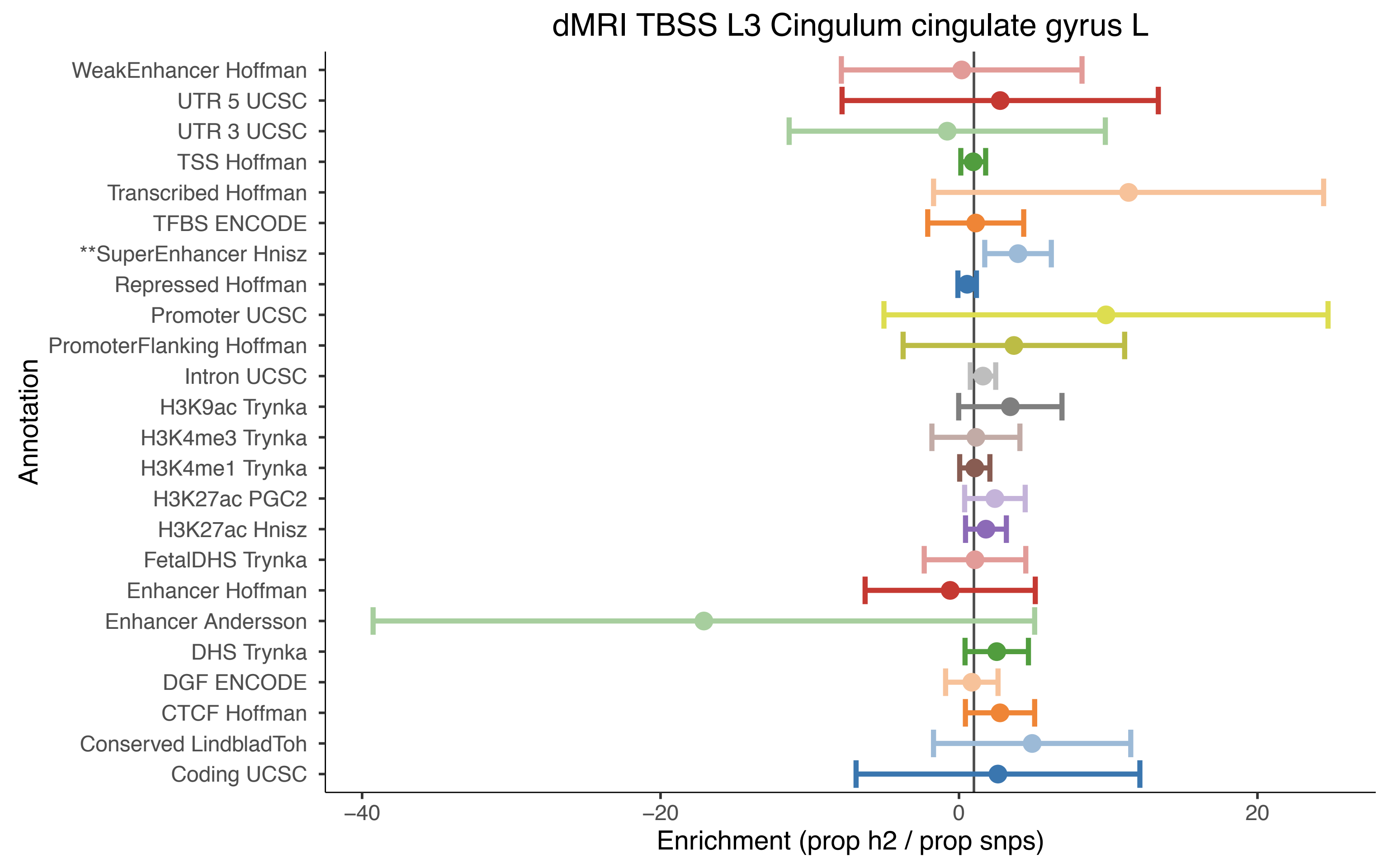
dMRI TBSS L3 Cingulum cingulate gyrus L
Annotation
WeakEnhancer Hoffman
UTR 5 UCSC
UTR 3 UCSC
TSS Hoffman
Transcribed Hoffman
TFBS ENCODE
**SuperEnhancer Hnisz
Repressed Hoffman
Promoter UCSC
PromoterFlanking Hoffman
Intron UCSC
H3K9ac Trynka
H3K4me3 Trynka
H3K4me1 Trynka
H3K27ac PGC2
H3K27ac Hnisz
FetalDHS Trynka
Enhancer Hoffman
Enhancer Andersson
DHS Trynka
DGF ENCODE
CTCF Hoffman
Conserved LindbladToh
Coding UCSC
−40
−20
0
20
Enrichment (prop h2 / prop snps)

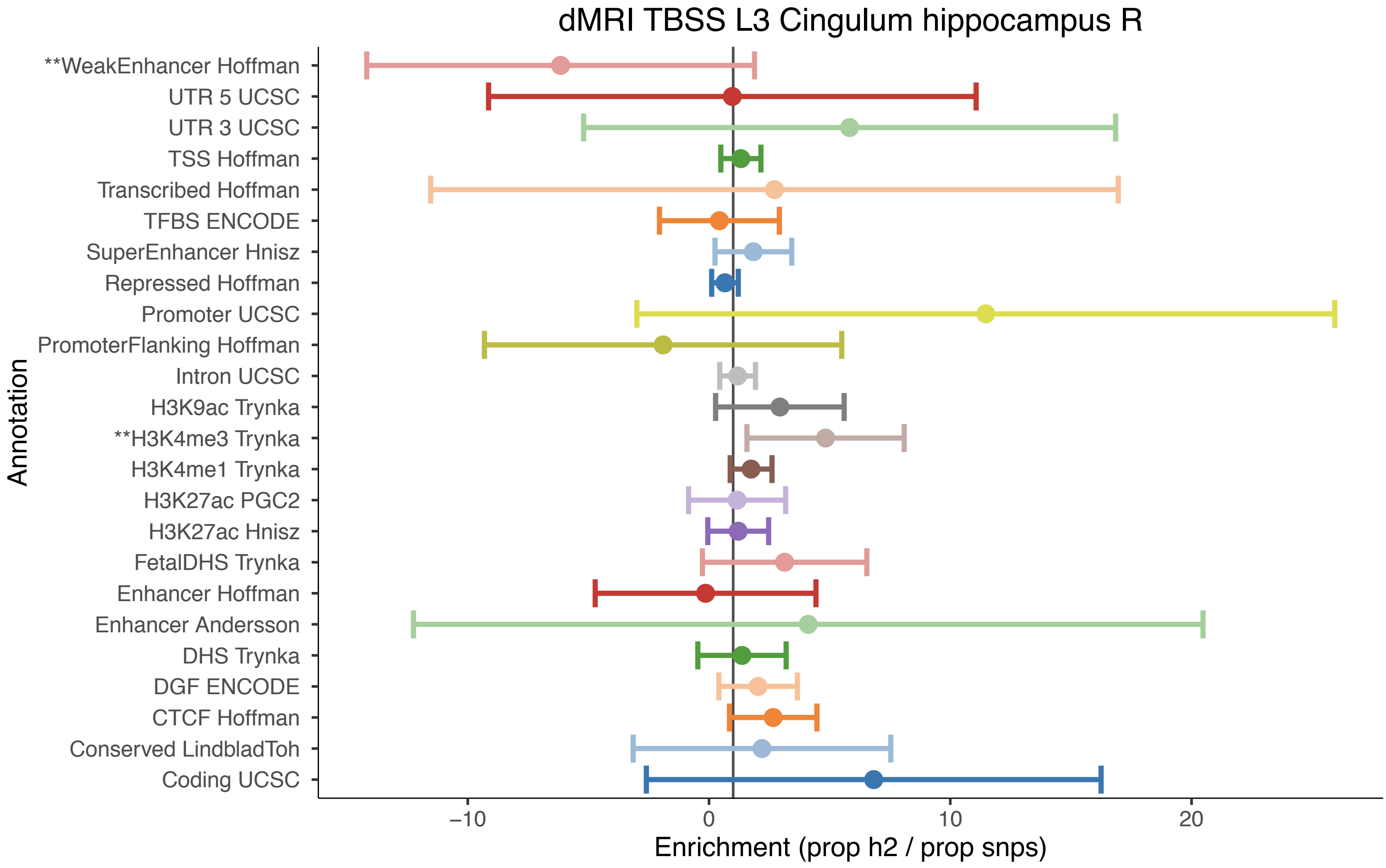

dMRI TBSS L3 Cingulum hippocampus R
**WeakEnhancer Hoffman
UTR 5 UCSC
UTR 3 UCSC
TSS Hoffman
Transcribed Hoffman
TFBS ENCODE
SuperEnhancer Hnisz
Repressed Hoffman
Promoter UCSC
PromoterFlanking Hoffman
Intron UCSC
H3K9ac Trynka
**H3K4me3 Trynka
H3K4me1 Trynka
H3K27ac PGC2
H3K27ac Hnisz
FetalDHS Trynka
Enhancer Hoffman
Enhancer Andersson
DHS Trynka
DGF ENCODE
CTCF Hoffman
Conserved LindbladToh
Coding UCSC
Annotation
−10
0
10
20
Enrichment (prop h2 / prop snps)

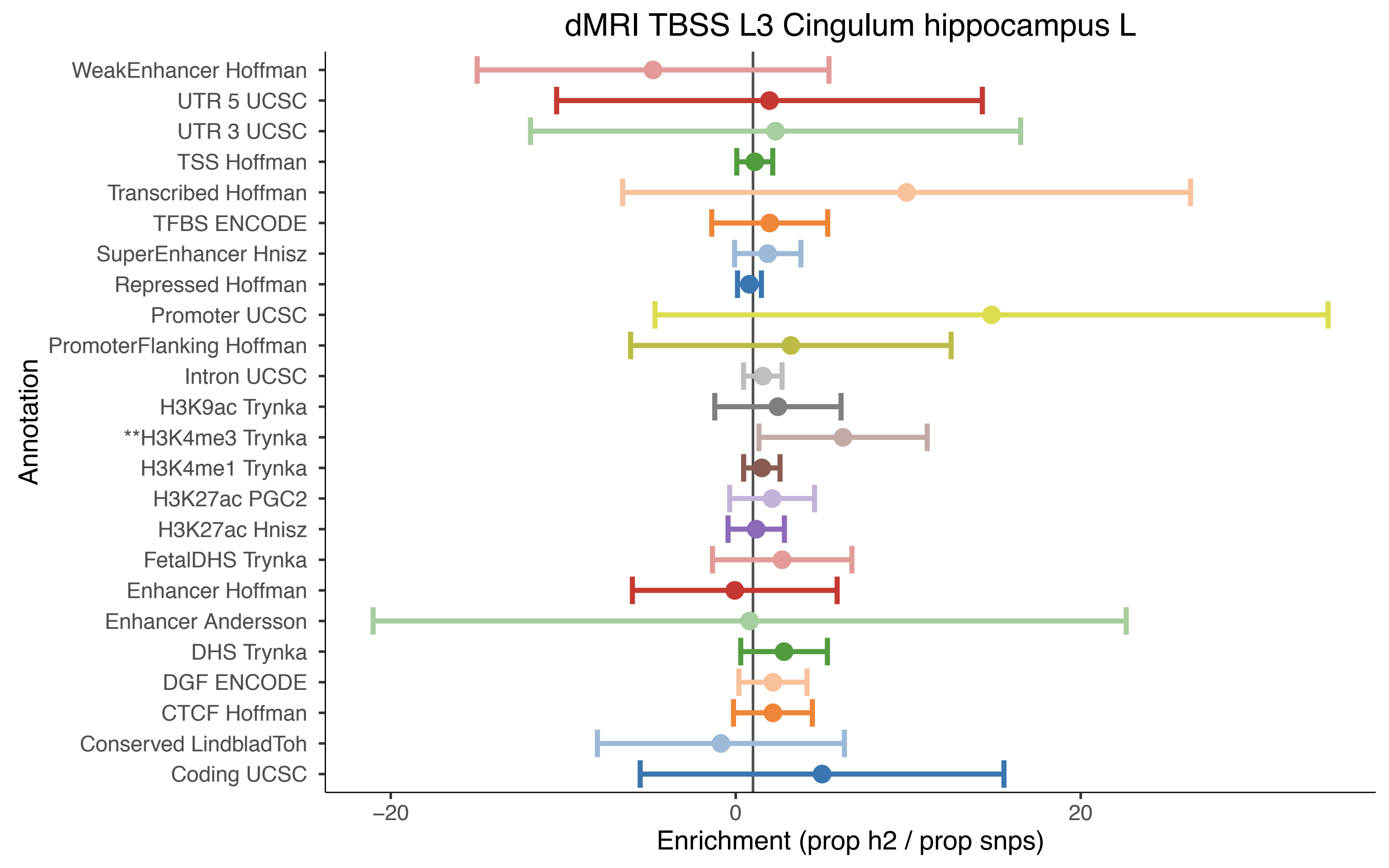
dMRI TBSS L3 Cingulum hippocampus L
WeakEnhancer Hoffman
UTR 5 UCSC
UTR 3 UCSC
TSS Hoffman
Transcribed Hoffman
TFBS ENCODE
SuperEnhancer Hnisz
Repressed Hoffman
Promoter UCSC
PromoterFlanking Hoffman
Intron UCSC
H3K9ac Trynka
**H3K4me3 Trynka
H3K4me1 Trynka
H3K27ac PGC2
H3K27ac Hnisz
FetalDHS Trynka
Enhancer Hoffman
Enhancer Andersson
DHS Trynka
DGF ENCODE
CTCF Hoffman
Conserved LindbladToh
Coding UCSC
Annotation
−20
0
20
Enrichment (prop h2 / prop snps)

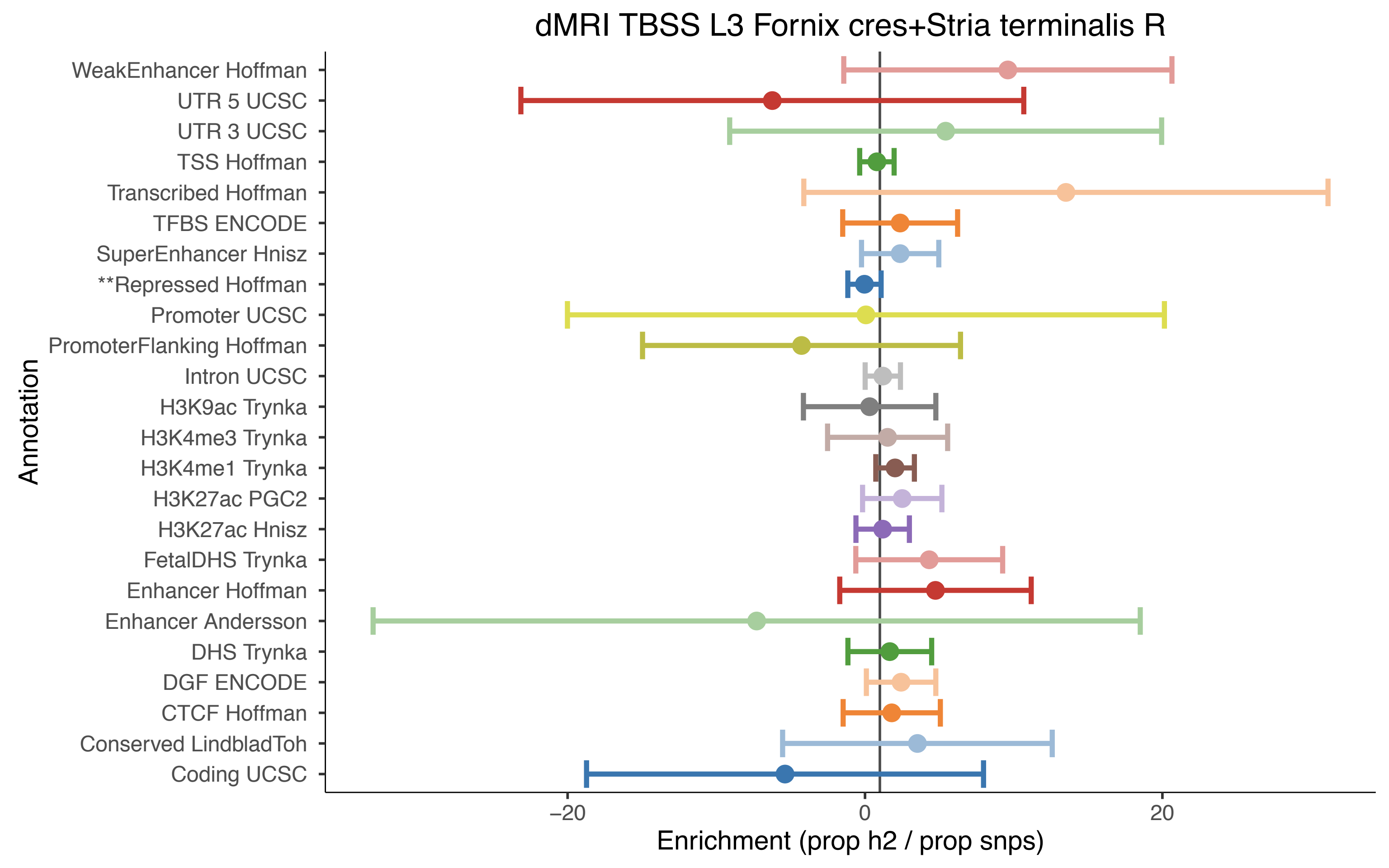

dMRI TBSS L3 Fornix cres+Stria terminalis R
WeakEnhancer Hoffman
UTR 5 UCSC
UTR 3 UCSC
TSS Hoffman
Transcribed Hoffman
TFBS ENCODE
SuperEnhancer Hnisz
**Repressed Hoffman
Promoter UCSC
PromoterFlanking Hoffman
Intron UCSC
H3K9ac Trynka
H3K4me3 Trynka
H3K4me1 Trynka
H3K27ac PGC2
H3K27ac Hnisz
FetalDHS Trynka
Enhancer Hoffman
Enhancer Andersson
DHS Trynka
DGF ENCODE
CTCF Hoffman
Conserved LindbladToh
Coding UCSC
Annotation
−20
0
20
Enrichment (prop h2 / prop snps)

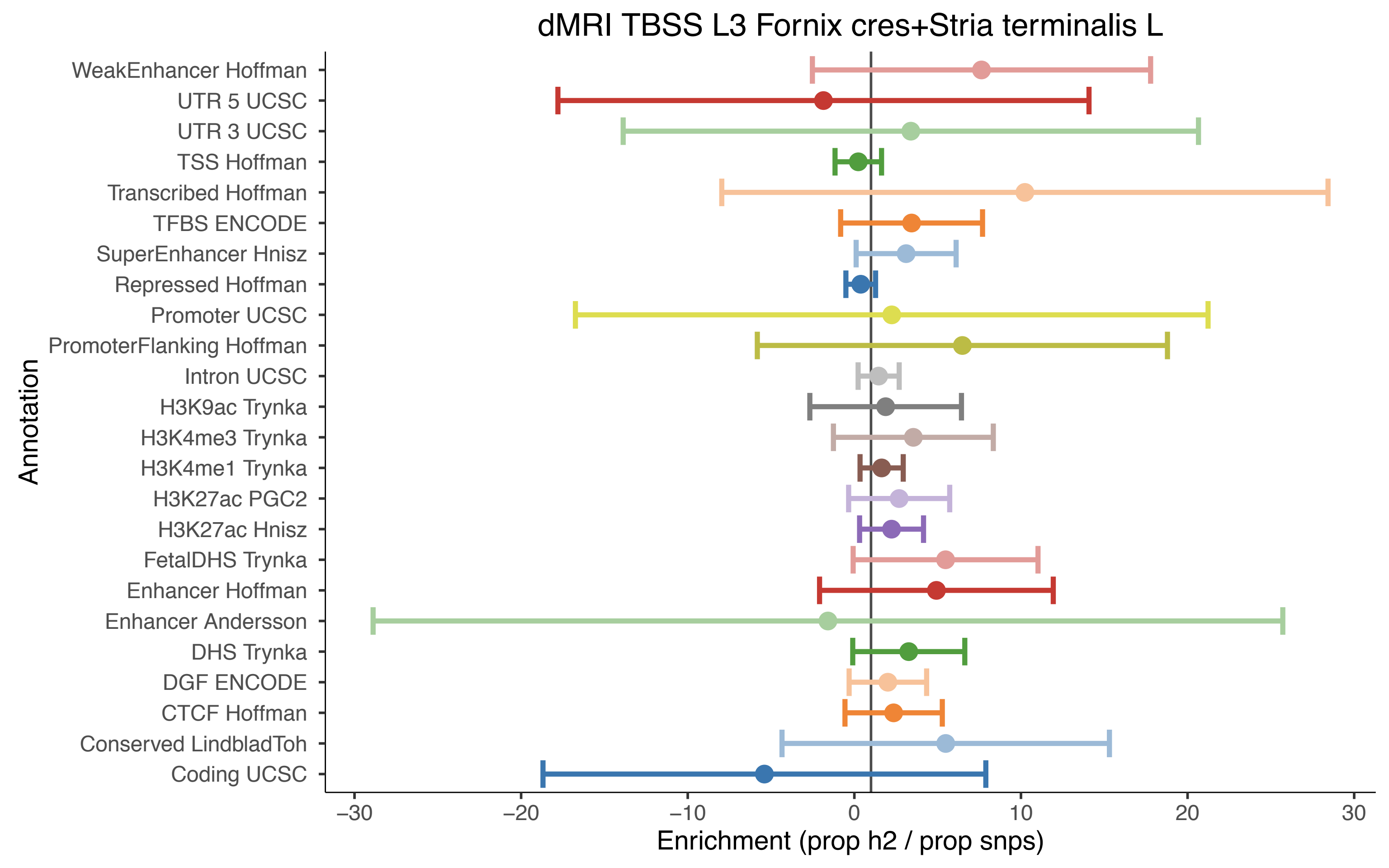
dMRI TBSS L3 Fornix cres+Stria terminalis L
WeakEnhancer Hoffman
UTR 5 UCSC
UTR 3 UCSC
TSS Hoffman
Transcribed Hoffman
TFBS ENCODE
SuperEnhancer Hnisz
Repressed Hoffman
Promoter UCSC
PromoterFlanking Hoffman
Intron UCSC
H3K9ac Trynka
H3K4me3 Trynka
H3K4me1 Trynka
H3K27ac PGC2
H3K27ac Hnisz
FetalDHS Trynka
Enhancer Hoffman
Enhancer Andersson
DHS Trynka
DGF ENCODE
CTCF Hoffman
Conserved LindbladToh
Coding UCSC
Annotation
−30
−20
−10
0
10
20
30
Enrichment (prop h2 / prop snps)

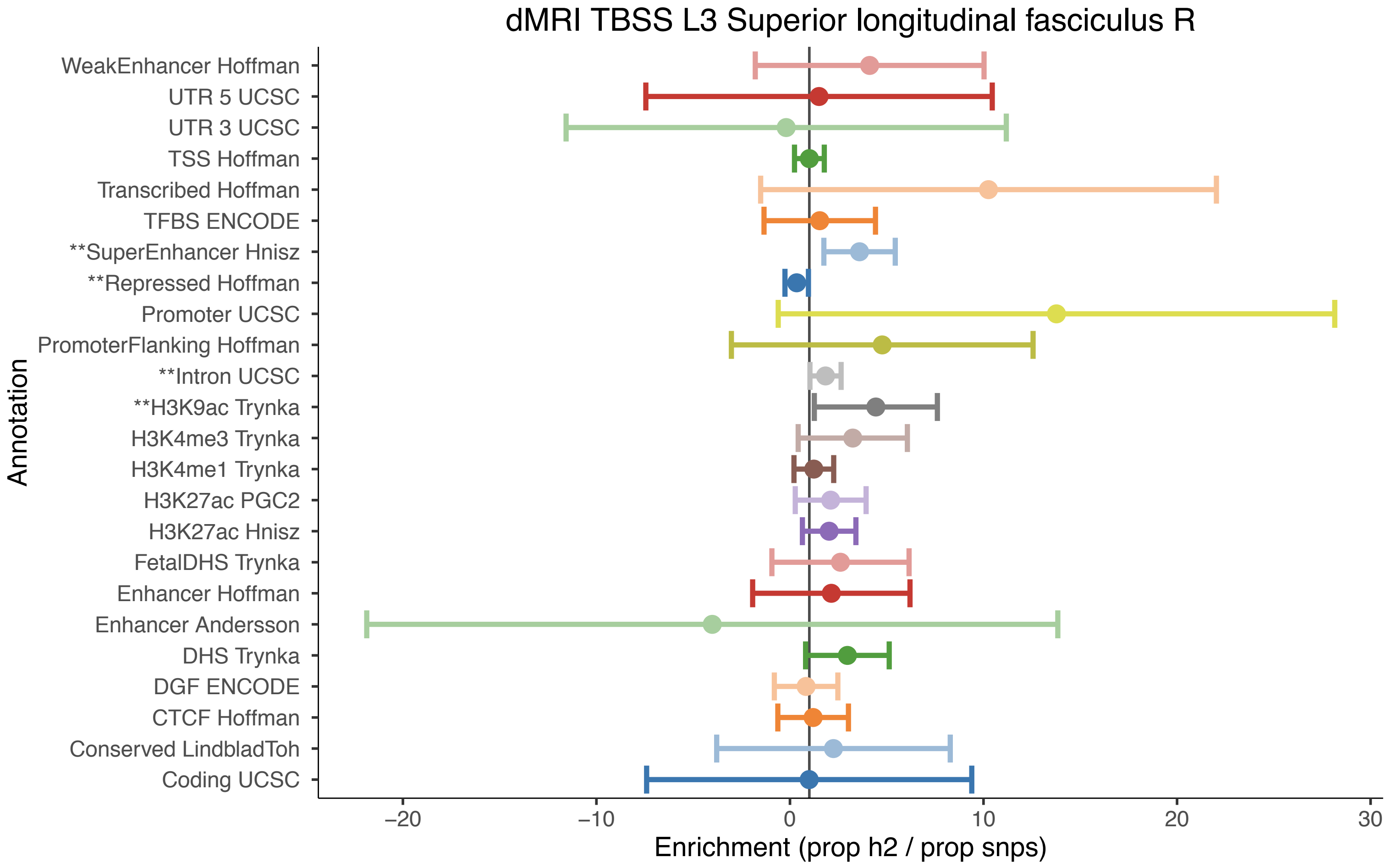
dMRI TBSS L3 Superior longitudinal fasciculus R
WeakEnhancer Hoffman
UTR 5 UCSC
UTR 3 UCSC
TSS Hoffman
Transcribed Hoffman
TFBS ENCODE
**SuperEnhancer Hnisz
**Repressed Hoffman
Promoter UCSC
PromoterFlanking Hoffman
**Intron UCSC
**H3K9ac Trynka
H3K4me3 Trynka
H3K4me1 Trynka
H3K27ac PGC2
H3K27ac Hnisz
FetalDHS Trynka
Enhancer Hoffman
Enhancer Andersson
DHS Trynka
DGF ENCODE
CTCF Hoffman
Conserved LindbladToh
Coding UCSC
Annotation
−20
−10
0
10
20
30
Enrichment (prop h2 / prop snps)

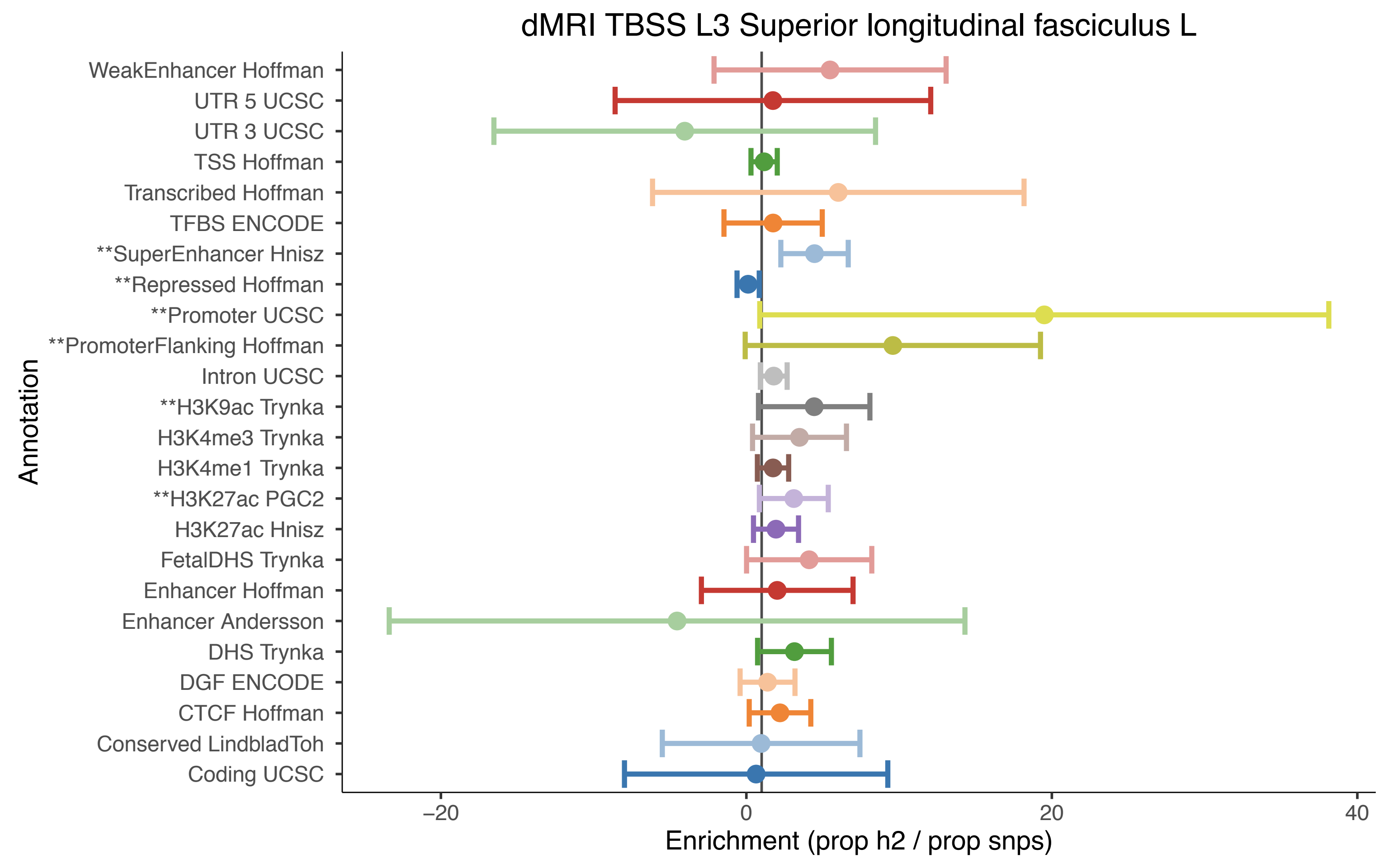

dMRI TBSS L3 Superior longitudinal fasciculus L
WeakEnhancer Hoffman
UTR 5 UCSC
UTR 3 UCSC
TSS Hoffman
Transcribed Hoffman
TFBS ENCODE
**SuperEnhancer Hnisz
**Repressed Hoffman
**Promoter UCSC
**PromoterFlanking Hoffman
Intron UCSC
**H3K9ac Trynka
H3K4me3 Trynka
H3K4me1 Trynka
**H3K27ac PGC2
H3K27ac Hnisz
FetalDHS Trynka
Enhancer Hoffman
Enhancer Andersson
DHS Trynka
DGF ENCODE
CTCF Hoffman
Conserved LindbladToh
Coding UCSC
Annotation
−20
0
20
40
Enrichment (prop h2 / prop snps)

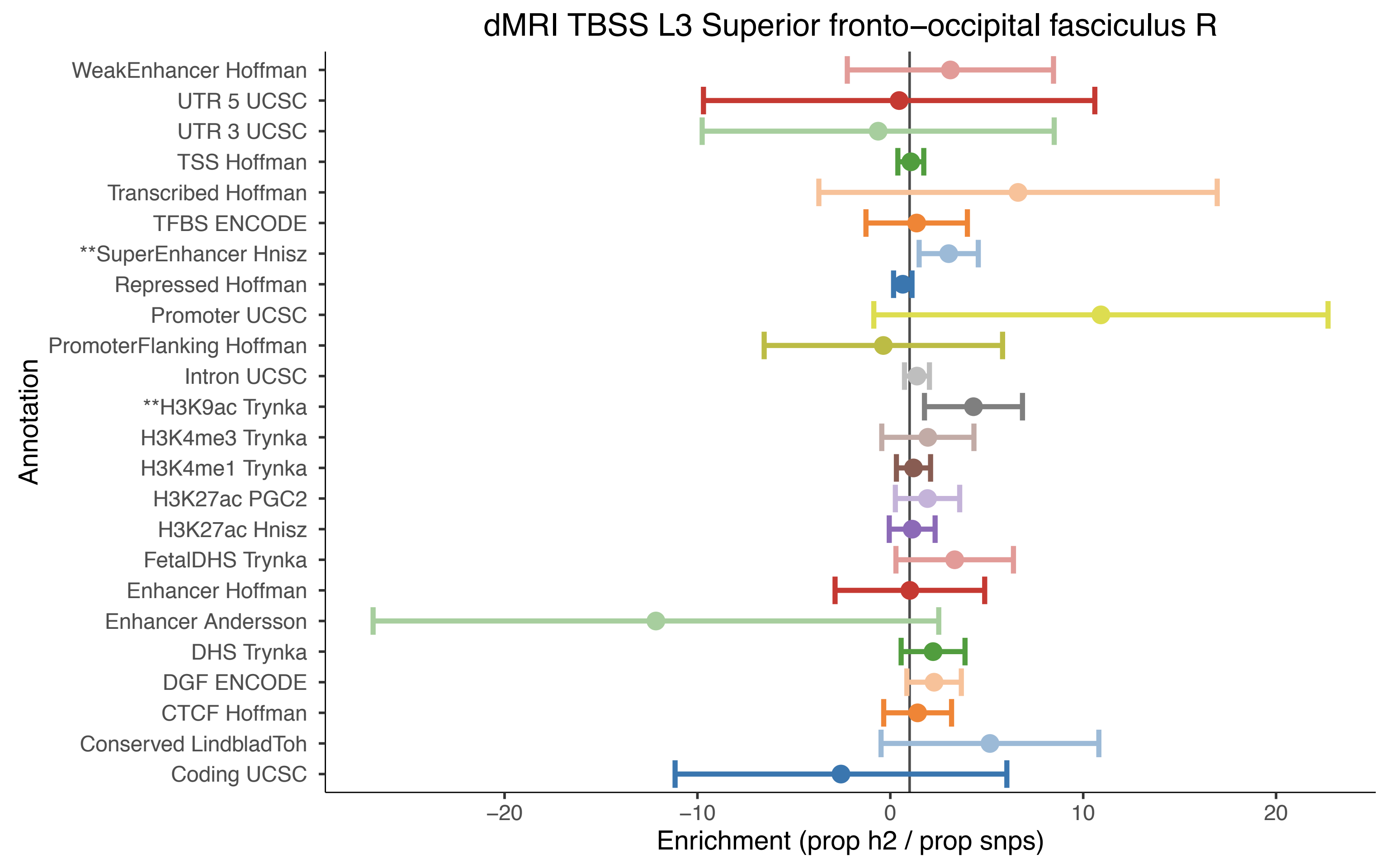

dMRI TBSS L3 Superior fronto−occipital fasciculus R
WeakEnhancer Hoffman
UTR 5 UCSC
UTR 3 UCSC
TSS Hoffman
Transcribed Hoffman
TFBS ENCODE
**SuperEnhancer Hnisz
Repressed Hoffman
Promoter UCSC
PromoterFlanking Hoffman
Intron UCSC
**H3K9ac Trynka
H3K4me3 Trynka
H3K4me1 Trynka
H3K27ac PGC2
H3K27ac Hnisz
FetalDHS Trynka
Enhancer Hoffman
Enhancer Andersson
DHS Trynka
DGF ENCODE
CTCF Hoffman
Conserved LindbladToh
Coding UCSC
Annotation
−20
−10
0
10
20
Enrichment (prop h2 / prop snps)

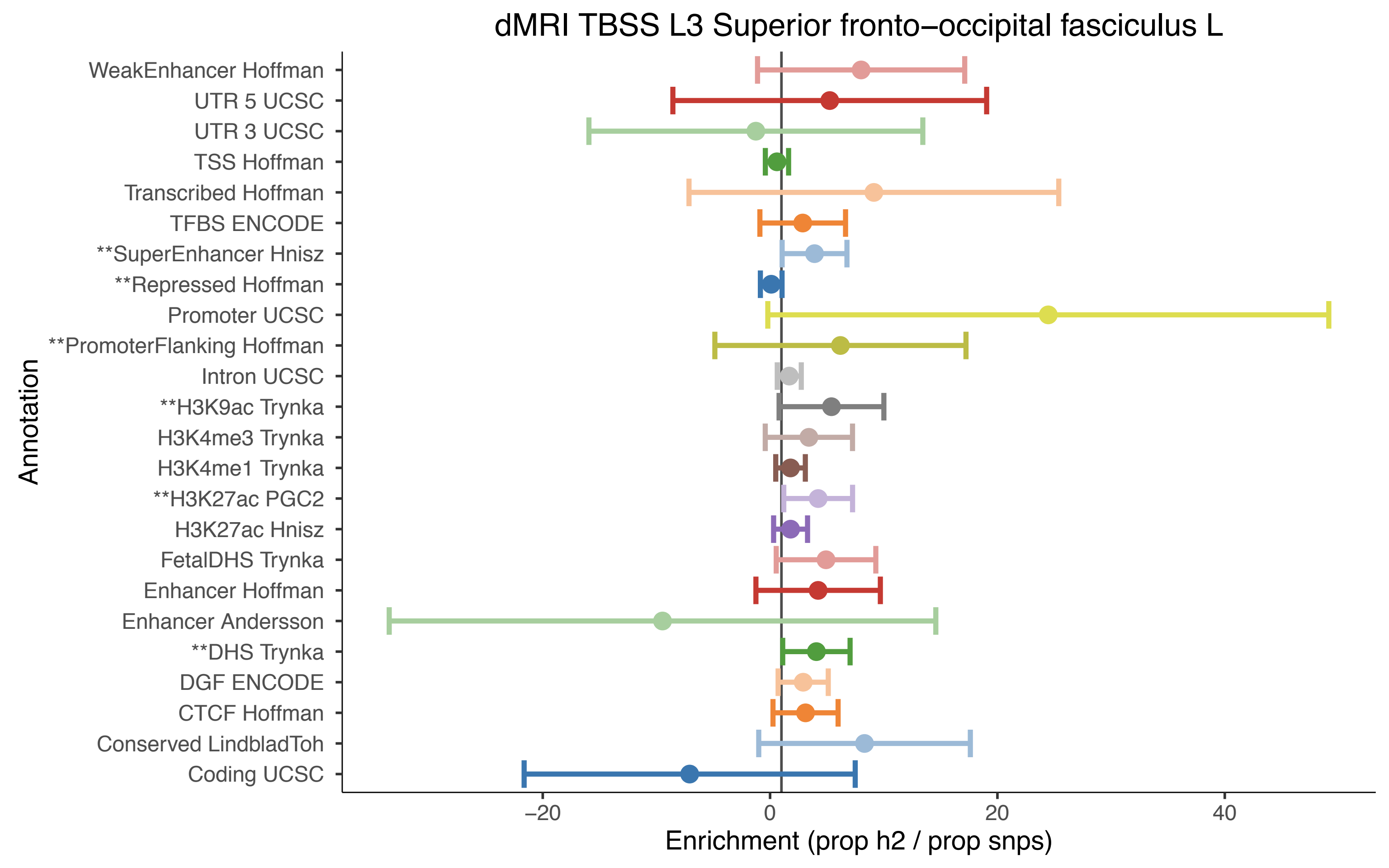

dMRI TBSS L3 Superior fronto−occipital fasciculus L
WeakEnhancer Hoffman
UTR 5 UCSC
UTR 3 UCSC
TSS Hoffman
Transcribed Hoffman
TFBS ENCODE
**SuperEnhancer Hnisz
**Repressed Hoffman
Promoter UCSC
**PromoterFlanking Hoffman
Intron UCSC
**H3K9ac Trynka
H3K4me3 Trynka
H3K4me1 Trynka
**H3K27ac PGC2
H3K27ac Hnisz
FetalDHS Trynka
Enhancer Hoffman
Enhancer Andersson
**DHS Trynka
DGF ENCODE
CTCF Hoffman
Conserved LindbladToh
Coding UCSC
Annotation
−20
0
20
40
Enrichment (prop h2 / prop snps)

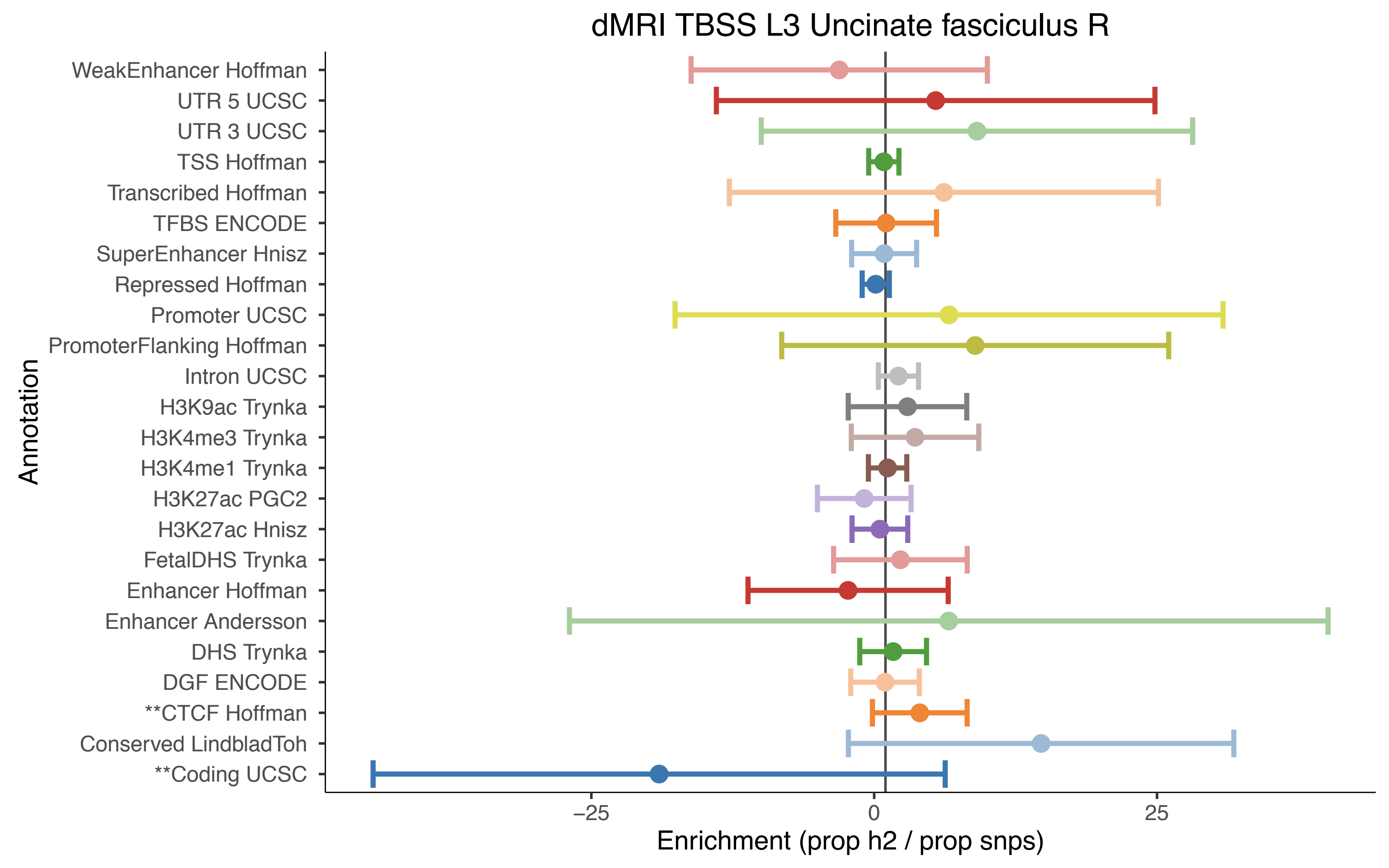

dMRI TBSS L3 Uncinate fasciculus R
WeakEnhancer Hoffman
UTR 5 UCSC
UTR 3 UCSC
TSS Hoffman
Transcribed Hoffman
TFBS ENCODE
SuperEnhancer Hnisz
Repressed Hoffman
Promoter UCSC
PromoterFlanking Hoffman
Intron UCSC
H3K9ac Trynka
H3K4me3 Trynka
H3K4me1 Trynka
H3K27ac PGC2
H3K27ac Hnisz
FetalDHS Trynka
Enhancer Hoffman
Enhancer Andersson
DHS Trynka
DGF ENCODE
**CTCF Hoffman
Conserved LindbladToh
**Coding UCSC
Annotation
−25
0
25
Enrichment (prop h2 / prop snps)

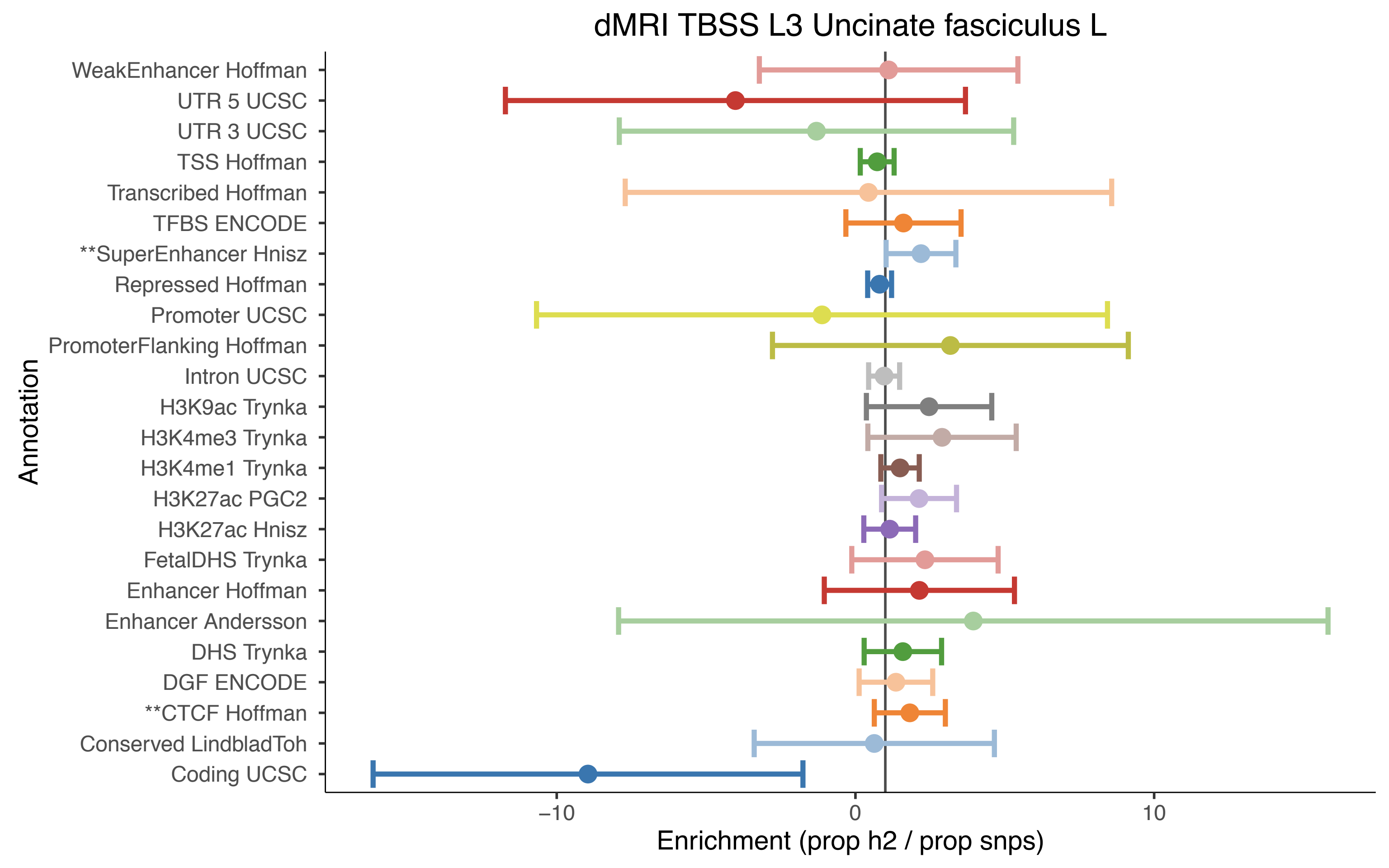
dMRI TBSS L3 Uncinate fasciculus L
WeakEnhancer Hoffman
UTR 5 UCSC
UTR 3 UCSC
TSS Hoffman
Transcribed Hoffman
TFBS ENCODE
**SuperEnhancer Hnisz
Repressed Hoffman
Promoter UCSC
PromoterFlanking Hoffman
Intron UCSC
H3K9ac Trynka
H3K4me3 Trynka
H3K4me1 Trynka
H3K27ac PGC2
H3K27ac Hnisz
FetalDHS Trynka
Enhancer Hoffman
Enhancer Andersson
DHS Trynka
DGF ENCODE
**CTCF Hoffman
Conserved LindbladToh
Coding UCSC
Annotation
−10
0
10
Enrichment (prop h2 / prop snps)

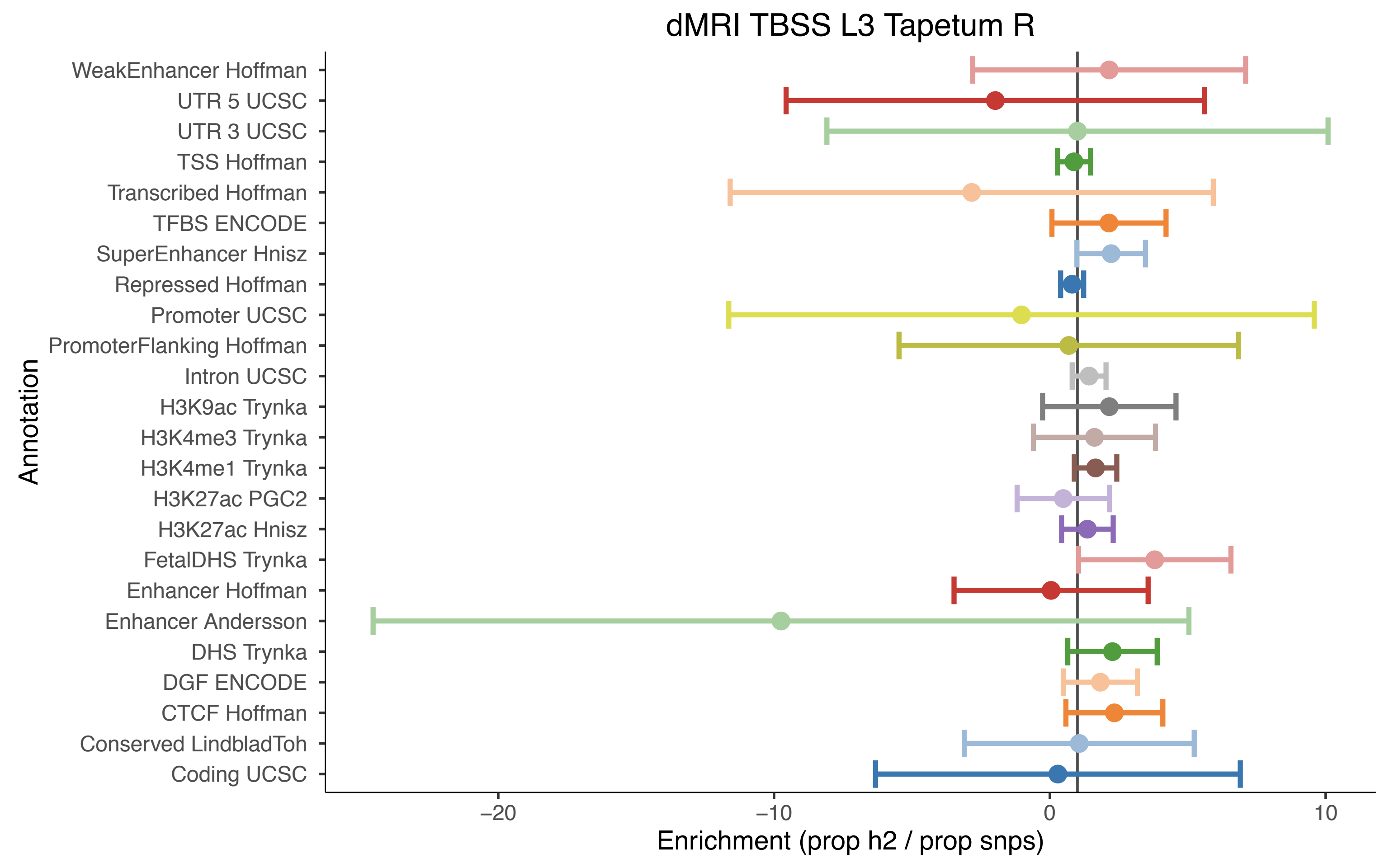

dMRI TBSS L3 Tapetum R
Annotation
WeakEnhancer Hoffman
UTR 5 UCSC
UTR 3 UCSC
TSS Hoffman
Transcribed Hoffman
TFBS ENCODE
SuperEnhancer Hnisz
Repressed Hoffman
Promoter UCSC
PromoterFlanking Hoffman
Intron UCSC
H3K9ac Trynka
H3K4me3 Trynka
H3K4me1 Trynka
H3K27ac PGC2
H3K27ac Hnisz
FetalDHS Trynka
Enhancer Hoffman
Enhancer Andersson
DHS Trynka
DGF ENCODE
CTCF Hoffman
Conserved LindbladToh
Coding UCSC
−20
−10
0
10
Enrichment (prop h2 / prop snps)

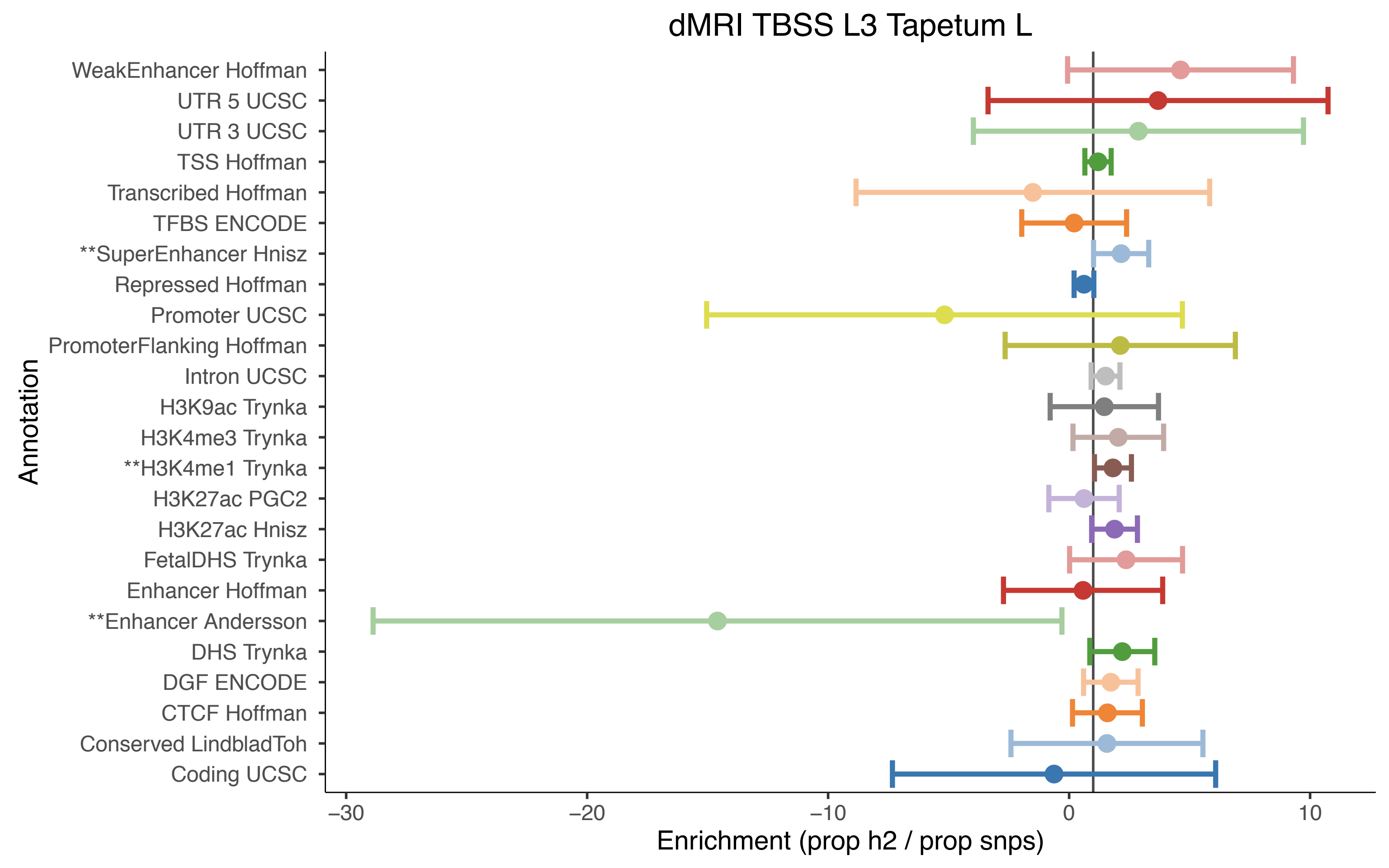

dMRI TBSS L3 Tapetum L
WeakEnhancer Hoffman
UTR 5 UCSC
UTR 3 UCSC
TSS Hoffman
Transcribed Hoffman
TFBS ENCODE
**SuperEnhancer Hnisz
Repressed Hoffman
Promoter UCSC
PromoterFlanking Hoffman
Intron UCSC
H3K9ac Trynka
H3K4me3 Trynka
**H3K4me1 Trynka
H3K27ac PGC2
H3K27ac Hnisz
FetalDHS Trynka
Enhancer Hoffman
**Enhancer Andersson
DHS Trynka
DGF ENCODE
CTCF Hoffman
Conserved LindbladToh
Coding UCSC
Annotation
−30
−20
−10
0
10
Enrichment (prop h2 / prop snps)

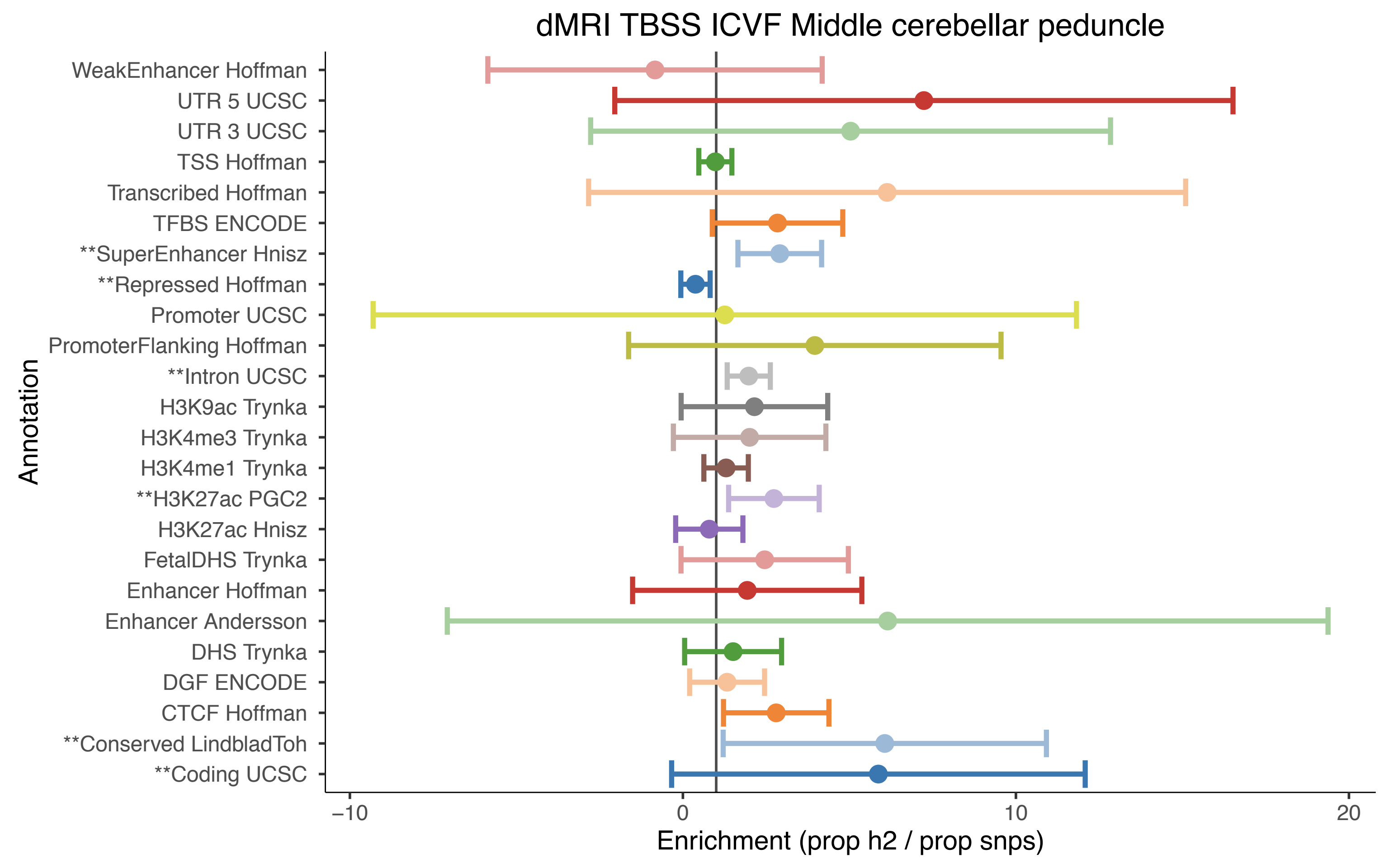
dMRI TBSS ICVF Middle cerebellar peduncle
WeakEnhancer Hoffman
UTR 5 UCSC
UTR 3 UCSC
TSS Hoffman
Transcribed Hoffman
TFBS ENCODE
**SuperEnhancer Hnisz
**Repressed Hoffman
Promoter UCSC
PromoterFlanking Hoffman
**Intron UCSC
H3K9ac Trynka
H3K4me3 Trynka
H3K4me1 Trynka
**H3K27ac PGC2
H3K27ac Hnisz
FetalDHS Trynka
Enhancer Hoffman
Enhancer Andersson
DHS Trynka
DGF ENCODE
CTCF Hoffman
**Conserved LindbladToh
**Coding UCSC
Annotation
−10
0
10
20
Enrichment (prop h2 / prop snps)

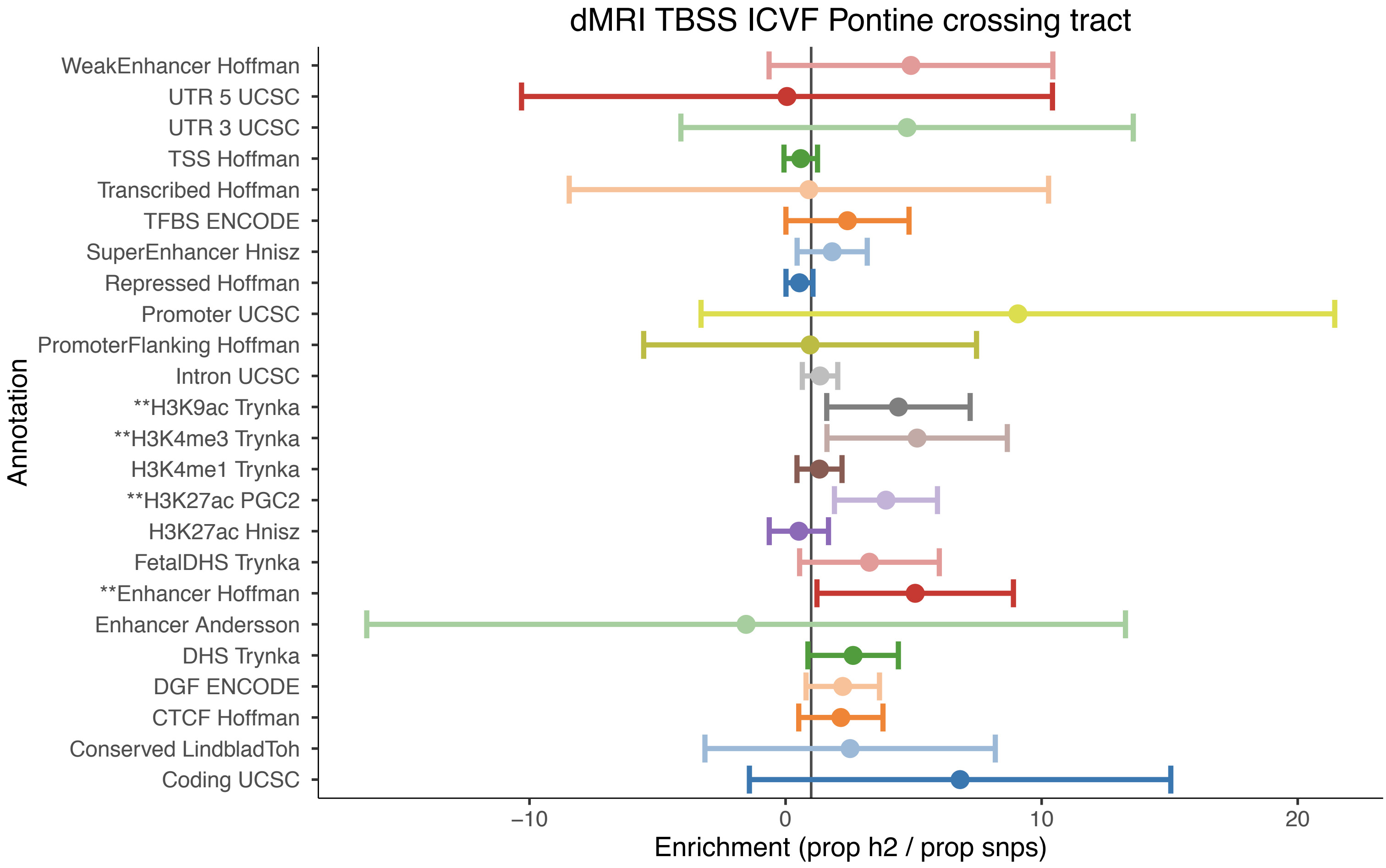
dMRI TBSS ICVF Pontine crossing tract
Annotation
WeakEnhancer Hoffman
UTR 5 UCSC
UTR 3 UCSC
TSS Hoffman
Transcribed Hoffman
TFBS ENCODE
SuperEnhancer Hnisz
Repressed Hoffman
Promoter UCSC
PromoterFlanking Hoffman
Intron UCSC
**H3K9ac Trynka
**H3K4me3 Trynka
H3K4me1 Trynka
**H3K27ac PGC2
H3K27ac Hnisz
FetalDHS Trynka
**Enhancer Hoffman
Enhancer Andersson
DHS Trynka
DGF ENCODE
CTCF Hoffman
Conserved LindbladToh
Coding UCSC
−10
0
10
20
Enrichment (prop h2 / prop snps)

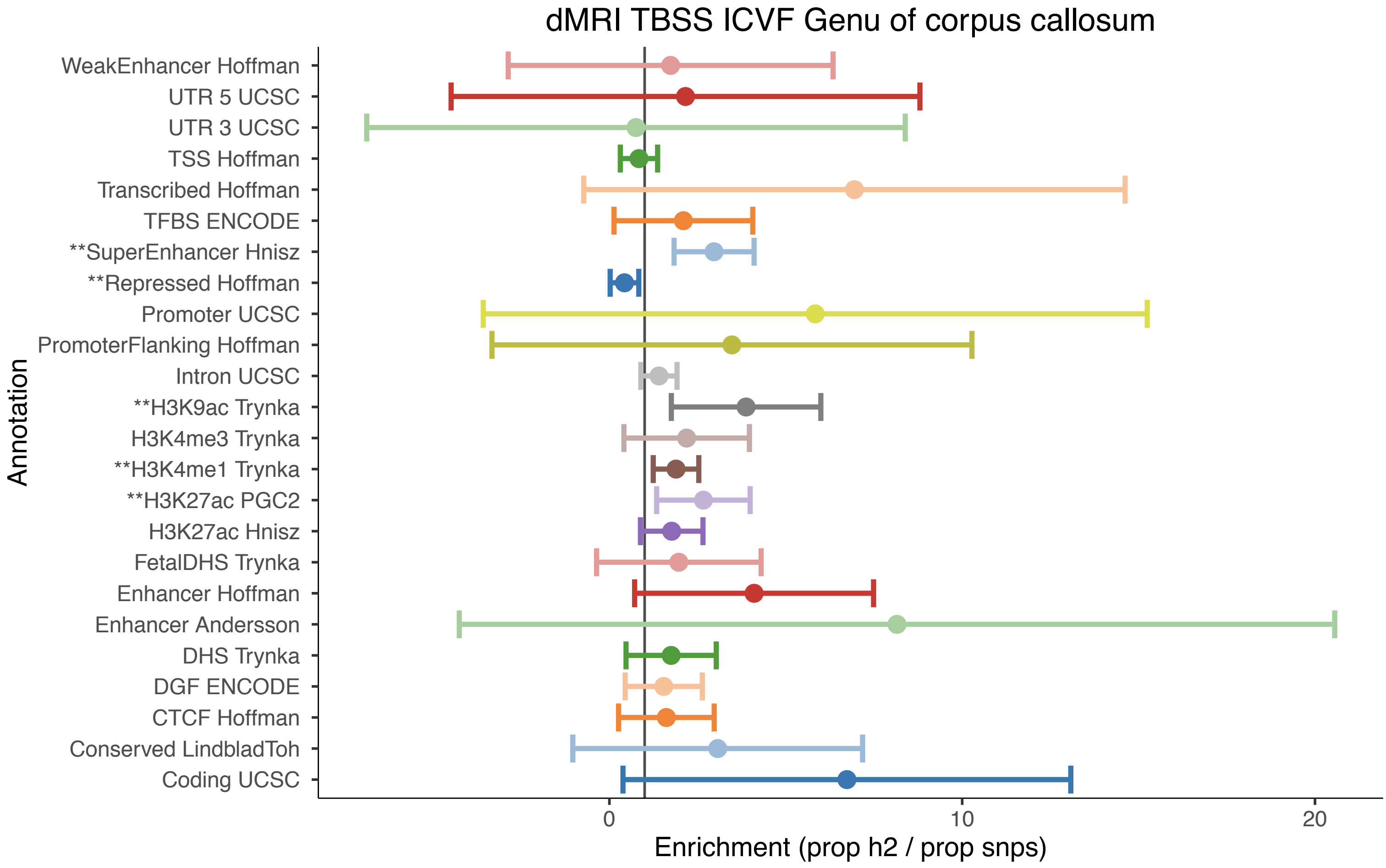

dMRI TBSS ICVF Genu of corpus callosum
WeakEnhancer Hoffman
UTR 5 UCSC
UTR 3 UCSC
TSS Hoffman
Transcribed Hoffman
TFBS ENCODE
**SuperEnhancer Hnisz
**Repressed Hoffman
Promoter UCSC
PromoterFlanking Hoffman
Intron UCSC
**H3K9ac Trynka
H3K4me3 Trynka
**H3K4me1 Trynka
**H3K27ac PGC2
H3K27ac Hnisz
FetalDHS Trynka
Enhancer Hoffman
Enhancer Andersson
DHS Trynka
DGF ENCODE
CTCF Hoffman
Conserved LindbladToh
Coding UCSC
Annotation
0
10
20
Enrichment (prop h2 / prop snps)

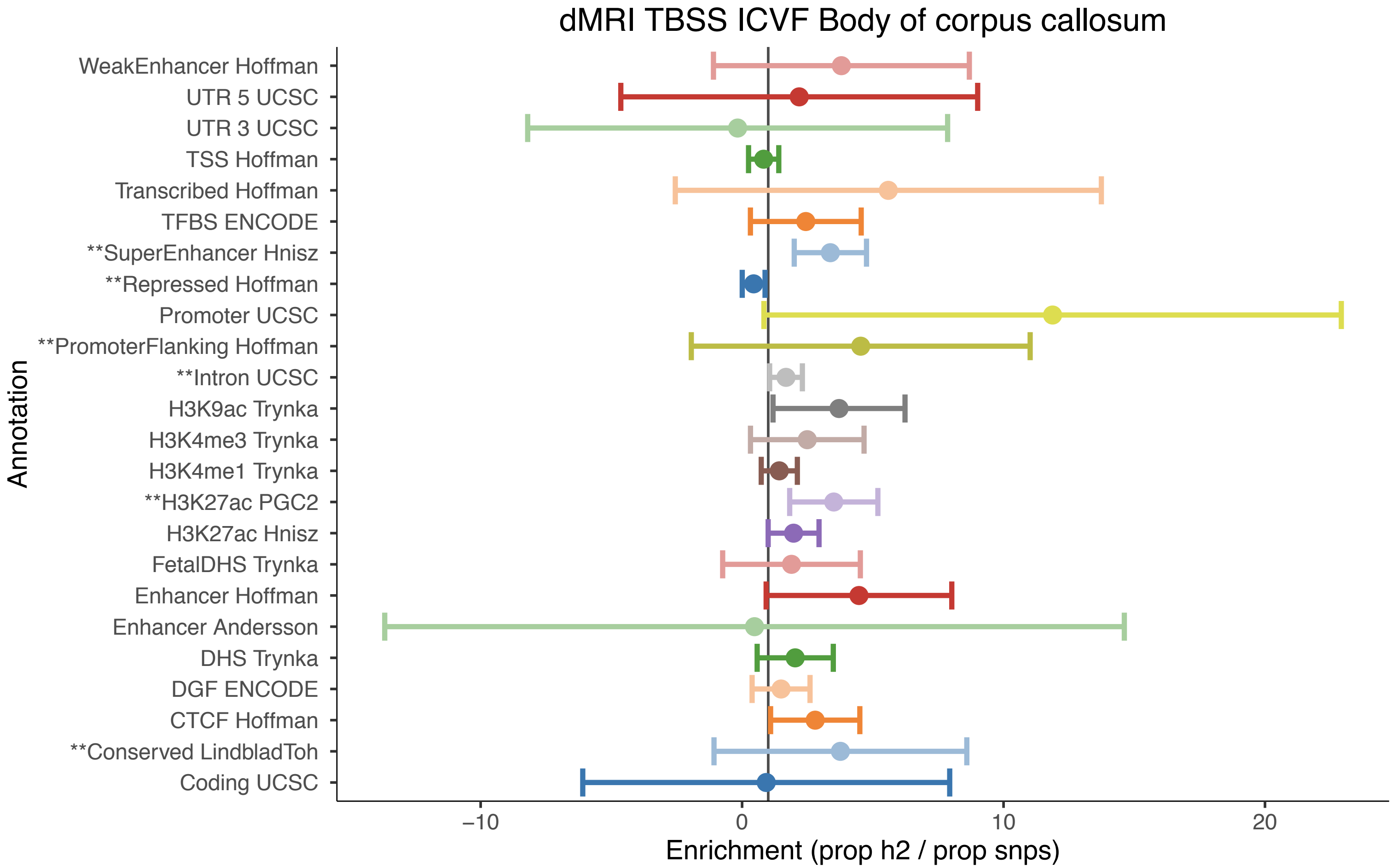

dMRI TBSS ICVF Body of corpus callosum
WeakEnhancer Hoffman
UTR 5 UCSC
UTR 3 UCSC
TSS Hoffman
Transcribed Hoffman
TFBS ENCODE
**SuperEnhancer Hnisz
**Repressed Hoffman
Promoter UCSC
**PromoterFlanking Hoffman
**Intron UCSC
H3K9ac Trynka
H3K4me3 Trynka
H3K4me1 Trynka
**H3K27ac PGC2
H3K27ac Hnisz
FetalDHS Trynka
Enhancer Hoffman
Enhancer Andersson
DHS Trynka
DGF ENCODE
CTCF Hoffman
**Conserved LindbladToh
Coding UCSC
Annotation
−10
0
10
20
Enrichment (prop h2 / prop snps)

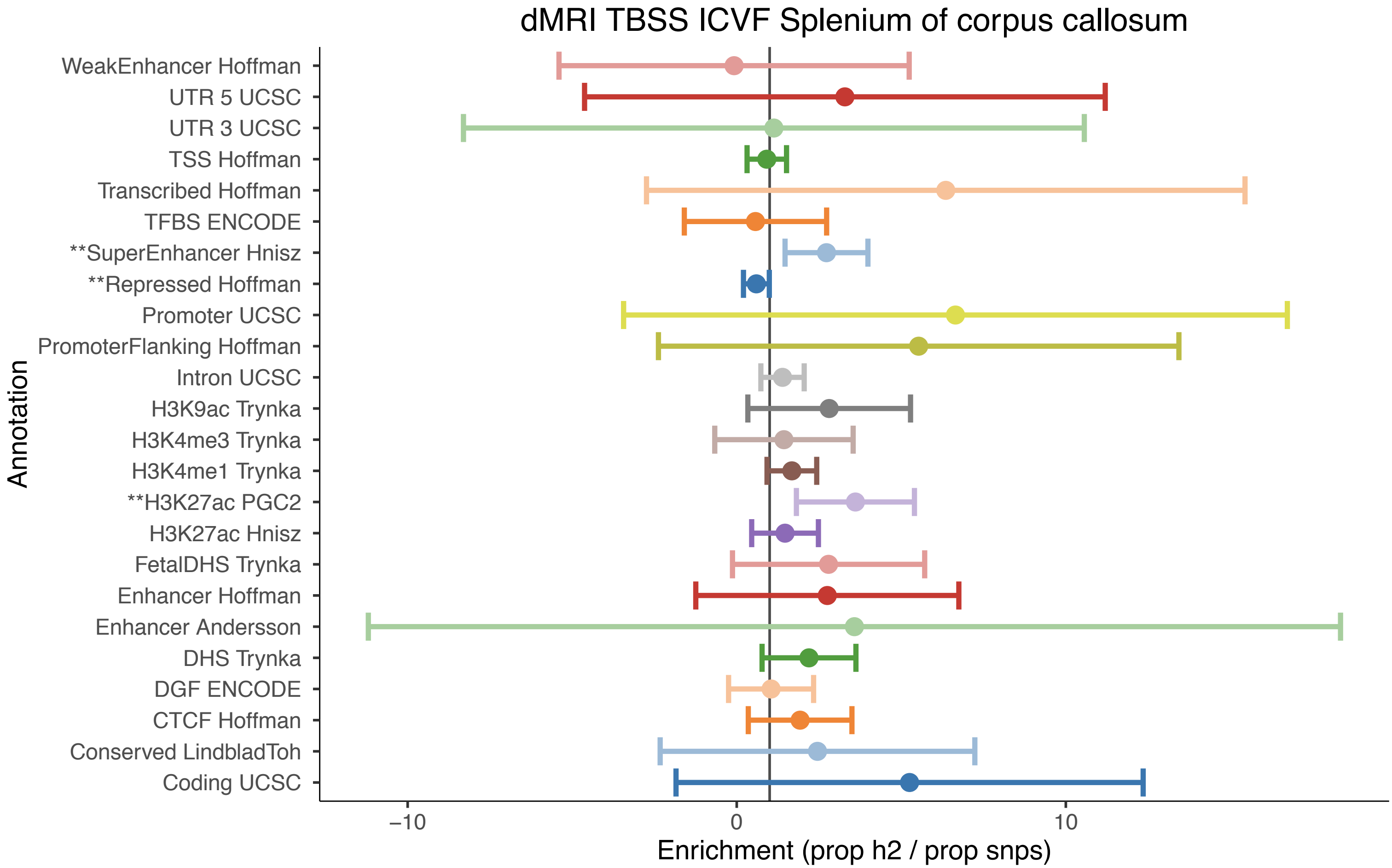

dMRI TBSS ICVF Splenium of corpus callosum
WeakEnhancer Hoffman
UTR 5 UCSC
UTR 3 UCSC
TSS Hoffman
Transcribed Hoffman
TFBS ENCODE
**SuperEnhancer Hnisz
**Repressed Hoffman
Promoter UCSC
PromoterFlanking Hoffman
Intron UCSC
H3K9ac Trynka
H3K4me3 Trynka
H3K4me1 Trynka
**H3K27ac PGC2
H3K27ac Hnisz
FetalDHS Trynka
Enhancer Hoffman
Enhancer Andersson
DHS Trynka
DGF ENCODE
CTCF Hoffman
Conserved LindbladToh
Coding UCSC
Annotation
−10
0
10
Enrichment (prop h2 / prop snps)

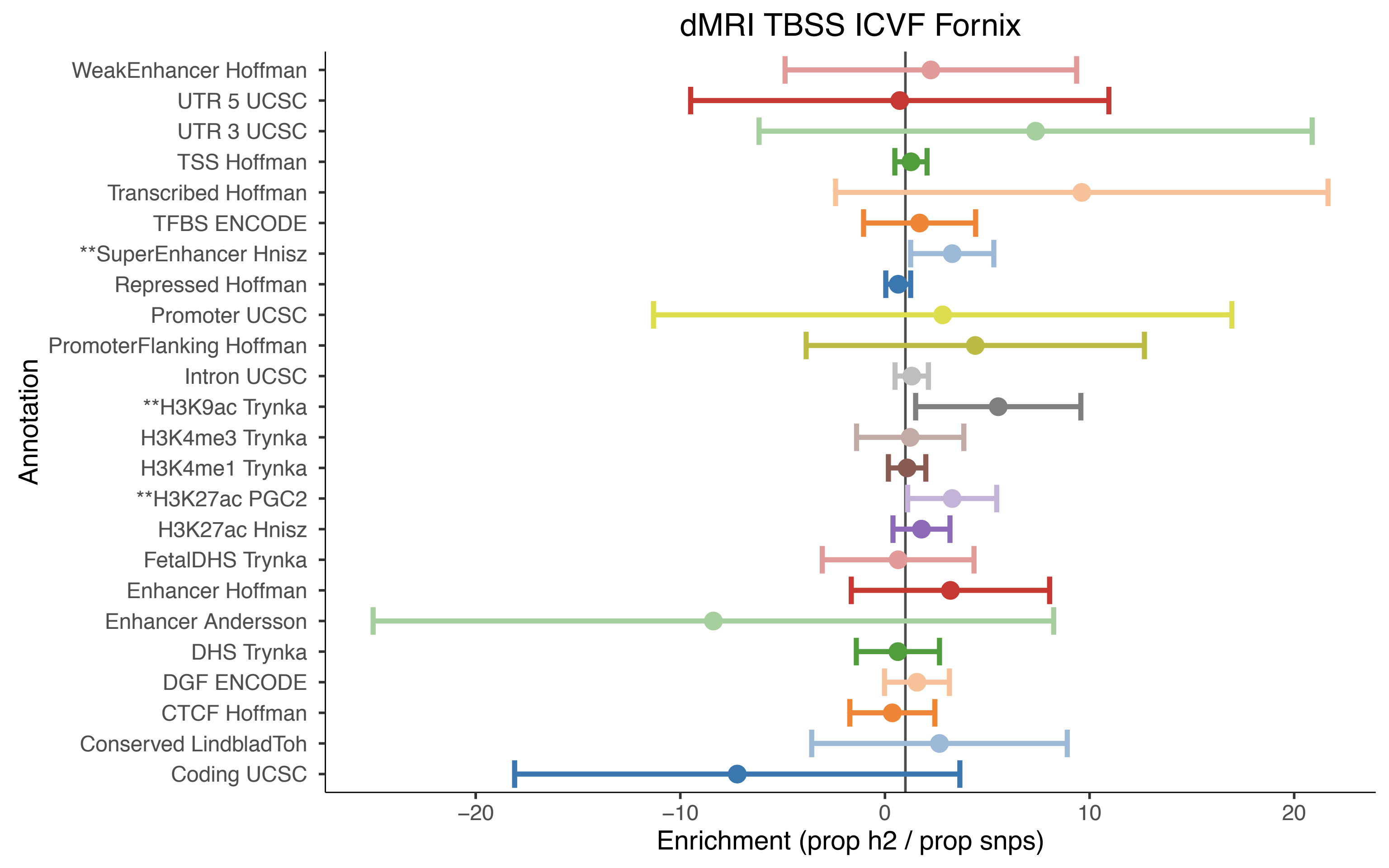
dMRI TBSS ICVF Fornix
WeakEnhancer Hoffman
UTR 5 UCSC
UTR 3 UCSC
TSS Hoffman
Transcribed Hoffman
TFBS ENCODE
**SuperEnhancer Hnisz
Repressed Hoffman
Promoter UCSC
PromoterFlanking Hoffman
Intron UCSC
**H3K9ac Trynka
H3K4me3 Trynka
H3K4me1 Trynka
**H3K27ac PGC2
H3K27ac Hnisz
FetalDHS Trynka
Enhancer Hoffman
Enhancer Andersson
DHS Trynka
DGF ENCODE
CTCF Hoffman
Conserved LindbladToh
Coding UCSC
Annotation
−20
−10
0
10
20
Enrichment (prop h2 / prop snps)

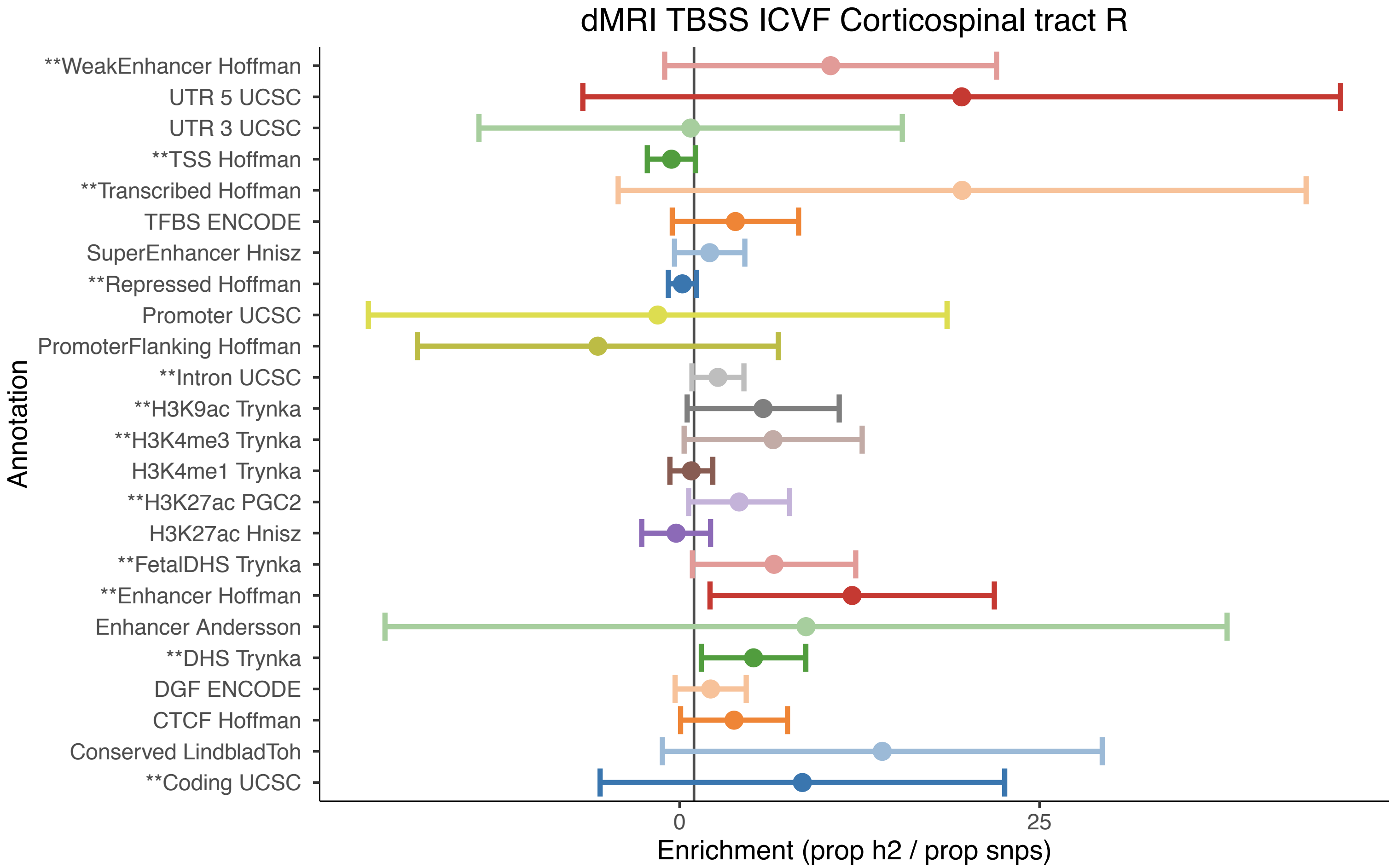

dMRI TBSS ICVF Corticospinal tract R
**WeakEnhancer Hoffman
UTR 5 UCSC
UTR 3 UCSC
**TSS Hoffman
**Transcribed Hoffman
TFBS ENCODE
SuperEnhancer Hnisz
**Repressed Hoffman
Promoter UCSC
PromoterFlanking Hoffman
**Intron UCSC
**H3K9ac Trynka
**H3K4me3 Trynka
H3K4me1 Trynka
**H3K27ac PGC2
H3K27ac Hnisz
**FetalDHS Trynka
**Enhancer Hoffman
Enhancer Andersson
**DHS Trynka
DGF ENCODE
CTCF Hoffman
Conserved LindbladToh
**Coding UCSC
Annotation
0
25
Enrichment (prop h2 / prop snps)

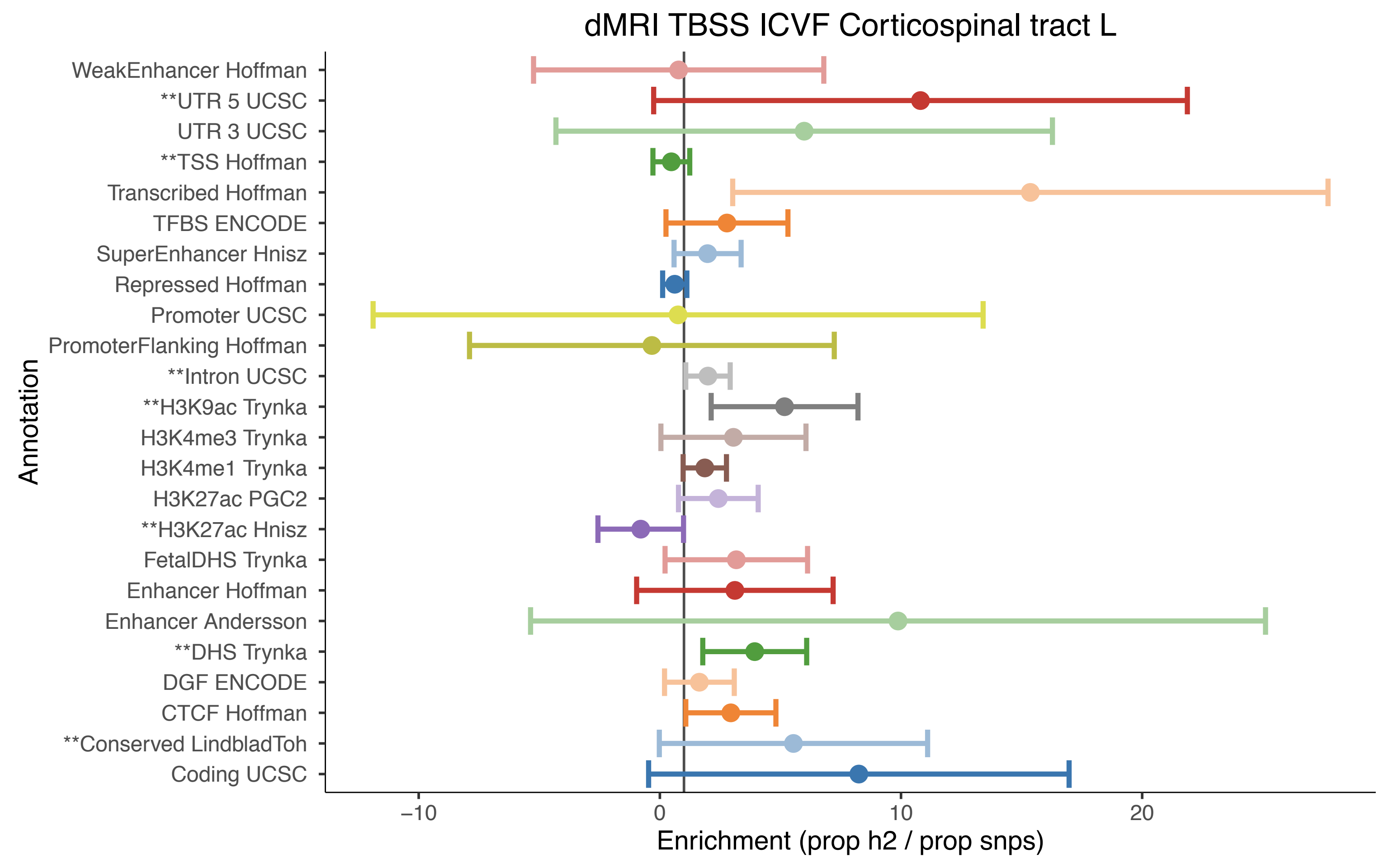
dMRI TBSS ICVF Corticospinal tract L
WeakEnhancer Hoffman
**UTR 5 UCSC
UTR 3 UCSC
**TSS Hoffman
Transcribed Hoffman
TFBS ENCODE
SuperEnhancer Hnisz
Repressed Hoffman
Promoter UCSC
PromoterFlanking Hoffman
**Intron UCSC
**H3K9ac Trynka
H3K4me3 Trynka
H3K4me1 Trynka
H3K27ac PGC2
**H3K27ac Hnisz
FetalDHS Trynka
Enhancer Hoffman
Enhancer Andersson
**DHS Trynka
DGF ENCODE
CTCF Hoffman
**Conserved LindbladToh
Coding UCSC
Annotation
−10
0
10
20
Enrichment (prop h2 / prop snps)

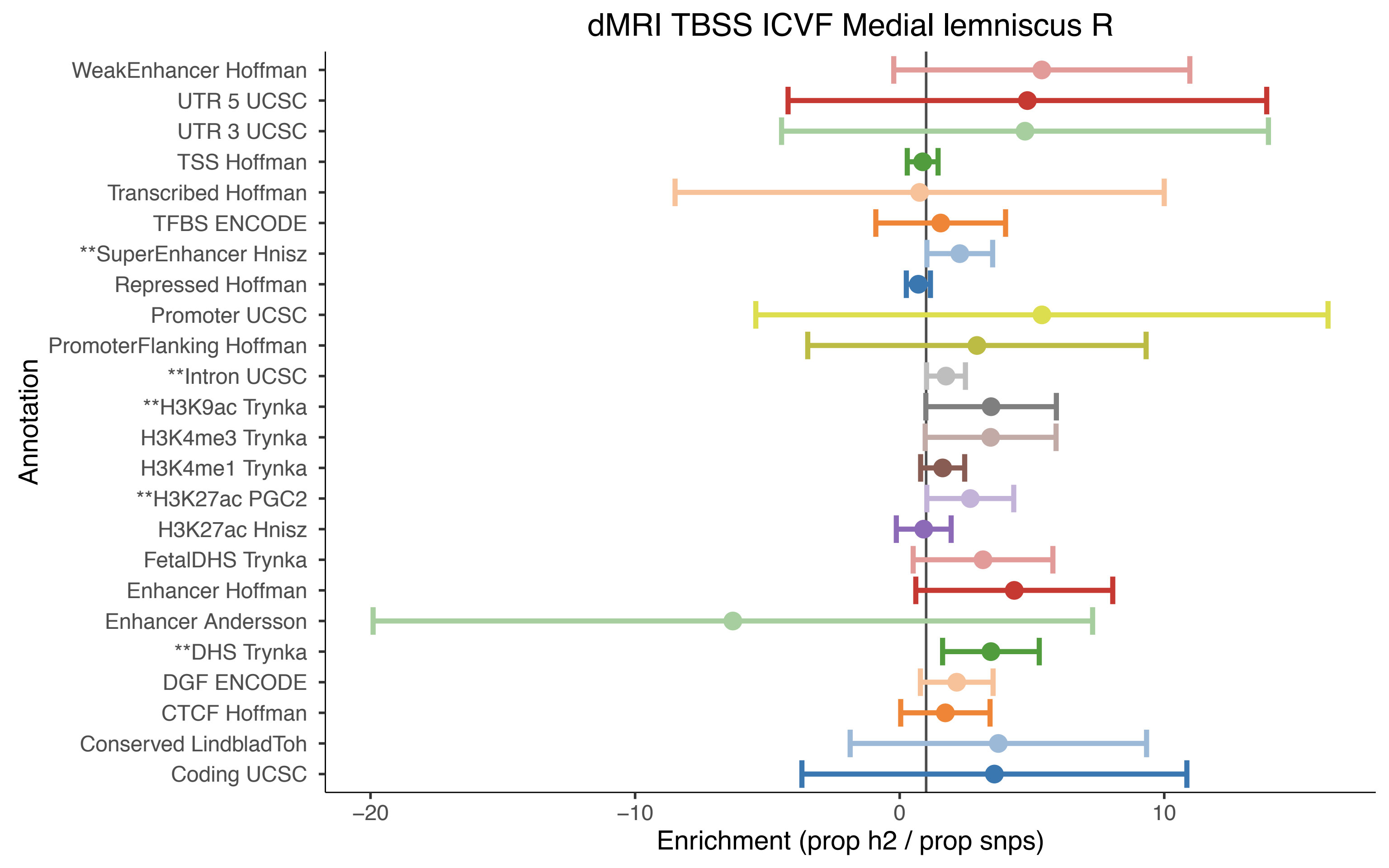

dMRI TBSS ICVF Medial lemniscus R
WeakEnhancer Hoffman
UTR 5 UCSC
UTR 3 UCSC
TSS Hoffman
Transcribed Hoffman
TFBS ENCODE
**SuperEnhancer Hnisz
Repressed Hoffman
Promoter UCSC
PromoterFlanking Hoffman
**Intron UCSC
**H3K9ac Trynka
H3K4me3 Trynka
H3K4me1 Trynka
**H3K27ac PGC2
H3K27ac Hnisz
FetalDHS Trynka
Enhancer Hoffman
Enhancer Andersson
**DHS Trynka
DGF ENCODE
CTCF Hoffman
Conserved LindbladToh
Coding UCSC
Annotation
−20
−10
0
10
Enrichment (prop h2 / prop snps)

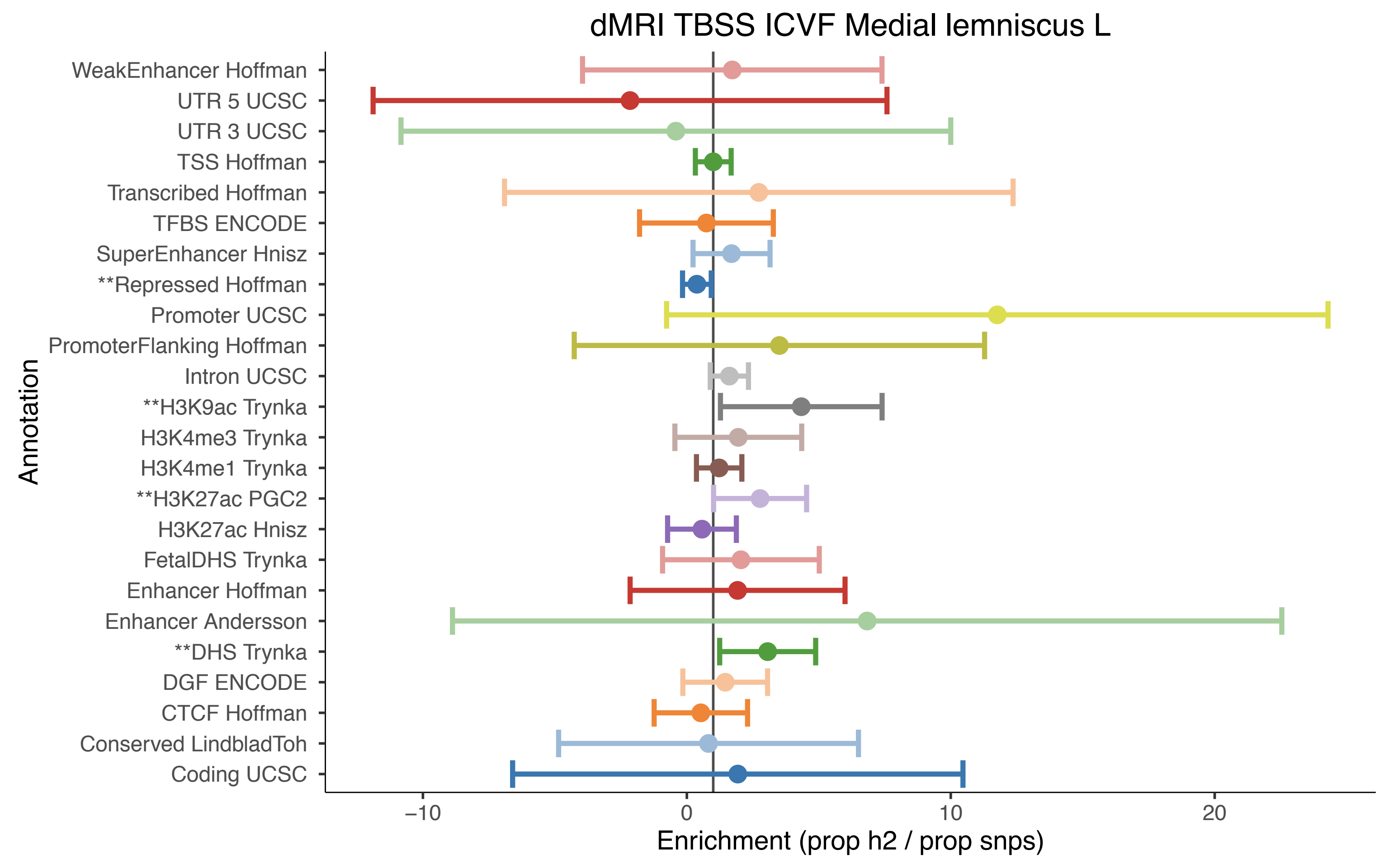

dMRI TBSS ICVF Medial lemniscus L
WeakEnhancer Hoffman
UTR 5 UCSC
UTR 3 UCSC
TSS Hoffman
Transcribed Hoffman
TFBS ENCODE
SuperEnhancer Hnisz
**Repressed Hoffman
Promoter UCSC
PromoterFlanking Hoffman
Intron UCSC
**H3K9ac Trynka
H3K4me3 Trynka
H3K4me1 Trynka
**H3K27ac PGC2
H3K27ac Hnisz
FetalDHS Trynka
Enhancer Hoffman
Enhancer Andersson
**DHS Trynka
DGF ENCODE
CTCF Hoffman
Conserved LindbladToh
Coding UCSC
Annotation
−10
0
10
20
Enrichment (prop h2 / prop snps)

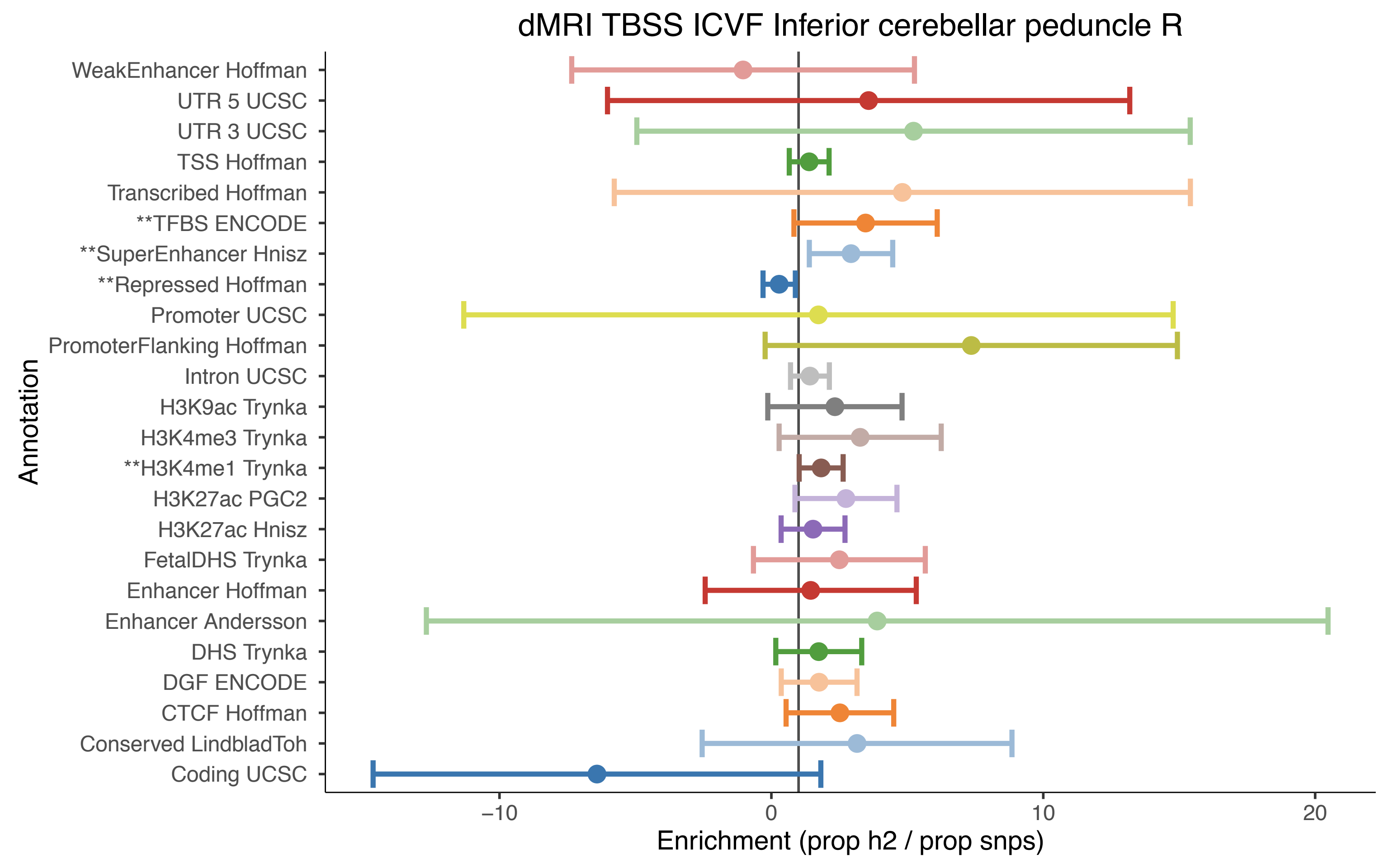
dMRI TBSS ICVF Inferior cerebellar peduncle R
WeakEnhancer Hoffman
UTR 5 UCSC
UTR 3 UCSC
TSS Hoffman
Transcribed Hoffman
**TFBS ENCODE
**SuperEnhancer Hnisz
**Repressed Hoffman
Promoter UCSC
PromoterFlanking Hoffman
Intron UCSC
H3K9ac Trynka
H3K4me3 Trynka
**H3K4me1 Trynka
H3K27ac PGC2
H3K27ac Hnisz
FetalDHS Trynka
Enhancer Hoffman
Enhancer Andersson
DHS Trynka
DGF ENCODE
CTCF Hoffman
Conserved LindbladToh
Coding UCSC
Annotation
−10
0
10
20
Enrichment (prop h2 / prop snps)

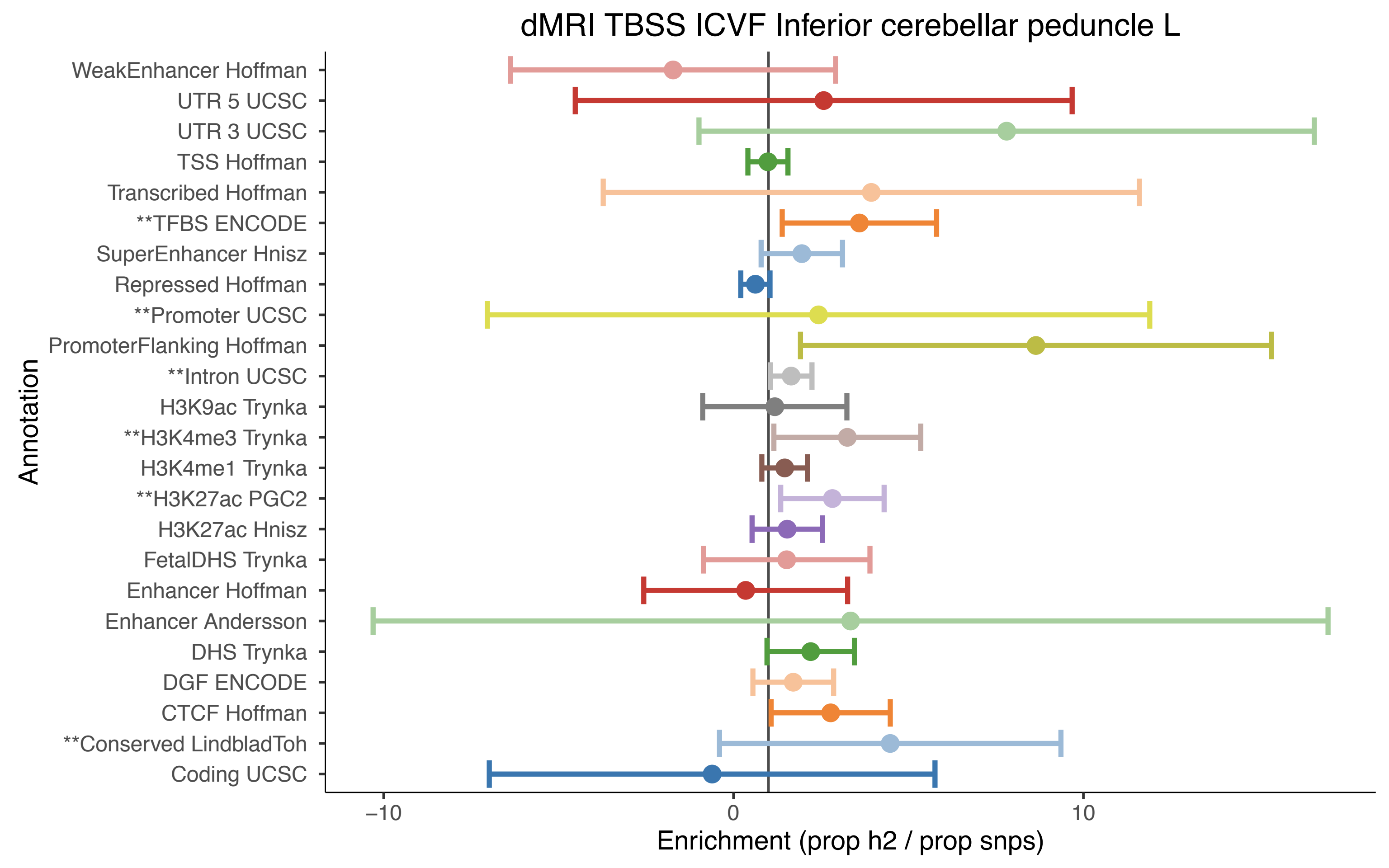

dMRI TBSS ICVF Inferior cerebellar peduncle L
WeakEnhancer Hoffman
UTR 5 UCSC
UTR 3 UCSC
TSS Hoffman
Transcribed Hoffman
**TFBS ENCODE
SuperEnhancer Hnisz
Repressed Hoffman
**Promoter UCSC
PromoterFlanking Hoffman
**Intron UCSC
H3K9ac Trynka
**H3K4me3 Trynka
H3K4me1 Trynka
**H3K27ac PGC2
H3K27ac Hnisz
FetalDHS Trynka
Enhancer Hoffman
Enhancer Andersson
DHS Trynka
DGF ENCODE
CTCF Hoffman
**Conserved LindbladToh
Coding UCSC
Annotation
−10
0
10
Enrichment (prop h2 / prop snps)

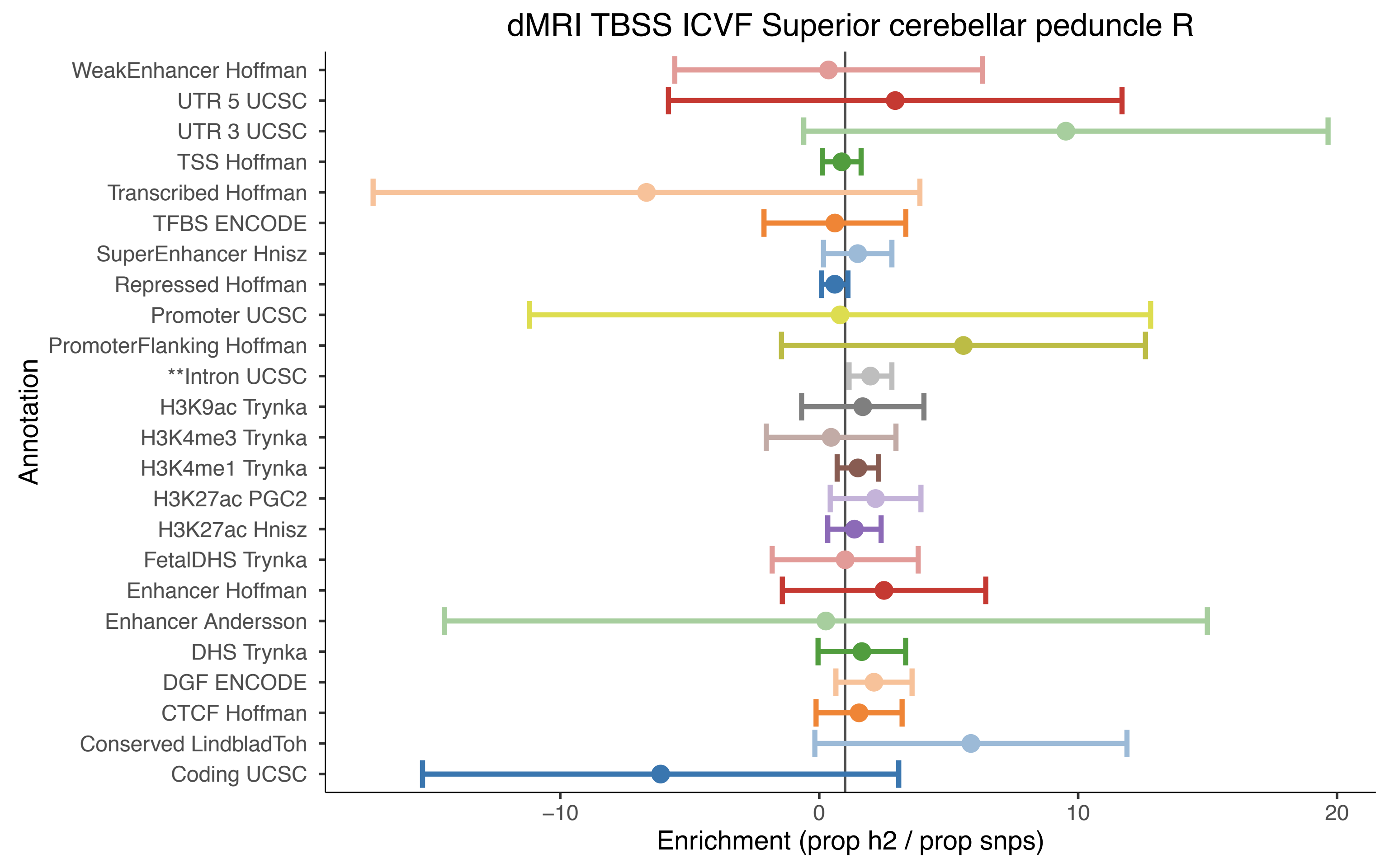

dMRI TBSS ICVF Superior cerebellar peduncle R
Annotation
WeakEnhancer Hoffman
UTR 5 UCSC
UTR 3 UCSC
TSS Hoffman
Transcribed Hoffman
TFBS ENCODE
SuperEnhancer Hnisz
Repressed Hoffman
Promoter UCSC
PromoterFlanking Hoffman
**Intron UCSC
H3K9ac Trynka
H3K4me3 Trynka
H3K4me1 Trynka
H3K27ac PGC2
H3K27ac Hnisz
FetalDHS Trynka
Enhancer Hoffman
Enhancer Andersson
DHS Trynka
DGF ENCODE
CTCF Hoffman
Conserved LindbladToh
Coding UCSC
−10
0
10
20
Enrichment (prop h2 / prop snps)

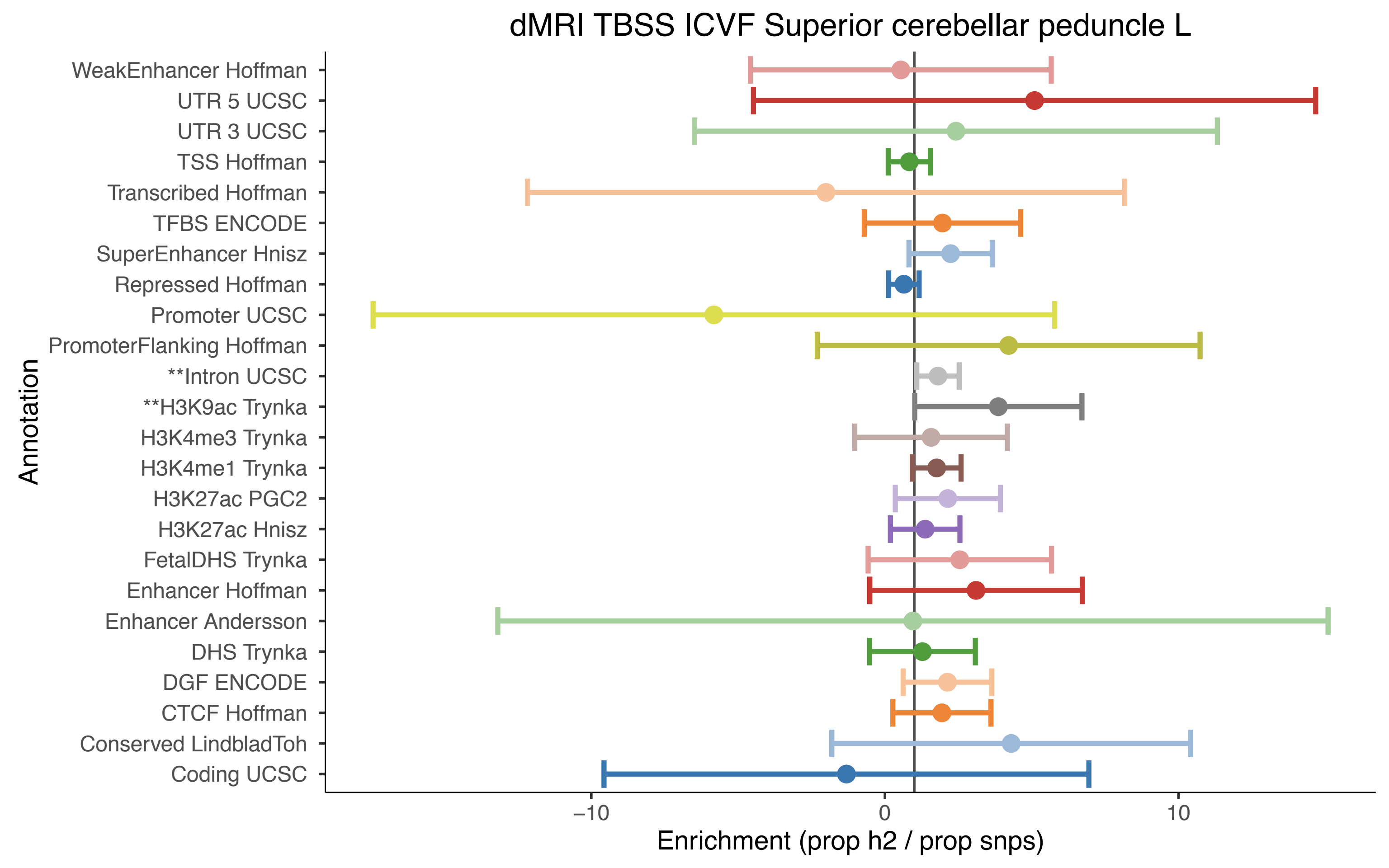

dMRI TBSS ICVF Superior cerebellar peduncle L
WeakEnhancer Hoffman
UTR 5 UCSC
UTR 3 UCSC
TSS Hoffman
Transcribed Hoffman
TFBS ENCODE
SuperEnhancer Hnisz
Repressed Hoffman
Promoter UCSC
PromoterFlanking Hoffman
**Intron UCSC
**H3K9ac Trynka
H3K4me3 Trynka
H3K4me1 Trynka
H3K27ac PGC2
H3K27ac Hnisz
FetalDHS Trynka
Enhancer Hoffman
Enhancer Andersson
DHS Trynka
DGF ENCODE
CTCF Hoffman
Conserved LindbladToh
Coding UCSC
Annotation
−10
0
10
Enrichment (prop h2 / prop snps)

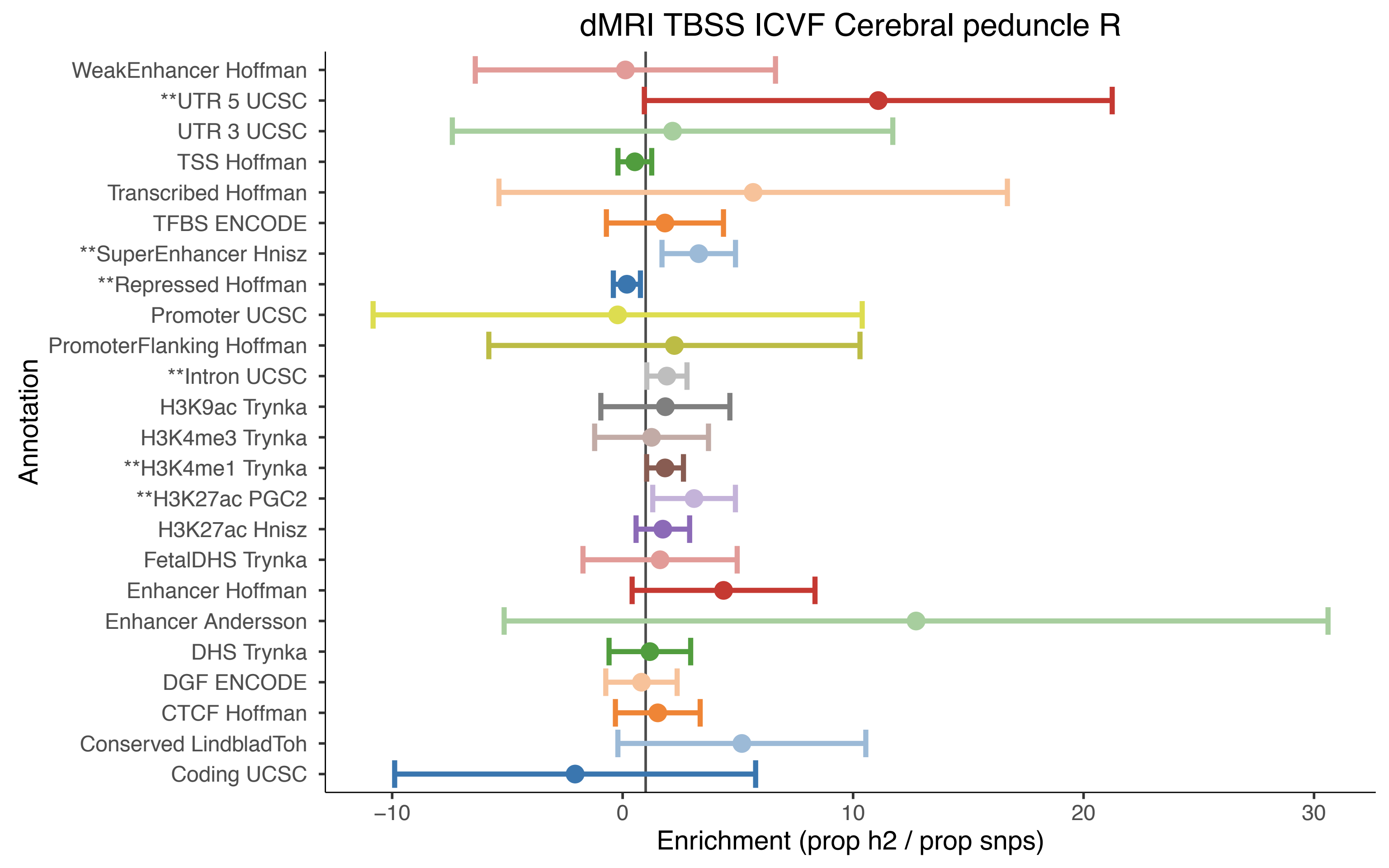
dMRI TBSS ICVF Cerebral peduncle R
WeakEnhancer Hoffman
**UTR 5 UCSC
UTR 3 UCSC
TSS Hoffman
Transcribed Hoffman
TFBS ENCODE
**SuperEnhancer Hnisz
**Repressed Hoffman
Promoter UCSC
PromoterFlanking Hoffman
**Intron UCSC
H3K9ac Trynka
H3K4me3 Trynka
**H3K4me1 Trynka
**H3K27ac PGC2
H3K27ac Hnisz
FetalDHS Trynka
Enhancer Hoffman
Enhancer Andersson
DHS Trynka
DGF ENCODE
CTCF Hoffman
Conserved LindbladToh
Coding UCSC
Annotation
−10
0
10
20
30
Enrichment (prop h2 / prop snps)

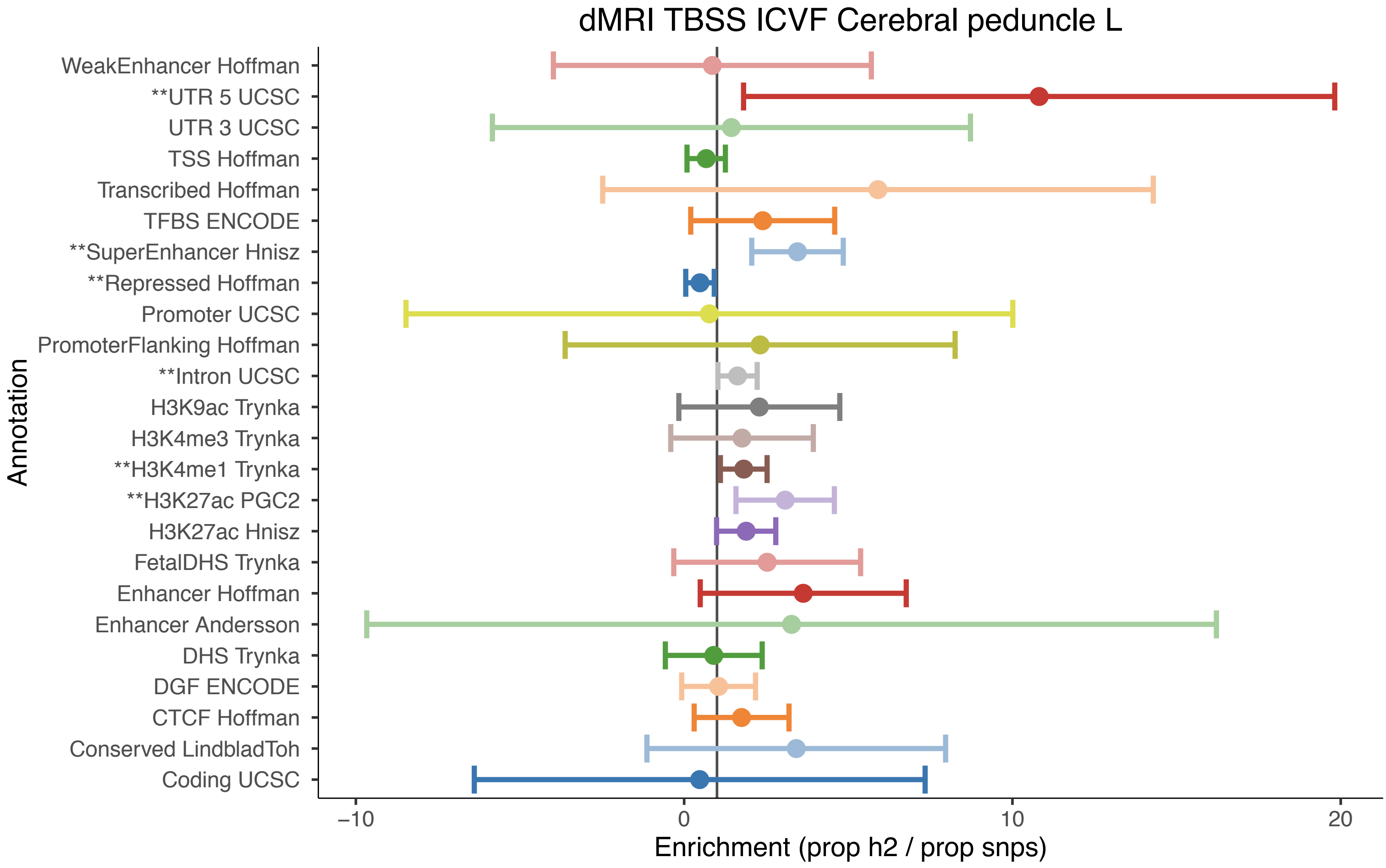

dMRI TBSS ICVF Cerebral peduncle L
WeakEnhancer Hoffman
**UTR 5 UCSC
UTR 3 UCSC
TSS Hoffman
Transcribed Hoffman
TFBS ENCODE
**SuperEnhancer Hnisz
**Repressed Hoffman
Promoter UCSC
PromoterFlanking Hoffman
**Intron UCSC
H3K9ac Trynka
H3K4me3 Trynka
**H3K4me1 Trynka
**H3K27ac PGC2
H3K27ac Hnisz
FetalDHS Trynka
Enhancer Hoffman
Enhancer Andersson
DHS Trynka
DGF ENCODE
CTCF Hoffman
Conserved LindbladToh
Coding UCSC
Annotation
−10
0
10
20
Enrichment (prop h2 / prop snps)

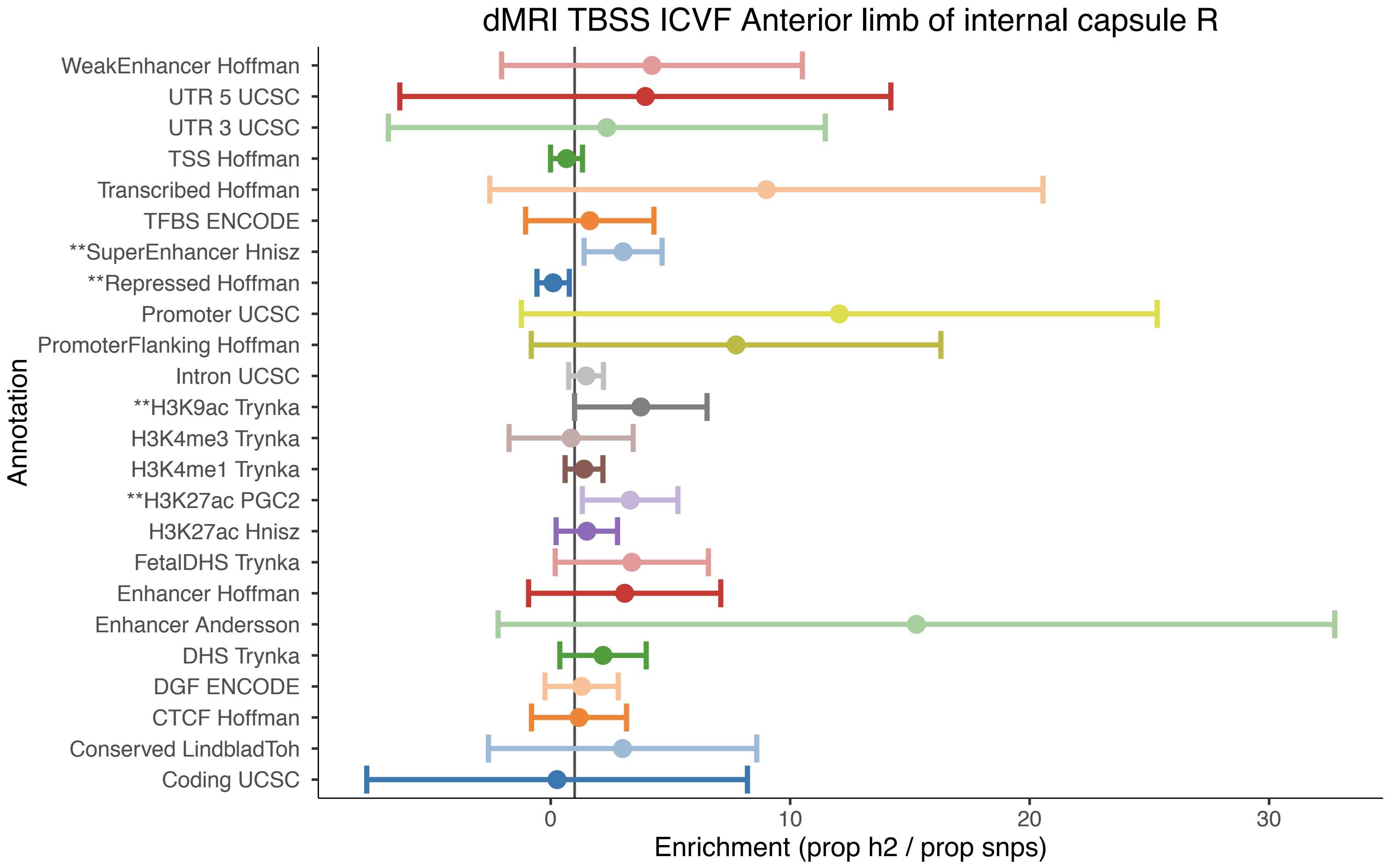
dMRI TBSS ICVF Anterior limb of internal capsule R
WeakEnhancer Hoffman
UTR 5 UCSC
UTR 3 UCSC
TSS Hoffman
Transcribed Hoffman
TFBS ENCODE
**SuperEnhancer Hnisz
**Repressed Hoffman
Promoter UCSC
PromoterFlanking Hoffman
Intron UCSC
**H3K9ac Trynka
H3K4me3 Trynka
H3K4me1 Trynka
**H3K27ac PGC2
H3K27ac Hnisz
FetalDHS Trynka
Enhancer Hoffman
Enhancer Andersson
DHS Trynka
DGF ENCODE
CTCF Hoffman
Conserved LindbladToh
Coding UCSC
Annotation
0
10
20
30
Enrichment (prop h2 / prop snps)

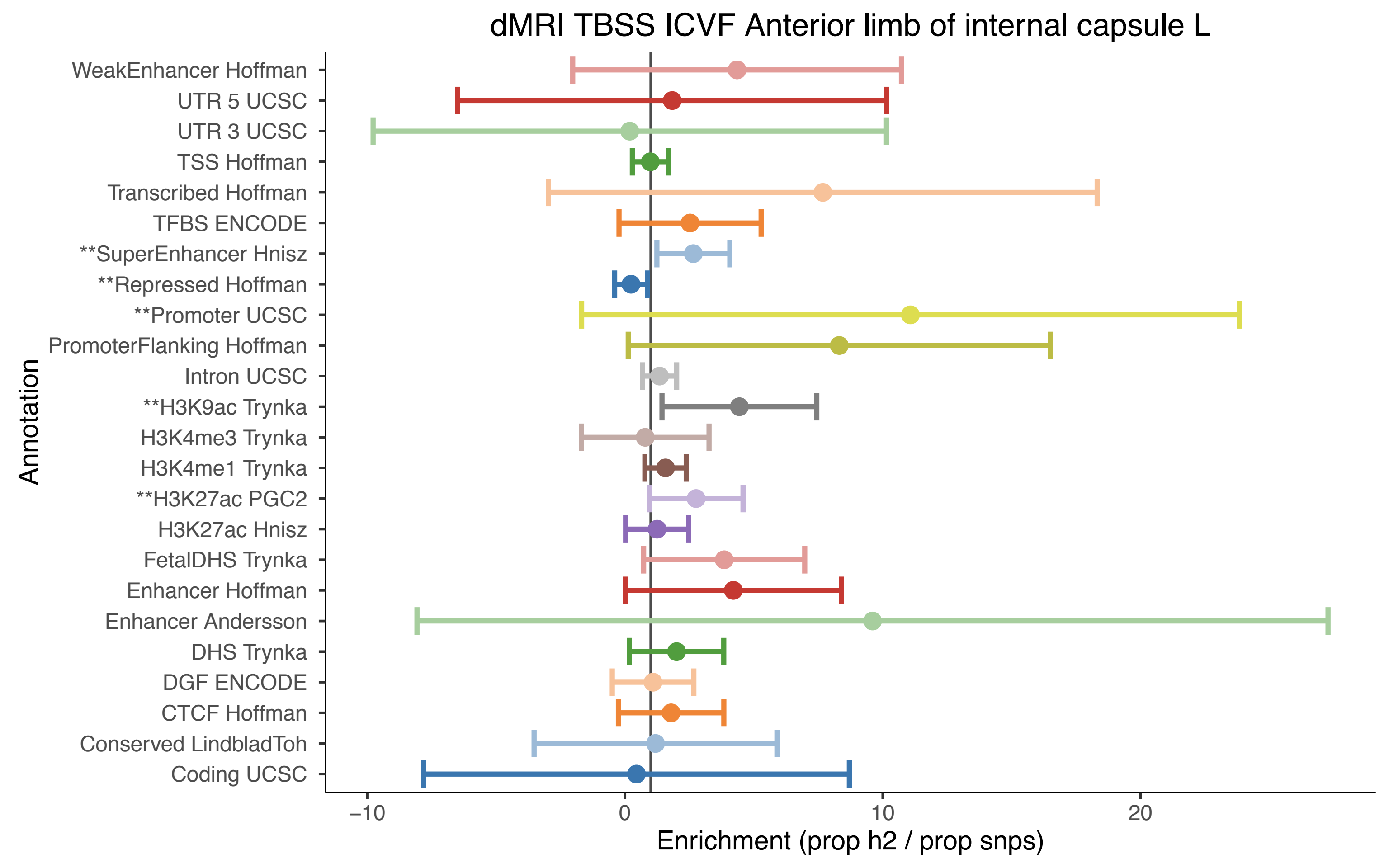

dMRI TBSS ICVF Anterior limb of internal capsule L
WeakEnhancer Hoffman
UTR 5 UCSC
UTR 3 UCSC
TSS Hoffman
Transcribed Hoffman
TFBS ENCODE
**SuperEnhancer Hnisz
**Repressed Hoffman
**Promoter UCSC
PromoterFlanking Hoffman
Intron UCSC
**H3K9ac Trynka
H3K4me3 Trynka
H3K4me1 Trynka
**H3K27ac PGC2
H3K27ac Hnisz
FetalDHS Trynka
Enhancer Hoffman
Enhancer Andersson
DHS Trynka
DGF ENCODE
CTCF Hoffman
Conserved LindbladToh
Coding UCSC
Annotation
−10
0
10
20
Enrichment (prop h2 / prop snps)

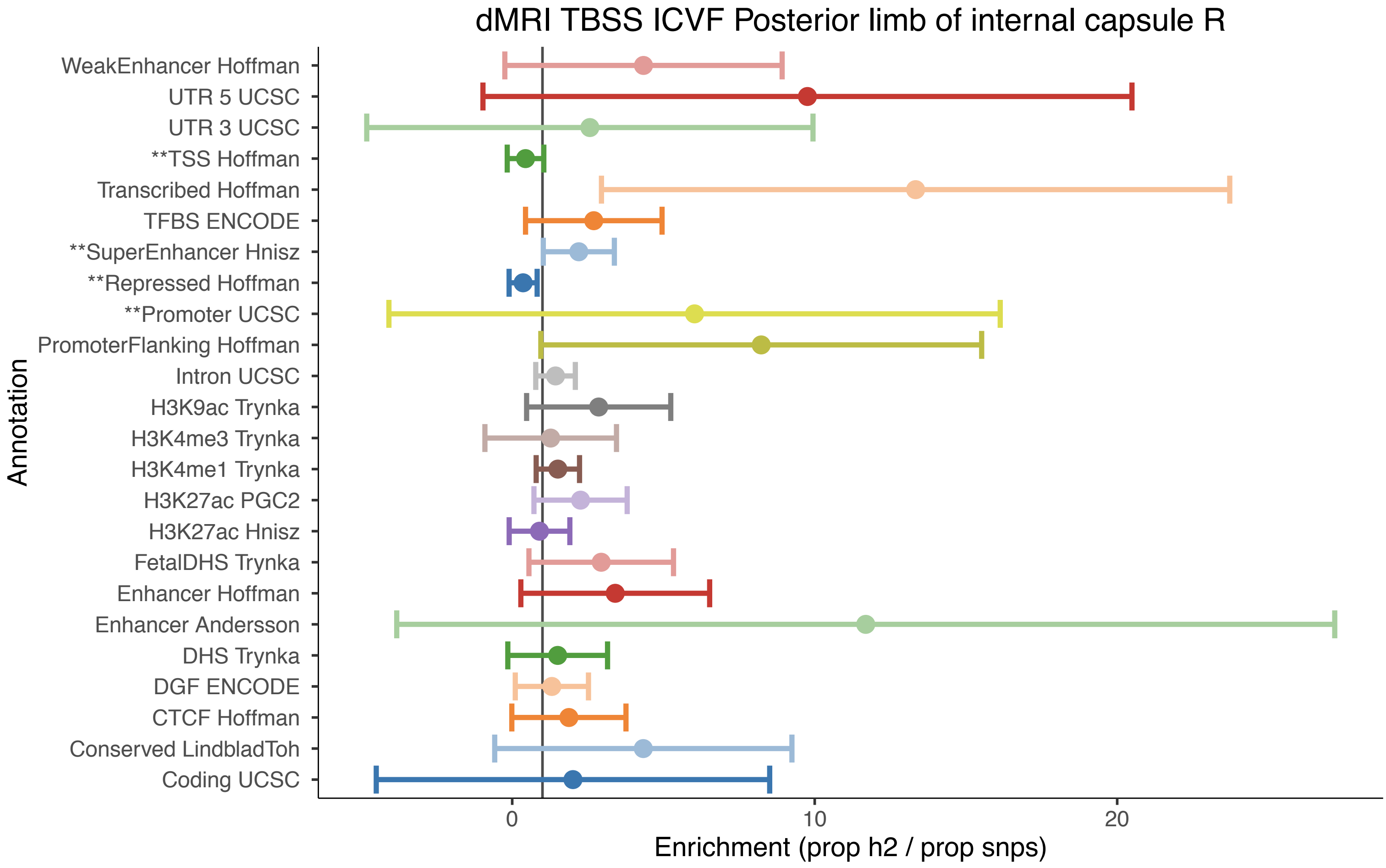
dMRI TBSS ICVF Posterior limb of internal capsule R
WeakEnhancer Hoffman
UTR 5 UCSC
UTR 3 UCSC
**TSS Hoffman
Transcribed Hoffman
TFBS ENCODE
**SuperEnhancer Hnisz
**Repressed Hoffman
**Promoter UCSC
PromoterFlanking Hoffman
Intron UCSC
H3K9ac Trynka
H3K4me3 Trynka
H3K4me1 Trynka
H3K27ac PGC2
H3K27ac Hnisz
FetalDHS Trynka
Enhancer Hoffman
Enhancer Andersson
DHS Trynka
DGF ENCODE
CTCF Hoffman
Conserved LindbladToh
Coding UCSC
Annotation
0
10
20
Enrichment (prop h2 / prop snps)

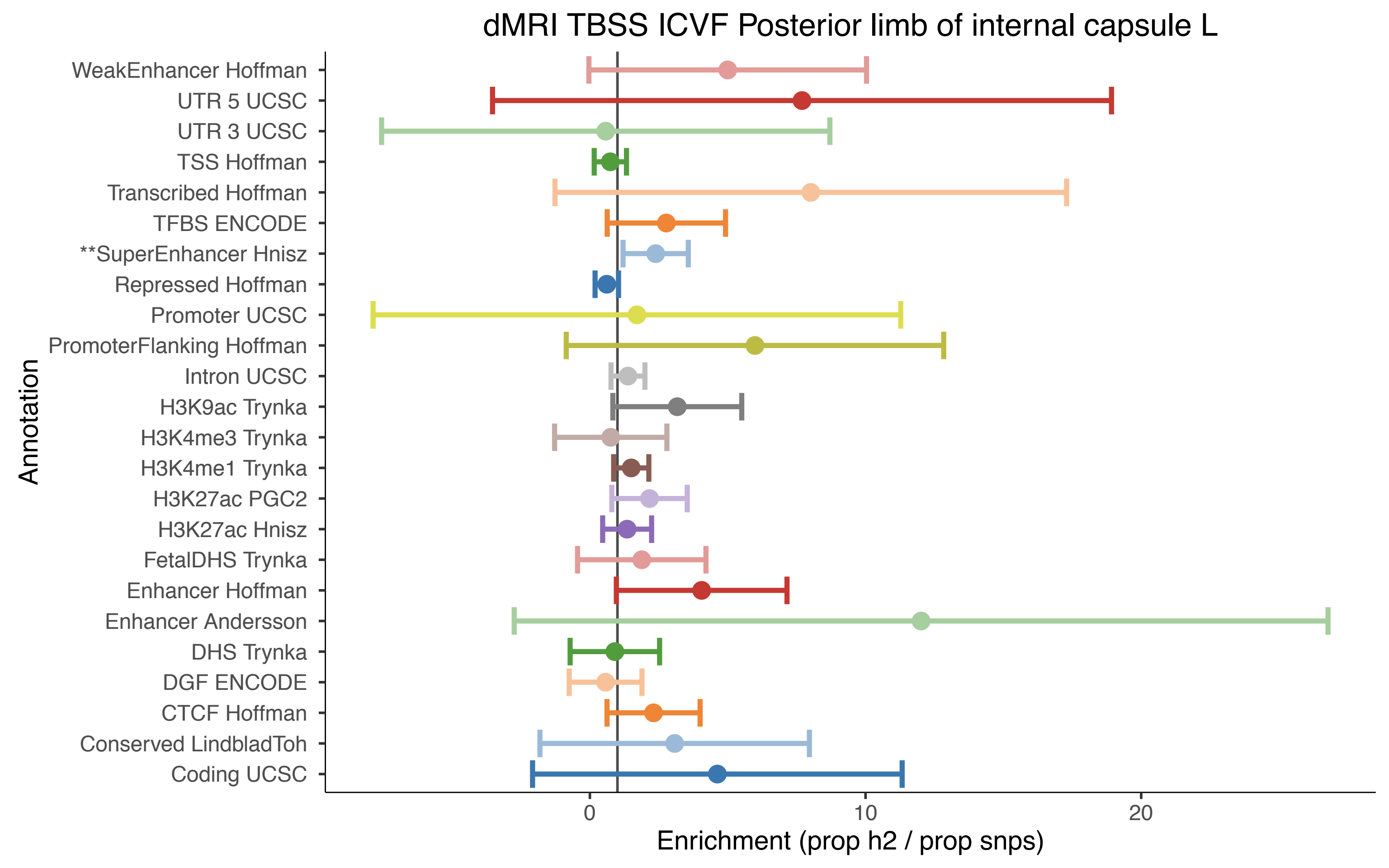
dMRI TBSS ICVF Posterior limb of internal capsule L
WeakEnhancer Hoffman
UTR 5 UCSC
UTR 3 UCSC
TSS Hoffman
Transcribed Hoffman
TFBS ENCODE
**SuperEnhancer Hnisz
Repressed Hoffman
Promoter UCSC
PromoterFlanking Hoffman
Intron UCSC
H3K9ac Trynka
H3K4me3 Trynka
H3K4me1 Trynka
H3K27ac PGC2
H3K27ac Hnisz
FetalDHS Trynka
Enhancer Hoffman
Enhancer Andersson
DHS Trynka
DGF ENCODE
CTCF Hoffman
Conserved LindbladToh
Coding UCSC
Annotation
0
10
20
Enrichment (prop h2 / prop snps)

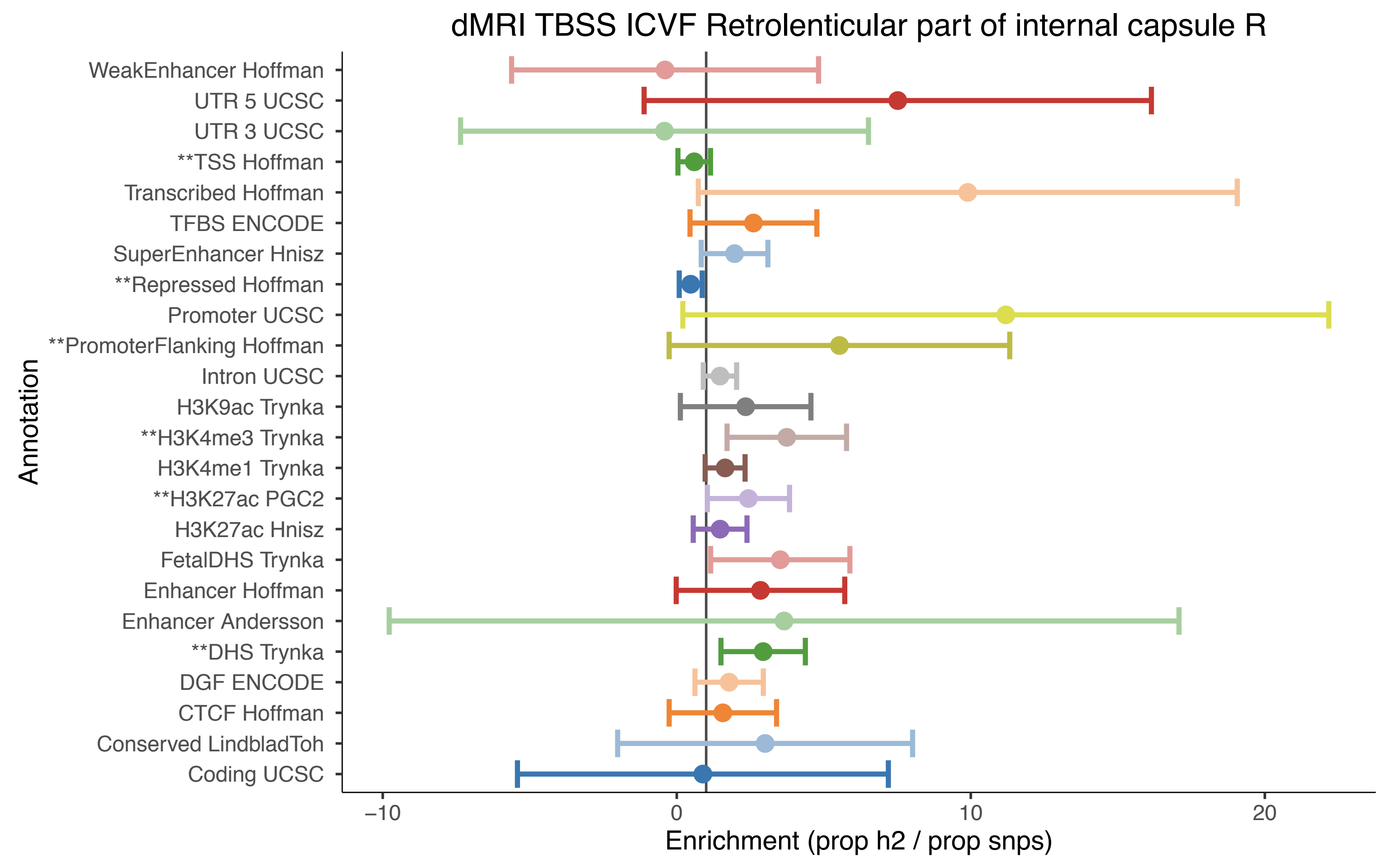

dMRI TBSS ICVF Retrolenticular part of internal capsule R
WeakEnhancer Hoffman
UTR 5 UCSC
UTR 3 UCSC
**TSS Hoffman
Transcribed Hoffman
TFBS ENCODE
SuperEnhancer Hnisz
**Repressed Hoffman
Promoter UCSC
**PromoterFlanking Hoffman
Intron UCSC
H3K9ac Trynka
**H3K4me3 Trynka
H3K4me1 Trynka
**H3K27ac PGC2
H3K27ac Hnisz
FetalDHS Trynka
Enhancer Hoffman
Enhancer Andersson
**DHS Trynka
DGF ENCODE
CTCF Hoffman
Conserved LindbladToh
Coding UCSC
Annotation
−10
0
10
20
Enrichment (prop h2 / prop snps)

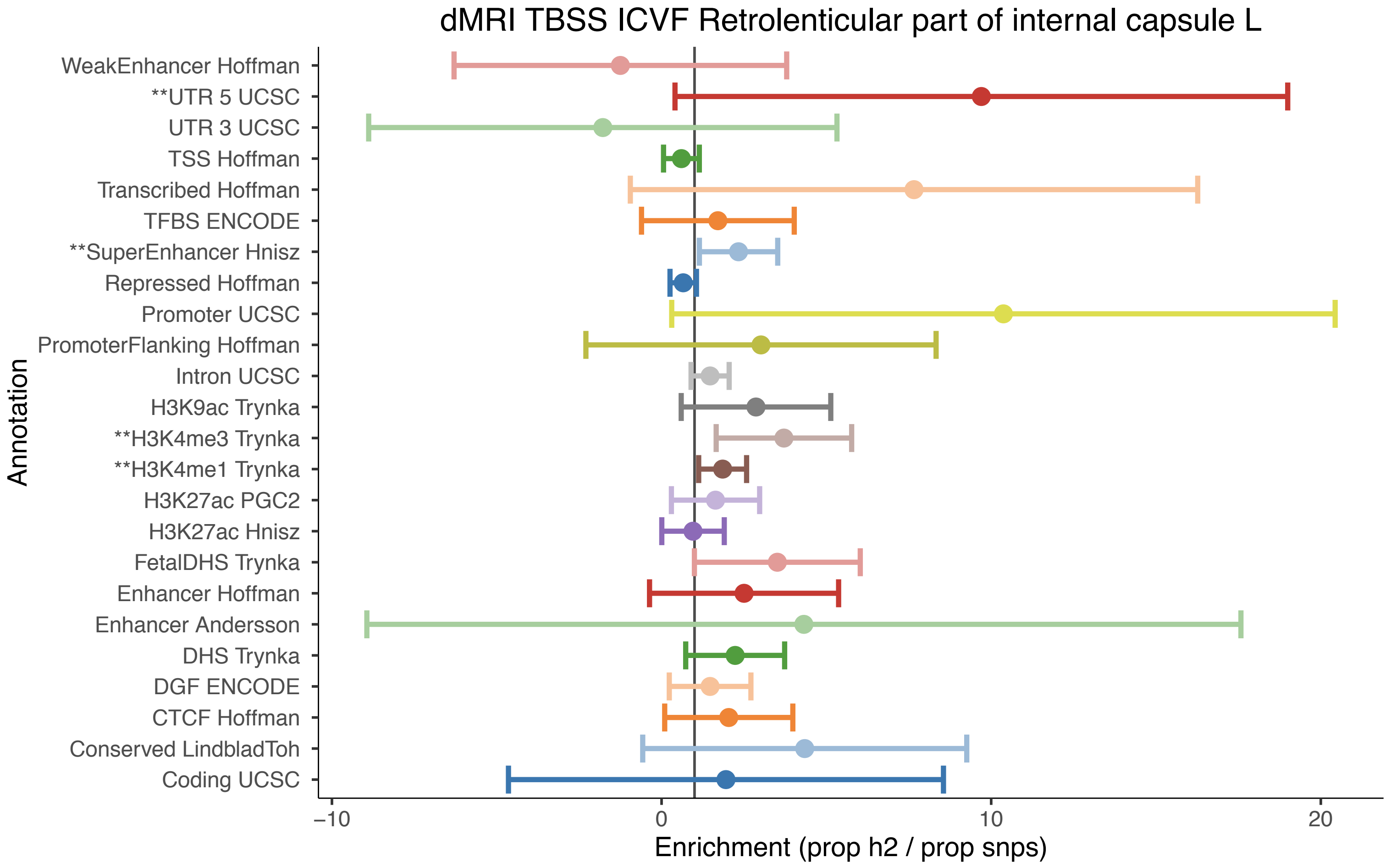
dMRI TBSS ICVF Retrolenticular part of internal capsule L
WeakEnhancer Hoffman
**UTR 5 UCSC
UTR 3 UCSC
TSS Hoffman
Transcribed Hoffman
TFBS ENCODE
**SuperEnhancer Hnisz
Repressed Hoffman
Promoter UCSC
PromoterFlanking Hoffman
Intron UCSC
H3K9ac Trynka
**H3K4me3 Trynka
**H3K4me1 Trynka
H3K27ac PGC2
H3K27ac Hnisz
FetalDHS Trynka
Enhancer Hoffman
Enhancer Andersson
DHS Trynka
DGF ENCODE
CTCF Hoffman
Conserved LindbladToh
Coding UCSC
Annotation
−10
0
10
20
Enrichment (prop h2 / prop snps)

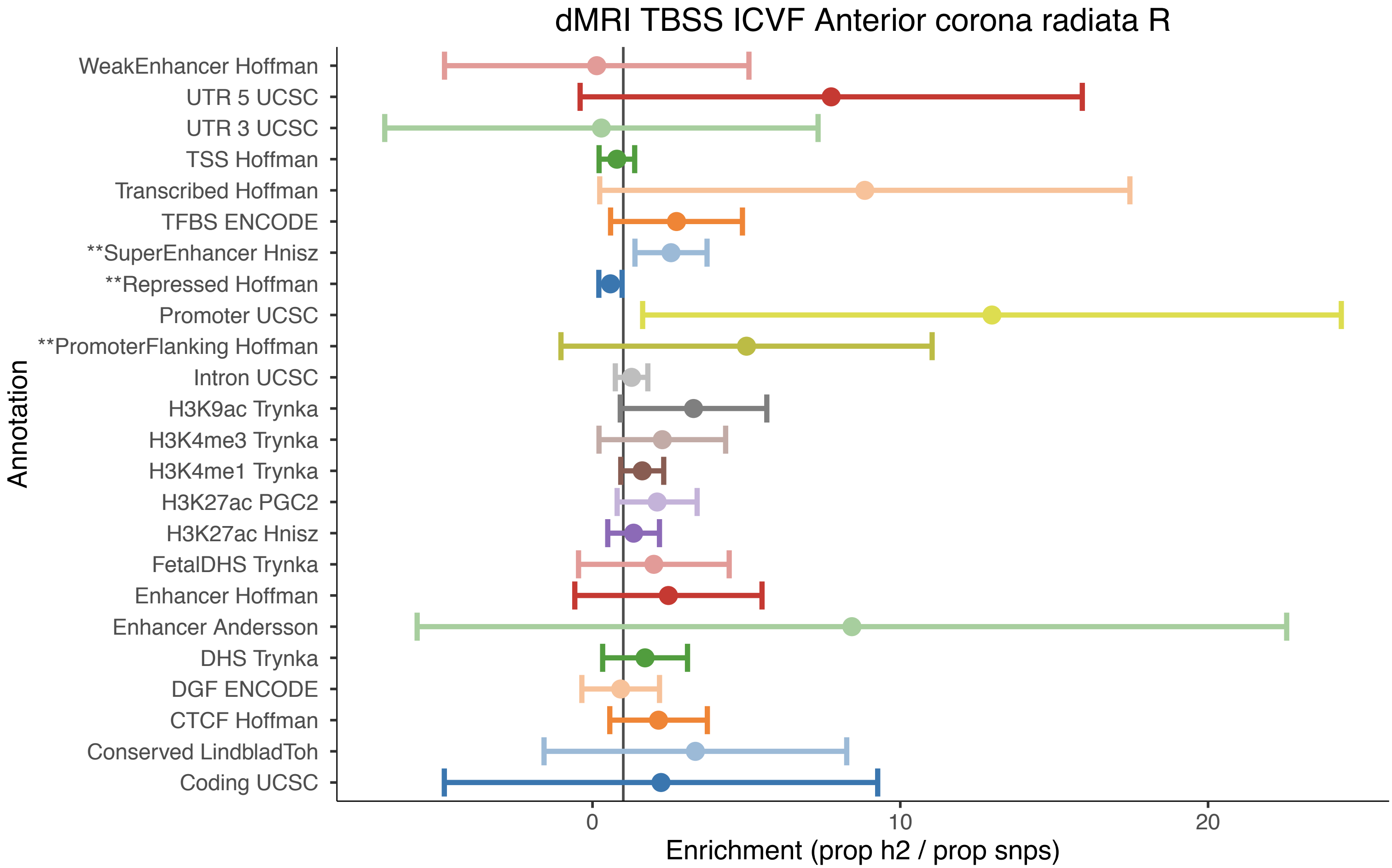
dMRI TBSS ICVF Anterior corona radiata R
Annotation
WeakEnhancer Hoffman
UTR 5 UCSC
UTR 3 UCSC
TSS Hoffman
Transcribed Hoffman
TFBS ENCODE
**SuperEnhancer Hnisz
**Repressed Hoffman
Promoter UCSC
**PromoterFlanking Hoffman
Intron UCSC
H3K9ac Trynka
H3K4me3 Trynka
H3K4me1 Trynka
H3K27ac PGC2
H3K27ac Hnisz
FetalDHS Trynka
Enhancer Hoffman
Enhancer Andersson
DHS Trynka
DGF ENCODE
CTCF Hoffman
Conserved LindbladToh
Coding UCSC
0
10
20
Enrichment (prop h2 / prop snps)

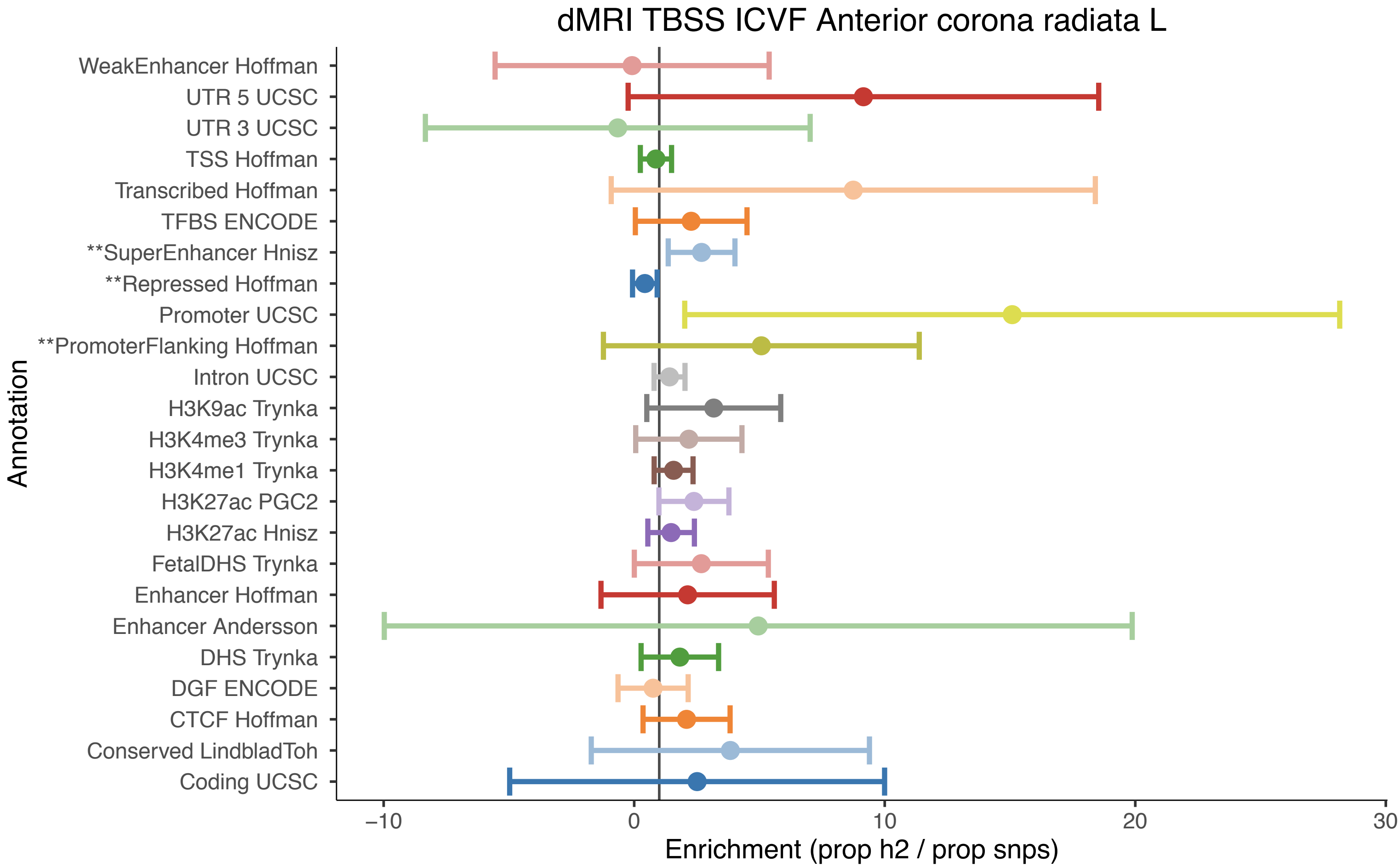
dMRI TBSS ICVF Anterior corona radiata L
WeakEnhancer Hoffman
UTR 5 UCSC
UTR 3 UCSC
TSS Hoffman
Transcribed Hoffman
TFBS ENCODE
**SuperEnhancer Hnisz
**Repressed Hoffman
Promoter UCSC
**PromoterFlanking Hoffman
Intron UCSC
H3K9ac Trynka
H3K4me3 Trynka
H3K4me1 Trynka
H3K27ac PGC2
H3K27ac Hnisz
FetalDHS Trynka
Enhancer Hoffman
Enhancer Andersson
DHS Trynka
DGF ENCODE
CTCF Hoffman
Conserved LindbladToh
Coding UCSC
Annotation
−10
0
10
20
30
Enrichment (prop h2 / prop snps)

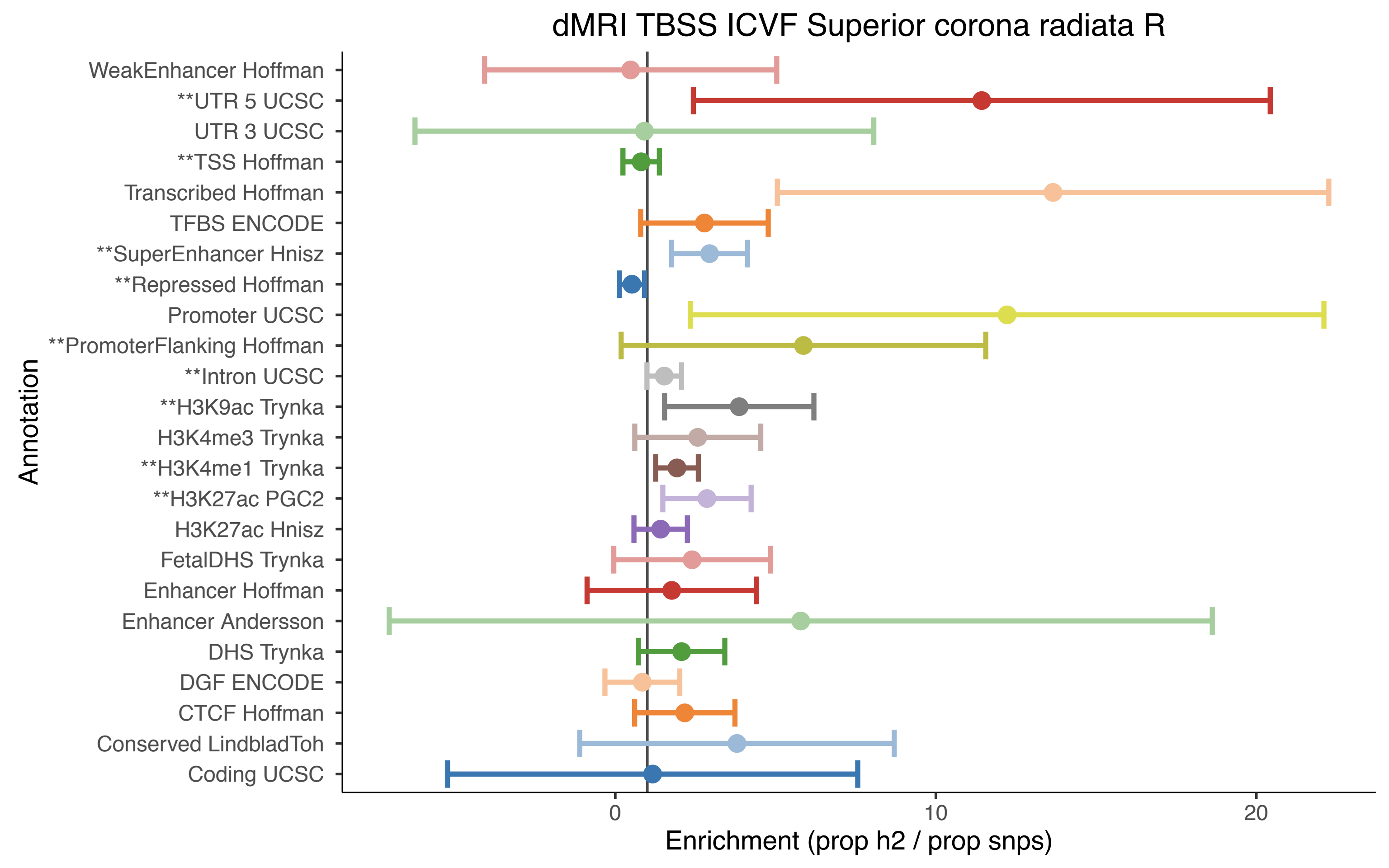

dMRI TBSS ICVF Superior corona radiata R
WeakEnhancer Hoffman
**UTR 5 UCSC
UTR 3 UCSC
**TSS Hoffman
Transcribed Hoffman
TFBS ENCODE
**SuperEnhancer Hnisz
**Repressed Hoffman
Promoter UCSC
**PromoterFlanking Hoffman
**Intron UCSC
**H3K9ac Trynka
H3K4me3 Trynka
**H3K4me1 Trynka
**H3K27ac PGC2
H3K27ac Hnisz
FetalDHS Trynka
Enhancer Hoffman
Enhancer Andersson
DHS Trynka
DGF ENCODE
CTCF Hoffman
Conserved LindbladToh
Coding UCSC
Annotation
0
10
20
Enrichment (prop h2 / prop snps)

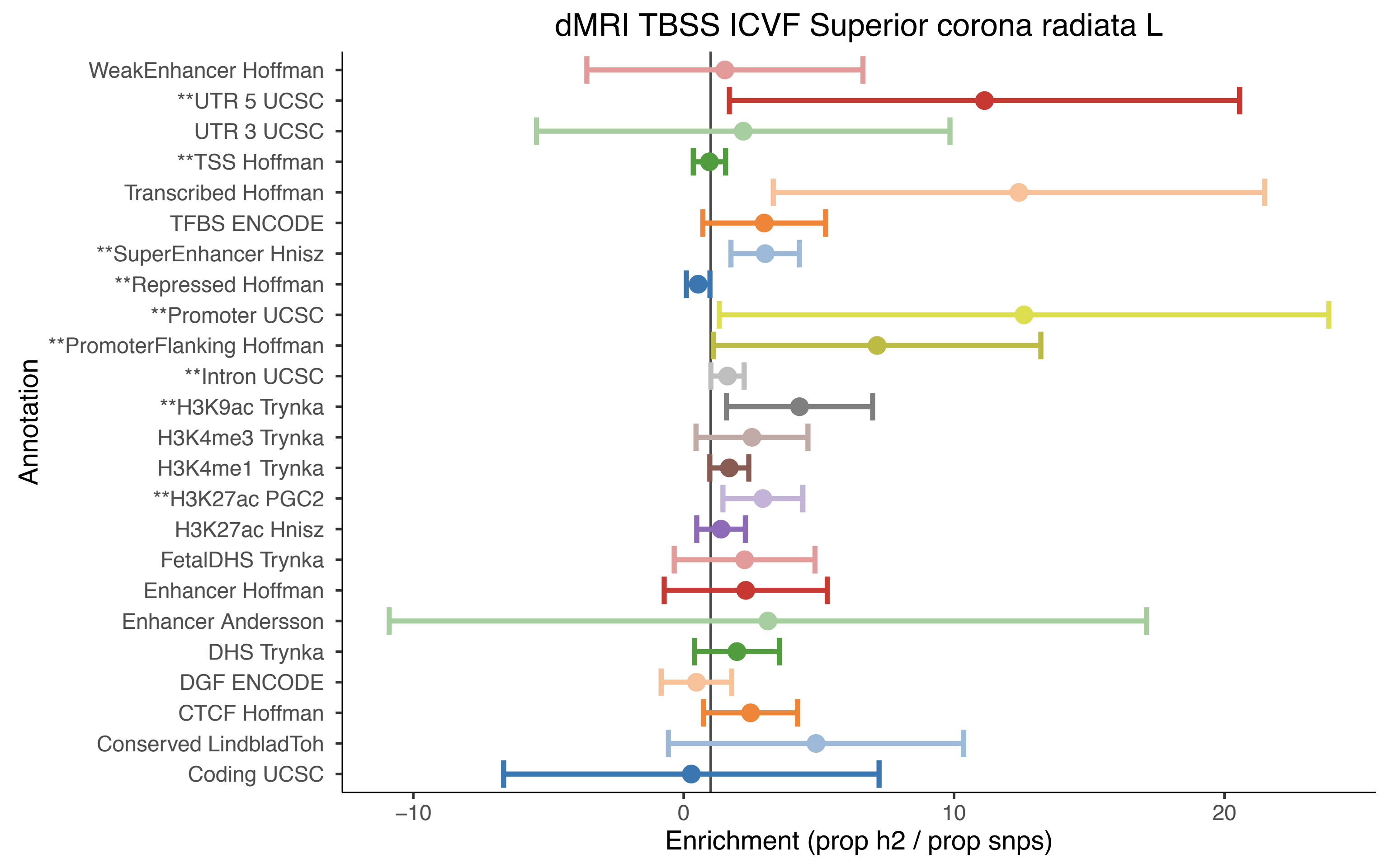

dMRI TBSS ICVF Superior corona radiata L
WeakEnhancer Hoffman
**UTR 5 UCSC
UTR 3 UCSC
**TSS Hoffman
Transcribed Hoffman
TFBS ENCODE
**SuperEnhancer Hnisz
**Repressed Hoffman
**Promoter UCSC
**PromoterFlanking Hoffman
**Intron UCSC
**H3K9ac Trynka
H3K4me3 Trynka
H3K4me1 Trynka
**H3K27ac PGC2
H3K27ac Hnisz
FetalDHS Trynka
Enhancer Hoffman
Enhancer Andersson
DHS Trynka
DGF ENCODE
CTCF Hoffman
Conserved LindbladToh
Coding UCSC
Annotation
−10
0
10
20
Enrichment (prop h2 / prop snps)

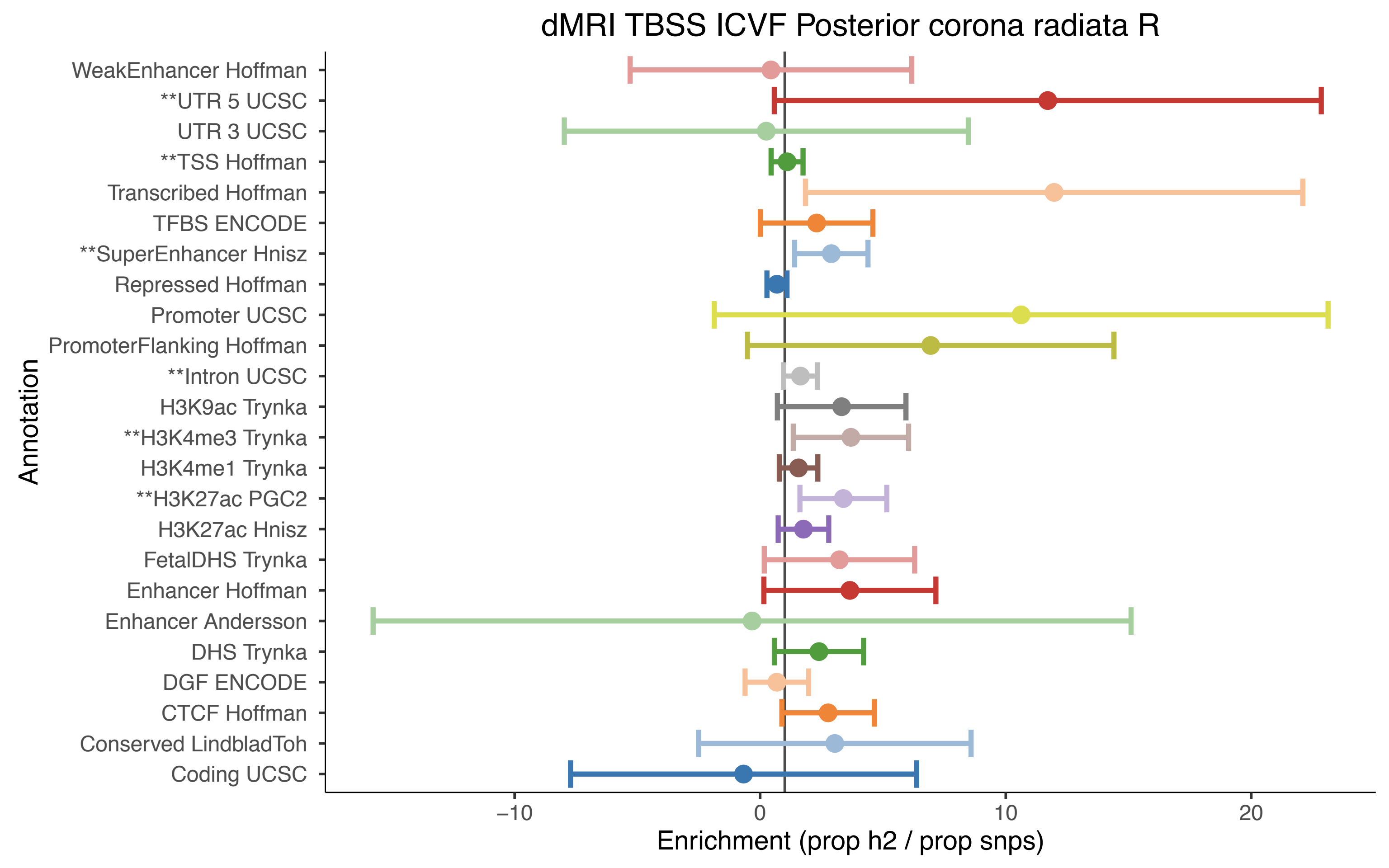

dMRI TBSS ICVF Posterior corona radiata R
WeakEnhancer Hoffman
**UTR 5 UCSC
UTR 3 UCSC
**TSS Hoffman
Transcribed Hoffman
TFBS ENCODE
**SuperEnhancer Hnisz
Repressed Hoffman
Promoter UCSC
PromoterFlanking Hoffman
**Intron UCSC
H3K9ac Trynka
**H3K4me3 Trynka
H3K4me1 Trynka
**H3K27ac PGC2
H3K27ac Hnisz
FetalDHS Trynka
Enhancer Hoffman
Enhancer Andersson
DHS Trynka
DGF ENCODE
CTCF Hoffman
Conserved LindbladToh
Coding UCSC
Annotation
−10
0
10
20
Enrichment (prop h2 / prop snps)

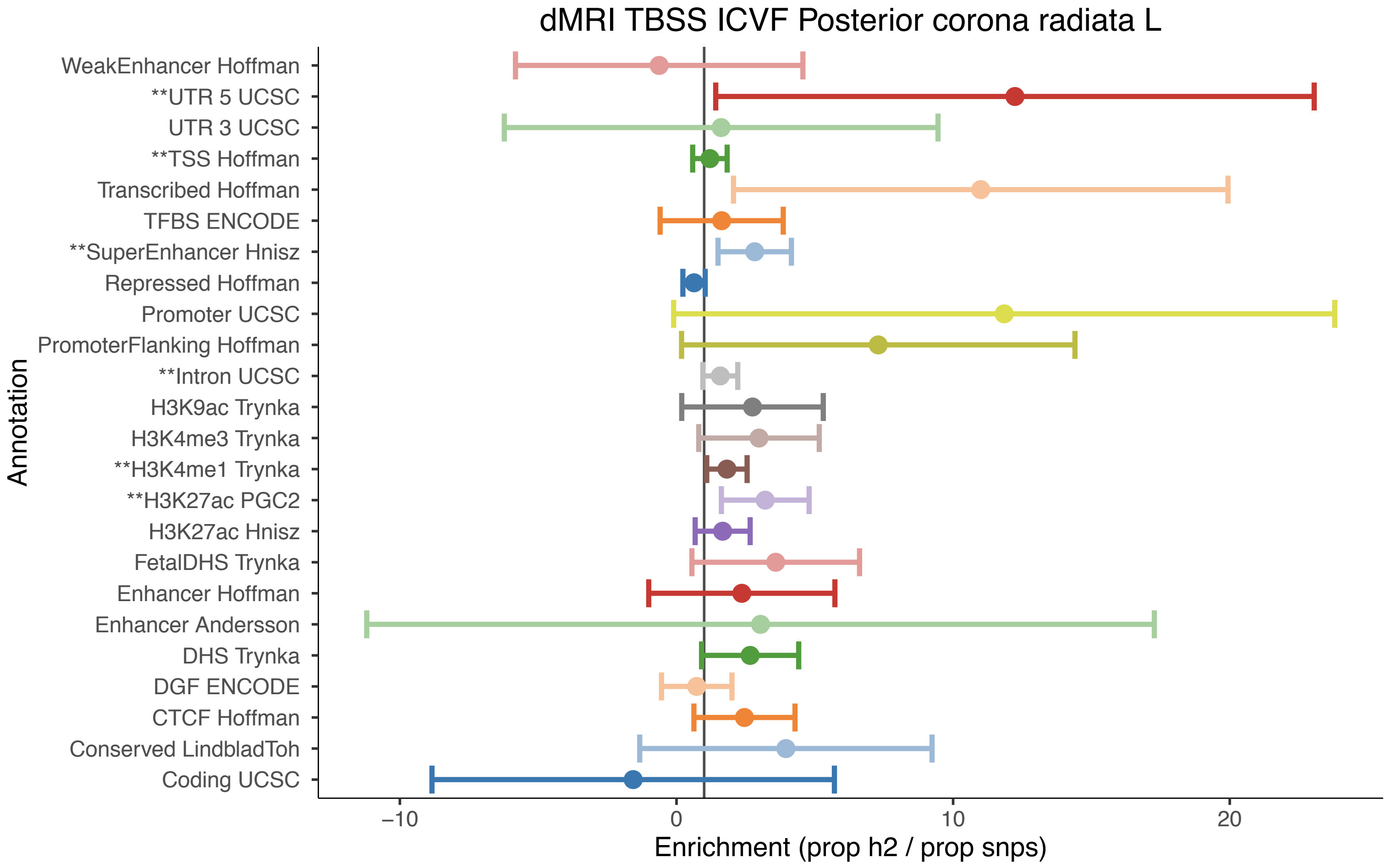

dMRI TBSS ICVF Posterior corona radiata L
WeakEnhancer Hoffman
**UTR 5 UCSC
UTR 3 UCSC
**TSS Hoffman
Transcribed Hoffman
TFBS ENCODE
**SuperEnhancer Hnisz
Repressed Hoffman
Promoter UCSC
PromoterFlanking Hoffman
**Intron UCSC
H3K9ac Trynka
H3K4me3 Trynka
**H3K4me1 Trynka
**H3K27ac PGC2
H3K27ac Hnisz
FetalDHS Trynka
Enhancer Hoffman
Enhancer Andersson
DHS Trynka
DGF ENCODE
CTCF Hoffman
Conserved LindbladToh
Coding UCSC
Annotation
−10
0
10
20
Enrichment (prop h2 / prop snps)

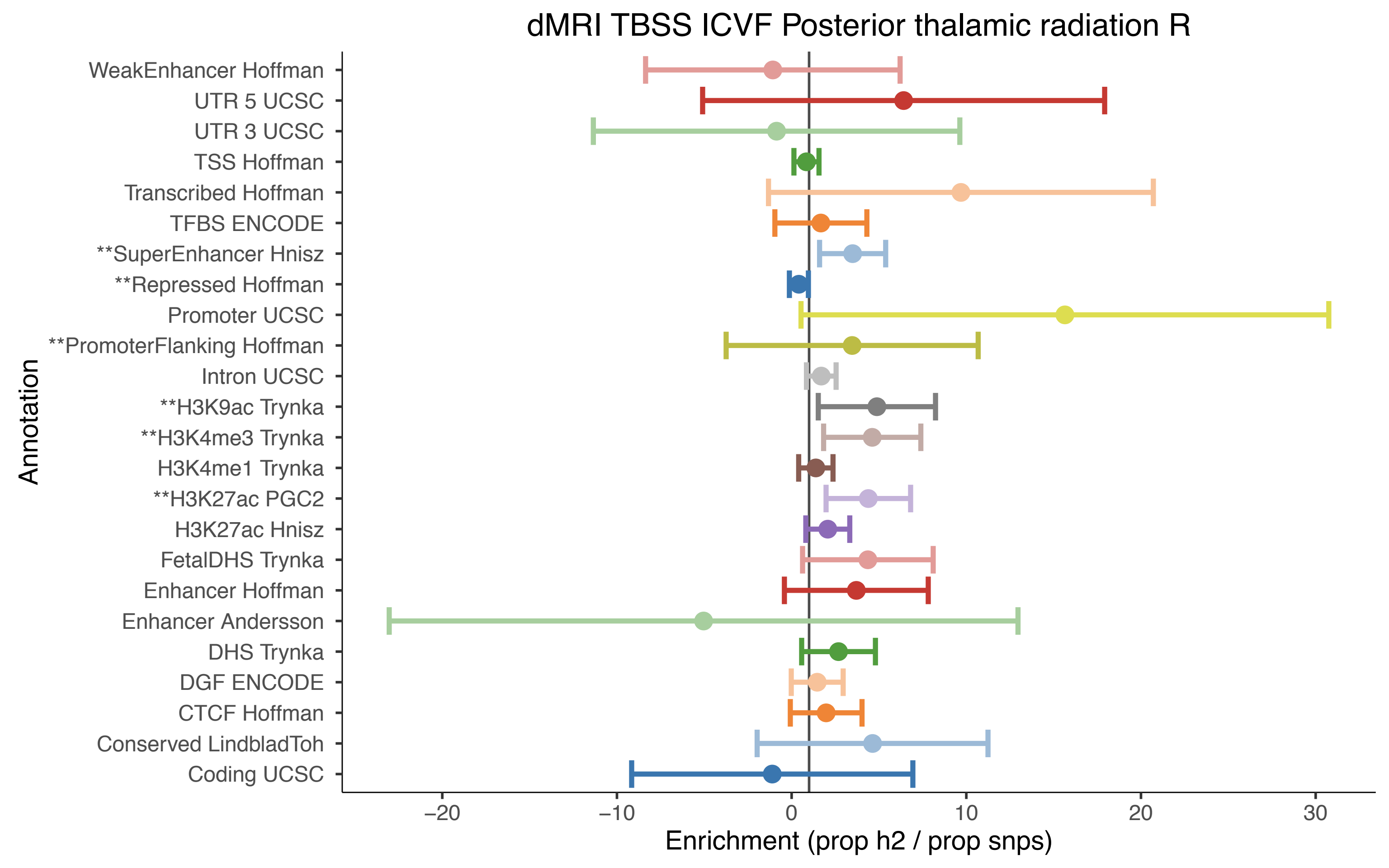
dMRI TBSS ICVF Posterior thalamic radiation R
Annotation
WeakEnhancer Hoffman
UTR 5 UCSC
UTR 3 UCSC
TSS Hoffman
Transcribed Hoffman
TFBS ENCODE
**SuperEnhancer Hnisz
**Repressed Hoffman
Promoter UCSC
**PromoterFlanking Hoffman
Intron UCSC
**H3K9ac Trynka
**H3K4me3 Trynka
H3K4me1 Trynka
**H3K27ac PGC2
H3K27ac Hnisz
FetalDHS Trynka
Enhancer Hoffman
Enhancer Andersson
DHS Trynka
DGF ENCODE
CTCF Hoffman
Conserved LindbladToh
Coding UCSC
−20
−10
0
10
20
30
Enrichment (prop h2 / prop snps)

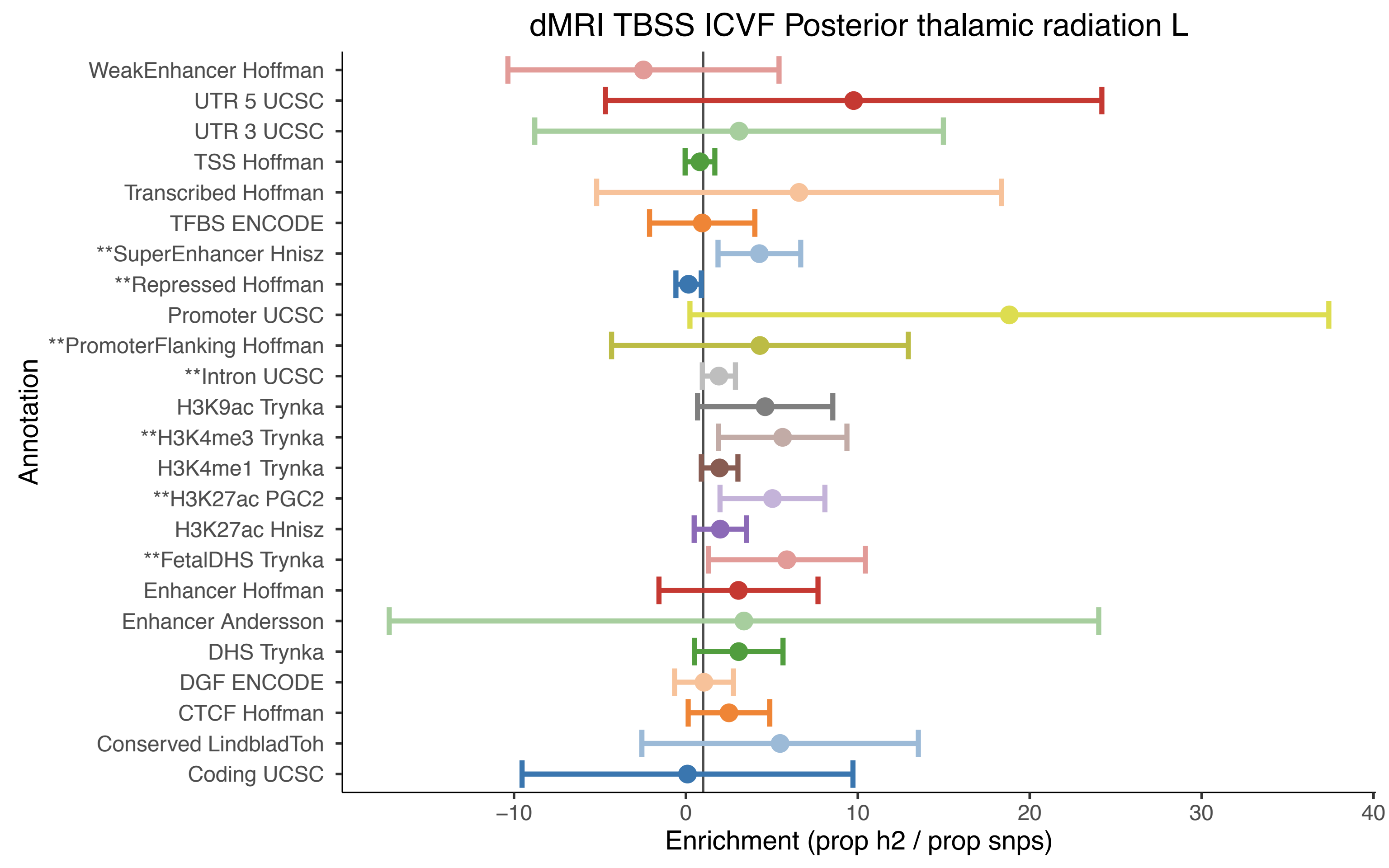

dMRI TBSS ICVF Posterior thalamic radiation L
WeakEnhancer Hoffman
UTR 5 UCSC
UTR 3 UCSC
TSS Hoffman
Transcribed Hoffman
TFBS ENCODE
**SuperEnhancer Hnisz
**Repressed Hoffman
Promoter UCSC
**PromoterFlanking Hoffman
**Intron UCSC
H3K9ac Trynka
**H3K4me3 Trynka
H3K4me1 Trynka
**H3K27ac PGC2
H3K27ac Hnisz
**FetalDHS Trynka
Enhancer Hoffman
Enhancer Andersson
DHS Trynka
DGF ENCODE
CTCF Hoffman
Conserved LindbladToh
Coding UCSC
Annotation
−10
0
10
20
30
40
Enrichment (prop h2 / prop snps)

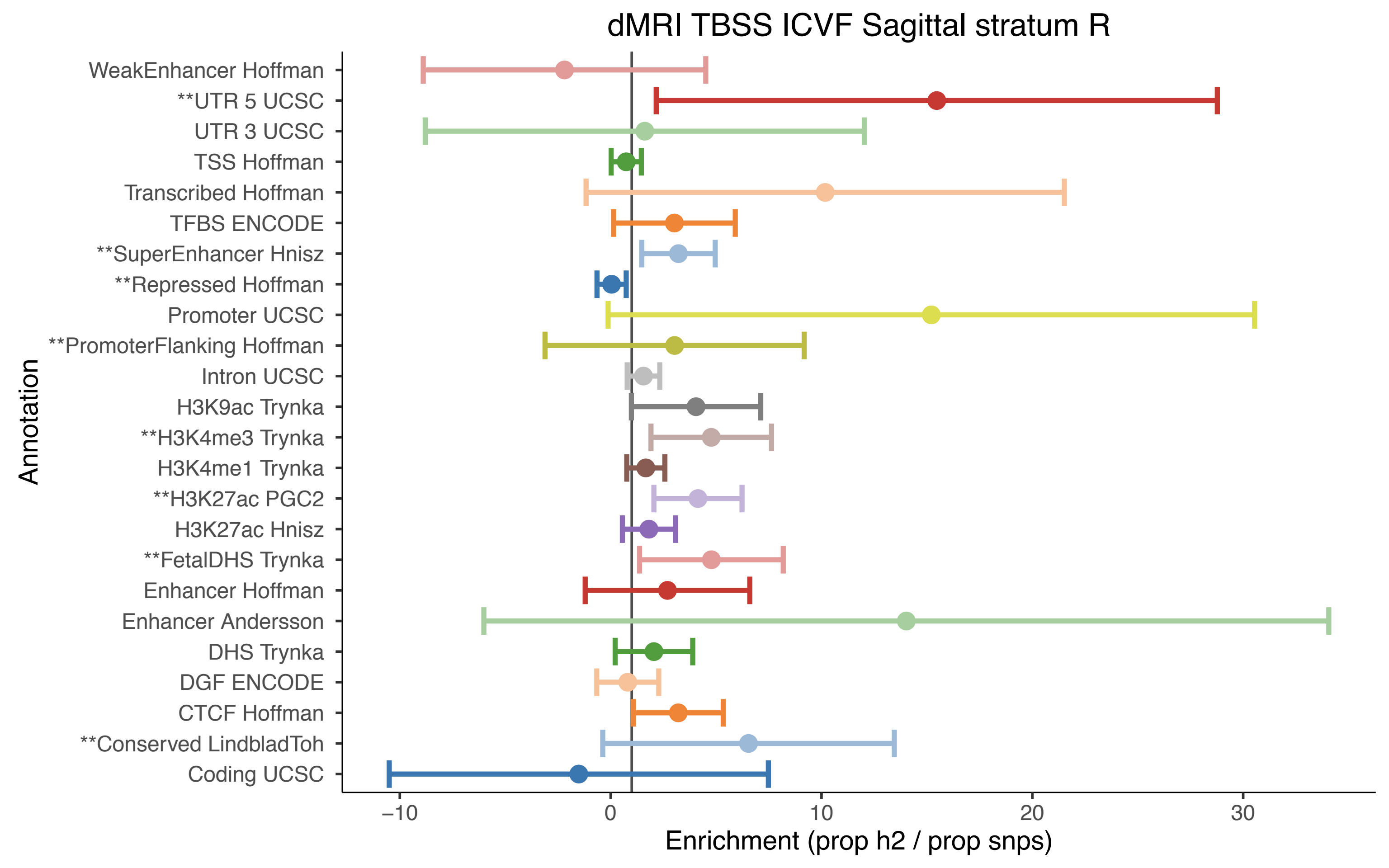

dMRI TBSS ICVF Sagittal stratum R
WeakEnhancer Hoffman
**UTR 5 UCSC
UTR 3 UCSC
TSS Hoffman
Transcribed Hoffman
TFBS ENCODE
**SuperEnhancer Hnisz
**Repressed Hoffman
Promoter UCSC
**PromoterFlanking Hoffman
Intron UCSC
H3K9ac Trynka
**H3K4me3 Trynka
H3K4me1 Trynka
**H3K27ac PGC2
H3K27ac Hnisz
**FetalDHS Trynka
Enhancer Hoffman
Enhancer Andersson
DHS Trynka
DGF ENCODE
CTCF Hoffman
**Conserved LindbladToh
Coding UCSC
Annotation
−10
0
10
20
30
Enrichment (prop h2 / prop snps)

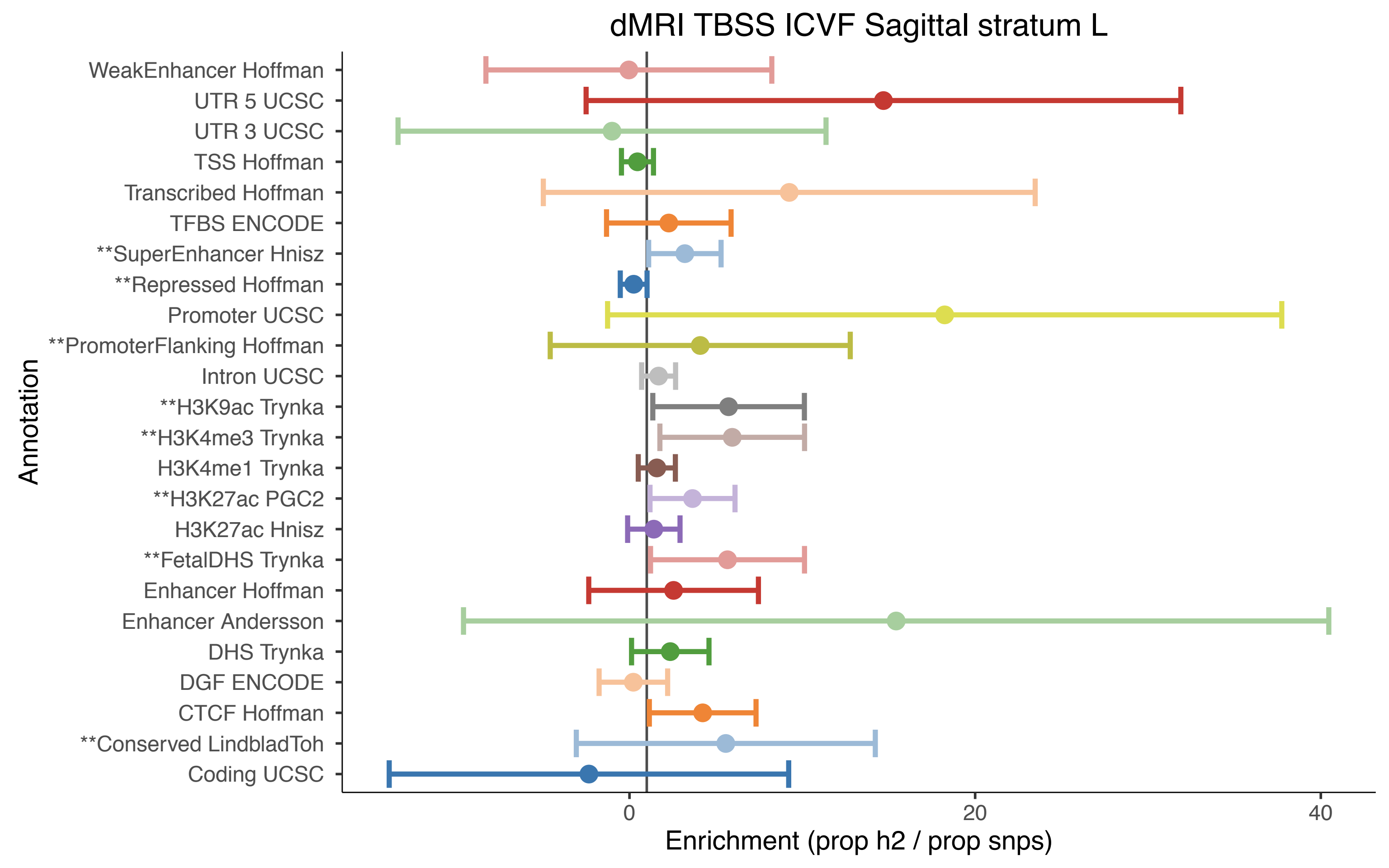

dMRI TBSS ICVF Sagittal stratum L
WeakEnhancer Hoffman
UTR 5 UCSC
UTR 3 UCSC
TSS Hoffman
Transcribed Hoffman
TFBS ENCODE
**SuperEnhancer Hnisz
**Repressed Hoffman
Promoter UCSC
**PromoterFlanking Hoffman
Intron UCSC
**H3K9ac Trynka
**H3K4me3 Trynka
H3K4me1 Trynka
**H3K27ac PGC2
H3K27ac Hnisz
**FetalDHS Trynka
Enhancer Hoffman
Enhancer Andersson
DHS Trynka
DGF ENCODE
CTCF Hoffman
**Conserved LindbladToh
Coding UCSC
Annotation
0
20
40
Enrichment (prop h2 / prop snps)

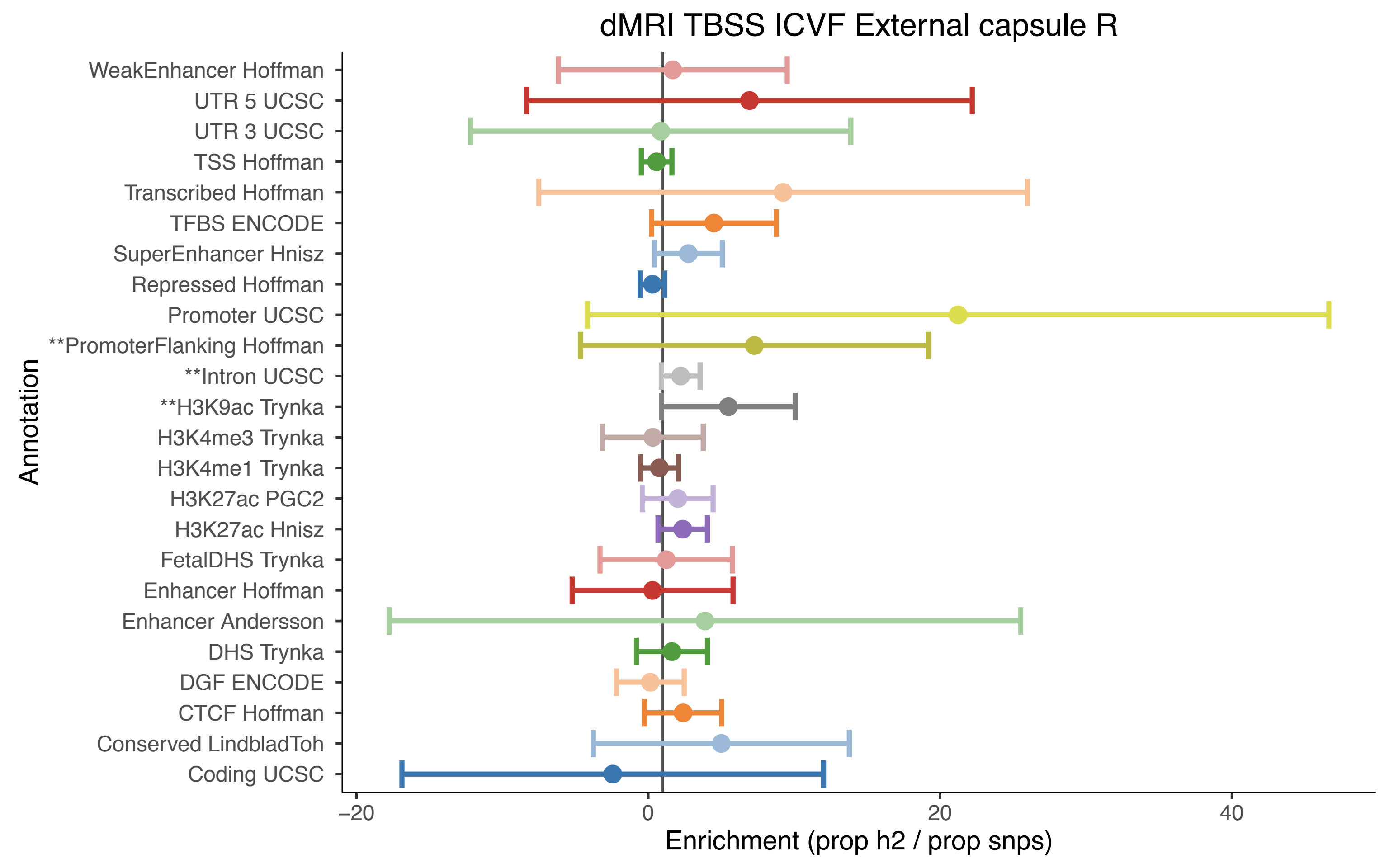
dMRI TBSS ICVF External capsule R
WeakEnhancer Hoffman
UTR 5 UCSC
UTR 3 UCSC
TSS Hoffman
Transcribed Hoffman
TFBS ENCODE
SuperEnhancer Hnisz
Repressed Hoffman
Promoter UCSC
**PromoterFlanking Hoffman
**Intron UCSC
**H3K9ac Trynka
H3K4me3 Trynka
H3K4me1 Trynka
H3K27ac PGC2
H3K27ac Hnisz
FetalDHS Trynka
Enhancer Hoffman
Enhancer Andersson
DHS Trynka
DGF ENCODE
CTCF Hoffman
Conserved LindbladToh
Coding UCSC
Annotation
−20
0
20
40
Enrichment (prop h2 / prop snps)

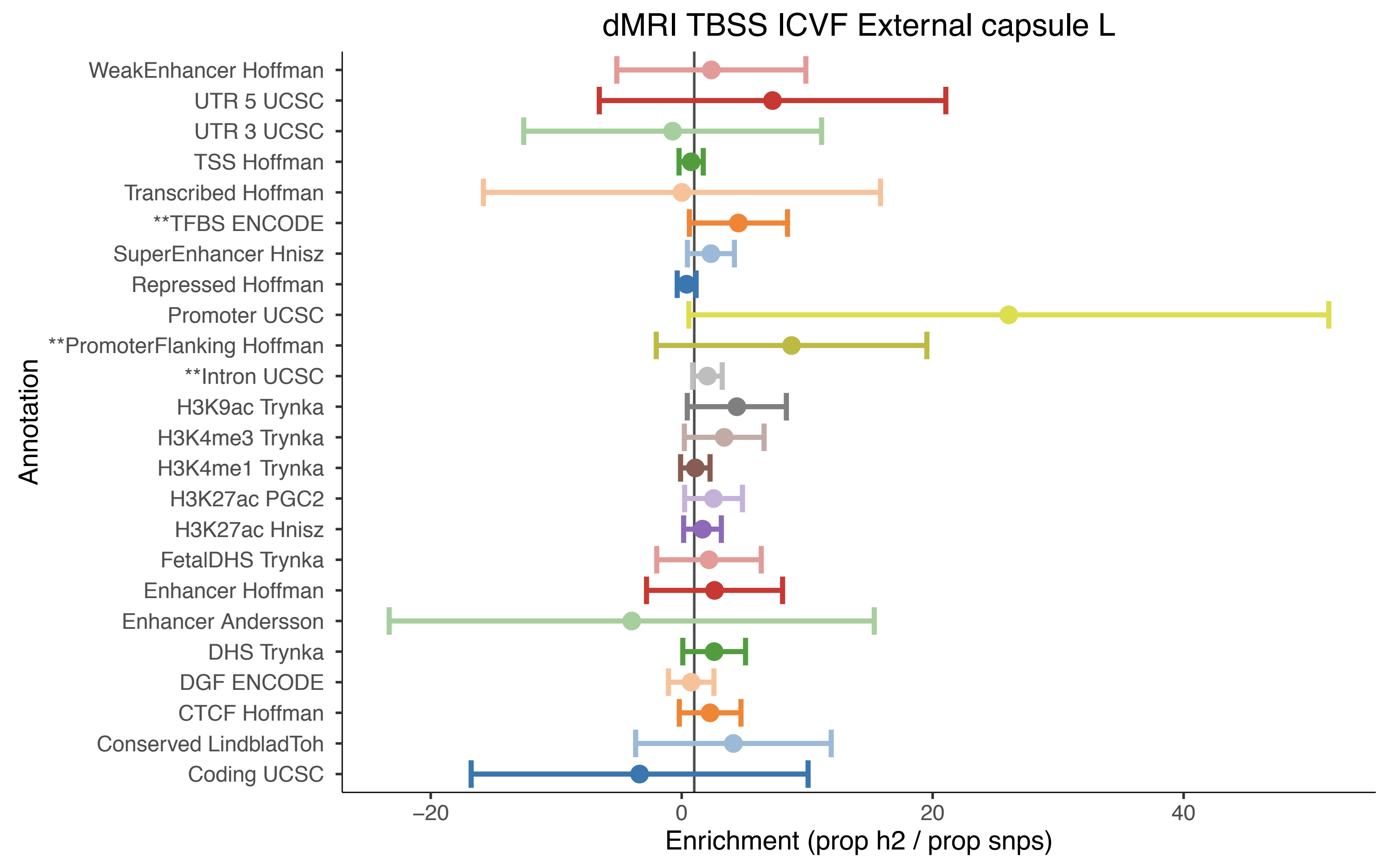
dMRI TBSS ICVF External capsule L
WeakEnhancer Hoffman
UTR 5 UCSC
UTR 3 UCSC
TSS Hoffman
Transcribed Hoffman
**TFBS ENCODE
SuperEnhancer Hnisz
Repressed Hoffman
Promoter UCSC
**PromoterFlanking Hoffman
**Intron UCSC
H3K9ac Trynka
H3K4me3 Trynka
H3K4me1 Trynka
H3K27ac PGC2
H3K27ac Hnisz
FetalDHS Trynka
Enhancer Hoffman
Enhancer Andersson
DHS Trynka
DGF ENCODE
CTCF Hoffman
Conserved LindbladToh
Coding UCSC
Annotation
−20
0
20
40
Enrichment (prop h2 / prop snps)

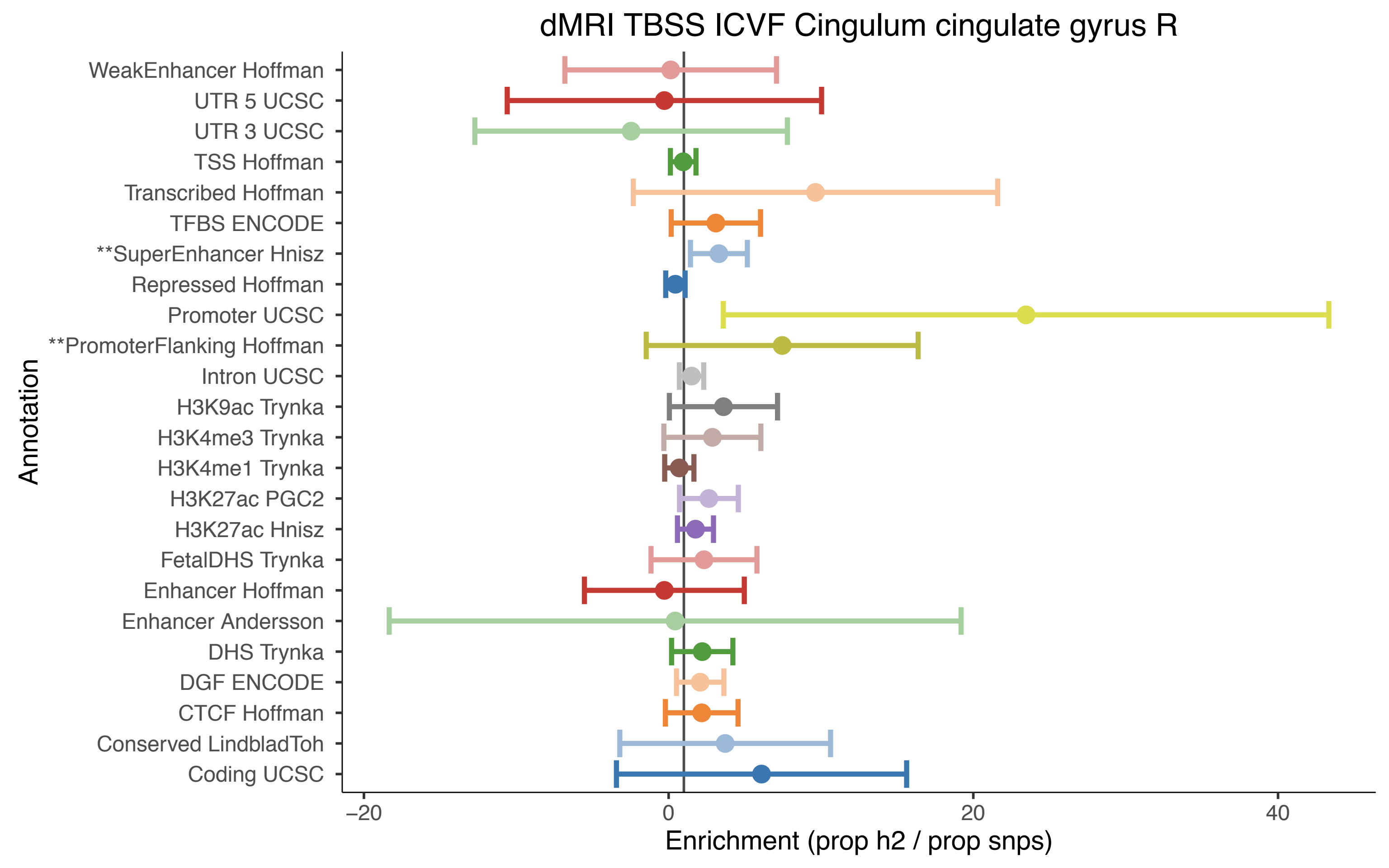

dMRI TBSS ICVF Cingulum cingulate gyrus R
WeakEnhancer Hoffman
UTR 5 UCSC
UTR 3 UCSC
TSS Hoffman
Transcribed Hoffman
TFBS ENCODE
**SuperEnhancer Hnisz
Repressed Hoffman
Promoter UCSC
**PromoterFlanking Hoffman
Intron UCSC
H3K9ac Trynka
H3K4me3 Trynka
H3K4me1 Trynka
H3K27ac PGC2
H3K27ac Hnisz
FetalDHS Trynka
Enhancer Hoffman
Enhancer Andersson
DHS Trynka
DGF ENCODE
CTCF Hoffman
Conserved LindbladToh
Coding UCSC
Annotation
−20
0
20
40
Enrichment (prop h2 / prop snps)

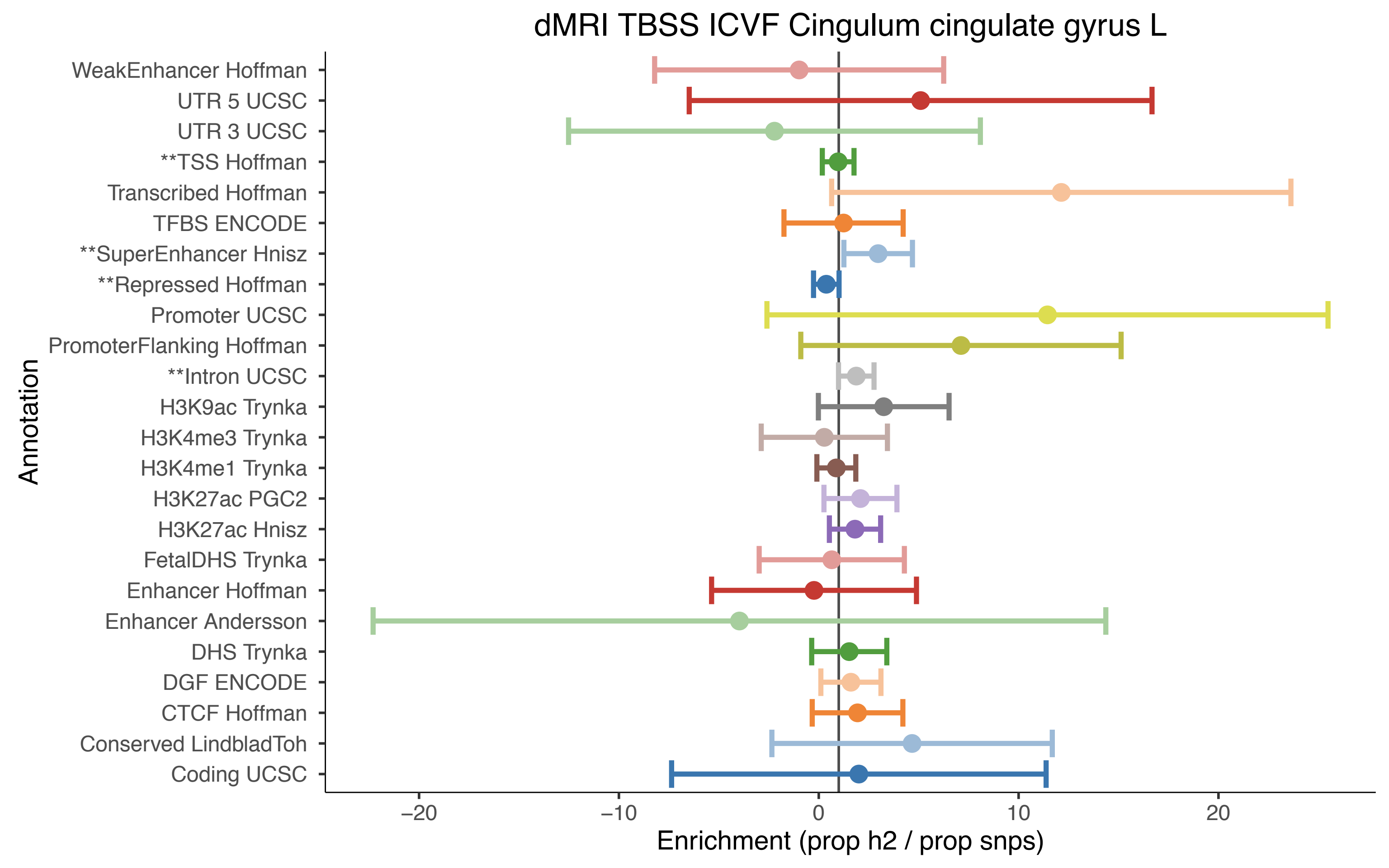
dMRI TBSS ICVF Cingulum cingulate gyrus L
WeakEnhancer Hoffman
UTR 5 UCSC
UTR 3 UCSC
**TSS Hoffman
Transcribed Hoffman
TFBS ENCODE
**SuperEnhancer Hnisz
**Repressed Hoffman
Promoter UCSC
PromoterFlanking Hoffman
**Intron UCSC
H3K9ac Trynka
H3K4me3 Trynka
H3K4me1 Trynka
H3K27ac PGC2
H3K27ac Hnisz
FetalDHS Trynka
Enhancer Hoffman
Enhancer Andersson
DHS Trynka
DGF ENCODE
CTCF Hoffman
Conserved LindbladToh
Coding UCSC
Annotation
−20
−10
0
10
20
Enrichment (prop h2 / prop snps)

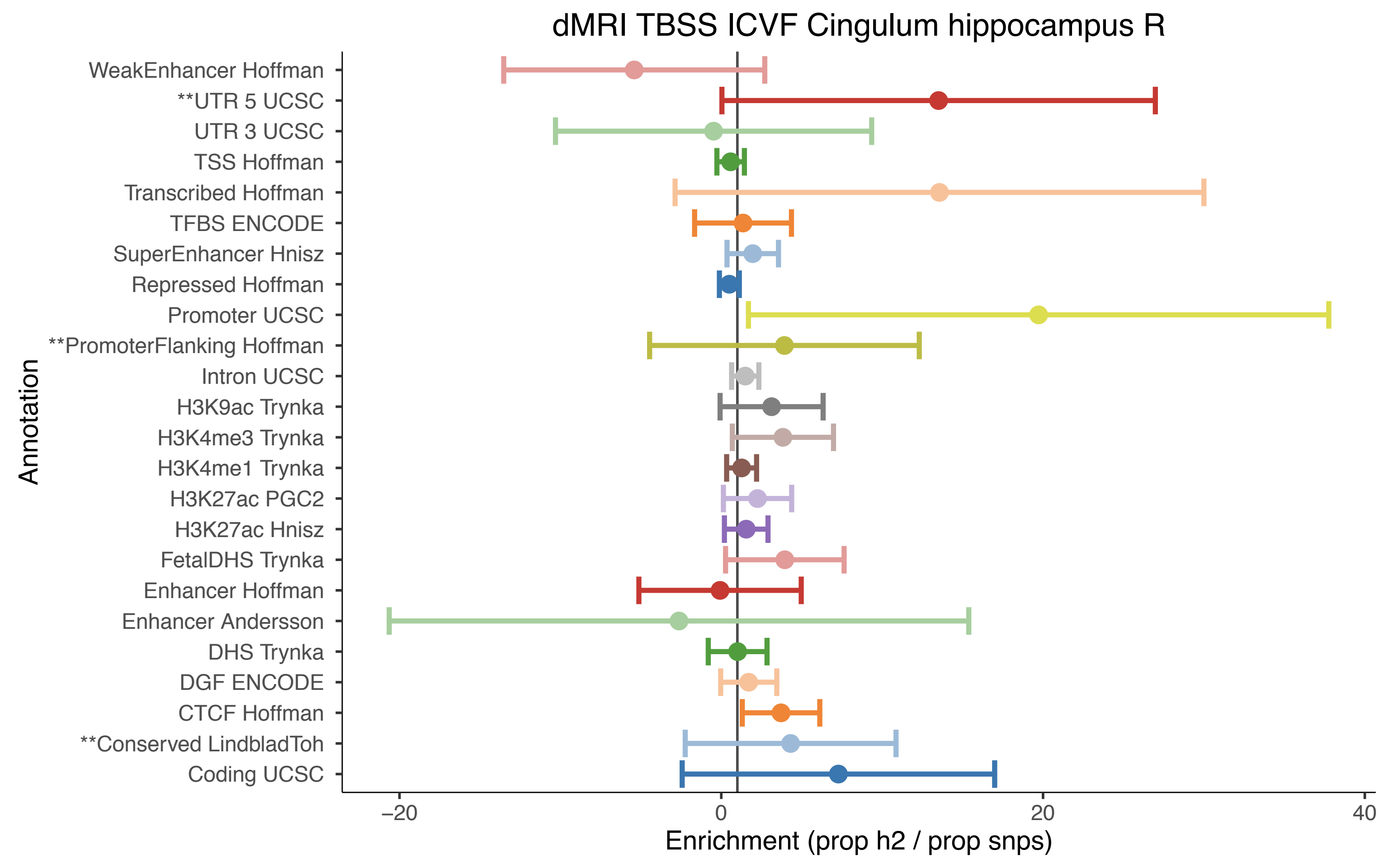

dMRI TBSS ICVF Cingulum hippocampus R
WeakEnhancer Hoffman
**UTR 5 UCSC
UTR 3 UCSC
TSS Hoffman
Transcribed Hoffman
TFBS ENCODE
SuperEnhancer Hnisz
Repressed Hoffman
Promoter UCSC
**PromoterFlanking Hoffman
Intron UCSC
H3K9ac Trynka
H3K4me3 Trynka
H3K4me1 Trynka
H3K27ac PGC2
H3K27ac Hnisz
FetalDHS Trynka
Enhancer Hoffman
Enhancer Andersson
DHS Trynka
DGF ENCODE
CTCF Hoffman
**Conserved LindbladToh
Coding UCSC
Annotation
−20
0
20
40
Enrichment (prop h2 / prop snps)

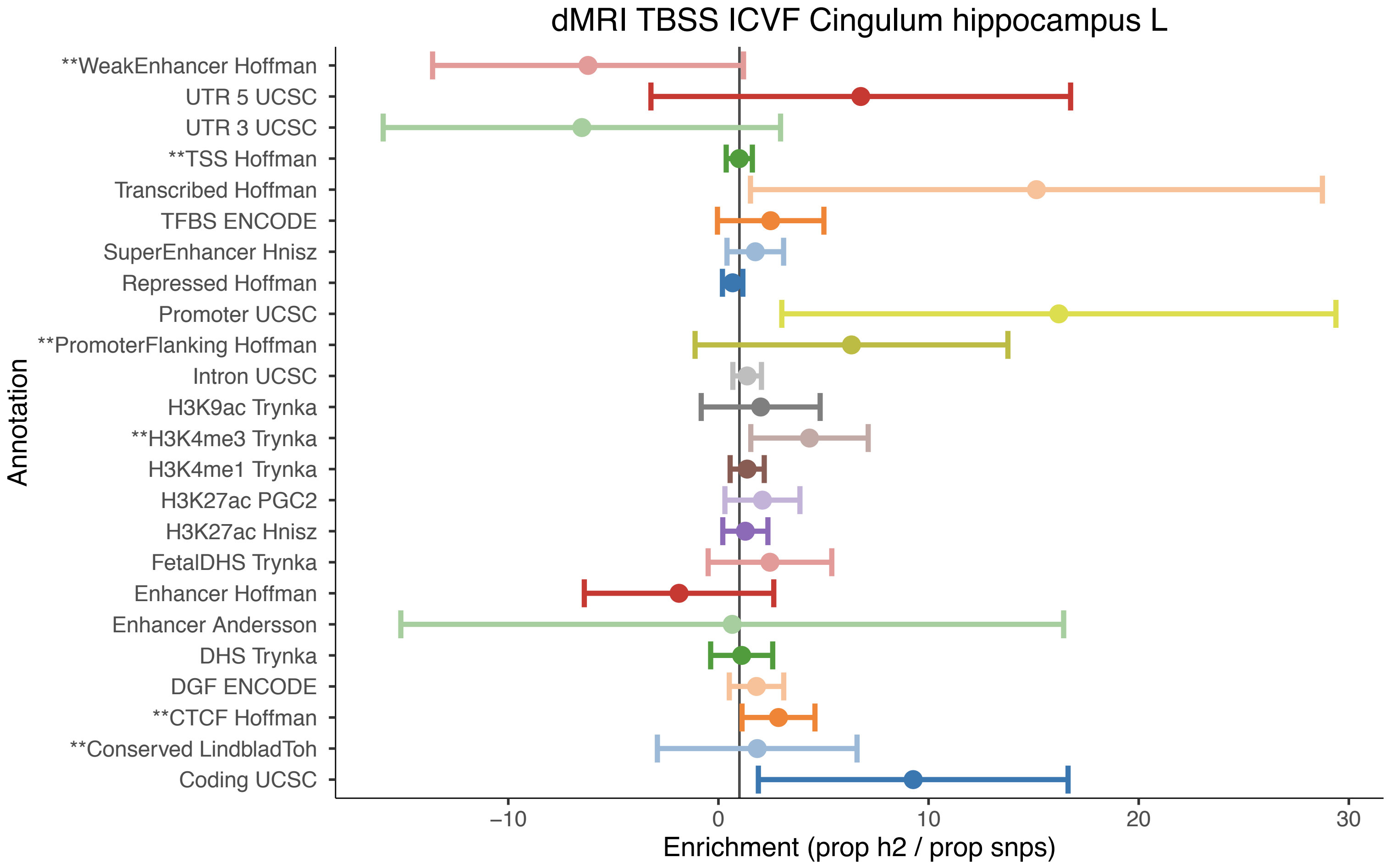
dMRI TBSS ICVF Cingulum hippocampus L
Annotation
**WeakEnhancer Hoffman
UTR 5 UCSC
UTR 3 UCSC
**TSS Hoffman
Transcribed Hoffman
TFBS ENCODE
SuperEnhancer Hnisz
Repressed Hoffman
Promoter UCSC
**PromoterFlanking Hoffman
Intron UCSC
H3K9ac Trynka
**H3K4me3 Trynka
H3K4me1 Trynka
H3K27ac PGC2
H3K27ac Hnisz
FetalDHS Trynka
Enhancer Hoffman
Enhancer Andersson
DHS Trynka
DGF ENCODE
**CTCF Hoffman
**Conserved LindbladToh
Coding UCSC
−10
0
10
20
30
Enrichment (prop h2 / prop snps)

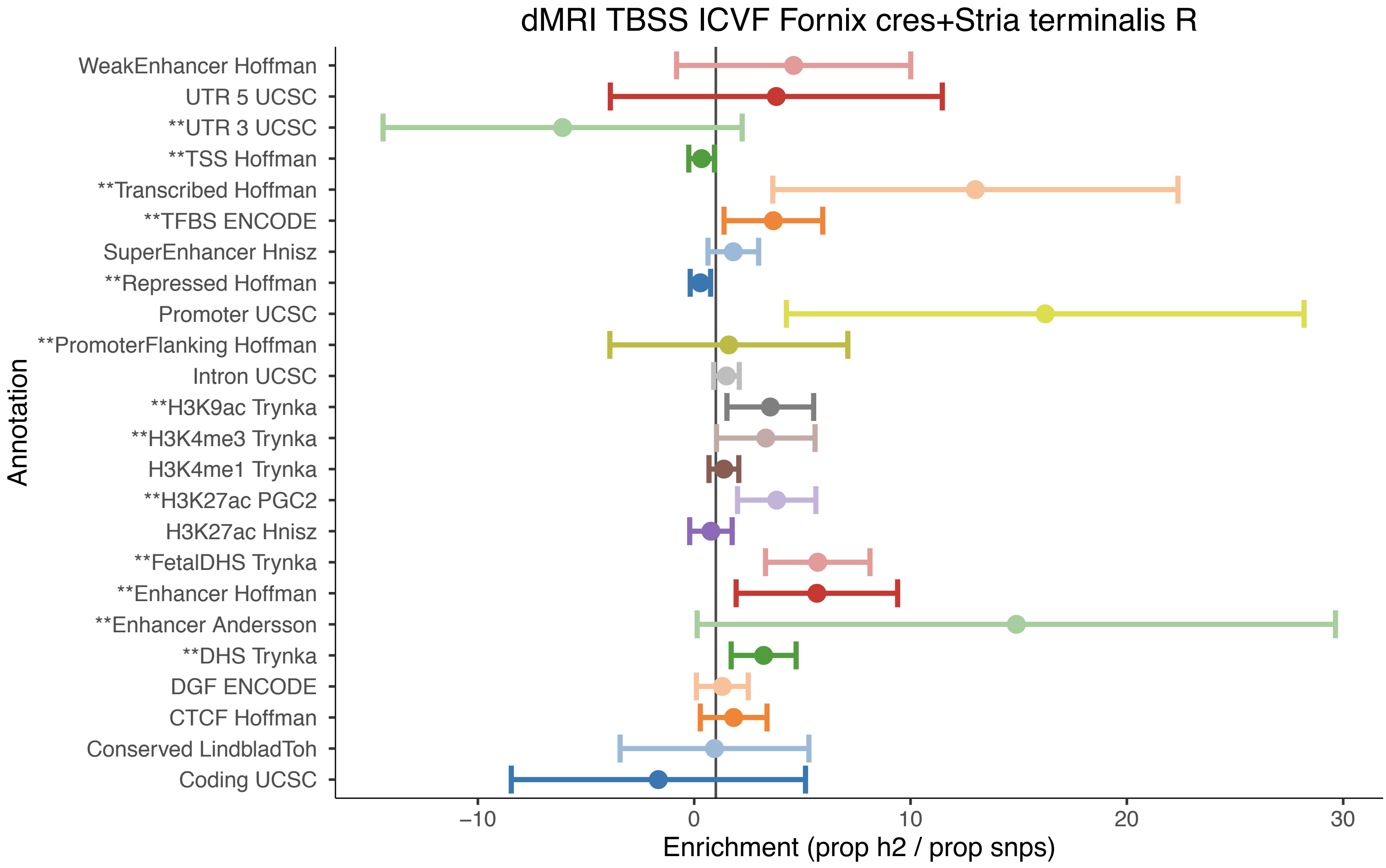
dMRI TBSS ICVF Fornix cres+Stria terminalis R
WeakEnhancer Hoffman
UTR 5 UCSC
**UTR 3 UCSC
**TSS Hoffman
**Transcribed Hoffman
**TFBS ENCODE
SuperEnhancer Hnisz
**Repressed Hoffman
Promoter UCSC
**PromoterFlanking Hoffman
Intron UCSC
**H3K9ac Trynka
**H3K4me3 Trynka
H3K4me1 Trynka
**H3K27ac PGC2
H3K27ac Hnisz
**FetalDHS Trynka
**Enhancer Hoffman
**Enhancer Andersson
**DHS Trynka
DGF ENCODE
CTCF Hoffman
Conserved LindbladToh
Coding UCSC
Annotation
−10
0
10
20
30
Enrichment (prop h2 / prop snps)

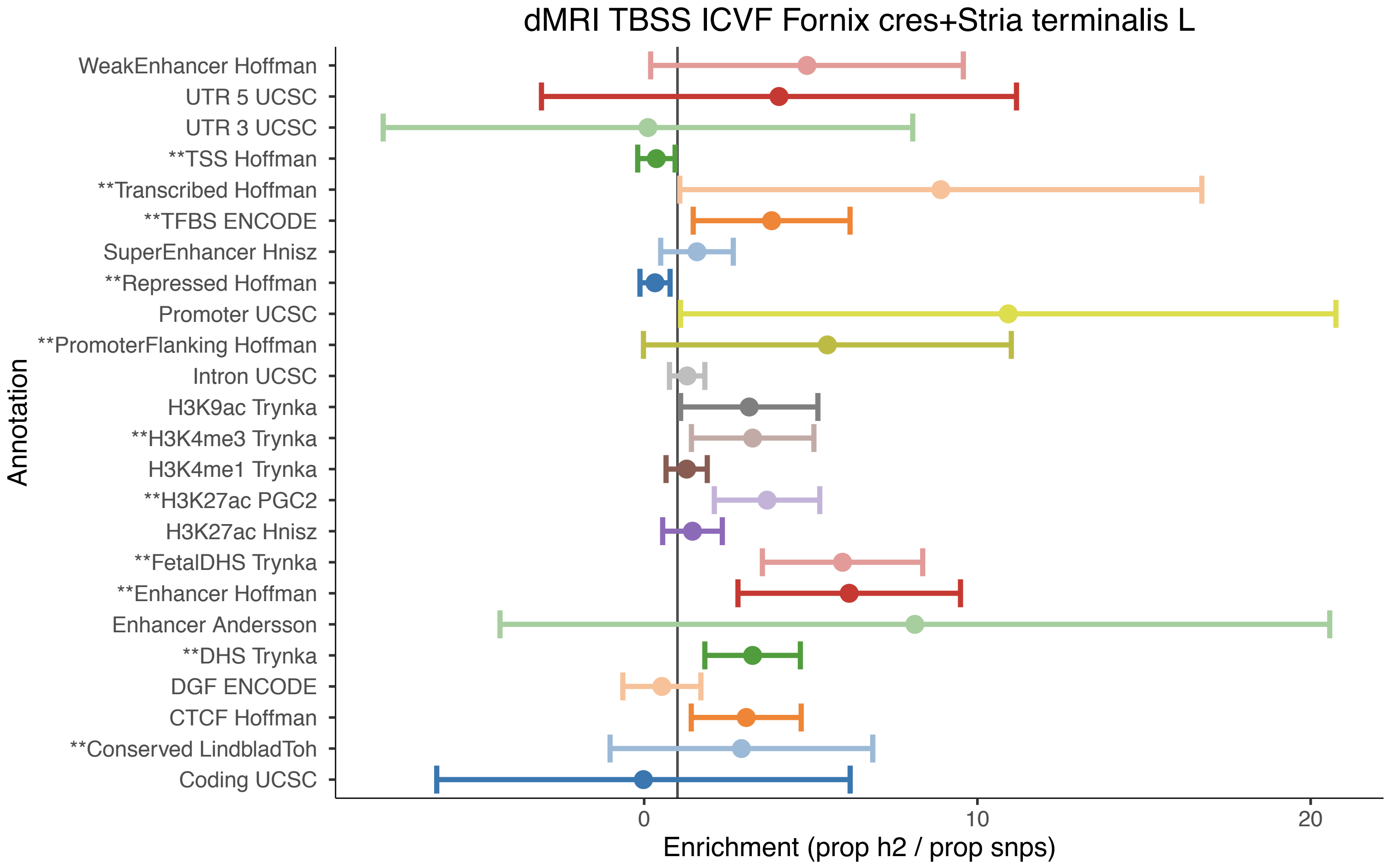

dMRI TBSS ICVF Fornix cres+Stria terminalis L
WeakEnhancer Hoffman
UTR 5 UCSC
UTR 3 UCSC
**TSS Hoffman
**Transcribed Hoffman
**TFBS ENCODE
SuperEnhancer Hnisz
**Repressed Hoffman
Promoter UCSC
**PromoterFlanking Hoffman
Intron UCSC
H3K9ac Trynka
**H3K4me3 Trynka
H3K4me1 Trynka
**H3K27ac PGC2
H3K27ac Hnisz
**FetalDHS Trynka
**Enhancer Hoffman
Enhancer Andersson
**DHS Trynka
DGF ENCODE
CTCF Hoffman
**Conserved LindbladToh
Coding UCSC
Annotation
0
10
20
Enrichment (prop h2 / prop snps)

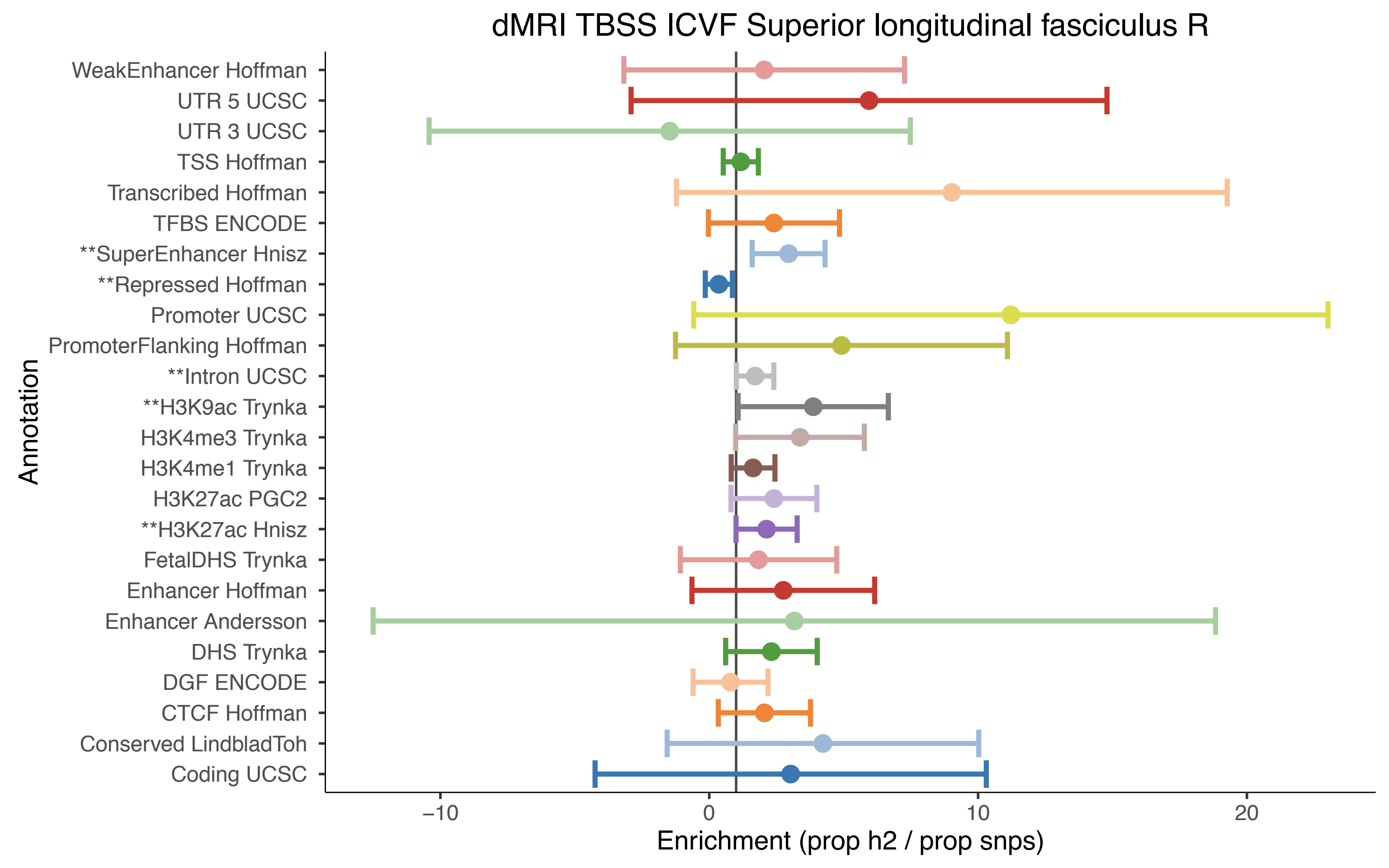
dMRI TBSS ICVF Superior longitudinal fasciculus R
WeakEnhancer Hoffman
UTR 5 UCSC
UTR 3 UCSC
TSS Hoffman
Transcribed Hoffman
TFBS ENCODE
**SuperEnhancer Hnisz
**Repressed Hoffman
Promoter UCSC
PromoterFlanking Hoffman
**Intron UCSC
**H3K9ac Trynka
H3K4me3 Trynka
H3K4me1 Trynka
H3K27ac PGC2
**H3K27ac Hnisz
FetalDHS Trynka
Enhancer Hoffman
Enhancer Andersson
DHS Trynka
DGF ENCODE
CTCF Hoffman
Conserved LindbladToh
Coding UCSC
Annotation
−10
0
10
20
Enrichment (prop h2 / prop snps)

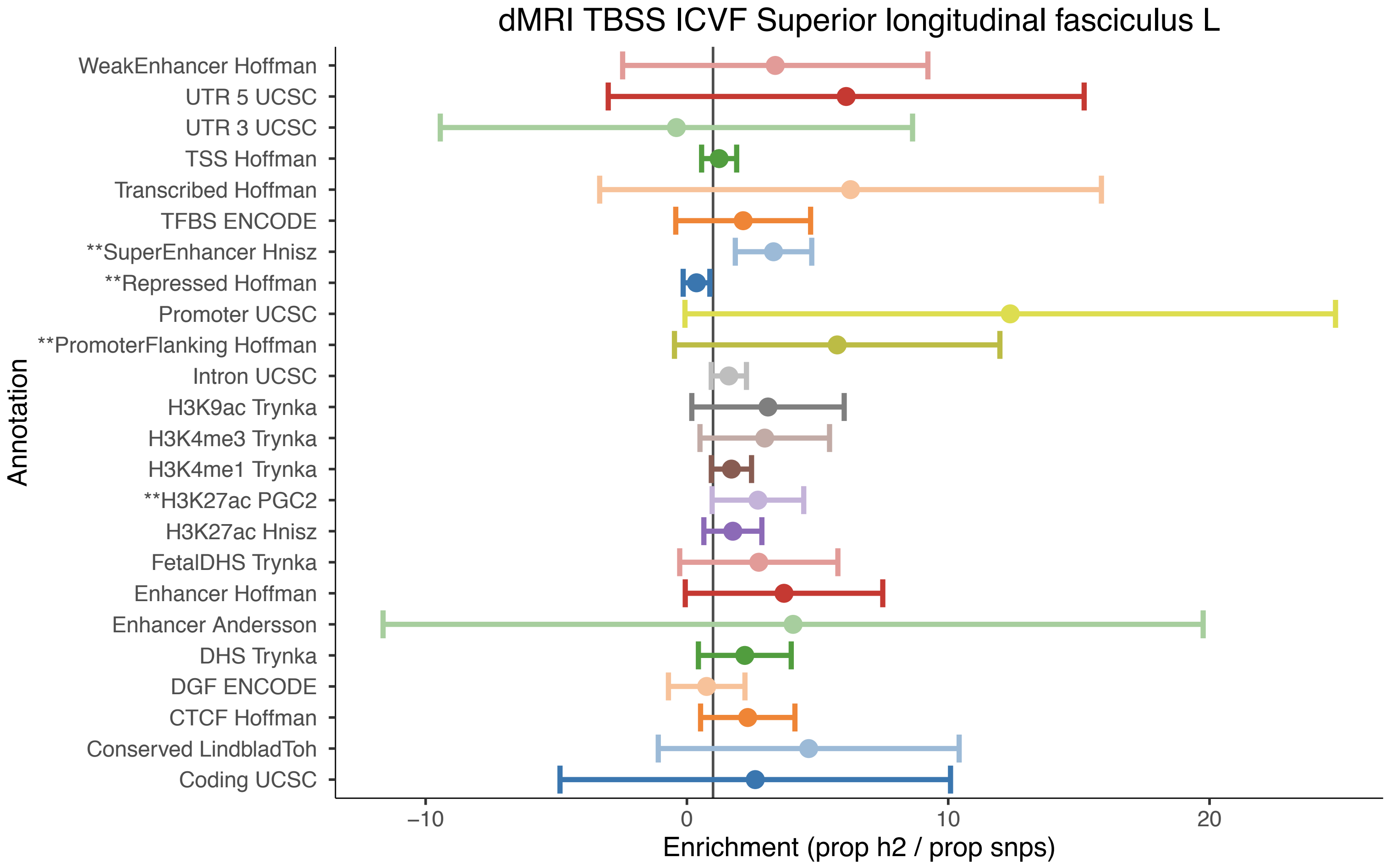
dMRI TBSS ICVF Superior longitudinal fasciculus L
WeakEnhancer Hoffman
UTR 5 UCSC
UTR 3 UCSC
TSS Hoffman
Transcribed Hoffman
TFBS ENCODE
**SuperEnhancer Hnisz
**Repressed Hoffman
Promoter UCSC
**PromoterFlanking Hoffman
Intron UCSC
H3K9ac Trynka
H3K4me3 Trynka
H3K4me1 Trynka
**H3K27ac PGC2
H3K27ac Hnisz
FetalDHS Trynka
Enhancer Hoffman
Enhancer Andersson
DHS Trynka
DGF ENCODE
CTCF Hoffman
Conserved LindbladToh
Coding UCSC
Annotation
−10
0
10
20
Enrichment (prop h2 / prop snps)

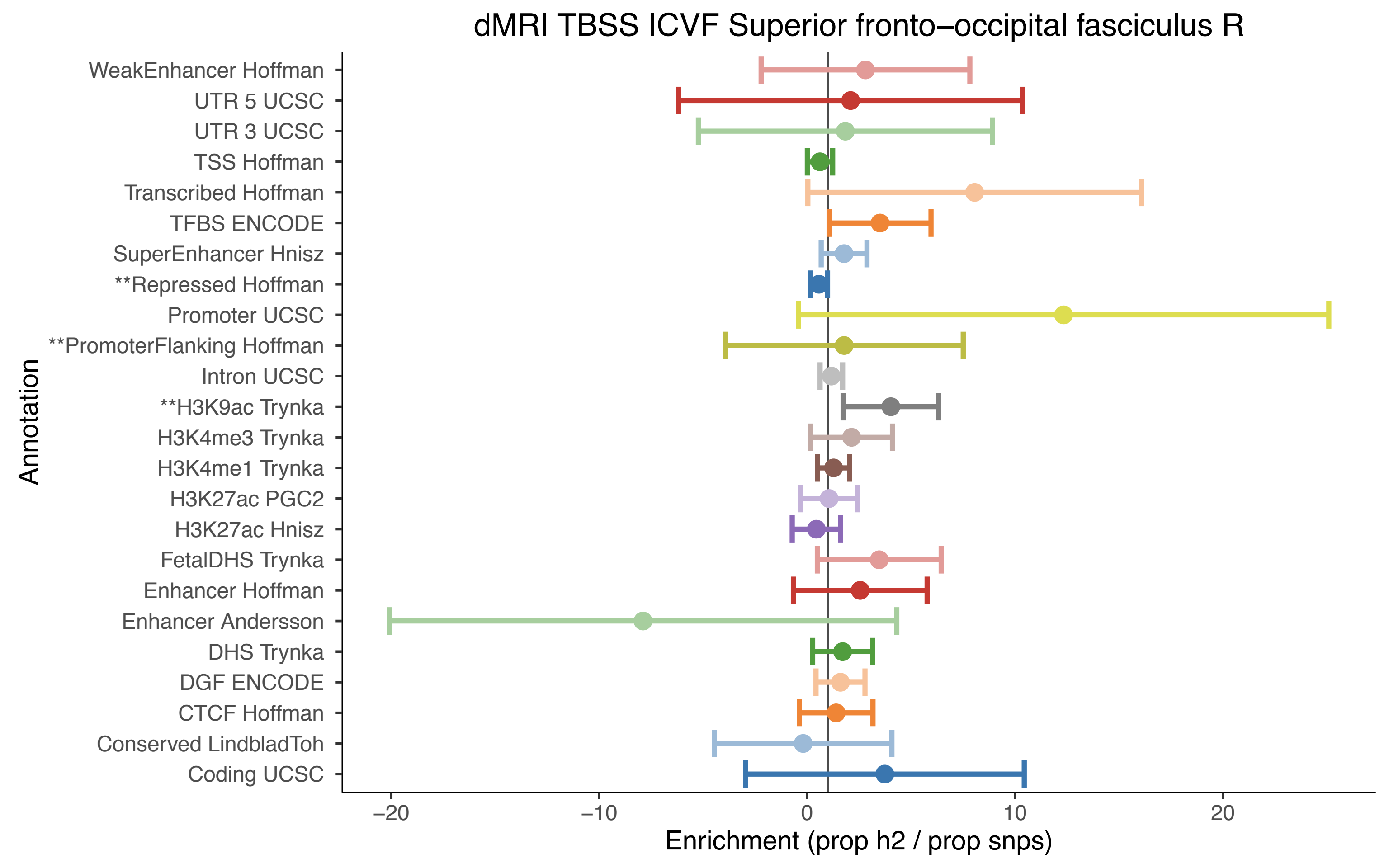

dMRI TBSS ICVF Superior fronto−occipital fasciculus R
WeakEnhancer Hoffman
UTR 5 UCSC
UTR 3 UCSC
TSS Hoffman
Transcribed Hoffman
TFBS ENCODE
SuperEnhancer Hnisz
**Repressed Hoffman
Promoter UCSC
**PromoterFlanking Hoffman
Intron UCSC
**H3K9ac Trynka
H3K4me3 Trynka
H3K4me1 Trynka
H3K27ac PGC2
H3K27ac Hnisz
FetalDHS Trynka
Enhancer Hoffman
Enhancer Andersson
DHS Trynka
DGF ENCODE
CTCF Hoffman
Conserved LindbladToh
Coding UCSC
Annotation
−20
−10
0
10
20
Enrichment (prop h2 / prop snps)

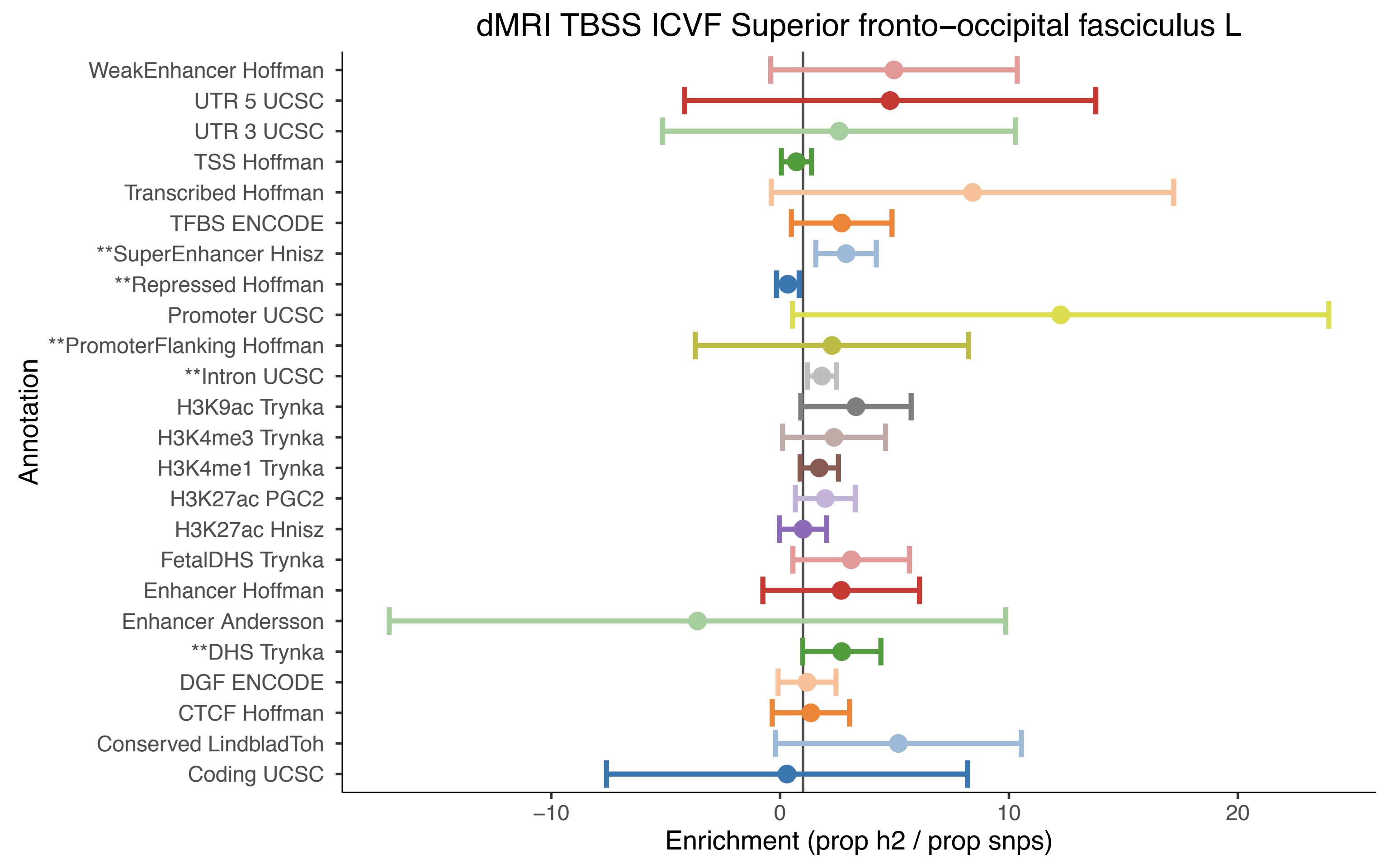

dMRI TBSS ICVF Superior fronto−occipital fasciculus L
WeakEnhancer Hoffman
UTR 5 UCSC
UTR 3 UCSC
TSS Hoffman
Transcribed Hoffman
TFBS ENCODE
**SuperEnhancer Hnisz
**Repressed Hoffman
Promoter UCSC
**PromoterFlanking Hoffman
**Intron UCSC
H3K9ac Trynka
H3K4me3 Trynka
H3K4me1 Trynka
H3K27ac PGC2
H3K27ac Hnisz
FetalDHS Trynka
Enhancer Hoffman
Enhancer Andersson
**DHS Trynka
DGF ENCODE
CTCF Hoffman
Conserved LindbladToh
Coding UCSC
Annotation
−10
0
10
20
Enrichment (prop h2 / prop snps)

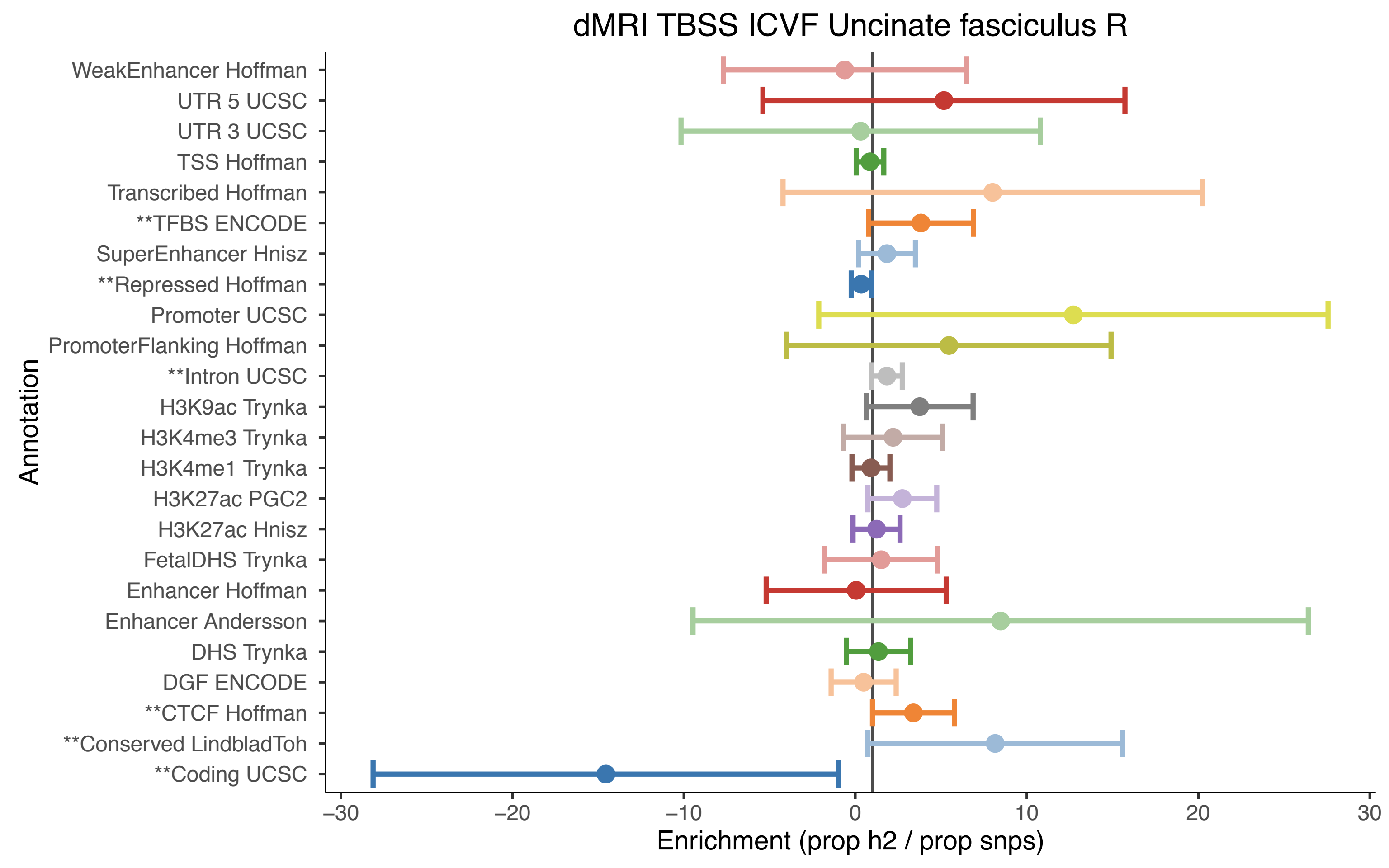
dMRI TBSS ICVF Uncinate fasciculus R
WeakEnhancer Hoffman
UTR 5 UCSC
UTR 3 UCSC
TSS Hoffman
Transcribed Hoffman
**TFBS ENCODE
SuperEnhancer Hnisz
**Repressed Hoffman
Promoter UCSC
PromoterFlanking Hoffman
**Intron UCSC
H3K9ac Trynka
H3K4me3 Trynka
H3K4me1 Trynka
H3K27ac PGC2
H3K27ac Hnisz
FetalDHS Trynka
Enhancer Hoffman
Enhancer Andersson
DHS Trynka
DGF ENCODE
**CTCF Hoffman
**Conserved LindbladToh
**Coding UCSC
Annotation
−30
−20
−10
0
10
20
30
Enrichment (prop h2 / prop snps)

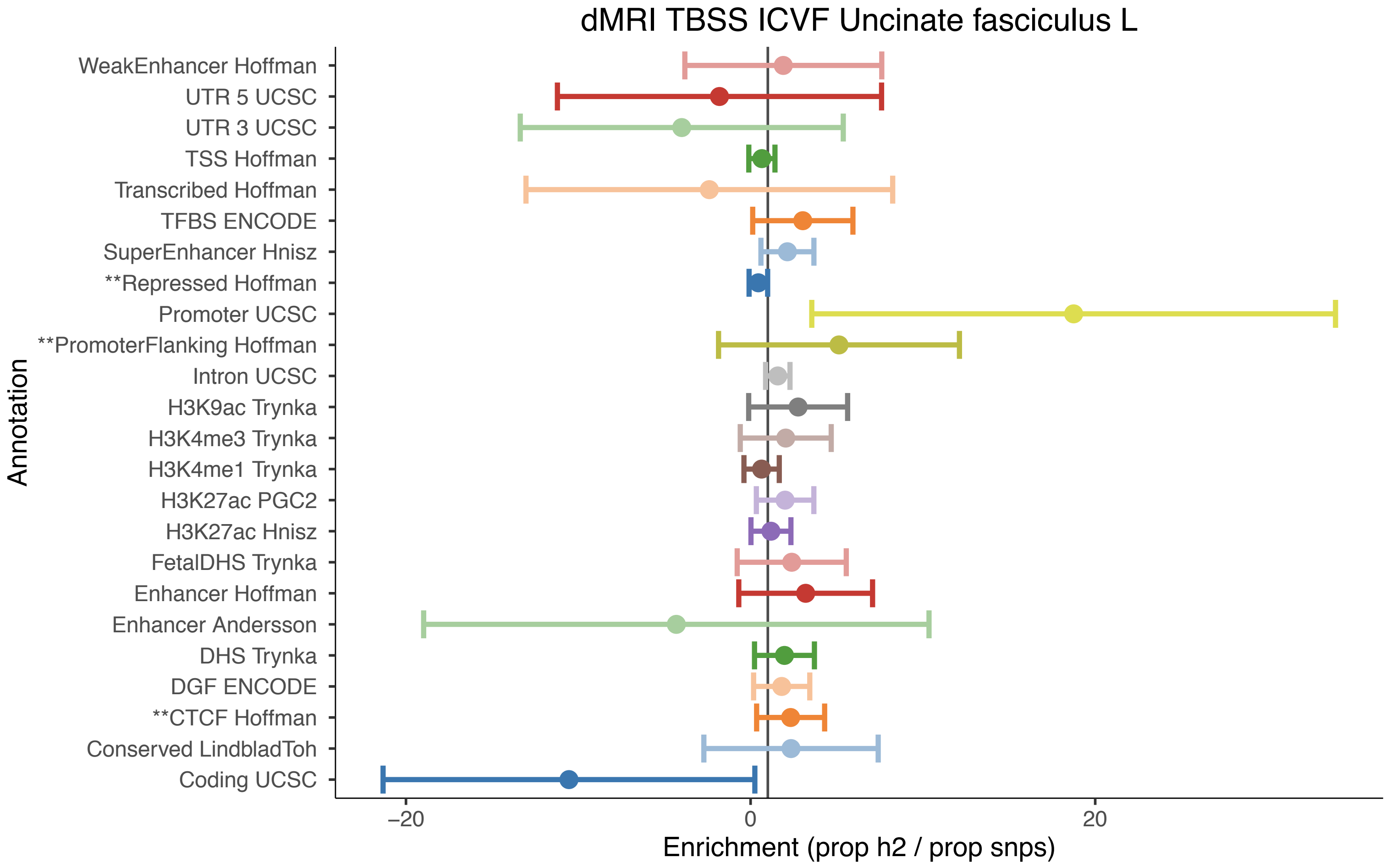
dMRI TBSS ICVF Uncinate fasciculus L
WeakEnhancer Hoffman
UTR 5 UCSC
UTR 3 UCSC
TSS Hoffman
Transcribed Hoffman
TFBS ENCODE
SuperEnhancer Hnisz
**Repressed Hoffman
Promoter UCSC
**PromoterFlanking Hoffman
Intron UCSC
H3K9ac Trynka
H3K4me3 Trynka
H3K4me1 Trynka
H3K27ac PGC2
H3K27ac Hnisz
FetalDHS Trynka
Enhancer Hoffman
Enhancer Andersson
DHS Trynka
DGF ENCODE
**CTCF Hoffman
Conserved LindbladToh
Coding UCSC
Annotation
−20
0
20
Enrichment (prop h2 / prop snps)

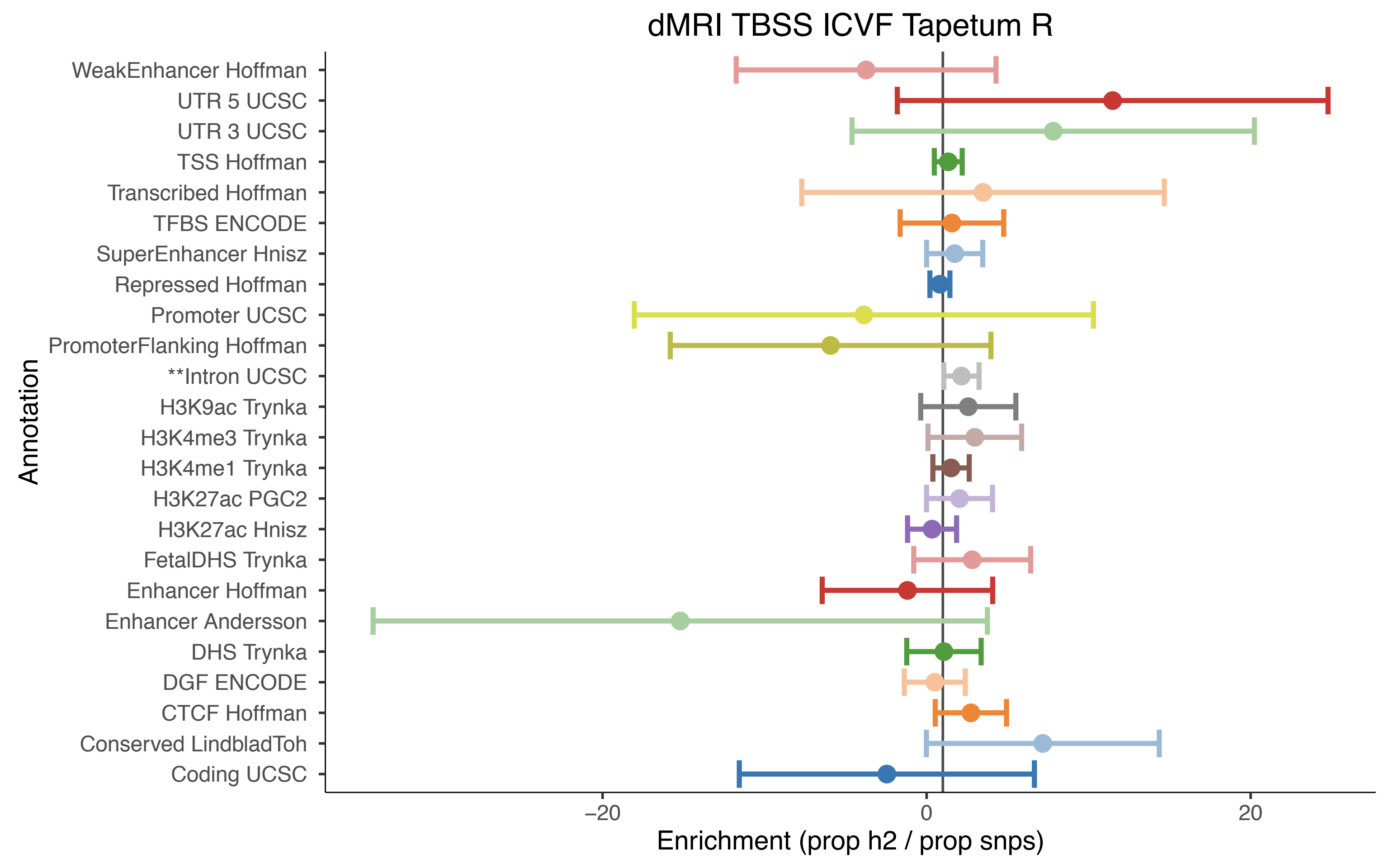
dMRI TBSS ICVF Tapetum R
WeakEnhancer Hoffman
UTR 5 UCSC
UTR 3 UCSC
TSS Hoffman
Transcribed Hoffman
TFBS ENCODE
SuperEnhancer Hnisz
Repressed Hoffman
Promoter UCSC
PromoterFlanking Hoffman
**Intron UCSC
H3K9ac Trynka
H3K4me3 Trynka
H3K4me1 Trynka
H3K27ac PGC2
H3K27ac Hnisz
FetalDHS Trynka
Enhancer Hoffman
Enhancer Andersson
DHS Trynka
DGF ENCODE
CTCF Hoffman
Conserved LindbladToh
Coding UCSC
Annotation
−20
0
20
Enrichment (prop h2 / prop snps)

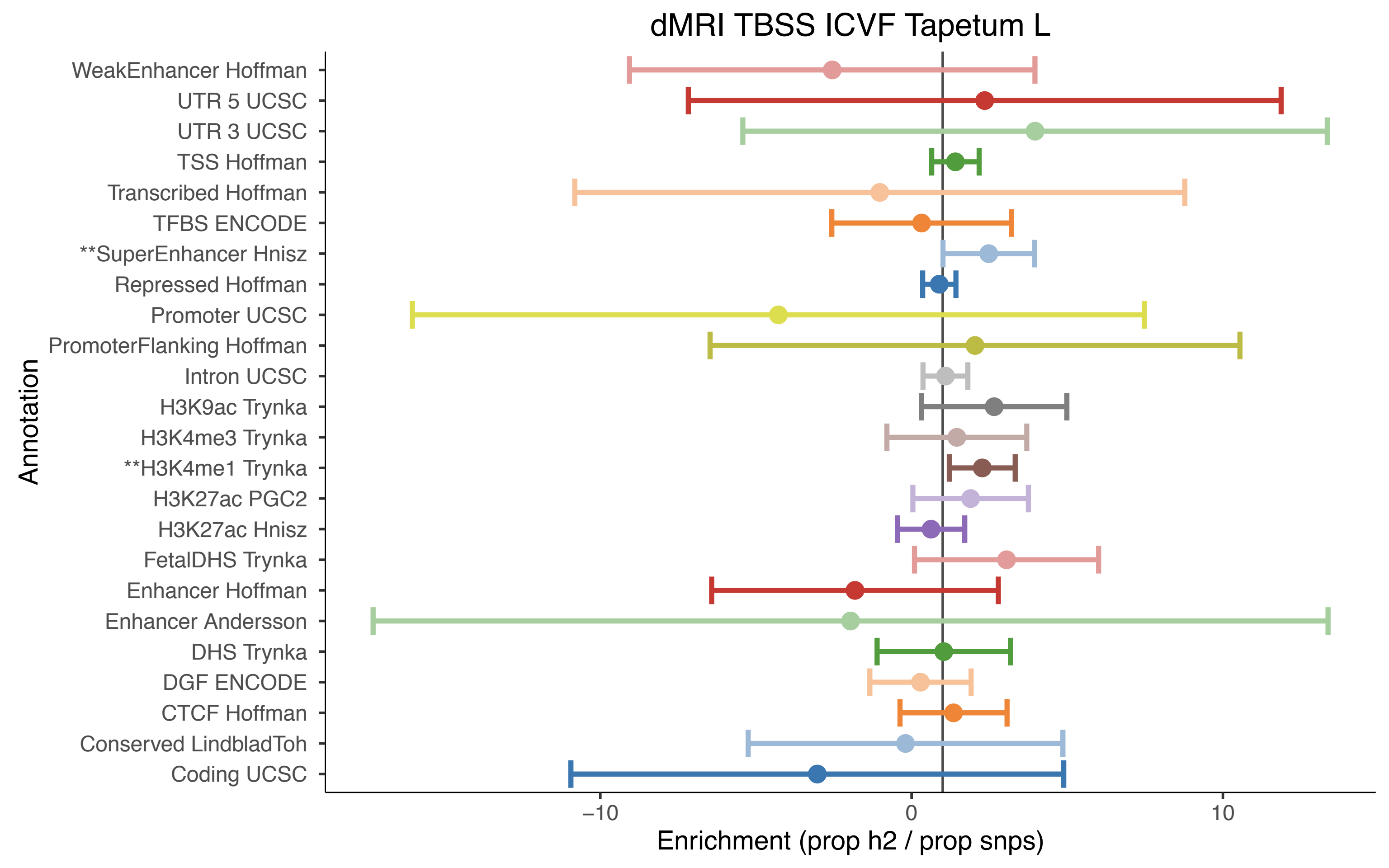
dMRI TBSS ICVF Tapetum L
WeakEnhancer Hoffman
UTR 5 UCSC
UTR 3 UCSC
TSS Hoffman
Transcribed Hoffman
TFBS ENCODE
**SuperEnhancer Hnisz
Repressed Hoffman
Promoter UCSC
PromoterFlanking Hoffman
Intron UCSC
H3K9ac Trynka
H3K4me3 Trynka
**H3K4me1 Trynka
H3K27ac PGC2
H3K27ac Hnisz
FetalDHS Trynka
Enhancer Hoffman
Enhancer Andersson
DHS Trynka
DGF ENCODE
CTCF Hoffman
Conserved LindbladToh
Coding UCSC
Annotation
−10
0
10
Enrichment (prop h2 / prop snps)

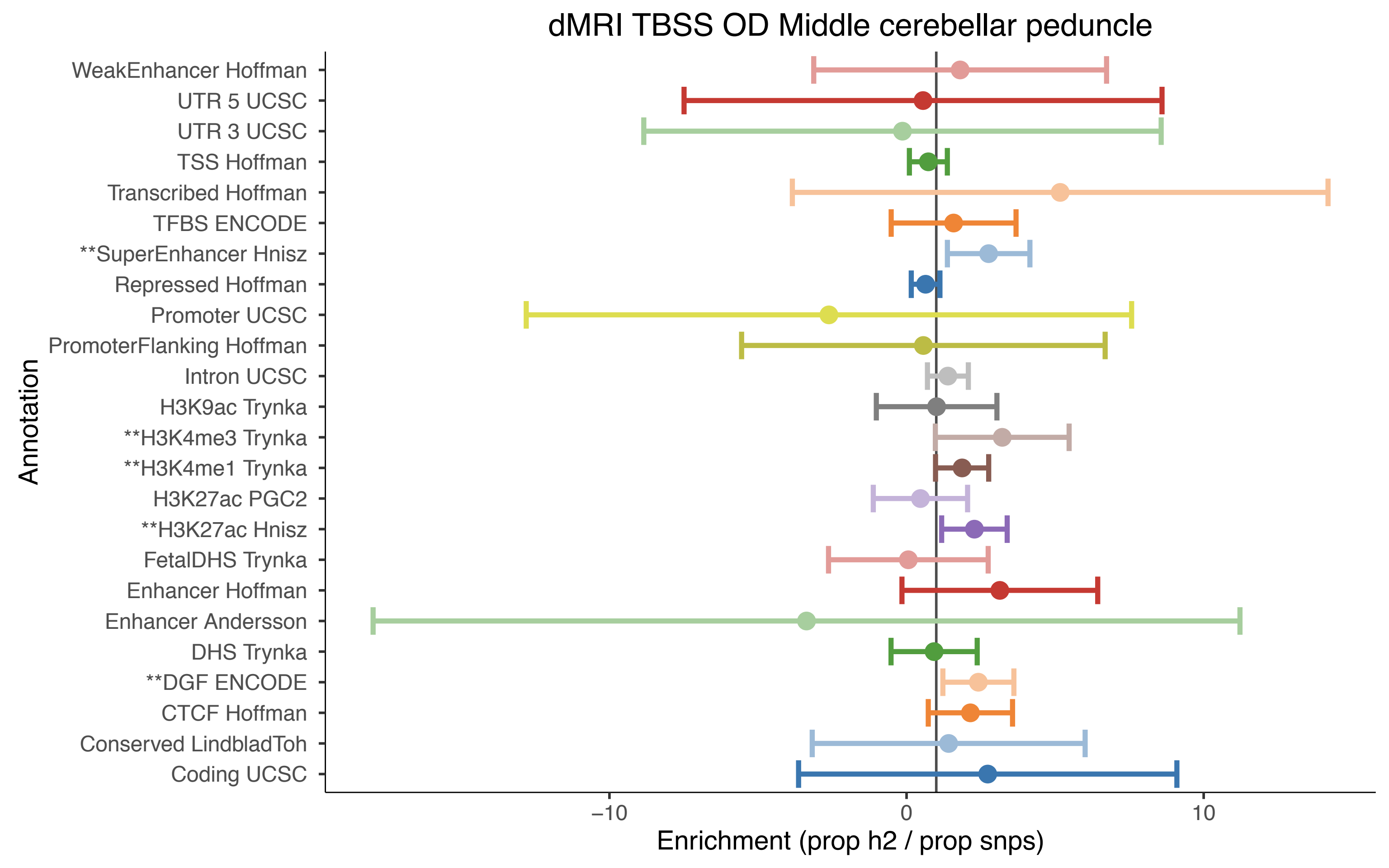

dMRI TBSS OD Middle cerebellar peduncle
WeakEnhancer Hoffman
UTR 5 UCSC
UTR 3 UCSC
TSS Hoffman
Transcribed Hoffman
TFBS ENCODE
**SuperEnhancer Hnisz
Repressed Hoffman
Promoter UCSC
PromoterFlanking Hoffman
Intron UCSC
H3K9ac Trynka
**H3K4me3 Trynka
**H3K4me1 Trynka
H3K27ac PGC2
**H3K27ac Hnisz
FetalDHS Trynka
Enhancer Hoffman
Enhancer Andersson
DHS Trynka
**DGF ENCODE
CTCF Hoffman
Conserved LindbladToh
Coding UCSC
Annotation
−10
0
10
Enrichment (prop h2 / prop snps)

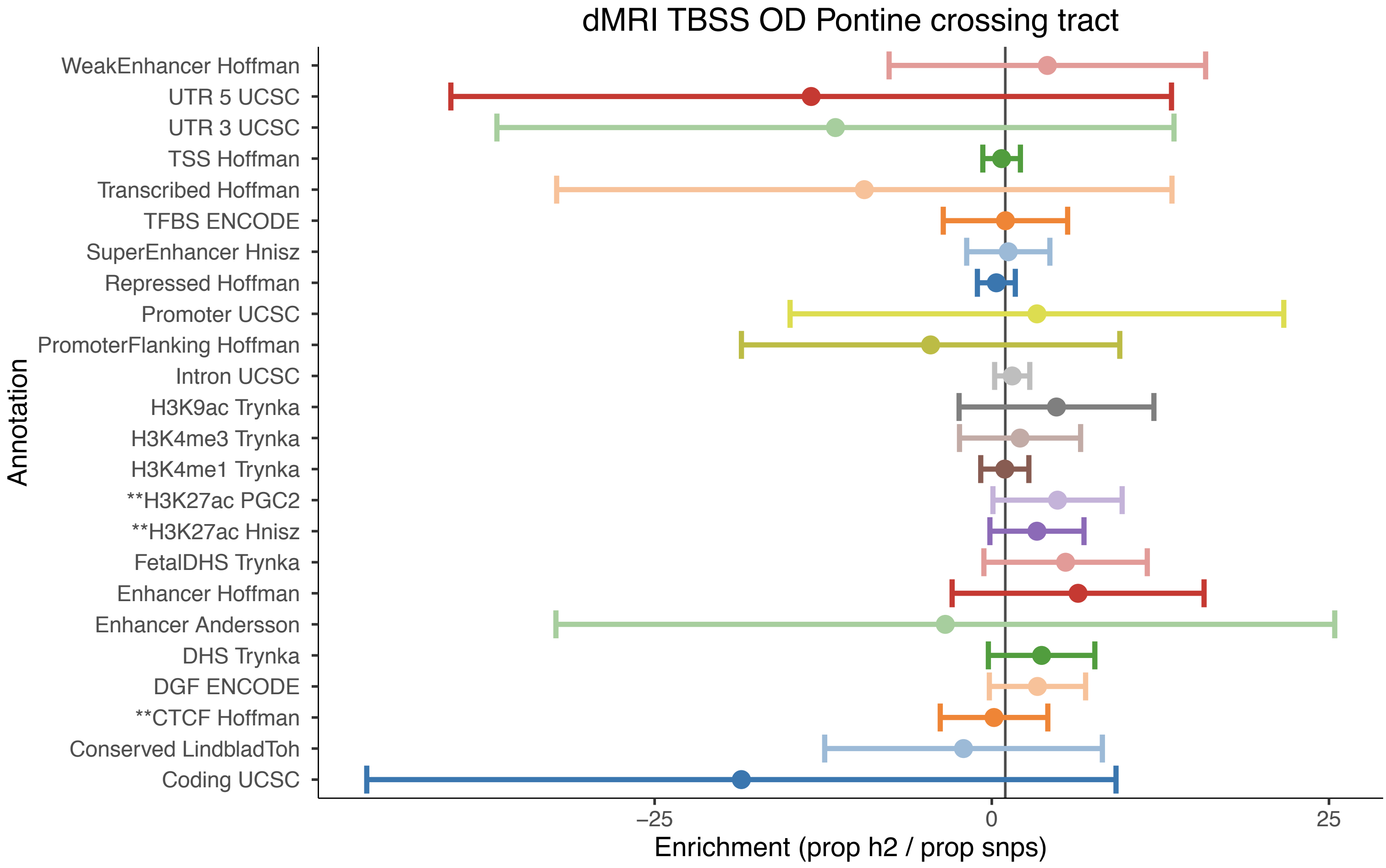
dMRI TBSS OD Pontine crossing tract
WeakEnhancer Hoffman
UTR 5 UCSC
UTR 3 UCSC
TSS Hoffman
Transcribed Hoffman
TFBS ENCODE
SuperEnhancer Hnisz
Repressed Hoffman
Promoter UCSC
PromoterFlanking Hoffman
Intron UCSC
H3K9ac Trynka
H3K4me3 Trynka
H3K4me1 Trynka
**H3K27ac PGC2
**H3K27ac Hnisz
FetalDHS Trynka
Enhancer Hoffman
Enhancer Andersson
DHS Trynka
DGF ENCODE
**CTCF Hoffman
Conserved LindbladToh
Coding UCSC
Annotation
−25
0
25
Enrichment (prop h2 / prop snps)

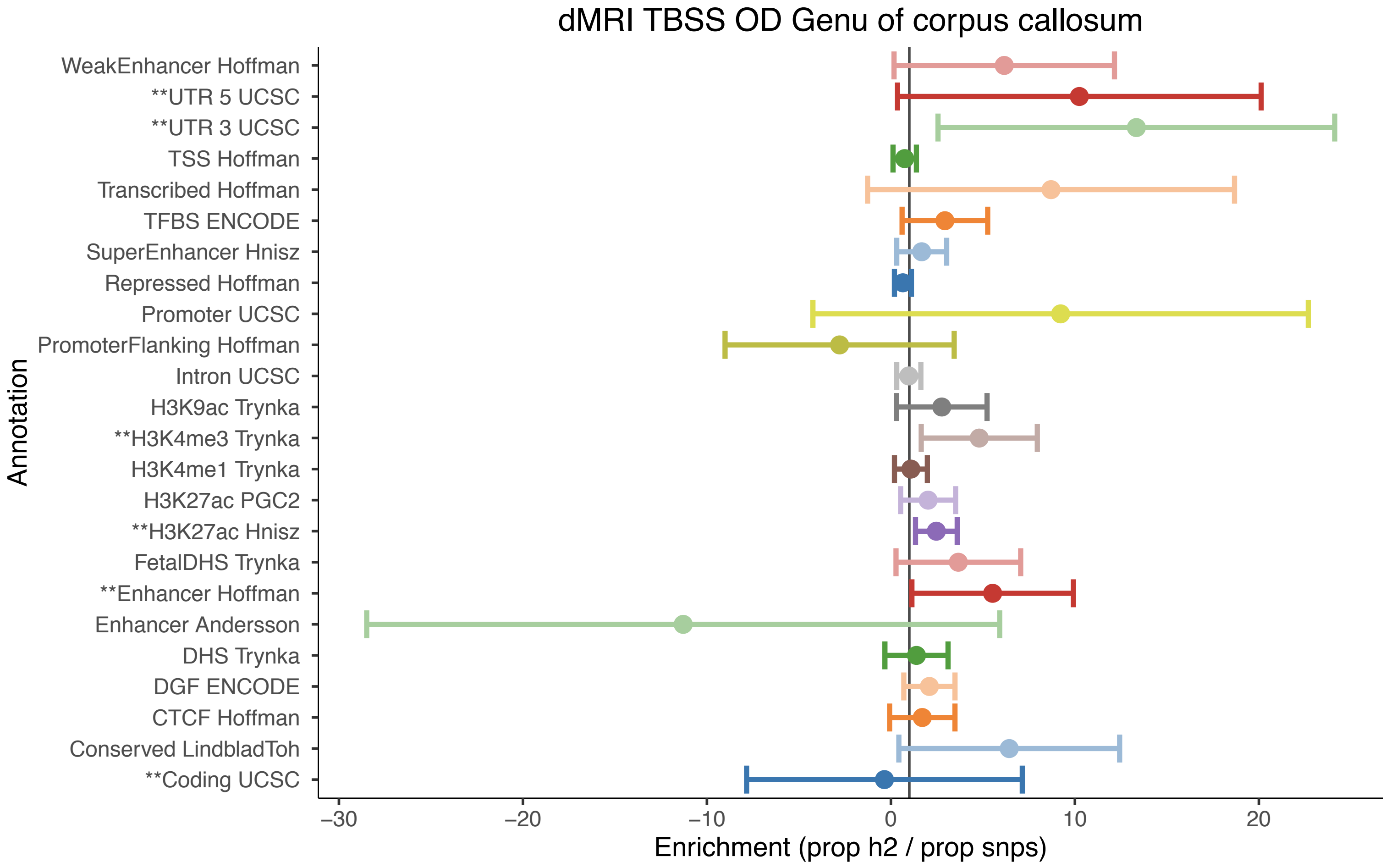

dMRI TBSS OD Genu of corpus callosum
WeakEnhancer Hoffman
**UTR 5 UCSC
**UTR 3 UCSC
TSS Hoffman
Transcribed Hoffman
TFBS ENCODE
SuperEnhancer Hnisz
Repressed Hoffman
Promoter UCSC
PromoterFlanking Hoffman
Intron UCSC
H3K9ac Trynka
**H3K4me3 Trynka
H3K4me1 Trynka
H3K27ac PGC2
**H3K27ac Hnisz
FetalDHS Trynka
**Enhancer Hoffman
Enhancer Andersson
DHS Trynka
DGF ENCODE
CTCF Hoffman
Conserved LindbladToh
**Coding UCSC
Annotation
−30
−20
−10
0
10
20
Enrichment (prop h2 / prop snps)

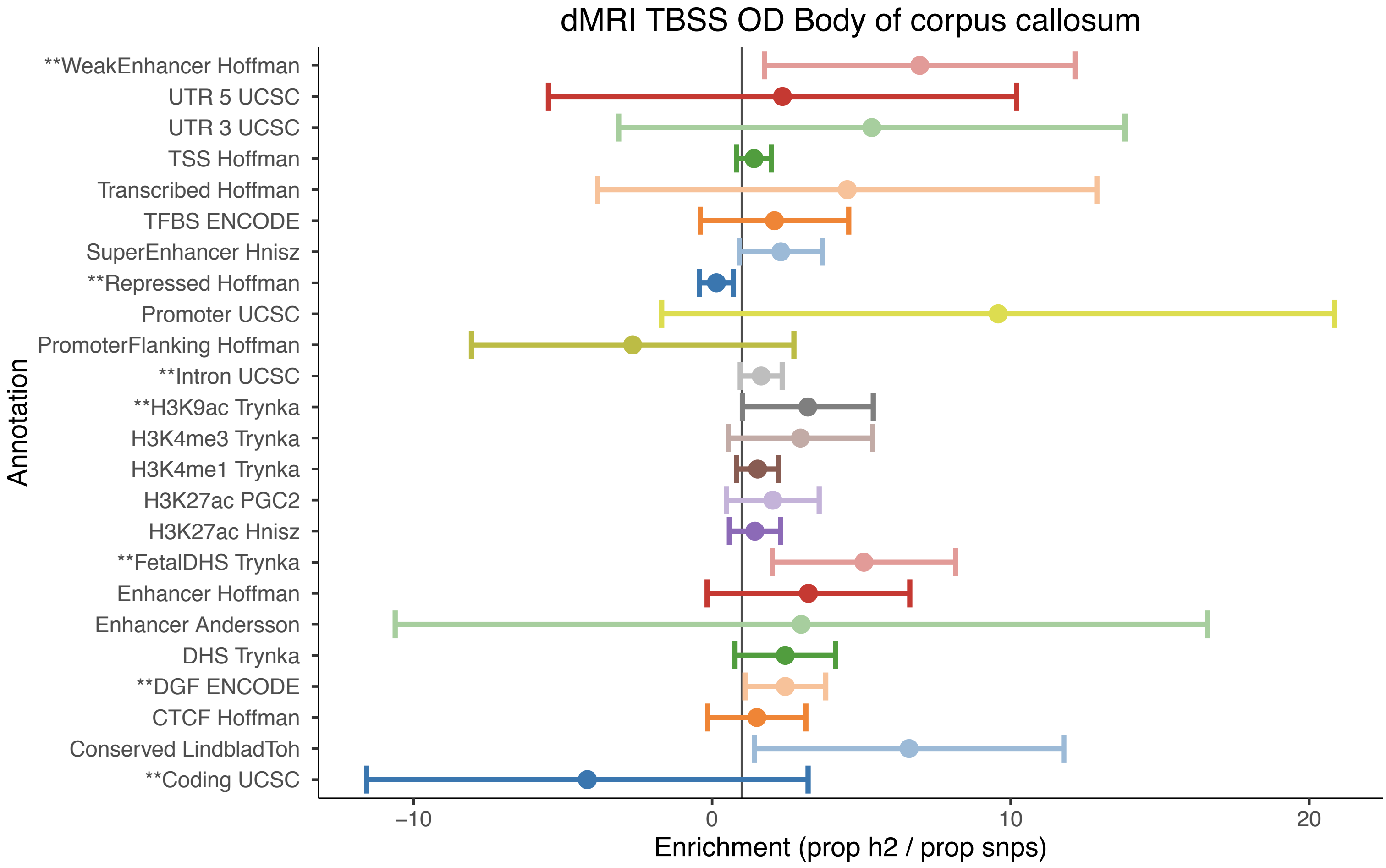

dMRI TBSS OD Body of corpus callosum
**WeakEnhancer Hoffman
UTR 5 UCSC
UTR 3 UCSC
TSS Hoffman
Transcribed Hoffman
TFBS ENCODE
SuperEnhancer Hnisz
**Repressed Hoffman
Promoter UCSC
PromoterFlanking Hoffman
**Intron UCSC
**H3K9ac Trynka
H3K4me3 Trynka
H3K4me1 Trynka
H3K27ac PGC2
H3K27ac Hnisz
**FetalDHS Trynka
Enhancer Hoffman
Enhancer Andersson
DHS Trynka
**DGF ENCODE
CTCF Hoffman
Conserved LindbladToh
**Coding UCSC
Annotation
−10
0
10
20
Enrichment (prop h2 / prop snps)

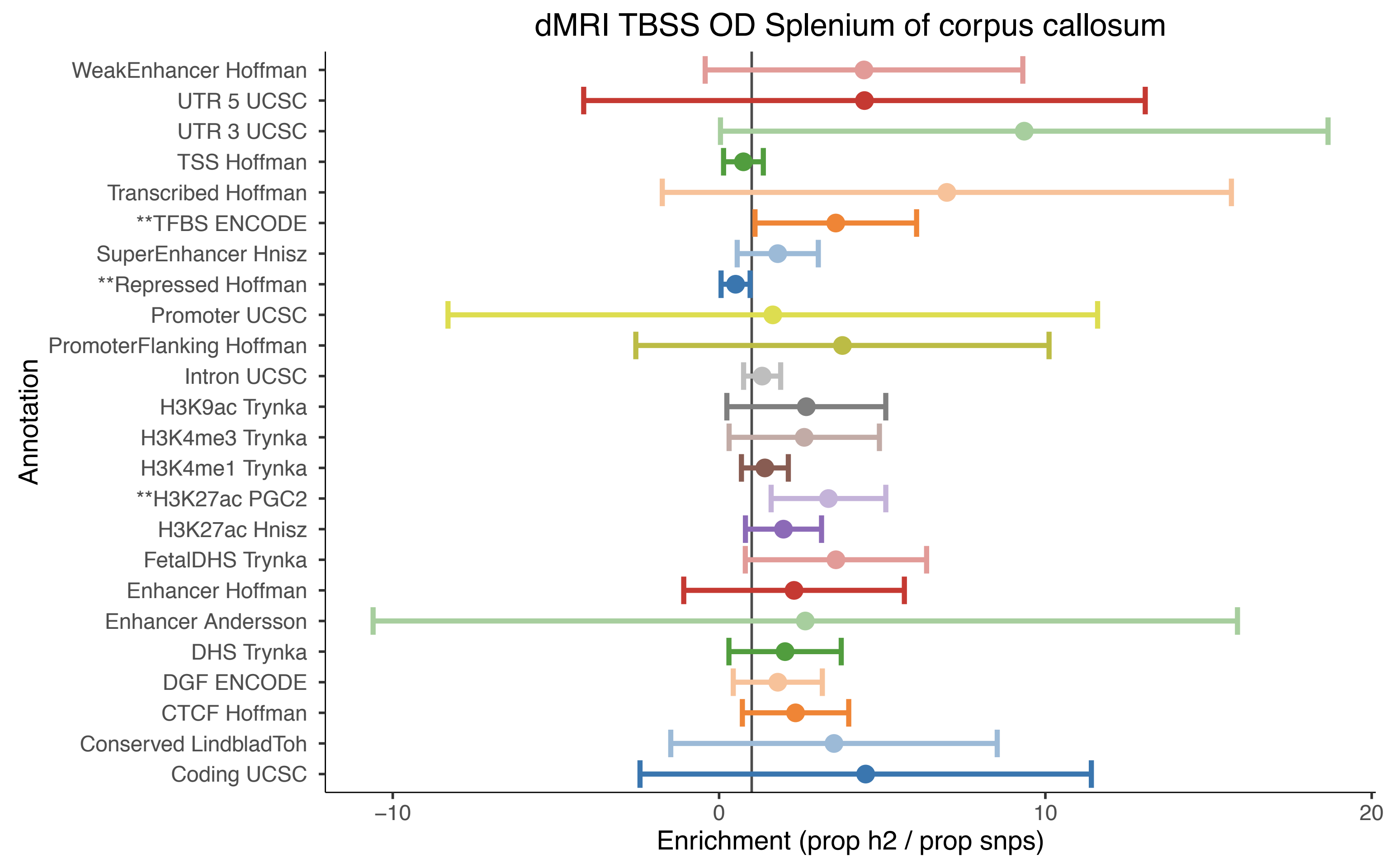
dMRI TBSS OD Splenium of corpus callosum
WeakEnhancer Hoffman
UTR 5 UCSC
UTR 3 UCSC
TSS Hoffman
Transcribed Hoffman
**TFBS ENCODE
SuperEnhancer Hnisz
**Repressed Hoffman
Promoter UCSC
PromoterFlanking Hoffman
Intron UCSC
H3K9ac Trynka
H3K4me3 Trynka
H3K4me1 Trynka
**H3K27ac PGC2
H3K27ac Hnisz
FetalDHS Trynka
Enhancer Hoffman
Enhancer Andersson
DHS Trynka
DGF ENCODE
CTCF Hoffman
Conserved LindbladToh
Coding UCSC
Annotation
−10
0
10
20
Enrichment (prop h2 / prop snps)

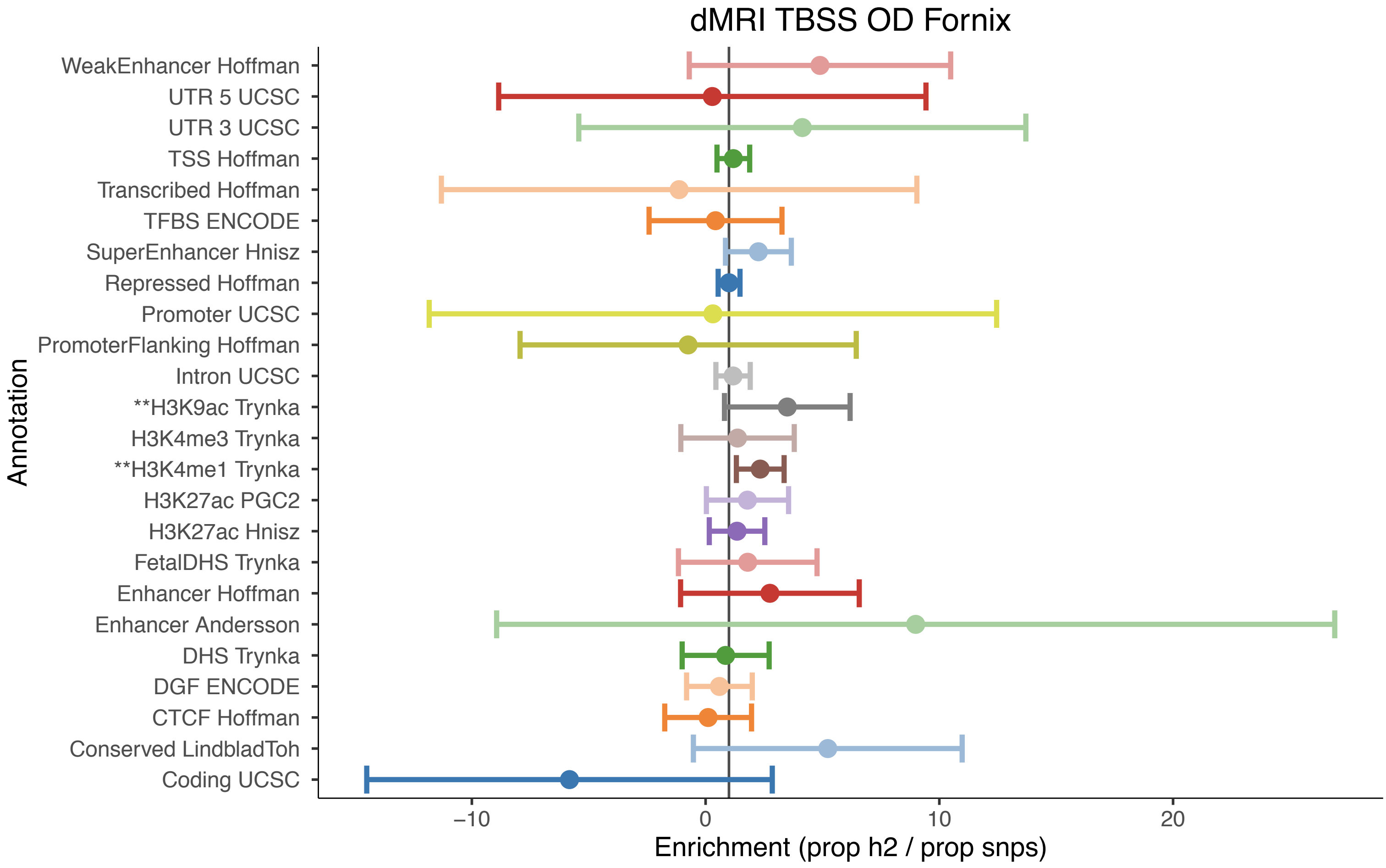

dMRI TBSS OD Fornix
Annotation
WeakEnhancer Hoffman
UTR 5 UCSC
UTR 3 UCSC
TSS Hoffman
Transcribed Hoffman
TFBS ENCODE
SuperEnhancer Hnisz
Repressed Hoffman
Promoter UCSC
PromoterFlanking Hoffman
Intron UCSC
**H3K9ac Trynka
H3K4me3 Trynka
**H3K4me1 Trynka
H3K27ac PGC2
H3K27ac Hnisz
FetalDHS Trynka
Enhancer Hoffman
Enhancer Andersson
DHS Trynka
DGF ENCODE
CTCF Hoffman
Conserved LindbladToh
Coding UCSC
−10
0
10
20
Enrichment (prop h2 / prop snps)

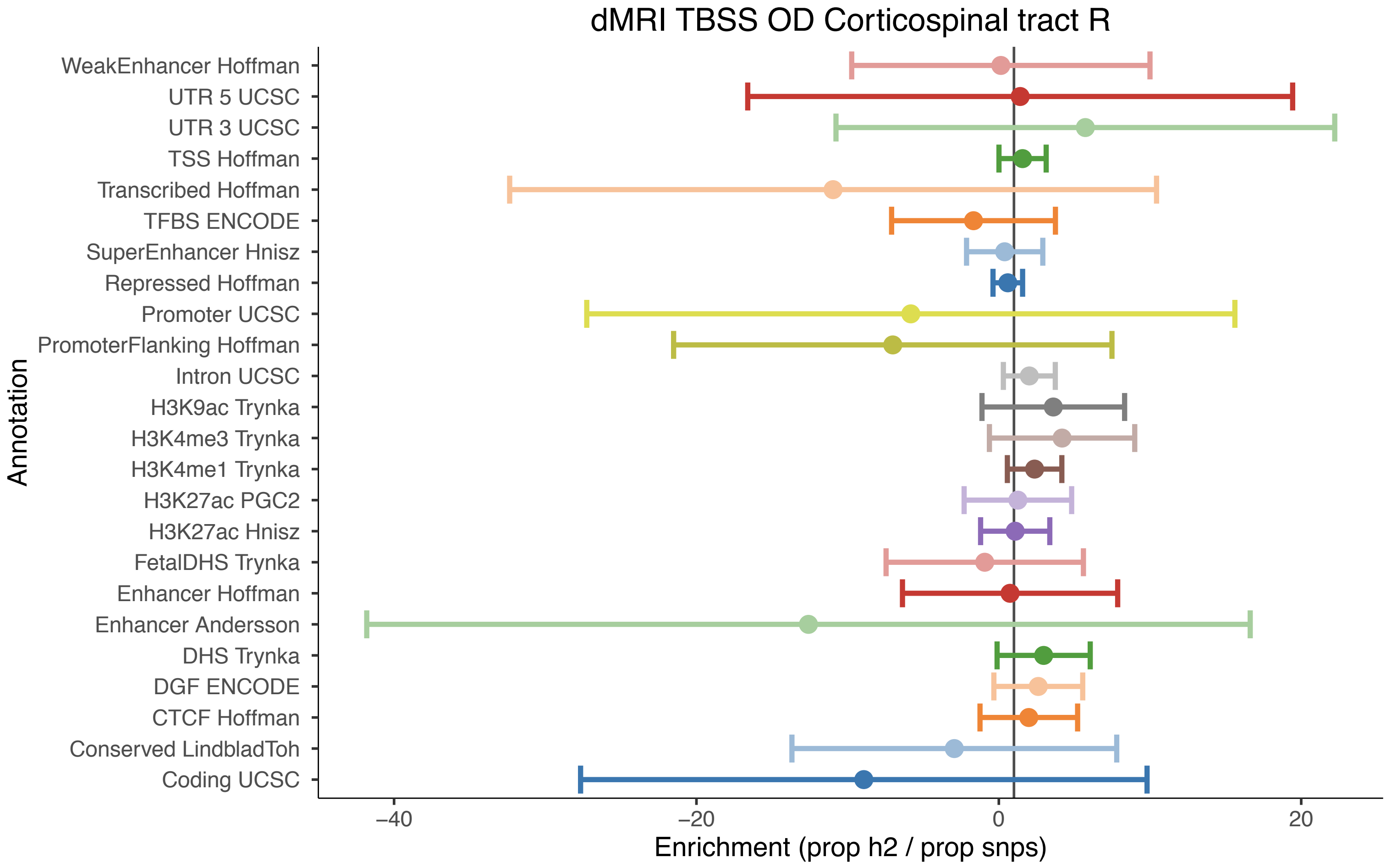

dMRI TBSS OD Corticospinal tract R
Annotation
WeakEnhancer Hoffman
UTR 5 UCSC
UTR 3 UCSC
TSS Hoffman
Transcribed Hoffman
TFBS ENCODE
SuperEnhancer Hnisz
Repressed Hoffman
Promoter UCSC
PromoterFlanking Hoffman
Intron UCSC
H3K9ac Trynka
H3K4me3 Trynka
H3K4me1 Trynka
H3K27ac PGC2
H3K27ac Hnisz
FetalDHS Trynka
Enhancer Hoffman
Enhancer Andersson
DHS Trynka
DGF ENCODE
CTCF Hoffman
Conserved LindbladToh
Coding UCSC
−40
−20
0
20
Enrichment (prop h2 / prop snps)

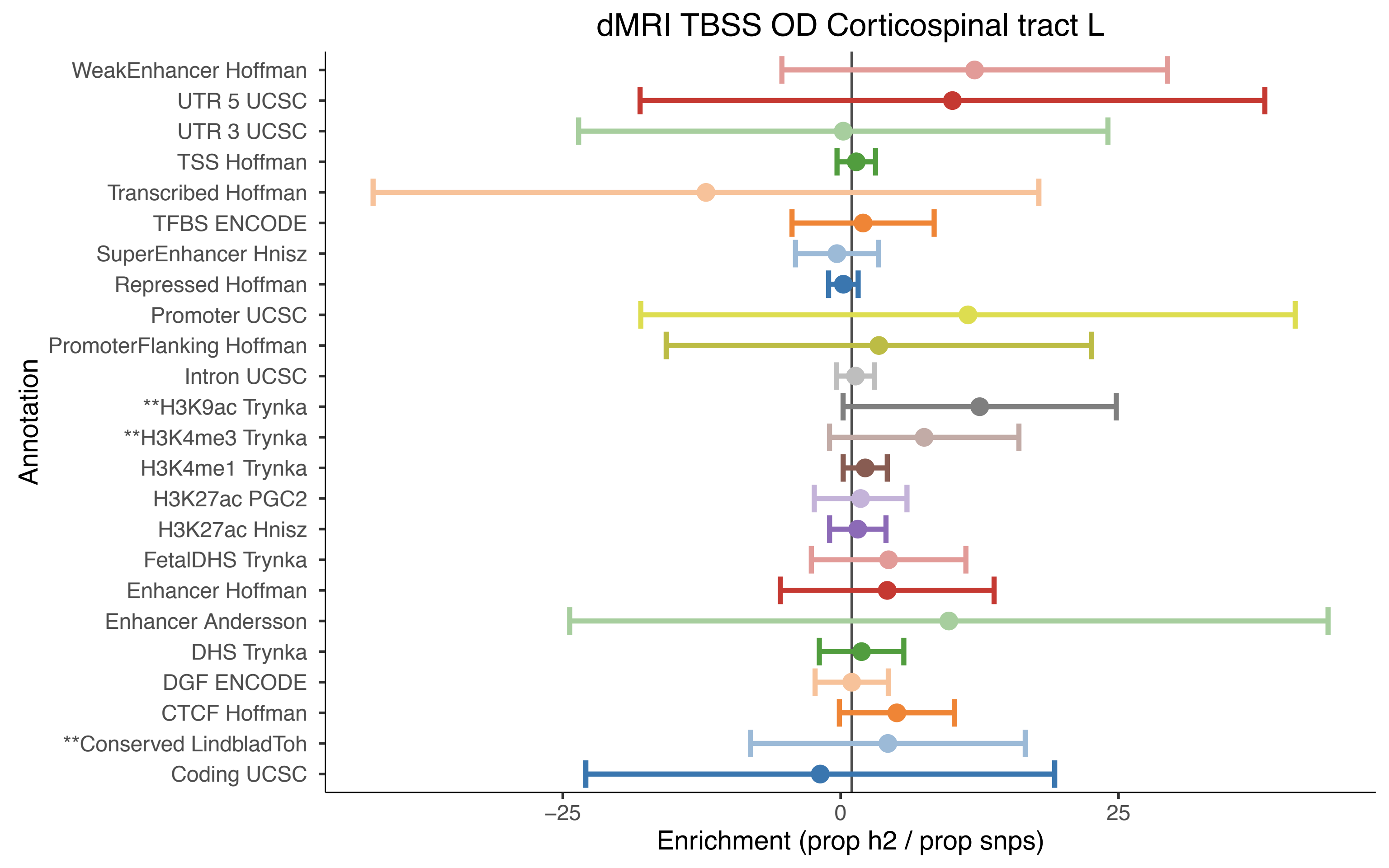
dMRI TBSS OD Corticospinal tract L
WeakEnhancer Hoffman
UTR 5 UCSC
UTR 3 UCSC
TSS Hoffman
Transcribed Hoffman
TFBS ENCODE
SuperEnhancer Hnisz
Repressed Hoffman
Promoter UCSC
PromoterFlanking Hoffman
Intron UCSC
**H3K9ac Trynka
**H3K4me3 Trynka
H3K4me1 Trynka
H3K27ac PGC2
H3K27ac Hnisz
FetalDHS Trynka
Enhancer Hoffman
Enhancer Andersson
DHS Trynka
DGF ENCODE
CTCF Hoffman
**Conserved LindbladToh
Coding UCSC
Annotation
−25
0
25
Enrichment (prop h2 / prop snps)

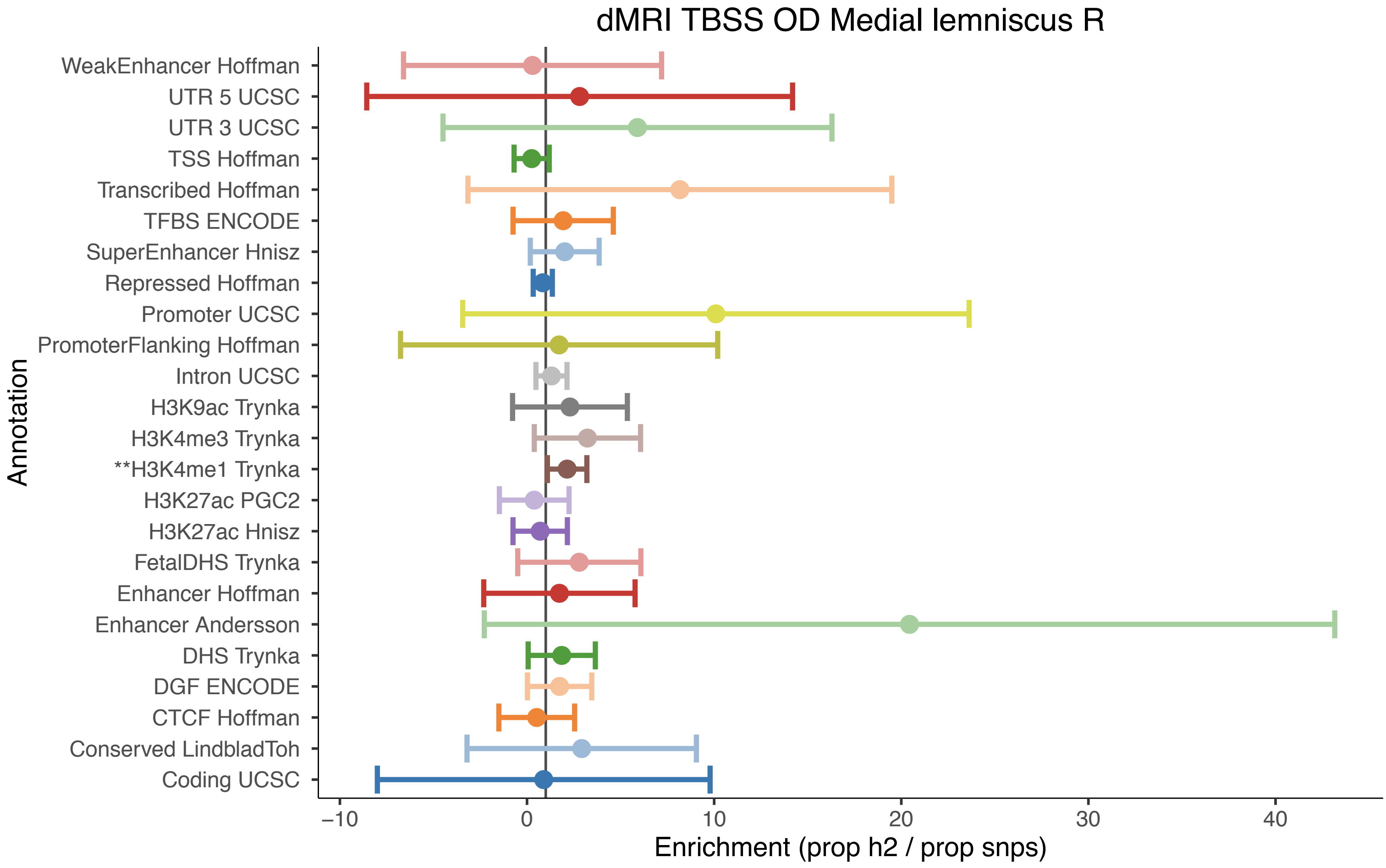
dMRI TBSS OD Medial lemniscus R
WeakEnhancer Hoffman
UTR 5 UCSC
UTR 3 UCSC
TSS Hoffman
Transcribed Hoffman
TFBS ENCODE
SuperEnhancer Hnisz
Repressed Hoffman
Promoter UCSC
PromoterFlanking Hoffman
Intron UCSC
H3K9ac Trynka
H3K4me3 Trynka
**H3K4me1 Trynka
H3K27ac PGC2
H3K27ac Hnisz
FetalDHS Trynka
Enhancer Hoffman
Enhancer Andersson
DHS Trynka
DGF ENCODE
CTCF Hoffman
Conserved LindbladToh
Coding UCSC
Annotation
−10
0
10
20
30
40
Enrichment (prop h2 / prop snps)

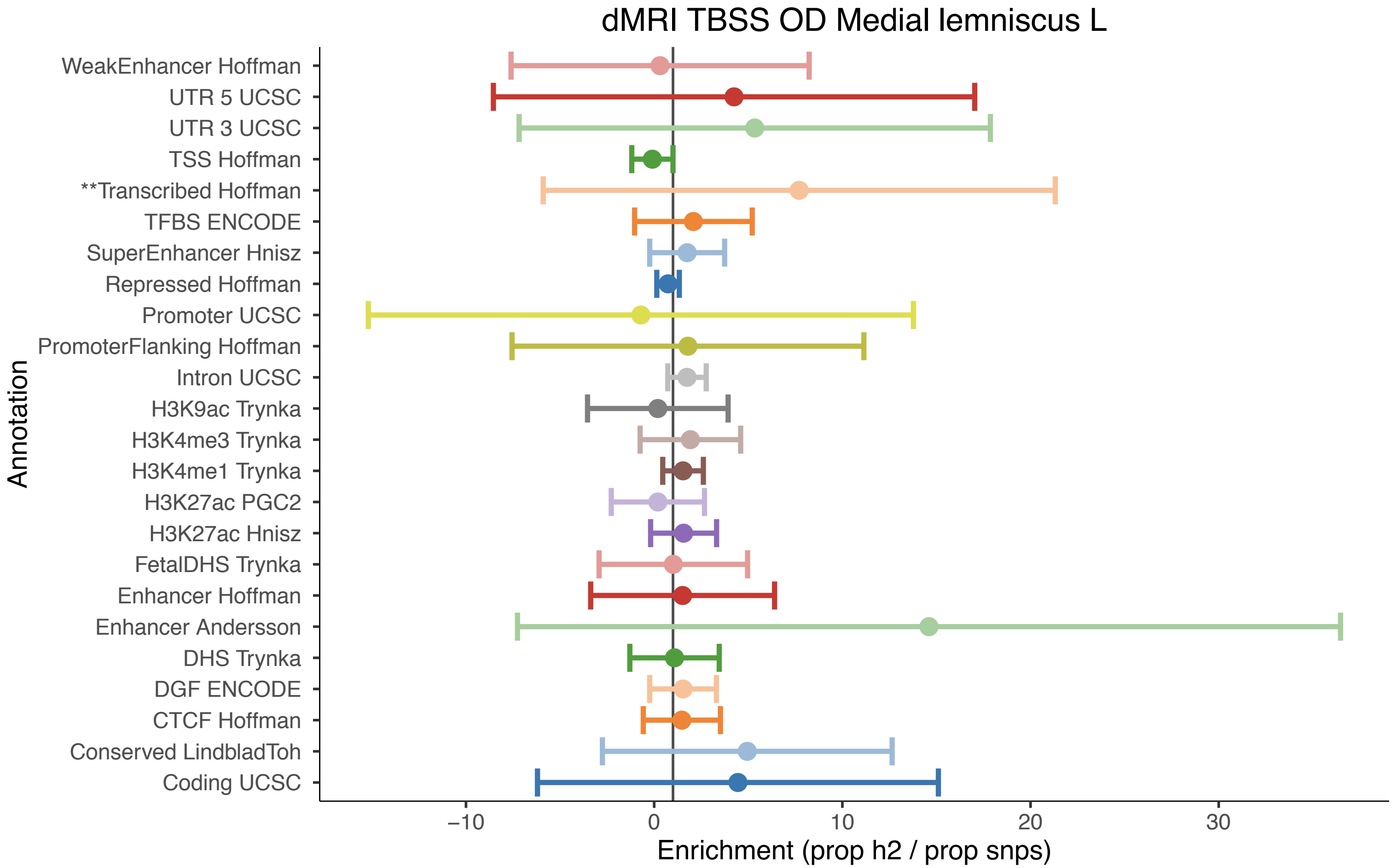

dMRI TBSS OD Medial lemniscus L
WeakEnhancer Hoffman
UTR 5 UCSC
UTR 3 UCSC
TSS Hoffman
**Transcribed Hoffman
TFBS ENCODE
SuperEnhancer Hnisz
Repressed Hoffman
Promoter UCSC
PromoterFlanking Hoffman
Intron UCSC
H3K9ac Trynka
H3K4me3 Trynka
H3K4me1 Trynka
H3K27ac PGC2
H3K27ac Hnisz
FetalDHS Trynka
Enhancer Hoffman
Enhancer Andersson
DHS Trynka
DGF ENCODE
CTCF Hoffman
Conserved LindbladToh
Coding UCSC
Annotation
−10
0
10
20
30
Enrichment (prop h2 / prop snps)

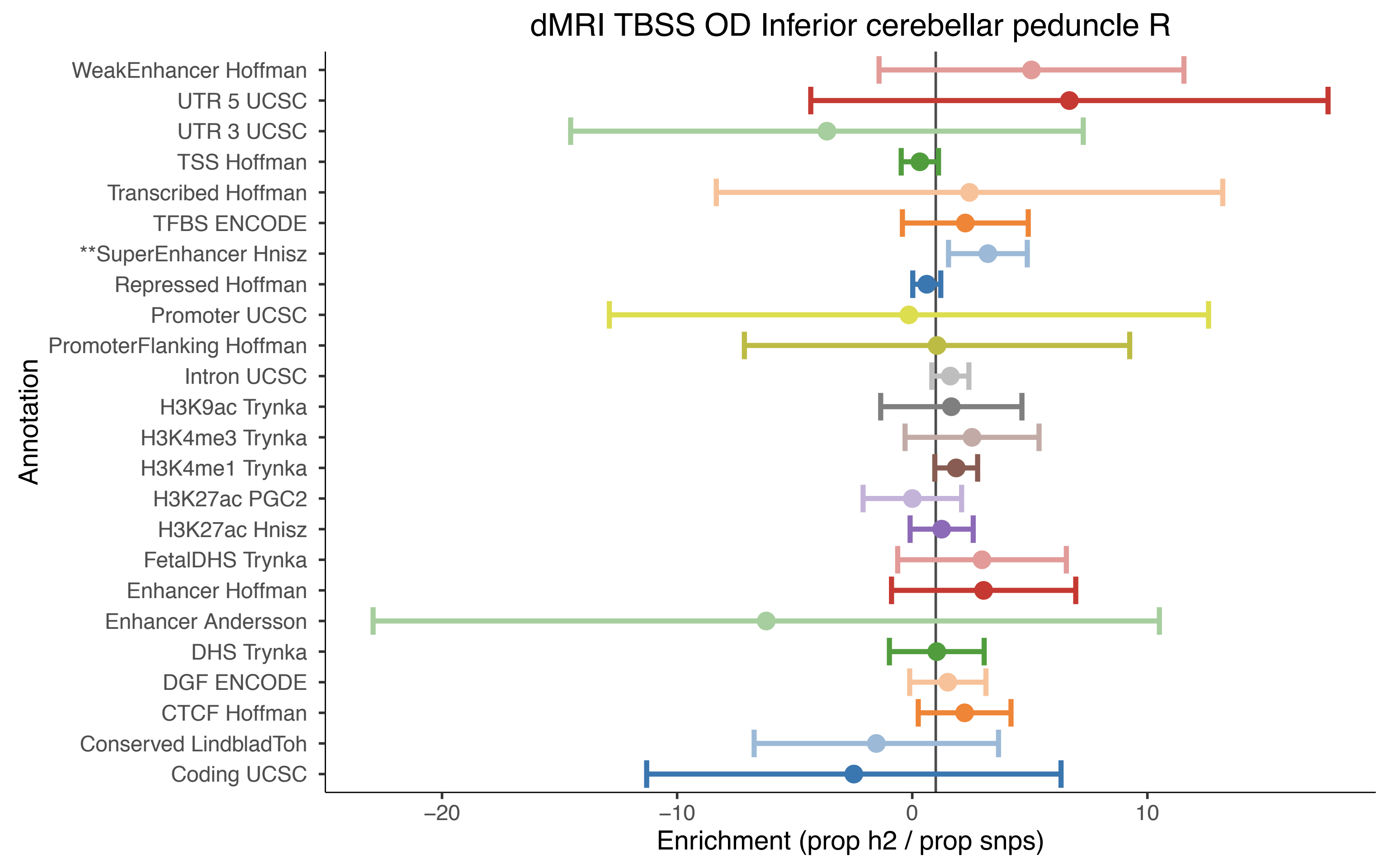
dMRI TBSS OD Inferior cerebellar peduncle R
WeakEnhancer Hoffman
UTR 5 UCSC
UTR 3 UCSC
TSS Hoffman
Transcribed Hoffman
TFBS ENCODE
**SuperEnhancer Hnisz
Repressed Hoffman
Promoter UCSC
PromoterFlanking Hoffman
Intron UCSC
H3K9ac Trynka
H3K4me3 Trynka
H3K4me1 Trynka
H3K27ac PGC2
H3K27ac Hnisz
FetalDHS Trynka
Enhancer Hoffman
Enhancer Andersson
DHS Trynka
DGF ENCODE
CTCF Hoffman
Conserved LindbladToh
Coding UCSC
Annotation
−20
−10
0
10
Enrichment (prop h2 / prop snps)

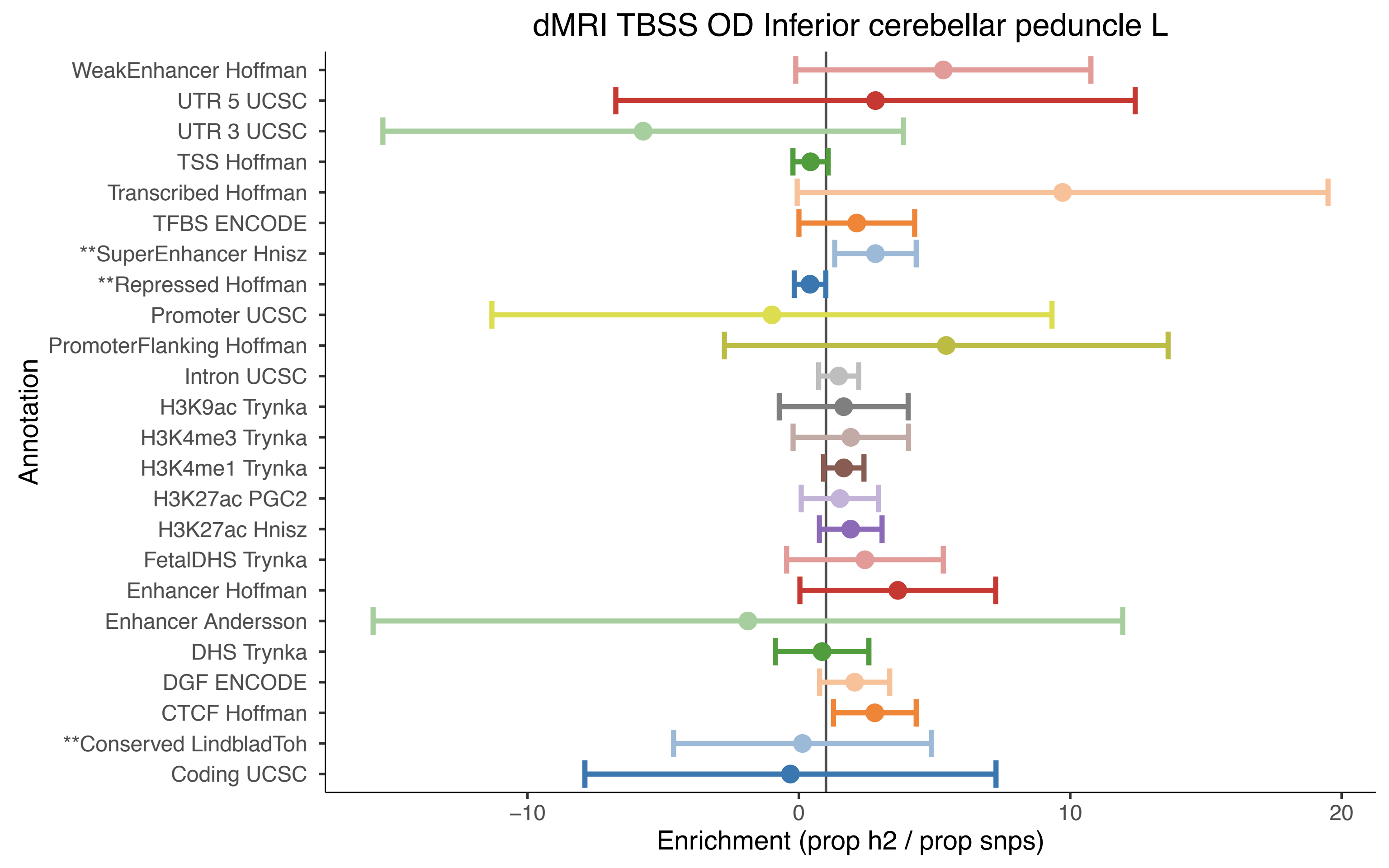

dMRI TBSS OD Inferior cerebellar peduncle L
WeakEnhancer Hoffman
UTR 5 UCSC
UTR 3 UCSC
TSS Hoffman
Transcribed Hoffman
TFBS ENCODE
**SuperEnhancer Hnisz
**Repressed Hoffman
Promoter UCSC
PromoterFlanking Hoffman
Intron UCSC
H3K9ac Trynka
H3K4me3 Trynka
H3K4me1 Trynka
H3K27ac PGC2
H3K27ac Hnisz
FetalDHS Trynka
Enhancer Hoffman
Enhancer Andersson
DHS Trynka
DGF ENCODE
CTCF Hoffman
**Conserved LindbladToh
Coding UCSC
Annotation
−10
0
10
20
Enrichment (prop h2 / prop snps)

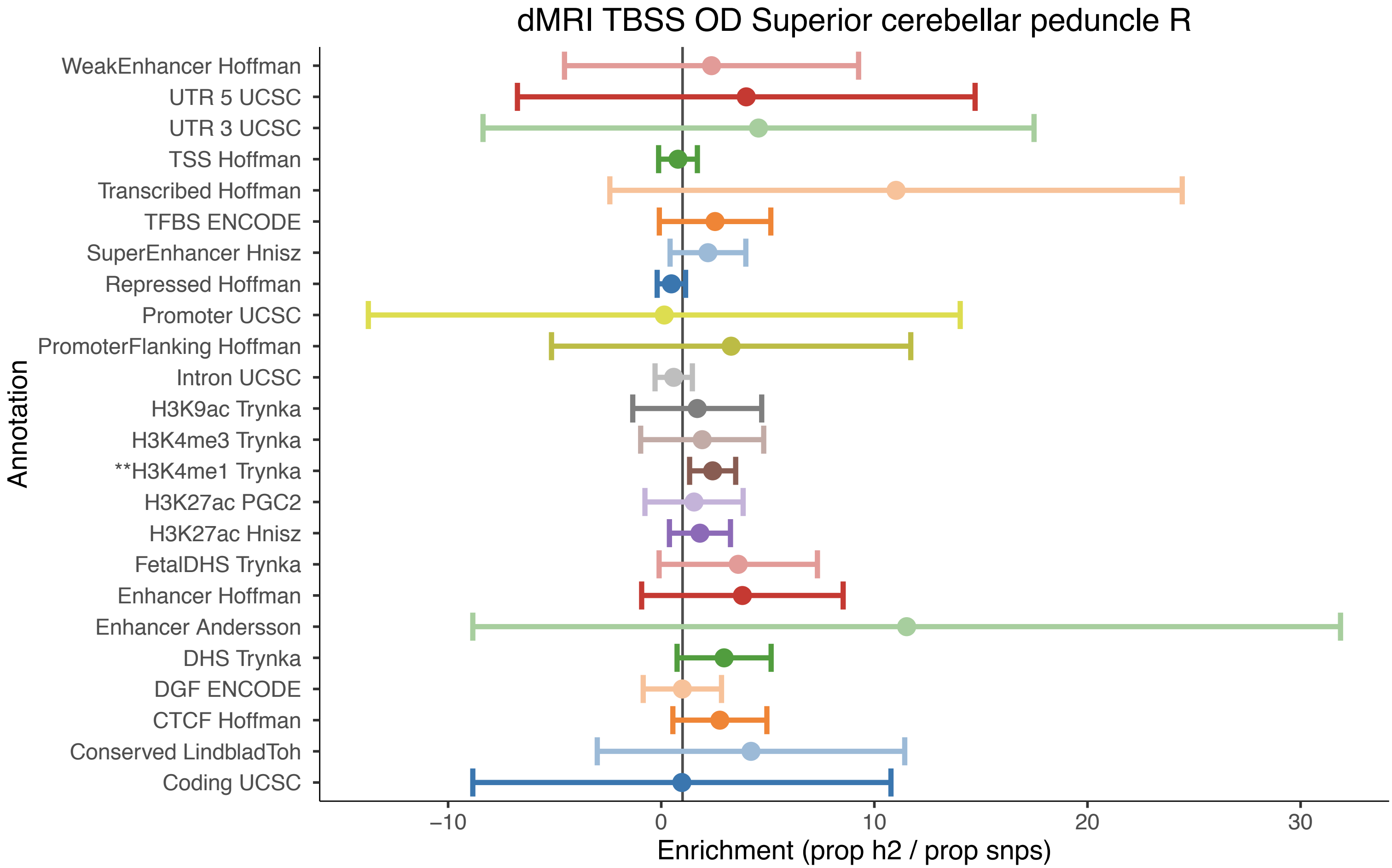

dMRI TBSS OD Superior cerebellar peduncle R
WeakEnhancer Hoffman
UTR 5 UCSC
UTR 3 UCSC
TSS Hoffman
Transcribed Hoffman
TFBS ENCODE
SuperEnhancer Hnisz
Repressed Hoffman
Promoter UCSC
PromoterFlanking Hoffman
Intron UCSC
H3K9ac Trynka
H3K4me3 Trynka
**H3K4me1 Trynka
H3K27ac PGC2
H3K27ac Hnisz
FetalDHS Trynka
Enhancer Hoffman
Enhancer Andersson
DHS Trynka
DGF ENCODE
CTCF Hoffman
Conserved LindbladToh
Coding UCSC
Annotation
−10
0
10
20
30
Enrichment (prop h2 / prop snps)

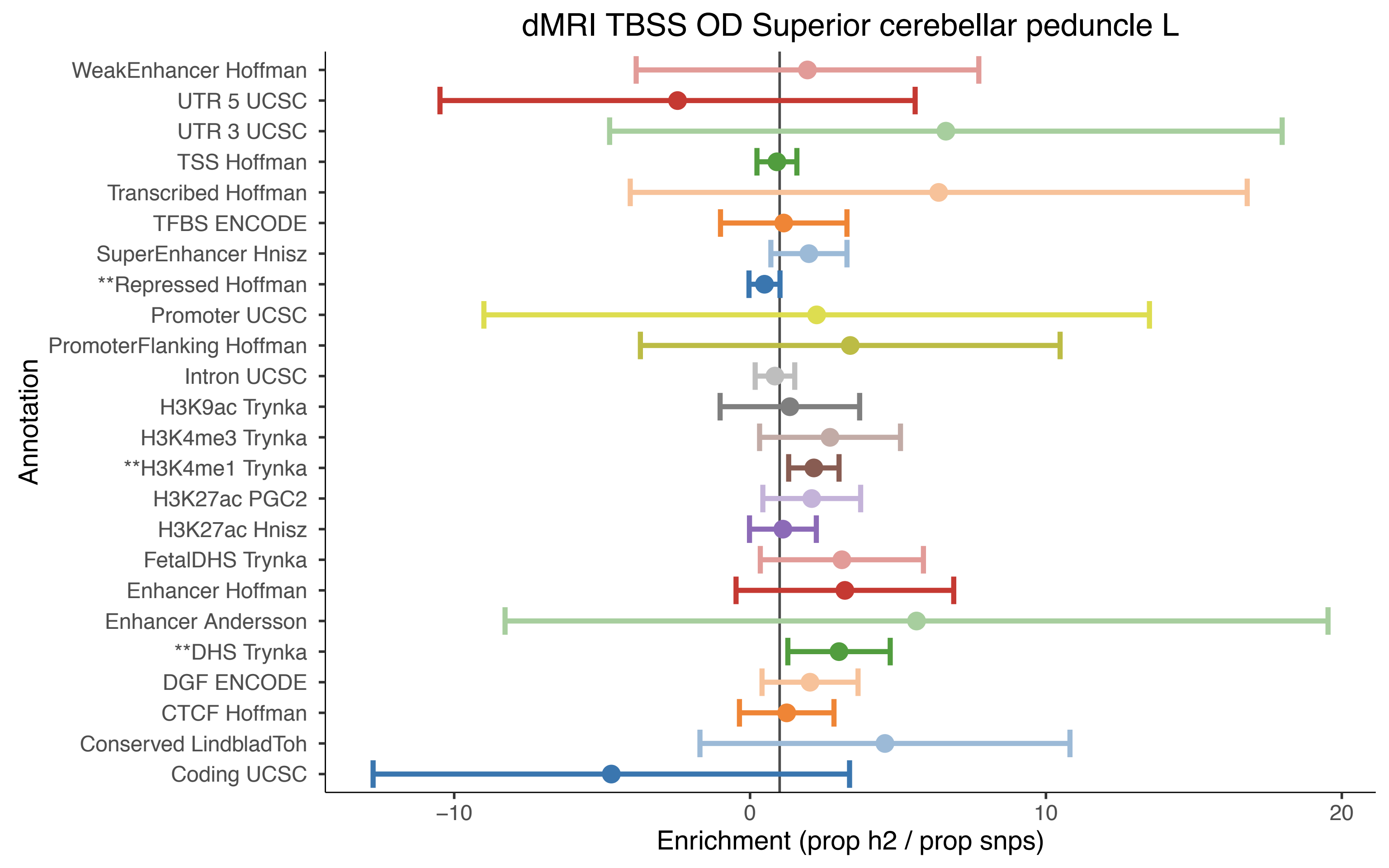

dMRI TBSS OD Superior cerebellar peduncle L
WeakEnhancer Hoffman
UTR 5 UCSC
UTR 3 UCSC
TSS Hoffman
Transcribed Hoffman
TFBS ENCODE
SuperEnhancer Hnisz
**Repressed Hoffman
Promoter UCSC
PromoterFlanking Hoffman
Intron UCSC
H3K9ac Trynka
H3K4me3 Trynka
**H3K4me1 Trynka
H3K27ac PGC2
H3K27ac Hnisz
FetalDHS Trynka
Enhancer Hoffman
Enhancer Andersson
**DHS Trynka
DGF ENCODE
CTCF Hoffman
Conserved LindbladToh
Coding UCSC
Annotation
−10
0
10
20
Enrichment (prop h2 / prop snps)

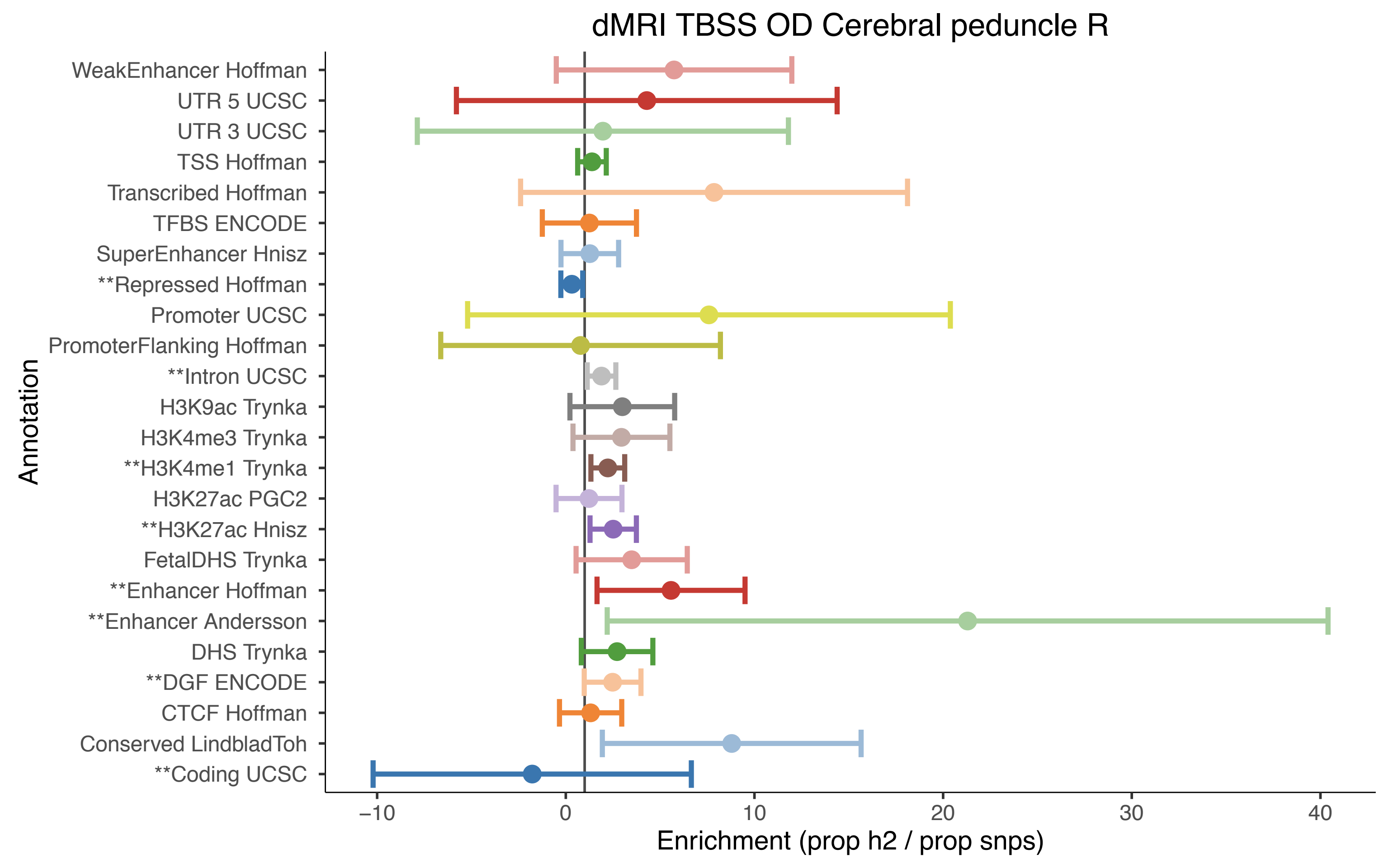

dMRI TBSS OD Cerebral peduncle R
WeakEnhancer Hoffman
UTR 5 UCSC
UTR 3 UCSC
TSS Hoffman
Transcribed Hoffman
TFBS ENCODE
SuperEnhancer Hnisz
**Repressed Hoffman
Promoter UCSC
PromoterFlanking Hoffman
**Intron UCSC
H3K9ac Trynka
H3K4me3 Trynka
**H3K4me1 Trynka
H3K27ac PGC2
**H3K27ac Hnisz
FetalDHS Trynka
**Enhancer Hoffman
**Enhancer Andersson
DHS Trynka
**DGF ENCODE
CTCF Hoffman
Conserved LindbladToh
**Coding UCSC
Annotation
−10
0
10
20
30
40
Enrichment (prop h2 / prop snps)

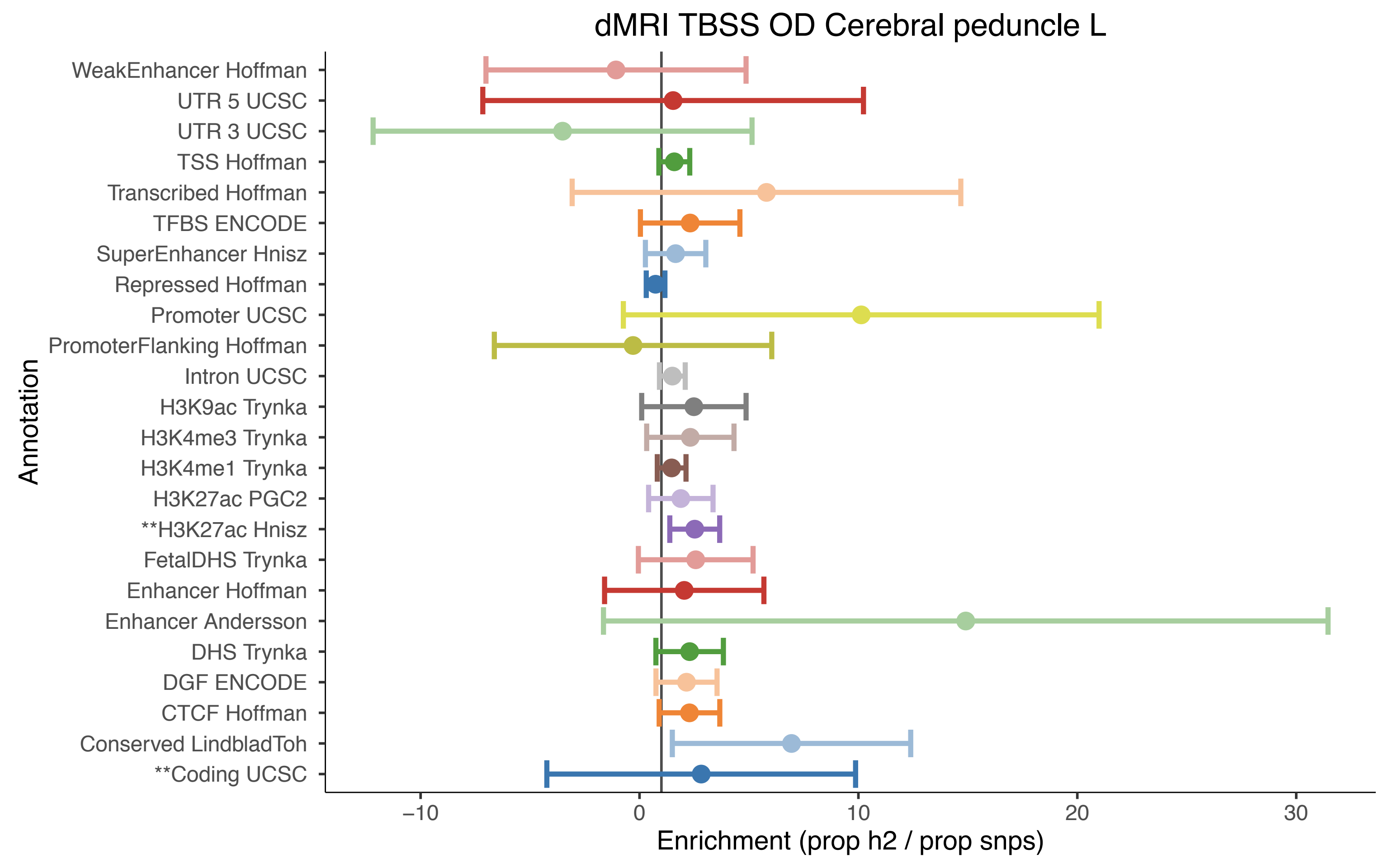

dMRI TBSS OD Cerebral peduncle L
WeakEnhancer Hoffman
UTR 5 UCSC
UTR 3 UCSC
TSS Hoffman
Transcribed Hoffman
TFBS ENCODE
SuperEnhancer Hnisz
Repressed Hoffman
Promoter UCSC
PromoterFlanking Hoffman
Intron UCSC
H3K9ac Trynka
H3K4me3 Trynka
H3K4me1 Trynka
H3K27ac PGC2
**H3K27ac Hnisz
FetalDHS Trynka
Enhancer Hoffman
Enhancer Andersson
DHS Trynka
DGF ENCODE
CTCF Hoffman
Conserved LindbladToh
**Coding UCSC
Annotation
−10
0
10
20
30
Enrichment (prop h2 / prop snps)

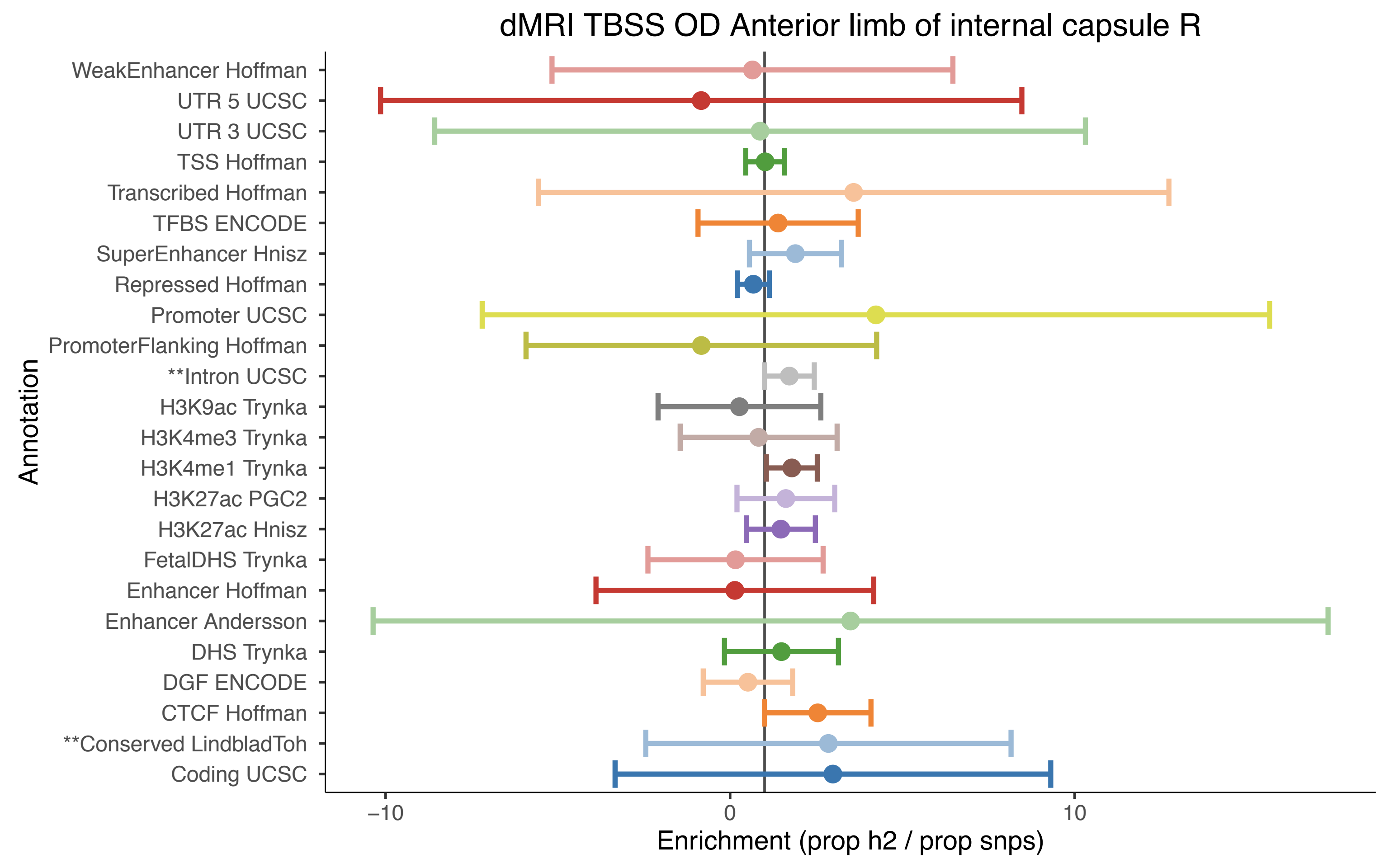
dMRI TBSS OD Anterior limb of internal capsule R
WeakEnhancer Hoffman
UTR 5 UCSC
UTR 3 UCSC
TSS Hoffman
Transcribed Hoffman
TFBS ENCODE
SuperEnhancer Hnisz
Repressed Hoffman
Promoter UCSC
PromoterFlanking Hoffman
**Intron UCSC
H3K9ac Trynka
H3K4me3 Trynka
H3K4me1 Trynka
H3K27ac PGC2
H3K27ac Hnisz
FetalDHS Trynka
Enhancer Hoffman
Enhancer Andersson
DHS Trynka
DGF ENCODE
CTCF Hoffman
**Conserved LindbladToh
Coding UCSC
Annotation
−10
0
10
Enrichment (prop h2 / prop snps)

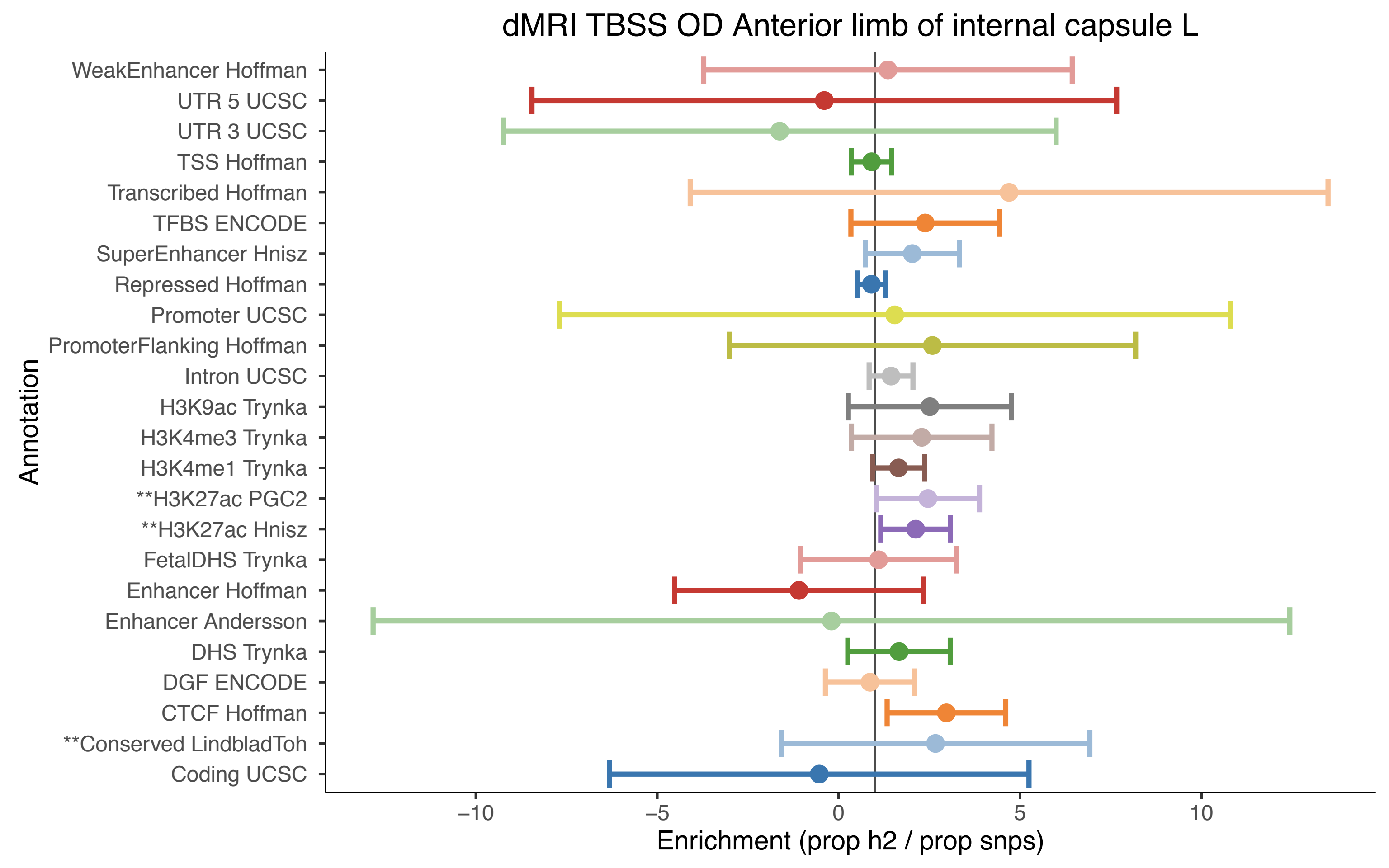
dMRI TBSS OD Anterior limb of internal capsule L
Annotation
WeakEnhancer Hoffman
UTR 5 UCSC
UTR 3 UCSC
TSS Hoffman
Transcribed Hoffman
TFBS ENCODE
SuperEnhancer Hnisz
Repressed Hoffman
Promoter UCSC
PromoterFlanking Hoffman
Intron UCSC
H3K9ac Trynka
H3K4me3 Trynka
H3K4me1 Trynka
**H3K27ac PGC2
**H3K27ac Hnisz
FetalDHS Trynka
Enhancer Hoffman
Enhancer Andersson
DHS Trynka
DGF ENCODE
CTCF Hoffman
**Conserved LindbladToh
Coding UCSC
−10
−5
0
5
10
Enrichment (prop h2 / prop snps)

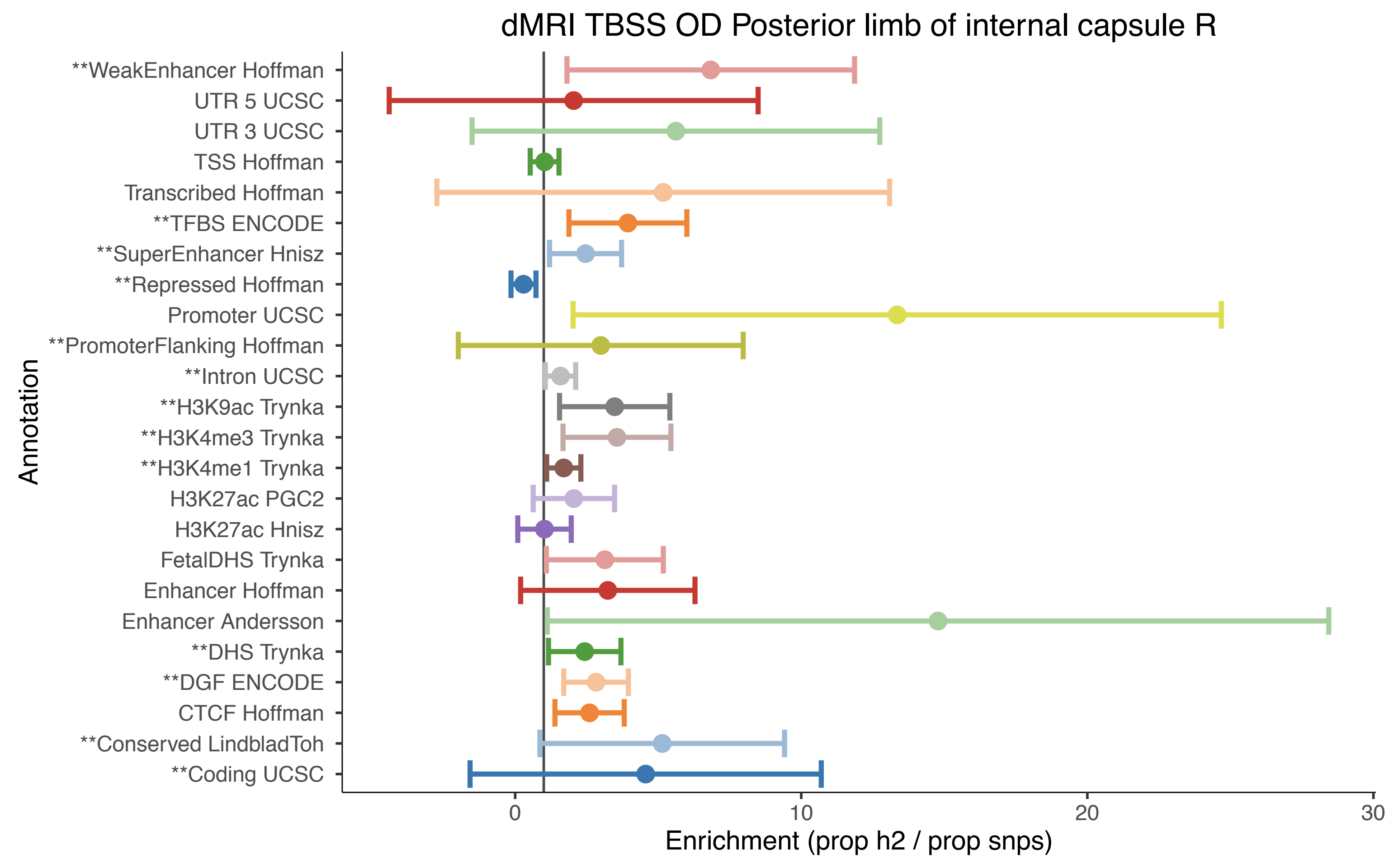

dMRI TBSS OD Posterior limb of internal capsule R
**WeakEnhancer Hoffman
UTR 5 UCSC
UTR 3 UCSC
TSS Hoffman
Transcribed Hoffman
**TFBS ENCODE
**SuperEnhancer Hnisz
**Repressed Hoffman
Promoter UCSC
**PromoterFlanking Hoffman
**Intron UCSC
**H3K9ac Trynka
**H3K4me3 Trynka
**H3K4me1 Trynka
H3K27ac PGC2
H3K27ac Hnisz
FetalDHS Trynka
Enhancer Hoffman
Enhancer Andersson
**DHS Trynka
**DGF ENCODE
CTCF Hoffman
**Conserved LindbladToh
**Coding UCSC
Annotation
0
10
20
30
Enrichment (prop h2 / prop snps)

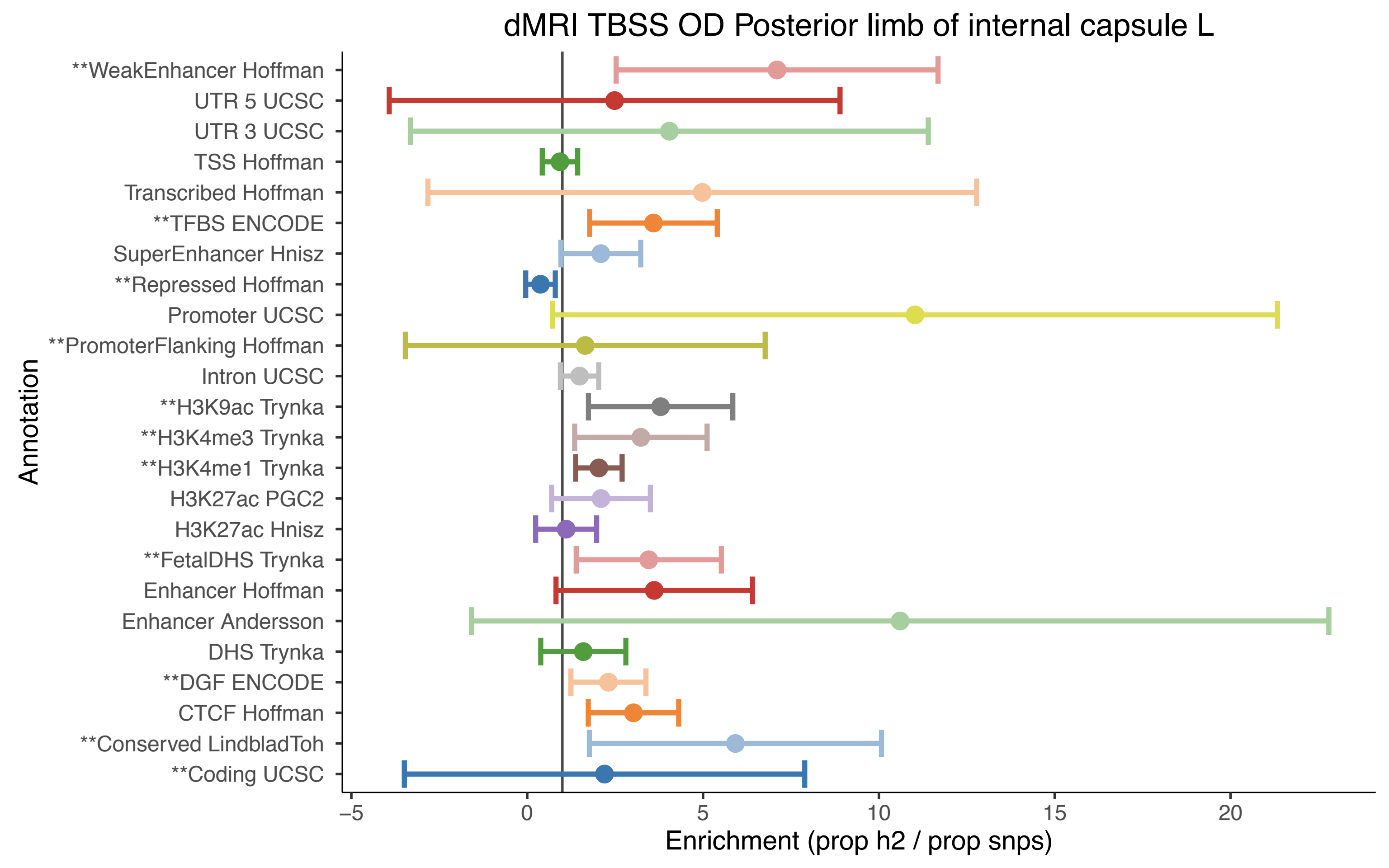

dMRI TBSS OD Posterior limb of internal capsule L
Annotation
**WeakEnhancer Hoffman
UTR 5 UCSC
UTR 3 UCSC
TSS Hoffman
Transcribed Hoffman
**TFBS ENCODE
SuperEnhancer Hnisz
**Repressed Hoffman
Promoter UCSC
**PromoterFlanking Hoffman
Intron UCSC
**H3K9ac Trynka
**H3K4me3 Trynka
**H3K4me1 Trynka
H3K27ac PGC2
H3K27ac Hnisz
**FetalDHS Trynka
Enhancer Hoffman
Enhancer Andersson
DHS Trynka
**DGF ENCODE
CTCF Hoffman
**Conserved LindbladToh
**Coding UCSC
−5
0
5
10
15
20
Enrichment (prop h2 / prop snps)

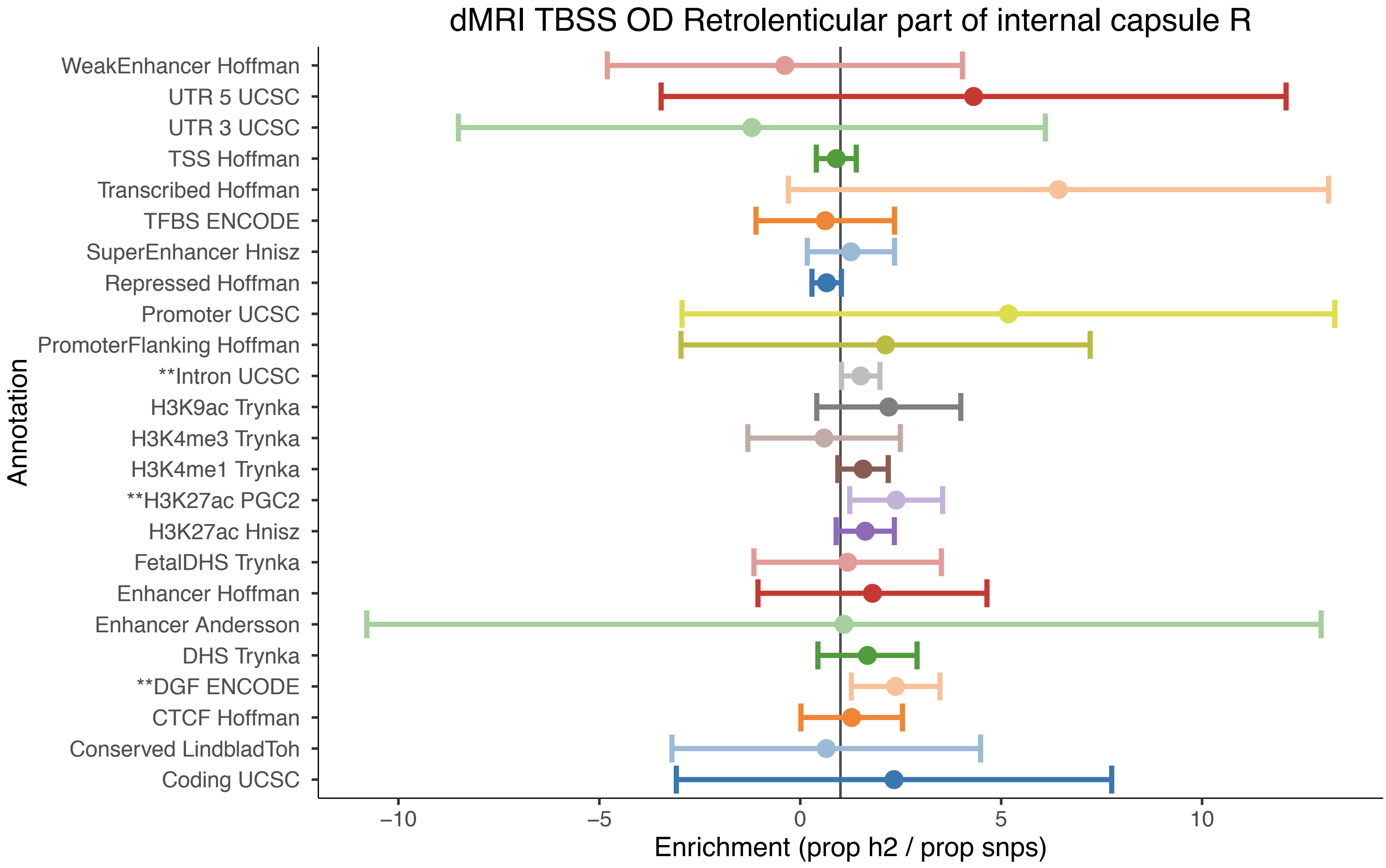

dMRI TBSS OD Retrolenticular part of internal capsule R
WeakEnhancer Hoffman
UTR 5 UCSC
UTR 3 UCSC
TSS Hoffman
Transcribed Hoffman
TFBS ENCODE
SuperEnhancer Hnisz
Repressed Hoffman
Promoter UCSC
PromoterFlanking Hoffman
**Intron UCSC
H3K9ac Trynka
H3K4me3 Trynka
H3K4me1 Trynka
**H3K27ac PGC2
H3K27ac Hnisz
FetalDHS Trynka
Enhancer Hoffman
Enhancer Andersson
DHS Trynka
**DGF ENCODE
CTCF Hoffman
Conserved LindbladToh
Coding UCSC
Annotation
−10
−5
0
5
10
Enrichment (prop h2 / prop snps)

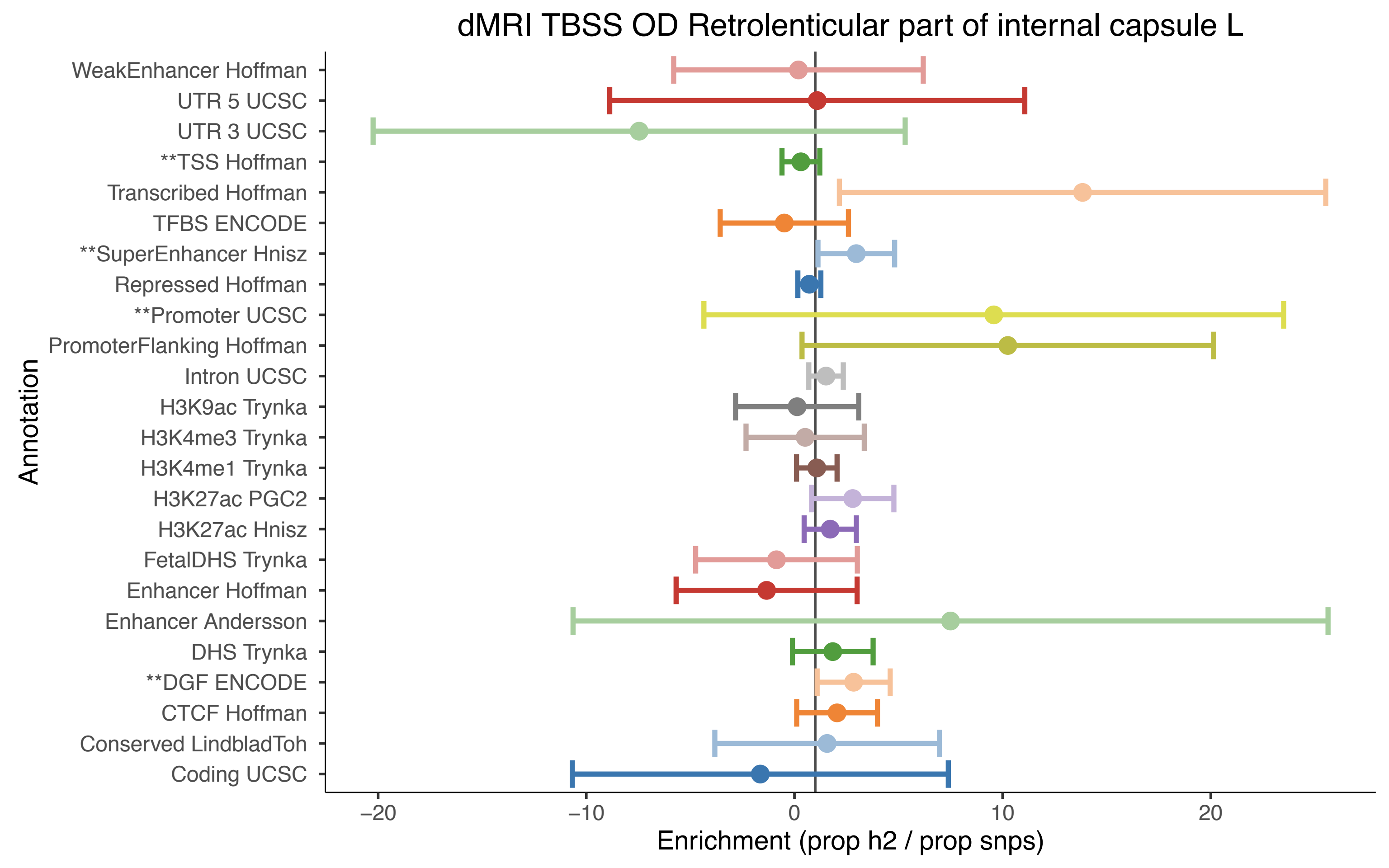

dMRI TBSS OD Retrolenticular part of internal capsule L
WeakEnhancer Hoffman
UTR 5 UCSC
UTR 3 UCSC
**TSS Hoffman
Transcribed Hoffman
TFBS ENCODE
**SuperEnhancer Hnisz
Repressed Hoffman
**Promoter UCSC
PromoterFlanking Hoffman
Intron UCSC
H3K9ac Trynka
H3K4me3 Trynka
H3K4me1 Trynka
H3K27ac PGC2
H3K27ac Hnisz
FetalDHS Trynka
Enhancer Hoffman
Enhancer Andersson
DHS Trynka
**DGF ENCODE
CTCF Hoffman
Conserved LindbladToh
Coding UCSC
Annotation
−20
−10
0
10
20
Enrichment (prop h2 / prop snps)

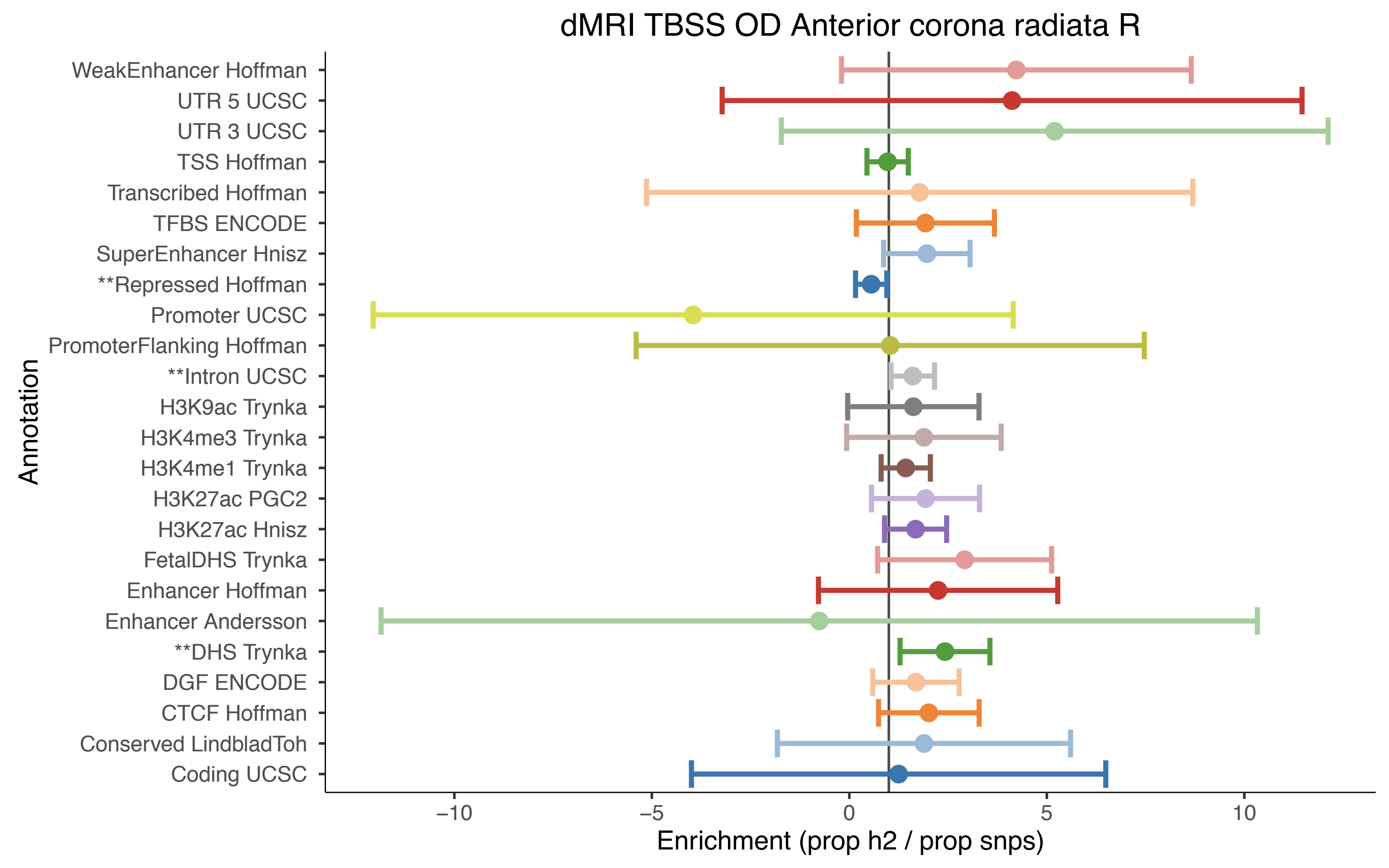

dMRI TBSS OD Anterior corona radiata R
WeakEnhancer Hoffman
UTR 5 UCSC
UTR 3 UCSC
TSS Hoffman
Transcribed Hoffman
TFBS ENCODE
SuperEnhancer Hnisz
**Repressed Hoffman
Promoter UCSC
PromoterFlanking Hoffman
**Intron UCSC
H3K9ac Trynka
H3K4me3 Trynka
H3K4me1 Trynka
H3K27ac PGC2
H3K27ac Hnisz
FetalDHS Trynka
Enhancer Hoffman
Enhancer Andersson
**DHS Trynka
DGF ENCODE
CTCF Hoffman
Conserved LindbladToh
Coding UCSC
Annotation
−10
−5
0
5
10
Enrichment (prop h2 / prop snps)

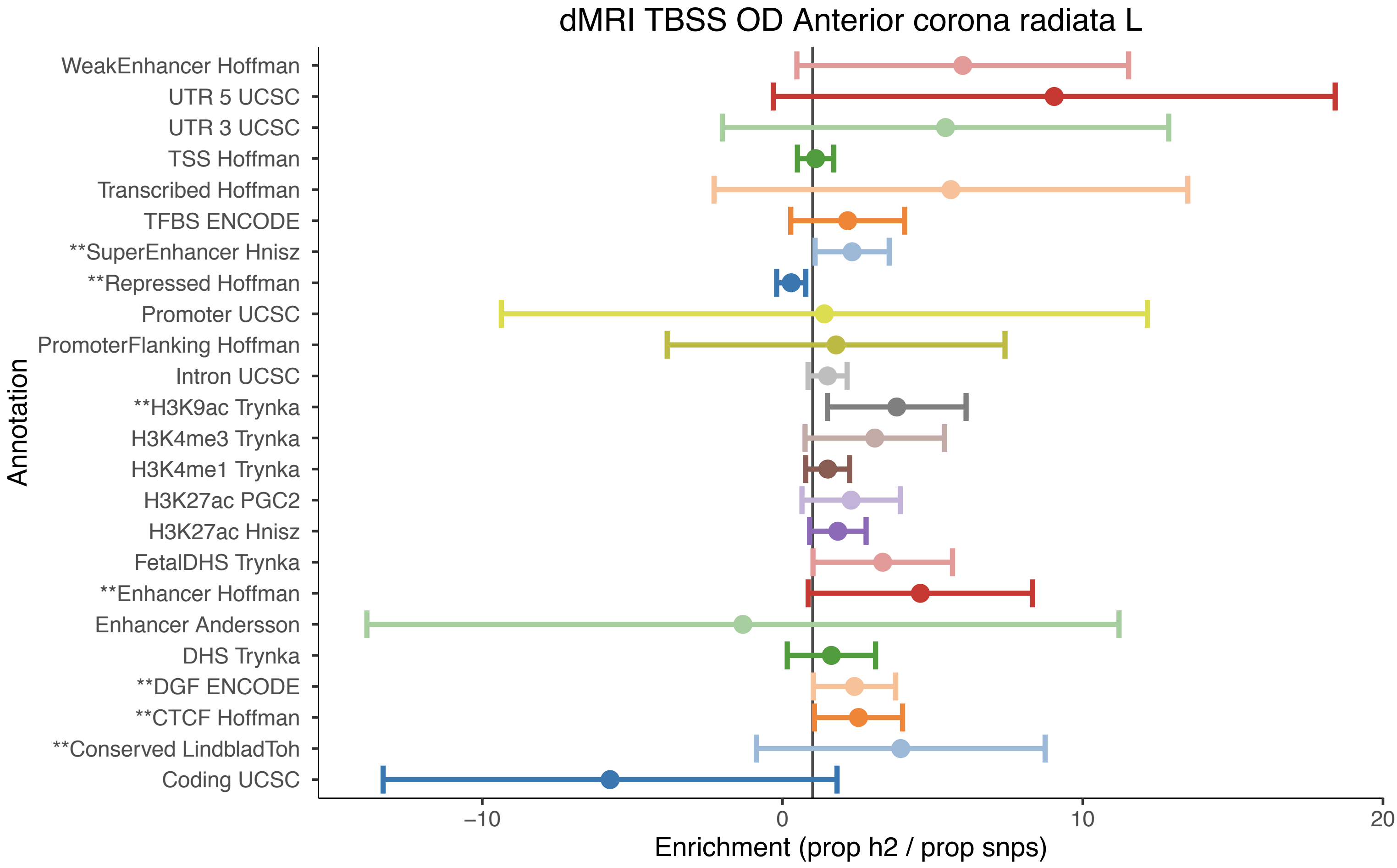

dMRI TBSS OD Anterior corona radiata L
WeakEnhancer Hoffman
UTR 5 UCSC
UTR 3 UCSC
TSS Hoffman
Transcribed Hoffman
TFBS ENCODE
**SuperEnhancer Hnisz
**Repressed Hoffman
Promoter UCSC
PromoterFlanking Hoffman
Intron UCSC
**H3K9ac Trynka
H3K4me3 Trynka
H3K4me1 Trynka
H3K27ac PGC2
H3K27ac Hnisz
FetalDHS Trynka
**Enhancer Hoffman
Enhancer Andersson
DHS Trynka
**DGF ENCODE
**CTCF Hoffman
**Conserved LindbladToh
Coding UCSC
Annotation
−10
0
10
20
Enrichment (prop h2 / prop snps)

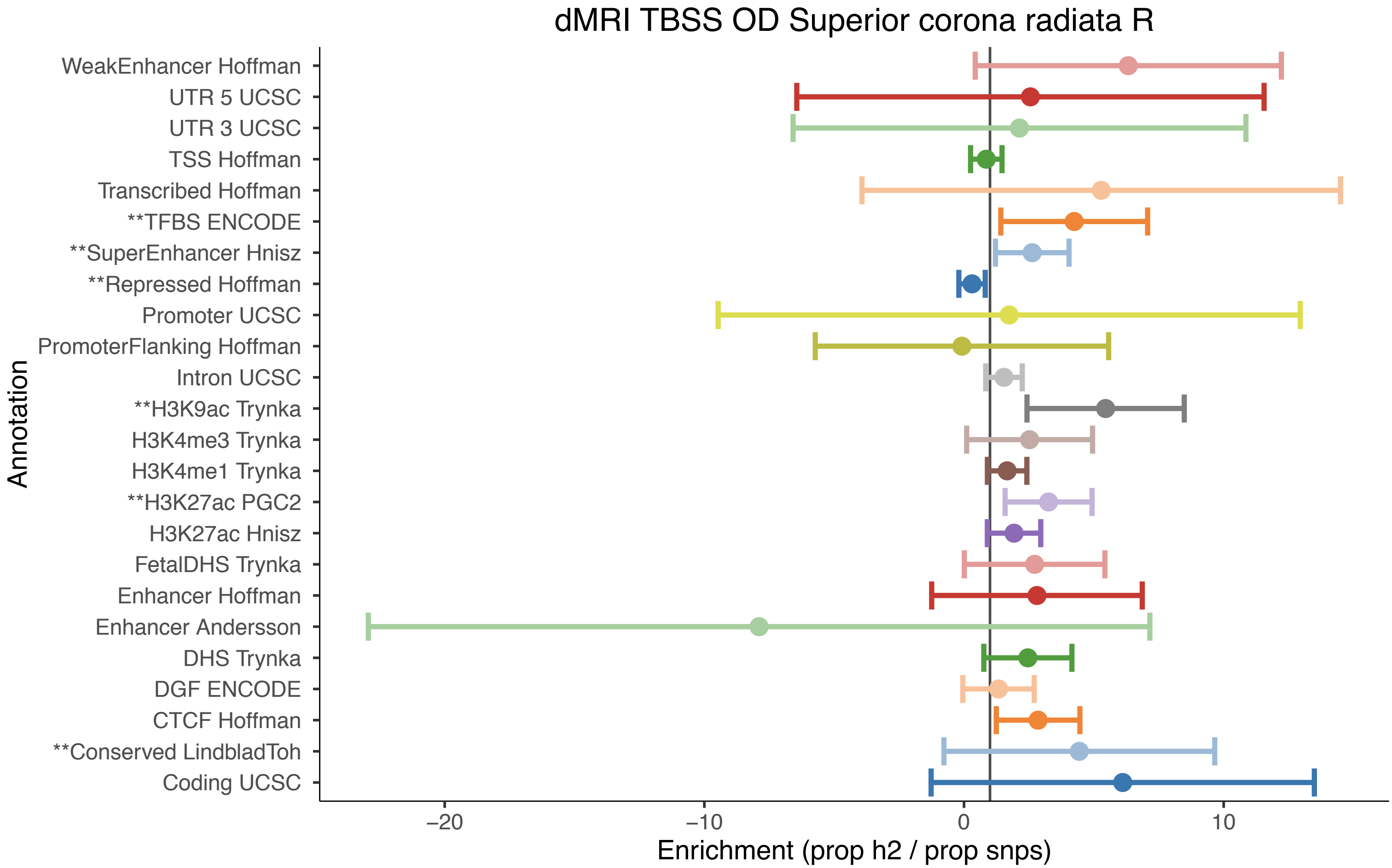
dMRI TBSS OD Superior corona radiata R
Annotation
WeakEnhancer Hoffman
UTR 5 UCSC
UTR 3 UCSC
TSS Hoffman
Transcribed Hoffman
**TFBS ENCODE
**SuperEnhancer Hnisz
**Repressed Hoffman
Promoter UCSC
PromoterFlanking Hoffman
Intron UCSC
**H3K9ac Trynka
H3K4me3 Trynka
H3K4me1 Trynka
**H3K27ac PGC2
H3K27ac Hnisz
FetalDHS Trynka
Enhancer Hoffman
Enhancer Andersson
DHS Trynka
DGF ENCODE
CTCF Hoffman
**Conserved LindbladToh
Coding UCSC
−20
−10
0
10
Enrichment (prop h2 / prop snps)

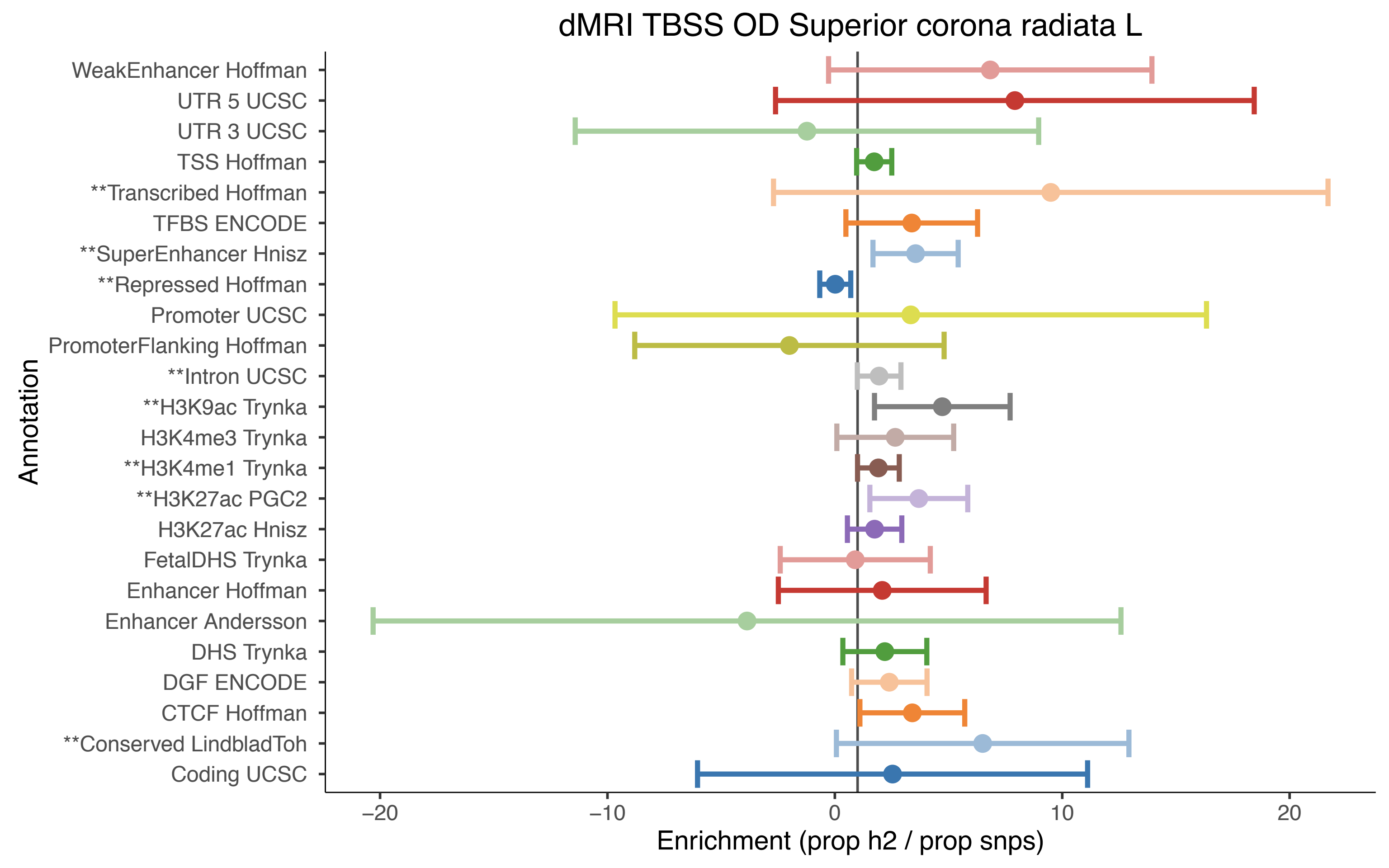

dMRI TBSS OD Superior corona radiata L
WeakEnhancer Hoffman
UTR 5 UCSC
UTR 3 UCSC
TSS Hoffman
**Transcribed Hoffman
TFBS ENCODE
**SuperEnhancer Hnisz
**Repressed Hoffman
Promoter UCSC
PromoterFlanking Hoffman
**Intron UCSC
**H3K9ac Trynka
H3K4me3 Trynka
**H3K4me1 Trynka
**H3K27ac PGC2
H3K27ac Hnisz
FetalDHS Trynka
Enhancer Hoffman
Enhancer Andersson
DHS Trynka
DGF ENCODE
CTCF Hoffman
**Conserved LindbladToh
Coding UCSC
Annotation
−20
−10
0
10
20
Enrichment (prop h2 / prop snps)

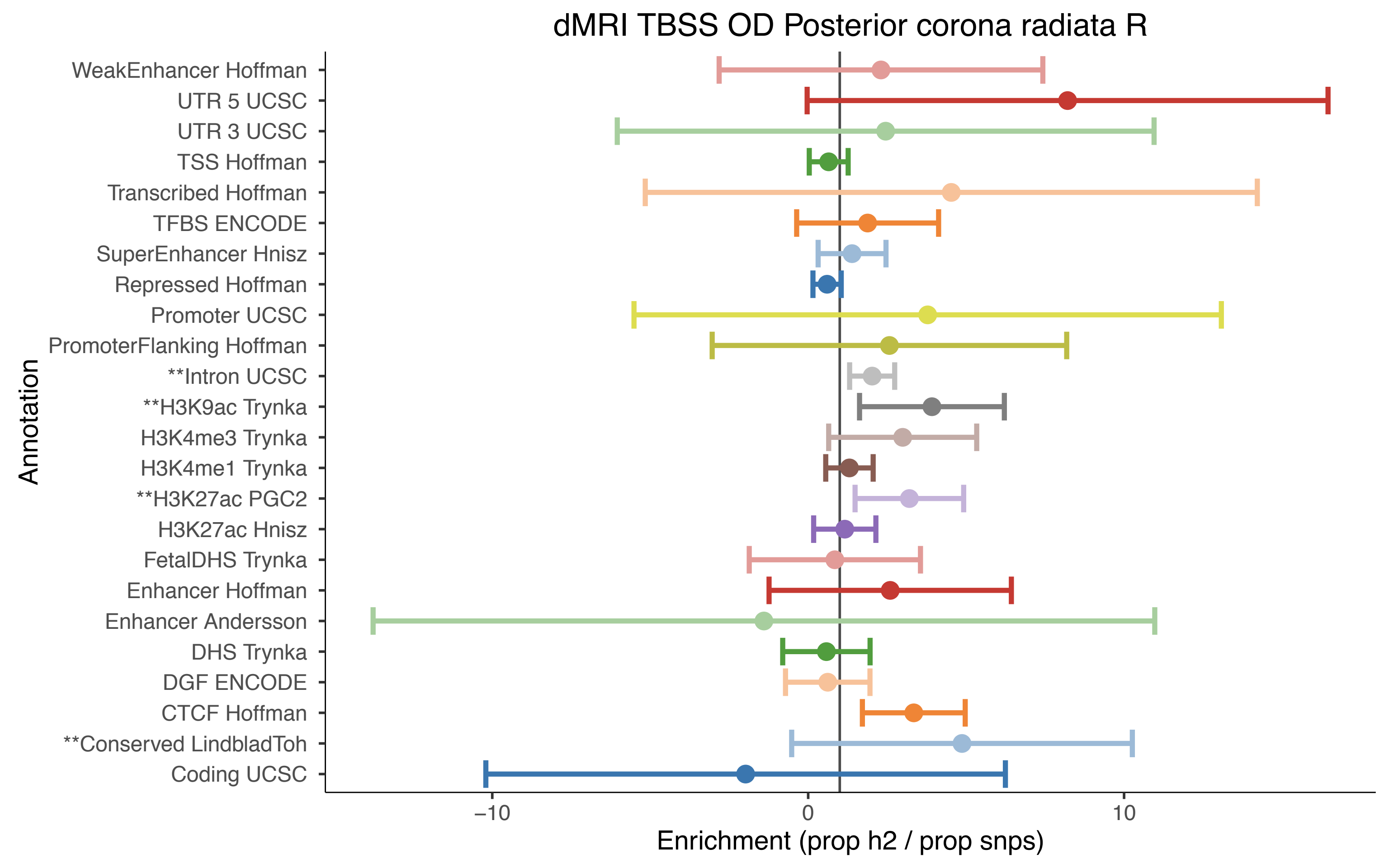
dMRI TBSS OD Posterior corona radiata R
WeakEnhancer Hoffman
UTR 5 UCSC
UTR 3 UCSC
TSS Hoffman
Transcribed Hoffman
TFBS ENCODE
SuperEnhancer Hnisz
Repressed Hoffman
Promoter UCSC
PromoterFlanking Hoffman
**Intron UCSC
**H3K9ac Trynka
H3K4me3 Trynka
H3K4me1 Trynka
**H3K27ac PGC2
H3K27ac Hnisz
FetalDHS Trynka
Enhancer Hoffman
Enhancer Andersson
DHS Trynka
DGF ENCODE
CTCF Hoffman
**Conserved LindbladToh
Coding UCSC
Annotation
−10
0
10
Enrichment (prop h2 / prop snps)

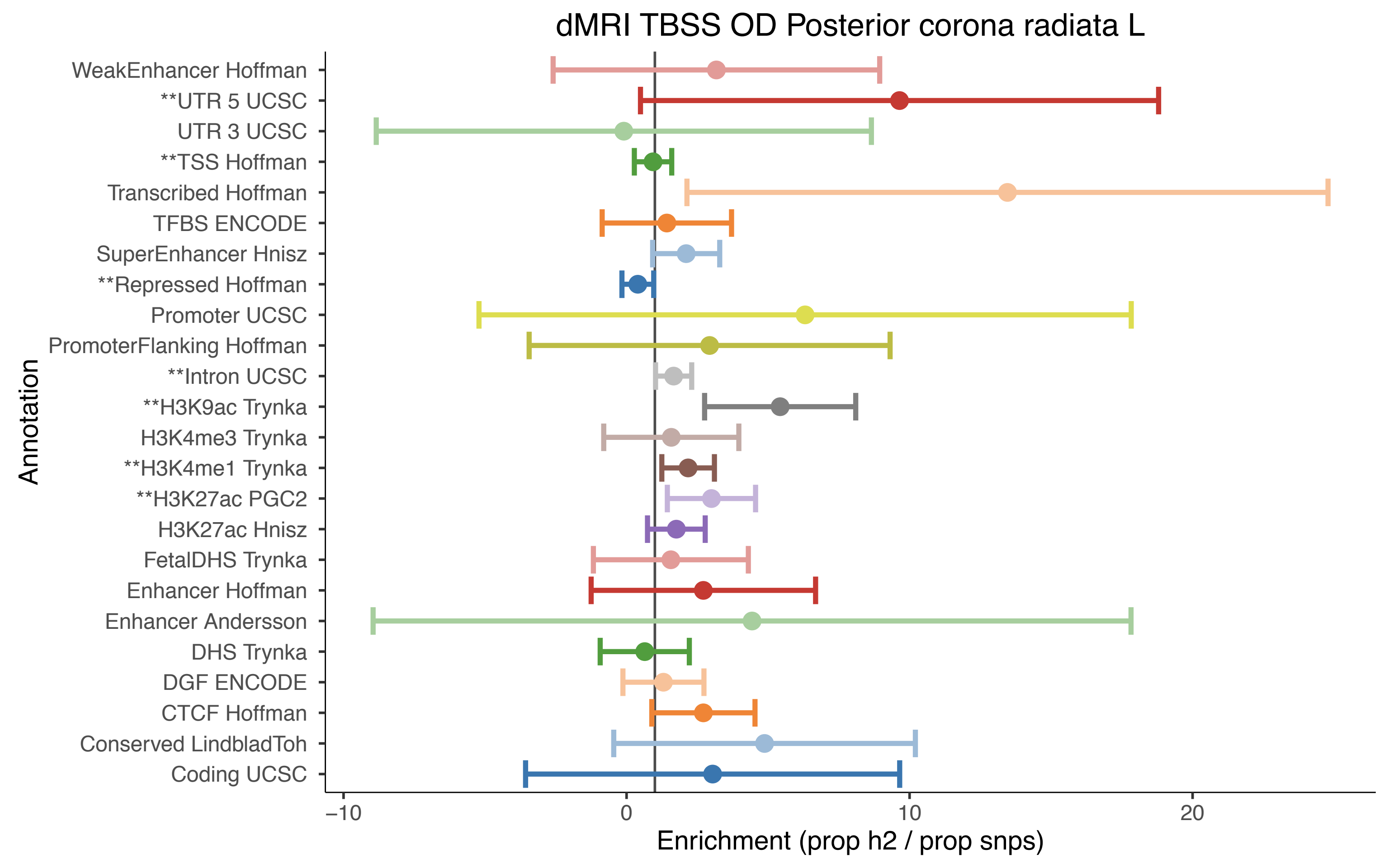
dMRI TBSS OD Posterior corona radiata L
WeakEnhancer Hoffman
**UTR 5 UCSC
UTR 3 UCSC
**TSS Hoffman
Transcribed Hoffman
TFBS ENCODE
SuperEnhancer Hnisz
**Repressed Hoffman
Promoter UCSC
PromoterFlanking Hoffman
**Intron UCSC
**H3K9ac Trynka
H3K4me3 Trynka
**H3K4me1 Trynka
**H3K27ac PGC2
H3K27ac Hnisz
FetalDHS Trynka
Enhancer Hoffman
Enhancer Andersson
DHS Trynka
DGF ENCODE
CTCF Hoffman
Conserved LindbladToh
Coding UCSC
Annotation
−10
0
10
20
Enrichment (prop h2 / prop snps)

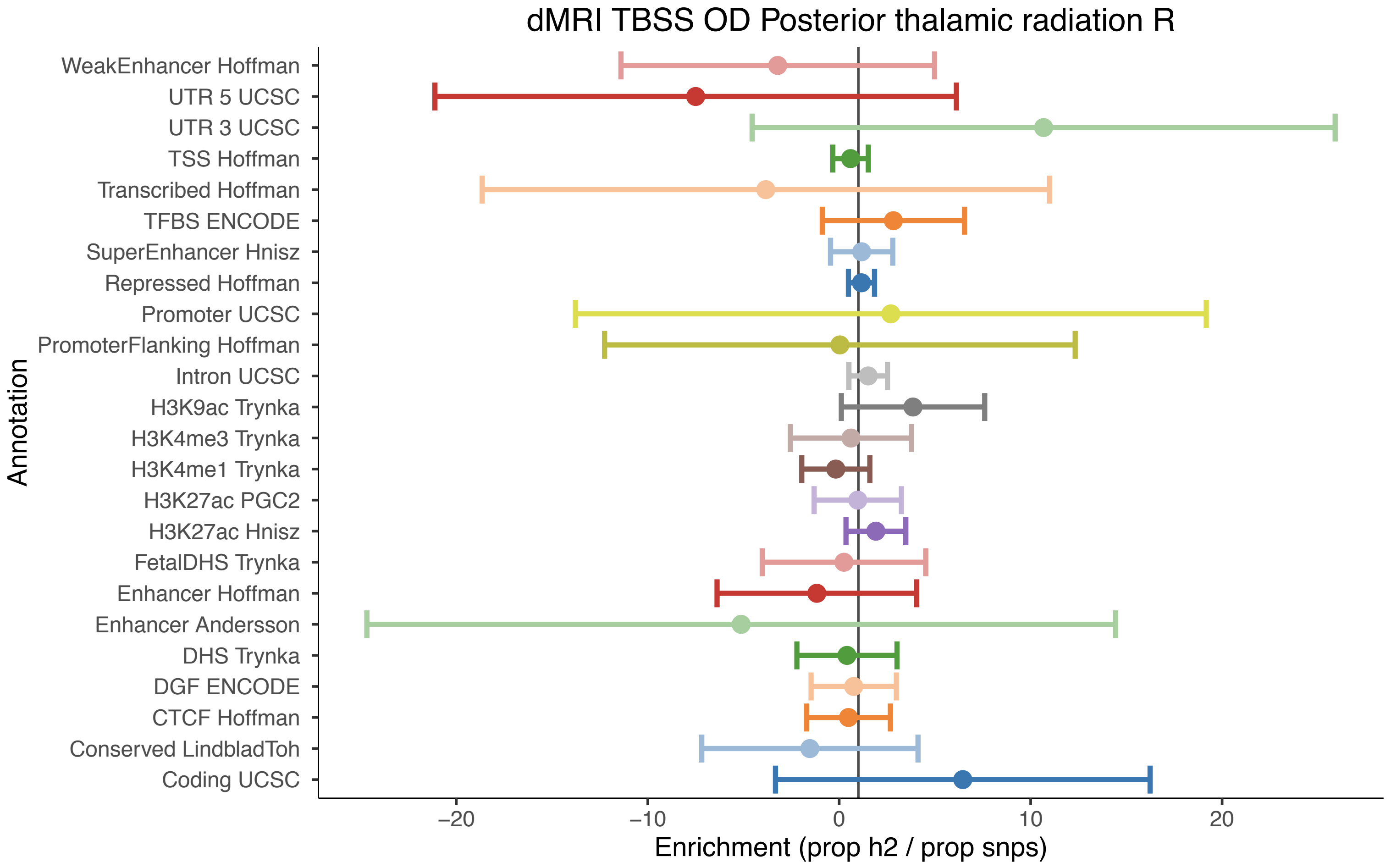

dMRI TBSS OD Posterior thalamic radiation R
WeakEnhancer Hoffman
UTR 5 UCSC
UTR 3 UCSC
TSS Hoffman
Transcribed Hoffman
TFBS ENCODE
SuperEnhancer Hnisz
Repressed Hoffman
Promoter UCSC
PromoterFlanking Hoffman
Intron UCSC
H3K9ac Trynka
H3K4me3 Trynka
H3K4me1 Trynka
H3K27ac PGC2
H3K27ac Hnisz
FetalDHS Trynka
Enhancer Hoffman
Enhancer Andersson
DHS Trynka
DGF ENCODE
CTCF Hoffman
Conserved LindbladToh
Coding UCSC
Annotation
−20
−10
0
10
20
Enrichment (prop h2 / prop snps)

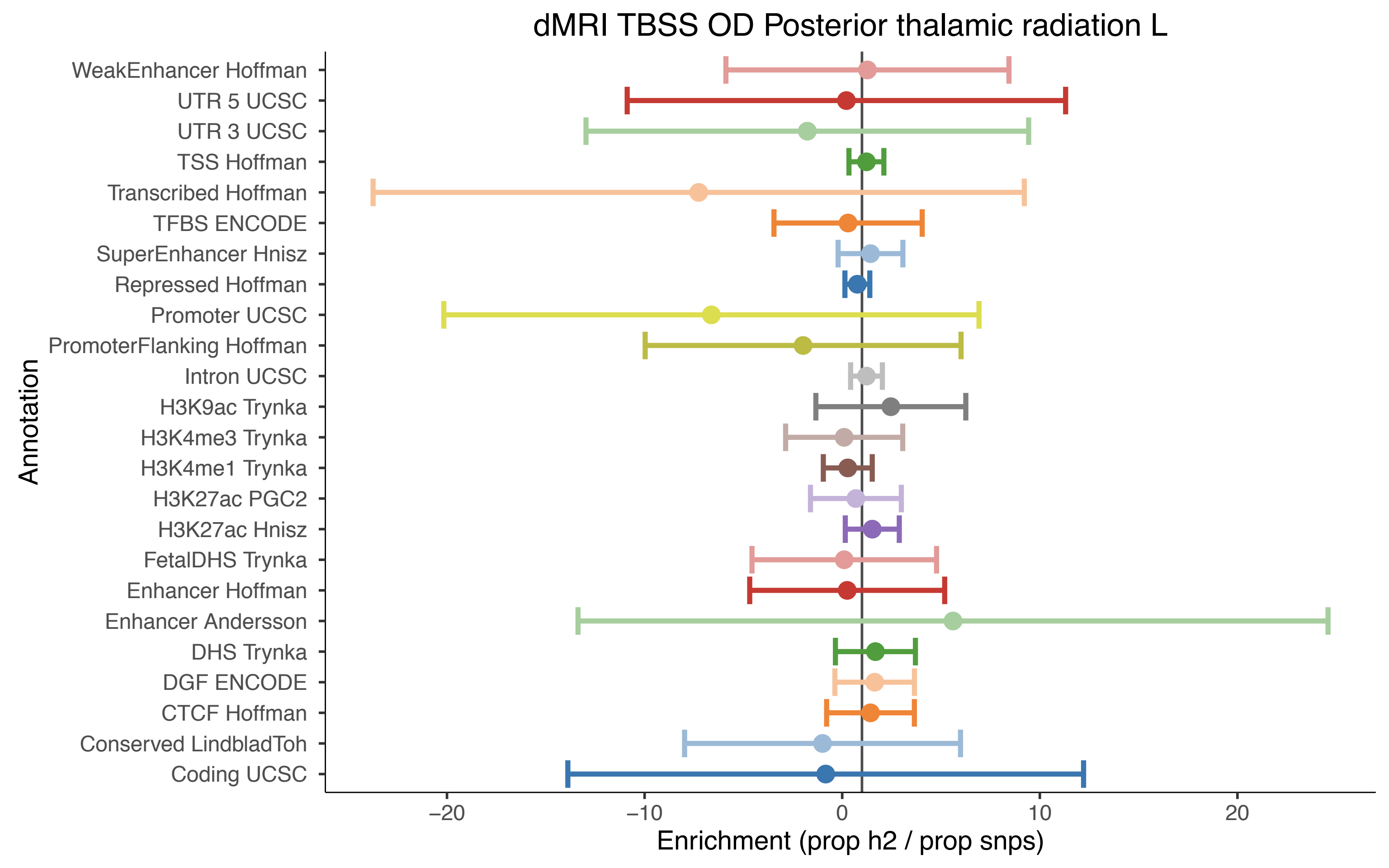

dMRI TBSS OD Posterior thalamic radiation L
WeakEnhancer Hoffman
UTR 5 UCSC
UTR 3 UCSC
TSS Hoffman
Transcribed Hoffman
TFBS ENCODE
SuperEnhancer Hnisz
Repressed Hoffman
Promoter UCSC
PromoterFlanking Hoffman
Intron UCSC
H3K9ac Trynka
H3K4me3 Trynka
H3K4me1 Trynka
H3K27ac PGC2
H3K27ac Hnisz
FetalDHS Trynka
Enhancer Hoffman
Enhancer Andersson
DHS Trynka
DGF ENCODE
CTCF Hoffman
Conserved LindbladToh
Coding UCSC
Annotation
−20
−10
0
10
20
Enrichment (prop h2 / prop snps)

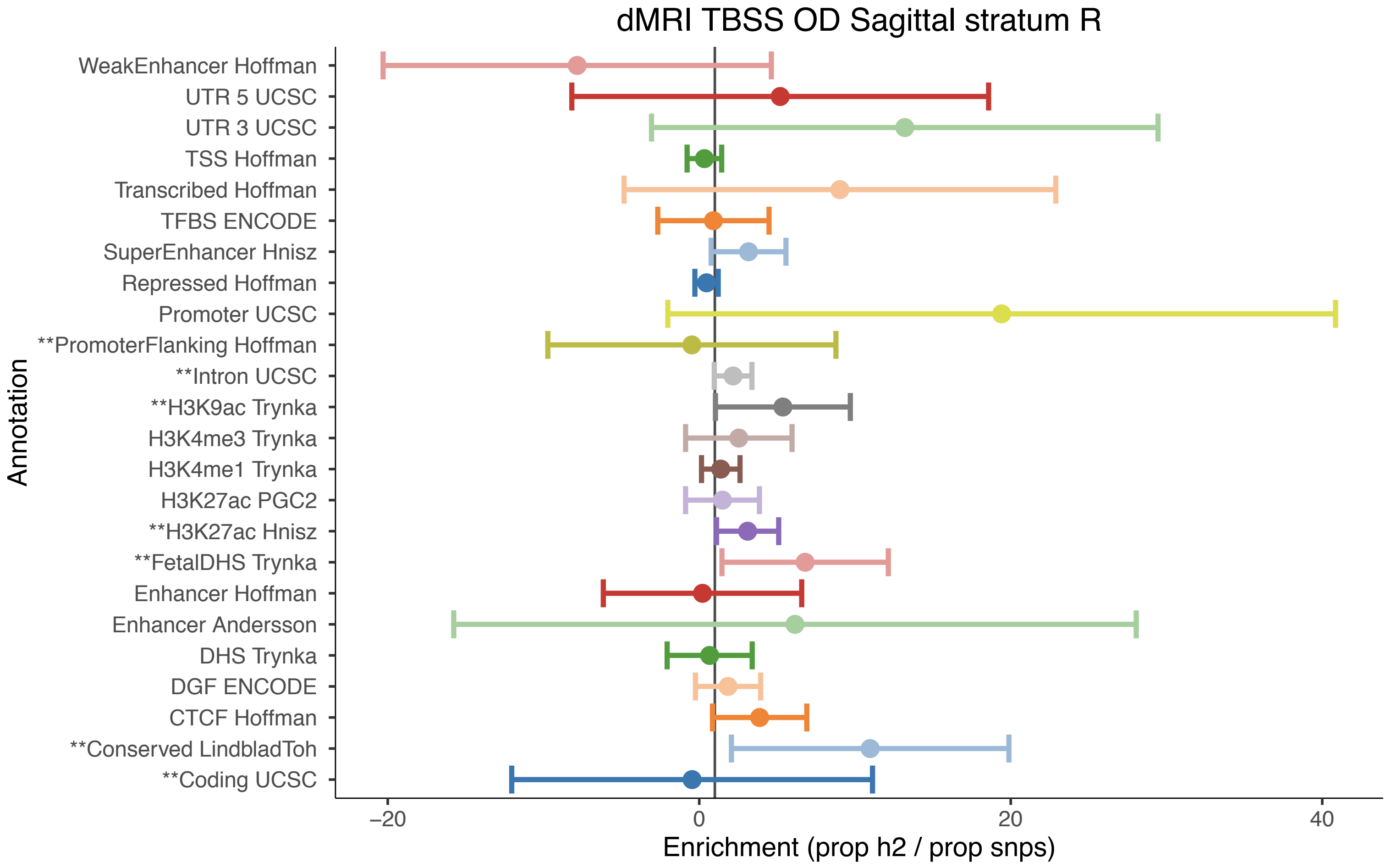
dMRI TBSS OD Sagittal stratum R
WeakEnhancer Hoffman
UTR 5 UCSC
UTR 3 UCSC
TSS Hoffman
Transcribed Hoffman
TFBS ENCODE
SuperEnhancer Hnisz
Repressed Hoffman
Promoter UCSC
**PromoterFlanking Hoffman
**Intron UCSC
**H3K9ac Trynka
H3K4me3 Trynka
H3K4me1 Trynka
H3K27ac PGC2
**H3K27ac Hnisz
**FetalDHS Trynka
Enhancer Hoffman
Enhancer Andersson
DHS Trynka
DGF ENCODE
CTCF Hoffman
**Conserved LindbladToh
**Coding UCSC
Annotation
−20
0
20
40
Enrichment (prop h2 / prop snps)

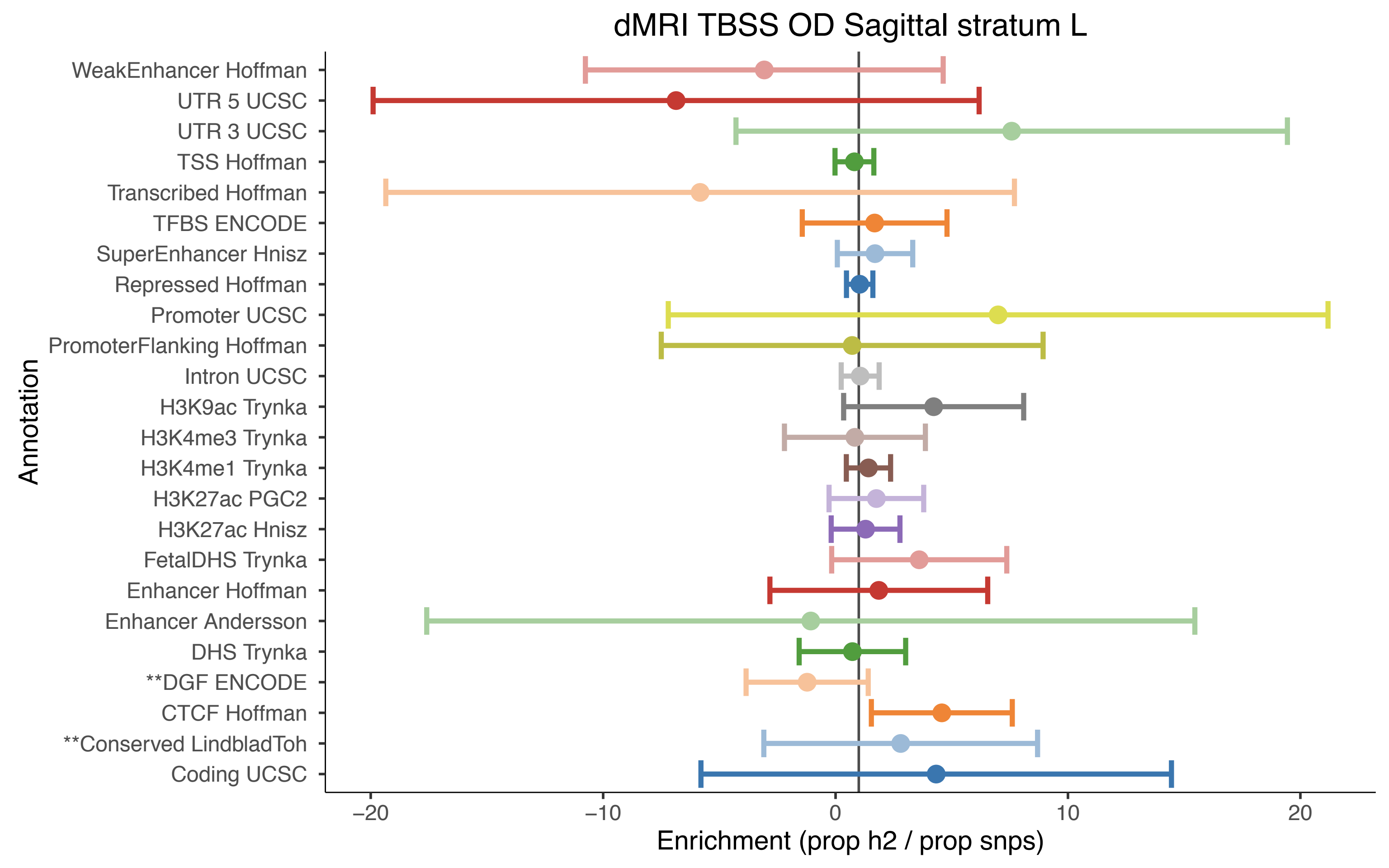

dMRI TBSS OD Sagittal stratum L
WeakEnhancer Hoffman
UTR 5 UCSC
UTR 3 UCSC
TSS Hoffman
Transcribed Hoffman
TFBS ENCODE
SuperEnhancer Hnisz
Repressed Hoffman
Promoter UCSC
PromoterFlanking Hoffman
Intron UCSC
H3K9ac Trynka
H3K4me3 Trynka
H3K4me1 Trynka
H3K27ac PGC2
H3K27ac Hnisz
FetalDHS Trynka
Enhancer Hoffman
Enhancer Andersson
DHS Trynka
**DGF ENCODE
CTCF Hoffman
**Conserved LindbladToh
Coding UCSC
Annotation
−20
−10
0
10
20
Enrichment (prop h2 / prop snps)

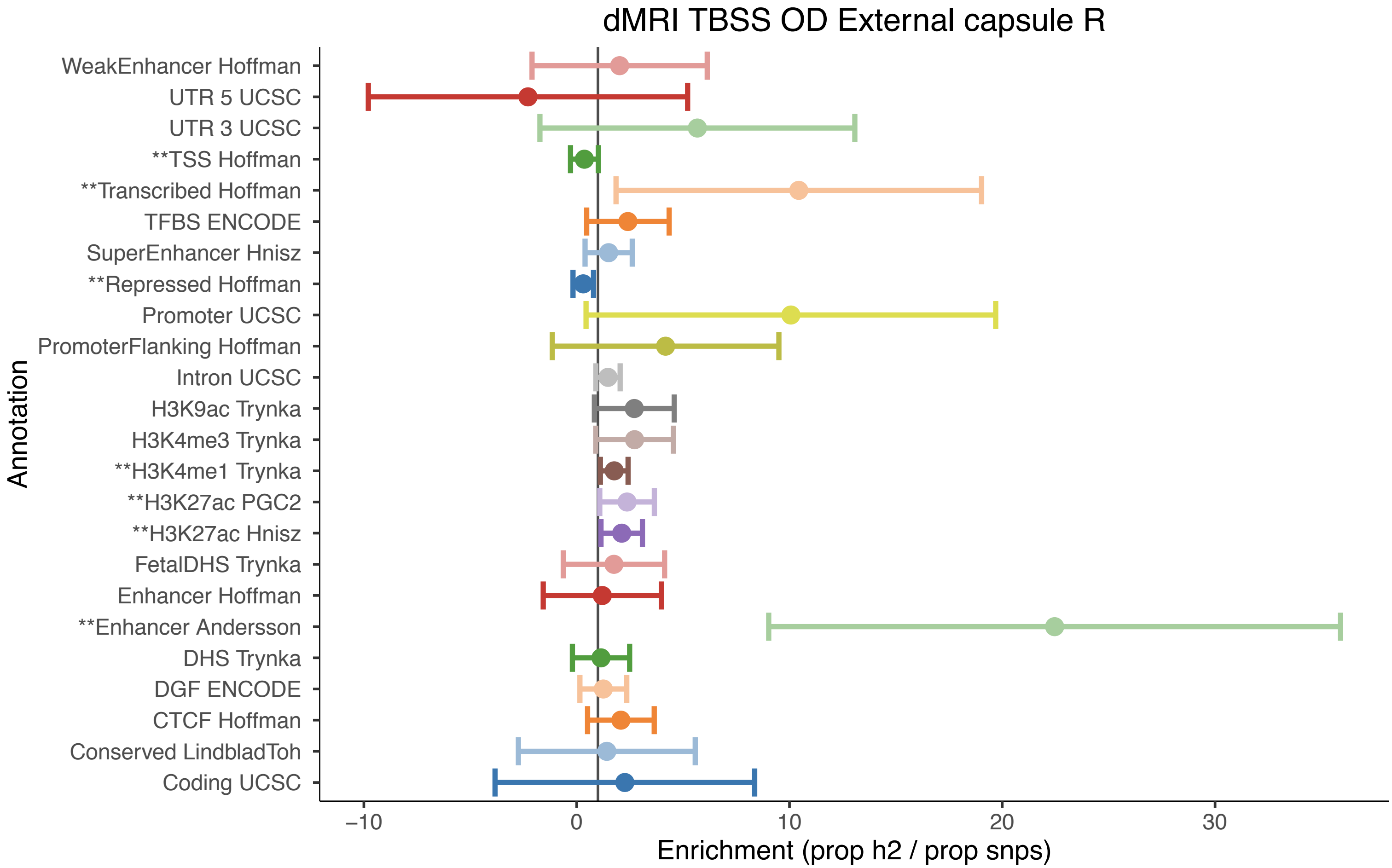

dMRI TBSS OD External capsule R
WeakEnhancer Hoffman
UTR 5 UCSC
UTR 3 UCSC
**TSS Hoffman
**Transcribed Hoffman
TFBS ENCODE
SuperEnhancer Hnisz
**Repressed Hoffman
Promoter UCSC
PromoterFlanking Hoffman
Intron UCSC
H3K9ac Trynka
H3K4me3 Trynka
**H3K4me1 Trynka
**H3K27ac PGC2
**H3K27ac Hnisz
FetalDHS Trynka
Enhancer Hoffman
**Enhancer Andersson
DHS Trynka
DGF ENCODE
CTCF Hoffman
Conserved LindbladToh
Coding UCSC
Annotation
−10
0
10
20
30
Enrichment (prop h2 / prop snps)

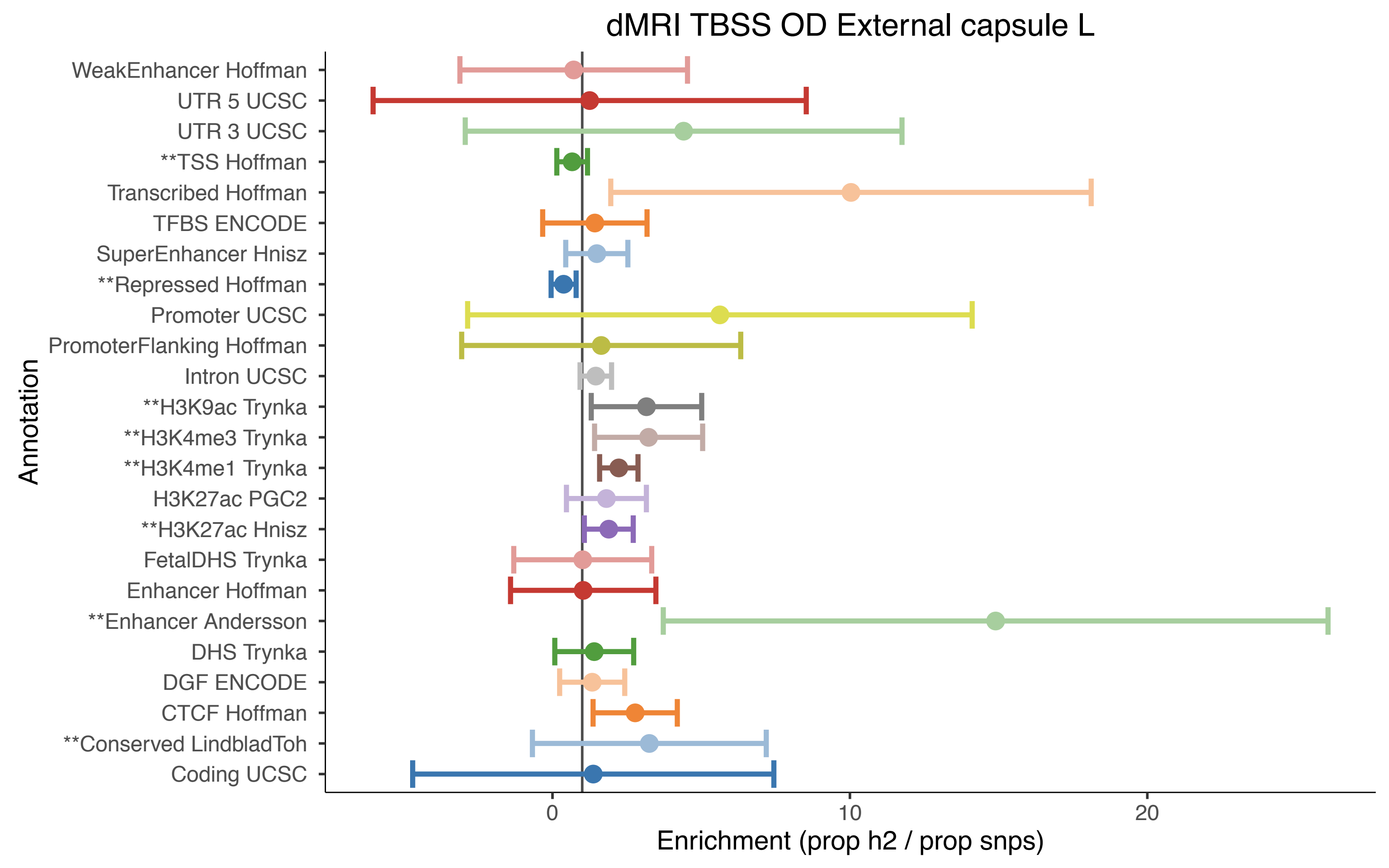

dMRI TBSS OD External capsule L
WeakEnhancer Hoffman
UTR 5 UCSC
UTR 3 UCSC
**TSS Hoffman
Transcribed Hoffman
TFBS ENCODE
SuperEnhancer Hnisz
**Repressed Hoffman
Promoter UCSC
PromoterFlanking Hoffman
Intron UCSC
**H3K9ac Trynka
**H3K4me3 Trynka
**H3K4me1 Trynka
H3K27ac PGC2
**H3K27ac Hnisz
FetalDHS Trynka
Enhancer Hoffman
**Enhancer Andersson
DHS Trynka
DGF ENCODE
CTCF Hoffman
**Conserved LindbladToh
Coding UCSC
Annotation
0
10
20
Enrichment (prop h2 / prop snps)

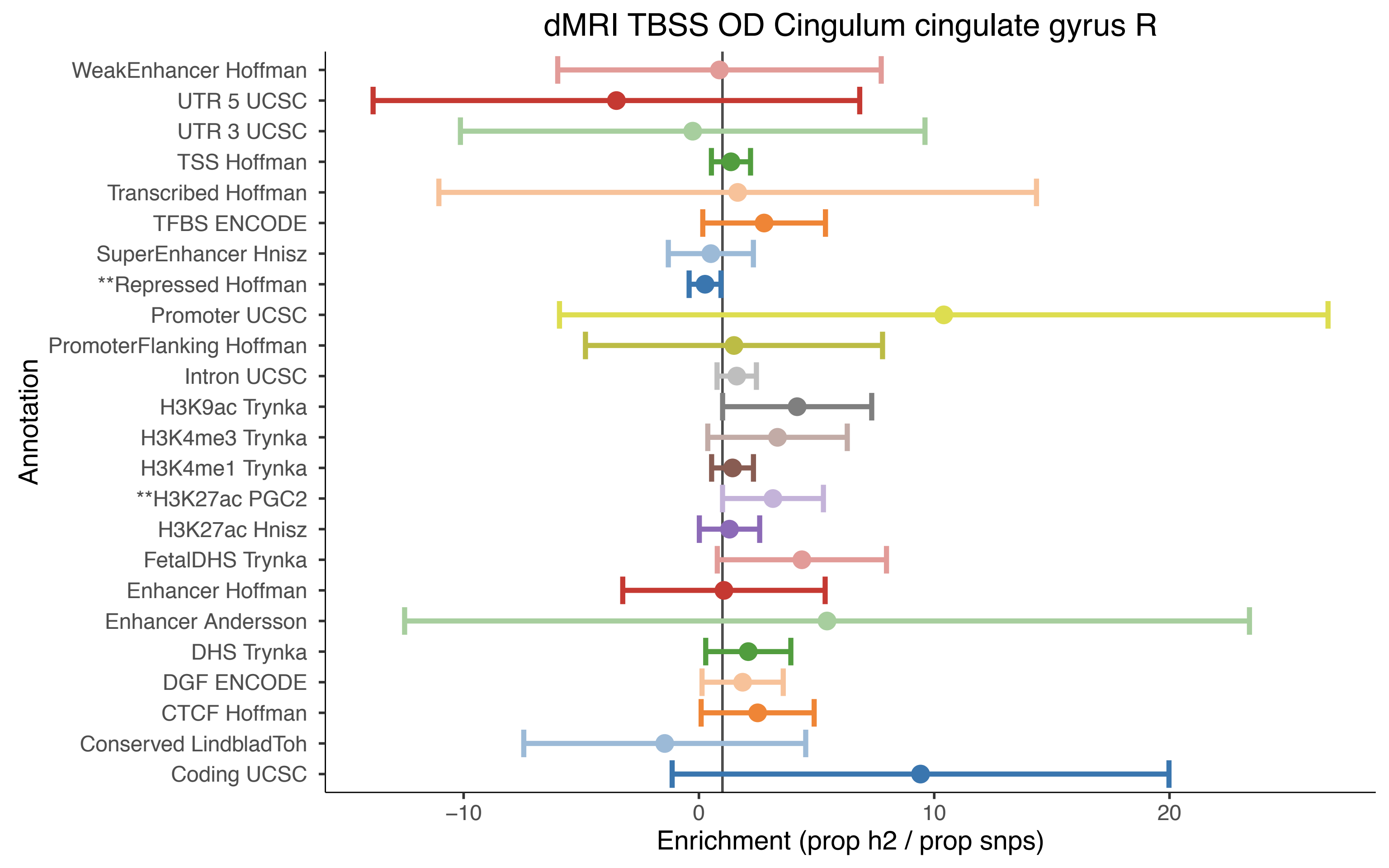
dMRI TBSS OD Cingulum cingulate gyrus R
WeakEnhancer Hoffman
UTR 5 UCSC
UTR 3 UCSC
TSS Hoffman
Transcribed Hoffman
TFBS ENCODE
SuperEnhancer Hnisz
**Repressed Hoffman
Promoter UCSC
PromoterFlanking Hoffman
Intron UCSC
H3K9ac Trynka
H3K4me3 Trynka
H3K4me1 Trynka
**H3K27ac PGC2
H3K27ac Hnisz
FetalDHS Trynka
Enhancer Hoffman
Enhancer Andersson
DHS Trynka
DGF ENCODE
CTCF Hoffman
Conserved LindbladToh
Coding UCSC
Annotation
−10
0
10
20
Enrichment (prop h2 / prop snps)

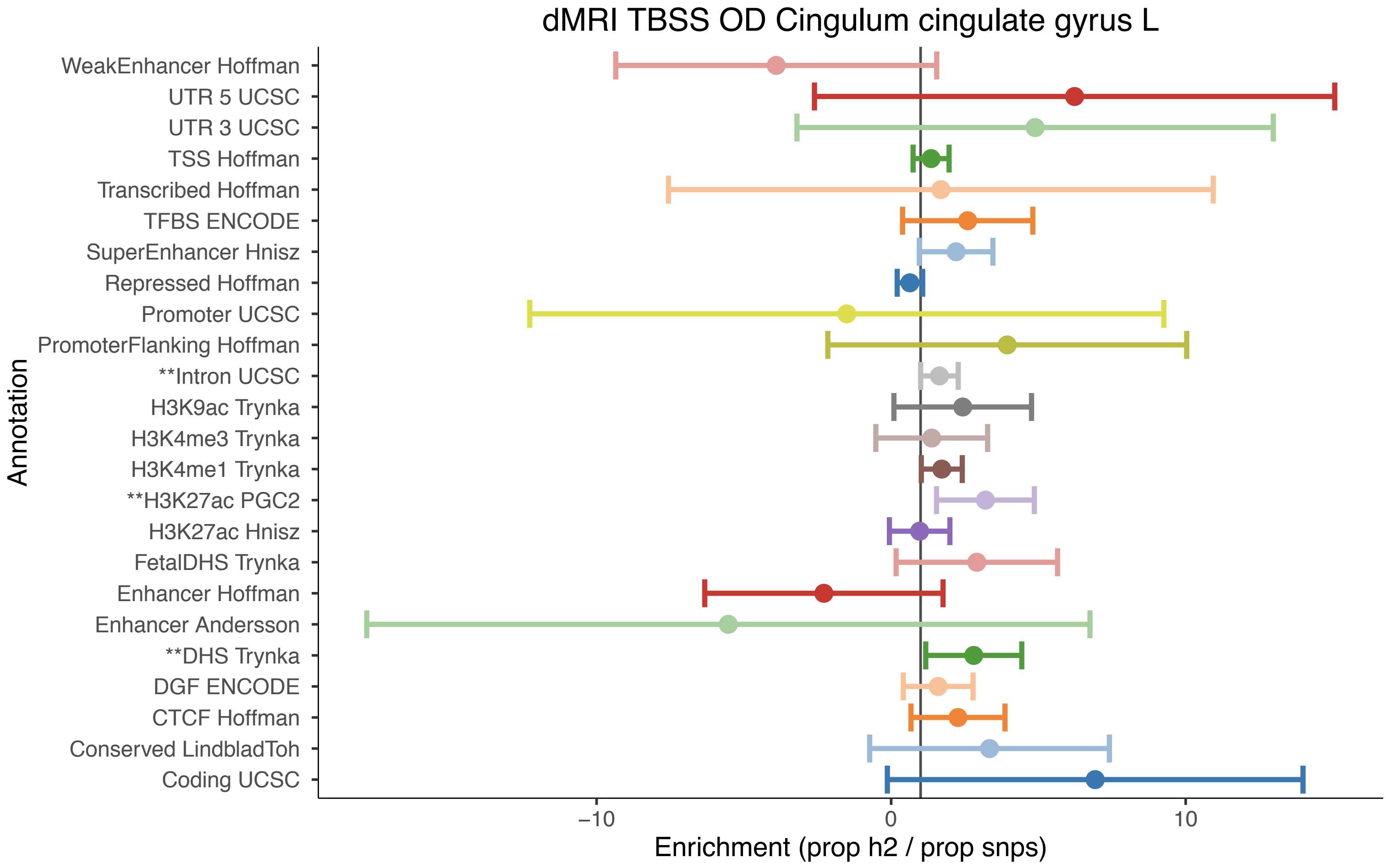
dMRI TBSS OD Cingulum cingulate gyrus L
WeakEnhancer Hoffman
UTR 5 UCSC
UTR 3 UCSC
TSS Hoffman
Transcribed Hoffman
TFBS ENCODE
SuperEnhancer Hnisz
Repressed Hoffman
Promoter UCSC
PromoterFlanking Hoffman
**Intron UCSC
H3K9ac Trynka
H3K4me3 Trynka
H3K4me1 Trynka
**H3K27ac PGC2
H3K27ac Hnisz
FetalDHS Trynka
Enhancer Hoffman
Enhancer Andersson
**DHS Trynka
DGF ENCODE
CTCF Hoffman
Conserved LindbladToh
Coding UCSC
Annotation
−10
0
10
Enrichment (prop h2 / prop snps)

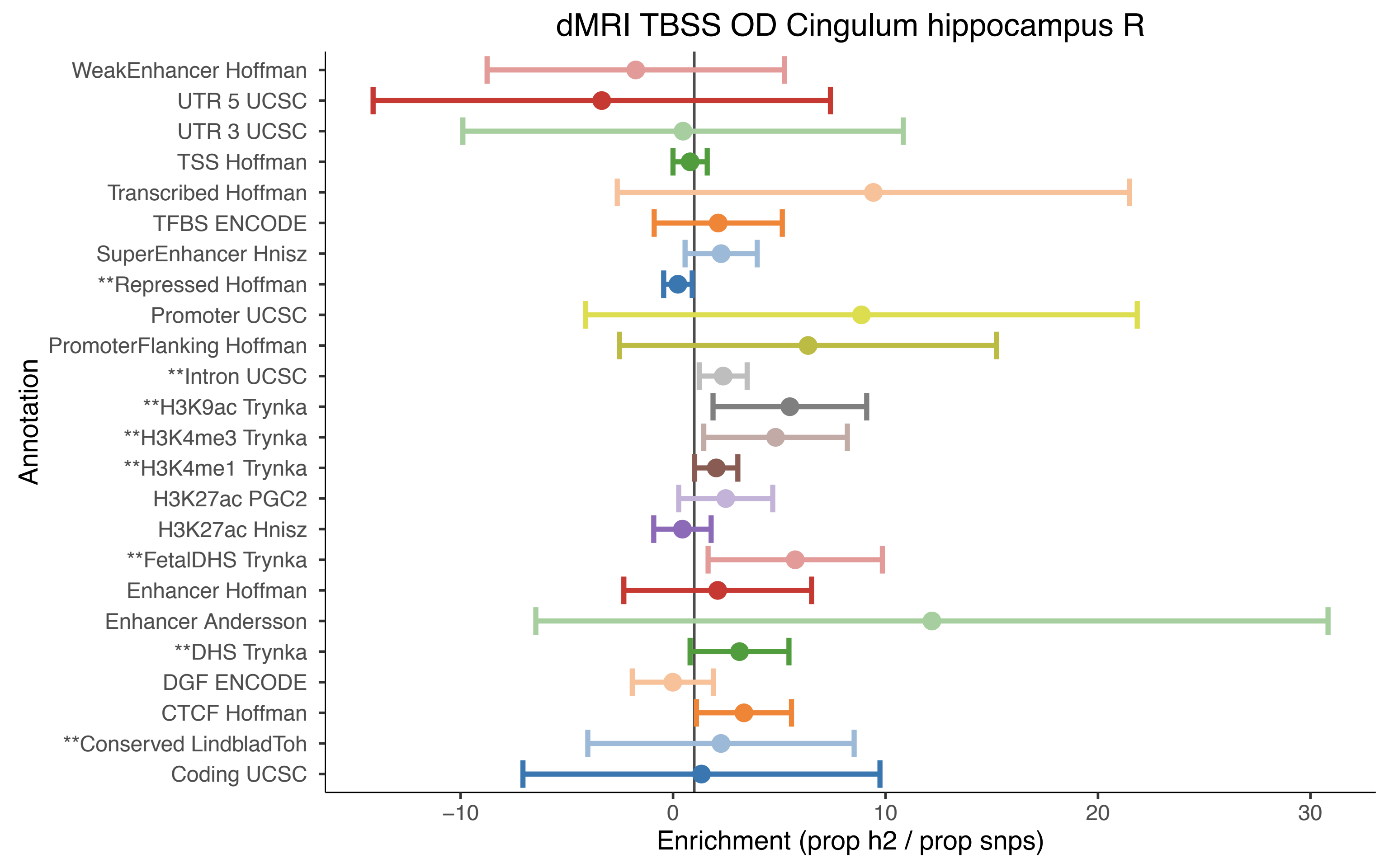

dMRI TBSS OD Cingulum hippocampus R
WeakEnhancer Hoffman
UTR 5 UCSC
UTR 3 UCSC
TSS Hoffman
Transcribed Hoffman
TFBS ENCODE
SuperEnhancer Hnisz
**Repressed Hoffman
Promoter UCSC
PromoterFlanking Hoffman
**Intron UCSC
**H3K9ac Trynka
**H3K4me3 Trynka
**H3K4me1 Trynka
H3K27ac PGC2
H3K27ac Hnisz
**FetalDHS Trynka
Enhancer Hoffman
Enhancer Andersson
**DHS Trynka
DGF ENCODE
CTCF Hoffman
**Conserved LindbladToh
Coding UCSC
Annotation
−10
0
10
20
30
Enrichment (prop h2 / prop snps)

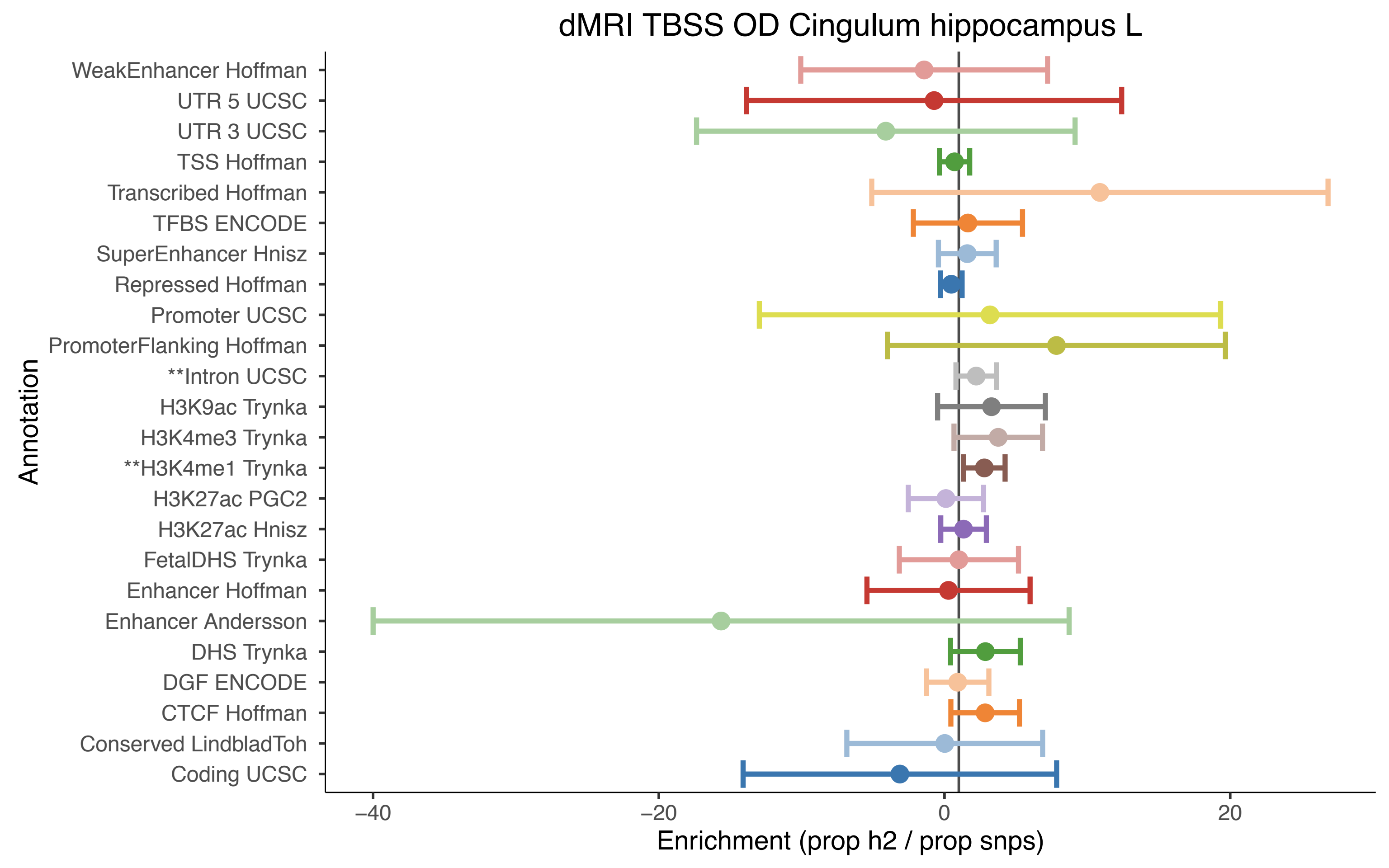

dMRI TBSS OD Cingulum hippocampus L
WeakEnhancer Hoffman
UTR 5 UCSC
UTR 3 UCSC
TSS Hoffman
Transcribed Hoffman
TFBS ENCODE
SuperEnhancer Hnisz
Repressed Hoffman
Promoter UCSC
PromoterFlanking Hoffman
**Intron UCSC
H3K9ac Trynka
H3K4me3 Trynka
**H3K4me1 Trynka
H3K27ac PGC2
H3K27ac Hnisz
FetalDHS Trynka
Enhancer Hoffman
Enhancer Andersson
DHS Trynka
DGF ENCODE
CTCF Hoffman
Conserved LindbladToh
Coding UCSC
Annotation
−40
−20
0
20
Enrichment (prop h2 / prop snps)

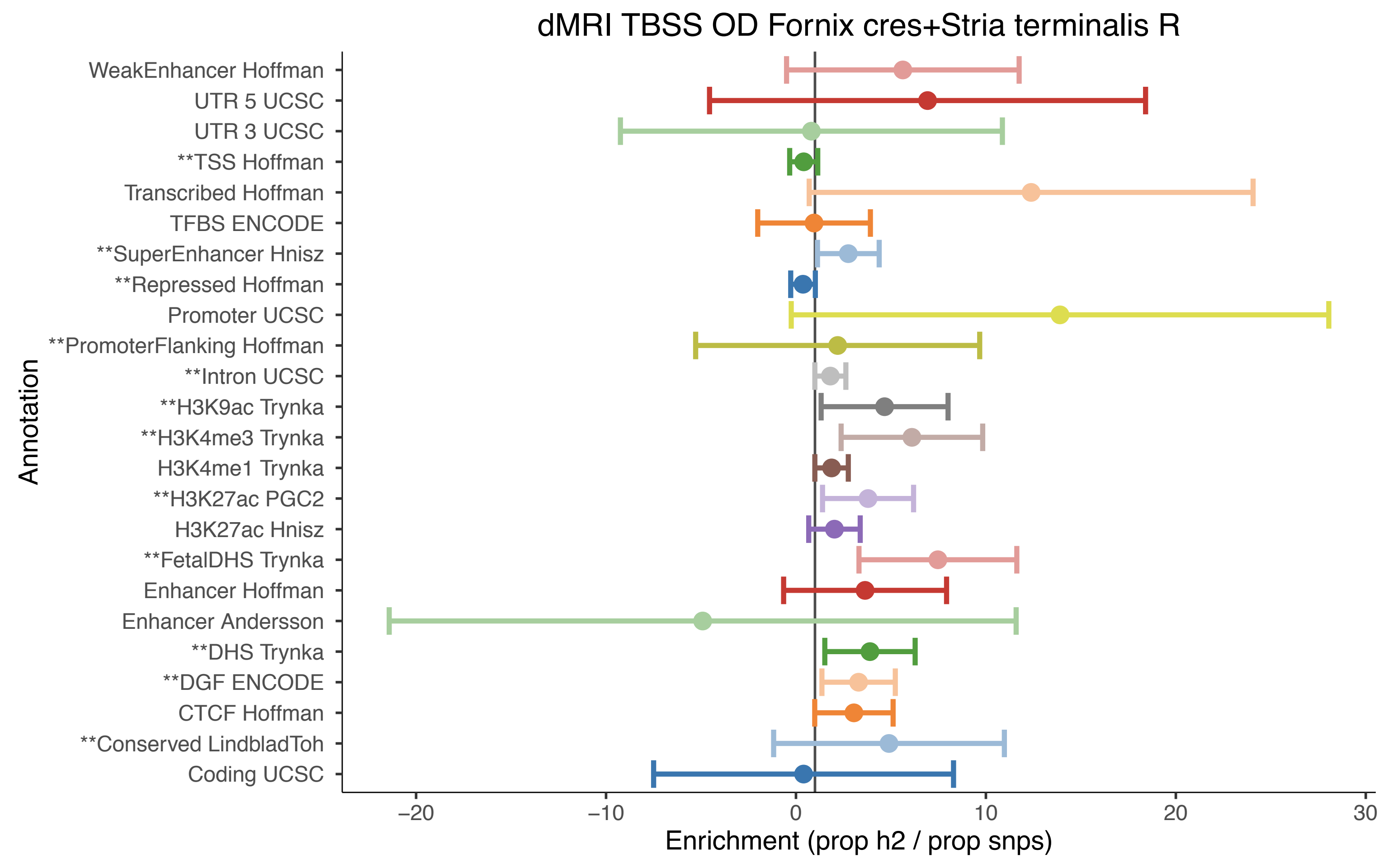

dMRI TBSS OD Fornix cres+Stria terminalis R
Annotation
WeakEnhancer Hoffman
UTR 5 UCSC
UTR 3 UCSC
**TSS Hoffman
Transcribed Hoffman
TFBS ENCODE
**SuperEnhancer Hnisz
**Repressed Hoffman
Promoter UCSC
**PromoterFlanking Hoffman
**Intron UCSC
**H3K9ac Trynka
**H3K4me3 Trynka
H3K4me1 Trynka
**H3K27ac PGC2
H3K27ac Hnisz
**FetalDHS Trynka
Enhancer Hoffman
Enhancer Andersson
**DHS Trynka
**DGF ENCODE
CTCF Hoffman
**Conserved LindbladToh
Coding UCSC
−20
−10
0
10
20
30
Enrichment (prop h2 / prop snps)

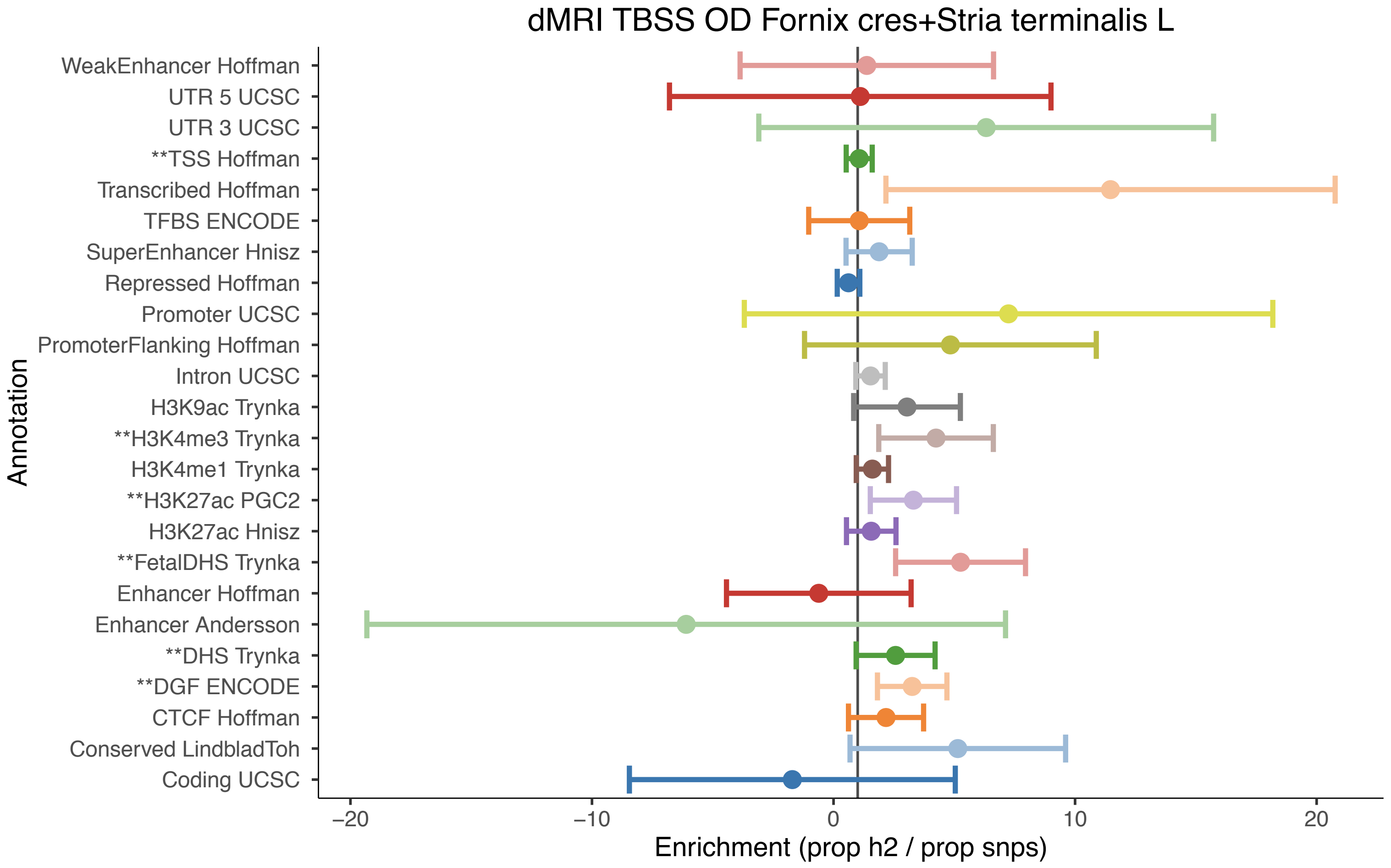

dMRI TBSS OD Fornix cres+Stria terminalis L
WeakEnhancer Hoffman
UTR 5 UCSC
UTR 3 UCSC
**TSS Hoffman
Transcribed Hoffman
TFBS ENCODE
SuperEnhancer Hnisz
Repressed Hoffman
Promoter UCSC
PromoterFlanking Hoffman
Intron UCSC
H3K9ac Trynka
**H3K4me3 Trynka
H3K4me1 Trynka
**H3K27ac PGC2
H3K27ac Hnisz
**FetalDHS Trynka
Enhancer Hoffman
Enhancer Andersson
**DHS Trynka
**DGF ENCODE
CTCF Hoffman
Conserved LindbladToh
Coding UCSC
Annotation
−20
−10
0
10
20
Enrichment (prop h2 / prop snps)

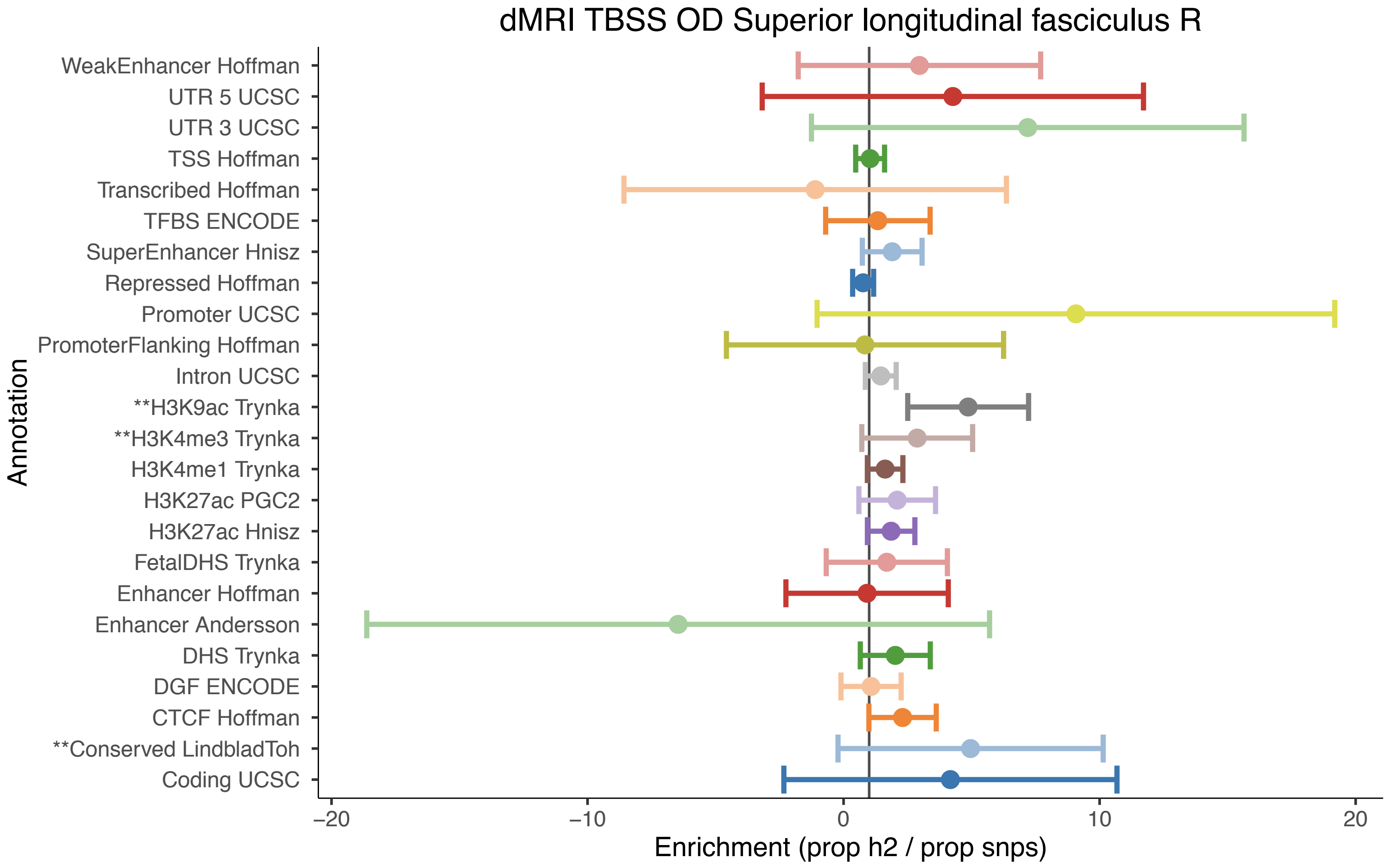
dMRI TBSS OD Superior longitudinal fasciculus R
WeakEnhancer Hoffman
UTR 5 UCSC
UTR 3 UCSC
TSS Hoffman
Transcribed Hoffman
TFBS ENCODE
SuperEnhancer Hnisz
Repressed Hoffman
Promoter UCSC
PromoterFlanking Hoffman
Intron UCSC
**H3K9ac Trynka
**H3K4me3 Trynka
H3K4me1 Trynka
H3K27ac PGC2
H3K27ac Hnisz
FetalDHS Trynka
Enhancer Hoffman
Enhancer Andersson
DHS Trynka
DGF ENCODE
CTCF Hoffman
**Conserved LindbladToh
Coding UCSC
Annotation
−20
−10
0
10
20
Enrichment (prop h2 / prop snps)

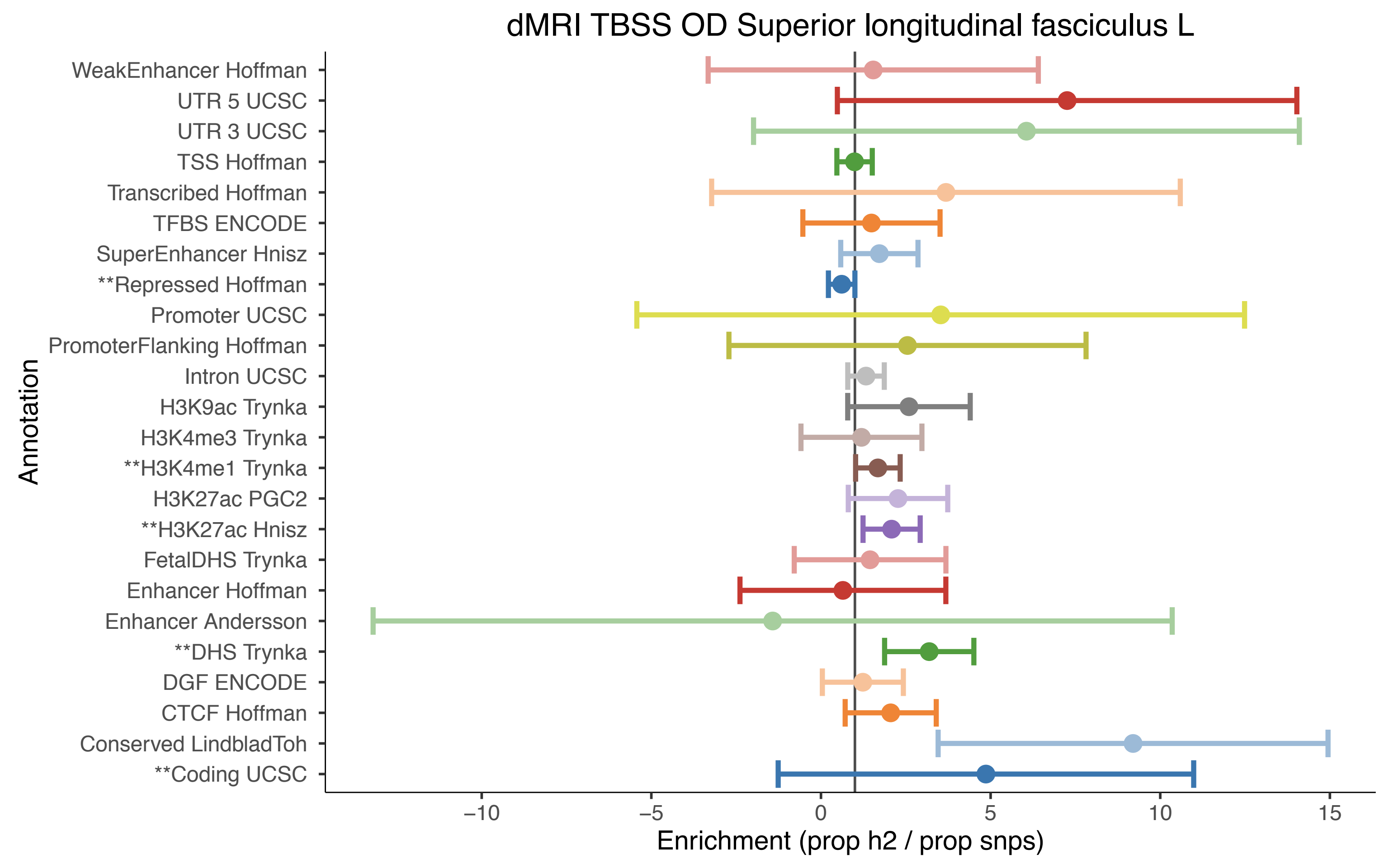

dMRI TBSS OD Superior longitudinal fasciculus L
WeakEnhancer Hoffman
UTR 5 UCSC
UTR 3 UCSC
TSS Hoffman
Transcribed Hoffman
TFBS ENCODE
SuperEnhancer Hnisz
**Repressed Hoffman
Promoter UCSC
PromoterFlanking Hoffman
Intron UCSC
H3K9ac Trynka
H3K4me3 Trynka
**H3K4me1 Trynka
H3K27ac PGC2
**H3K27ac Hnisz
FetalDHS Trynka
Enhancer Hoffman
Enhancer Andersson
**DHS Trynka
DGF ENCODE
CTCF Hoffman
Conserved LindbladToh
**Coding UCSC
Annotation
−10
−5
0
5
10
15
Enrichment (prop h2 / prop snps)

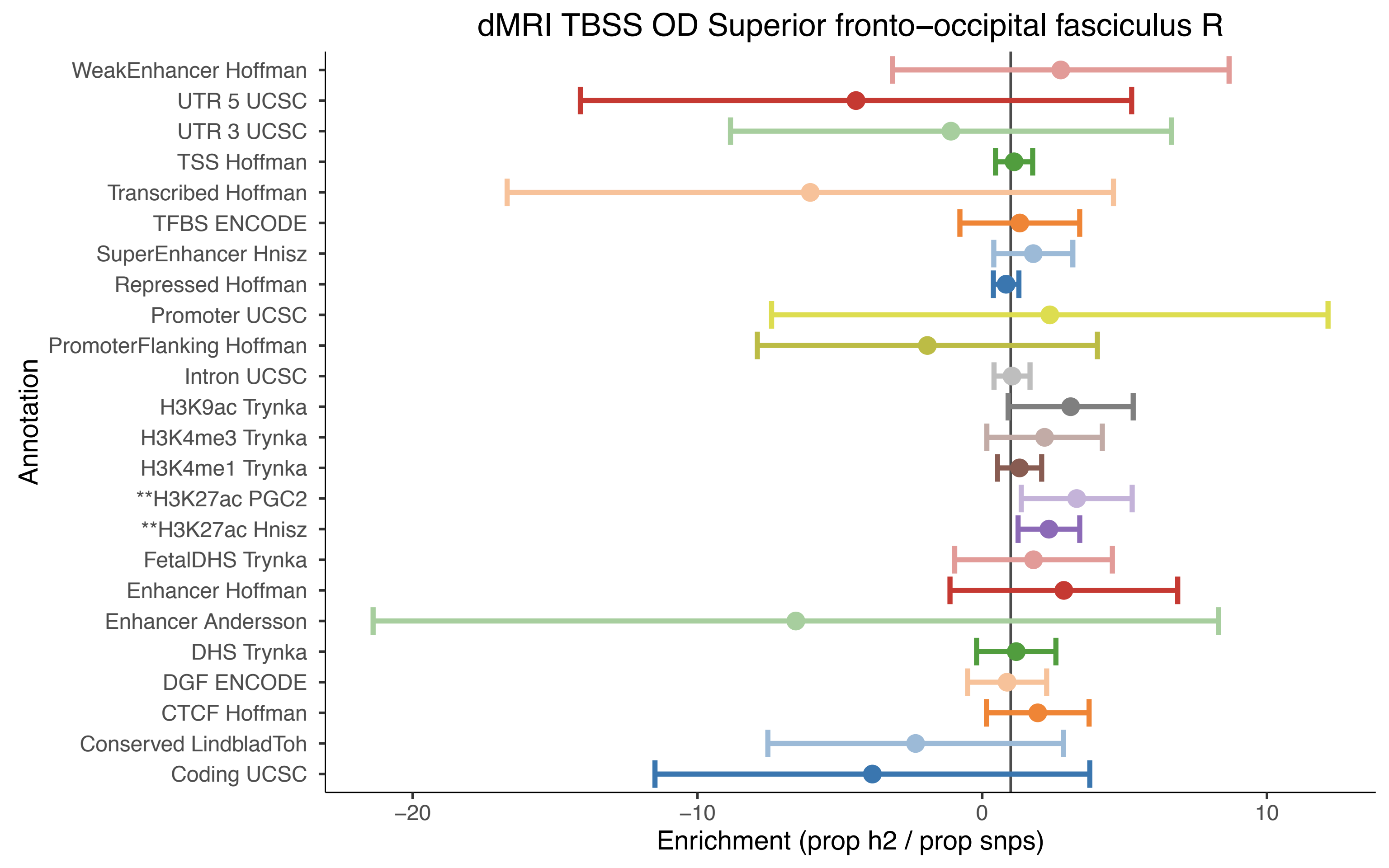
dMRI TBSS OD Superior fronto−occipital fasciculus R
WeakEnhancer Hoffman
UTR 5 UCSC
UTR 3 UCSC
TSS Hoffman
Transcribed Hoffman
TFBS ENCODE
SuperEnhancer Hnisz
Repressed Hoffman
Promoter UCSC
PromoterFlanking Hoffman
Intron UCSC
H3K9ac Trynka
H3K4me3 Trynka
H3K4me1 Trynka
**H3K27ac PGC2
**H3K27ac Hnisz
FetalDHS Trynka
Enhancer Hoffman
Enhancer Andersson
DHS Trynka
DGF ENCODE
CTCF Hoffman
Conserved LindbladToh
Coding UCSC
Annotation
−20
−10
0
10
Enrichment (prop h2 / prop snps)

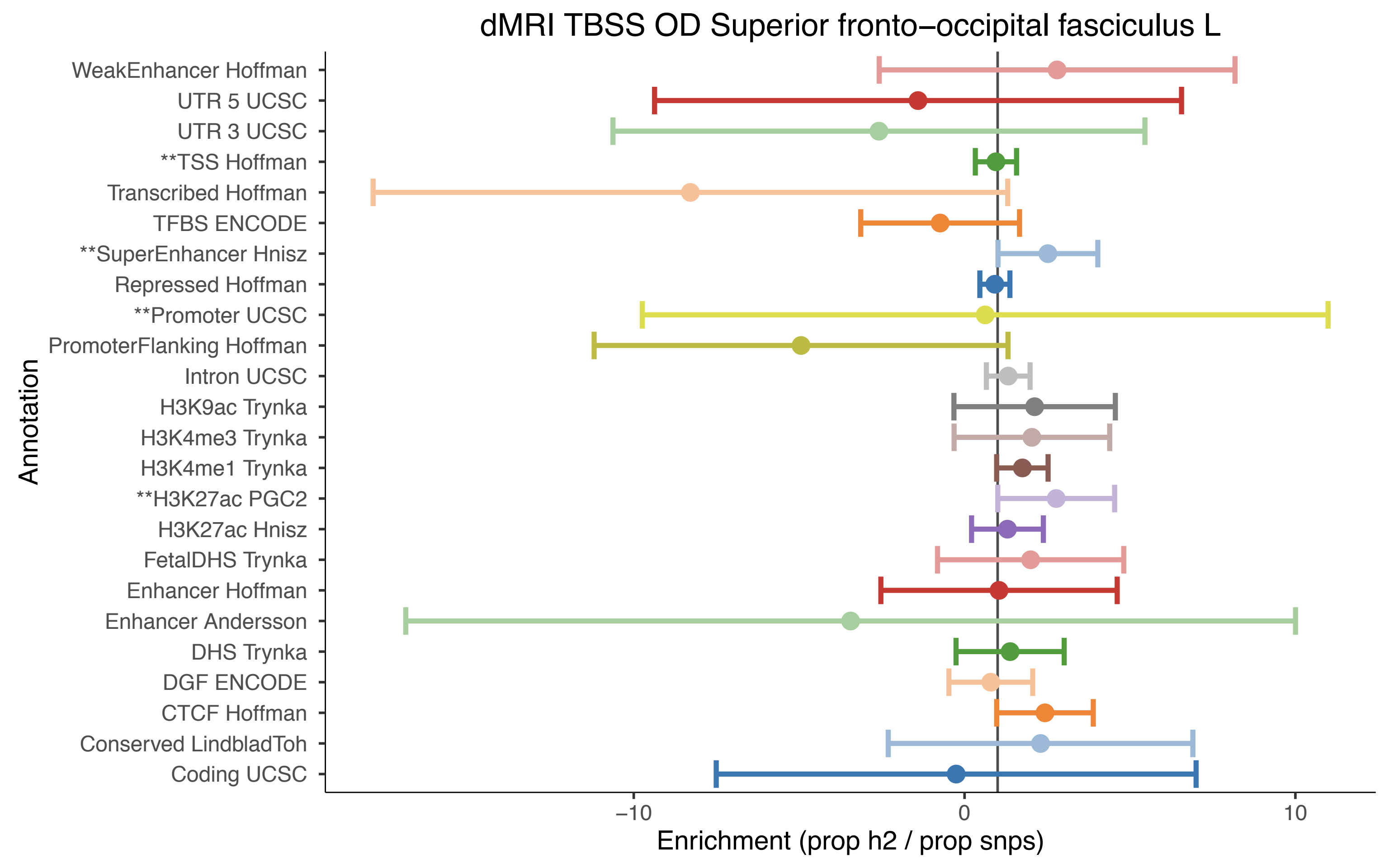

dMRI TBSS OD Superior fronto−occipital fasciculus L
WeakEnhancer Hoffman
UTR 5 UCSC
UTR 3 UCSC
**TSS Hoffman
Transcribed Hoffman
TFBS ENCODE
**SuperEnhancer Hnisz
Repressed Hoffman
**Promoter UCSC
PromoterFlanking Hoffman
Intron UCSC
H3K9ac Trynka
H3K4me3 Trynka
H3K4me1 Trynka
**H3K27ac PGC2
H3K27ac Hnisz
FetalDHS Trynka
Enhancer Hoffman
Enhancer Andersson
DHS Trynka
DGF ENCODE
CTCF Hoffman
Conserved LindbladToh
Coding UCSC
Annotation
−10
0
10
Enrichment (prop h2 / prop snps)

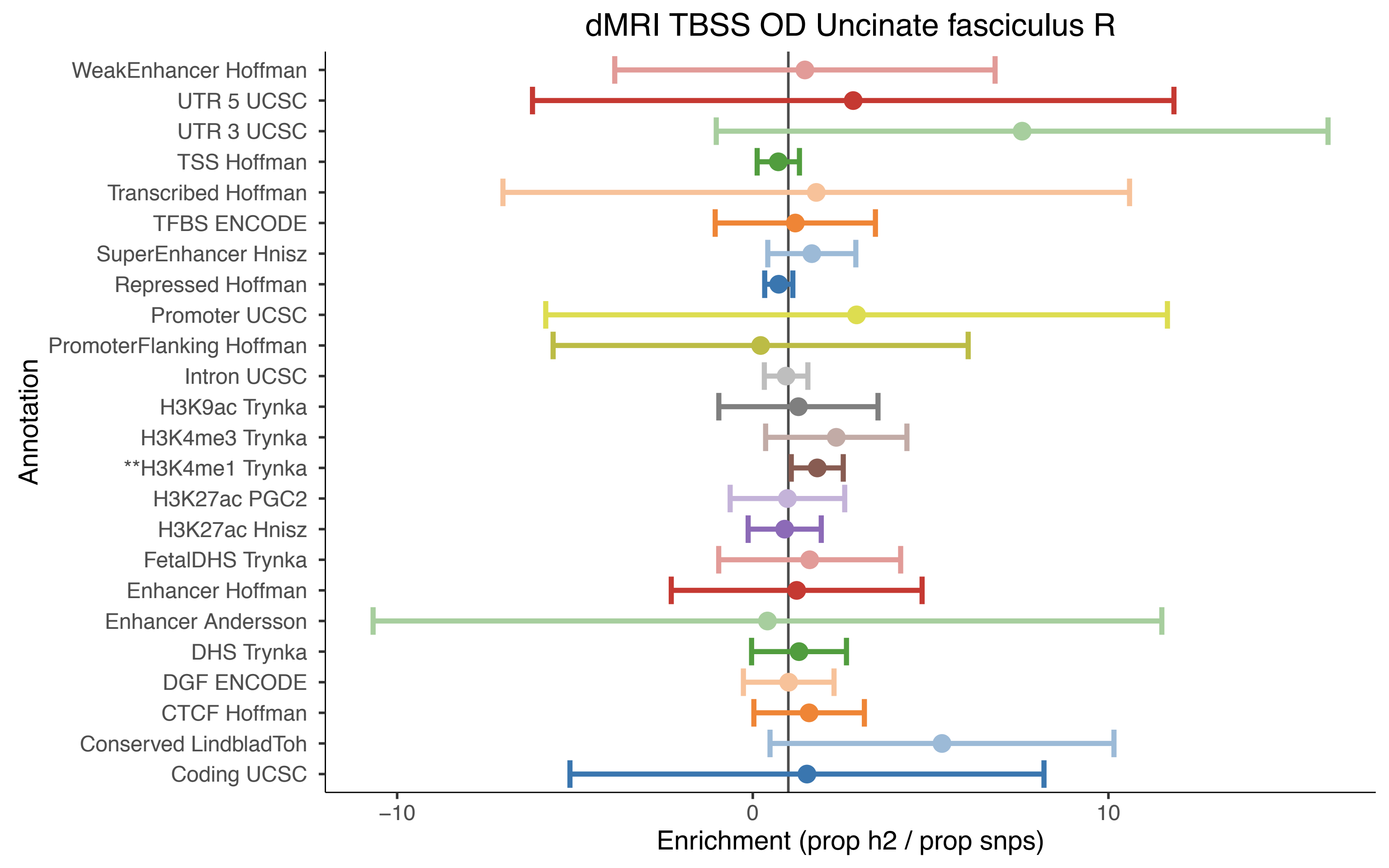

dMRI TBSS OD Uncinate fasciculus R
Annotation
WeakEnhancer Hoffman
UTR 5 UCSC
UTR 3 UCSC
TSS Hoffman
Transcribed Hoffman
TFBS ENCODE
SuperEnhancer Hnisz
Repressed Hoffman
Promoter UCSC
PromoterFlanking Hoffman
Intron UCSC
H3K9ac Trynka
H3K4me3 Trynka
**H3K4me1 Trynka
H3K27ac PGC2
H3K27ac Hnisz
FetalDHS Trynka
Enhancer Hoffman
Enhancer Andersson
DHS Trynka
DGF ENCODE
CTCF Hoffman
Conserved LindbladToh
Coding UCSC
−10
0
10
Enrichment (prop h2 / prop snps)

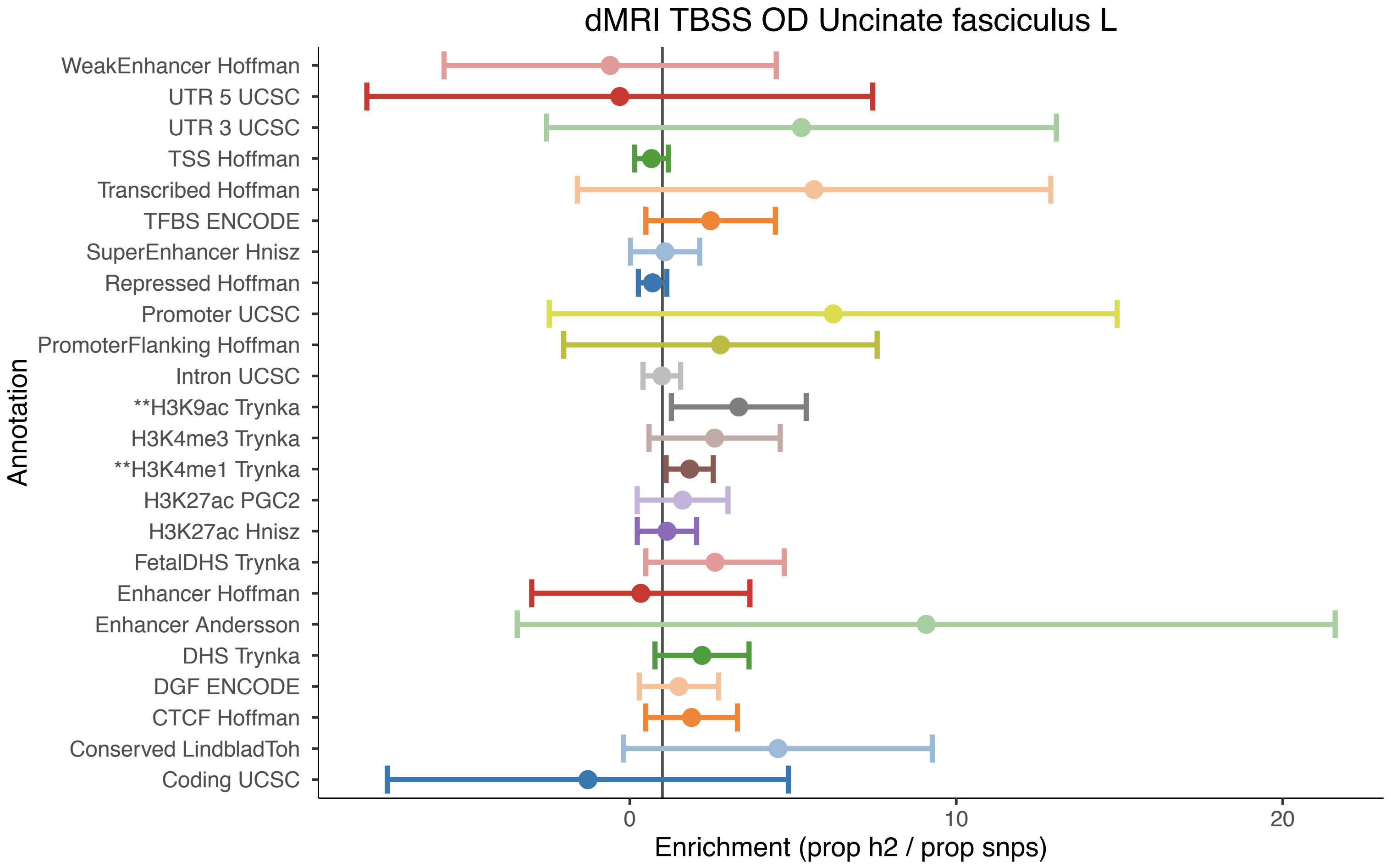
dMRI TBSS OD Uncinate fasciculus L
WeakEnhancer Hoffman
UTR 5 UCSC
UTR 3 UCSC
TSS Hoffman
Transcribed Hoffman
TFBS ENCODE
SuperEnhancer Hnisz
Repressed Hoffman
Promoter UCSC
PromoterFlanking Hoffman
Intron UCSC
**H3K9ac Trynka
H3K4me3 Trynka
**H3K4me1 Trynka
H3K27ac PGC2
H3K27ac Hnisz
FetalDHS Trynka
Enhancer Hoffman
Enhancer Andersson
DHS Trynka
DGF ENCODE
CTCF Hoffman
Conserved LindbladToh
Coding UCSC
Annotation
0
10
20
Enrichment (prop h2 / prop snps)

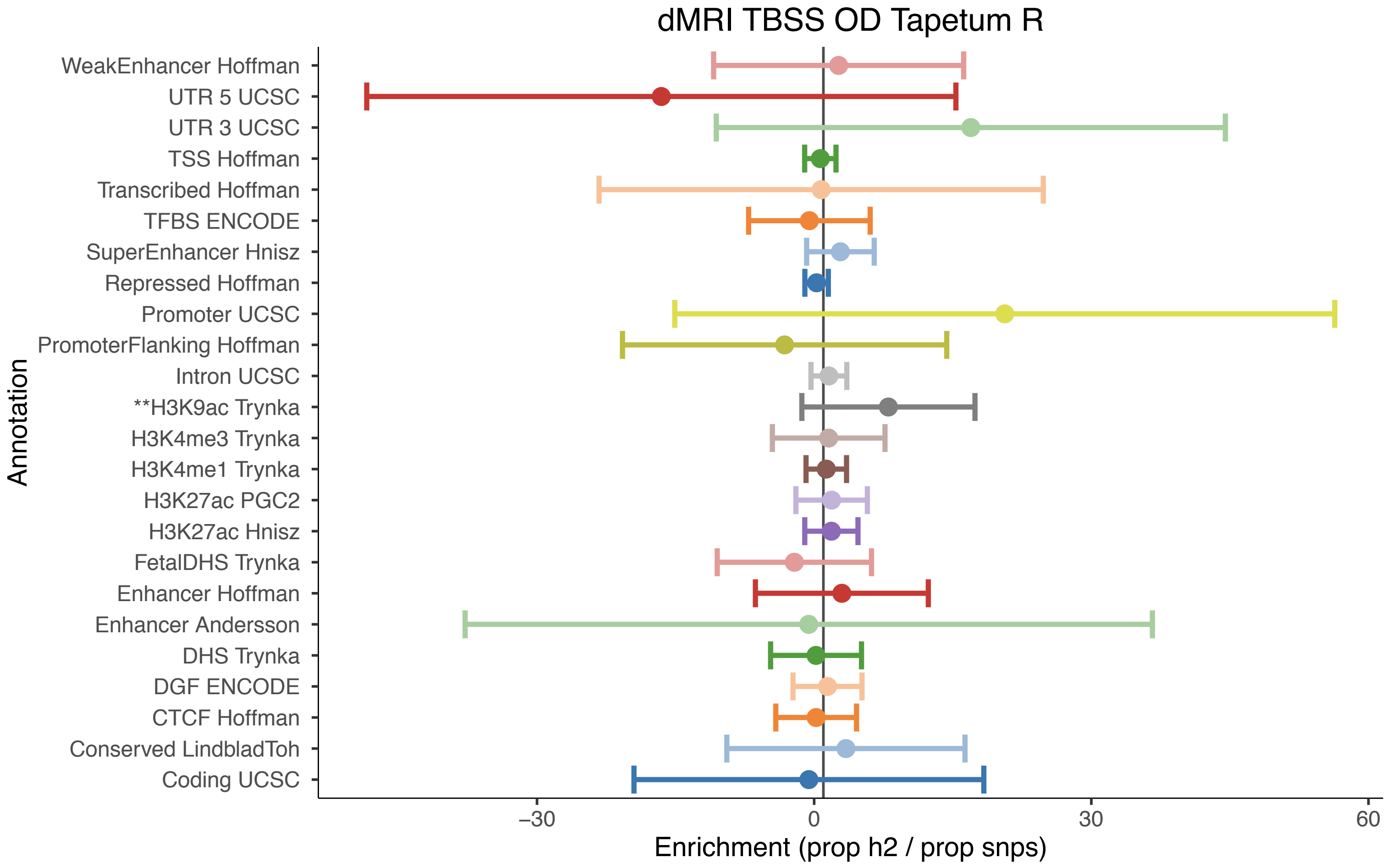
dMRI TBSS OD Tapetum R
WeakEnhancer Hoffman
UTR 5 UCSC
UTR 3 UCSC
TSS Hoffman
Transcribed Hoffman
TFBS ENCODE
SuperEnhancer Hnisz
Repressed Hoffman
Promoter UCSC
PromoterFlanking Hoffman
Intron UCSC
**H3K9ac Trynka
H3K4me3 Trynka
H3K4me1 Trynka
H3K27ac PGC2
H3K27ac Hnisz
FetalDHS Trynka
Enhancer Hoffman
Enhancer Andersson
DHS Trynka
DGF ENCODE
CTCF Hoffman
Conserved LindbladToh
Coding UCSC
Annotation
−30
0
30
60
Enrichment (prop h2 / prop snps)

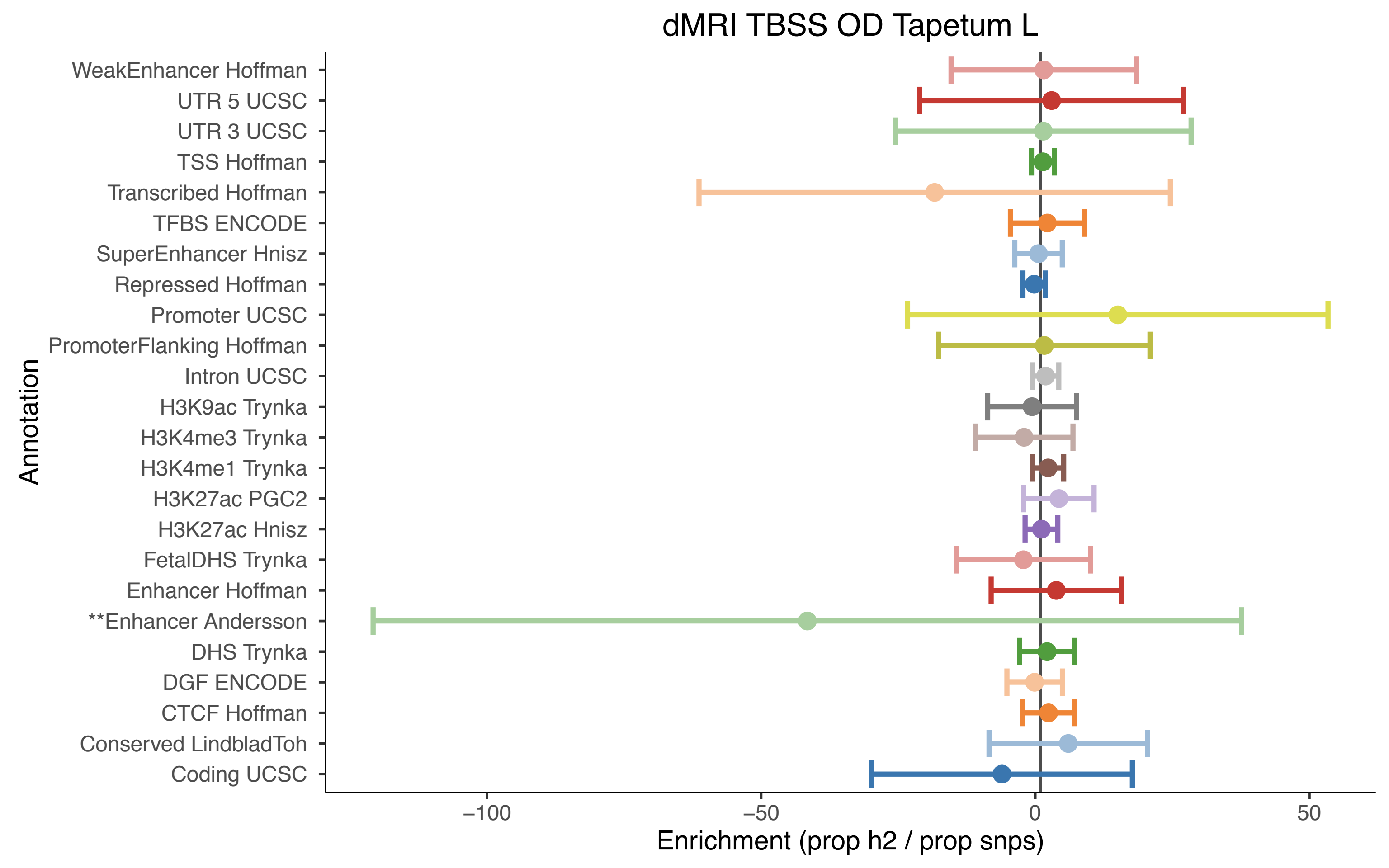

dMRI TBSS OD Tapetum L
WeakEnhancer Hoffman
UTR 5 UCSC
UTR 3 UCSC
TSS Hoffman
Transcribed Hoffman
TFBS ENCODE
SuperEnhancer Hnisz
Repressed Hoffman
Promoter UCSC
PromoterFlanking Hoffman
Intron UCSC
H3K9ac Trynka
H3K4me3 Trynka
H3K4me1 Trynka
H3K27ac PGC2
H3K27ac Hnisz
FetalDHS Trynka
Enhancer Hoffman
**Enhancer Andersson
DHS Trynka
DGF ENCODE
CTCF Hoffman
Conserved LindbladToh
Coding UCSC
Annotation
−100
−50
0
50
Enrichment (prop h2 / prop snps)

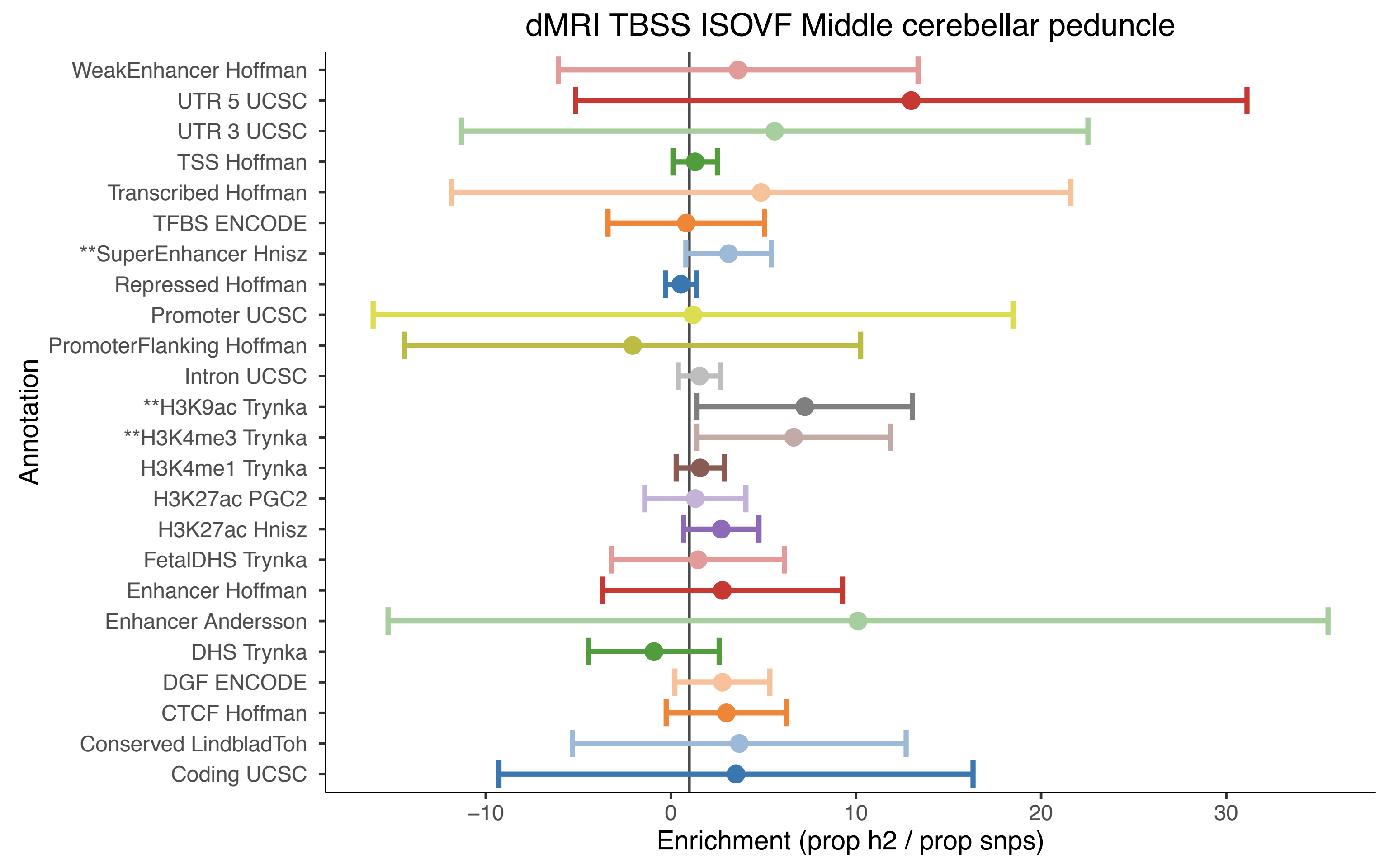
dMRI TBSS ISOVF Middle cerebellar peduncle
Annotation
WeakEnhancer Hoffman
UTR 5 UCSC
UTR 3 UCSC
TSS Hoffman
Transcribed Hoffman
TFBS ENCODE
**SuperEnhancer Hnisz
Repressed Hoffman
Promoter UCSC
PromoterFlanking Hoffman
Intron UCSC
**H3K9ac Trynka
**H3K4me3 Trynka
H3K4me1 Trynka
H3K27ac PGC2
H3K27ac Hnisz
FetalDHS Trynka
Enhancer Hoffman
Enhancer Andersson
DHS Trynka
DGF ENCODE
CTCF Hoffman
Conserved LindbladToh
Coding UCSC
−10
0
10
20
30
Enrichment (prop h2 / prop snps)

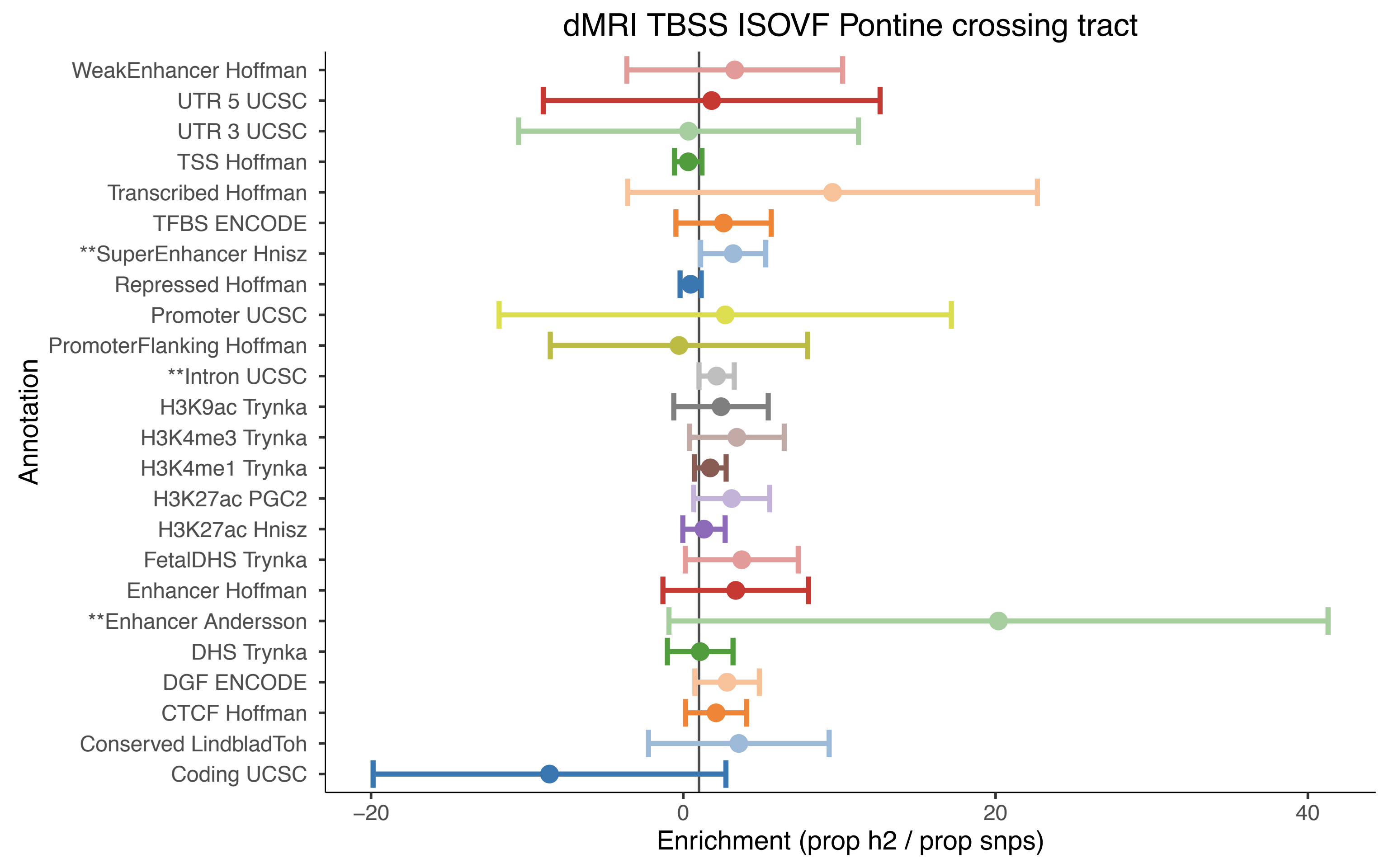
dMRI TBSS ISOVF Pontine crossing tract
WeakEnhancer Hoffman
UTR 5 UCSC
UTR 3 UCSC
TSS Hoffman
Transcribed Hoffman
TFBS ENCODE
**SuperEnhancer Hnisz
Repressed Hoffman
Promoter UCSC
PromoterFlanking Hoffman
**Intron UCSC
H3K9ac Trynka
H3K4me3 Trynka
H3K4me1 Trynka
H3K27ac PGC2
H3K27ac Hnisz
FetalDHS Trynka
Enhancer Hoffman
**Enhancer Andersson
DHS Trynka
DGF ENCODE
CTCF Hoffman
Conserved LindbladToh
Coding UCSC
Annotation
−20
0
20
40
Enrichment (prop h2 / prop snps)

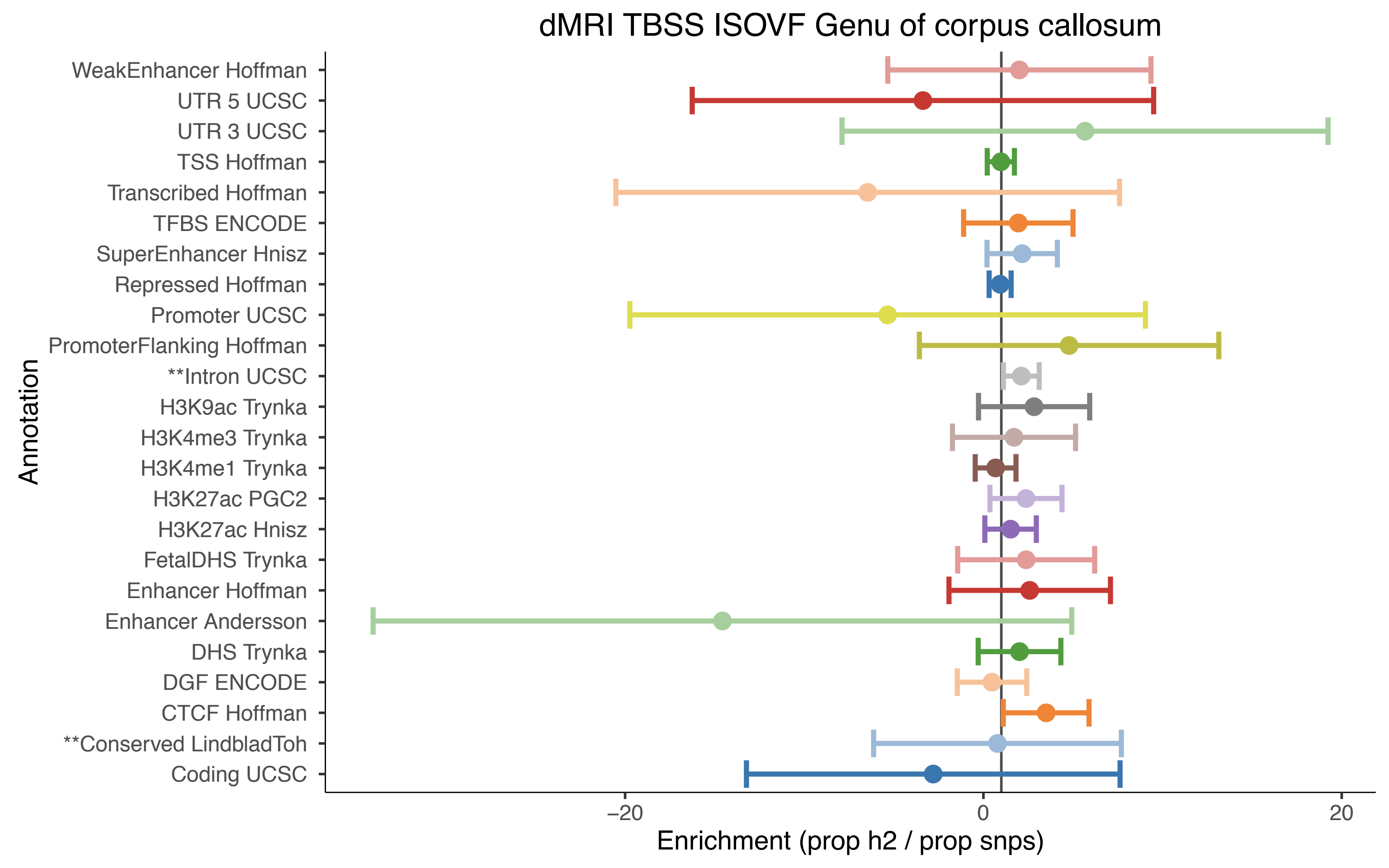

dMRI TBSS ISOVF Genu of corpus callosum
WeakEnhancer Hoffman
UTR 5 UCSC
UTR 3 UCSC
TSS Hoffman
Transcribed Hoffman
TFBS ENCODE
SuperEnhancer Hnisz
Repressed Hoffman
Promoter UCSC
PromoterFlanking Hoffman
**Intron UCSC
H3K9ac Trynka
H3K4me3 Trynka
H3K4me1 Trynka
H3K27ac PGC2
H3K27ac Hnisz
FetalDHS Trynka
Enhancer Hoffman
Enhancer Andersson
DHS Trynka
DGF ENCODE
CTCF Hoffman
**Conserved LindbladToh
Coding UCSC
Annotation
−20
0
20
Enrichment (prop h2 / prop snps)

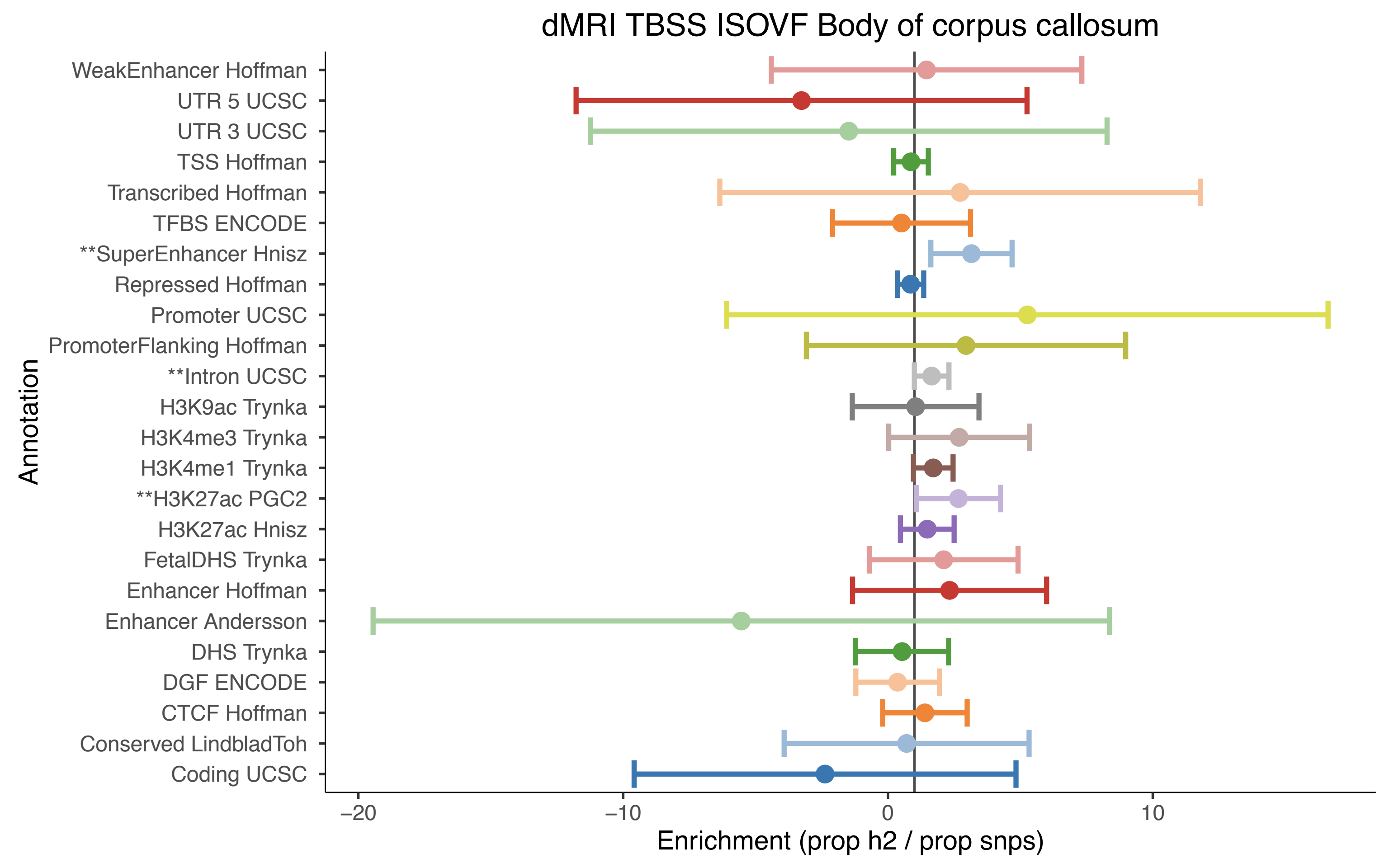
dMRI TBSS ISOVF Body of corpus callosum
WeakEnhancer Hoffman
UTR 5 UCSC
UTR 3 UCSC
TSS Hoffman
Transcribed Hoffman
TFBS ENCODE
**SuperEnhancer Hnisz
Repressed Hoffman
Promoter UCSC
PromoterFlanking Hoffman
**Intron UCSC
H3K9ac Trynka
H3K4me3 Trynka
H3K4me1 Trynka
**H3K27ac PGC2
H3K27ac Hnisz
FetalDHS Trynka
Enhancer Hoffman
Enhancer Andersson
DHS Trynka
DGF ENCODE
CTCF Hoffman
Conserved LindbladToh
Coding UCSC
Annotation
−20
−10
0
10
Enrichment (prop h2 / prop snps)

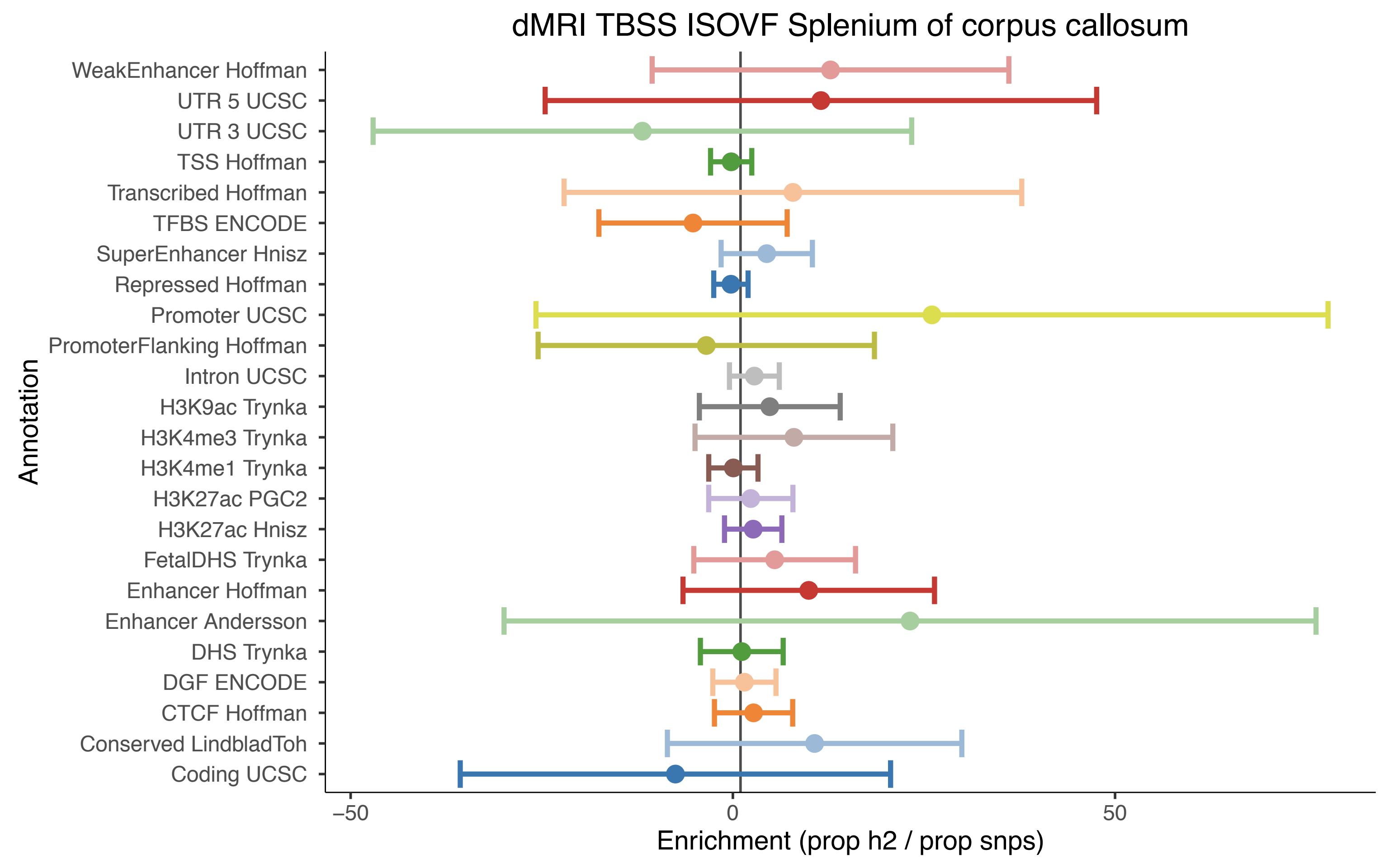

dMRI TBSS ISOVF Splenium of corpus callosum
WeakEnhancer Hoffman
UTR 5 UCSC
UTR 3 UCSC
TSS Hoffman
Transcribed Hoffman
TFBS ENCODE
SuperEnhancer Hnisz
Repressed Hoffman
Promoter UCSC
PromoterFlanking Hoffman
Intron UCSC
H3K9ac Trynka
H3K4me3 Trynka
H3K4me1 Trynka
H3K27ac PGC2
H3K27ac Hnisz
FetalDHS Trynka
Enhancer Hoffman
Enhancer Andersson
DHS Trynka
DGF ENCODE
CTCF Hoffman
Conserved LindbladToh
Coding UCSC
Annotation
−50
0
50
Enrichment (prop h2 / prop snps)

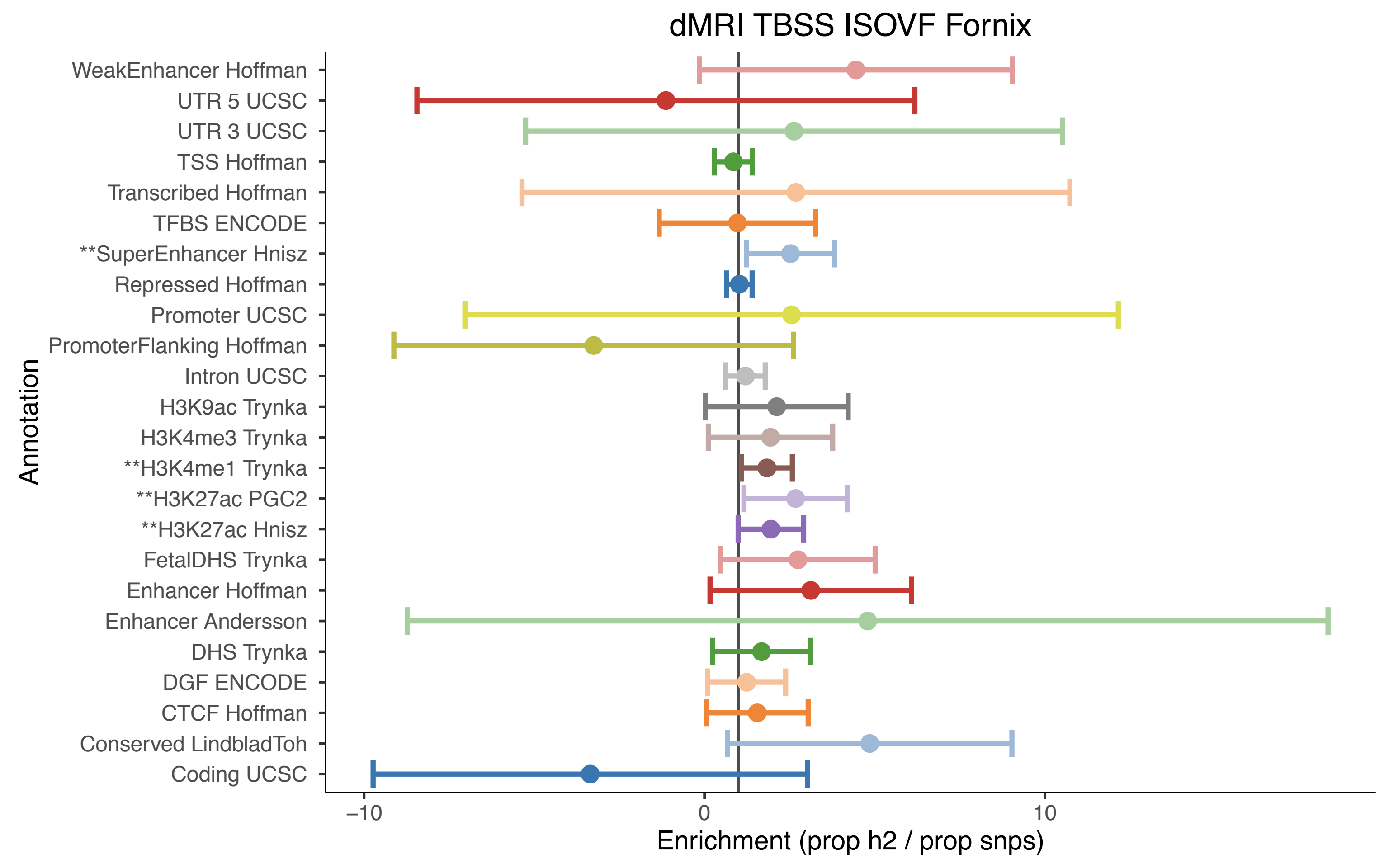
dMRI TBSS ISOVF Fornix
WeakEnhancer Hoffman
UTR 5 UCSC
UTR 3 UCSC
TSS Hoffman
Transcribed Hoffman
TFBS ENCODE
**SuperEnhancer Hnisz
Repressed Hoffman
Promoter UCSC
PromoterFlanking Hoffman
Intron UCSC
H3K9ac Trynka
H3K4me3 Trynka
**H3K4me1 Trynka
**H3K27ac PGC2
**H3K27ac Hnisz
FetalDHS Trynka
Enhancer Hoffman
Enhancer Andersson
DHS Trynka
DGF ENCODE
CTCF Hoffman
Conserved LindbladToh
Coding UCSC
Annotation
−10
0
10
Enrichment (prop h2 / prop snps)

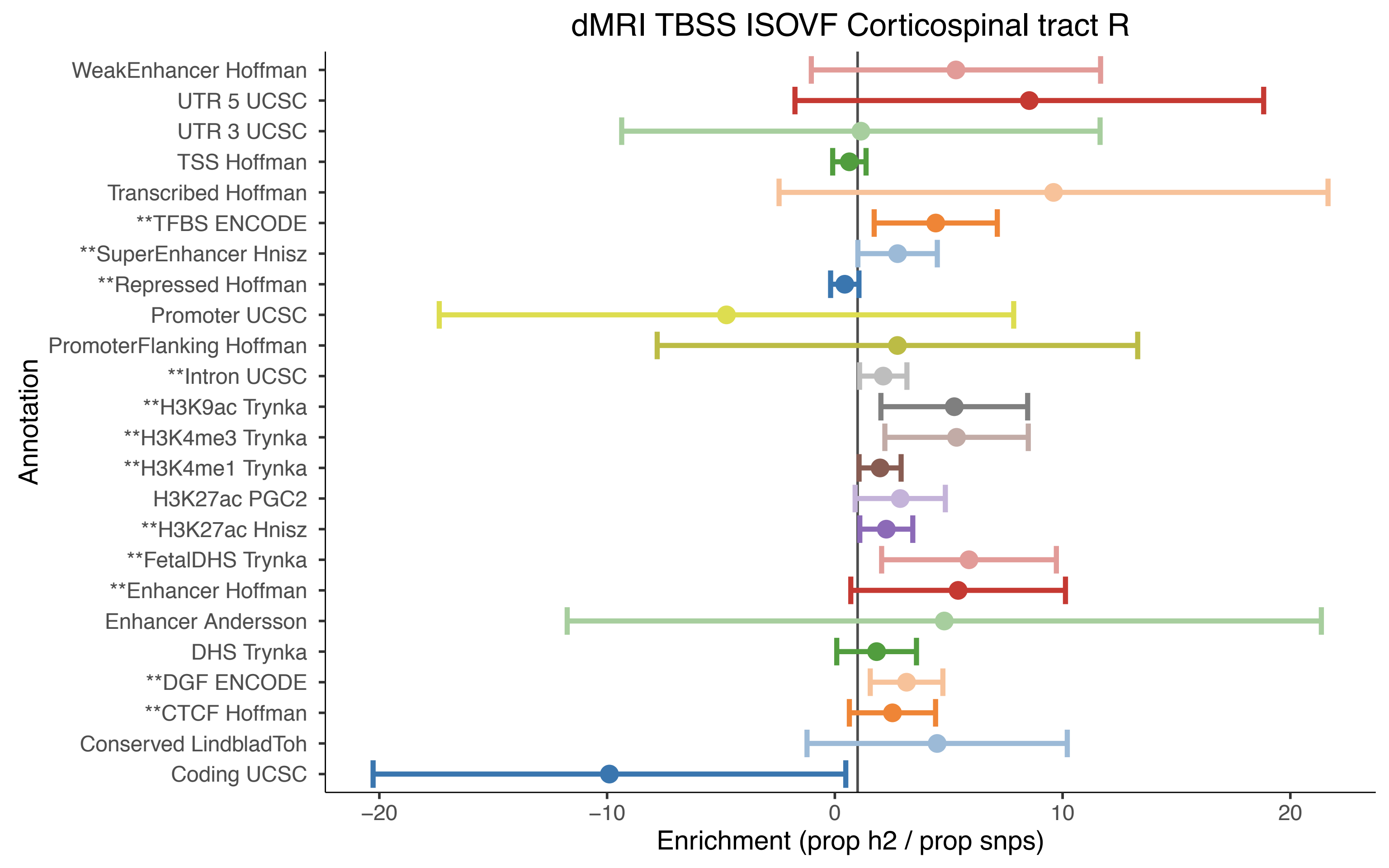

dMRI TBSS ISOVF Corticospinal tract R
WeakEnhancer Hoffman
UTR 5 UCSC
UTR 3 UCSC
TSS Hoffman
Transcribed Hoffman
**TFBS ENCODE
**SuperEnhancer Hnisz
**Repressed Hoffman
Promoter UCSC
PromoterFlanking Hoffman
**Intron UCSC
**H3K9ac Trynka
**H3K4me3 Trynka
**H3K4me1 Trynka
H3K27ac PGC2
**H3K27ac Hnisz
**FetalDHS Trynka
**Enhancer Hoffman
Enhancer Andersson
DHS Trynka
**DGF ENCODE
**CTCF Hoffman
Conserved LindbladToh
Coding UCSC
Annotation
−20
−10
0
10
20
Enrichment (prop h2 / prop snps)

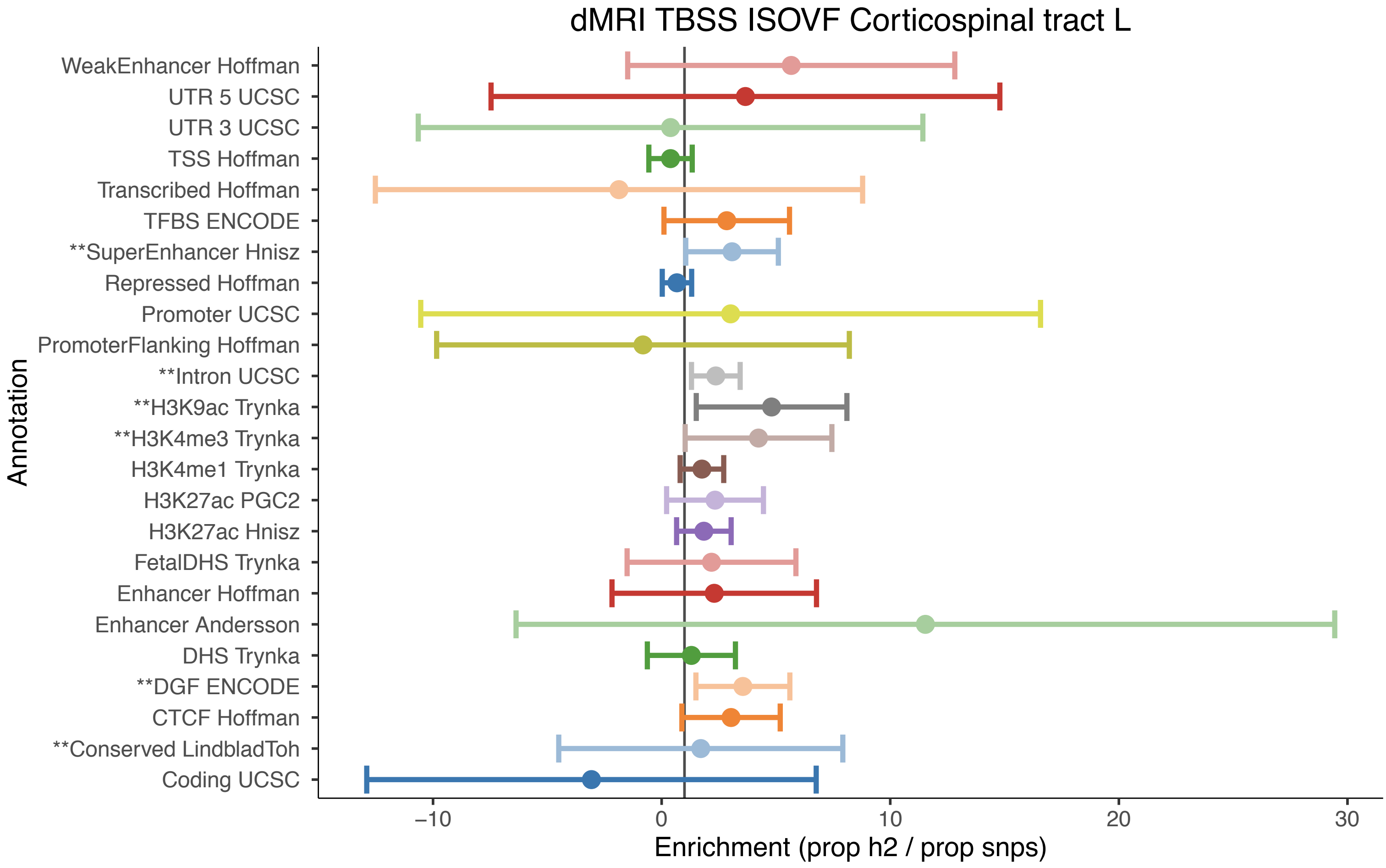
dMRI TBSS ISOVF Corticospinal tract L
WeakEnhancer Hoffman
UTR 5 UCSC
UTR 3 UCSC
TSS Hoffman
Transcribed Hoffman
TFBS ENCODE
**SuperEnhancer Hnisz
Repressed Hoffman
Promoter UCSC
PromoterFlanking Hoffman
**Intron UCSC
**H3K9ac Trynka
**H3K4me3 Trynka
H3K4me1 Trynka
H3K27ac PGC2
H3K27ac Hnisz
FetalDHS Trynka
Enhancer Hoffman
Enhancer Andersson
DHS Trynka
**DGF ENCODE
CTCF Hoffman
**Conserved LindbladToh
Coding UCSC
Annotation
−10
0
10
20
30
Enrichment (prop h2 / prop snps)

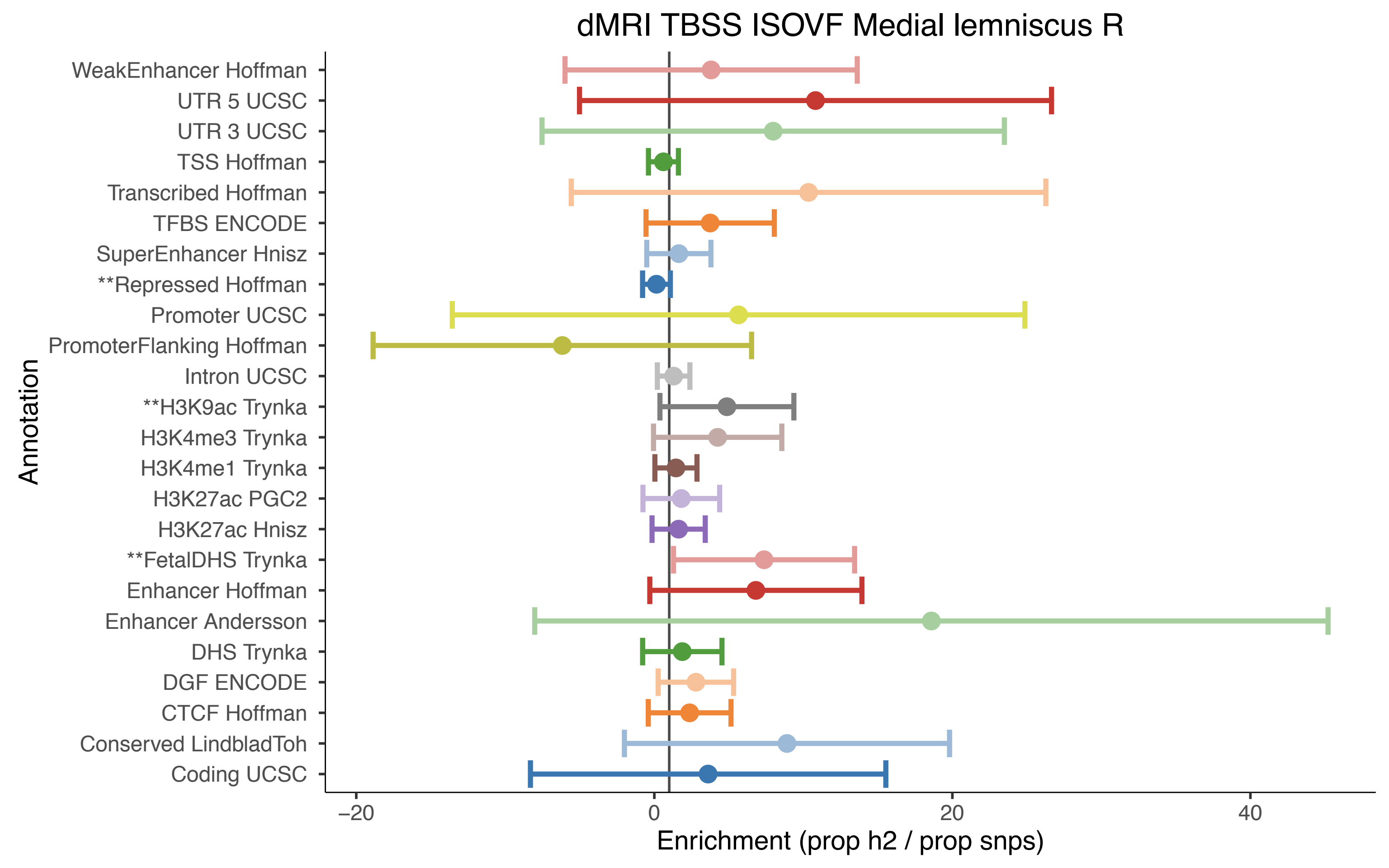
dMRI TBSS ISOVF Medial lemniscus R
WeakEnhancer Hoffman
UTR 5 UCSC
UTR 3 UCSC
TSS Hoffman
Transcribed Hoffman
TFBS ENCODE
SuperEnhancer Hnisz
**Repressed Hoffman
Promoter UCSC
PromoterFlanking Hoffman
Intron UCSC
**H3K9ac Trynka
H3K4me3 Trynka
H3K4me1 Trynka
H3K27ac PGC2
H3K27ac Hnisz
**FetalDHS Trynka
Enhancer Hoffman
Enhancer Andersson
DHS Trynka
DGF ENCODE
CTCF Hoffman
Conserved LindbladToh
Coding UCSC
Annotation
−20
0
20
40
Enrichment (prop h2 / prop snps)

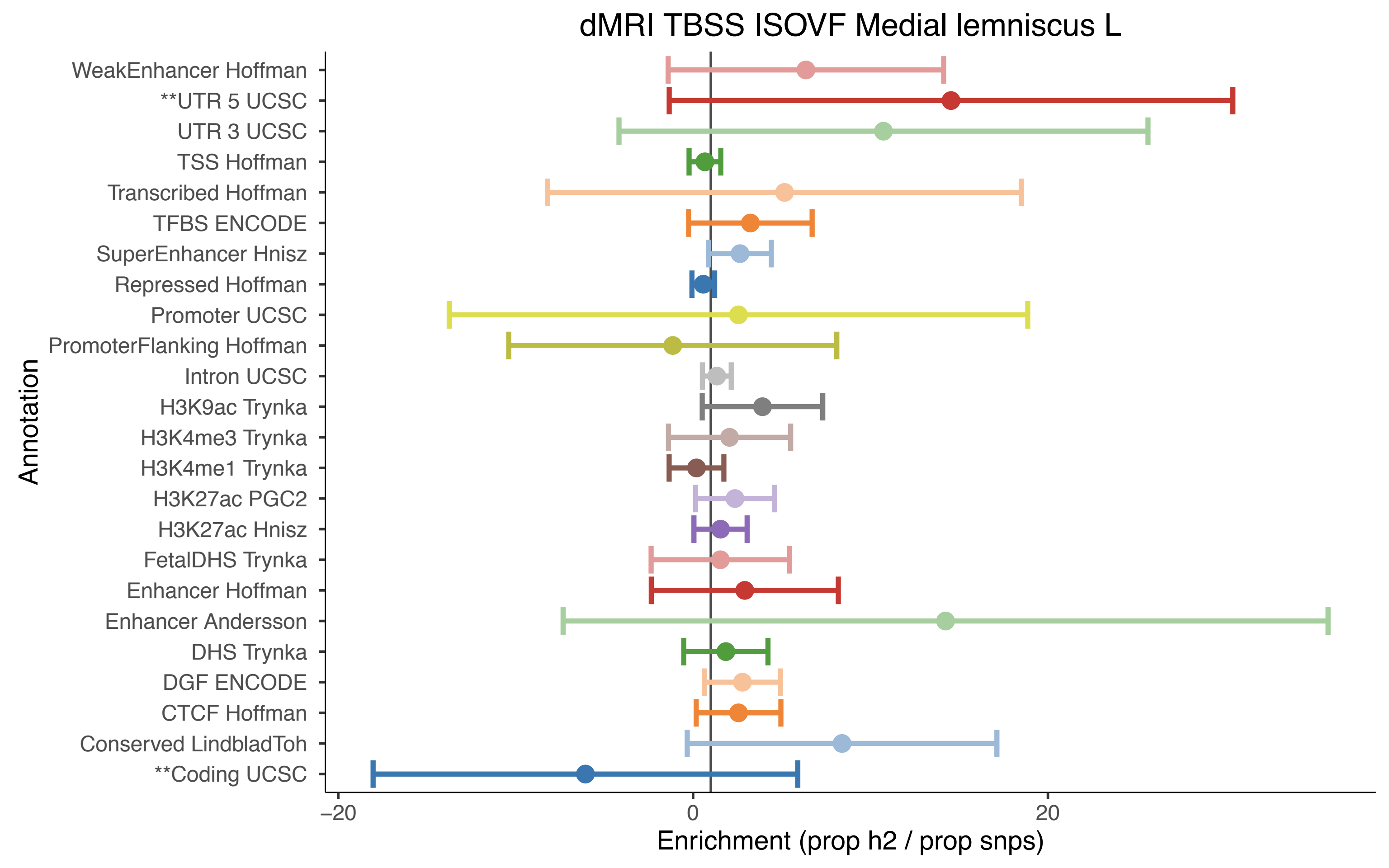

dMRI TBSS ISOVF Medial lemniscus L
WeakEnhancer Hoffman
**UTR 5 UCSC
UTR 3 UCSC
TSS Hoffman
Transcribed Hoffman
TFBS ENCODE
SuperEnhancer Hnisz
Repressed Hoffman
Promoter UCSC
PromoterFlanking Hoffman
Intron UCSC
H3K9ac Trynka
H3K4me3 Trynka
H3K4me1 Trynka
H3K27ac PGC2
H3K27ac Hnisz
FetalDHS Trynka
Enhancer Hoffman
Enhancer Andersson
DHS Trynka
DGF ENCODE
CTCF Hoffman
Conserved LindbladToh
**Coding UCSC
Annotation
−20
0
20
Enrichment (prop h2 / prop snps)

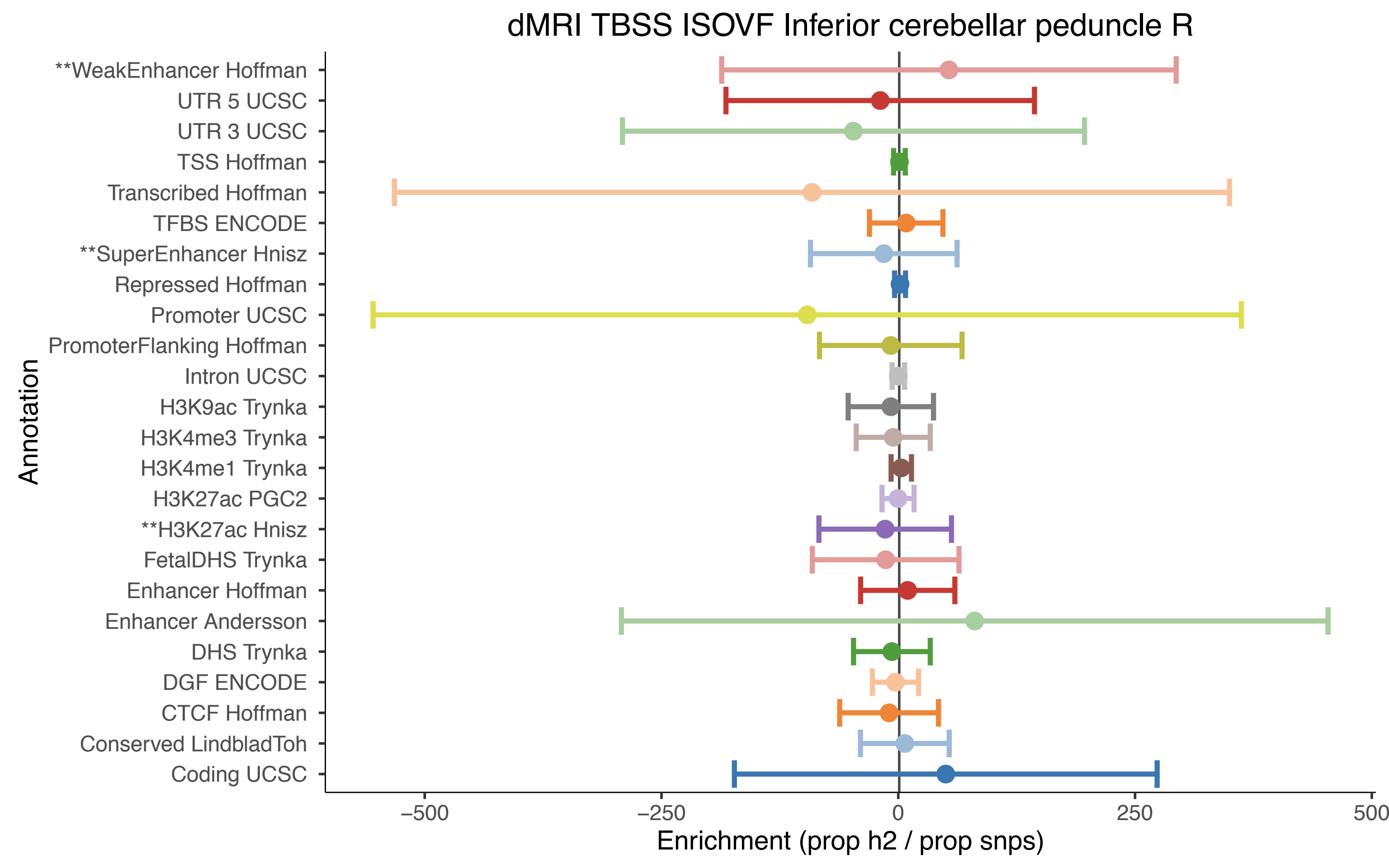

dMRI TBSS ISOVF Inferior cerebellar peduncle R
**WeakEnhancer Hoffman
UTR 5 UCSC
UTR 3 UCSC
TSS Hoffman
Transcribed Hoffman
TFBS ENCODE
**SuperEnhancer Hnisz
Repressed Hoffman
Promoter UCSC
PromoterFlanking Hoffman
Intron UCSC
H3K9ac Trynka
H3K4me3 Trynka
H3K4me1 Trynka
H3K27ac PGC2
**H3K27ac Hnisz
FetalDHS Trynka
Enhancer Hoffman
Enhancer Andersson
DHS Trynka
DGF ENCODE
CTCF Hoffman
Conserved LindbladToh
Coding UCSC
Annotation
−500
−250
0
250
500
Enrichment (prop h2 / prop snps)

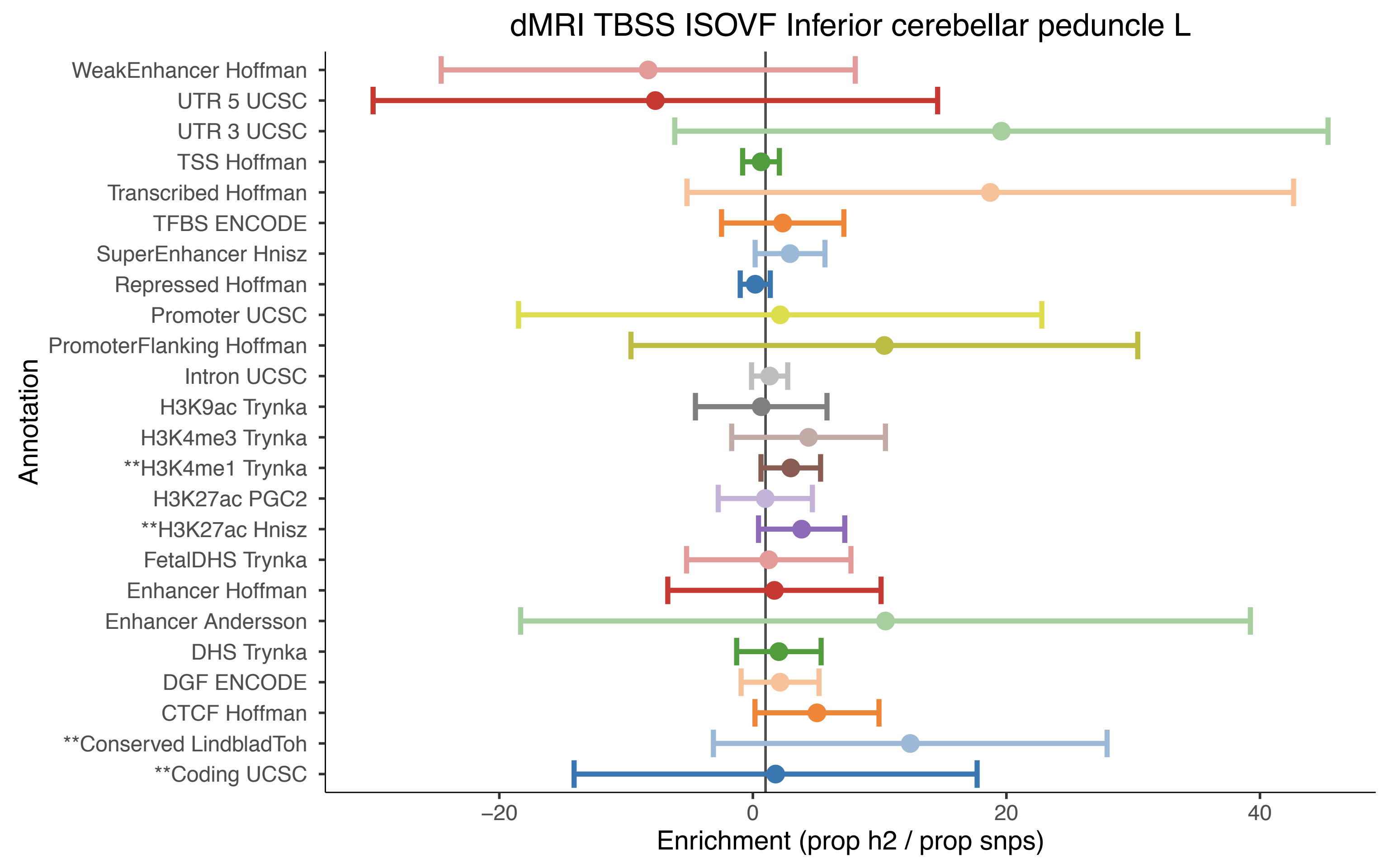
dMRI TBSS ISOVF Inferior cerebellar peduncle L
WeakEnhancer Hoffman
UTR 5 UCSC
UTR 3 UCSC
TSS Hoffman
Transcribed Hoffman
TFBS ENCODE
SuperEnhancer Hnisz
Repressed Hoffman
Promoter UCSC
PromoterFlanking Hoffman
Intron UCSC
H3K9ac Trynka
H3K4me3 Trynka
**H3K4me1 Trynka
H3K27ac PGC2
**H3K27ac Hnisz
FetalDHS Trynka
Enhancer Hoffman
Enhancer Andersson
DHS Trynka
DGF ENCODE
CTCF Hoffman
**Conserved LindbladToh
**Coding UCSC
Annotation
−20
0
20
40
Enrichment (prop h2 / prop snps)

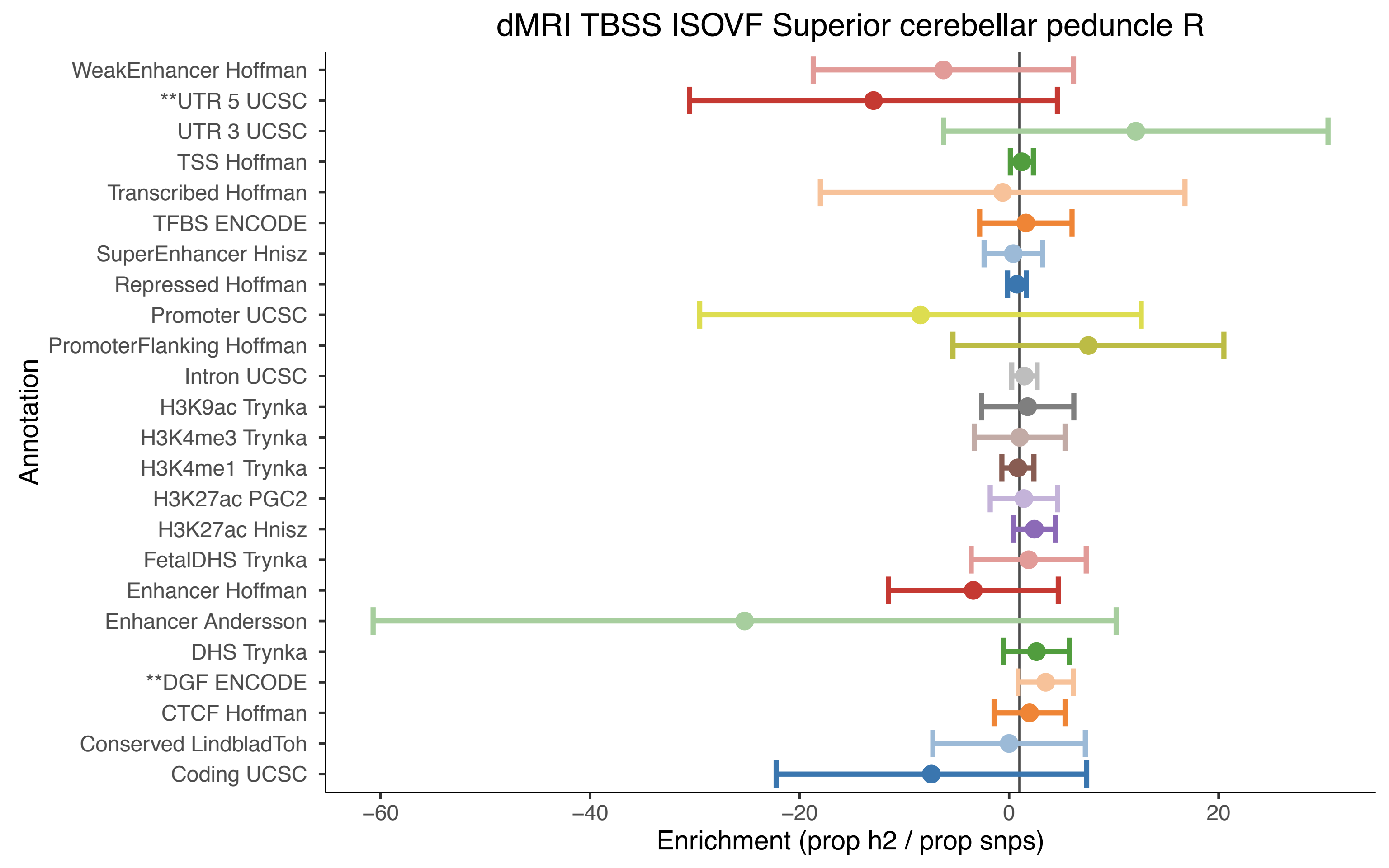

dMRI TBSS ISOVF Superior cerebellar peduncle R
WeakEnhancer Hoffman
**UTR 5 UCSC
UTR 3 UCSC
TSS Hoffman
Transcribed Hoffman
TFBS ENCODE
SuperEnhancer Hnisz
Repressed Hoffman
Promoter UCSC
PromoterFlanking Hoffman
Intron UCSC
H3K9ac Trynka
H3K4me3 Trynka
H3K4me1 Trynka
H3K27ac PGC2
H3K27ac Hnisz
FetalDHS Trynka
Enhancer Hoffman
Enhancer Andersson
DHS Trynka
**DGF ENCODE
CTCF Hoffman
Conserved LindbladToh
Coding UCSC
Annotation
−60
−40
−20
0
20
Enrichment (prop h2 / prop snps)

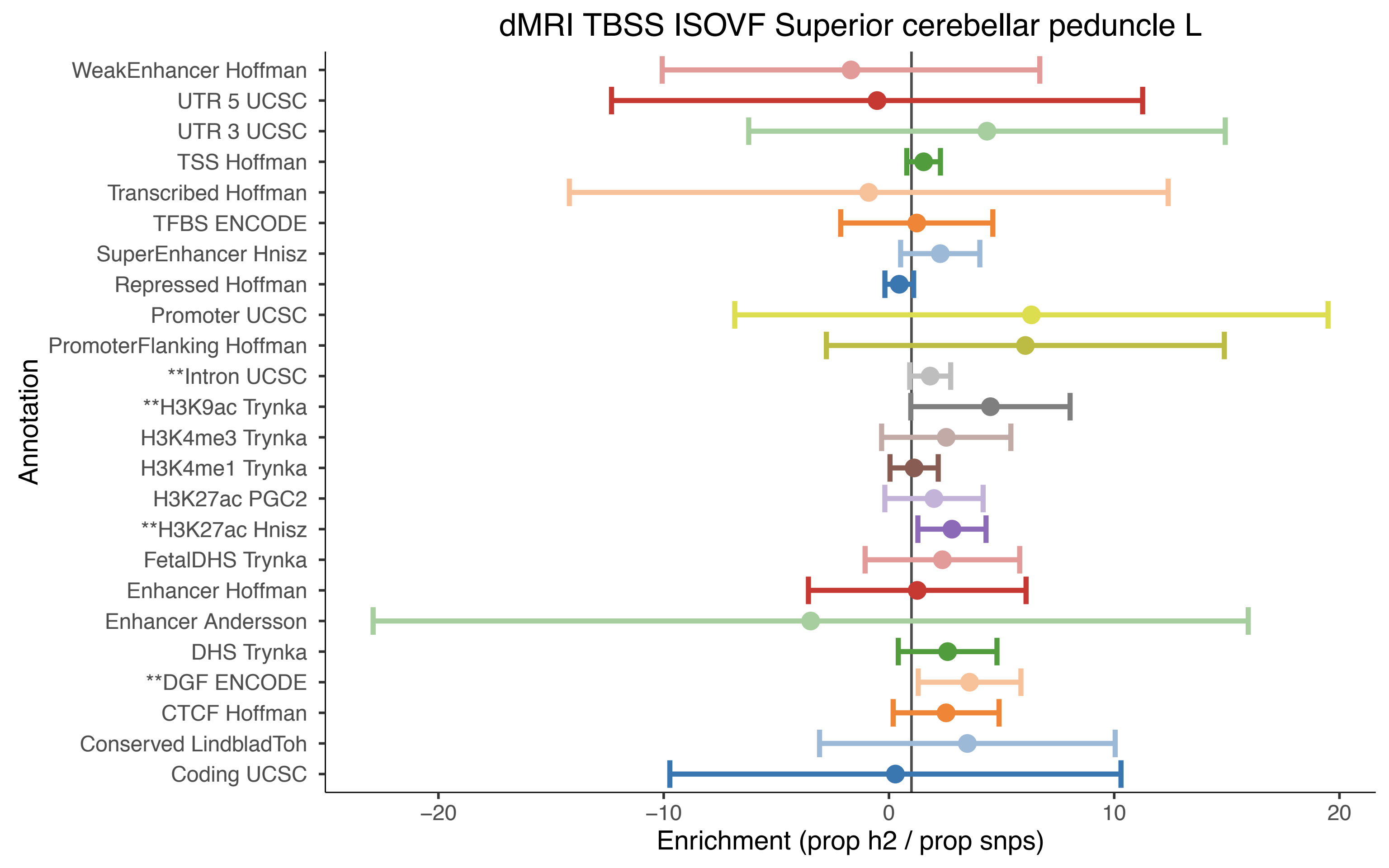
dMRI TBSS ISOVF Superior cerebellar peduncle L
WeakEnhancer Hoffman
UTR 5 UCSC
UTR 3 UCSC
TSS Hoffman
Transcribed Hoffman
TFBS ENCODE
SuperEnhancer Hnisz
Repressed Hoffman
Promoter UCSC
PromoterFlanking Hoffman
**Intron UCSC
**H3K9ac Trynka
H3K4me3 Trynka
H3K4me1 Trynka
H3K27ac PGC2
**H3K27ac Hnisz
FetalDHS Trynka
Enhancer Hoffman
Enhancer Andersson
DHS Trynka
**DGF ENCODE
CTCF Hoffman
Conserved LindbladToh
Coding UCSC
Annotation
−20
−10
0
10
20
Enrichment (prop h2 / prop snps)

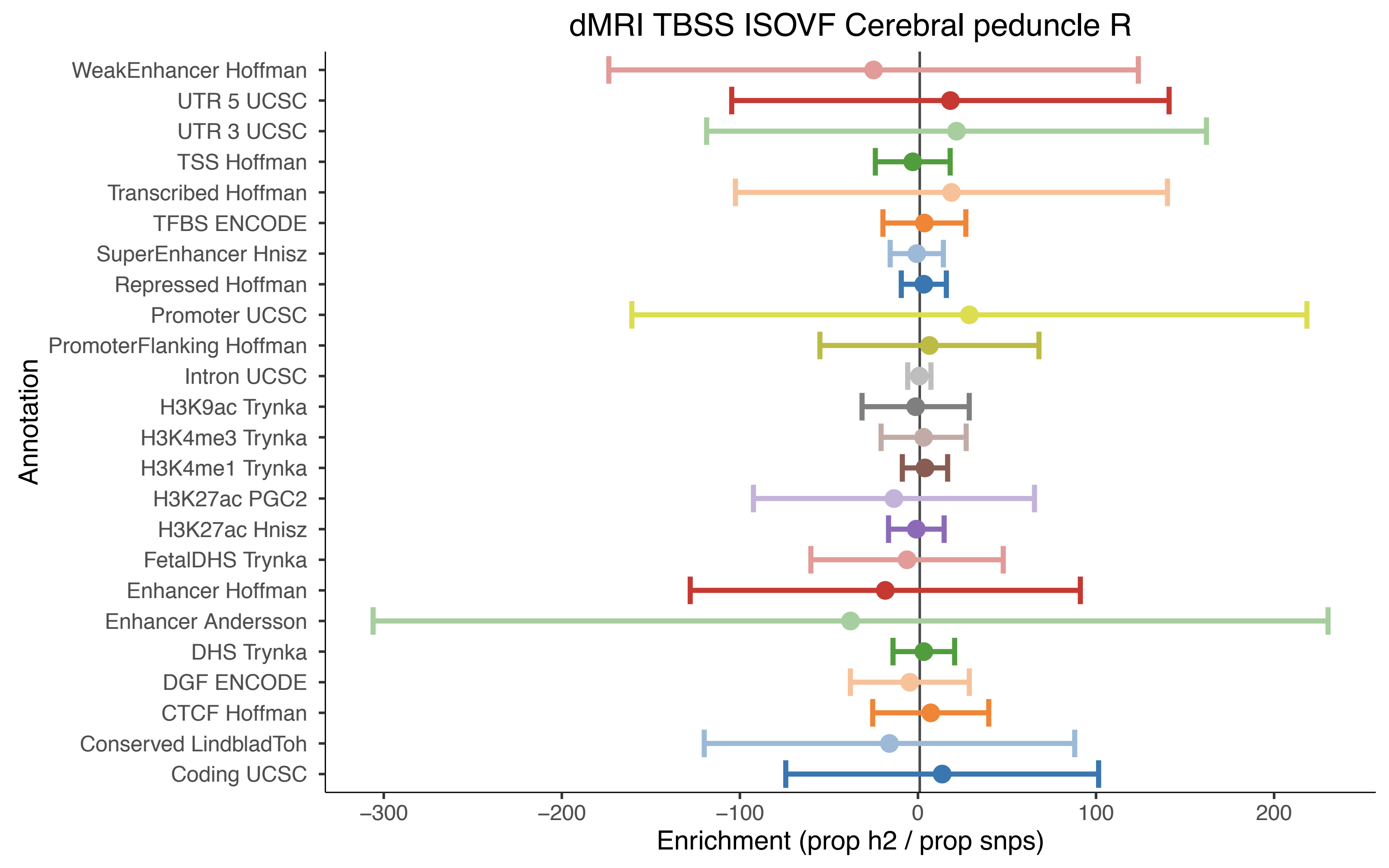

dMRI TBSS ISOVF Cerebral peduncle R
Annotation
WeakEnhancer Hoffman
UTR 5 UCSC
UTR 3 UCSC
TSS Hoffman
Transcribed Hoffman
TFBS ENCODE
SuperEnhancer Hnisz
Repressed Hoffman
Promoter UCSC
PromoterFlanking Hoffman
Intron UCSC
H3K9ac Trynka
H3K4me3 Trynka
H3K4me1 Trynka
H3K27ac PGC2
H3K27ac Hnisz
FetalDHS Trynka
Enhancer Hoffman
Enhancer Andersson
DHS Trynka
DGF ENCODE
CTCF Hoffman
Conserved LindbladToh
Coding UCSC
−300
−200
−100
0
100
200
Enrichment (prop h2 / prop snps)

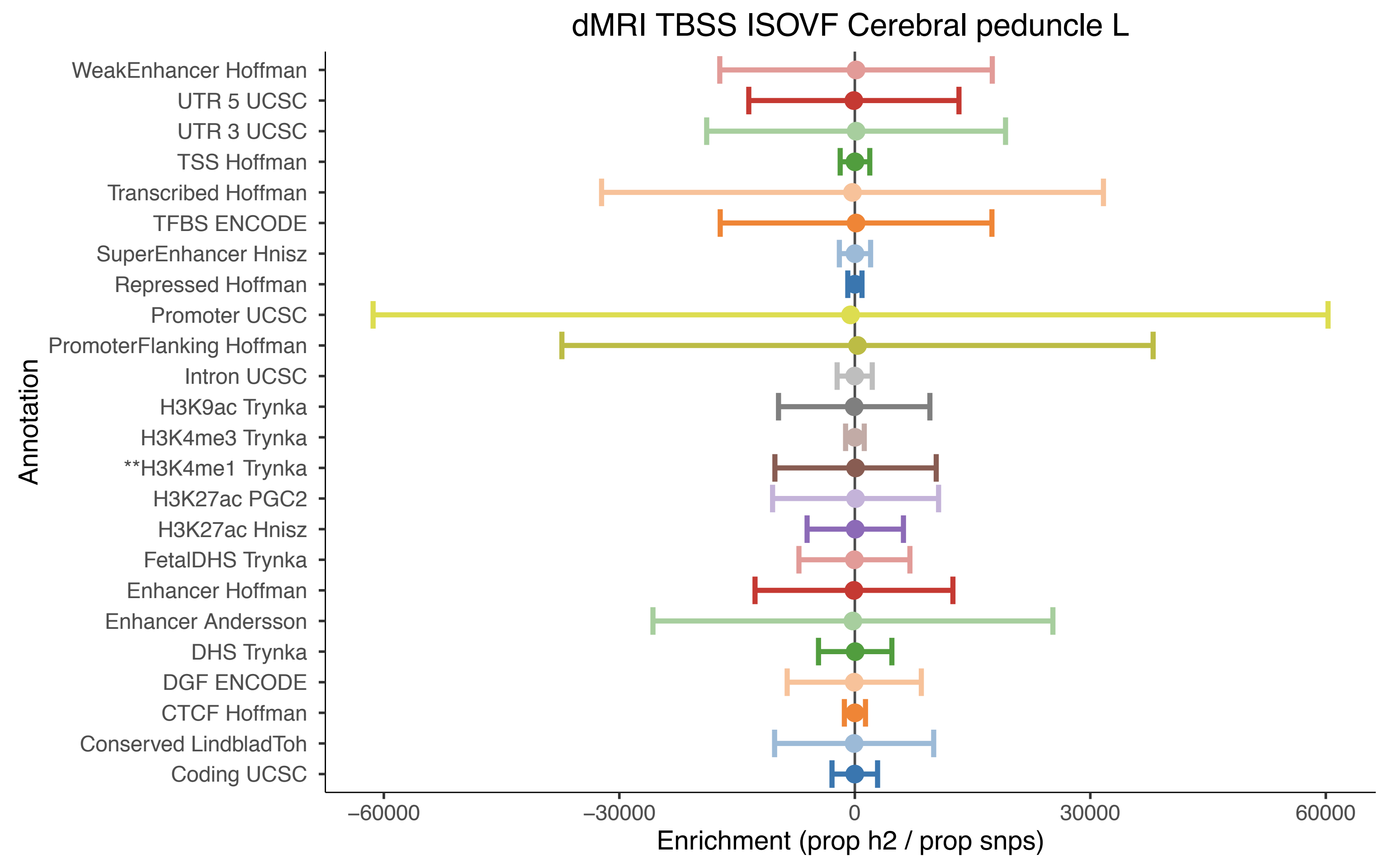

dMRI TBSS ISOVF Cerebral peduncle L
WeakEnhancer Hoffman
UTR 5 UCSC
UTR 3 UCSC
TSS Hoffman
Transcribed Hoffman
TFBS ENCODE
SuperEnhancer Hnisz
Repressed Hoffman
Promoter UCSC
PromoterFlanking Hoffman
Intron UCSC
H3K9ac Trynka
H3K4me3 Trynka
**H3K4me1 Trynka
H3K27ac PGC2
H3K27ac Hnisz
FetalDHS Trynka
Enhancer Hoffman
Enhancer Andersson
DHS Trynka
DGF ENCODE
CTCF Hoffman
Conserved LindbladToh
Coding UCSC
Annotation
−60000
−30000
0
30000
60000
Enrichment (prop h2 / prop snps)

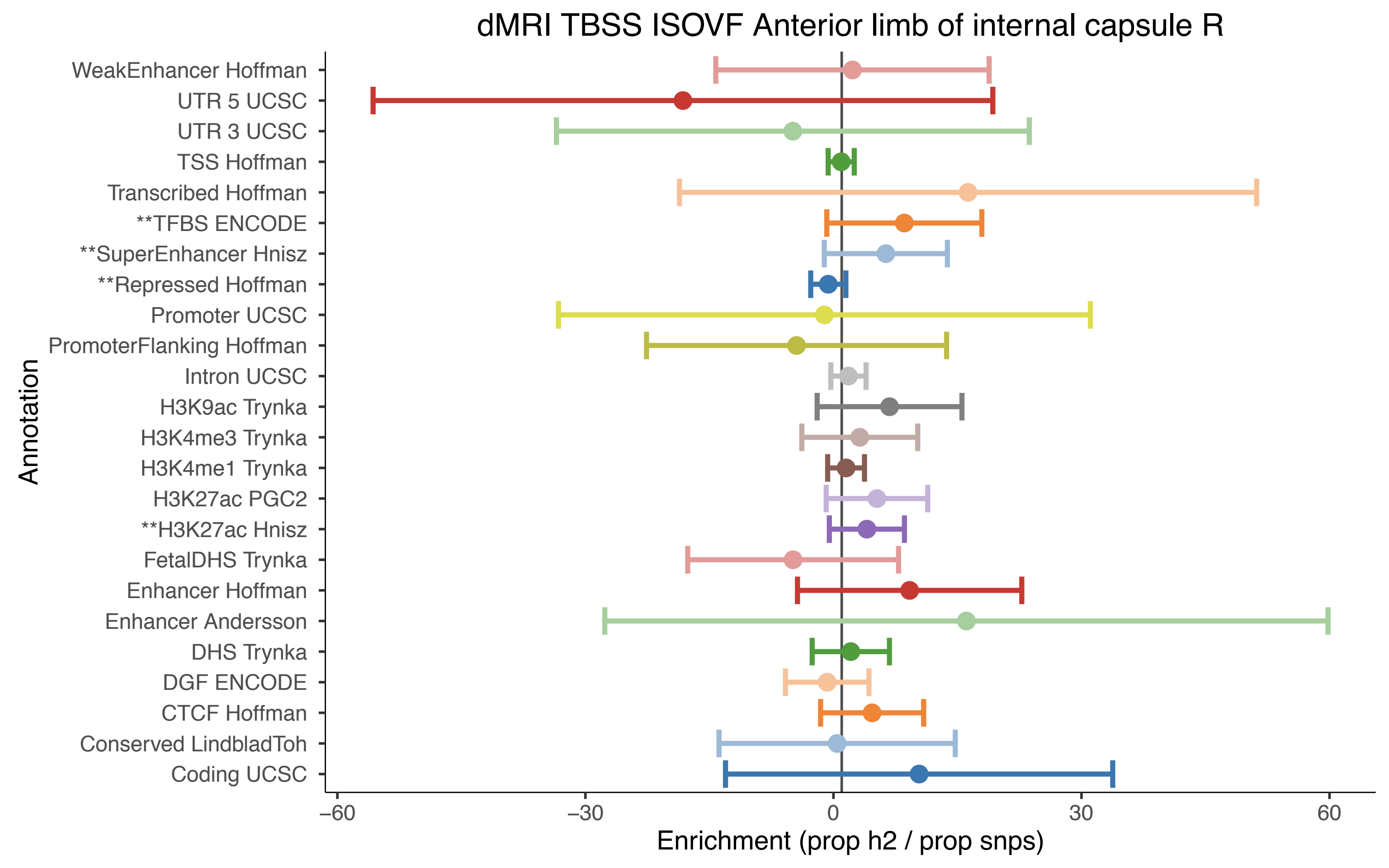
dMRI TBSS ISOVF Anterior limb of internal capsule R
WeakEnhancer Hoffman
UTR 5 UCSC
UTR 3 UCSC
TSS Hoffman
Transcribed Hoffman
**TFBS ENCODE
**SuperEnhancer Hnisz
**Repressed Hoffman
Promoter UCSC
PromoterFlanking Hoffman
Intron UCSC
H3K9ac Trynka
H3K4me3 Trynka
H3K4me1 Trynka
H3K27ac PGC2
**H3K27ac Hnisz
FetalDHS Trynka
Enhancer Hoffman
Enhancer Andersson
DHS Trynka
DGF ENCODE
CTCF Hoffman
Conserved LindbladToh
Coding UCSC
Annotation
−60
−30
0
30
60
Enrichment (prop h2 / prop snps)

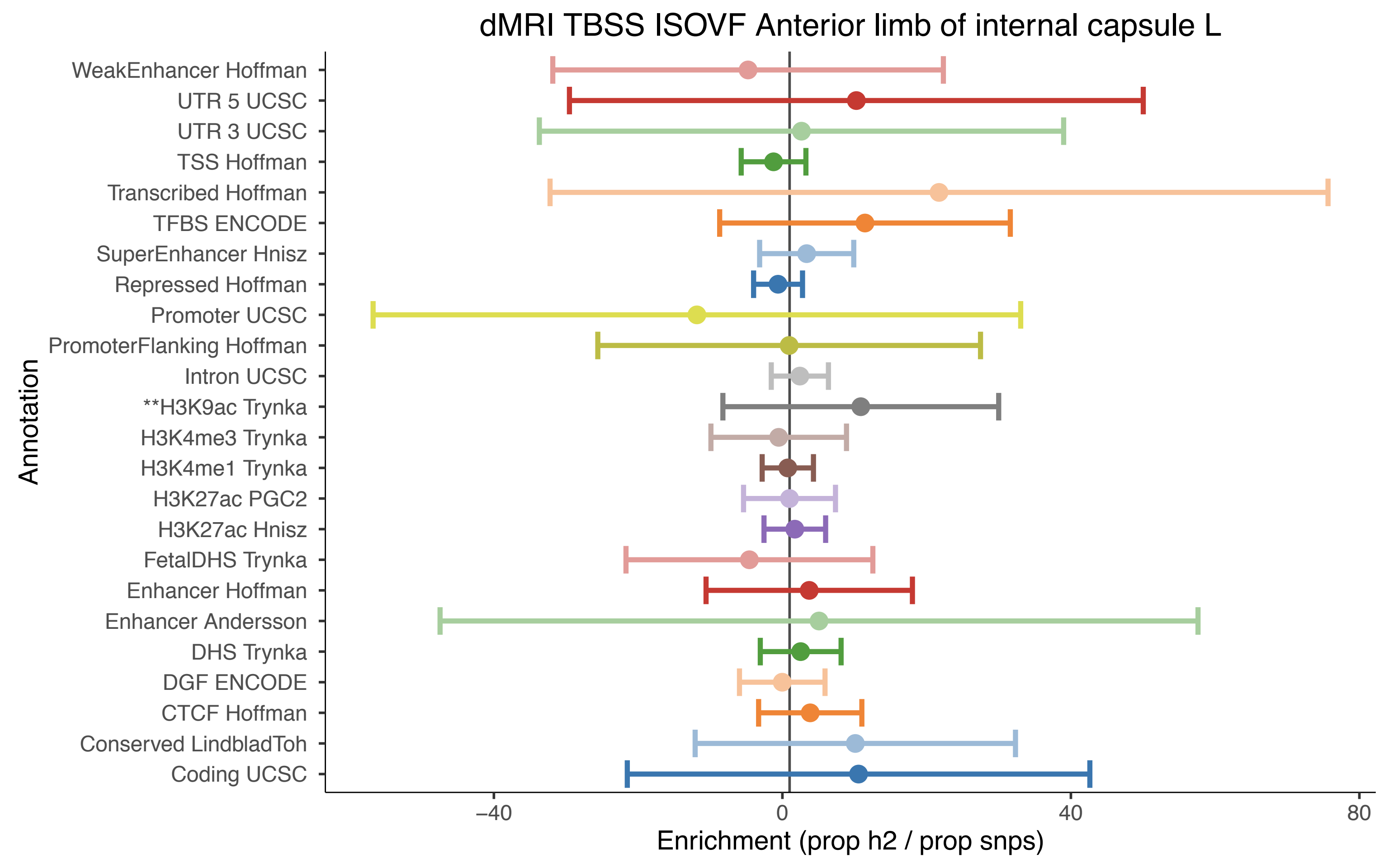
dMRI TBSS ISOVF Anterior limb of internal capsule L
WeakEnhancer Hoffman
UTR 5 UCSC
UTR 3 UCSC
TSS Hoffman
Transcribed Hoffman
TFBS ENCODE
SuperEnhancer Hnisz
Repressed Hoffman
Promoter UCSC
PromoterFlanking Hoffman
Intron UCSC
**H3K9ac Trynka
H3K4me3 Trynka
H3K4me1 Trynka
H3K27ac PGC2
H3K27ac Hnisz
FetalDHS Trynka
Enhancer Hoffman
Enhancer Andersson
DHS Trynka
DGF ENCODE
CTCF Hoffman
Conserved LindbladToh
Coding UCSC
Annotation
−40
0
40
80
Enrichment (prop h2 / prop snps)

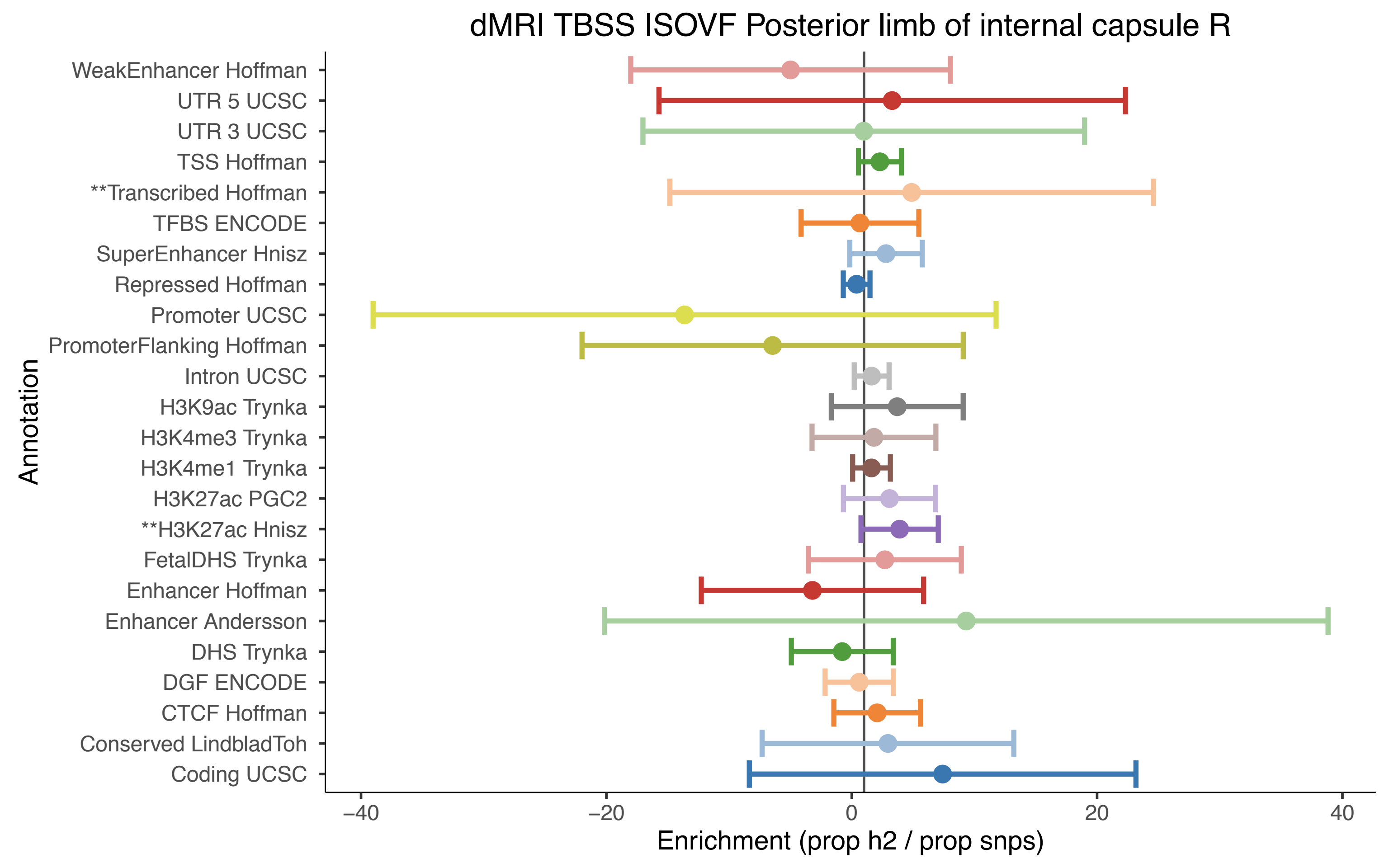

dMRI TBSS ISOVF Posterior limb of internal capsule R
WeakEnhancer Hoffman
UTR 5 UCSC
UTR 3 UCSC
TSS Hoffman
**Transcribed Hoffman
TFBS ENCODE
SuperEnhancer Hnisz
Repressed Hoffman
Promoter UCSC
PromoterFlanking Hoffman
Intron UCSC
H3K9ac Trynka
H3K4me3 Trynka
H3K4me1 Trynka
H3K27ac PGC2
**H3K27ac Hnisz
FetalDHS Trynka
Enhancer Hoffman
Enhancer Andersson
DHS Trynka
DGF ENCODE
CTCF Hoffman
Conserved LindbladToh
Coding UCSC
Annotation
−40
−20
0
20
40
Enrichment (prop h2 / prop snps)

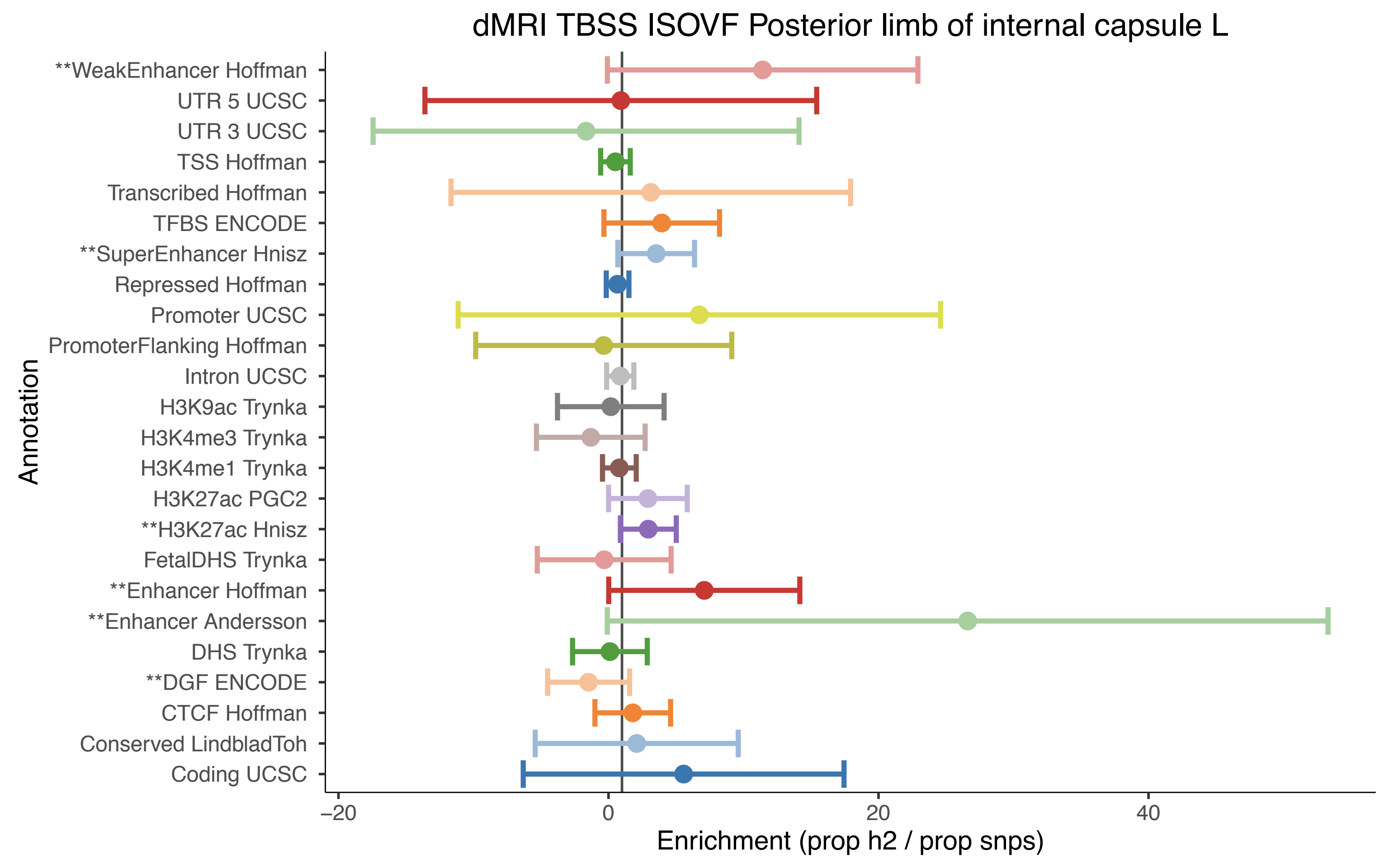

dMRI TBSS ISOVF Posterior limb of internal capsule L
**WeakEnhancer Hoffman
UTR 5 UCSC
UTR 3 UCSC
TSS Hoffman
Transcribed Hoffman
TFBS ENCODE
**SuperEnhancer Hnisz
Repressed Hoffman
Promoter UCSC
PromoterFlanking Hoffman
Intron UCSC
H3K9ac Trynka
H3K4me3 Trynka
H3K4me1 Trynka
H3K27ac PGC2
**H3K27ac Hnisz
FetalDHS Trynka
**Enhancer Hoffman
**Enhancer Andersson
DHS Trynka
**DGF ENCODE
CTCF Hoffman
Conserved LindbladToh
Coding UCSC
Annotation
−20
0
20
40
Enrichment (prop h2 / prop snps)

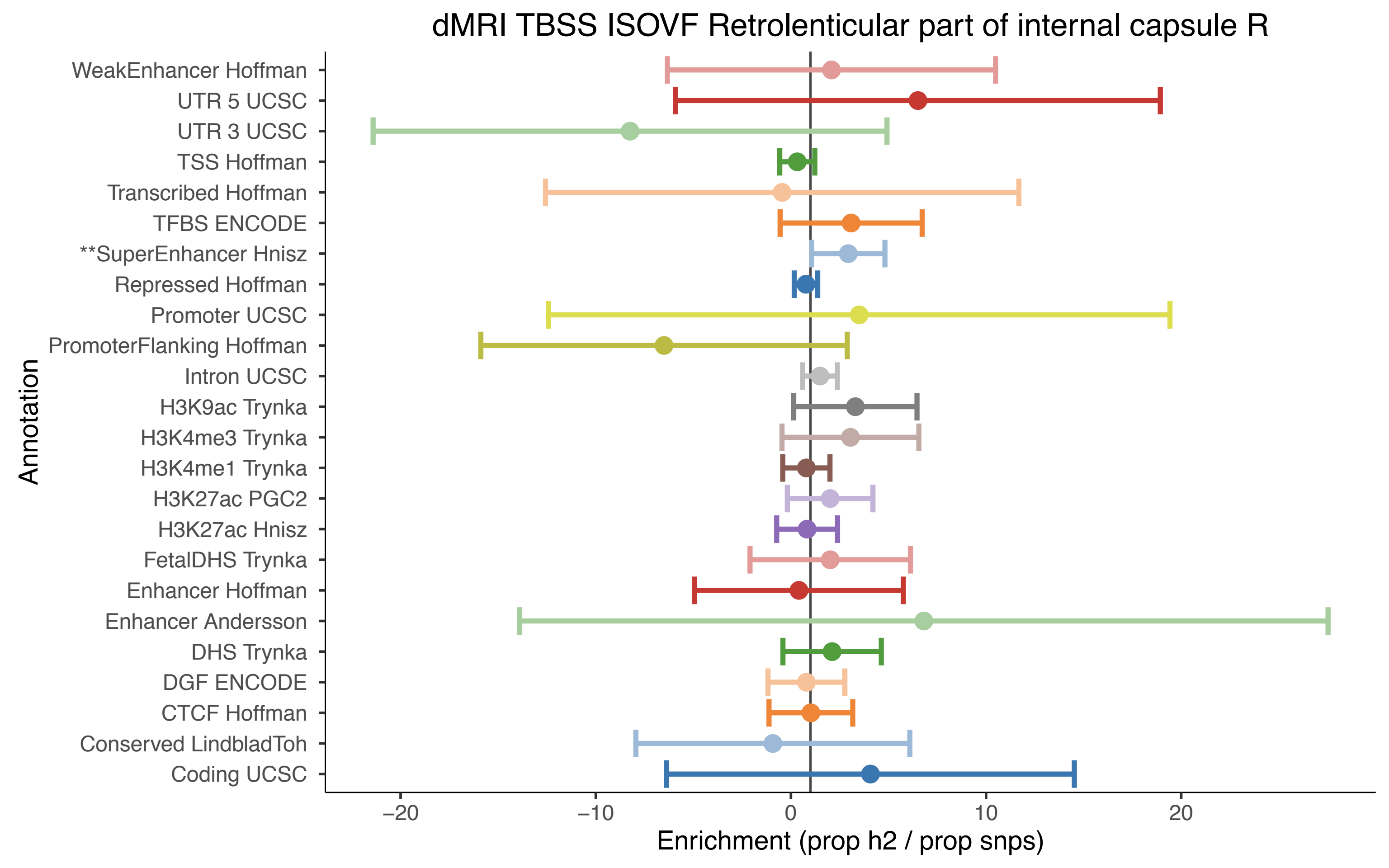
dMRI TBSS ISOVF Retrolenticular part of internal capsule R
WeakEnhancer Hoffman
UTR 5 UCSC
UTR 3 UCSC
TSS Hoffman
Transcribed Hoffman
TFBS ENCODE
**SuperEnhancer Hnisz
Repressed Hoffman
Promoter UCSC
PromoterFlanking Hoffman
Intron UCSC
H3K9ac Trynka
H3K4me3 Trynka
H3K4me1 Trynka
H3K27ac PGC2
H3K27ac Hnisz
FetalDHS Trynka
Enhancer Hoffman
Enhancer Andersson
DHS Trynka
DGF ENCODE
CTCF Hoffman
Conserved LindbladToh
Coding UCSC
Annotation
−20
−10
0
10
20
Enrichment (prop h2 / prop snps)

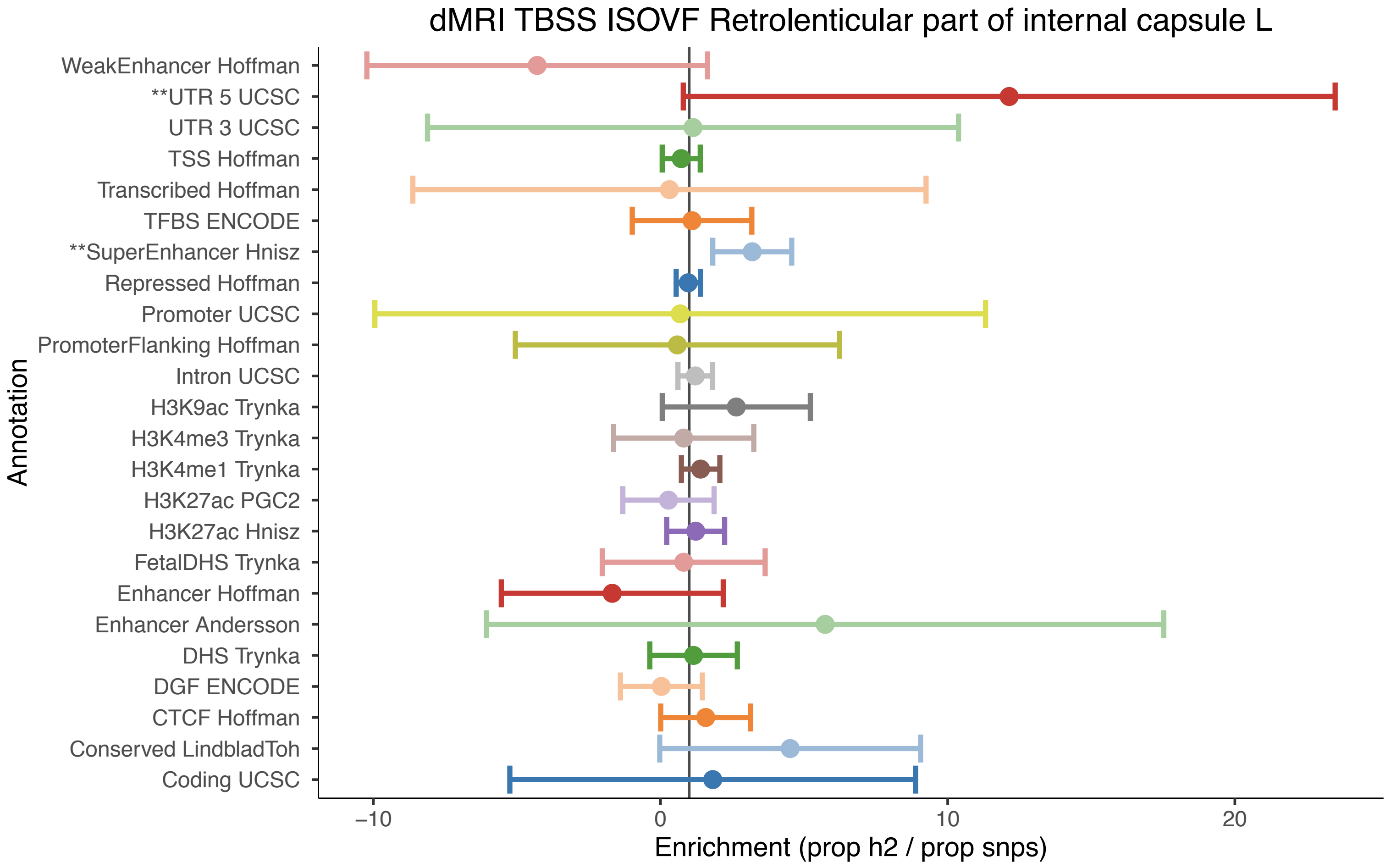

dMRI TBSS ISOVF Retrolenticular part of internal capsule L
WeakEnhancer Hoffman
**UTR 5 UCSC
UTR 3 UCSC
TSS Hoffman
Transcribed Hoffman
TFBS ENCODE
**SuperEnhancer Hnisz
Repressed Hoffman
Promoter UCSC
PromoterFlanking Hoffman
Intron UCSC
H3K9ac Trynka
H3K4me3 Trynka
H3K4me1 Trynka
H3K27ac PGC2
H3K27ac Hnisz
FetalDHS Trynka
Enhancer Hoffman
Enhancer Andersson
DHS Trynka
DGF ENCODE
CTCF Hoffman
Conserved LindbladToh
Coding UCSC
Annotation
−10
0
10
20
Enrichment (prop h2 / prop snps)

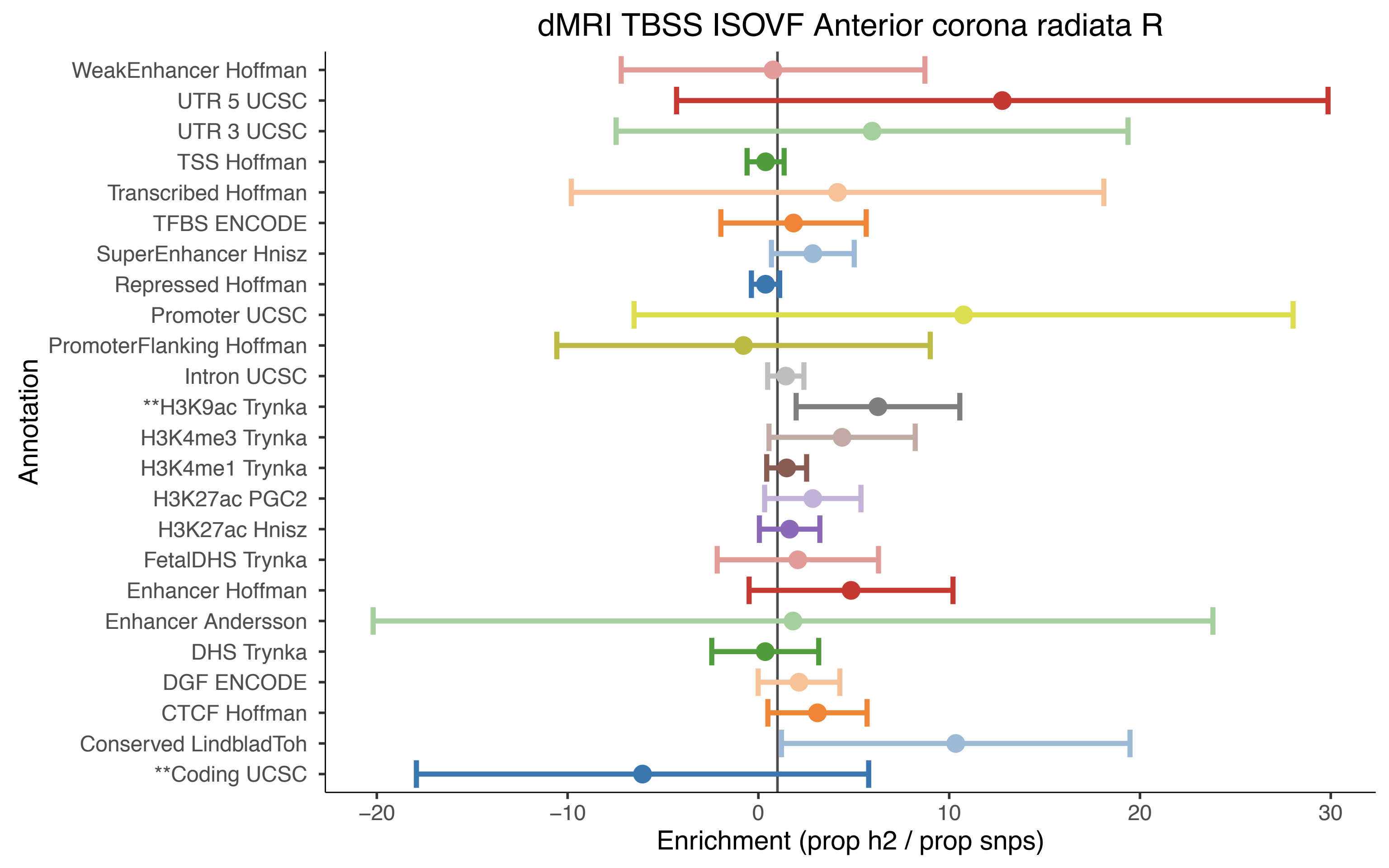

dMRI TBSS ISOVF Anterior corona radiata R
WeakEnhancer Hoffman
UTR 5 UCSC
UTR 3 UCSC
TSS Hoffman
Transcribed Hoffman
TFBS ENCODE
SuperEnhancer Hnisz
Repressed Hoffman
Promoter UCSC
PromoterFlanking Hoffman
Intron UCSC
**H3K9ac Trynka
H3K4me3 Trynka
H3K4me1 Trynka
H3K27ac PGC2
H3K27ac Hnisz
FetalDHS Trynka
Enhancer Hoffman
Enhancer Andersson
DHS Trynka
DGF ENCODE
CTCF Hoffman
Conserved LindbladToh
**Coding UCSC
Annotation
−20
−10
0
10
20
30
Enrichment (prop h2 / prop snps)

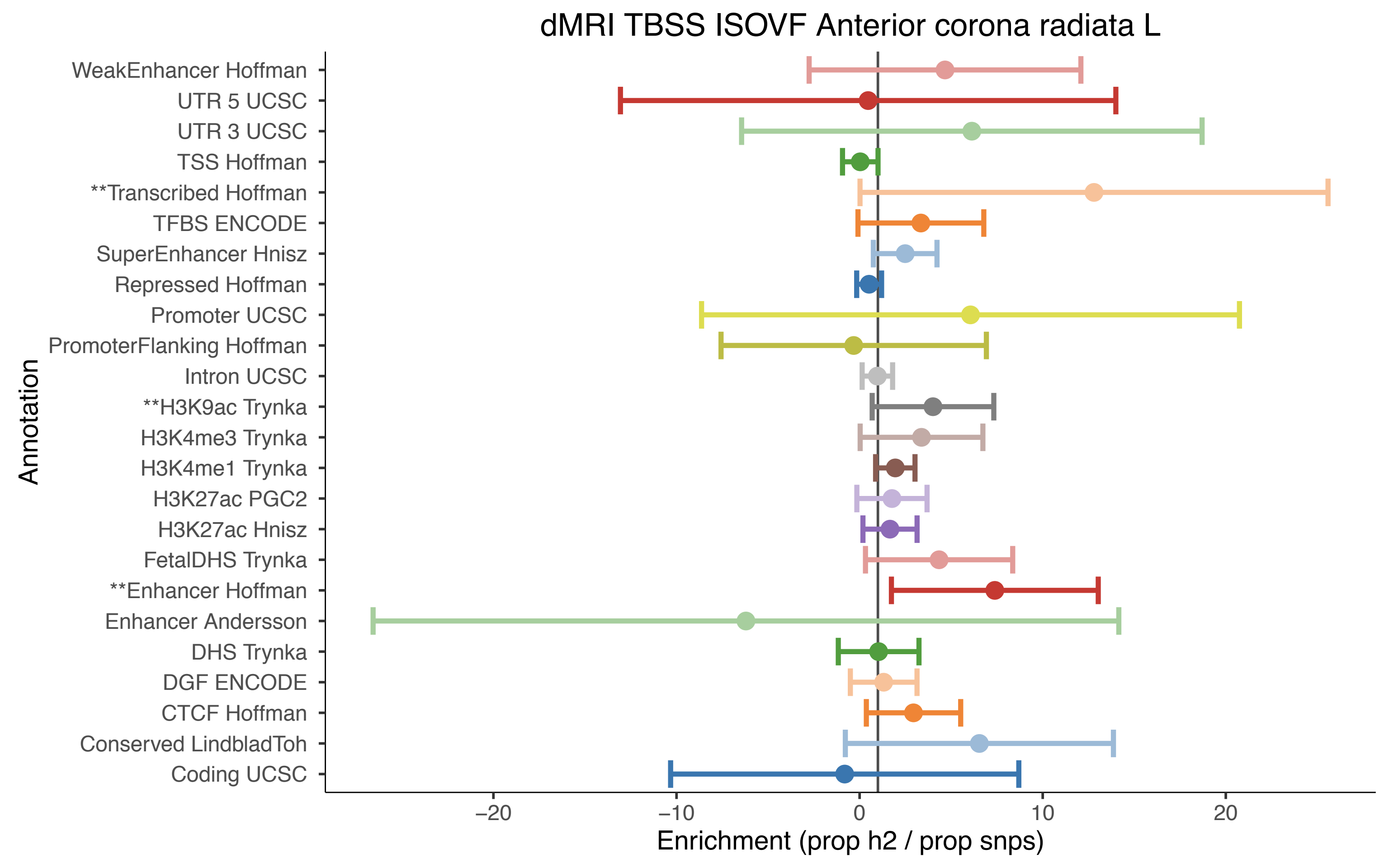
dMRI TBSS ISOVF Anterior corona radiata L
WeakEnhancer Hoffman
UTR 5 UCSC
UTR 3 UCSC
TSS Hoffman
**Transcribed Hoffman
TFBS ENCODE
SuperEnhancer Hnisz
Repressed Hoffman
Promoter UCSC
PromoterFlanking Hoffman
Intron UCSC
**H3K9ac Trynka
H3K4me3 Trynka
H3K4me1 Trynka
H3K27ac PGC2
H3K27ac Hnisz
FetalDHS Trynka
**Enhancer Hoffman
Enhancer Andersson
DHS Trynka
DGF ENCODE
CTCF Hoffman
Conserved LindbladToh
Coding UCSC
Annotation
−20
−10
0
10
20
Enrichment (prop h2 / prop snps)

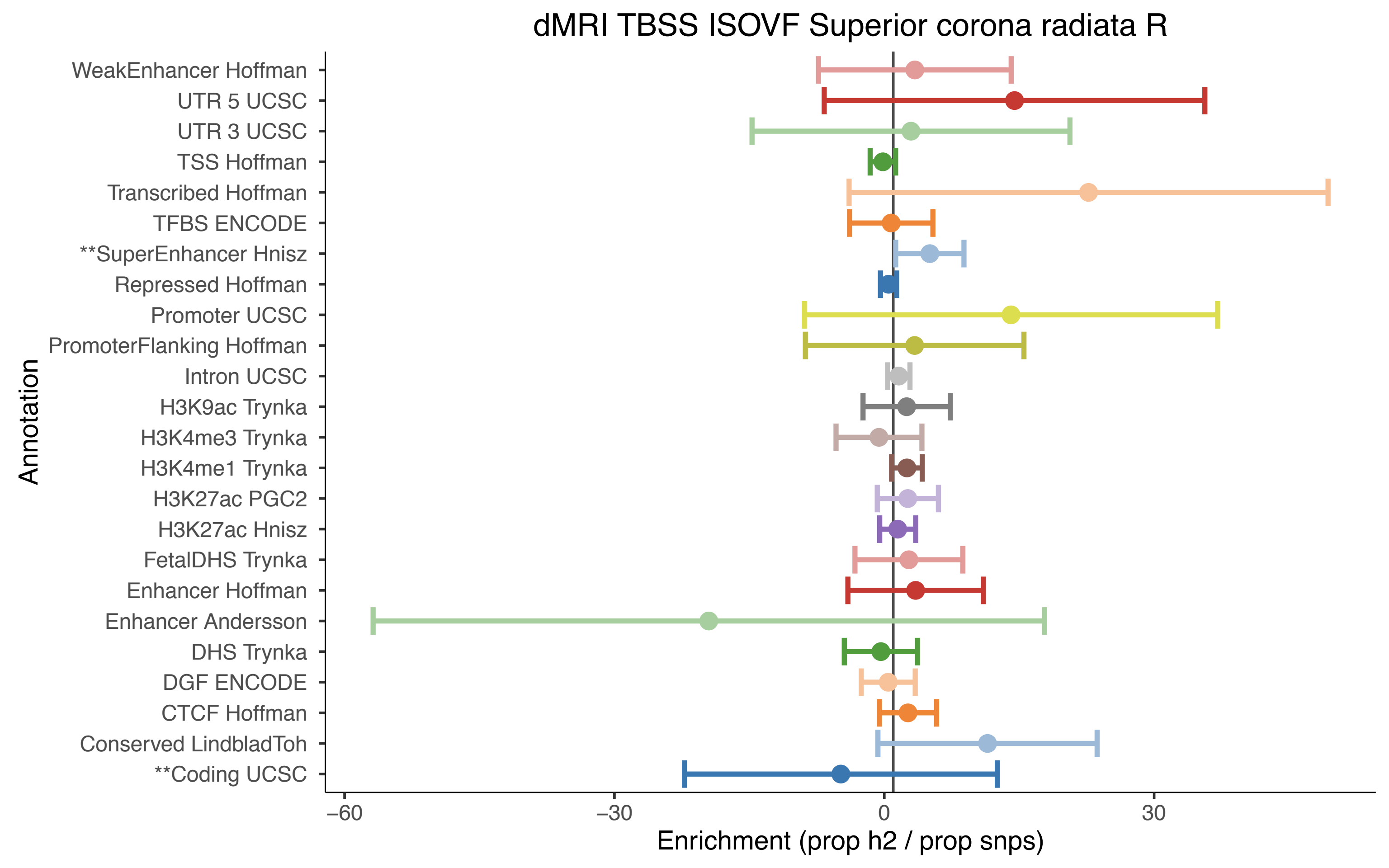
dMRI TBSS ISOVF Superior corona radiata R
WeakEnhancer Hoffman
UTR 5 UCSC
UTR 3 UCSC
TSS Hoffman
Transcribed Hoffman
TFBS ENCODE
**SuperEnhancer Hnisz
Repressed Hoffman
Promoter UCSC
PromoterFlanking Hoffman
Intron UCSC
H3K9ac Trynka
H3K4me3 Trynka
H3K4me1 Trynka
H3K27ac PGC2
H3K27ac Hnisz
FetalDHS Trynka
Enhancer Hoffman
Enhancer Andersson
DHS Trynka
DGF ENCODE
CTCF Hoffman
Conserved LindbladToh
**Coding UCSC
Annotation
−60
−30
0
30
Enrichment (prop h2 / prop snps)

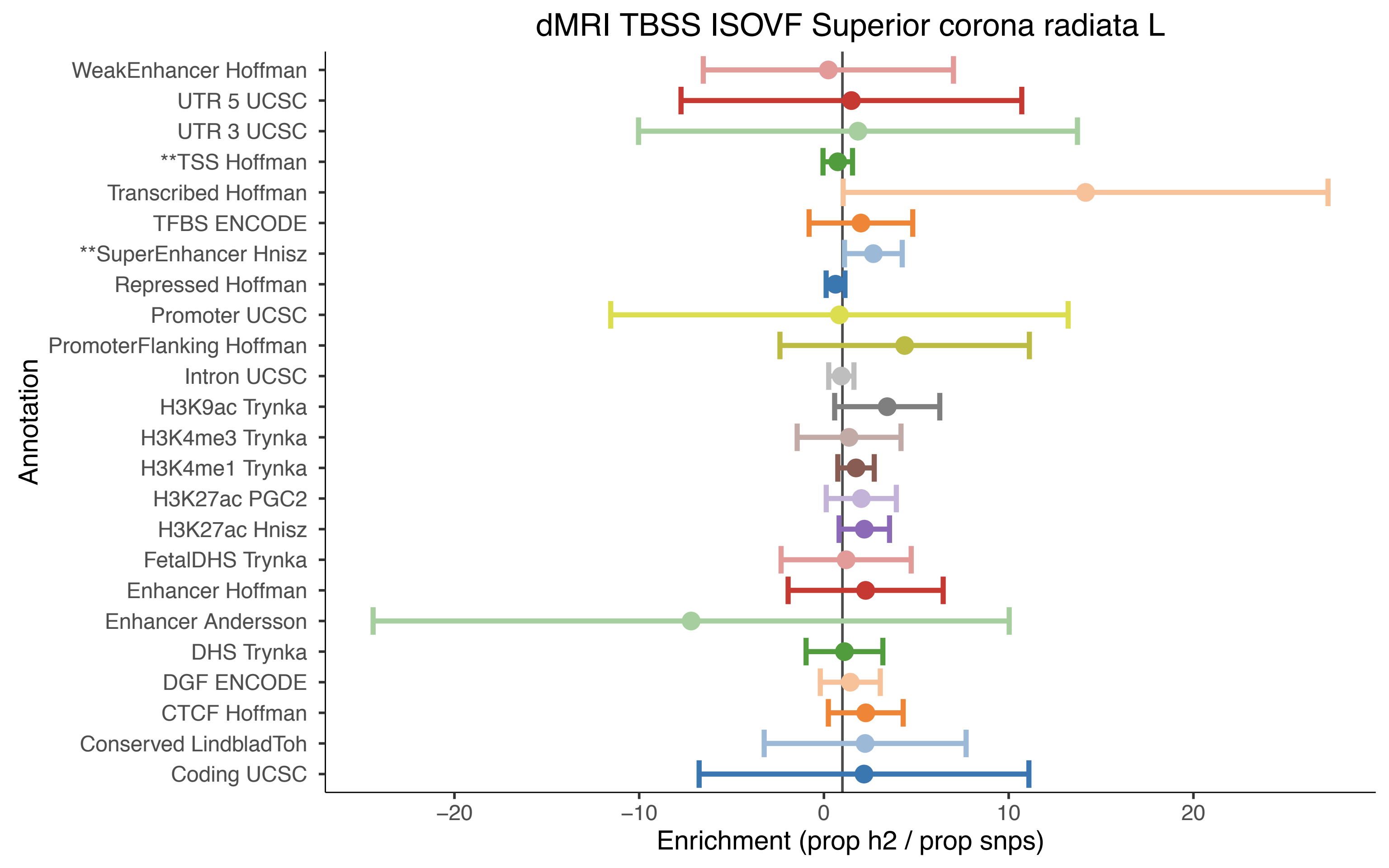

dMRI TBSS ISOVF Superior corona radiata L
WeakEnhancer Hoffman
UTR 5 UCSC
UTR 3 UCSC
**TSS Hoffman
Transcribed Hoffman
TFBS ENCODE
**SuperEnhancer Hnisz
Repressed Hoffman
Promoter UCSC
PromoterFlanking Hoffman
Intron UCSC
H3K9ac Trynka
H3K4me3 Trynka
H3K4me1 Trynka
H3K27ac PGC2
H3K27ac Hnisz
FetalDHS Trynka
Enhancer Hoffman
Enhancer Andersson
DHS Trynka
DGF ENCODE
CTCF Hoffman
Conserved LindbladToh
Coding UCSC
Annotation
−20
−10
0
10
20
Enrichment (prop h2 / prop snps)

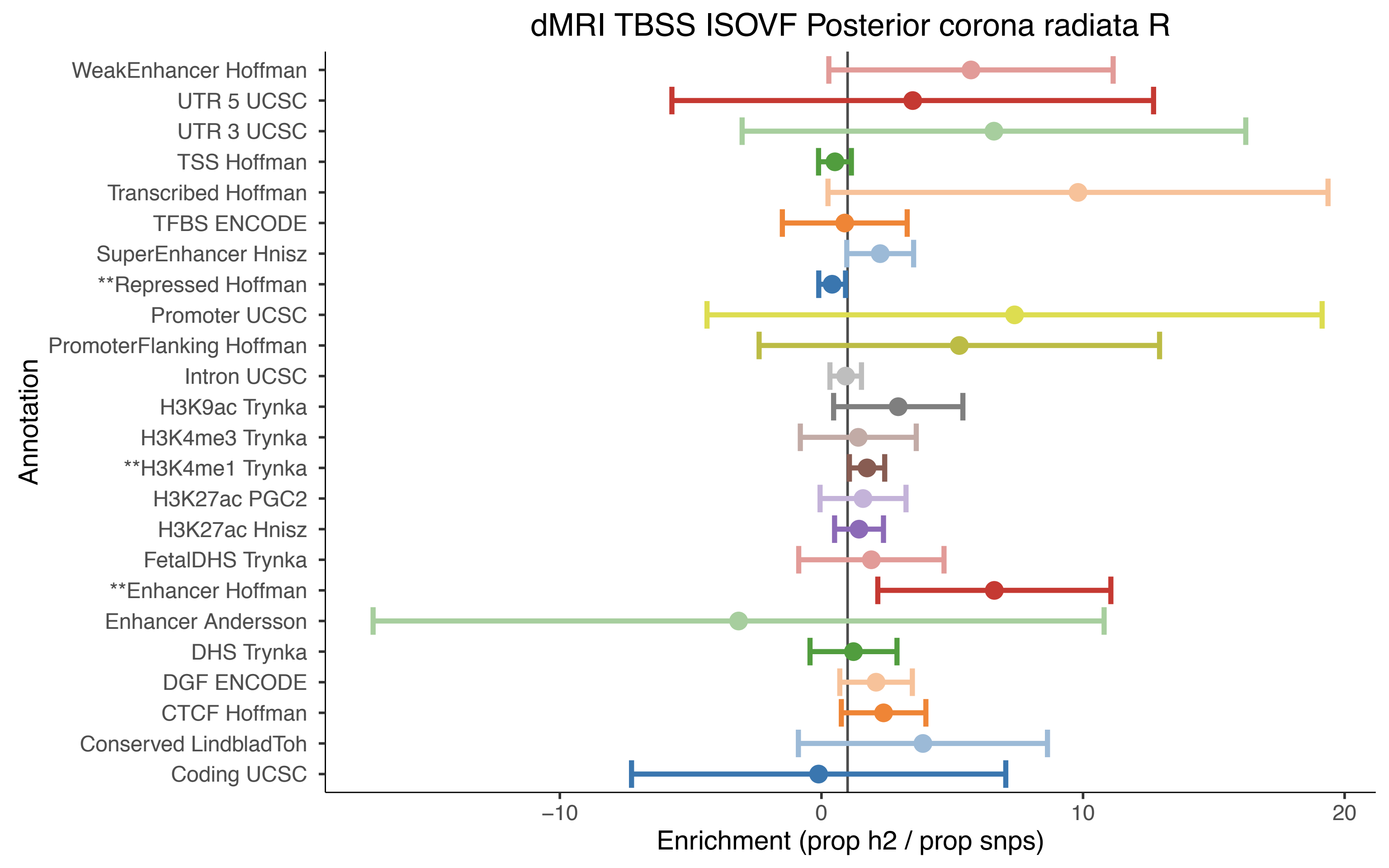
dMRI TBSS ISOVF Posterior corona radiata R
WeakEnhancer Hoffman
UTR 5 UCSC
UTR 3 UCSC
TSS Hoffman
Transcribed Hoffman
TFBS ENCODE
SuperEnhancer Hnisz
**Repressed Hoffman
Promoter UCSC
PromoterFlanking Hoffman
Intron UCSC
H3K9ac Trynka
H3K4me3 Trynka
**H3K4me1 Trynka
H3K27ac PGC2
H3K27ac Hnisz
FetalDHS Trynka
**Enhancer Hoffman
Enhancer Andersson
DHS Trynka
DGF ENCODE
CTCF Hoffman
Conserved LindbladToh
Coding UCSC
Annotation
−10
0
10
20
Enrichment (prop h2 / prop snps)

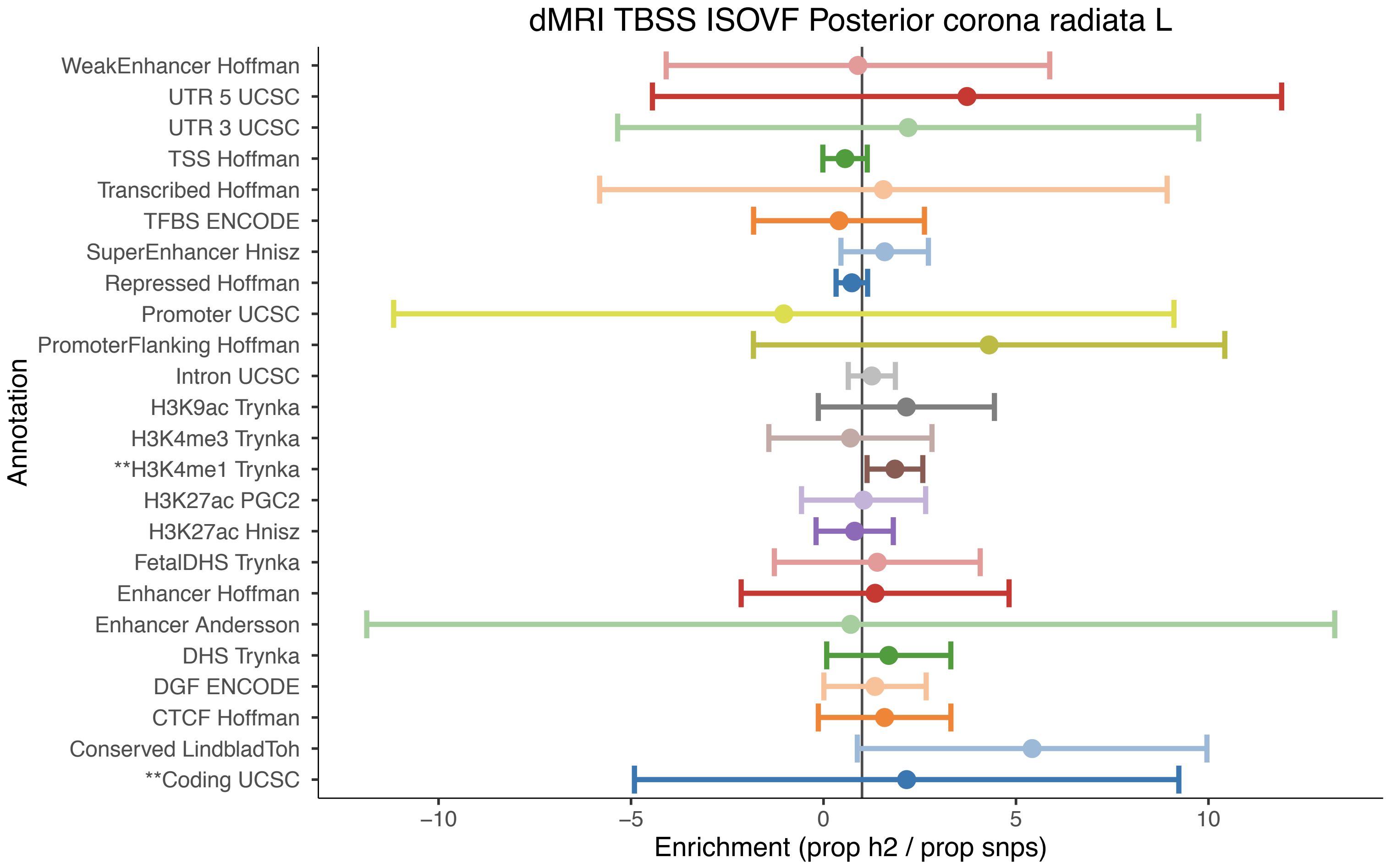
dMRI TBSS ISOVF Posterior corona radiata L
WeakEnhancer Hoffman
UTR 5 UCSC
UTR 3 UCSC
TSS Hoffman
Transcribed Hoffman
TFBS ENCODE
SuperEnhancer Hnisz
Repressed Hoffman
Promoter UCSC
PromoterFlanking Hoffman
Intron UCSC
H3K9ac Trynka
H3K4me3 Trynka
**H3K4me1 Trynka
H3K27ac PGC2
H3K27ac Hnisz
FetalDHS Trynka
Enhancer Hoffman
Enhancer Andersson
DHS Trynka
DGF ENCODE
CTCF Hoffman
Conserved LindbladToh
**Coding UCSC
Annotation
−10
−5
0
5
10
Enrichment (prop h2 / prop snps)

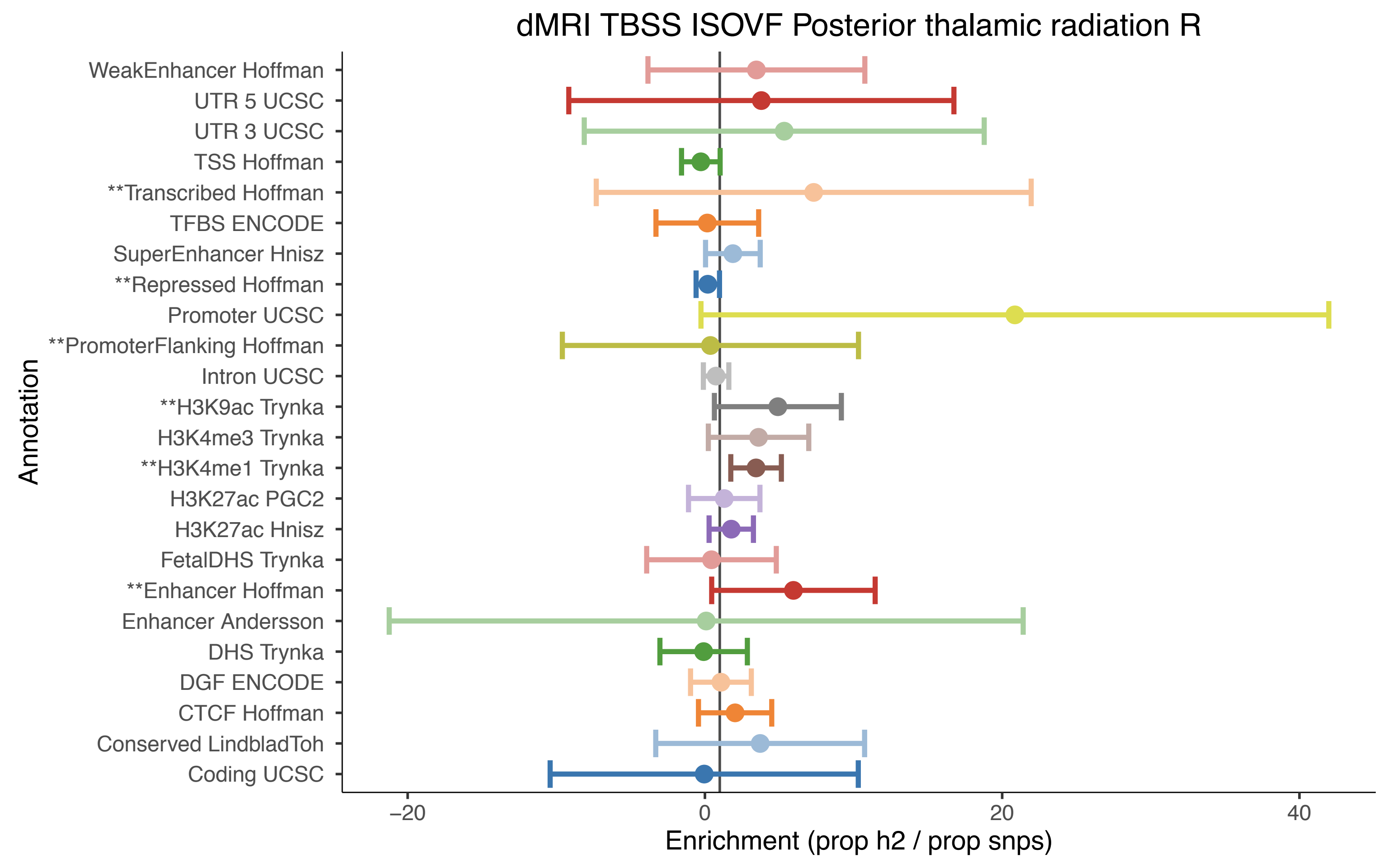
dMRI TBSS ISOVF Posterior thalamic radiation R
WeakEnhancer Hoffman
UTR 5 UCSC
UTR 3 UCSC
TSS Hoffman
**Transcribed Hoffman
TFBS ENCODE
SuperEnhancer Hnisz
**Repressed Hoffman
Promoter UCSC
**PromoterFlanking Hoffman
Intron UCSC
**H3K9ac Trynka
H3K4me3 Trynka
**H3K4me1 Trynka
H3K27ac PGC2
H3K27ac Hnisz
FetalDHS Trynka
**Enhancer Hoffman
Enhancer Andersson
DHS Trynka
DGF ENCODE
CTCF Hoffman
Conserved LindbladToh
Coding UCSC
Annotation
−20
0
20
40
Enrichment (prop h2 / prop snps)

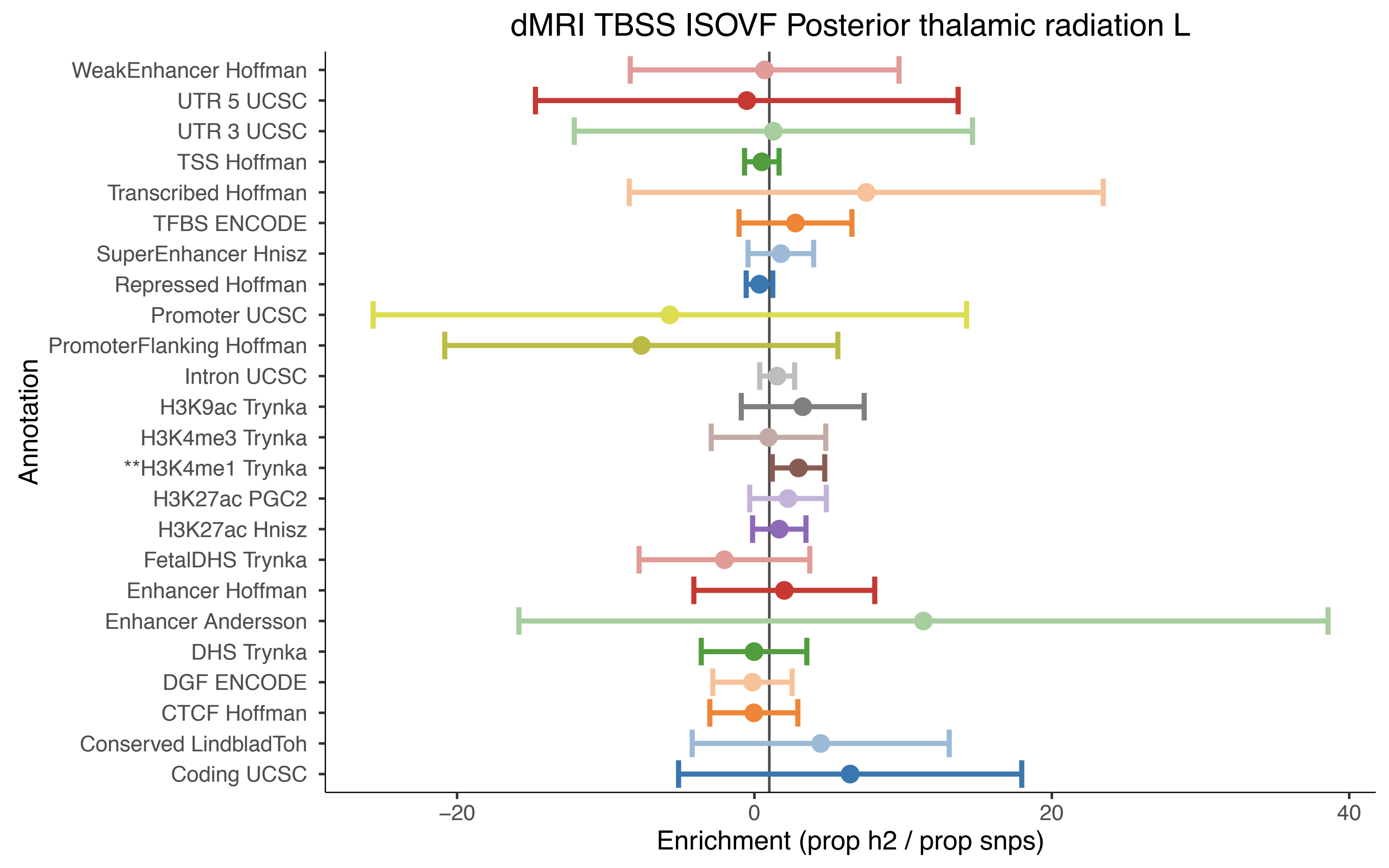

dMRI TBSS ISOVF Posterior thalamic radiation L
WeakEnhancer Hoffman
UTR 5 UCSC
UTR 3 UCSC
TSS Hoffman
Transcribed Hoffman
TFBS ENCODE
SuperEnhancer Hnisz
Repressed Hoffman
Promoter UCSC
PromoterFlanking Hoffman
Intron UCSC
H3K9ac Trynka
H3K4me3 Trynka
**H3K4me1 Trynka
H3K27ac PGC2
H3K27ac Hnisz
FetalDHS Trynka
Enhancer Hoffman
Enhancer Andersson
DHS Trynka
DGF ENCODE
CTCF Hoffman
Conserved LindbladToh
Coding UCSC
Annotation
−20
0
20
40
Enrichment (prop h2 / prop snps)

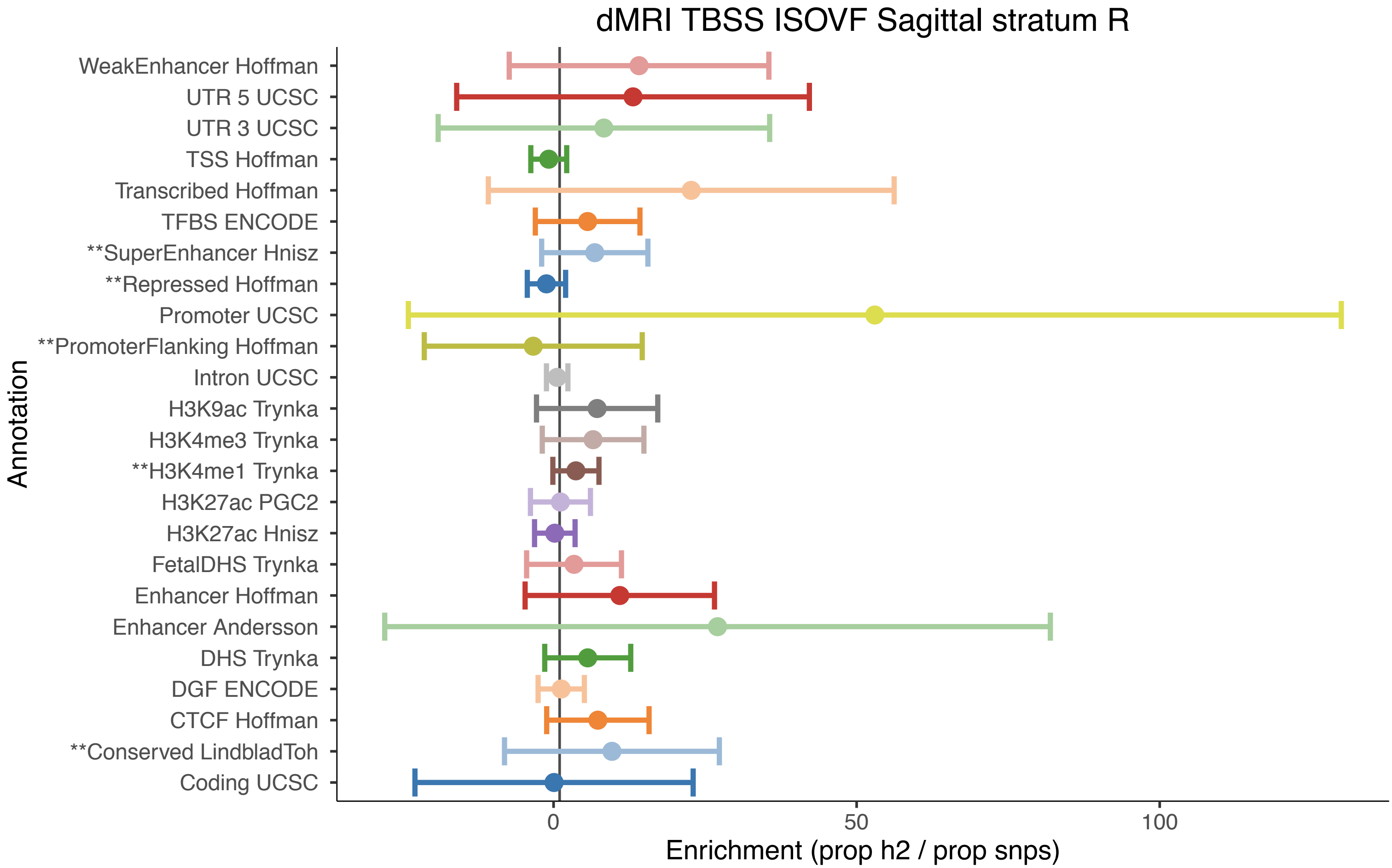
dMRI TBSS ISOVF Sagittal stratum R
WeakEnhancer Hoffman
UTR 5 UCSC
UTR 3 UCSC
TSS Hoffman
Transcribed Hoffman
TFBS ENCODE
**SuperEnhancer Hnisz
**Repressed Hoffman
Promoter UCSC
**PromoterFlanking Hoffman
Intron UCSC
H3K9ac Trynka
H3K4me3 Trynka
**H3K4me1 Trynka
H3K27ac PGC2
H3K27ac Hnisz
FetalDHS Trynka
Enhancer Hoffman
Enhancer Andersson
DHS Trynka
DGF ENCODE
CTCF Hoffman
**Conserved LindbladToh
Coding UCSC
Annotation
0
50
100
Enrichment (prop h2 / prop snps)

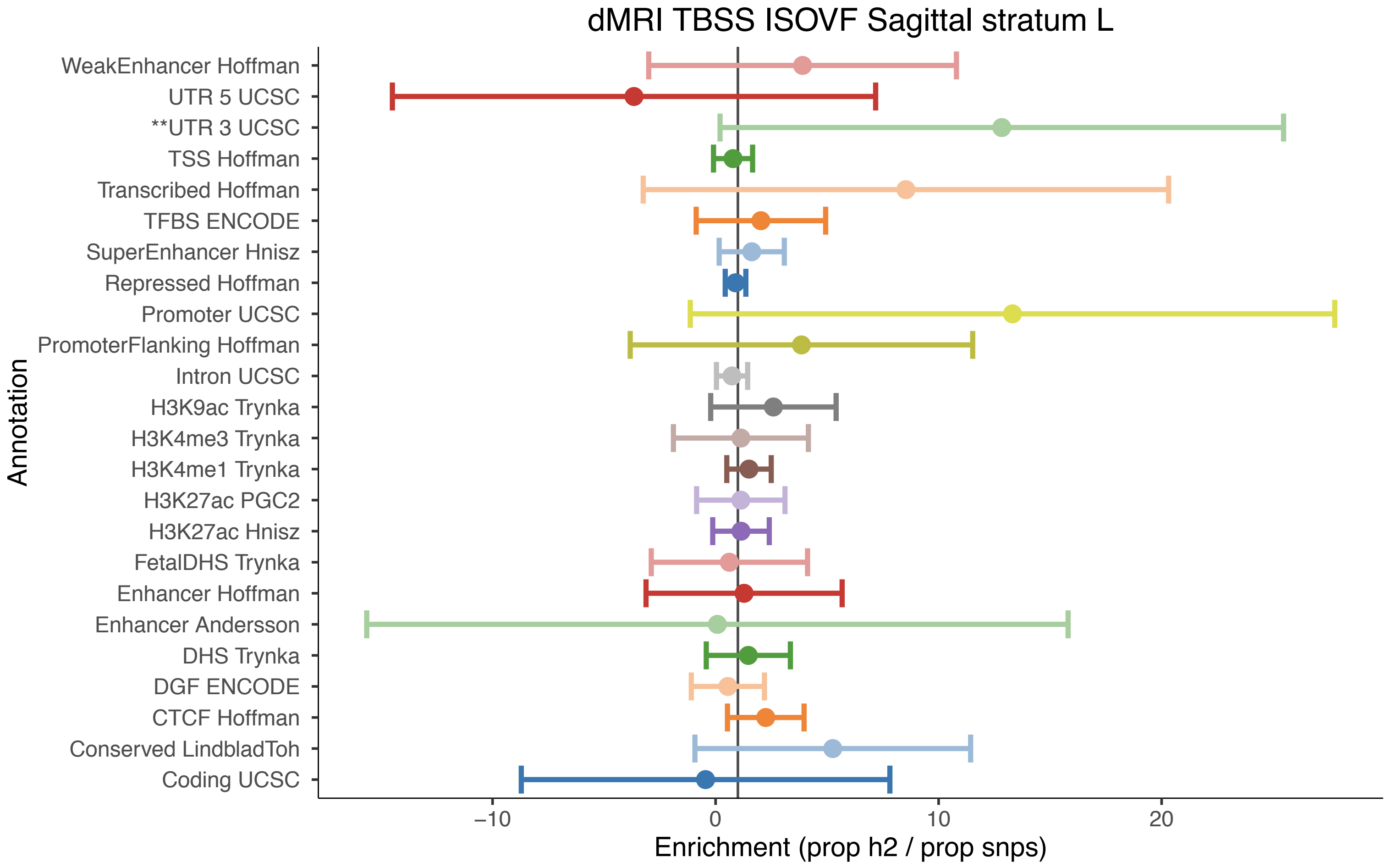

dMRI TBSS ISOVF Sagittal stratum L
WeakEnhancer Hoffman
UTR 5 UCSC
**UTR 3 UCSC
TSS Hoffman
Transcribed Hoffman
TFBS ENCODE
SuperEnhancer Hnisz
Repressed Hoffman
Promoter UCSC
PromoterFlanking Hoffman
Intron UCSC
H3K9ac Trynka
H3K4me3 Trynka
H3K4me1 Trynka
H3K27ac PGC2
H3K27ac Hnisz
FetalDHS Trynka
Enhancer Hoffman
Enhancer Andersson
DHS Trynka
DGF ENCODE
CTCF Hoffman
Conserved LindbladToh
Coding UCSC
Annotation
−10
0
10
20
Enrichment (prop h2 / prop snps)

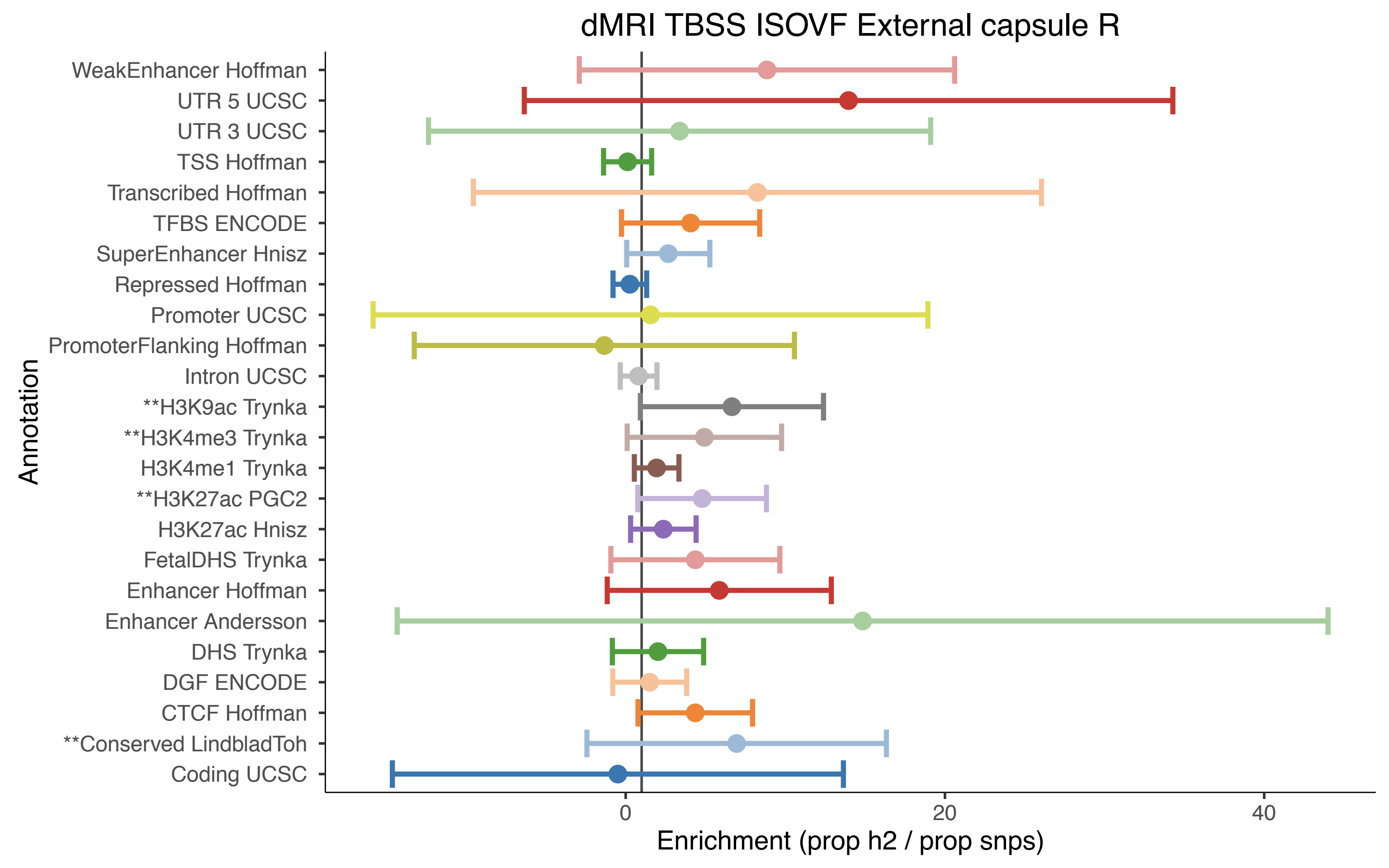
dMRI TBSS ISOVF External capsule R
WeakEnhancer Hoffman
UTR 5 UCSC
UTR 3 UCSC
TSS Hoffman
Transcribed Hoffman
TFBS ENCODE
SuperEnhancer Hnisz
Repressed Hoffman
Promoter UCSC
PromoterFlanking Hoffman
Intron UCSC
**H3K9ac Trynka
**H3K4me3 Trynka
H3K4me1 Trynka
**H3K27ac PGC2
H3K27ac Hnisz
FetalDHS Trynka
Enhancer Hoffman
Enhancer Andersson
DHS Trynka
DGF ENCODE
CTCF Hoffman
**Conserved LindbladToh
Coding UCSC
Annotation
0
20
40
Enrichment (prop h2 / prop snps)

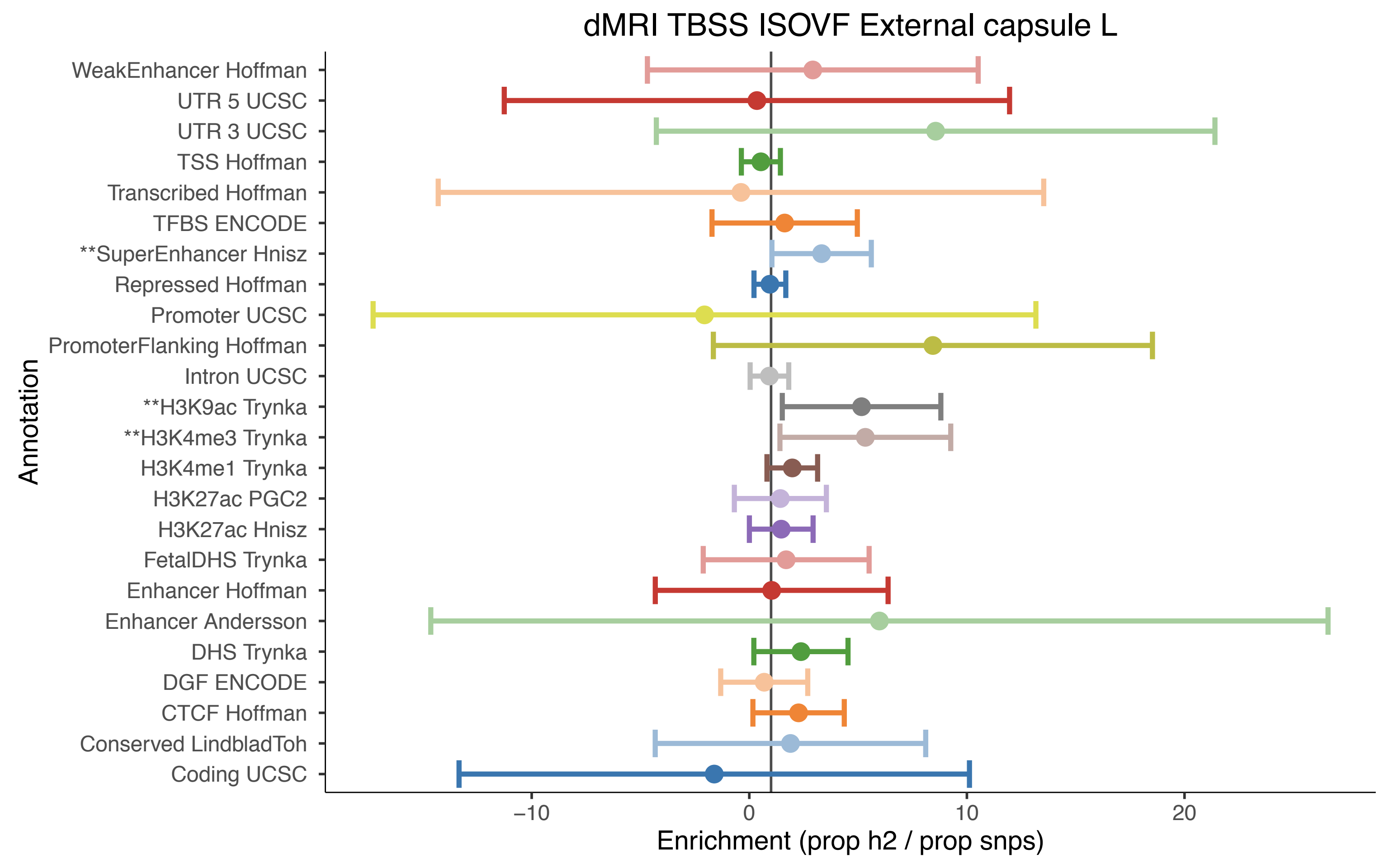

dMRI TBSS ISOVF External capsule L
WeakEnhancer Hoffman
UTR 5 UCSC
UTR 3 UCSC
TSS Hoffman
Transcribed Hoffman
TFBS ENCODE
**SuperEnhancer Hnisz
Repressed Hoffman
Promoter UCSC
PromoterFlanking Hoffman
Intron UCSC
**H3K9ac Trynka
**H3K4me3 Trynka
H3K4me1 Trynka
H3K27ac PGC2
H3K27ac Hnisz
FetalDHS Trynka
Enhancer Hoffman
Enhancer Andersson
DHS Trynka
DGF ENCODE
CTCF Hoffman
Conserved LindbladToh
Coding UCSC
Annotation
−10
0
10
20
Enrichment (prop h2 / prop snps)

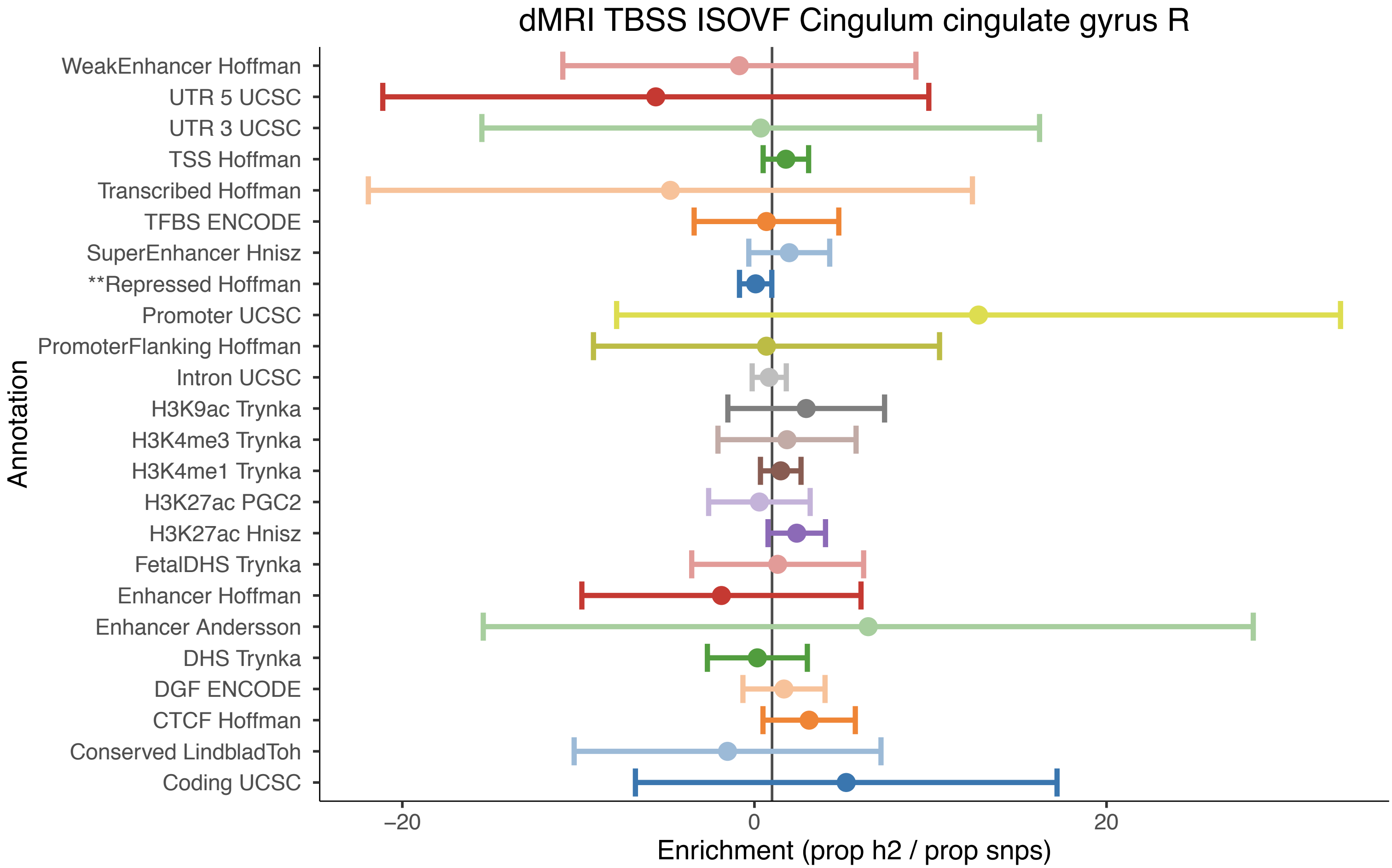
dMRI TBSS ISOVF Cingulum cingulate gyrus R
WeakEnhancer Hoffman
UTR 5 UCSC
UTR 3 UCSC
TSS Hoffman
Transcribed Hoffman
TFBS ENCODE
SuperEnhancer Hnisz
**Repressed Hoffman
Promoter UCSC
PromoterFlanking Hoffman
Intron UCSC
H3K9ac Trynka
H3K4me3 Trynka
H3K4me1 Trynka
H3K27ac PGC2
H3K27ac Hnisz
FetalDHS Trynka
Enhancer Hoffman
Enhancer Andersson
DHS Trynka
DGF ENCODE
CTCF Hoffman
Conserved LindbladToh
Coding UCSC
Annotation
−20
0
20
Enrichment (prop h2 / prop snps)

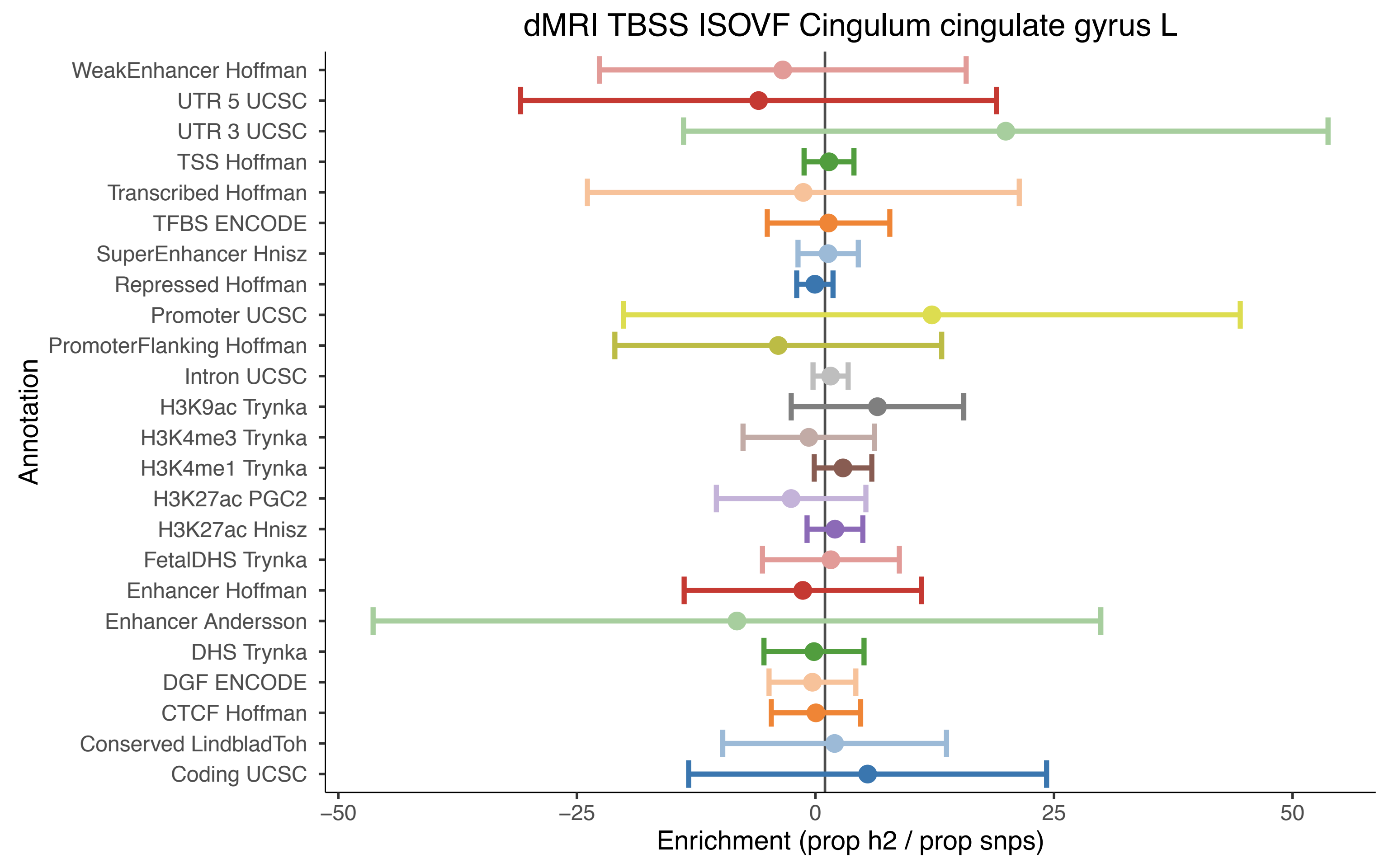
dMRI TBSS ISOVF Cingulum cingulate gyrus L
WeakEnhancer Hoffman
UTR 5 UCSC
UTR 3 UCSC
TSS Hoffman
Transcribed Hoffman
TFBS ENCODE
SuperEnhancer Hnisz
Repressed Hoffman
Promoter UCSC
PromoterFlanking Hoffman
Intron UCSC
H3K9ac Trynka
H3K4me3 Trynka
H3K4me1 Trynka
H3K27ac PGC2
H3K27ac Hnisz
FetalDHS Trynka
Enhancer Hoffman
Enhancer Andersson
DHS Trynka
DGF ENCODE
CTCF Hoffman
Conserved LindbladToh
Coding UCSC
Annotation
−50
−25
0
25
50
Enrichment (prop h2 / prop snps)

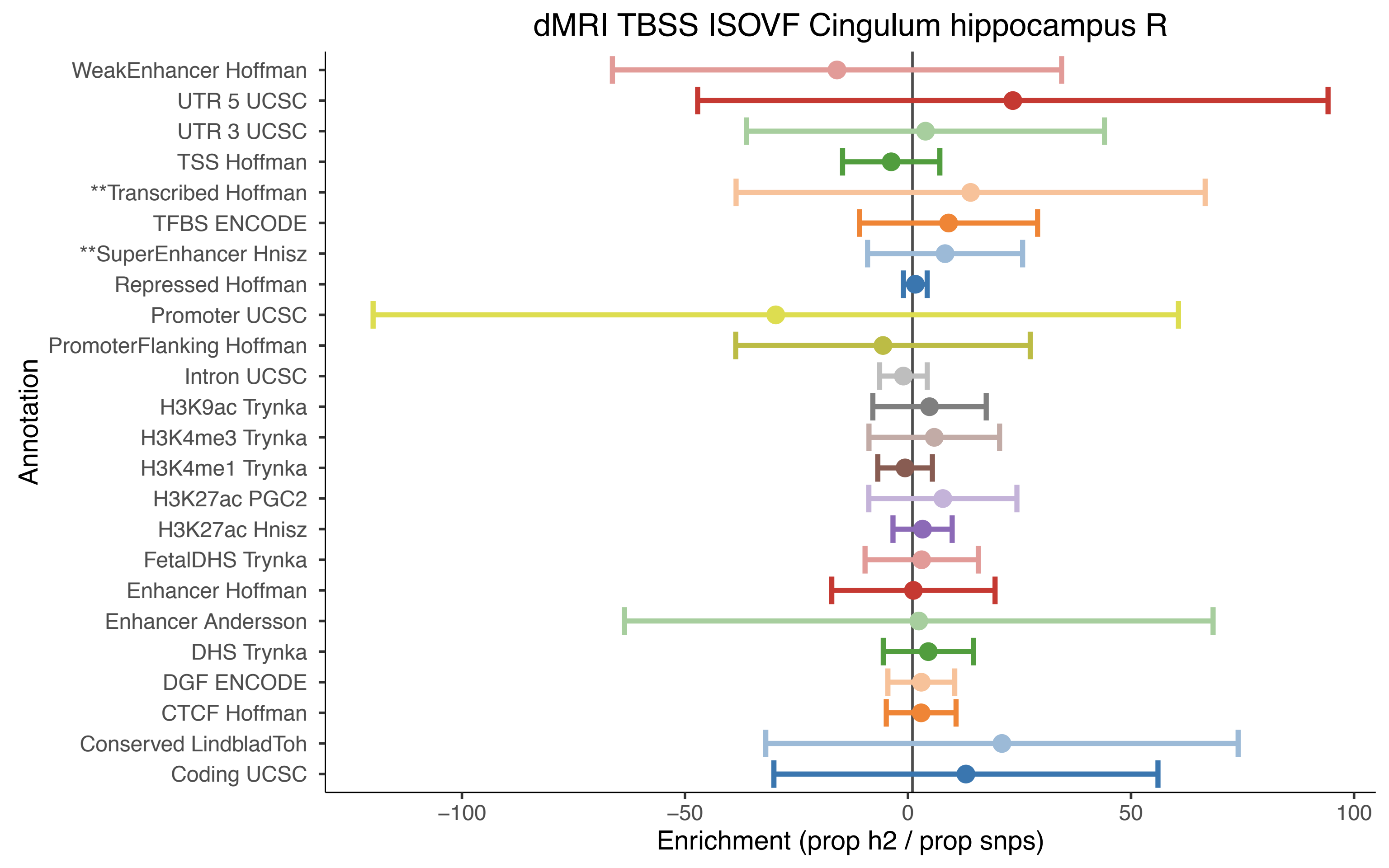
dMRI TBSS ISOVF Cingulum hippocampus R
WeakEnhancer Hoffman
UTR 5 UCSC
UTR 3 UCSC
TSS Hoffman
**Transcribed Hoffman
TFBS ENCODE
**SuperEnhancer Hnisz
Repressed Hoffman
Promoter UCSC
PromoterFlanking Hoffman
Intron UCSC
H3K9ac Trynka
H3K4me3 Trynka
H3K4me1 Trynka
H3K27ac PGC2
H3K27ac Hnisz
FetalDHS Trynka
Enhancer Hoffman
Enhancer Andersson
DHS Trynka
DGF ENCODE
CTCF Hoffman
Conserved LindbladToh
Coding UCSC
Annotation
−100
−50
0
50
100
Enrichment (prop h2 / prop snps)

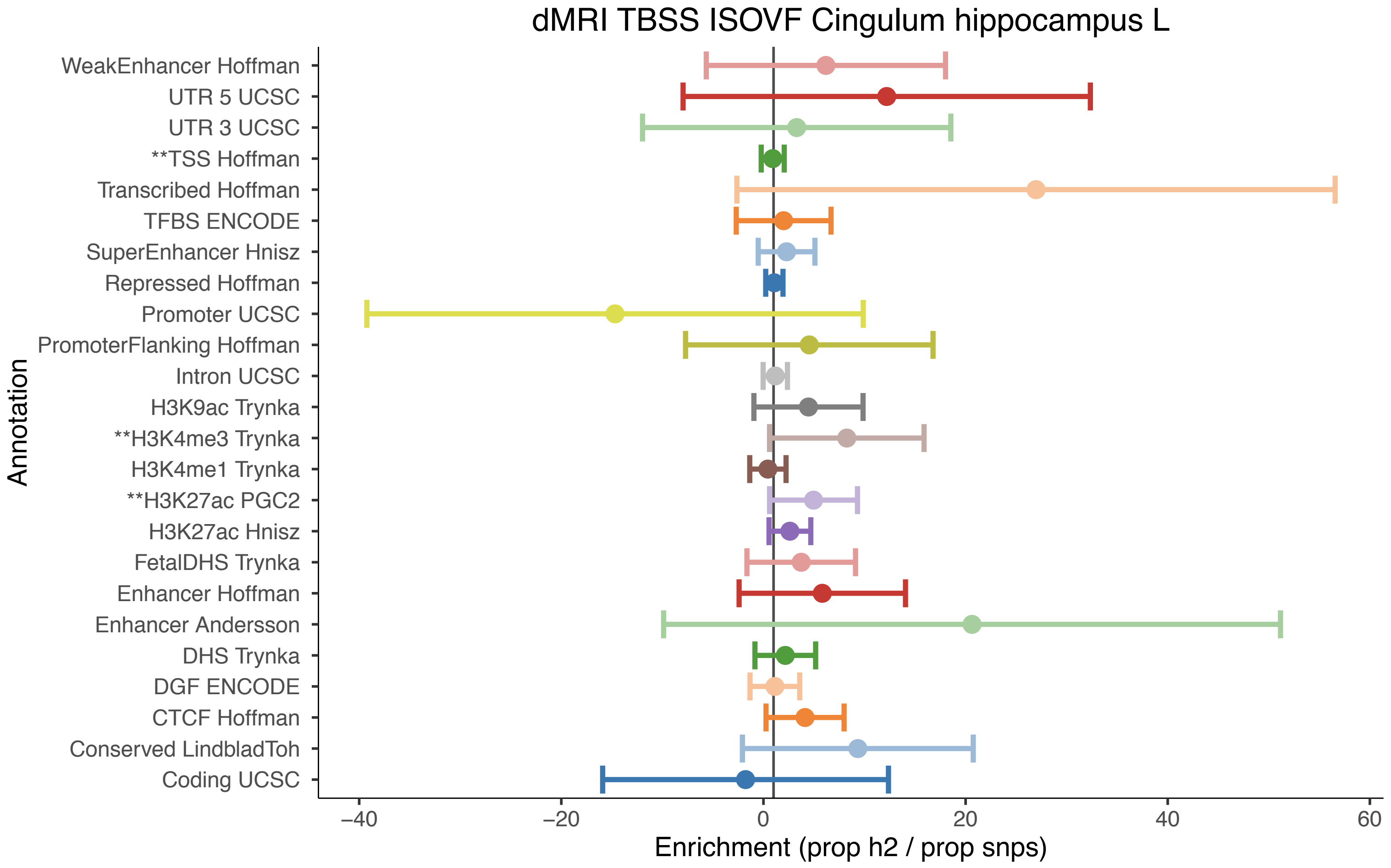
dMRI TBSS ISOVF Cingulum hippocampus L
WeakEnhancer Hoffman
UTR 5 UCSC
UTR 3 UCSC
**TSS Hoffman
Transcribed Hoffman
TFBS ENCODE
SuperEnhancer Hnisz
Repressed Hoffman
Promoter UCSC
PromoterFlanking Hoffman
Intron UCSC
H3K9ac Trynka
**H3K4me3 Trynka
H3K4me1 Trynka
**H3K27ac PGC2
H3K27ac Hnisz
FetalDHS Trynka
Enhancer Hoffman
Enhancer Andersson
DHS Trynka
DGF ENCODE
CTCF Hoffman
Conserved LindbladToh
Coding UCSC
Annotation
−40
−20
0
20
40
60
Enrichment (prop h2 / prop snps)

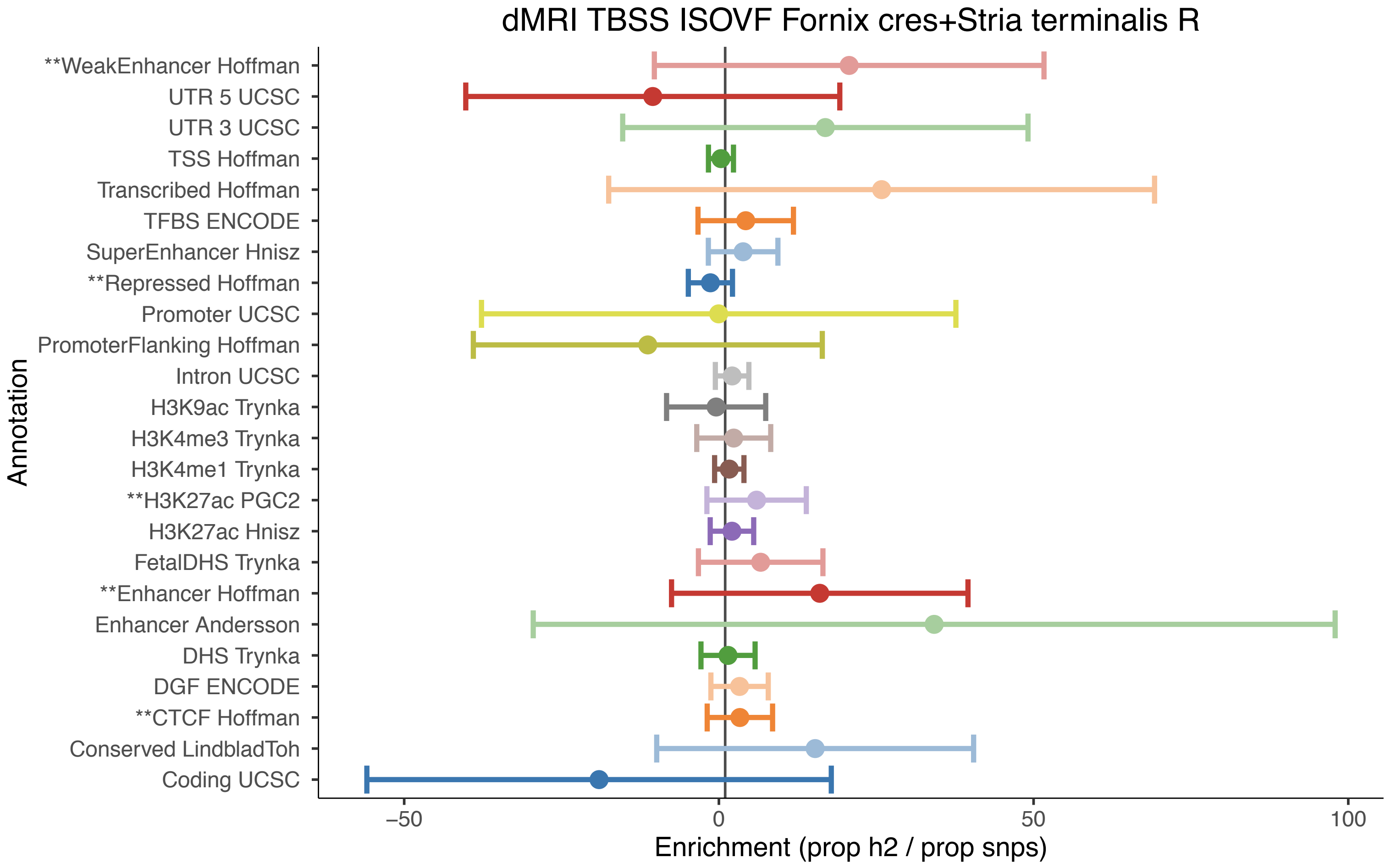

dMRI TBSS ISOVF Fornix cres+Stria terminalis R
**WeakEnhancer Hoffman
UTR 5 UCSC
UTR 3 UCSC
TSS Hoffman
Transcribed Hoffman
TFBS ENCODE
SuperEnhancer Hnisz
**Repressed Hoffman
Promoter UCSC
PromoterFlanking Hoffman
Intron UCSC
H3K9ac Trynka
H3K4me3 Trynka
H3K4me1 Trynka
**H3K27ac PGC2
H3K27ac Hnisz
FetalDHS Trynka
**Enhancer Hoffman
Enhancer Andersson
DHS Trynka
DGF ENCODE
**CTCF Hoffman
Conserved LindbladToh
Coding UCSC
Annotation
−50
0
50
100
Enrichment (prop h2 / prop snps)

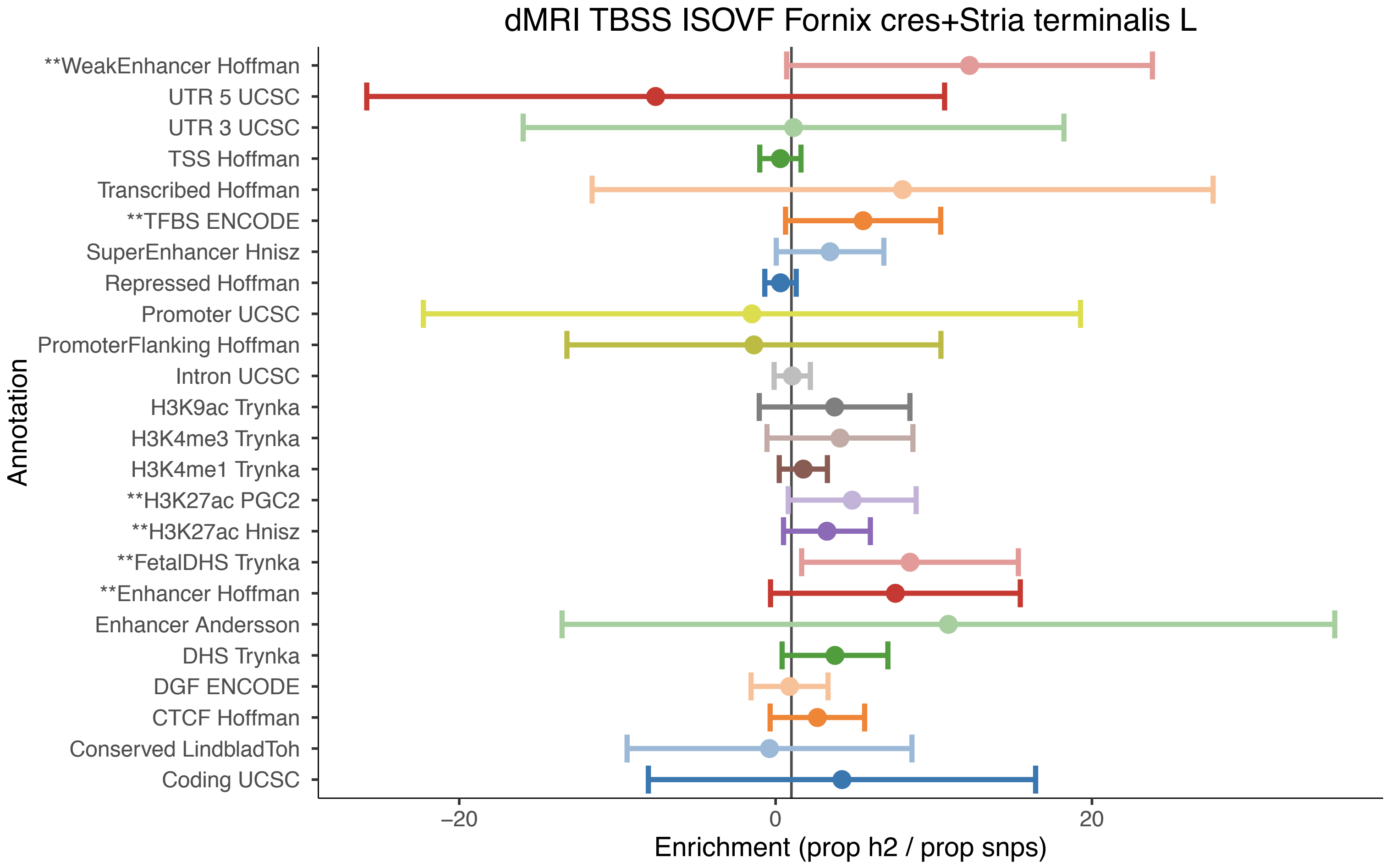

dMRI TBSS ISOVF Fornix cres+Stria terminalis L
**WeakEnhancer Hoffman
UTR 5 UCSC
UTR 3 UCSC
TSS Hoffman
Transcribed Hoffman
**TFBS ENCODE
SuperEnhancer Hnisz
Repressed Hoffman
Promoter UCSC
PromoterFlanking Hoffman
Intron UCSC
H3K9ac Trynka
H3K4me3 Trynka
H3K4me1 Trynka
**H3K27ac PGC2
**H3K27ac Hnisz
**FetalDHS Trynka
**Enhancer Hoffman
Enhancer Andersson
DHS Trynka
DGF ENCODE
CTCF Hoffman
Conserved LindbladToh
Coding UCSC
Annotation
−20
0
20
Enrichment (prop h2 / prop snps)

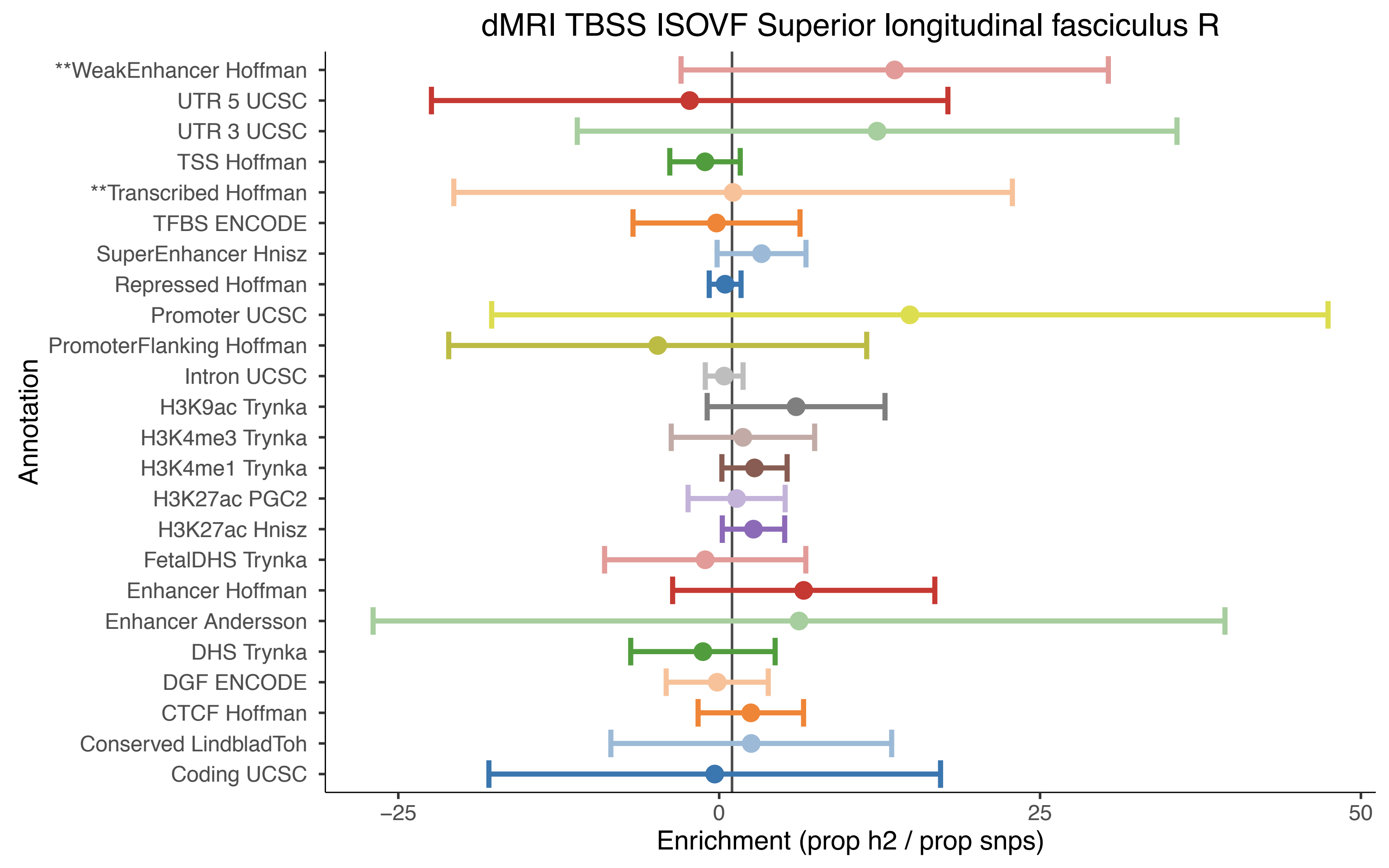

dMRI TBSS ISOVF Superior longitudinal fasciculus R
**WeakEnhancer Hoffman
UTR 5 UCSC
UTR 3 UCSC
TSS Hoffman
**Transcribed Hoffman
TFBS ENCODE
SuperEnhancer Hnisz
Repressed Hoffman
Promoter UCSC
PromoterFlanking Hoffman
Intron UCSC
H3K9ac Trynka
H3K4me3 Trynka
H3K4me1 Trynka
H3K27ac PGC2
H3K27ac Hnisz
FetalDHS Trynka
Enhancer Hoffman
Enhancer Andersson
DHS Trynka
DGF ENCODE
CTCF Hoffman
Conserved LindbladToh
Coding UCSC
Annotation
−25
0
25
50
Enrichment (prop h2 / prop snps)

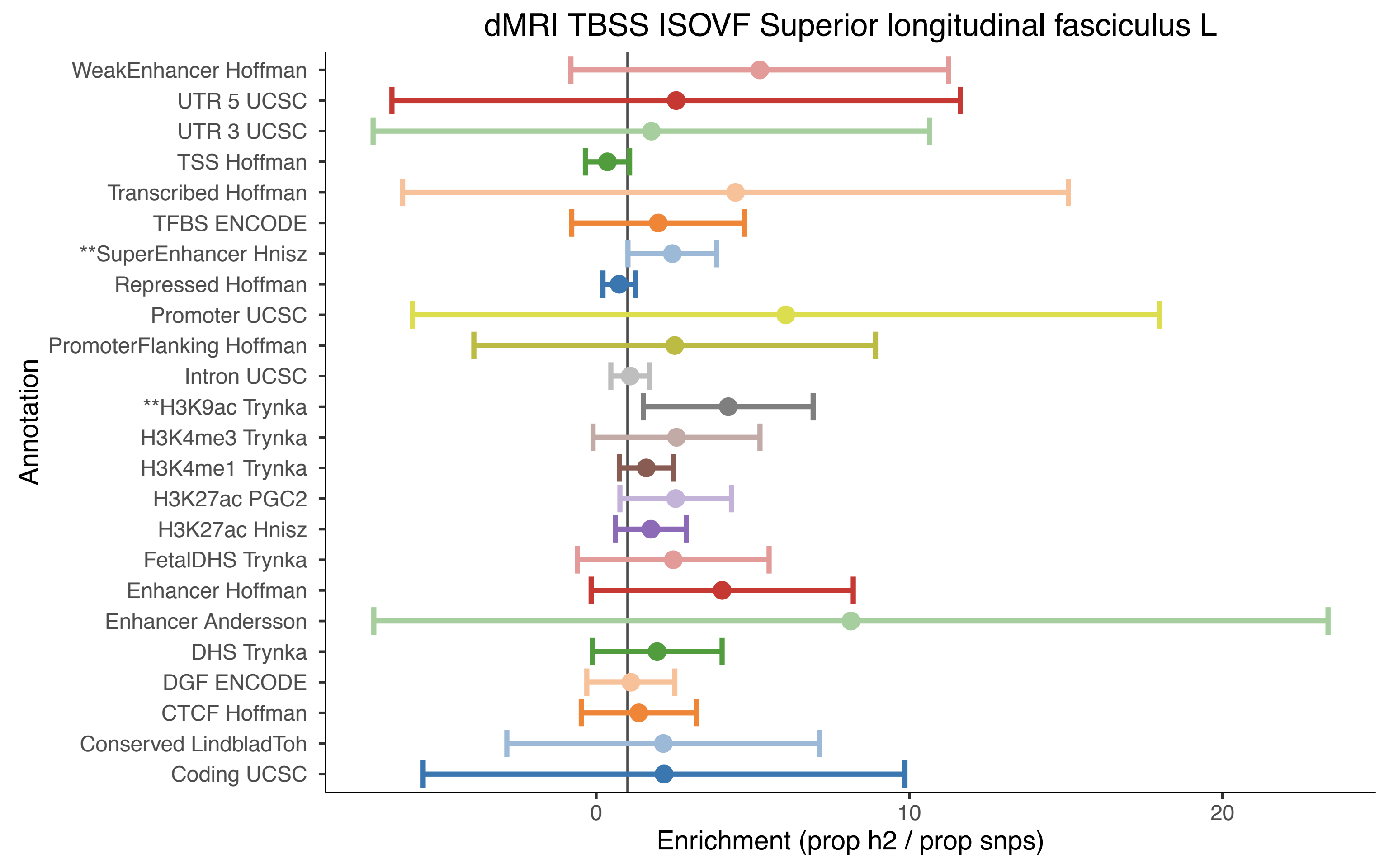

dMRI TBSS ISOVF Superior longitudinal fasciculus L
WeakEnhancer Hoffman
UTR 5 UCSC
UTR 3 UCSC
TSS Hoffman
Transcribed Hoffman
TFBS ENCODE
**SuperEnhancer Hnisz
Repressed Hoffman
Promoter UCSC
PromoterFlanking Hoffman
Intron UCSC
**H3K9ac Trynka
H3K4me3 Trynka
H3K4me1 Trynka
H3K27ac PGC2
H3K27ac Hnisz
FetalDHS Trynka
Enhancer Hoffman
Enhancer Andersson
DHS Trynka
DGF ENCODE
CTCF Hoffman
Conserved LindbladToh
Coding UCSC
Annotation
0
10
20
Enrichment (prop h2 / prop snps)

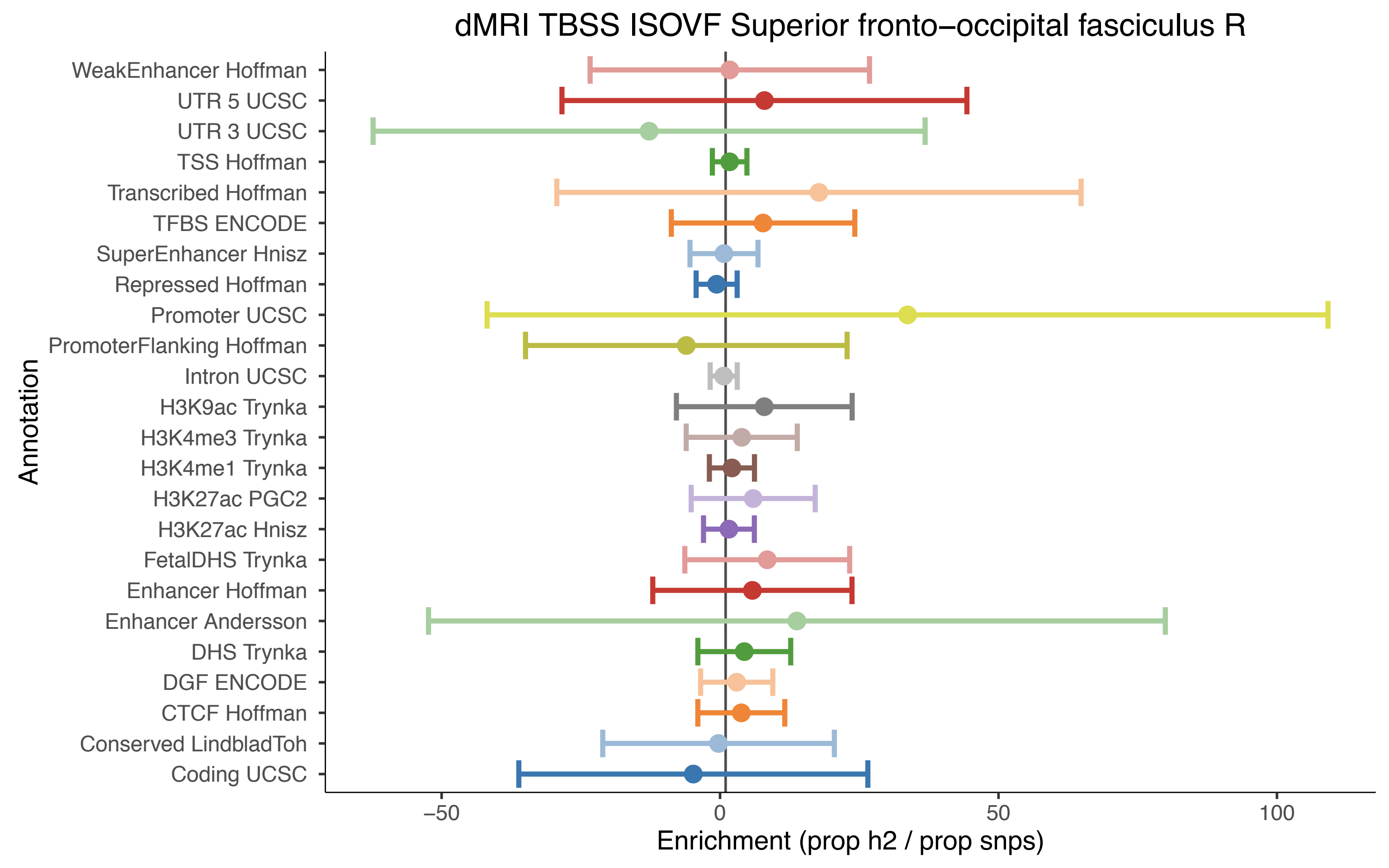
dMRI TBSS ISOVF Superior fronto−occipital fasciculus R
WeakEnhancer Hoffman
UTR 5 UCSC
UTR 3 UCSC
TSS Hoffman
Transcribed Hoffman
TFBS ENCODE
SuperEnhancer Hnisz
Repressed Hoffman
Promoter UCSC
PromoterFlanking Hoffman
Intron UCSC
H3K9ac Trynka
H3K4me3 Trynka
H3K4me1 Trynka
H3K27ac PGC2
H3K27ac Hnisz
FetalDHS Trynka
Enhancer Hoffman
Enhancer Andersson
DHS Trynka
DGF ENCODE
CTCF Hoffman
Conserved LindbladToh
Coding UCSC
Annotation
−50
0
50
100
Enrichment (prop h2 / prop snps)

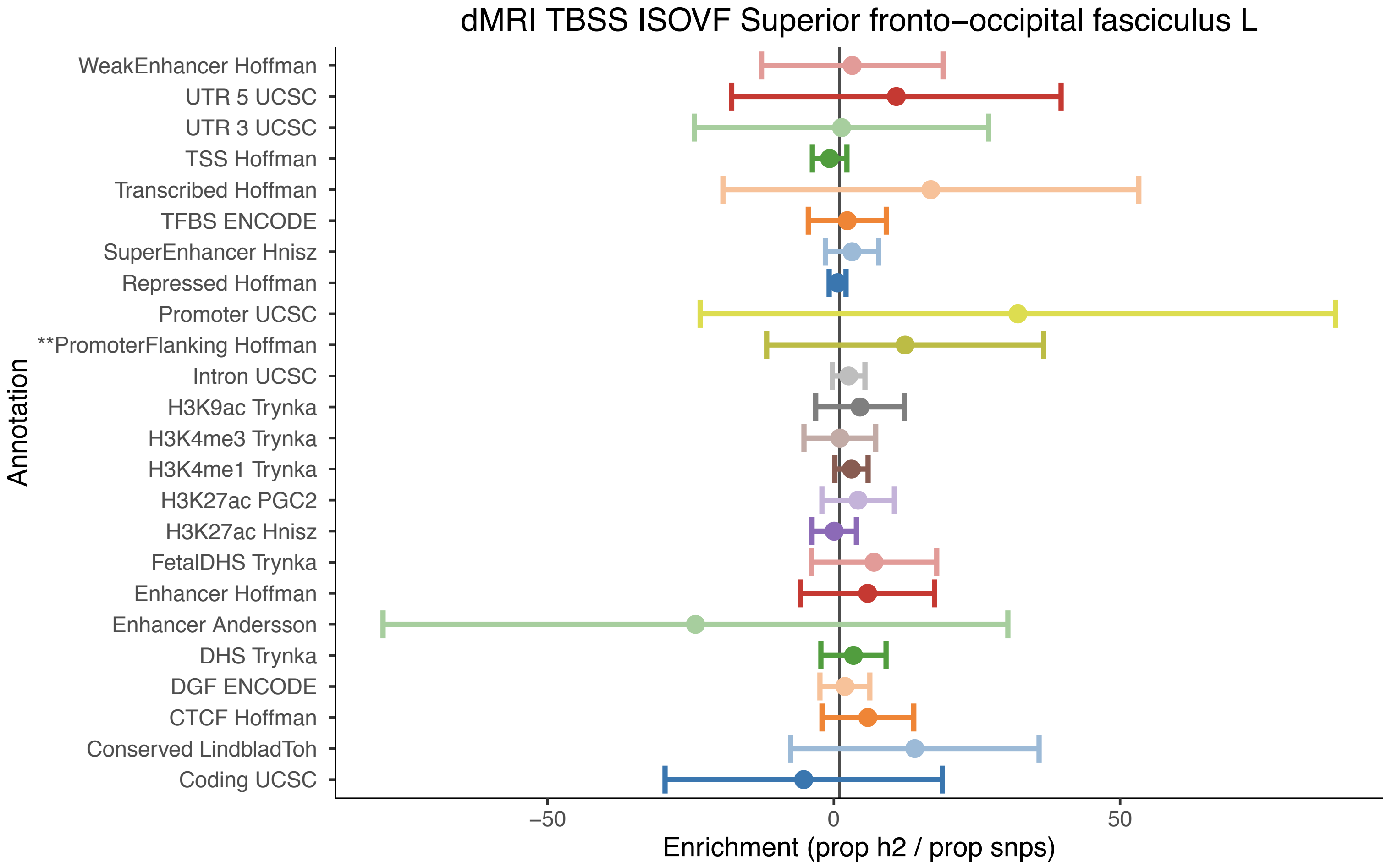

dMRI TBSS ISOVF Superior fronto−occipital fasciculus L
WeakEnhancer Hoffman
UTR 5 UCSC
UTR 3 UCSC
TSS Hoffman
Transcribed Hoffman
TFBS ENCODE
SuperEnhancer Hnisz
Repressed Hoffman
Promoter UCSC
**PromoterFlanking Hoffman
Intron UCSC
H3K9ac Trynka
H3K4me3 Trynka
H3K4me1 Trynka
H3K27ac PGC2
H3K27ac Hnisz
FetalDHS Trynka
Enhancer Hoffman
Enhancer Andersson
DHS Trynka
DGF ENCODE
CTCF Hoffman
Conserved LindbladToh
Coding UCSC
Annotation
−50
0
50
Enrichment (prop h2 / prop snps)

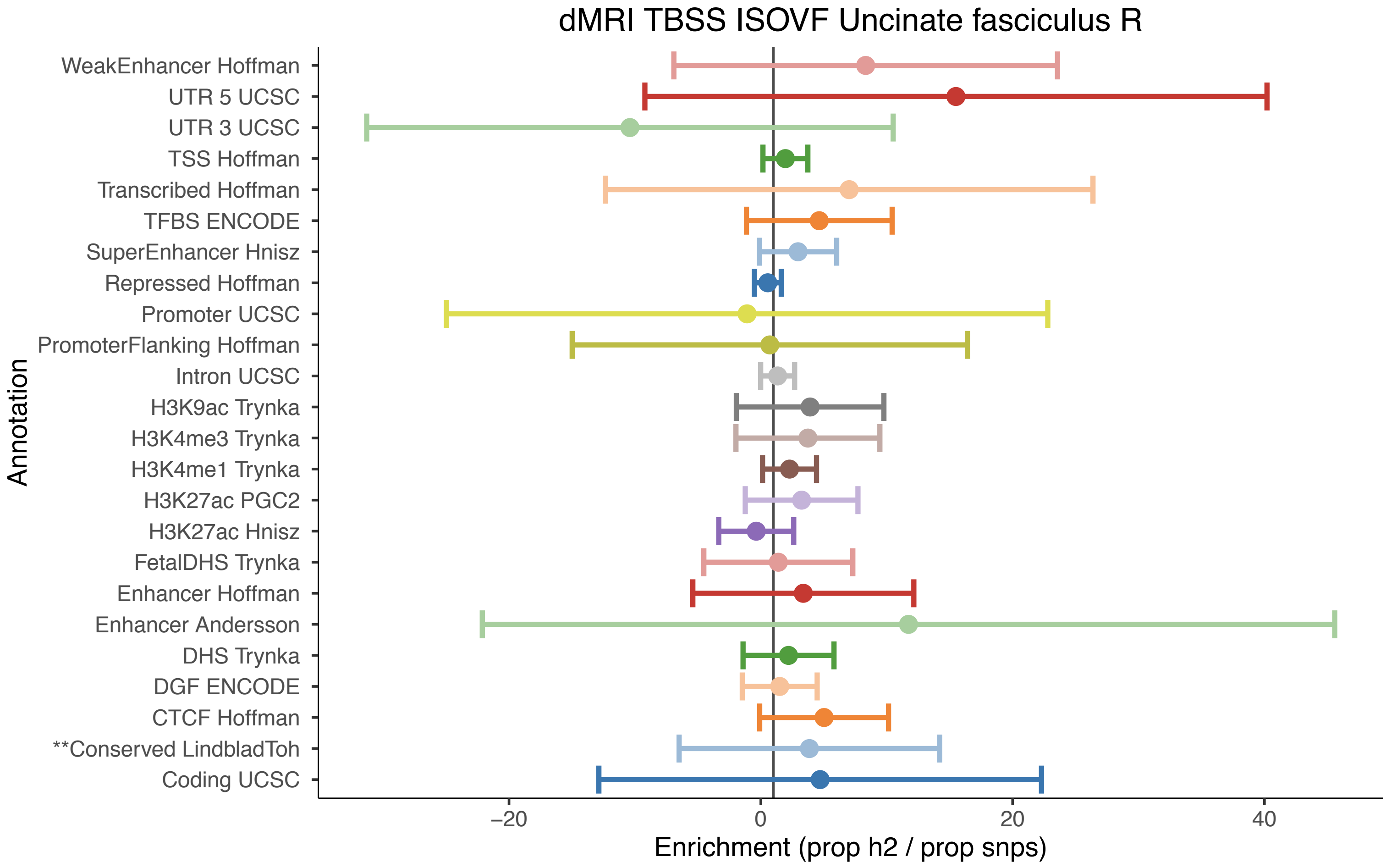

dMRI TBSS ISOVF Uncinate fasciculus R
WeakEnhancer Hoffman
UTR 5 UCSC
UTR 3 UCSC
TSS Hoffman
Transcribed Hoffman
TFBS ENCODE
SuperEnhancer Hnisz
Repressed Hoffman
Promoter UCSC
PromoterFlanking Hoffman
Intron UCSC
H3K9ac Trynka
H3K4me3 Trynka
H3K4me1 Trynka
H3K27ac PGC2
H3K27ac Hnisz
FetalDHS Trynka
Enhancer Hoffman
Enhancer Andersson
DHS Trynka
DGF ENCODE
CTCF Hoffman
**Conserved LindbladToh
Coding UCSC
Annotation
−20
0
20
40
Enrichment (prop h2 / prop snps)

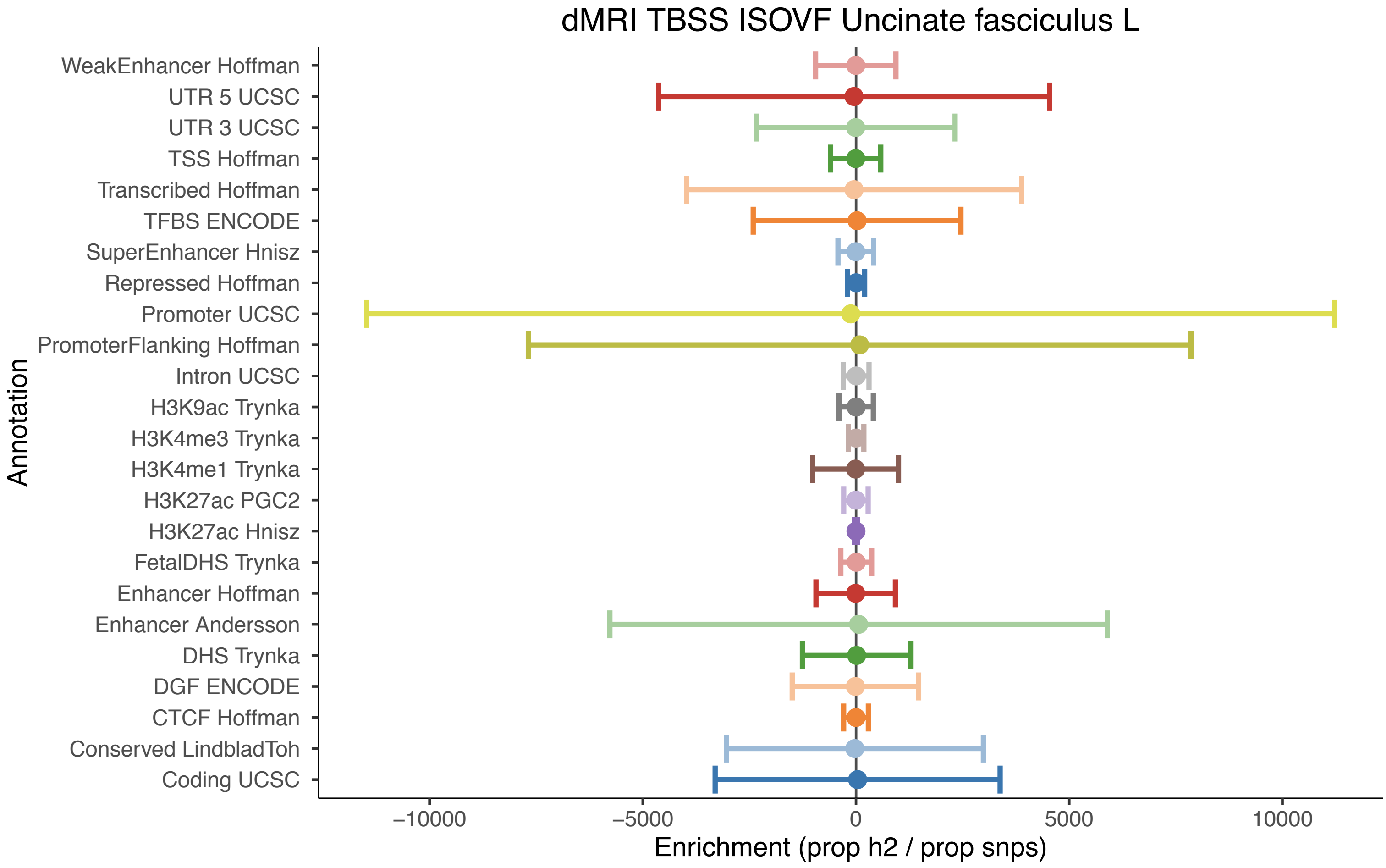
dMRI TBSS ISOVF Uncinate fasciculus L
Annotation
WeakEnhancer Hoffman
UTR 5 UCSC
UTR 3 UCSC
TSS Hoffman
Transcribed Hoffman
TFBS ENCODE
SuperEnhancer Hnisz
Repressed Hoffman
Promoter UCSC
PromoterFlanking Hoffman
Intron UCSC
H3K9ac Trynka
H3K4me3 Trynka
H3K4me1 Trynka
H3K27ac PGC2
H3K27ac Hnisz
FetalDHS Trynka
Enhancer Hoffman
Enhancer Andersson
DHS Trynka
DGF ENCODE
CTCF Hoffman
Conserved LindbladToh
Coding UCSC
−10000
−5000
0
5000
10000
Enrichment (prop h2 / prop snps)

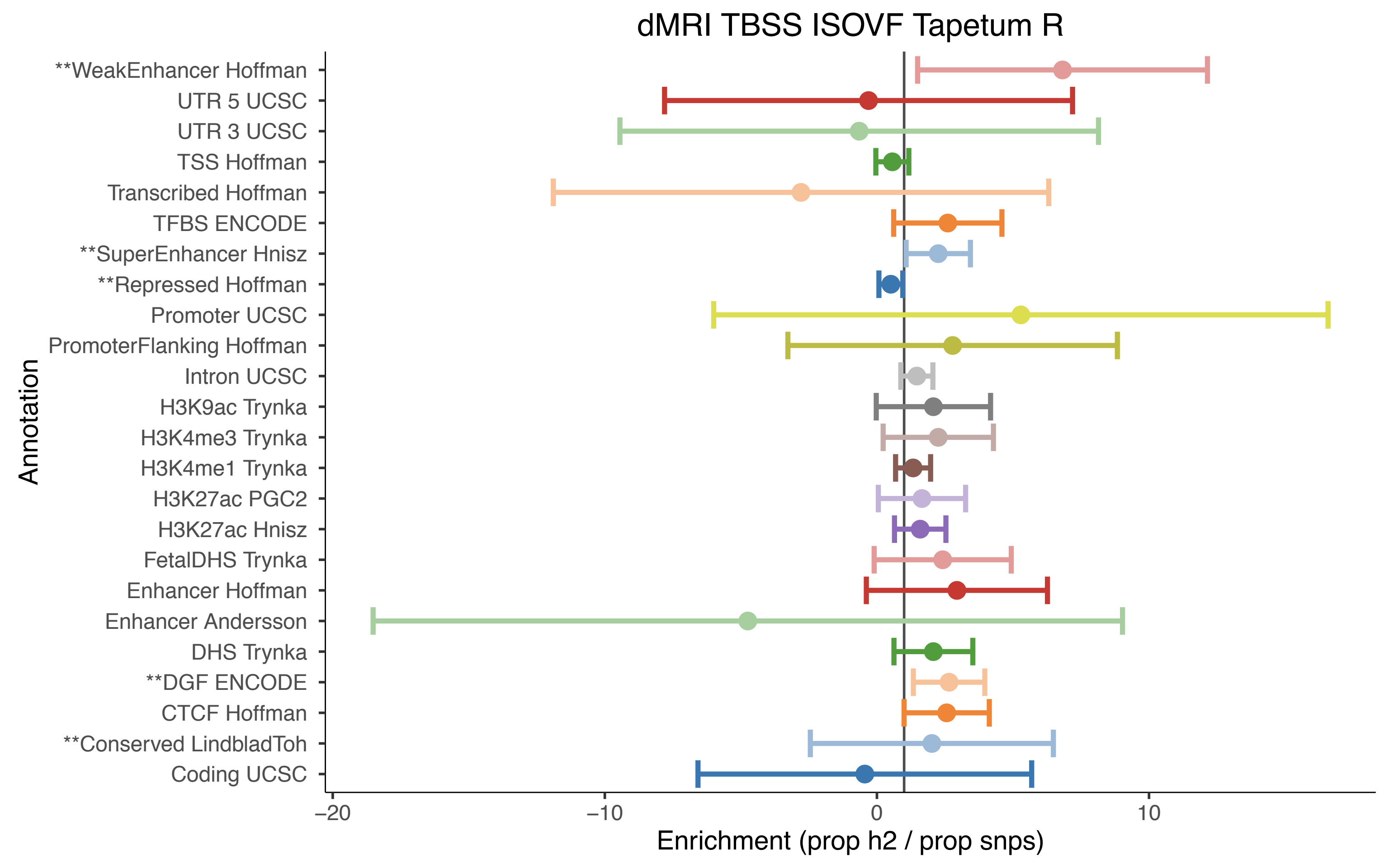

dMRI TBSS ISOVF Tapetum R
**WeakEnhancer Hoffman
UTR 5 UCSC
UTR 3 UCSC
TSS Hoffman
Transcribed Hoffman
TFBS ENCODE
**SuperEnhancer Hnisz
**Repressed Hoffman
Promoter UCSC
PromoterFlanking Hoffman
Intron UCSC
H3K9ac Trynka
H3K4me3 Trynka
H3K4me1 Trynka
H3K27ac PGC2
H3K27ac Hnisz
FetalDHS Trynka
Enhancer Hoffman
Enhancer Andersson
DHS Trynka
**DGF ENCODE
CTCF Hoffman
**Conserved LindbladToh
Coding UCSC
Annotation
−20
−10
0
10
Enrichment (prop h2 / prop snps)

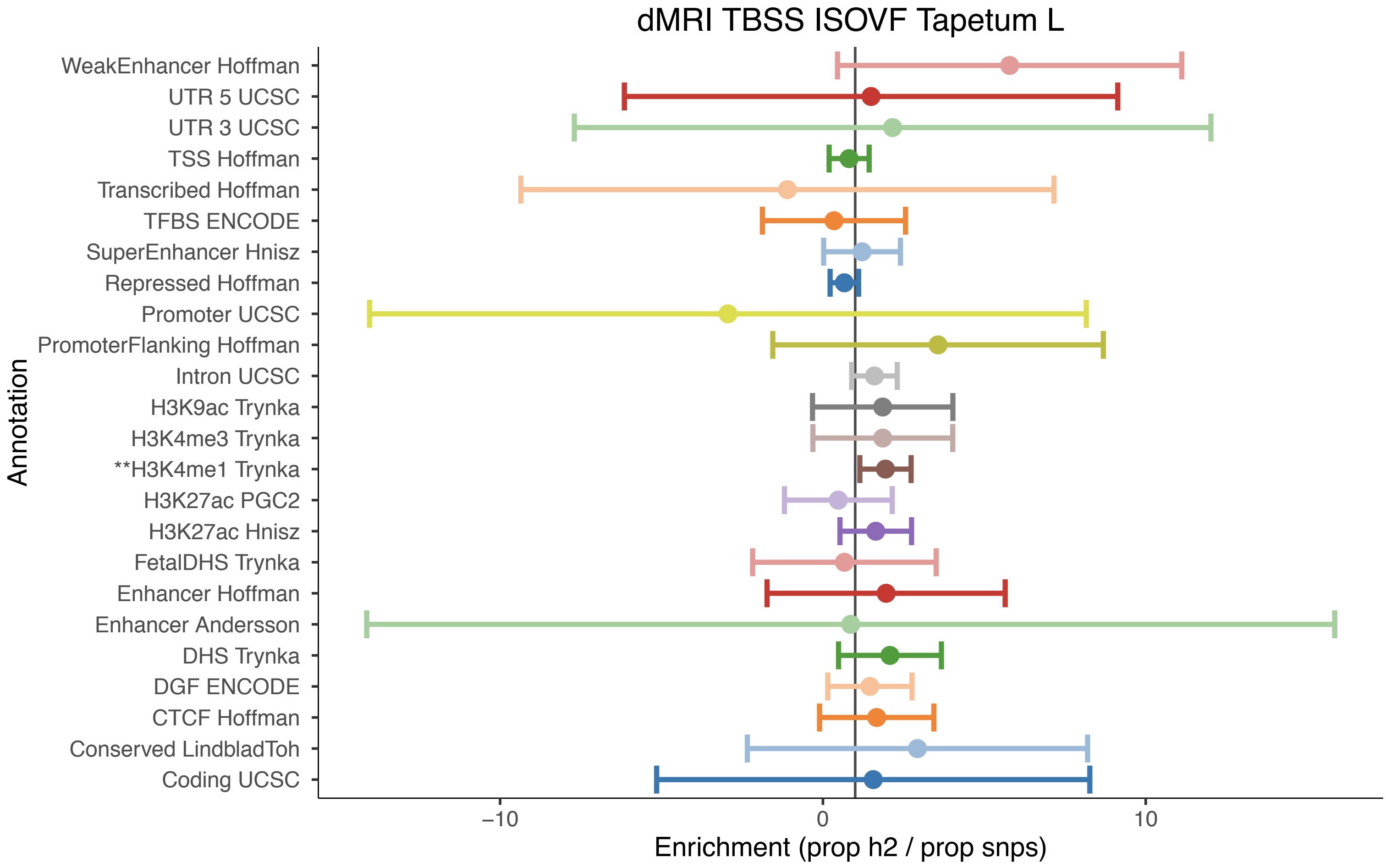

dMRI TBSS ISOVF Tapetum L
WeakEnhancer Hoffman
UTR 5 UCSC
UTR 3 UCSC
TSS Hoffman
Transcribed Hoffman
TFBS ENCODE
SuperEnhancer Hnisz
Repressed Hoffman
Promoter UCSC
PromoterFlanking Hoffman
Intron UCSC
H3K9ac Trynka
H3K4me3 Trynka
**H3K4me1 Trynka
H3K27ac PGC2
H3K27ac Hnisz
FetalDHS Trynka
Enhancer Hoffman
Enhancer Andersson
DHS Trynka
DGF ENCODE
CTCF Hoffman
Conserved LindbladToh
Coding UCSC
Annotation
−10
0
10
Enrichment (prop h2 / prop snps)

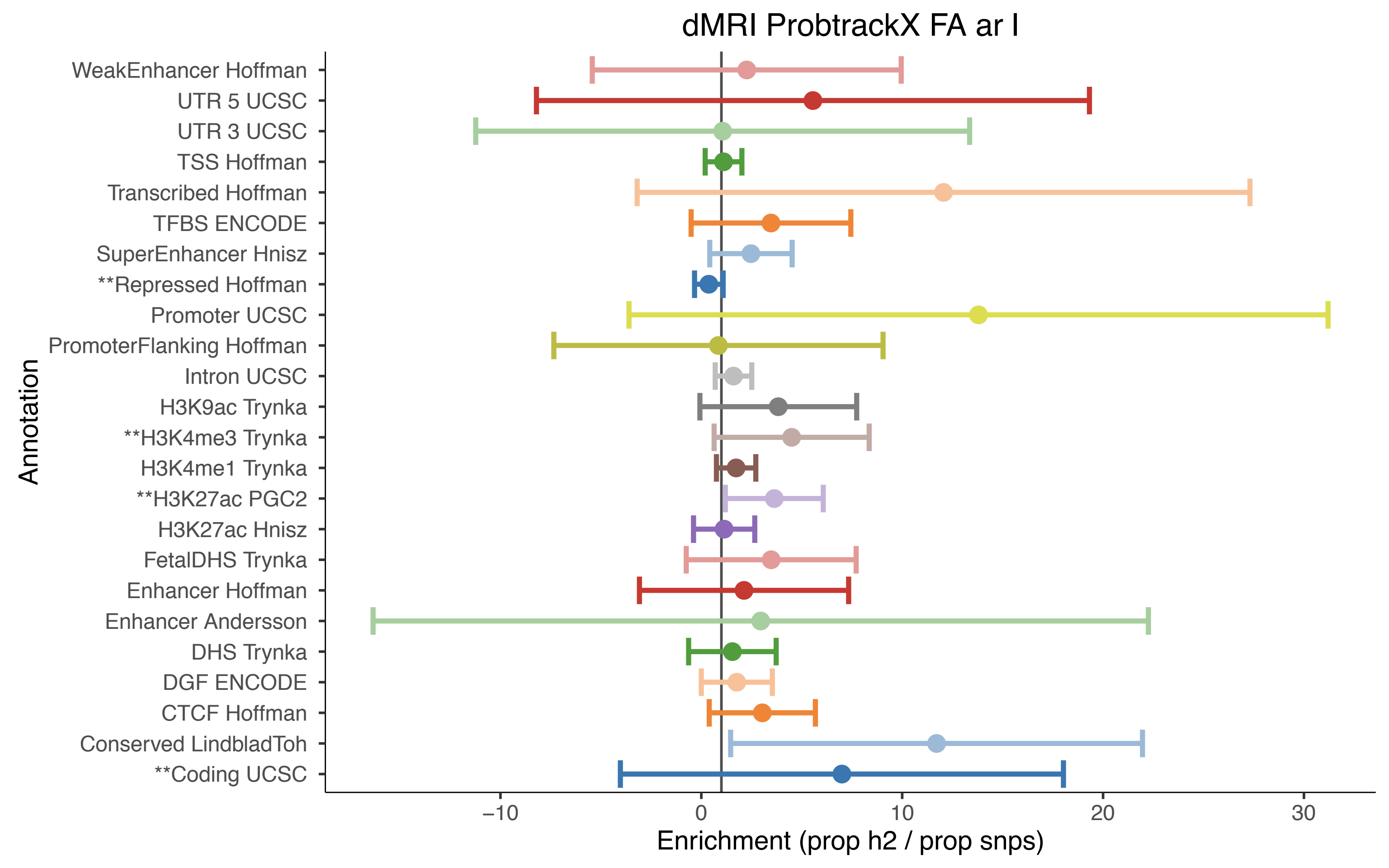
dMRI ProbtrackX FA ar l
WeakEnhancer Hoffman
UTR 5 UCSC
UTR 3 UCSC
TSS Hoffman
Transcribed Hoffman
TFBS ENCODE
SuperEnhancer Hnisz
**Repressed Hoffman
Promoter UCSC
PromoterFlanking Hoffman
Intron UCSC
H3K9ac Trynka
**H3K4me3 Trynka
H3K4me1 Trynka
**H3K27ac PGC2
H3K27ac Hnisz
FetalDHS Trynka
Enhancer Hoffman
Enhancer Andersson
DHS Trynka
DGF ENCODE
CTCF Hoffman
Conserved LindbladToh
**Coding UCSC
Annotation
−10
0
10
20
30
Enrichment (prop h2 / prop snps)

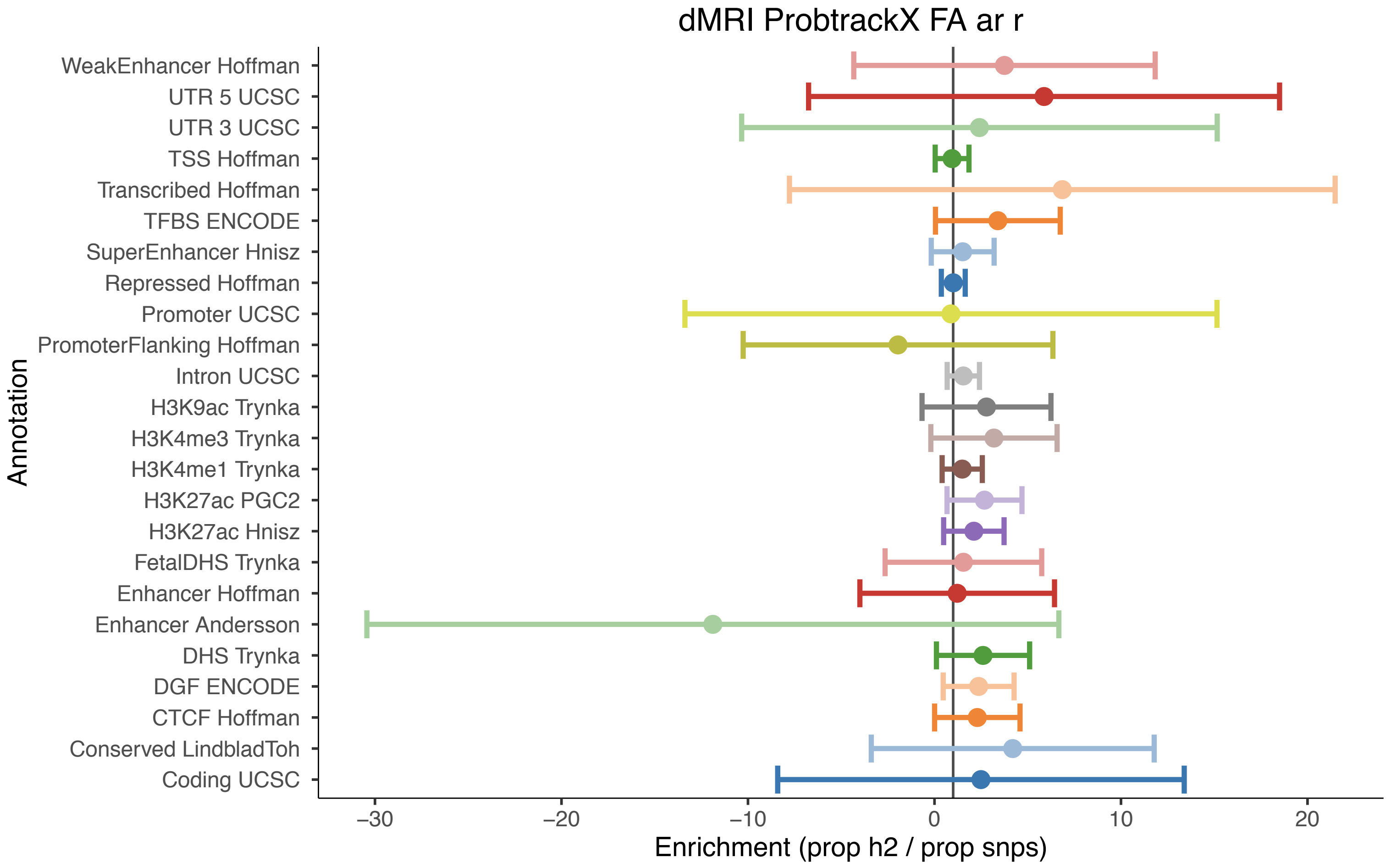
dMRI ProbtrackX FA ar r
WeakEnhancer Hoffman
UTR 5 UCSC
UTR 3 UCSC
TSS Hoffman
Transcribed Hoffman
TFBS ENCODE
SuperEnhancer Hnisz
Repressed Hoffman
Promoter UCSC
PromoterFlanking Hoffman
Intron UCSC
H3K9ac Trynka
H3K4me3 Trynka
H3K4me1 Trynka
H3K27ac PGC2
H3K27ac Hnisz
FetalDHS Trynka
Enhancer Hoffman
Enhancer Andersson
DHS Trynka
DGF ENCODE
CTCF Hoffman
Conserved LindbladToh
Coding UCSC
Annotation
−30
−20
−10
0
10
20
Enrichment (prop h2 / prop snps)

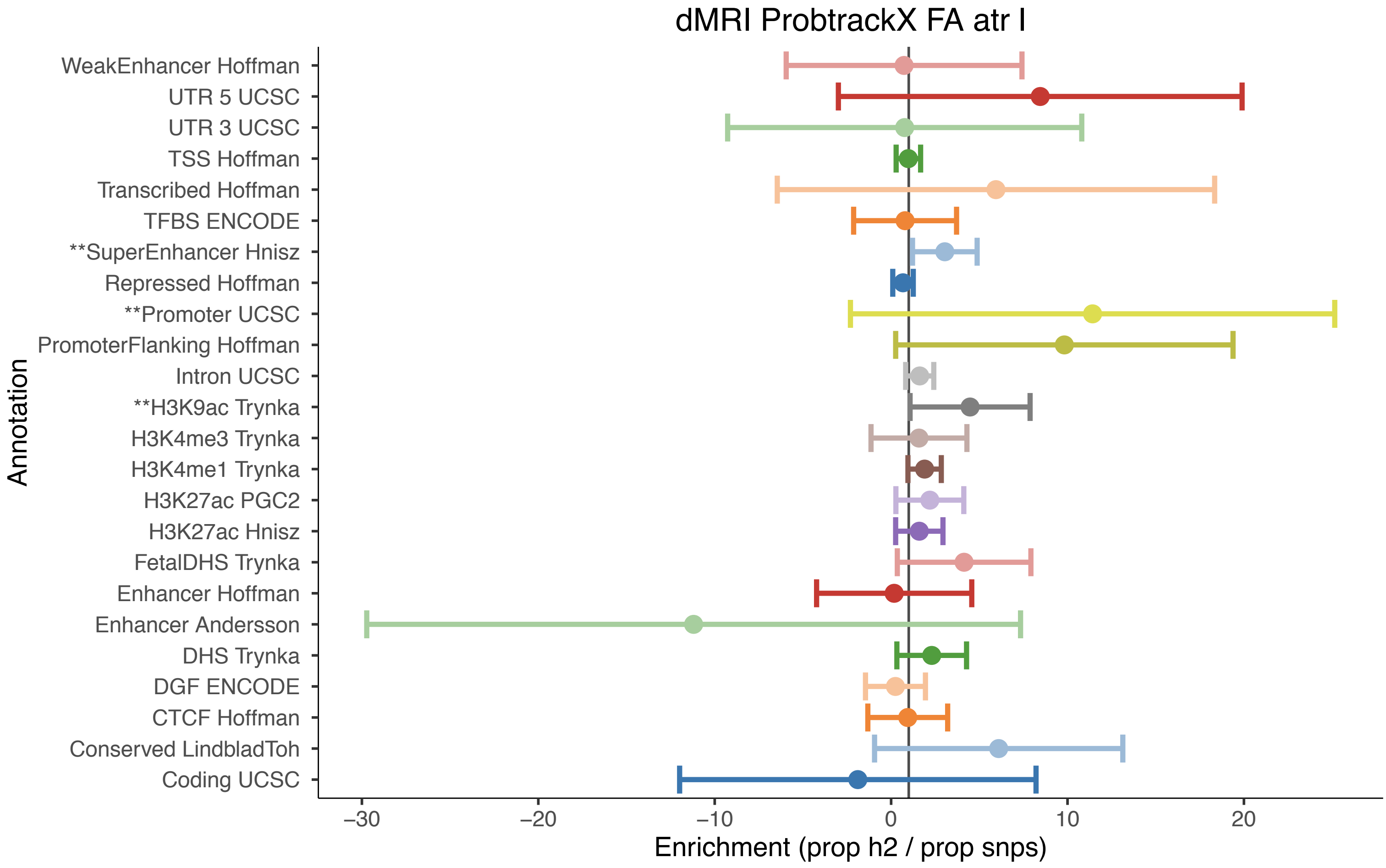
dMRI ProbtrackX FA atr l
WeakEnhancer Hoffman
UTR 5 UCSC
UTR 3 UCSC
TSS Hoffman
Transcribed Hoffman
TFBS ENCODE
**SuperEnhancer Hnisz
Repressed Hoffman
**Promoter UCSC
PromoterFlanking Hoffman
Intron UCSC
**H3K9ac Trynka
H3K4me3 Trynka
H3K4me1 Trynka
H3K27ac PGC2
H3K27ac Hnisz
FetalDHS Trynka
Enhancer Hoffman
Enhancer Andersson
DHS Trynka
DGF ENCODE
CTCF Hoffman
Conserved LindbladToh
Coding UCSC
Annotation
−30
−20
−10
0
10
20
Enrichment (prop h2 / prop snps)

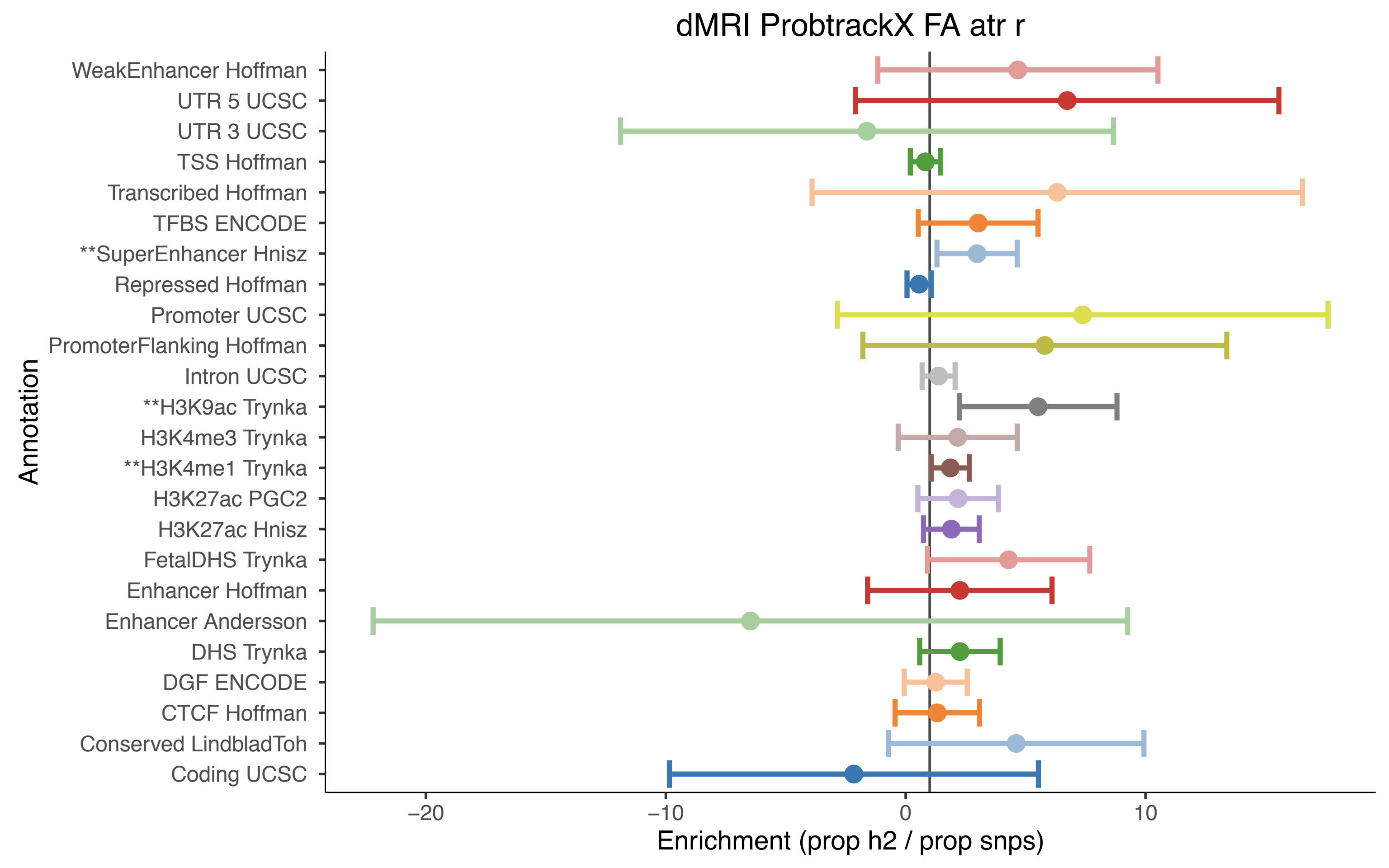

dMRI ProbtrackX FA atr r
WeakEnhancer Hoffman
UTR 5 UCSC
UTR 3 UCSC
TSS Hoffman
Transcribed Hoffman
TFBS ENCODE
**SuperEnhancer Hnisz
Repressed Hoffman
Promoter UCSC
PromoterFlanking Hoffman
Intron UCSC
**H3K9ac Trynka
H3K4me3 Trynka
**H3K4me1 Trynka
H3K27ac PGC2
H3K27ac Hnisz
FetalDHS Trynka
Enhancer Hoffman
Enhancer Andersson
DHS Trynka
DGF ENCODE
CTCF Hoffman
Conserved LindbladToh
Coding UCSC
Annotation
−20
−10
0
10
Enrichment (prop h2 / prop snps)

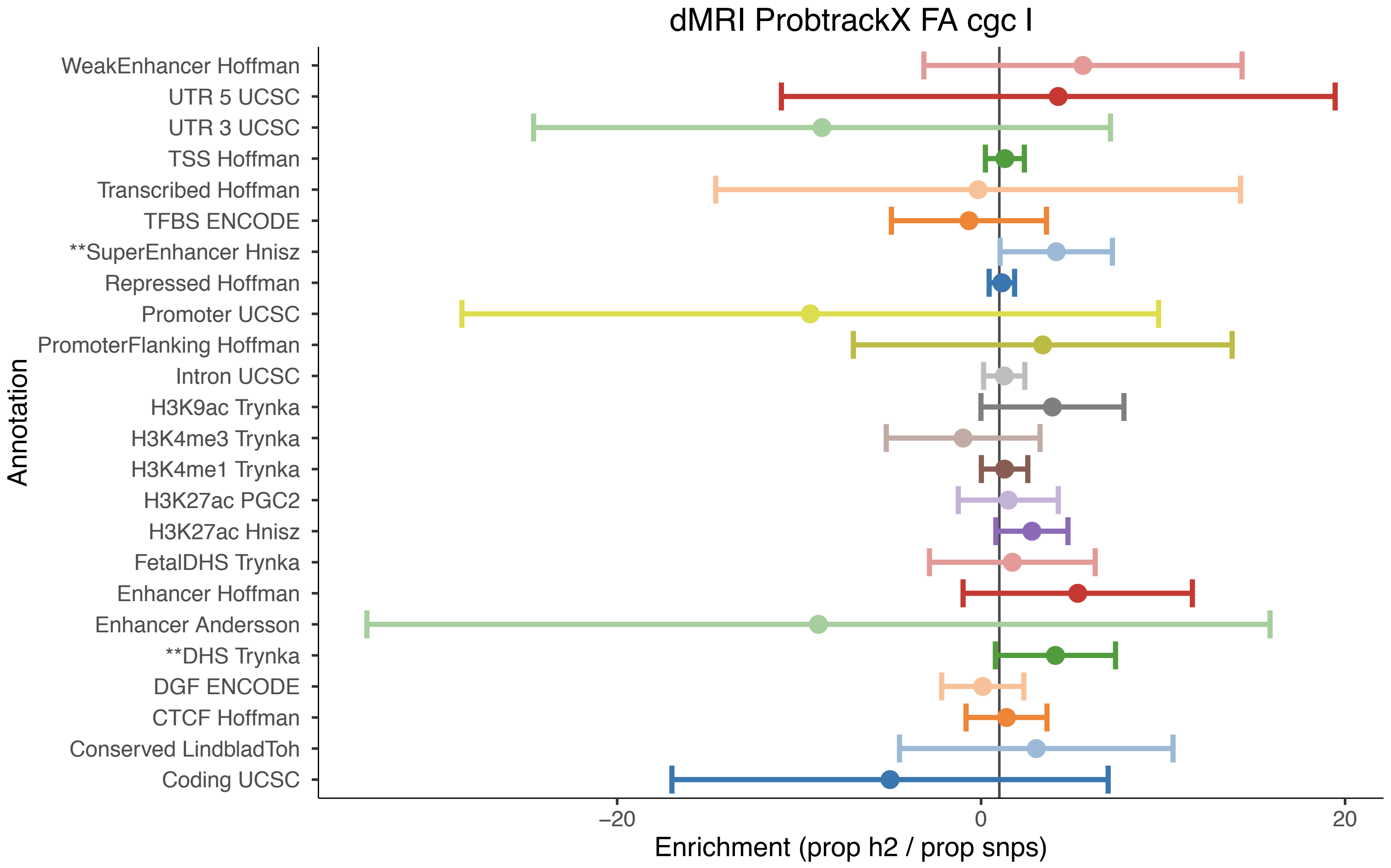

dMRI ProbtrackX FA cgc l
WeakEnhancer Hoffman
UTR 5 UCSC
UTR 3 UCSC
TSS Hoffman
Transcribed Hoffman
TFBS ENCODE
**SuperEnhancer Hnisz
Repressed Hoffman
Promoter UCSC
PromoterFlanking Hoffman
Intron UCSC
H3K9ac Trynka
H3K4me3 Trynka
H3K4me1 Trynka
H3K27ac PGC2
H3K27ac Hnisz
FetalDHS Trynka
Enhancer Hoffman
Enhancer Andersson
**DHS Trynka
DGF ENCODE
CTCF Hoffman
Conserved LindbladToh
Coding UCSC
Annotation
−20
0
20
Enrichment (prop h2 / prop snps)

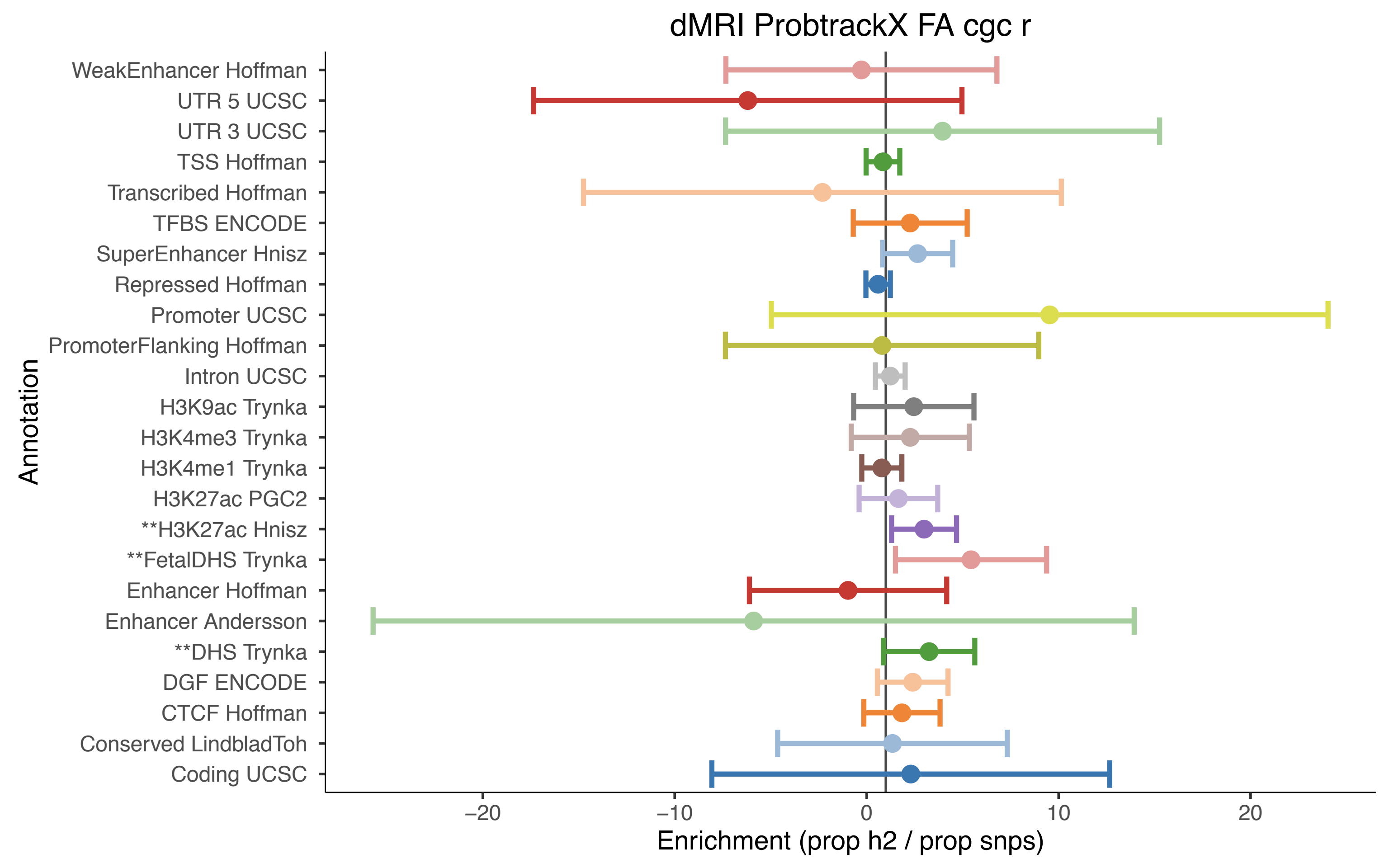
dMRI ProbtrackX FA cgc r
Annotation
WeakEnhancer Hoffman
UTR 5 UCSC
UTR 3 UCSC
TSS Hoffman
Transcribed Hoffman
TFBS ENCODE
SuperEnhancer Hnisz
Repressed Hoffman
Promoter UCSC
PromoterFlanking Hoffman
Intron UCSC
H3K9ac Trynka
H3K4me3 Trynka
H3K4me1 Trynka
H3K27ac PGC2
**H3K27ac Hnisz
**FetalDHS Trynka
Enhancer Hoffman
Enhancer Andersson
**DHS Trynka
DGF ENCODE
CTCF Hoffman
Conserved LindbladToh
Coding UCSC
−20
−10
0
10
20
Enrichment (prop h2 / prop snps)

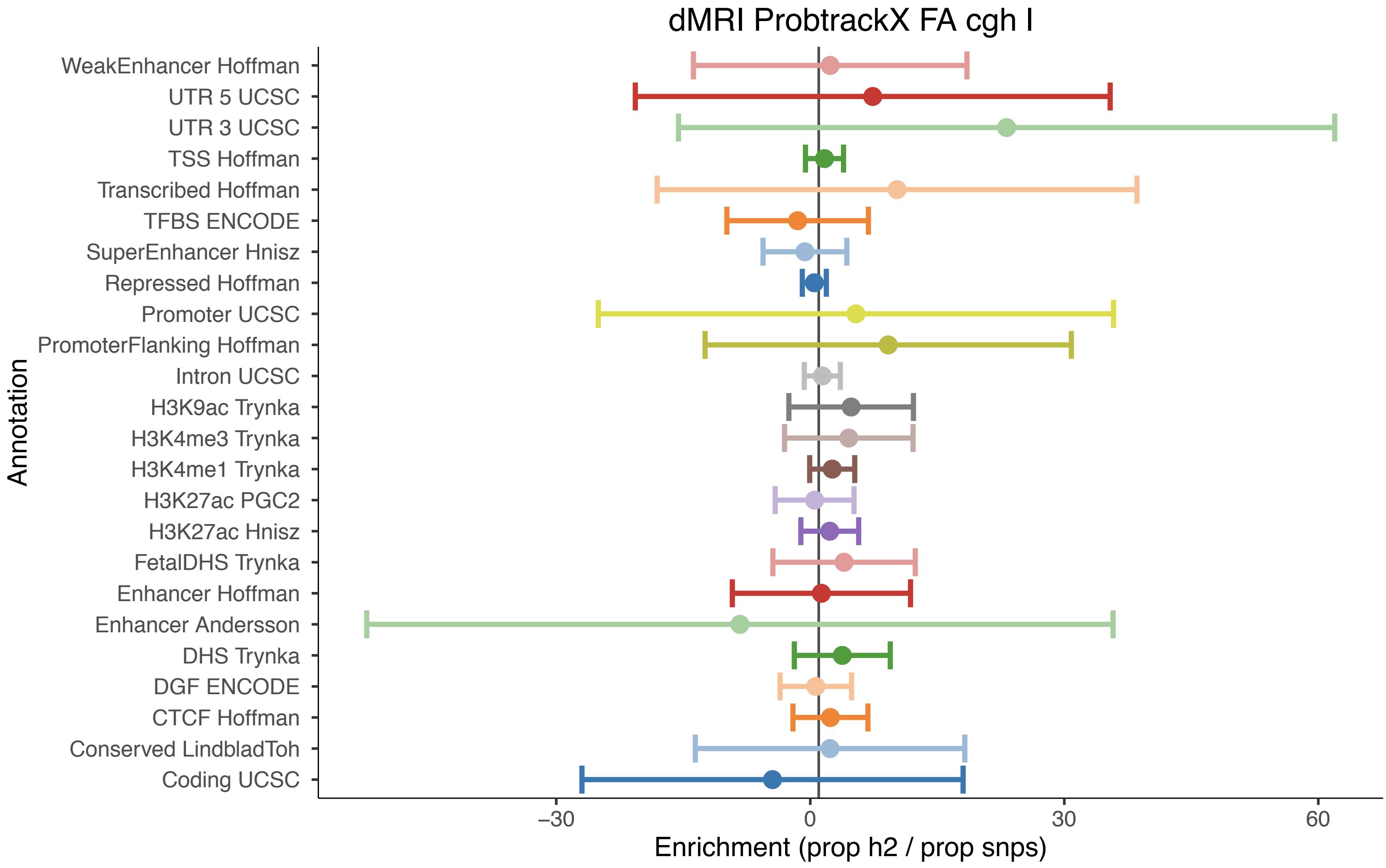
dMRI ProbtrackX FA cgh l
Annotation
WeakEnhancer Hoffman
UTR 5 UCSC
UTR 3 UCSC
TSS Hoffman
Transcribed Hoffman
TFBS ENCODE
SuperEnhancer Hnisz
Repressed Hoffman
Promoter UCSC
PromoterFlanking Hoffman
Intron UCSC
H3K9ac Trynka
H3K4me3 Trynka
H3K4me1 Trynka
H3K27ac PGC2
H3K27ac Hnisz
FetalDHS Trynka
Enhancer Hoffman
Enhancer Andersson
DHS Trynka
DGF ENCODE
CTCF Hoffman
Conserved LindbladToh
Coding UCSC
–30
0
30
60
Enrichment (prop h2 / prop snps)

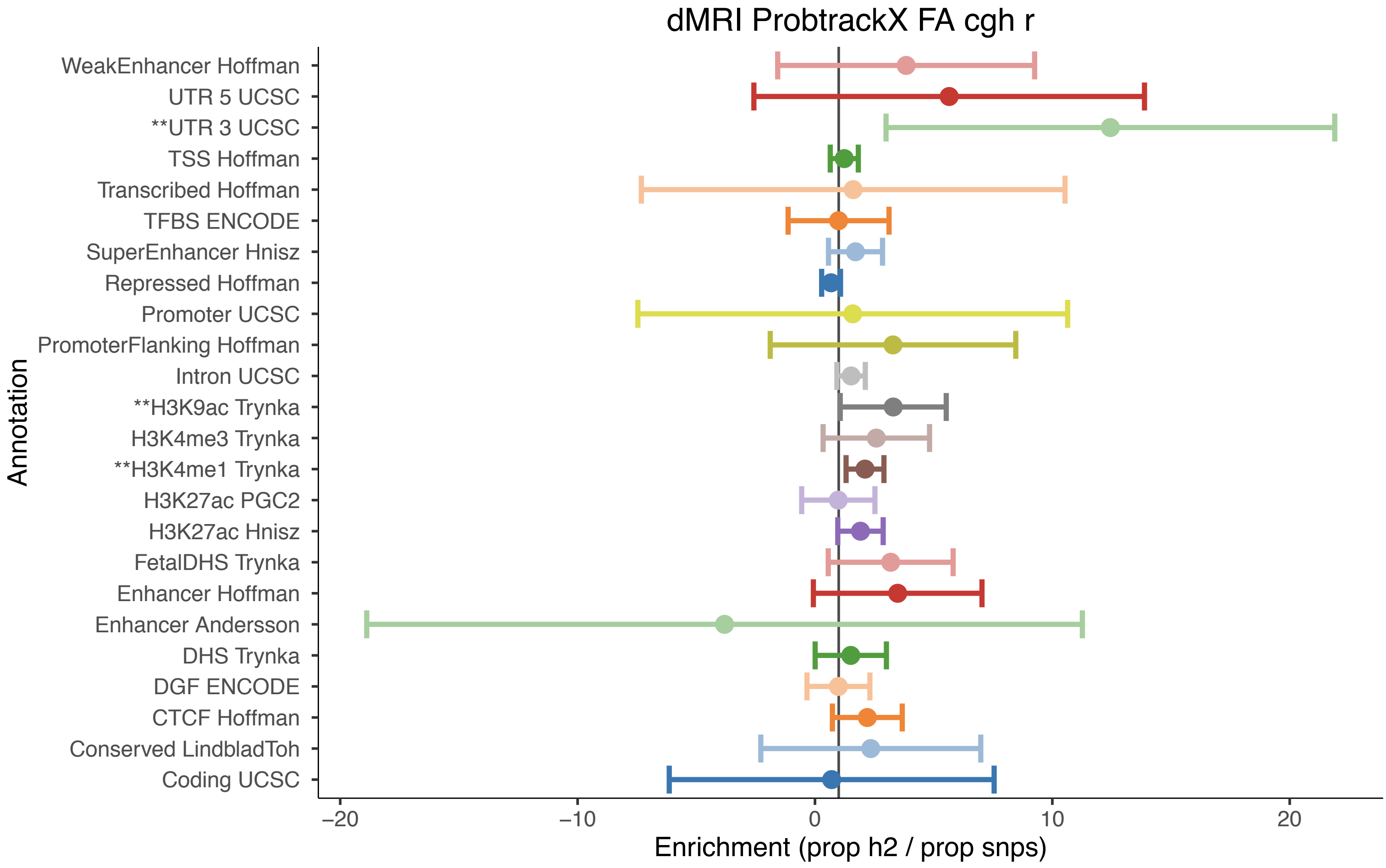
dMRI ProbtrackX FA cgh r
WeakEnhancer Hoffman
UTR 5 UCSC
**UTR 3 UCSC
TSS Hoffman
Transcribed Hoffman
TFBS ENCODE
SuperEnhancer Hnisz
Repressed Hoffman
Promoter UCSC
PromoterFlanking Hoffman
Intron UCSC
**H3K9ac Trynka
H3K4me3 Trynka
**H3K4me1 Trynka
H3K27ac PGC2
H3K27ac Hnisz
FetalDHS Trynka
Enhancer Hoffman
Enhancer Andersson
DHS Trynka
DGF ENCODE
CTCF Hoffman
Conserved LindbladToh
Coding UCSC
Annotation
−20
−10
0
10
20
Enrichment (prop h2 / prop snps)

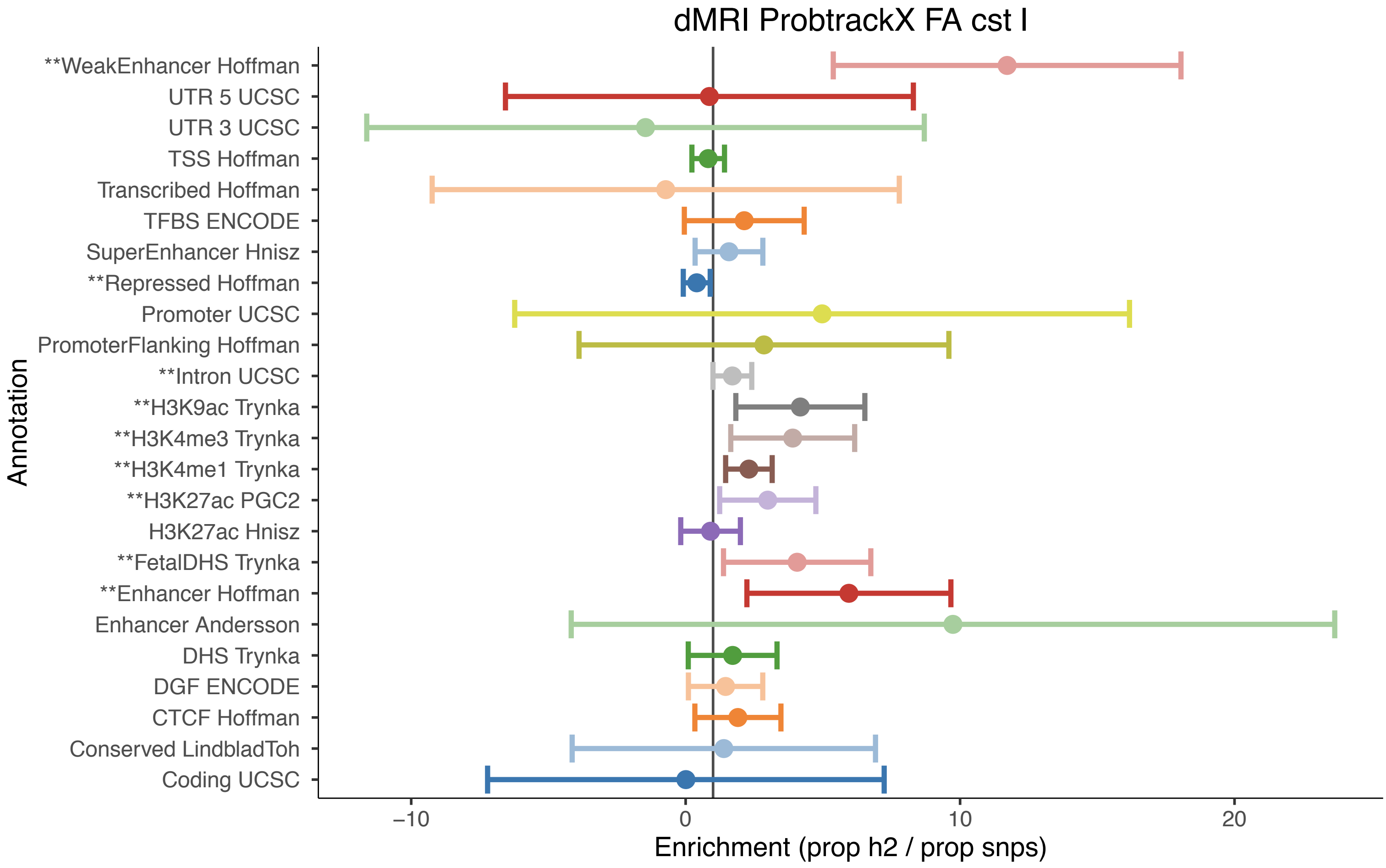

dMRI ProbtrackX FA cst l
**WeakEnhancer Hoffman
UTR 5 UCSC
UTR 3 UCSC
TSS Hoffman
Transcribed Hoffman
TFBS ENCODE
SuperEnhancer Hnisz
**Repressed Hoffman
Promoter UCSC
PromoterFlanking Hoffman
**Intron UCSC
**H3K9ac Trynka
**H3K4me3 Trynka
**H3K4me1 Trynka
**H3K27ac PGC2
H3K27ac Hnisz
**FetalDHS Trynka
**Enhancer Hoffman
Enhancer Andersson
DHS Trynka
DGF ENCODE
CTCF Hoffman
Conserved LindbladToh
Coding UCSC
Annotation
−10
0
10
20
Enrichment (prop h2 / prop snps)

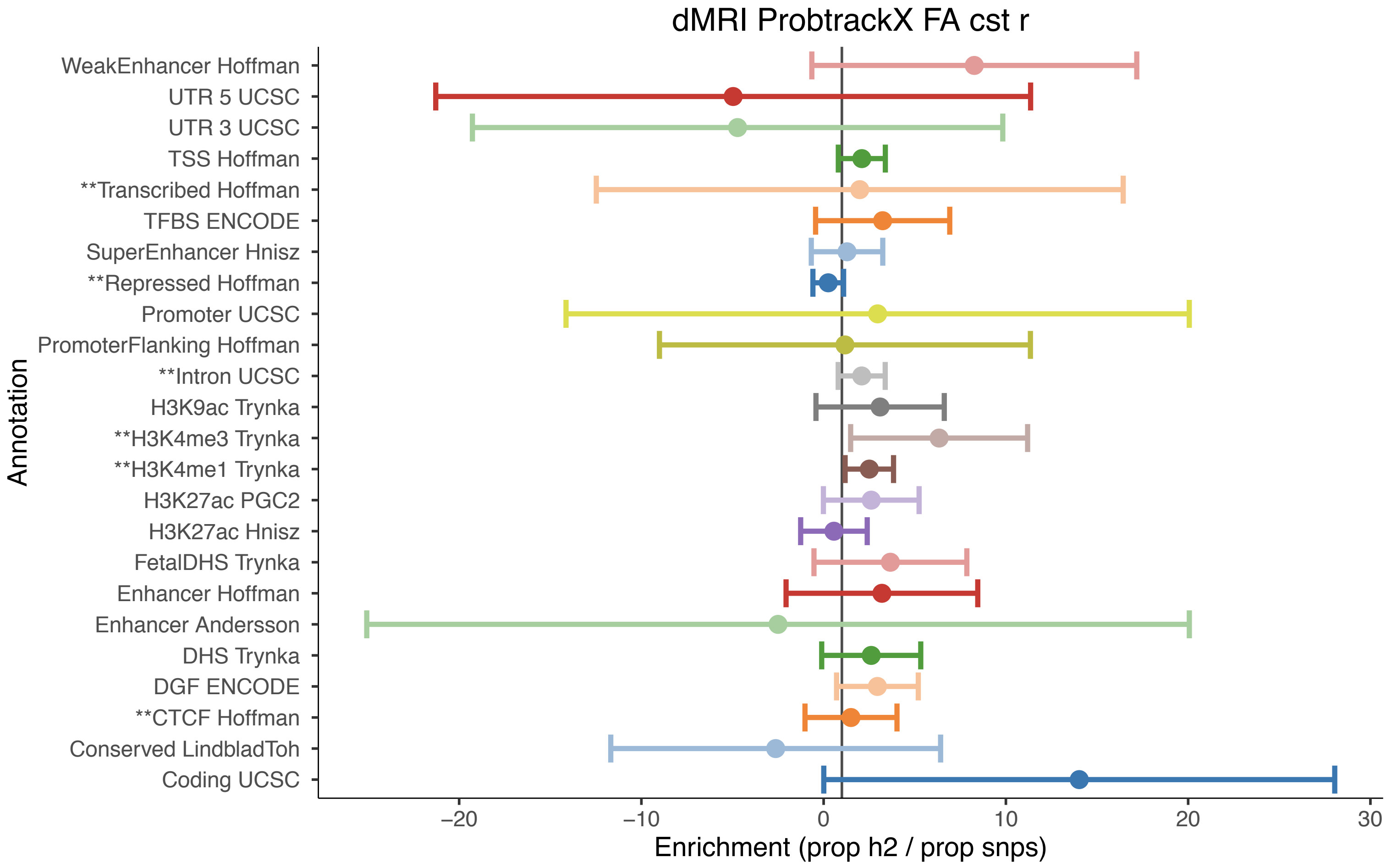
dMRI ProbtrackX FA cst r
WeakEnhancer Hoffman
UTR 5 UCSC
UTR 3 UCSC
TSS Hoffman
**Transcribed Hoffman
TFBS ENCODE
SuperEnhancer Hnisz
**Repressed Hoffman
Promoter UCSC
PromoterFlanking Hoffman
**Intron UCSC
H3K9ac Trynka
**H3K4me3 Trynka
**H3K4me1 Trynka
H3K27ac PGC2
H3K27ac Hnisz
FetalDHS Trynka
Enhancer Hoffman
Enhancer Andersson
DHS Trynka
DGF ENCODE
**CTCF Hoffman
Conserved LindbladToh
Coding UCSC
Annotation
−20
−10
0
10
20
30
Enrichment (prop h2 / prop snps)

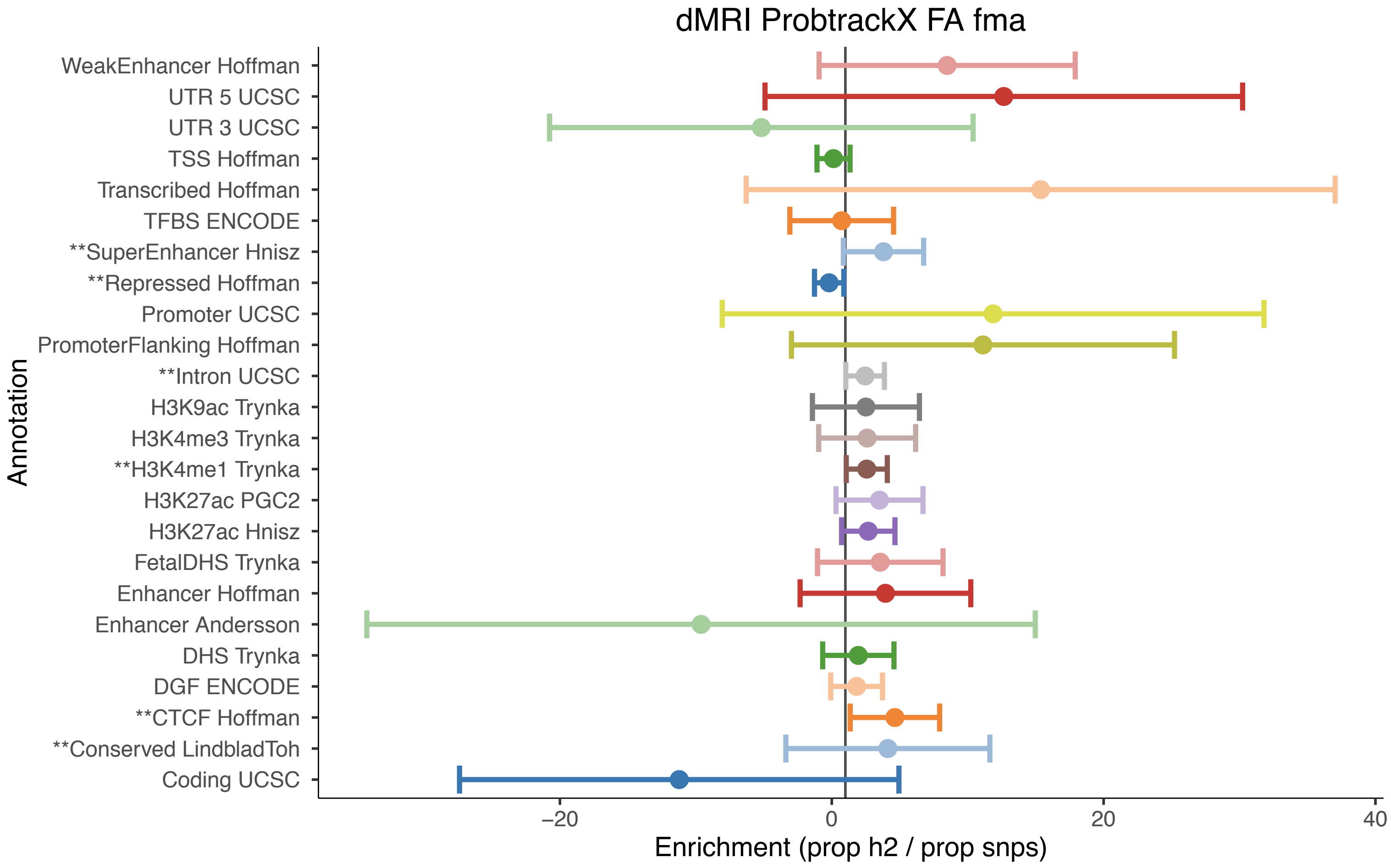

dMRI ProbtrackX FA fma
WeakEnhancer Hoffman
UTR 5 UCSC
UTR 3 UCSC
TSS Hoffman
Transcribed Hoffman
TFBS ENCODE
**SuperEnhancer Hnisz
**Repressed Hoffman
Promoter UCSC
PromoterFlanking Hoffman
**Intron UCSC
H3K9ac Trynka
H3K4me3 Trynka
**H3K4me1 Trynka
H3K27ac PGC2
H3K27ac Hnisz
FetalDHS Trynka
Enhancer Hoffman
Enhancer Andersson
DHS Trynka
DGF ENCODE
**CTCF Hoffman
**Conserved LindbladToh
Coding UCSC
Annotation
−20
0
20
40
Enrichment (prop h2 / prop snps)

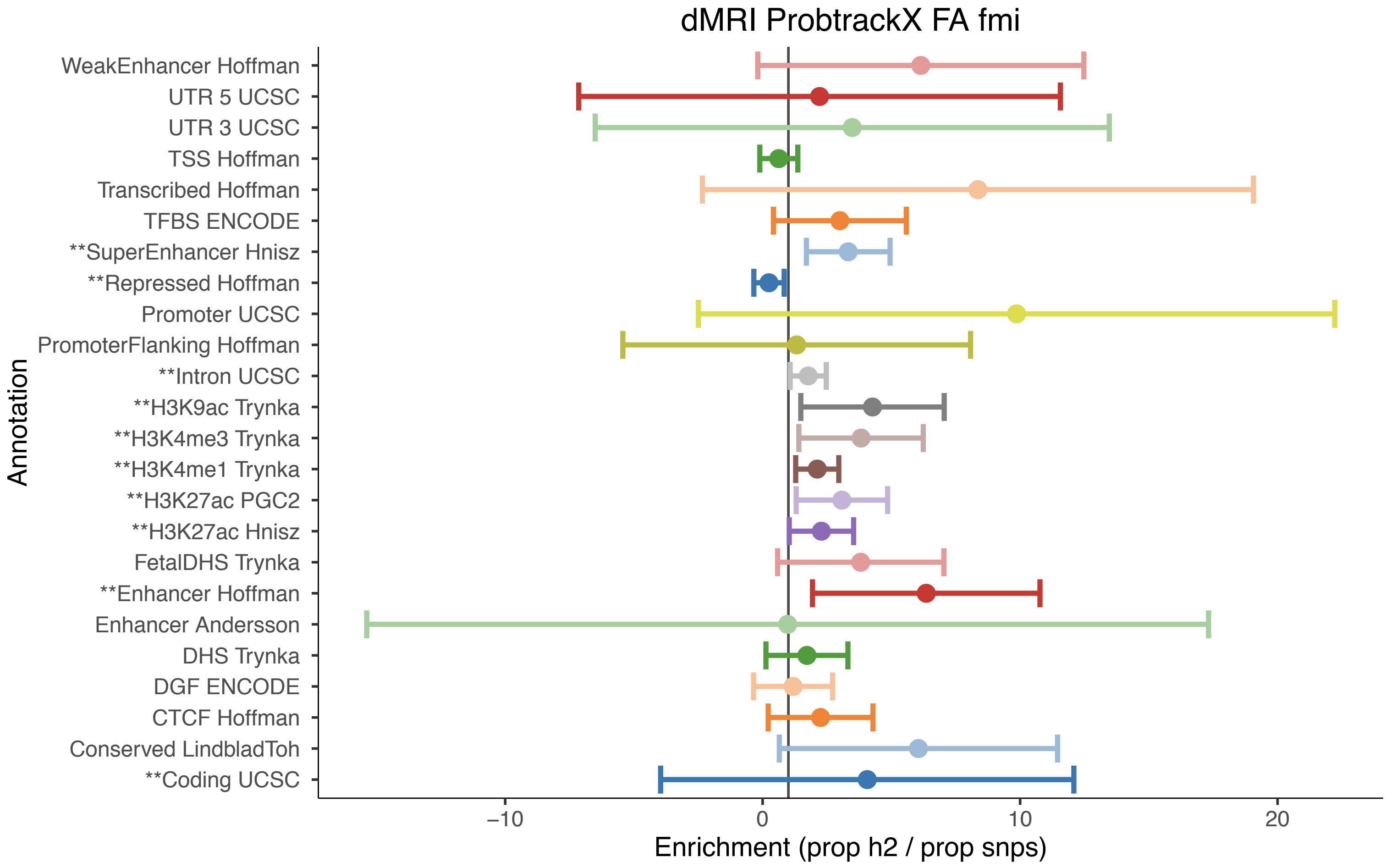
dMRI ProbtrackX FA fmi
WeakEnhancer Hoffman
UTR 5 UCSC
UTR 3 UCSC
TSS Hoffman
Transcribed Hoffman
TFBS ENCODE
**SuperEnhancer Hnisz
**Repressed Hoffman
Promoter UCSC
PromoterFlanking Hoffman
**Intron UCSC
**H3K9ac Trynka
**H3K4me3 Trynka
**H3K4me1 Trynka
**H3K27ac PGC2
**H3K27ac Hnisz
FetalDHS Trynka
**Enhancer Hoffman
Enhancer Andersson
DHS Trynka
DGF ENCODE
CTCF Hoffman
Conserved LindbladToh
**Coding UCSC
Annotation
−10
0
10
20
Enrichment (prop h2 / prop snps)

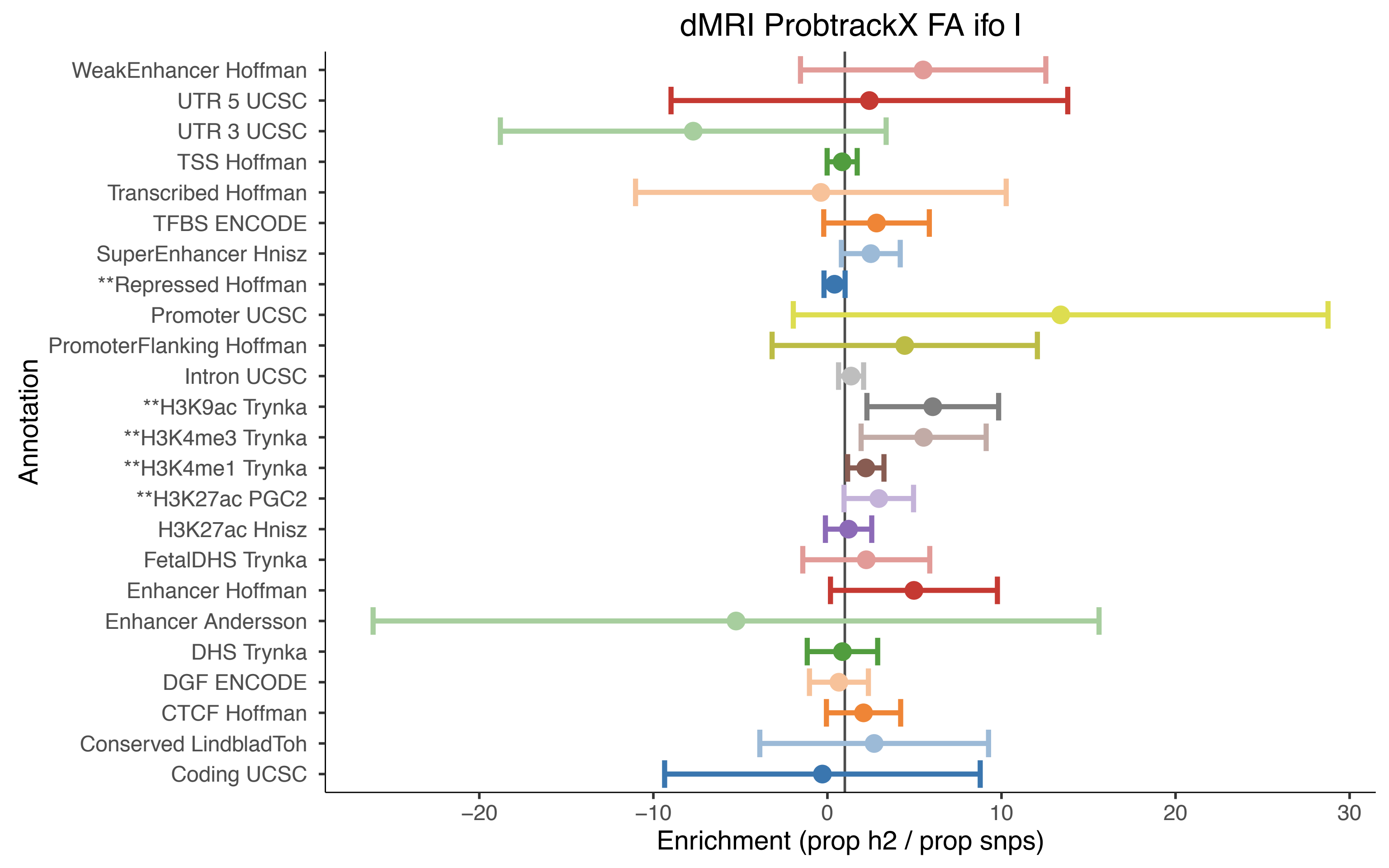

dMRI ProbtrackX FA ifo l
WeakEnhancer Hoffman
UTR 5 UCSC
UTR 3 UCSC
TSS Hoffman
Transcribed Hoffman
TFBS ENCODE
SuperEnhancer Hnisz
**Repressed Hoffman
Promoter UCSC
PromoterFlanking Hoffman
Intron UCSC
**H3K9ac Trynka
**H3K4me3 Trynka
**H3K4me1 Trynka
**H3K27ac PGC2
H3K27ac Hnisz
FetalDHS Trynka
Enhancer Hoffman
Enhancer Andersson
DHS Trynka
DGF ENCODE
CTCF Hoffman
Conserved LindbladToh
Coding UCSC
Annotation
−20
−10
0
10
20
30
Enrichment (prop h2 / prop snps)

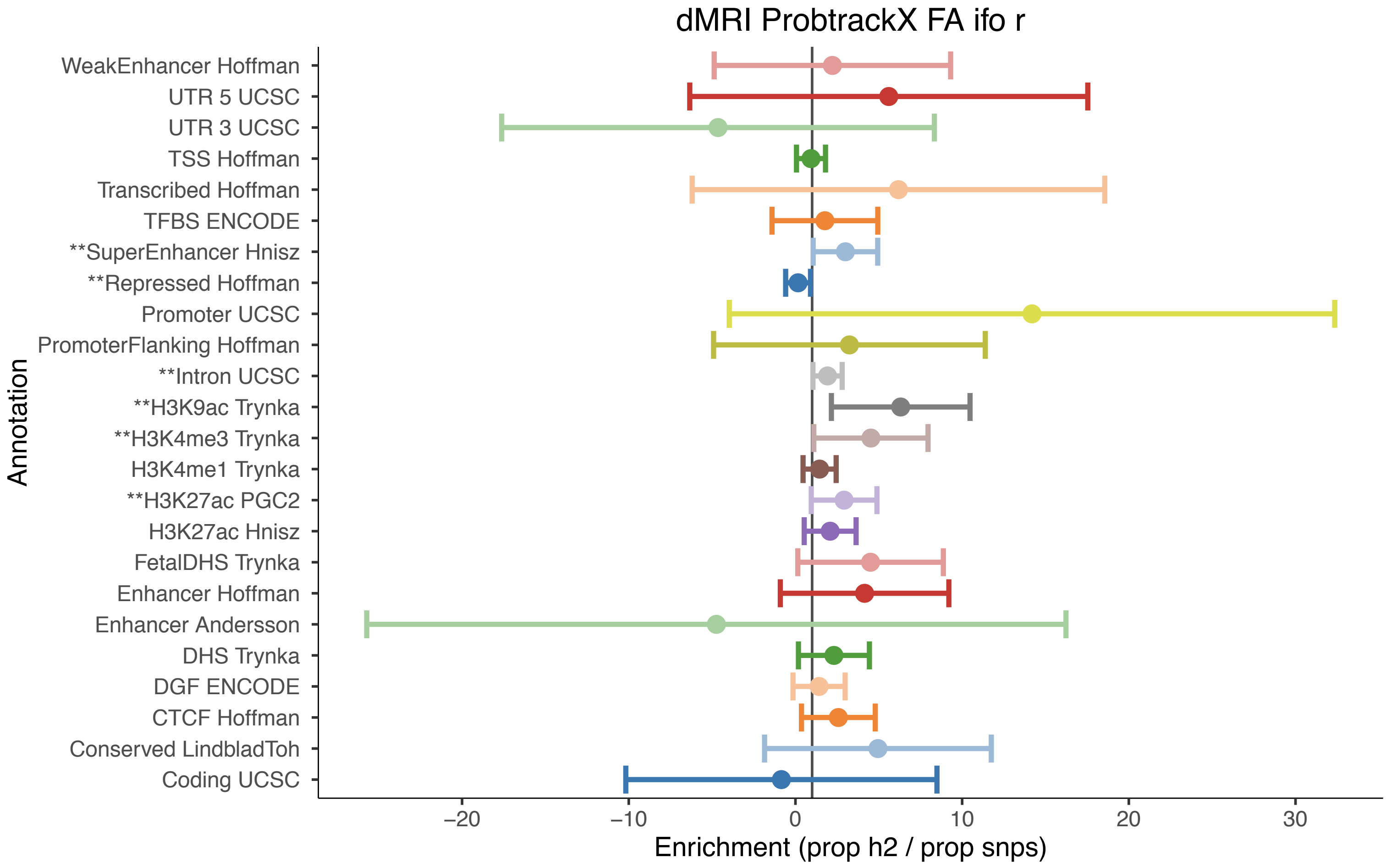
dMRI ProbtrackX FA ifo r
WeakEnhancer Hoffman
UTR 5 UCSC
UTR 3 UCSC
TSS Hoffman
Transcribed Hoffman
TFBS ENCODE
**SuperEnhancer Hnisz
**Repressed Hoffman
Promoter UCSC
PromoterFlanking Hoffman
**Intron UCSC
**H3K9ac Trynka
**H3K4me3 Trynka
H3K4me1 Trynka
**H3K27ac PGC2
H3K27ac Hnisz
FetalDHS Trynka
Enhancer Hoffman
Enhancer Andersson
DHS Trynka
DGF ENCODE
CTCF Hoffman
Conserved LindbladToh
Coding UCSC
Annotation
−20
−10
0
10
20
30
Enrichment (prop h2 / prop snps)

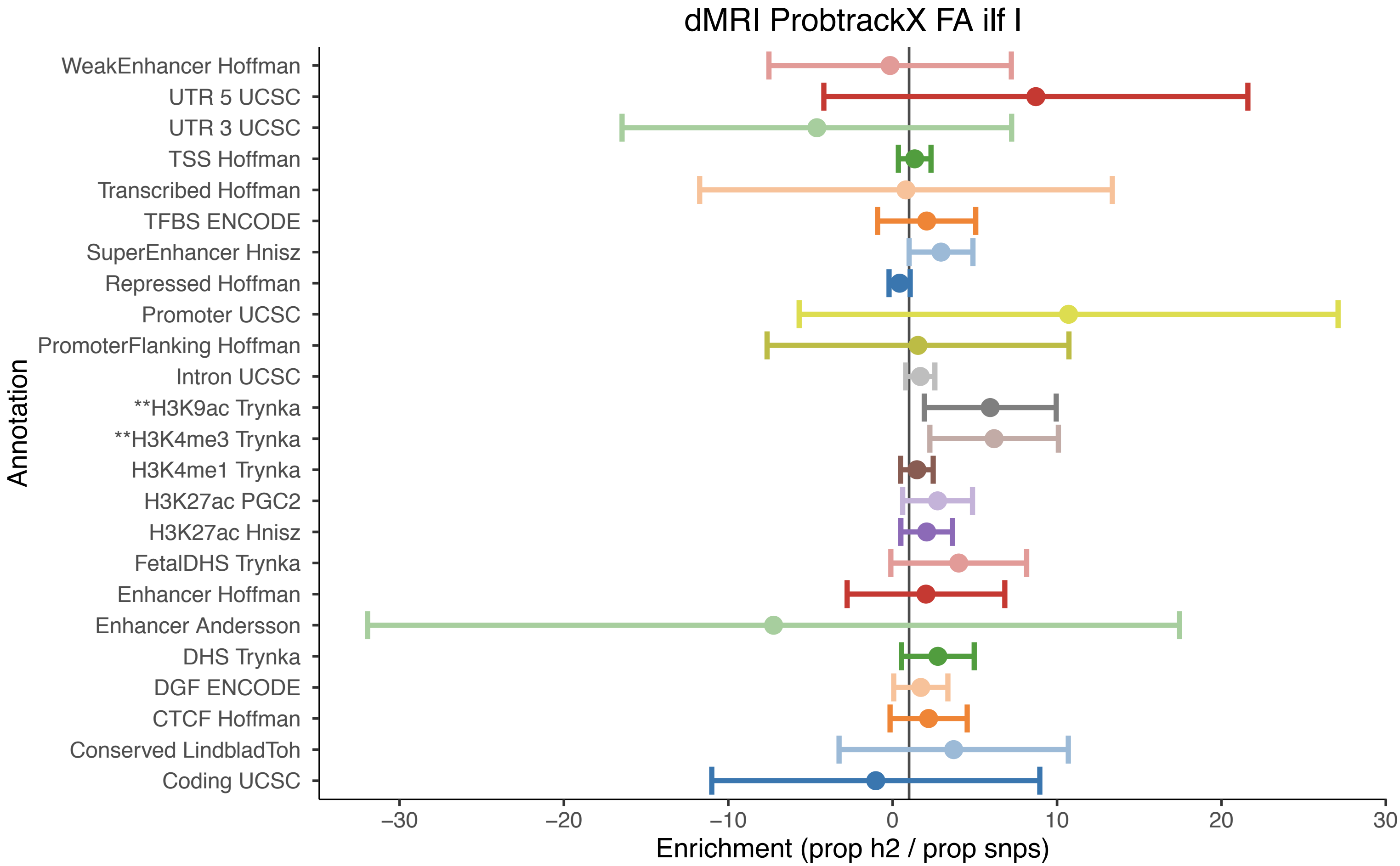
dMRI ProbtrackX FA ilf l
WeakEnhancer Hoffman
UTR 5 UCSC
UTR 3 UCSC
TSS Hoffman
Transcribed Hoffman
TFBS ENCODE
SuperEnhancer Hnisz
Repressed Hoffman
Promoter UCSC
PromoterFlanking Hoffman
Intron UCSC
**H3K9ac Trynka
**H3K4me3 Trynka
H3K4me1 Trynka
H3K27ac PGC2
H3K27ac Hnisz
FetalDHS Trynka
Enhancer Hoffman
Enhancer Andersson
DHS Trynka
DGF ENCODE
CTCF Hoffman
Conserved LindbladToh
Coding UCSC
Annotation
−30
−20
−10
0
10
20
30
Enrichment (prop h2 / prop snps)

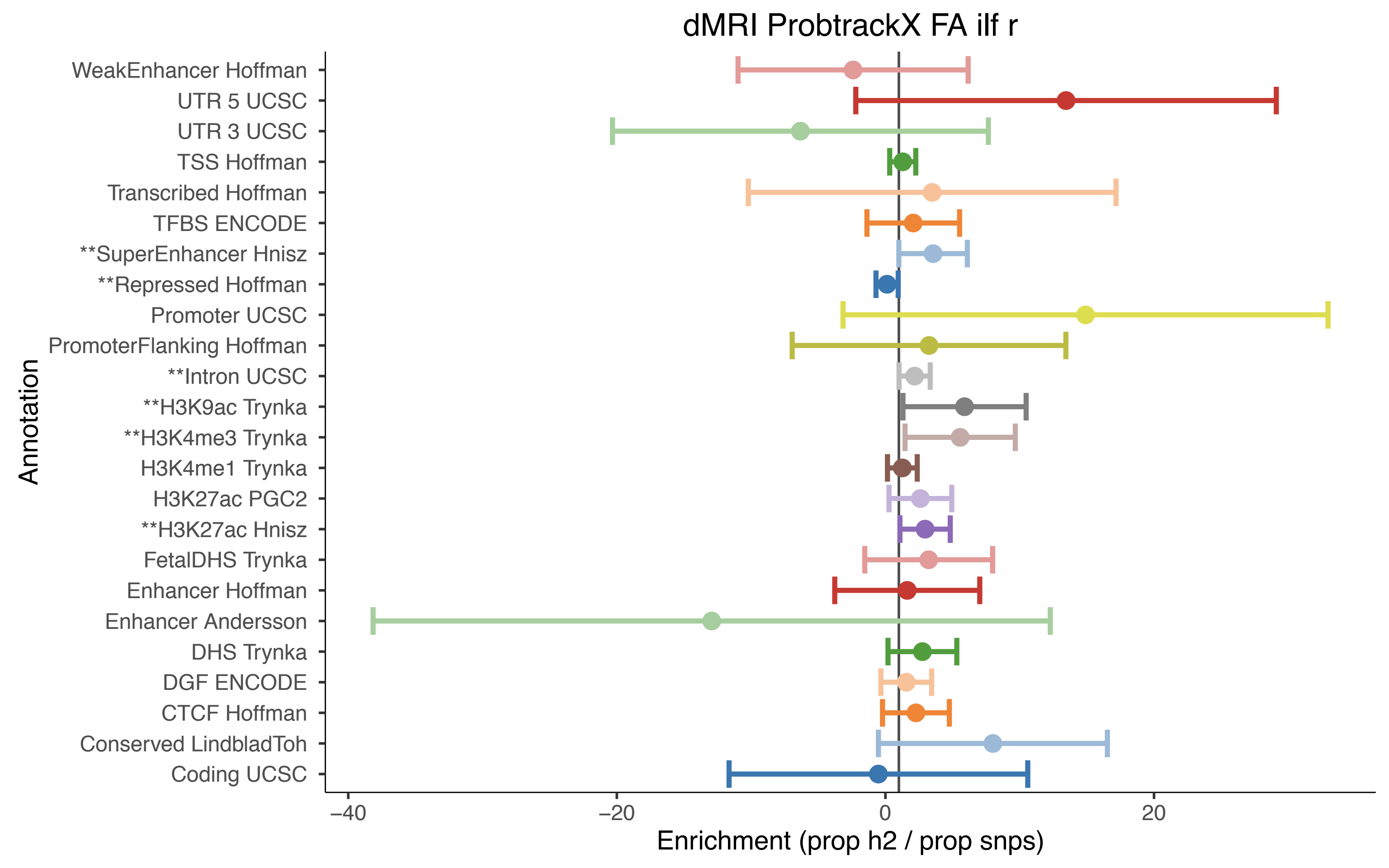
dMRI ProbtrackX FA ilf r
WeakEnhancer Hoffman
UTR 5 UCSC
UTR 3 UCSC
TSS Hoffman
Transcribed Hoffman
TFBS ENCODE
**SuperEnhancer Hnisz
**Repressed Hoffman
Promoter UCSC
PromoterFlanking Hoffman
**Intron UCSC
**H3K9ac Trynka
**H3K4me3 Trynka
H3K4me1 Trynka
H3K27ac PGC2
**H3K27ac Hnisz
FetalDHS Trynka
Enhancer Hoffman
Enhancer Andersson
DHS Trynka
DGF ENCODE
CTCF Hoffman
Conserved LindbladToh
Coding UCSC
Annotation
−40
−20
0
20
Enrichment (prop h2 / prop snps)

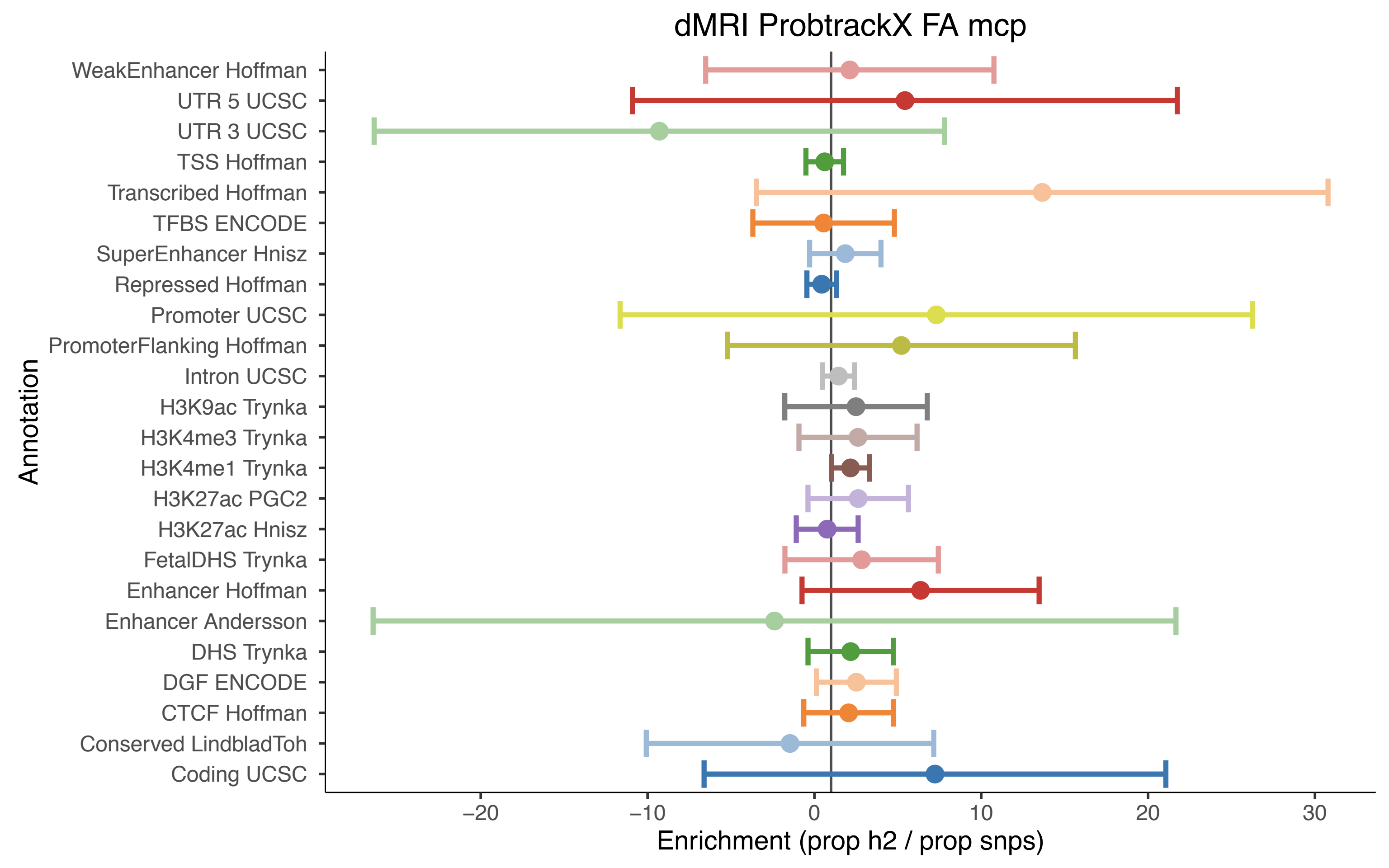
dMRI ProbtrackX FA mcp
Annotation
WeakEnhancer Hoffman
UTR 5 UCSC
UTR 3 UCSC
TSS Hoffman
Transcribed Hoffman
TFBS ENCODE
SuperEnhancer Hnisz
Repressed Hoffman
Promoter UCSC
PromoterFlanking Hoffman
Intron UCSC
H3K9ac Trynka
H3K4me3 Trynka
H3K4me1 Trynka
H3K27ac PGC2
H3K27ac Hnisz
FetalDHS Trynka
Enhancer Hoffman
Enhancer Andersson
DHS Trynka
DGF ENCODE
CTCF Hoffman
Conserved LindbladToh
Coding UCSC
−20
−10
0
10
20
30
Enrichment (prop h2 / prop snps)

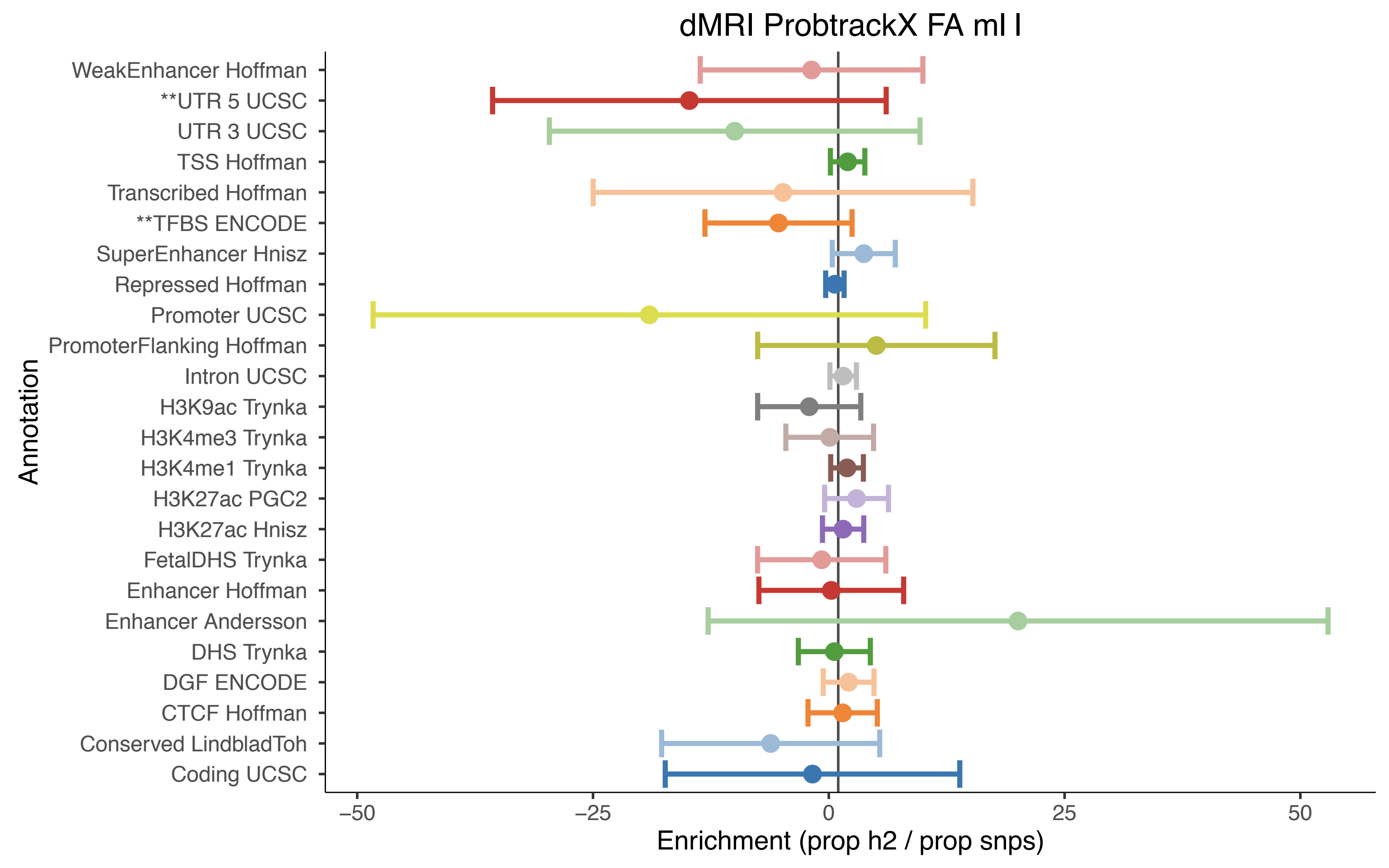

dMRI ProbtrackX FA ml l
WeakEnhancer Hoffman
**UTR 5 UCSC
UTR 3 UCSC
TSS Hoffman
Transcribed Hoffman
**TFBS ENCODE
SuperEnhancer Hnisz
Repressed Hoffman
Promoter UCSC
PromoterFlanking Hoffman
Intron UCSC
H3K9ac Trynka
H3K4me3 Trynka
H3K4me1 Trynka
H3K27ac PGC2
H3K27ac Hnisz
FetalDHS Trynka
Enhancer Hoffman
Enhancer Andersson
DHS Trynka
DGF ENCODE
CTCF Hoffman
Conserved LindbladToh
Coding UCSC
Annotation
−50
−25
0
25
50
Enrichment (prop h2 / prop snps)

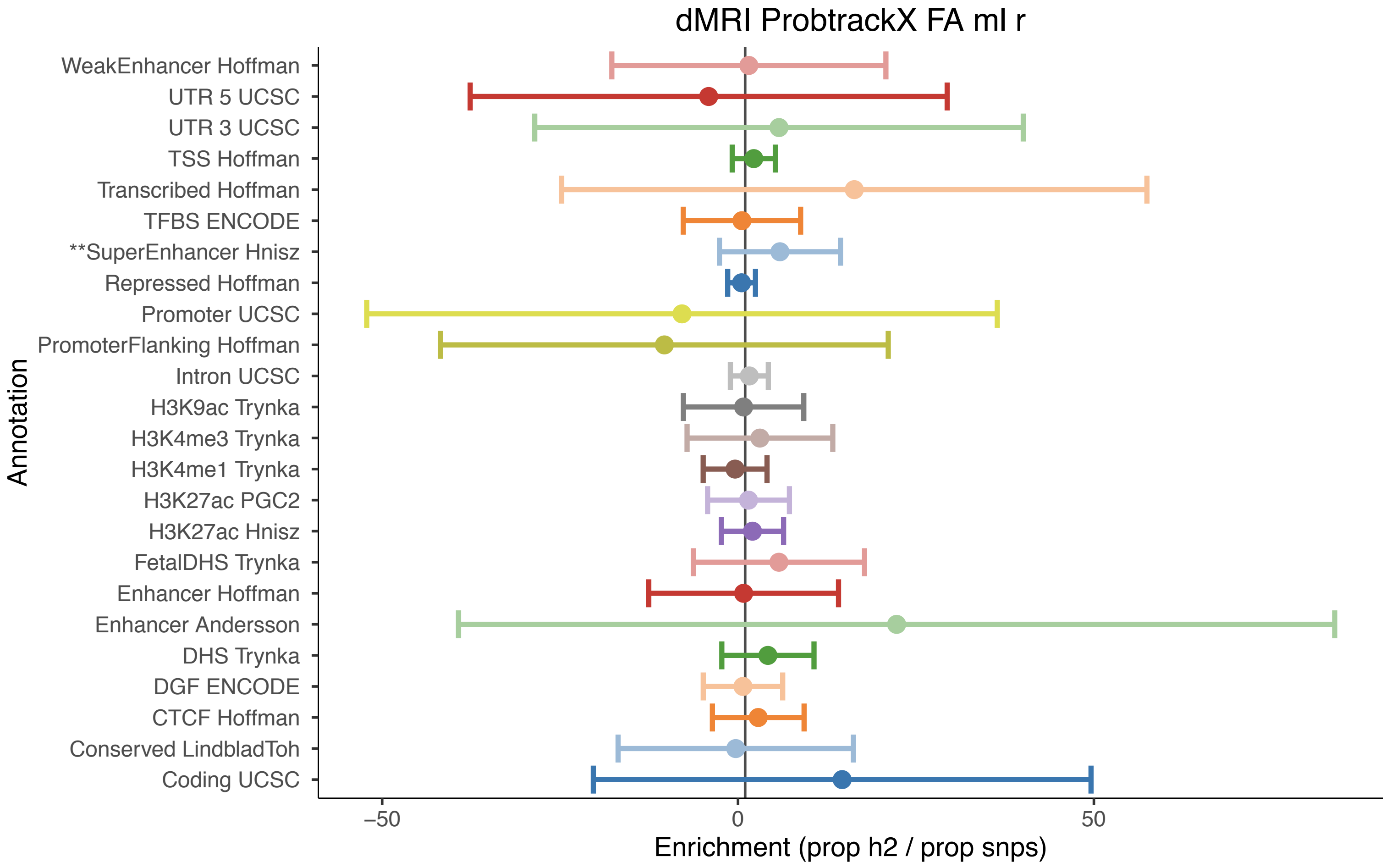

dMRI ProbtrackX FA ml r
WeakEnhancer Hoffman
UTR 5 UCSC
UTR 3 UCSC
TSS Hoffman
Transcribed Hoffman
TFBS ENCODE
**SuperEnhancer Hnisz
Repressed Hoffman
Promoter UCSC
PromoterFlanking Hoffman
Intron UCSC
H3K9ac Trynka
H3K4me3 Trynka
H3K4me1 Trynka
H3K27ac PGC2
H3K27ac Hnisz
FetalDHS Trynka
Enhancer Hoffman
Enhancer Andersson
DHS Trynka
DGF ENCODE
CTCF Hoffman
Conserved LindbladToh
Coding UCSC
Annotation
−50
0
50
Enrichment (prop h2 / prop snps)

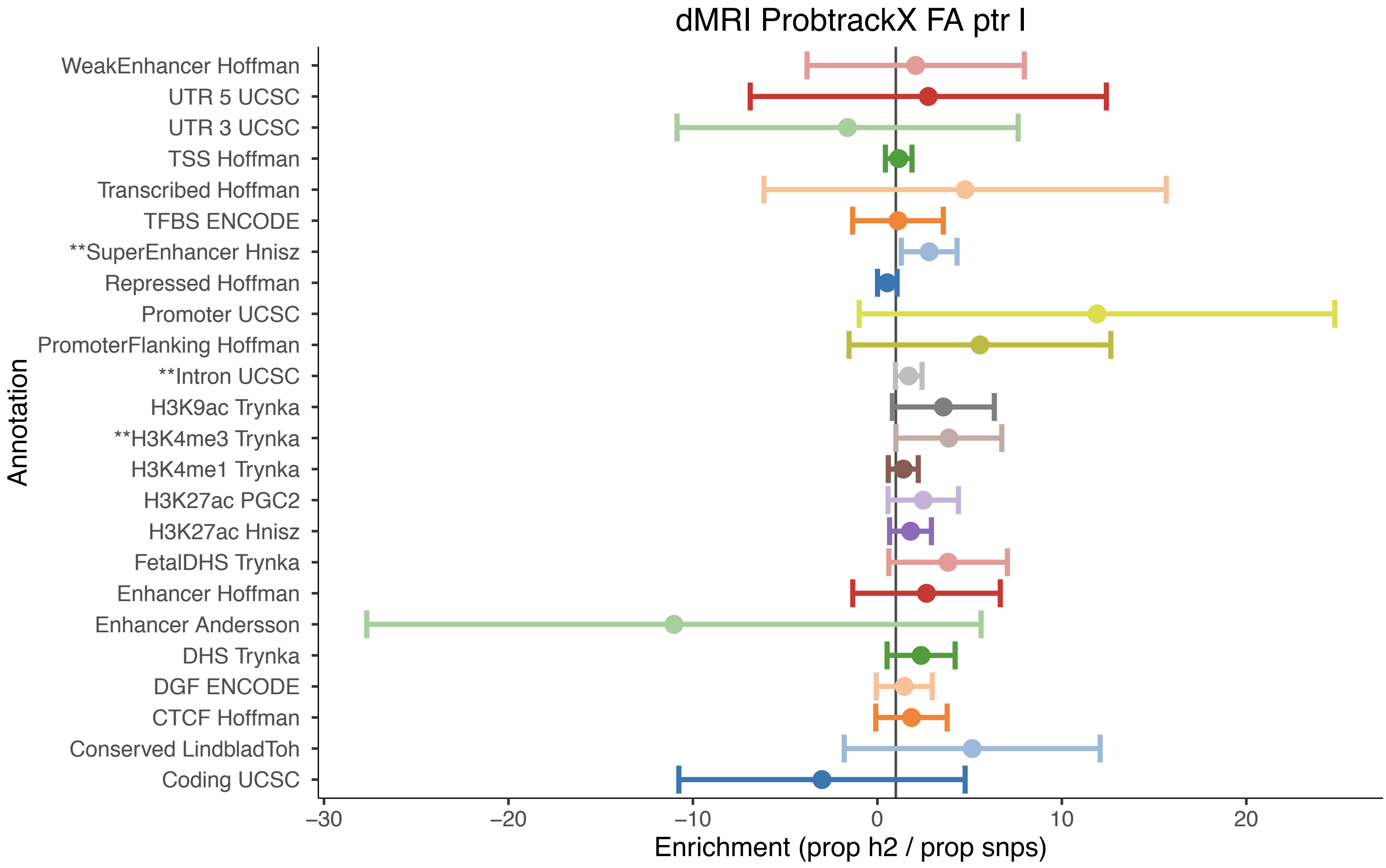
dMRI ProbtrackX FA ptr I
WeakEnhancer Hoffman
UTR 5 UCSC
UTR 3 UCSC
TSS Hoffman
Transcribed Hoffman
TFBS ENCODE
**SuperEnhancer Hnisz
Repressed Hoffman
Promoter UCSC
PromoterFlanking Hoffman
**Intron UCSC
H3K9ac Trynka
**H3K4me3 Trynka
H3K4me1 Trynka
H3K27ac PGC2
H3K27ac Hnisz
FetalDHS Trynka
Enhancer Hoffman
Enhancer Andersson
DHS Trynka
DGF ENCODE
CTCF Hoffman
Conserved LindbladToh
Coding UCSC
Annotation
−30
−20
−10
0
10
20
Enrichment (prop h2 / prop snps)

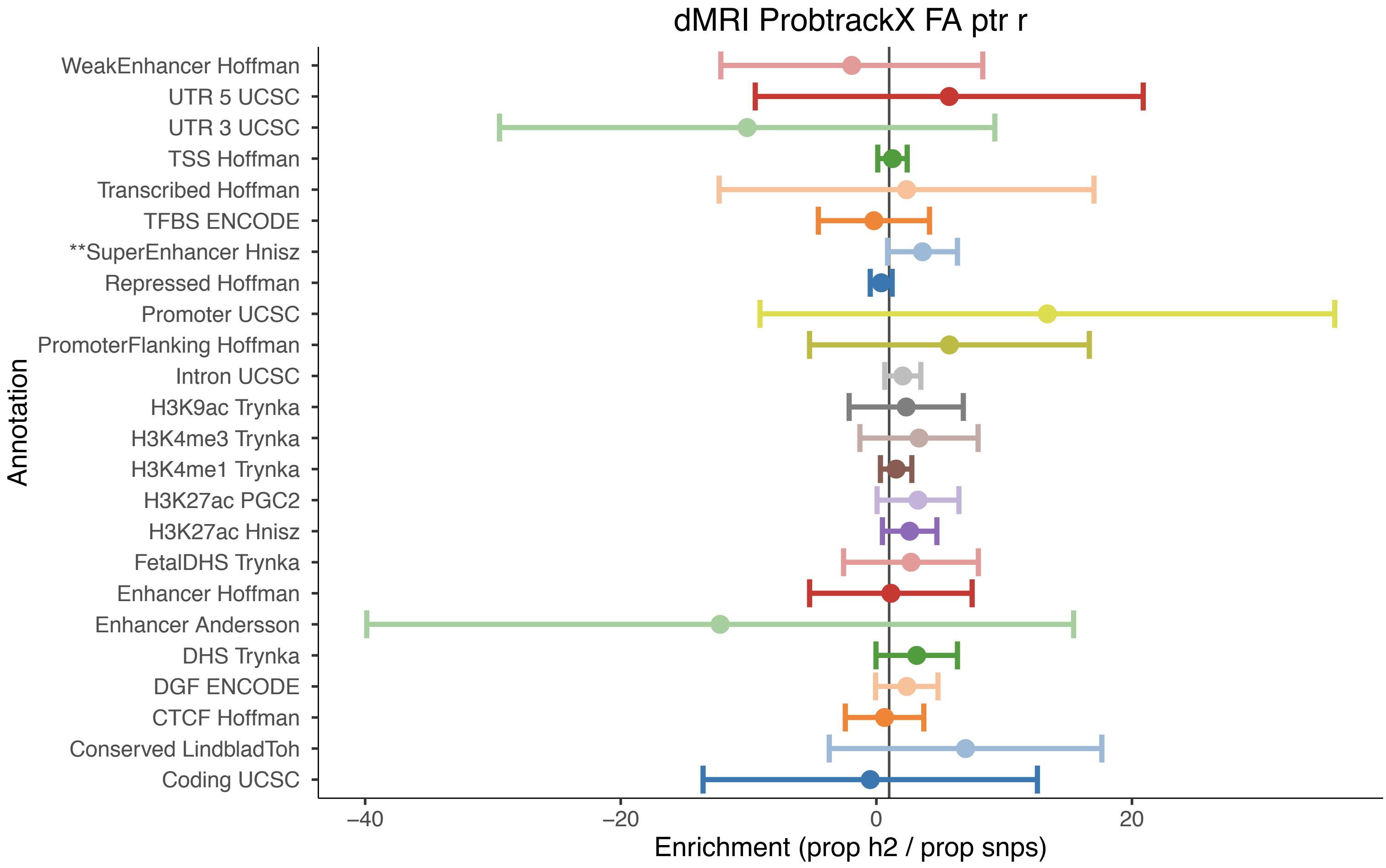
dMRI ProbtrackX FA ptr r
WeakEnhancer Hoffman
UTR 5 UCSC
UTR 3 UCSC
TSS Hoffman
Transcribed Hoffman
TFBS ENCODE
**SuperEnhancer Hnisz
Repressed Hoffman
Promoter UCSC
PromoterFlanking Hoffman
Intron UCSC
H3K9ac Trynka
H3K4me3 Trynka
H3K4me1 Trynka
H3K27ac PGC2
H3K27ac Hnisz
FetalDHS Trynka
Enhancer Hoffman
Enhancer Andersson
DHS Trynka
DGF ENCODE
CTCF Hoffman
Conserved LindbladToh
Coding UCSC
Annotation
−40
−20
0
20
Enrichment (prop h2 / prop snps)

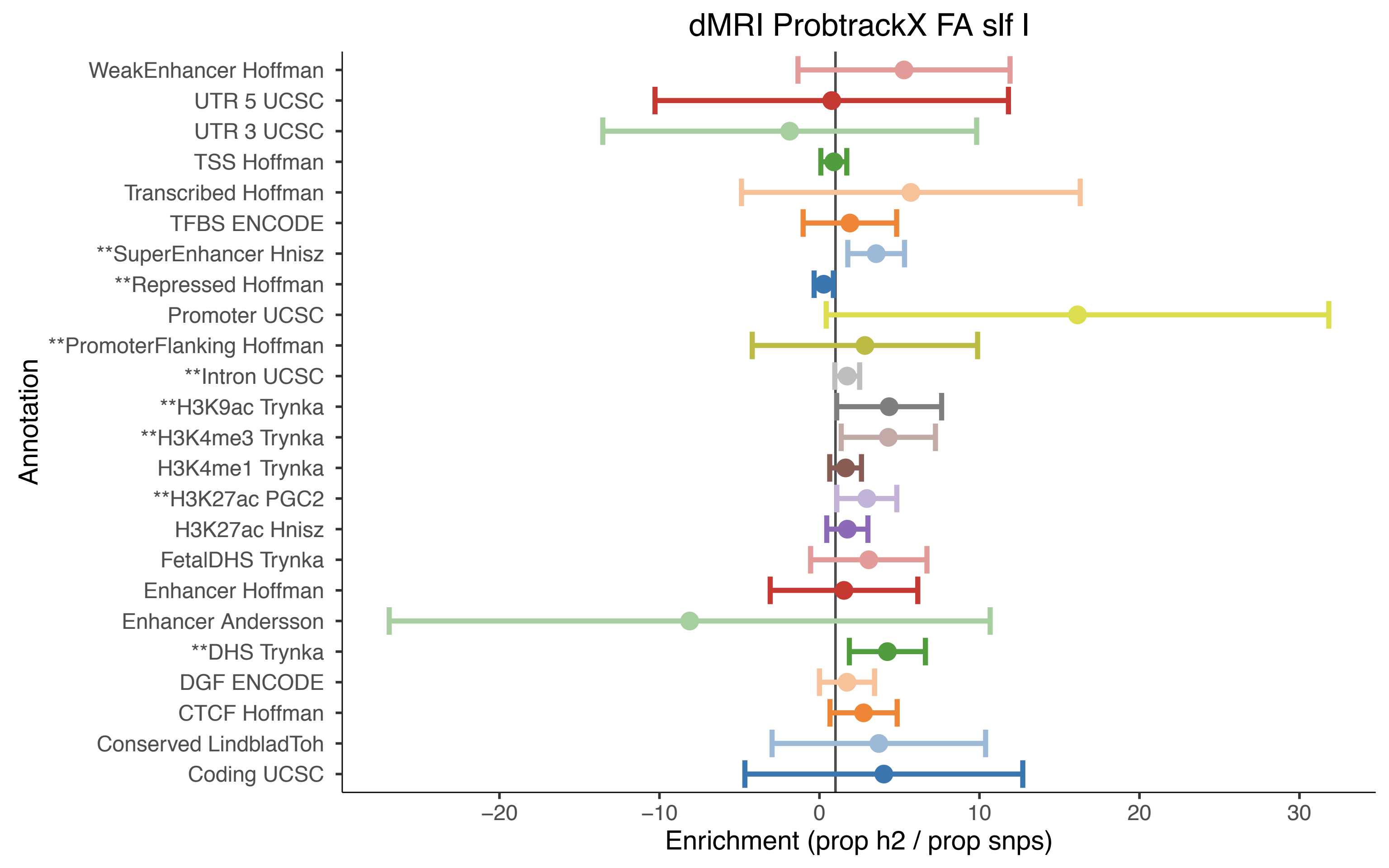

dMRI ProbtrackX FA slf I
WeakEnhancer Hoffman
UTR 5 UCSC
UTR 3 UCSC
TSS Hoffman
Transcribed Hoffman
TFBS ENCODE
**SuperEnhancer Hnisz
**Repressed Hoffman
Promoter UCSC
**PromoterFlanking Hoffman
**Intron UCSC
**H3K9ac Trynka
**H3K4me3 Trynka
H3K4me1 Trynka
**H3K27ac PGC2
H3K27ac Hnisz
FetalDHS Trynka
Enhancer Hoffman
Enhancer Andersson
**DHS Trynka
DGF ENCODE
CTCF Hoffman
Conserved LindbladToh
Coding UCSC
Annotation
−20
−10
0
10
20
30
Enrichment (prop h2 / prop snps)

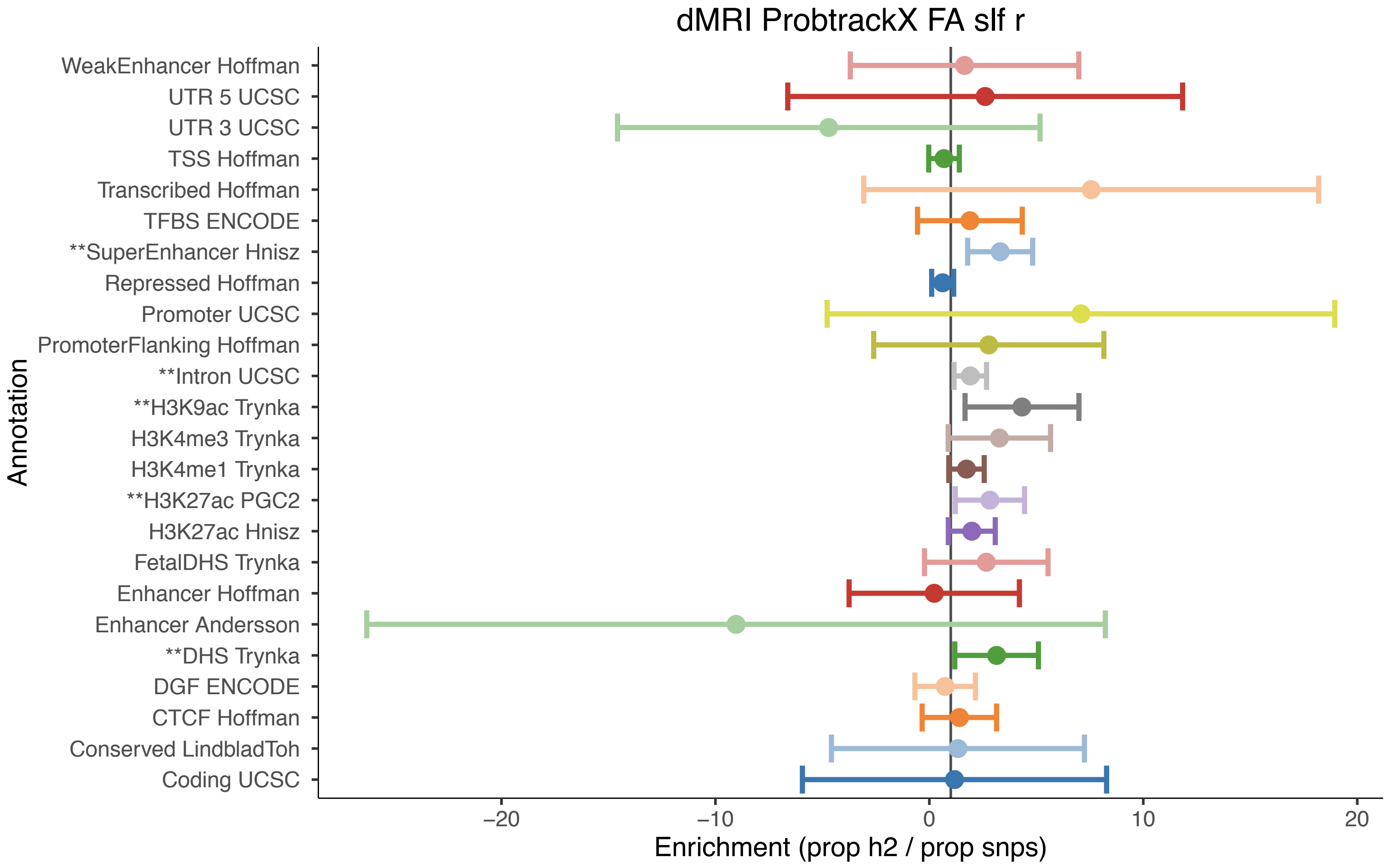

dMRI ProbtrackX FA slf r
WeakEnhancer Hoffman
UTR 5 UCSC
UTR 3 UCSC
TSS Hoffman
Transcribed Hoffman
TFBS ENCODE
**SuperEnhancer Hnisz
Repressed Hoffman
Promoter UCSC
PromoterFlanking Hoffman
**Intron UCSC
**H3K9ac Trynka
H3K4me3 Trynka
H3K4me1 Trynka
**H3K27ac PGC2
H3K27ac Hnisz
FetalDHS Trynka
Enhancer Hoffman
Enhancer Andersson
**DHS Trynka
DGF ENCODE
CTCF Hoffman
Conserved LindbladToh
Coding UCSC
Annotation
−20
−10
0
10
20
Enrichment (prop h2 / prop snps)

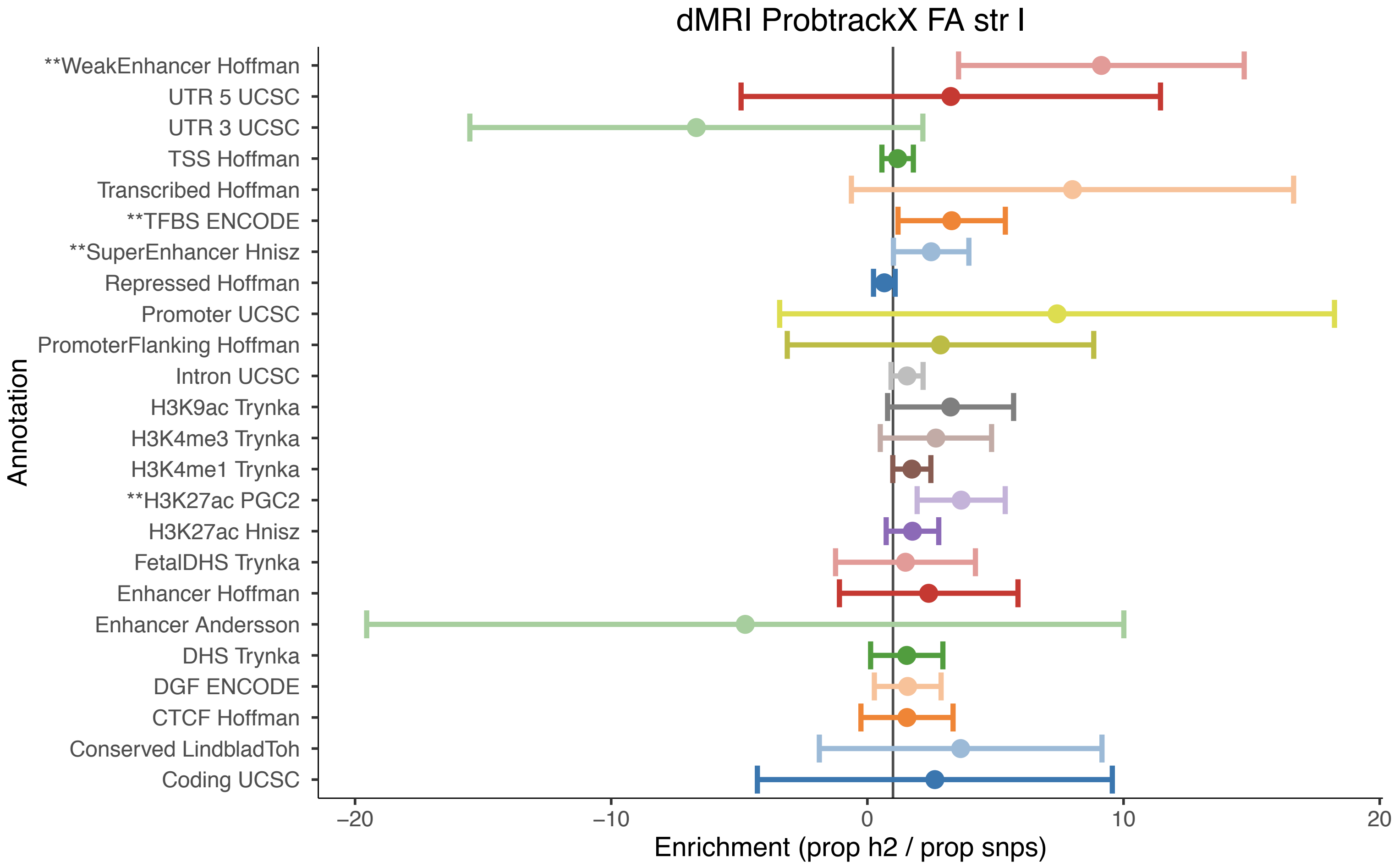
dMRI ProbtrackX FA str I
**WeakEnhancer Hoffman
UTR 5 UCSC
UTR 3 UCSC
TSS Hoffman
Transcribed Hoffman
**TFBS ENCODE
**SuperEnhancer Hnisz
Repressed Hoffman
Promoter UCSC
PromoterFlanking Hoffman
Intron UCSC
H3K9ac Trynka
H3K4me3 Trynka
H3K4me1 Trynka
**H3K27ac PGC2
H3K27ac Hnisz
FetalDHS Trynka
Enhancer Hoffman
Enhancer Andersson
DHS Trynka
DGF ENCODE
CTCF Hoffman
Conserved LindbladToh
Coding UCSC
Annotation
−20
−10
0
10
20
Enrichment (prop h2 / prop snps)

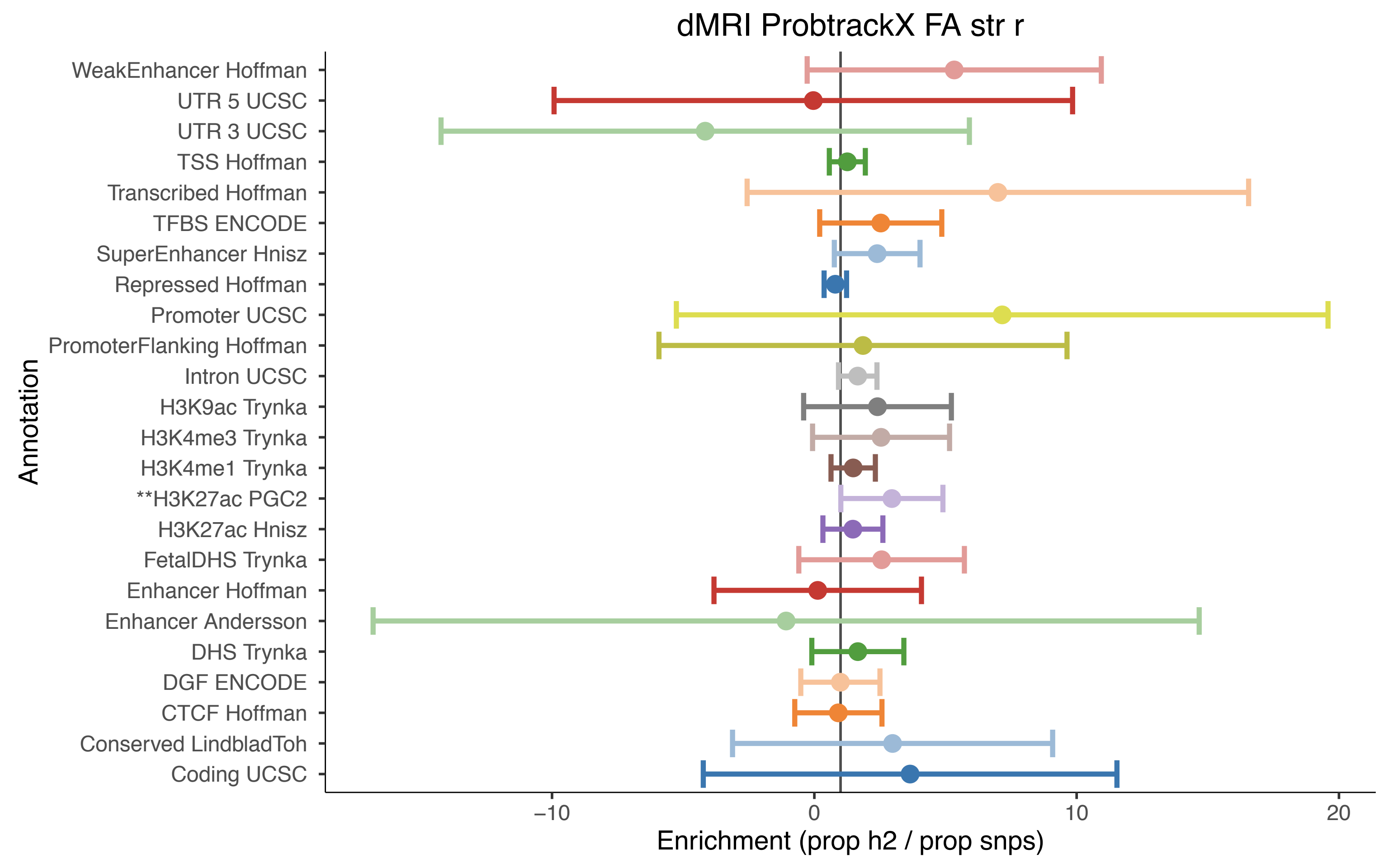

dMRI ProbtrackX FA str r
WeakEnhancer Hoffman
UTR 5 UCSC
UTR 3 UCSC
TSS Hoffman
Transcribed Hoffman
TFBS ENCODE
SuperEnhancer Hnisz
Repressed Hoffman
Promoter UCSC
PromoterFlanking Hoffman
Intron UCSC
H3K9ac Trynka
H3K4me3 Trynka
H3K4me1 Trynka
**H3K27ac PGC2
H3K27ac Hnisz
FetalDHS Trynka
Enhancer Hoffman
Enhancer Andersson
DHS Trynka
DGF ENCODE
CTCF Hoffman
Conserved LindbladToh
Coding UCSC
Annotation
−10
0
10
20
Enrichment (prop h2 / prop snps)

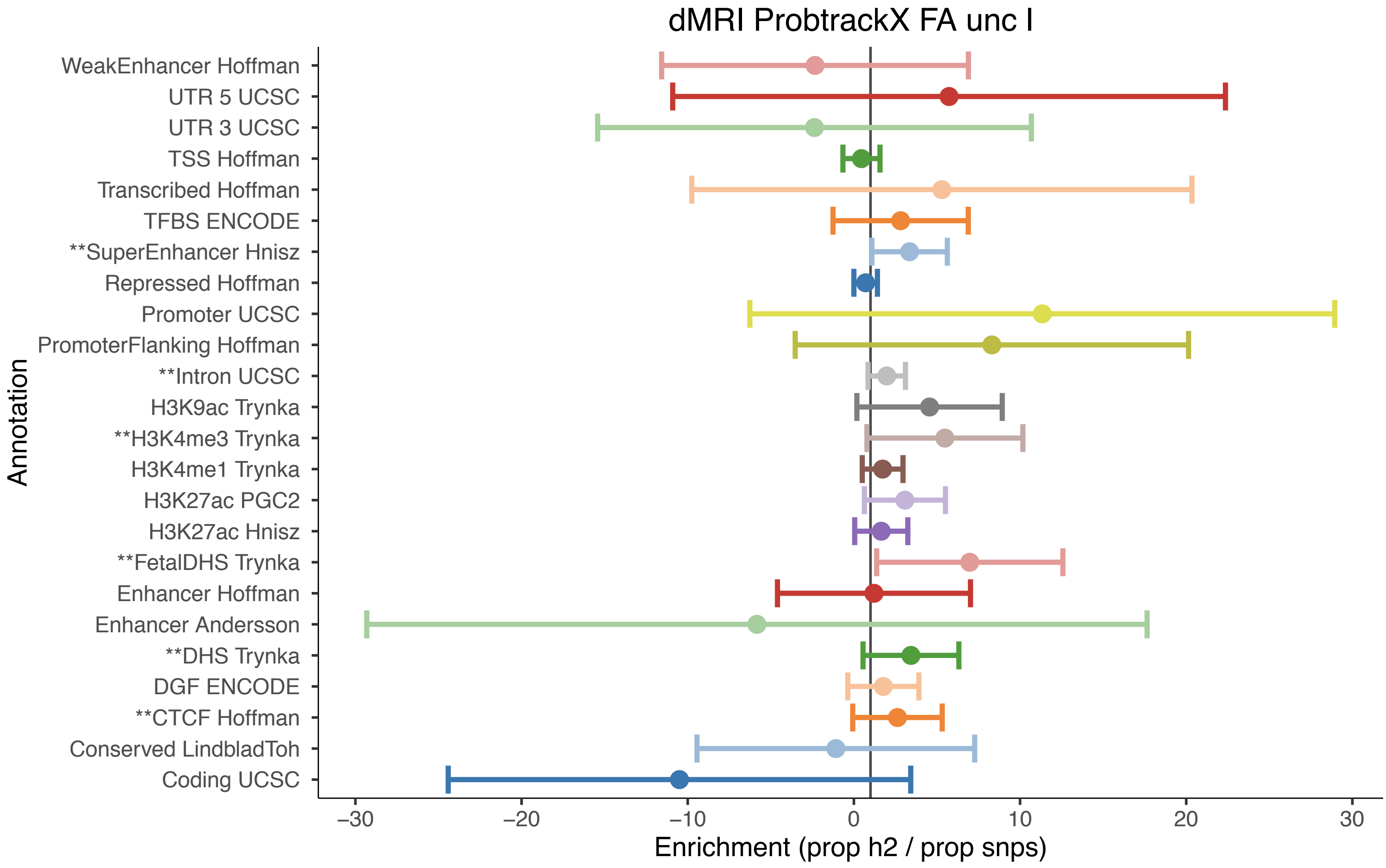

dMRI ProbtrackX FA unc I
WeakEnhancer Hoffman
UTR 5 UCSC
UTR 3 UCSC
TSS Hoffman
Transcribed Hoffman
TFBS ENCODE
**SuperEnhancer Hnisz
Repressed Hoffman
Promoter UCSC
PromoterFlanking Hoffman
**Intron UCSC
H3K9ac Trynka
**H3K4me3 Trynka
H3K4me1 Trynka
H3K27ac PGC2
H3K27ac Hnisz
**FetalDHS Trynka
Enhancer Hoffman
Enhancer Andersson
**DHS Trynka
DGF ENCODE
**CTCF Hoffman
Conserved LindbladToh
Coding UCSC
Annotation
−30
−20
−10
0
10
20
30
Enrichment (prop h2 / prop snps)

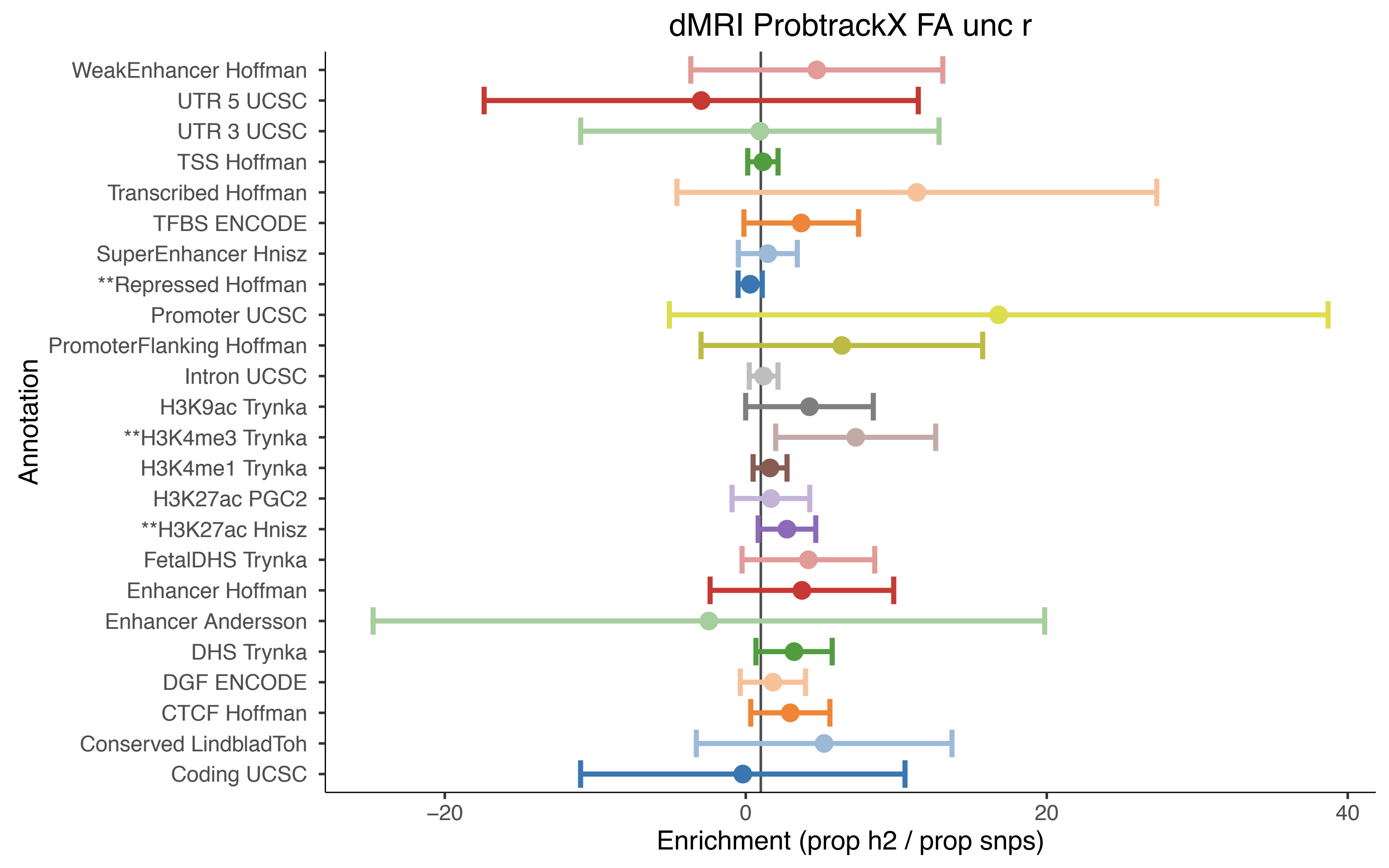
dMRI ProbtrackX FA unc r
WeakEnhancer Hoffman
UTR 5 UCSC
UTR 3 UCSC
TSS Hoffman
Transcribed Hoffman
TFBS ENCODE
SuperEnhancer Hnisz
**Repressed Hoffman
Promoter UCSC
PromoterFlanking Hoffman
Intron UCSC
H3K9ac Trynka
**H3K4me3 Trynka
H3K4me1 Trynka
H3K27ac PGC2
**H3K27ac Hnisz
FetalDHS Trynka
Enhancer Hoffman
Enhancer Andersson
DHS Trynka
DGF ENCODE
CTCF Hoffman
Conserved LindbladToh
Coding UCSC
Annotation
−20
0
20
40
Enrichment (prop h2 / prop snps)

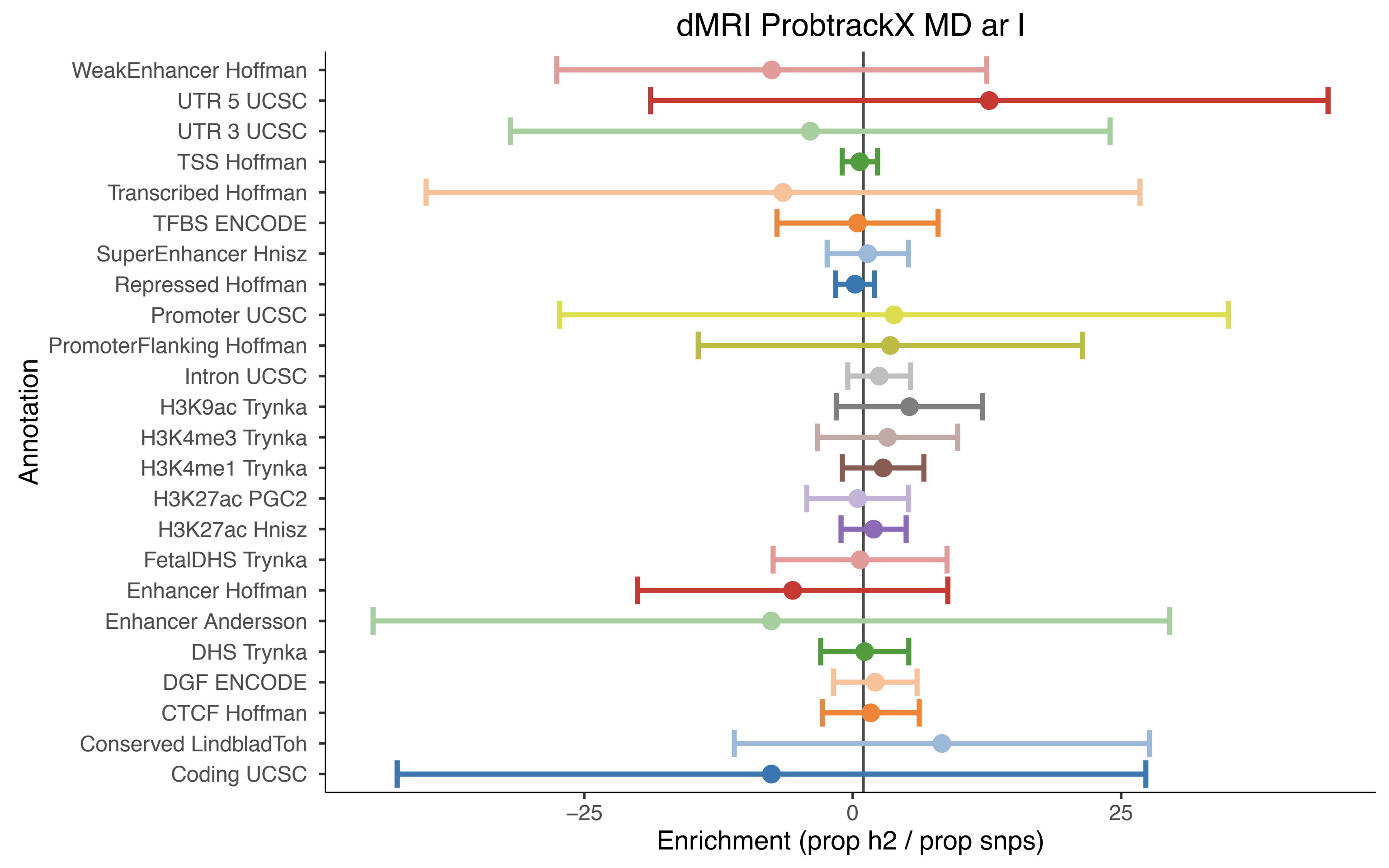
dMRI ProbtrackX MD ar l
Annotation
WeakEnhancer Hoffman
UTR 5 UCSC
UTR 3 UCSC
TSS Hoffman
Transcribed Hoffman
TFBS ENCODE
SuperEnhancer Hnisz
Repressed Hoffman
Promoter UCSC
PromoterFlanking Hoffman
Intron UCSC
H3K9ac Trynka
H3K4me3 Trynka
H3K4me1 Trynka
H3K27ac PGC2
H3K27ac Hnisz
FetalDHS Trynka
Enhancer Hoffman
Enhancer Andersson
DHS Trynka
DGF ENCODE
CTCF Hoffman
Conserved LindbladToh
Coding UCSC
−25
0
25
Enrichment (prop h2 / prop snps)

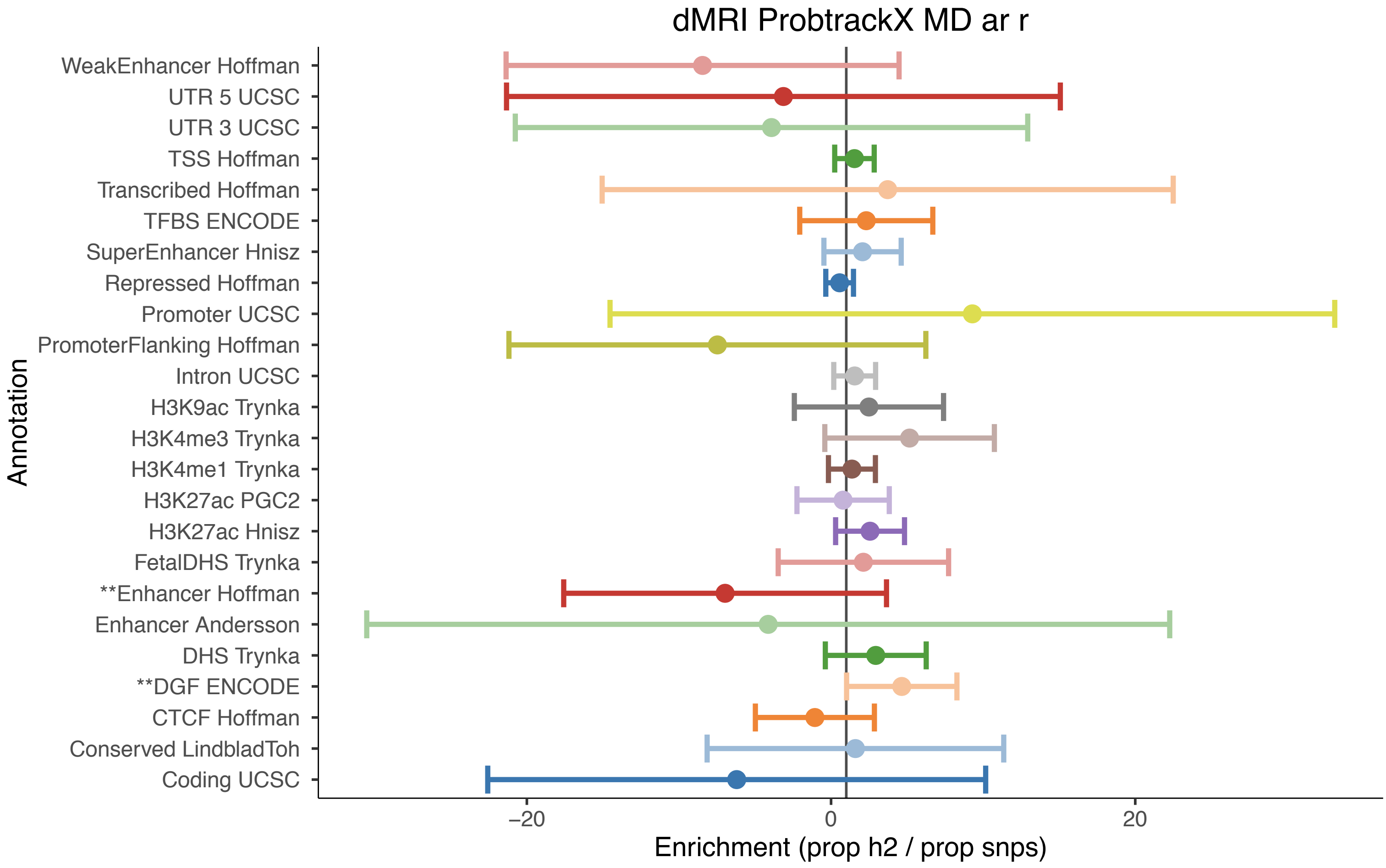

dMRI ProbtrackX MD ar r
WeakEnhancer Hoffman
UTR 5 UCSC
UTR 3 UCSC
TSS Hoffman
Transcribed Hoffman
TFBS ENCODE
SuperEnhancer Hnisz
Repressed Hoffman
Promoter UCSC
PromoterFlanking Hoffman
Intron UCSC
H3K9ac Trynka
H3K4me3 Trynka
H3K4me1 Trynka
H3K27ac PGC2
H3K27ac Hnisz
FetalDHS Trynka
**Enhancer Hoffman
Enhancer Andersson
DHS Trynka
**DGF ENCODE
CTCF Hoffman
Conserved LindbladToh
Coding UCSC
Annotation
−20
0
20
Enrichment (prop h2 / prop snps)

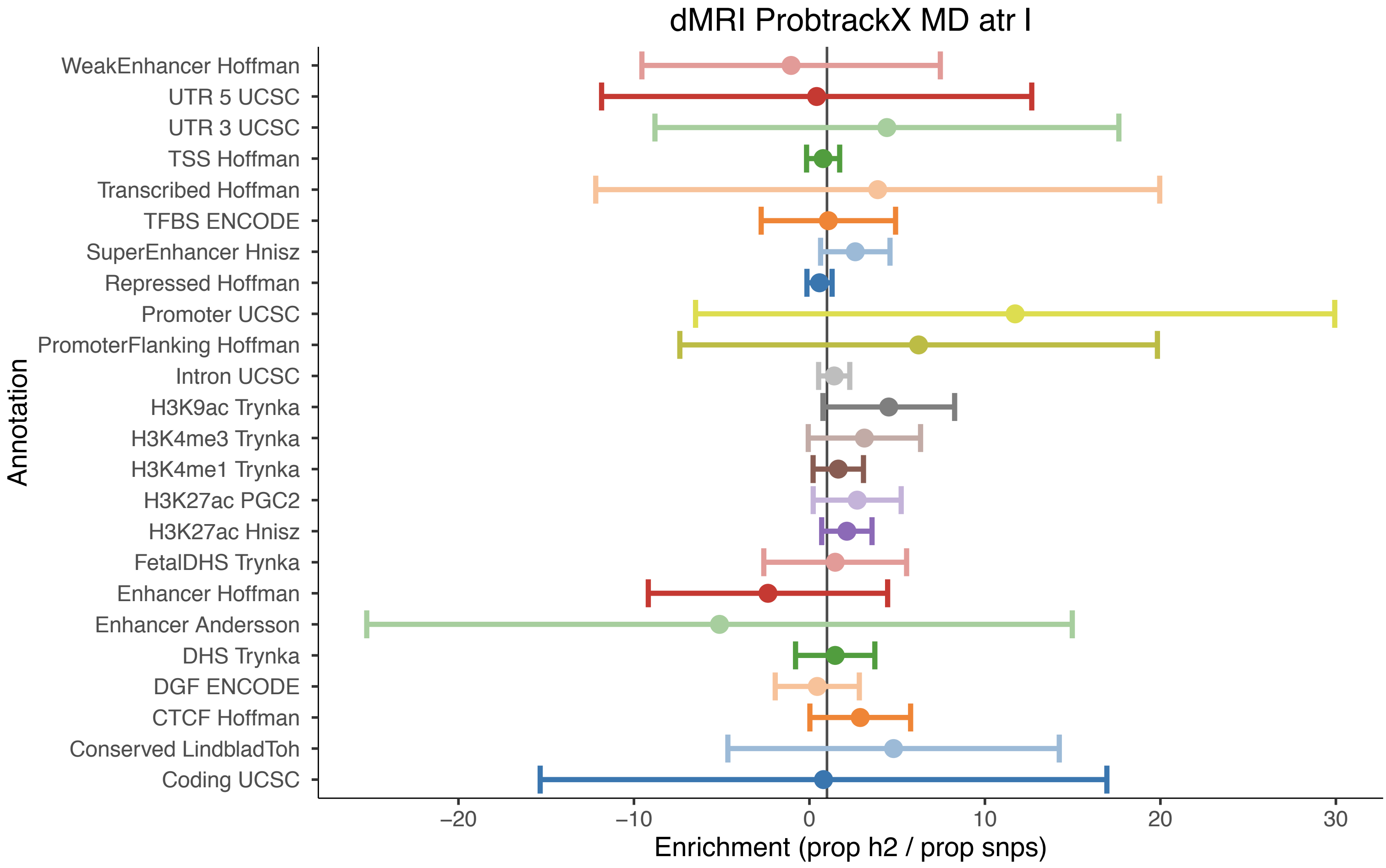

dMRI ProbtrackX MD atr l
Annotation
WeakEnhancer Hoffman
UTR 5 UCSC
UTR 3 UCSC
TSS Hoffman
Transcribed Hoffman
TFBS ENCODE
SuperEnhancer Hnisz
Repressed Hoffman
Promoter UCSC
PromoterFlanking Hoffman
Intron UCSC
H3K9ac Trynka
H3K4me3 Trynka
H3K4me1 Trynka
H3K27ac PGC2
H3K27ac Hnisz
FetalDHS Trynka
Enhancer Hoffman
Enhancer Andersson
DHS Trynka
DGF ENCODE
CTCF Hoffman
Conserved LindbladToh
Coding UCSC
−20
−10
0
10
20
30
Enrichment (prop h2 / prop snps)

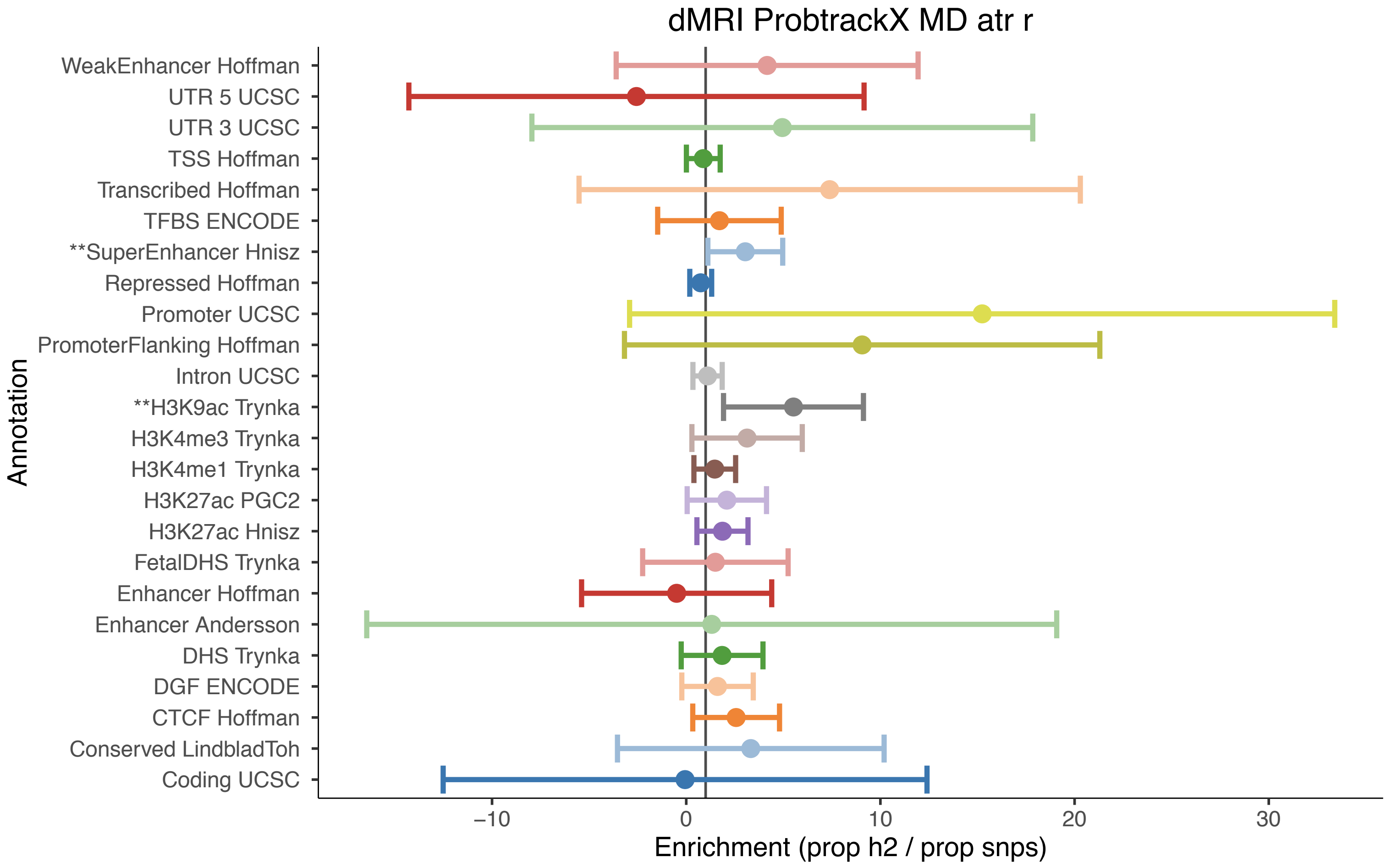

dMRI ProbtrackX MD atr r
WeakEnhancer Hoffman
UTR 5 UCSC
UTR 3 UCSC
TSS Hoffman
Transcribed Hoffman
TFBS ENCODE
**SuperEnhancer Hnisz
Repressed Hoffman
Promoter UCSC
PromoterFlanking Hoffman
Intron UCSC
**H3K9ac Trynka
H3K4me3 Trynka
H3K4me1 Trynka
H3K27ac PGC2
H3K27ac Hnisz
FetalDHS Trynka
Enhancer Hoffman
Enhancer Andersson
DHS Trynka
DGF ENCODE
CTCF Hoffman
Conserved LindbladToh
Coding UCSC
Annotation
−10
0
10
20
30
Enrichment (prop h2 / prop snps)

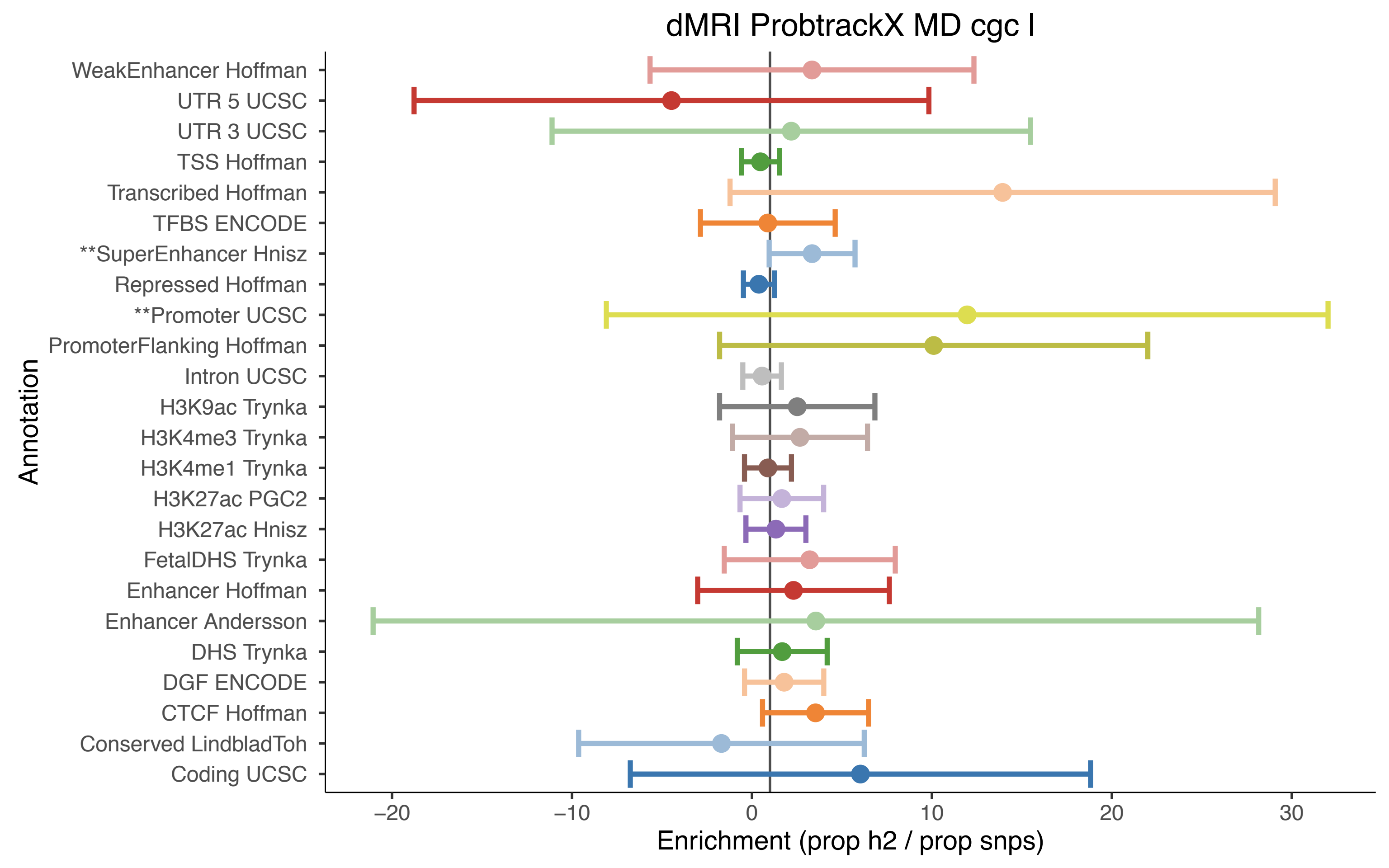
dMRI ProbtrackX MD cgc I
WeakEnhancer Hoffman
UTR 5 UCSC
UTR 3 UCSC
TSS Hoffman
Transcribed Hoffman
TFBS ENCODE
**SuperEnhancer Hnisz
Repressed Hoffman
**Promoter UCSC
PromoterFlanking Hoffman
Intron UCSC
H3K9ac Trynka
H3K4me3 Trynka
H3K4me1 Trynka
H3K27ac PGC2
H3K27ac Hnisz
FetalDHS Trynka
Enhancer Hoffman
Enhancer Andersson
DHS Trynka
DGF ENCODE
CTCF Hoffman
Conserved LindbladToh
Coding UCSC
Annotation
−20
−10
0
10
20
30
Enrichment (prop h2 / prop snps)

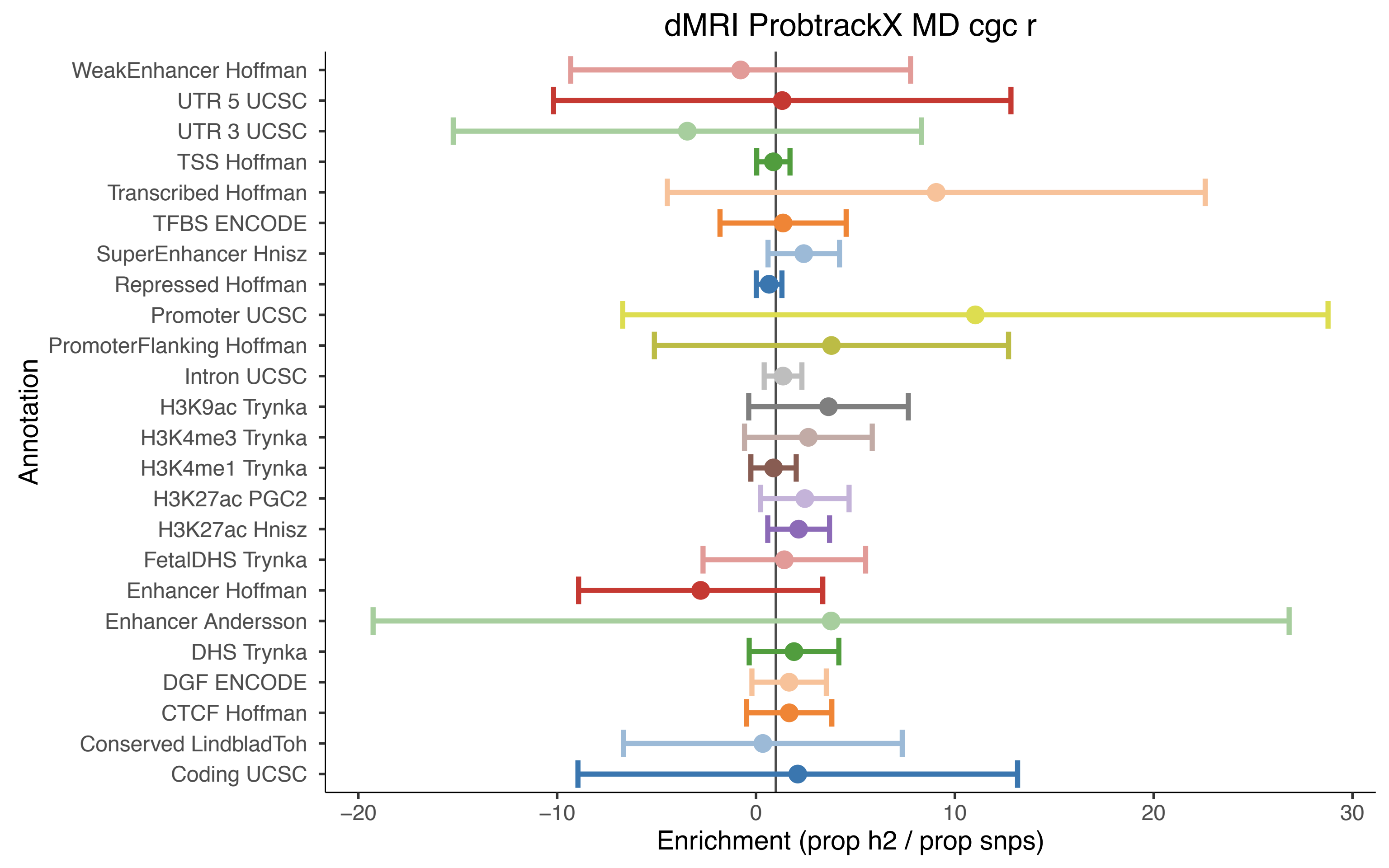
dMRI ProbtrackX MD cgc r
WeakEnhancer Hoffman
UTR 5 UCSC
UTR 3 UCSC
TSS Hoffman
Transcribed Hoffman
TFBS ENCODE
SuperEnhancer Hnisz
Repressed Hoffman
Promoter UCSC
PromoterFlanking Hoffman
Intron UCSC
H3K9ac Trynka
H3K4me3 Trynka
H3K4me1 Trynka
H3K27ac PGC2
H3K27ac Hnisz
FetalDHS Trynka
Enhancer Hoffman
Enhancer Andersson
DHS Trynka
DGF ENCODE
CTCF Hoffman
Conserved LindbladToh
Coding UCSC
Annotation
−20
−10
0
10
20
30
Enrichment (prop h2 / prop snps)

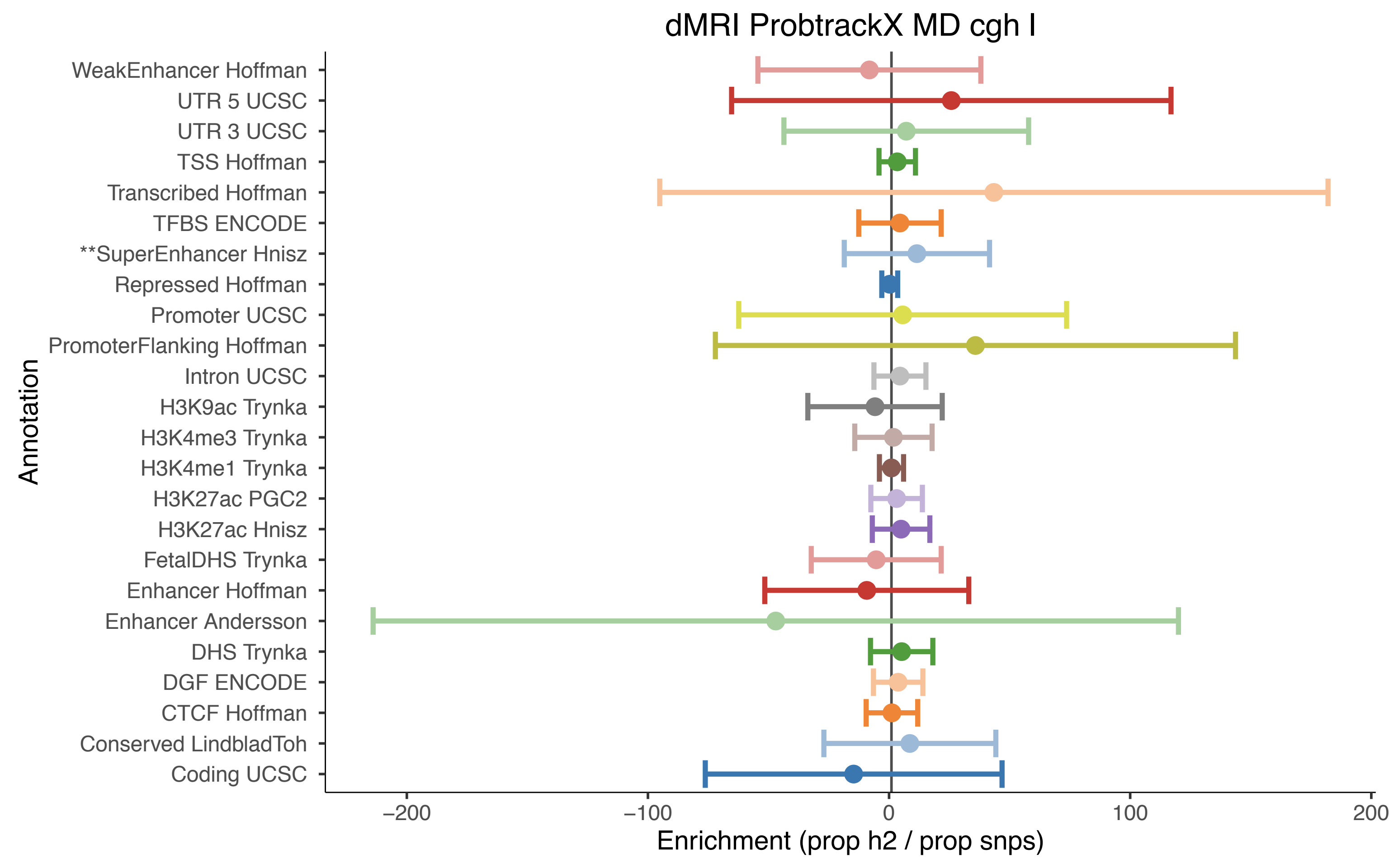
dMRI ProbtrackX MD cgh l
Annotation
WeakEnhancer Hoffman
UTR 5 UCSC
UTR 3 UCSC
TSS Hoffman
Transcribed Hoffman
TFBS ENCODE
**SuperEnhancer Hnisz
Repressed Hoffman
Promoter UCSC
PromoterFlanking Hoffman
Intron UCSC
H3K9ac Trynka
H3K4me3 Trynka
H3K4me1 Trynka
H3K27ac PGC2
H3K27ac Hnisz
FetalDHS Trynka
Enhancer Hoffman
Enhancer Andersson
DHS Trynka
DGF ENCODE
CTCF Hoffman
Conserved LindbladToh
Coding UCSC
−200
−100
0
100
200
Enrichment (prop h2 / prop snps)

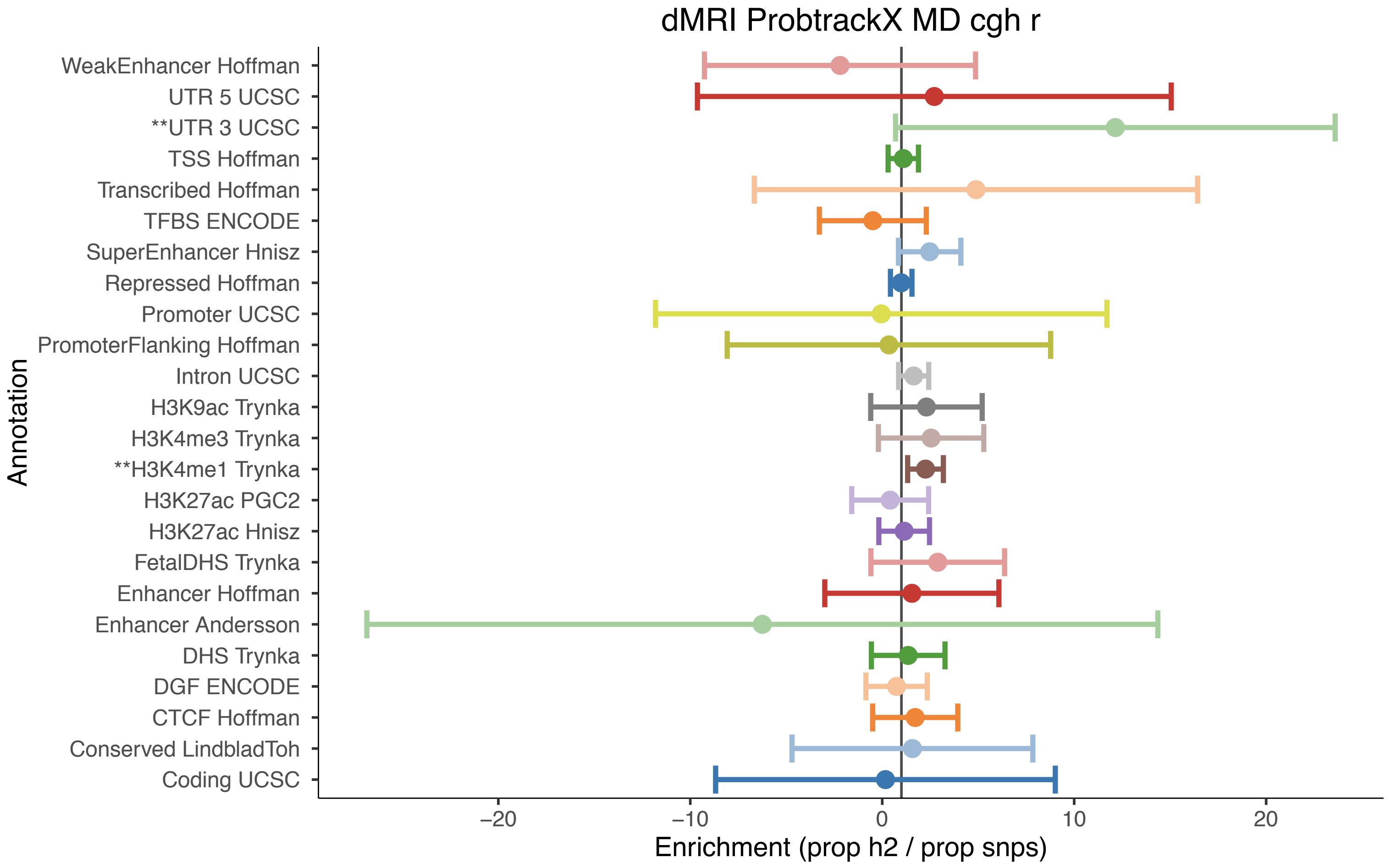

dMRI ProbtrackX MD cgh r
WeakEnhancer Hoffman
UTR 5 UCSC
**UTR 3 UCSC
TSS Hoffman
Transcribed Hoffman
TFBS ENCODE
SuperEnhancer Hnisz
Repressed Hoffman
Promoter UCSC
PromoterFlanking Hoffman
Intron UCSC
H3K9ac Trynka
H3K4me3 Trynka
**H3K4me1 Trynka
H3K27ac PGC2
H3K27ac Hnisz
FetalDHS Trynka
Enhancer Hoffman
Enhancer Andersson
DHS Trynka
DGF ENCODE
CTCF Hoffman
Conserved LindbladToh
Coding UCSC
Annotation
−20
−10
0
10
20
Enrichment (prop h2 / prop snps)

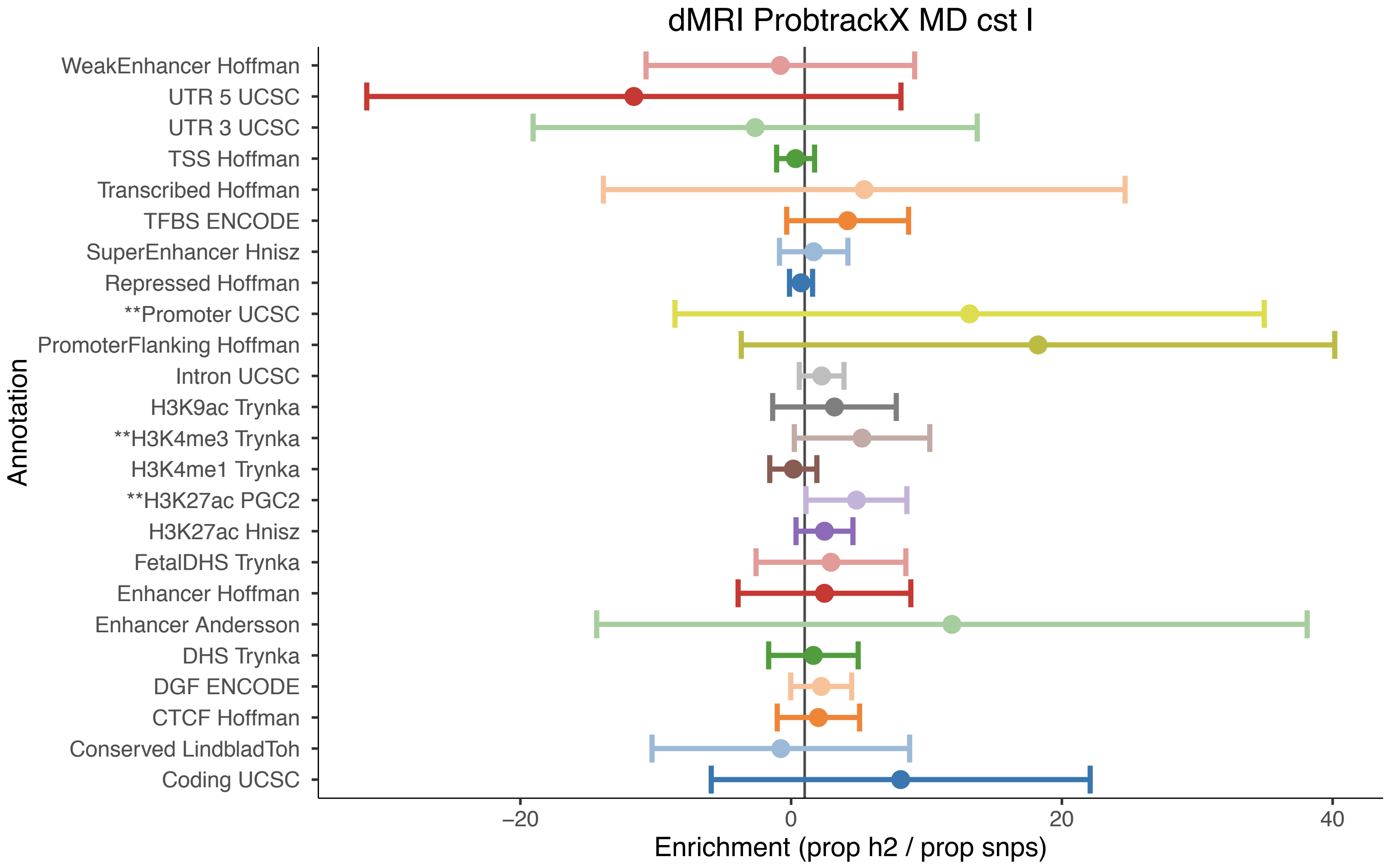
dMRI ProbtrackX MD cst l
WeakEnhancer Hoffman
UTR 5 UCSC
UTR 3 UCSC
TSS Hoffman
Transcribed Hoffman
TFBS ENCODE
SuperEnhancer Hnisz
Repressed Hoffman
**Promoter UCSC
PromoterFlanking Hoffman
Intron UCSC
H3K9ac Trynka
**H3K4me3 Trynka
H3K4me1 Trynka
**H3K27ac PGC2
H3K27ac Hnisz
FetalDHS Trynka
Enhancer Hoffman
Enhancer Andersson
DHS Trynka
DGF ENCODE
CTCF Hoffman
Conserved LindbladToh
Coding UCSC
Annotation
−20
0
20
40
Enrichment (prop h2 / prop snps)

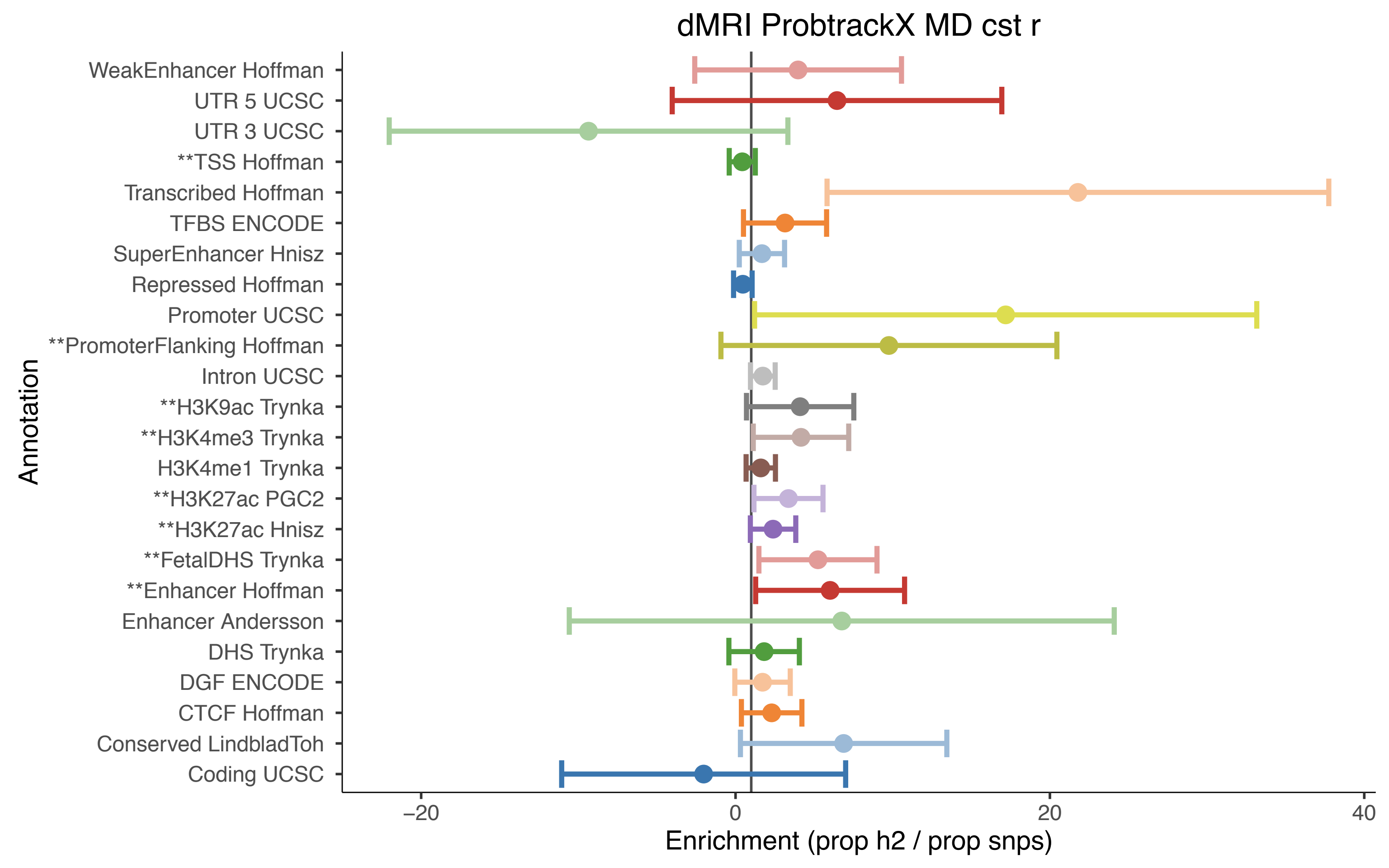

dMRI ProbtrackX MD cst r
Annotation
WeakEnhancer Hoffman
UTR 5 UCSC
UTR 3 UCSC
**TSS Hoffman
Transcribed Hoffman
TFBS ENCODE
SuperEnhancer Hnisz
Repressed Hoffman
Promoter UCSC
**PromoterFlanking Hoffman
Intron UCSC
**H3K9ac Trynka
**H3K4me3 Trynka
H3K4me1 Trynka
**H3K27ac PGC2
**H3K27ac Hnisz
**FetalDHS Trynka
**Enhancer Hoffman
Enhancer Andersson
DHS Trynka
DGF ENCODE
CTCF Hoffman
Conserved LindbladToh
Coding UCSC
−20
0
20
40
Enrichment (prop h2 / prop snps)

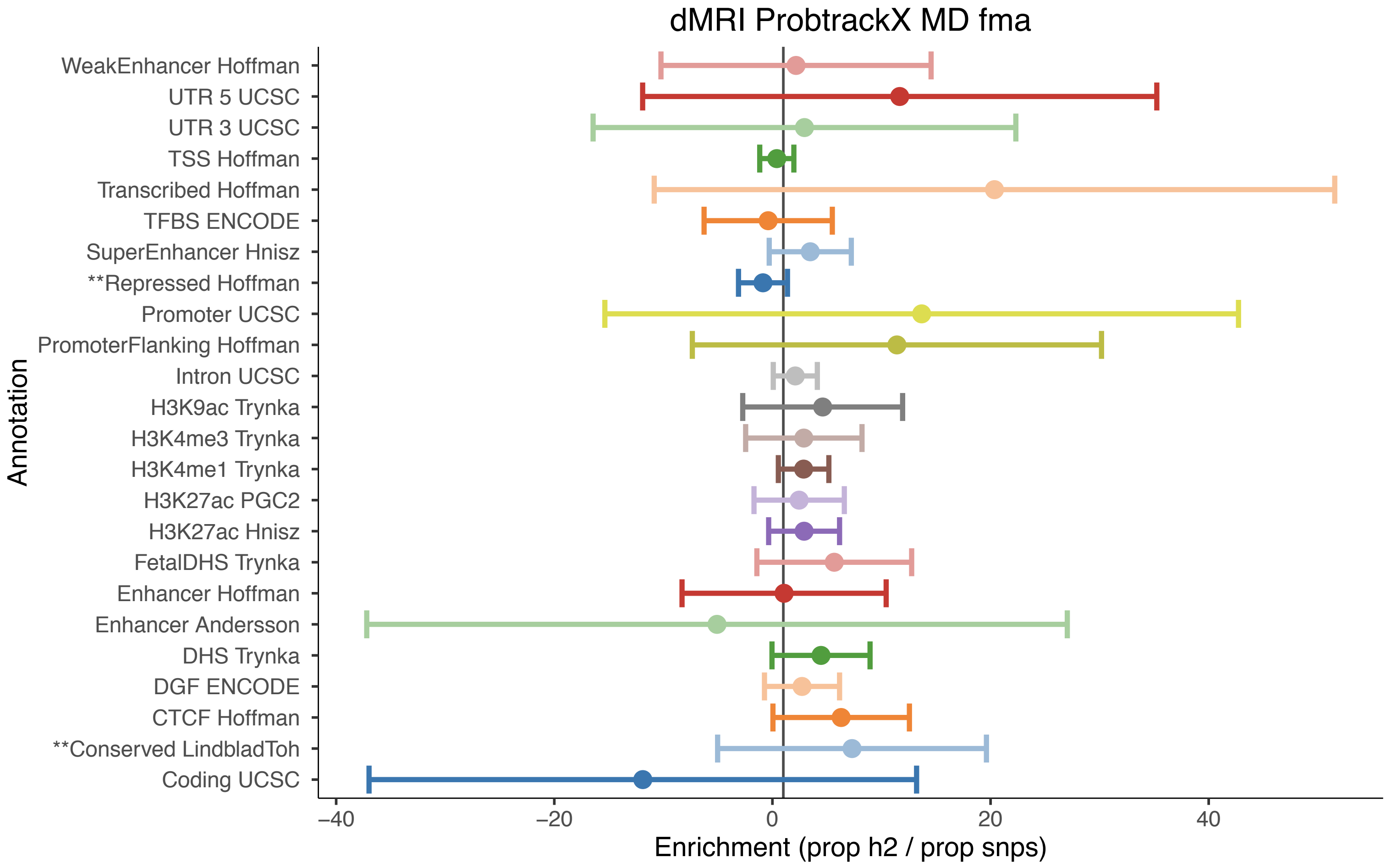

dMRI ProbtrackX MD fma
WeakEnhancer Hoffman
UTR 5 UCSC
UTR 3 UCSC
TSS Hoffman
Transcribed Hoffman
TFBS ENCODE
SuperEnhancer Hnisz
**Repressed Hoffman
Promoter UCSC
PromoterFlanking Hoffman
Intron UCSC
H3K9ac Trynka
H3K4me3 Trynka
H3K4me1 Trynka
H3K27ac PGC2
H3K27ac Hnisz
FetalDHS Trynka
Enhancer Hoffman
Enhancer Andersson
DHS Trynka
DGF ENCODE
CTCF Hoffman
**Conserved LindbladToh
Coding UCSC
Annotation
−40
−20
0
20
40
Enrichment (prop h2 / prop snps)

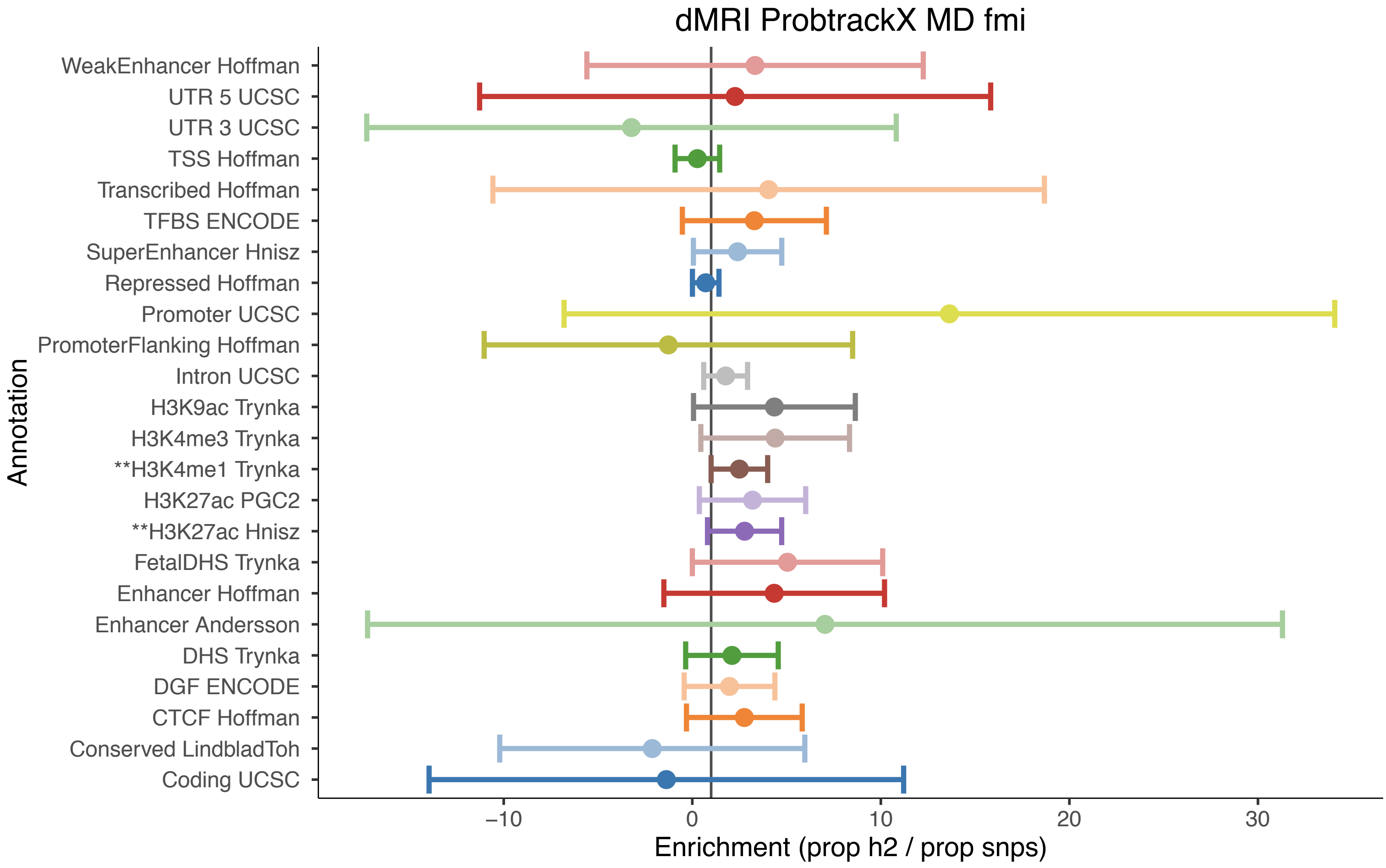

dMRI ProbtrackX MD fmi
Annotation
WeakEnhancer Hoffman
UTR 5 UCSC
UTR 3 UCSC
TSS Hoffman
Transcribed Hoffman
TFBS ENCODE
SuperEnhancer Hnisz
Repressed Hoffman
Promoter UCSC
PromoterFlanking Hoffman
Intron UCSC
H3K9ac Trynka
H3K4me3 Trynka
**H3K4me1 Trynka
H3K27ac PGC2
**H3K27ac Hnisz
FetalDHS Trynka
Enhancer Hoffman
Enhancer Andersson
DHS Trynka
DGF ENCODE
CTCF Hoffman
Conserved LindbladToh
Coding UCSC
−10
0
10
20
30
Enrichment (prop h2 / prop snps)

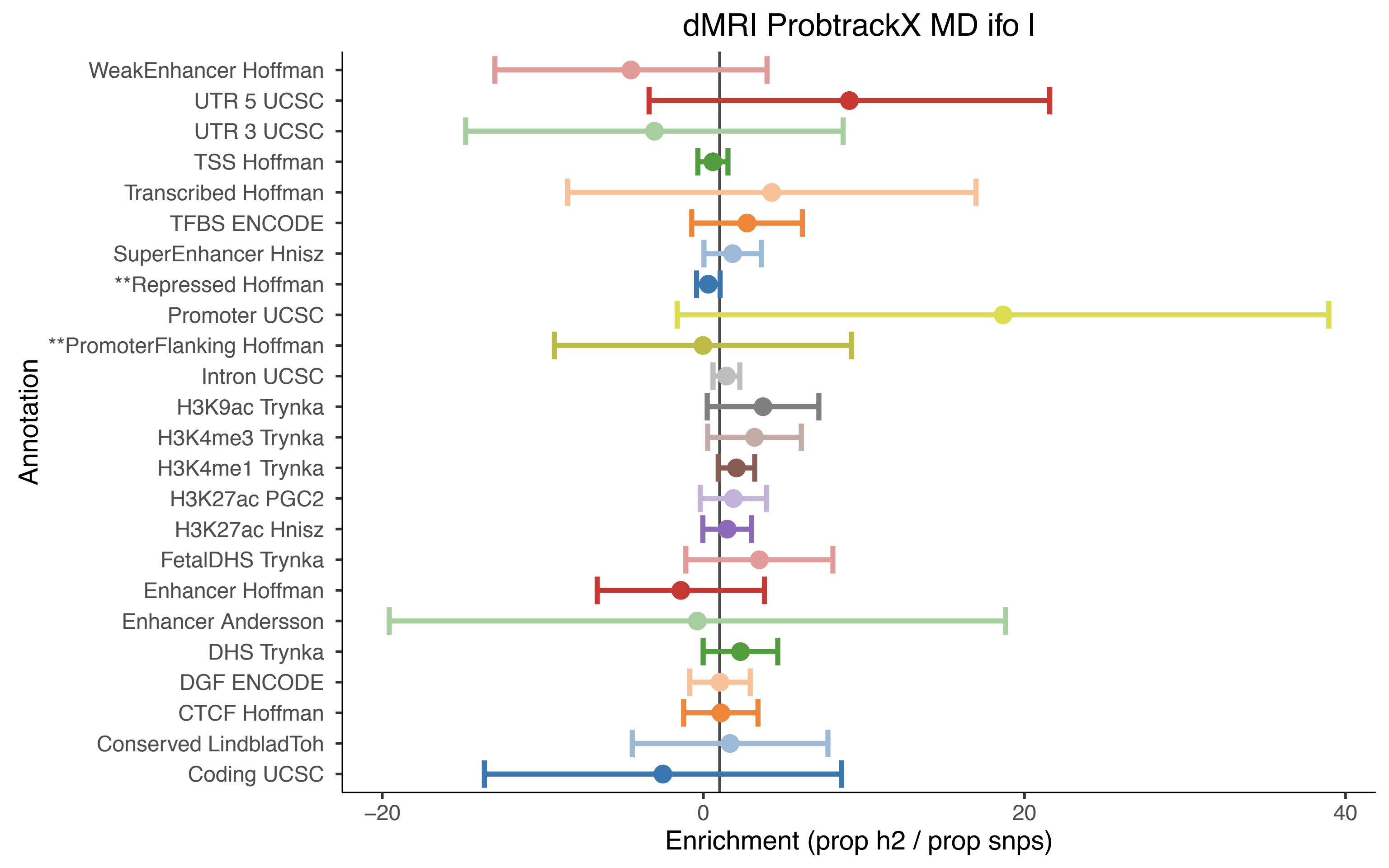

dMRI ProbtrackX MD ifo l
WeakEnhancer Hoffman
UTR 5 UCSC
UTR 3 UCSC
TSS Hoffman
Transcribed Hoffman
TFBS ENCODE
SuperEnhancer Hnisz
**Repressed Hoffman
Promoter UCSC
**PromoterFlanking Hoffman
Intron UCSC
H3K9ac Trynka
H3K4me3 Trynka
H3K4me1 Trynka
H3K27ac PGC2
H3K27ac Hnisz
FetalDHS Trynka
Enhancer Hoffman
Enhancer Andersson
DHS Trynka
DGF ENCODE
CTCF Hoffman
Conserved LindbladToh
Coding UCSC
Annotation
−20
0
20
40
Enrichment (prop h2 / prop snps)

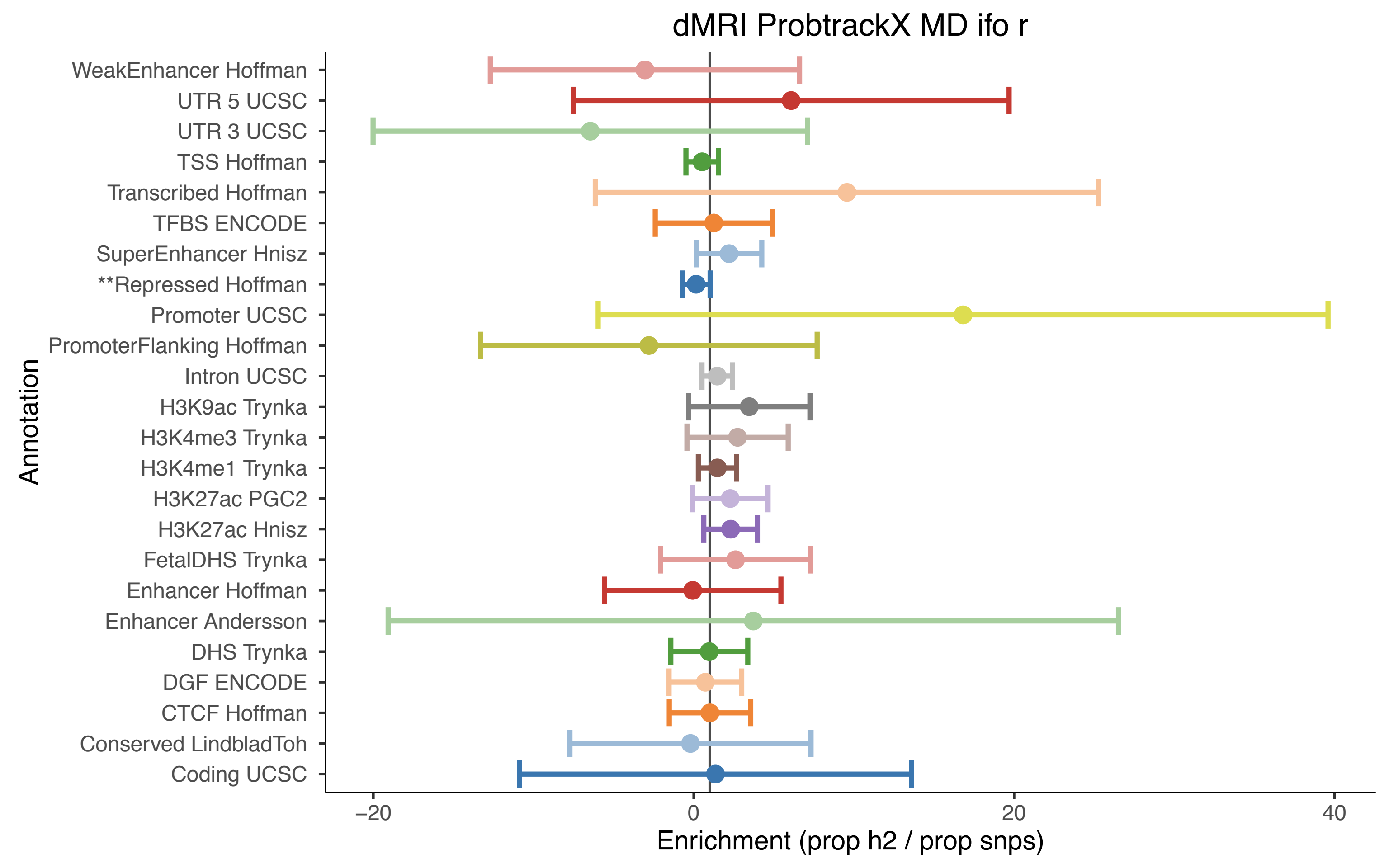

dMRI ProbtrackX MD ifo r
WeakEnhancer Hoffman
UTR 5 UCSC
UTR 3 UCSC
TSS Hoffman
Transcribed Hoffman
TFBS ENCODE
SuperEnhancer Hnisz
**Repressed Hoffman
Promoter UCSC
PromoterFlanking Hoffman
Intron UCSC
H3K9ac Trynka
H3K4me3 Trynka
H3K4me1 Trynka
H3K27ac PGC2
H3K27ac Hnisz
FetalDHS Trynka
Enhancer Hoffman
Enhancer Andersson
DHS Trynka
DGF ENCODE
CTCF Hoffman
Conserved LindbladToh
Coding UCSC
Annotation
−20
0
20
40
Enrichment (prop h2 / prop snps)

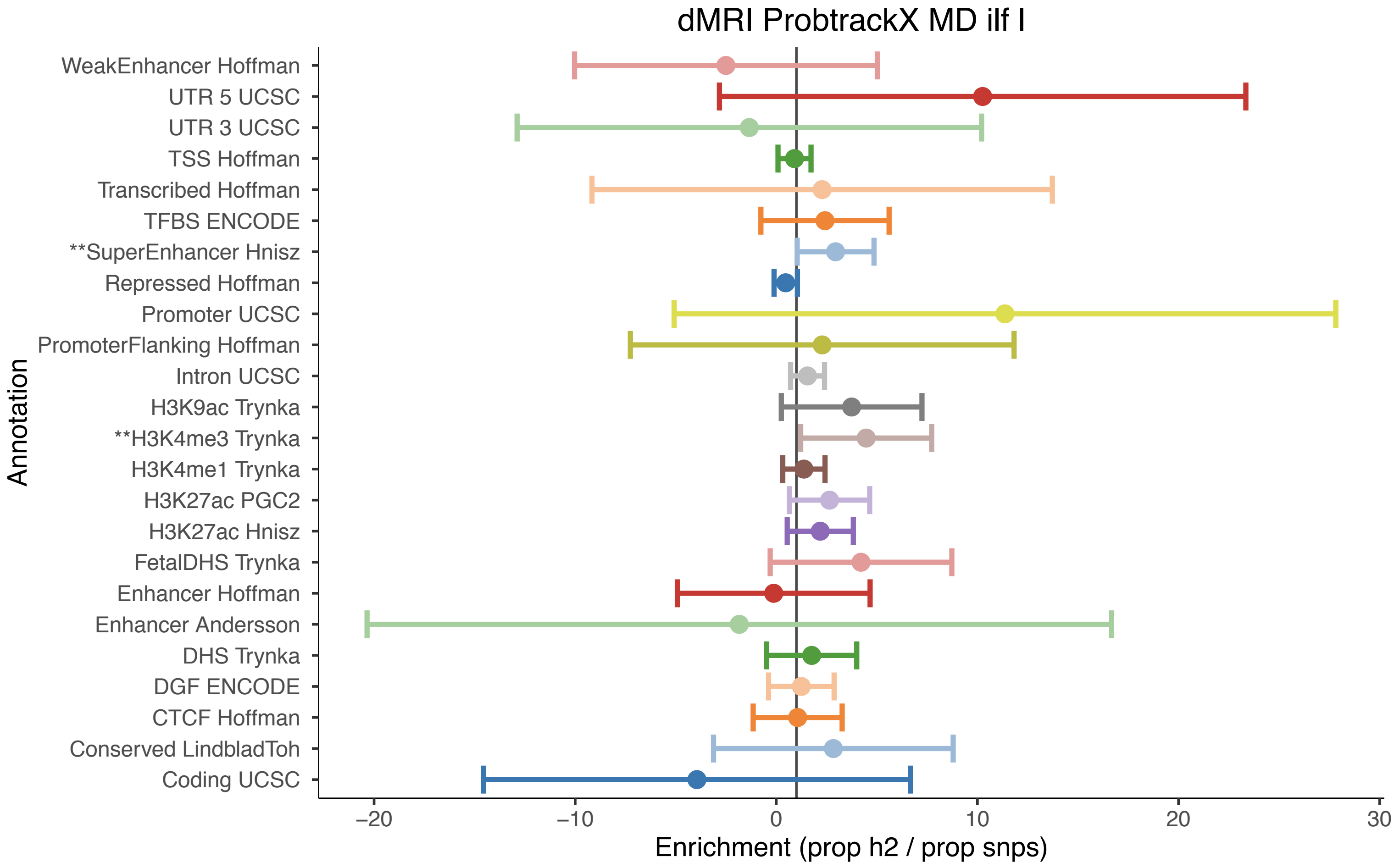

dMRI ProbtrackX MD ilf l
Annotation
WeakEnhancer Hoffman
UTR 5 UCSC
UTR 3 UCSC
TSS Hoffman
Transcribed Hoffman
TFBS ENCODE
**SuperEnhancer Hnisz
Repressed Hoffman
Promoter UCSC
PromoterFlanking Hoffman
Intron UCSC
H3K9ac Trynka
**H3K4me3 Trynka
H3K4me1 Trynka
H3K27ac PGC2
H3K27ac Hnisz
FetalDHS Trynka
Enhancer Hoffman
Enhancer Andersson
DHS Trynka
DGF ENCODE
CTCF Hoffman
Conserved LindbladToh
Coding UCSC
−20
−10
0
10
20
30
Enrichment (prop h2 / prop snps)

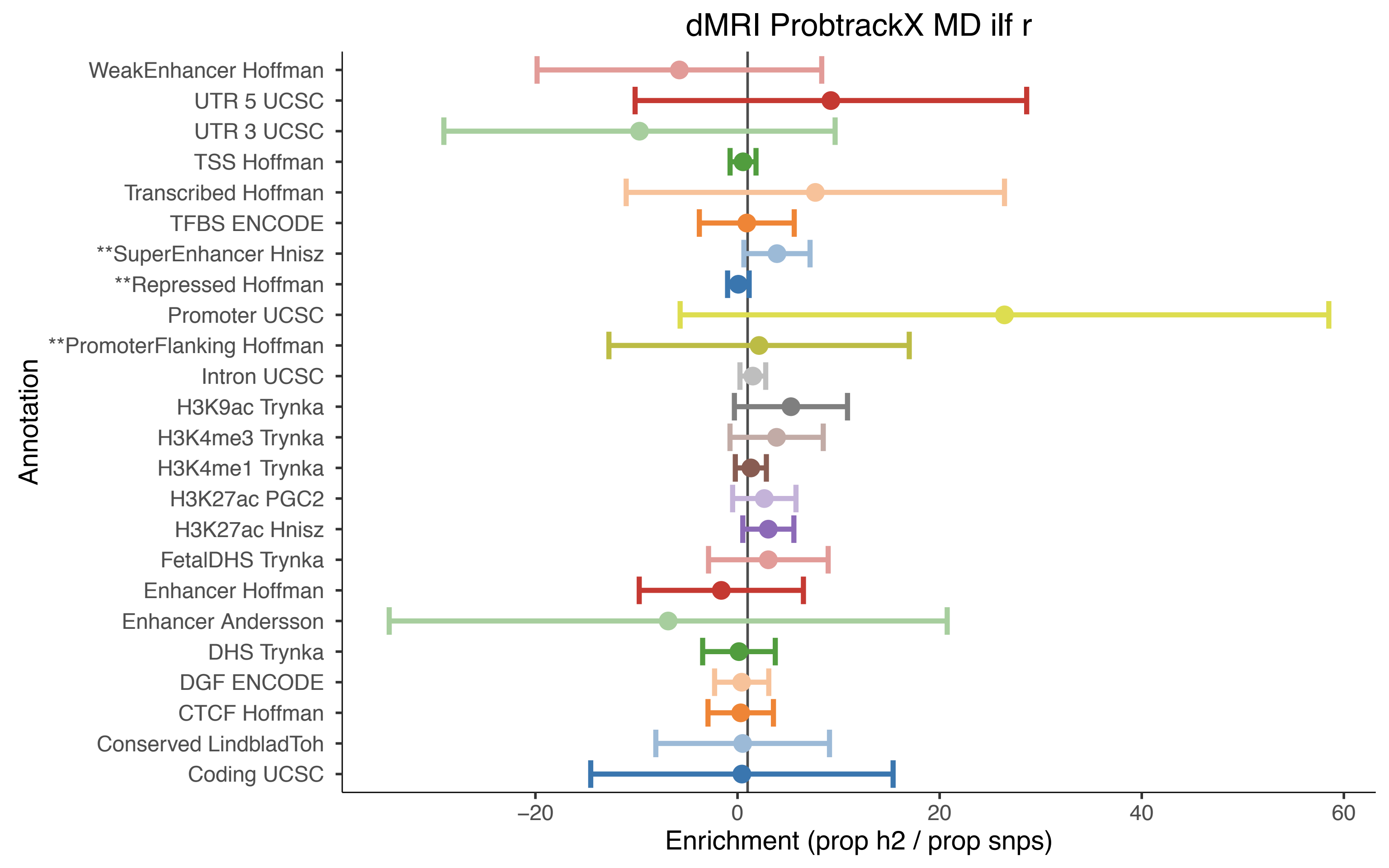

dMRI ProbtrackX MD ilf r
WeakEnhancer Hoffman
UTR 5 UCSC
UTR 3 UCSC
TSS Hoffman
Transcribed Hoffman
TFBS ENCODE
**SuperEnhancer Hnisz
**Repressed Hoffman
Promoter UCSC
**PromoterFlanking Hoffman
Intron UCSC
H3K9ac Trynka
H3K4me3 Trynka
H3K4me1 Trynka
H3K27ac PGC2
H3K27ac Hnisz
FetalDHS Trynka
Enhancer Hoffman
Enhancer Andersson
DHS Trynka
DGF ENCODE
CTCF Hoffman
Conserved LindbladToh
Coding UCSC
Annotation
−20
0
20
40
60
Enrichment (prop h2 / prop snps)

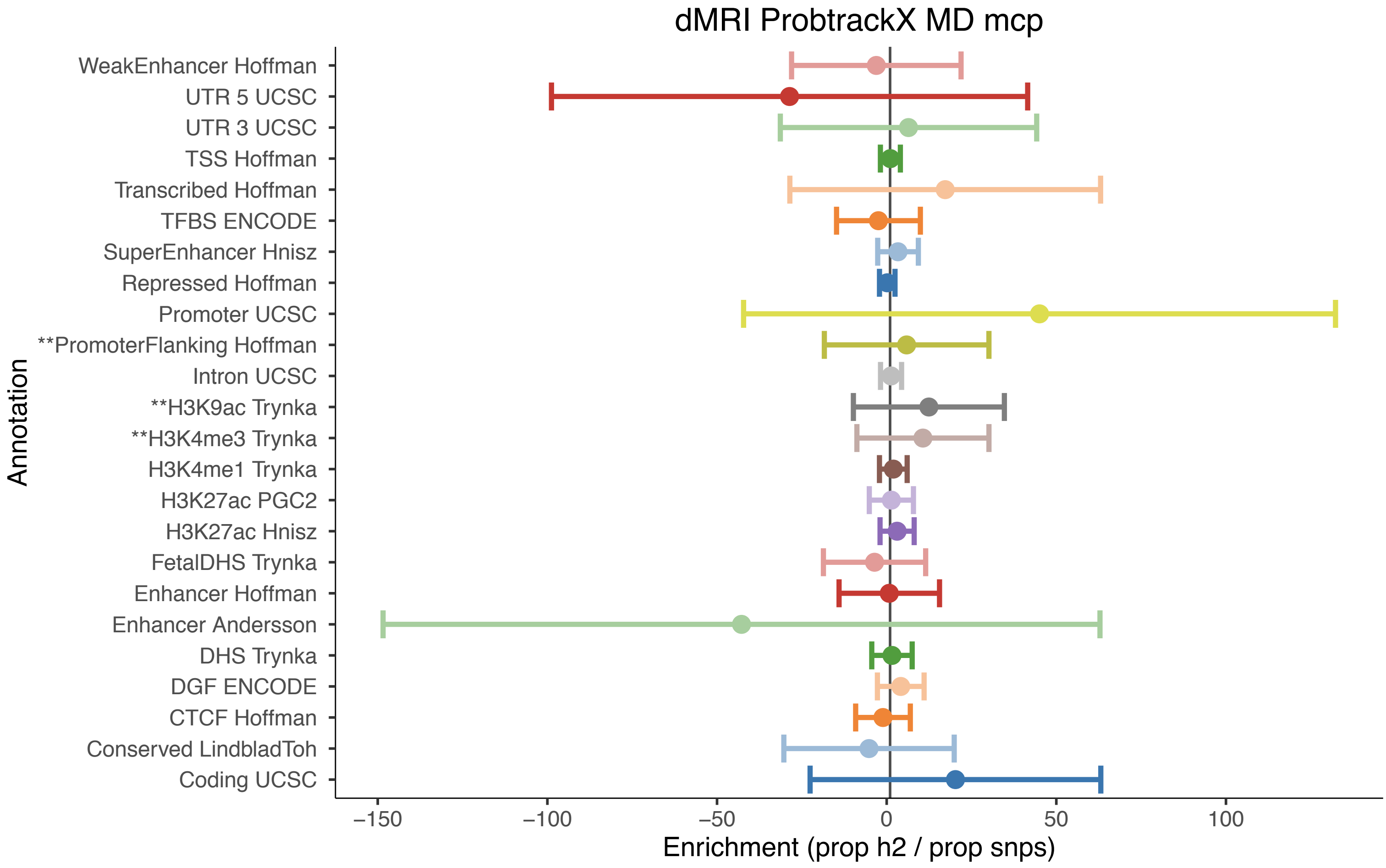

dMRI ProbtrackX MD mcp
Annotation
WeakEnhancer Hoffman
UTR 5 UCSC
UTR 3 UCSC
TSS Hoffman
Transcribed Hoffman
TFBS ENCODE
SuperEnhancer Hnisz
Repressed Hoffman
Promoter UCSC
**PromoterFlanking Hoffman
Intron UCSC
**H3K9ac Trynka
**H3K4me3 Trynka
H3K4me1 Trynka
H3K27ac PGC2
H3K27ac Hnisz
FetalDHS Trynka
Enhancer Hoffman
Enhancer Andersson
DHS Trynka
DGF ENCODE
CTCF Hoffman
Conserved LindbladToh
Coding UCSC
−150
−100
−50
0
50
100
Enrichment (prop h2 / prop snps)

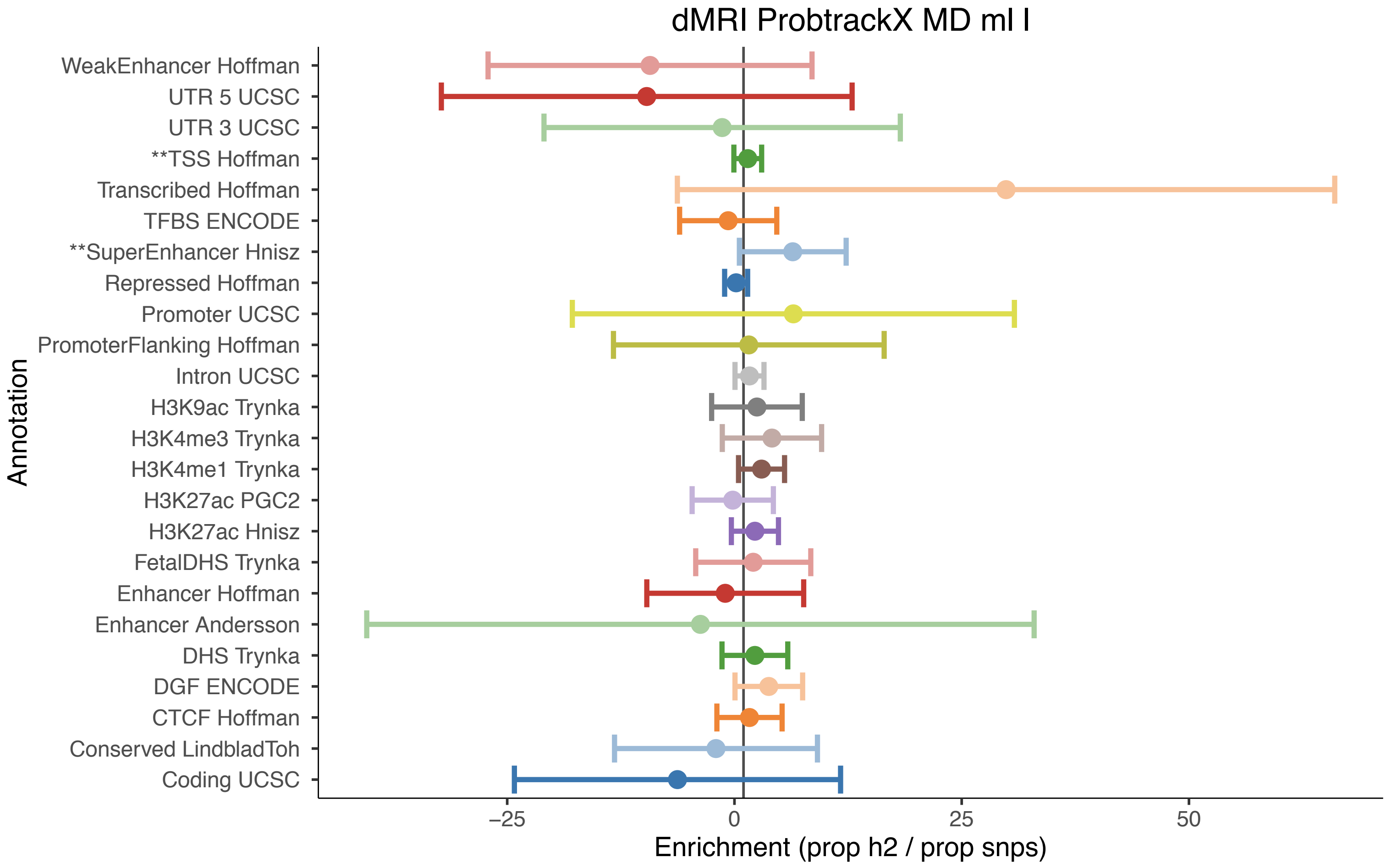
dMRI ProbtrackX MD ml I
WeakEnhancer Hoffman
UTR 5 UCSC
UTR 3 UCSC
**TSS Hoffman
Transcribed Hoffman
TFBS ENCODE
**SuperEnhancer Hnisz
Repressed Hoffman
Promoter UCSC
PromoterFlanking Hoffman
Intron UCSC
H3K9ac Trynka
H3K4me3 Trynka
H3K4me1 Trynka
H3K27ac PGC2
H3K27ac Hnisz
FetalDHS Trynka
Enhancer Hoffman
Enhancer Andersson
DHS Trynka
DGF ENCODE
CTCF Hoffman
Conserved LindbladToh
Coding UCSC
Annotation
−25
0
25
50
Enrichment (prop h2 / prop snps)

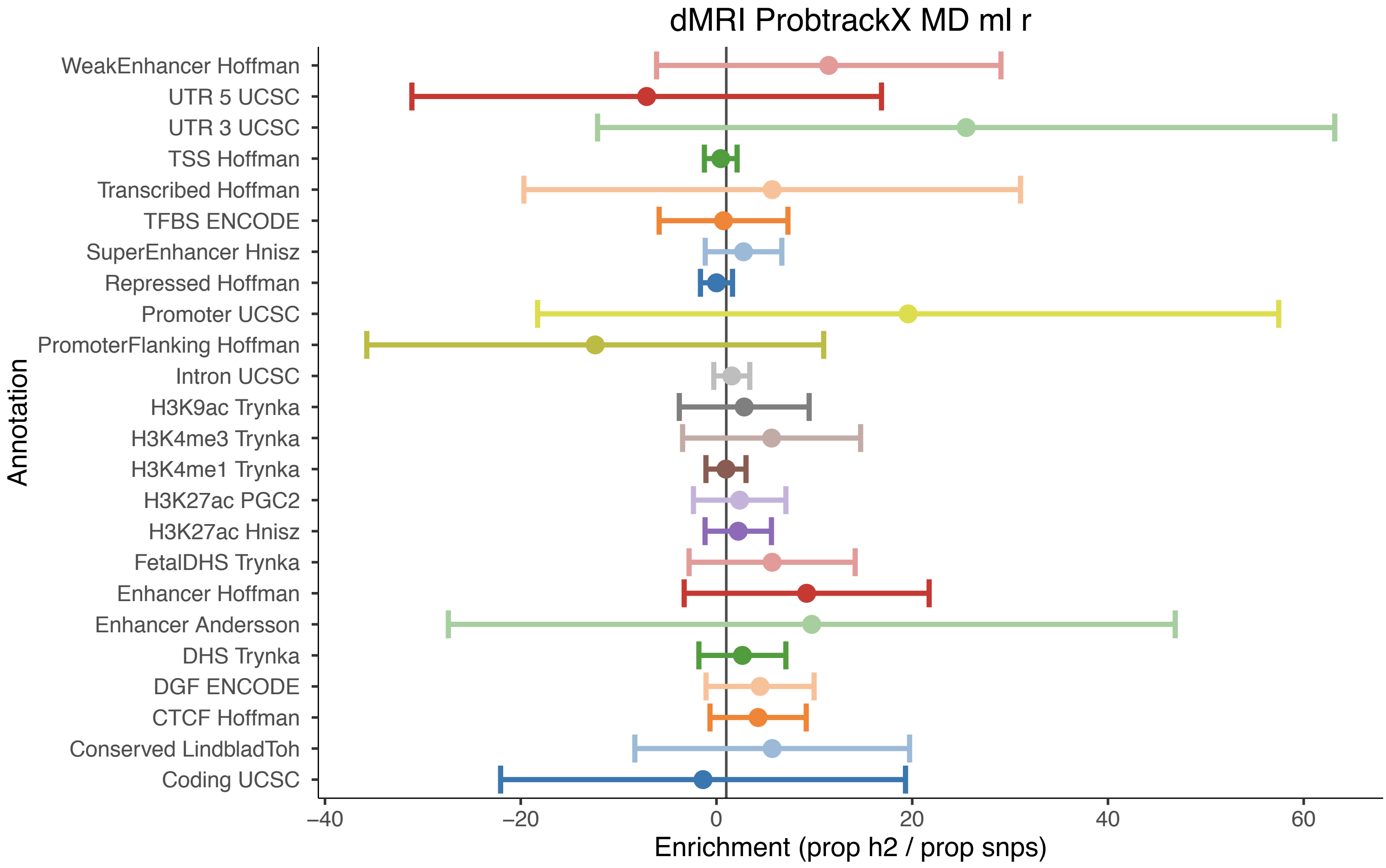

dMRI ProbtrackX MD ml r
Annotation
WeakEnhancer Hoffman
UTR 5 UCSC
UTR 3 UCSC
TSS Hoffman
Transcribed Hoffman
TFBS ENCODE
SuperEnhancer Hnisz
Repressed Hoffman
Promoter UCSC
PromoterFlanking Hoffman
Intron UCSC
H3K9ac Trynka
H3K4me3 Trynka
H3K4me1 Trynka
H3K27ac PGC2
H3K27ac Hnisz
FetalDHS Trynka
Enhancer Hoffman
Enhancer Andersson
DHS Trynka
DGF ENCODE
CTCF Hoffman
Conserved LindbladToh
Coding UCSC
−40
−20
0
20
40
60
Enrichment (prop h2 / prop snps)

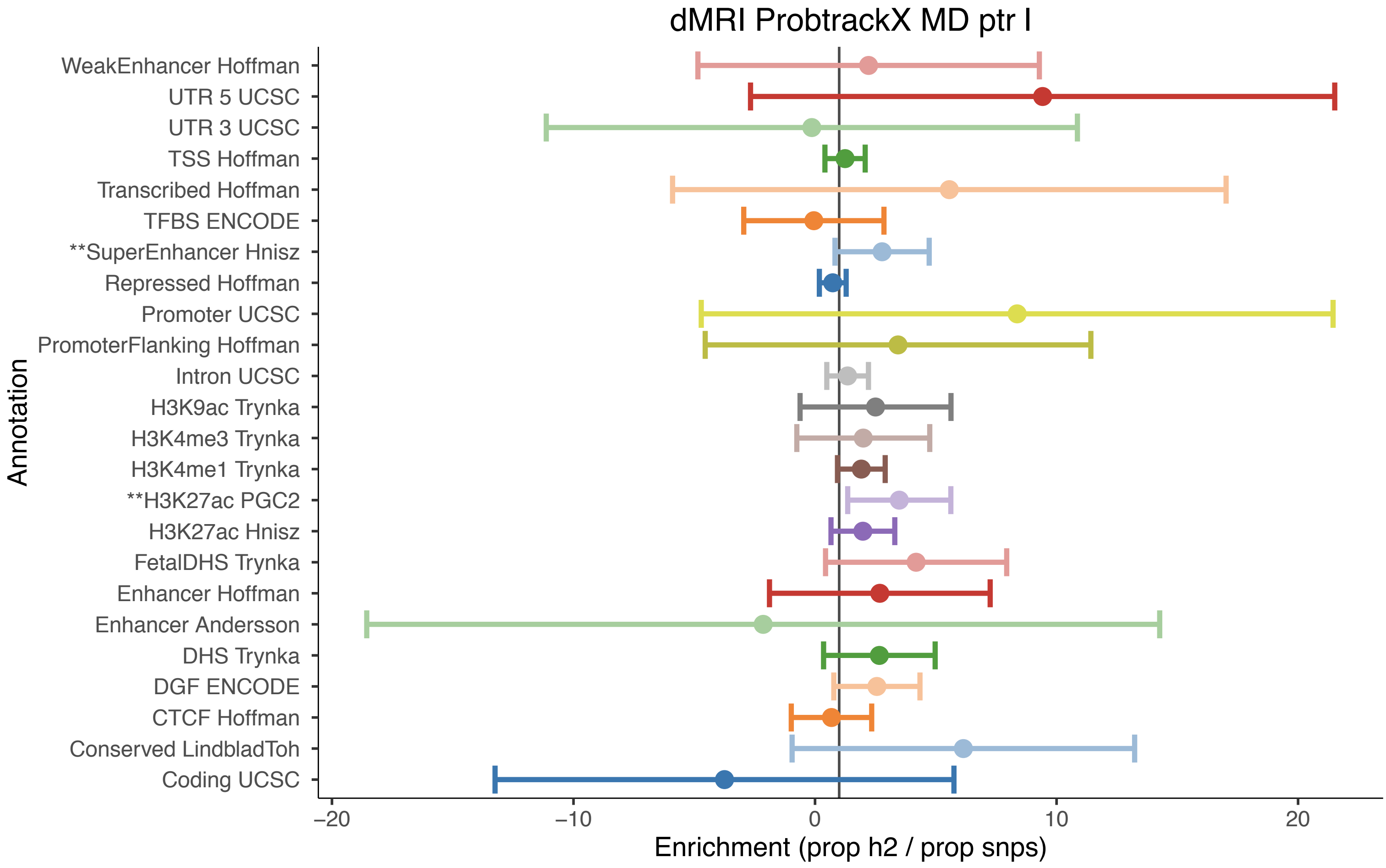

dMRI ProbtrackX MD ptr I
Annotation
WeakEnhancer Hoffman
UTR 5 UCSC
UTR 3 UCSC
TSS Hoffman
Transcribed Hoffman
TFBS ENCODE
**SuperEnhancer Hnisz
Repressed Hoffman
Promoter UCSC
PromoterFlanking Hoffman
Intron UCSC
H3K9ac Trynka
H3K4me3 Trynka
H3K4me1 Trynka
**H3K27ac PGC2
H3K27ac Hnisz
FetalDHS Trynka
Enhancer Hoffman
Enhancer Andersson
DHS Trynka
DGF ENCODE
CTCF Hoffman
Conserved LindbladToh
Coding UCSC
−20
−10
0
10
20
Enrichment (prop h2 / prop snps)

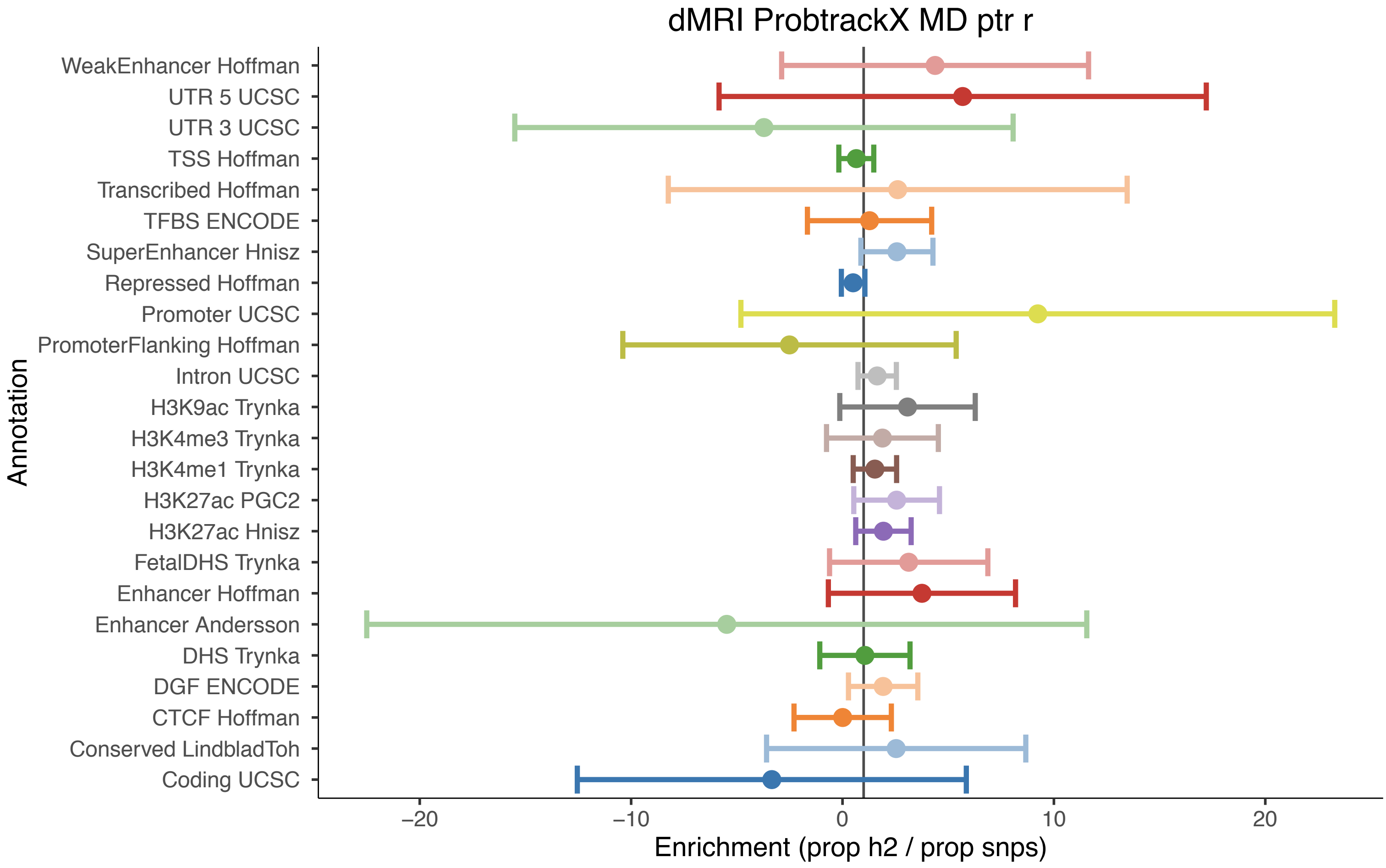
dMRI ProbtrackX MD ptr r
WeakEnhancer Hoffman
UTR 5 UCSC
UTR 3 UCSC
TSS Hoffman
Transcribed Hoffman
TFBS ENCODE
SuperEnhancer Hnisz
Repressed Hoffman
Promoter UCSC
PromoterFlanking Hoffman
Intron UCSC
H3K9ac Trynka
H3K4me3 Trynka
H3K4me1 Trynka
H3K27ac PGC2
H3K27ac Hnisz
FetalDHS Trynka
Enhancer Hoffman
Enhancer Andersson
DHS Trynka
DGF ENCODE
CTCF Hoffman
Conserved LindbladToh
Coding UCSC
Annotation
−20
−10
0
10
20
Enrichment (prop h2 / prop snps)

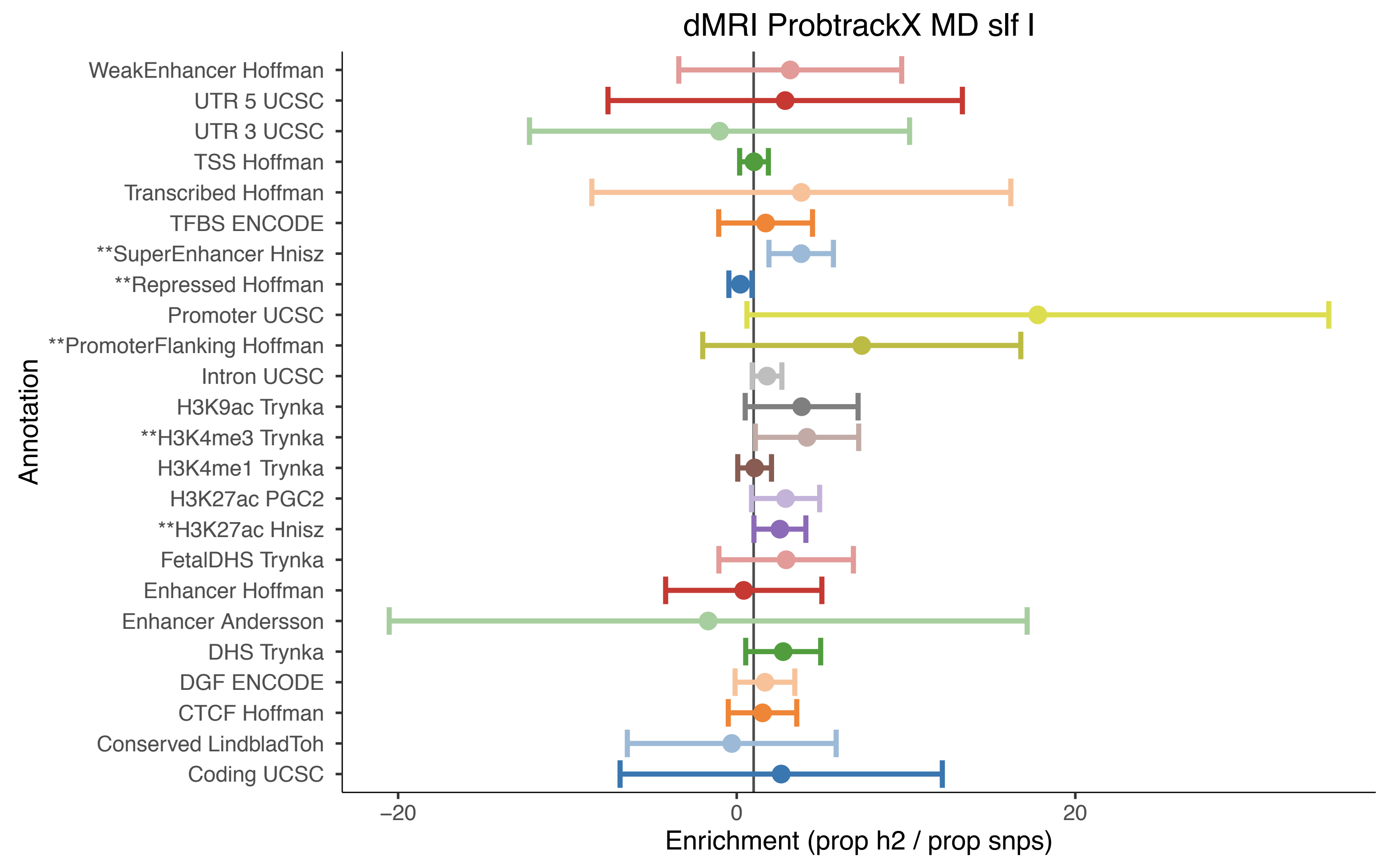
dMRI ProbtrackX MD slf I
WeakEnhancer Hoffman
UTR 5 UCSC
UTR 3 UCSC
TSS Hoffman
Transcribed Hoffman
TFBS ENCODE
**SuperEnhancer Hnisz
**Repressed Hoffman
Promoter UCSC
**PromoterFlanking Hoffman
Intron UCSC
H3K9ac Trynka
**H3K4me3 Trynka
H3K4me1 Trynka
H3K27ac PGC2
**H3K27ac Hnisz
FetalDHS Trynka
Enhancer Hoffman
Enhancer Andersson
DHS Trynka
DGF ENCODE
CTCF Hoffman
Conserved LindbladToh
Coding UCSC
Annotation
−20
0
20
Enrichment (prop h2 / prop snps)

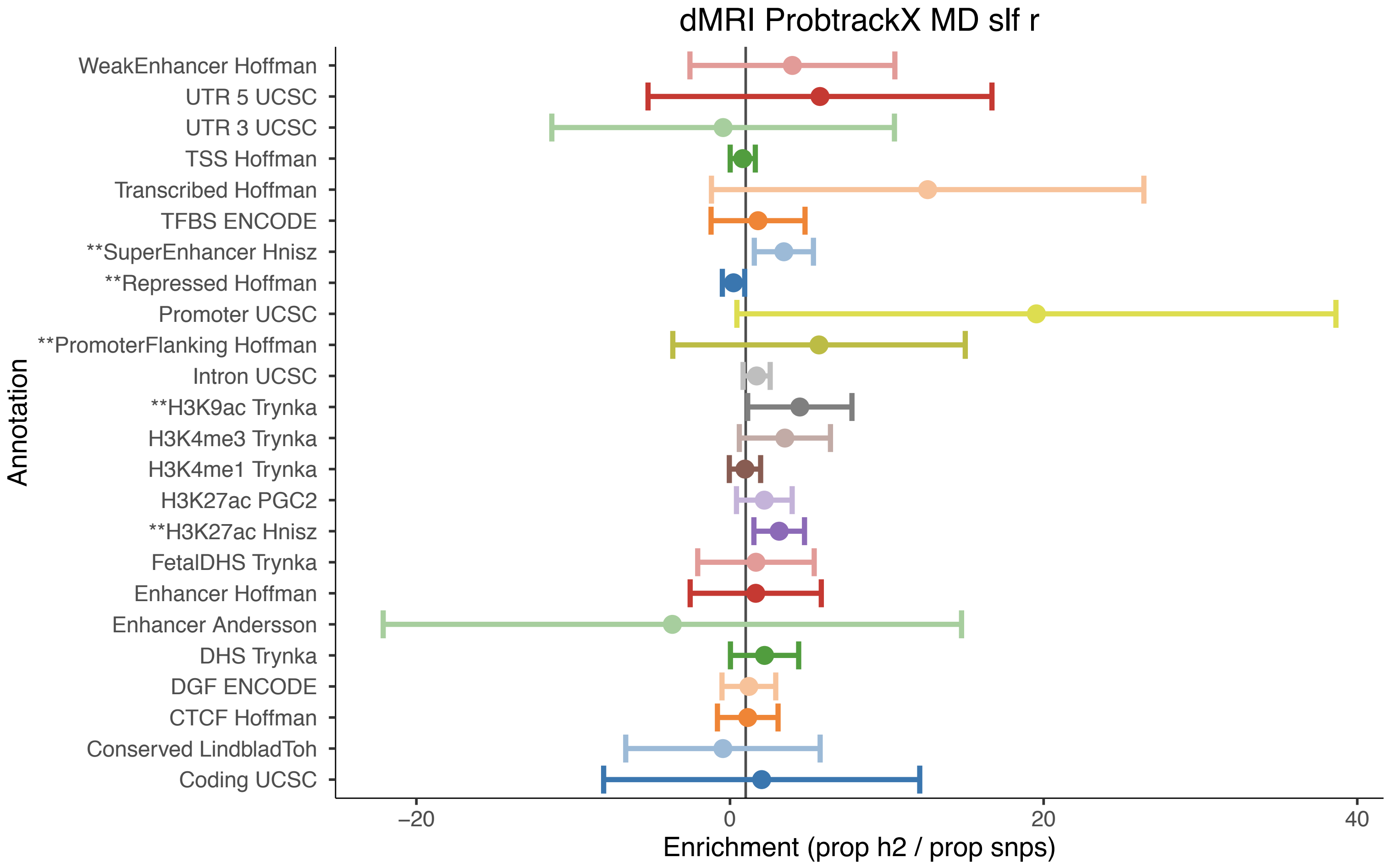

dMRI ProbtrackX MD slf r
WeakEnhancer Hoffman
UTR 5 UCSC
UTR 3 UCSC
TSS Hoffman
Transcribed Hoffman
TFBS ENCODE
**SuperEnhancer Hnisz
**Repressed Hoffman
Promoter UCSC
**PromoterFlanking Hoffman
Intron UCSC
**H3K9ac Trynka
H3K4me3 Trynka
H3K4me1 Trynka
H3K27ac PGC2
**H3K27ac Hnisz
FetalDHS Trynka
Enhancer Hoffman
Enhancer Andersson
DHS Trynka
DGF ENCODE
CTCF Hoffman
Conserved LindbladToh
Coding UCSC
Annotation
−20
0
20
40
Enrichment (prop h2 / prop snps)

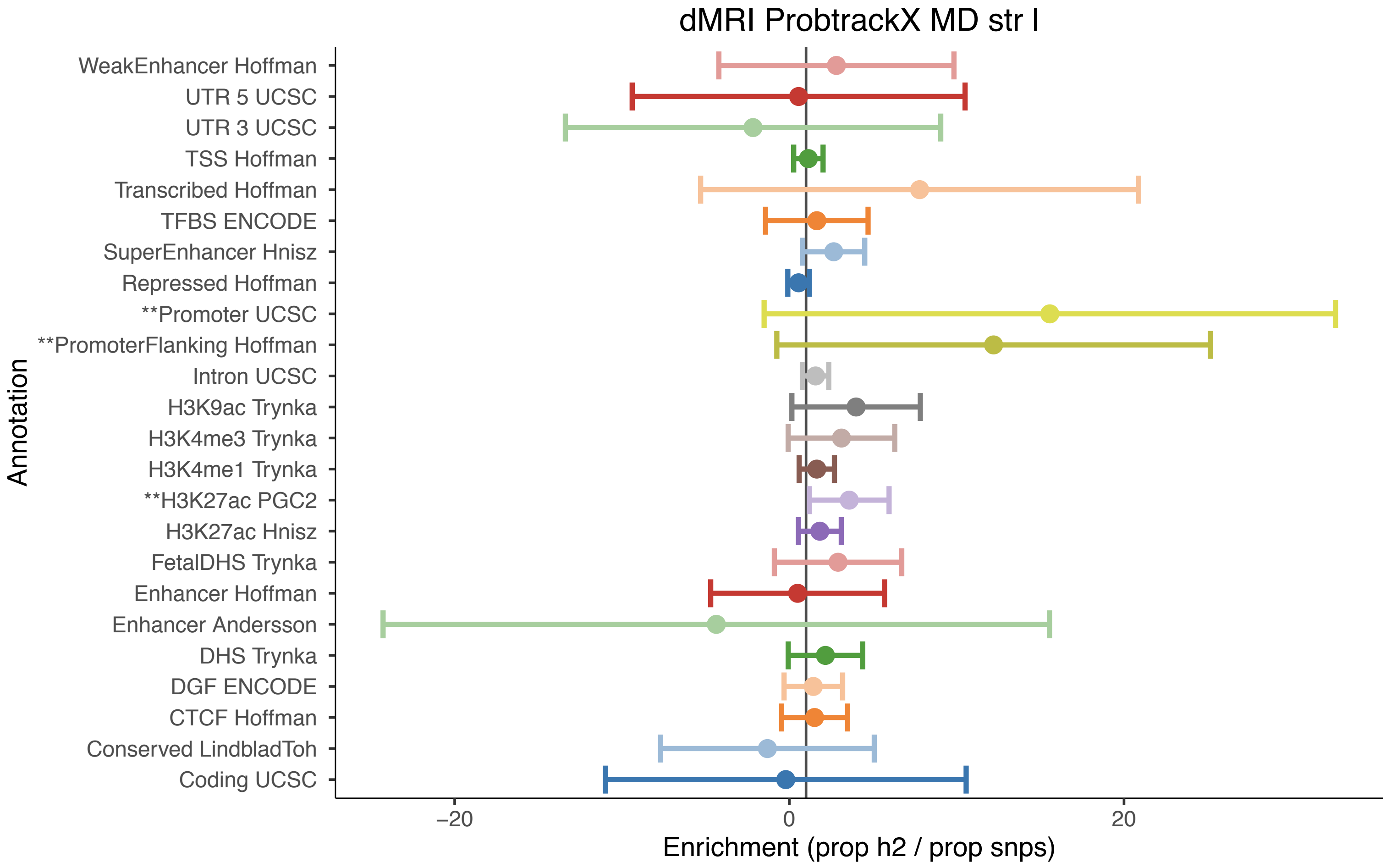

dMRI ProbtrackX MD str I
WeakEnhancer Hoffman
UTR 5 UCSC
UTR 3 UCSC
TSS Hoffman
Transcribed Hoffman
TFBS ENCODE
SuperEnhancer Hnisz
Repressed Hoffman
**Promoter UCSC
**PromoterFlanking Hoffman
Intron UCSC
H3K9ac Trynka
H3K4me3 Trynka
H3K4me1 Trynka
**H3K27ac PGC2
H3K27ac Hnisz
FetalDHS Trynka
Enhancer Hoffman
Enhancer Andersson
DHS Trynka
DGF ENCODE
CTCF Hoffman
Conserved LindbladToh
Coding UCSC
Annotation
−20
0
20
Enrichment (prop h2 / prop snps)

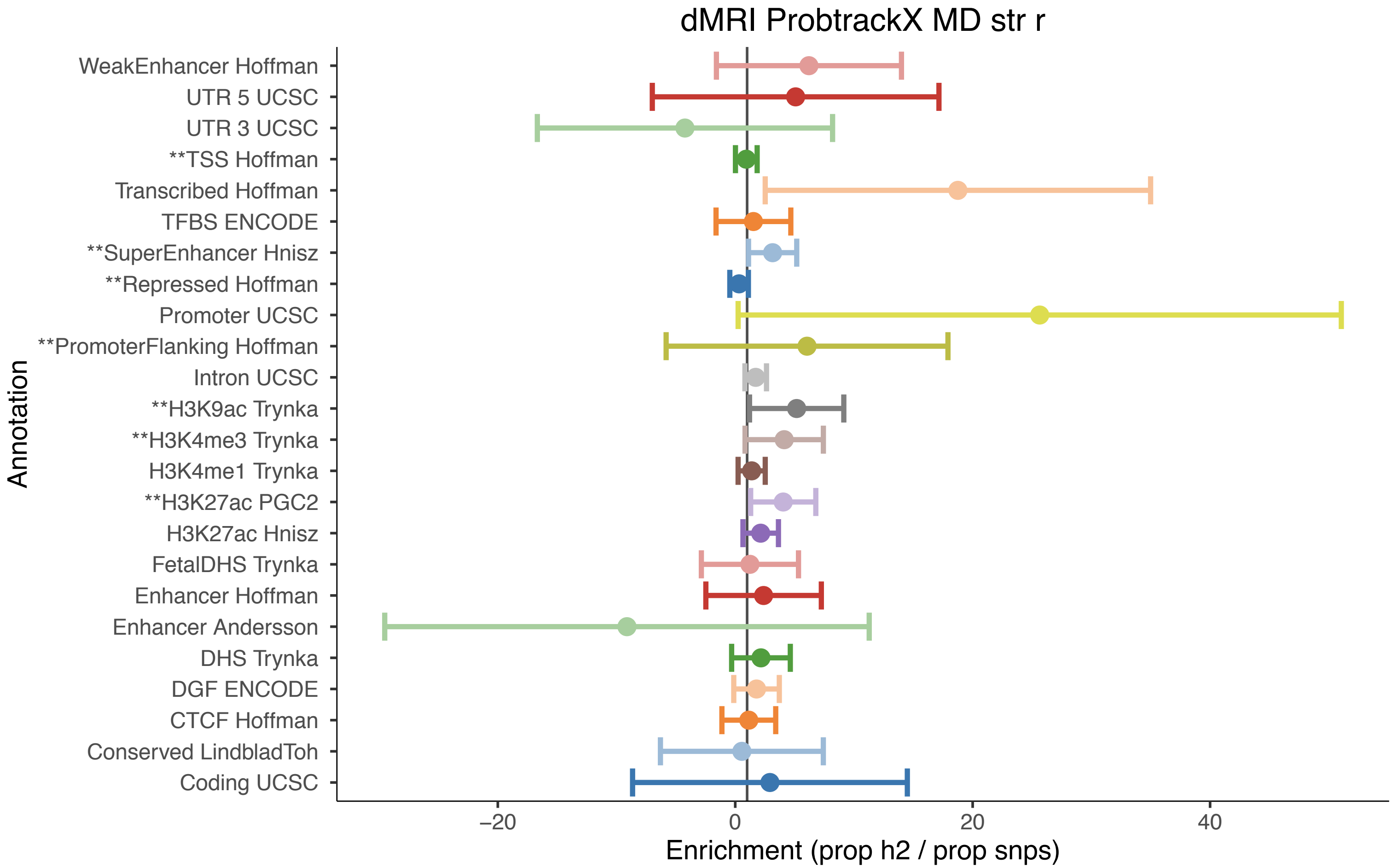
dMRI ProbtrackX MD str r
WeakEnhancer Hoffman
UTR 5 UCSC
UTR 3 UCSC
**TSS Hoffman
Transcribed Hoffman
TFBS ENCODE
**SuperEnhancer Hnisz
**Repressed Hoffman
Promoter UCSC
**PromoterFlanking Hoffman
Intron UCSC
**H3K9ac Trynka
**H3K4me3 Trynka
H3K4me1 Trynka
**H3K27ac PGC2
H3K27ac Hnisz
FetalDHS Trynka
Enhancer Hoffman
Enhancer Andersson
DHS Trynka
DGF ENCODE
CTCF Hoffman
Conserved LindbladToh
Coding UCSC
Annotation
−20
0
20
40
Enrichment (prop h2 / prop snps)

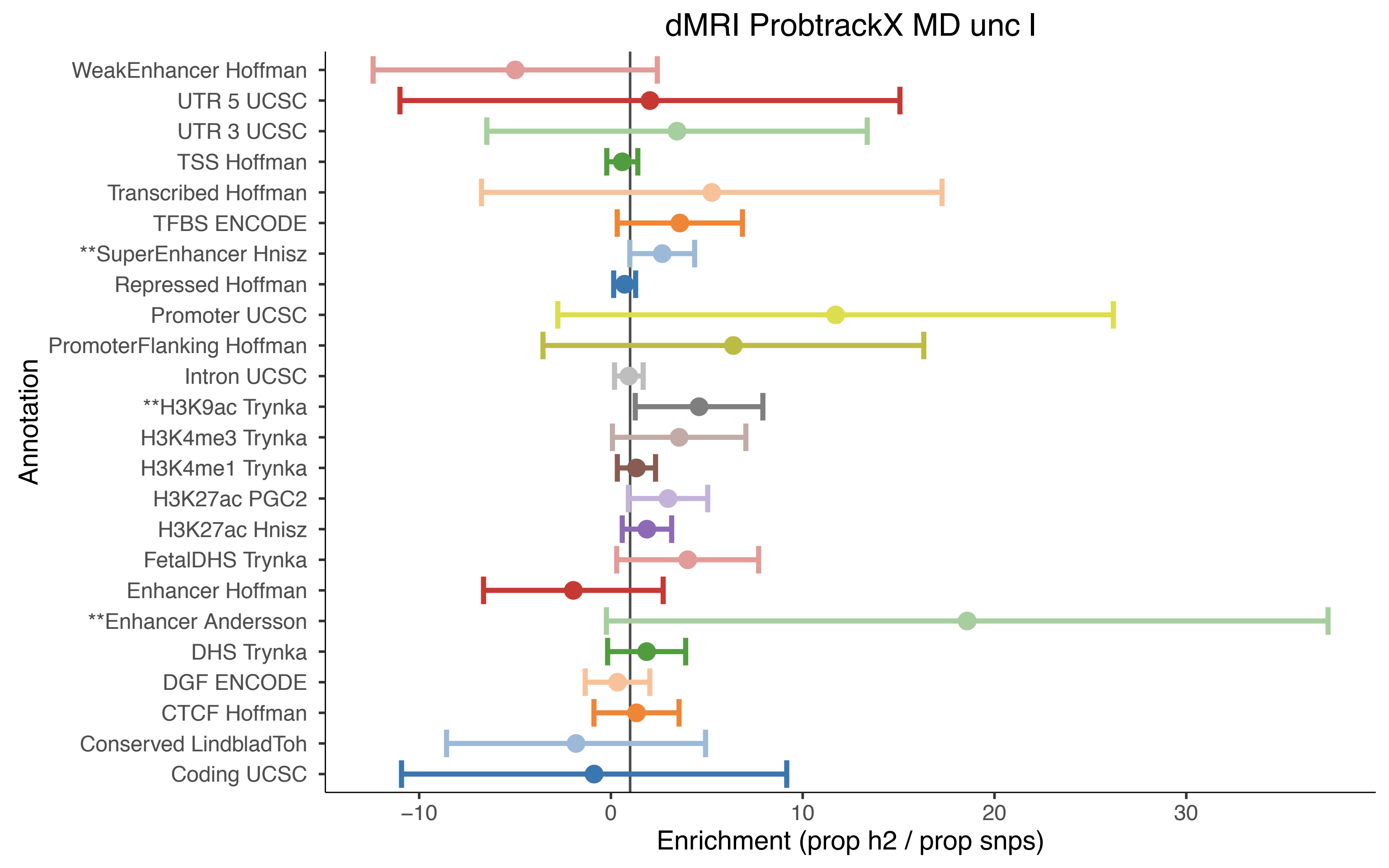

dMRI ProbtrackX MD unc I
WeakEnhancer Hoffman
UTR 5 UCSC
UTR 3 UCSC
TSS Hoffman
Transcribed Hoffman
TFBS ENCODE
**SuperEnhancer Hnisz
Repressed Hoffman
Promoter UCSC
PromoterFlanking Hoffman
Intron UCSC
**H3K9ac Trynka
H3K4me3 Trynka
H3K4me1 Trynka
H3K27ac PGC2
H3K27ac Hnisz
FetalDHS Trynka
Enhancer Hoffman
**Enhancer Andersson
DHS Trynka
DGF ENCODE
CTCF Hoffman
Conserved LindbladToh
Coding UCSC
Annotation
−10
0
10
20
30
Enrichment (prop h2 / prop snps)

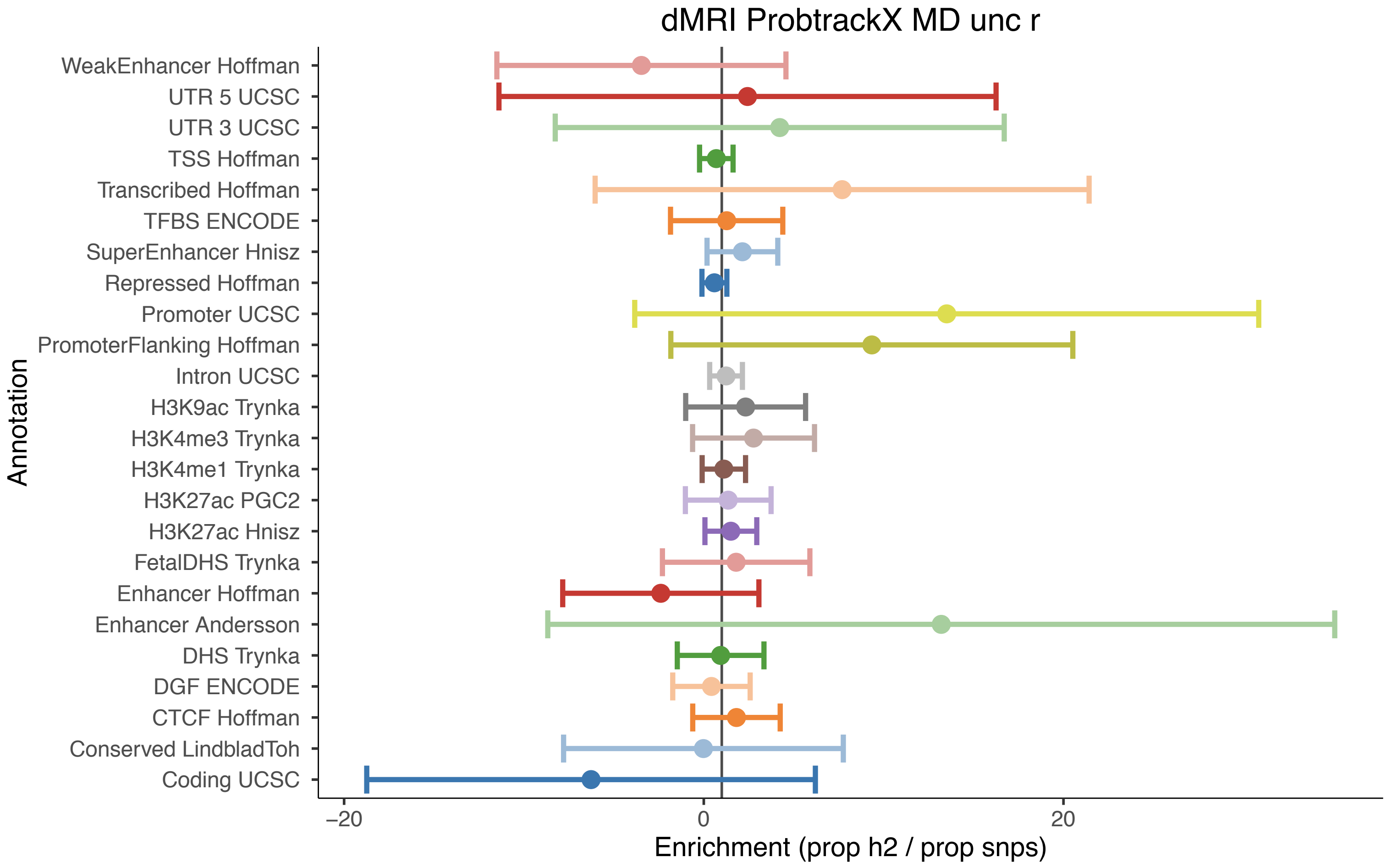
dMRI ProbtrackX MD unc r
WeakEnhancer Hoffman
UTR 5 UCSC
UTR 3 UCSC
TSS Hoffman
Transcribed Hoffman
TFBS ENCODE
SuperEnhancer Hnisz
Repressed Hoffman
Promoter UCSC
PromoterFlanking Hoffman
Intron UCSC
H3K9ac Trynka
H3K4me3 Trynka
H3K4me1 Trynka
H3K27ac PGC2
H3K27ac Hnisz
FetalDHS Trynka
Enhancer Hoffman
Enhancer Andersson
DHS Trynka
DGF ENCODE
CTCF Hoffman
Conserved LindbladToh
Coding UCSC
Annotation
−20
0
20
Enrichment (prop h2 / prop snps)

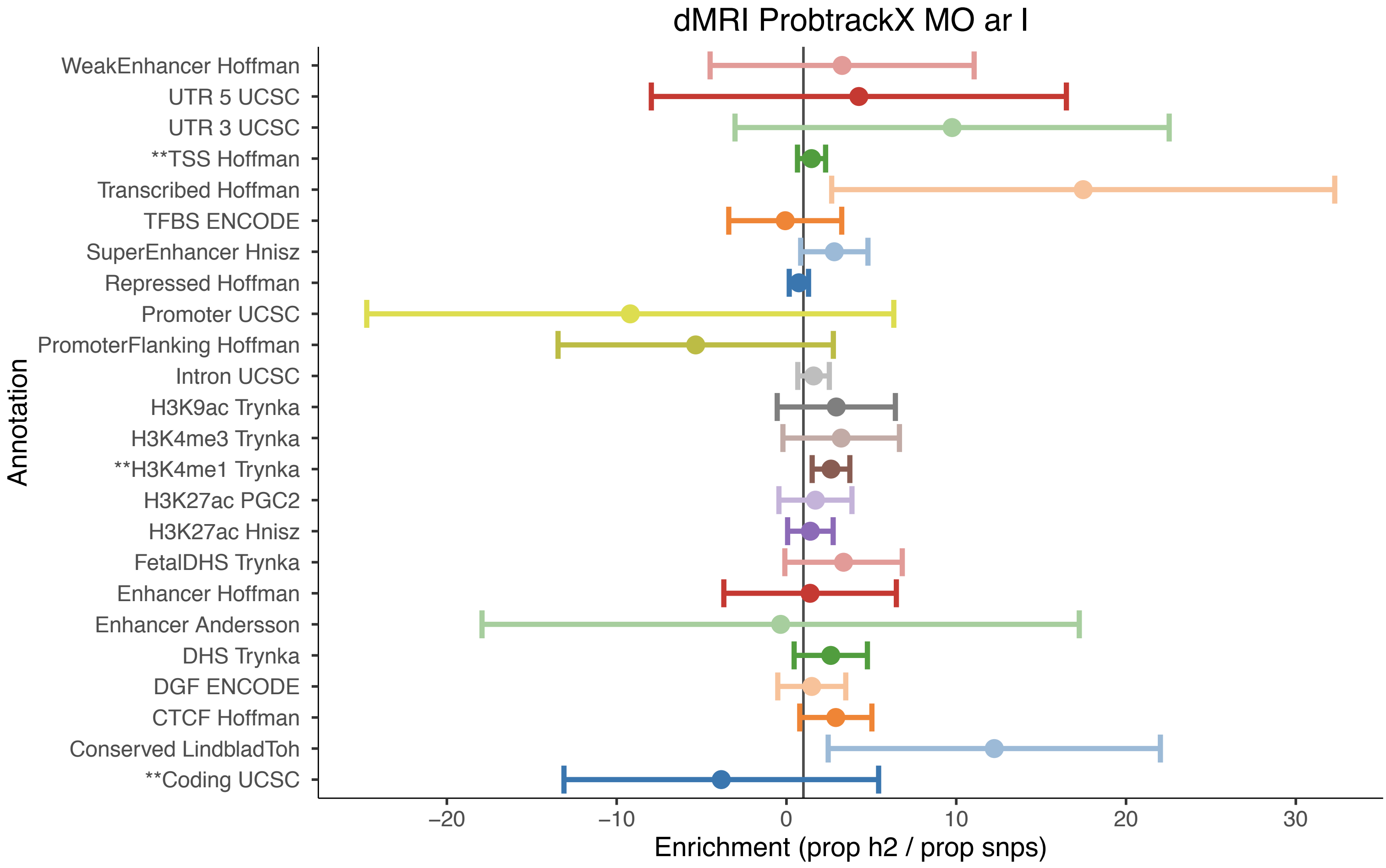
dMRI ProbtrackX MO ar l
WeakEnhancer Hoffman
UTR 5 UCSC
UTR 3 UCSC
**TSS Hoffman
Transcribed Hoffman
TFBS ENCODE
SuperEnhancer Hnisz
Repressed Hoffman
Promoter UCSC
PromoterFlanking Hoffman
Intron UCSC
H3K9ac Trynka
H3K4me3 Trynka
**H3K4me1 Trynka
H3K27ac PGC2
H3K27ac Hnisz
FetalDHS Trynka
Enhancer Hoffman
Enhancer Andersson
DHS Trynka
DGF ENCODE
CTCF Hoffman
Conserved LindbladToh
**Coding UCSC
Annotation
−20
−10
0
10
20
30
Enrichment (prop h2 / prop snps)

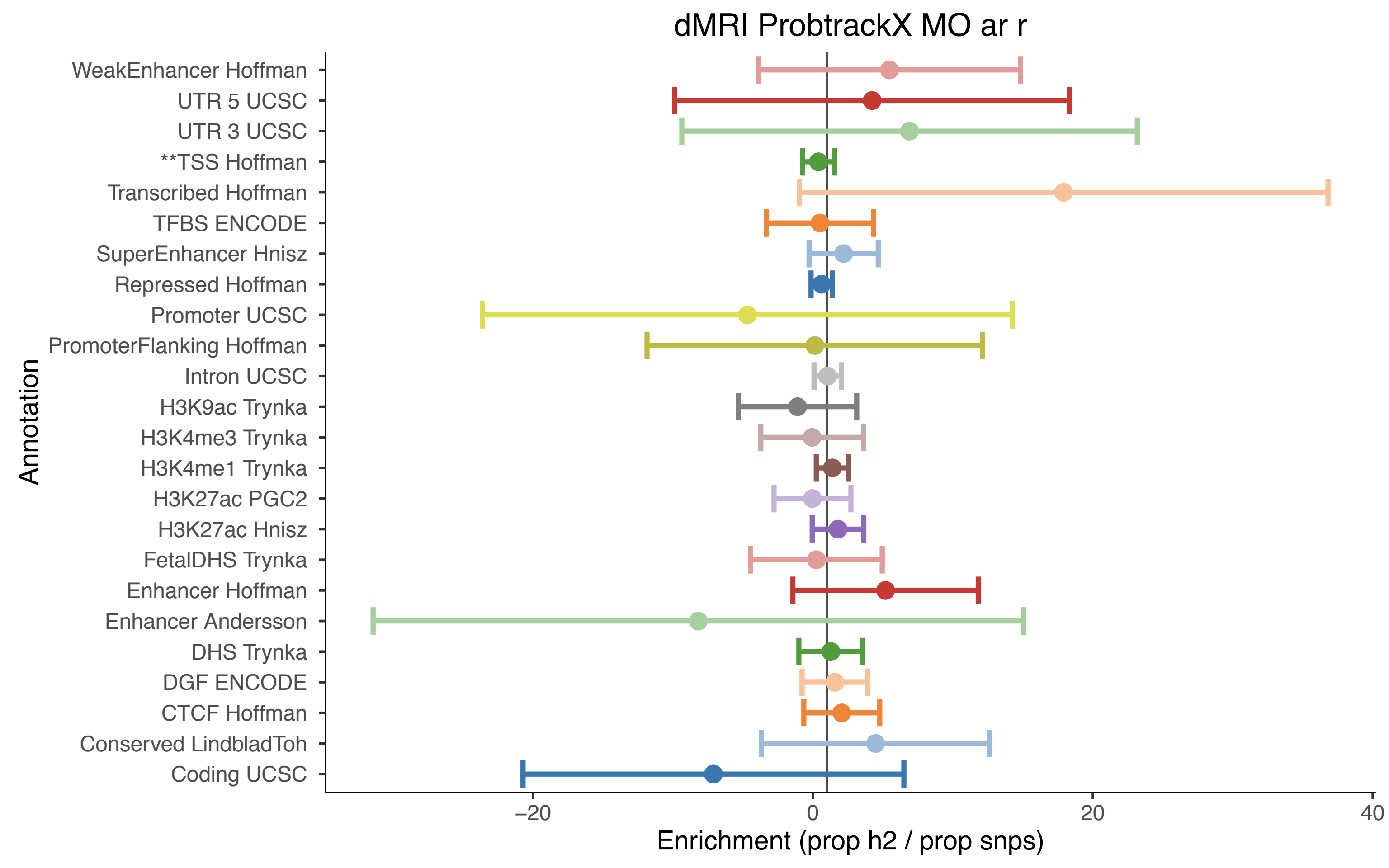
dMRI ProbtrackX MO ar r
WeakEnhancer Hoffman
UTR 5 UCSC
UTR 3 UCSC
**TSS Hoffman
Transcribed Hoffman
TFBS ENCODE
SuperEnhancer Hnisz
Repressed Hoffman
Promoter UCSC
PromoterFlanking Hoffman
Intron UCSC
H3K9ac Trynka
H3K4me3 Trynka
H3K4me1 Trynka
H3K27ac PGC2
H3K27ac Hnisz
FetalDHS Trynka
Enhancer Hoffman
Enhancer Andersson
DHS Trynka
DGF ENCODE
CTCF Hoffman
Conserved LindbladToh
Coding UCSC
Annotation
−20
0
20
40
Enrichment (prop h2 / prop snps)

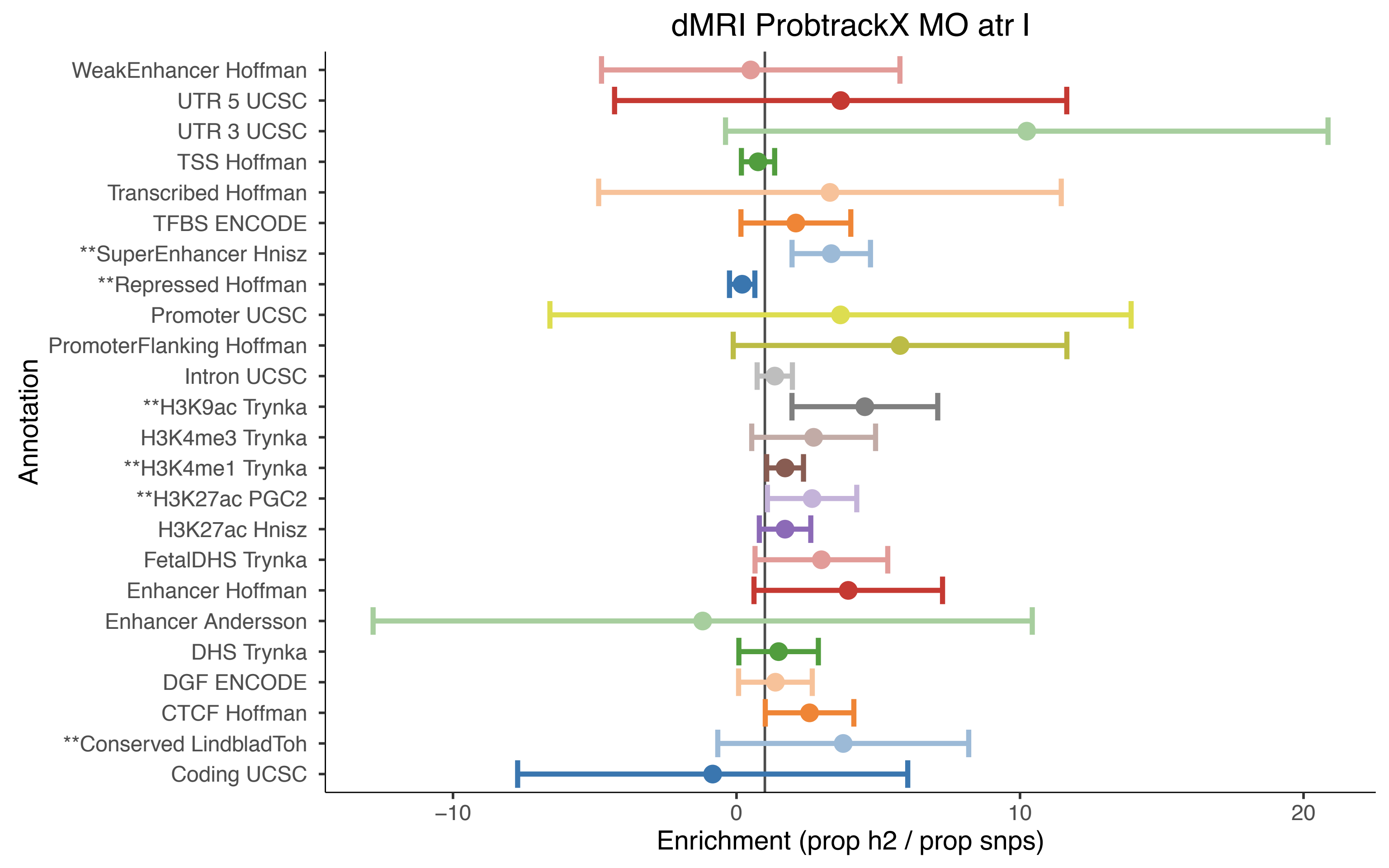

dMRI ProbtrackX MO atr l
WeakEnhancer Hoffman
UTR 5 UCSC
UTR 3 UCSC
TSS Hoffman
Transcribed Hoffman
TFBS ENCODE
**SuperEnhancer Hnisz
**Repressed Hoffman
Promoter UCSC
PromoterFlanking Hoffman
Intron UCSC
**H3K9ac Trynka
H3K4me3 Trynka
**H3K4me1 Trynka
**H3K27ac PGC2
H3K27ac Hnisz
FetalDHS Trynka
Enhancer Hoffman
Enhancer Andersson
DHS Trynka
DGF ENCODE
CTCF Hoffman
**Conserved LindbladToh
Coding UCSC
Annotation
−10
0
10
20
Enrichment (prop h2 / prop snps)

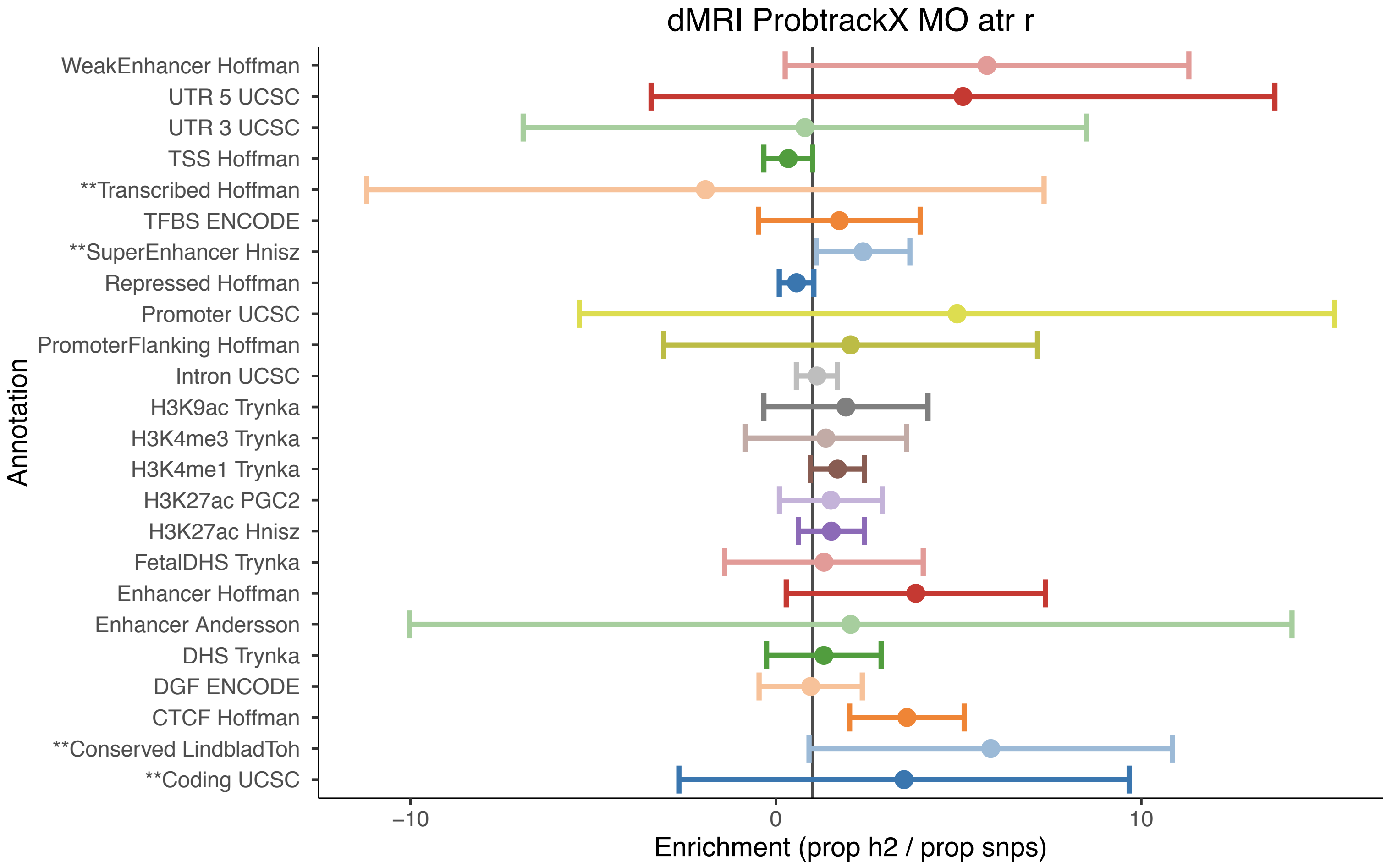
dMRI ProbtrackX MO atr r
WeakEnhancer Hoffman
UTR 5 UCSC
UTR 3 UCSC
TSS Hoffman
**Transcribed Hoffman
TFBS ENCODE
**SuperEnhancer Hnisz
Repressed Hoffman
Promoter UCSC
PromoterFlanking Hoffman
Intron UCSC
H3K9ac Trynka
H3K4me3 Trynka
H3K4me1 Trynka
H3K27ac PGC2
H3K27ac Hnisz
FetalDHS Trynka
Enhancer Hoffman
Enhancer Andersson
DHS Trynka
DGF ENCODE
CTCF Hoffman
**Conserved LindbladToh
**Coding UCSC
Annotation
−10
0
10
Enrichment (prop h2 / prop snps)

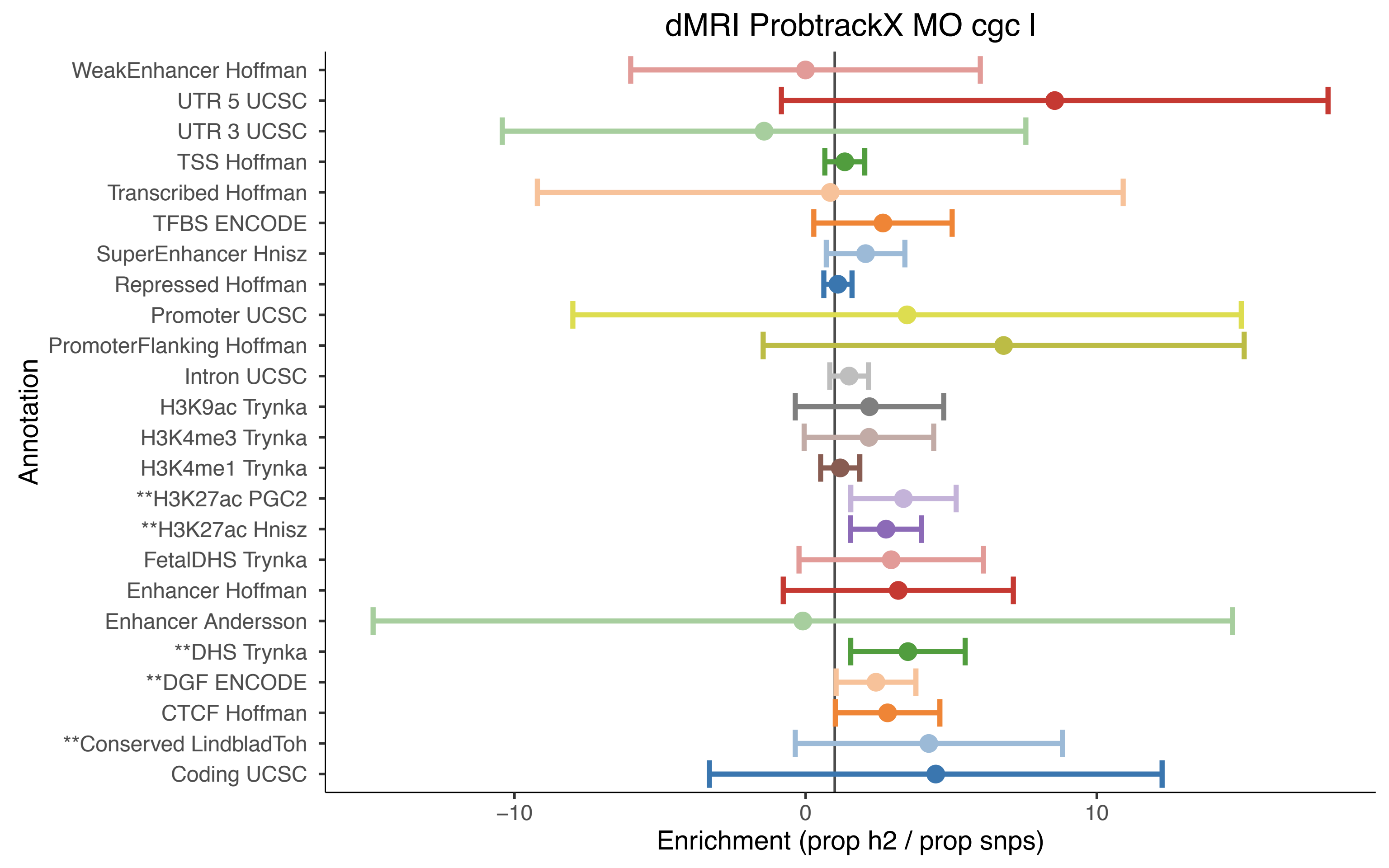
dMRI ProbtrackX MO cgc I
WeakEnhancer Hoffman
UTR 5 UCSC
UTR 3 UCSC
TSS Hoffman
Transcribed Hoffman
TFBS ENCODE
SuperEnhancer Hnisz
Repressed Hoffman
Promoter UCSC
PromoterFlanking Hoffman
Intron UCSC
H3K9ac Trynka
H3K4me3 Trynka
H3K4me1 Trynka
**H3K27ac PGC2
**H3K27ac Hnisz
FetalDHS Trynka
Enhancer Hoffman
Enhancer Andersson
**DHS Trynka
**DGF ENCODE
CTCF Hoffman
**Conserved LindbladToh
Coding UCSC
Annotation
−10
0
10
Enrichment (prop h2 / prop snps)

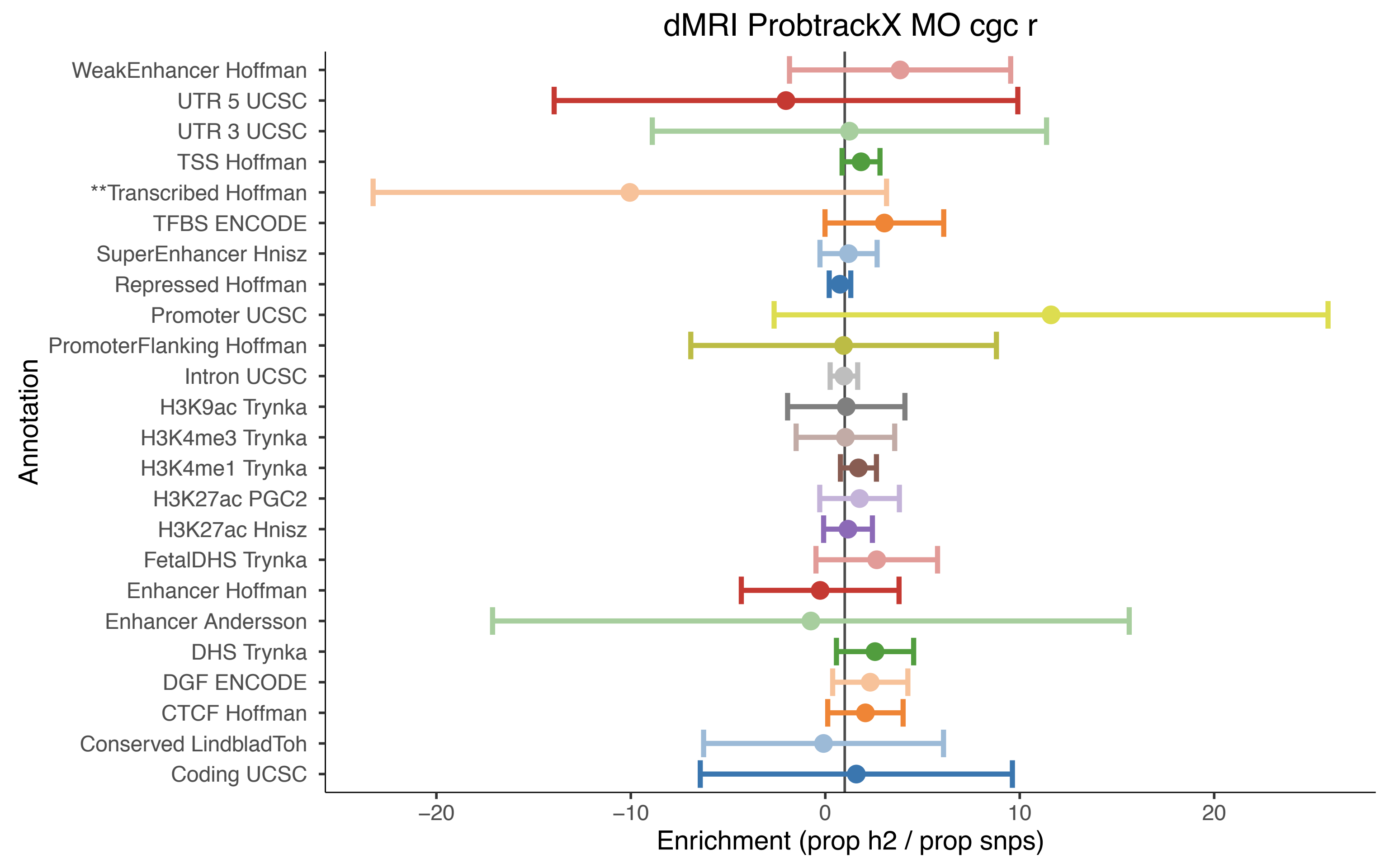

dMRI ProbtrackX MO cgc r
Annotation
WeakEnhancer Hoffman
UTR 5 UCSC
UTR 3 UCSC
TSS Hoffman
**Transcribed Hoffman
TFBS ENCODE
SuperEnhancer Hnisz
Repressed Hoffman
Promoter UCSC
PromoterFlanking Hoffman
Intron UCSC
H3K9ac Trynka
H3K4me3 Trynka
H3K4me1 Trynka
H3K27ac PGC2
H3K27ac Hnisz
FetalDHS Trynka
Enhancer Hoffman
Enhancer Andersson
DHS Trynka
DGF ENCODE
CTCF Hoffman
Conserved LindbladToh
Coding UCSC
−20
−10
0
10
20
Enrichment (prop h2 / prop snps)

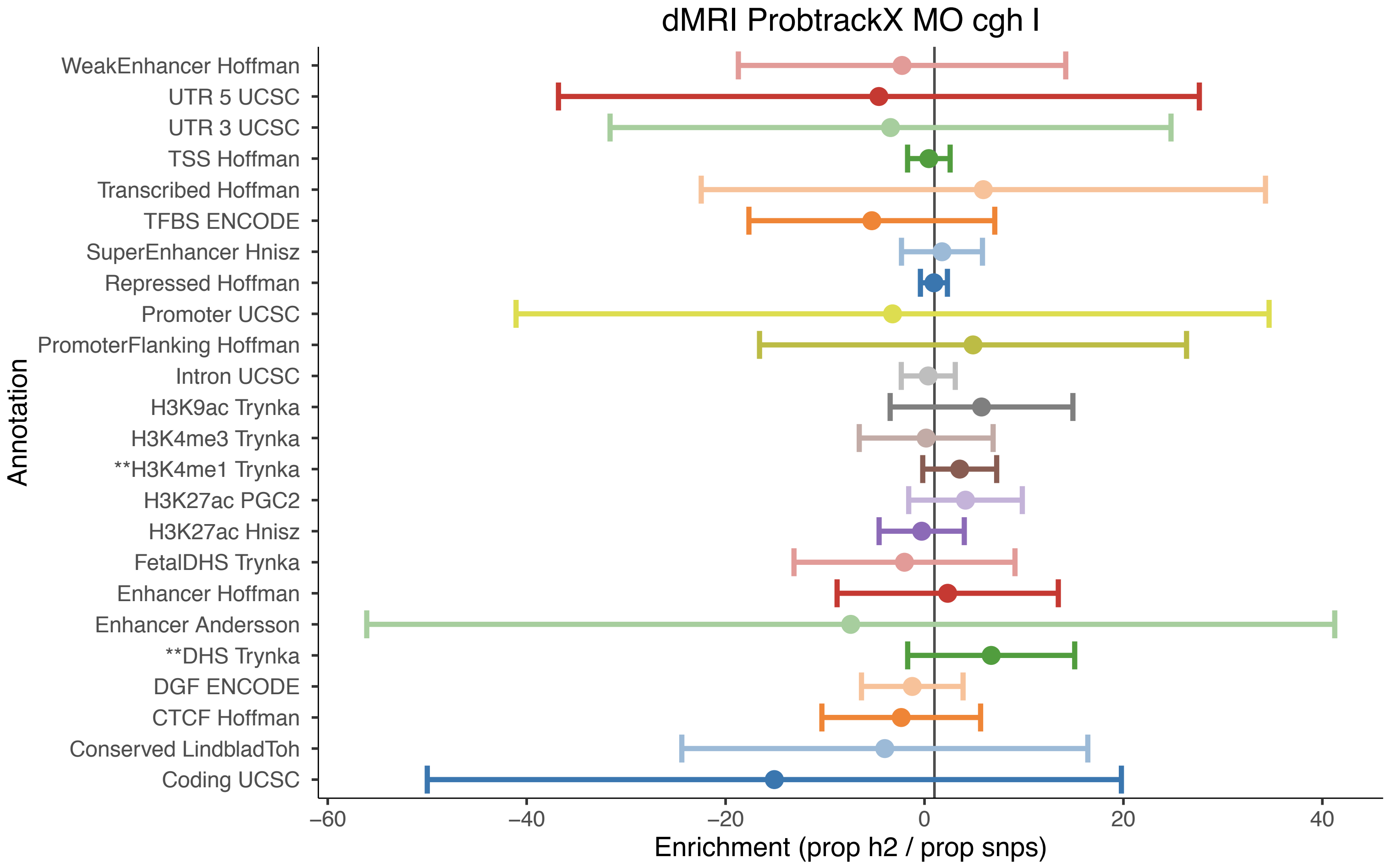

dMRI ProbtrackX MO cgh I
WeakEnhancer Hoffman
UTR 5 UCSC
UTR 3 UCSC
TSS Hoffman
Transcribed Hoffman
TFBS ENCODE
SuperEnhancer Hnisz
Repressed Hoffman
Promoter UCSC
PromoterFlanking Hoffman
Intron UCSC
H3K9ac Trynka
H3K4me3 Trynka
**H3K4me1 Trynka
H3K27ac PGC2
H3K27ac Hnisz
FetalDHS Trynka
Enhancer Hoffman
Enhancer Andersson
**DHS Trynka
DGF ENCODE
CTCF Hoffman
Conserved LindbladToh
Coding UCSC
Annotation
−60
−40
−20
0
20
40
Enrichment (prop h2 / prop snps)

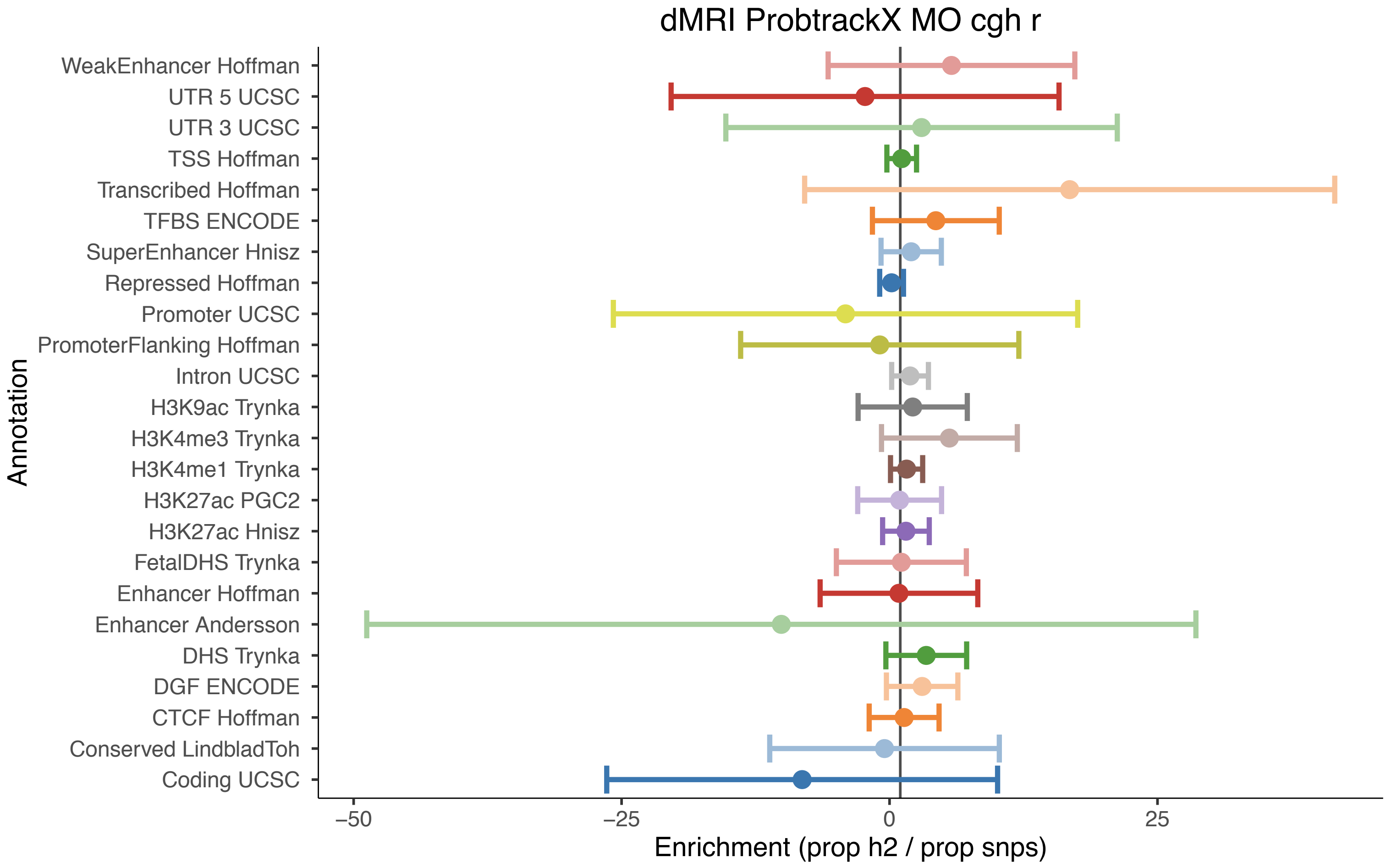

dMRI ProbtrackX MO cgh r
WeakEnhancer Hoffman
UTR 5 UCSC
UTR 3 UCSC
TSS Hoffman
Transcribed Hoffman
TFBS ENCODE
SuperEnhancer Hnisz
Repressed Hoffman
Promoter UCSC
PromoterFlanking Hoffman
Intron UCSC
H3K9ac Trynka
H3K4me3 Trynka
H3K4me1 Trynka
H3K27ac PGC2
H3K27ac Hnisz
FetalDHS Trynka
Enhancer Hoffman
Enhancer Andersson
DHS Trynka
DGF ENCODE
CTCF Hoffman
Conserved LindbladToh
Coding UCSC
Annotation
−50
−25
0
25
Enrichment (prop h2 / prop snps)

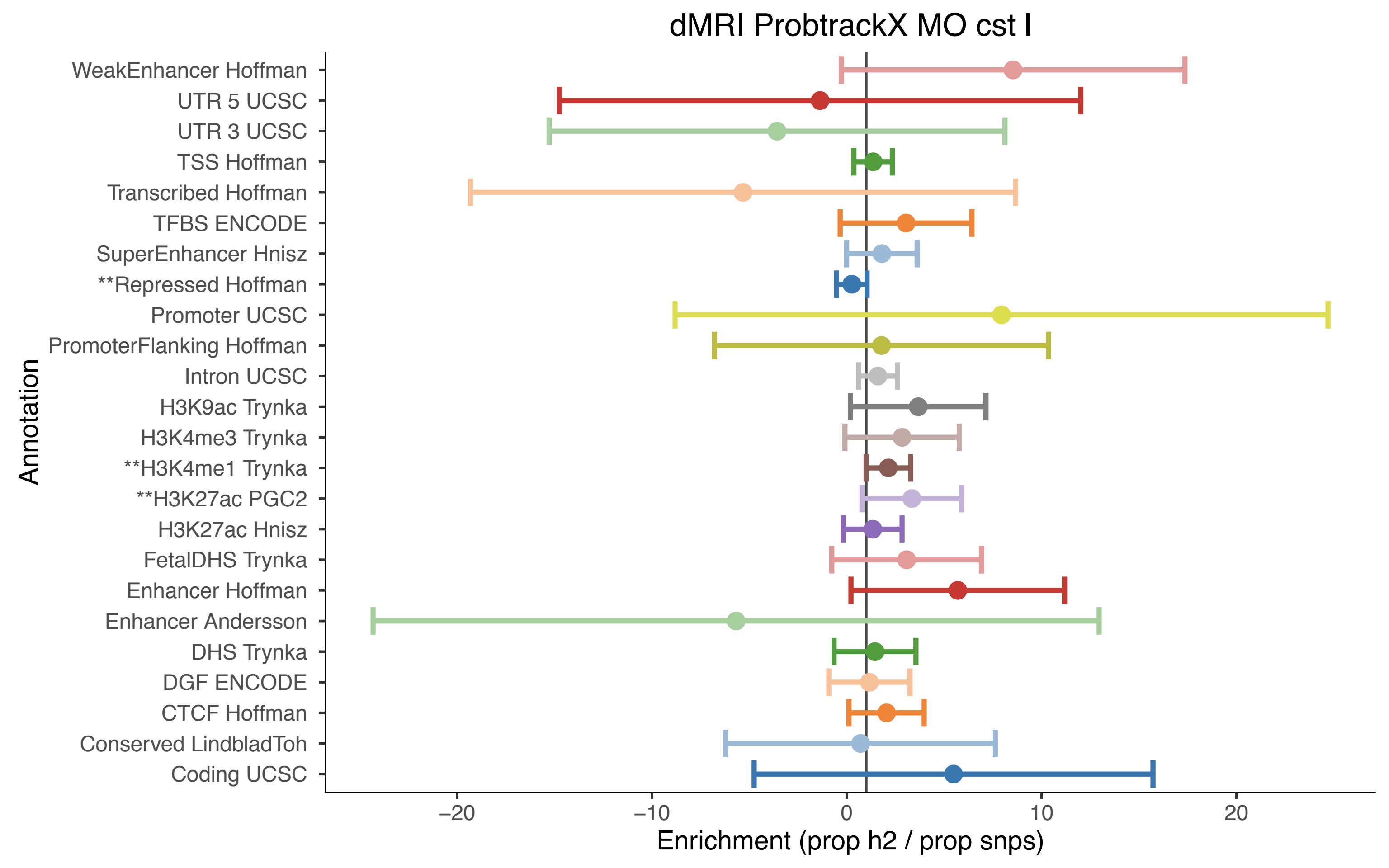

dMRI ProbtrackX MO cst l
WeakEnhancer Hoffman
UTR 5 UCSC
UTR 3 UCSC
TSS Hoffman
Transcribed Hoffman
TFBS ENCODE
SuperEnhancer Hnisz
**Repressed Hoffman
Promoter UCSC
PromoterFlanking Hoffman
Intron UCSC
H3K9ac Trynka
H3K4me3 Trynka
**H3K4me1 Trynka
**H3K27ac PGC2
H3K27ac Hnisz
FetalDHS Trynka
Enhancer Hoffman
Enhancer Andersson
DHS Trynka
DGF ENCODE
CTCF Hoffman
Conserved LindbladToh
Coding UCSC
Annotation
−20
−10
0
10
20
Enrichment (prop h2 / prop snps)

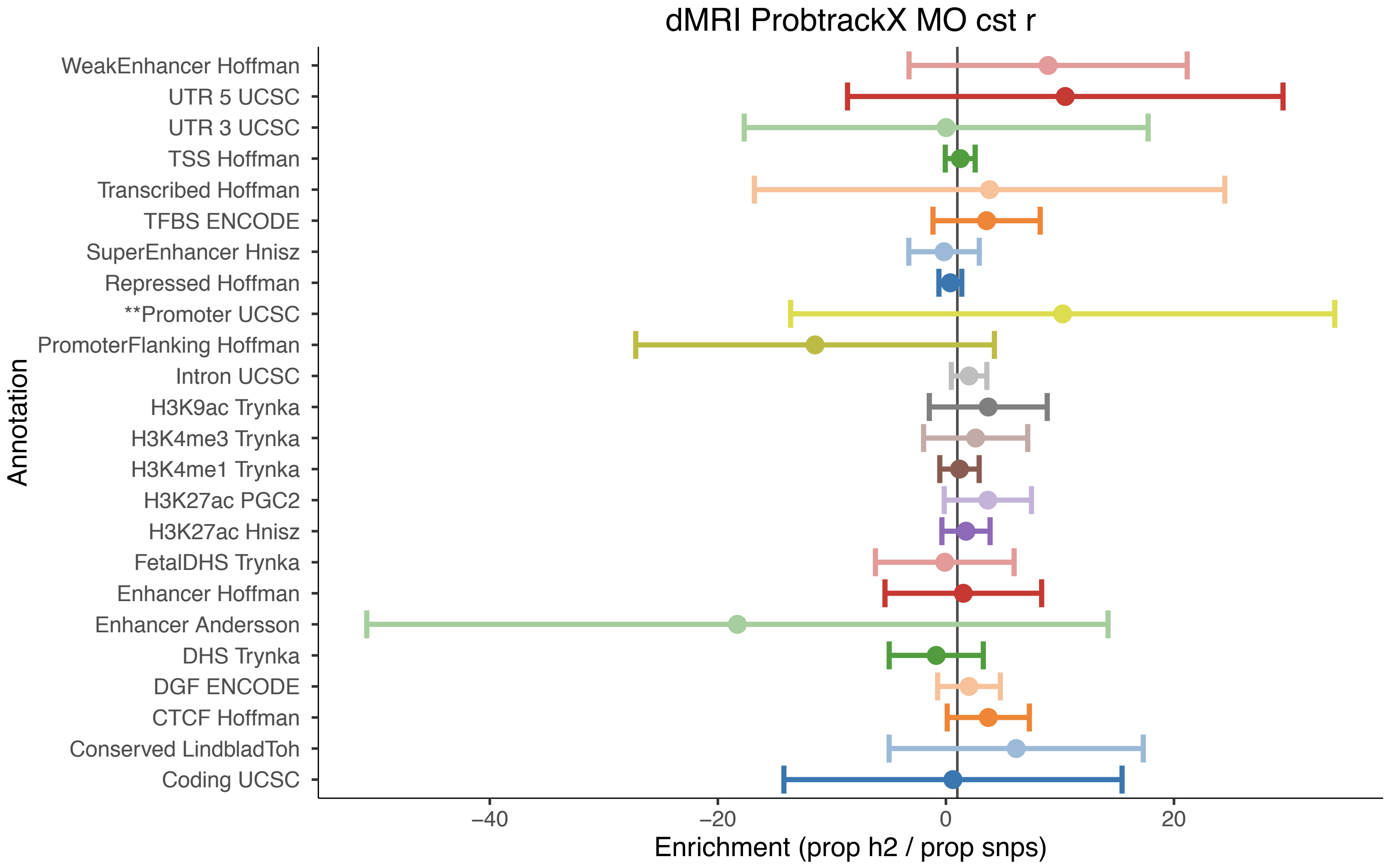

dMRI ProbtrackX MO cst r
WeakEnhancer Hoffman
UTR 5 UCSC
UTR 3 UCSC
TSS Hoffman
Transcribed Hoffman
TFBS ENCODE
SuperEnhancer Hnisz
Repressed Hoffman
**Promoter UCSC
PromoterFlanking Hoffman
Intron UCSC
H3K9ac Trynka
H3K4me3 Trynka
H3K4me1 Trynka
H3K27ac PGC2
H3K27ac Hnisz
FetalDHS Trynka
Enhancer Hoffman
Enhancer Andersson
DHS Trynka
DGF ENCODE
CTCF Hoffman
Conserved LindbladToh
Coding UCSC
Annotation
−40
−20
0
20
Enrichment (prop h2 / prop snps)

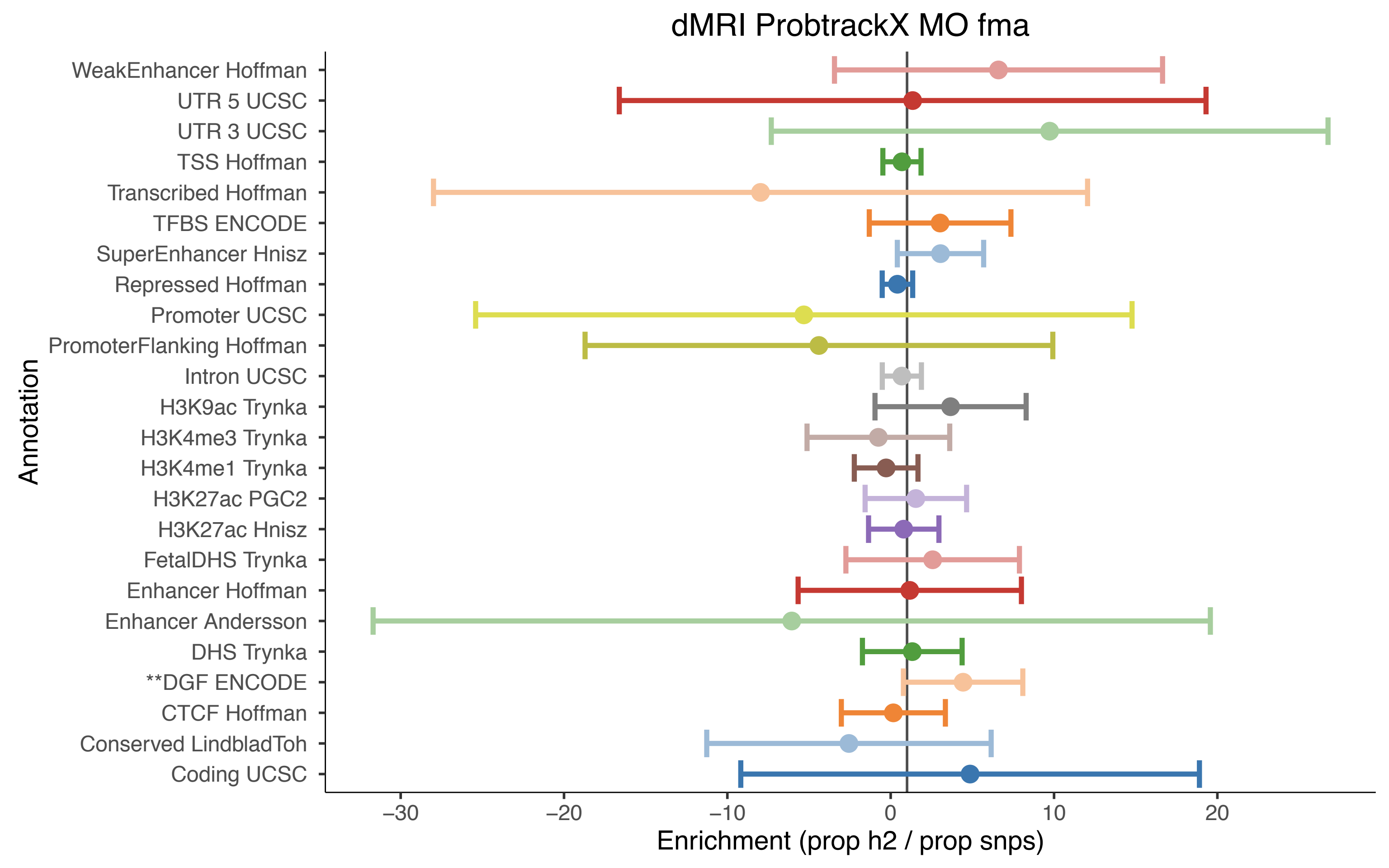
dMRI ProbtrackX MO fma
WeakEnhancer Hoffman
UTR 5 UCSC
UTR 3 UCSC
TSS Hoffman
Transcribed Hoffman
TFBS ENCODE
SuperEnhancer Hnisz
Repressed Hoffman
Promoter UCSC
PromoterFlanking Hoffman
Intron UCSC
H3K9ac Trynka
H3K4me3 Trynka
H3K4me1 Trynka
H3K27ac PGC2
H3K27ac Hnisz
FetalDHS Trynka
Enhancer Hoffman
Enhancer Andersson
DHS Trynka
**DGF ENCODE
CTCF Hoffman
Conserved LindbladToh
Coding UCSC
Annotation
−30
−20
−10
0
10
20
Enrichment (prop h2 / prop snps)

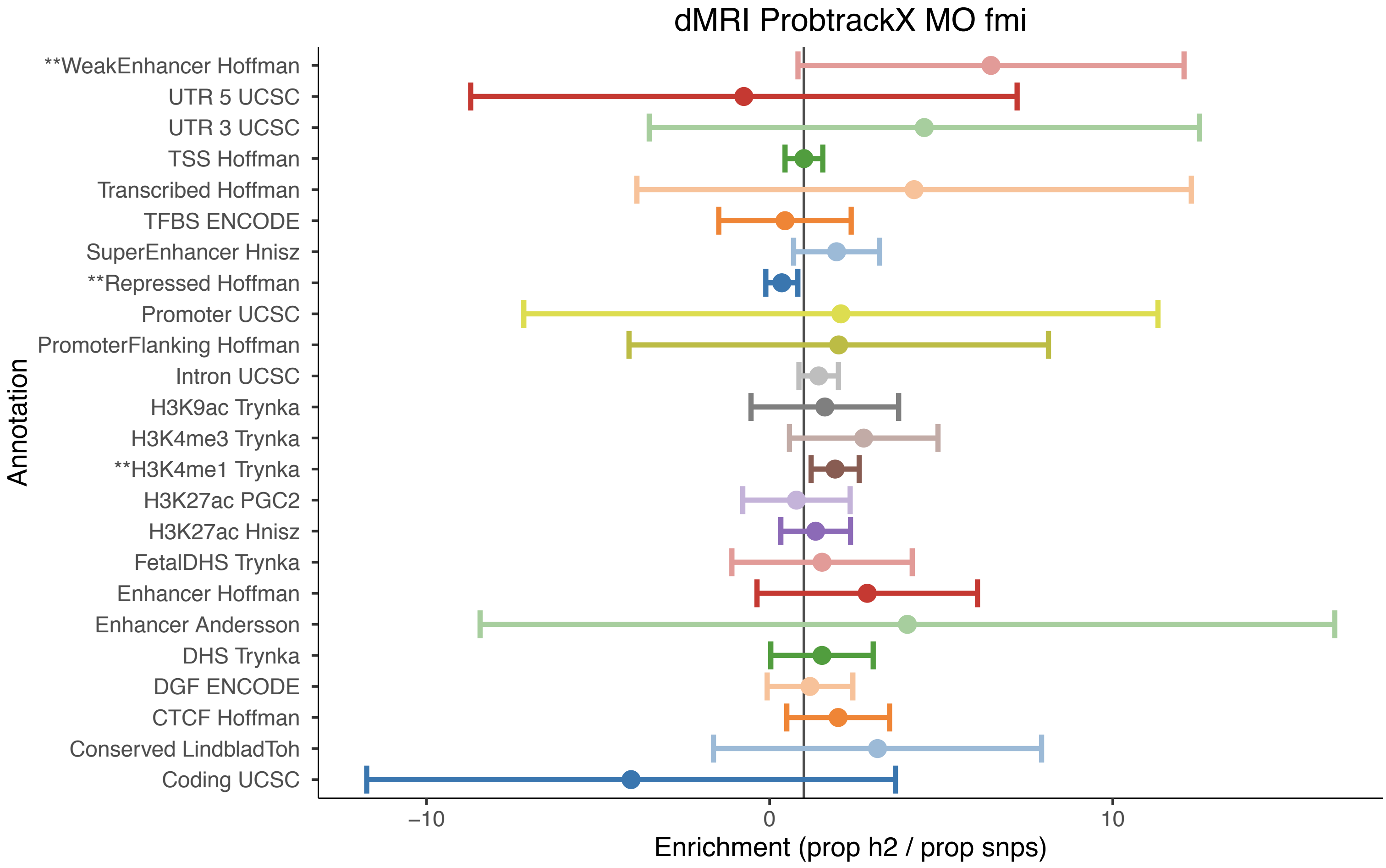

dMRI ProbtrackX MO fmi
**WeakEnhancer Hoffman
UTR 5 UCSC
UTR 3 UCSC
TSS Hoffman
Transcribed Hoffman
TFBS ENCODE
SuperEnhancer Hnisz
**Repressed Hoffman
Promoter UCSC
PromoterFlanking Hoffman
Intron UCSC
H3K9ac Trynka
H3K4me3 Trynka
**H3K4me1 Trynka
H3K27ac PGC2
H3K27ac Hnisz
FetalDHS Trynka
Enhancer Hoffman
Enhancer Andersson
DHS Trynka
DGF ENCODE
CTCF Hoffman
Conserved LindbladToh
Coding UCSC
Annotation
−10
0
10
Enrichment (prop h2 / prop snps)

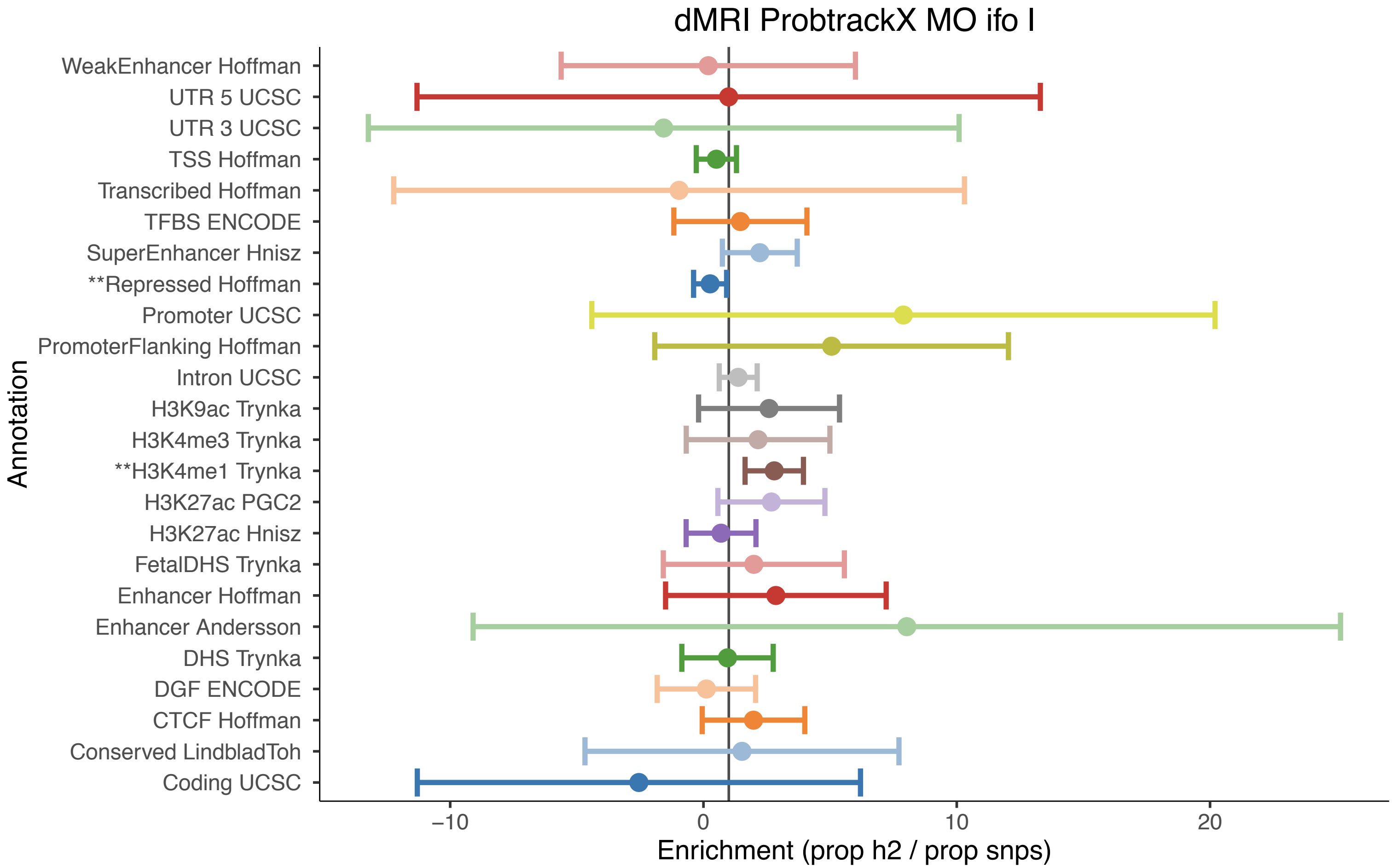

dMRI ProbtrackX MO ifo l
WeakEnhancer Hoffman
UTR 5 UCSC
UTR 3 UCSC
TSS Hoffman
Transcribed Hoffman
TFBS ENCODE
SuperEnhancer Hnisz
**Repressed Hoffman
Promoter UCSC
PromoterFlanking Hoffman
Intron UCSC
H3K9ac Trynka
H3K4me3 Trynka
**H3K4me1 Trynka
H3K27ac PGC2
H3K27ac Hnisz
FetalDHS Trynka
Enhancer Hoffman
Enhancer Andersson
DHS Trynka
DGF ENCODE
CTCF Hoffman
Conserved LindbladToh
Coding UCSC
Annotation
−10
0
10
20
Enrichment (prop h2 / prop snps)

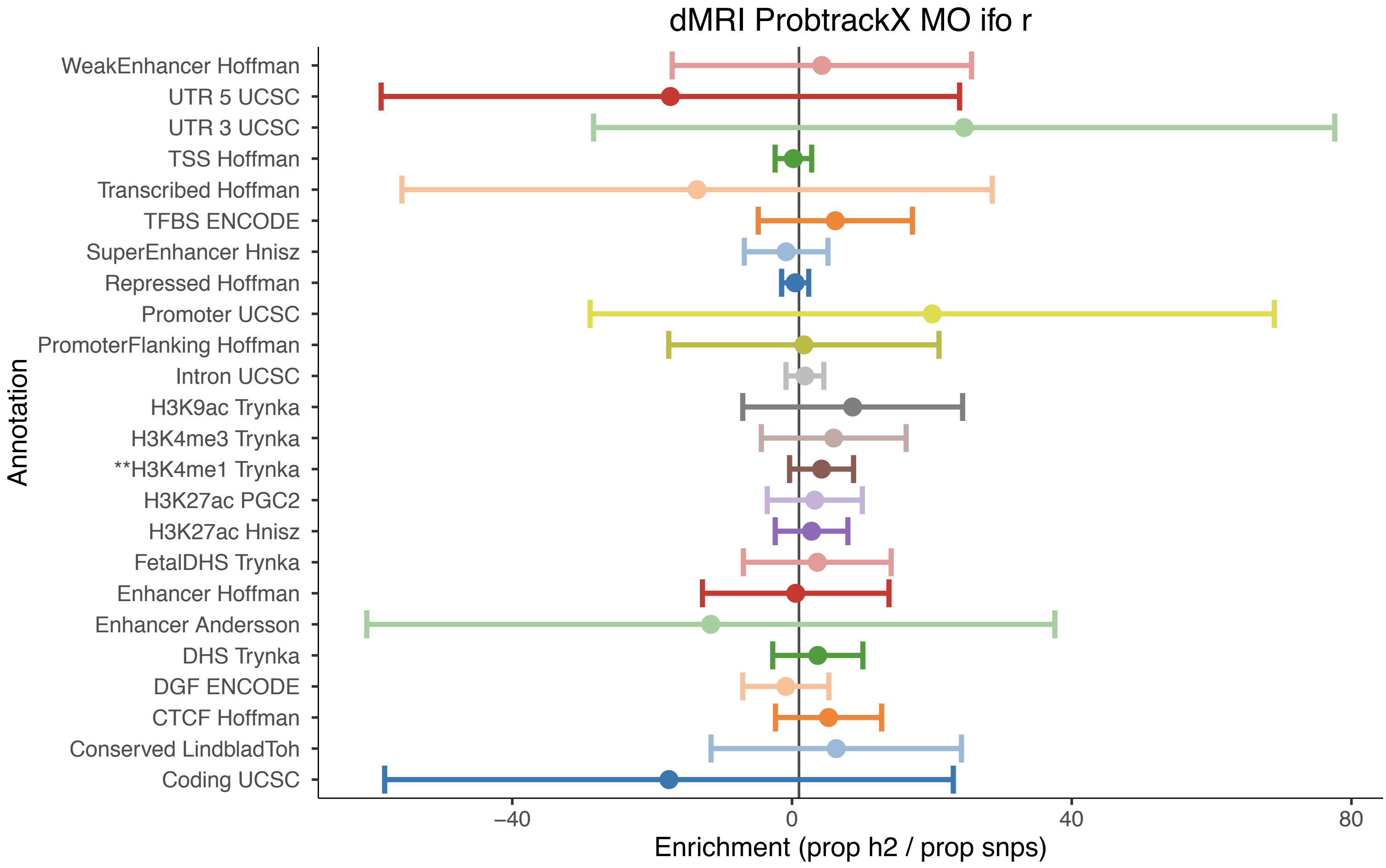

dMRI ProbtrackX MO ifo r
WeakEnhancer Hoffman
UTR 5 UCSC
UTR 3 UCSC
TSS Hoffman
Transcribed Hoffman
TFBS ENCODE
SuperEnhancer Hnisz
Repressed Hoffman
Promoter UCSC
PromoterFlanking Hoffman
Intron UCSC
H3K9ac Trynka
H3K4me3 Trynka
**H3K4me1 Trynka
H3K27ac PGC2
H3K27ac Hnisz
FetalDHS Trynka
Enhancer Hoffman
Enhancer Andersson
DHS Trynka
DGF ENCODE
CTCF Hoffman
Conserved LindbladToh
Coding UCSC
Annotation
−40
0
40
80
Enrichment (prop h2 / prop snps)

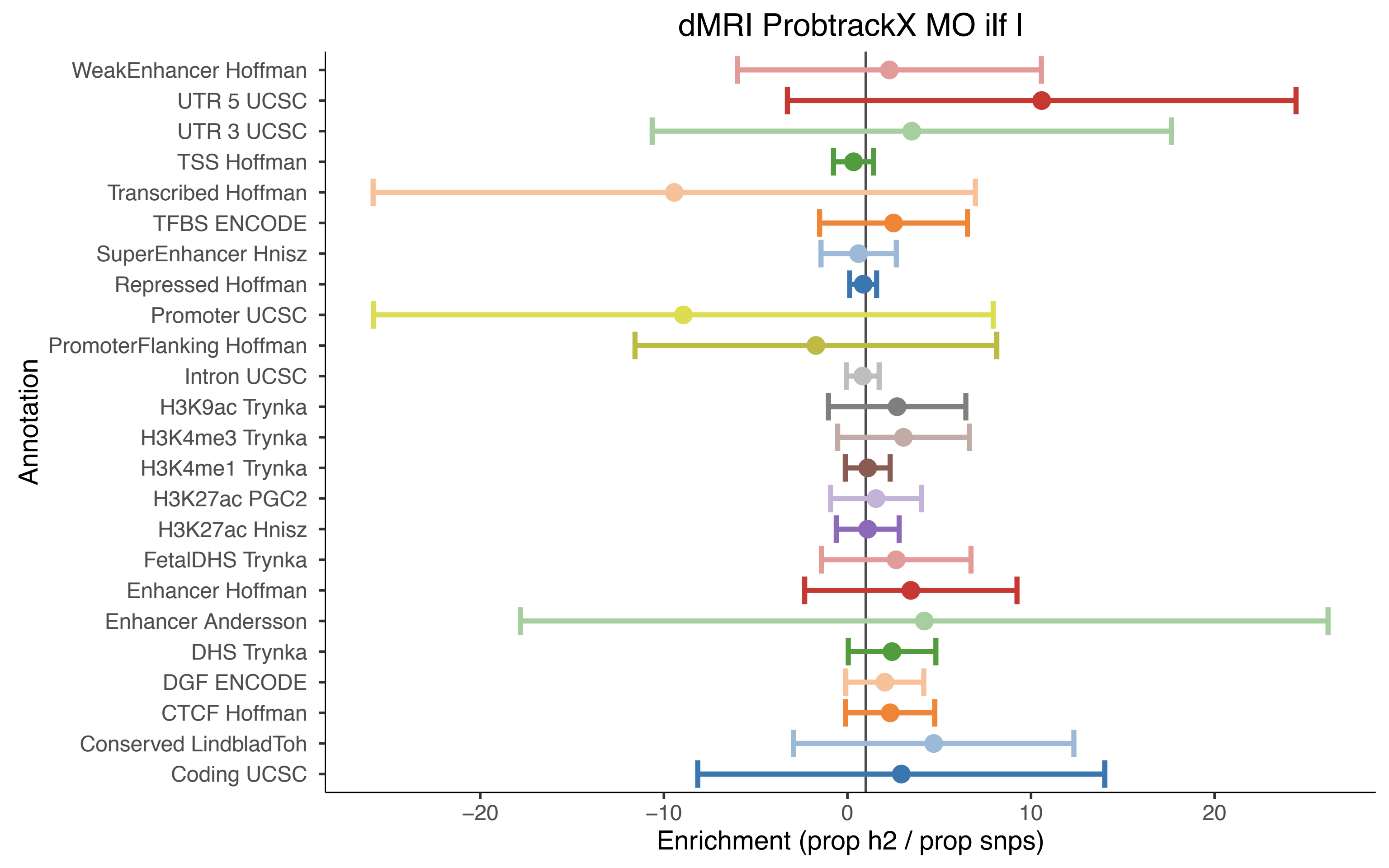

dMRI ProbtrackX MO ilf l
Annotation
WeakEnhancer Hoffman
UTR 5 UCSC
UTR 3 UCSC
TSS Hoffman
Transcribed Hoffman
TFBS ENCODE
SuperEnhancer Hnisz
Repressed Hoffman
Promoter UCSC
PromoterFlanking Hoffman
Intron UCSC
H3K9ac Trynka
H3K4me3 Trynka
H3K4me1 Trynka
H3K27ac PGC2
H3K27ac Hnisz
FetalDHS Trynka
Enhancer Hoffman
Enhancer Andersson
DHS Trynka
DGF ENCODE
CTCF Hoffman
Conserved LindbladToh
Coding UCSC
−20
−10
0
10
20
Enrichment (prop h2 / prop snps)

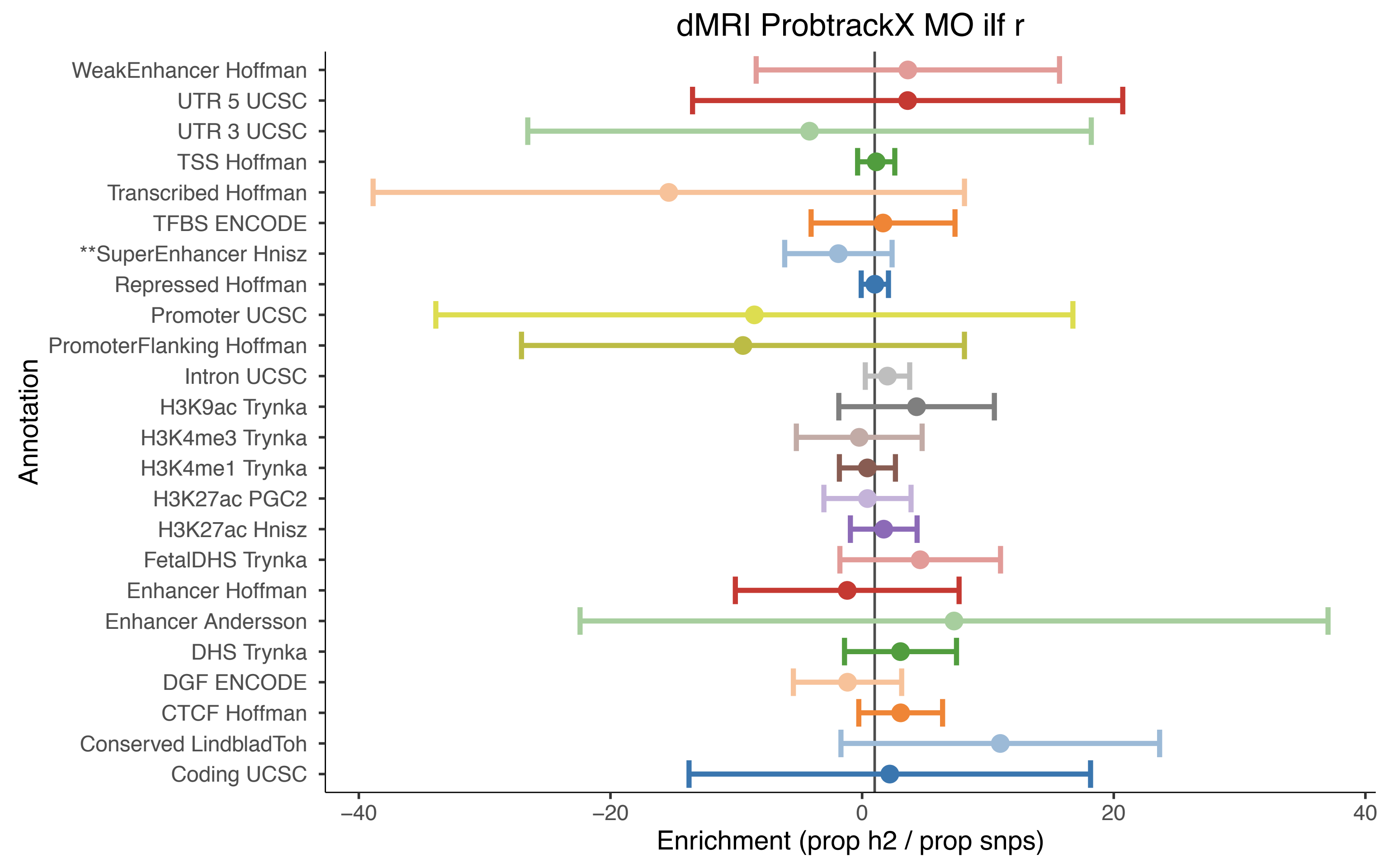
dMRI ProbtrackX MO ilf r
WeakEnhancer Hoffman
UTR 5 UCSC
UTR 3 UCSC
TSS Hoffman
Transcribed Hoffman
TFBS ENCODE
**SuperEnhancer Hnisz
Repressed Hoffman
Promoter UCSC
PromoterFlanking Hoffman
Intron UCSC
H3K9ac Trynka
H3K4me3 Trynka
H3K4me1 Trynka
H3K27ac PGC2
H3K27ac Hnisz
FetalDHS Trynka
Enhancer Hoffman
Enhancer Andersson
DHS Trynka
DGF ENCODE
CTCF Hoffman
Conserved LindbladToh
Coding UCSC
Annotation
−40
−20
0
20
40
Enrichment (prop h2 / prop snps)

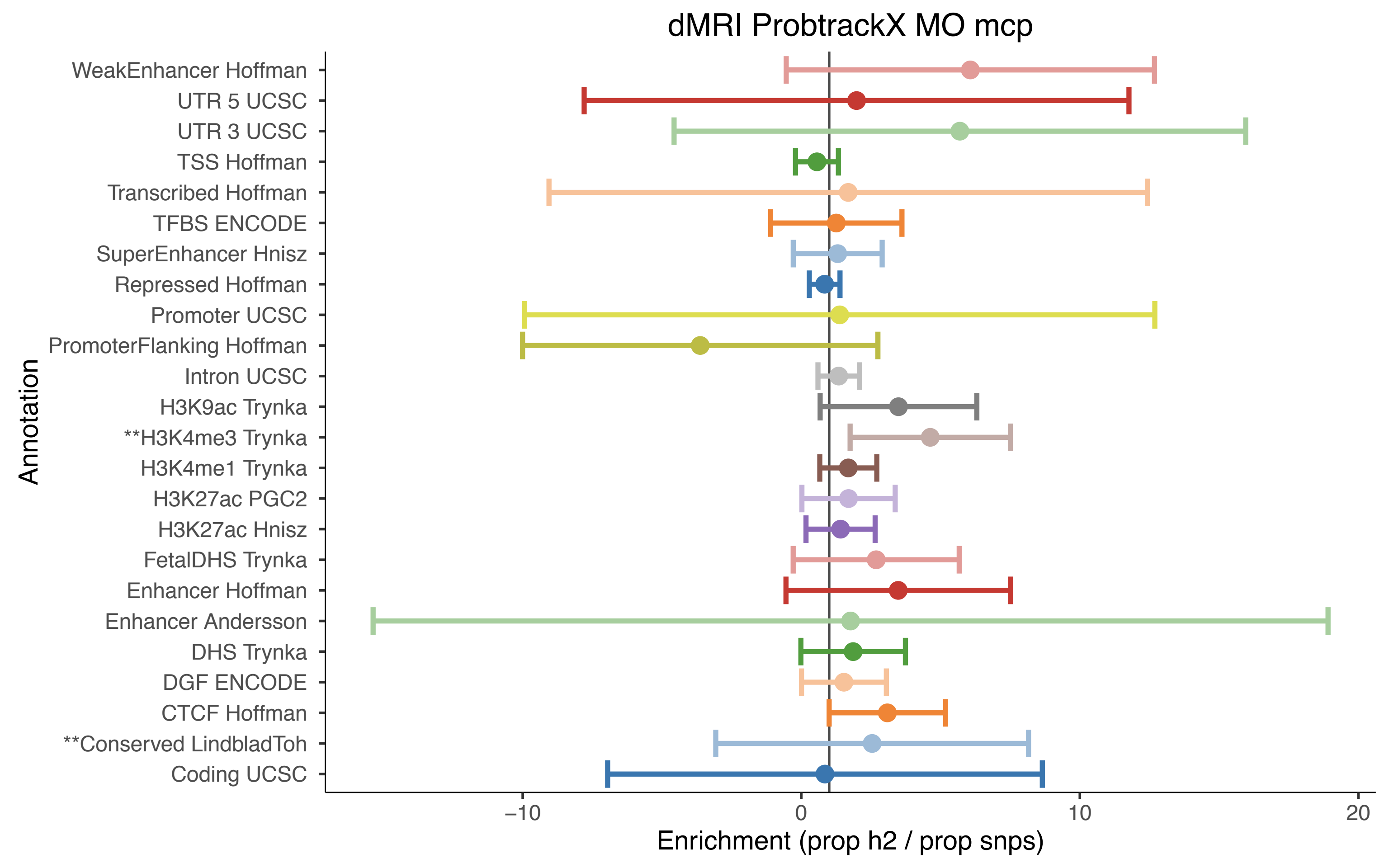
dMRI ProbtrackX MO mcp
WeakEnhancer Hoffman
UTR 5 UCSC
UTR 3 UCSC
TSS Hoffman
Transcribed Hoffman
TFBS ENCODE
SuperEnhancer Hnisz
Repressed Hoffman
Promoter UCSC
PromoterFlanking Hoffman
Intron UCSC
H3K9ac Trynka
**H3K4me3 Trynka
H3K4me1 Trynka
H3K27ac PGC2
H3K27ac Hnisz
FetalDHS Trynka
Enhancer Hoffman
Enhancer Andersson
DHS Trynka
DGF ENCODE
CTCF Hoffman
**Conserved LindbladToh
Coding UCSC
Annotation
−10
0
10
20
Enrichment (prop h2 / prop snps)

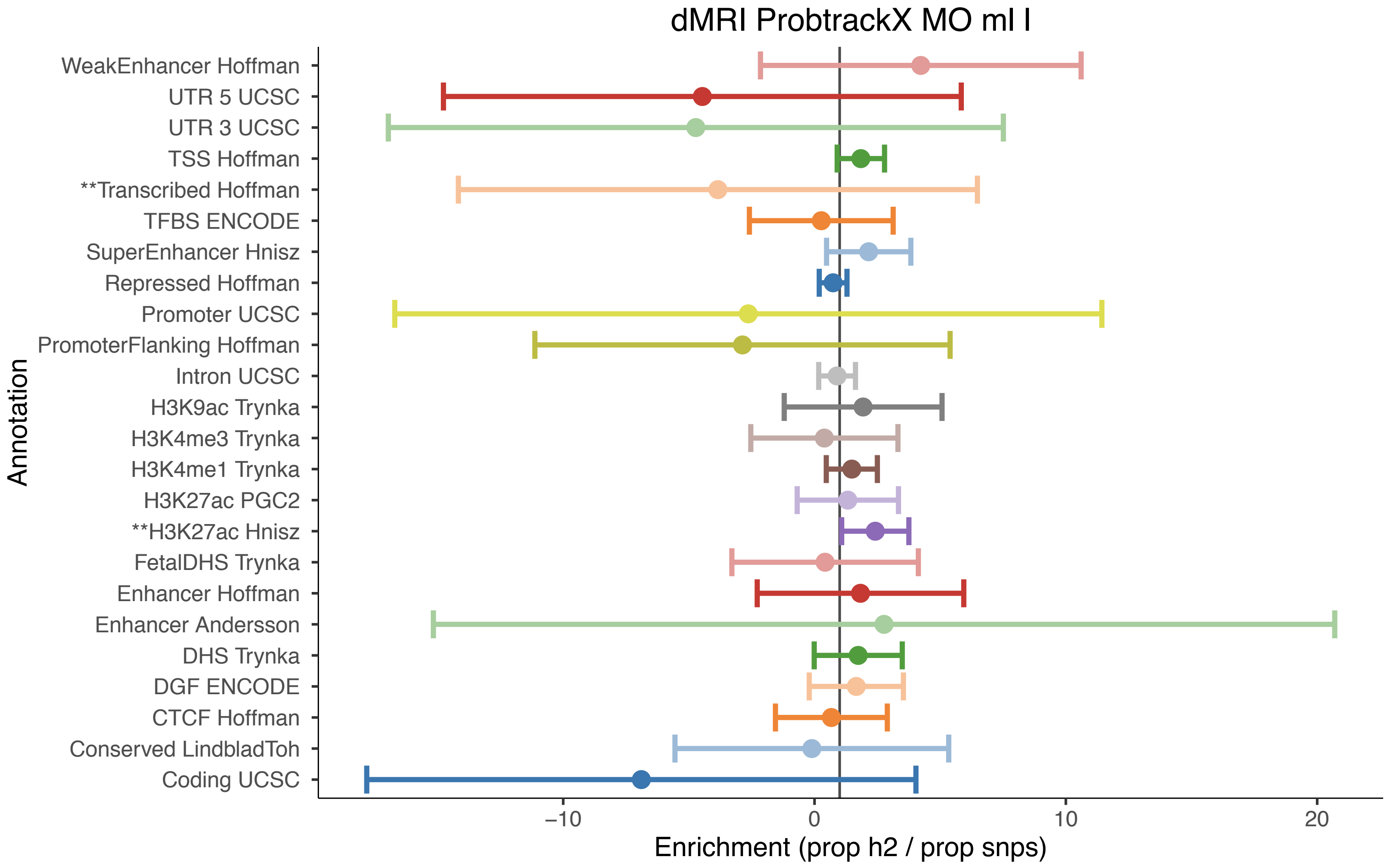

dMRI ProbtrackX MO ml I
WeakEnhancer Hoffman
UTR 5 UCSC
UTR 3 UCSC
TSS Hoffman
**Transcribed Hoffman
TFBS ENCODE
SuperEnhancer Hnisz
Repressed Hoffman
Promoter UCSC
PromoterFlanking Hoffman
Intron UCSC
H3K9ac Trynka
H3K4me3 Trynka
H3K4me1 Trynka
H3K27ac PGC2
**H3K27ac Hnisz
FetalDHS Trynka
Enhancer Hoffman
Enhancer Andersson
DHS Trynka
DGF ENCODE
CTCF Hoffman
Conserved LindbladToh
Coding UCSC
Annotation
−10
0
10
20
Enrichment (prop h2 / prop snps)

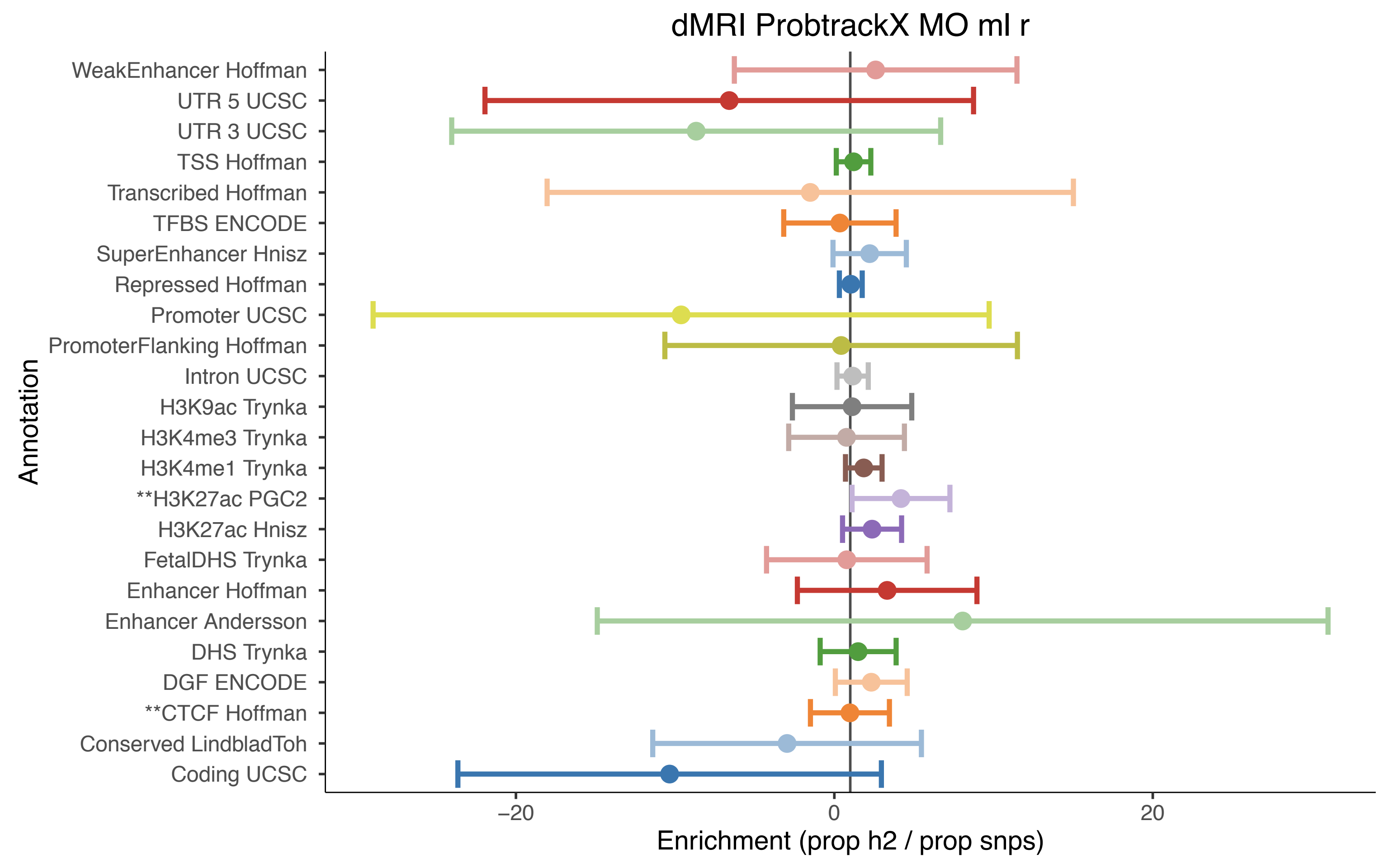

dMRI ProbtrackX MO ml r
WeakEnhancer Hoffman
UTR 5 UCSC
UTR 3 UCSC
TSS Hoffman
Transcribed Hoffman
TFBS ENCODE
SuperEnhancer Hnisz
Repressed Hoffman
Promoter UCSC
PromoterFlanking Hoffman
Intron UCSC
H3K9ac Trynka
H3K4me3 Trynka
H3K4me1 Trynka
**H3K27ac PGC2
H3K27ac Hnisz
FetalDHS Trynka
Enhancer Hoffman
Enhancer Andersson
DHS Trynka
DGF ENCODE
**CTCF Hoffman
Conserved LindbladToh
Coding UCSC
Annotation
−20
0
20
Enrichment (prop h2 / prop snps)

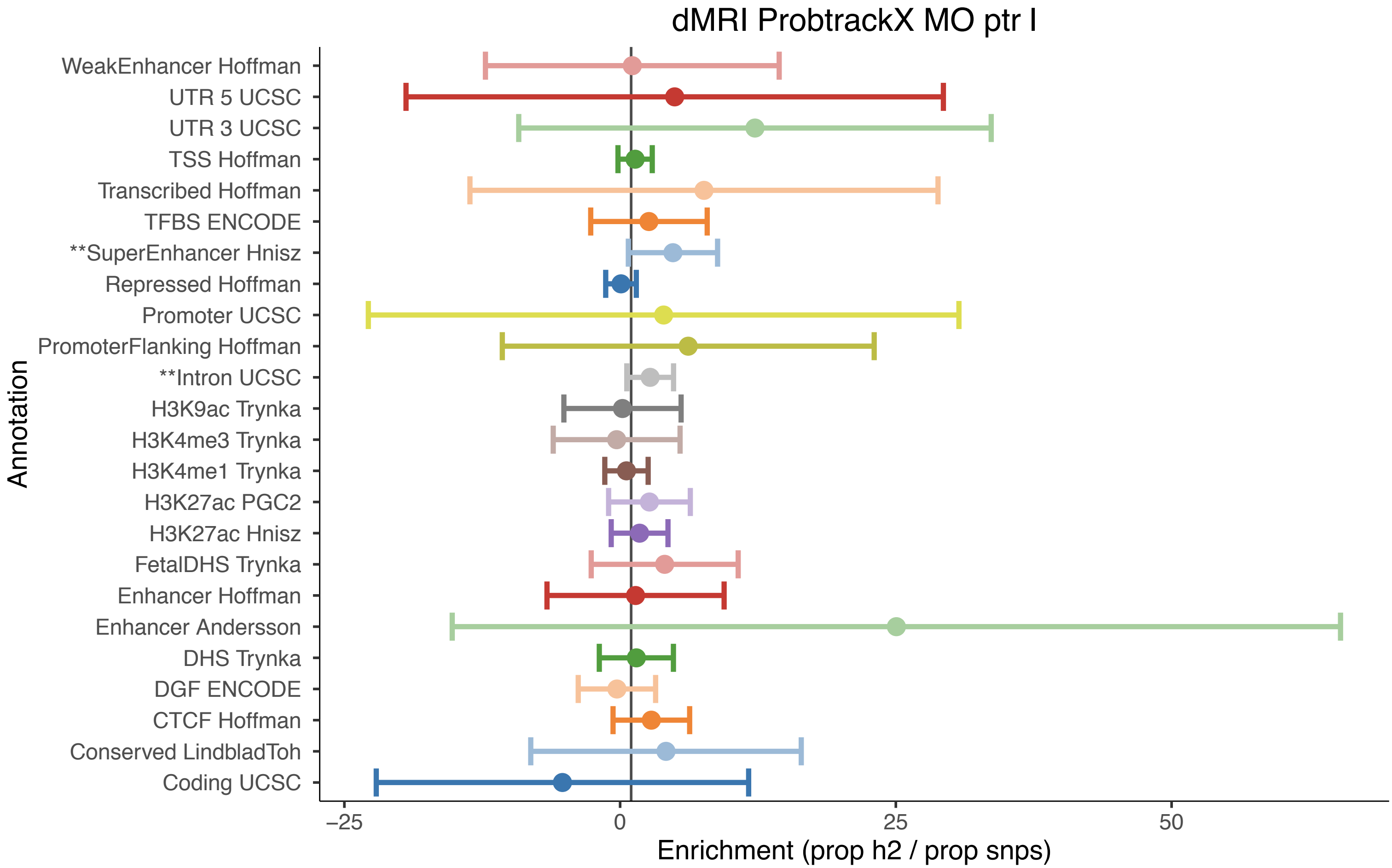
dMRI ProbtrackX MO ptr l
WeakEnhancer Hoffman
UTR 5 UCSC
UTR 3 UCSC
TSS Hoffman
Transcribed Hoffman
TFBS ENCODE
**SuperEnhancer Hnisz
Repressed Hoffman
Promoter UCSC
PromoterFlanking Hoffman
**Intron UCSC
H3K9ac Trynka
H3K4me3 Trynka
H3K4me1 Trynka
H3K27ac PGC2
H3K27ac Hnisz
FetalDHS Trynka
Enhancer Hoffman
Enhancer Andersson
DHS Trynka
DGF ENCODE
CTCF Hoffman
Conserved LindbladToh
Coding UCSC
Annotation
−25
0
25
50
Enrichment (prop h2 / prop snps)

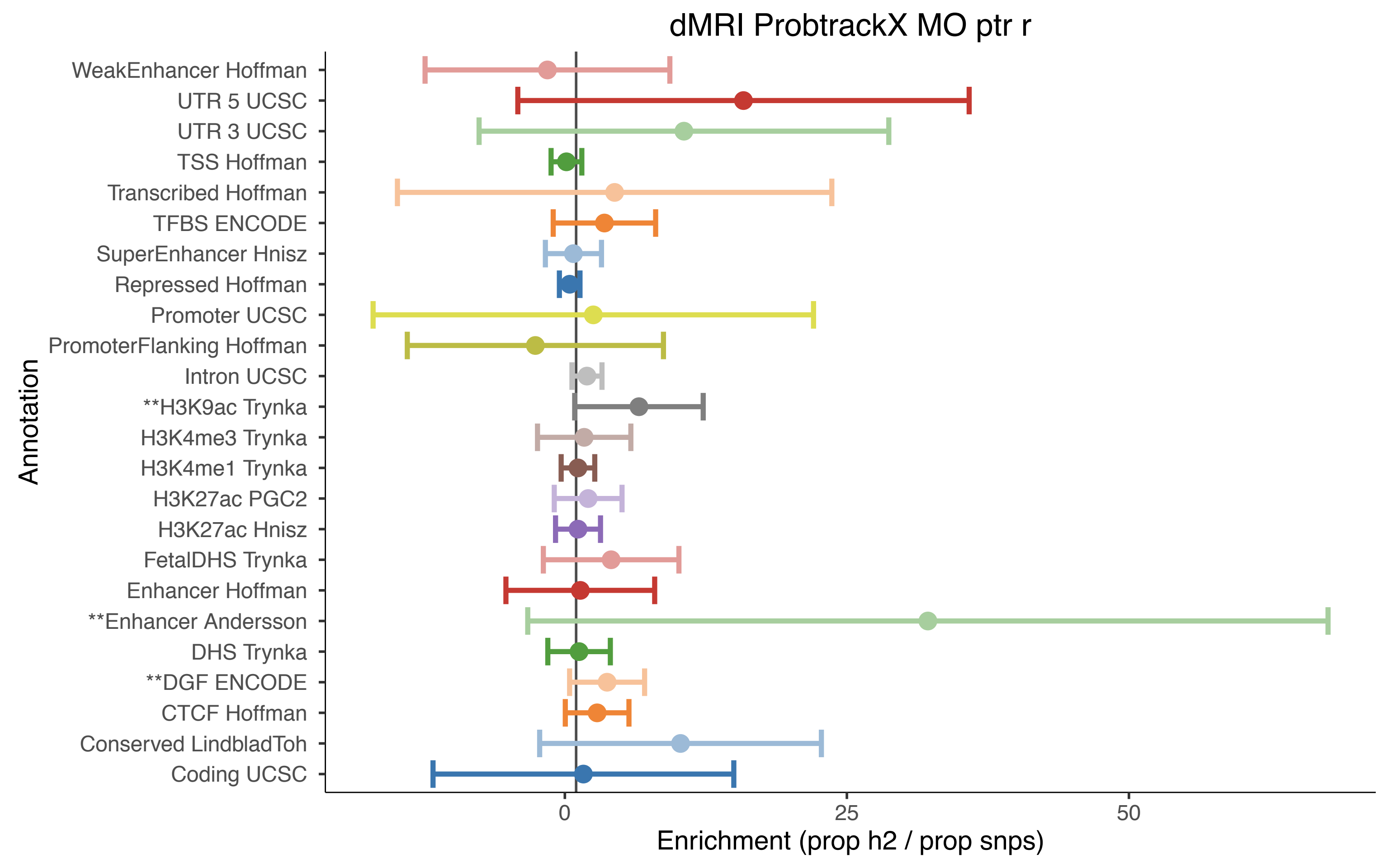

dMRI ProbtrackX MO ptr r
Annotation
WeakEnhancer Hoffman
UTR 5 UCSC
UTR 3 UCSC
TSS Hoffman
Transcribed Hoffman
TFBS ENCODE
SuperEnhancer Hnisz
Repressed Hoffman
Promoter UCSC
PromoterFlanking Hoffman
Intron UCSC
**H3K9ac Trynka
H3K4me3 Trynka
H3K4me1 Trynka
H3K27ac PGC2
H3K27ac Hnisz
FetalDHS Trynka
Enhancer Hoffman
**Enhancer Andersson
DHS Trynka
**DGF ENCODE
CTCF Hoffman
Conserved LindbladToh
Coding UCSC
0
25
50
Enrichment (prop h2 / prop snps)

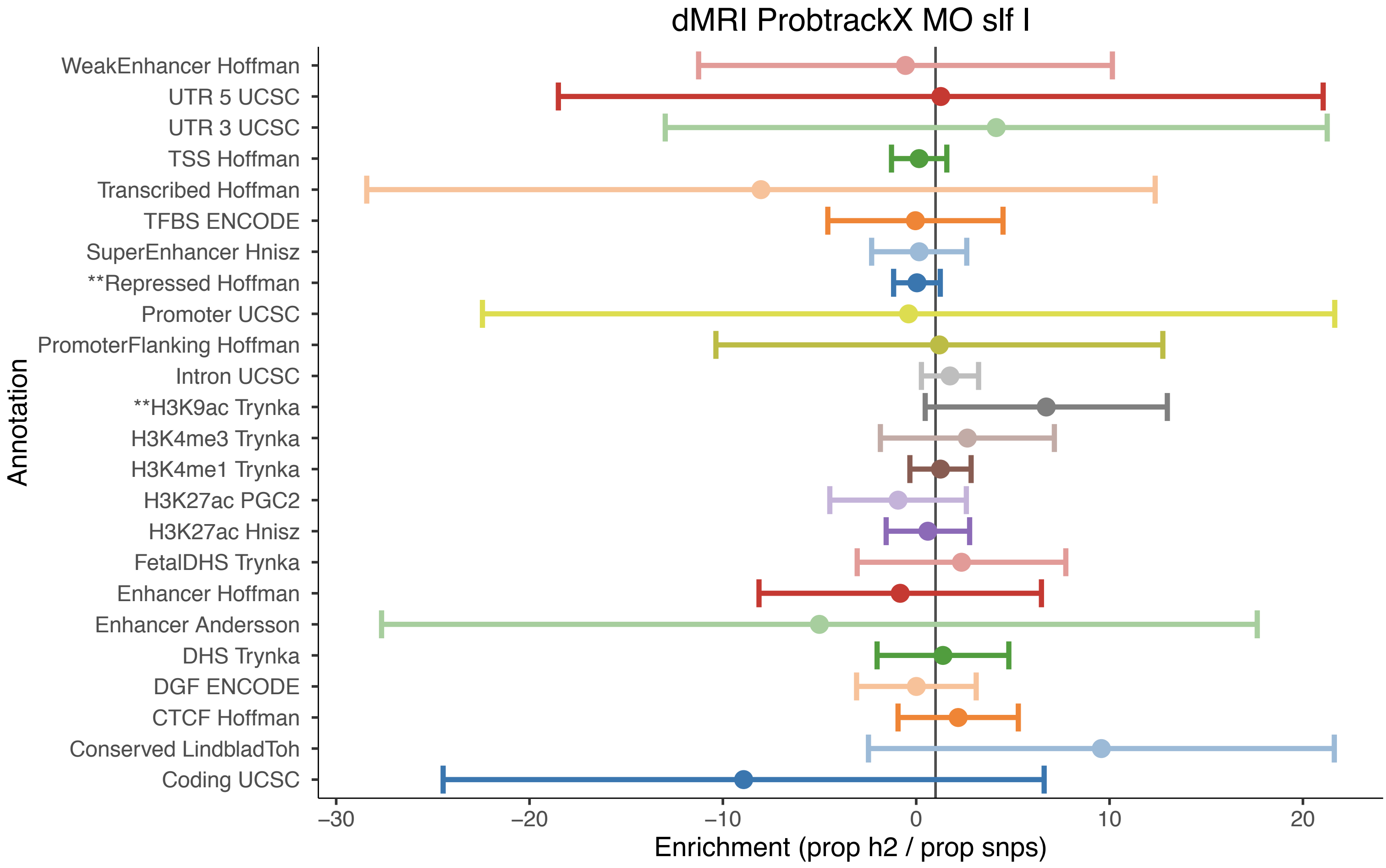
dMRI ProbtrackX MO slf I
WeakEnhancer Hoffman
UTR 5 UCSC
UTR 3 UCSC
TSS Hoffman
Transcribed Hoffman
TFBS ENCODE
SuperEnhancer Hnisz
**Repressed Hoffman
Promoter UCSC
PromoterFlanking Hoffman
Intron UCSC
**H3K9ac Trynka
H3K4me3 Trynka
H3K4me1 Trynka
H3K27ac PGC2
H3K27ac Hnisz
FetalDHS Trynka
Enhancer Hoffman
Enhancer Andersson
DHS Trynka
DGF ENCODE
CTCF Hoffman
Conserved LindbladToh
Coding UCSC
Annotation
−30
−20
−10
0
10
20
Enrichment (prop h2 / prop snps)

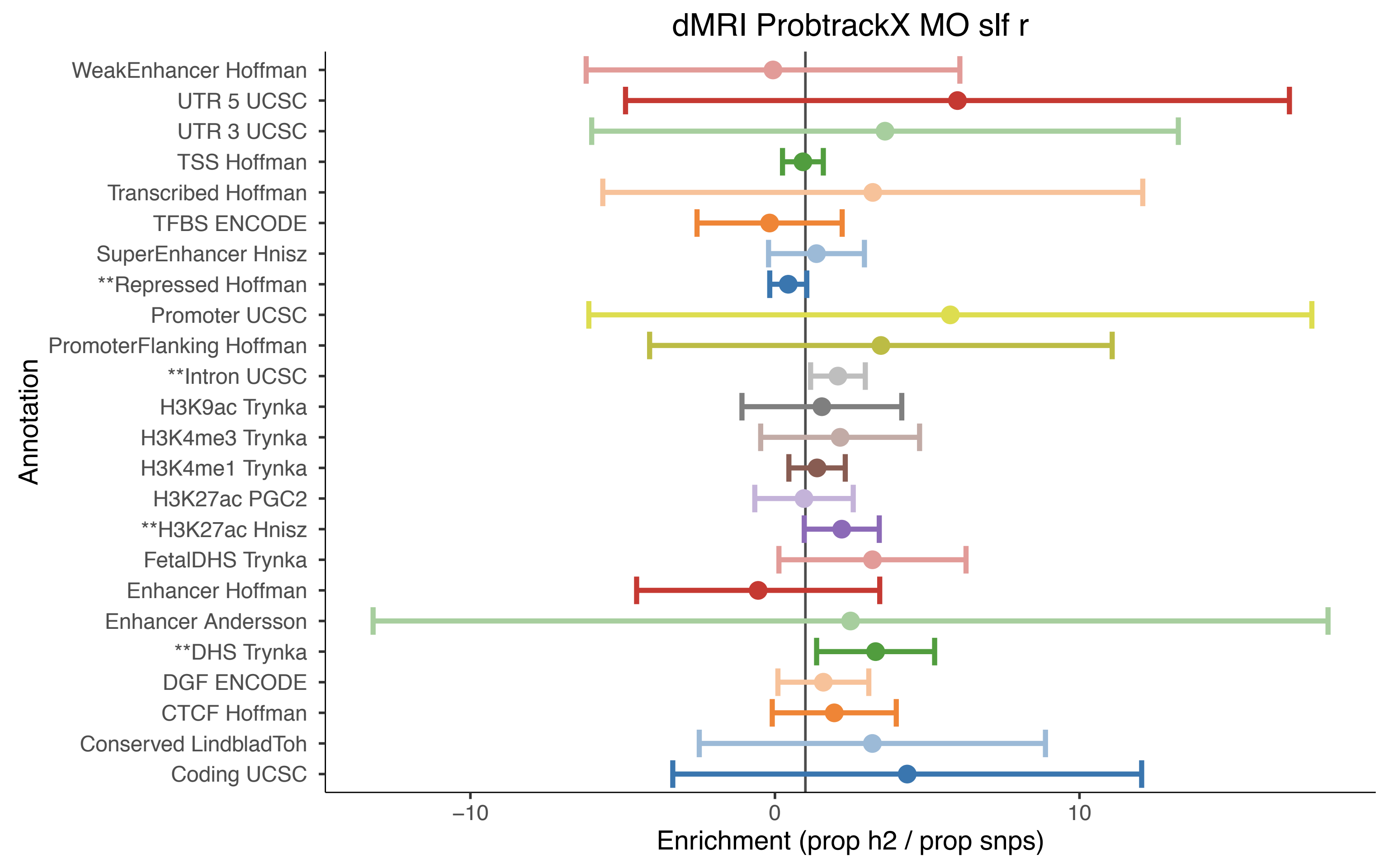

dMRI ProbtrackX MO slf r
WeakEnhancer Hoffman
UTR 5 UCSC
UTR 3 UCSC
TSS Hoffman
Transcribed Hoffman
TFBS ENCODE
SuperEnhancer Hnisz
**Repressed Hoffman
Promoter UCSC
PromoterFlanking Hoffman
**Intron UCSC
H3K9ac Trynka
H3K4me3 Trynka
H3K4me1 Trynka
H3K27ac PGC2
**H3K27ac Hnisz
FetalDHS Trynka
Enhancer Hoffman
Enhancer Andersson
**DHS Trynka
DGF ENCODE
CTCF Hoffman
Conserved LindbladToh
Coding UCSC
Annotation
−10
0
10
Enrichment (prop h2 / prop snps)

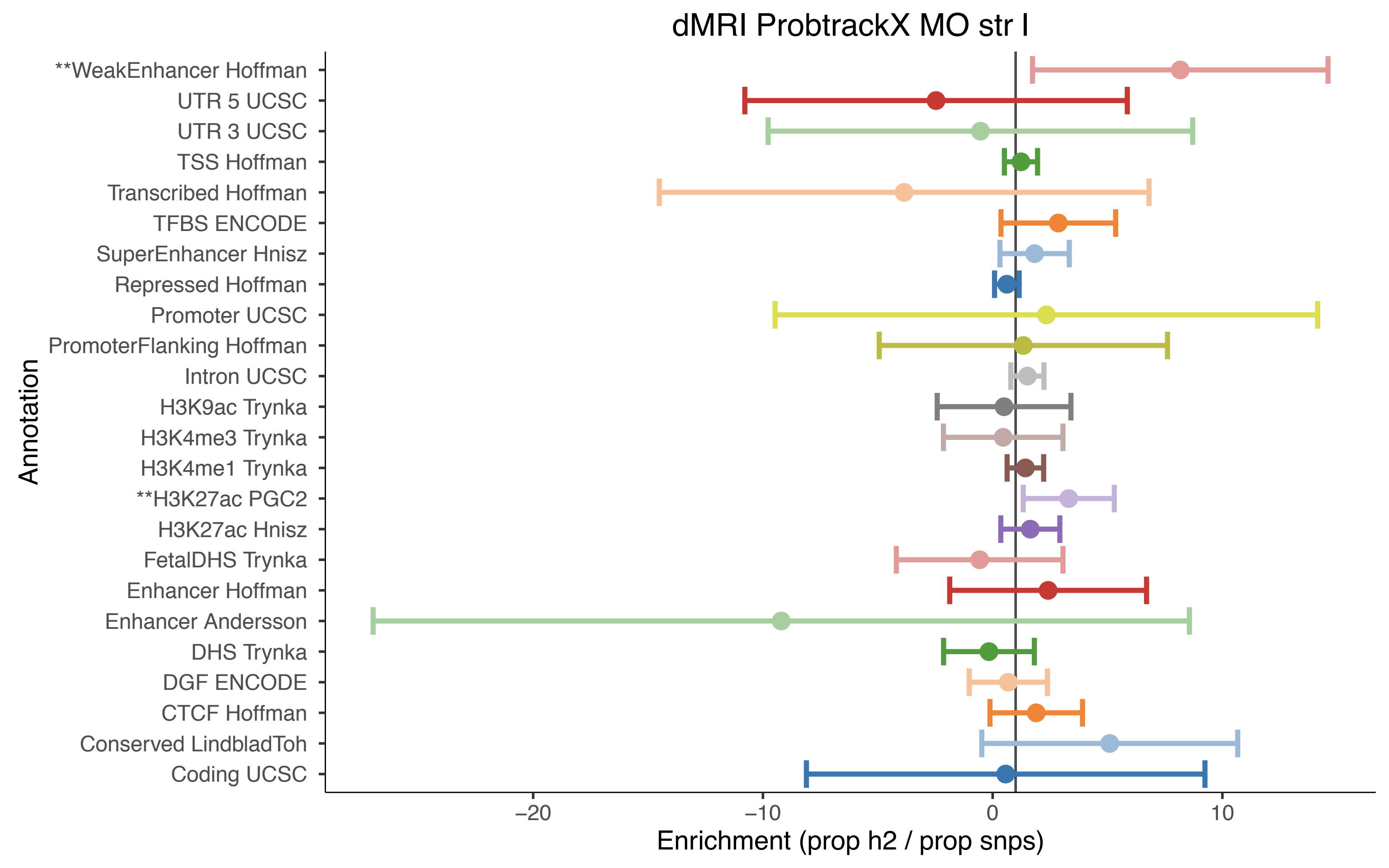

dMRI ProbtrackX MO str I
**WeakEnhancer Hoffman
UTR 5 UCSC
UTR 3 UCSC
TSS Hoffman
Transcribed Hoffman
TFBS ENCODE
SuperEnhancer Hnisz
Repressed Hoffman
Promoter UCSC
PromoterFlanking Hoffman
Intron UCSC
H3K9ac Trynka
H3K4me3 Trynka
H3K4me1 Trynka
**H3K27ac PGC2
H3K27ac Hnisz
FetalDHS Trynka
Enhancer Hoffman
Enhancer Andersson
DHS Trynka
DGF ENCODE
CTCF Hoffman
Conserved LindbladToh
Coding UCSC
Annotation
−20
−10
0
10
Enrichment (prop h2 / prop snps)

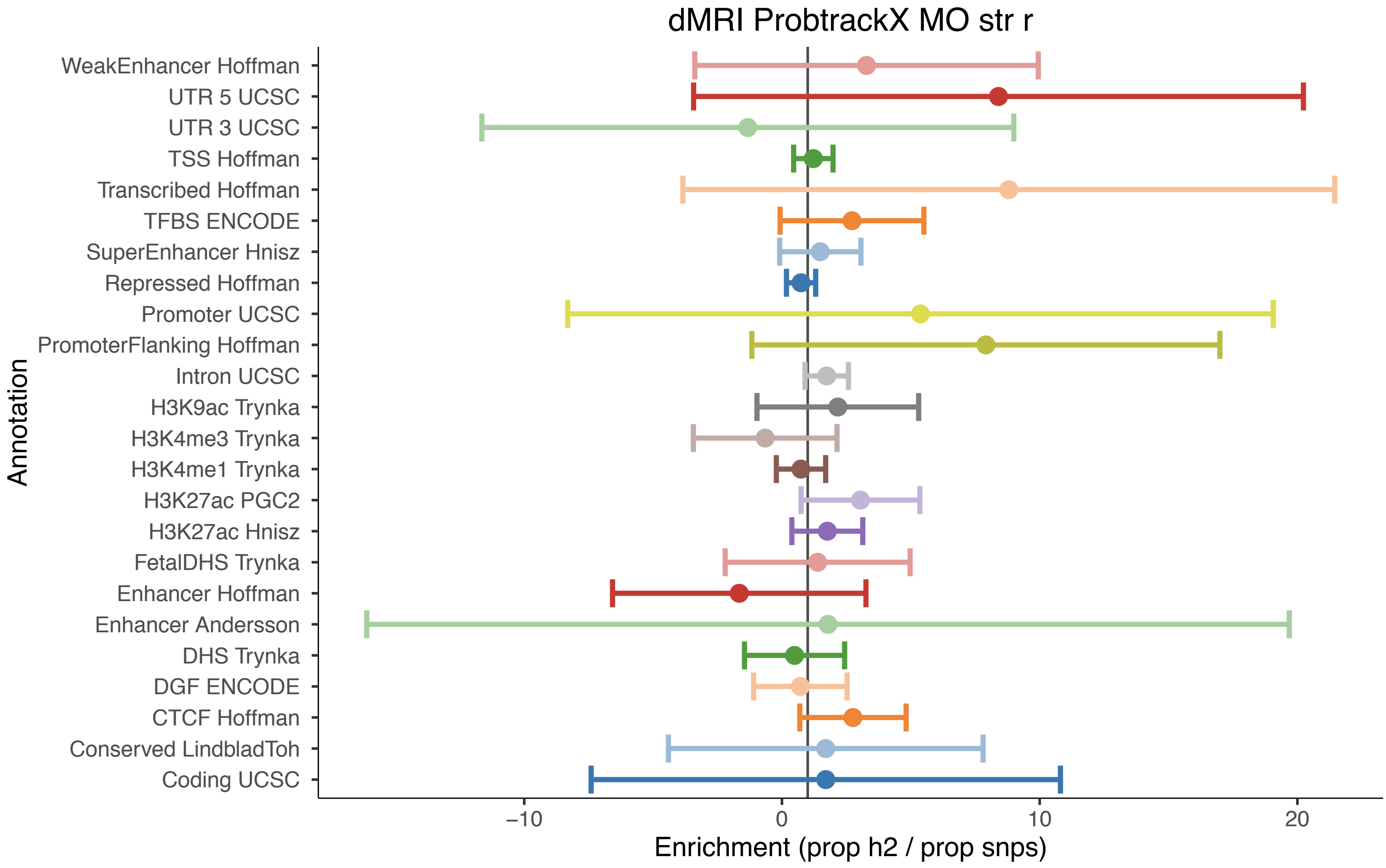
dMRI ProbtrackX MO str r
WeakEnhancer Hoffman
UTR 5 UCSC
UTR 3 UCSC
TSS Hoffman
Transcribed Hoffman
TFBS ENCODE
SuperEnhancer Hnisz
Repressed Hoffman
Promoter UCSC
PromoterFlanking Hoffman
Intron UCSC
H3K9ac Trynka
H3K4me3 Trynka
H3K4me1 Trynka
H3K27ac PGC2
H3K27ac Hnisz
FetalDHS Trynka
Enhancer Hoffman
Enhancer Andersson
DHS Trynka
DGF ENCODE
CTCF Hoffman
Conserved LindbladToh
Coding UCSC
Annotation
−10
0
10
20
Enrichment (prop h2 / prop snps)

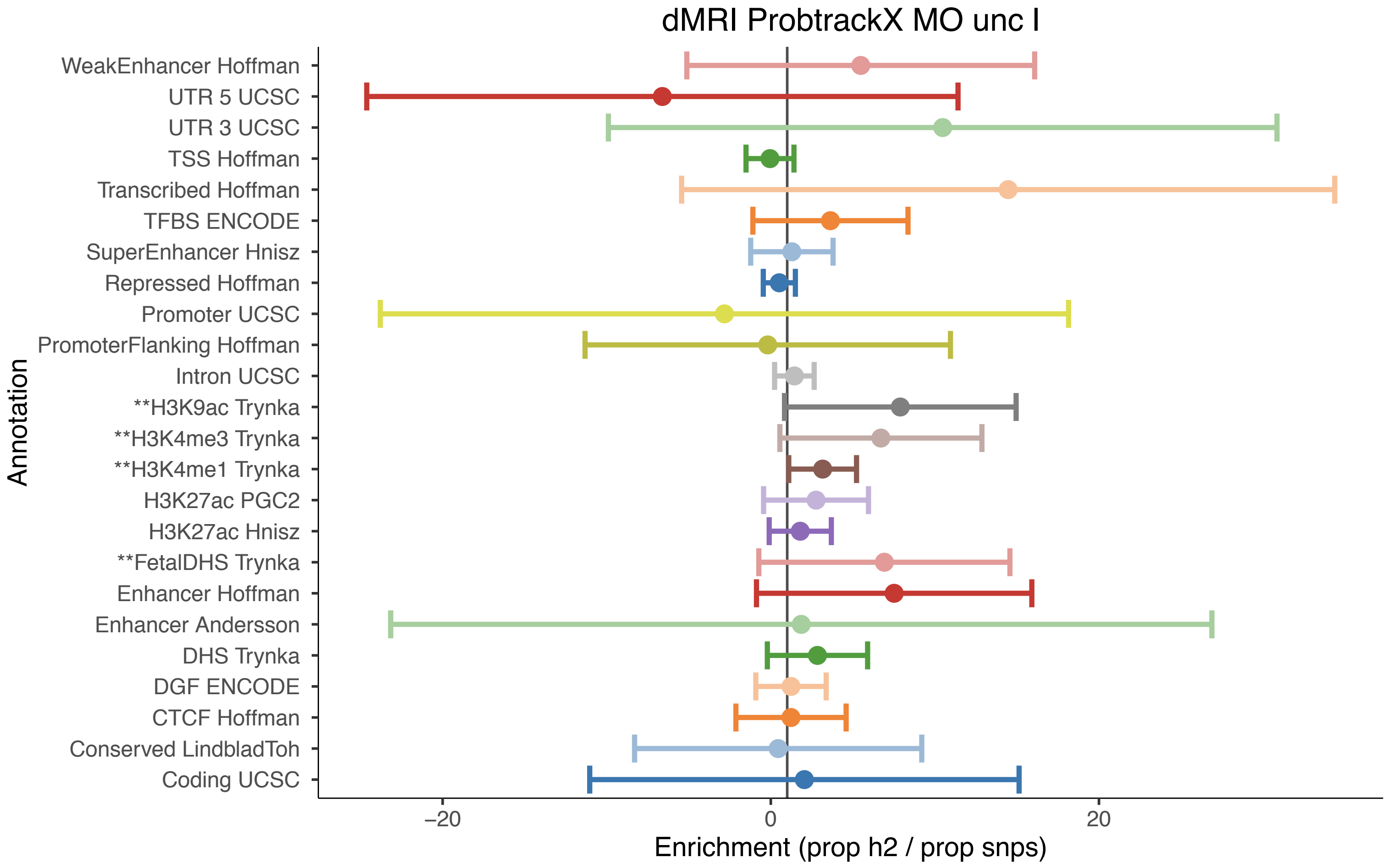
dMRI ProbtrackX MO unc I
WeakEnhancer Hoffman
UTR 5 UCSC
UTR 3 UCSC
TSS Hoffman
Transcribed Hoffman
TFBS ENCODE
SuperEnhancer Hnisz
Repressed Hoffman
Promoter UCSC
PromoterFlanking Hoffman
Intron UCSC
**H3K9ac Trynka
**H3K4me3 Trynka
**H3K4me1 Trynka
H3K27ac PGC2
H3K27ac Hnisz
**FetalDHS Trynka
Enhancer Hoffman
Enhancer Andersson
DHS Trynka
DGF ENCODE
CTCF Hoffman
Conserved LindbladToh
Coding UCSC
Annotation
−20
0
20
Enrichment (prop h2 / prop snps)

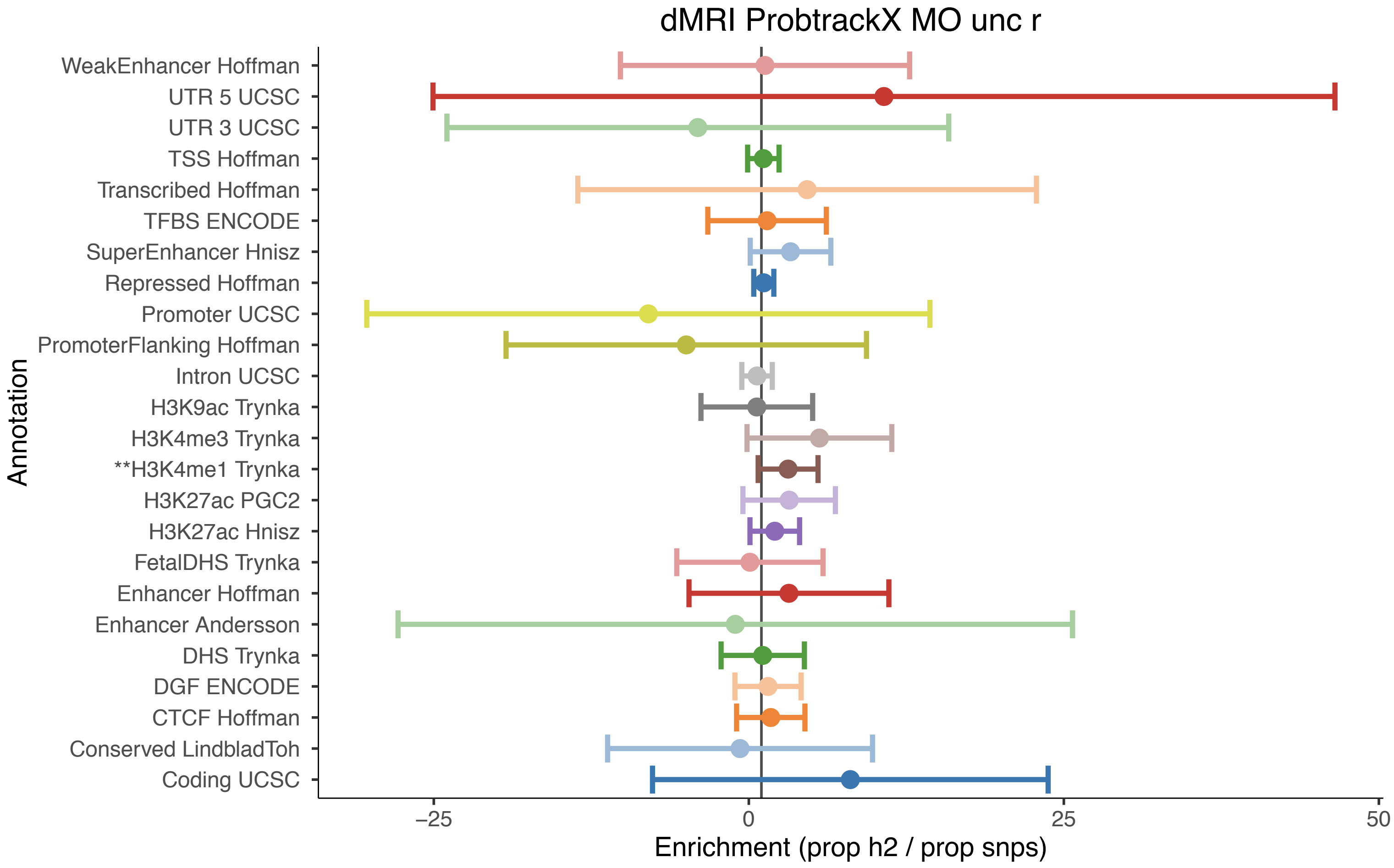

dMRI ProbtrackX MO unc r
WeakEnhancer Hoffman
UTR 5 UCSC
UTR 3 UCSC
TSS Hoffman
Transcribed Hoffman
TFBS ENCODE
SuperEnhancer Hnisz
Repressed Hoffman
Promoter UCSC
PromoterFlanking Hoffman
Intron UCSC
H3K9ac Trynka
H3K4me3 Trynka
**H3K4me1 Trynka
H3K27ac PGC2
H3K27ac Hnisz
FetalDHS Trynka
Enhancer Hoffman
Enhancer Andersson
DHS Trynka
DGF ENCODE
CTCF Hoffman
Conserved LindbladToh
Coding UCSC
Annotation
−25
0
25
50
Enrichment (prop h2 / prop snps)

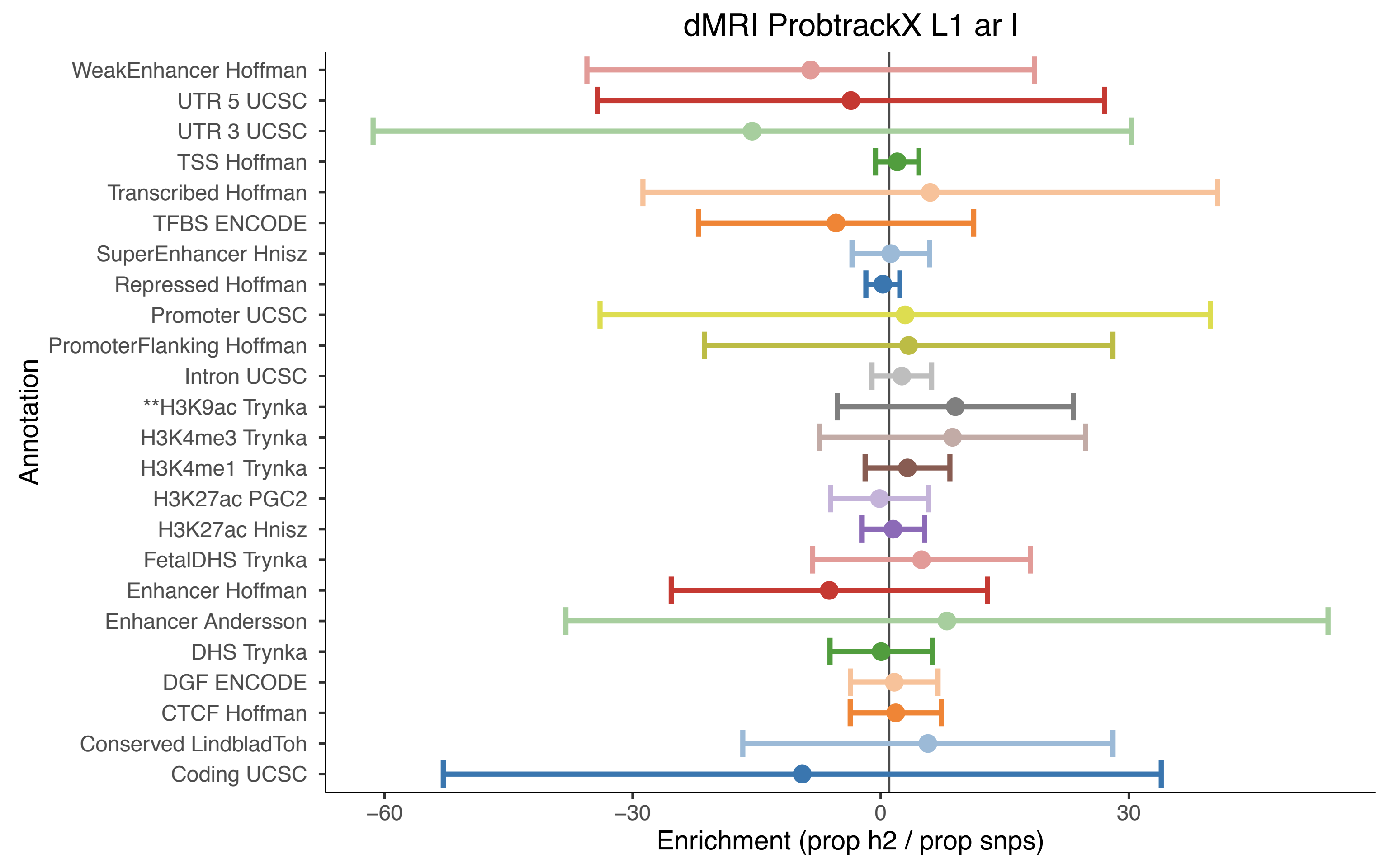

dMRI ProbtrackX L1 ar I
WeakEnhancer Hoffman
UTR 5 UCSC
UTR 3 UCSC
TSS Hoffman
Transcribed Hoffman
TFBS ENCODE
SuperEnhancer Hnisz
Repressed Hoffman
Promoter UCSC
PromoterFlanking Hoffman
Intron UCSC
**H3K9ac Trynka
H3K4me3 Trynka
H3K4me1 Trynka
H3K27ac PGC2
H3K27ac Hnisz
FetalDHS Trynka
Enhancer Hoffman
Enhancer Andersson
DHS Trynka
DGF ENCODE
CTCF Hoffman
Conserved LindbladToh
Coding UCSC
Annotation
−60
−30
0
30
Enrichment (prop h2 / prop snps)

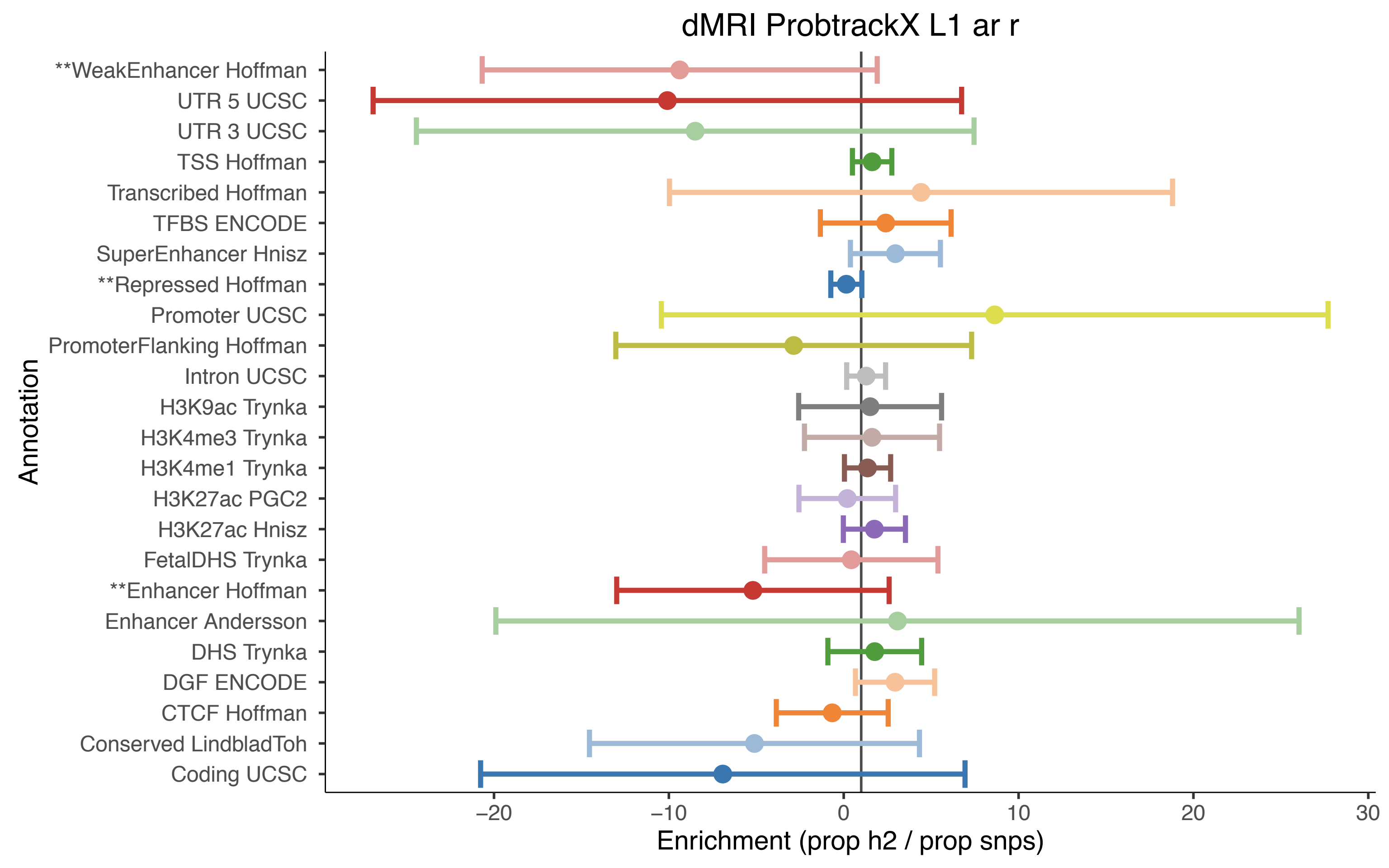
dMRI ProbtrackX L1 ar r
**WeakEnhancer Hoffman
UTR 5 UCSC
UTR 3 UCSC
TSS Hoffman
Transcribed Hoffman
TFBS ENCODE
SuperEnhancer Hnisz
**Repressed Hoffman
Promoter UCSC
PromoterFlanking Hoffman
Intron UCSC
H3K9ac Trynka
H3K4me3 Trynka
H3K4me1 Trynka
H3K27ac PGC2
H3K27ac Hnisz
FetalDHS Trynka
**Enhancer Hoffman
Enhancer Andersson
DHS Trynka
DGF ENCODE
CTCF Hoffman
Conserved LindbladToh
Coding UCSC
Annotation
−20
−10
0
10
20
30
Enrichment (prop h2 / prop snps)

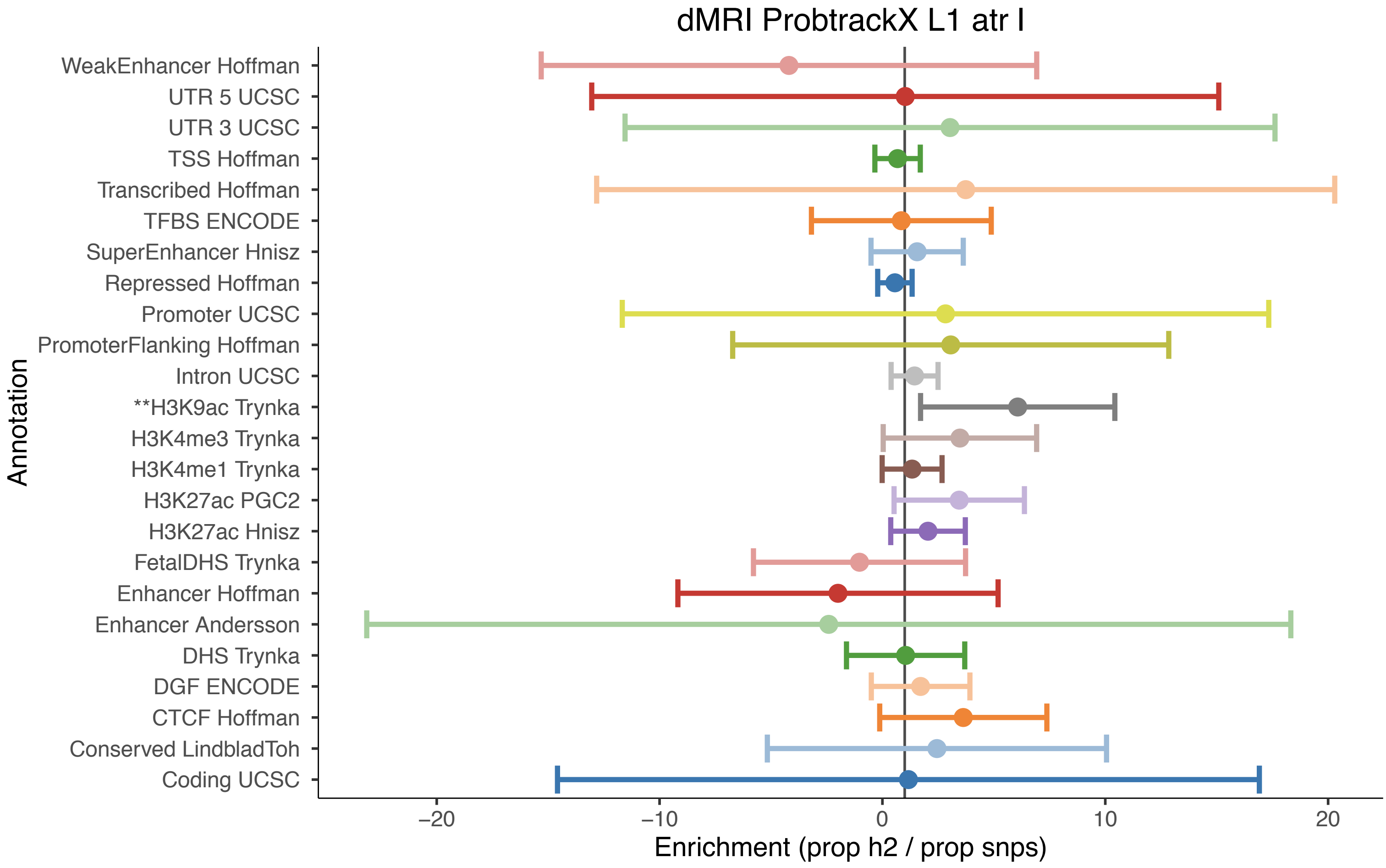
dMRI ProbtrackX L1 atr l
WeakEnhancer Hoffman
UTR 5 UCSC
UTR 3 UCSC
TSS Hoffman
Transcribed Hoffman
TFBS ENCODE
SuperEnhancer Hnisz
Repressed Hoffman
Promoter UCSC
PromoterFlanking Hoffman
Intron UCSC
**H3K9ac Trynka
H3K4me3 Trynka
H3K4me1 Trynka
H3K27ac PGC2
H3K27ac Hnisz
FetalDHS Trynka
Enhancer Hoffman
Enhancer Andersson
DHS Trynka
DGF ENCODE
CTCF Hoffman
Conserved LindbladToh
Coding UCSC
Annotation
−20
−10
0
10
20
Enrichment (prop h2 / prop snps)

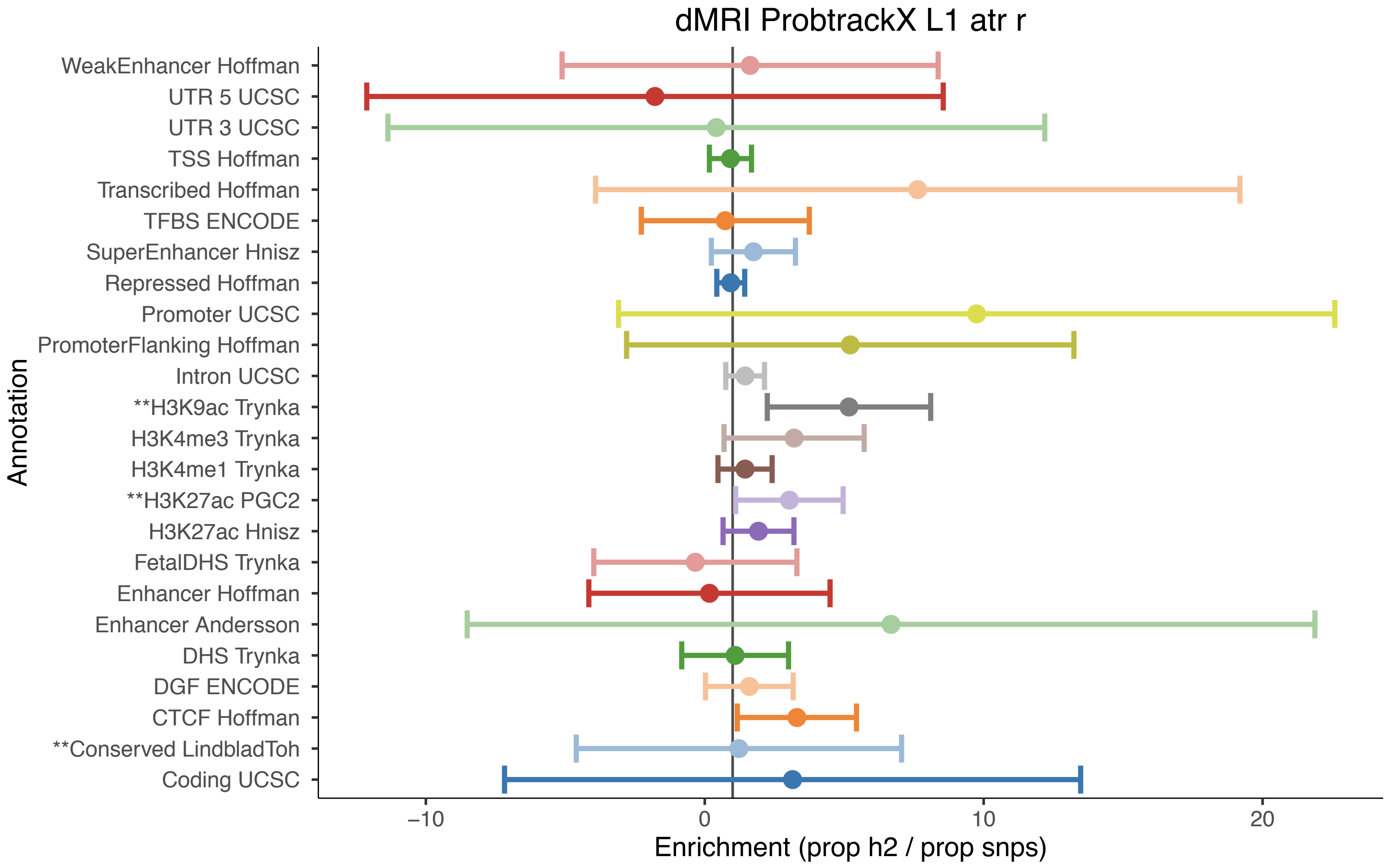
dMRI ProbtrackX L1 atr r
WeakEnhancer Hoffman
UTR 5 UCSC
UTR 3 UCSC
TSS Hoffman
Transcribed Hoffman
TFBS ENCODE
SuperEnhancer Hnisz
Repressed Hoffman
Promoter UCSC
PromoterFlanking Hoffman
Intron UCSC
**H3K9ac Trynka
H3K4me3 Trynka
H3K4me1 Trynka
**H3K27ac PGC2
H3K27ac Hnisz
FetalDHS Trynka
Enhancer Hoffman
Enhancer Andersson
DHS Trynka
DGF ENCODE
CTCF Hoffman
**Conserved LindbladToh
Coding UCSC
Annotation
−10
0
10
20
Enrichment (prop h2 / prop snps)

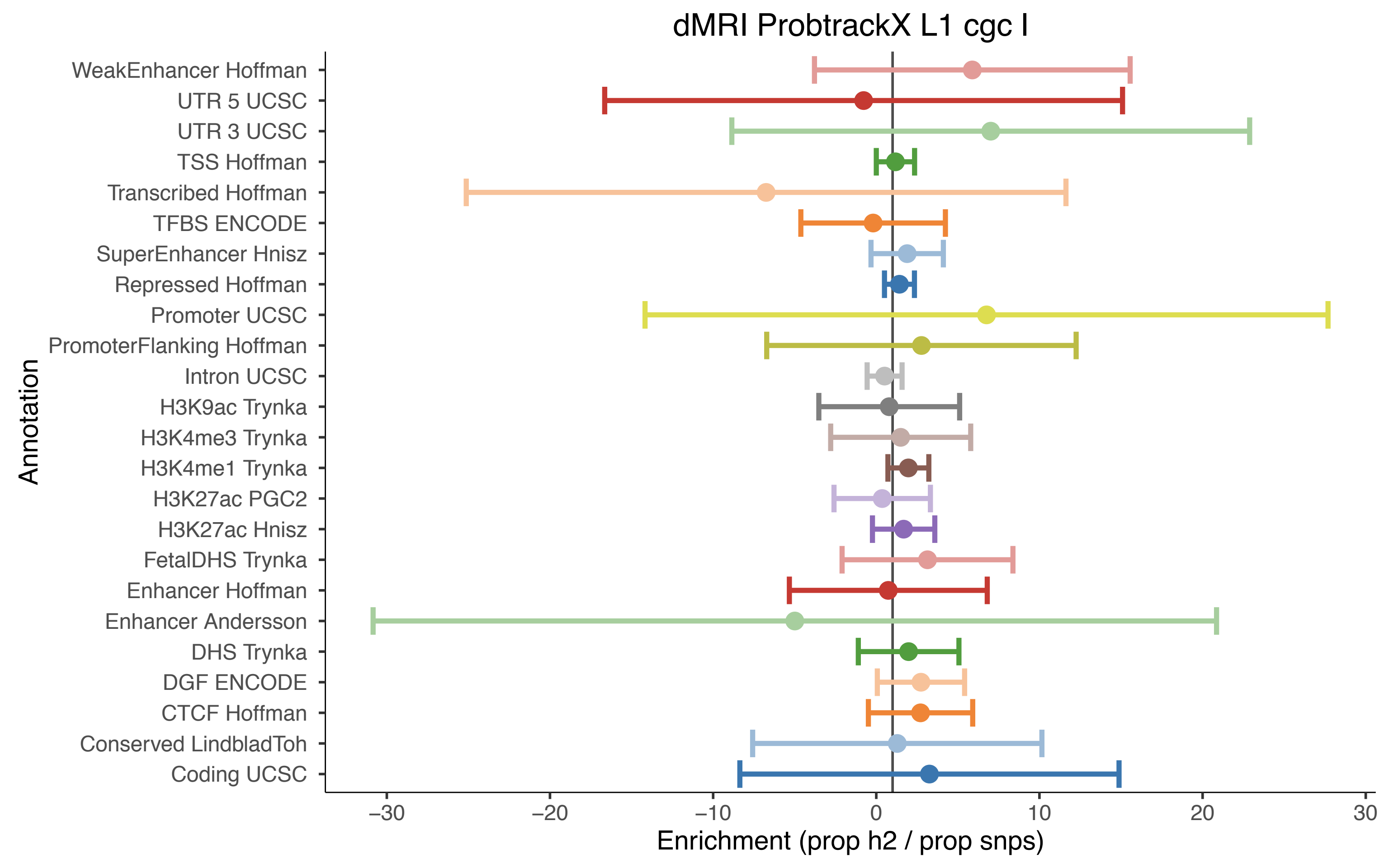

dMRI ProbtrackX L1 cgc I
WeakEnhancer Hoffman
UTR 5 UCSC
UTR 3 UCSC
TSS Hoffman
Transcribed Hoffman
TFBS ENCODE
SuperEnhancer Hnisz
Repressed Hoffman
Promoter UCSC
PromoterFlanking Hoffman
Intron UCSC
H3K9ac Trynka
H3K4me3 Trynka
H3K4me1 Trynka
H3K27ac PGC2
H3K27ac Hnisz
FetalDHS Trynka
Enhancer Hoffman
Enhancer Andersson
DHS Trynka
DGF ENCODE
CTCF Hoffman
Conserved LindbladToh
Coding UCSC
Annotation
−30
−20
−10
0
10
20
30
Enrichment (prop h2 / prop snps)

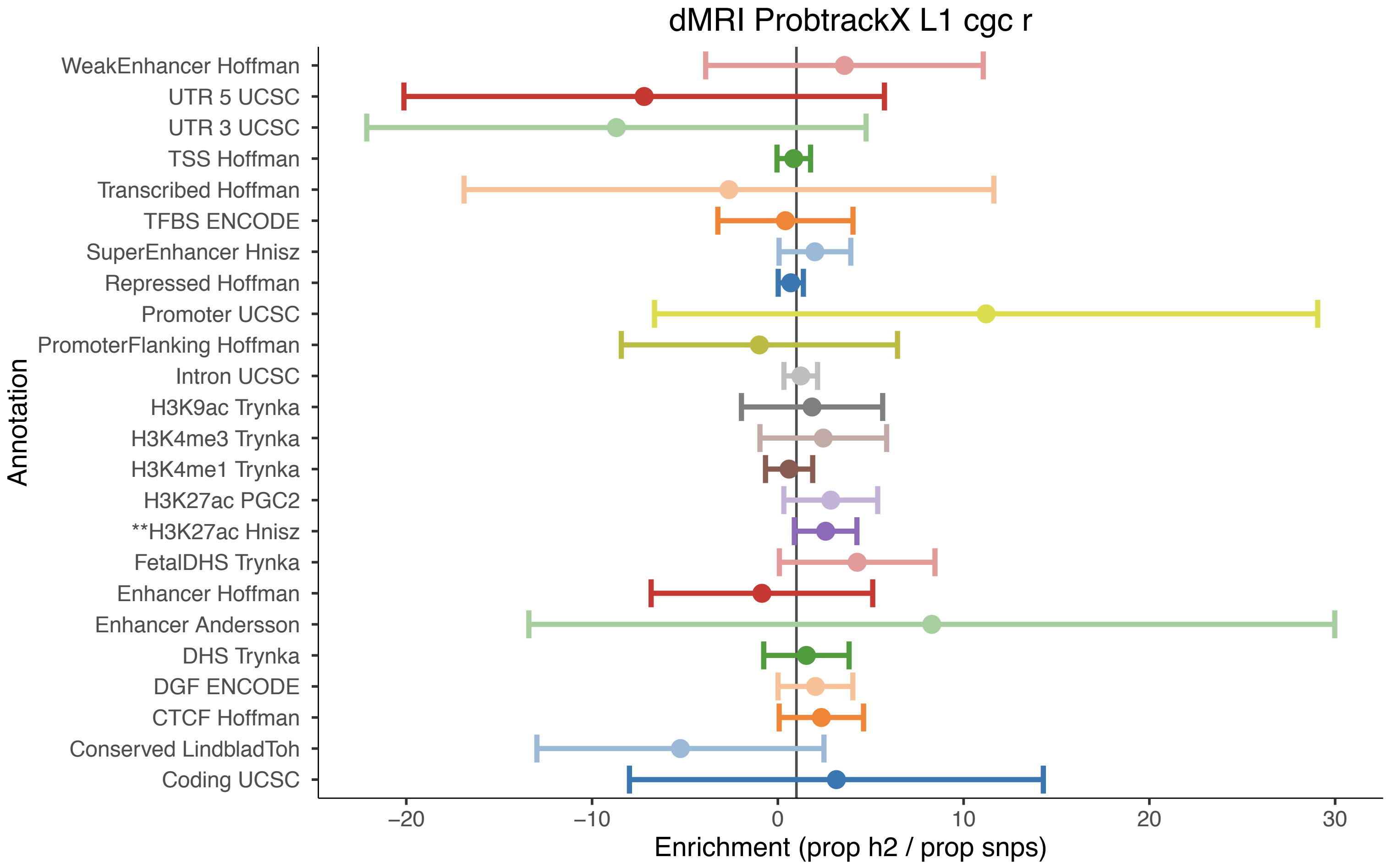
dMRI ProbtrackX L1 cgc r
WeakEnhancer Hoffman
UTR 5 UCSC
UTR 3 UCSC
TSS Hoffman
Transcribed Hoffman
TFBS ENCODE
SuperEnhancer Hnisz
Repressed Hoffman
Promoter UCSC
PromoterFlanking Hoffman
Intron UCSC
H3K9ac Trynka
H3K4me3 Trynka
H3K4me1 Trynka
H3K27ac PGC2
**H3K27ac Hnisz
FetalDHS Trynka
Enhancer Hoffman
Enhancer Andersson
DHS Trynka
DGF ENCODE
CTCF Hoffman
Conserved LindbladToh
Coding UCSC
Annotation
−20
−10
0
10
20
30
Enrichment (prop h2 / prop snps)

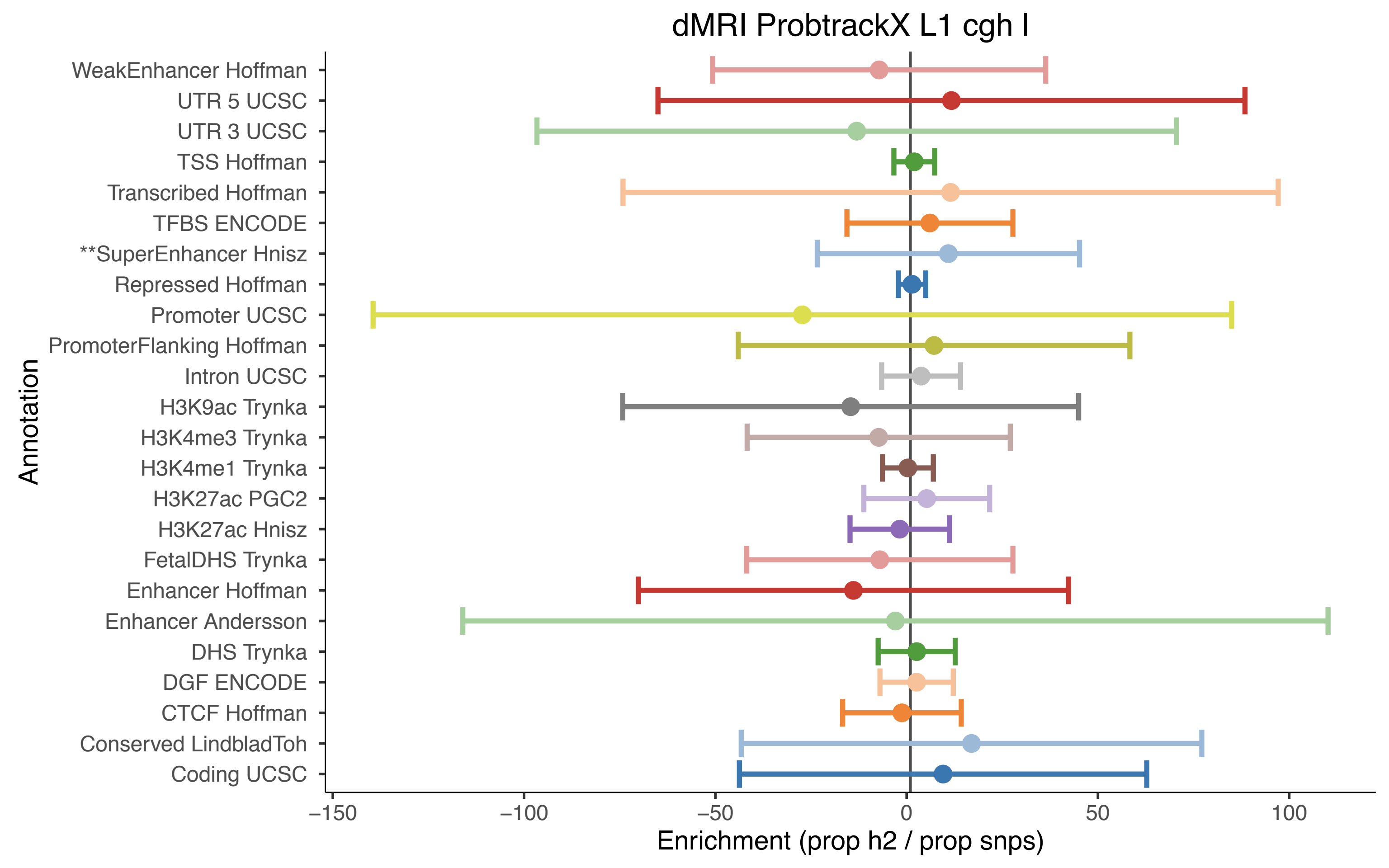

dMRI ProbtrackX L1 cgh I
WeakEnhancer Hoffman
UTR 5 UCSC
UTR 3 UCSC
TSS Hoffman
Transcribed Hoffman
TFBS ENCODE
**SuperEnhancer Hnisz
Repressed Hoffman
Promoter UCSC
PromoterFlanking Hoffman
Intron UCSC
H3K9ac Trynka
H3K4me3 Trynka
H3K4me1 Trynka
H3K27ac PGC2
H3K27ac Hnisz
FetalDHS Trynka
Enhancer Hoffman
Enhancer Andersson
DHS Trynka
DGF ENCODE
CTCF Hoffman
Conserved LindbladToh
Coding UCSC
Annotation
−150
−100
−50
0
50
100
Enrichment (prop h2 / prop snps)

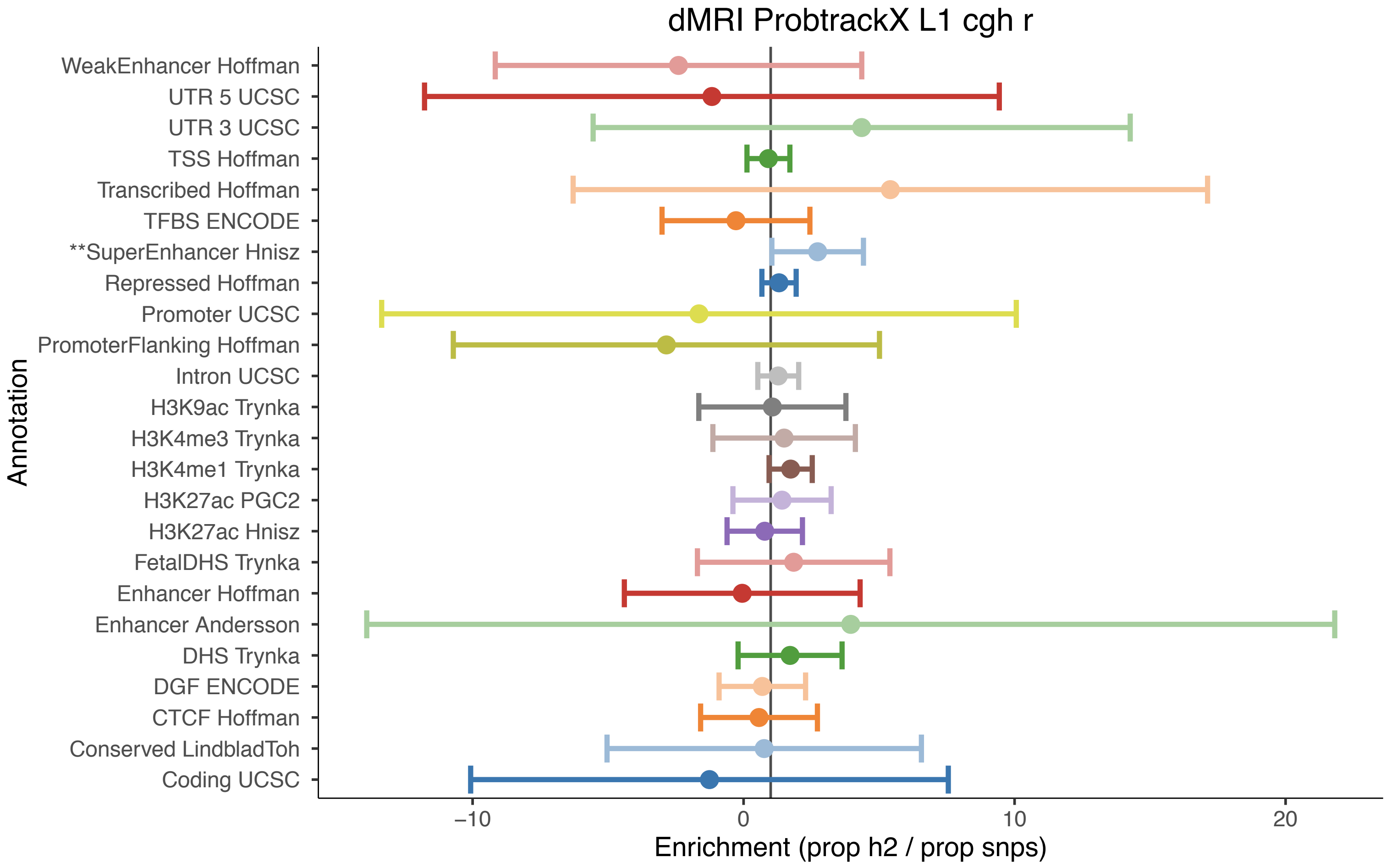
dMRI ProbtrackX L1 cgh r
WeakEnhancer Hoffman
UTR 5 UCSC
UTR 3 UCSC
TSS Hoffman
Transcribed Hoffman
TFBS ENCODE
**SuperEnhancer Hnisz
Repressed Hoffman
Promoter UCSC
PromoterFlanking Hoffman
Intron UCSC
H3K9ac Trynka
H3K4me3 Trynka
H3K4me1 Trynka
H3K27ac PGC2
H3K27ac Hnisz
FetalDHS Trynka
Enhancer Hoffman
Enhancer Andersson
DHS Trynka
DGF ENCODE
CTCF Hoffman
Conserved LindbladToh
Coding UCSC
Annotation
−10
0
10
20
Enrichment (prop h2 / prop snps)

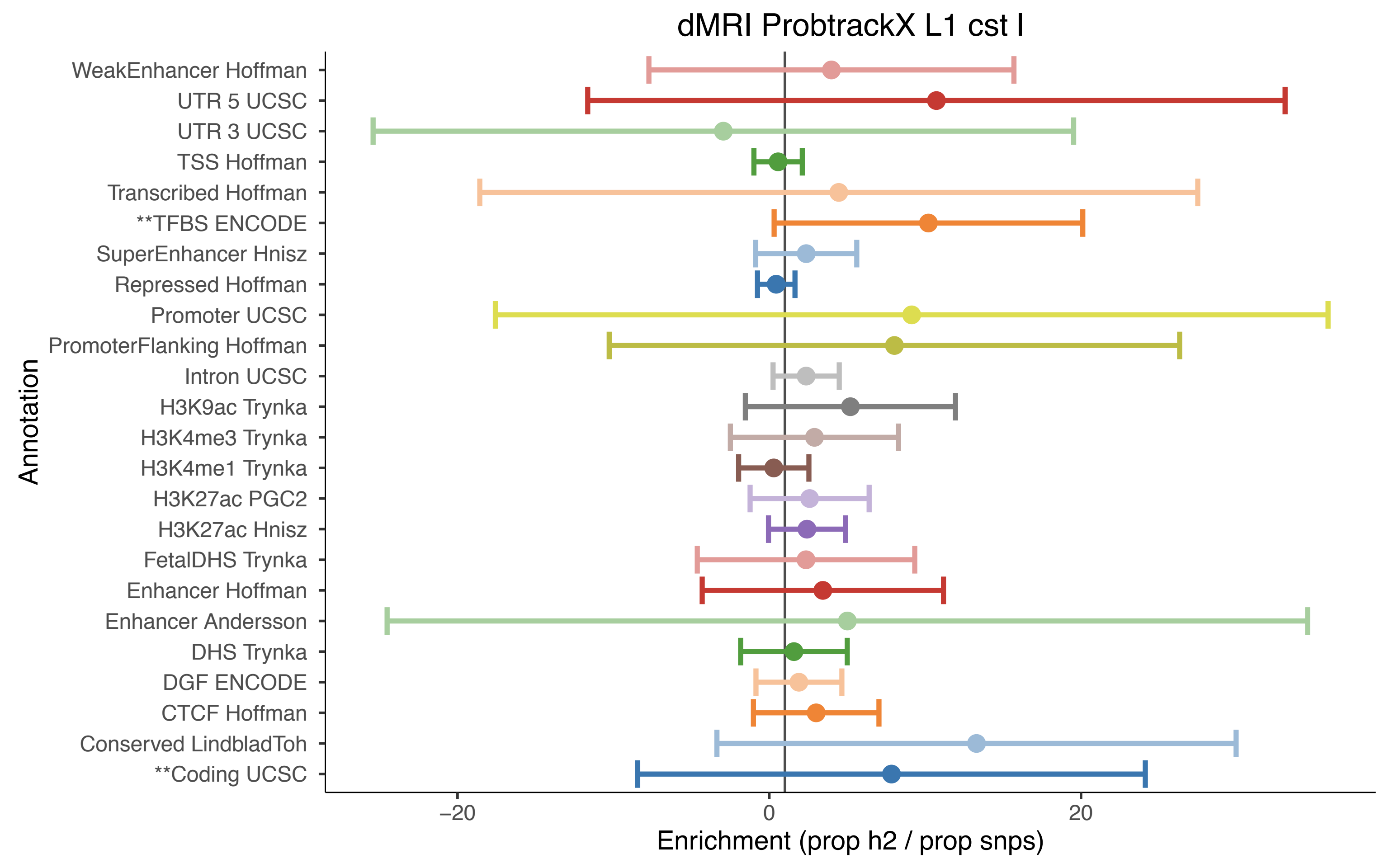

dMRI ProbtrackX L1 cst l
WeakEnhancer Hoffman
UTR 5 UCSC
UTR 3 UCSC
TSS Hoffman
Transcribed Hoffman
**TFBS ENCODE
SuperEnhancer Hnisz
Repressed Hoffman
Promoter UCSC
PromoterFlanking Hoffman
Intron UCSC
H3K9ac Trynka
H3K4me3 Trynka
H3K4me1 Trynka
H3K27ac PGC2
H3K27ac Hnisz
FetalDHS Trynka
Enhancer Hoffman
Enhancer Andersson
DHS Trynka
DGF ENCODE
CTCF Hoffman
Conserved LindbladToh
**Coding UCSC
Annotation
−20
0
20
Enrichment (prop h2 / prop snps)

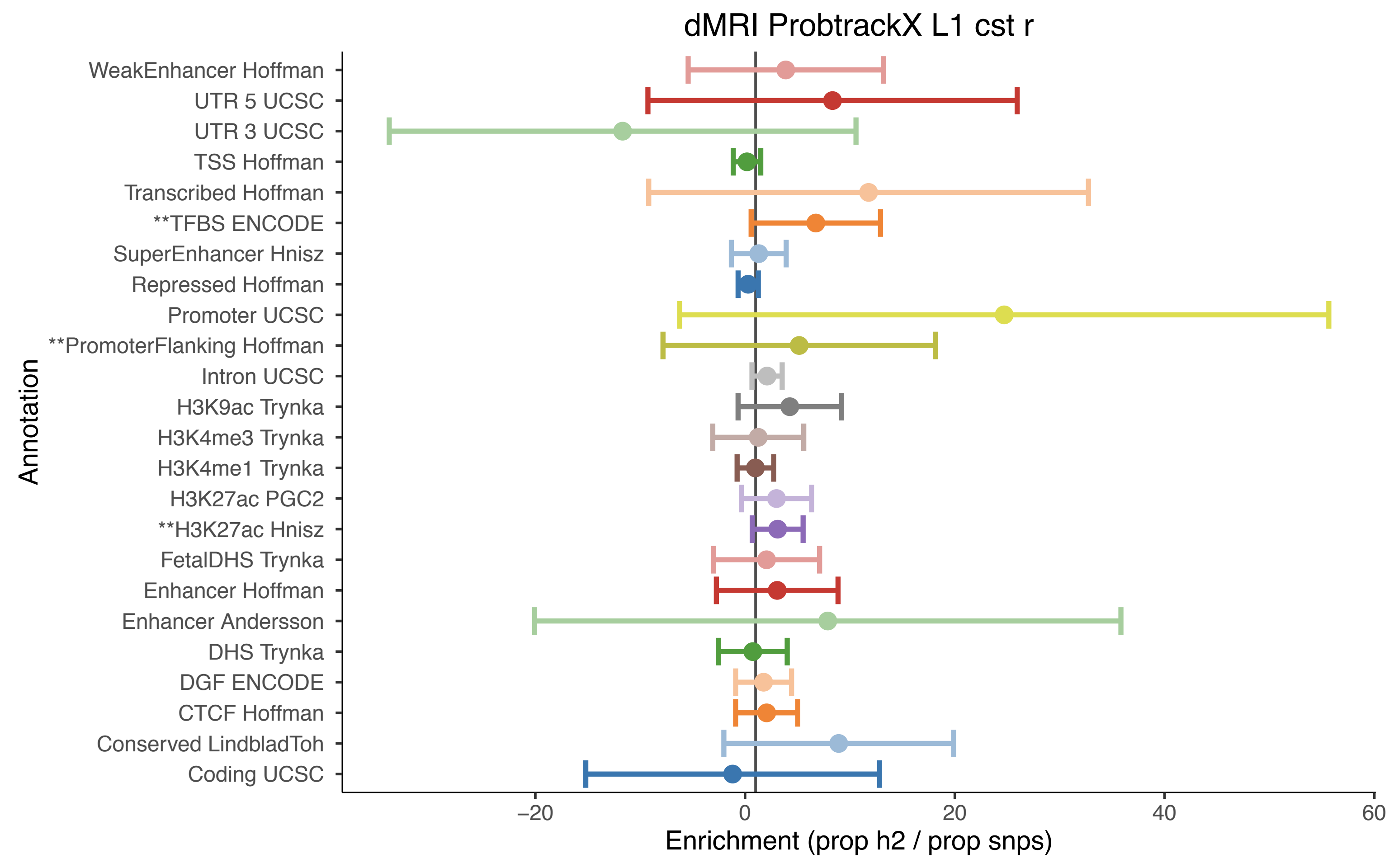

dMRI ProbtrackX L1 cst r
WeakEnhancer Hoffman
UTR 5 UCSC
UTR 3 UCSC
TSS Hoffman
Transcribed Hoffman
**TFBS ENCODE
SuperEnhancer Hnisz
Repressed Hoffman
Promoter UCSC
**PromoterFlanking Hoffman
Intron UCSC
H3K9ac Trynka
H3K4me3 Trynka
H3K4me1 Trynka
H3K27ac PGC2
**H3K27ac Hnisz
FetalDHS Trynka
Enhancer Hoffman
Enhancer Andersson
DHS Trynka
DGF ENCODE
CTCF Hoffman
Conserved LindbladToh
Coding UCSC
Annotation
−20
0
20
40
60
Enrichment (prop h2 / prop snps)

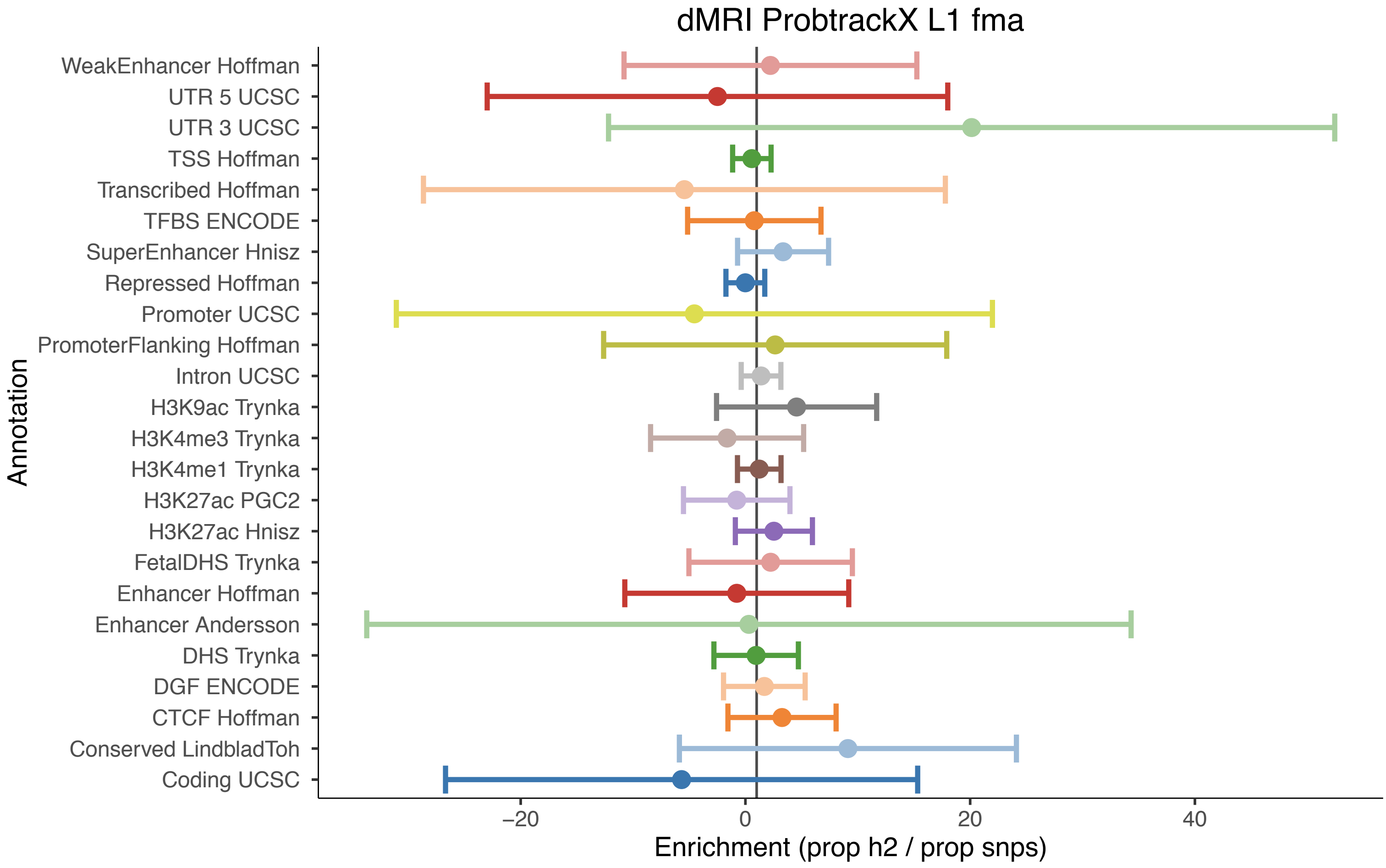

dMRI ProbtrackX L1 fma
Annotation
WeakEnhancer Hoffman
UTR 5 UCSC
UTR 3 UCSC
TSS Hoffman
Transcribed Hoffman
TFBS ENCODE
SuperEnhancer Hnisz
Repressed Hoffman
Promoter UCSC
PromoterFlanking Hoffman
Intron UCSC
H3K9ac Trynka
H3K4me3 Trynka
H3K4me1 Trynka
H3K27ac PGC2
H3K27ac Hnisz
FetalDHS Trynka
Enhancer Hoffman
Enhancer Andersson
DHS Trynka
DGF ENCODE
CTCF Hoffman
Conserved LindbladToh
Coding UCSC
−20
0
20
40
Enrichment (prop h2 / prop snps)

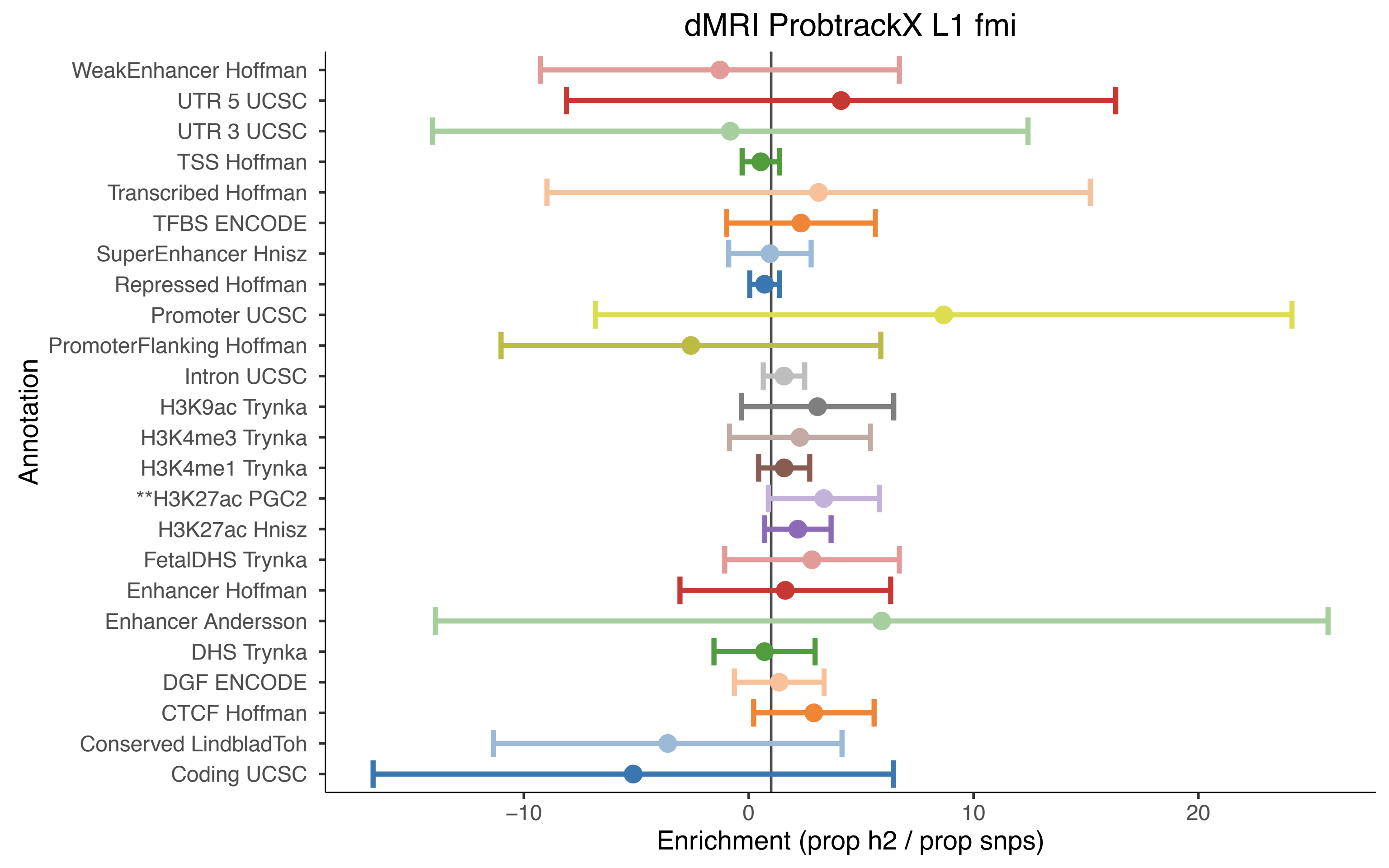
dMRI ProbtrackX L1 fmi
Annotation
WeakEnhancer Hoffman
UTR 5 UCSC
UTR 3 UCSC
TSS Hoffman
Transcribed Hoffman
TFBS ENCODE
SuperEnhancer Hnisz
Repressed Hoffman
Promoter UCSC
PromoterFlanking Hoffman
Intron UCSC
H3K9ac Trynka
H3K4me3 Trynka
H3K4me1 Trynka
**H3K27ac PGC2
H3K27ac Hnisz
FetalDHS Trynka
Enhancer Hoffman
Enhancer Andersson
DHS Trynka
DGF ENCODE
CTCF Hoffman
Conserved LindbladToh
Coding UCSC
−10
0
10
20
Enrichment (prop h2 / prop snps)

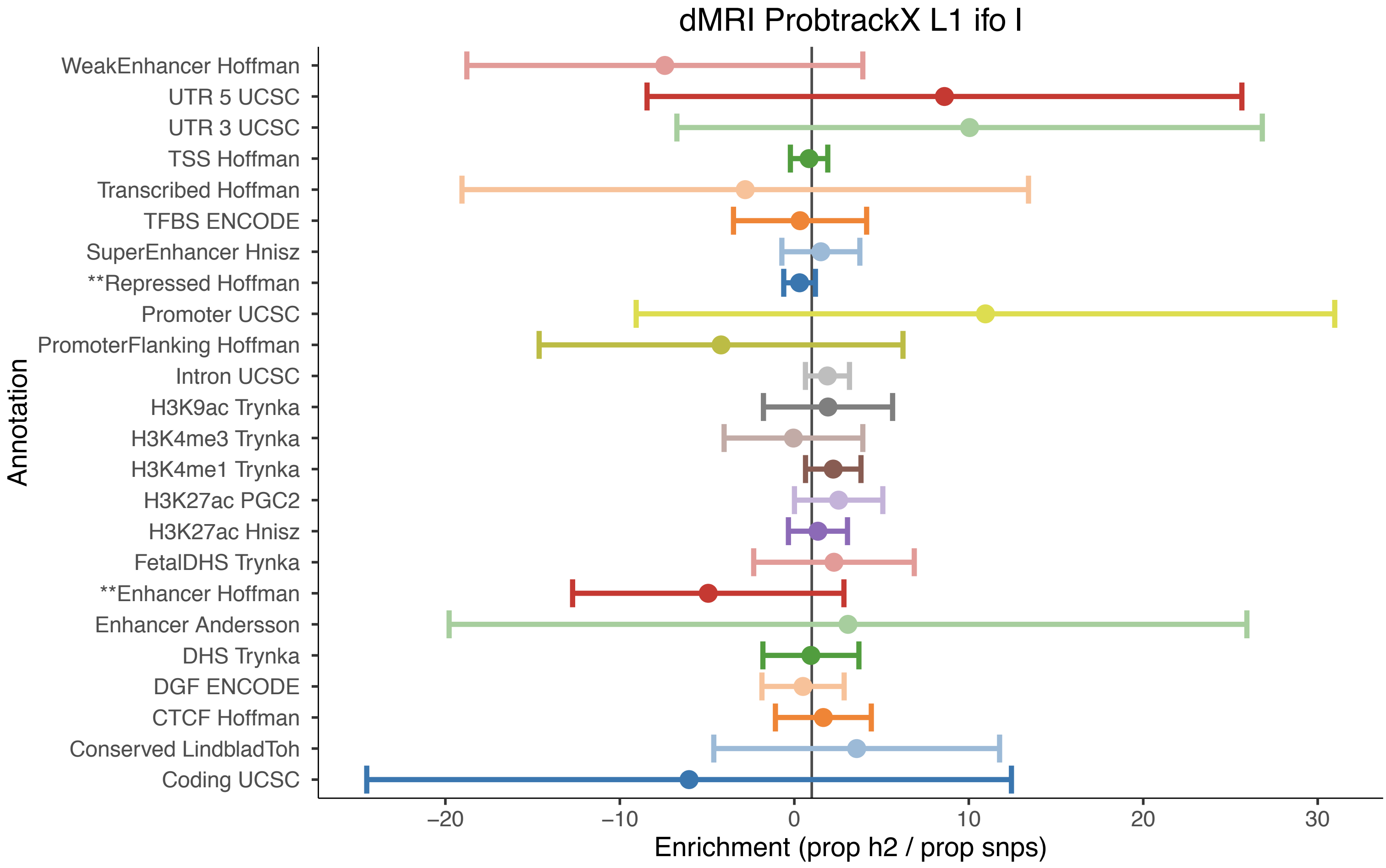

dMRI ProbtrackX L1 ifo l
WeakEnhancer Hoffman
UTR 5 UCSC
UTR 3 UCSC
TSS Hoffman
Transcribed Hoffman
TFBS ENCODE
SuperEnhancer Hnisz
**Repressed Hoffman
Promoter UCSC
PromoterFlanking Hoffman
Intron UCSC
H3K9ac Trynka
H3K4me3 Trynka
H3K4me1 Trynka
H3K27ac PGC2
H3K27ac Hnisz
FetalDHS Trynka
**Enhancer Hoffman
Enhancer Andersson
DHS Trynka
DGF ENCODE
CTCF Hoffman
Conserved LindbladToh
Coding UCSC
Annotation
−20
−10
0
10
20
30
Enrichment (prop h2 / prop snps)

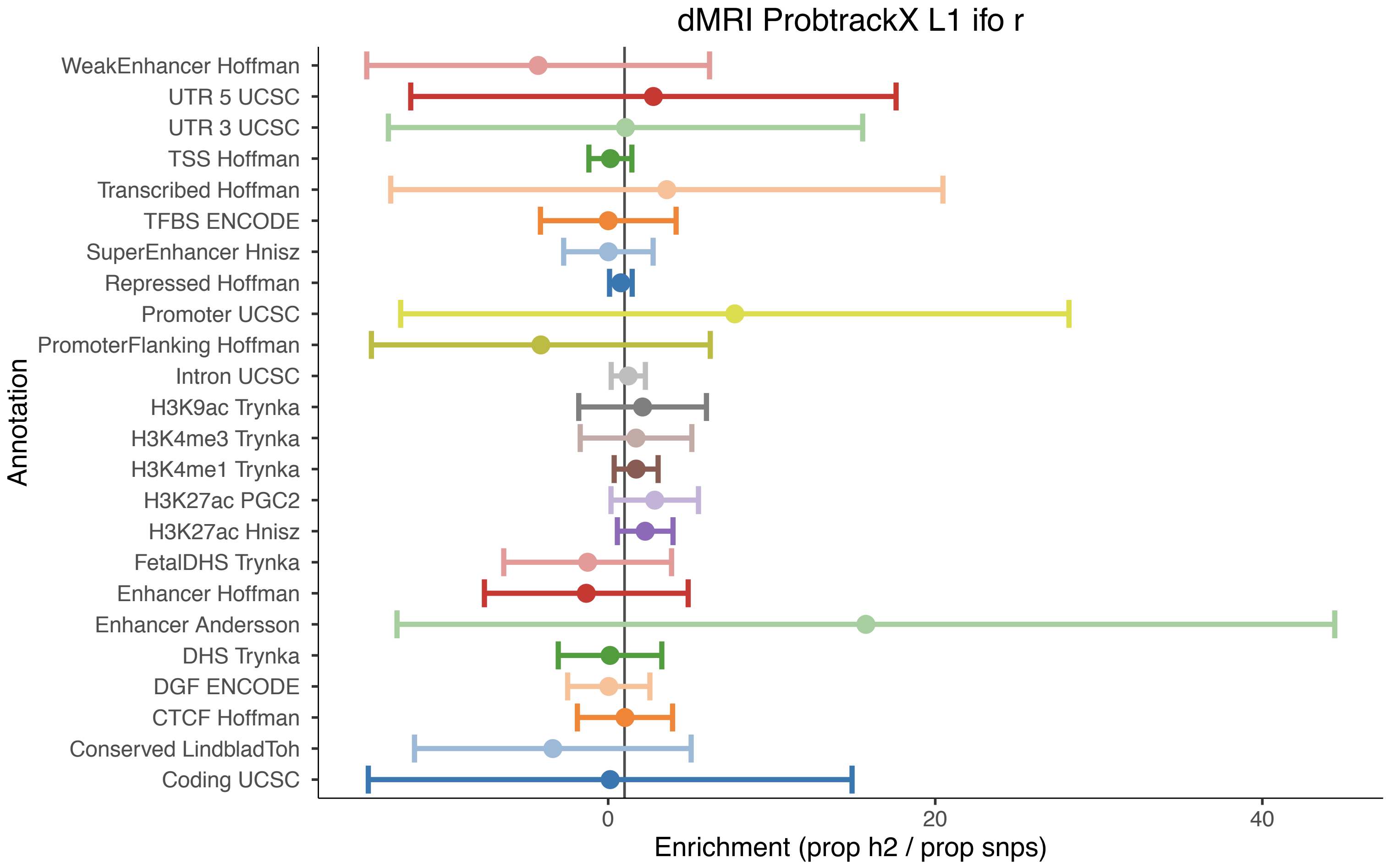
dMRI ProbtrackX L1 ifo r
Annotation
WeakEnhancer Hoffman
UTR 5 UCSC
UTR 3 UCSC
TSS Hoffman
Transcribed Hoffman
TFBS ENCODE
SuperEnhancer Hnisz
Repressed Hoffman
Promoter UCSC
PromoterFlanking Hoffman
Intron UCSC
H3K9ac Trynka
H3K4me3 Trynka
H3K4me1 Trynka
H3K27ac PGC2
H3K27ac Hnisz
FetalDHS Trynka
Enhancer Hoffman
Enhancer Andersson
DHS Trynka
DGF ENCODE
CTCF Hoffman
Conserved LindbladToh
Coding UCSC
0
20
40
Enrichment (prop h2 / prop snps)

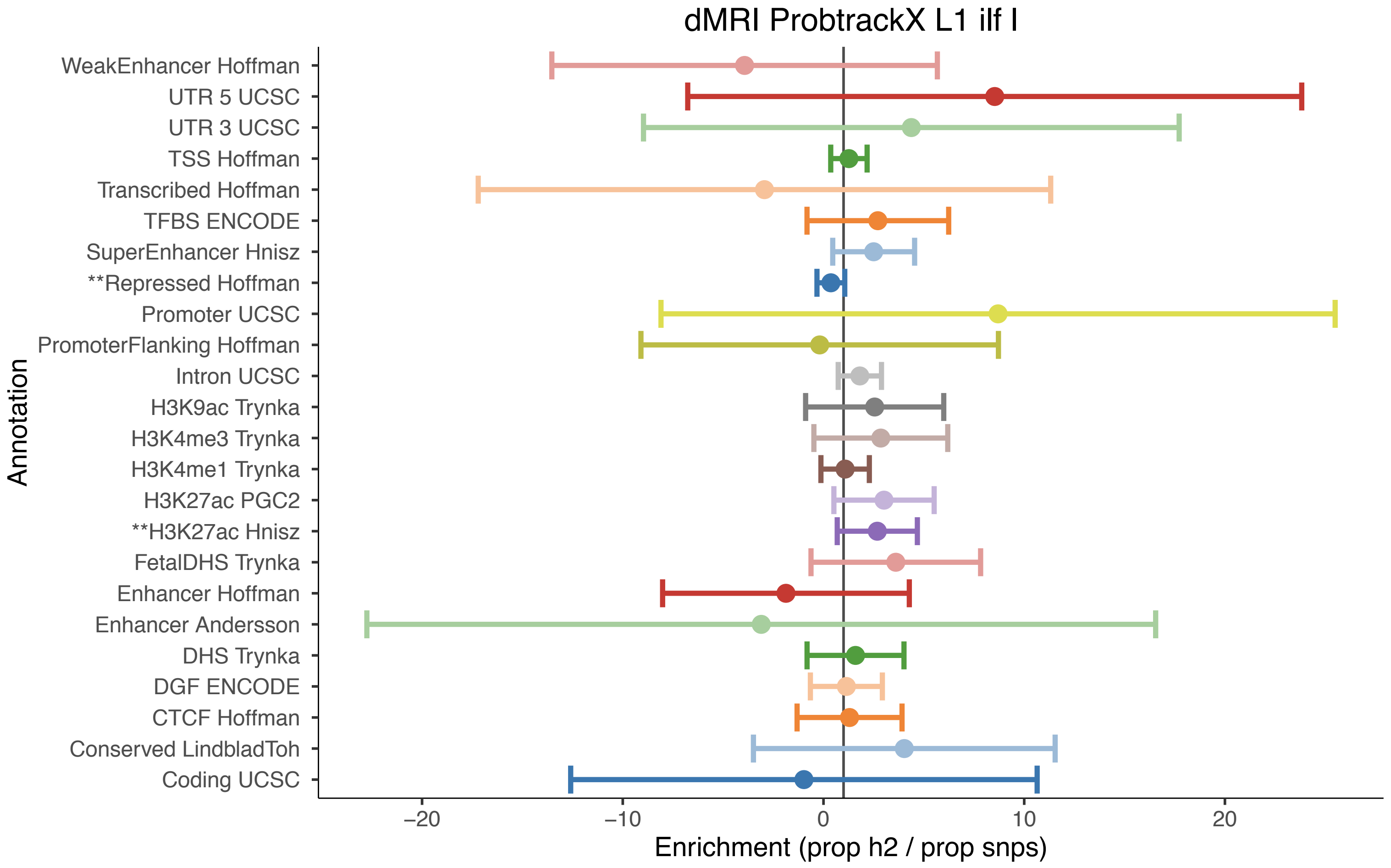
dMRI ProbtrackX L1 ilf l
WeakEnhancer Hoffman
UTR 5 UCSC
UTR 3 UCSC
TSS Hoffman
Transcribed Hoffman
TFBS ENCODE
SuperEnhancer Hnisz
**Repressed Hoffman
Promoter UCSC
PromoterFlanking Hoffman
Intron UCSC
H3K9ac Trynka
H3K4me3 Trynka
H3K4me1 Trynka
H3K27ac PGC2
**H3K27ac Hnisz
FetalDHS Trynka
Enhancer Hoffman
Enhancer Andersson
DHS Trynka
DGF ENCODE
CTCF Hoffman
Conserved LindbladToh
Coding UCSC
Annotation
−20
−10
0
10
20
Enrichment (prop h2 / prop snps)

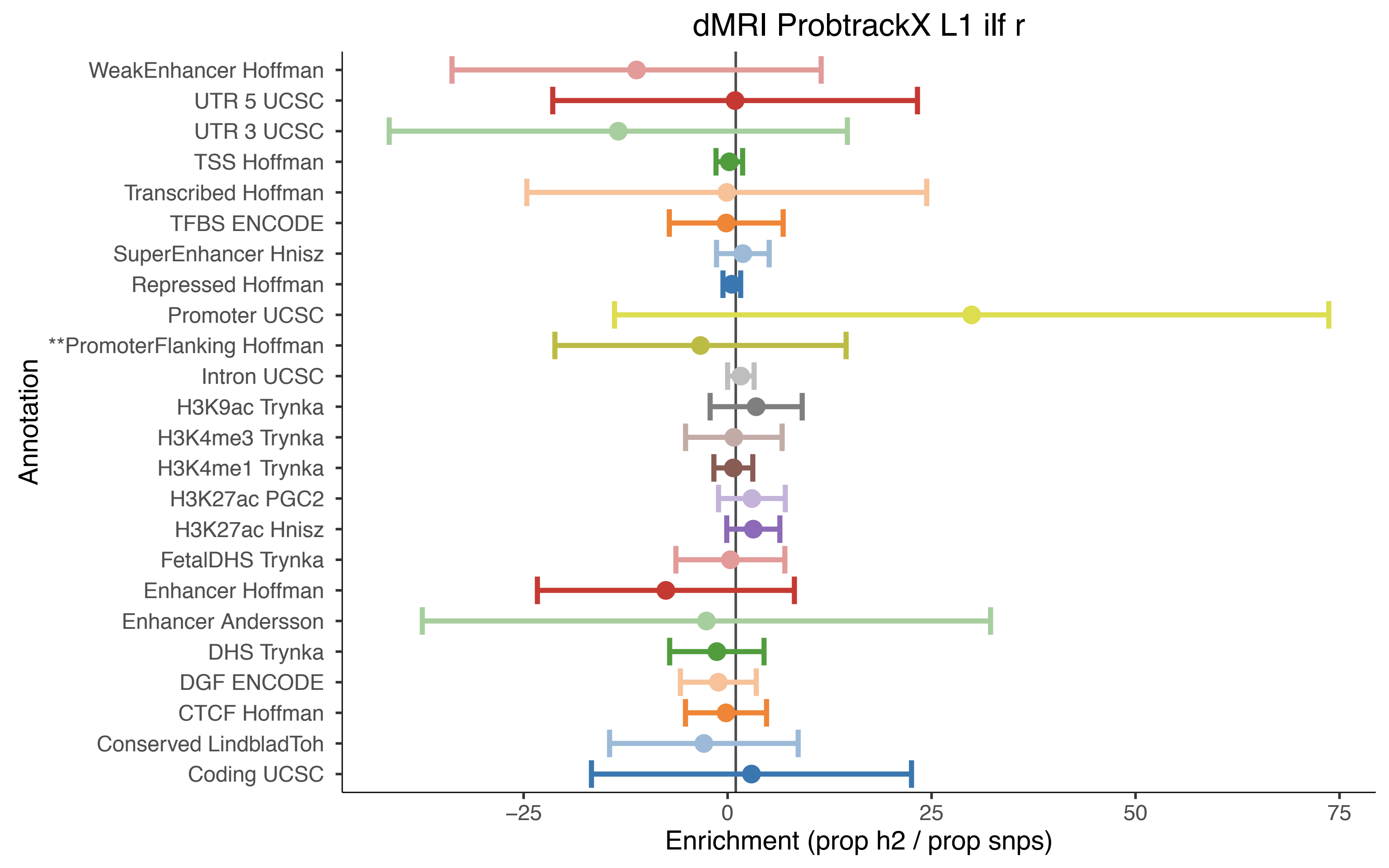
dMRI ProbtrackX L1 ilf r
WeakEnhancer Hoffman
UTR 5 UCSC
UTR 3 UCSC
TSS Hoffman
Transcribed Hoffman
TFBS ENCODE
SuperEnhancer Hnisz
Repressed Hoffman
Promoter UCSC
**PromoterFlanking Hoffman
Intron UCSC
H3K9ac Trynka
H3K4me3 Trynka
H3K4me1 Trynka
H3K27ac PGC2
H3K27ac Hnisz
FetalDHS Trynka
Enhancer Hoffman
Enhancer Andersson
DHS Trynka
DGF ENCODE
CTCF Hoffman
Conserved LindbladToh
Coding UCSC
Annotation
−25
0
25
50
75
Enrichment (prop h2 / prop snps)

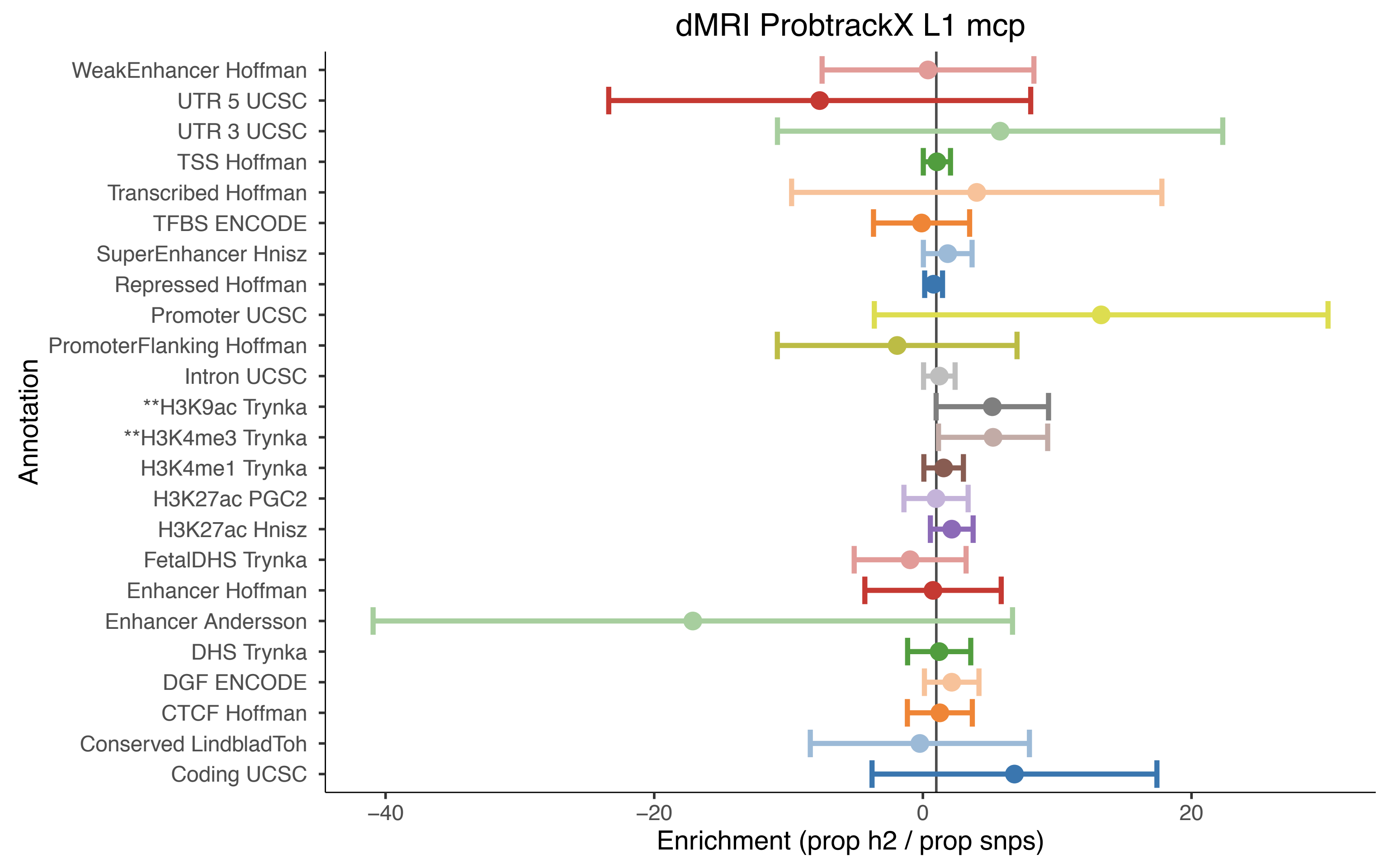

dMRI ProbtrackX L1 mcp
WeakEnhancer Hoffman
UTR 5 UCSC
UTR 3 UCSC
TSS Hoffman
Transcribed Hoffman
TFBS ENCODE
SuperEnhancer Hnisz
Repressed Hoffman
Promoter UCSC
PromoterFlanking Hoffman
Intron UCSC
**H3K9ac Trynka
**H3K4me3 Trynka
H3K4me1 Trynka
H3K27ac PGC2
H3K27ac Hnisz
FetalDHS Trynka
Enhancer Hoffman
Enhancer Andersson
DHS Trynka
DGF ENCODE
CTCF Hoffman
Conserved LindbladToh
Coding UCSC
Annotation
−40
−20
0
20
Enrichment (prop h2 / prop snps)

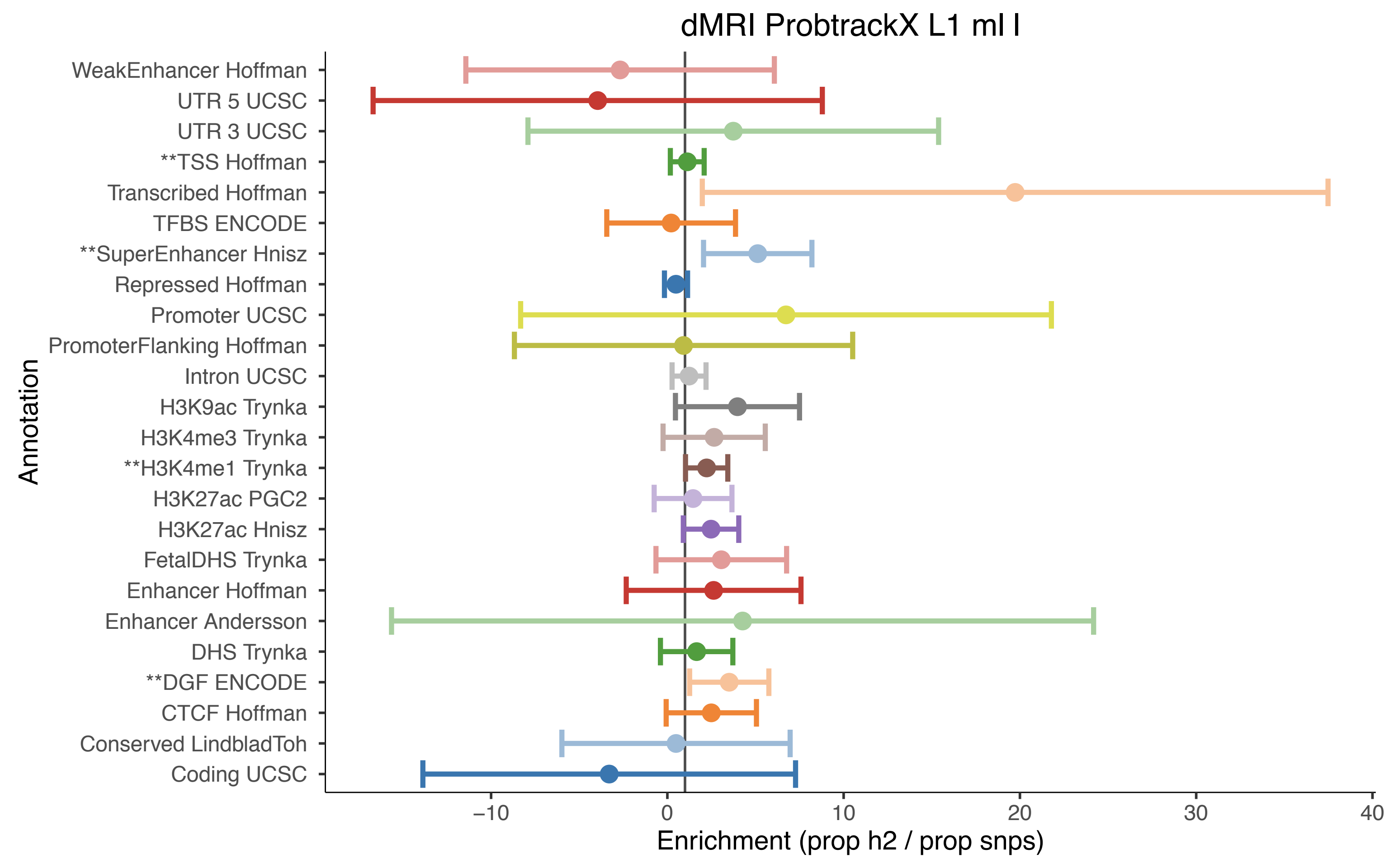

dMRI ProbtrackX L1 ml I
WeakEnhancer Hoffman
UTR 5 UCSC
UTR 3 UCSC
**TSS Hoffman
Transcribed Hoffman
TFBS ENCODE
**SuperEnhancer Hnisz
Repressed Hoffman
Promoter UCSC
PromoterFlanking Hoffman
Intron UCSC
H3K9ac Trynka
H3K4me3 Trynka
**H3K4me1 Trynka
H3K27ac PGC2
H3K27ac Hnisz
FetalDHS Trynka
Enhancer Hoffman
Enhancer Andersson
DHS Trynka
**DGF ENCODE
CTCF Hoffman
Conserved LindbladToh
Coding UCSC
Annotation
−10
0
10
20
30
40
Enrichment (prop h2 / prop snps)

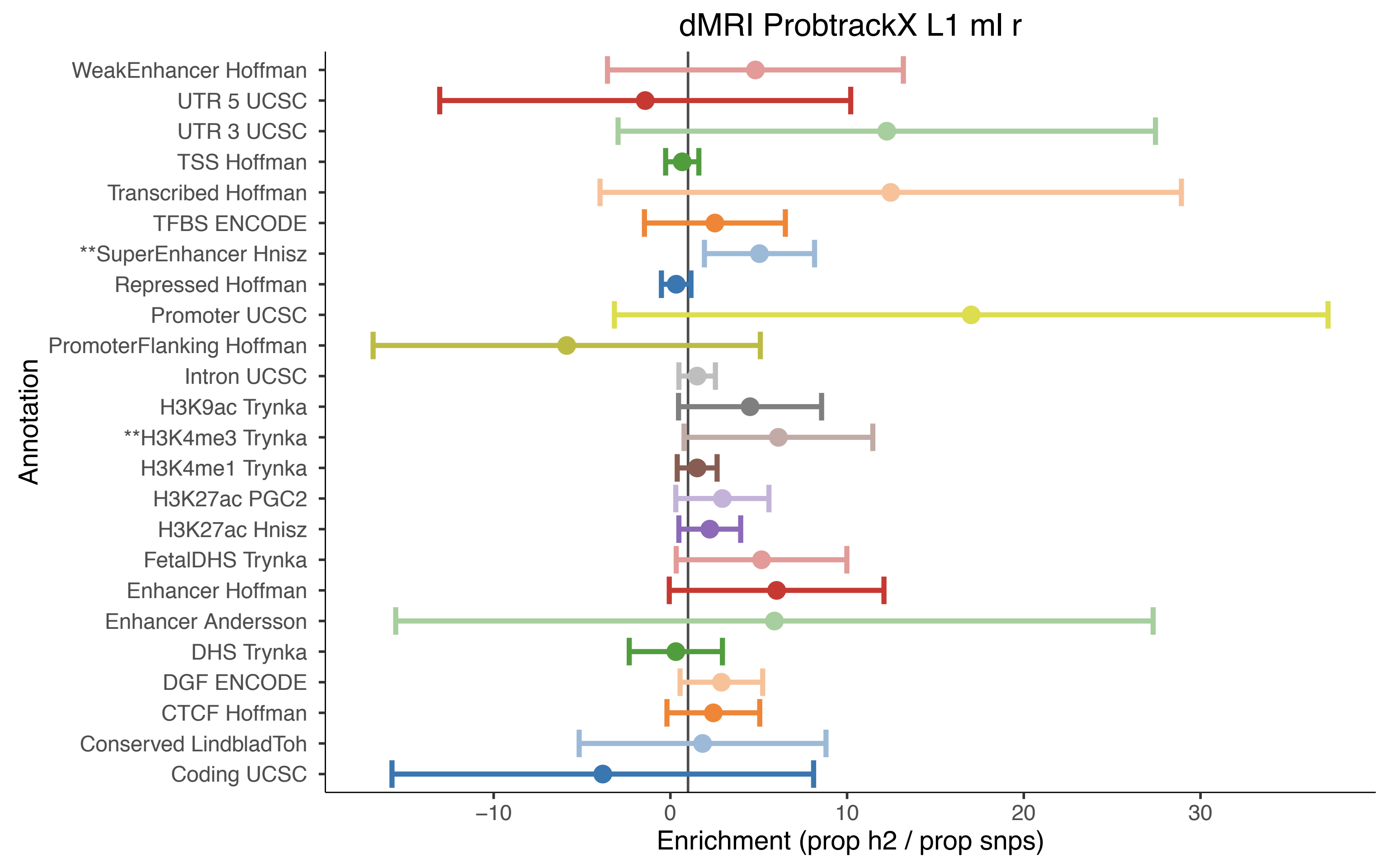
dMRI ProbtrackX L1 ml r
WeakEnhancer Hoffman
UTR 5 UCSC
UTR 3 UCSC
TSS Hoffman
Transcribed Hoffman
TFBS ENCODE
**SuperEnhancer Hnisz
Repressed Hoffman
Promoter UCSC
PromoterFlanking Hoffman
Intron UCSC
H3K9ac Trynka
**H3K4me3 Trynka
H3K4me1 Trynka
H3K27ac PGC2
H3K27ac Hnisz
FetalDHS Trynka
Enhancer Hoffman
Enhancer Andersson
DHS Trynka
DGF ENCODE
CTCF Hoffman
Conserved LindbladToh
Coding UCSC
Annotation
−10
0
10
20
30
Enrichment (prop h2 / prop snps)

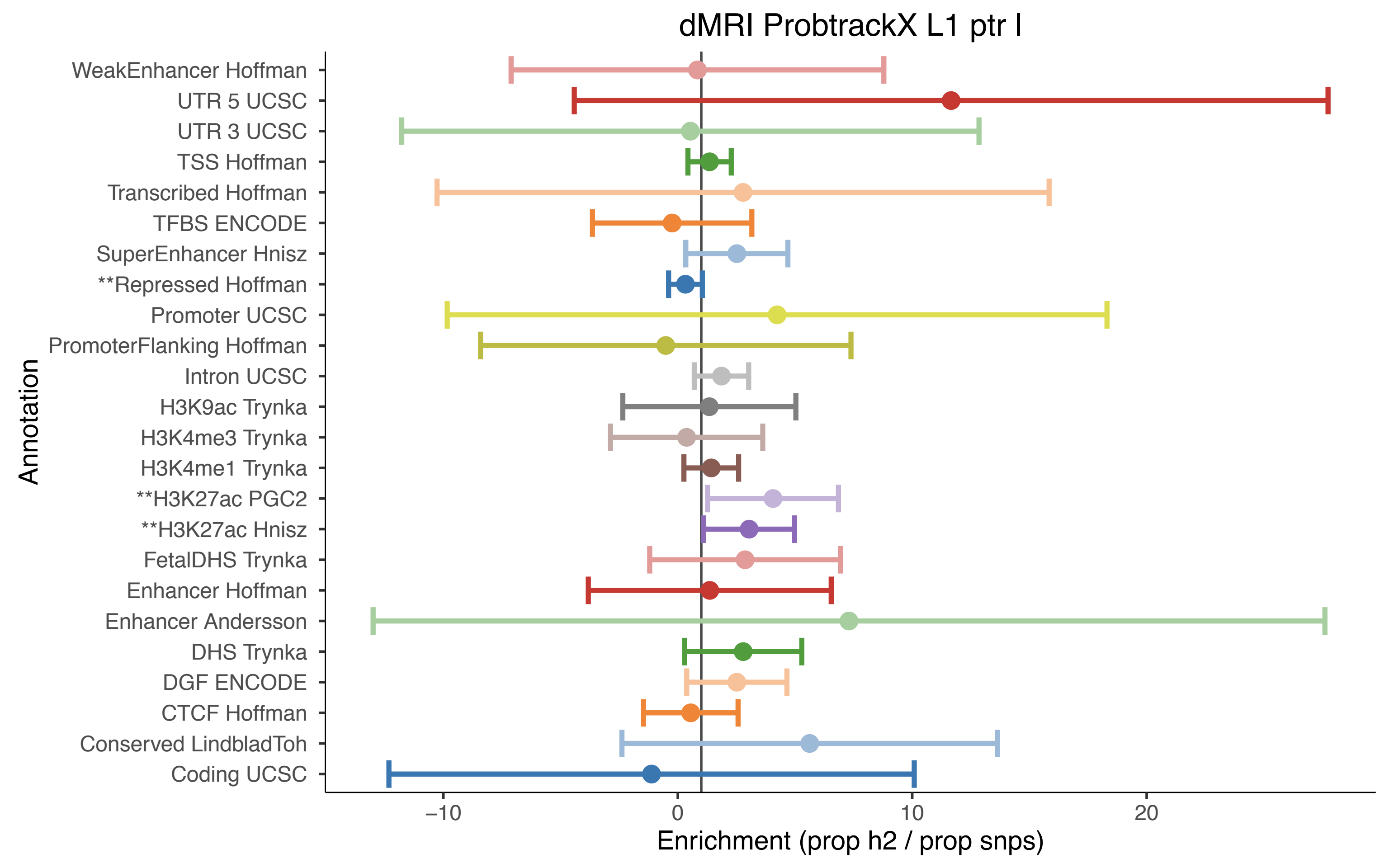
dMRI ProbtrackX L1 ptr I
WeakEnhancer Hoffman
UTR 5 UCSC
UTR 3 UCSC
TSS Hoffman
Transcribed Hoffman
TFBS ENCODE
SuperEnhancer Hnisz
**Repressed Hoffman
Promoter UCSC
PromoterFlanking Hoffman
Intron UCSC
H3K9ac Trynka
H3K4me3 Trynka
H3K4me1 Trynka
**H3K27ac PGC2
**H3K27ac Hnisz
FetalDHS Trynka
Enhancer Hoffman
Enhancer Andersson
DHS Trynka
DGF ENCODE
CTCF Hoffman
Conserved LindbladToh
Coding UCSC
Annotation
−10
0
10
20
Enrichment (prop h2 / prop snps)

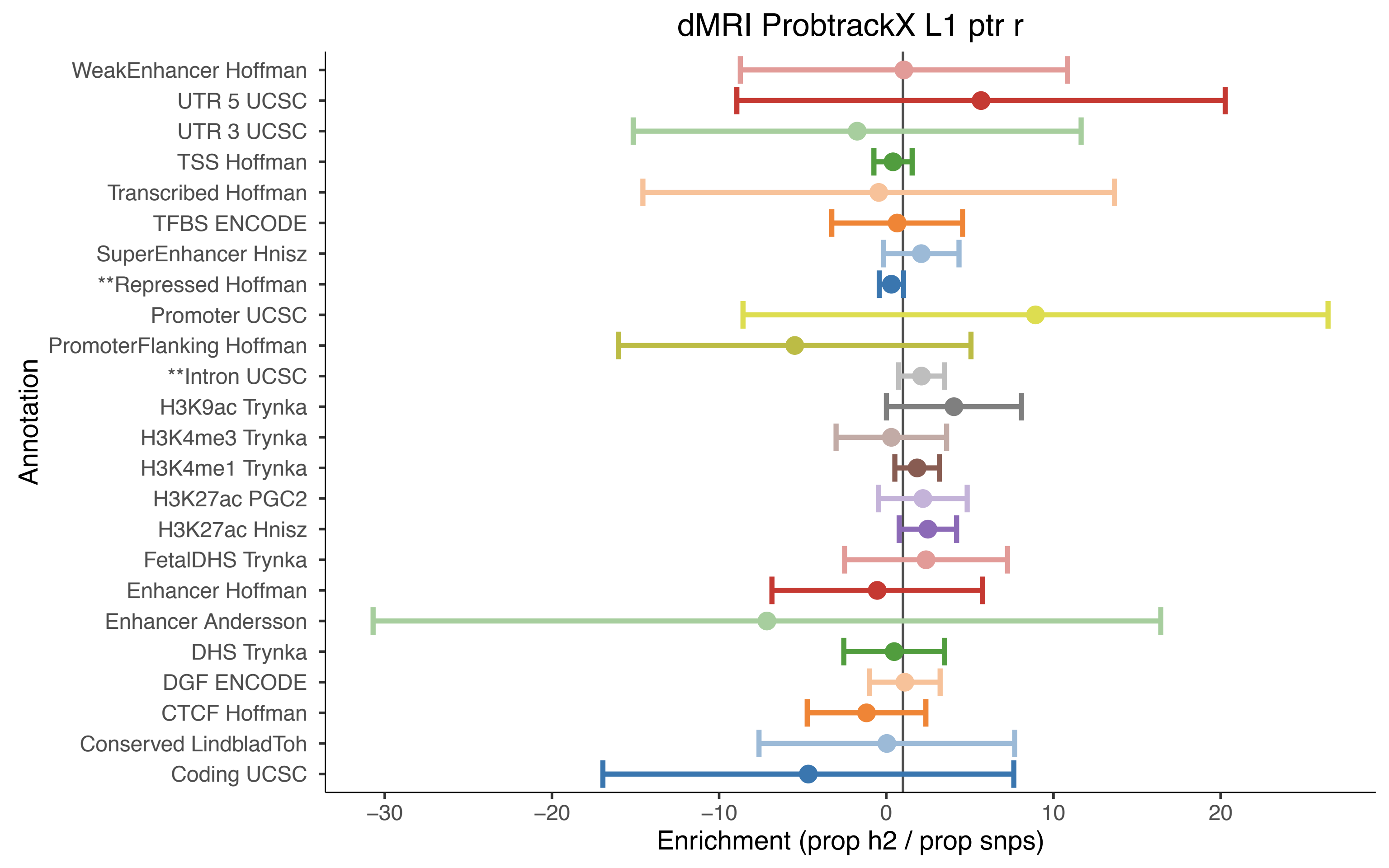
dMRI ProbtrackX L1 ptr r
WeakEnhancer Hoffman
UTR 5 UCSC
UTR 3 UCSC
TSS Hoffman
Transcribed Hoffman
TFBS ENCODE
SuperEnhancer Hnisz
**Repressed Hoffman
Promoter UCSC
PromoterFlanking Hoffman
**Intron UCSC
H3K9ac Trynka
H3K4me3 Trynka
H3K4me1 Trynka
H3K27ac PGC2
H3K27ac Hnisz
FetalDHS Trynka
Enhancer Hoffman
Enhancer Andersson
DHS Trynka
DGF ENCODE
CTCF Hoffman
Conserved LindbladToh
Coding UCSC
Annotation
−30
−20
−10
0
10
20
Enrichment (prop h2 / prop snps)

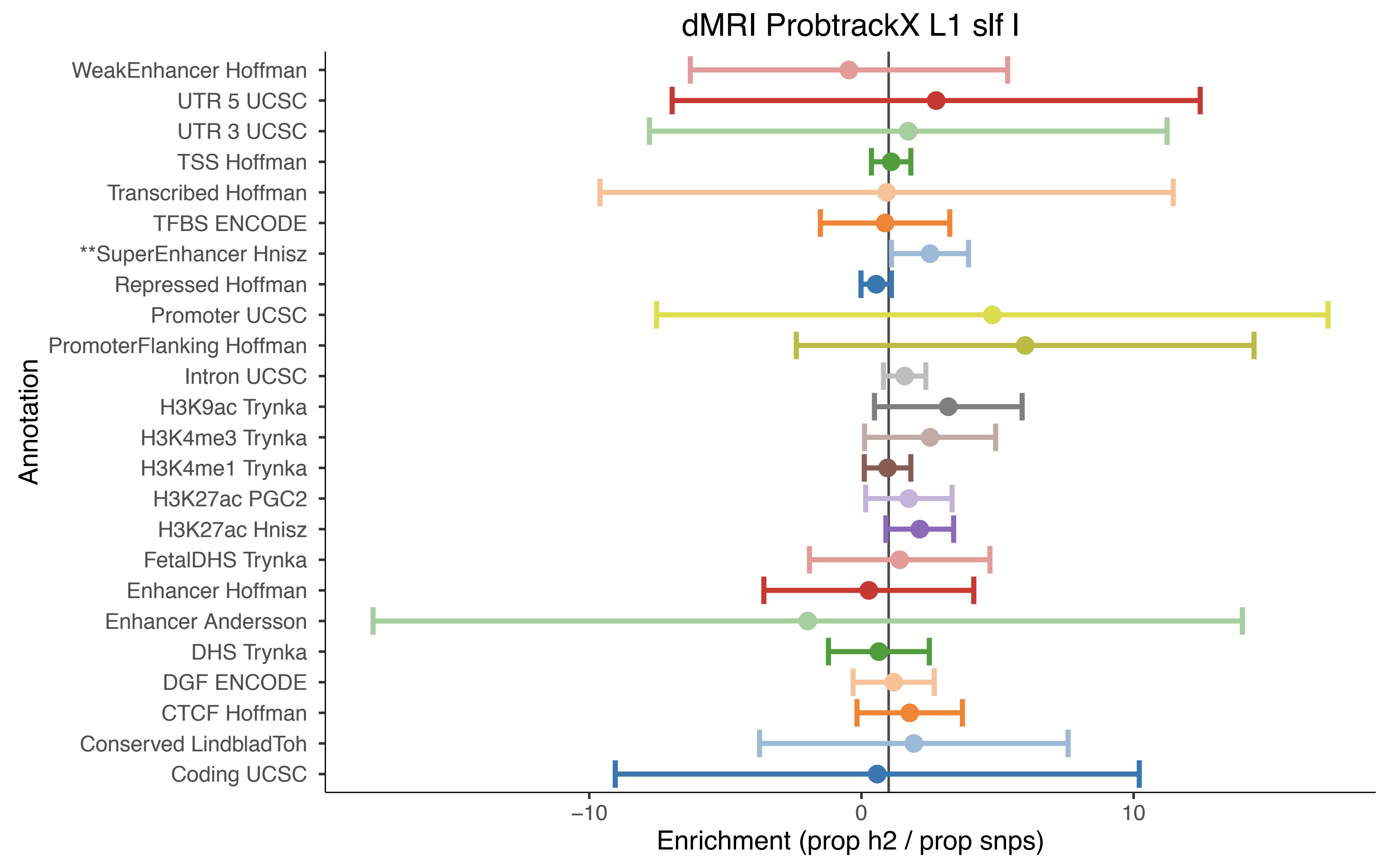

dMRI ProbtrackX L1 slf I
WeakEnhancer Hoffman
UTR 5 UCSC
UTR 3 UCSC
TSS Hoffman
Transcribed Hoffman
TFBS ENCODE
**SuperEnhancer Hnisz
Repressed Hoffman
Promoter UCSC
PromoterFlanking Hoffman
Intron UCSC
H3K9ac Trynka
H3K4me3 Trynka
H3K4me1 Trynka
H3K27ac PGC2
H3K27ac Hnisz
FetalDHS Trynka
Enhancer Hoffman
Enhancer Andersson
DHS Trynka
DGF ENCODE
CTCF Hoffman
Conserved LindbladToh
Coding UCSC
Annotation
−10
0
10
Enrichment (prop h2 / prop snps)

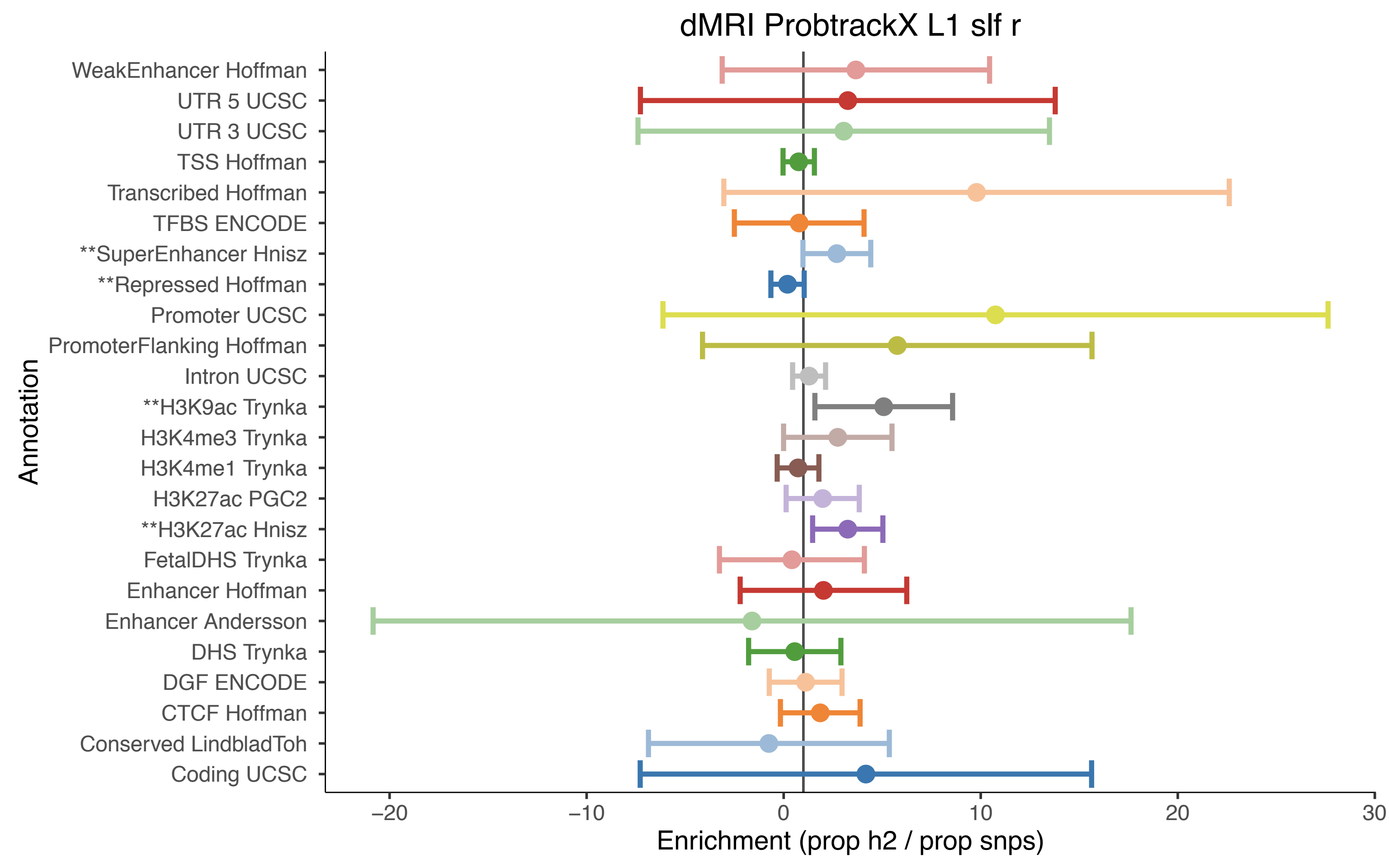
dMRI ProbtrackX L1 slf r
WeakEnhancer Hoffman
UTR 5 UCSC
UTR 3 UCSC
TSS Hoffman
Transcribed Hoffman
TFBS ENCODE
**SuperEnhancer Hnisz
**Repressed Hoffman
Promoter UCSC
PromoterFlanking Hoffman
Intron UCSC
**H3K9ac Trynka
H3K4me3 Trynka
H3K4me1 Trynka
H3K27ac PGC2
**H3K27ac Hnisz
FetalDHS Trynka
Enhancer Hoffman
Enhancer Andersson
DHS Trynka
DGF ENCODE
CTCF Hoffman
Conserved LindbladToh
Coding UCSC
Annotation
−20
−10
0
10
20
30
Enrichment (prop h2 / prop snps)

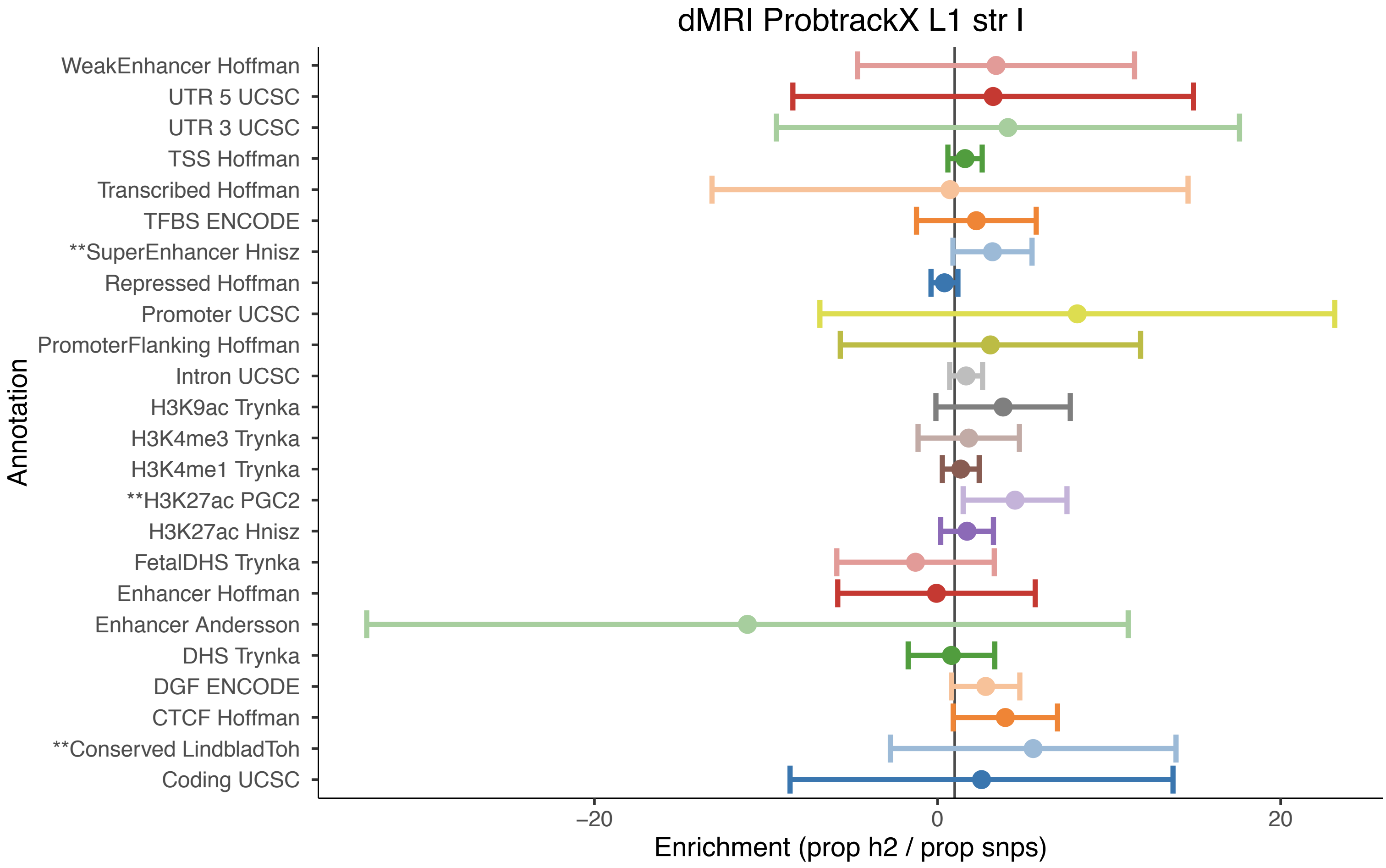

dMRI ProbtrackX L1 str I
WeakEnhancer Hoffman
UTR 5 UCSC
UTR 3 UCSC
TSS Hoffman
Transcribed Hoffman
TFBS ENCODE
**SuperEnhancer Hnisz
Repressed Hoffman
Promoter UCSC
PromoterFlanking Hoffman
Intron UCSC
H3K9ac Trynka
H3K4me3 Trynka
H3K4me1 Trynka
**H3K27ac PGC2
H3K27ac Hnisz
FetalDHS Trynka
Enhancer Hoffman
Enhancer Andersson
DHS Trynka
DGF ENCODE
CTCF Hoffman
**Conserved LindbladToh
Coding UCSC
Annotation
−20
0
20
Enrichment (prop h2 / prop snps)

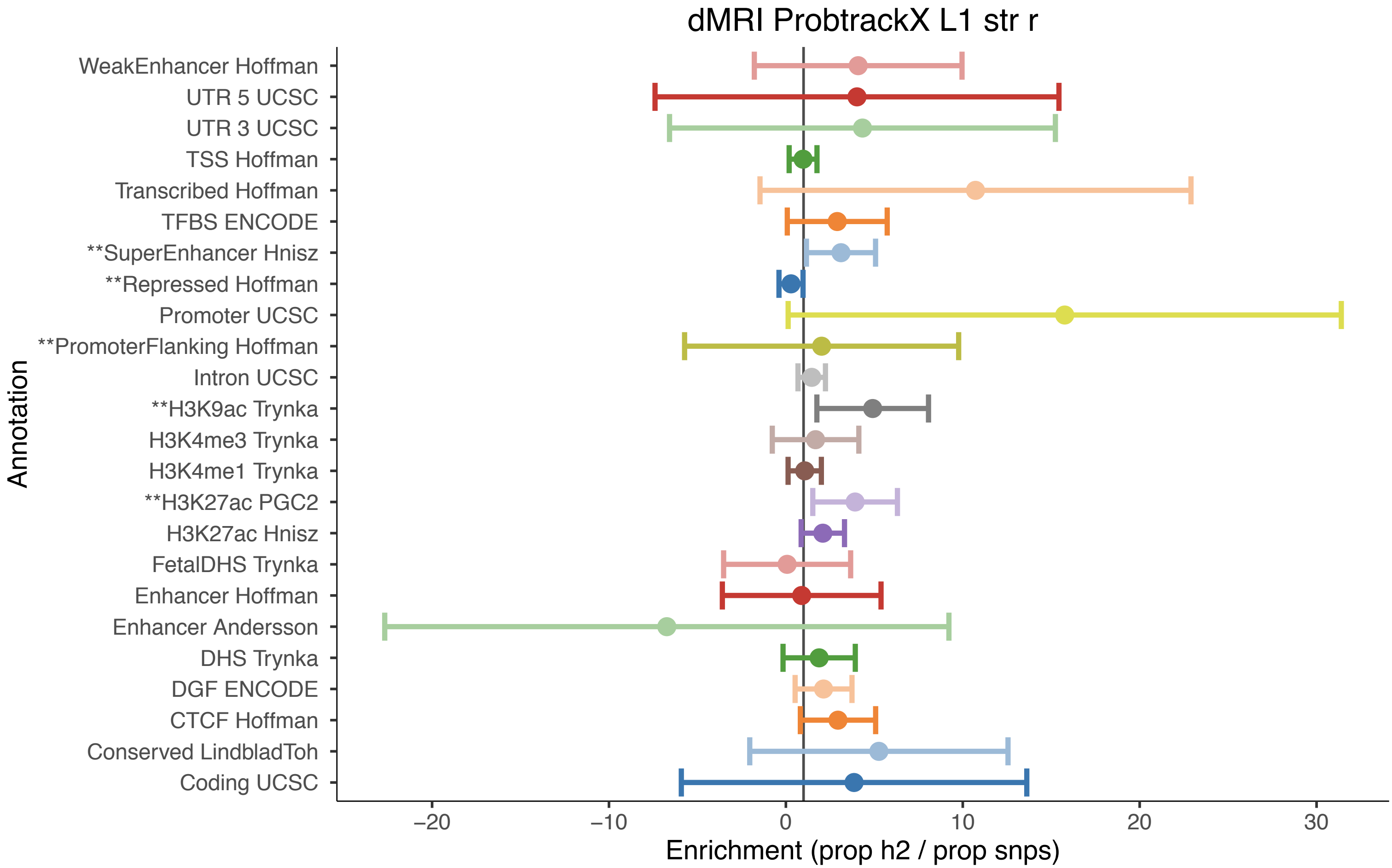
dMRI ProbtrackX L1 str r
WeakEnhancer Hoffman
UTR 5 UCSC
UTR 3 UCSC
TSS Hoffman
Transcribed Hoffman
TFBS ENCODE
**SuperEnhancer Hnisz
**Repressed Hoffman
Promoter UCSC
**PromoterFlanking Hoffman
Intron UCSC
**H3K9ac Trynka
H3K4me3 Trynka
H3K4me1 Trynka
**H3K27ac PGC2
H3K27ac Hnisz
FetalDHS Trynka
Enhancer Hoffman
Enhancer Andersson
DHS Trynka
DGF ENCODE
CTCF Hoffman
Conserved LindbladToh
Coding UCSC
Annotation
−20
−10
0
10
20
30
Enrichment (prop h2 / prop snps)

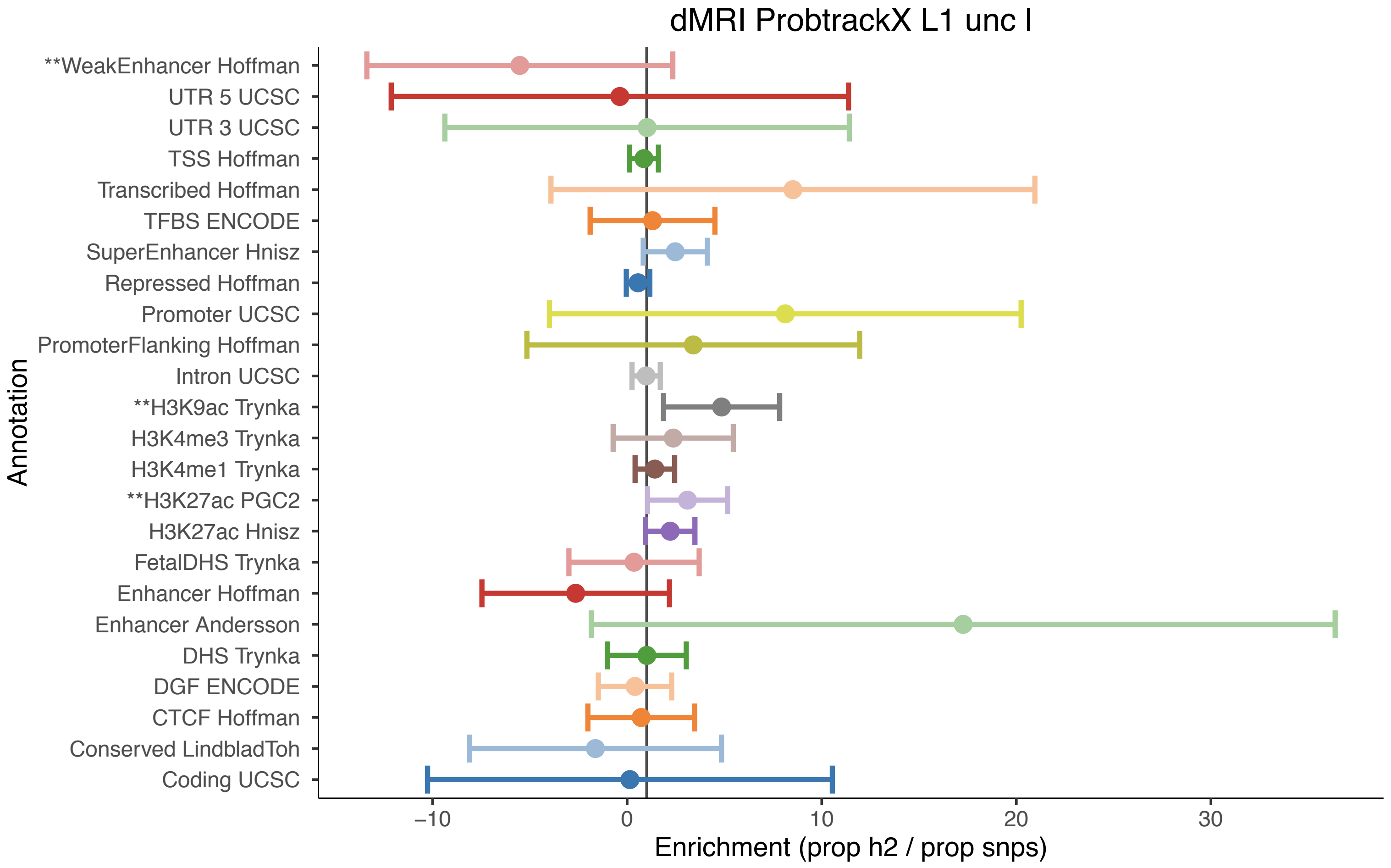

dMRI ProbtrackX L1 unc I
**WeakEnhancer Hoffman
UTR 5 UCSC
UTR 3 UCSC
TSS Hoffman
Transcribed Hoffman
TFBS ENCODE
SuperEnhancer Hnisz
Repressed Hoffman
Promoter UCSC
PromoterFlanking Hoffman
Intron UCSC
**H3K9ac Trynka
H3K4me3 Trynka
H3K4me1 Trynka
**H3K27ac PGC2
H3K27ac Hnisz
FetalDHS Trynka
Enhancer Hoffman
Enhancer Andersson
DHS Trynka
DGF ENCODE
CTCF Hoffman
Conserved LindbladToh
Coding UCSC
Annotation
−10
0
10
20
30
Enrichment (prop h2 / prop snps)

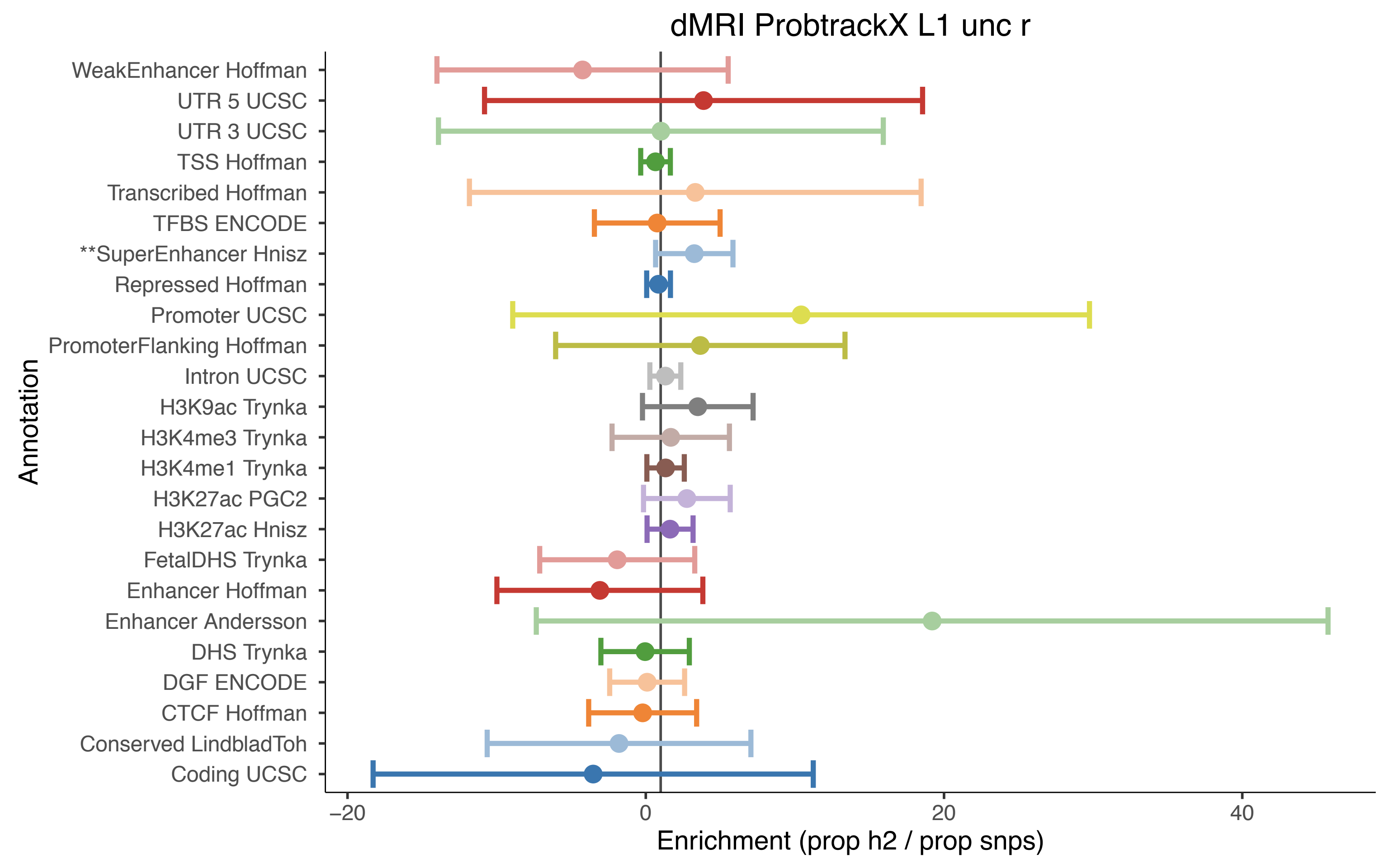

dMRI ProbtrackX L1 unc r
Annotation
WeakEnhancer Hoffman
UTR 5 UCSC
UTR 3 UCSC
TSS Hoffman
Transcribed Hoffman
TFBS ENCODE
**SuperEnhancer Hnisz
Repressed Hoffman
Promoter UCSC
PromoterFlanking Hoffman
Intron UCSC
H3K9ac Trynka
H3K4me3 Trynka
H3K4me1 Trynka
H3K27ac PGC2
H3K27ac Hnisz
FetalDHS Trynka
Enhancer Hoffman
Enhancer Andersson
DHS Trynka
DGF ENCODE
CTCF Hoffman
Conserved LindbladToh
Coding UCSC
−20
0
20
40
Enrichment (prop h2 / prop snps)

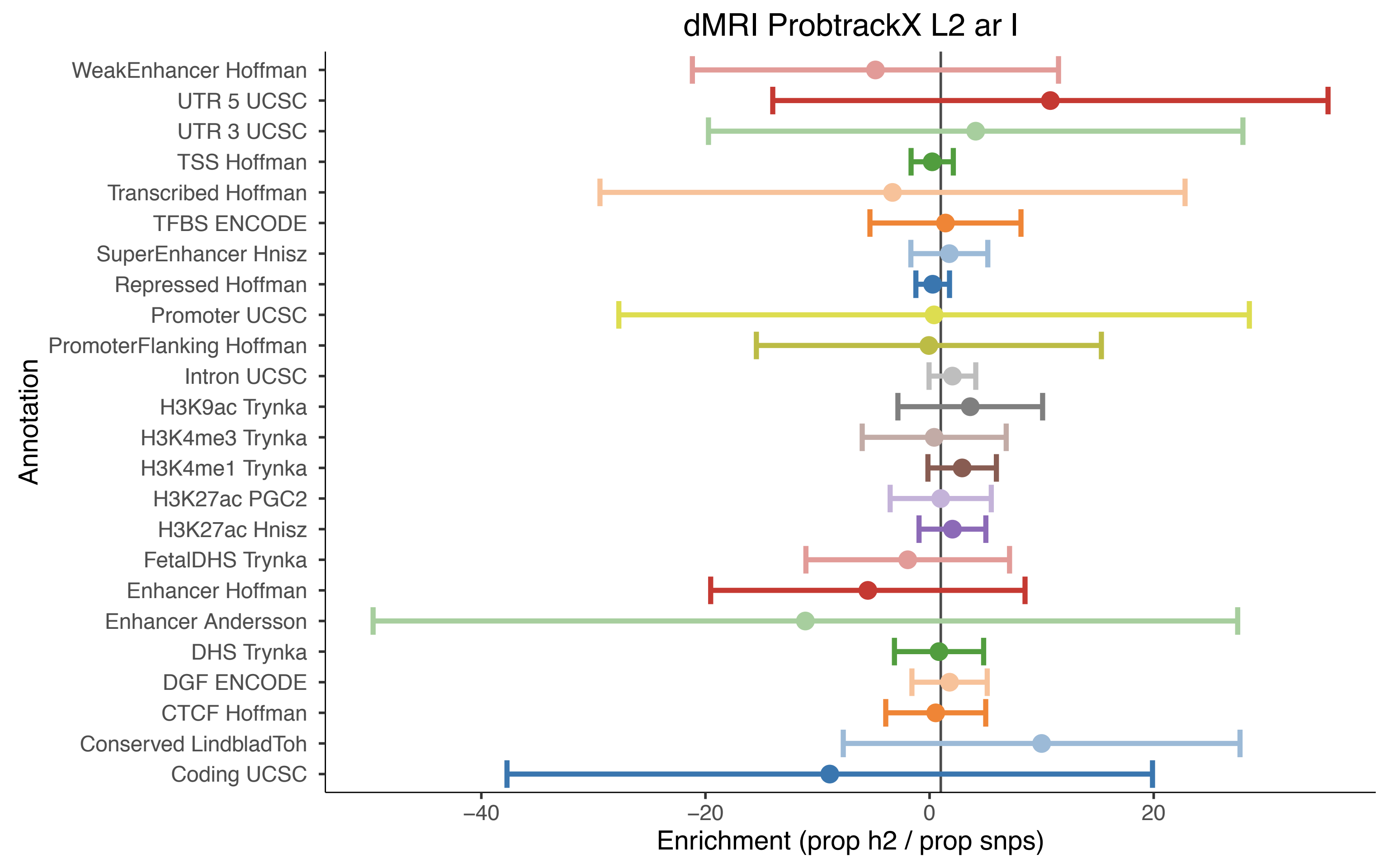
dMRI ProbtrackX L2 ar I
WeakEnhancer Hoffman
UTR 5 UCSC
UTR 3 UCSC
TSS Hoffman
Transcribed Hoffman
TFBS ENCODE
SuperEnhancer Hnisz
Repressed Hoffman
Promoter UCSC
PromoterFlanking Hoffman
Intron UCSC
H3K9ac Trynka
H3K4me3 Trynka
H3K4me1 Trynka
H3K27ac PGC2
H3K27ac Hnisz
FetalDHS Trynka
Enhancer Hoffman
Enhancer Andersson
DHS Trynka
DGF ENCODE
CTCF Hoffman
Conserved LindbladToh
Coding UCSC
Annotation
−40
−20
0
20
Enrichment (prop h2 / prop snps)

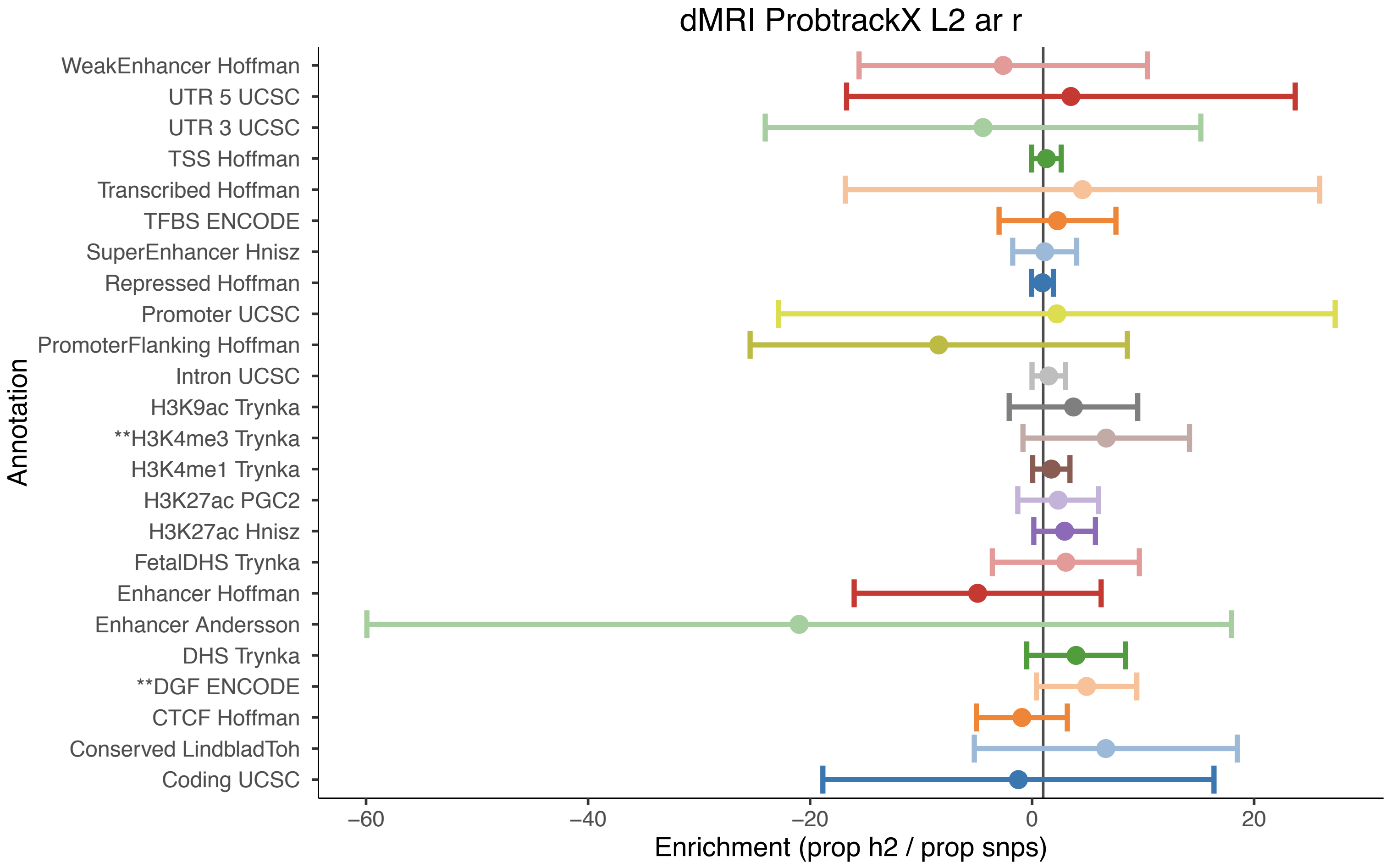
dMRI ProbtrackX L2 ar r
WeakEnhancer Hoffman
UTR 5 UCSC
UTR 3 UCSC
TSS Hoffman
Transcribed Hoffman
TFBS ENCODE
SuperEnhancer Hnisz
Repressed Hoffman
Promoter UCSC
PromoterFlanking Hoffman
Intron UCSC
H3K9ac Trynka
**H3K4me3 Trynka
H3K4me1 Trynka
H3K27ac PGC2
H3K27ac Hnisz
FetalDHS Trynka
Enhancer Hoffman
Enhancer Andersson
DHS Trynka
**DGF ENCODE
CTCF Hoffman
Conserved LindbladToh
Coding UCSC
Annotation
−60
−40
−20
0
20
Enrichment (prop h2 / prop snps)

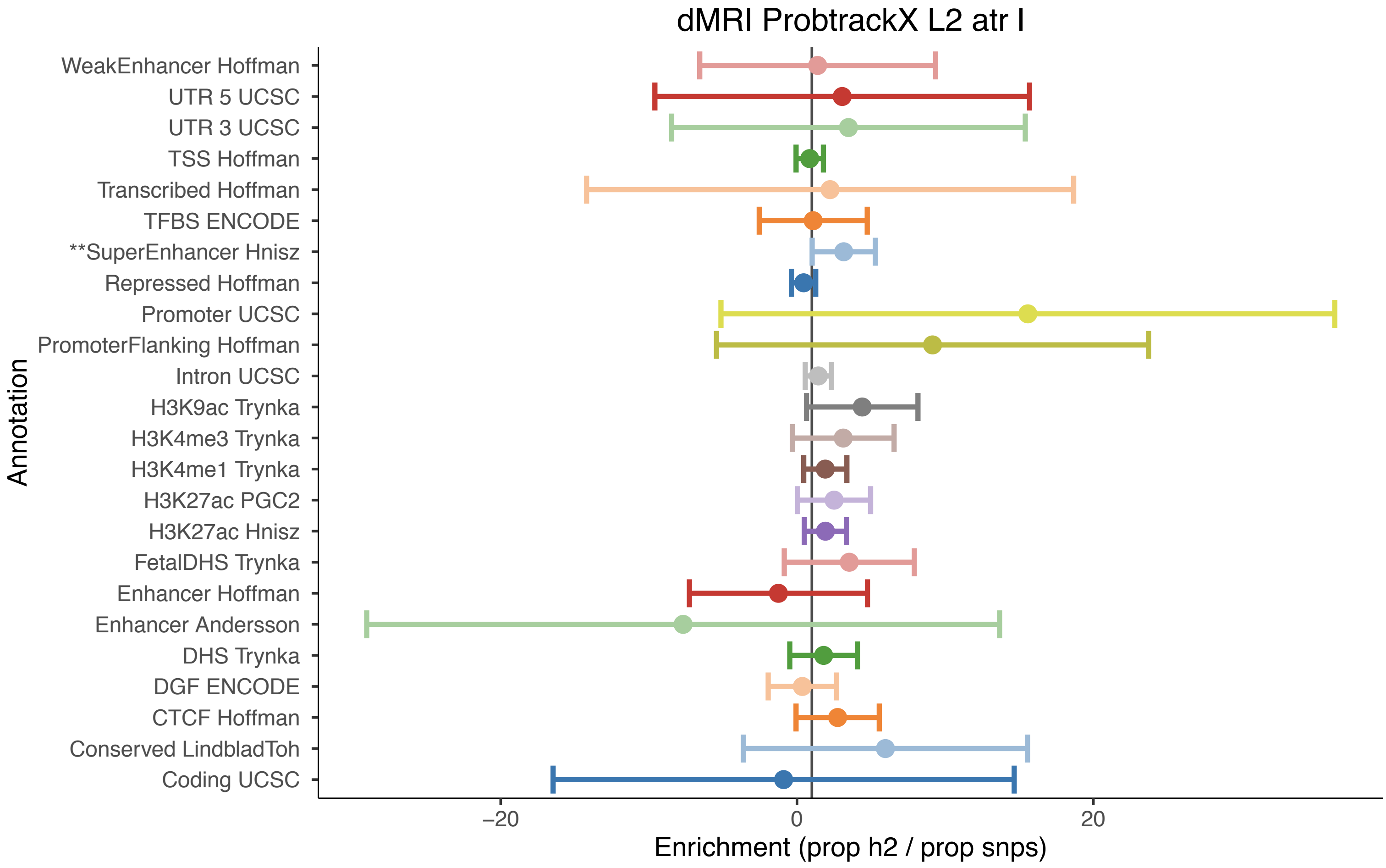
dMRI ProbtrackX L2 atr l
WeakEnhancer Hoffman
UTR 5 UCSC
UTR 3 UCSC
TSS Hoffman
Transcribed Hoffman
TFBS ENCODE
**SuperEnhancer Hnisz
Repressed Hoffman
Promoter UCSC
PromoterFlanking Hoffman
Intron UCSC
H3K9ac Trynka
H3K4me3 Trynka
H3K4me1 Trynka
H3K27ac PGC2
H3K27ac Hnisz
FetalDHS Trynka
Enhancer Hoffman
Enhancer Andersson
DHS Trynka
DGF ENCODE
CTCF Hoffman
Conserved LindbladToh
Coding UCSC
Annotation
−20
0
20
Enrichment (prop h2 / prop snps)

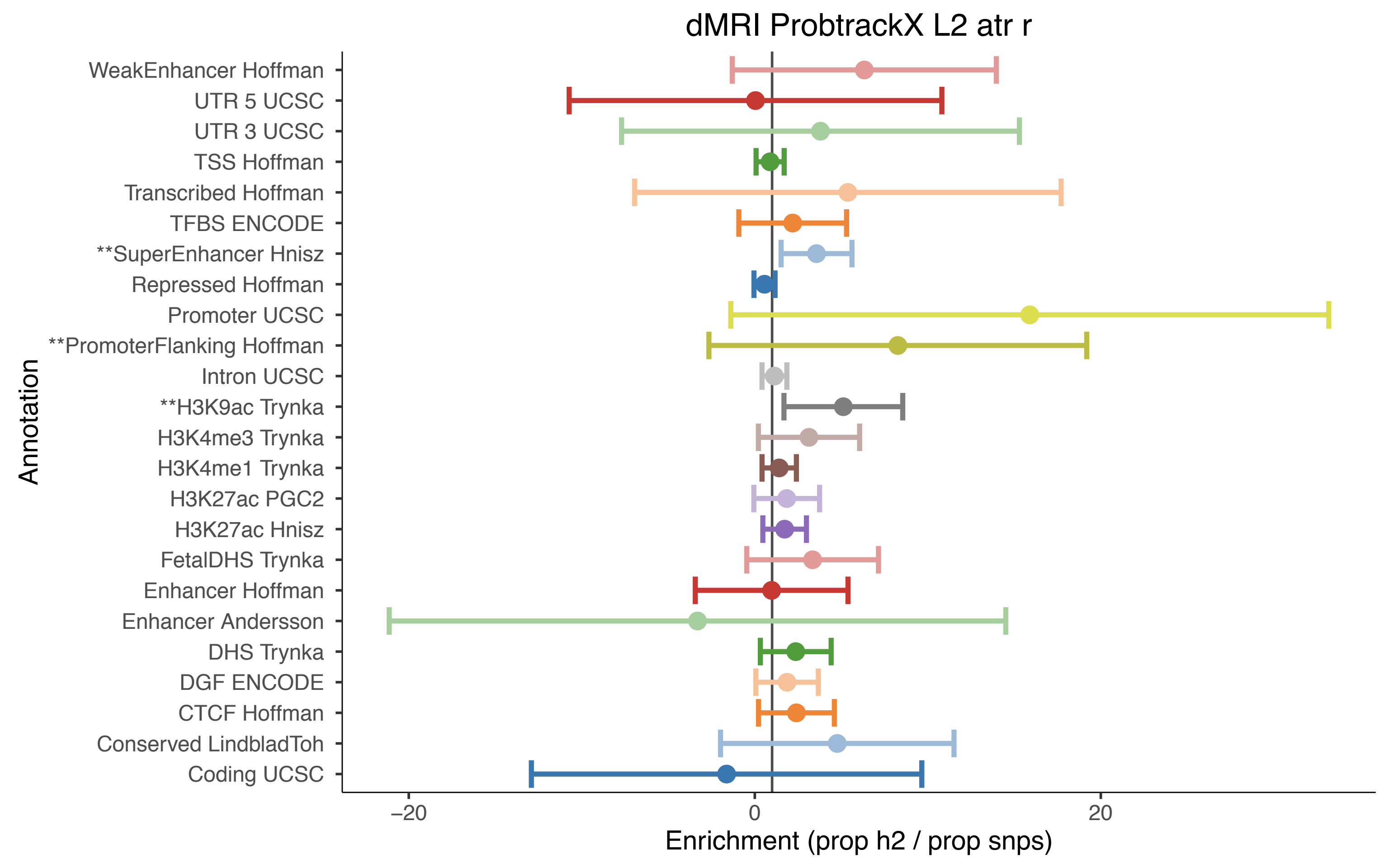

dMRI ProbtrackX L2 atr r
WeakEnhancer Hoffman
UTR 5 UCSC
UTR 3 UCSC
TSS Hoffman
Transcribed Hoffman
TFBS ENCODE
**SuperEnhancer Hnisz
Repressed Hoffman
Promoter UCSC
**PromoterFlanking Hoffman
Intron UCSC
**H3K9ac Trynka
H3K4me3 Trynka
H3K4me1 Trynka
H3K27ac PGC2
H3K27ac Hnisz
FetalDHS Trynka
Enhancer Hoffman
Enhancer Andersson
DHS Trynka
DGF ENCODE
CTCF Hoffman
Conserved LindbladToh
Coding UCSC
Annotation
−20
0
20
Enrichment (prop h2 / prop snps)

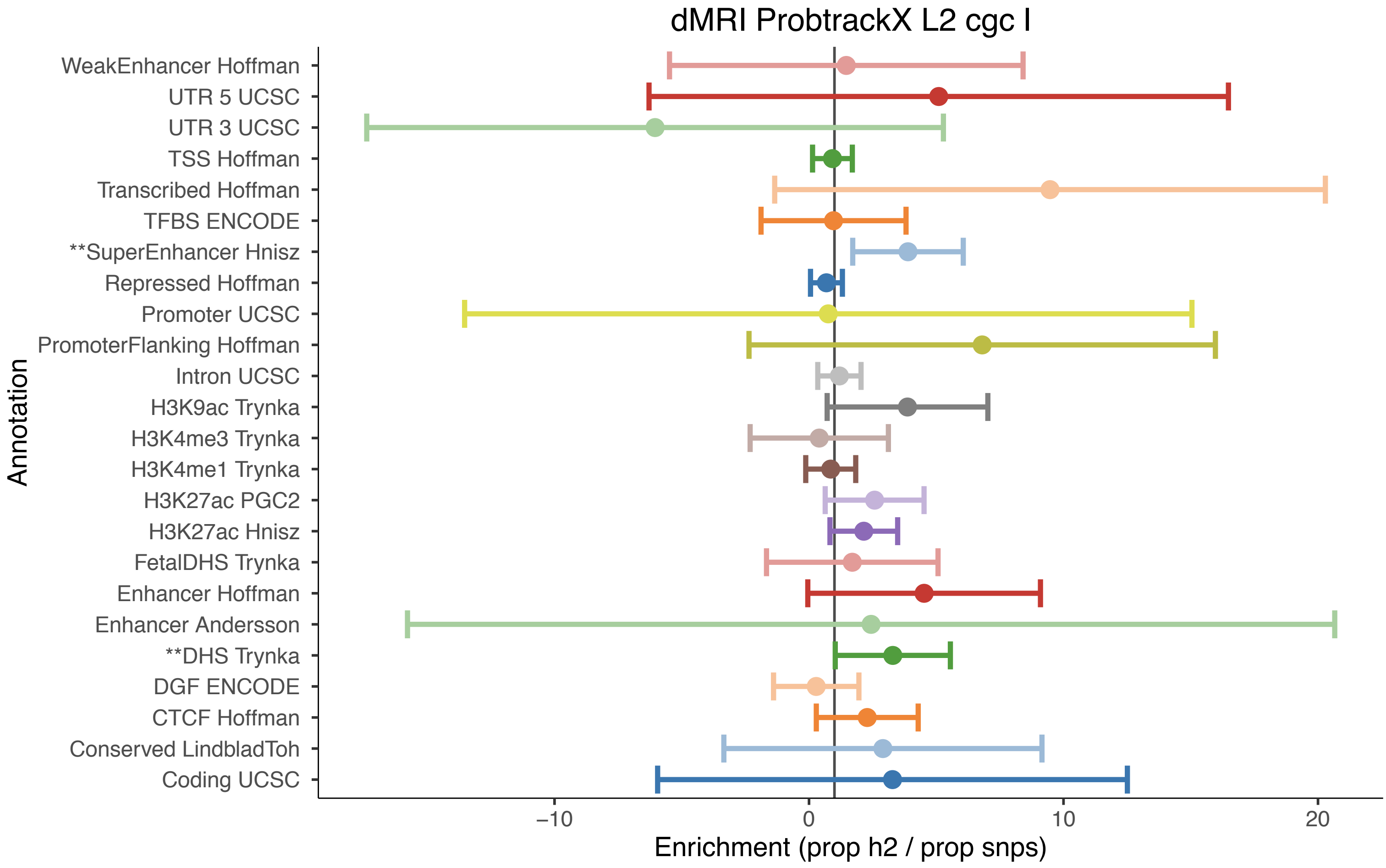
dMRI ProbtrackX L2 cgc I
WeakEnhancer Hoffman
UTR 5 UCSC
UTR 3 UCSC
TSS Hoffman
Transcribed Hoffman
TFBS ENCODE
**SuperEnhancer Hnisz
Repressed Hoffman
Promoter UCSC
PromoterFlanking Hoffman
Intron UCSC
H3K9ac Trynka
H3K4me3 Trynka
H3K4me1 Trynka
H3K27ac PGC2
H3K27ac Hnisz
FetalDHS Trynka
Enhancer Hoffman
Enhancer Andersson
**DHS Trynka
DGF ENCODE
CTCF Hoffman
Conserved LindbladToh
Coding UCSC
Annotation
−10
0
10
20
Enrichment (prop h2 / prop snps)

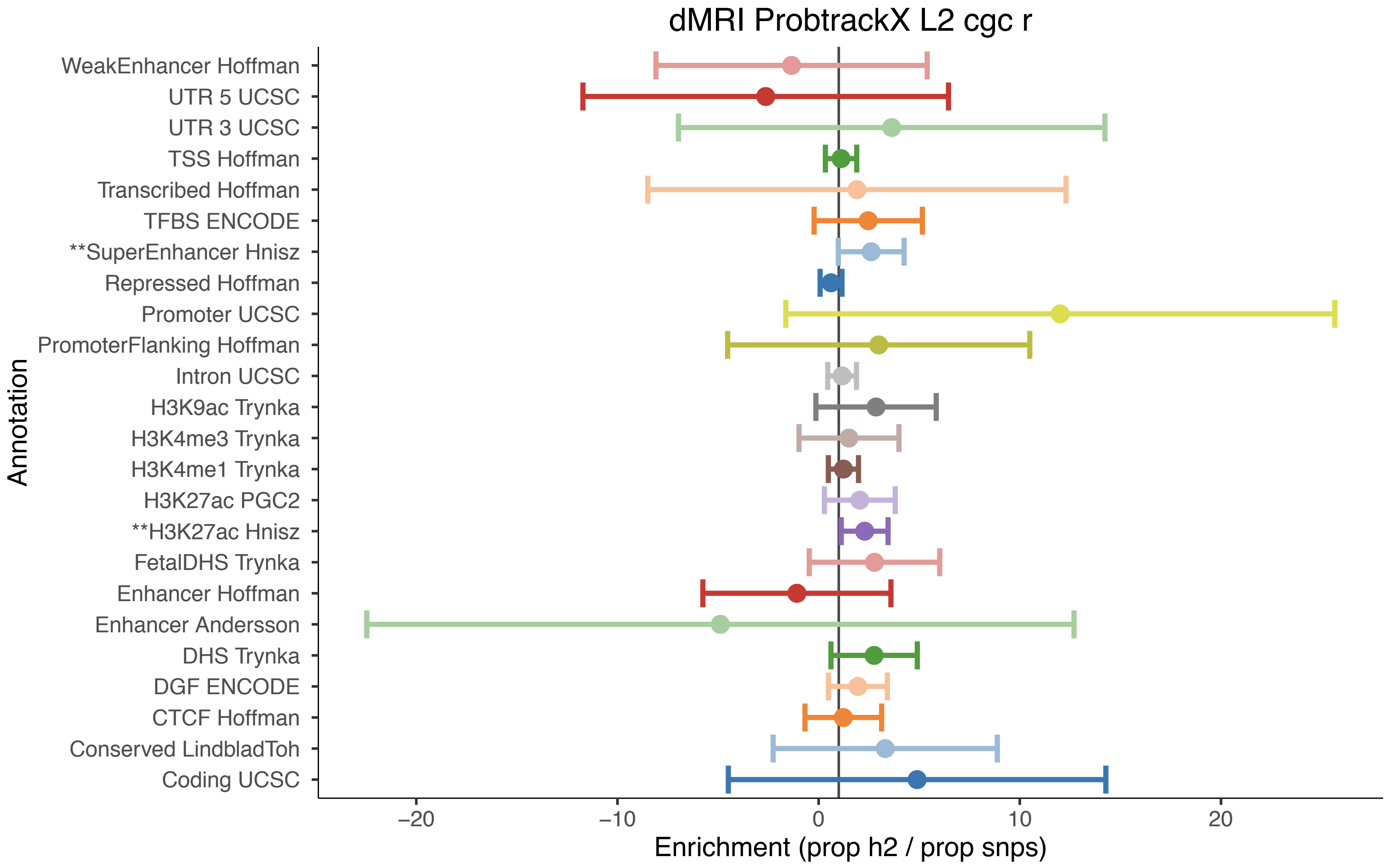
dMRI ProbtrackX L2 cgc r
Annotation
WeakEnhancer Hoffman
UTR 5 UCSC
UTR 3 UCSC
TSS Hoffman
Transcribed Hoffman
TFBS ENCODE
**SuperEnhancer Hnisz
Repressed Hoffman
Promoter UCSC
PromoterFlanking Hoffman
Intron UCSC
H3K9ac Trynka
H3K4me3 Trynka
H3K4me1 Trynka
H3K27ac PGC2
**H3K27ac Hnisz
FetalDHS Trynka
Enhancer Hoffman
Enhancer Andersson
DHS Trynka
DGF ENCODE
CTCF Hoffman
Conserved LindbladToh
Coding UCSC
−20
−10
0
10
20
Enrichment (prop h2 / prop snps)

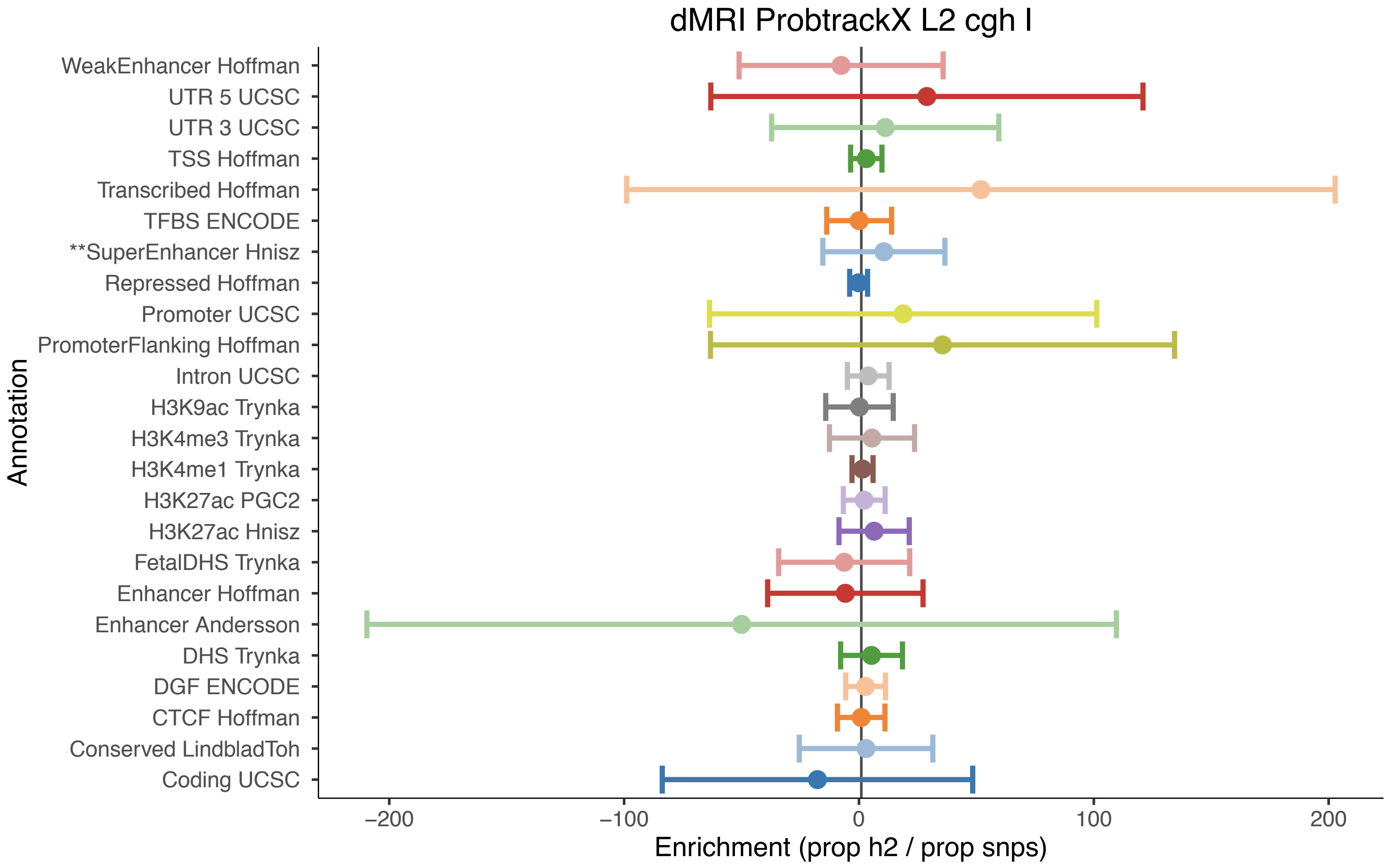
dMRI ProbtrackX L2 cgh I
WeakEnhancer Hoffman
UTR 5 UCSC
UTR 3 UCSC
TSS Hoffman
Transcribed Hoffman
TFBS ENCODE
**SuperEnhancer Hnisz
Repressed Hoffman
Promoter UCSC
PromoterFlanking Hoffman
Intron UCSC
H3K9ac Trynka
H3K4me3 Trynka
H3K4me1 Trynka
H3K27ac PGC2
H3K27ac Hnisz
FetalDHS Trynka
Enhancer Hoffman
Enhancer Andersson
DHS Trynka
DGF ENCODE
CTCF Hoffman
Conserved LindbladToh
Coding UCSC
Annotation
−200
−100
0
100
200
Enrichment (prop h2 / prop snps)

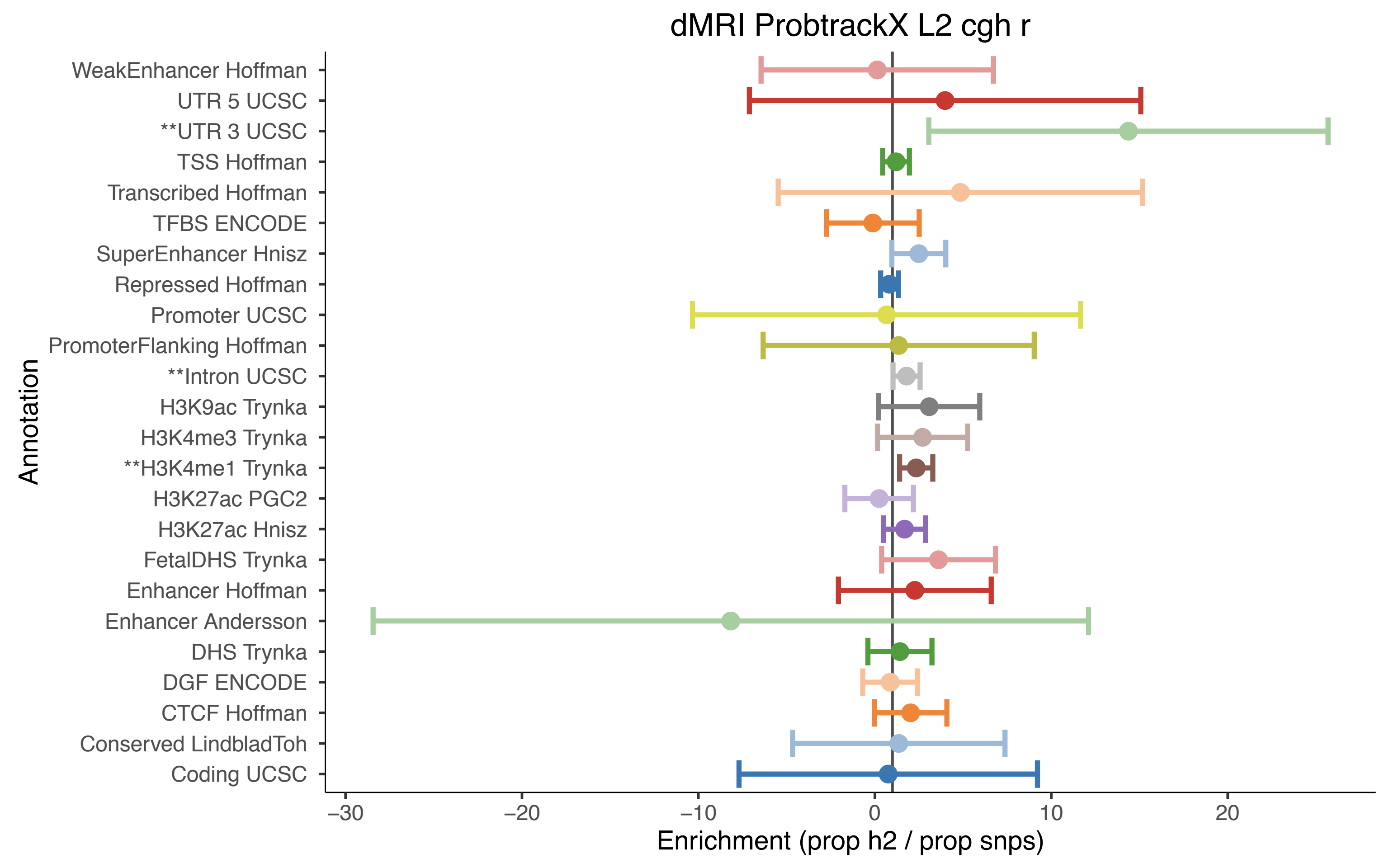

dMRI ProbtrackX L2 cgh r
WeakEnhancer Hoffman
UTR 5 UCSC
**UTR 3 UCSC
TSS Hoffman
Transcribed Hoffman
TFBS ENCODE
SuperEnhancer Hnisz
Repressed Hoffman
Promoter UCSC
PromoterFlanking Hoffman
**Intron UCSC
H3K9ac Trynka
H3K4me3 Trynka
**H3K4me1 Trynka
H3K27ac PGC2
H3K27ac Hnisz
FetalDHS Trynka
Enhancer Hoffman
Enhancer Andersson
DHS Trynka
DGF ENCODE
CTCF Hoffman
Conserved LindbladToh
Coding UCSC
Annotation
−30
−20
−10
0
10
20
Enrichment (prop h2 / prop snps)

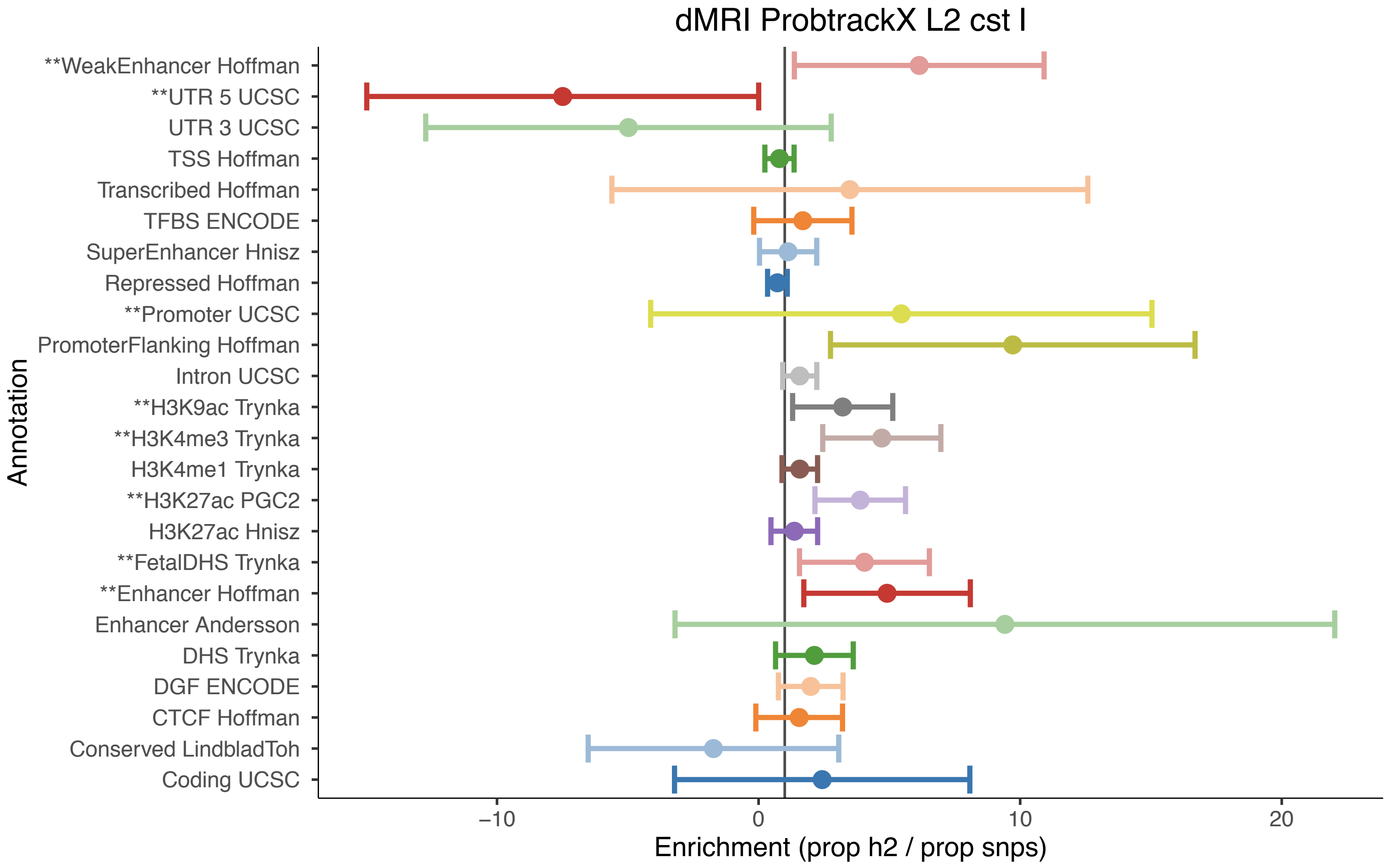

dMRI ProbtrackX L2 cst l
**WeakEnhancer Hoffman
**UTR 5 UCSC
UTR 3 UCSC
TSS Hoffman
Transcribed Hoffman
TFBS ENCODE
SuperEnhancer Hnisz
Repressed Hoffman
**Promoter UCSC
PromoterFlanking Hoffman
Intron UCSC
**H3K9ac Trynka
**H3K4me3 Trynka
H3K4me1 Trynka
**H3K27ac PGC2
H3K27ac Hnisz
**FetalDHS Trynka
**Enhancer Hoffman
Enhancer Andersson
DHS Trynka
DGF ENCODE
CTCF Hoffman
Conserved LindbladToh
Coding UCSC
Annotation
−10
0
10
20
Enrichment (prop h2 / prop snps)

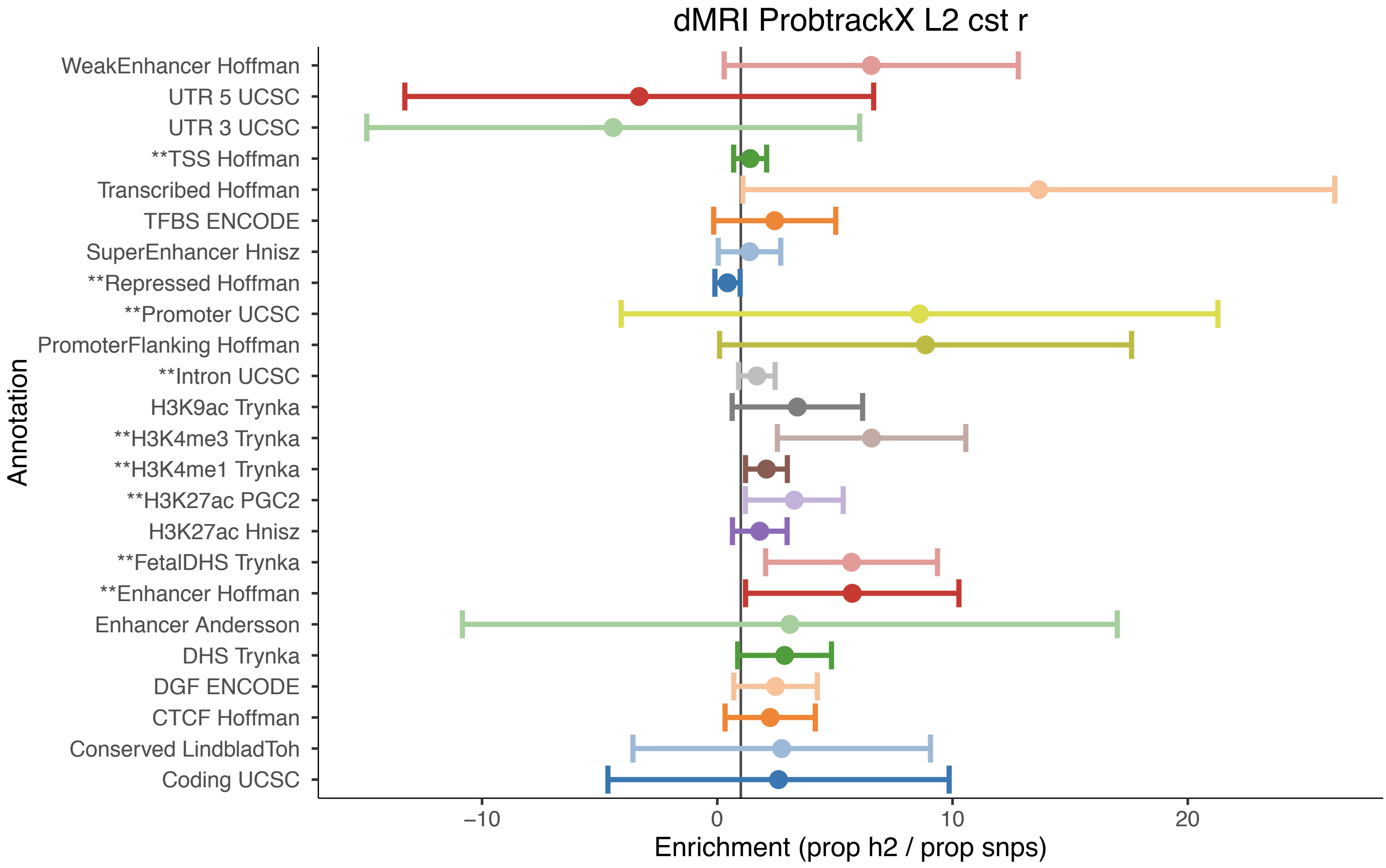
dMRI ProbtrackX L2 cst r
WeakEnhancer Hoffman
UTR 5 UCSC
UTR 3 UCSC
**TSS Hoffman
Transcribed Hoffman
TFBS ENCODE
SuperEnhancer Hnisz
**Repressed Hoffman
**Promoter UCSC
PromoterFlanking Hoffman
**Intron UCSC
H3K9ac Trynka
**H3K4me3 Trynka
**H3K4me1 Trynka
**H3K27ac PGC2
H3K27ac Hnisz
**FetalDHS Trynka
**Enhancer Hoffman
Enhancer Andersson
DHS Trynka
DGF ENCODE
CTCF Hoffman
Conserved LindbladToh
Coding UCSC
Annotation
−10
0
10
20
Enrichment (prop h2 / prop snps)

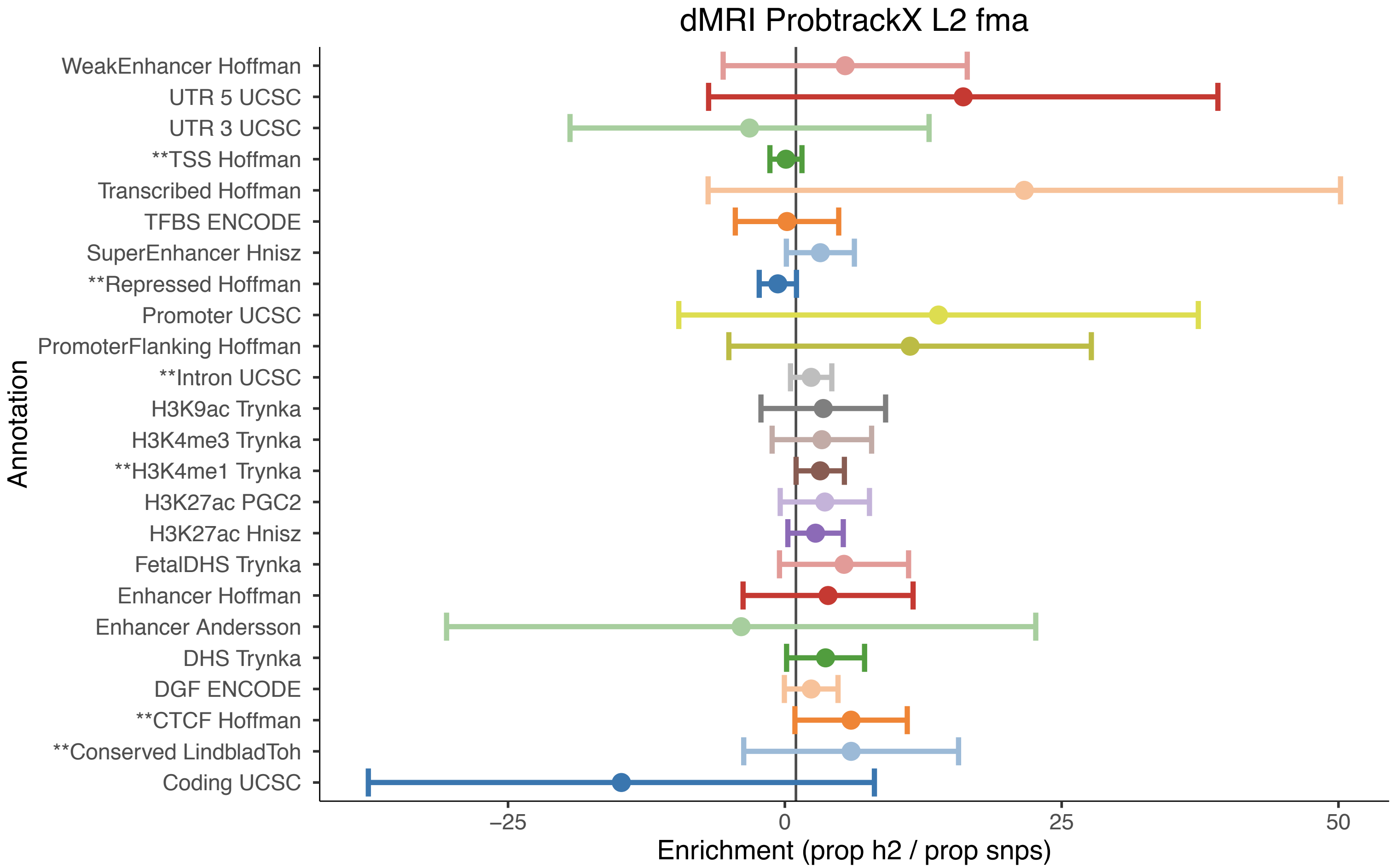
dMRI ProbtrackX L2 fma
WeakEnhancer Hoffman
UTR 5 UCSC
UTR 3 UCSC
**TSS Hoffman
Transcribed Hoffman
TFBS ENCODE
SuperEnhancer Hnisz
**Repressed Hoffman
Promoter UCSC
PromoterFlanking Hoffman
**Intron UCSC
H3K9ac Trynka
H3K4me3 Trynka
**H3K4me1 Trynka
H3K27ac PGC2
H3K27ac Hnisz
FetalDHS Trynka
Enhancer Hoffman
Enhancer Andersson
DHS Trynka
DGF ENCODE
**CTCF Hoffman
**Conserved LindbladToh
Coding UCSC
Annotation
−25
0
25
50
Enrichment (prop h2 / prop snps)

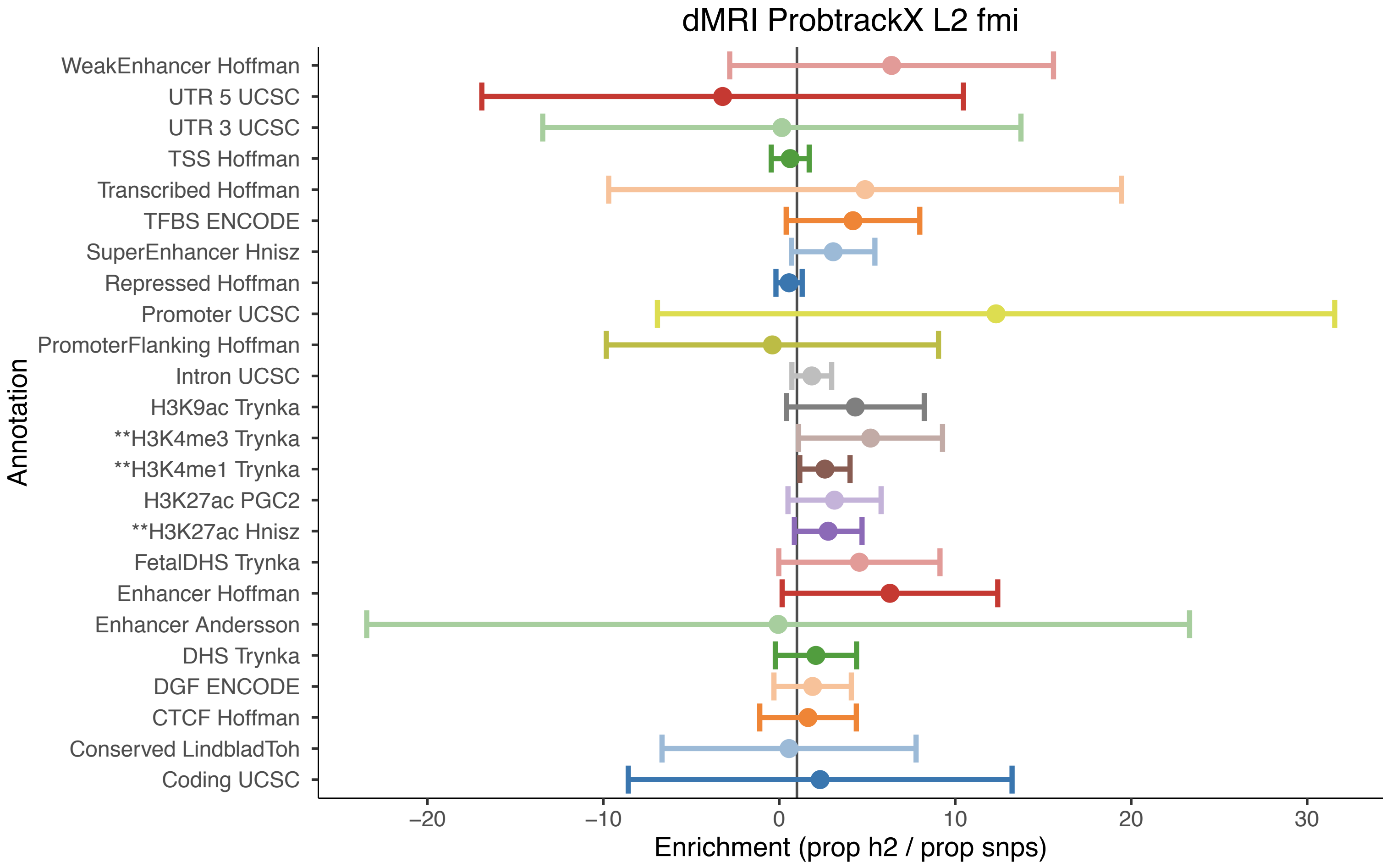

dMRI ProbtrackX L2 fmi
WeakEnhancer Hoffman
UTR 5 UCSC
UTR 3 UCSC
TSS Hoffman
Transcribed Hoffman
TFBS ENCODE
SuperEnhancer Hnisz
Repressed Hoffman
Promoter UCSC
PromoterFlanking Hoffman
Intron UCSC
H3K9ac Trynka
**H3K4me3 Trynka
**H3K4me1 Trynka
H3K27ac PGC2
**H3K27ac Hnisz
FetalDHS Trynka
Enhancer Hoffman
Enhancer Andersson
DHS Trynka
DGF ENCODE
CTCF Hoffman
Conserved LindbladToh
Coding UCSC
Annotation
−20
−10
0
10
20
30
Enrichment (prop h2 / prop snps)

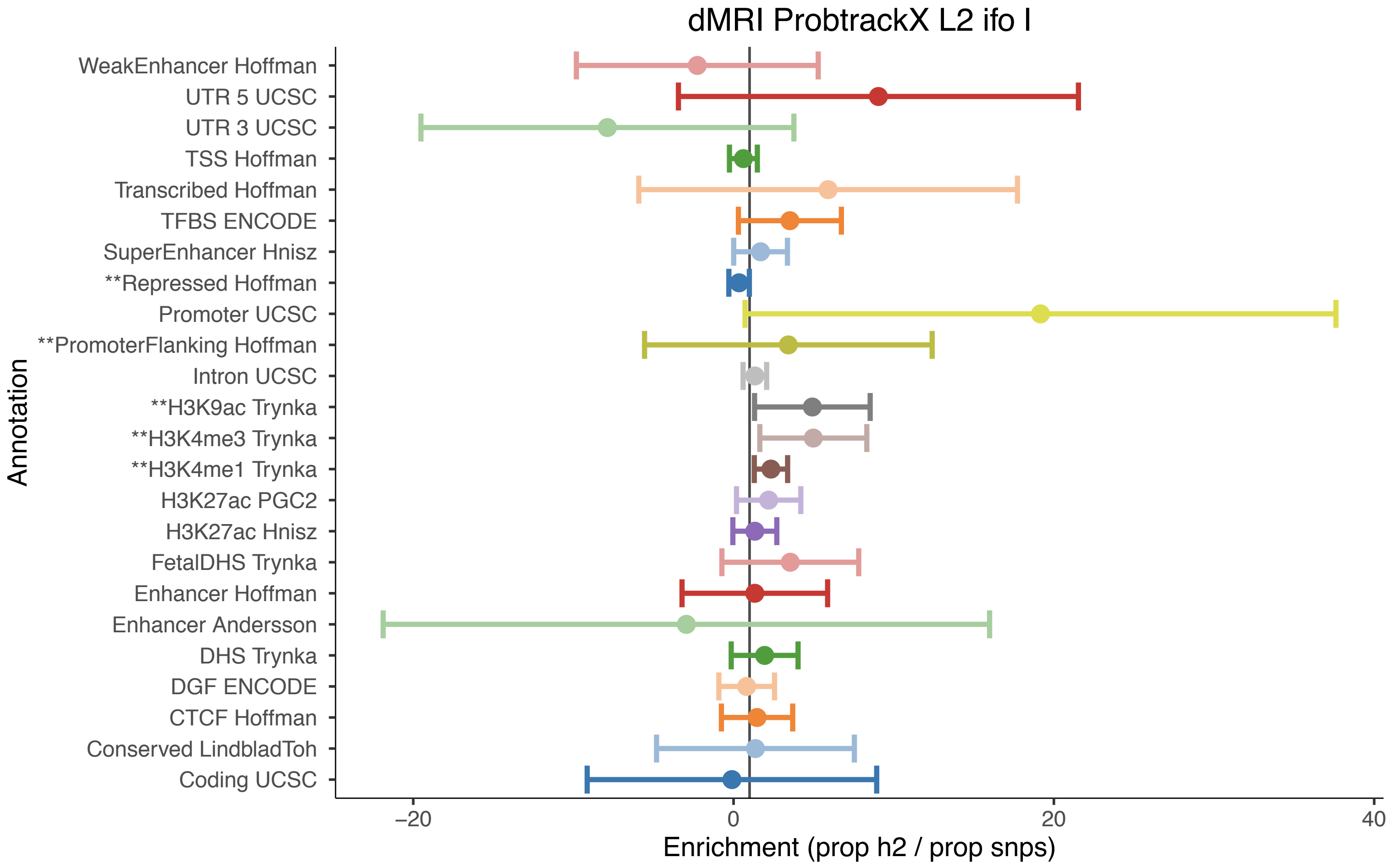

dMRI ProbtrackX L2 ifo l
WeakEnhancer Hoffman
UTR 5 UCSC
UTR 3 UCSC
TSS Hoffman
Transcribed Hoffman
TFBS ENCODE
SuperEnhancer Hnisz
**Repressed Hoffman
Promoter UCSC
**PromoterFlanking Hoffman
Intron UCSC
**H3K9ac Trynka
**H3K4me3 Trynka
**H3K4me1 Trynka
H3K27ac PGC2
H3K27ac Hnisz
FetalDHS Trynka
Enhancer Hoffman
Enhancer Andersson
DHS Trynka
DGF ENCODE
CTCF Hoffman
Conserved LindbladToh
Coding UCSC
Annotation
−20
0
20
40
Enrichment (prop h2 / prop snps)

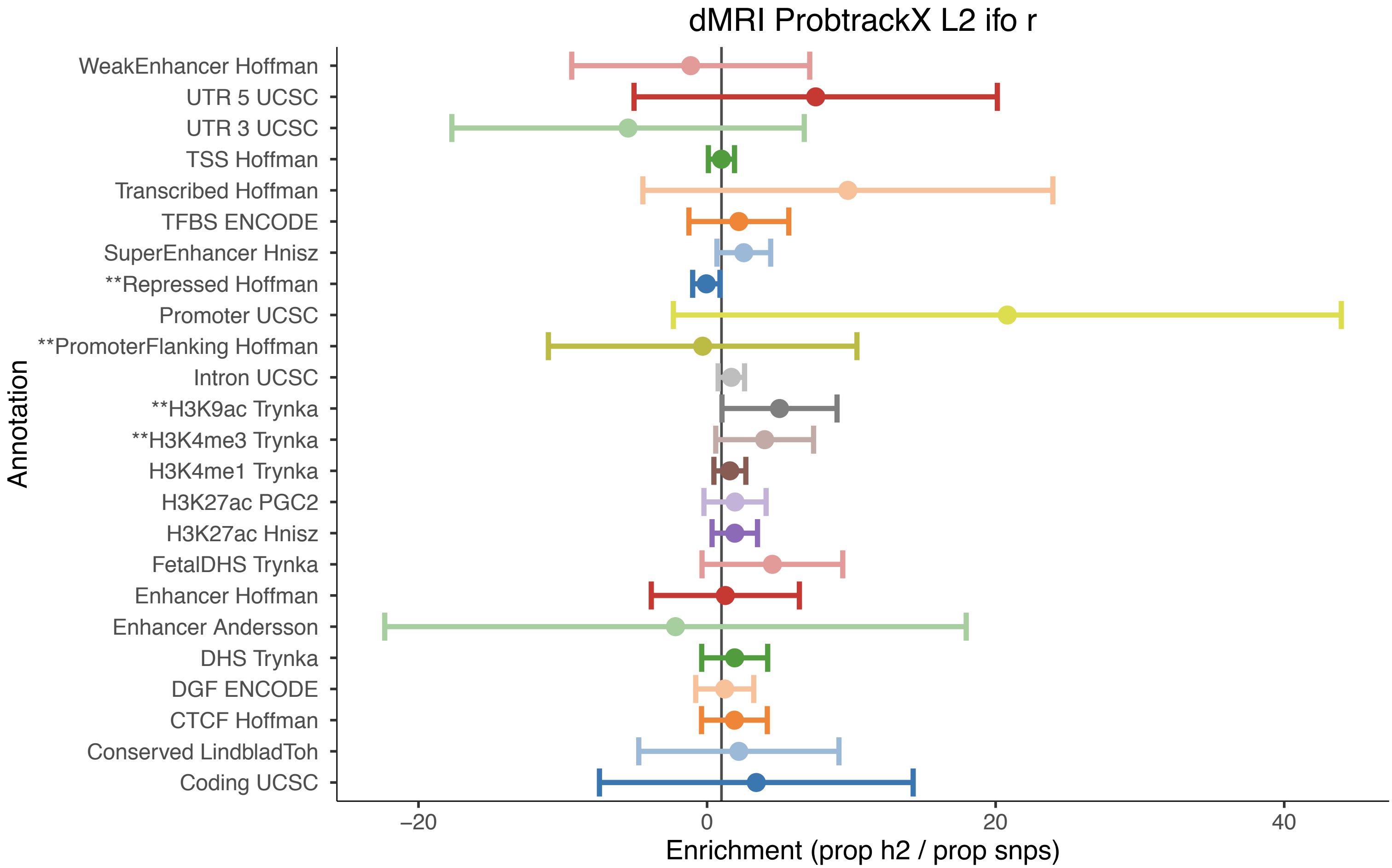

dMRI ProbtrackX L2 ifo r
WeakEnhancer Hoffman
UTR 5 UCSC
UTR 3 UCSC
TSS Hoffman
Transcribed Hoffman
TFBS ENCODE
SuperEnhancer Hnisz
**Repressed Hoffman
Promoter UCSC
**PromoterFlanking Hoffman
Intron UCSC
**H3K9ac Trynka
**H3K4me3 Trynka
H3K4me1 Trynka
H3K27ac PGC2
H3K27ac Hnisz
FetalDHS Trynka
Enhancer Hoffman
Enhancer Andersson
DHS Trynka
DGF ENCODE
CTCF Hoffman
Conserved LindbladToh
Coding UCSC
Annotation
−20
0
20
40
Enrichment (prop h2 / prop snps)

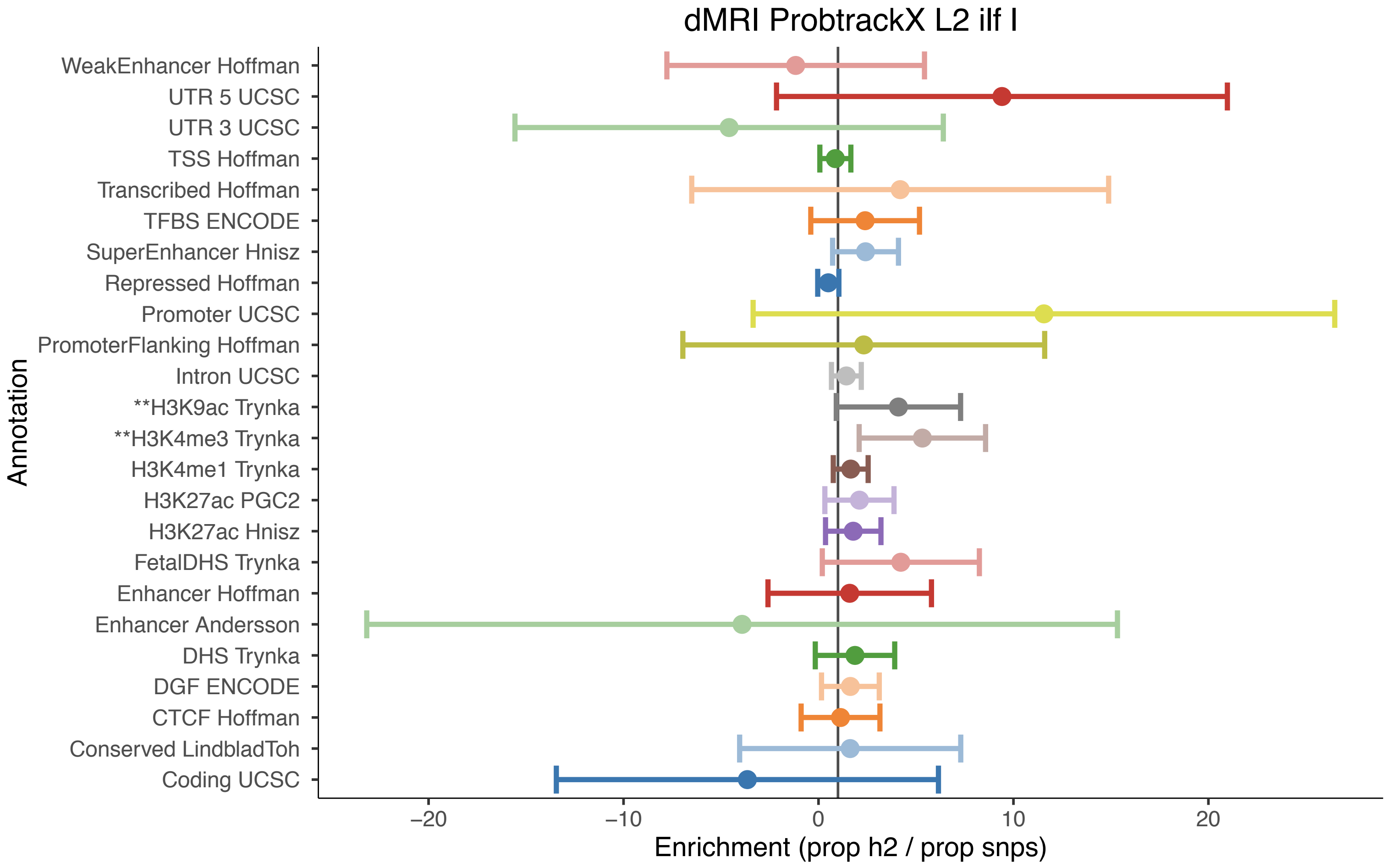
dMRI ProbtrackX L2 ilf l
WeakEnhancer Hoffman
UTR 5 UCSC
UTR 3 UCSC
TSS Hoffman
Transcribed Hoffman
TFBS ENCODE
SuperEnhancer Hnisz
Repressed Hoffman
Promoter UCSC
PromoterFlanking Hoffman
Intron UCSC
**H3K9ac Trynka
**H3K4me3 Trynka
H3K4me1 Trynka
H3K27ac PGC2
H3K27ac Hnisz
FetalDHS Trynka
Enhancer Hoffman
Enhancer Andersson
DHS Trynka
DGF ENCODE
CTCF Hoffman
Conserved LindbladToh
Coding UCSC
Annotation
−20
−10
0
10
20
Enrichment (prop h2 / prop snps)

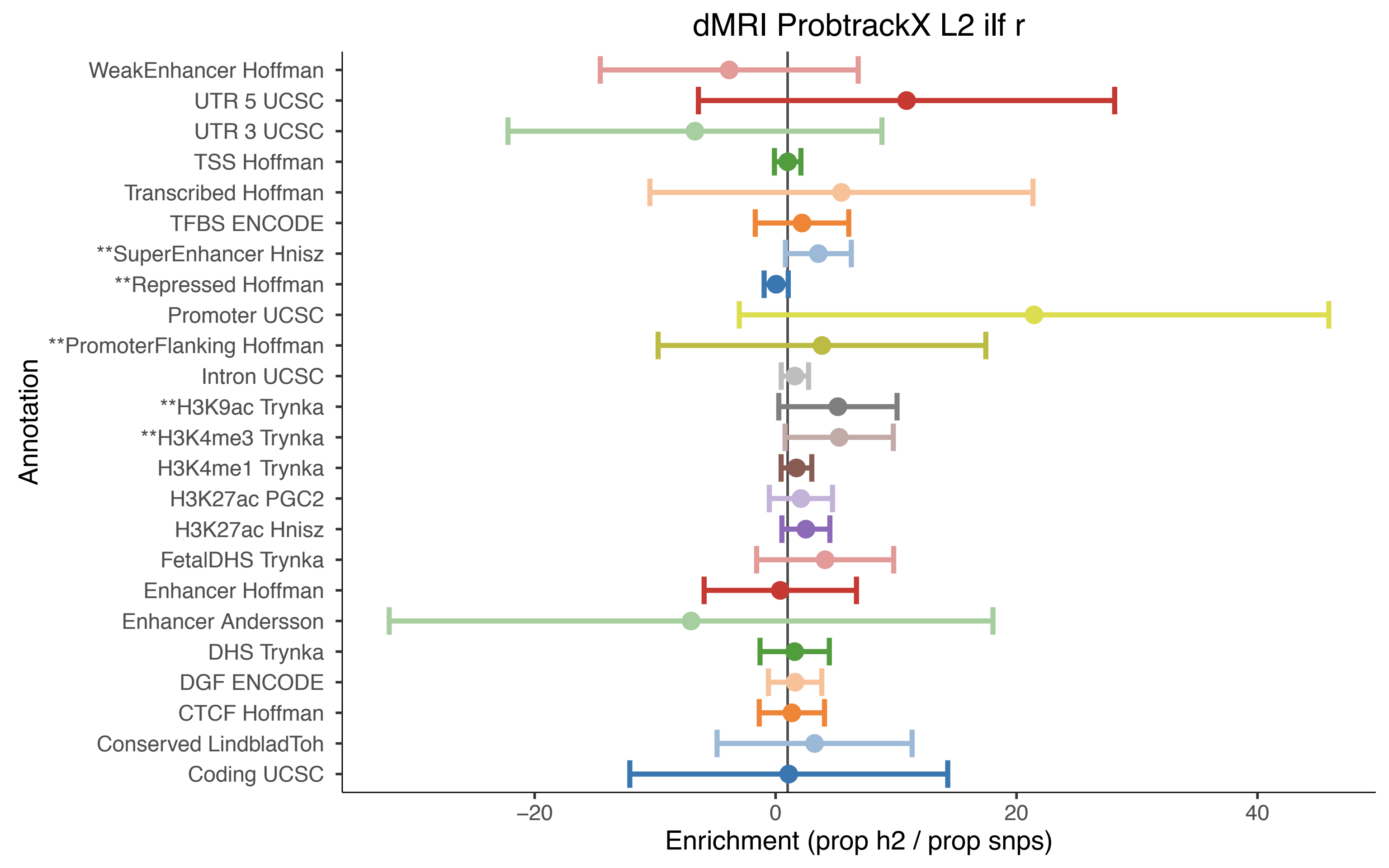

dMRI ProbtrackX L2 ilf r
Annotation
WeakEnhancer Hoffman
UTR 5 UCSC
UTR 3 UCSC
TSS Hoffman
Transcribed Hoffman
TFBS ENCODE
**SuperEnhancer Hnisz
**Repressed Hoffman
Promoter UCSC
**PromoterFlanking Hoffman
Intron UCSC
**H3K9ac Trynka
**H3K4me3 Trynka
H3K4me1 Trynka
H3K27ac PGC2
H3K27ac Hnisz
FetalDHS Trynka
Enhancer Hoffman
Enhancer Andersson
DHS Trynka
DGF ENCODE
CTCF Hoffman
Conserved LindbladToh
Coding UCSC
−20
0
20
40
Enrichment (prop h2 / prop snps)

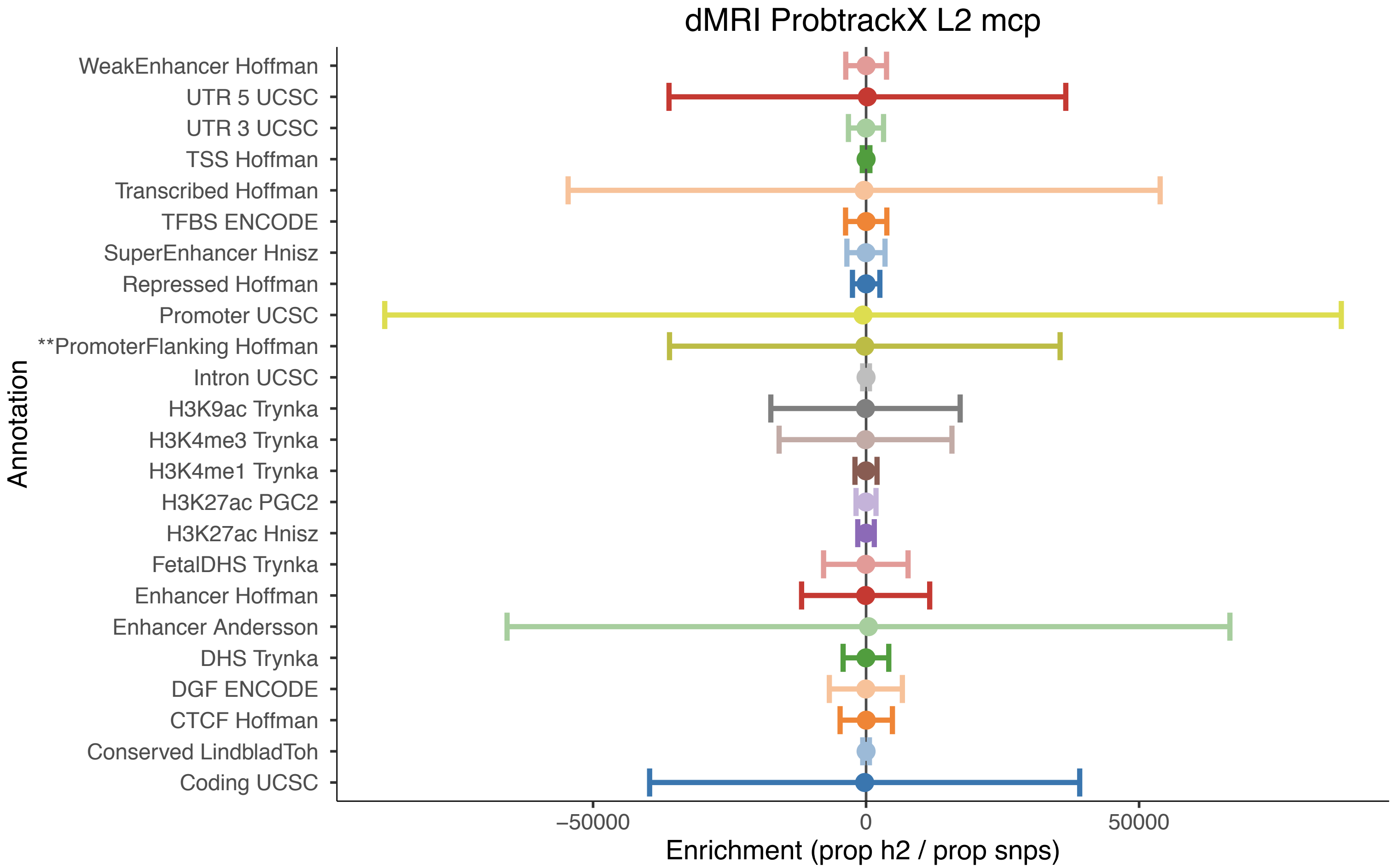
dMRI ProbtrackX L2 mcp
Annotation
WeakEnhancer Hoffman
UTR 5 UCSC
UTR 3 UCSC
TSS Hoffman
Transcribed Hoffman
TFBS ENCODE
SuperEnhancer Hnisz
Repressed Hoffman
Promoter UCSC
**PromoterFlanking Hoffman
Intron UCSC
H3K9ac Trynka
H3K4me3 Trynka
H3K4me1 Trynka
H3K27ac PGC2
H3K27ac Hnisz
FetalDHS Trynka
Enhancer Hoffman
Enhancer Andersson
DHS Trynka
DGF ENCODE
CTCF Hoffman
Conserved LindbladToh
Coding UCSC
−50000
0
50000
Enrichment (prop h2 / prop snps)

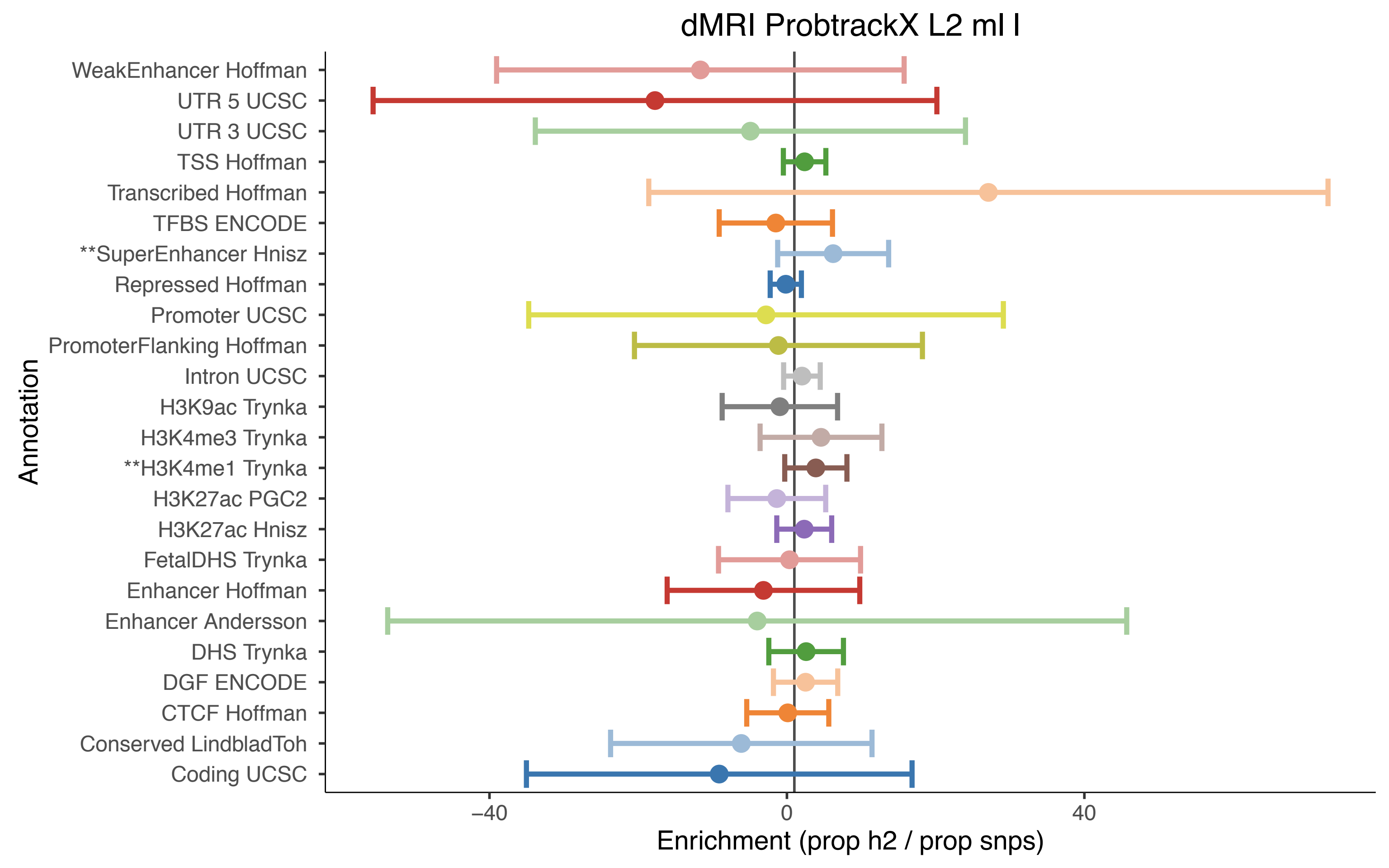

dMRI ProbtrackX L2 ml l
WeakEnhancer Hoffman
UTR 5 UCSC
UTR 3 UCSC
TSS Hoffman
Transcribed Hoffman
TFBS ENCODE
**SuperEnhancer Hnisz
Repressed Hoffman
Promoter UCSC
PromoterFlanking Hoffman
Intron UCSC
H3K9ac Trynka
H3K4me3 Trynka
**H3K4me1 Trynka
H3K27ac PGC2
H3K27ac Hnisz
FetalDHS Trynka
Enhancer Hoffman
Enhancer Andersson
DHS Trynka
DGF ENCODE
CTCF Hoffman
Conserved LindbladToh
Coding UCSC
Annotation
−40
0
40
Enrichment (prop h2 / prop snps)

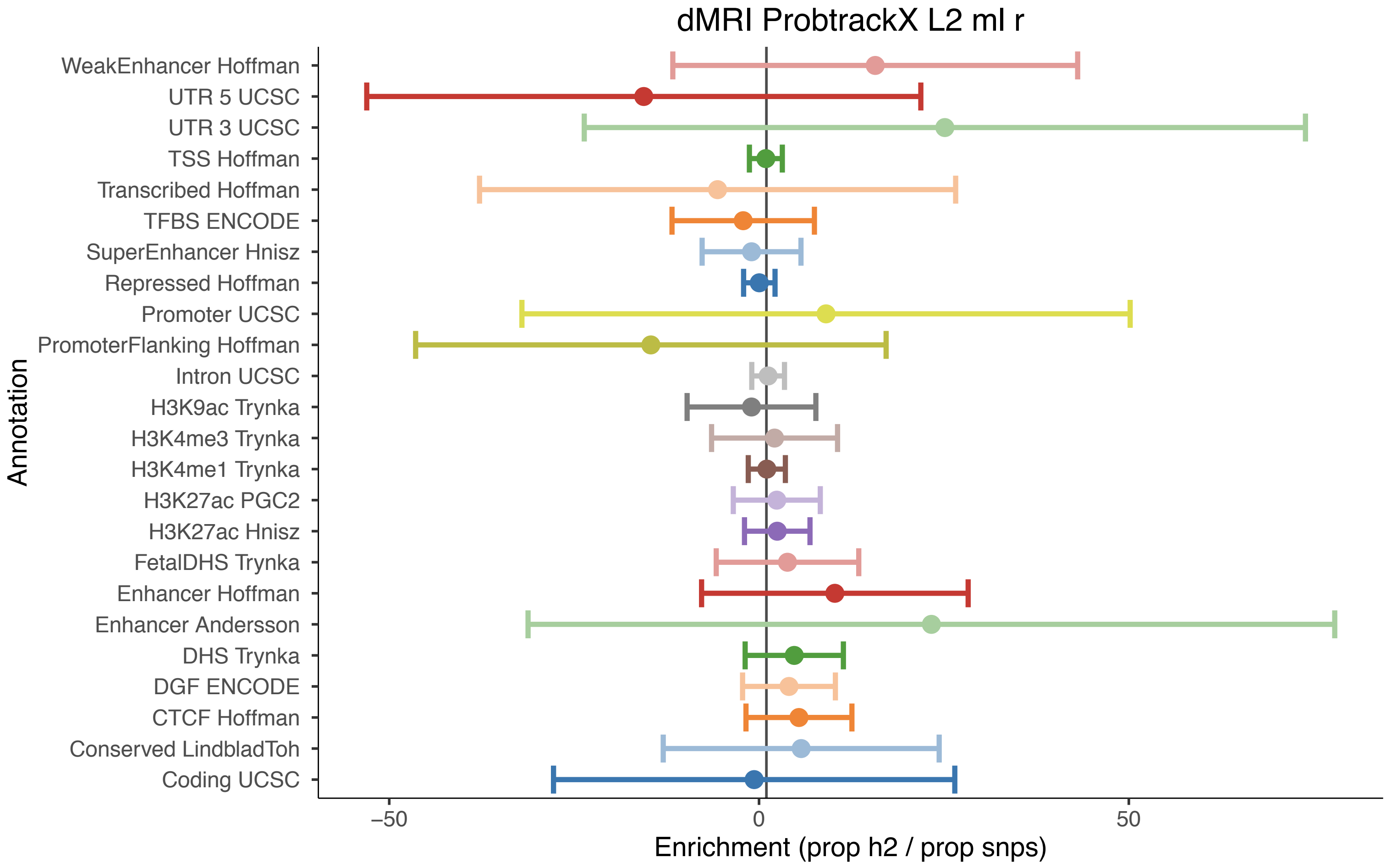

dMRI ProbtrackX L2 ml r
Annotation
WeakEnhancer Hoffman
UTR 5 UCSC
UTR 3 UCSC
TSS Hoffman
Transcribed Hoffman
TFBS ENCODE
SuperEnhancer Hnisz
Repressed Hoffman
Promoter UCSC
PromoterFlanking Hoffman
Intron UCSC
H3K9ac Trynka
H3K4me3 Trynka
H3K4me1 Trynka
H3K27ac PGC2
H3K27ac Hnisz
FetalDHS Trynka
Enhancer Hoffman
Enhancer Andersson
DHS Trynka
DGF ENCODE
CTCF Hoffman
Conserved LindbladToh
Coding UCSC
−50
0
50
Enrichment (prop h2 / prop snps)

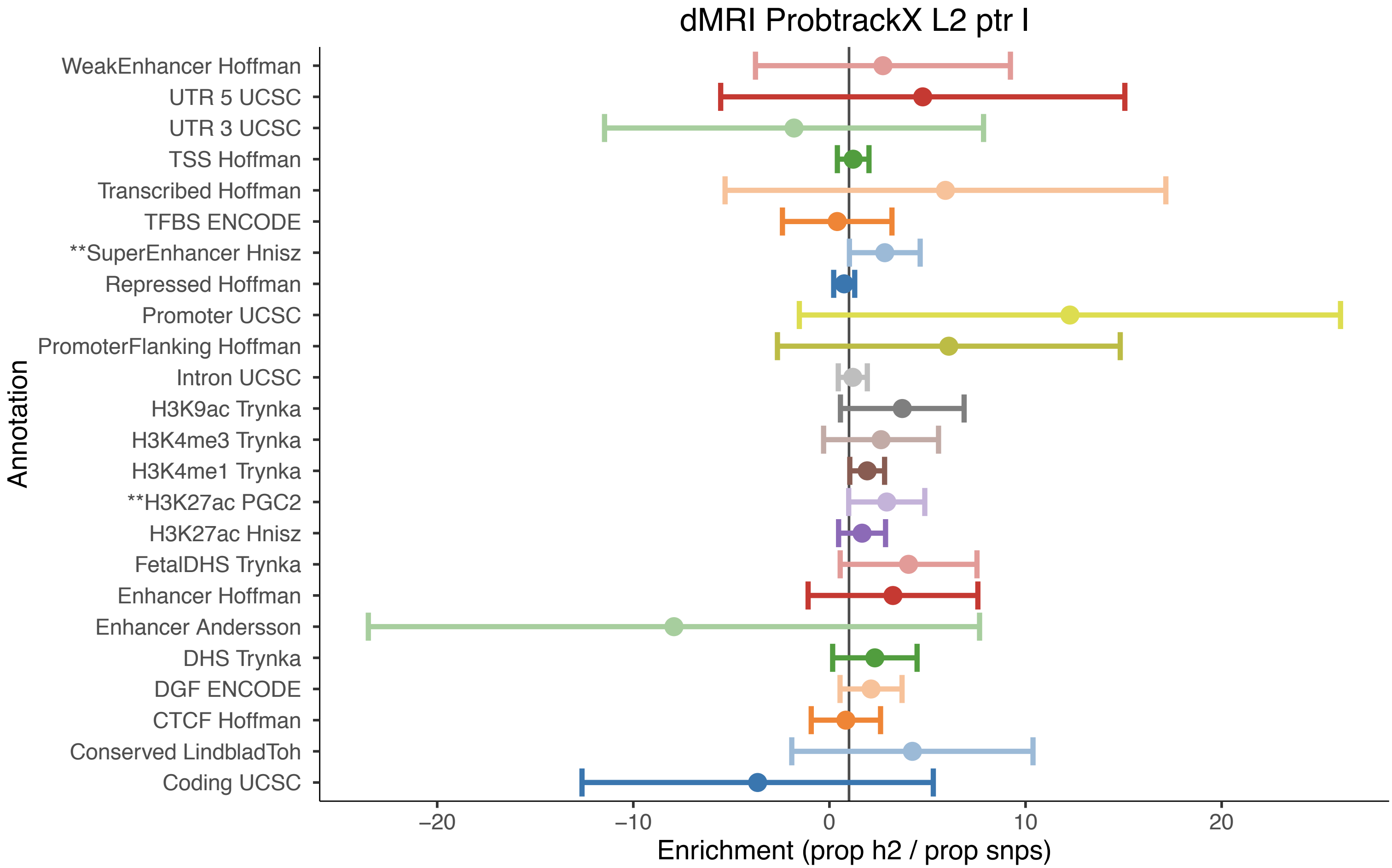
dMRI ProbtrackX L2 ptr I
WeakEnhancer Hoffman
UTR 5 UCSC
UTR 3 UCSC
TSS Hoffman
Transcribed Hoffman
TFBS ENCODE
**SuperEnhancer Hnisz
Repressed Hoffman
Promoter UCSC
PromoterFlanking Hoffman
Intron UCSC
H3K9ac Trynka
H3K4me3 Trynka
H3K4me1 Trynka
**H3K27ac PGC2
H3K27ac Hnisz
FetalDHS Trynka
Enhancer Hoffman
Enhancer Andersson
DHS Trynka
DGF ENCODE
CTCF Hoffman
Conserved LindbladToh
Coding UCSC
Annotation
−20
−10
0
10
20
Enrichment (prop h2 / prop snps)

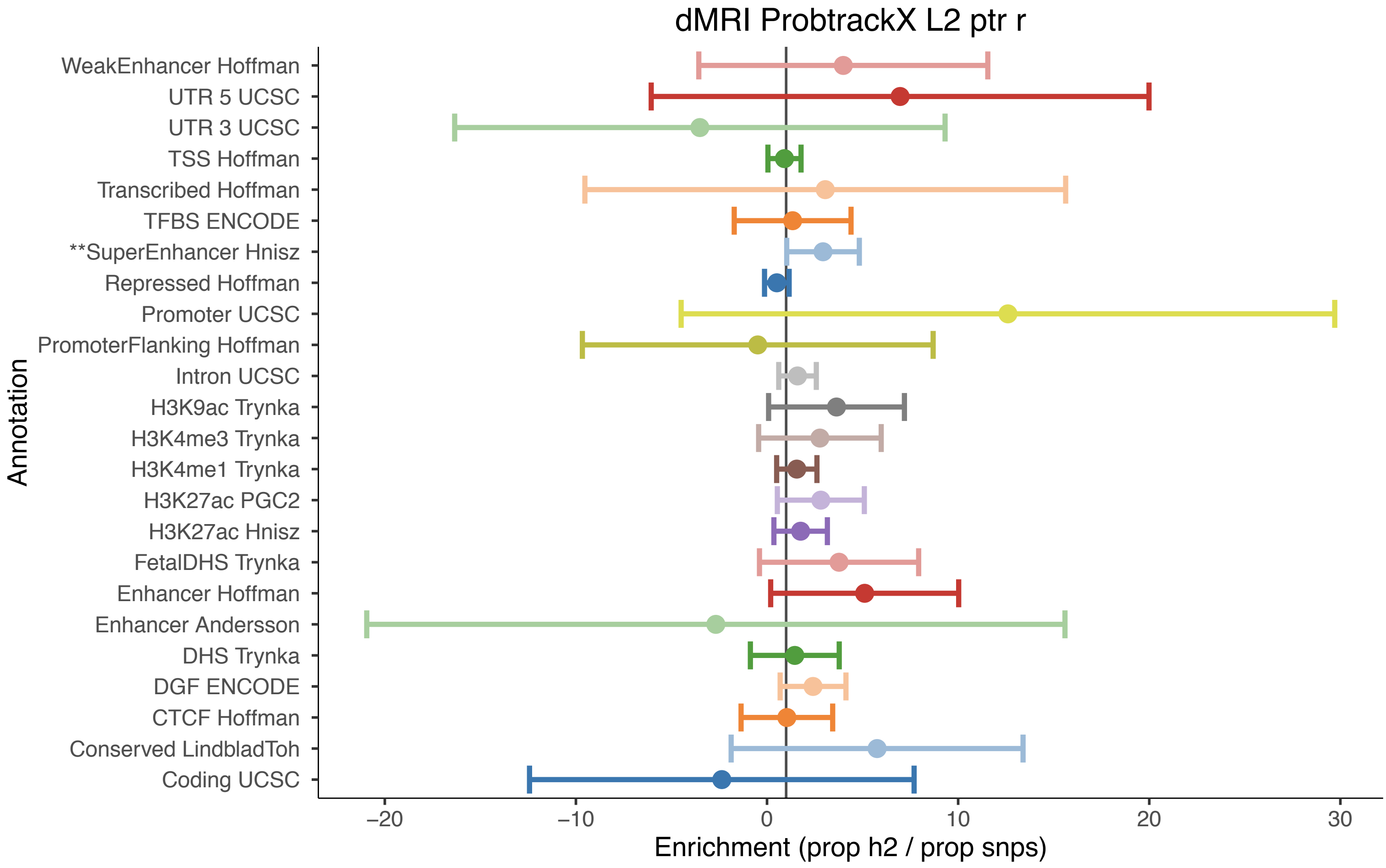
dMRI ProbtrackX L2 ptr r
WeakEnhancer Hoffman
UTR 5 UCSC
UTR 3 UCSC
TSS Hoffman
Transcribed Hoffman
TFBS ENCODE
**SuperEnhancer Hnisz
Repressed Hoffman
Promoter UCSC
PromoterFlanking Hoffman
Intron UCSC
H3K9ac Trynka
H3K4me3 Trynka
H3K4me1 Trynka
H3K27ac PGC2
H3K27ac Hnisz
FetalDHS Trynka
Enhancer Hoffman
Enhancer Andersson
DHS Trynka
DGF ENCODE
CTCF Hoffman
Conserved LindbladToh
Coding UCSC
Annotation
−20
−10
0
10
20
30
Enrichment (prop h2 / prop snps)

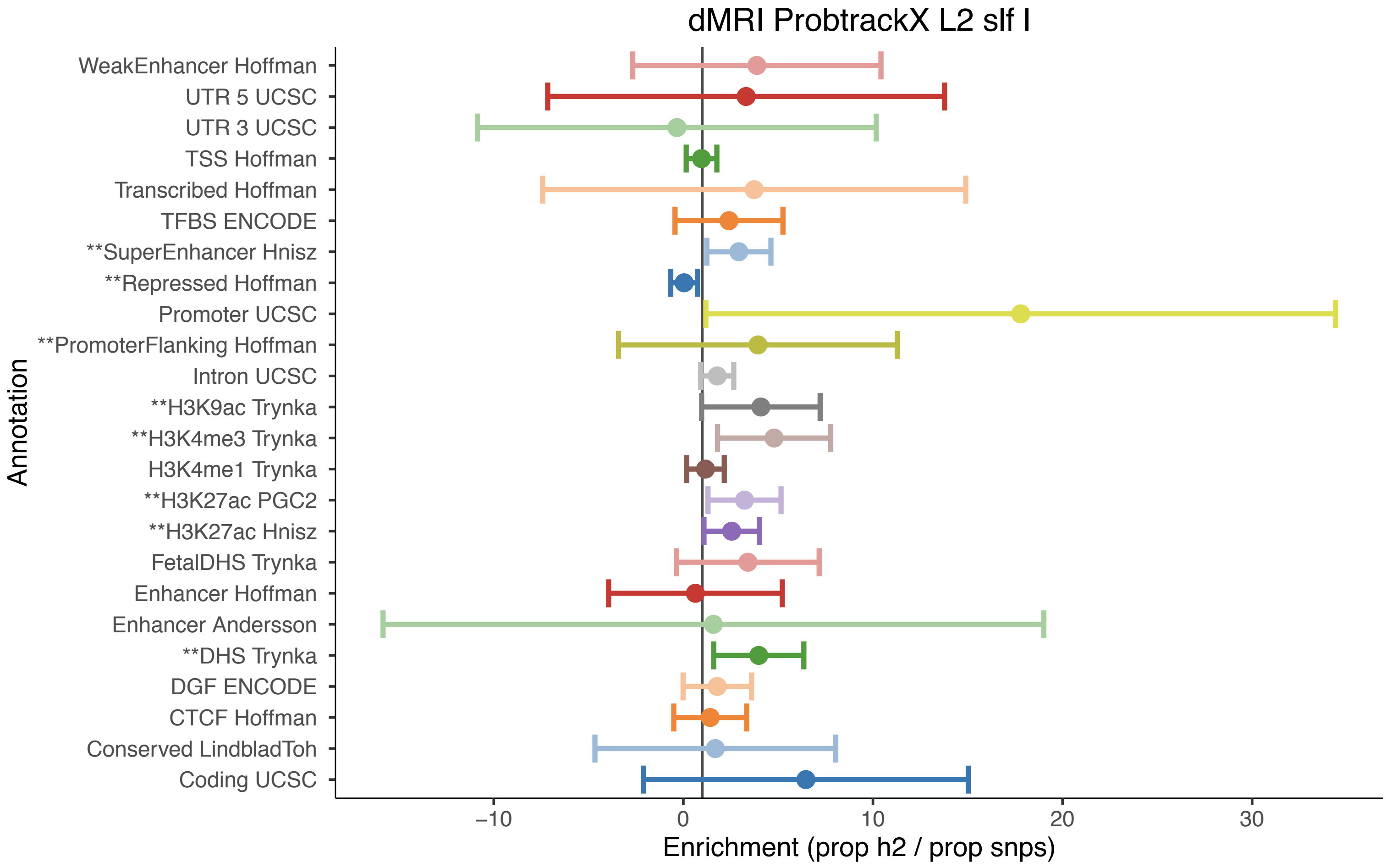
dMRI ProbtrackX L2 slf I
WeakEnhancer Hoffman
UTR 5 UCSC
UTR 3 UCSC
TSS Hoffman
Transcribed Hoffman
TFBS ENCODE
**SuperEnhancer Hnisz
**Repressed Hoffman
Promoter UCSC
**PromoterFlanking Hoffman
Intron UCSC
**H3K9ac Trynka
**H3K4me3 Trynka
H3K4me1 Trynka
**H3K27ac PGC2
**H3K27ac Hnisz
FetalDHS Trynka
Enhancer Hoffman
Enhancer Andersson
**DHS Trynka
DGF ENCODE
CTCF Hoffman
Conserved LindbladToh
Coding UCSC
Annotation
−10
0
10
20
30
Enrichment (prop h2 / prop snps)

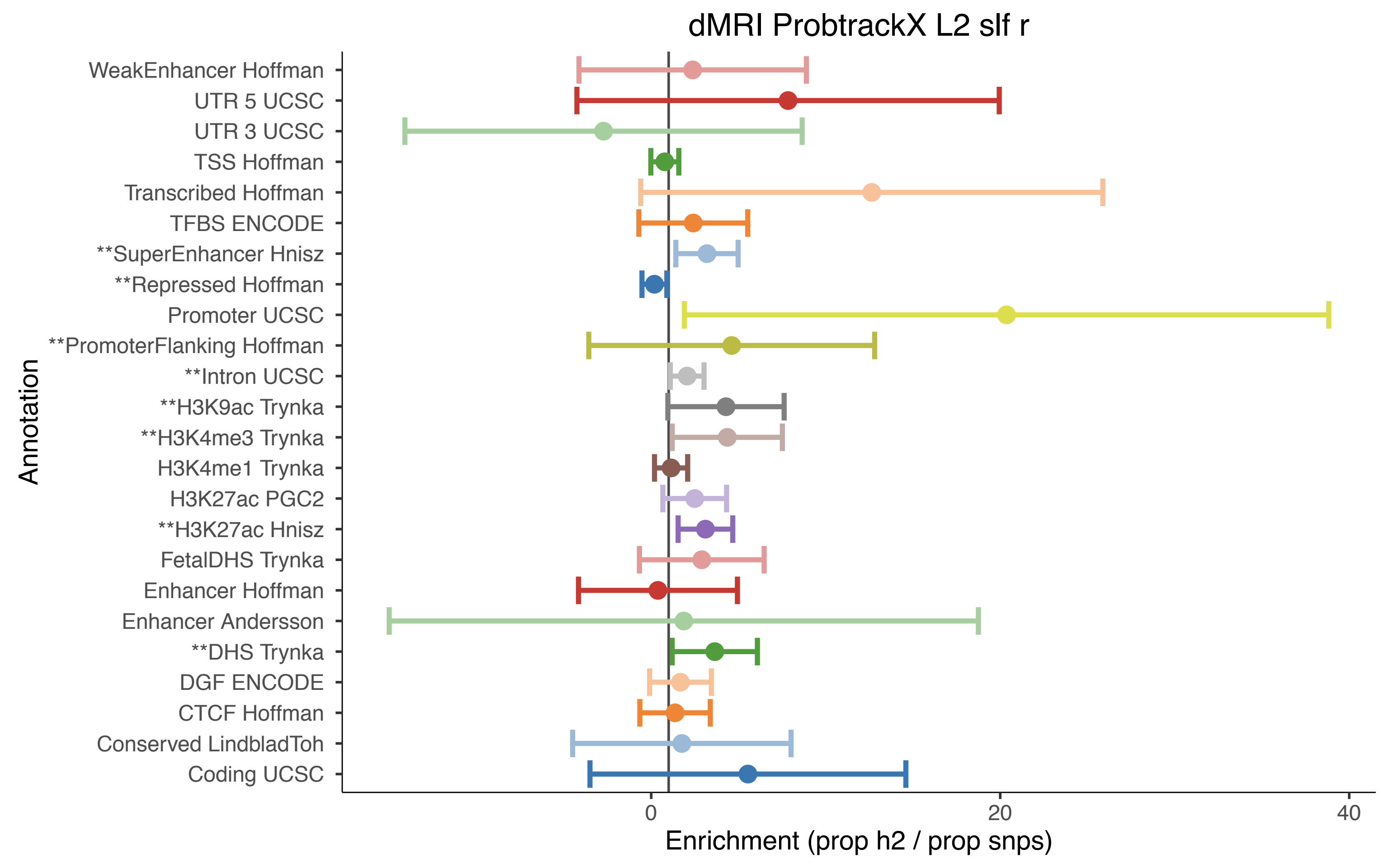

dMRI ProbtrackX L2 slf r
WeakEnhancer Hoffman
UTR 5 UCSC
UTR 3 UCSC
TSS Hoffman
Transcribed Hoffman
TFBS ENCODE
**SuperEnhancer Hnisz
**Repressed Hoffman
Promoter UCSC
**PromoterFlanking Hoffman
**Intron UCSC
**H3K9ac Trynka
**H3K4me3 Trynka
H3K4me1 Trynka
H3K27ac PGC2
**H3K27ac Hnisz
FetalDHS Trynka
Enhancer Hoffman
Enhancer Andersson
**DHS Trynka
DGF ENCODE
CTCF Hoffman
Conserved LindbladToh
Coding UCSC
Annotation
0
20
40
Enrichment (prop h2 / prop snps)

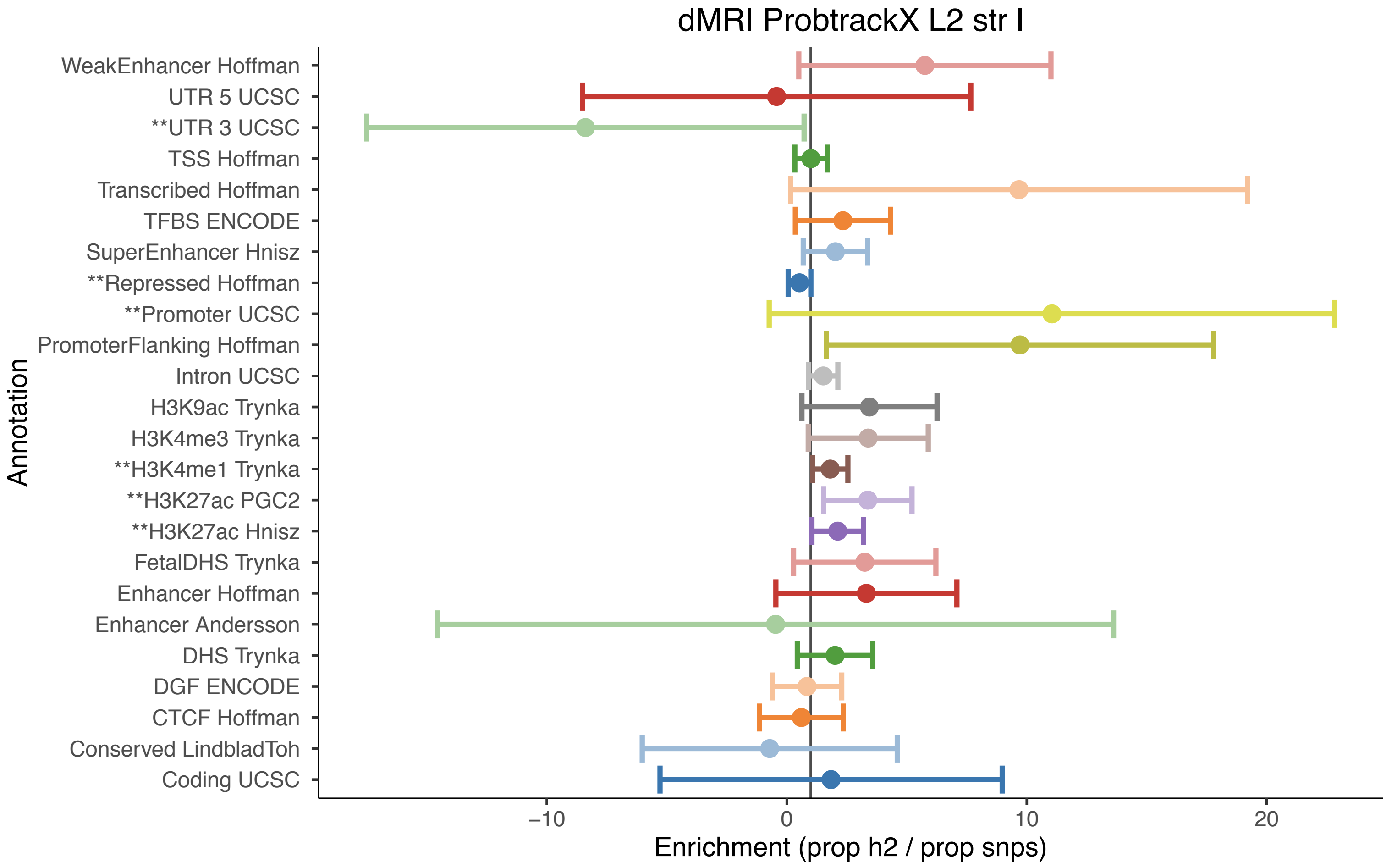
dMRI ProbtrackX L2 str I
Annotation
WeakEnhancer Hoffman
UTR 5 UCSC
**UTR 3 UCSC
TSS Hoffman
Transcribed Hoffman
TFBS ENCODE
SuperEnhancer Hnisz
**Repressed Hoffman
**Promoter UCSC
PromoterFlanking Hoffman
Intron UCSC
H3K9ac Trynka
H3K4me3 Trynka
**H3K4me1 Trynka
**H3K27ac PGC2
**H3K27ac Hnisz
FetalDHS Trynka
Enhancer Hoffman
Enhancer Andersson
DHS Trynka
DGF ENCODE
CTCF Hoffman
Conserved LindbladToh
Coding UCSC
−10
0
10
20
Enrichment (prop h2 / prop snps)

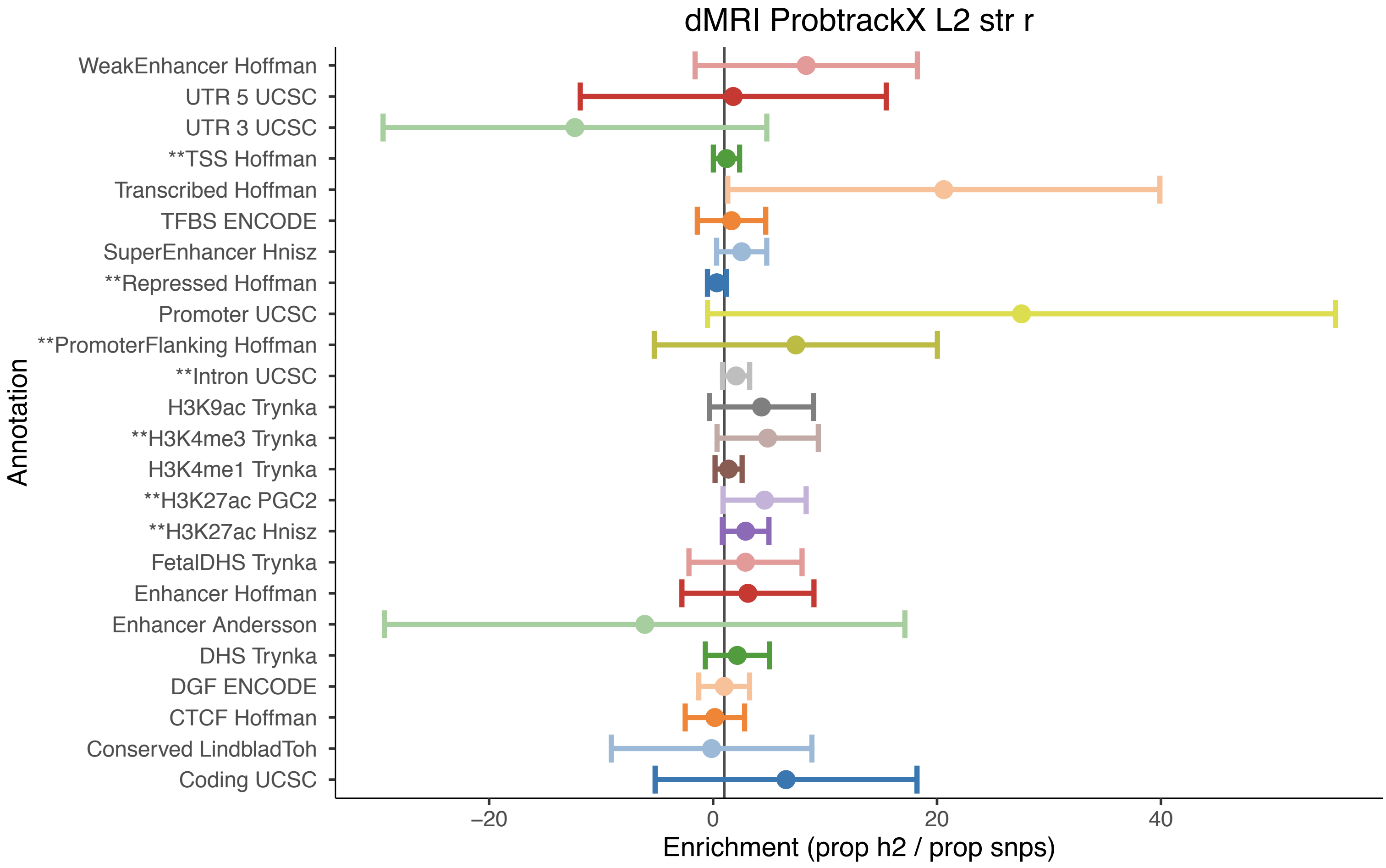
dMRI ProbtrackX L2 str r
WeakEnhancer Hoffman
UTR 5 UCSC
UTR 3 UCSC
**TSS Hoffman
Transcribed Hoffman
TFBS ENCODE
SuperEnhancer Hnisz
**Repressed Hoffman
Promoter UCSC
**PromoterFlanking Hoffman
**Intron UCSC
H3K9ac Trynka
**H3K4me3 Trynka
H3K4me1 Trynka
**H3K27ac PGC2
**H3K27ac Hnisz
FetalDHS Trynka
Enhancer Hoffman
Enhancer Andersson
DHS Trynka
DGF ENCODE
CTCF Hoffman
Conserved LindbladToh
Coding UCSC
Annotation
−20
0
20
40
Enrichment (prop h2 / prop snps)

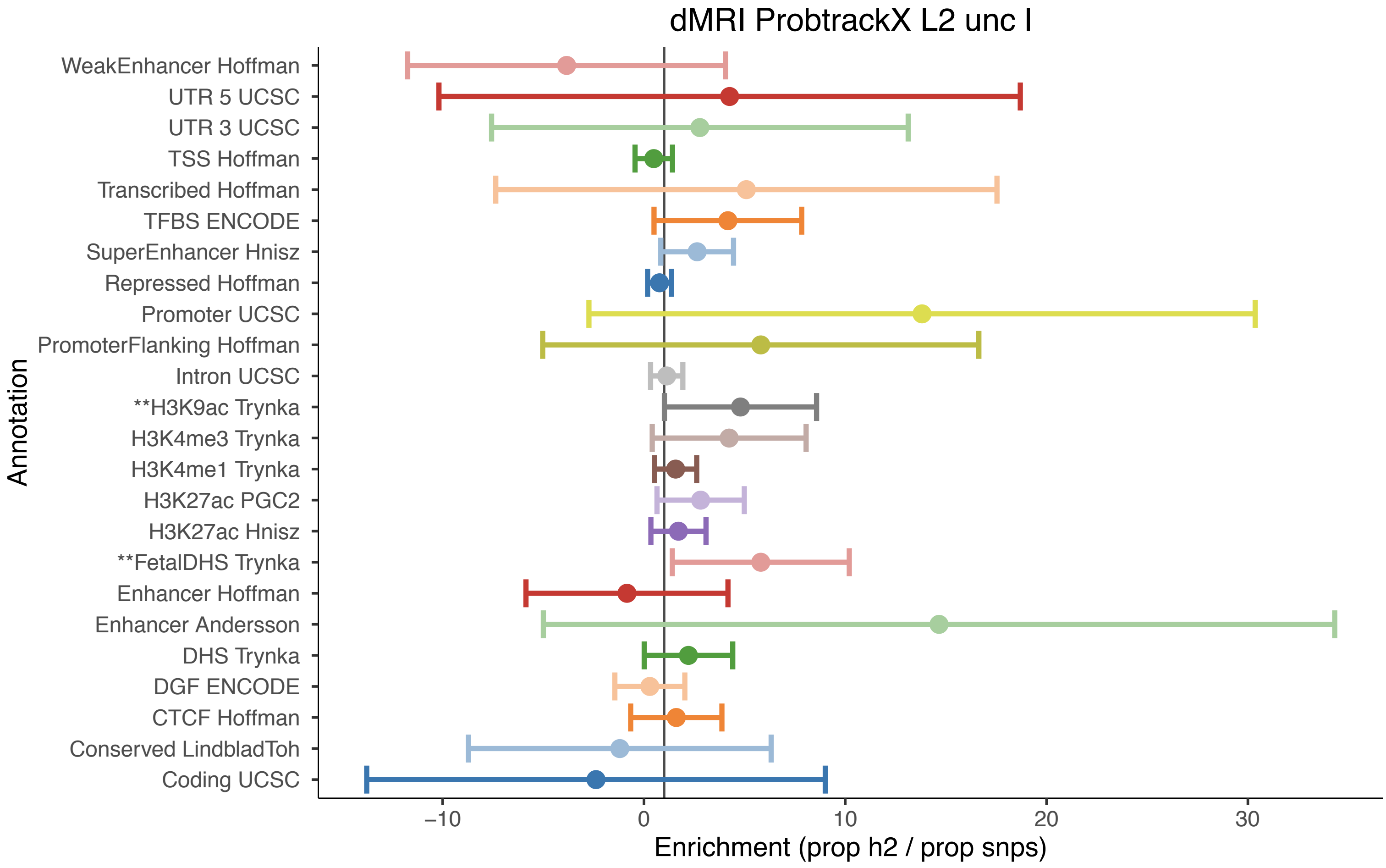
dMRI ProbtrackX L2 unc I
WeakEnhancer Hoffman
UTR 5 UCSC
UTR 3 UCSC
TSS Hoffman
Transcribed Hoffman
TFBS ENCODE
SuperEnhancer Hnisz
Repressed Hoffman
Promoter UCSC
PromoterFlanking Hoffman
Intron UCSC
**H3K9ac Trynka
H3K4me3 Trynka
H3K4me1 Trynka
H3K27ac PGC2
H3K27ac Hnisz
**FetalDHS Trynka
Enhancer Hoffman
Enhancer Andersson
DHS Trynka
DGF ENCODE
CTCF Hoffman
Conserved LindbladToh
Coding UCSC
Annotation
−10
0
10
20
30
Enrichment (prop h2 / prop snps)

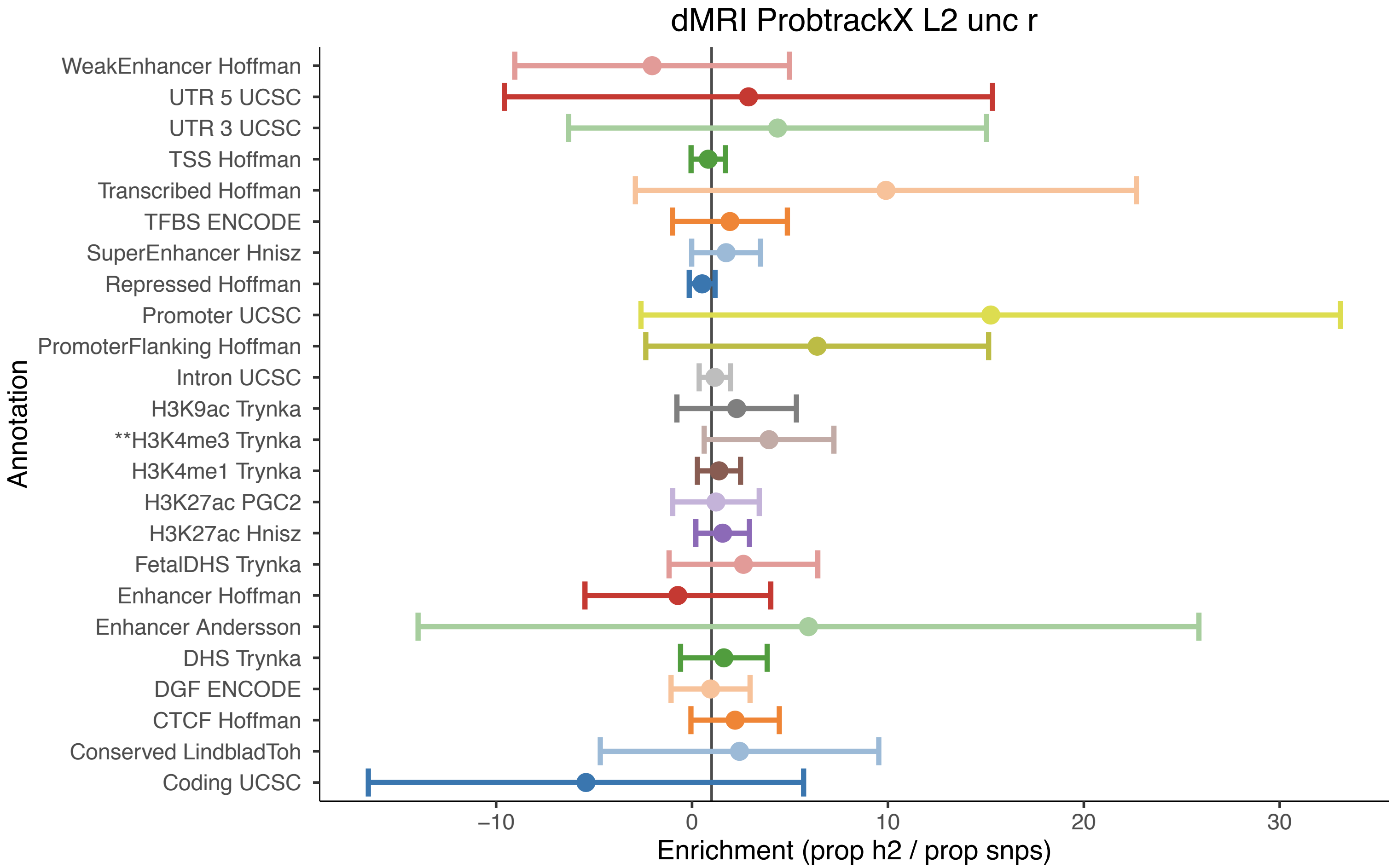
dMRI ProbtrackX L2 unc r
Annotation
WeakEnhancer Hoffman
UTR 5 UCSC
UTR 3 UCSC
TSS Hoffman
Transcribed Hoffman
TFBS ENCODE
SuperEnhancer Hnisz
Repressed Hoffman
Promoter UCSC
PromoterFlanking Hoffman
Intron UCSC
H3K9ac Trynka
**H3K4me3 Trynka
H3K4me1 Trynka
H3K27ac PGC2
H3K27ac Hnisz
FetalDHS Trynka
Enhancer Hoffman
Enhancer Andersson
DHS Trynka
DGF ENCODE
CTCF Hoffman
Conserved LindbladToh
Coding UCSC
−10
0
10
20
30
Enrichment (prop h2 / prop snps)

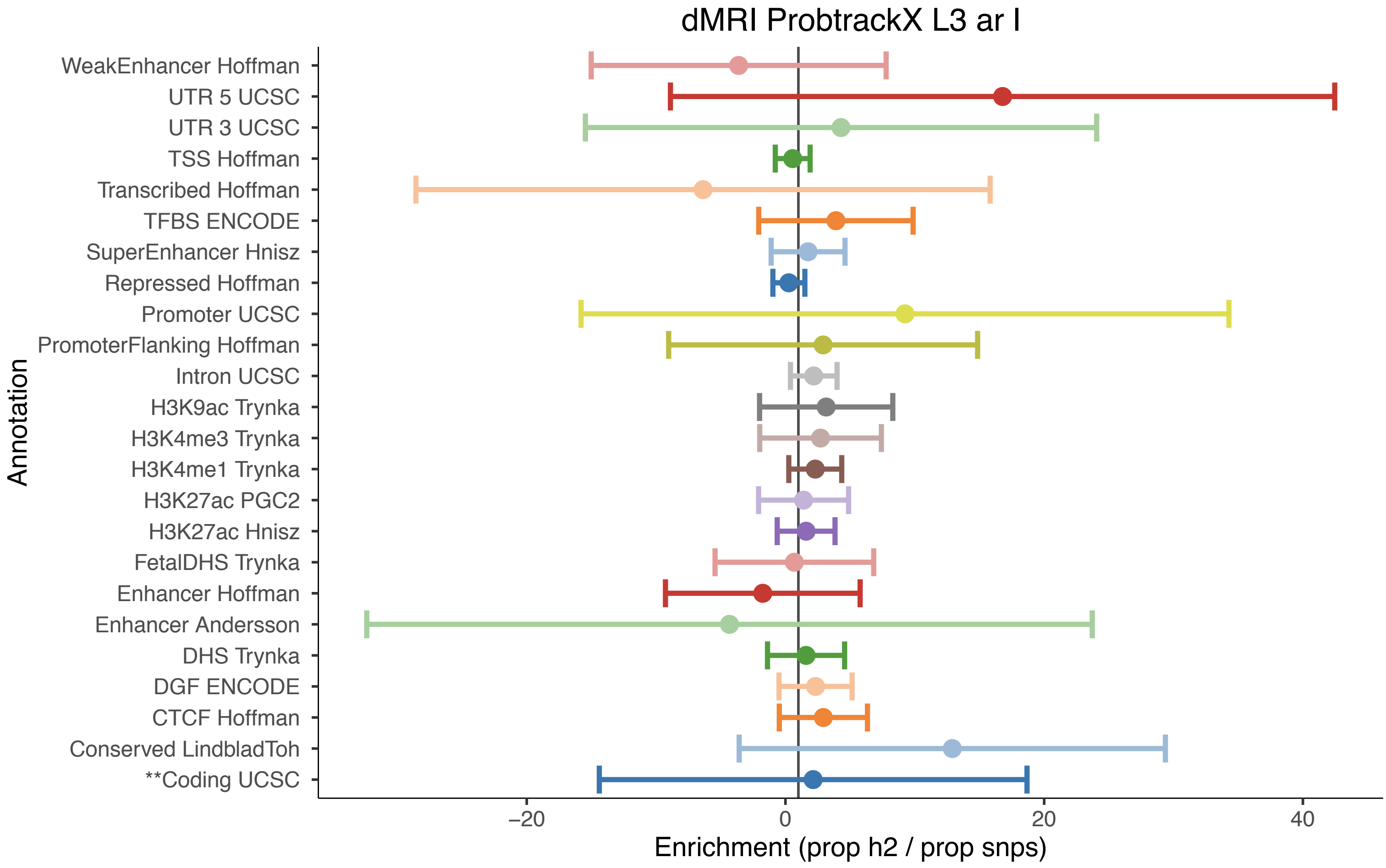

dMRI ProbtrackX L3 ar l
Annotation
WeakEnhancer Hoffman
UTR 5 UCSC
UTR 3 UCSC
TSS Hoffman
Transcribed Hoffman
TFBS ENCODE
SuperEnhancer Hnisz
Repressed Hoffman
Promoter UCSC
PromoterFlanking Hoffman
Intron UCSC
H3K9ac Trynka
H3K4me3 Trynka
H3K4me1 Trynka
H3K27ac PGC2
H3K27ac Hnisz
FetalDHS Trynka
Enhancer Hoffman
Enhancer Andersson
DHS Trynka
DGF ENCODE
CTCF Hoffman
Conserved LindbladToh
**Coding UCSC
−20
0
20
40
Enrichment (prop h2 / prop snps)

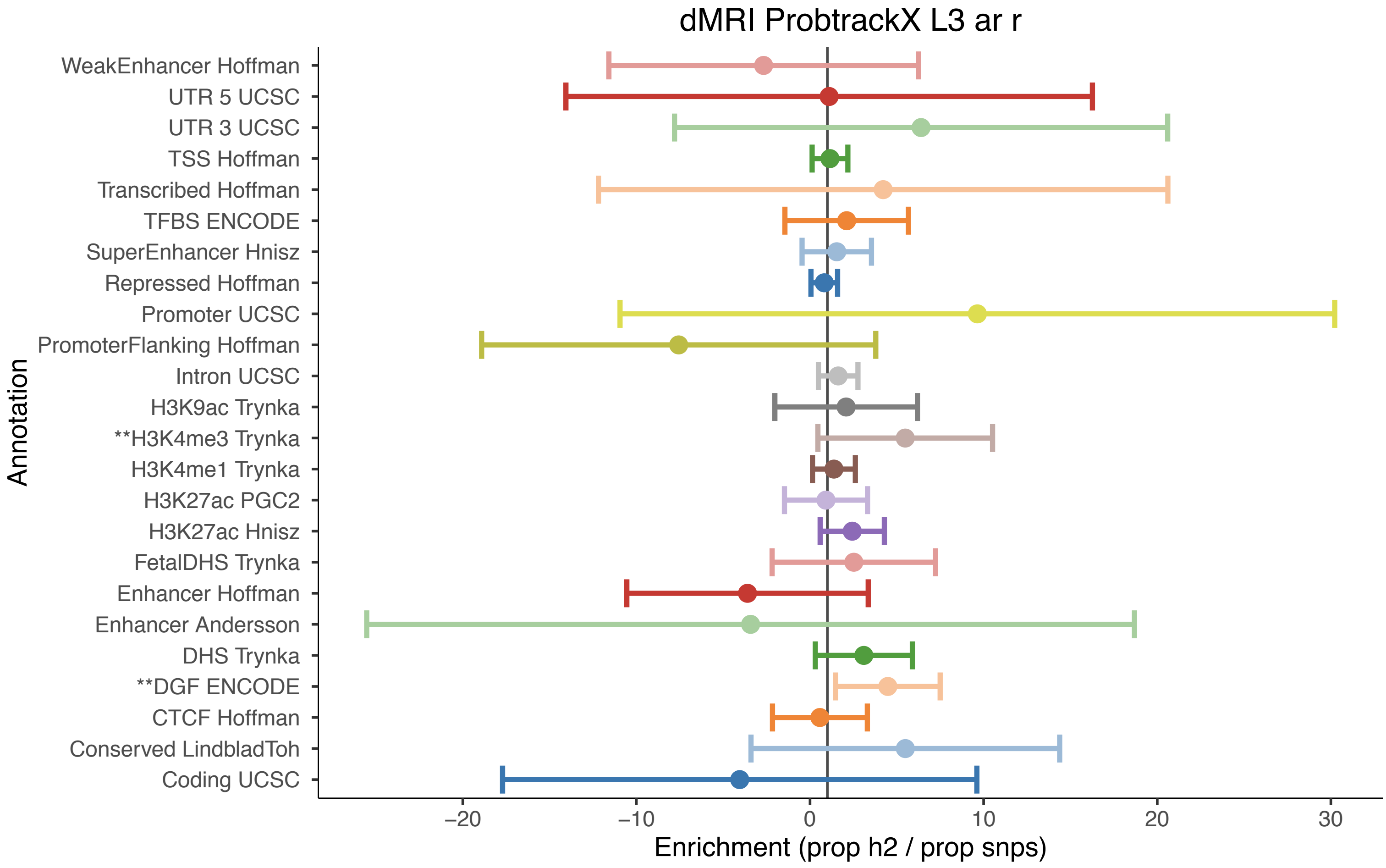
dMRI ProbtrackX L3 ar r
WeakEnhancer Hoffman
UTR 5 UCSC
UTR 3 UCSC
TSS Hoffman
Transcribed Hoffman
TFBS ENCODE
SuperEnhancer Hnisz
Repressed Hoffman
Promoter UCSC
PromoterFlanking Hoffman
Intron UCSC
H3K9ac Trynka
**H3K4me3 Trynka
H3K4me1 Trynka
H3K27ac PGC2
H3K27ac Hnisz
FetalDHS Trynka
Enhancer Hoffman
Enhancer Andersson
DHS Trynka
**DGF ENCODE
CTCF Hoffman
Conserved LindbladToh
Coding UCSC
Annotation
−20
−10
0
10
20
30
Enrichment (prop h2 / prop snps)

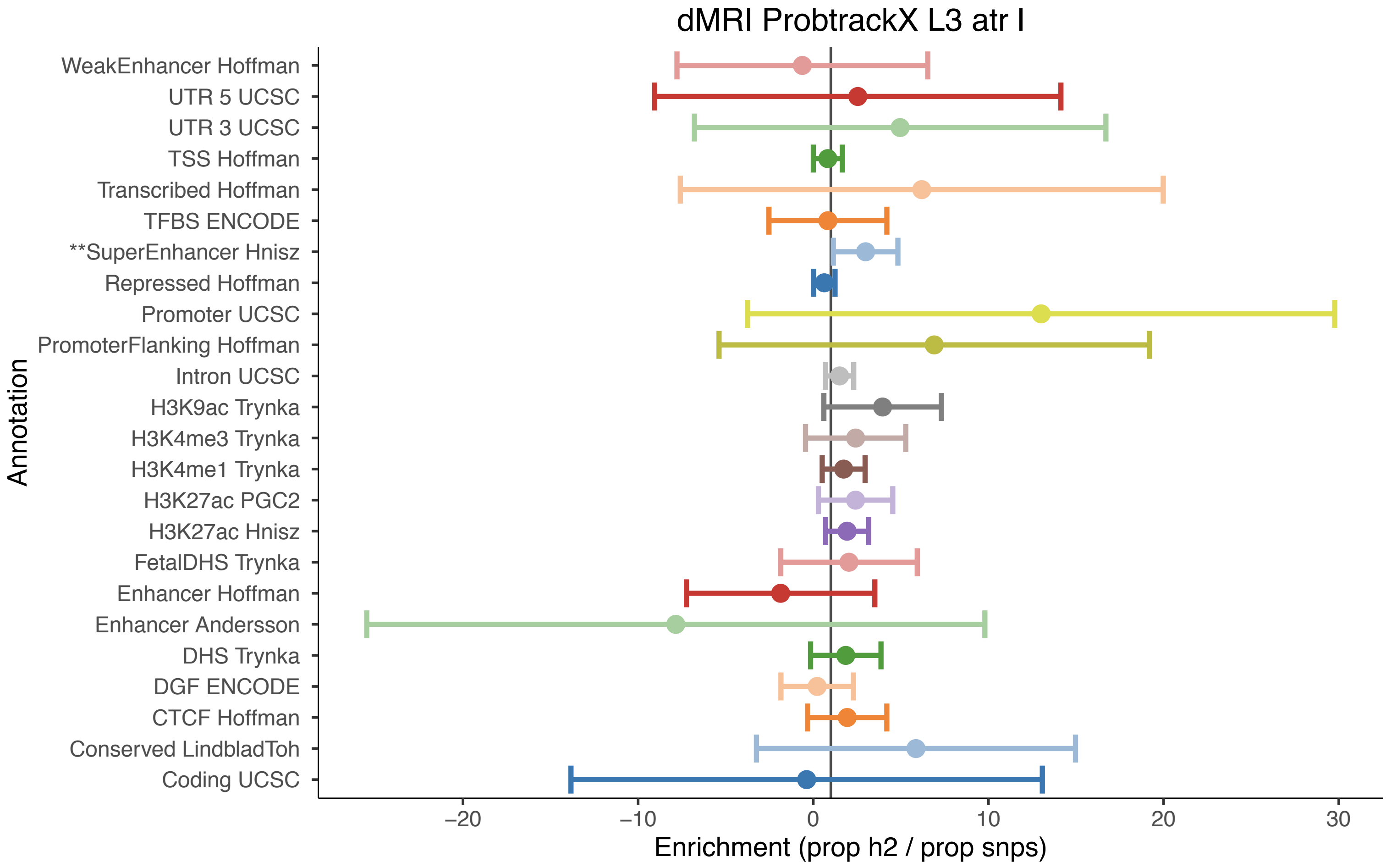
dMRI ProbtrackX L3 atr l
Annotation
WeakEnhancer Hoffman
UTR 5 UCSC
UTR 3 UCSC
TSS Hoffman
Transcribed Hoffman
TFBS ENCODE
**SuperEnhancer Hnisz
Repressed Hoffman
Promoter UCSC
PromoterFlanking Hoffman
Intron UCSC
H3K9ac Trynka
H3K4me3 Trynka
H3K4me1 Trynka
H3K27ac PGC2
H3K27ac Hnisz
FetalDHS Trynka
Enhancer Hoffman
Enhancer Andersson
DHS Trynka
DGF ENCODE
CTCF Hoffman
Conserved LindbladToh
Coding UCSC
−20
−10
0
10
20
30
Enrichment (prop h2 / prop snps)

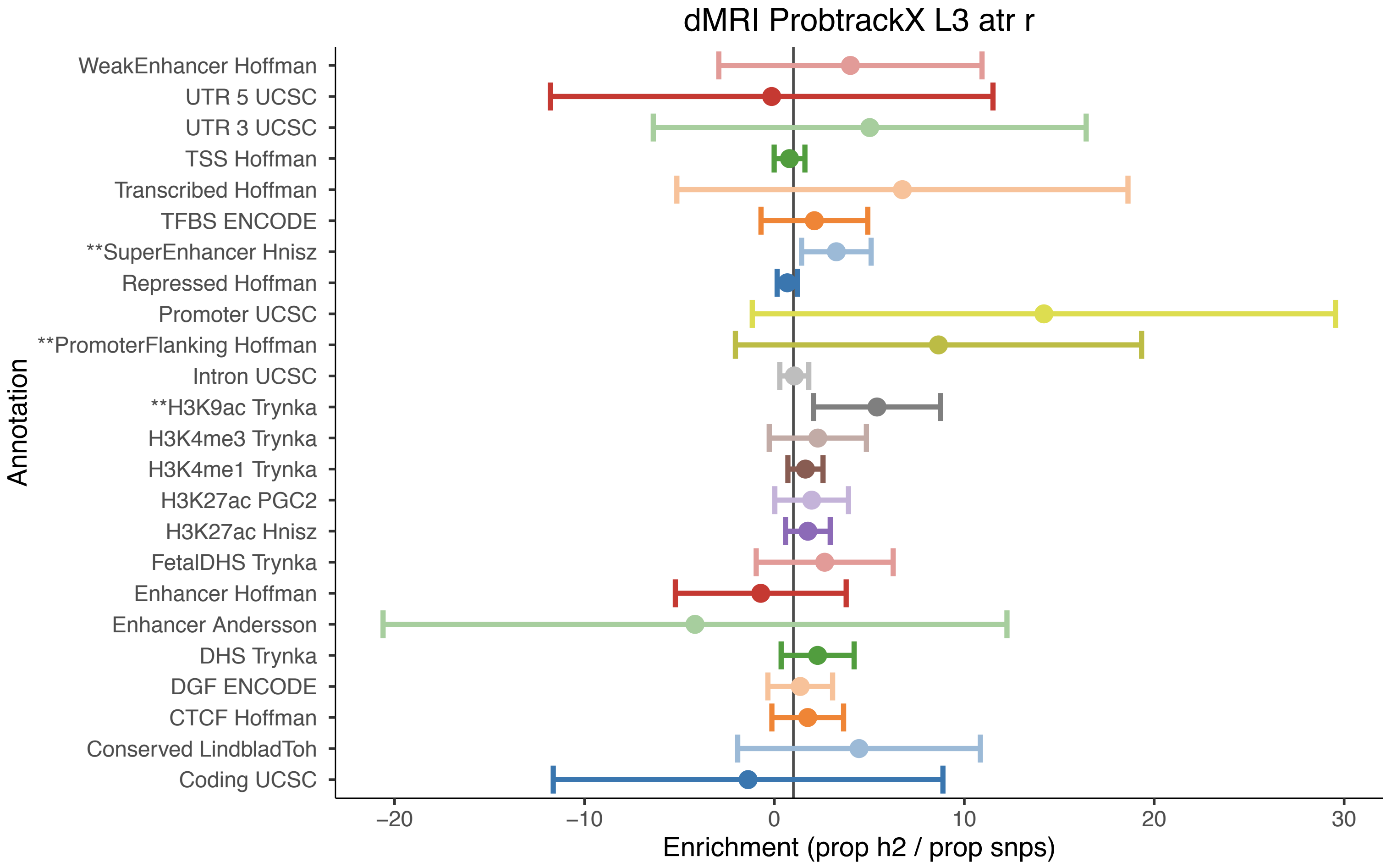

dMRI ProbtrackX L3 atr r
WeakEnhancer Hoffman
UTR 5 UCSC
UTR 3 UCSC
TSS Hoffman
Transcribed Hoffman
TFBS ENCODE
**SuperEnhancer Hnisz
Repressed Hoffman
Promoter UCSC
**PromoterFlanking Hoffman
Intron UCSC
**H3K9ac Trynka
H3K4me3 Trynka
H3K4me1 Trynka
H3K27ac PGC2
H3K27ac Hnisz
FetalDHS Trynka
Enhancer Hoffman
Enhancer Andersson
DHS Trynka
DGF ENCODE
CTCF Hoffman
Conserved LindbladToh
Coding UCSC
Annotation
−20
−10
0
10
20
30
Enrichment (prop h2 / prop snps)

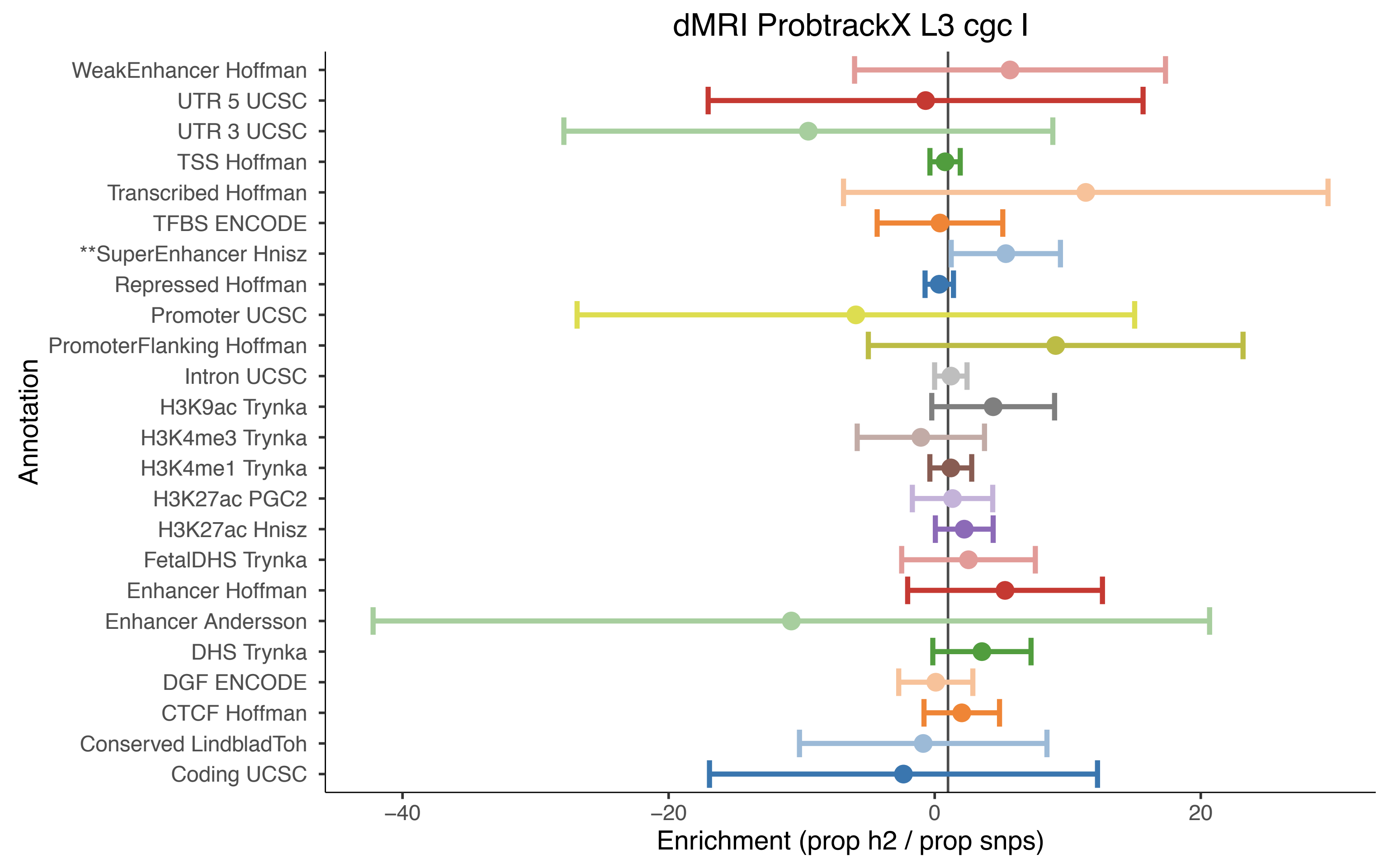
dMRI ProbtrackX L3 cgc l
Annotation
WeakEnhancer Hoffman
UTR 5 UCSC
UTR 3 UCSC
TSS Hoffman
Transcribed Hoffman
TFBS ENCODE
**SuperEnhancer Hnisz
Repressed Hoffman
Promoter UCSC
PromoterFlanking Hoffman
Intron UCSC
H3K9ac Trynka
H3K4me3 Trynka
H3K4me1 Trynka
H3K27ac PGC2
H3K27ac Hnisz
FetalDHS Trynka
Enhancer Hoffman
Enhancer Andersson
DHS Trynka
DGF ENCODE
CTCF Hoffman
Conserved LindbladToh
Coding UCSC
−40
−20
0
20
Enrichment (prop h2 / prop snps)

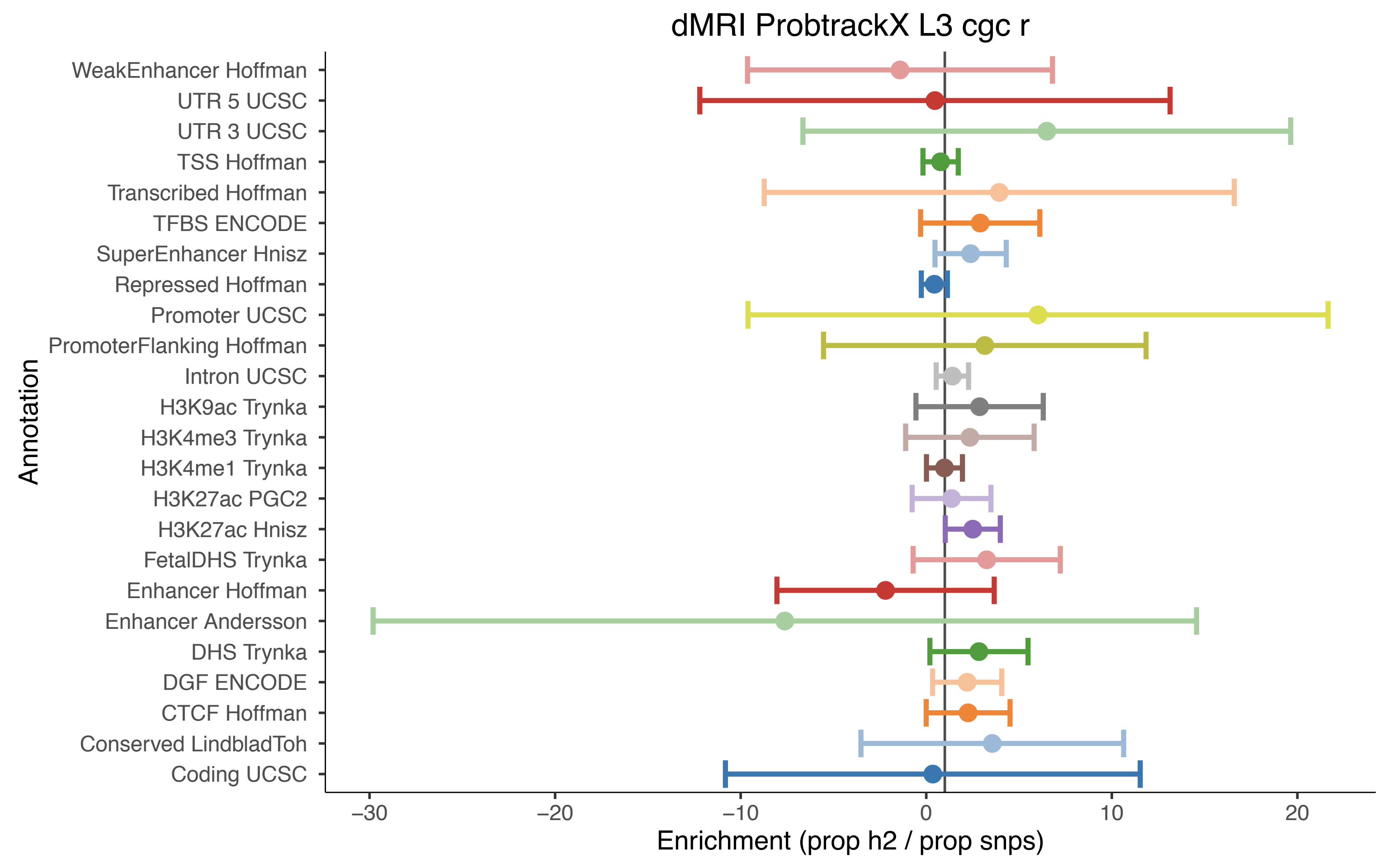
dMRI ProbtrackX L3 cgc r
Annotation
WeakEnhancer Hoffman
UTR 5 UCSC
UTR 3 UCSC
TSS Hoffman
Transcribed Hoffman
TFBS ENCODE
SuperEnhancer Hnisz
Repressed Hoffman
Promoter UCSC
PromoterFlanking Hoffman
Intron UCSC
H3K9ac Trynka
H3K4me3 Trynka
H3K4me1 Trynka
H3K27ac PGC2
H3K27ac Hnisz
FetalDHS Trynka
Enhancer Hoffman
Enhancer Andersson
DHS Trynka
DGF ENCODE
CTCF Hoffman
Conserved LindbladToh
Coding UCSC
−30
−20
−10
0
10
20
Enrichment (prop h2 / prop snps)

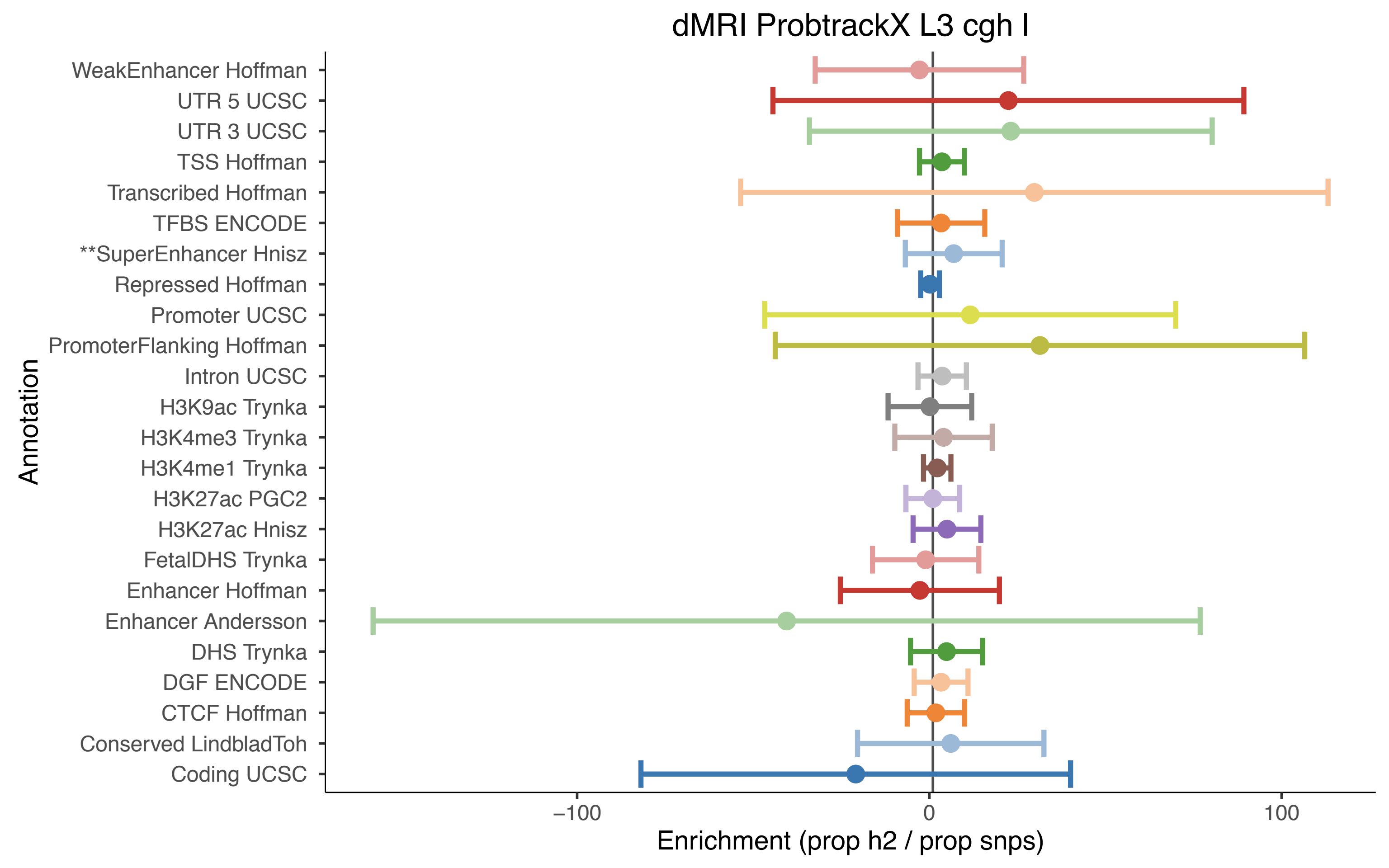
dMRI ProbtrackX L3 cgh I
WeakEnhancer Hoffman
UTR 5 UCSC
UTR 3 UCSC
TSS Hoffman
Transcribed Hoffman
TFBS ENCODE
**SuperEnhancer Hnisz
Repressed Hoffman
Promoter UCSC
PromoterFlanking Hoffman
Intron UCSC
H3K9ac Trynka
H3K4me3 Trynka
H3K4me1 Trynka
H3K27ac PGC2
H3K27ac Hnisz
FetalDHS Trynka
Enhancer Hoffman
Enhancer Andersson
DHS Trynka
DGF ENCODE
CTCF Hoffman
Conserved LindbladToh
Coding UCSC
Annotation
−100
0
100
Enrichment (prop h2 / prop snps)

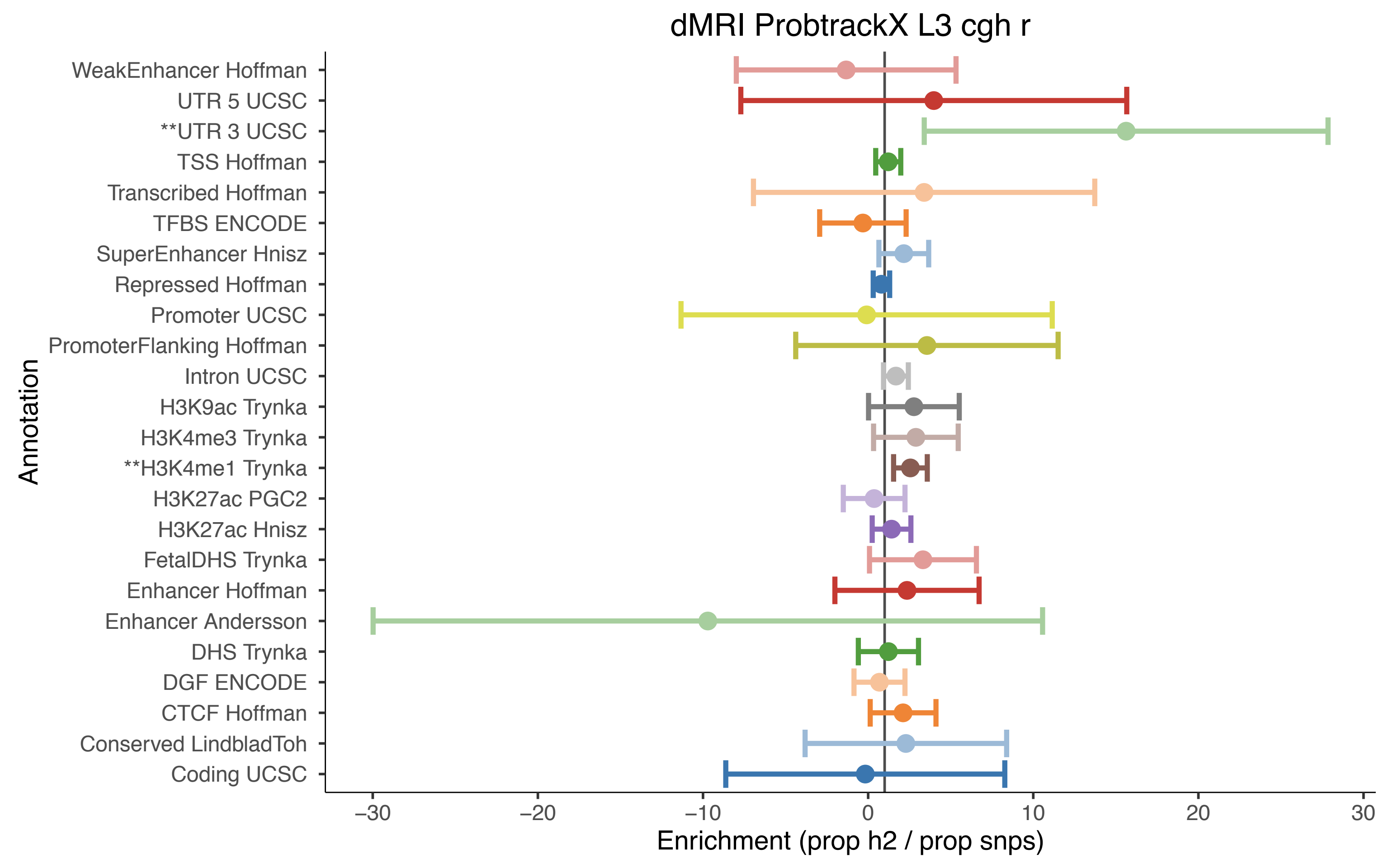
dMRI ProbtrackX L3 cgh r
WeakEnhancer Hoffman
UTR 5 UCSC
**UTR 3 UCSC
TSS Hoffman
Transcribed Hoffman
TFBS ENCODE
SuperEnhancer Hnisz
Repressed Hoffman
Promoter UCSC
PromoterFlanking Hoffman
Intron UCSC
H3K9ac Trynka
H3K4me3 Trynka
**H3K4me1 Trynka
H3K27ac PGC2
H3K27ac Hnisz
FetalDHS Trynka
Enhancer Hoffman
Enhancer Andersson
DHS Trynka
DGF ENCODE
CTCF Hoffman
Conserved LindbladToh
Coding UCSC
Annotation
−30
−20
−10
0
10
20
30
Enrichment (prop h2 / prop snps)

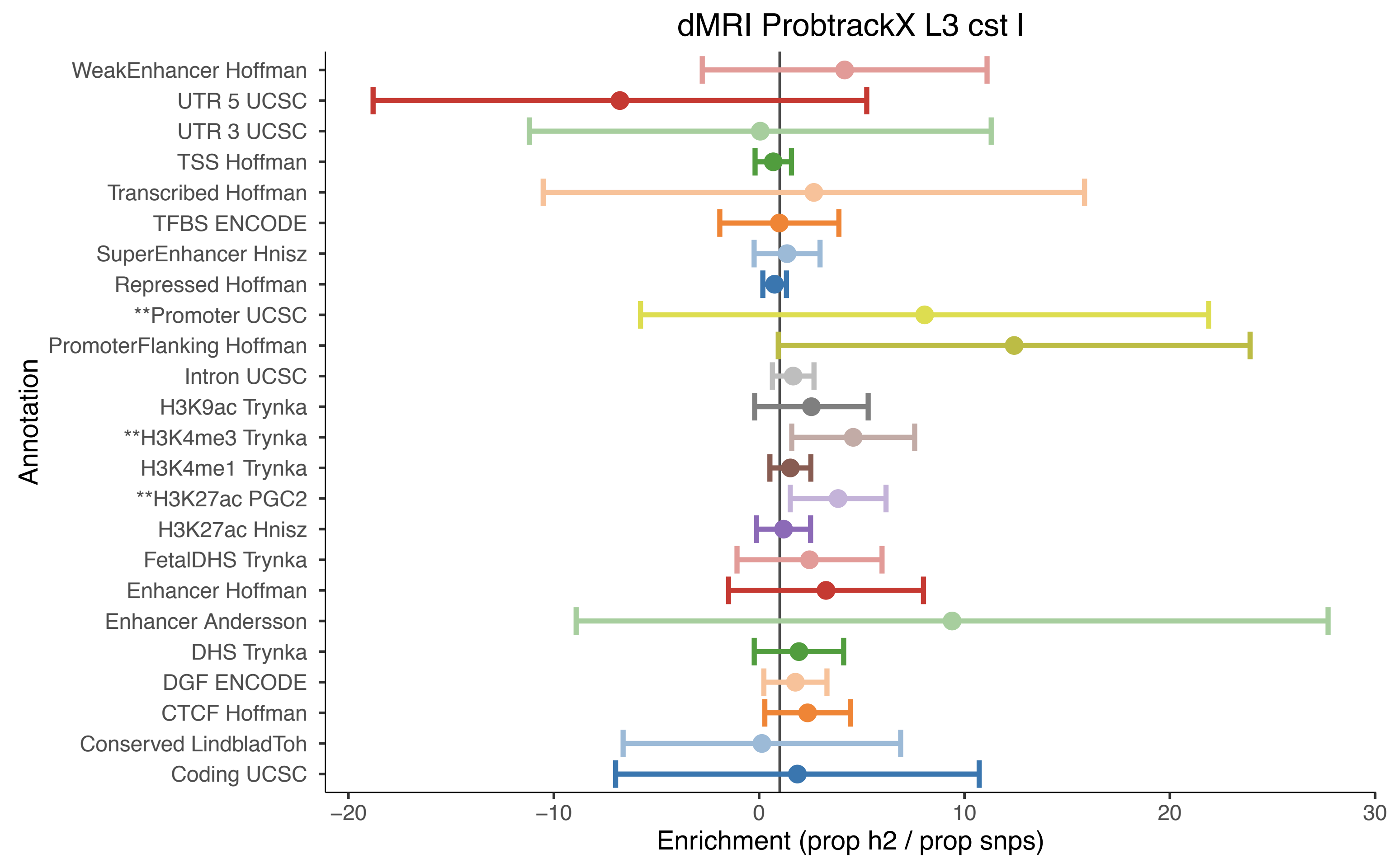
dMRI ProbtrackX L3 cst l
WeakEnhancer Hoffman
UTR 5 UCSC
UTR 3 UCSC
TSS Hoffman
Transcribed Hoffman
TFBS ENCODE
SuperEnhancer Hnisz
Repressed Hoffman
**Promoter UCSC
PromoterFlanking Hoffman
Intron UCSC
H3K9ac Trynka
**H3K4me3 Trynka
H3K4me1 Trynka
**H3K27ac PGC2
H3K27ac Hnisz
FetalDHS Trynka
Enhancer Hoffman
Enhancer Andersson
DHS Trynka
DGF ENCODE
CTCF Hoffman
Conserved LindbladToh
Coding UCSC
Annotation
−20
−10
0
10
20
30
Enrichment (prop h2 / prop snps)

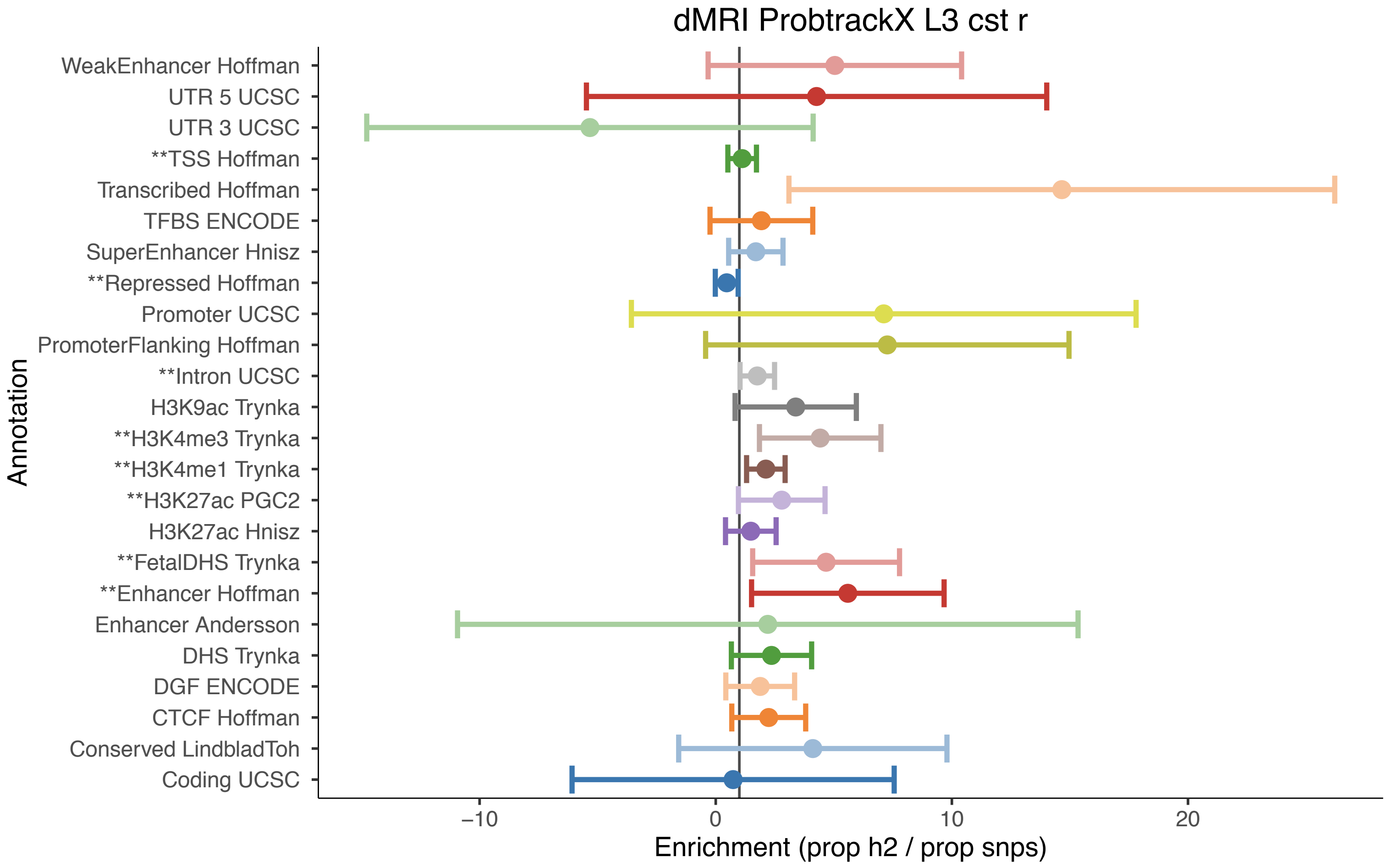

dMRI ProbtrackX L3 cst r
Annotation
WeakEnhancer Hoffman
UTR 5 UCSC
UTR 3 UCSC
**TSS Hoffman
Transcribed Hoffman
TFBS ENCODE
SuperEnhancer Hnisz
**Repressed Hoffman
Promoter UCSC
PromoterFlanking Hoffman
**Intron UCSC
H3K9ac Trynka
**H3K4me3 Trynka
**H3K4me1 Trynka
**H3K27ac PGC2
H3K27ac Hnisz
**FetalDHS Trynka
**Enhancer Hoffman
Enhancer Andersson
DHS Trynka
DGF ENCODE
CTCF Hoffman
Conserved LindbladToh
Coding UCSC
−10
0
10
20
Enrichment (prop h2 / prop snps)

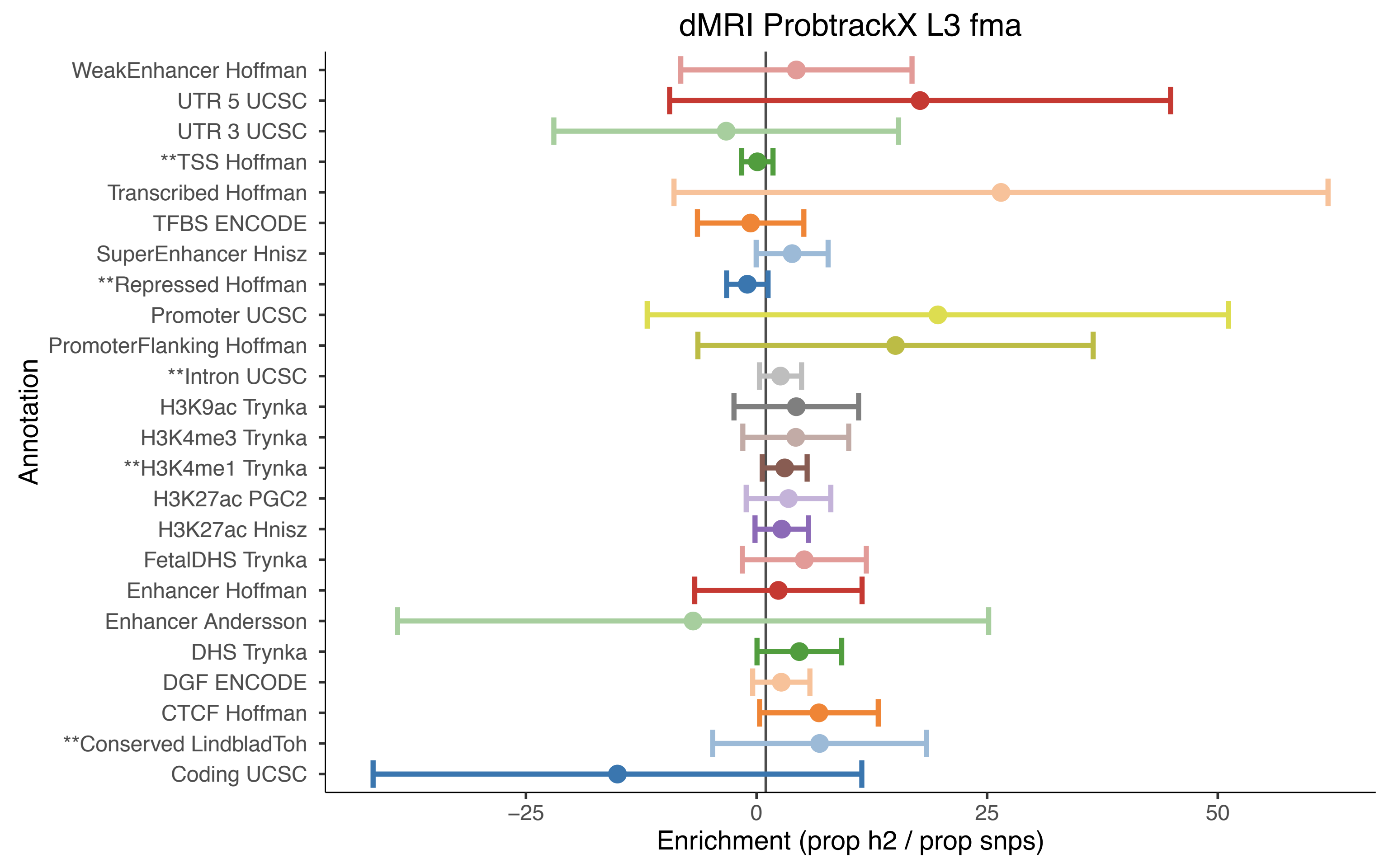
dMRI ProbtrackX L3 fma
WeakEnhancer Hoffman
UTR 5 UCSC
UTR 3 UCSC
**TSS Hoffman
Transcribed Hoffman
TFBS ENCODE
SuperEnhancer Hnisz
**Repressed Hoffman
Promoter UCSC
PromoterFlanking Hoffman
**Intron UCSC
H3K9ac Trynka
H3K4me3 Trynka
**H3K4me1 Trynka
H3K27ac PGC2
H3K27ac Hnisz
FetalDHS Trynka
Enhancer Hoffman
Enhancer Andersson
DHS Trynka
DGF ENCODE
CTCF Hoffman
**Conserved LindbladToh
Coding UCSC
Annotation
−25
0
25
50
Enrichment (prop h2 / prop snps)

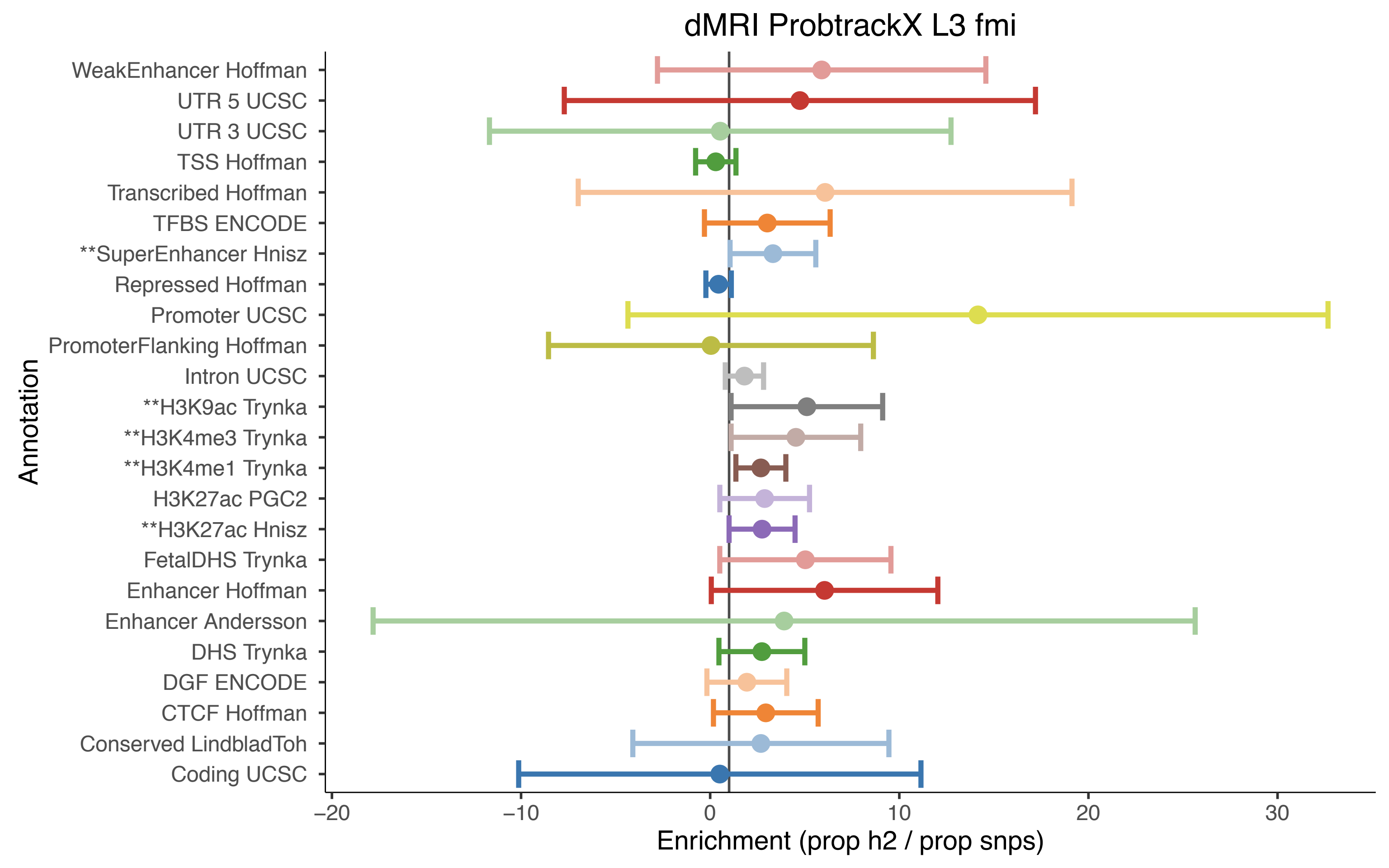

dMRI ProbtrackX L3 fmi
Annotation
WeakEnhancer Hoffman
UTR 5 UCSC
UTR 3 UCSC
TSS Hoffman
Transcribed Hoffman
TFBS ENCODE
**SuperEnhancer Hnisz
Repressed Hoffman
Promoter UCSC
PromoterFlanking Hoffman
Intron UCSC
**H3K9ac Trynka
**H3K4me3 Trynka
**H3K4me1 Trynka
H3K27ac PGC2
**H3K27ac Hnisz
FetalDHS Trynka
Enhancer Hoffman
Enhancer Andersson
DHS Trynka
DGF ENCODE
CTCF Hoffman
Conserved LindbladToh
Coding UCSC
−20
−10
0
10
20
30
Enrichment (prop h2 / prop snps)

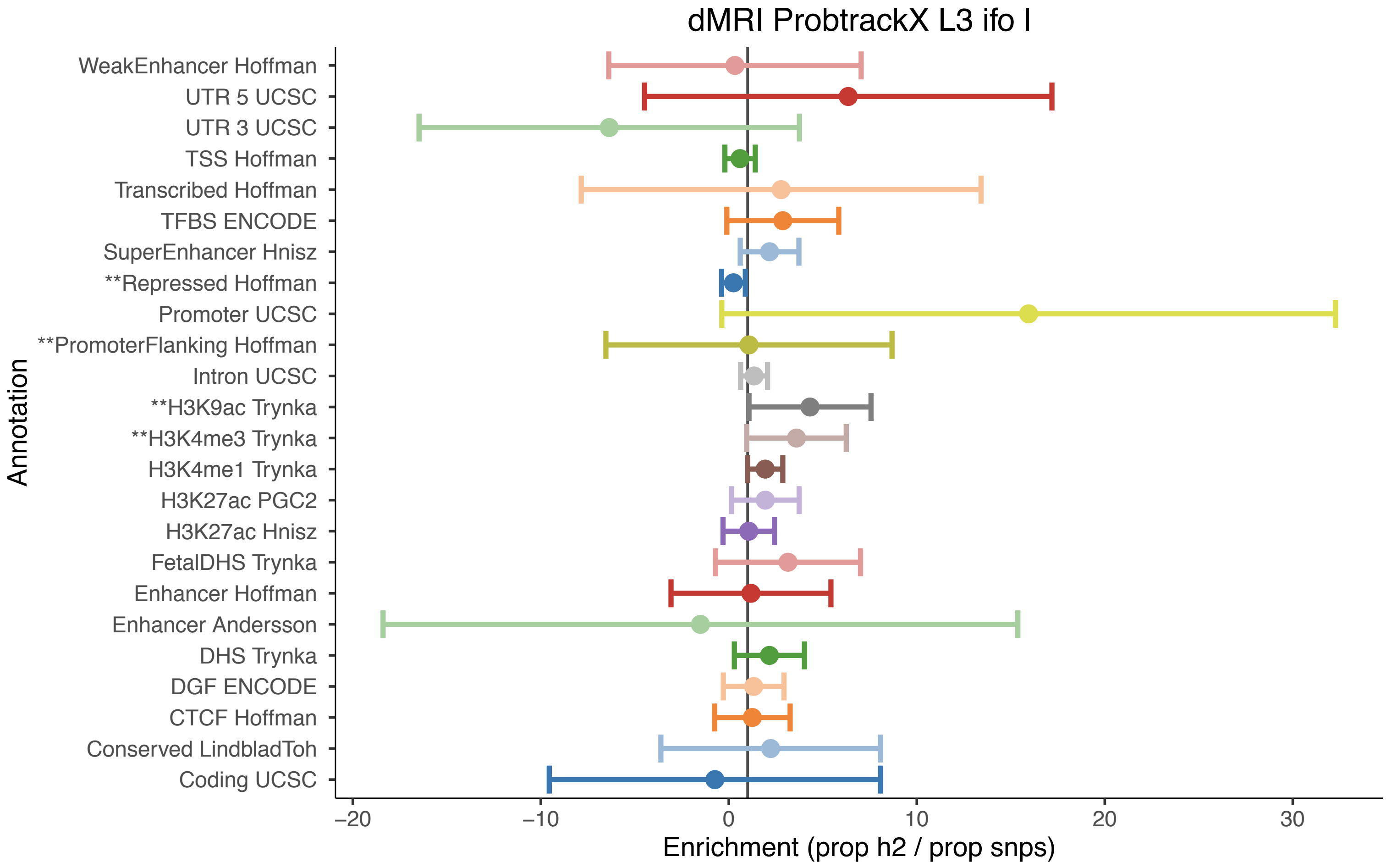
dMRI ProbtrackX L3 ifo l
Annotation
WeakEnhancer Hoffman
UTR 5 UCSC
UTR 3 UCSC
TSS Hoffman
Transcribed Hoffman
TFBS ENCODE
SuperEnhancer Hnisz
**Repressed Hoffman
Promoter UCSC
**PromoterFlanking Hoffman
Intron UCSC
**H3K9ac Trynka
**H3K4me3 Trynka
H3K4me1 Trynka
H3K27ac PGC2
H3K27ac Hnisz
FetalDHS Trynka
Enhancer Hoffman
Enhancer Andersson
DHS Trynka
DGF ENCODE
CTCF Hoffman
Conserved LindbladToh
Coding UCSC
−20
−10
0
10
20
30
Enrichment (prop h2 / prop snps)

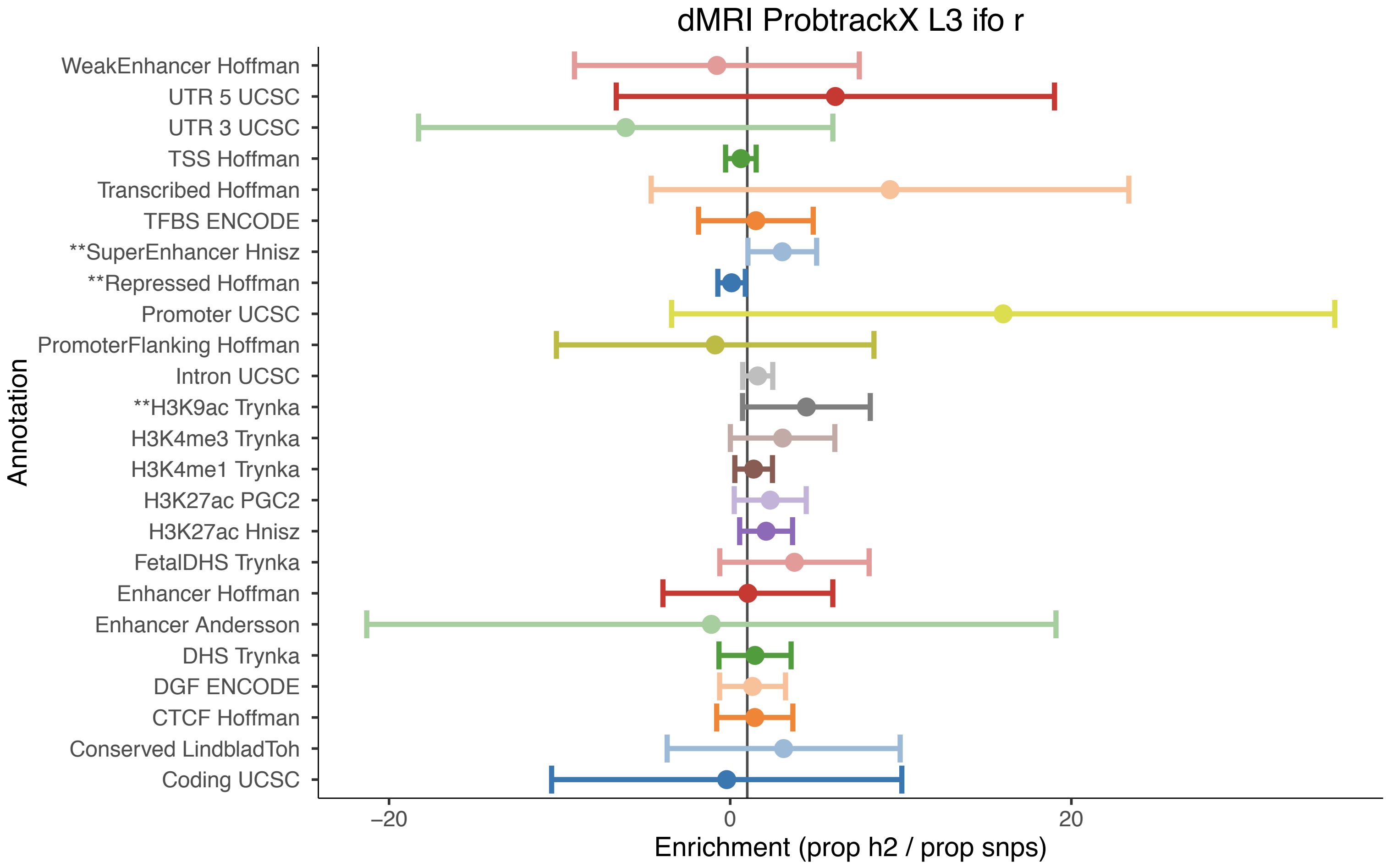

dMRI ProbtrackX L3 ifo r
WeakEnhancer Hoffman
UTR 5 UCSC
UTR 3 UCSC
TSS Hoffman
Transcribed Hoffman
TFBS ENCODE
**SuperEnhancer Hnisz
**Repressed Hoffman
Promoter UCSC
PromoterFlanking Hoffman
Intron UCSC
**H3K9ac Trynka
H3K4me3 Trynka
H3K4me1 Trynka
H3K27ac PGC2
H3K27ac Hnisz
FetalDHS Trynka
Enhancer Hoffman
Enhancer Andersson
DHS Trynka
DGF ENCODE
CTCF Hoffman
Conserved LindbladToh
Coding UCSC
Annotation
−20
0
20
Enrichment (prop h2 / prop snps)

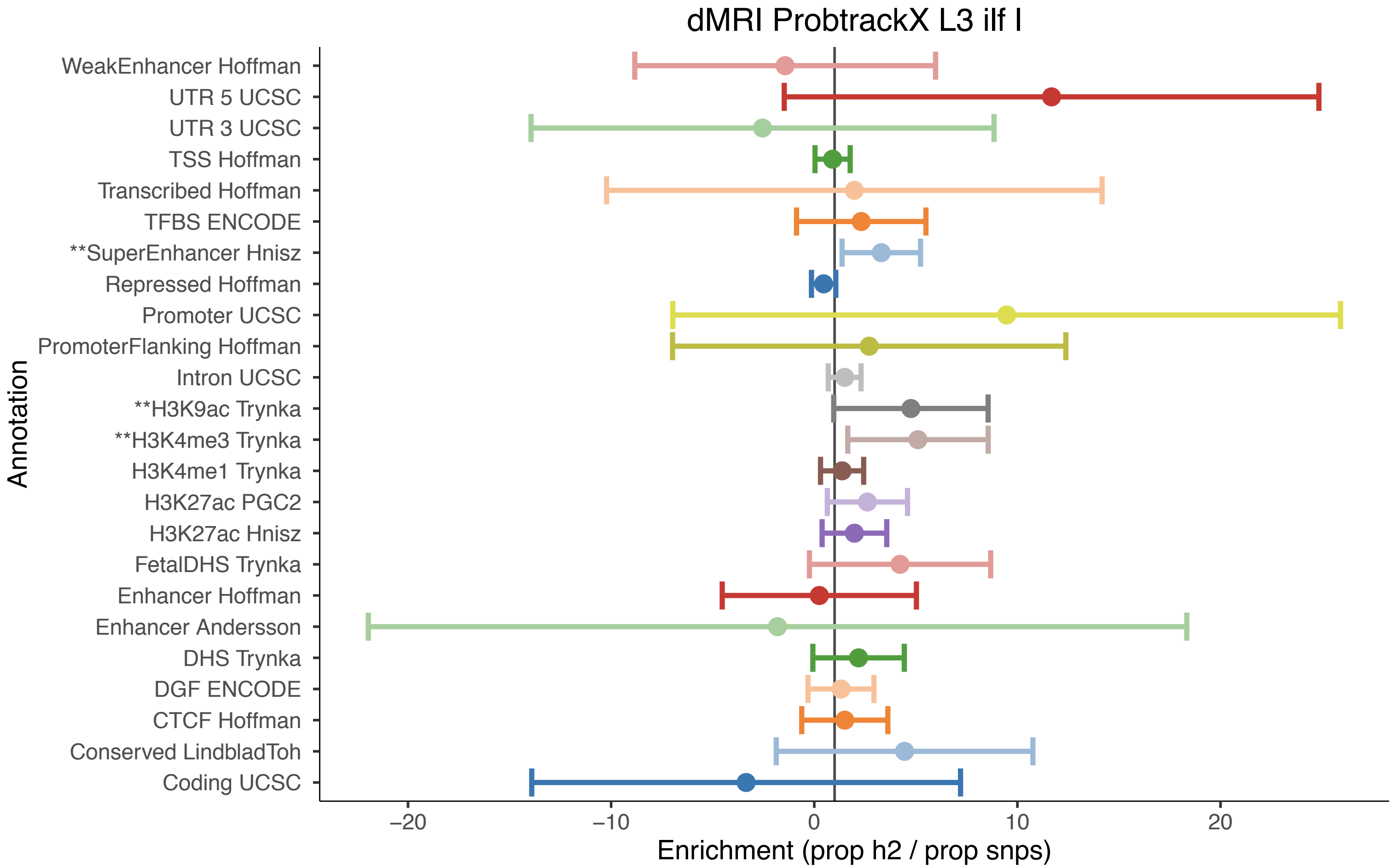
dMRI ProbtrackX L3 ilf l
WeakEnhancer Hoffman
UTR 5 UCSC
UTR 3 UCSC
TSS Hoffman
Transcribed Hoffman
TFBS ENCODE
**SuperEnhancer Hnisz
Repressed Hoffman
Promoter UCSC
PromoterFlanking Hoffman
Intron UCSC
**H3K9ac Trynka
**H3K4me3 Trynka
H3K4me1 Trynka
H3K27ac PGC2
H3K27ac Hnisz
FetalDHS Trynka
Enhancer Hoffman
Enhancer Andersson
DHS Trynka
DGF ENCODE
CTCF Hoffman
Conserved LindbladToh
Coding UCSC
Annotation
−20
−10
0
10
20
Enrichment (prop h2 / prop snps)

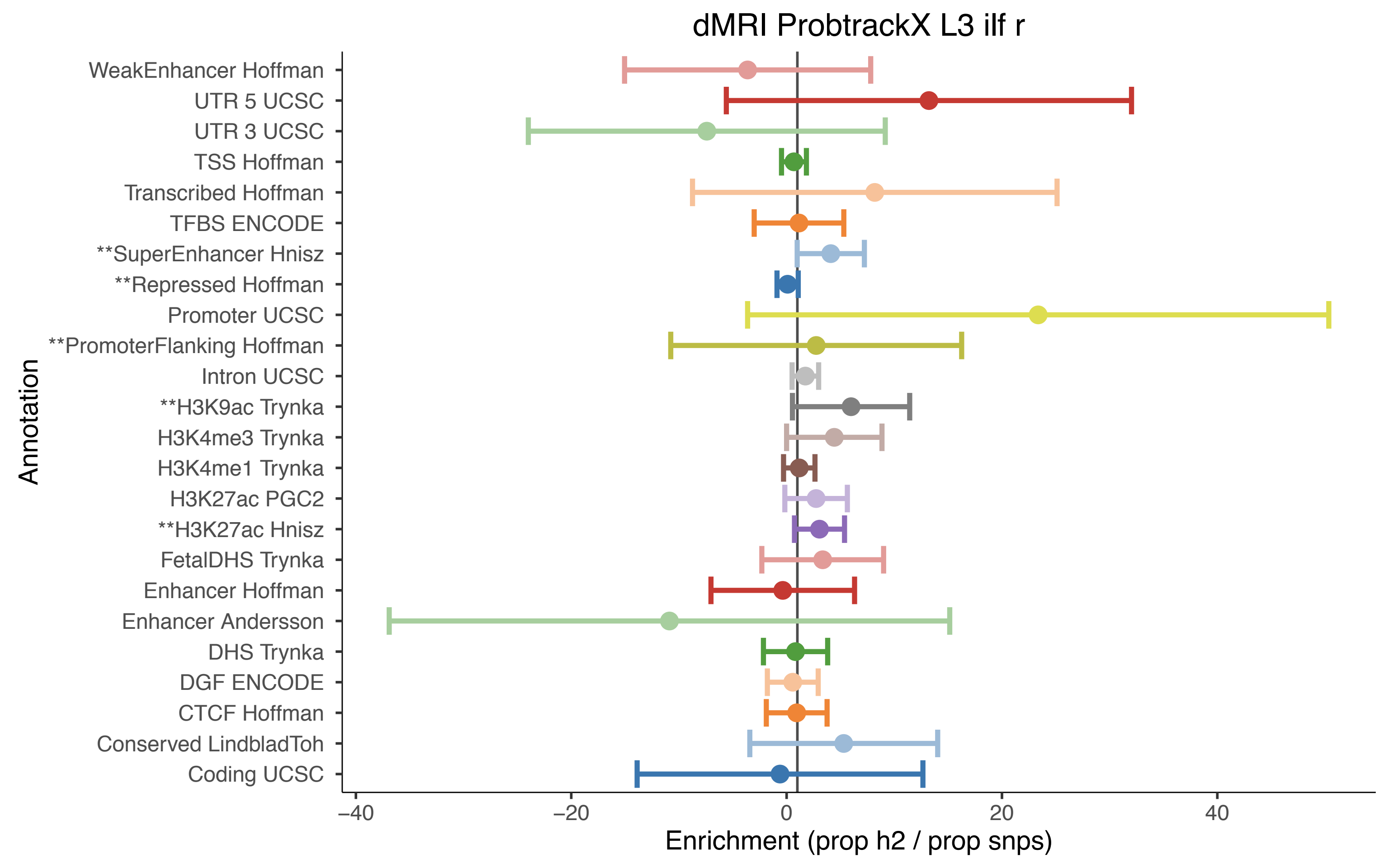
dMRI ProbtrackX L3 ilf r
WeakEnhancer Hoffman
UTR 5 UCSC
UTR 3 UCSC
TSS Hoffman
Transcribed Hoffman
TFBS ENCODE
**SuperEnhancer Hnisz
**Repressed Hoffman
Promoter UCSC
**PromoterFlanking Hoffman
Intron UCSC
**H3K9ac Trynka
H3K4me3 Trynka
H3K4me1 Trynka
H3K27ac PGC2
**H3K27ac Hnisz
FetalDHS Trynka
Enhancer Hoffman
Enhancer Andersson
DHS Trynka
DGF ENCODE
CTCF Hoffman
Conserved LindbladToh
Coding UCSC
Annotation
−40
−20
0
20
40
Enrichment (prop h2 / prop snps)

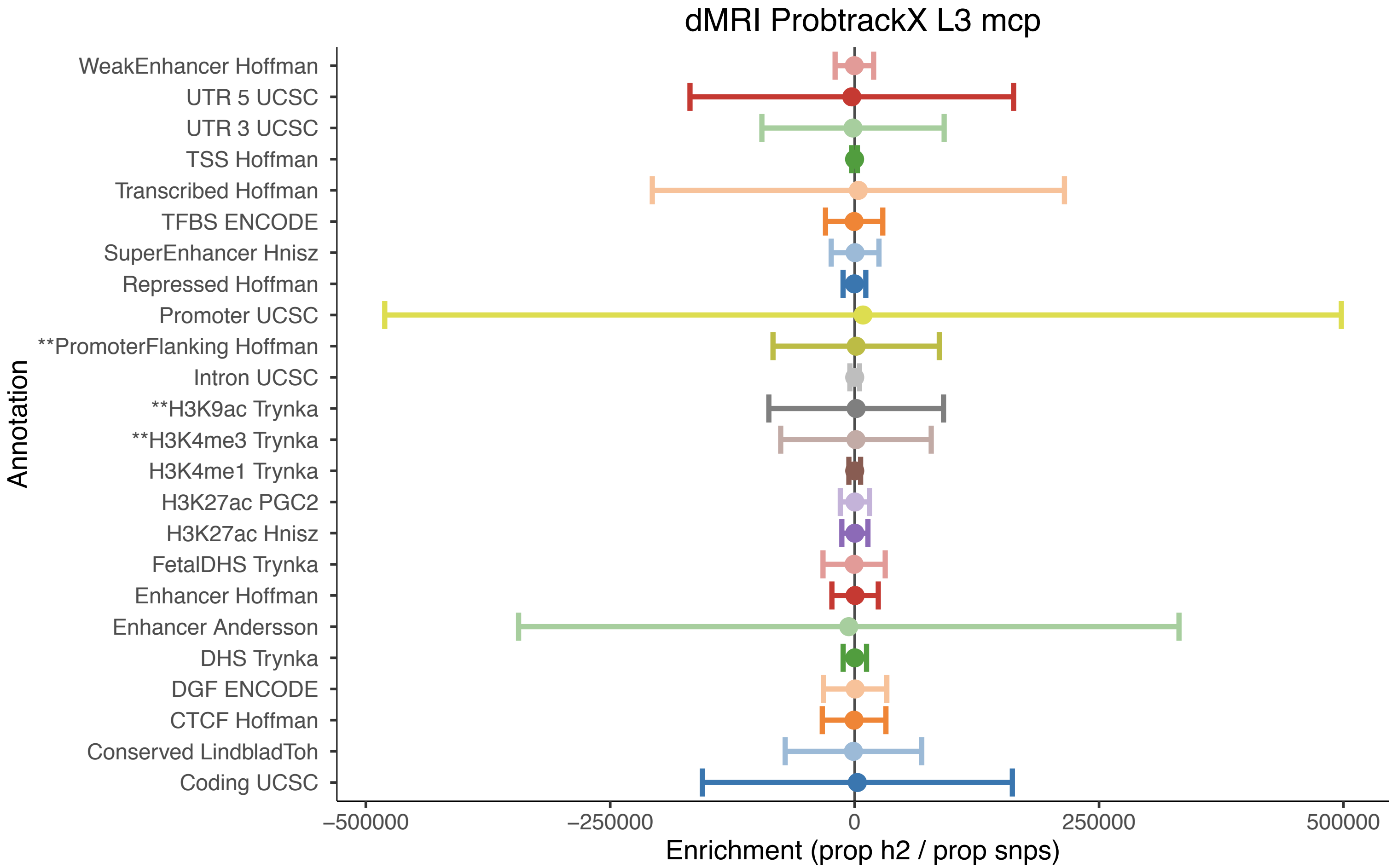
dMRI ProbtrackX L3 mcp
Annotation
WeakEnhancer Hoffman
UTR 5 UCSC
UTR 3 UCSC
TSS Hoffman
Transcribed Hoffman
TFBS ENCODE
SuperEnhancer Hnisz
Repressed Hoffman
Promoter UCSC
**PromoterFlanking Hoffman
Intron UCSC
**H3K9ac Trynka
**H3K4me3 Trynka
H3K4me1 Trynka
H3K27ac PGC2
H3K27ac Hnisz
FetalDHS Trynka
Enhancer Hoffman
Enhancer Andersson
DHS Trynka
DGF ENCODE
CTCF Hoffman
Conserved LindbladToh
Coding UCSC
−500000
−250000
0
250000
500000
Enrichment (prop h2 / prop snps)

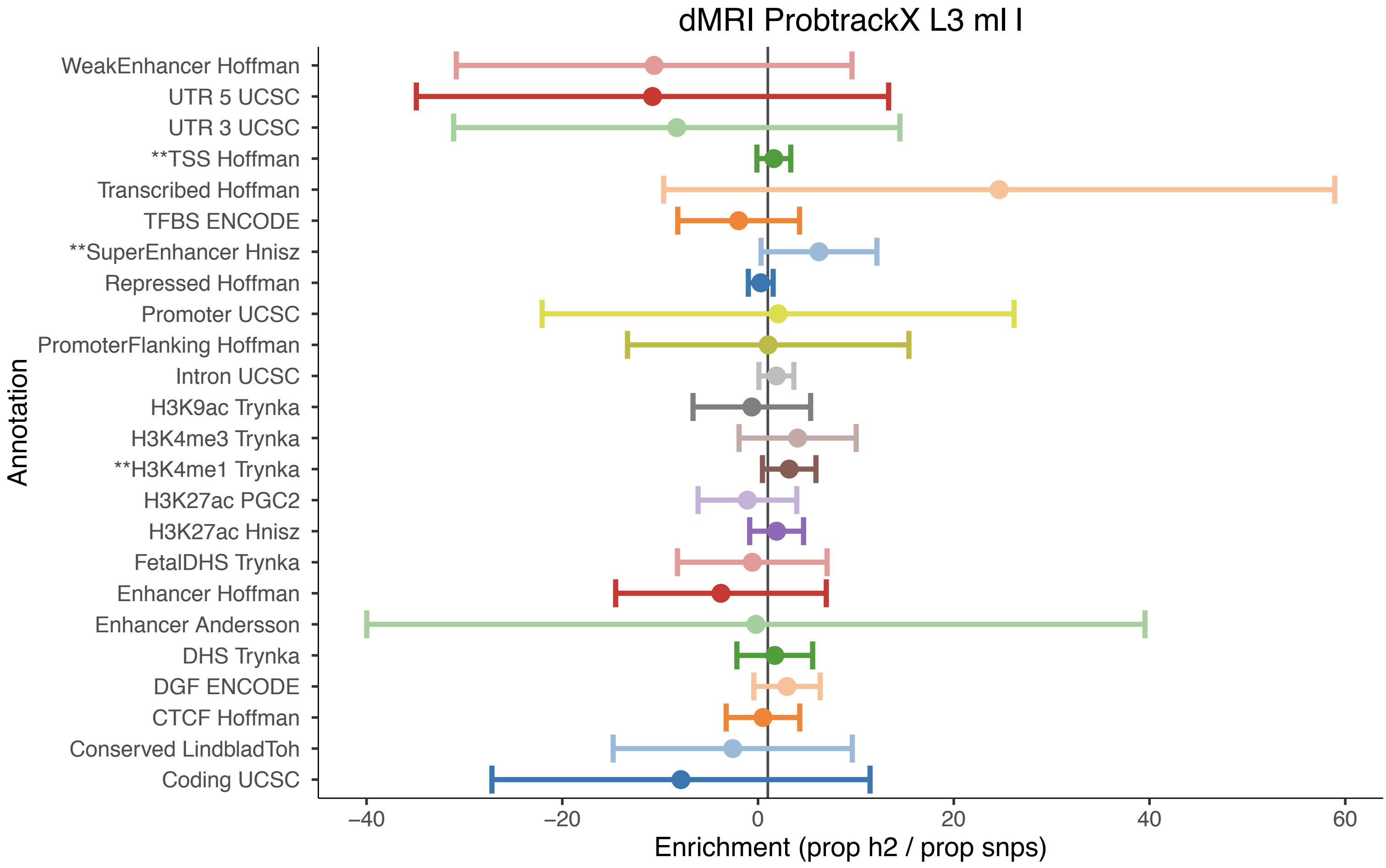

dMRI ProbtrackX L3 ml l
WeakEnhancer Hoffman
UTR 5 UCSC
UTR 3 UCSC
**TSS Hoffman
Transcribed Hoffman
TFBS ENCODE
**SuperEnhancer Hnisz
Repressed Hoffman
Promoter UCSC
PromoterFlanking Hoffman
Intron UCSC
H3K9ac Trynka
H3K4me3 Trynka
**H3K4me1 Trynka
H3K27ac PGC2
H3K27ac Hnisz
FetalDHS Trynka
Enhancer Hoffman
Enhancer Andersson
DHS Trynka
DGF ENCODE
CTCF Hoffman
Conserved LindbladToh
Coding UCSC
Annotation
−40
−20
0
20
40
60
Enrichment (prop h2 / prop snps)

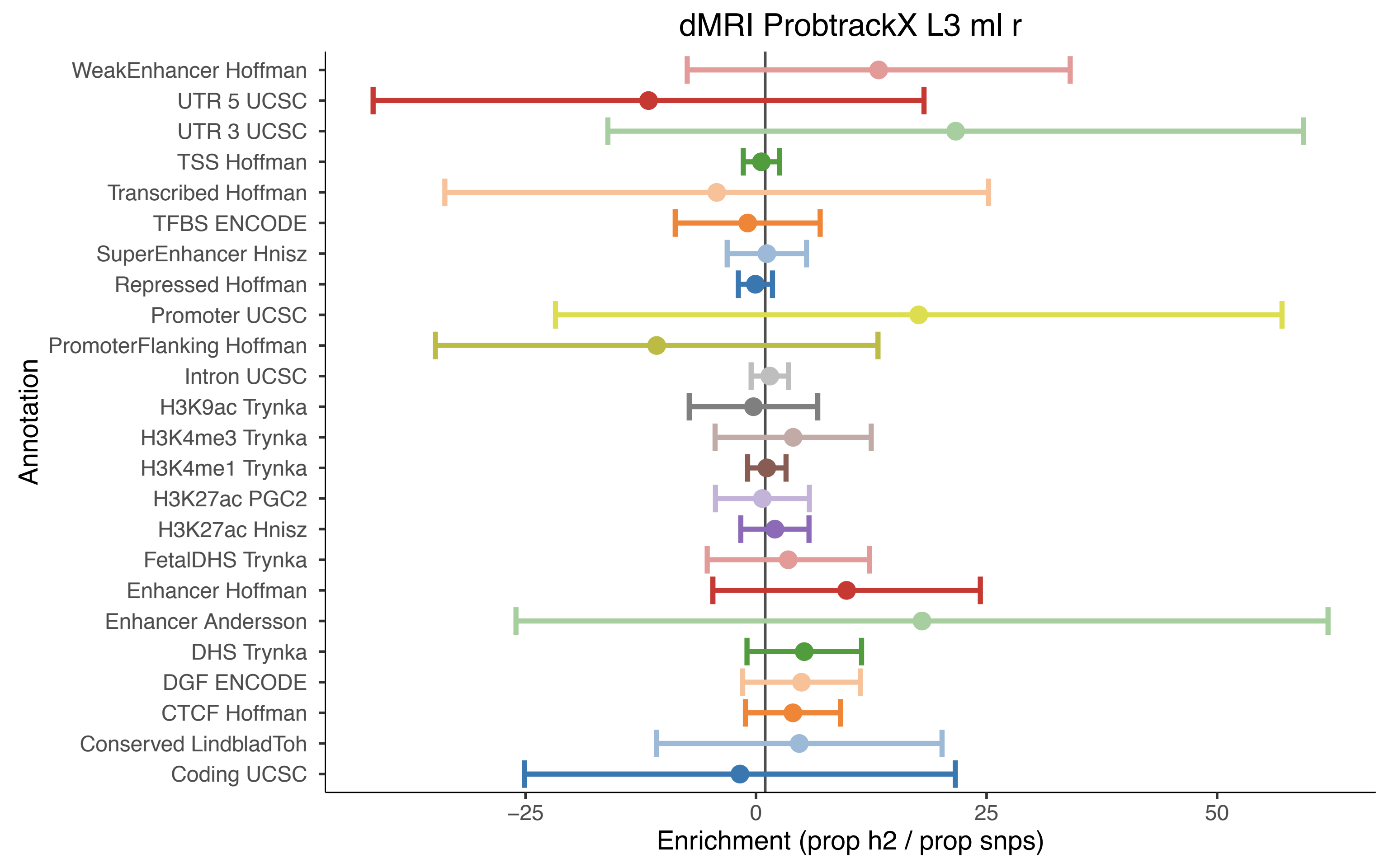

dMRI ProbtrackX L3 ml r
WeakEnhancer Hoffman
UTR 5 UCSC
UTR 3 UCSC
TSS Hoffman
Transcribed Hoffman
TFBS ENCODE
SuperEnhancer Hnisz
Repressed Hoffman
Promoter UCSC
PromoterFlanking Hoffman
Intron UCSC
H3K9ac Trynka
H3K4me3 Trynka
H3K4me1 Trynka
H3K27ac PGC2
H3K27ac Hnisz
FetalDHS Trynka
Enhancer Hoffman
Enhancer Andersson
DHS Trynka
DGF ENCODE
CTCF Hoffman
Conserved LindbladToh
Coding UCSC
Annotation
−25
0
25
50
Enrichment (prop h2 / prop snps)

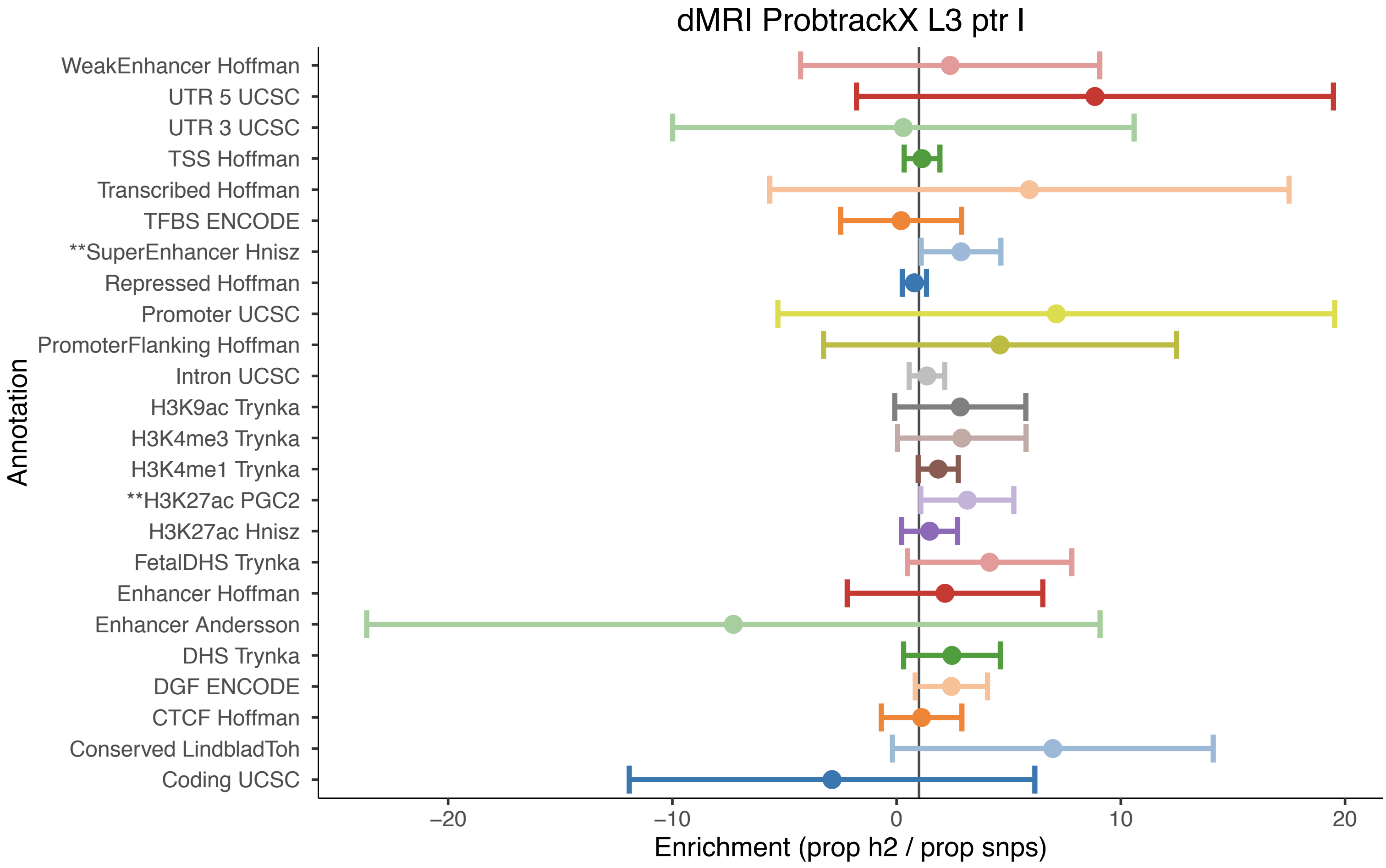
dMRI ProbtrackX L3 ptr I
Annotation
WeakEnhancer Hoffman
UTR 5 UCSC
UTR 3 UCSC
TSS Hoffman
Transcribed Hoffman
TFBS ENCODE
**SuperEnhancer Hnisz
Repressed Hoffman
Promoter UCSC
PromoterFlanking Hoffman
Intron UCSC
H3K9ac Trynka
H3K4me3 Trynka
H3K4me1 Trynka
**H3K27ac PGC2
H3K27ac Hnisz
FetalDHS Trynka
Enhancer Hoffman
Enhancer Andersson
DHS Trynka
DGF ENCODE
CTCF Hoffman
Conserved LindbladToh
Coding UCSC
−20
−10
0
10
20
Enrichment (prop h2 / prop snps)

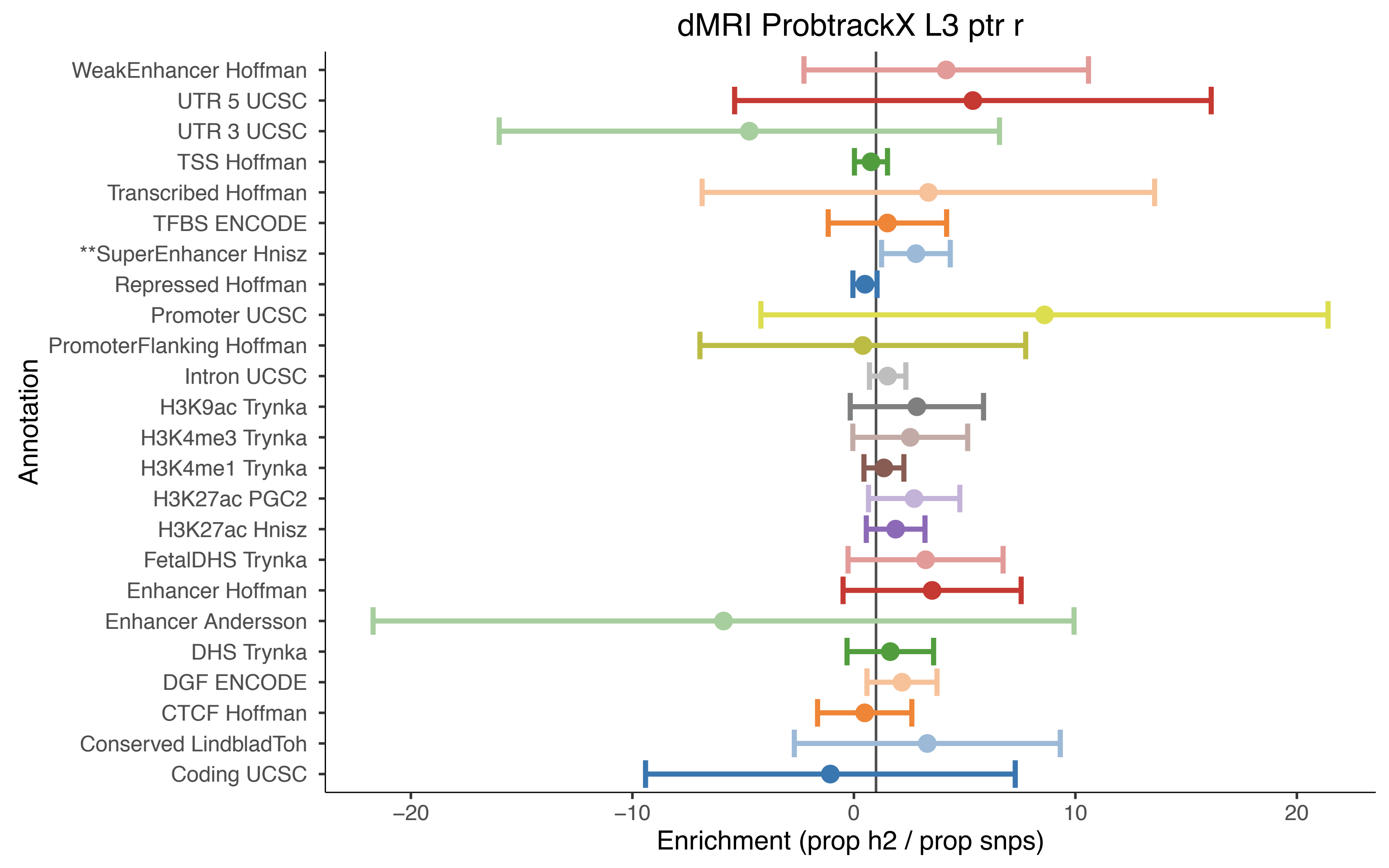

dMRI ProbtrackX L3 ptr r
WeakEnhancer Hoffman
UTR 5 UCSC
UTR 3 UCSC
TSS Hoffman
Transcribed Hoffman
TFBS ENCODE
**SuperEnhancer Hnisz
Repressed Hoffman
Promoter UCSC
PromoterFlanking Hoffman
Intron UCSC
H3K9ac Trynka
H3K4me3 Trynka
H3K4me1 Trynka
H3K27ac PGC2
H3K27ac Hnisz
FetalDHS Trynka
Enhancer Hoffman
Enhancer Andersson
DHS Trynka
DGF ENCODE
CTCF Hoffman
Conserved LindbladToh
Coding UCSC
Annotation
−20
−10
0
10
20
Enrichment (prop h2 / prop snps)

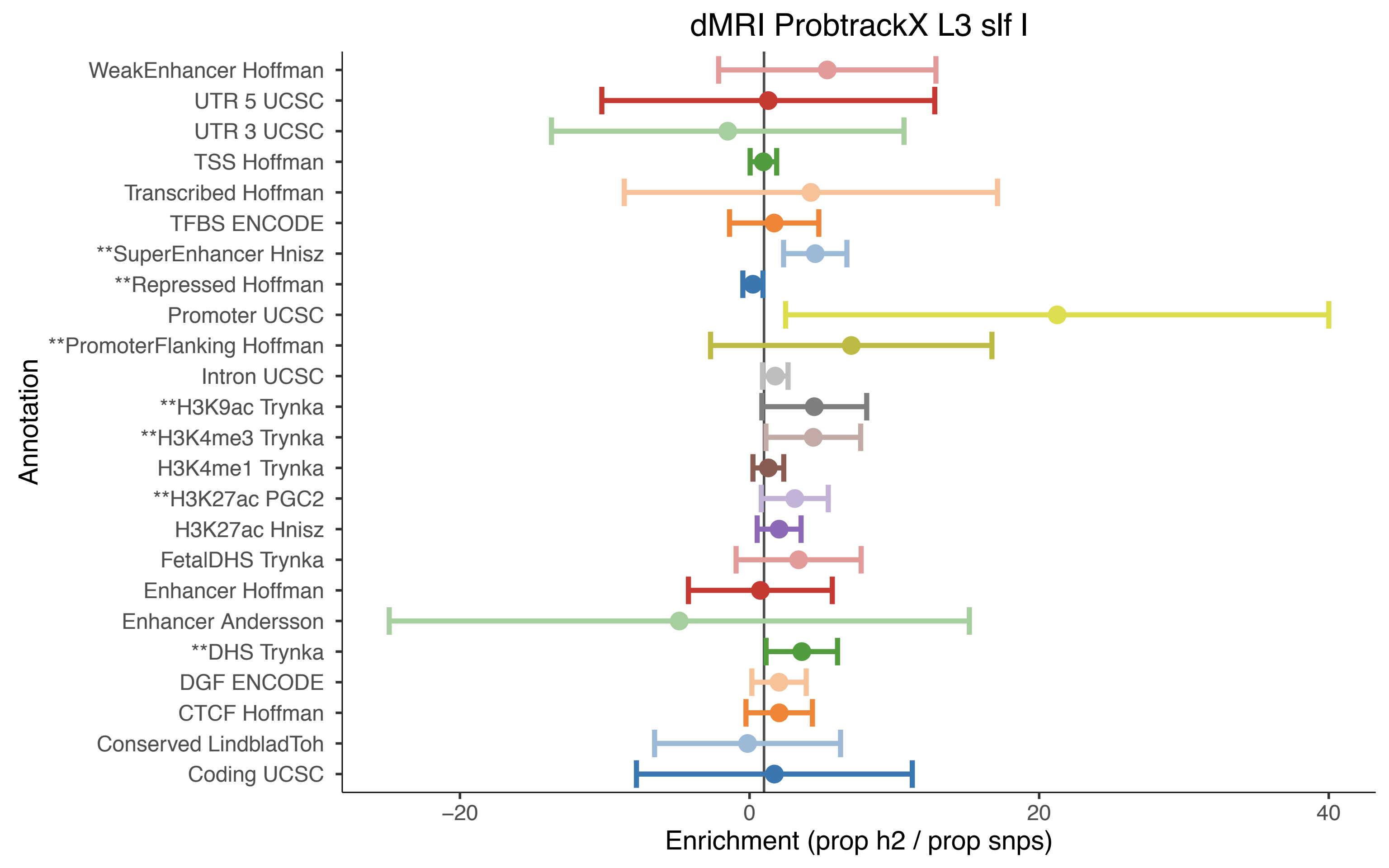
dMRI ProbtrackX L3 slf I
WeakEnhancer Hoffman
UTR 5 UCSC
UTR 3 UCSC
TSS Hoffman
Transcribed Hoffman
TFBS ENCODE
**SuperEnhancer Hnisz
**Repressed Hoffman
Promoter UCSC
**PromoterFlanking Hoffman
Intron UCSC
**H3K9ac Trynka
**H3K4me3 Trynka
H3K4me1 Trynka
**H3K27ac PGC2
H3K27ac Hnisz
FetalDHS Trynka
Enhancer Hoffman
Enhancer Andersson
**DHS Trynka
DGF ENCODE
CTCF Hoffman
Conserved LindbladToh
Coding UCSC
Annotation
−20
0
20
40
Enrichment (prop h2 / prop snps)

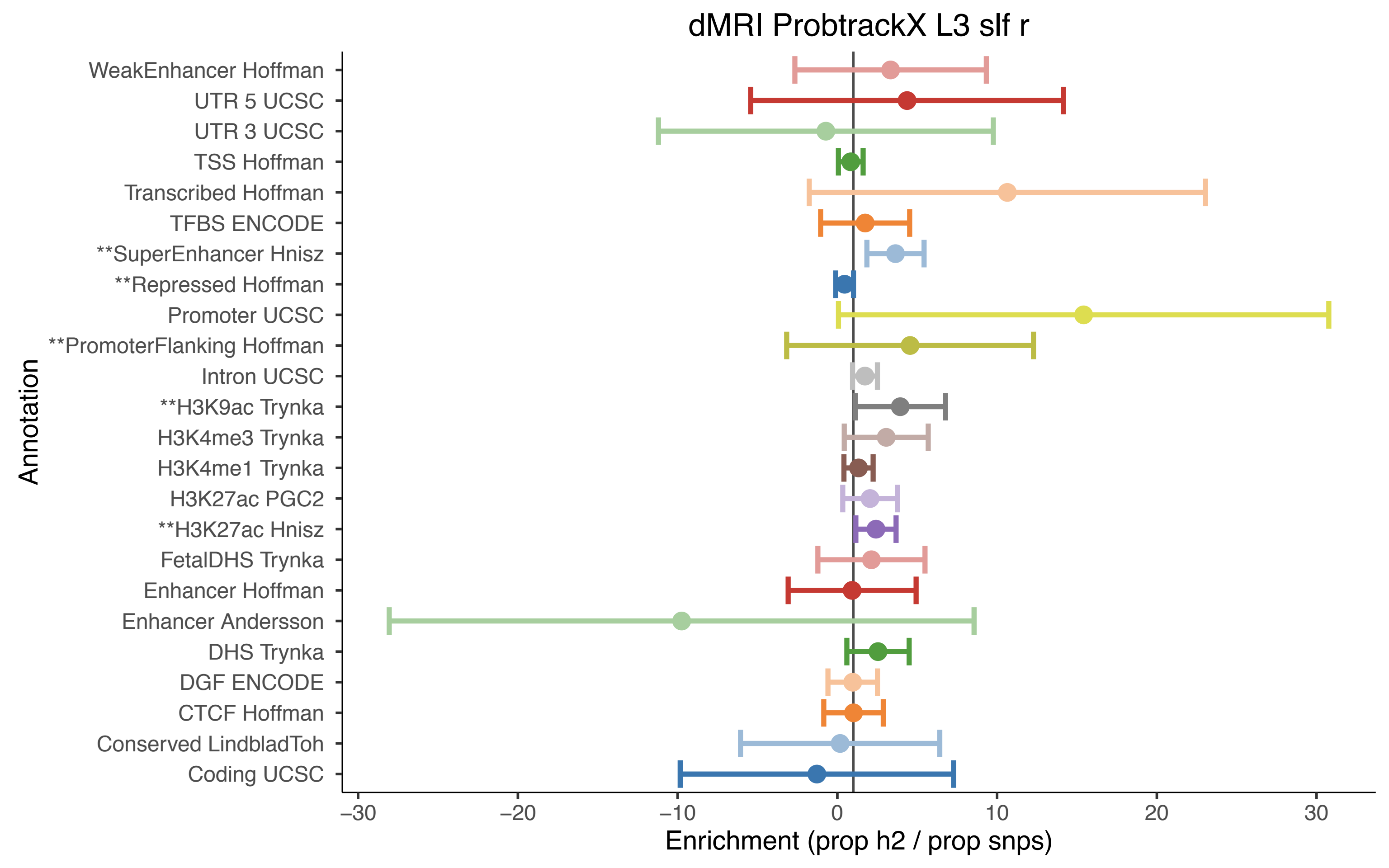

dMRI ProbtrackX L3 slf r
WeakEnhancer Hoffman
UTR 5 UCSC
UTR 3 UCSC
TSS Hoffman
Transcribed Hoffman
TFBS ENCODE
**SuperEnhancer Hnisz
**Repressed Hoffman
Promoter UCSC
**PromoterFlanking Hoffman
Intron UCSC
**H3K9ac Trynka
H3K4me3 Trynka
H3K4me1 Trynka
H3K27ac PGC2
**H3K27ac Hnisz
FetalDHS Trynka
Enhancer Hoffman
Enhancer Andersson
DHS Trynka
DGF ENCODE
CTCF Hoffman
Conserved LindbladToh
Coding UCSC
Annotation
−30
−20
−10
0
10
20
30
Enrichment (prop h2 / prop snps)

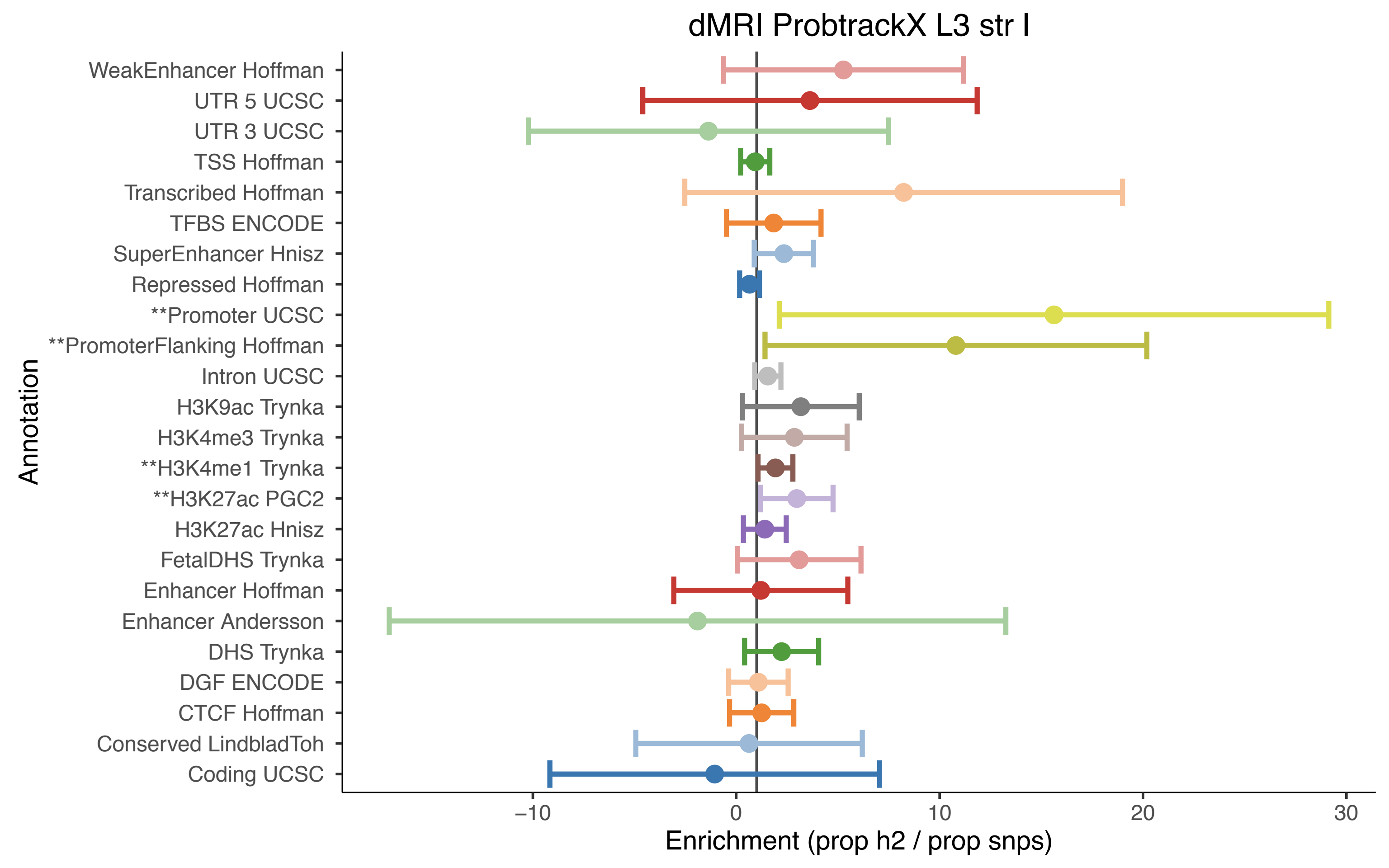

dMRI ProbtrackX L3 str I
Annotation
WeakEnhancer Hoffman
UTR 5 UCSC
UTR 3 UCSC
TSS Hoffman
Transcribed Hoffman
TFBS ENCODE
SuperEnhancer Hnisz
Repressed Hoffman
**Promoter UCSC
**PromoterFlanking Hoffman
Intron UCSC
H3K9ac Trynka
H3K4me3 Trynka
**H3K4me1 Trynka
**H3K27ac PGC2
H3K27ac Hnisz
FetalDHS Trynka
Enhancer Hoffman
Enhancer Andersson
DHS Trynka
DGF ENCODE
CTCF Hoffman
Conserved LindbladToh
Coding UCSC
−10
0
10
20
30
Enrichment (prop h2 / prop snps)

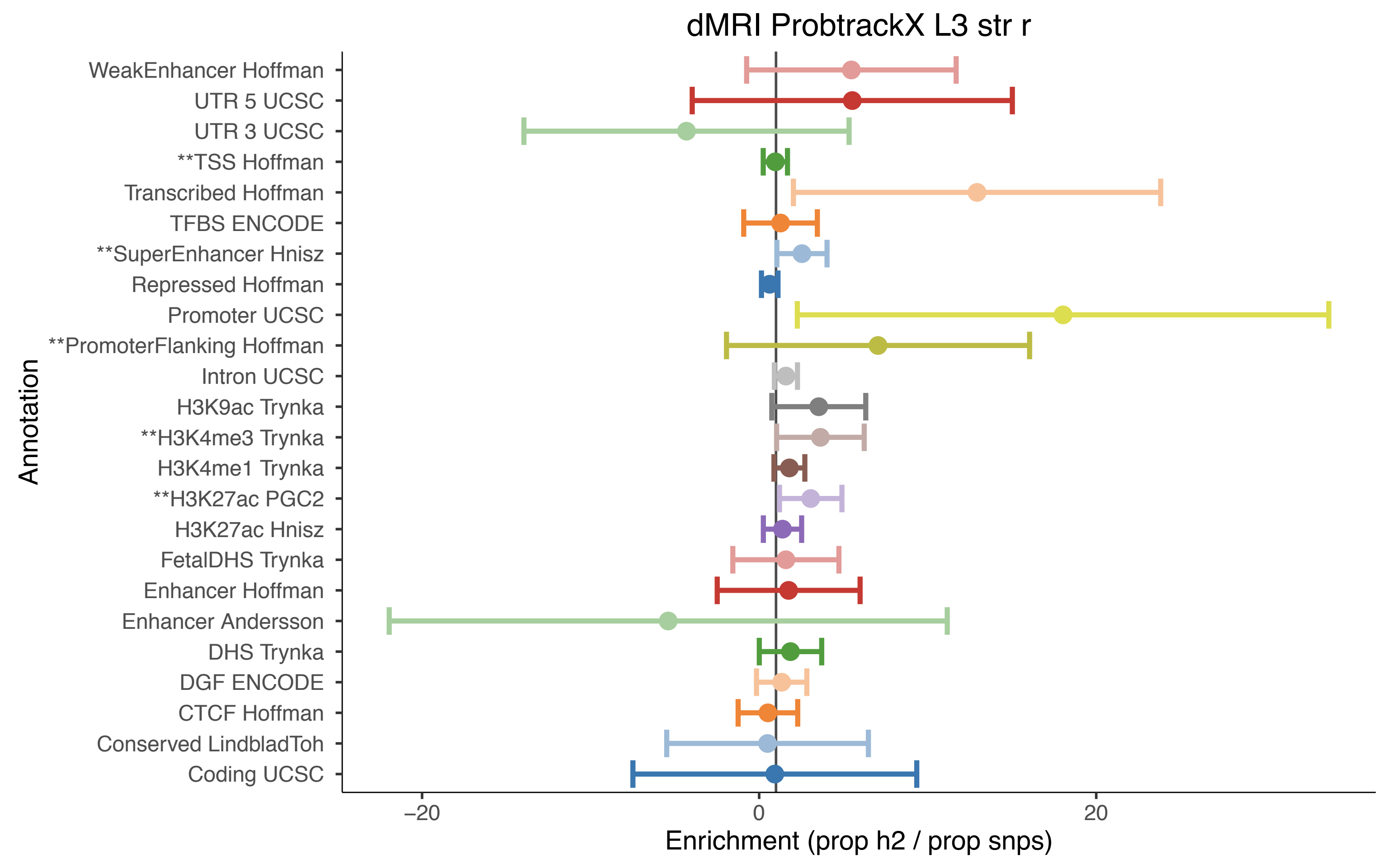

dMRI ProbtrackX L3 str r
WeakEnhancer Hoffman
UTR 5 UCSC
UTR 3 UCSC
**TSS Hoffman
Transcribed Hoffman
TFBS ENCODE
**SuperEnhancer Hnisz
Repressed Hoffman
Promoter UCSC
**PromoterFlanking Hoffman
Intron UCSC
H3K9ac Trynka
**H3K4me3 Trynka
H3K4me1 Trynka
**H3K27ac PGC2
H3K27ac Hnisz
FetalDHS Trynka
Enhancer Hoffman
Enhancer Andersson
DHS Trynka
DGF ENCODE
CTCF Hoffman
Conserved LindbladToh
Coding UCSC
Annotation
−20
0
20
Enrichment (prop h2 / prop snps)

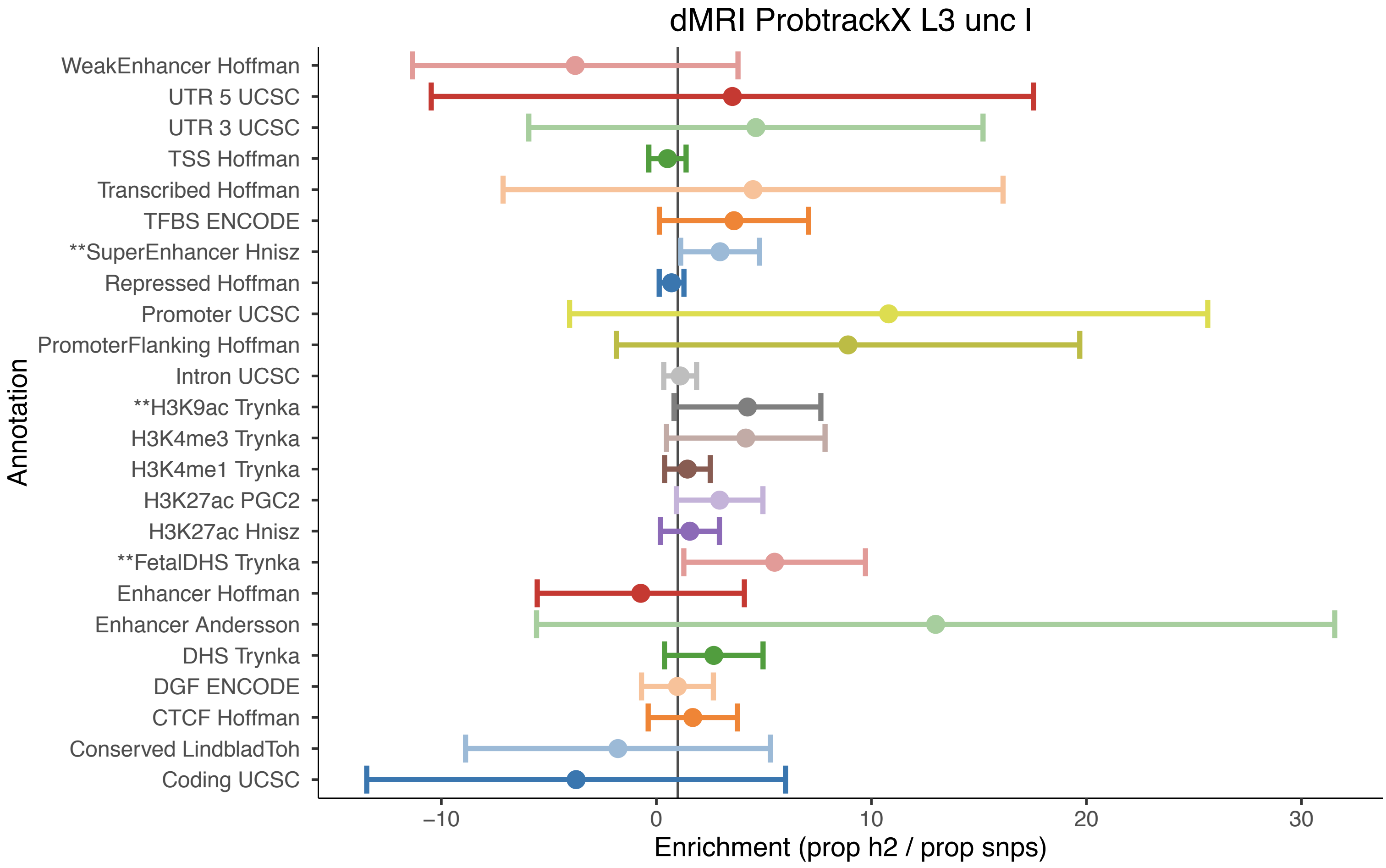

dMRI ProbtrackX L3 unc I
WeakEnhancer Hoffman
UTR 5 UCSC
UTR 3 UCSC
TSS Hoffman
Transcribed Hoffman
TFBS ENCODE
**SuperEnhancer Hnisz
Repressed Hoffman
Promoter UCSC
PromoterFlanking Hoffman
Intron UCSC
**H3K9ac Trynka
H3K4me3 Trynka
H3K4me1 Trynka
H3K27ac PGC2
H3K27ac Hnisz
**FetalDHS Trynka
Enhancer Hoffman
Enhancer Andersson
DHS Trynka
DGF ENCODE
CTCF Hoffman
Conserved LindbladToh
Coding UCSC
Annotation
−10
0
10
20
30
Enrichment (prop h2 / prop snps)

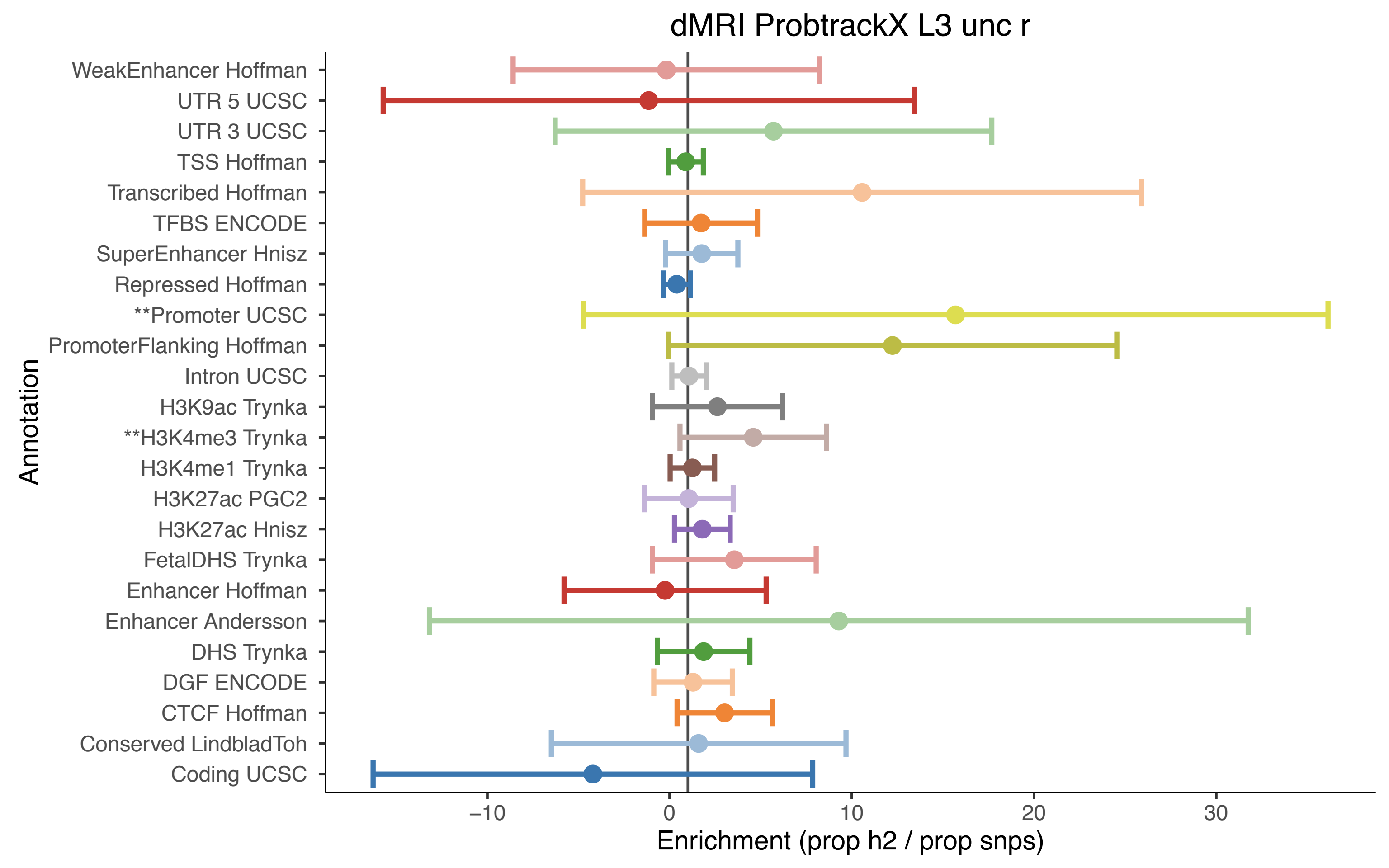
dMRI ProbtrackX L3 unc r
WeakEnhancer Hoffman
UTR 5 UCSC
UTR 3 UCSC
TSS Hoffman
Transcribed Hoffman
TFBS ENCODE
SuperEnhancer Hnisz
Repressed Hoffman
**Promoter UCSC
PromoterFlanking Hoffman
Intron UCSC
H3K9ac Trynka
**H3K4me3 Trynka
H3K4me1 Trynka
H3K27ac PGC2
H3K27ac Hnisz
FetalDHS Trynka
Enhancer Hoffman
Enhancer Andersson
DHS Trynka
DGF ENCODE
CTCF Hoffman
Conserved LindbladToh
Coding UCSC
Annotation
−10
0
10
20
30
Enrichment (prop h2 / prop snps)

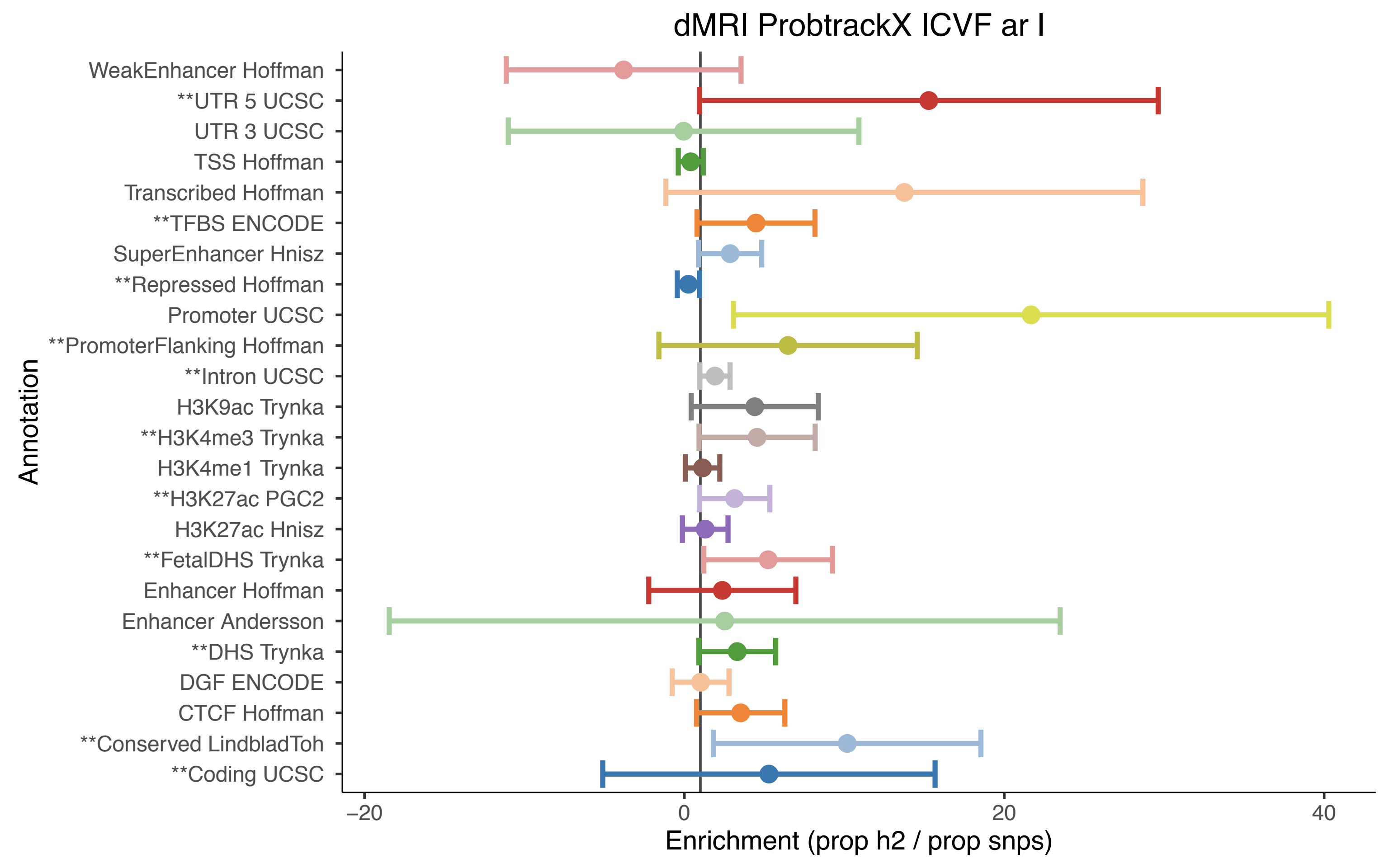

dMRI ProbtrackX ICVF ar l
WeakEnhancer Hoffman
**UTR 5 UCSC
UTR 3 UCSC
TSS Hoffman
Transcribed Hoffman
**TFBS ENCODE
SuperEnhancer Hnisz
**Repressed Hoffman
Promoter UCSC
**PromoterFlanking Hoffman
**Intron UCSC
H3K9ac Trynka
**H3K4me3 Trynka
H3K4me1 Trynka
**H3K27ac PGC2
H3K27ac Hnisz
**FetalDHS Trynka
Enhancer Hoffman
Enhancer Andersson
**DHS Trynka
DGF ENCODE
CTCF Hoffman
**Conserved LindbladToh
**Coding UCSC
Annotation
−20
0
20
40
Enrichment (prop h2 / prop snps)

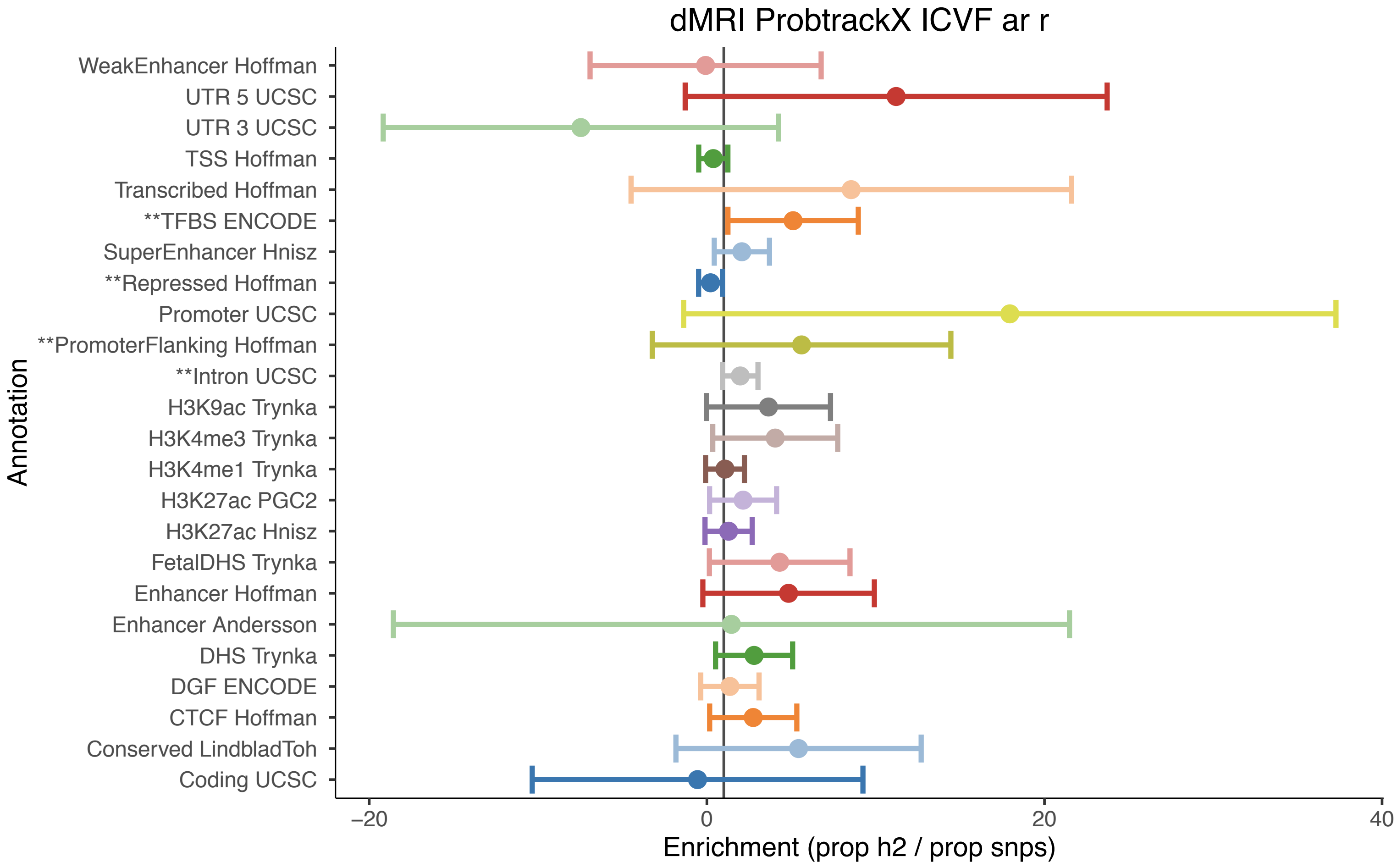

dMRI ProbtrackX ICVF ar r
Annotation
WeakEnhancer Hoffman
UTR 5 UCSC
UTR 3 UCSC
TSS Hoffman
Transcribed Hoffman
**TFBS ENCODE
SuperEnhancer Hnisz
**Repressed Hoffman
Promoter UCSC
**PromoterFlanking Hoffman
**Intron UCSC
H3K9ac Trynka
H3K4me3 Trynka
H3K4me1 Trynka
H3K27ac PGC2
H3K27ac Hnisz
FetalDHS Trynka
Enhancer Hoffman
Enhancer Andersson
DHS Trynka
DGF ENCODE
CTCF Hoffman
Conserved LindbladToh
Coding UCSC
−20
0
20
40
Enrichment (prop h2 / prop snps)

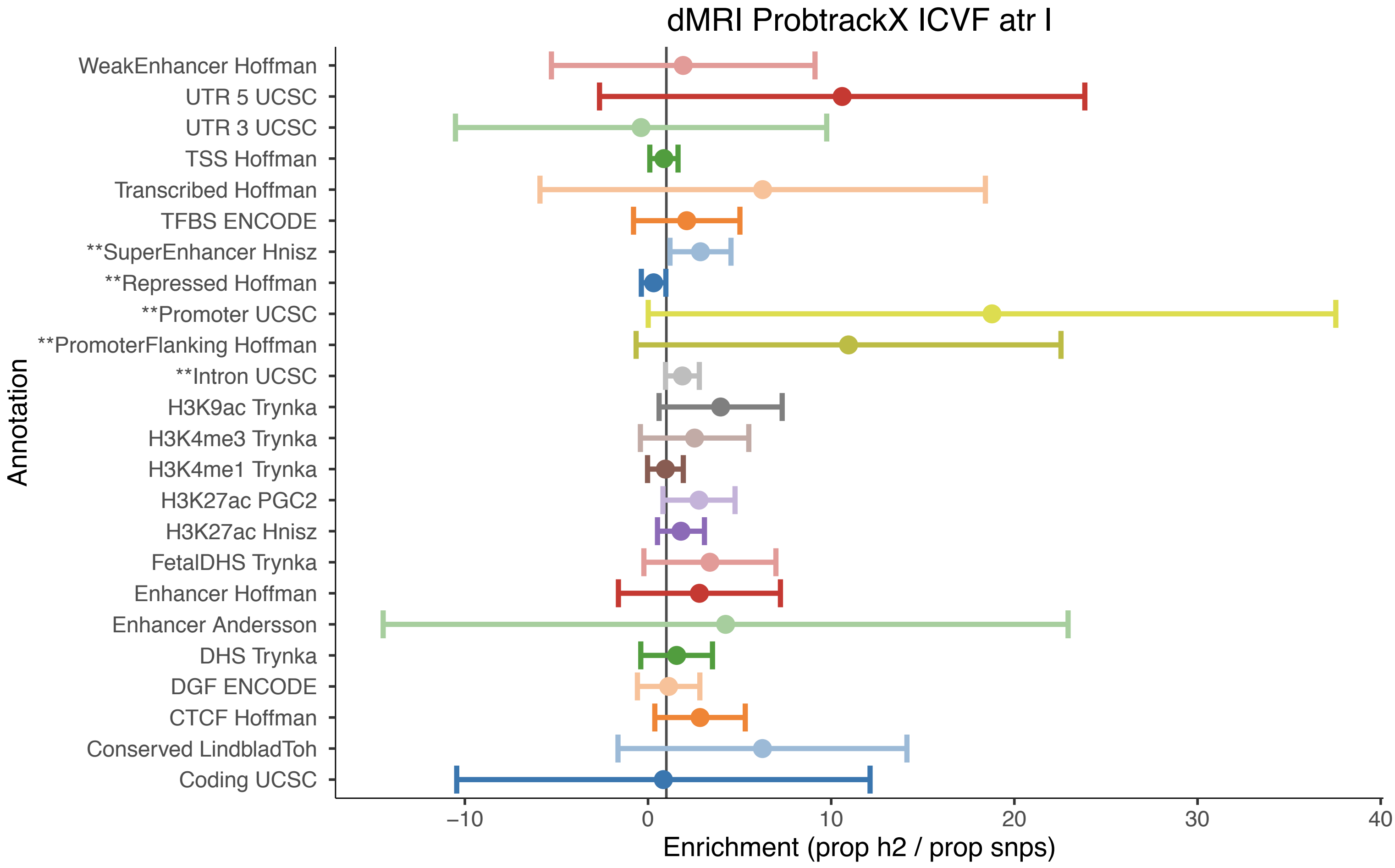

dMRI ProbtrackX ICVF atr l
WeakEnhancer Hoffman
UTR 5 UCSC
UTR 3 UCSC
TSS Hoffman
Transcribed Hoffman
TFBS ENCODE
**SuperEnhancer Hnisz
**Repressed Hoffman
**Promoter UCSC
**PromoterFlanking Hoffman
**Intron UCSC
H3K9ac Trynka
H3K4me3 Trynka
H3K4me1 Trynka
H3K27ac PGC2
H3K27ac Hnisz
FetalDHS Trynka
Enhancer Hoffman
Enhancer Andersson
DHS Trynka
DGF ENCODE
CTCF Hoffman
Conserved LindbladToh
Coding UCSC
Annotation
−10
0
10
20
30
40
Enrichment (prop h2 / prop snps)

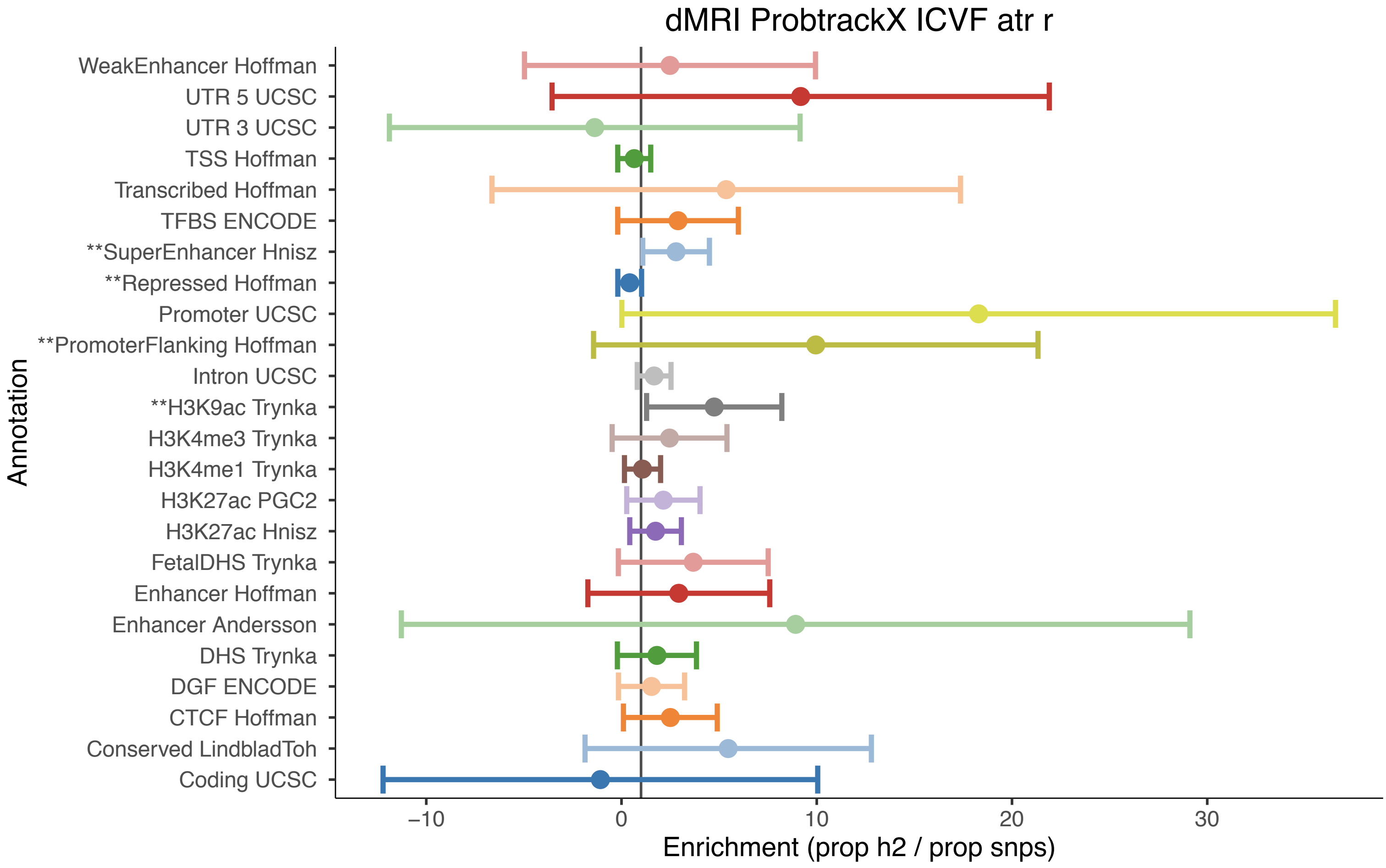

dMRI ProbtrackX ICVF atr r
Annotation
WeakEnhancer Hoffman
UTR 5 UCSC
UTR 3 UCSC
TSS Hoffman
Transcribed Hoffman
TFBS ENCODE
**SuperEnhancer Hnisz
**Repressed Hoffman
Promoter UCSC
**PromoterFlanking Hoffman
Intron UCSC
**H3K9ac Trynka
H3K4me3 Trynka
H3K4me1 Trynka
H3K27ac PGC2
H3K27ac Hnisz
FetalDHS Trynka
Enhancer Hoffman
Enhancer Andersson
DHS Trynka
DGF ENCODE
CTCF Hoffman
Conserved LindbladToh
Coding UCSC
−10
0
10
20
30
Enrichment (prop h2 / prop snps)

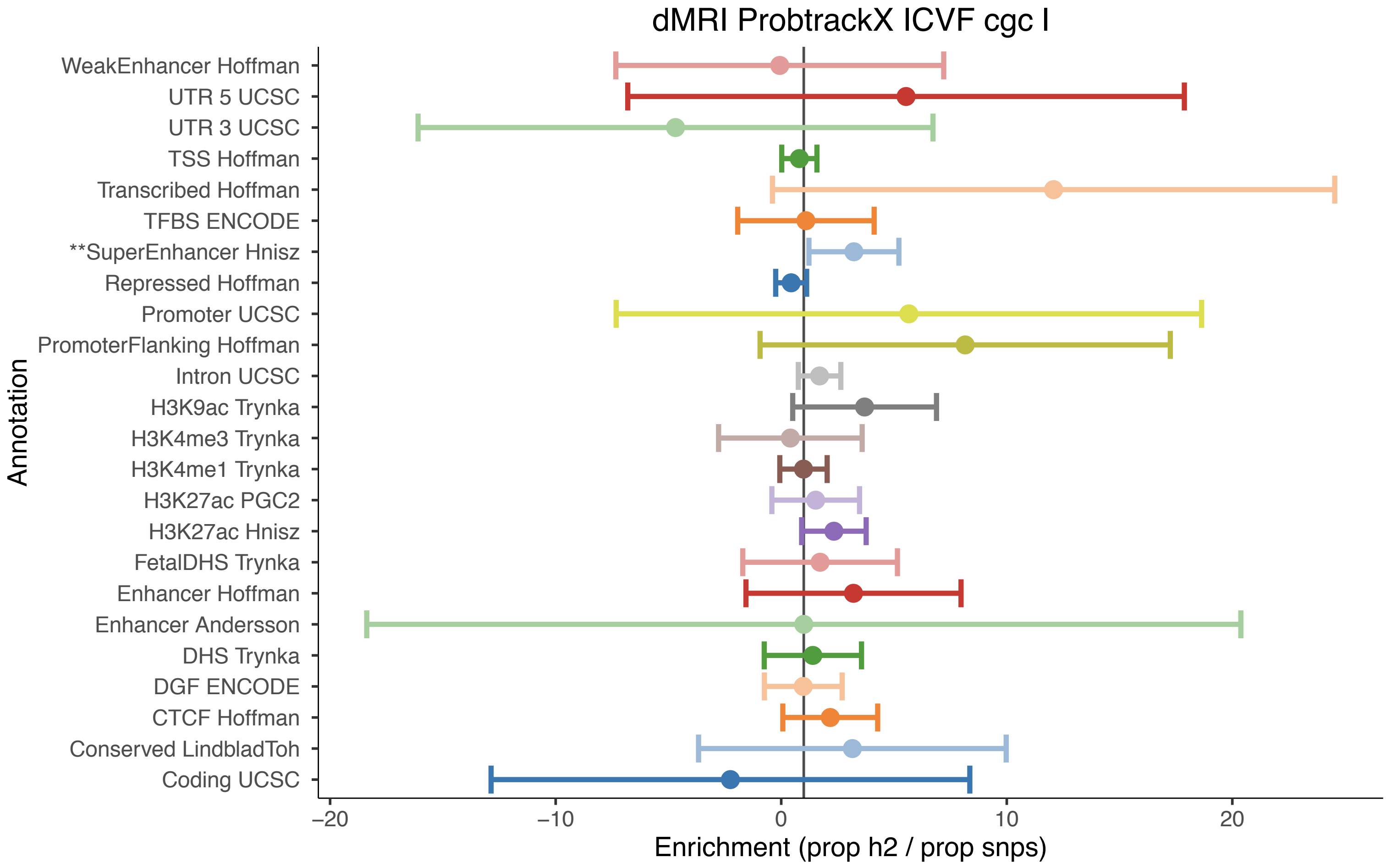
dMRI ProbtrackX ICVF cgc l
WeakEnhancer Hoffman
UTR 5 UCSC
UTR 3 UCSC
TSS Hoffman
Transcribed Hoffman
TFBS ENCODE
**SuperEnhancer Hnisz
Repressed Hoffman
Promoter UCSC
PromoterFlanking Hoffman
Intron UCSC
H3K9ac Trynka
H3K4me3 Trynka
H3K4me1 Trynka
H3K27ac PGC2
H3K27ac Hnisz
FetalDHS Trynka
Enhancer Hoffman
Enhancer Andersson
DHS Trynka
DGF ENCODE
CTCF Hoffman
Conserved LindbladToh
Coding UCSC
Annotation
−20
−10
0
10
20
Enrichment (prop h2 / prop snps)

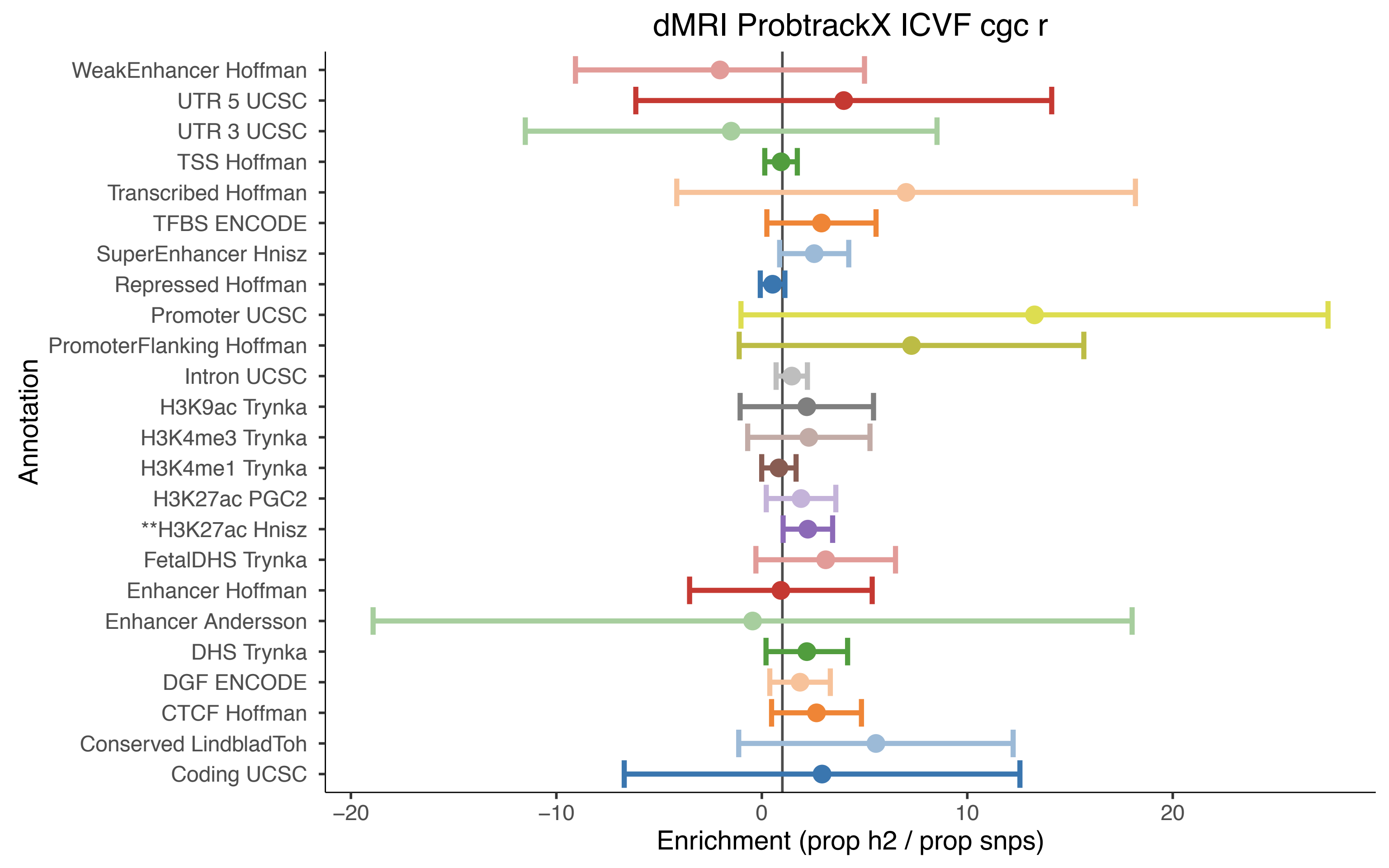
dMRI ProbtrackX ICVF cgc r
Annotation
WeakEnhancer Hoffman
UTR 5 UCSC
UTR 3 UCSC
TSS Hoffman
Transcribed Hoffman
TFBS ENCODE
SuperEnhancer Hnisz
Repressed Hoffman
Promoter UCSC
PromoterFlanking Hoffman
Intron UCSC
H3K9ac Trynka
H3K4me3 Trynka
H3K4me1 Trynka
H3K27ac PGC2
**H3K27ac Hnisz
FetalDHS Trynka
Enhancer Hoffman
Enhancer Andersson
DHS Trynka
DGF ENCODE
CTCF Hoffman
Conserved LindbladToh
Coding UCSC
−20
−10
0
10
20
Enrichment (prop h2 / prop snps)

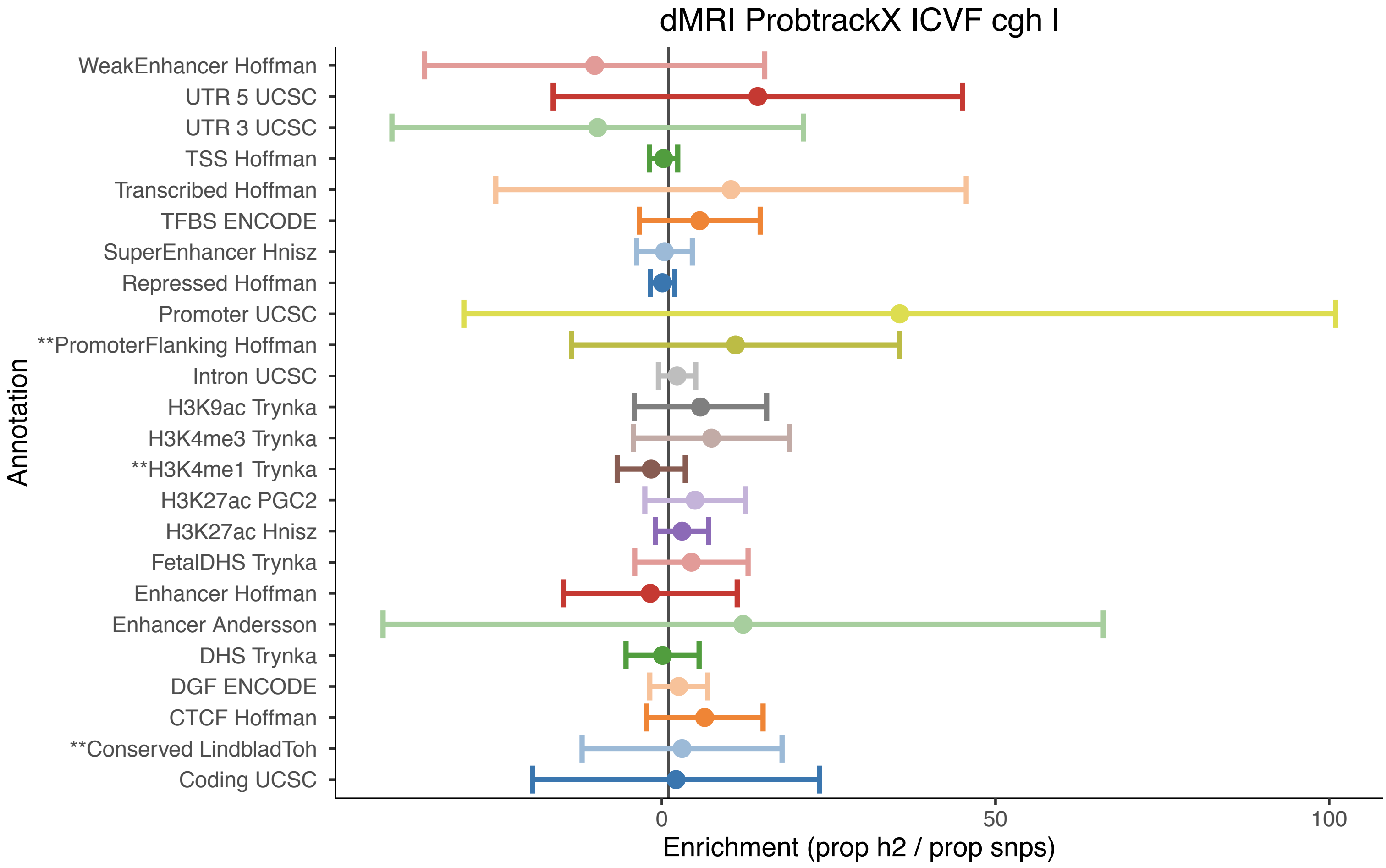

dMRI ProbtrackX ICVF cgh l
WeakEnhancer Hoffman
UTR 5 UCSC
UTR 3 UCSC
TSS Hoffman
Transcribed Hoffman
TFBS ENCODE
SuperEnhancer Hnisz
Repressed Hoffman
Promoter UCSC
**PromoterFlanking Hoffman
Intron UCSC
H3K9ac Trynka
H3K4me3 Trynka
**H3K4me1 Trynka
H3K27ac PGC2
H3K27ac Hnisz
FetalDHS Trynka
Enhancer Hoffman
Enhancer Andersson
DHS Trynka
DGF ENCODE
CTCF Hoffman
**Conserved LindbladToh
Coding UCSC
Annotation
0
50
100
Enrichment (prop h2 / prop snps)

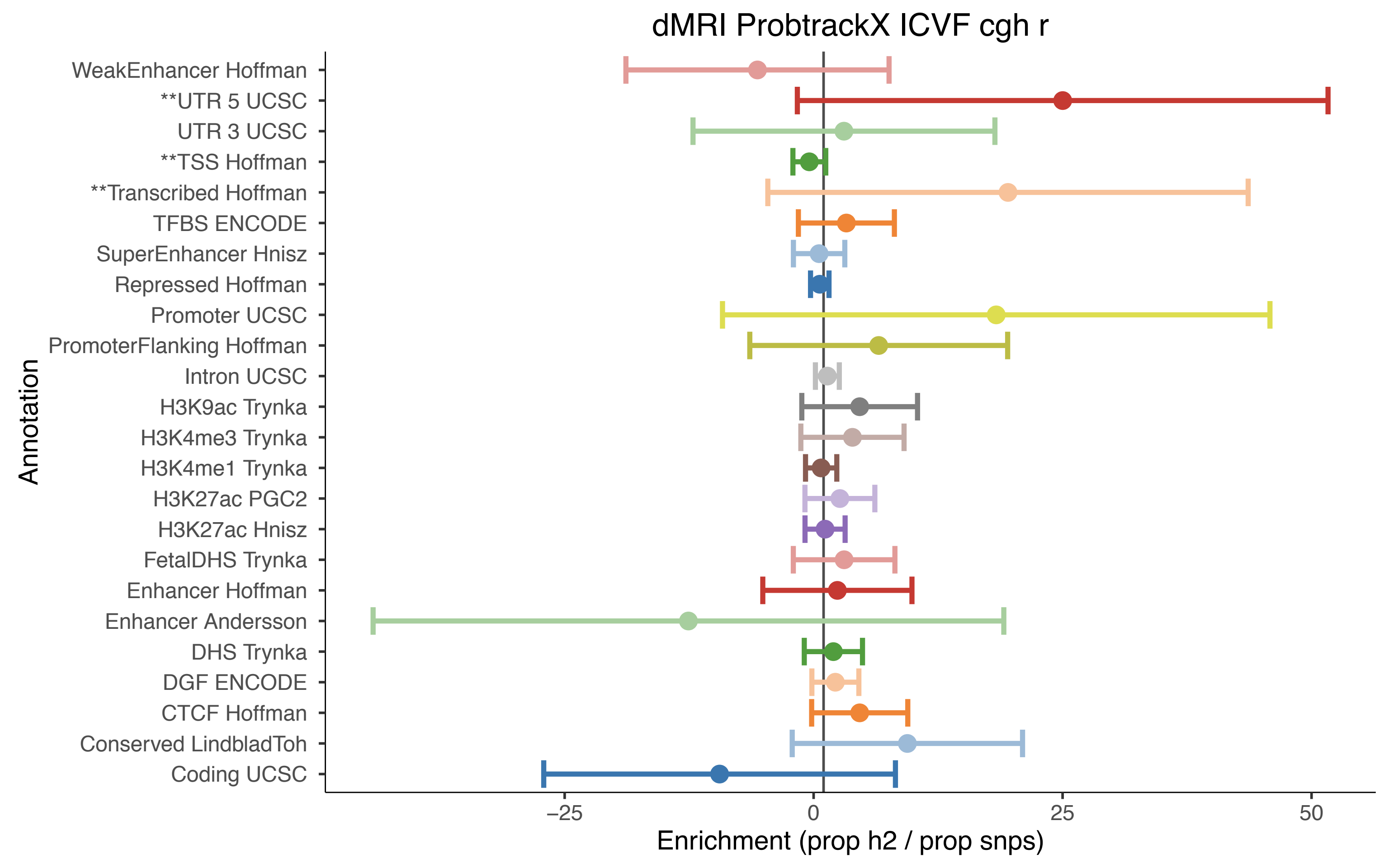

dMRI ProbtrackX ICVF cgh r
Annotation
WeakEnhancer Hoffman
**UTR 5 UCSC
UTR 3 UCSC
**TSS Hoffman
**Transcribed Hoffman
TFBS ENCODE
SuperEnhancer Hnisz
Repressed Hoffman
Promoter UCSC
PromoterFlanking Hoffman
Intron UCSC
H3K9ac Trynka
H3K4me3 Trynka
H3K4me1 Trynka
H3K27ac PGC2
H3K27ac Hnisz
FetalDHS Trynka
Enhancer Hoffman
Enhancer Andersson
DHS Trynka
DGF ENCODE
CTCF Hoffman
Conserved LindbladToh
Coding UCSC
−25
0
25
50
Enrichment (prop h2 / prop snps)

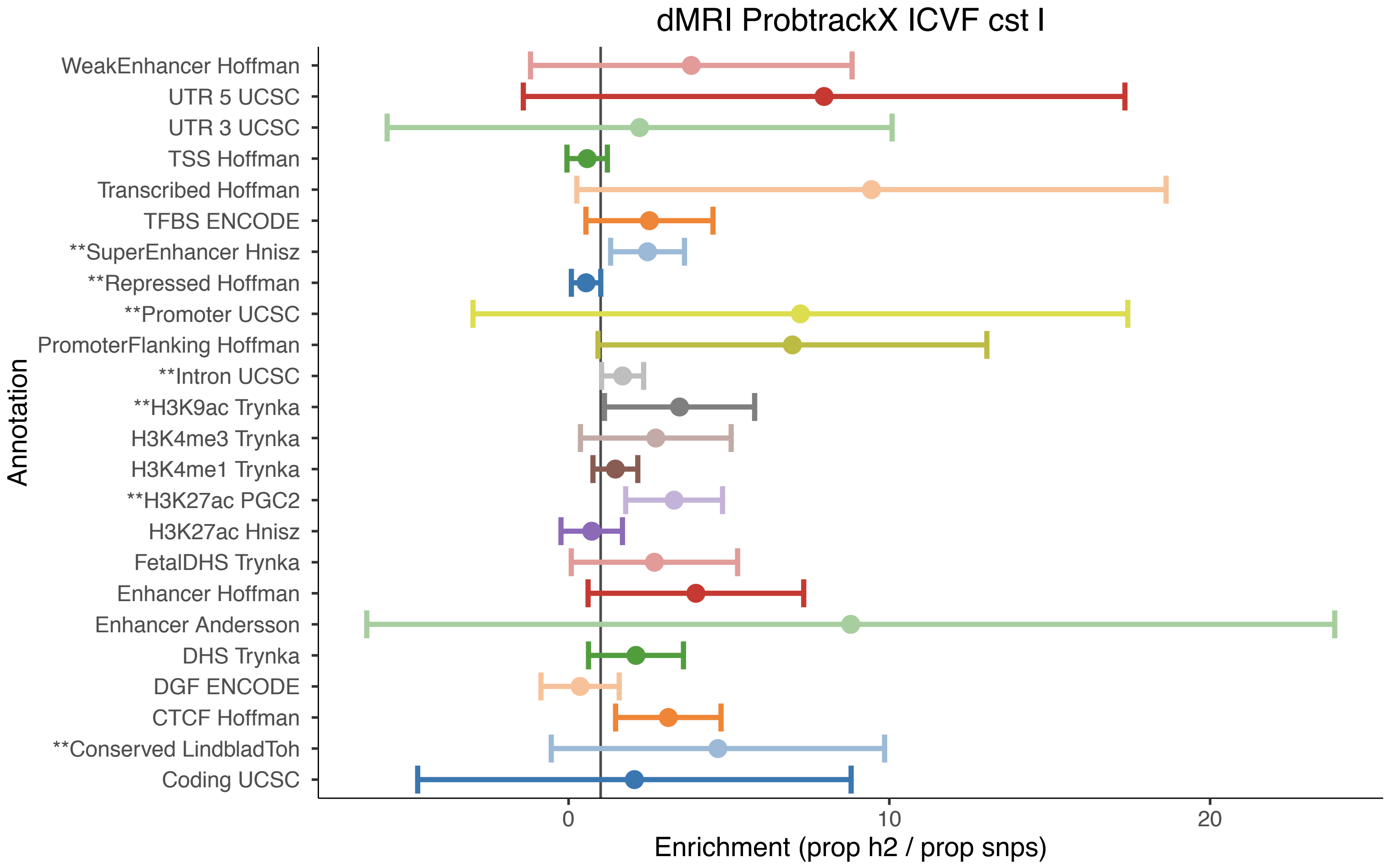

dMRI ProbtrackX ICVF cst l
WeakEnhancer Hoffman
UTR 5 UCSC
UTR 3 UCSC
TSS Hoffman
Transcribed Hoffman
TFBS ENCODE
**SuperEnhancer Hnisz
**Repressed Hoffman
**Promoter UCSC
PromoterFlanking Hoffman
**Intron UCSC
**H3K9ac Trynka
H3K4me3 Trynka
H3K4me1 Trynka
**H3K27ac PGC2
H3K27ac Hnisz
FetalDHS Trynka
Enhancer Hoffman
Enhancer Andersson
DHS Trynka
DGF ENCODE
CTCF Hoffman
**Conserved LindbladToh
Coding UCSC
Annotation
0
10
20
Enrichment (prop h2 / prop snps)

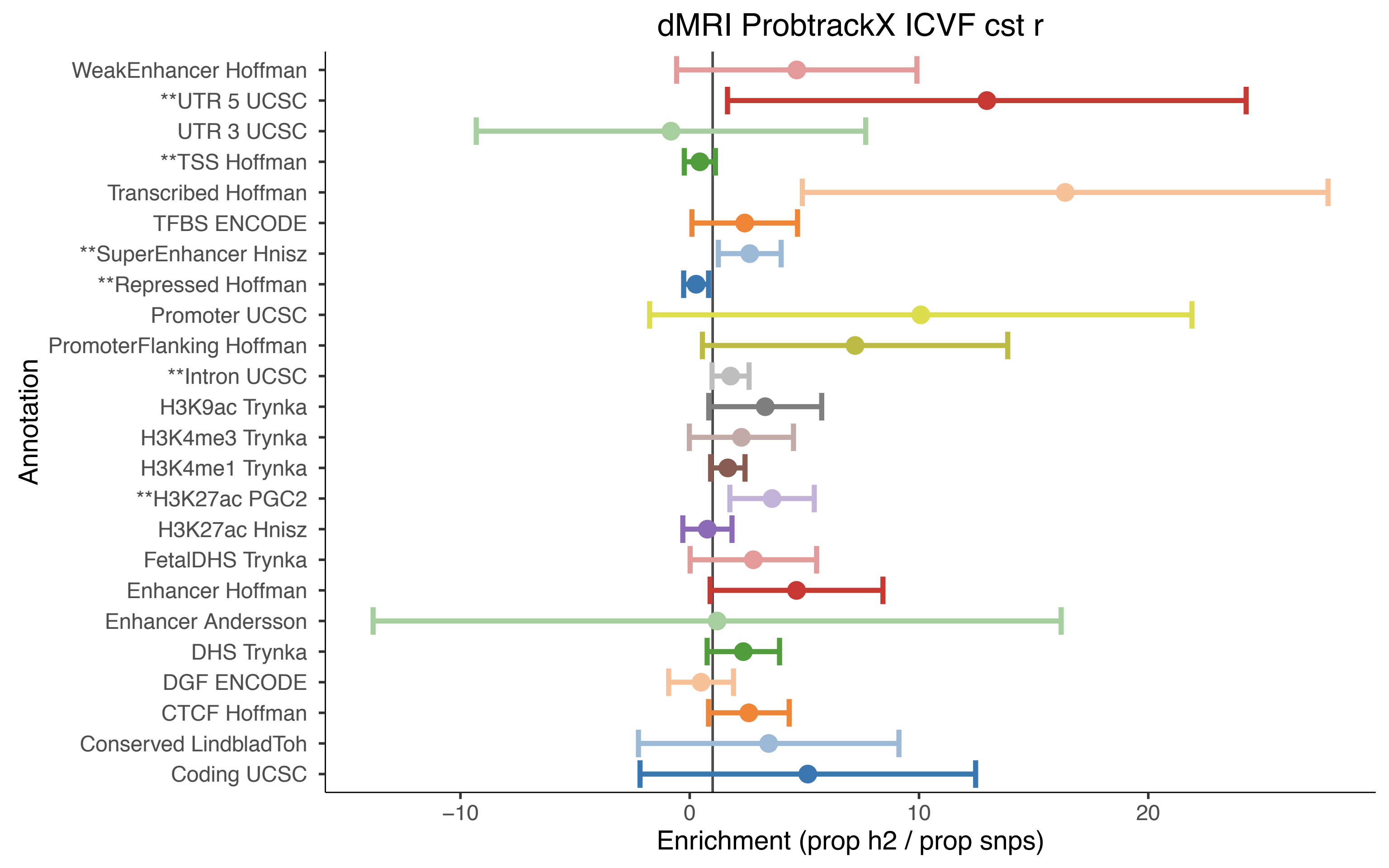

dMRI ProbtrackX ICVF cst r
WeakEnhancer Hoffman
**UTR 5 UCSC
UTR 3 UCSC
**TSS Hoffman
Transcribed Hoffman
TFBS ENCODE
**SuperEnhancer Hnisz
**Repressed Hoffman
Promoter UCSC
PromoterFlanking Hoffman
**Intron UCSC
H3K9ac Trynka
H3K4me3 Trynka
H3K4me1 Trynka
**H3K27ac PGC2
H3K27ac Hnisz
FetalDHS Trynka
Enhancer Hoffman
Enhancer Andersson
DHS Trynka
DGF ENCODE
CTCF Hoffman
Conserved LindbladToh
Coding UCSC
Annotation
−10
0
10
20
Enrichment (prop h2 / prop snps)

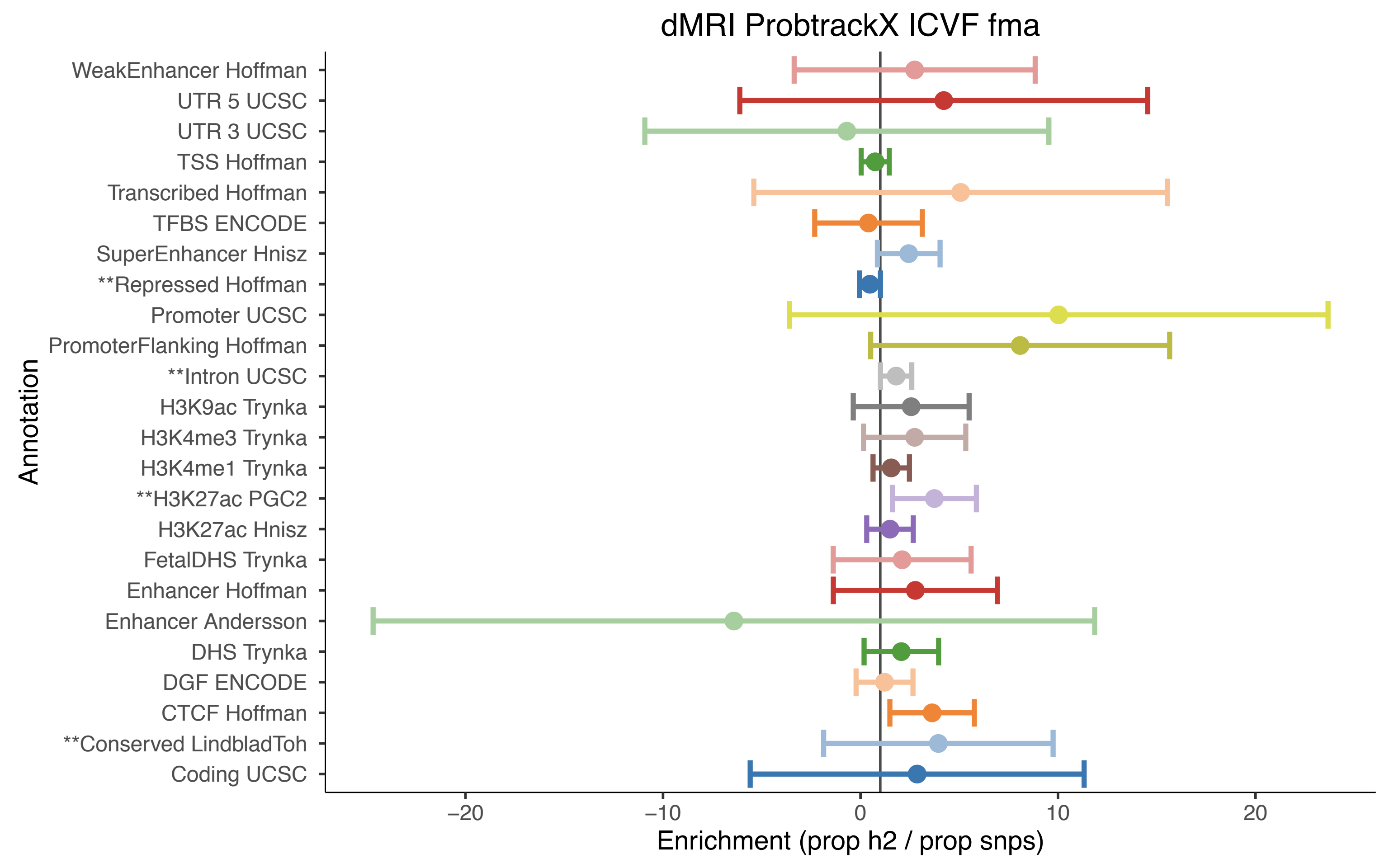

dMRI ProbtrackX ICVF fma
Annotation
WeakEnhancer Hoffman
UTR 5 UCSC
UTR 3 UCSC
TSS Hoffman
Transcribed Hoffman
TFBS ENCODE
SuperEnhancer Hnisz
**Repressed Hoffman
Promoter UCSC
PromoterFlanking Hoffman
**Intron UCSC
H3K9ac Trynka
H3K4me3 Trynka
H3K4me1 Trynka
**H3K27ac PGC2
H3K27ac Hnisz
FetalDHS Trynka
Enhancer Hoffman
Enhancer Andersson
DHS Trynka
DGF ENCODE
CTCF Hoffman
**Conserved LindbladToh
Coding UCSC
−20
−10
0
10
20
Enrichment (prop h2 / prop snps)

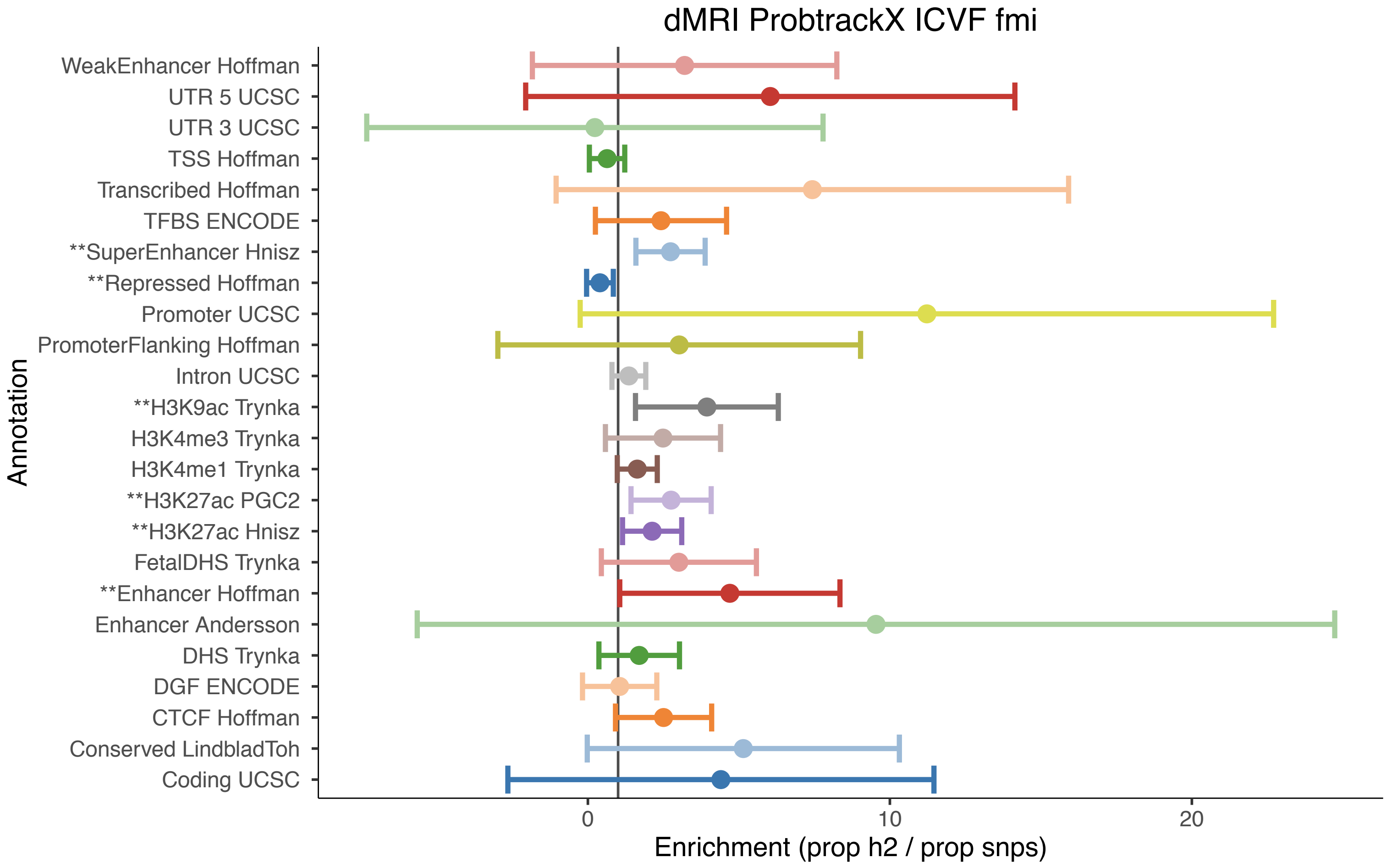

dMRI ProbtrackX ICVF fmi
WeakEnhancer Hoffman
UTR 5 UCSC
UTR 3 UCSC
TSS Hoffman
Transcribed Hoffman
TFBS ENCODE
**SuperEnhancer Hnisz
**Repressed Hoffman
Promoter UCSC
PromoterFlanking Hoffman
Intron UCSC
**H3K9ac Trynka
H3K4me3 Trynka
H3K4me1 Trynka
**H3K27ac PGC2
**H3K27ac Hnisz
FetalDHS Trynka
**Enhancer Hoffman
Enhancer Andersson
DHS Trynka
DGF ENCODE
CTCF Hoffman
Conserved LindbladToh
Coding UCSC
Annotation
0
10
20
Enrichment (prop h2 / prop snps)

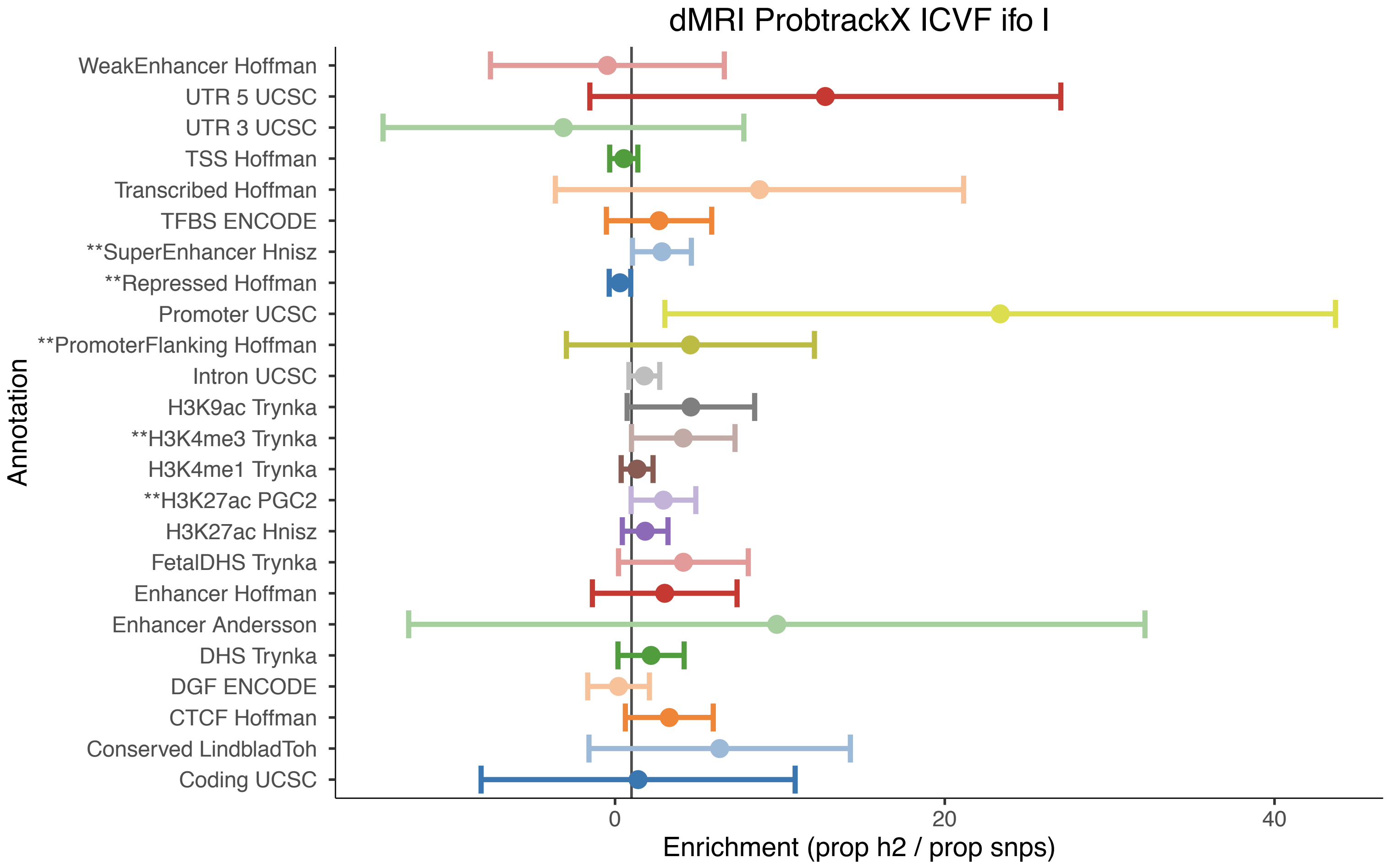
dMRI ProbtrackX ICVF ifo l
WeakEnhancer Hoffman
UTR 5 UCSC
UTR 3 UCSC
TSS Hoffman
Transcribed Hoffman
TFBS ENCODE
**SuperEnhancer Hnisz
**Repressed Hoffman
Promoter UCSC
**PromoterFlanking Hoffman
Intron UCSC
H3K9ac Trynka
**H3K4me3 Trynka
H3K4me1 Trynka
**H3K27ac PGC2
H3K27ac Hnisz
FetalDHS Trynka
Enhancer Hoffman
Enhancer Andersson
DHS Trynka
DGF ENCODE
CTCF Hoffman
Conserved LindbladToh
Coding UCSC
Annotation
0
20
40
Enrichment (prop h2 / prop snps)

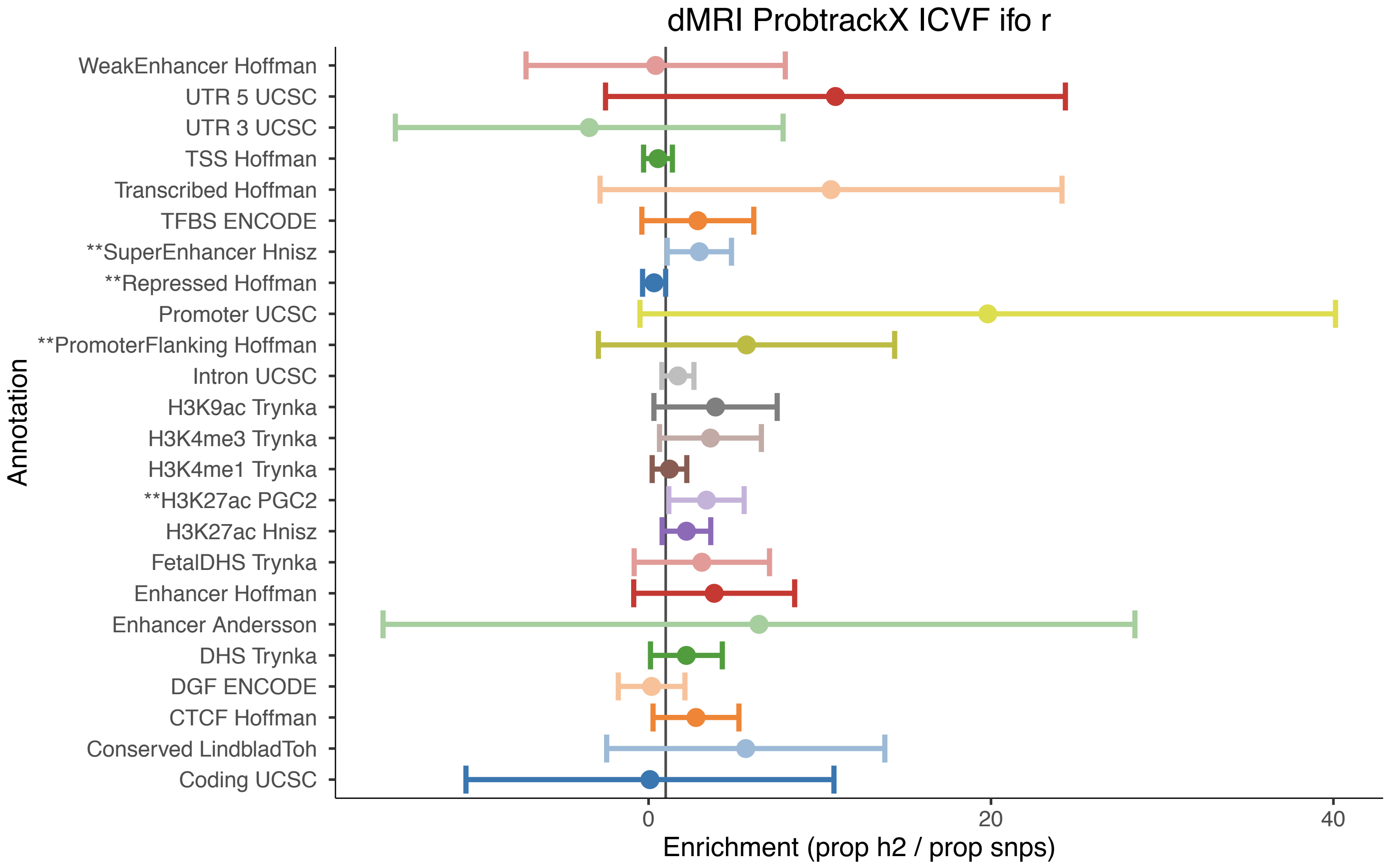

dMRI ProbtrackX ICVF ifo r
WeakEnhancer Hoffman
UTR 5 UCSC
UTR 3 UCSC
TSS Hoffman
Transcribed Hoffman
TFBS ENCODE
**SuperEnhancer Hnisz
**Repressed Hoffman
Promoter UCSC
**PromoterFlanking Hoffman
Intron UCSC
H3K9ac Trynka
H3K4me3 Trynka
H3K4me1 Trynka
**H3K27ac PGC2
H3K27ac Hnisz
FetalDHS Trynka
Enhancer Hoffman
Enhancer Andersson
DHS Trynka
DGF ENCODE
CTCF Hoffman
Conserved LindbladToh
Coding UCSC
Annotation
0
20
40
Enrichment (prop h2 / prop snps)

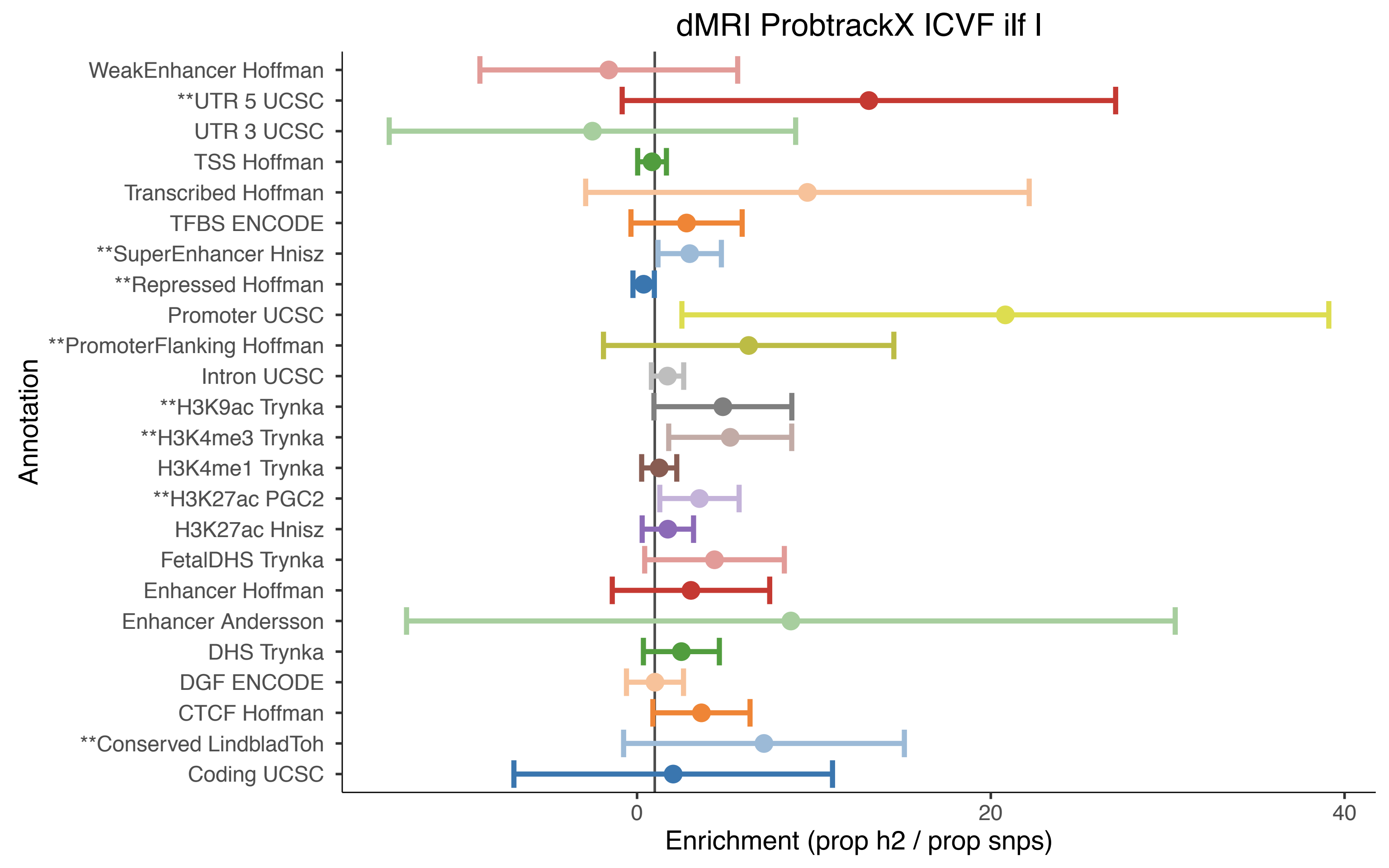
dMRI ProbtrackX ICVF ilf l
WeakEnhancer Hoffman
**UTR 5 UCSC
UTR 3 UCSC
TSS Hoffman
Transcribed Hoffman
TFBS ENCODE
**SuperEnhancer Hnisz
**Repressed Hoffman
Promoter UCSC
**PromoterFlanking Hoffman
Intron UCSC
**H3K9ac Trynka
**H3K4me3 Trynka
H3K4me1 Trynka
**H3K27ac PGC2
H3K27ac Hnisz
FetalDHS Trynka
Enhancer Hoffman
Enhancer Andersson
DHS Trynka
DGF ENCODE
CTCF Hoffman
**Conserved LindbladToh
Coding UCSC
Annotation
0
20
40
Enrichment (prop h2 / prop snps)

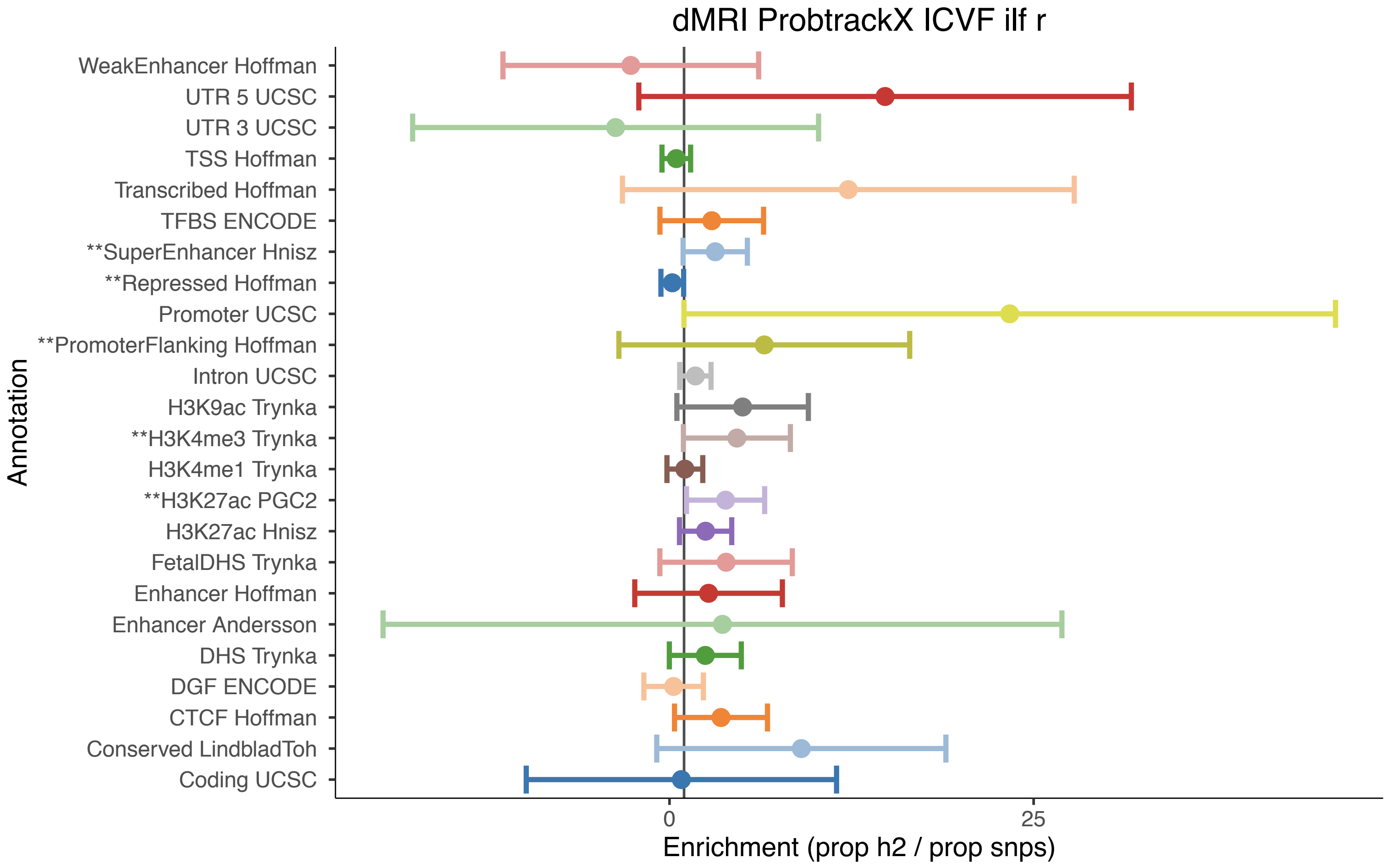
dMRI ProbtrackX ICVF ilf r
WeakEnhancer Hoffman
UTR 5 UCSC
UTR 3 UCSC
TSS Hoffman
Transcribed Hoffman
TFBS ENCODE
**SuperEnhancer Hnisz
**Repressed Hoffman
Promoter UCSC
**PromoterFlanking Hoffman
Intron UCSC
H3K9ac Trynka
**H3K4me3 Trynka
H3K4me1 Trynka
**H3K27ac PGC2
H3K27ac Hnisz
FetalDHS Trynka
Enhancer Hoffman
Enhancer Andersson
DHS Trynka
DGF ENCODE
CTCF Hoffman
Conserved LindbladToh
Coding UCSC
Annotation
0
25
Enrichment (prop h2 / prop snps)

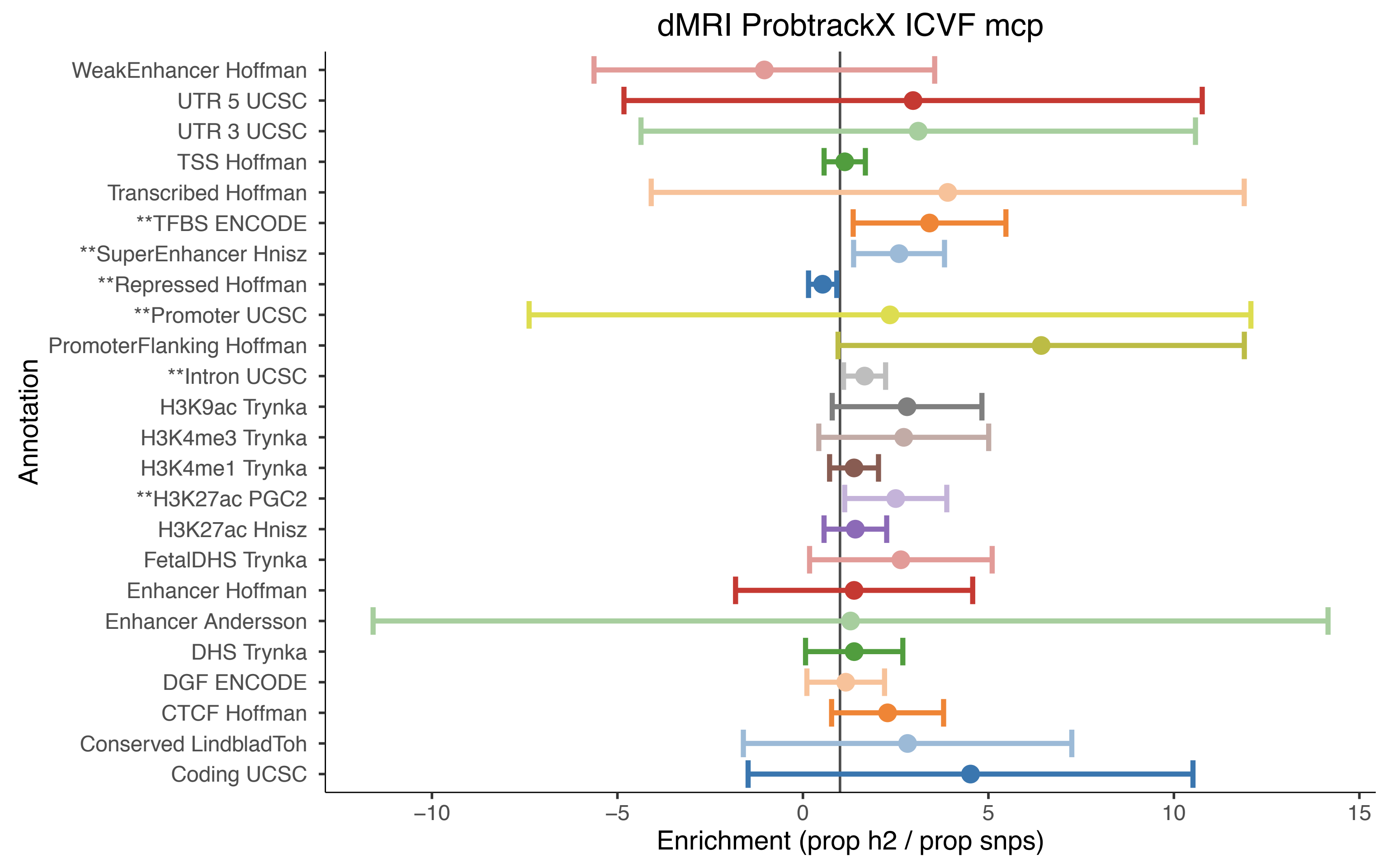

dMRI ProbtrackX ICVF mcp
WeakEnhancer Hoffman
UTR 5 UCSC
UTR 3 UCSC
TSS Hoffman
Transcribed Hoffman
**TFBS ENCODE
**SuperEnhancer Hnisz
**Repressed Hoffman
**Promoter UCSC
PromoterFlanking Hoffman
**Intron UCSC
H3K9ac Trynka
H3K4me3 Trynka
H3K4me1 Trynka
**H3K27ac PGC2
H3K27ac Hnisz
FetalDHS Trynka
Enhancer Hoffman
Enhancer Andersson
DHS Trynka
DGF ENCODE
CTCF Hoffman
Conserved LindbladToh
Coding UCSC
Annotation
−10
−5
0
5
10
15
Enrichment (prop h2 / prop snps)

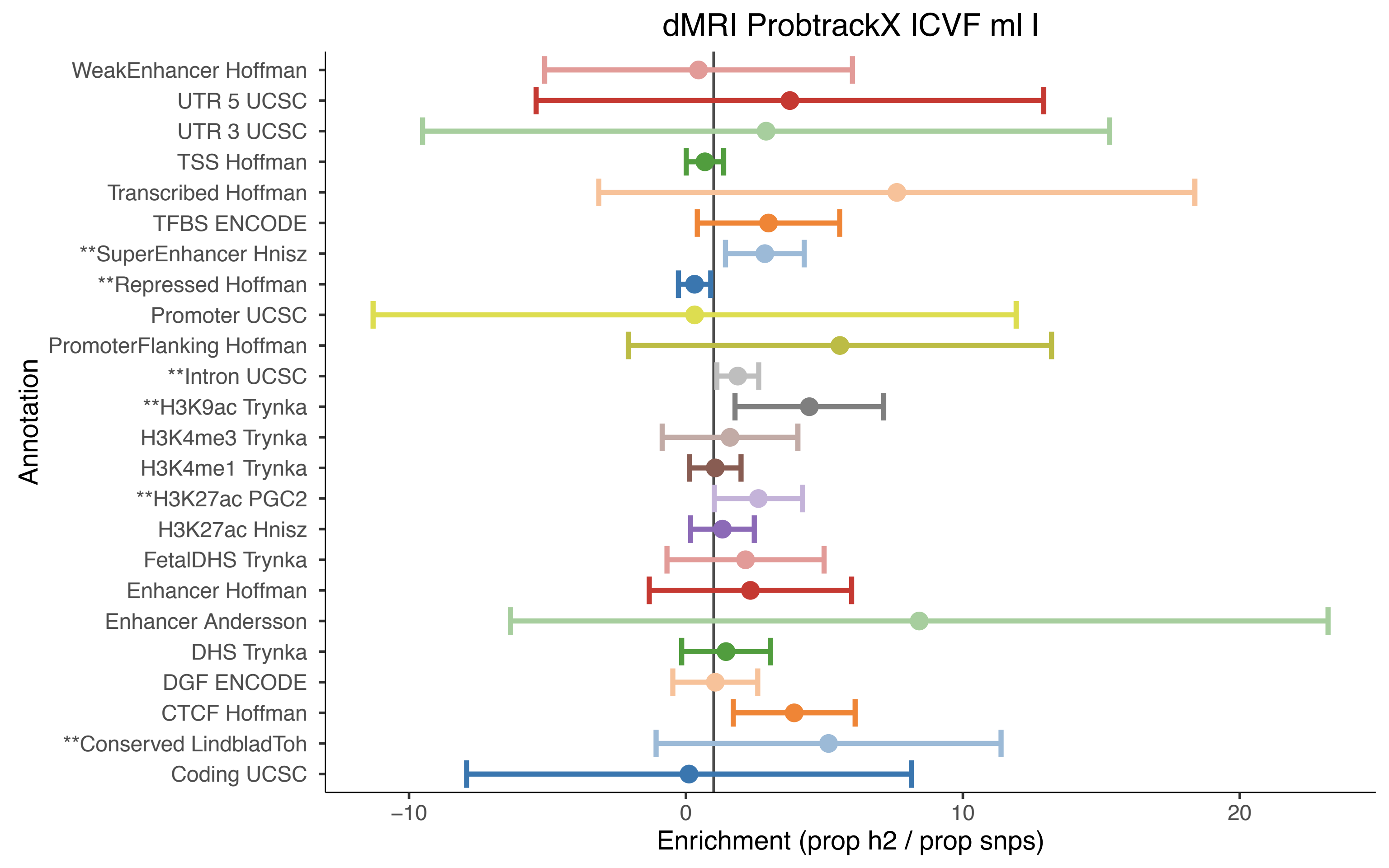

dMRI ProbtrackX ICVF ml l
WeakEnhancer Hoffman
UTR 5 UCSC
UTR 3 UCSC
TSS Hoffman
Transcribed Hoffman
TFBS ENCODE
**SuperEnhancer Hnisz
**Repressed Hoffman
Promoter UCSC
PromoterFlanking Hoffman
**Intron UCSC
**H3K9ac Trynka
H3K4me3 Trynka
H3K4me1 Trynka
**H3K27ac PGC2
H3K27ac Hnisz
FetalDHS Trynka
Enhancer Hoffman
Enhancer Andersson
DHS Trynka
DGF ENCODE
CTCF Hoffman
**Conserved LindbladToh
Coding UCSC
Annotation
−10
0
10
20
Enrichment (prop h2 / prop snps)

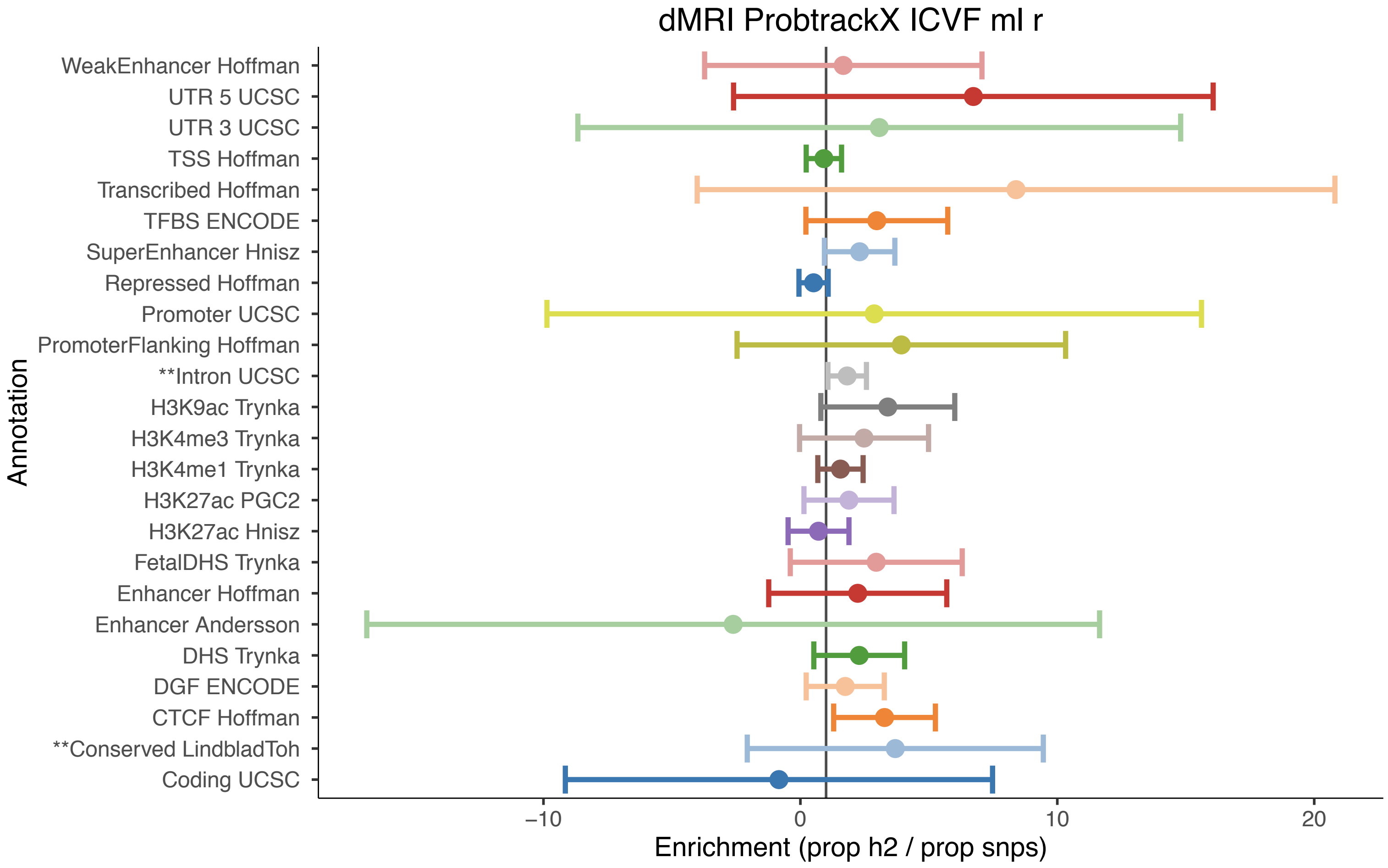

dMRI ProbtrackX ICVF ml r
WeakEnhancer Hoffman
UTR 5 UCSC
UTR 3 UCSC
TSS Hoffman
Transcribed Hoffman
TFBS ENCODE
SuperEnhancer Hnisz
Repressed Hoffman
Promoter UCSC
PromoterFlanking Hoffman
**Intron UCSC
H3K9ac Trynka
H3K4me3 Trynka
H3K4me1 Trynka
H3K27ac PGC2
H3K27ac Hnisz
FetalDHS Trynka
Enhancer Hoffman
Enhancer Andersson
DHS Trynka
DGF ENCODE
CTCF Hoffman
**Conserved LindbladToh
Coding UCSC
Annotation
−10
0
10
20
Enrichment (prop h2 / prop snps)

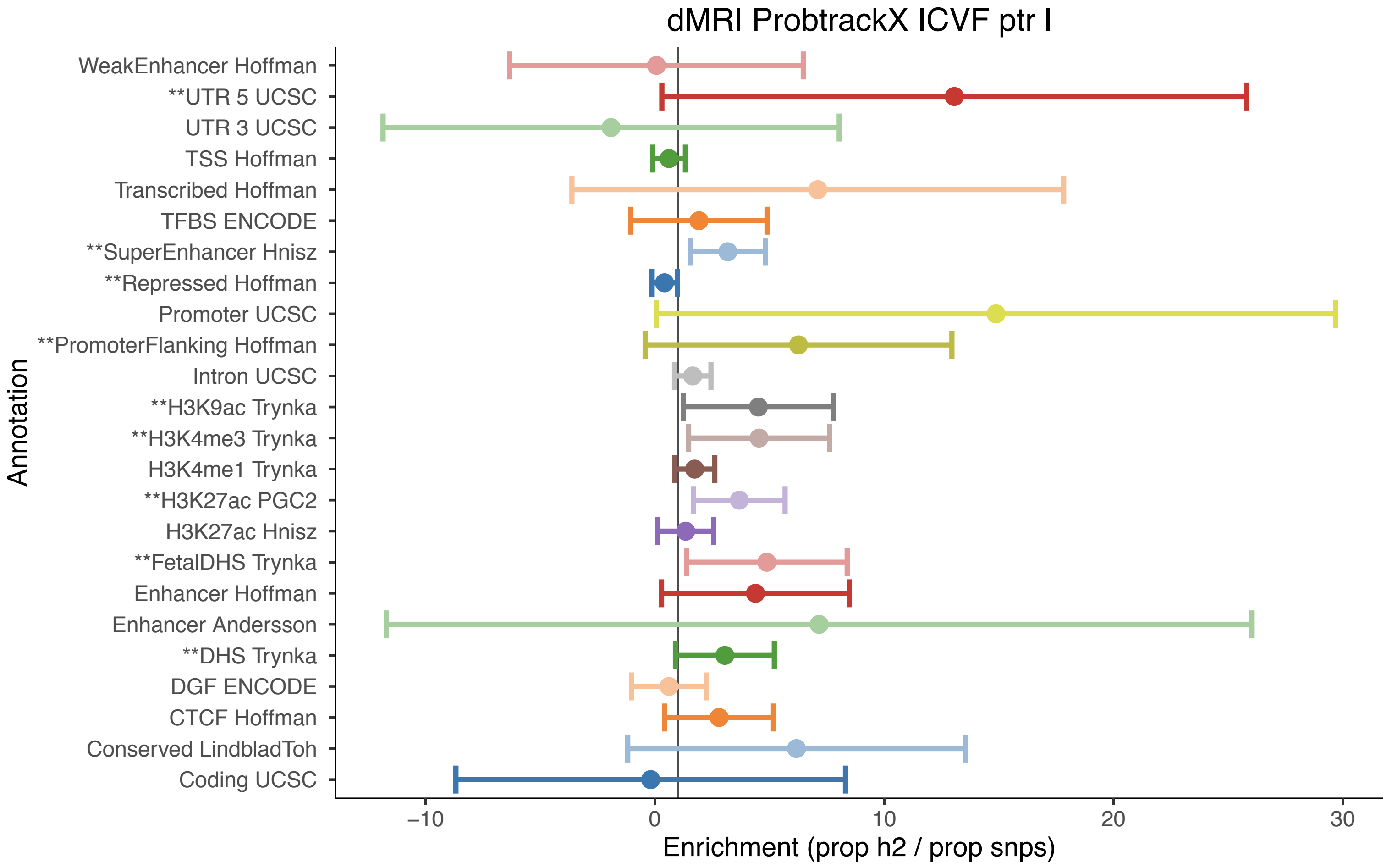
dMRI ProbtrackX ICVF ptr l
Annotation
WeakEnhancer Hoffman
**UTR 5 UCSC
UTR 3 UCSC
TSS Hoffman
Transcribed Hoffman
TFBS ENCODE
**SuperEnhancer Hnisz
**Repressed Hoffman
Promoter UCSC
**PromoterFlanking Hoffman
Intron UCSC
**H3K9ac Trynka
**H3K4me3 Trynka
H3K4me1 Trynka
**H3K27ac PGC2
H3K27ac Hnisz
**FetalDHS Trynka
Enhancer Hoffman
Enhancer Andersson
**DHS Trynka
DGF ENCODE
CTCF Hoffman
Conserved LindbladToh
Coding UCSC
−10
0
10
20
30
Enrichment (prop h2 / prop snps)

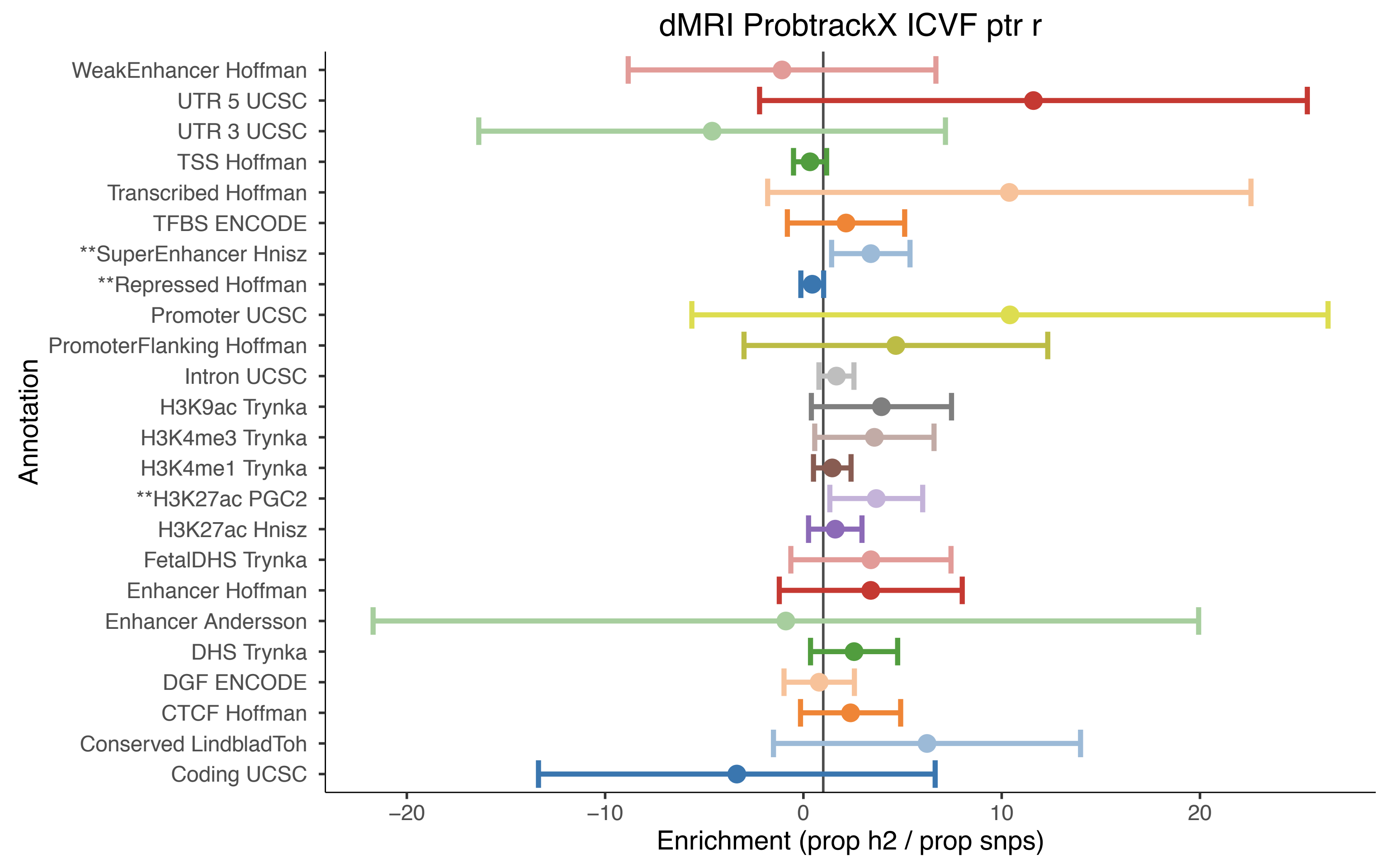

dMRI ProbtrackX ICVF ptr r
WeakEnhancer Hoffman
UTR 5 UCSC
UTR 3 UCSC
TSS Hoffman
Transcribed Hoffman
TFBS ENCODE
**SuperEnhancer Hnisz
**Repressed Hoffman
Promoter UCSC
PromoterFlanking Hoffman
Intron UCSC
H3K9ac Trynka
H3K4me3 Trynka
H3K4me1 Trynka
**H3K27ac PGC2
H3K27ac Hnisz
FetalDHS Trynka
Enhancer Hoffman
Enhancer Andersson
DHS Trynka
DGF ENCODE
CTCF Hoffman
Conserved LindbladToh
Coding UCSC
Annotation
−20
−10
0
10
20
Enrichment (prop h2 / prop snps)

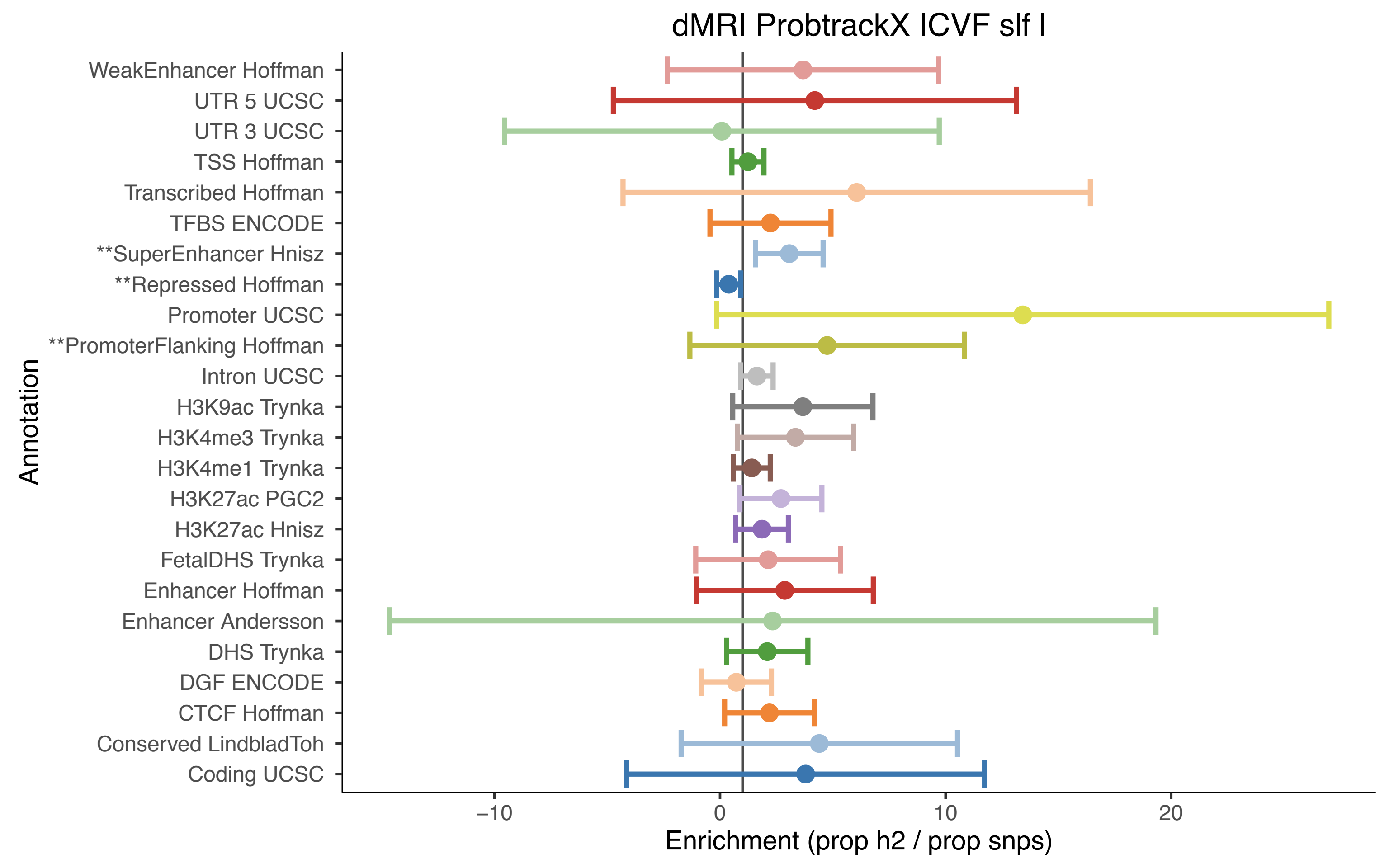

dMRI ProbtrackX ICVF slf I
WeakEnhancer Hoffman
UTR 5 UCSC
UTR 3 UCSC
TSS Hoffman
Transcribed Hoffman
TFBS ENCODE
**SuperEnhancer Hnisz
**Repressed Hoffman
Promoter UCSC
**PromoterFlanking Hoffman
Intron UCSC
H3K9ac Trynka
H3K4me3 Trynka
H3K4me1 Trynka
H3K27ac PGC2
H3K27ac Hnisz
FetalDHS Trynka
Enhancer Hoffman
Enhancer Andersson
DHS Trynka
DGF ENCODE
CTCF Hoffman
Conserved LindbladToh
Coding UCSC
Annotation
−10
0
10
20
Enrichment (prop h2 / prop snps)

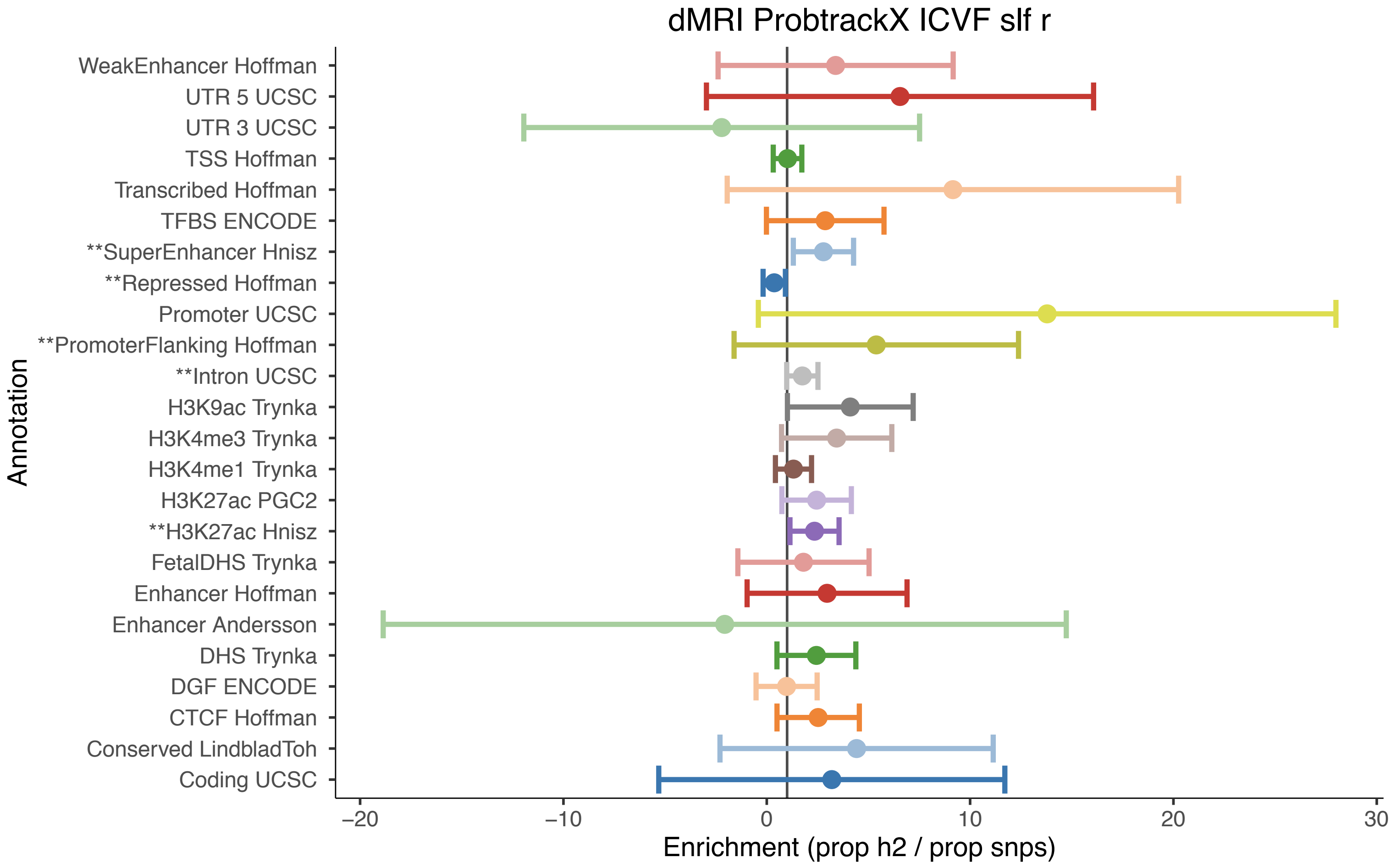

dMRI ProbtrackX ICVF slf r
WeakEnhancer Hoffman
UTR 5 UCSC
UTR 3 UCSC
TSS Hoffman
Transcribed Hoffman
TFBS ENCODE
**SuperEnhancer Hnisz
**Repressed Hoffman
Promoter UCSC
**PromoterFlanking Hoffman
**Intron UCSC
H3K9ac Trynka
H3K4me3 Trynka
H3K4me1 Trynka
H3K27ac PGC2
**H3K27ac Hnisz
FetalDHS Trynka
Enhancer Hoffman
Enhancer Andersson
DHS Trynka
DGF ENCODE
CTCF Hoffman
Conserved LindbladToh
Coding UCSC
Annotation
−20
−10
0
10
20
30
Enrichment (prop h2 / prop snps)

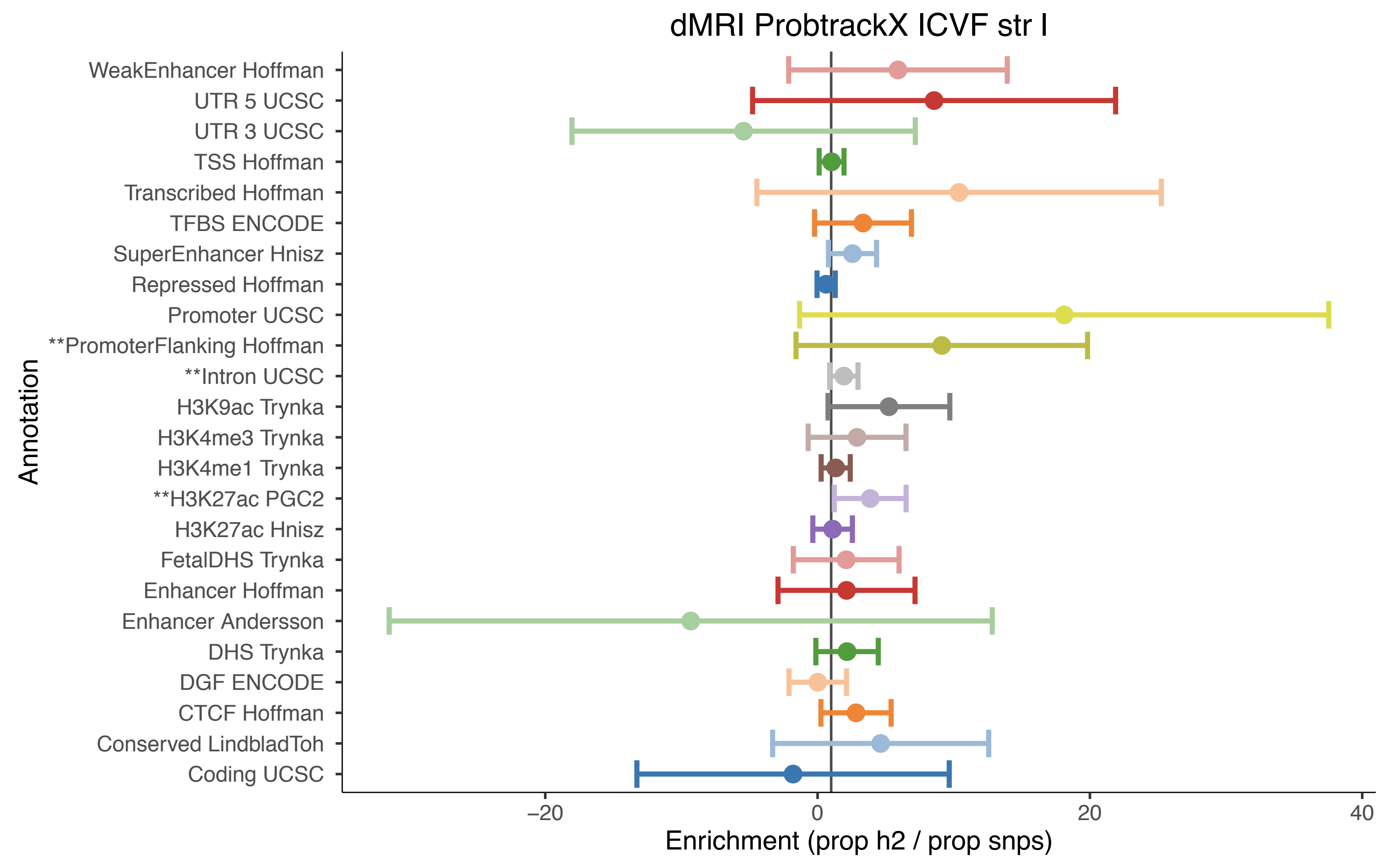

dMRI ProbtrackX ICVF str l
WeakEnhancer Hoffman
UTR 5 UCSC
UTR 3 UCSC
TSS Hoffman
Transcribed Hoffman
TFBS ENCODE
SuperEnhancer Hnisz
Repressed Hoffman
Promoter UCSC
**PromoterFlanking Hoffman
**Intron UCSC
H3K9ac Trynka
H3K4me3 Trynka
H3K4me1 Trynka
**H3K27ac PGC2
H3K27ac Hnisz
FetalDHS Trynka
Enhancer Hoffman
Enhancer Andersson
DHS Trynka
DGF ENCODE
CTCF Hoffman
Conserved LindbladToh
Coding UCSC
Annotation
−20
0
20
40
Enrichment (prop h2 / prop snps)

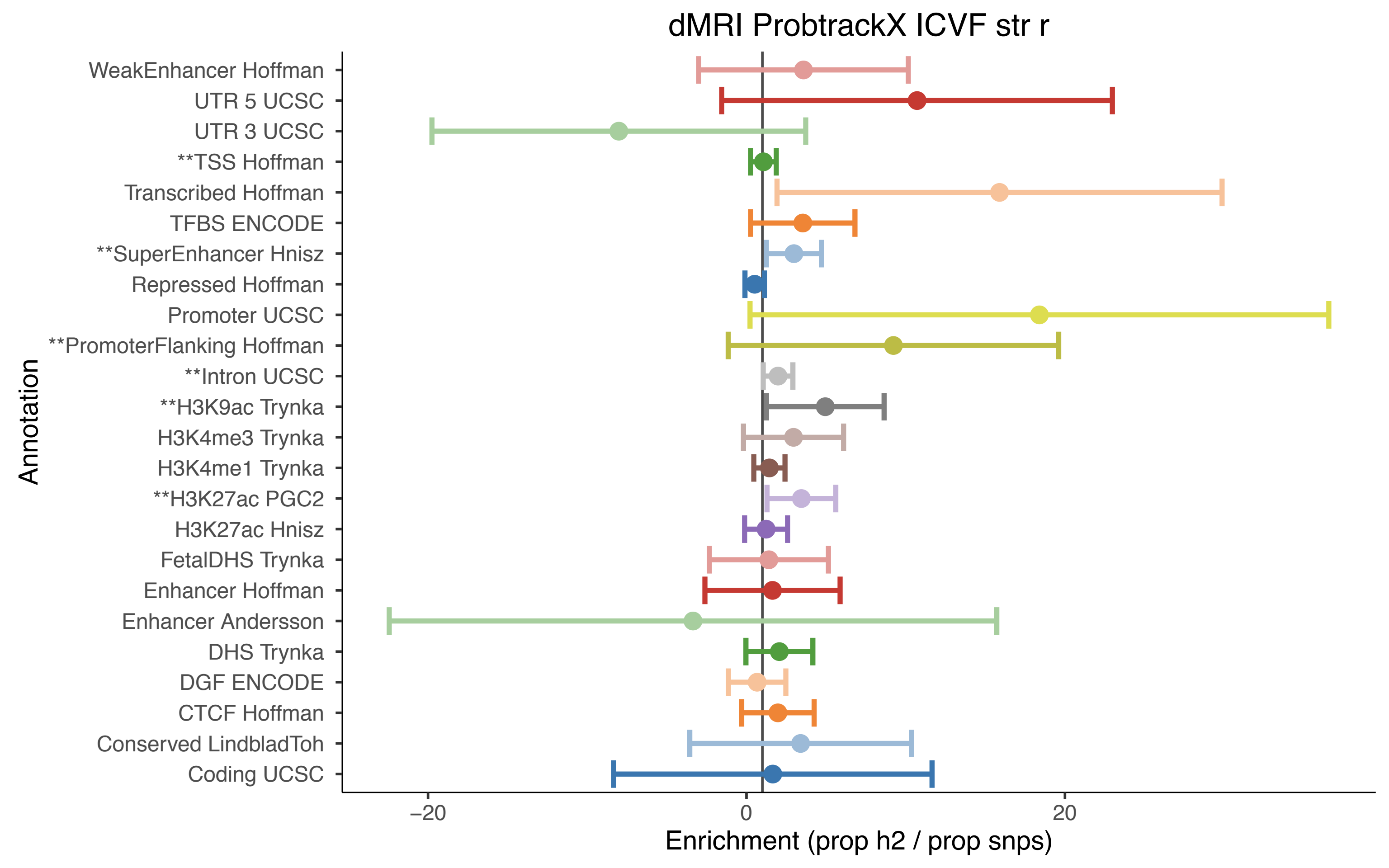

dMRI ProbtrackX ICVF str r
WeakEnhancer Hoffman
UTR 5 UCSC
UTR 3 UCSC
**TSS Hoffman
Transcribed Hoffman
TFBS ENCODE
**SuperEnhancer Hnisz
Repressed Hoffman
Promoter UCSC
**PromoterFlanking Hoffman
**Intron UCSC
**H3K9ac Trynka
H3K4me3 Trynka
H3K4me1 Trynka
**H3K27ac PGC2
H3K27ac Hnisz
FetalDHS Trynka
Enhancer Hoffman
Enhancer Andersson
DHS Trynka
DGF ENCODE
CTCF Hoffman
Conserved LindbladToh
Coding UCSC
Annotation
−20
0
20
Enrichment (prop h2 / prop snps)

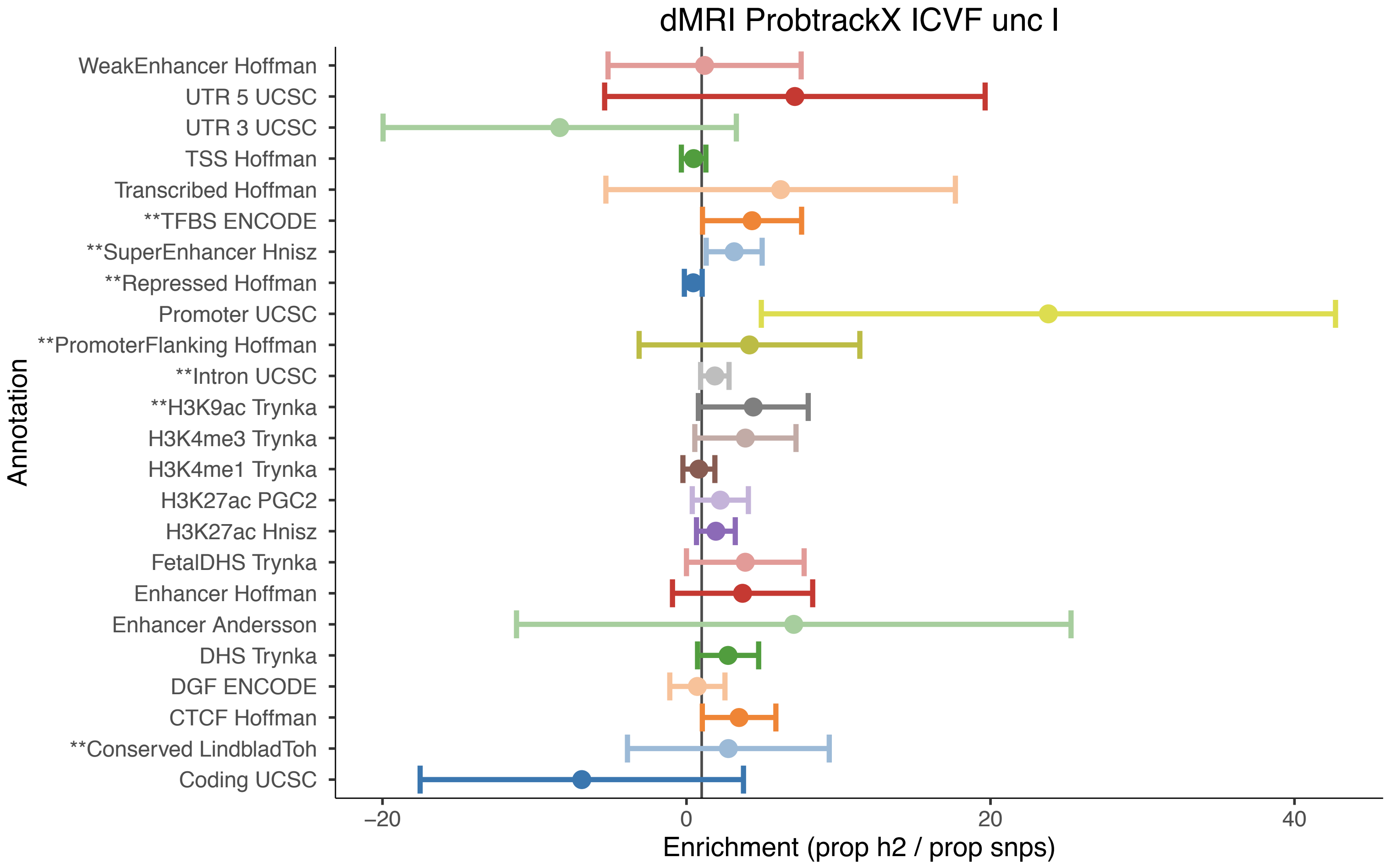

dMRI ProbtrackX ICVF unc l
WeakEnhancer Hoffman
UTR 5 UCSC
UTR 3 UCSC
TSS Hoffman
Transcribed Hoffman
**TFBS ENCODE
**SuperEnhancer Hnisz
**Repressed Hoffman
Promoter UCSC
**PromoterFlanking Hoffman
**Intron UCSC
**H3K9ac Trynka
H3K4me3 Trynka
H3K4me1 Trynka
H3K27ac PGC2
H3K27ac Hnisz
FetalDHS Trynka
Enhancer Hoffman
Enhancer Andersson
DHS Trynka
DGF ENCODE
CTCF Hoffman
**Conserved LindbladToh
Coding UCSC
Annotation
−20
0
20
40
Enrichment (prop h2 / prop snps)

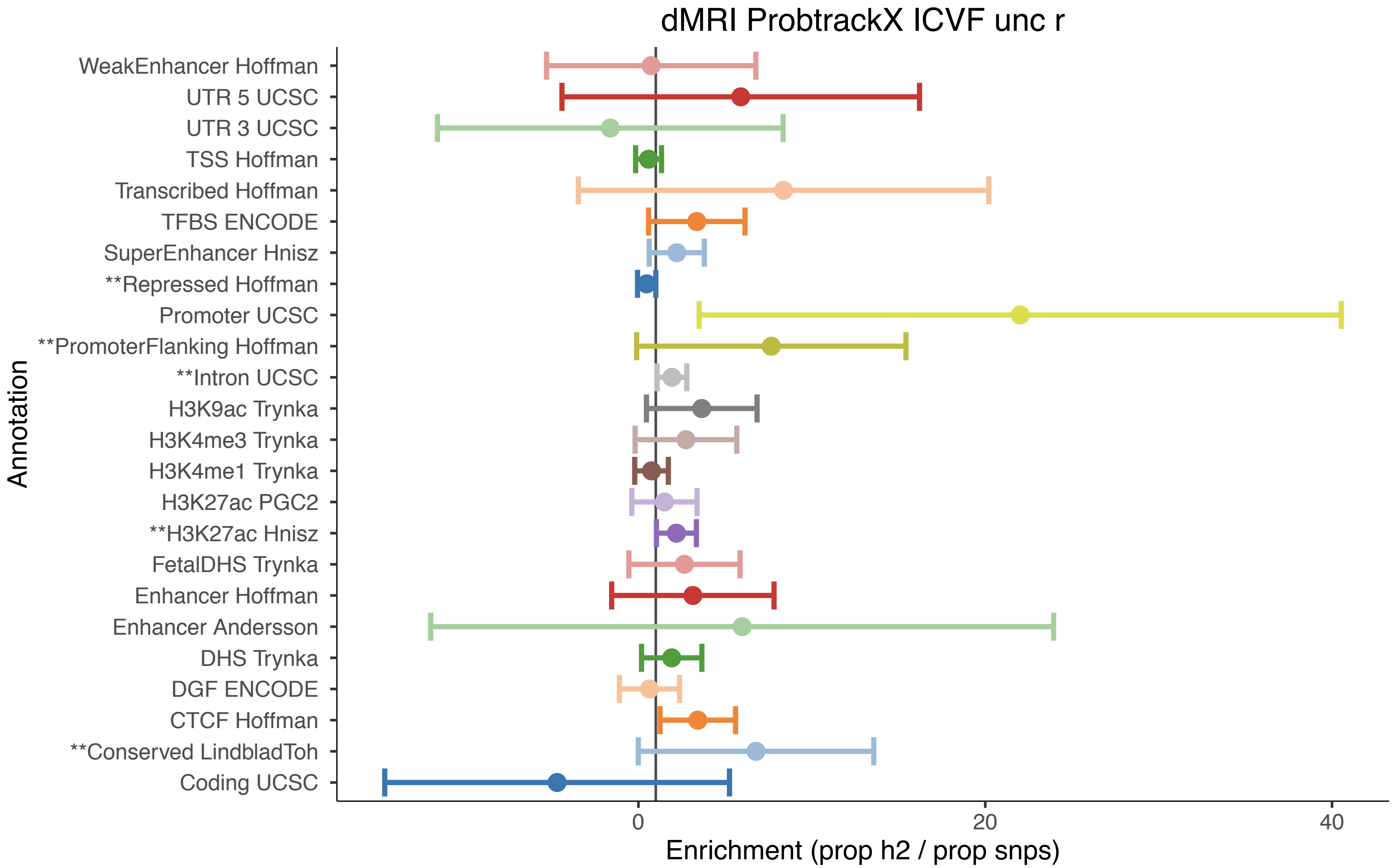
dMRI ProbtrackX ICVF unc r
WeakEnhancer Hoffman
UTR 5 UCSC
UTR 3 UCSC
TSS Hoffman
Transcribed Hoffman
TFBS ENCODE
SuperEnhancer Hnisz
**Repressed Hoffman
Promoter UCSC
**PromoterFlanking Hoffman
**Intron UCSC
H3K9ac Trynka
H3K4me3 Trynka
H3K4me1 Trynka
H3K27ac PGC2
**H3K27ac Hnisz
FetalDHS Trynka
Enhancer Hoffman
Enhancer Andersson
DHS Trynka
DGF ENCODE
CTCF Hoffman
**Conserved LindbladToh
Coding UCSC
Annotation
0
20
40
Enrichment (prop h2 / prop snps)

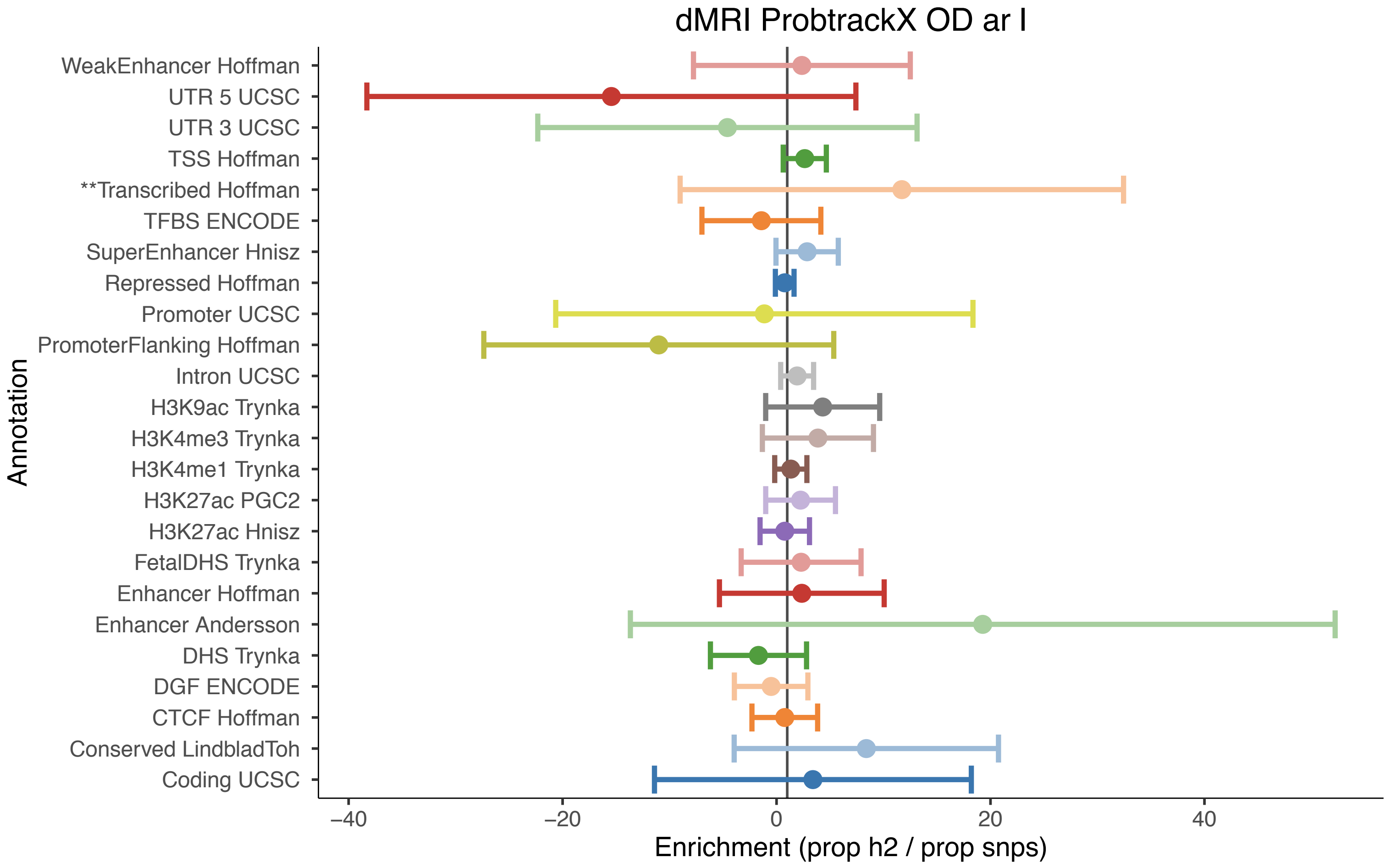
dMRI ProbtrackX OD ar l
Annotation
WeakEnhancer Hoffman
UTR 5 UCSC
UTR 3 UCSC
TSS Hoffman
**Transcribed Hoffman
TFBS ENCODE
SuperEnhancer Hnisz
Repressed Hoffman
Promoter UCSC
PromoterFlanking Hoffman
Intron UCSC
H3K9ac Trynka
H3K4me3 Trynka
H3K4me1 Trynka
H3K27ac PGC2
H3K27ac Hnisz
FetalDHS Trynka
Enhancer Hoffman
Enhancer Andersson
DHS Trynka
DGF ENCODE
CTCF Hoffman
Conserved LindbladToh
Coding UCSC
−40
−20
0
20
40
Enrichment (prop h2 / prop snps)

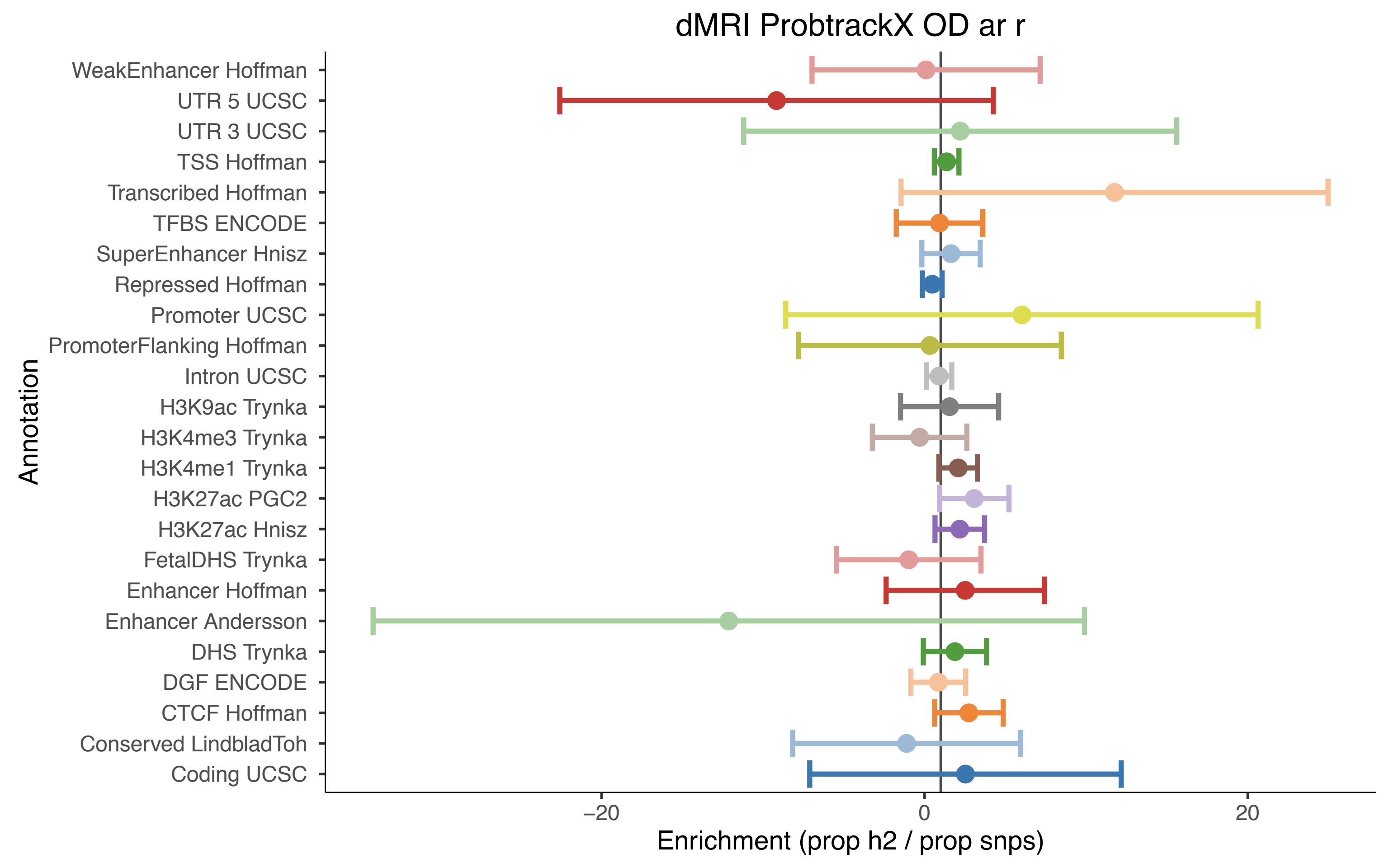
dMRI ProbtrackX OD ar r
WeakEnhancer Hoffman
UTR 5 UCSC
UTR 3 UCSC
TSS Hoffman
Transcribed Hoffman
TFBS ENCODE
SuperEnhancer Hnisz
Repressed Hoffman
Promoter UCSC
PromoterFlanking Hoffman
Intron UCSC
H3K9ac Trynka
H3K4me3 Trynka
H3K4me1 Trynka
H3K27ac PGC2
H3K27ac Hnisz
FetalDHS Trynka
Enhancer Hoffman
Enhancer Andersson
DHS Trynka
DGF ENCODE
CTCF Hoffman
Conserved LindbladToh
Coding UCSC
Annotation
−20
0
20
Enrichment (prop h2 / prop snps)

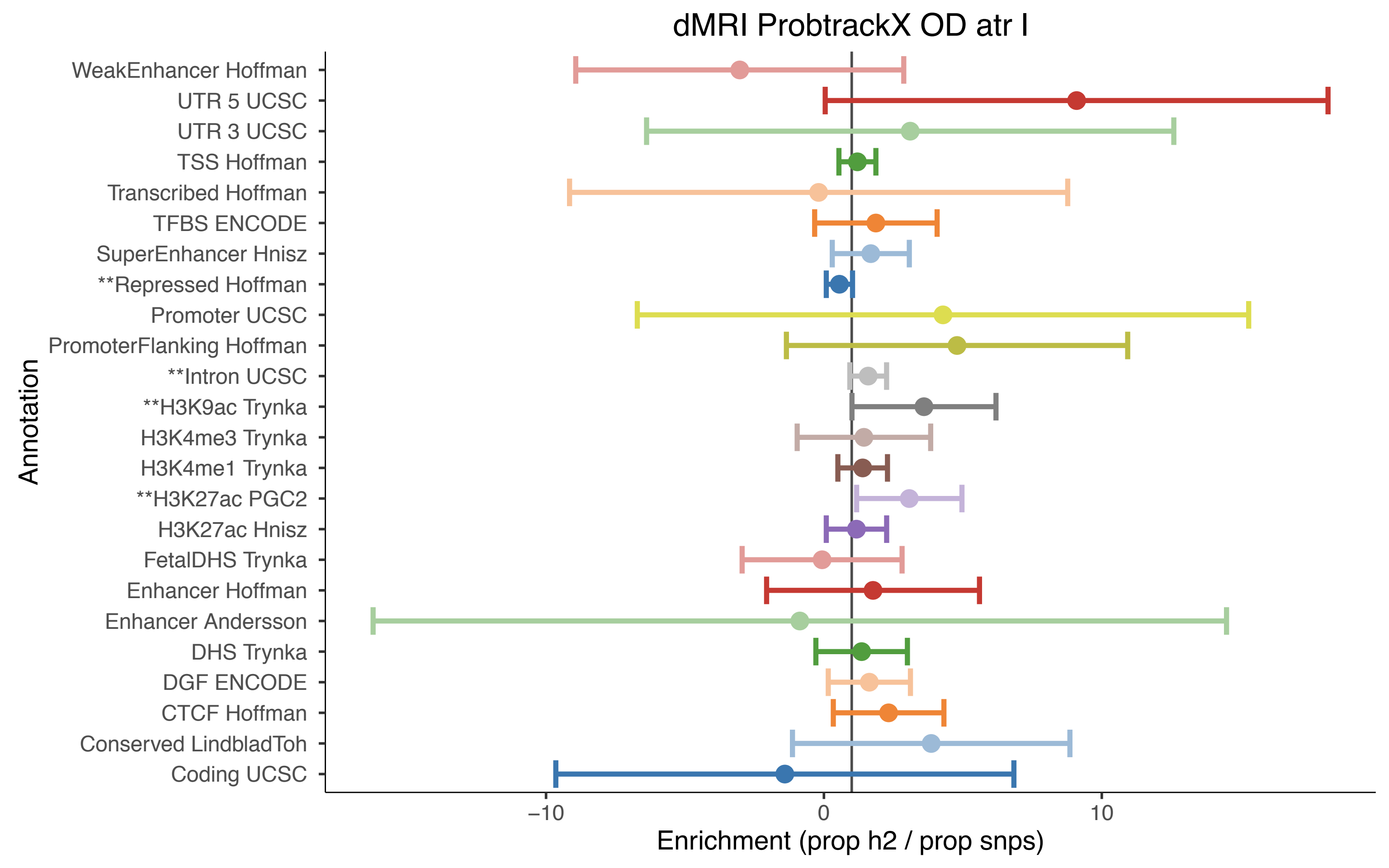

dMRI ProbtrackX OD atr l
WeakEnhancer Hoffman
UTR 5 UCSC
UTR 3 UCSC
TSS Hoffman
Transcribed Hoffman
TFBS ENCODE
SuperEnhancer Hnisz
**Repressed Hoffman
Promoter UCSC
PromoterFlanking Hoffman
**Intron UCSC
**H3K9ac Trynka
H3K4me3 Trynka
H3K4me1 Trynka
**H3K27ac PGC2
H3K27ac Hnisz
FetalDHS Trynka
Enhancer Hoffman
Enhancer Andersson
DHS Trynka
DGF ENCODE
CTCF Hoffman
Conserved LindbladToh
Coding UCSC
Annotation
−10
0
10
Enrichment (prop h2 / prop snps)

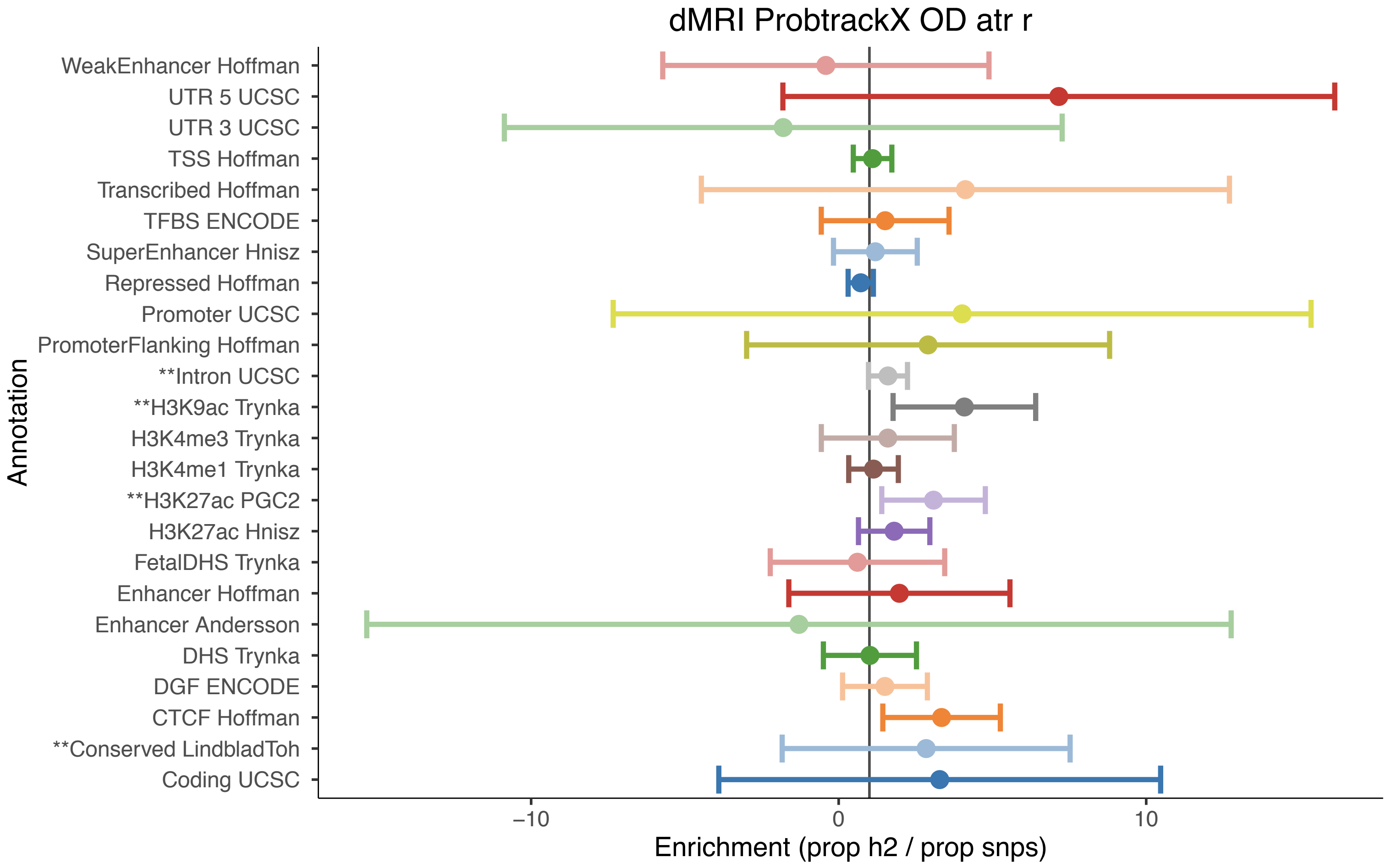

dMRI ProbtrackX OD atr r
Annotation
WeakEnhancer Hoffman
UTR 5 UCSC
UTR 3 UCSC
TSS Hoffman
Transcribed Hoffman
TFBS ENCODE
SuperEnhancer Hnisz
Repressed Hoffman
Promoter UCSC
PromoterFlanking Hoffman
**Intron UCSC
**H3K9ac Trynka
H3K4me3 Trynka
H3K4me1 Trynka
**H3K27ac PGC2
H3K27ac Hnisz
FetalDHS Trynka
Enhancer Hoffman
Enhancer Andersson
DHS Trynka
DGF ENCODE
CTCF Hoffman
**Conserved LindbladToh
Coding UCSC
−10
0
10
Enrichment (prop h2 / prop snps)

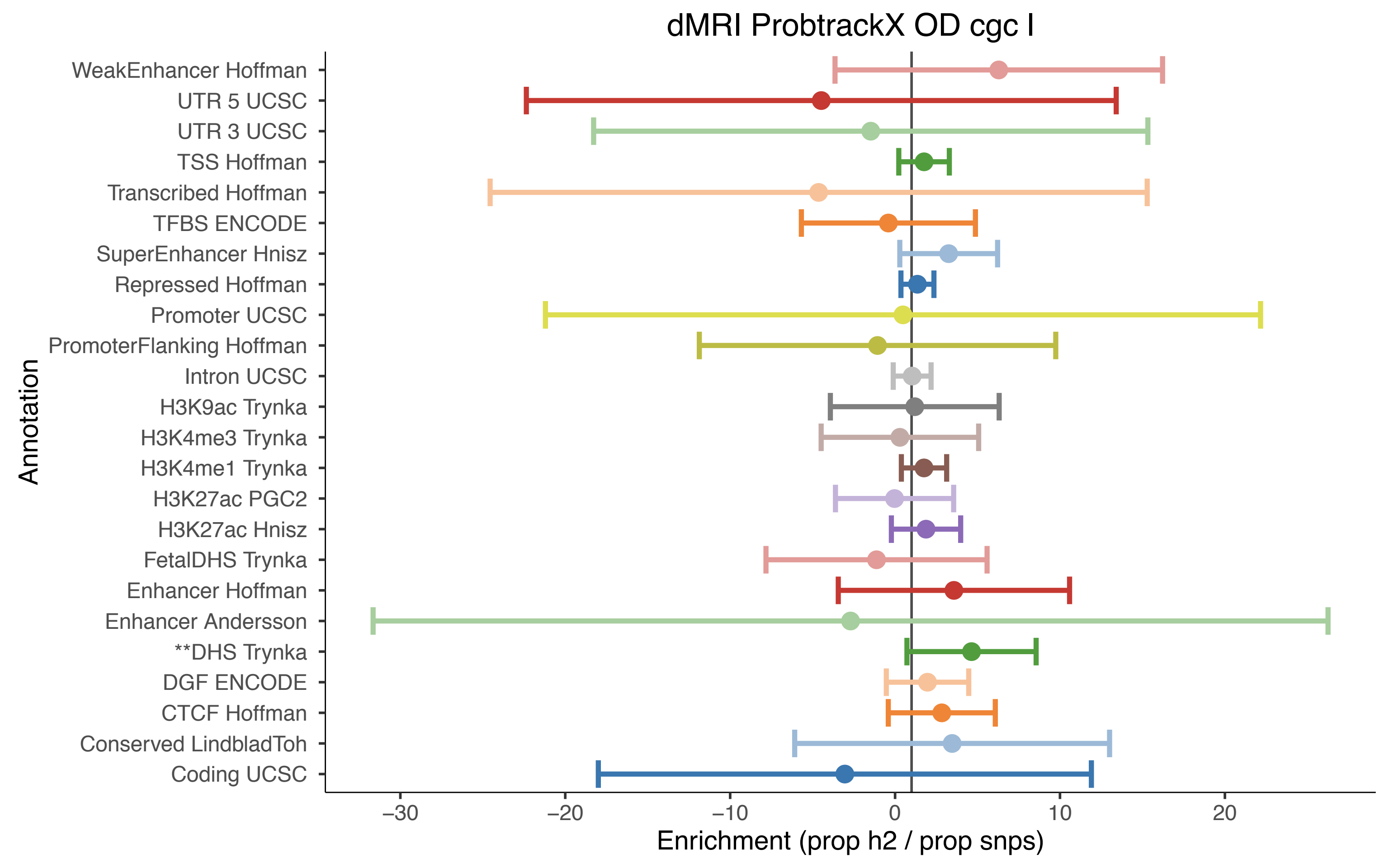

dMRI ProbtrackX OD cgc I
WeakEnhancer Hoffman
UTR 5 UCSC
UTR 3 UCSC
TSS Hoffman
Transcribed Hoffman
TFBS ENCODE
SuperEnhancer Hnisz
Repressed Hoffman
Promoter UCSC
PromoterFlanking Hoffman
Intron UCSC
H3K9ac Trynka
H3K4me3 Trynka
H3K4me1 Trynka
H3K27ac PGC2
H3K27ac Hnisz
FetalDHS Trynka
Enhancer Hoffman
Enhancer Andersson
**DHS Trynka
DGF ENCODE
CTCF Hoffman
Conserved LindbladToh
Coding UCSC
Annotation
−30
−20
−10
0
10
20
Enrichment (prop h2 / prop snps)

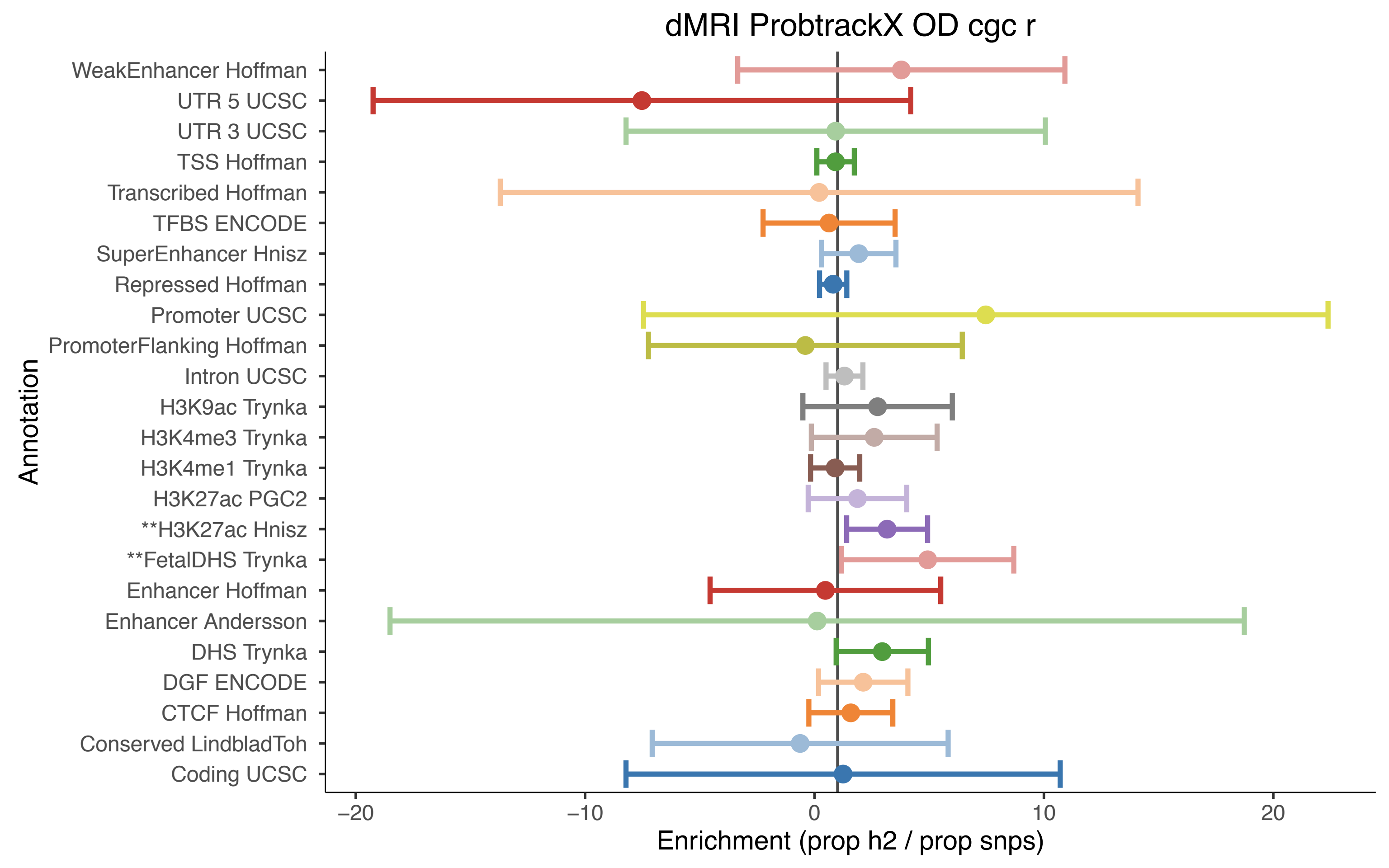
dMRI ProbtrackX OD cgc r
WeakEnhancer Hoffman
UTR 5 UCSC
UTR 3 UCSC
TSS Hoffman
Transcribed Hoffman
TFBS ENCODE
SuperEnhancer Hnisz
Repressed Hoffman
Promoter UCSC
PromoterFlanking Hoffman
Intron UCSC
H3K9ac Trynka
H3K4me3 Trynka
H3K4me1 Trynka
H3K27ac PGC2
**H3K27ac Hnisz
**FetalDHS Trynka
Enhancer Hoffman
Enhancer Andersson
DHS Trynka
DGF ENCODE
CTCF Hoffman
Conserved LindbladToh
Coding UCSC
Annotation
−20
−10
0
10
20
Enrichment (prop h2 / prop snps)

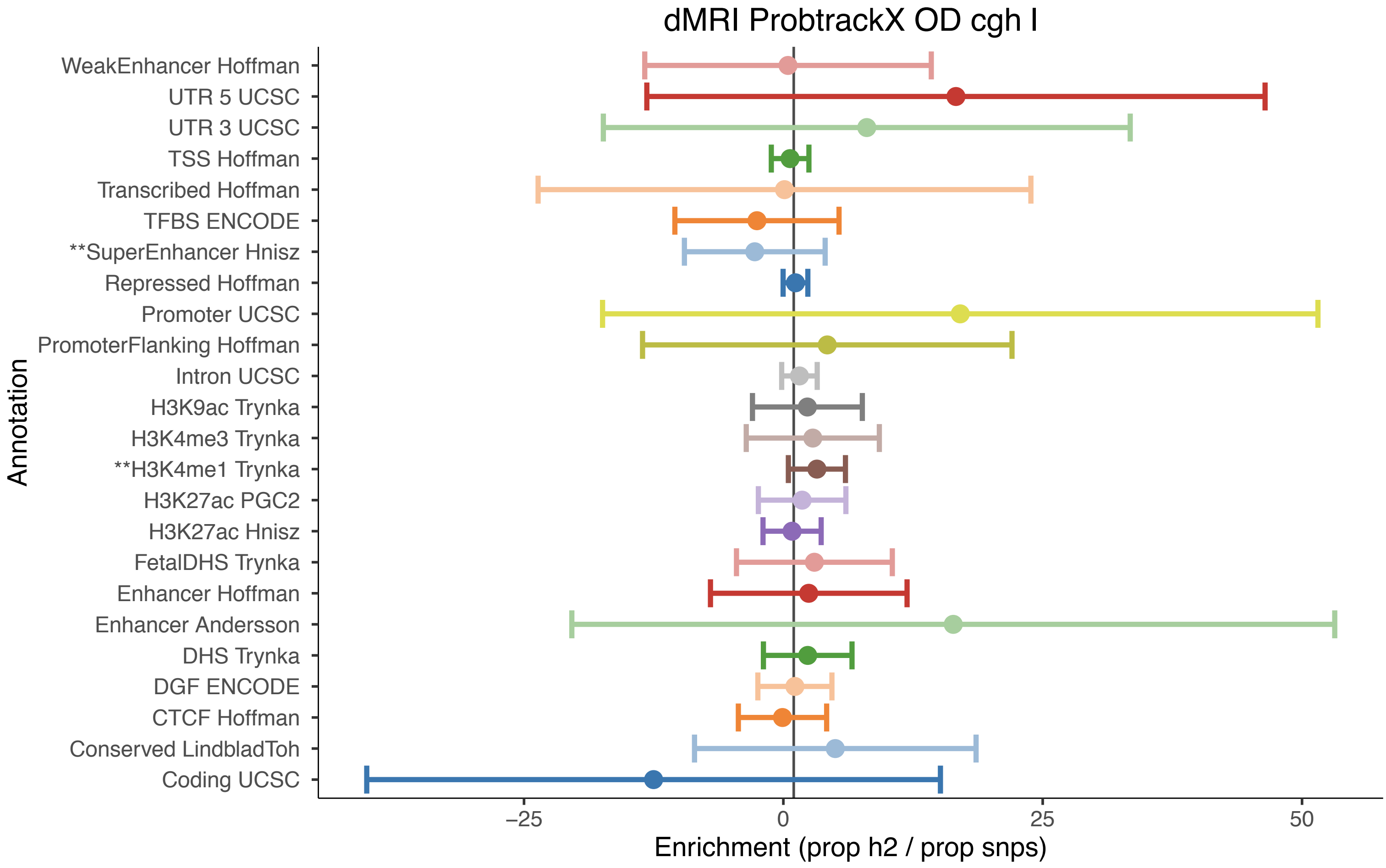
dMRI ProbtrackX OD cgh l
WeakEnhancer Hoffman
UTR 5 UCSC
UTR 3 UCSC
TSS Hoffman
Transcribed Hoffman
TFBS ENCODE
**SuperEnhancer Hnisz
Repressed Hoffman
Promoter UCSC
PromoterFlanking Hoffman
Intron UCSC
H3K9ac Trynka
H3K4me3 Trynka
**H3K4me1 Trynka
H3K27ac PGC2
H3K27ac Hnisz
FetalDHS Trynka
Enhancer Hoffman
Enhancer Andersson
DHS Trynka
DGF ENCODE
CTCF Hoffman
Conserved LindbladToh
Coding UCSC
Annotation
–25
0
25
50
Enrichment (prop h2 / prop snps)

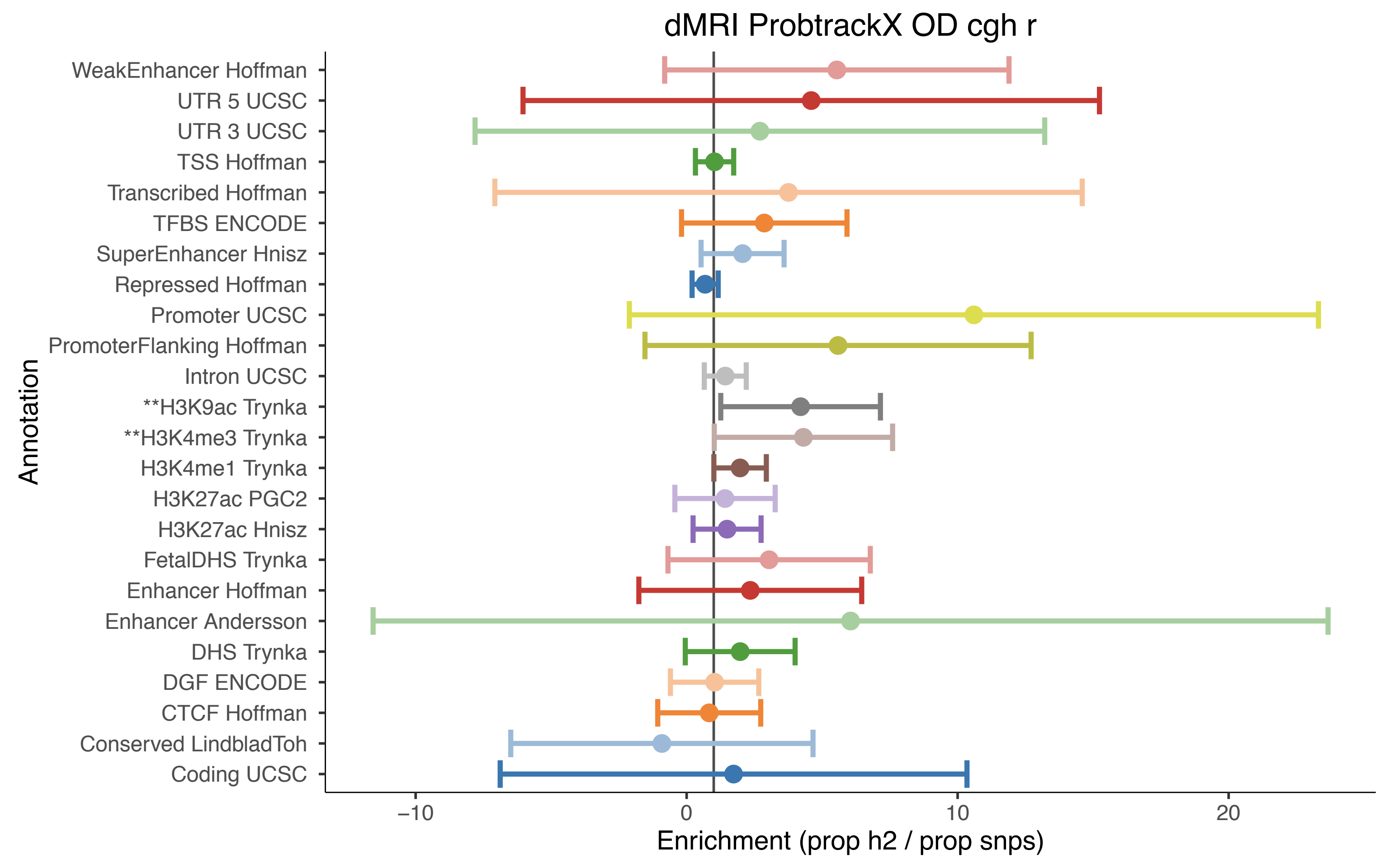
dMRI ProbtrackX OD cgh r
Annotation
WeakEnhancer Hoffman
UTR 5 UCSC
UTR 3 UCSC
TSS Hoffman
Transcribed Hoffman
TFBS ENCODE
SuperEnhancer Hnisz
Repressed Hoffman
Promoter UCSC
PromoterFlanking Hoffman
Intron UCSC
**H3K9ac Trynka
**H3K4me3 Trynka
H3K4me1 Trynka
H3K27ac PGC2
H3K27ac Hnisz
FetalDHS Trynka
Enhancer Hoffman
Enhancer Andersson
DHS Trynka
DGF ENCODE
CTCF Hoffman
Conserved LindbladToh
Coding UCSC
–10
0
10
20
Enrichment (prop h2 / prop snps)

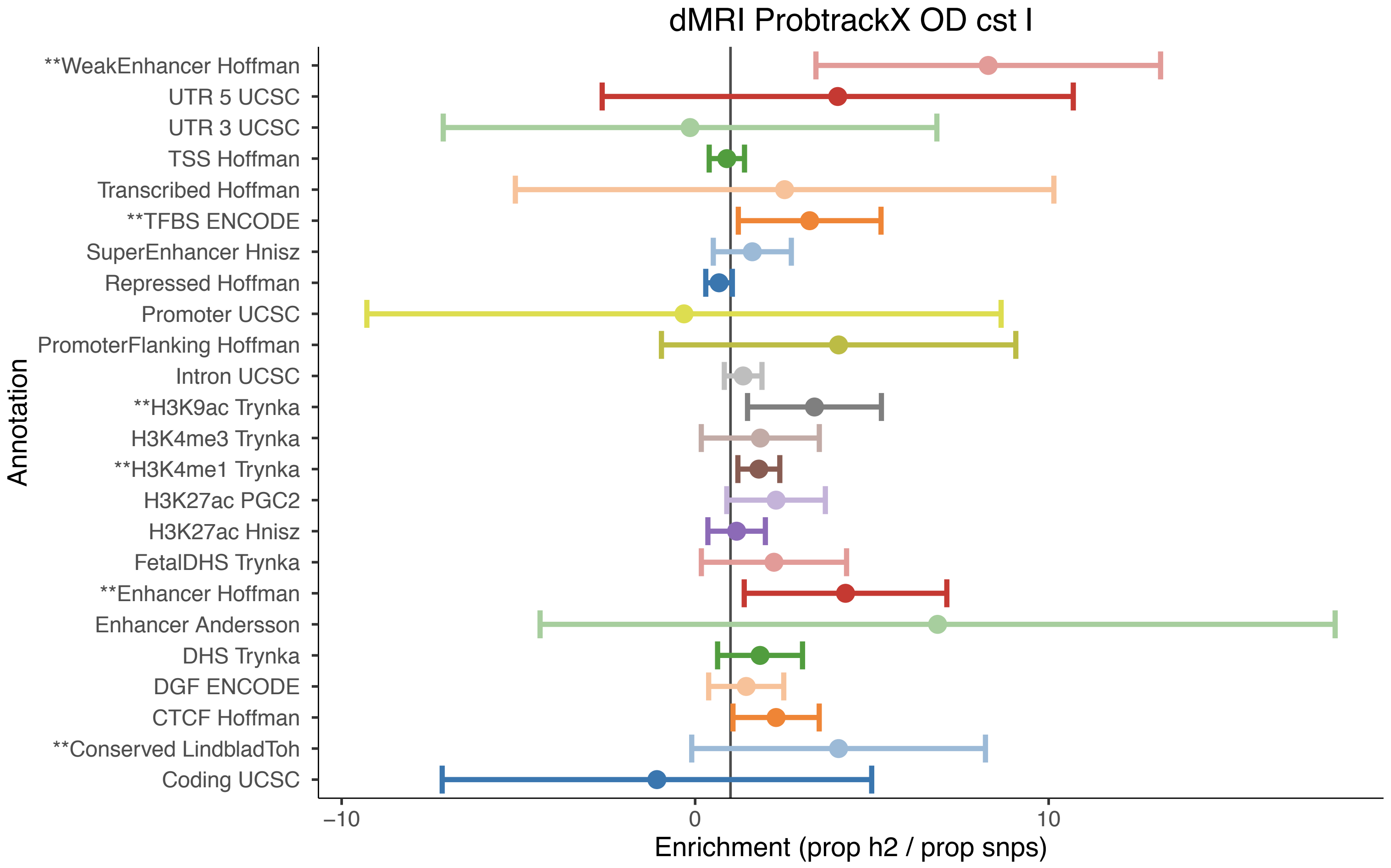

dMRI ProbtrackX OD cst l
**WeakEnhancer Hoffman
UTR 5 UCSC
UTR 3 UCSC
TSS Hoffman
Transcribed Hoffman
**TFBS ENCODE
SuperEnhancer Hnisz
Repressed Hoffman
Promoter UCSC
PromoterFlanking Hoffman
Intron UCSC
**H3K9ac Trynka
H3K4me3 Trynka
**H3K4me1 Trynka
H3K27ac PGC2
H3K27ac Hnisz
FetalDHS Trynka
**Enhancer Hoffman
Enhancer Andersson
DHS Trynka
DGF ENCODE
CTCF Hoffman
**Conserved LindbladToh
Coding UCSC
Annotation
−10
0
10
Enrichment (prop h2 / prop snps)

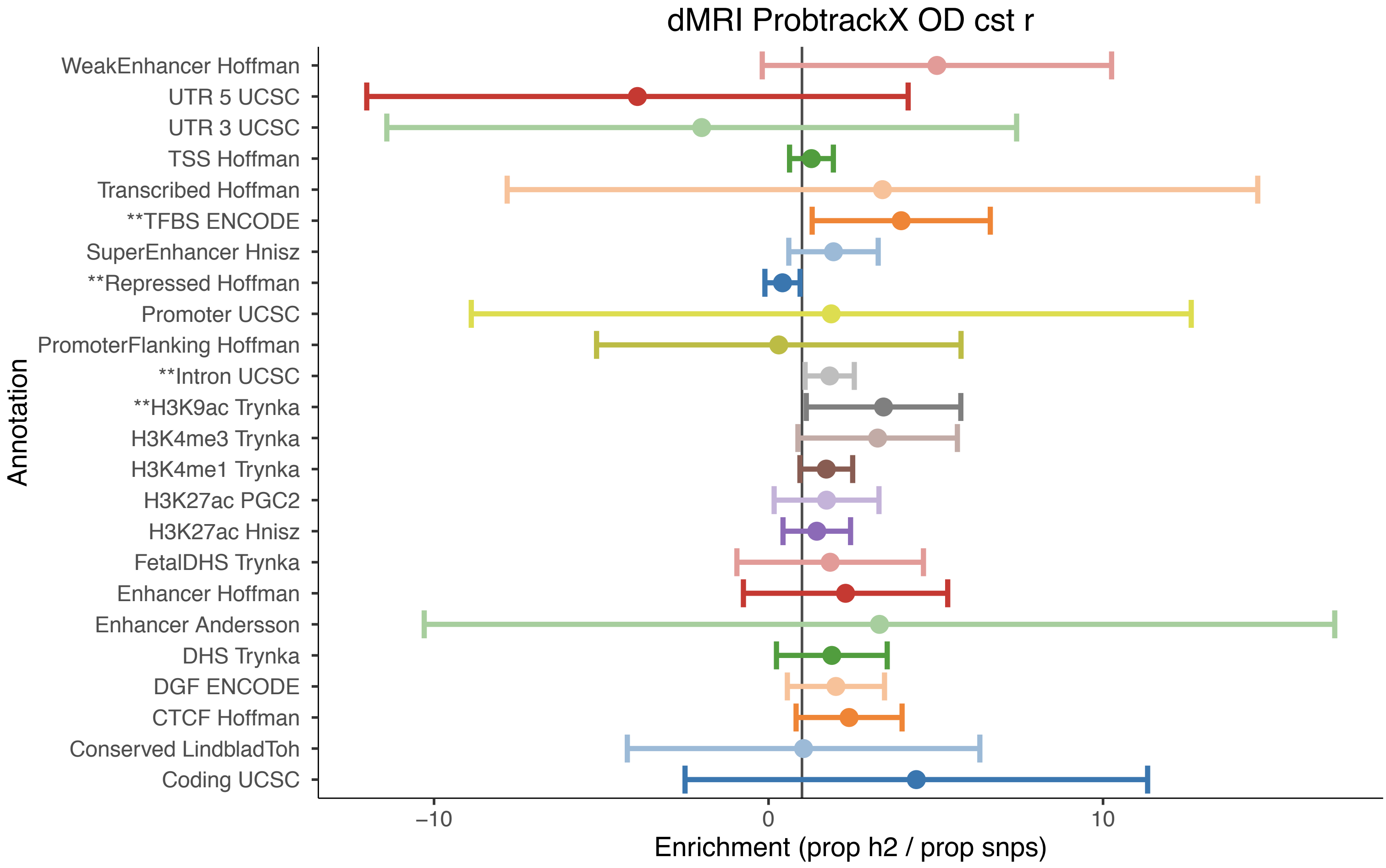

dMRI ProbtrackX OD cst r
WeakEnhancer Hoffman
UTR 5 UCSC
UTR 3 UCSC
TSS Hoffman
Transcribed Hoffman
**TFBS ENCODE
SuperEnhancer Hnisz
**Repressed Hoffman
Promoter UCSC
PromoterFlanking Hoffman
**Intron UCSC
**H3K9ac Trynka
H3K4me3 Trynka
H3K4me1 Trynka
H3K27ac PGC2
H3K27ac Hnisz
FetalDHS Trynka
Enhancer Hoffman
Enhancer Andersson
DHS Trynka
DGF ENCODE
CTCF Hoffman
Conserved LindbladToh
Coding UCSC
Annotation
−10
0
10
Enrichment (prop h2 / prop snps)

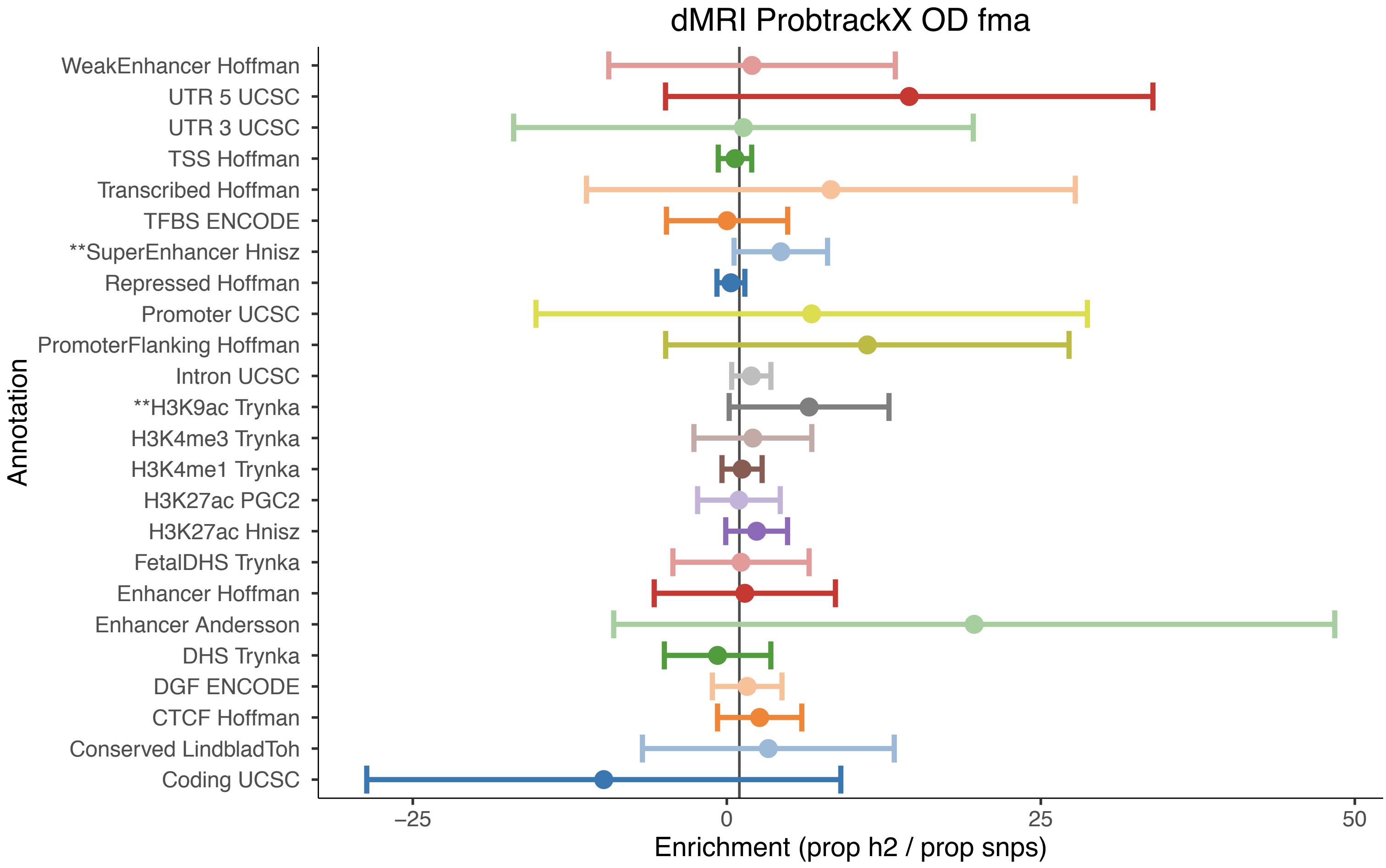

dMRI ProbtrackX OD fma
WeakEnhancer Hoffman
UTR 5 UCSC
UTR 3 UCSC
TSS Hoffman
Transcribed Hoffman
TFBS ENCODE
**SuperEnhancer Hnisz
Repressed Hoffman
Promoter UCSC
PromoterFlanking Hoffman
Intron UCSC
**H3K9ac Trynka
H3K4me3 Trynka
H3K4me1 Trynka
H3K27ac PGC2
H3K27ac Hnisz
FetalDHS Trynka
Enhancer Hoffman
Enhancer Andersson
DHS Trynka
DGF ENCODE
CTCF Hoffman
Conserved LindbladToh
Coding UCSC
Annotation
−25
0
25
50
Enrichment (prop h2 / prop snps)

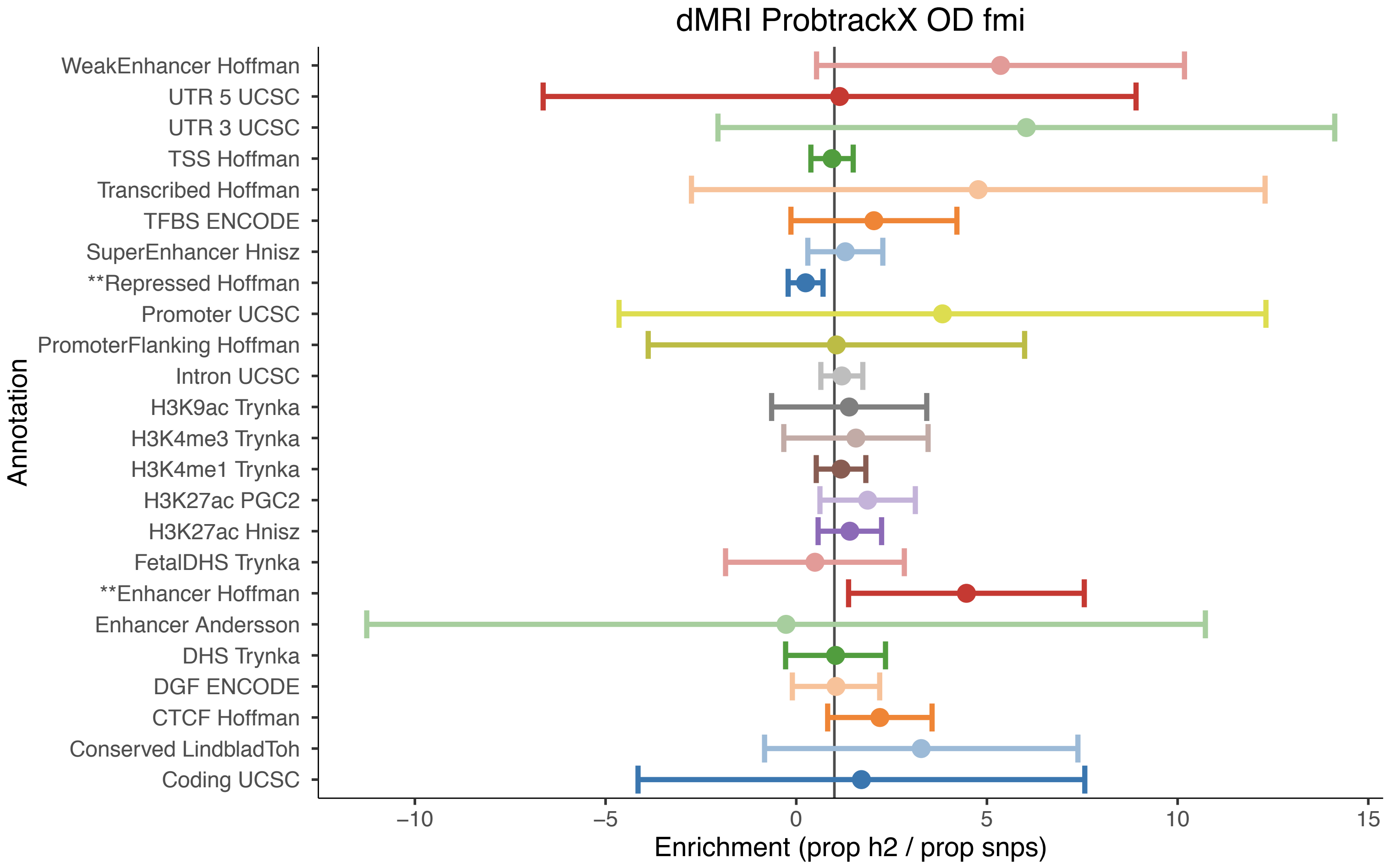
dMRI ProbtrackX OD fmi
WeakEnhancer Hoffman
UTR 5 UCSC
UTR 3 UCSC
TSS Hoffman
Transcribed Hoffman
TFBS ENCODE
SuperEnhancer Hnisz
**Repressed Hoffman
Promoter UCSC
PromoterFlanking Hoffman
Intron UCSC
H3K9ac Trynka
H3K4me3 Trynka
H3K4me1 Trynka
H3K27ac PGC2
H3K27ac Hnisz
FetalDHS Trynka
**Enhancer Hoffman
Enhancer Andersson
DHS Trynka
DGF ENCODE
CTCF Hoffman
Conserved LindbladToh
Coding UCSC
Annotation
−10
−5
0
5
10
15
Enrichment (prop h2 / prop snps)

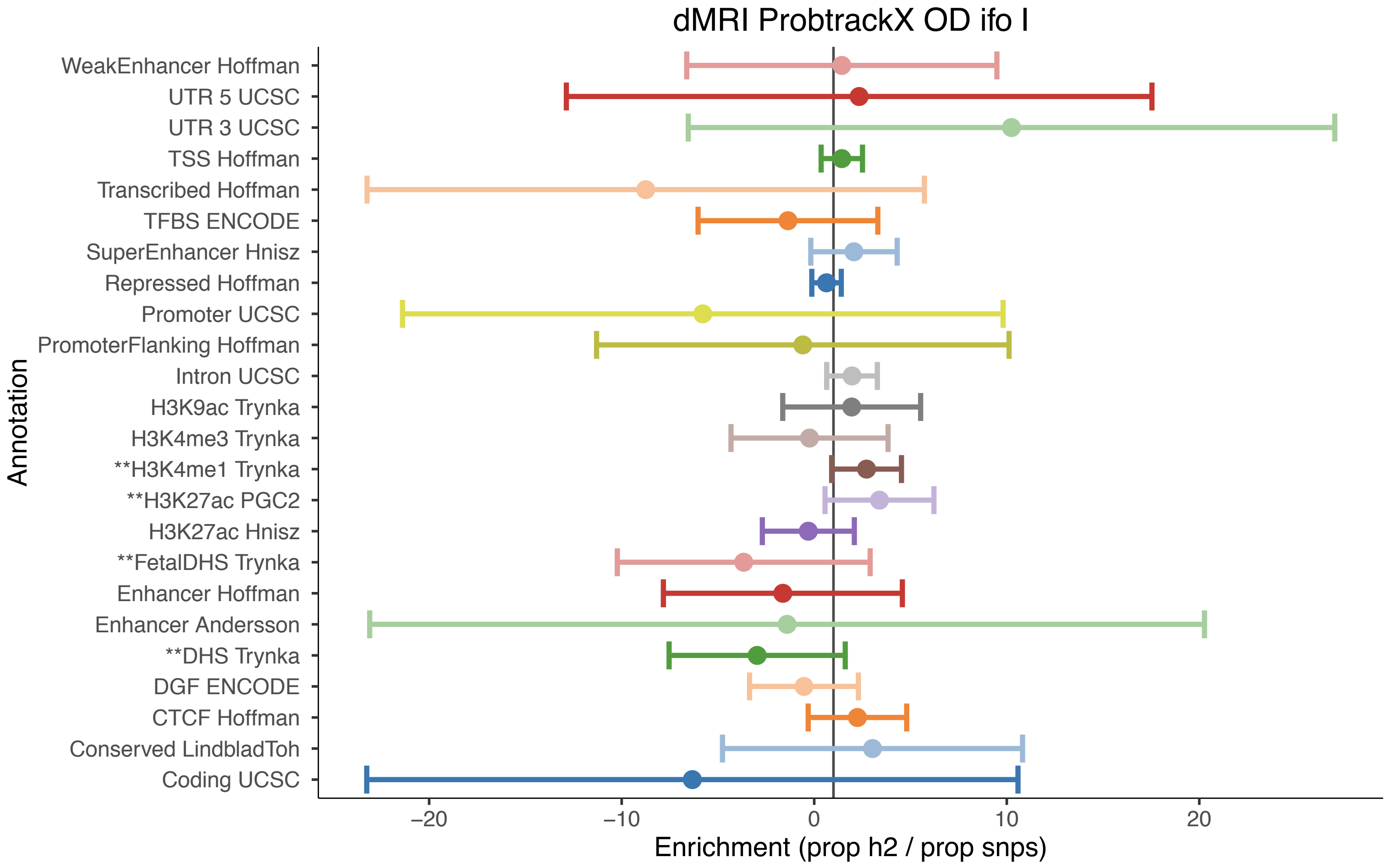

dMRI ProbtrackX OD ifo l
Annotation
WeakEnhancer Hoffman
UTR 5 UCSC
UTR 3 UCSC
TSS Hoffman
Transcribed Hoffman
TFBS ENCODE
SuperEnhancer Hnisz
Repressed Hoffman
Promoter UCSC
PromoterFlanking Hoffman
Intron UCSC
H3K9ac Trynka
H3K4me3 Trynka
**H3K4me1 Trynka
**H3K27ac PGC2
H3K27ac Hnisz
**FetalDHS Trynka
Enhancer Hoffman
Enhancer Andersson
**DHS Trynka
DGF ENCODE
CTCF Hoffman
Conserved LindbladToh
Coding UCSC
−20
−10
0
10
20
Enrichment (prop h2 / prop snps)

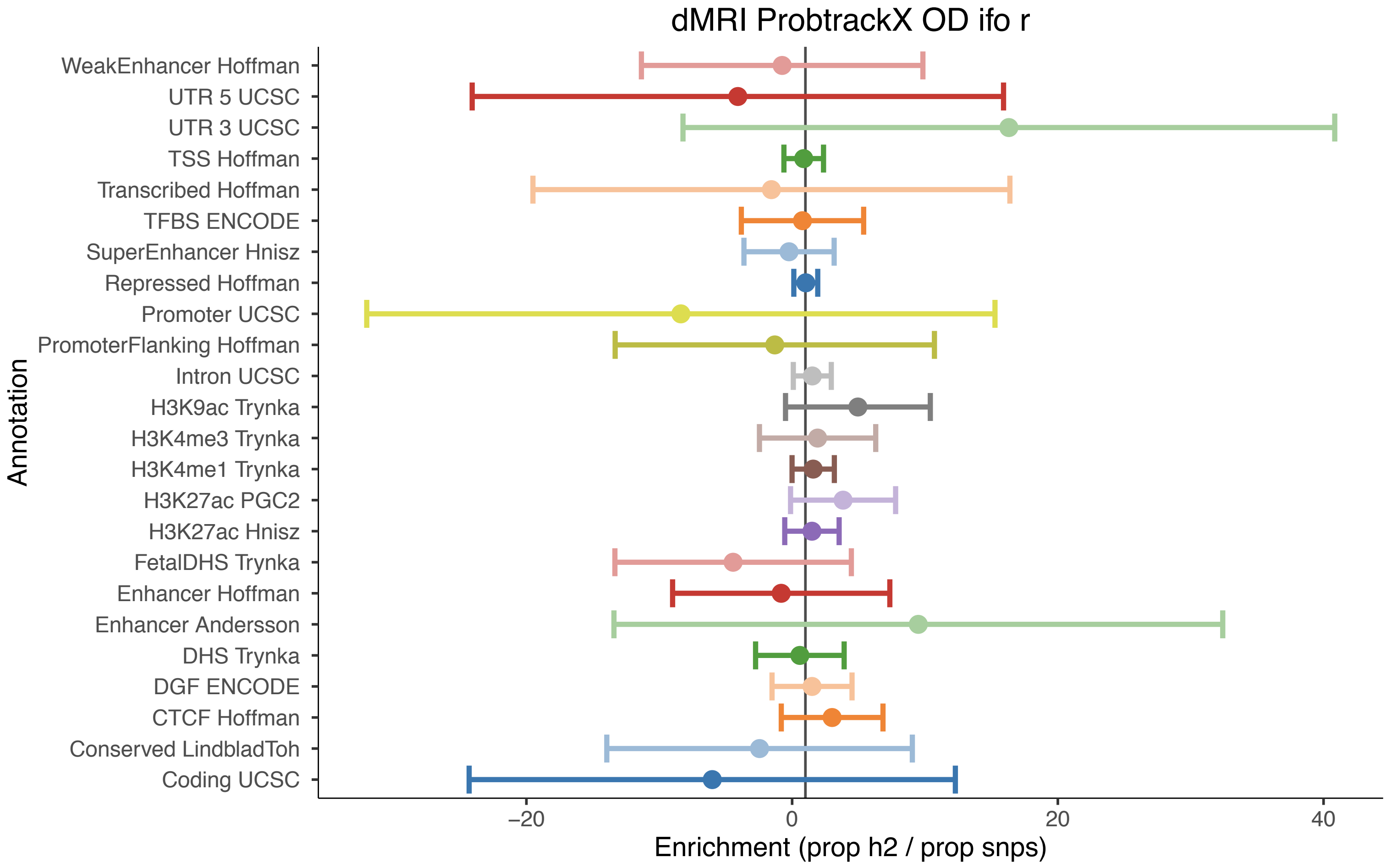
dMRI ProbtrackX OD ifo r
WeakEnhancer Hoffman
UTR 5 UCSC
UTR 3 UCSC
TSS Hoffman
Transcribed Hoffman
TFBS ENCODE
SuperEnhancer Hnisz
Repressed Hoffman
Promoter UCSC
PromoterFlanking Hoffman
Intron UCSC
H3K9ac Trynka
H3K4me3 Trynka
H3K4me1 Trynka
H3K27ac PGC2
H3K27ac Hnisz
FetalDHS Trynka
Enhancer Hoffman
Enhancer Andersson
DHS Trynka
DGF ENCODE
CTCF Hoffman
Conserved LindbladToh
Coding UCSC
Annotation
−20
0
20
40
Enrichment (prop h2 / prop snps)

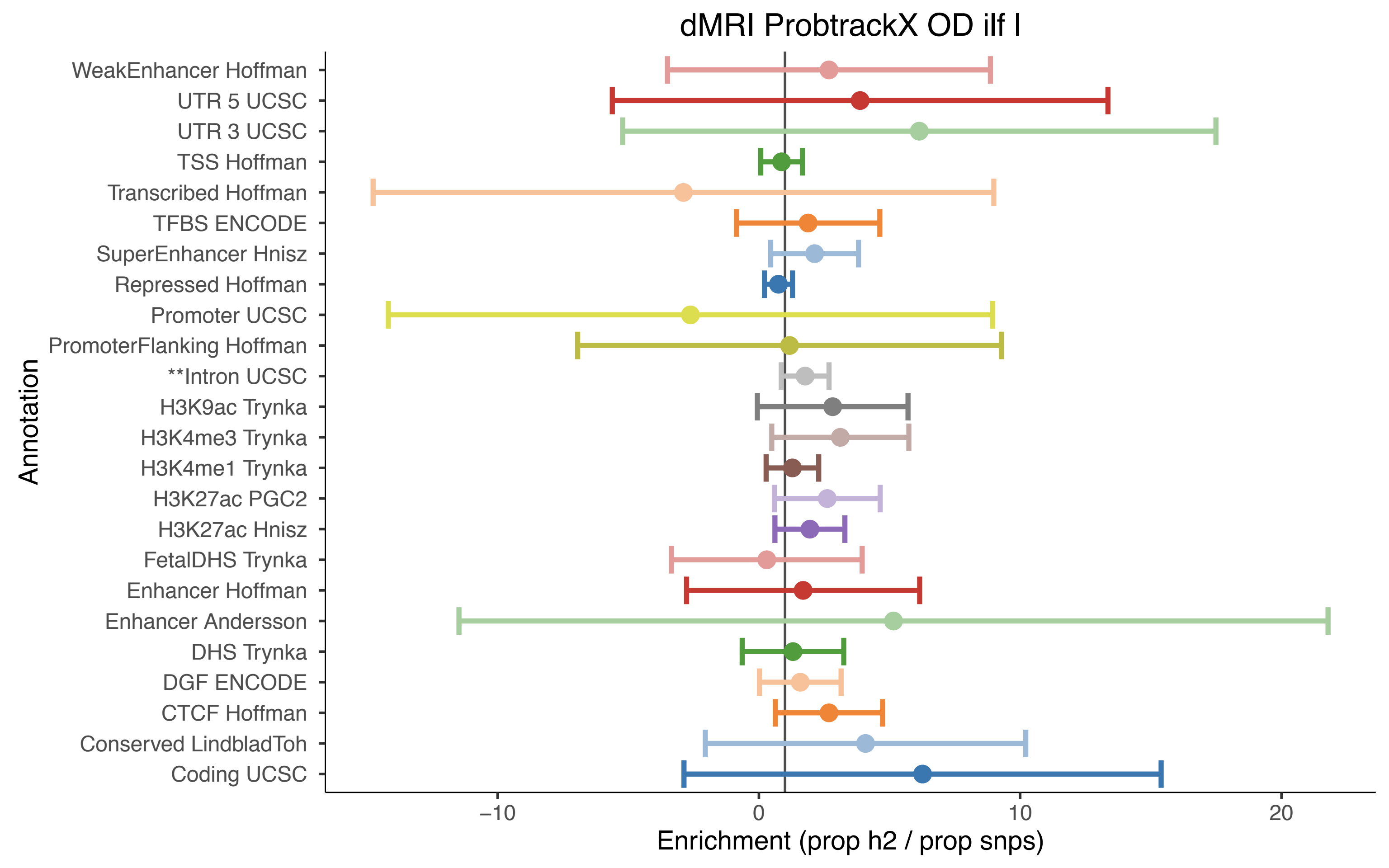
dMRI ProbtrackX OD ilf l
WeakEnhancer Hoffman
UTR 5 UCSC
UTR 3 UCSC
TSS Hoffman
Transcribed Hoffman
TFBS ENCODE
SuperEnhancer Hnisz
Repressed Hoffman
Promoter UCSC
PromoterFlanking Hoffman
**Intron UCSC
H3K9ac Trynka
H3K4me3 Trynka
H3K4me1 Trynka
H3K27ac PGC2
H3K27ac Hnisz
FetalDHS Trynka
Enhancer Hoffman
Enhancer Andersson
DHS Trynka
DGF ENCODE
CTCF Hoffman
Conserved LindbladToh
Coding UCSC
Annotation
−10
0
10
20
Enrichment (prop h2 / prop snps)

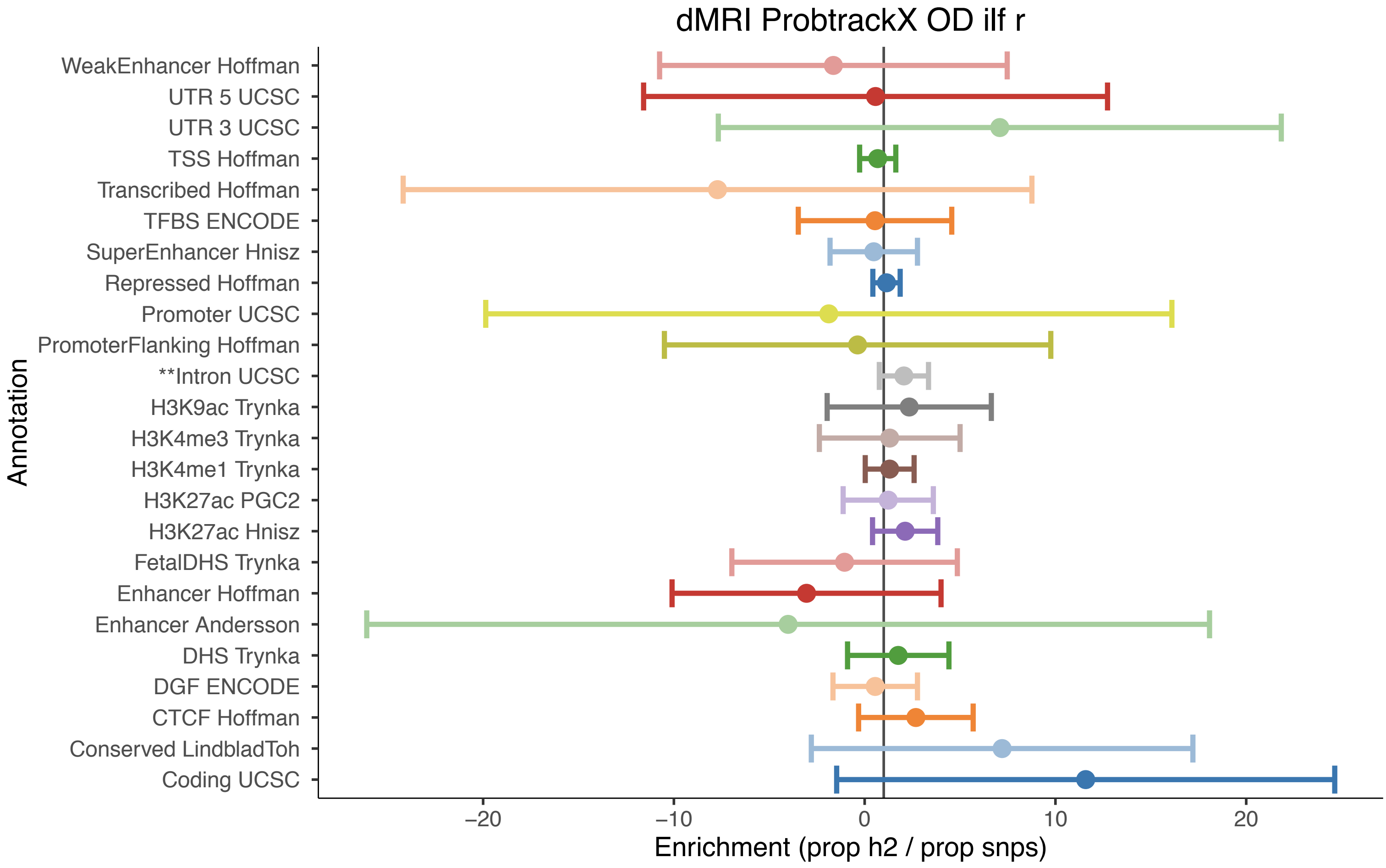
dMRI ProbtrackX OD ilf r
Annotation
WeakEnhancer Hoffman
UTR 5 UCSC
UTR 3 UCSC
TSS Hoffman
Transcribed Hoffman
TFBS ENCODE
SuperEnhancer Hnisz
Repressed Hoffman
Promoter UCSC
PromoterFlanking Hoffman
**Intron UCSC
H3K9ac Trynka
H3K4me3 Trynka
H3K4me1 Trynka
H3K27ac PGC2
H3K27ac Hnisz
FetalDHS Trynka
Enhancer Hoffman
Enhancer Andersson
DHS Trynka
DGF ENCODE
CTCF Hoffman
Conserved LindbladToh
Coding UCSC
−20
−10
0
10
20
Enrichment (prop h2 / prop snps)

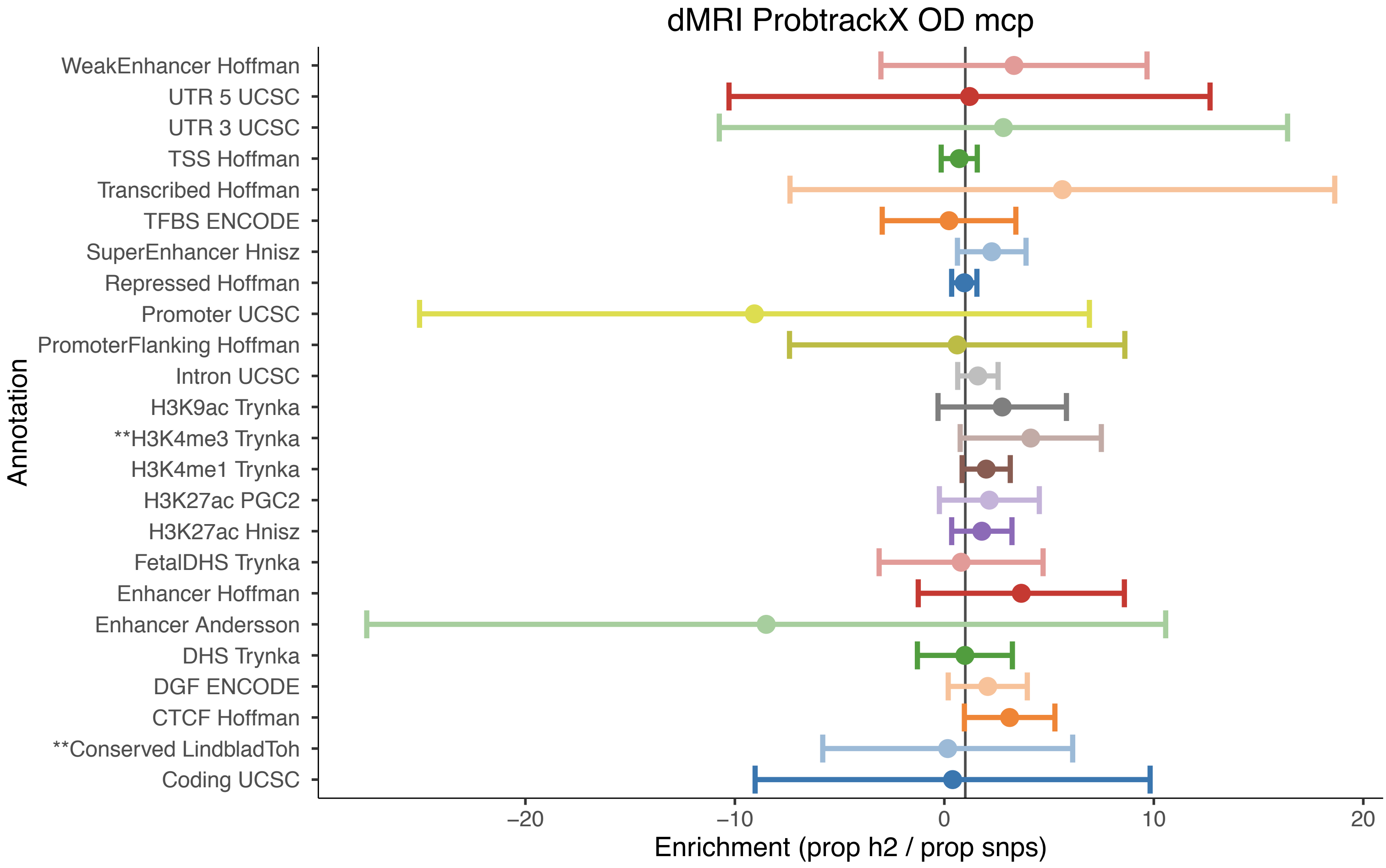

dMRI ProbtrackX OD mcp
WeakEnhancer Hoffman
UTR 5 UCSC
UTR 3 UCSC
TSS Hoffman
Transcribed Hoffman
TFBS ENCODE
SuperEnhancer Hnisz
Repressed Hoffman
Promoter UCSC
PromoterFlanking Hoffman
Intron UCSC
H3K9ac Trynka
**H3K4me3 Trynka
H3K4me1 Trynka
H3K27ac PGC2
H3K27ac Hnisz
FetalDHS Trynka
Enhancer Hoffman
Enhancer Andersson
DHS Trynka
DGF ENCODE
CTCF Hoffman
**Conserved LindbladToh
Coding UCSC
Annotation
−20
−10
0
10
20
Enrichment (prop h2 / prop snps)

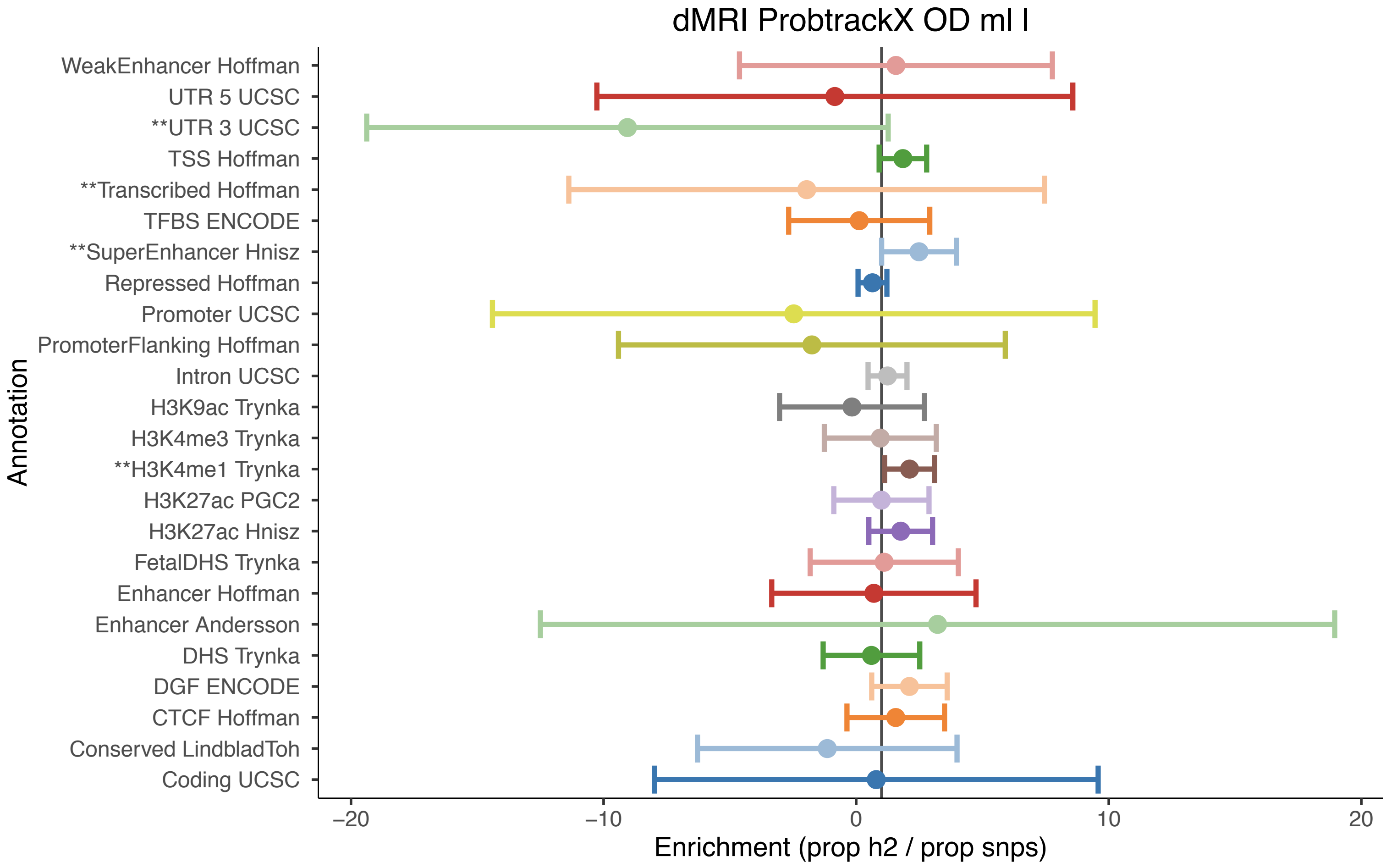
dMRI ProbtrackX OD ml l
WeakEnhancer Hoffman
UTR 5 UCSC
**UTR 3 UCSC
TSS Hoffman
**Transcribed Hoffman
TFBS ENCODE
**SuperEnhancer Hnisz
Repressed Hoffman
Promoter UCSC
PromoterFlanking Hoffman
Intron UCSC
H3K9ac Trynka
H3K4me3 Trynka
**H3K4me1 Trynka
H3K27ac PGC2
H3K27ac Hnisz
FetalDHS Trynka
Enhancer Hoffman
Enhancer Andersson
DHS Trynka
DGF ENCODE
CTCF Hoffman
Conserved LindbladToh
Coding UCSC
Annotation
−20
−10
0
10
20
Enrichment (prop h2 / prop snps)

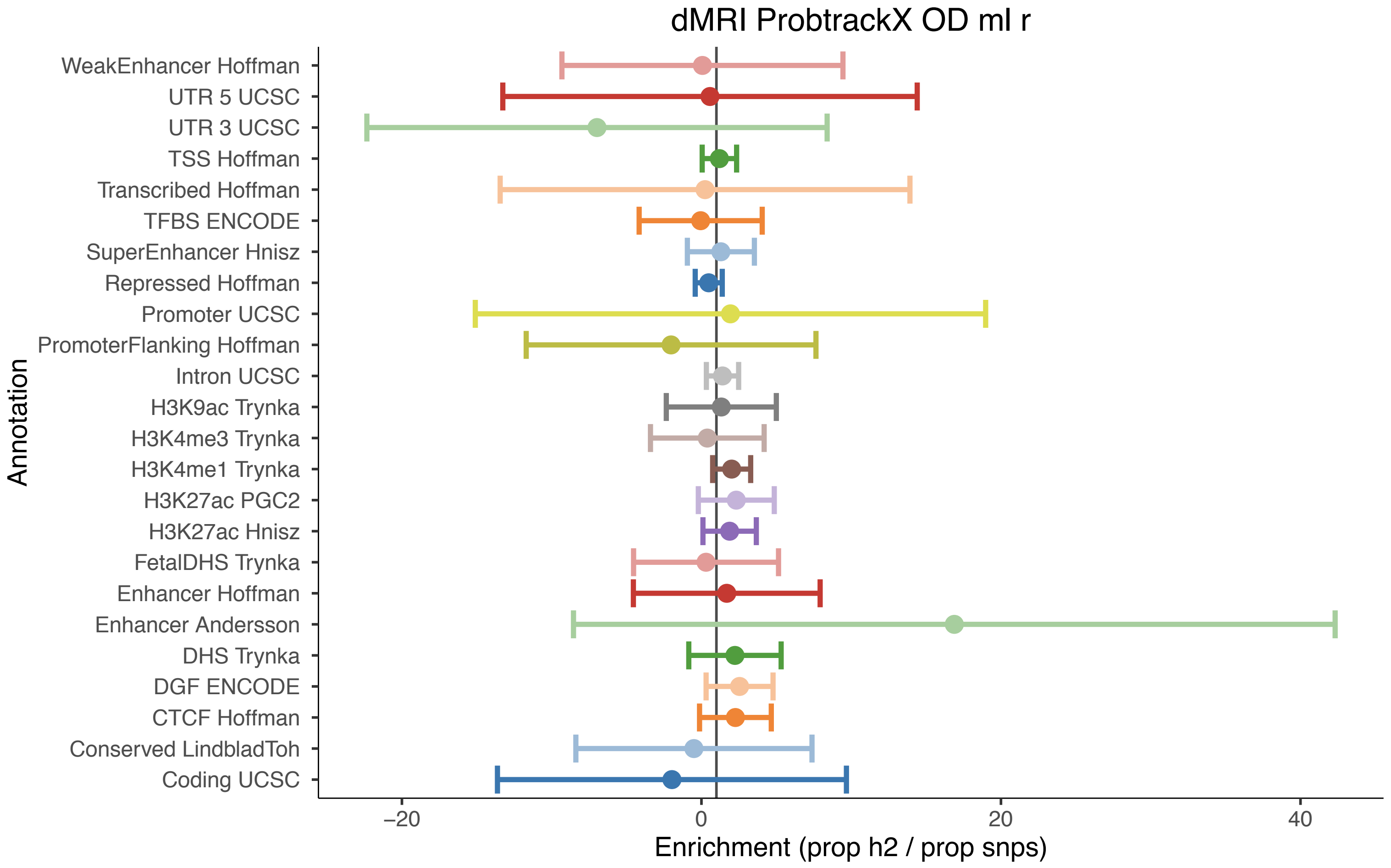
dMRI ProbtrackX OD ml r
Annotation
WeakEnhancer Hoffman
UTR 5 UCSC
UTR 3 UCSC
TSS Hoffman
Transcribed Hoffman
TFBS ENCODE
SuperEnhancer Hnisz
Repressed Hoffman
Promoter UCSC
PromoterFlanking Hoffman
Intron UCSC
H3K9ac Trynka
H3K4me3 Trynka
H3K4me1 Trynka
H3K27ac PGC2
H3K27ac Hnisz
FetalDHS Trynka
Enhancer Hoffman
Enhancer Andersson
DHS Trynka
DGF ENCODE
CTCF Hoffman
Conserved LindbladToh
Coding UCSC
−20
0
20
40
Enrichment (prop h2 / prop snps)

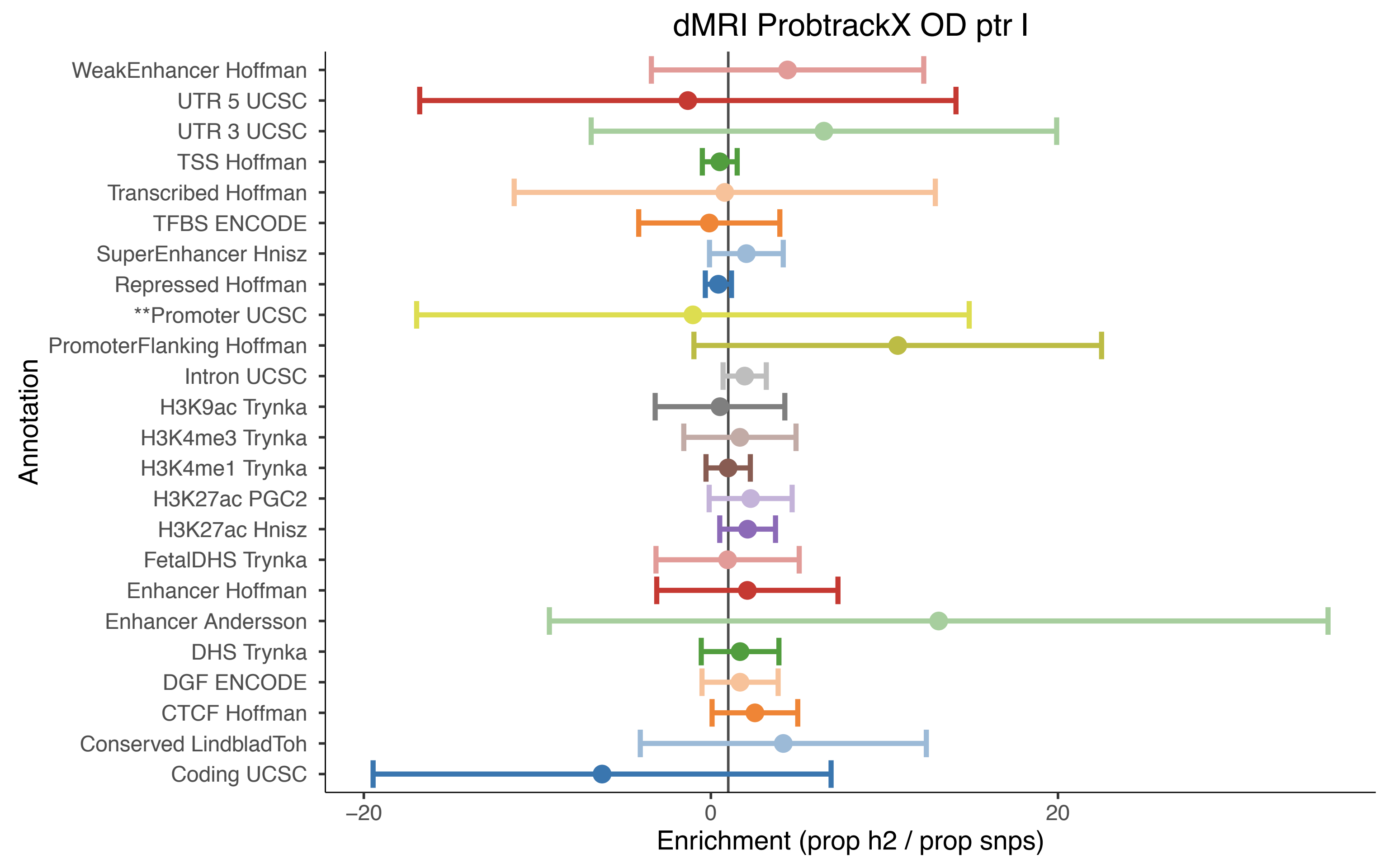

dMRI ProbtrackX OD ptr l
WeakEnhancer Hoffman
UTR 5 UCSC
UTR 3 UCSC
TSS Hoffman
Transcribed Hoffman
TFBS ENCODE
SuperEnhancer Hnisz
Repressed Hoffman
**Promoter UCSC
PromoterFlanking Hoffman
Intron UCSC
H3K9ac Trynka
H3K4me3 Trynka
H3K4me1 Trynka
H3K27ac PGC2
H3K27ac Hnisz
FetalDHS Trynka
Enhancer Hoffman
Enhancer Andersson
DHS Trynka
DGF ENCODE
CTCF Hoffman
Conserved LindbladToh
Coding UCSC
Annotation
−20
0
20
Enrichment (prop h2 / prop snps)

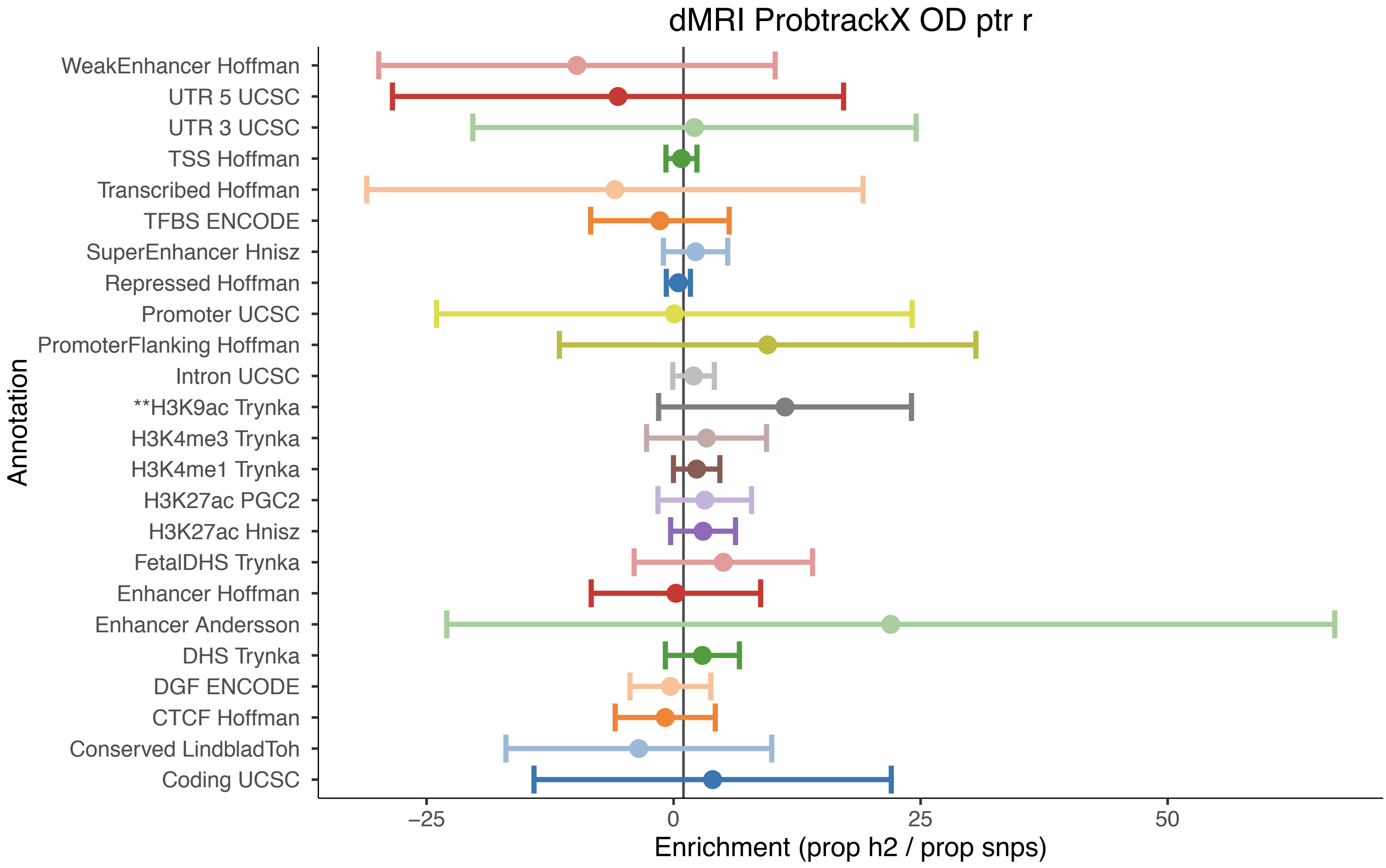
dMRI ProbtrackX OD ptr r
WeakEnhancer Hoffman
UTR 5 UCSC
UTR 3 UCSC
TSS Hoffman
Transcribed Hoffman
TFBS ENCODE
SuperEnhancer Hnisz
Repressed Hoffman
Promoter UCSC
PromoterFlanking Hoffman
Intron UCSC
**H3K9ac Trynka
H3K4me3 Trynka
H3K4me1 Trynka
H3K27ac PGC2
H3K27ac Hnisz
FetalDHS Trynka
Enhancer Hoffman
Enhancer Andersson
DHS Trynka
DGF ENCODE
CTCF Hoffman
Conserved LindbladToh
Coding UCSC
Annotation
−25
0
25
50
Enrichment (prop h2 / prop snps)

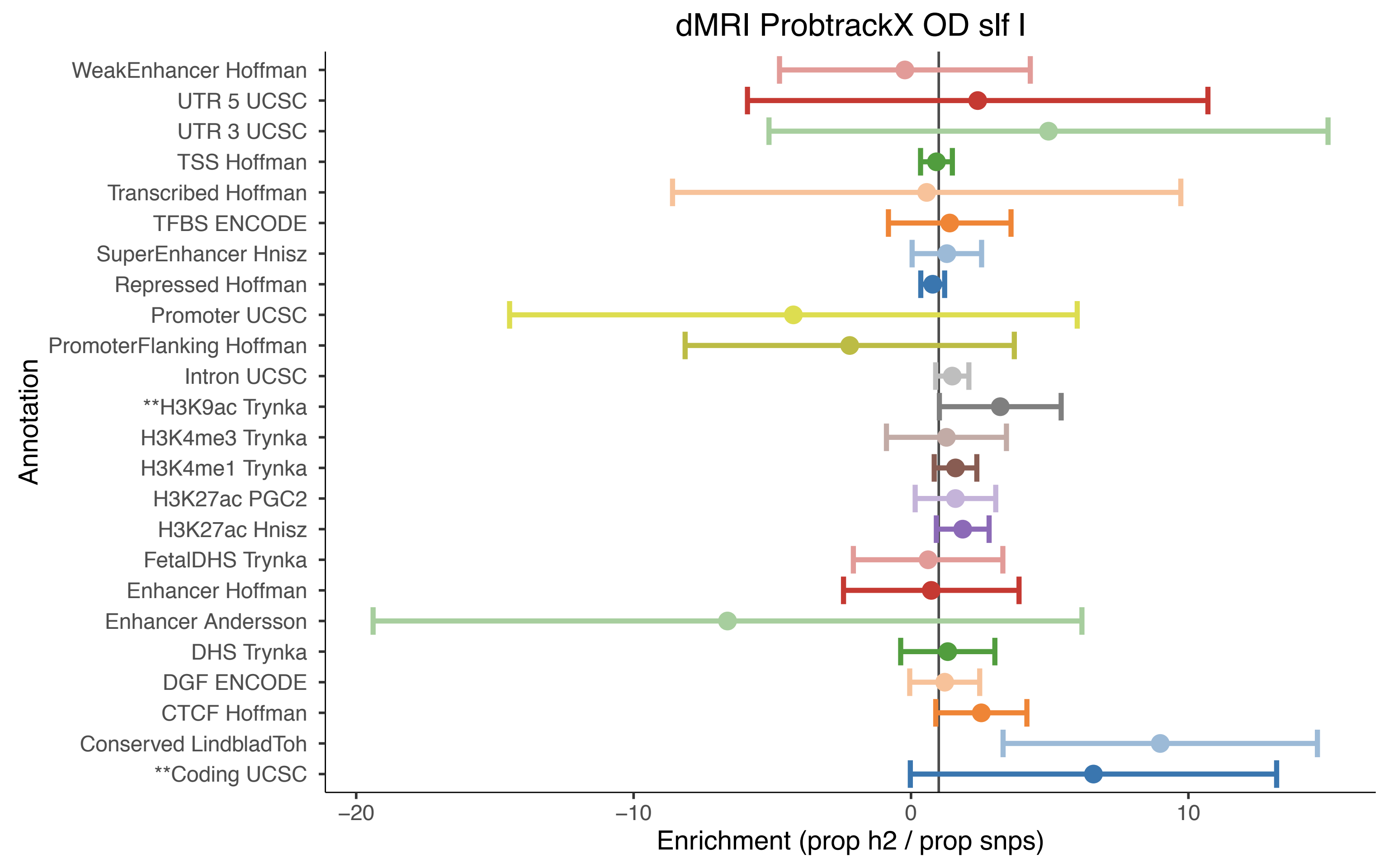
dMRI ProbtrackX OD slf I
Annotation
WeakEnhancer Hoffman
UTR 5 UCSC
UTR 3 UCSC
TSS Hoffman
Transcribed Hoffman
TFBS ENCODE
SuperEnhancer Hnisz
Repressed Hoffman
Promoter UCSC
PromoterFlanking Hoffman
Intron UCSC
**H3K9ac Trynka
H3K4me3 Trynka
H3K4me1 Trynka
H3K27ac PGC2
H3K27ac Hnisz
FetalDHS Trynka
Enhancer Hoffman
Enhancer Andersson
DHS Trynka
DGF ENCODE
CTCF Hoffman
Conserved LindbladToh
**Coding UCSC
−20
−10
0
10
Enrichment (prop h2 / prop snps)

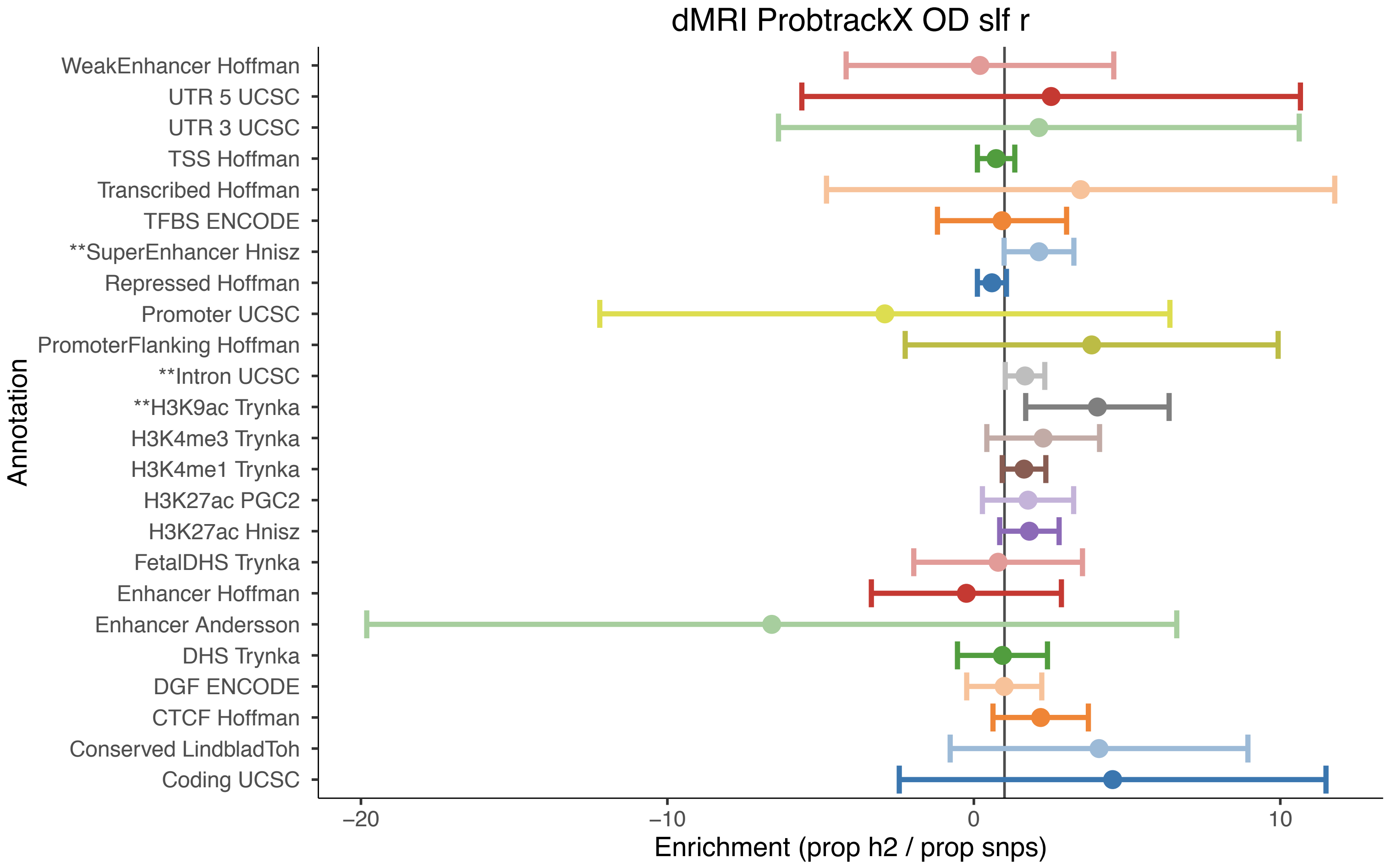
dMRI ProbtrackX OD slf r
WeakEnhancer Hoffman
UTR 5 UCSC
UTR 3 UCSC
TSS Hoffman
Transcribed Hoffman
TFBS ENCODE
**SuperEnhancer Hnisz
Repressed Hoffman
Promoter UCSC
PromoterFlanking Hoffman
**Intron UCSC
**H3K9ac Trynka
H3K4me3 Trynka
H3K4me1 Trynka
H3K27ac PGC2
H3K27ac Hnisz
FetalDHS Trynka
Enhancer Hoffman
Enhancer Andersson
DHS Trynka
DGF ENCODE
CTCF Hoffman
Conserved LindbladToh
Coding UCSC
Annotation
−20
−10
0
10
Enrichment (prop h2 / prop snps)

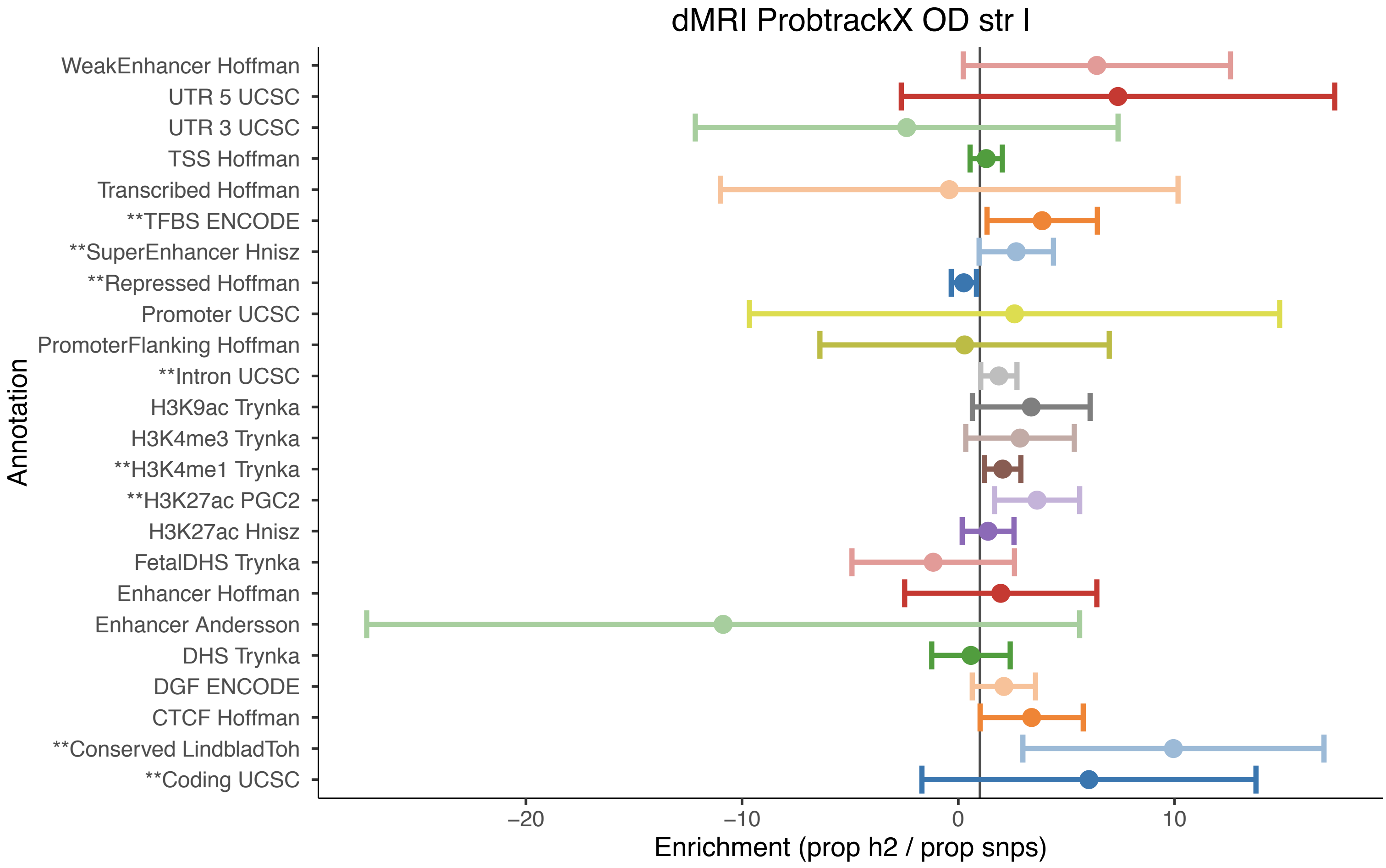

dMRI ProbtrackX OD str I
WeakEnhancer Hoffman
UTR 5 UCSC
UTR 3 UCSC
TSS Hoffman
Transcribed Hoffman
**TFBS ENCODE
**SuperEnhancer Hnisz
**Repressed Hoffman
Promoter UCSC
PromoterFlanking Hoffman
**Intron UCSC
H3K9ac Trynka
H3K4me3 Trynka
**H3K4me1 Trynka
**H3K27ac PGC2
H3K27ac Hnisz
FetalDHS Trynka
Enhancer Hoffman
Enhancer Andersson
DHS Trynka
DGF ENCODE
CTCF Hoffman
**Conserved LindbladToh
**Coding UCSC
Annotation
−20
−10
0
10
Enrichment (prop h2 / prop snps)

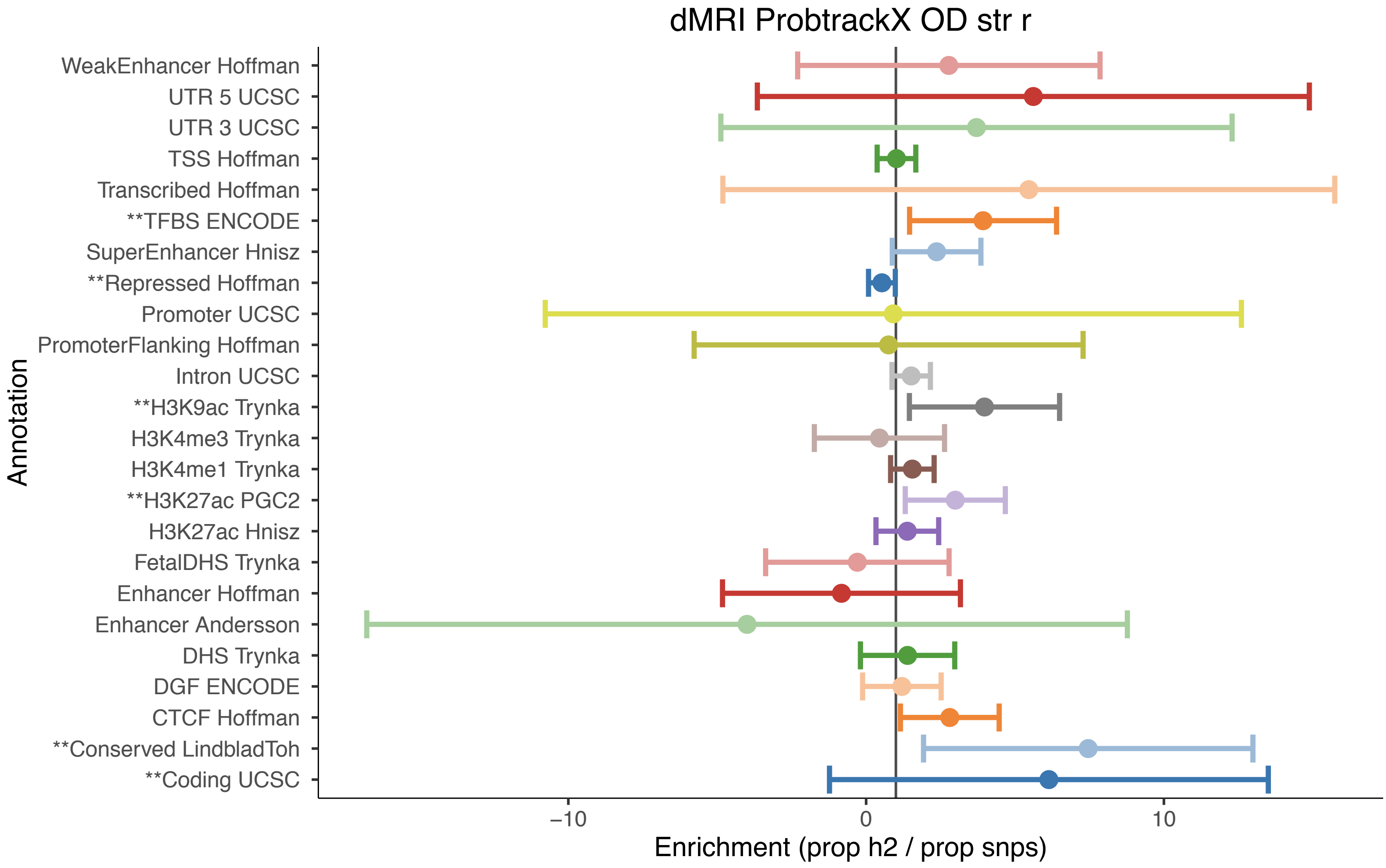

dMRI ProbtrackX OD str r
WeakEnhancer Hoffman
UTR 5 UCSC
UTR 3 UCSC
TSS Hoffman
Transcribed Hoffman
**TFBS ENCODE
SuperEnhancer Hnisz
**Repressed Hoffman
Promoter UCSC
PromoterFlanking Hoffman
Intron UCSC
**H3K9ac Trynka
H3K4me3 Trynka
H3K4me1 Trynka
**H3K27ac PGC2
H3K27ac Hnisz
FetalDHS Trynka
Enhancer Hoffman
Enhancer Andersson
DHS Trynka
DGF ENCODE
CTCF Hoffman
**Conserved LindbladToh
**Coding UCSC
Annotation
−10
0
10
Enrichment (prop h2 / prop snps)

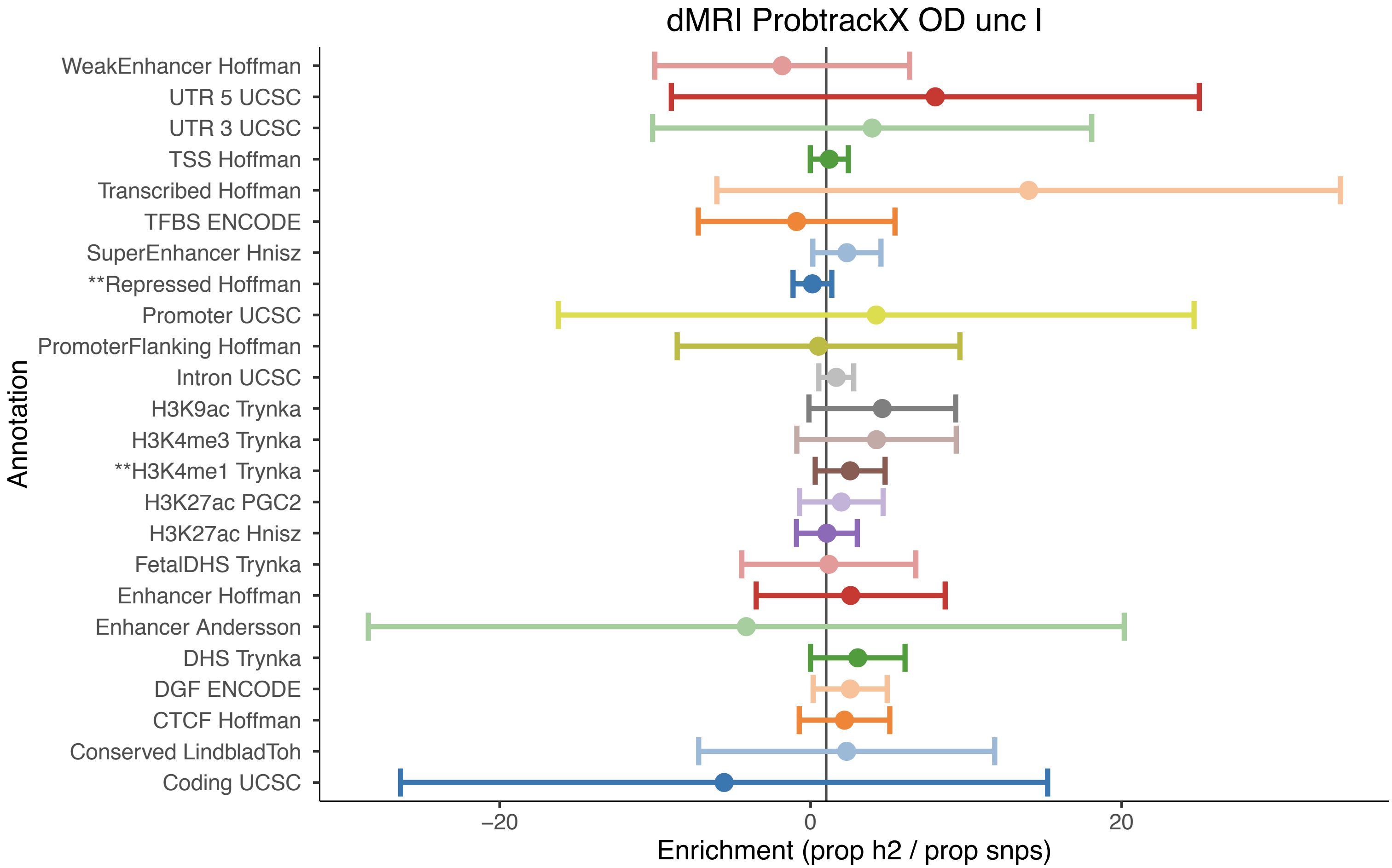
dMRI ProbtrackX OD unc I
WeakEnhancer Hoffman
UTR 5 UCSC
UTR 3 UCSC
TSS Hoffman
Transcribed Hoffman
TFBS ENCODE
SuperEnhancer Hnisz
**Repressed Hoffman
Promoter UCSC
PromoterFlanking Hoffman
Intron UCSC
H3K9ac Trynka
H3K4me3 Trynka
**H3K4me1 Trynka
H3K27ac PGC2
H3K27ac Hnisz
FetalDHS Trynka
Enhancer Hoffman
Enhancer Andersson
DHS Trynka
DGF ENCODE
CTCF Hoffman
Conserved LindbladToh
Coding UCSC
Annotation
−20
0
20
Enrichment (prop h2 / prop snps)

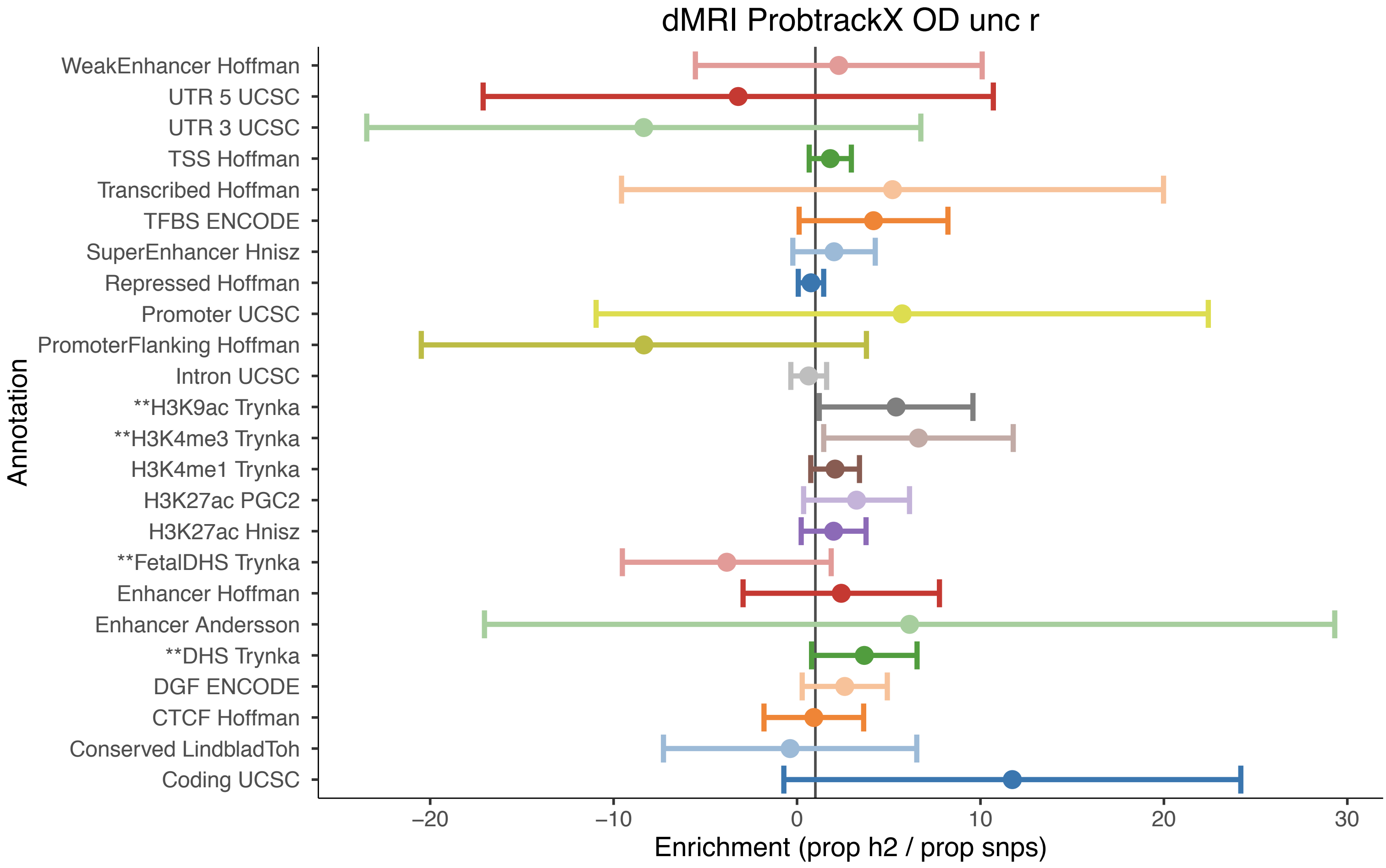
dMRI ProbtrackX OD unc r
WeakEnhancer Hoffman
UTR 5 UCSC
UTR 3 UCSC
TSS Hoffman
Transcribed Hoffman
TFBS ENCODE
SuperEnhancer Hnisz
Repressed Hoffman
Promoter UCSC
PromoterFlanking Hoffman
Intron UCSC
**H3K9ac Trynka
**H3K4me3 Trynka
H3K4me1 Trynka
H3K27ac PGC2
H3K27ac Hnisz
**FetalDHS Trynka
Enhancer Hoffman
Enhancer Andersson
**DHS Trynka
DGF ENCODE
CTCF Hoffman
Conserved LindbladToh
Coding UCSC
Annotation
−20
−10
0
10
20
30
Enrichment (prop h2 / prop snps)

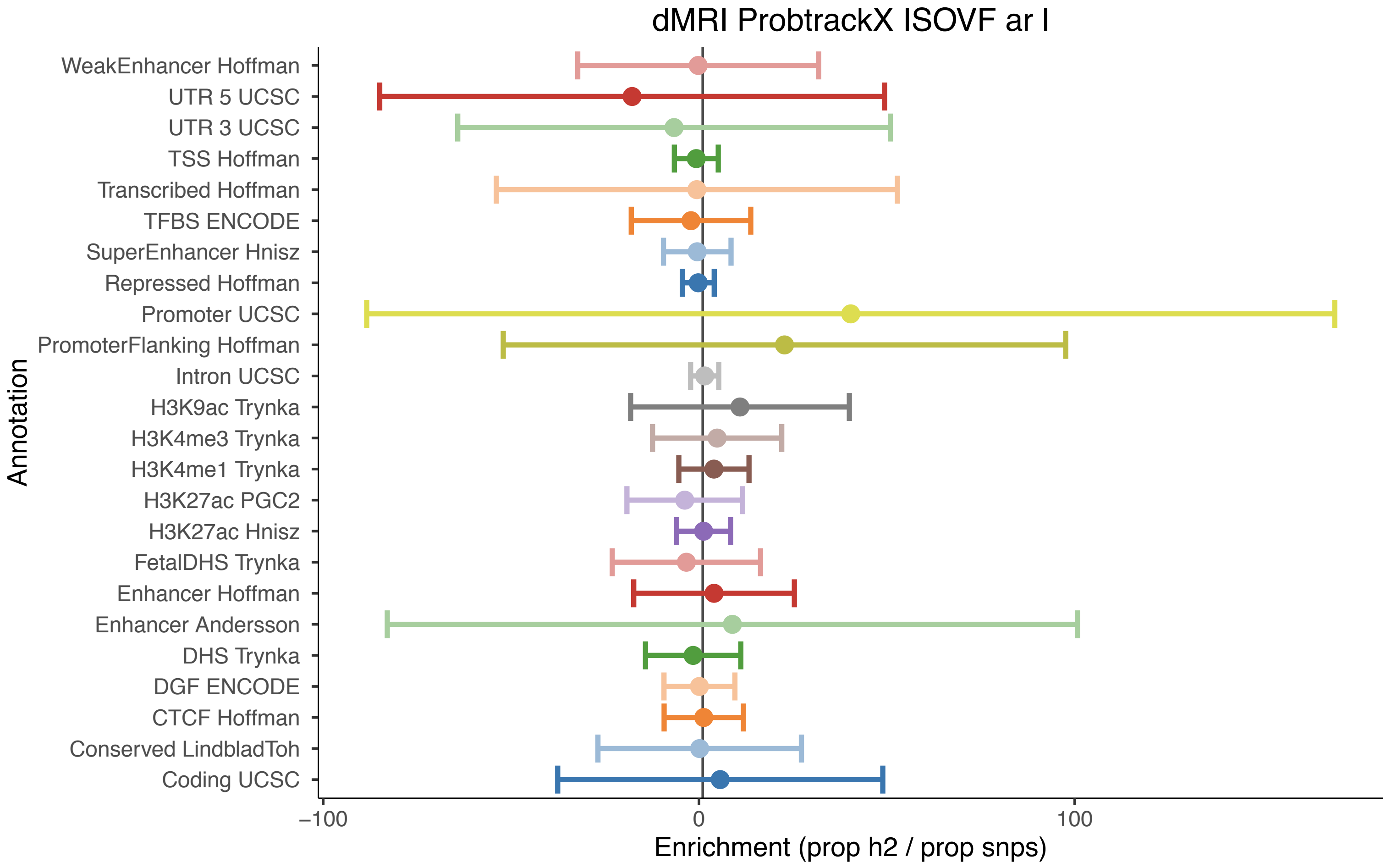

dMRI ProbtrackX ISOVF ar l
WeakEnhancer Hoffman
UTR 5 UCSC
UTR 3 UCSC
TSS Hoffman
Transcribed Hoffman
TFBS ENCODE
SuperEnhancer Hnisz
Repressed Hoffman
Promoter UCSC
PromoterFlanking Hoffman
Intron UCSC
H3K9ac Trynka
H3K4me3 Trynka
H3K4me1 Trynka
H3K27ac PGC2
H3K27ac Hnisz
FetalDHS Trynka
Enhancer Hoffman
Enhancer Andersson
DHS Trynka
DGF ENCODE
CTCF Hoffman
Conserved LindbladToh
Coding UCSC
Annotation
−100
0
100
Enrichment (prop h2 / prop snps)

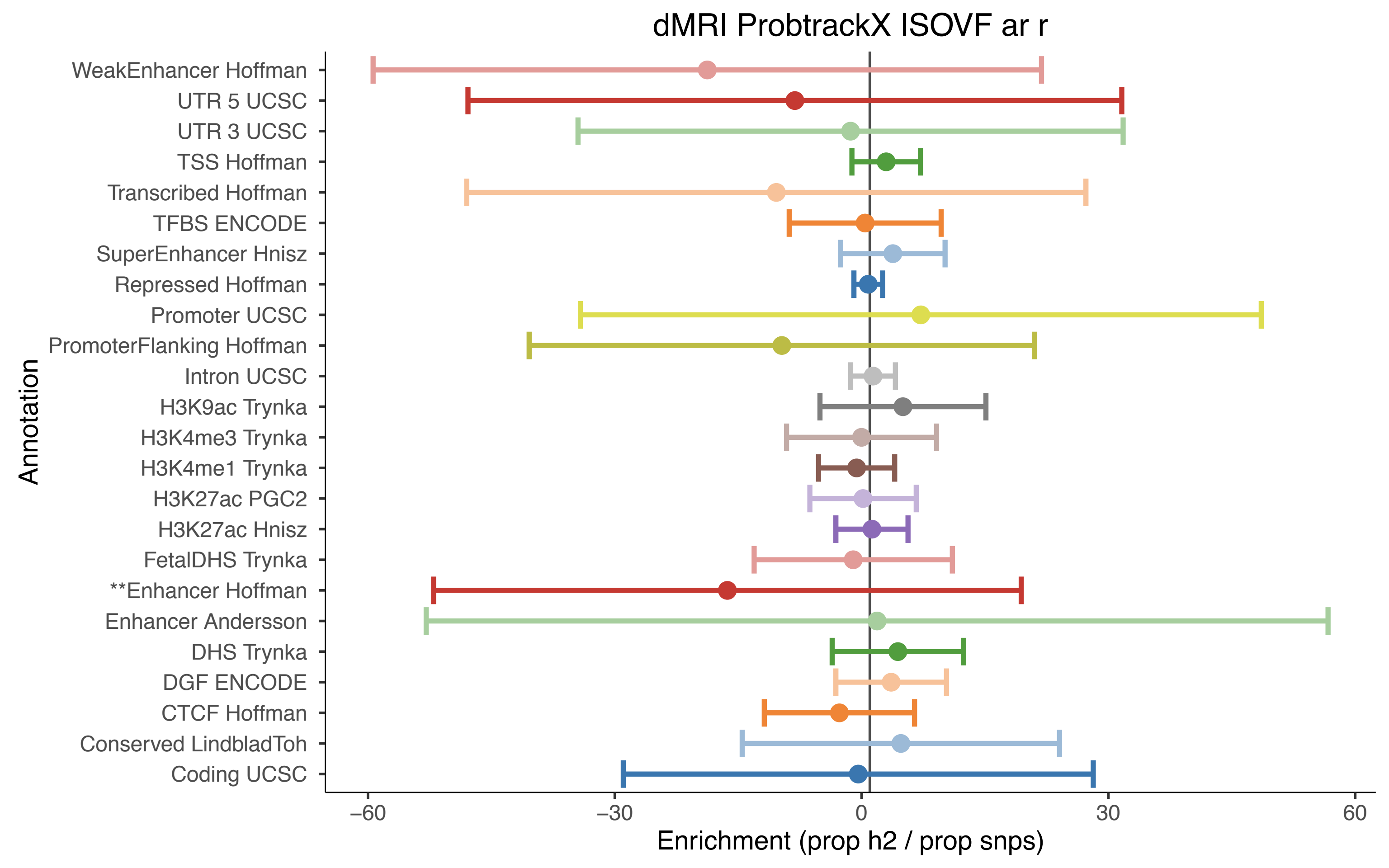

dMRI ProbtrackX ISOVF ar r
WeakEnhancer Hoffman
UTR 5 UCSC
UTR 3 UCSC
TSS Hoffman
Transcribed Hoffman
TFBS ENCODE
SuperEnhancer Hnisz
Repressed Hoffman
Promoter UCSC
PromoterFlanking Hoffman
Intron UCSC
H3K9ac Trynka
H3K4me3 Trynka
H3K4me1 Trynka
H3K27ac PGC2
H3K27ac Hnisz
FetalDHS Trynka
**Enhancer Hoffman
Enhancer Andersson
DHS Trynka
DGF ENCODE
CTCF Hoffman
Conserved LindbladToh
Coding UCSC
Annotation
−60
−30
0
30
60
Enrichment (prop h2 / prop snps)

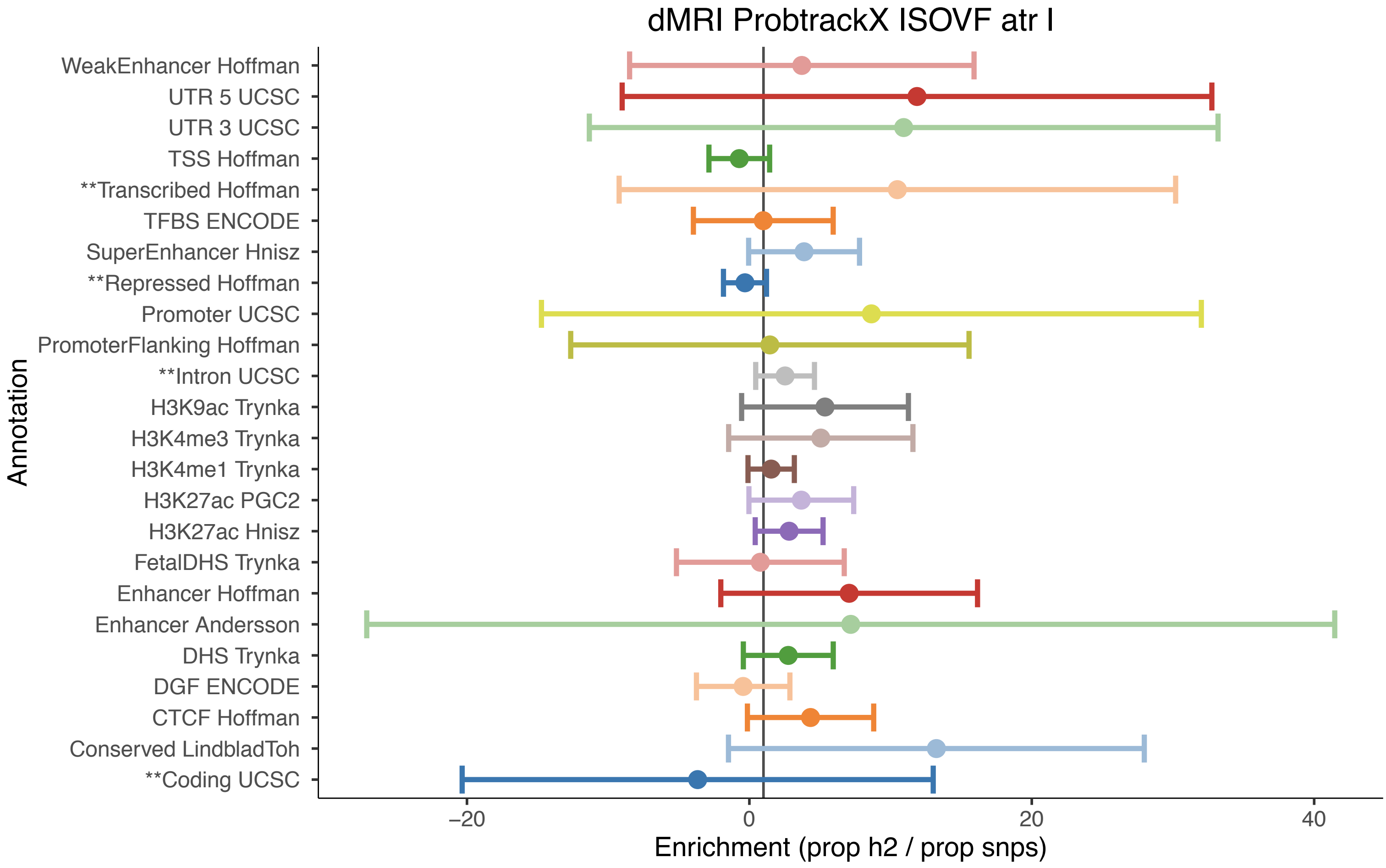

dMRI ProbtrackX ISOVF atr l
Annotation
WeakEnhancer Hoffman
UTR 5 UCSC
UTR 3 UCSC
TSS Hoffman
**Transcribed Hoffman
TFBS ENCODE
SuperEnhancer Hnisz
**Repressed Hoffman
Promoter UCSC
PromoterFlanking Hoffman
**Intron UCSC
H3K9ac Trynka
H3K4me3 Trynka
H3K4me1 Trynka
H3K27ac PGC2
H3K27ac Hnisz
FetalDHS Trynka
Enhancer Hoffman
Enhancer Andersson
DHS Trynka
DGF ENCODE
CTCF Hoffman
Conserved LindbladToh
**Coding UCSC
−20
0
20
40
Enrichment (prop h2 / prop snps)

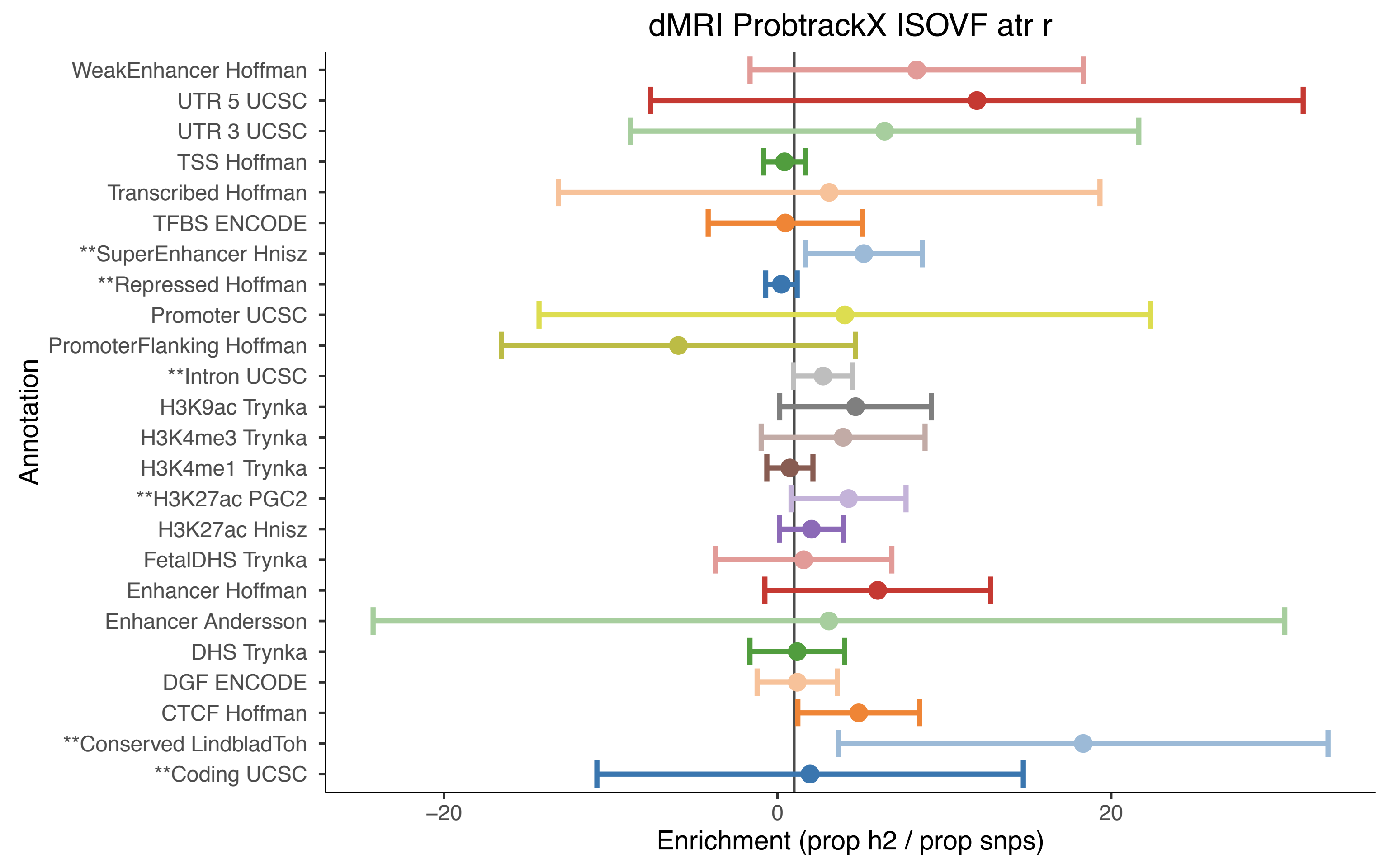
dMRI ProbtrackX ISOVF atr r
WeakEnhancer Hoffman
UTR 5 UCSC
UTR 3 UCSC
TSS Hoffman
Transcribed Hoffman
TFBS ENCODE
**SuperEnhancer Hnisz
**Repressed Hoffman
Promoter UCSC
PromoterFlanking Hoffman
**Intron UCSC
H3K9ac Trynka
H3K4me3 Trynka
H3K4me1 Trynka
**H3K27ac PGC2
H3K27ac Hnisz
FetalDHS Trynka
Enhancer Hoffman
Enhancer Andersson
DHS Trynka
DGF ENCODE
CTCF Hoffman
**Conserved LindbladToh
**Coding UCSC
Annotation
−20
0
20
Enrichment (prop h2 / prop snps)

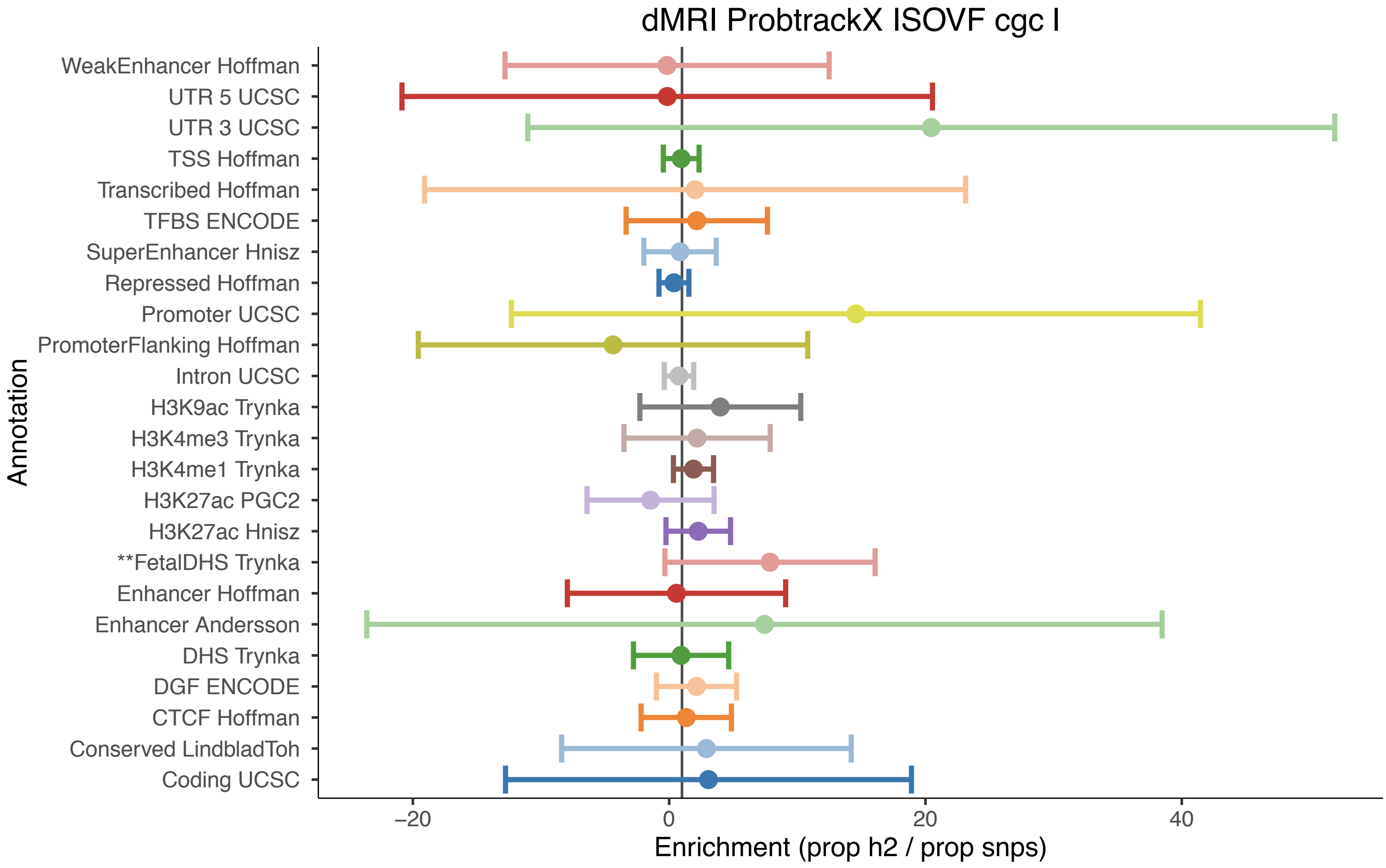

dMRI ProbtrackX ISOVF cgc I
WeakEnhancer Hoffman
UTR 5 UCSC
UTR 3 UCSC
TSS Hoffman
Transcribed Hoffman
TFBS ENCODE
SuperEnhancer Hnisz
Repressed Hoffman
Promoter UCSC
PromoterFlanking Hoffman
Intron UCSC
H3K9ac Trynka
H3K4me3 Trynka
H3K4me1 Trynka
H3K27ac PGC2
H3K27ac Hnisz
**FetalDHS Trynka
Enhancer Hoffman
Enhancer Andersson
DHS Trynka
DGF ENCODE
CTCF Hoffman
Conserved LindbladToh
Coding UCSC
Annotation
−20
0
20
40
Enrichment (prop h2 / prop snps)

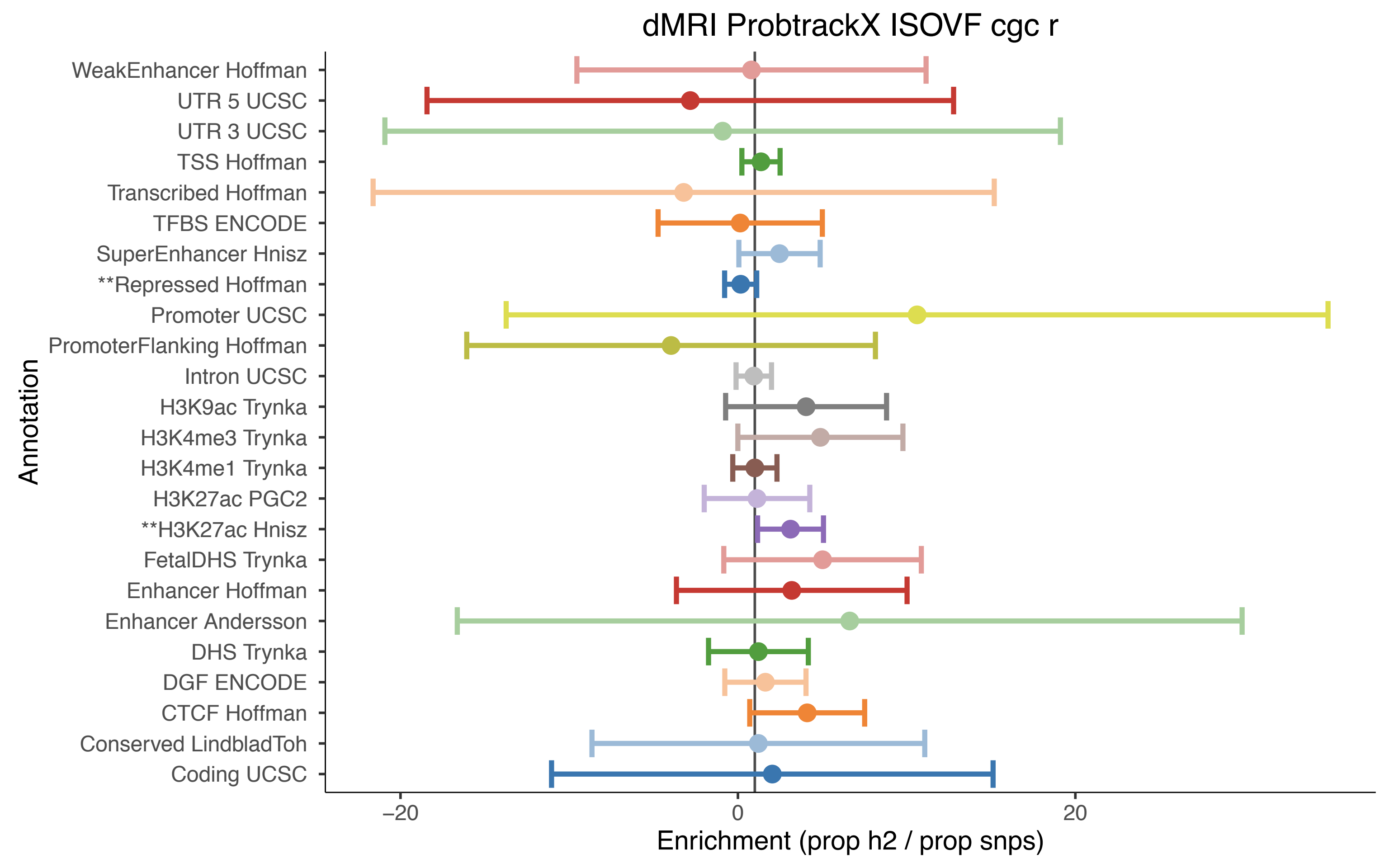

dMRI ProbtrackX ISOVF cgc r
WeakEnhancer Hoffman
UTR 5 UCSC
UTR 3 UCSC
TSS Hoffman
Transcribed Hoffman
TFBS ENCODE
SuperEnhancer Hnisz
**Repressed Hoffman
Promoter UCSC
PromoterFlanking Hoffman
Intron UCSC
H3K9ac Trynka
H3K4me3 Trynka
H3K4me1 Trynka
H3K27ac PGC2
**H3K27ac Hnisz
FetalDHS Trynka
Enhancer Hoffman
Enhancer Andersson
DHS Trynka
DGF ENCODE
CTCF Hoffman
Conserved LindbladToh
Coding UCSC
Annotation
−20
0
20
Enrichment (prop h2 / prop snps)

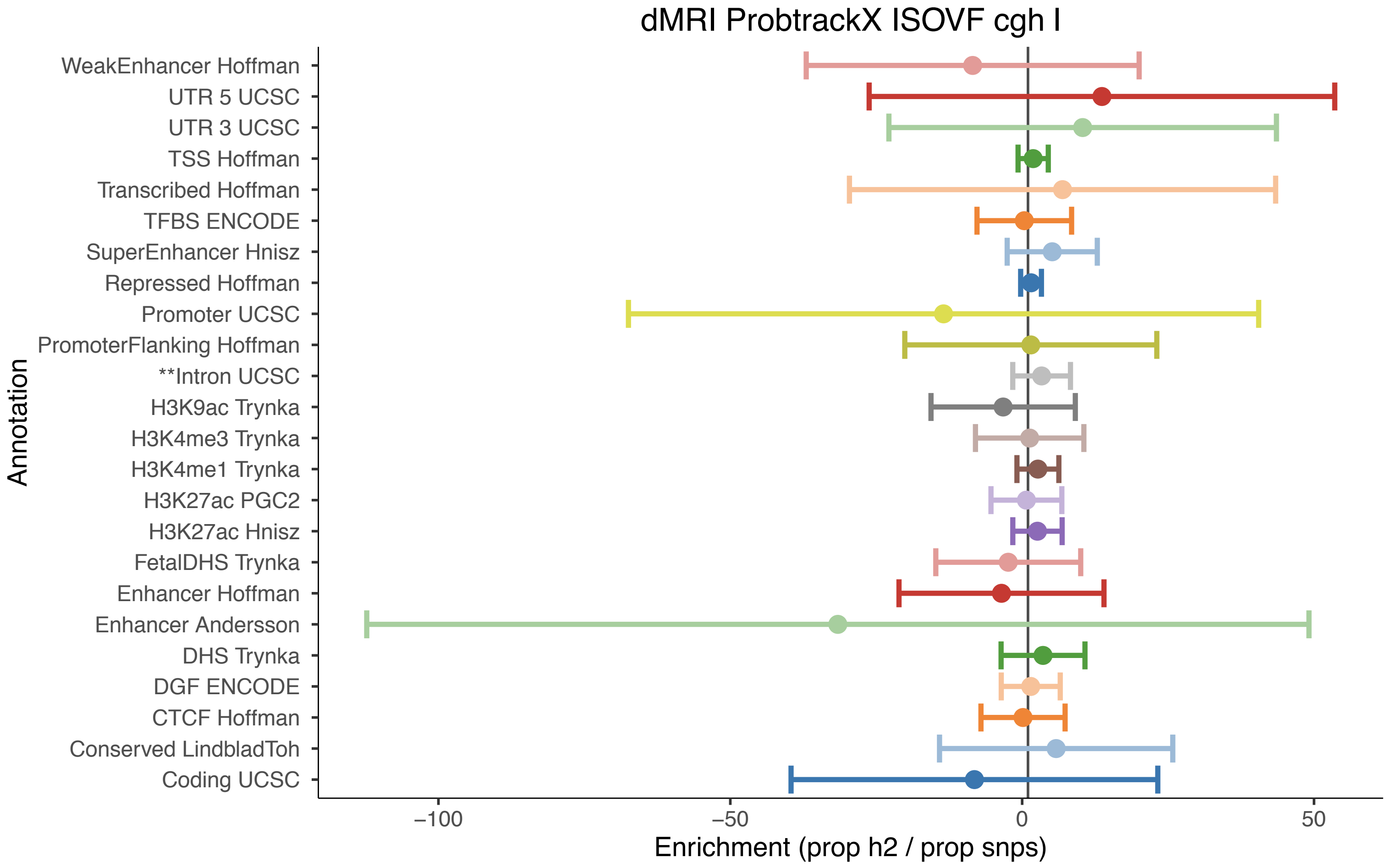

dMRI ProbtrackX ISOVF cgh l
WeakEnhancer Hoffman
UTR 5 UCSC
UTR 3 UCSC
TSS Hoffman
Transcribed Hoffman
TFBS ENCODE
SuperEnhancer Hnisz
Repressed Hoffman
Promoter UCSC
PromoterFlanking Hoffman
**Intron UCSC
H3K9ac Trynka
H3K4me3 Trynka
H3K4me1 Trynka
H3K27ac PGC2
H3K27ac Hnisz
FetalDHS Trynka
Enhancer Hoffman
Enhancer Andersson
DHS Trynka
DGF ENCODE
CTCF Hoffman
Conserved LindbladToh
Coding UCSC
Annotation
−100
−50
0
50
Enrichment (prop h2 / prop snps)

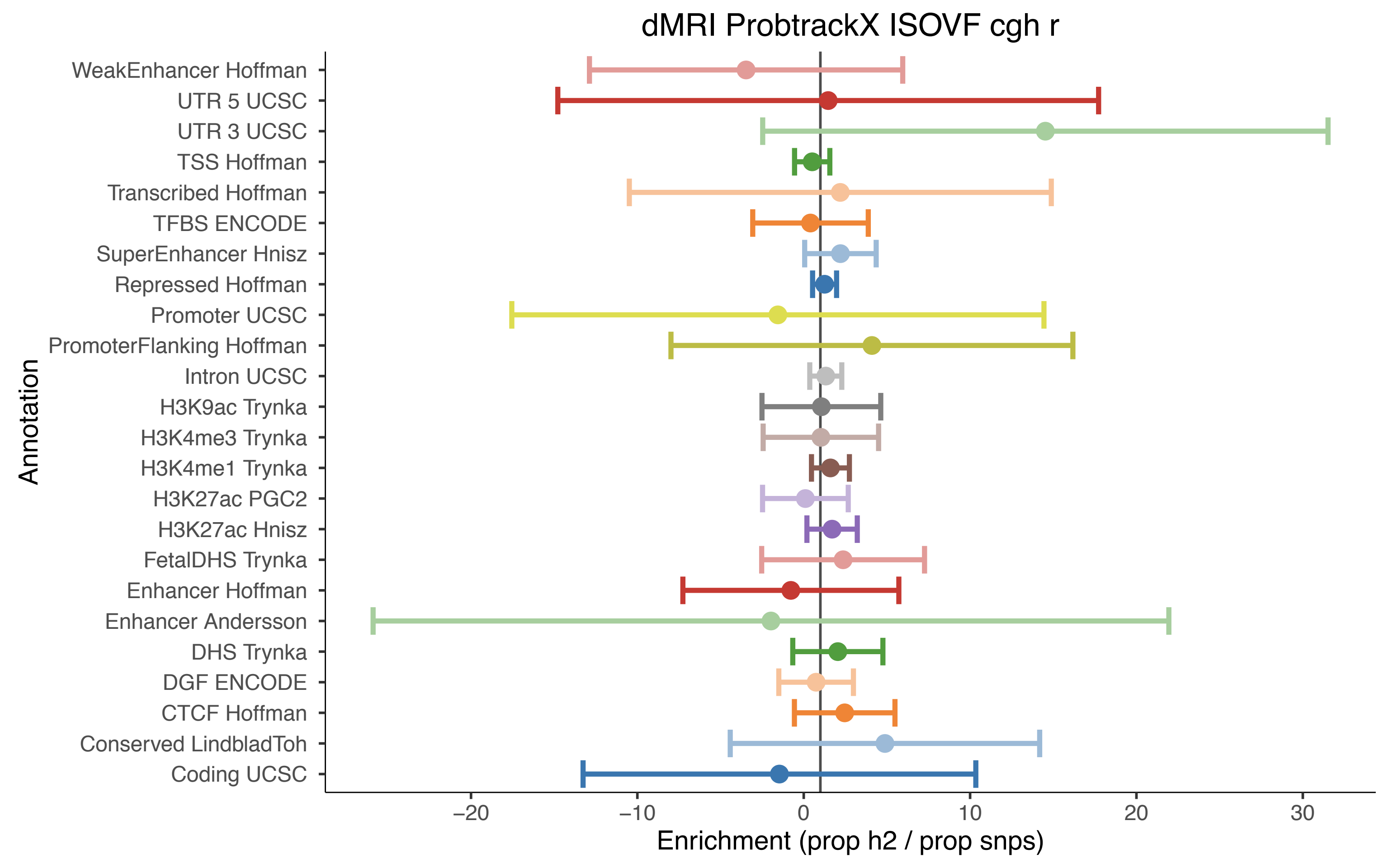

dMRI ProbtrackX ISOVF cgh r
Annotation
WeakEnhancer Hoffman
UTR 5 UCSC
UTR 3 UCSC
TSS Hoffman
Transcribed Hoffman
TFBS ENCODE
SuperEnhancer Hnisz
Repressed Hoffman
Promoter UCSC
PromoterFlanking Hoffman
Intron UCSC
H3K9ac Trynka
H3K4me3 Trynka
H3K4me1 Trynka
H3K27ac PGC2
H3K27ac Hnisz
FetalDHS Trynka
Enhancer Hoffman
Enhancer Andersson
DHS Trynka
DGF ENCODE
CTCF Hoffman
Conserved LindbladToh
Coding UCSC
−20
−10
0
10
20
30
Enrichment (prop h2 / prop snps)

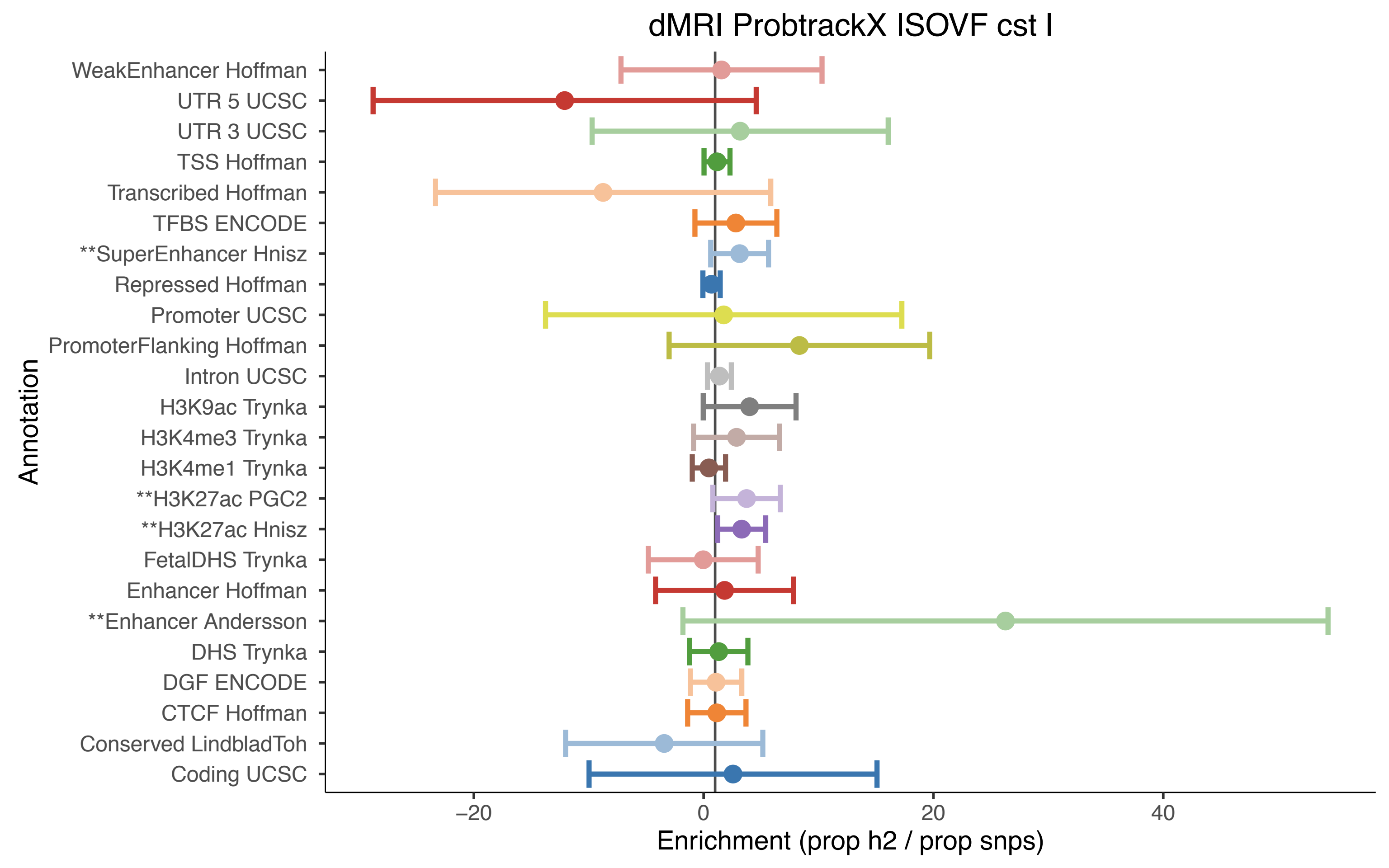
dMRI ProbtrackX ISOVF cst l
Annotation
WeakEnhancer Hoffman
UTR 5 UCSC
UTR 3 UCSC
TSS Hoffman
Transcribed Hoffman
TFBS ENCODE
**SuperEnhancer Hnisz
Repressed Hoffman
Promoter UCSC
PromoterFlanking Hoffman
Intron UCSC
H3K9ac Trynka
H3K4me3 Trynka
H3K4me1 Trynka
**H3K27ac PGC2
**H3K27ac Hnisz
FetalDHS Trynka
Enhancer Hoffman
**Enhancer Andersson
DHS Trynka
DGF ENCODE
CTCF Hoffman
Conserved LindbladToh
Coding UCSC
−20
0
20
40
Enrichment (prop h2 / prop snps)

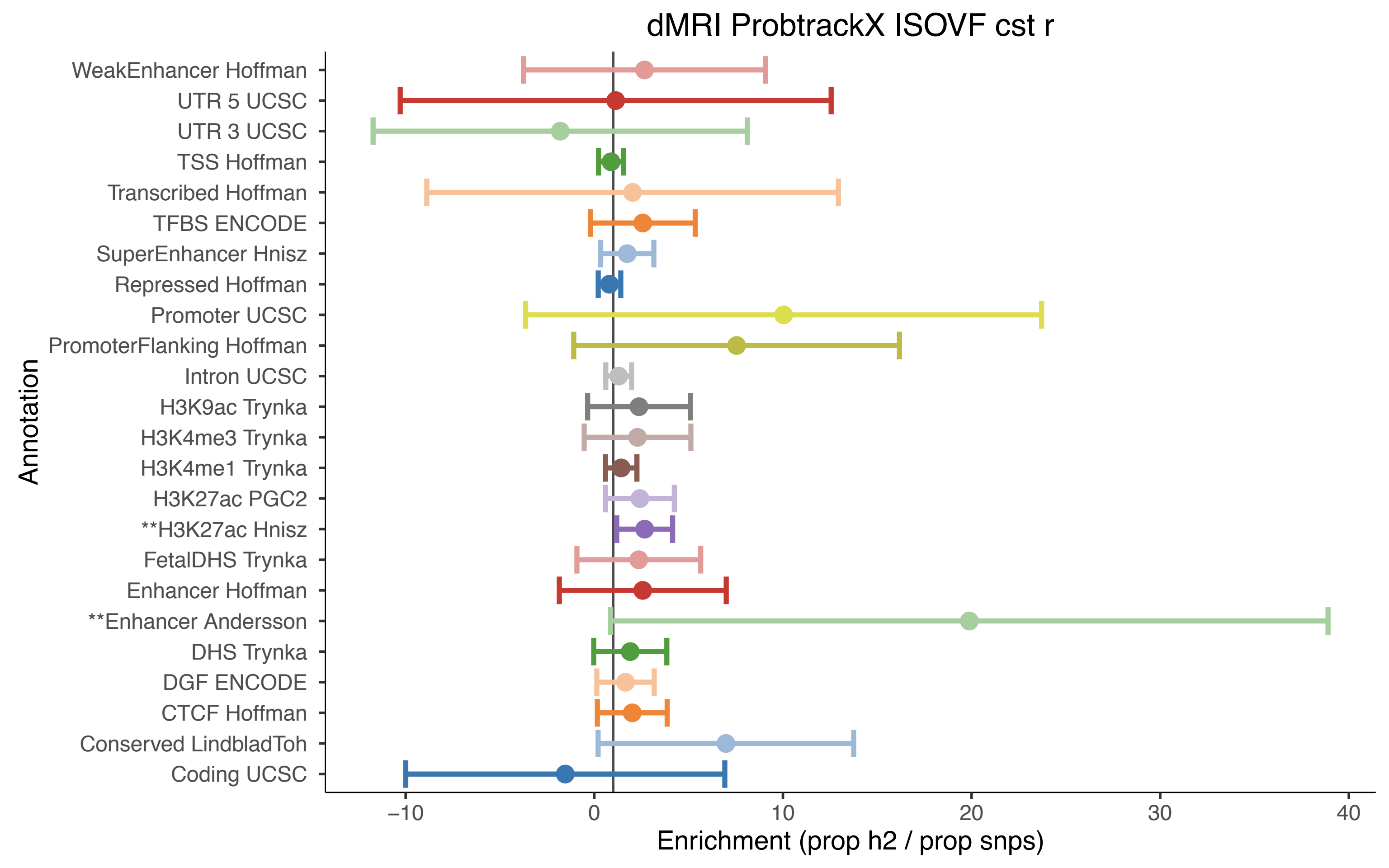

dMRI ProbtrackX ISOVF cst r
WeakEnhancer Hoffman
UTR 5 UCSC
UTR 3 UCSC
TSS Hoffman
Transcribed Hoffman
TFBS ENCODE
SuperEnhancer Hnisz
Repressed Hoffman
Promoter UCSC
PromoterFlanking Hoffman
Intron UCSC
H3K9ac Trynka
H3K4me3 Trynka
H3K4me1 Trynka
H3K27ac PGC2
**H3K27ac Hnisz
FetalDHS Trynka
Enhancer Hoffman
**Enhancer Andersson
DHS Trynka
DGF ENCODE
CTCF Hoffman
Conserved LindbladToh
Coding UCSC
Annotation
−10
0
10
20
30
40
Enrichment (prop h2 / prop snps)

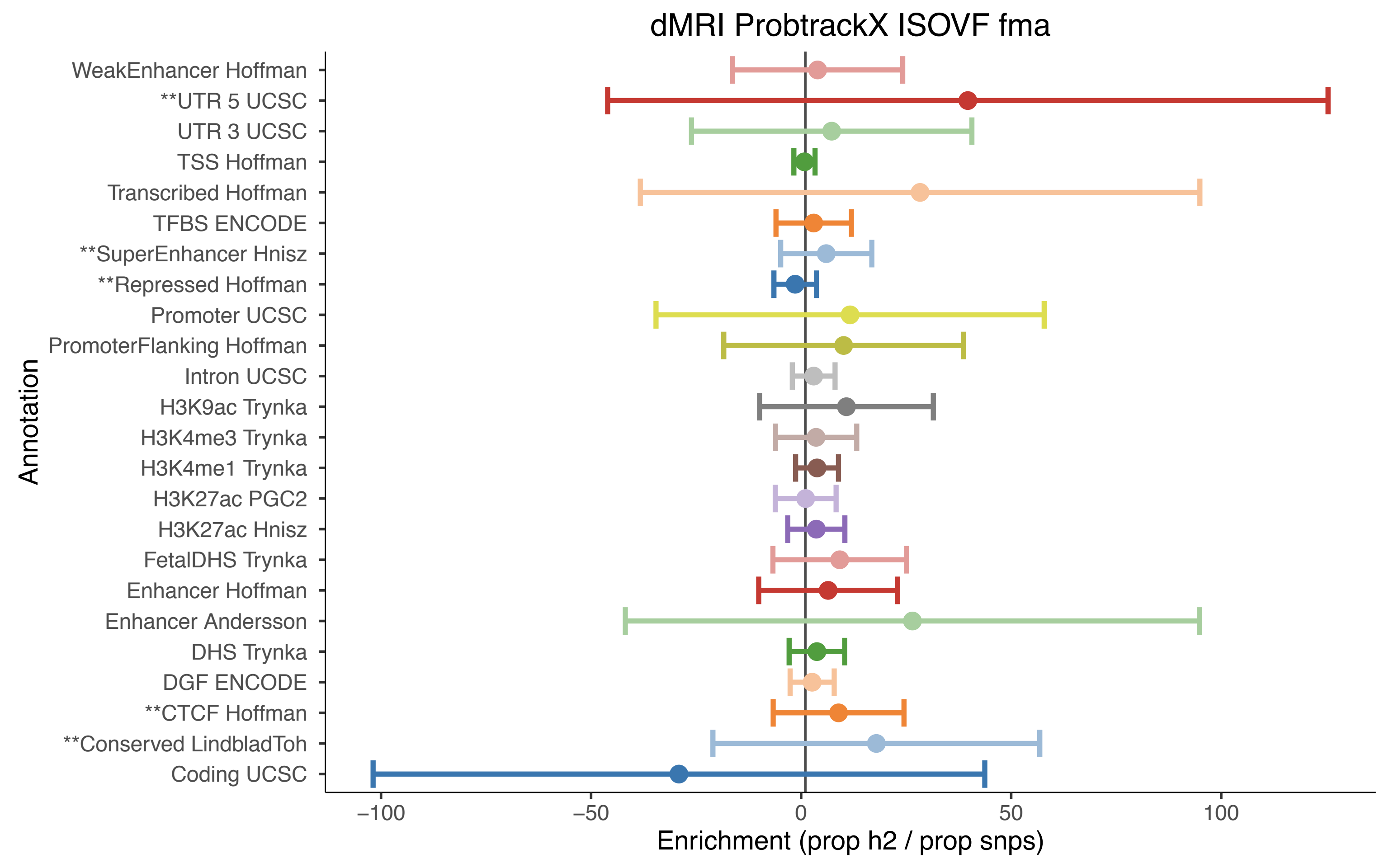

dMRI ProbtrackX ISOVF fma
WeakEnhancer Hoffman
**UTR 5 UCSC
UTR 3 UCSC
TSS Hoffman
Transcribed Hoffman
TFBS ENCODE
**SuperEnhancer Hnisz
**Repressed Hoffman
Promoter UCSC
PromoterFlanking Hoffman
Intron UCSC
H3K9ac Trynka
H3K4me3 Trynka
H3K4me1 Trynka
H3K27ac PGC2
H3K27ac Hnisz
FetalDHS Trynka
Enhancer Hoffman
Enhancer Andersson
DHS Trynka
DGF ENCODE
**CTCF Hoffman
**Conserved LindbladToh
Coding UCSC
Annotation
−100
−50
0
50
100
Enrichment (prop h2 / prop snps)

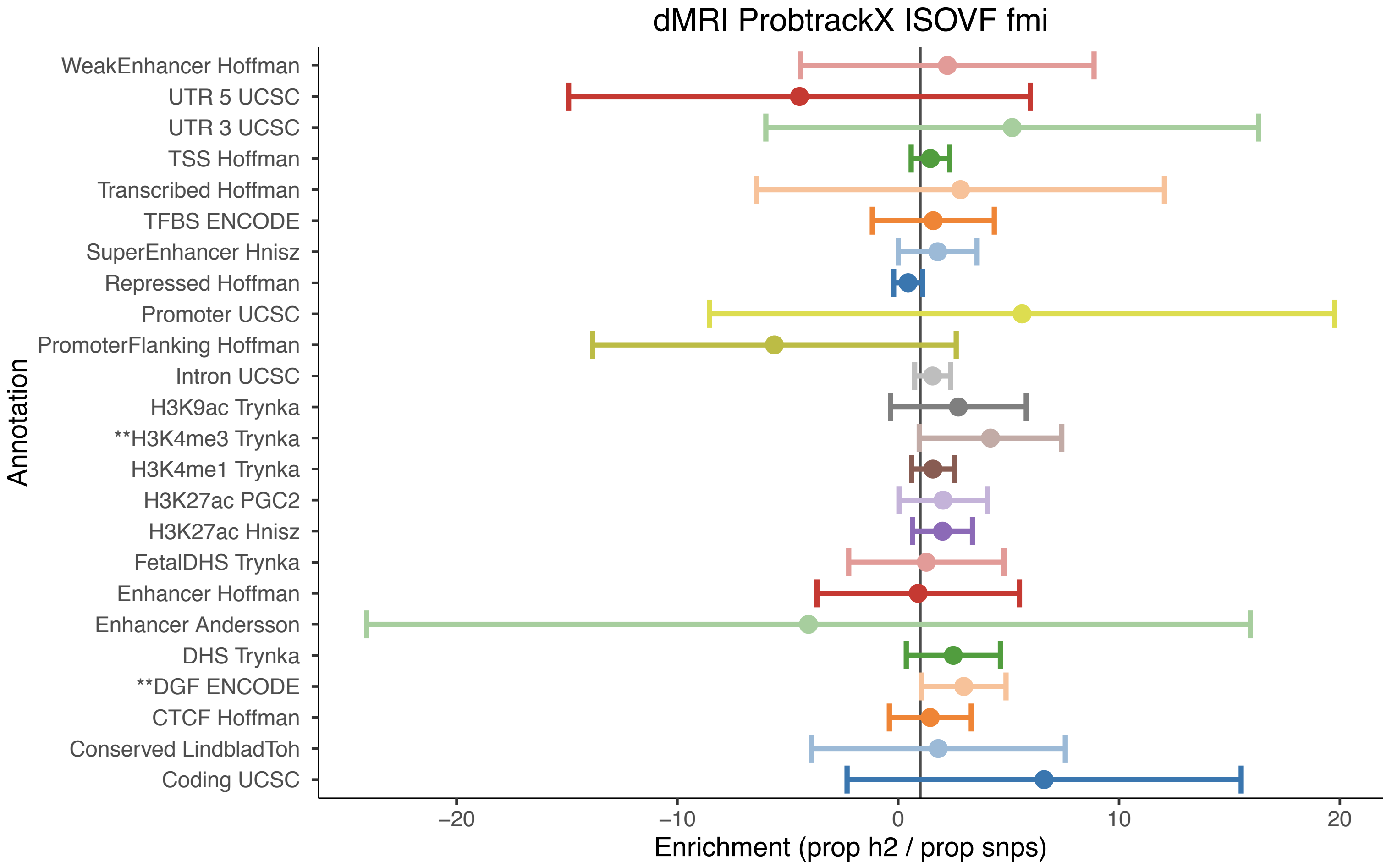
dMRI ProbtrackX ISOVF fmi
Annotation
WeakEnhancer Hoffman
UTR 5 UCSC
UTR 3 UCSC
TSS Hoffman
Transcribed Hoffman
TFBS ENCODE
SuperEnhancer Hnisz
Repressed Hoffman
Promoter UCSC
PromoterFlanking Hoffman
Intron UCSC
H3K9ac Trynka
**H3K4me3 Trynka
H3K4me1 Trynka
H3K27ac PGC2
H3K27ac Hnisz
FetalDHS Trynka
Enhancer Hoffman
Enhancer Andersson
DHS Trynka
**DGF ENCODE
CTCF Hoffman
Conserved LindbladToh
Coding UCSC
−20
−10
0
10
20
Enrichment (prop h2 / prop snps)

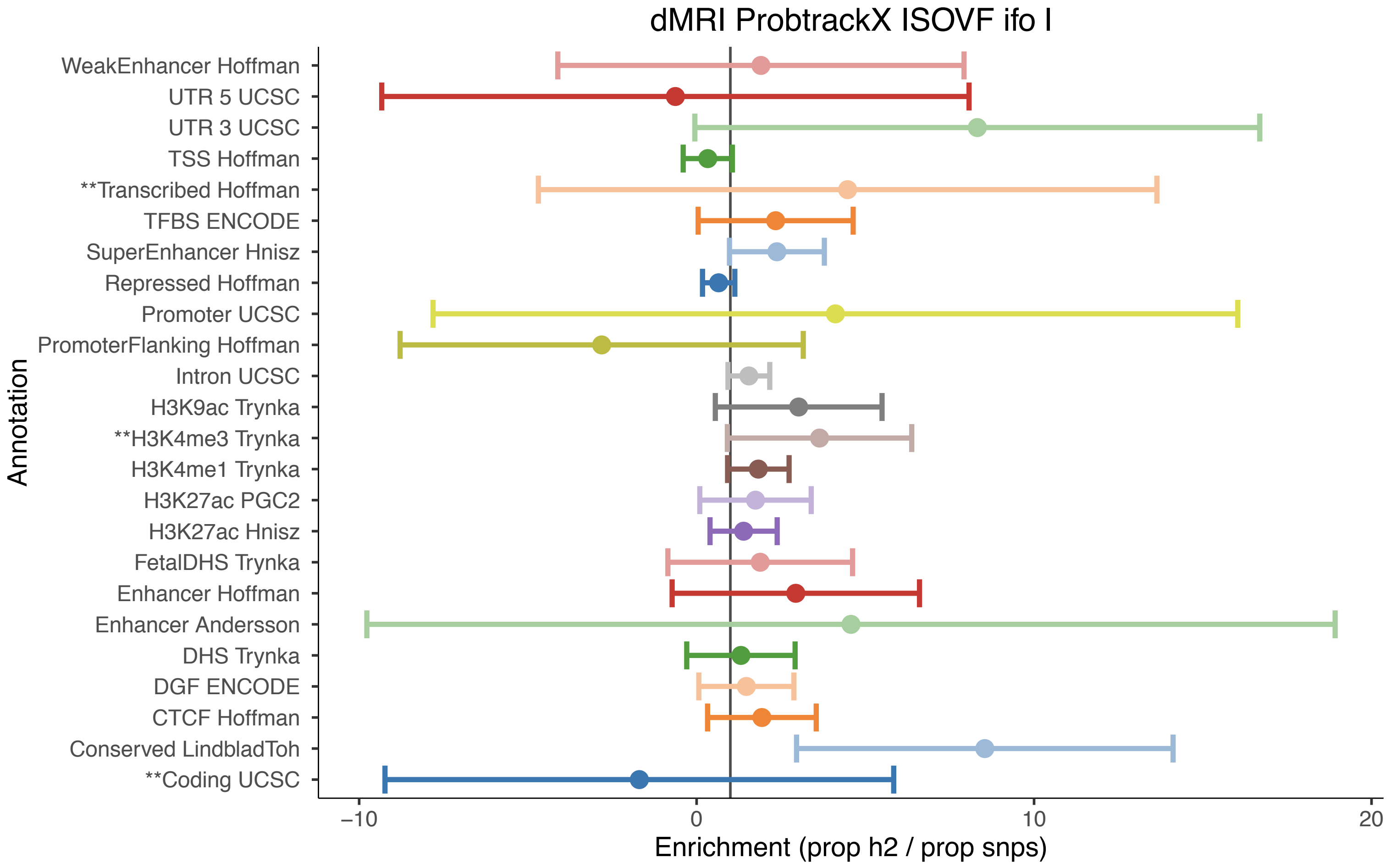

dMRI ProbtrackX ISOVF ifo l
WeakEnhancer Hoffman
UTR 5 UCSC
UTR 3 UCSC
TSS Hoffman
**Transcribed Hoffman
TFBS ENCODE
SuperEnhancer Hnisz
Repressed Hoffman
Promoter UCSC
PromoterFlanking Hoffman
Intron UCSC
H3K9ac Trynka
**H3K4me3 Trynka
H3K4me1 Trynka
H3K27ac PGC2
H3K27ac Hnisz
FetalDHS Trynka
Enhancer Hoffman
Enhancer Andersson
DHS Trynka
DGF ENCODE
CTCF Hoffman
Conserved LindbladToh
**Coding UCSC
Annotation
−10
0
10
20
Enrichment (prop h2 / prop snps)

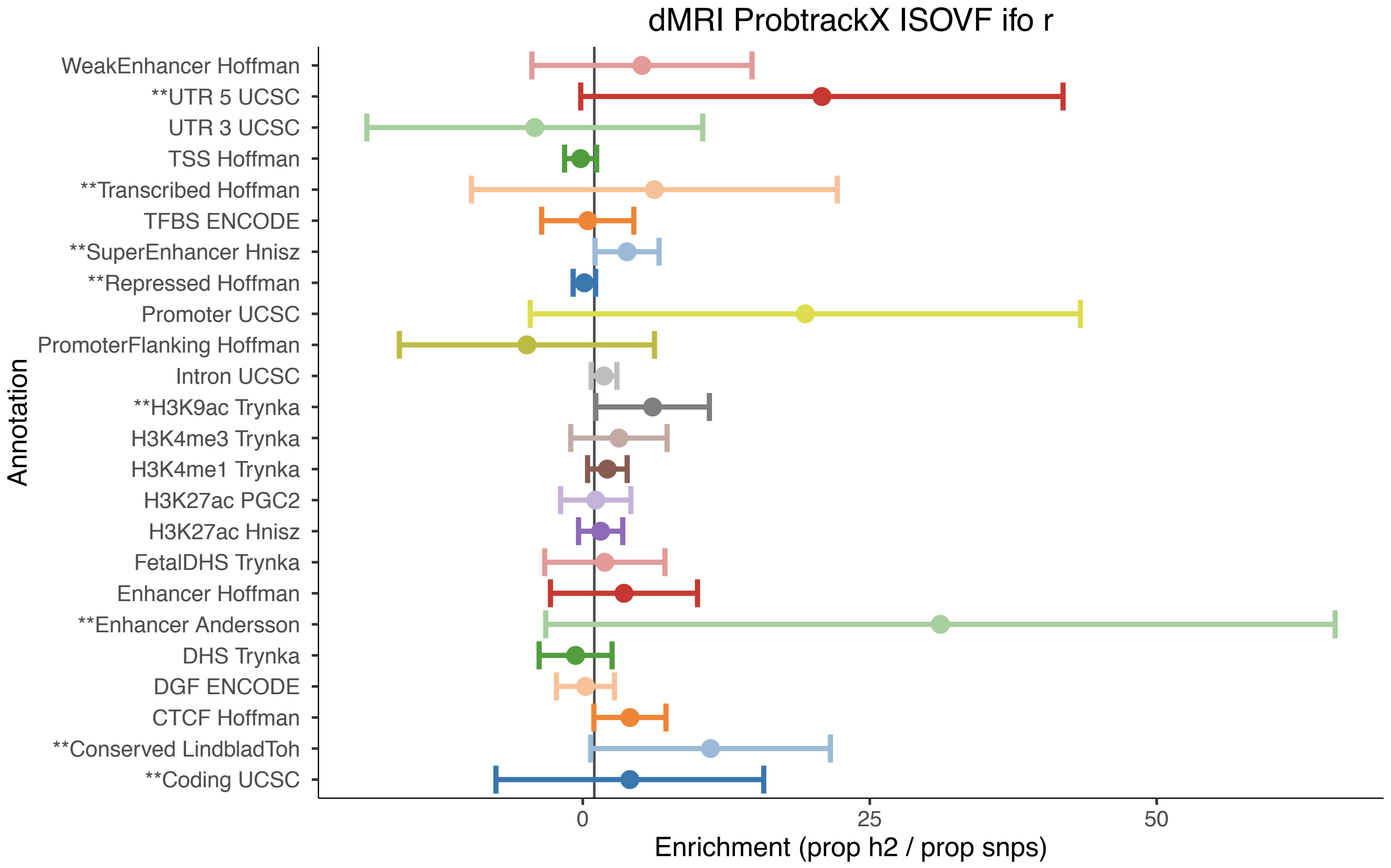
dMRI ProbtrackX ISOVF ifo r
WeakEnhancer Hoffman
**UTR 5 UCSC
UTR 3 UCSC
TSS Hoffman
**Transcribed Hoffman
TFBS ENCODE
**SuperEnhancer Hnisz
**Repressed Hoffman
Promoter UCSC
PromoterFlanking Hoffman
Intron UCSC
**H3K9ac Trynka
H3K4me3 Trynka
H3K4me1 Trynka
H3K27ac PGC2
H3K27ac Hnisz
FetalDHS Trynka
Enhancer Hoffman
**Enhancer Andersson
DHS Trynka
DGF ENCODE
CTCF Hoffman
**Conserved LindbladToh
**Coding UCSC
Annotation
0
25
50
Enrichment (prop h2 / prop snps)

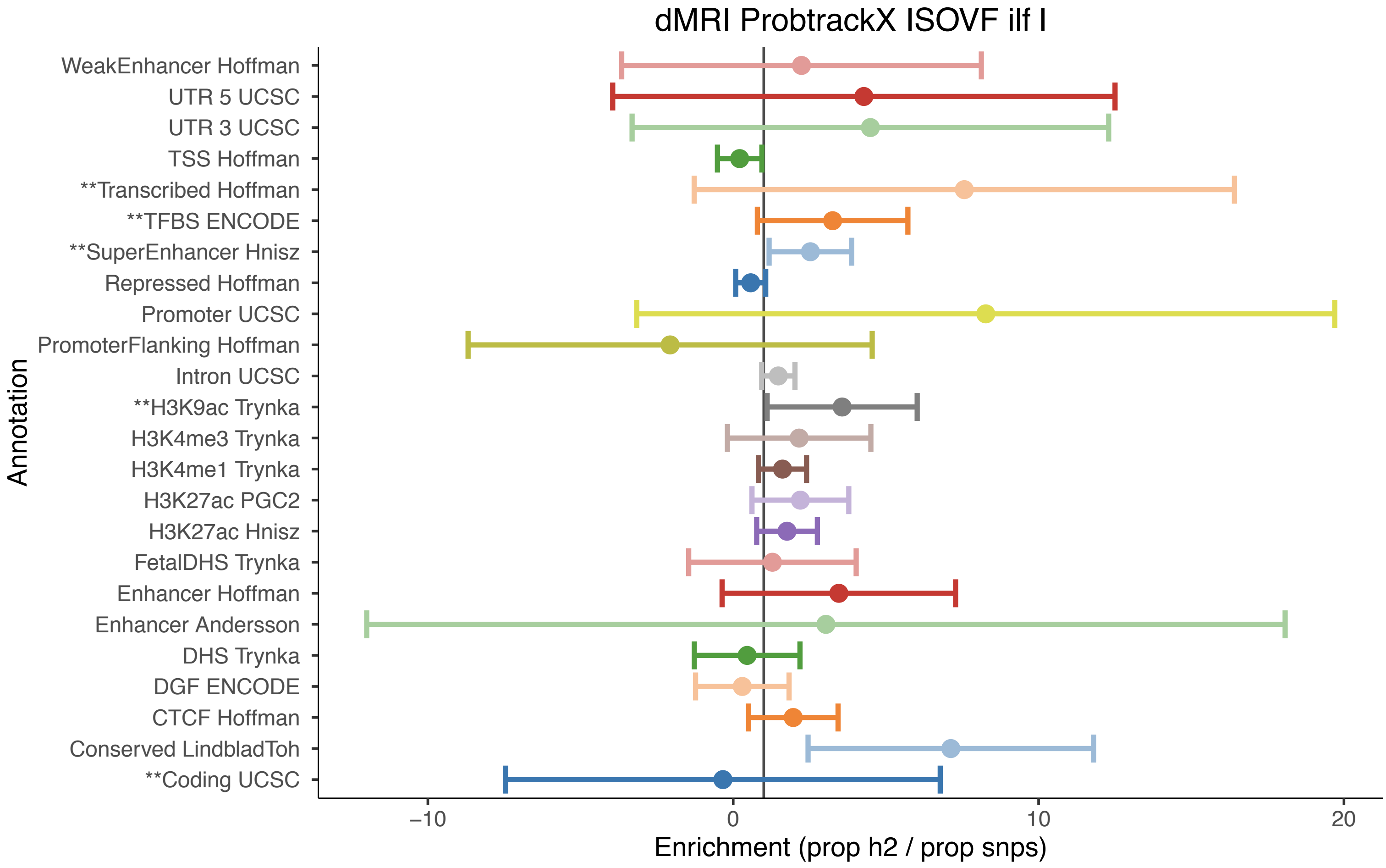
dMRI ProbtrackX ISOVF ilf l
WeakEnhancer Hoffman
UTR 5 UCSC
UTR 3 UCSC
TSS Hoffman
**Transcribed Hoffman
**TFBS ENCODE
**SuperEnhancer Hnisz
Repressed Hoffman
Promoter UCSC
PromoterFlanking Hoffman
Intron UCSC
**H3K9ac Trynka
H3K4me3 Trynka
H3K4me1 Trynka
H3K27ac PGC2
H3K27ac Hnisz
FetalDHS Trynka
Enhancer Hoffman
Enhancer Andersson
DHS Trynka
DGF ENCODE
CTCF Hoffman
Conserved LindbladToh
**Coding UCSC
Annotation
−10
0
10
20
Enrichment (prop h2 / prop snps)

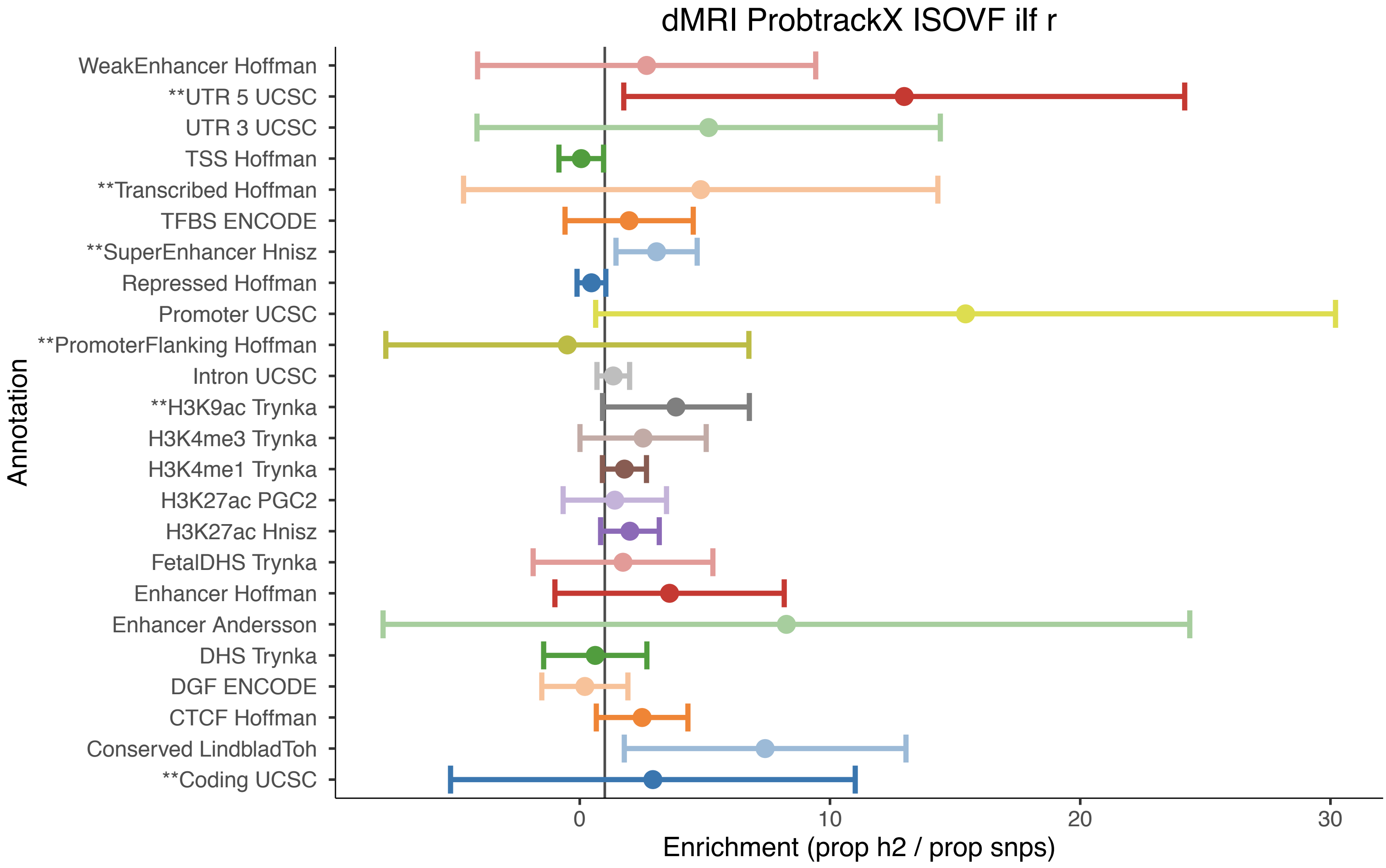

dMRI ProbtrackX ISOVF ilf r
WeakEnhancer Hoffman
**UTR 5 UCSC
UTR 3 UCSC
TSS Hoffman
**Transcribed Hoffman
TFBS ENCODE
**SuperEnhancer Hnisz
Repressed Hoffman
Promoter UCSC
**PromoterFlanking Hoffman
Intron UCSC
**H3K9ac Trynka
H3K4me3 Trynka
H3K4me1 Trynka
H3K27ac PGC2
H3K27ac Hnisz
FetalDHS Trynka
Enhancer Hoffman
Enhancer Andersson
DHS Trynka
DGF ENCODE
CTCF Hoffman
Conserved LindbladToh
**Coding UCSC
Annotation
0
10
20
30
Enrichment (prop h2 / prop snps)

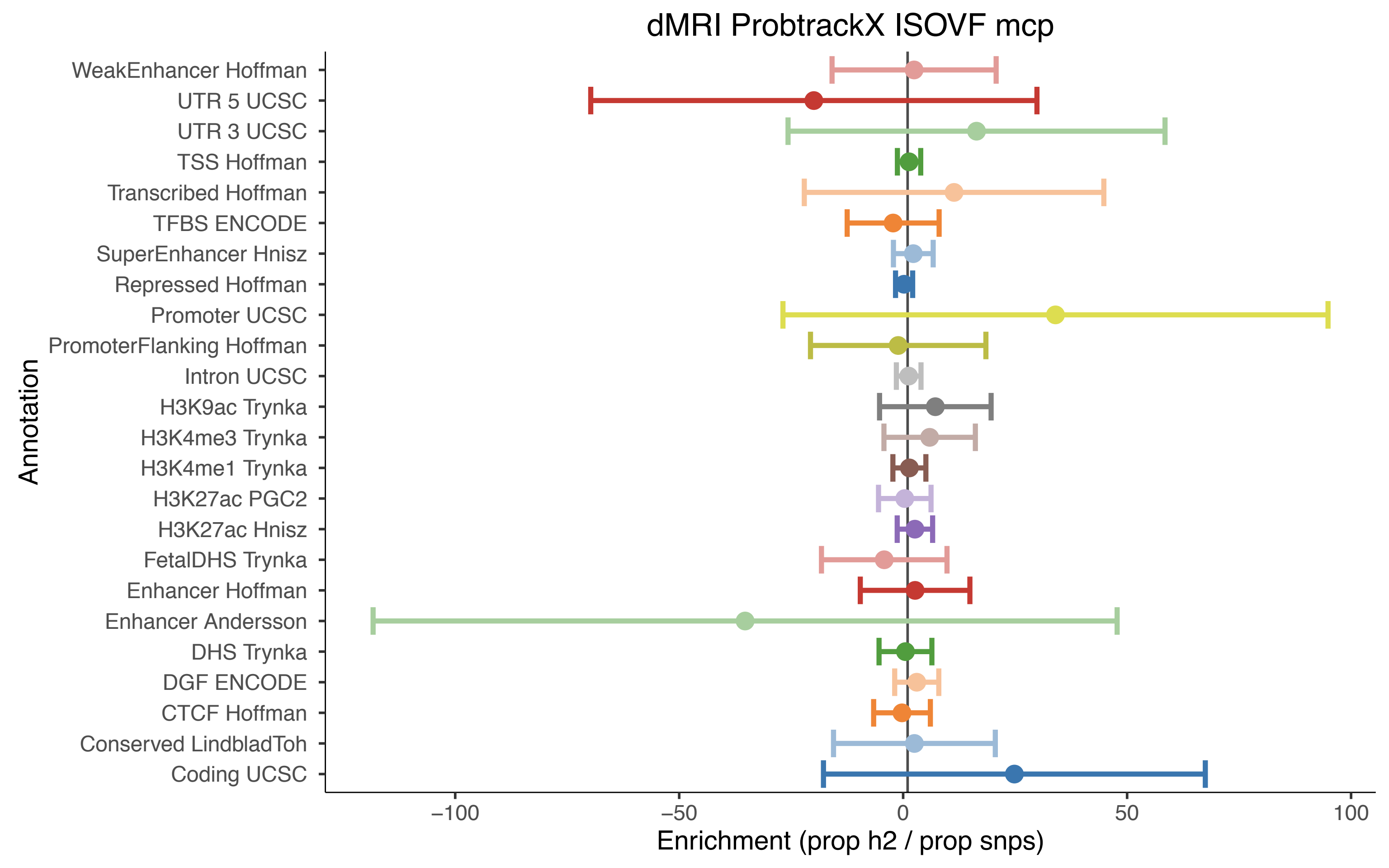

dMRI ProbtrackX ISOVF mcp
WeakEnhancer Hoffman
UTR 5 UCSC
UTR 3 UCSC
TSS Hoffman
Transcribed Hoffman
TFBS ENCODE
SuperEnhancer Hnisz
Repressed Hoffman
Promoter UCSC
PromoterFlanking Hoffman
Intron UCSC
H3K9ac Trynka
H3K4me3 Trynka
H3K4me1 Trynka
H3K27ac PGC2
H3K27ac Hnisz
FetalDHS Trynka
Enhancer Hoffman
Enhancer Andersson
DHS Trynka
DGF ENCODE
CTCF Hoffman
Conserved LindbladToh
Coding UCSC
Annotation
−100
−50
0
50
100
Enrichment (prop h2 / prop snps)

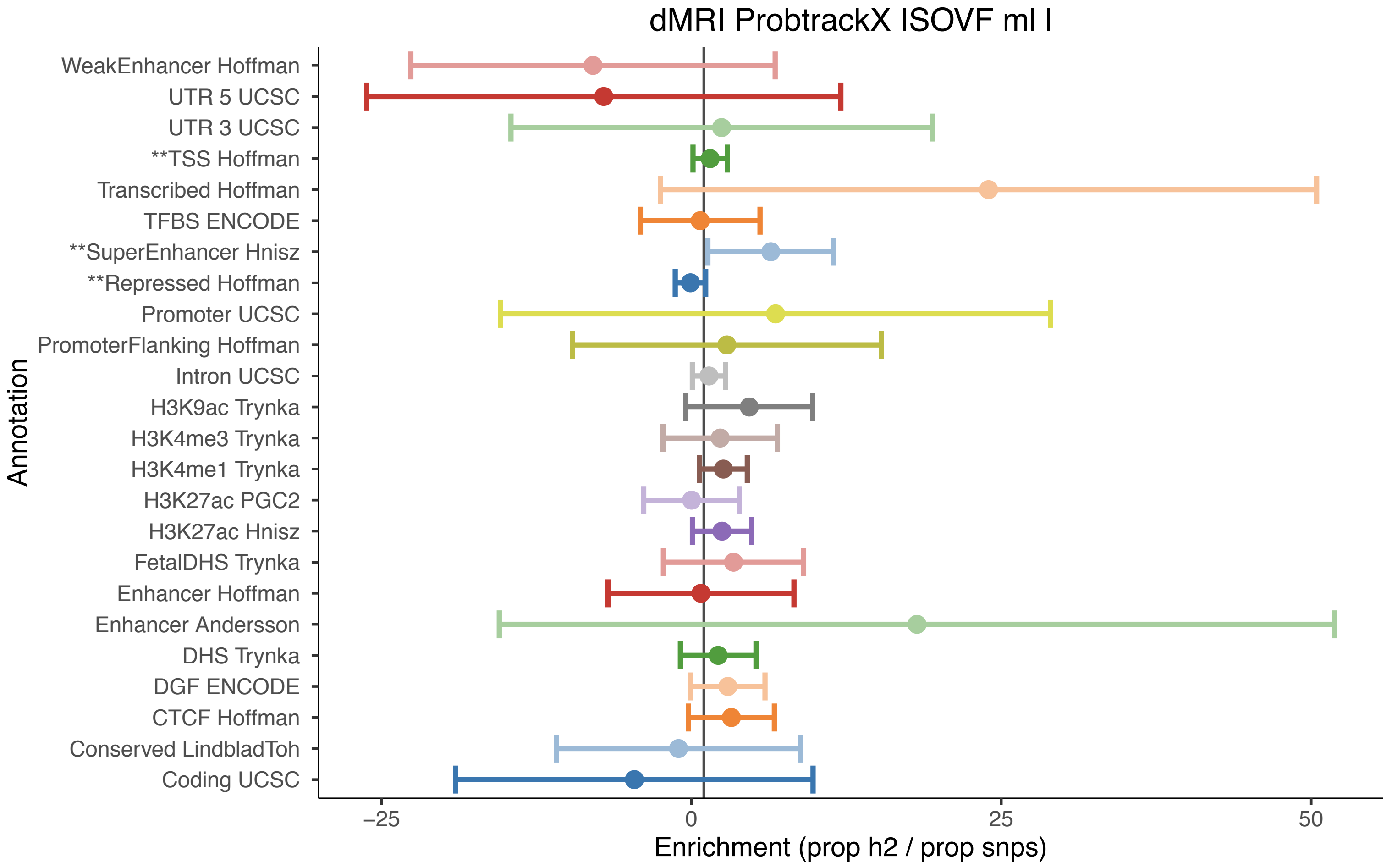
dMRI ProbtrackX ISOVF ml l
WeakEnhancer Hoffman
UTR 5 UCSC
UTR 3 UCSC
**TSS Hoffman
Transcribed Hoffman
TFBS ENCODE
**SuperEnhancer Hnisz
**Repressed Hoffman
Promoter UCSC
PromoterFlanking Hoffman
Intron UCSC
H3K9ac Trynka
H3K4me3 Trynka
H3K4me1 Trynka
H3K27ac PGC2
H3K27ac Hnisz
FetalDHS Trynka
Enhancer Hoffman
Enhancer Andersson
DHS Trynka
DGF ENCODE
CTCF Hoffman
Conserved LindbladToh
Coding UCSC
Annotation
−25
0
25
50
Enrichment (prop h2 / prop snps)

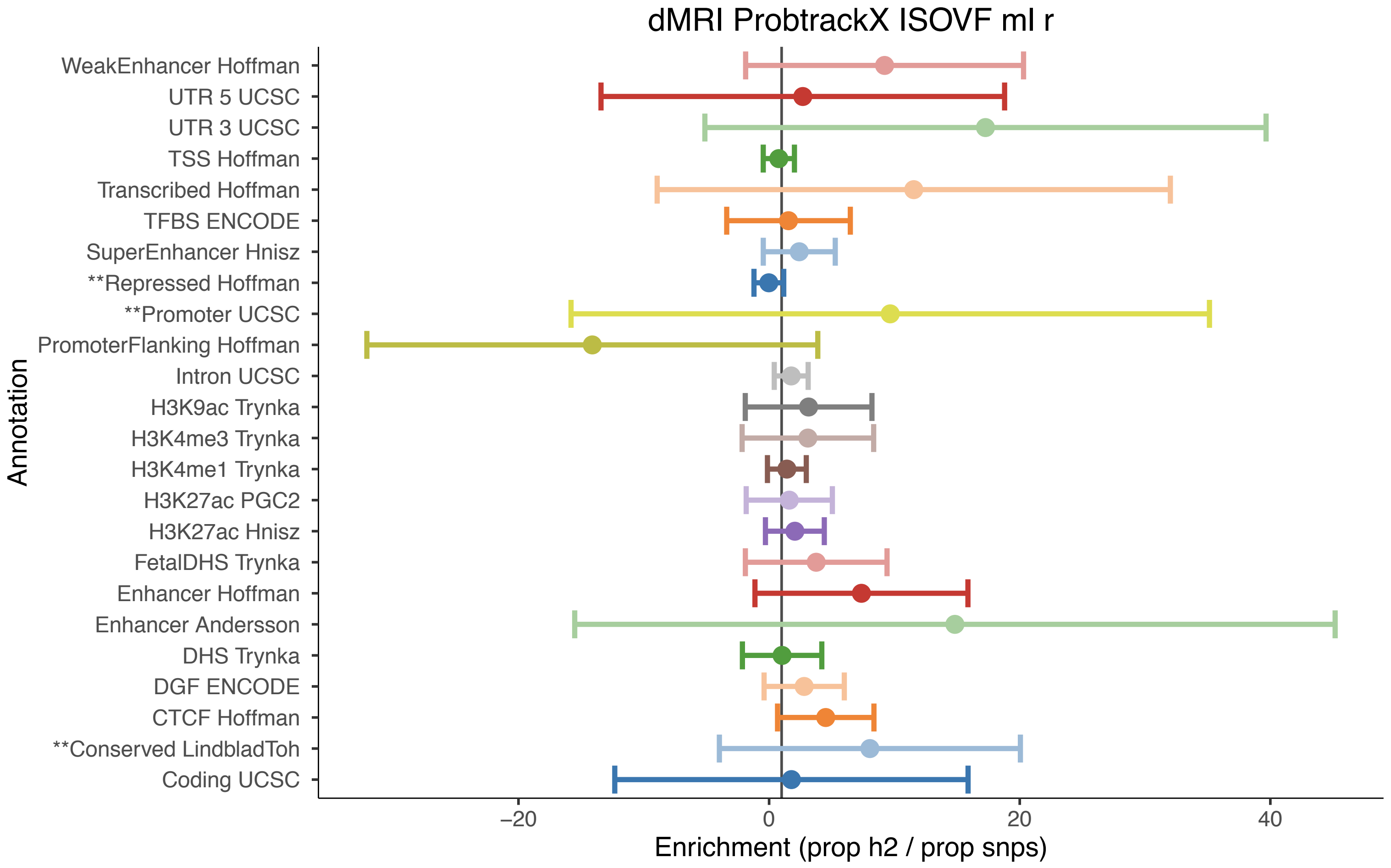
dMRI ProbtrackX ISOVF ml r
WeakEnhancer Hoffman
UTR 5 UCSC
UTR 3 UCSC
TSS Hoffman
Transcribed Hoffman
TFBS ENCODE
SuperEnhancer Hnisz
**Repressed Hoffman
**Promoter UCSC
PromoterFlanking Hoffman
Intron UCSC
H3K9ac Trynka
H3K4me3 Trynka
H3K4me1 Trynka
H3K27ac PGC2
H3K27ac Hnisz
FetalDHS Trynka
Enhancer Hoffman
Enhancer Andersson
DHS Trynka
DGF ENCODE
CTCF Hoffman
**Conserved LindbladToh
Coding UCSC
Annotation
−20
0
20
40
Enrichment (prop h2 / prop snps)

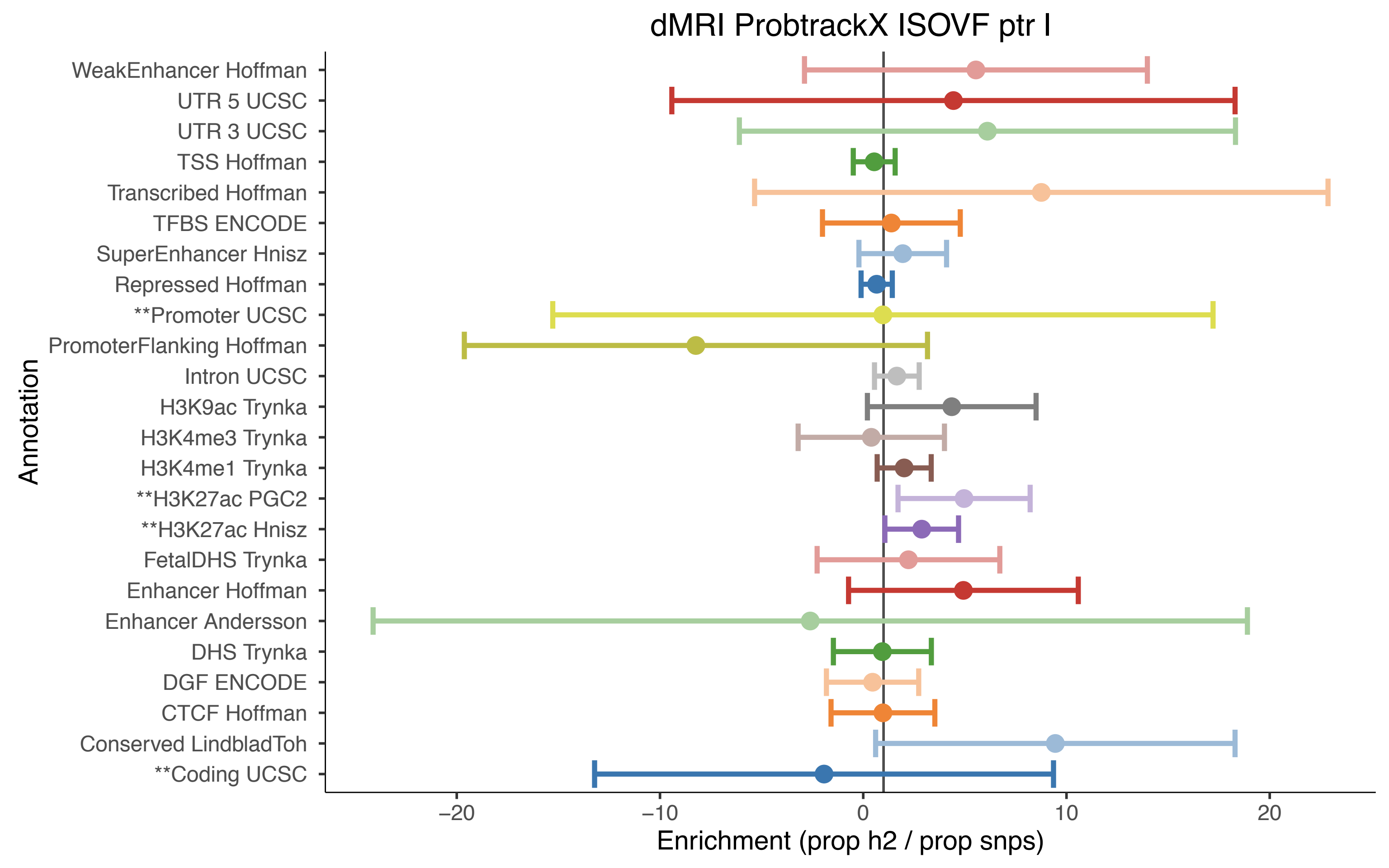

dMRI ProbtrackX ISOVF ptr I
WeakEnhancer Hoffman
UTR 5 UCSC
UTR 3 UCSC
TSS Hoffman
Transcribed Hoffman
TFBS ENCODE
SuperEnhancer Hnisz
Repressed Hoffman
**Promoter UCSC
PromoterFlanking Hoffman
Intron UCSC
H3K9ac Trynka
H3K4me3 Trynka
H3K4me1 Trynka
**H3K27ac PGC2
**H3K27ac Hnisz
FetalDHS Trynka
Enhancer Hoffman
Enhancer Andersson
DHS Trynka
DGF ENCODE
CTCF Hoffman
Conserved LindbladToh
**Coding UCSC
Annotation
−20
−10
0
10
20
Enrichment (prop h2 / prop snps)

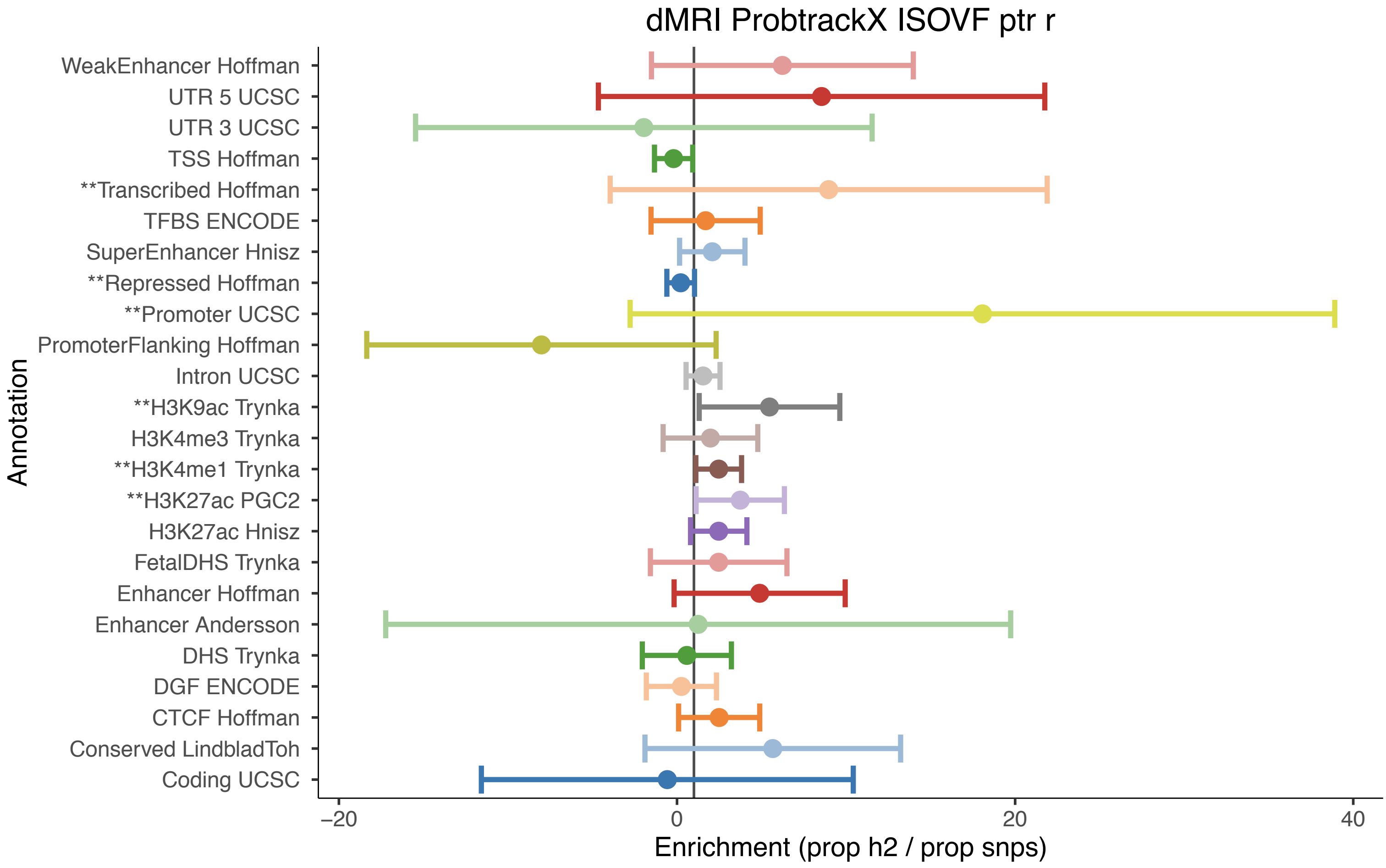

dMRI ProbtrackX ISOVF ptr r
WeakEnhancer Hoffman
UTR 5 UCSC
UTR 3 UCSC
TSS Hoffman
**Transcribed Hoffman
TFBS ENCODE
SuperEnhancer Hnisz
**Repressed Hoffman
**Promoter UCSC
PromoterFlanking Hoffman
Intron UCSC
**H3K9ac Trynka
H3K4me3 Trynka
**H3K4me1 Trynka
**H3K27ac PGC2
H3K27ac Hnisz
FetalDHS Trynka
Enhancer Hoffman
Enhancer Andersson
DHS Trynka
DGF ENCODE
CTCF Hoffman
Conserved LindbladToh
Coding UCSC
Annotation
−20
0
20
40
Enrichment (prop h2 / prop snps)

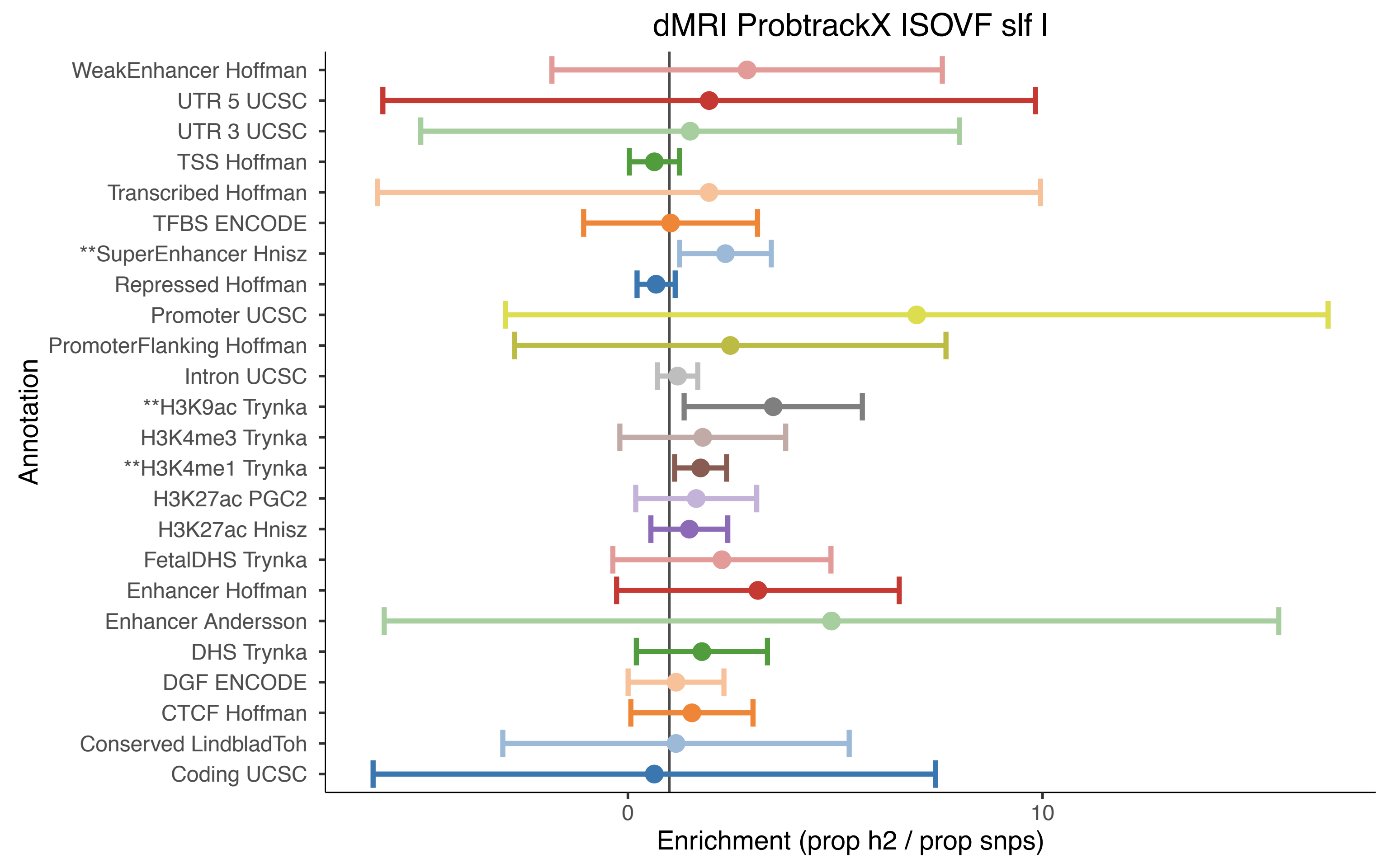
dMRI ProbtrackX ISOVF slf l
WeakEnhancer Hoffman
UTR 5 UCSC
UTR 3 UCSC
TSS Hoffman
Transcribed Hoffman
TFBS ENCODE
**SuperEnhancer Hnisz
Repressed Hoffman
Promoter UCSC
PromoterFlanking Hoffman
Intron UCSC
**H3K9ac Trynka
H3K4me3 Trynka
**H3K4me1 Trynka
H3K27ac PGC2
H3K27ac Hnisz
FetalDHS Trynka
Enhancer Hoffman
Enhancer Andersson
DHS Trynka
DGF ENCODE
CTCF Hoffman
Conserved LindbladToh
Coding UCSC
Annotation
0
10
Enrichment (prop h2 / prop snps)

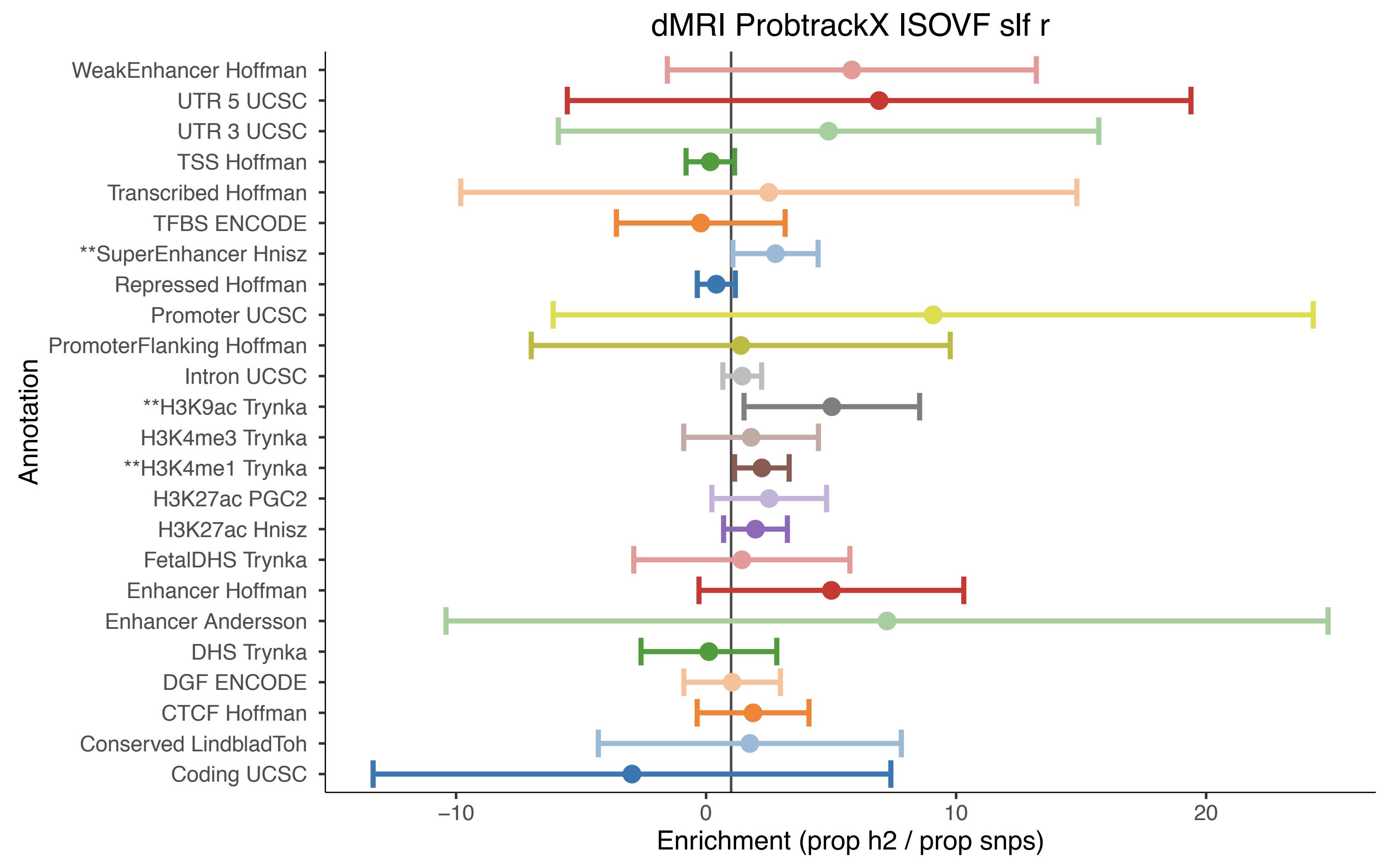

dMRI ProbtrackX ISOVF slf r
WeakEnhancer Hoffman
UTR 5 UCSC
UTR 3 UCSC
TSS Hoffman
Transcribed Hoffman
TFBS ENCODE
**SuperEnhancer Hnisz
Repressed Hoffman
Promoter UCSC
PromoterFlanking Hoffman
Intron UCSC
**H3K9ac Trynka
H3K4me3 Trynka
**H3K4me1 Trynka
H3K27ac PGC2
H3K27ac Hnisz
FetalDHS Trynka
Enhancer Hoffman
Enhancer Andersson
DHS Trynka
DGF ENCODE
CTCF Hoffman
Conserved LindbladToh
Coding UCSC
Annotation
−10
0
10
20
Enrichment (prop h2 / prop snps)

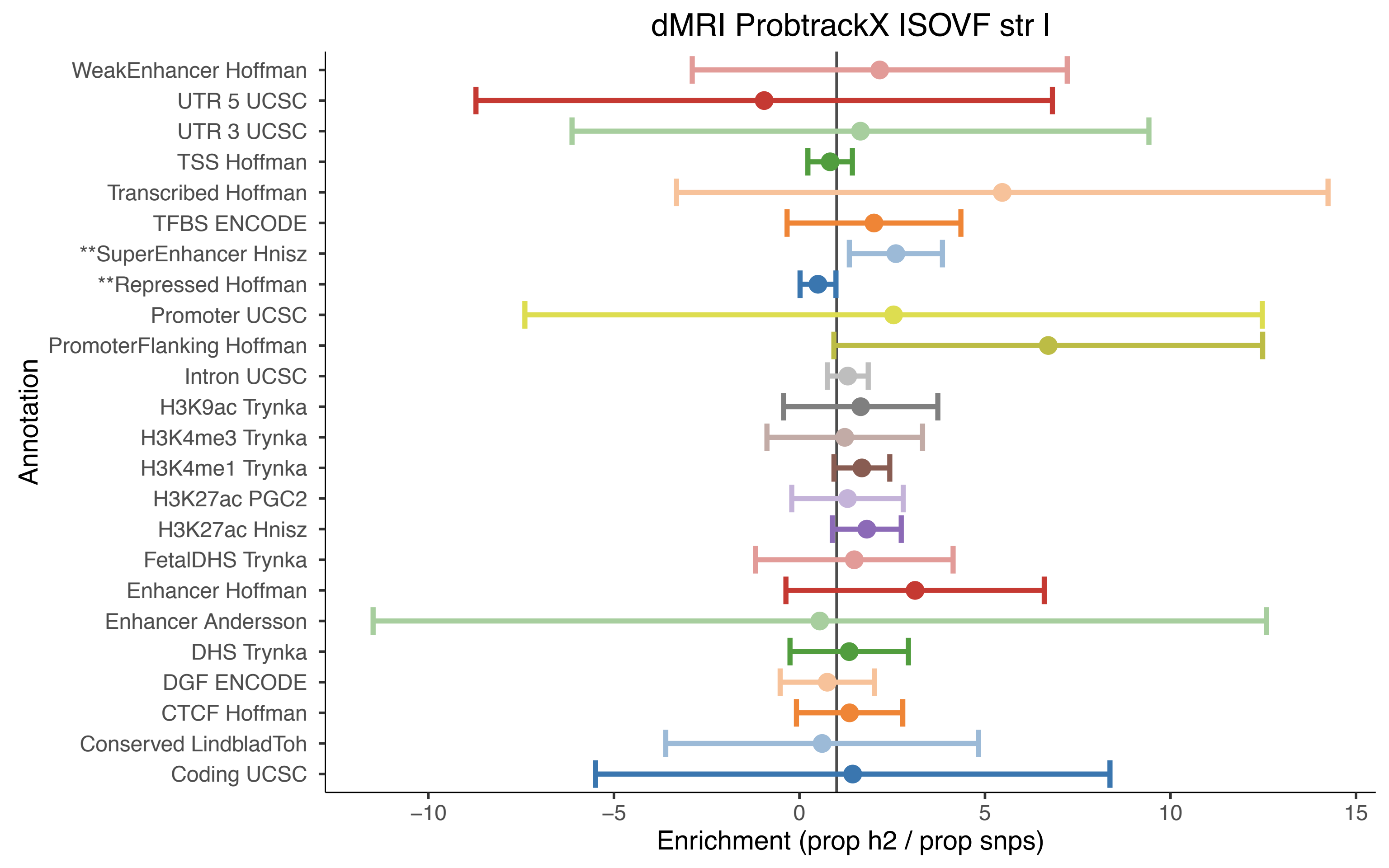

dMRI ProbtrackX ISOVF str I
WeakEnhancer Hoffman
UTR 5 UCSC
UTR 3 UCSC
TSS Hoffman
Transcribed Hoffman
TFBS ENCODE
**SuperEnhancer Hnisz
**Repressed Hoffman
Promoter UCSC
PromoterFlanking Hoffman
Intron UCSC
H3K9ac Trynka
H3K4me3 Trynka
H3K4me1 Trynka
H3K27ac PGC2
H3K27ac Hnisz
FetalDHS Trynka
Enhancer Hoffman
Enhancer Andersson
DHS Trynka
DGF ENCODE
CTCF Hoffman
Conserved LindbladToh
Coding UCSC
Annotation
−10
−5
0
5
10
15
Enrichment (prop h2 / prop snps)

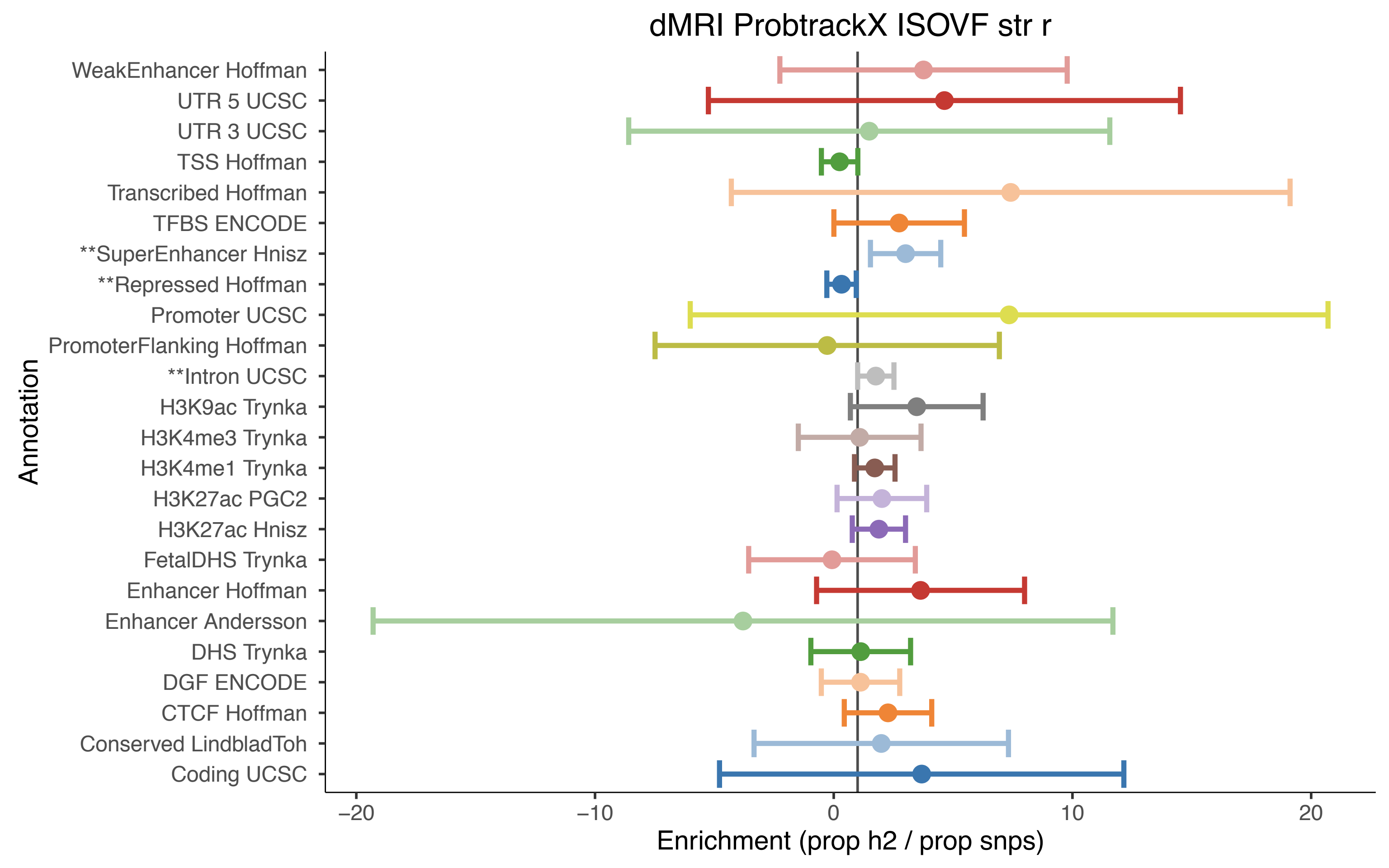

dMRI ProbtrackX ISOVF str r
WeakEnhancer Hoffman
UTR 5 UCSC
UTR 3 UCSC
TSS Hoffman
Transcribed Hoffman
TFBS ENCODE
**SuperEnhancer Hnisz
**Repressed Hoffman
Promoter UCSC
PromoterFlanking Hoffman
**Intron UCSC
H3K9ac Trynka
H3K4me3 Trynka
H3K4me1 Trynka
H3K27ac PGC2
H3K27ac Hnisz
FetalDHS Trynka
Enhancer Hoffman
Enhancer Andersson
DHS Trynka
DGF ENCODE
CTCF Hoffman
Conserved LindbladToh
Coding UCSC
Annotation
−20
−10
0
10
20
Enrichment (prop h2 / prop snps)

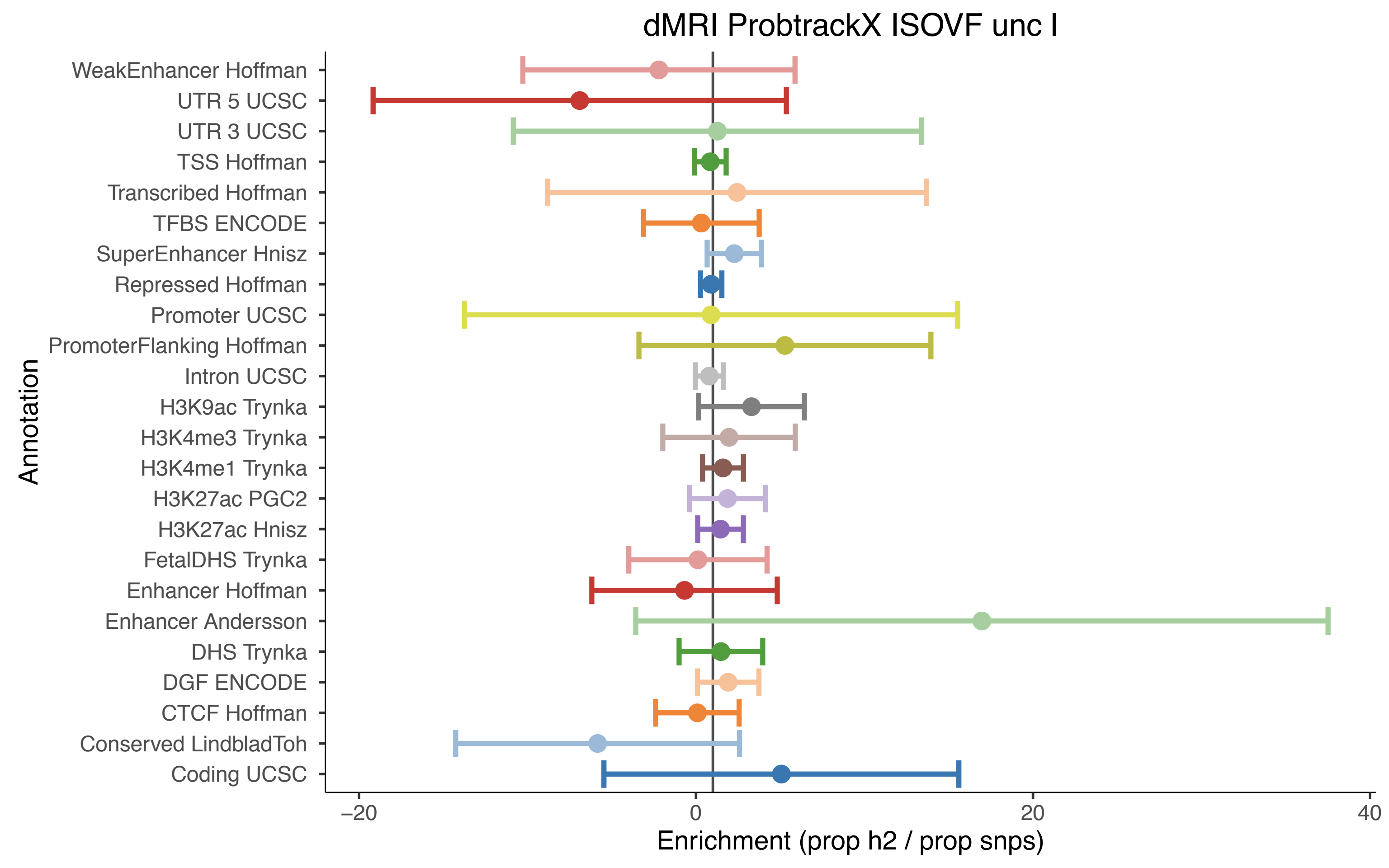
dMRI ProbtrackX ISOVF unc I
WeakEnhancer Hoffman
UTR 5 UCSC
UTR 3 UCSC
TSS Hoffman
Transcribed Hoffman
TFBS ENCODE
SuperEnhancer Hnisz
Repressed Hoffman
Promoter UCSC
PromoterFlanking Hoffman
Intron UCSC
H3K9ac Trynka
H3K4me3 Trynka
H3K4me1 Trynka
H3K27ac PGC2
H3K27ac Hnisz
FetalDHS Trynka
Enhancer Hoffman
Enhancer Andersson
DHS Trynka
DGF ENCODE
CTCF Hoffman
Conserved LindbladToh
Coding UCSC
Annotation
−20
0
20
40
Enrichment (prop h2 / prop snps)

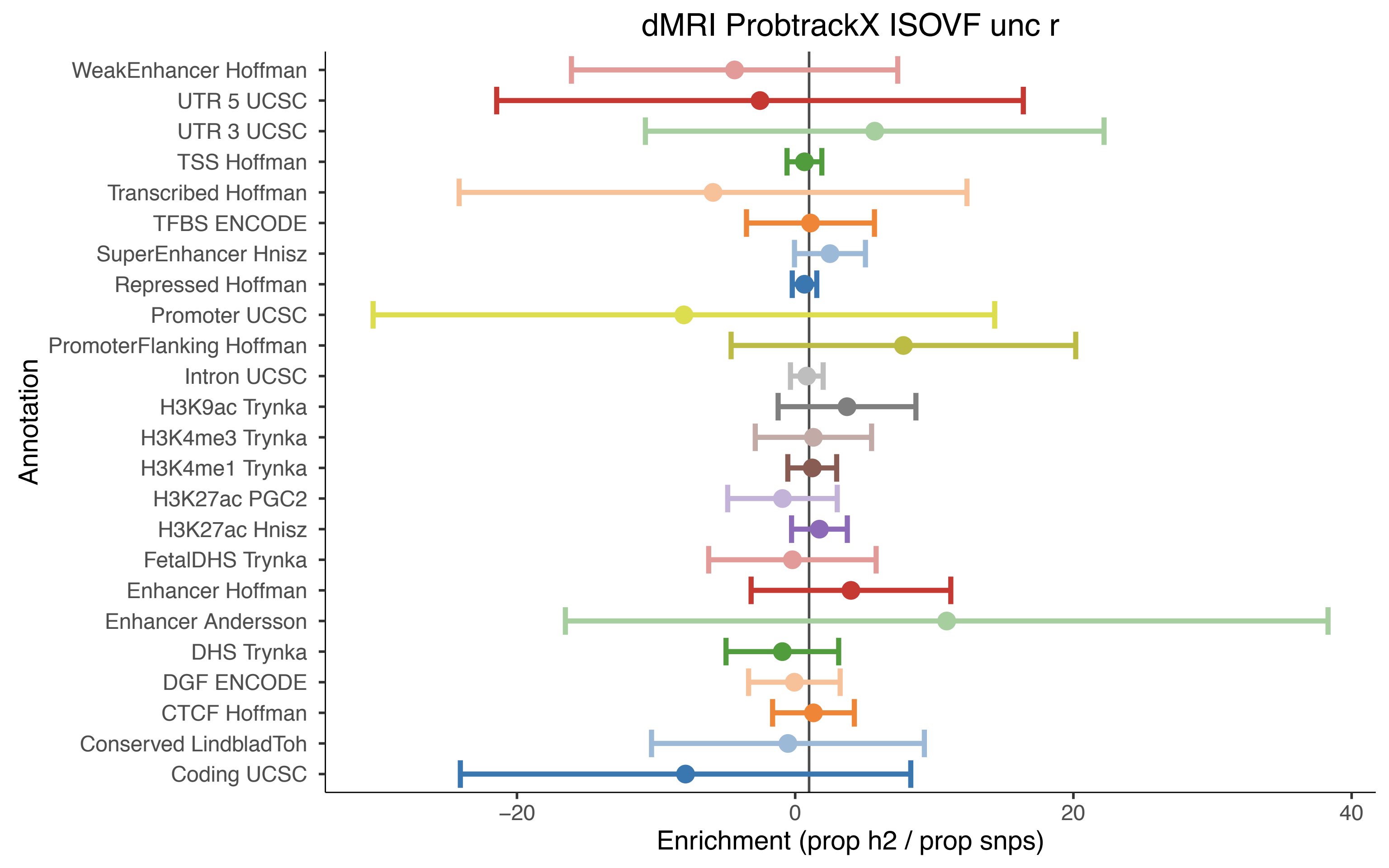

dMRI ProbtrackX ISOVF unc r
WeakEnhancer Hoffman
UTR 5 UCSC
UTR 3 UCSC
TSS Hoffman
Transcribed Hoffman
TFBS ENCODE
SuperEnhancer Hnisz
Repressed Hoffman
Promoter UCSC
PromoterFlanking Hoffman
Intron UCSC
H3K9ac Trynka
H3K4me3 Trynka
H3K4me1 Trynka
H3K27ac PGC2
H3K27ac Hnisz
FetalDHS Trynka
Enhancer Hoffman
Enhancer Andersson
DHS Trynka
DGF ENCODE
CTCF Hoffman
Conserved LindbladToh
Coding UCSC
Annotation
−20
0
20
40
Enrichment (prop h2 / prop snps)

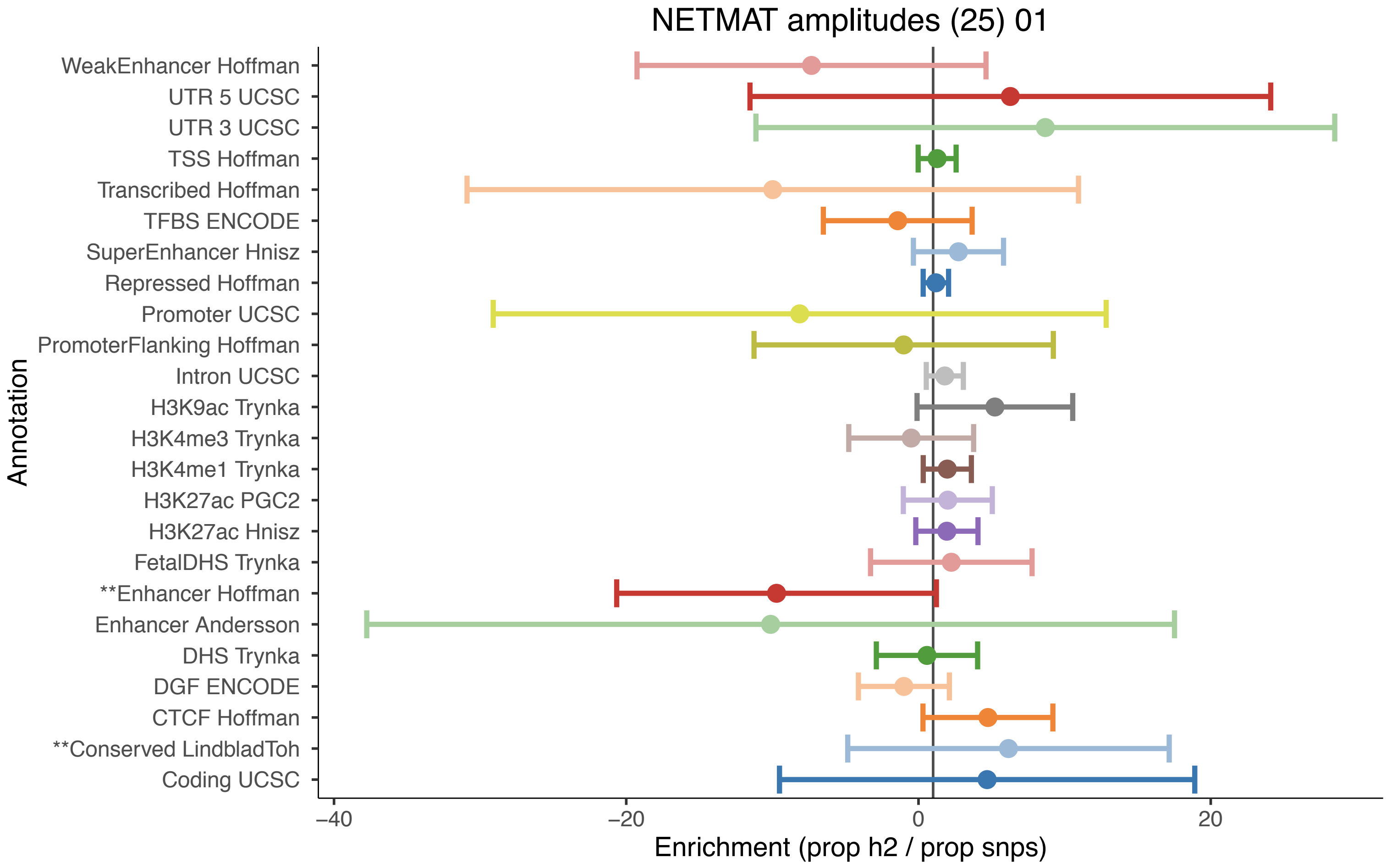
NETMAT amplitudes (25) 01
WeakEnhancer Hoffman
UTR 5 UCSC
UTR 3 UCSC
TSS Hoffman
Transcribed Hoffman
TFBS ENCODE
SuperEnhancer Hnisz
Repressed Hoffman
Promoter UCSC
PromoterFlanking Hoffman
Intron UCSC
H3K9ac Trynka
H3K4me3 Trynka
H3K4me1 Trynka
H3K27ac PGC2
H3K27ac Hnisz
FetalDHS Trynka
**Enhancer Hoffman
Enhancer Andersson
DHS Trynka
DGF ENCODE
CTCF Hoffman
**Conserved LindbladToh
Coding UCSC
Annotation
−40
−20
0
20
Enrichment (prop h2 / prop snps)

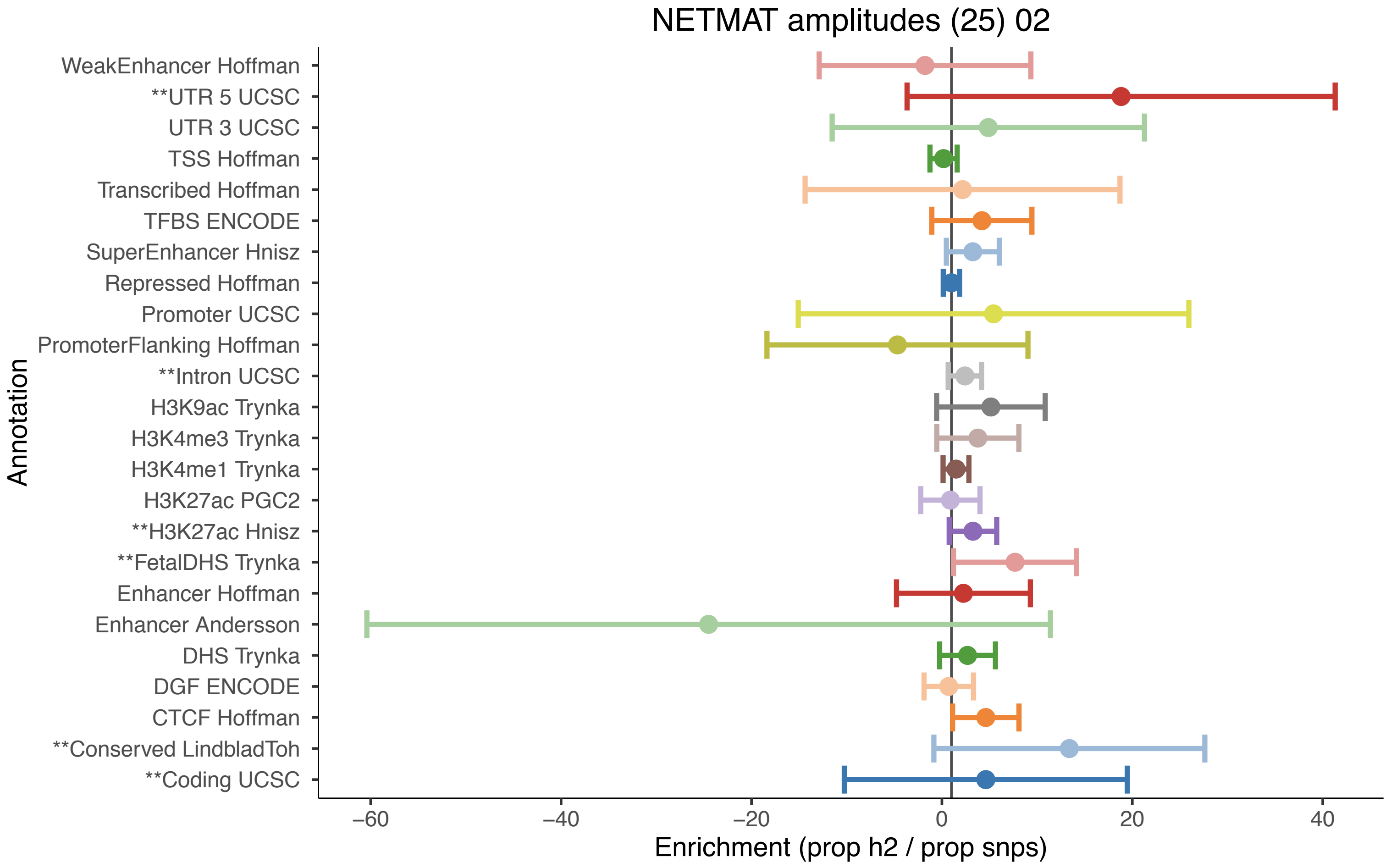
NETMAT amplitudes (25) 02
WeakEnhancer Hoffman
**UTR 5 UCSC
UTR 3 UCSC
TSS Hoffman
Transcribed Hoffman
TFBS ENCODE
SuperEnhancer Hnisz
Repressed Hoffman
Promoter UCSC
PromoterFlanking Hoffman
**Intron UCSC
H3K9ac Trynka
H3K4me3 Trynka
H3K4me1 Trynka
H3K27ac PGC2
**H3K27ac Hnisz
**FetalDHS Trynka
Enhancer Hoffman
Enhancer Andersson
DHS Trynka
DGF ENCODE
CTCF Hoffman
**Conserved LindbladToh
**Coding UCSC
Annotation
−60
−40
−20
0
20
40
Enrichment (prop h2 / prop snps)

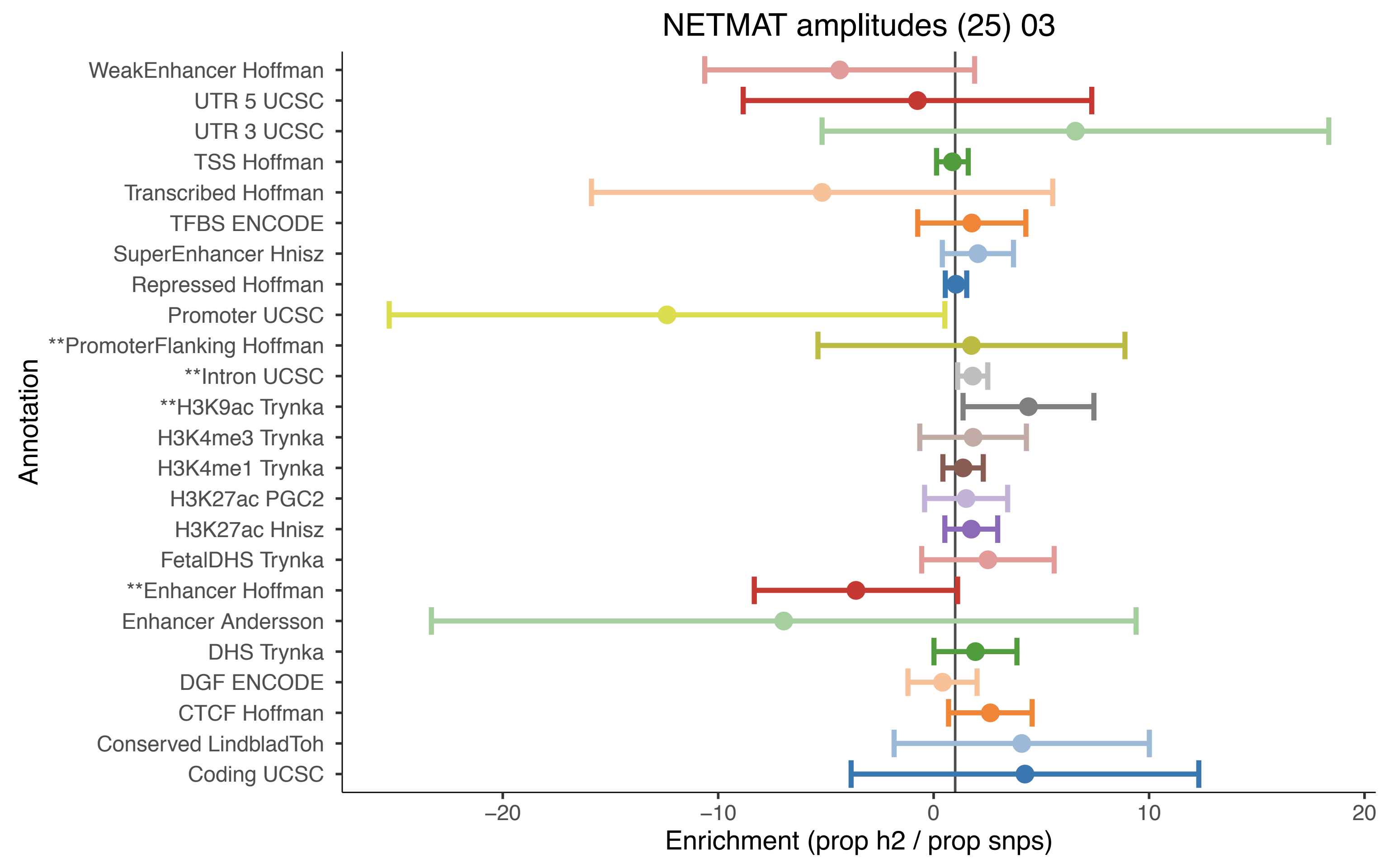

NETMAT amplitudes (25) 03
Annotation
WeakEnhancer Hoffman
UTR 5 UCSC
UTR 3 UCSC
TSS Hoffman
Transcribed Hoffman
TFBS ENCODE
SuperEnhancer Hnisz
Repressed Hoffman
Promoter UCSC
**PromoterFlanking Hoffman
**Intron UCSC
**H3K9ac Trynka
H3K4me3 Trynka
H3K4me1 Trynka
H3K27ac PGC2
H3K27ac Hnisz
FetalDHS Trynka
**Enhancer Hoffman
Enhancer Andersson
DHS Trynka
DGF ENCODE
CTCF Hoffman
Conserved LindbladToh
Coding UCSC
−20
−10
0
10
20
Enrichment (prop h2 / prop snps)

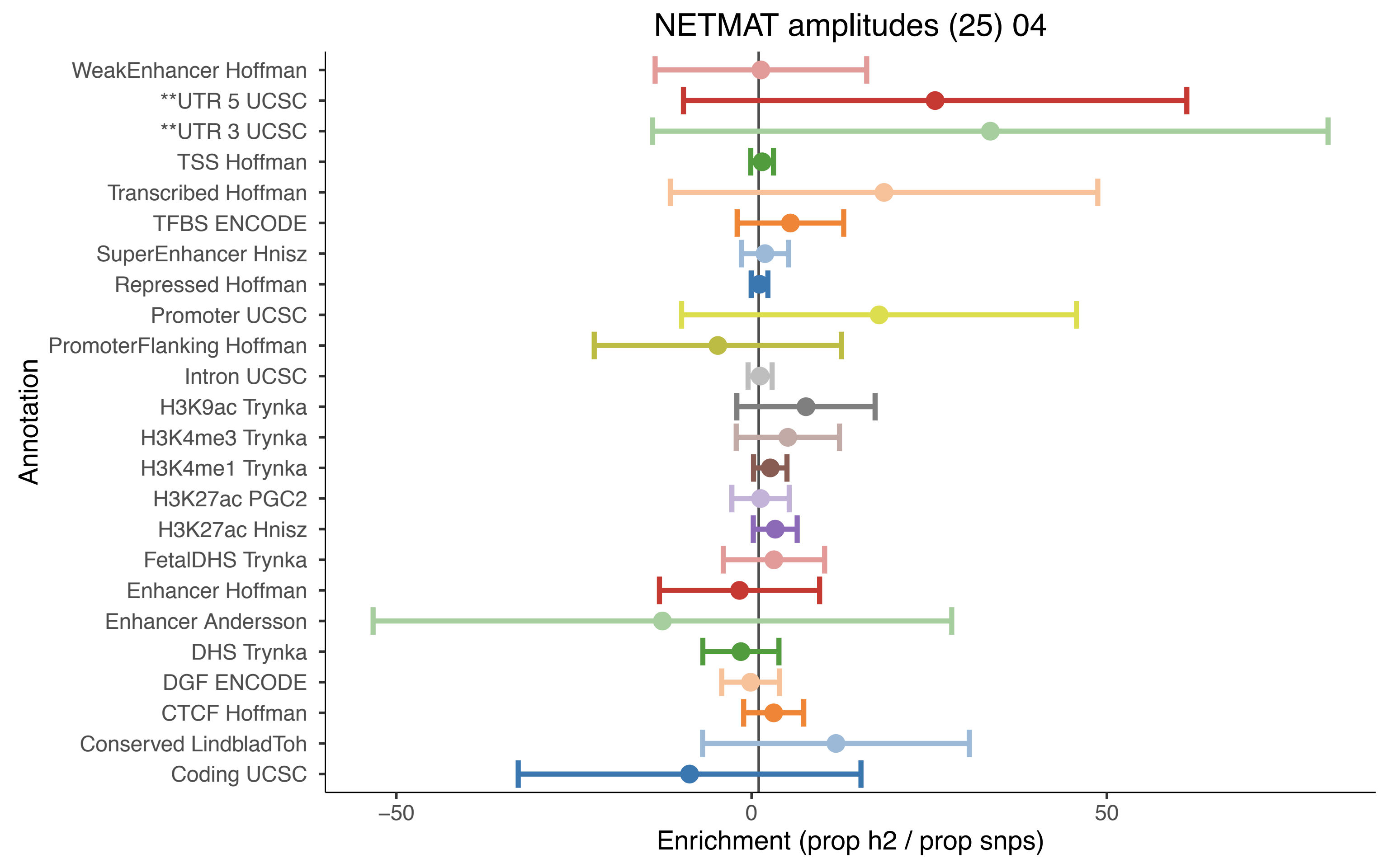

NETMAT amplitudes (25) 04
Annotation
WeakEnhancer Hoffman
**UTR 5 UCSC
**UTR 3 UCSC
TSS Hoffman
Transcribed Hoffman
TFBS ENCODE
SuperEnhancer Hnisz
Repressed Hoffman
Promoter UCSC
PromoterFlanking Hoffman
Intron UCSC
H3K9ac Trynka
H3K4me3 Trynka
H3K4me1 Trynka
H3K27ac PGC2
H3K27ac Hnisz
FetalDHS Trynka
Enhancer Hoffman
Enhancer Andersson
DHS Trynka
DGF ENCODE
CTCF Hoffman
Conserved LindbladToh
Coding UCSC
−50
0
50
Enrichment (prop h2 / prop snps)

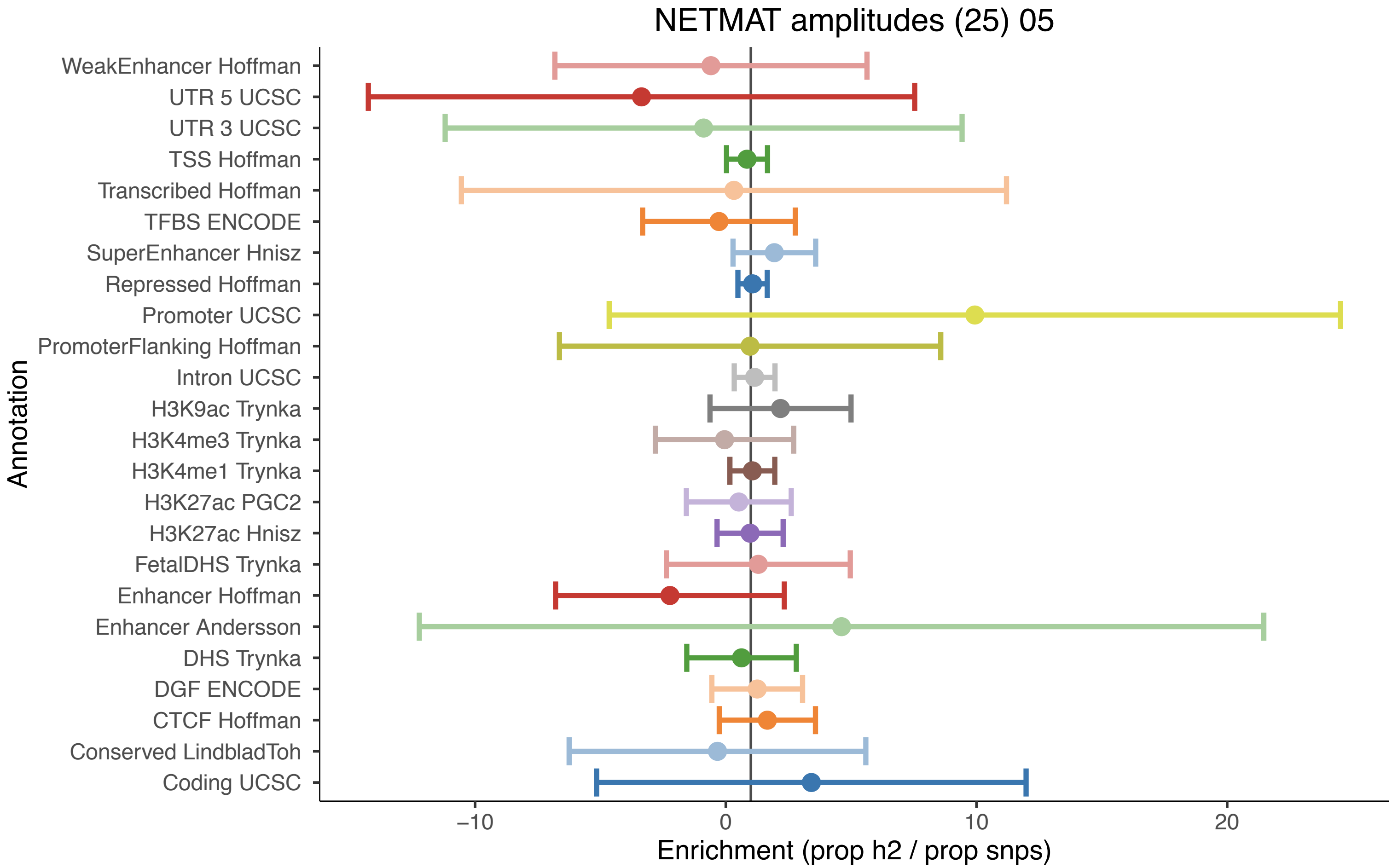

NETMAT amplitudes (25) 05
WeakEnhancer Hoffman
UTR 5 UCSC
UTR 3 UCSC
TSS Hoffman
Transcribed Hoffman
TFBS ENCODE
SuperEnhancer Hnisz
Repressed Hoffman
Promoter UCSC
PromoterFlanking Hoffman
Intron UCSC
H3K9ac Trynka
H3K4me3 Trynka
H3K4me1 Trynka
H3K27ac PGC2
H3K27ac Hnisz
FetalDHS Trynka
Enhancer Hoffman
Enhancer Andersson
DHS Trynka
DGF ENCODE
CTCF Hoffman
Conserved LindbladToh
Coding UCSC
Annotation
−10
0
10
20
Enrichment (prop h2 / prop snps)

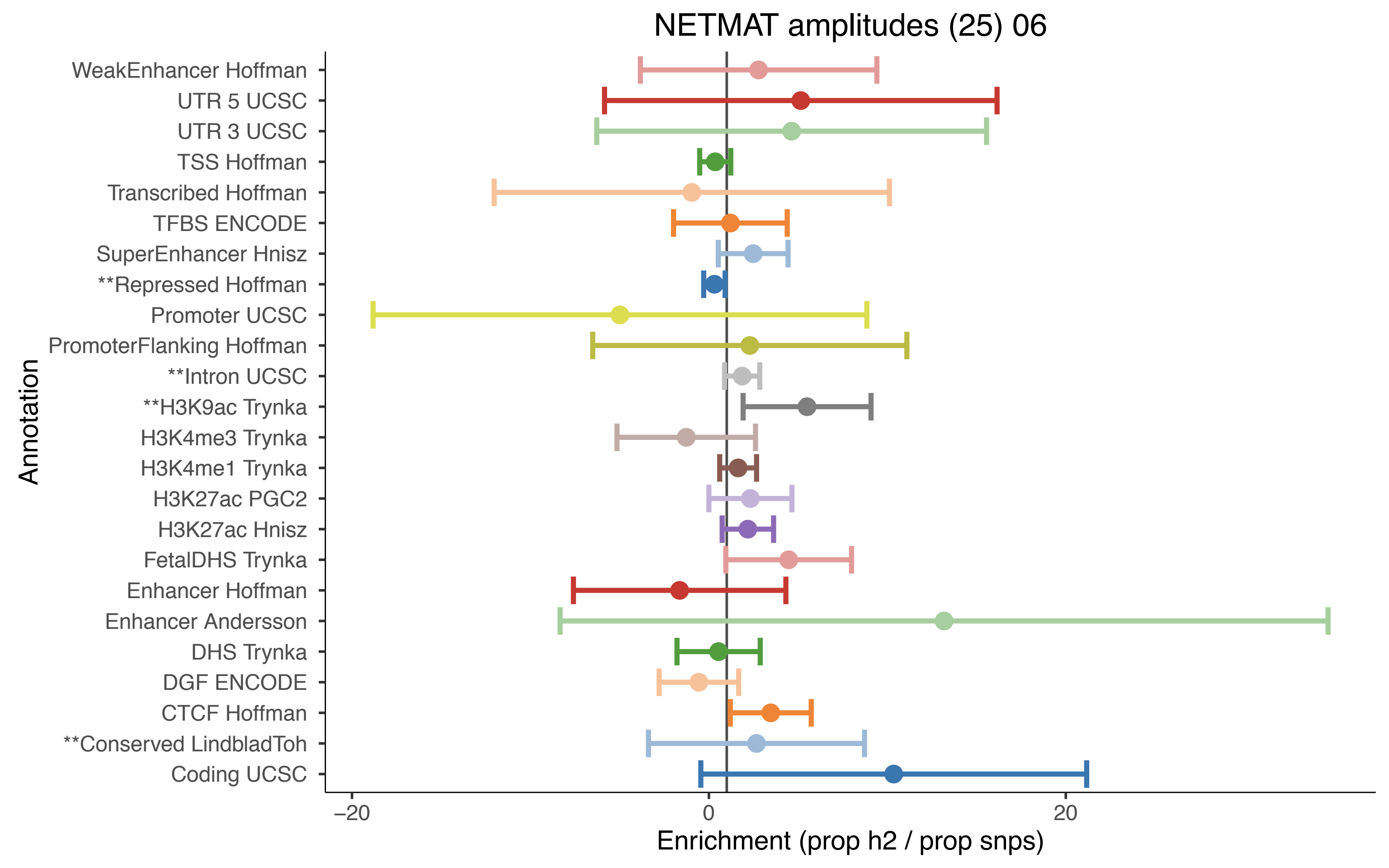

NETMAT amplitudes (25) 06
WeakEnhancer Hoffman
UTR 5 UCSC
UTR 3 UCSC
TSS Hoffman
Transcribed Hoffman
TFBS ENCODE
SuperEnhancer Hnisz
**Repressed Hoffman
Promoter UCSC
PromoterFlanking Hoffman
**Intron UCSC
**H3K9ac Trynka
H3K4me3 Trynka
H3K4me1 Trynka
H3K27ac PGC2
H3K27ac Hnisz
FetalDHS Trynka
Enhancer Hoffman
Enhancer Andersson
DHS Trynka
DGF ENCODE
CTCF Hoffman
**Conserved LindbladToh
Coding UCSC
Annotation
−20
0
20
Enrichment (prop h2 / prop snps)

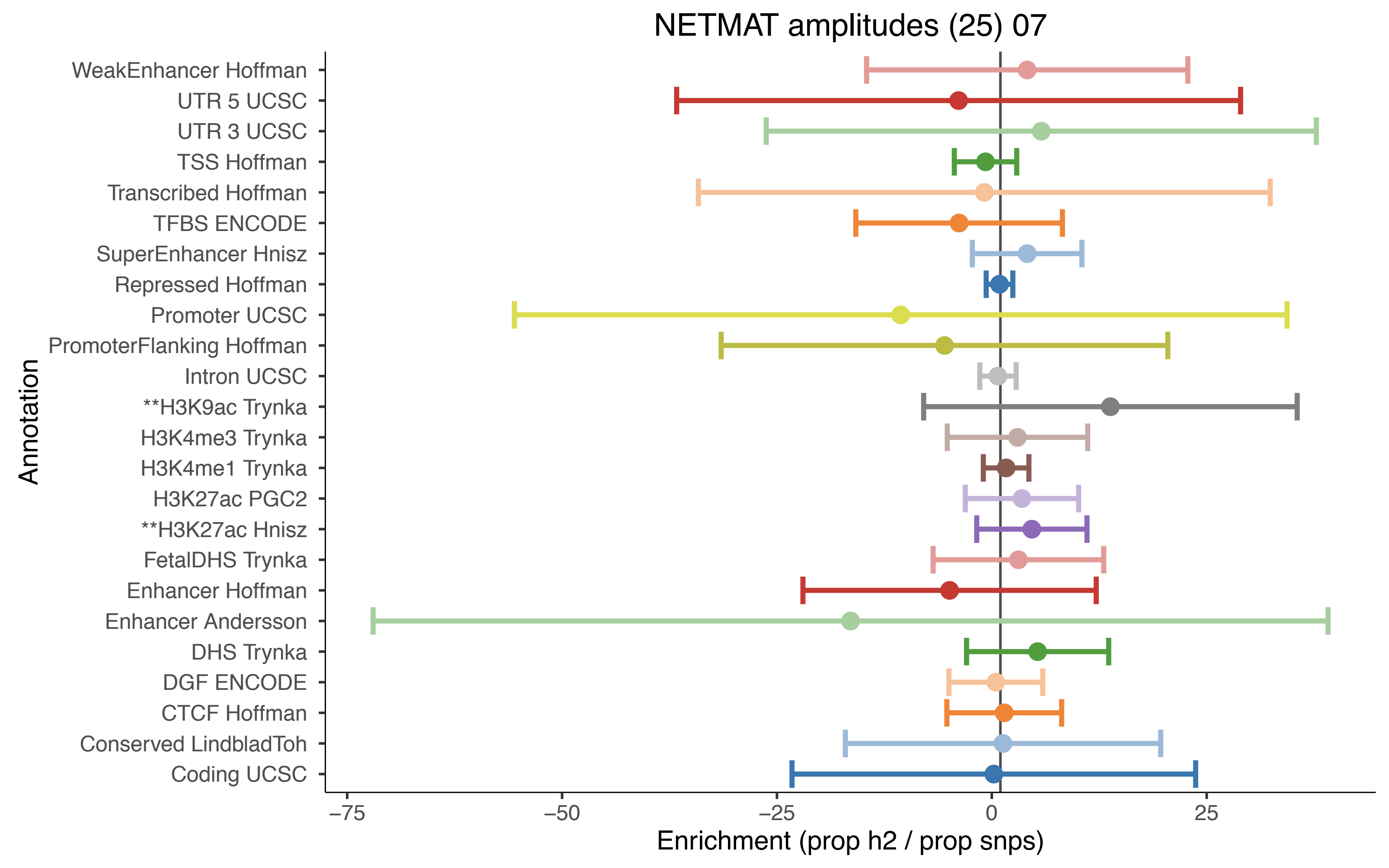

NETMAT amplitudes (25) 07
WeakEnhancer Hoffman
UTR 5 UCSC
UTR 3 UCSC
TSS Hoffman
Transcribed Hoffman
TFBS ENCODE
SuperEnhancer Hnisz
Repressed Hoffman
Promoter UCSC
PromoterFlanking Hoffman
Intron UCSC
**H3K9ac Trynka
H3K4me3 Trynka
H3K4me1 Trynka
H3K27ac PGC2
**H3K27ac Hnisz
FetalDHS Trynka
Enhancer Hoffman
Enhancer Andersson
DHS Trynka
DGF ENCODE
CTCF Hoffman
Conserved LindbladToh
Coding UCSC
Annotation
−75
−50
−25
0
25
Enrichment (prop h2 / prop snps)

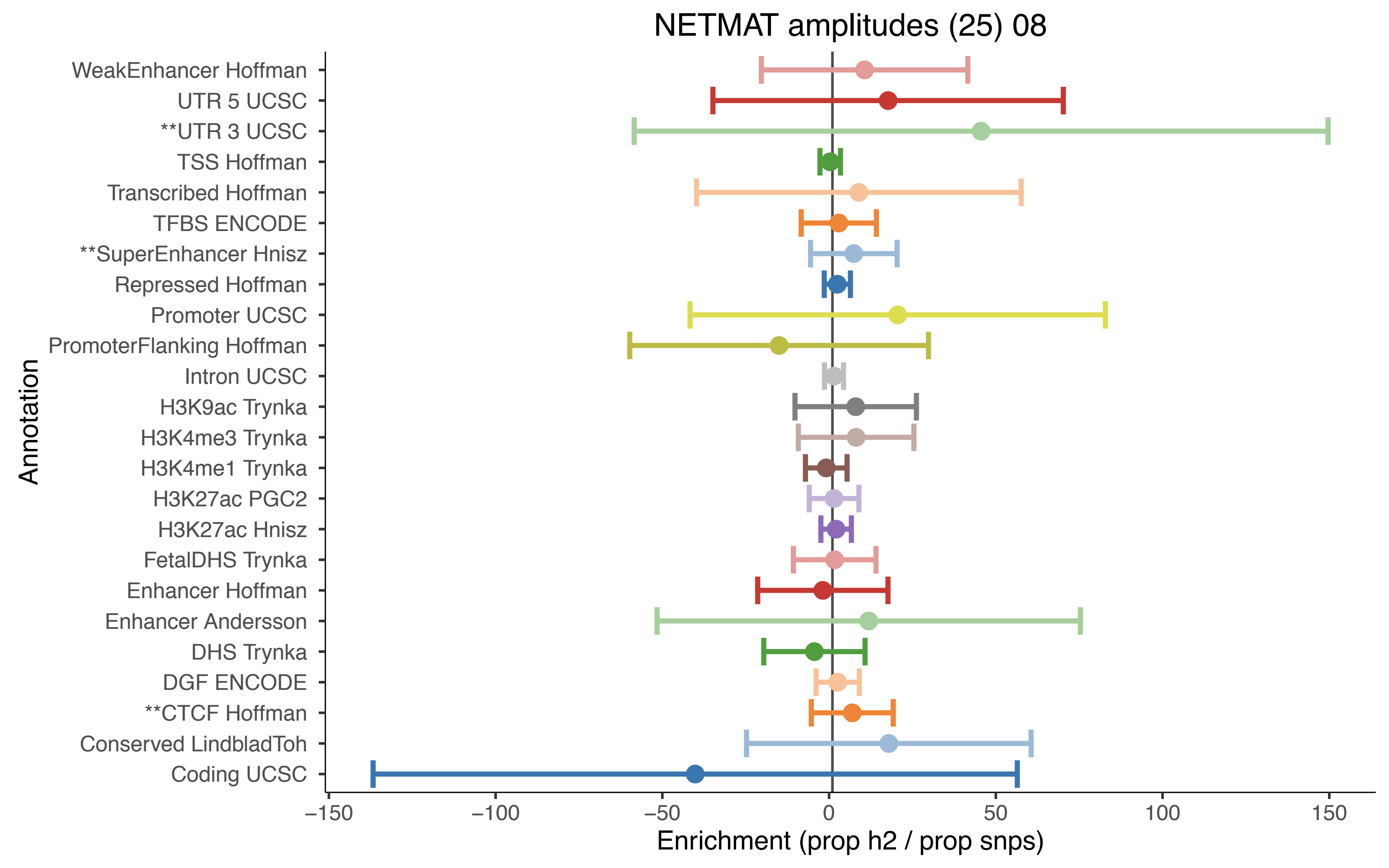

NETMAT amplitudes (25) 08
WeakEnhancer Hoffman
UTR 5 UCSC
**UTR 3 UCSC
TSS Hoffman
Transcribed Hoffman
TFBS ENCODE
**SuperEnhancer Hnisz
Repressed Hoffman
Promoter UCSC
PromoterFlanking Hoffman
Intron UCSC
H3K9ac Trynka
H3K4me3 Trynka
H3K4me1 Trynka
H3K27ac PGC2
H3K27ac Hnisz
FetalDHS Trynka
Enhancer Hoffman
Enhancer Andersson
DHS Trynka
DGF ENCODE
**CTCF Hoffman
Conserved LindbladToh
Coding UCSC
Annotation
−150
−100
−50
0
50
100
150
Enrichment (prop h2 / prop snps)

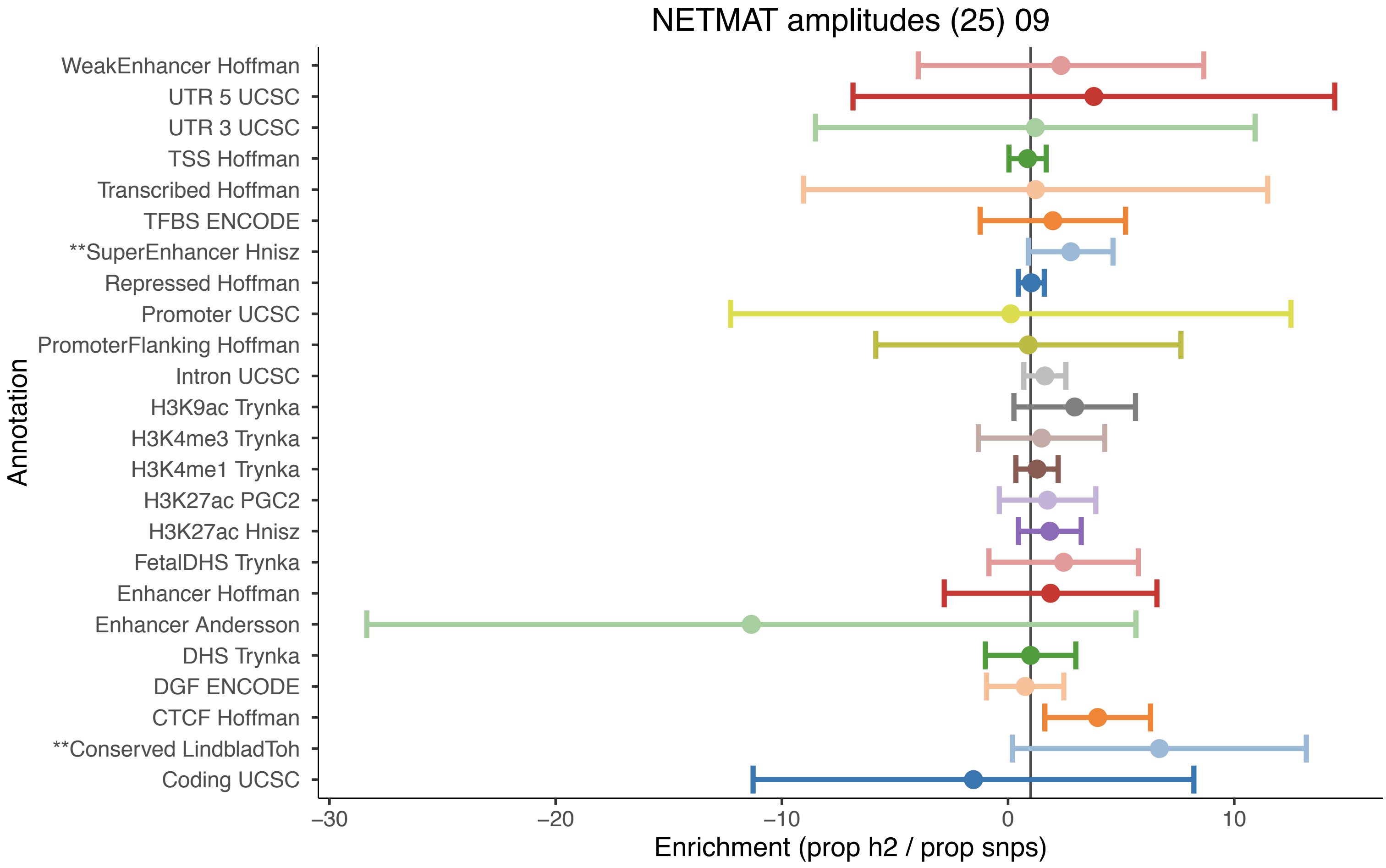

NETMAT amplitudes (25) 09
WeakEnhancer Hoffman
UTR 5 UCSC
UTR 3 UCSC
TSS Hoffman
Transcribed Hoffman
TFBS ENCODE
**SuperEnhancer Hnisz
Repressed Hoffman
Promoter UCSC
PromoterFlanking Hoffman
Intron UCSC
H3K9ac Trynka
H3K4me3 Trynka
H3K4me1 Trynka
H3K27ac PGC2
H3K27ac Hnisz
FetalDHS Trynka
Enhancer Hoffman
Enhancer Andersson
DHS Trynka
DGF ENCODE
CTCF Hoffman
**Conserved LindbladToh
Coding UCSC
Annotation
−30
−20
−10
0
10
Enrichment (prop h2 / prop snps)

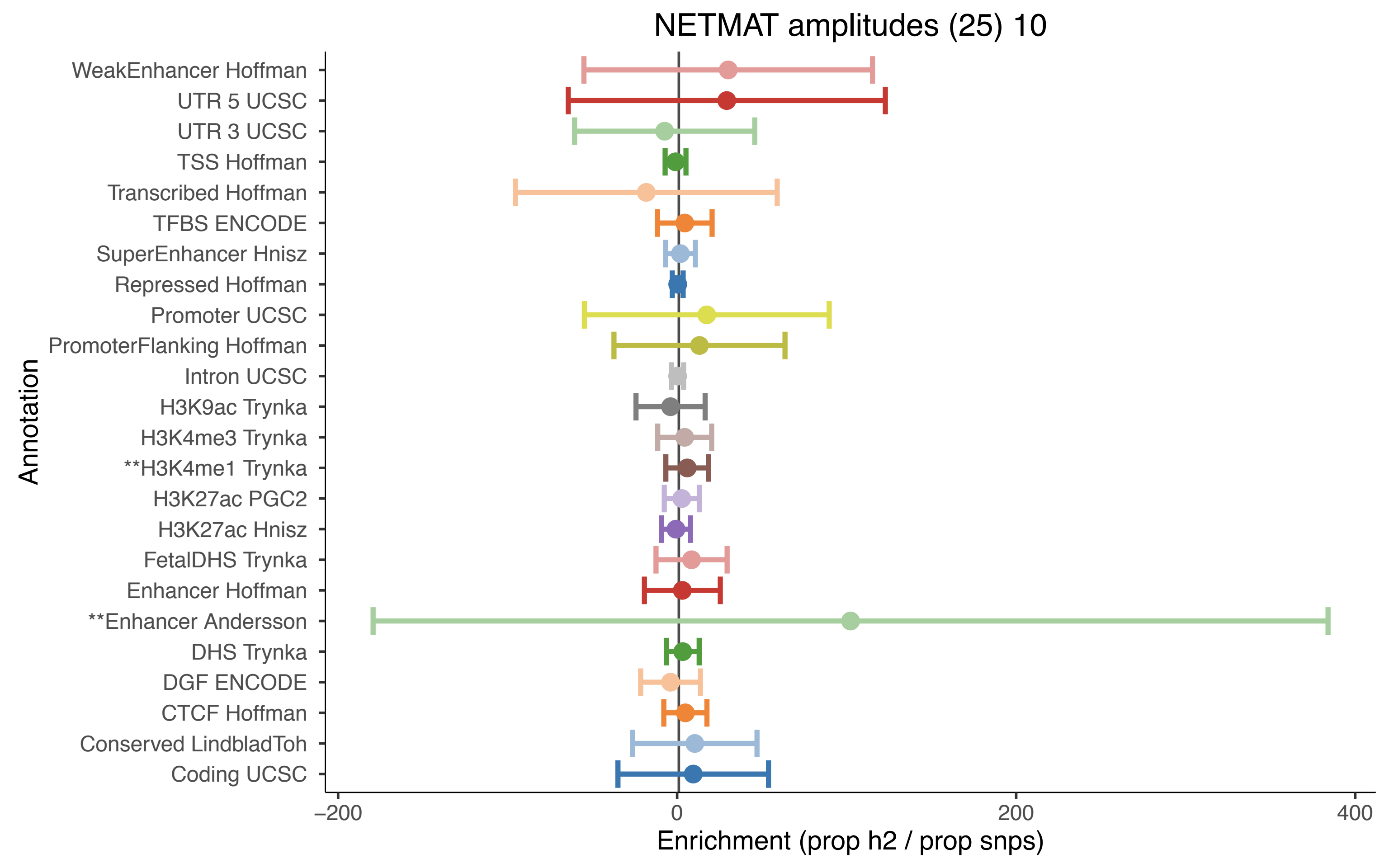

NETMAT amplitudes (25) 10
WeakEnhancer Hoffman
UTR 5 UCSC
UTR 3 UCSC
TSS Hoffman
Transcribed Hoffman
TFBS ENCODE
SuperEnhancer Hnisz
Repressed Hoffman
Promoter UCSC
PromoterFlanking Hoffman
Intron UCSC
H3K9ac Trynka
H3K4me3 Trynka
**H3K4me1 Trynka
H3K27ac PGC2
H3K27ac Hnisz
FetalDHS Trynka
Enhancer Hoffman
**Enhancer Andersson
DHS Trynka
DGF ENCODE
CTCF Hoffman
Conserved LindbladToh
Coding UCSC
Annotation
−200
0
200
400
Enrichment (prop h2 / prop snps)

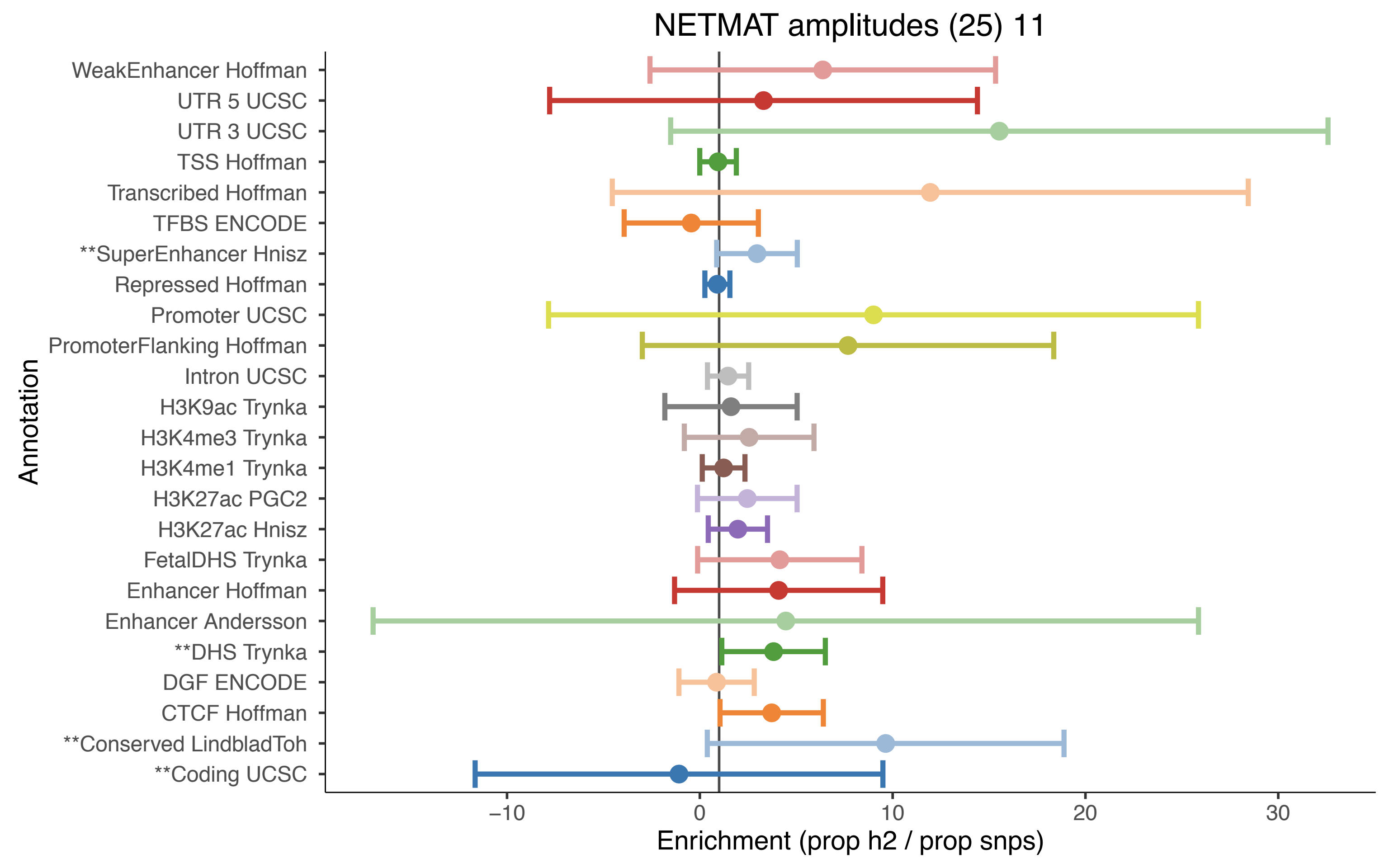

NETMAT amplitudes (25) 11
WeakEnhancer Hoffman
UTR 5 UCSC
UTR 3 UCSC
TSS Hoffman
Transcribed Hoffman
TFBS ENCODE
**SuperEnhancer Hnisz
Repressed Hoffman
Promoter UCSC
PromoterFlanking Hoffman
Intron UCSC
H3K9ac Trynka
H3K4me3 Trynka
H3K4me1 Trynka
H3K27ac PGC2
H3K27ac Hnisz
FetalDHS Trynka
Enhancer Hoffman
Enhancer Andersson
**DHS Trynka
DGF ENCODE
CTCF Hoffman
**Conserved LindbladToh
**Coding UCSC
Annotation
−10
0
10
20
30
Enrichment (prop h2 / prop snps)

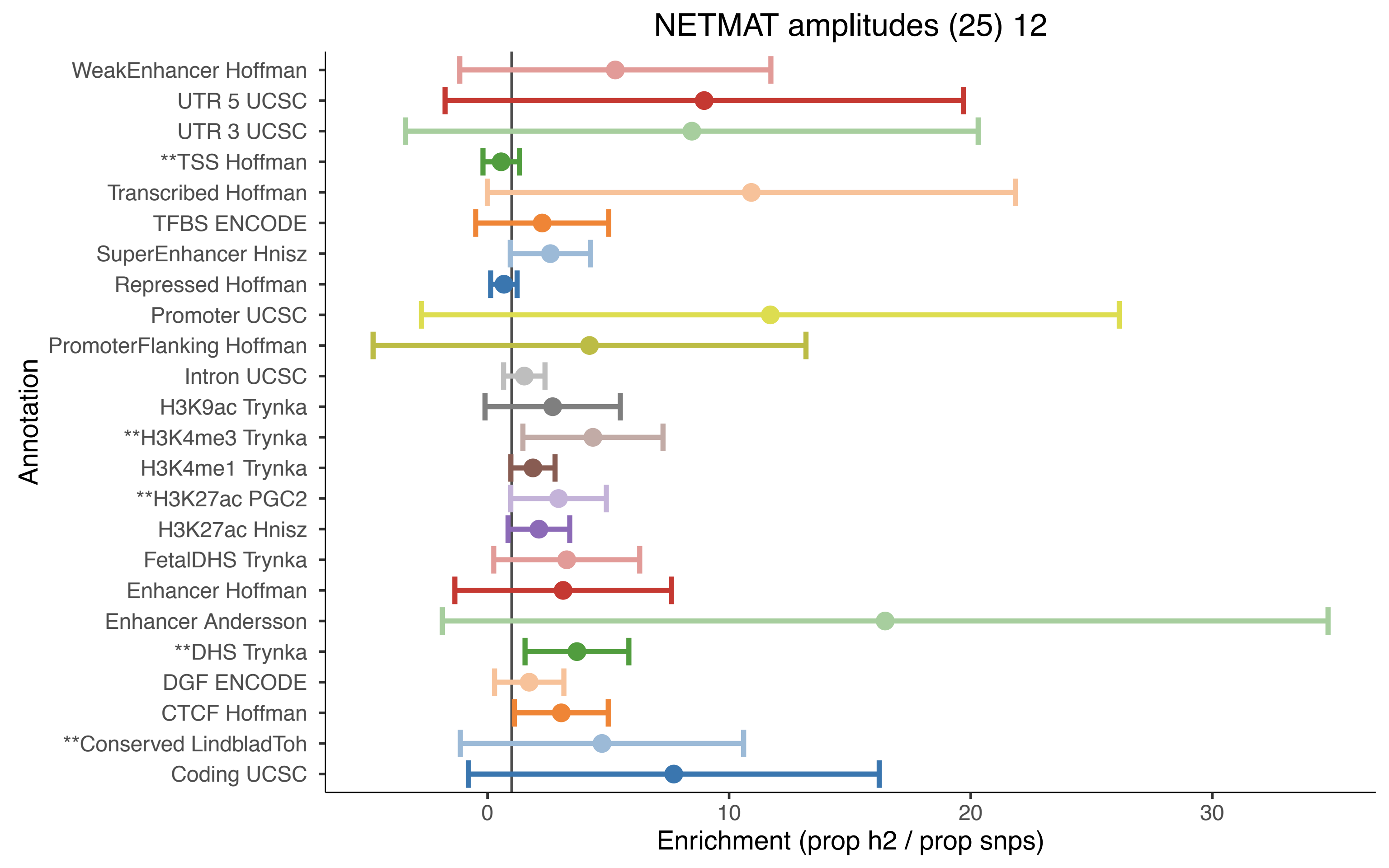

NETMAT amplitudes (25) 12
WeakEnhancer Hoffman
UTR 5 UCSC
UTR 3 UCSC
**TSS Hoffman
Transcribed Hoffman
TFBS ENCODE
SuperEnhancer Hnisz
Repressed Hoffman
Promoter UCSC
PromoterFlanking Hoffman
Intron UCSC
H3K9ac Trynka
**H3K4me3 Trynka
H3K4me1 Trynka
**H3K27ac PGC2
H3K27ac Hnisz
FetalDHS Trynka
Enhancer Hoffman
Enhancer Andersson
**DHS Trynka
DGF ENCODE
CTCF Hoffman
**Conserved LindbladToh
Coding UCSC
Annotation
0
10
20
30
Enrichment (prop h2 / prop snps)

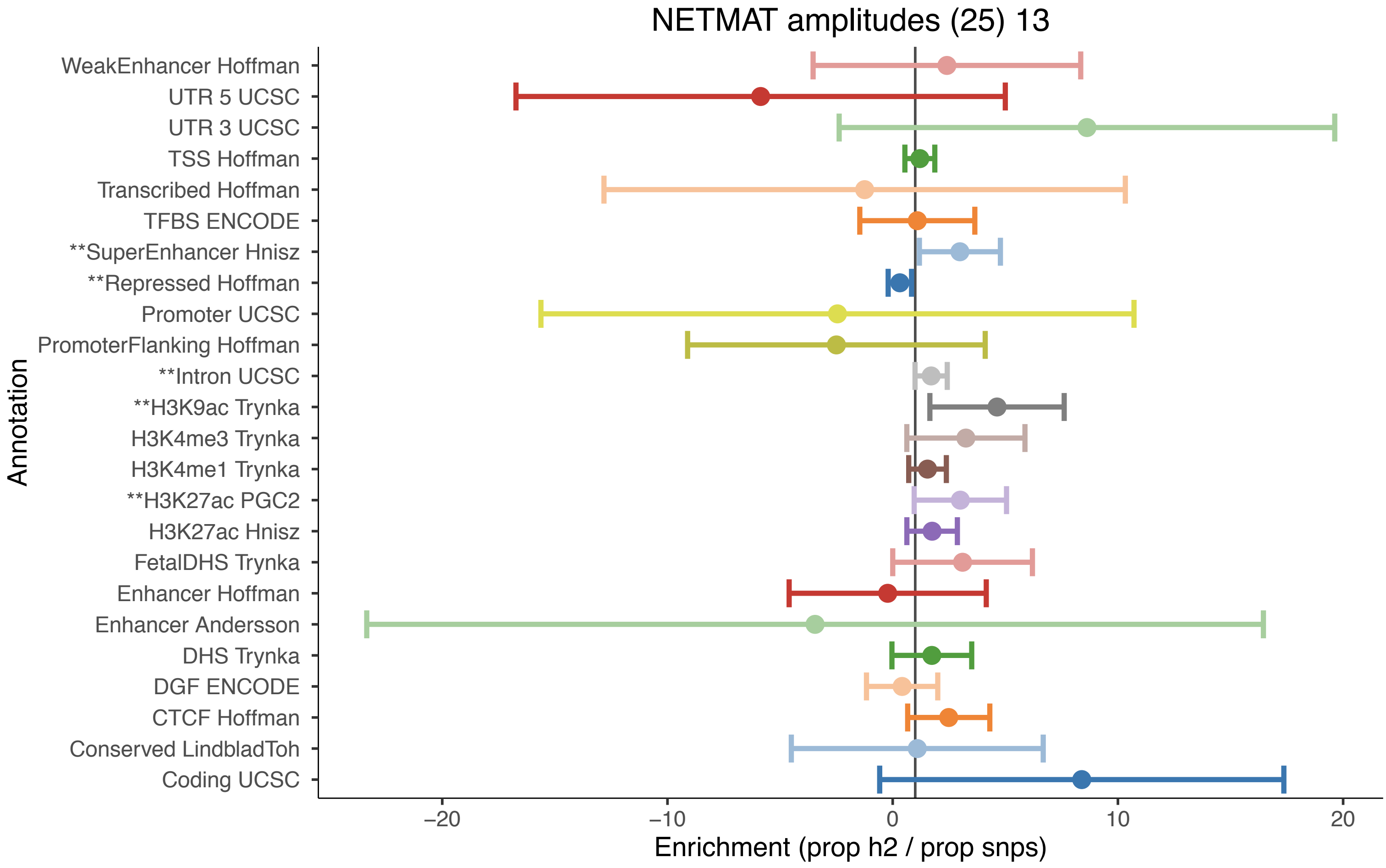
NETMAT amplitudes (25) 13
WeakEnhancer Hoffman
UTR 5 UCSC
UTR 3 UCSC
TSS Hoffman
Transcribed Hoffman
TFBS ENCODE
**SuperEnhancer Hnisz
**Repressed Hoffman
Promoter UCSC
PromoterFlanking Hoffman
**Intron UCSC
**H3K9ac Trynka
H3K4me3 Trynka
H3K4me1 Trynka
**H3K27ac PGC2
H3K27ac Hnisz
FetalDHS Trynka
Enhancer Hoffman
Enhancer Andersson
DHS Trynka
DGF ENCODE
CTCF Hoffman
Conserved LindbladToh
Coding UCSC
Annotation
−20
−10
0
10
20
Enrichment (prop h2 / prop snps)

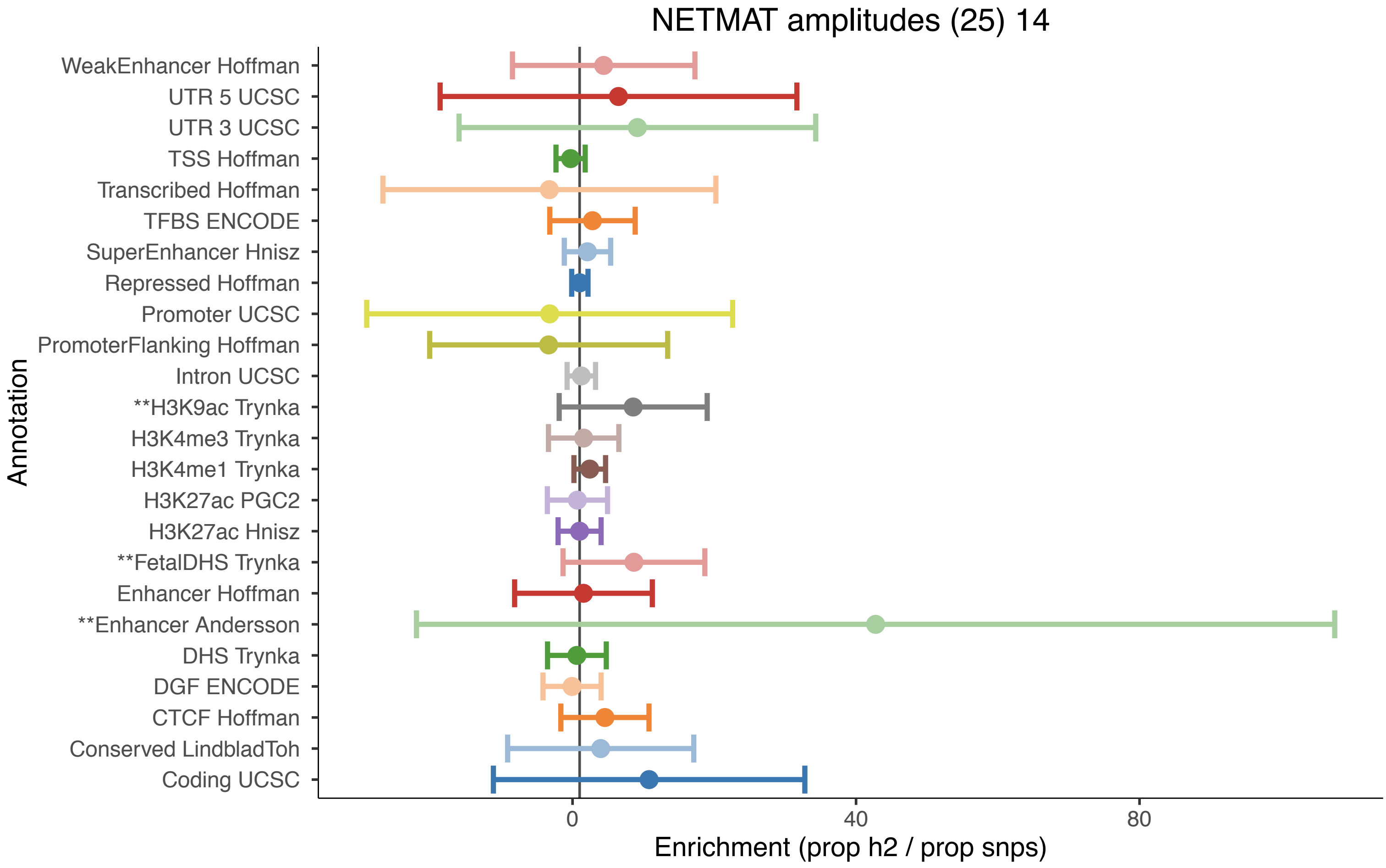

NETMAT amplitudes (25) 14
WeakEnhancer Hoffman
UTR 5 UCSC
UTR 3 UCSC
TSS Hoffman
Transcribed Hoffman
TFBS ENCODE
SuperEnhancer Hnisz
Repressed Hoffman
Promoter UCSC
PromoterFlanking Hoffman
Intron UCSC
**H3K9ac Trynka
H3K4me3 Trynka
H3K4me1 Trynka
H3K27ac PGC2
H3K27ac Hnisz
**FetalDHS Trynka
Enhancer Hoffman
**Enhancer Andersson
DHS Trynka
DGF ENCODE
CTCF Hoffman
Conserved LindbladToh
Coding UCSC
Annotation
0
40
80
Enrichment (prop h2 / prop snps)

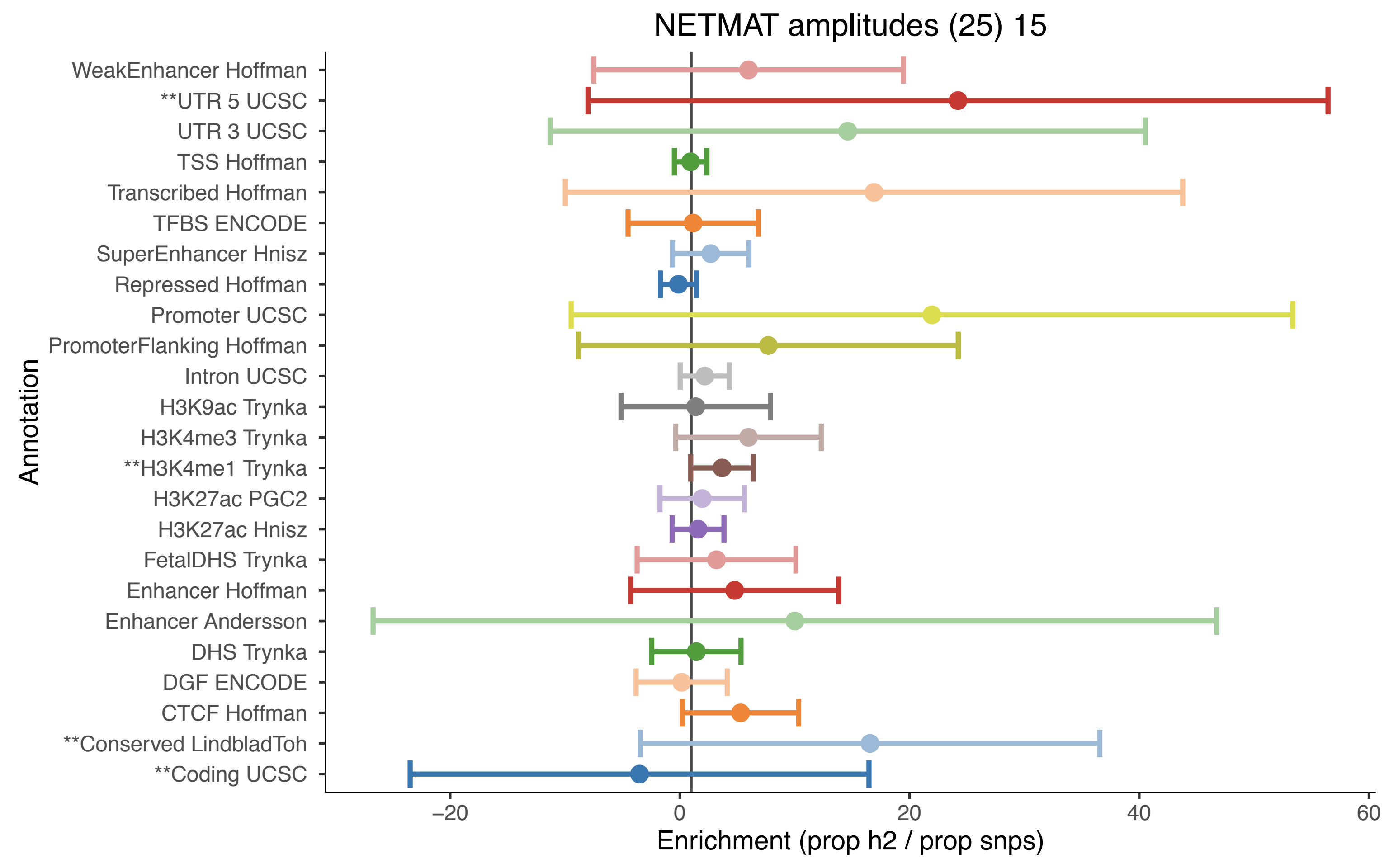

NETMAT amplitudes (25) 15
WeakEnhancer Hoffman
**UTR 5 UCSC
UTR 3 UCSC
TSS Hoffman
Transcribed Hoffman
TFBS ENCODE
SuperEnhancer Hnisz
Repressed Hoffman
Promoter UCSC
PromoterFlanking Hoffman
Intron UCSC
H3K9ac Trynka
H3K4me3 Trynka
**H3K4me1 Trynka
H3K27ac PGC2
H3K27ac Hnisz
FetalDHS Trynka
Enhancer Hoffman
Enhancer Andersson
DHS Trynka
DGF ENCODE
CTCF Hoffman
**Conserved LindbladToh
**Coding UCSC
Annotation
−20
0
20
40
60
Enrichment (prop h2 / prop snps)

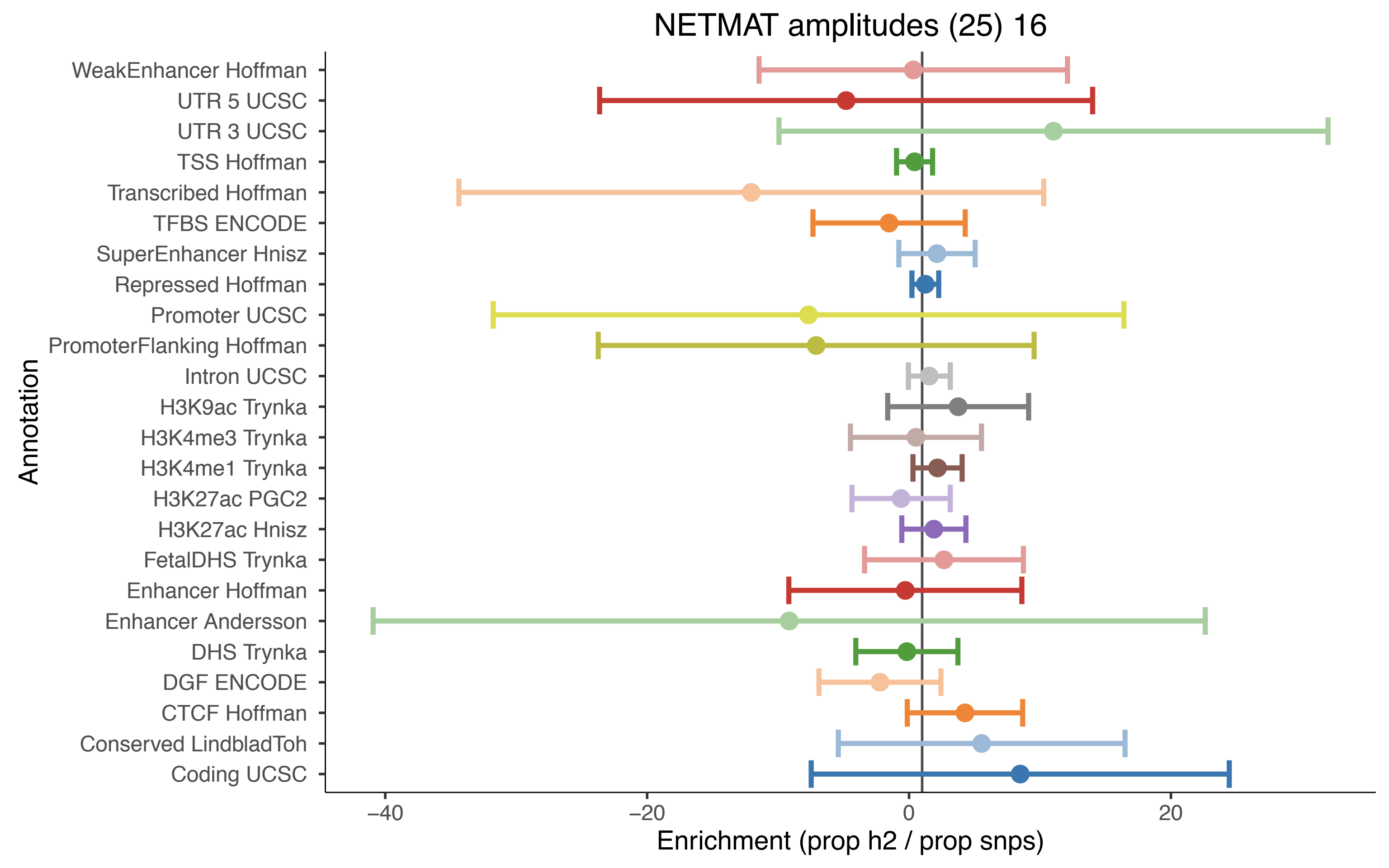
NETMAT amplitudes (25) 16
WeakEnhancer Hoffman
UTR 5 UCSC
UTR 3 UCSC
TSS Hoffman
Transcribed Hoffman
TFBS ENCODE
SuperEnhancer Hnisz
Repressed Hoffman
Promoter UCSC
PromoterFlanking Hoffman
Intron UCSC
H3K9ac Trynka
H3K4me3 Trynka
H3K4me1 Trynka
H3K27ac PGC2
H3K27ac Hnisz
FetalDHS Trynka
Enhancer Hoffman
Enhancer Andersson
DHS Trynka
DGF ENCODE
CTCF Hoffman
Conserved LindbladToh
Coding UCSC
Annotation
−40
−20
0
20
Enrichment (prop h2 / prop snps)

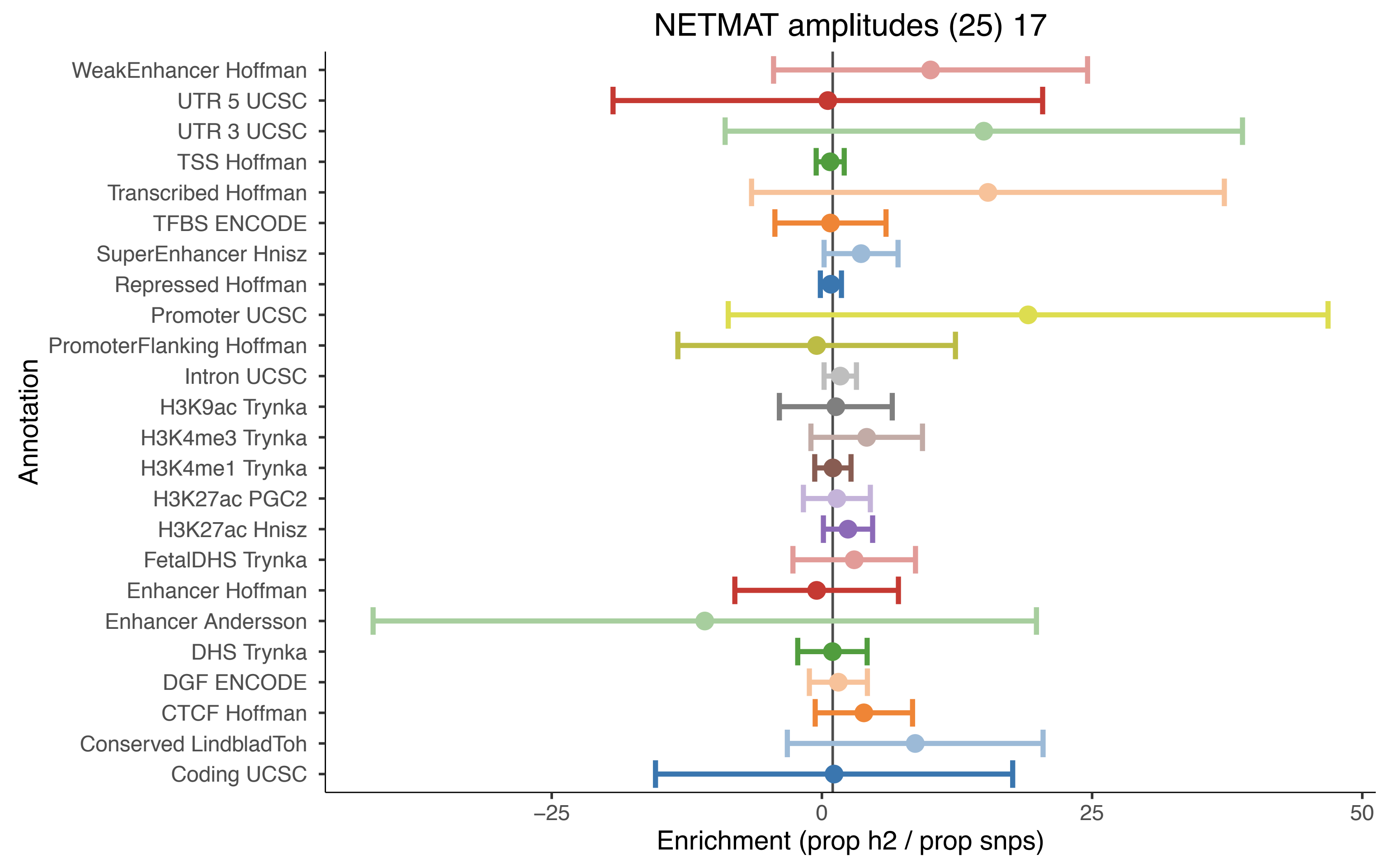
NETMAT amplitudes (25) 17
WeakEnhancer Hoffman
UTR 5 UCSC
UTR 3 UCSC
TSS Hoffman
Transcribed Hoffman
TFBS ENCODE
SuperEnhancer Hnisz
Repressed Hoffman
Promoter UCSC
PromoterFlanking Hoffman
Intron UCSC
H3K9ac Trynka
H3K4me3 Trynka
H3K4me1 Trynka
H3K27ac PGC2
H3K27ac Hnisz
FetalDHS Trynka
Enhancer Hoffman
Enhancer Andersson
DHS Trynka
DGF ENCODE
CTCF Hoffman
Conserved LindbladToh
Coding UCSC
Annotation
−25
0
25
50
Enrichment (prop h2 / prop snps)

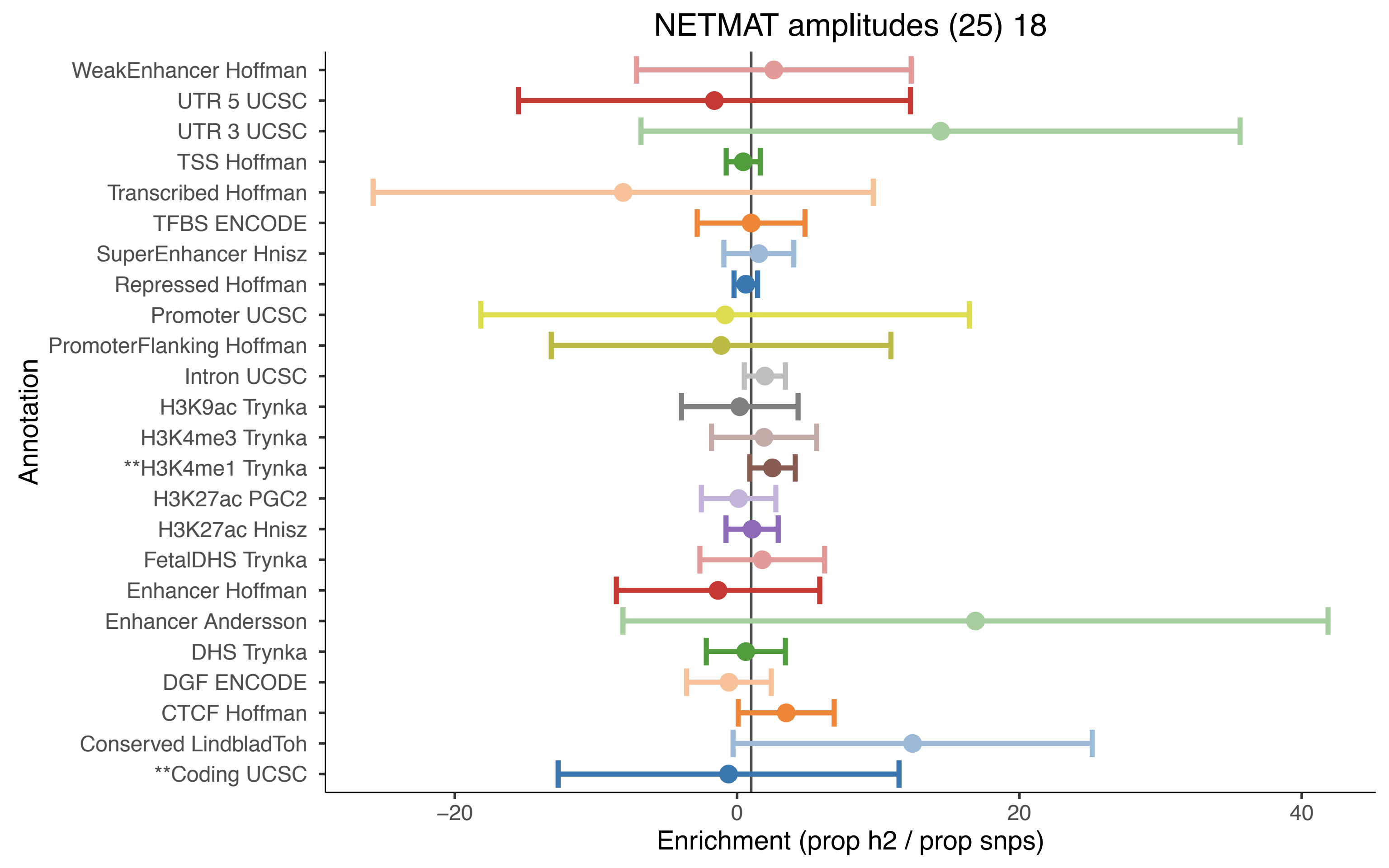

NETMAT amplitudes (25) 18
WeakEnhancer Hoffman
UTR 5 UCSC
UTR 3 UCSC
TSS Hoffman
Transcribed Hoffman
TFBS ENCODE
SuperEnhancer Hnisz
Repressed Hoffman
Promoter UCSC
PromoterFlanking Hoffman
Intron UCSC
H3K9ac Trynka
H3K4me3 Trynka
**H3K4me1 Trynka
H3K27ac PGC2
H3K27ac Hnisz
FetalDHS Trynka
Enhancer Hoffman
Enhancer Andersson
DHS Trynka
DGF ENCODE
CTCF Hoffman
Conserved LindbladToh
**Coding UCSC
Annotation
−20
0
20
40
Enrichment (prop h2 / prop snps)

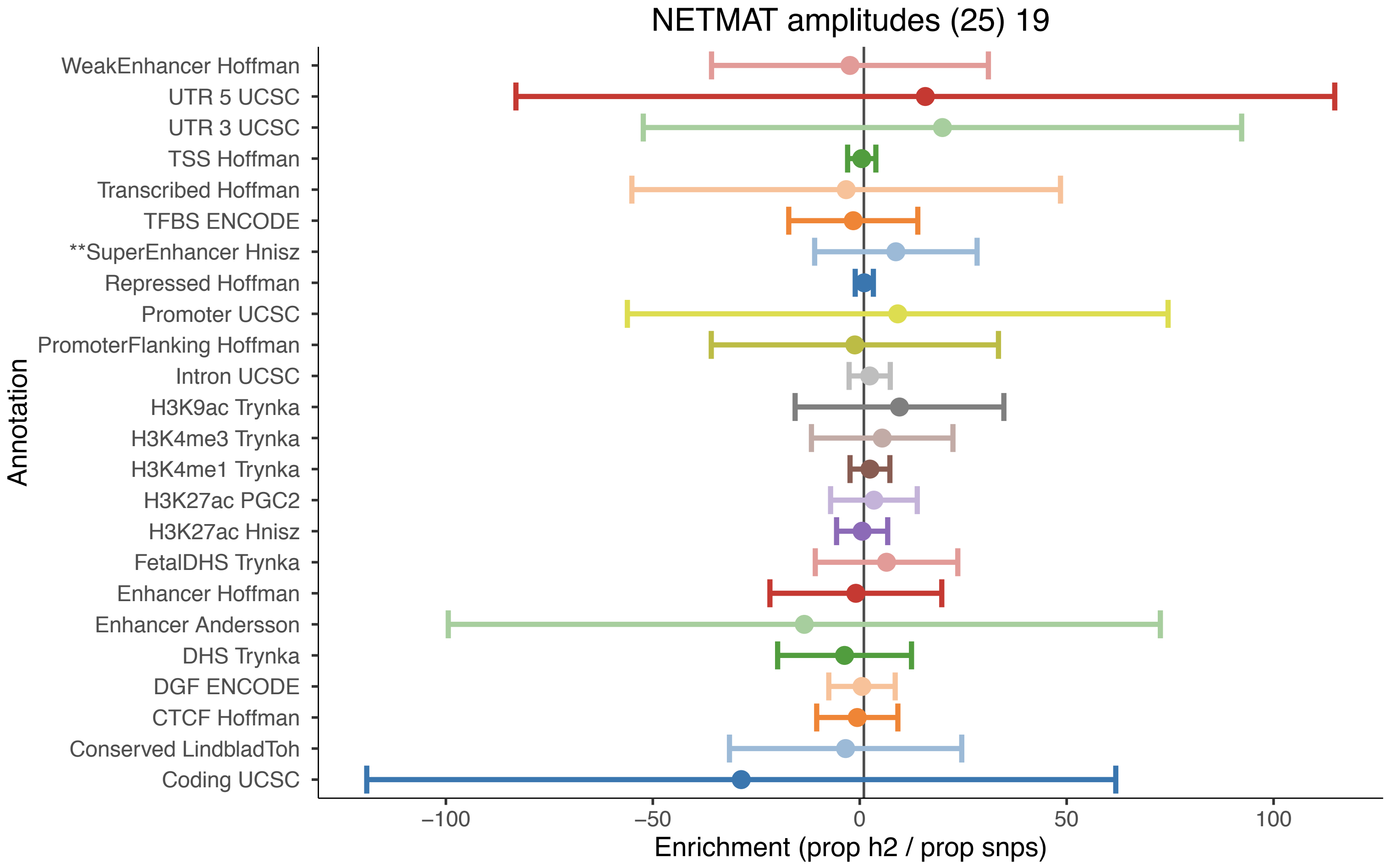
NETMAT amplitudes (25) 19
WeakEnhancer Hoffman
UTR 5 UCSC
UTR 3 UCSC
TSS Hoffman
Transcribed Hoffman
TFBS ENCODE
**SuperEnhancer Hnisz
Repressed Hoffman
Promoter UCSC
PromoterFlanking Hoffman
Intron UCSC
H3K9ac Trynka
H3K4me3 Trynka
H3K4me1 Trynka
H3K27ac PGC2
H3K27ac Hnisz
FetalDHS Trynka
Enhancer Hoffman
Enhancer Andersson
DHS Trynka
DGF ENCODE
CTCF Hoffman
Conserved LindbladToh
Coding UCSC
Annotation
−100
−50
0
50
100
Enrichment (prop h2 / prop snps)

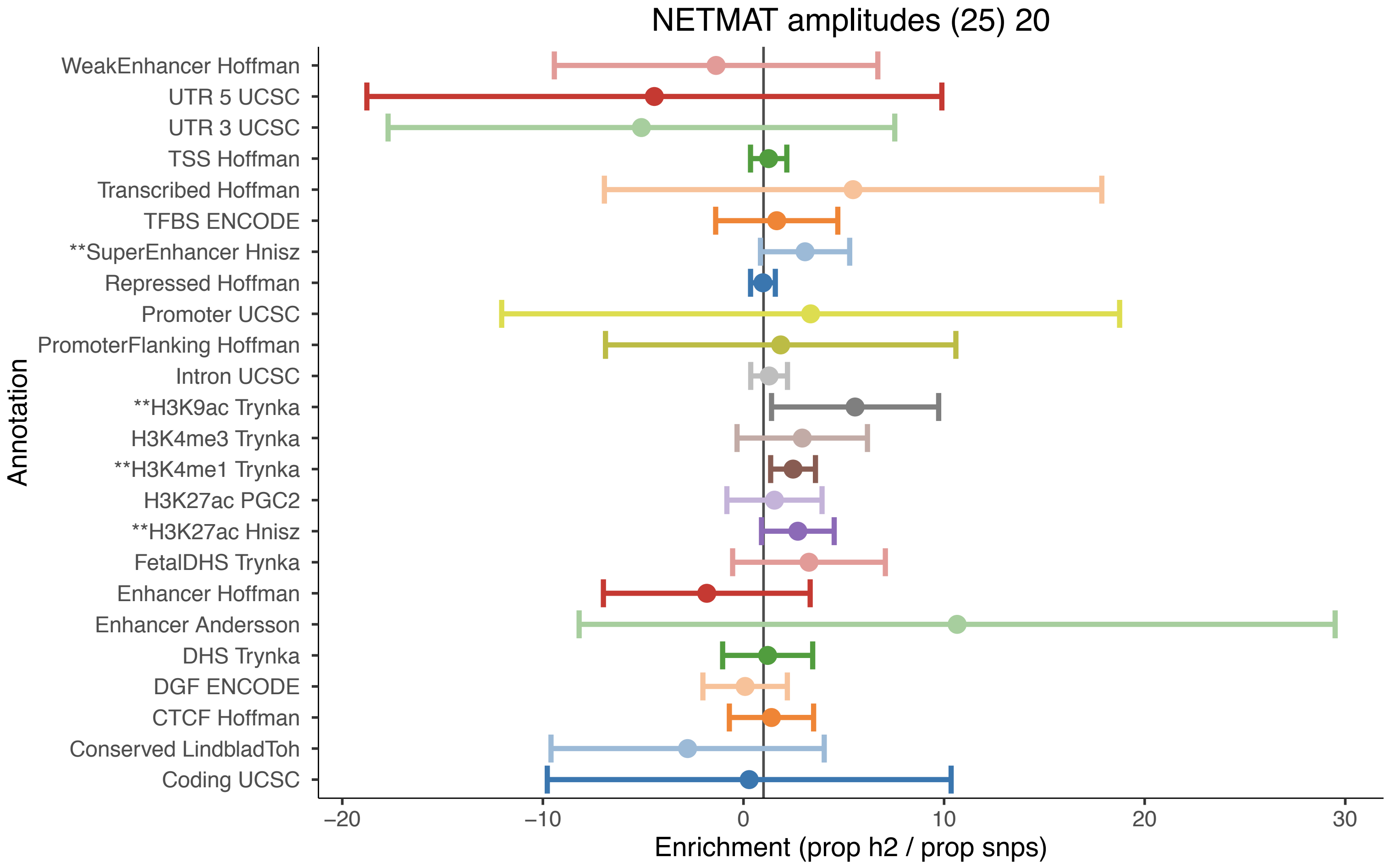
NETMAT amplitudes (25) 20
WeakEnhancer Hoffman
UTR 5 UCSC
UTR 3 UCSC
TSS Hoffman
Transcribed Hoffman
TFBS ENCODE
**SuperEnhancer Hnisz
Repressed Hoffman
Promoter UCSC
PromoterFlanking Hoffman
Intron UCSC
**H3K9ac Trynka
H3K4me3 Trynka
**H3K4me1 Trynka
H3K27ac PGC2
**H3K27ac Hnisz
FetalDHS Trynka
Enhancer Hoffman
Enhancer Andersson
DHS Trynka
DGF ENCODE
CTCF Hoffman
Conserved LindbladToh
Coding UCSC
Annotation
−20
−10
0
10
20
30
Enrichment (prop h2 / prop snps)

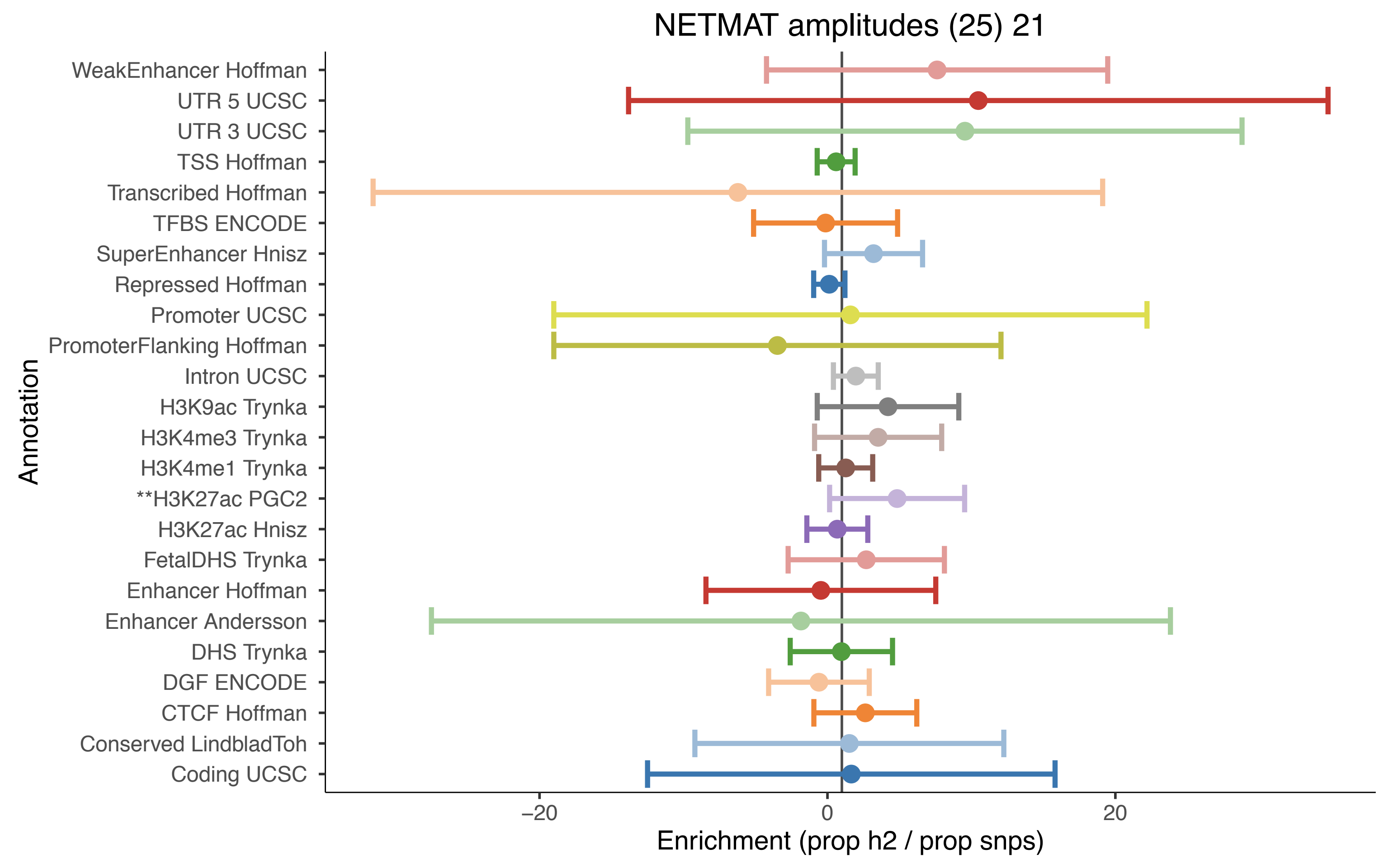
NETMAT amplitudes (25) 21
WeakEnhancer Hoffman
UTR 5 UCSC
UTR 3 UCSC
TSS Hoffman
Transcribed Hoffman
TFBS ENCODE
SuperEnhancer Hnisz
Repressed Hoffman
Promoter UCSC
PromoterFlanking Hoffman
Intron UCSC
H3K9ac Trynka
H3K4me3 Trynka
H3K4me1 Trynka
**H3K27ac PGC2
H3K27ac Hnisz
FetalDHS Trynka
Enhancer Hoffman
Enhancer Andersson
DHS Trynka
DGF ENCODE
CTCF Hoffman
Conserved LindbladToh
Coding UCSC
Annotation
−20
0
20
Enrichment (prop h2 / prop snps)

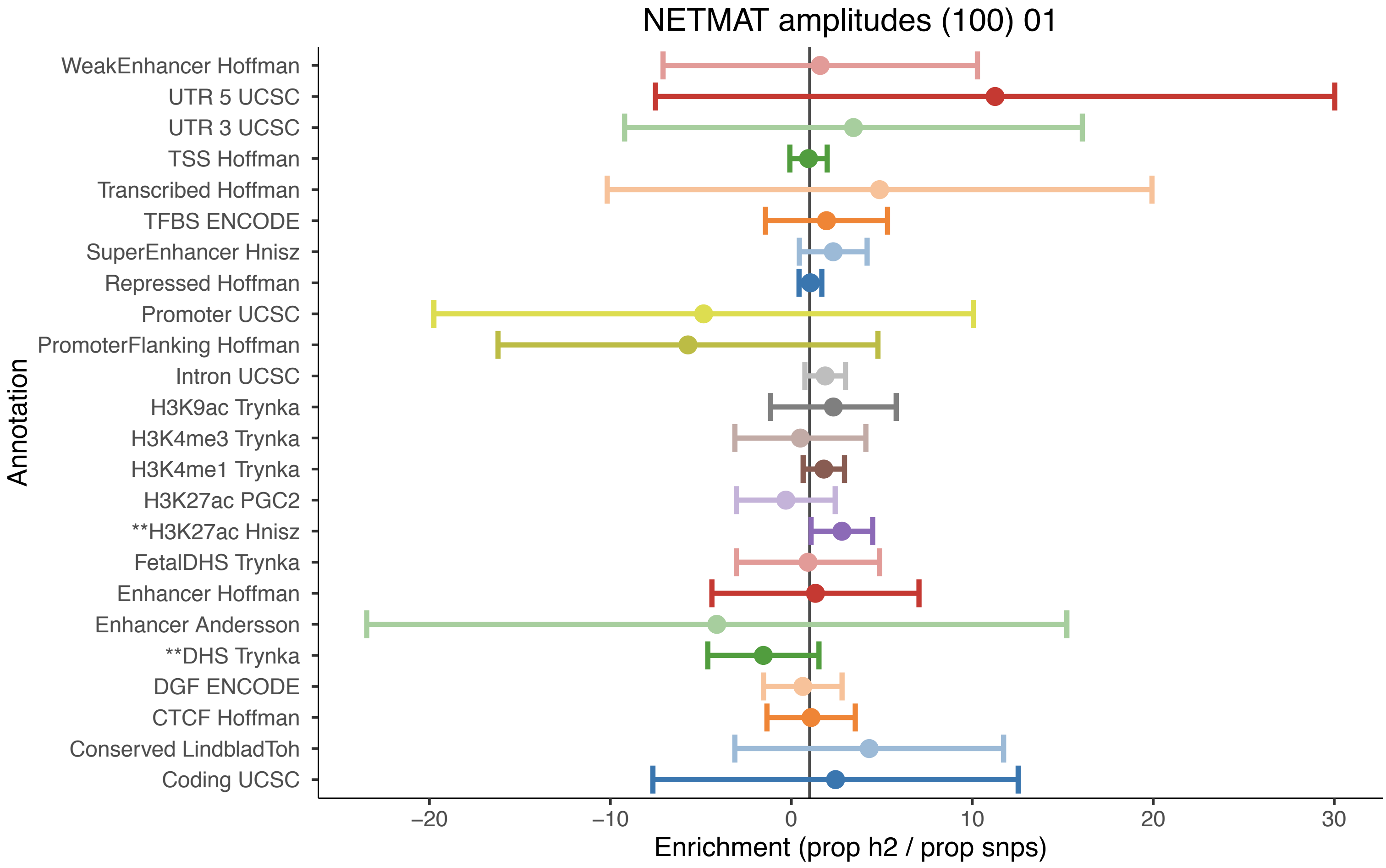
NETMAT amplitudes (100) 01
Annotation
WeakEnhancer Hoffman
UTR 5 UCSC
UTR 3 UCSC
TSS Hoffman
Transcribed Hoffman
TFBS ENCODE
SuperEnhancer Hnisz
Repressed Hoffman
Promoter UCSC
PromoterFlanking Hoffman
Intron UCSC
H3K9ac Trynka
H3K4me3 Trynka
H3K4me1 Trynka
H3K27ac PGC2
**H3K27ac Hnisz
FetalDHS Trynka
Enhancer Hoffman
Enhancer Andersson
**DHS Trynka
DGF ENCODE
CTCF Hoffman
Conserved LindbladToh
Coding UCSC
−20
−10
0
10
20
30
Enrichment (prop h2 / prop snps)

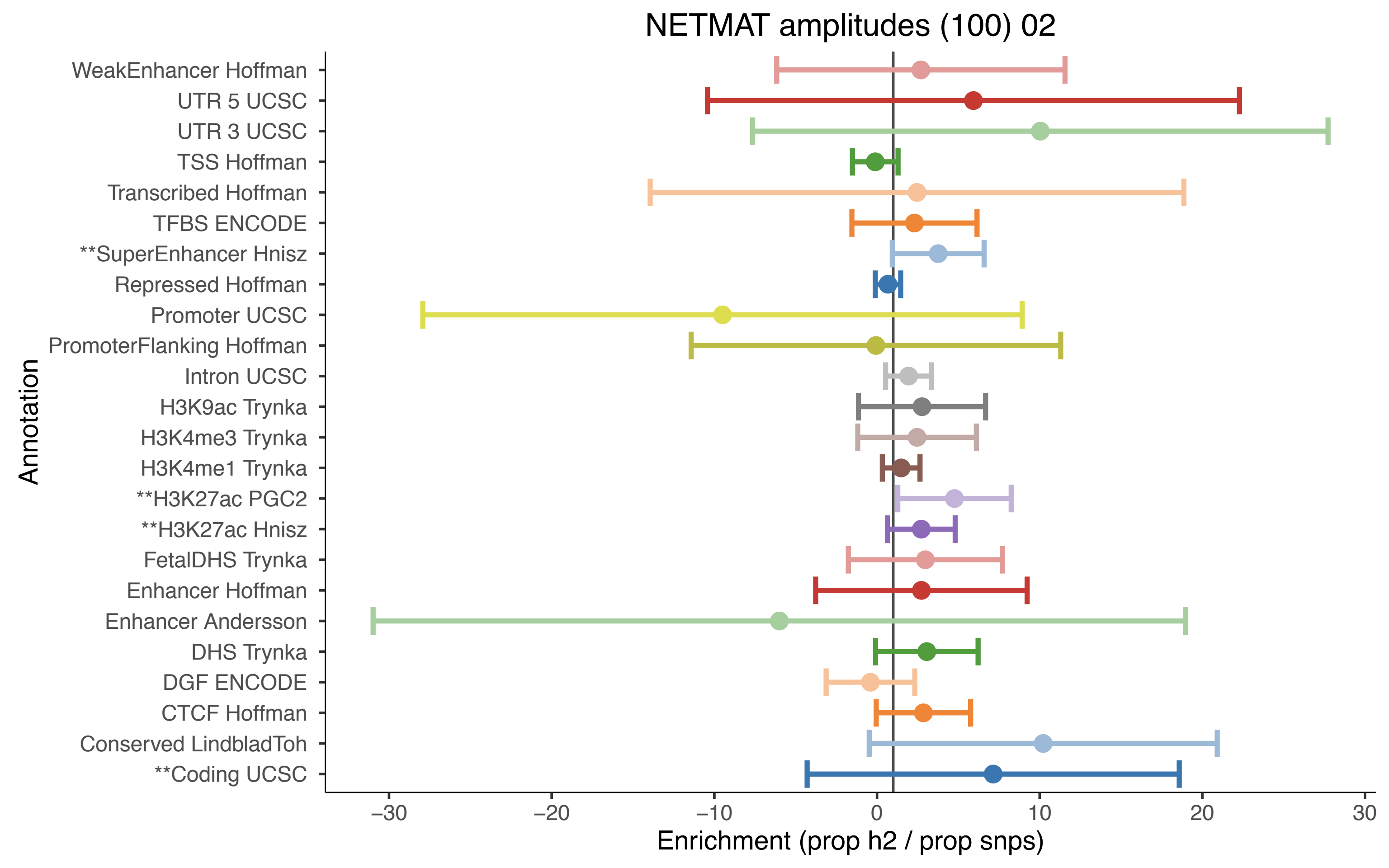
NETMAT amplitudes (100) 02
WeakEnhancer Hoffman
UTR 5 UCSC
UTR 3 UCSC
TSS Hoffman
Transcribed Hoffman
TFBS ENCODE
**SuperEnhancer Hnisz
Repressed Hoffman
Promoter UCSC
PromoterFlanking Hoffman
Intron UCSC
H3K9ac Trynka
H3K4me3 Trynka
H3K4me1 Trynka
**H3K27ac PGC2
**H3K27ac Hnisz
FetalDHS Trynka
Enhancer Hoffman
Enhancer Andersson
DHS Trynka
DGF ENCODE
CTCF Hoffman
Conserved LindbladToh
**Coding UCSC
Annotation
−30
−20
−10
0
10
20
30
Enrichment (prop h2 / prop snps)

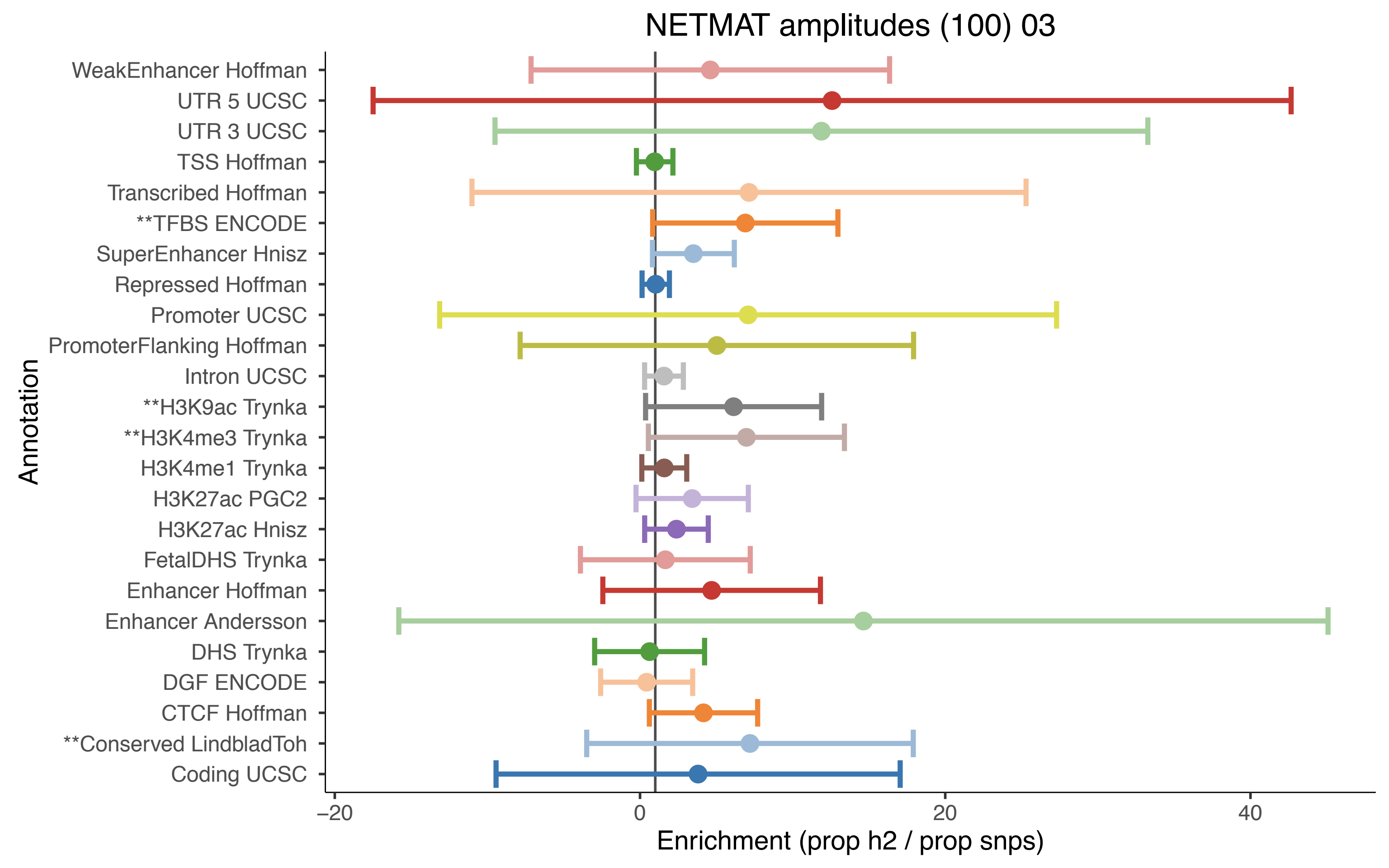

NETMAT amplitudes (100) 03
Annotation
WeakEnhancer Hoffman
UTR 5 UCSC
UTR 3 UCSC
TSS Hoffman
Transcribed Hoffman
**TFBS ENCODE
SuperEnhancer Hnisz
Repressed Hoffman
Promoter UCSC
PromoterFlanking Hoffman
Intron UCSC
**H3K9ac Trynka
**H3K4me3 Trynka
H3K4me1 Trynka
H3K27ac PGC2
H3K27ac Hnisz
FetalDHS Trynka
Enhancer Hoffman
Enhancer Andersson
DHS Trynka
DGF ENCODE
CTCF Hoffman
**Conserved LindbladToh
Coding UCSC
–20
0
20
40
Enrichment (prop h2 / prop snps)

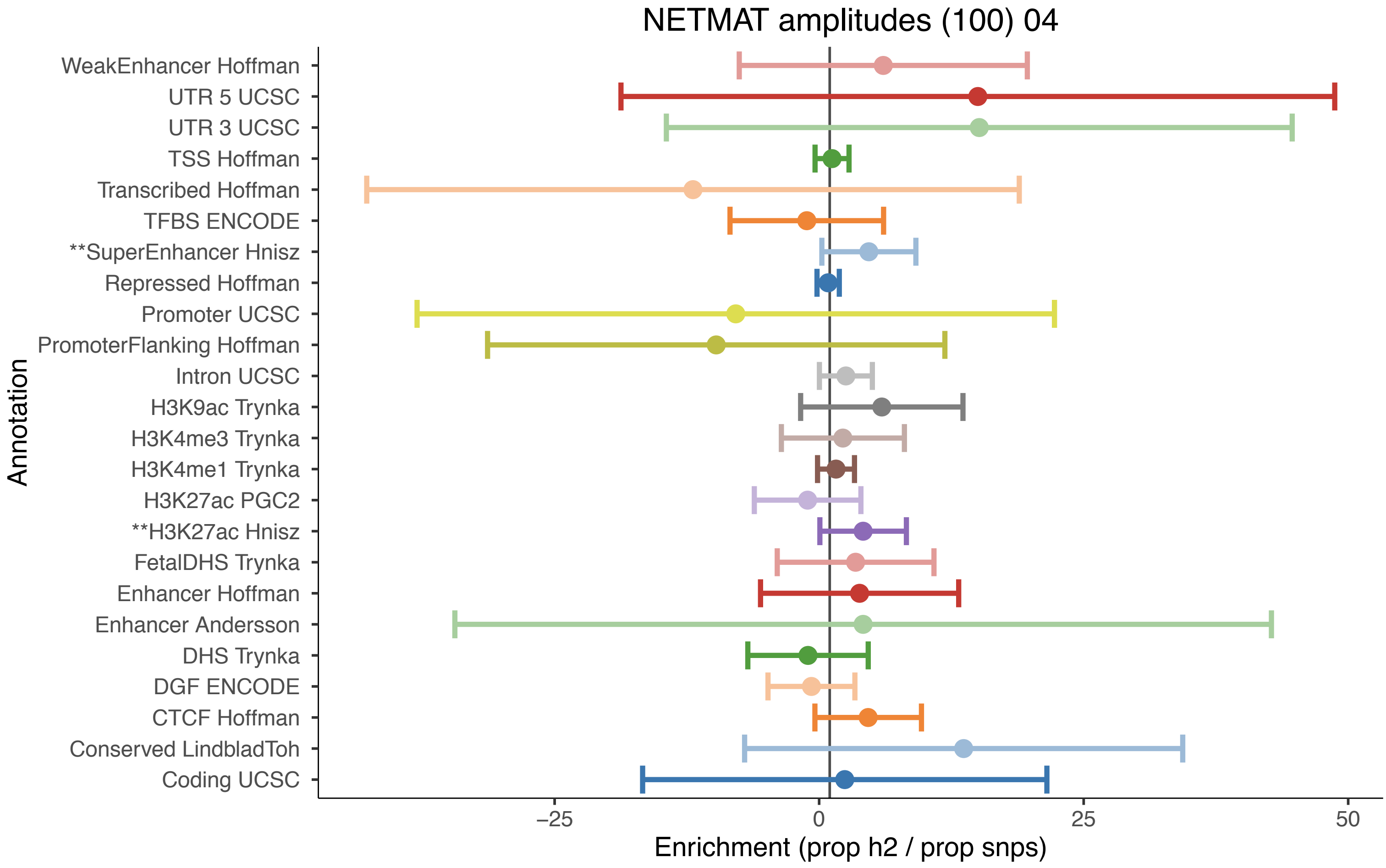

NETMAT amplitudes (100) 04
WeakEnhancer Hoffman
UTR 5 UCSC
UTR 3 UCSC
TSS Hoffman
Transcribed Hoffman
TFBS ENCODE
**SuperEnhancer Hnisz
Repressed Hoffman
Promoter UCSC
PromoterFlanking Hoffman
Intron UCSC
H3K9ac Trynka
H3K4me3 Trynka
H3K4me1 Trynka
H3K27ac PGC2
**H3K27ac Hnisz
FetalDHS Trynka
Enhancer Hoffman
Enhancer Andersson
DHS Trynka
DGF ENCODE
CTCF Hoffman
Conserved LindbladToh
Coding UCSC
Annotation
−25
0
25
50
Enrichment (prop h2 / prop snps)

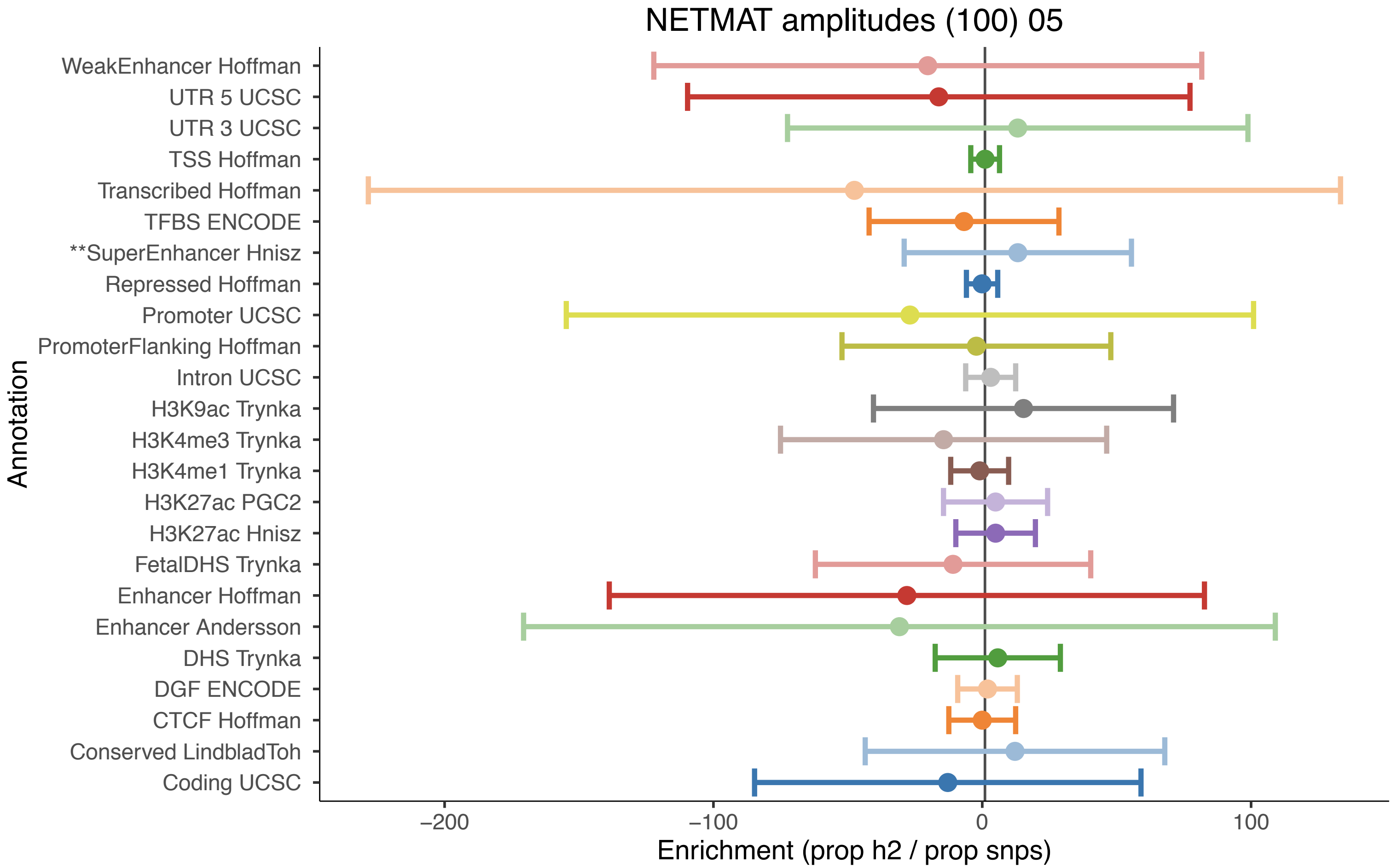

NETMAT amplitudes (100) 05
WeakEnhancer Hoffman
UTR 5 UCSC
UTR 3 UCSC
TSS Hoffman
Transcribed Hoffman
TFBS ENCODE
**SuperEnhancer Hnisz
Repressed Hoffman
Promoter UCSC
PromoterFlanking Hoffman
Intron UCSC
H3K9ac Trynka
H3K4me3 Trynka
H3K4me1 Trynka
H3K27ac PGC2
H3K27ac Hnisz
FetalDHS Trynka
Enhancer Hoffman
Enhancer Andersson
DHS Trynka
DGF ENCODE
CTCF Hoffman
Conserved LindbladToh
Coding UCSC
Annotation
−200
−100
0
100
Enrichment (prop h2 / prop snps)

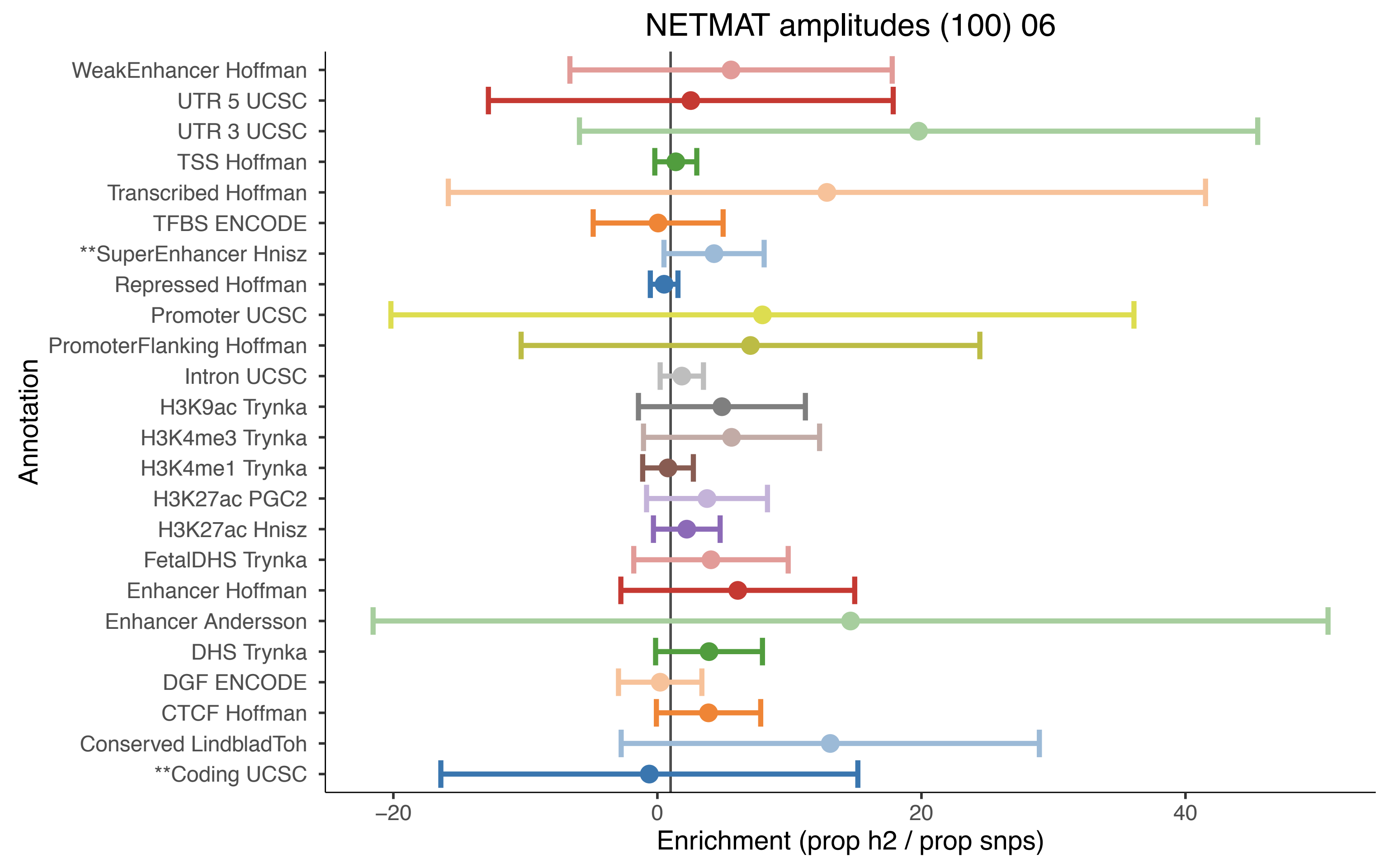

NETMAT amplitudes (100) 06
WeakEnhancer Hoffman
UTR 5 UCSC
UTR 3 UCSC
TSS Hoffman
Transcribed Hoffman
TFBS ENCODE
**SuperEnhancer Hnisz
Repressed Hoffman
Promoter UCSC
PromoterFlanking Hoffman
Intron UCSC
H3K9ac Trynka
H3K4me3 Trynka
H3K4me1 Trynka
H3K27ac PGC2
H3K27ac Hnisz
FetalDHS Trynka
Enhancer Hoffman
Enhancer Andersson
DHS Trynka
DGF ENCODE
CTCF Hoffman
Conserved LindbladToh
**Coding UCSC
Annotation
−20
0
20
40
Enrichment (prop h2 / prop snps)

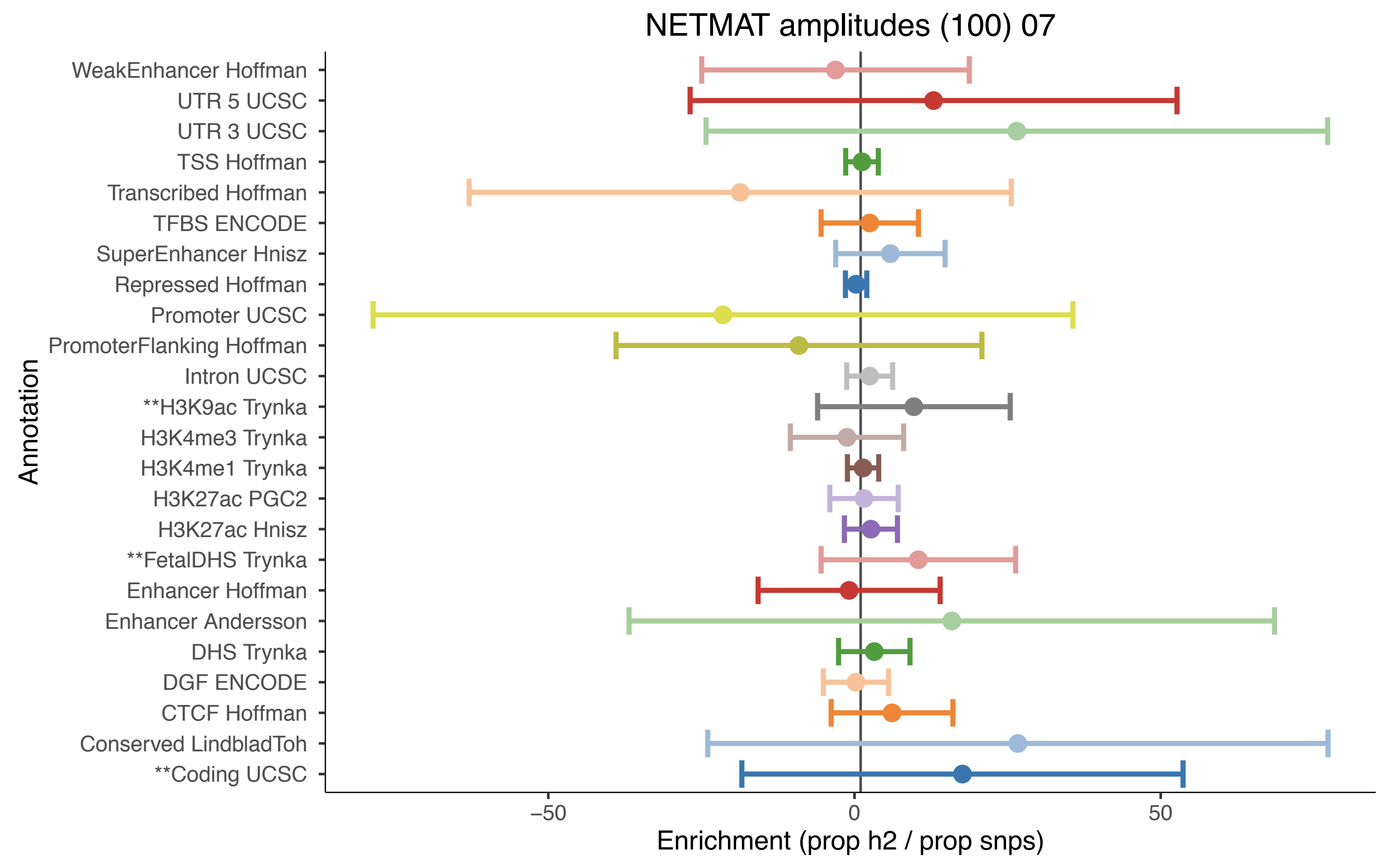

NETMAT amplitudes (100) 07
WeakEnhancer Hoffman
UTR 5 UCSC
UTR 3 UCSC
TSS Hoffman
Transcribed Hoffman
TFBS ENCODE
SuperEnhancer Hnisz
Repressed Hoffman
Promoter UCSC
PromoterFlanking Hoffman
Intron UCSC
**H3K9ac Trynka
H3K4me3 Trynka
H3K4me1 Trynka
H3K27ac PGC2
H3K27ac Hnisz
**FetalDHS Trynka
Enhancer Hoffman
Enhancer Andersson
DHS Trynka
DGF ENCODE
CTCF Hoffman
Conserved LindbladToh
**Coding UCSC
Annotation
−50
0
50
Enrichment (prop h2 / prop snps)

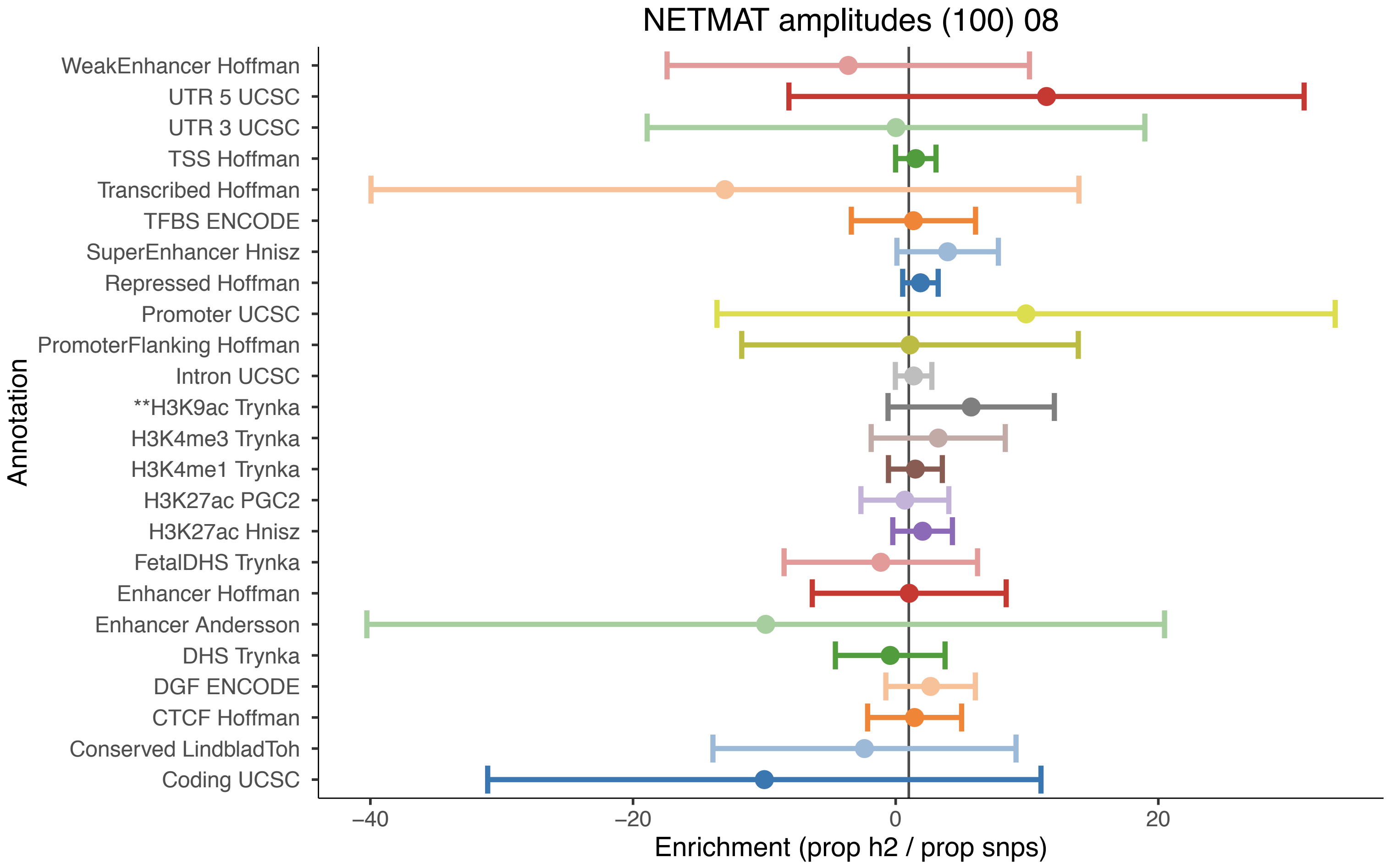
NETMAT amplitudes (100) 08
WeakEnhancer Hoffman
UTR 5 UCSC
UTR 3 UCSC
TSS Hoffman
Transcribed Hoffman
TFBS ENCODE
SuperEnhancer Hnisz
Repressed Hoffman
Promoter UCSC
PromoterFlanking Hoffman
Intron UCSC
**H3K9ac Trynka
H3K4me3 Trynka
H3K4me1 Trynka
H3K27ac PGC2
H3K27ac Hnisz
FetalDHS Trynka
Enhancer Hoffman
Enhancer Andersson
DHS Trynka
DGF ENCODE
CTCF Hoffman
Conserved LindbladToh
Coding UCSC
Annotation
−40
−20
0
20
Enrichment (prop h2 / prop snps)

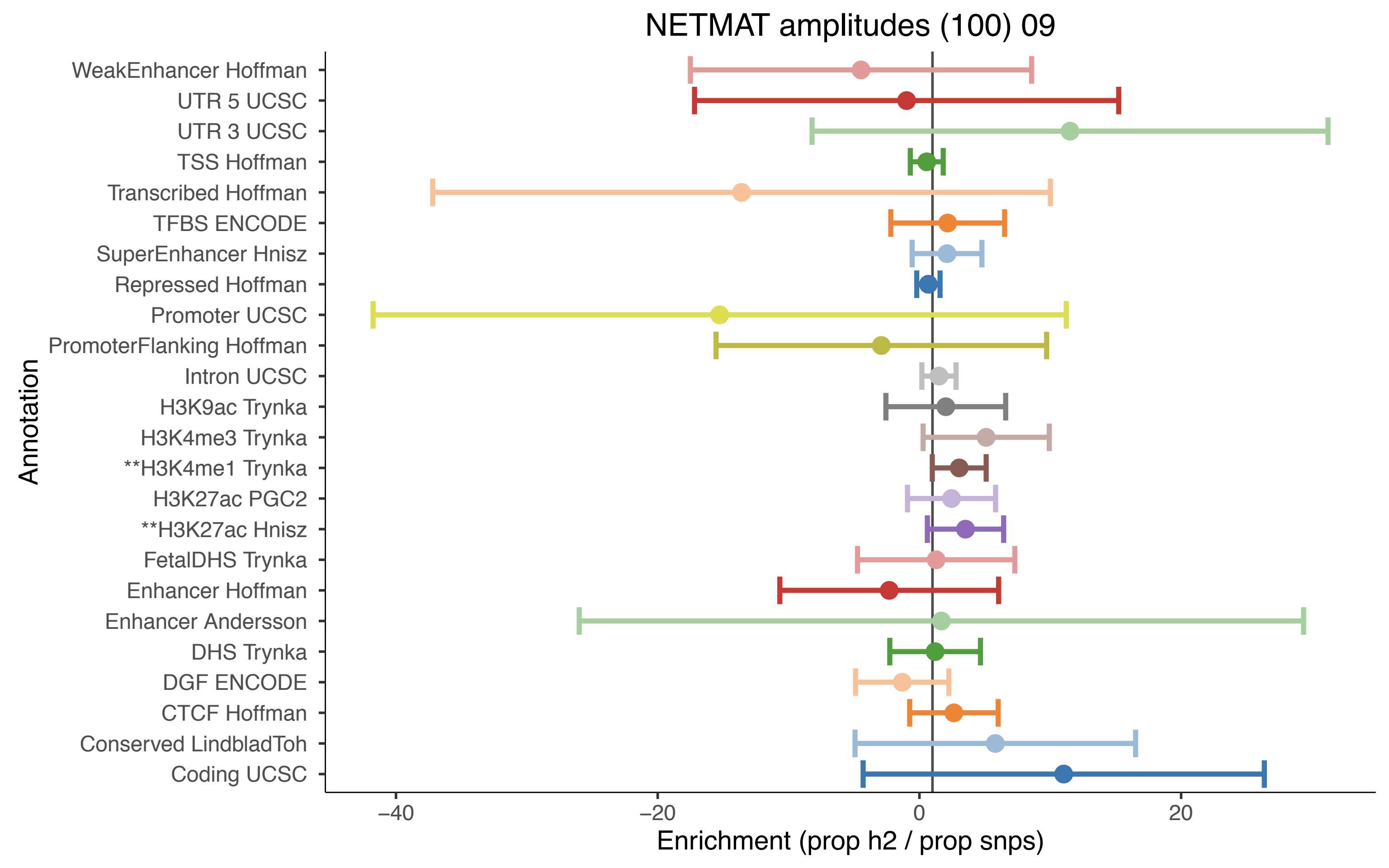
NETMAT amplitudes (100) 09
WeakEnhancer Hoffman
UTR 5 UCSC
UTR 3 UCSC
TSS Hoffman
Transcribed Hoffman
TFBS ENCODE
SuperEnhancer Hnisz
Repressed Hoffman
Promoter UCSC
PromoterFlanking Hoffman
Intron UCSC
H3K9ac Trynka
H3K4me3 Trynka
**H3K4me1 Trynka
H3K27ac PGC2
**H3K27ac Hnisz
FetalDHS Trynka
Enhancer Hoffman
Enhancer Andersson
DHS Trynka
DGF ENCODE
CTCF Hoffman
Conserved LindbladToh
Coding UCSC
Annotation
−40
−20
0
20
Enrichment (prop h2 / prop snps)

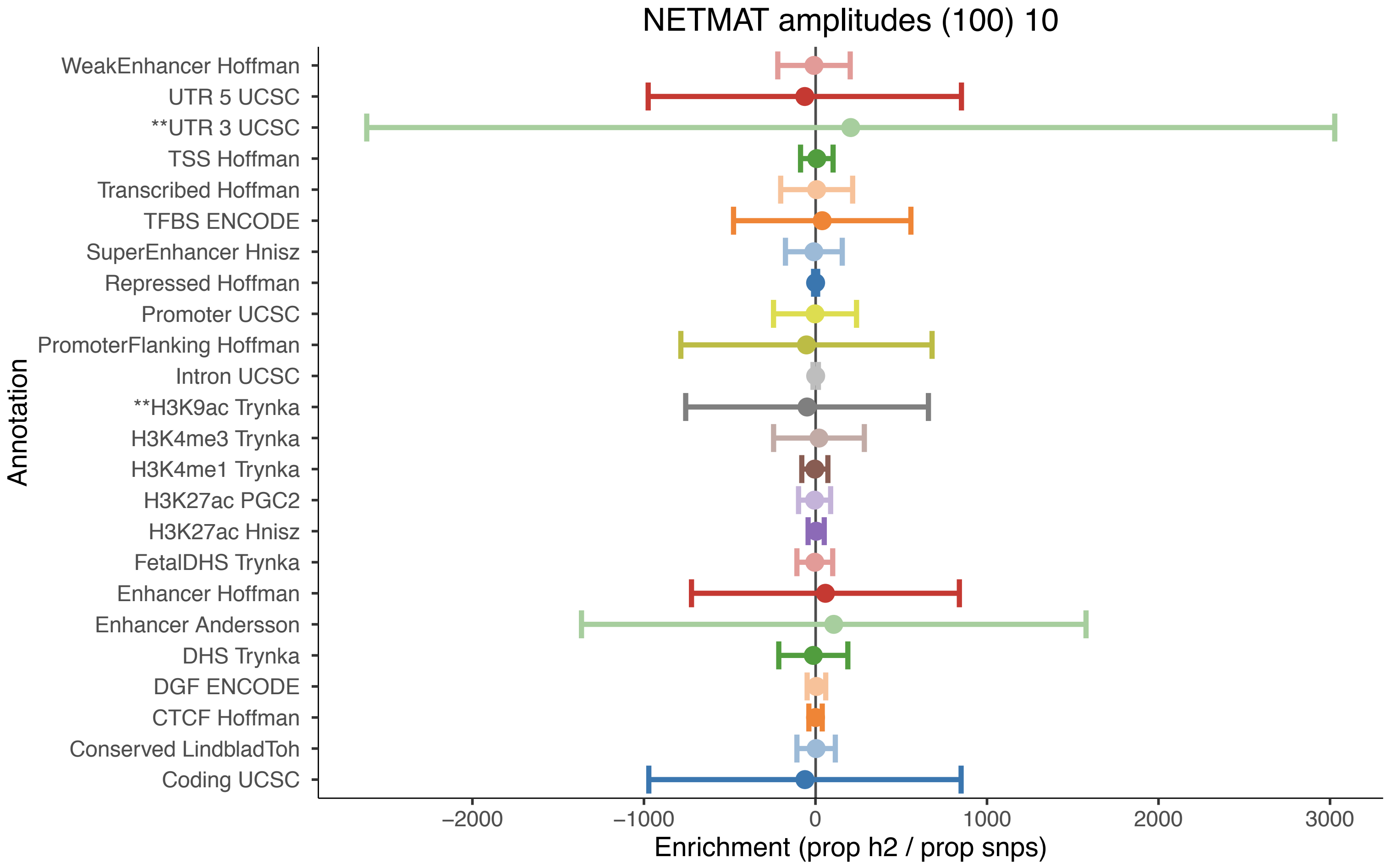
NETMAT amplitudes (100) 10
WeakEnhancer Hoffman
UTR 5 UCSC
**UTR 3 UCSC
TSS Hoffman
Transcribed Hoffman
TFBS ENCODE
SuperEnhancer Hnisz
Repressed Hoffman
Promoter UCSC
PromoterFlanking Hoffman
Intron UCSC
**H3K9ac Trynka
H3K4me3 Trynka
H3K4me1 Trynka
H3K27ac PGC2
H3K27ac Hnisz
FetalDHS Trynka
Enhancer Hoffman
Enhancer Andersson
DHS Trynka
DGF ENCODE
CTCF Hoffman
Conserved LindbladToh
Coding UCSC
Annotation
−2000
−1000
0
1000
2000
3000
Enrichment (prop h2 / prop snps)

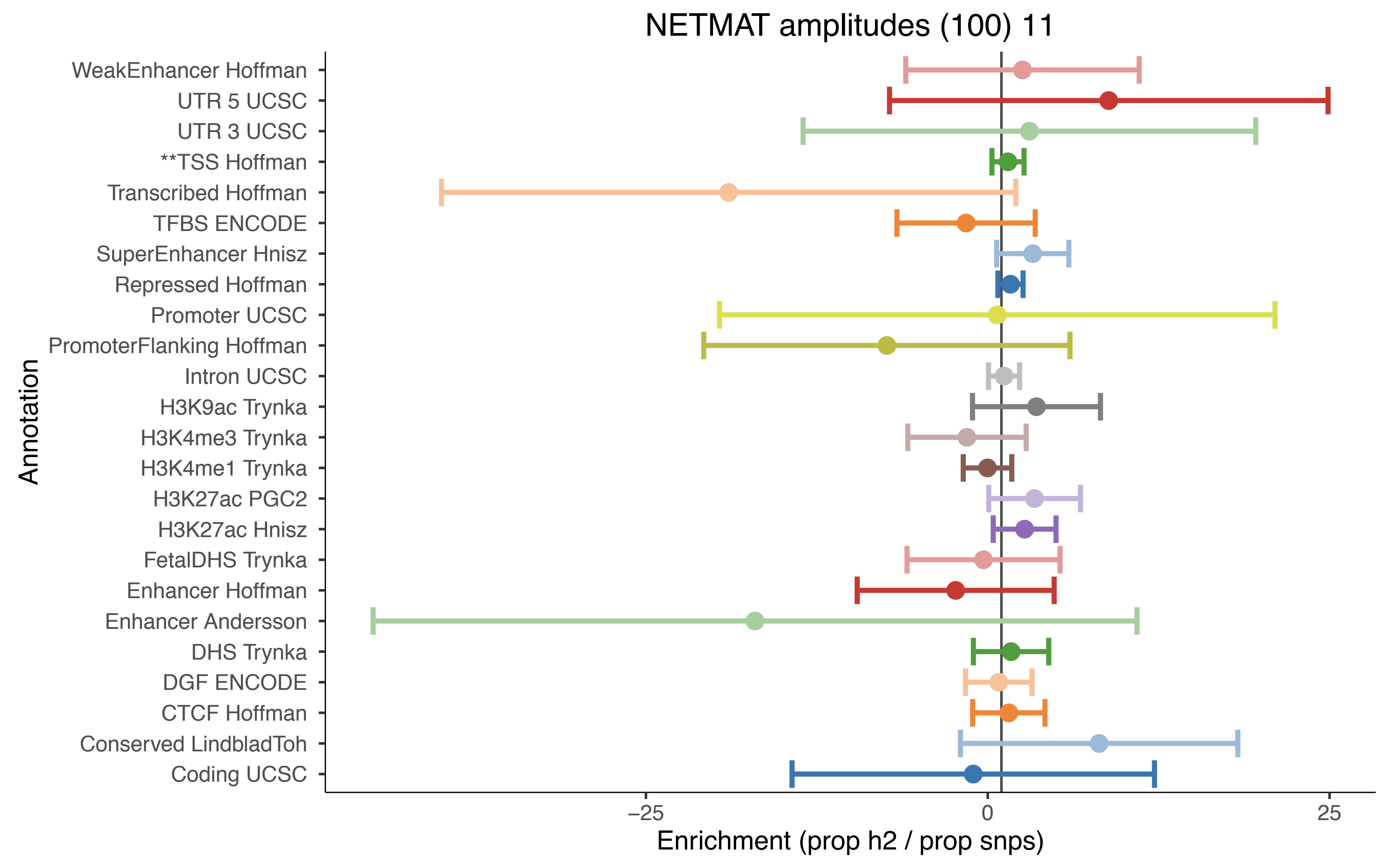

NETMAT amplitudes (100) 11
WeakEnhancer Hoffman
UTR 5 UCSC
UTR 3 UCSC
**TSS Hoffman
Transcribed Hoffman
TFBS ENCODE
SuperEnhancer Hnisz
Repressed Hoffman
Promoter UCSC
PromoterFlanking Hoffman
Intron UCSC
H3K9ac Trynka
H3K4me3 Trynka
H3K4me1 Trynka
H3K27ac PGC2
H3K27ac Hnisz
FetalDHS Trynka
Enhancer Hoffman
Enhancer Andersson
DHS Trynka
DGF ENCODE
CTCF Hoffman
Conserved LindbladToh
Coding UCSC
Annotation
−25
0
25
Enrichment (prop h2 / prop snps)

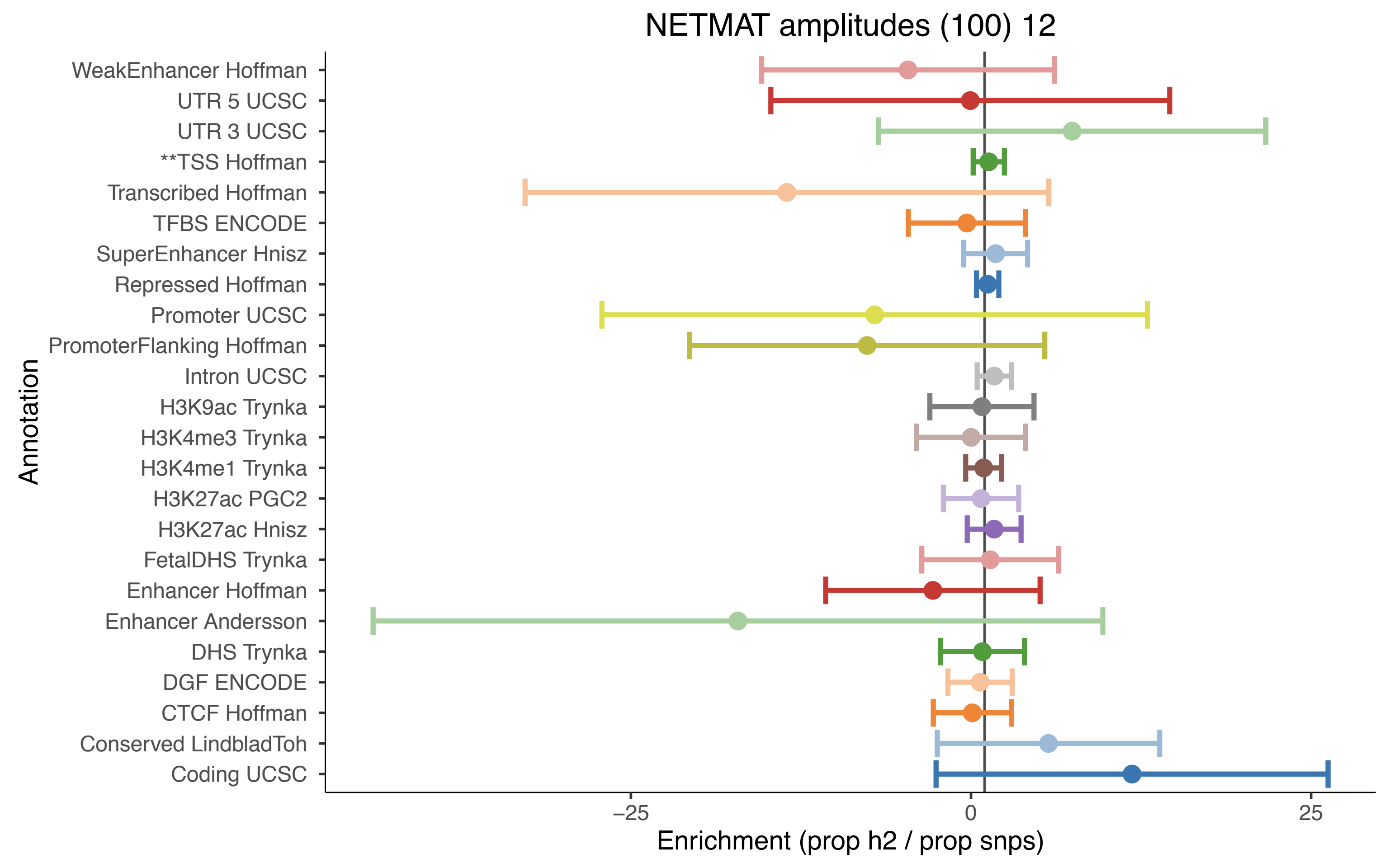

NETMAT amplitudes (100) 12
Annotation
WeakEnhancer Hoffman
UTR 5 UCSC
UTR 3 UCSC
**TSS Hoffman
Transcribed Hoffman
TFBS ENCODE
SuperEnhancer Hnisz
Repressed Hoffman
Promoter UCSC
PromoterFlanking Hoffman
Intron UCSC
H3K9ac Trynka
H3K4me3 Trynka
H3K4me1 Trynka
H3K27ac PGC2
H3K27ac Hnisz
FetalDHS Trynka
Enhancer Hoffman
Enhancer Andersson
DHS Trynka
DGF ENCODE
CTCF Hoffman
Conserved LindbladToh
Coding UCSC
−25
0
25
Enrichment (prop h2 / prop snps)

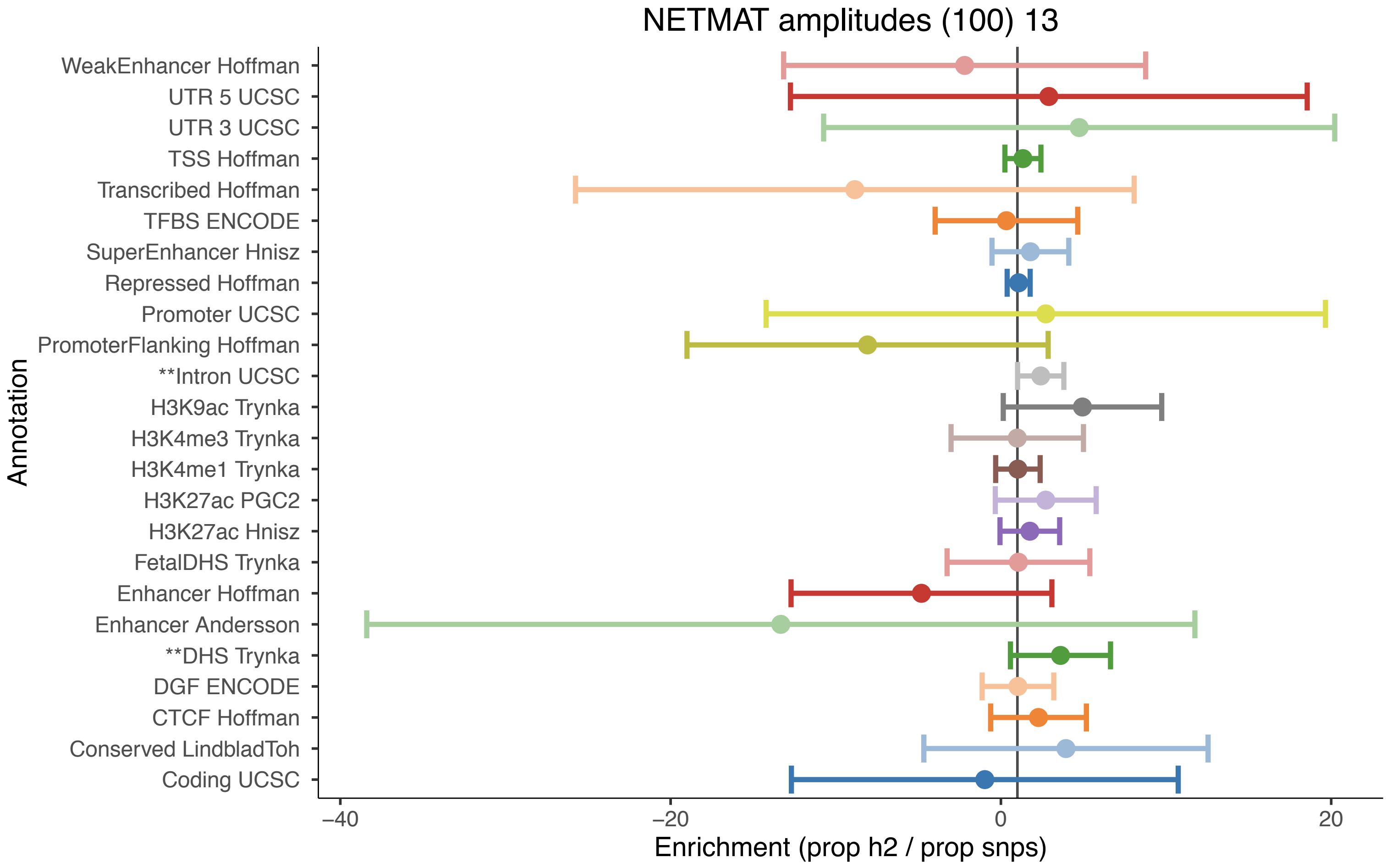

NETMAT amplitudes (100) 13
WeakEnhancer Hoffman
UTR 5 UCSC
UTR 3 UCSC
TSS Hoffman
Transcribed Hoffman
TFBS ENCODE
SuperEnhancer Hnisz
Repressed Hoffman
Promoter UCSC
PromoterFlanking Hoffman
**Intron UCSC
H3K9ac Trynka
H3K4me3 Trynka
H3K4me1 Trynka
H3K27ac PGC2
H3K27ac Hnisz
FetalDHS Trynka
Enhancer Hoffman
Enhancer Andersson
**DHS Trynka
DGF ENCODE
CTCF Hoffman
Conserved LindbladToh
Coding UCSC
Annotation
−40
−20
0
20
Enrichment (prop h2 / prop snps)

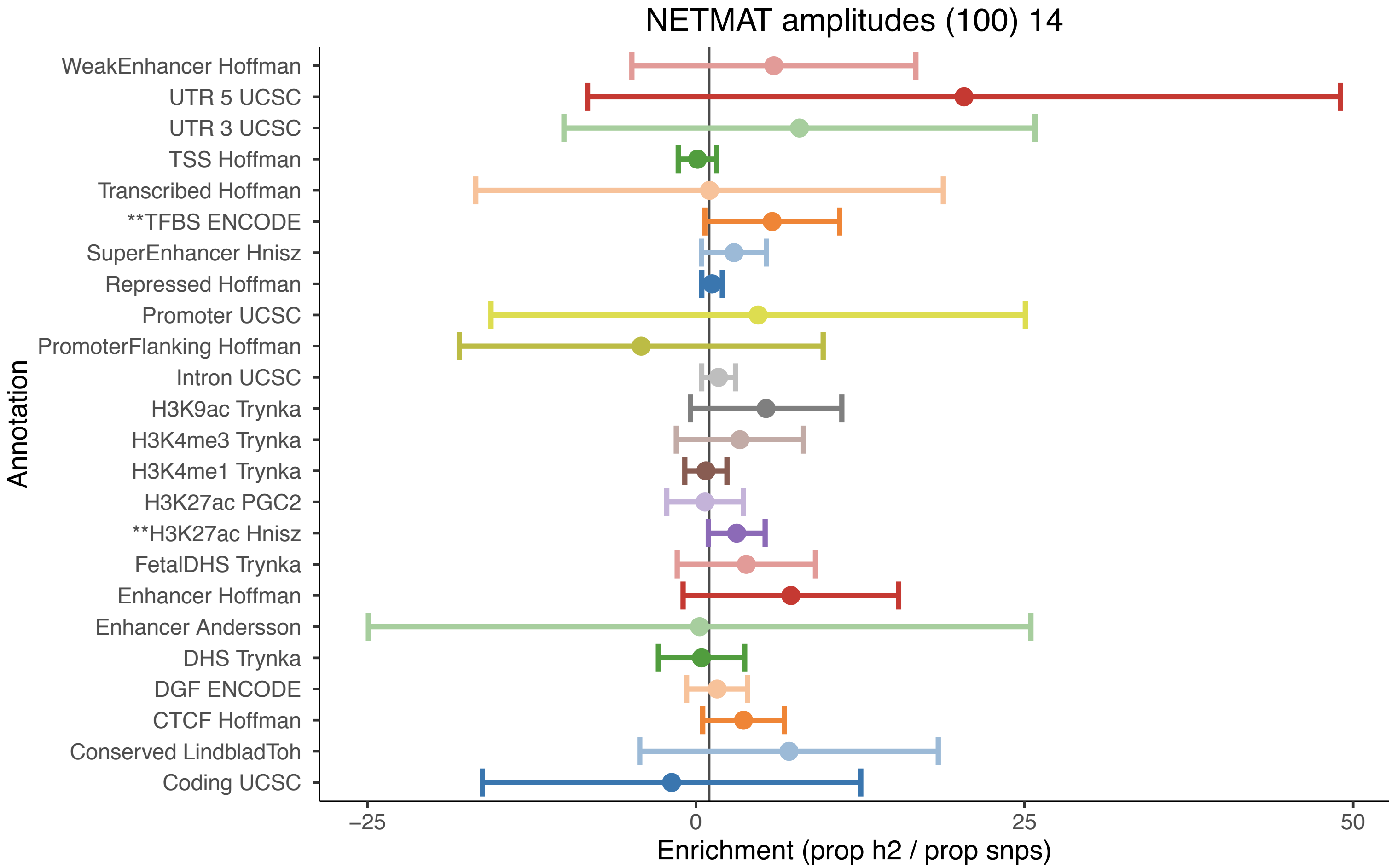

NETMAT amplitudes (100) 14
WeakEnhancer Hoffman
UTR 5 UCSC
UTR 3 UCSC
TSS Hoffman
Transcribed Hoffman
**TFBS ENCODE
SuperEnhancer Hnisz
Repressed Hoffman
Promoter UCSC
PromoterFlanking Hoffman
Intron UCSC
H3K9ac Trynka
H3K4me3 Trynka
H3K4me1 Trynka
H3K27ac PGC2
**H3K27ac Hnisz
FetalDHS Trynka
Enhancer Hoffman
Enhancer Andersson
DHS Trynka
DGF ENCODE
CTCF Hoffman
Conserved LindbladToh
Coding UCSC
Annotation
−25
0
25
50
Enrichment (prop h2 / prop snps)

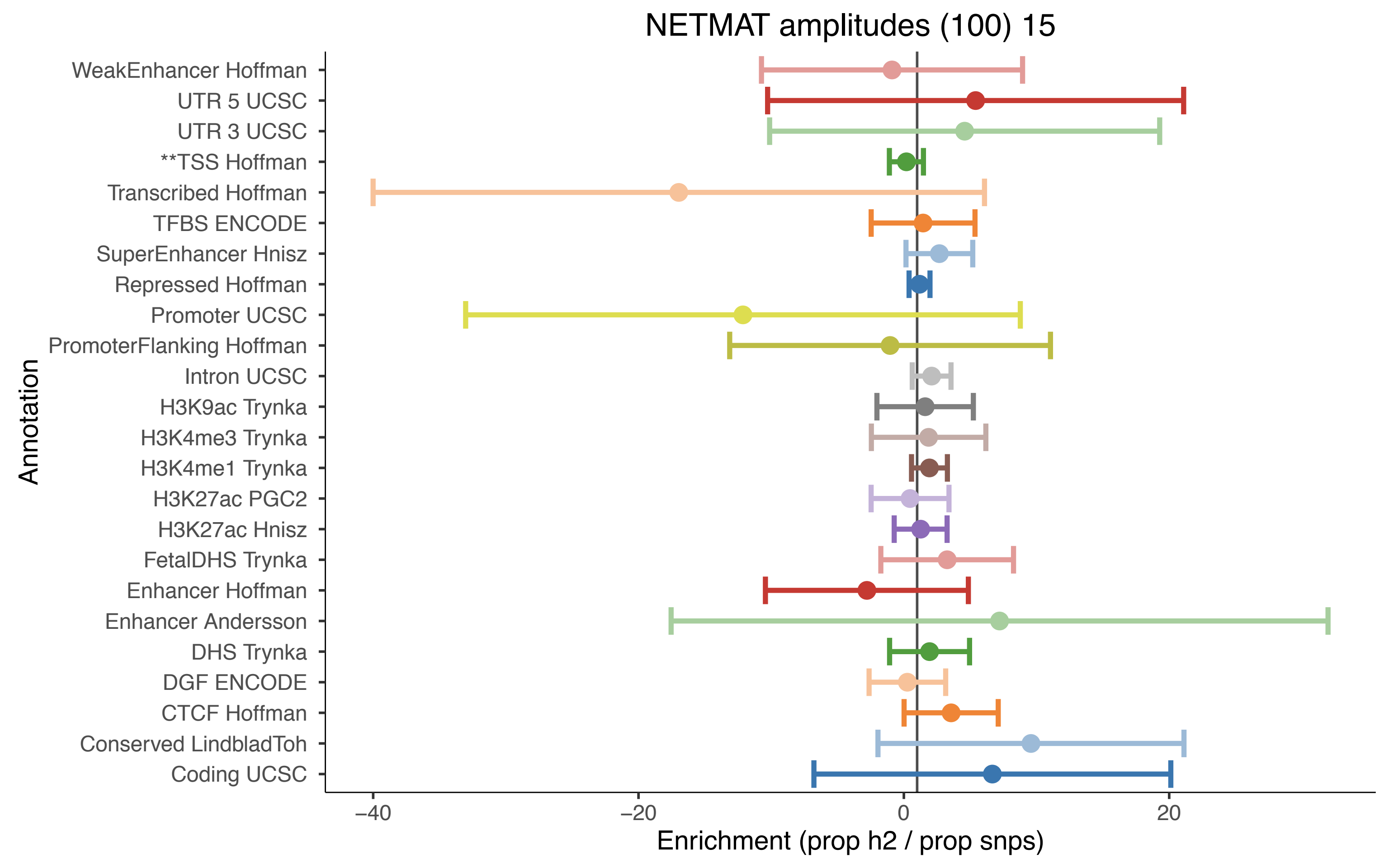

NETMAT amplitudes (100) 15
WeakEnhancer Hoffman
UTR 5 UCSC
UTR 3 UCSC
**TSS Hoffman
Transcribed Hoffman
TFBS ENCODE
SuperEnhancer Hnisz
Repressed Hoffman
Promoter UCSC
PromoterFlanking Hoffman
Intron UCSC
H3K9ac Trynka
H3K4me3 Trynka
H3K4me1 Trynka
H3K27ac PGC2
H3K27ac Hnisz
FetalDHS Trynka
Enhancer Hoffman
Enhancer Andersson
DHS Trynka
DGF ENCODE
CTCF Hoffman
Conserved LindbladToh
Coding UCSC
Annotation
−40
−20
0
20
Enrichment (prop h2 / prop snps)

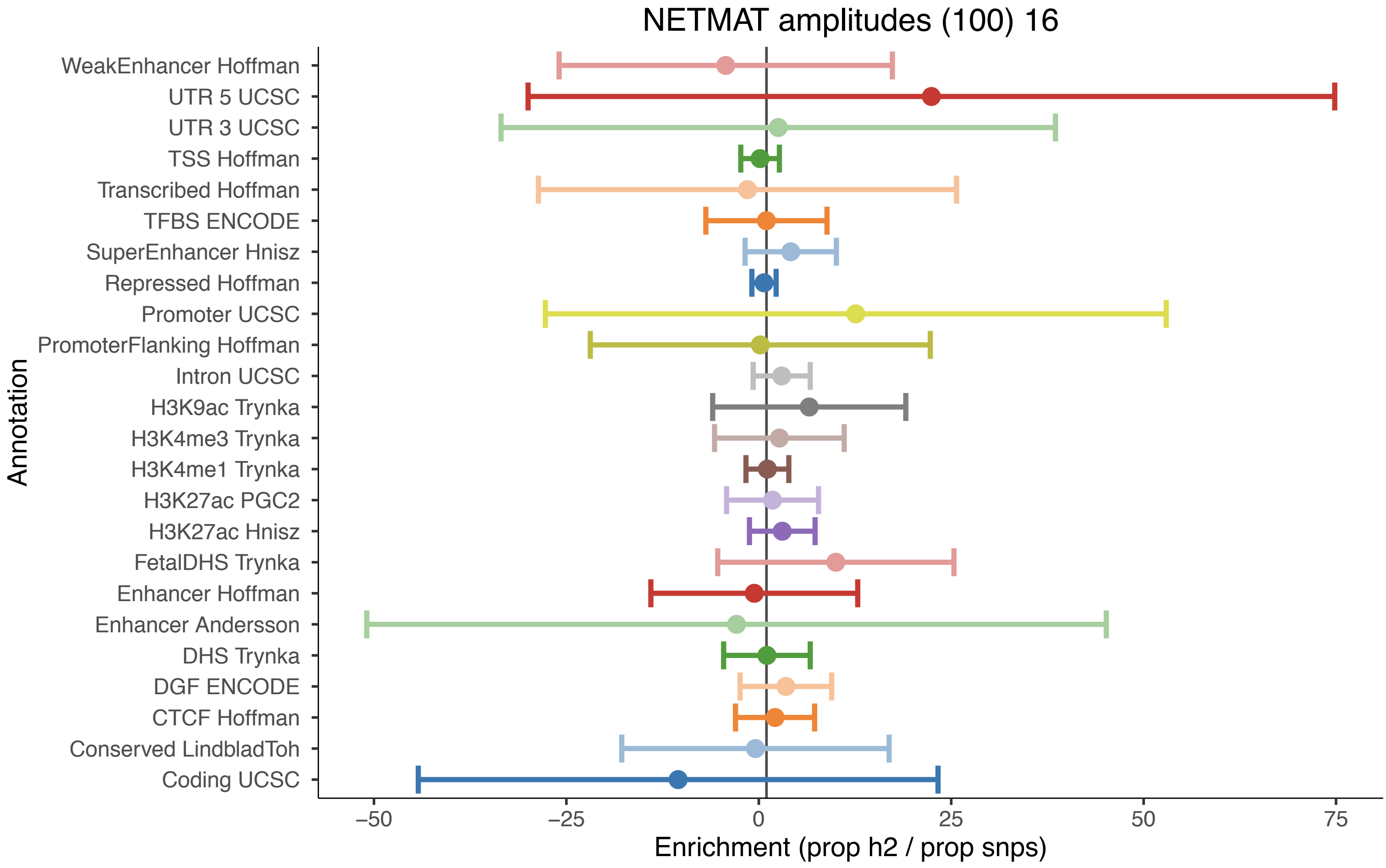

NETMAT amplitudes (100) 16
Annotation
WeakEnhancer Hoffman
UTR 5 UCSC
UTR 3 UCSC
TSS Hoffman
Transcribed Hoffman
TFBS ENCODE
SuperEnhancer Hnisz
Repressed Hoffman
Promoter UCSC
PromoterFlanking Hoffman
Intron UCSC
H3K9ac Trynka
H3K4me3 Trynka
H3K4me1 Trynka
H3K27ac PGC2
H3K27ac Hnisz
FetalDHS Trynka
Enhancer Hoffman
Enhancer Andersson
DHS Trynka
DGF ENCODE
CTCF Hoffman
Conserved LindbladToh
Coding UCSC
−50
−25
0
25
50
75
Enrichment (prop h2 / prop snps)

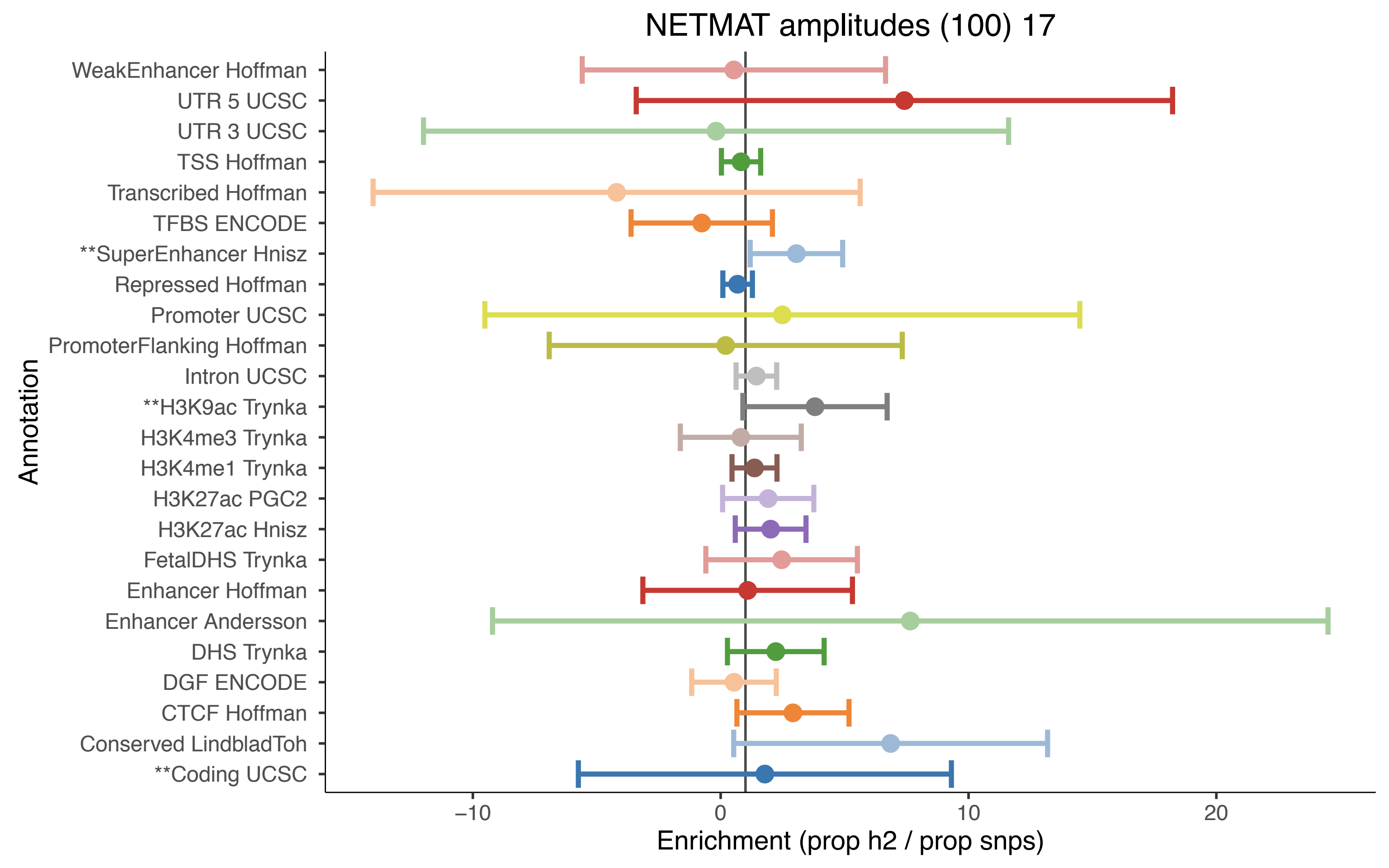

NETMAT amplitudes (100) 17
WeakEnhancer Hoffman
UTR 5 UCSC
UTR 3 UCSC
TSS Hoffman
Transcribed Hoffman
TFBS ENCODE
**SuperEnhancer Hnisz
Repressed Hoffman
Promoter UCSC
PromoterFlanking Hoffman
Intron UCSC
**H3K9ac Trynka
H3K4me3 Trynka
H3K4me1 Trynka
H3K27ac PGC2
H3K27ac Hnisz
FetalDHS Trynka
Enhancer Hoffman
Enhancer Andersson
DHS Trynka
DGF ENCODE
CTCF Hoffman
Conserved LindbladToh
**Coding UCSC
Annotation
−10
0
10
20
Enrichment (prop h2 / prop snps)

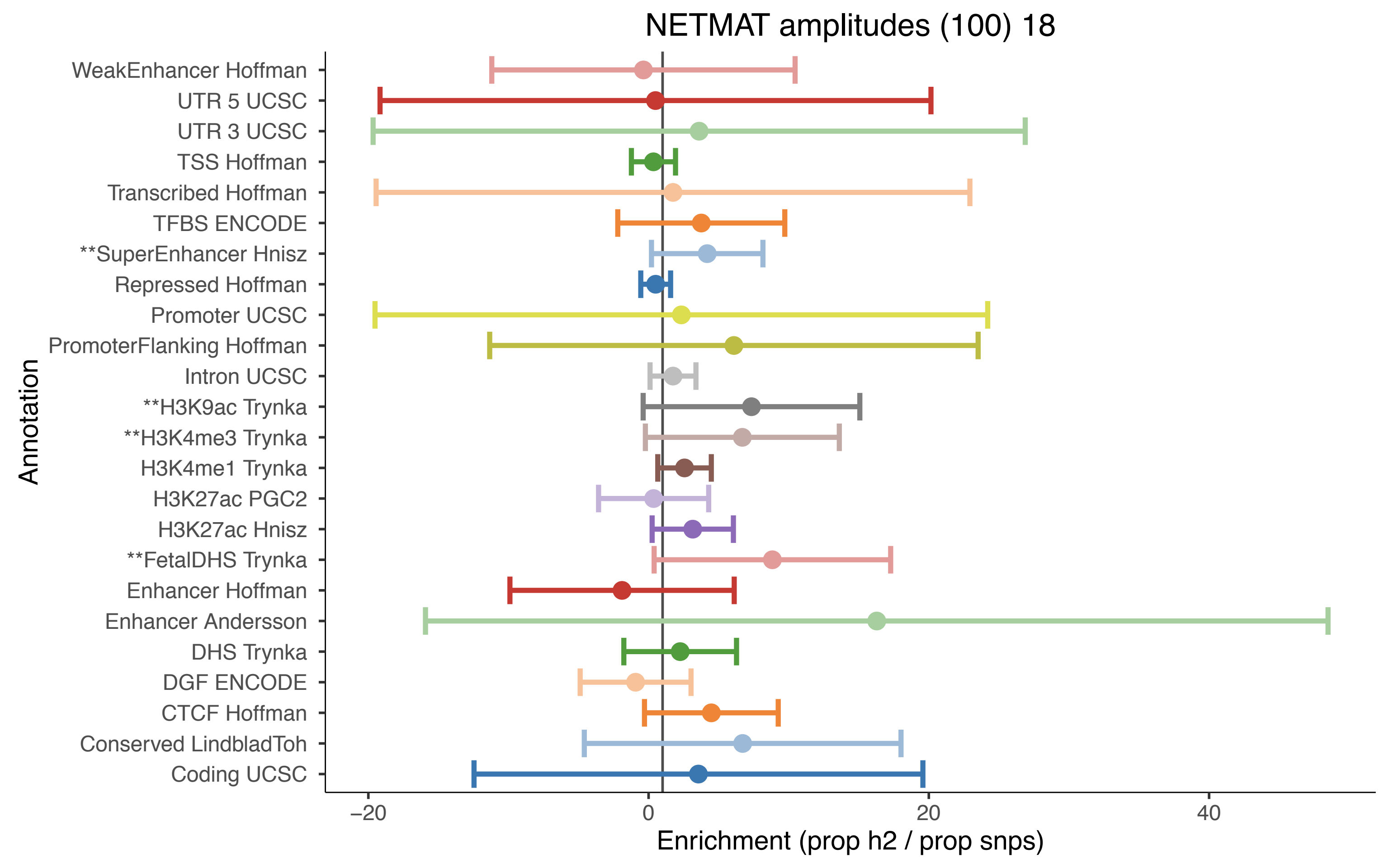
NETMAT amplitudes (100) 18
WeakEnhancer Hoffman
UTR 5 UCSC
UTR 3 UCSC
TSS Hoffman
Transcribed Hoffman
TFBS ENCODE
**SuperEnhancer Hnisz
Repressed Hoffman
Promoter UCSC
PromoterFlanking Hoffman
Intron UCSC
**H3K9ac Trynka
**H3K4me3 Trynka
H3K4me1 Trynka
H3K27ac PGC2
H3K27ac Hnisz
**FetalDHS Trynka
Enhancer Hoffman
Enhancer Andersson
DHS Trynka
DGF ENCODE
CTCF Hoffman
Conserved LindbladToh
Coding UCSC
Annotation
−20
0
20
40
Enrichment (prop h2 / prop snps)

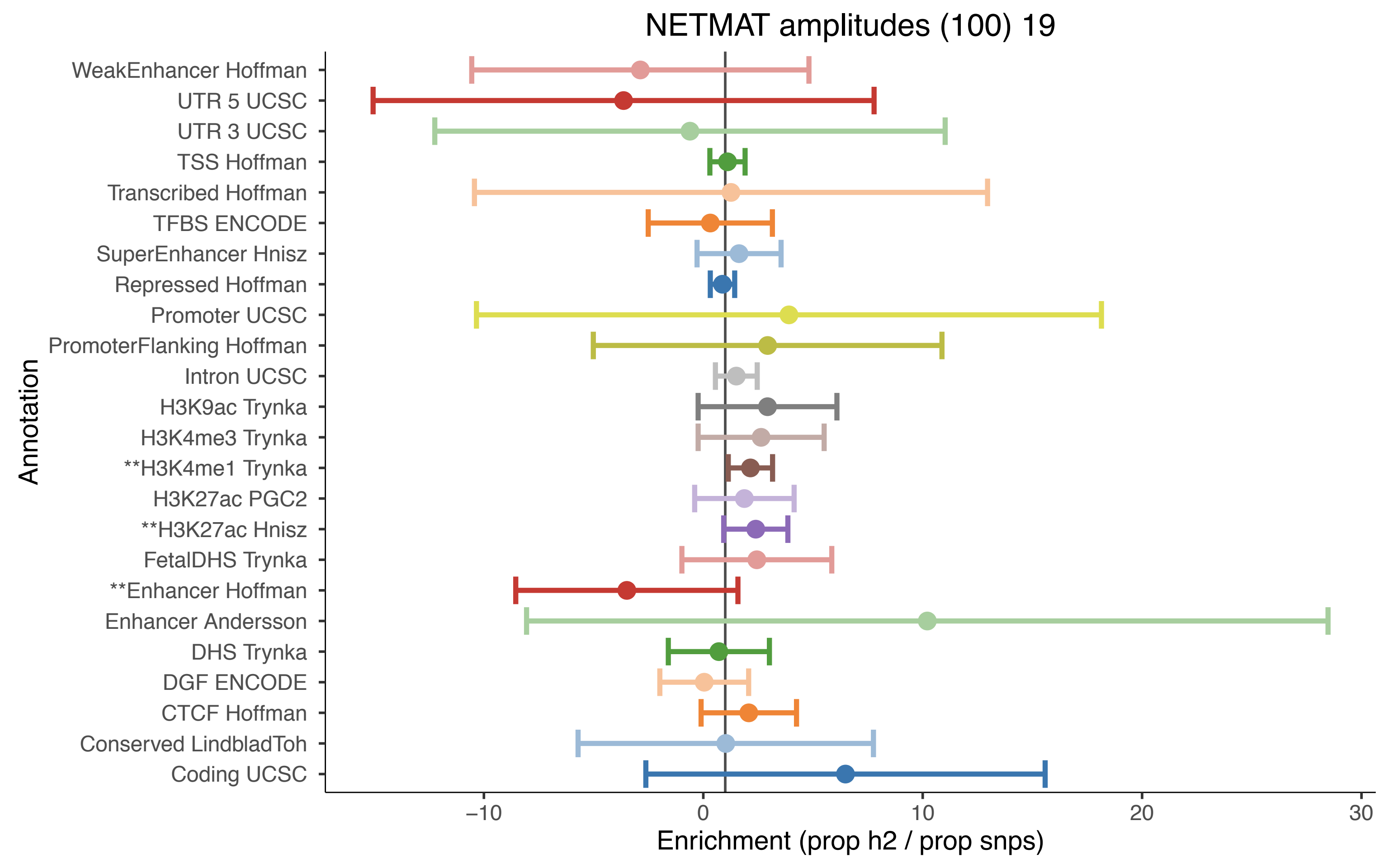
NETMAT amplitudes (100) 19
WeakEnhancer Hoffman
UTR 5 UCSC
UTR 3 UCSC
TSS Hoffman
Transcribed Hoffman
TFBS ENCODE
SuperEnhancer Hnisz
Repressed Hoffman
Promoter UCSC
PromoterFlanking Hoffman
Intron UCSC
H3K9ac Trynka
H3K4me3 Trynka
**H3K4me1 Trynka
H3K27ac PGC2
**H3K27ac Hnisz
FetalDHS Trynka
**Enhancer Hoffman
Enhancer Andersson
DHS Trynka
DGF ENCODE
CTCF Hoffman
Conserved LindbladToh
Coding UCSC
Annotation
−10
0
10
20
30
Enrichment (prop h2 / prop snps)

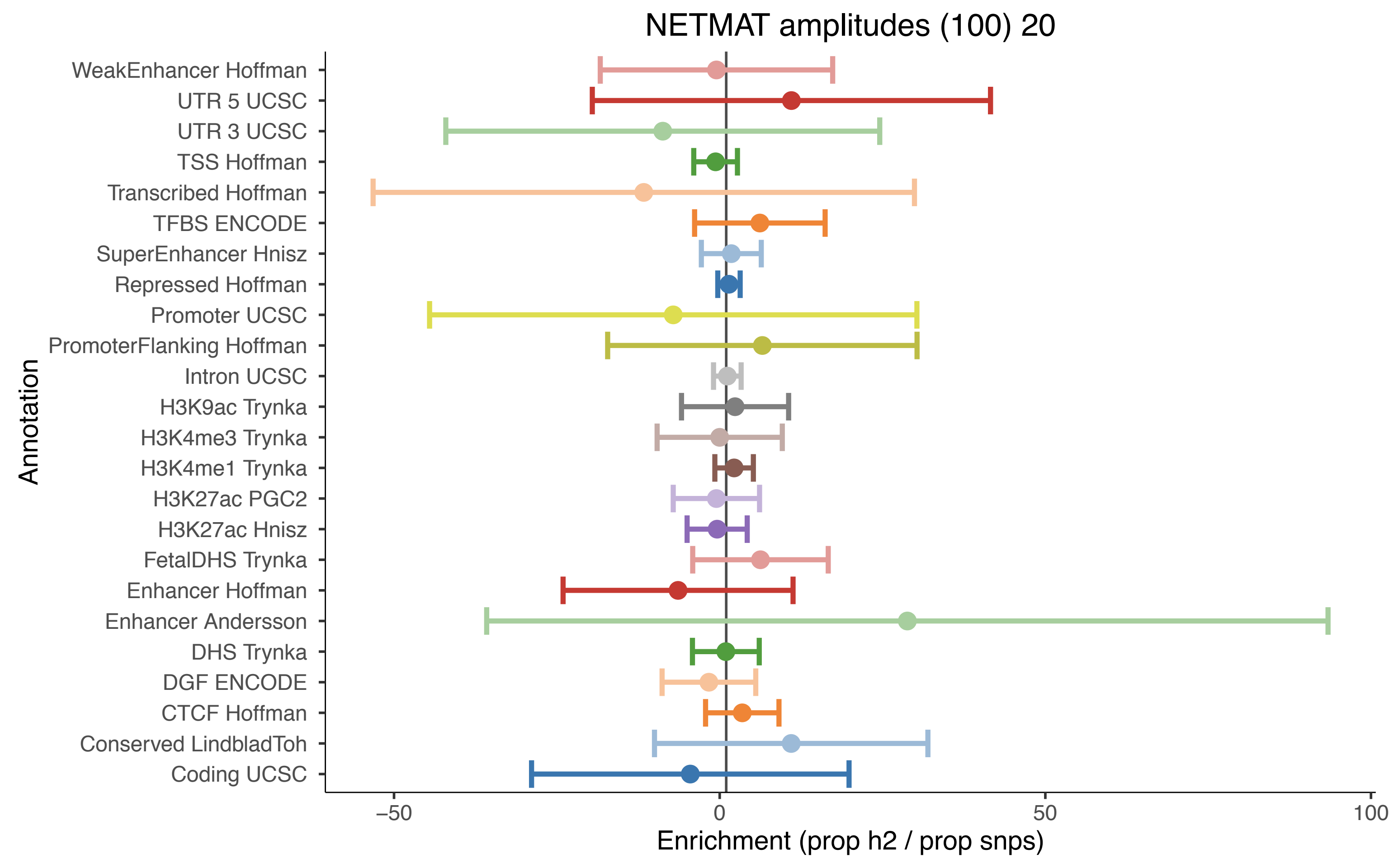
NETMAT amplitudes (100) 20
WeakEnhancer Hoffman
UTR 5 UCSC
UTR 3 UCSC
TSS Hoffman
Transcribed Hoffman
TFBS ENCODE
SuperEnhancer Hnisz
Repressed Hoffman
Promoter UCSC
PromoterFlanking Hoffman
Intron UCSC
H3K9ac Trynka
H3K4me3 Trynka
H3K4me1 Trynka
H3K27ac PGC2
H3K27ac Hnisz
FetalDHS Trynka
Enhancer Hoffman
Enhancer Andersson
DHS Trynka
DGF ENCODE
CTCF Hoffman
Conserved LindbladToh
Coding UCSC
Annotation
−50
0
50
100
Enrichment (prop h2 / prop snps)

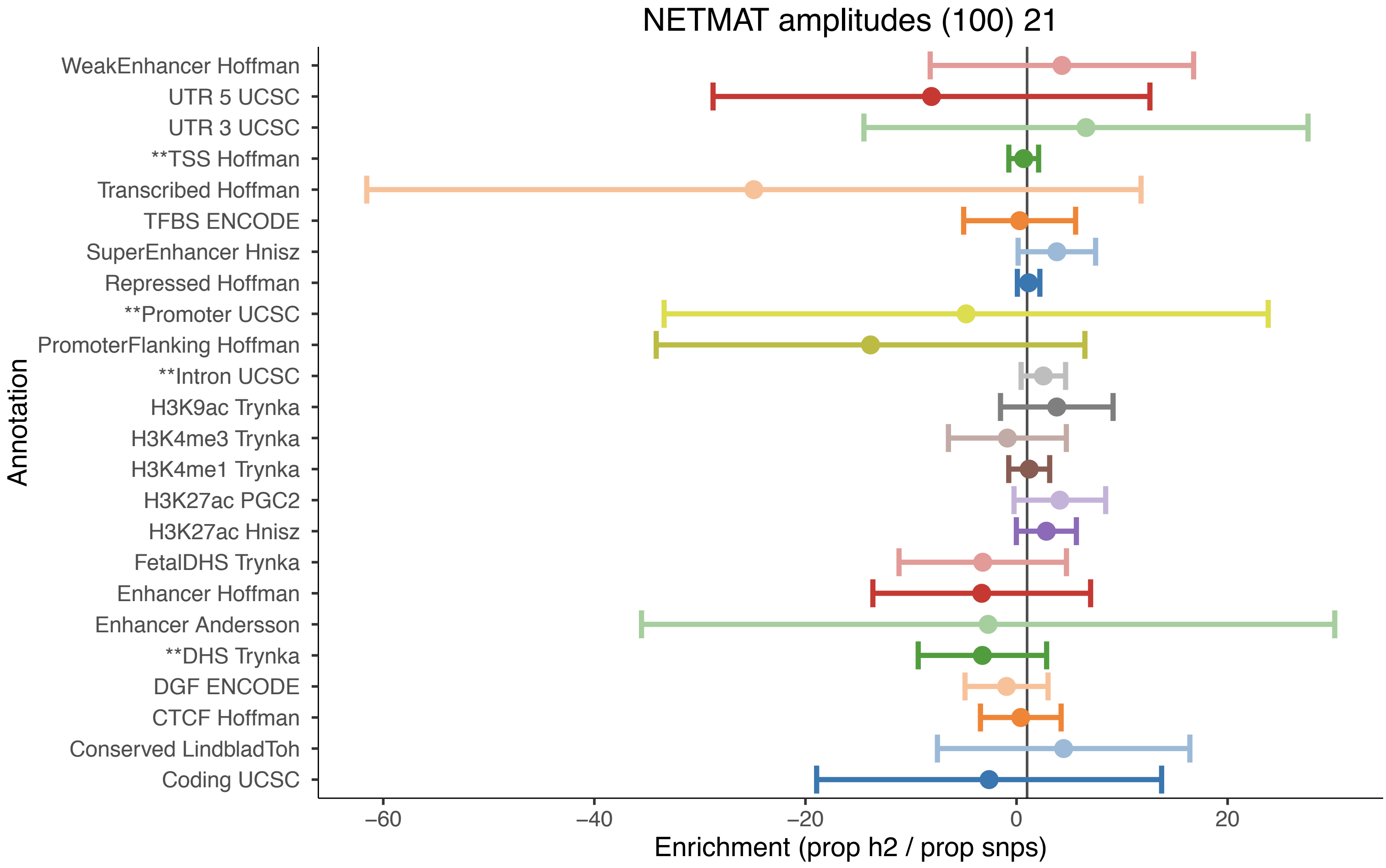

NETMAT amplitudes (100) 21
WeakEnhancer Hoffman
UTR 5 UCSC
UTR 3 UCSC
**TSS Hoffman
Transcribed Hoffman
TFBS ENCODE
SuperEnhancer Hnisz
Repressed Hoffman
**Promoter UCSC
PromoterFlanking Hoffman
**Intron UCSC
H3K9ac Trynka
H3K4me3 Trynka
H3K4me1 Trynka
H3K27ac PGC2
H3K27ac Hnisz
FetalDHS Trynka
Enhancer Hoffman
Enhancer Andersson
**DHS Trynka
DGF ENCODE
CTCF Hoffman
Conserved LindbladToh
Coding UCSC
Annotation
−60
−40
−20
0
20
Enrichment (prop h2 / prop snps)

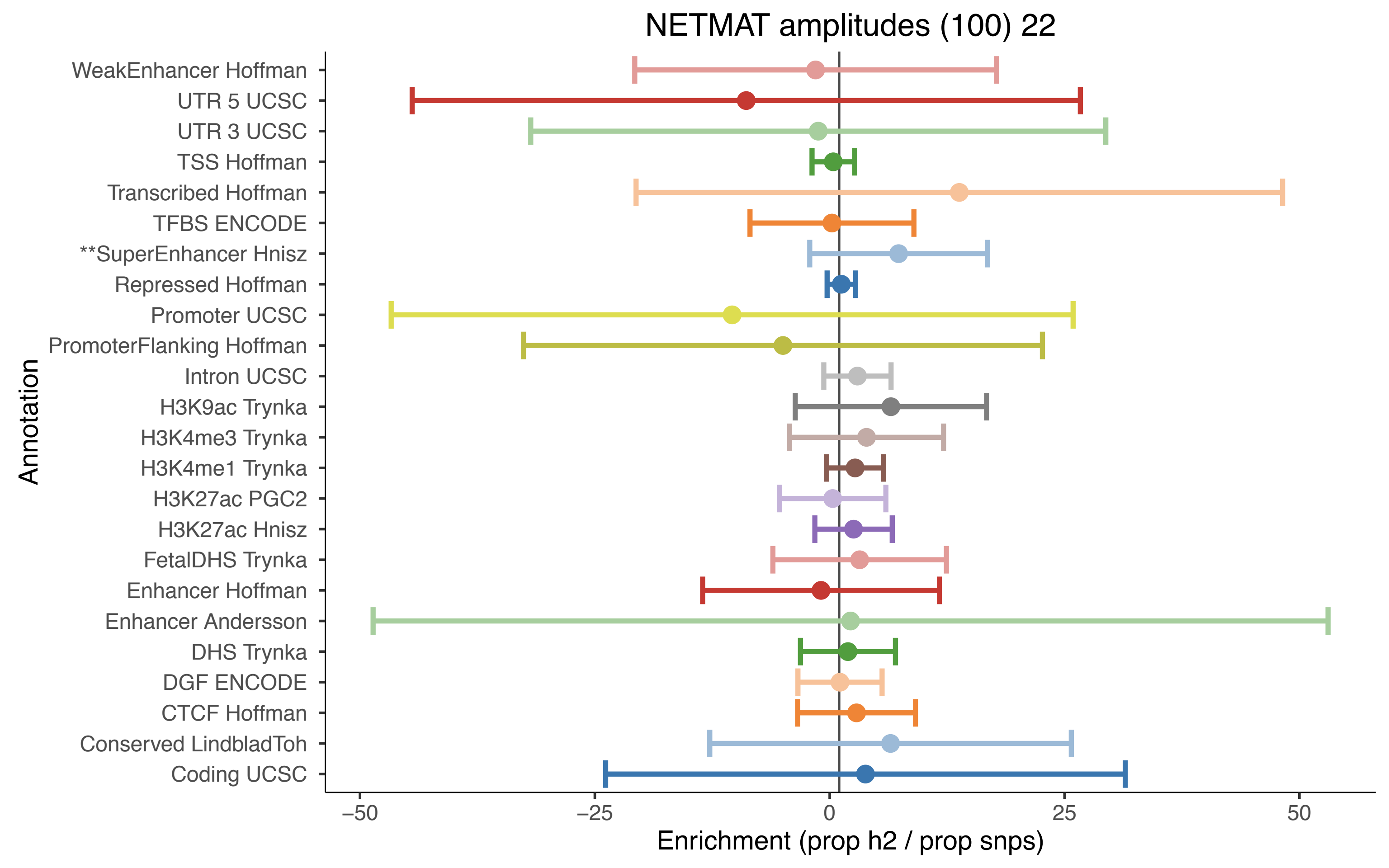
NETMAT amplitudes (100) 22
WeakEnhancer Hoffman
UTR 5 UCSC
UTR 3 UCSC
TSS Hoffman
Transcribed Hoffman
TFBS ENCODE
**SuperEnhancer Hnisz
Repressed Hoffman
Promoter UCSC
PromoterFlanking Hoffman
Intron UCSC
H3K9ac Trynka
H3K4me3 Trynka
H3K4me1 Trynka
H3K27ac PGC2
H3K27ac Hnisz
FetalDHS Trynka
Enhancer Hoffman
Enhancer Andersson
DHS Trynka
DGF ENCODE
CTCF Hoffman
Conserved LindbladToh
Coding UCSC
Annotation
−50
−25
0
25
50
Enrichment (prop h2 / prop snps)

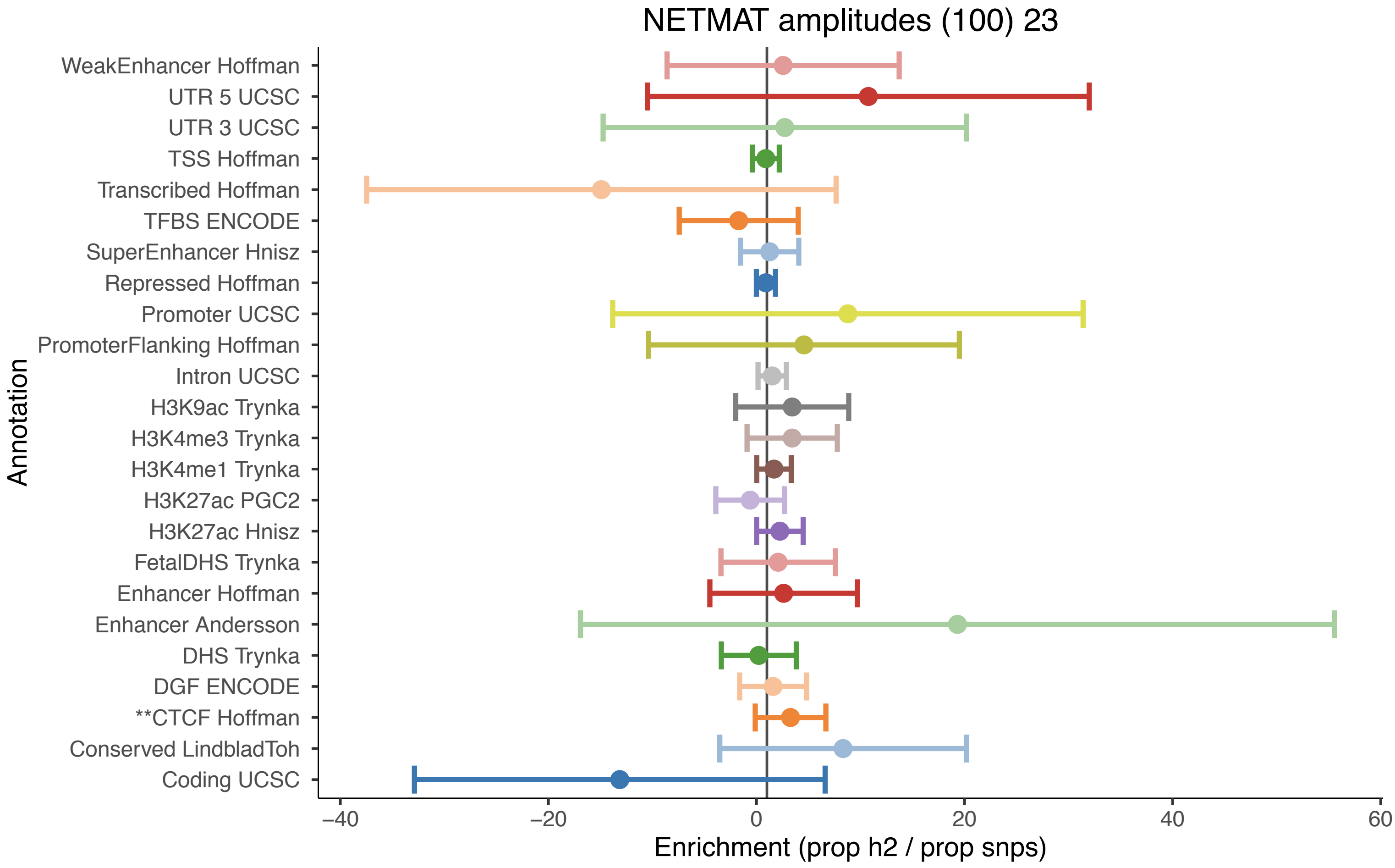
NETMAT amplitudes (100) 23
WeakEnhancer Hoffman
UTR 5 UCSC
UTR 3 UCSC
TSS Hoffman
Transcribed Hoffman
TFBS ENCODE
SuperEnhancer Hnisz
Repressed Hoffman
Promoter UCSC
PromoterFlanking Hoffman
Intron UCSC
H3K9ac Trynka
H3K4me3 Trynka
H3K4me1 Trynka
H3K27ac PGC2
H3K27ac Hnisz
FetalDHS Trynka
Enhancer Hoffman
Enhancer Andersson
DHS Trynka
DGF ENCODE
**CTCF Hoffman
Conserved LindbladToh
Coding UCSC
Annotation
−40
−20
0
20
40
60
Enrichment (prop h2 / prop snps)

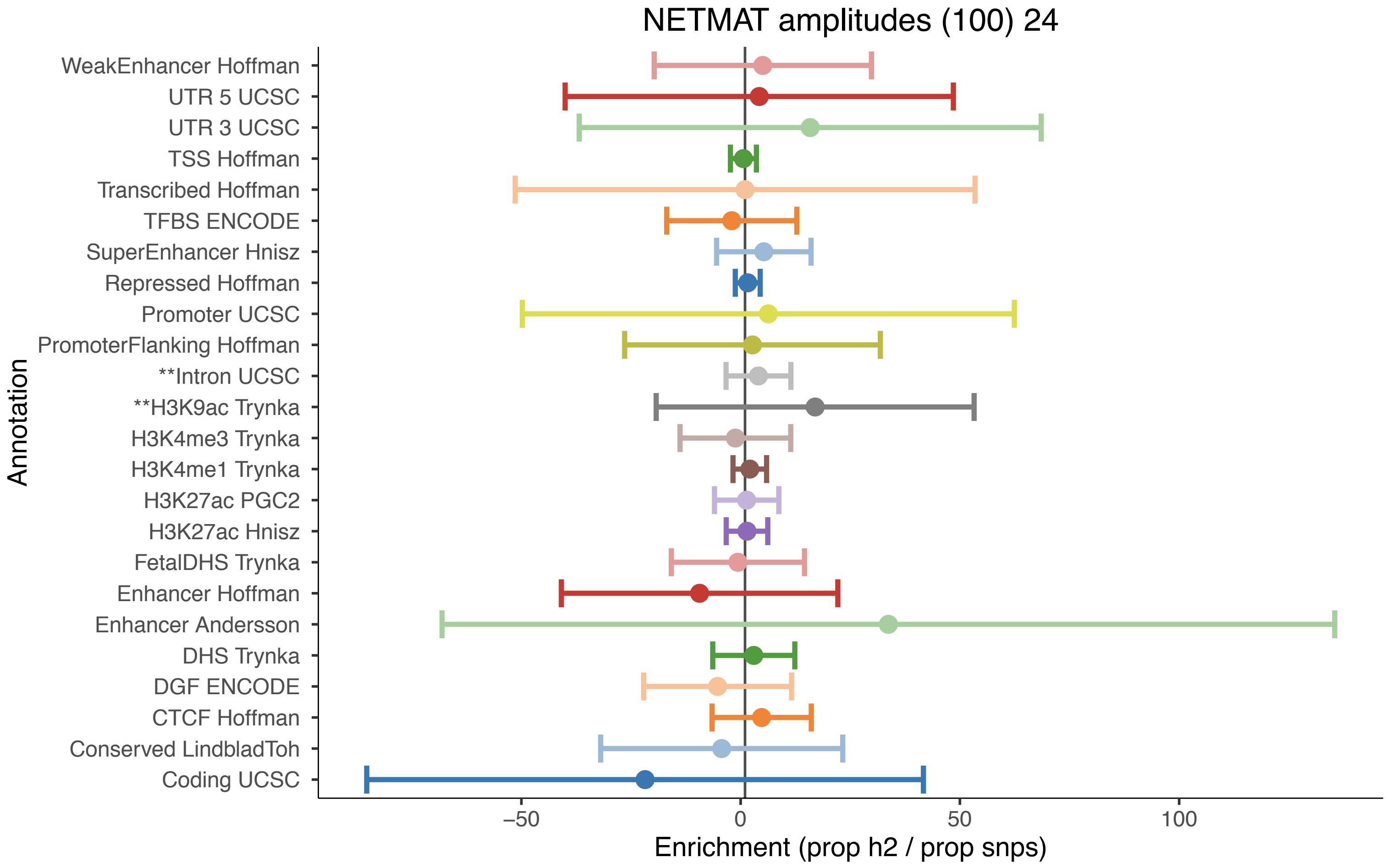
NETMAT amplitudes (100) 24
WeakEnhancer Hoffman
UTR 5 UCSC
UTR 3 UCSC
TSS Hoffman
Transcribed Hoffman
TFBS ENCODE
SuperEnhancer Hnisz
Repressed Hoffman
Promoter UCSC
PromoterFlanking Hoffman
**Intron UCSC
**H3K9ac Trynka
H3K4me3 Trynka
H3K4me1 Trynka
H3K27ac PGC2
H3K27ac Hnisz
FetalDHS Trynka
Enhancer Hoffman
Enhancer Andersson
DHS Trynka
DGF ENCODE
CTCF Hoffman
Conserved LindbladToh
Coding UCSC
Annotation
−50
0
50
100
Enrichment (prop h2 / prop snps)

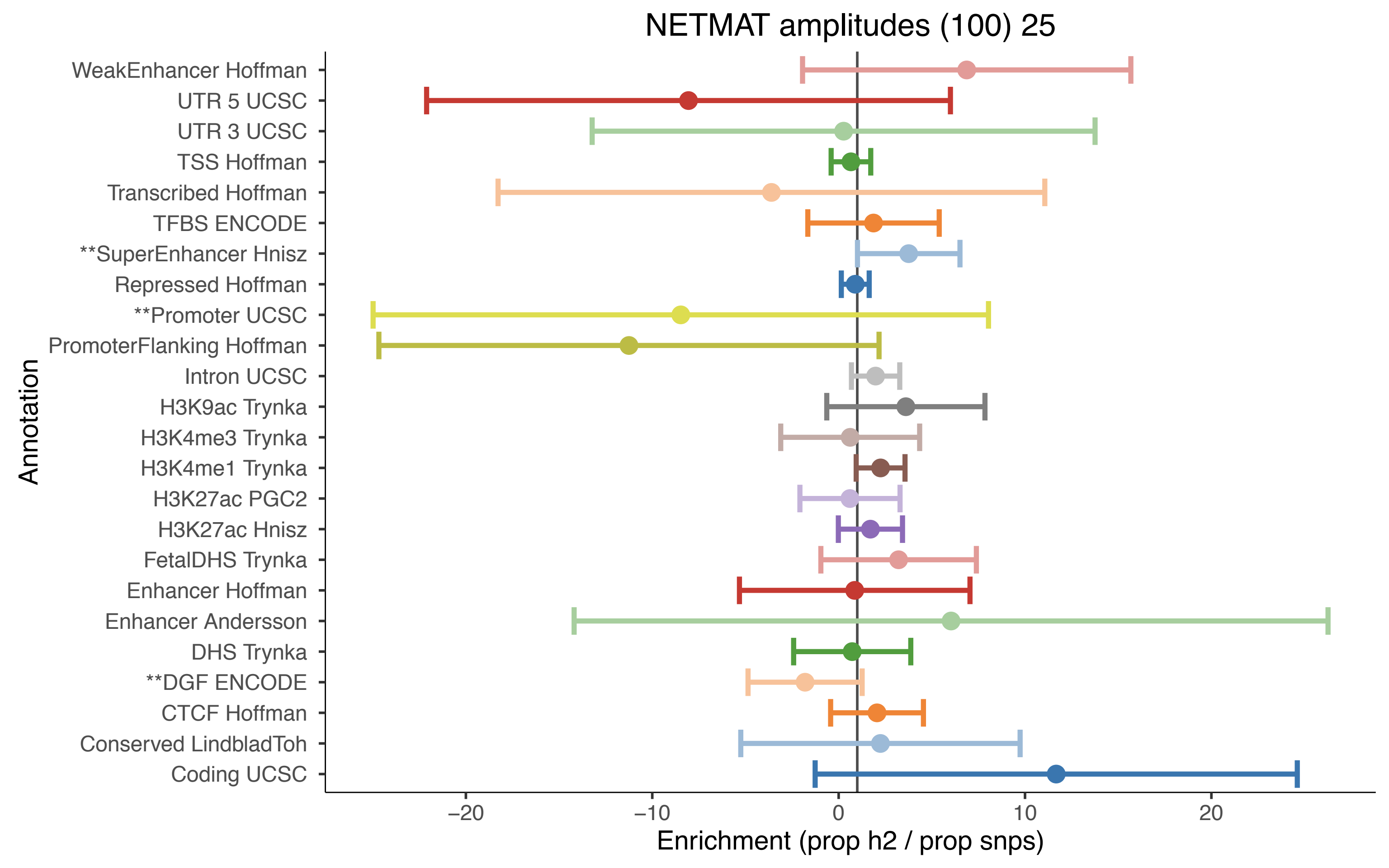

NETMAT amplitudes (100) 25
WeakEnhancer Hoffman
UTR 5 UCSC
UTR 3 UCSC
TSS Hoffman
Transcribed Hoffman
TFBS ENCODE
**SuperEnhancer Hnisz
Repressed Hoffman
**Promoter UCSC
PromoterFlanking Hoffman
Intron UCSC
H3K9ac Trynka
H3K4me3 Trynka
H3K4me1 Trynka
H3K27ac PGC2
H3K27ac Hnisz
FetalDHS Trynka
Enhancer Hoffman
Enhancer Andersson
DHS Trynka
**DGF ENCODE
CTCF Hoffman
Conserved LindbladToh
Coding UCSC
Annotation
−20
−10
0
10
20
Enrichment (prop h2 / prop snps)

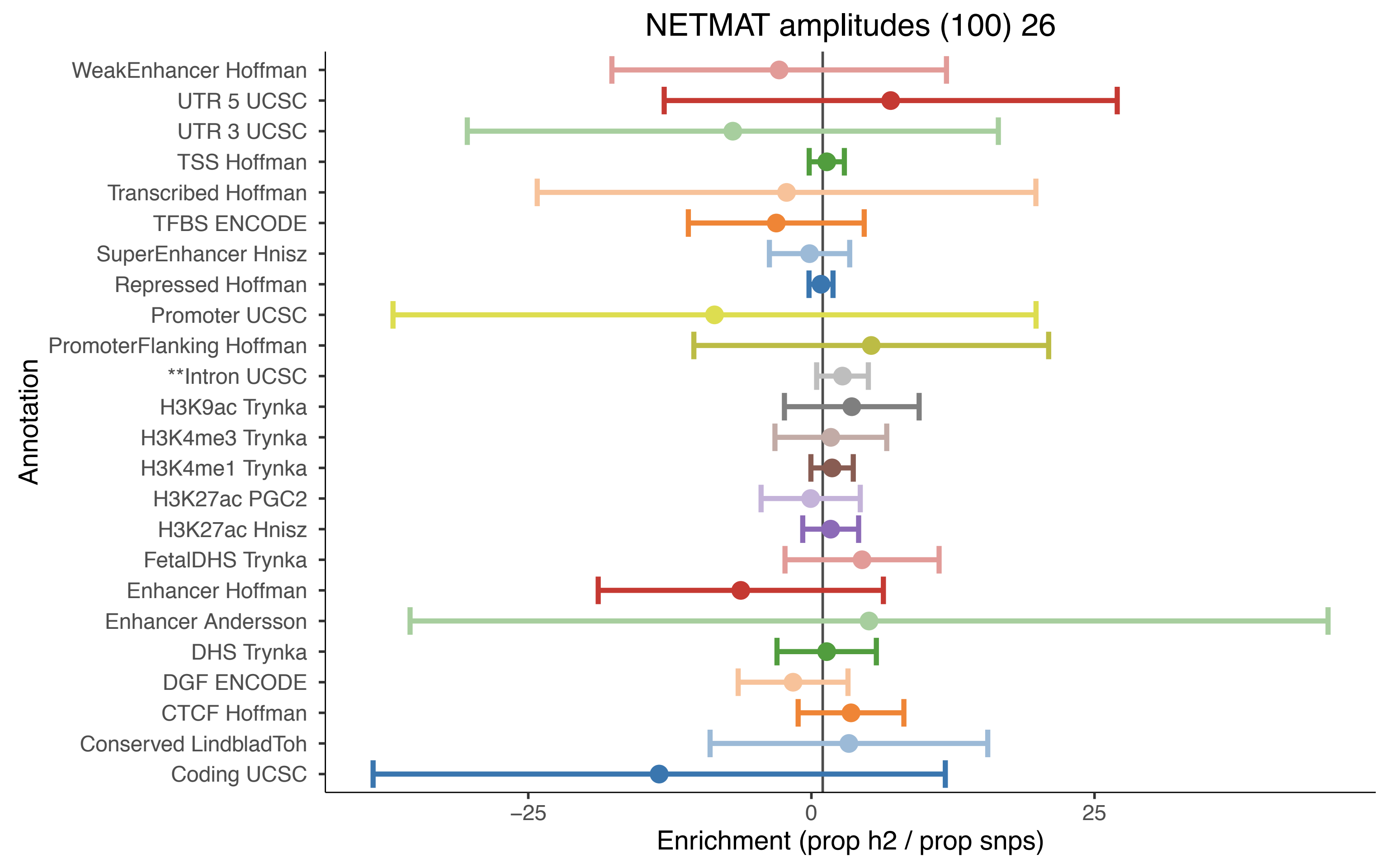
NETMAT amplitudes (100) 26
WeakEnhancer Hoffman
UTR 5 UCSC
UTR 3 UCSC
TSS Hoffman
Transcribed Hoffman
TFBS ENCODE
SuperEnhancer Hnisz
Repressed Hoffman
Promoter UCSC
PromoterFlanking Hoffman
**Intron UCSC
H3K9ac Trynka
H3K4me3 Trynka
H3K4me1 Trynka
H3K27ac PGC2
H3K27ac Hnisz
FetalDHS Trynka
Enhancer Hoffman
Enhancer Andersson
DHS Trynka
DGF ENCODE
CTCF Hoffman
Conserved LindbladToh
Coding UCSC
Annotation
−25
0
25
Enrichment (prop h2 / prop snps)

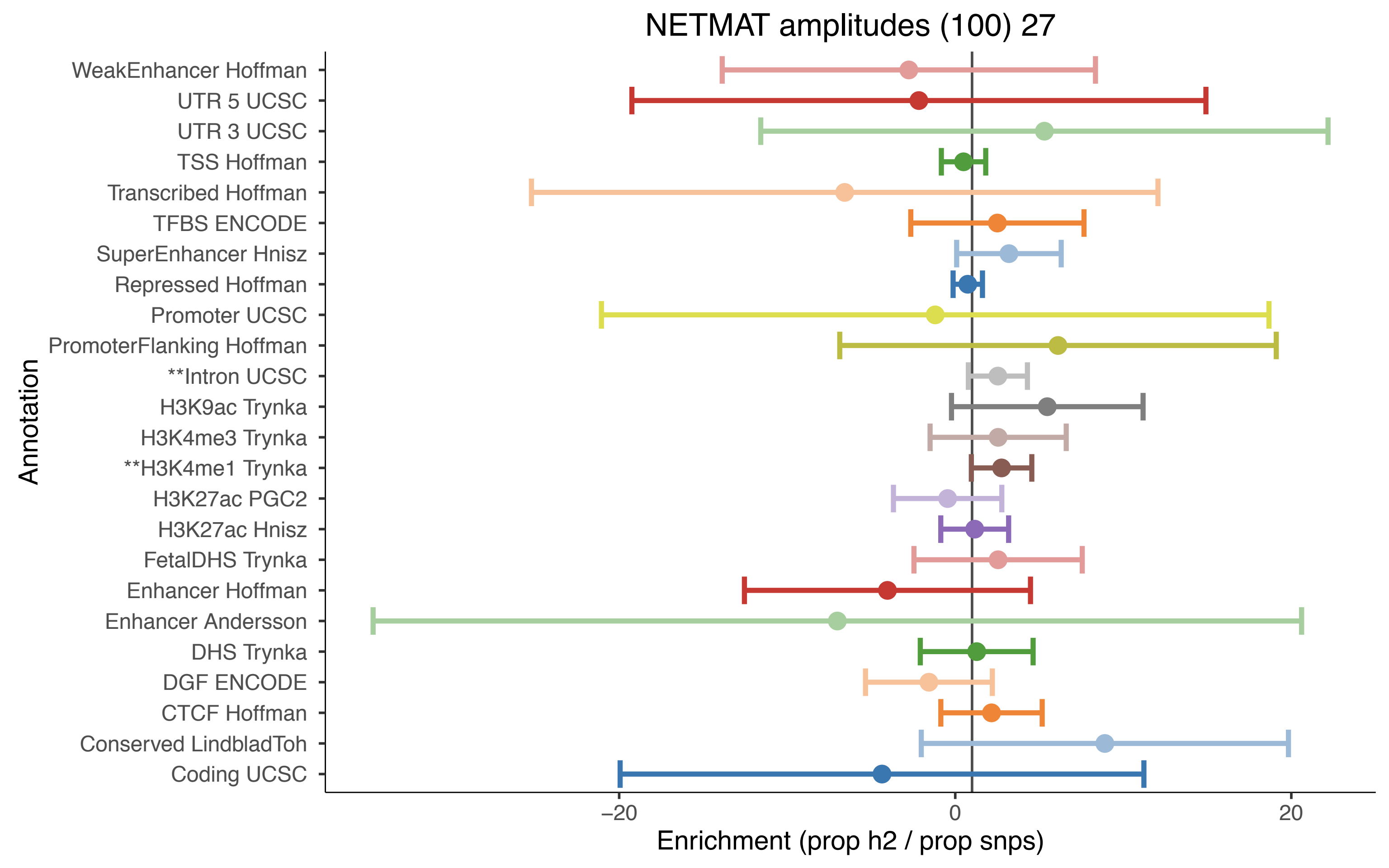
NETMAT amplitudes (100) 27
WeakEnhancer Hoffman
UTR 5 UCSC
UTR 3 UCSC
TSS Hoffman
Transcribed Hoffman
TFBS ENCODE
SuperEnhancer Hnisz
Repressed Hoffman
Promoter UCSC
PromoterFlanking Hoffman
**Intron UCSC
H3K9ac Trynka
H3K4me3 Trynka
**H3K4me1 Trynka
H3K27ac PGC2
H3K27ac Hnisz
FetalDHS Trynka
Enhancer Hoffman
Enhancer Andersson
DHS Trynka
DGF ENCODE
CTCF Hoffman
Conserved LindbladToh
Coding UCSC
Annotation
−20
0
20
Enrichment (prop h2 / prop snps)

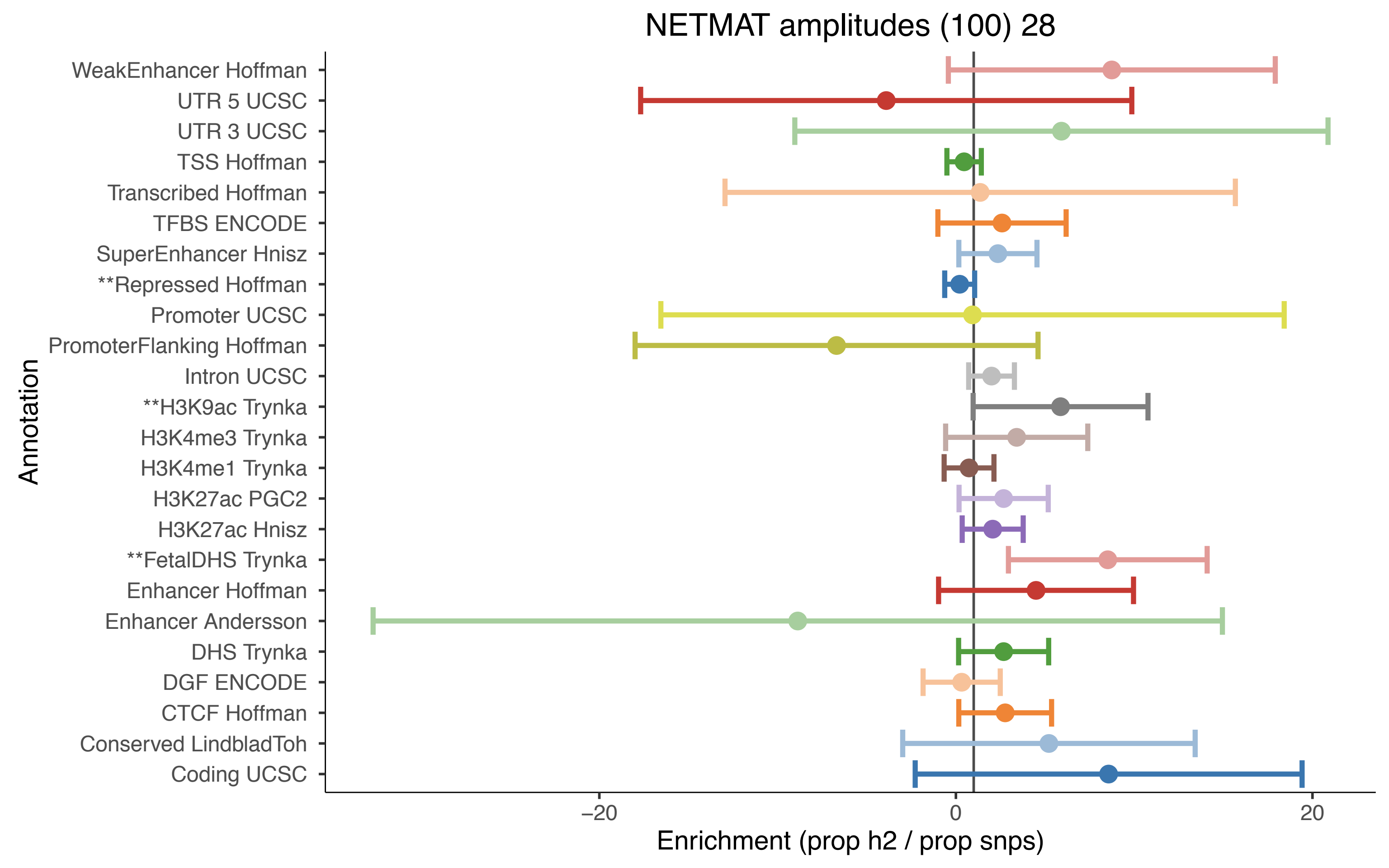

NETMAT amplitudes (100) 28
WeakEnhancer Hoffman
UTR 5 UCSC
UTR 3 UCSC
TSS Hoffman
Transcribed Hoffman
TFBS ENCODE
SuperEnhancer Hnisz
**Repressed Hoffman
Promoter UCSC
PromoterFlanking Hoffman
Intron UCSC
**H3K9ac Trynka
H3K4me3 Trynka
H3K4me1 Trynka
H3K27ac PGC2
H3K27ac Hnisz
**FetalDHS Trynka
Enhancer Hoffman
Enhancer Andersson
DHS Trynka
DGF ENCODE
CTCF Hoffman
Conserved LindbladToh
Coding UCSC
Annotation
−20
0
20
Enrichment (prop h2 / prop snps)

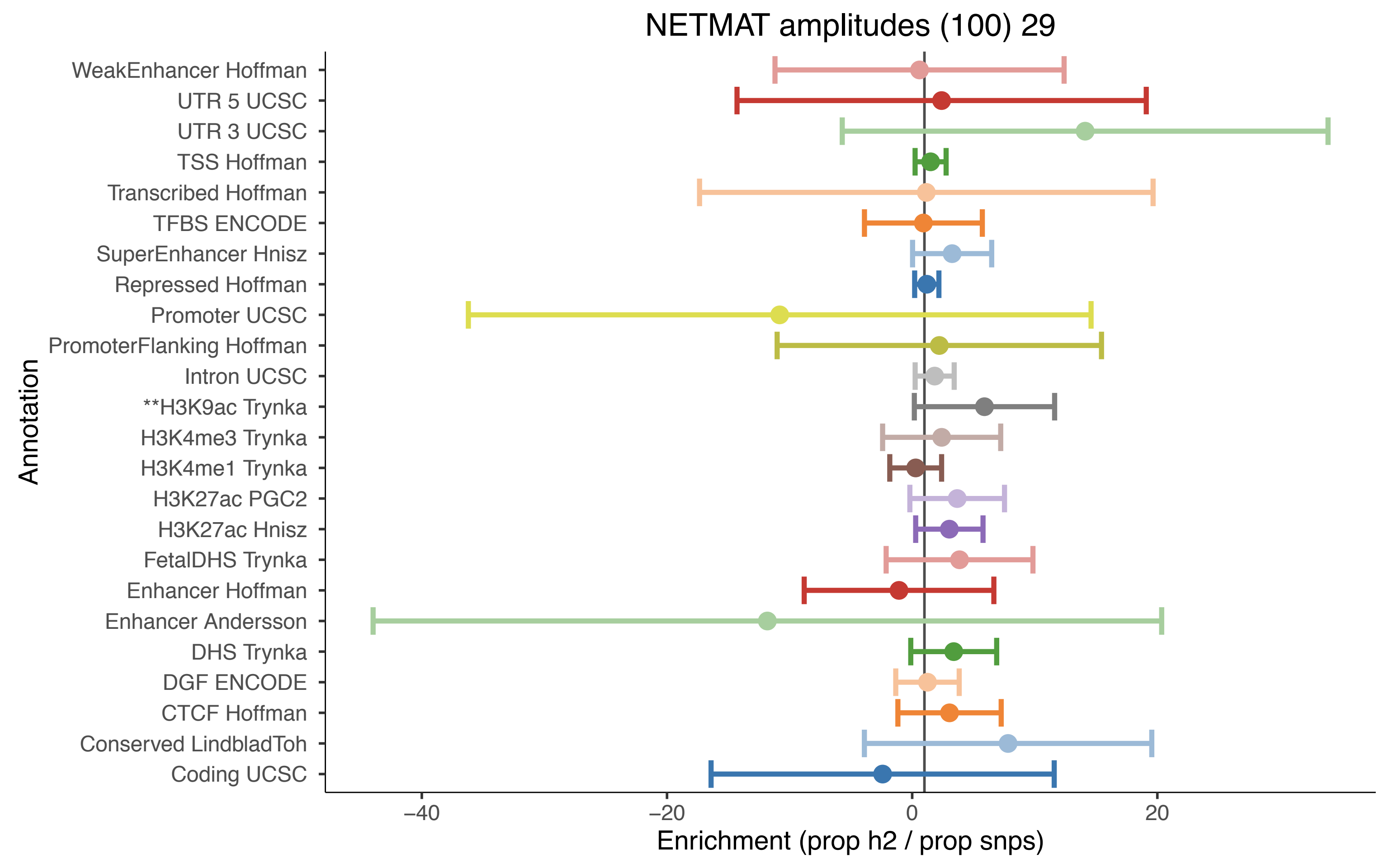
NETMAT amplitudes (100) 29
Annotation
WeakEnhancer Hoffman
UTR 5 UCSC
UTR 3 UCSC
TSS Hoffman
Transcribed Hoffman
TFBS ENCODE
SuperEnhancer Hnisz
Repressed Hoffman
Promoter UCSC
PromoterFlanking Hoffman
Intron UCSC
**H3K9ac Trynka
H3K4me3 Trynka
H3K4me1 Trynka
H3K27ac PGC2
H3K27ac Hnisz
FetalDHS Trynka
Enhancer Hoffman
Enhancer Andersson
DHS Trynka
DGF ENCODE
CTCF Hoffman
Conserved LindbladToh
Coding UCSC
−40
−20
0
20
Enrichment (prop h2 / prop snps)

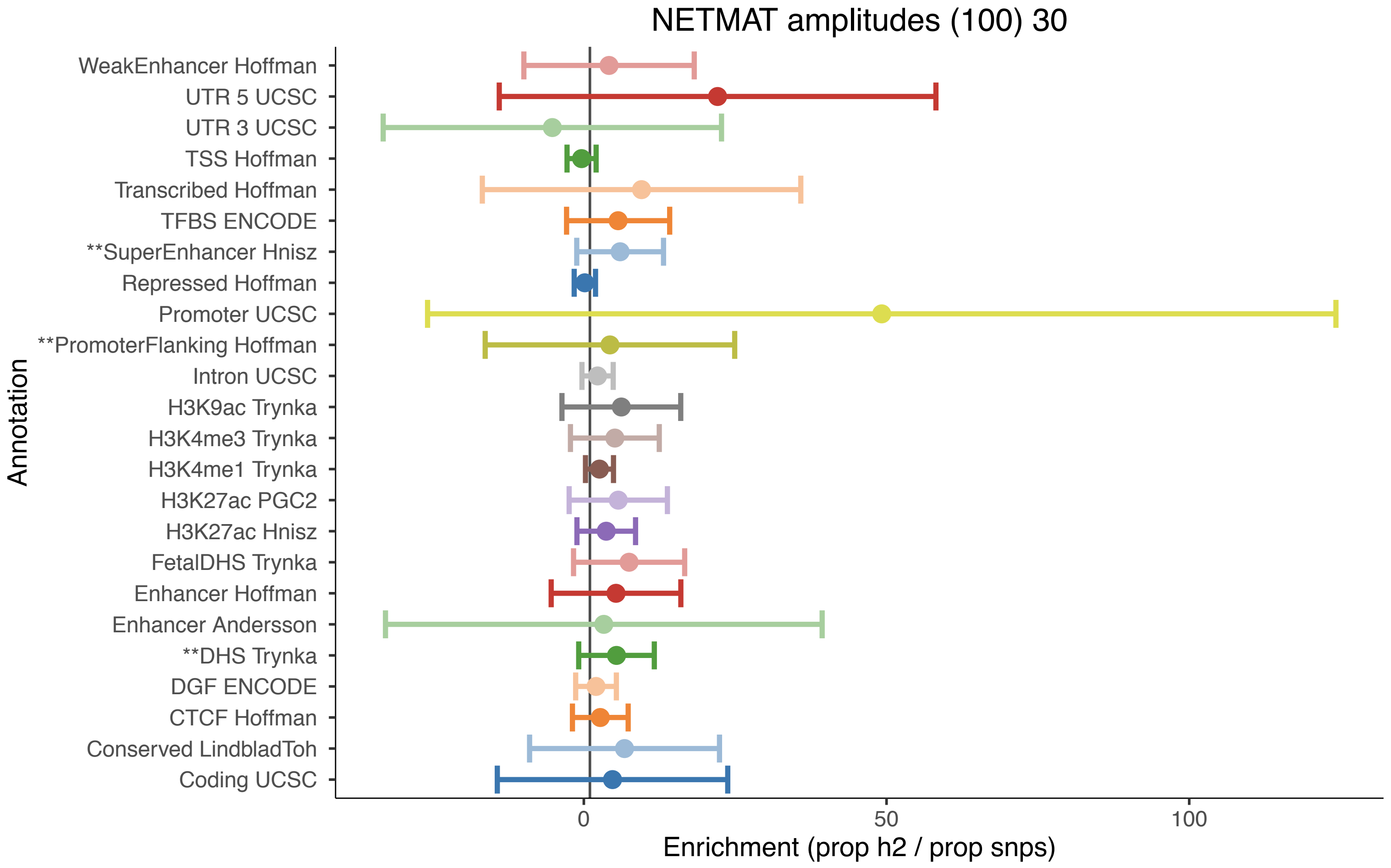
NETMAT amplitudes (100) 30
WeakEnhancer Hoffman
UTR 5 UCSC
UTR 3 UCSC
TSS Hoffman
Transcribed Hoffman
TFBS ENCODE
**SuperEnhancer Hnisz
Repressed Hoffman
Promoter UCSC
**PromoterFlanking Hoffman
Intron UCSC
H3K9ac Trynka
H3K4me3 Trynka
H3K4me1 Trynka
H3K27ac PGC2
H3K27ac Hnisz
FetalDHS Trynka
Enhancer Hoffman
Enhancer Andersson
**DHS Trynka
DGF ENCODE
CTCF Hoffman
Conserved LindbladToh
Coding UCSC
Annotation
0
50
100
Enrichment (prop h2 / prop snps)

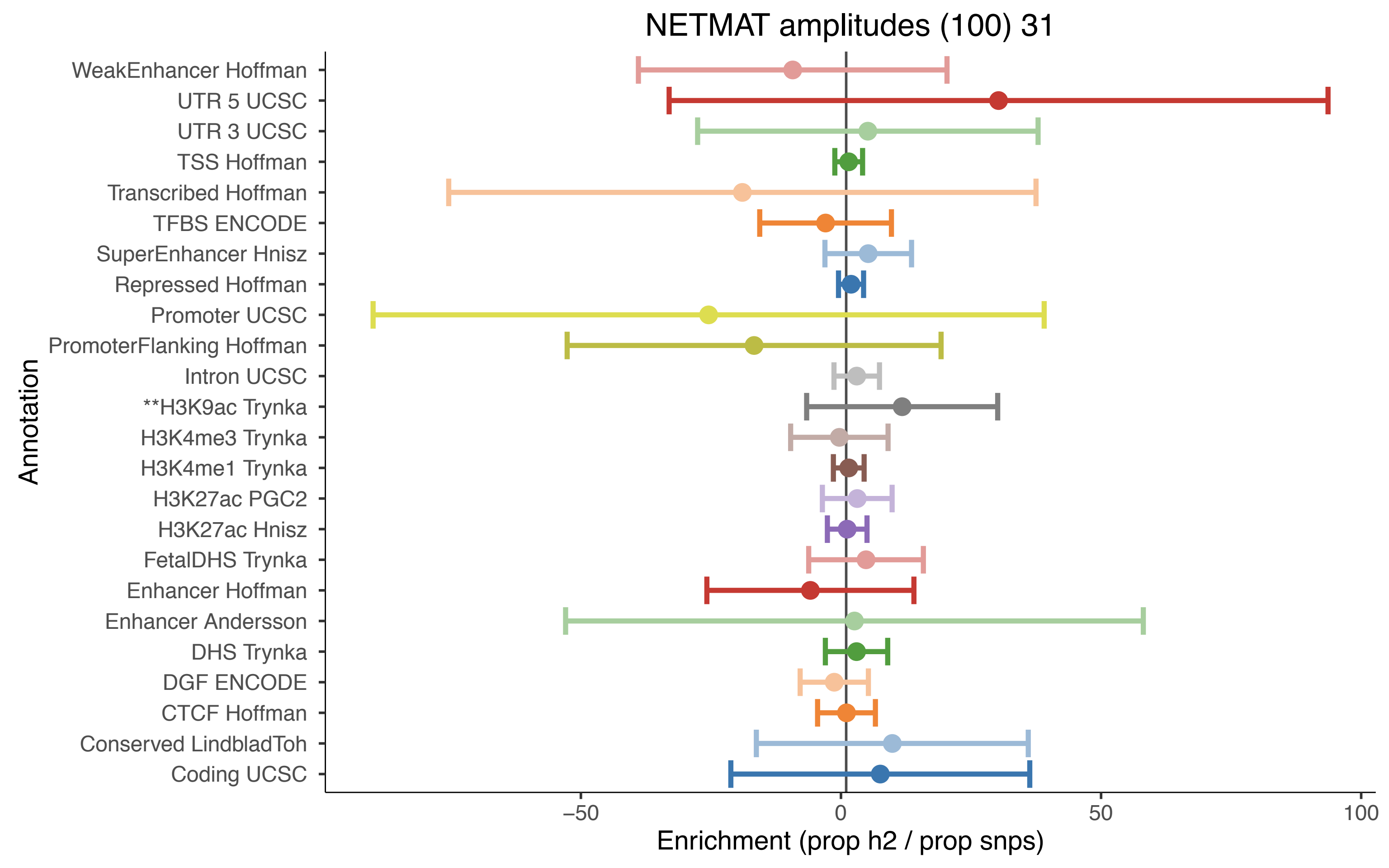

NETMAT amplitudes (100) 31
WeakEnhancer Hoffman
UTR 5 UCSC
UTR 3 UCSC
TSS Hoffman
Transcribed Hoffman
TFBS ENCODE
SuperEnhancer Hnisz
Repressed Hoffman
Promoter UCSC
PromoterFlanking Hoffman
Intron UCSC
**H3K9ac Trynka
H3K4me3 Trynka
H3K4me1 Trynka
H3K27ac PGC2
H3K27ac Hnisz
FetalDHS Trynka
Enhancer Hoffman
Enhancer Andersson
DHS Trynka
DGF ENCODE
CTCF Hoffman
Conserved LindbladToh
Coding UCSC
Annotation
−50
0
50
100
Enrichment (prop h2 / prop snps)

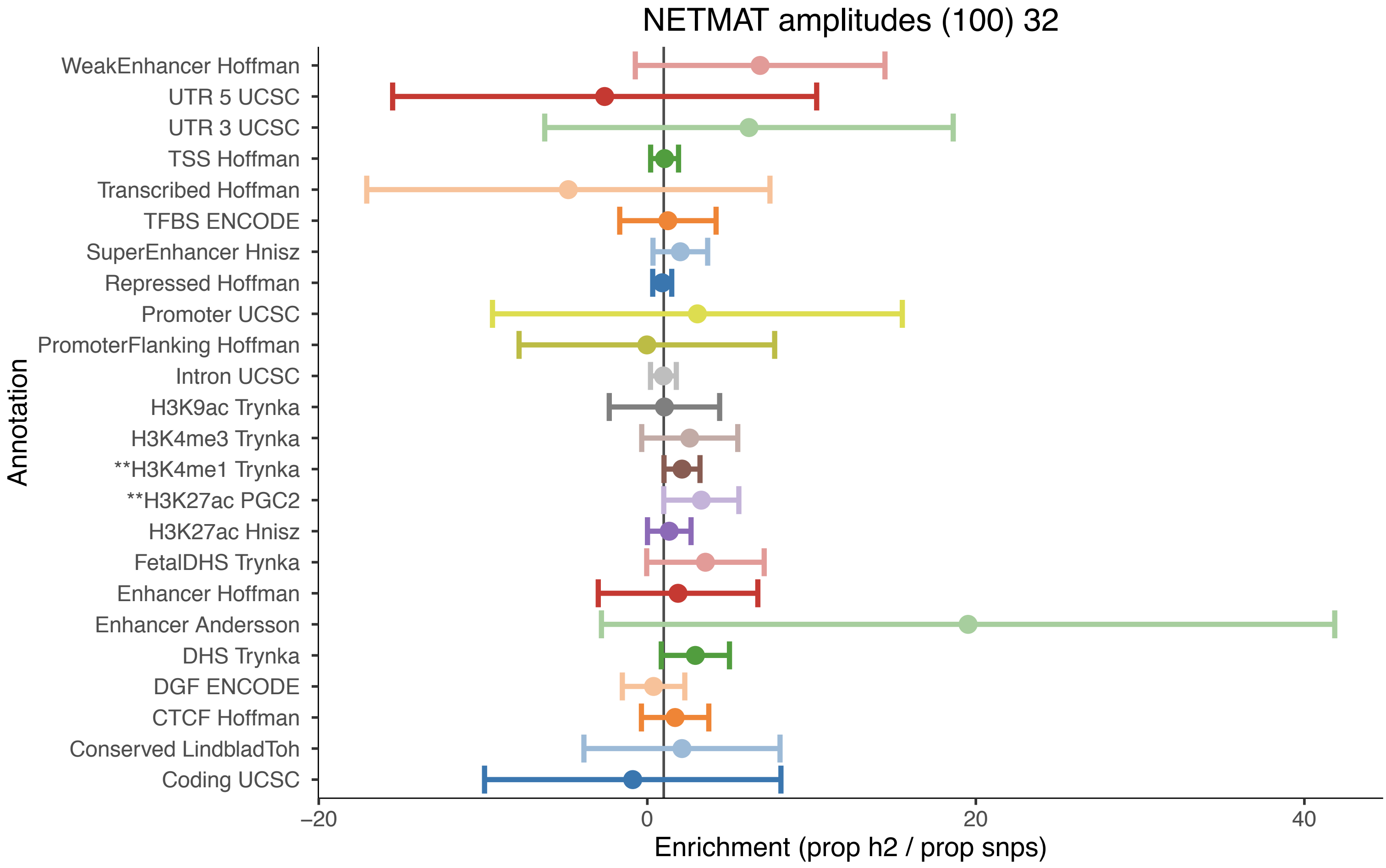

NETMAT amplitudes (100) 32
WeakEnhancer Hoffman
UTR 5 UCSC
UTR 3 UCSC
TSS Hoffman
Transcribed Hoffman
TFBS ENCODE
SuperEnhancer Hnisz
Repressed Hoffman
Promoter UCSC
PromoterFlanking Hoffman
Intron UCSC
H3K9ac Trynka
H3K4me3 Trynka
**H3K4me1 Trynka
**H3K27ac PGC2
H3K27ac Hnisz
FetalDHS Trynka
Enhancer Hoffman
Enhancer Andersson
DHS Trynka
DGF ENCODE
CTCF Hoffman
Conserved LindbladToh
Coding UCSC
Annotation
−20
0
20
40
Enrichment (prop h2 / prop snps)

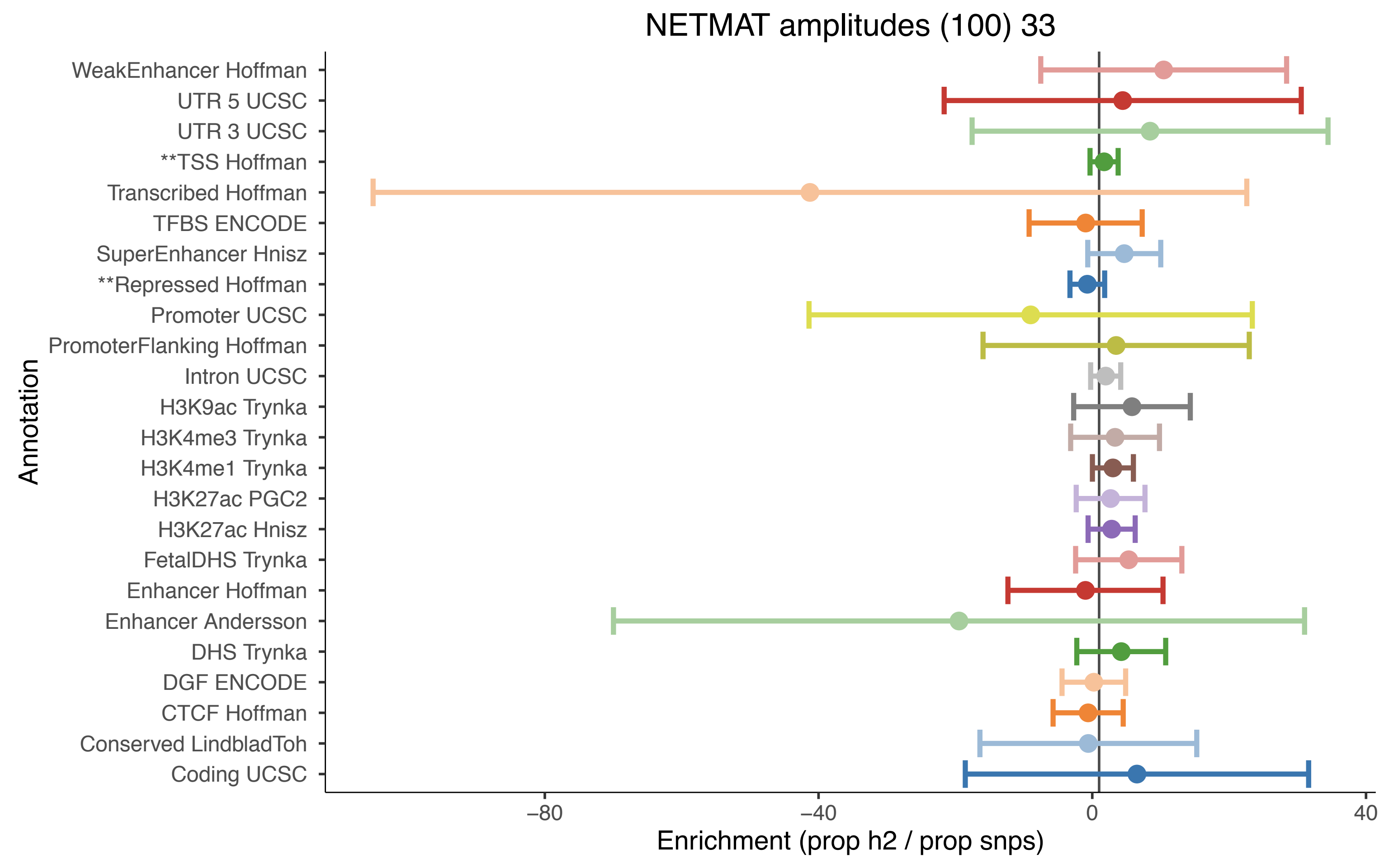
NETMAT amplitudes (100) 33
WeakEnhancer Hoffman
UTR 5 UCSC
UTR 3 UCSC
**TSS Hoffman
Transcribed Hoffman
TFBS ENCODE
SuperEnhancer Hnisz
**Repressed Hoffman
Promoter UCSC
PromoterFlanking Hoffman
Intron UCSC
H3K9ac Trynka
H3K4me3 Trynka
H3K4me1 Trynka
H3K27ac PGC2
H3K27ac Hnisz
FetalDHS Trynka
Enhancer Hoffman
Enhancer Andersson
DHS Trynka
DGF ENCODE
CTCF Hoffman
Conserved LindbladToh
Coding UCSC
Annotation
−80
−40
0
40
Enrichment (prop h2 / prop snps)

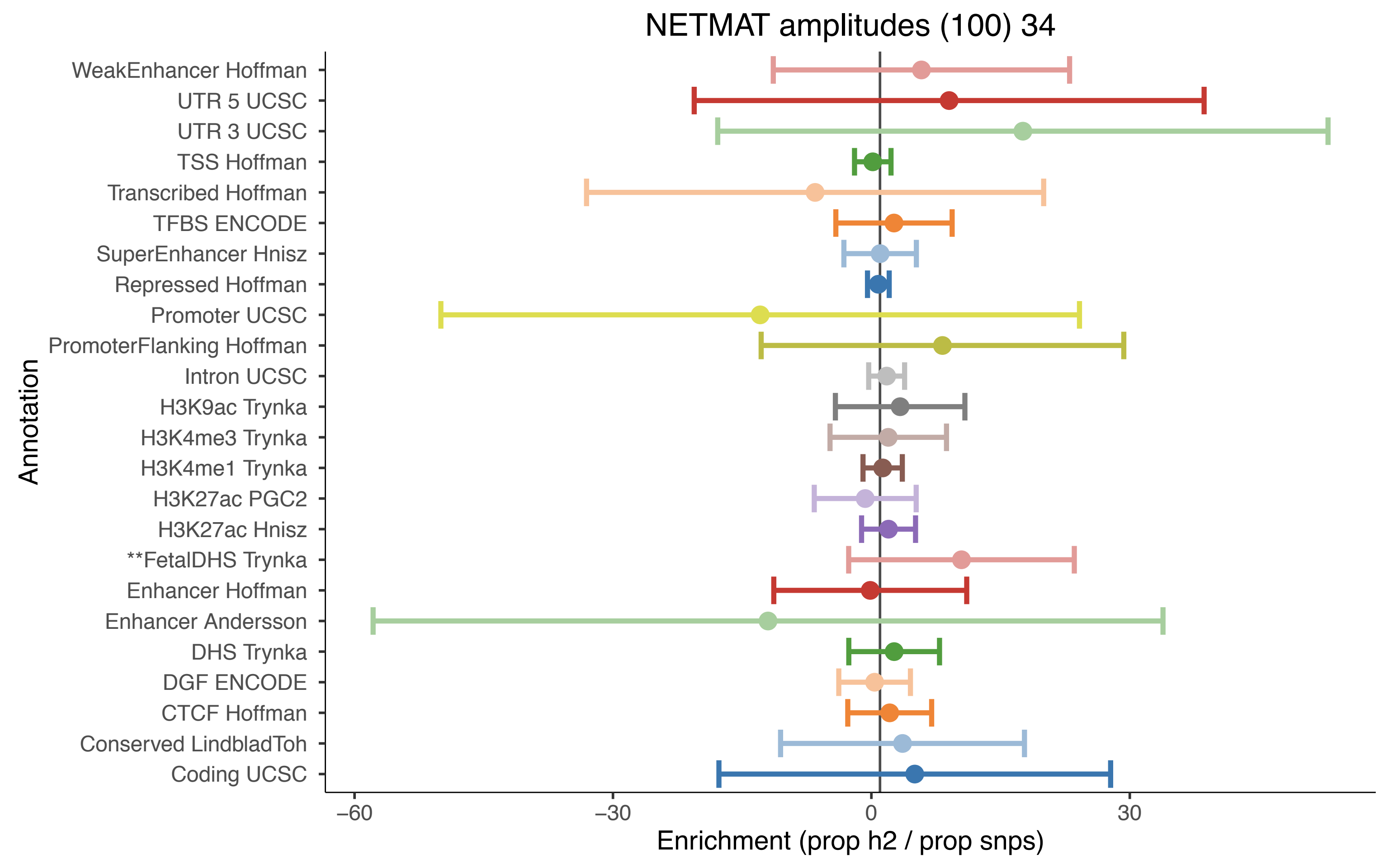

NETMAT amplitudes (100) 34
WeakEnhancer Hoffman
UTR 5 UCSC
UTR 3 UCSC
TSS Hoffman
Transcribed Hoffman
TFBS ENCODE
SuperEnhancer Hnisz
Repressed Hoffman
Promoter UCSC
PromoterFlanking Hoffman
Intron UCSC
H3K9ac Trynka
H3K4me3 Trynka
H3K4me1 Trynka
H3K27ac PGC2
H3K27ac Hnisz
**FetalDHS Trynka
Enhancer Hoffman
Enhancer Andersson
DHS Trynka
DGF ENCODE
CTCF Hoffman
Conserved LindbladToh
Coding UCSC
Annotation
−60
−30
0
30
Enrichment (prop h2 / prop snps)

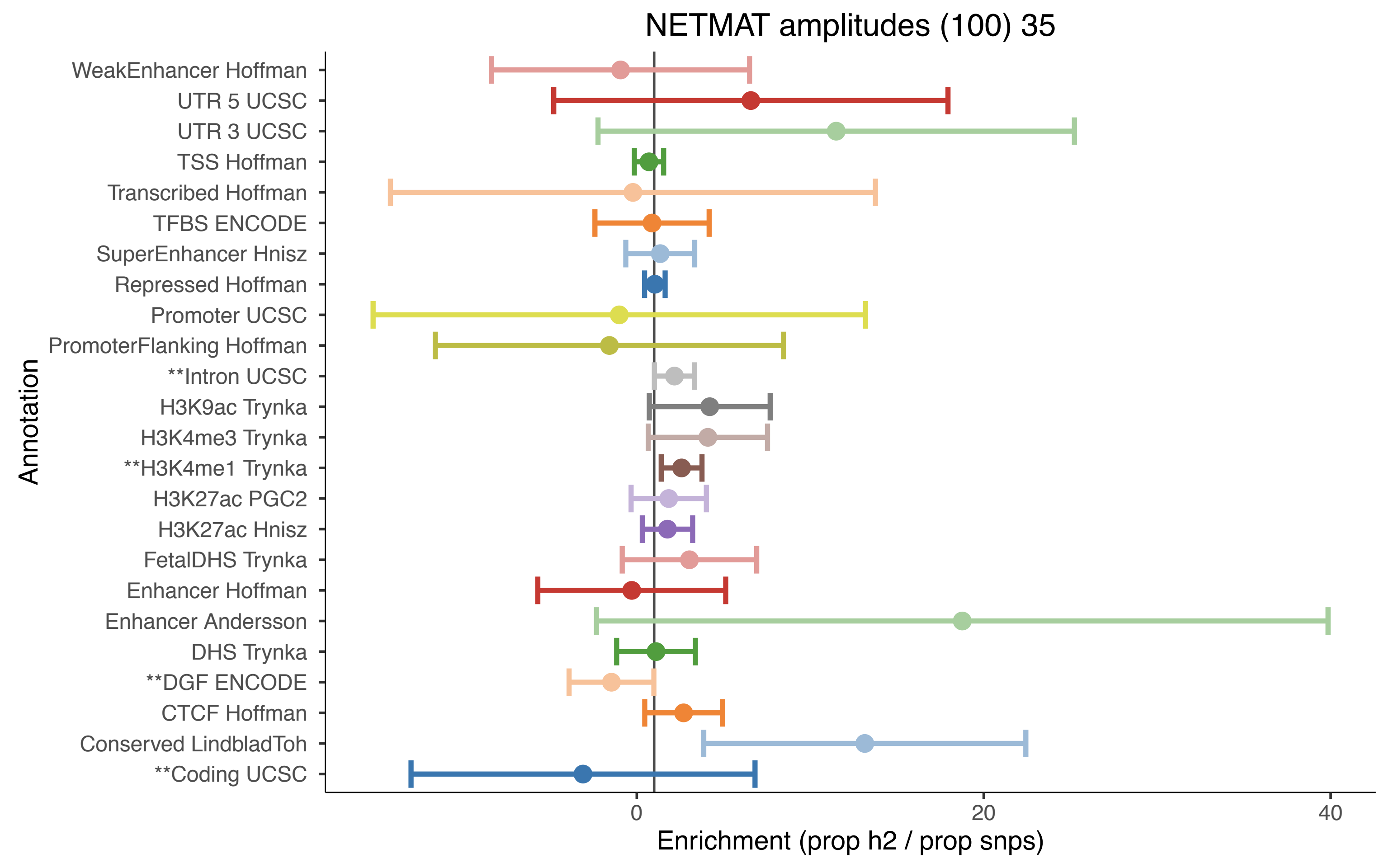

NETMAT amplitudes (100) 35
WeakEnhancer Hoffman
UTR 5 UCSC
UTR 3 UCSC
TSS Hoffman
Transcribed Hoffman
TFBS ENCODE
SuperEnhancer Hnisz
Repressed Hoffman
Promoter UCSC
PromoterFlanking Hoffman
**Intron UCSC
H3K9ac Trynka
H3K4me3 Trynka
**H3K4me1 Trynka
H3K27ac PGC2
H3K27ac Hnisz
FetalDHS Trynka
Enhancer Hoffman
Enhancer Andersson
DHS Trynka
**DGF ENCODE
CTCF Hoffman
Conserved LindbladToh
**Coding UCSC
Annotation
0
20
40
Enrichment (prop h2 / prop snps)

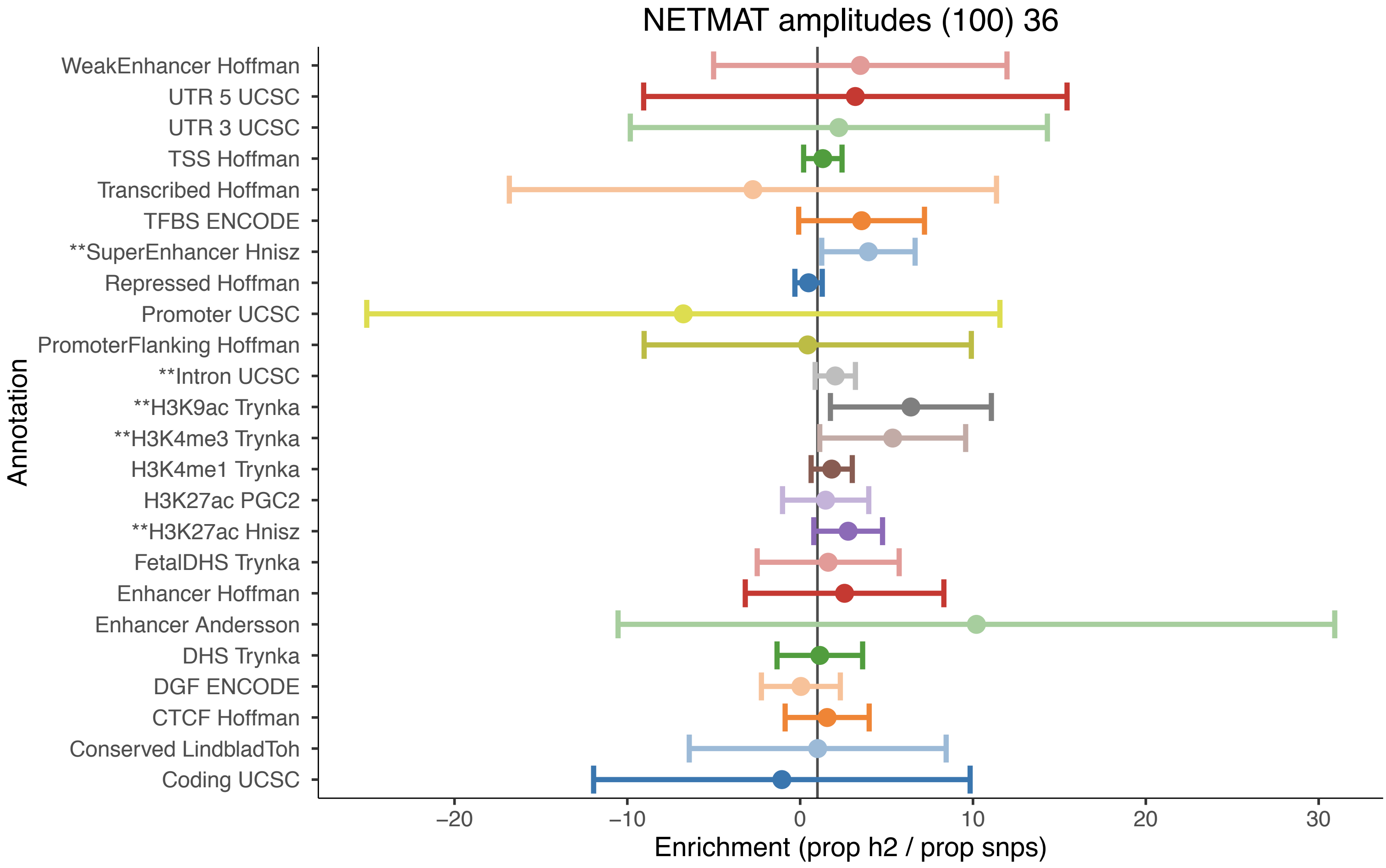
NETMAT amplitudes (100) 36
WeakEnhancer Hoffman
UTR 5 UCSC
UTR 3 UCSC
TSS Hoffman
Transcribed Hoffman
TFBS ENCODE
**SuperEnhancer Hnisz
Repressed Hoffman
Promoter UCSC
PromoterFlanking Hoffman
**Intron UCSC
**H3K9ac Trynka
**H3K4me3 Trynka
H3K4me1 Trynka
H3K27ac PGC2
**H3K27ac Hnisz
FetalDHS Trynka
Enhancer Hoffman
Enhancer Andersson
DHS Trynka
DGF ENCODE
CTCF Hoffman
Conserved LindbladToh
Coding UCSC
Annotation
−20
−10
0
10
20
30
Enrichment (prop h2 / prop snps)

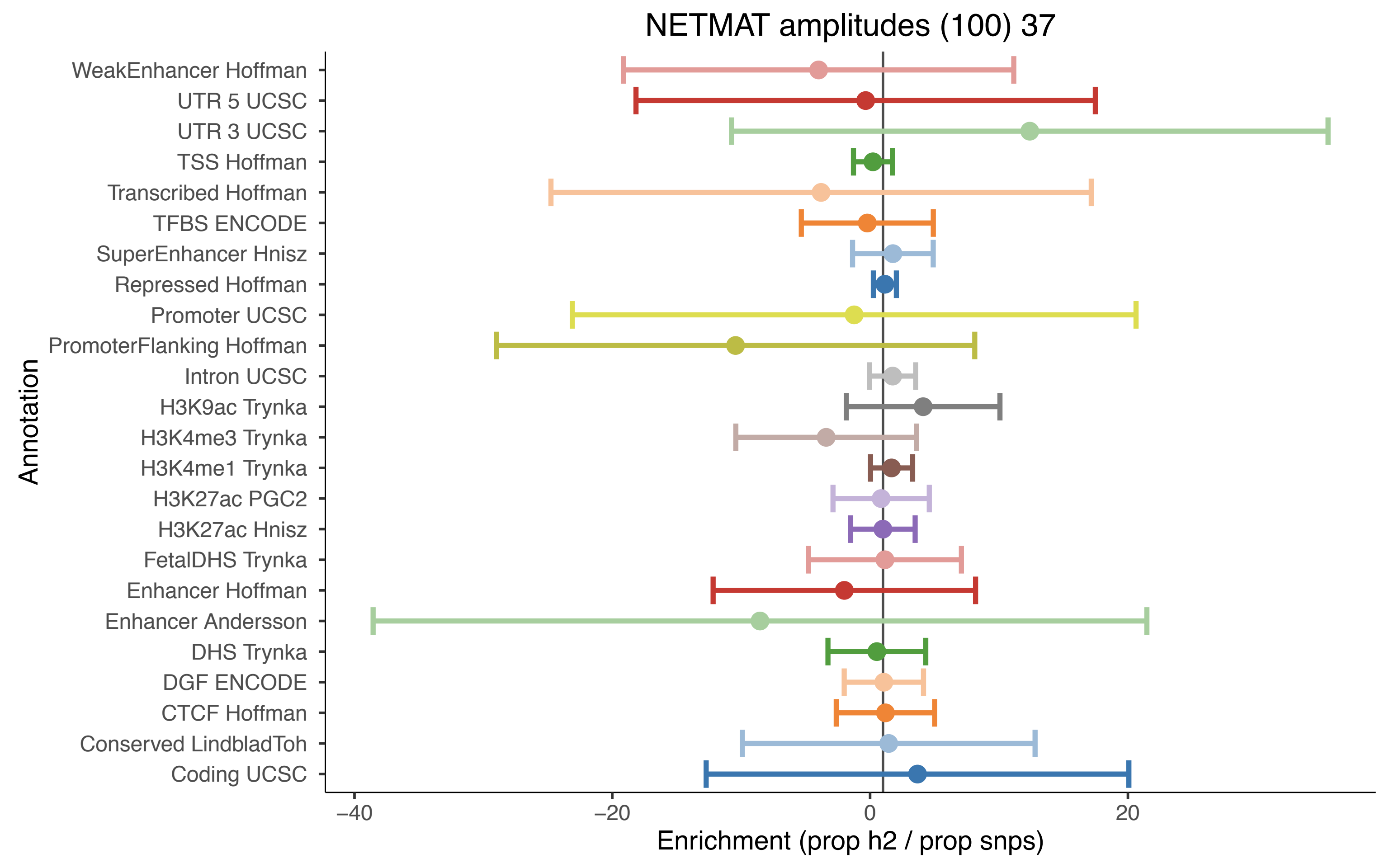

NETMAT amplitudes (100) 37
WeakEnhancer Hoffman
UTR 5 UCSC
UTR 3 UCSC
TSS Hoffman
Transcribed Hoffman
TFBS ENCODE
SuperEnhancer Hnisz
Repressed Hoffman
Promoter UCSC
PromoterFlanking Hoffman
Intron UCSC
H3K9ac Trynka
H3K4me3 Trynka
H3K4me1 Trynka
H3K27ac PGC2
H3K27ac Hnisz
FetalDHS Trynka
Enhancer Hoffman
Enhancer Andersson
DHS Trynka
DGF ENCODE
CTCF Hoffman
Conserved LindbladToh
Coding UCSC
Annotation
−40
−20
0
20
Enrichment (prop h2 / prop snps)

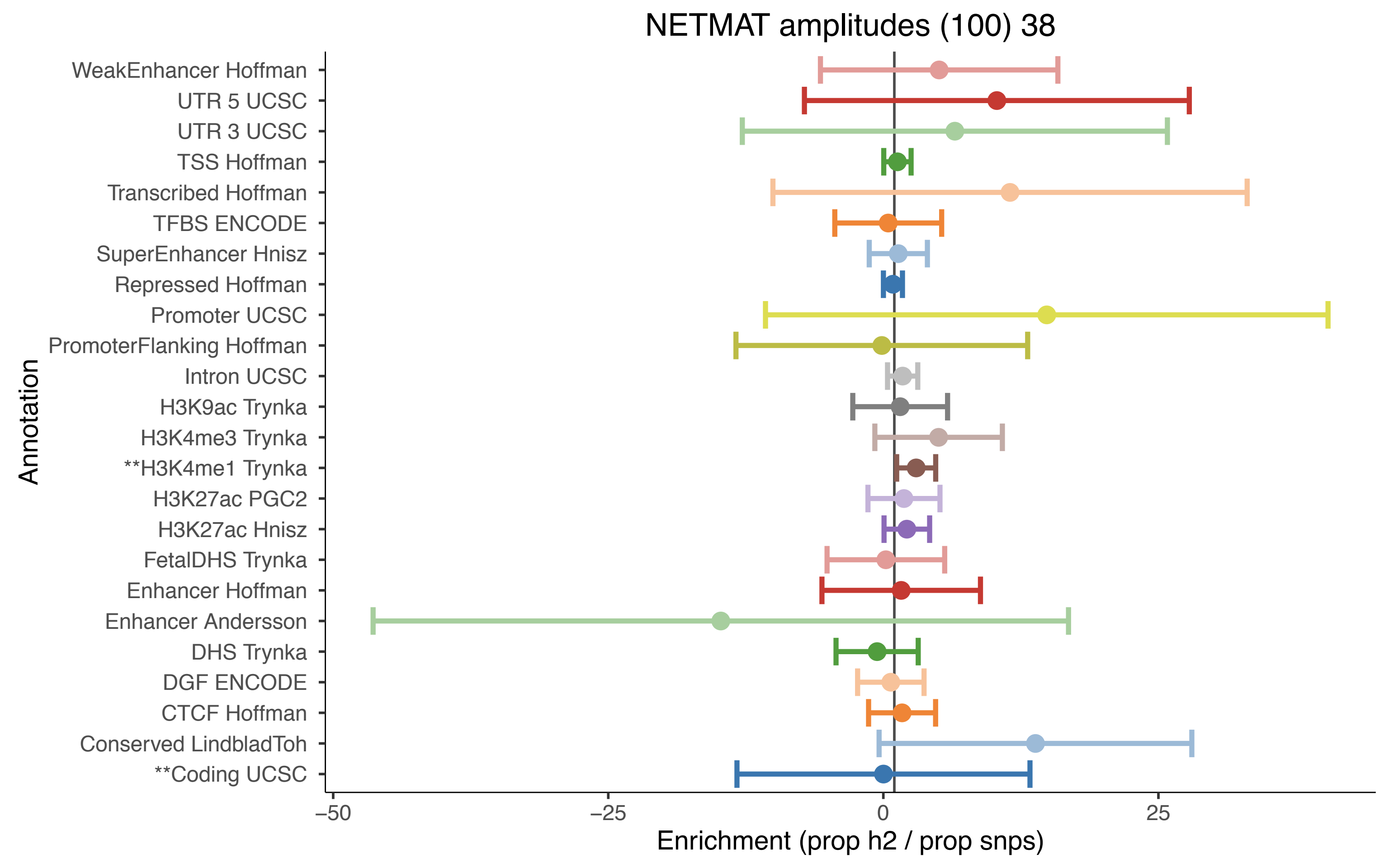
NETMAT amplitudes (100) 38
WeakEnhancer Hoffman
UTR 5 UCSC
UTR 3 UCSC
TSS Hoffman
Transcribed Hoffman
TFBS ENCODE
SuperEnhancer Hnisz
Repressed Hoffman
Promoter UCSC
PromoterFlanking Hoffman
Intron UCSC
H3K9ac Trynka
H3K4me3 Trynka
**H3K4me1 Trynka
H3K27ac PGC2
H3K27ac Hnisz
FetalDHS Trynka
Enhancer Hoffman
Enhancer Andersson
DHS Trynka
DGF ENCODE
CTCF Hoffman
Conserved LindbladToh
**Coding UCSC
Annotation
−50
−25
0
25
Enrichment (prop h2 / prop snps)

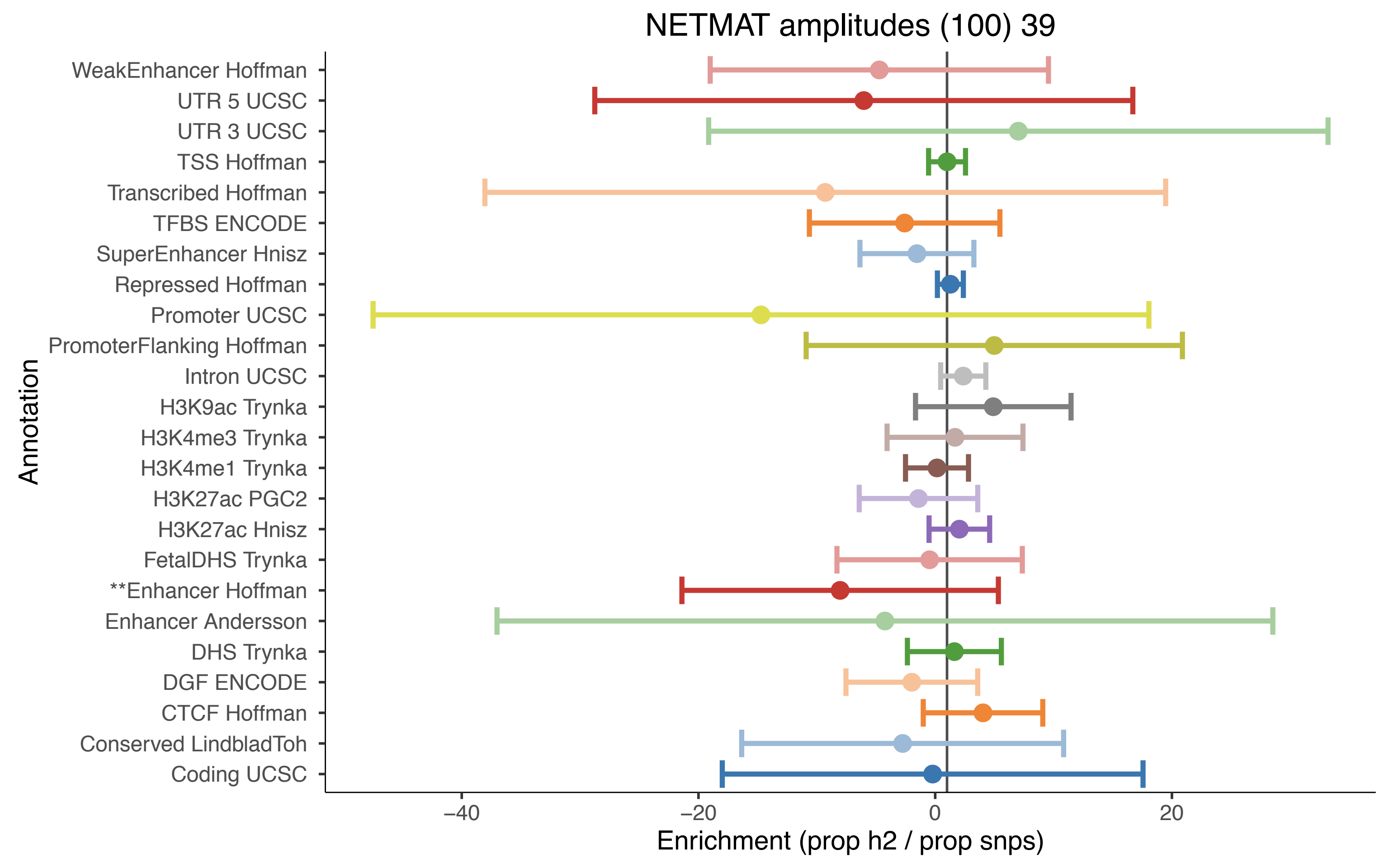
NETMAT amplitudes (100) 39
WeakEnhancer Hoffman
UTR 5 UCSC
UTR 3 UCSC
TSS Hoffman
Transcribed Hoffman
TFBS ENCODE
SuperEnhancer Hnisz
Repressed Hoffman
Promoter UCSC
PromoterFlanking Hoffman
Intron UCSC
H3K9ac Trynka
H3K4me3 Trynka
H3K4me1 Trynka
H3K27ac PGC2
H3K27ac Hnisz
FetalDHS Trynka
**Enhancer Hoffman
Enhancer Andersson
DHS Trynka
DGF ENCODE
CTCF Hoffman
Conserved LindbladToh
Coding UCSC
Annotation
−40
−20
0
20
Enrichment (prop h2 / prop snps)

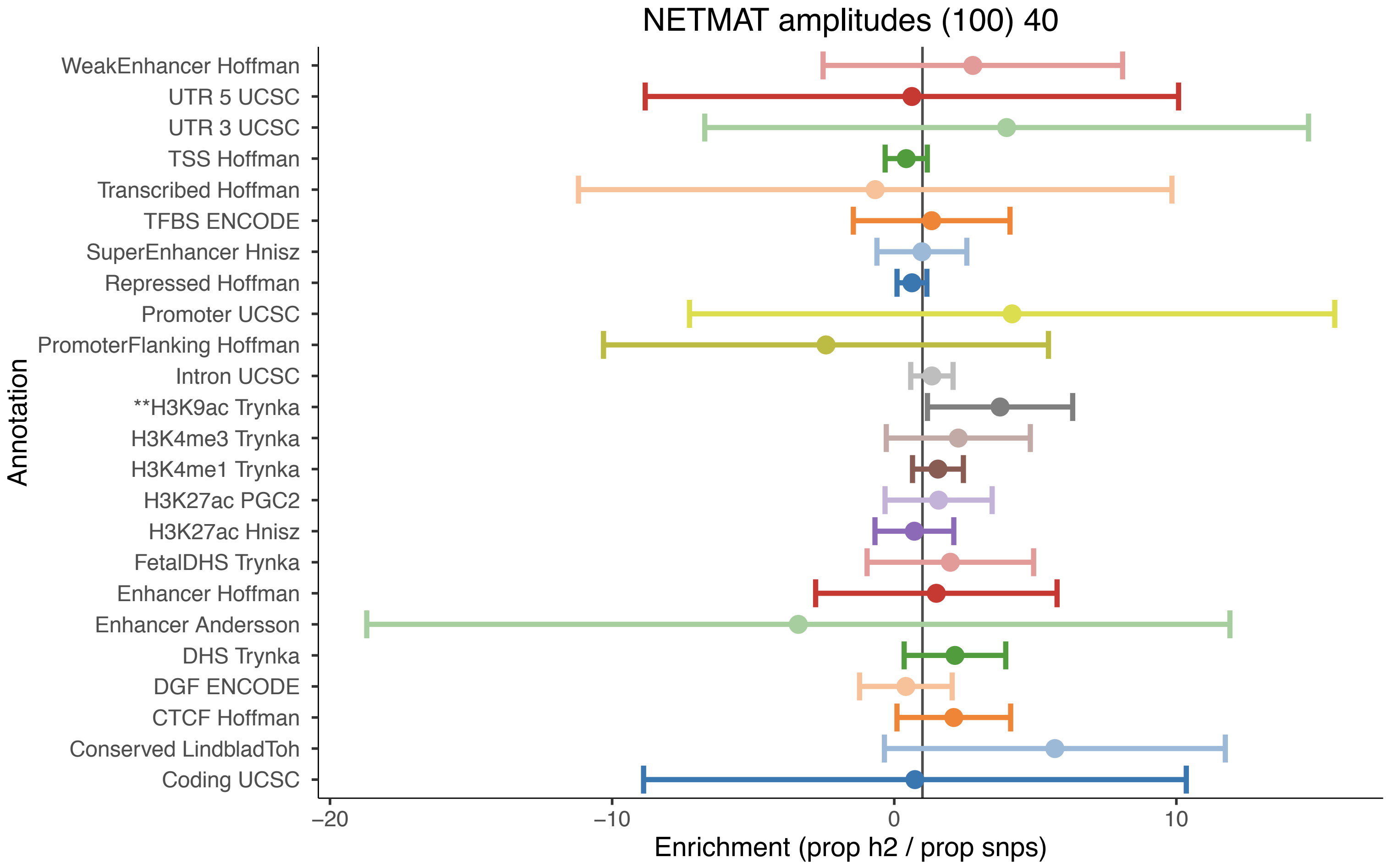

NETMAT amplitudes (100) 40
WeakEnhancer Hoffman
UTR 5 UCSC
UTR 3 UCSC
TSS Hoffman
Transcribed Hoffman
TFBS ENCODE
SuperEnhancer Hnisz
Repressed Hoffman
Promoter UCSC
PromoterFlanking Hoffman
Intron UCSC
**H3K9ac Trynka
H3K4me3 Trynka
H3K4me1 Trynka
H3K27ac PGC2
H3K27ac Hnisz
FetalDHS Trynka
Enhancer Hoffman
Enhancer Andersson
DHS Trynka
DGF ENCODE
CTCF Hoffman
Conserved LindbladToh
Coding UCSC
Annotation
−20
−10
0
10
Enrichment (prop h2 / prop snps)

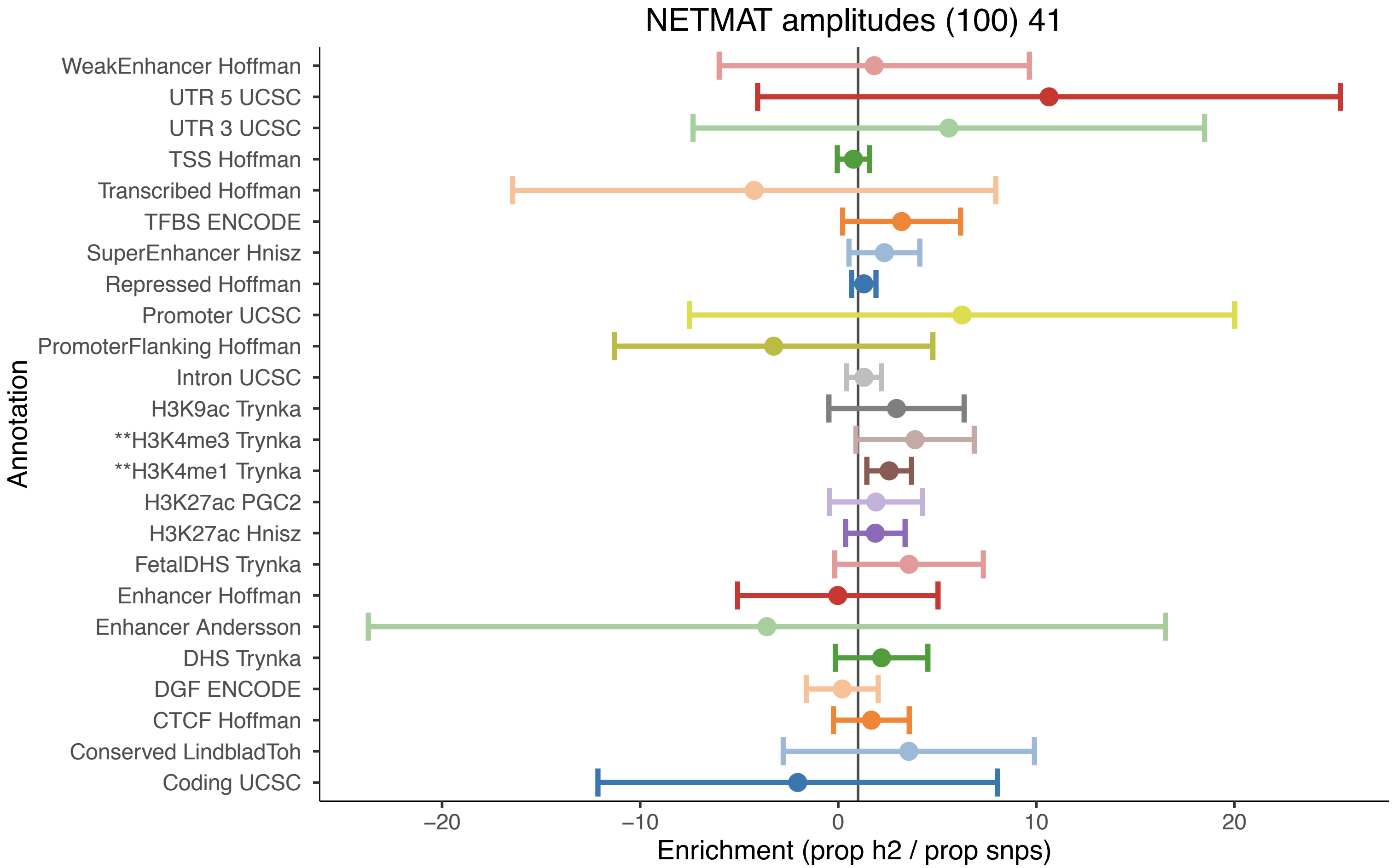

NETMAT amplitudes (100) 41
Annotation
WeakEnhancer Hoffman
UTR 5 UCSC
UTR 3 UCSC
TSS Hoffman
Transcribed Hoffman
TFBS ENCODE
SuperEnhancer Hnisz
Repressed Hoffman
Promoter UCSC
PromoterFlanking Hoffman
Intron UCSC
H3K9ac Trynka
**H3K4me3 Trynka
**H3K4me1 Trynka
H3K27ac PGC2
H3K27ac Hnisz
FetalDHS Trynka
Enhancer Hoffman
Enhancer Andersson
DHS Trynka
DGF ENCODE
CTCF Hoffman
Conserved LindbladToh
Coding UCSC
−20
−10
0
10
20
Enrichment (prop h2 / prop snps)

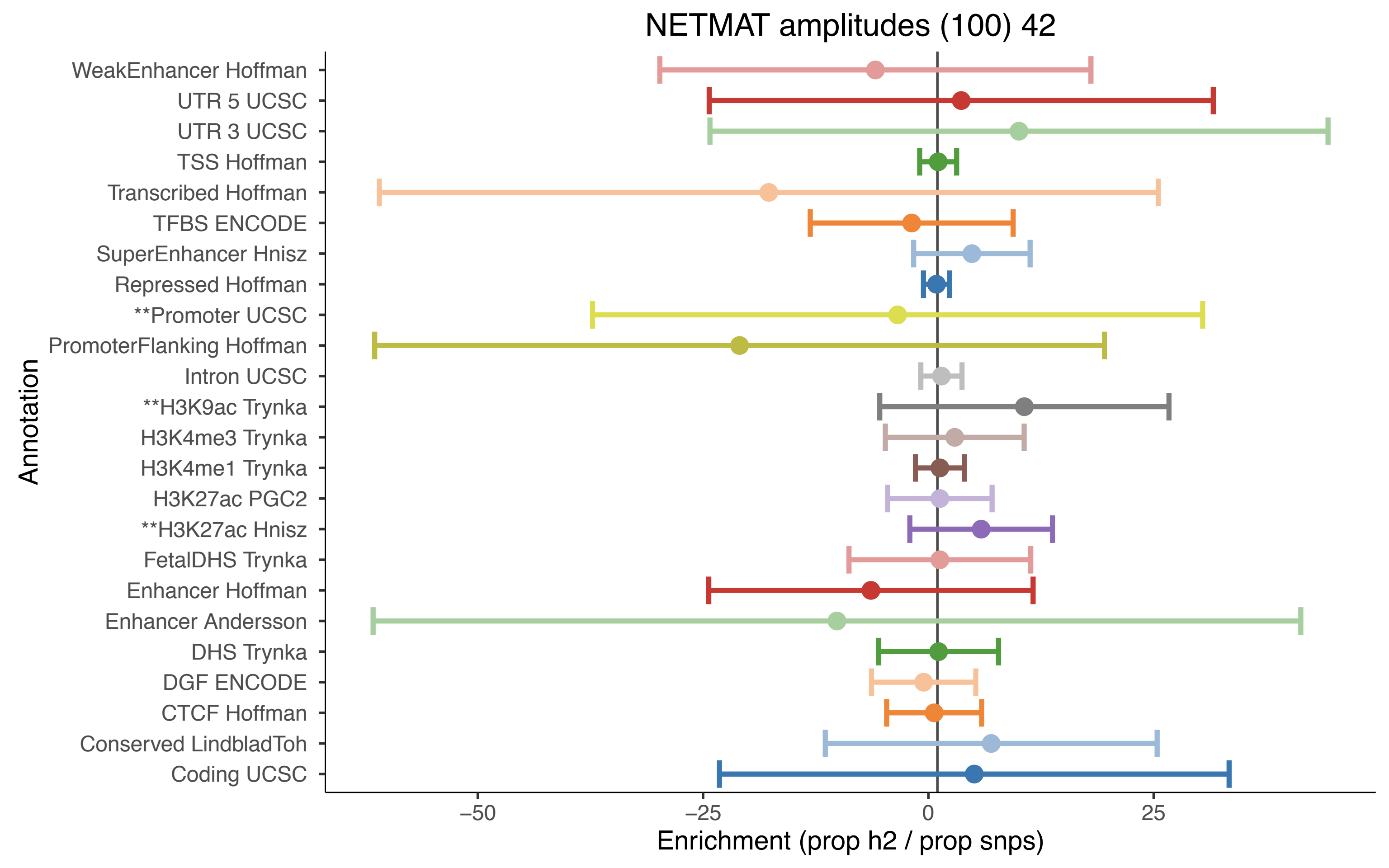
NETMAT amplitudes (100) 42
WeakEnhancer Hoffman
UTR 5 UCSC
UTR 3 UCSC
TSS Hoffman
Transcribed Hoffman
TFBS ENCODE
SuperEnhancer Hnisz
Repressed Hoffman
**Promoter UCSC
PromoterFlanking Hoffman
Intron UCSC
**H3K9ac Trynka
H3K4me3 Trynka
H3K4me1 Trynka
H3K27ac PGC2
**H3K27ac Hnisz
FetalDHS Trynka
Enhancer Hoffman
Enhancer Andersson
DHS Trynka
DGF ENCODE
CTCF Hoffman
Conserved LindbladToh
Coding UCSC
Annotation
−50
−25
0
25
Enrichment (prop h2 / prop snps)

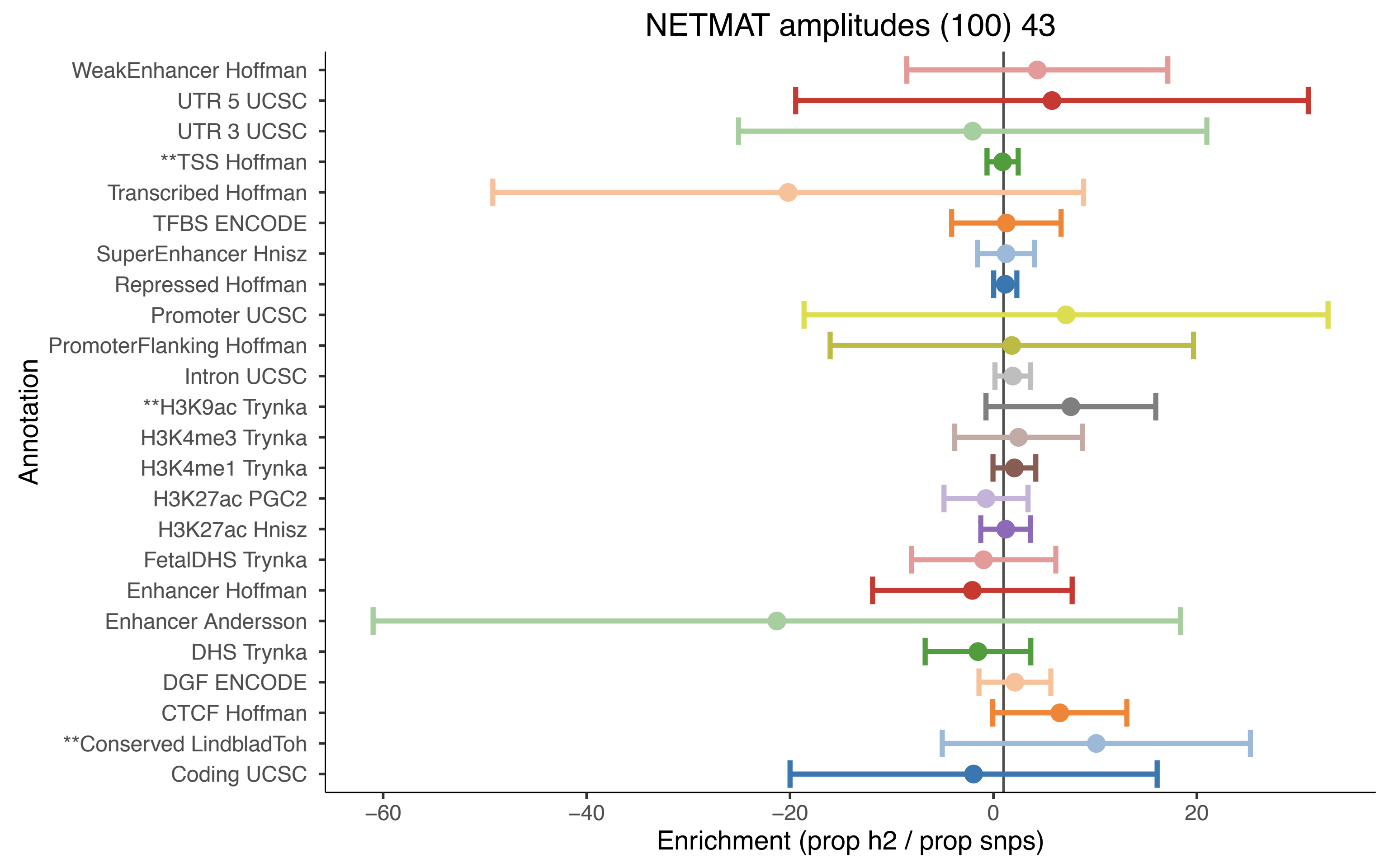
NETMAT amplitudes (100) 43
WeakEnhancer Hoffman
UTR 5 UCSC
UTR 3 UCSC
**TSS Hoffman
Transcribed Hoffman
TFBS ENCODE
SuperEnhancer Hnisz
Repressed Hoffman
Promoter UCSC
PromoterFlanking Hoffman
Intron UCSC
**H3K9ac Trynka
H3K4me3 Trynka
H3K4me1 Trynka
H3K27ac PGC2
H3K27ac Hnisz
FetalDHS Trynka
Enhancer Hoffman
Enhancer Andersson
DHS Trynka
DGF ENCODE
CTCF Hoffman
**Conserved LindbladToh
Coding UCSC
Annotation
−60
−40
−20
0
20
Enrichment (prop h2 / prop snps)

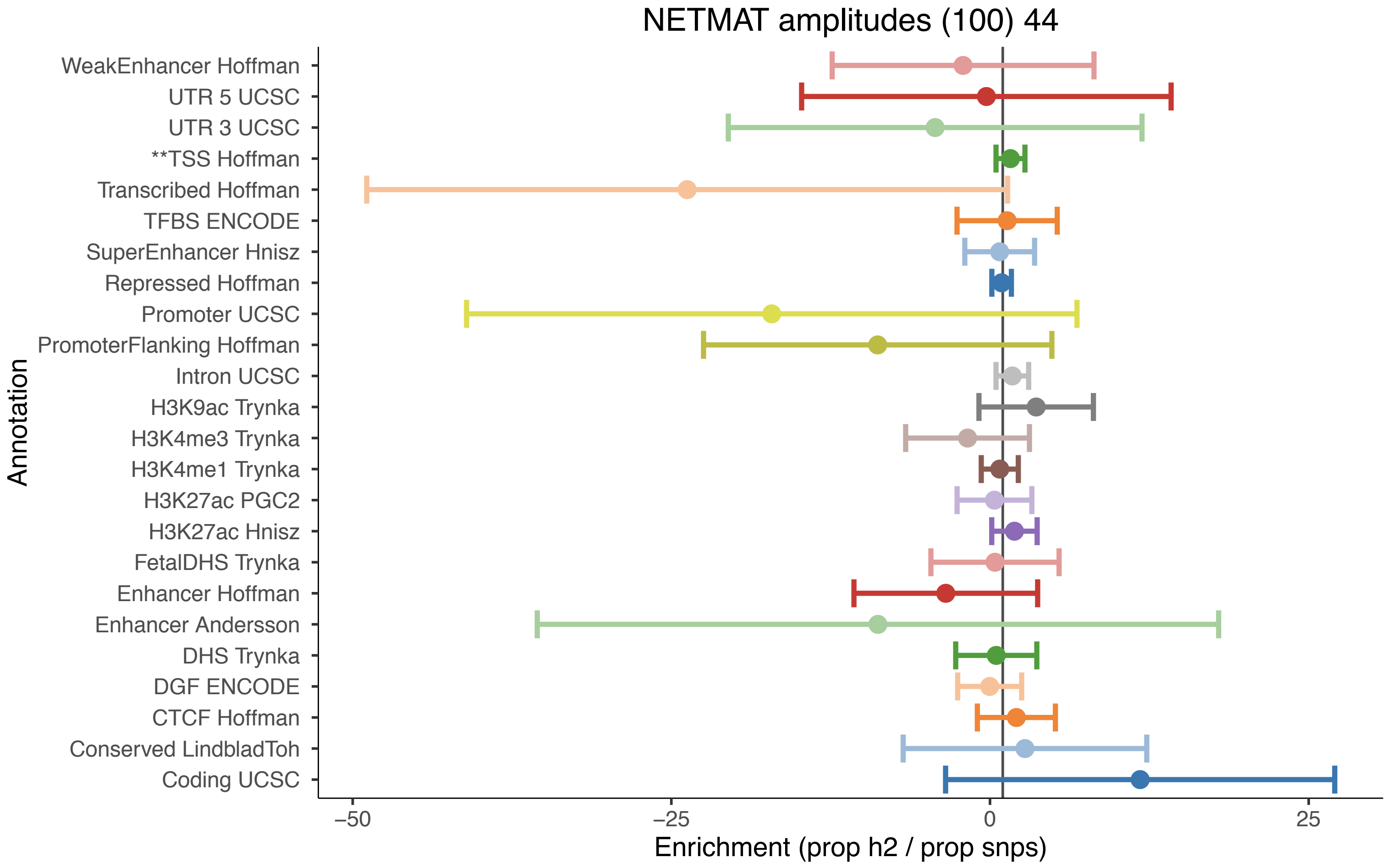

NETMAT amplitudes (100) 44
WeakEnhancer Hoffman
UTR 5 UCSC
UTR 3 UCSC
**TSS Hoffman
Transcribed Hoffman
TFBS ENCODE
SuperEnhancer Hnisz
Repressed Hoffman
Promoter UCSC
PromoterFlanking Hoffman
Intron UCSC
H3K9ac Trynka
H3K4me3 Trynka
H3K4me1 Trynka
H3K27ac PGC2
H3K27ac Hnisz
FetalDHS Trynka
Enhancer Hoffman
Enhancer Andersson
DHS Trynka
DGF ENCODE
CTCF Hoffman
Conserved LindbladToh
Coding UCSC
Annotation
−50
−25
0
25
Enrichment (prop h2 / prop snps)

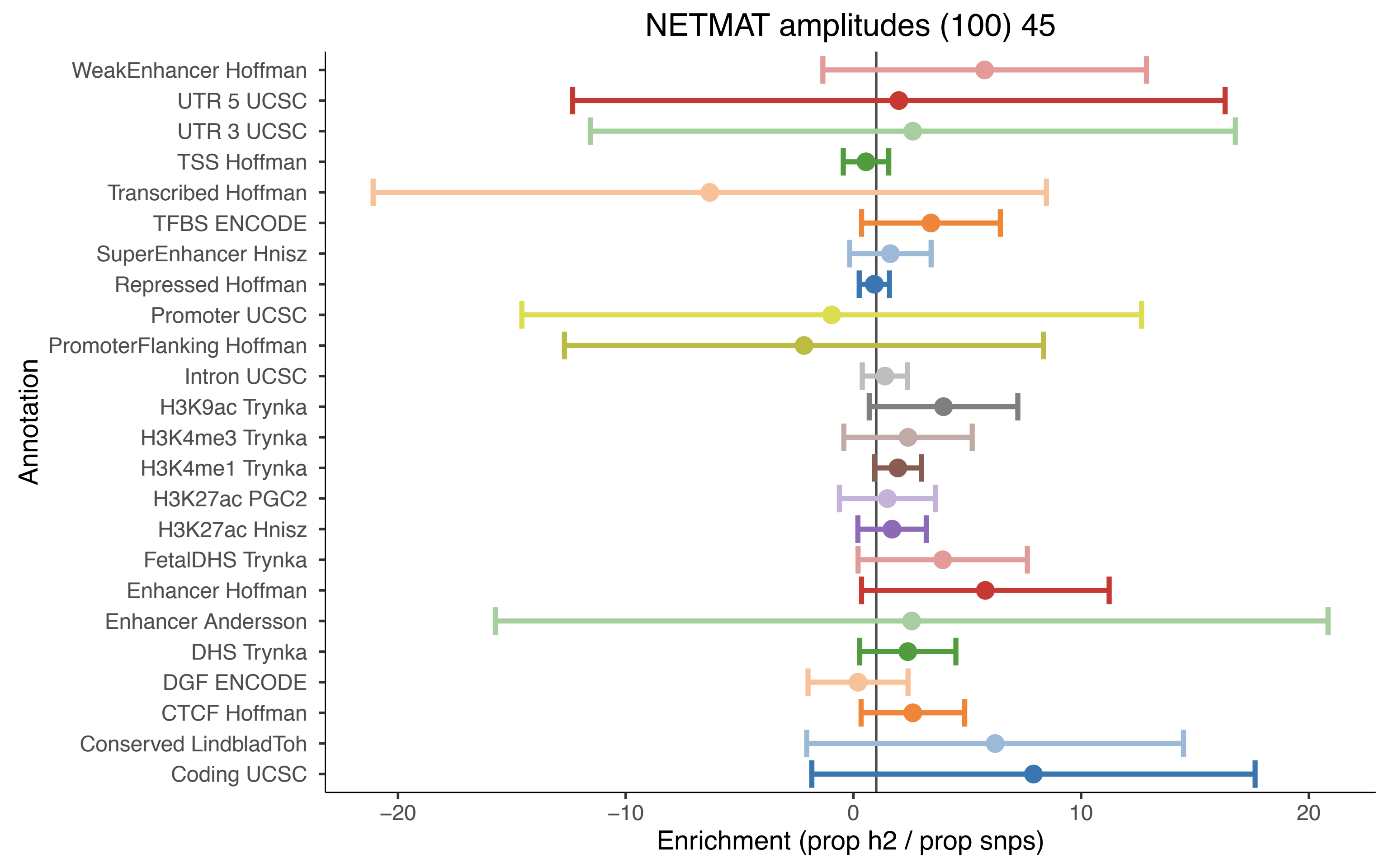
NETMAT amplitudes (100) 45
WeakEnhancer Hoffman
UTR 5 UCSC
UTR 3 UCSC
TSS Hoffman
Transcribed Hoffman
TFBS ENCODE
SuperEnhancer Hnisz
Repressed Hoffman
Promoter UCSC
PromoterFlanking Hoffman
Intron UCSC
H3K9ac Trynka
H3K4me3 Trynka
H3K4me1 Trynka
H3K27ac PGC2
H3K27ac Hnisz
FetalDHS Trynka
Enhancer Hoffman
Enhancer Andersson
DHS Trynka
DGF ENCODE
CTCF Hoffman
Conserved LindbladToh
Coding UCSC
Annotation
−20
−10
0
10
20
Enrichment (prop h2 / prop snps)

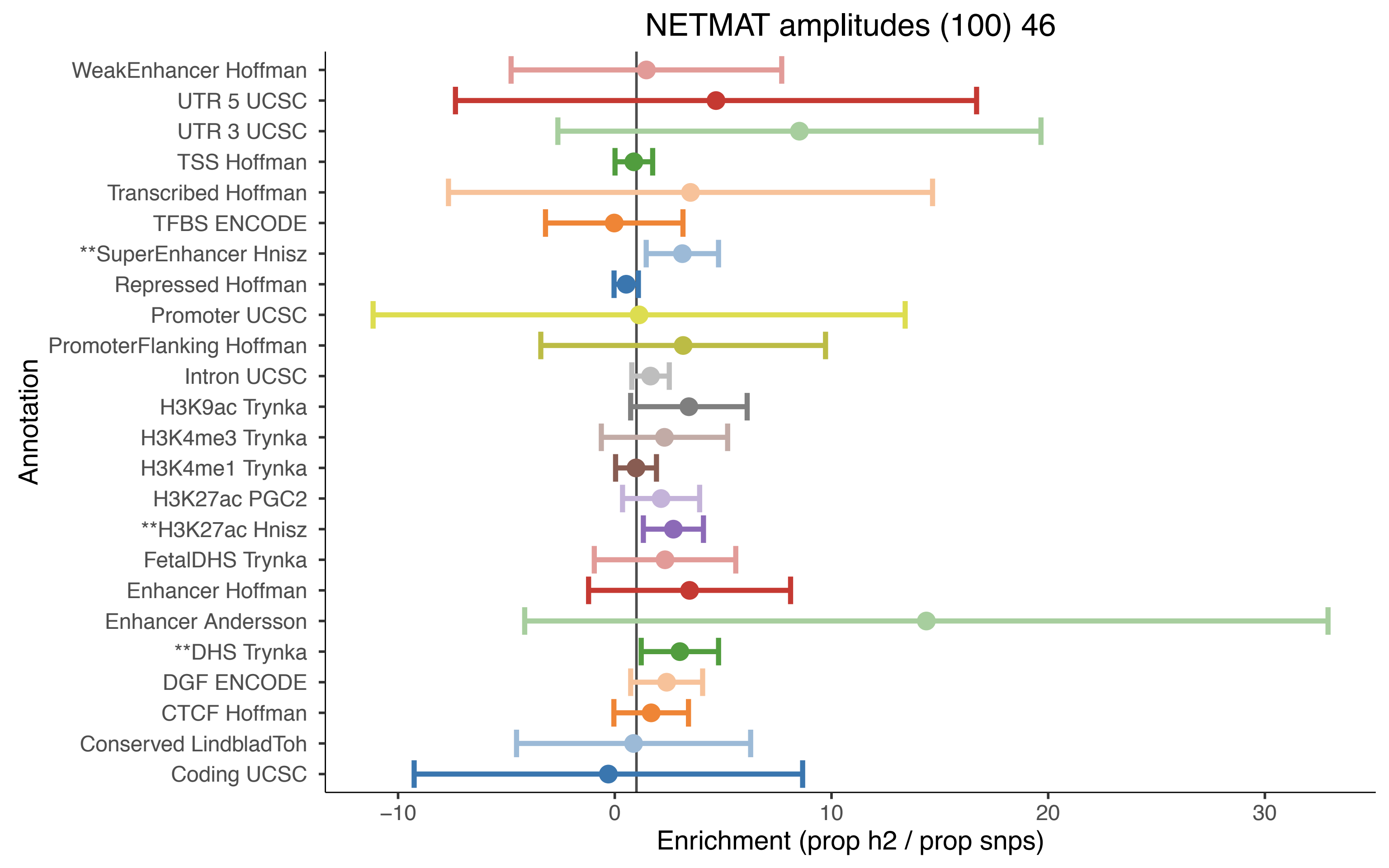
NETMAT amplitudes (100) 46
WeakEnhancer Hoffman
UTR 5 UCSC
UTR 3 UCSC
TSS Hoffman
Transcribed Hoffman
TFBS ENCODE
**SuperEnhancer Hnisz
Repressed Hoffman
Promoter UCSC
PromoterFlanking Hoffman
Intron UCSC
H3K9ac Trynka
H3K4me3 Trynka
H3K4me1 Trynka
H3K27ac PGC2
**H3K27ac Hnisz
FetalDHS Trynka
Enhancer Hoffman
Enhancer Andersson
**DHS Trynka
DGF ENCODE
CTCF Hoffman
Conserved LindbladToh
Coding UCSC
Annotation
−10
0
10
20
30
Enrichment (prop h2 / prop snps)

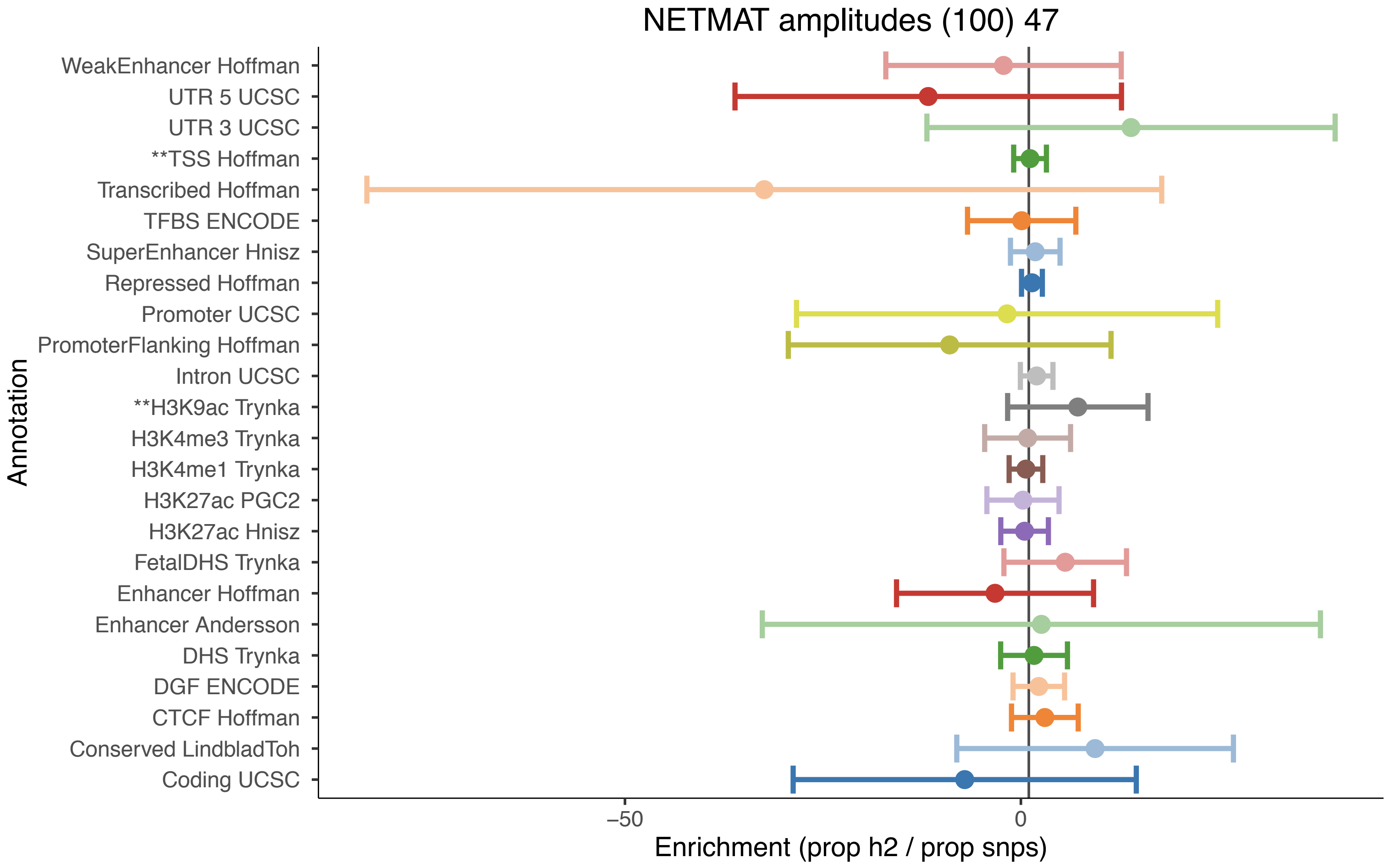
NETMAT amplitudes (100) 47
WeakEnhancer Hoffman
UTR 5 UCSC
UTR 3 UCSC
**TSS Hoffman
Transcribed Hoffman
TFBS ENCODE
SuperEnhancer Hnisz
Repressed Hoffman
Promoter UCSC
PromoterFlanking Hoffman
Intron UCSC
**H3K9ac Trynka
H3K4me3 Trynka
H3K4me1 Trynka
H3K27ac PGC2
H3K27ac Hnisz
FetalDHS Trynka
Enhancer Hoffman
Enhancer Andersson
DHS Trynka
DGF ENCODE
CTCF Hoffman
Conserved LindbladToh
Coding UCSC
Annotation
−50
0
Enrichment (prop h2 / prop snps)

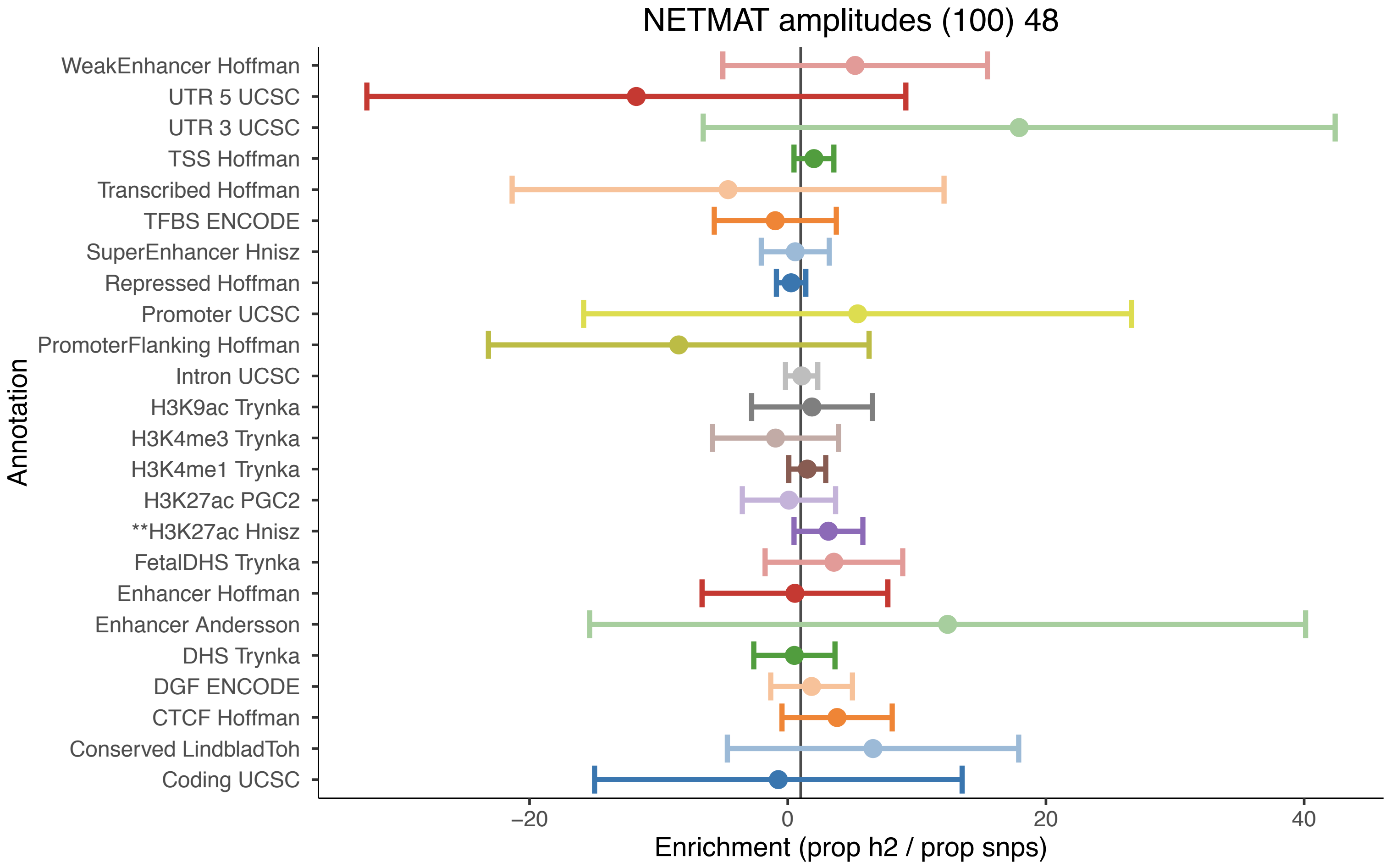
NETMAT amplitudes (100) 48
Annotation
WeakEnhancer Hoffman
UTR 5 UCSC
UTR 3 UCSC
TSS Hoffman
Transcribed Hoffman
TFBS ENCODE
SuperEnhancer Hnisz
Repressed Hoffman
Promoter UCSC
PromoterFlanking Hoffman
Intron UCSC
H3K9ac Trynka
H3K4me3 Trynka
H3K4me1 Trynka
H3K27ac PGC2
**H3K27ac Hnisz
FetalDHS Trynka
Enhancer Hoffman
Enhancer Andersson
DHS Trynka
DGF ENCODE
CTCF Hoffman
Conserved LindbladToh
Coding UCSC
−20
0
20
40
Enrichment (prop h2 / prop snps)

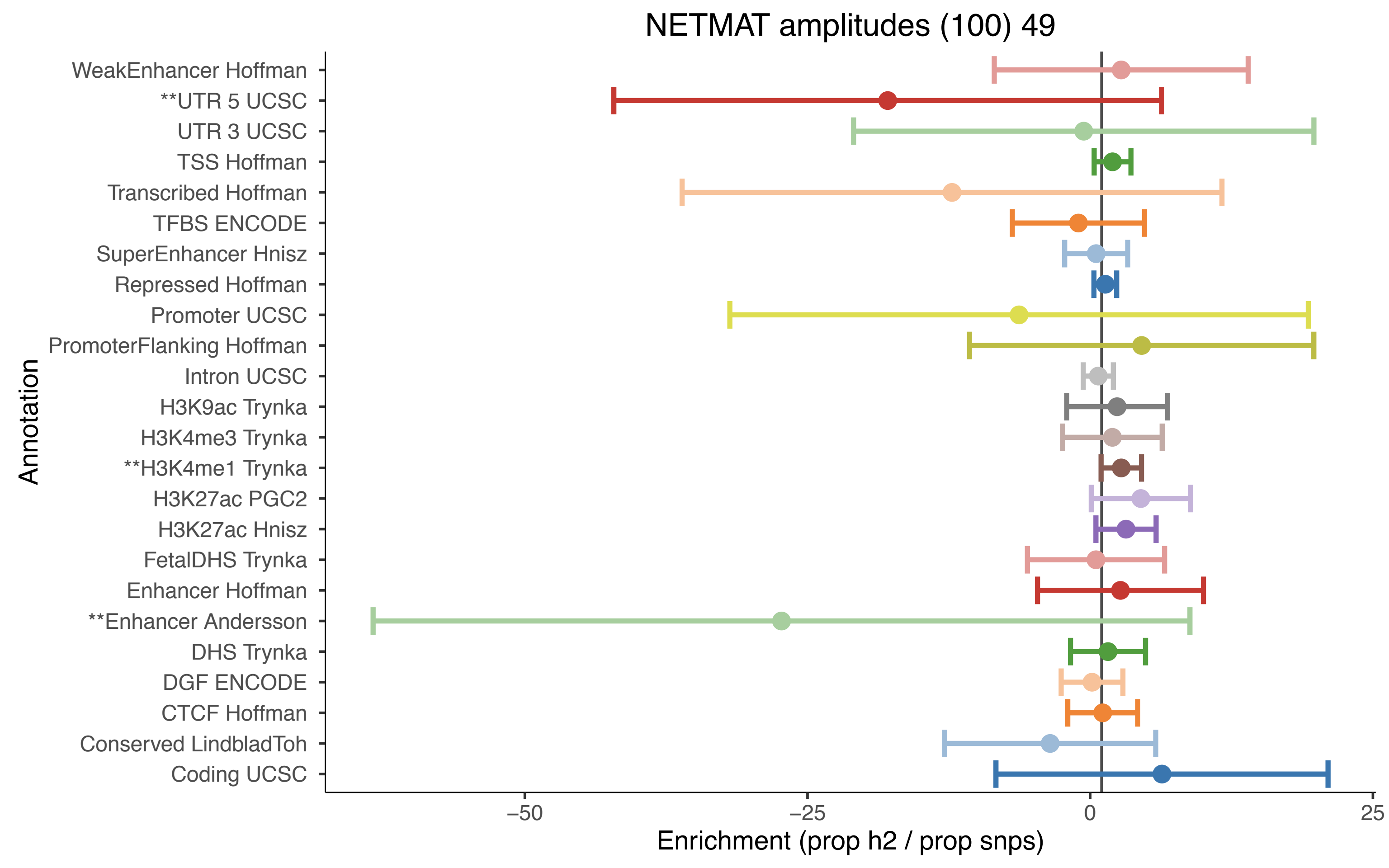
NETMAT amplitudes (100) 49
Annotation
WeakEnhancer Hoffman
**UTR 5 UCSC
UTR 3 UCSC
TSS Hoffman
Transcribed Hoffman
TFBS ENCODE
SuperEnhancer Hnisz
Repressed Hoffman
Promoter UCSC
PromoterFlanking Hoffman
Intron UCSC
H3K9ac Trynka
H3K4me3 Trynka
**H3K4me1 Trynka
H3K27ac PGC2
H3K27ac Hnisz
FetalDHS Trynka
Enhancer Hoffman
**Enhancer Andersson
DHS Trynka
DGF ENCODE
CTCF Hoffman
Conserved LindbladToh
Coding UCSC
−50
−25
0
25
Enrichment (prop h2 / prop snps)

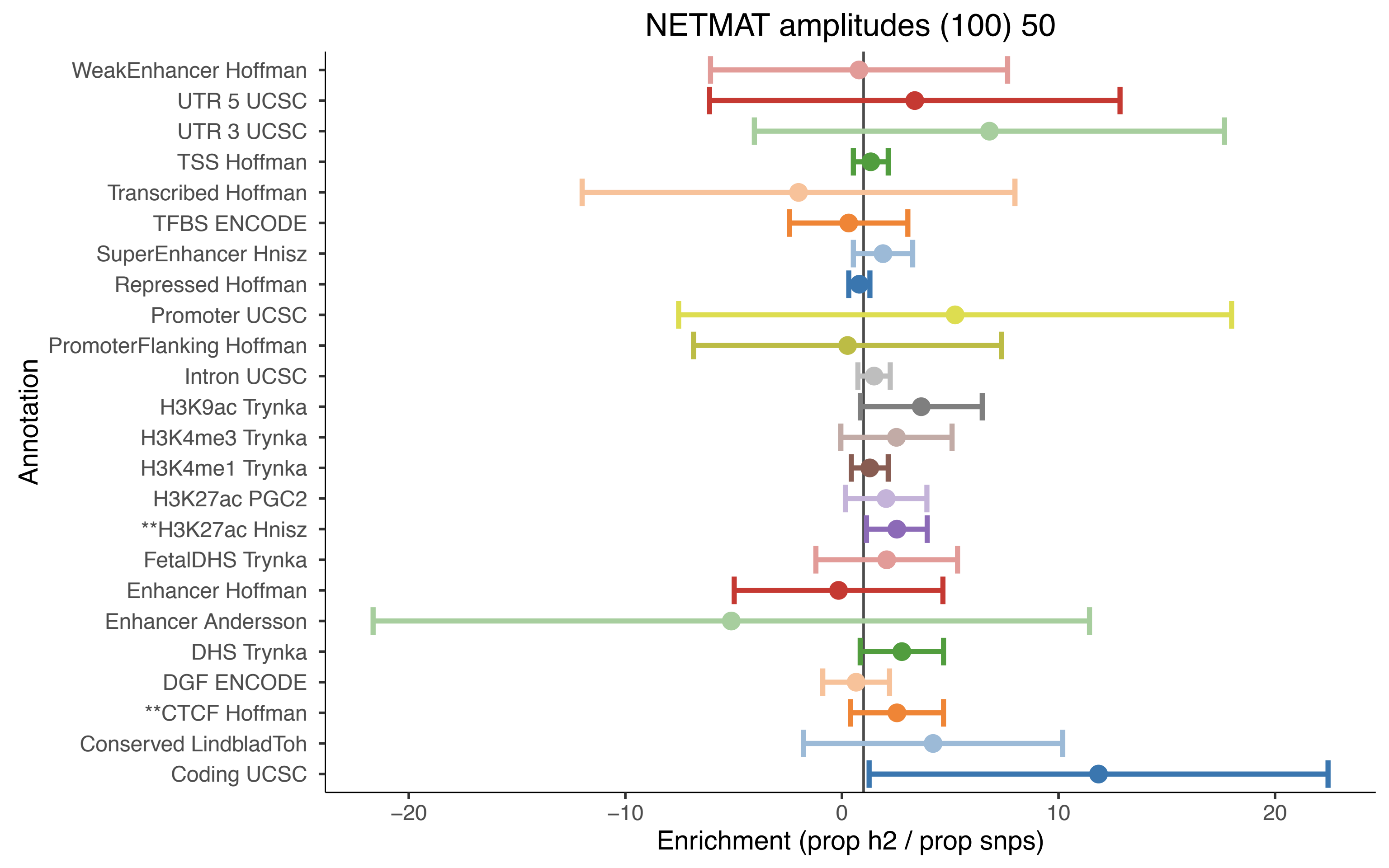

NETMAT amplitudes (100) 50
WeakEnhancer Hoffman
UTR 5 UCSC
UTR 3 UCSC
TSS Hoffman
Transcribed Hoffman
TFBS ENCODE
SuperEnhancer Hnisz
Repressed Hoffman
Promoter UCSC
PromoterFlanking Hoffman
Intron UCSC
H3K9ac Trynka
H3K4me3 Trynka
H3K4me1 Trynka
H3K27ac PGC2
**H3K27ac Hnisz
FetalDHS Trynka
Enhancer Hoffman
Enhancer Andersson
DHS Trynka
DGF ENCODE
**CTCF Hoffman
Conserved LindbladToh
Coding UCSC
Annotation
−20
−10
0
10
20
Enrichment (prop h2 / prop snps)

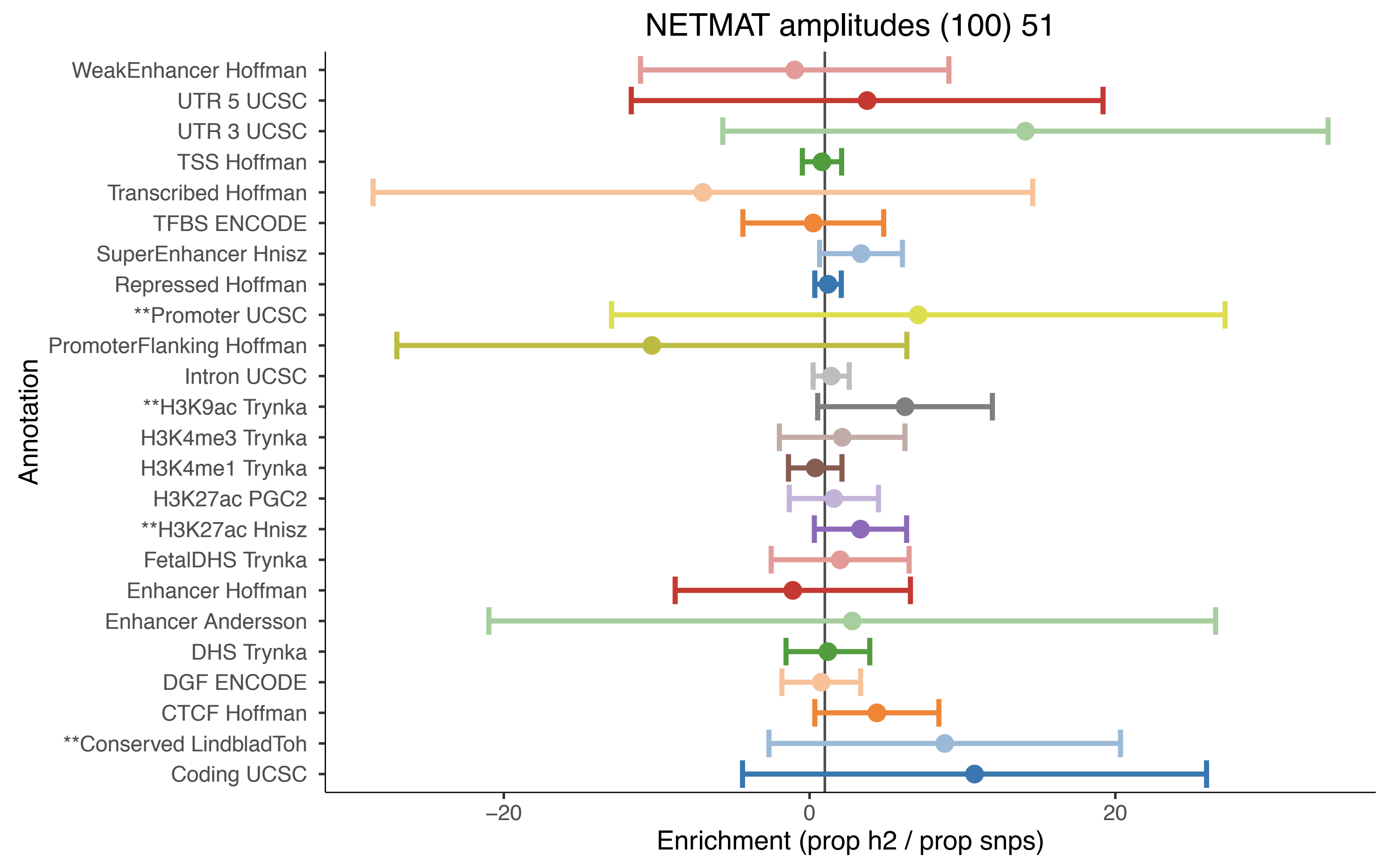
NETMAT amplitudes (100) 51
WeakEnhancer Hoffman
UTR 5 UCSC
UTR 3 UCSC
TSS Hoffman
Transcribed Hoffman
TFBS ENCODE
SuperEnhancer Hnisz
Repressed Hoffman
**Promoter UCSC
PromoterFlanking Hoffman
Intron UCSC
**H3K9ac Trynka
H3K4me3 Trynka
H3K4me1 Trynka
H3K27ac PGC2
**H3K27ac Hnisz
FetalDHS Trynka
Enhancer Hoffman
Enhancer Andersson
DHS Trynka
DGF ENCODE
CTCF Hoffman
**Conserved LindbladToh
Coding UCSC
Annotation
−20
0
20
Enrichment (prop h2 / prop snps)

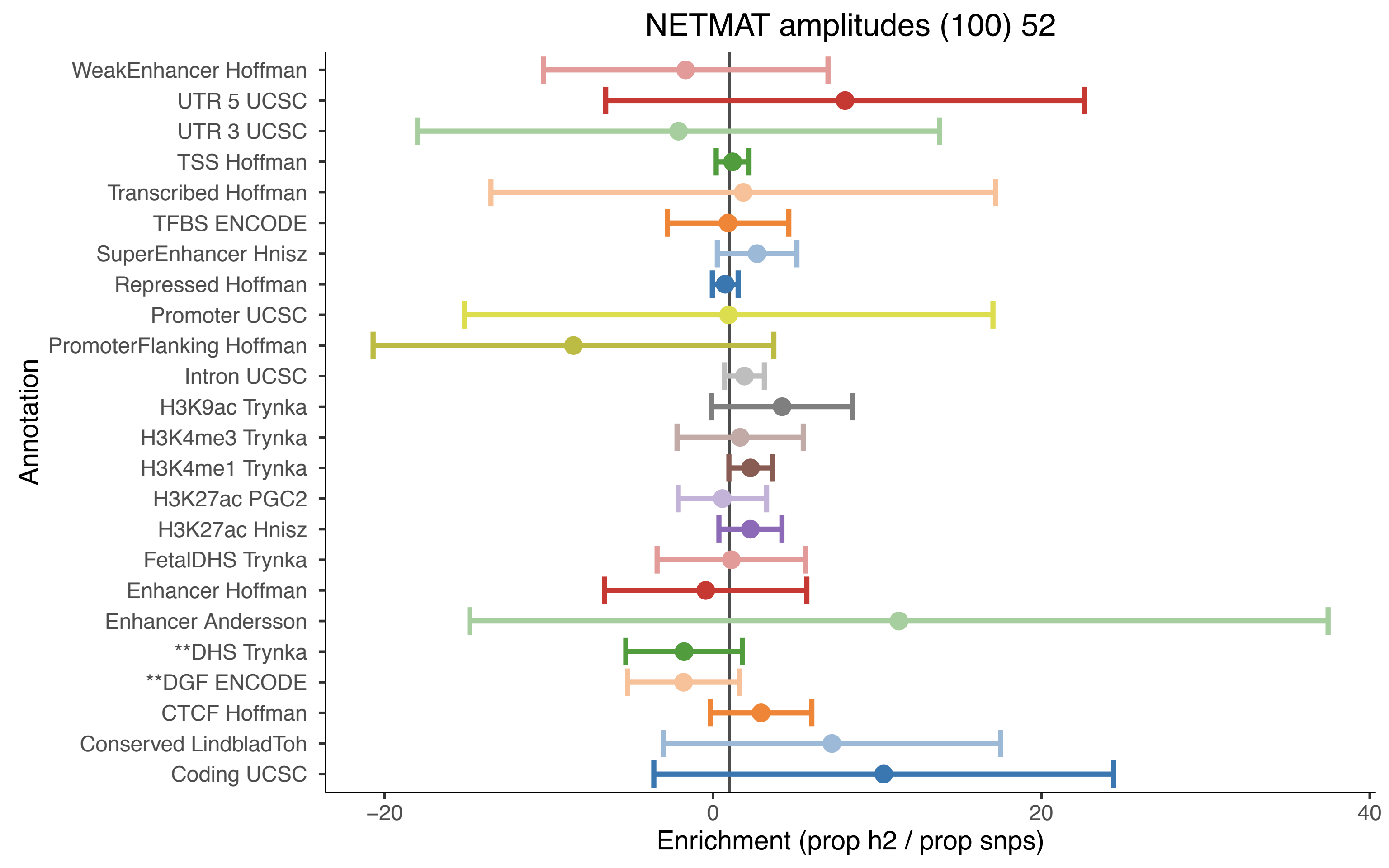
NETMAT amplitudes (100) 52
WeakEnhancer Hoffman
UTR 5 UCSC
UTR 3 UCSC
TSS Hoffman
Transcribed Hoffman
TFBS ENCODE
SuperEnhancer Hnisz
Repressed Hoffman
Promoter UCSC
PromoterFlanking Hoffman
Intron UCSC
H3K9ac Trynka
H3K4me3 Trynka
H3K4me1 Trynka
H3K27ac PGC2
H3K27ac Hnisz
FetalDHS Trynka
Enhancer Hoffman
Enhancer Andersson
**DHS Trynka
**DGF ENCODE
CTCF Hoffman
Conserved LindbladToh
Coding UCSC
Annotation
−20
0
20
40
Enrichment (prop h2 / prop snps)

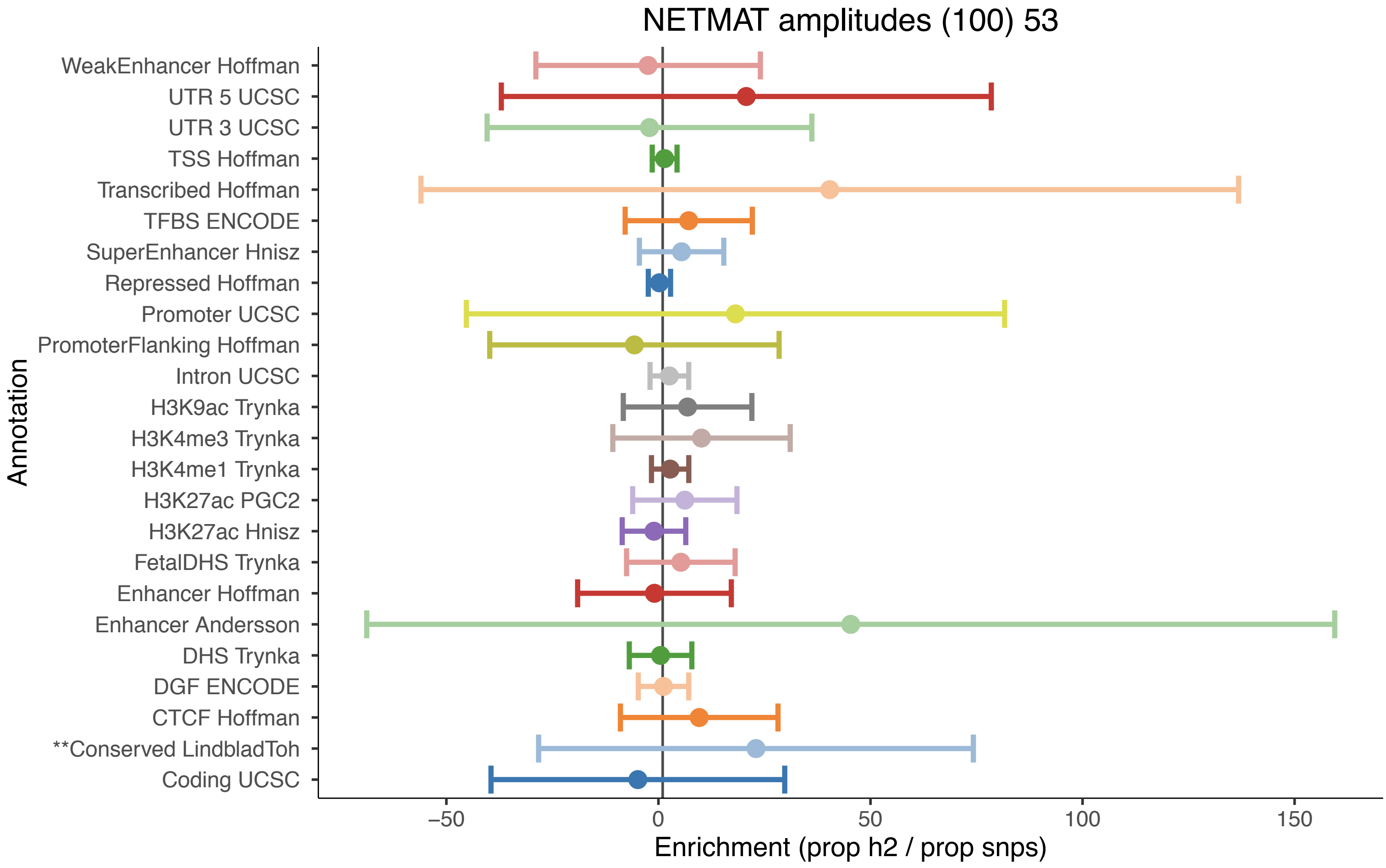

NETMAT amplitudes (100) 53
WeakEnhancer Hoffman
UTR 5 UCSC
UTR 3 UCSC
TSS Hoffman
Transcribed Hoffman
TFBS ENCODE
SuperEnhancer Hnisz
Repressed Hoffman
Promoter UCSC
PromoterFlanking Hoffman
Intron UCSC
H3K9ac Trynka
H3K4me3 Trynka
H3K4me1 Trynka
H3K27ac PGC2
H3K27ac Hnisz
FetalDHS Trynka
Enhancer Hoffman
Enhancer Andersson
DHS Trynka
DGF ENCODE
CTCF Hoffman
**Conserved LindbladToh
Coding UCSC
Annotation
−50
0
50
100
150
Enrichment (prop h2 / prop snps)

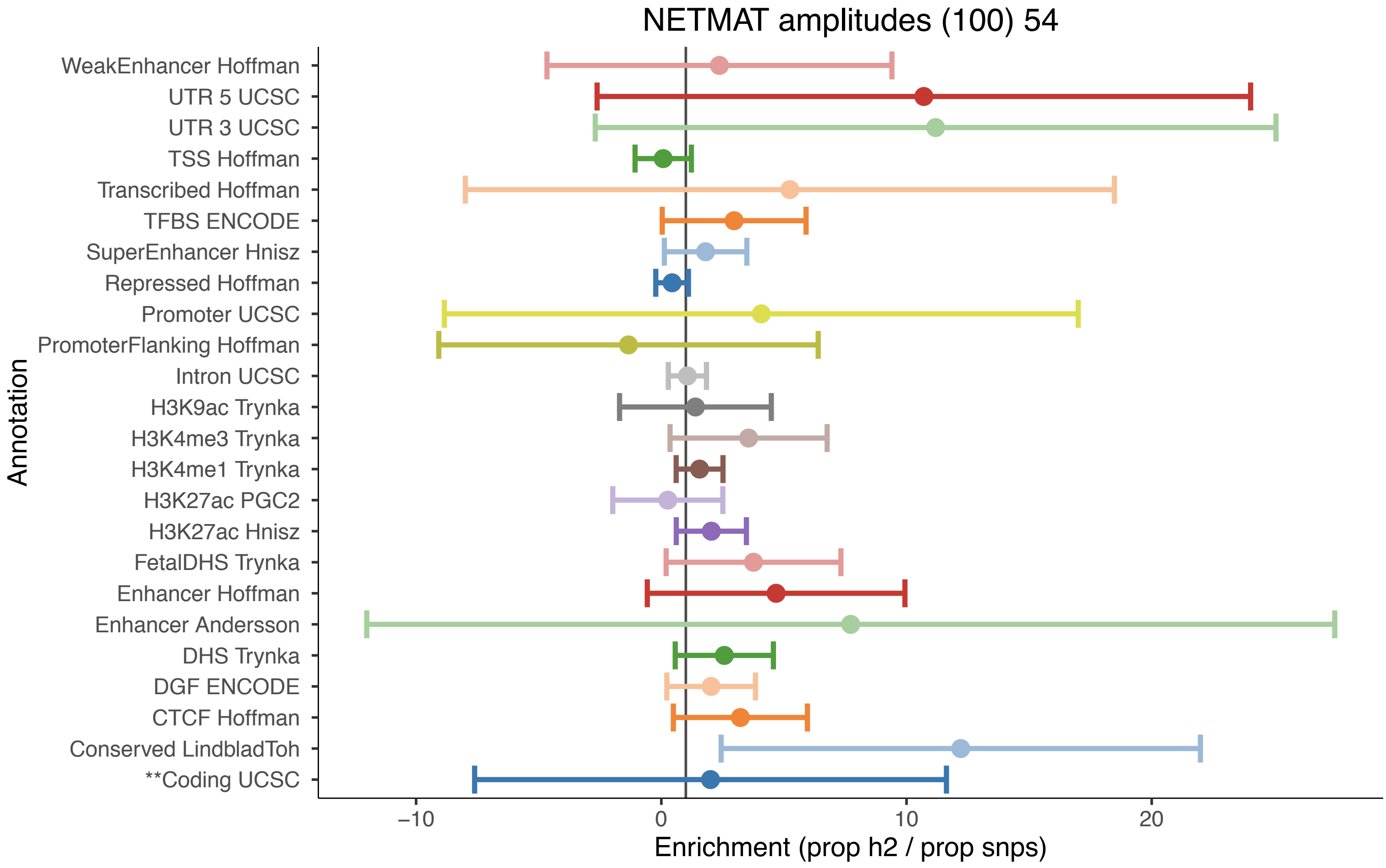

NETMAT amplitudes (100) 54
WeakEnhancer Hoffman
UTR 5 UCSC
UTR 3 UCSC
TSS Hoffman
Transcribed Hoffman
TFBS ENCODE
SuperEnhancer Hnisz
Repressed Hoffman
Promoter UCSC
PromoterFlanking Hoffman
Intron UCSC
H3K9ac Trynka
H3K4me3 Trynka
H3K4me1 Trynka
H3K27ac PGC2
H3K27ac Hnisz
FetalDHS Trynka
Enhancer Hoffman
Enhancer Andersson
DHS Trynka
DGF ENCODE
CTCF Hoffman
Conserved LindbladToh
**Coding UCSC
Annotation
−10
0
10
20
Enrichment (prop h2 / prop snps)

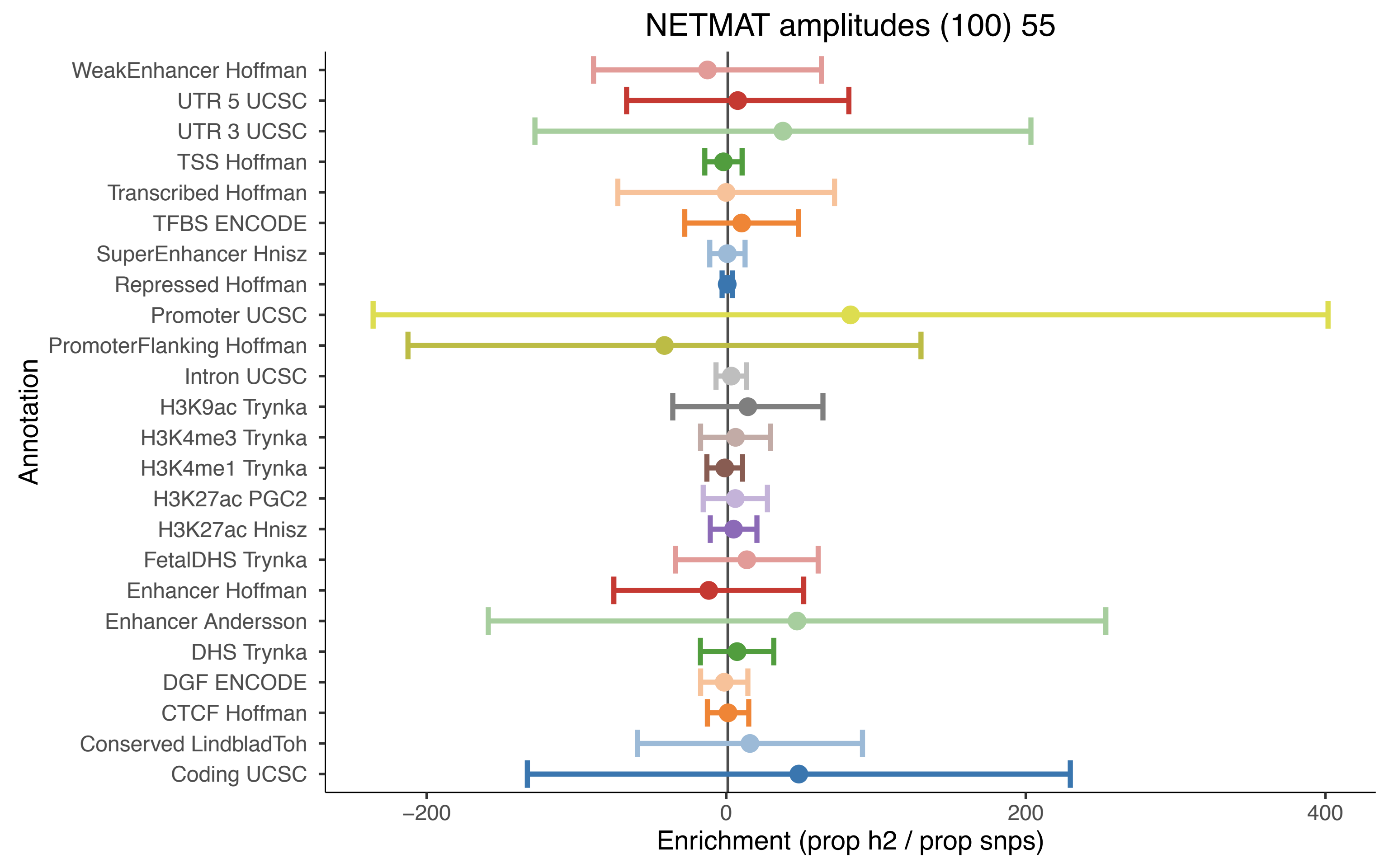
NETMAT amplitudes (100) 55
Annotation
WeakEnhancer Hoffman
UTR 5 UCSC
UTR 3 UCSC
TSS Hoffman
Transcribed Hoffman
TFBS ENCODE
SuperEnhancer Hnisz
Repressed Hoffman
Promoter UCSC
PromoterFlanking Hoffman
Intron UCSC
H3K9ac Trynka
H3K4me3 Trynka
H3K4me1 Trynka
H3K27ac PGC2
H3K27ac Hnisz
FetalDHS Trynka
Enhancer Hoffman
Enhancer Andersson
DHS Trynka
DGF ENCODE
CTCF Hoffman
Conserved LindbladToh
Coding UCSC
−200
0
200
400
Enrichment (prop h2 / prop snps)

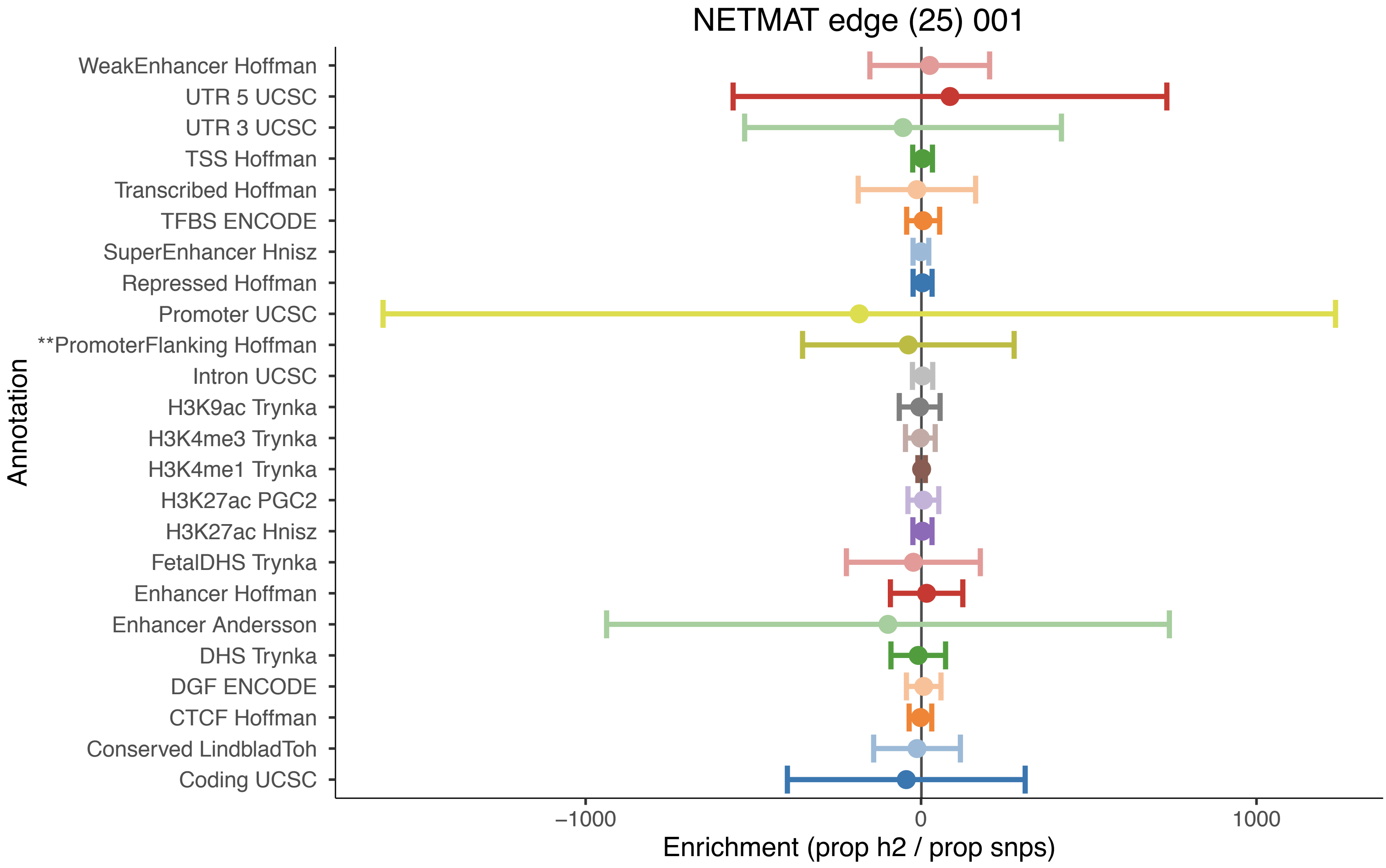

NETMAT edge (25) 001
WeakEnhancer Hoffman
UTR 5 UCSC
UTR 3 UCSC
TSS Hoffman
Transcribed Hoffman
TFBS ENCODE
SuperEnhancer Hnisz
Repressed Hoffman
Promoter UCSC
**PromoterFlanking Hoffman
Intron UCSC
H3K9ac Trynka
H3K4me3 Trynka
H3K4me1 Trynka
H3K27ac PGC2
H3K27ac Hnisz
FetalDHS Trynka
Enhancer Hoffman
Enhancer Andersson
DHS Trynka
DGF ENCODE
CTCF Hoffman
Conserved LindbladToh
Coding UCSC
Annotation
−1000
0
1000
Enrichment (prop h2 / prop snps)

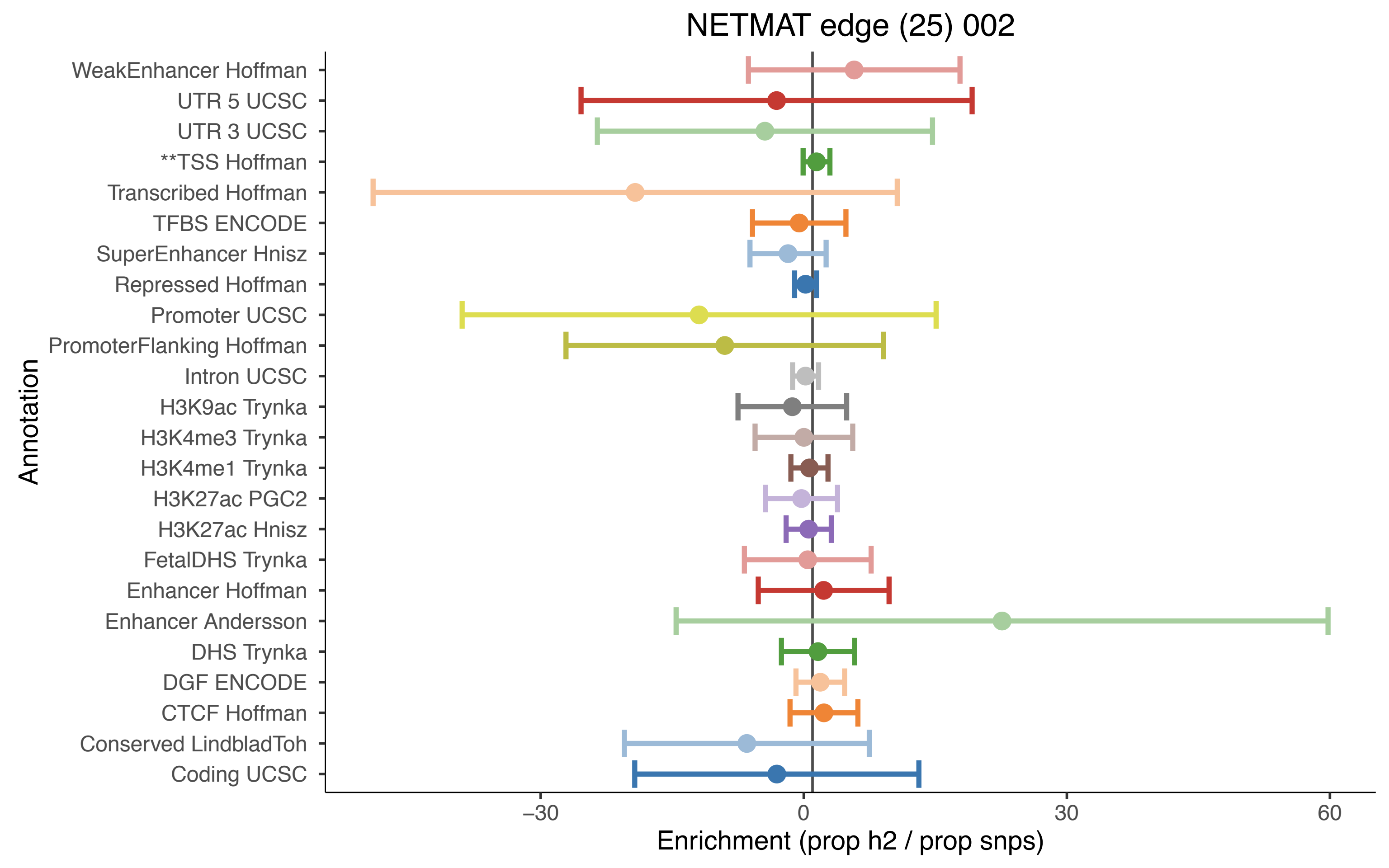

NETMAT edge (25) 002
WeakEnhancer Hoffman
UTR 5 UCSC
UTR 3 UCSC
**TSS Hoffman
Transcribed Hoffman
TFBS ENCODE
SuperEnhancer Hnisz
Repressed Hoffman
Promoter UCSC
PromoterFlanking Hoffman
Intron UCSC
H3K9ac Trynka
H3K4me3 Trynka
H3K4me1 Trynka
H3K27ac PGC2
H3K27ac Hnisz
FetalDHS Trynka
Enhancer Hoffman
Enhancer Andersson
DHS Trynka
DGF ENCODE
CTCF Hoffman
Conserved LindbladToh
Coding UCSC
Annotation
−30
0
30
60
Enrichment (prop h2 / prop snps)

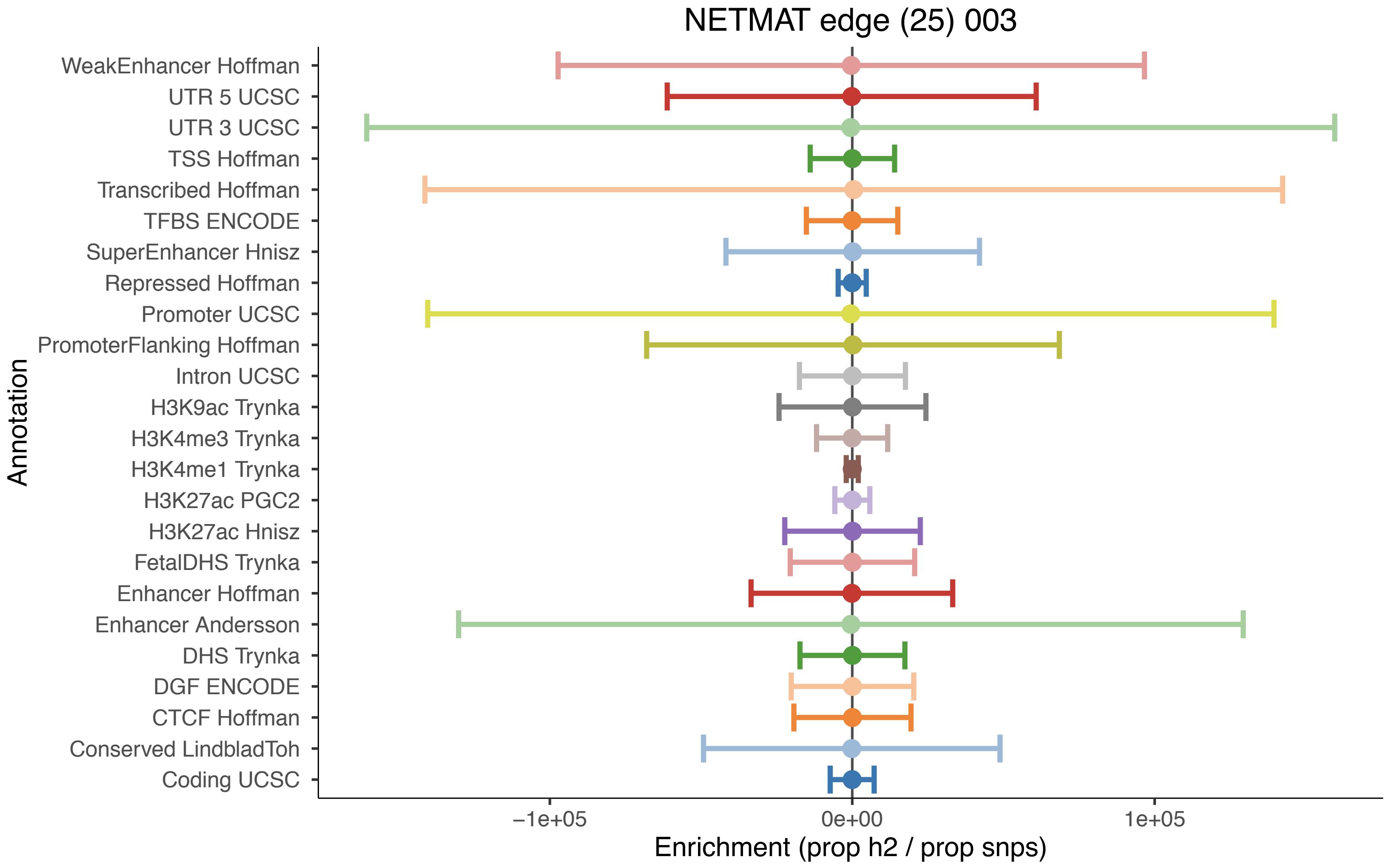

NETMAT edge (25) 003
WeakEnhancer Hoffman
UTR 5 UCSC
UTR 3 UCSC
TSS Hoffman
Transcribed Hoffman
TFBS ENCODE
SuperEnhancer Hnisz
Repressed Hoffman
Promoter UCSC
PromoterFlanking Hoffman
Intron UCSC
H3K9ac Trynka
H3K4me3 Trynka
H3K4me1 Trynka
H3K27ac PGC2
H3K27ac Hnisz
FetalDHS Trynka
Enhancer Hoffman
Enhancer Andersson
DHS Trynka
DGF ENCODE
CTCF Hoffman
Conserved LindbladToh
Coding UCSC
Annotation
−1e+05
0e+00
1e+05
Enrichment (prop h2 / prop snps)

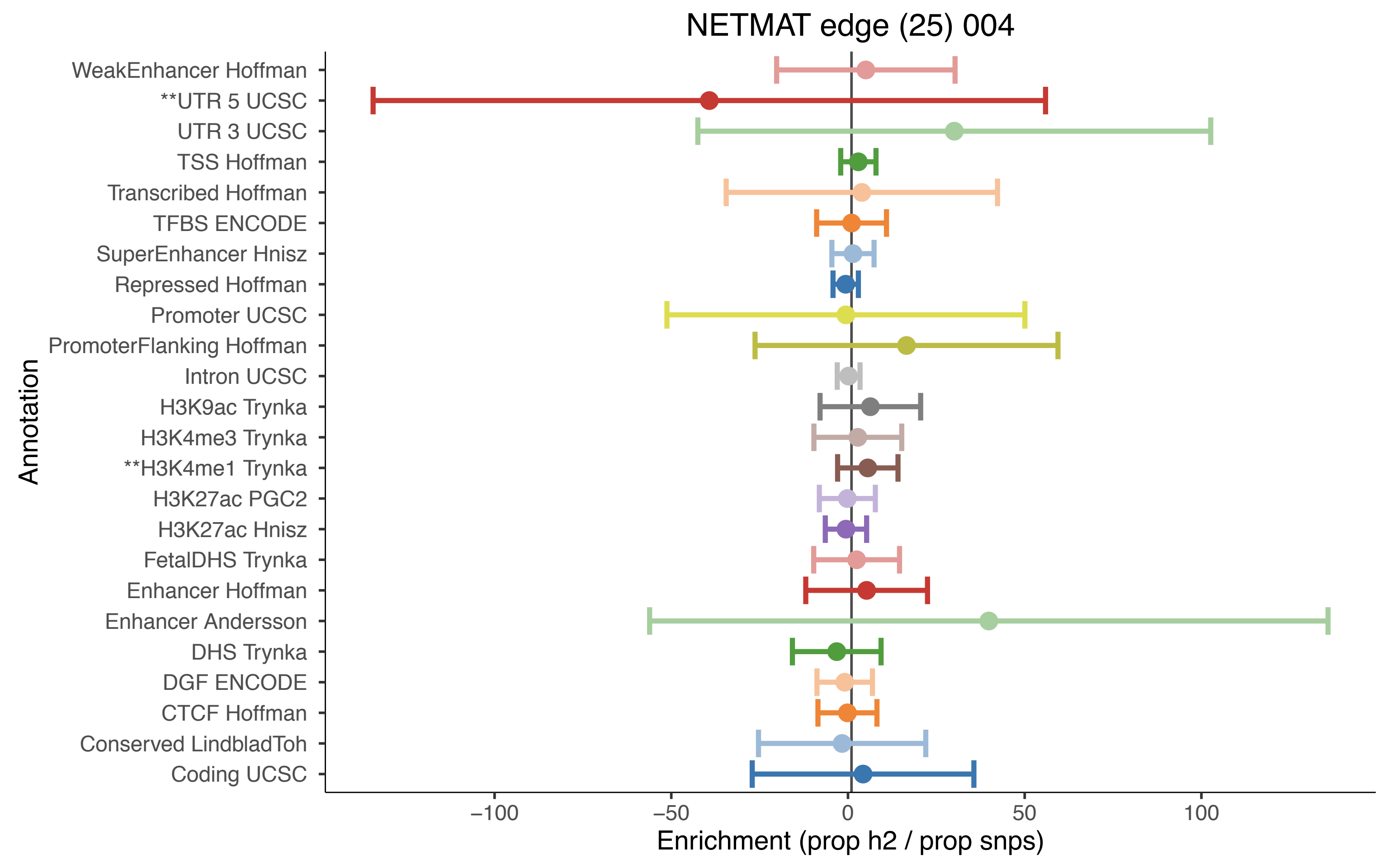
NETMAT edge (25) 004
WeakEnhancer Hoffman
**UTR 5 UCSC
UTR 3 UCSC
TSS Hoffman
Transcribed Hoffman
TFBS ENCODE
SuperEnhancer Hnisz
Repressed Hoffman
Promoter UCSC
PromoterFlanking Hoffman
Intron UCSC
H3K9ac Trynka
H3K4me3 Trynka
**H3K4me1 Trynka
H3K27ac PGC2
H3K27ac Hnisz
FetalDHS Trynka
Enhancer Hoffman
Enhancer Andersson
DHS Trynka
DGF ENCODE
CTCF Hoffman
Conserved LindbladToh
Coding UCSC
Annotation
−100
−50
0
50
100
Enrichment (prop h2 / prop snps)

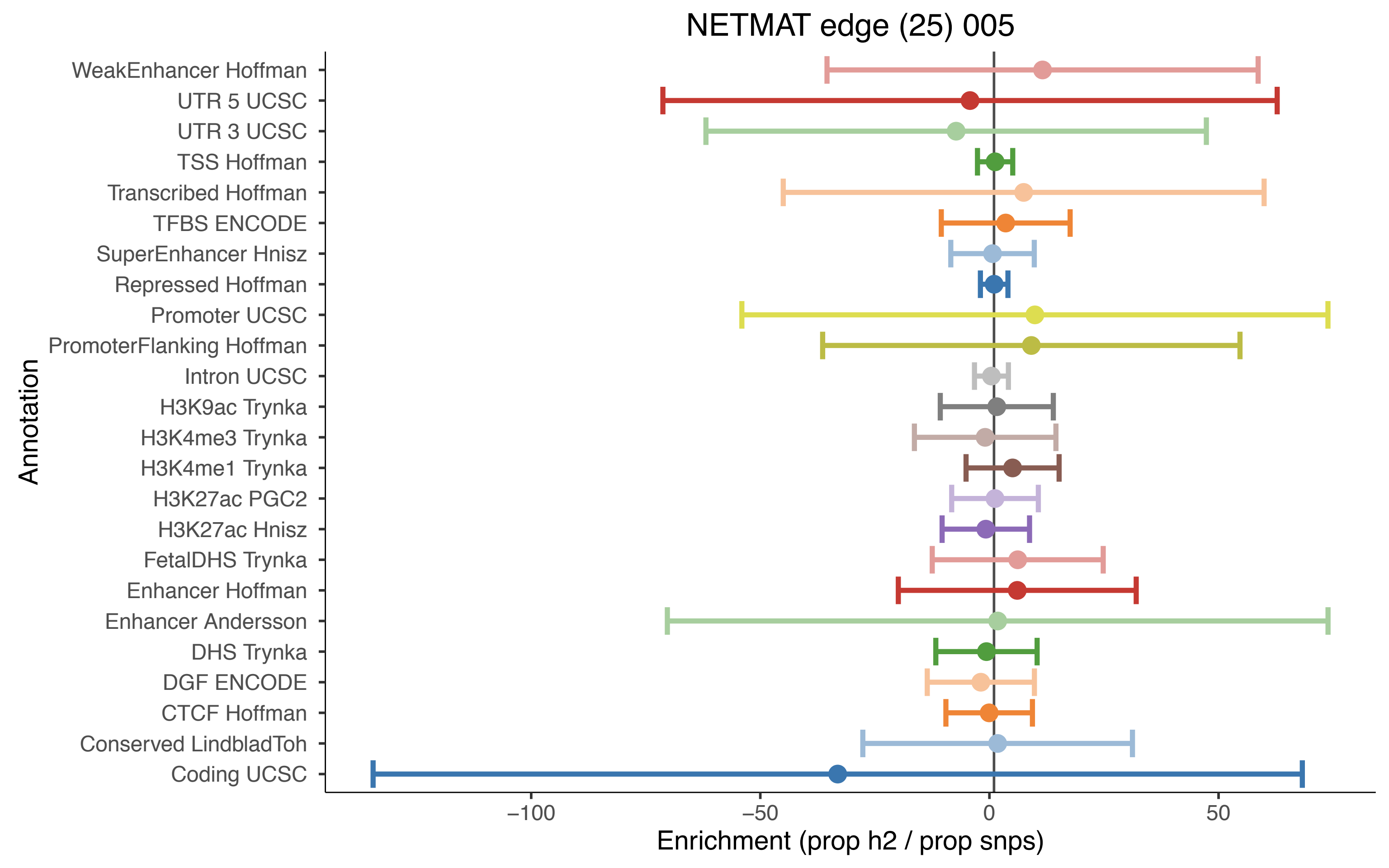

NETMAT edge (25) 005
WeakEnhancer Hoffman
UTR 5 UCSC
UTR 3 UCSC
TSS Hoffman
Transcribed Hoffman
TFBS ENCODE
SuperEnhancer Hnisz
Repressed Hoffman
Promoter UCSC
PromoterFlanking Hoffman
Intron UCSC
H3K9ac Trynka
H3K4me3 Trynka
H3K4me1 Trynka
H3K27ac PGC2
H3K27ac Hnisz
FetalDHS Trynka
Enhancer Hoffman
Enhancer Andersson
DHS Trynka
DGF ENCODE
CTCF Hoffman
Conserved LindbladToh
Coding UCSC
Annotation
−100
−50
0
50
Enrichment (prop h2 / prop snps)

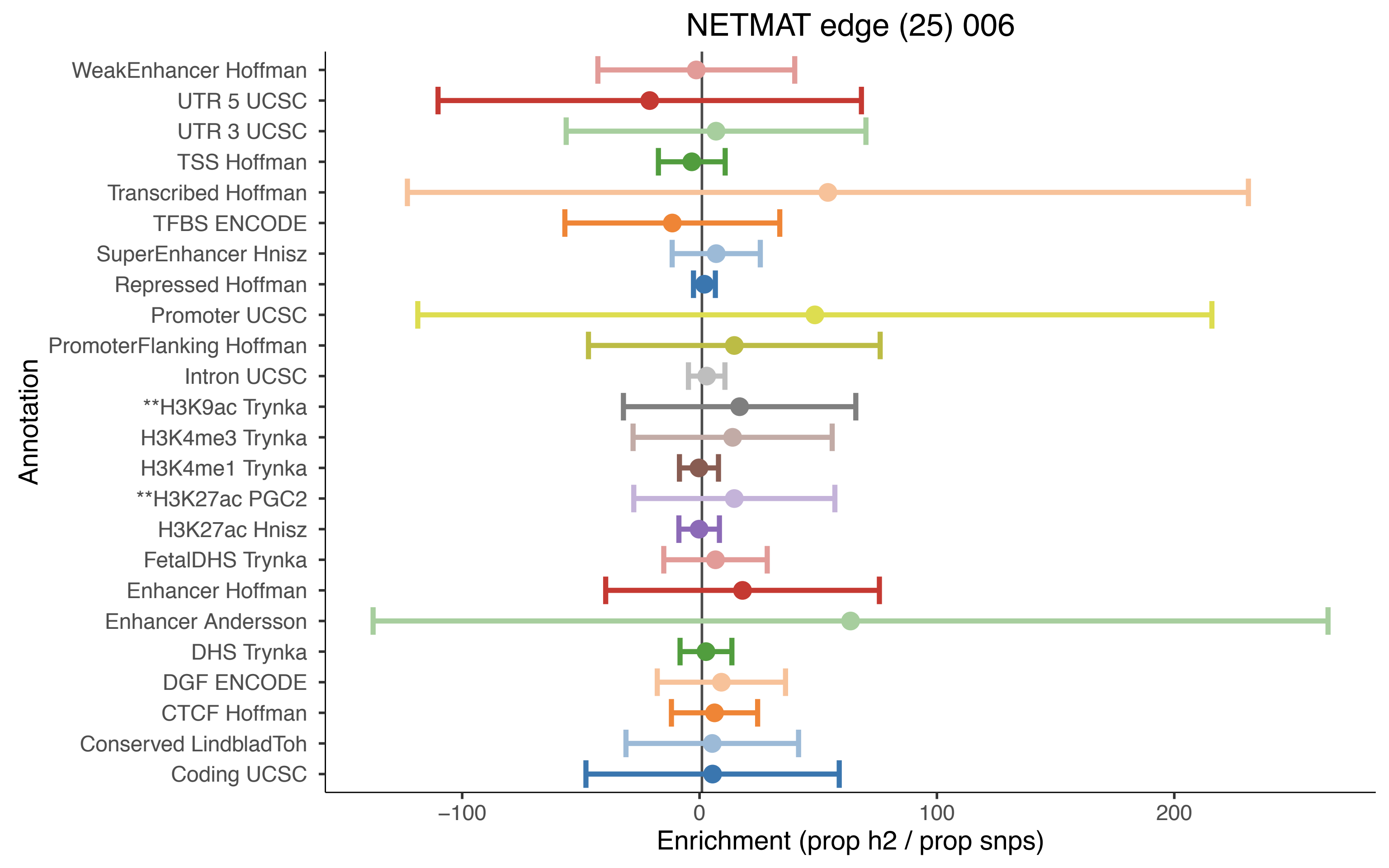
NETMAT edge (25) 006
WeakEnhancer Hoffman
UTR 5 UCSC
UTR 3 UCSC
TSS Hoffman
Transcribed Hoffman
TFBS ENCODE
SuperEnhancer Hnisz
Repressed Hoffman
Promoter UCSC
PromoterFlanking Hoffman
Intron UCSC
**H3K9ac Trynka
H3K4me3 Trynka
H3K4me1 Trynka
**H3K27ac PGC2
H3K27ac Hnisz
FetalDHS Trynka
Enhancer Hoffman
Enhancer Andersson
DHS Trynka
DGF ENCODE
CTCF Hoffman
Conserved LindbladToh
Coding UCSC
Annotation
−100
0
100
200
Enrichment (prop h2 / prop snps)

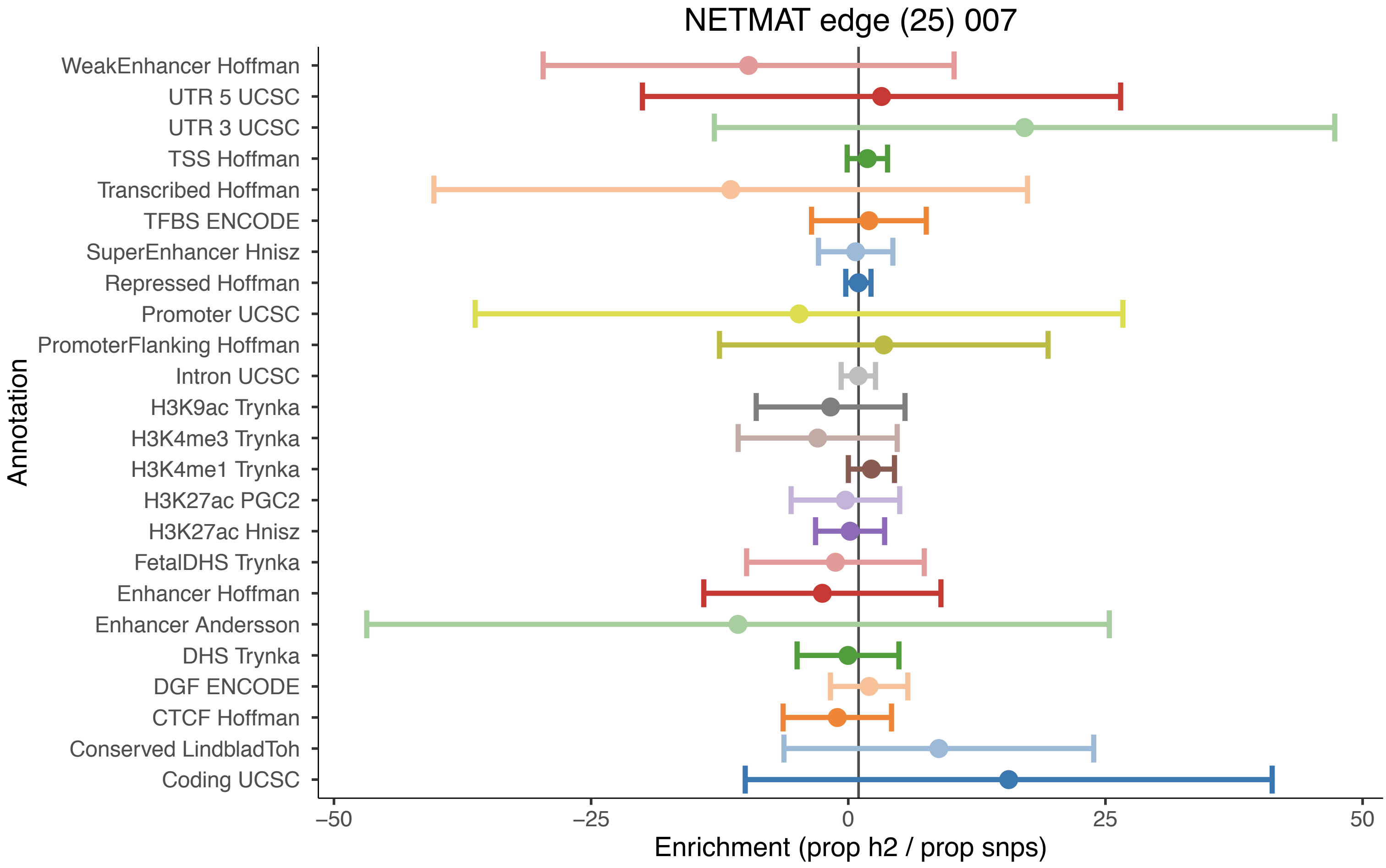

NETMAT edge (25) 007
WeakEnhancer Hoffman
UTR 5 UCSC
UTR 3 UCSC
TSS Hoffman
Transcribed Hoffman
TFBS ENCODE
SuperEnhancer Hnisz
Repressed Hoffman
Promoter UCSC
PromoterFlanking Hoffman
Intron UCSC
H3K9ac Trynka
H3K4me3 Trynka
H3K4me1 Trynka
H3K27ac PGC2
H3K27ac Hnisz
FetalDHS Trynka
Enhancer Hoffman
Enhancer Andersson
DHS Trynka
DGF ENCODE
CTCF Hoffman
Conserved LindbladToh
Coding UCSC
Annotation
−50
−25
0
25
50
Enrichment (prop h2 / prop snps)

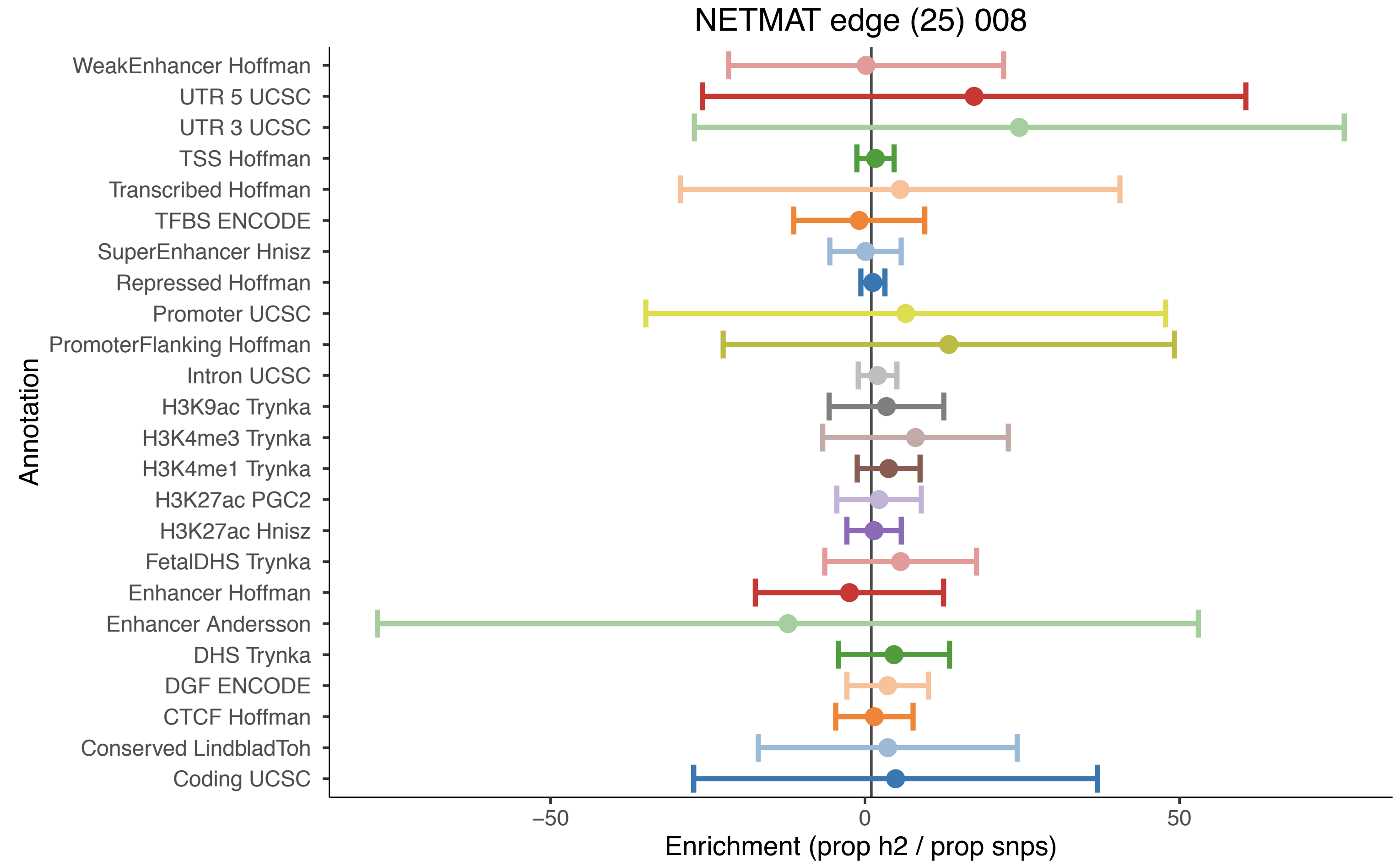

NETMAT edge (25) 008
WeakEnhancer Hoffman
UTR 5 UCSC
UTR 3 UCSC
TSS Hoffman
Transcribed Hoffman
TFBS ENCODE
SuperEnhancer Hnisz
Repressed Hoffman
Promoter UCSC
PromoterFlanking Hoffman
Intron UCSC
H3K9ac Trynka
H3K4me3 Trynka
H3K4me1 Trynka
H3K27ac PGC2
H3K27ac Hnisz
FetalDHS Trynka
Enhancer Hoffman
Enhancer Andersson
DHS Trynka
DGF ENCODE
CTCF Hoffman
Conserved LindbladToh
Coding UCSC
Annotation
−50
0
50
Enrichment (prop h2 / prop snps)

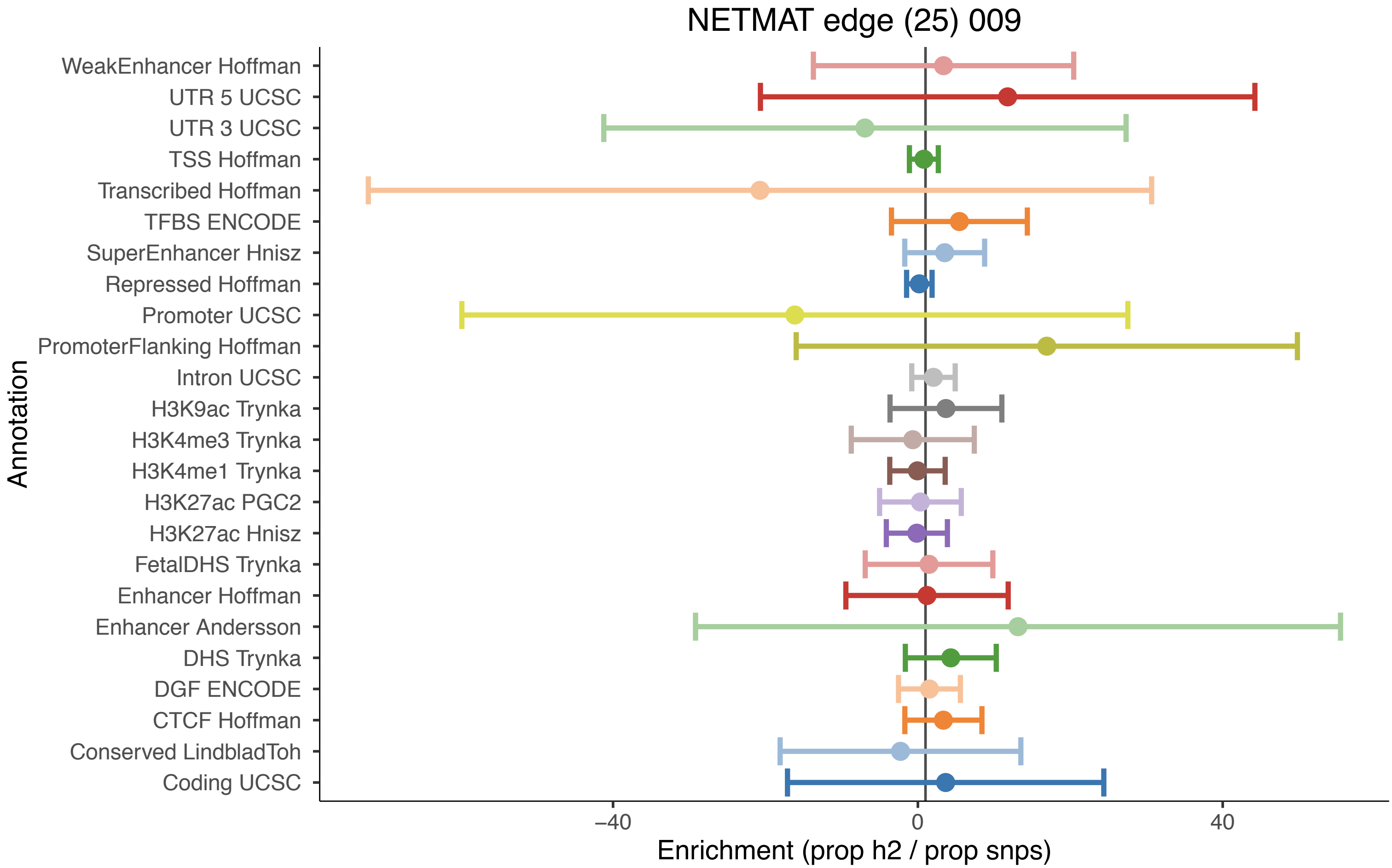

NETMAT edge (25) 009
WeakEnhancer Hoffman
UTR 5 UCSC
UTR 3 UCSC
TSS Hoffman
Transcribed Hoffman
TFBS ENCODE
SuperEnhancer Hnisz
Repressed Hoffman
Promoter UCSC
PromoterFlanking Hoffman
Intron UCSC
H3K9ac Trynka
H3K4me3 Trynka
H3K4me1 Trynka
H3K27ac PGC2
H3K27ac Hnisz
FetalDHS Trynka
Enhancer Hoffman
Enhancer Andersson
DHS Trynka
DGF ENCODE
CTCF Hoffman
Conserved LindbladToh
Coding UCSC
Annotation
−40
0
40
Enrichment (prop h2 / prop snps)

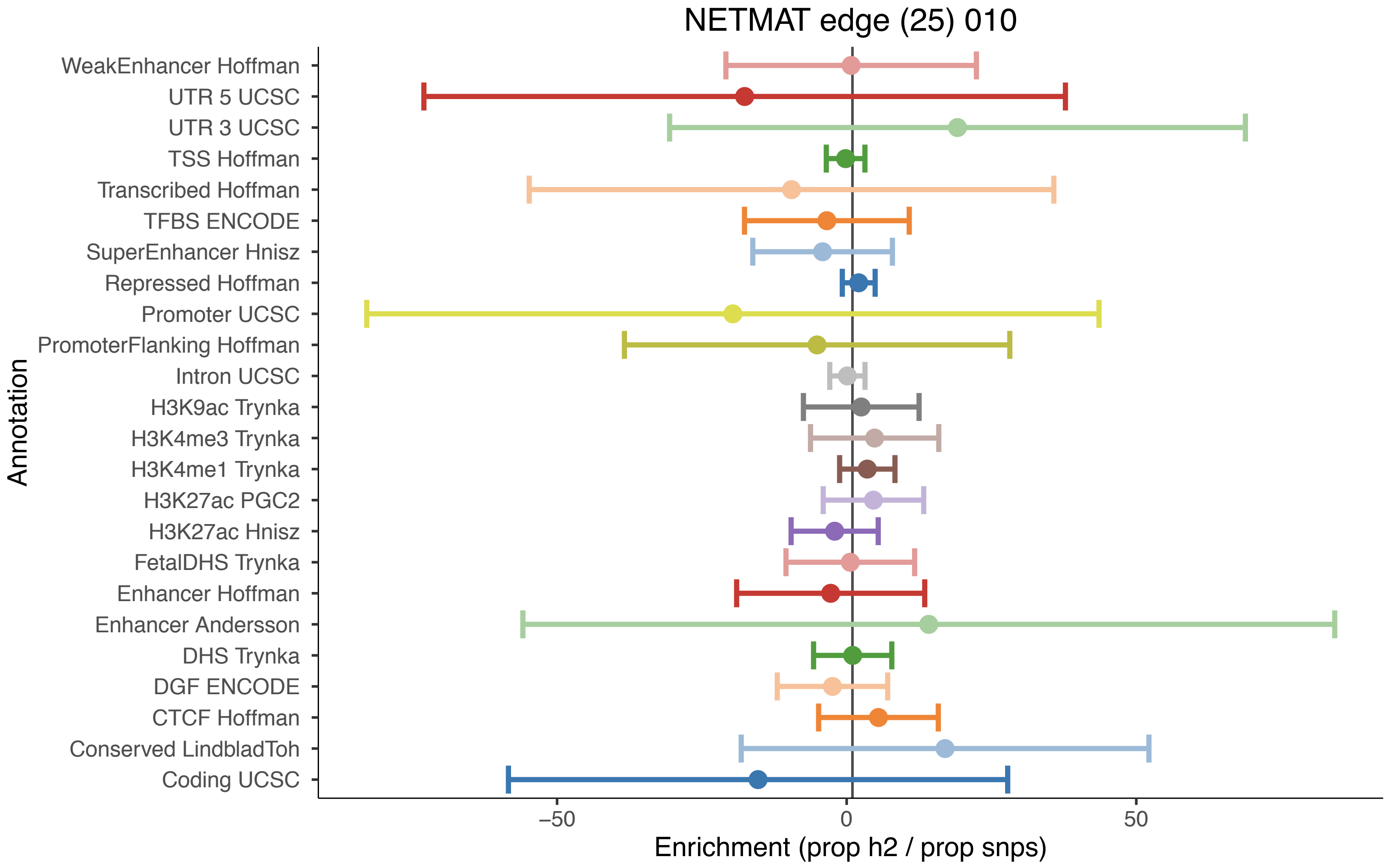
NETMAT edge (25) 010
WeakEnhancer Hoffman
UTR 5 UCSC
UTR 3 UCSC
TSS Hoffman
Transcribed Hoffman
TFBS ENCODE
SuperEnhancer Hnisz
Repressed Hoffman
Promoter UCSC
PromoterFlanking Hoffman
Intron UCSC
H3K9ac Trynka
H3K4me3 Trynka
H3K4me1 Trynka
H3K27ac PGC2
H3K27ac Hnisz
FetalDHS Trynka
Enhancer Hoffman
Enhancer Andersson
DHS Trynka
DGF ENCODE
CTCF Hoffman
Conserved LindbladToh
Coding UCSC
Annotation
−50
0
50
Enrichment (prop h2 / prop snps)

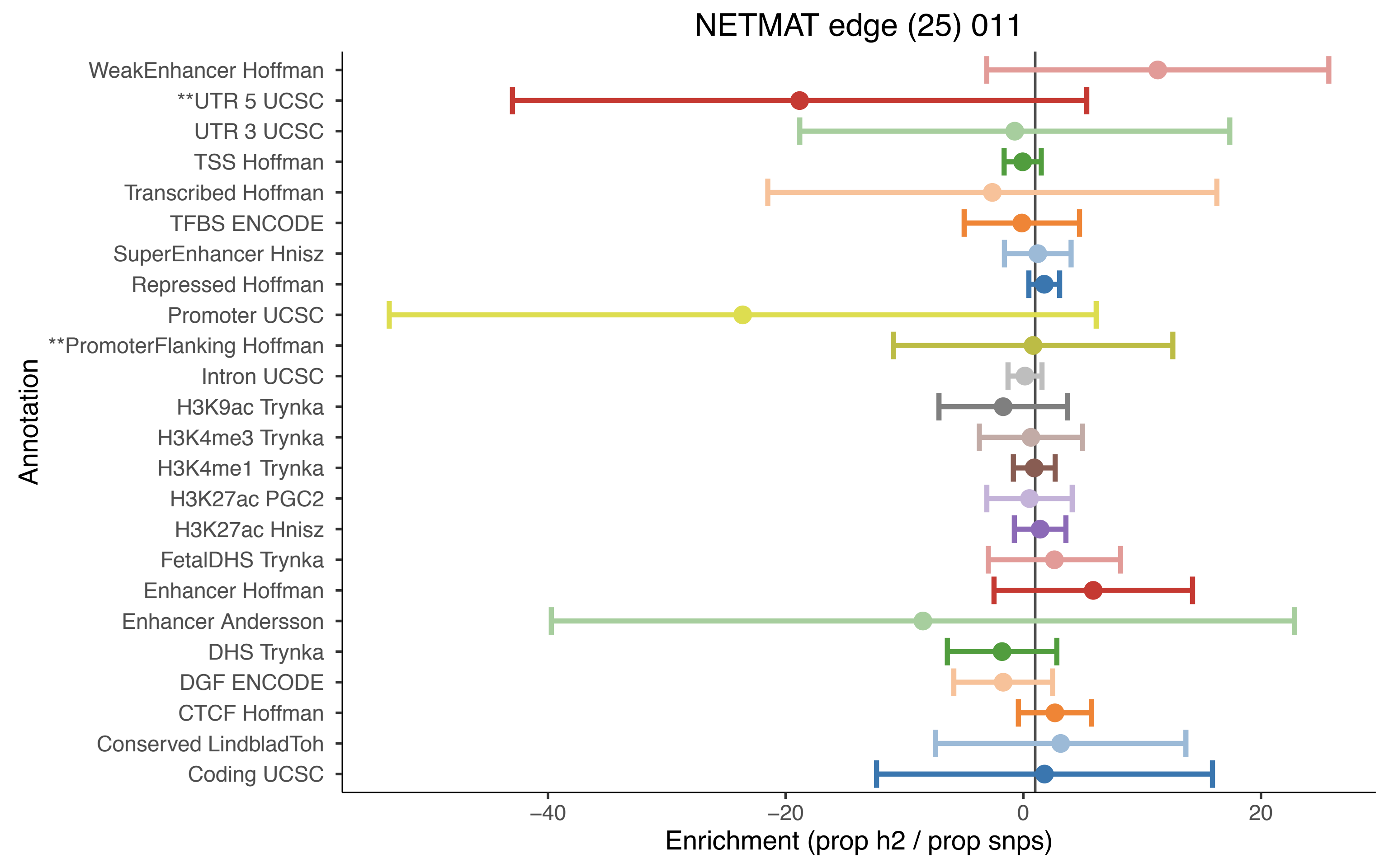

NETMAT edge (25) 011
WeakEnhancer Hoffman
**UTR 5 UCSC
UTR 3 UCSC
TSS Hoffman
Transcribed Hoffman
TFBS ENCODE
SuperEnhancer Hnisz
Repressed Hoffman
Promoter UCSC
**PromoterFlanking Hoffman
Intron UCSC
H3K9ac Trynka
H3K4me3 Trynka
H3K4me1 Trynka
H3K27ac PGC2
H3K27ac Hnisz
FetalDHS Trynka
Enhancer Hoffman
Enhancer Andersson
DHS Trynka
DGF ENCODE
CTCF Hoffman
Conserved LindbladToh
Coding UCSC
Annotation
−40
−20
0
20
Enrichment (prop h2 / prop snps)

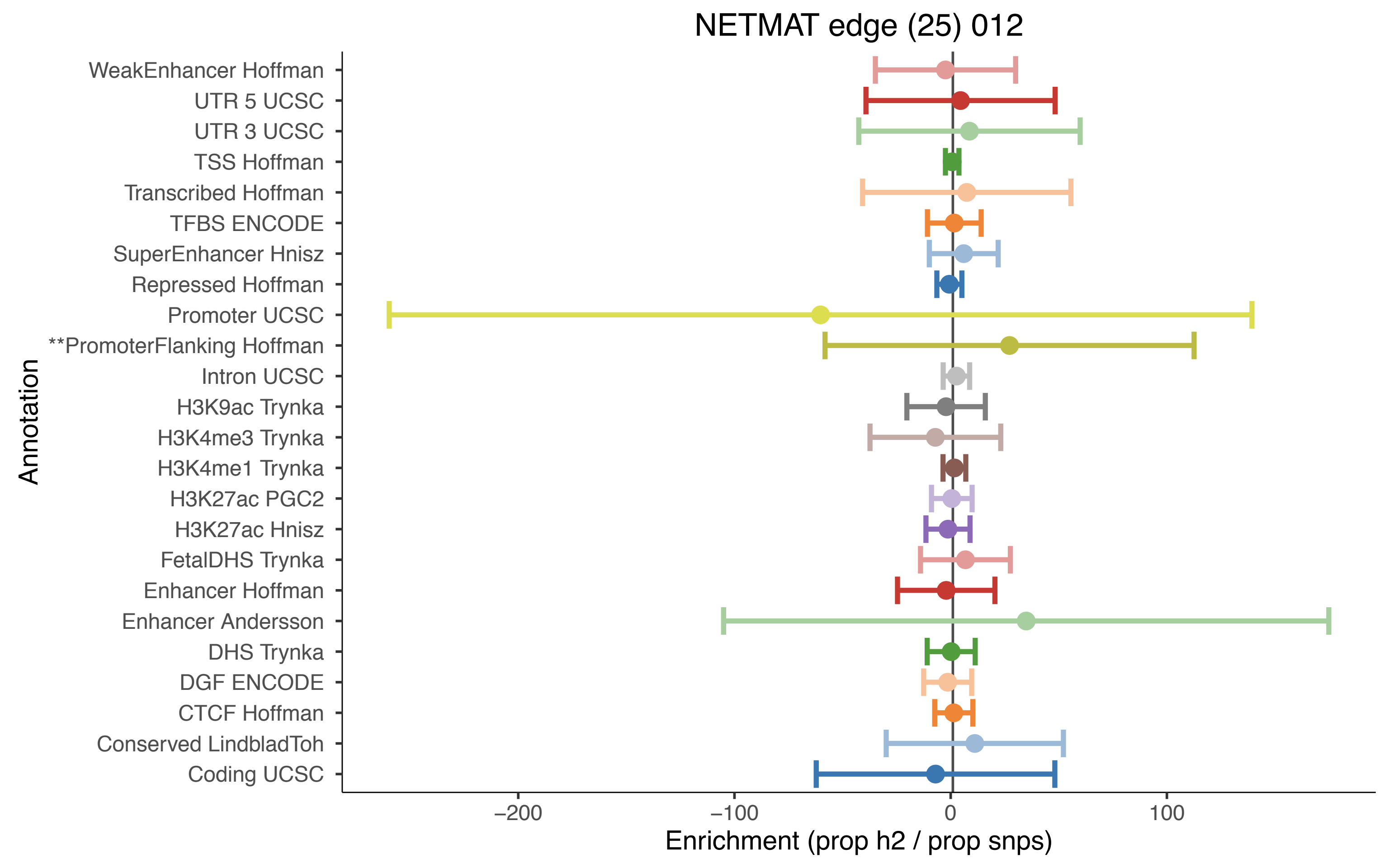

NETMAT edge (25) 012
WeakEnhancer Hoffman
UTR 5 UCSC
UTR 3 UCSC
TSS Hoffman
Transcribed Hoffman
TFBS ENCODE
SuperEnhancer Hnisz
Repressed Hoffman
Promoter UCSC
**PromoterFlanking Hoffman
Intron UCSC
H3K9ac Trynka
H3K4me3 Trynka
H3K4me1 Trynka
H3K27ac PGC2
H3K27ac Hnisz
FetalDHS Trynka
Enhancer Hoffman
Enhancer Andersson
DHS Trynka
DGF ENCODE
CTCF Hoffman
Conserved LindbladToh
Coding UCSC
Annotation
−200
−100
0
100
Enrichment (prop h2 / prop snps)

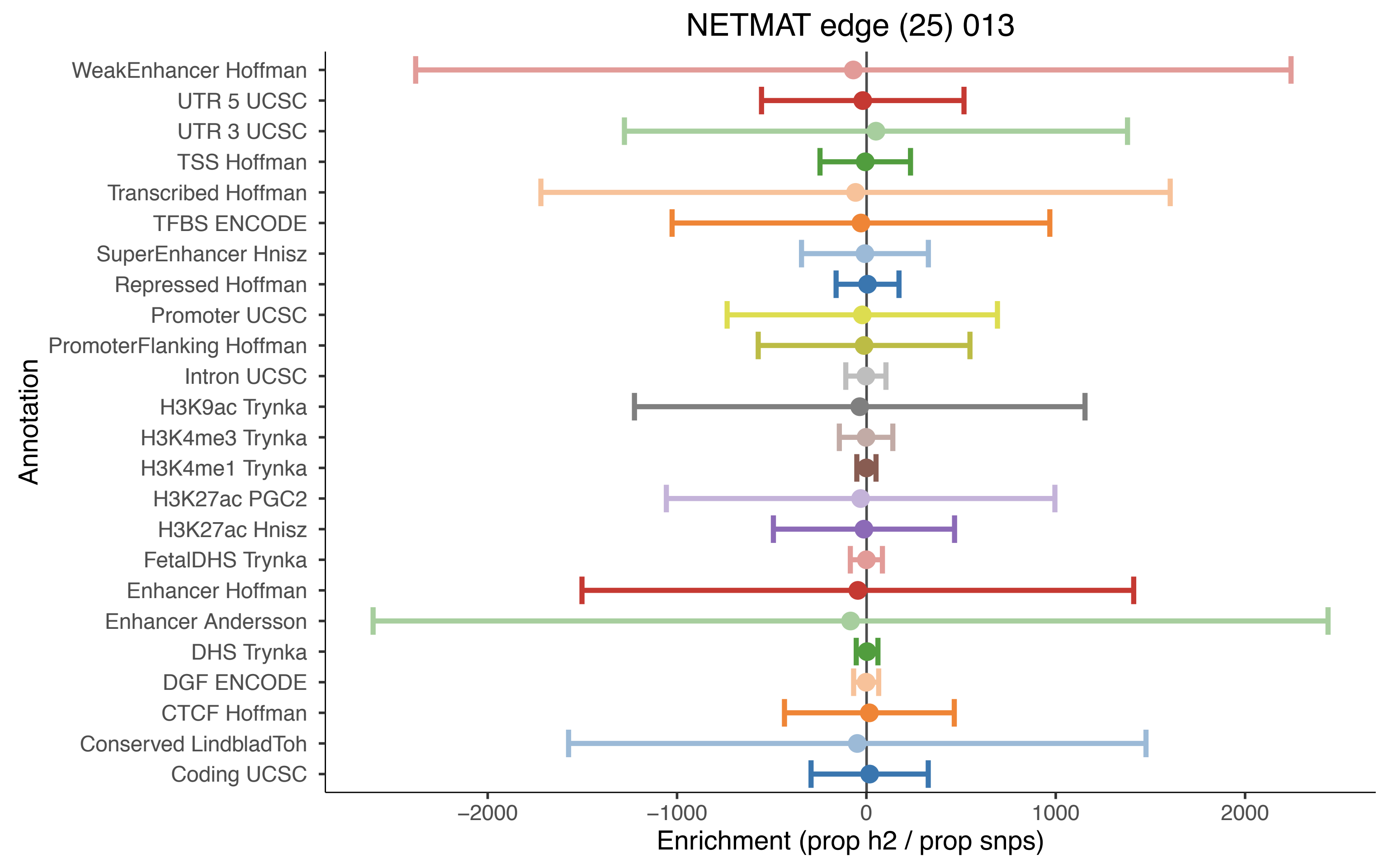

NETMAT edge (25) 013
Annotation
WeakEnhancer Hoffman
UTR 5 UCSC
UTR 3 UCSC
TSS Hoffman
Transcribed Hoffman
TFBS ENCODE
SuperEnhancer Hnisz
Repressed Hoffman
Promoter UCSC
PromoterFlanking Hoffman
Intron UCSC
H3K9ac Trynka
H3K4me3 Trynka
H3K4me1 Trynka
H3K27ac PGC2
H3K27ac Hnisz
FetalDHS Trynka
Enhancer Hoffman
Enhancer Andersson
DHS Trynka
DGF ENCODE
CTCF Hoffman
Conserved LindbladToh
Coding UCSC
−2000
−1000
0
1000
2000
Enrichment (prop h2 / prop snps)

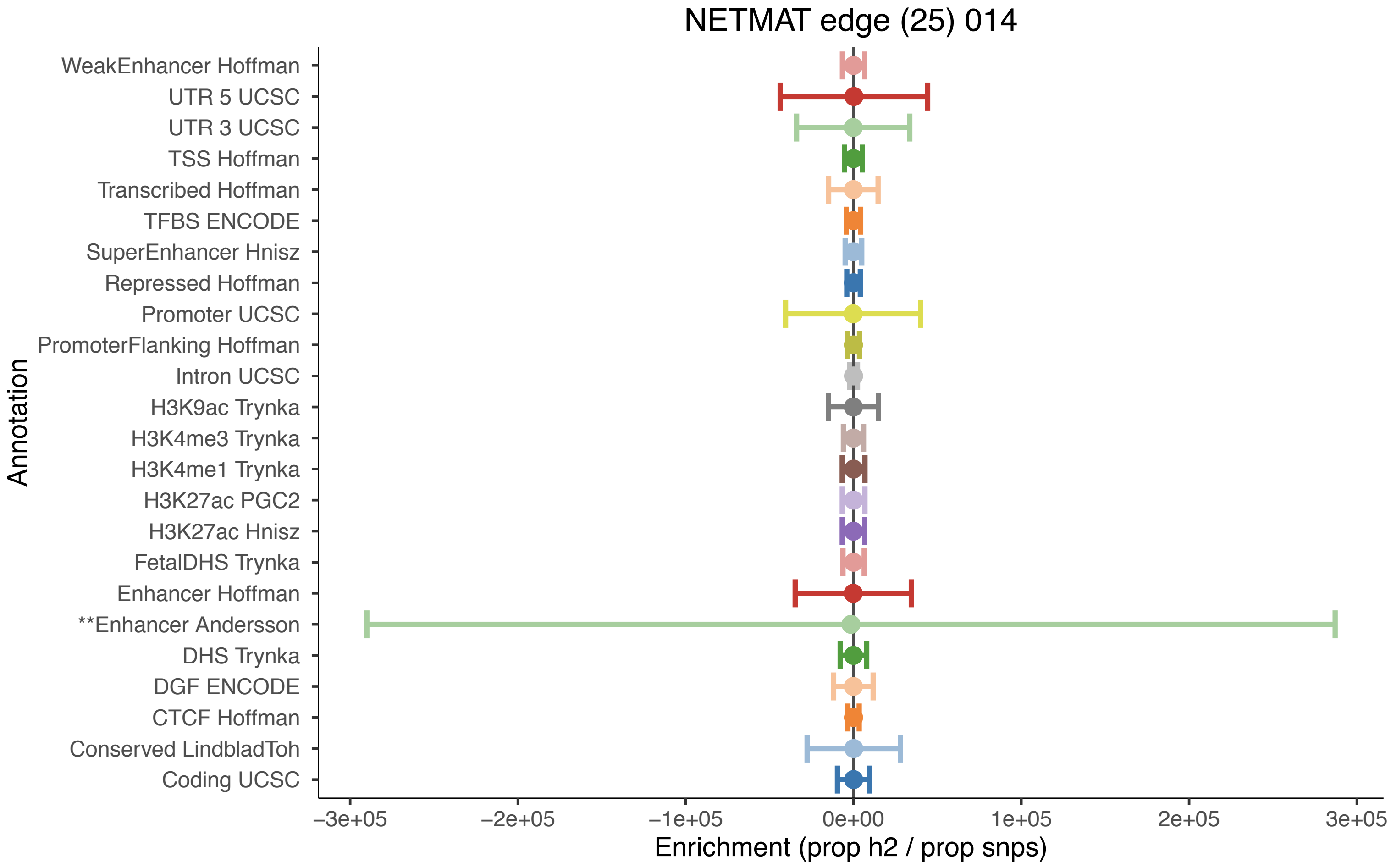

NETMAT edge (25) 014
WeakEnhancer Hoffman
UTR 5 UCSC
UTR 3 UCSC
TSS Hoffman
Transcribed Hoffman
TFBS ENCODE
SuperEnhancer Hnisz
Repressed Hoffman
Promoter UCSC
PromoterFlanking Hoffman
Intron UCSC
H3K9ac Trynka
H3K4me3 Trynka
H3K4me1 Trynka
H3K27ac PGC2
H3K27ac Hnisz
FetalDHS Trynka
Enhancer Hoffman
**Enhancer Andersson
DHS Trynka
DGF ENCODE
CTCF Hoffman
Conserved LindbladToh
Coding UCSC
Annotation
−3e+05
−2e+05
−1e+05
0e+00
1e+05
2e+05
3e+05
Enrichment (prop h2 / prop snps)

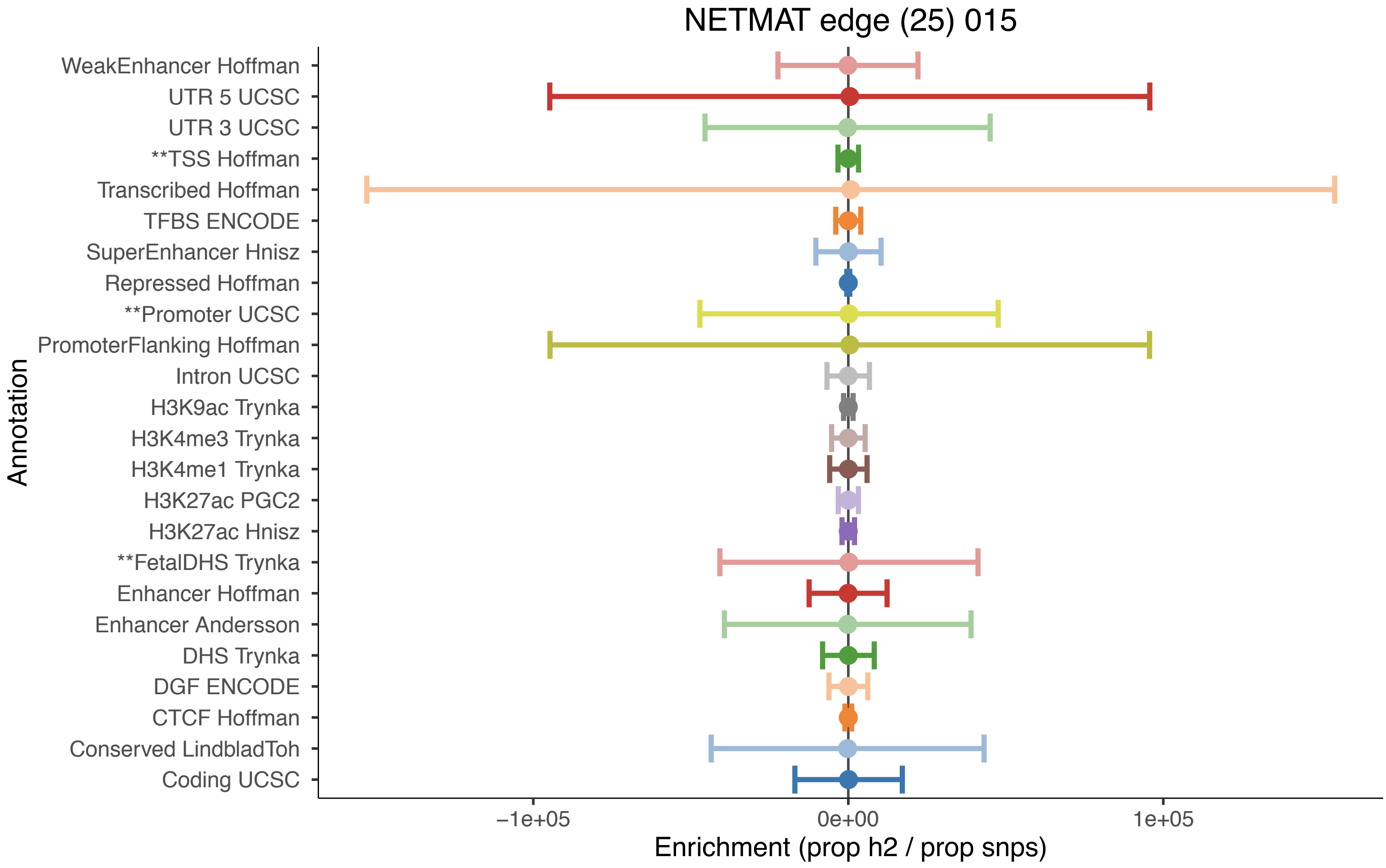
NETMAT edge (25) 015
WeakEnhancer Hoffman
UTR 5 UCSC
UTR 3 UCSC
**TSS Hoffman
Transcribed Hoffman
TFBS ENCODE
SuperEnhancer Hnisz
Repressed Hoffman
**Promoter UCSC
PromoterFlanking Hoffman
Intron UCSC
H3K9ac Trynka
H3K4me3 Trynka
H3K4me1 Trynka
H3K27ac PGC2
H3K27ac Hnisz
**FetalDHS Trynka
Enhancer Hoffman
Enhancer Andersson
DHS Trynka
DGF ENCODE
CTCF Hoffman
Conserved LindbladToh
Coding UCSC
Annotation
−1e+05
0e+00
1e+05
Enrichment (prop h2 / prop snps)

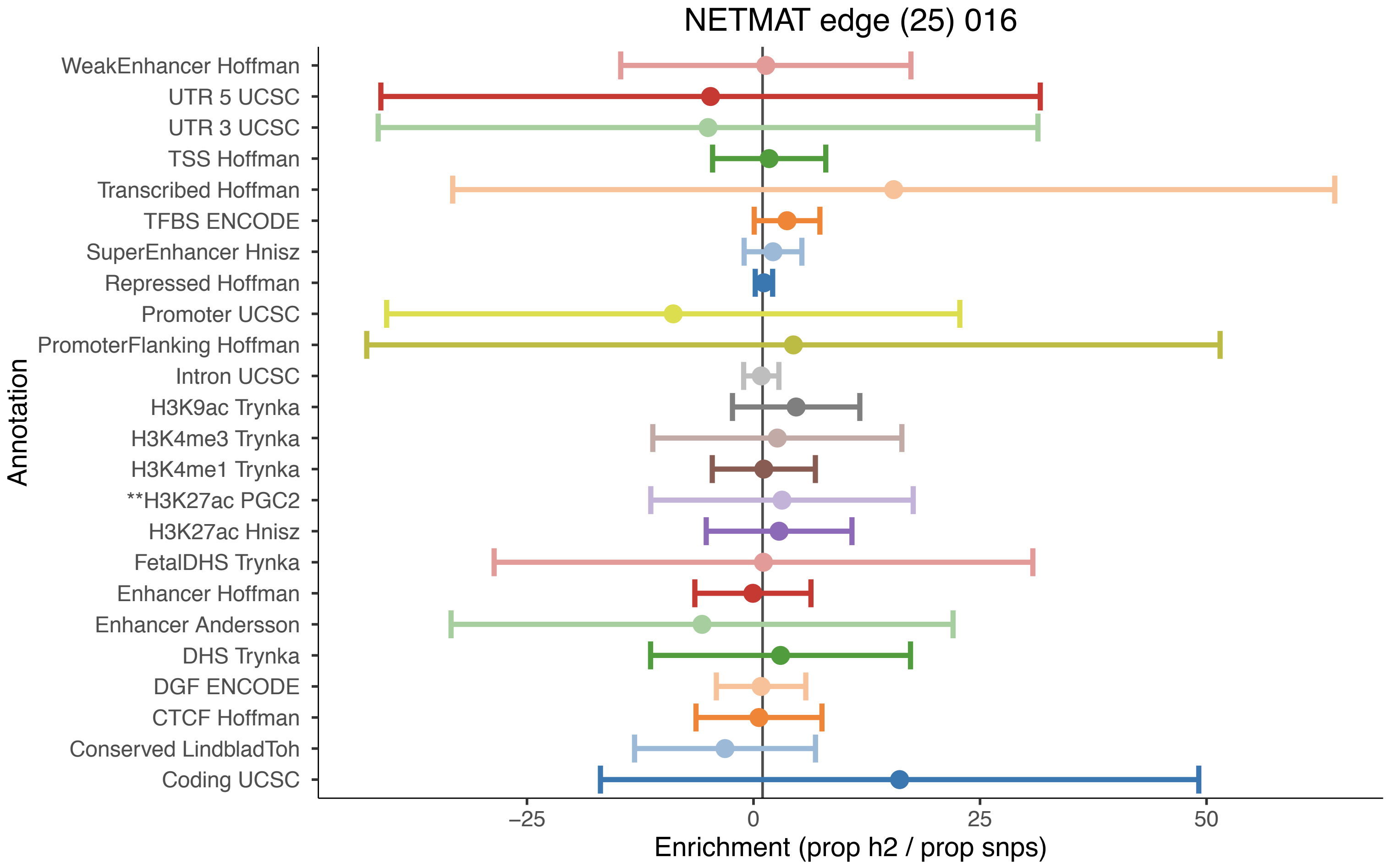

NETMAT edge (25) 016
WeakEnhancer Hoffman
UTR 5 UCSC
UTR 3 UCSC
TSS Hoffman
Transcribed Hoffman
TFBS ENCODE
SuperEnhancer Hnisz
Repressed Hoffman
Promoter UCSC
PromoterFlanking Hoffman
Intron UCSC
H3K9ac Trynka
H3K4me3 Trynka
H3K4me1 Trynka
**H3K27ac PGC2
H3K27ac Hnisz
FetalDHS Trynka
Enhancer Hoffman
Enhancer Andersson
DHS Trynka
DGF ENCODE
CTCF Hoffman
Conserved LindbladToh
Coding UCSC
Annotation
−25
0
25
50
Enrichment (prop h2 / prop snps)

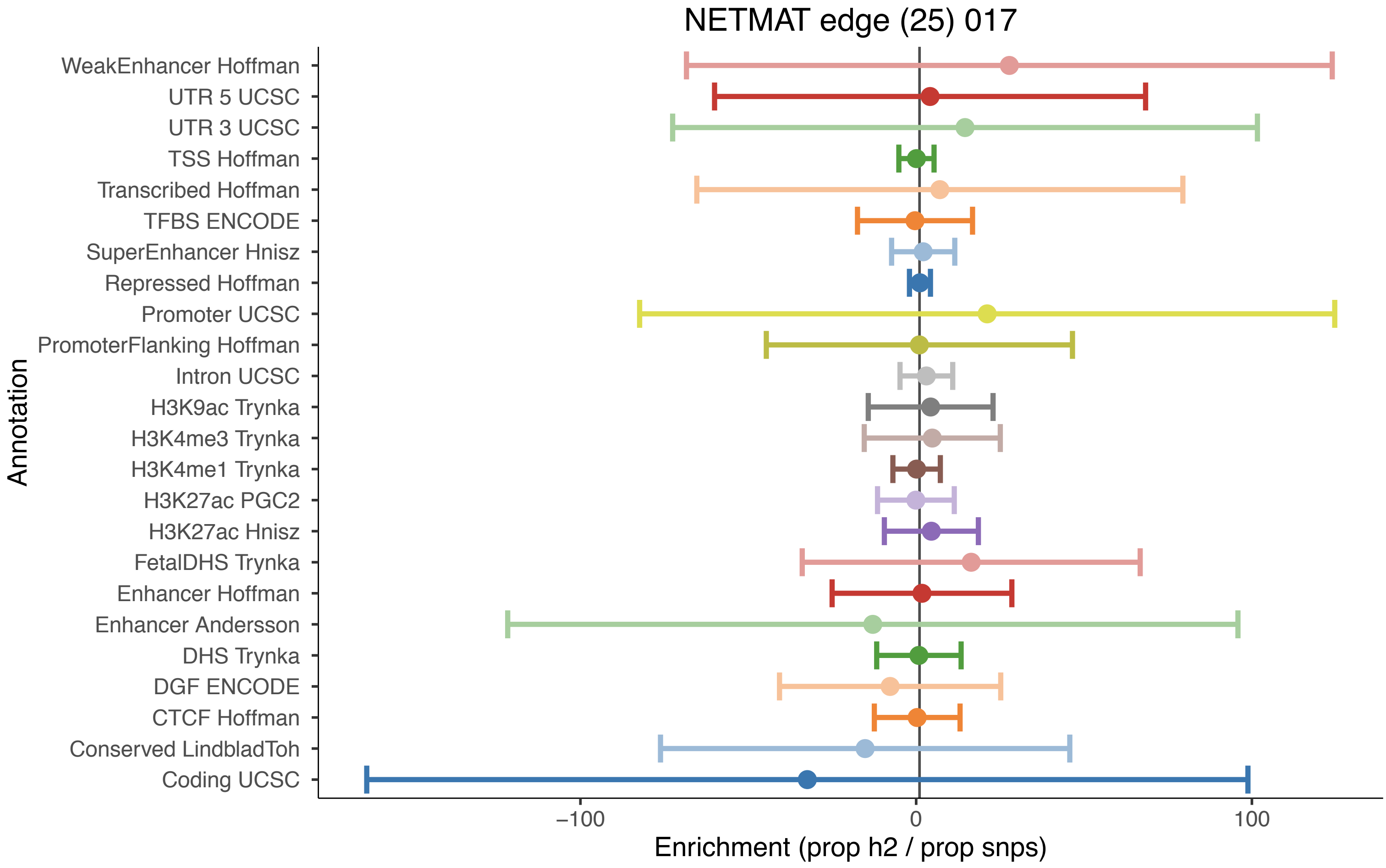

NETMAT edge (25) 017
WeakEnhancer Hoffman
UTR 5 UCSC
UTR 3 UCSC
TSS Hoffman
Transcribed Hoffman
TFBS ENCODE
SuperEnhancer Hnisz
Repressed Hoffman
Promoter UCSC
PromoterFlanking Hoffman
Intron UCSC
H3K9ac Trynka
H3K4me3 Trynka
H3K4me1 Trynka
H3K27ac PGC2
H3K27ac Hnisz
FetalDHS Trynka
Enhancer Hoffman
Enhancer Andersson
DHS Trynka
DGF ENCODE
CTCF Hoffman
Conserved LindbladToh
Coding UCSC
Annotation
−100
0
100
Enrichment (prop h2 / prop snps)

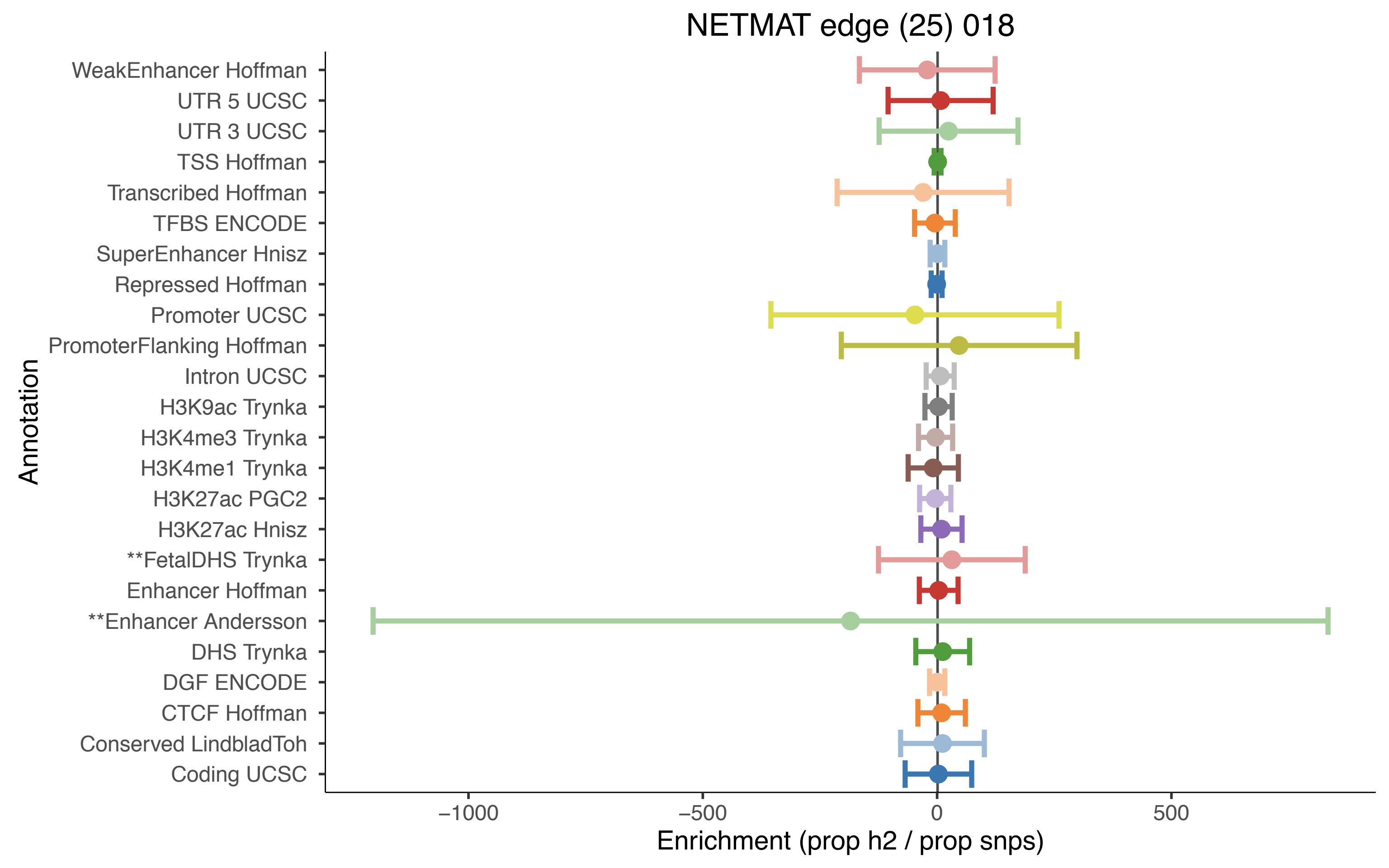
NETMAT edge (25) 018
Annotation
WeakEnhancer Hoffman
UTR 5 UCSC
UTR 3 UCSC
TSS Hoffman
Transcribed Hoffman
TFBS ENCODE
SuperEnhancer Hnisz
Repressed Hoffman
Promoter UCSC
PromoterFlanking Hoffman
Intron UCSC
H3K9ac Trynka
H3K4me3 Trynka
H3K4me1 Trynka
H3K27ac PGC2
H3K27ac Hnisz
**FetalDHS Trynka
Enhancer Hoffman
**Enhancer Andersson
DHS Trynka
DGF ENCODE
CTCF Hoffman
Conserved LindbladToh
Coding UCSC
−1000
−500
0
500
Enrichment (prop h2 / prop snps)

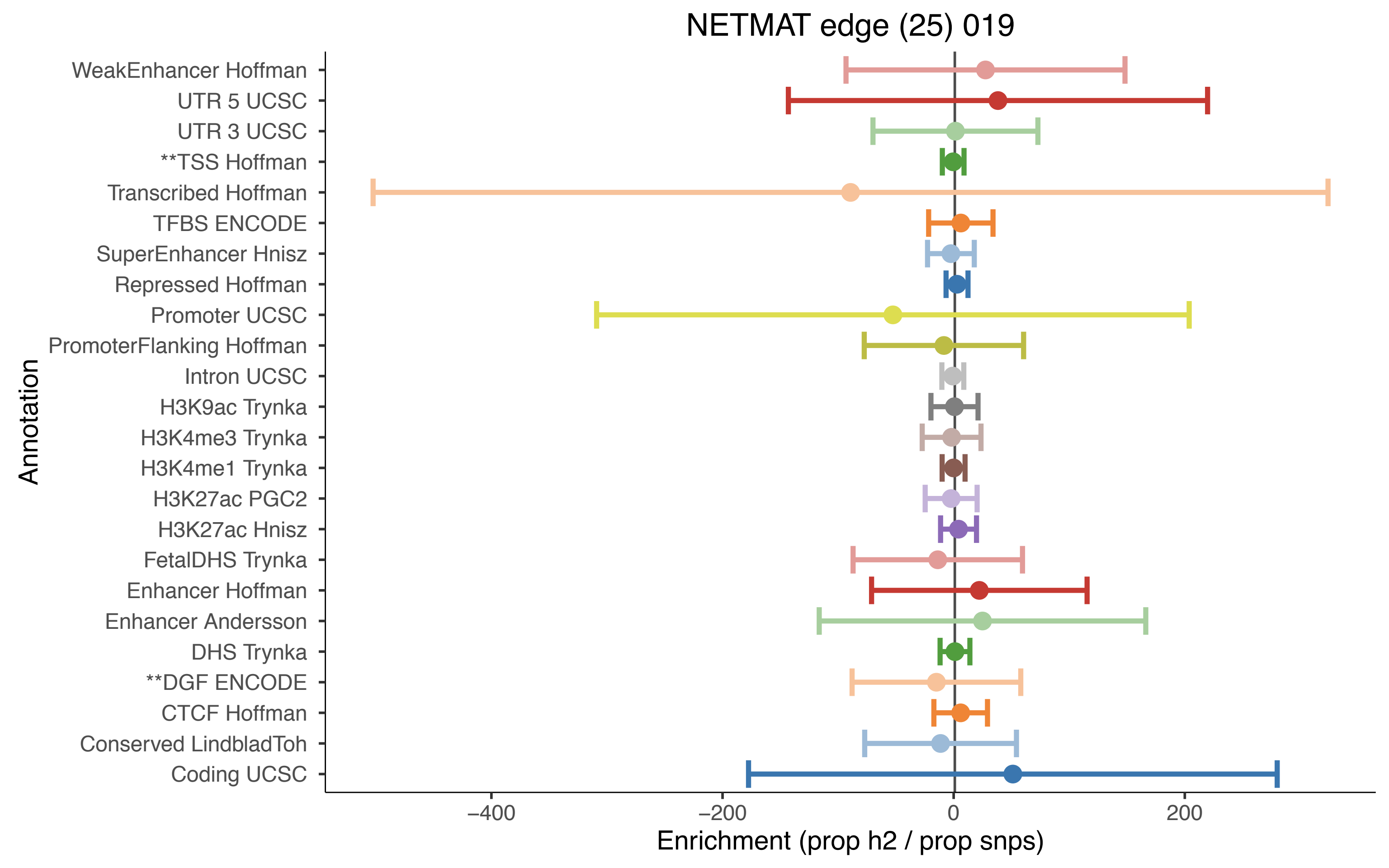

NETMAT edge (25) 019
WeakEnhancer Hoffman
UTR 5 UCSC
UTR 3 UCSC
**TSS Hoffman
Transcribed Hoffman
TFBS ENCODE
SuperEnhancer Hnisz
Repressed Hoffman
Promoter UCSC
PromoterFlanking Hoffman
Intron UCSC
H3K9ac Trynka
H3K4me3 Trynka
H3K4me1 Trynka
H3K27ac PGC2
H3K27ac Hnisz
FetalDHS Trynka
Enhancer Hoffman
Enhancer Andersson
DHS Trynka
**DGF ENCODE
CTCF Hoffman
Conserved LindbladToh
Coding UCSC
Annotation
−400
−200
0
200
Enrichment (prop h2 / prop snps)

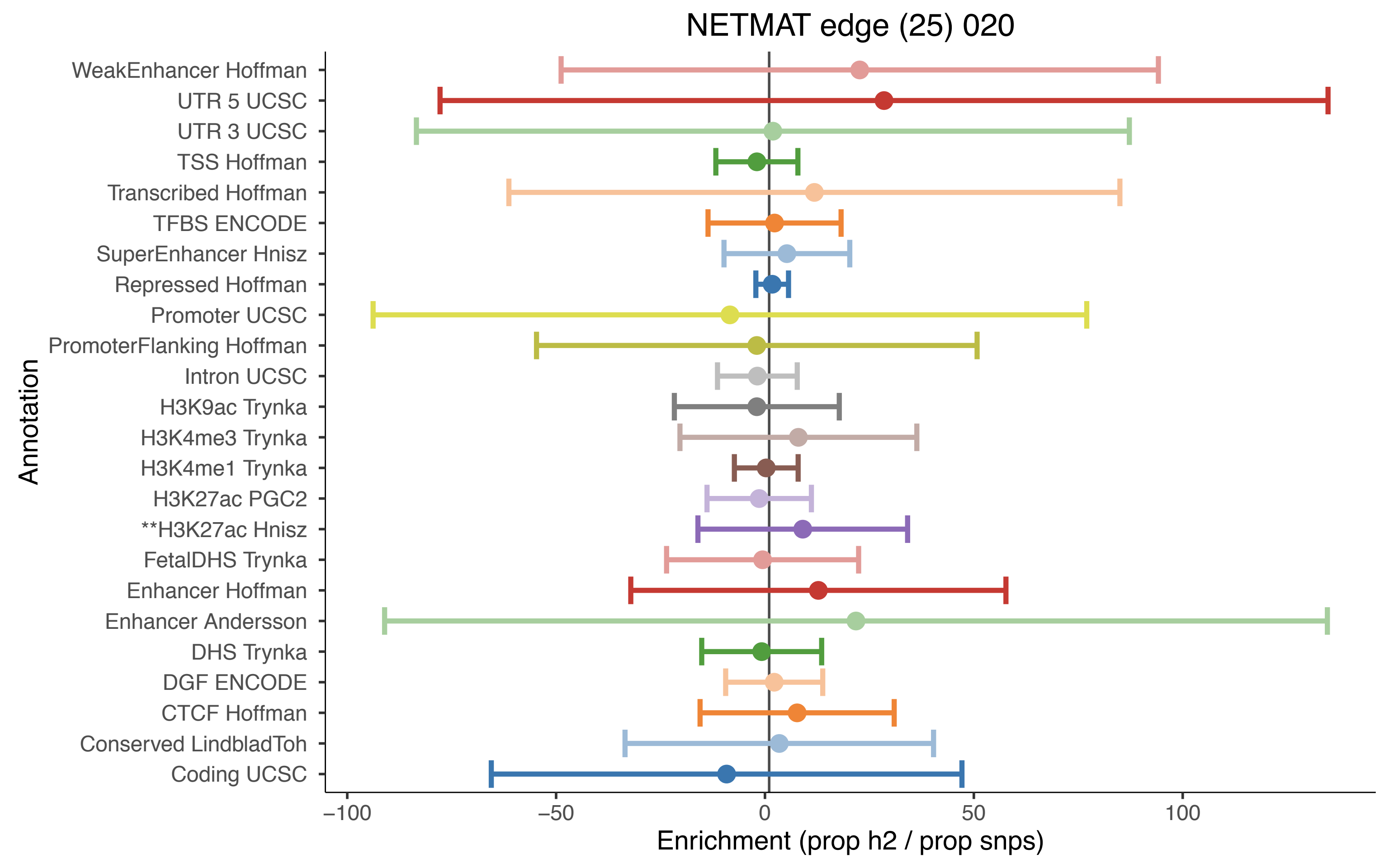

NETMAT edge (25) 020
WeakEnhancer Hoffman
UTR 5 UCSC
UTR 3 UCSC
TSS Hoffman
Transcribed Hoffman
TFBS ENCODE
SuperEnhancer Hnisz
Repressed Hoffman
Promoter UCSC
PromoterFlanking Hoffman
Intron UCSC
H3K9ac Trynka
H3K4me3 Trynka
H3K4me1 Trynka
H3K27ac PGC2
**H3K27ac Hnisz
FetalDHS Trynka
Enhancer Hoffman
Enhancer Andersson
DHS Trynka
DGF ENCODE
CTCF Hoffman
Conserved LindbladToh
Coding UCSC
Annotation
−100
−50
0
50
100
Enrichment (prop h2 / prop snps)

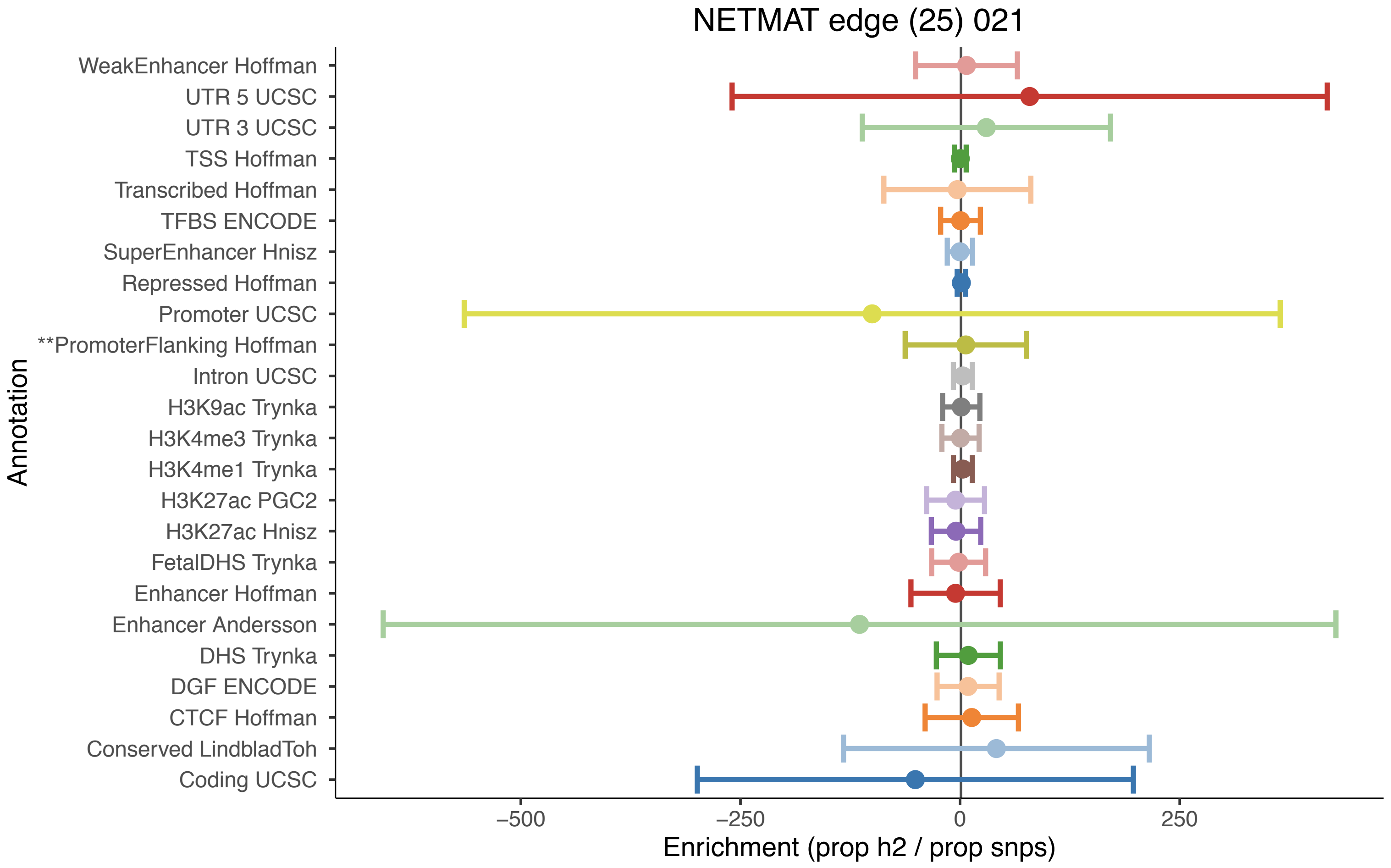
NETMAT edge (25) 021
WeakEnhancer Hoffman
UTR 5 UCSC
UTR 3 UCSC
TSS Hoffman
Transcribed Hoffman
TFBS ENCODE
SuperEnhancer Hnisz
Repressed Hoffman
Promoter UCSC
**PromoterFlanking Hoffman
Intron UCSC
H3K9ac Trynka
H3K4me3 Trynka
H3K4me1 Trynka
H3K27ac PGC2
H3K27ac Hnisz
FetalDHS Trynka
Enhancer Hoffman
Enhancer Andersson
DHS Trynka
DGF ENCODE
CTCF Hoffman
Conserved LindbladToh
Coding UCSC
Annotation
−500
−250
0
250
Enrichment (prop h2 / prop snps)

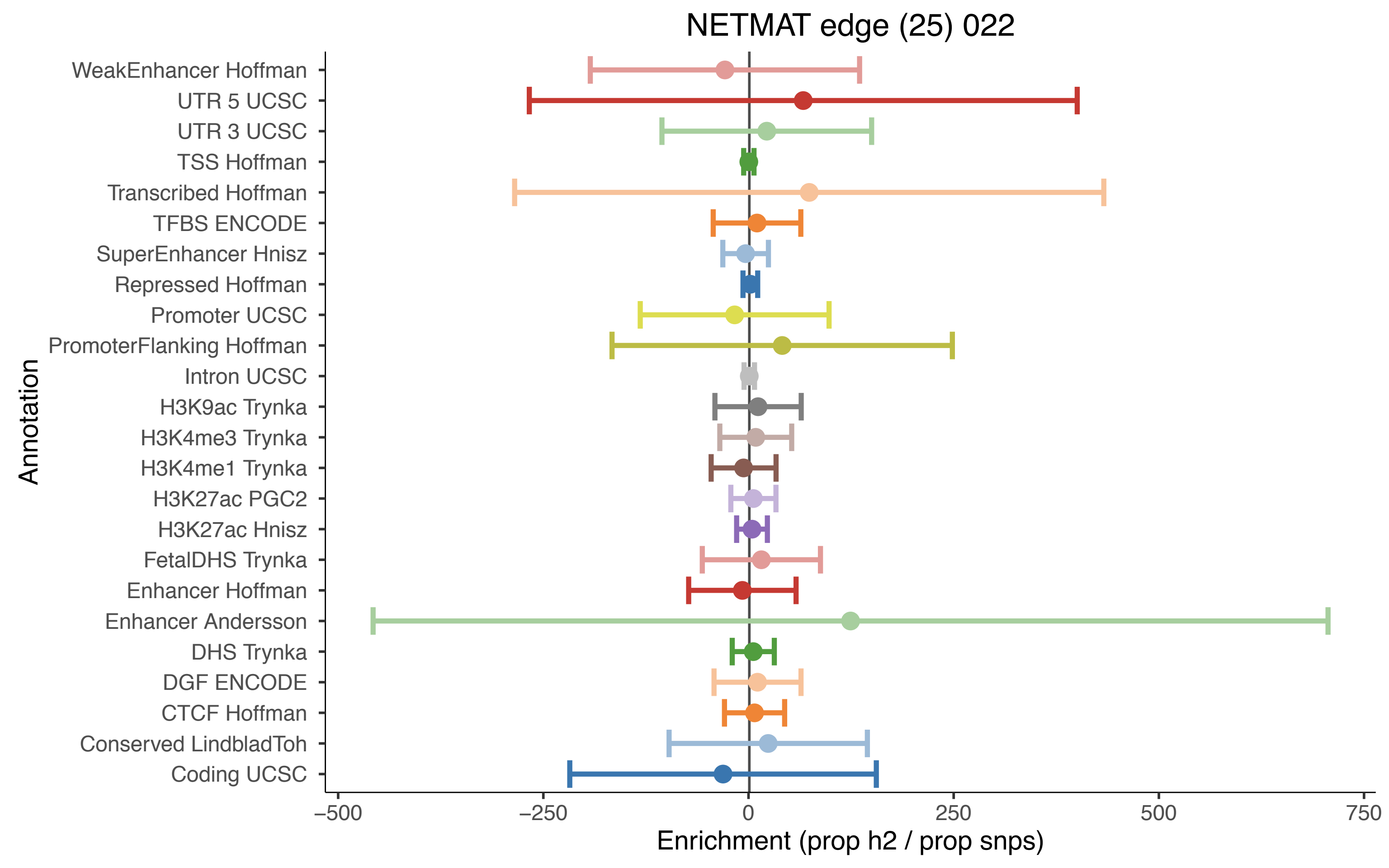

NETMAT edge (25) 022
Annotation
WeakEnhancer Hoffman
UTR 5 UCSC
UTR 3 UCSC
TSS Hoffman
Transcribed Hoffman
TFBS ENCODE
SuperEnhancer Hnisz
Repressed Hoffman
Promoter UCSC
PromoterFlanking Hoffman
Intron UCSC
H3K9ac Trynka
H3K4me3 Trynka
H3K4me1 Trynka
H3K27ac PGC2
H3K27ac Hnisz
FetalDHS Trynka
Enhancer Hoffman
Enhancer Andersson
DHS Trynka
DGF ENCODE
CTCF Hoffman
Conserved LindbladToh
Coding UCSC
−500
−250
0
250
500
750
Enrichment (prop h2 / prop snps)

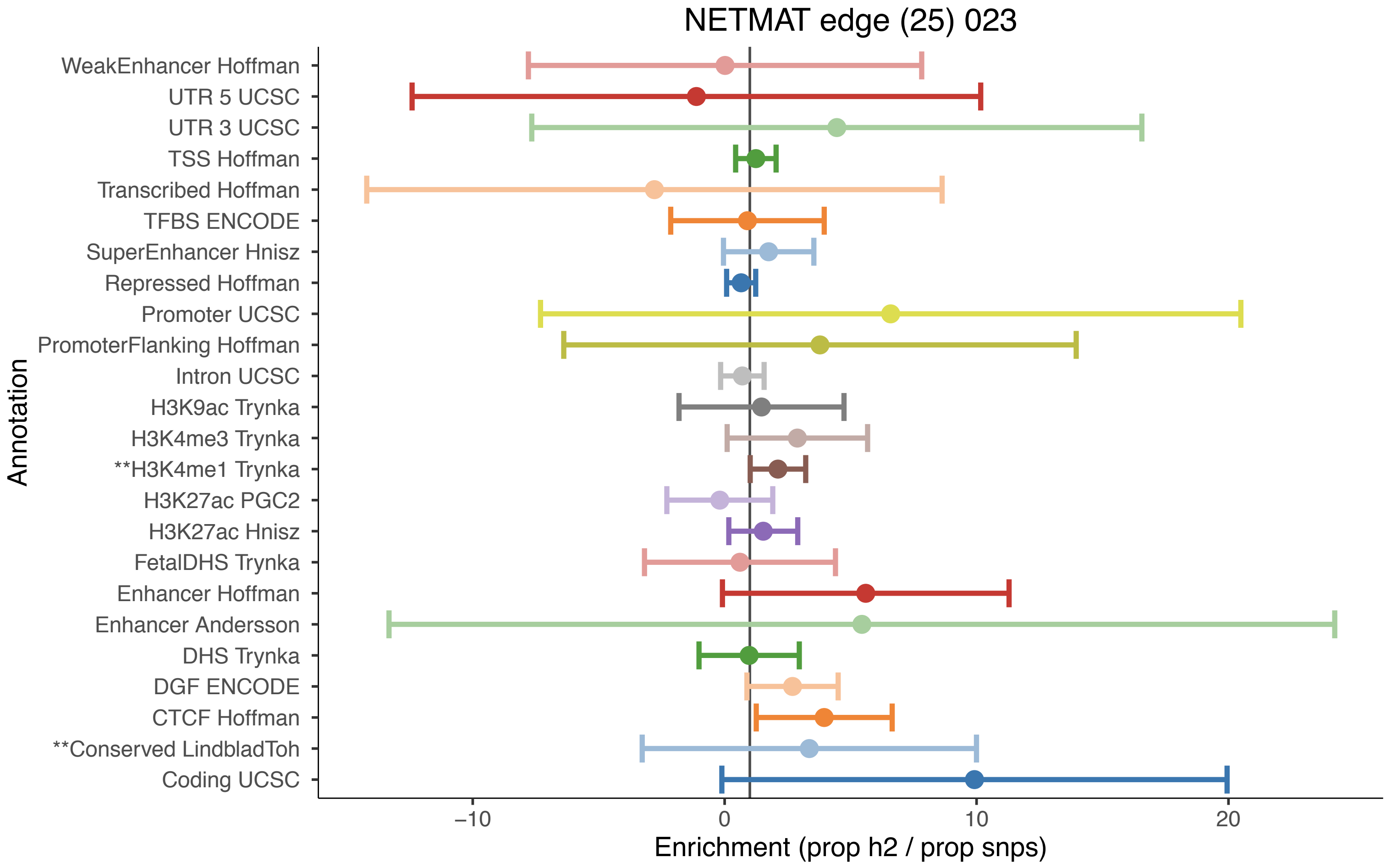

NETMAT edge (25) 023
WeakEnhancer Hoffman
UTR 5 UCSC
UTR 3 UCSC
TSS Hoffman
Transcribed Hoffman
TFBS ENCODE
SuperEnhancer Hnisz
Repressed Hoffman
Promoter UCSC
PromoterFlanking Hoffman
Intron UCSC
H3K9ac Trynka
H3K4me3 Trynka
**H3K4me1 Trynka
H3K27ac PGC2
H3K27ac Hnisz
FetalDHS Trynka
Enhancer Hoffman
Enhancer Andersson
DHS Trynka
DGF ENCODE
CTCF Hoffman
**Conserved LindbladToh
Coding UCSC
Annotation
−10
0
10
20
Enrichment (prop h2 / prop snps)

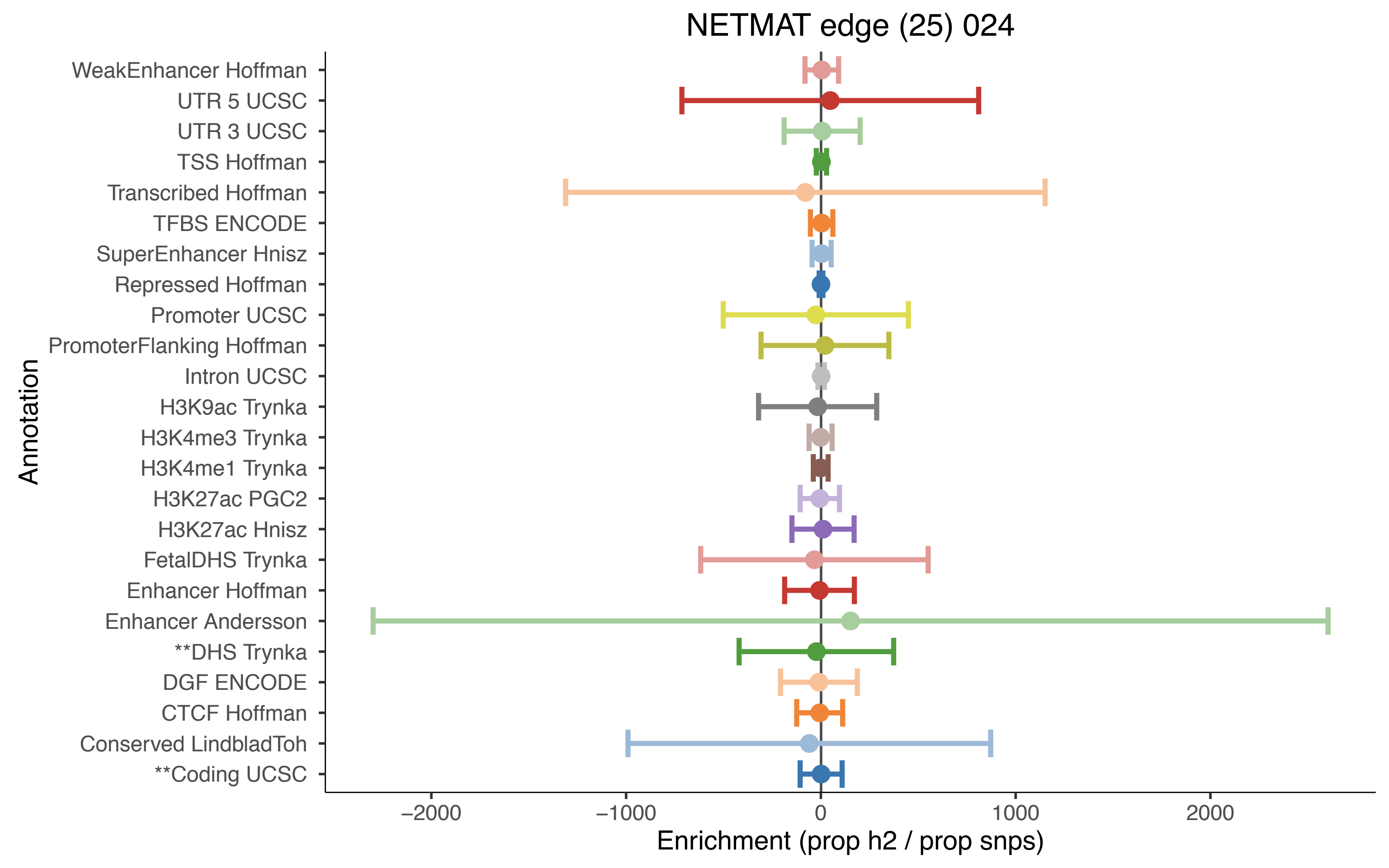

NETMAT edge (25) 024
Annotation
WeakEnhancer Hoffman
UTR 5 UCSC
UTR 3 UCSC
TSS Hoffman
Transcribed Hoffman
TFBS ENCODE
SuperEnhancer Hnisz
Repressed Hoffman
Promoter UCSC
PromoterFlanking Hoffman
Intron UCSC
H3K9ac Trynka
H3K4me3 Trynka
H3K4me1 Trynka
H3K27ac PGC2
H3K27ac Hnisz
FetalDHS Trynka
Enhancer Hoffman
Enhancer Andersson
**DHS Trynka
DGF ENCODE
CTCF Hoffman
Conserved LindbladToh
**Coding UCSC
−2000
−1000
0
1000
2000
Enrichment (prop h2 / prop snps)

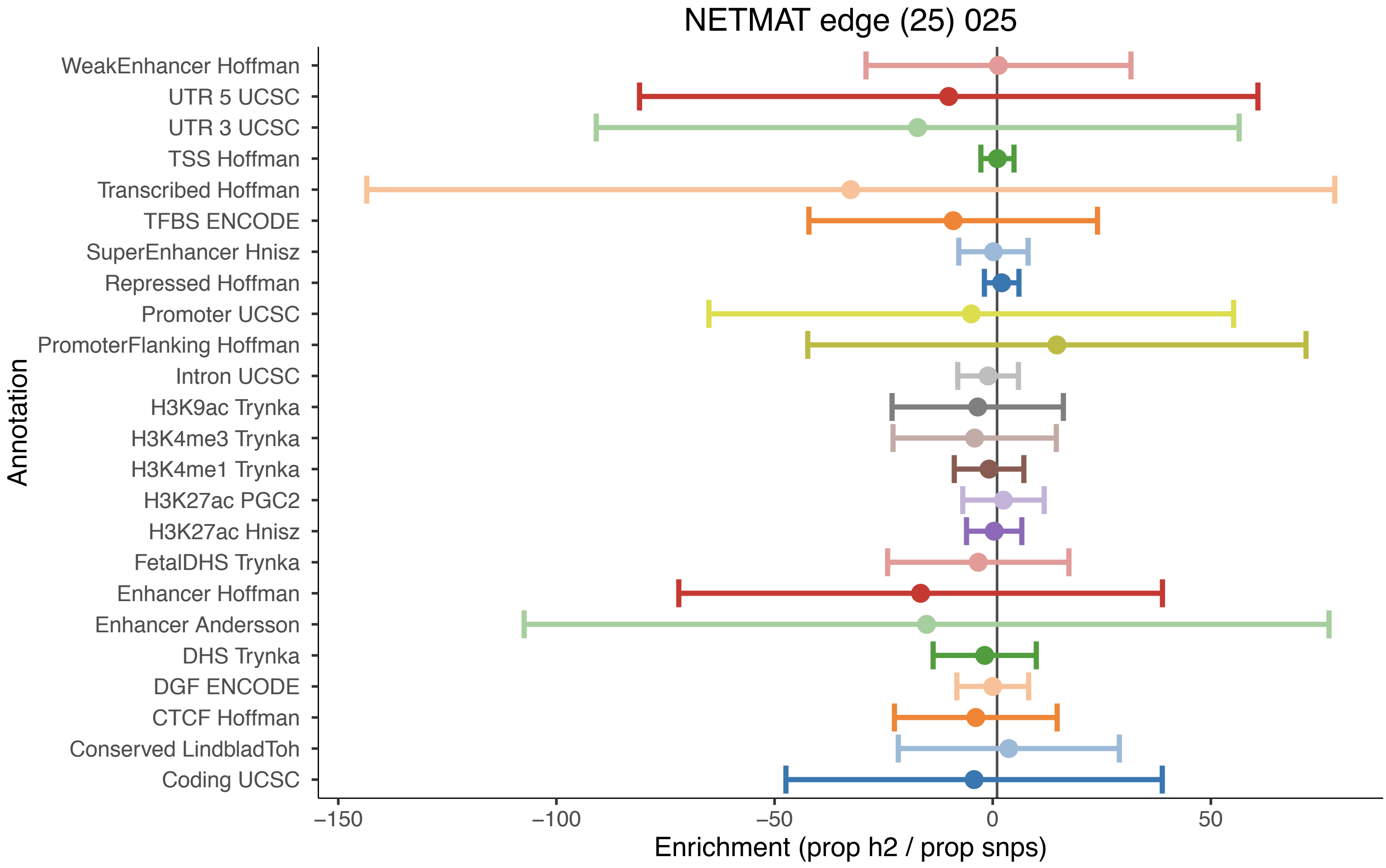

NETMAT edge (25) 025
Annotation
WeakEnhancer Hoffman
UTR 5 UCSC
UTR 3 UCSC
TSS Hoffman
Transcribed Hoffman
TFBS ENCODE
SuperEnhancer Hnisz
Repressed Hoffman
Promoter UCSC
PromoterFlanking Hoffman
Intron UCSC
H3K9ac Trynka
H3K4me3 Trynka
H3K4me1 Trynka
H3K27ac PGC2
H3K27ac Hnisz
FetalDHS Trynka
Enhancer Hoffman
Enhancer Andersson
DHS Trynka
DGF ENCODE
CTCF Hoffman
Conserved LindbladToh
Coding UCSC
−150
−100
−50
0
50
Enrichment (prop h2 / prop snps)

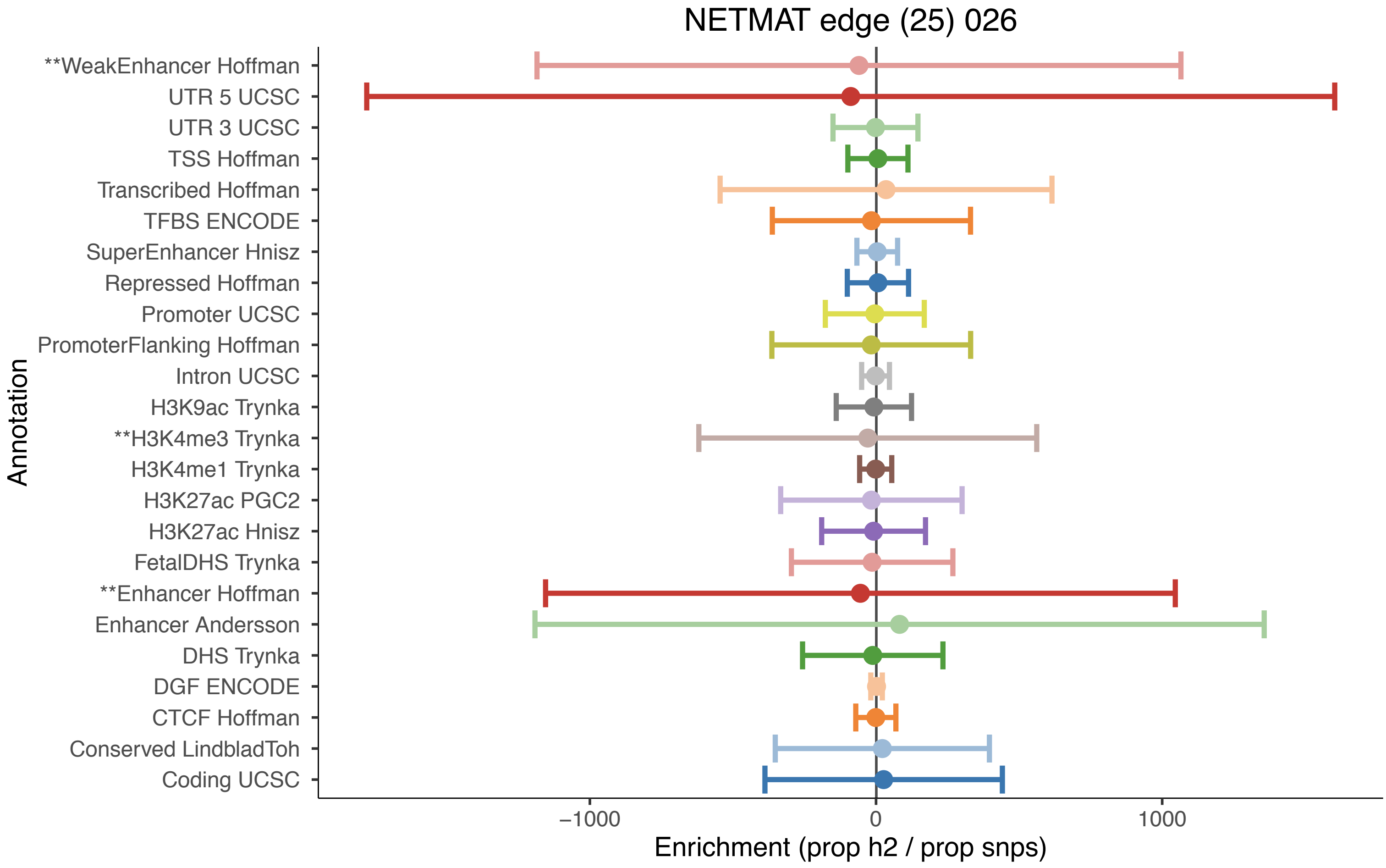

NETMAT edge (25) 026
**WeakEnhancer Hoffman
UTR 5 UCSC
UTR 3 UCSC
TSS Hoffman
Transcribed Hoffman
TFBS ENCODE
SuperEnhancer Hnisz
Repressed Hoffman
Promoter UCSC
PromoterFlanking Hoffman
Intron UCSC
H3K9ac Trynka
**H3K4me3 Trynka
H3K4me1 Trynka
H3K27ac PGC2
H3K27ac Hnisz
FetalDHS Trynka
**Enhancer Hoffman
Enhancer Andersson
DHS Trynka
DGF ENCODE
CTCF Hoffman
Conserved LindbladToh
Coding UCSC
Annotation
−1000
0
1000
Enrichment (prop h2 / prop snps)

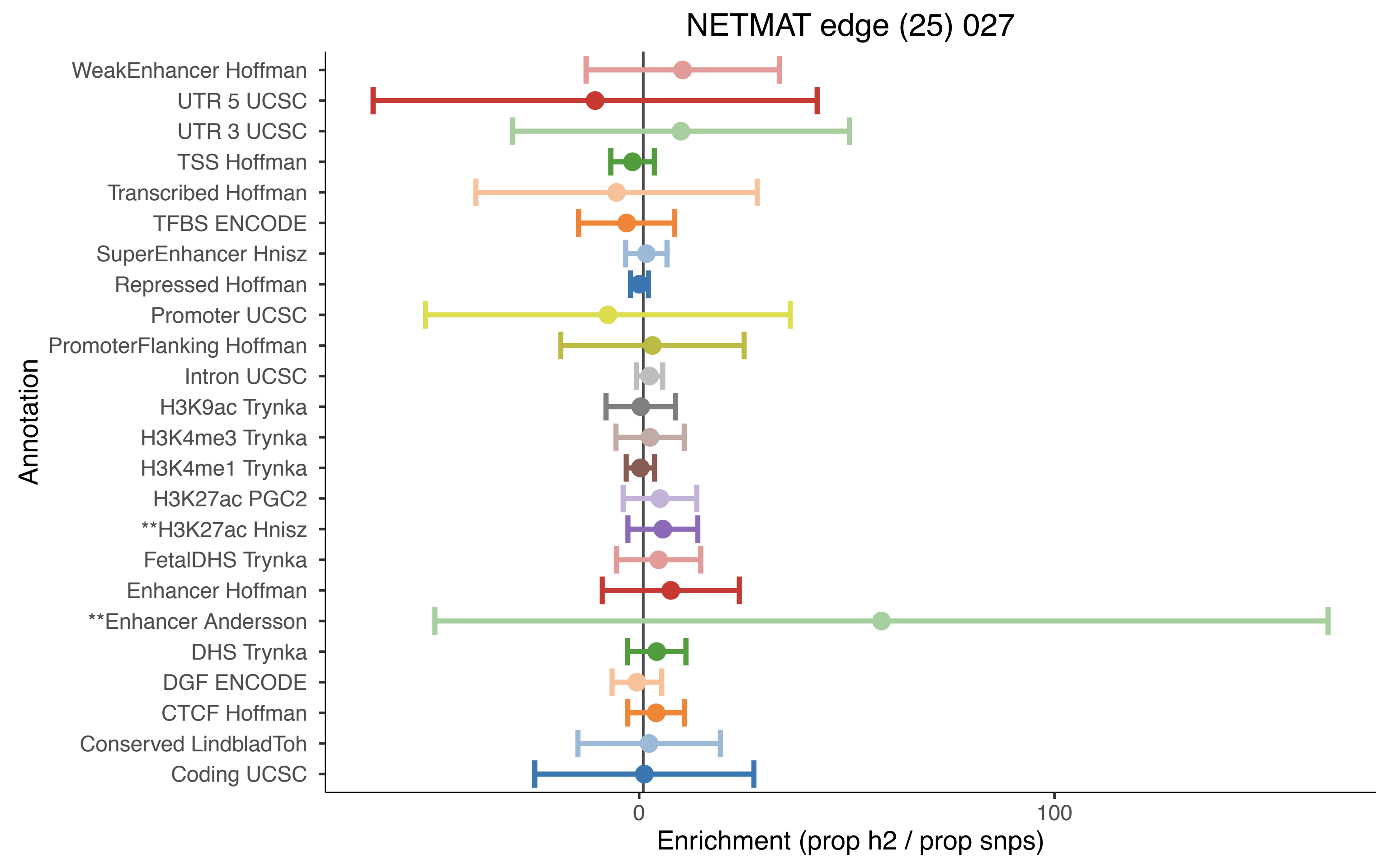

NETMAT edge (25) 027
WeakEnhancer Hoffman
UTR 5 UCSC
UTR 3 UCSC
TSS Hoffman
Transcribed Hoffman
TFBS ENCODE
SuperEnhancer Hnisz
Repressed Hoffman
Promoter UCSC
PromoterFlanking Hoffman
Intron UCSC
H3K9ac Trynka
H3K4me3 Trynka
H3K4me1 Trynka
H3K27ac PGC2
**H3K27ac Hnisz
FetalDHS Trynka
Enhancer Hoffman
**Enhancer Andersson
DHS Trynka
DGF ENCODE
CTCF Hoffman
Conserved LindbladToh
Coding UCSC
Annotation
0
100
Enrichment (prop h2 / prop snps)

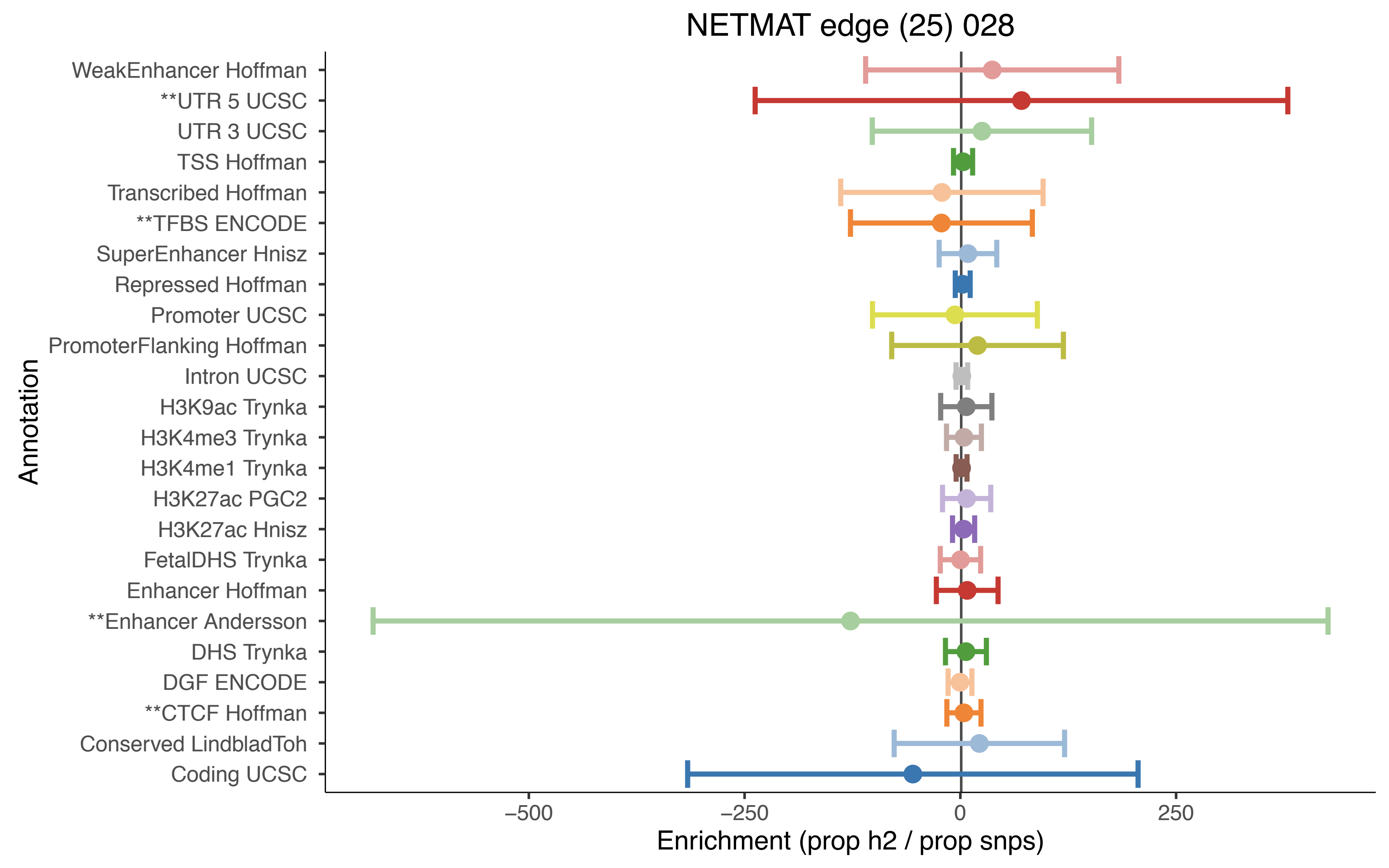

NETMAT edge (25) 028
WeakEnhancer Hoffman
**UTR 5 UCSC
UTR 3 UCSC
TSS Hoffman
Transcribed Hoffman
**TFBS ENCODE
SuperEnhancer Hnisz
Repressed Hoffman
Promoter UCSC
PromoterFlanking Hoffman
Intron UCSC
H3K9ac Trynka
H3K4me3 Trynka
H3K4me1 Trynka
H3K27ac PGC2
H3K27ac Hnisz
FetalDHS Trynka
Enhancer Hoffman
**Enhancer Andersson
DHS Trynka
DGF ENCODE
**CTCF Hoffman
Conserved LindbladToh
Coding UCSC
Annotation
−500
−250
0
250
Enrichment (prop h2 / prop snps)

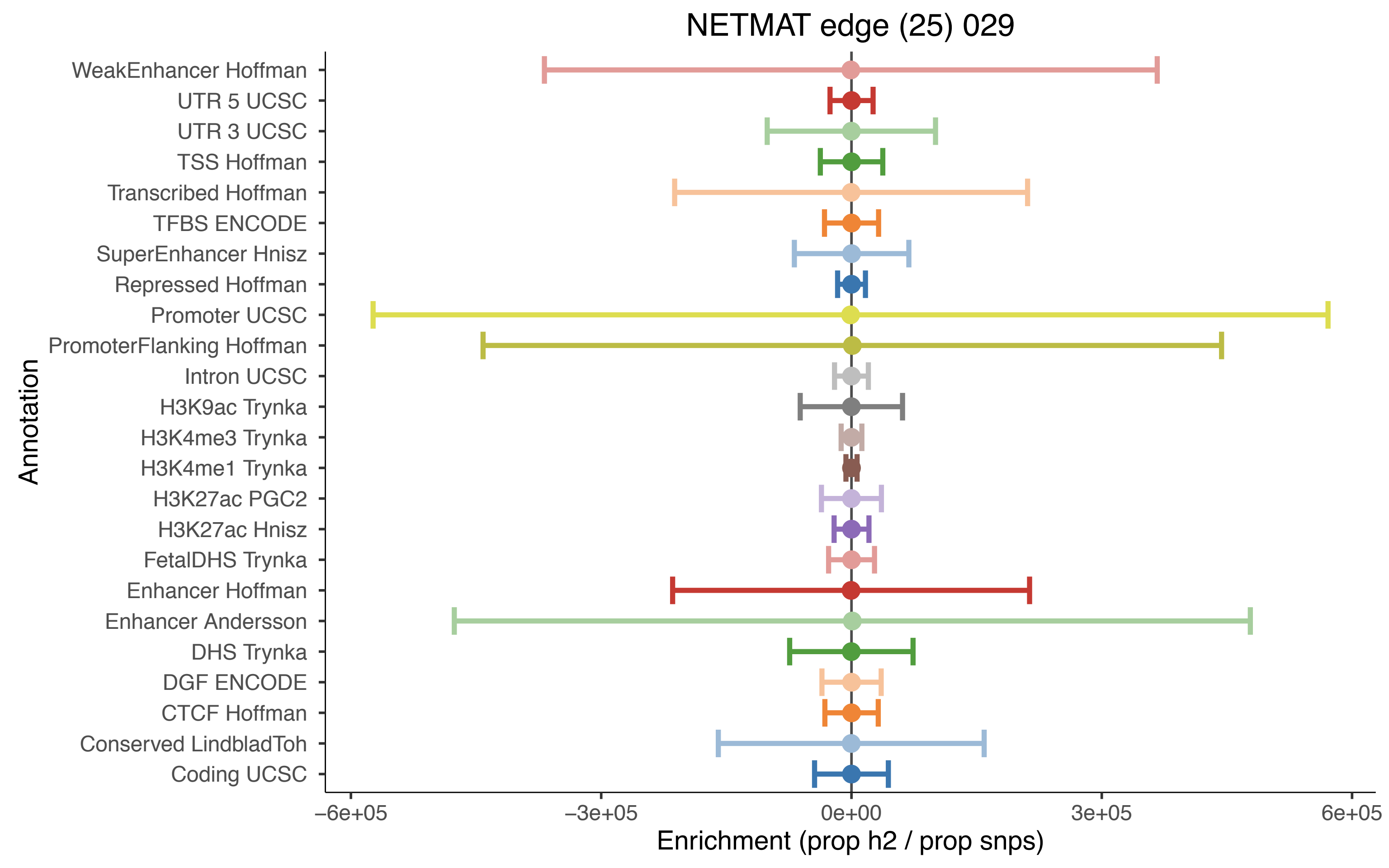

NETMAT edge (25) 029
Annotation
WeakEnhancer Hoffman
UTR 5 UCSC
UTR 3 UCSC
TSS Hoffman
Transcribed Hoffman
TFBS ENCODE
SuperEnhancer Hnisz
Repressed Hoffman
Promoter UCSC
PromoterFlanking Hoffman
Intron UCSC
H3K9ac Trynka
H3K4me3 Trynka
H3K4me1 Trynka
H3K27ac PGC2
H3K27ac Hnisz
FetalDHS Trynka
Enhancer Hoffman
Enhancer Andersson
DHS Trynka
DGF ENCODE
CTCF Hoffman
Conserved LindbladToh
Coding UCSC
−6e+05
−3e+05
0e+00
3e+05
6e+05
Enrichment (prop h2 / prop snps)

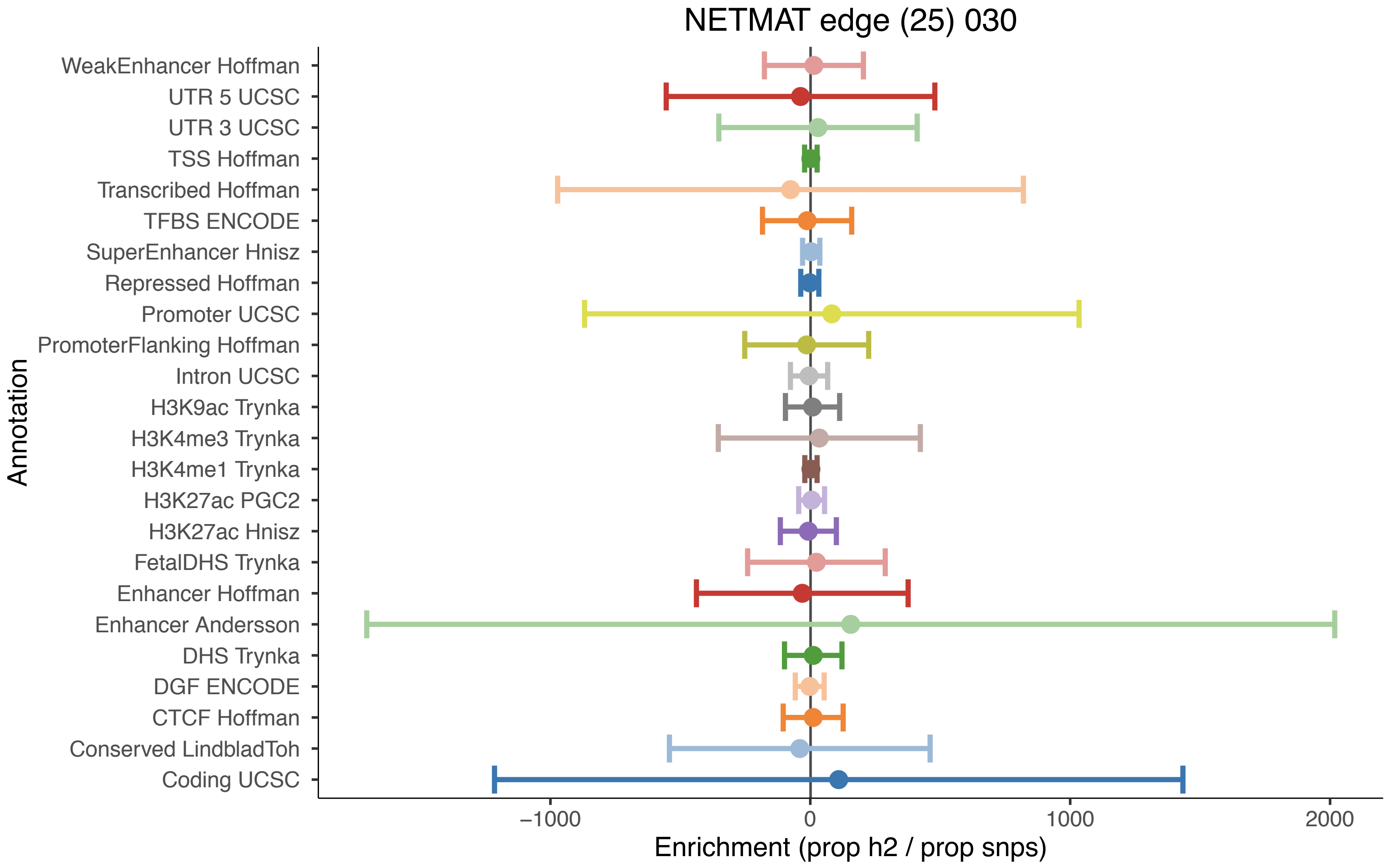

NETMAT edge (25) 030
Annotation
WeakEnhancer Hoffman
UTR 5 UCSC
UTR 3 UCSC
TSS Hoffman
Transcribed Hoffman
TFBS ENCODE
SuperEnhancer Hnisz
Repressed Hoffman
Promoter UCSC
PromoterFlanking Hoffman
Intron UCSC
H3K9ac Trynka
H3K4me3 Trynka
H3K4me1 Trynka
H3K27ac PGC2
H3K27ac Hnisz
FetalDHS Trynka
Enhancer Hoffman
Enhancer Andersson
DHS Trynka
DGF ENCODE
CTCF Hoffman
Conserved LindbladToh
Coding UCSC
−1000
0
1000
2000
Enrichment (prop h2 / prop snps)

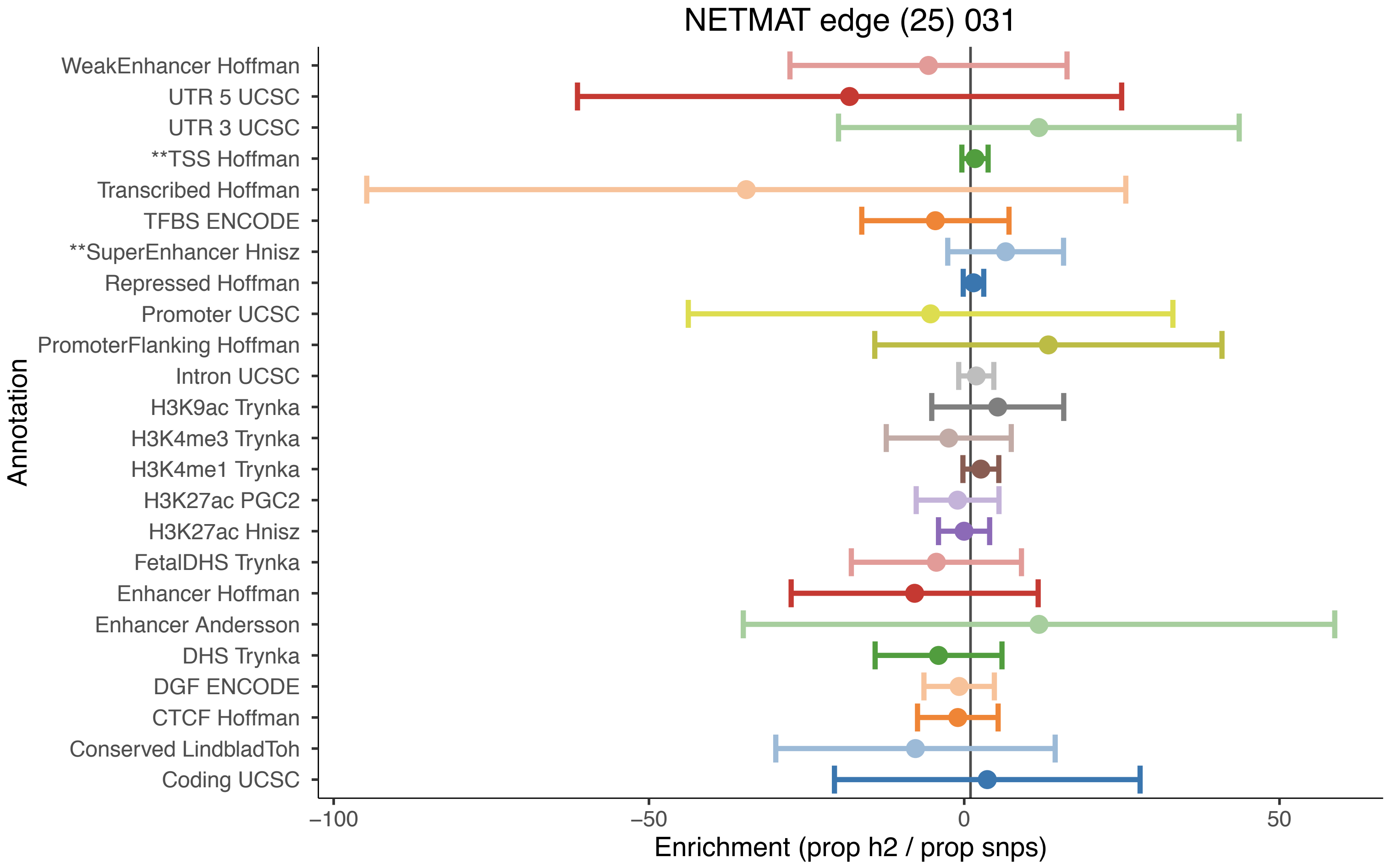

NETMAT edge (25) 031
WeakEnhancer Hoffman
UTR 5 UCSC
UTR 3 UCSC
**TSS Hoffman
Transcribed Hoffman
TFBS ENCODE
**SuperEnhancer Hnisz
Repressed Hoffman
Promoter UCSC
PromoterFlanking Hoffman
Intron UCSC
H3K9ac Trynka
H3K4me3 Trynka
H3K4me1 Trynka
H3K27ac PGC2
H3K27ac Hnisz
FetalDHS Trynka
Enhancer Hoffman
Enhancer Andersson
DHS Trynka
DGF ENCODE
CTCF Hoffman
Conserved LindbladToh
Coding UCSC
Annotation
−100
−50
0
50
Enrichment (prop h2 / prop snps)

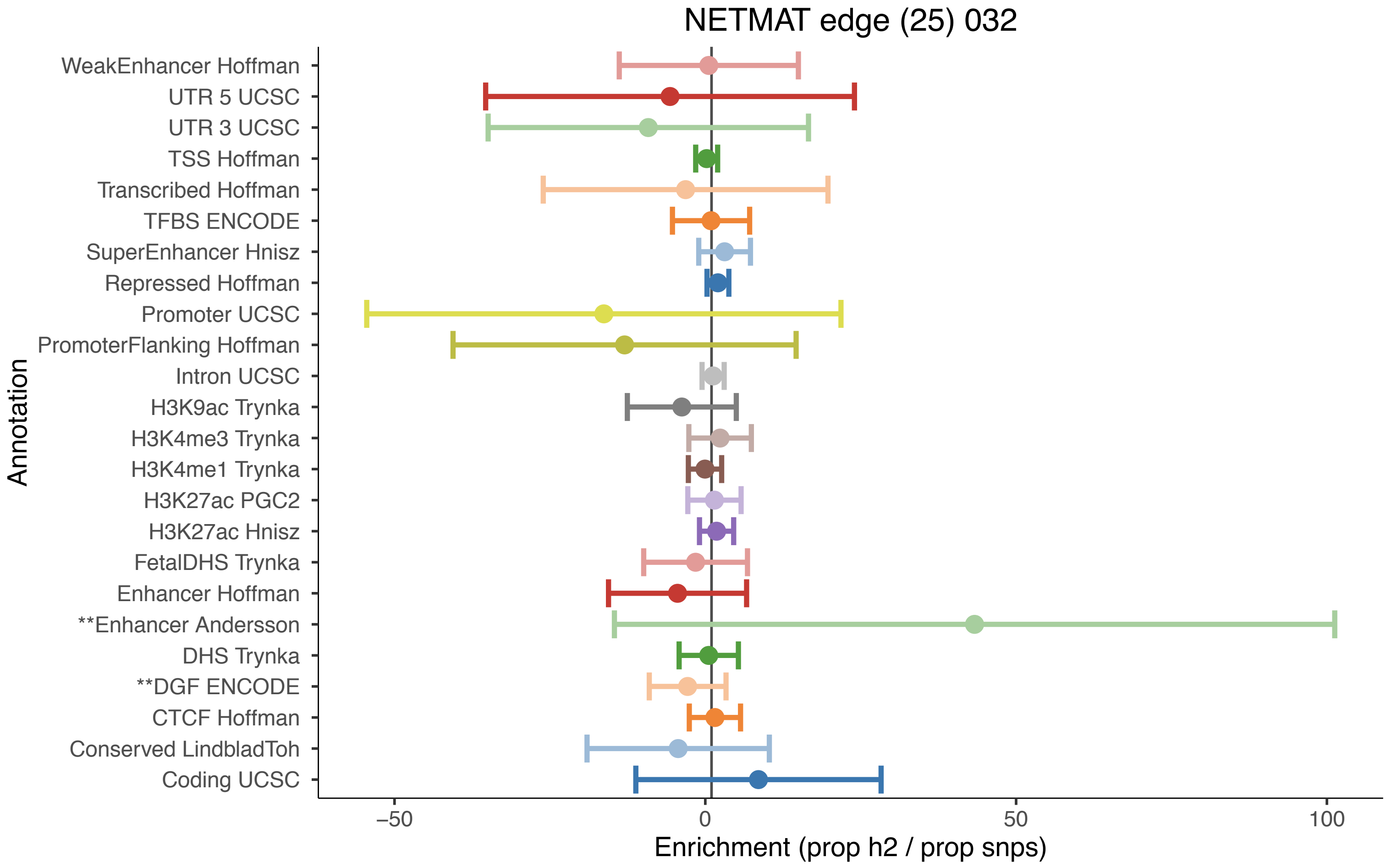

NETMAT edge (25) 032
Annotation
WeakEnhancer Hoffman
UTR 5 UCSC
UTR 3 UCSC
TSS Hoffman
Transcribed Hoffman
TFBS ENCODE
SuperEnhancer Hnisz
Repressed Hoffman
Promoter UCSC
PromoterFlanking Hoffman
Intron UCSC
H3K9ac Trynka
H3K4me3 Trynka
H3K4me1 Trynka
H3K27ac PGC2
H3K27ac Hnisz
FetalDHS Trynka
Enhancer Hoffman
**Enhancer Andersson
DHS Trynka
**DGF ENCODE
CTCF Hoffman
Conserved LindbladToh
Coding UCSC
−50
0
50
100
Enrichment (prop h2 / prop snps)

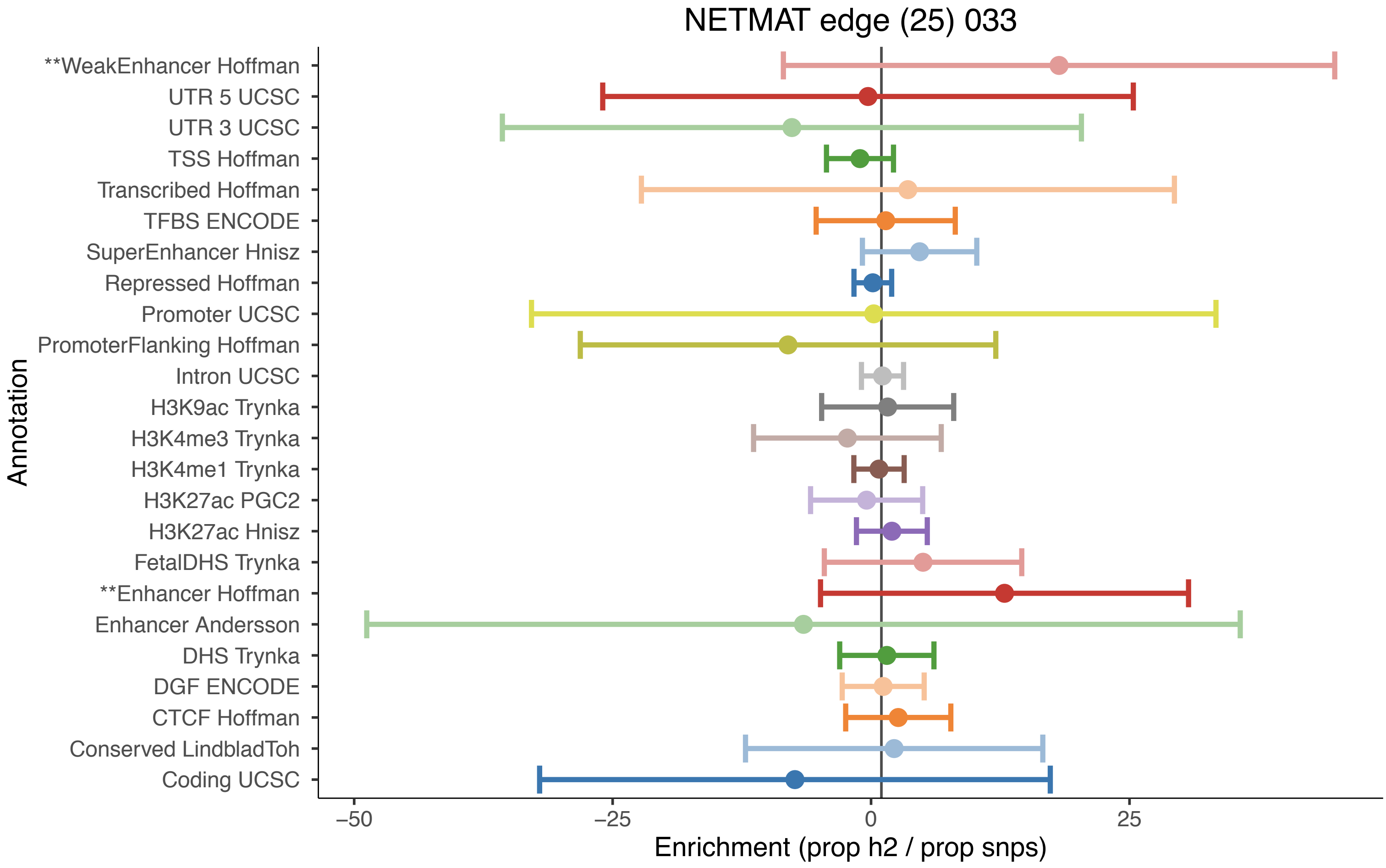
NETMAT edge (25) 033
**WeakEnhancer Hoffman
UTR 5 UCSC
UTR 3 UCSC
TSS Hoffman
Transcribed Hoffman
TFBS ENCODE
SuperEnhancer Hnisz
Repressed Hoffman
Promoter UCSC
PromoterFlanking Hoffman
Intron UCSC
H3K9ac Trynka
H3K4me3 Trynka
H3K4me1 Trynka
H3K27ac PGC2
H3K27ac Hnisz
FetalDHS Trynka
**Enhancer Hoffman
Enhancer Andersson
DHS Trynka
DGF ENCODE
CTCF Hoffman
Conserved LindbladToh
Coding UCSC
Annotation
−50
−25
0
25
Enrichment (prop h2 / prop snps)

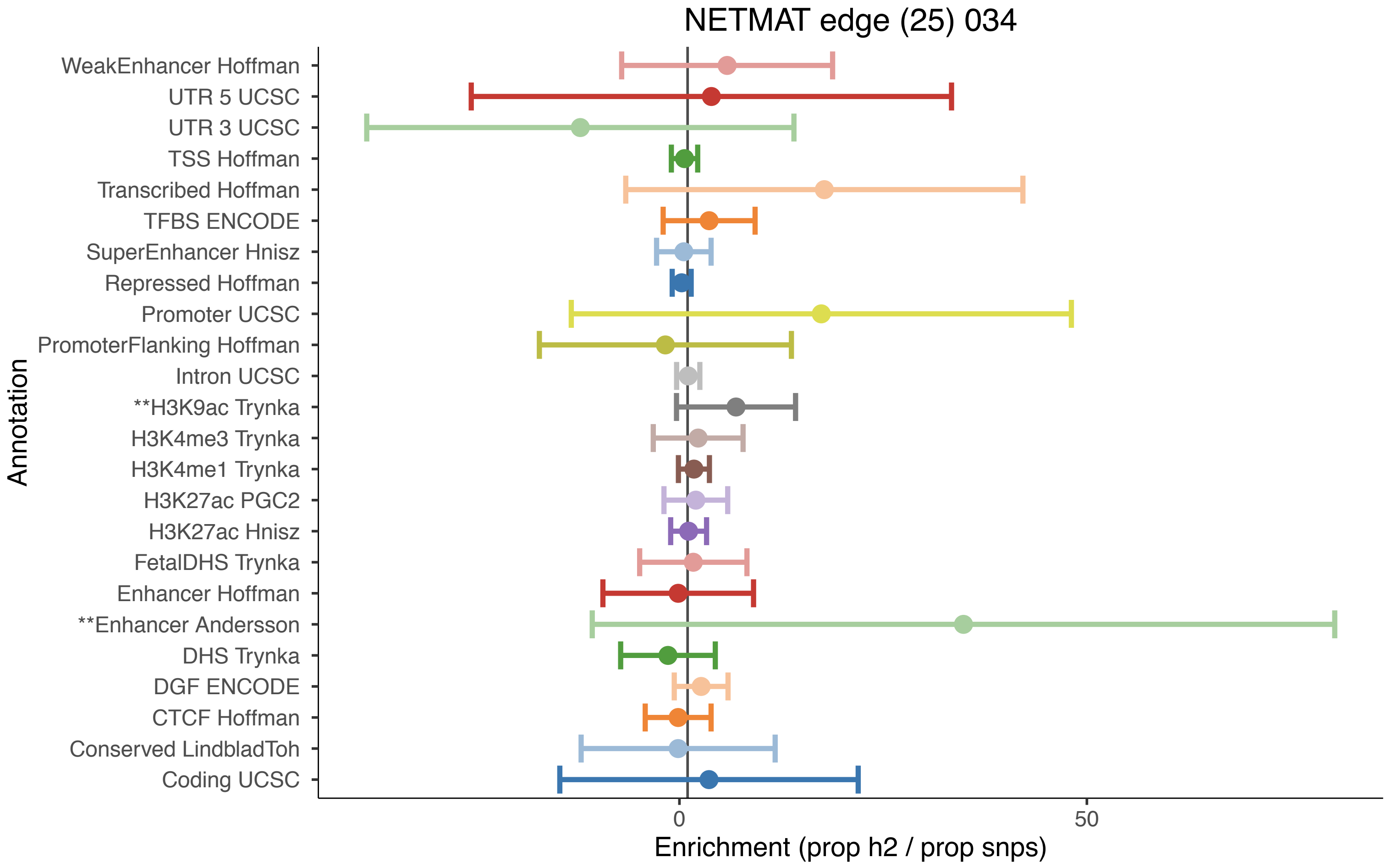
NETMAT edge (25) 034
WeakEnhancer Hoffman
UTR 5 UCSC
UTR 3 UCSC
TSS Hoffman
Transcribed Hoffman
TFBS ENCODE
SuperEnhancer Hnisz
Repressed Hoffman
Promoter UCSC
PromoterFlanking Hoffman
Intron UCSC
**H3K9ac Trynka
H3K4me3 Trynka
H3K4me1 Trynka
H3K27ac PGC2
H3K27ac Hnisz
FetalDHS Trynka
Enhancer Hoffman
**Enhancer Andersson
DHS Trynka
DGF ENCODE
CTCF Hoffman
Conserved LindbladToh
Coding UCSC
Annotation
0
50
Enrichment (prop h2 / prop snps)

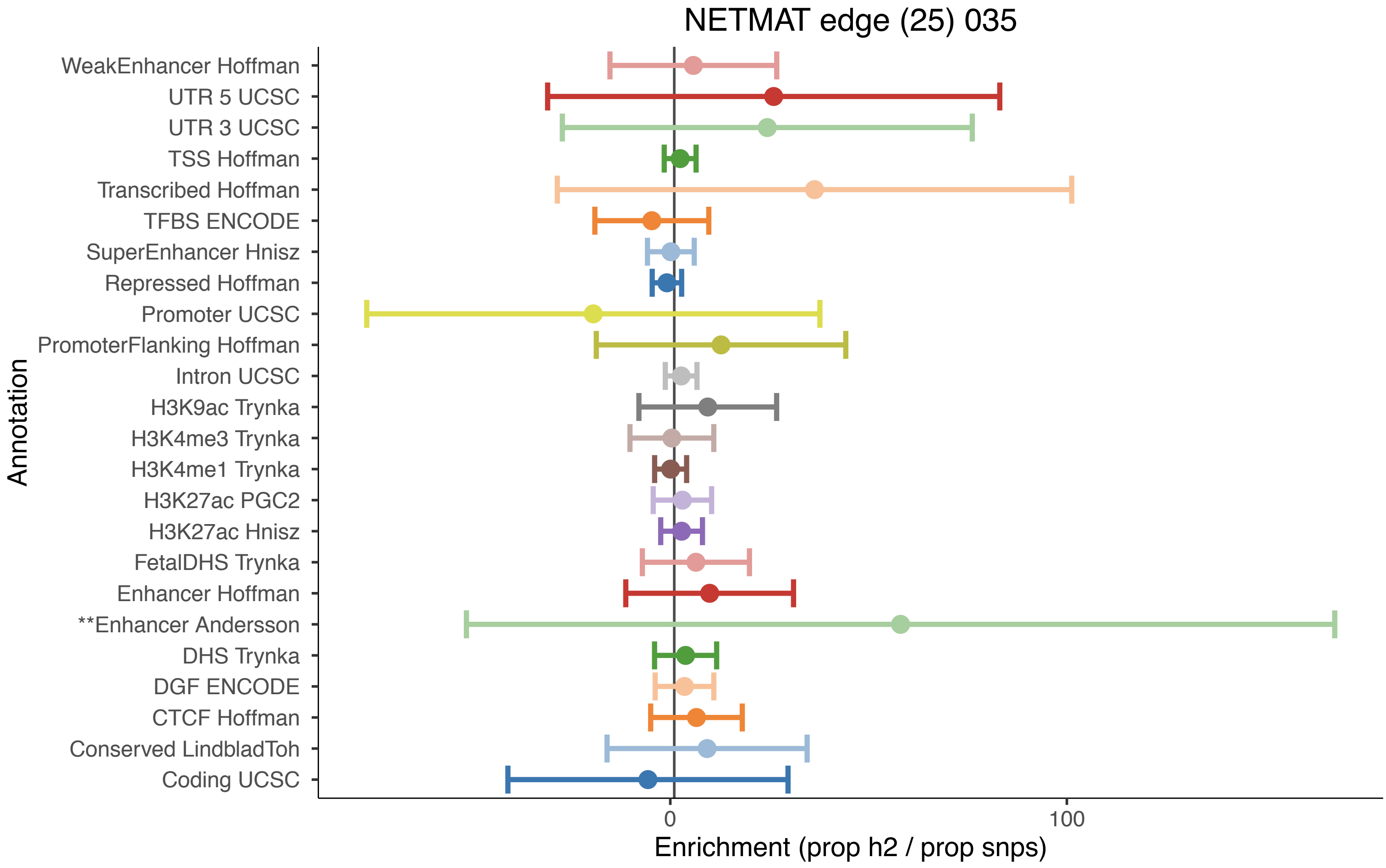
NETMAT edge (25) 035
WeakEnhancer Hoffman
UTR 5 UCSC
UTR 3 UCSC
TSS Hoffman
Transcribed Hoffman
TFBS ENCODE
SuperEnhancer Hnisz
Repressed Hoffman
Promoter UCSC
PromoterFlanking Hoffman
Intron UCSC
H3K9ac Trynka
H3K4me3 Trynka
H3K4me1 Trynka
H3K27ac PGC2
H3K27ac Hnisz
FetalDHS Trynka
Enhancer Hoffman
**Enhancer Andersson
DHS Trynka
DGF ENCODE
CTCF Hoffman
Conserved LindbladToh
Coding UCSC
Annotation
0
100
Enrichment (prop h2 / prop snps)

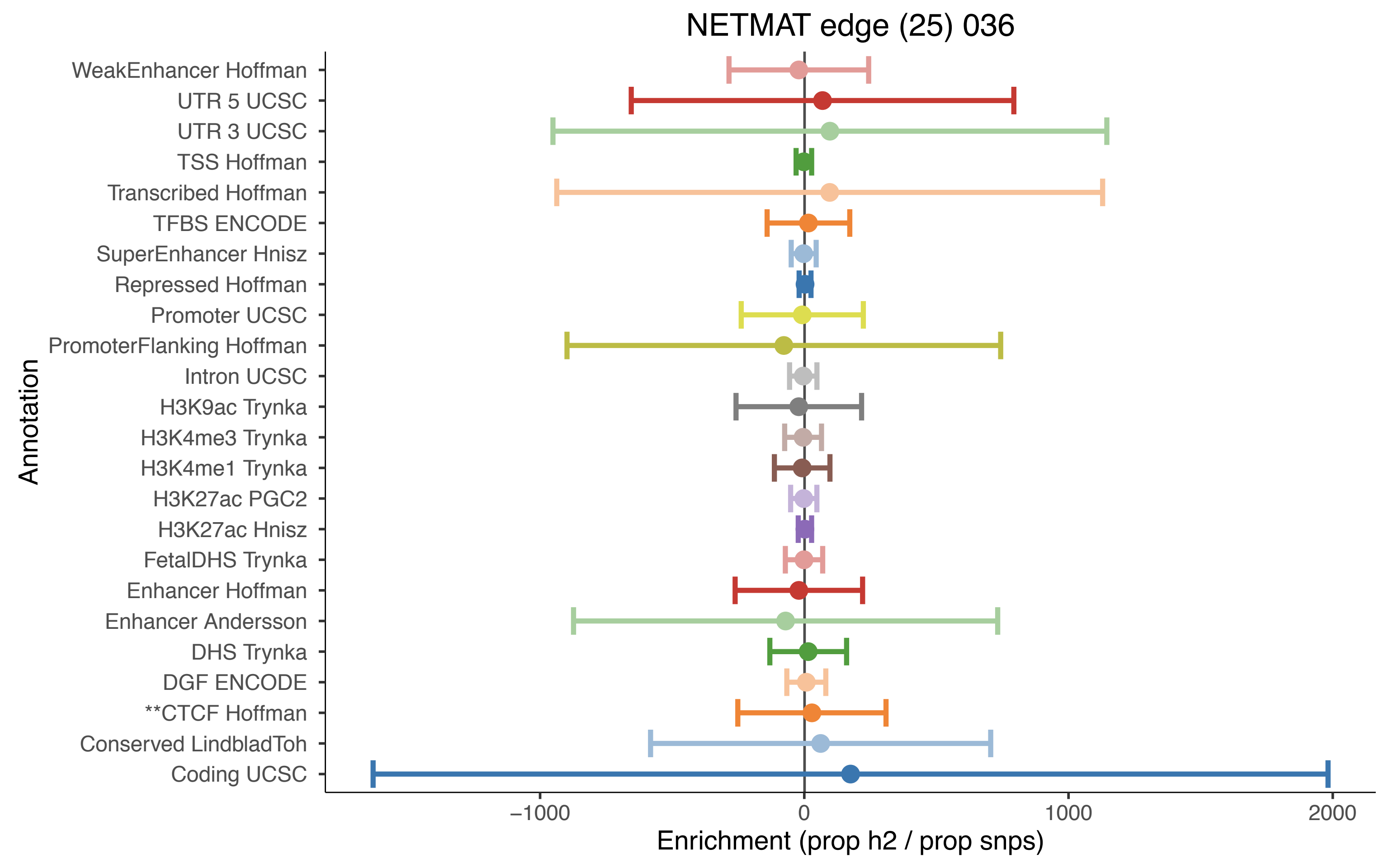

NETMAT edge (25) 036
WeakEnhancer Hoffman
UTR 5 UCSC
UTR 3 UCSC
TSS Hoffman
Transcribed Hoffman
TFBS ENCODE
SuperEnhancer Hnisz
Repressed Hoffman
Promoter UCSC
PromoterFlanking Hoffman
Intron UCSC
H3K9ac Trynka
H3K4me3 Trynka
H3K4me1 Trynka
H3K27ac PGC2
H3K27ac Hnisz
FetalDHS Trynka
Enhancer Hoffman
Enhancer Andersson
DHS Trynka
DGF ENCODE
**CTCF Hoffman
Conserved LindbladToh
Coding UCSC
Annotation
−1000
0
1000
2000
Enrichment (prop h2 / prop snps)

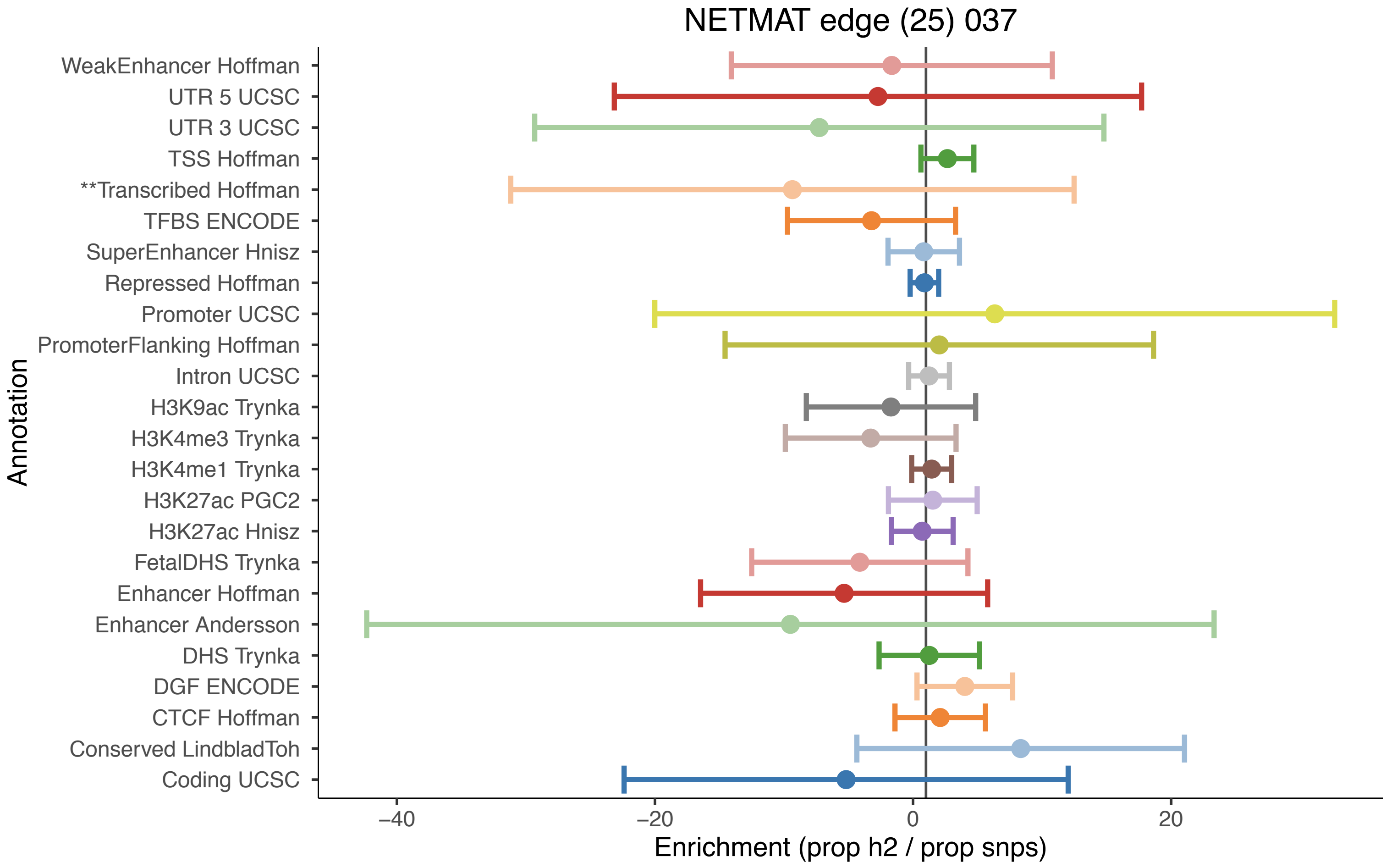
NETMAT edge (25) 037
WeakEnhancer Hoffman
UTR 5 UCSC
UTR 3 UCSC
TSS Hoffman
**Transcribed Hoffman
TFBS ENCODE
SuperEnhancer Hnisz
Repressed Hoffman
Promoter UCSC
PromoterFlanking Hoffman
Intron UCSC
H3K9ac Trynka
H3K4me3 Trynka
H3K4me1 Trynka
H3K27ac PGC2
H3K27ac Hnisz
FetalDHS Trynka
Enhancer Hoffman
Enhancer Andersson
DHS Trynka
DGF ENCODE
CTCF Hoffman
Conserved LindbladToh
Coding UCSC
Annotation
−40
−20
0
20
Enrichment (prop h2 / prop snps)

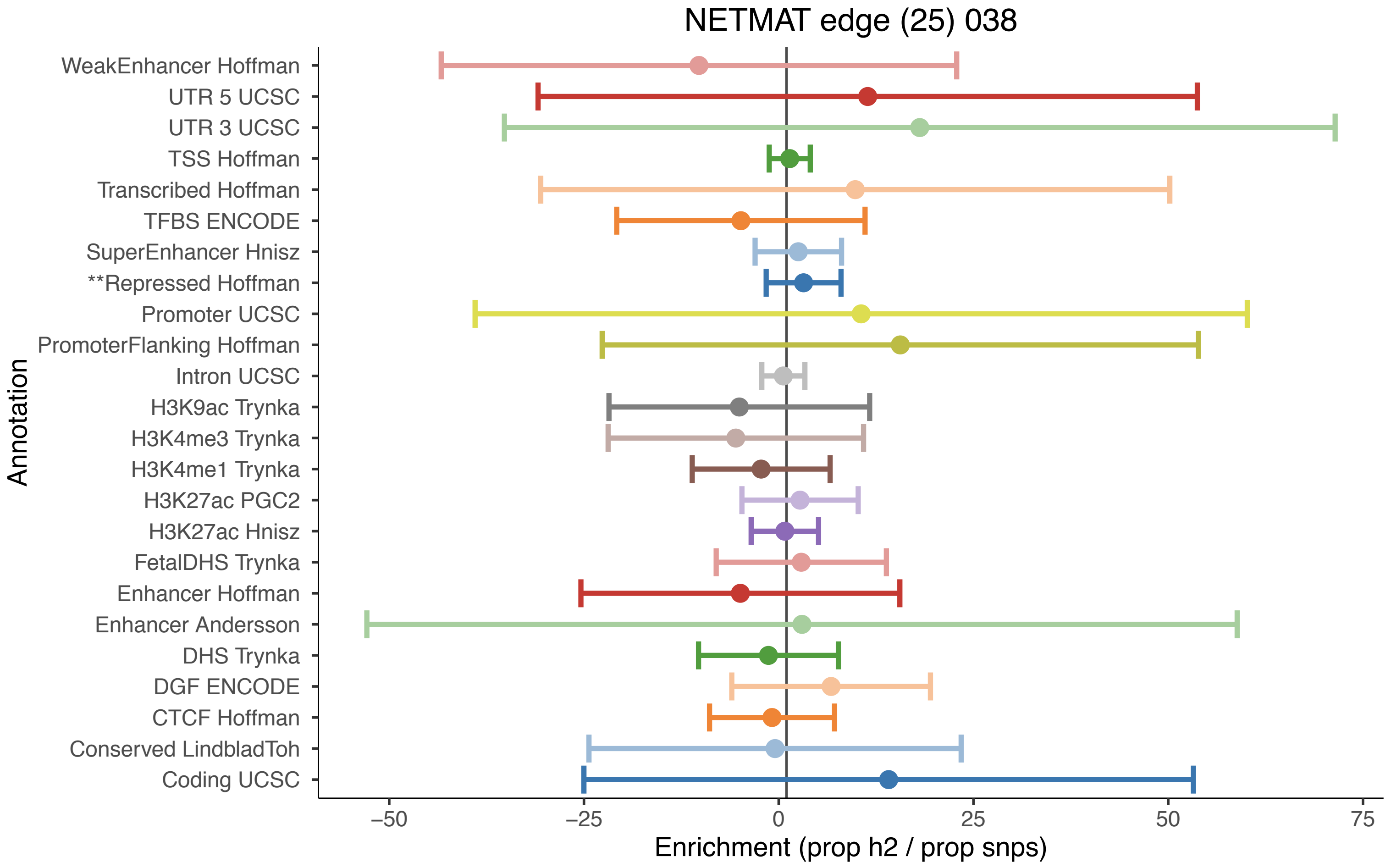
NETMAT edge (25) 038
Annotation
WeakEnhancer Hoffman
UTR 5 UCSC
UTR 3 UCSC
TSS Hoffman
Transcribed Hoffman
TFBS ENCODE
SuperEnhancer Hnisz
**Repressed Hoffman
Promoter UCSC
PromoterFlanking Hoffman
Intron UCSC
H3K9ac Trynka
H3K4me3 Trynka
H3K4me1 Trynka
H3K27ac PGC2
H3K27ac Hnisz
FetalDHS Trynka
Enhancer Hoffman
Enhancer Andersson
DHS Trynka
DGF ENCODE
CTCF Hoffman
Conserved LindbladToh
Coding UCSC
−50
−25
0
25
50
75
Enrichment (prop h2 / prop snps)

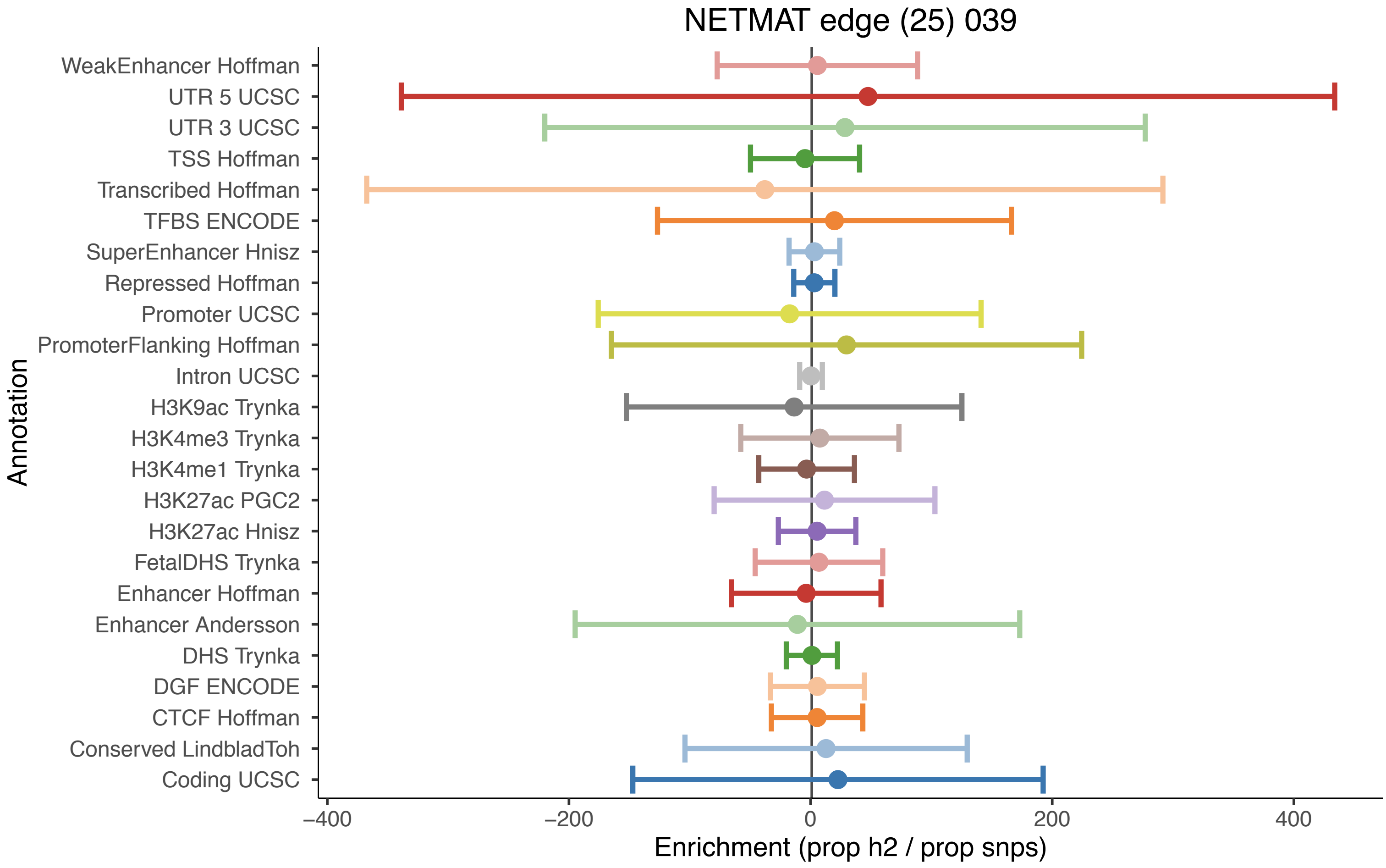

NETMAT edge (25) 039
WeakEnhancer Hoffman
UTR 5 UCSC
UTR 3 UCSC
TSS Hoffman
Transcribed Hoffman
TFBS ENCODE
SuperEnhancer Hnisz
Repressed Hoffman
Promoter UCSC
PromoterFlanking Hoffman
Intron UCSC
H3K9ac Trynka
H3K4me3 Trynka
H3K4me1 Trynka
H3K27ac PGC2
H3K27ac Hnisz
FetalDHS Trynka
Enhancer Hoffman
Enhancer Andersson
DHS Trynka
DGF ENCODE
CTCF Hoffman
Conserved LindbladToh
Coding UCSC
Annotation
−400
−200
0
200
400
Enrichment (prop h2 / prop snps)

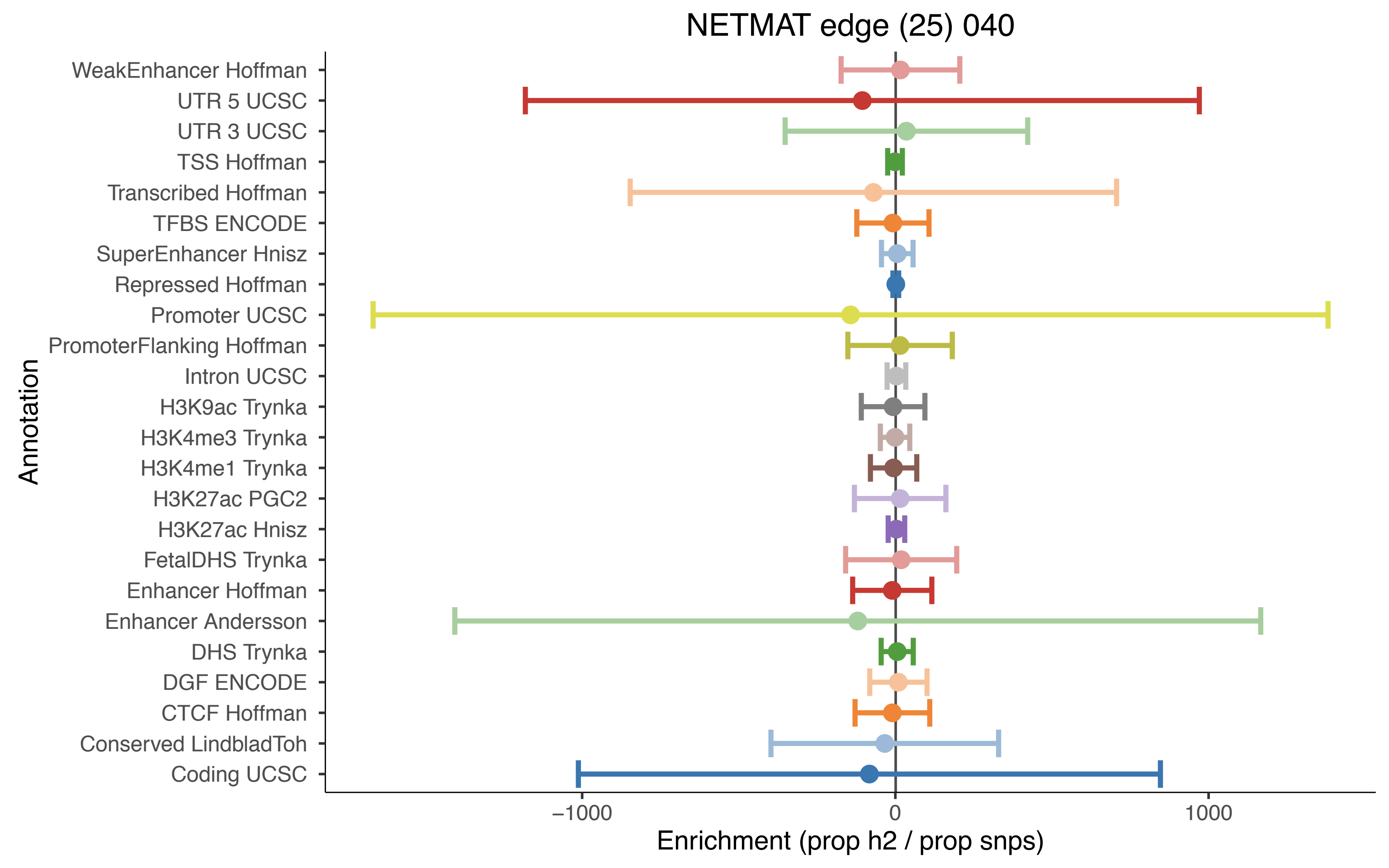

NETMAT edge (25) 040
WeakEnhancer Hoffman
UTR 5 UCSC
UTR 3 UCSC
TSS Hoffman
Transcribed Hoffman
TFBS ENCODE
SuperEnhancer Hnisz
Repressed Hoffman
Promoter UCSC
PromoterFlanking Hoffman
Intron UCSC
H3K9ac Trynka
H3K4me3 Trynka
H3K4me1 Trynka
H3K27ac PGC2
H3K27ac Hnisz
FetalDHS Trynka
Enhancer Hoffman
Enhancer Andersson
DHS Trynka
DGF ENCODE
CTCF Hoffman
Conserved LindbladToh
Coding UCSC
Annotation
−1000
0
1000
Enrichment (prop h2 / prop snps)

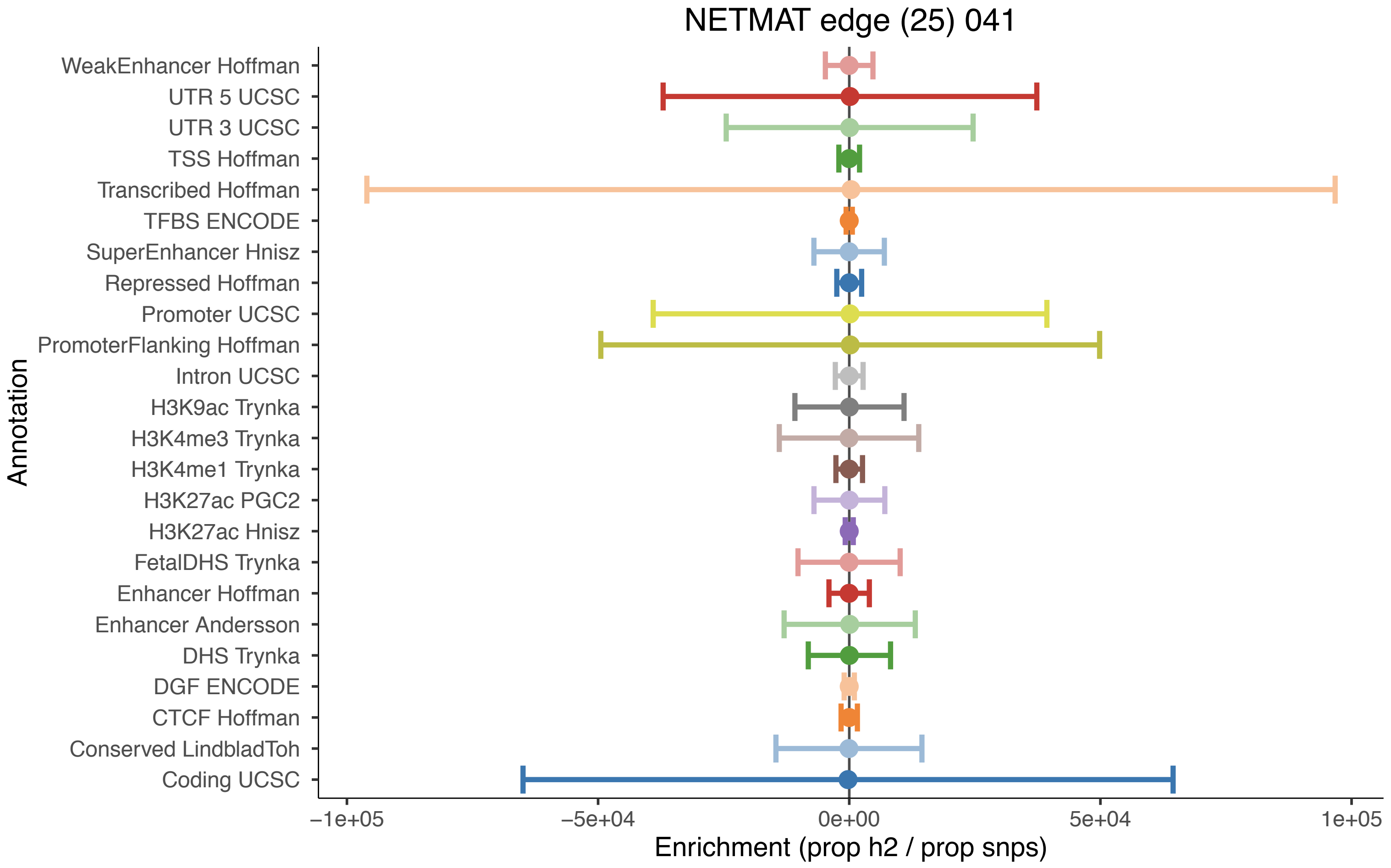

NETMAT edge (25) 041
WeakEnhancer Hoffman
UTR 5 UCSC
UTR 3 UCSC
TSS Hoffman
Transcribed Hoffman
TFBS ENCODE
SuperEnhancer Hnisz
Repressed Hoffman
Promoter UCSC
PromoterFlanking Hoffman
Intron UCSC
H3K9ac Trynka
H3K4me3 Trynka
H3K4me1 Trynka
H3K27ac PGC2
H3K27ac Hnisz
FetalDHS Trynka
Enhancer Hoffman
Enhancer Andersson
DHS Trynka
DGF ENCODE
CTCF Hoffman
Conserved LindbladToh
Coding UCSC
Annotation
−1e+05
−5e+04
0e+00
5e+04
1e+05
Enrichment (prop h2 / prop snps)

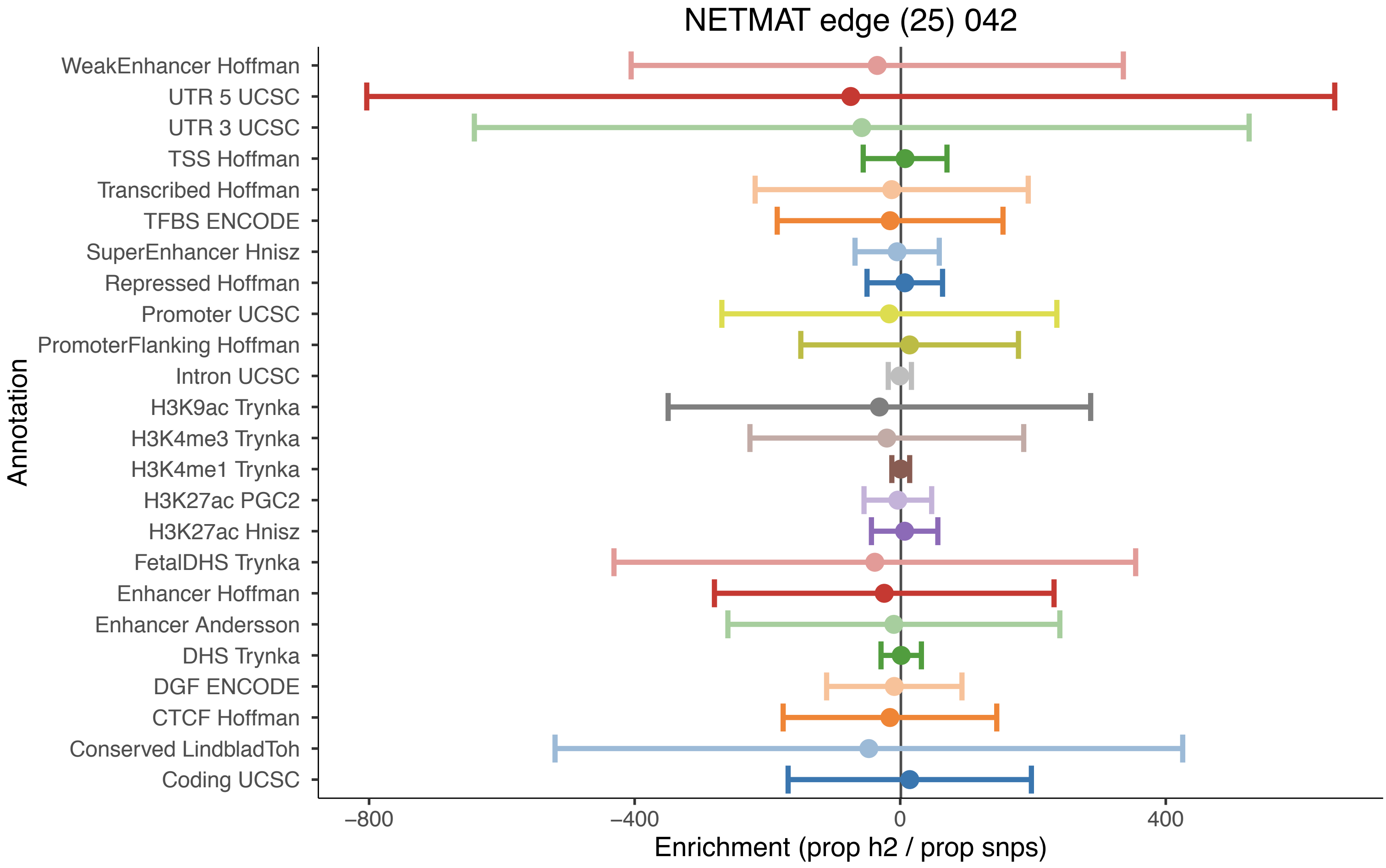

NETMAT edge (25) 042
WeakEnhancer Hoffman
UTR 5 UCSC
UTR 3 UCSC
TSS Hoffman
Transcribed Hoffman
TFBS ENCODE
SuperEnhancer Hnisz
Repressed Hoffman
Promoter UCSC
PromoterFlanking Hoffman
Intron UCSC
H3K9ac Trynka
H3K4me3 Trynka
H3K4me1 Trynka
H3K27ac PGC2
H3K27ac Hnisz
FetalDHS Trynka
Enhancer Hoffman
Enhancer Andersson
DHS Trynka
DGF ENCODE
CTCF Hoffman
Conserved LindbladToh
Coding UCSC
Annotation
−800
−400
0
400
Enrichment (prop h2 / prop snps)

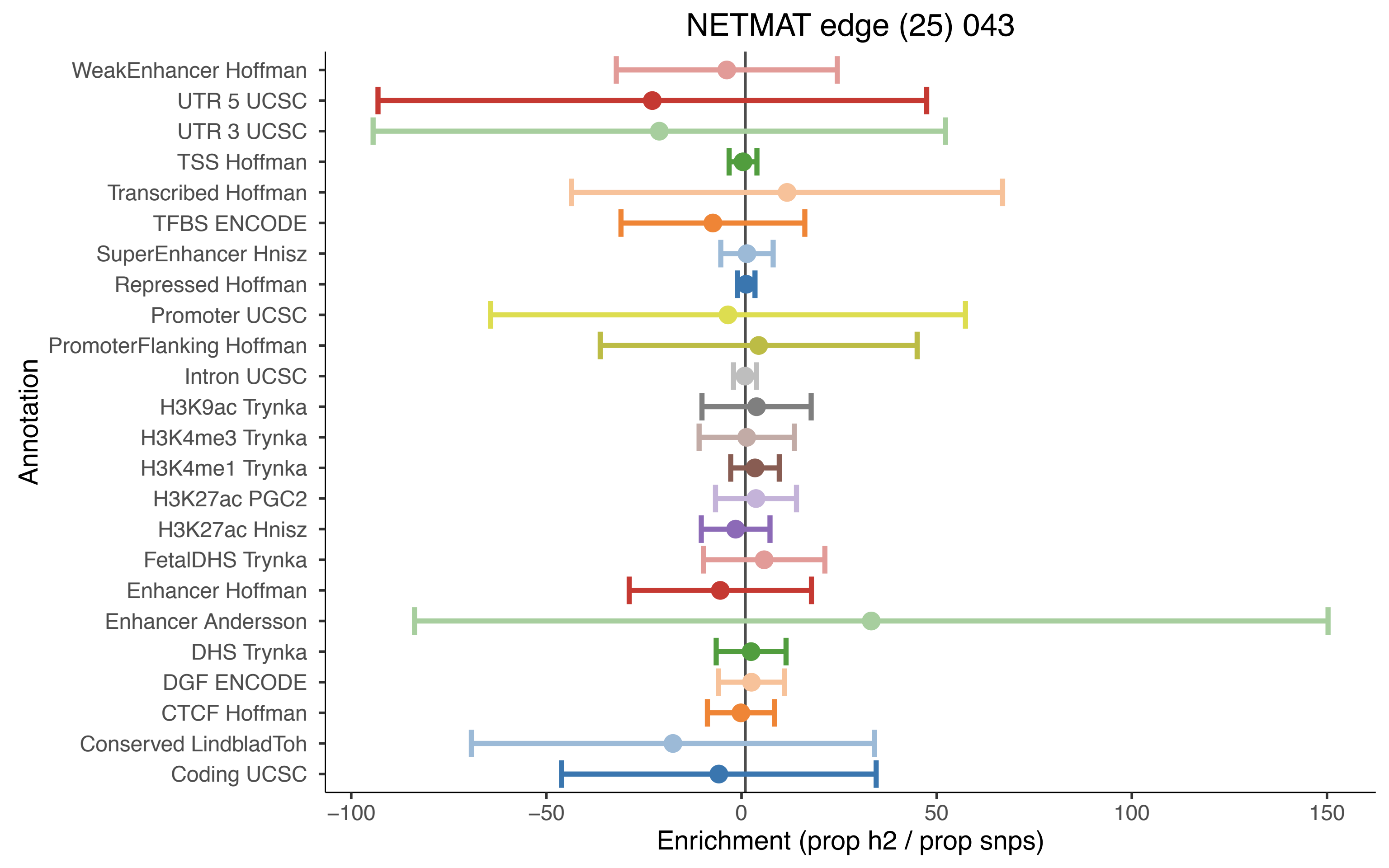
NETMAT edge (25) 043
Annotation
WeakEnhancer Hoffman
UTR 5 UCSC
UTR 3 UCSC
TSS Hoffman
Transcribed Hoffman
TFBS ENCODE
SuperEnhancer Hnisz
Repressed Hoffman
Promoter UCSC
PromoterFlanking Hoffman
Intron UCSC
H3K9ac Trynka
H3K4me3 Trynka
H3K4me1 Trynka
H3K27ac PGC2
H3K27ac Hnisz
FetalDHS Trynka
Enhancer Hoffman
Enhancer Andersson
DHS Trynka
DGF ENCODE
CTCF Hoffman
Conserved LindbladToh
Coding UCSC
−100
−50
0
50
100
150
Enrichment (prop h2 / prop snps)

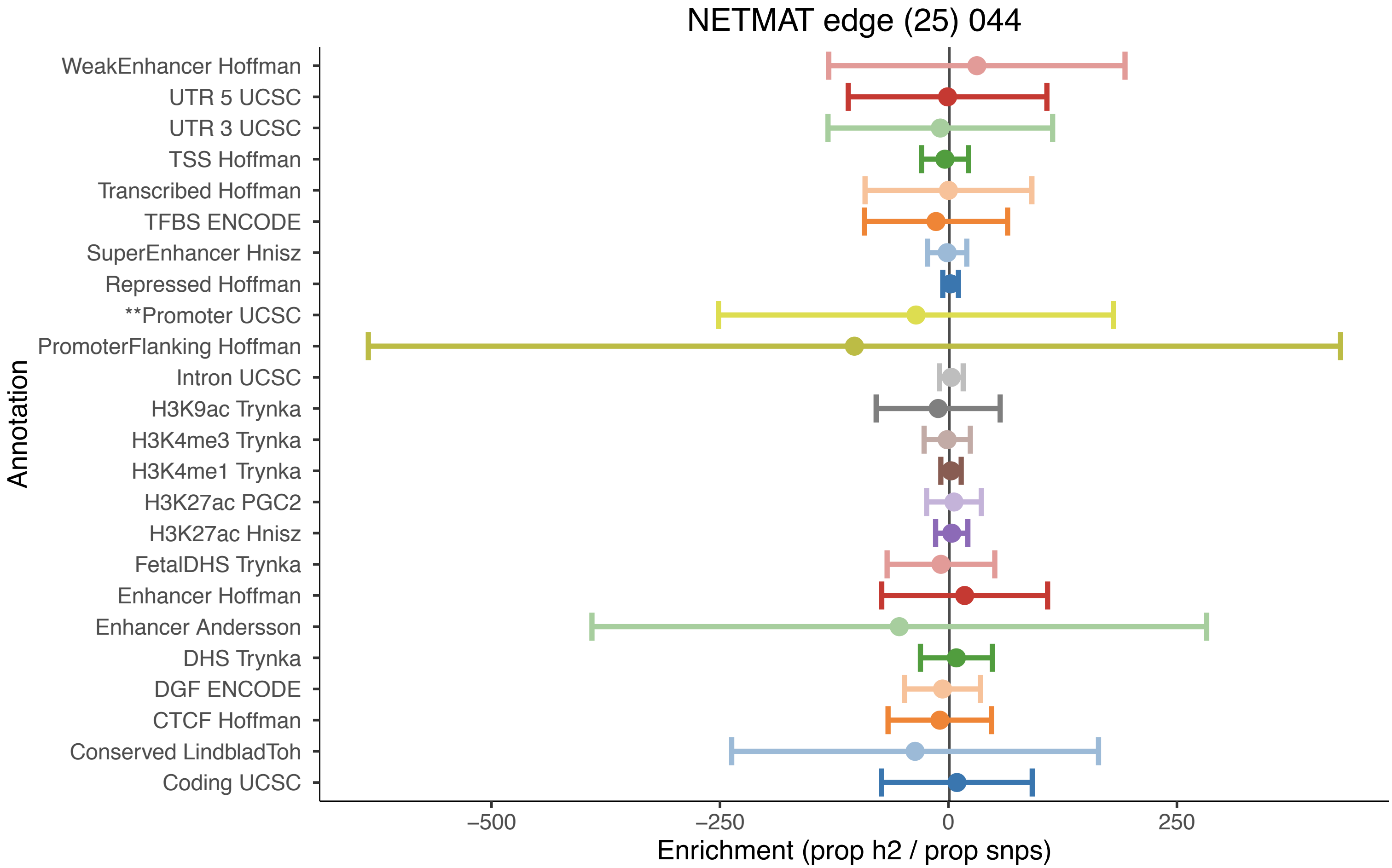
NETMAT edge (25) 044
WeakEnhancer Hoffman
UTR 5 UCSC
UTR 3 UCSC
TSS Hoffman
Transcribed Hoffman
TFBS ENCODE
SuperEnhancer Hnisz
Repressed Hoffman
**Promoter UCSC
PromoterFlanking Hoffman
Intron UCSC
H3K9ac Trynka
H3K4me3 Trynka
H3K4me1 Trynka
H3K27ac PGC2
H3K27ac Hnisz
FetalDHS Trynka
Enhancer Hoffman
Enhancer Andersson
DHS Trynka
DGF ENCODE
CTCF Hoffman
Conserved LindbladToh
Coding UCSC
Annotation
−500
−250
0
250
Enrichment (prop h2 / prop snps)

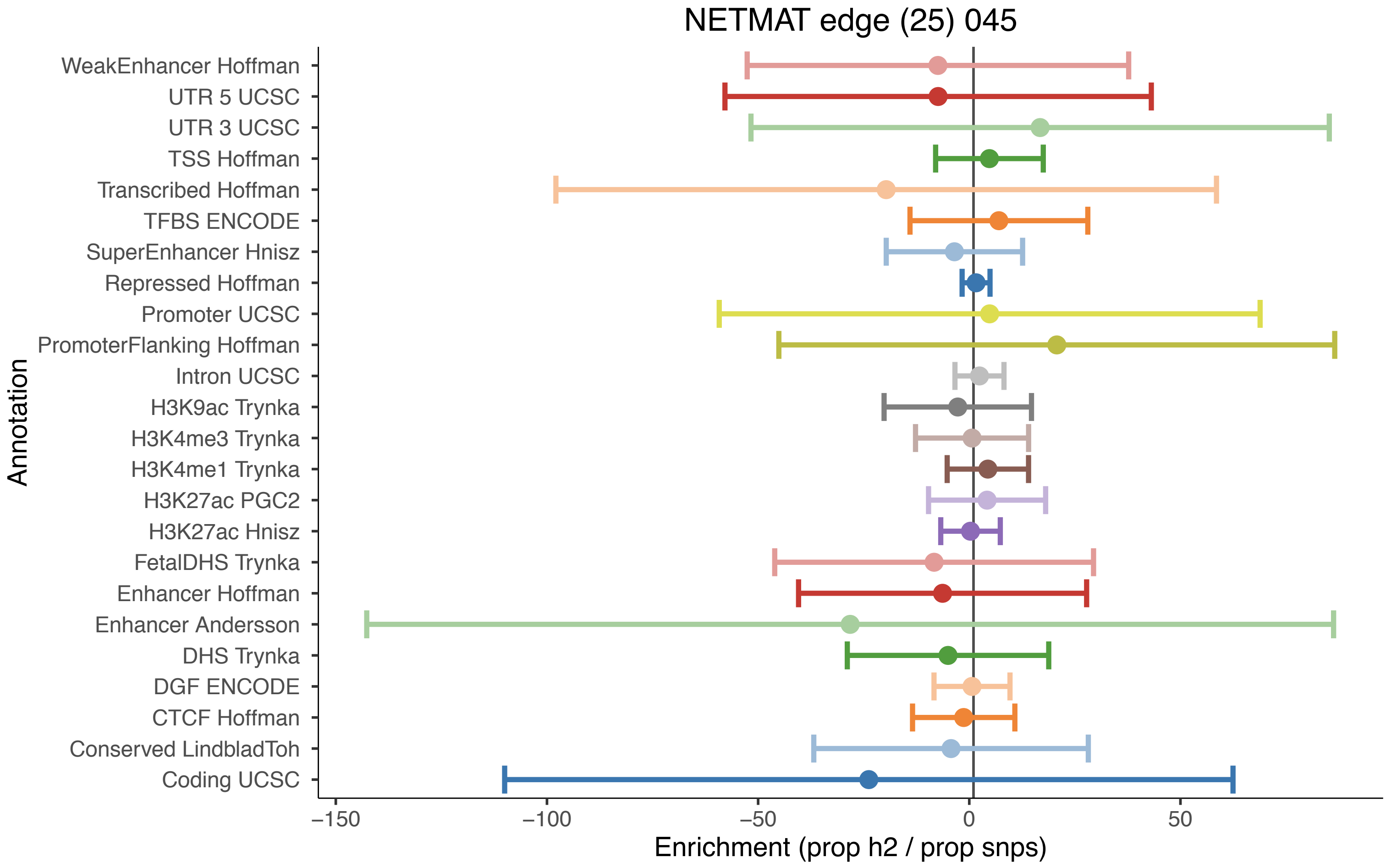
NETMAT edge (25) 045
Annotation
WeakEnhancer Hoffman
UTR 5 UCSC
UTR 3 UCSC
TSS Hoffman
Transcribed Hoffman
TFBS ENCODE
SuperEnhancer Hnisz
Repressed Hoffman
Promoter UCSC
PromoterFlanking Hoffman
Intron UCSC
H3K9ac Trynka
H3K4me3 Trynka
H3K4me1 Trynka
H3K27ac PGC2
H3K27ac Hnisz
FetalDHS Trynka
Enhancer Hoffman
Enhancer Andersson
DHS Trynka
DGF ENCODE
CTCF Hoffman
Conserved LindbladToh
Coding UCSC
−150
−100
−50
0
50
Enrichment (prop h2 / prop snps)

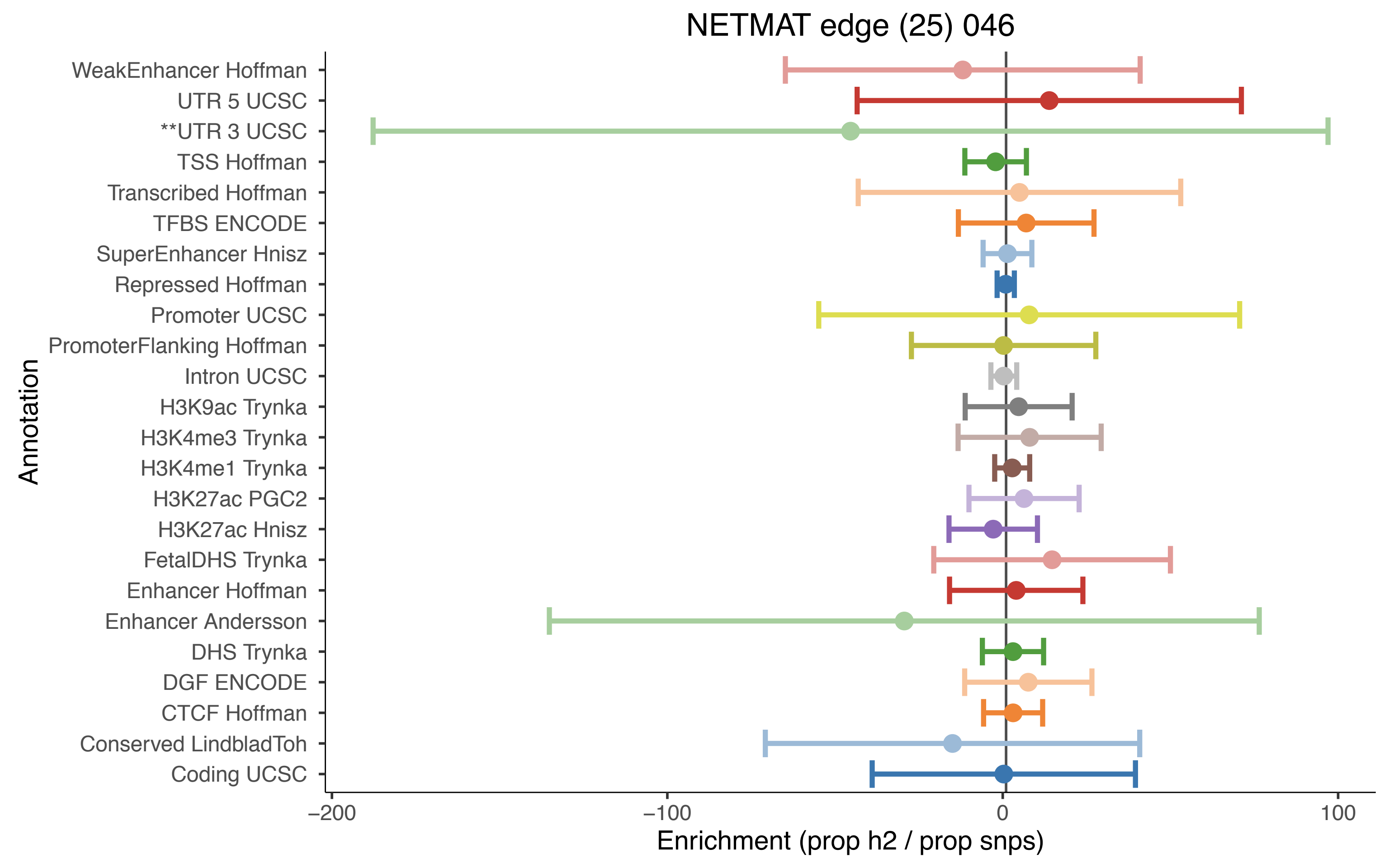

NETMAT edge (25) 046
WeakEnhancer Hoffman
UTR 5 UCSC
**UTR 3 UCSC
TSS Hoffman
Transcribed Hoffman
TFBS ENCODE
SuperEnhancer Hnisz
Repressed Hoffman
Promoter UCSC
PromoterFlanking Hoffman
Intron UCSC
H3K9ac Trynka
H3K4me3 Trynka
H3K4me1 Trynka
H3K27ac PGC2
H3K27ac Hnisz
FetalDHS Trynka
Enhancer Hoffman
Enhancer Andersson
DHS Trynka
DGF ENCODE
CTCF Hoffman
Conserved LindbladToh
Coding UCSC
Annotation
−200
−100
0
100
Enrichment (prop h2 / prop snps)

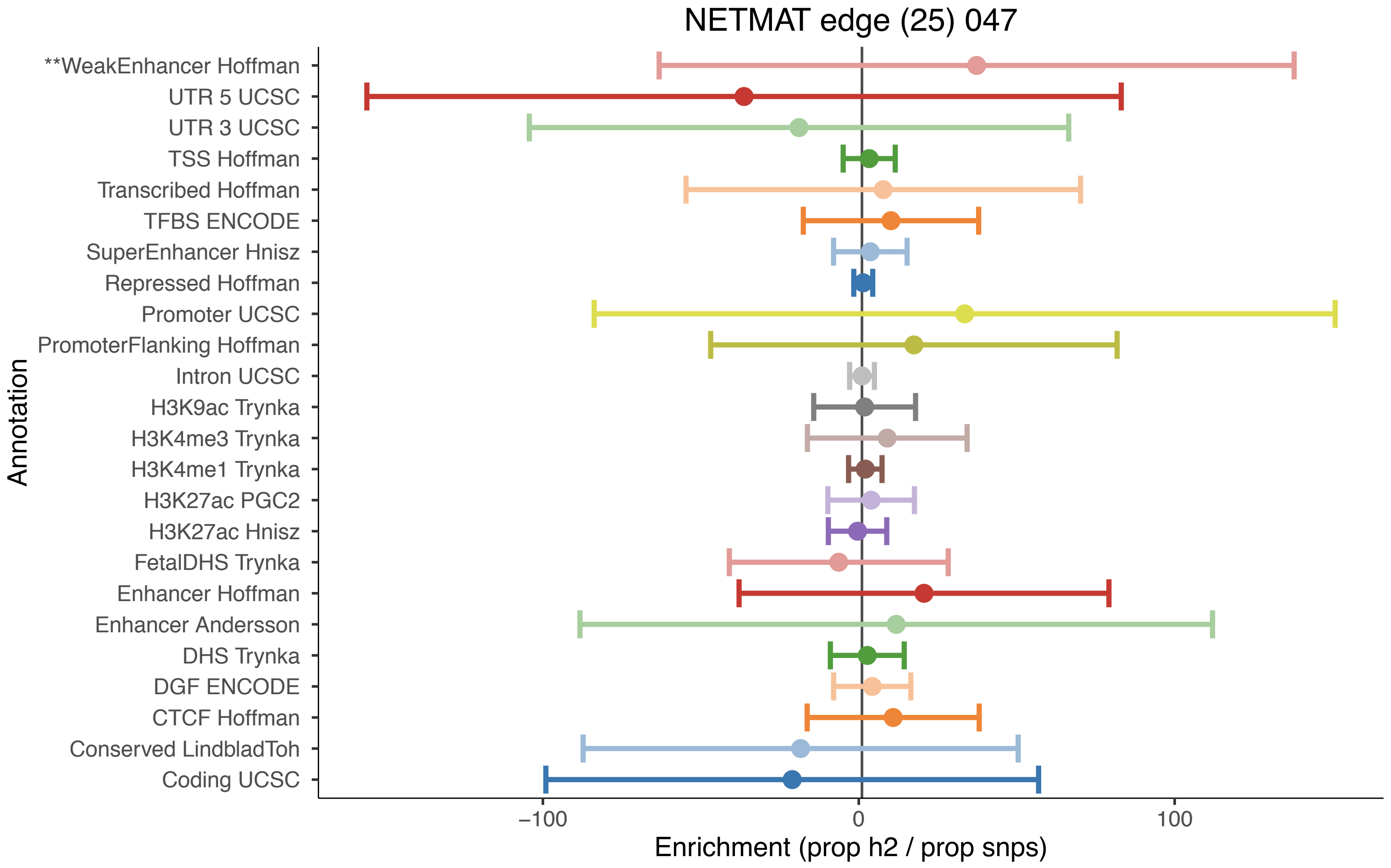
NETMAT edge (25) 047
**WeakEnhancer Hoffman
UTR 5 UCSC
UTR 3 UCSC
TSS Hoffman
Transcribed Hoffman
TFBS ENCODE
SuperEnhancer Hnisz
Repressed Hoffman
Promoter UCSC
PromoterFlanking Hoffman
Intron UCSC
H3K9ac Trynka
H3K4me3 Trynka
H3K4me1 Trynka
H3K27ac PGC2
H3K27ac Hnisz
FetalDHS Trynka
Enhancer Hoffman
Enhancer Andersson
DHS Trynka
DGF ENCODE
CTCF Hoffman
Conserved LindbladToh
Coding UCSC
Annotation
−100
0
100
Enrichment (prop h2 / prop snps)

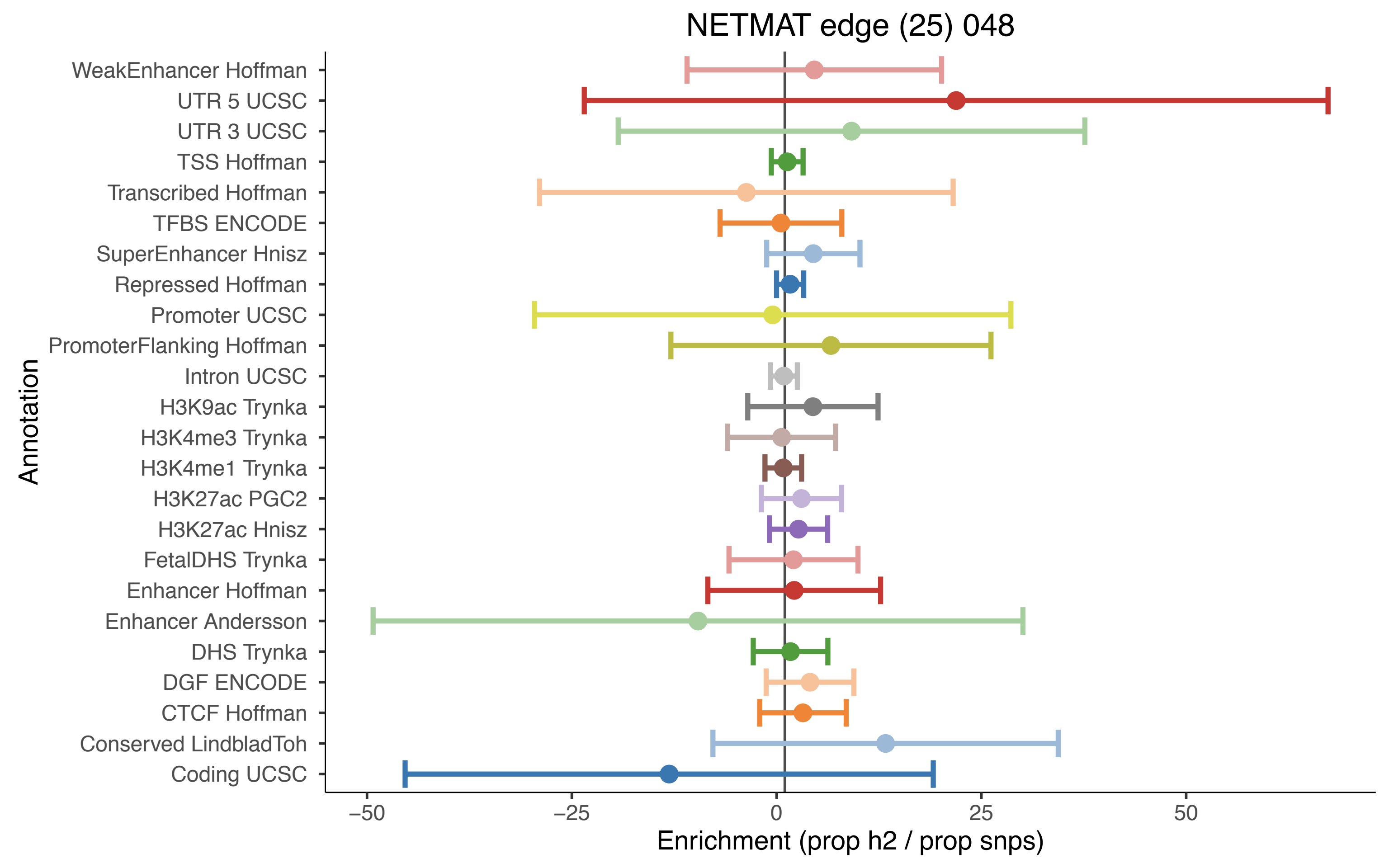

NETMAT edge (25) 048
WeakEnhancer Hoffman
UTR 5 UCSC
UTR 3 UCSC
TSS Hoffman
Transcribed Hoffman
TFBS ENCODE
SuperEnhancer Hnisz
Repressed Hoffman
Promoter UCSC
PromoterFlanking Hoffman
Intron UCSC
H3K9ac Trynka
H3K4me3 Trynka
H3K4me1 Trynka
H3K27ac PGC2
H3K27ac Hnisz
FetalDHS Trynka
Enhancer Hoffman
Enhancer Andersson
DHS Trynka
DGF ENCODE
CTCF Hoffman
Conserved LindbladToh
Coding UCSC
Annotation
−50
−25
0
25
50
Enrichment (prop h2 / prop snps)

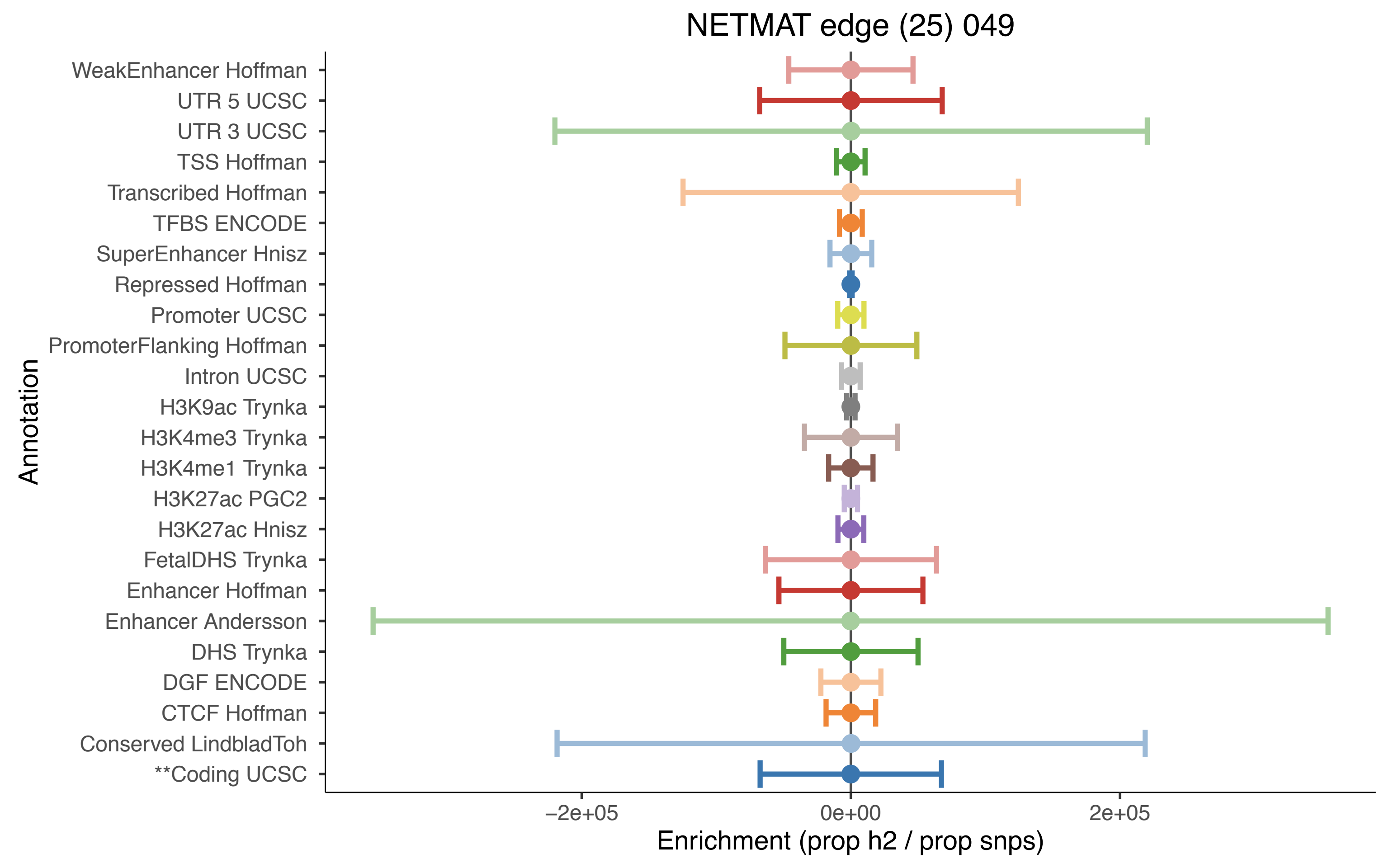

NETMAT edge (25) 049
WeakEnhancer Hoffman
UTR 5 UCSC
UTR 3 UCSC
TSS Hoffman
Transcribed Hoffman
TFBS ENCODE
SuperEnhancer Hnisz
Repressed Hoffman
Promoter UCSC
PromoterFlanking Hoffman
Intron UCSC
H3K9ac Trynka
H3K4me3 Trynka
H3K4me1 Trynka
H3K27ac PGC2
H3K27ac Hnisz
FetalDHS Trynka
Enhancer Hoffman
Enhancer Andersson
DHS Trynka
DGF ENCODE
CTCF Hoffman
Conserved LindbladToh
**Coding UCSC
Annotation
−2e+05
0e+00
2e+05
Enrichment (prop h2 / prop snps)

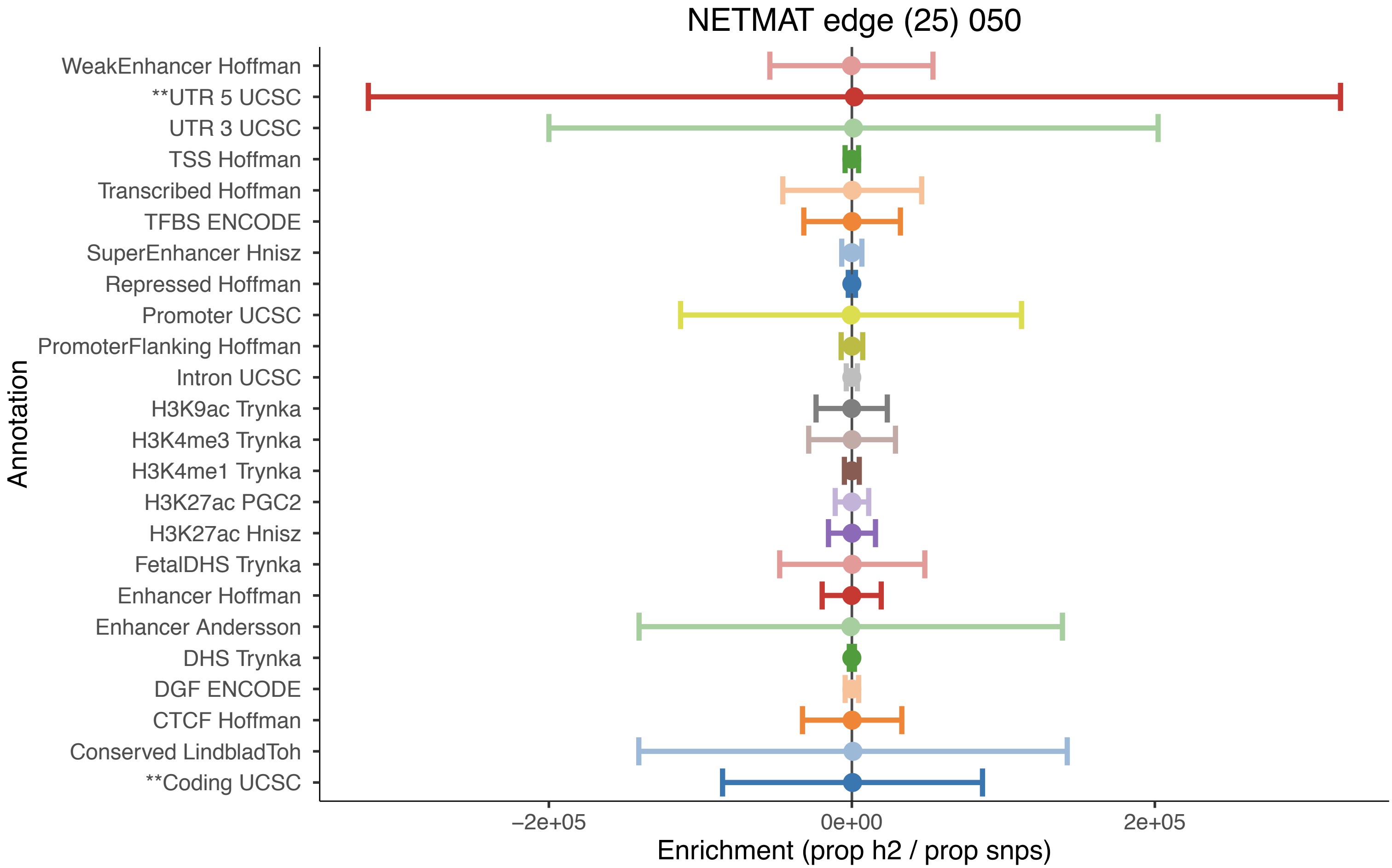
NETMAT edge (25) 050
WeakEnhancer Hoffman
**UTR 5 UCSC
UTR 3 UCSC
TSS Hoffman
Transcribed Hoffman
TFBS ENCODE
SuperEnhancer Hnisz
Repressed Hoffman
Promoter UCSC
PromoterFlanking Hoffman
Intron UCSC
H3K9ac Trynka
H3K4me3 Trynka
H3K4me1 Trynka
H3K27ac PGC2
H3K27ac Hnisz
FetalDHS Trynka
Enhancer Hoffman
Enhancer Andersson
DHS Trynka
DGF ENCODE
CTCF Hoffman
Conserved LindbladToh
**Coding UCSC
Annotation
−2e+05
0e+00
2e+05
Enrichment (prop h2 / prop snps)

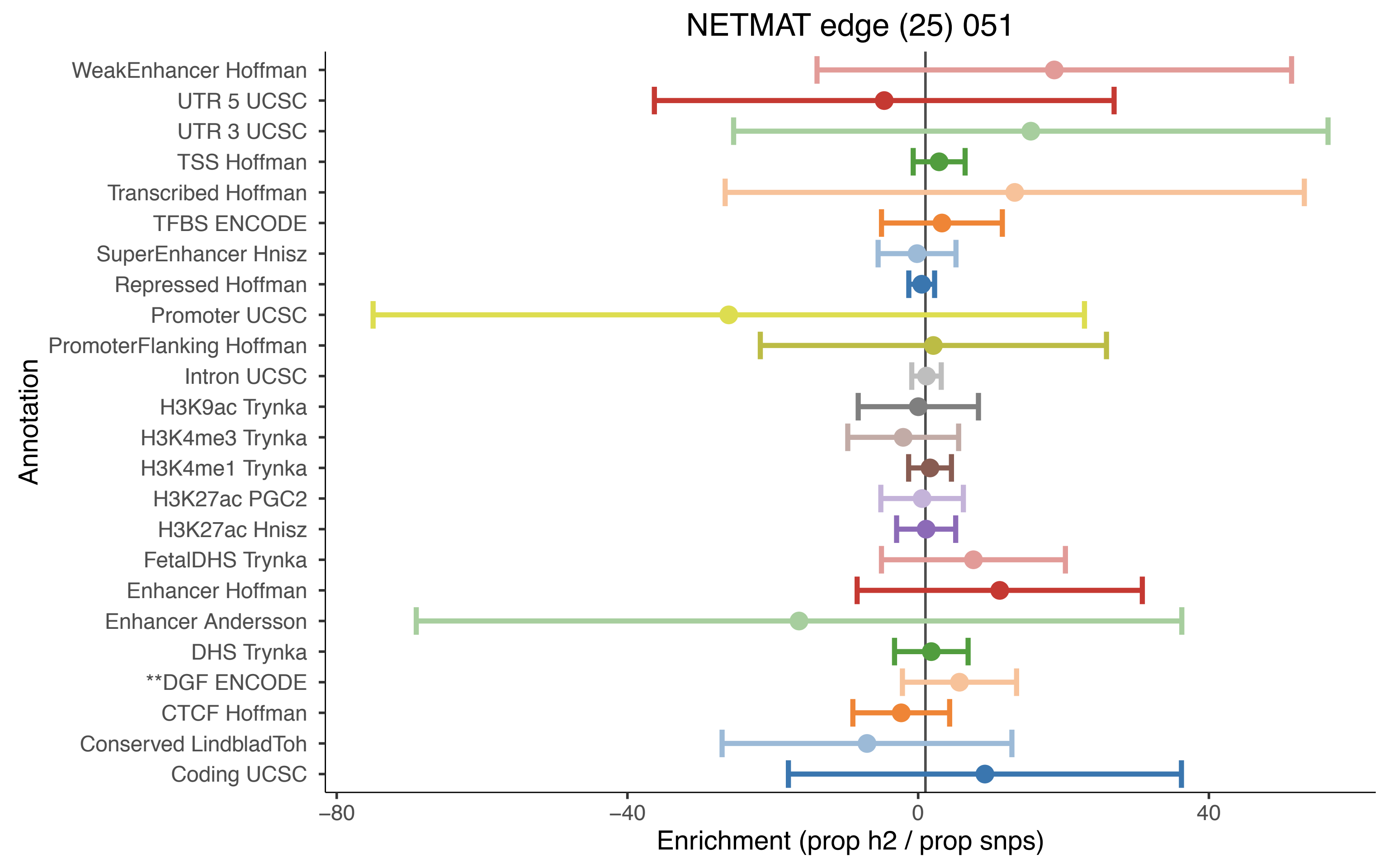

NETMAT edge (25) 051
WeakEnhancer Hoffman
UTR 5 UCSC
UTR 3 UCSC
TSS Hoffman
Transcribed Hoffman
TFBS ENCODE
SuperEnhancer Hnisz
Repressed Hoffman
Promoter UCSC
PromoterFlanking Hoffman
Intron UCSC
H3K9ac Trynka
H3K4me3 Trynka
H3K4me1 Trynka
H3K27ac PGC2
H3K27ac Hnisz
FetalDHS Trynka
Enhancer Hoffman
Enhancer Andersson
DHS Trynka
**DGF ENCODE
CTCF Hoffman
Conserved LindbladToh
Coding UCSC
Annotation
−80
−40
0
40
Enrichment (prop h2 / prop snps)

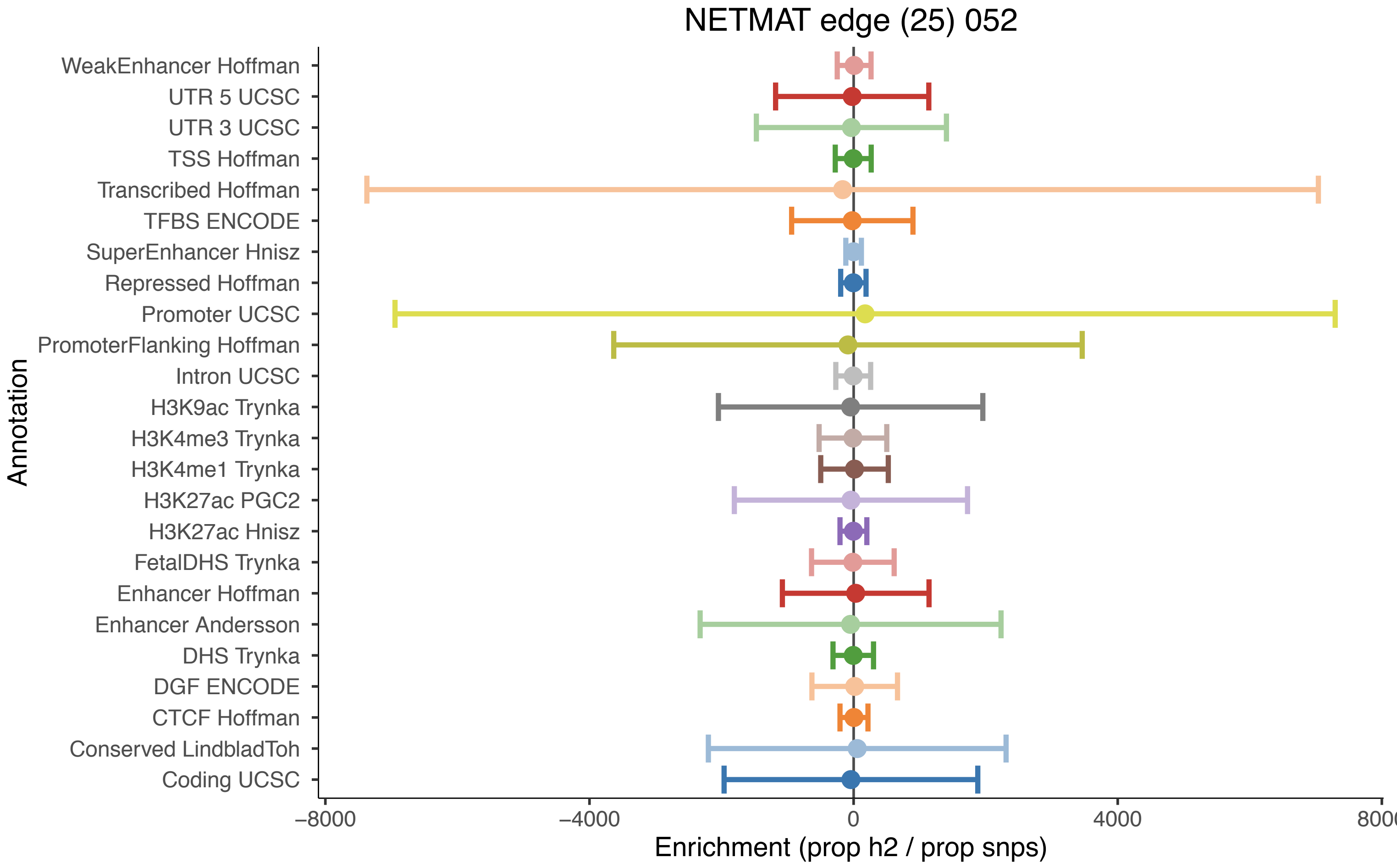

NETMAT edge (25) 052
WeakEnhancer Hoffman
UTR 5 UCSC
UTR 3 UCSC
TSS Hoffman
Transcribed Hoffman
TFBS ENCODE
SuperEnhancer Hnisz
Repressed Hoffman
Promoter UCSC
PromoterFlanking Hoffman
Intron UCSC
H3K9ac Trynka
H3K4me3 Trynka
H3K4me1 Trynka
H3K27ac PGC2
H3K27ac Hnisz
FetalDHS Trynka
Enhancer Hoffman
Enhancer Andersson
DHS Trynka
DGF ENCODE
CTCF Hoffman
Conserved LindbladToh
Coding UCSC
Annotation
−8000
−4000
0
4000
Enrichment (prop h2 / prop snps)

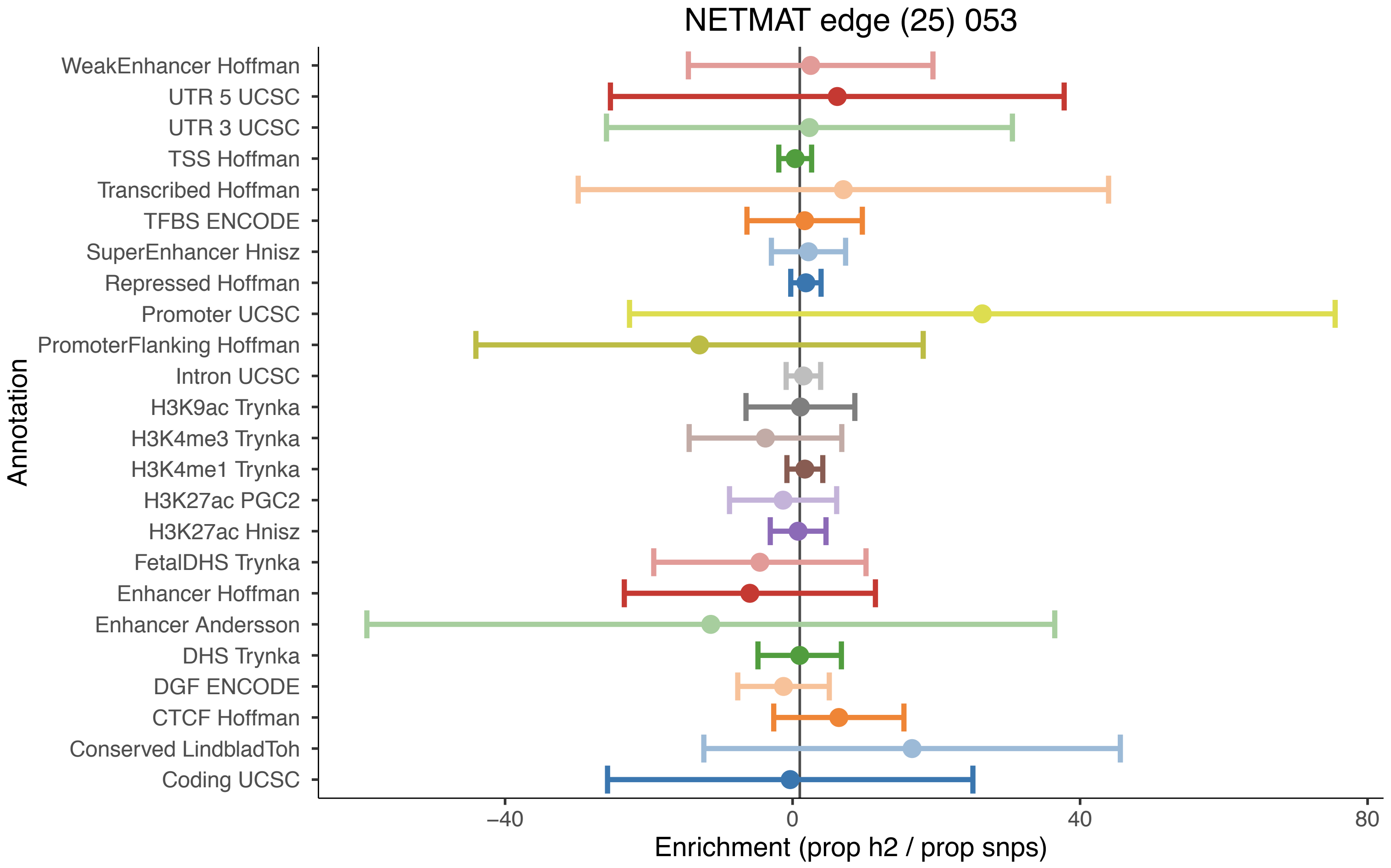
NETMAT edge (25) 053
Annotation
WeakEnhancer Hoffman
UTR 5 UCSC
UTR 3 UCSC
TSS Hoffman
Transcribed Hoffman
TFBS ENCODE
SuperEnhancer Hnisz
Repressed Hoffman
Promoter UCSC
PromoterFlanking Hoffman
Intron UCSC
H3K9ac Trynka
H3K4me3 Trynka
H3K4me1 Trynka
H3K27ac PGC2
H3K27ac Hnisz
FetalDHS Trynka
Enhancer Hoffman
Enhancer Andersson
DHS Trynka
DGF ENCODE
CTCF Hoffman
Conserved LindbladToh
Coding UCSC
−40
0
40
80
Enrichment (prop h2 / prop snps)

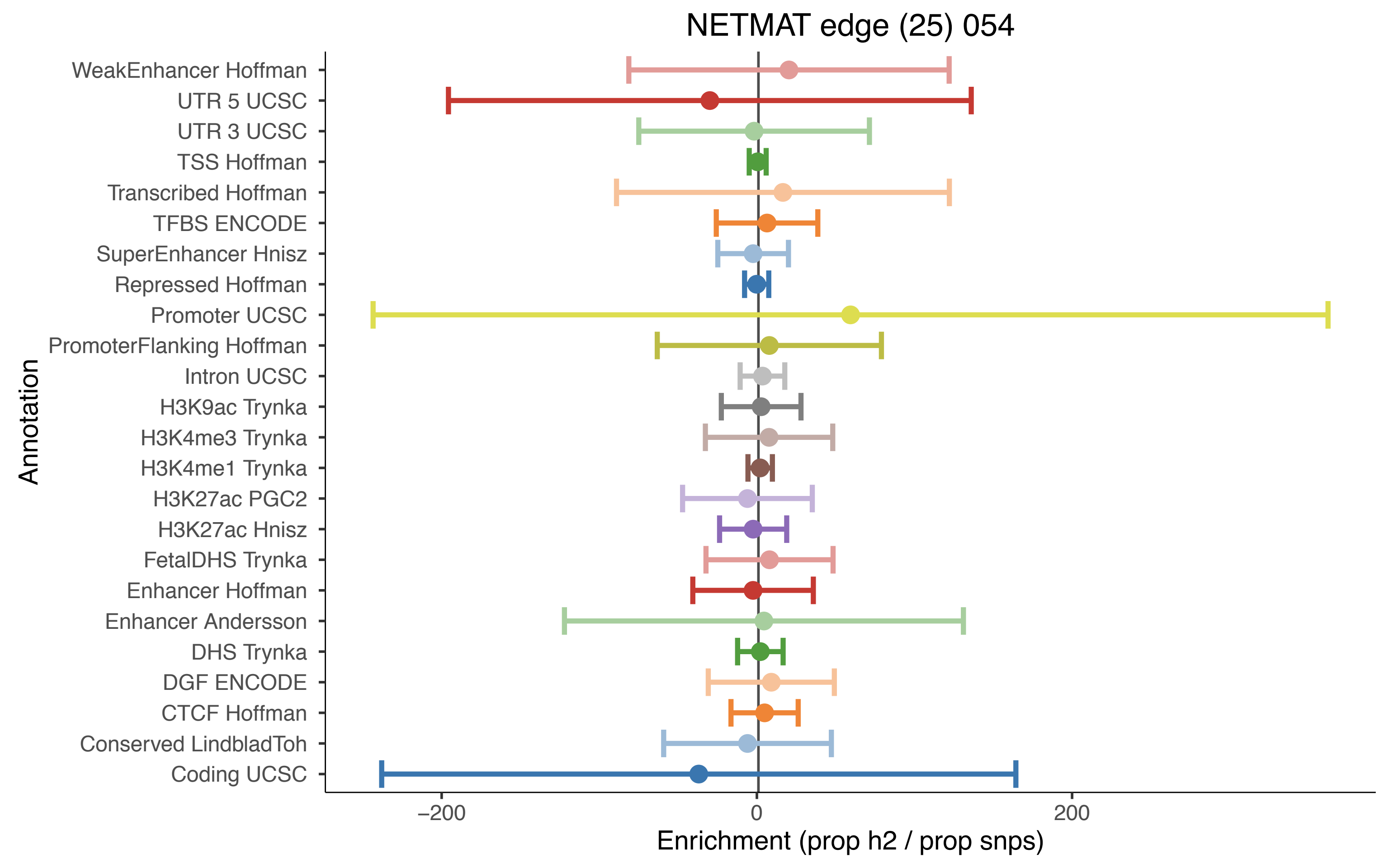

NETMAT edge (25) 054
WeakEnhancer Hoffman
UTR 5 UCSC
UTR 3 UCSC
TSS Hoffman
Transcribed Hoffman
TFBS ENCODE
SuperEnhancer Hnisz
Repressed Hoffman
Promoter UCSC
PromoterFlanking Hoffman
Intron UCSC
H3K9ac Trynka
H3K4me3 Trynka
H3K4me1 Trynka
H3K27ac PGC2
H3K27ac Hnisz
FetalDHS Trynka
Enhancer Hoffman
Enhancer Andersson
DHS Trynka
DGF ENCODE
CTCF Hoffman
Conserved LindbladToh
Coding UCSC
Annotation
−200
0
200
Enrichment (prop h2 / prop snps)

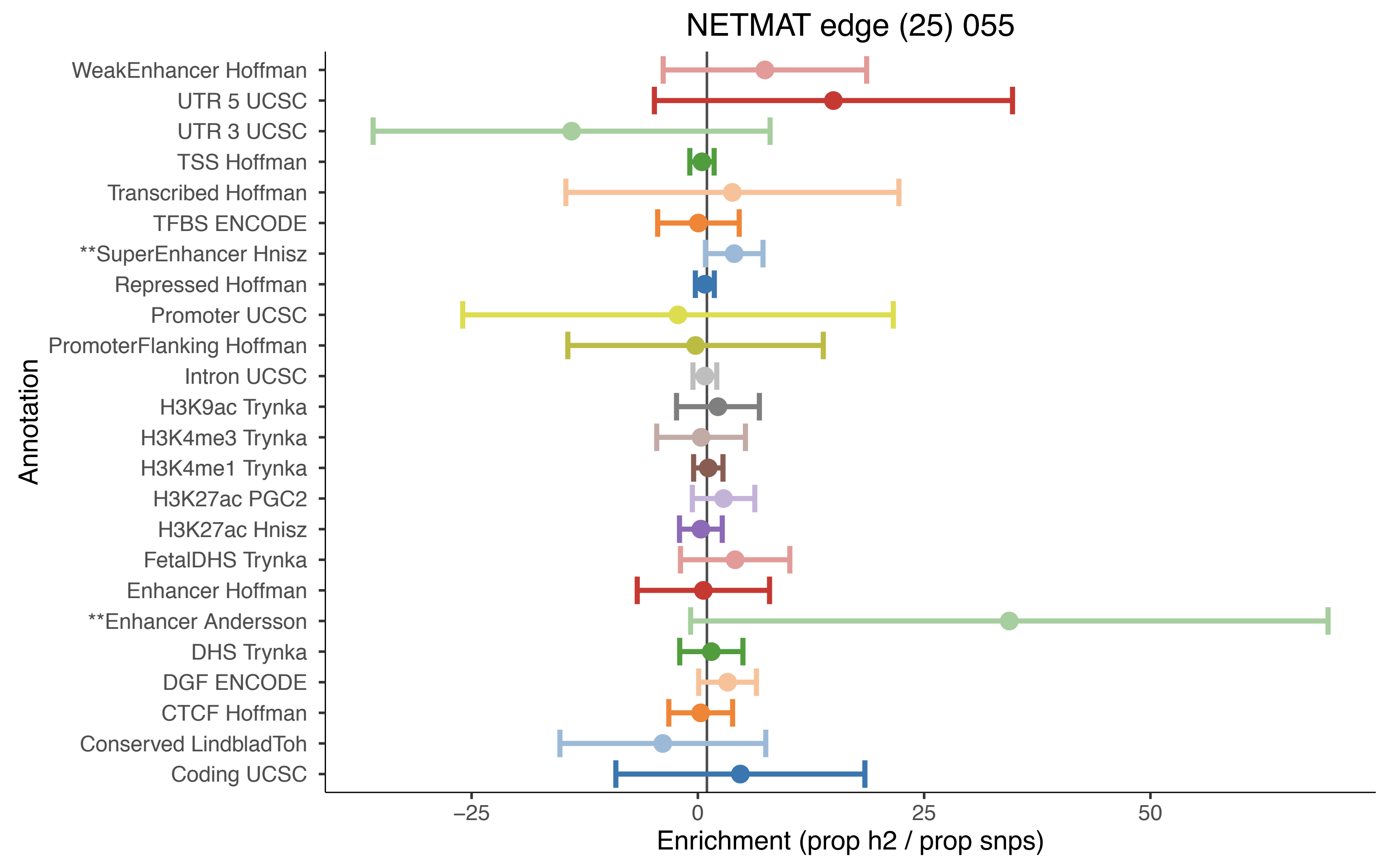

NETMAT edge (25) 055
WeakEnhancer Hoffman
UTR 5 UCSC
UTR 3 UCSC
TSS Hoffman
Transcribed Hoffman
TFBS ENCODE
**SuperEnhancer Hnisz
Repressed Hoffman
Promoter UCSC
PromoterFlanking Hoffman
Intron UCSC
H3K9ac Trynka
H3K4me3 Trynka
H3K4me1 Trynka
H3K27ac PGC2
H3K27ac Hnisz
FetalDHS Trynka
Enhancer Hoffman
**Enhancer Andersson
DHS Trynka
DGF ENCODE
CTCF Hoffman
Conserved LindbladToh
Coding UCSC
Annotation
−25
0
25
50
Enrichment (prop h2 / prop snps)

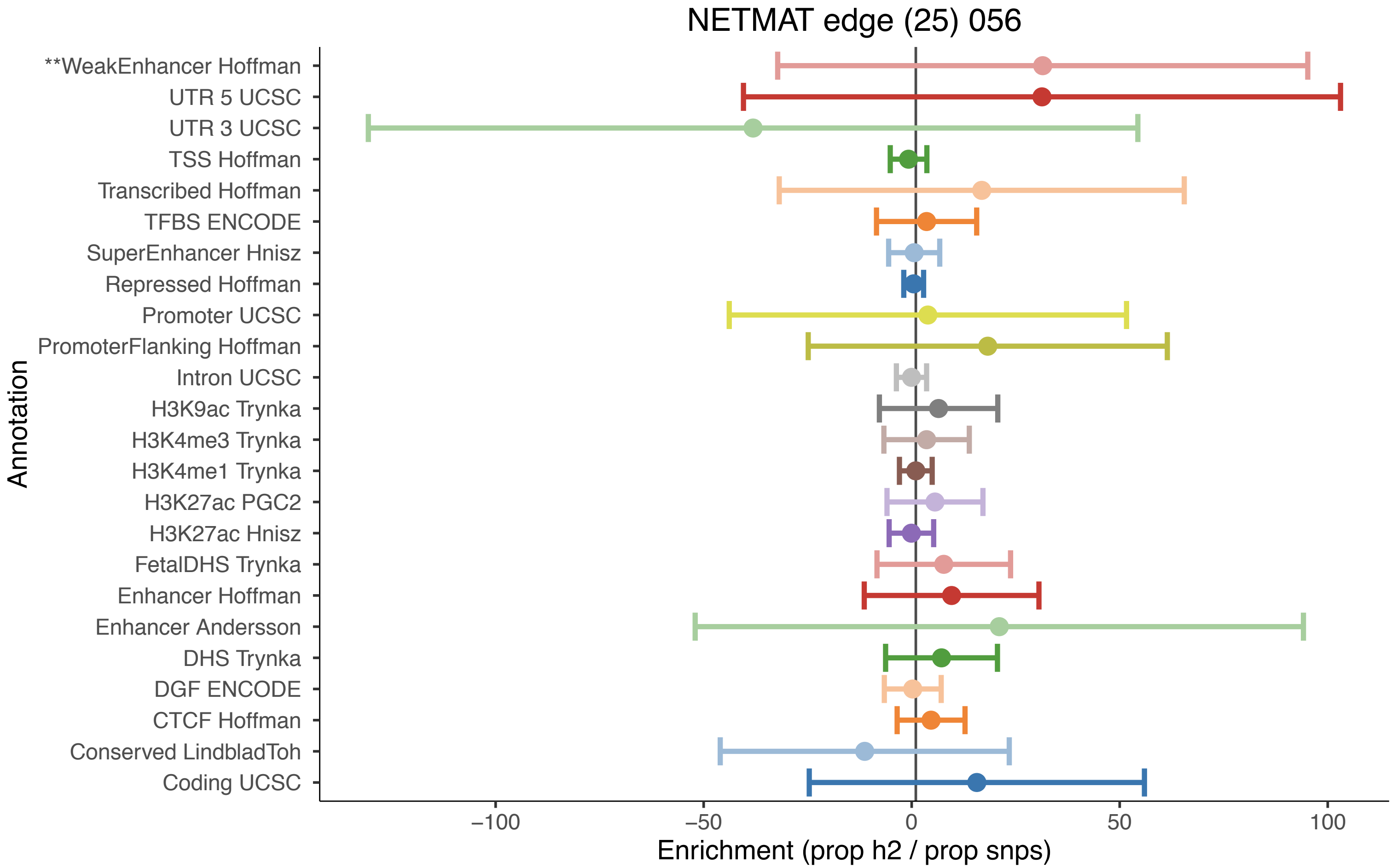

NETMAT edge (25) 056
**WeakEnhancer Hoffman
UTR 5 UCSC
UTR 3 UCSC
TSS Hoffman
Transcribed Hoffman
TFBS ENCODE
SuperEnhancer Hnisz
Repressed Hoffman
Promoter UCSC
PromoterFlanking Hoffman
Intron UCSC
H3K9ac Trynka
H3K4me3 Trynka
H3K4me1 Trynka
H3K27ac PGC2
H3K27ac Hnisz
FetalDHS Trynka
Enhancer Hoffman
Enhancer Andersson
DHS Trynka
DGF ENCODE
CTCF Hoffman
Conserved LindbladToh
Coding UCSC
Annotation
−100
−50
0
50
100
Enrichment (prop h2 / prop snps)

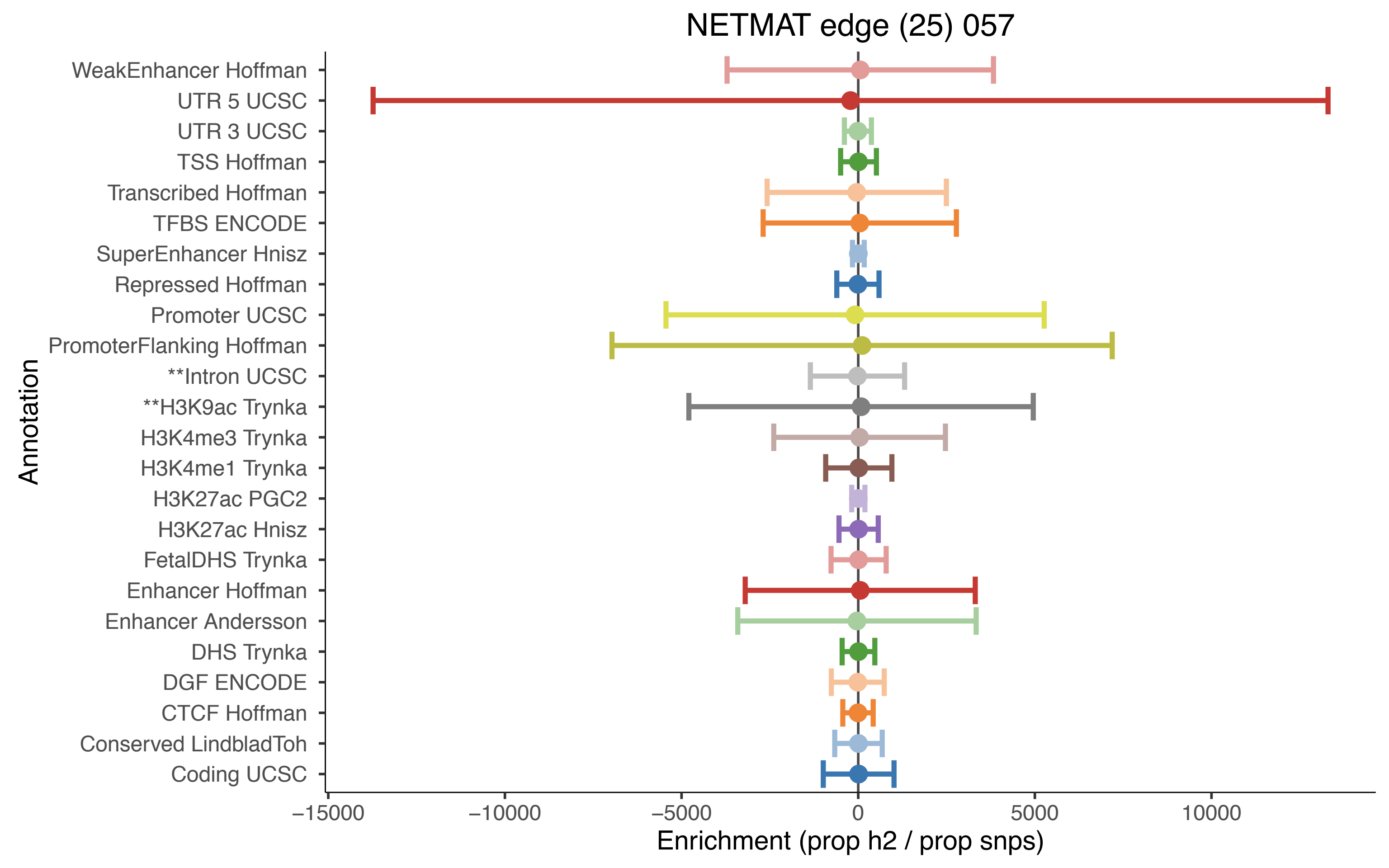

NETMAT edge (25) 057
WeakEnhancer Hoffman
UTR 5 UCSC
UTR 3 UCSC
TSS Hoffman
Transcribed Hoffman
TFBS ENCODE
SuperEnhancer Hnisz
Repressed Hoffman
Promoter UCSC
PromoterFlanking Hoffman
**Intron UCSC
**H3K9ac Trynka
H3K4me3 Trynka
H3K4me1 Trynka
H3K27ac PGC2
H3K27ac Hnisz
FetalDHS Trynka
Enhancer Hoffman
Enhancer Andersson
DHS Trynka
DGF ENCODE
CTCF Hoffman
Conserved LindbladToh
Coding UCSC
Annotation
−15000
−10000
−5000
0
5000
10000
Enrichment (prop h2 / prop snps)

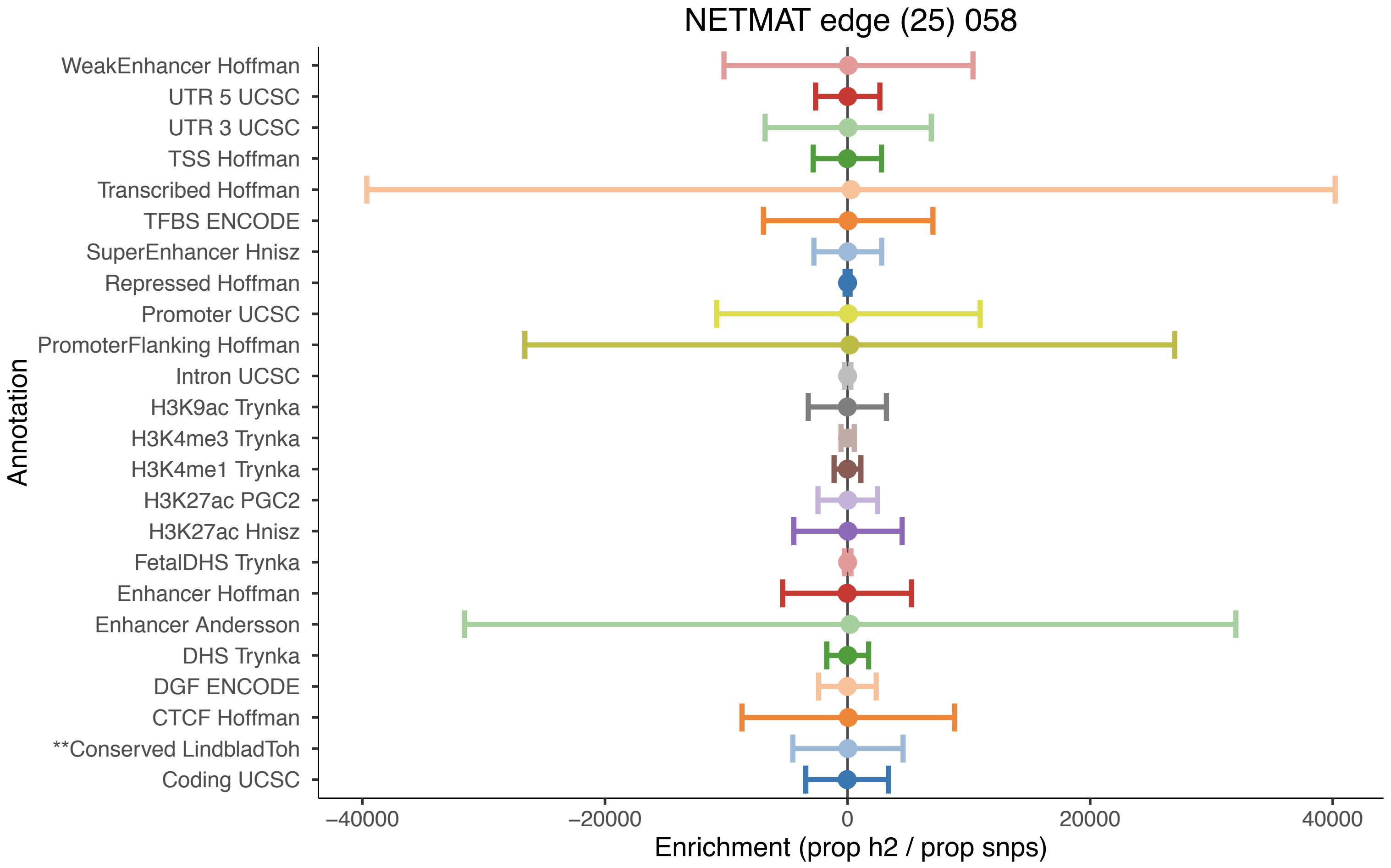

NETMAT edge (25) 058
WeakEnhancer Hoffman
UTR 5 UCSC
UTR 3 UCSC
TSS Hoffman
Transcribed Hoffman
TFBS ENCODE
SuperEnhancer Hnisz
Repressed Hoffman
Promoter UCSC
PromoterFlanking Hoffman
Intron UCSC
H3K9ac Trynka
H3K4me3 Trynka
H3K4me1 Trynka
H3K27ac PGC2
H3K27ac Hnisz
FetalDHS Trynka
Enhancer Hoffman
Enhancer Andersson
DHS Trynka
DGF ENCODE
CTCF Hoffman
**Conserved LindbladToh
Coding UCSC
Annotation
−40000
−20000
0
20000
40000
Enrichment (prop h2 / prop snps)

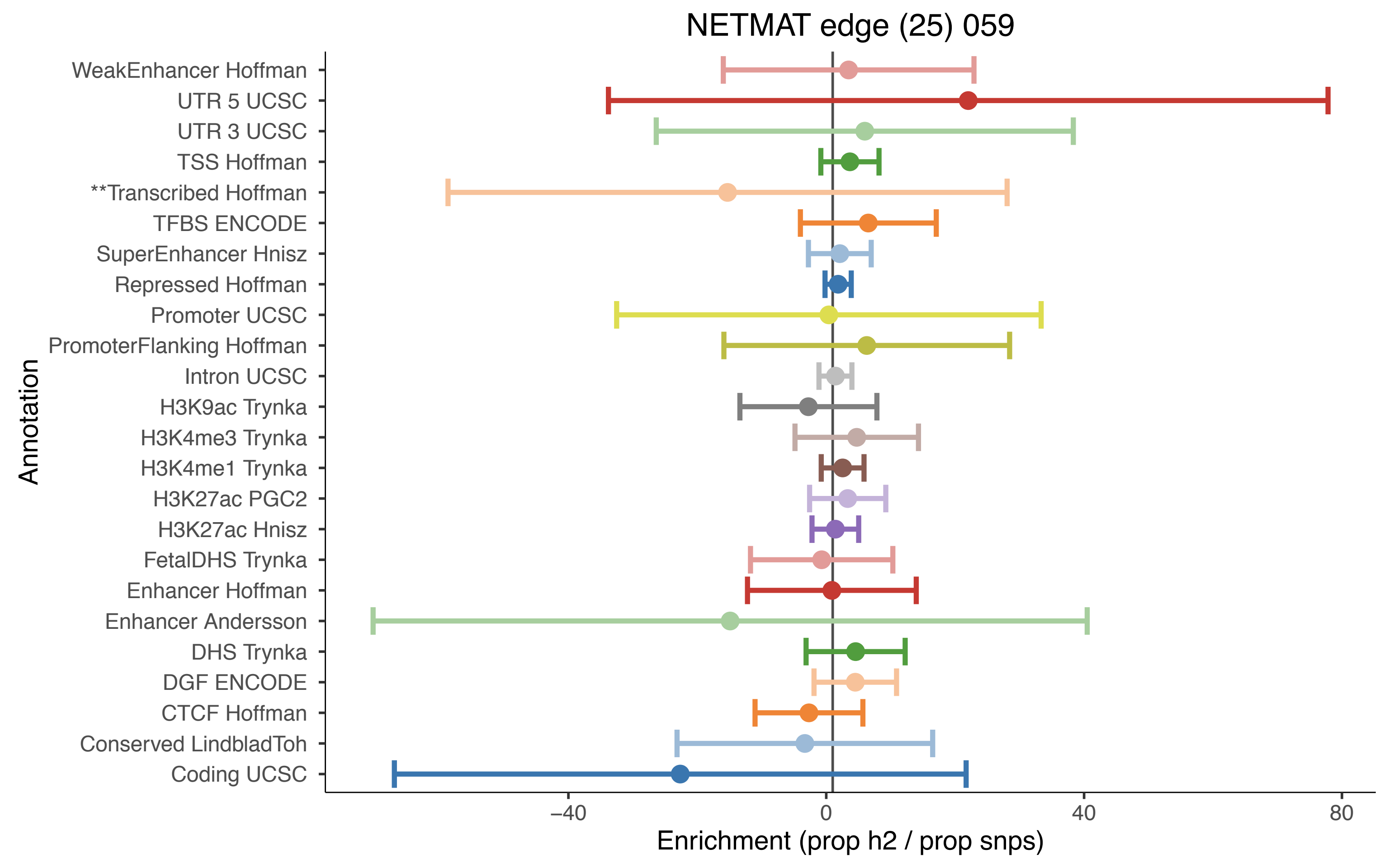
NETMAT edge (25) 059
WeakEnhancer Hoffman
UTR 5 UCSC
UTR 3 UCSC
TSS Hoffman
**Transcribed Hoffman
TFBS ENCODE
SuperEnhancer Hnisz
Repressed Hoffman
Promoter UCSC
PromoterFlanking Hoffman
Intron UCSC
H3K9ac Trynka
H3K4me3 Trynka
H3K4me1 Trynka
H3K27ac PGC2
H3K27ac Hnisz
FetalDHS Trynka
Enhancer Hoffman
Enhancer Andersson
DHS Trynka
DGF ENCODE
CTCF Hoffman
Conserved LindbladToh
Coding UCSC
Annotation
−40
0
40
80
Enrichment (prop h2 / prop snps)

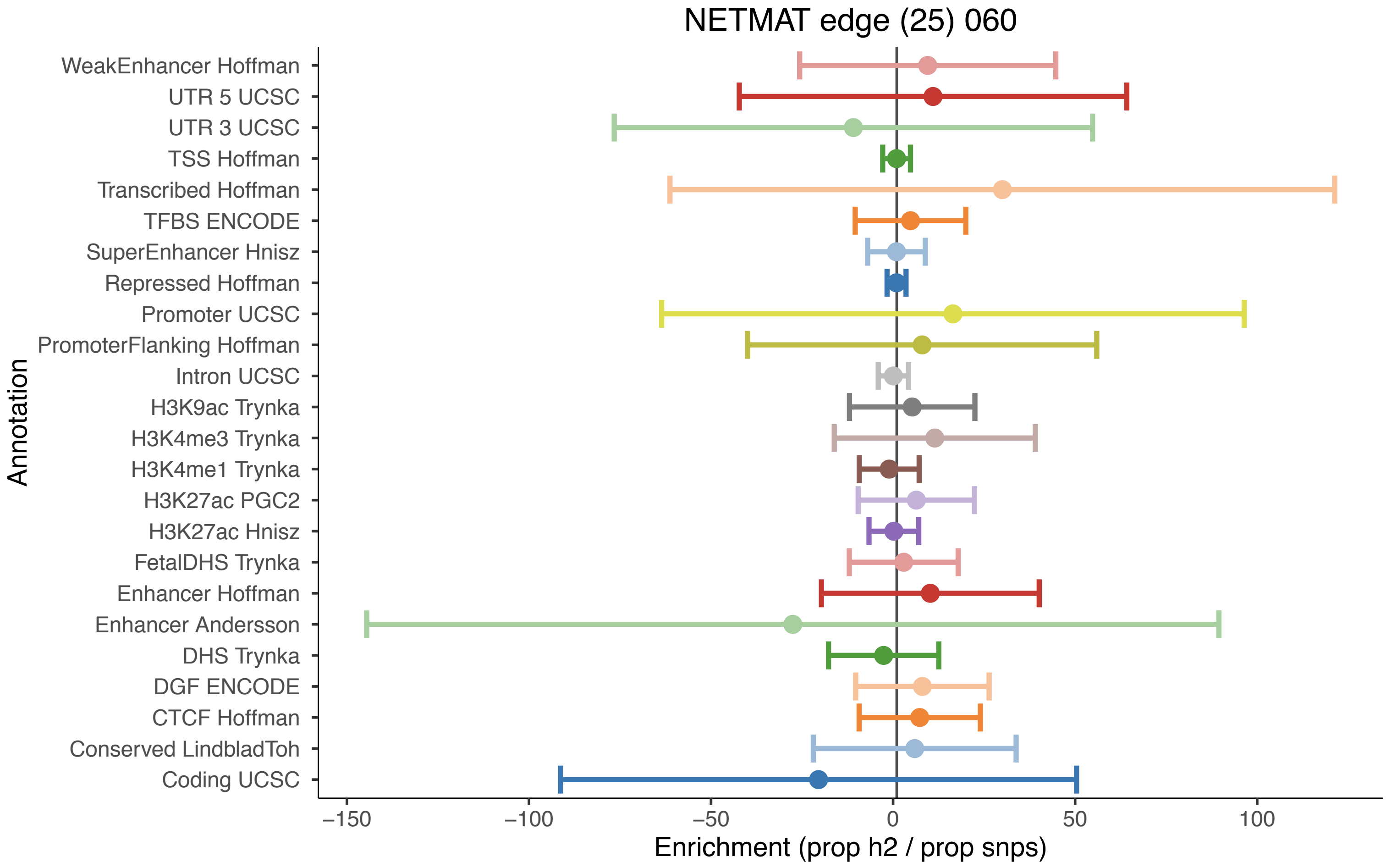
NETMAT edge (25) 060
Annotation
WeakEnhancer Hoffman
UTR 5 UCSC
UTR 3 UCSC
TSS Hoffman
Transcribed Hoffman
TFBS ENCODE
SuperEnhancer Hnisz
Repressed Hoffman
Promoter UCSC
PromoterFlanking Hoffman
Intron UCSC
H3K9ac Trynka
H3K4me3 Trynka
H3K4me1 Trynka
H3K27ac PGC2
H3K27ac Hnisz
FetalDHS Trynka
Enhancer Hoffman
Enhancer Andersson
DHS Trynka
DGF ENCODE
CTCF Hoffman
Conserved LindbladToh
Coding UCSC
−150
−100
−50
0
50
100
Enrichment (prop h2 / prop snps)

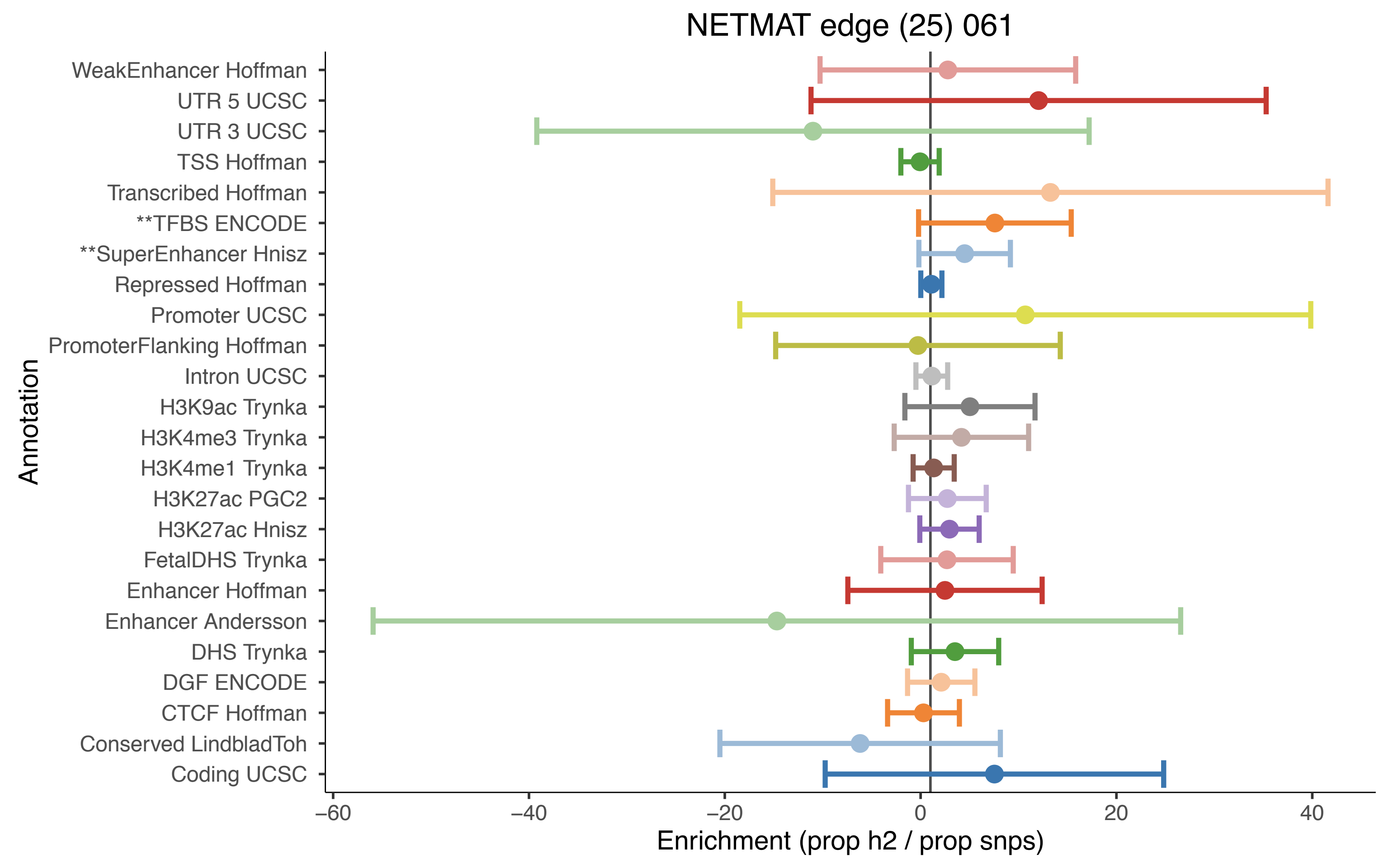

NETMAT edge (25) 061
WeakEnhancer Hoffman
UTR 5 UCSC
UTR 3 UCSC
TSS Hoffman
Transcribed Hoffman
**TFBS ENCODE
**SuperEnhancer Hnisz
Repressed Hoffman
Promoter UCSC
PromoterFlanking Hoffman
Intron UCSC
H3K9ac Trynka
H3K4me3 Trynka
H3K4me1 Trynka
H3K27ac PGC2
H3K27ac Hnisz
FetalDHS Trynka
Enhancer Hoffman
Enhancer Andersson
DHS Trynka
DGF ENCODE
CTCF Hoffman
Conserved LindbladToh
Coding UCSC
Annotation
−60
−40
−20
0
20
40
Enrichment (prop h2 / prop snps)

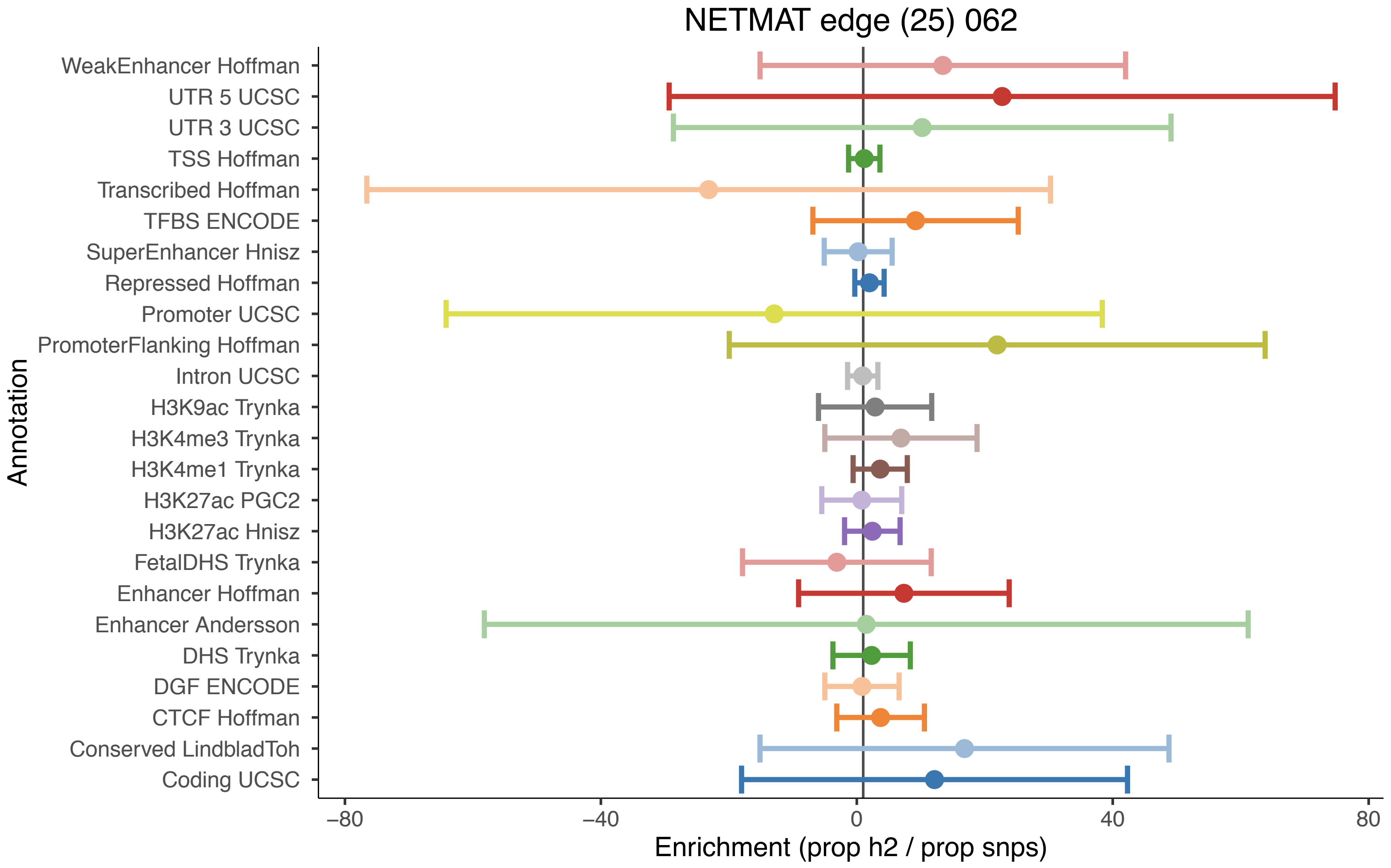
NETMAT edge (25) 062
WeakEnhancer Hoffman
UTR 5 UCSC
UTR 3 UCSC
TSS Hoffman
Transcribed Hoffman
TFBS ENCODE
SuperEnhancer Hnisz
Repressed Hoffman
Promoter UCSC
PromoterFlanking Hoffman
Intron UCSC
H3K9ac Trynka
H3K4me3 Trynka
H3K4me1 Trynka
H3K27ac PGC2
H3K27ac Hnisz
FetalDHS Trynka
Enhancer Hoffman
Enhancer Andersson
DHS Trynka
DGF ENCODE
CTCF Hoffman
Conserved LindbladToh
Coding UCSC
Annotation
−80
−40
0
40
80
Enrichment (prop h2 / prop snps)

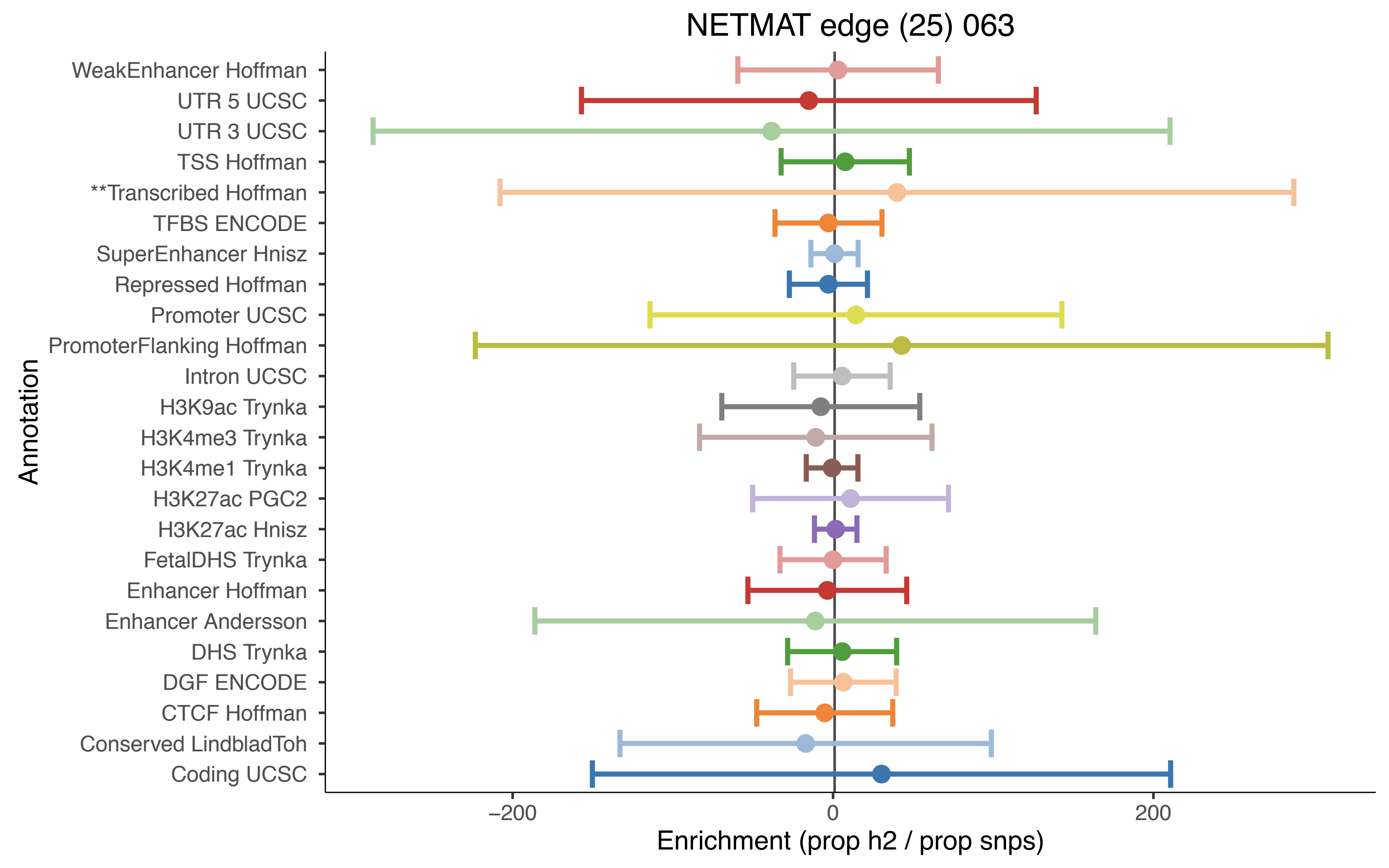
NETMAT edge (25) 063
WeakEnhancer Hoffman
UTR 5 UCSC
UTR 3 UCSC
TSS Hoffman
**Transcribed Hoffman
TFBS ENCODE
SuperEnhancer Hnisz
Repressed Hoffman
Promoter UCSC
PromoterFlanking Hoffman
Intron UCSC
H3K9ac Trynka
H3K4me3 Trynka
H3K4me1 Trynka
H3K27ac PGC2
H3K27ac Hnisz
FetalDHS Trynka
Enhancer Hoffman
Enhancer Andersson
DHS Trynka
DGF ENCODE
CTCF Hoffman
Conserved LindbladToh
Coding UCSC
Annotation
−200
0
200
Enrichment (prop h2 / prop snps)

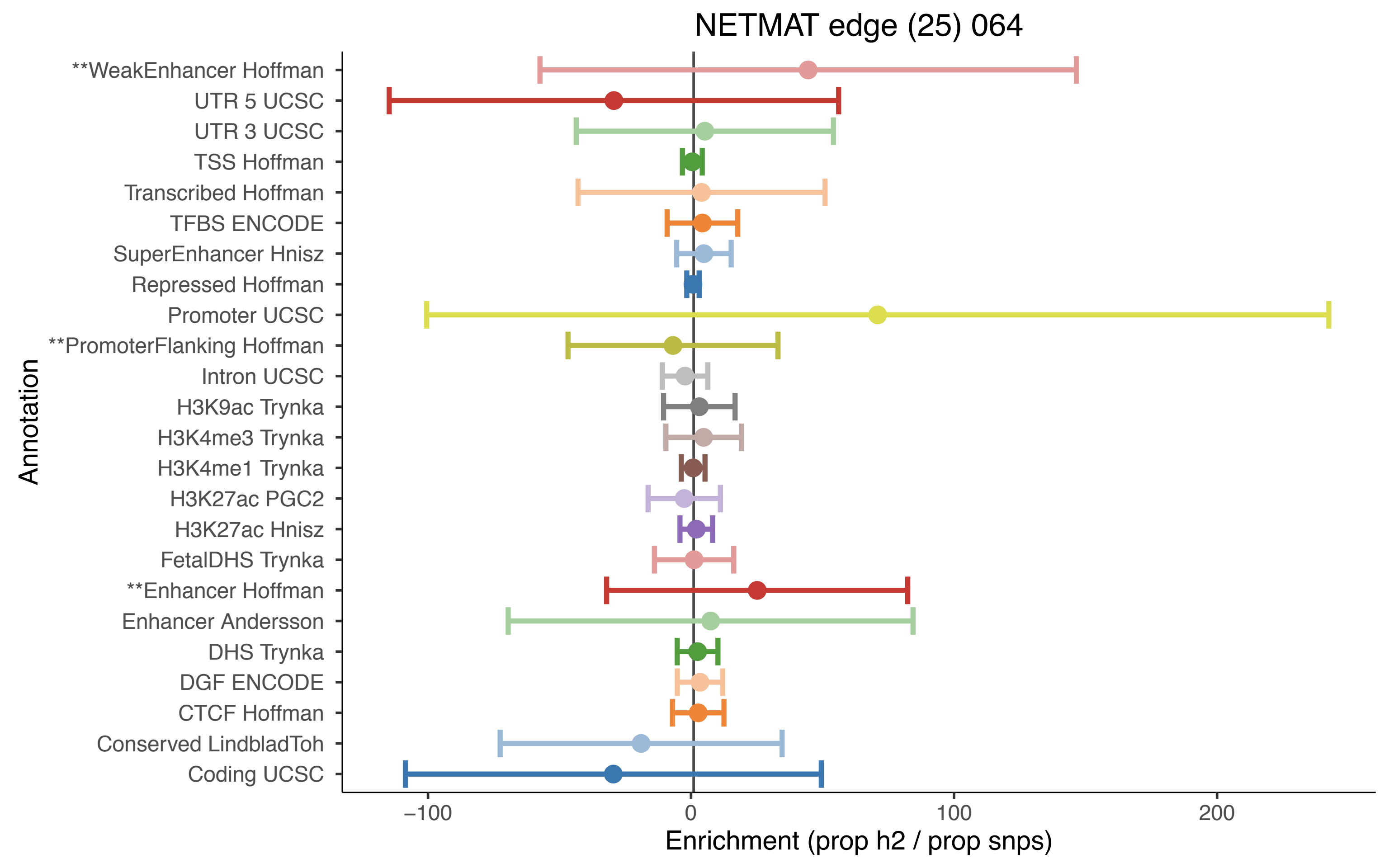

NETMAT edge (25) 064
**WeakEnhancer Hoffman
UTR 5 UCSC
UTR 3 UCSC
TSS Hoffman
Transcribed Hoffman
TFBS ENCODE
SuperEnhancer Hnisz
Repressed Hoffman
Promoter UCSC
**PromoterFlanking Hoffman
Intron UCSC
H3K9ac Trynka
H3K4me3 Trynka
H3K4me1 Trynka
H3K27ac PGC2
H3K27ac Hnisz
FetalDHS Trynka
**Enhancer Hoffman
Enhancer Andersson
DHS Trynka
DGF ENCODE
CTCF Hoffman
Conserved LindbladToh
Coding UCSC
Annotation
−100
0
100
200
Enrichment (prop h2 / prop snps)

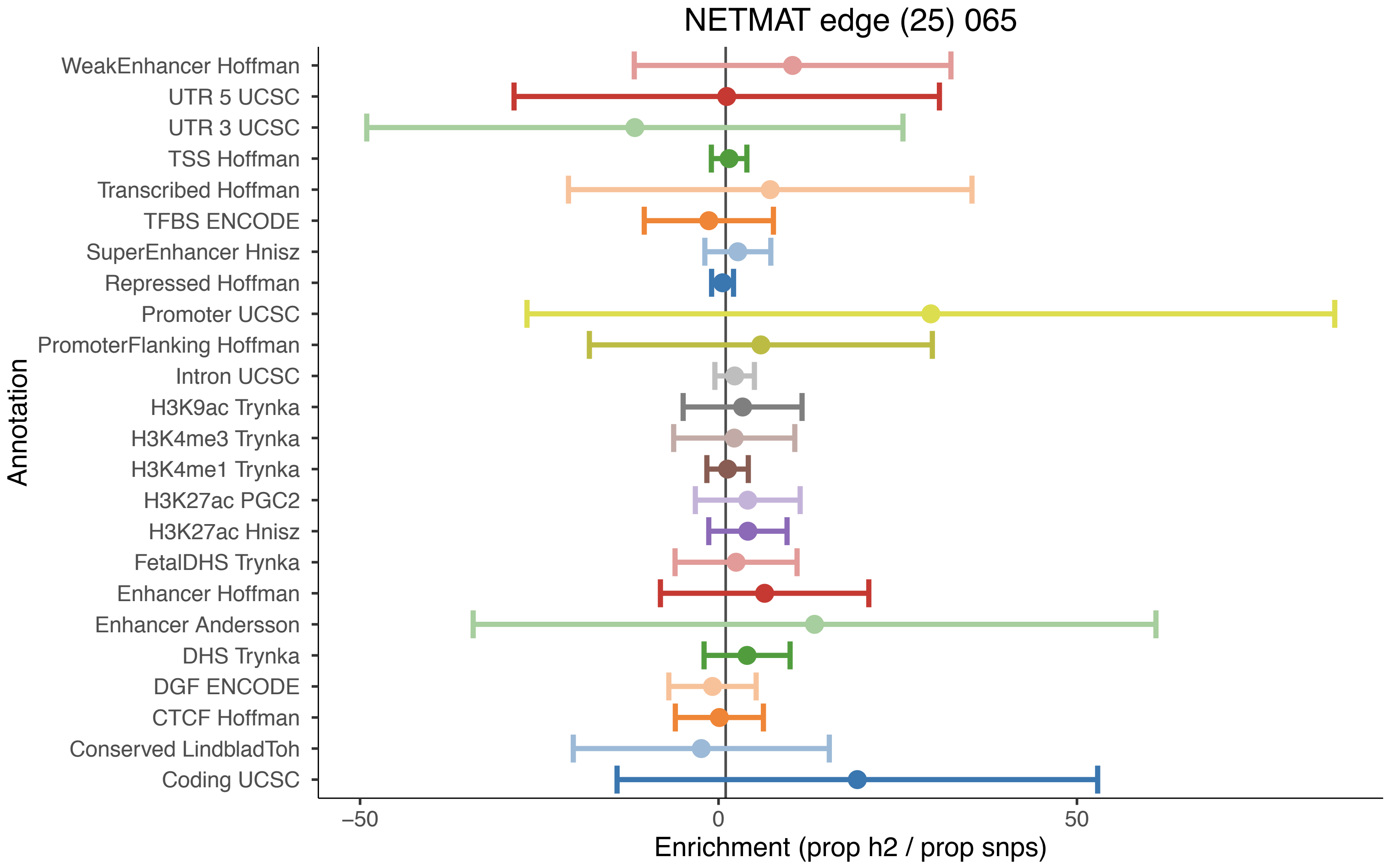

NETMAT edge (25) 065
Annotation
WeakEnhancer Hoffman
UTR 5 UCSC
UTR 3 UCSC
TSS Hoffman
Transcribed Hoffman
TFBS ENCODE
SuperEnhancer Hnisz
Repressed Hoffman
Promoter UCSC
PromoterFlanking Hoffman
Intron UCSC
H3K9ac Trynka
H3K4me3 Trynka
H3K4me1 Trynka
H3K27ac PGC2
H3K27ac Hnisz
FetalDHS Trynka
Enhancer Hoffman
Enhancer Andersson
DHS Trynka
DGF ENCODE
CTCF Hoffman
Conserved LindbladToh
Coding UCSC
−50
0
50
Enrichment (prop h2 / prop snps)

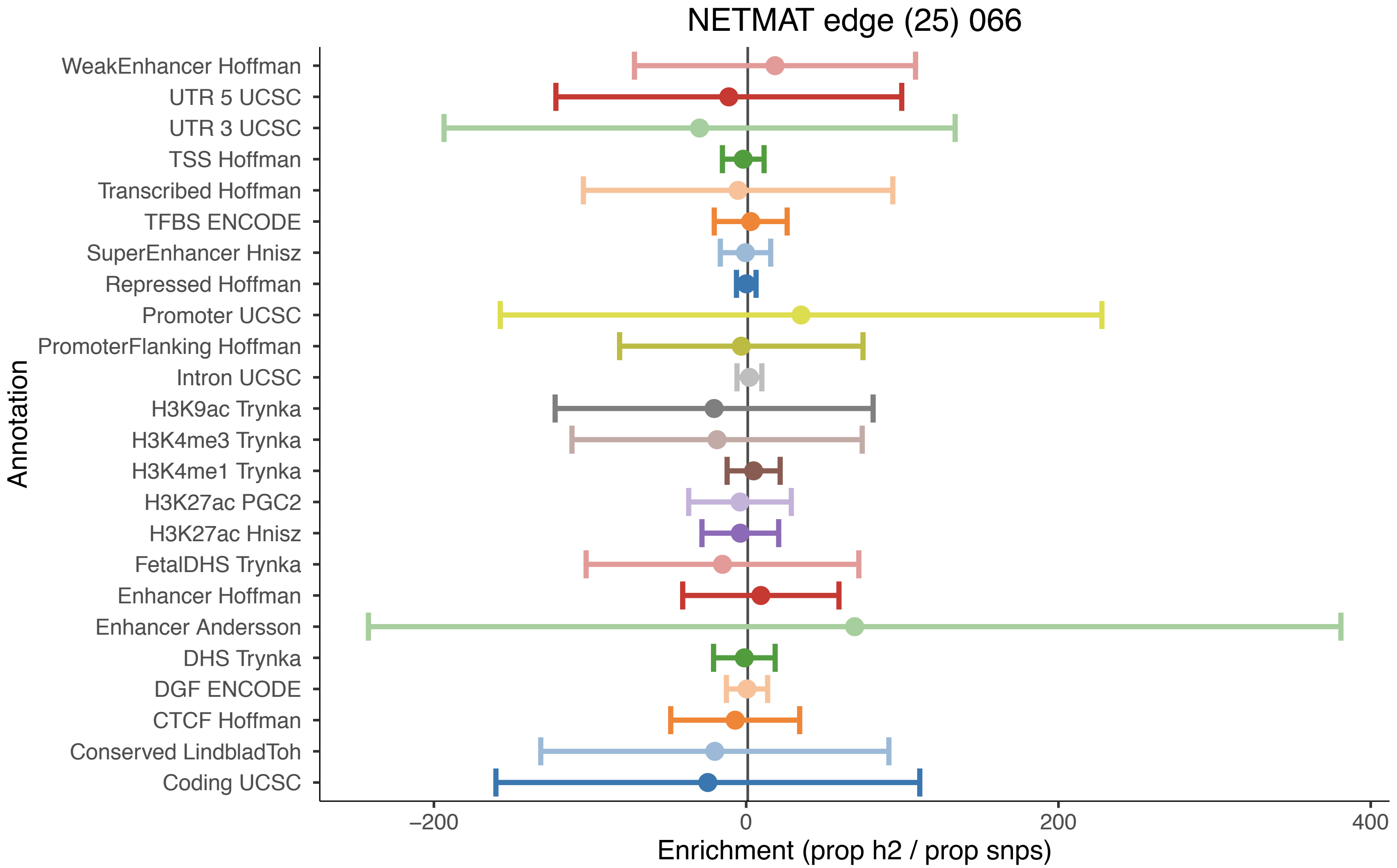
NETMAT edge (25) 066
WeakEnhancer Hoffman
UTR 5 UCSC
UTR 3 UCSC
TSS Hoffman
Transcribed Hoffman
TFBS ENCODE
SuperEnhancer Hnisz
Repressed Hoffman
Promoter UCSC
PromoterFlanking Hoffman
Intron UCSC
H3K9ac Trynka
H3K4me3 Trynka
H3K4me1 Trynka
H3K27ac PGC2
H3K27ac Hnisz
FetalDHS Trynka
Enhancer Hoffman
Enhancer Andersson
DHS Trynka
DGF ENCODE
CTCF Hoffman
Conserved LindbladToh
Coding UCSC
Annotation
−200
0
200
400
Enrichment (prop h2 / prop snps)

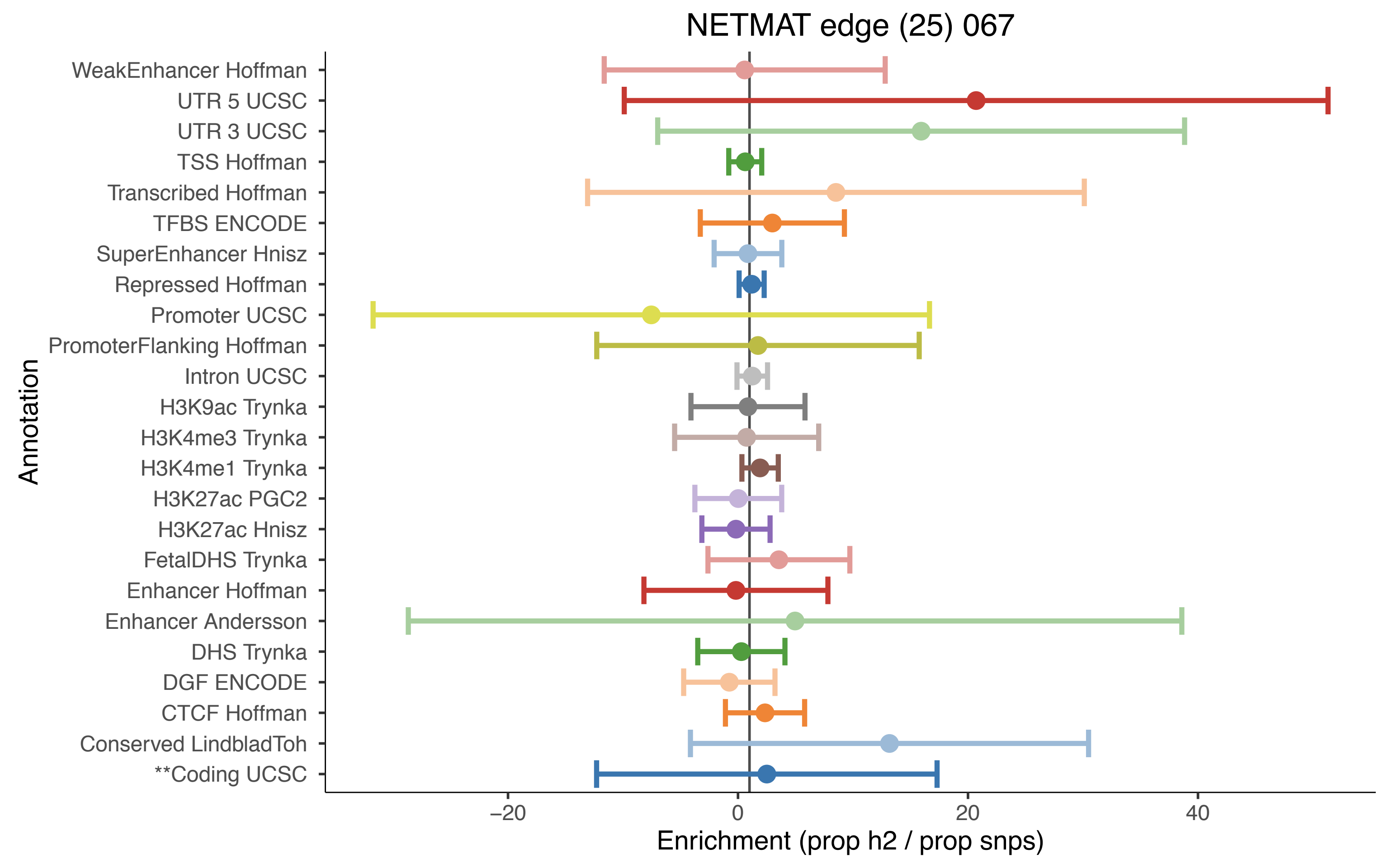
NETMAT edge (25) 067
Annotation
WeakEnhancer Hoffman
UTR 5 UCSC
UTR 3 UCSC
TSS Hoffman
Transcribed Hoffman
TFBS ENCODE
SuperEnhancer Hnisz
Repressed Hoffman
Promoter UCSC
PromoterFlanking Hoffman
Intron UCSC
H3K9ac Trynka
H3K4me3 Trynka
H3K4me1 Trynka
H3K27ac PGC2
H3K27ac Hnisz
FetalDHS Trynka
Enhancer Hoffman
Enhancer Andersson
DHS Trynka
DGF ENCODE
CTCF Hoffman
Conserved LindbladToh
**Coding UCSC
−20
0
20
40
Enrichment (prop h2 / prop snps)

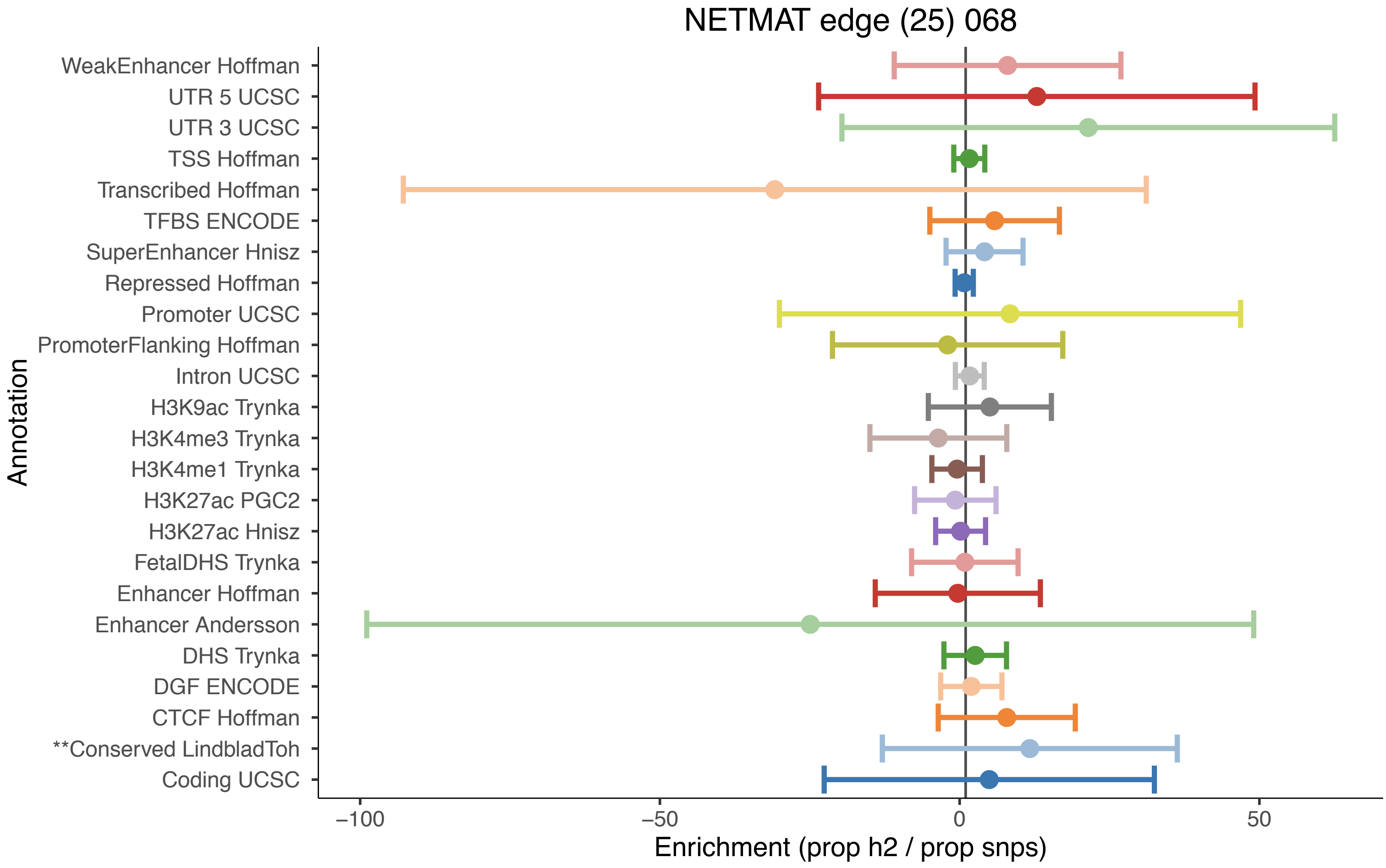
NETMAT edge (25) 068
WeakEnhancer Hoffman
UTR 5 UCSC
UTR 3 UCSC
TSS Hoffman
Transcribed Hoffman
TFBS ENCODE
SuperEnhancer Hnisz
Repressed Hoffman
Promoter UCSC
PromoterFlanking Hoffman
Intron UCSC
H3K9ac Trynka
H3K4me3 Trynka
H3K4me1 Trynka
H3K27ac PGC2
H3K27ac Hnisz
FetalDHS Trynka
Enhancer Hoffman
Enhancer Andersson
DHS Trynka
DGF ENCODE
CTCF Hoffman
**Conserved LindbladToh
Coding UCSC
Annotation
−100
−50
0
50
Enrichment (prop h2 / prop snps)

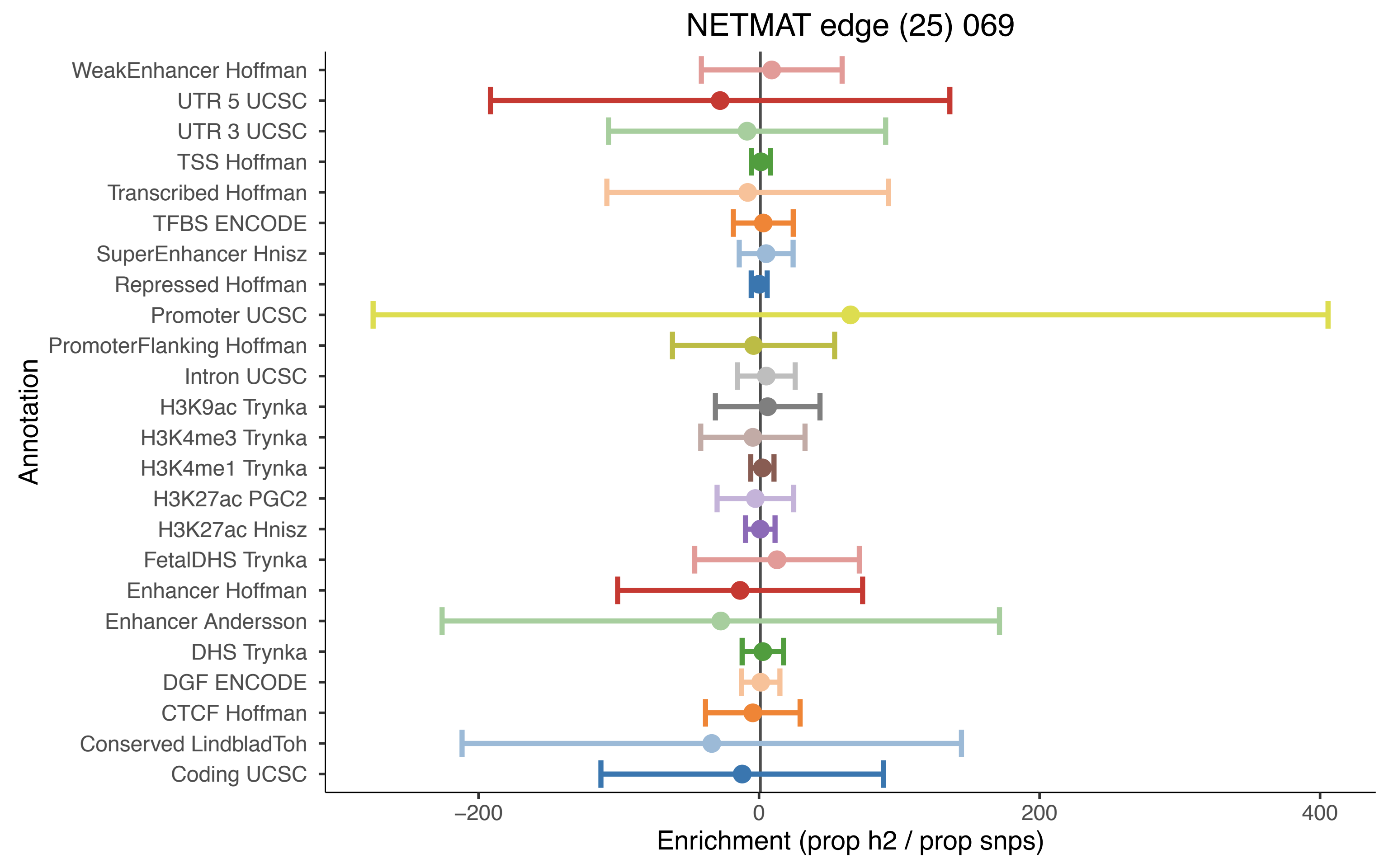
NETMAT edge (25) 069
Annotation
WeakEnhancer Hoffman
UTR 5 UCSC
UTR 3 UCSC
TSS Hoffman
Transcribed Hoffman
TFBS ENCODE
SuperEnhancer Hnisz
Repressed Hoffman
Promoter UCSC
PromoterFlanking Hoffman
Intron UCSC
H3K9ac Trynka
H3K4me3 Trynka
H3K4me1 Trynka
H3K27ac PGC2
H3K27ac Hnisz
FetalDHS Trynka
Enhancer Hoffman
Enhancer Andersson
DHS Trynka
DGF ENCODE
CTCF Hoffman
Conserved LindbladToh
Coding UCSC
−200
0
200
400
Enrichment (prop h2 / prop snps)

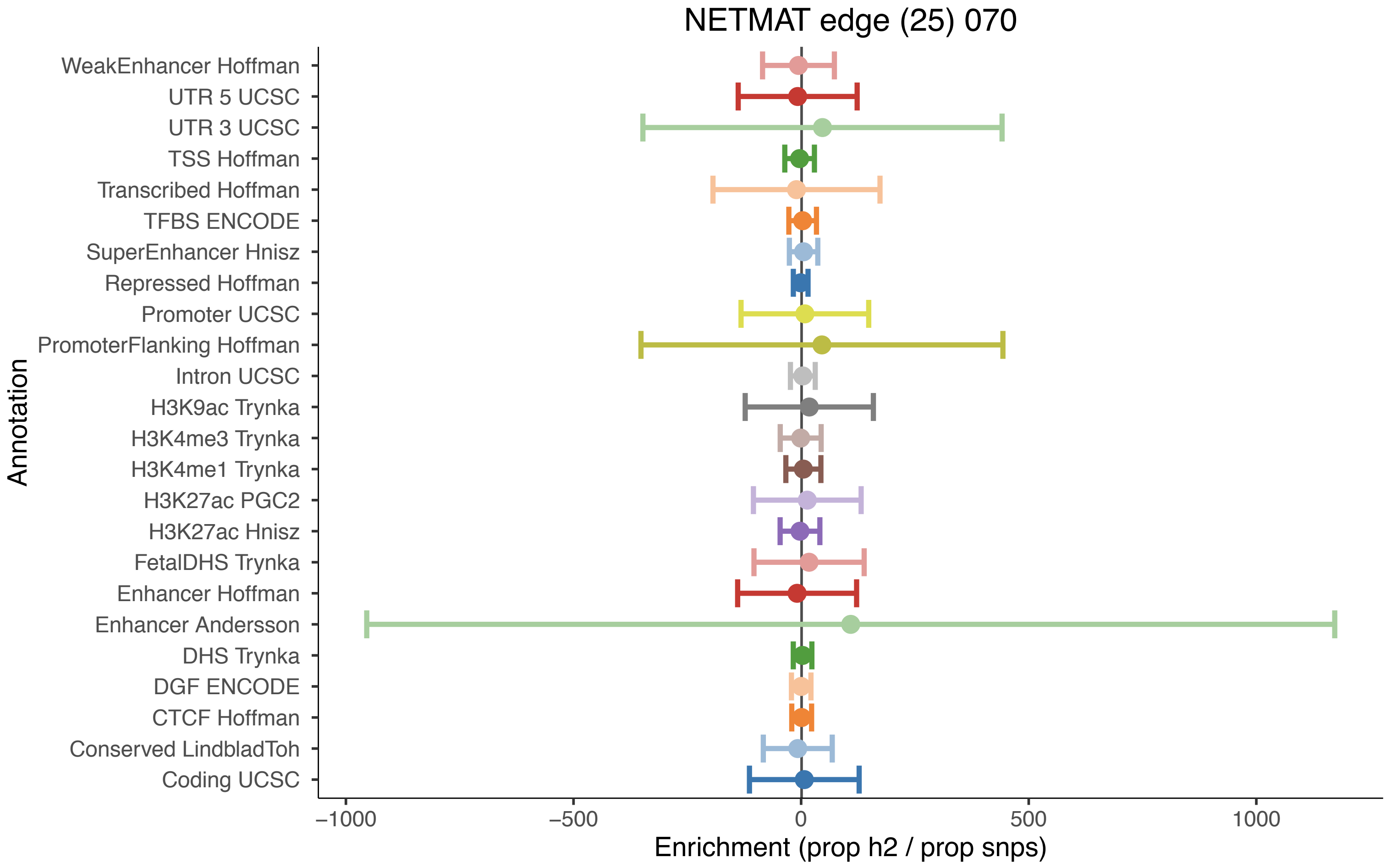
NETMAT edge (25) 070
WeakEnhancer Hoffman
UTR 5 UCSC
UTR 3 UCSC
TSS Hoffman
Transcribed Hoffman
TFBS ENCODE
SuperEnhancer Hnisz
Repressed Hoffman
Promoter UCSC
PromoterFlanking Hoffman
Intron UCSC
H3K9ac Trynka
H3K4me3 Trynka
H3K4me1 Trynka
H3K27ac PGC2
H3K27ac Hnisz
FetalDHS Trynka
Enhancer Hoffman
Enhancer Andersson
DHS Trynka
DGF ENCODE
CTCF Hoffman
Conserved LindbladToh
Coding UCSC
Annotation
−1000
−500
0
500
1000
Enrichment (prop h2 / prop snps)

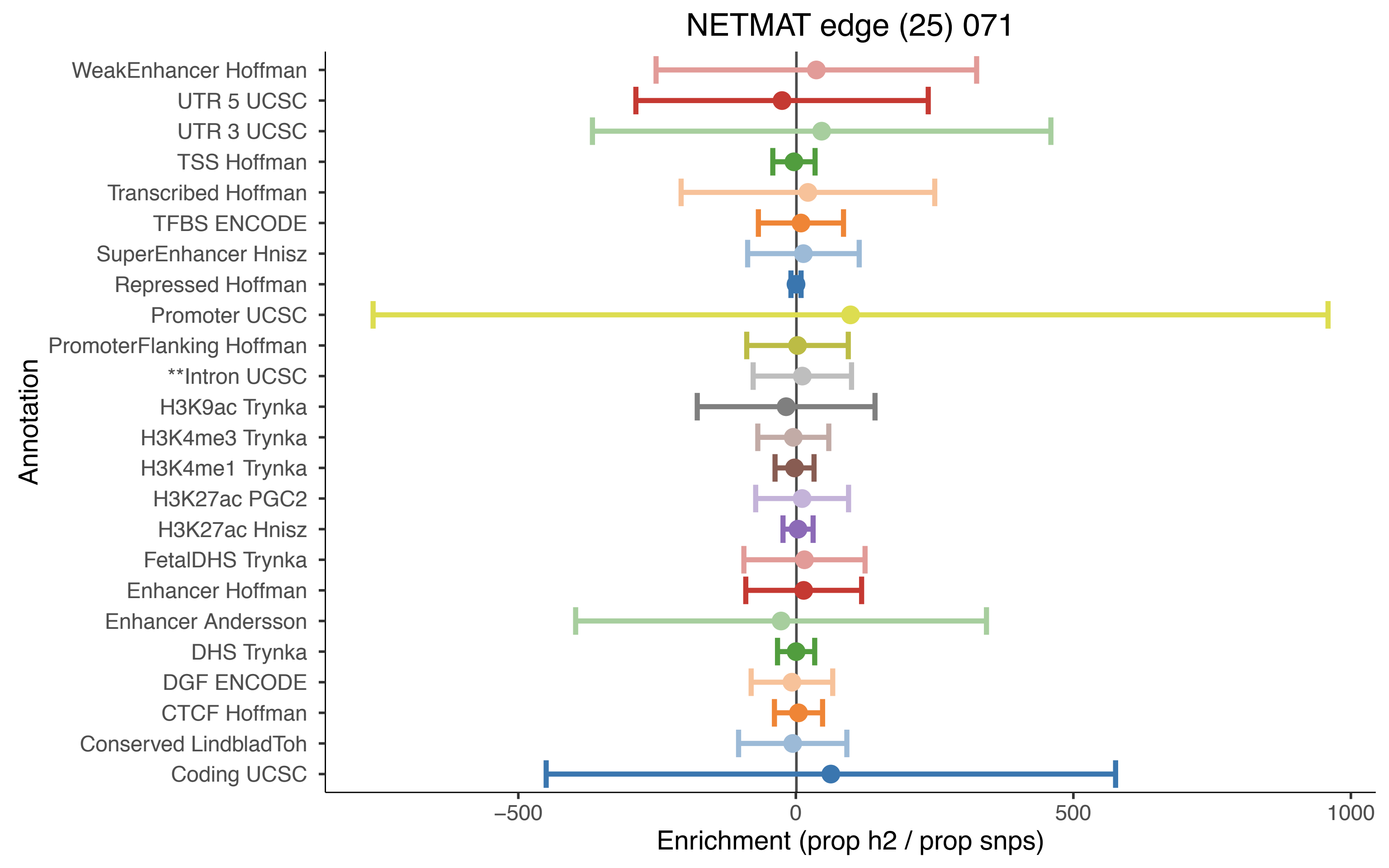
NETMAT edge (25) 071
WeakEnhancer Hoffman
UTR 5 UCSC
UTR 3 UCSC
TSS Hoffman
Transcribed Hoffman
TFBS ENCODE
SuperEnhancer Hnisz
Repressed Hoffman
Promoter UCSC
PromoterFlanking Hoffman
**Intron UCSC
H3K9ac Trynka
H3K4me3 Trynka
H3K4me1 Trynka
H3K27ac PGC2
H3K27ac Hnisz
FetalDHS Trynka
Enhancer Hoffman
Enhancer Andersson
DHS Trynka
DGF ENCODE
CTCF Hoffman
Conserved LindbladToh
Coding UCSC
Annotation
−500
0
500
1000
Enrichment (prop h2 / prop snps)

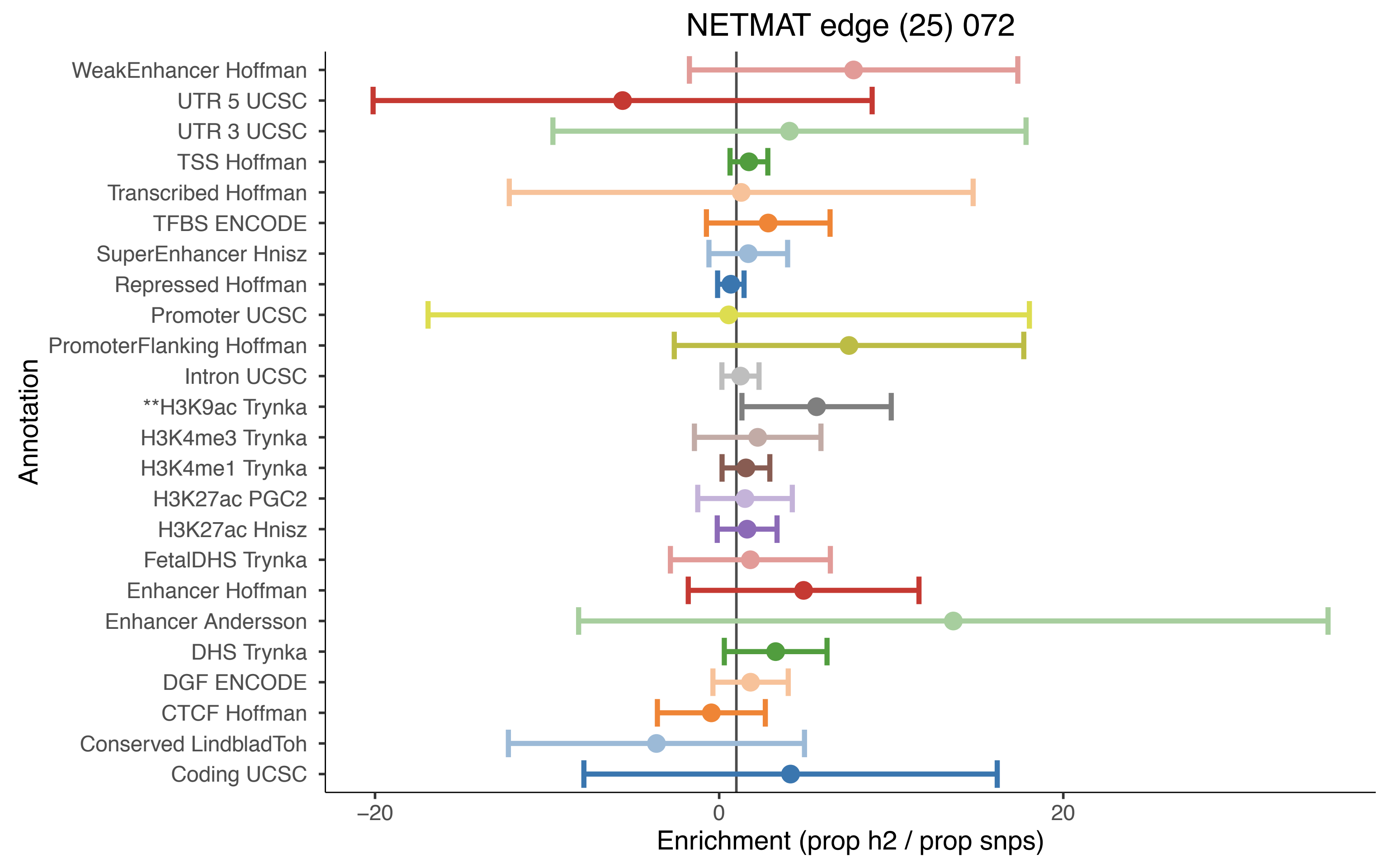

NETMAT edge (25) 072
Annotation
WeakEnhancer Hoffman
UTR 5 UCSC
UTR 3 UCSC
TSS Hoffman
Transcribed Hoffman
TFBS ENCODE
SuperEnhancer Hnisz
Repressed Hoffman
Promoter UCSC
PromoterFlanking Hoffman
Intron UCSC
**H3K9ac Trynka
H3K4me3 Trynka
H3K4me1 Trynka
H3K27ac PGC2
H3K27ac Hnisz
FetalDHS Trynka
Enhancer Hoffman
Enhancer Andersson
DHS Trynka
DGF ENCODE
CTCF Hoffman
Conserved LindbladToh
Coding UCSC
−20
0
20
Enrichment (prop h2 / prop snps)

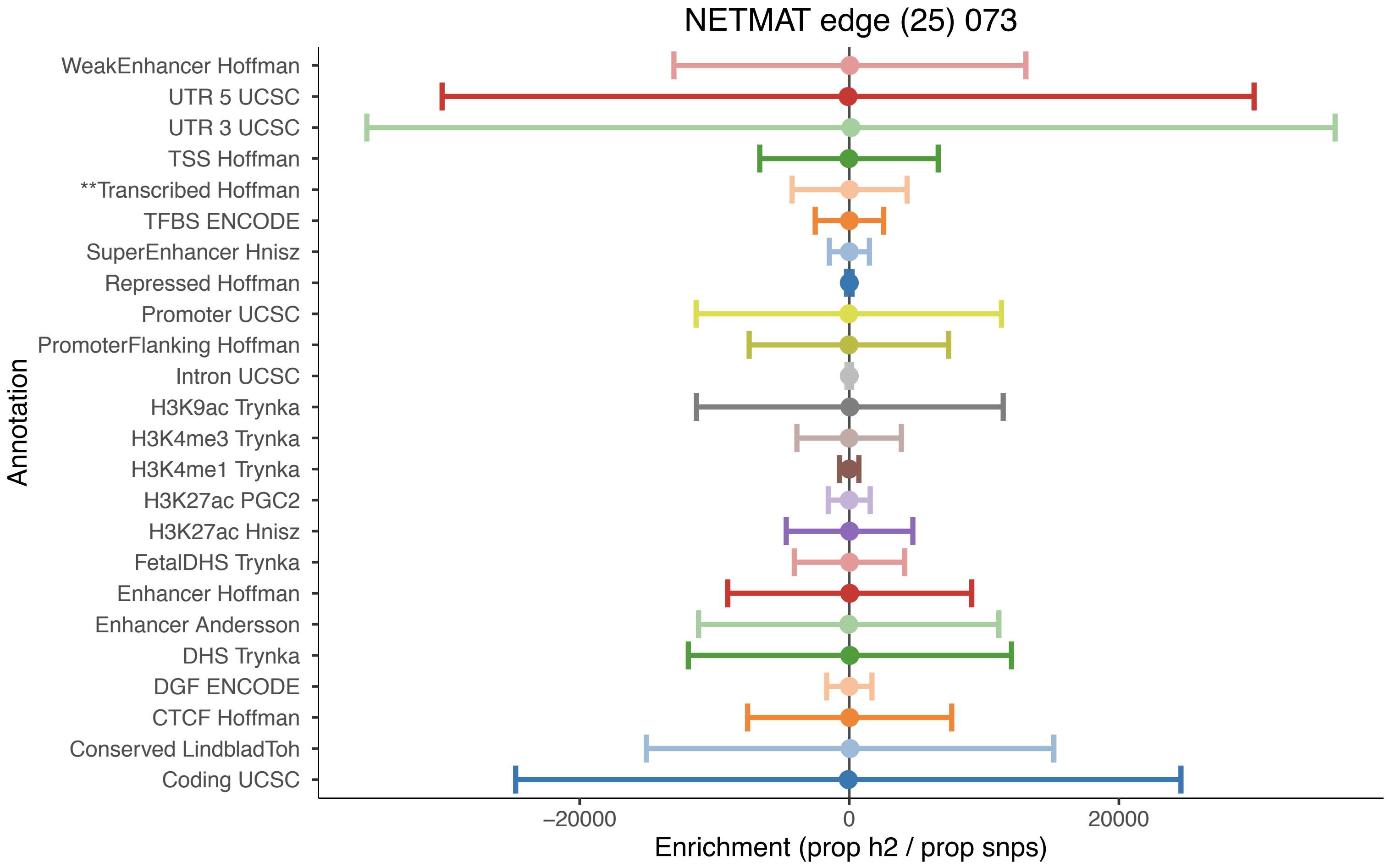
NETMAT edge (25) 073
WeakEnhancer Hoffman
UTR 5 UCSC
UTR 3 UCSC
TSS Hoffman
**Transcribed Hoffman
TFBS ENCODE
SuperEnhancer Hnisz
Repressed Hoffman
Promoter UCSC
PromoterFlanking Hoffman
Intron UCSC
H3K9ac Trynka
H3K4me3 Trynka
H3K4me1 Trynka
H3K27ac PGC2
H3K27ac Hnisz
FetalDHS Trynka
Enhancer Hoffman
Enhancer Andersson
DHS Trynka
DGF ENCODE
CTCF Hoffman
Conserved LindbladToh
Coding UCSC
Annotation
−20000
0
20000
Enrichment (prop h2 / prop snps)

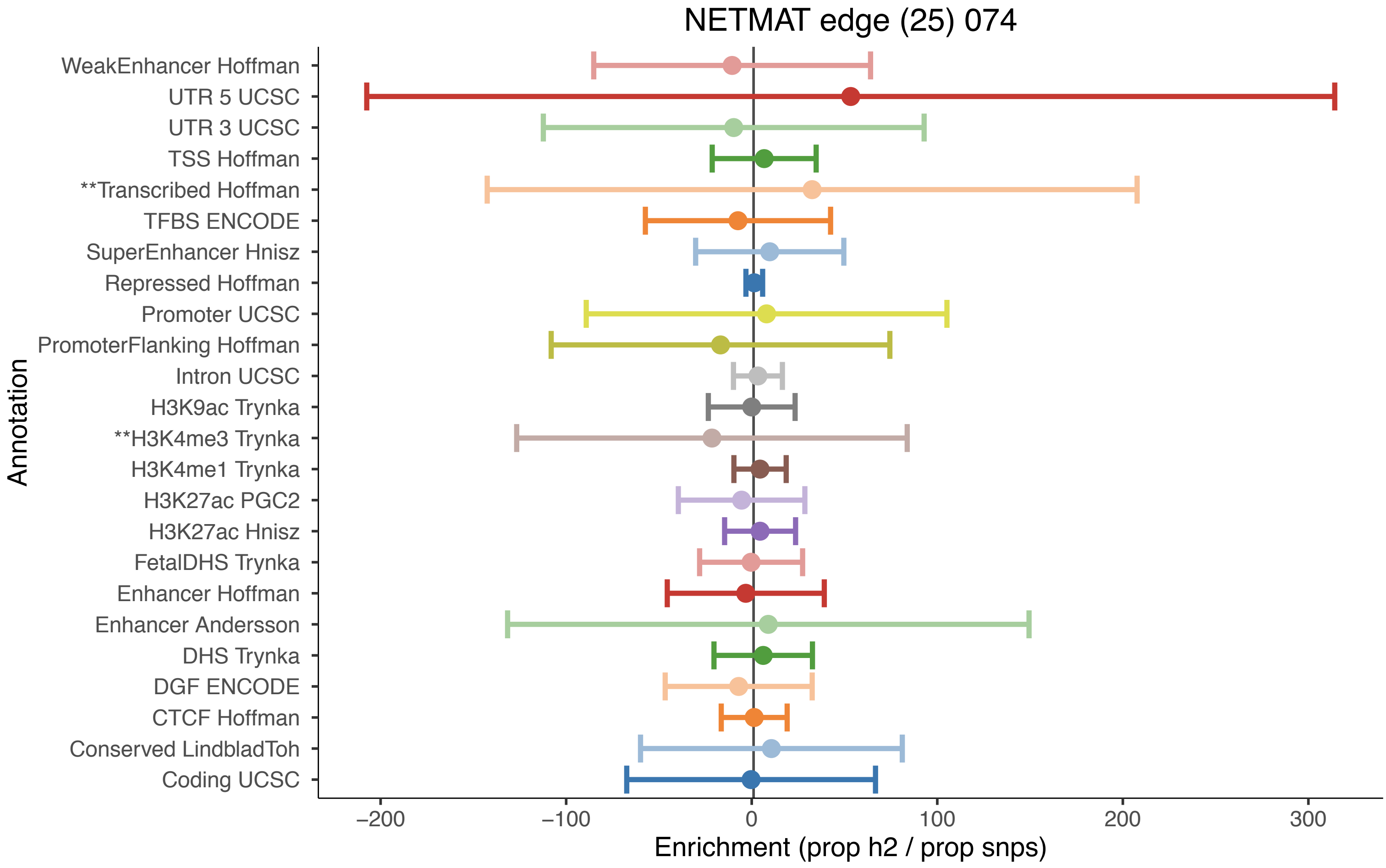
NETMAT edge (25) 074
Annotation
WeakEnhancer Hoffman
UTR 5 UCSC
UTR 3 UCSC
TSS Hoffman
**Transcribed Hoffman
TFBS ENCODE
SuperEnhancer Hnisz
Repressed Hoffman
Promoter UCSC
PromoterFlanking Hoffman
Intron UCSC
H3K9ac Trynka
**H3K4me3 Trynka
H3K4me1 Trynka
H3K27ac PGC2
H3K27ac Hnisz
FetalDHS Trynka
Enhancer Hoffman
Enhancer Andersson
DHS Trynka
DGF ENCODE
CTCF Hoffman
Conserved LindbladToh
Coding UCSC
−200
−100
0
100
200
300
Enrichment (prop h2 / prop snps)

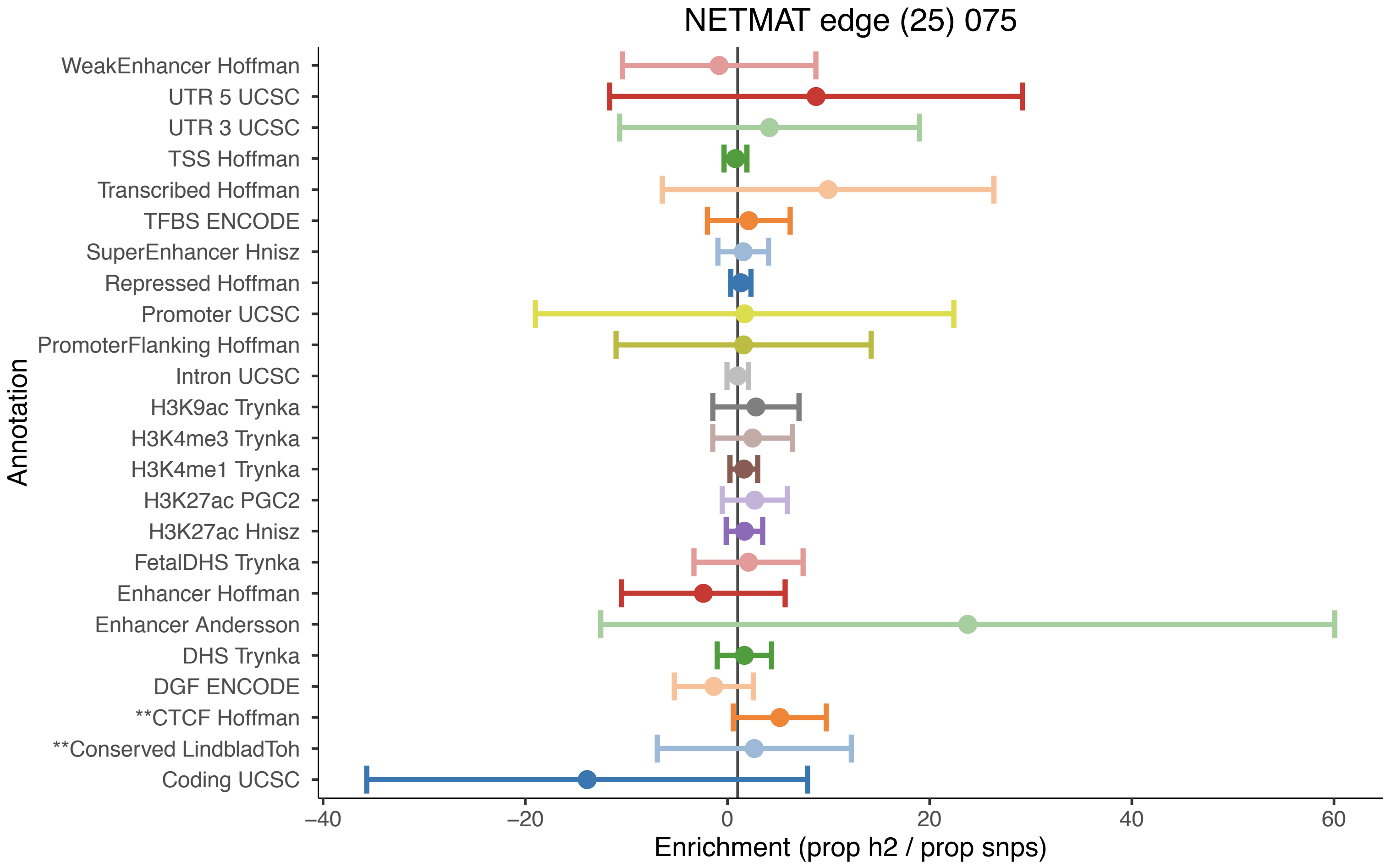
NETMAT edge (25) 075
Annotation
WeakEnhancer Hoffman
UTR 5 UCSC
UTR 3 UCSC
TSS Hoffman
Transcribed Hoffman
TFBS ENCODE
SuperEnhancer Hnisz
Repressed Hoffman
Promoter UCSC
PromoterFlanking Hoffman
Intron UCSC
H3K9ac Trynka
H3K4me3 Trynka
H3K4me1 Trynka
H3K27ac PGC2
H3K27ac Hnisz
FetalDHS Trynka
Enhancer Hoffman
Enhancer Andersson
DHS Trynka
DGF ENCODE
**CTCF Hoffman
**Conserved LindbladToh
Coding UCSC
−40
−20
0
20
40
60
Enrichment (prop h2 / prop snps)

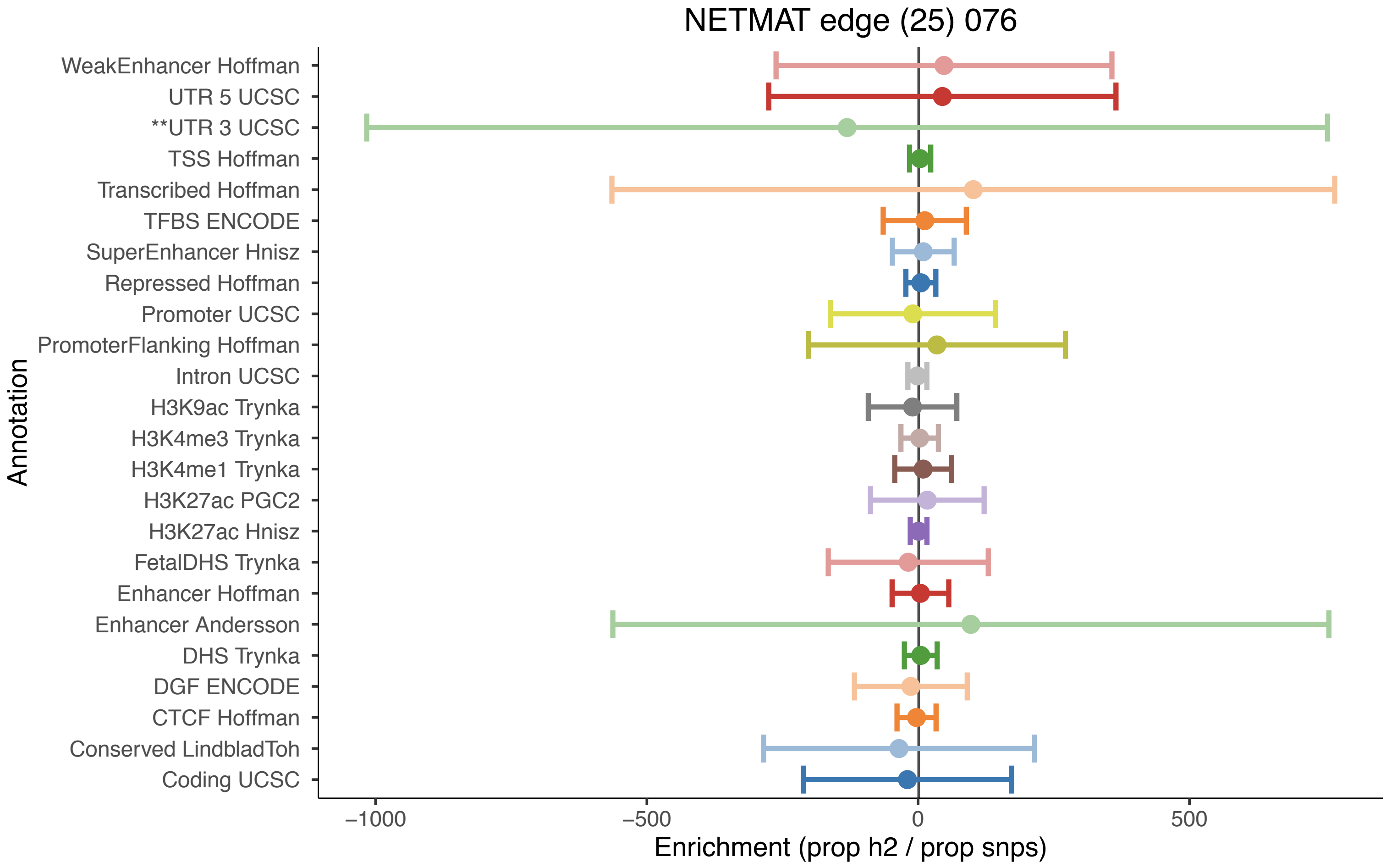
NETMAT edge (25) 076
WeakEnhancer Hoffman
UTR 5 UCSC
**UTR 3 UCSC
TSS Hoffman
Transcribed Hoffman
TFBS ENCODE
SuperEnhancer Hnisz
Repressed Hoffman
Promoter UCSC
PromoterFlanking Hoffman
Intron UCSC
H3K9ac Trynka
H3K4me3 Trynka
H3K4me1 Trynka
H3K27ac PGC2
H3K27ac Hnisz
FetalDHS Trynka
Enhancer Hoffman
Enhancer Andersson
DHS Trynka
DGF ENCODE
CTCF Hoffman
Conserved LindbladToh
Coding UCSC
Annotation
−1000
−500
0
500
Enrichment (prop h2 / prop snps)

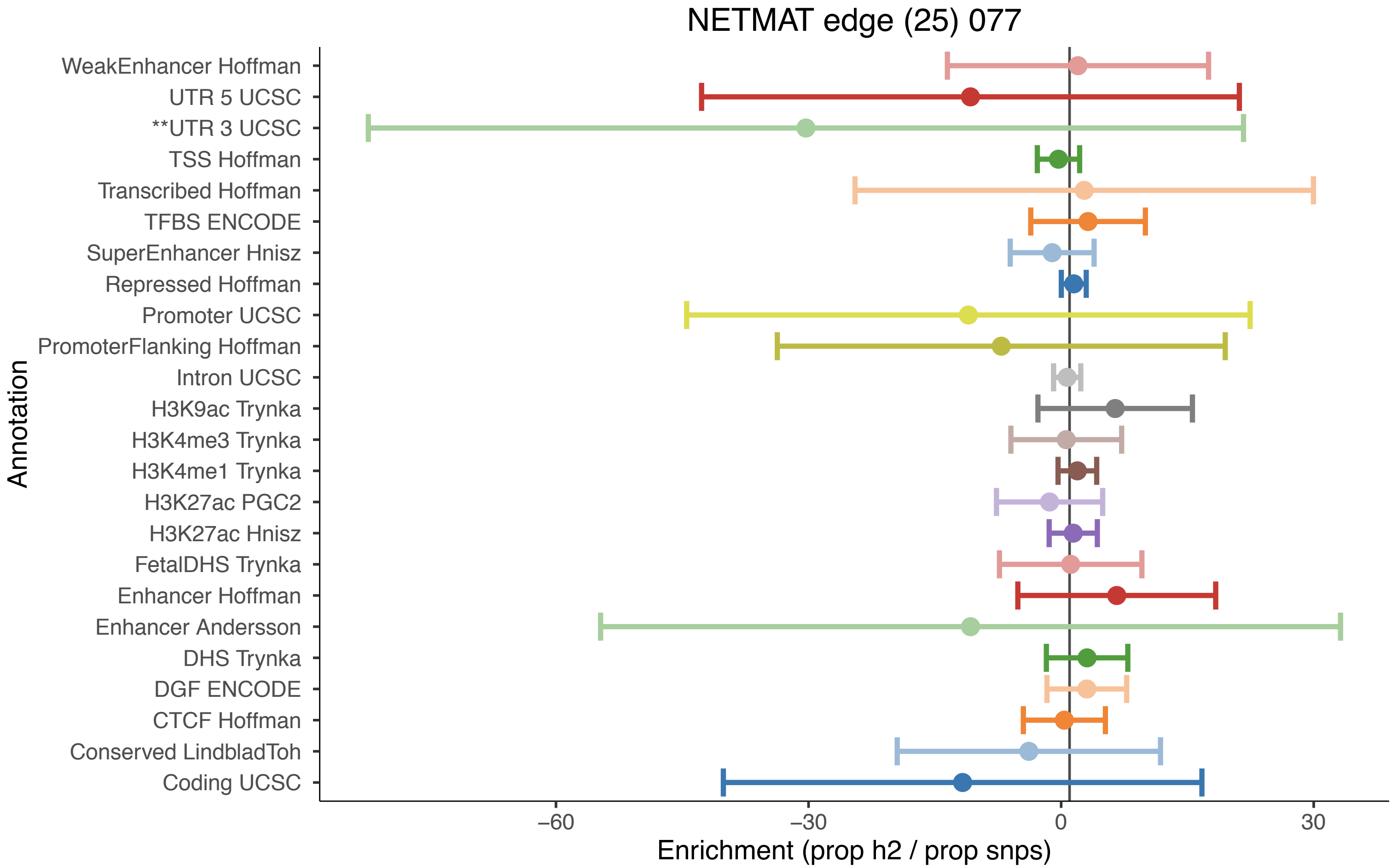
NETMAT edge (25) 077
WeakEnhancer Hoffman
UTR 5 UCSC
**UTR 3 UCSC
TSS Hoffman
Transcribed Hoffman
TFBS ENCODE
SuperEnhancer Hnisz
Repressed Hoffman
Promoter UCSC
PromoterFlanking Hoffman
Intron UCSC
H3K9ac Trynka
H3K4me3 Trynka
H3K4me1 Trynka
H3K27ac PGC2
H3K27ac Hnisz
FetalDHS Trynka
Enhancer Hoffman
Enhancer Andersson
DHS Trynka
DGF ENCODE
CTCF Hoffman
Conserved LindbladToh
Coding UCSC
Annotation
−60
−30
0
30
Enrichment (prop h2 / prop snps)

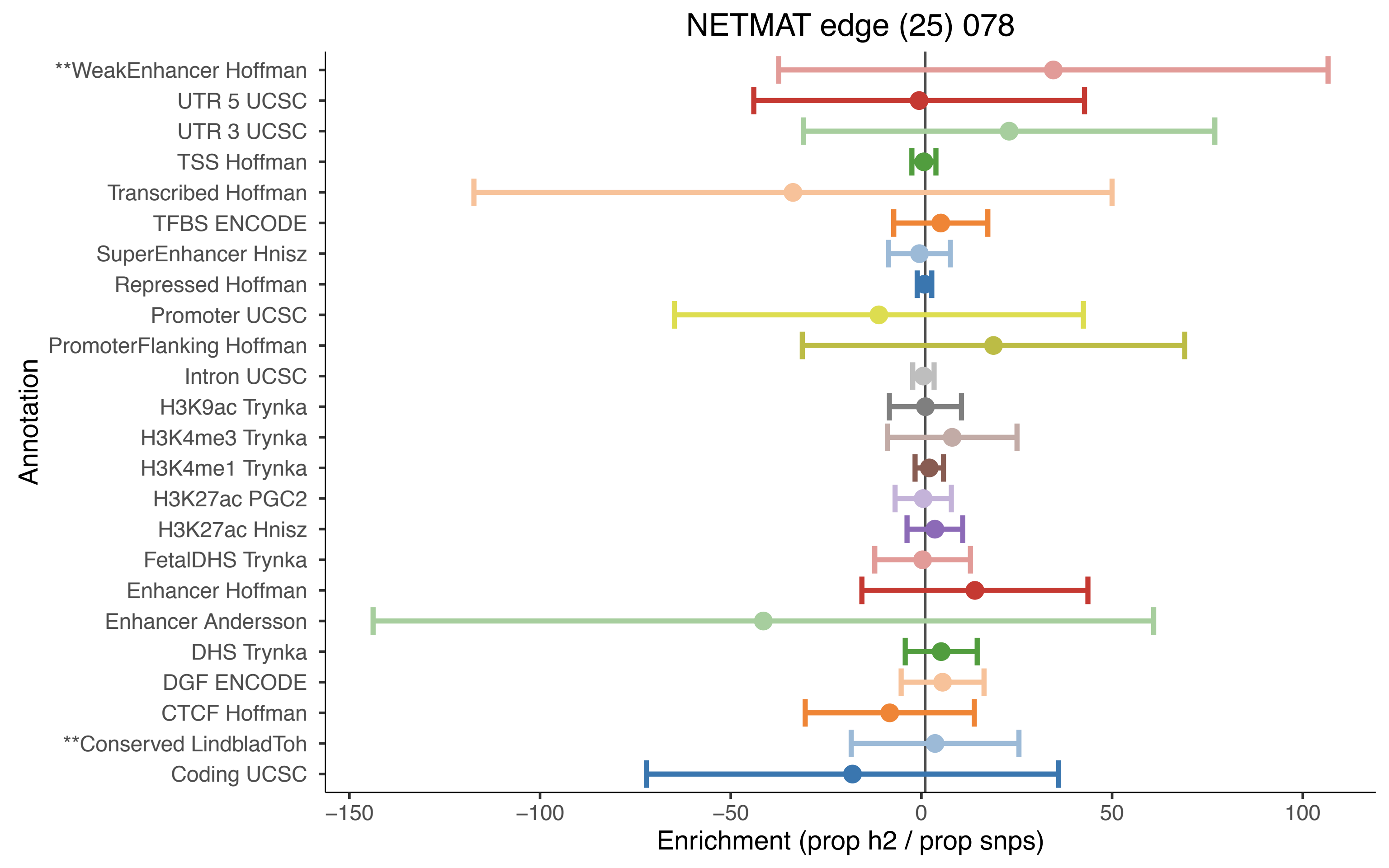

NETMAT edge (25) 078
Annotation
**WeakEnhancer Hoffman
UTR 5 UCSC
UTR 3 UCSC
TSS Hoffman
Transcribed Hoffman
TFBS ENCODE
SuperEnhancer Hnisz
Repressed Hoffman
Promoter UCSC
PromoterFlanking Hoffman
Intron UCSC
H3K9ac Trynka
H3K4me3 Trynka
H3K4me1 Trynka
H3K27ac PGC2
H3K27ac Hnisz
FetalDHS Trynka
Enhancer Hoffman
Enhancer Andersson
DHS Trynka
DGF ENCODE
CTCF Hoffman
**Conserved LindbladToh
Coding UCSC
−150
−100
−50
0
50
100
Enrichment (prop h2 / prop snps)

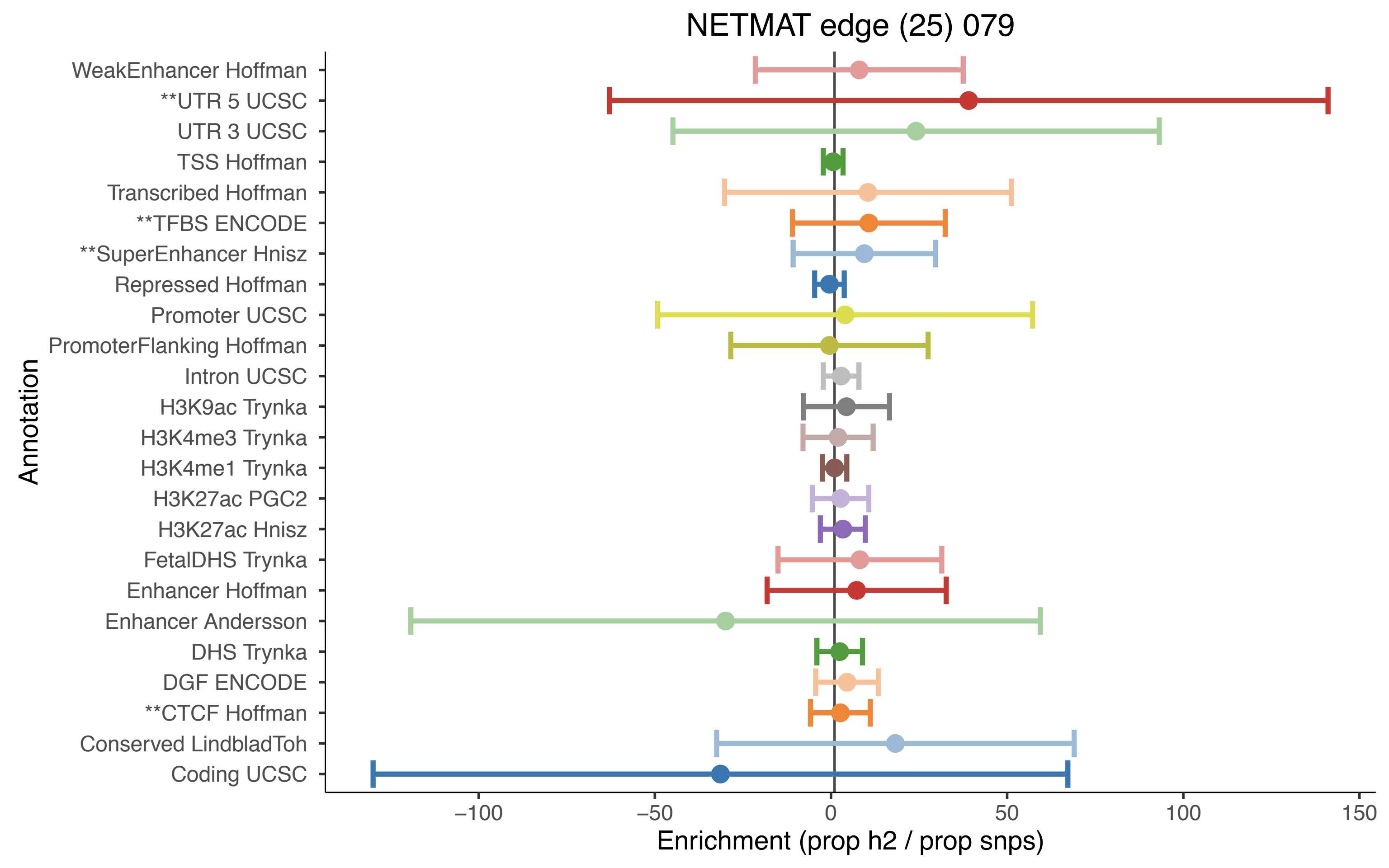
NETMAT edge (25) 079
WeakEnhancer Hoffman
**UTR 5 UCSC
UTR 3 UCSC
TSS Hoffman
Transcribed Hoffman
**TFBS ENCODE
**SuperEnhancer Hnisz
Repressed Hoffman
Promoter UCSC
PromoterFlanking Hoffman
Intron UCSC
H3K9ac Trynka
H3K4me3 Trynka
H3K4me1 Trynka
H3K27ac PGC2
H3K27ac Hnisz
FetalDHS Trynka
Enhancer Hoffman
Enhancer Andersson
DHS Trynka
DGF ENCODE
**CTCF Hoffman
Conserved LindbladToh
Coding UCSC
Annotation
−100
−50
0
50
100
150
Enrichment (prop h2 / prop snps)

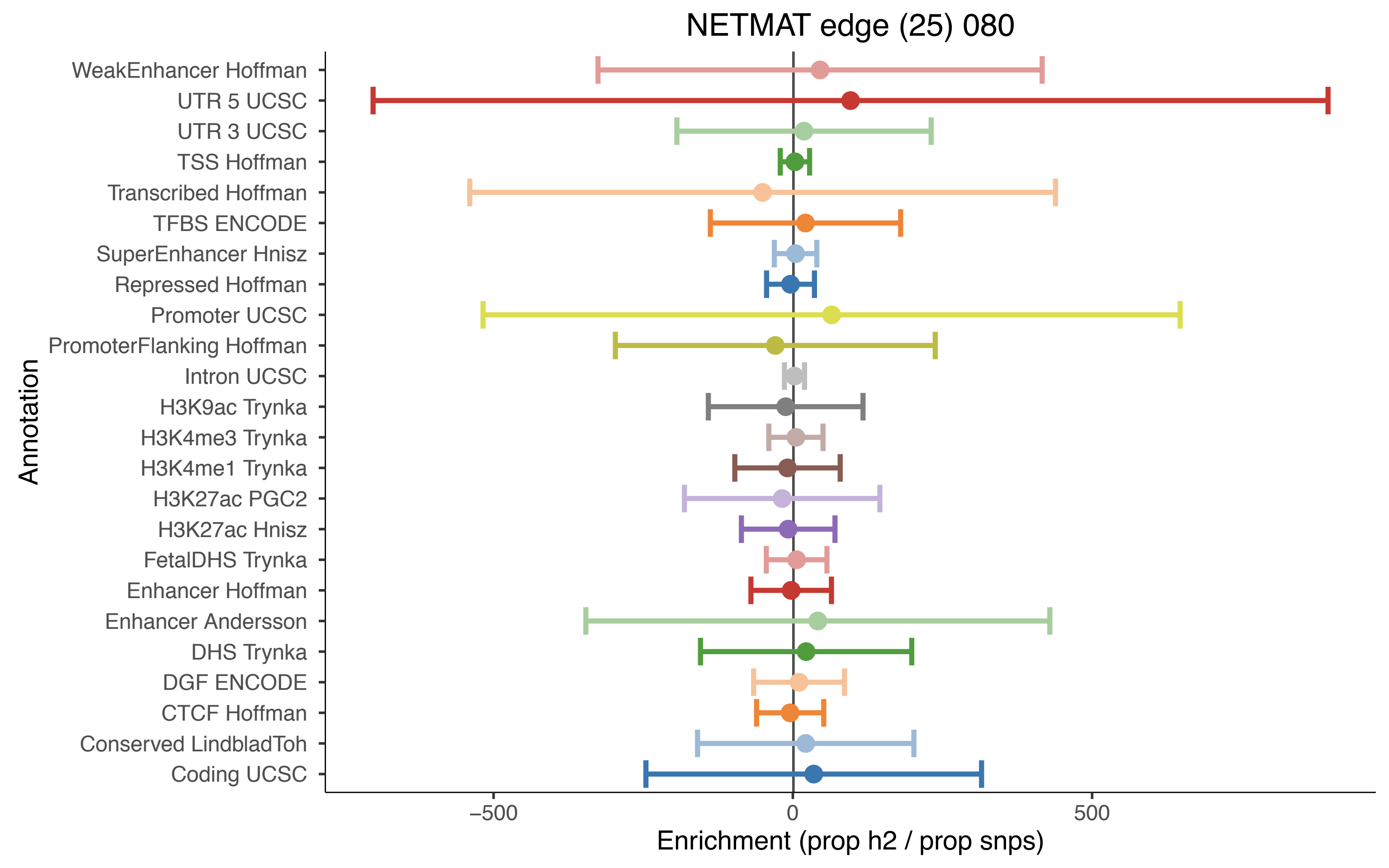
NETMAT edge (25) 080
Annotation
WeakEnhancer Hoffman
UTR 5 UCSC
UTR 3 UCSC
TSS Hoffman
Transcribed Hoffman
TFBS ENCODE
SuperEnhancer Hnisz
Repressed Hoffman
Promoter UCSC
PromoterFlanking Hoffman
Intron UCSC
H3K9ac Trynka
H3K4me3 Trynka
H3K4me1 Trynka
H3K27ac PGC2
H3K27ac Hnisz
FetalDHS Trynka
Enhancer Hoffman
Enhancer Andersson
DHS Trynka
DGF ENCODE
CTCF Hoffman
Conserved LindbladToh
Coding UCSC
−500
0
500
Enrichment (prop h2 / prop snps)

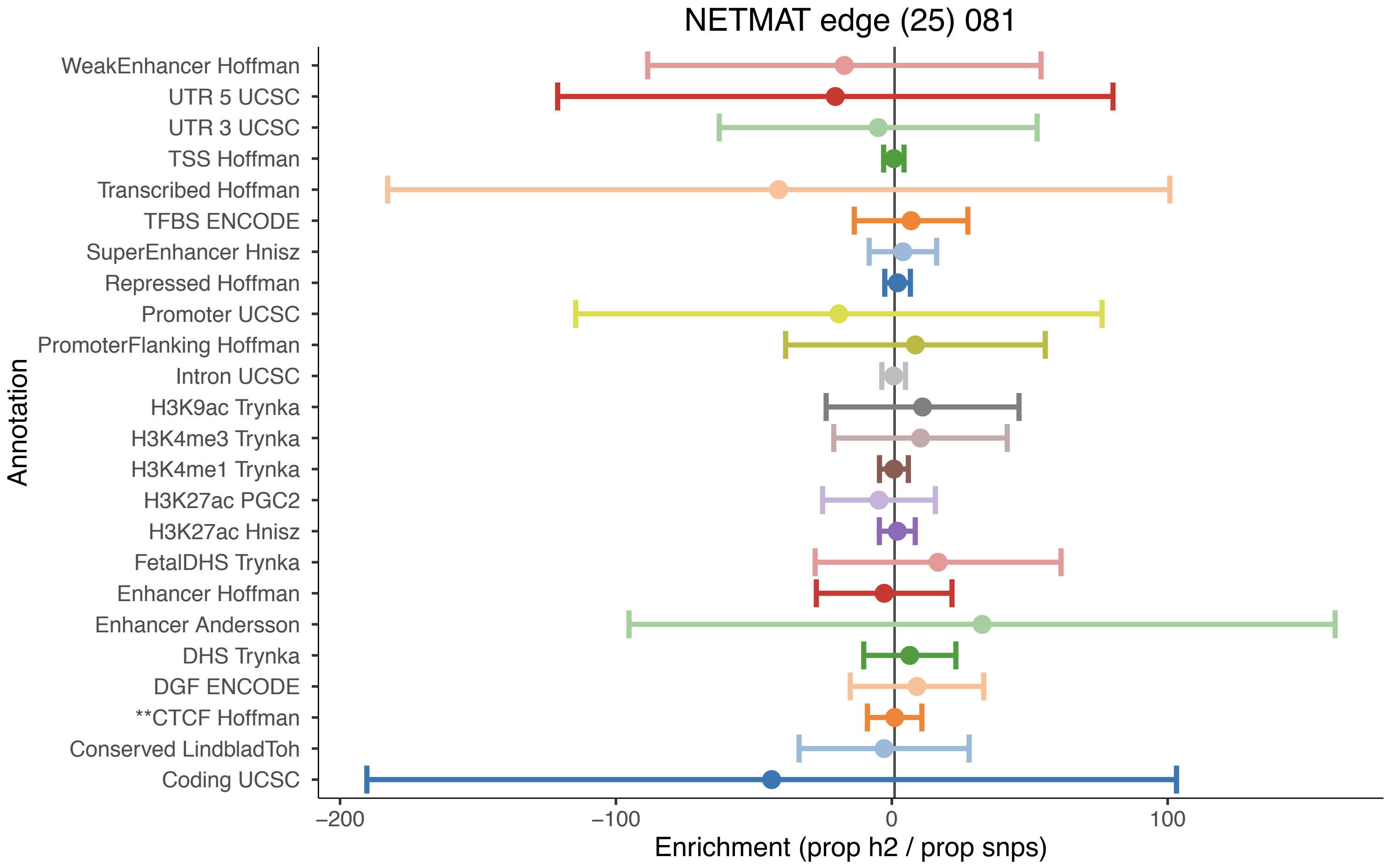

NETMAT edge (25) 081
Annotation
WeakEnhancer Hoffman
UTR 5 UCSC
UTR 3 UCSC
TSS Hoffman
Transcribed Hoffman
TFBS ENCODE
SuperEnhancer Hnisz
Repressed Hoffman
Promoter UCSC
PromoterFlanking Hoffman
Intron UCSC
H3K9ac Trynka
H3K4me3 Trynka
H3K4me1 Trynka
H3K27ac PGC2
H3K27ac Hnisz
FetalDHS Trynka
Enhancer Hoffman
Enhancer Andersson
DHS Trynka
DGF ENCODE
**CTCF Hoffman
Conserved LindbladToh
Coding UCSC
−200
−100
0
100
Enrichment (prop h2 / prop snps)

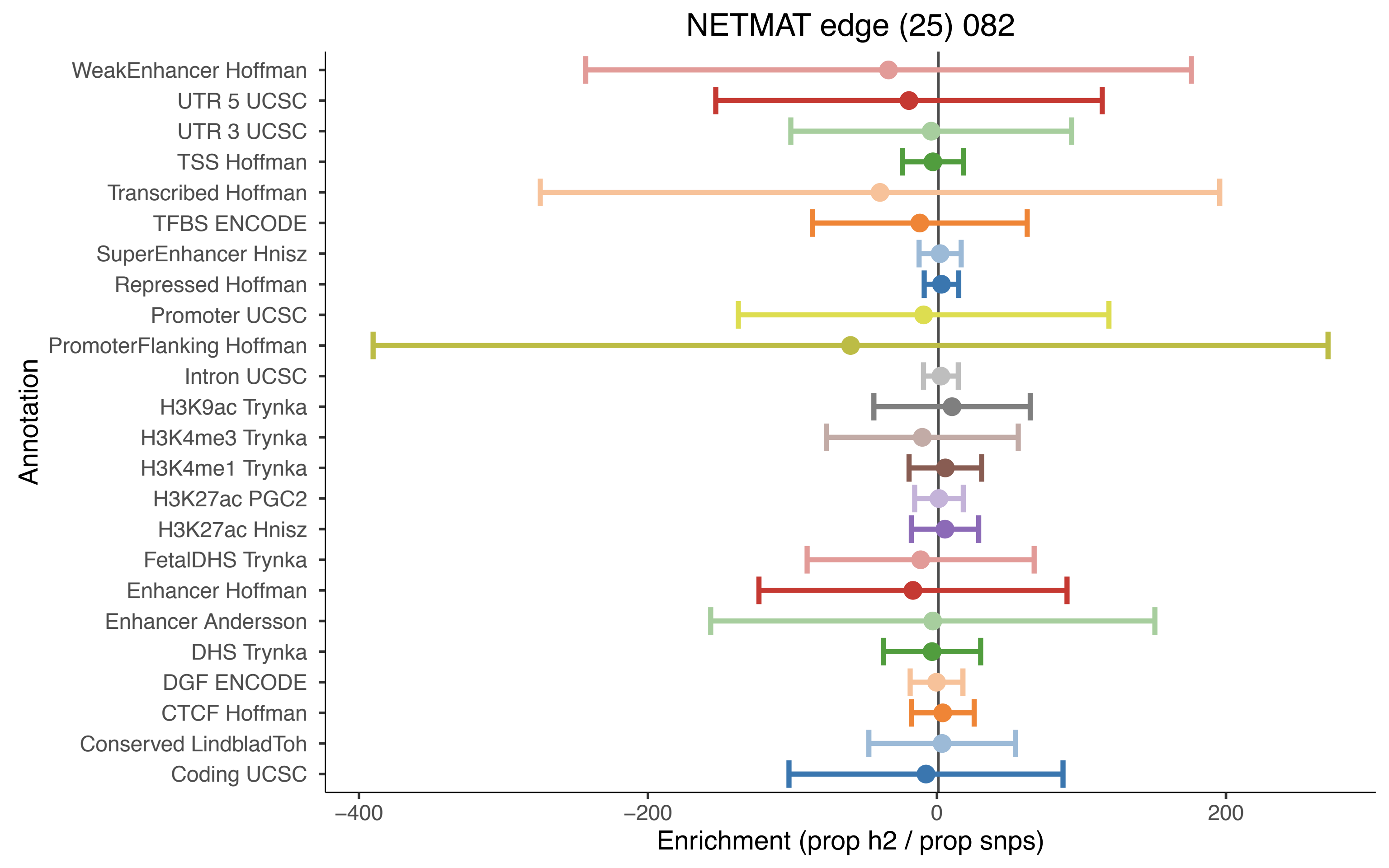

NETMAT edge (25) 082
WeakEnhancer Hoffman
UTR 5 UCSC
UTR 3 UCSC
TSS Hoffman
Transcribed Hoffman
TFBS ENCODE
SuperEnhancer Hnisz
Repressed Hoffman
Promoter UCSC
PromoterFlanking Hoffman
Intron UCSC
H3K9ac Trynka
H3K4me3 Trynka
H3K4me1 Trynka
H3K27ac PGC2
H3K27ac Hnisz
FetalDHS Trynka
Enhancer Hoffman
Enhancer Andersson
DHS Trynka
DGF ENCODE
CTCF Hoffman
Conserved LindbladToh
Coding UCSC
Annotation
−400
−200
0
200
Enrichment (prop h2 / prop snps)

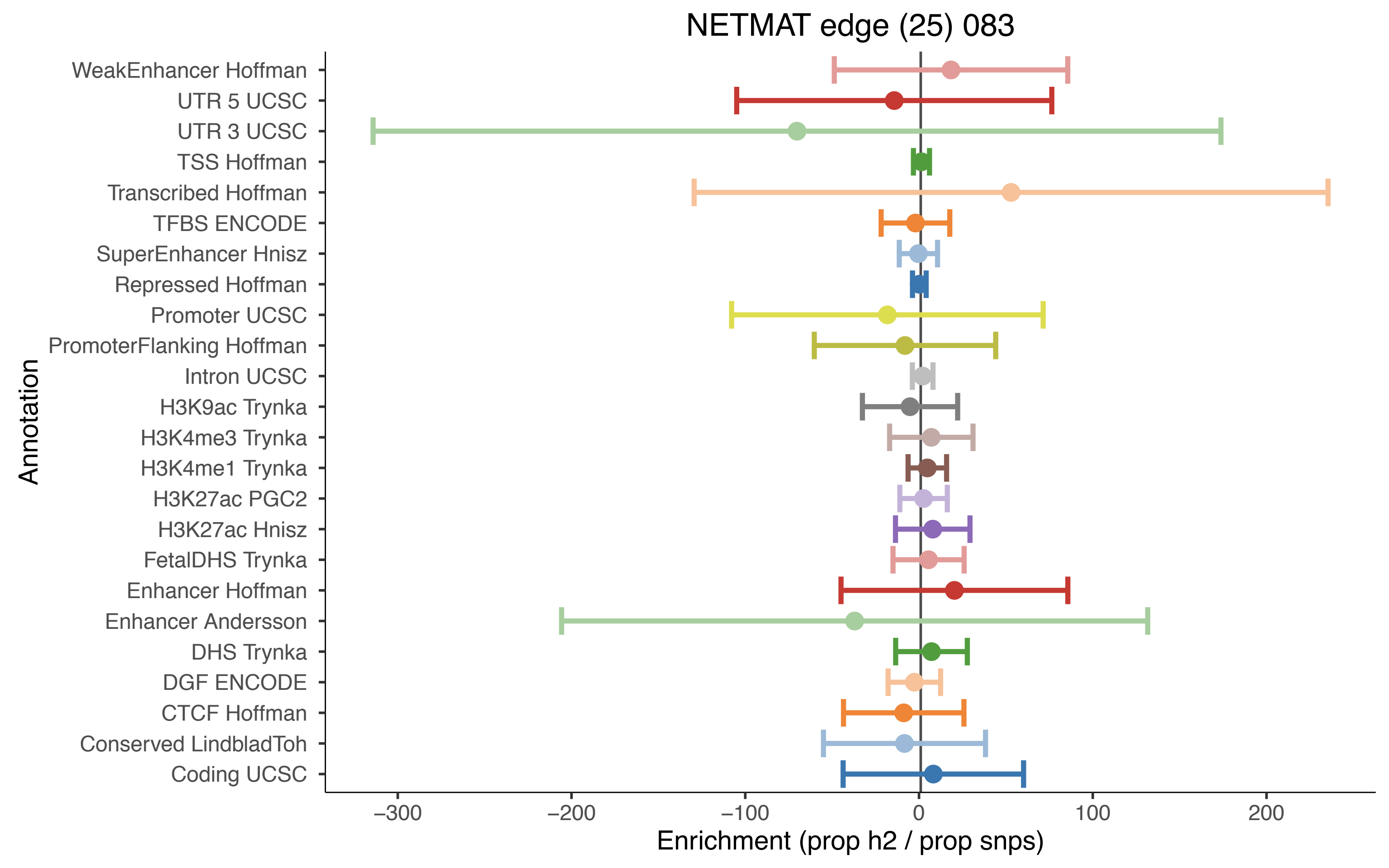
NETMAT edge (25) 083
WeakEnhancer Hoffman
UTR 5 UCSC
UTR 3 UCSC
TSS Hoffman
Transcribed Hoffman
TFBS ENCODE
SuperEnhancer Hnisz
Repressed Hoffman
Promoter UCSC
PromoterFlanking Hoffman
Intron UCSC
H3K9ac Trynka
H3K4me3 Trynka
H3K4me1 Trynka
H3K27ac PGC2
H3K27ac Hnisz
FetalDHS Trynka
Enhancer Hoffman
Enhancer Andersson
DHS Trynka
DGF ENCODE
CTCF Hoffman
Conserved LindbladToh
Coding UCSC
Annotation
−300
−200
−100
0
100
200
Enrichment (prop h2 / prop snps)

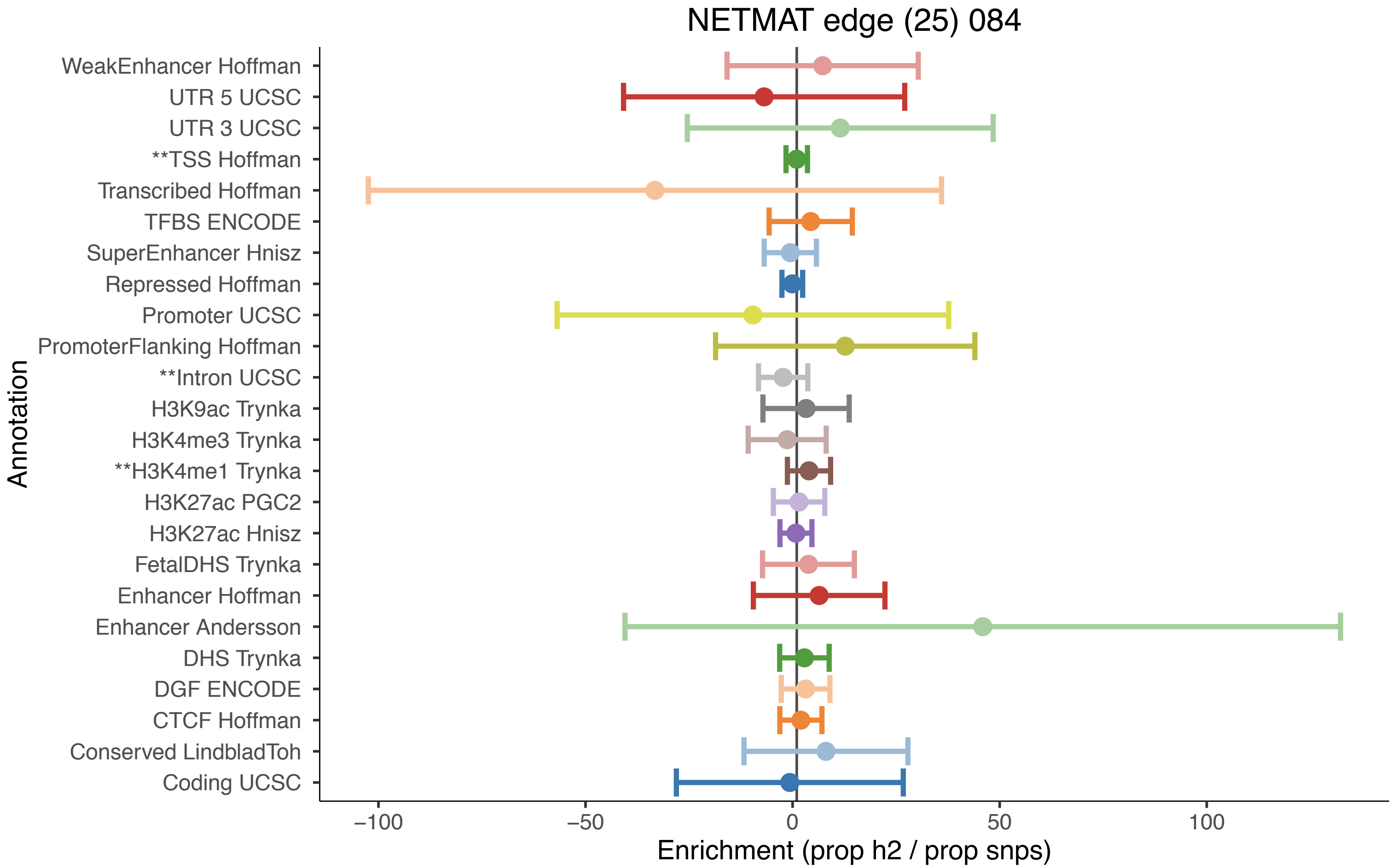

NETMAT edge (25) 084
Annotation
WeakEnhancer Hoffman
UTR 5 UCSC
UTR 3 UCSC
**TSS Hoffman
Transcribed Hoffman
TFBS ENCODE
SuperEnhancer Hnisz
Repressed Hoffman
Promoter UCSC
PromoterFlanking Hoffman
**Intron UCSC
H3K9ac Trynka
H3K4me3 Trynka
**H3K4me1 Trynka
H3K27ac PGC2
H3K27ac Hnisz
FetalDHS Trynka
Enhancer Hoffman
Enhancer Andersson
DHS Trynka
DGF ENCODE
CTCF Hoffman
Conserved LindbladToh
Coding UCSC
−100
−50
0
50
100
Enrichment (prop h2 / prop snps)

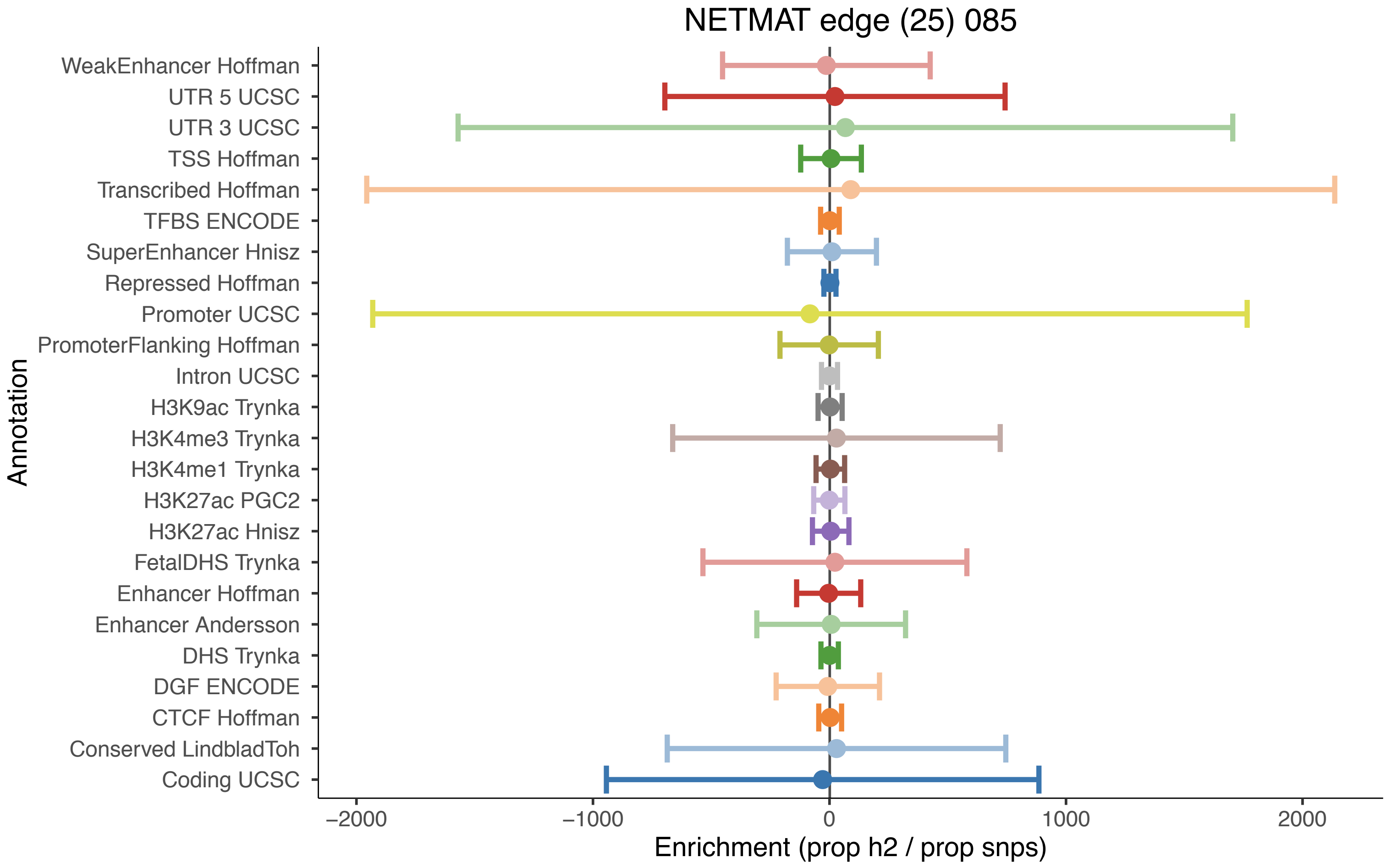

NETMAT edge (25) 085
WeakEnhancer Hoffman
UTR 5 UCSC
UTR 3 UCSC
TSS Hoffman
Transcribed Hoffman
TFBS ENCODE
SuperEnhancer Hnisz
Repressed Hoffman
Promoter UCSC
PromoterFlanking Hoffman
Intron UCSC
H3K9ac Trynka
H3K4me3 Trynka
H3K4me1 Trynka
H3K27ac PGC2
H3K27ac Hnisz
FetalDHS Trynka
Enhancer Hoffman
Enhancer Andersson
DHS Trynka
DGF ENCODE
CTCF Hoffman
Conserved LindbladToh
Coding UCSC
Annotation
−2000
−1000
0
1000
2000
Enrichment (prop h2 / prop snps)

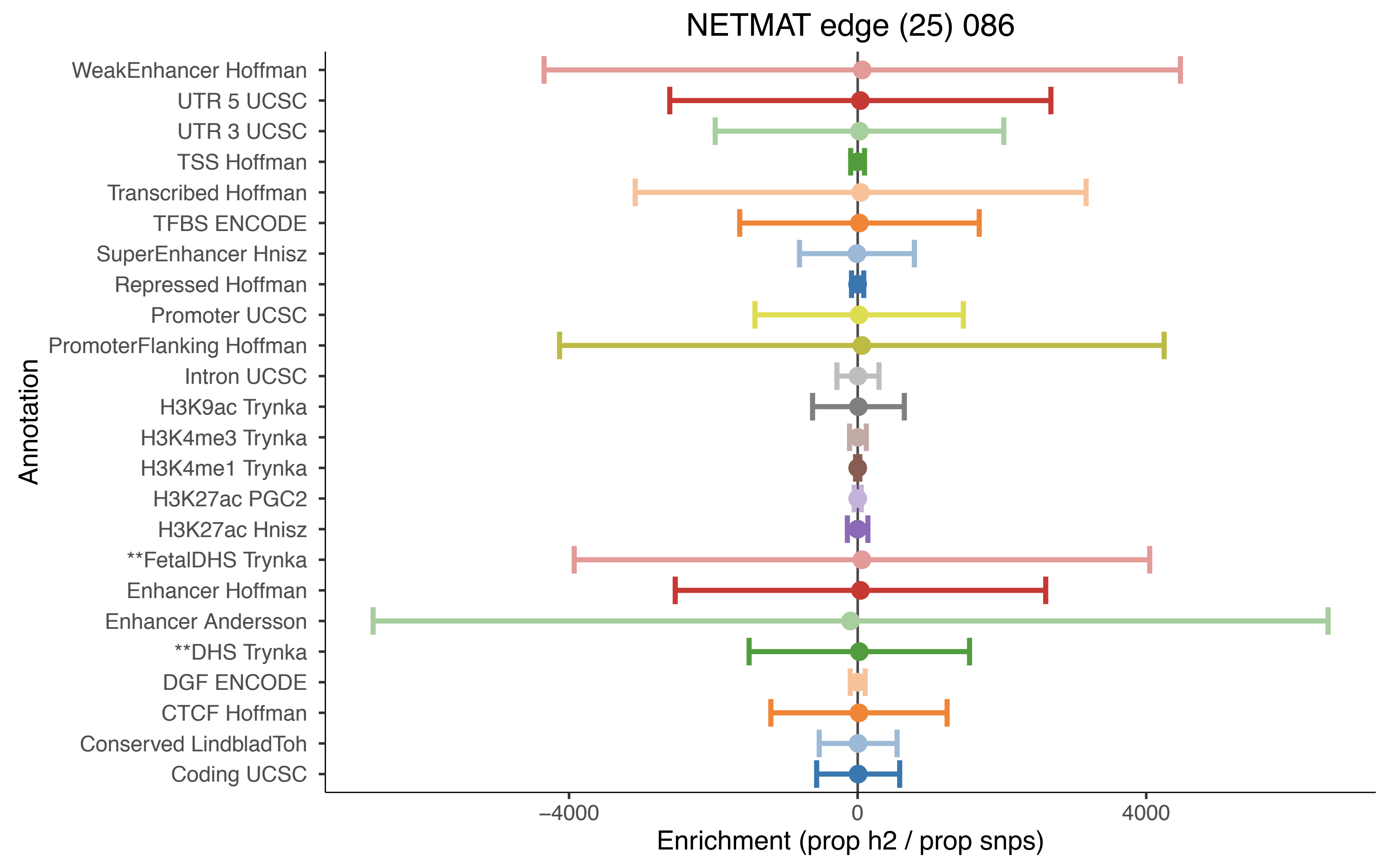

NETMAT edge (25) 086
WeakEnhancer Hoffman
UTR 5 UCSC
UTR 3 UCSC
TSS Hoffman
Transcribed Hoffman
TFBS ENCODE
SuperEnhancer Hnisz
Repressed Hoffman
Promoter UCSC
PromoterFlanking Hoffman
Intron UCSC
H3K9ac Trynka
H3K4me3 Trynka
H3K4me1 Trynka
H3K27ac PGC2
H3K27ac Hnisz
**FetalDHS Trynka
Enhancer Hoffman
Enhancer Andersson
**DHS Trynka
DGF ENCODE
CTCF Hoffman
Conserved LindbladToh
Coding UCSC
Annotation
−4000
0
4000
Enrichment (prop h2 / prop snps)

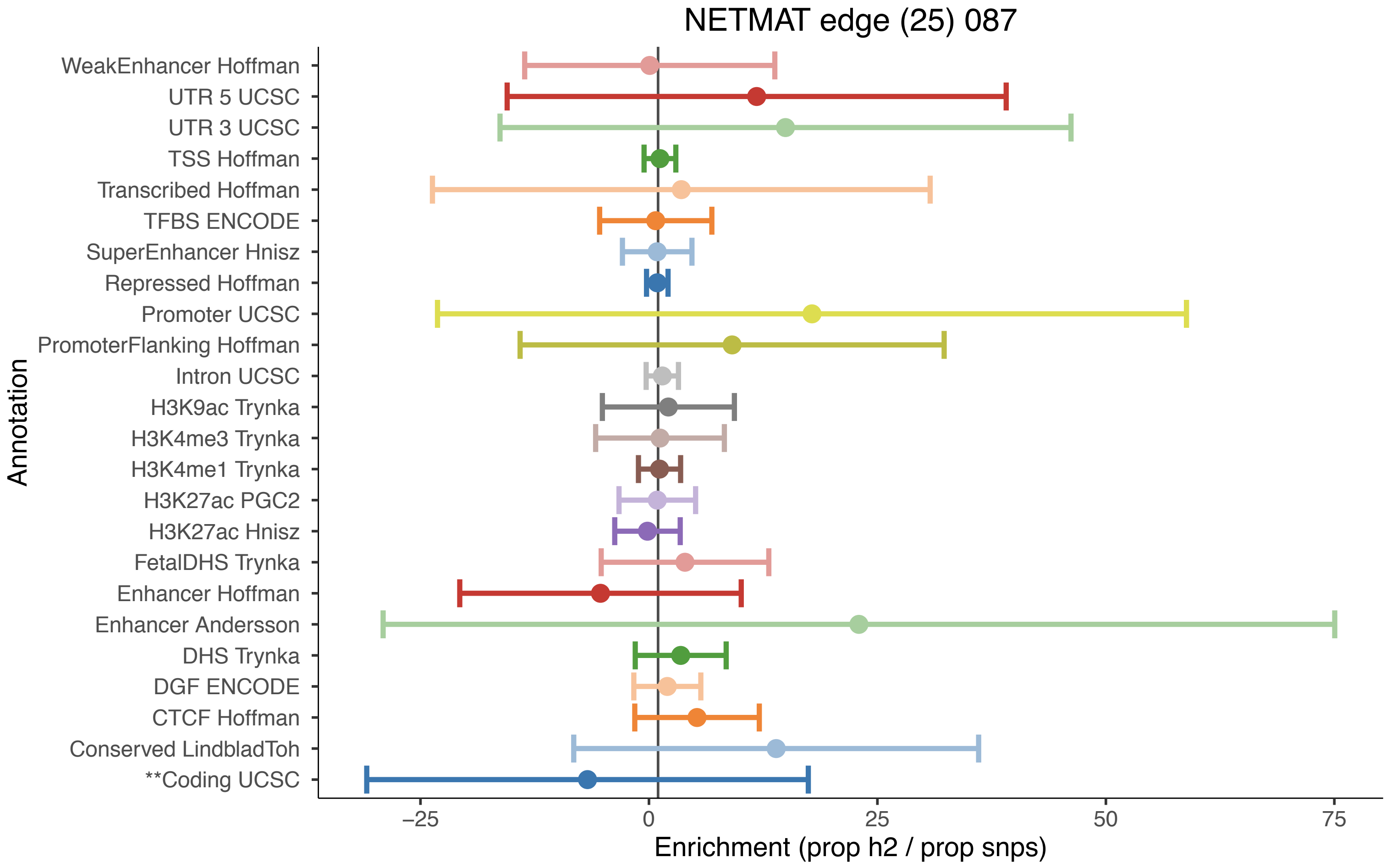
NETMAT edge (25) 087
WeakEnhancer Hoffman
UTR 5 UCSC
UTR 3 UCSC
TSS Hoffman
Transcribed Hoffman
TFBS ENCODE
SuperEnhancer Hnisz
Repressed Hoffman
Promoter UCSC
PromoterFlanking Hoffman
Intron UCSC
H3K9ac Trynka
H3K4me3 Trynka
H3K4me1 Trynka
H3K27ac PGC2
H3K27ac Hnisz
FetalDHS Trynka
Enhancer Hoffman
Enhancer Andersson
DHS Trynka
DGF ENCODE
CTCF Hoffman
Conserved LindbladToh
**Coding UCSC
Annotation
−25
0
25
50
75
Enrichment (prop h2 / prop snps)

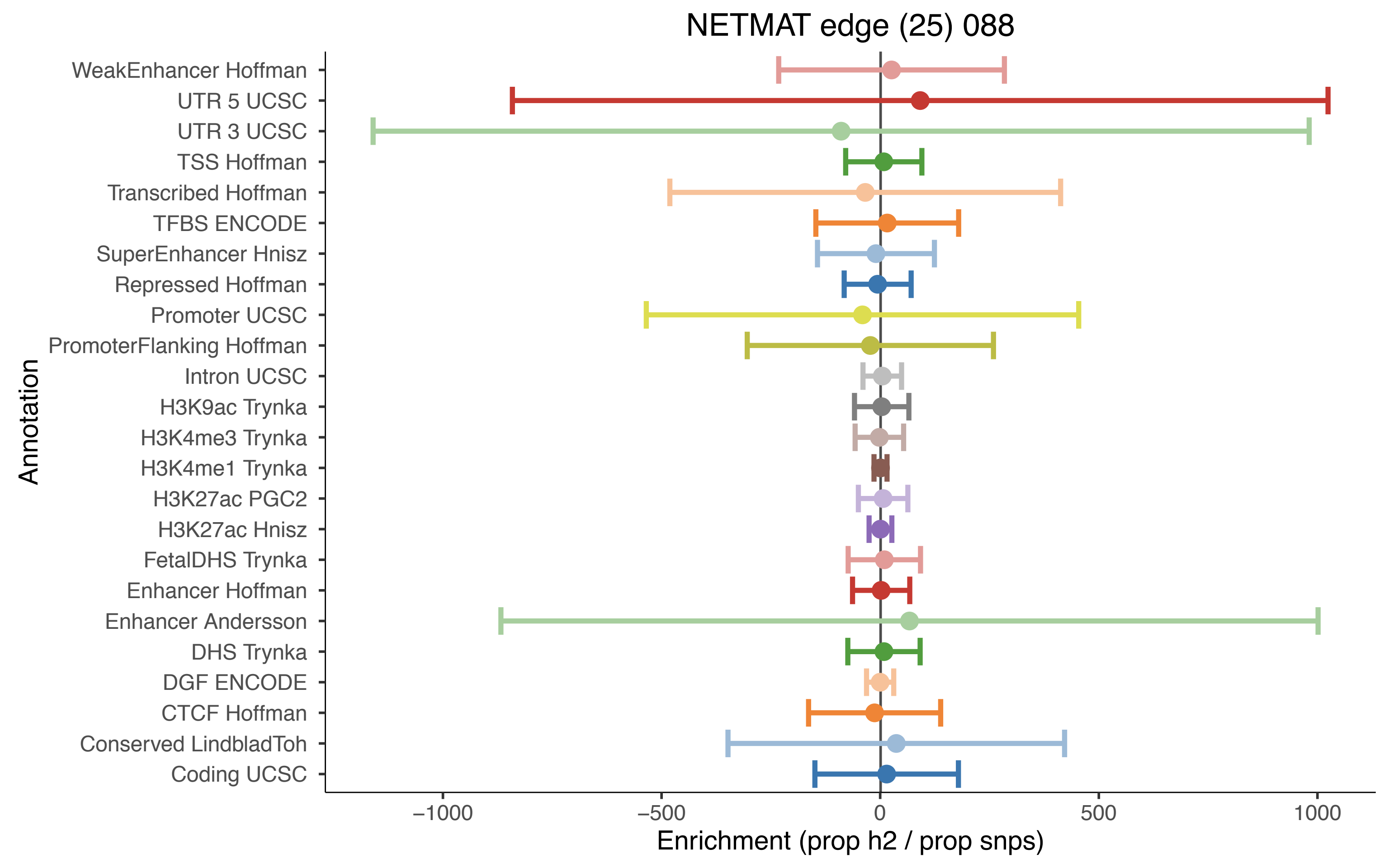

NETMAT edge (25) 088
Annotation
WeakEnhancer Hoffman
UTR 5 UCSC
UTR 3 UCSC
TSS Hoffman
Transcribed Hoffman
TFBS ENCODE
SuperEnhancer Hnisz
Repressed Hoffman
Promoter UCSC
PromoterFlanking Hoffman
Intron UCSC
H3K9ac Trynka
H3K4me3 Trynka
H3K4me1 Trynka
H3K27ac PGC2
H3K27ac Hnisz
FetalDHS Trynka
Enhancer Hoffman
Enhancer Andersson
DHS Trynka
DGF ENCODE
CTCF Hoffman
Conserved LindbladToh
Coding UCSC
−1000
−500
0
500
1000
Enrichment (prop h2 / prop snps)

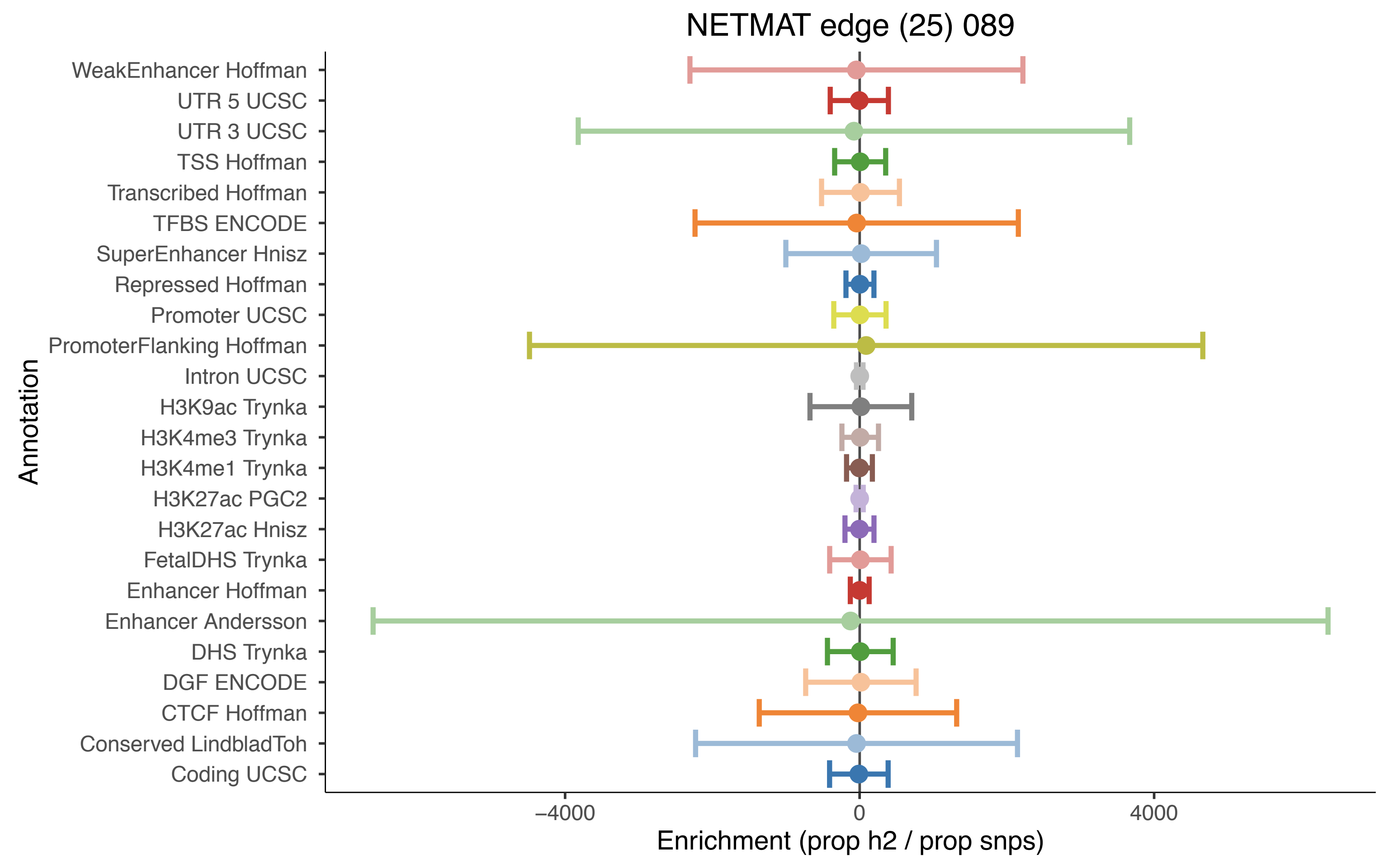

NETMAT edge (25) 089
Annotation
WeakEnhancer Hoffman
UTR 5 UCSC
UTR 3 UCSC
TSS Hoffman
Transcribed Hoffman
TFBS ENCODE
SuperEnhancer Hnisz
Repressed Hoffman
Promoter UCSC
PromoterFlanking Hoffman
Intron UCSC
H3K9ac Trynka
H3K4me3 Trynka
H3K4me1 Trynka
H3K27ac PGC2
H3K27ac Hnisz
FetalDHS Trynka
Enhancer Hoffman
Enhancer Andersson
DHS Trynka
DGF ENCODE
CTCF Hoffman
Conserved LindbladToh
Coding UCSC
−4000
0
4000
Enrichment (prop h2 / prop snps)

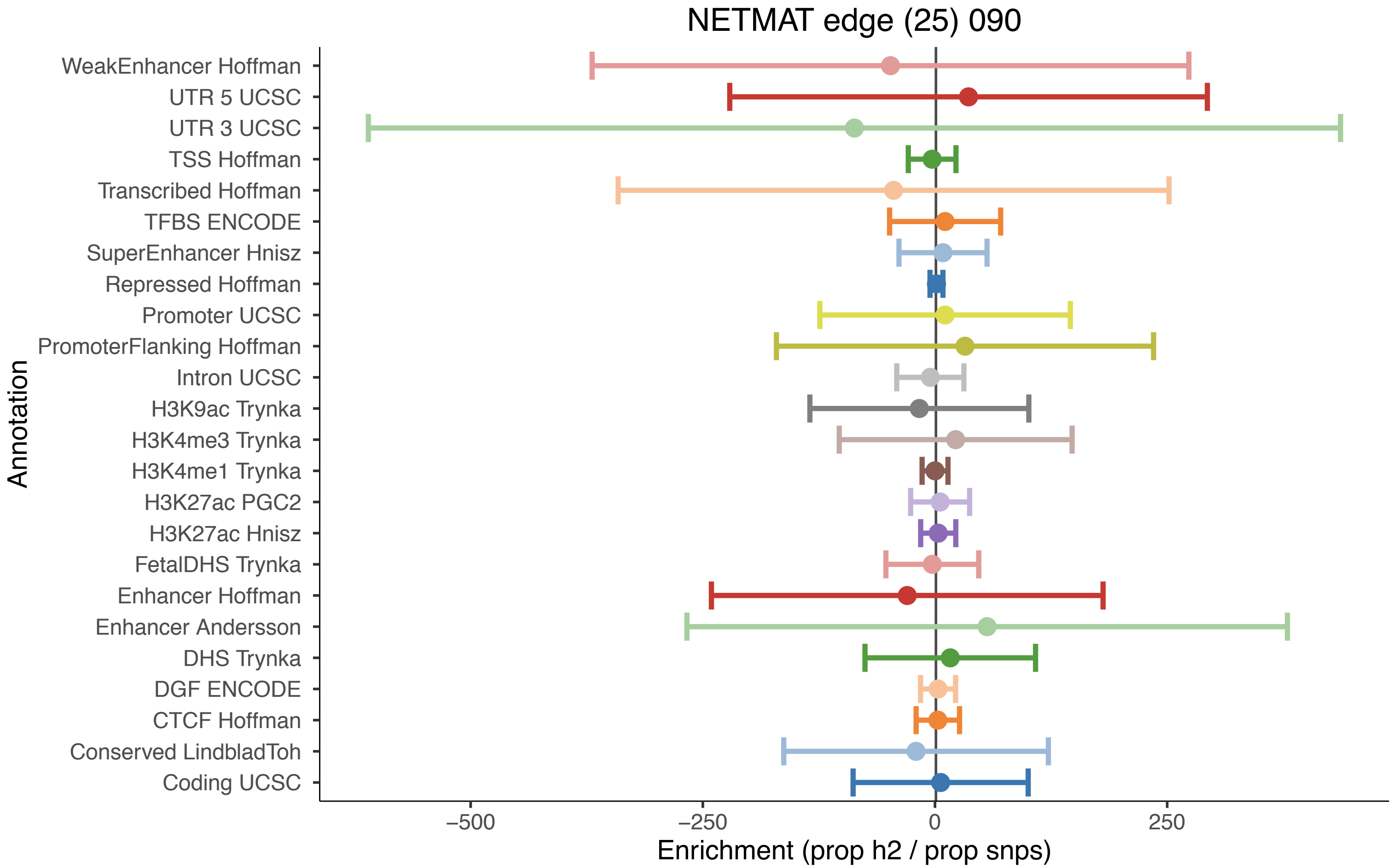
NETMAT edge (25) 090
Annotation
WeakEnhancer Hoffman
UTR 5 UCSC
UTR 3 UCSC
TSS Hoffman
Transcribed Hoffman
TFBS ENCODE
SuperEnhancer Hnisz
Repressed Hoffman
Promoter UCSC
PromoterFlanking Hoffman
Intron UCSC
H3K9ac Trynka
H3K4me3 Trynka
H3K4me1 Trynka
H3K27ac PGC2
H3K27ac Hnisz
FetalDHS Trynka
Enhancer Hoffman
Enhancer Andersson
DHS Trynka
DGF ENCODE
CTCF Hoffman
Conserved LindbladToh
Coding UCSC
−500
−250
0
250
Enrichment (prop h2 / prop snps)

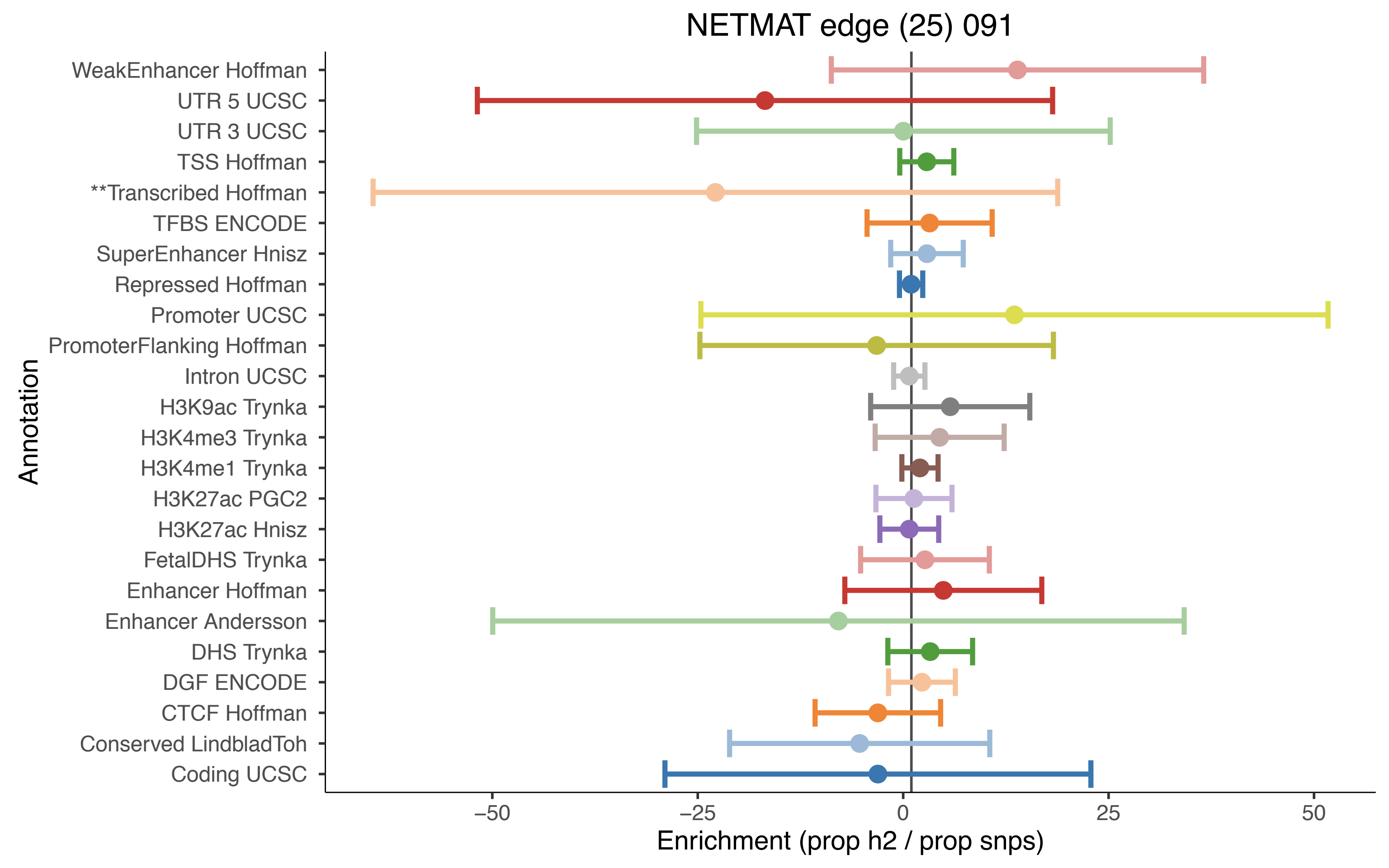
NETMAT edge (25) 091
WeakEnhancer Hoffman
UTR 5 UCSC
UTR 3 UCSC
TSS Hoffman
**Transcribed Hoffman
TFBS ENCODE
SuperEnhancer Hnisz
Repressed Hoffman
Promoter UCSC
PromoterFlanking Hoffman
Intron UCSC
H3K9ac Trynka
H3K4me3 Trynka
H3K4me1 Trynka
H3K27ac PGC2
H3K27ac Hnisz
FetalDHS Trynka
Enhancer Hoffman
Enhancer Andersson
DHS Trynka
DGF ENCODE
CTCF Hoffman
Conserved LindbladToh
Coding UCSC
Annotation
−50
−25
0
25
50
Enrichment (prop h2 / prop snps)

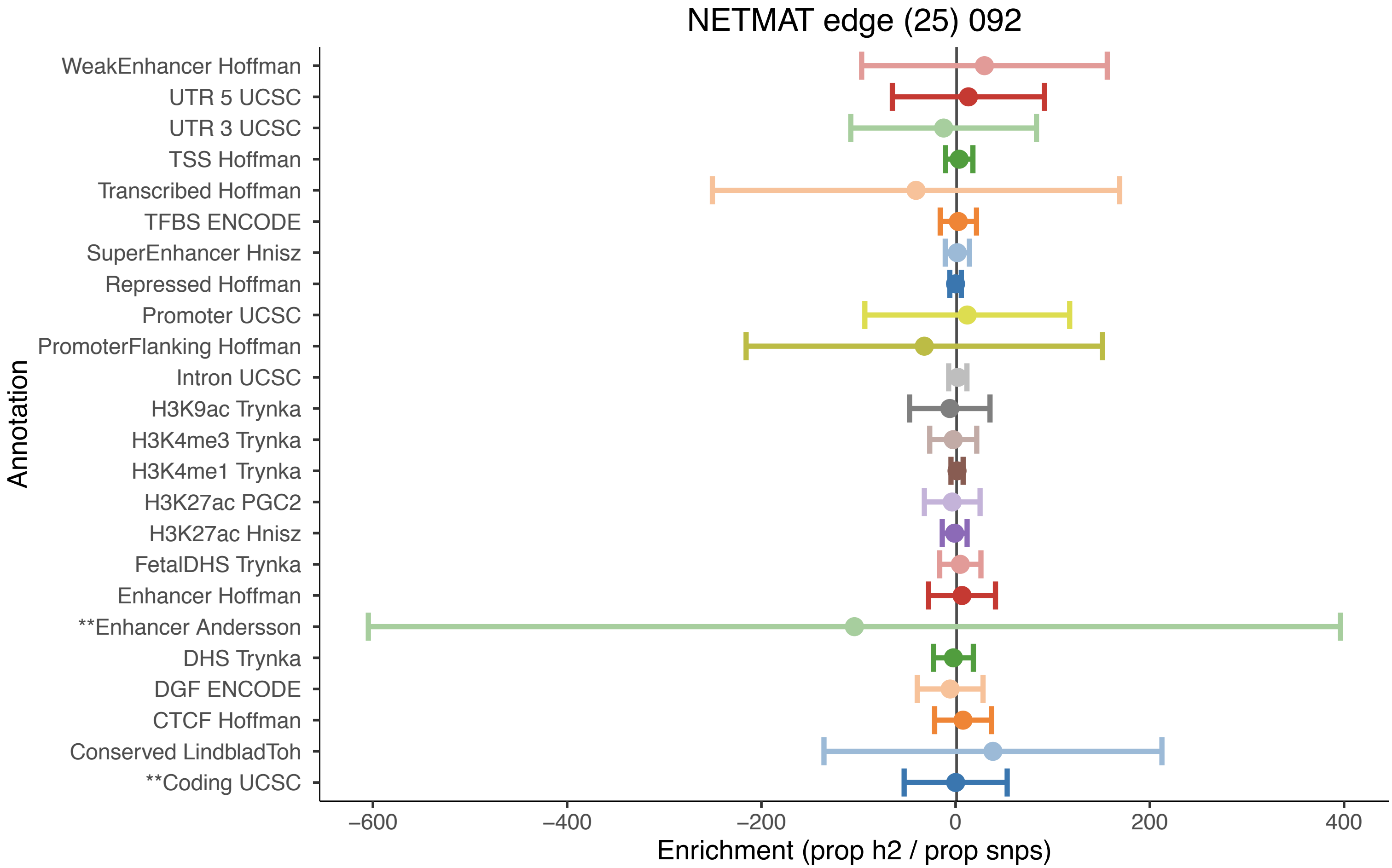
NETMAT edge (25) 092
WeakEnhancer Hoffman
UTR 5 UCSC
UTR 3 UCSC
TSS Hoffman
Transcribed Hoffman
TFBS ENCODE
SuperEnhancer Hnisz
Repressed Hoffman
Promoter UCSC
PromoterFlanking Hoffman
Intron UCSC
H3K9ac Trynka
H3K4me3 Trynka
H3K4me1 Trynka
H3K27ac PGC2
H3K27ac Hnisz
FetalDHS Trynka
Enhancer Hoffman
**Enhancer Andersson
DHS Trynka
DGF ENCODE
CTCF Hoffman
Conserved LindbladToh
**Coding UCSC
Annotation
−600
−400
−200
0
200
400
Enrichment (prop h2 / prop snps)

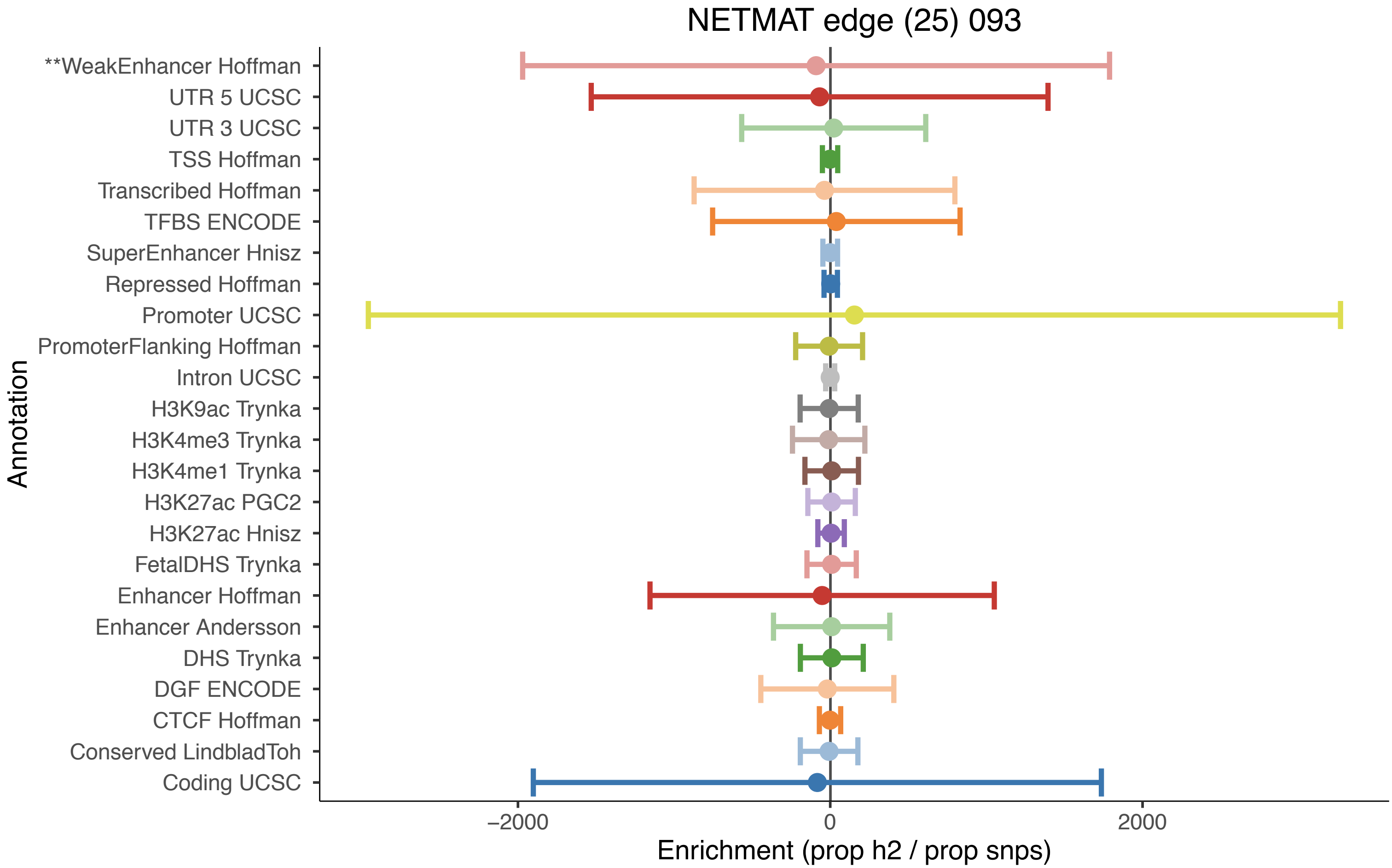
NETMAT edge (25) 093
**WeakEnhancer Hoffman
UTR 5 UCSC
UTR 3 UCSC
TSS Hoffman
Transcribed Hoffman
TFBS ENCODE
SuperEnhancer Hnisz
Repressed Hoffman
Promoter UCSC
PromoterFlanking Hoffman
Intron UCSC
H3K9ac Trynka
H3K4me3 Trynka
H3K4me1 Trynka
H3K27ac PGC2
H3K27ac Hnisz
FetalDHS Trynka
Enhancer Hoffman
Enhancer Andersson
DHS Trynka
DGF ENCODE
CTCF Hoffman
Conserved LindbladToh
Coding UCSC
Annotation
−2000
0
2000
Enrichment (prop h2 / prop snps)

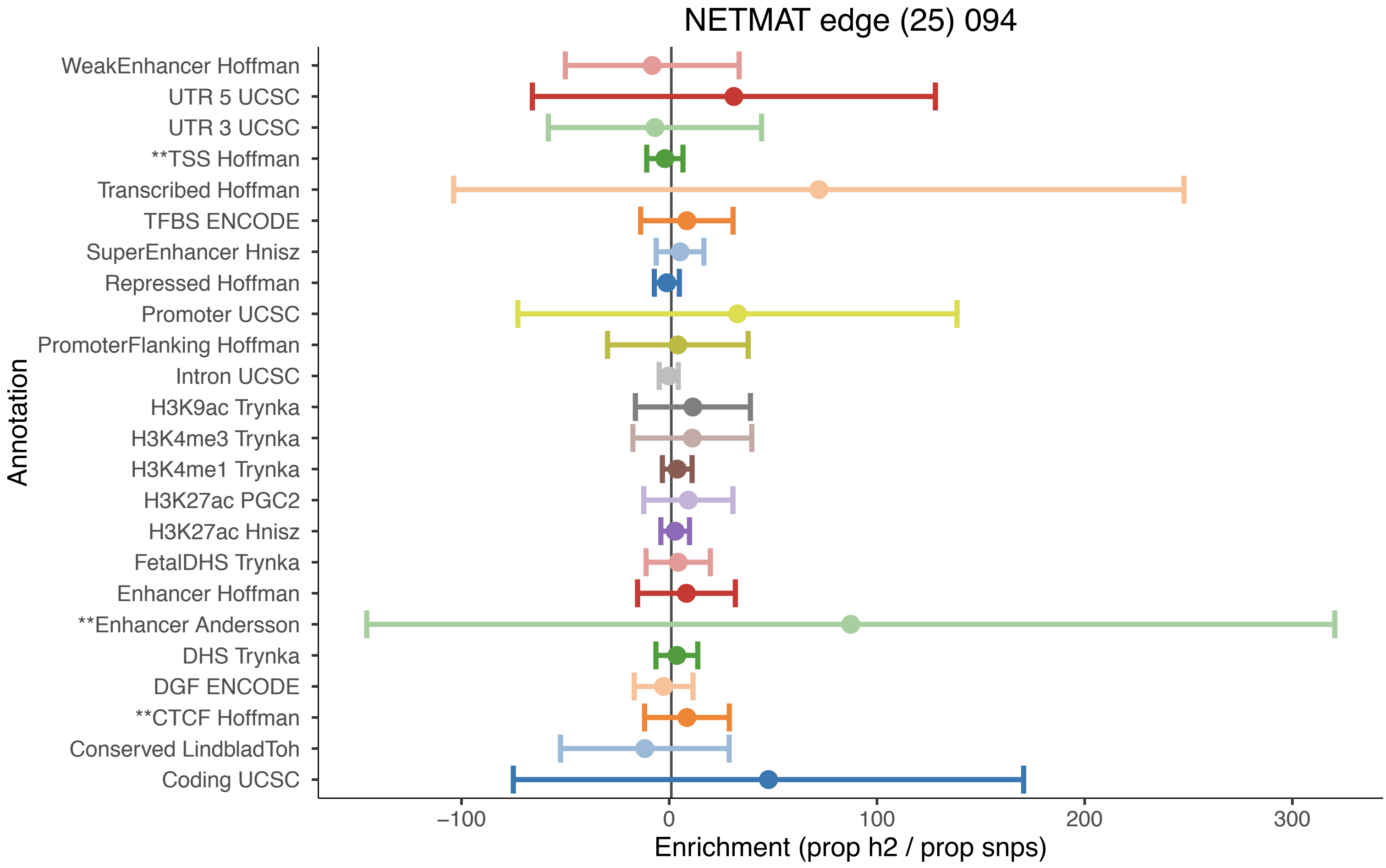

NETMAT edge (25) 094
Annotation
WeakEnhancer Hoffman
UTR 5 UCSC
UTR 3 UCSC
**TSS Hoffman
Transcribed Hoffman
TFBS ENCODE
SuperEnhancer Hnisz
Repressed Hoffman
Promoter UCSC
PromoterFlanking Hoffman
Intron UCSC
H3K9ac Trynka
H3K4me3 Trynka
H3K4me1 Trynka
H3K27ac PGC2
H3K27ac Hnisz
FetalDHS Trynka
Enhancer Hoffman
**Enhancer Andersson
DHS Trynka
DGF ENCODE
**CTCF Hoffman
Conserved LindbladToh
Coding UCSC
−100
0
100
200
300
Enrichment (prop h2 / prop snps)

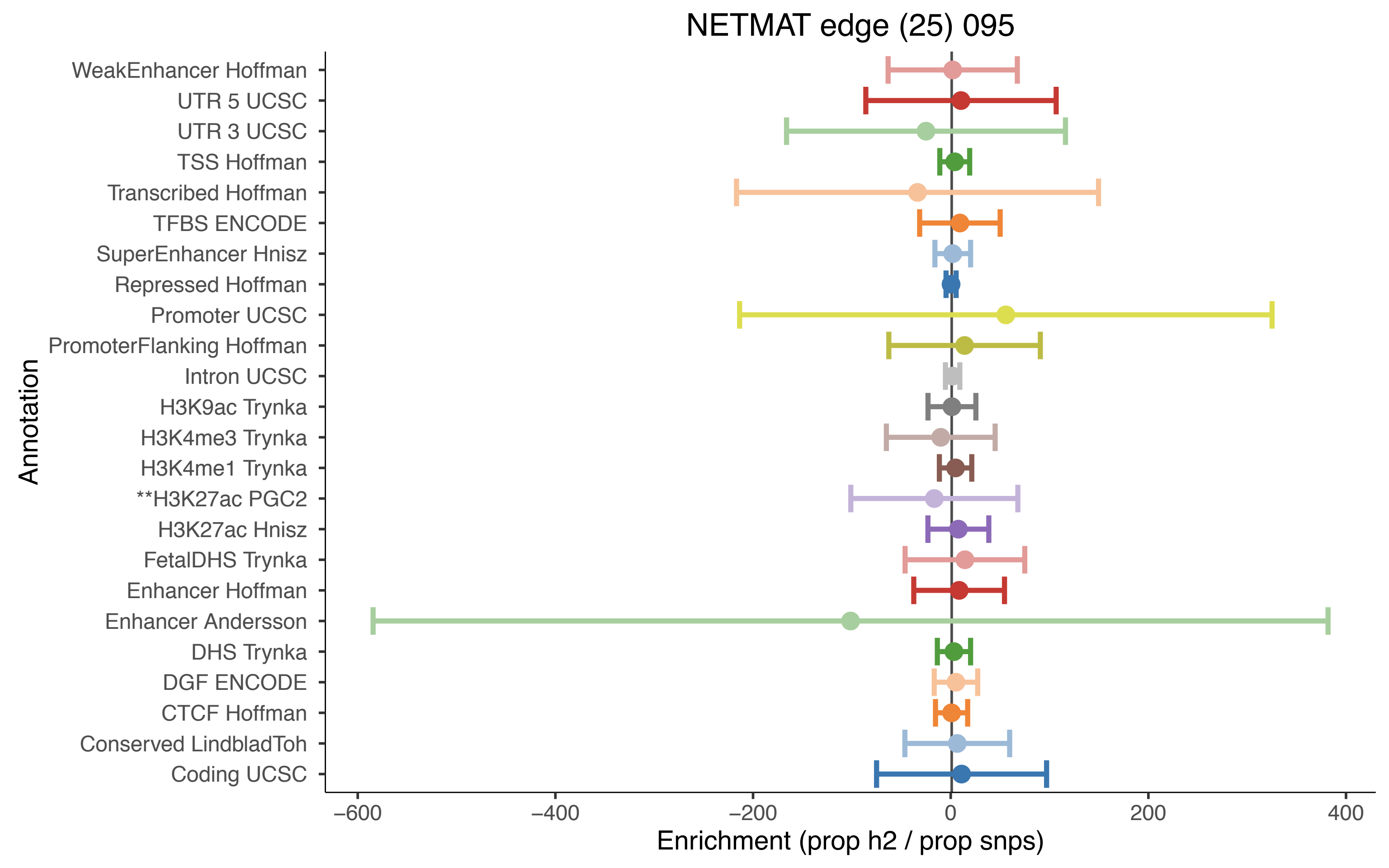

NETMAT edge (25) 095
Annotation
WeakEnhancer Hoffman
UTR 5 UCSC
UTR 3 UCSC
TSS Hoffman
Transcribed Hoffman
TFBS ENCODE
SuperEnhancer Hnisz
Repressed Hoffman
Promoter UCSC
PromoterFlanking Hoffman
Intron UCSC
H3K9ac Trynka
H3K4me3 Trynka
H3K4me1 Trynka
**H3K27ac PGC2
H3K27ac Hnisz
FetalDHS Trynka
Enhancer Hoffman
Enhancer Andersson
DHS Trynka
DGF ENCODE
CTCF Hoffman
Conserved LindbladToh
Coding UCSC
−600
−400
−200
0
200
400
Enrichment (prop h2 / prop snps)

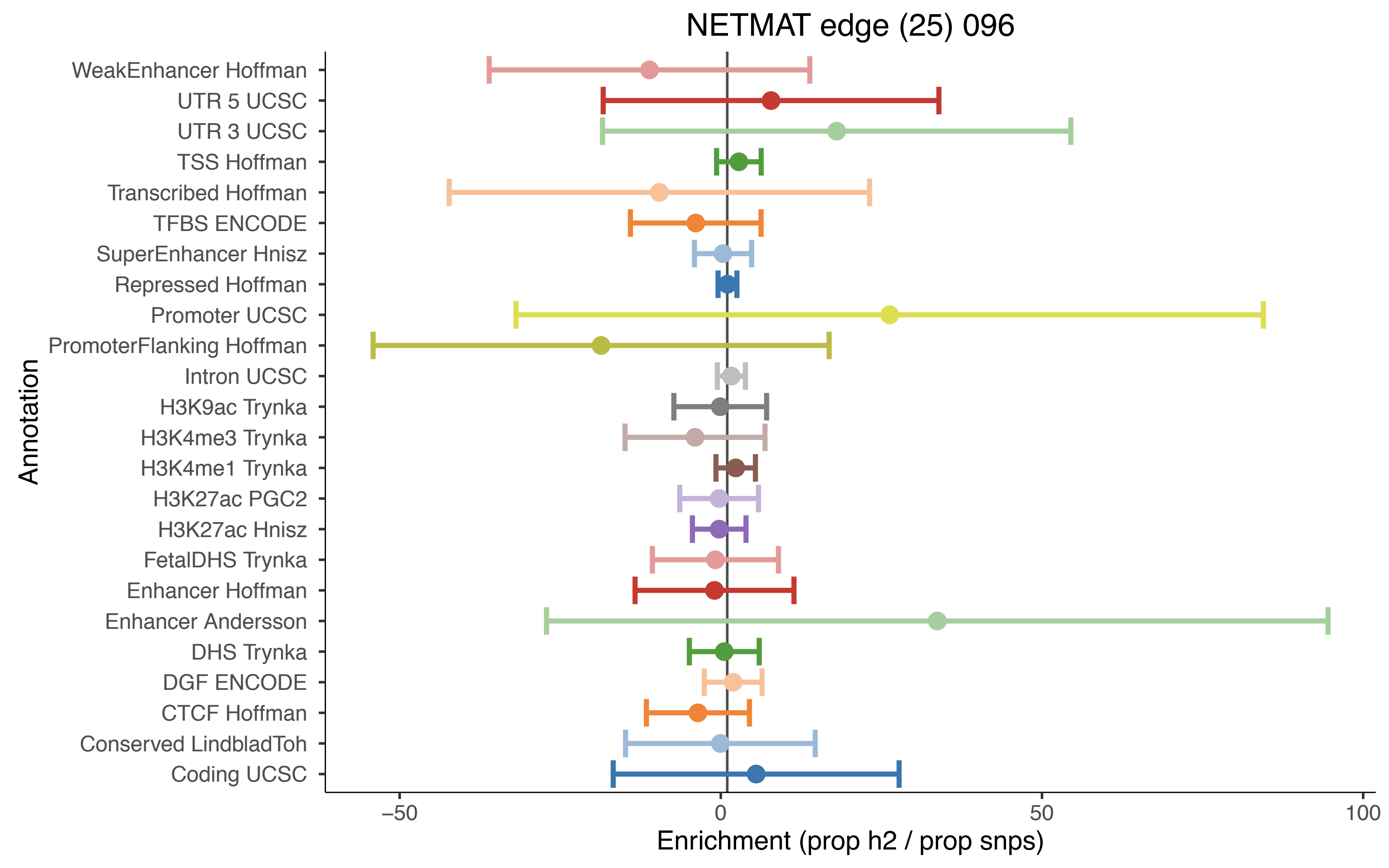

NETMAT edge (25) 096
Annotation
WeakEnhancer Hoffman
UTR 5 UCSC
UTR 3 UCSC
TSS Hoffman
Transcribed Hoffman
TFBS ENCODE
SuperEnhancer Hnisz
Repressed Hoffman
Promoter UCSC
PromoterFlanking Hoffman
Intron UCSC
H3K9ac Trynka
H3K4me3 Trynka
H3K4me1 Trynka
H3K27ac PGC2
H3K27ac Hnisz
FetalDHS Trynka
Enhancer Hoffman
Enhancer Andersson
DHS Trynka
DGF ENCODE
CTCF Hoffman
Conserved LindbladToh
Coding UCSC
−50
0
50
100
Enrichment (prop h2 / prop snps)

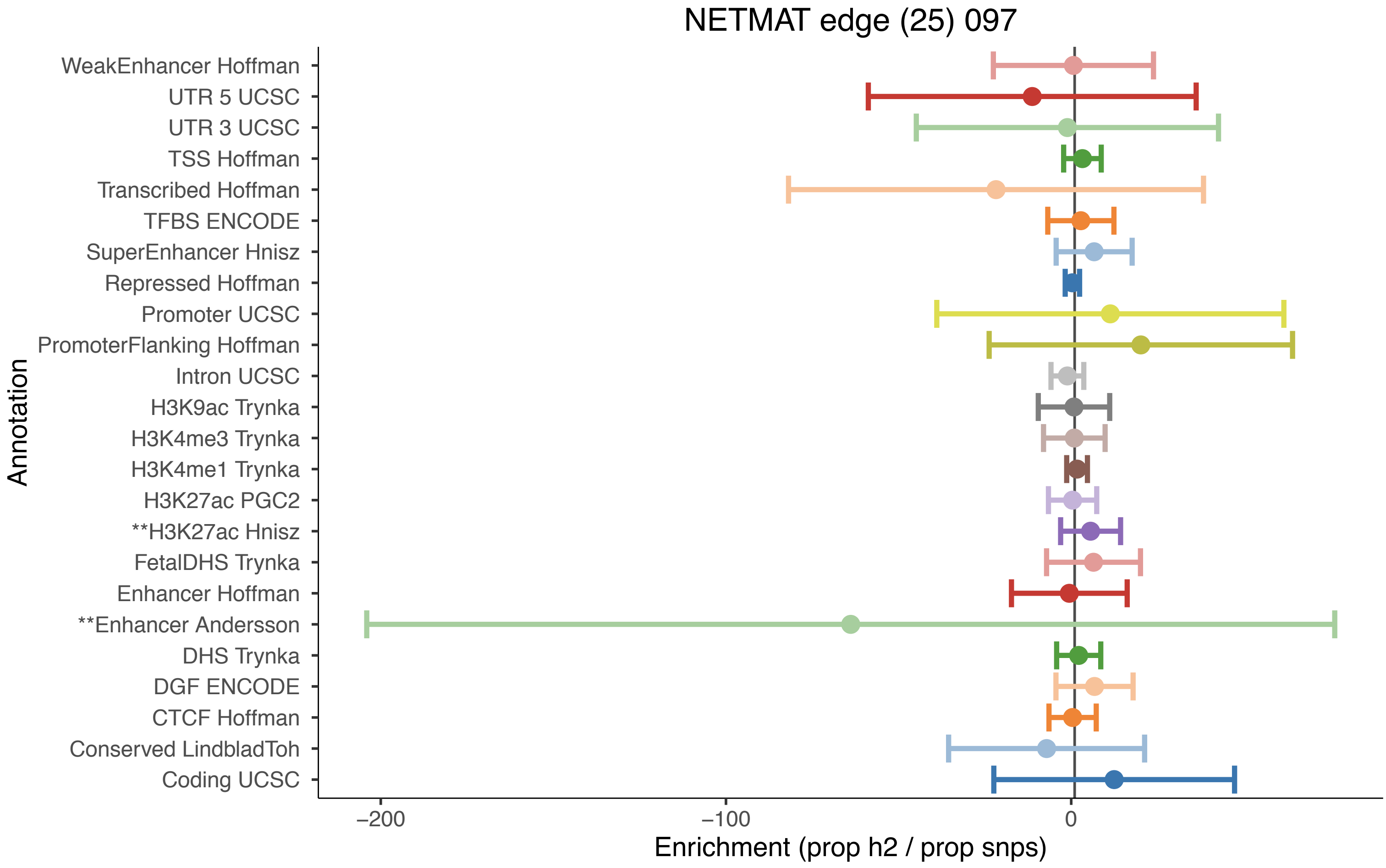

NETMAT edge (25) 097
Annotation
WeakEnhancer Hoffman
UTR 5 UCSC
UTR 3 UCSC
TSS Hoffman
Transcribed Hoffman
TFBS ENCODE
SuperEnhancer Hnisz
Repressed Hoffman
Promoter UCSC
PromoterFlanking Hoffman
Intron UCSC
H3K9ac Trynka
H3K4me3 Trynka
H3K4me1 Trynka
H3K27ac PGC2
**H3K27ac Hnisz
FetalDHS Trynka
Enhancer Hoffman
**Enhancer Andersson
DHS Trynka
DGF ENCODE
CTCF Hoffman
Conserved LindbladToh
Coding UCSC
−200
−100
0
Enrichment (prop h2 / prop snps)

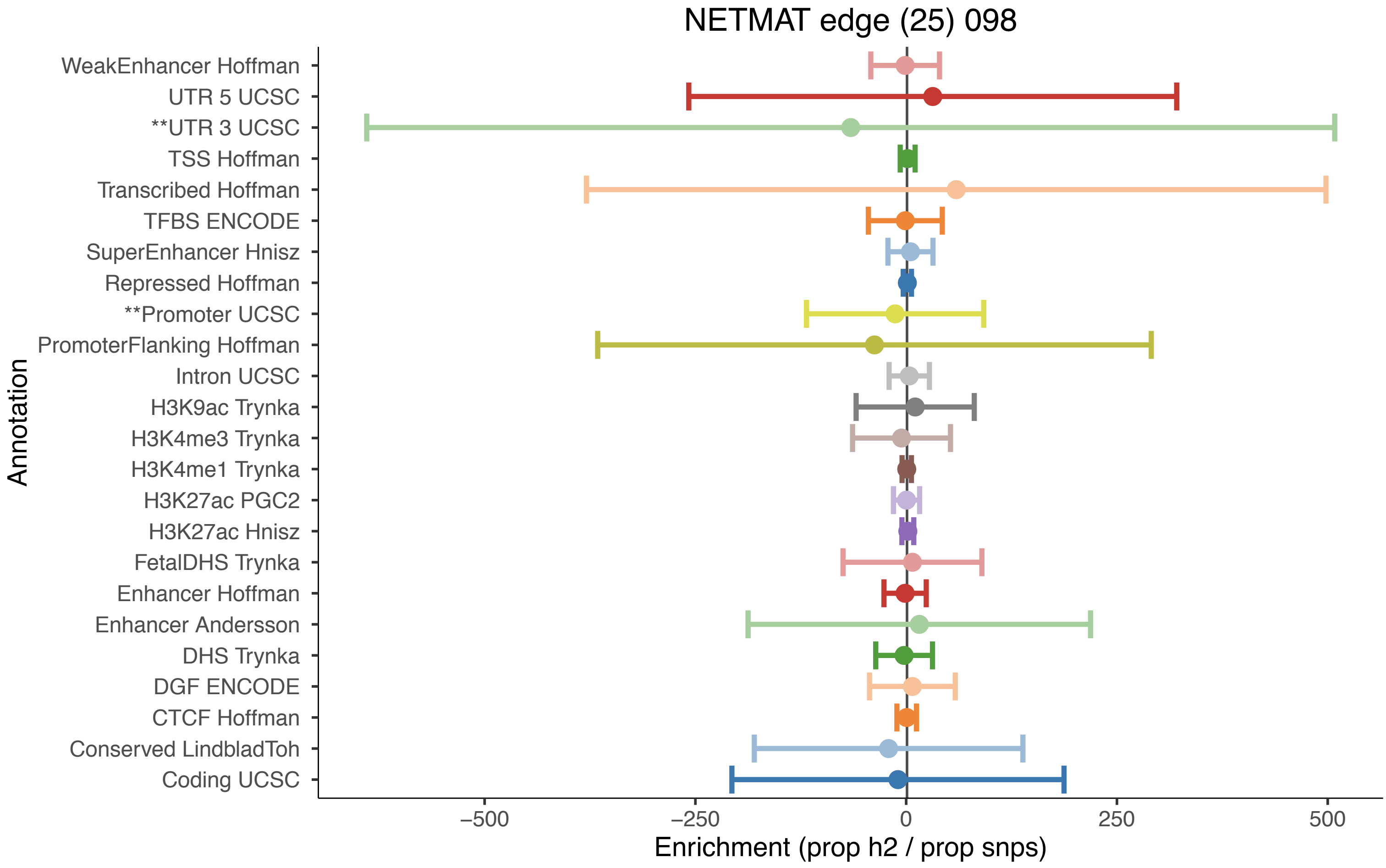

NETMAT edge (25) 098
Annotation
WeakEnhancer Hoffman
UTR 5 UCSC
**UTR 3 UCSC
TSS Hoffman
Transcribed Hoffman
TFBS ENCODE
SuperEnhancer Hnisz
Repressed Hoffman
**Promoter UCSC
PromoterFlanking Hoffman
Intron UCSC
H3K9ac Trynka
H3K4me3 Trynka
H3K4me1 Trynka
H3K27ac PGC2
H3K27ac Hnisz
FetalDHS Trynka
Enhancer Hoffman
Enhancer Andersson
DHS Trynka
DGF ENCODE
CTCF Hoffman
Conserved LindbladToh
Coding UCSC
−500
−250
0
250
500
Enrichment (prop h2 / prop snps)

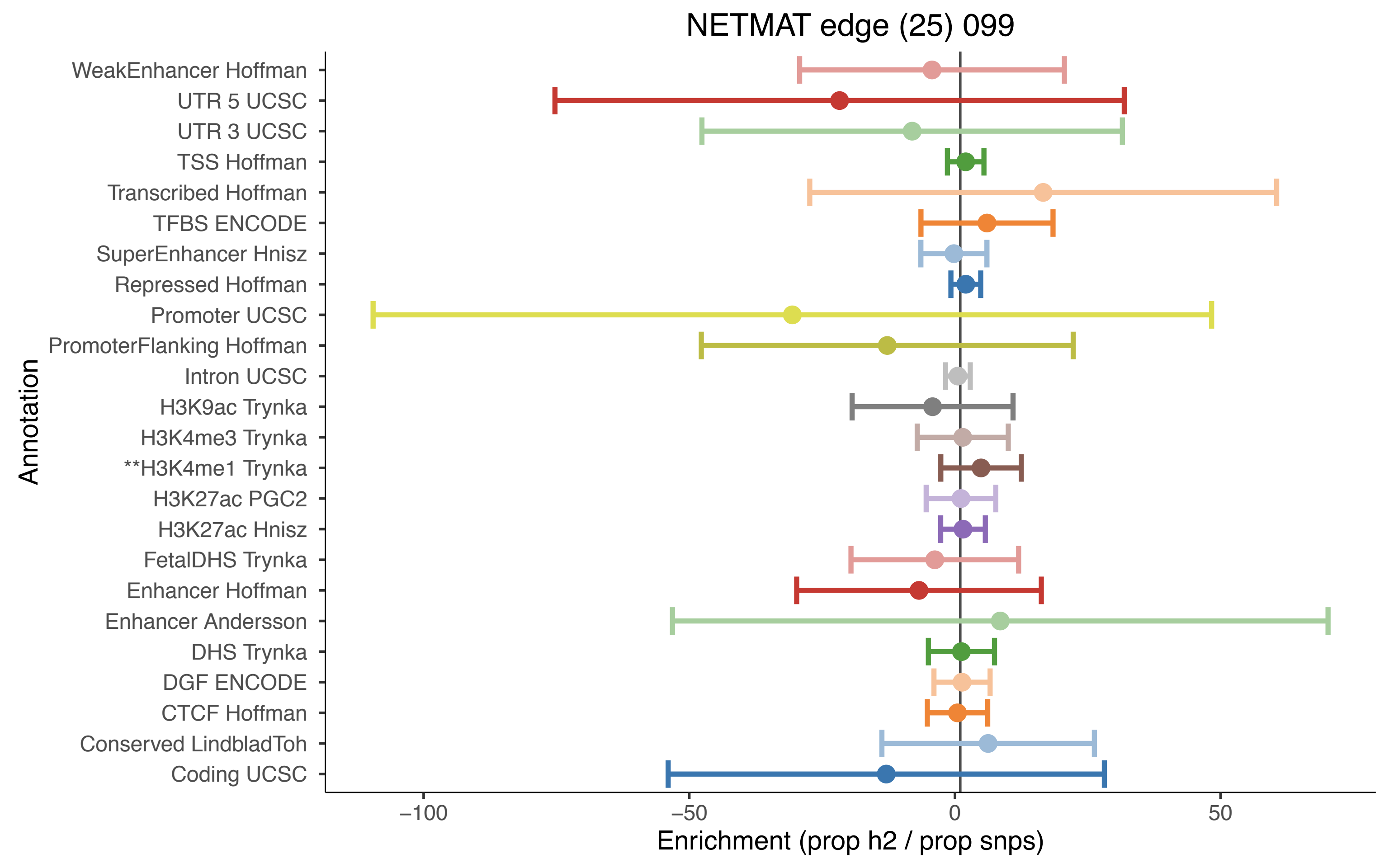

NETMAT edge (25) 099
Annotation
WeakEnhancer Hoffman
UTR 5 UCSC
UTR 3 UCSC
TSS Hoffman
Transcribed Hoffman
TFBS ENCODE
SuperEnhancer Hnisz
Repressed Hoffman
Promoter UCSC
PromoterFlanking Hoffman
Intron UCSC
H3K9ac Trynka
H3K4me3 Trynka
**H3K4me1 Trynka
H3K27ac PGC2
H3K27ac Hnisz
FetalDHS Trynka
Enhancer Hoffman
Enhancer Andersson
DHS Trynka
DGF ENCODE
CTCF Hoffman
Conserved LindbladToh
Coding UCSC
−100
−50
0
50
Enrichment (prop h2 / prop snps)

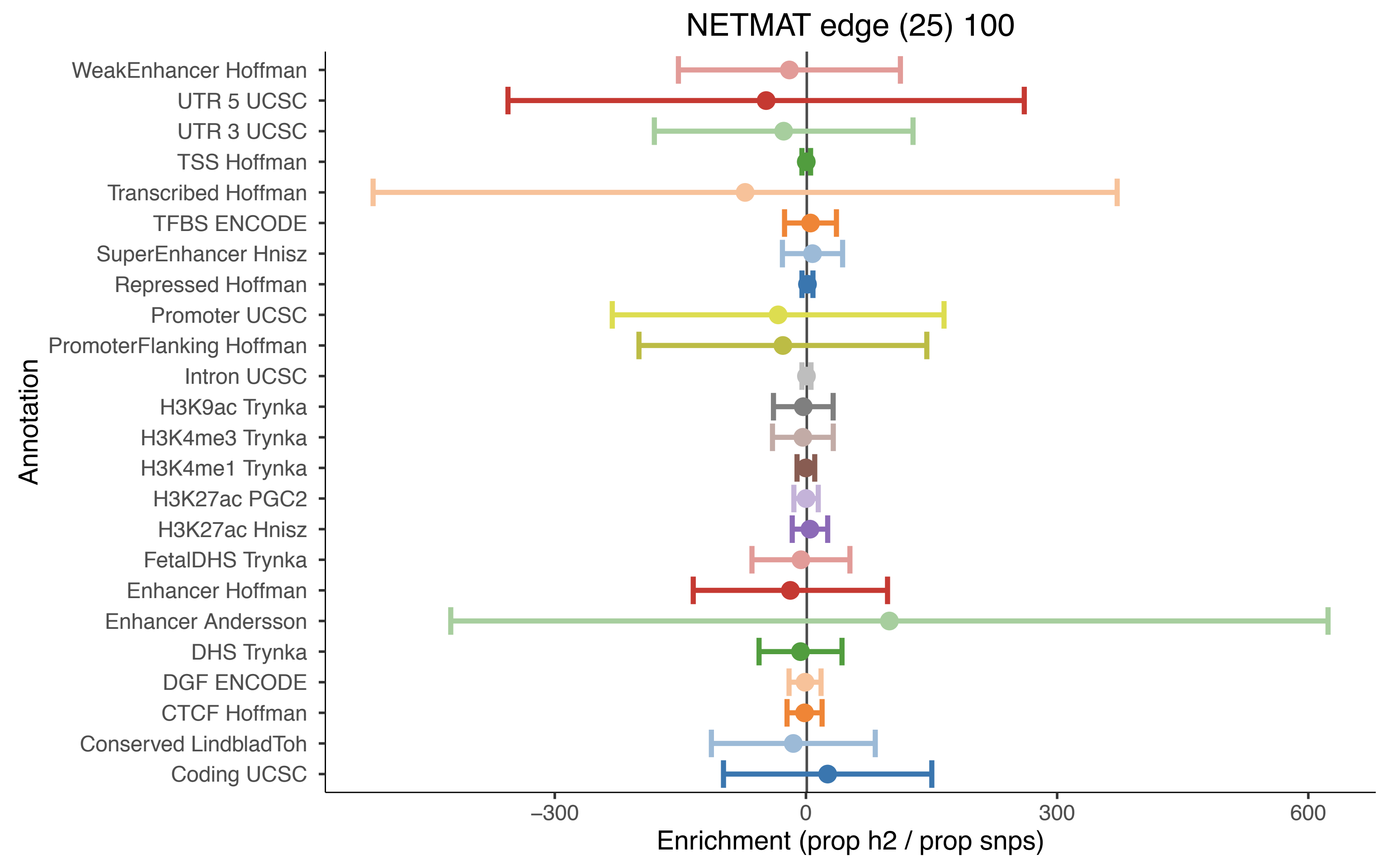
NETMAT edge (25) 100
WeakEnhancer Hoffman
UTR 5 UCSC
UTR 3 UCSC
TSS Hoffman
Transcribed Hoffman
TFBS ENCODE
SuperEnhancer Hnisz
Repressed Hoffman
Promoter UCSC
PromoterFlanking Hoffman
Intron UCSC
H3K9ac Trynka
H3K4me3 Trynka
H3K4me1 Trynka
H3K27ac PGC2
H3K27ac Hnisz
FetalDHS Trynka
Enhancer Hoffman
Enhancer Andersson
DHS Trynka
DGF ENCODE
CTCF Hoffman
Conserved LindbladToh
Coding UCSC
Annotation
−300
0
300
600
Enrichment (prop h2 / prop snps)

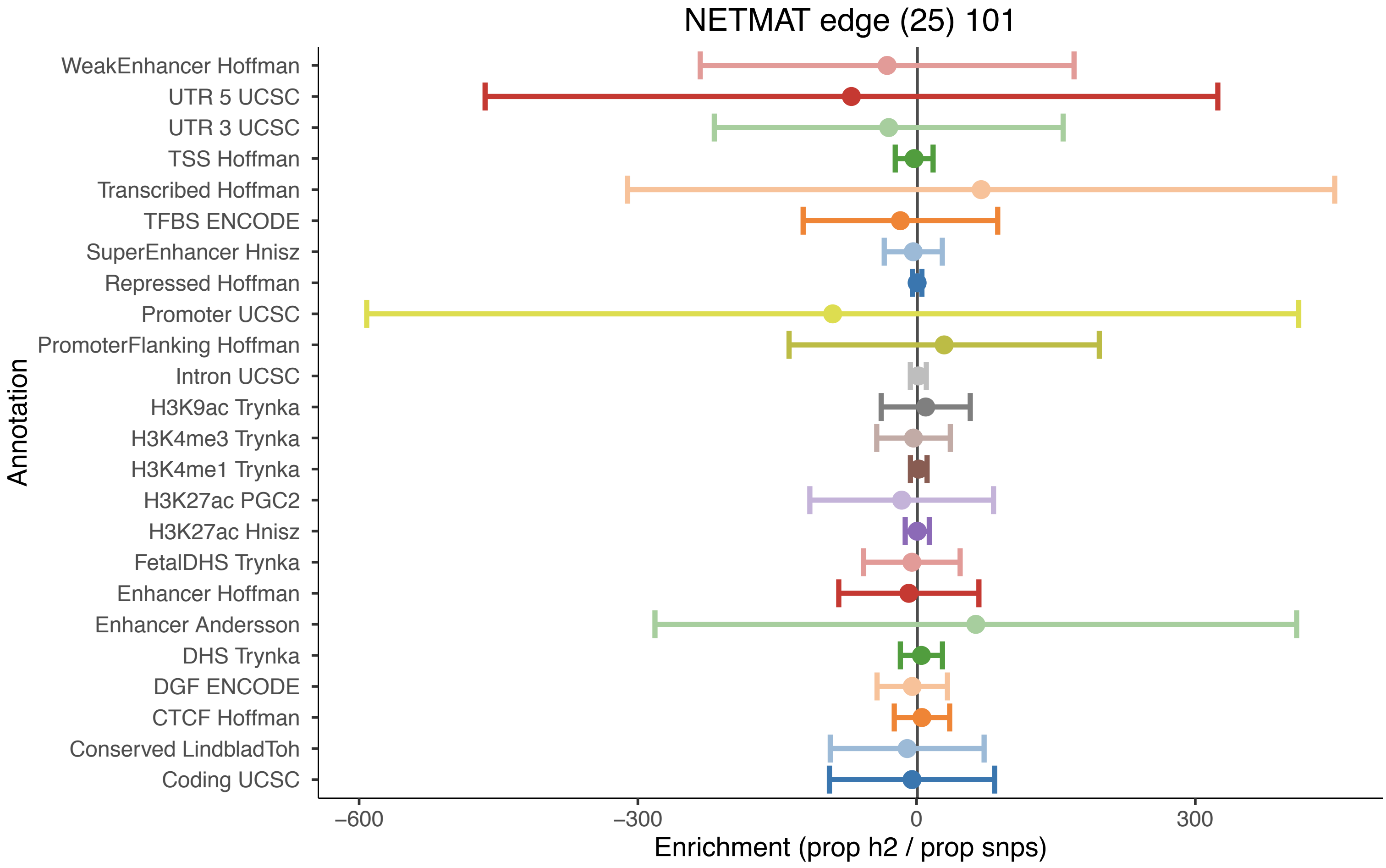
NETMAT edge (25) 101
WeakEnhancer Hoffman
UTR 5 UCSC
UTR 3 UCSC
TSS Hoffman
Transcribed Hoffman
TFBS ENCODE
SuperEnhancer Hnisz
Repressed Hoffman
Promoter UCSC
PromoterFlanking Hoffman
Intron UCSC
H3K9ac Trynka
H3K4me3 Trynka
H3K4me1 Trynka
H3K27ac PGC2
H3K27ac Hnisz
FetalDHS Trynka
Enhancer Hoffman
Enhancer Andersson
DHS Trynka
DGF ENCODE
CTCF Hoffman
Conserved LindbladToh
Coding UCSC
Annotation
−600
−300
0
300
Enrichment (prop h2 / prop snps)

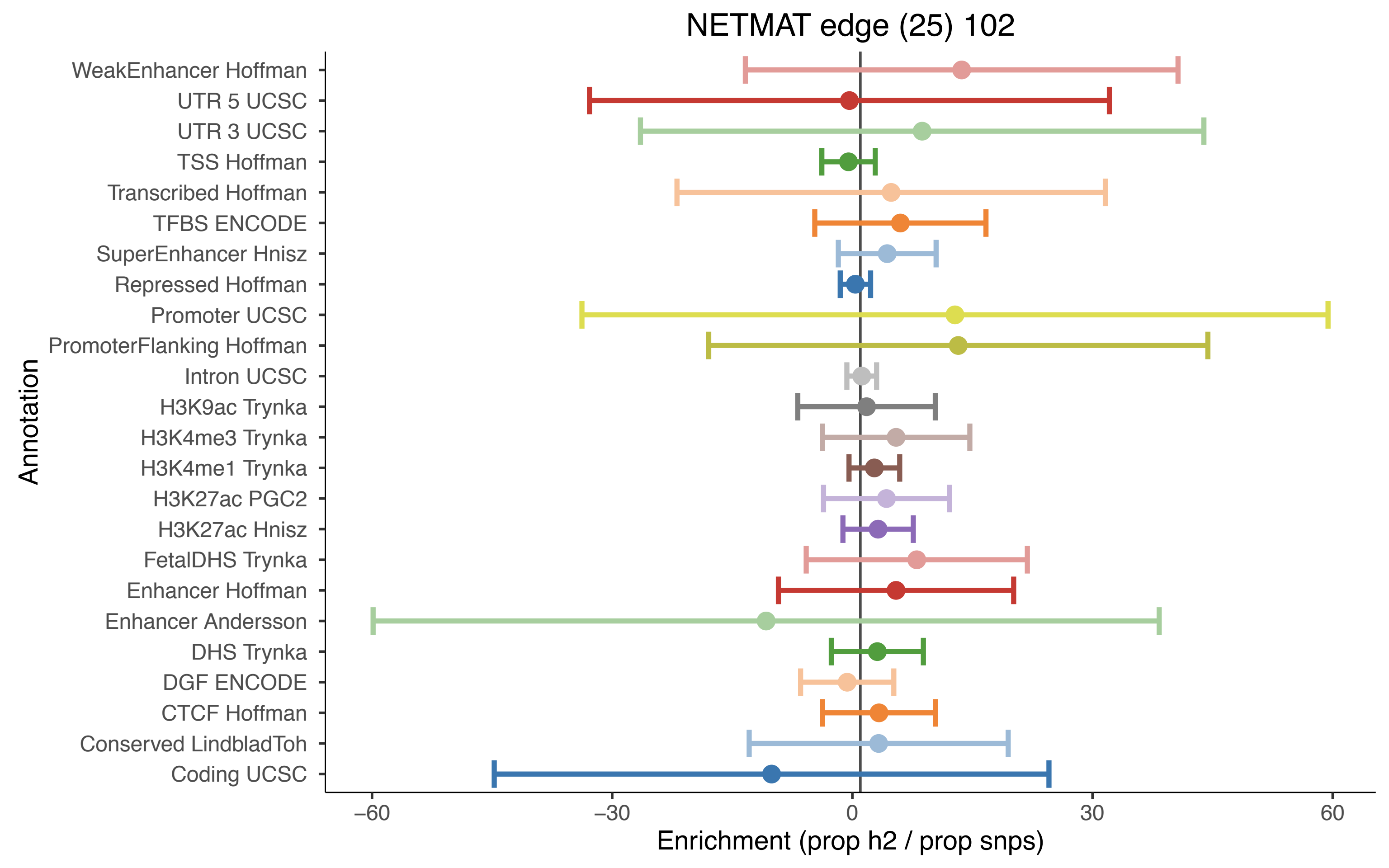

NETMAT edge (25) 102
WeakEnhancer Hoffman
UTR 5 UCSC
UTR 3 UCSC
TSS Hoffman
Transcribed Hoffman
TFBS ENCODE
SuperEnhancer Hnisz
Repressed Hoffman
Promoter UCSC
PromoterFlanking Hoffman
Intron UCSC
H3K9ac Trynka
H3K4me3 Trynka
H3K4me1 Trynka
H3K27ac PGC2
H3K27ac Hnisz
FetalDHS Trynka
Enhancer Hoffman
Enhancer Andersson
DHS Trynka
DGF ENCODE
CTCF Hoffman
Conserved LindbladToh
Coding UCSC
Annotation
−60
−30
0
30
60
Enrichment (prop h2 / prop snps)

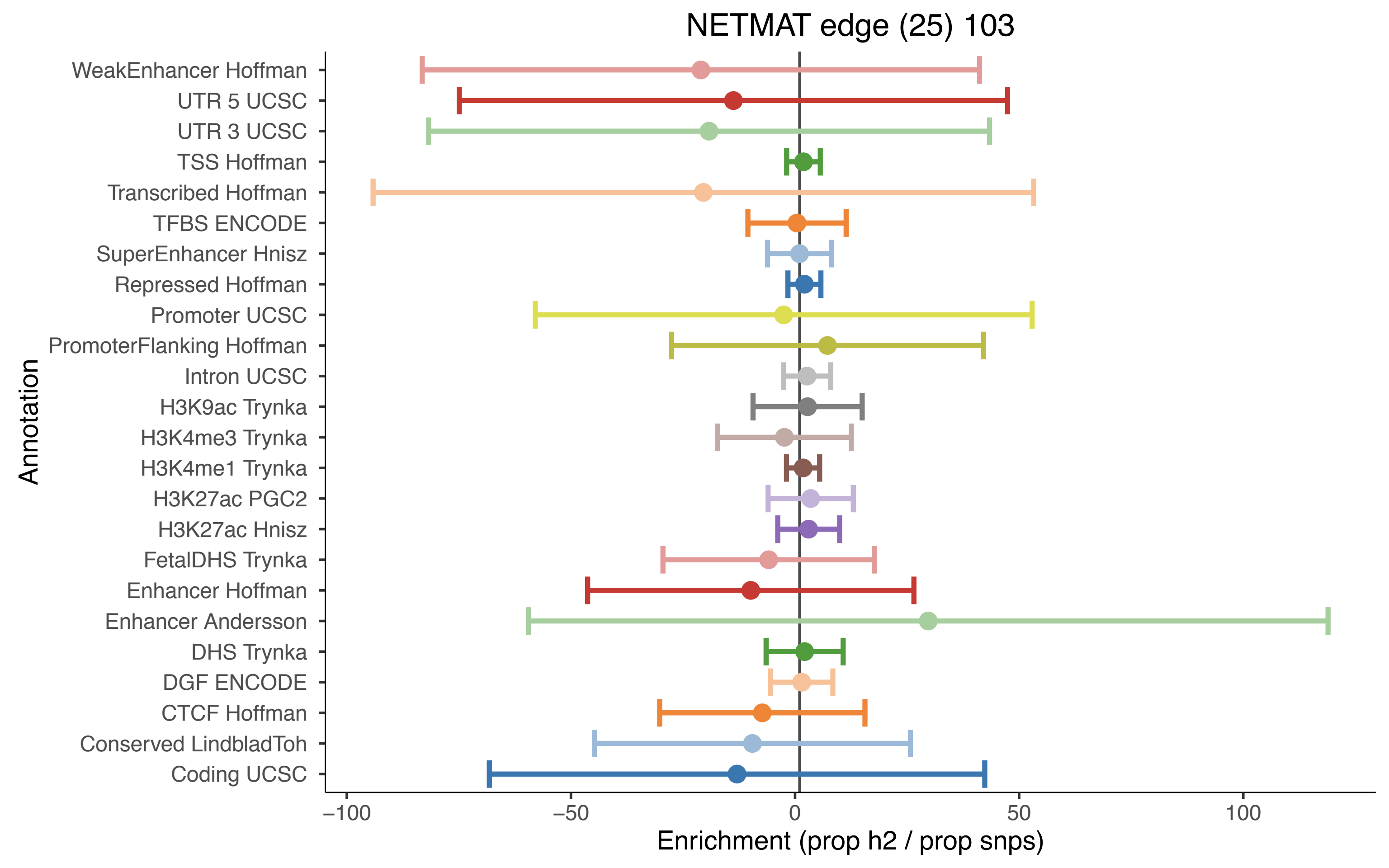
NETMAT edge (25) 103
Annotation
WeakEnhancer Hoffman
UTR 5 UCSC
UTR 3 UCSC
TSS Hoffman
Transcribed Hoffman
TFBS ENCODE
SuperEnhancer Hnisz
Repressed Hoffman
Promoter UCSC
PromoterFlanking Hoffman
Intron UCSC
H3K9ac Trynka
H3K4me3 Trynka
H3K4me1 Trynka
H3K27ac PGC2
H3K27ac Hnisz
FetalDHS Trynka
Enhancer Hoffman
Enhancer Andersson
DHS Trynka
DGF ENCODE
CTCF Hoffman
Conserved LindbladToh
Coding UCSC
−100
−50
0
50
100
Enrichment (prop h2 / prop snps)

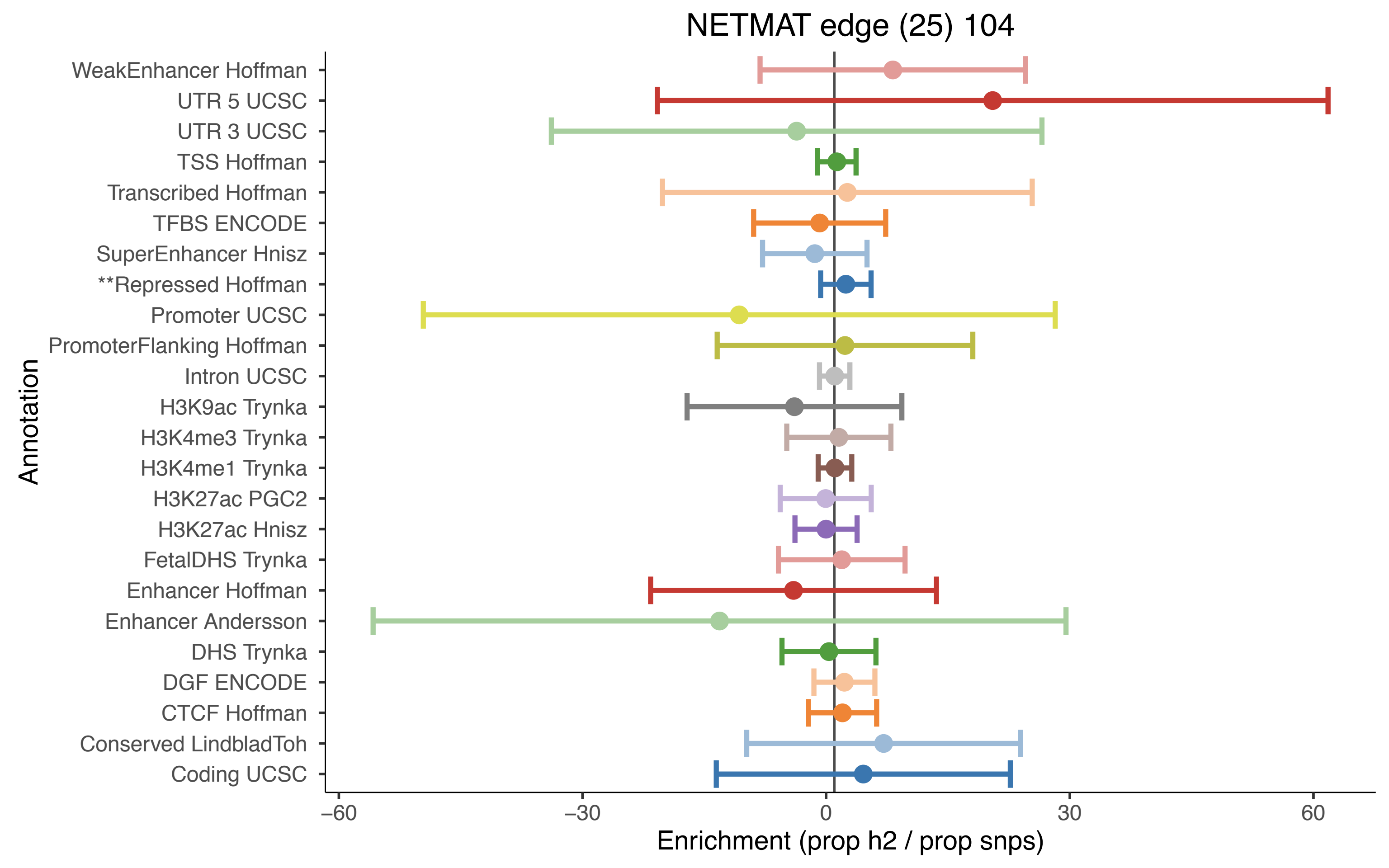

NETMAT edge (25) 104
WeakEnhancer Hoffman
UTR 5 UCSC
UTR 3 UCSC
TSS Hoffman
Transcribed Hoffman
TFBS ENCODE
SuperEnhancer Hnisz
**Repressed Hoffman
Promoter UCSC
PromoterFlanking Hoffman
Intron UCSC
H3K9ac Trynka
H3K4me3 Trynka
H3K4me1 Trynka
H3K27ac PGC2
H3K27ac Hnisz
FetalDHS Trynka
Enhancer Hoffman
Enhancer Andersson
DHS Trynka
DGF ENCODE
CTCF Hoffman
Conserved LindbladToh
Coding UCSC
Annotation
−60
−30
0
30
60
Enrichment (prop h2 / prop snps)

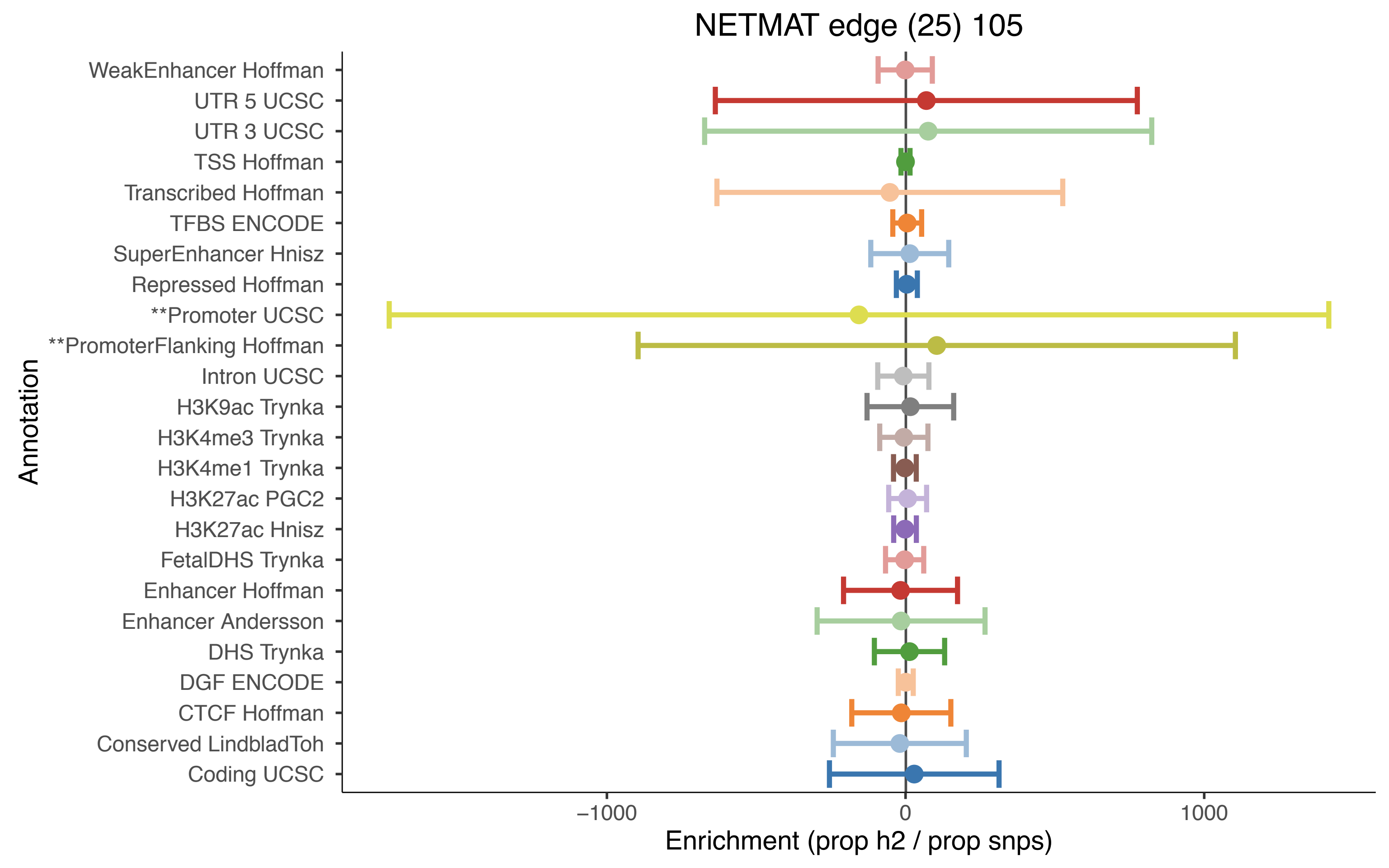

NETMAT edge (25) 105
WeakEnhancer Hoffman
UTR 5 UCSC
UTR 3 UCSC
TSS Hoffman
Transcribed Hoffman
TFBS ENCODE
SuperEnhancer Hnisz
Repressed Hoffman
**Promoter UCSC
**PromoterFlanking Hoffman
Intron UCSC
H3K9ac Trynka
H3K4me3 Trynka
H3K4me1 Trynka
H3K27ac PGC2
H3K27ac Hnisz
FetalDHS Trynka
Enhancer Hoffman
Enhancer Andersson
DHS Trynka
DGF ENCODE
CTCF Hoffman
Conserved LindbladToh
Coding UCSC
Annotation
−1000
0
1000
Enrichment (prop h2 / prop snps)

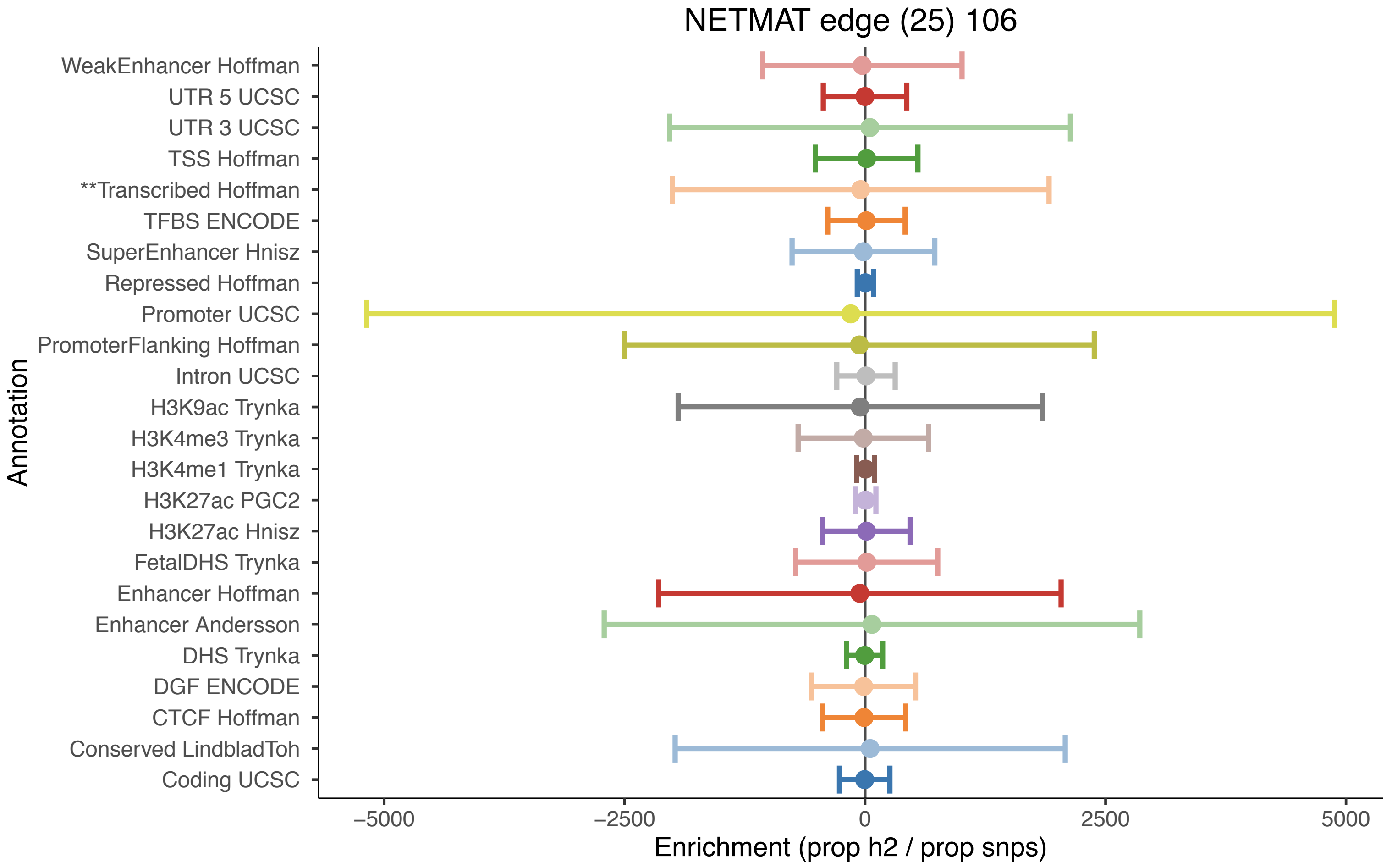

NETMAT edge (25) 106
Annotation
WeakEnhancer Hoffman
UTR 5 UCSC
UTR 3 UCSC
TSS Hoffman
**Transcribed Hoffman
TFBS ENCODE
SuperEnhancer Hnisz
Repressed Hoffman
Promoter UCSC
PromoterFlanking Hoffman
Intron UCSC
H3K9ac Trynka
H3K4me3 Trynka
H3K4me1 Trynka
H3K27ac PGC2
H3K27ac Hnisz
FetalDHS Trynka
Enhancer Hoffman
Enhancer Andersson
DHS Trynka
DGF ENCODE
CTCF Hoffman
Conserved LindbladToh
Coding UCSC
−5000
−2500
0
2500
5000
Enrichment (prop h2 / prop snps)

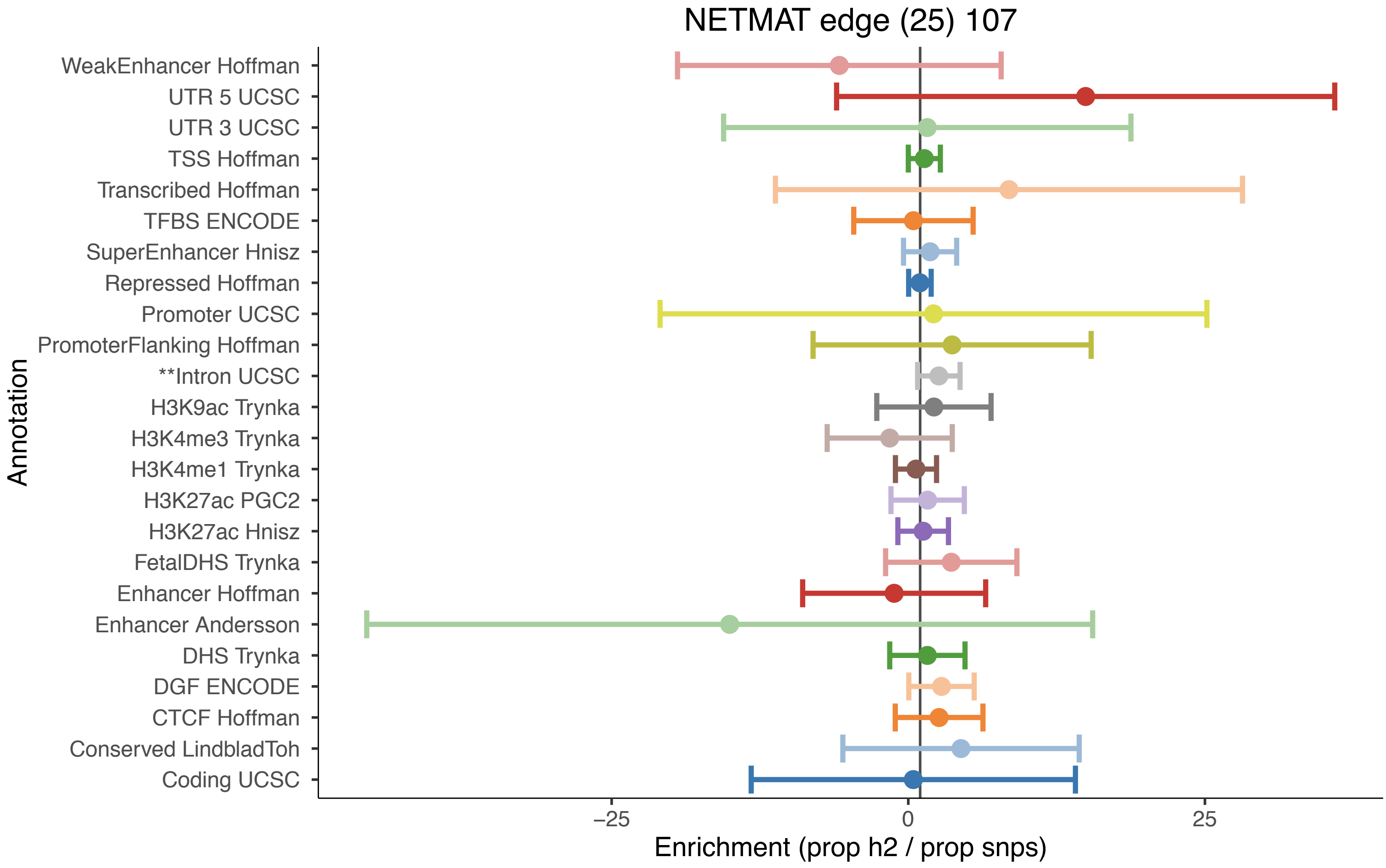

NETMAT edge (25) 107
WeakEnhancer Hoffman
UTR 5 UCSC
UTR 3 UCSC
TSS Hoffman
Transcribed Hoffman
TFBS ENCODE
SuperEnhancer Hnisz
Repressed Hoffman
Promoter UCSC
PromoterFlanking Hoffman
**Intron UCSC
H3K9ac Trynka
H3K4me3 Trynka
H3K4me1 Trynka
H3K27ac PGC2
H3K27ac Hnisz
FetalDHS Trynka
Enhancer Hoffman
Enhancer Andersson
DHS Trynka
DGF ENCODE
CTCF Hoffman
Conserved LindbladToh
Coding UCSC
Annotation
−25
0
25
Enrichment (prop h2 / prop snps)

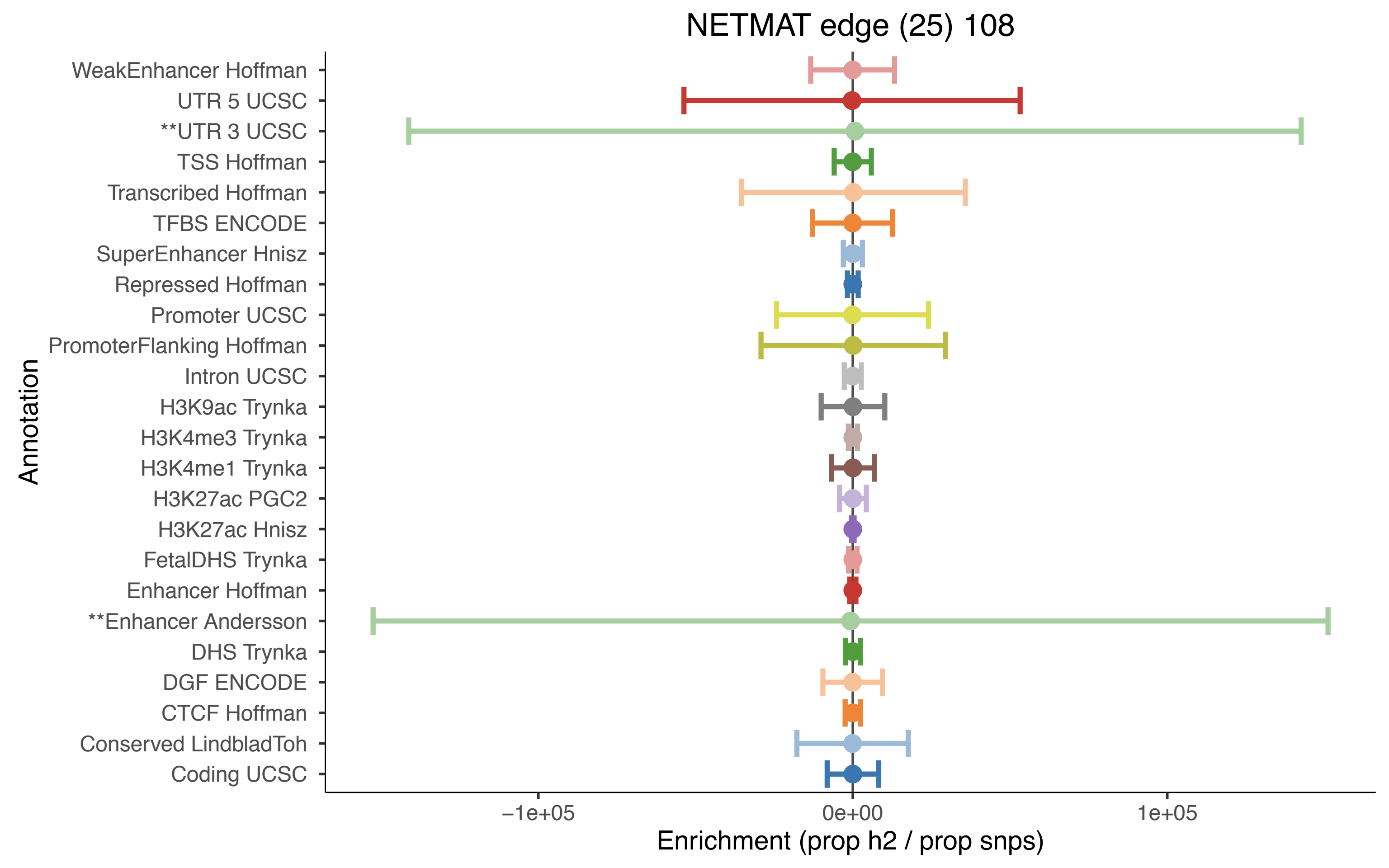
NETMAT edge (25) 108
WeakEnhancer Hoffman
UTR 5 UCSC
**UTR 3 UCSC
TSS Hoffman
Transcribed Hoffman
TFBS ENCODE
SuperEnhancer Hnisz
Repressed Hoffman
Promoter UCSC
PromoterFlanking Hoffman
Intron UCSC
H3K9ac Trynka
H3K4me3 Trynka
H3K4me1 Trynka
H3K27ac PGC2
H3K27ac Hnisz
FetalDHS Trynka
Enhancer Hoffman
**Enhancer Andersson
DHS Trynka
DGF ENCODE
CTCF Hoffman
Conserved LindbladToh
Coding UCSC
Annotation
−1e+05
0e+00
1e+05
Enrichment (prop h2 / prop snps)

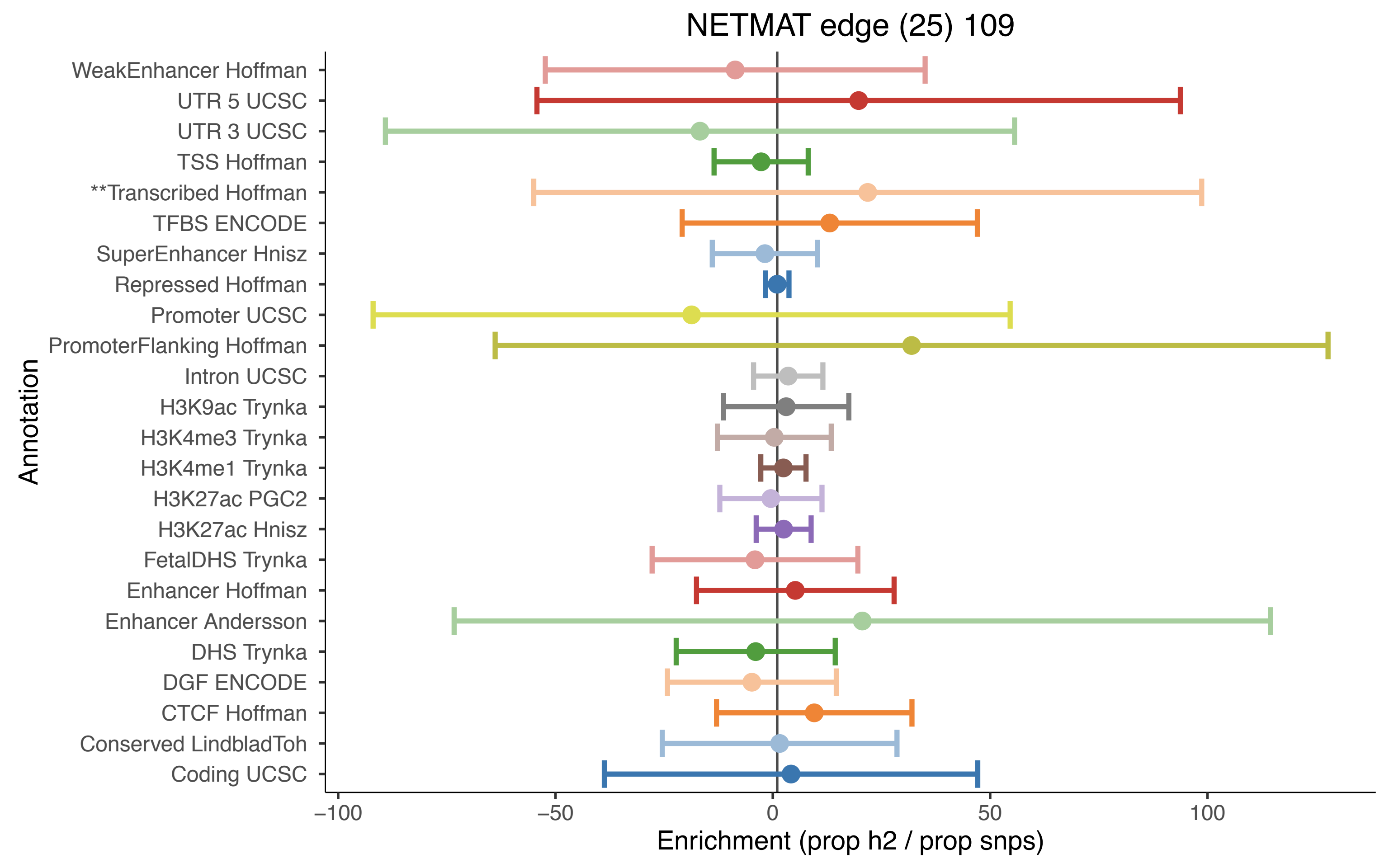

NETMAT edge (25) 109
WeakEnhancer Hoffman
UTR 5 UCSC
UTR 3 UCSC
TSS Hoffman
**Transcribed Hoffman
TFBS ENCODE
SuperEnhancer Hnisz
Repressed Hoffman
Promoter UCSC
PromoterFlanking Hoffman
Intron UCSC
H3K9ac Trynka
H3K4me3 Trynka
H3K4me1 Trynka
H3K27ac PGC2
H3K27ac Hnisz
FetalDHS Trynka
Enhancer Hoffman
Enhancer Andersson
DHS Trynka
DGF ENCODE
CTCF Hoffman
Conserved LindbladToh
Coding UCSC
Annotation
−100
−50
0
50
100
Enrichment (prop h2 / prop snps)

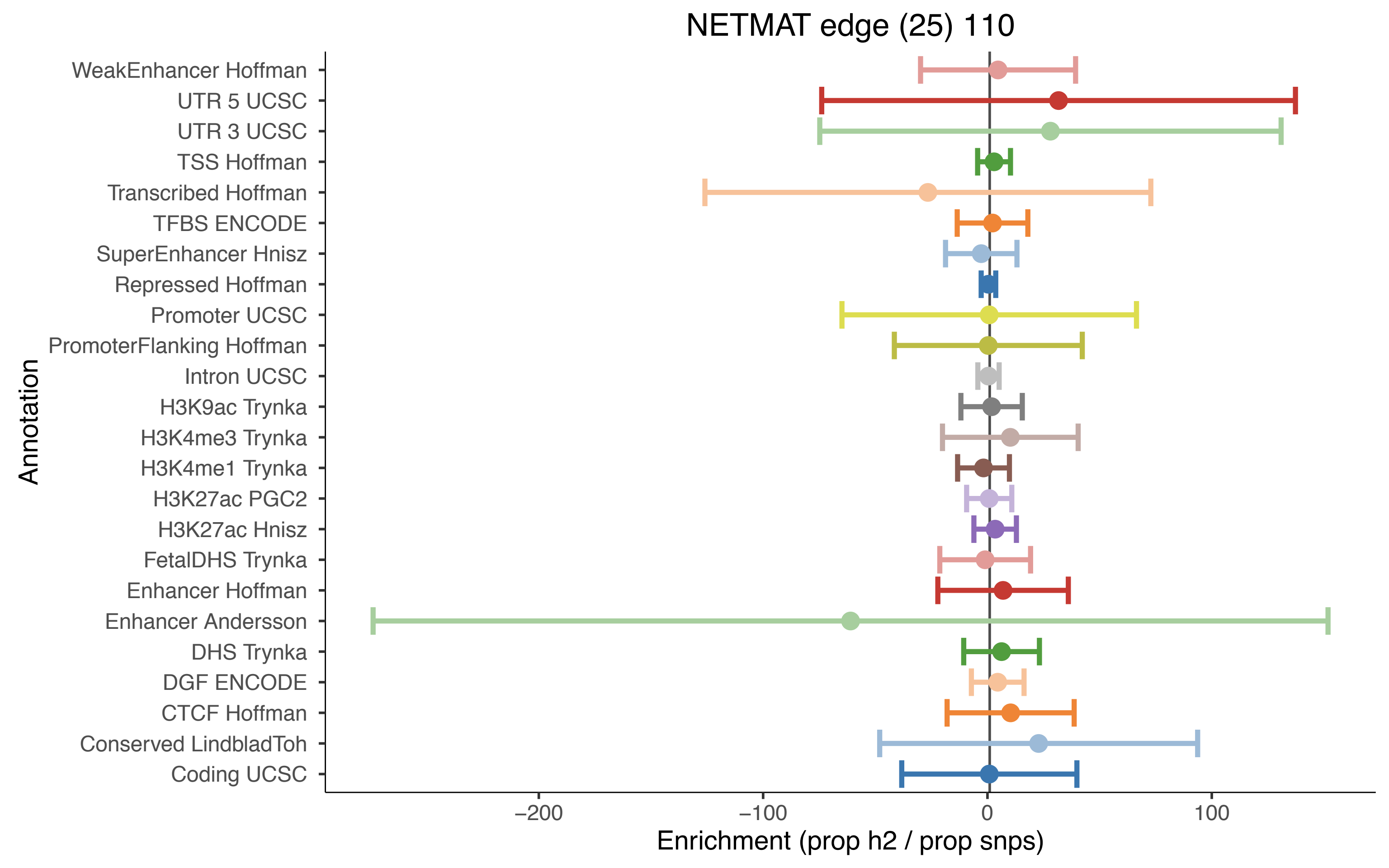
NETMAT edge (25) 110
WeakEnhancer Hoffman
UTR 5 UCSC
UTR 3 UCSC
TSS Hoffman
Transcribed Hoffman
TFBS ENCODE
SuperEnhancer Hnisz
Repressed Hoffman
Promoter UCSC
PromoterFlanking Hoffman
Intron UCSC
H3K9ac Trynka
H3K4me3 Trynka
H3K4me1 Trynka
H3K27ac PGC2
H3K27ac Hnisz
FetalDHS Trynka
Enhancer Hoffman
Enhancer Andersson
DHS Trynka
DGF ENCODE
CTCF Hoffman
Conserved LindbladToh
Coding UCSC
Annotation
−200
−100
0
100
Enrichment (prop h2 / prop snps)

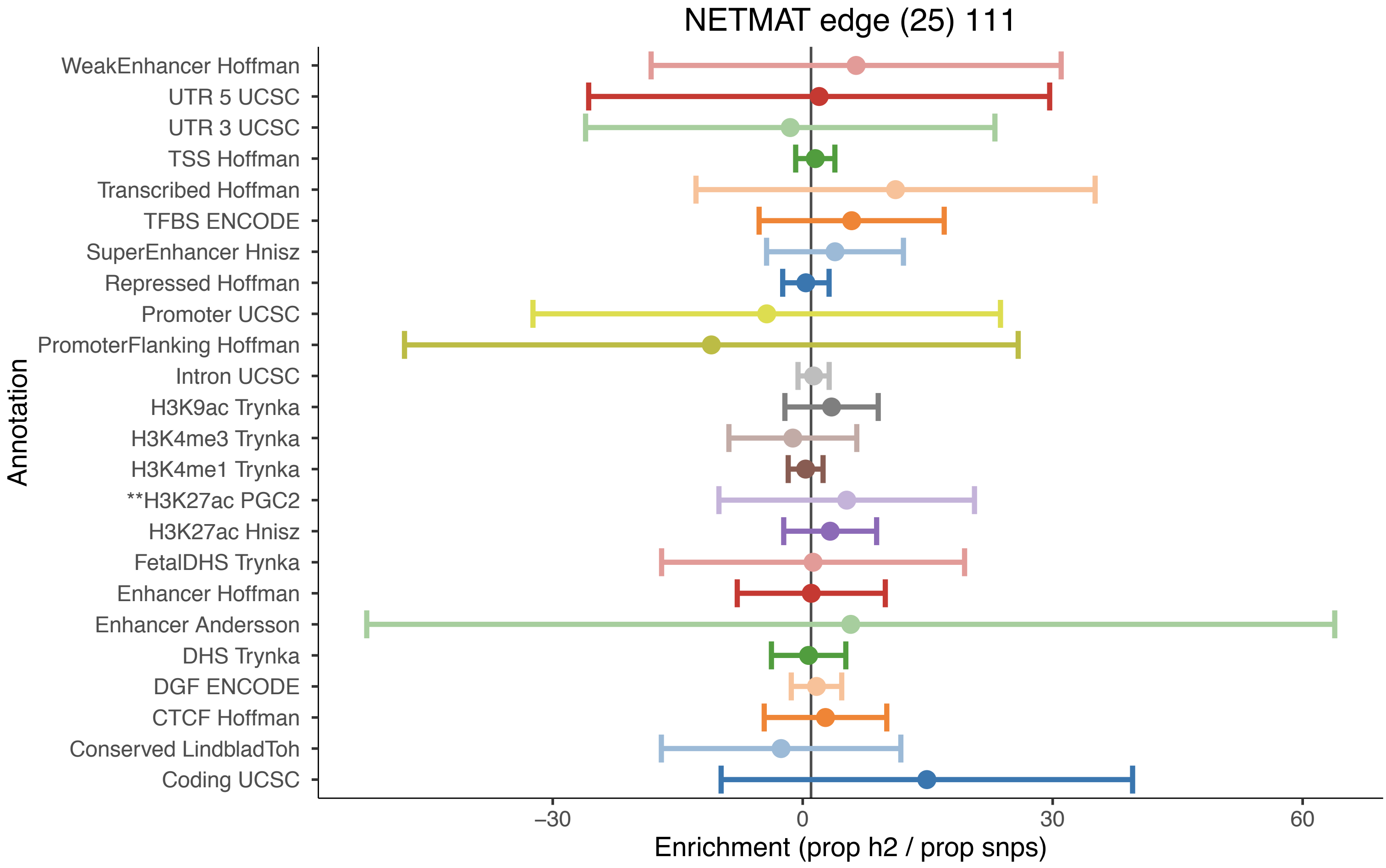
NETMAT edge (25) 111
WeakEnhancer Hoffman
UTR 5 UCSC
UTR 3 UCSC
TSS Hoffman
Transcribed Hoffman
TFBS ENCODE
SuperEnhancer Hnisz
Repressed Hoffman
Promoter UCSC
PromoterFlanking Hoffman
Intron UCSC
H3K9ac Trynka
H3K4me3 Trynka
H3K4me1 Trynka
**H3K27ac PGC2
H3K27ac Hnisz
FetalDHS Trynka
Enhancer Hoffman
Enhancer Andersson
DHS Trynka
DGF ENCODE
CTCF Hoffman
Conserved LindbladToh
Coding UCSC
Annotation
−30
0
30
60
Enrichment (prop h2 / prop snps)

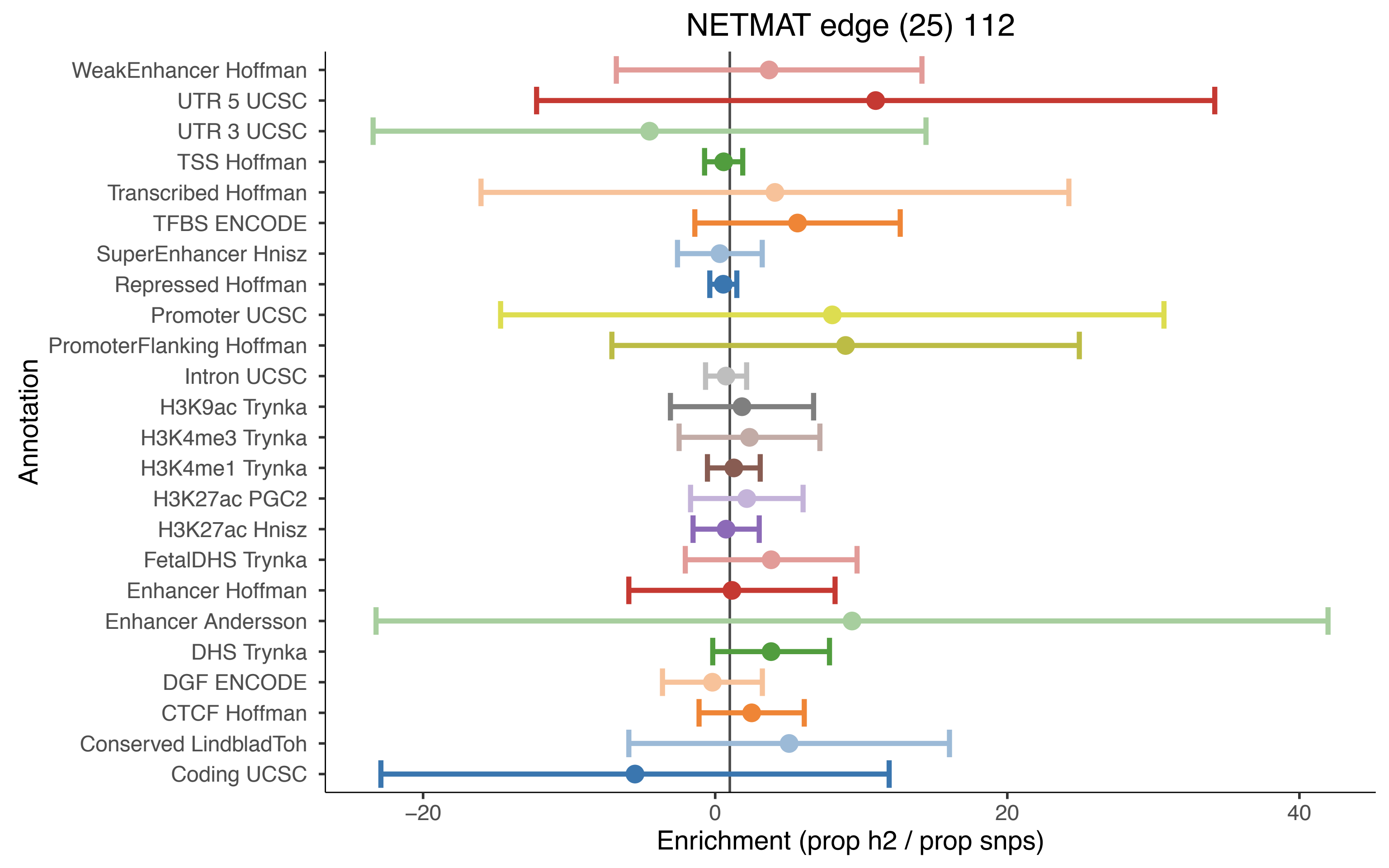

NETMAT edge (25) 112
WeakEnhancer Hoffman
UTR 5 UCSC
UTR 3 UCSC
TSS Hoffman
Transcribed Hoffman
TFBS ENCODE
SuperEnhancer Hnisz
Repressed Hoffman
Promoter UCSC
PromoterFlanking Hoffman
Intron UCSC
H3K9ac Trynka
H3K4me3 Trynka
H3K4me1 Trynka
H3K27ac PGC2
H3K27ac Hnisz
FetalDHS Trynka
Enhancer Hoffman
Enhancer Andersson
DHS Trynka
DGF ENCODE
CTCF Hoffman
Conserved LindbladToh
Coding UCSC
Annotation
−20
0
20
40
Enrichment (prop h2 / prop snps)

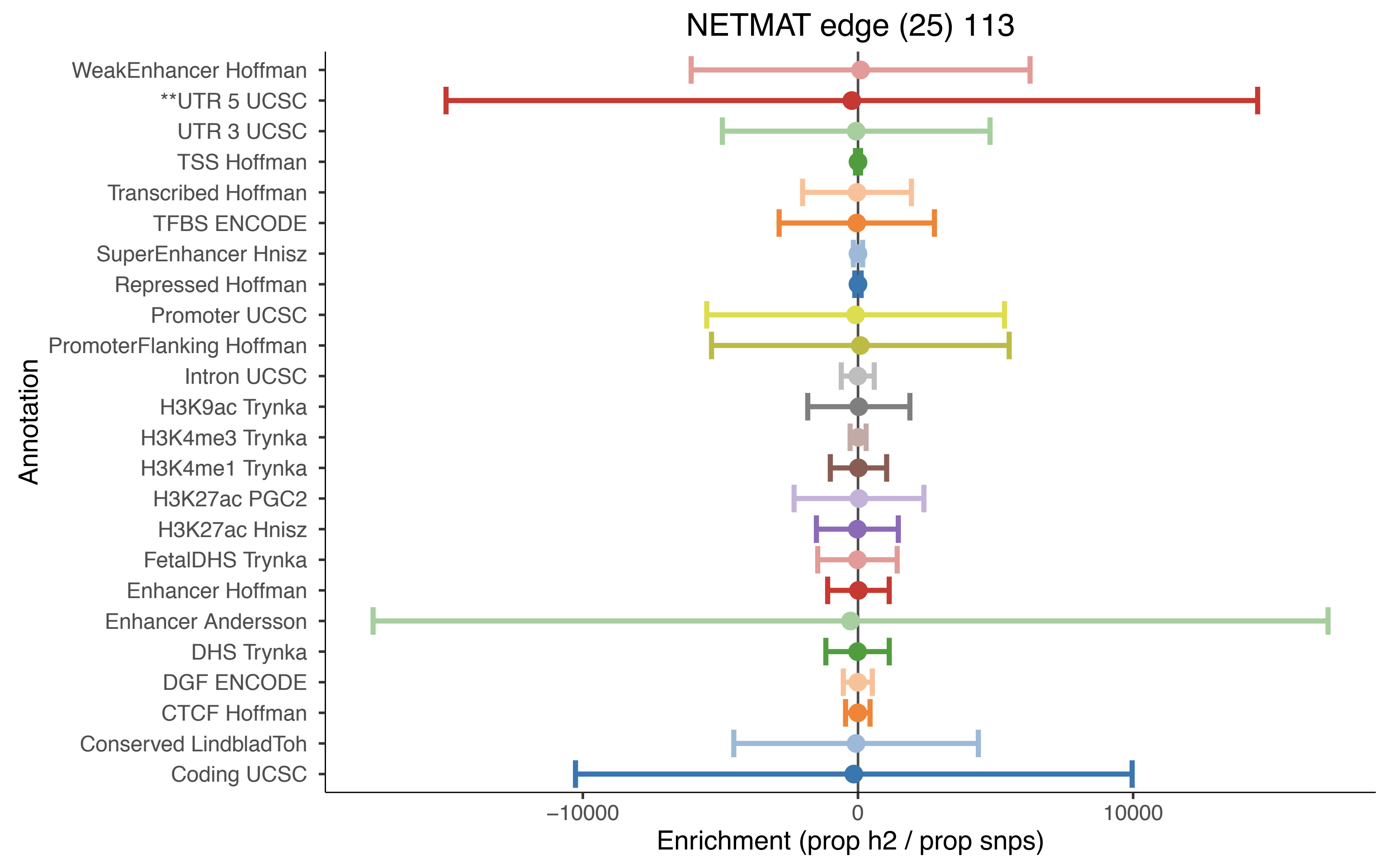

NETMAT edge (25) 113
WeakEnhancer Hoffman
**UTR 5 UCSC
UTR 3 UCSC
TSS Hoffman
Transcribed Hoffman
TFBS ENCODE
SuperEnhancer Hnisz
Repressed Hoffman
Promoter UCSC
PromoterFlanking Hoffman
Intron UCSC
H3K9ac Trynka
H3K4me3 Trynka
H3K4me1 Trynka
H3K27ac PGC2
H3K27ac Hnisz
FetalDHS Trynka
Enhancer Hoffman
Enhancer Andersson
DHS Trynka
DGF ENCODE
CTCF Hoffman
Conserved LindbladToh
Coding UCSC
Annotation
−10000
0
10000
Enrichment (prop h2 / prop snps)

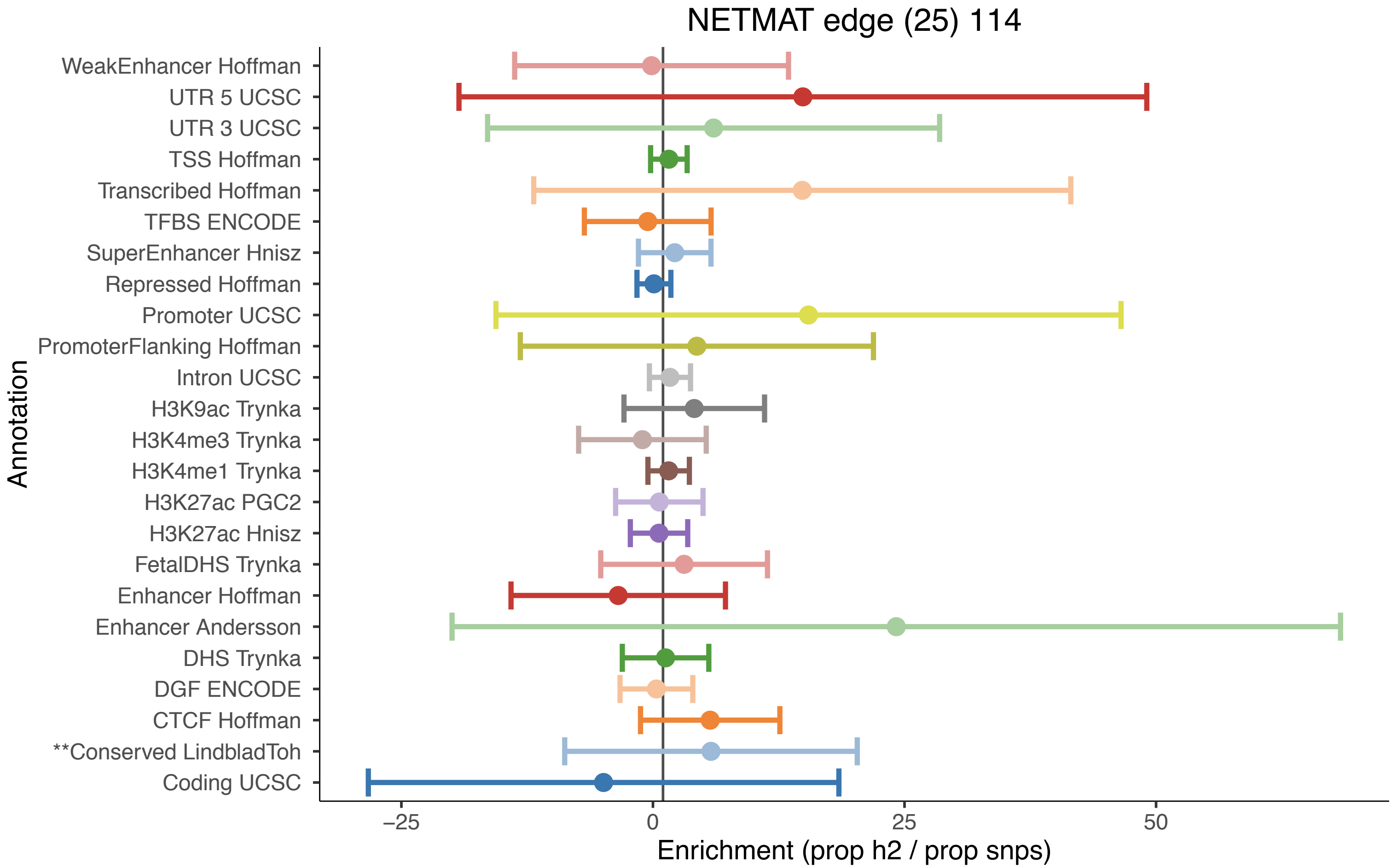

NETMAT edge (25) 114
WeakEnhancer Hoffman
UTR 5 UCSC
UTR 3 UCSC
TSS Hoffman
Transcribed Hoffman
TFBS ENCODE
SuperEnhancer Hnisz
Repressed Hoffman
Promoter UCSC
PromoterFlanking Hoffman
Intron UCSC
H3K9ac Trynka
H3K4me3 Trynka
H3K4me1 Trynka
H3K27ac PGC2
H3K27ac Hnisz
FetalDHS Trynka
Enhancer Hoffman
Enhancer Andersson
DHS Trynka
DGF ENCODE
CTCF Hoffman
**Conserved LindbladToh
Coding UCSC
Annotation
−25
0
25
50
Enrichment (prop h2 / prop snps)

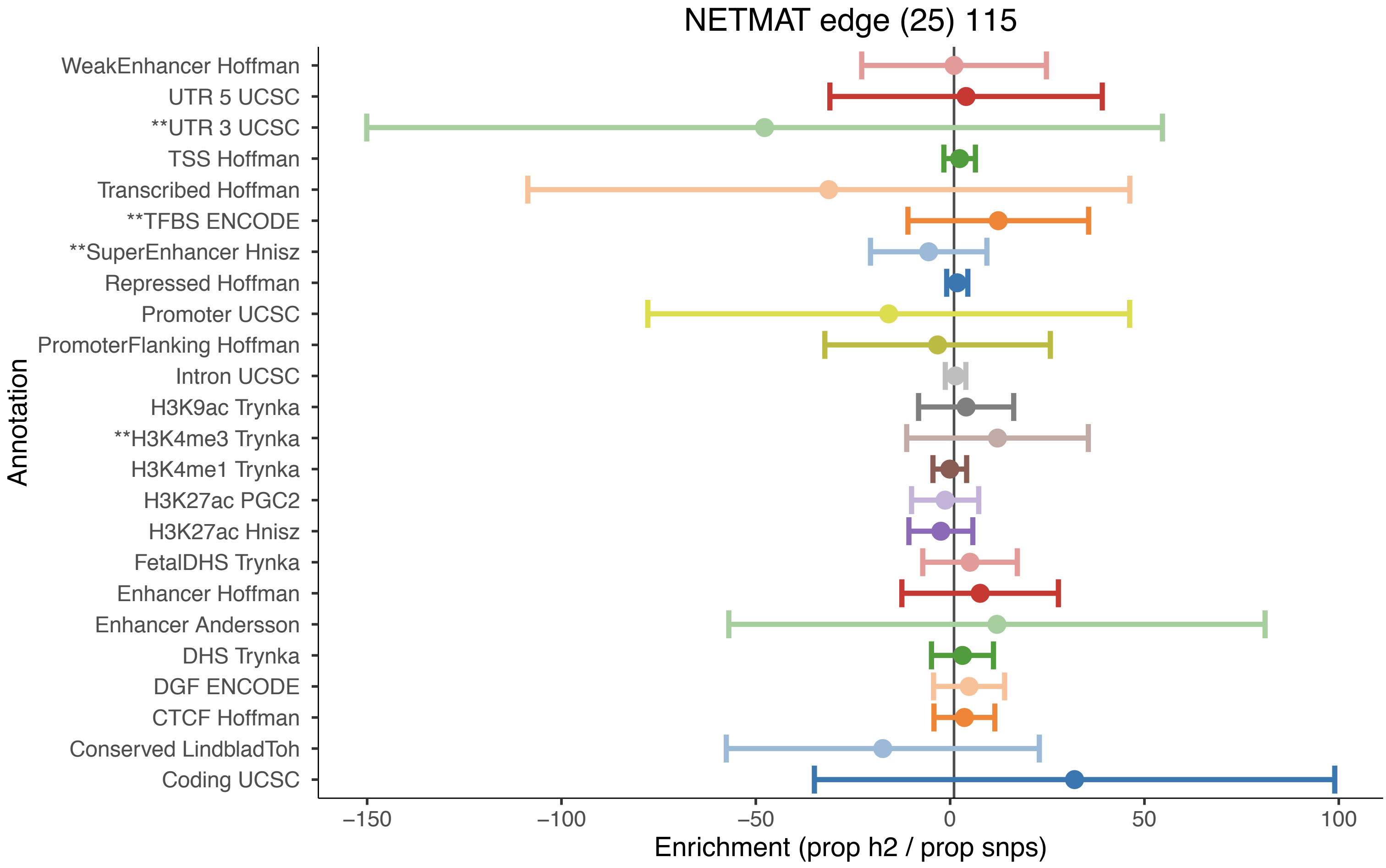
NETMAT edge (25) 115
WeakEnhancer Hoffman
UTR 5 UCSC
**UTR 3 UCSC
TSS Hoffman
Transcribed Hoffman
**TFBS ENCODE
**SuperEnhancer Hnisz
Repressed Hoffman
Promoter UCSC
PromoterFlanking Hoffman
Intron UCSC
H3K9ac Trynka
**H3K4me3 Trynka
H3K4me1 Trynka
H3K27ac PGC2
H3K27ac Hnisz
FetalDHS Trynka
Enhancer Hoffman
Enhancer Andersson
DHS Trynka
DGF ENCODE
CTCF Hoffman
Conserved LindbladToh
Coding UCSC
Annotation
−150
−100
−50
0
50
100
Enrichment (prop h2 / prop snps)

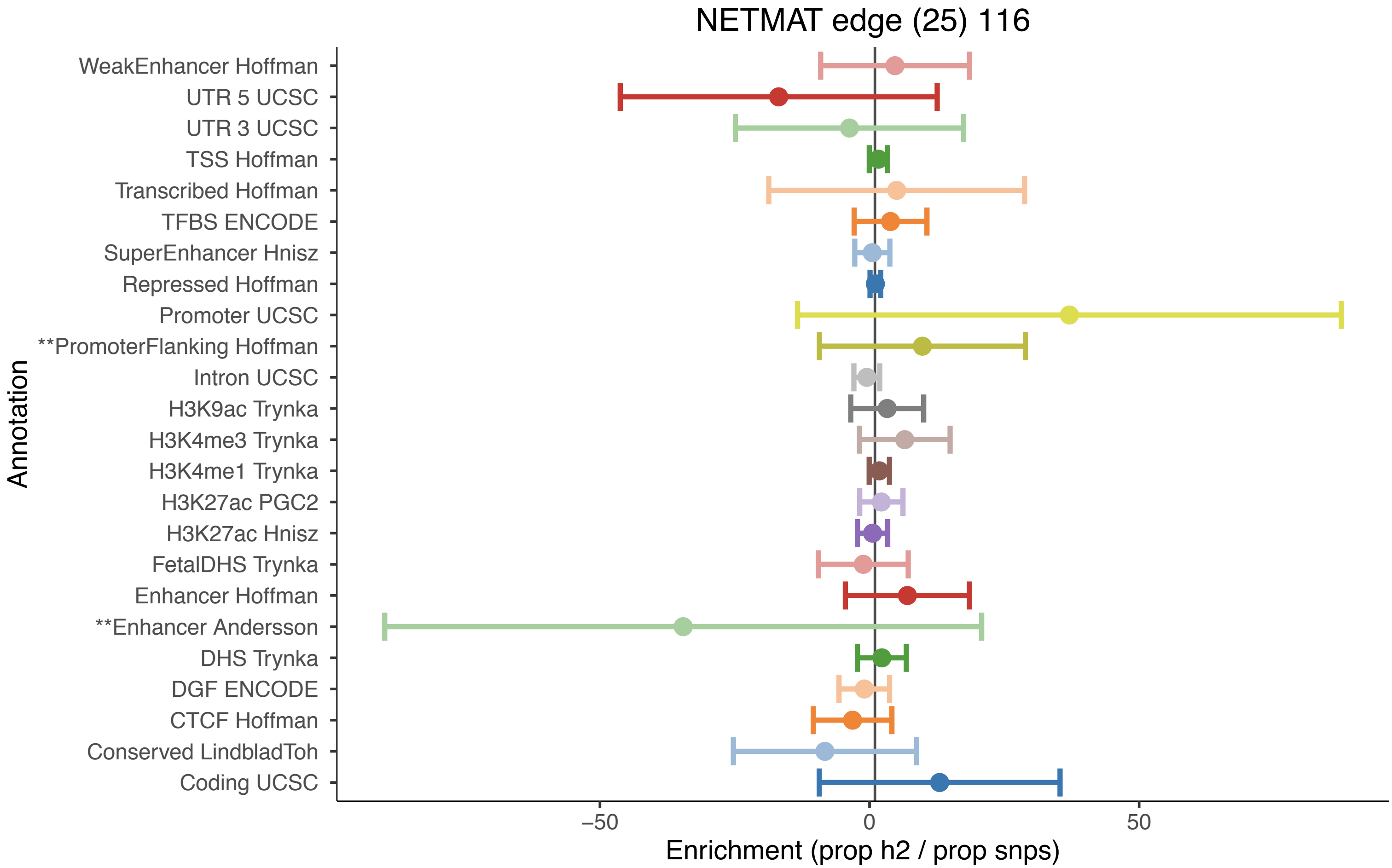
NETMAT edge (25) 116
WeakEnhancer Hoffman
UTR 5 UCSC
UTR 3 UCSC
TSS Hoffman
Transcribed Hoffman
TFBS ENCODE
SuperEnhancer Hnisz
Repressed Hoffman
Promoter UCSC
**PromoterFlanking Hoffman
Intron UCSC
H3K9ac Trynka
H3K4me3 Trynka
H3K4me1 Trynka
H3K27ac PGC2
H3K27ac Hnisz
FetalDHS Trynka
Enhancer Hoffman
**Enhancer Andersson
DHS Trynka
DGF ENCODE
CTCF Hoffman
Conserved LindbladToh
Coding UCSC
Annotation
−50
0
50
Enrichment (prop h2 / prop snps)

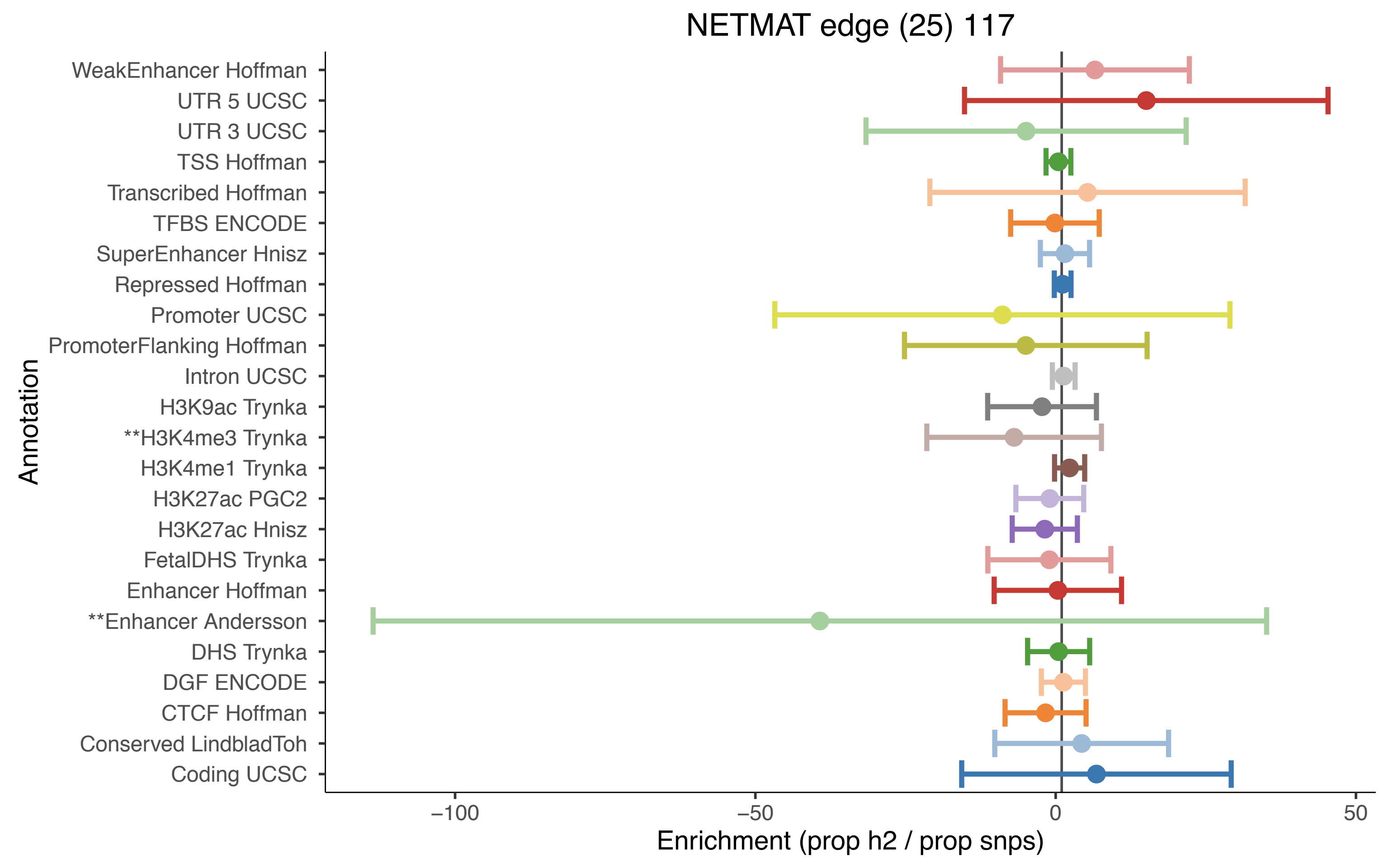
NETMAT edge (25) 117
WeakEnhancer Hoffman
UTR 5 UCSC
UTR 3 UCSC
TSS Hoffman
Transcribed Hoffman
TFBS ENCODE
SuperEnhancer Hnisz
Repressed Hoffman
Promoter UCSC
PromoterFlanking Hoffman
Intron UCSC
H3K9ac Trynka
**H3K4me3 Trynka
H3K4me1 Trynka
H3K27ac PGC2
H3K27ac Hnisz
FetalDHS Trynka
Enhancer Hoffman
**Enhancer Andersson
DHS Trynka
DGF ENCODE
CTCF Hoffman
Conserved LindbladToh
Coding UCSC
Annotation
−100
−50
0
50
Enrichment (prop h2 / prop snps)

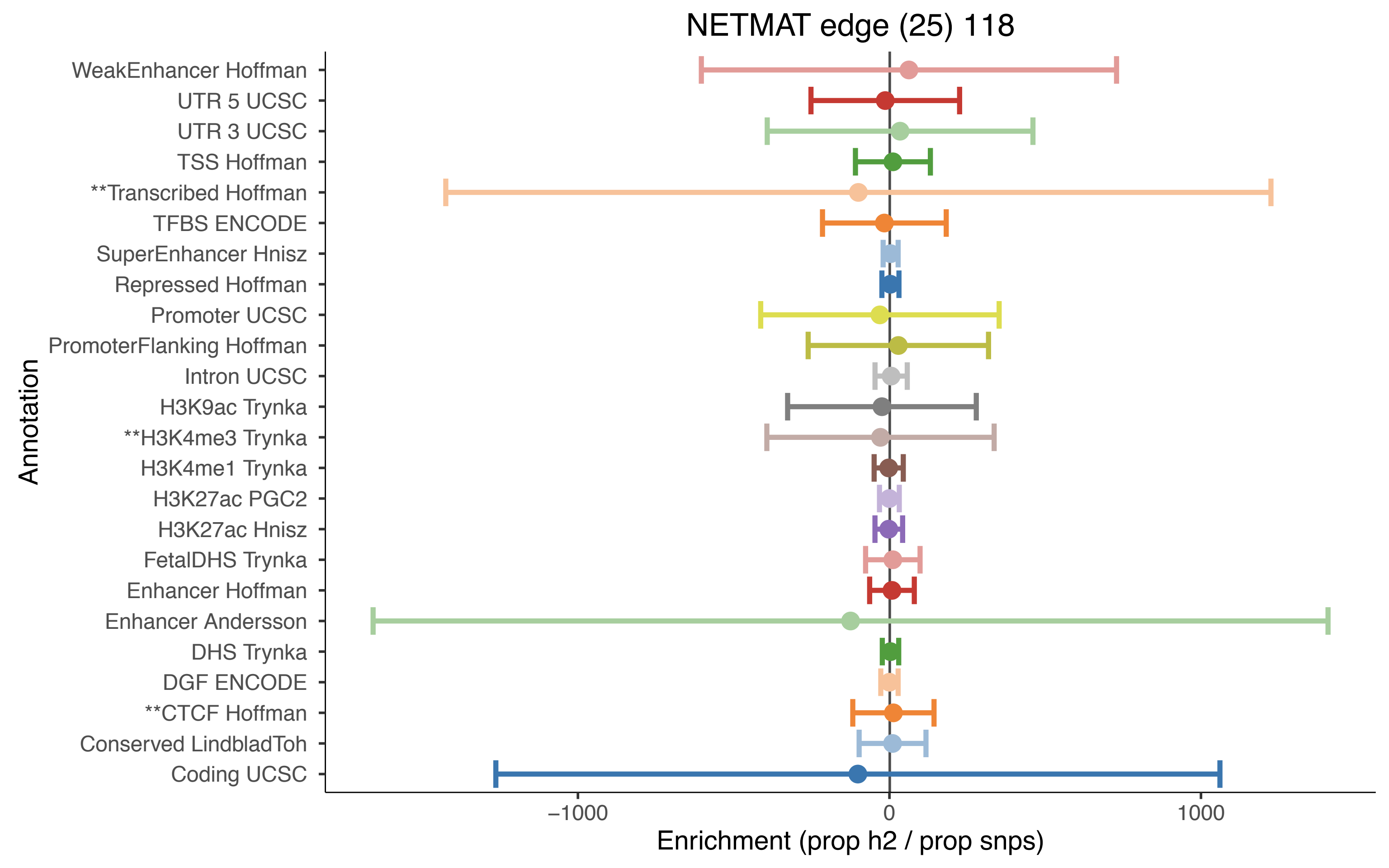
NETMAT edge (25) 118
WeakEnhancer Hoffman
UTR 5 UCSC
UTR 3 UCSC
TSS Hoffman
**Transcribed Hoffman
TFBS ENCODE
SuperEnhancer Hnisz
Repressed Hoffman
Promoter UCSC
PromoterFlanking Hoffman
Intron UCSC
H3K9ac Trynka
**H3K4me3 Trynka
H3K4me1 Trynka
H3K27ac PGC2
H3K27ac Hnisz
FetalDHS Trynka
Enhancer Hoffman
Enhancer Andersson
DHS Trynka
DGF ENCODE
**CTCF Hoffman
Conserved LindbladToh
Coding UCSC
Annotation
−1000
0
1000
Enrichment (prop h2 / prop snps)

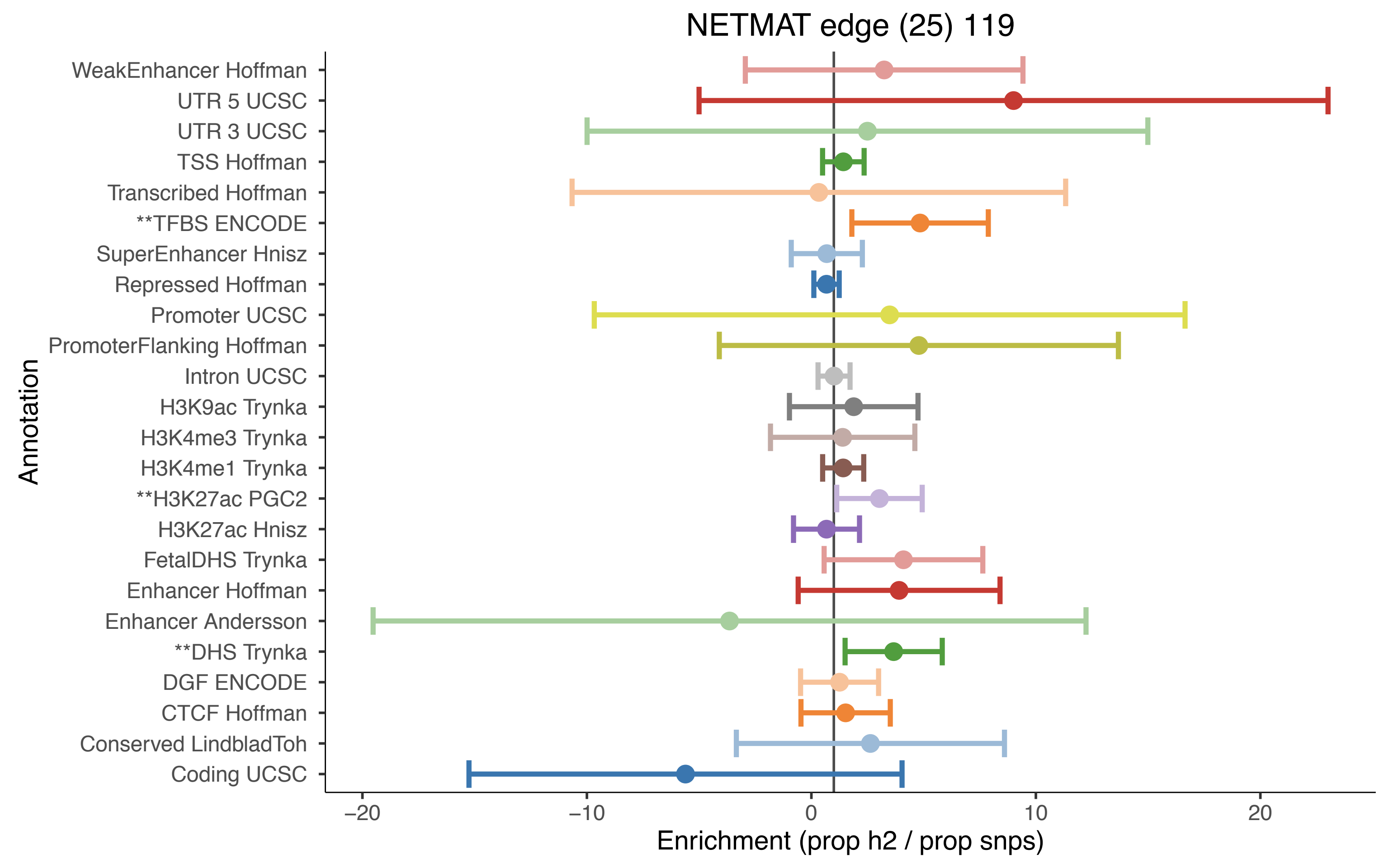
NETMAT edge (25) 119
WeakEnhancer Hoffman
UTR 5 UCSC
UTR 3 UCSC
TSS Hoffman
Transcribed Hoffman
**TFBS ENCODE
SuperEnhancer Hnisz
Repressed Hoffman
Promoter UCSC
PromoterFlanking Hoffman
Intron UCSC
H3K9ac Trynka
H3K4me3 Trynka
H3K4me1 Trynka
**H3K27ac PGC2
H3K27ac Hnisz
FetalDHS Trynka
Enhancer Hoffman
Enhancer Andersson
**DHS Trynka
DGF ENCODE
CTCF Hoffman
Conserved LindbladToh
Coding UCSC
Annotation
−20
−10
0
10
20
Enrichment (prop h2 / prop snps)

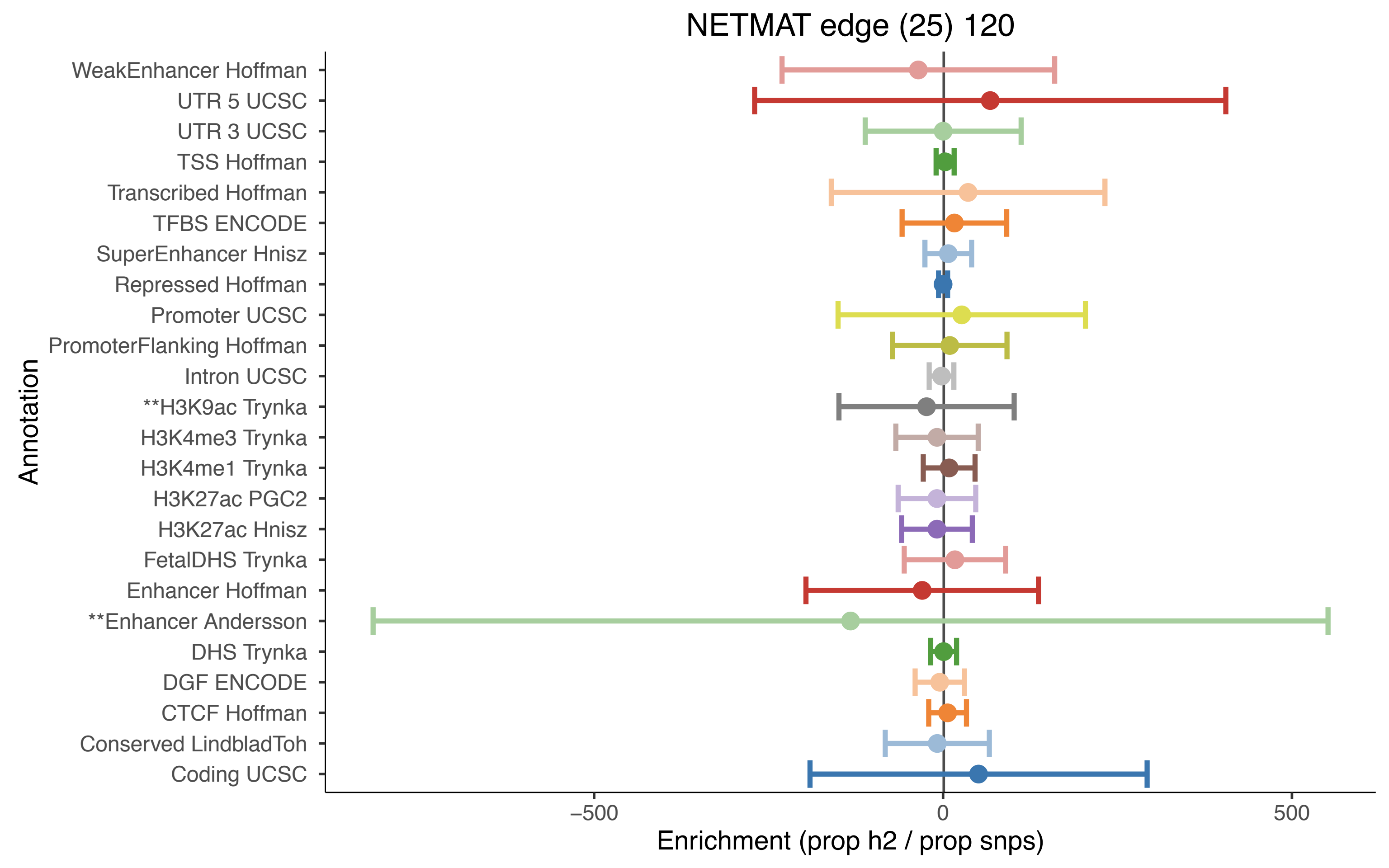
NETMAT edge (25) 120
Annotation
WeakEnhancer Hoffman
UTR 5 UCSC
UTR 3 UCSC
TSS Hoffman
Transcribed Hoffman
TFBS ENCODE
SuperEnhancer Hnisz
Repressed Hoffman
Promoter UCSC
PromoterFlanking Hoffman
Intron UCSC
**H3K9ac Trynka
H3K4me3 Trynka
H3K4me1 Trynka
H3K27ac PGC2
H3K27ac Hnisz
FetalDHS Trynka
Enhancer Hoffman
**Enhancer Andersson
DHS Trynka
DGF ENCODE
CTCF Hoffman
Conserved LindbladToh
Coding UCSC
−500
0
500
Enrichment (prop h2 / prop snps)

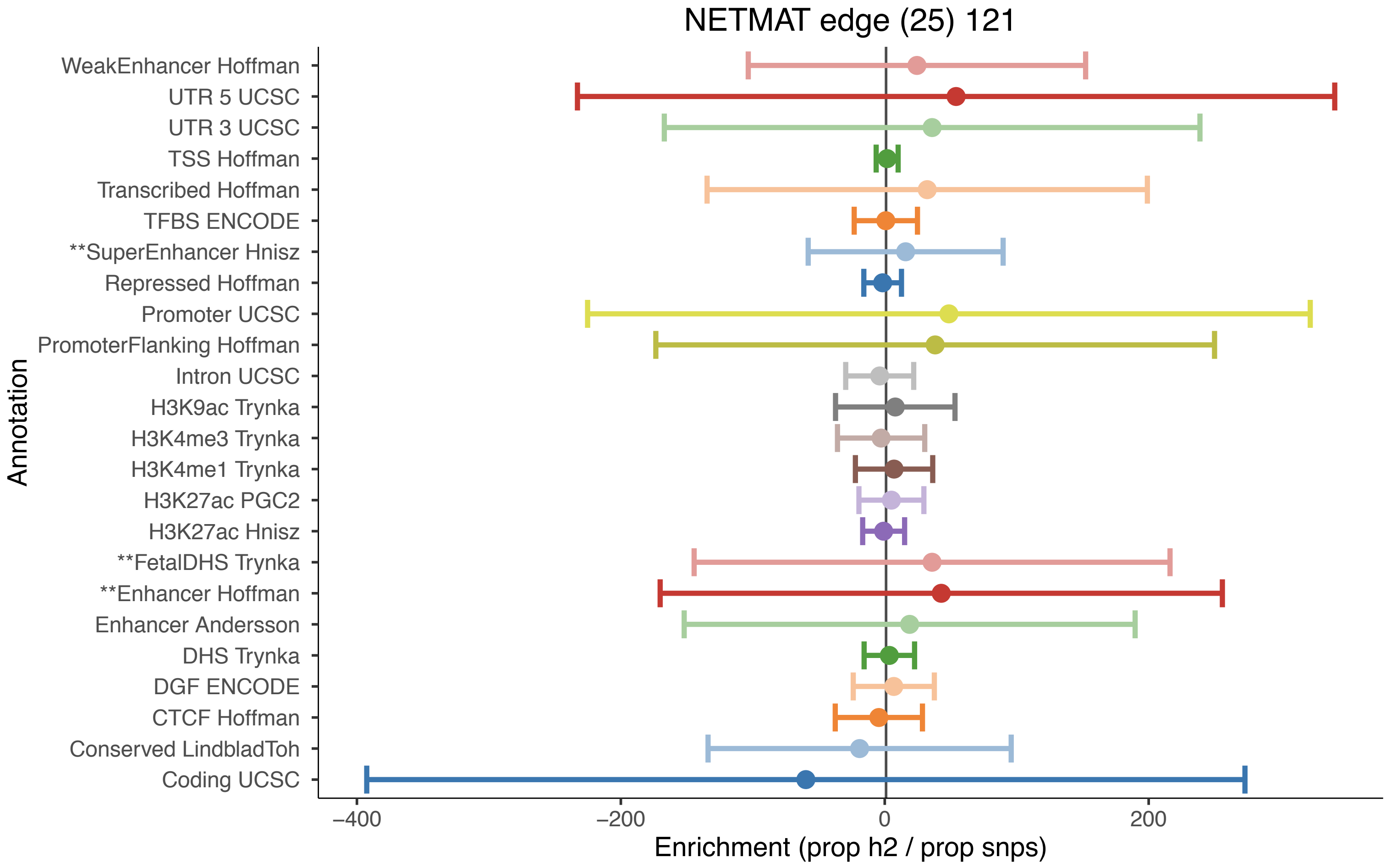

NETMAT edge (25) 121
WeakEnhancer Hoffman
UTR 5 UCSC
UTR 3 UCSC
TSS Hoffman
Transcribed Hoffman
TFBS ENCODE
**SuperEnhancer Hnisz
Repressed Hoffman
Promoter UCSC
PromoterFlanking Hoffman
Intron UCSC
H3K9ac Trynka
H3K4me3 Trynka
H3K4me1 Trynka
H3K27ac PGC2
H3K27ac Hnisz
**FetalDHS Trynka
**Enhancer Hoffman
Enhancer Andersson
DHS Trynka
DGF ENCODE
CTCF Hoffman
Conserved LindbladToh
Coding UCSC
Annotation
−400
−200
0
200
Enrichment (prop h2 / prop snps)

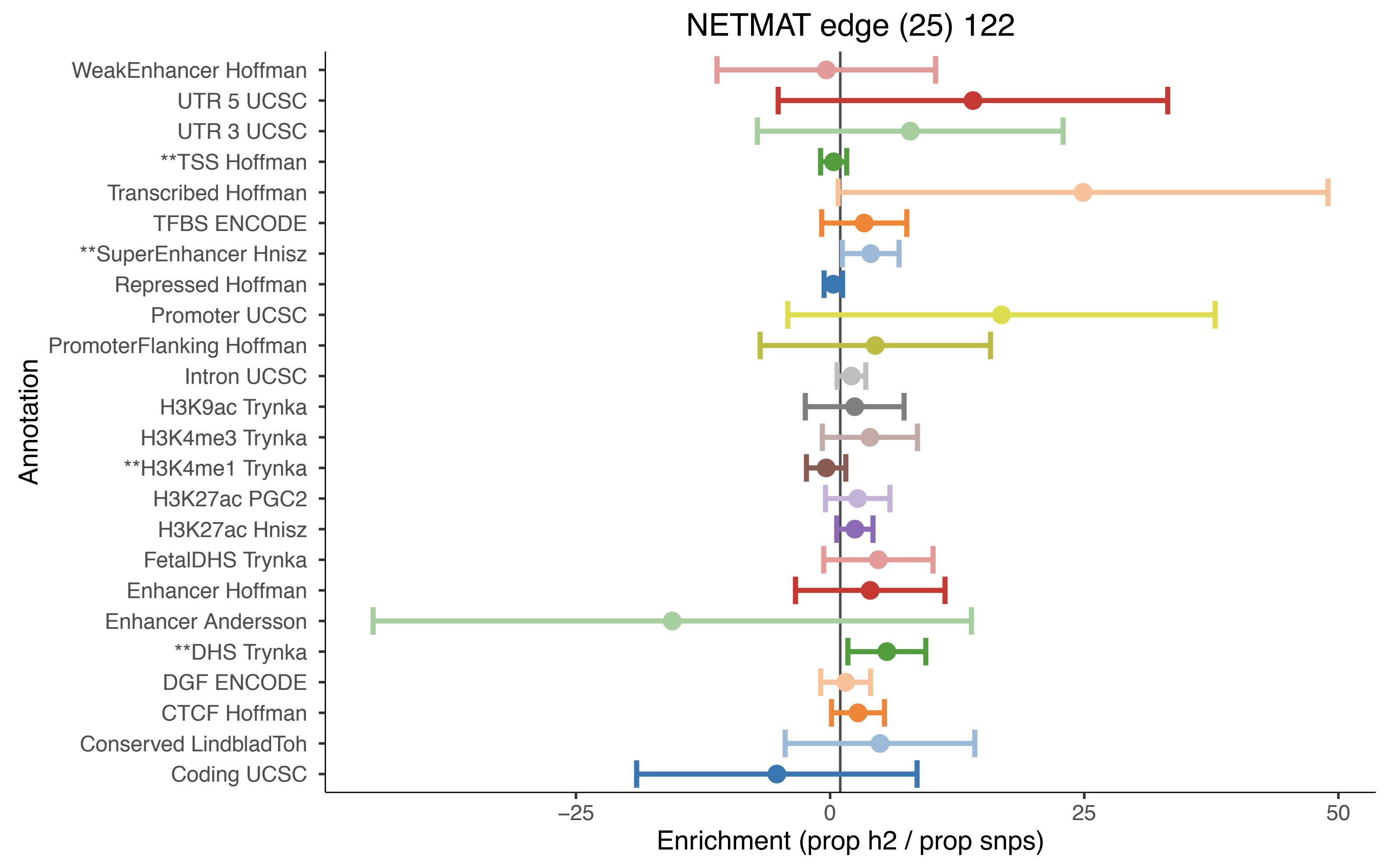
NETMAT edge (25) 122
WeakEnhancer Hoffman
UTR 5 UCSC
UTR 3 UCSC
**TSS Hoffman
Transcribed Hoffman
TFBS ENCODE
**SuperEnhancer Hnisz
Repressed Hoffman
Promoter UCSC
PromoterFlanking Hoffman
Intron UCSC
H3K9ac Trynka
H3K4me3 Trynka
**H3K4me1 Trynka
H3K27ac PGC2
H3K27ac Hnisz
FetalDHS Trynka
Enhancer Hoffman
Enhancer Andersson
**DHS Trynka
DGF ENCODE
CTCF Hoffman
Conserved LindbladToh
Coding UCSC
Annotation
−25
0
25
50
Enrichment (prop h2 / prop snps)

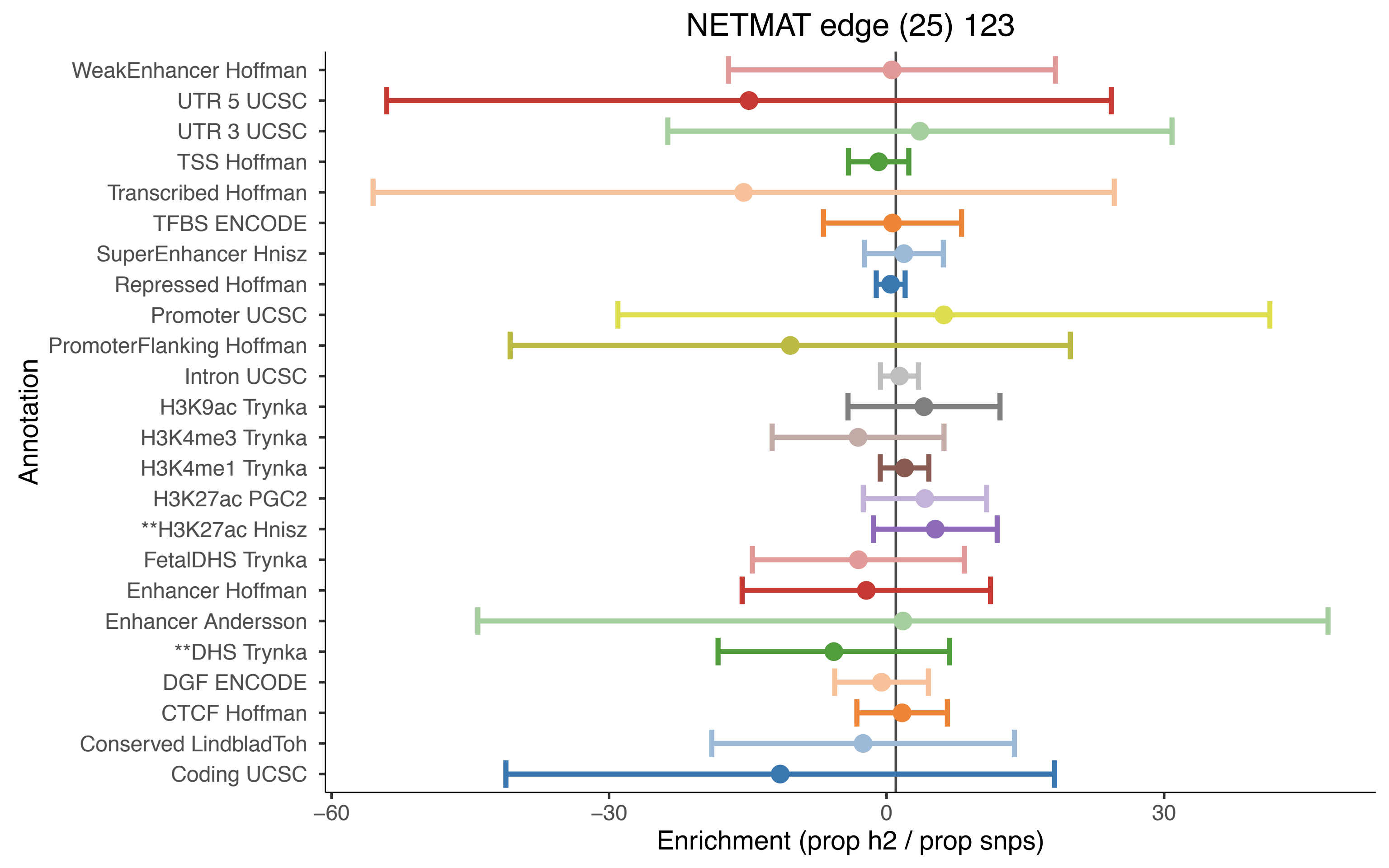

NETMAT edge (25) 123
WeakEnhancer Hoffman
UTR 5 UCSC
UTR 3 UCSC
TSS Hoffman
Transcribed Hoffman
TFBS ENCODE
SuperEnhancer Hnisz
Repressed Hoffman
Promoter UCSC
PromoterFlanking Hoffman
Intron UCSC
H3K9ac Trynka
H3K4me3 Trynka
H3K4me1 Trynka
H3K27ac PGC2
**H3K27ac Hnisz
FetalDHS Trynka
Enhancer Hoffman
Enhancer Andersson
**DHS Trynka
DGF ENCODE
CTCF Hoffman
Conserved LindbladToh
Coding UCSC
Annotation
−60
−30
0
30
Enrichment (prop h2 / prop snps)

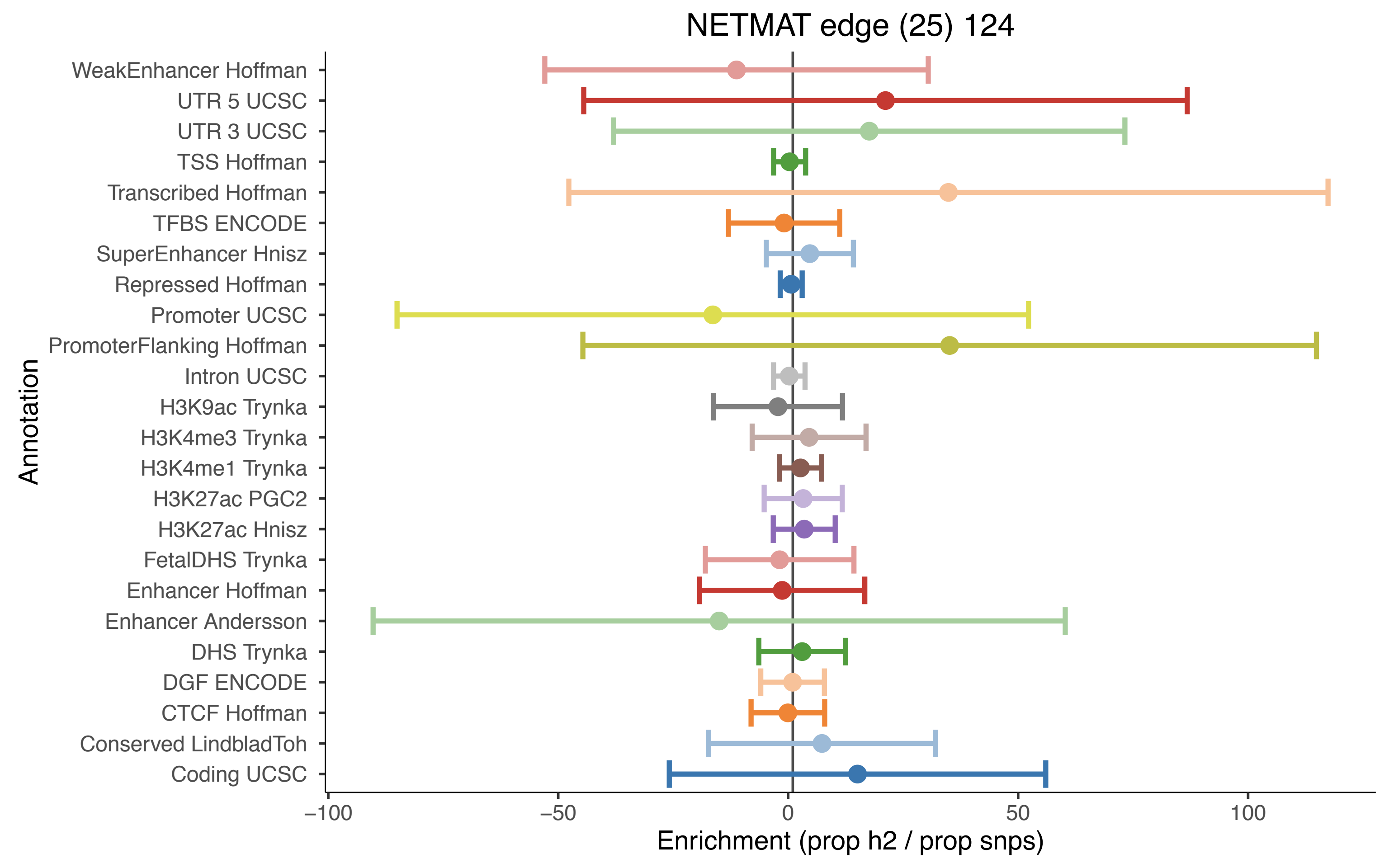
NETMAT edge (25) 124
WeakEnhancer Hoffman
UTR 5 UCSC
UTR 3 UCSC
TSS Hoffman
Transcribed Hoffman
TFBS ENCODE
SuperEnhancer Hnisz
Repressed Hoffman
Promoter UCSC
PromoterFlanking Hoffman
Intron UCSC
H3K9ac Trynka
H3K4me3 Trynka
H3K4me1 Trynka
H3K27ac PGC2
H3K27ac Hnisz
FetalDHS Trynka
Enhancer Hoffman
Enhancer Andersson
DHS Trynka
DGF ENCODE
CTCF Hoffman
Conserved LindbladToh
Coding UCSC
Annotation
−100
−50
0
50
100
Enrichment (prop h2 / prop snps)

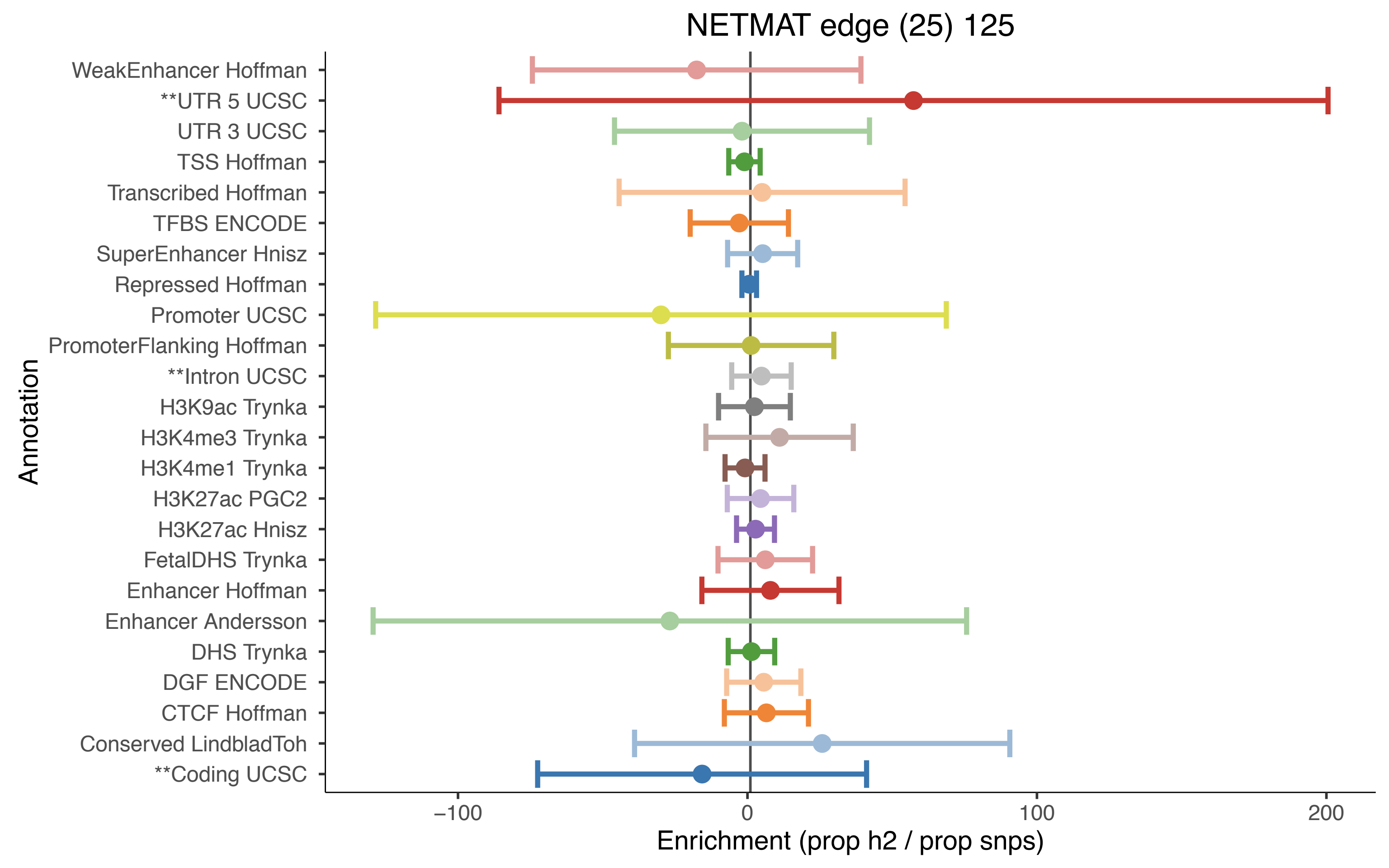

NETMAT edge (25) 125
WeakEnhancer Hoffman
**UTR 5 UCSC
UTR 3 UCSC
TSS Hoffman
Transcribed Hoffman
TFBS ENCODE
SuperEnhancer Hnisz
Repressed Hoffman
Promoter UCSC
PromoterFlanking Hoffman
**Intron UCSC
H3K9ac Trynka
H3K4me3 Trynka
H3K4me1 Trynka
H3K27ac PGC2
H3K27ac Hnisz
FetalDHS Trynka
Enhancer Hoffman
Enhancer Andersson
DHS Trynka
DGF ENCODE
CTCF Hoffman
Conserved LindbladToh
**Coding UCSC
Annotation
−100
0
100
200
Enrichment (prop h2 / prop snps)

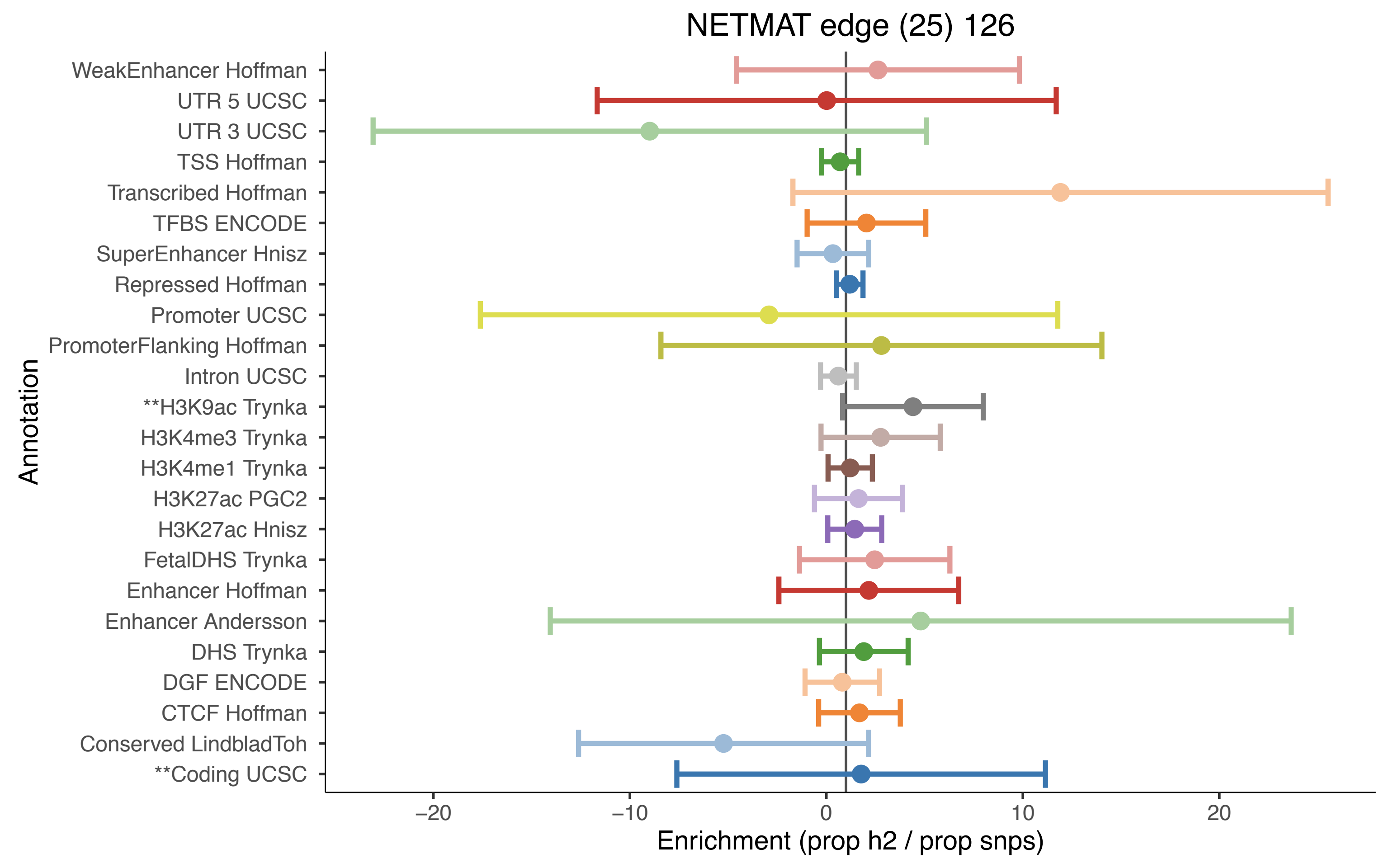
NETMAT edge (25) 126
WeakEnhancer Hoffman
UTR 5 UCSC
UTR 3 UCSC
TSS Hoffman
Transcribed Hoffman
TFBS ENCODE
SuperEnhancer Hnisz
Repressed Hoffman
Promoter UCSC
PromoterFlanking Hoffman
Intron UCSC
**H3K9ac Trynka
H3K4me3 Trynka
H3K4me1 Trynka
H3K27ac PGC2
H3K27ac Hnisz
FetalDHS Trynka
Enhancer Hoffman
Enhancer Andersson
DHS Trynka
DGF ENCODE
CTCF Hoffman
Conserved LindbladToh
**Coding UCSC
Annotation
−20
−10
0
10
20
Enrichment (prop h2 / prop snps)

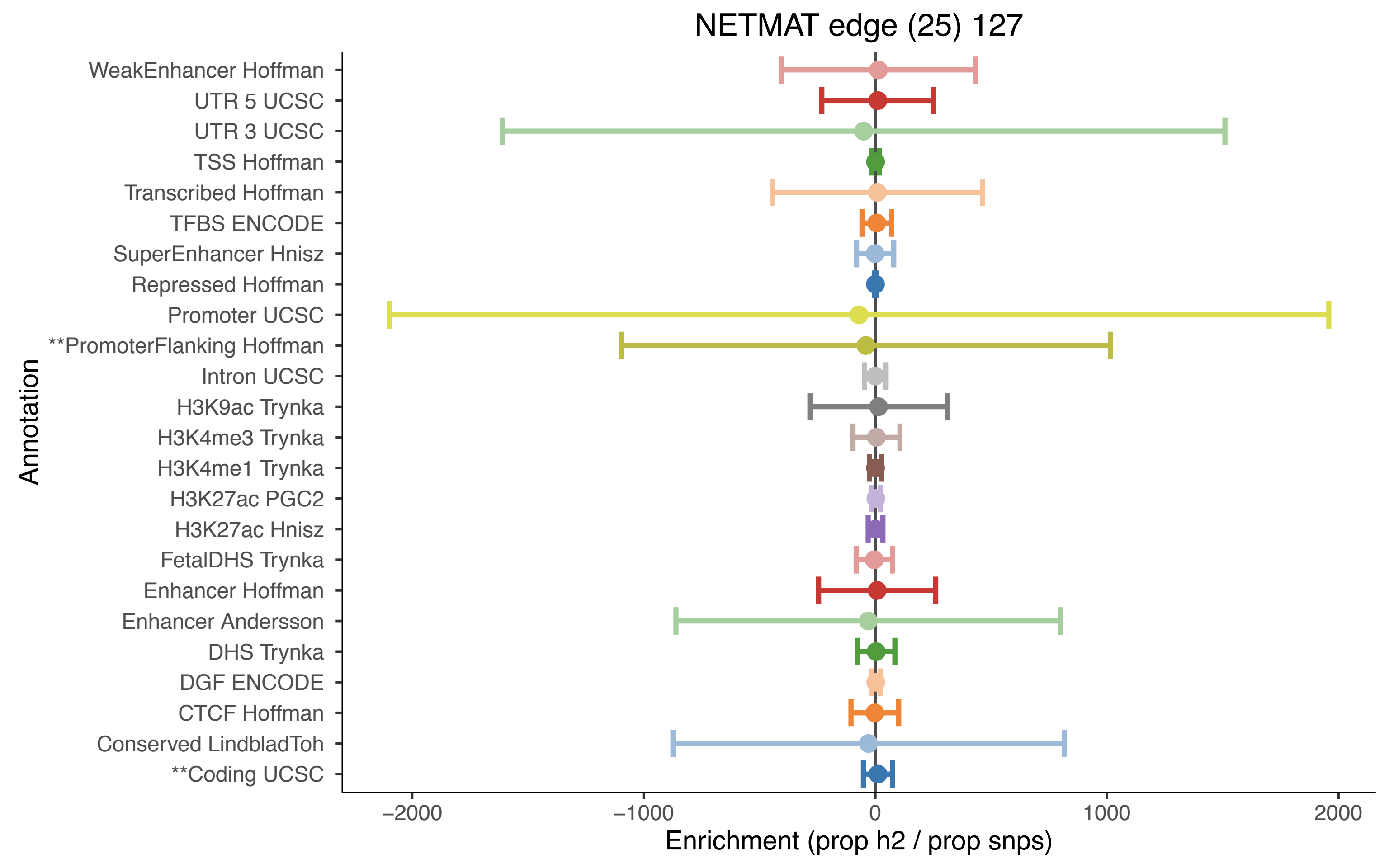

NETMAT edge (25) 127
WeakEnhancer Hoffman
UTR 5 UCSC
UTR 3 UCSC
TSS Hoffman
Transcribed Hoffman
TFBS ENCODE
SuperEnhancer Hnisz
Repressed Hoffman
Promoter UCSC
**PromoterFlanking Hoffman
Intron UCSC
H3K9ac Trynka
H3K4me3 Trynka
H3K4me1 Trynka
H3K27ac PGC2
H3K27ac Hnisz
FetalDHS Trynka
Enhancer Hoffman
Enhancer Andersson
DHS Trynka
DGF ENCODE
CTCF Hoffman
Conserved LindbladToh
**Coding UCSC
Annotation
−2000
−1000
0
1000
2000
Enrichment (prop h2 / prop snps)

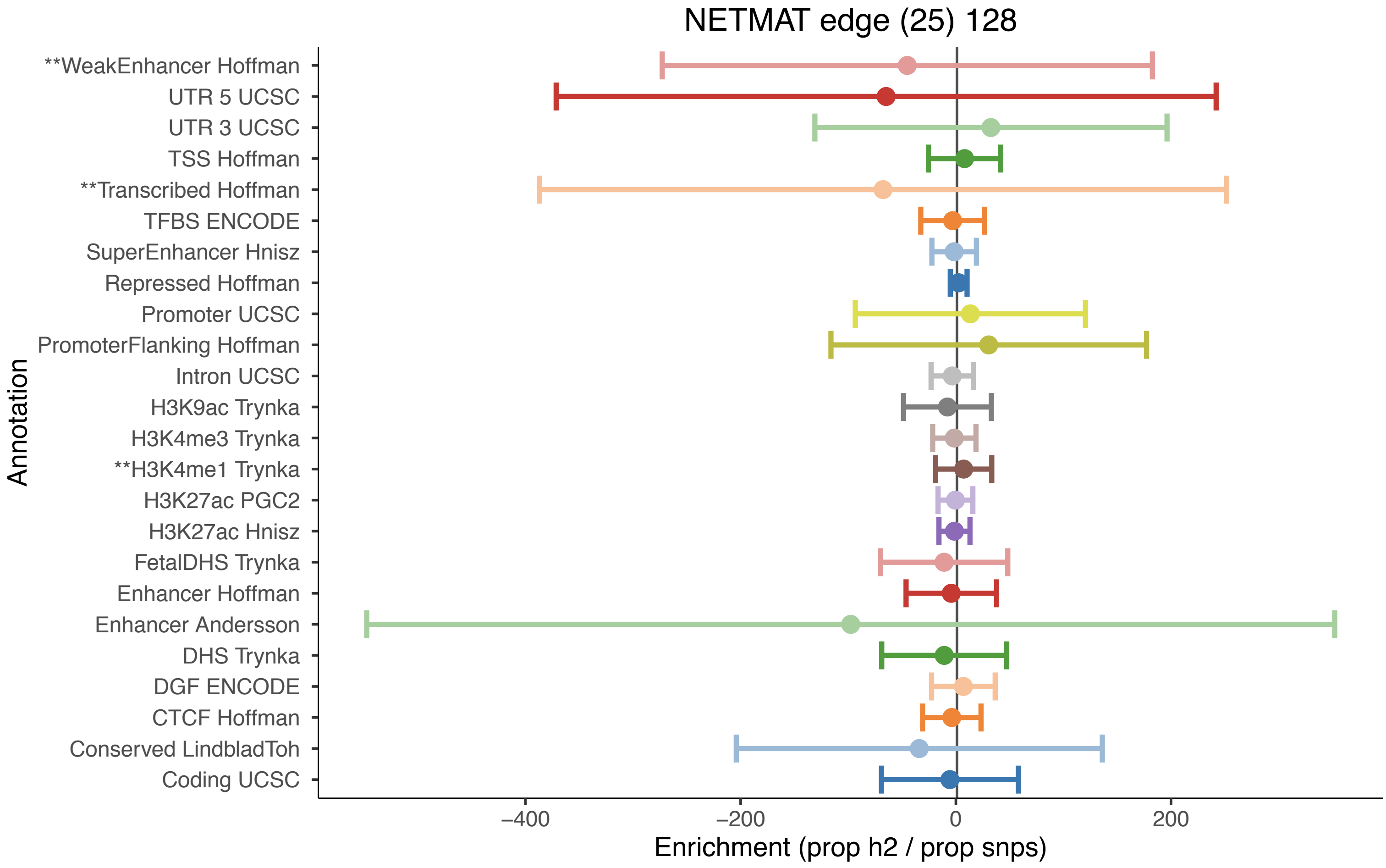

NETMAT edge (25) 128
Annotation
**WeakEnhancer Hoffman
UTR 5 UCSC
UTR 3 UCSC
TSS Hoffman
**Transcribed Hoffman
TFBS ENCODE
SuperEnhancer Hnisz
Repressed Hoffman
Promoter UCSC
PromoterFlanking Hoffman
Intron UCSC
H3K9ac Trynka
H3K4me3 Trynka
**H3K4me1 Trynka
H3K27ac PGC2
H3K27ac Hnisz
FetalDHS Trynka
Enhancer Hoffman
Enhancer Andersson
DHS Trynka
DGF ENCODE
CTCF Hoffman
Conserved LindbladToh
Coding UCSC
−400
−200
0
200
Enrichment (prop h2 / prop snps)

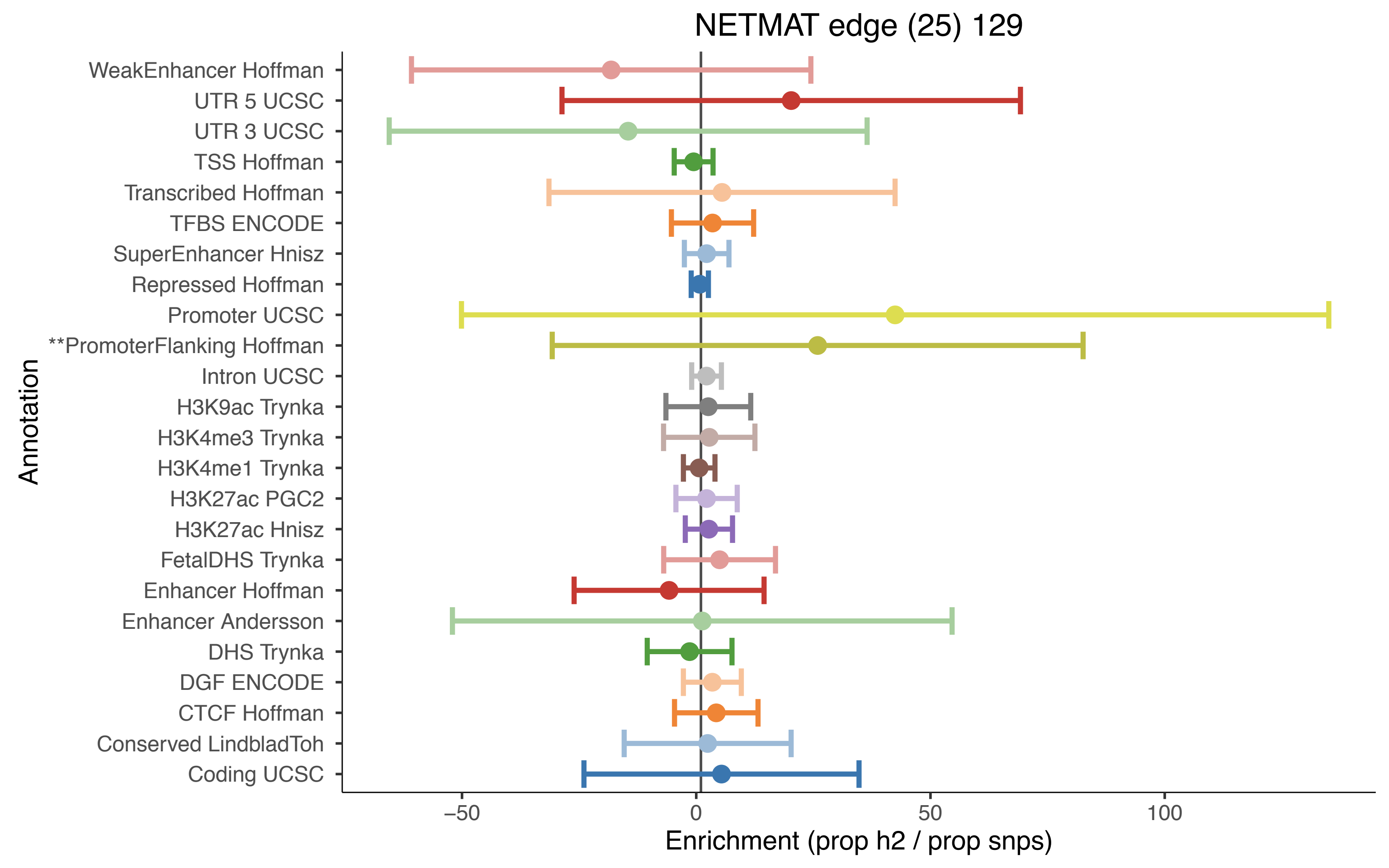

NETMAT edge (25) 129
WeakEnhancer Hoffman
UTR 5 UCSC
UTR 3 UCSC
TSS Hoffman
Transcribed Hoffman
TFBS ENCODE
SuperEnhancer Hnisz
Repressed Hoffman
Promoter UCSC
**PromoterFlanking Hoffman
Intron UCSC
H3K9ac Trynka
H3K4me3 Trynka
H3K4me1 Trynka
H3K27ac PGC2
H3K27ac Hnisz
FetalDHS Trynka
Enhancer Hoffman
Enhancer Andersson
DHS Trynka
DGF ENCODE
CTCF Hoffman
Conserved LindbladToh
Coding UCSC
Annotation
−50
0
50
100
Enrichment (prop h2 / prop snps)

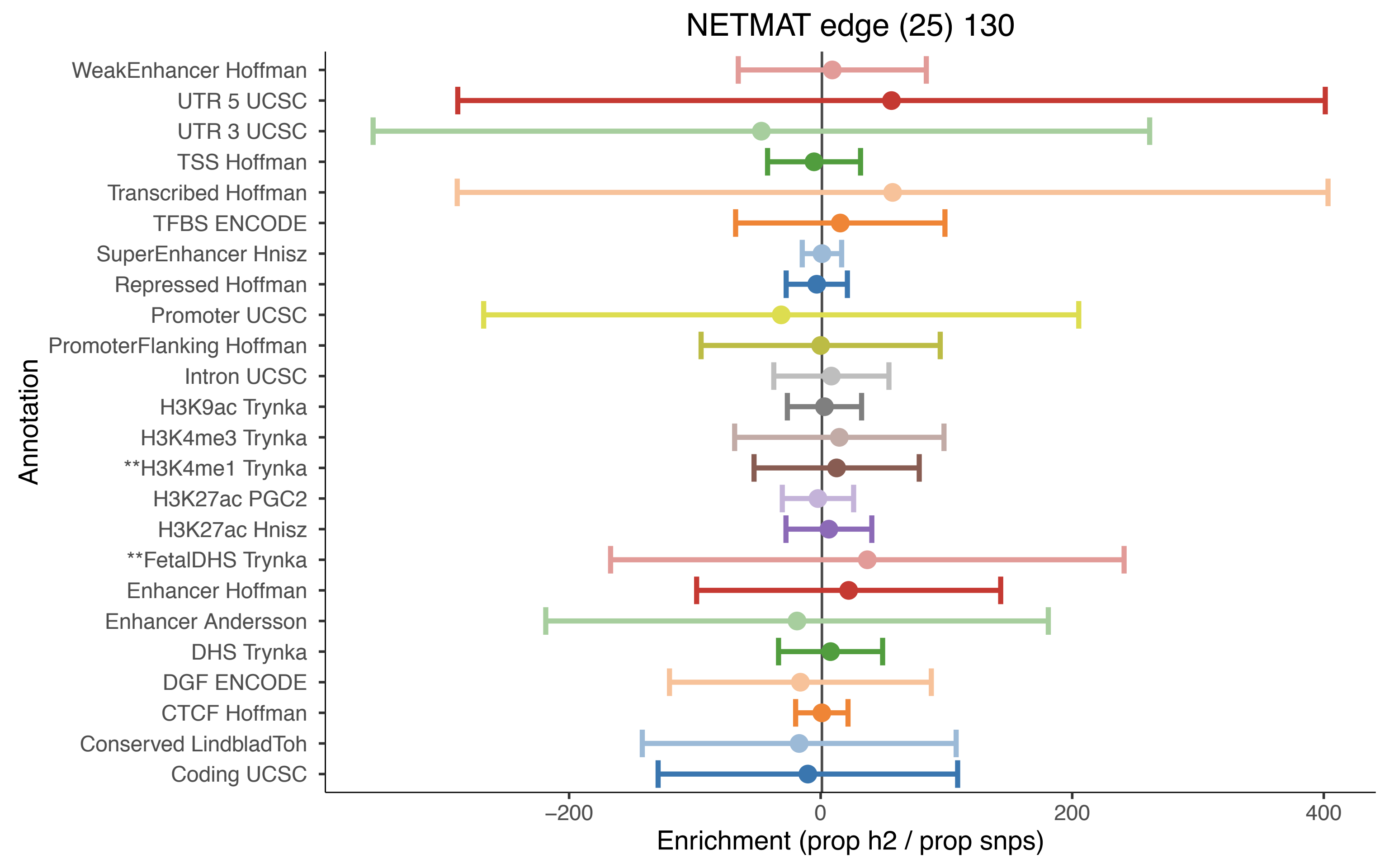

NETMAT edge (25) 130
WeakEnhancer Hoffman
UTR 5 UCSC
UTR 3 UCSC
TSS Hoffman
Transcribed Hoffman
TFBS ENCODE
SuperEnhancer Hnisz
Repressed Hoffman
Promoter UCSC
PromoterFlanking Hoffman
Intron UCSC
H3K9ac Trynka
H3K4me3 Trynka
**H3K4me1 Trynka
H3K27ac PGC2
H3K27ac Hnisz
**FetalDHS Trynka
Enhancer Hoffman
Enhancer Andersson
DHS Trynka
DGF ENCODE
CTCF Hoffman
Conserved LindbladToh
Coding UCSC
Annotation
−200
0
200
400
Enrichment (prop h2 / prop snps)

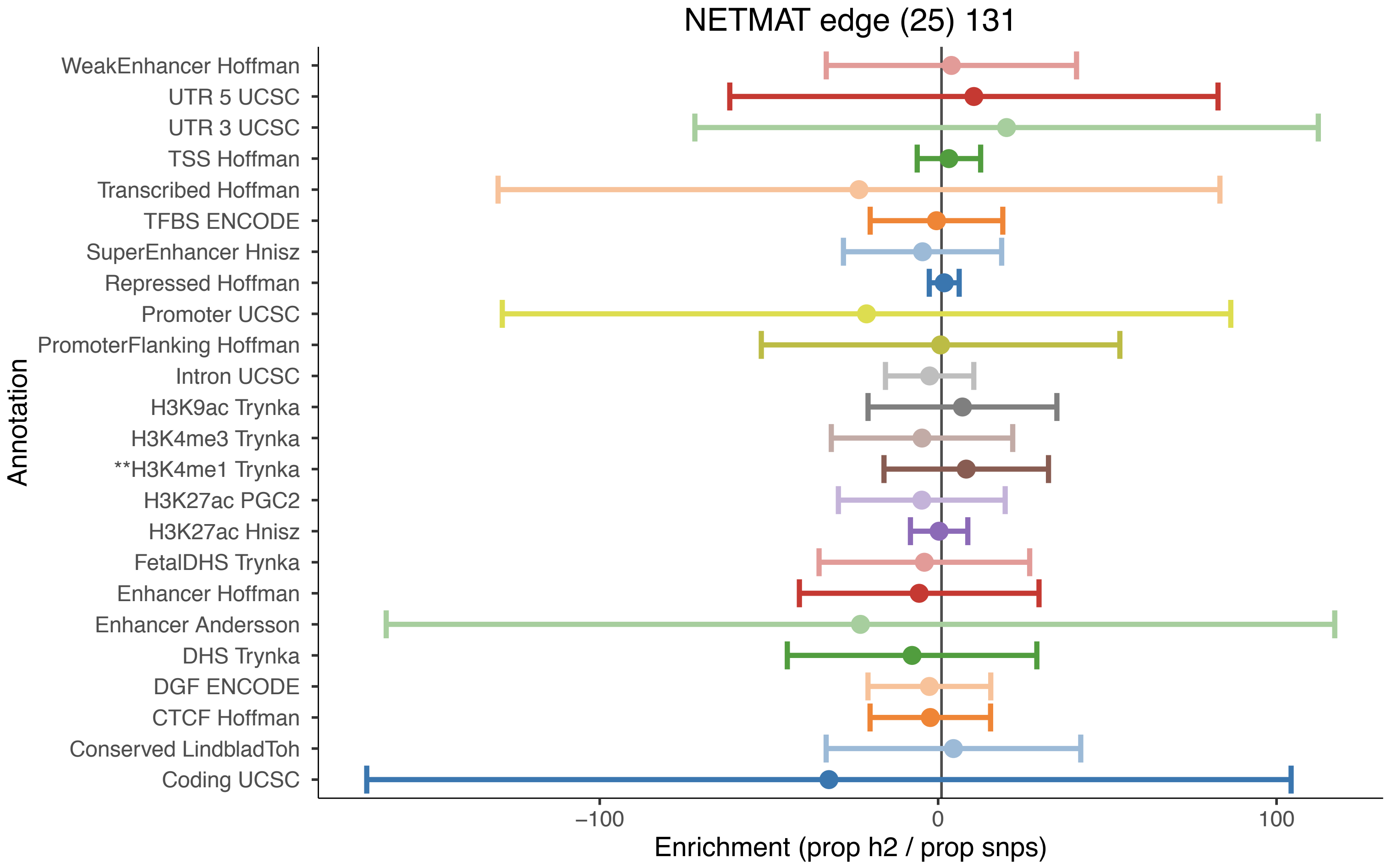
NETMAT edge (25) 131
Annotation
WeakEnhancer Hoffman
UTR 5 UCSC
UTR 3 UCSC
TSS Hoffman
Transcribed Hoffman
TFBS ENCODE
SuperEnhancer Hnisz
Repressed Hoffman
Promoter UCSC
PromoterFlanking Hoffman
Intron UCSC
H3K9ac Trynka
H3K4me3 Trynka
**H3K4me1 Trynka
H3K27ac PGC2
H3K27ac Hnisz
FetalDHS Trynka
Enhancer Hoffman
Enhancer Andersson
DHS Trynka
DGF ENCODE
CTCF Hoffman
Conserved LindbladToh
Coding UCSC
−100
0
100
Enrichment (prop h2 / prop snps)

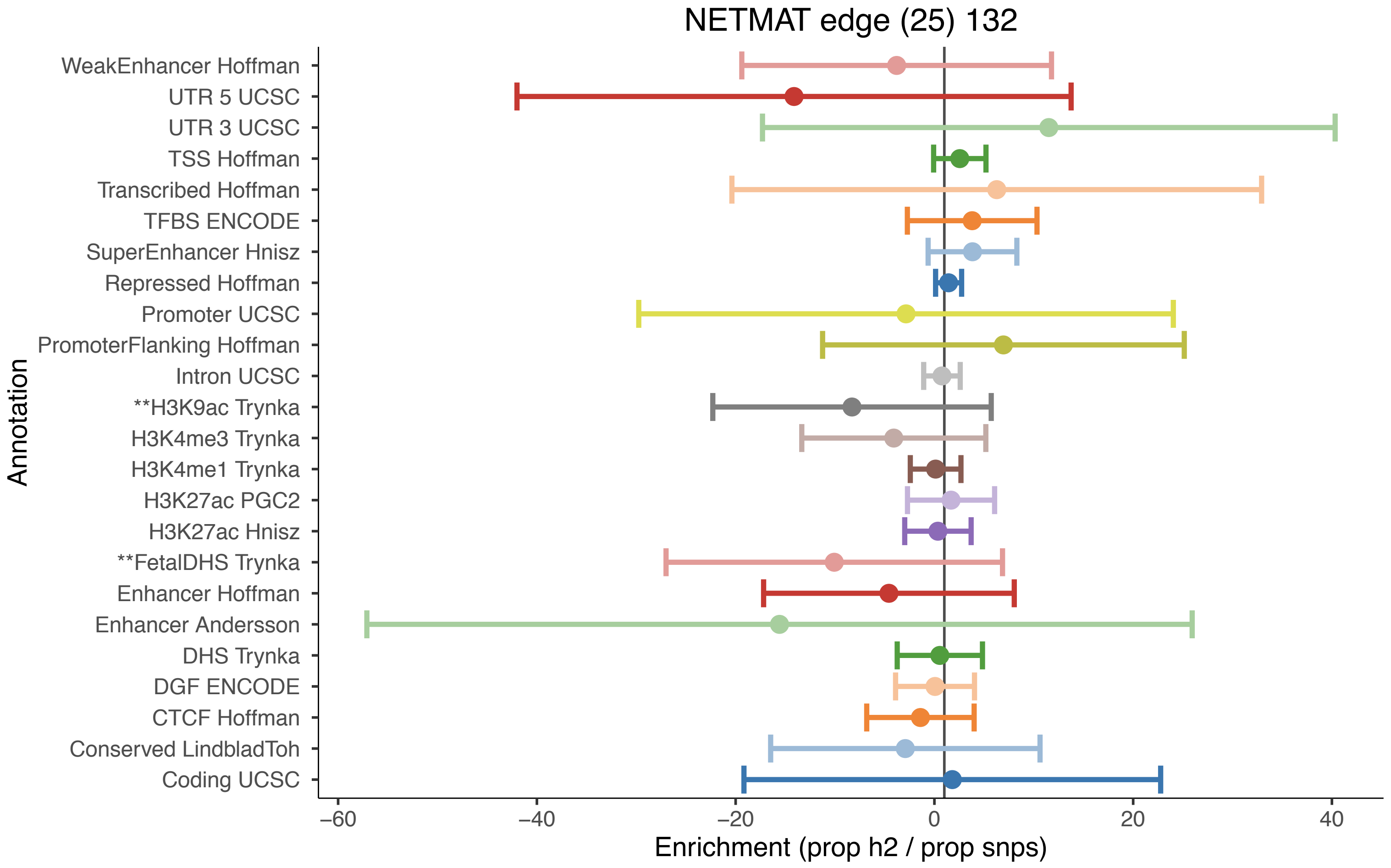
NETMAT edge (25) 132
WeakEnhancer Hoffman
UTR 5 UCSC
UTR 3 UCSC
TSS Hoffman
Transcribed Hoffman
TFBS ENCODE
SuperEnhancer Hnisz
Repressed Hoffman
Promoter UCSC
PromoterFlanking Hoffman
Intron UCSC
**H3K9ac Trynka
H3K4me3 Trynka
H3K4me1 Trynka
H3K27ac PGC2
H3K27ac Hnisz
**FetalDHS Trynka
Enhancer Hoffman
Enhancer Andersson
DHS Trynka
DGF ENCODE
CTCF Hoffman
Conserved LindbladToh
Coding UCSC
Annotation
−60
−40
−20
0
20
40
Enrichment (prop h2 / prop snps)

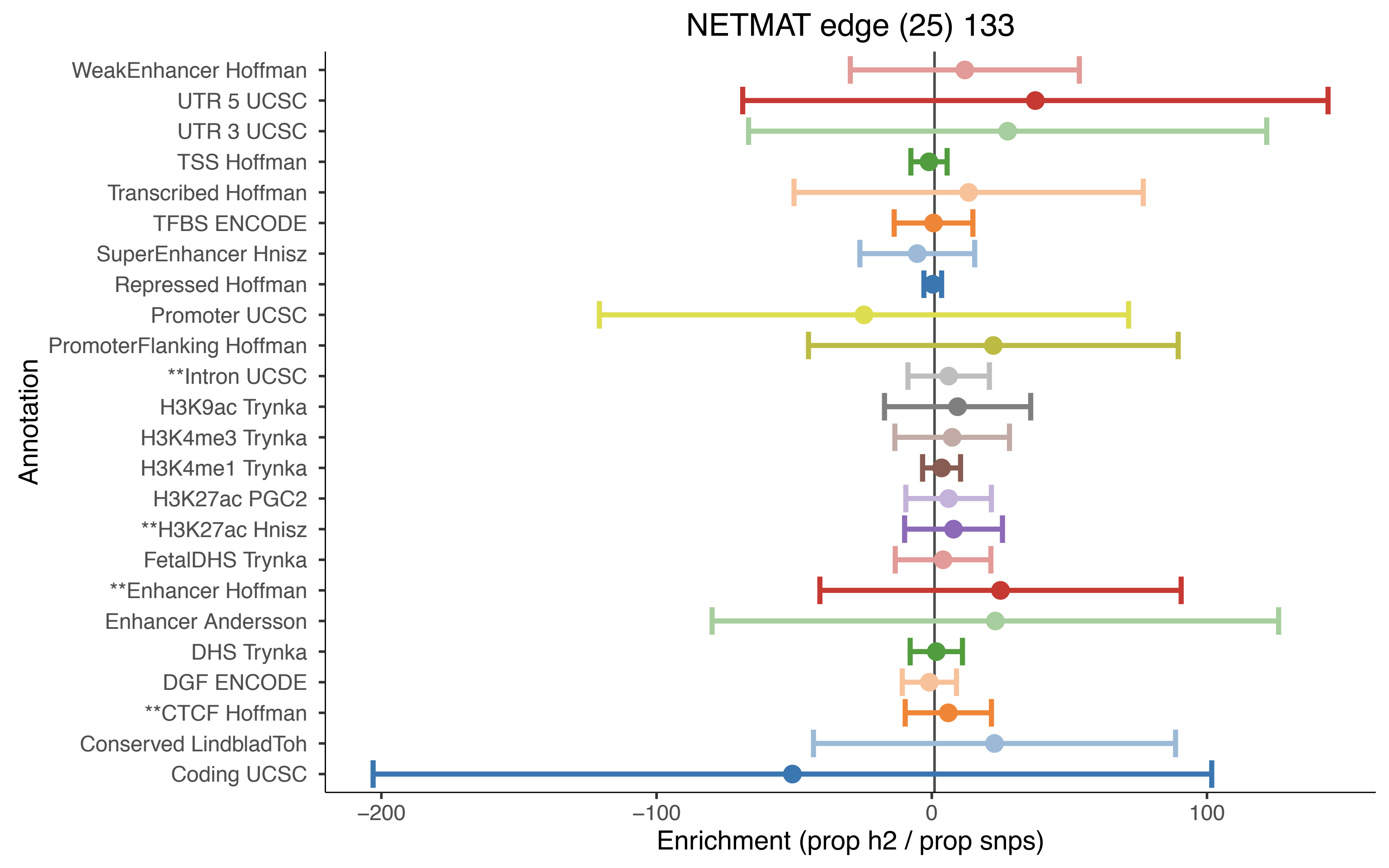

NETMAT edge (25) 133
WeakEnhancer Hoffman
UTR 5 UCSC
UTR 3 UCSC
TSS Hoffman
Transcribed Hoffman
TFBS ENCODE
SuperEnhancer Hnisz
Repressed Hoffman
Promoter UCSC
PromoterFlanking Hoffman
**Intron UCSC
H3K9ac Trynka
H3K4me3 Trynka
H3K4me1 Trynka
H3K27ac PGC2
**H3K27ac Hnisz
FetalDHS Trynka
**Enhancer Hoffman
Enhancer Andersson
DHS Trynka
DGF ENCODE
**CTCF Hoffman
Conserved LindbladToh
Coding UCSC
Annotation
−200
−100
0
100
Enrichment (prop h2 / prop snps)

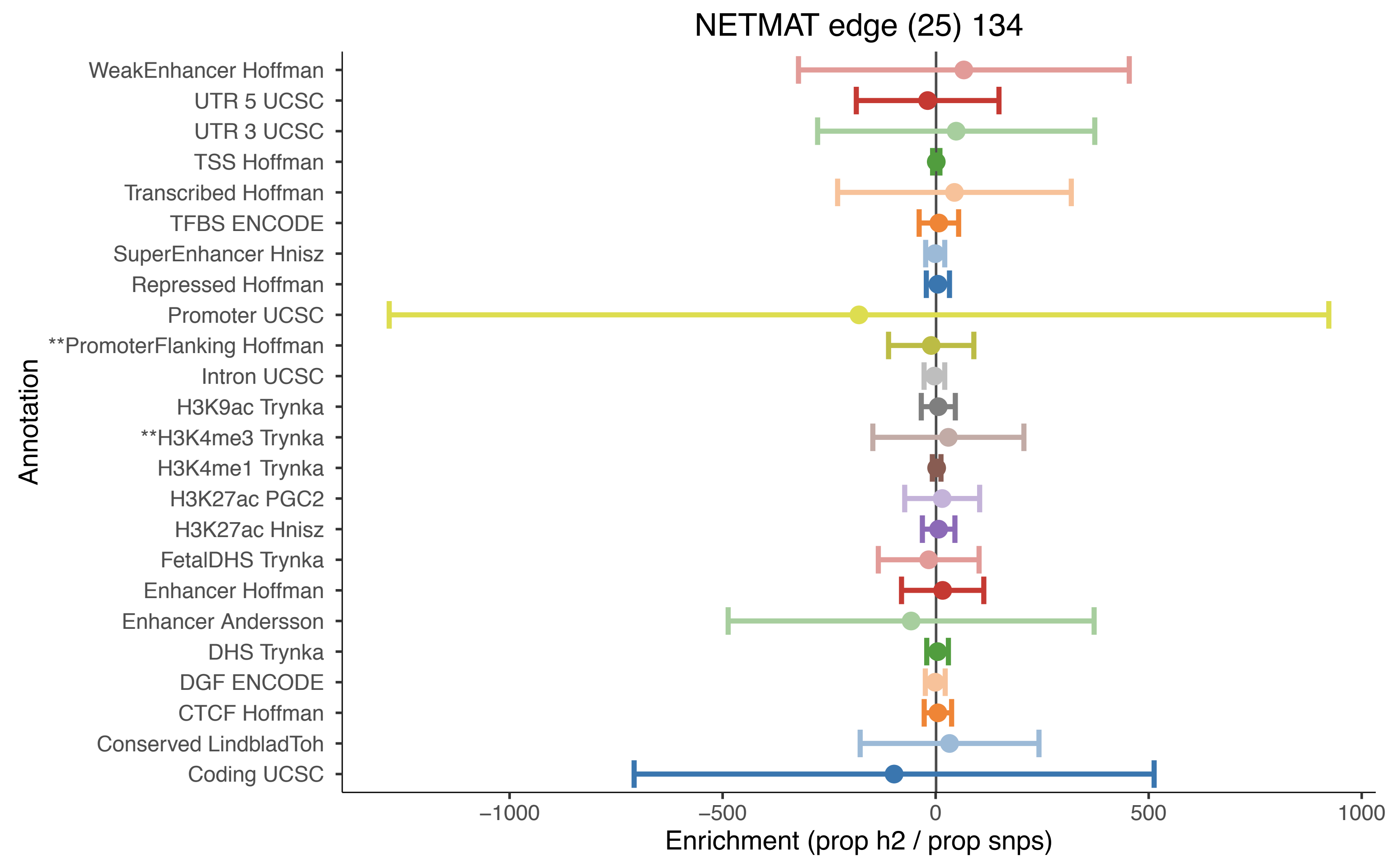

NETMAT edge (25) 134
WeakEnhancer Hoffman
UTR 5 UCSC
UTR 3 UCSC
TSS Hoffman
Transcribed Hoffman
TFBS ENCODE
SuperEnhancer Hnisz
Repressed Hoffman
Promoter UCSC
**PromoterFlanking Hoffman
Intron UCSC
H3K9ac Trynka
**H3K4me3 Trynka
H3K4me1 Trynka
H3K27ac PGC2
H3K27ac Hnisz
FetalDHS Trynka
Enhancer Hoffman
Enhancer Andersson
DHS Trynka
DGF ENCODE
CTCF Hoffman
Conserved LindbladToh
Coding UCSC
Annotation
−1000
−500
0
500
1000
Enrichment (prop h2 / prop snps)

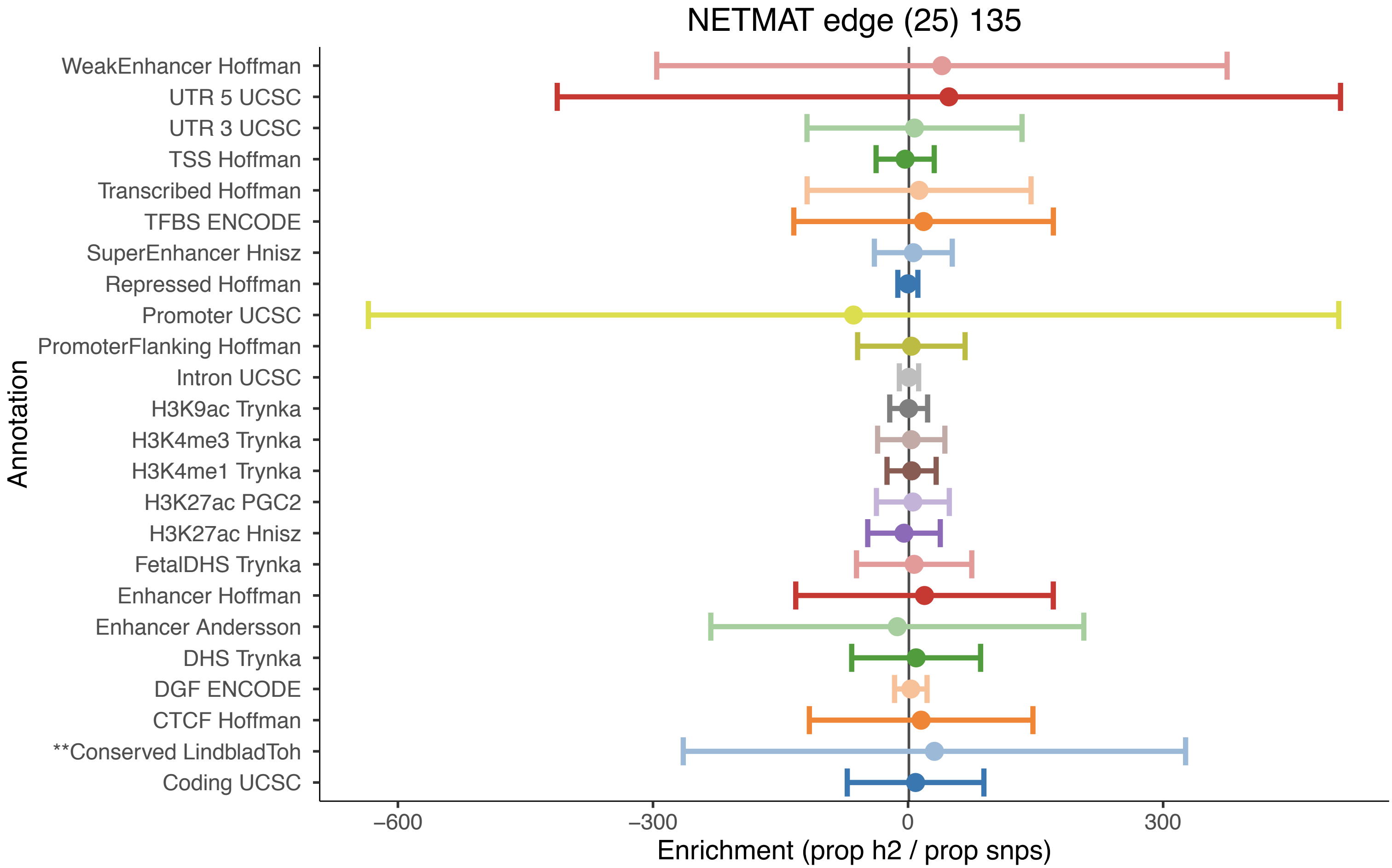

NETMAT edge (25) 135
WeakEnhancer Hoffman
UTR 5 UCSC
UTR 3 UCSC
TSS Hoffman
Transcribed Hoffman
TFBS ENCODE
SuperEnhancer Hnisz
Repressed Hoffman
Promoter UCSC
PromoterFlanking Hoffman
Intron UCSC
H3K9ac Trynka
H3K4me3 Trynka
H3K4me1 Trynka
H3K27ac PGC2
H3K27ac Hnisz
FetalDHS Trynka
Enhancer Hoffman
Enhancer Andersson
DHS Trynka
DGF ENCODE
CTCF Hoffman
**Conserved LindbladToh
Coding UCSC
Annotation
−600
−300
0
300
Enrichment (prop h2 / prop snps)

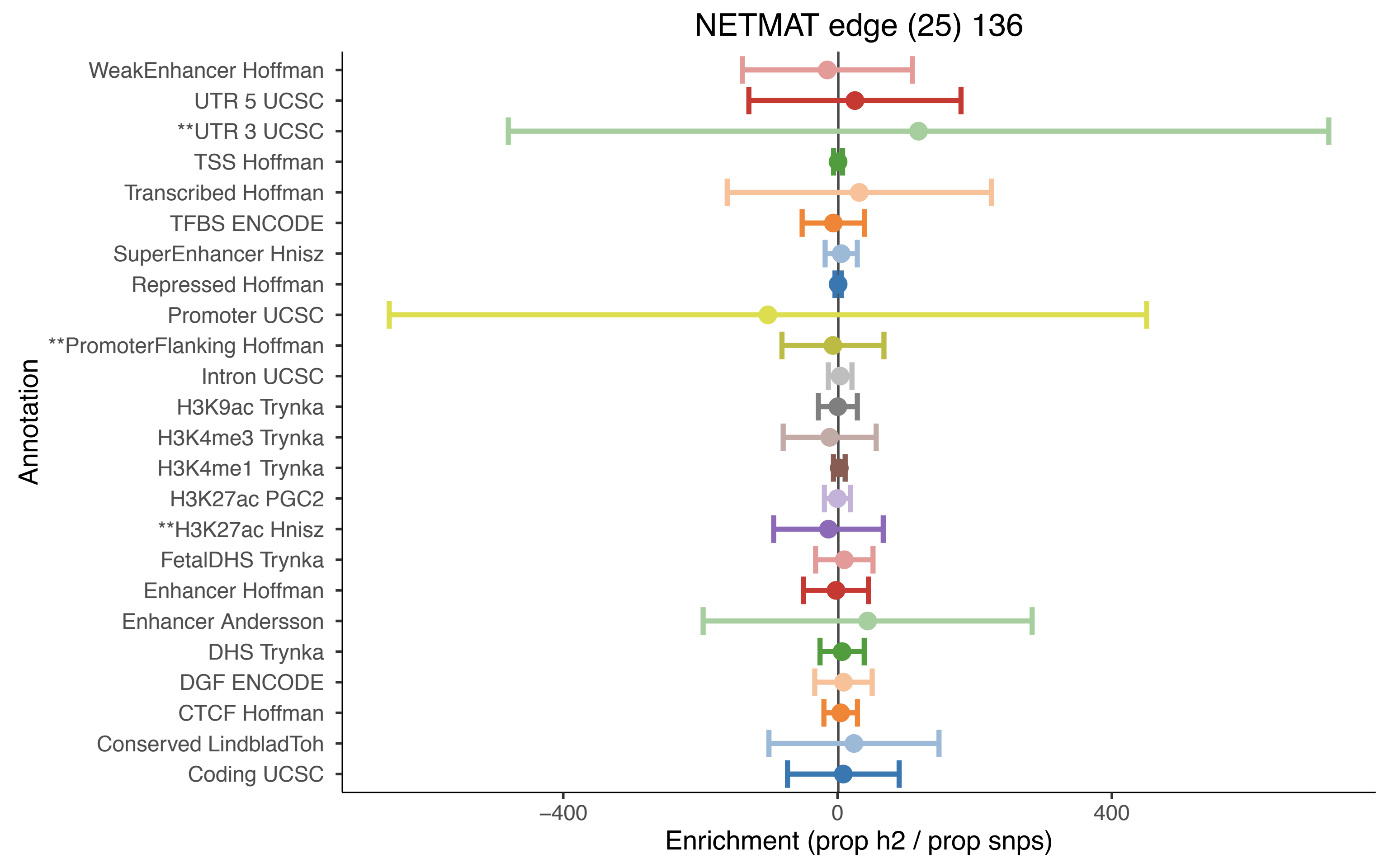
NETMAT edge (25) 136
WeakEnhancer Hoffman
UTR 5 UCSC
**UTR 3 UCSC
TSS Hoffman
Transcribed Hoffman
TFBS ENCODE
SuperEnhancer Hnisz
Repressed Hoffman
Promoter UCSC
**PromoterFlanking Hoffman
Intron UCSC
H3K9ac Trynka
H3K4me3 Trynka
H3K4me1 Trynka
H3K27ac PGC2
**H3K27ac Hnisz
FetalDHS Trynka
Enhancer Hoffman
Enhancer Andersson
DHS Trynka
DGF ENCODE
CTCF Hoffman
Conserved LindbladToh
Coding UCSC
Annotation
−400
0
400
Enrichment (prop h2 / prop snps)

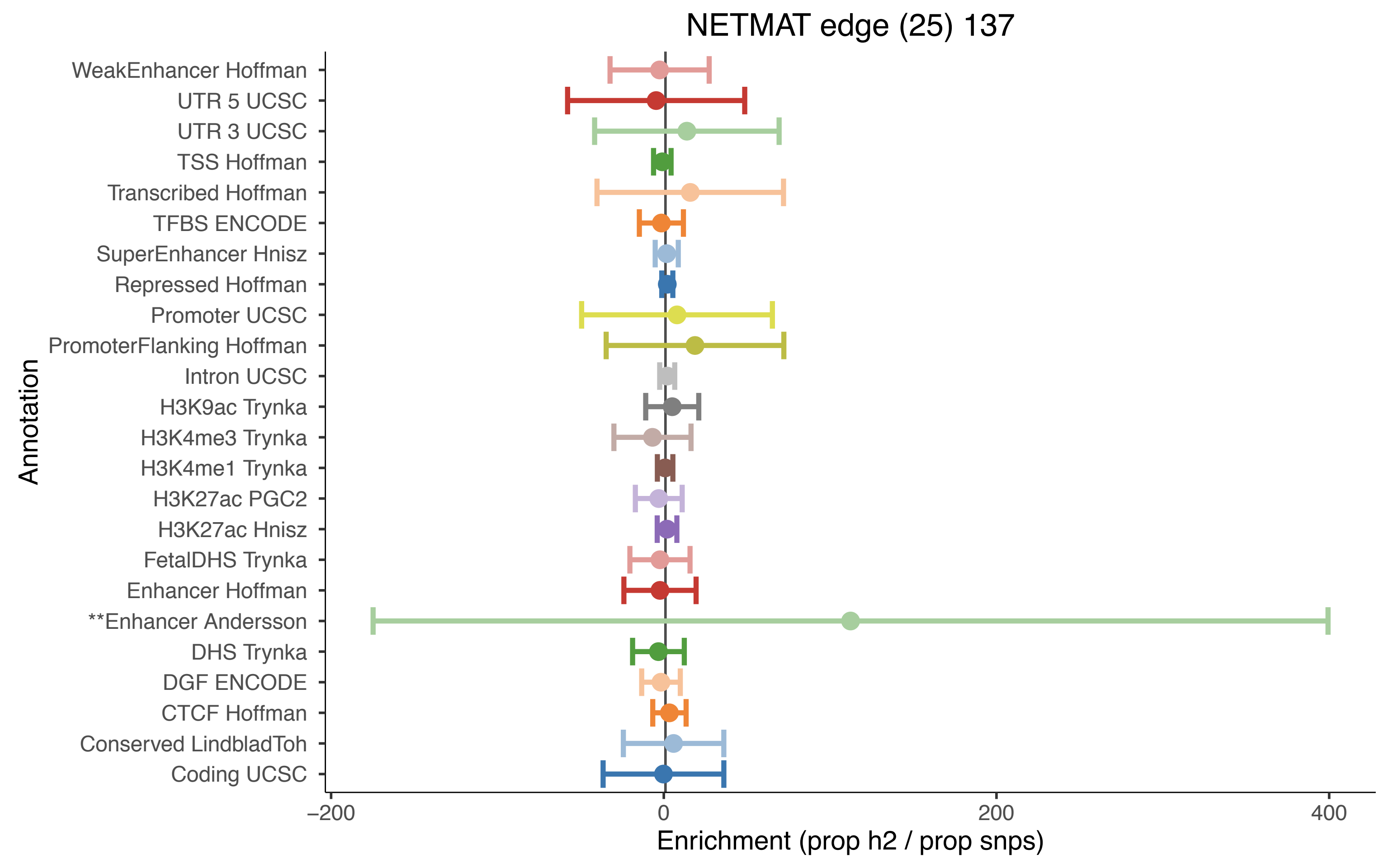

NETMAT edge (25) 137
Annotation
WeakEnhancer Hoffman
UTR 5 UCSC
UTR 3 UCSC
TSS Hoffman
Transcribed Hoffman
TFBS ENCODE
SuperEnhancer Hnisz
Repressed Hoffman
Promoter UCSC
PromoterFlanking Hoffman
Intron UCSC
H3K9ac Trynka
H3K4me3 Trynka
H3K4me1 Trynka
H3K27ac PGC2
H3K27ac Hnisz
FetalDHS Trynka
Enhancer Hoffman
**Enhancer Andersson
DHS Trynka
DGF ENCODE
CTCF Hoffman
Conserved LindbladToh
Coding UCSC
−200
0
200
400
Enrichment (prop h2 / prop snps)

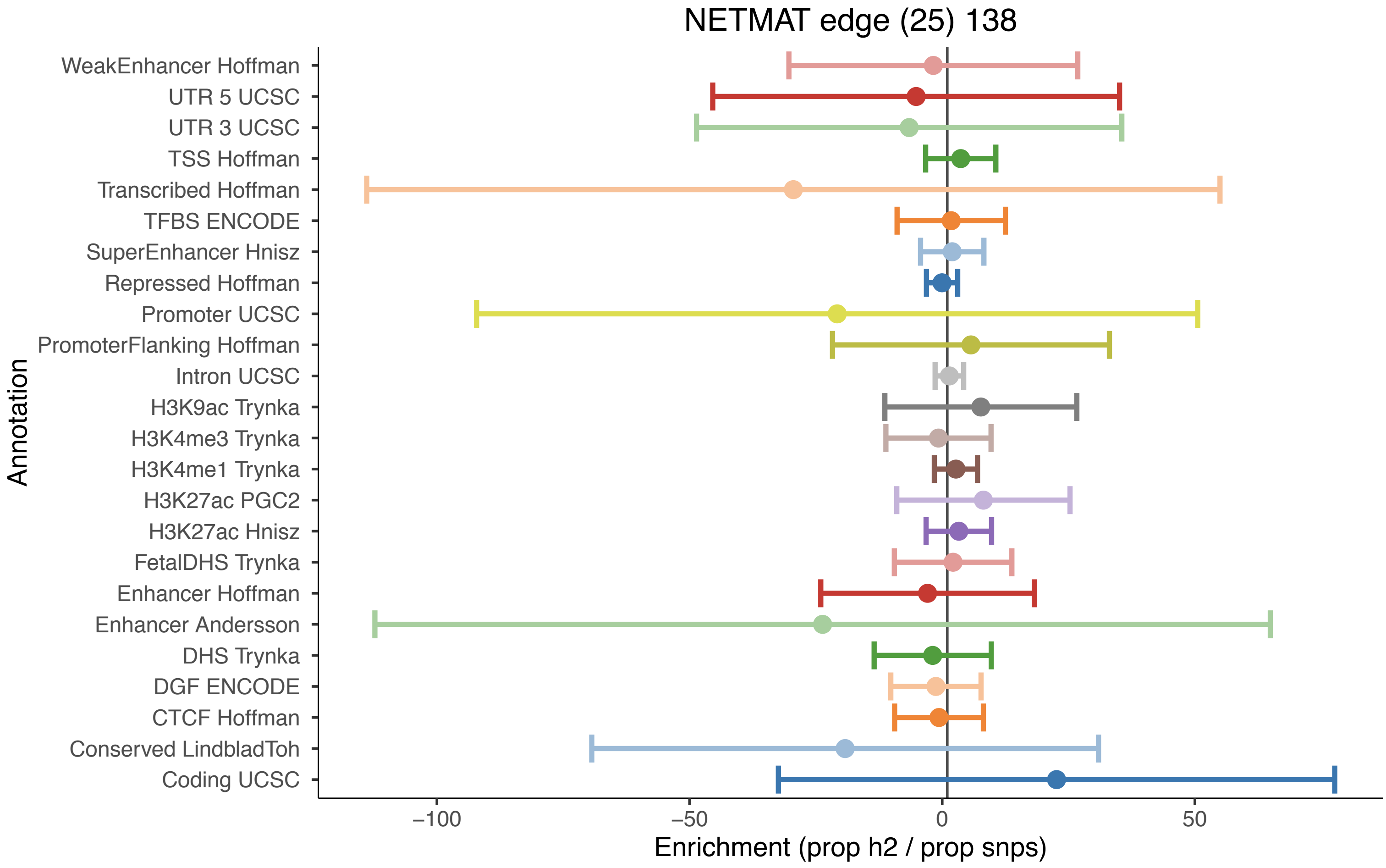

NETMAT edge (25) 138
WeakEnhancer Hoffman
UTR 5 UCSC
UTR 3 UCSC
TSS Hoffman
Transcribed Hoffman
TFBS ENCODE
SuperEnhancer Hnisz
Repressed Hoffman
Promoter UCSC
PromoterFlanking Hoffman
Intron UCSC
H3K9ac Trynka
H3K4me3 Trynka
H3K4me1 Trynka
H3K27ac PGC2
H3K27ac Hnisz
FetalDHS Trynka
Enhancer Hoffman
Enhancer Andersson
DHS Trynka
DGF ENCODE
CTCF Hoffman
Conserved LindbladToh
Coding UCSC
Annotation
−100
−50
0
50
Enrichment (prop h2 / prop snps)

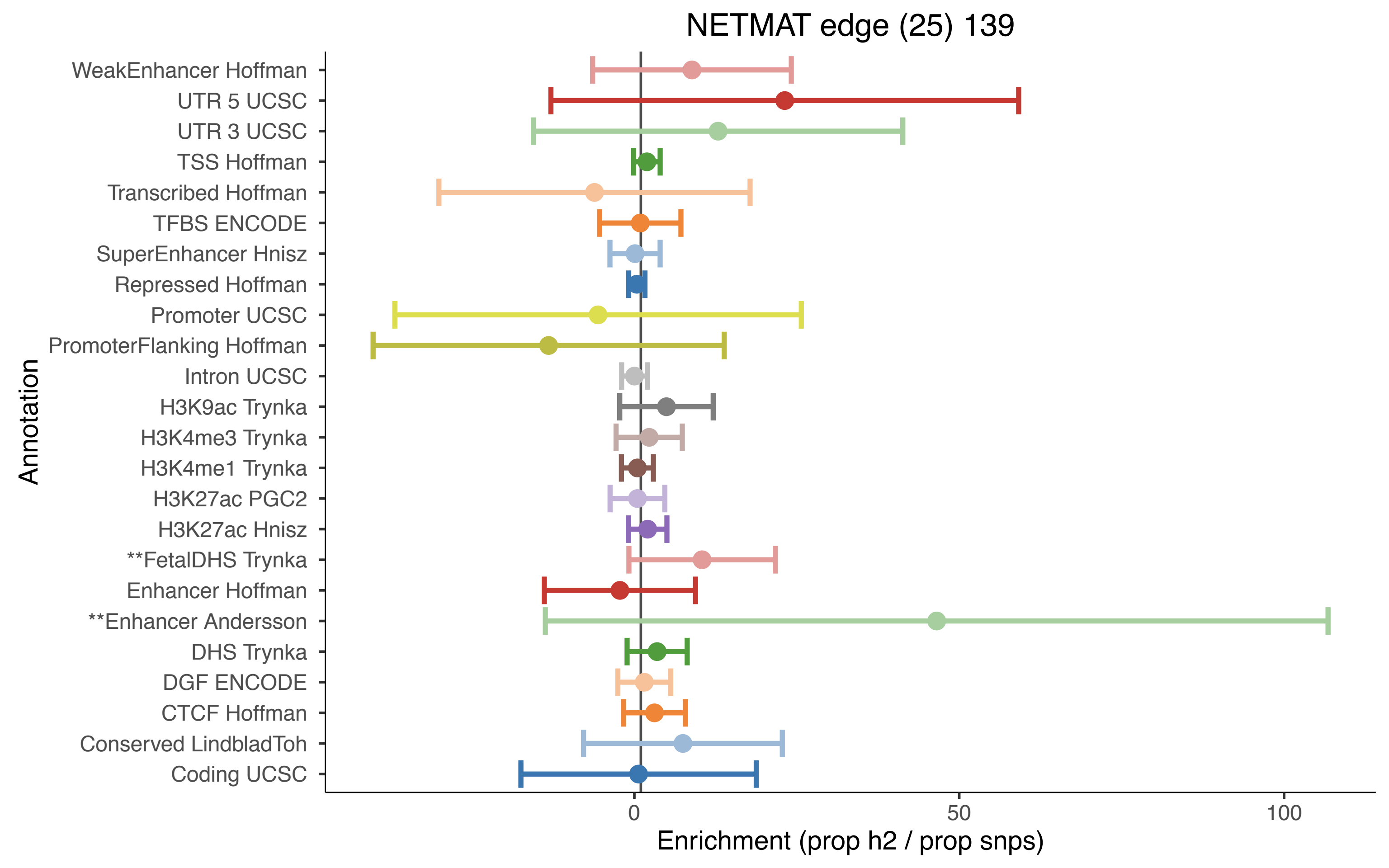

NETMAT edge (25) 139
WeakEnhancer Hoffman
UTR 5 UCSC
UTR 3 UCSC
TSS Hoffman
Transcribed Hoffman
TFBS ENCODE
SuperEnhancer Hnisz
Repressed Hoffman
Promoter UCSC
PromoterFlanking Hoffman
Intron UCSC
H3K9ac Trynka
H3K4me3 Trynka
H3K4me1 Trynka
H3K27ac PGC2
H3K27ac Hnisz
**FetalDHS Trynka
Enhancer Hoffman
**Enhancer Andersson
DHS Trynka
DGF ENCODE
CTCF Hoffman
Conserved LindbladToh
Coding UCSC
Annotation
0
50
100
Enrichment (prop h2 / prop snps)

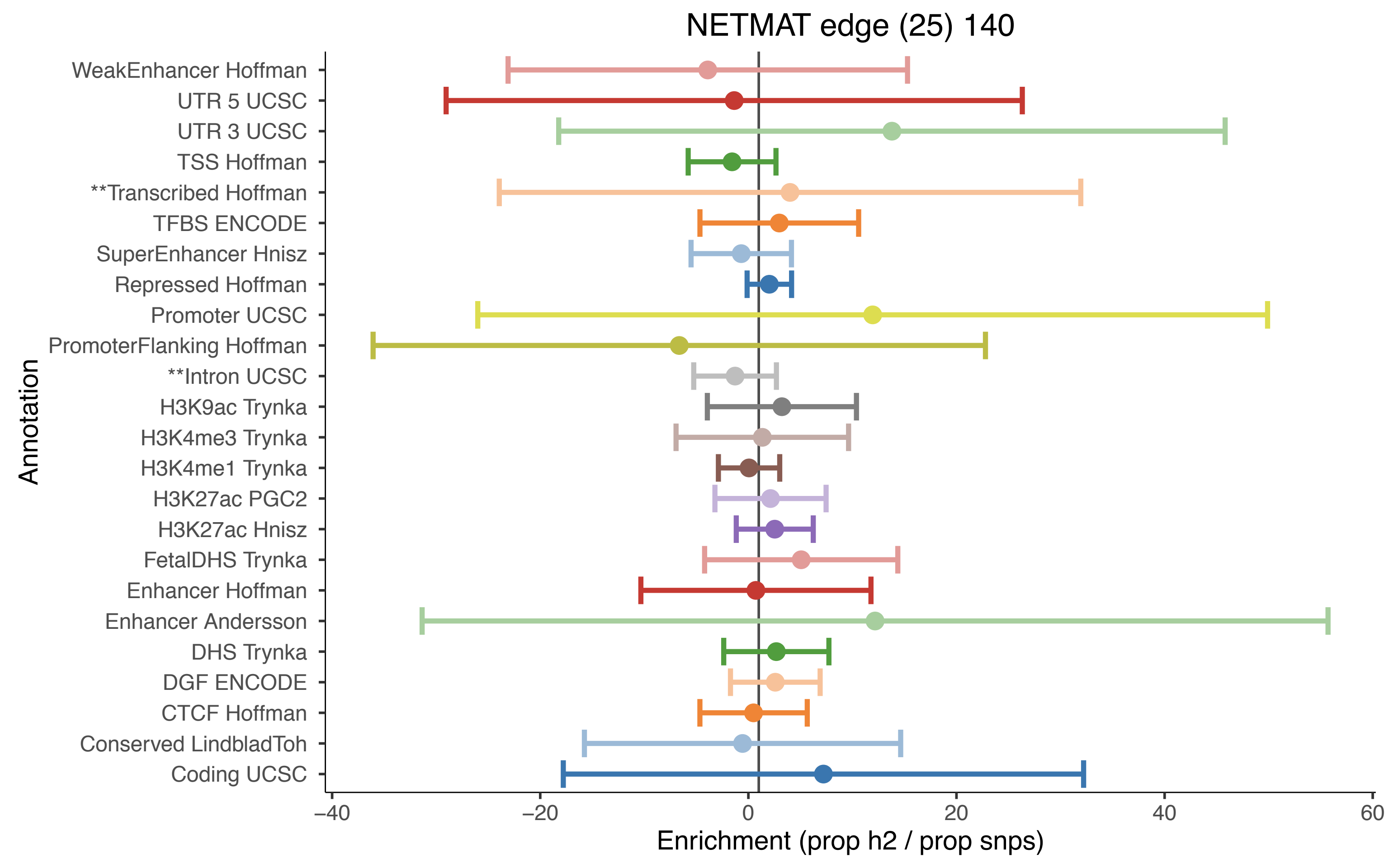

NETMAT edge (25) 140
WeakEnhancer Hoffman
UTR 5 UCSC
UTR 3 UCSC
TSS Hoffman
**Transcribed Hoffman
TFBS ENCODE
SuperEnhancer Hnisz
Repressed Hoffman
Promoter UCSC
PromoterFlanking Hoffman
**Intron UCSC
H3K9ac Trynka
H3K4me3 Trynka
H3K4me1 Trynka
H3K27ac PGC2
H3K27ac Hnisz
FetalDHS Trynka
Enhancer Hoffman
Enhancer Andersson
DHS Trynka
DGF ENCODE
CTCF Hoffman
Conserved LindbladToh
Coding UCSC
Annotation
−40
−20
0
20
40
60
Enrichment (prop h2 / prop snps)

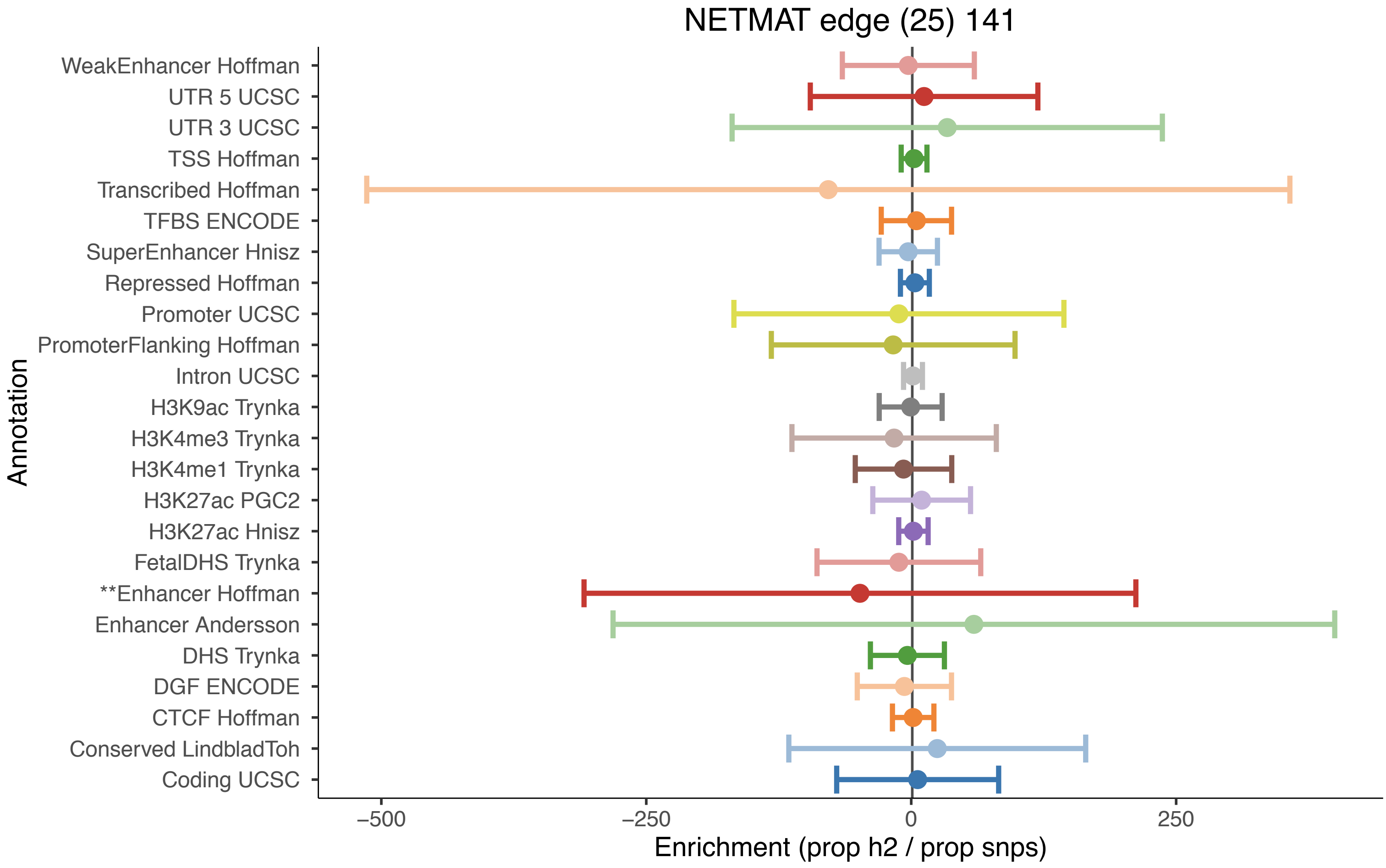

NETMAT edge (25) 141
WeakEnhancer Hoffman
UTR 5 UCSC
UTR 3 UCSC
TSS Hoffman
Transcribed Hoffman
TFBS ENCODE
SuperEnhancer Hnisz
Repressed Hoffman
Promoter UCSC
PromoterFlanking Hoffman
Intron UCSC
H3K9ac Trynka
H3K4me3 Trynka
H3K4me1 Trynka
H3K27ac PGC2
H3K27ac Hnisz
FetalDHS Trynka
**Enhancer Hoffman
Enhancer Andersson
DHS Trynka
DGF ENCODE
CTCF Hoffman
Conserved LindbladToh
Coding UCSC
Annotation
−500
−250
0
250
Enrichment (prop h2 / prop snps)

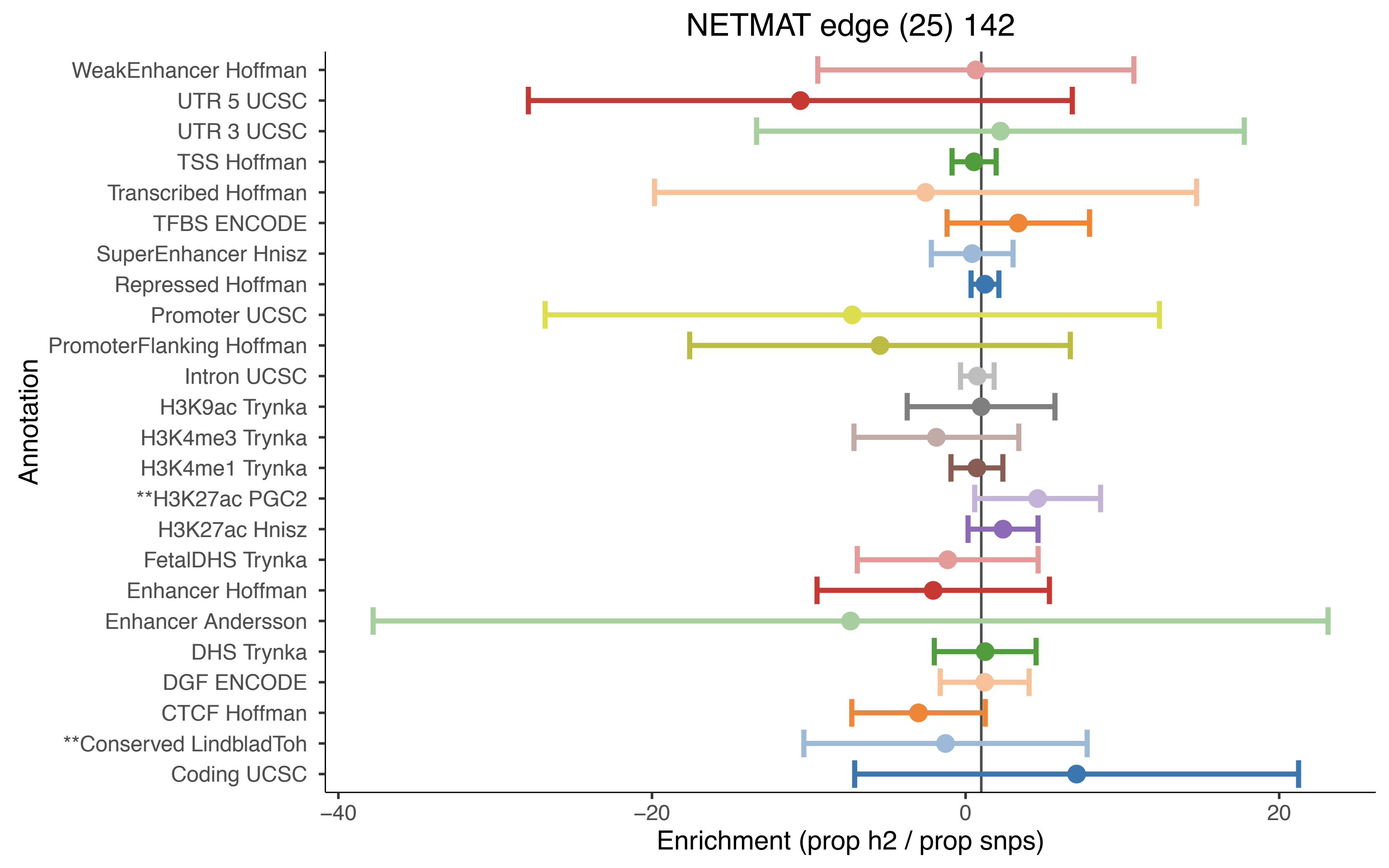

NETMAT edge (25) 142
Annotation
WeakEnhancer Hoffman
UTR 5 UCSC
UTR 3 UCSC
TSS Hoffman
Transcribed Hoffman
TFBS ENCODE
SuperEnhancer Hnisz
Repressed Hoffman
Promoter UCSC
PromoterFlanking Hoffman
Intron UCSC
H3K9ac Trynka
H3K4me3 Trynka
H3K4me1 Trynka
**H3K27ac PGC2
H3K27ac Hnisz
FetalDHS Trynka
Enhancer Hoffman
Enhancer Andersson
DHS Trynka
DGF ENCODE
CTCF Hoffman
**Conserved LindbladToh
Coding UCSC
−40
−20
0
20
Enrichment (prop h2 / prop snps)

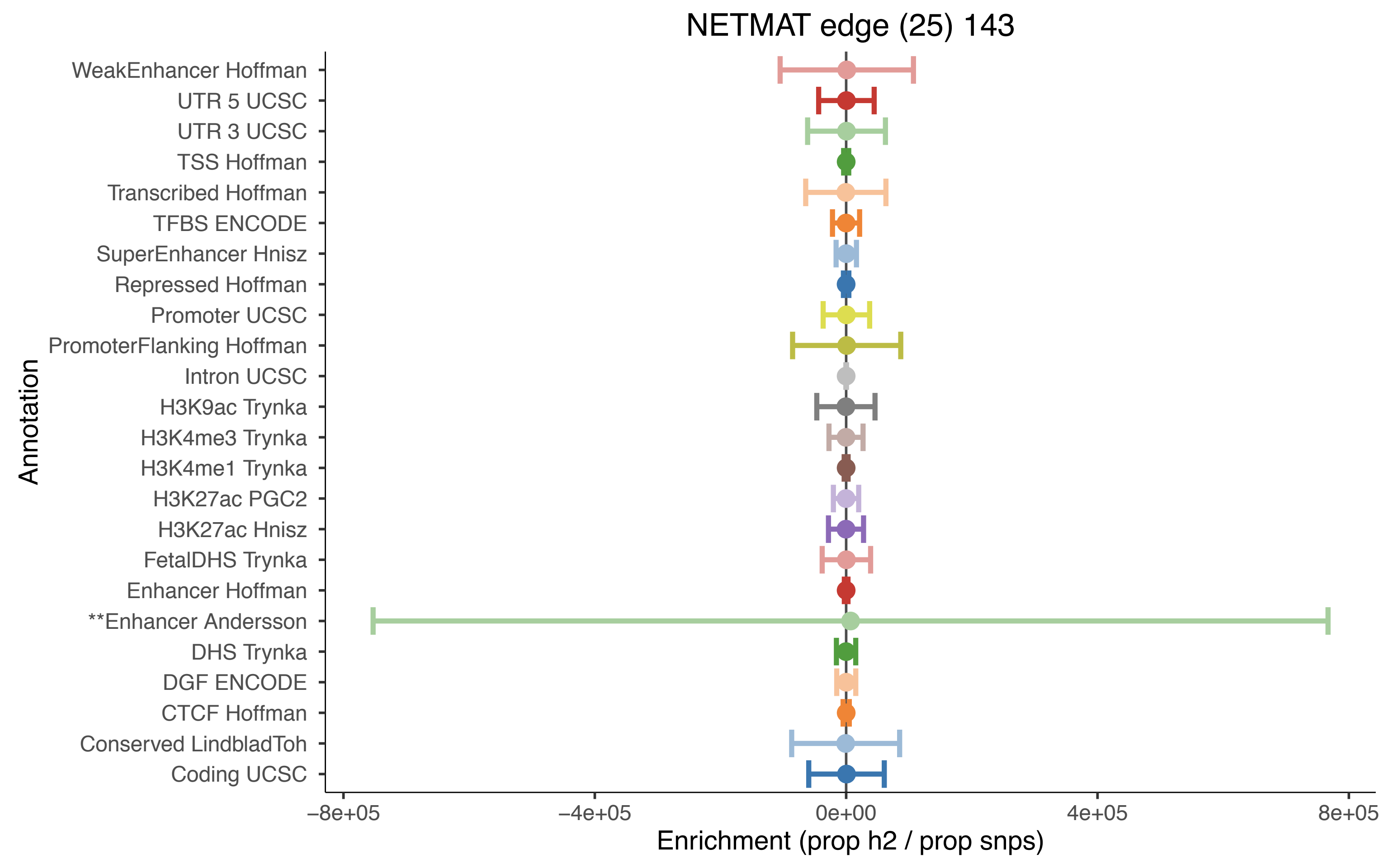
NETMAT edge (25) 143
WeakEnhancer Hoffman
UTR 5 UCSC
UTR 3 UCSC
TSS Hoffman
Transcribed Hoffman
TFBS ENCODE
SuperEnhancer Hnisz
Repressed Hoffman
Promoter UCSC
PromoterFlanking Hoffman
Intron UCSC
H3K9ac Trynka
H3K4me3 Trynka
H3K4me1 Trynka
H3K27ac PGC2
H3K27ac Hnisz
FetalDHS Trynka
Enhancer Hoffman
**Enhancer Andersson
DHS Trynka
DGF ENCODE
CTCF Hoffman
Conserved LindbladToh
Coding UCSC
Annotation
−8e+05
−4e+05
0e+00
4e+05
8e+05
Enrichment (prop h2 / prop snps)

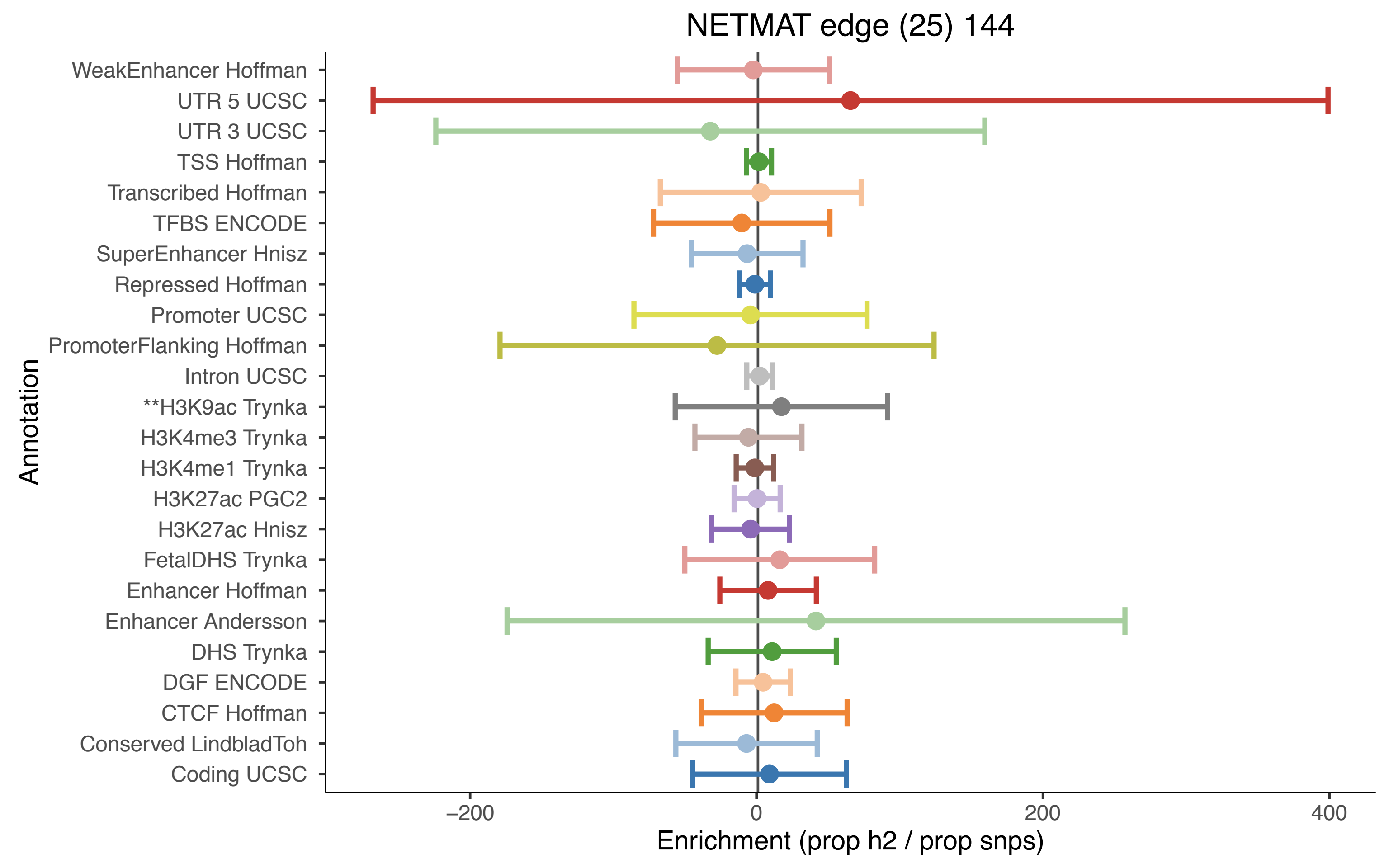
NETMAT edge (25) 144
WeakEnhancer Hoffman
UTR 5 UCSC
UTR 3 UCSC
TSS Hoffman
Transcribed Hoffman
TFBS ENCODE
SuperEnhancer Hnisz
Repressed Hoffman
Promoter UCSC
PromoterFlanking Hoffman
Intron UCSC
**H3K9ac Trynka
H3K4me3 Trynka
H3K4me1 Trynka
H3K27ac PGC2
H3K27ac Hnisz
FetalDHS Trynka
Enhancer Hoffman
Enhancer Andersson
DHS Trynka
DGF ENCODE
CTCF Hoffman
Conserved LindbladToh
Coding UCSC
Annotation
−200
0
200
400
Enrichment (prop h2 / prop snps)

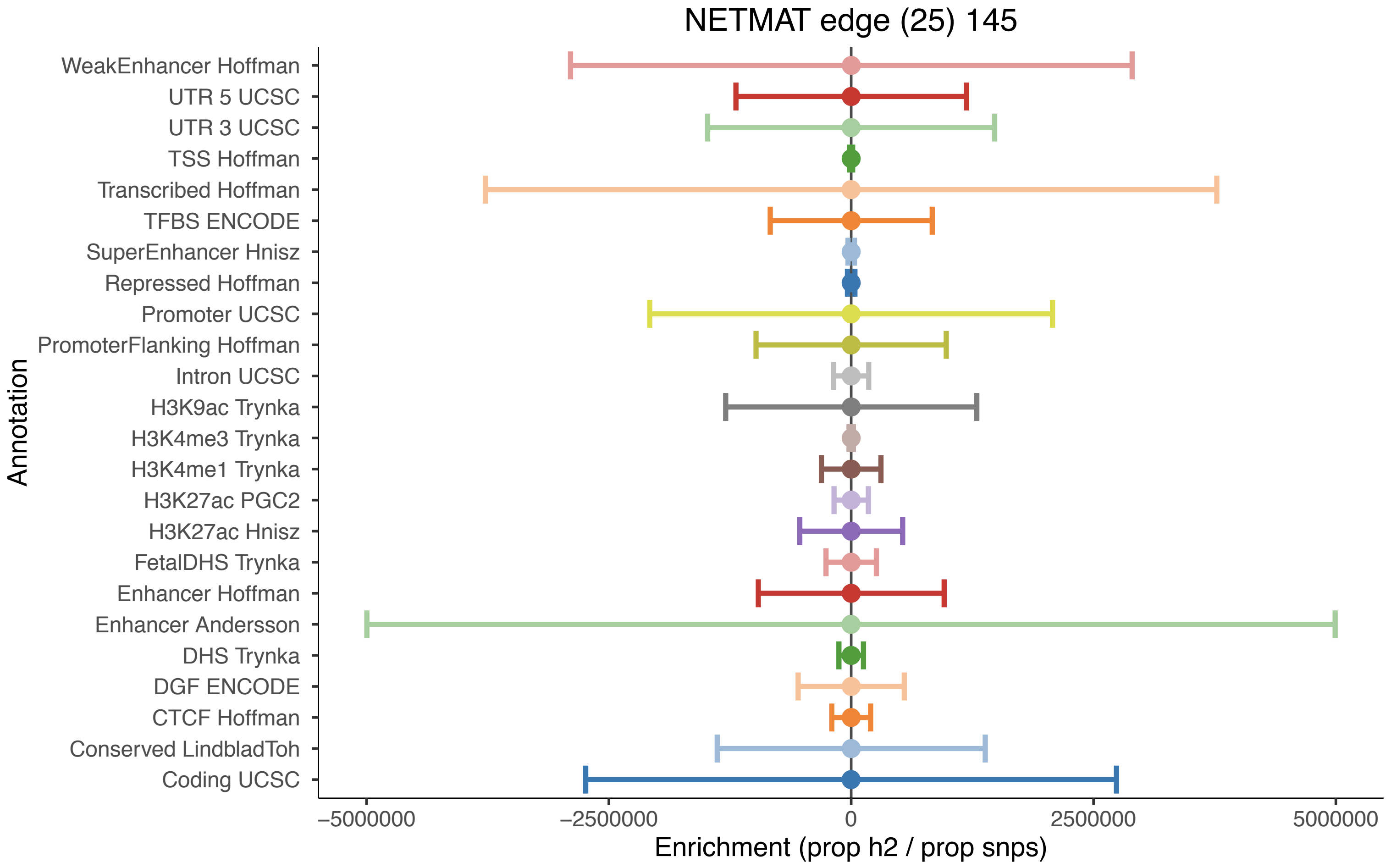

NETMAT edge (25) 145
WeakEnhancer Hoffman
UTR 5 UCSC
UTR 3 UCSC
TSS Hoffman
Transcribed Hoffman
TFBS ENCODE
SuperEnhancer Hnisz
Repressed Hoffman
Promoter UCSC
PromoterFlanking Hoffman
Intron UCSC
H3K9ac Trynka
H3K4me3 Trynka
H3K4me1 Trynka
H3K27ac PGC2
H3K27ac Hnisz
FetalDHS Trynka
Enhancer Hoffman
Enhancer Andersson
DHS Trynka
DGF ENCODE
CTCF Hoffman
Conserved LindbladToh
Coding UCSC
Annotation
−5000000
−2500000
0
2500000
5000000
Enrichment (prop h2 / prop snps)

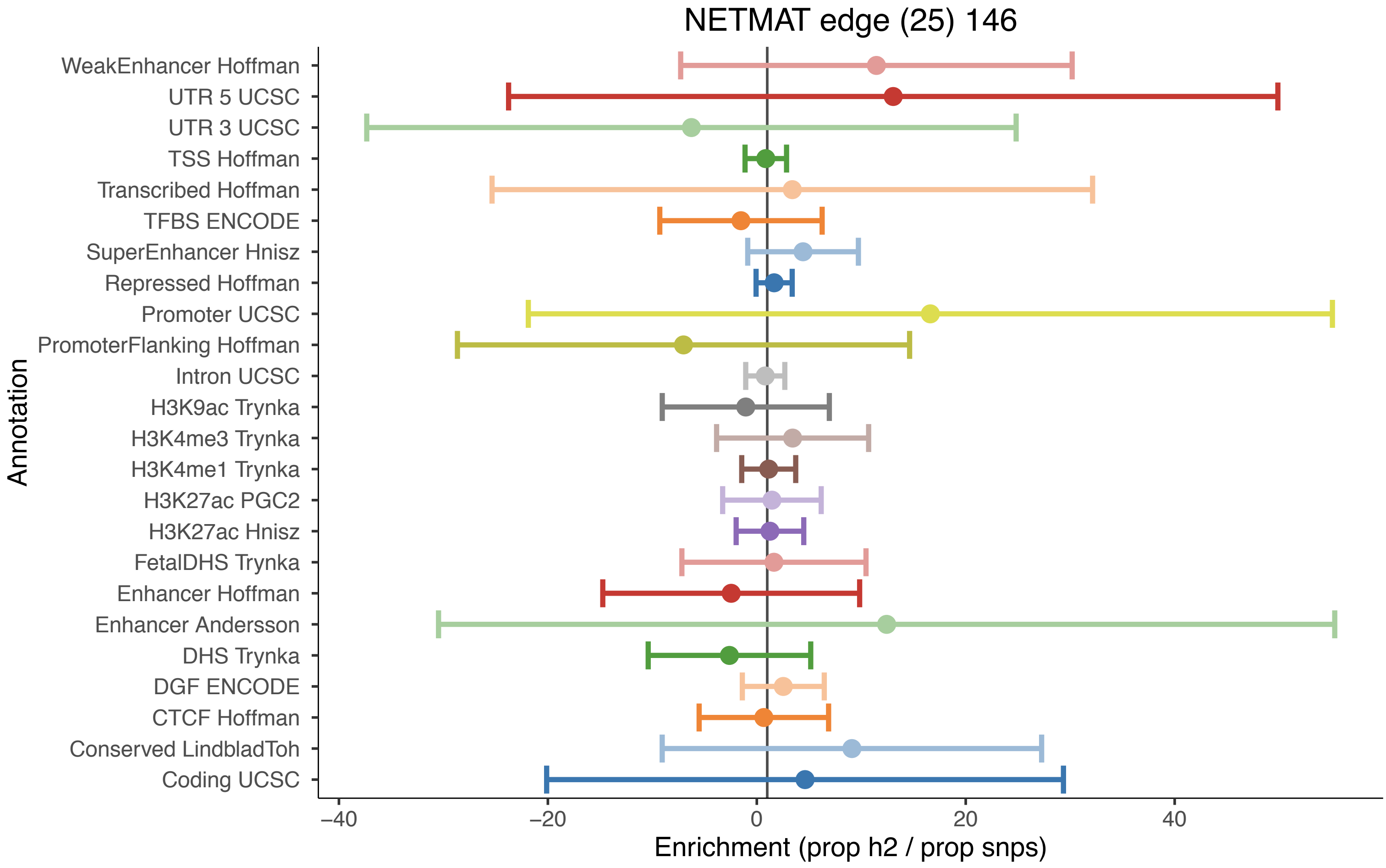
NETMAT edge (25) 146
WeakEnhancer Hoffman
UTR 5 UCSC
UTR 3 UCSC
TSS Hoffman
Transcribed Hoffman
TFBS ENCODE
SuperEnhancer Hnisz
Repressed Hoffman
Promoter UCSC
PromoterFlanking Hoffman
Intron UCSC
H3K9ac Trynka
H3K4me3 Trynka
H3K4me1 Trynka
H3K27ac PGC2
H3K27ac Hnisz
FetalDHS Trynka
Enhancer Hoffman
Enhancer Andersson
DHS Trynka
DGF ENCODE
CTCF Hoffman
Conserved LindbladToh
Coding UCSC
Annotation
−40
−20
0
20
40
Enrichment (prop h2 / prop snps)

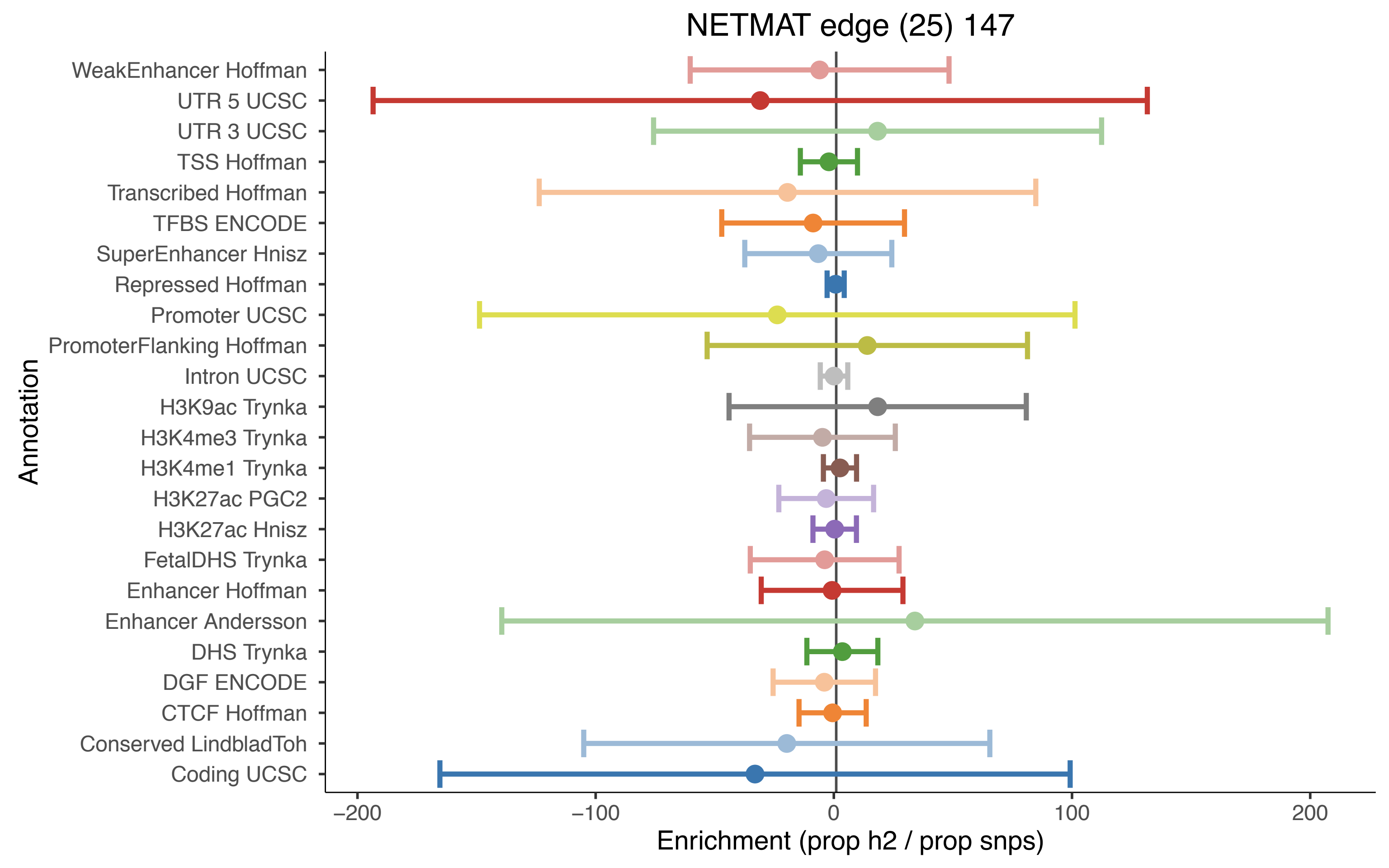

NETMAT edge (25) 147
WeakEnhancer Hoffman
UTR 5 UCSC
UTR 3 UCSC
TSS Hoffman
Transcribed Hoffman
TFBS ENCODE
SuperEnhancer Hnisz
Repressed Hoffman
Promoter UCSC
PromoterFlanking Hoffman
Intron UCSC
H3K9ac Trynka
H3K4me3 Trynka
H3K4me1 Trynka
H3K27ac PGC2
H3K27ac Hnisz
FetalDHS Trynka
Enhancer Hoffman
Enhancer Andersson
DHS Trynka
DGF ENCODE
CTCF Hoffman
Conserved LindbladToh
Coding UCSC
Annotation
−200
−100
0
100
200
Enrichment (prop h2 / prop snps)

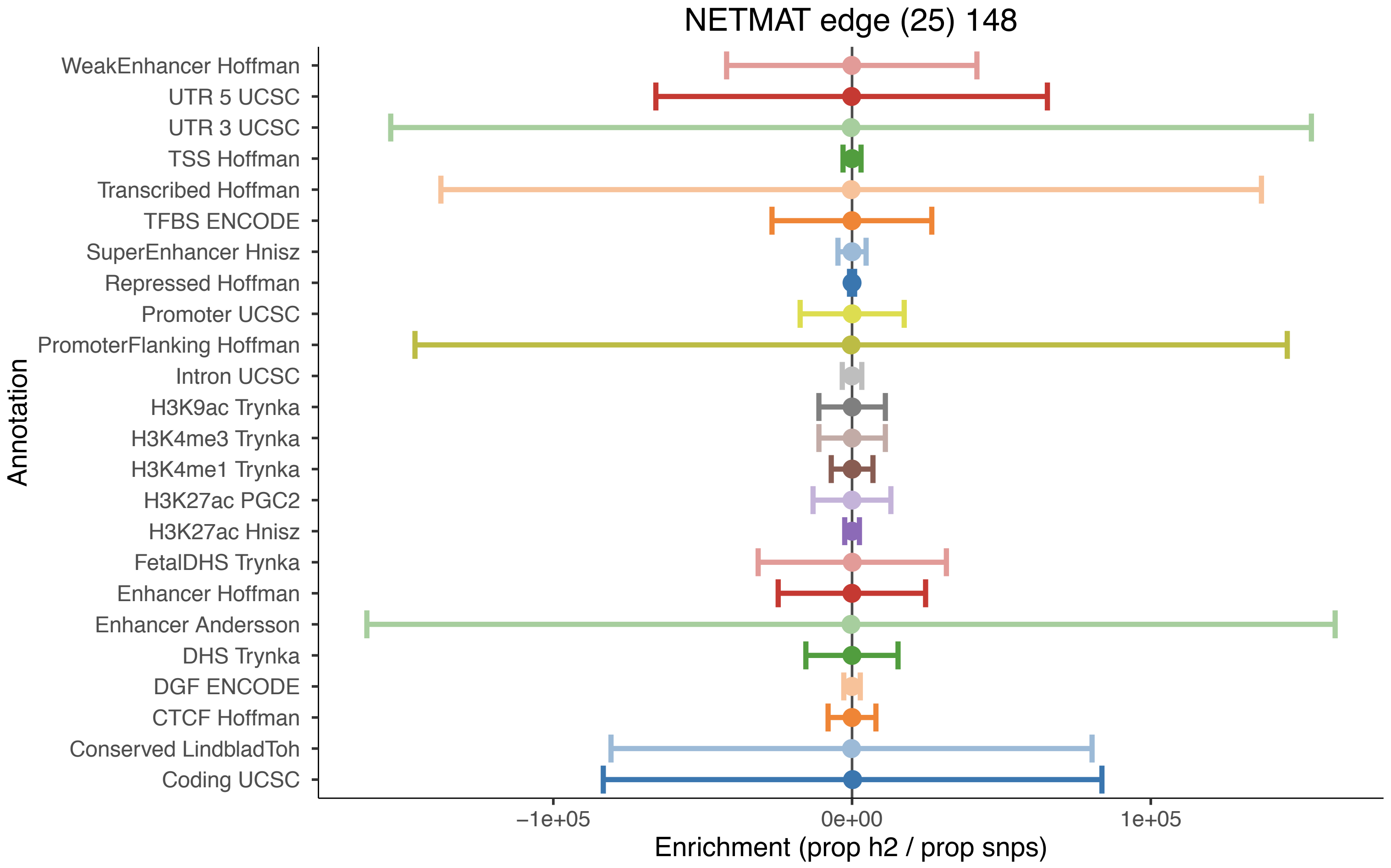
NETMAT edge (25) 148
WeakEnhancer Hoffman
UTR 5 UCSC
UTR 3 UCSC
TSS Hoffman
Transcribed Hoffman
TFBS ENCODE
SuperEnhancer Hnisz
Repressed Hoffman
Promoter UCSC
PromoterFlanking Hoffman
Intron UCSC
H3K9ac Trynka
H3K4me3 Trynka
H3K4me1 Trynka
H3K27ac PGC2
H3K27ac Hnisz
FetalDHS Trynka
Enhancer Hoffman
Enhancer Andersson
DHS Trynka
DGF ENCODE
CTCF Hoffman
Conserved LindbladToh
Coding UCSC
Annotation
−1e+05
0e+00
1e+05
Enrichment (prop h2 / prop snps)

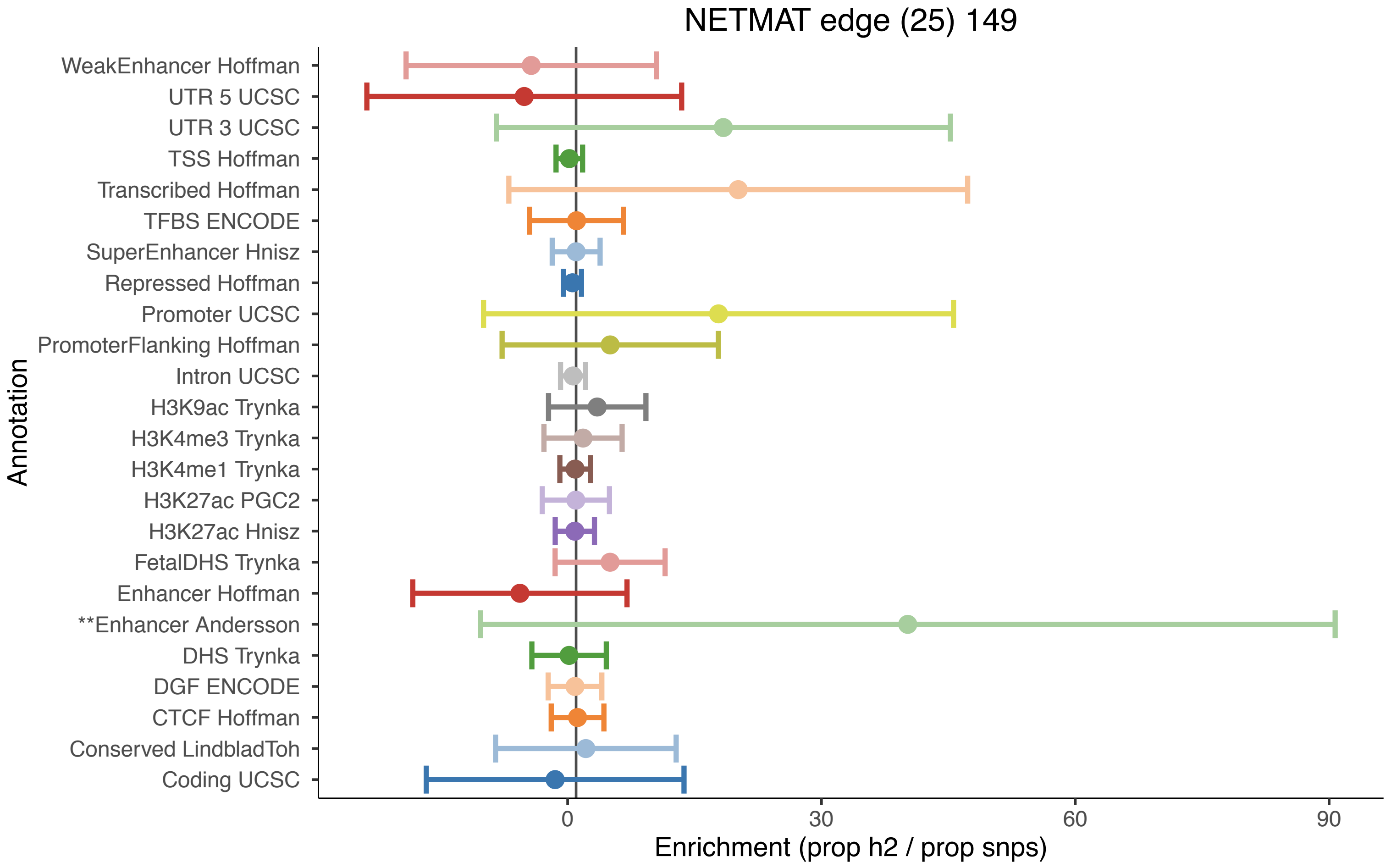
NETMAT edge (25) 149
Annotation
WeakEnhancer Hoffman
UTR 5 UCSC
UTR 3 UCSC
TSS Hoffman
Transcribed Hoffman
TFBS ENCODE
SuperEnhancer Hnisz
Repressed Hoffman
Promoter UCSC
PromoterFlanking Hoffman
Intron UCSC
H3K9ac Trynka
H3K4me3 Trynka
H3K4me1 Trynka
H3K27ac PGC2
H3K27ac Hnisz
FetalDHS Trynka
Enhancer Hoffman
**Enhancer Andersson
DHS Trynka
DGF ENCODE
CTCF Hoffman
Conserved LindbladToh
Coding UCSC
0
30
60
90
Enrichment (prop h2 / prop snps)

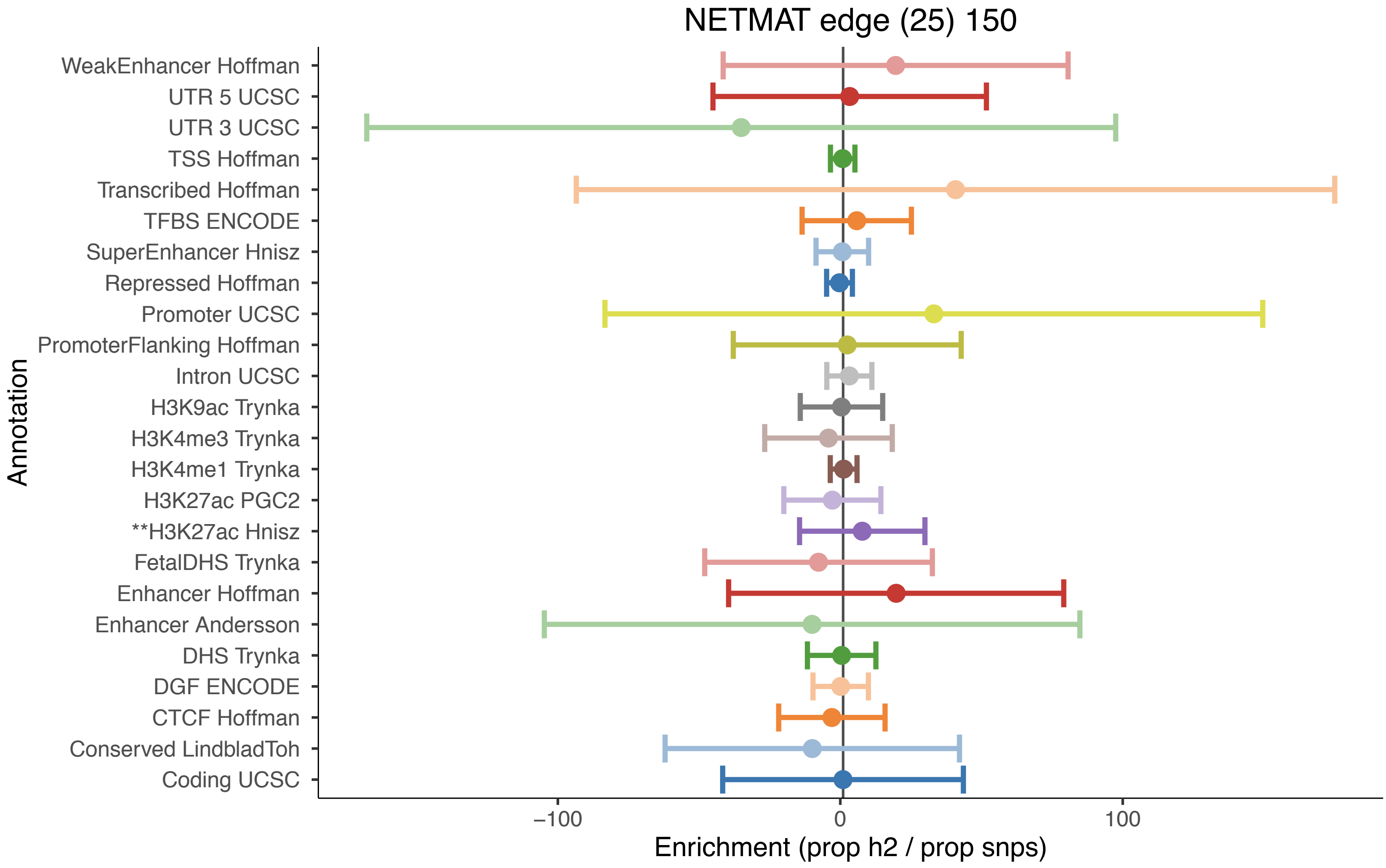

NETMAT edge (25) 150
Annotation
WeakEnhancer Hoffman
UTR 5 UCSC
UTR 3 UCSC
TSS Hoffman
Transcribed Hoffman
TFBS ENCODE
SuperEnhancer Hnisz
Repressed Hoffman
Promoter UCSC
PromoterFlanking Hoffman
Intron UCSC
H3K9ac Trynka
H3K4me3 Trynka
H3K4me1 Trynka
H3K27ac PGC2
**H3K27ac Hnisz
FetalDHS Trynka
Enhancer Hoffman
Enhancer Andersson
DHS Trynka
DGF ENCODE
CTCF Hoffman
Conserved LindbladToh
Coding UCSC
−100
0
100
Enrichment (prop h2 / prop snps)

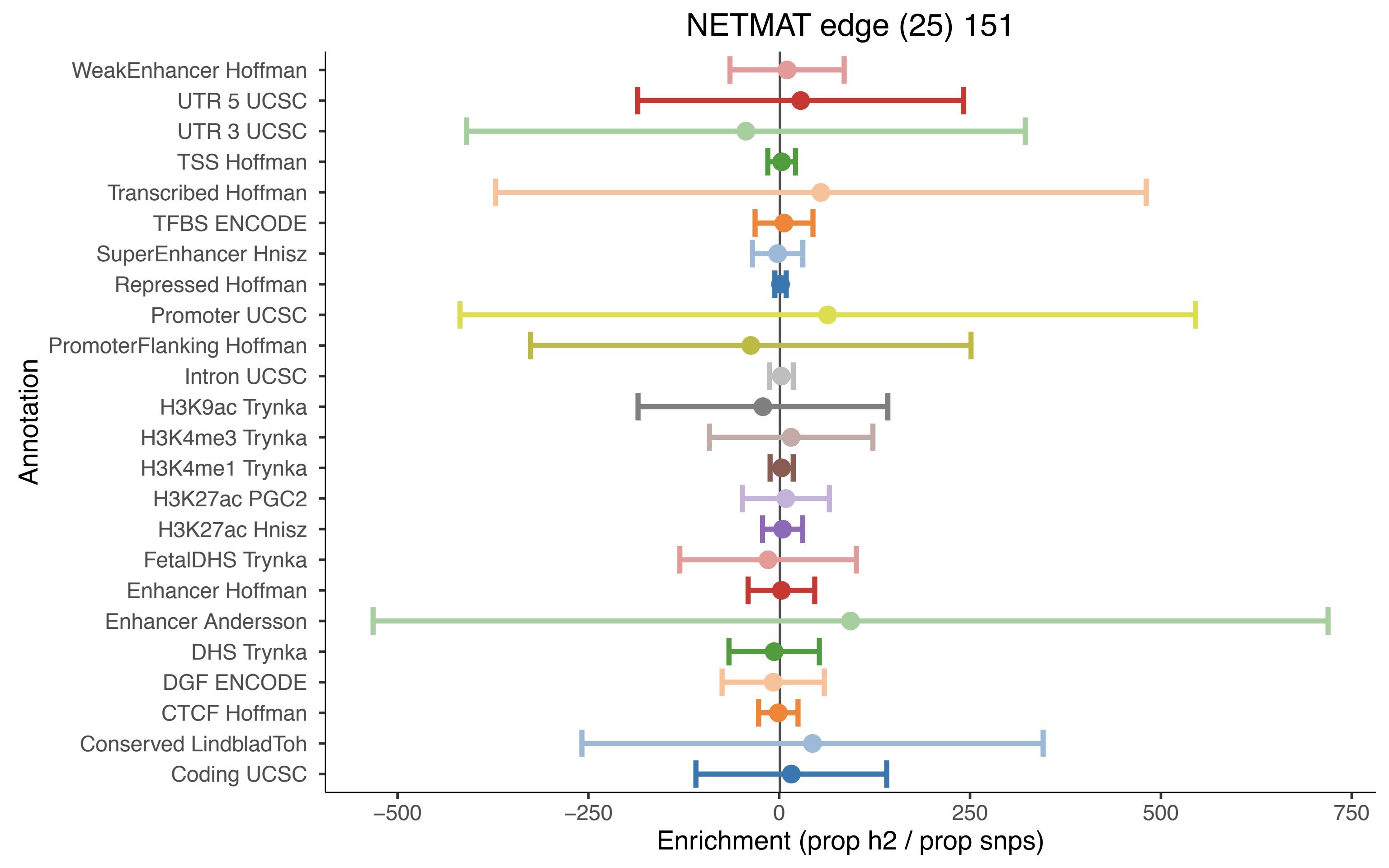

NETMAT edge (25) 151
WeakEnhancer Hoffman
UTR 5 UCSC
UTR 3 UCSC
TSS Hoffman
Transcribed Hoffman
TFBS ENCODE
SuperEnhancer Hnisz
Repressed Hoffman
Promoter UCSC
PromoterFlanking Hoffman
Intron UCSC
H3K9ac Trynka
H3K4me3 Trynka
H3K4me1 Trynka
H3K27ac PGC2
H3K27ac Hnisz
FetalDHS Trynka
Enhancer Hoffman
Enhancer Andersson
DHS Trynka
DGF ENCODE
CTCF Hoffman
Conserved LindbladToh
Coding UCSC
Annotation
−500
−250
0
250
500
750
Enrichment (prop h2 / prop snps)

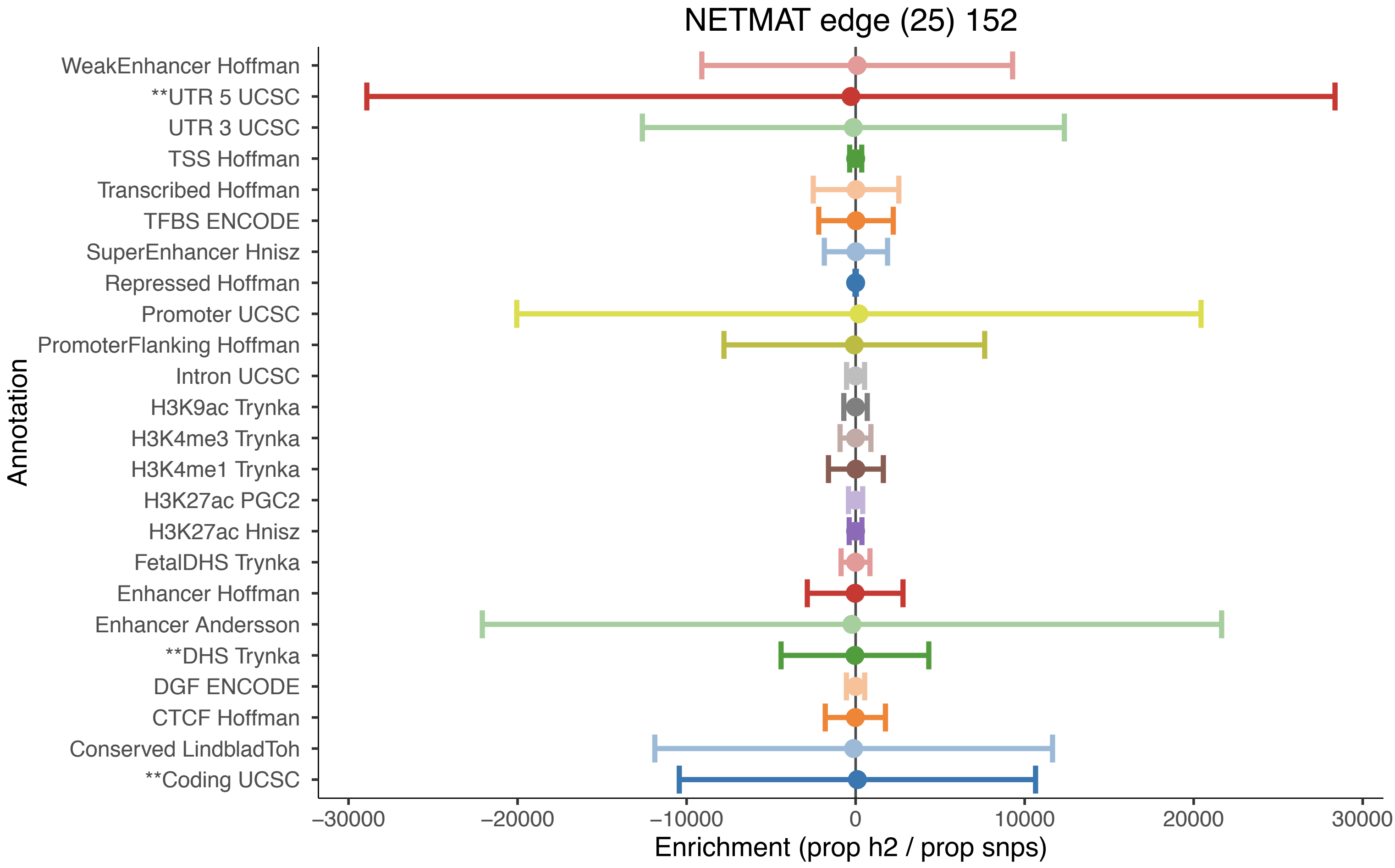

NETMAT edge (25) 152
WeakEnhancer Hoffman
**UTR 5 UCSC
UTR 3 UCSC
TSS Hoffman
Transcribed Hoffman
TFBS ENCODE
SuperEnhancer Hnisz
Repressed Hoffman
Promoter UCSC
PromoterFlanking Hoffman
Intron UCSC
H3K9ac Trynka
H3K4me3 Trynka
H3K4me1 Trynka
H3K27ac PGC2
H3K27ac Hnisz
FetalDHS Trynka
Enhancer Hoffman
Enhancer Andersson
**DHS Trynka
DGF ENCODE
CTCF Hoffman
Conserved LindbladToh
**Coding UCSC
Annotation
−30000
−20000
−10000
0
10000
20000
30000
Enrichment (prop h2 / prop snps)

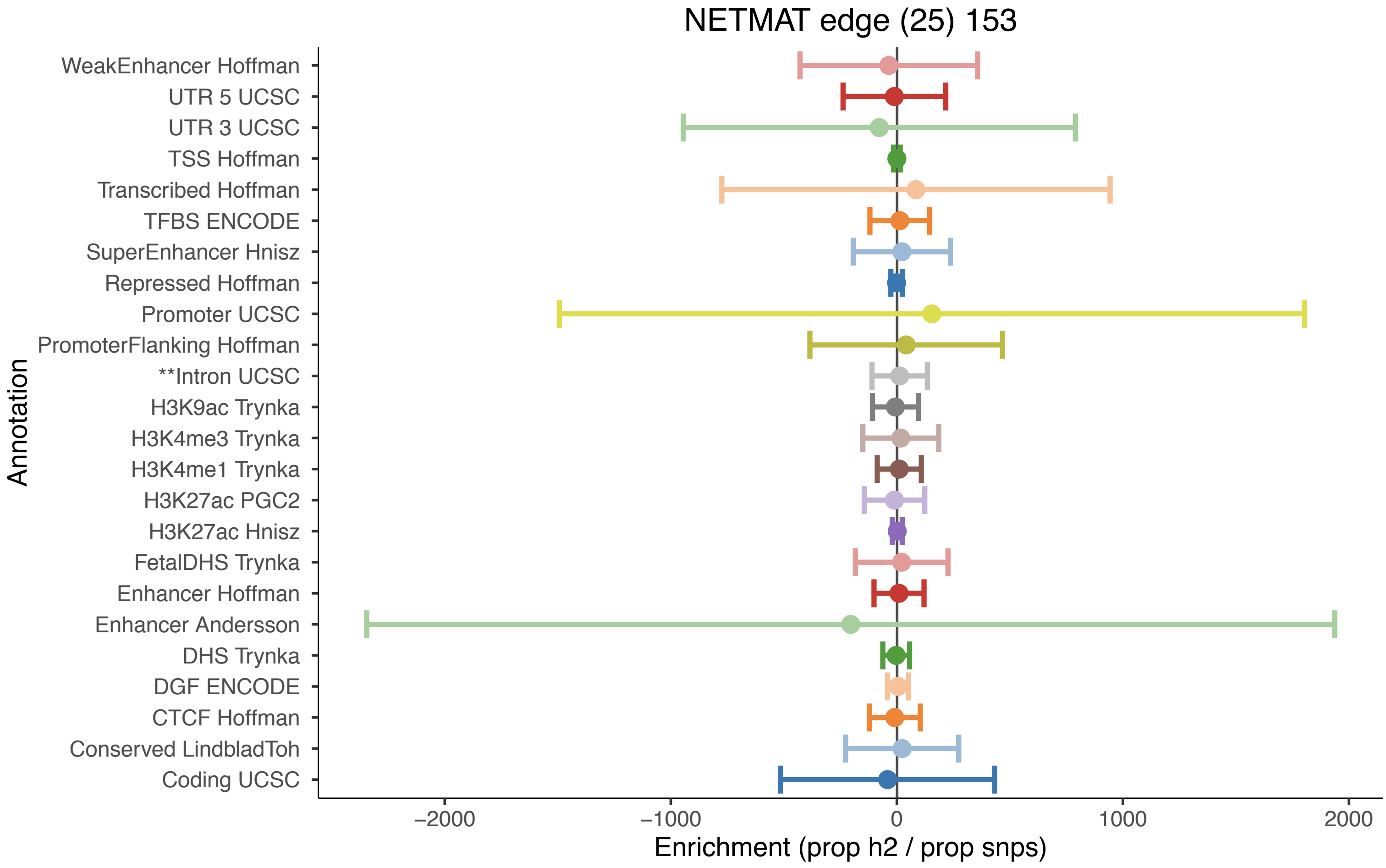
NETMAT edge (25) 153
Annotation
WeakEnhancer Hoffman
UTR 5 UCSC
UTR 3 UCSC
TSS Hoffman
Transcribed Hoffman
TFBS ENCODE
SuperEnhancer Hnisz
Repressed Hoffman
Promoter UCSC
PromoterFlanking Hoffman
**Intron UCSC
H3K9ac Trynka
H3K4me3 Trynka
H3K4me1 Trynka
H3K27ac PGC2
H3K27ac Hnisz
FetalDHS Trynka
Enhancer Hoffman
Enhancer Andersson
DHS Trynka
DGF ENCODE
CTCF Hoffman
Conserved LindbladToh
Coding UCSC
−2000
−1000
0
1000
2000
Enrichment (prop h2 / prop snps)

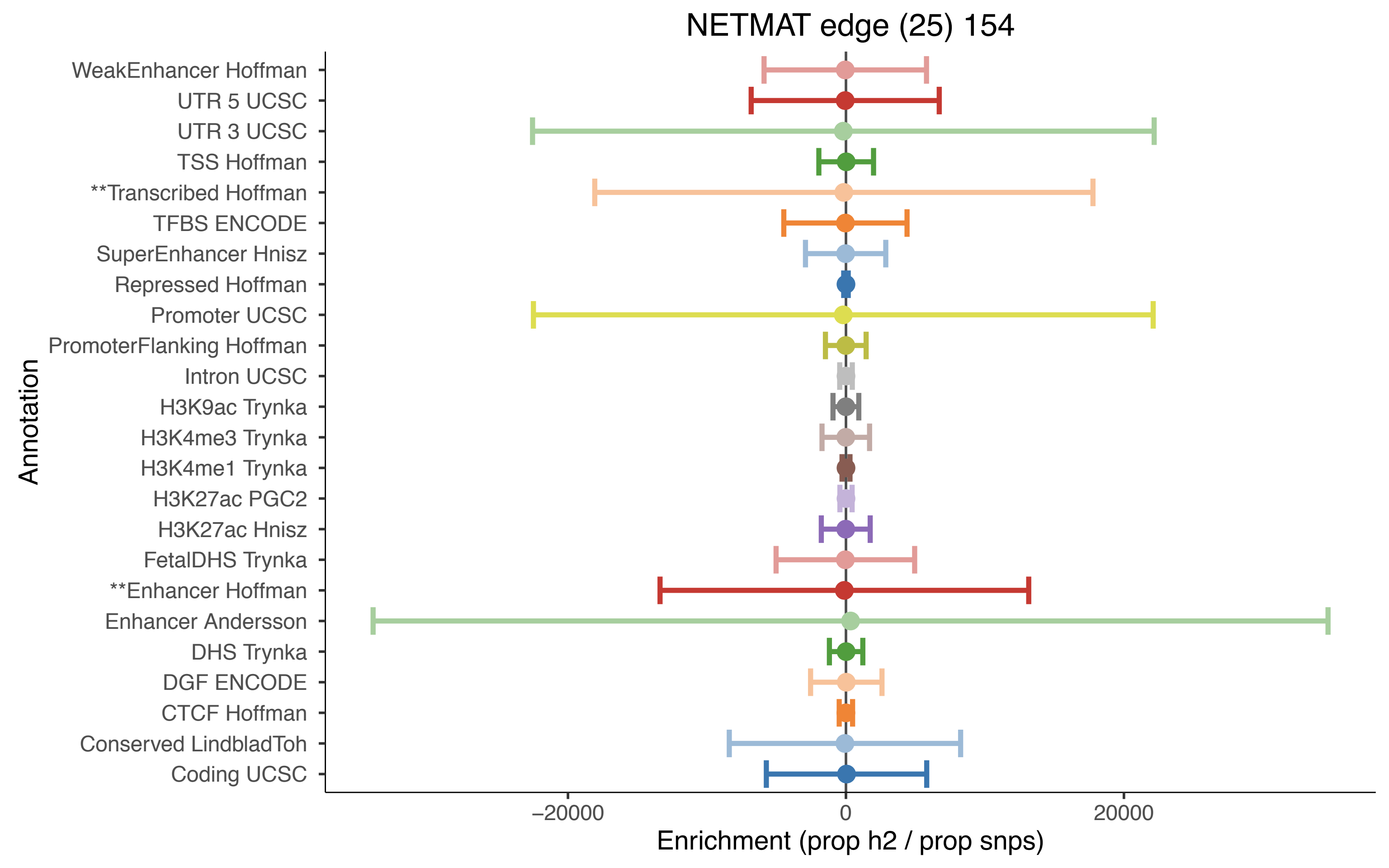
NETMAT edge (25) 154
WeakEnhancer Hoffman
UTR 5 UCSC
UTR 3 UCSC
TSS Hoffman
**Transcribed Hoffman
TFBS ENCODE
SuperEnhancer Hnisz
Repressed Hoffman
Promoter UCSC
PromoterFlanking Hoffman
Intron UCSC
H3K9ac Trynka
H3K4me3 Trynka
H3K4me1 Trynka
H3K27ac PGC2
H3K27ac Hnisz
FetalDHS Trynka
**Enhancer Hoffman
Enhancer Andersson
DHS Trynka
DGF ENCODE
CTCF Hoffman
Conserved LindbladToh
Coding UCSC
Annotation
−20000
0
20000
Enrichment (prop h2 / prop snps)

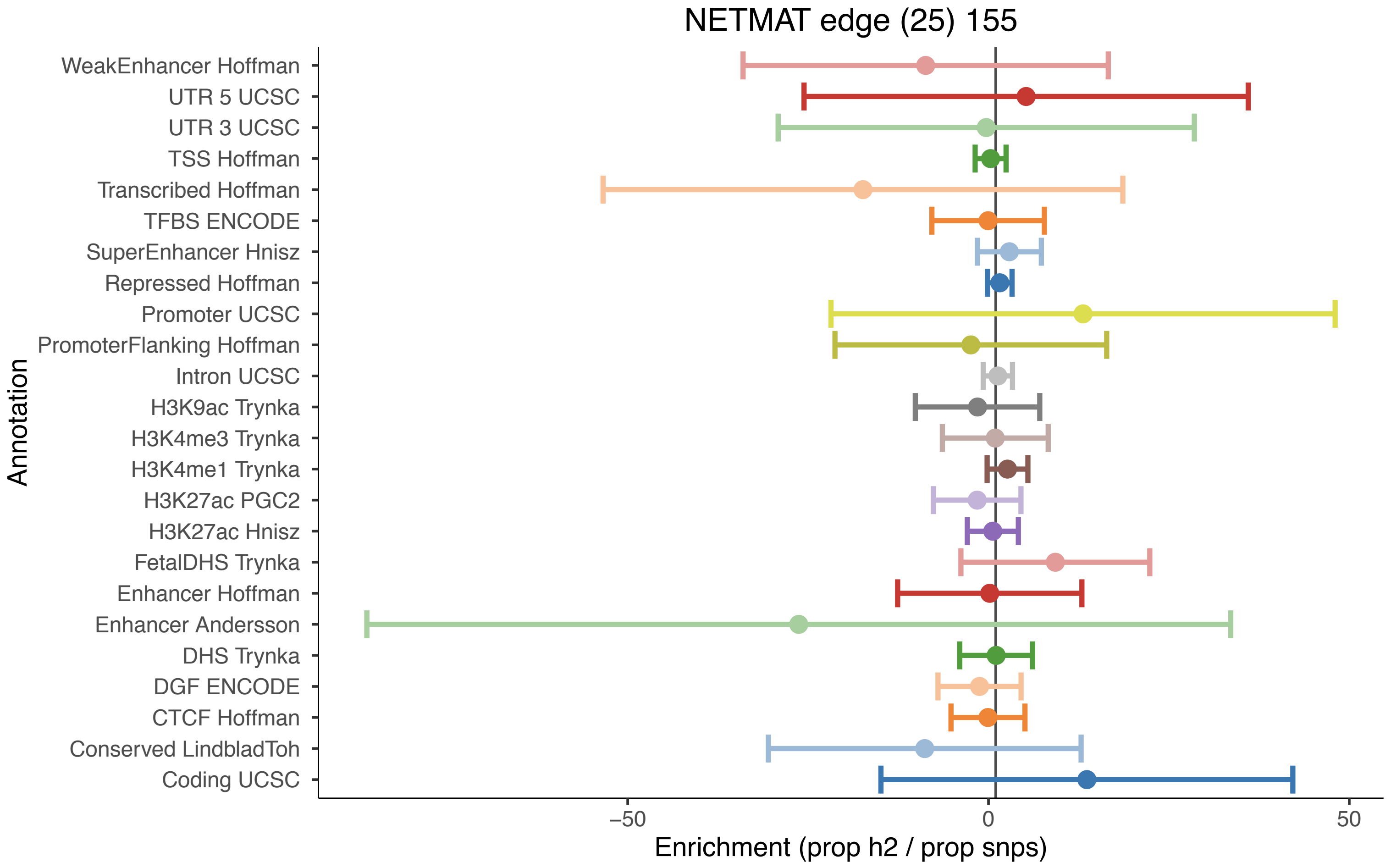

NETMAT edge (25) 155
WeakEnhancer Hoffman
UTR 5 UCSC
UTR 3 UCSC
TSS Hoffman
Transcribed Hoffman
TFBS ENCODE
SuperEnhancer Hnisz
Repressed Hoffman
Promoter UCSC
PromoterFlanking Hoffman
Intron UCSC
H3K9ac Trynka
H3K4me3 Trynka
H3K4me1 Trynka
H3K27ac PGC2
H3K27ac Hnisz
FetalDHS Trynka
Enhancer Hoffman
Enhancer Andersson
DHS Trynka
DGF ENCODE
CTCF Hoffman
Conserved LindbladToh
Coding UCSC
Annotation
−50
0
50
Enrichment (prop h2 / prop snps)

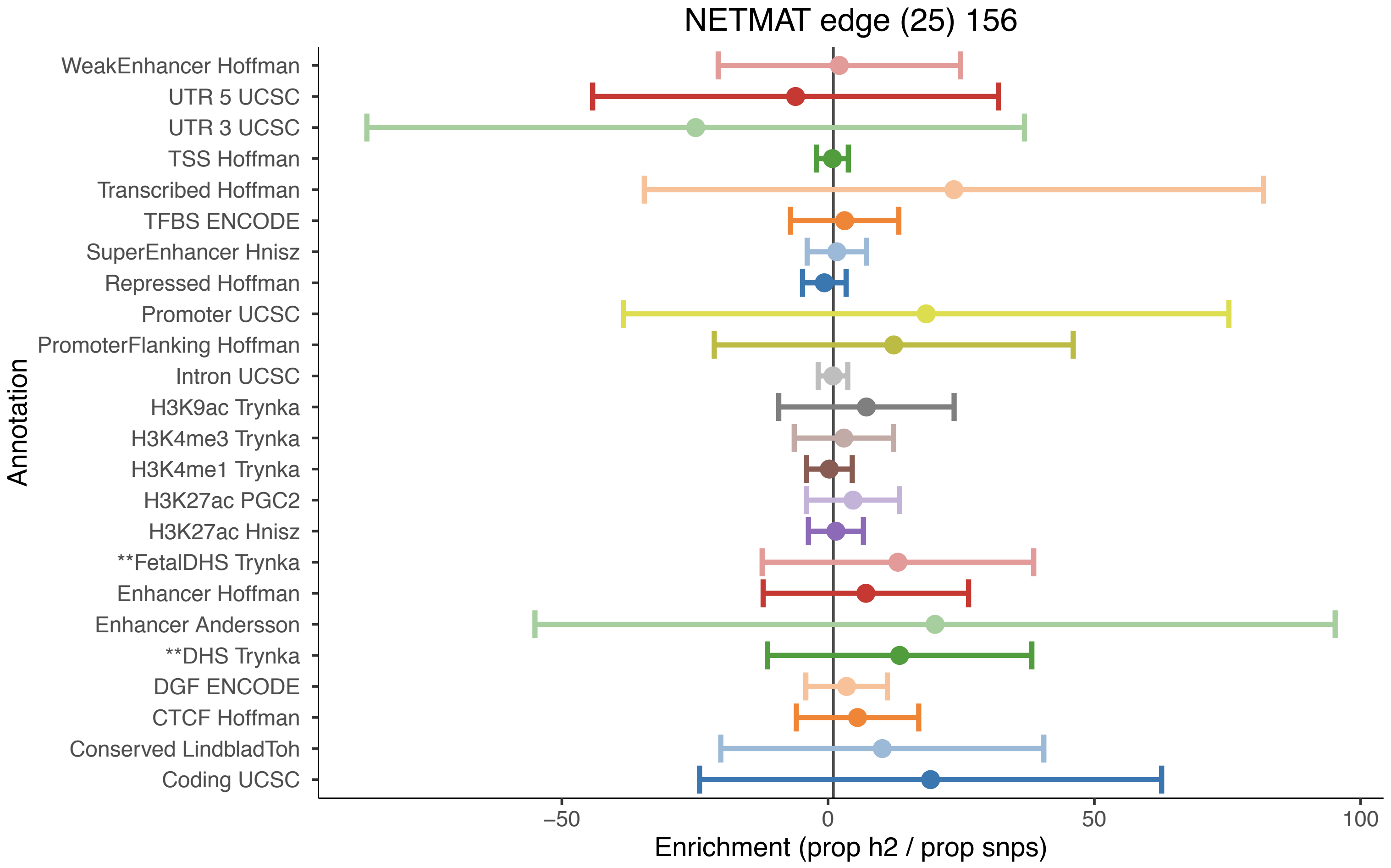
NETMAT edge (25) 156
WeakEnhancer Hoffman
UTR 5 UCSC
UTR 3 UCSC
TSS Hoffman
Transcribed Hoffman
TFBS ENCODE
SuperEnhancer Hnisz
Repressed Hoffman
Promoter UCSC
PromoterFlanking Hoffman
Intron UCSC
H3K9ac Trynka
H3K4me3 Trynka
H3K4me1 Trynka
H3K27ac PGC2
H3K27ac Hnisz
**FetalDHS Trynka
Enhancer Hoffman
Enhancer Andersson
**DHS Trynka
DGF ENCODE
CTCF Hoffman
Conserved LindbladToh
Coding UCSC
Annotation
−50
0
50
100
Enrichment (prop h2 / prop snps)

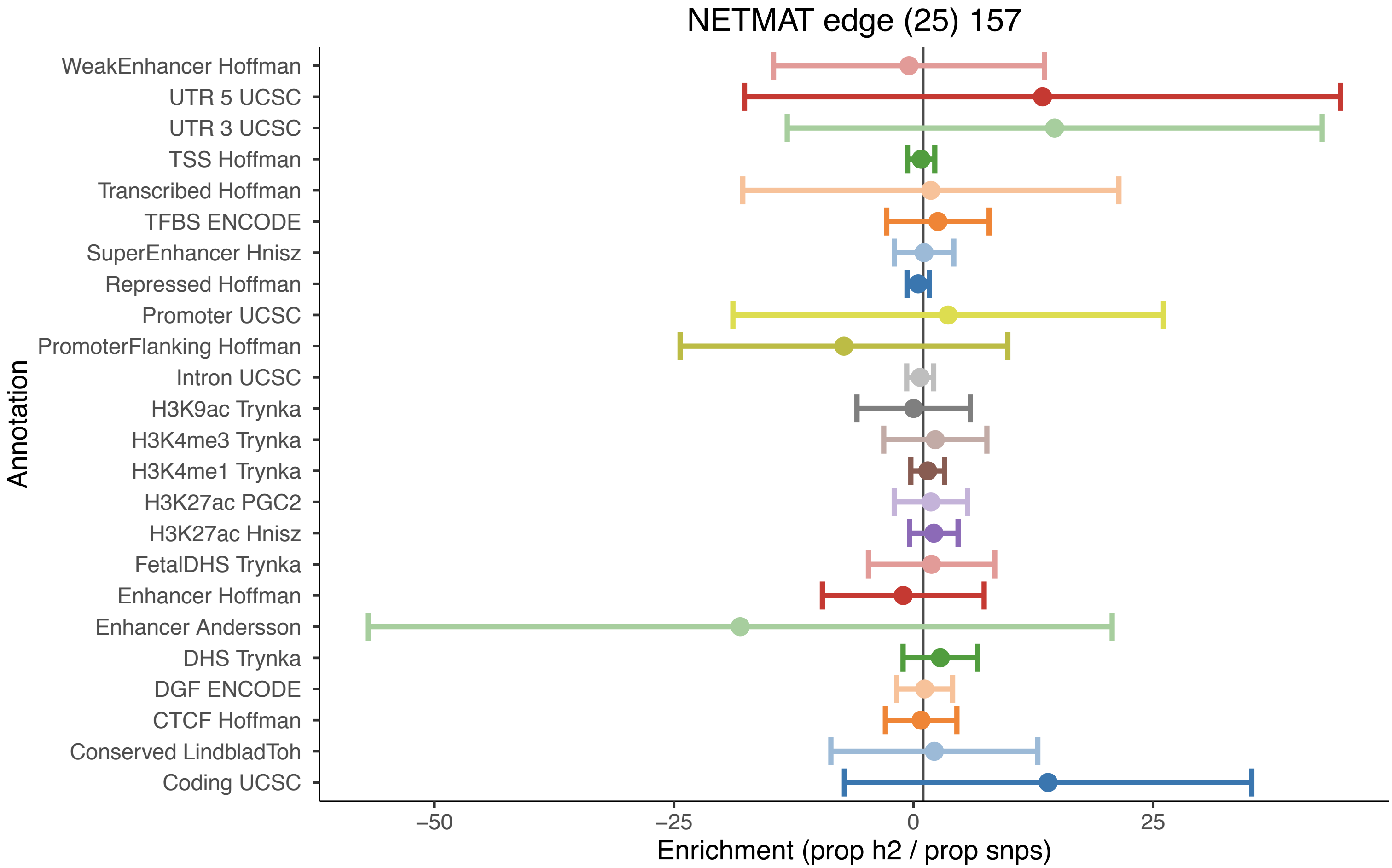
NETMAT edge (25) 157
WeakEnhancer Hoffman
UTR 5 UCSC
UTR 3 UCSC
TSS Hoffman
Transcribed Hoffman
TFBS ENCODE
SuperEnhancer Hnisz
Repressed Hoffman
Promoter UCSC
PromoterFlanking Hoffman
Intron UCSC
H3K9ac Trynka
H3K4me3 Trynka
H3K4me1 Trynka
H3K27ac PGC2
H3K27ac Hnisz
FetalDHS Trynka
Enhancer Hoffman
Enhancer Andersson
DHS Trynka
DGF ENCODE
CTCF Hoffman
Conserved LindbladToh
Coding UCSC
Annotation
−50
−25
0
25
Enrichment (prop h2 / prop snps)

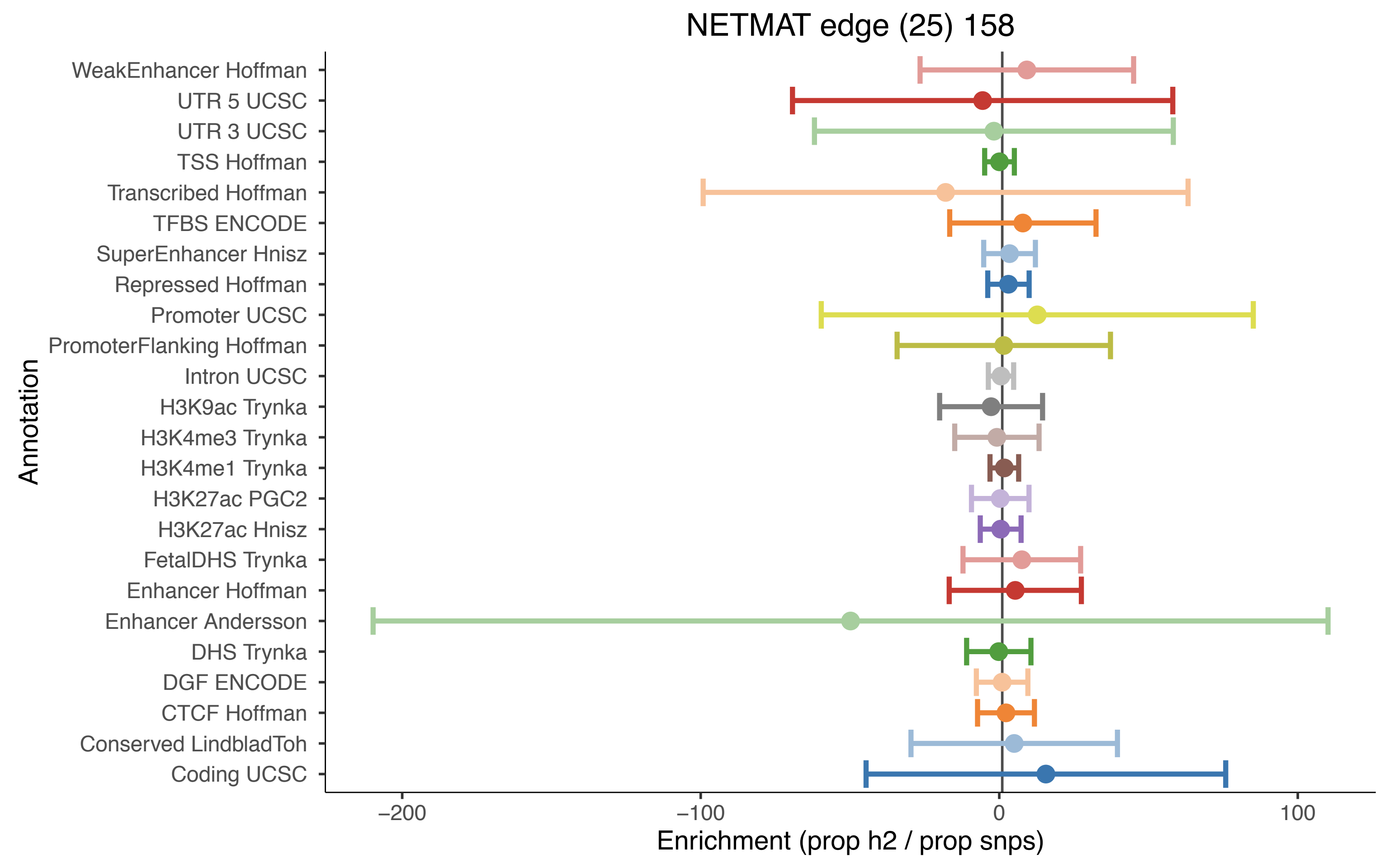

NETMAT edge (25) 158
Annotation
WeakEnhancer Hoffman
UTR 5 UCSC
UTR 3 UCSC
TSS Hoffman
Transcribed Hoffman
TFBS ENCODE
SuperEnhancer Hnisz
Repressed Hoffman
Promoter UCSC
PromoterFlanking Hoffman
Intron UCSC
H3K9ac Trynka
H3K4me3 Trynka
H3K4me1 Trynka
H3K27ac PGC2
H3K27ac Hnisz
FetalDHS Trynka
Enhancer Hoffman
Enhancer Andersson
DHS Trynka
DGF ENCODE
CTCF Hoffman
Conserved LindbladToh
Coding UCSC
−200
−100
0
100
Enrichment (prop h2 / prop snps)

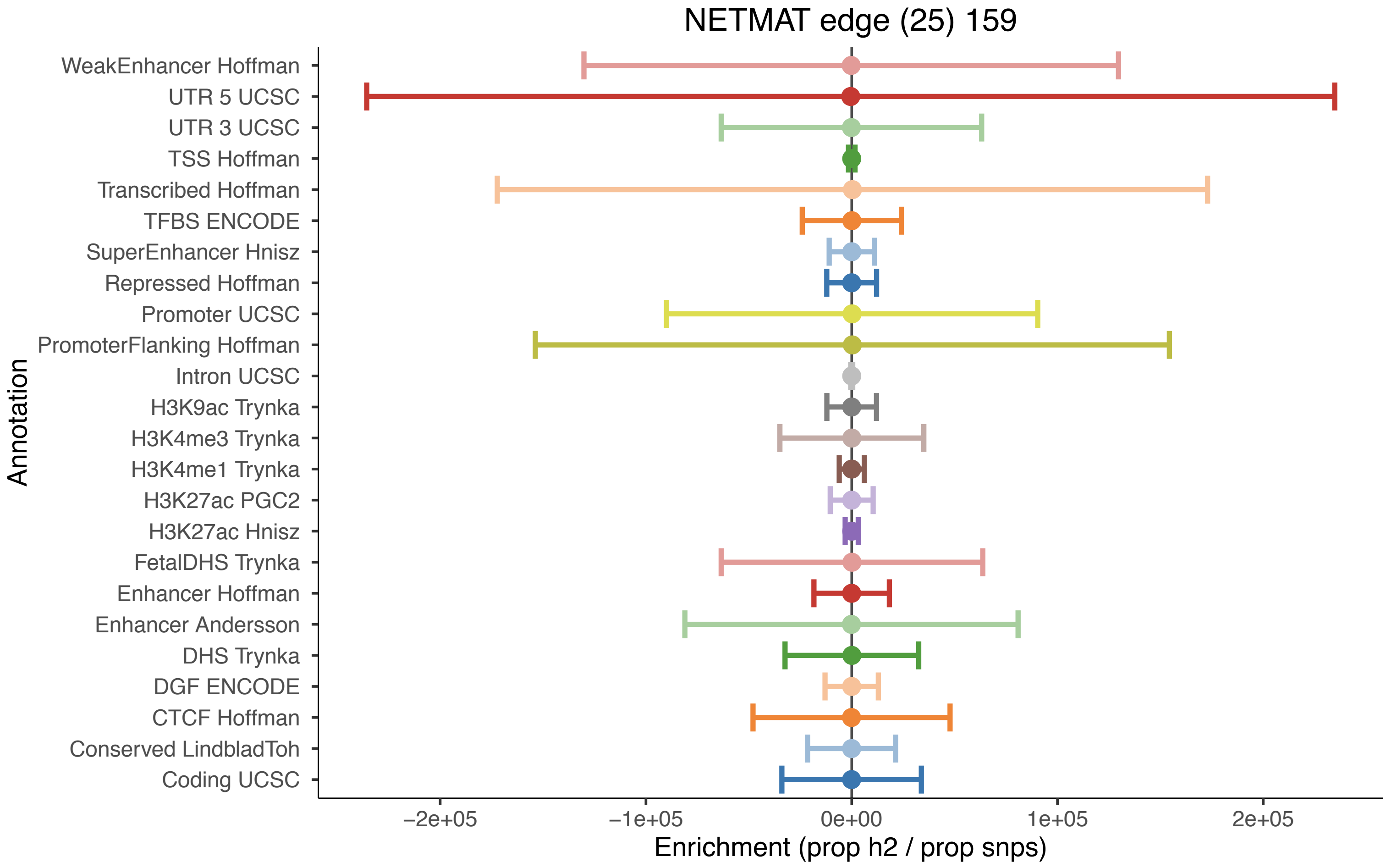
NETMAT edge (25) 159
WeakEnhancer Hoffman
UTR 5 UCSC
UTR 3 UCSC
TSS Hoffman
Transcribed Hoffman
TFBS ENCODE
SuperEnhancer Hnisz
Repressed Hoffman
Promoter UCSC
PromoterFlanking Hoffman
Intron UCSC
H3K9ac Trynka
H3K4me3 Trynka
H3K4me1 Trynka
H3K27ac PGC2
H3K27ac Hnisz
FetalDHS Trynka
Enhancer Hoffman
Enhancer Andersson
DHS Trynka
DGF ENCODE
CTCF Hoffman
Conserved LindbladToh
Coding UCSC
Annotation
−2e+05
−1e+05
0e+00
1e+05
2e+05
Enrichment (prop h2 / prop snps)

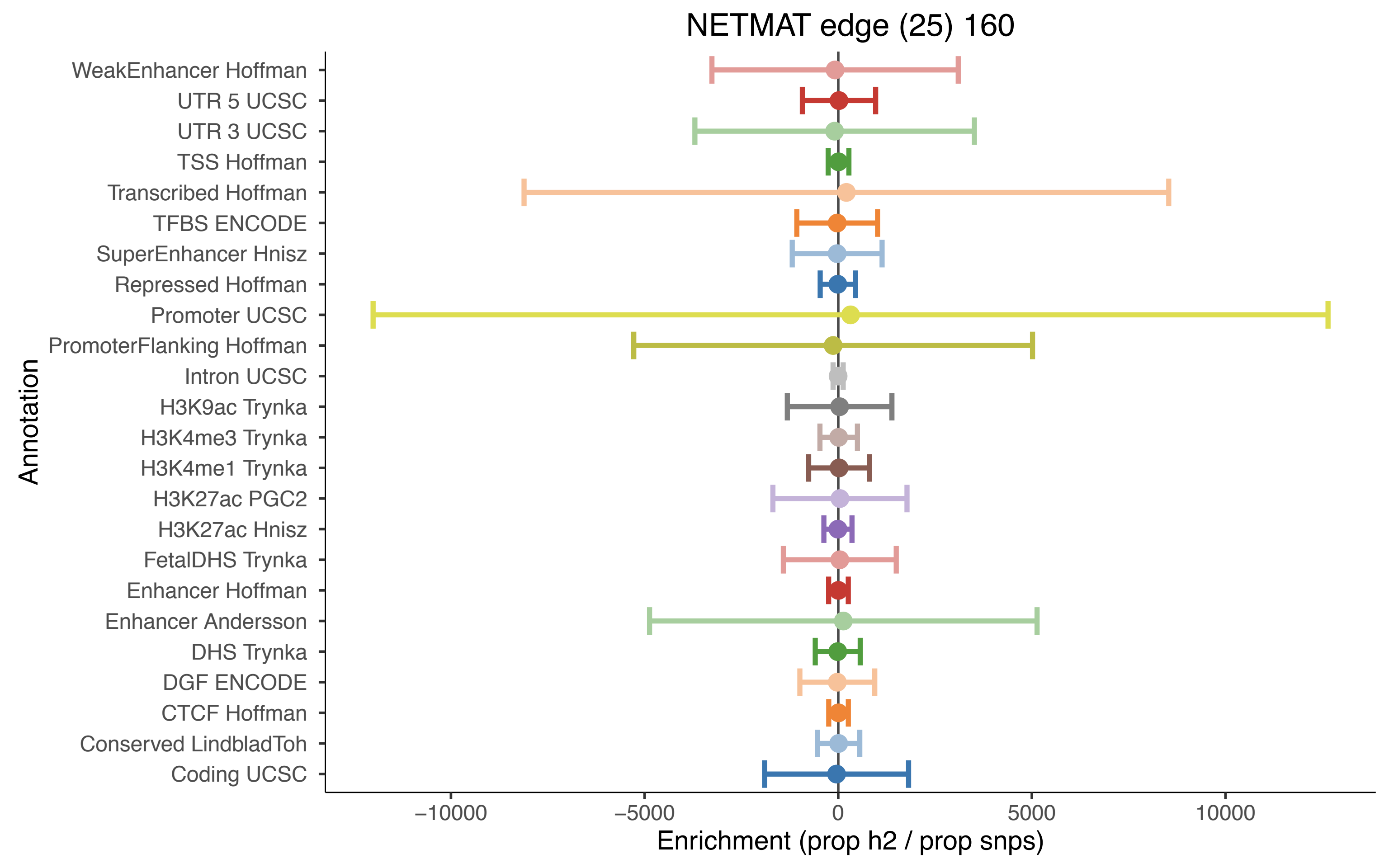

NETMAT edge (25) 160
WeakEnhancer Hoffman
UTR 5 UCSC
UTR 3 UCSC
TSS Hoffman
Transcribed Hoffman
TFBS ENCODE
SuperEnhancer Hnisz
Repressed Hoffman
Promoter UCSC
PromoterFlanking Hoffman
Intron UCSC
H3K9ac Trynka
H3K4me3 Trynka
H3K4me1 Trynka
H3K27ac PGC2
H3K27ac Hnisz
FetalDHS Trynka
Enhancer Hoffman
Enhancer Andersson
DHS Trynka
DGF ENCODE
CTCF Hoffman
Conserved LindbladToh
Coding UCSC
Annotation
−10000
−5000
0
5000
10000
Enrichment (prop h2 / prop snps)

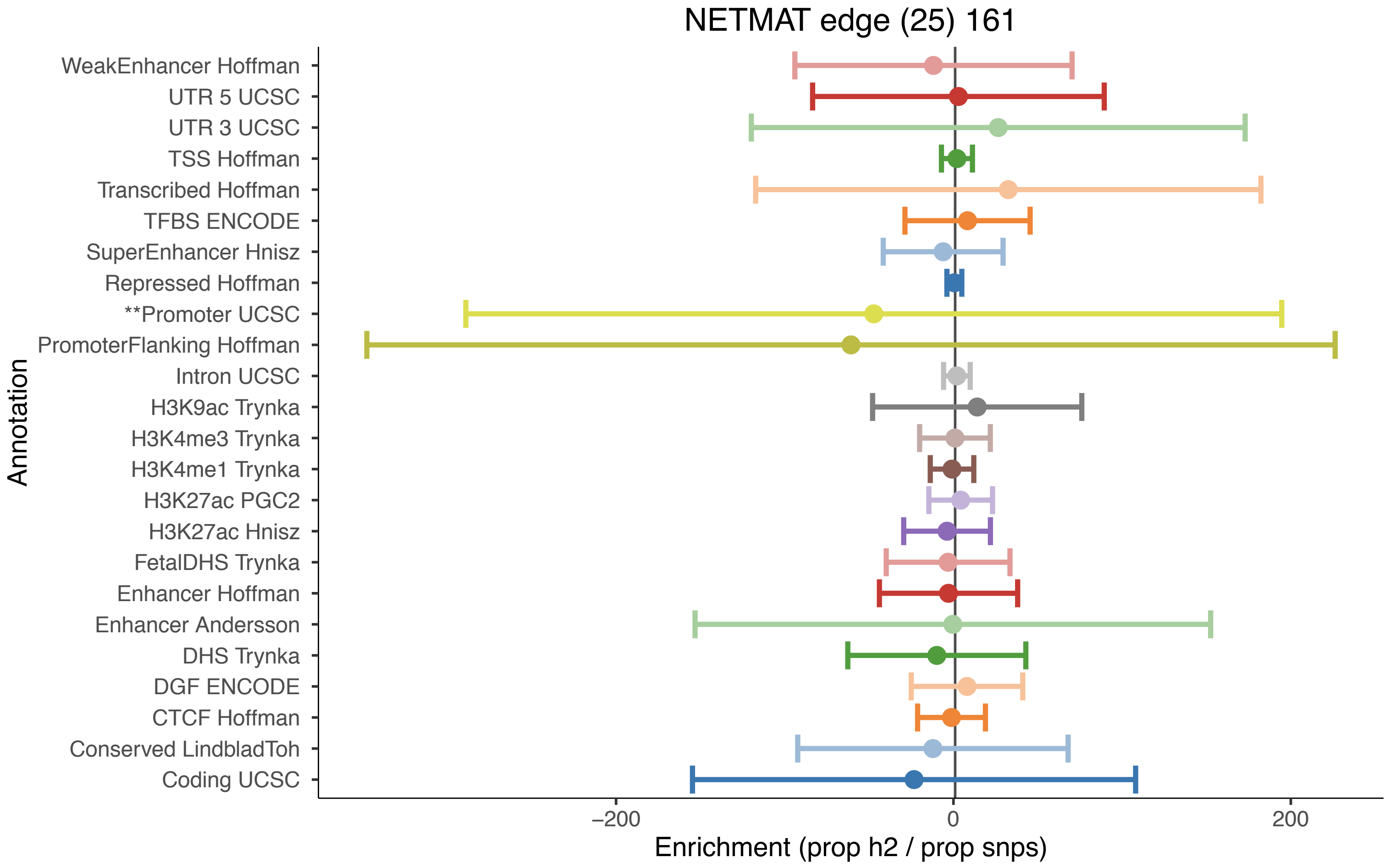

NETMAT edge (25) 161
WeakEnhancer Hoffman
UTR 5 UCSC
UTR 3 UCSC
TSS Hoffman
Transcribed Hoffman
TFBS ENCODE
SuperEnhancer Hnisz
Repressed Hoffman
**Promoter UCSC
PromoterFlanking Hoffman
Intron UCSC
H3K9ac Trynka
H3K4me3 Trynka
H3K4me1 Trynka
H3K27ac PGC2
H3K27ac Hnisz
FetalDHS Trynka
Enhancer Hoffman
Enhancer Andersson
DHS Trynka
DGF ENCODE
CTCF Hoffman
Conserved LindbladToh
Coding UCSC
Annotation
−200
0
200
Enrichment (prop h2 / prop snps)

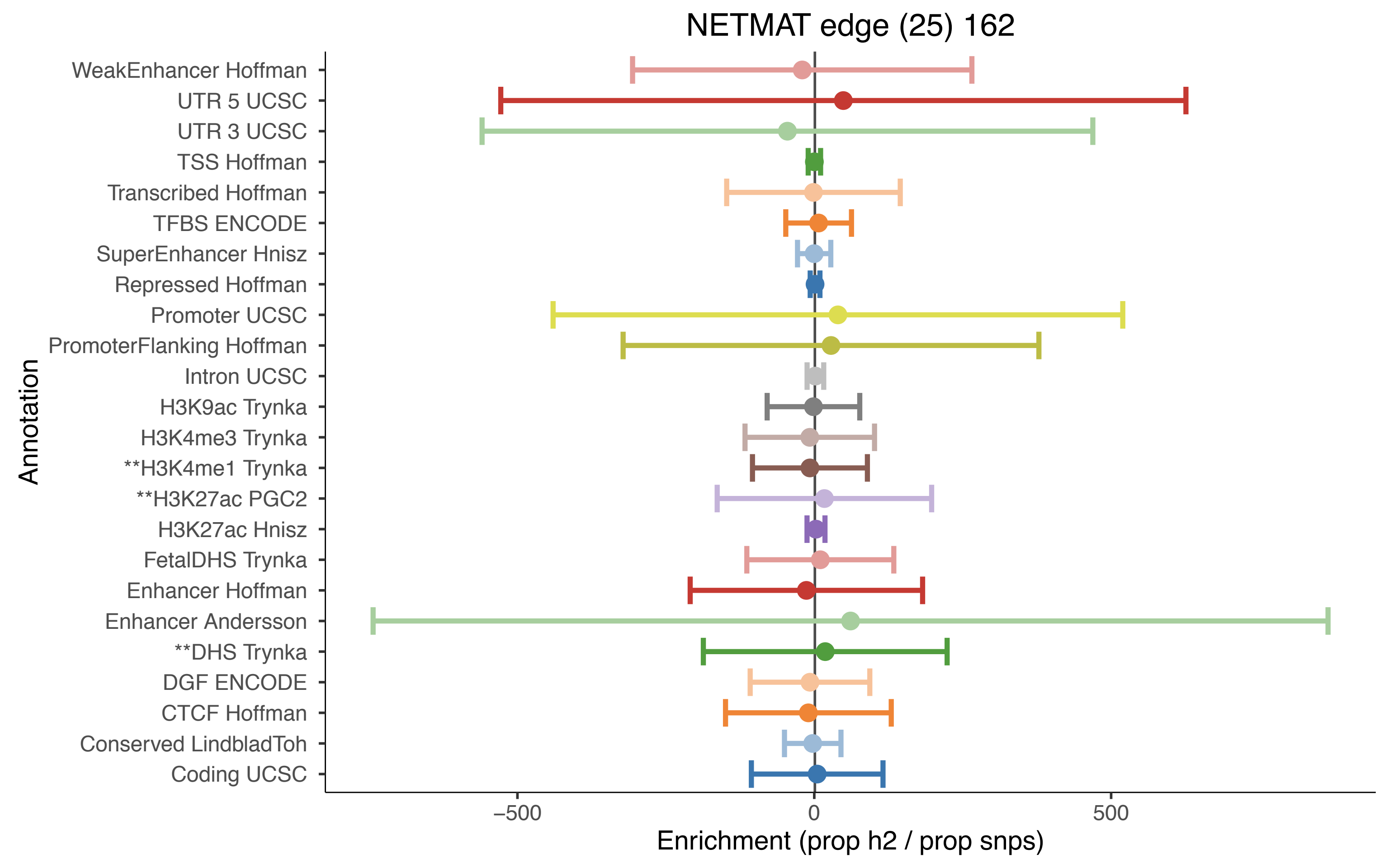
NETMAT edge (25) 162
Annotation
WeakEnhancer Hoffman
UTR 5 UCSC
UTR 3 UCSC
TSS Hoffman
Transcribed Hoffman
TFBS ENCODE
SuperEnhancer Hnisz
Repressed Hoffman
Promoter UCSC
PromoterFlanking Hoffman
Intron UCSC
H3K9ac Trynka
H3K4me3 Trynka
**H3K4me1 Trynka
**H3K27ac PGC2
H3K27ac Hnisz
FetalDHS Trynka
Enhancer Hoffman
Enhancer Andersson
**DHS Trynka
DGF ENCODE
CTCF Hoffman
Conserved LindbladToh
Coding UCSC
−500
0
500
Enrichment (prop h2 / prop snps)

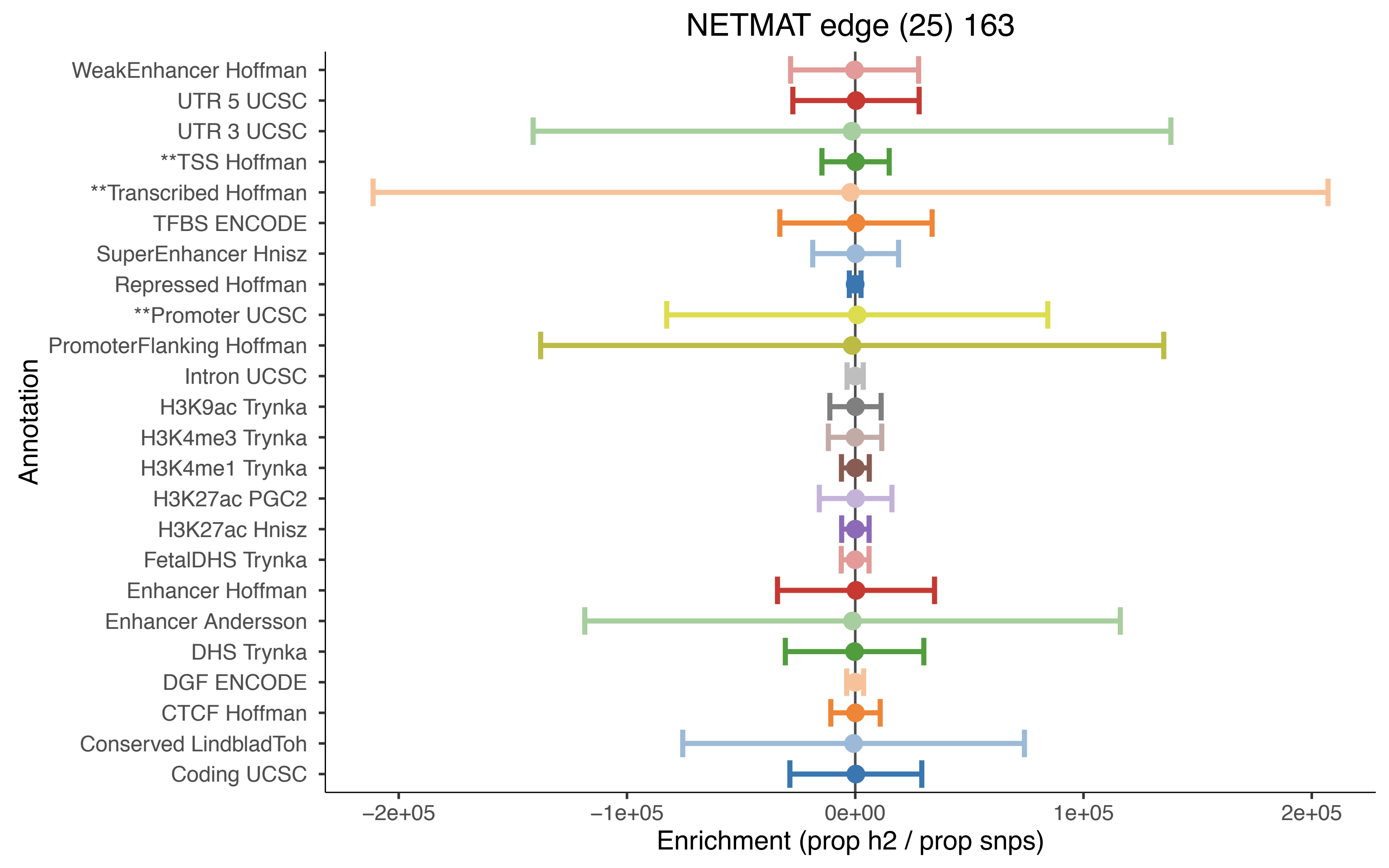
NETMAT edge (25) 163
WeakEnhancer Hoffman
UTR 5 UCSC
UTR 3 UCSC
**TSS Hoffman
**Transcribed Hoffman
TFBS ENCODE
SuperEnhancer Hnisz
Repressed Hoffman
**Promoter UCSC
PromoterFlanking Hoffman
Intron UCSC
H3K9ac Trynka
H3K4me3 Trynka
H3K4me1 Trynka
H3K27ac PGC2
H3K27ac Hnisz
FetalDHS Trynka
Enhancer Hoffman
Enhancer Andersson
DHS Trynka
DGF ENCODE
CTCF Hoffman
Conserved LindbladToh
Coding UCSC
Annotation
−2e+05
−1e+05
0e+00
1e+05
2e+05
Enrichment (prop h2 / prop snps)

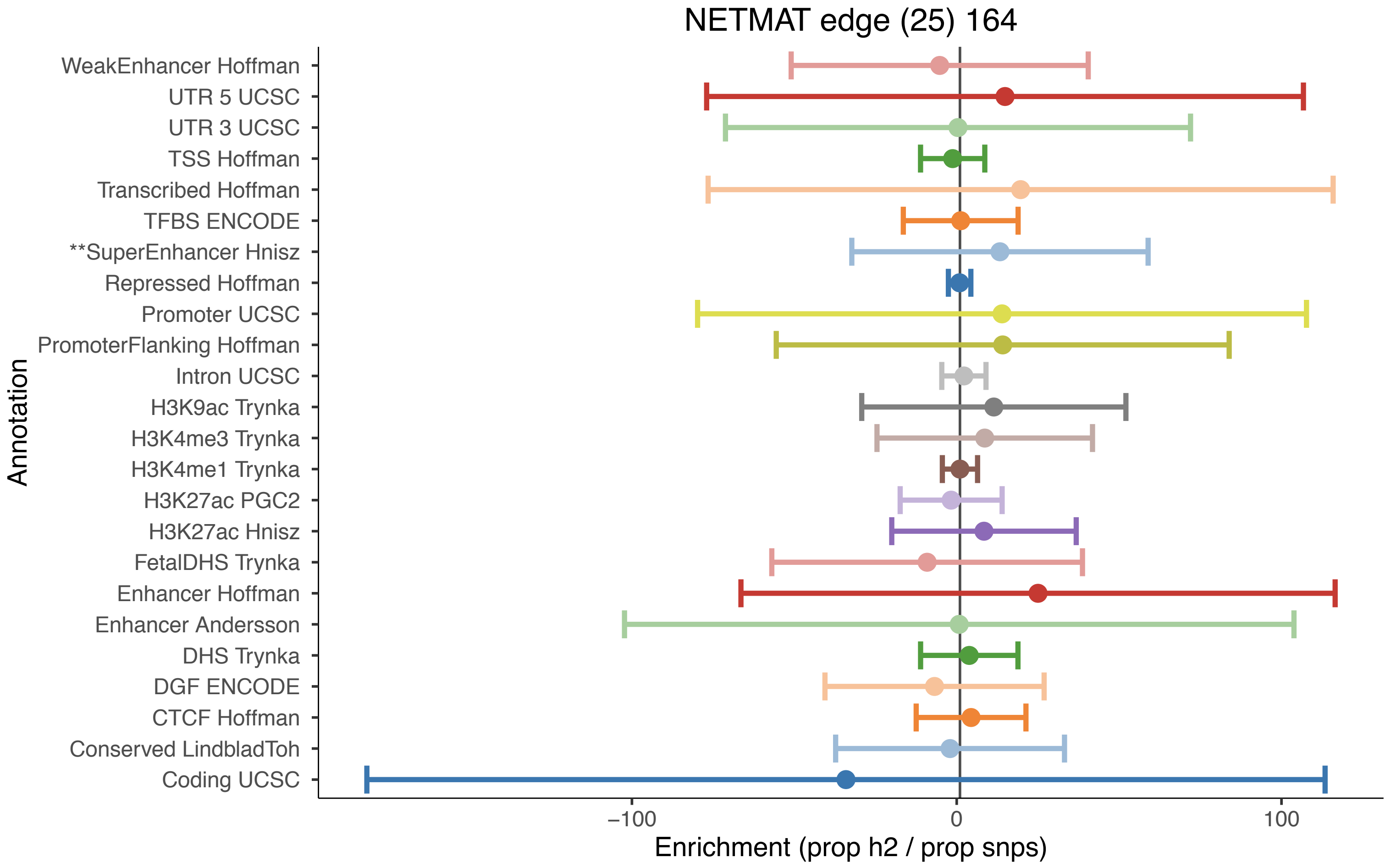

NETMAT edge (25) 164
WeakEnhancer Hoffman
UTR 5 UCSC
UTR 3 UCSC
TSS Hoffman
Transcribed Hoffman
TFBS ENCODE
**SuperEnhancer Hnisz
Repressed Hoffman
Promoter UCSC
PromoterFlanking Hoffman
Intron UCSC
H3K9ac Trynka
H3K4me3 Trynka
H3K4me1 Trynka
H3K27ac PGC2
H3K27ac Hnisz
FetalDHS Trynka
Enhancer Hoffman
Enhancer Andersson
DHS Trynka
DGF ENCODE
CTCF Hoffman
Conserved LindbladToh
Coding UCSC
Annotation
−100
0
100
Enrichment (prop h2 / prop snps)

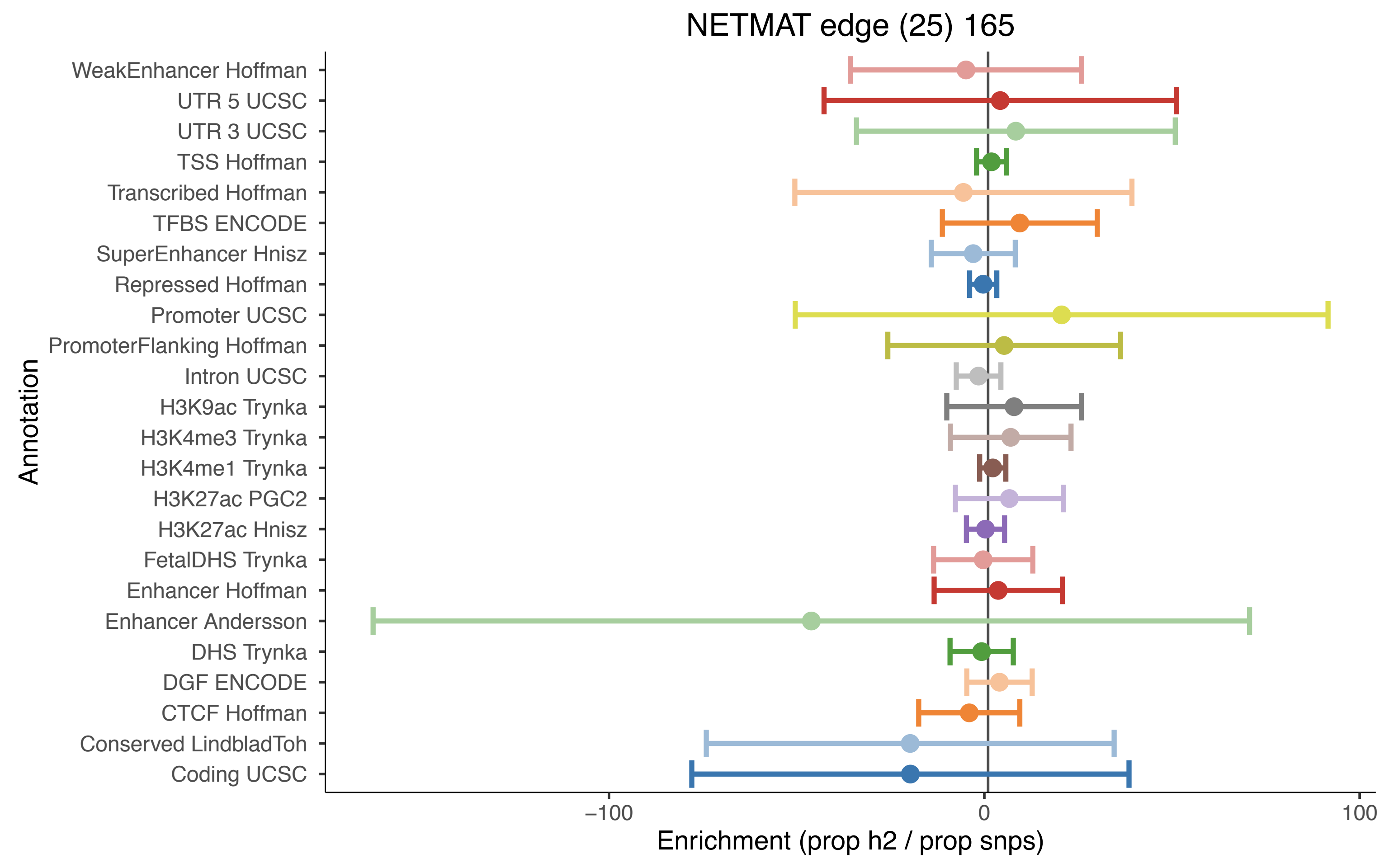

NETMAT edge (25) 165
WeakEnhancer Hoffman
UTR 5 UCSC
UTR 3 UCSC
TSS Hoffman
Transcribed Hoffman
TFBS ENCODE
SuperEnhancer Hnisz
Repressed Hoffman
Promoter UCSC
PromoterFlanking Hoffman
Intron UCSC
H3K9ac Trynka
H3K4me3 Trynka
H3K4me1 Trynka
H3K27ac PGC2
H3K27ac Hnisz
FetalDHS Trynka
Enhancer Hoffman
Enhancer Andersson
DHS Trynka
DGF ENCODE
CTCF Hoffman
Conserved LindbladToh
Coding UCSC
Annotation
−100
0
100
Enrichment (prop h2 / prop snps)

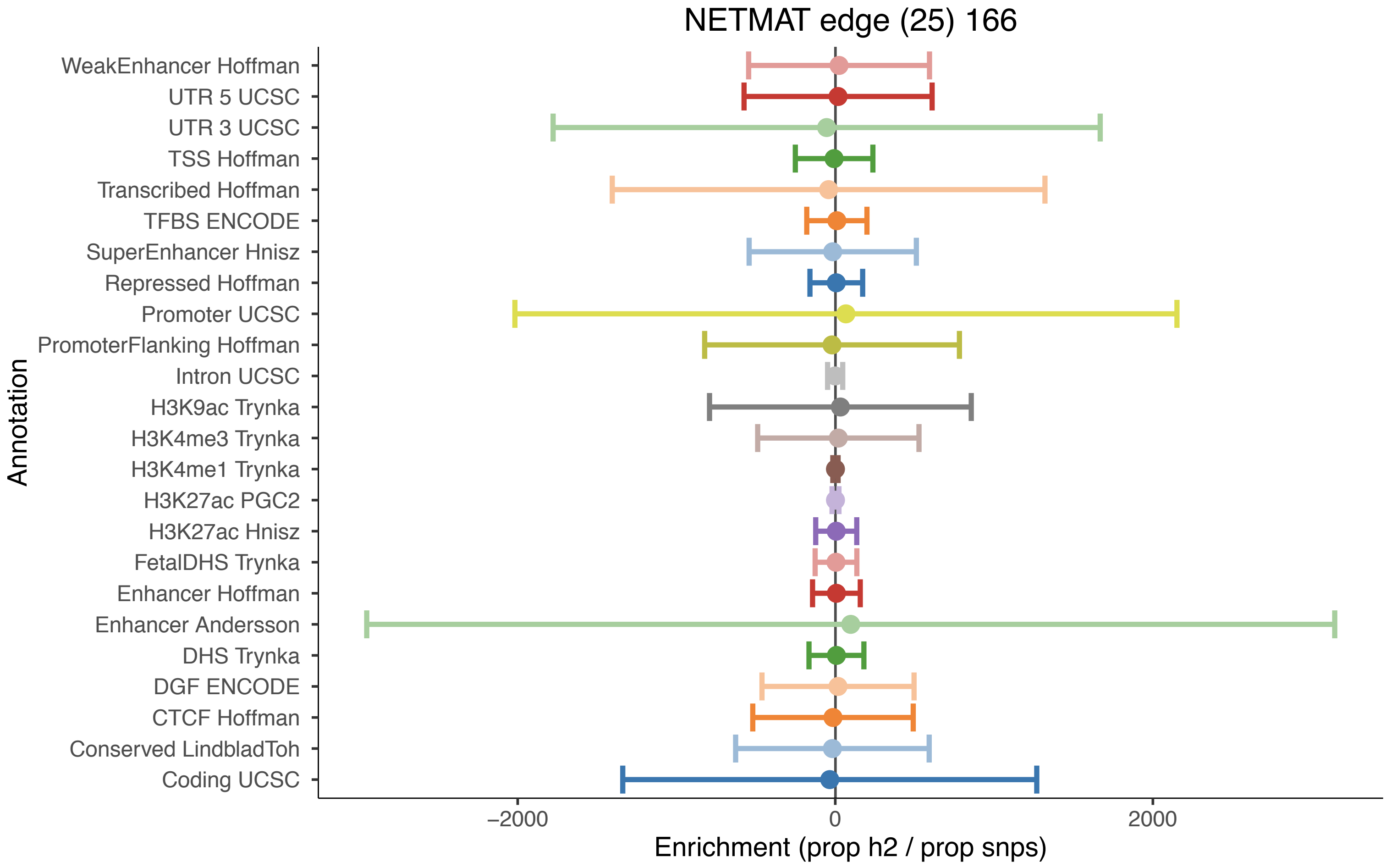

NETMAT edge (25) 166
Annotation
WeakEnhancer Hoffman
UTR 5 UCSC
UTR 3 UCSC
TSS Hoffman
Transcribed Hoffman
TFBS ENCODE
SuperEnhancer Hnisz
Repressed Hoffman
Promoter UCSC
PromoterFlanking Hoffman
Intron UCSC
H3K9ac Trynka
H3K4me3 Trynka
H3K4me1 Trynka
H3K27ac PGC2
H3K27ac Hnisz
FetalDHS Trynka
Enhancer Hoffman
Enhancer Andersson
DHS Trynka
DGF ENCODE
CTCF Hoffman
Conserved LindbladToh
Coding UCSC
−2000
0
2000
Enrichment (prop h2 / prop snps)

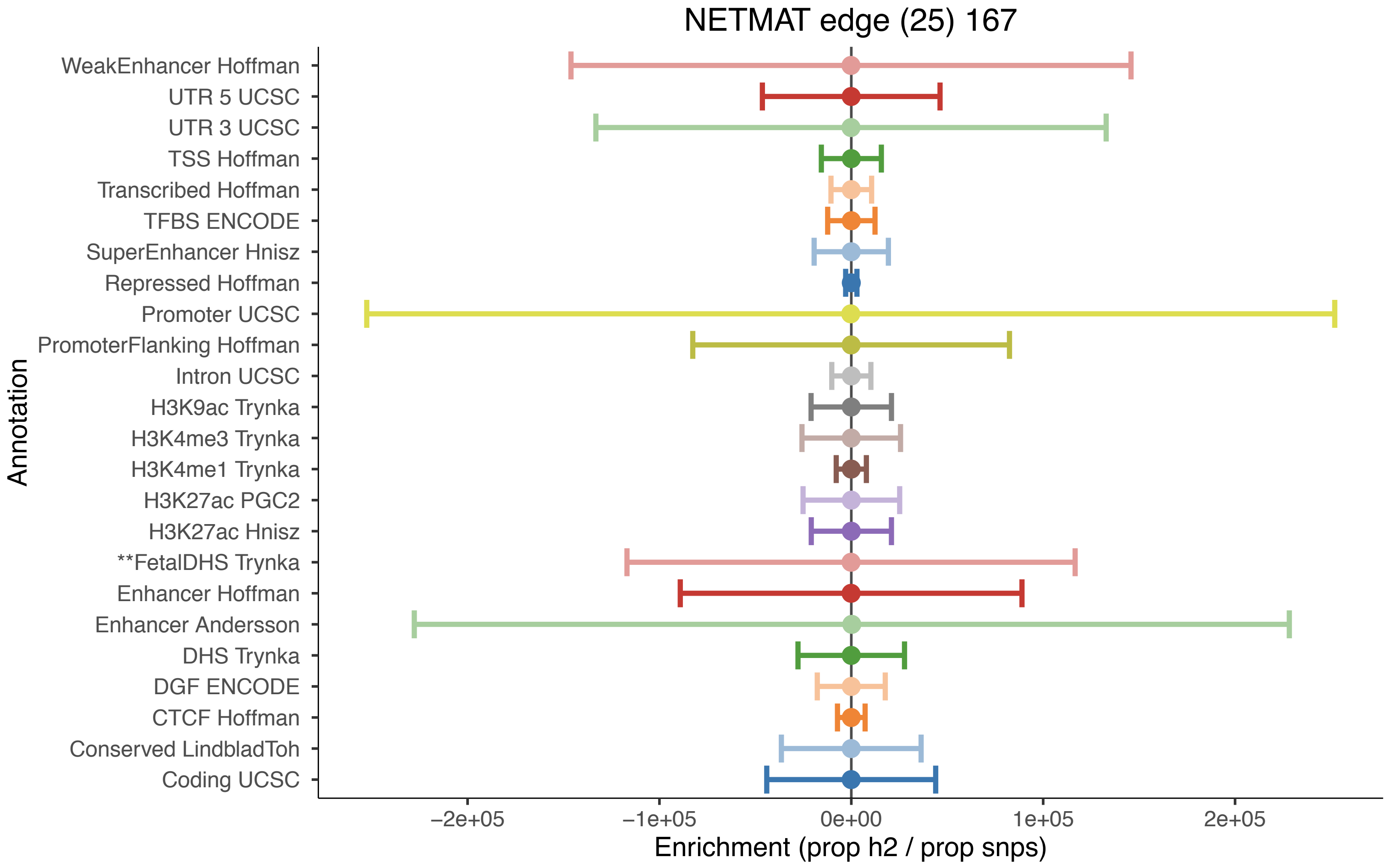
NETMAT edge (25) 167
WeakEnhancer Hoffman
UTR 5 UCSC
UTR 3 UCSC
TSS Hoffman
Transcribed Hoffman
TFBS ENCODE
SuperEnhancer Hnisz
Repressed Hoffman
Promoter UCSC
PromoterFlanking Hoffman
Intron UCSC
H3K9ac Trynka
H3K4me3 Trynka
H3K4me1 Trynka
H3K27ac PGC2
H3K27ac Hnisz
**FetalDHS Trynka
Enhancer Hoffman
Enhancer Andersson
DHS Trynka
DGF ENCODE
CTCF Hoffman
Conserved LindbladToh
Coding UCSC
Annotation
−2e+05
−1e+05
0e+00
1e+05
2e+05
Enrichment (prop h2 / prop snps)

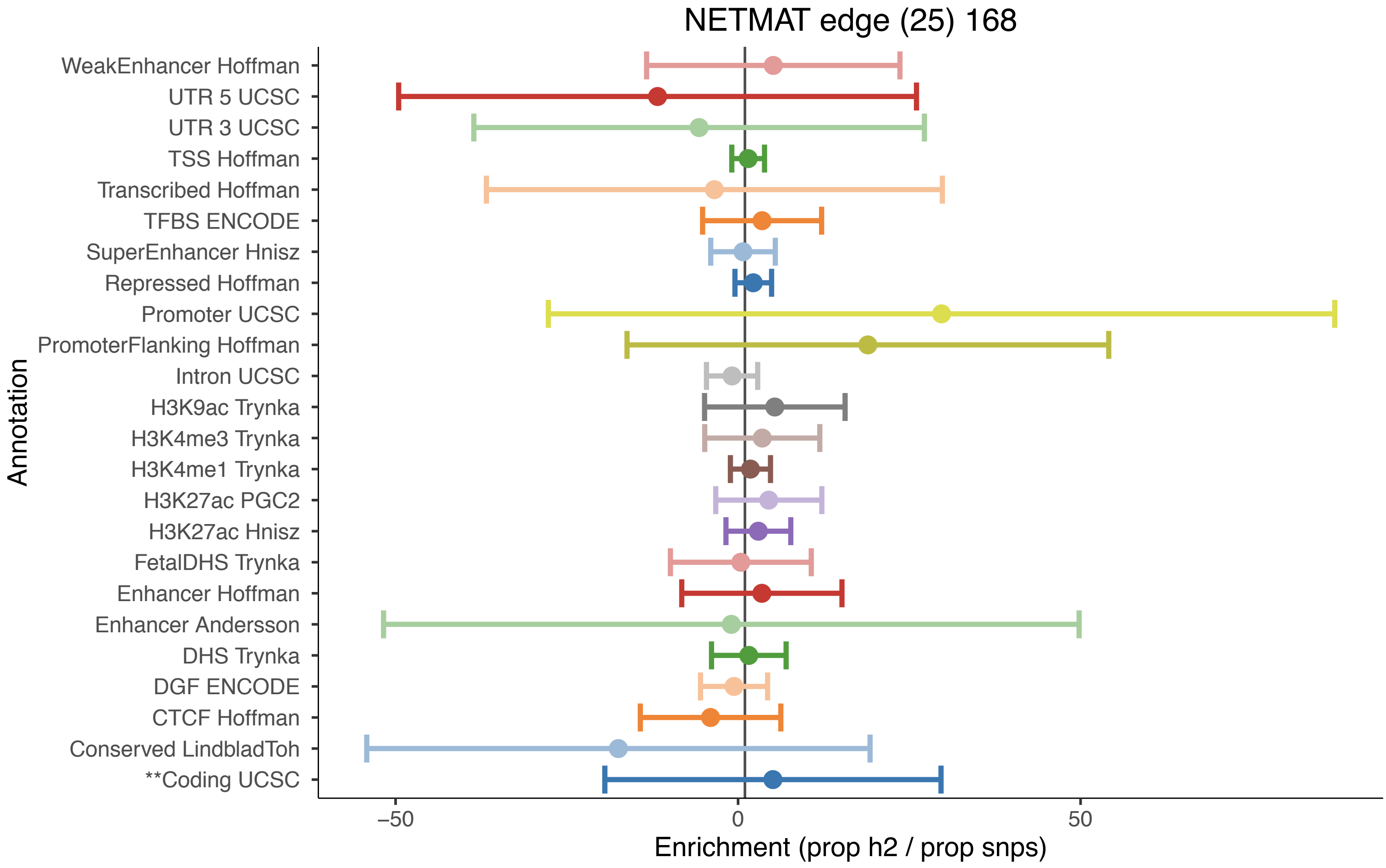

NETMAT edge (25) 168
WeakEnhancer Hoffman
UTR 5 UCSC
UTR 3 UCSC
TSS Hoffman
Transcribed Hoffman
TFBS ENCODE
SuperEnhancer Hnisz
Repressed Hoffman
Promoter UCSC
PromoterFlanking Hoffman
Intron UCSC
H3K9ac Trynka
H3K4me3 Trynka
H3K4me1 Trynka
H3K27ac PGC2
H3K27ac Hnisz
FetalDHS Trynka
Enhancer Hoffman
Enhancer Andersson
DHS Trynka
DGF ENCODE
CTCF Hoffman
Conserved LindbladToh
**Coding UCSC
Annotation
−50
0
50
Enrichment (prop h2 / prop snps)

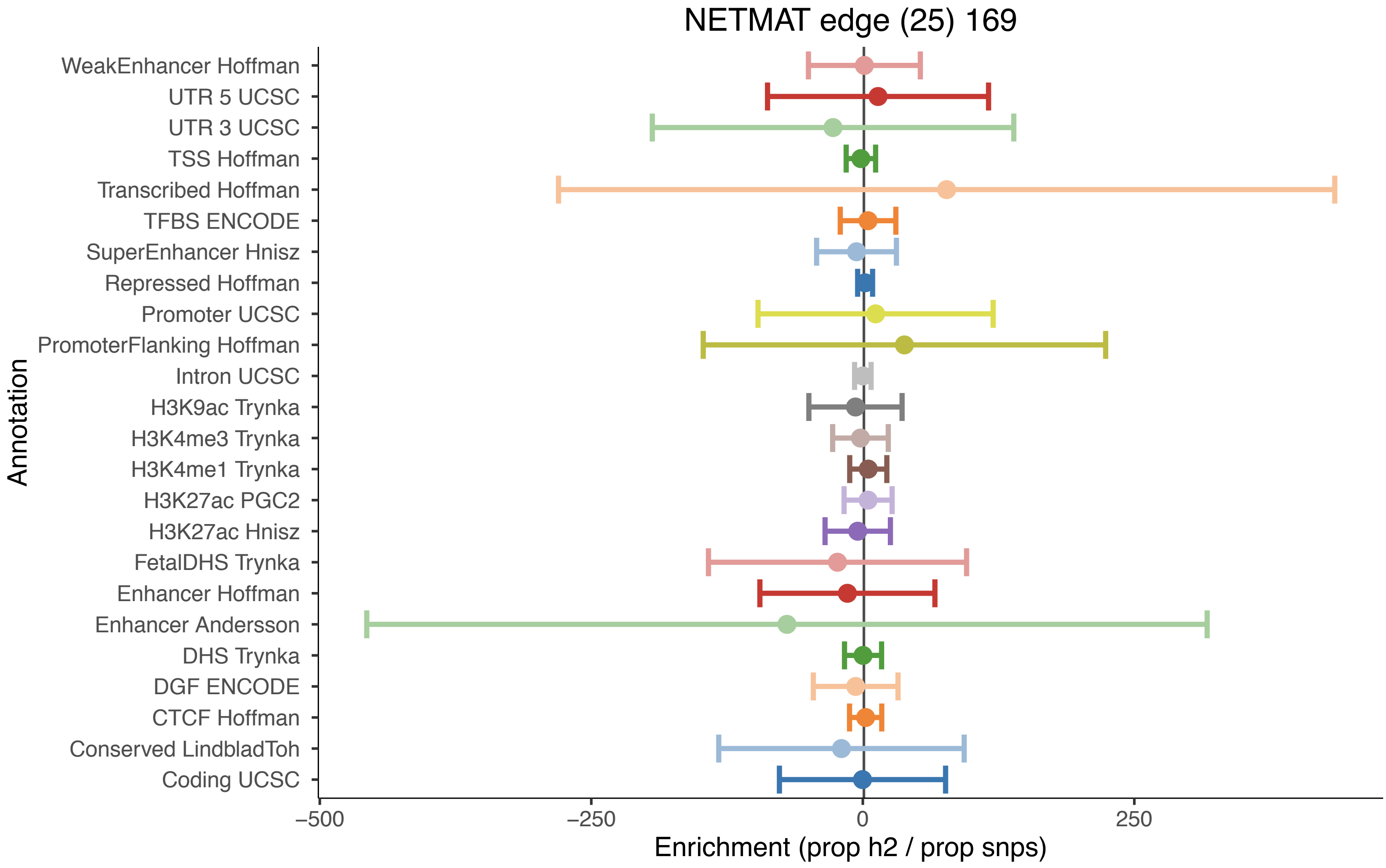

NETMAT edge (25) 169
WeakEnhancer Hoffman
UTR 5 UCSC
UTR 3 UCSC
TSS Hoffman
Transcribed Hoffman
TFBS ENCODE
SuperEnhancer Hnisz
Repressed Hoffman
Promoter UCSC
PromoterFlanking Hoffman
Intron UCSC
H3K9ac Trynka
H3K4me3 Trynka
H3K4me1 Trynka
H3K27ac PGC2
H3K27ac Hnisz
FetalDHS Trynka
Enhancer Hoffman
Enhancer Andersson
DHS Trynka
DGF ENCODE
CTCF Hoffman
Conserved LindbladToh
Coding UCSC
Annotation
−500
−250
0
250
Enrichment (prop h2 / prop snps)

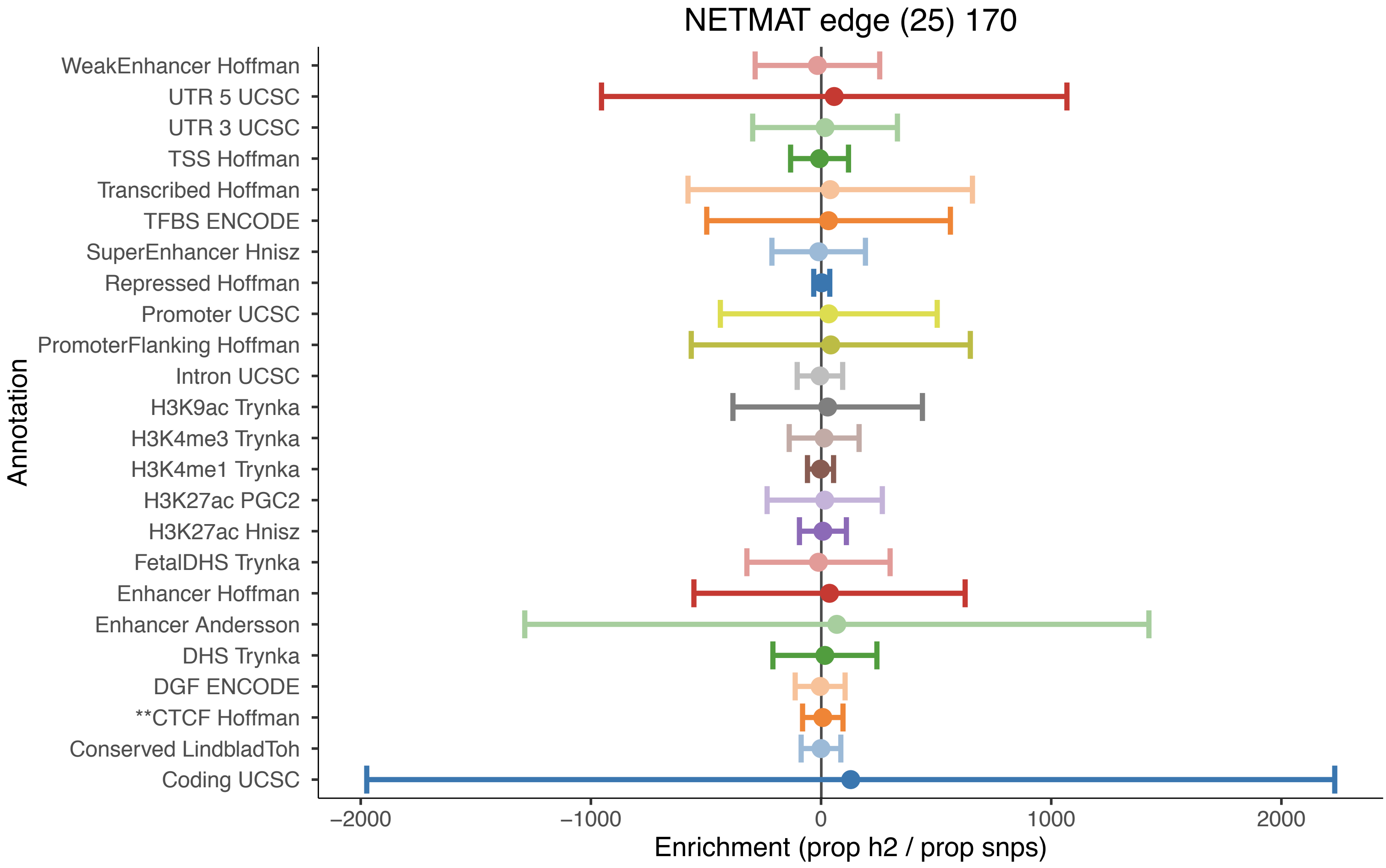
NETMAT edge (25) 170
WeakEnhancer Hoffman
UTR 5 UCSC
UTR 3 UCSC
TSS Hoffman
Transcribed Hoffman
TFBS ENCODE
SuperEnhancer Hnisz
Repressed Hoffman
Promoter UCSC
PromoterFlanking Hoffman
Intron UCSC
H3K9ac Trynka
H3K4me3 Trynka
H3K4me1 Trynka
H3K27ac PGC2
H3K27ac Hnisz
FetalDHS Trynka
Enhancer Hoffman
Enhancer Andersson
DHS Trynka
DGF ENCODE
**CTCF Hoffman
Conserved LindbladToh
Coding UCSC
Annotation
−2000
−1000
0
1000
2000
Enrichment (prop h2 / prop snps)

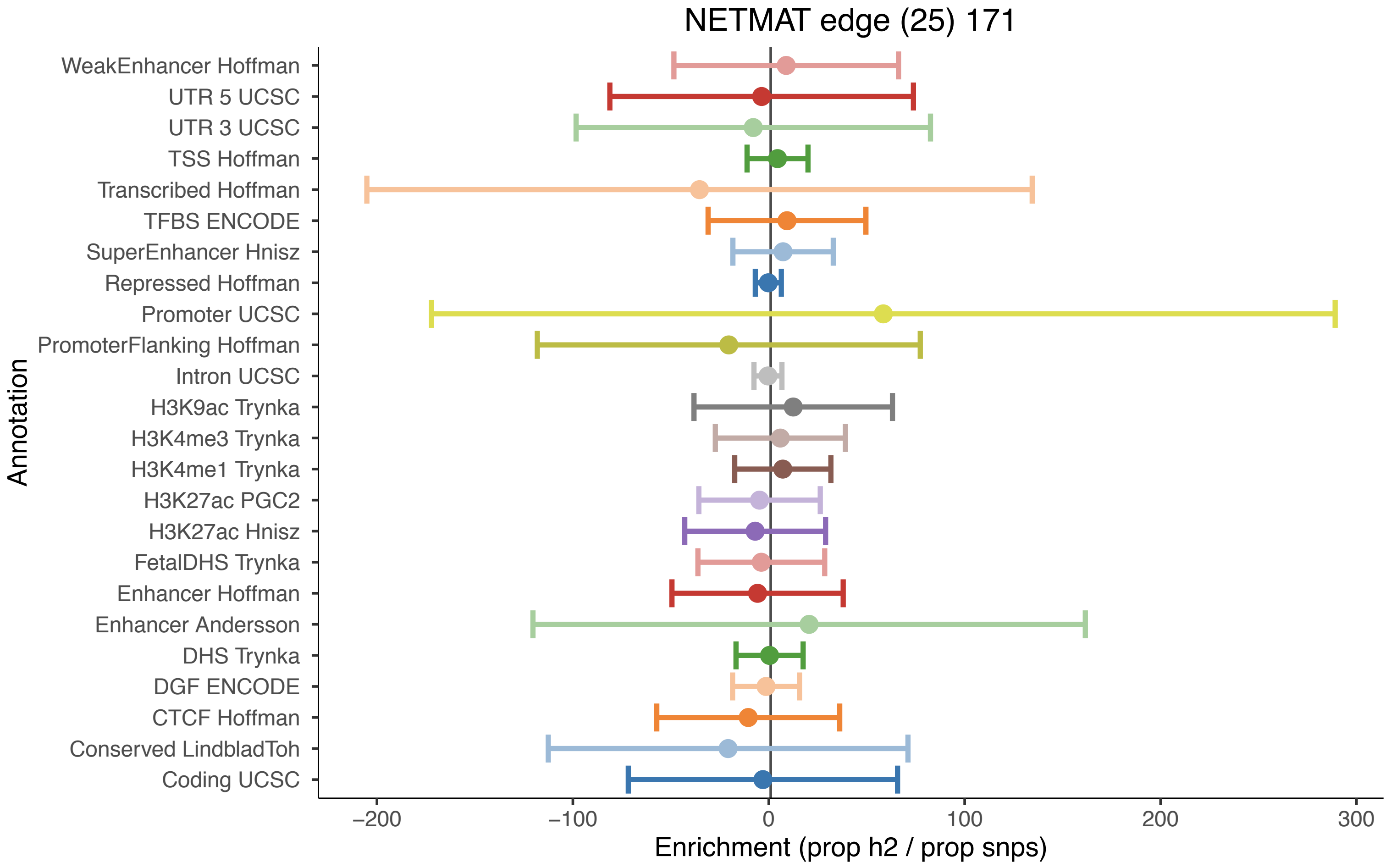
NETMAT edge (25) 171
WeakEnhancer Hoffman
UTR 5 UCSC
UTR 3 UCSC
TSS Hoffman
Transcribed Hoffman
TFBS ENCODE
SuperEnhancer Hnisz
Repressed Hoffman
Promoter UCSC
PromoterFlanking Hoffman
Intron UCSC
H3K9ac Trynka
H3K4me3 Trynka
H3K4me1 Trynka
H3K27ac PGC2
H3K27ac Hnisz
FetalDHS Trynka
Enhancer Hoffman
Enhancer Andersson
DHS Trynka
DGF ENCODE
CTCF Hoffman
Conserved LindbladToh
Coding UCSC
Annotation
−200
−100
0
100
200
300
Enrichment (prop h2 / prop snps)

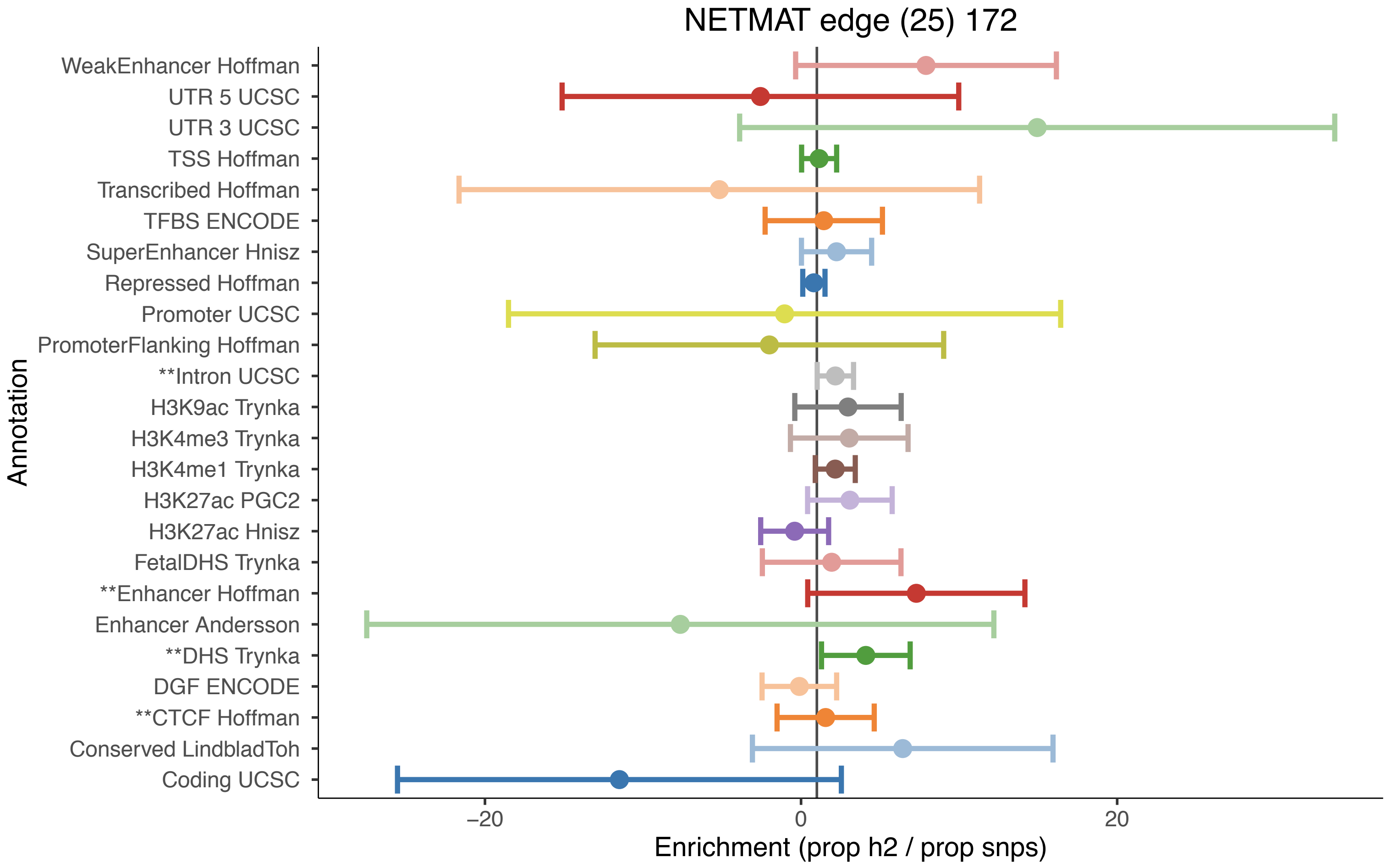
NETMAT edge (25) 172
WeakEnhancer Hoffman
UTR 5 UCSC
UTR 3 UCSC
TSS Hoffman
Transcribed Hoffman
TFBS ENCODE
SuperEnhancer Hnisz
Repressed Hoffman
Promoter UCSC
PromoterFlanking Hoffman
**Intron UCSC
H3K9ac Trynka
H3K4me3 Trynka
H3K4me1 Trynka
H3K27ac PGC2
H3K27ac Hnisz
FetalDHS Trynka
**Enhancer Hoffman
Enhancer Andersson
**DHS Trynka
DGF ENCODE
**CTCF Hoffman
Conserved LindbladToh
Coding UCSC
Annotation
−20
0
20
Enrichment (prop h2 / prop snps)

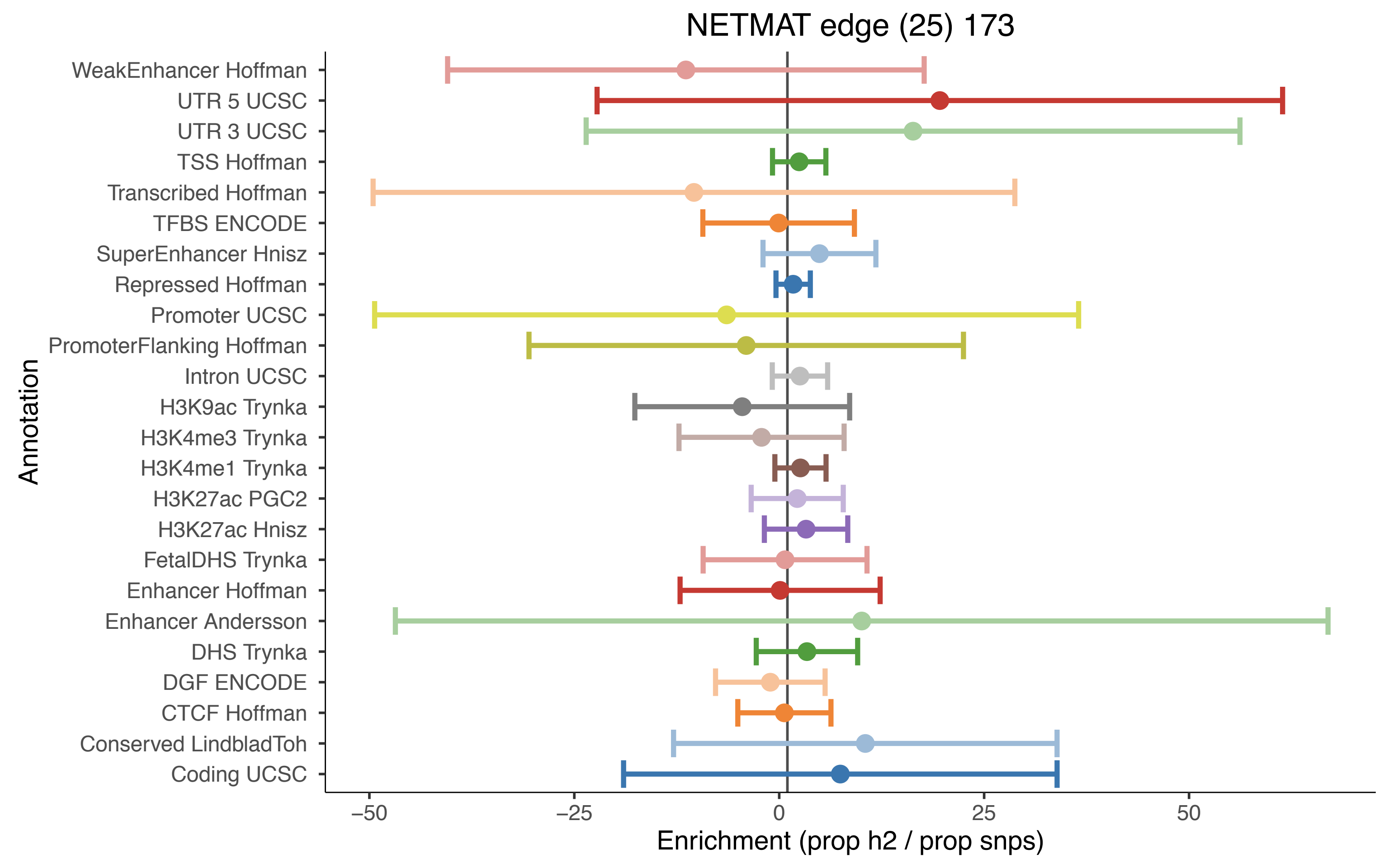
NETMAT edge (25) 173
WeakEnhancer Hoffman
UTR 5 UCSC
UTR 3 UCSC
TSS Hoffman
Transcribed Hoffman
TFBS ENCODE
SuperEnhancer Hnisz
Repressed Hoffman
Promoter UCSC
PromoterFlanking Hoffman
Intron UCSC
H3K9ac Trynka
H3K4me3 Trynka
H3K4me1 Trynka
H3K27ac PGC2
H3K27ac Hnisz
FetalDHS Trynka
Enhancer Hoffman
Enhancer Andersson
DHS Trynka
DGF ENCODE
CTCF Hoffman
Conserved LindbladToh
Coding UCSC
Annotation
−50
−25
0
25
50
Enrichment (prop h2 / prop snps)

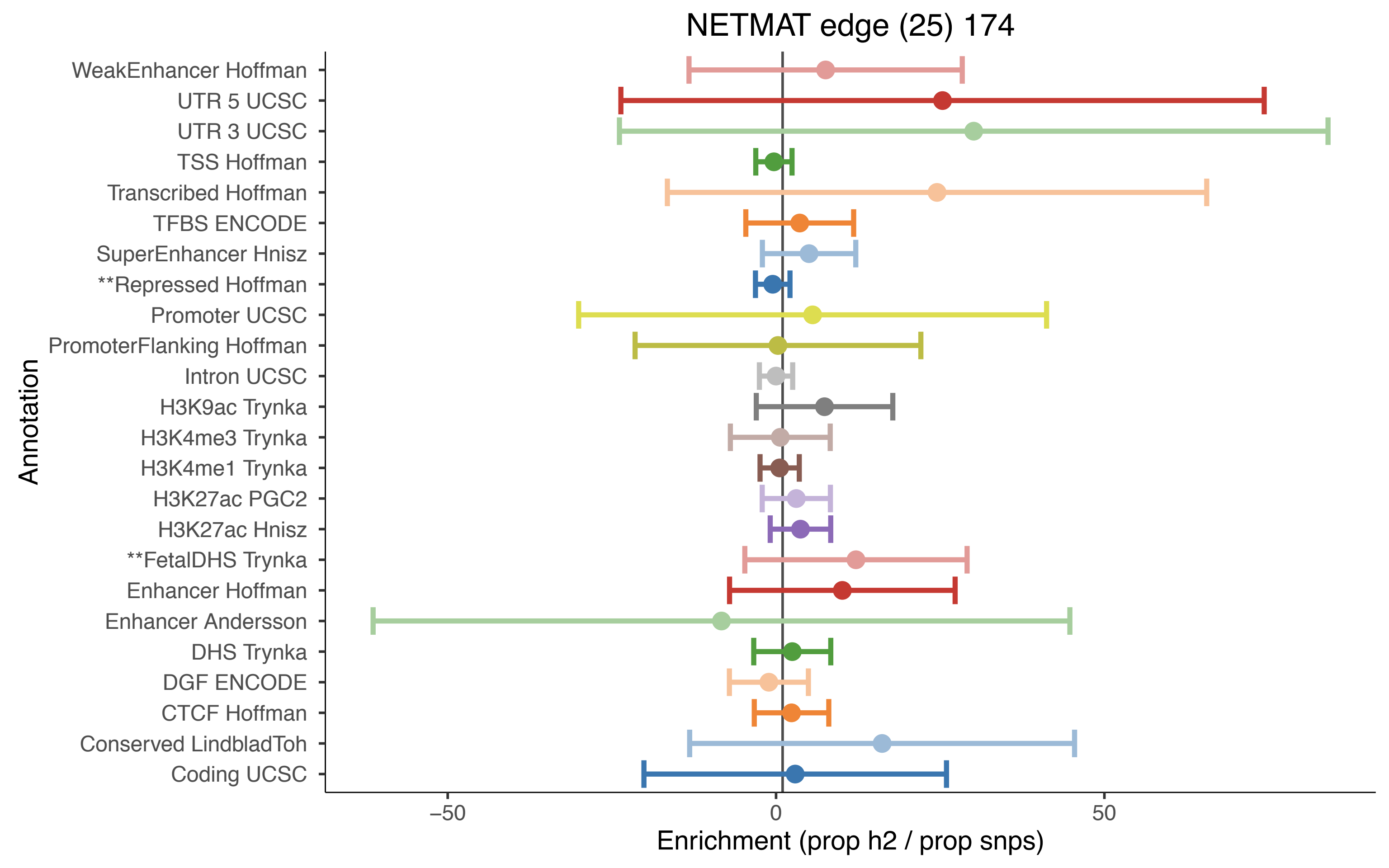
NETMAT edge (25) 174
WeakEnhancer Hoffman
UTR 5 UCSC
UTR 3 UCSC
TSS Hoffman
Transcribed Hoffman
TFBS ENCODE
SuperEnhancer Hnisz
**Repressed Hoffman
Promoter UCSC
PromoterFlanking Hoffman
Intron UCSC
H3K9ac Trynka
H3K4me3 Trynka
H3K4me1 Trynka
H3K27ac PGC2
H3K27ac Hnisz
**FetalDHS Trynka
Enhancer Hoffman
Enhancer Andersson
DHS Trynka
DGF ENCODE
CTCF Hoffman
Conserved LindbladToh
Coding UCSC
Annotation
−50
0
50
Enrichment (prop h2 / prop snps)

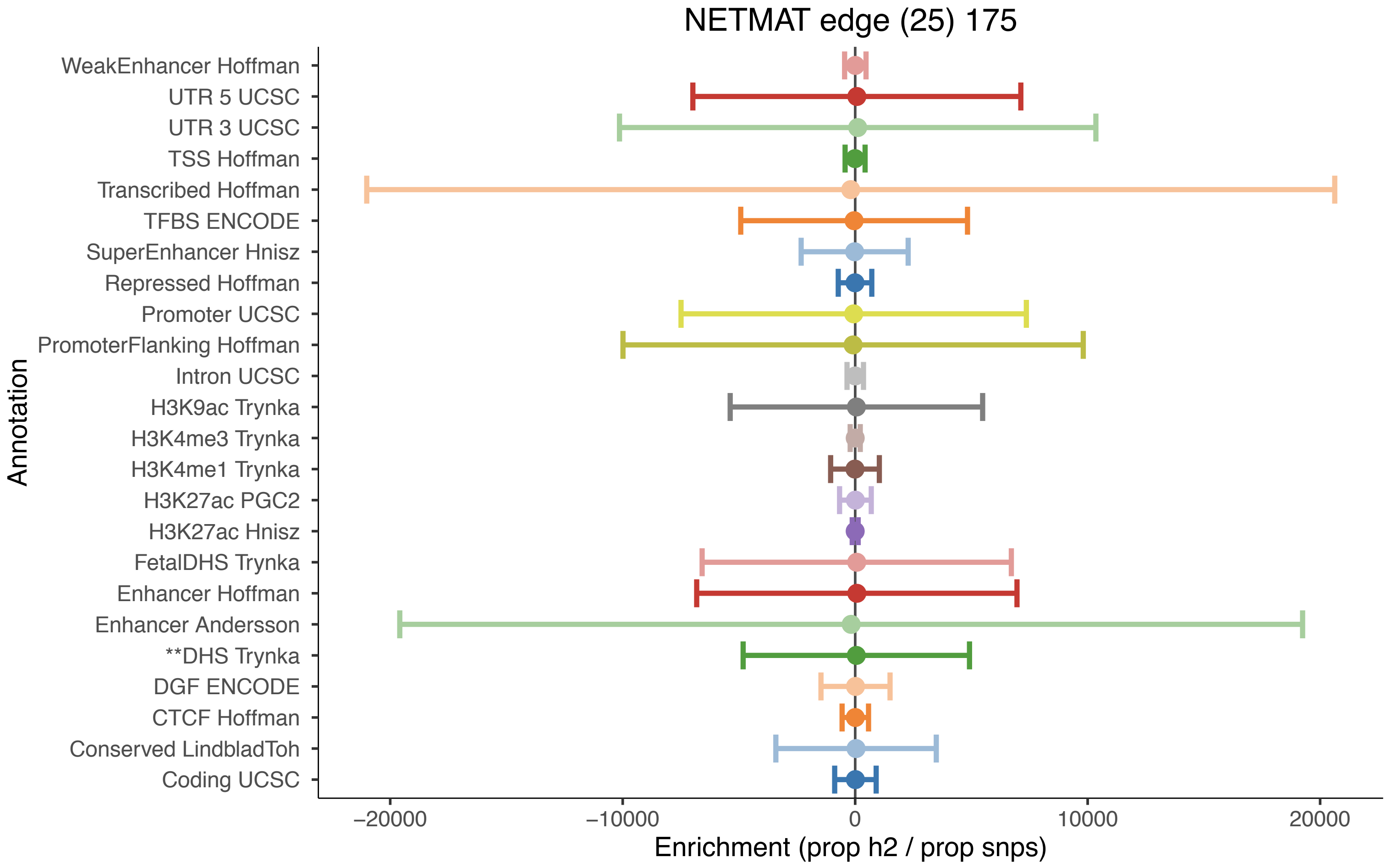

NETMAT edge (25) 175
WeakEnhancer Hoffman
UTR 5 UCSC
UTR 3 UCSC
TSS Hoffman
Transcribed Hoffman
TFBS ENCODE
SuperEnhancer Hnisz
Repressed Hoffman
Promoter UCSC
PromoterFlanking Hoffman
Intron UCSC
H3K9ac Trynka
H3K4me3 Trynka
H3K4me1 Trynka
H3K27ac PGC2
H3K27ac Hnisz
FetalDHS Trynka
Enhancer Hoffman
Enhancer Andersson
**DHS Trynka
DGF ENCODE
CTCF Hoffman
Conserved LindbladToh
Coding UCSC
Annotation
−20000
−10000
0
10000
20000
Enrichment (prop h2 / prop snps)

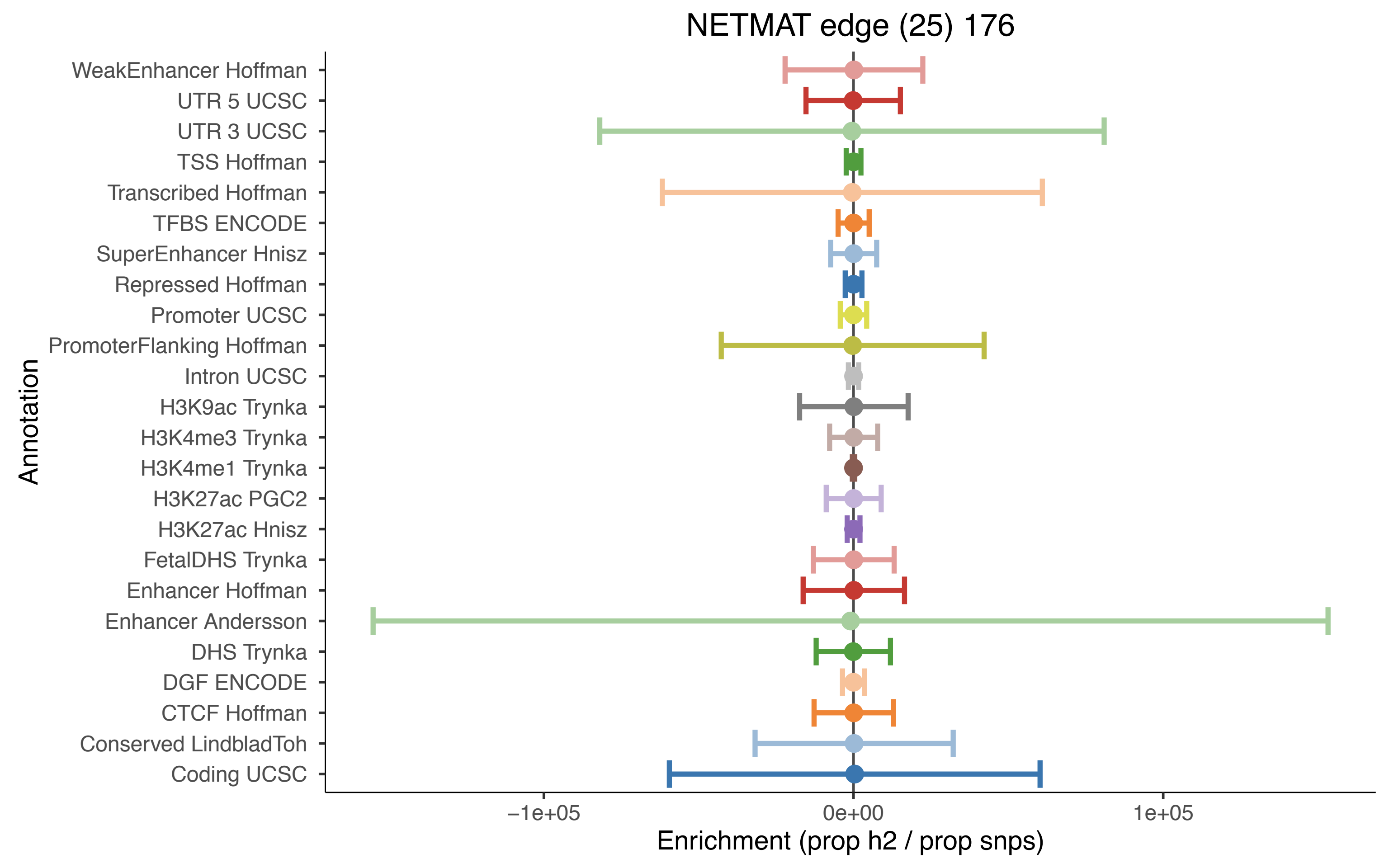
NETMAT edge (25) 176
WeakEnhancer Hoffman
UTR 5 UCSC
UTR 3 UCSC
TSS Hoffman
Transcribed Hoffman
TFBS ENCODE
SuperEnhancer Hnisz
Repressed Hoffman
Promoter UCSC
PromoterFlanking Hoffman
Intron UCSC
H3K9ac Trynka
H3K4me3 Trynka
H3K4me1 Trynka
H3K27ac PGC2
H3K27ac Hnisz
FetalDHS Trynka
Enhancer Hoffman
Enhancer Andersson
DHS Trynka
DGF ENCODE
CTCF Hoffman
Conserved LindbladToh
Coding UCSC
Annotation
−1e+05
0e+00
1e+05
Enrichment (prop h2 / prop snps)

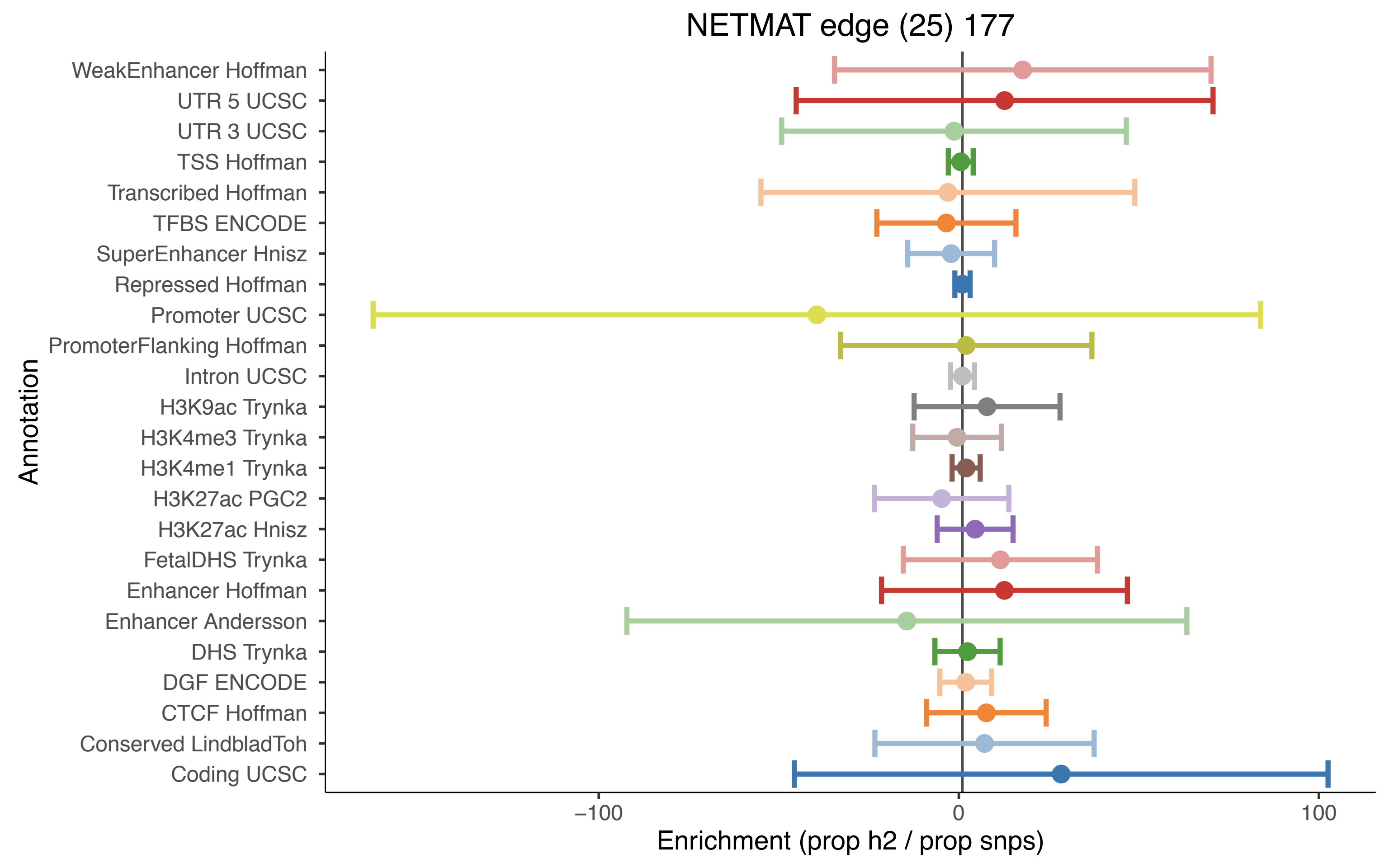
NETMAT edge (25) 177
Annotation
WeakEnhancer Hoffman
UTR 5 UCSC
UTR 3 UCSC
TSS Hoffman
Transcribed Hoffman
TFBS ENCODE
SuperEnhancer Hnisz
Repressed Hoffman
Promoter UCSC
PromoterFlanking Hoffman
Intron UCSC
H3K9ac Trynka
H3K4me3 Trynka
H3K4me1 Trynka
H3K27ac PGC2
H3K27ac Hnisz
FetalDHS Trynka
Enhancer Hoffman
Enhancer Andersson
DHS Trynka
DGF ENCODE
CTCF Hoffman
Conserved LindbladToh
Coding UCSC
−100
0
100
Enrichment (prop h2 / prop snps)

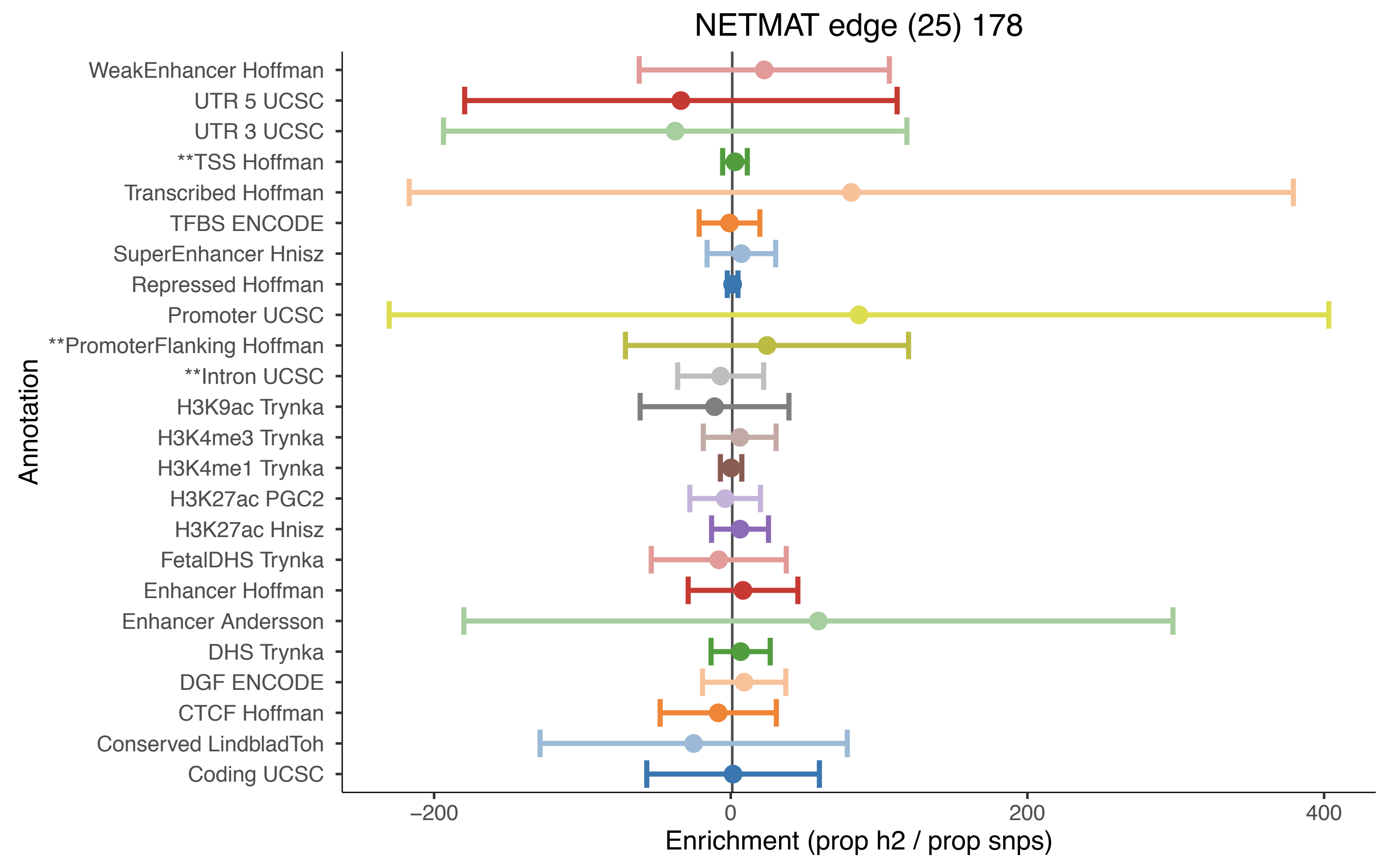

NETMAT edge (25) 178
WeakEnhancer Hoffman
UTR 5 UCSC
UTR 3 UCSC
**TSS Hoffman
Transcribed Hoffman
TFBS ENCODE
SuperEnhancer Hnisz
Repressed Hoffman
Promoter UCSC
**PromoterFlanking Hoffman
**Intron UCSC
H3K9ac Trynka
H3K4me3 Trynka
H3K4me1 Trynka
H3K27ac PGC2
H3K27ac Hnisz
FetalDHS Trynka
Enhancer Hoffman
Enhancer Andersson
DHS Trynka
DGF ENCODE
CTCF Hoffman
Conserved LindbladToh
Coding UCSC
Annotation
−200
0
200
400
Enrichment (prop h2 / prop snps)

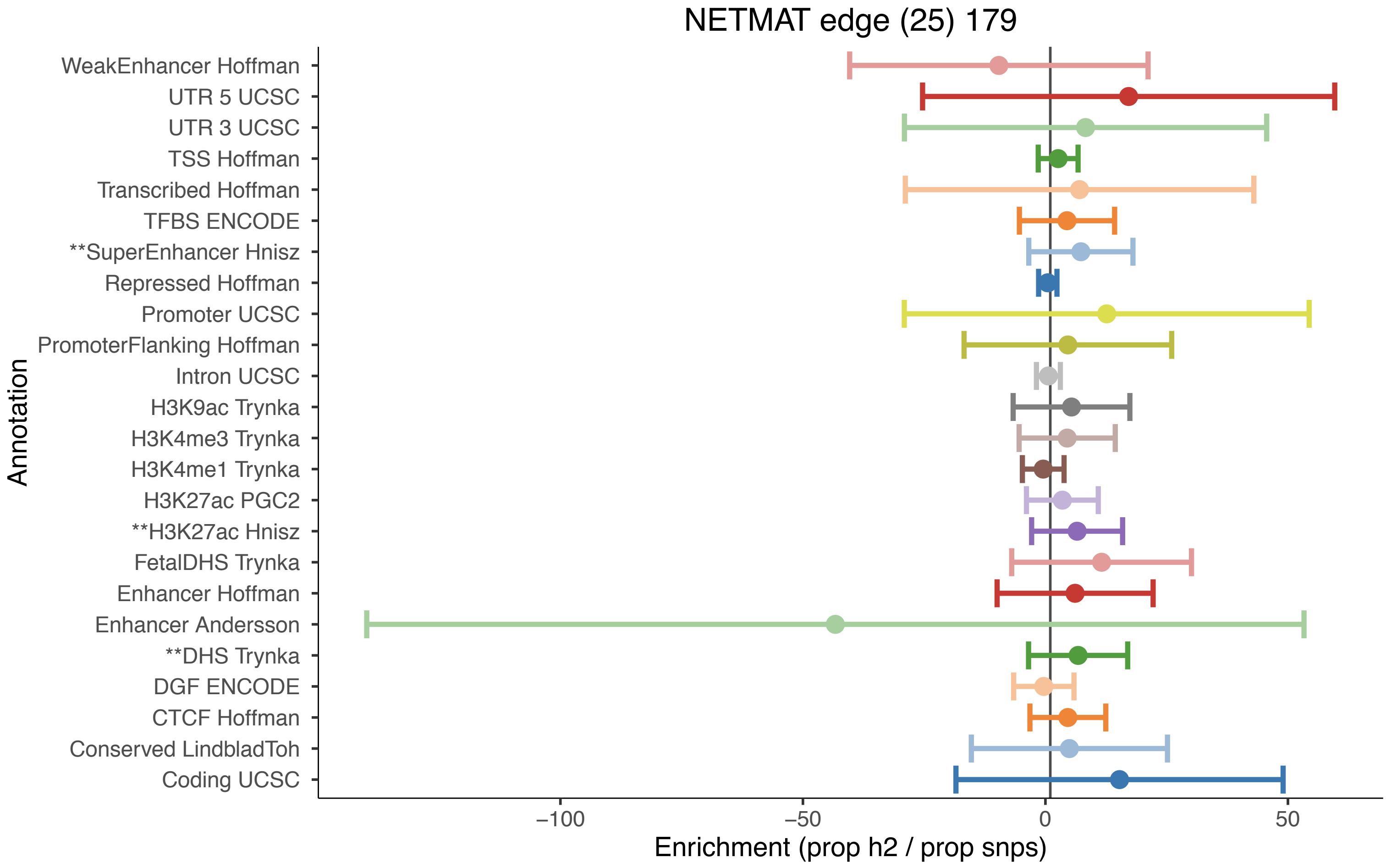

NETMAT edge (25) 179
WeakEnhancer Hoffman
UTR 5 UCSC
UTR 3 UCSC
TSS Hoffman
Transcribed Hoffman
TFBS ENCODE
**SuperEnhancer Hnisz
Repressed Hoffman
Promoter UCSC
PromoterFlanking Hoffman
Intron UCSC
H3K9ac Trynka
H3K4me3 Trynka
H3K4me1 Trynka
H3K27ac PGC2
**H3K27ac Hnisz
FetalDHS Trynka
Enhancer Hoffman
Enhancer Andersson
**DHS Trynka
DGF ENCODE
CTCF Hoffman
Conserved LindbladToh
Coding UCSC
Annotation
−100
−50
0
50
Enrichment (prop h2 / prop snps)

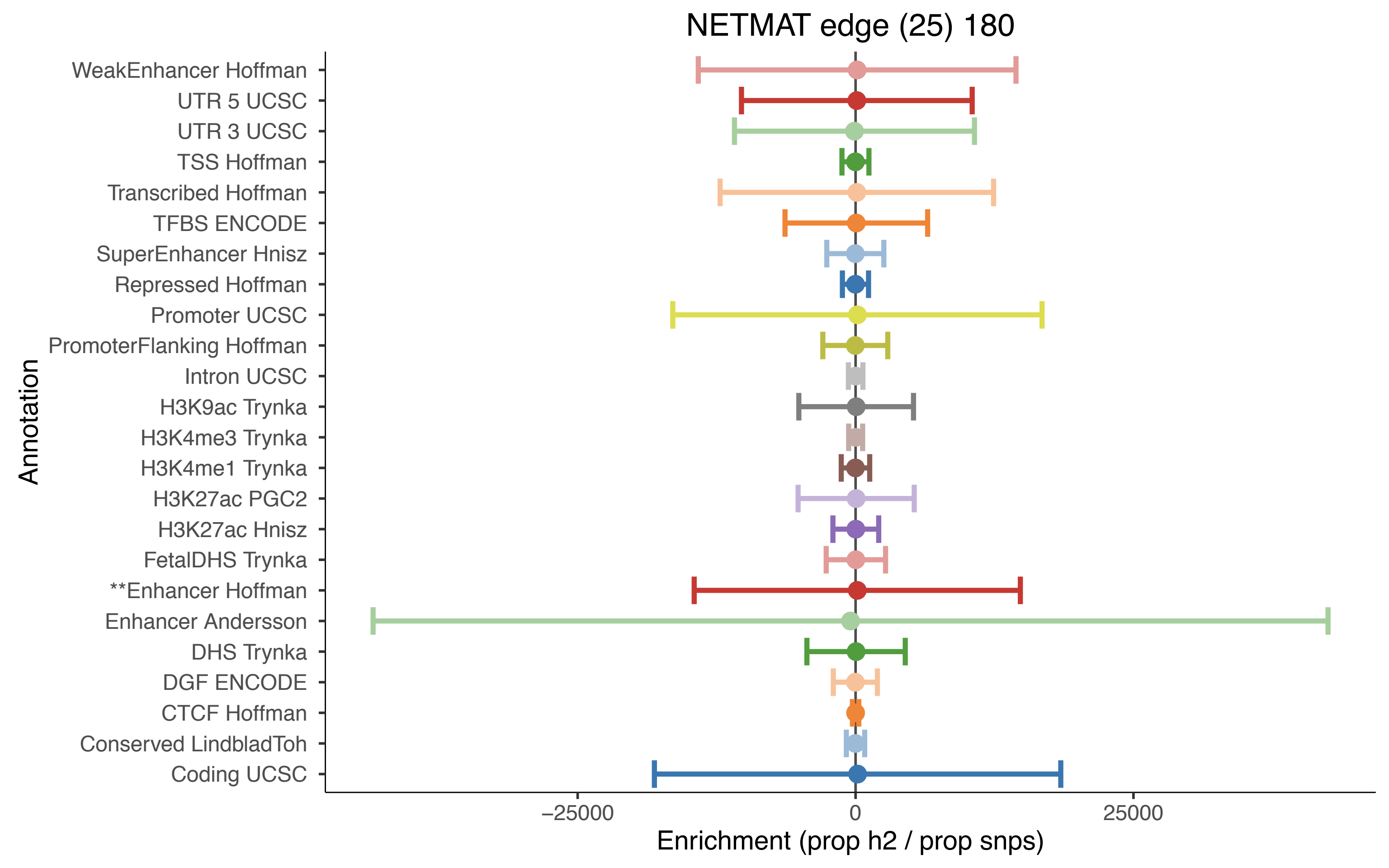
NETMAT edge (25) 180
WeakEnhancer Hoffman
UTR 5 UCSC
UTR 3 UCSC
TSS Hoffman
Transcribed Hoffman
TFBS ENCODE
SuperEnhancer Hnisz
Repressed Hoffman
Promoter UCSC
PromoterFlanking Hoffman
Intron UCSC
H3K9ac Trynka
H3K4me3 Trynka
H3K4me1 Trynka
H3K27ac PGC2
H3K27ac Hnisz
FetalDHS Trynka
**Enhancer Hoffman
Enhancer Andersson
DHS Trynka
DGF ENCODE
CTCF Hoffman
Conserved LindbladToh
Coding UCSC
Annotation
−25000
0
25000
Enrichment (prop h2 / prop snps)

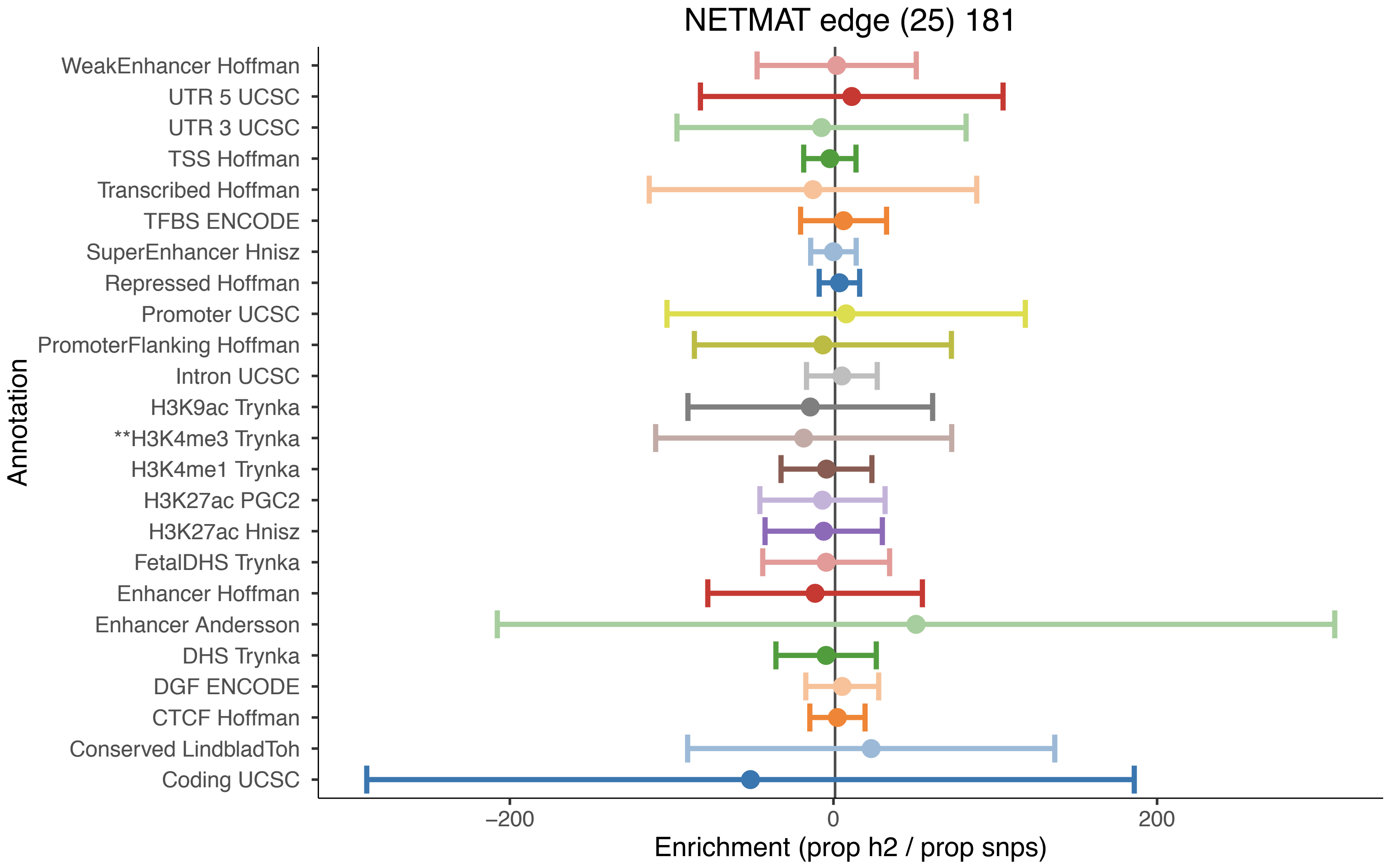
NETMAT edge (25) 181
WeakEnhancer Hoffman
UTR 5 UCSC
UTR 3 UCSC
TSS Hoffman
Transcribed Hoffman
TFBS ENCODE
SuperEnhancer Hnisz
Repressed Hoffman
Promoter UCSC
PromoterFlanking Hoffman
Intron UCSC
H3K9ac Trynka
**H3K4me3 Trynka
H3K4me1 Trynka
H3K27ac PGC2
H3K27ac Hnisz
FetalDHS Trynka
Enhancer Hoffman
Enhancer Andersson
DHS Trynka
DGF ENCODE
CTCF Hoffman
Conserved LindbladToh
Coding UCSC
Annotation
−200
0
200
Enrichment (prop h2 / prop snps)

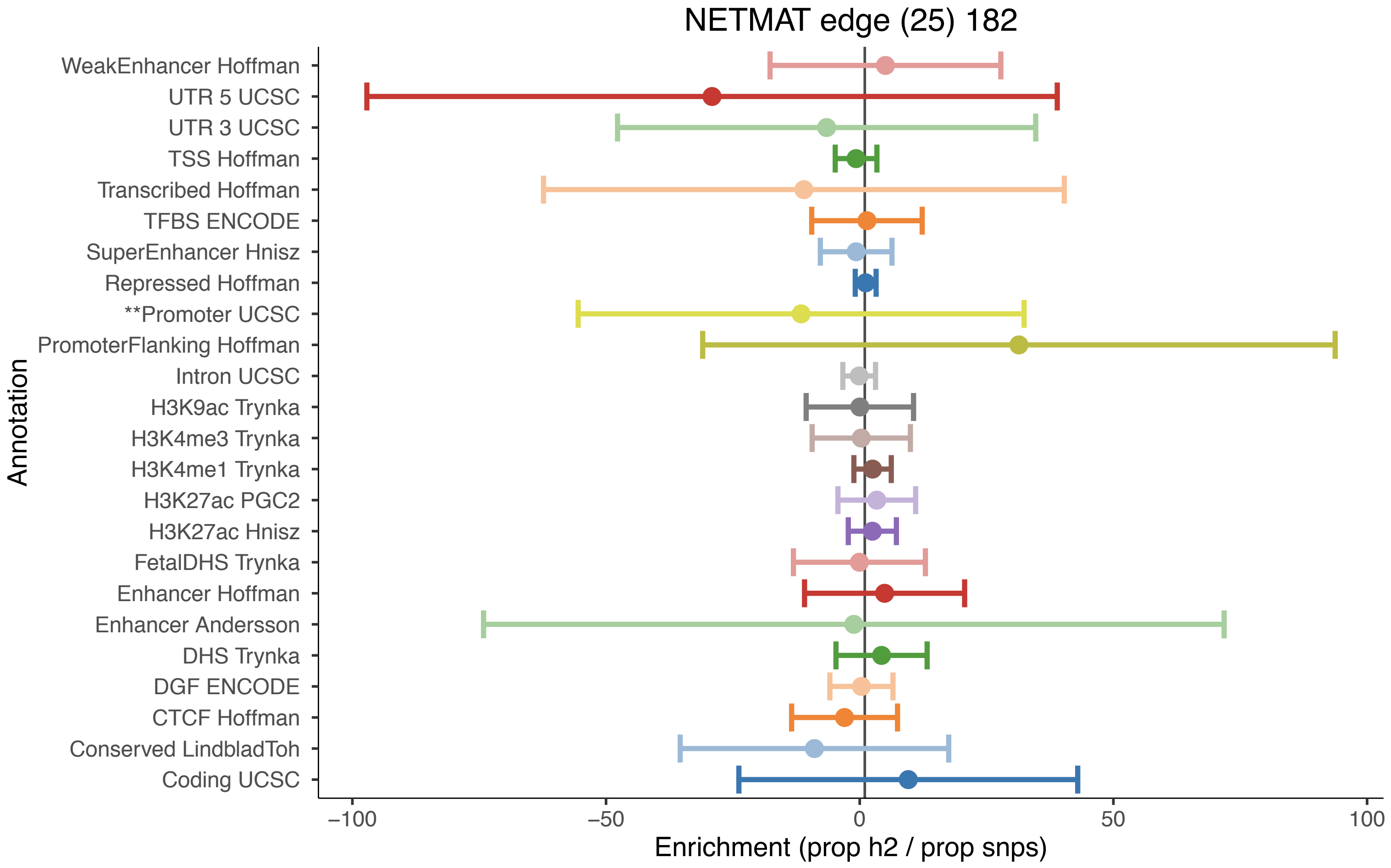

NETMAT edge (25) 182
WeakEnhancer Hoffman
UTR 5 UCSC
UTR 3 UCSC
TSS Hoffman
Transcribed Hoffman
TFBS ENCODE
SuperEnhancer Hnisz
Repressed Hoffman
**Promoter UCSC
PromoterFlanking Hoffman
Intron UCSC
H3K9ac Trynka
H3K4me3 Trynka
H3K4me1 Trynka
H3K27ac PGC2
H3K27ac Hnisz
FetalDHS Trynka
Enhancer Hoffman
Enhancer Andersson
DHS Trynka
DGF ENCODE
CTCF Hoffman
Conserved LindbladToh
Coding UCSC
Annotation
−100
−50
0
50
100
Enrichment (prop h2 / prop snps)

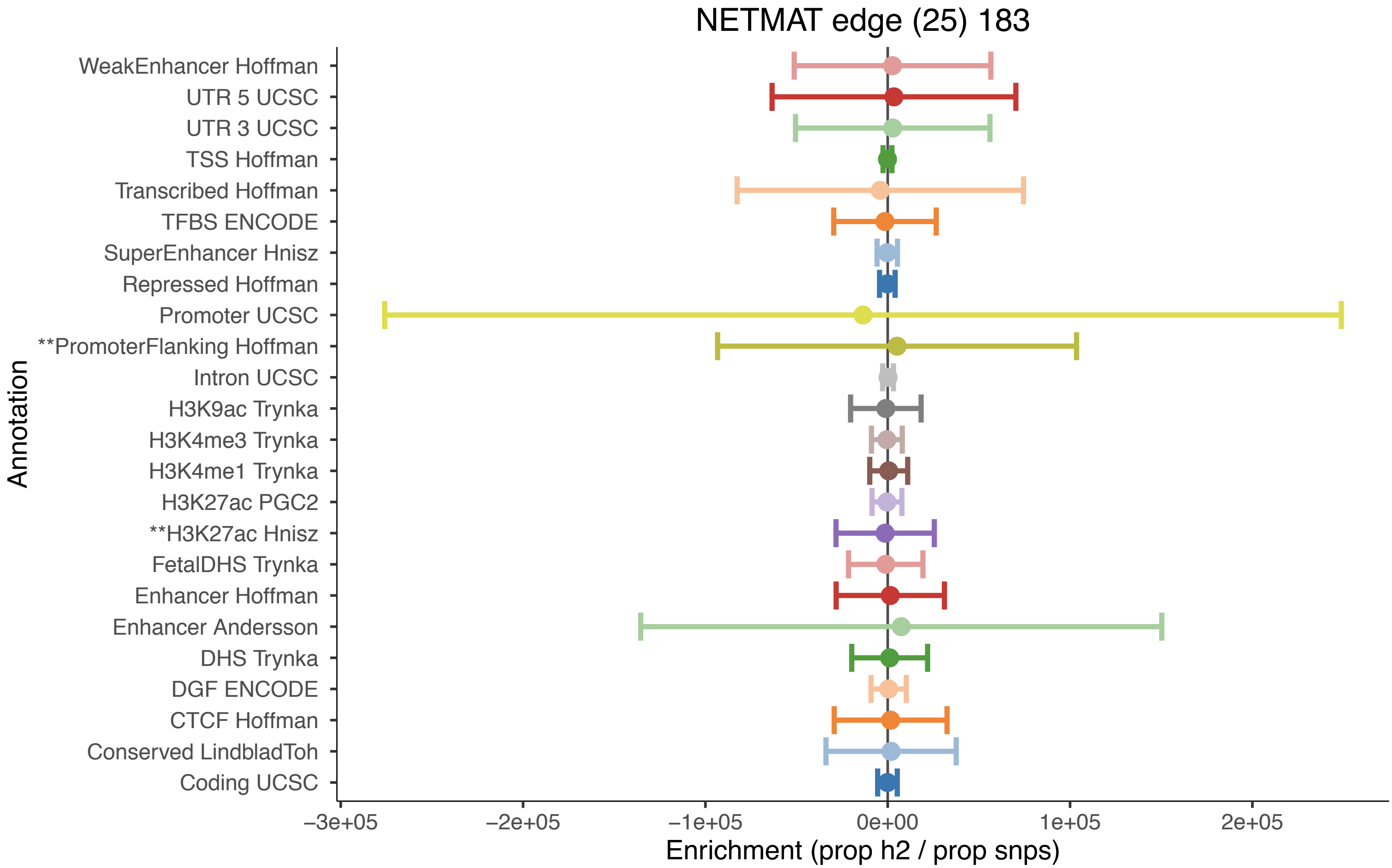

NETMAT edge (25) 183
Annotation
WeakEnhancer Hoffman
UTR 5 UCSC
UTR 3 UCSC
TSS Hoffman
Transcribed Hoffman
TFBS ENCODE
SuperEnhancer Hnisz
Repressed Hoffman
Promoter UCSC
**PromoterFlanking Hoffman
Intron UCSC
H3K9ac Trynka
H3K4me3 Trynka
H3K4me1 Trynka
H3K27ac PGC2
**H3K27ac Hnisz
FetalDHS Trynka
Enhancer Hoffman
Enhancer Andersson
DHS Trynka
DGF ENCODE
CTCF Hoffman
Conserved LindbladToh
Coding UCSC
−3e+05
−2e+05
−1e+05
0e+00
1e+05
2e+05
Enrichment (prop h2 / prop snps)

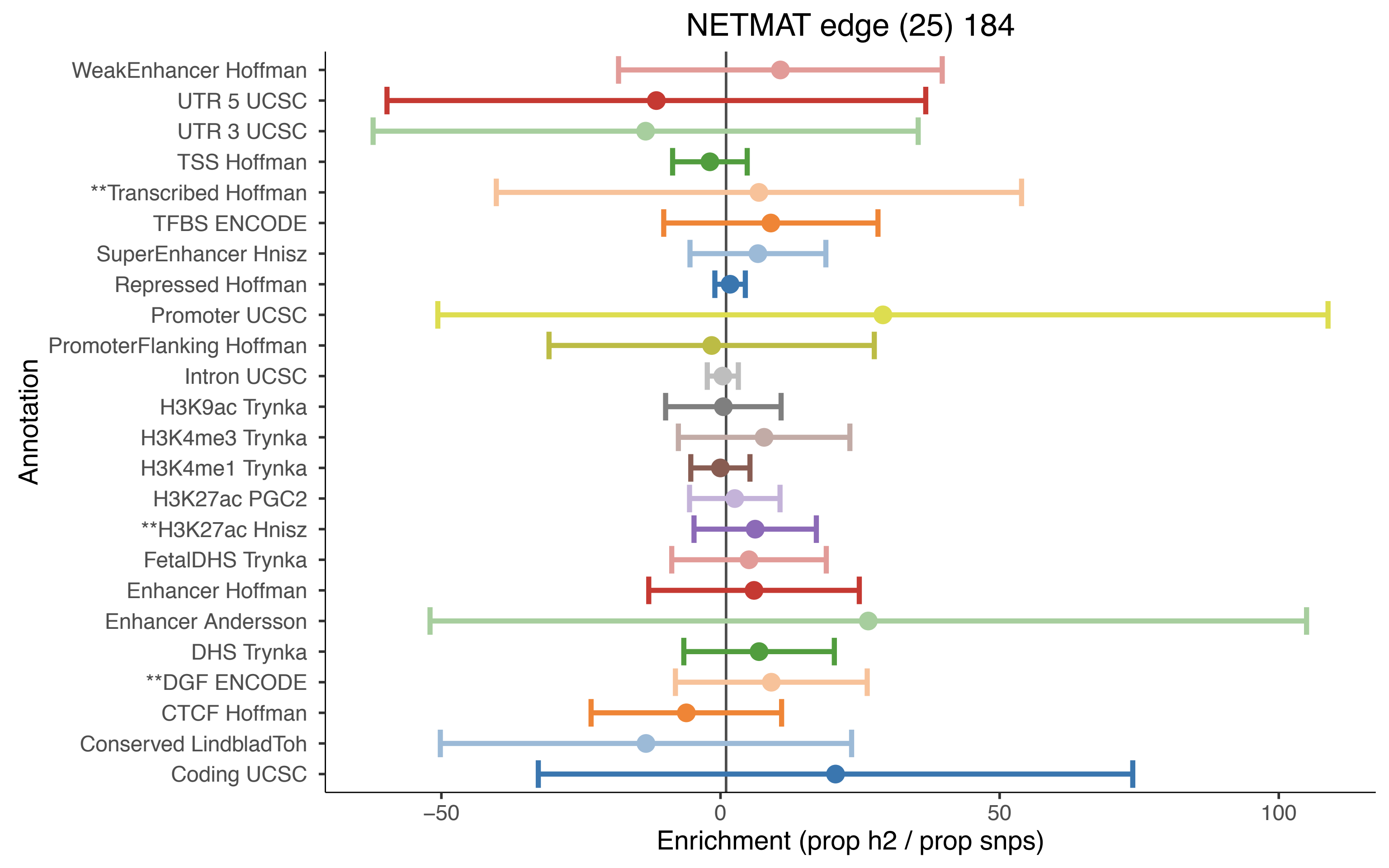
NETMAT edge (25) 184
WeakEnhancer Hoffman
UTR 5 UCSC
UTR 3 UCSC
TSS Hoffman
**Transcribed Hoffman
TFBS ENCODE
SuperEnhancer Hnisz
Repressed Hoffman
Promoter UCSC
PromoterFlanking Hoffman
Intron UCSC
H3K9ac Trynka
H3K4me3 Trynka
H3K4me1 Trynka
H3K27ac PGC2
**H3K27ac Hnisz
FetalDHS Trynka
Enhancer Hoffman
Enhancer Andersson
DHS Trynka
**DGF ENCODE
CTCF Hoffman
Conserved LindbladToh
Coding UCSC
Annotation
−50
0
50
100
Enrichment (prop h2 / prop snps)

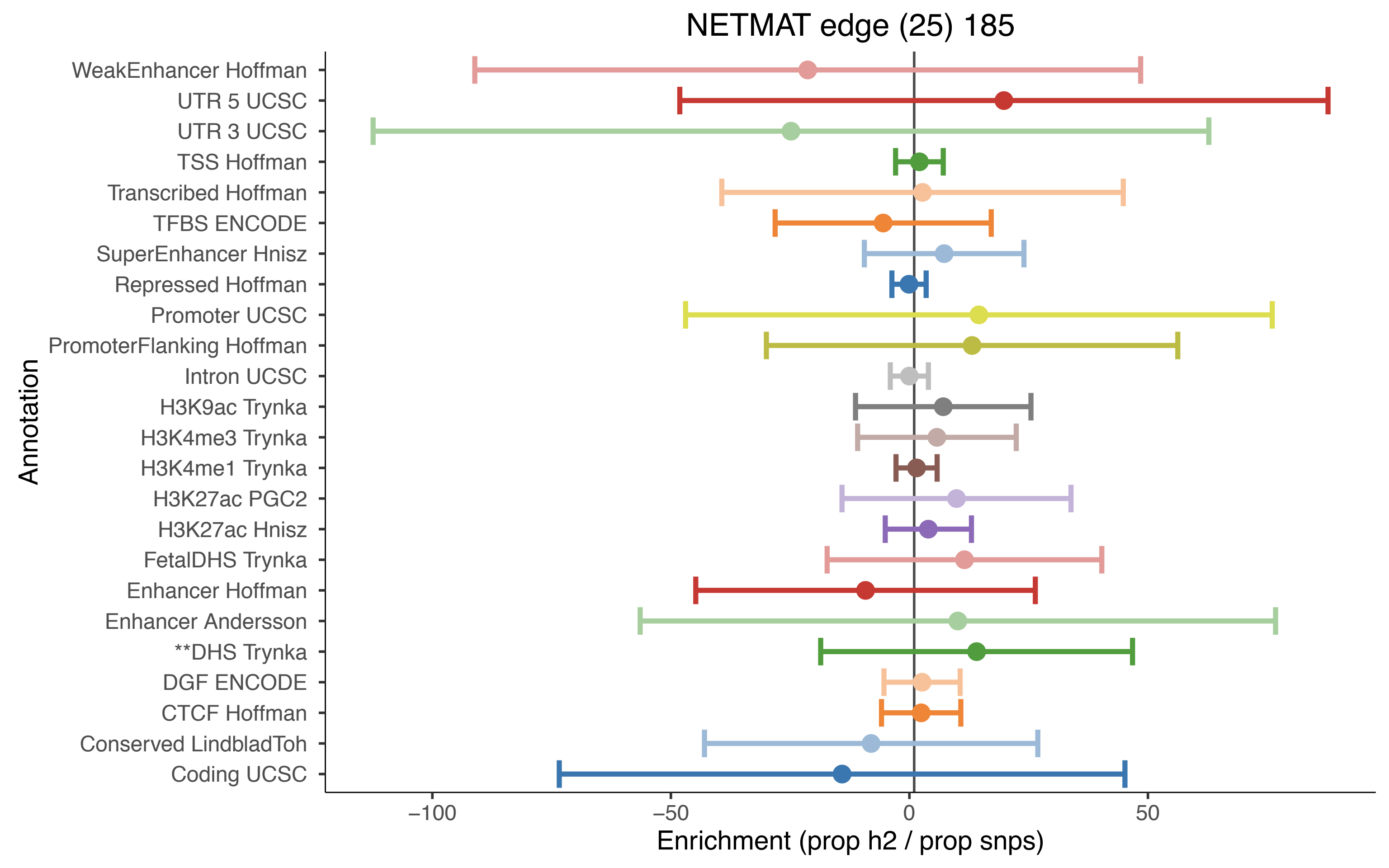
NETMAT edge (25) 185
Annotation
WeakEnhancer Hoffman
UTR 5 UCSC
UTR 3 UCSC
TSS Hoffman
Transcribed Hoffman
TFBS ENCODE
SuperEnhancer Hnisz
Repressed Hoffman
Promoter UCSC
PromoterFlanking Hoffman
Intron UCSC
H3K9ac Trynka
H3K4me3 Trynka
H3K4me1 Trynka
H3K27ac PGC2
H3K27ac Hnisz
FetalDHS Trynka
Enhancer Hoffman
Enhancer Andersson
**DHS Trynka
DGF ENCODE
CTCF Hoffman
Conserved LindbladToh
Coding UCSC
−100
−50
0
50
Enrichment (prop h2 / prop snps)

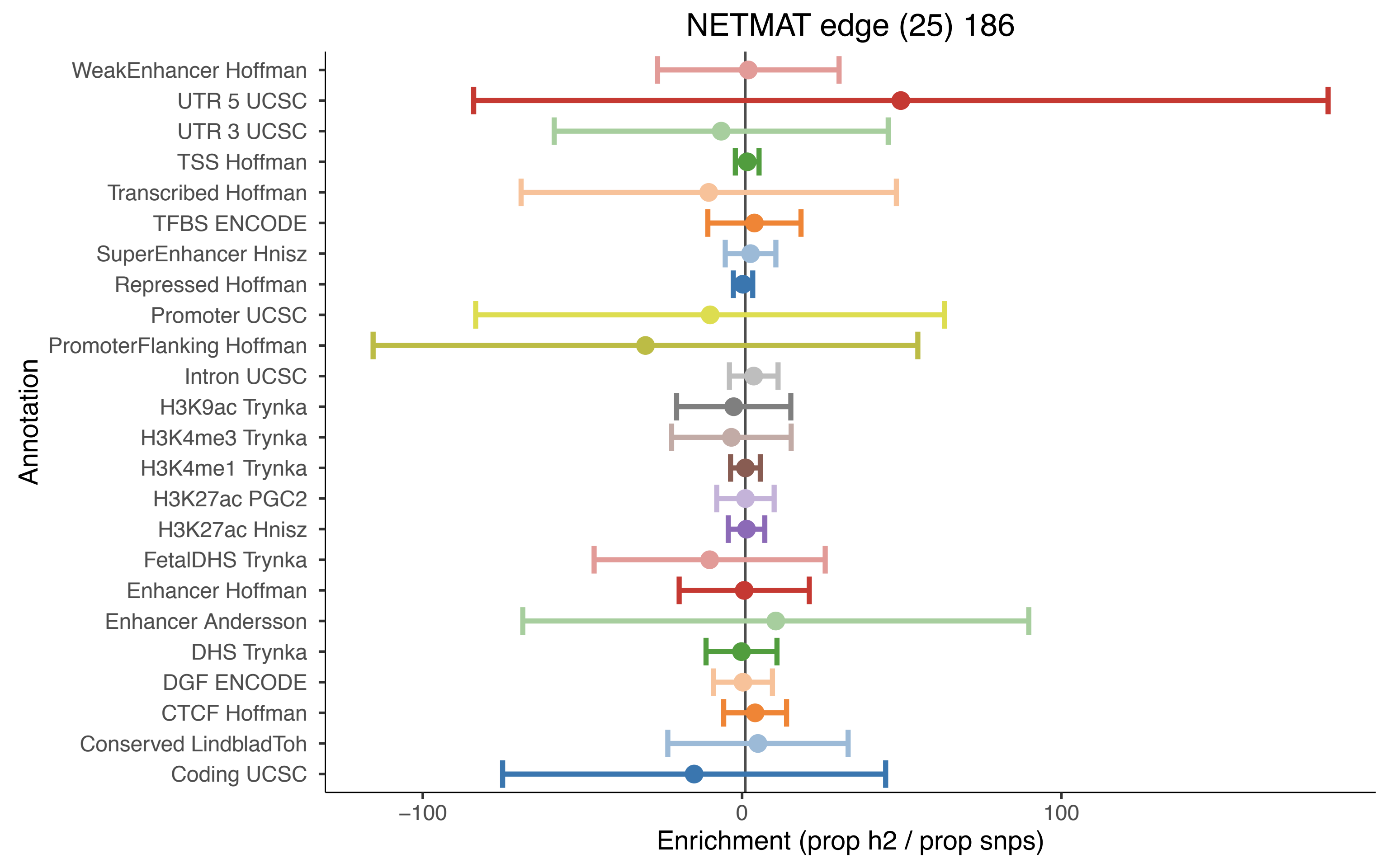

NETMAT edge (25) 186
Annotation
WeakEnhancer Hoffman
UTR 5 UCSC
UTR 3 UCSC
TSS Hoffman
Transcribed Hoffman
TFBS ENCODE
SuperEnhancer Hnisz
Repressed Hoffman
Promoter UCSC
PromoterFlanking Hoffman
Intron UCSC
H3K9ac Trynka
H3K4me3 Trynka
H3K4me1 Trynka
H3K27ac PGC2
H3K27ac Hnisz
FetalDHS Trynka
Enhancer Hoffman
Enhancer Andersson
DHS Trynka
DGF ENCODE
CTCF Hoffman
Conserved LindbladToh
Coding UCSC
−100
0
100
Enrichment (prop h2 / prop snps)

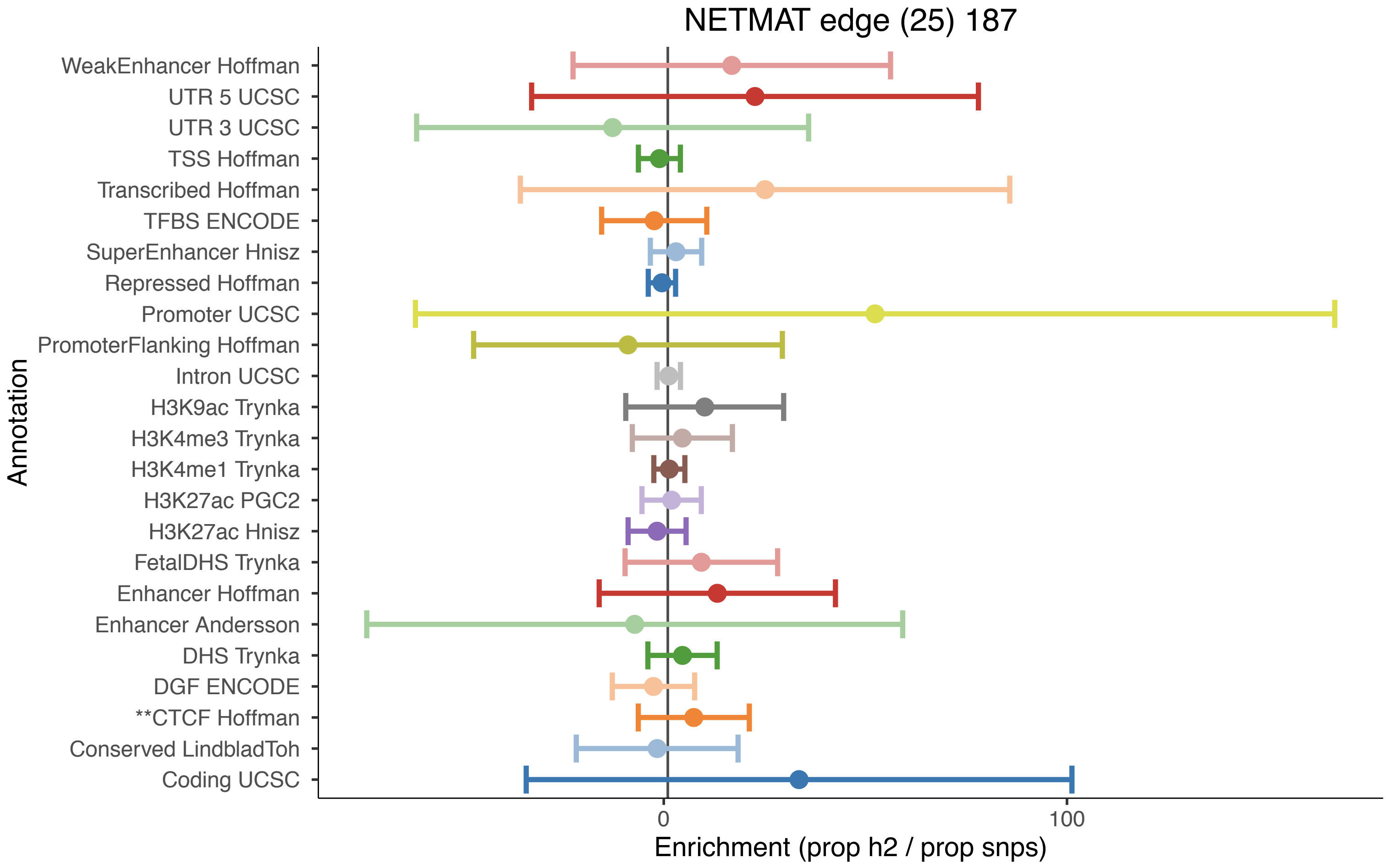

NETMAT edge (25) 187
WeakEnhancer Hoffman
UTR 5 UCSC
UTR 3 UCSC
TSS Hoffman
Transcribed Hoffman
TFBS ENCODE
SuperEnhancer Hnisz
Repressed Hoffman
Promoter UCSC
PromoterFlanking Hoffman
Intron UCSC
H3K9ac Trynka
H3K4me3 Trynka
H3K4me1 Trynka
H3K27ac PGC2
H3K27ac Hnisz
FetalDHS Trynka
Enhancer Hoffman
Enhancer Andersson
DHS Trynka
DGF ENCODE
**CTCF Hoffman
Conserved LindbladToh
Coding UCSC
Annotation
0
100
Enrichment (prop h2 / prop snps)

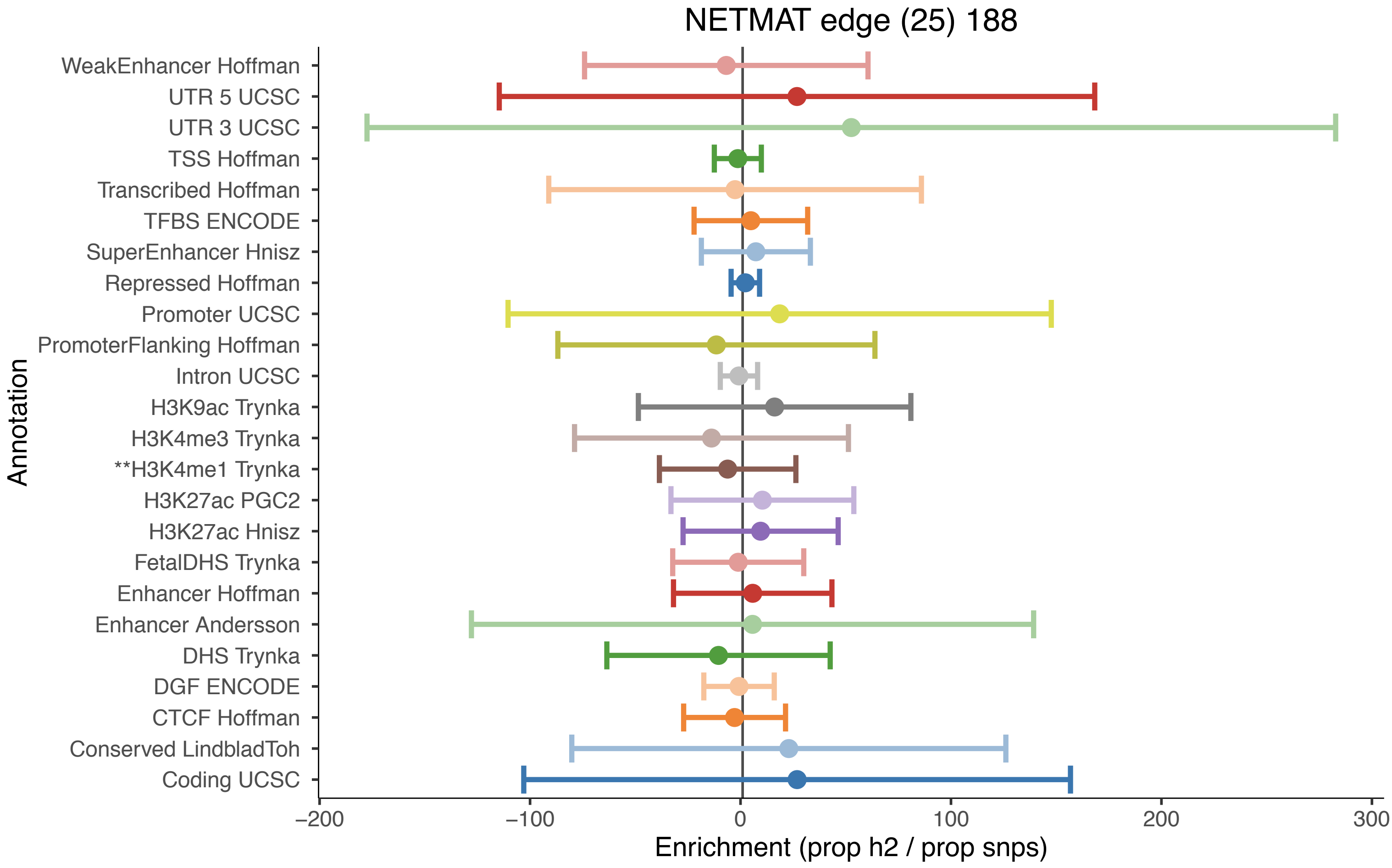

NETMAT edge (25) 188
Annotation
WeakEnhancer Hoffman
UTR 5 UCSC
UTR 3 UCSC
TSS Hoffman
Transcribed Hoffman
TFBS ENCODE
SuperEnhancer Hnisz
Repressed Hoffman
Promoter UCSC
PromoterFlanking Hoffman
Intron UCSC
H3K9ac Trynka
H3K4me3 Trynka
**H3K4me1 Trynka
H3K27ac PGC2
H3K27ac Hnisz
FetalDHS Trynka
Enhancer Hoffman
Enhancer Andersson
DHS Trynka
DGF ENCODE
CTCF Hoffman
Conserved LindbladToh
Coding UCSC
−200
−100
0
100
200
300
Enrichment (prop h2 / prop snps)

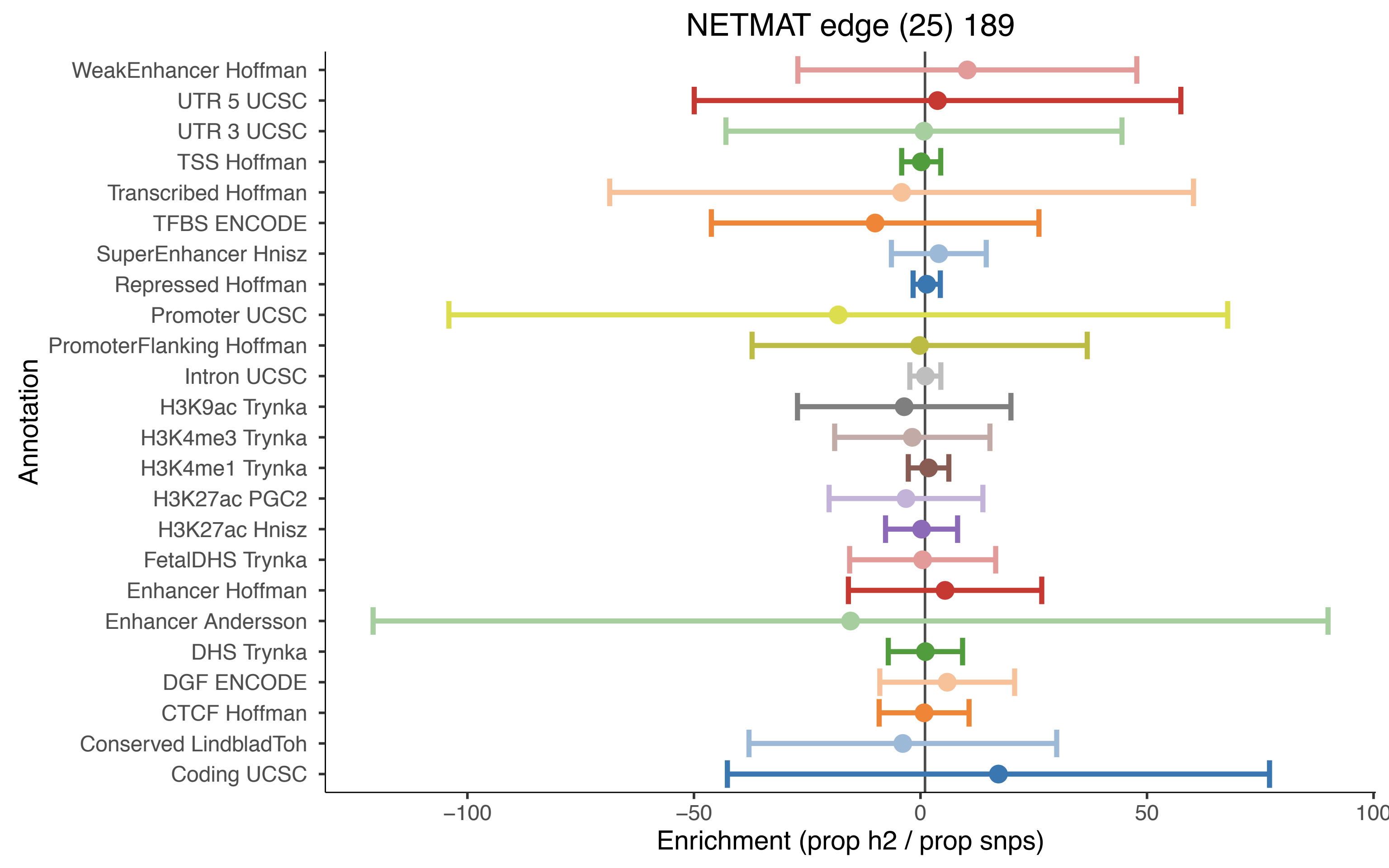

NETMAT edge (25) 189
WeakEnhancer Hoffman
UTR 5 UCSC
UTR 3 UCSC
TSS Hoffman
Transcribed Hoffman
TFBS ENCODE
SuperEnhancer Hnisz
Repressed Hoffman
Promoter UCSC
PromoterFlanking Hoffman
Intron UCSC
H3K9ac Trynka
H3K4me3 Trynka
H3K4me1 Trynka
H3K27ac PGC2
H3K27ac Hnisz
FetalDHS Trynka
Enhancer Hoffman
Enhancer Andersson
DHS Trynka
DGF ENCODE
CTCF Hoffman
Conserved LindbladToh
Coding UCSC
Annotation
−100
−50
0
50
100
Enrichment (prop h2 / prop snps)

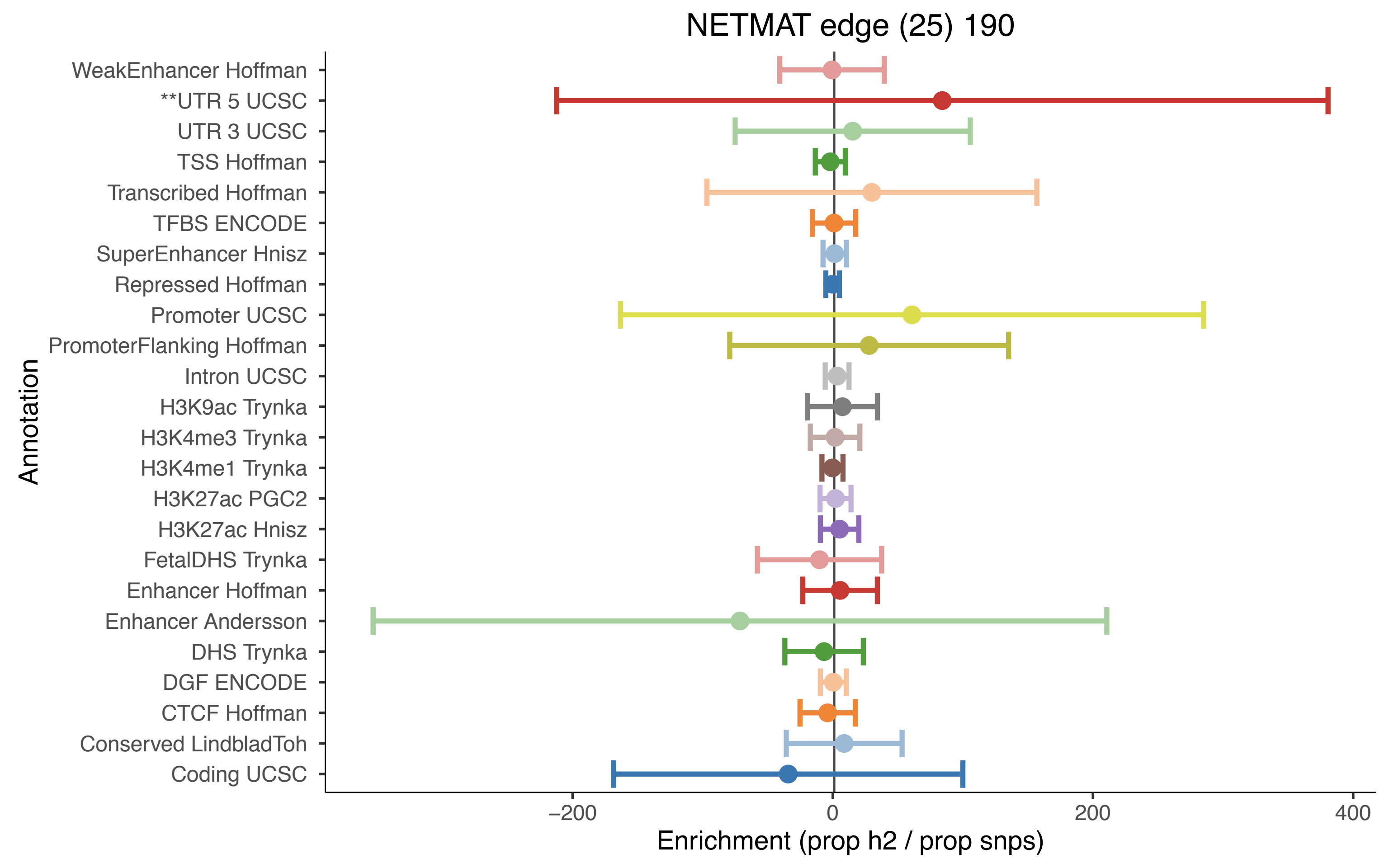
NETMAT edge (25) 190
WeakEnhancer Hoffman
**UTR 5 UCSC
UTR 3 UCSC
TSS Hoffman
Transcribed Hoffman
TFBS ENCODE
SuperEnhancer Hnisz
Repressed Hoffman
Promoter UCSC
PromoterFlanking Hoffman
Intron UCSC
H3K9ac Trynka
H3K4me3 Trynka
H3K4me1 Trynka
H3K27ac PGC2
H3K27ac Hnisz
FetalDHS Trynka
Enhancer Hoffman
Enhancer Andersson
DHS Trynka
DGF ENCODE
CTCF Hoffman
Conserved LindbladToh
Coding UCSC
Annotation
−200
0
200
400
Enrichment (prop h2 / prop snps)

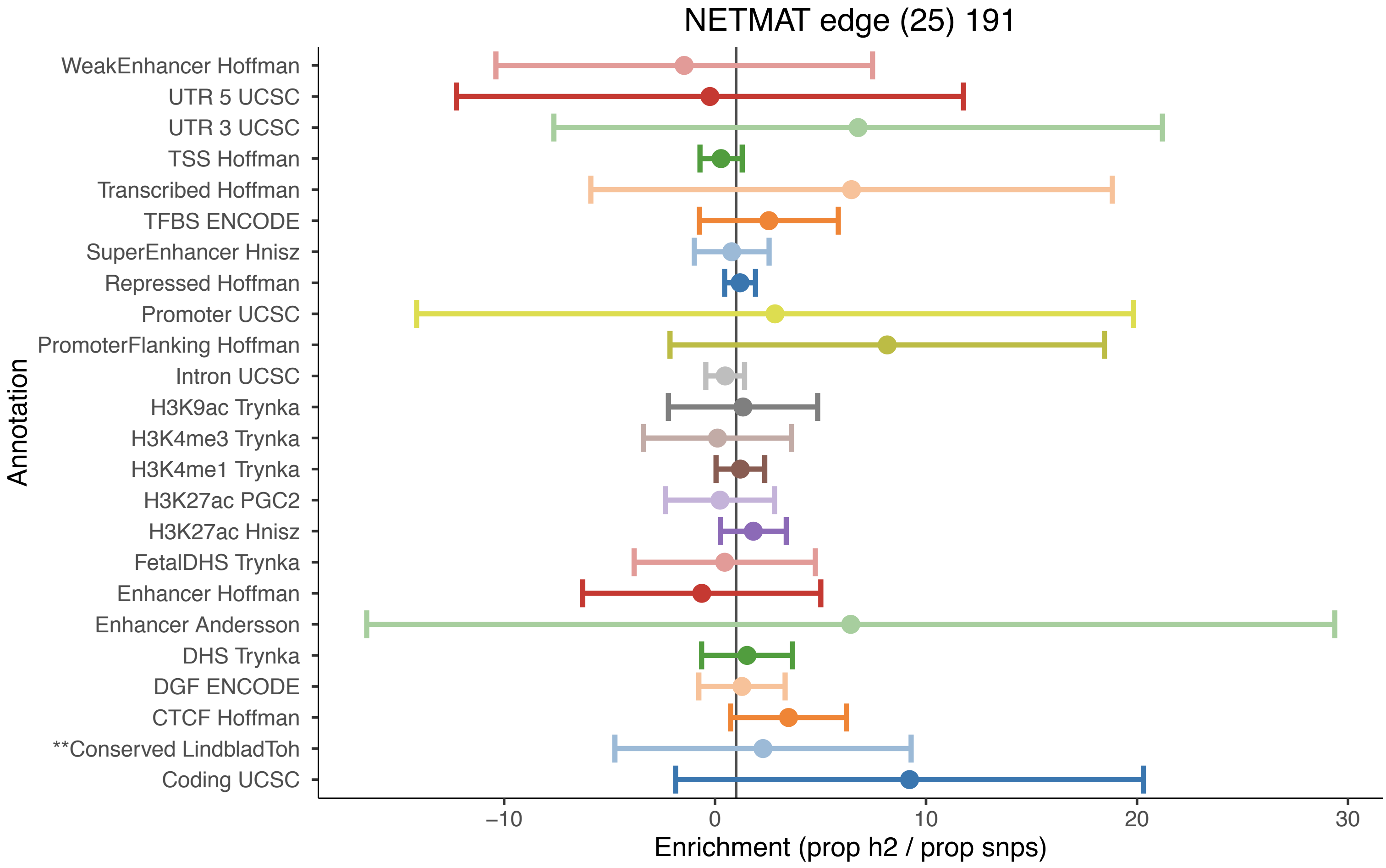
NETMAT edge (25) 191
WeakEnhancer Hoffman
UTR 5 UCSC
UTR 3 UCSC
TSS Hoffman
Transcribed Hoffman
TFBS ENCODE
SuperEnhancer Hnisz
Repressed Hoffman
Promoter UCSC
PromoterFlanking Hoffman
Intron UCSC
H3K9ac Trynka
H3K4me3 Trynka
H3K4me1 Trynka
H3K27ac PGC2
H3K27ac Hnisz
FetalDHS Trynka
Enhancer Hoffman
Enhancer Andersson
DHS Trynka
DGF ENCODE
CTCF Hoffman
**Conserved LindbladToh
Coding UCSC
Annotation
−10
0
10
20
30
Enrichment (prop h2 / prop snps)

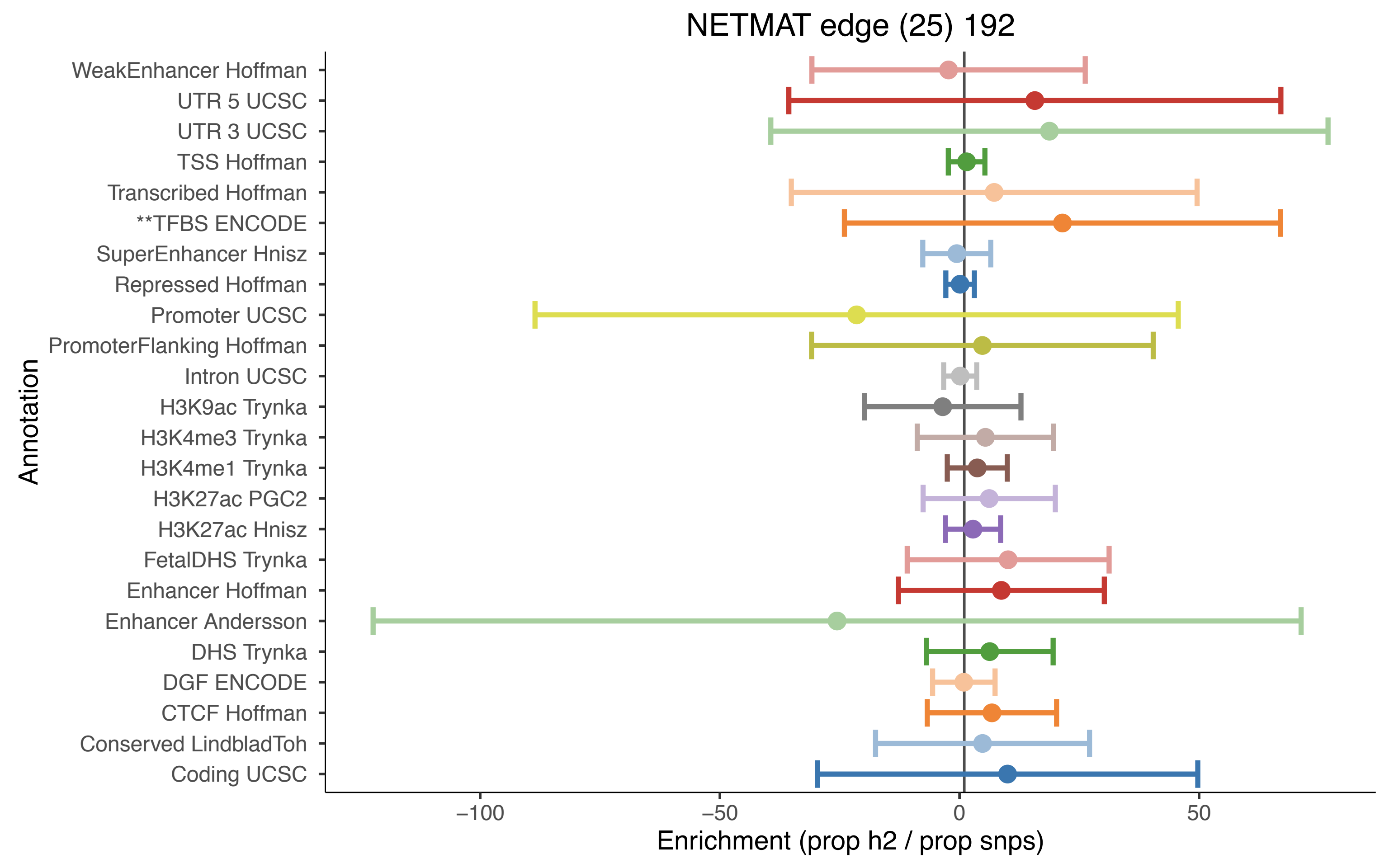
NETMAT edge (25) 192
Annotation
WeakEnhancer Hoffman
UTR 5 UCSC
UTR 3 UCSC
TSS Hoffman
Transcribed Hoffman
**TFBS ENCODE
SuperEnhancer Hnisz
Repressed Hoffman
Promoter UCSC
PromoterFlanking Hoffman
Intron UCSC
H3K9ac Trynka
H3K4me3 Trynka
H3K4me1 Trynka
H3K27ac PGC2
H3K27ac Hnisz
FetalDHS Trynka
Enhancer Hoffman
Enhancer Andersson
DHS Trynka
DGF ENCODE
CTCF Hoffman
Conserved LindbladToh
Coding UCSC
−100
−50
0
50
Enrichment (prop h2 / prop snps)

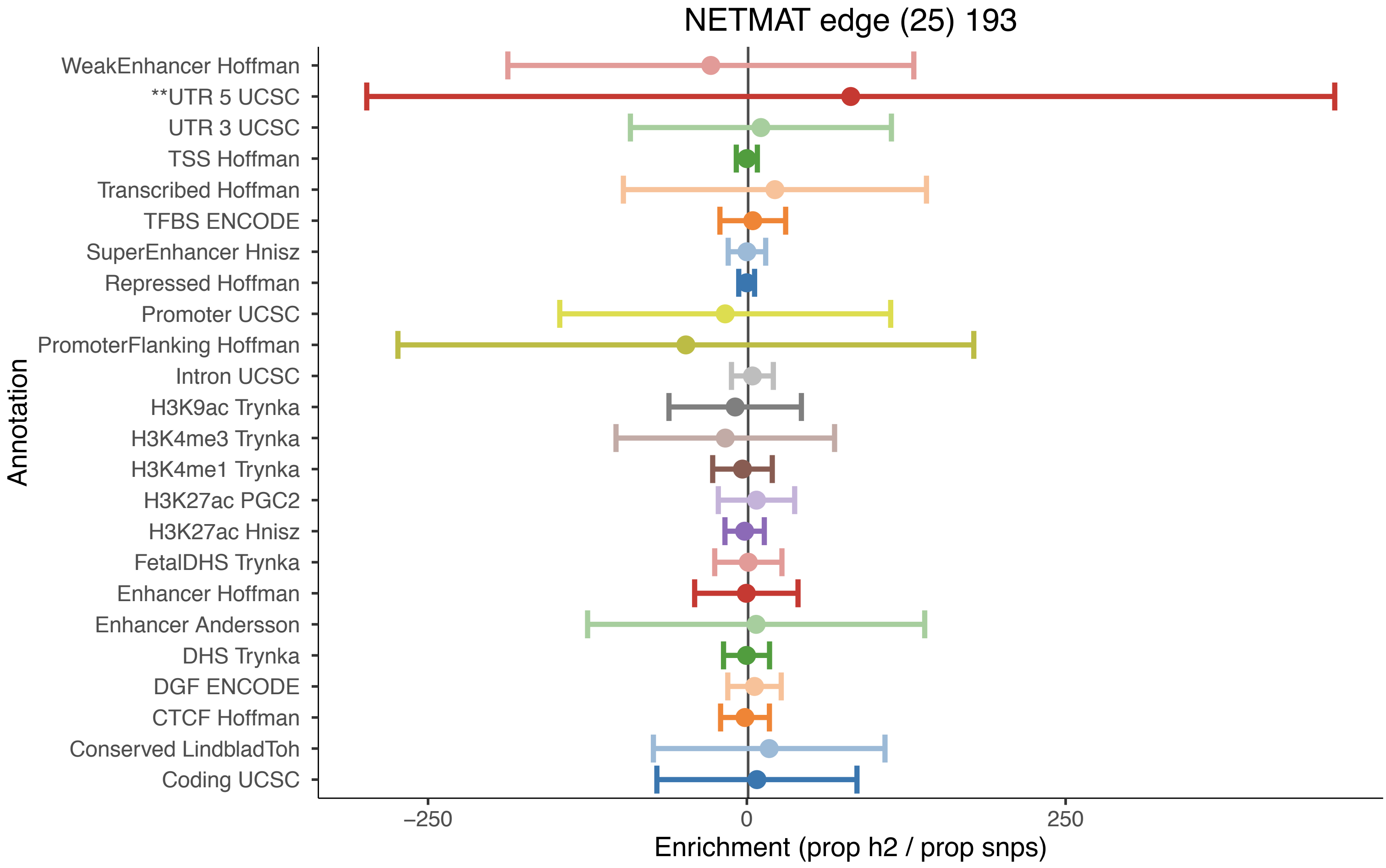
NETMAT edge (25) 193
WeakEnhancer Hoffman
**UTR 5 UCSC
UTR 3 UCSC
TSS Hoffman
Transcribed Hoffman
TFBS ENCODE
SuperEnhancer Hnisz
Repressed Hoffman
Promoter UCSC
PromoterFlanking Hoffman
Intron UCSC
H3K9ac Trynka
H3K4me3 Trynka
H3K4me1 Trynka
H3K27ac PGC2
H3K27ac Hnisz
FetalDHS Trynka
Enhancer Hoffman
Enhancer Andersson
DHS Trynka
DGF ENCODE
CTCF Hoffman
Conserved LindbladToh
Coding UCSC
Annotation
−250
0
250
Enrichment (prop h2 / prop snps)

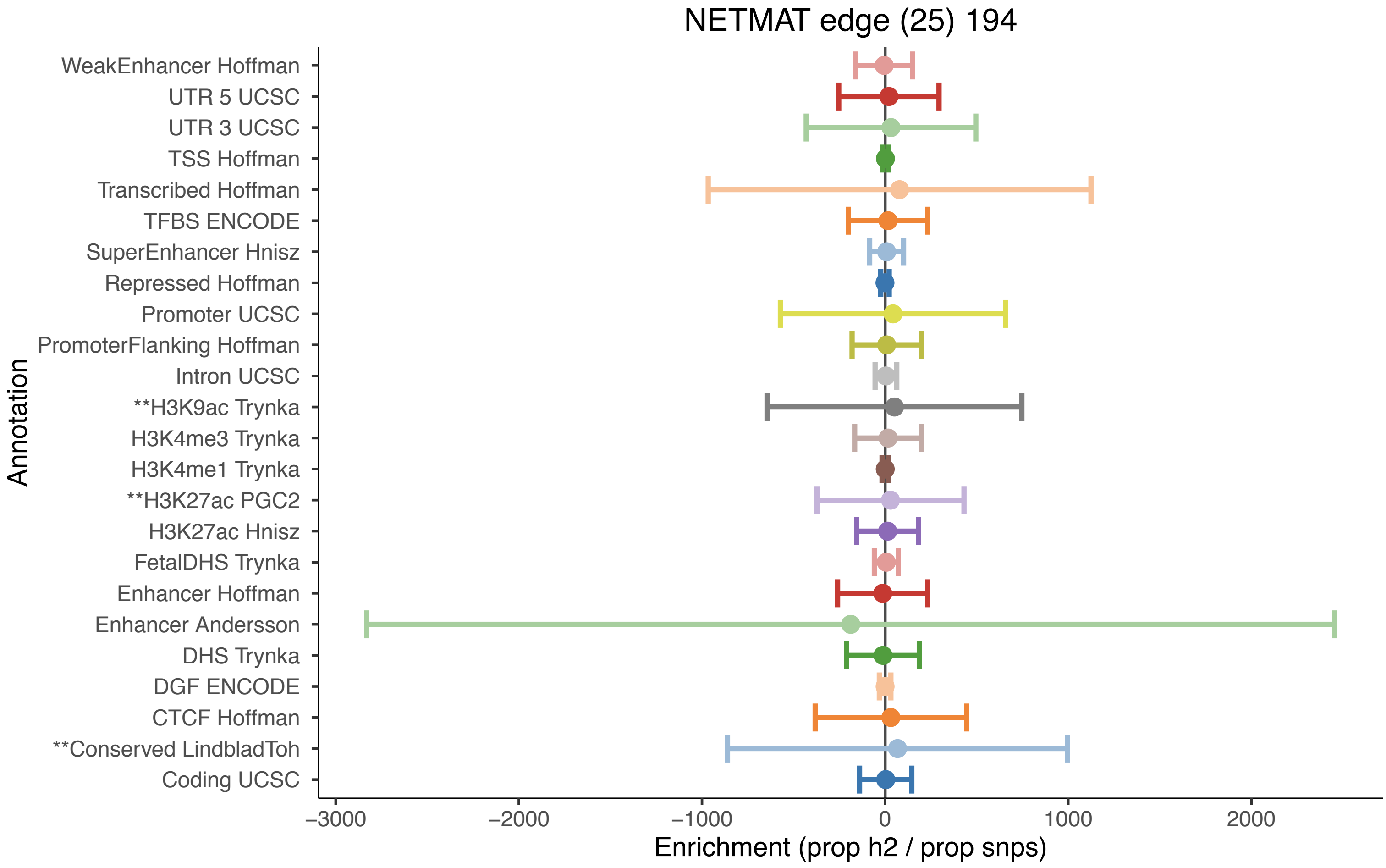

NETMAT edge (25) 194
WeakEnhancer Hoffman
UTR 5 UCSC
UTR 3 UCSC
TSS Hoffman
Transcribed Hoffman
TFBS ENCODE
SuperEnhancer Hnisz
Repressed Hoffman
Promoter UCSC
PromoterFlanking Hoffman
Intron UCSC
**H3K9ac Trynka
H3K4me3 Trynka
H3K4me1 Trynka
**H3K27ac PGC2
H3K27ac Hnisz
FetalDHS Trynka
Enhancer Hoffman
Enhancer Andersson
DHS Trynka
DGF ENCODE
CTCF Hoffman
**Conserved LindbladToh
Coding UCSC
Annotation
−3000
−2000
−1000
0
1000
2000
Enrichment (prop h2 / prop snps)

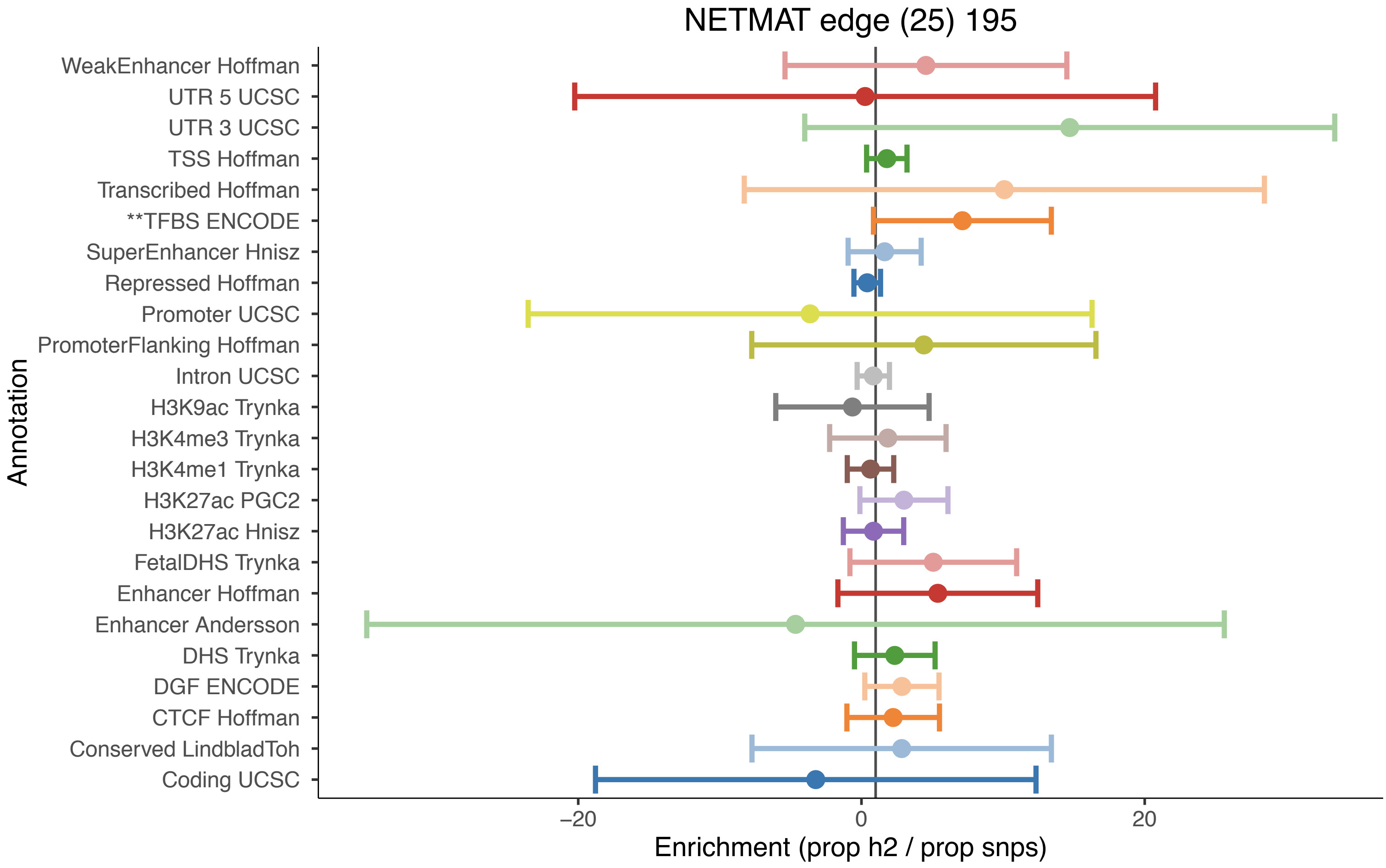
NETMAT edge (25) 195
WeakEnhancer Hoffman
UTR 5 UCSC
UTR 3 UCSC
TSS Hoffman
Transcribed Hoffman
**TFBS ENCODE
SuperEnhancer Hnisz
Repressed Hoffman
Promoter UCSC
PromoterFlanking Hoffman
Intron UCSC
H3K9ac Trynka
H3K4me3 Trynka
H3K4me1 Trynka
H3K27ac PGC2
H3K27ac Hnisz
FetalDHS Trynka
Enhancer Hoffman
Enhancer Andersson
DHS Trynka
DGF ENCODE
CTCF Hoffman
Conserved LindbladToh
Coding UCSC
Annotation
−20
0
20
Enrichment (prop h2 / prop snps)

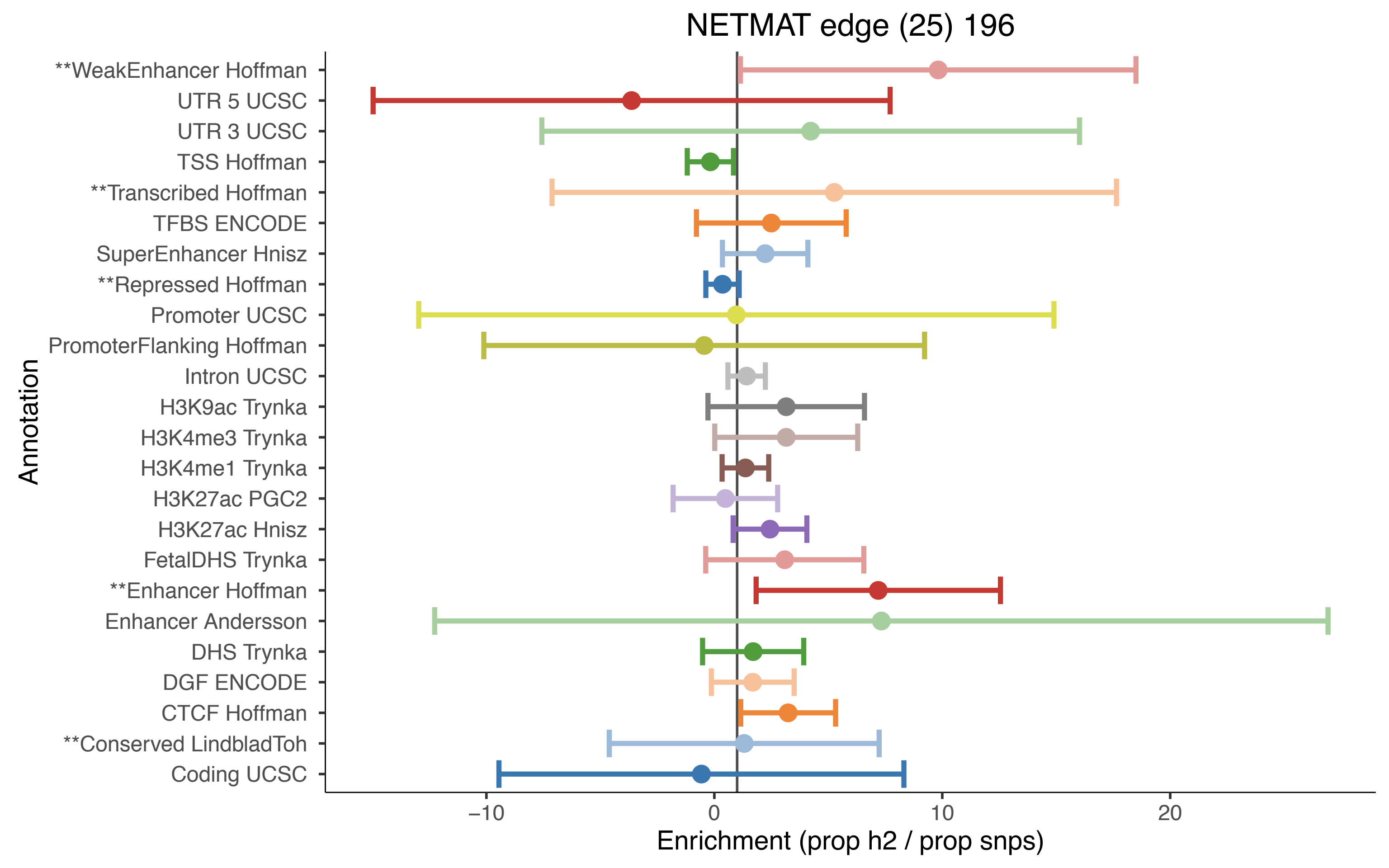
NETMAT edge (25) 196
**WeakEnhancer Hoffman
UTR 5 UCSC
UTR 3 UCSC
TSS Hoffman
**Transcribed Hoffman
TFBS ENCODE
SuperEnhancer Hnisz
**Repressed Hoffman
Promoter UCSC
PromoterFlanking Hoffman
Intron UCSC
H3K9ac Trynka
H3K4me3 Trynka
H3K4me1 Trynka
H3K27ac PGC2
H3K27ac Hnisz
FetalDHS Trynka
**Enhancer Hoffman
Enhancer Andersson
DHS Trynka
DGF ENCODE
CTCF Hoffman
**Conserved LindbladToh
Coding UCSC
Annotation
−10
0
10
20
Enrichment (prop h2 / prop snps)

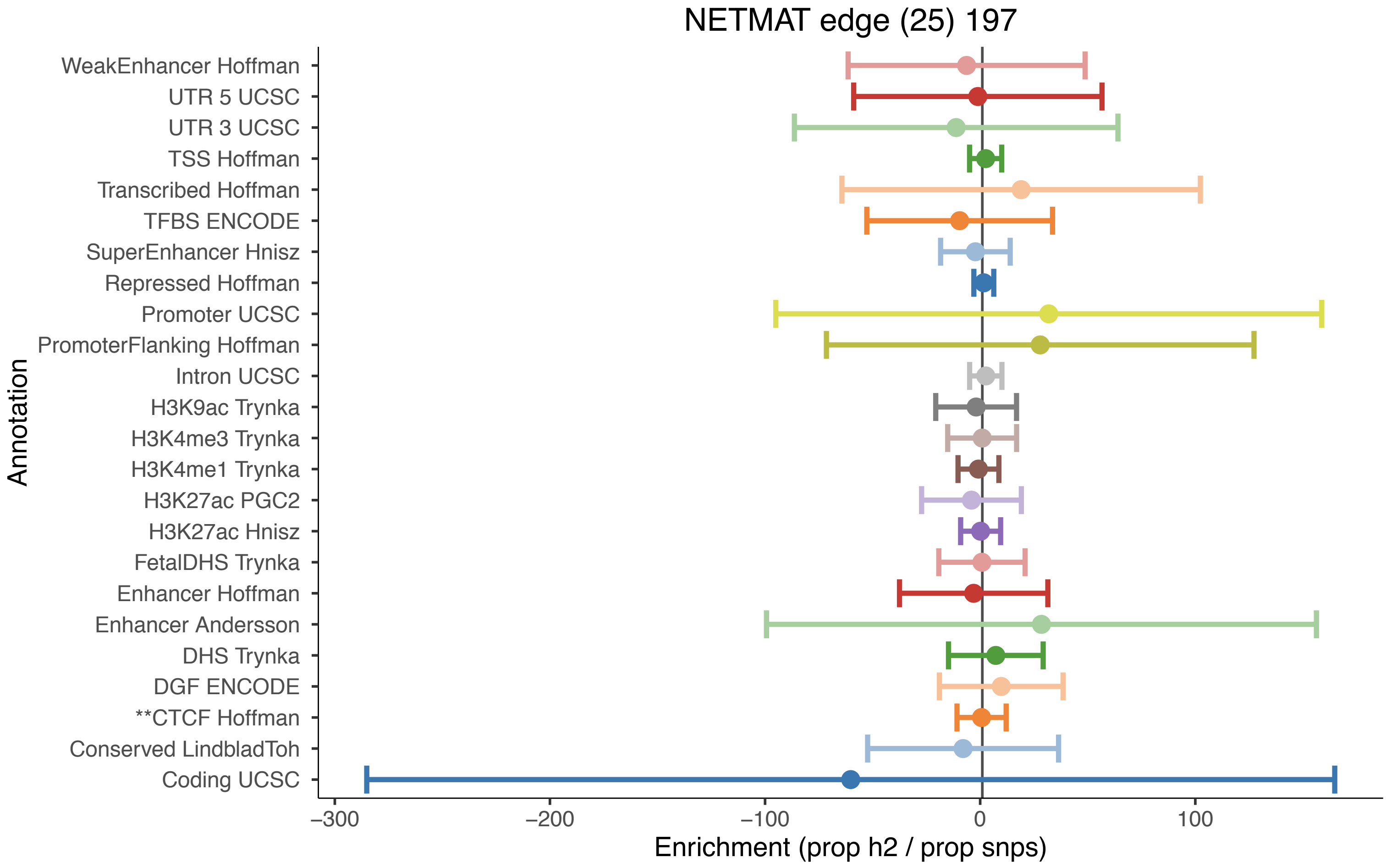
NETMAT edge (25) 197
Annotation
WeakEnhancer Hoffman
UTR 5 UCSC
UTR 3 UCSC
TSS Hoffman
Transcribed Hoffman
TFBS ENCODE
SuperEnhancer Hnisz
Repressed Hoffman
Promoter UCSC
PromoterFlanking Hoffman
Intron UCSC
H3K9ac Trynka
H3K4me3 Trynka
H3K4me1 Trynka
H3K27ac PGC2
H3K27ac Hnisz
FetalDHS Trynka
Enhancer Hoffman
Enhancer Andersson
DHS Trynka
DGF ENCODE
**CTCF Hoffman
Conserved LindbladToh
Coding UCSC
−300
−200
−100
0
100
Enrichment (prop h2 / prop snps)

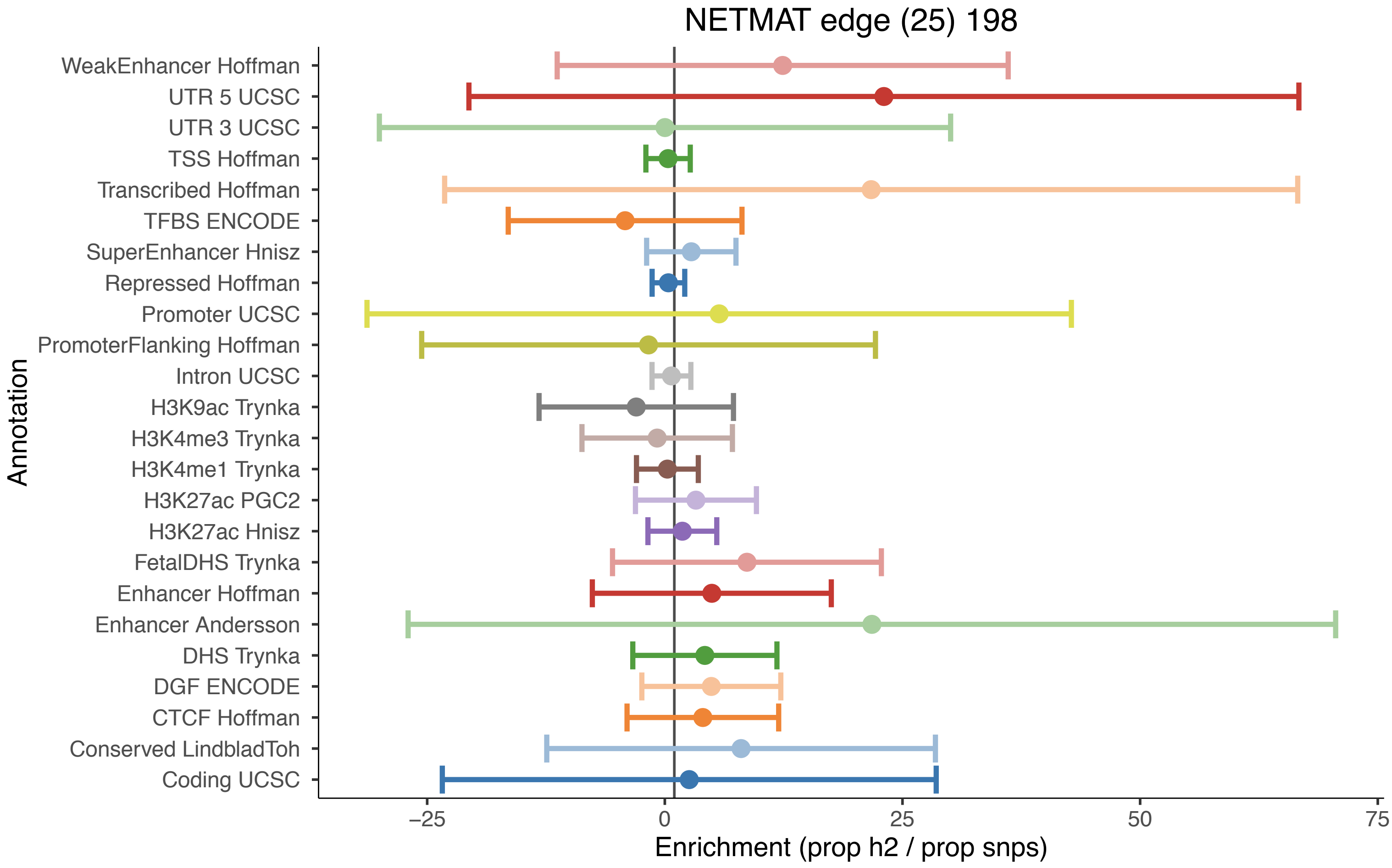
NETMAT edge (25) 198
WeakEnhancer Hoffman
UTR 5 UCSC
UTR 3 UCSC
TSS Hoffman
Transcribed Hoffman
TFBS ENCODE
SuperEnhancer Hnisz
Repressed Hoffman
Promoter UCSC
PromoterFlanking Hoffman
Intron UCSC
H3K9ac Trynka
H3K4me3 Trynka
H3K4me1 Trynka
H3K27ac PGC2
H3K27ac Hnisz
FetalDHS Trynka
Enhancer Hoffman
Enhancer Andersson
DHS Trynka
DGF ENCODE
CTCF Hoffman
Conserved LindbladToh
Coding UCSC
Annotation
−25
0
25
50
75
Enrichment (prop h2 / prop snps)

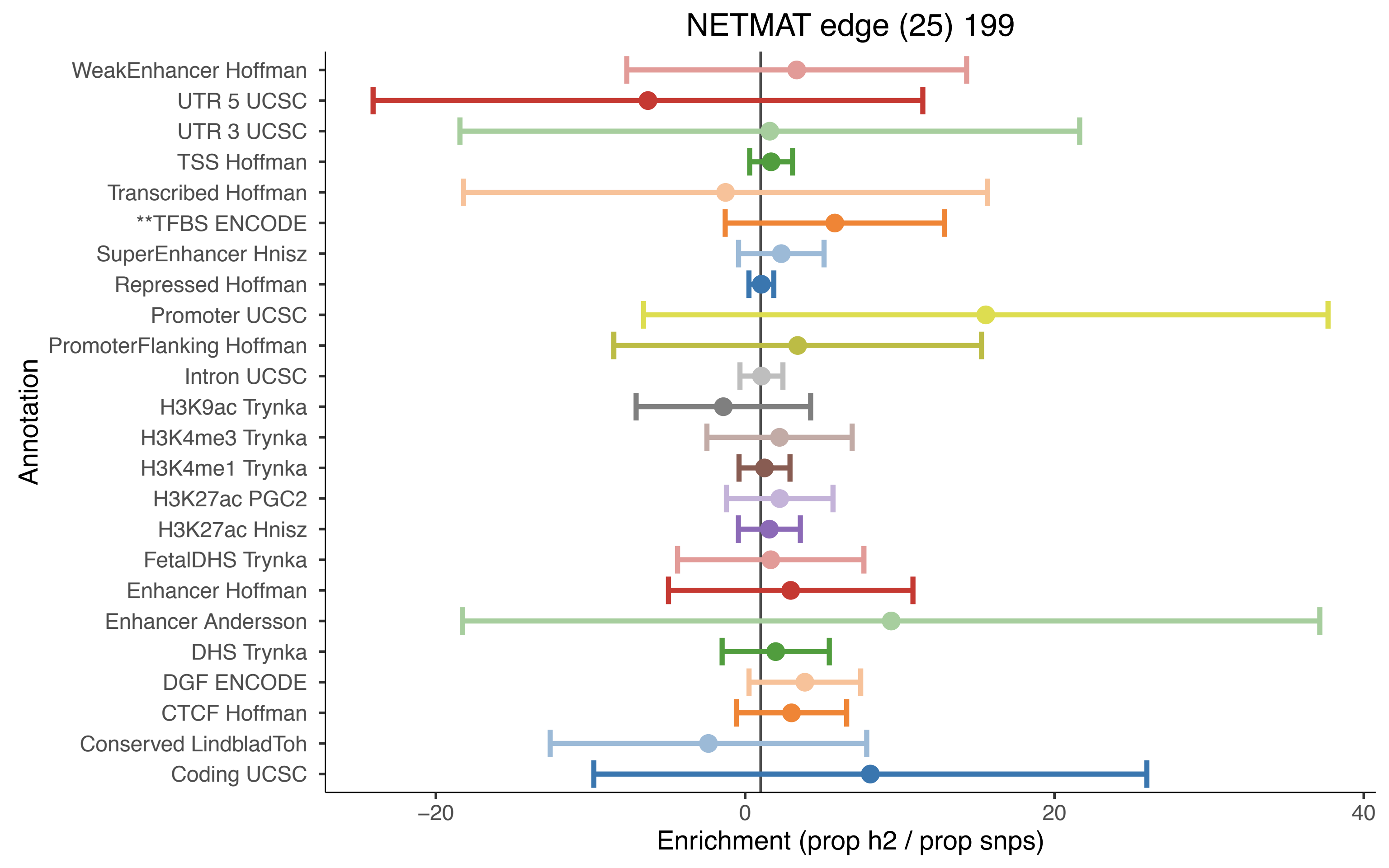
NETMAT edge (25) 199
WeakEnhancer Hoffman
UTR 5 UCSC
UTR 3 UCSC
TSS Hoffman
Transcribed Hoffman
**TFBS ENCODE
SuperEnhancer Hnisz
Repressed Hoffman
Promoter UCSC
PromoterFlanking Hoffman
Intron UCSC
H3K9ac Trynka
H3K4me3 Trynka
H3K4me1 Trynka
H3K27ac PGC2
H3K27ac Hnisz
FetalDHS Trynka
Enhancer Hoffman
Enhancer Andersson
DHS Trynka
DGF ENCODE
CTCF Hoffman
Conserved LindbladToh
Coding UCSC
Annotation
−20
0
20
40
Enrichment (prop h2 / prop snps)

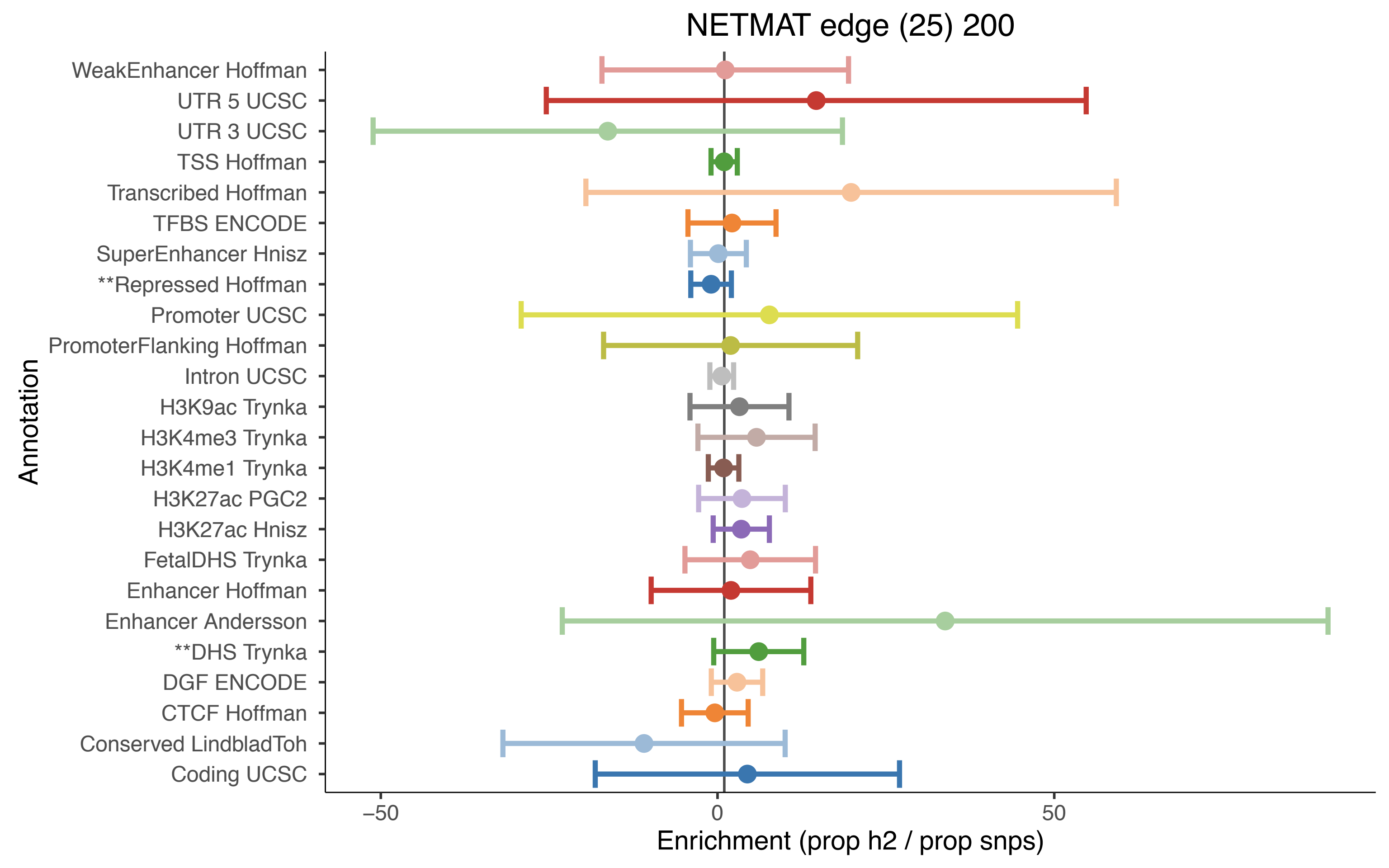
NETMAT edge (25) 200
WeakEnhancer Hoffman
UTR 5 UCSC
UTR 3 UCSC
TSS Hoffman
Transcribed Hoffman
TFBS ENCODE
SuperEnhancer Hnisz
**Repressed Hoffman
Promoter UCSC
PromoterFlanking Hoffman
Intron UCSC
H3K9ac Trynka
H3K4me3 Trynka
H3K4me1 Trynka
H3K27ac PGC2
H3K27ac Hnisz
FetalDHS Trynka
Enhancer Hoffman
Enhancer Andersson
**DHS Trynka
DGF ENCODE
CTCF Hoffman
Conserved LindbladToh
Coding UCSC
Annotation
−50
0
50
Enrichment (prop h2 / prop snps)

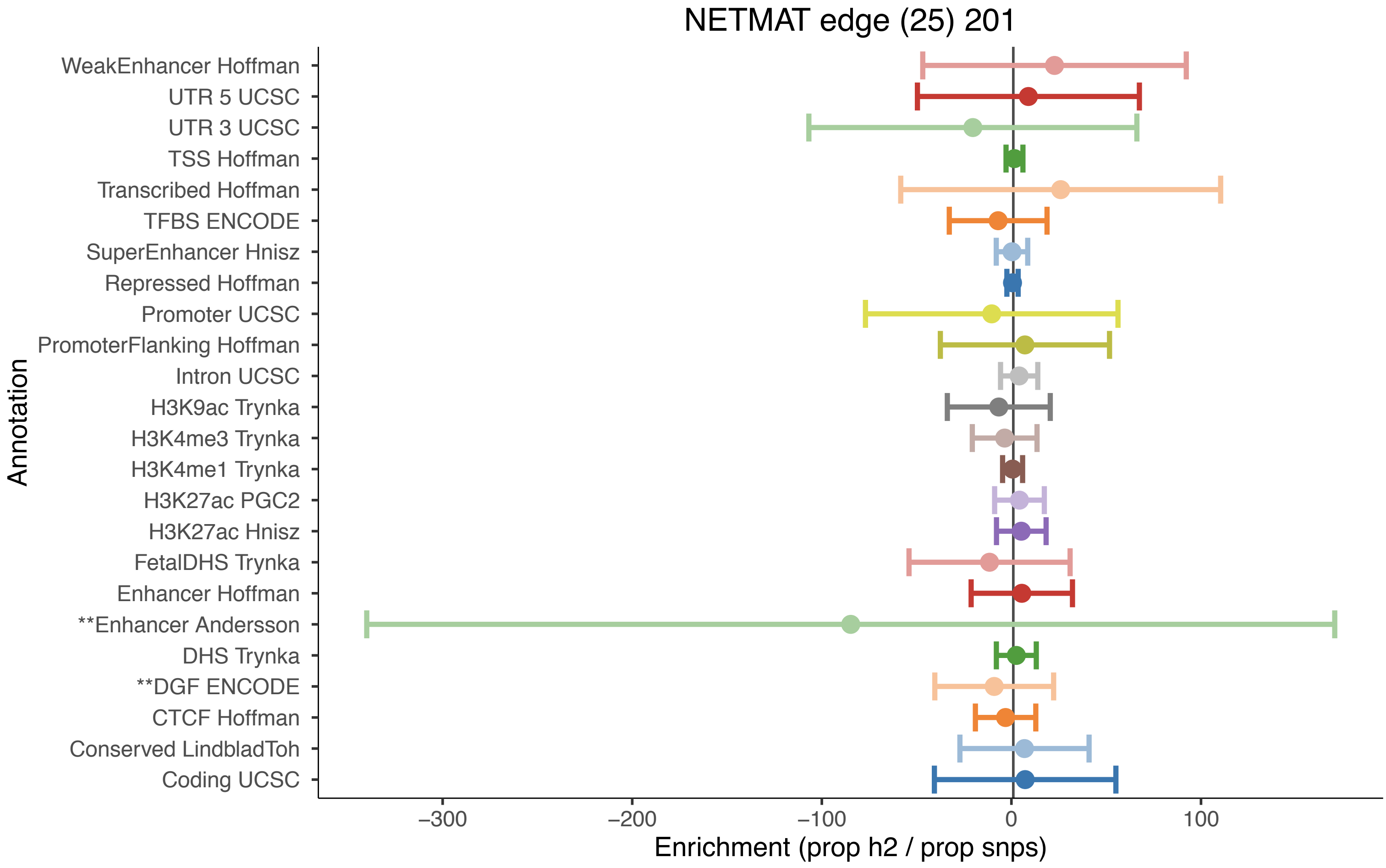
NETMAT edge (25) 201
Annotation
WeakEnhancer Hoffman
UTR 5 UCSC
UTR 3 UCSC
TSS Hoffman
Transcribed Hoffman
TFBS ENCODE
SuperEnhancer Hnisz
Repressed Hoffman
Promoter UCSC
PromoterFlanking Hoffman
Intron UCSC
H3K9ac Trynka
H3K4me3 Trynka
H3K4me1 Trynka
H3K27ac PGC2
H3K27ac Hnisz
FetalDHS Trynka
Enhancer Hoffman
**Enhancer Andersson
DHS Trynka
**DGF ENCODE
CTCF Hoffman
Conserved LindbladToh
Coding UCSC
−300
−200
−100
0
100
Enrichment (prop h2 / prop snps)

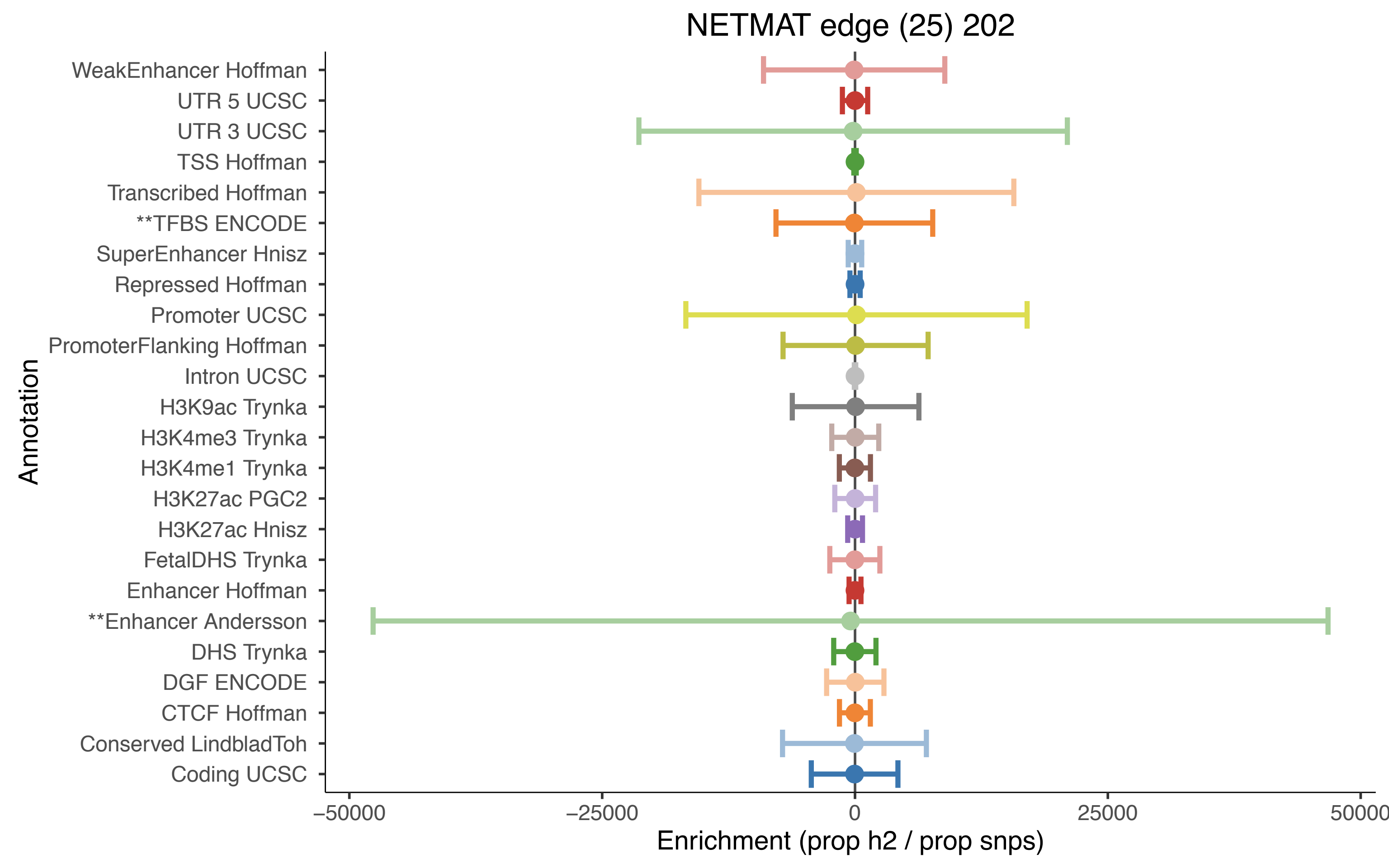
NETMAT edge (25) 202
Annotation
WeakEnhancer Hoffman
UTR 5 UCSC
UTR 3 UCSC
TSS Hoffman
Transcribed Hoffman
**TFBS ENCODE
SuperEnhancer Hnisz
Repressed Hoffman
Promoter UCSC
PromoterFlanking Hoffman
Intron UCSC
H3K9ac Trynka
H3K4me3 Trynka
H3K4me1 Trynka
H3K27ac PGC2
H3K27ac Hnisz
FetalDHS Trynka
Enhancer Hoffman
**Enhancer Andersson
DHS Trynka
DGF ENCODE
CTCF Hoffman
Conserved LindbladToh
Coding UCSC
−50000
−25000
0
25000
50000
Enrichment (prop h2 / prop snps)

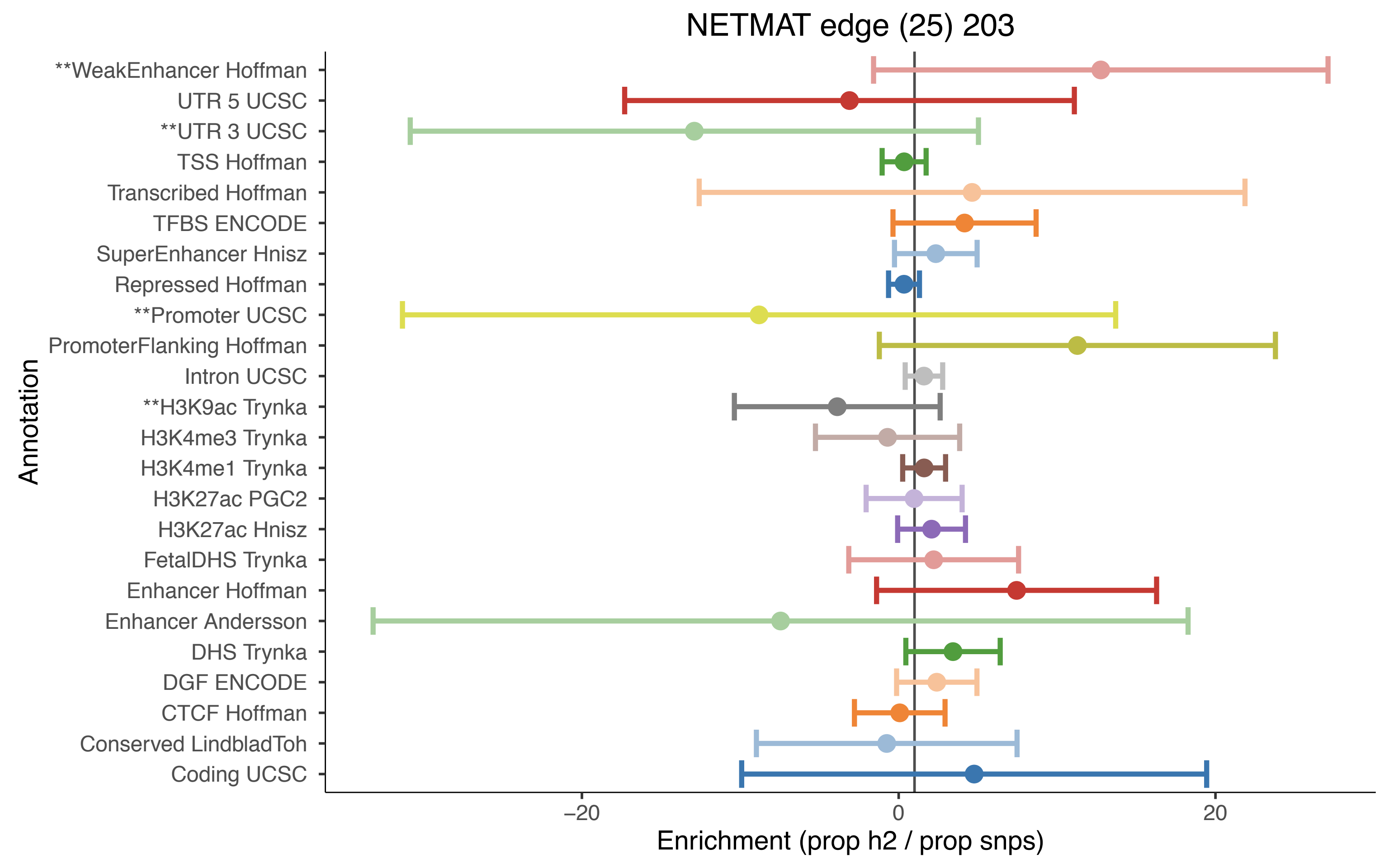
NETMAT edge (25) 203
**WeakEnhancer Hoffman
UTR 5 UCSC
**UTR 3 UCSC
TSS Hoffman
Transcribed Hoffman
TFBS ENCODE
SuperEnhancer Hnisz
Repressed Hoffman
**Promoter UCSC
PromoterFlanking Hoffman
Intron UCSC
**H3K9ac Trynka
H3K4me3 Trynka
H3K4me1 Trynka
H3K27ac PGC2
H3K27ac Hnisz
FetalDHS Trynka
Enhancer Hoffman
Enhancer Andersson
DHS Trynka
DGF ENCODE
CTCF Hoffman
Conserved LindbladToh
Coding UCSC
Annotation
−20
0
20
Enrichment (prop h2 / prop snps)

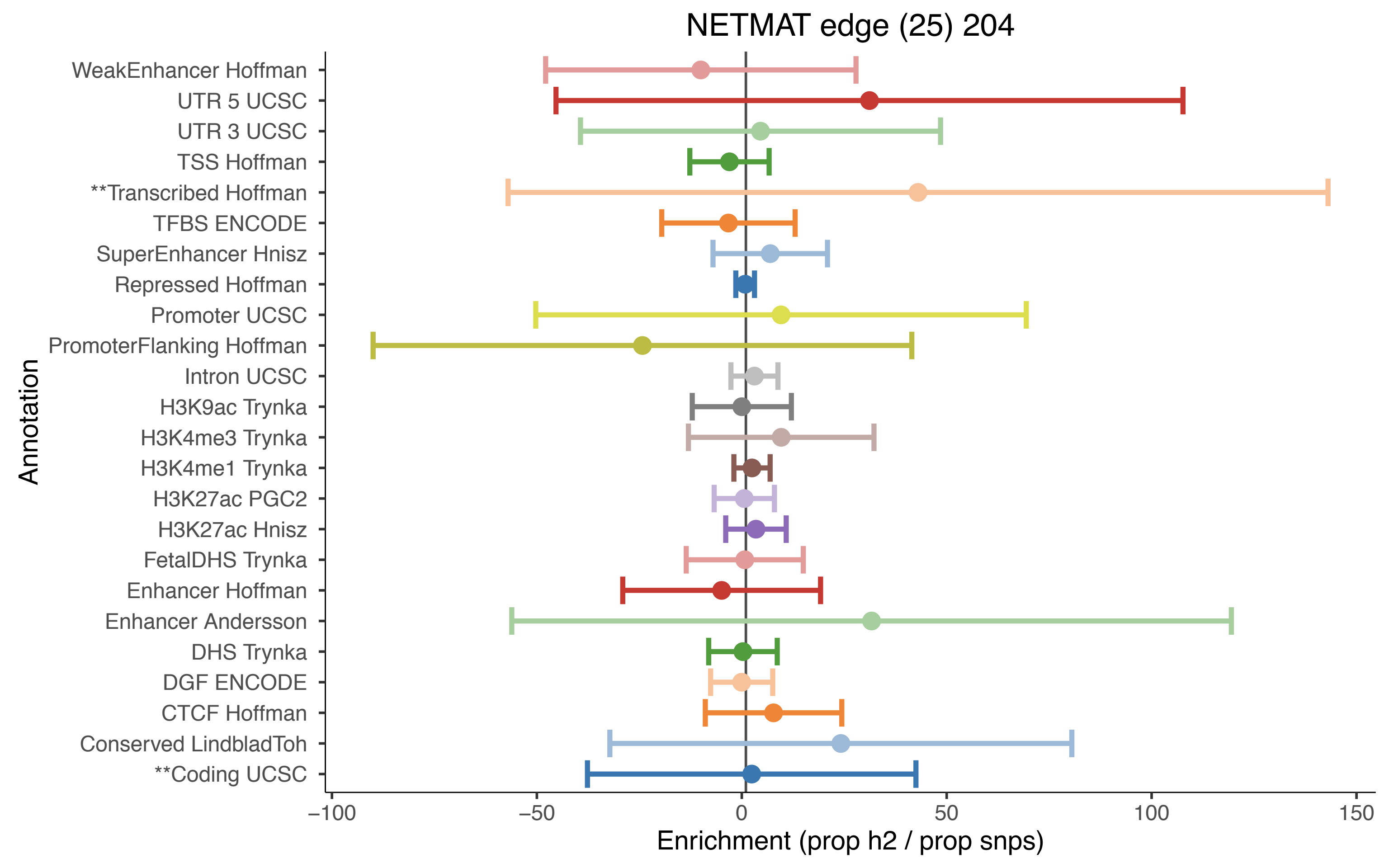

NETMAT edge (25) 204
WeakEnhancer Hoffman
UTR 5 UCSC
UTR 3 UCSC
TSS Hoffman
**Transcribed Hoffman
TFBS ENCODE
SuperEnhancer Hnisz
Repressed Hoffman
Promoter UCSC
PromoterFlanking Hoffman
Intron UCSC
H3K9ac Trynka
H3K4me3 Trynka
H3K4me1 Trynka
H3K27ac PGC2
H3K27ac Hnisz
FetalDHS Trynka
Enhancer Hoffman
Enhancer Andersson
DHS Trynka
DGF ENCODE
CTCF Hoffman
Conserved LindbladToh
**Coding UCSC
Annotation
−100
−50
0
50
100
150
Enrichment (prop h2 / prop snps)

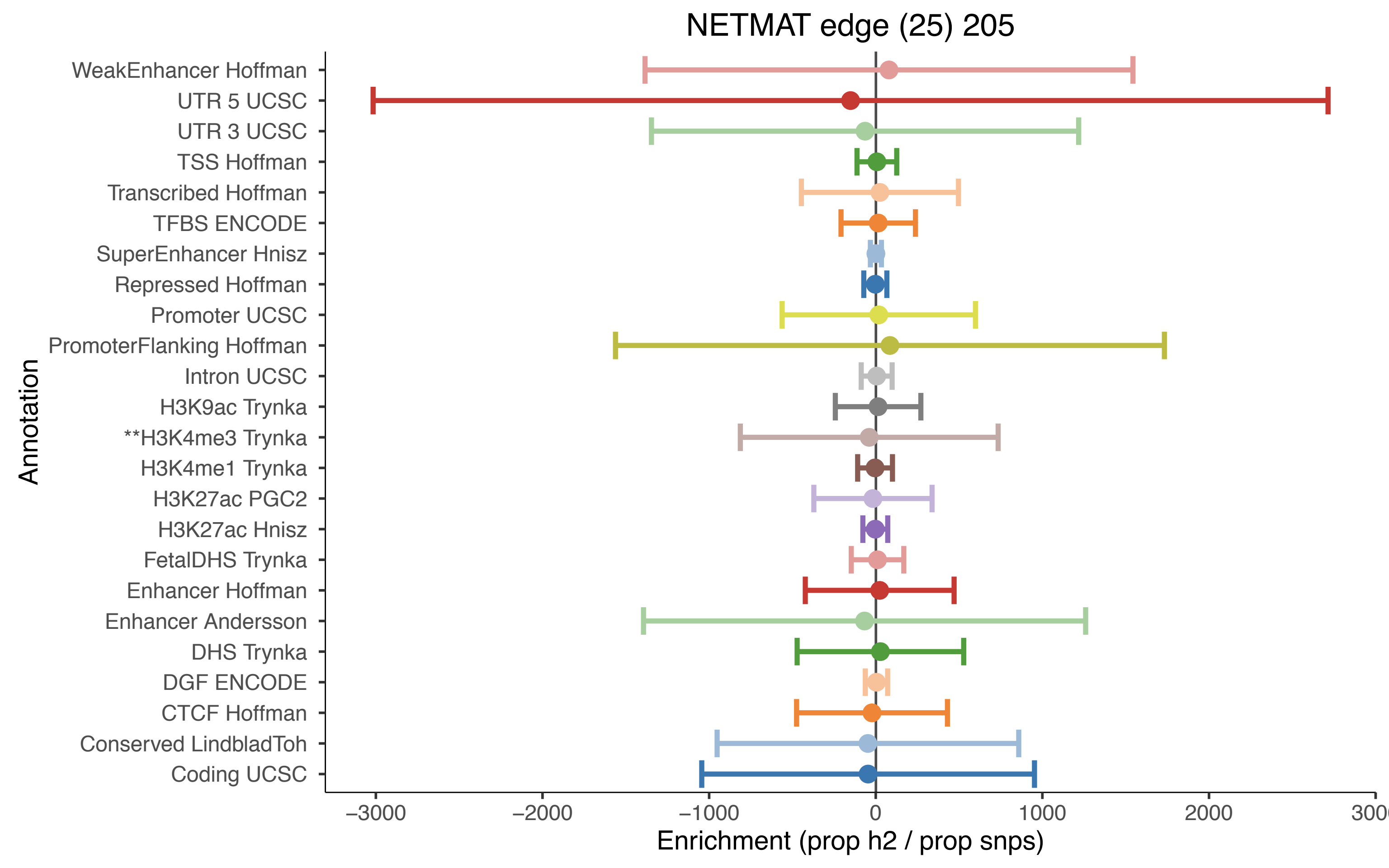

NETMAT edge (25) 205
WeakEnhancer Hoffman
UTR 5 UCSC
UTR 3 UCSC
TSS Hoffman
Transcribed Hoffman
TFBS ENCODE
SuperEnhancer Hnisz
Repressed Hoffman
Promoter UCSC
PromoterFlanking Hoffman
Intron UCSC
H3K9ac Trynka
**H3K4me3 Trynka
H3K4me1 Trynka
H3K27ac PGC2
H3K27ac Hnisz
FetalDHS Trynka
Enhancer Hoffman
Enhancer Andersson
DHS Trynka
DGF ENCODE
CTCF Hoffman
Conserved LindbladToh
Coding UCSC
Annotation
−3000
−2000
−1000
0
1000
2000
Enrichment (prop h2 / prop snps)

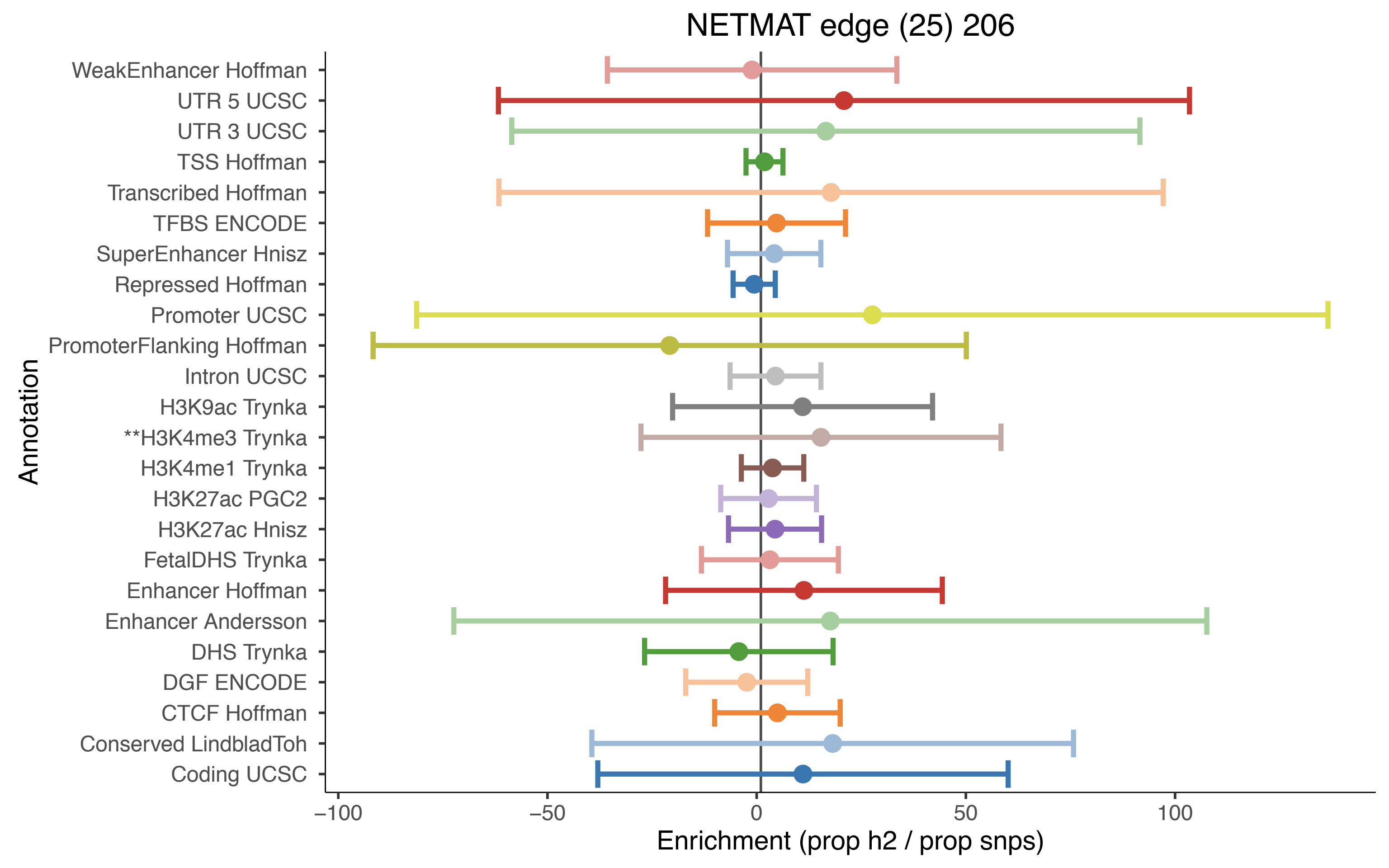

NETMAT edge (25) 206
WeakEnhancer Hoffman
UTR 5 UCSC
UTR 3 UCSC
TSS Hoffman
Transcribed Hoffman
TFBS ENCODE
SuperEnhancer Hnisz
Repressed Hoffman
Promoter UCSC
PromoterFlanking Hoffman
Intron UCSC
H3K9ac Trynka
**H3K4me3 Trynka
H3K4me1 Trynka
H3K27ac PGC2
H3K27ac Hnisz
FetalDHS Trynka
Enhancer Hoffman
Enhancer Andersson
DHS Trynka
DGF ENCODE
CTCF Hoffman
Conserved LindbladToh
Coding UCSC
Annotation
−100
−50
0
50
100
Enrichment (prop h2 / prop snps)

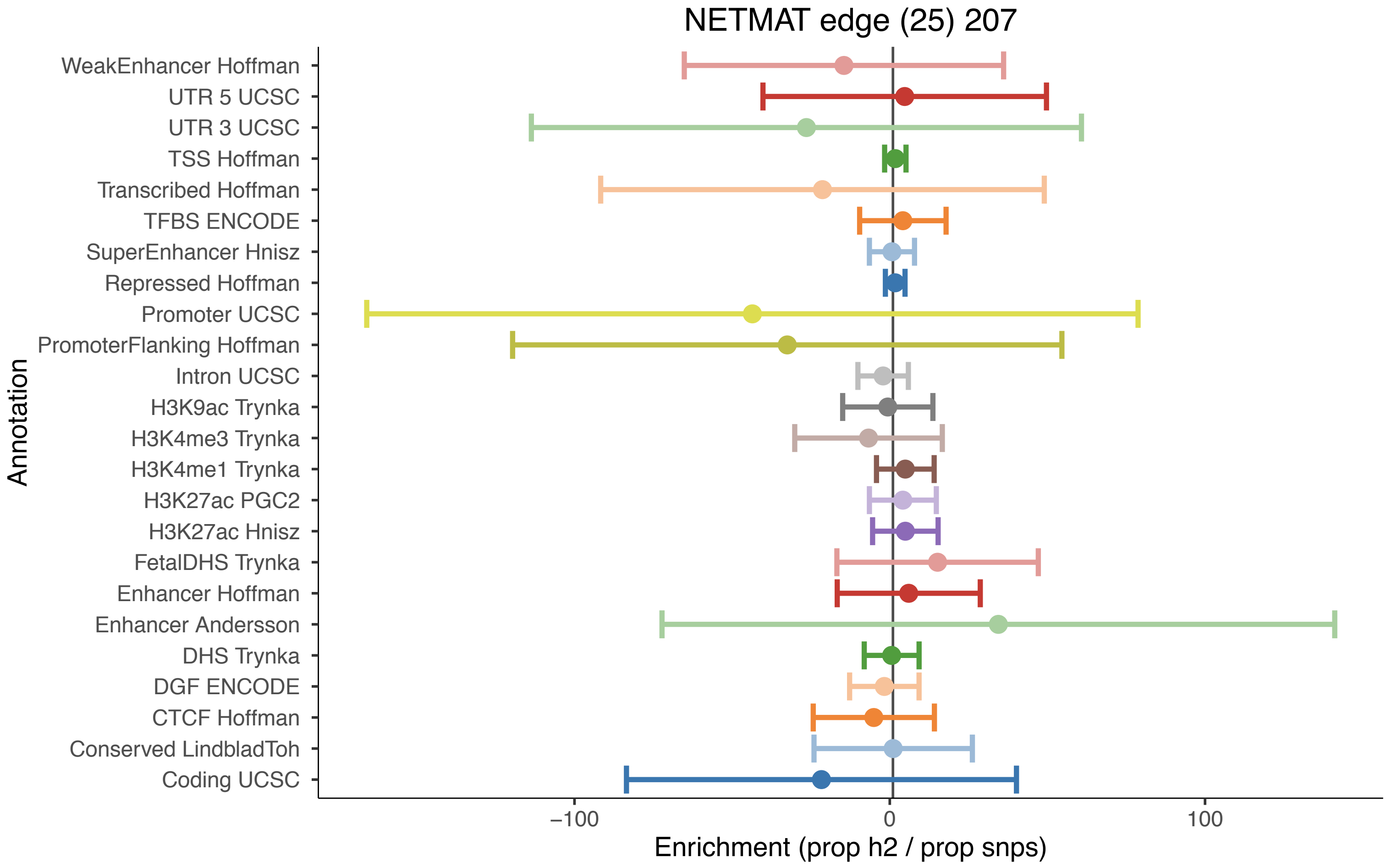
NETMAT edge (25) 207
WeakEnhancer Hoffman
UTR 5 UCSC
UTR 3 UCSC
TSS Hoffman
Transcribed Hoffman
TFBS ENCODE
SuperEnhancer Hnisz
Repressed Hoffman
Promoter UCSC
PromoterFlanking Hoffman
Intron UCSC
H3K9ac Trynka
H3K4me3 Trynka
H3K4me1 Trynka
H3K27ac PGC2
H3K27ac Hnisz
FetalDHS Trynka
Enhancer Hoffman
Enhancer Andersson
DHS Trynka
DGF ENCODE
CTCF Hoffman
Conserved LindbladToh
Coding UCSC
Annotation
−100
0
100
Enrichment (prop h2 / prop snps)

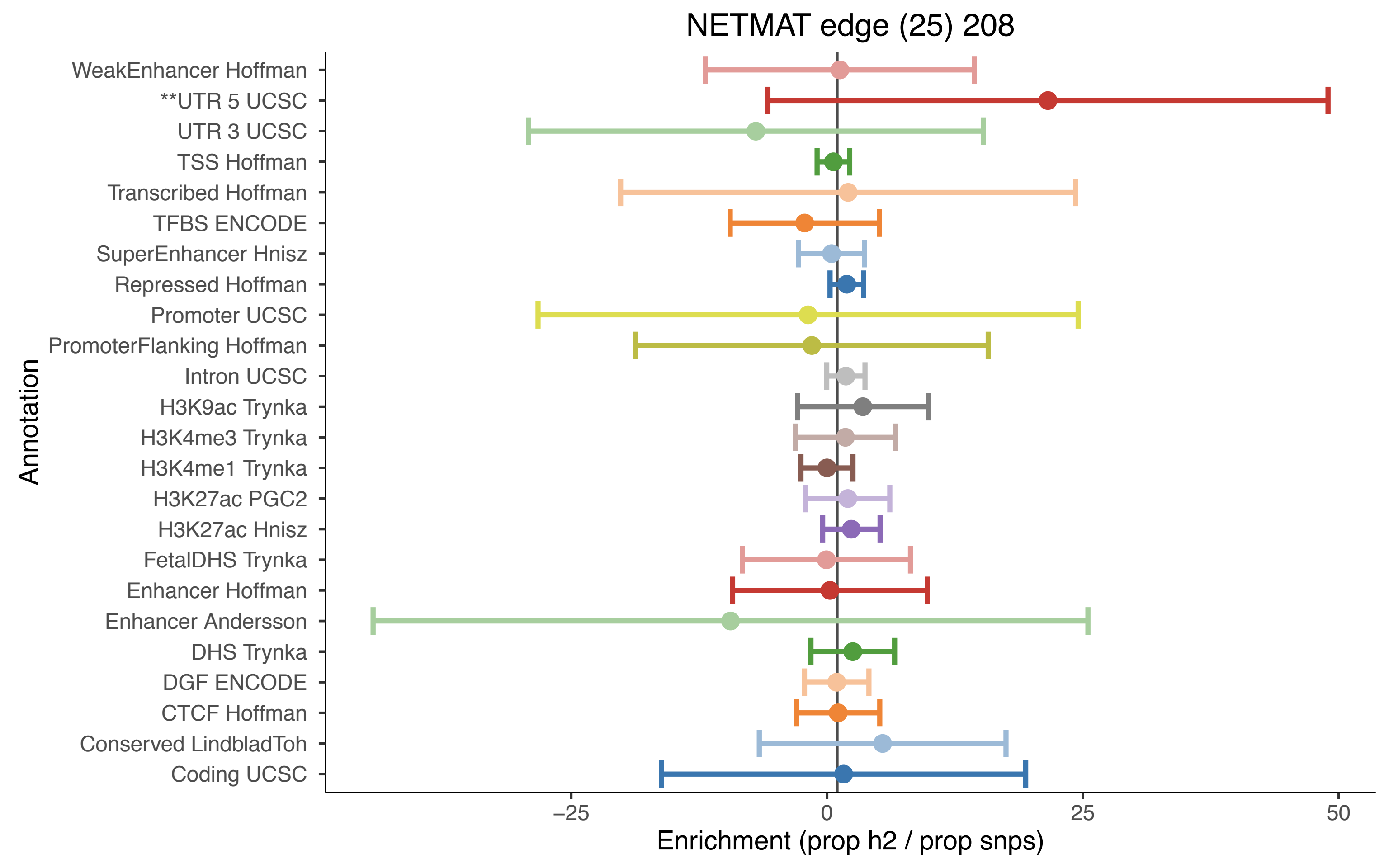
NETMAT edge (25) 208
WeakEnhancer Hoffman
**UTR 5 UCSC
UTR 3 UCSC
TSS Hoffman
Transcribed Hoffman
TFBS ENCODE
SuperEnhancer Hnisz
Repressed Hoffman
Promoter UCSC
PromoterFlanking Hoffman
Intron UCSC
H3K9ac Trynka
H3K4me3 Trynka
H3K4me1 Trynka
H3K27ac PGC2
H3K27ac Hnisz
FetalDHS Trynka
Enhancer Hoffman
Enhancer Andersson
DHS Trynka
DGF ENCODE
CTCF Hoffman
Conserved LindbladToh
Coding UCSC
Annotation
−25
0
25
50
Enrichment (prop h2 / prop snps)

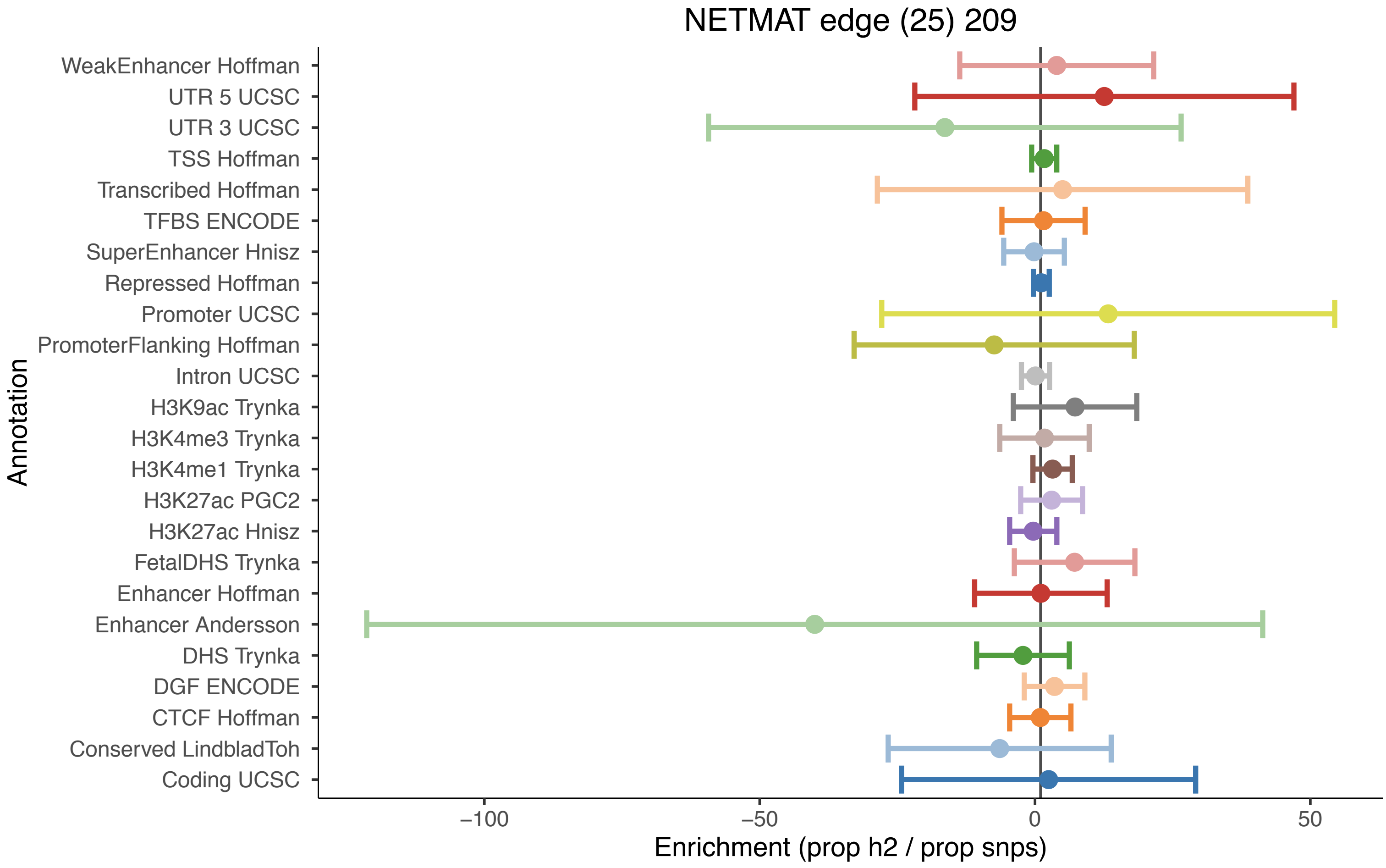
NETMAT edge (25) 209
WeakEnhancer Hoffman
UTR 5 UCSC
UTR 3 UCSC
TSS Hoffman
Transcribed Hoffman
TFBS ENCODE
SuperEnhancer Hnisz
Repressed Hoffman
Promoter UCSC
PromoterFlanking Hoffman
Intron UCSC
H3K9ac Trynka
H3K4me3 Trynka
H3K4me1 Trynka
H3K27ac PGC2
H3K27ac Hnisz
FetalDHS Trynka
Enhancer Hoffman
Enhancer Andersson
DHS Trynka
DGF ENCODE
CTCF Hoffman
Conserved LindbladToh
Coding UCSC
Annotation
−100
−50
0
50
Enrichment (prop h2 / prop snps)

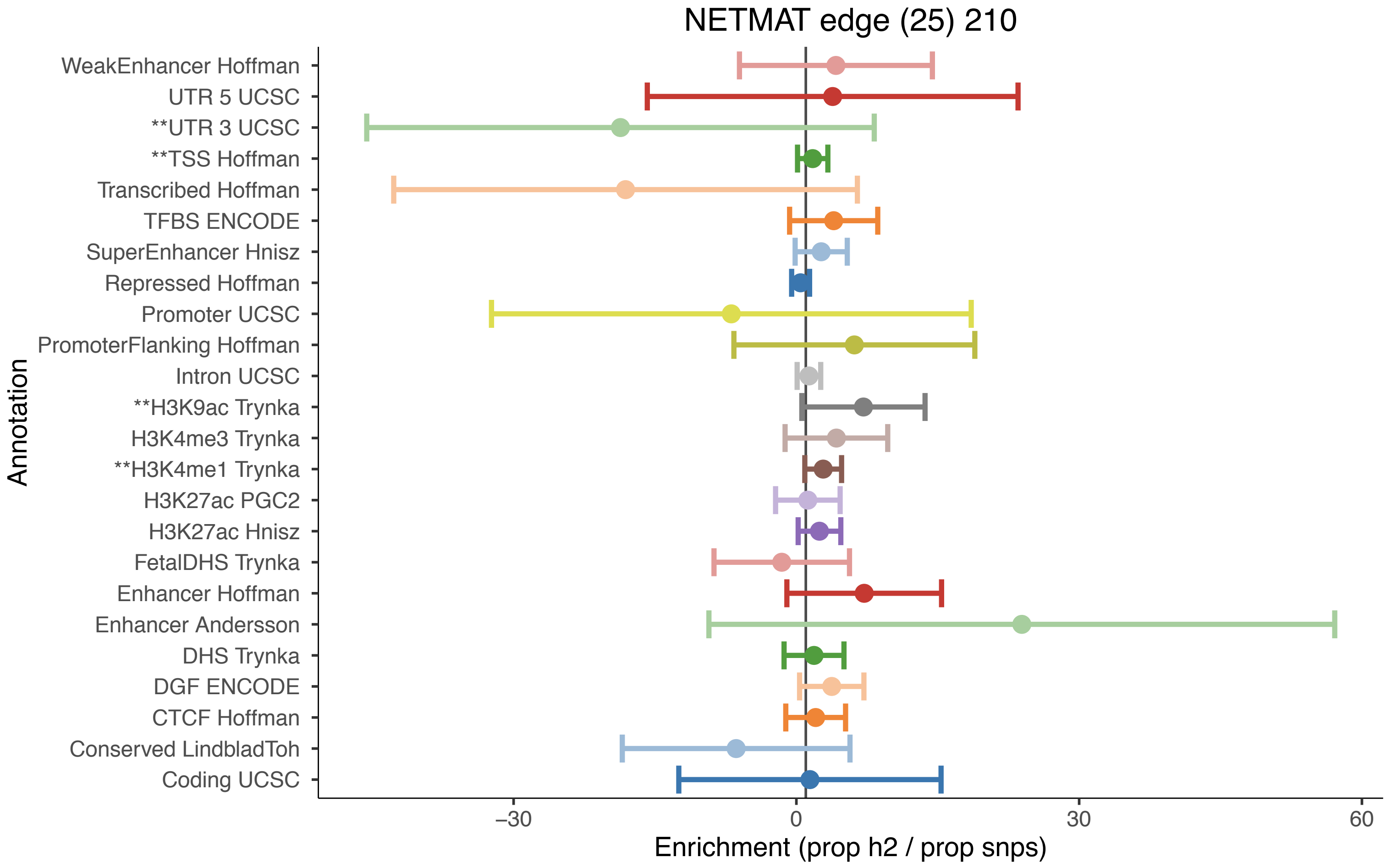

NETMAT edge (25) 210
WeakEnhancer Hoffman
UTR 5 UCSC
**UTR 3 UCSC
**TSS Hoffman
Transcribed Hoffman
TFBS ENCODE
SuperEnhancer Hnisz
Repressed Hoffman
Promoter UCSC
PromoterFlanking Hoffman
Intron UCSC
**H3K9ac Trynka
H3K4me3 Trynka
**H3K4me1 Trynka
H3K27ac PGC2
H3K27ac Hnisz
FetalDHS Trynka
Enhancer Hoffman
Enhancer Andersson
DHS Trynka
DGF ENCODE
CTCF Hoffman
Conserved LindbladToh
Coding UCSC
Annotation
–30
0
30
60
Enrichment (prop h2 / prop snps)

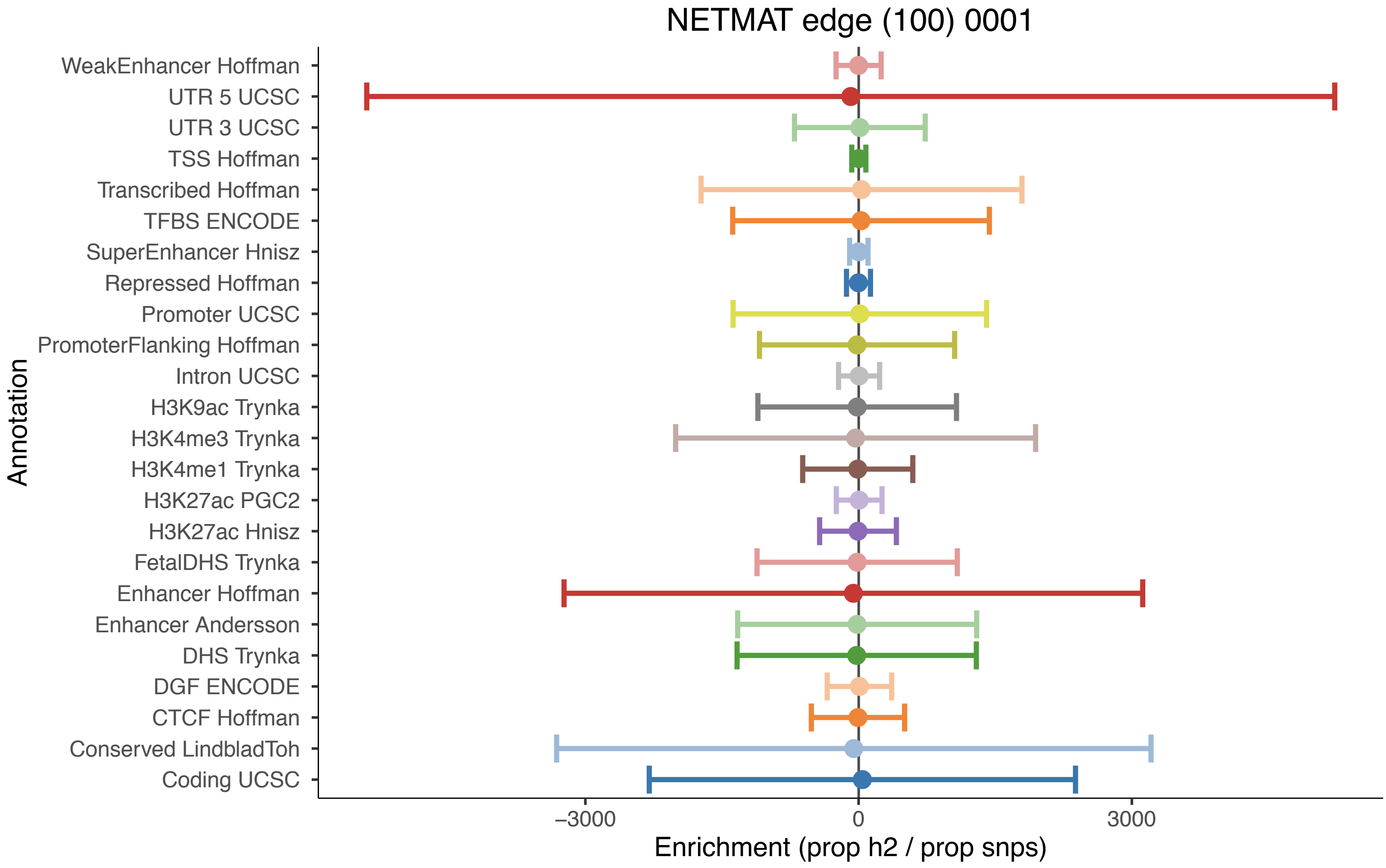
NETMAT edge (100) 0001
Annotation
WeakEnhancer Hoffman
UTR 5 UCSC
UTR 3 UCSC
TSS Hoffman
Transcribed Hoffman
TFBS ENCODE
SuperEnhancer Hnisz
Repressed Hoffman
Promoter UCSC
PromoterFlanking Hoffman
Intron UCSC
H3K9ac Trynka
H3K4me3 Trynka
H3K4me1 Trynka
H3K27ac PGC2
H3K27ac Hnisz
FetalDHS Trynka
Enhancer Hoffman
Enhancer Andersson
DHS Trynka
DGF ENCODE
CTCF Hoffman
Conserved LindbladToh
Coding UCSC
–3000
0
3000
Enrichment (prop h2 / prop snps)

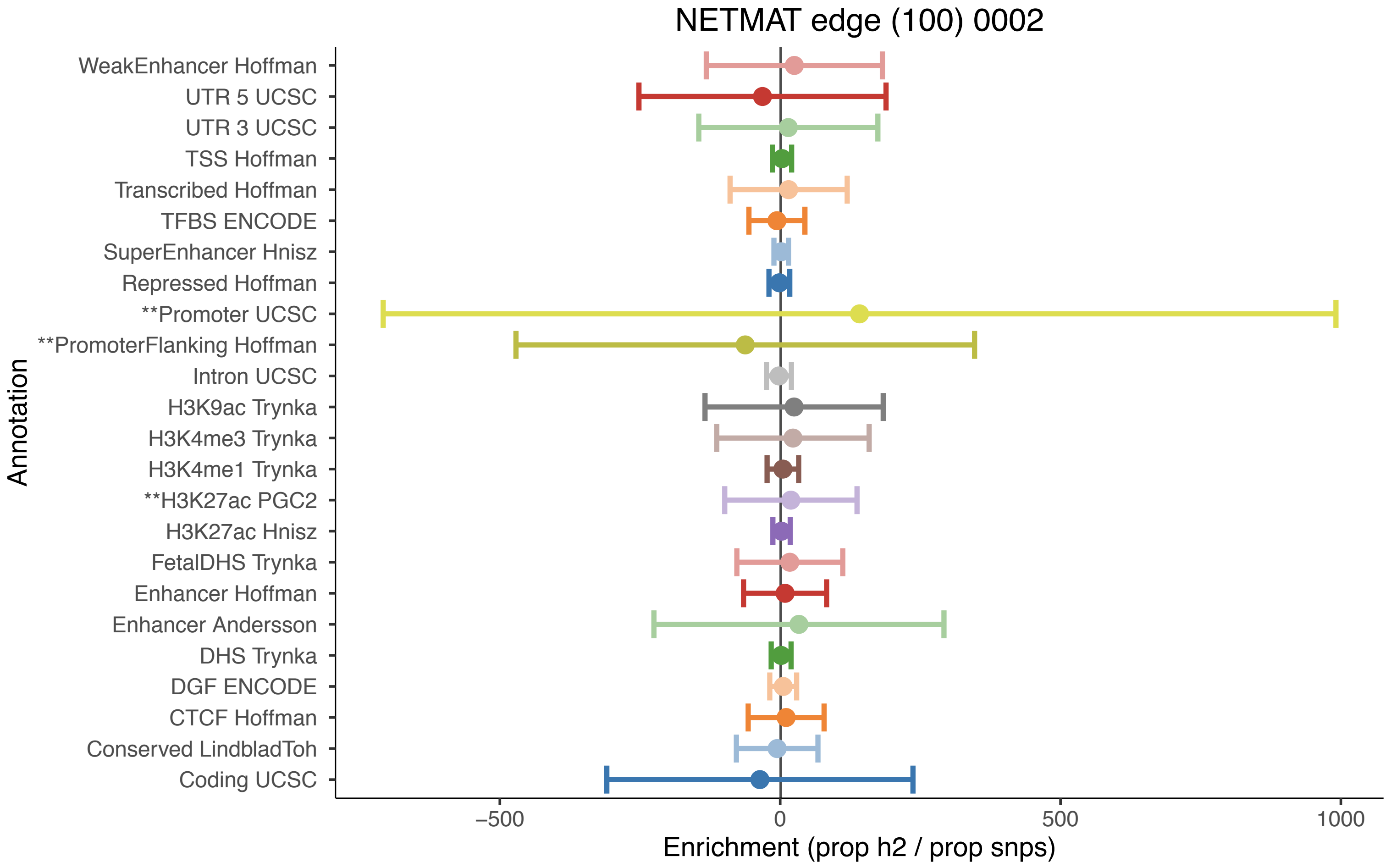
NETMAT edge (100) 0002
WeakEnhancer Hoffman
UTR 5 UCSC
UTR 3 UCSC
TSS Hoffman
Transcribed Hoffman
TFBS ENCODE
SuperEnhancer Hnisz
Repressed Hoffman
**Promoter UCSC
**PromoterFlanking Hoffman
Intron UCSC
H3K9ac Trynka
H3K4me3 Trynka
H3K4me1 Trynka
**H3K27ac PGC2
H3K27ac Hnisz
FetalDHS Trynka
Enhancer Hoffman
Enhancer Andersson
DHS Trynka
DGF ENCODE
CTCF Hoffman
Conserved LindbladToh
Coding UCSC
Annotation
−500
0
500
1000
Enrichment (prop h2 / prop snps)

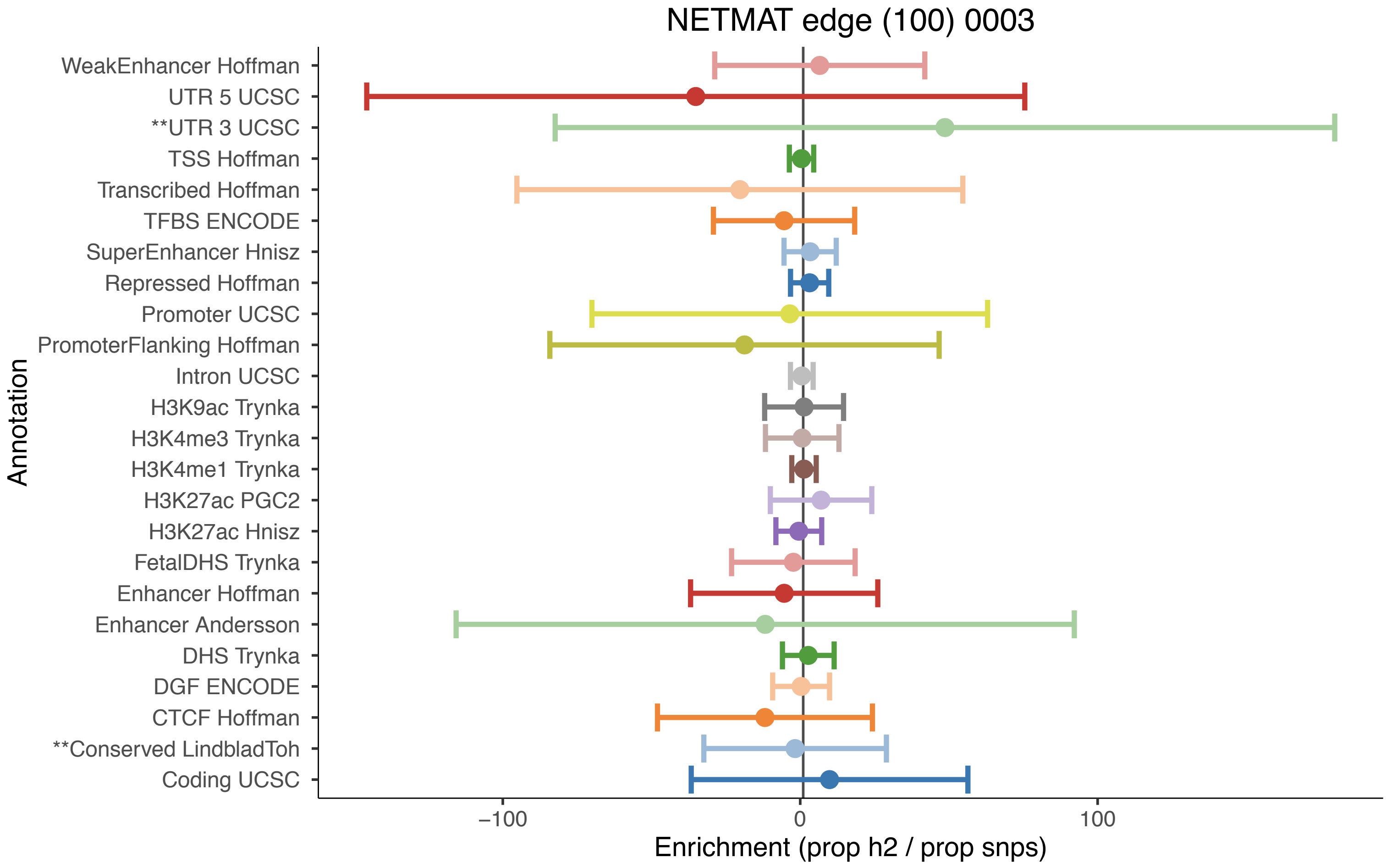
NETMAT edge (100) 0003
WeakEnhancer Hoffman
UTR 5 UCSC
**UTR 3 UCSC
TSS Hoffman
Transcribed Hoffman
TFBS ENCODE
SuperEnhancer Hnisz
Repressed Hoffman
Promoter UCSC
PromoterFlanking Hoffman
Intron UCSC
H3K9ac Trynka
H3K4me3 Trynka
H3K4me1 Trynka
H3K27ac PGC2
H3K27ac Hnisz
FetalDHS Trynka
Enhancer Hoffman
Enhancer Andersson
DHS Trynka
DGF ENCODE
CTCF Hoffman
**Conserved LindbladToh
Coding UCSC
Annotation
−100
0
100
Enrichment (prop h2 / prop snps)

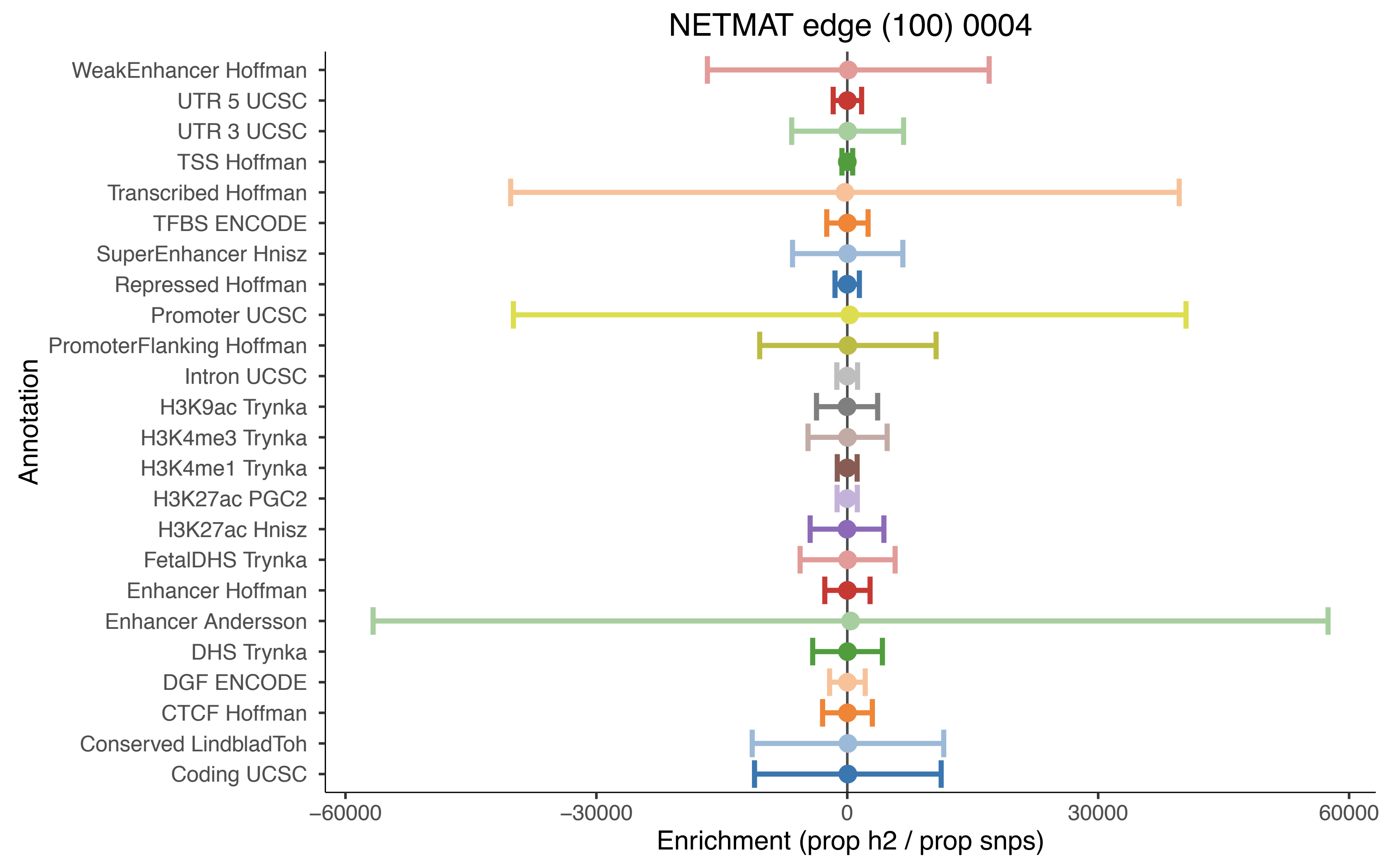

NETMAT edge (100) 0004
Annotation
WeakEnhancer Hoffman
UTR 5 UCSC
UTR 3 UCSC
TSS Hoffman
Transcribed Hoffman
TFBS ENCODE
SuperEnhancer Hnisz
Repressed Hoffman
Promoter UCSC
PromoterFlanking Hoffman
Intron UCSC
H3K9ac Trynka
H3K4me3 Trynka
H3K4me1 Trynka
H3K27ac PGC2
H3K27ac Hnisz
FetalDHS Trynka
Enhancer Hoffman
Enhancer Andersson
DHS Trynka
DGF ENCODE
CTCF Hoffman
Conserved LindbladToh
Coding UCSC
−60000
−30000
0
30000
60000
Enrichment (prop h2 / prop snps)

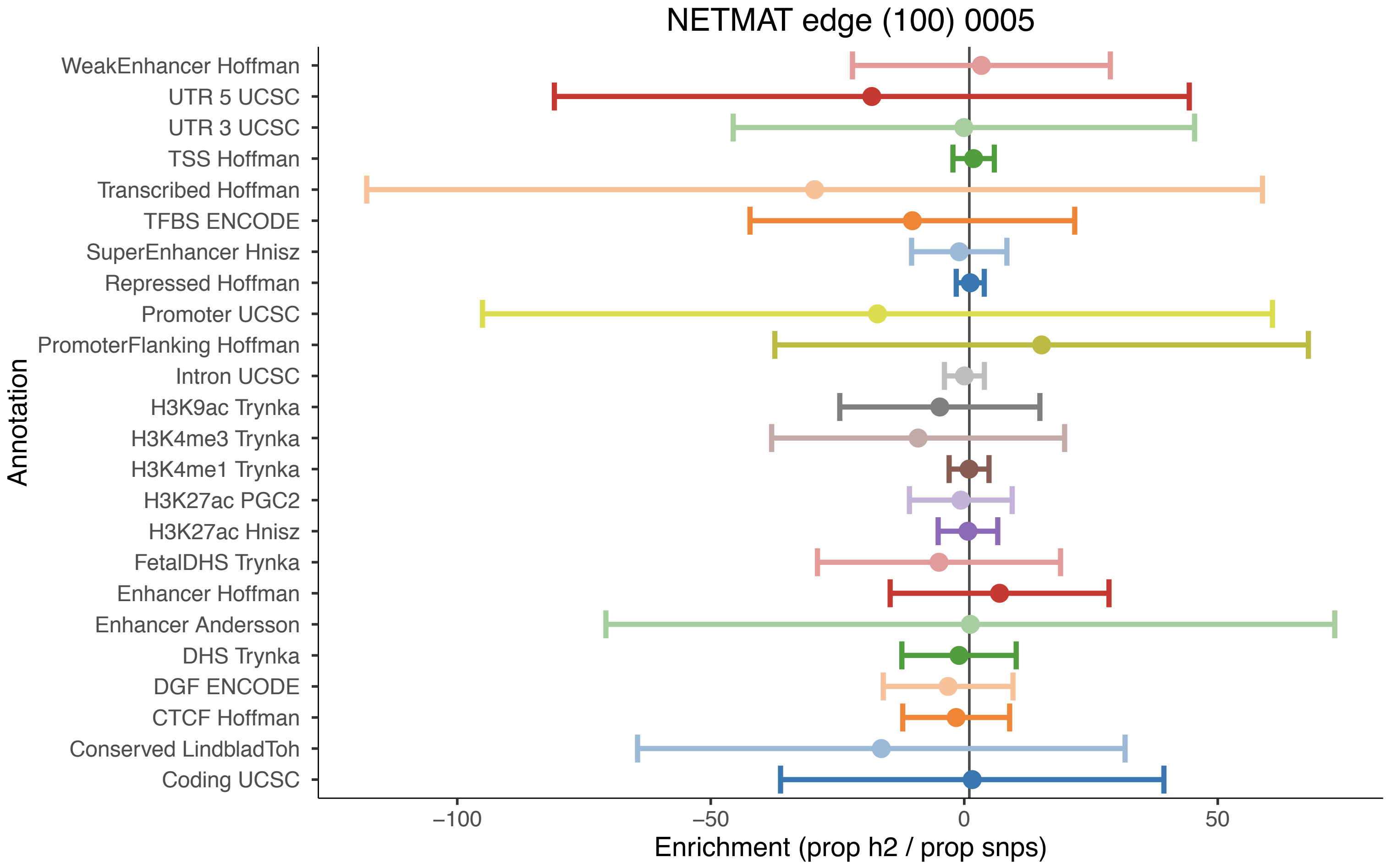
NETMAT edge (100) 0005
WeakEnhancer Hoffman
UTR 5 UCSC
UTR 3 UCSC
TSS Hoffman
Transcribed Hoffman
TFBS ENCODE
SuperEnhancer Hnisz
Repressed Hoffman
Promoter UCSC
PromoterFlanking Hoffman
Intron UCSC
H3K9ac Trynka
H3K4me3 Trynka
H3K4me1 Trynka
H3K27ac PGC2
H3K27ac Hnisz
FetalDHS Trynka
Enhancer Hoffman
Enhancer Andersson
DHS Trynka
DGF ENCODE
CTCF Hoffman
Conserved LindbladToh
Coding UCSC
Annotation
−100
−50
0
50
Enrichment (prop h2 / prop snps)

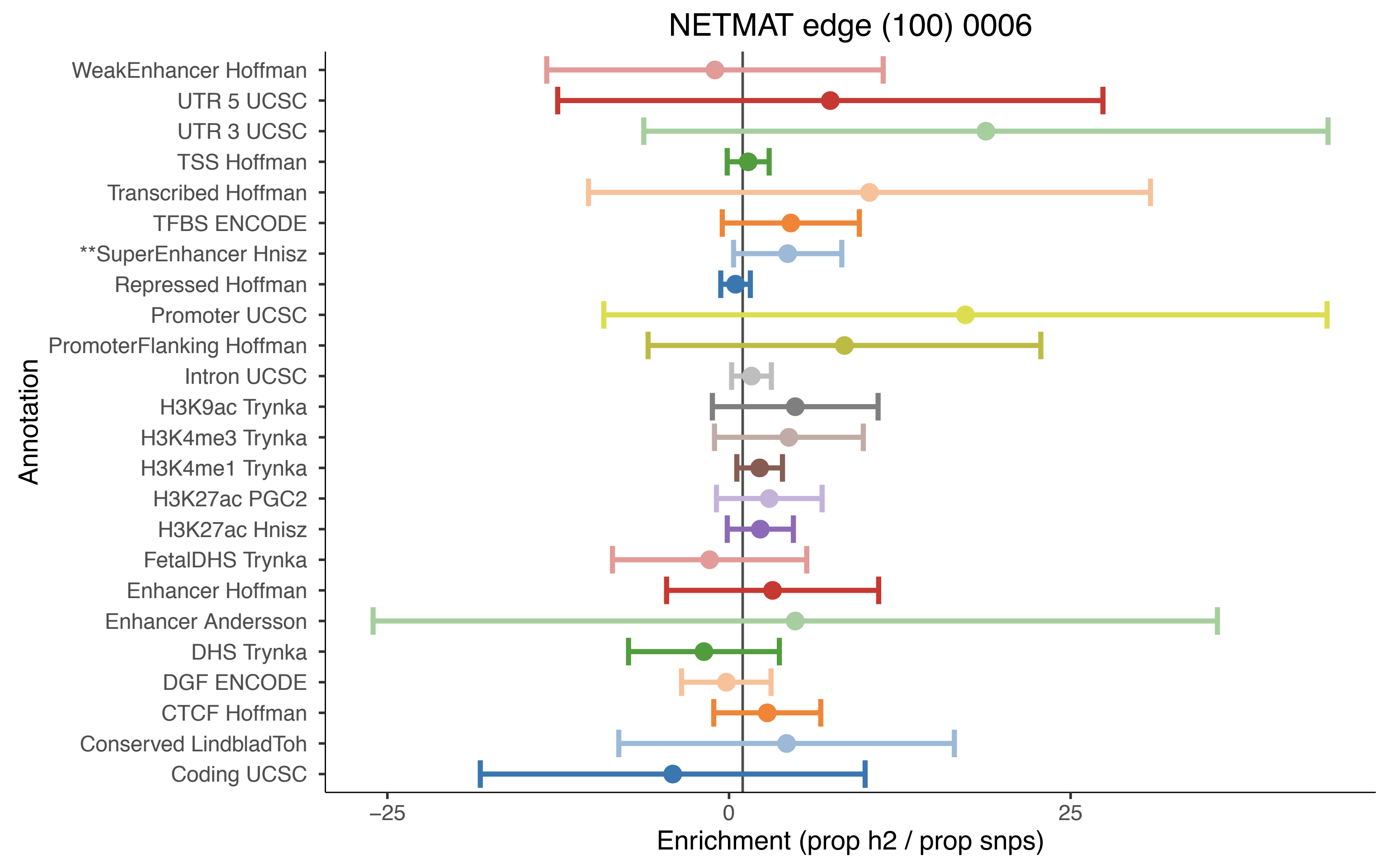
NETMAT edge (100) 0006
WeakEnhancer Hoffman
UTR 5 UCSC
UTR 3 UCSC
TSS Hoffman
Transcribed Hoffman
TFBS ENCODE
**SuperEnhancer Hnisz
Repressed Hoffman
Promoter UCSC
PromoterFlanking Hoffman
Intron UCSC
H3K9ac Trynka
H3K4me3 Trynka
H3K4me1 Trynka
H3K27ac PGC2
H3K27ac Hnisz
FetalDHS Trynka
Enhancer Hoffman
Enhancer Andersson
DHS Trynka
DGF ENCODE
CTCF Hoffman
Conserved LindbladToh
Coding UCSC
Annotation
−25
0
25
Enrichment (prop h2 / prop snps)

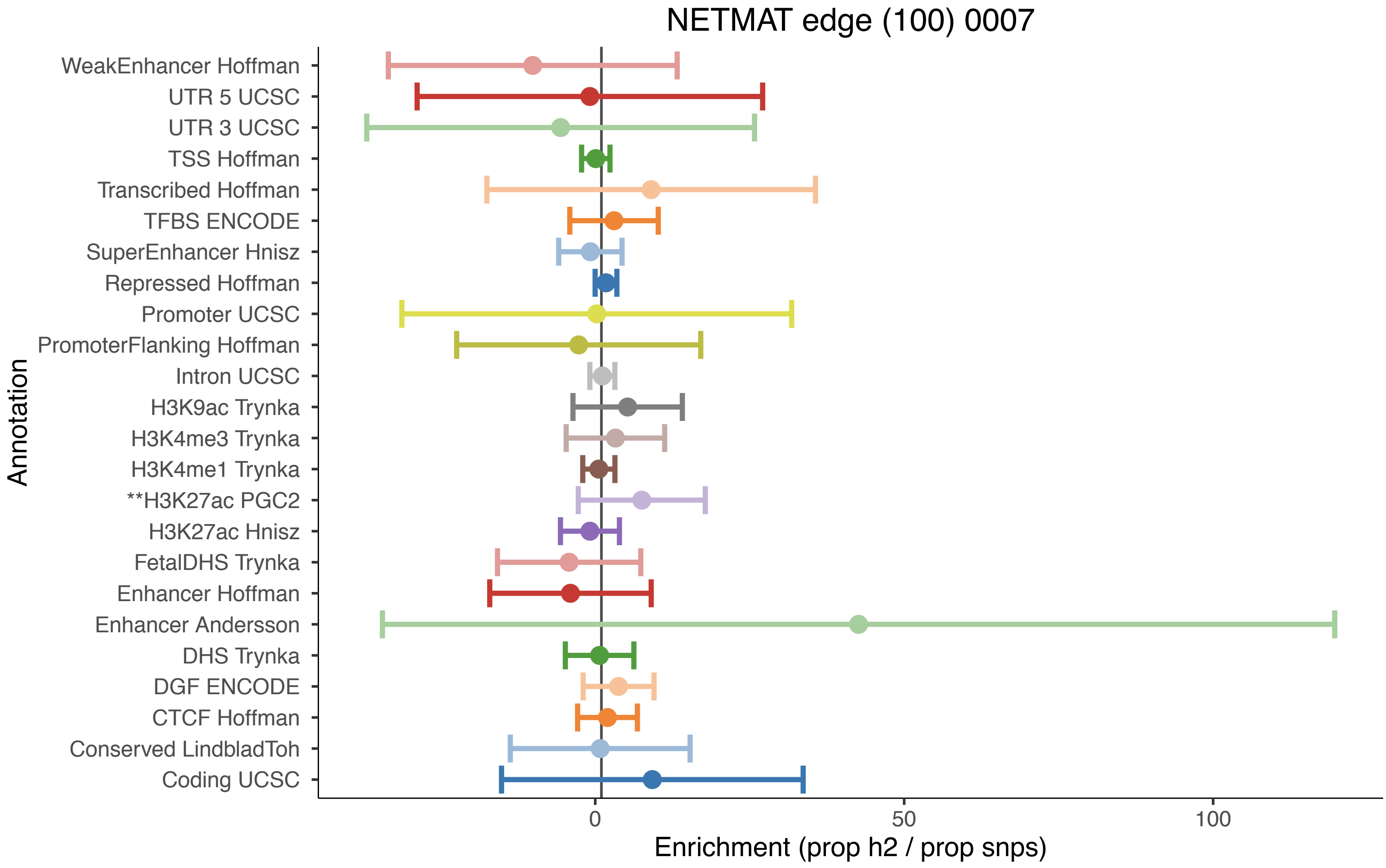

NETMAT edge (100) 0007
WeakEnhancer Hoffman
UTR 5 UCSC
UTR 3 UCSC
TSS Hoffman
Transcribed Hoffman
TFBS ENCODE
SuperEnhancer Hnisz
Repressed Hoffman
Promoter UCSC
PromoterFlanking Hoffman
Intron UCSC
H3K9ac Trynka
H3K4me3 Trynka
H3K4me1 Trynka
**H3K27ac PGC2
H3K27ac Hnisz
FetalDHS Trynka
Enhancer Hoffman
Enhancer Andersson
DHS Trynka
DGF ENCODE
CTCF Hoffman
Conserved LindbladToh
Coding UCSC
Annotation
0
50
100
Enrichment (prop h2 / prop snps)

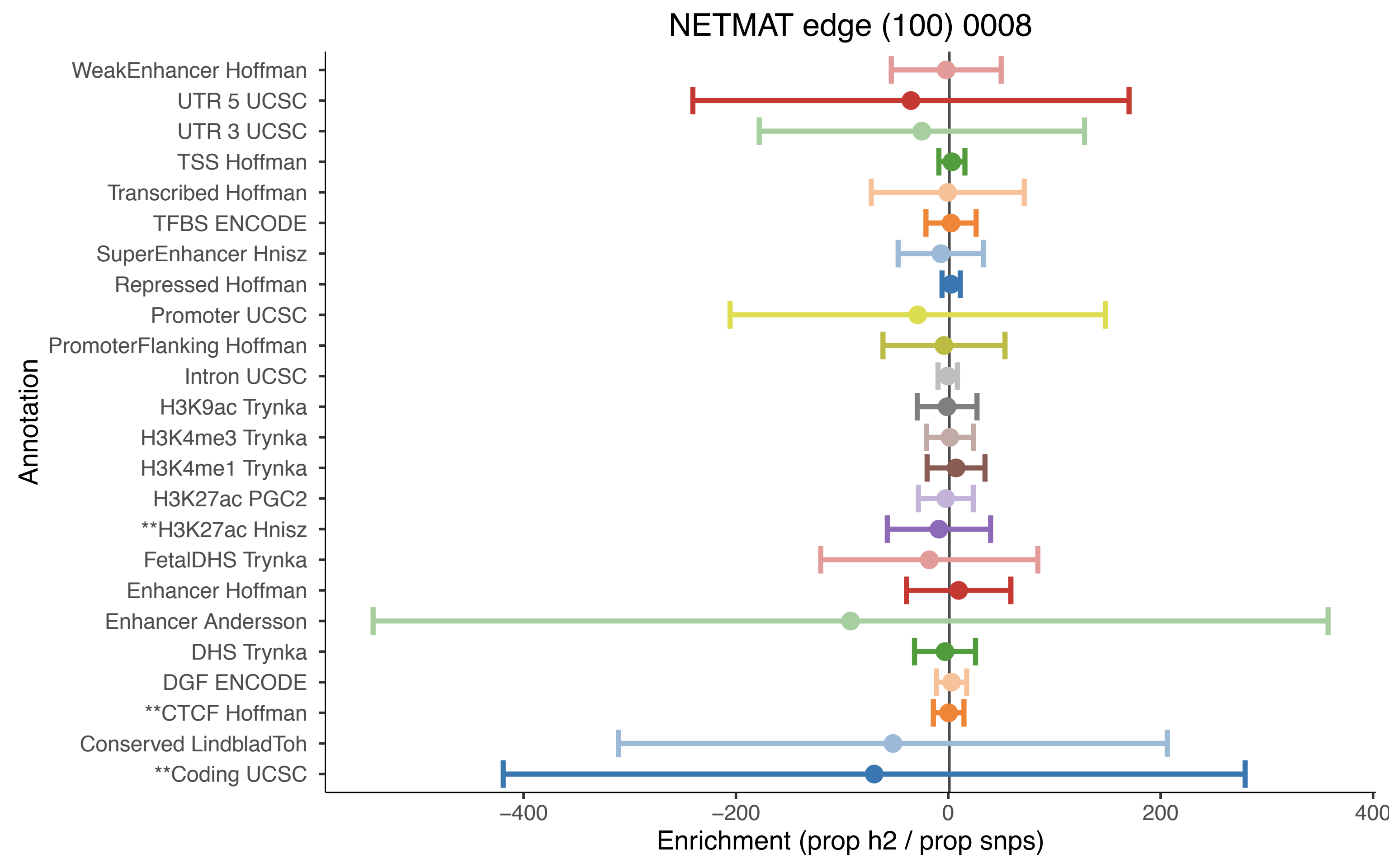

NETMAT edge (100) 0008
Annotation
WeakEnhancer Hoffman
UTR 5 UCSC
UTR 3 UCSC
TSS Hoffman
Transcribed Hoffman
TFBS ENCODE
SuperEnhancer Hnisz
Repressed Hoffman
Promoter UCSC
PromoterFlanking Hoffman
Intron UCSC
H3K9ac Trynka
H3K4me3 Trynka
H3K4me1 Trynka
H3K27ac PGC2
**H3K27ac Hnisz
FetalDHS Trynka
Enhancer Hoffman
Enhancer Andersson
DHS Trynka
DGF ENCODE
**CTCF Hoffman
Conserved LindbladToh
**Coding UCSC
−400
−200
0
200
400
Enrichment (prop h2 / prop snps)

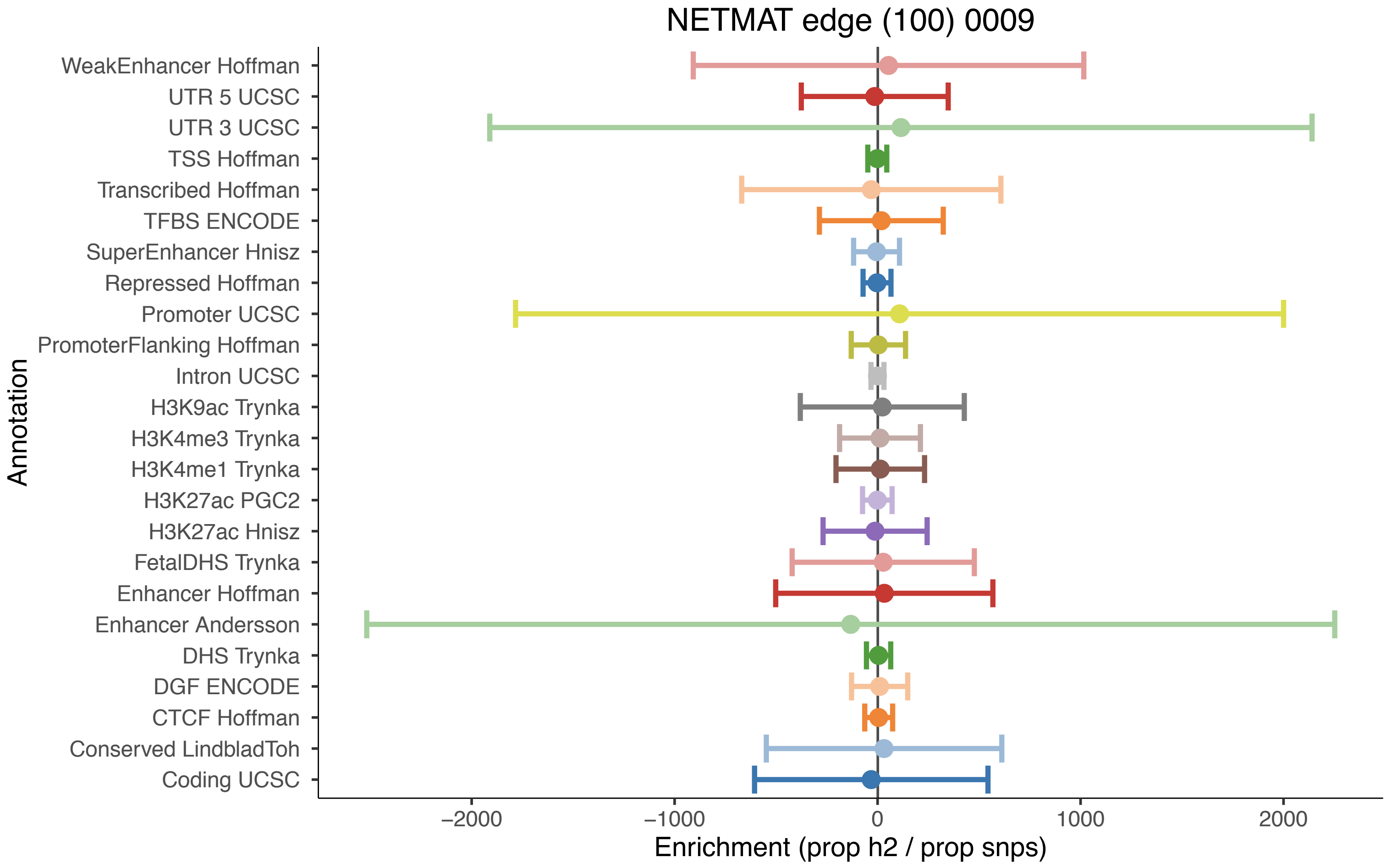

NETMAT edge (100) 0009
WeakEnhancer Hoffman
UTR 5 UCSC
UTR 3 UCSC
TSS Hoffman
Transcribed Hoffman
TFBS ENCODE
SuperEnhancer Hnisz
Repressed Hoffman
Promoter UCSC
PromoterFlanking Hoffman
Intron UCSC
H3K9ac Trynka
H3K4me3 Trynka
H3K4me1 Trynka
H3K27ac PGC2
H3K27ac Hnisz
FetalDHS Trynka
Enhancer Hoffman
Enhancer Andersson
DHS Trynka
DGF ENCODE
CTCF Hoffman
Conserved LindbladToh
Coding UCSC
Annotation
−2000
−1000
0
1000
2000
Enrichment (prop h2 / prop snps)

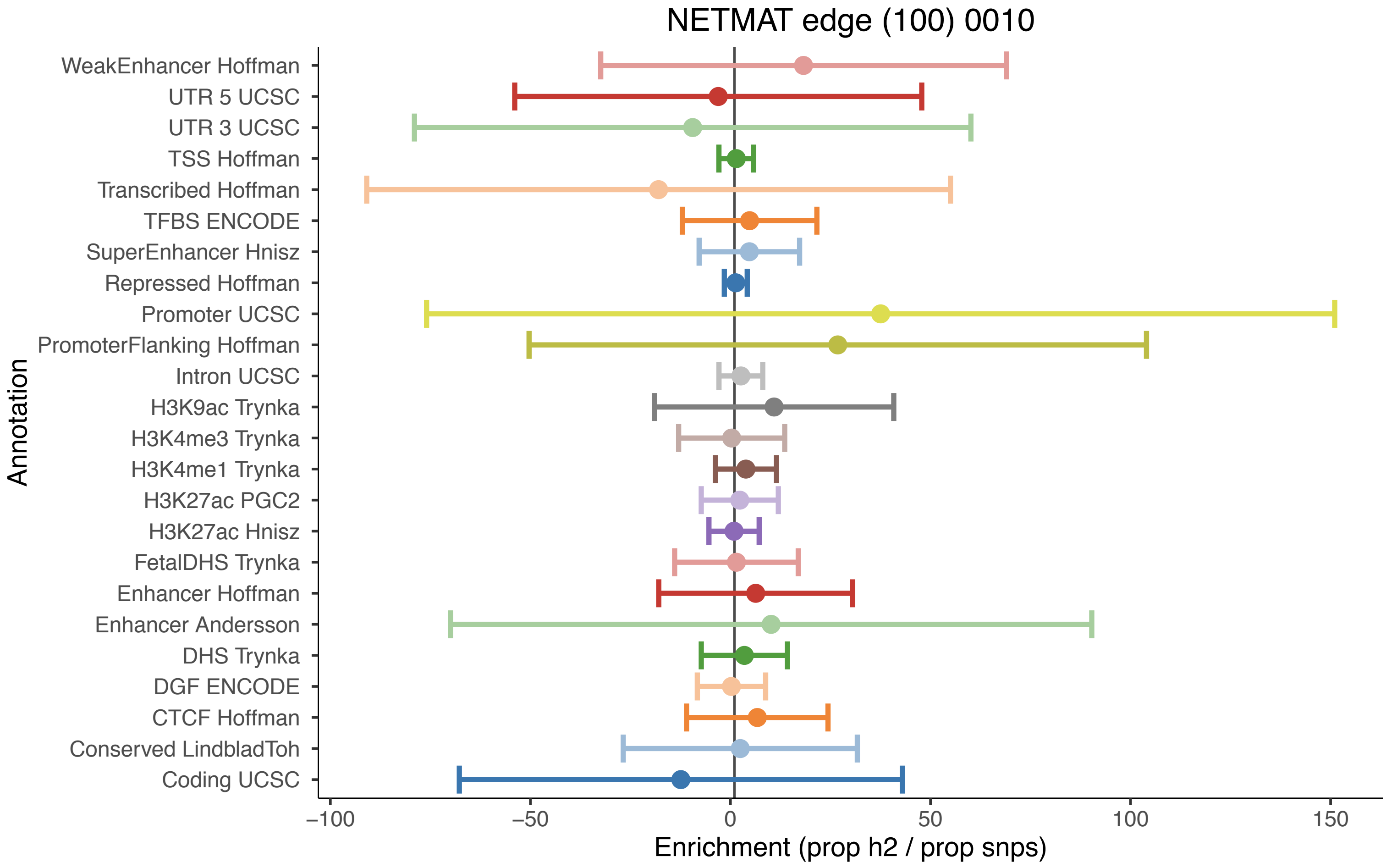
NETMAT edge (100) 0010
Annotation
WeakEnhancer Hoffman
UTR 5 UCSC
UTR 3 UCSC
TSS Hoffman
Transcribed Hoffman
TFBS ENCODE
SuperEnhancer Hnisz
Repressed Hoffman
Promoter UCSC
PromoterFlanking Hoffman
Intron UCSC
H3K9ac Trynka
H3K4me3 Trynka
H3K4me1 Trynka
H3K27ac PGC2
H3K27ac Hnisz
FetalDHS Trynka
Enhancer Hoffman
Enhancer Andersson
DHS Trynka
DGF ENCODE
CTCF Hoffman
Conserved LindbladToh
Coding UCSC
−100
−50
0
50
100
150
Enrichment (prop h2 / prop snps)

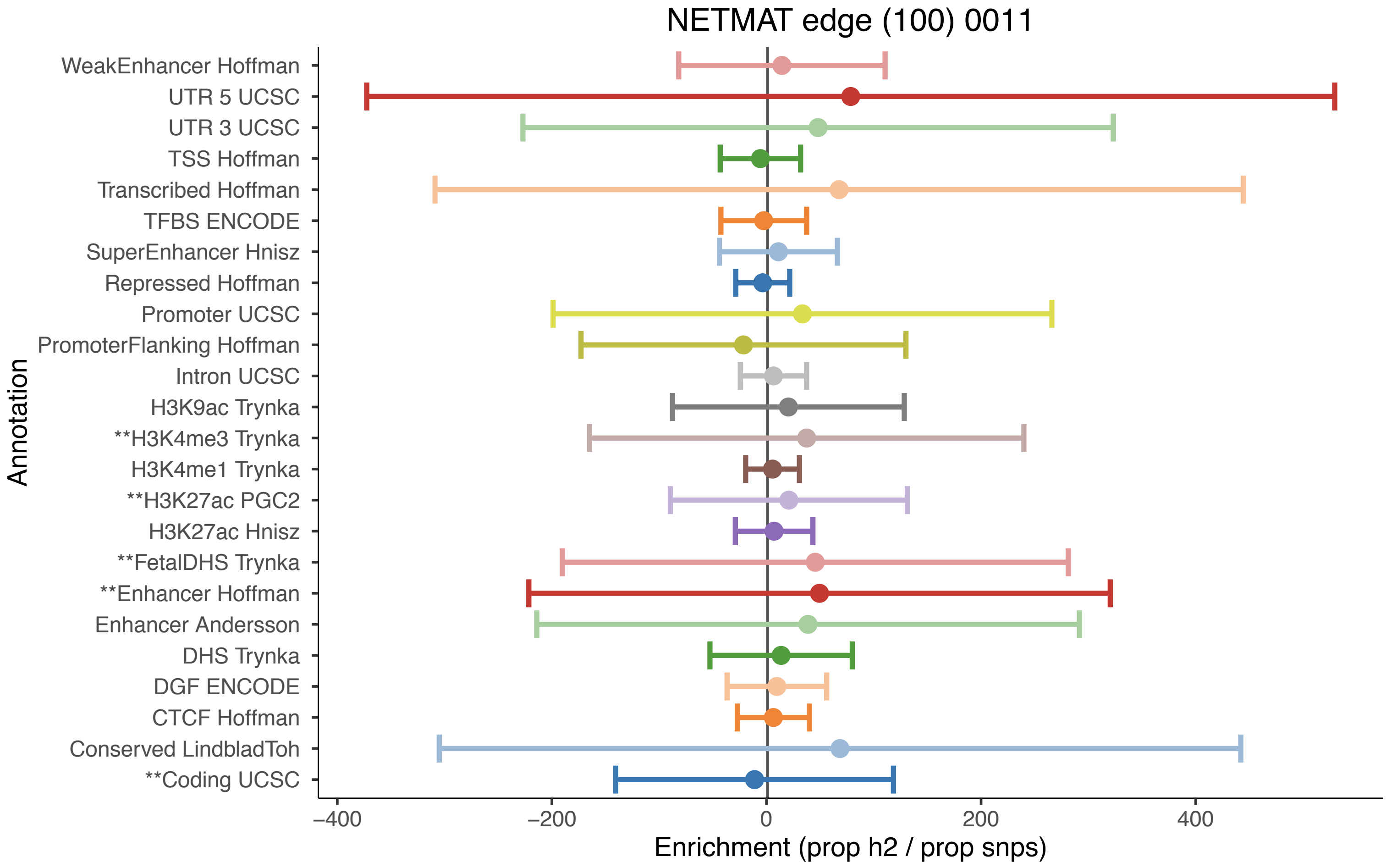
NETMAT edge (100) 0011
WeakEnhancer Hoffman
UTR 5 UCSC
UTR 3 UCSC
TSS Hoffman
Transcribed Hoffman
TFBS ENCODE
SuperEnhancer Hnisz
Repressed Hoffman
Promoter UCSC
PromoterFlanking Hoffman
Intron UCSC
H3K9ac Trynka
**H3K4me3 Trynka
H3K4me1 Trynka
**H3K27ac PGC2
H3K27ac Hnisz
**FetalDHS Trynka
**Enhancer Hoffman
Enhancer Andersson
DHS Trynka
DGF ENCODE
CTCF Hoffman
Conserved LindbladToh
**Coding UCSC
Annotation
−400
−200
0
200
400
Enrichment (prop h2 / prop snps)

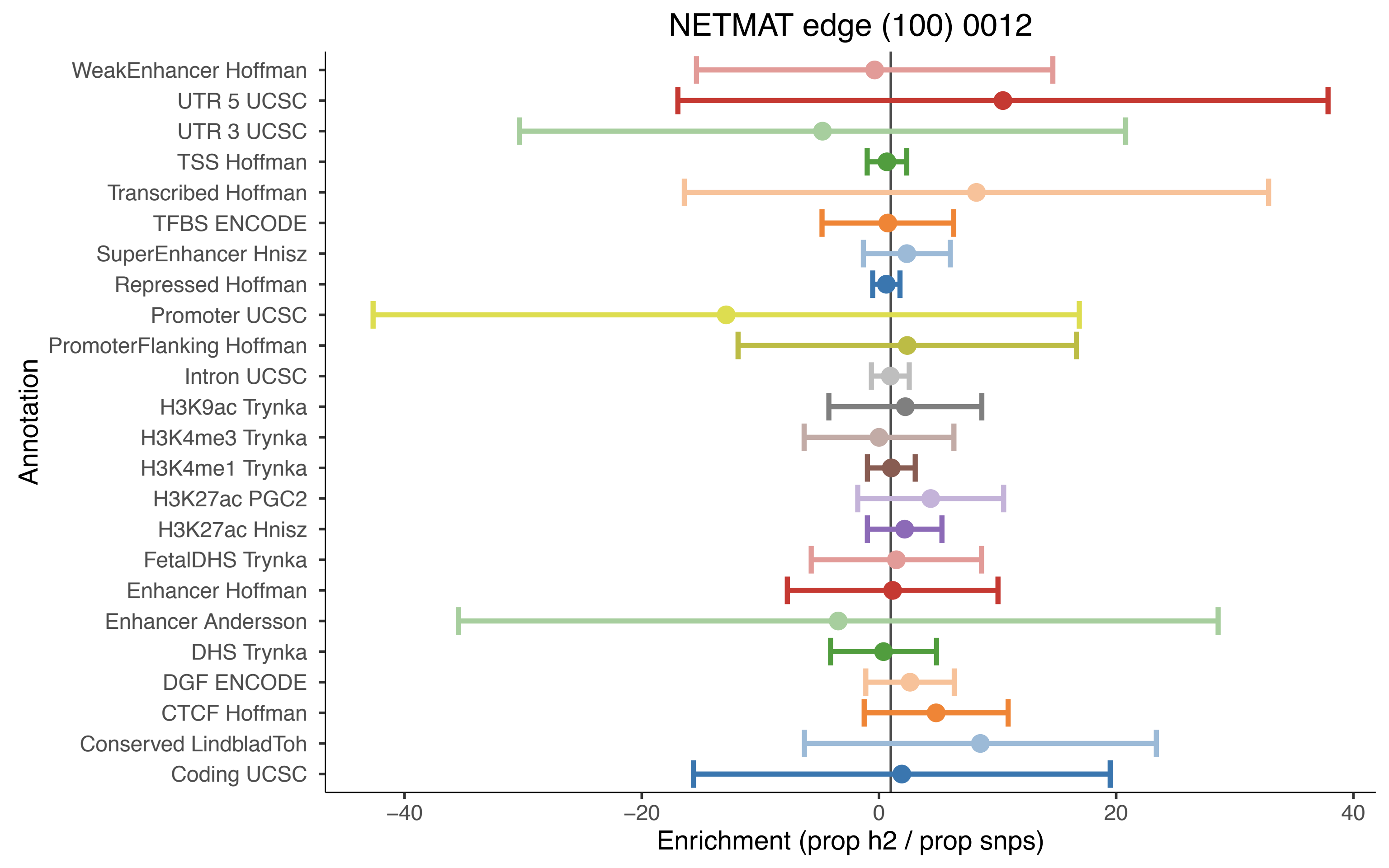

NETMAT edge (100) 0012
WeakEnhancer Hoffman
UTR 5 UCSC
UTR 3 UCSC
TSS Hoffman
Transcribed Hoffman
TFBS ENCODE
SuperEnhancer Hnisz
Repressed Hoffman
Promoter UCSC
PromoterFlanking Hoffman
Intron UCSC
H3K9ac Trynka
H3K4me3 Trynka
H3K4me1 Trynka
H3K27ac PGC2
H3K27ac Hnisz
FetalDHS Trynka
Enhancer Hoffman
Enhancer Andersson
DHS Trynka
DGF ENCODE
CTCF Hoffman
Conserved LindbladToh
Coding UCSC
Annotation
−40
−20
0
20
40
Enrichment (prop h2 / prop snps)

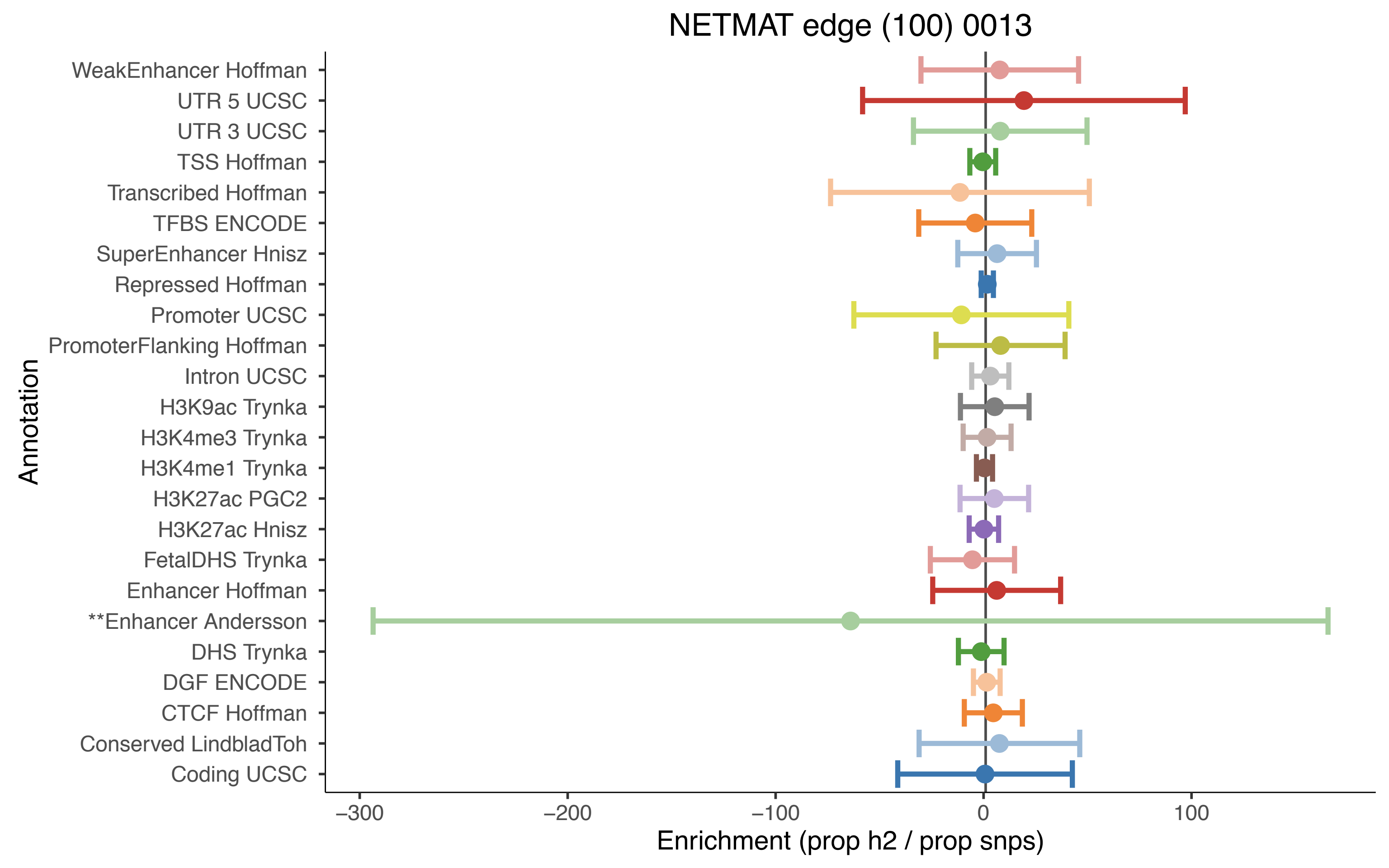
NETMAT edge (100) 0013
Annotation
WeakEnhancer Hoffman
UTR 5 UCSC
UTR 3 UCSC
TSS Hoffman
Transcribed Hoffman
TFBS ENCODE
SuperEnhancer Hnisz
Repressed Hoffman
Promoter UCSC
PromoterFlanking Hoffman
Intron UCSC
H3K9ac Trynka
H3K4me3 Trynka
H3K4me1 Trynka
H3K27ac PGC2
H3K27ac Hnisz
FetalDHS Trynka
Enhancer Hoffman
**Enhancer Andersson
DHS Trynka
DGF ENCODE
CTCF Hoffman
Conserved LindbladToh
Coding UCSC
−300
−200
−100
0
100
Enrichment (prop h2 / prop snps)

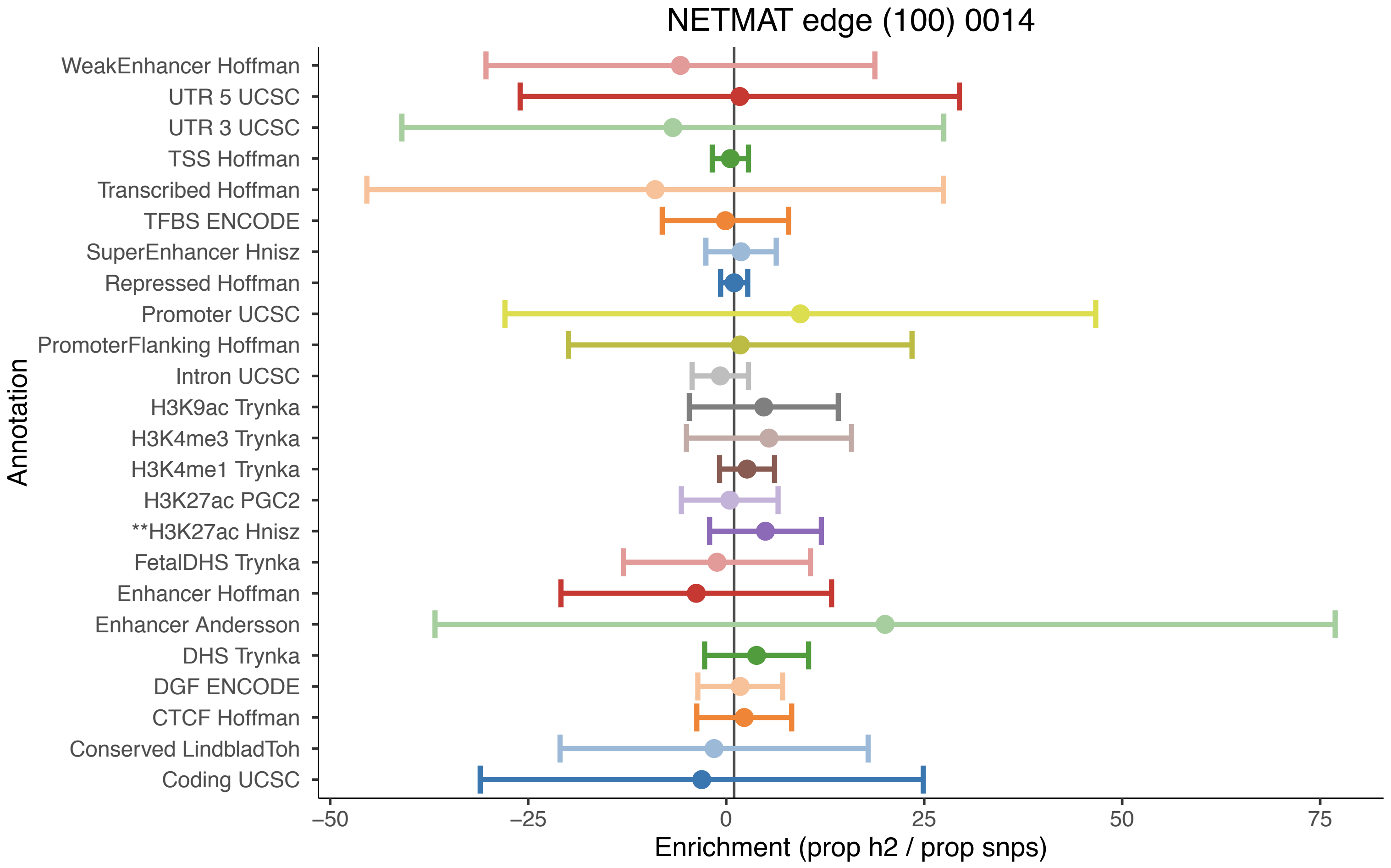

NETMAT edge (100) 0014
Annotation
WeakEnhancer Hoffman
UTR 5 UCSC
UTR 3 UCSC
TSS Hoffman
Transcribed Hoffman
TFBS ENCODE
SuperEnhancer Hnisz
Repressed Hoffman
Promoter UCSC
PromoterFlanking Hoffman
Intron UCSC
H3K9ac Trynka
H3K4me3 Trynka
H3K4me1 Trynka
H3K27ac PGC2
**H3K27ac Hnisz
FetalDHS Trynka
Enhancer Hoffman
Enhancer Andersson
DHS Trynka
DGF ENCODE
CTCF Hoffman
Conserved LindbladToh
Coding UCSC
−50
−25
0
25
50
75
Enrichment (prop h2 / prop snps)

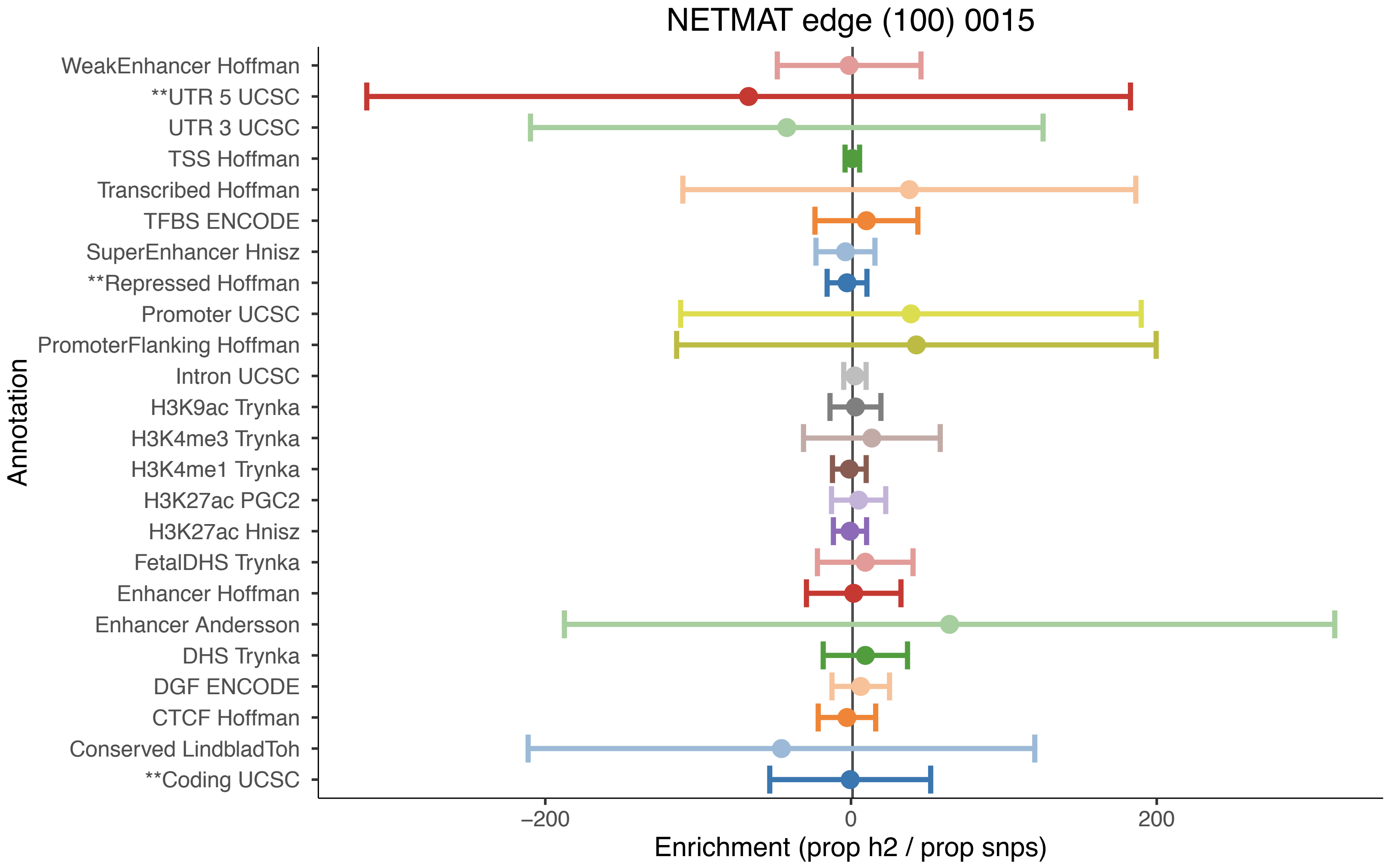

NETMAT edge (100) 0015
WeakEnhancer Hoffman
**UTR 5 UCSC
UTR 3 UCSC
TSS Hoffman
Transcribed Hoffman
TFBS ENCODE
SuperEnhancer Hnisz
**Repressed Hoffman
Promoter UCSC
PromoterFlanking Hoffman
Intron UCSC
H3K9ac Trynka
H3K4me3 Trynka
H3K4me1 Trynka
H3K27ac PGC2
H3K27ac Hnisz
FetalDHS Trynka
Enhancer Hoffman
Enhancer Andersson
DHS Trynka
DGF ENCODE
CTCF Hoffman
Conserved LindbladToh
**Coding UCSC
Annotation
−200
0
200
Enrichment (prop h2 / prop snps)

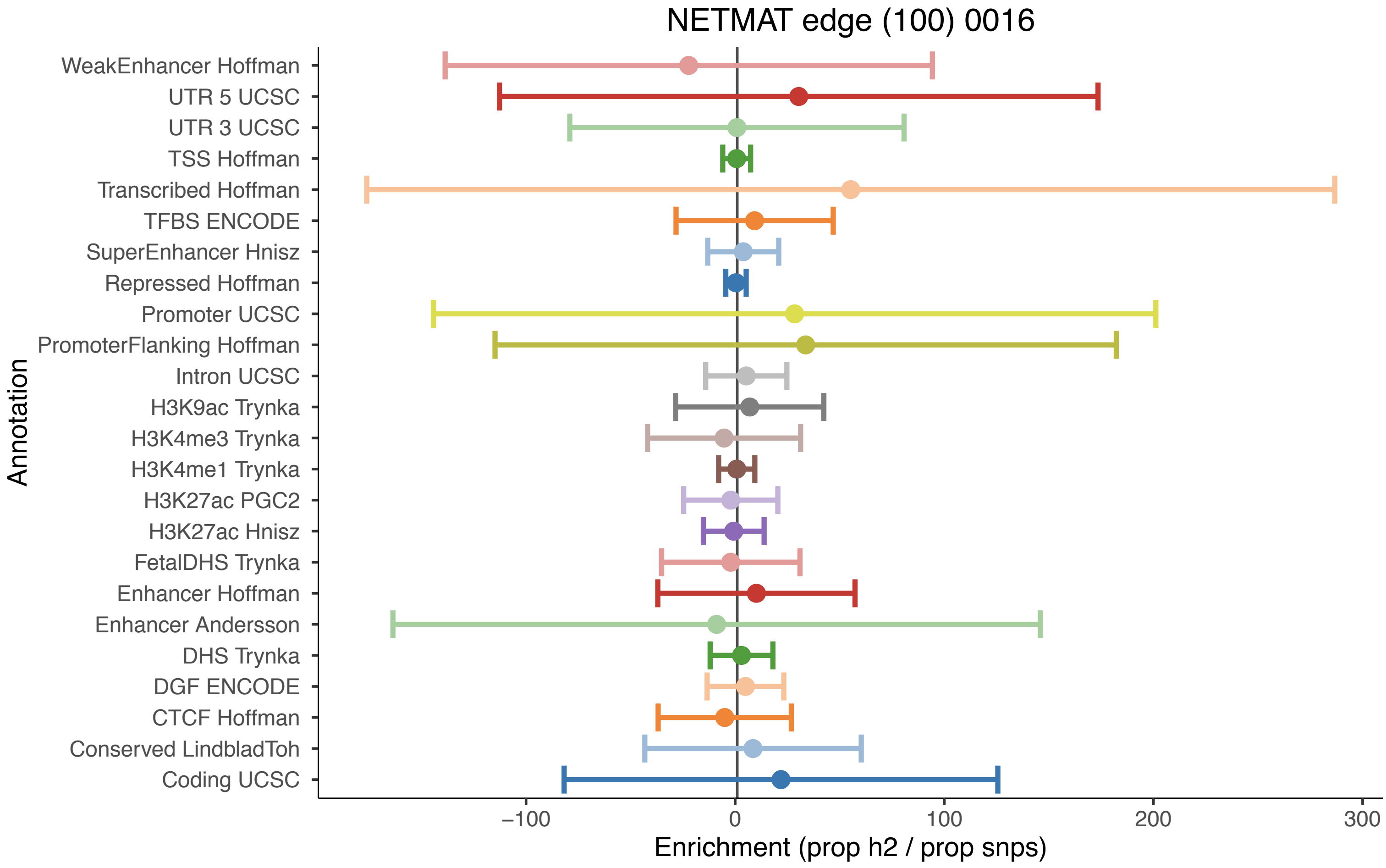
NETMAT edge (100) 0016
WeakEnhancer Hoffman
UTR 5 UCSC
UTR 3 UCSC
TSS Hoffman
Transcribed Hoffman
TFBS ENCODE
SuperEnhancer Hnisz
Repressed Hoffman
Promoter UCSC
PromoterFlanking Hoffman
Intron UCSC
H3K9ac Trynka
H3K4me3 Trynka
H3K4me1 Trynka
H3K27ac PGC2
H3K27ac Hnisz
FetalDHS Trynka
Enhancer Hoffman
Enhancer Andersson
DHS Trynka
DGF ENCODE
CTCF Hoffman
Conserved LindbladToh
Coding UCSC
Annotation
−100
0
100
200
300
Enrichment (prop h2 / prop snps)

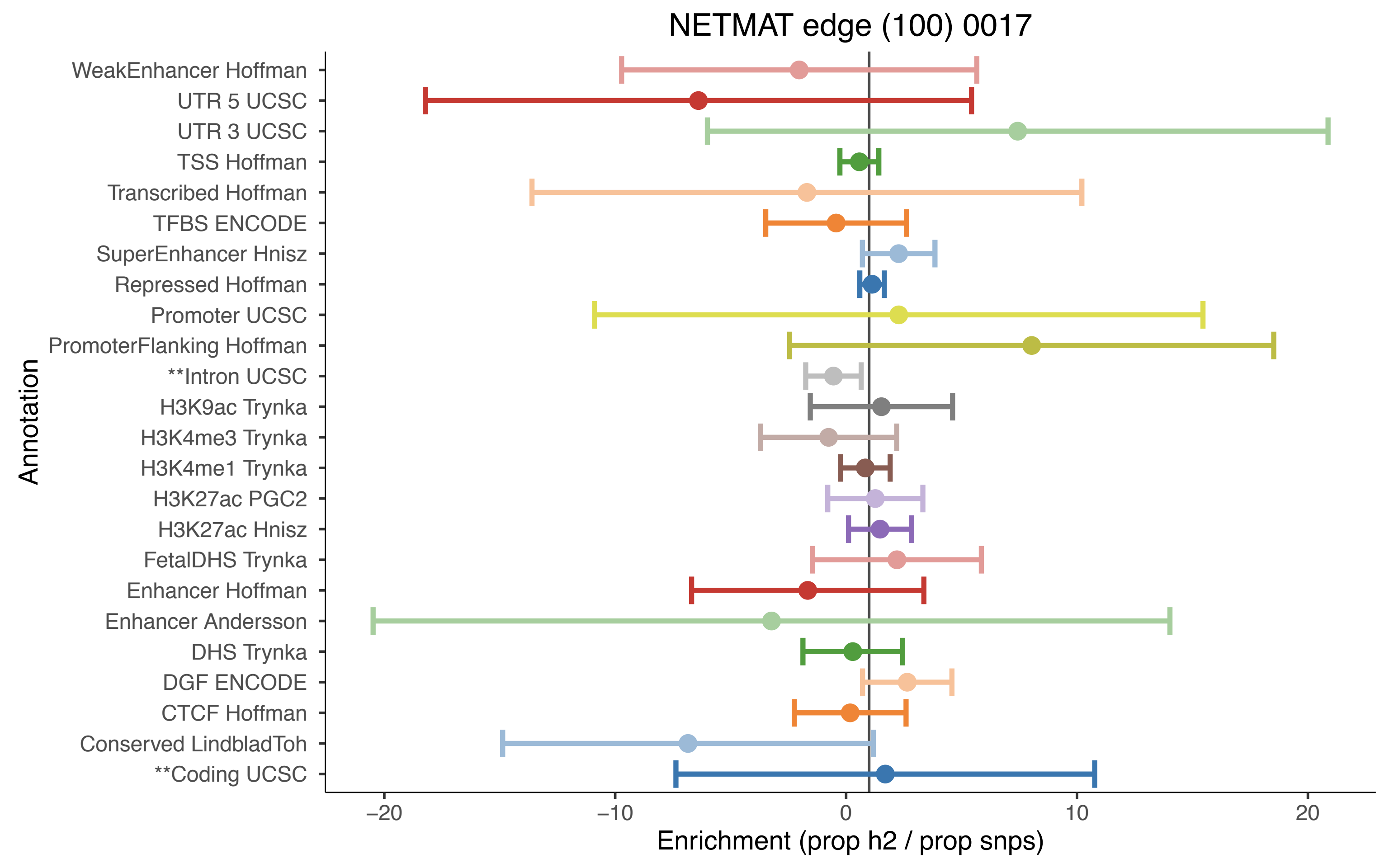

NETMAT edge (100) 0017
WeakEnhancer Hoffman
UTR 5 UCSC
UTR 3 UCSC
TSS Hoffman
Transcribed Hoffman
TFBS ENCODE
SuperEnhancer Hnisz
Repressed Hoffman
Promoter UCSC
PromoterFlanking Hoffman
**Intron UCSC
H3K9ac Trynka
H3K4me3 Trynka
H3K4me1 Trynka
H3K27ac PGC2
H3K27ac Hnisz
FetalDHS Trynka
Enhancer Hoffman
Enhancer Andersson
DHS Trynka
DGF ENCODE
CTCF Hoffman
Conserved LindbladToh
**Coding UCSC
Annotation
−20
−10
0
10
20
Enrichment (prop h2 / prop snps)

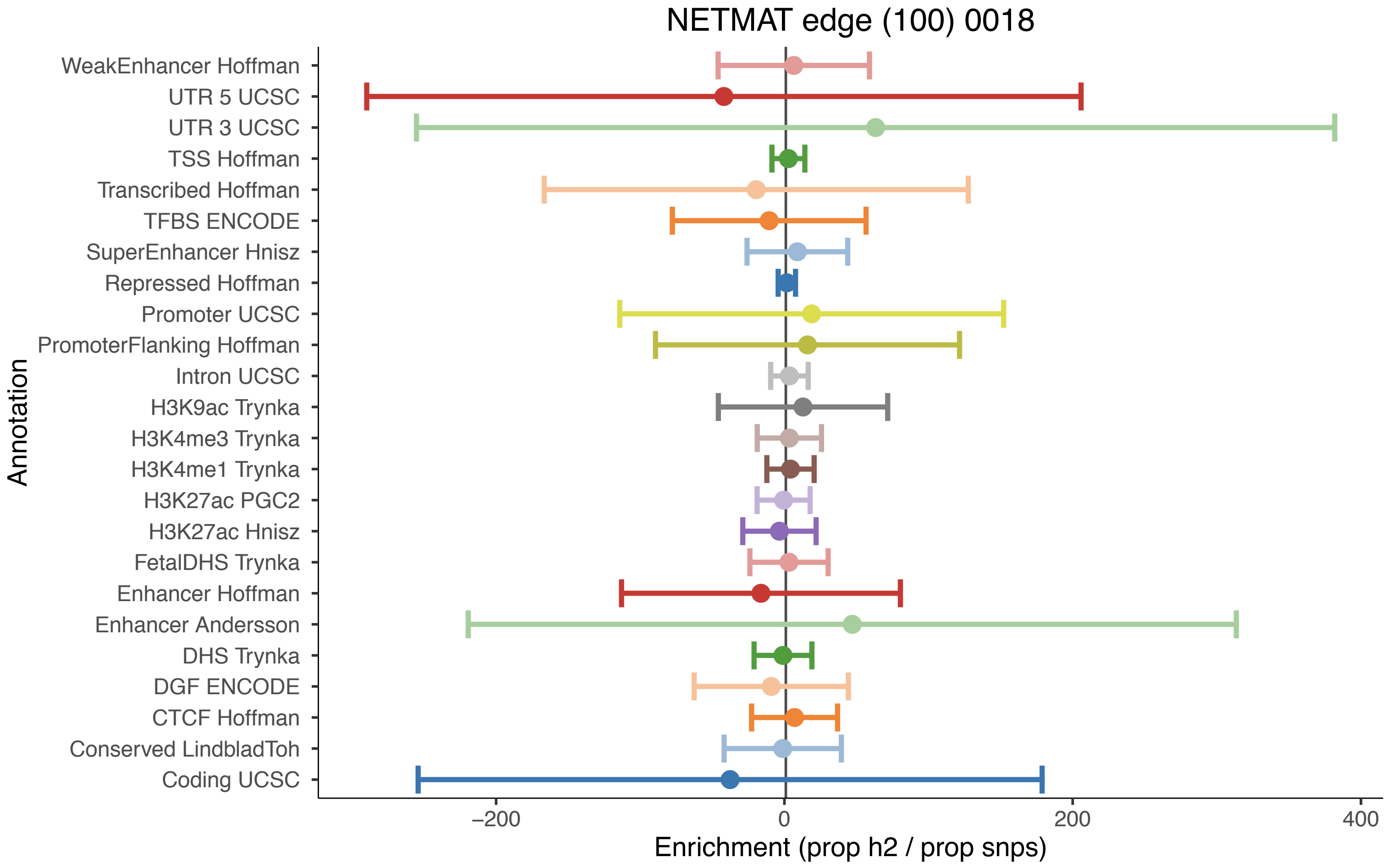

NETMAT edge (100) 0018
Annotation
WeakEnhancer Hoffman
UTR 5 UCSC
UTR 3 UCSC
TSS Hoffman
Transcribed Hoffman
TFBS ENCODE
SuperEnhancer Hnisz
Repressed Hoffman
Promoter UCSC
PromoterFlanking Hoffman
Intron UCSC
H3K9ac Trynka
H3K4me3 Trynka
H3K4me1 Trynka
H3K27ac PGC2
H3K27ac Hnisz
FetalDHS Trynka
Enhancer Hoffman
Enhancer Andersson
DHS Trynka
DGF ENCODE
CTCF Hoffman
Conserved LindbladToh
Coding UCSC
−200
0
200
400
Enrichment (prop h2 / prop snps)

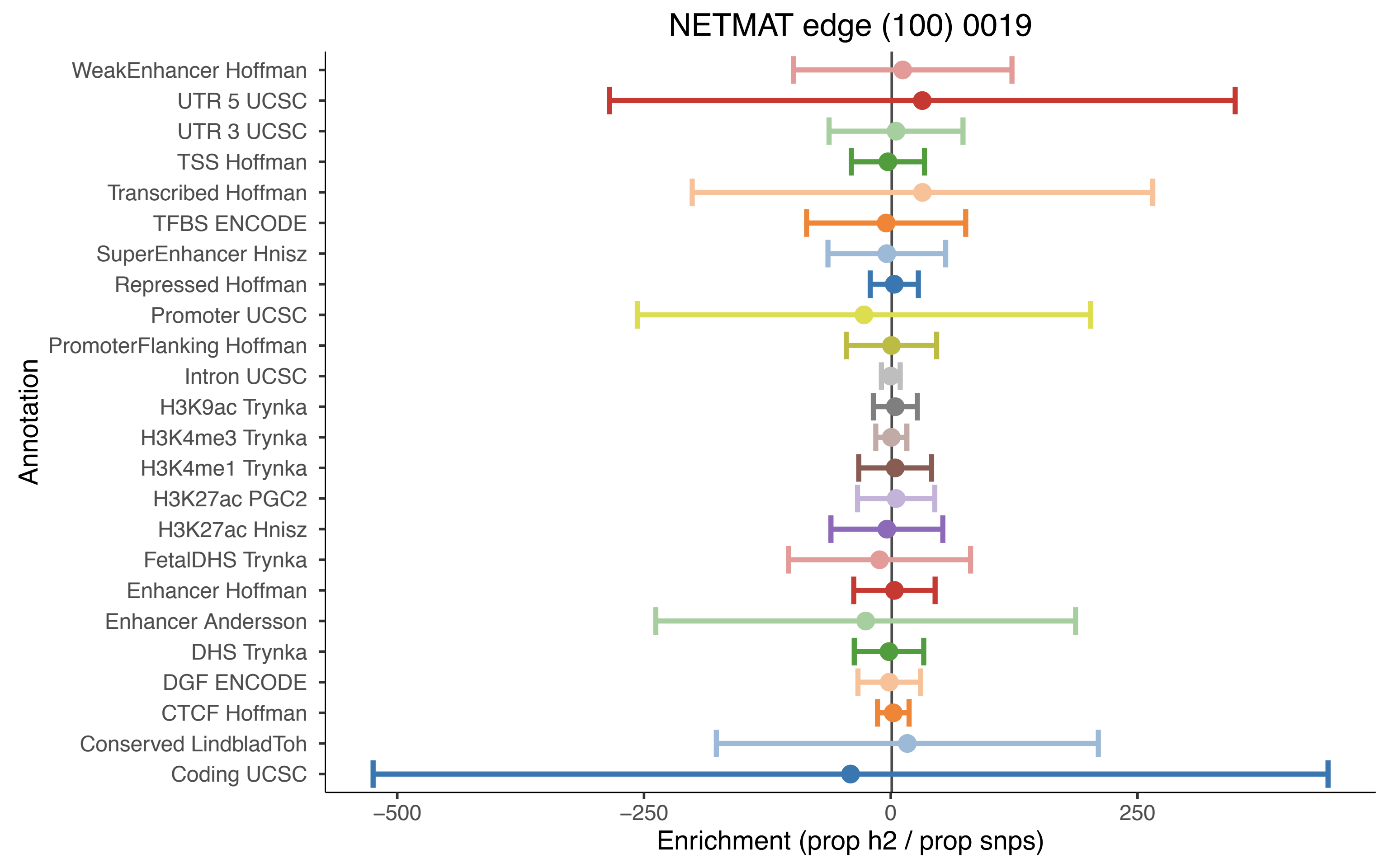
NETMAT edge (100) 0019
Annotation
WeakEnhancer Hoffman
UTR 5 UCSC
UTR 3 UCSC
TSS Hoffman
Transcribed Hoffman
TFBS ENCODE
SuperEnhancer Hnisz
Repressed Hoffman
Promoter UCSC
PromoterFlanking Hoffman
Intron UCSC
H3K9ac Trynka
H3K4me3 Trynka
H3K4me1 Trynka
H3K27ac PGC2
H3K27ac Hnisz
FetalDHS Trynka
Enhancer Hoffman
Enhancer Andersson
DHS Trynka
DGF ENCODE
CTCF Hoffman
Conserved LindbladToh
Coding UCSC
−500
−250
0
250
Enrichment (prop h2 / prop snps)

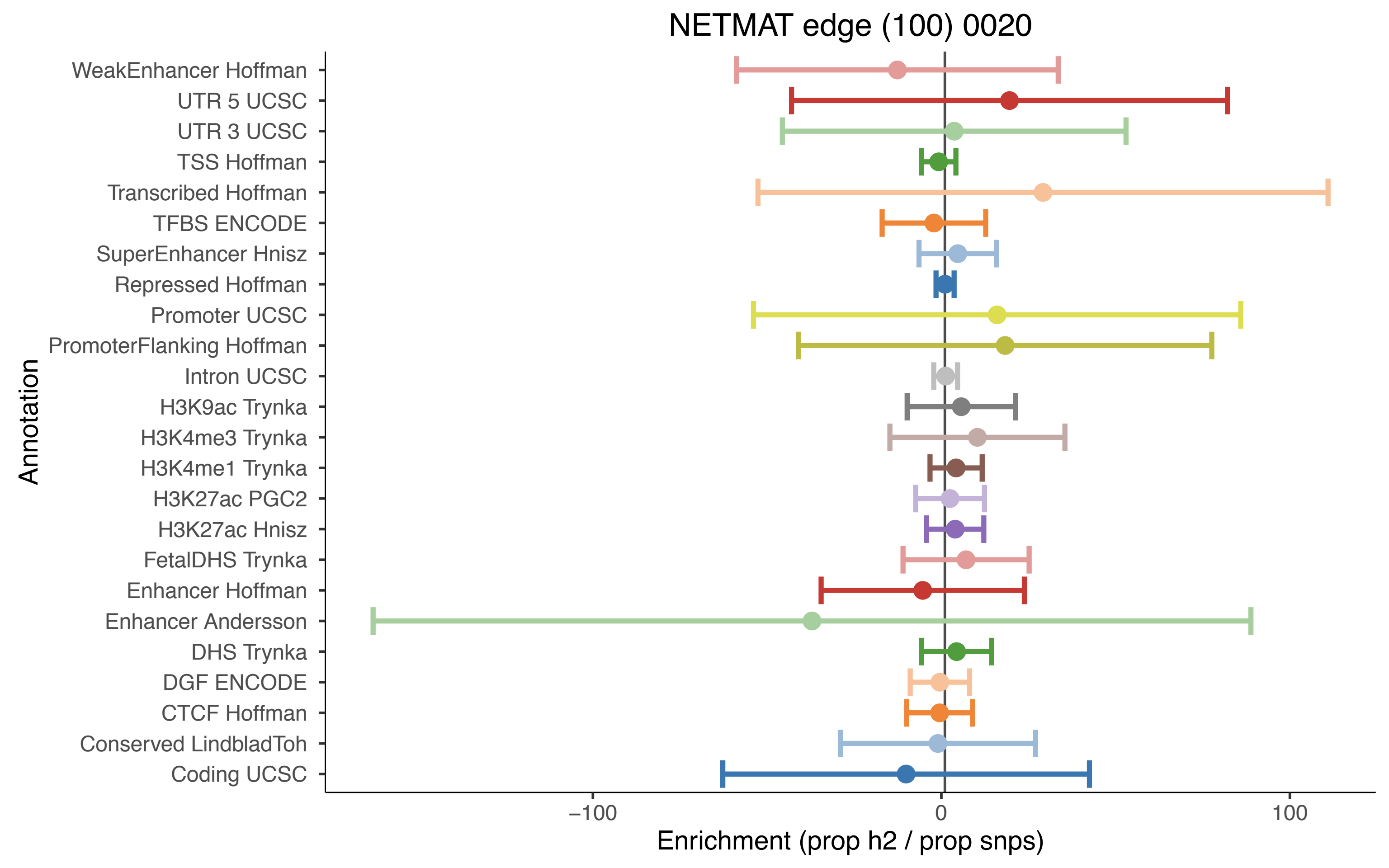
NETMAT edge (100) 0020
WeakEnhancer Hoffman
UTR 5 UCSC
UTR 3 UCSC
TSS Hoffman
Transcribed Hoffman
TFBS ENCODE
SuperEnhancer Hnisz
Repressed Hoffman
Promoter UCSC
PromoterFlanking Hoffman
Intron UCSC
H3K9ac Trynka
H3K4me3 Trynka
H3K4me1 Trynka
H3K27ac PGC2
H3K27ac Hnisz
FetalDHS Trynka
Enhancer Hoffman
Enhancer Andersson
DHS Trynka
DGF ENCODE
CTCF Hoffman
Conserved LindbladToh
Coding UCSC
Annotation
−100
0
100
Enrichment (prop h2 / prop snps)

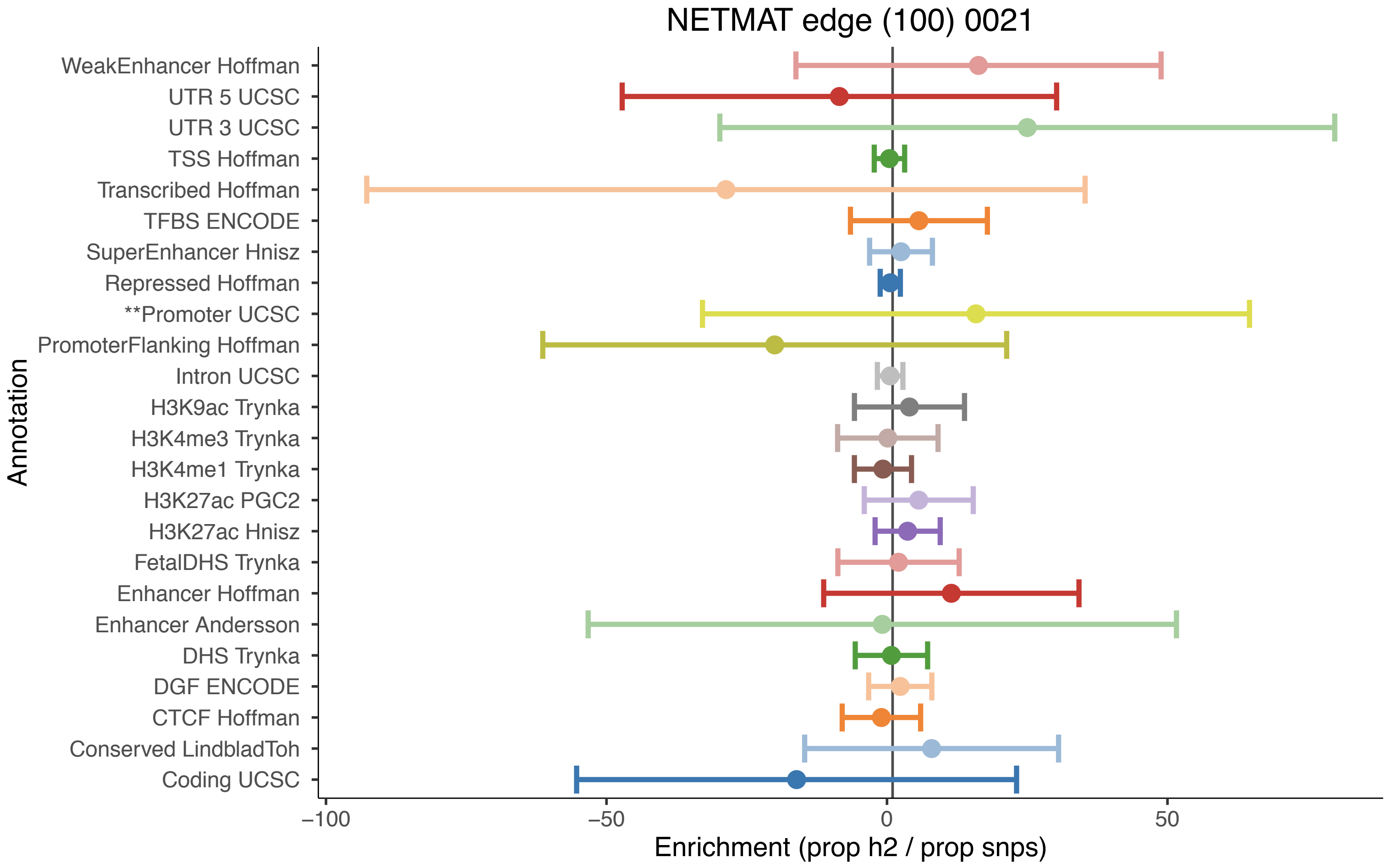
NETMAT edge (100) 0021
WeakEnhancer Hoffman
UTR 5 UCSC
UTR 3 UCSC
TSS Hoffman
Transcribed Hoffman
TFBS ENCODE
SuperEnhancer Hnisz
Repressed Hoffman
**Promoter UCSC
PromoterFlanking Hoffman
Intron UCSC
H3K9ac Trynka
H3K4me3 Trynka
H3K4me1 Trynka
H3K27ac PGC2
H3K27ac Hnisz
FetalDHS Trynka
Enhancer Hoffman
Enhancer Andersson
DHS Trynka
DGF ENCODE
CTCF Hoffman
Conserved LindbladToh
Coding UCSC
Annotation
−100
−50
0
50
Enrichment (prop h2 / prop snps)

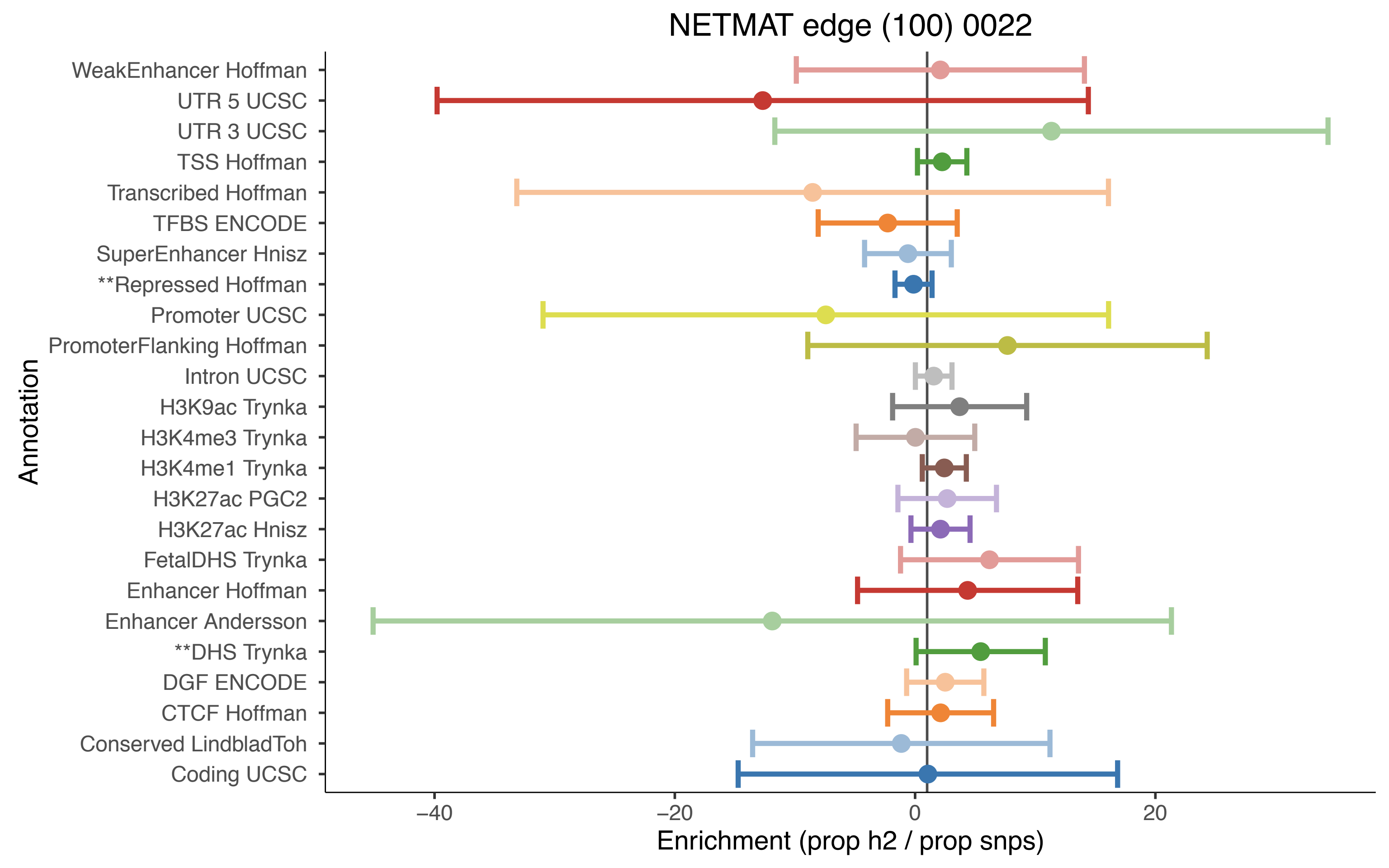
NETMAT edge (100) 0022
WeakEnhancer Hoffman
UTR 5 UCSC
UTR 3 UCSC
TSS Hoffman
Transcribed Hoffman
TFBS ENCODE
SuperEnhancer Hnisz
**Repressed Hoffman
Promoter UCSC
PromoterFlanking Hoffman
Intron UCSC
H3K9ac Trynka
H3K4me3 Trynka
H3K4me1 Trynka
H3K27ac PGC2
H3K27ac Hnisz
FetalDHS Trynka
Enhancer Hoffman
Enhancer Andersson
**DHS Trynka
DGF ENCODE
CTCF Hoffman
Conserved LindbladToh
Coding UCSC
Annotation
−40
−20
0
20
Enrichment (prop h2 / prop snps)

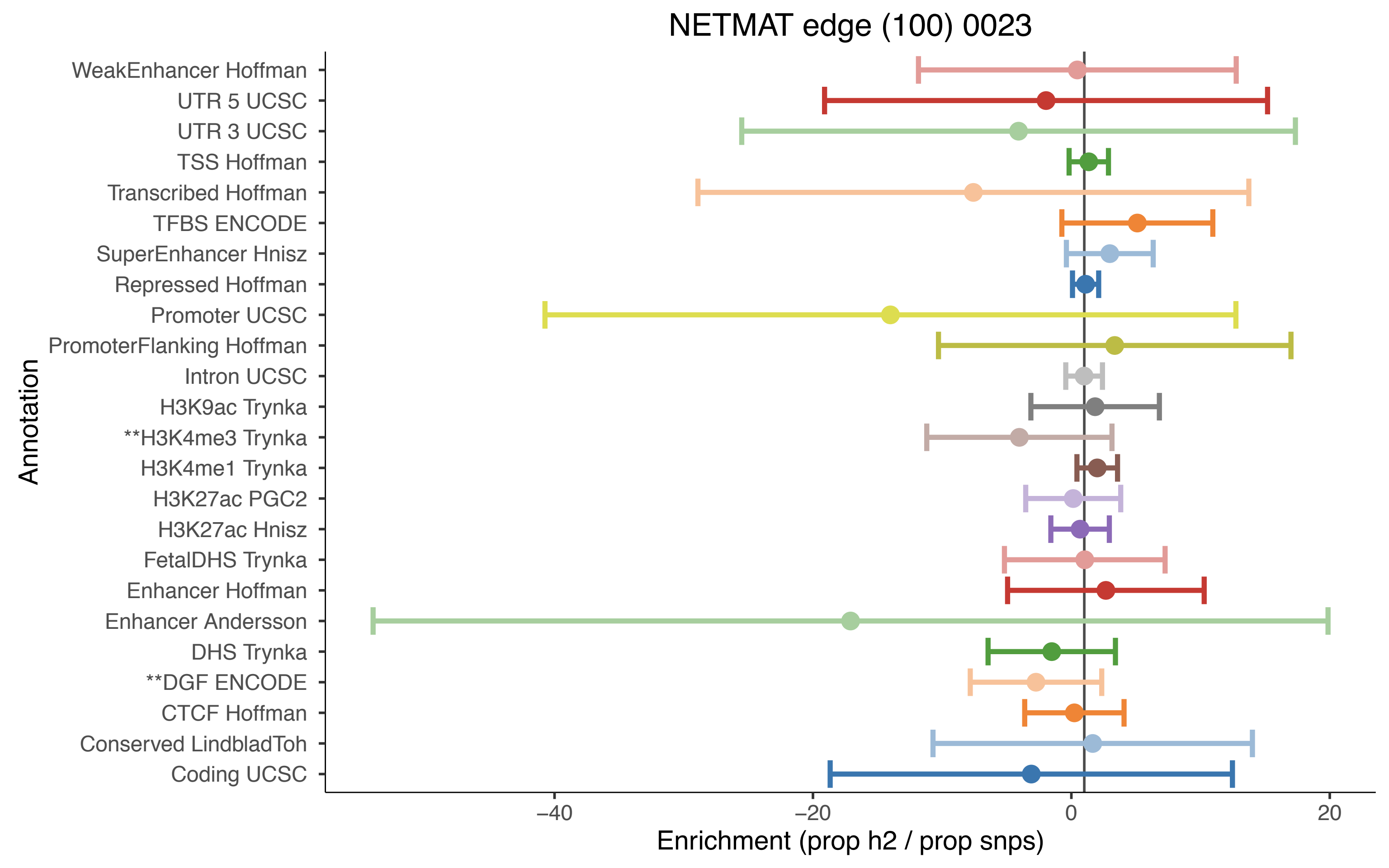
NETMAT edge (100) 0023
Annotation
WeakEnhancer Hoffman
UTR 5 UCSC
UTR 3 UCSC
TSS Hoffman
Transcribed Hoffman
TFBS ENCODE
SuperEnhancer Hnisz
Repressed Hoffman
Promoter UCSC
PromoterFlanking Hoffman
Intron UCSC
H3K9ac Trynka
**H3K4me3 Trynka
H3K4me1 Trynka
H3K27ac PGC2
H3K27ac Hnisz
FetalDHS Trynka
Enhancer Hoffman
Enhancer Andersson
DHS Trynka
**DGF ENCODE
CTCF Hoffman
Conserved LindbladToh
Coding UCSC
−40
−20
0
20
Enrichment (prop h2 / prop snps)

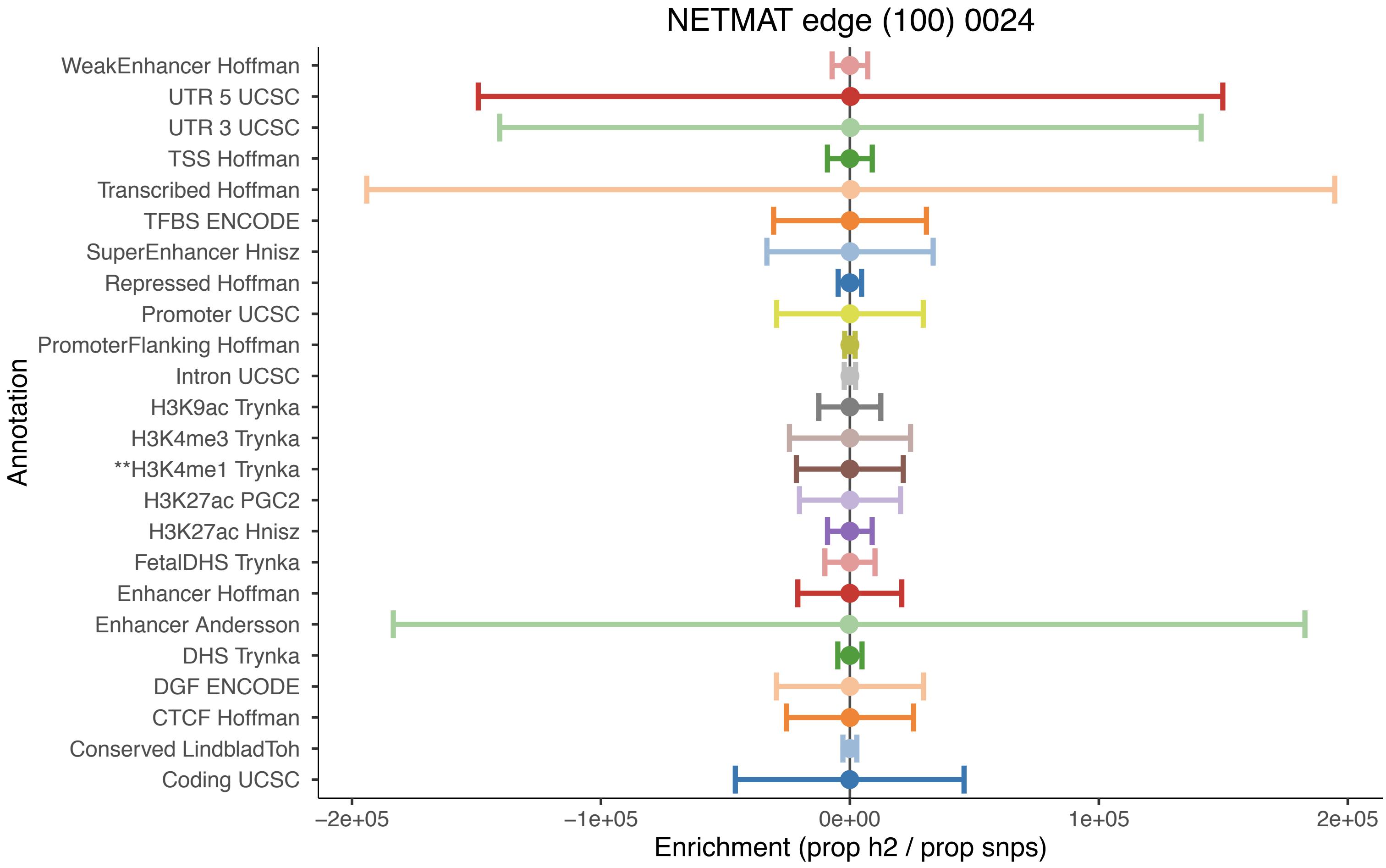
NETMAT edge (100) 0024
WeakEnhancer Hoffman
UTR 5 UCSC
UTR 3 UCSC
TSS Hoffman
Transcribed Hoffman
TFBS ENCODE
SuperEnhancer Hnisz
Repressed Hoffman
Promoter UCSC
PromoterFlanking Hoffman
Intron UCSC
H3K9ac Trynka
H3K4me3 Trynka
**H3K4me1 Trynka
H3K27ac PGC2
H3K27ac Hnisz
FetalDHS Trynka
Enhancer Hoffman
Enhancer Andersson
DHS Trynka
DGF ENCODE
CTCF Hoffman
Conserved LindbladToh
Coding UCSC
Annotation
−2e+05
−1e+05
0e+00
1e+05
2e+05
Enrichment (prop h2 / prop snps)

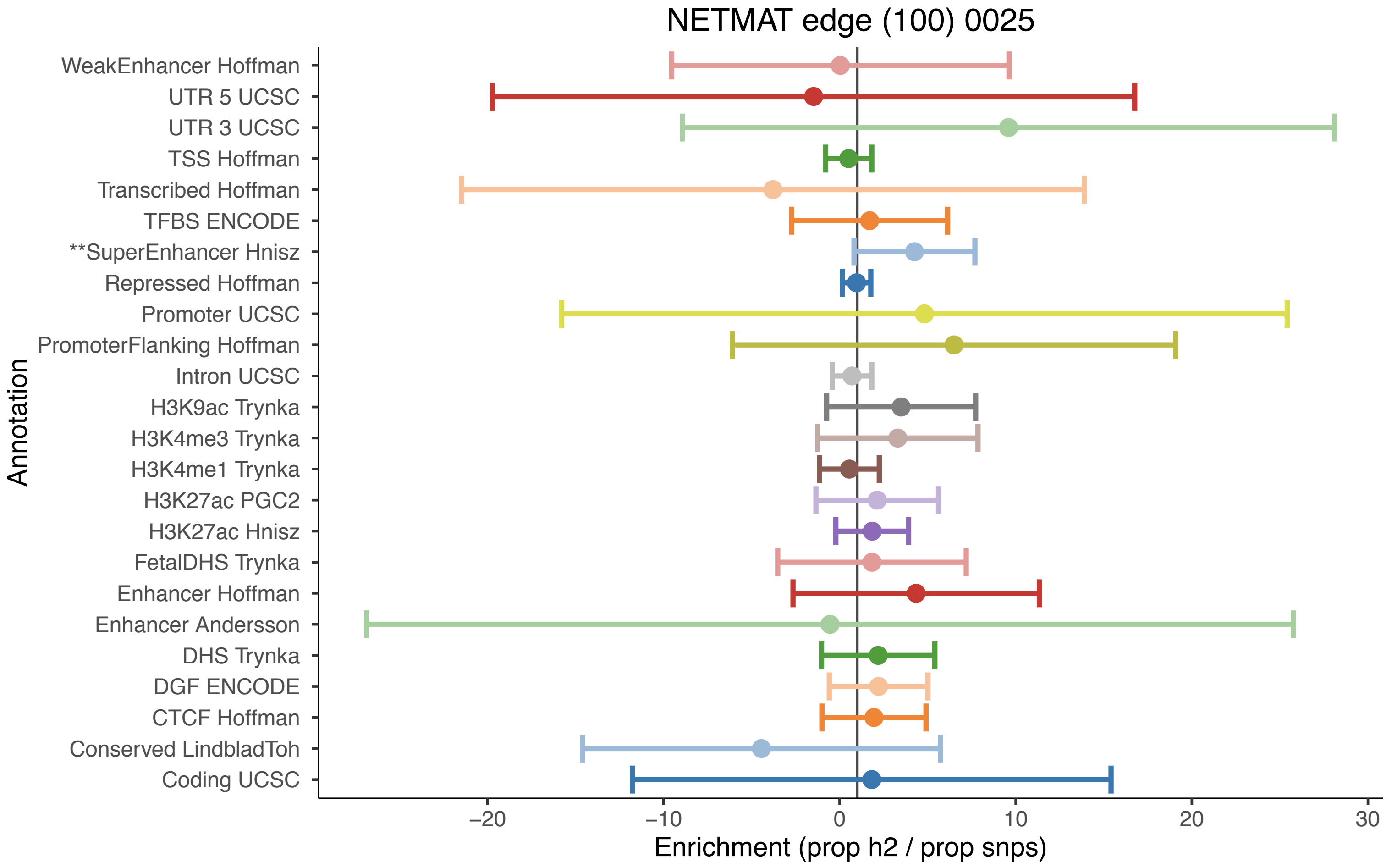
NETMAT edge (100) 0025
WeakEnhancer Hoffman
UTR 5 UCSC
UTR 3 UCSC
TSS Hoffman
Transcribed Hoffman
TFBS ENCODE
**SuperEnhancer Hnisz
Repressed Hoffman
Promoter UCSC
PromoterFlanking Hoffman
Intron UCSC
H3K9ac Trynka
H3K4me3 Trynka
H3K4me1 Trynka
H3K27ac PGC2
H3K27ac Hnisz
FetalDHS Trynka
Enhancer Hoffman
Enhancer Andersson
DHS Trynka
DGF ENCODE
CTCF Hoffman
Conserved LindbladToh
Coding UCSC
Annotation
−20
−10
0
10
20
30
Enrichment (prop h2 / prop snps)

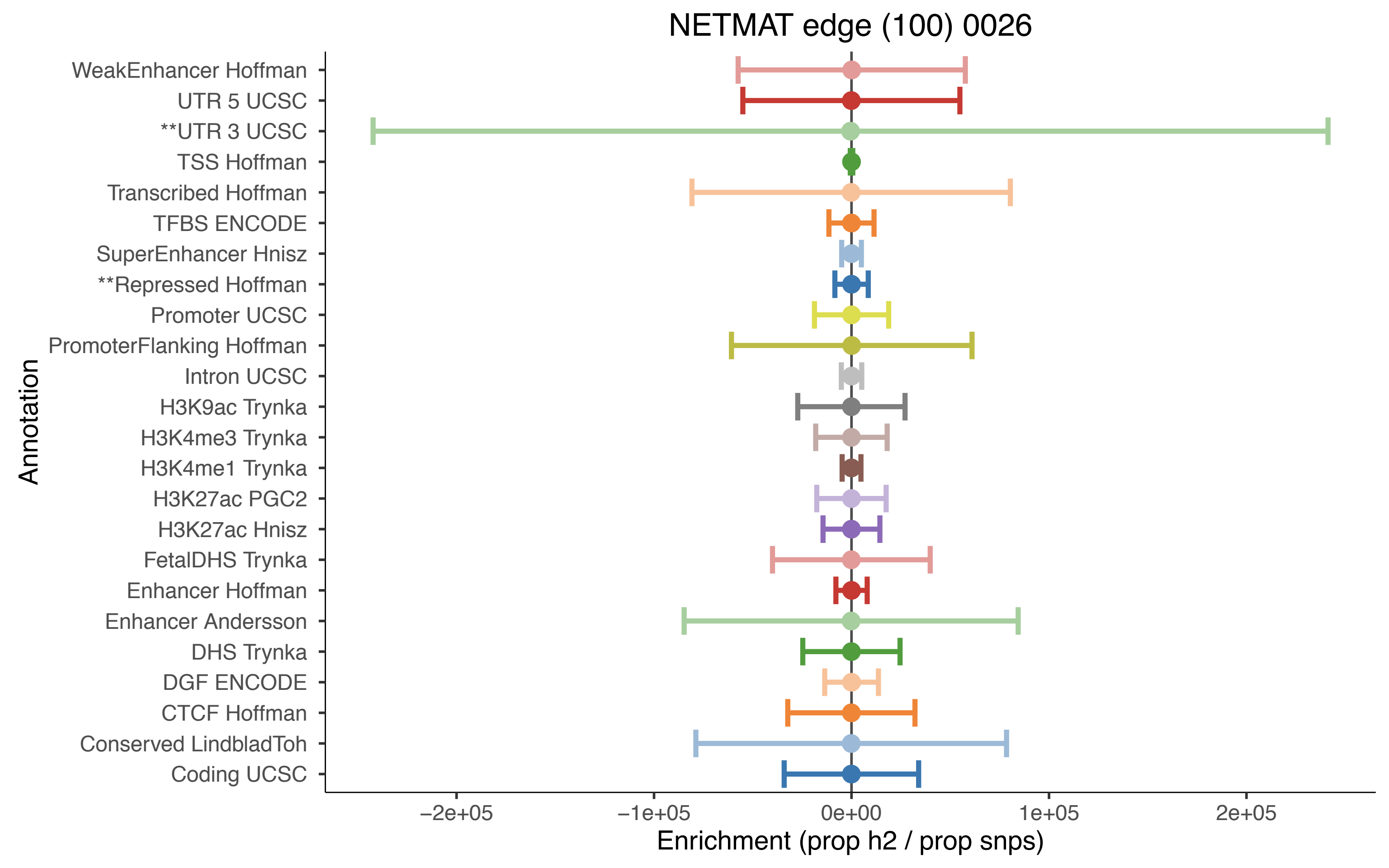
NETMAT edge (100) 0026
WeakEnhancer Hoffman
UTR 5 UCSC
**UTR 3 UCSC
TSS Hoffman
Transcribed Hoffman
TFBS ENCODE
SuperEnhancer Hnisz
**Repressed Hoffman
Promoter UCSC
PromoterFlanking Hoffman
Intron UCSC
H3K9ac Trynka
H3K4me3 Trynka
H3K4me1 Trynka
H3K27ac PGC2
H3K27ac Hnisz
FetalDHS Trynka
Enhancer Hoffman
Enhancer Andersson
DHS Trynka
DGF ENCODE
CTCF Hoffman
Conserved LindbladToh
Coding UCSC
Annotation
−2e+05
−1e+05
0e+00
1e+05
2e+05
Enrichment (prop h2 / prop snps)

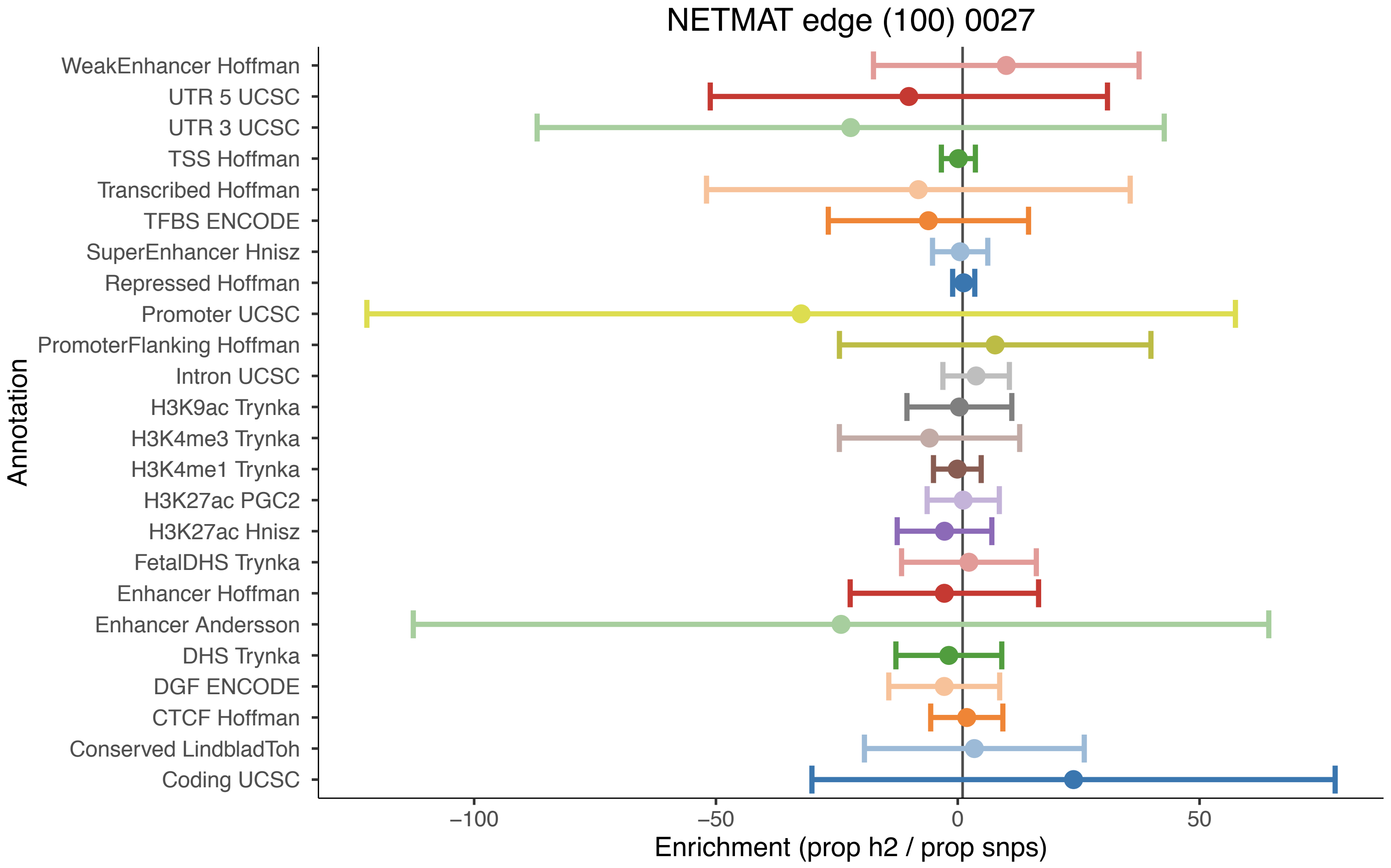
NETMAT edge (100) 0027
WeakEnhancer Hoffman
UTR 5 UCSC
UTR 3 UCSC
TSS Hoffman
Transcribed Hoffman
TFBS ENCODE
SuperEnhancer Hnisz
Repressed Hoffman
Promoter UCSC
PromoterFlanking Hoffman
Intron UCSC
H3K9ac Trynka
H3K4me3 Trynka
H3K4me1 Trynka
H3K27ac PGC2
H3K27ac Hnisz
FetalDHS Trynka
Enhancer Hoffman
Enhancer Andersson
DHS Trynka
DGF ENCODE
CTCF Hoffman
Conserved LindbladToh
Coding UCSC
Annotation
−100
−50
0
50
Enrichment (prop h2 / prop snps)

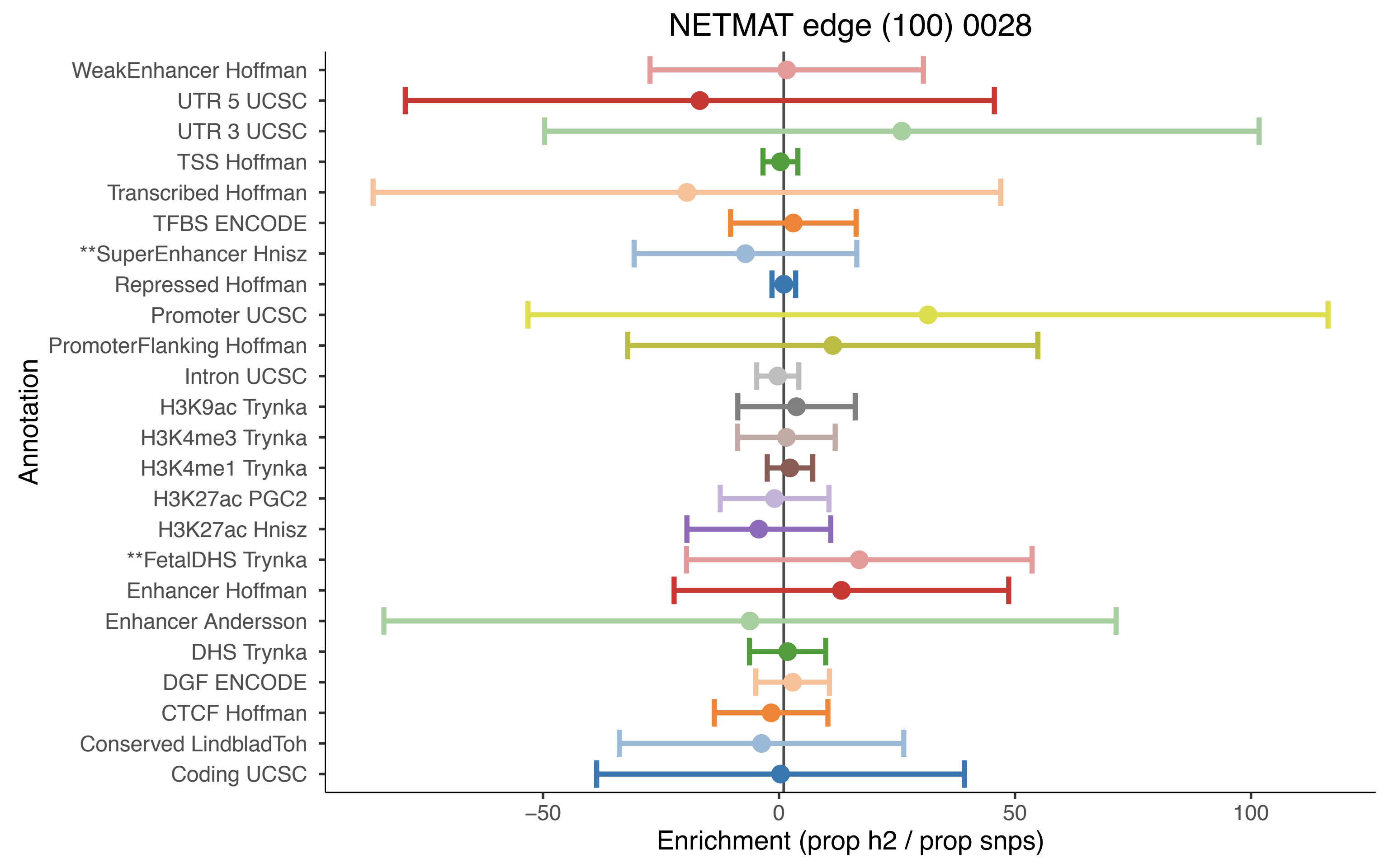

NETMAT edge (100) 0028
WeakEnhancer Hoffman
UTR 5 UCSC
UTR 3 UCSC
TSS Hoffman
Transcribed Hoffman
TFBS ENCODE
**SuperEnhancer Hnisz
Repressed Hoffman
Promoter UCSC
PromoterFlanking Hoffman
Intron UCSC
H3K9ac Trynka
H3K4me3 Trynka
H3K4me1 Trynka
H3K27ac PGC2
H3K27ac Hnisz
**FetalDHS Trynka
Enhancer Hoffman
Enhancer Andersson
DHS Trynka
DGF ENCODE
CTCF Hoffman
Conserved LindbladToh
Coding UCSC
Annotation
−50
0
50
100
Enrichment (prop h2 / prop snps)

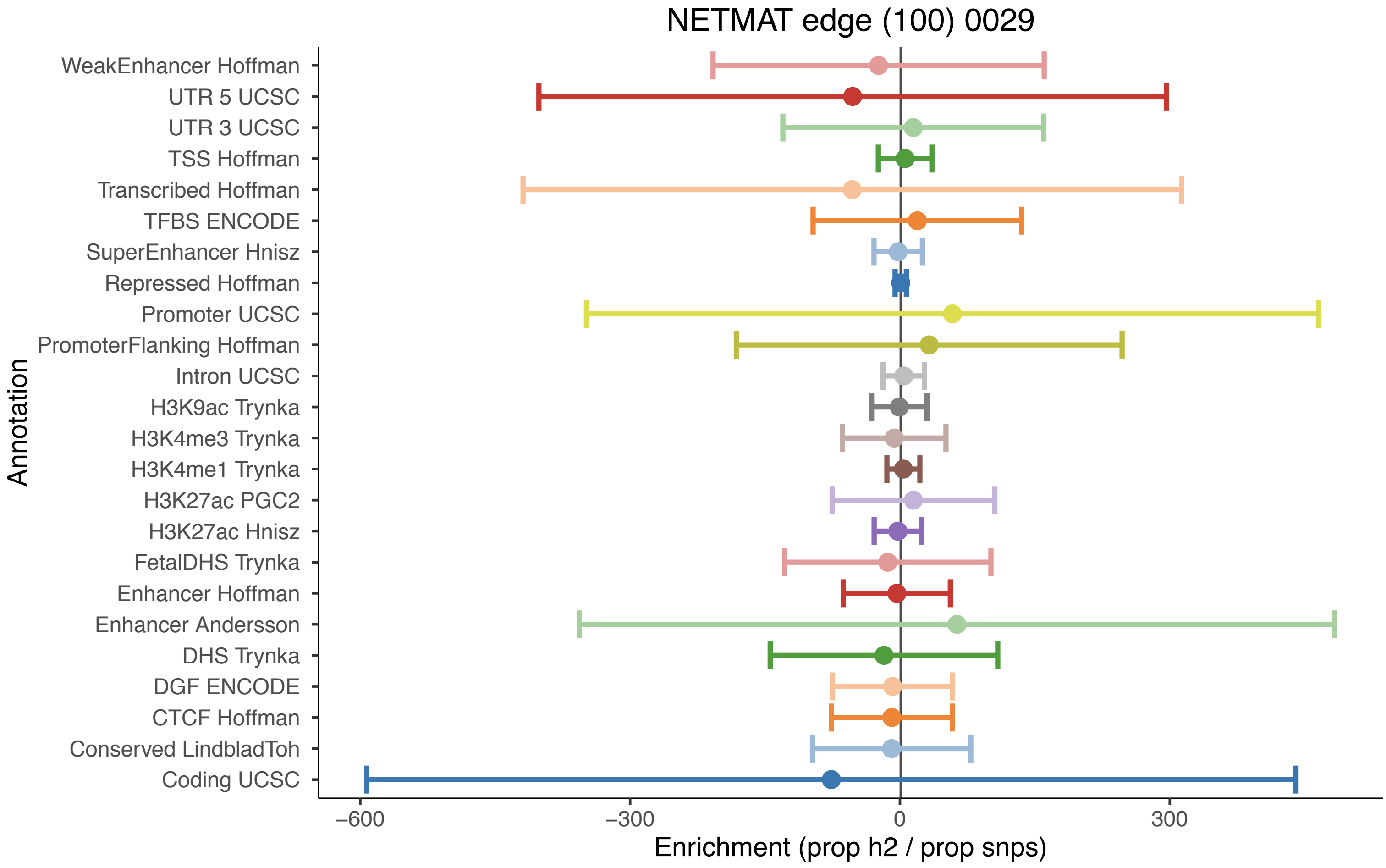

NETMAT edge (100) 0029
Annotation
WeakEnhancer Hoffman
UTR 5 UCSC
UTR 3 UCSC
TSS Hoffman
Transcribed Hoffman
TFBS ENCODE
SuperEnhancer Hnisz
Repressed Hoffman
Promoter UCSC
PromoterFlanking Hoffman
Intron UCSC
H3K9ac Trynka
H3K4me3 Trynka
H3K4me1 Trynka
H3K27ac PGC2
H3K27ac Hnisz
FetalDHS Trynka
Enhancer Hoffman
Enhancer Andersson
DHS Trynka
DGF ENCODE
CTCF Hoffman
Conserved LindbladToh
Coding UCSC
−600
−300
0
300
Enrichment (prop h2 / prop snps)

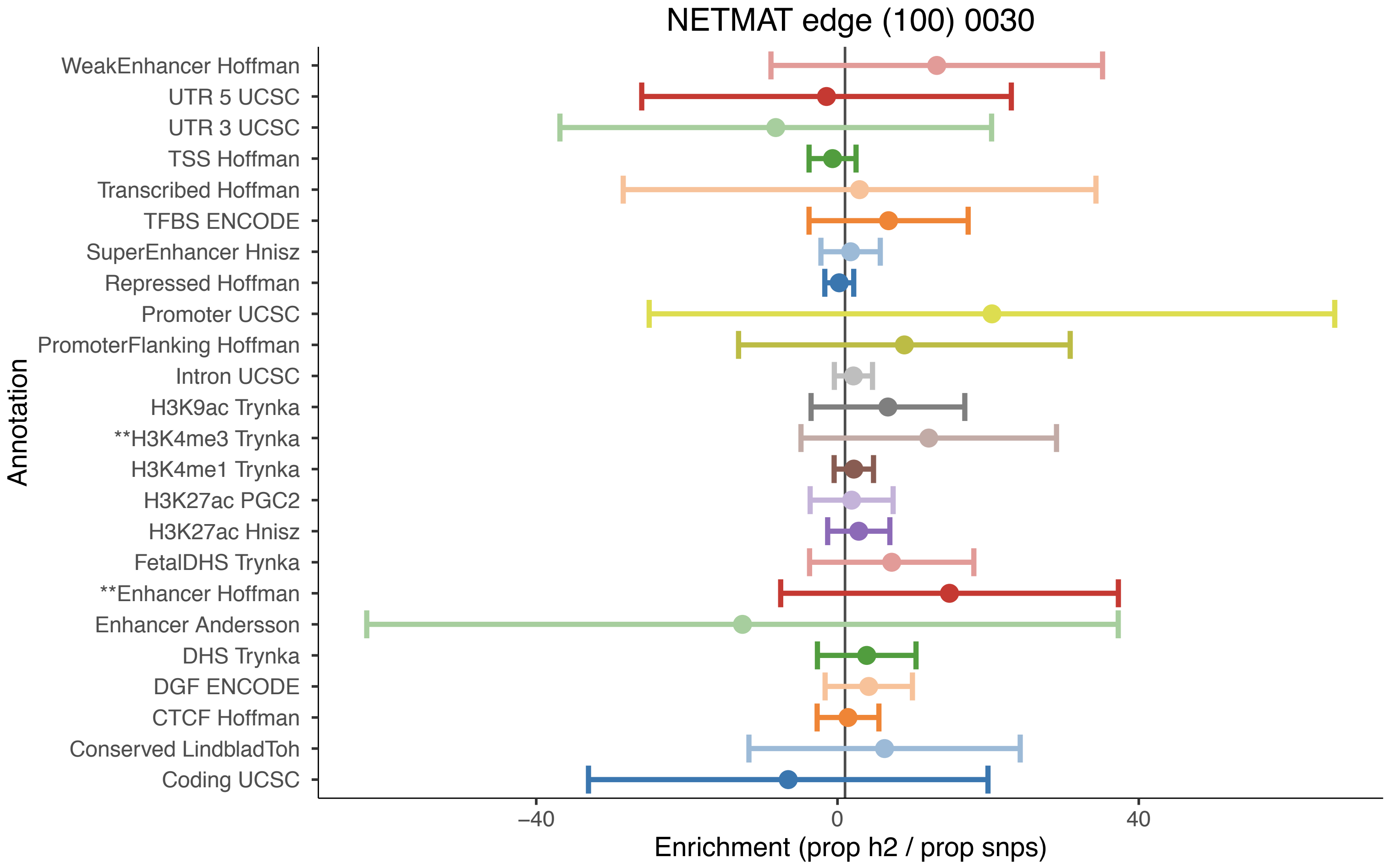
NETMAT edge (100) 0030
Annotation
WeakEnhancer Hoffman
UTR 5 UCSC
UTR 3 UCSC
TSS Hoffman
Transcribed Hoffman
TFBS ENCODE
SuperEnhancer Hnisz
Repressed Hoffman
Promoter UCSC
PromoterFlanking Hoffman
Intron UCSC
H3K9ac Trynka
**H3K4me3 Trynka
H3K4me1 Trynka
H3K27ac PGC2
H3K27ac Hnisz
FetalDHS Trynka
**Enhancer Hoffman
Enhancer Andersson
DHS Trynka
DGF ENCODE
CTCF Hoffman
Conserved LindbladToh
Coding UCSC
−40
0
40
Enrichment (prop h2 / prop snps)

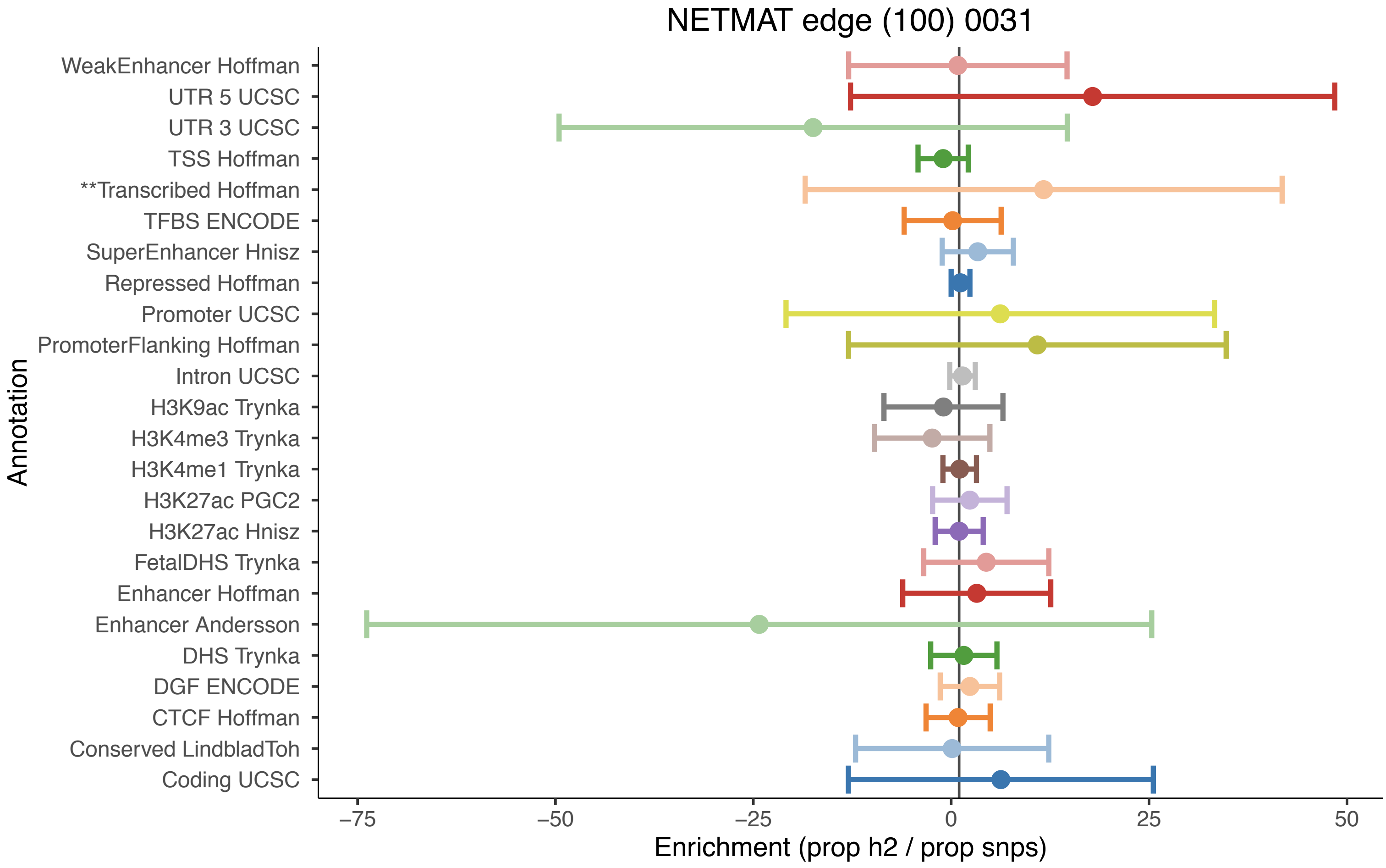

NETMAT edge (100) 0031
WeakEnhancer Hoffman
UTR 5 UCSC
UTR 3 UCSC
TSS Hoffman
**Transcribed Hoffman
TFBS ENCODE
SuperEnhancer Hnisz
Repressed Hoffman
Promoter UCSC
PromoterFlanking Hoffman
Intron UCSC
H3K9ac Trynka
H3K4me3 Trynka
H3K4me1 Trynka
H3K27ac PGC2
H3K27ac Hnisz
FetalDHS Trynka
Enhancer Hoffman
Enhancer Andersson
DHS Trynka
DGF ENCODE
CTCF Hoffman
Conserved LindbladToh
Coding UCSC
Annotation
−75
−50
−25
0
25
50
Enrichment (prop h2 / prop snps)

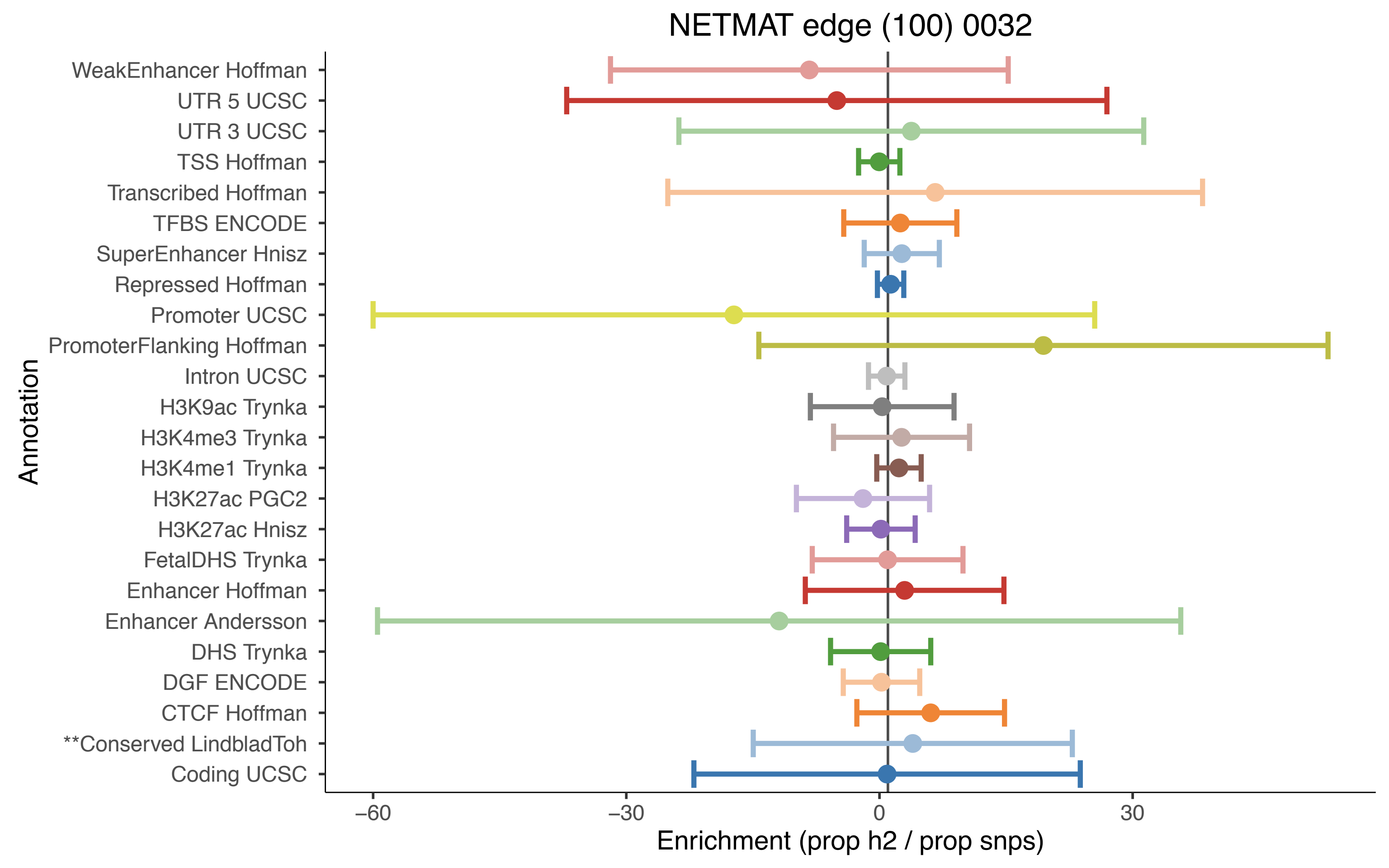
NETMAT edge (100) 0032
WeakEnhancer Hoffman
UTR 5 UCSC
UTR 3 UCSC
TSS Hoffman
Transcribed Hoffman
TFBS ENCODE
SuperEnhancer Hnisz
Repressed Hoffman
Promoter UCSC
PromoterFlanking Hoffman
Intron UCSC
H3K9ac Trynka
H3K4me3 Trynka
H3K4me1 Trynka
H3K27ac PGC2
H3K27ac Hnisz
FetalDHS Trynka
Enhancer Hoffman
Enhancer Andersson
DHS Trynka
DGF ENCODE
CTCF Hoffman
**Conserved LindbladToh
Coding UCSC
Annotation
−60
−30
0
30
Enrichment (prop h2 / prop snps)

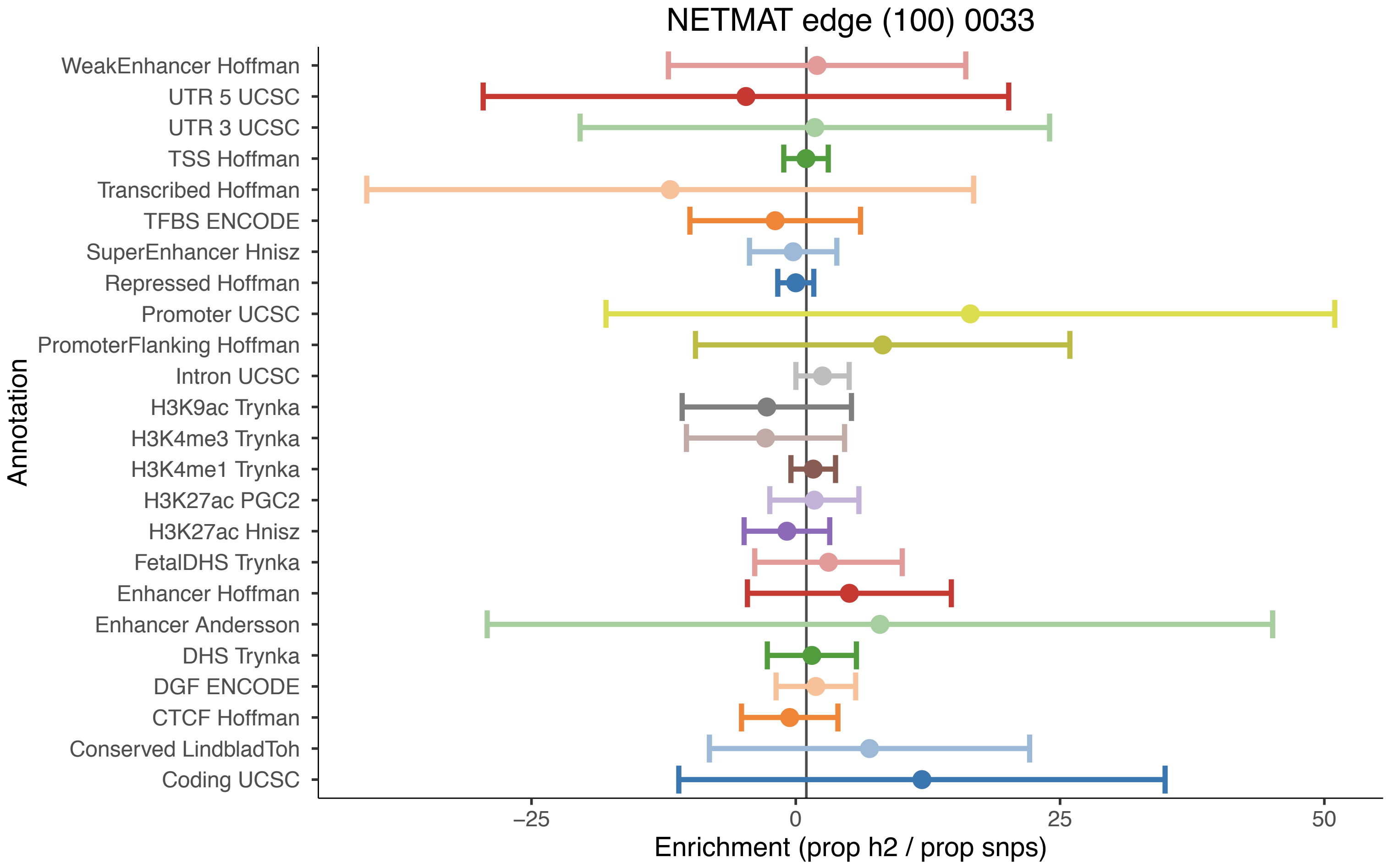
NETMAT edge (100) 0033
WeakEnhancer Hoffman
UTR 5 UCSC
UTR 3 UCSC
TSS Hoffman
Transcribed Hoffman
TFBS ENCODE
SuperEnhancer Hnisz
Repressed Hoffman
Promoter UCSC
PromoterFlanking Hoffman
Intron UCSC
H3K9ac Trynka
H3K4me3 Trynka
H3K4me1 Trynka
H3K27ac PGC2
H3K27ac Hnisz
FetalDHS Trynka
Enhancer Hoffman
Enhancer Andersson
DHS Trynka
DGF ENCODE
CTCF Hoffman
Conserved LindbladToh
Coding UCSC
Annotation
−25
0
25
50
Enrichment (prop h2 / prop snps)

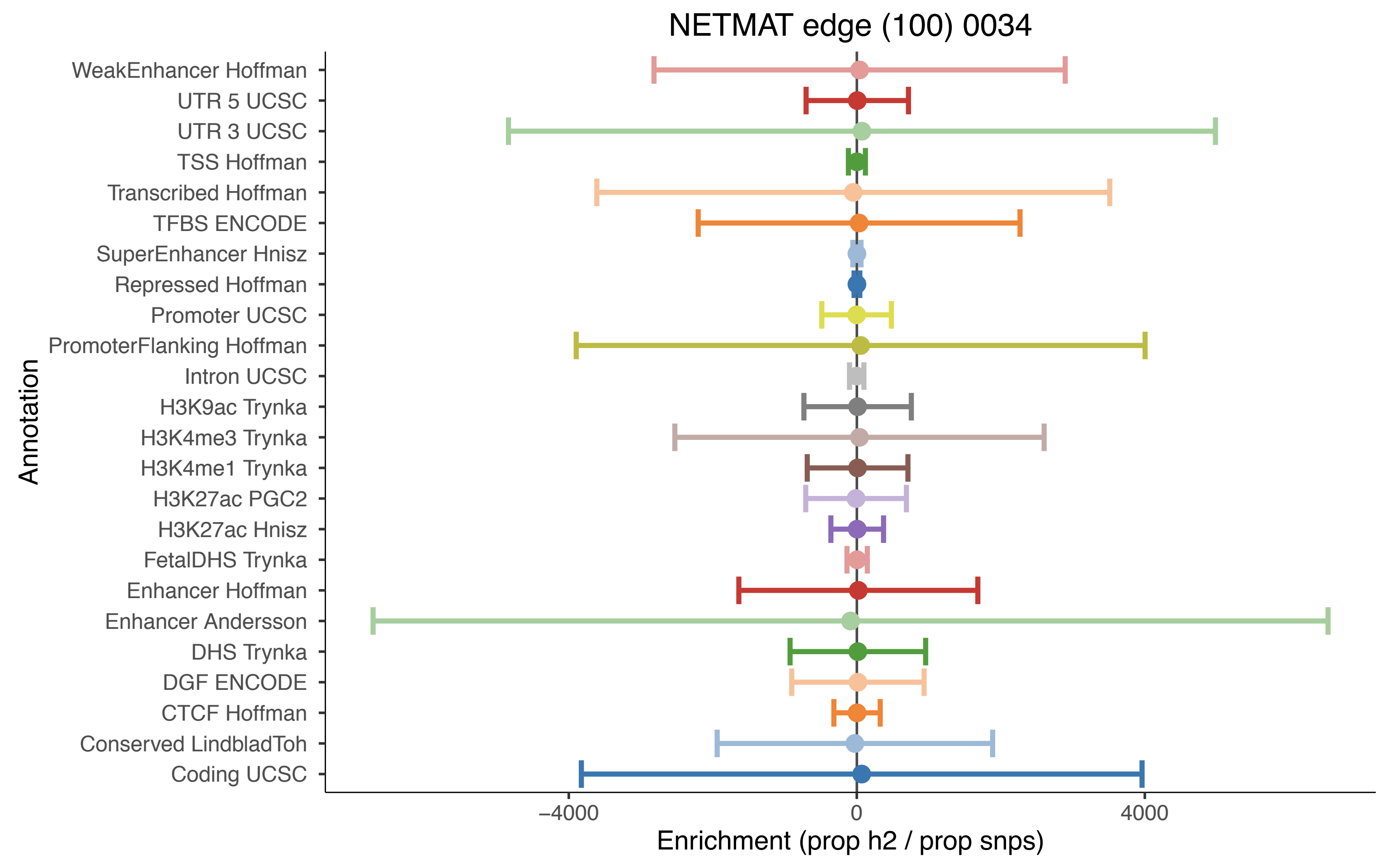
NETMAT edge (100) 0034
WeakEnhancer Hoffman
UTR 5 UCSC
UTR 3 UCSC
TSS Hoffman
Transcribed Hoffman
TFBS ENCODE
SuperEnhancer Hnisz
Repressed Hoffman
Promoter UCSC
PromoterFlanking Hoffman
Intron UCSC
H3K9ac Trynka
H3K4me3 Trynka
H3K4me1 Trynka
H3K27ac PGC2
H3K27ac Hnisz
FetalDHS Trynka
Enhancer Hoffman
Enhancer Andersson
DHS Trynka
DGF ENCODE
CTCF Hoffman
Conserved LindbladToh
Coding UCSC
Annotation
−4000
0
4000
Enrichment (prop h2 / prop snps)

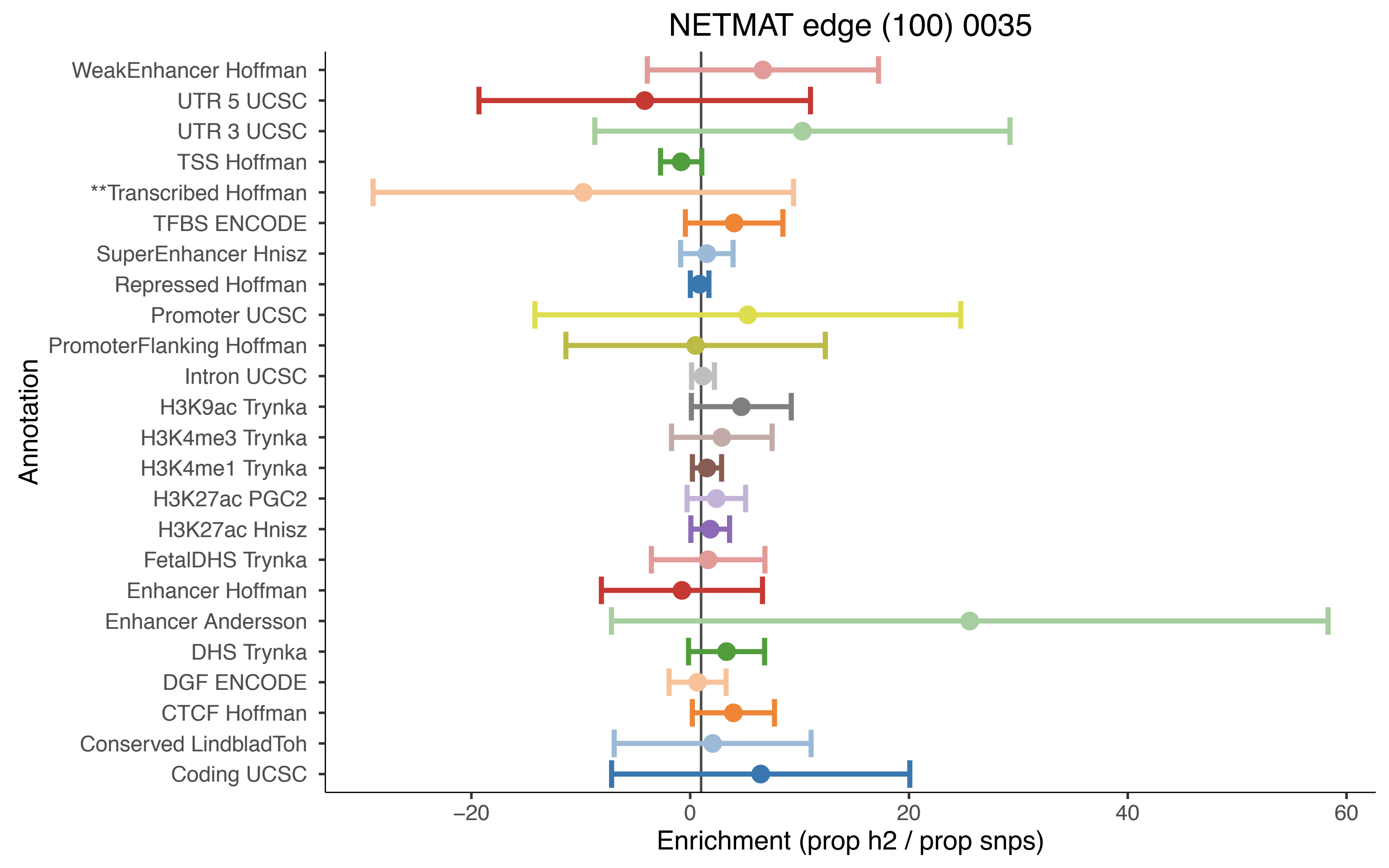

NETMAT edge (100) 0035
WeakEnhancer Hoffman
UTR 5 UCSC
UTR 3 UCSC
TSS Hoffman
**Transcribed Hoffman
TFBS ENCODE
SuperEnhancer Hnisz
Repressed Hoffman
Promoter UCSC
PromoterFlanking Hoffman
Intron UCSC
H3K9ac Trynka
H3K4me3 Trynka
H3K4me1 Trynka
H3K27ac PGC2
H3K27ac Hnisz
FetalDHS Trynka
Enhancer Hoffman
Enhancer Andersson
DHS Trynka
DGF ENCODE
CTCF Hoffman
Conserved LindbladToh
Coding UCSC
Annotation
−20
0
20
40
60
Enrichment (prop h2 / prop snps)

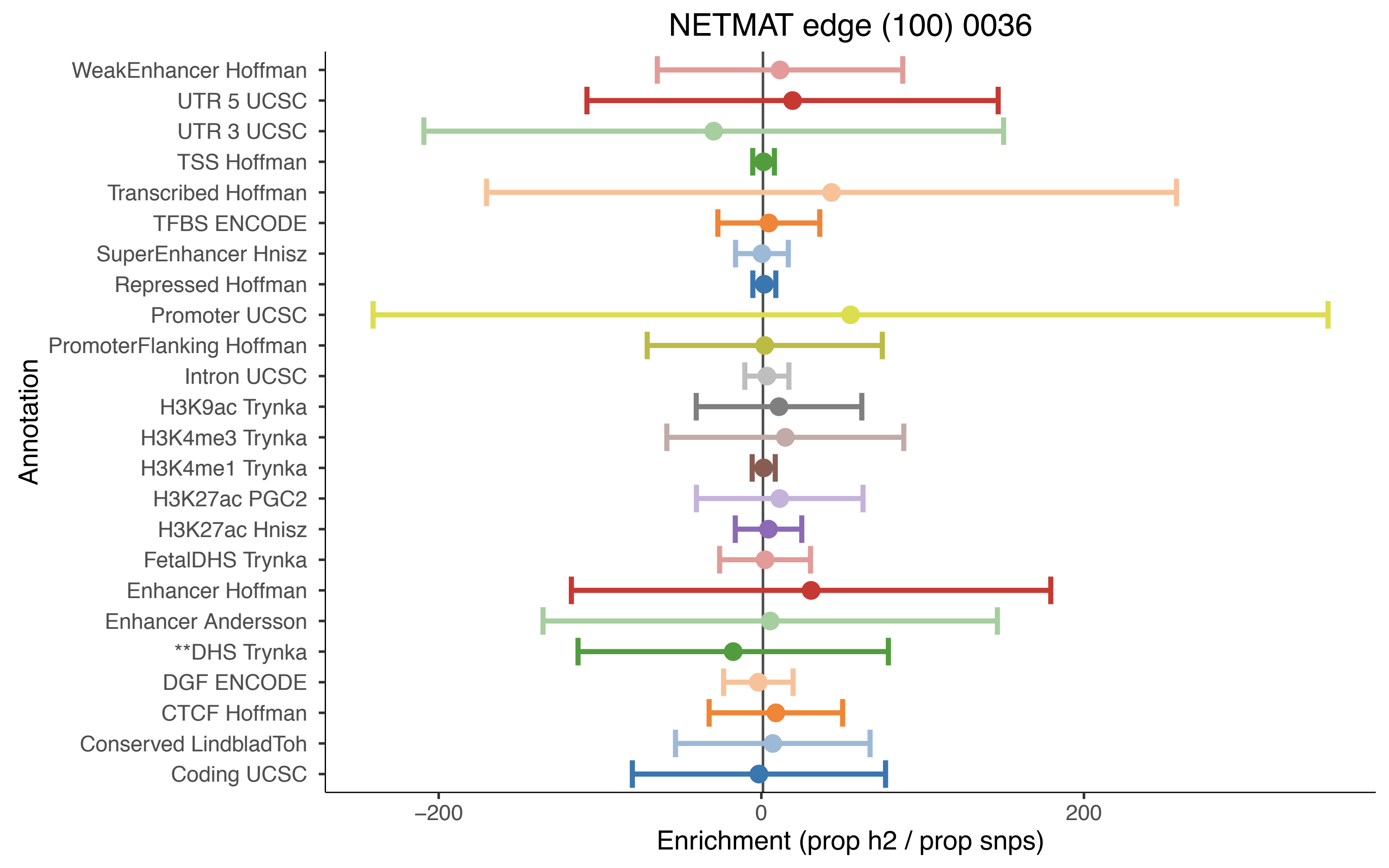

NETMAT edge (100) 0036
WeakEnhancer Hoffman
UTR 5 UCSC
UTR 3 UCSC
TSS Hoffman
Transcribed Hoffman
TFBS ENCODE
SuperEnhancer Hnisz
Repressed Hoffman
Promoter UCSC
PromoterFlanking Hoffman
Intron UCSC
H3K9ac Trynka
H3K4me3 Trynka
H3K4me1 Trynka
H3K27ac PGC2
H3K27ac Hnisz
FetalDHS Trynka
Enhancer Hoffman
Enhancer Andersson
**DHS Trynka
DGF ENCODE
CTCF Hoffman
Conserved LindbladToh
Coding UCSC
Annotation
−200
0
200
Enrichment (prop h2 / prop snps)

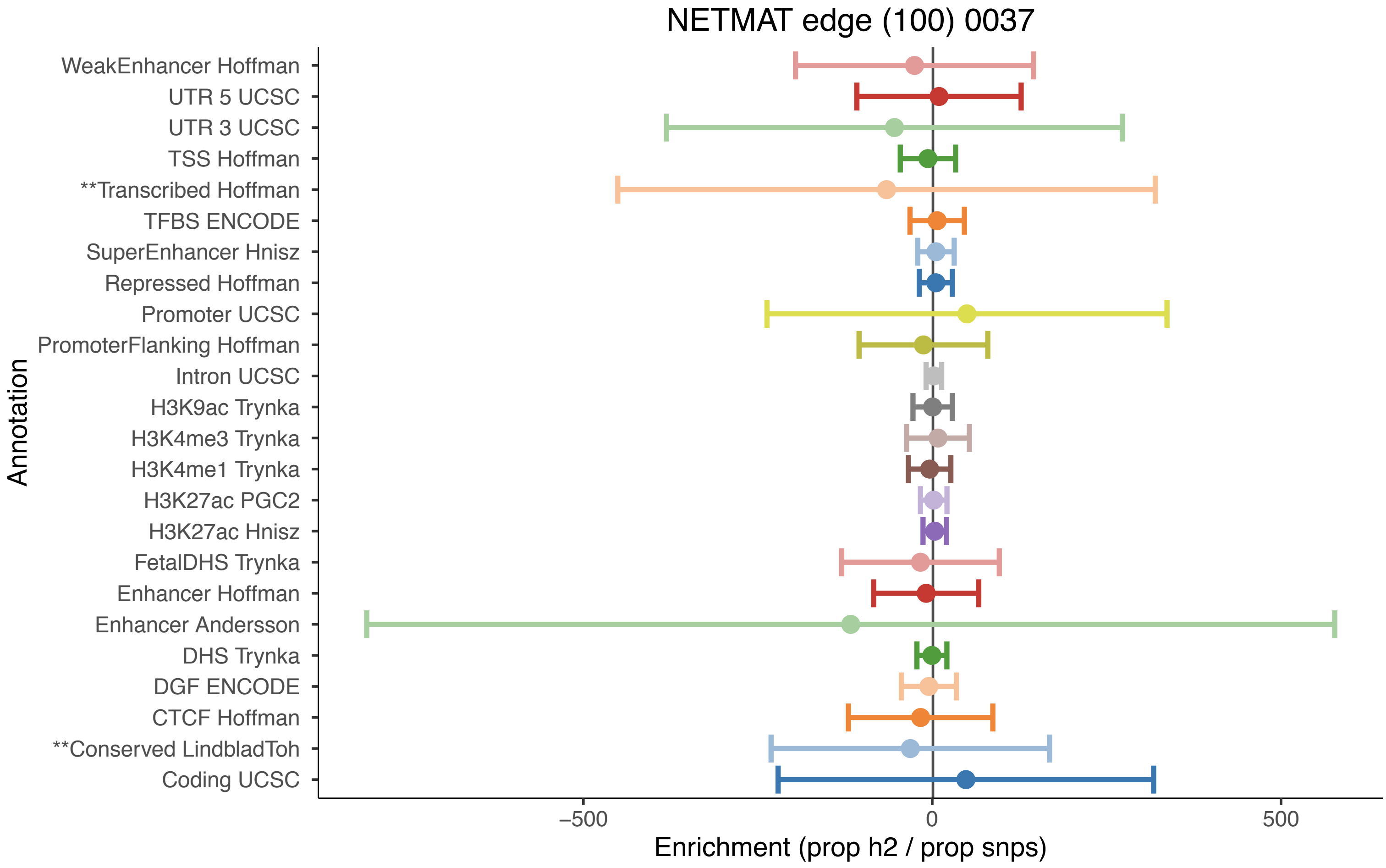

NETMAT edge (100) 0037
WeakEnhancer Hoffman
UTR 5 UCSC
UTR 3 UCSC
TSS Hoffman
**Transcribed Hoffman
TFBS ENCODE
SuperEnhancer Hnisz
Repressed Hoffman
Promoter UCSC
PromoterFlanking Hoffman
Intron UCSC
H3K9ac Trynka
H3K4me3 Trynka
H3K4me1 Trynka
H3K27ac PGC2
H3K27ac Hnisz
FetalDHS Trynka
Enhancer Hoffman
Enhancer Andersson
DHS Trynka
DGF ENCODE
CTCF Hoffman
**Conserved LindbladToh
Coding UCSC
Annotation
−500
0
500
Enrichment (prop h2 / prop snps)

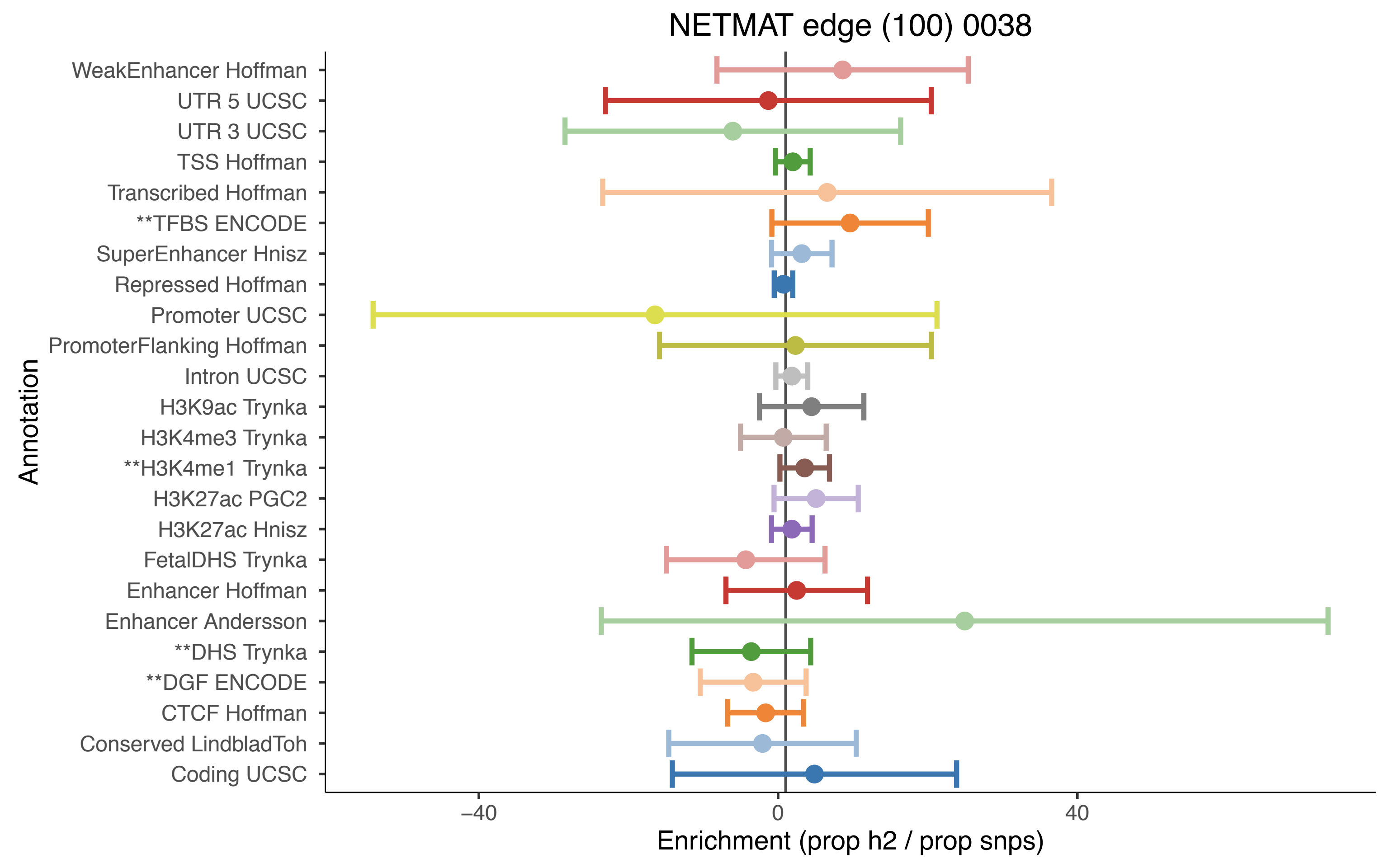

NETMAT edge (100) 0038
WeakEnhancer Hoffman
UTR 5 UCSC
UTR 3 UCSC
TSS Hoffman
Transcribed Hoffman
**TFBS ENCODE
SuperEnhancer Hnisz
Repressed Hoffman
Promoter UCSC
PromoterFlanking Hoffman
Intron UCSC
H3K9ac Trynka
H3K4me3 Trynka
**H3K4me1 Trynka
H3K27ac PGC2
H3K27ac Hnisz
FetalDHS Trynka
Enhancer Hoffman
Enhancer Andersson
**DHS Trynka
**DGF ENCODE
CTCF Hoffman
Conserved LindbladToh
Coding UCSC
Annotation
−40
0
40
Enrichment (prop h2 / prop snps)

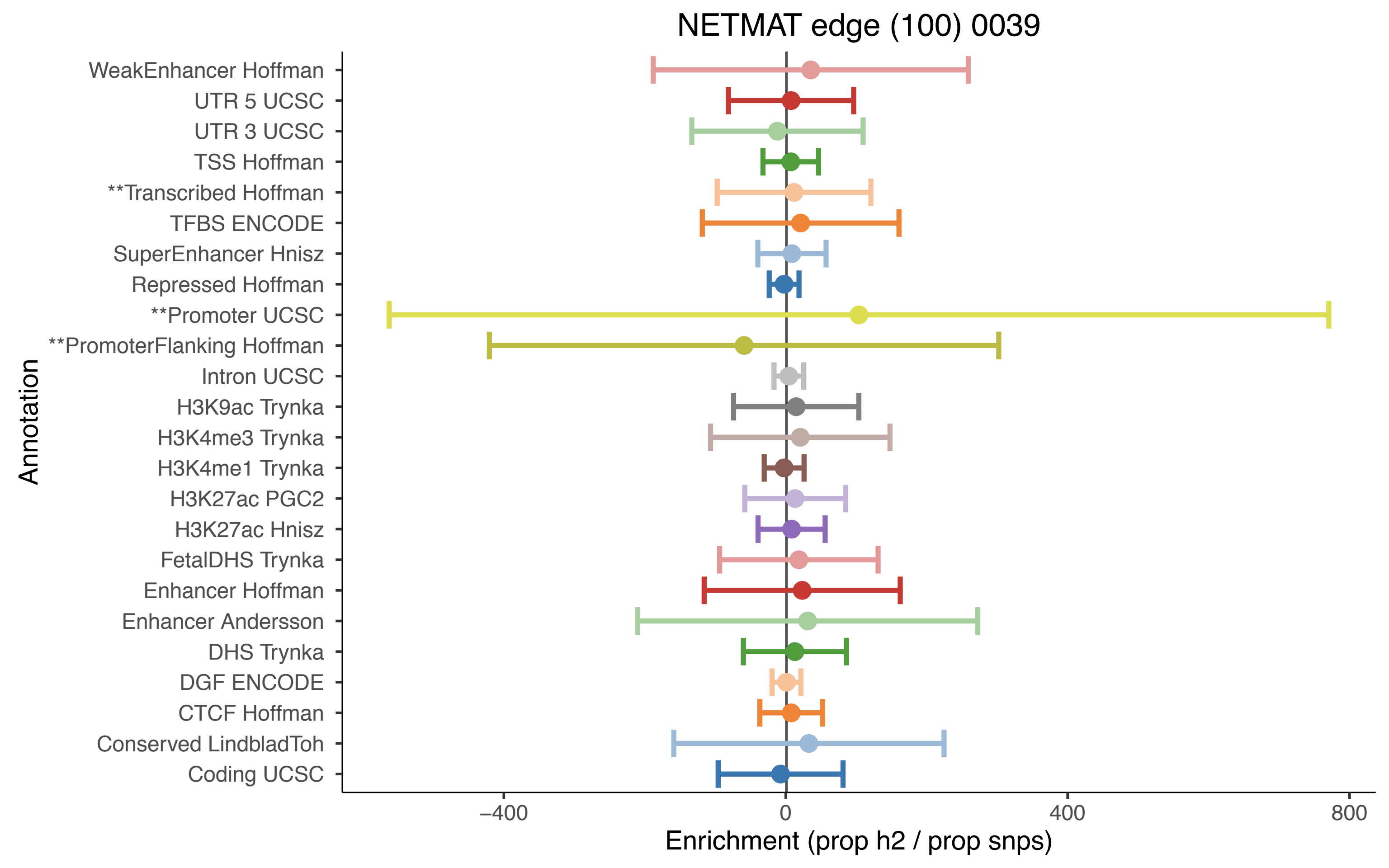

NETMAT edge (100) 0039
WeakEnhancer Hoffman
UTR 5 UCSC
UTR 3 UCSC
TSS Hoffman
**Transcribed Hoffman
TFBS ENCODE
SuperEnhancer Hnisz
Repressed Hoffman
**Promoter UCSC
**PromoterFlanking Hoffman
Intron UCSC
H3K9ac Trynka
H3K4me3 Trynka
H3K4me1 Trynka
H3K27ac PGC2
H3K27ac Hnisz
FetalDHS Trynka
Enhancer Hoffman
Enhancer Andersson
DHS Trynka
DGF ENCODE
CTCF Hoffman
Conserved LindbladToh
Coding UCSC
Annotation
−400
0
400
800
Enrichment (prop h2 / prop snps)

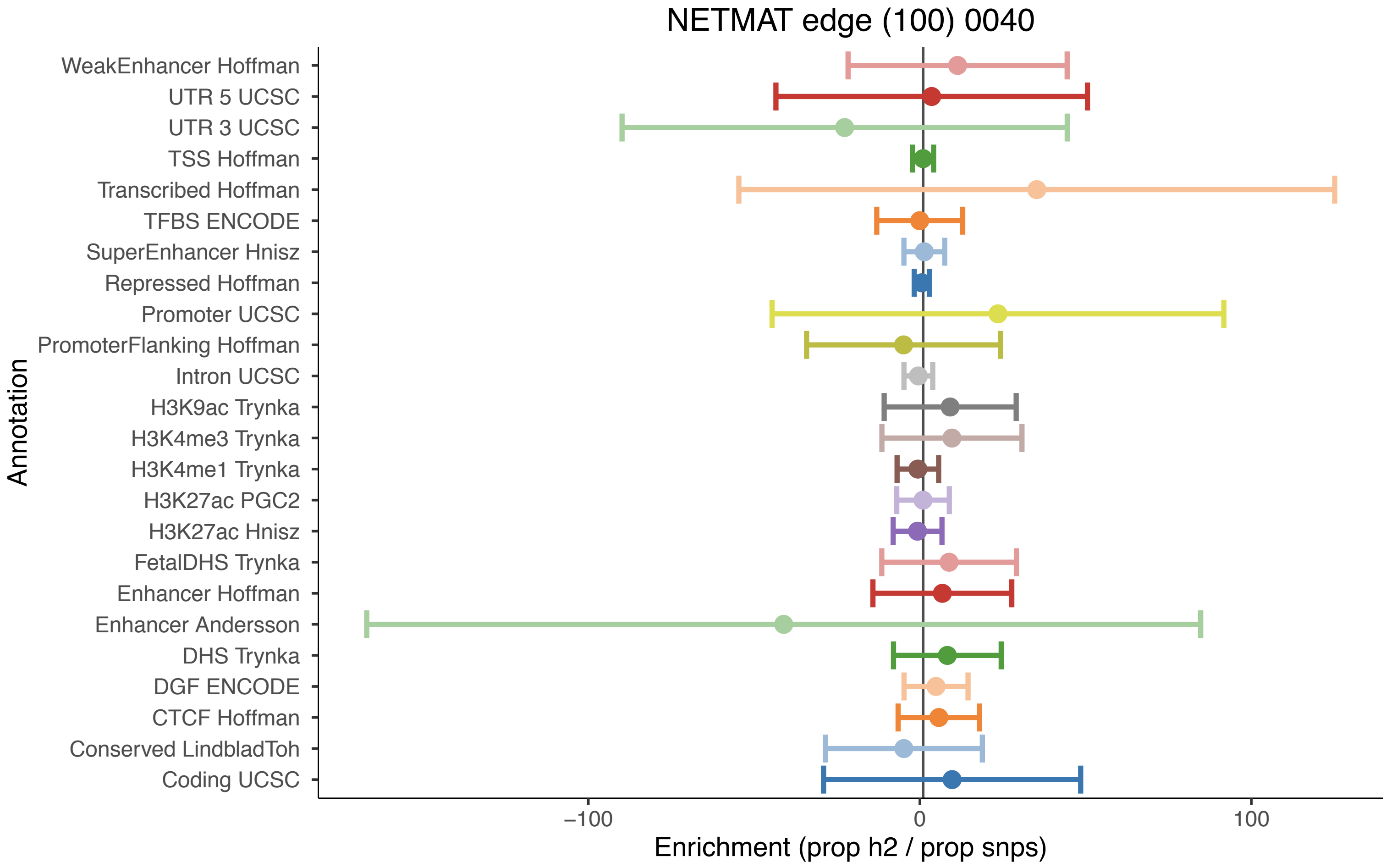
NETMAT edge (100) 0040
Annotation
WeakEnhancer Hoffman
UTR 5 UCSC
UTR 3 UCSC
TSS Hoffman
Transcribed Hoffman
TFBS ENCODE
SuperEnhancer Hnisz
Repressed Hoffman
Promoter UCSC
PromoterFlanking Hoffman
Intron UCSC
H3K9ac Trynka
H3K4me3 Trynka
H3K4me1 Trynka
H3K27ac PGC2
H3K27ac Hnisz
FetalDHS Trynka
Enhancer Hoffman
Enhancer Andersson
DHS Trynka
DGF ENCODE
CTCF Hoffman
Conserved LindbladToh
Coding UCSC
−100
0
100
Enrichment (prop h2 / prop snps)

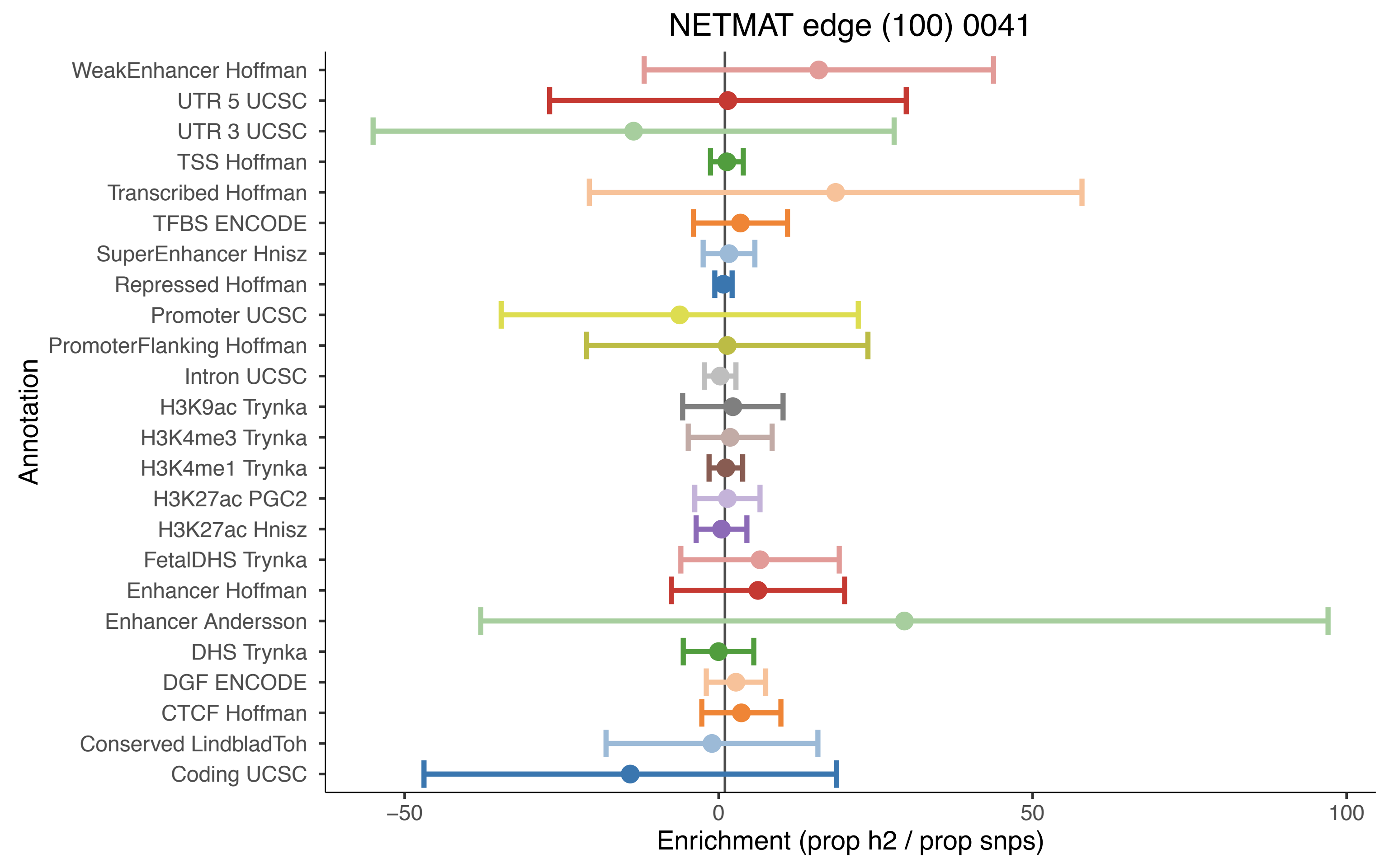
NETMAT edge (100) 0041
Annotation
WeakEnhancer Hoffman
UTR 5 UCSC
UTR 3 UCSC
TSS Hoffman
Transcribed Hoffman
TFBS ENCODE
SuperEnhancer Hnisz
Repressed Hoffman
Promoter UCSC
PromoterFlanking Hoffman
Intron UCSC
H3K9ac Trynka
H3K4me3 Trynka
H3K4me1 Trynka
H3K27ac PGC2
H3K27ac Hnisz
FetalDHS Trynka
Enhancer Hoffman
Enhancer Andersson
DHS Trynka
DGF ENCODE
CTCF Hoffman
Conserved LindbladToh
Coding UCSC
−50
0
50
100
Enrichment (prop h2 / prop snps)

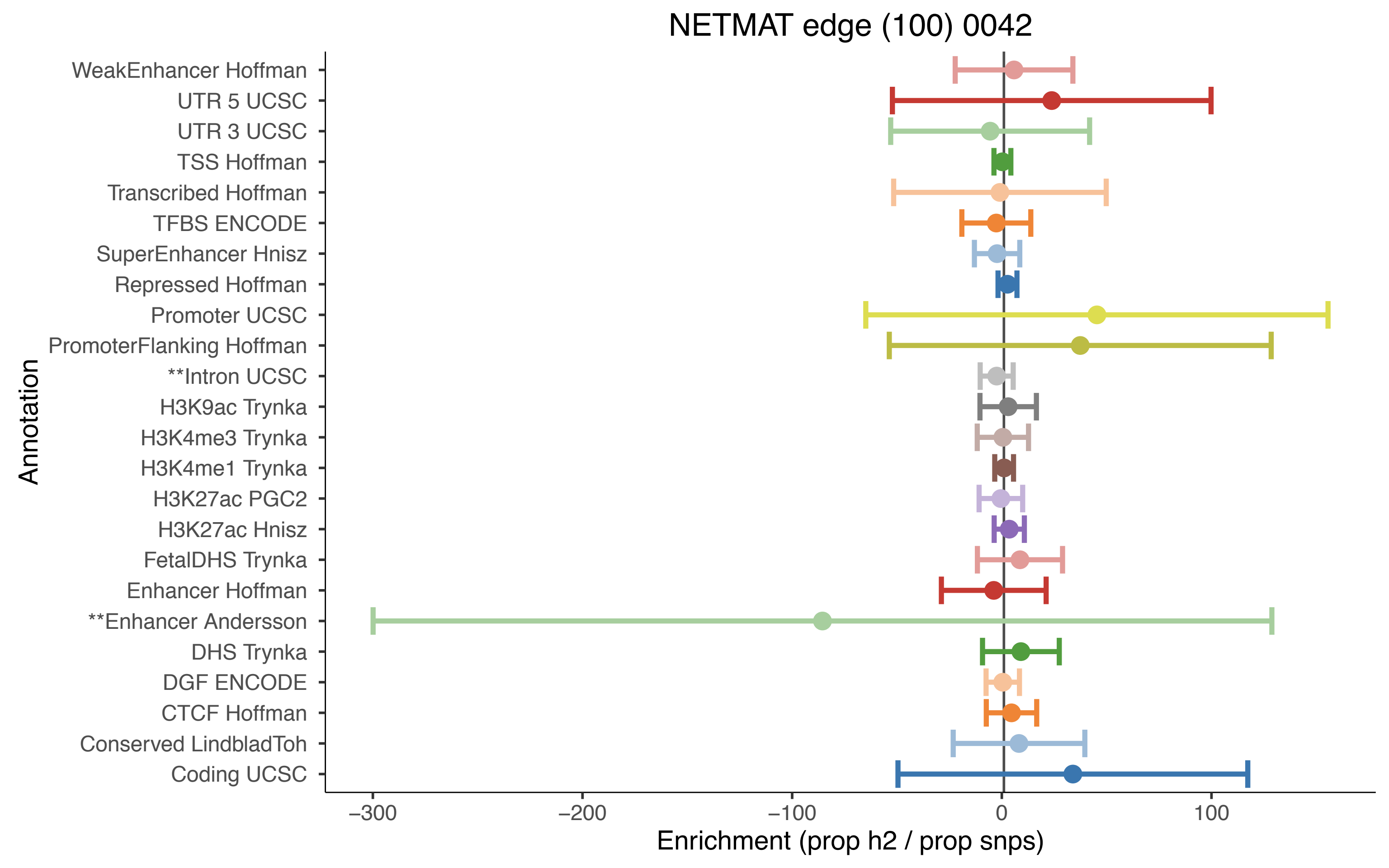

NETMAT edge (100) 0042
WeakEnhancer Hoffman
UTR 5 UCSC
UTR 3 UCSC
TSS Hoffman
Transcribed Hoffman
TFBS ENCODE
SuperEnhancer Hnisz
Repressed Hoffman
Promoter UCSC
PromoterFlanking Hoffman
**Intron UCSC
H3K9ac Trynka
H3K4me3 Trynka
H3K4me1 Trynka
H3K27ac PGC2
H3K27ac Hnisz
FetalDHS Trynka
Enhancer Hoffman
**Enhancer Andersson
DHS Trynka
DGF ENCODE
CTCF Hoffman
Conserved LindbladToh
Coding UCSC
Annotation
−300
−200
−100
0
100
Enrichment (prop h2 / prop snps)

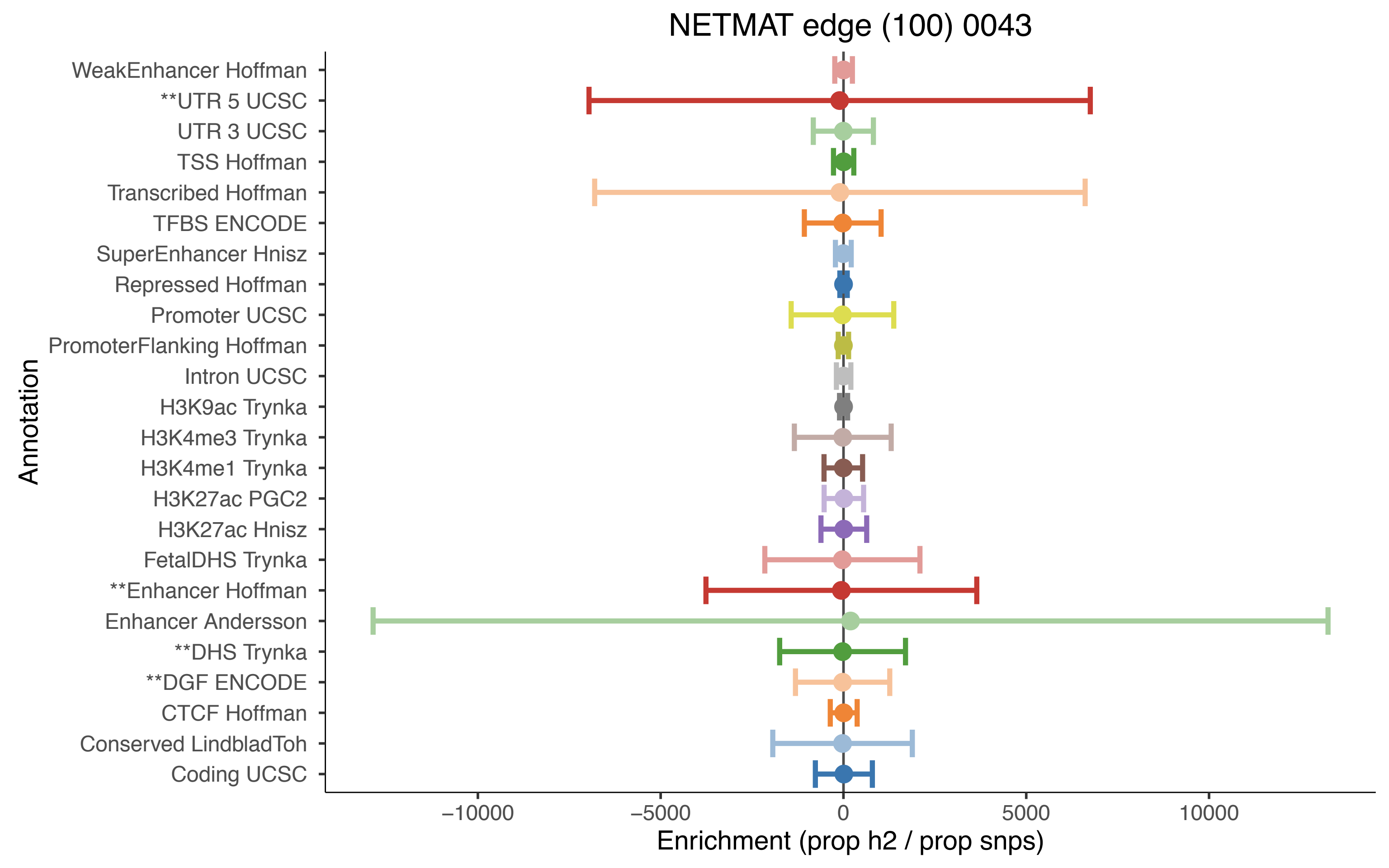

NETMAT edge (100) 0043
WeakEnhancer Hoffman
**UTR 5 UCSC
UTR 3 UCSC
TSS Hoffman
Transcribed Hoffman
TFBS ENCODE
SuperEnhancer Hnisz
Repressed Hoffman
Promoter UCSC
PromoterFlanking Hoffman
Intron UCSC
H3K9ac Trynka
H3K4me3 Trynka
H3K4me1 Trynka
H3K27ac PGC2
H3K27ac Hnisz
FetalDHS Trynka
**Enhancer Hoffman
Enhancer Andersson
**DHS Trynka
**DGF ENCODE
CTCF Hoffman
Conserved LindbladToh
Coding UCSC
Annotation
−10000
−5000
0
5000
10000
Enrichment (prop h2 / prop snps)

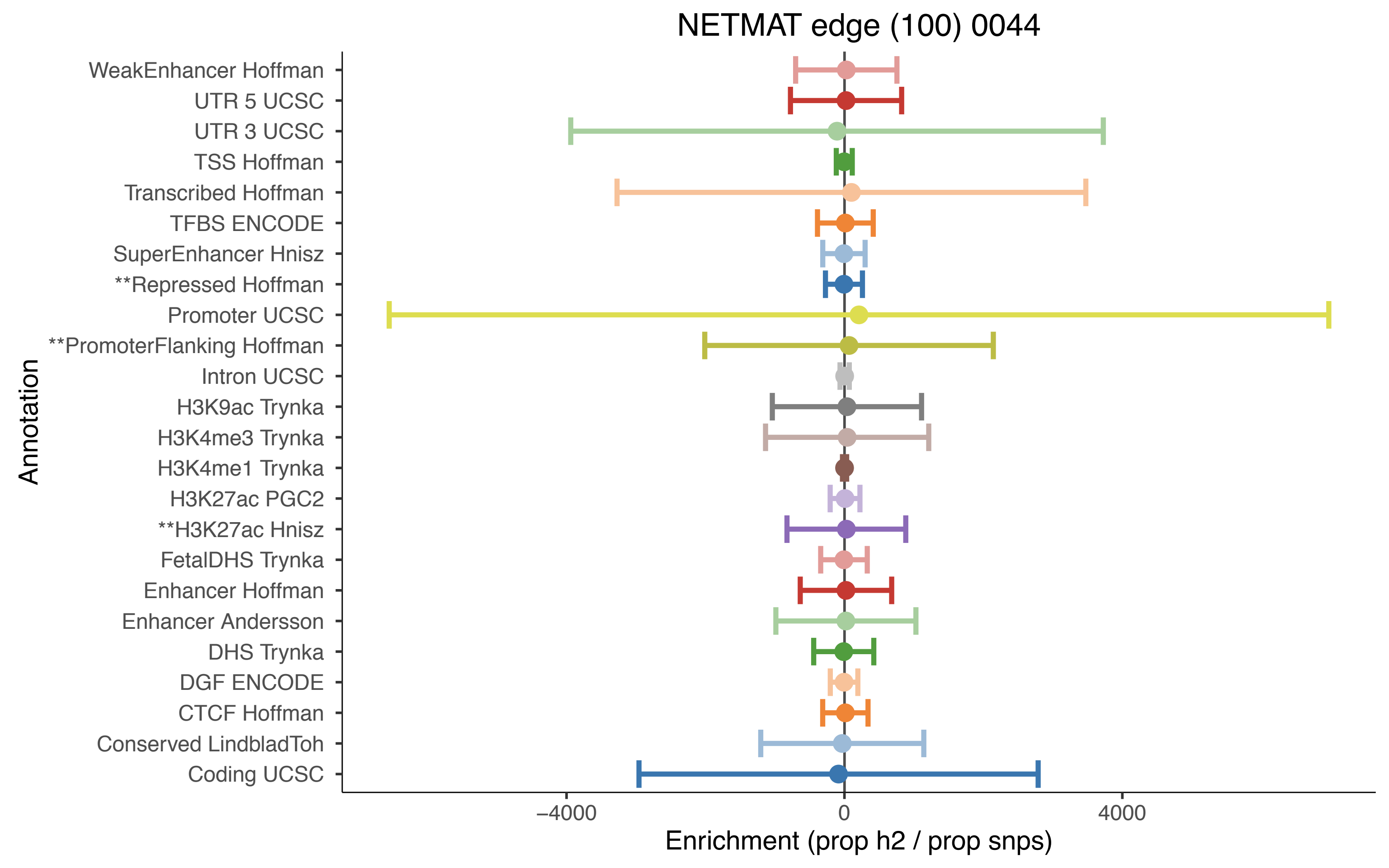

NETMAT edge (100) 0044
Annotation
WeakEnhancer Hoffman
UTR 5 UCSC
UTR 3 UCSC
TSS Hoffman
Transcribed Hoffman
TFBS ENCODE
SuperEnhancer Hnisz
**Repressed Hoffman
Promoter UCSC
**PromoterFlanking Hoffman
Intron UCSC
H3K9ac Trynka
H3K4me3 Trynka
H3K4me1 Trynka
H3K27ac PGC2
**H3K27ac Hnisz
FetalDHS Trynka
Enhancer Hoffman
Enhancer Andersson
DHS Trynka
DGF ENCODE
CTCF Hoffman
Conserved LindbladToh
Coding UCSC
−4000
0
4000
Enrichment (prop h2 / prop snps)

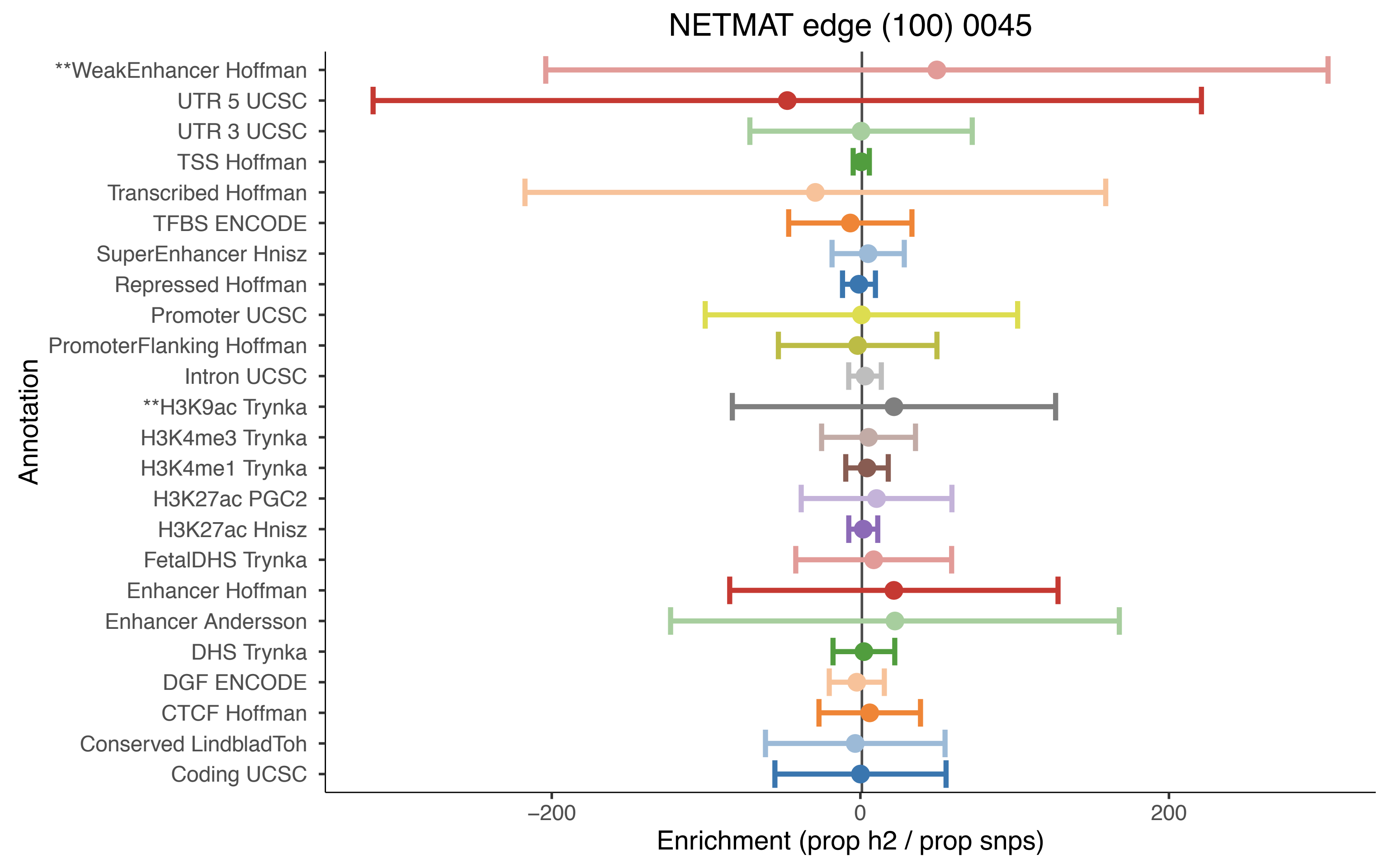
NETMAT edge (100) 0045
**WeakEnhancer Hoffman
UTR 5 UCSC
UTR 3 UCSC
TSS Hoffman
Transcribed Hoffman
TFBS ENCODE
SuperEnhancer Hnisz
Repressed Hoffman
Promoter UCSC
PromoterFlanking Hoffman
Intron UCSC
**H3K9ac Trynka
H3K4me3 Trynka
H3K4me1 Trynka
H3K27ac PGC2
H3K27ac Hnisz
FetalDHS Trynka
Enhancer Hoffman
Enhancer Andersson
DHS Trynka
DGF ENCODE
CTCF Hoffman
Conserved LindbladToh
Coding UCSC
Annotation
−200
0
200
Enrichment (prop h2 / prop snps)

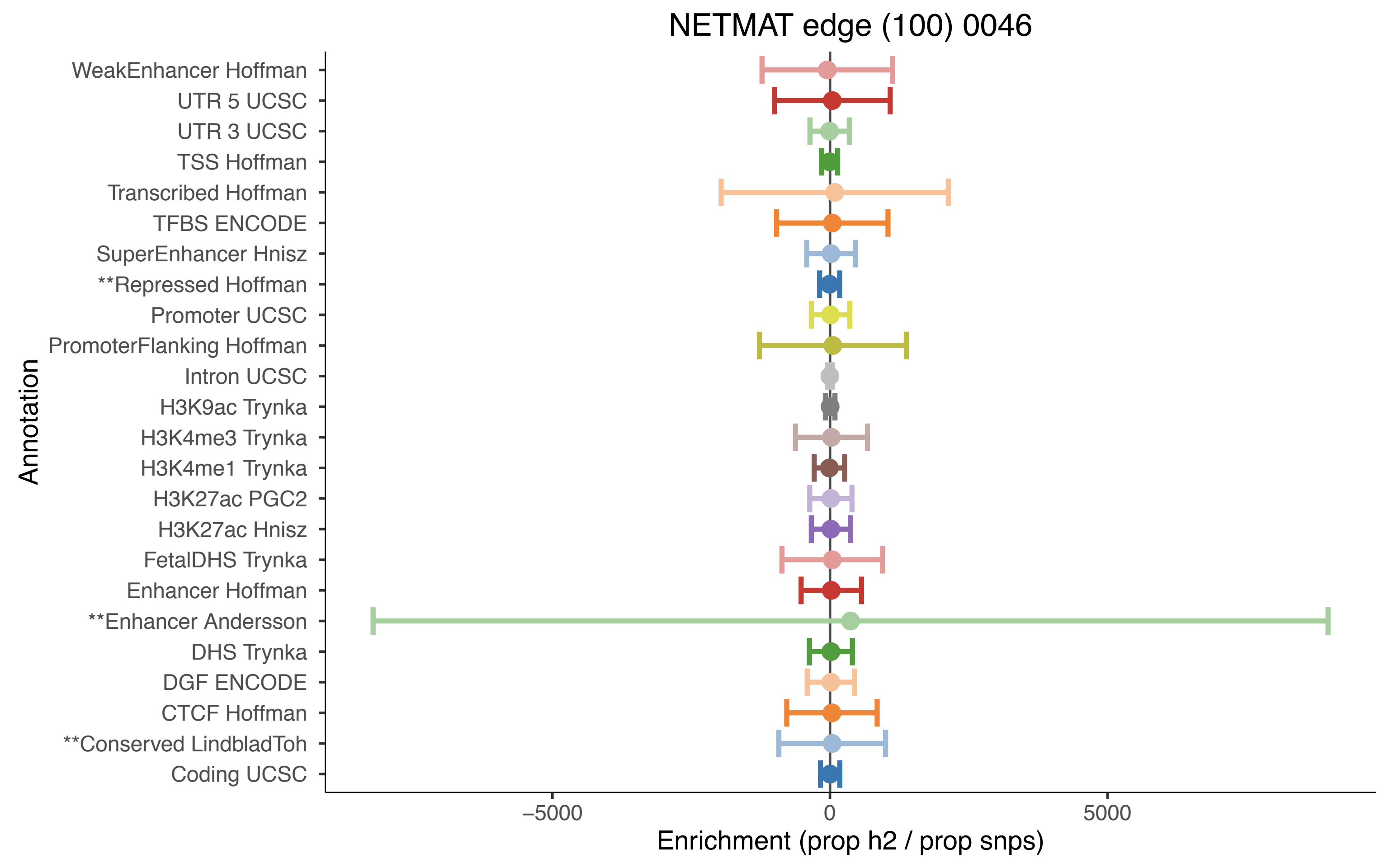
NETMAT edge (100) 0046
Annotation
WeakEnhancer Hoffman
UTR 5 UCSC
UTR 3 UCSC
TSS Hoffman
Transcribed Hoffman
TFBS ENCODE
SuperEnhancer Hnisz
**Repressed Hoffman
Promoter UCSC
PromoterFlanking Hoffman
Intron UCSC
H3K9ac Trynka
H3K4me3 Trynka
H3K4me1 Trynka
H3K27ac PGC2
H3K27ac Hnisz
FetalDHS Trynka
Enhancer Hoffman
**Enhancer Andersson
DHS Trynka
DGF ENCODE
CTCF Hoffman
**Conserved LindbladToh
Coding UCSC
−5000
0
5000
Enrichment (prop h2 / prop snps)

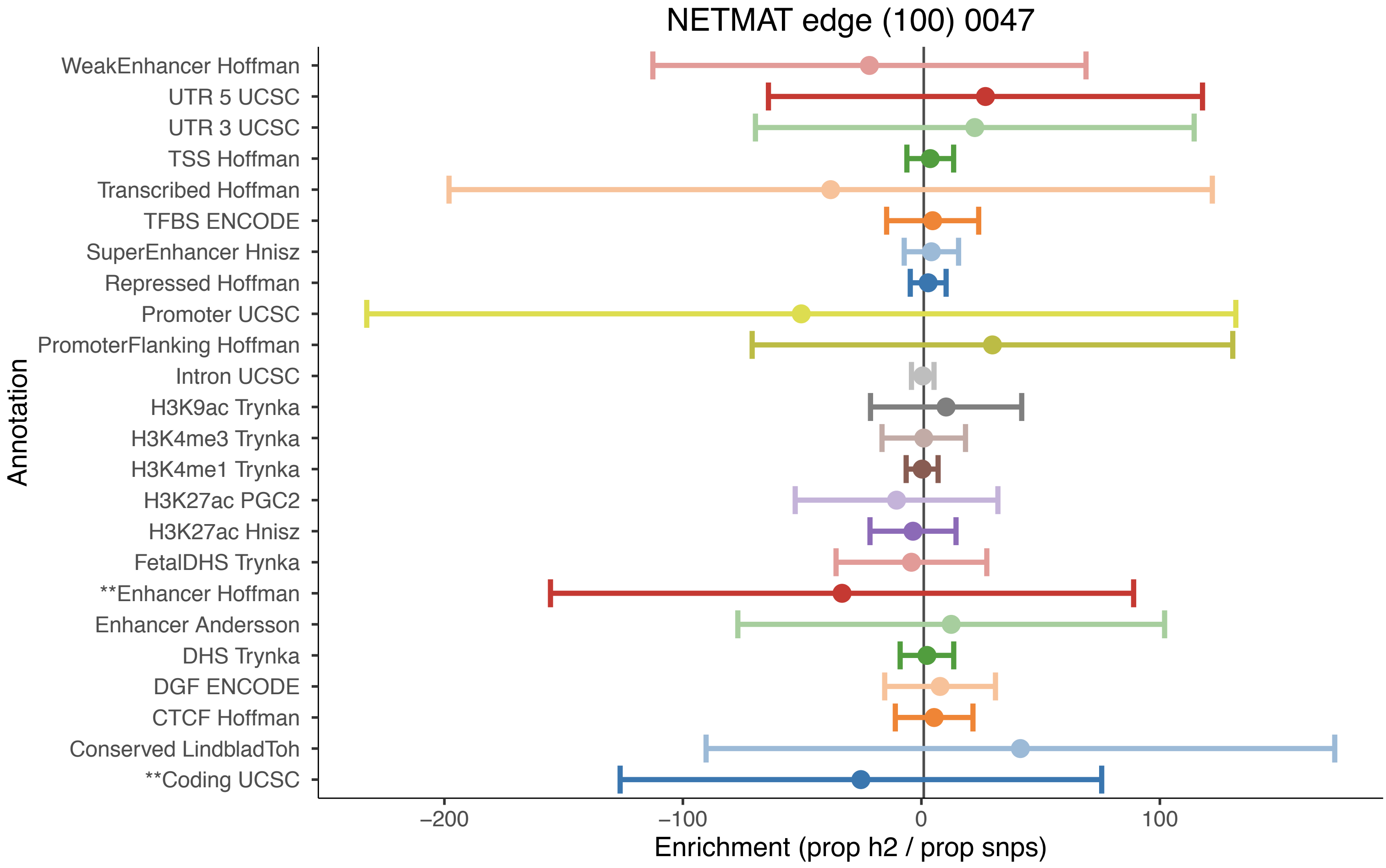

NETMAT edge (100) 0047
Annotation
WeakEnhancer Hoffman
UTR 5 UCSC
UTR 3 UCSC
TSS Hoffman
Transcribed Hoffman
TFBS ENCODE
SuperEnhancer Hnisz
Repressed Hoffman
Promoter UCSC
PromoterFlanking Hoffman
Intron UCSC
H3K9ac Trynka
H3K4me3 Trynka
H3K4me1 Trynka
H3K27ac PGC2
H3K27ac Hnisz
FetalDHS Trynka
**Enhancer Hoffman
Enhancer Andersson
DHS Trynka
DGF ENCODE
CTCF Hoffman
Conserved LindbladToh
**Coding UCSC
−200
−100
0
100
Enrichment (prop h2 / prop snps)

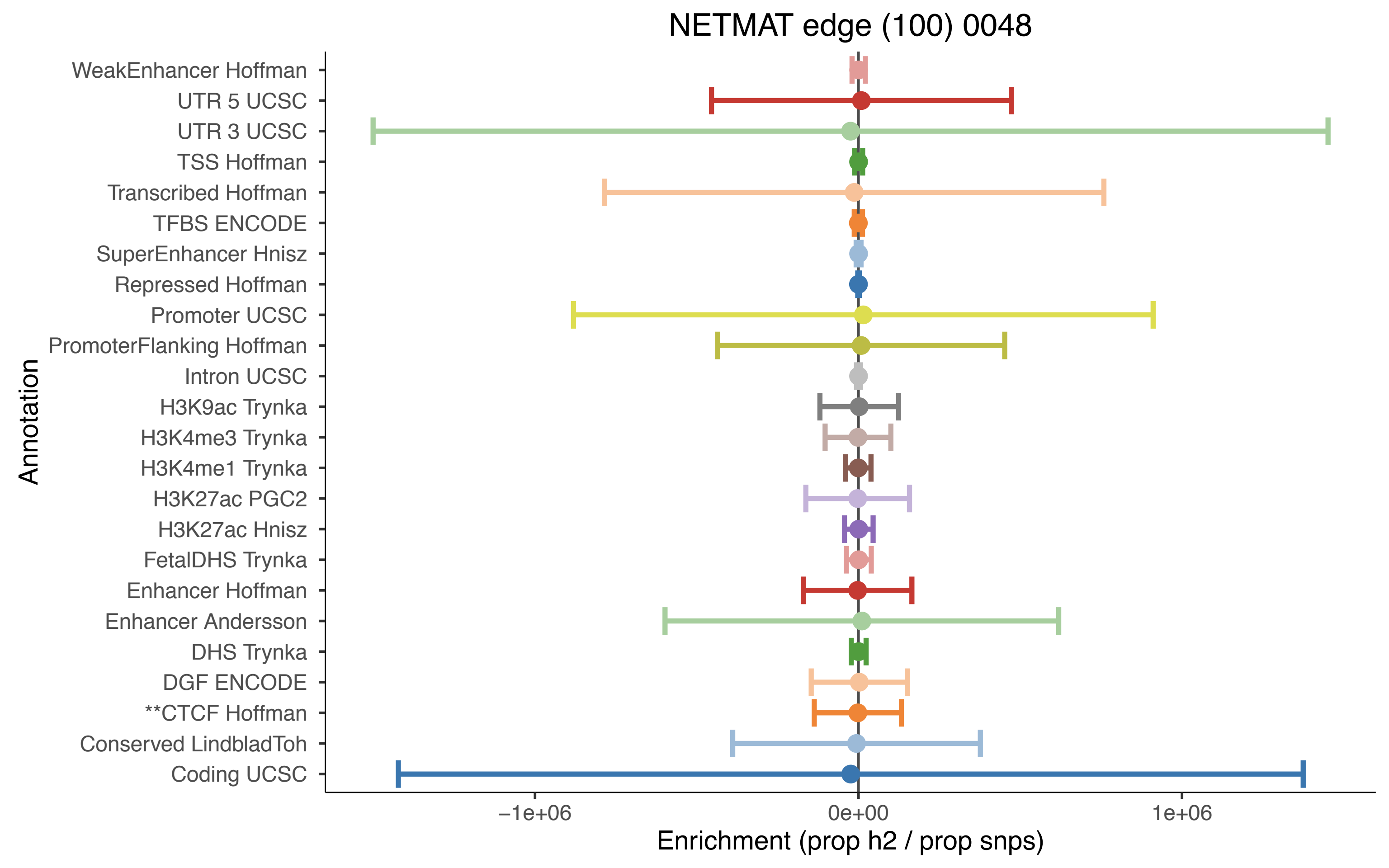
NETMAT edge (100) 0048
Annotation
WeakEnhancer Hoffman
UTR 5 UCSC
UTR 3 UCSC
TSS Hoffman
Transcribed Hoffman
TFBS ENCODE
SuperEnhancer Hnisz
Repressed Hoffman
Promoter UCSC
PromoterFlanking Hoffman
Intron UCSC
H3K9ac Trynka
H3K4me3 Trynka
H3K4me1 Trynka
H3K27ac PGC2
H3K27ac Hnisz
FetalDHS Trynka
Enhancer Hoffman
Enhancer Andersson
DHS Trynka
DGF ENCODE
**CTCF Hoffman
Conserved LindbladToh
Coding UCSC
−1e+06
0e+00
1e+06
Enrichment (prop h2 / prop snps)

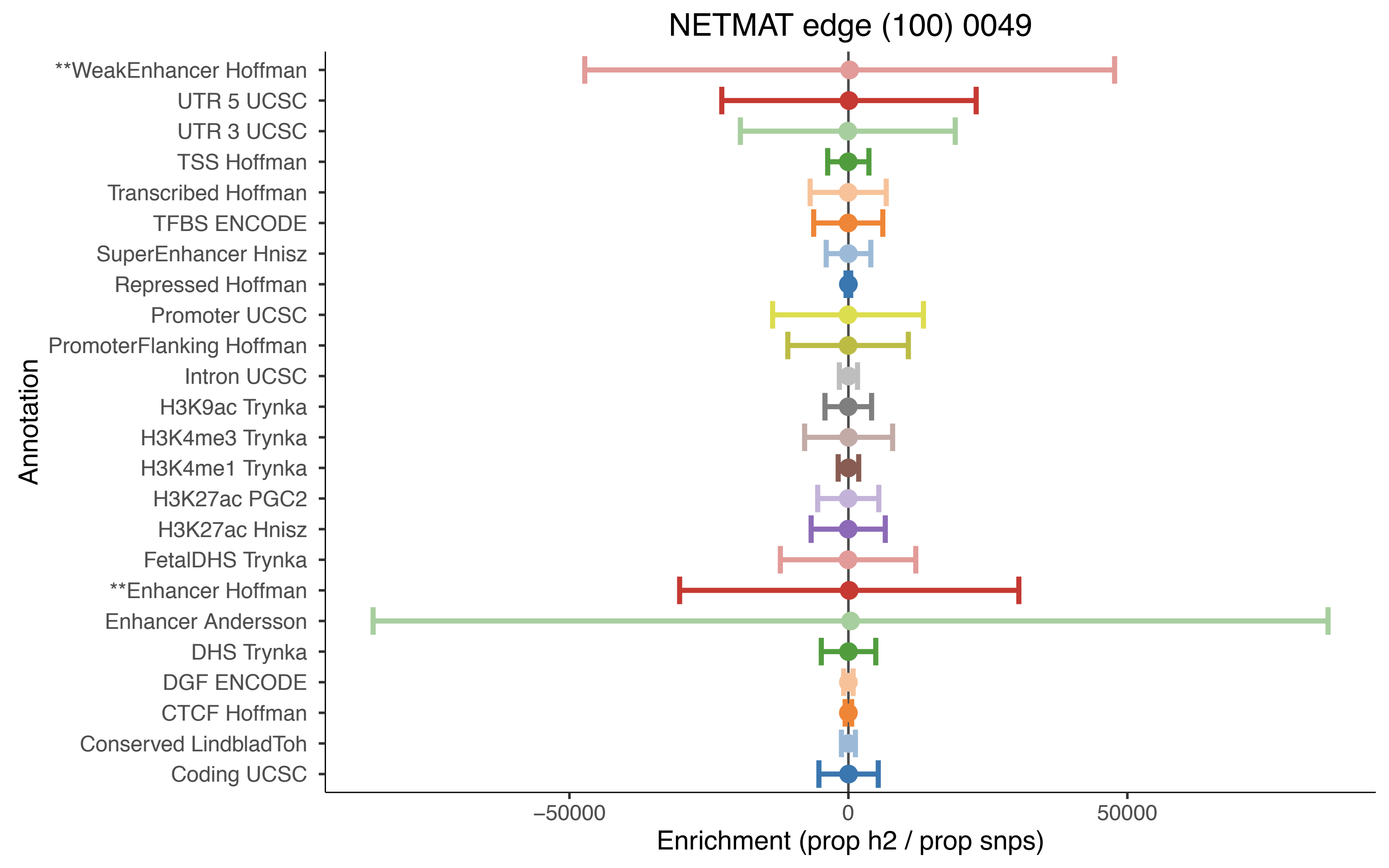

NETMAT edge (100) 0049
Annotation
**WeakEnhancer Hoffman
UTR 5 UCSC
UTR 3 UCSC
TSS Hoffman
Transcribed Hoffman
TFBS ENCODE
SuperEnhancer Hnisz
Repressed Hoffman
Promoter UCSC
PromoterFlanking Hoffman
Intron UCSC
H3K9ac Trynka
H3K4me3 Trynka
H3K4me1 Trynka
H3K27ac PGC2
H3K27ac Hnisz
FetalDHS Trynka
**Enhancer Hoffman
Enhancer Andersson
DHS Trynka
DGF ENCODE
CTCF Hoffman
Conserved LindbladToh
Coding UCSC
−50000
0
50000
Enrichment (prop h2 / prop snps)

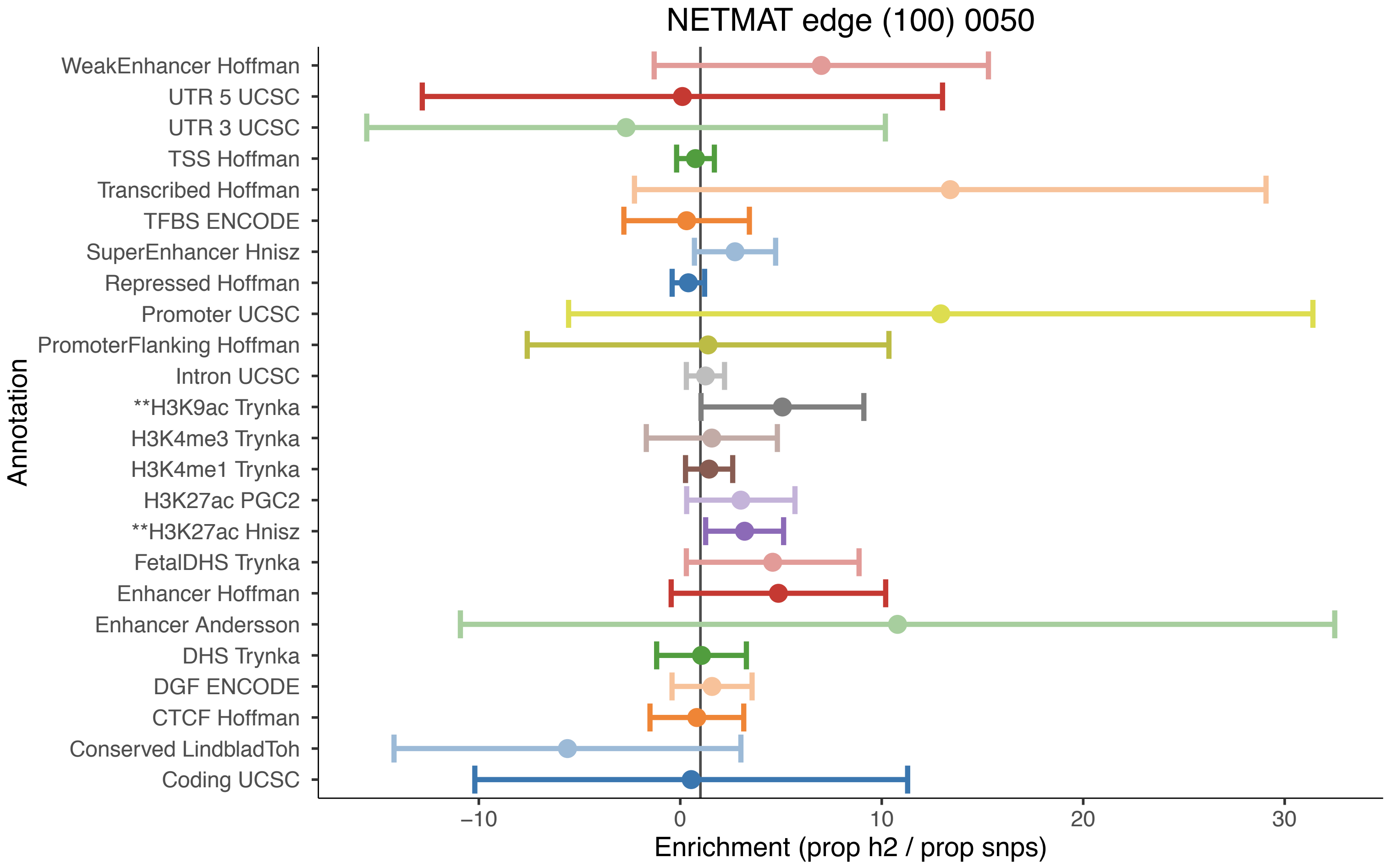
NETMAT edge (100) 0050
WeakEnhancer Hoffman
UTR 5 UCSC
UTR 3 UCSC
TSS Hoffman
Transcribed Hoffman
TFBS ENCODE
SuperEnhancer Hnisz
Repressed Hoffman
Promoter UCSC
PromoterFlanking Hoffman
Intron UCSC
**H3K9ac Trynka
H3K4me3 Trynka
H3K4me1 Trynka
H3K27ac PGC2
**H3K27ac Hnisz
FetalDHS Trynka
Enhancer Hoffman
Enhancer Andersson
DHS Trynka
DGF ENCODE
CTCF Hoffman
Conserved LindbladToh
Coding UCSC
Annotation
−10
0
10
20
30
Enrichment (prop h2 / prop snps)

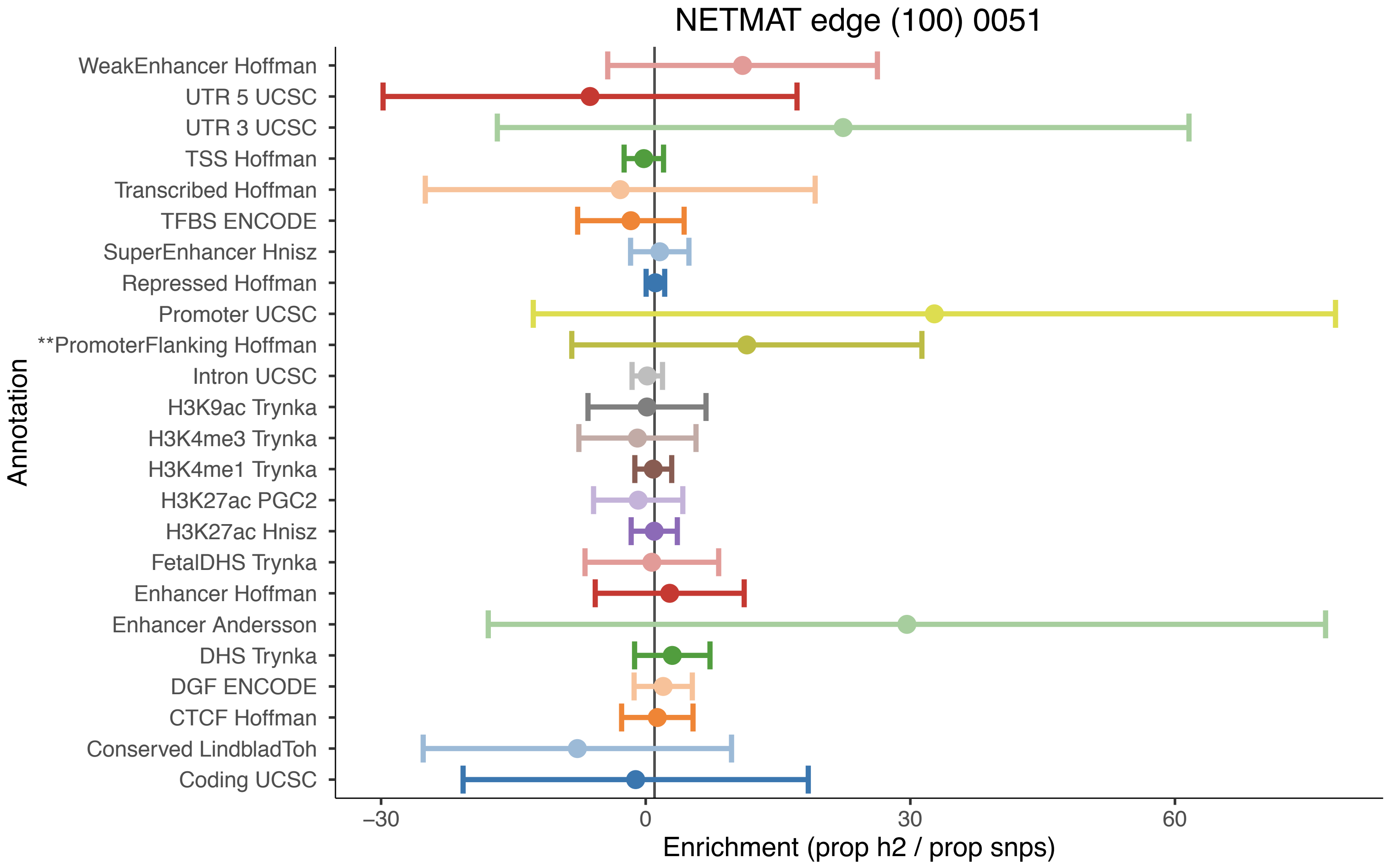
NETMAT edge (100) 0051
WeakEnhancer Hoffman
UTR 5 UCSC
UTR 3 UCSC
TSS Hoffman
Transcribed Hoffman
TFBS ENCODE
SuperEnhancer Hnisz
Repressed Hoffman
Promoter UCSC
**PromoterFlanking Hoffman
Intron UCSC
H3K9ac Trynka
H3K4me3 Trynka
H3K4me1 Trynka
H3K27ac PGC2
H3K27ac Hnisz
FetalDHS Trynka
Enhancer Hoffman
Enhancer Andersson
DHS Trynka
DGF ENCODE
CTCF Hoffman
Conserved LindbladToh
Coding UCSC
Annotation
−30
0
30
60
Enrichment (prop h2 / prop snps)

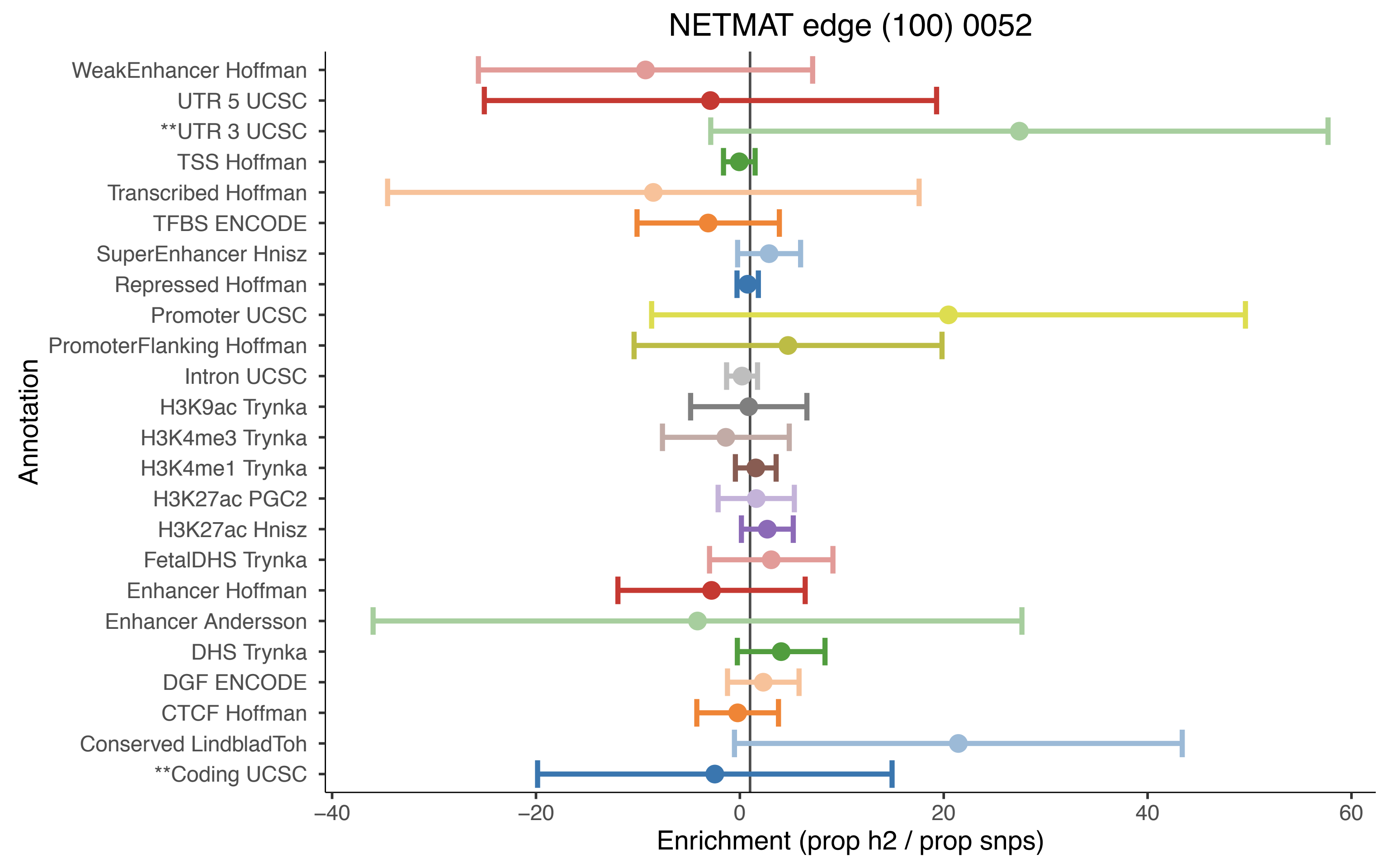

NETMAT edge (100) 0052
WeakEnhancer Hoffman
UTR 5 UCSC
**UTR 3 UCSC
TSS Hoffman
Transcribed Hoffman
TFBS ENCODE
SuperEnhancer Hnisz
Repressed Hoffman
Promoter UCSC
PromoterFlanking Hoffman
Intron UCSC
H3K9ac Trynka
H3K4me3 Trynka
H3K4me1 Trynka
H3K27ac PGC2
H3K27ac Hnisz
FetalDHS Trynka
Enhancer Hoffman
Enhancer Andersson
DHS Trynka
DGF ENCODE
CTCF Hoffman
Conserved LindbladToh
**Coding UCSC
Annotation
−40
−20
0
20
40
60
Enrichment (prop h2 / prop snps)

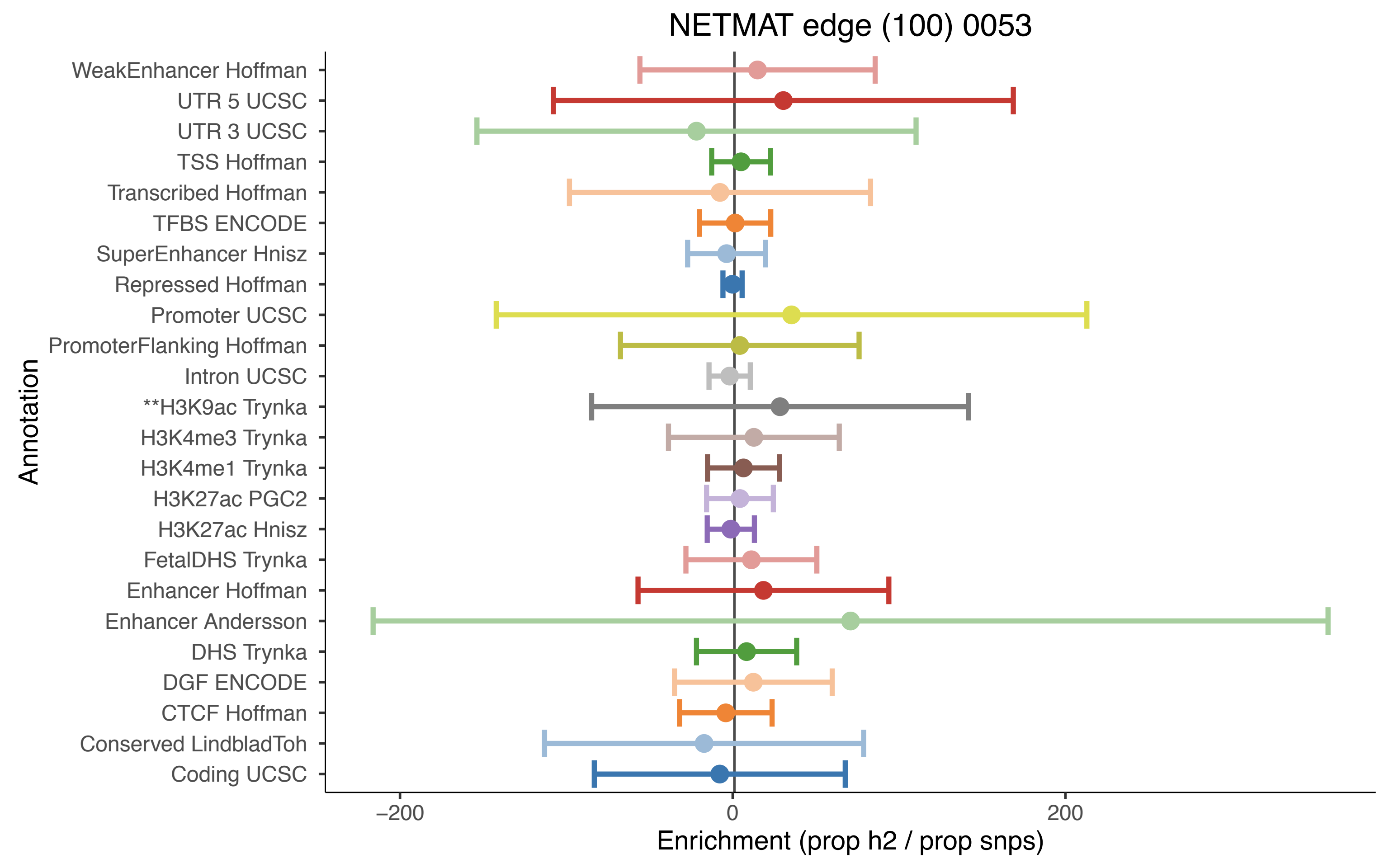
NETMAT edge (100) 0053
WeakEnhancer Hoffman
UTR 5 UCSC
UTR 3 UCSC
TSS Hoffman
Transcribed Hoffman
TFBS ENCODE
SuperEnhancer Hnisz
Repressed Hoffman
Promoter UCSC
PromoterFlanking Hoffman
Intron UCSC
**H3K9ac Trynka
H3K4me3 Trynka
H3K4me1 Trynka
H3K27ac PGC2
H3K27ac Hnisz
FetalDHS Trynka
Enhancer Hoffman
Enhancer Andersson
DHS Trynka
DGF ENCODE
CTCF Hoffman
Conserved LindbladToh
Coding UCSC
Annotation
−200
0
200
Enrichment (prop h2 / prop snps)

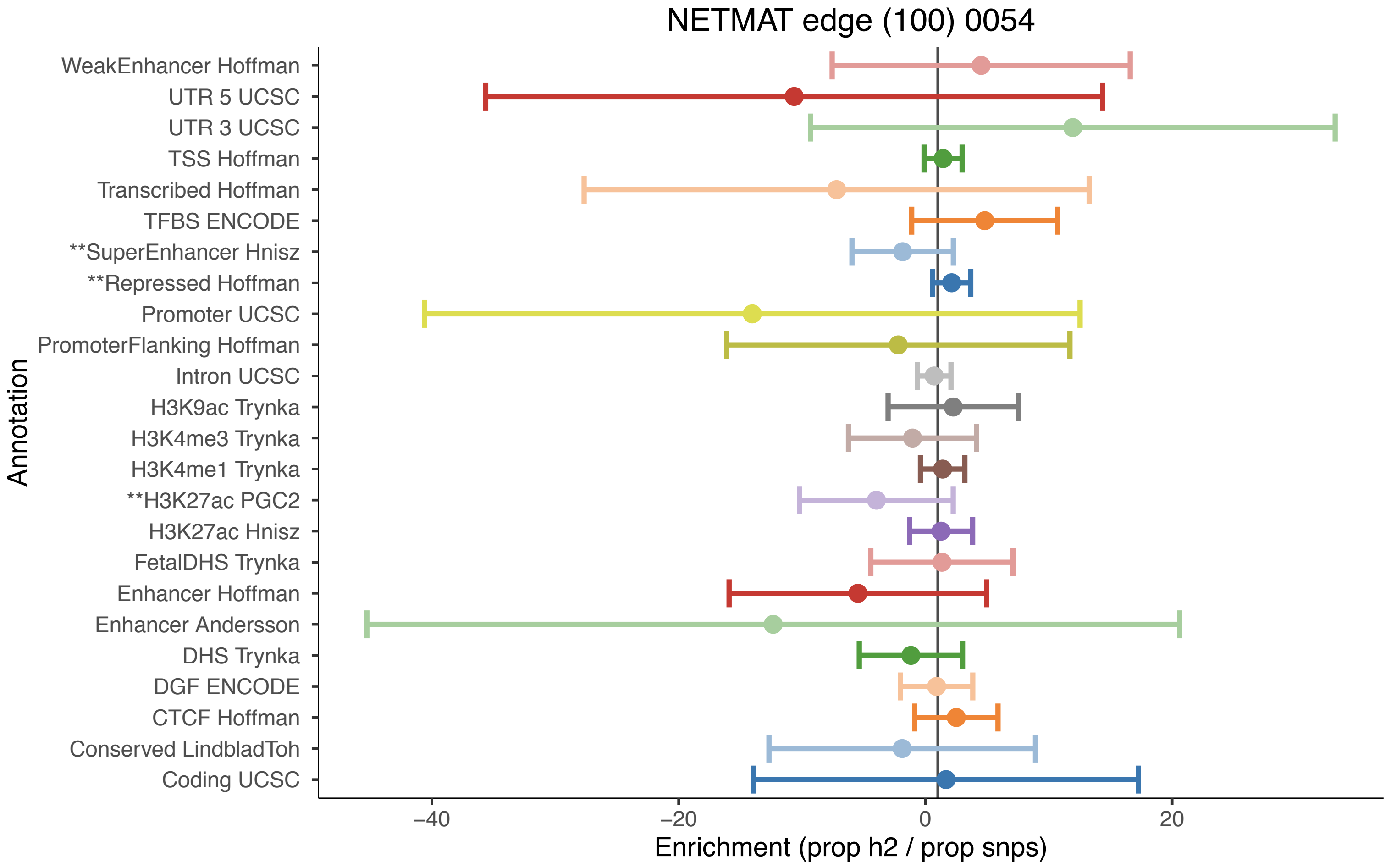
NETMAT edge (100) 0054
WeakEnhancer Hoffman
UTR 5 UCSC
UTR 3 UCSC
TSS Hoffman
Transcribed Hoffman
TFBS ENCODE
**SuperEnhancer Hnisz
**Repressed Hoffman
Promoter UCSC
PromoterFlanking Hoffman
Intron UCSC
H3K9ac Trynka
H3K4me3 Trynka
H3K4me1 Trynka
**H3K27ac PGC2
H3K27ac Hnisz
FetalDHS Trynka
Enhancer Hoffman
Enhancer Andersson
DHS Trynka
DGF ENCODE
CTCF Hoffman
Conserved LindbladToh
Coding UCSC
Annotation
−40
−20
0
20
Enrichment (prop h2 / prop snps)

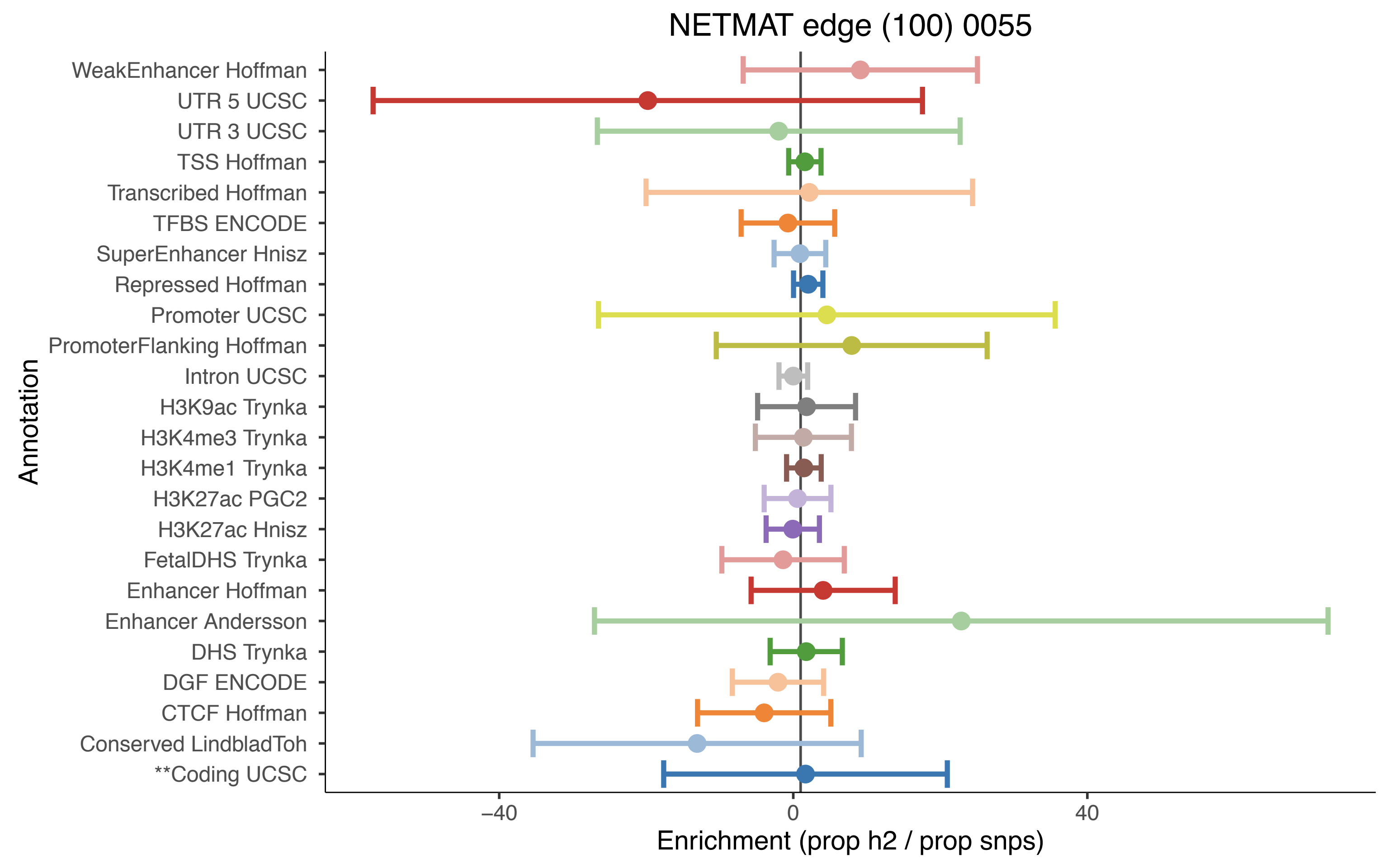
NETMAT edge (100) 0055
WeakEnhancer Hoffman
UTR 5 UCSC
UTR 3 UCSC
TSS Hoffman
Transcribed Hoffman
TFBS ENCODE
SuperEnhancer Hnisz
Repressed Hoffman
Promoter UCSC
PromoterFlanking Hoffman
Intron UCSC
H3K9ac Trynka
H3K4me3 Trynka
H3K4me1 Trynka
H3K27ac PGC2
H3K27ac Hnisz
FetalDHS Trynka
Enhancer Hoffman
Enhancer Andersson
DHS Trynka
DGF ENCODE
CTCF Hoffman
Conserved LindbladToh
**Coding UCSC
Annotation
−40
0
40
Enrichment (prop h2 / prop snps)

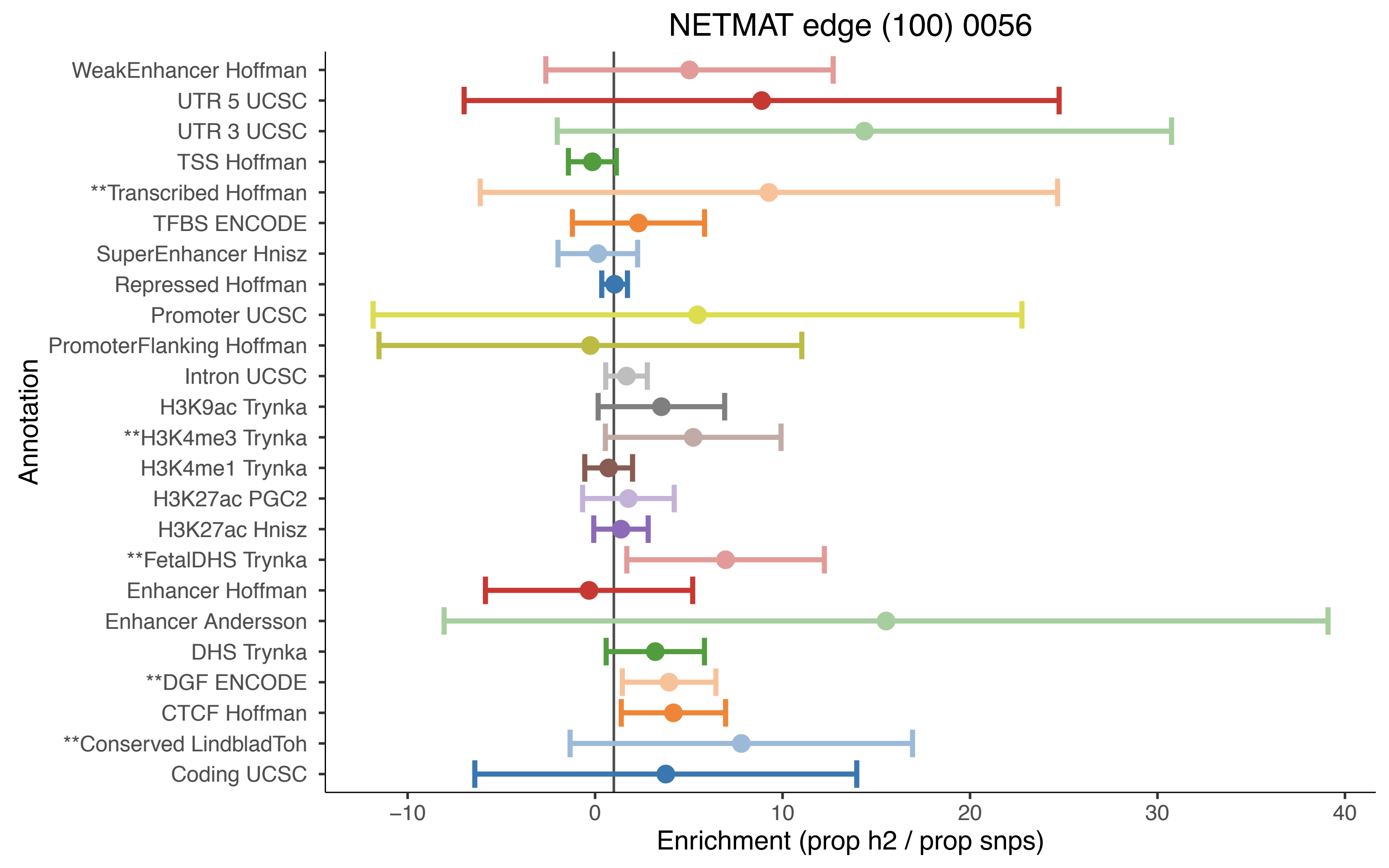
NETMAT edge (100) 0056
WeakEnhancer Hoffman
UTR 5 UCSC
UTR 3 UCSC
TSS Hoffman
**Transcribed Hoffman
TFBS ENCODE
SuperEnhancer Hnisz
Repressed Hoffman
Promoter UCSC
PromoterFlanking Hoffman
Intron UCSC
H3K9ac Trynka
**H3K4me3 Trynka
H3K4me1 Trynka
H3K27ac PGC2
H3K27ac Hnisz
**FetalDHS Trynka
Enhancer Hoffman
Enhancer Andersson
DHS Trynka
**DGF ENCODE
CTCF Hoffman
**Conserved LindbladToh
Coding UCSC
Annotation
−10
0
10
20
30
40
Enrichment (prop h2 / prop snps)

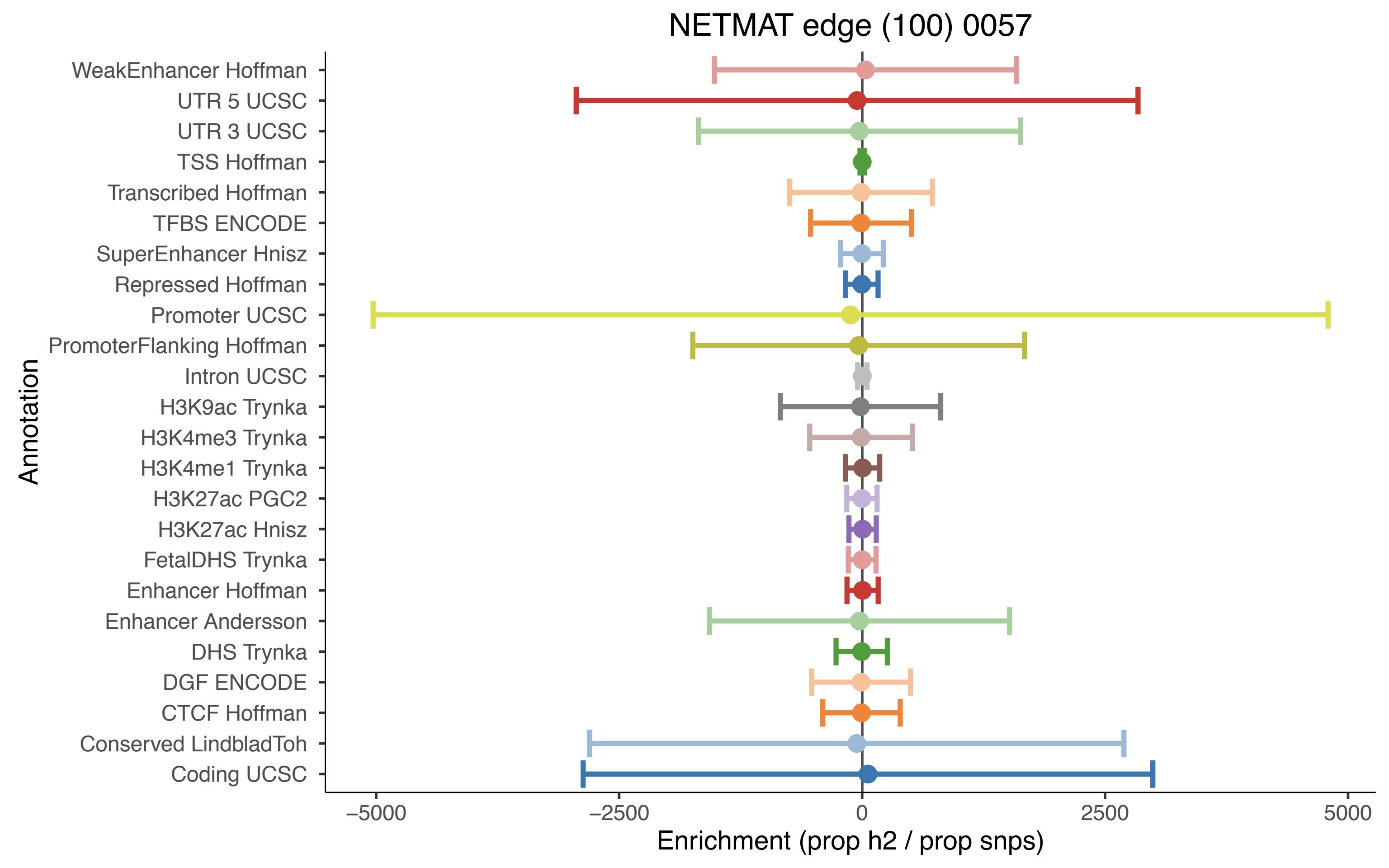

NETMAT edge (100) 0057
Annotation
WeakEnhancer Hoffman
UTR 5 UCSC
UTR 3 UCSC
TSS Hoffman
Transcribed Hoffman
TFBS ENCODE
SuperEnhancer Hnisz
Repressed Hoffman
Promoter UCSC
PromoterFlanking Hoffman
Intron UCSC
H3K9ac Trynka
H3K4me3 Trynka
H3K4me1 Trynka
H3K27ac PGC2
H3K27ac Hnisz
FetalDHS Trynka
Enhancer Hoffman
Enhancer Andersson
DHS Trynka
DGF ENCODE
CTCF Hoffman
Conserved LindbladToh
Coding UCSC
−5000
−2500
0
2500
5000
Enrichment (prop h2 / prop snps)

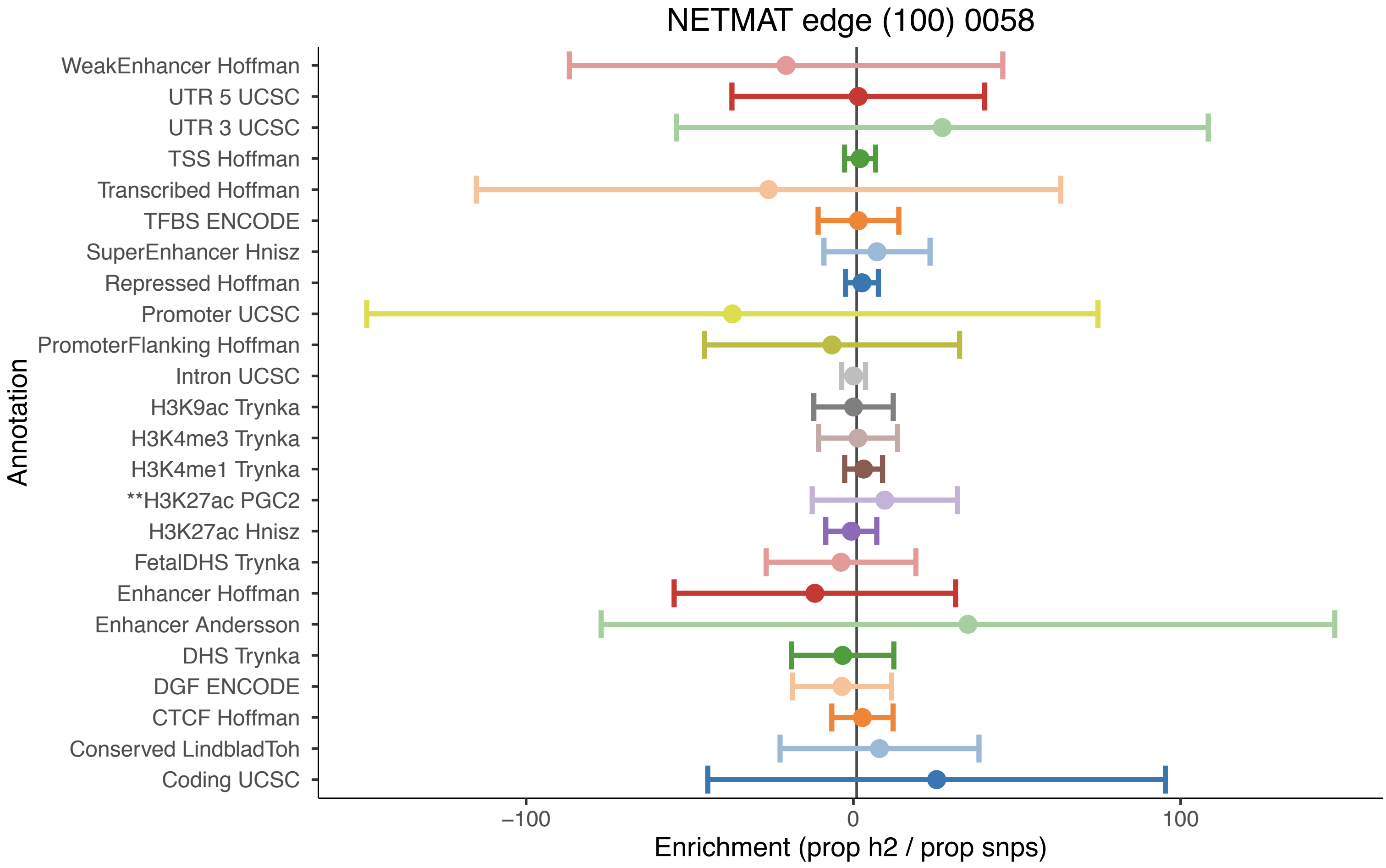

NETMAT edge (100) 0058
WeakEnhancer Hoffman
UTR 5 UCSC
UTR 3 UCSC
TSS Hoffman
Transcribed Hoffman
TFBS ENCODE
SuperEnhancer Hnisz
Repressed Hoffman
Promoter UCSC
PromoterFlanking Hoffman
Intron UCSC
H3K9ac Trynka
H3K4me3 Trynka
H3K4me1 Trynka
**H3K27ac PGC2
H3K27ac Hnisz
FetalDHS Trynka
Enhancer Hoffman
Enhancer Andersson
DHS Trynka
DGF ENCODE
CTCF Hoffman
Conserved LindbladToh
Coding UCSC
Annotation
−100
0
100
Enrichment (prop h2 / prop snps)

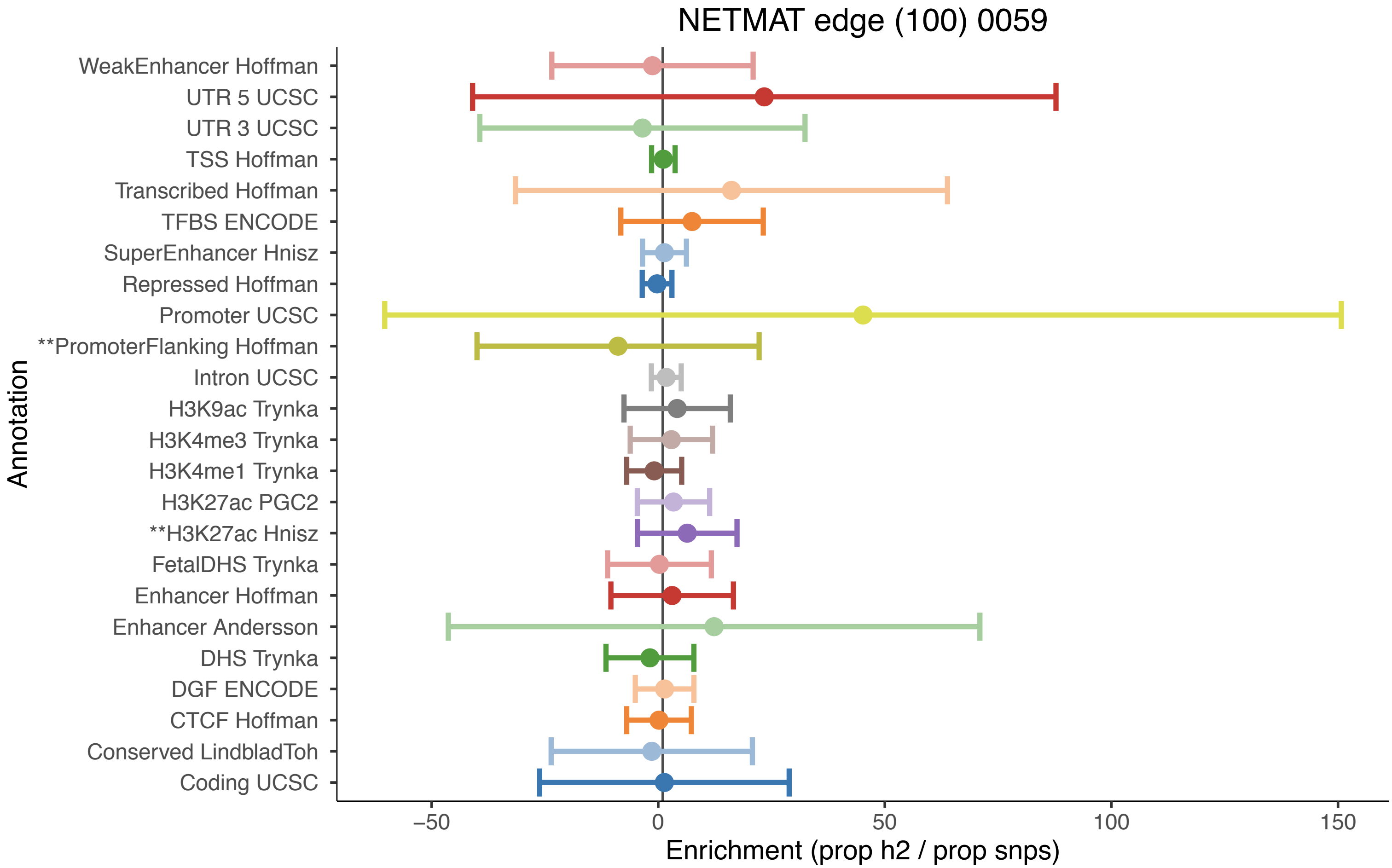

NETMAT edge (100) 0059
WeakEnhancer Hoffman
UTR 5 UCSC
UTR 3 UCSC
TSS Hoffman
Transcribed Hoffman
TFBS ENCODE
SuperEnhancer Hnisz
Repressed Hoffman
Promoter UCSC
**PromoterFlanking Hoffman
Intron UCSC
H3K9ac Trynka
H3K4me3 Trynka
H3K4me1 Trynka
H3K27ac PGC2
**H3K27ac Hnisz
FetalDHS Trynka
Enhancer Hoffman
Enhancer Andersson
DHS Trynka
DGF ENCODE
CTCF Hoffman
Conserved LindbladToh
Coding UCSC
Annotation
−50
0
50
100
150
Enrichment (prop h2 / prop snps)

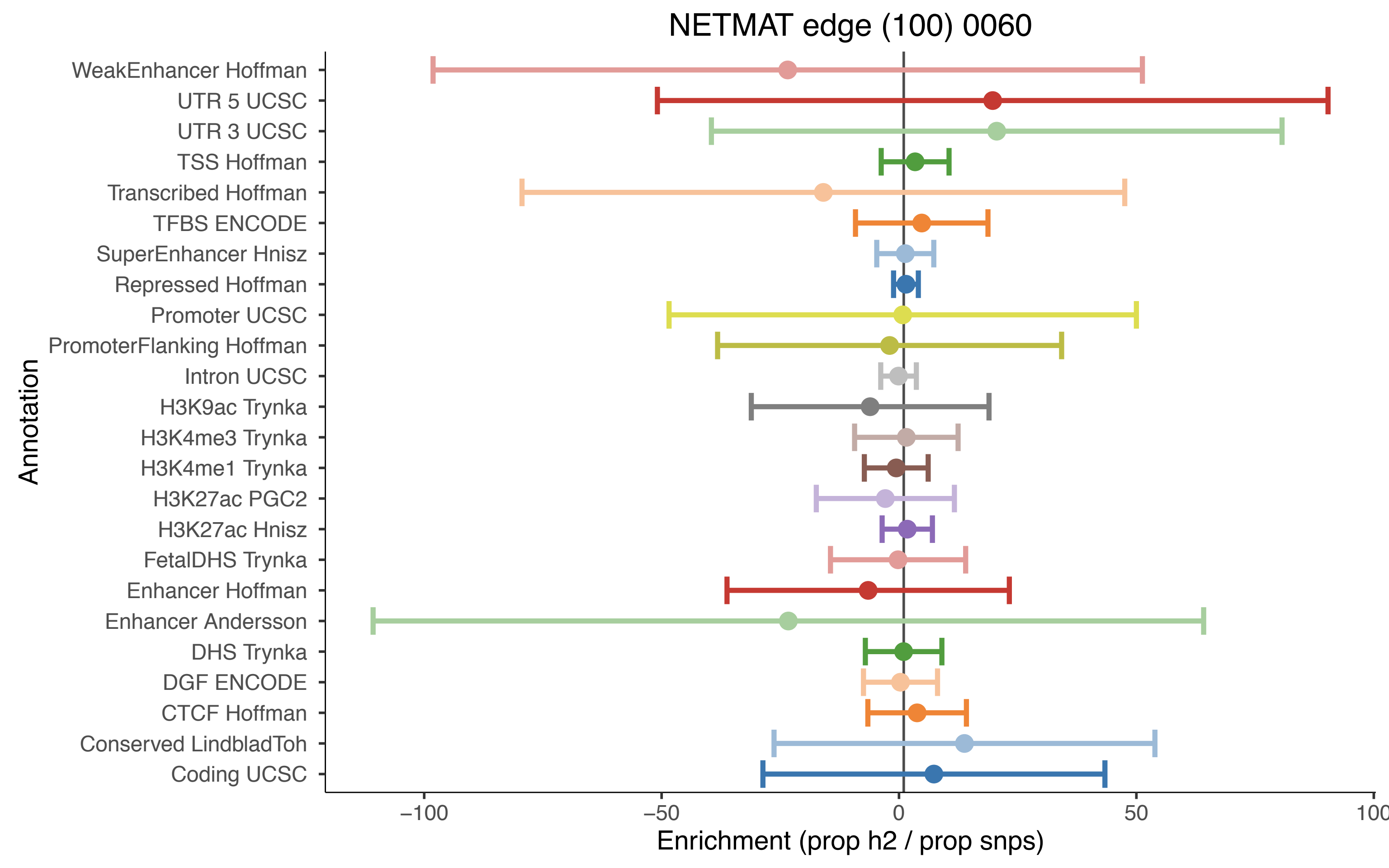

NETMAT edge (100) 0060
Annotation
WeakEnhancer Hoffman
UTR 5 UCSC
UTR 3 UCSC
TSS Hoffman
Transcribed Hoffman
TFBS ENCODE
SuperEnhancer Hnisz
Repressed Hoffman
Promoter UCSC
PromoterFlanking Hoffman
Intron UCSC
H3K9ac Trynka
H3K4me3 Trynka
H3K4me1 Trynka
H3K27ac PGC2
H3K27ac Hnisz
FetalDHS Trynka
Enhancer Hoffman
Enhancer Andersson
DHS Trynka
DGF ENCODE
CTCF Hoffman
Conserved LindbladToh
Coding UCSC
−100
−50
0
50
100
Enrichment (prop h2 / prop snps)

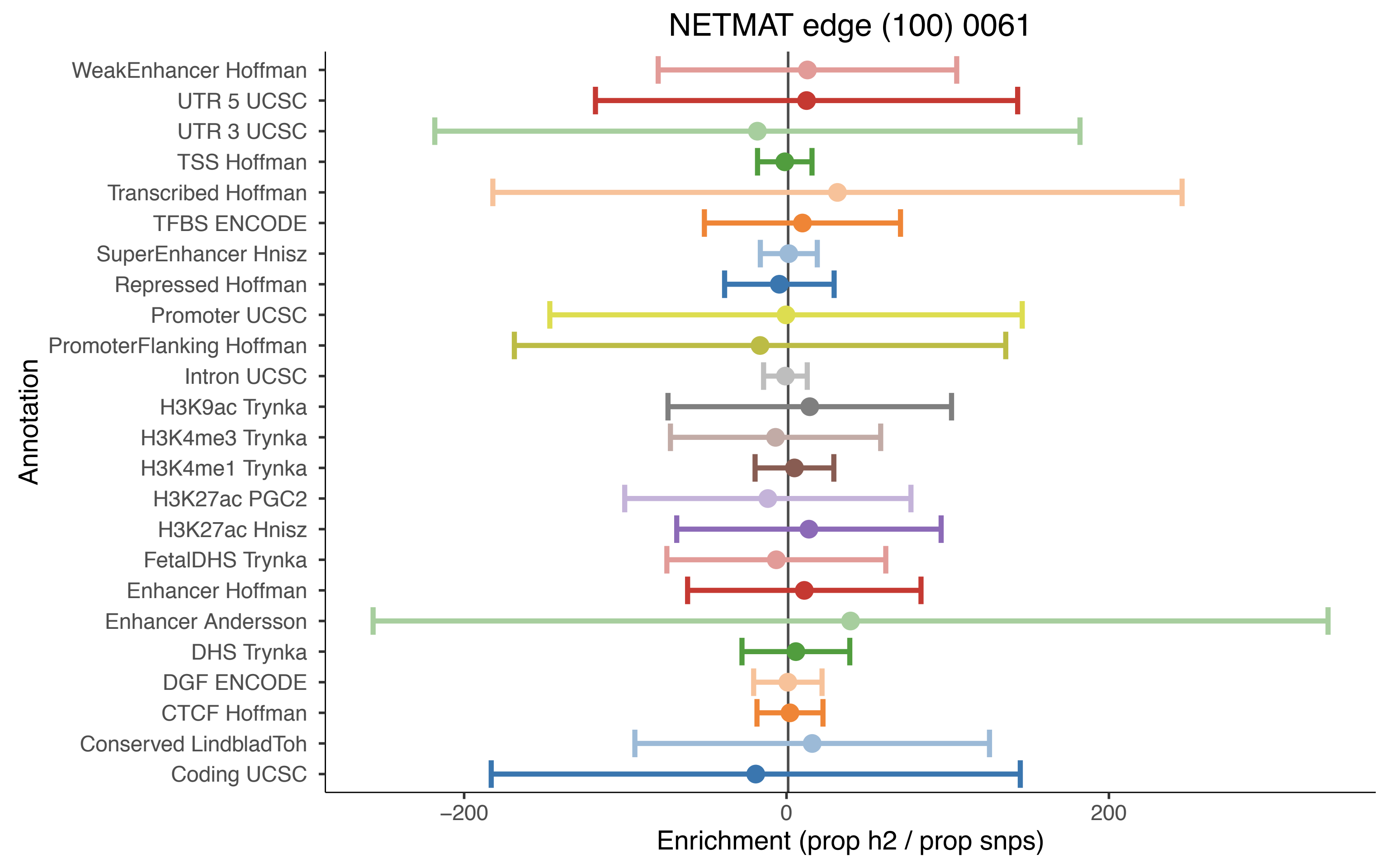

NETMAT edge (100) 0061
WeakEnhancer Hoffman
UTR 5 UCSC
UTR 3 UCSC
TSS Hoffman
Transcribed Hoffman
TFBS ENCODE
SuperEnhancer Hnisz
Repressed Hoffman
Promoter UCSC
PromoterFlanking Hoffman
Intron UCSC
H3K9ac Trynka
H3K4me3 Trynka
H3K4me1 Trynka
H3K27ac PGC2
H3K27ac Hnisz
FetalDHS Trynka
Enhancer Hoffman
Enhancer Andersson
DHS Trynka
DGF ENCODE
CTCF Hoffman
Conserved LindbladToh
Coding UCSC
Annotation
−200
0
200
Enrichment (prop h2 / prop snps)

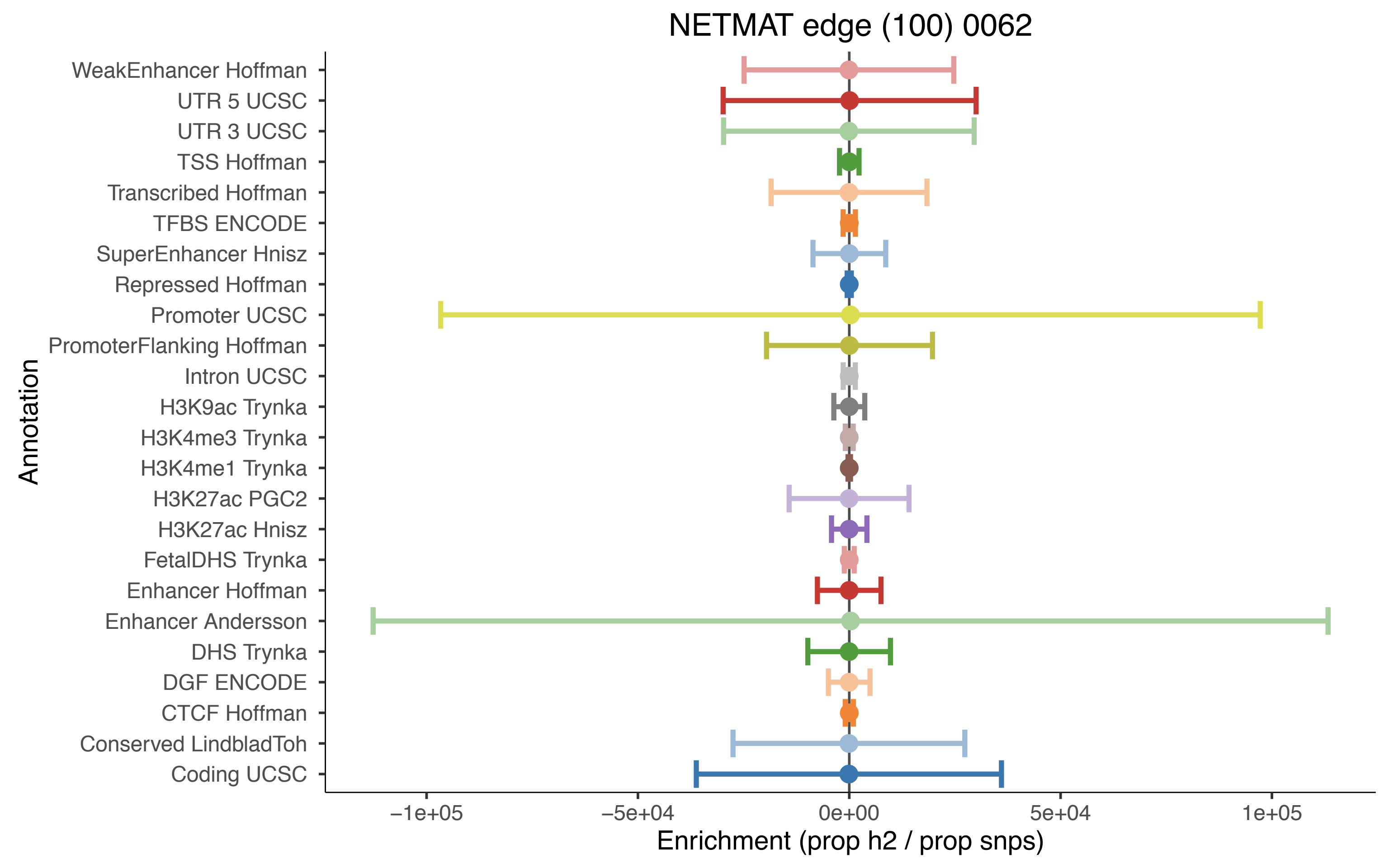
NETMAT edge (100) 0062
WeakEnhancer Hoffman
UTR 5 UCSC
UTR 3 UCSC
TSS Hoffman
Transcribed Hoffman
TFBS ENCODE
SuperEnhancer Hnisz
Repressed Hoffman
Promoter UCSC
PromoterFlanking Hoffman
Intron UCSC
H3K9ac Trynka
H3K4me3 Trynka
H3K4me1 Trynka
H3K27ac PGC2
H3K27ac Hnisz
FetalDHS Trynka
Enhancer Hoffman
Enhancer Andersson
DHS Trynka
DGF ENCODE
CTCF Hoffman
Conserved LindbladToh
Coding UCSC
Annotation
−1e+05
−5e+04
0e+00
5e+04
1e+05
Enrichment (prop h2 / prop snps)

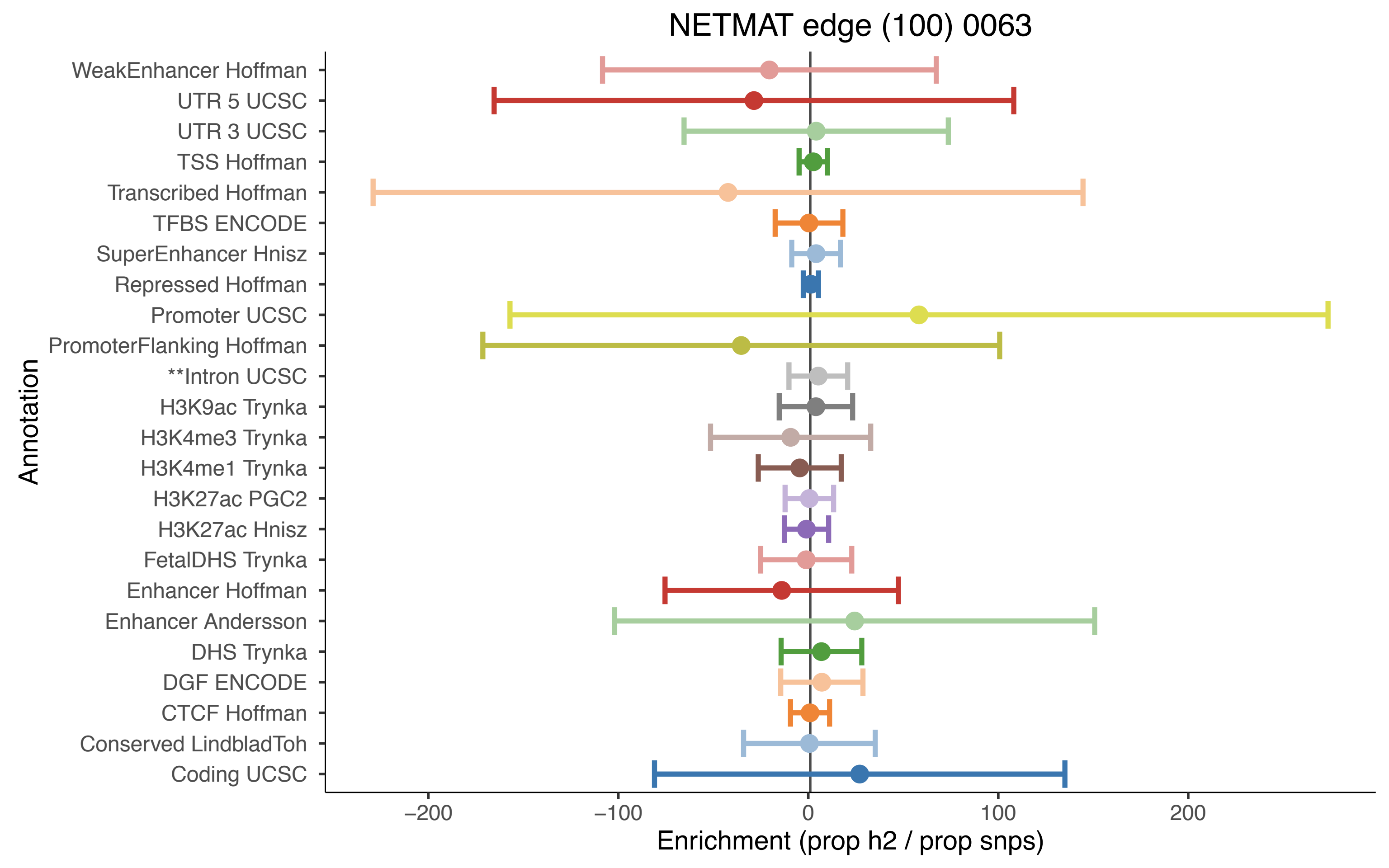
NETMAT edge (100) 0063
WeakEnhancer Hoffman
UTR 5 UCSC
UTR 3 UCSC
TSS Hoffman
Transcribed Hoffman
TFBS ENCODE
SuperEnhancer Hnisz
Repressed Hoffman
Promoter UCSC
PromoterFlanking Hoffman
**Intron UCSC
H3K9ac Trynka
H3K4me3 Trynka
H3K4me1 Trynka
H3K27ac PGC2
H3K27ac Hnisz
FetalDHS Trynka
Enhancer Hoffman
Enhancer Andersson
DHS Trynka
DGF ENCODE
CTCF Hoffman
Conserved LindbladToh
Coding UCSC
Annotation
−200
−100
0
100
200
Enrichment (prop h2 / prop snps)

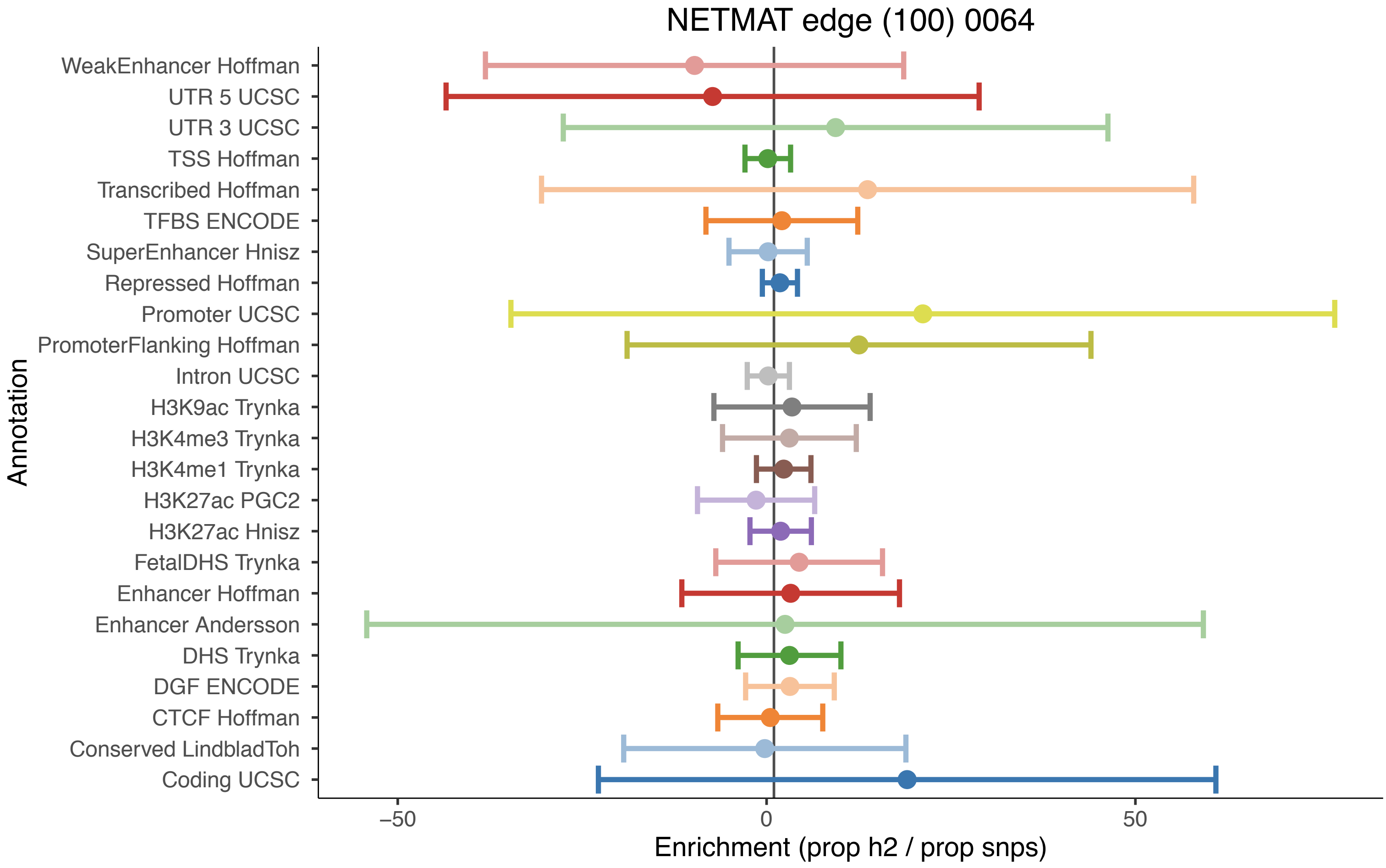
NETMAT edge (100) 0064
Annotation
WeakEnhancer Hoffman
UTR 5 UCSC
UTR 3 UCSC
TSS Hoffman
Transcribed Hoffman
TFBS ENCODE
SuperEnhancer Hnisz
Repressed Hoffman
Promoter UCSC
PromoterFlanking Hoffman
Intron UCSC
H3K9ac Trynka
H3K4me3 Trynka
H3K4me1 Trynka
H3K27ac PGC2
H3K27ac Hnisz
FetalDHS Trynka
Enhancer Hoffman
Enhancer Andersson
DHS Trynka
DGF ENCODE
CTCF Hoffman
Conserved LindbladToh
Coding UCSC
−50
0
50
Enrichment (prop h2 / prop snps)

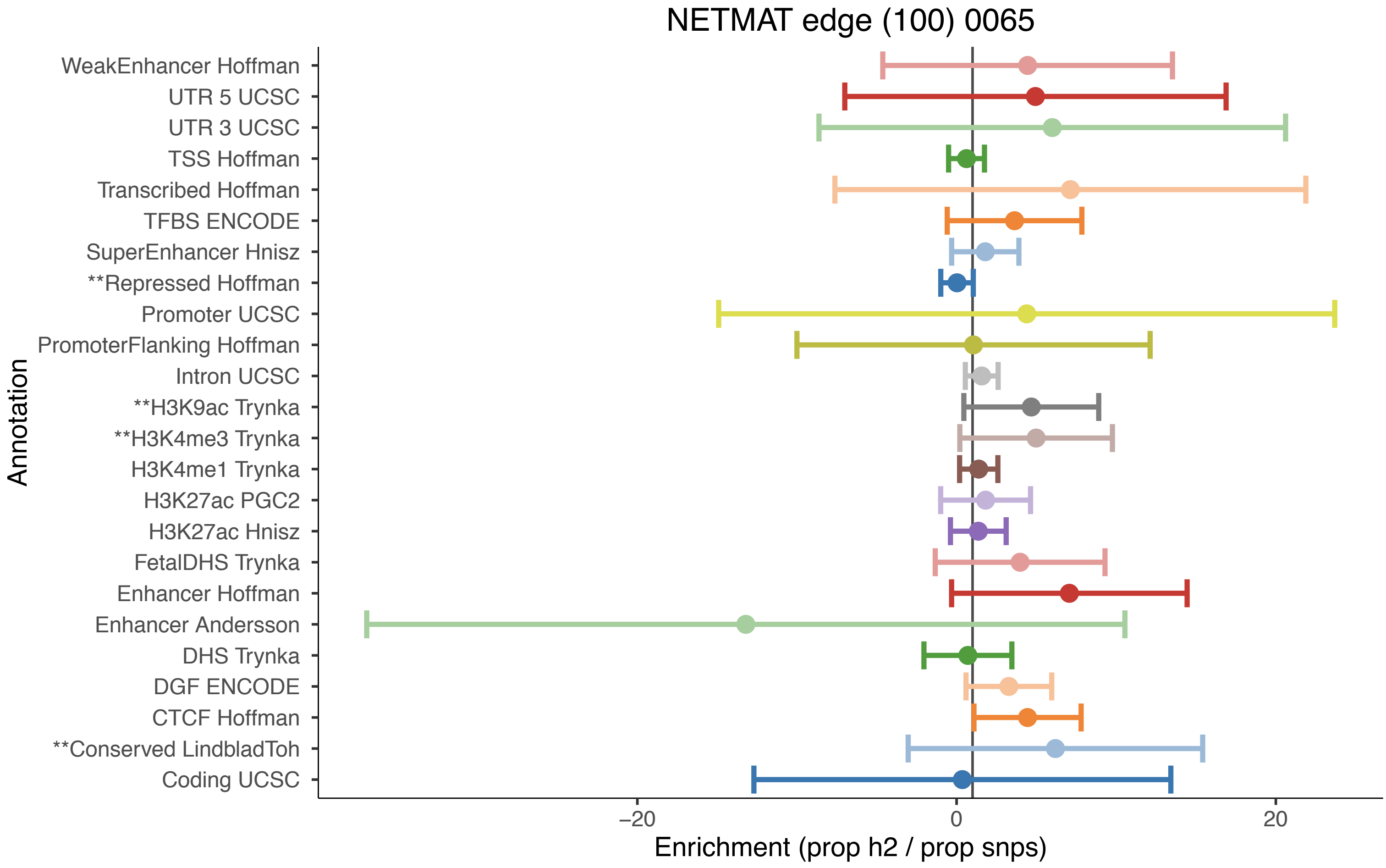

NETMAT edge (100) 0065
Annotation
WeakEnhancer Hoffman
UTR 5 UCSC
UTR 3 UCSC
TSS Hoffman
Transcribed Hoffman
TFBS ENCODE
SuperEnhancer Hnisz
**Repressed Hoffman
Promoter UCSC
PromoterFlanking Hoffman
Intron UCSC
**H3K9ac Trynka
**H3K4me3 Trynka
H3K4me1 Trynka
H3K27ac PGC2
H3K27ac Hnisz
FetalDHS Trynka
Enhancer Hoffman
Enhancer Andersson
DHS Trynka
DGF ENCODE
CTCF Hoffman
**Conserved LindbladToh
Coding UCSC
−20
0
20
Enrichment (prop h2 / prop snps)

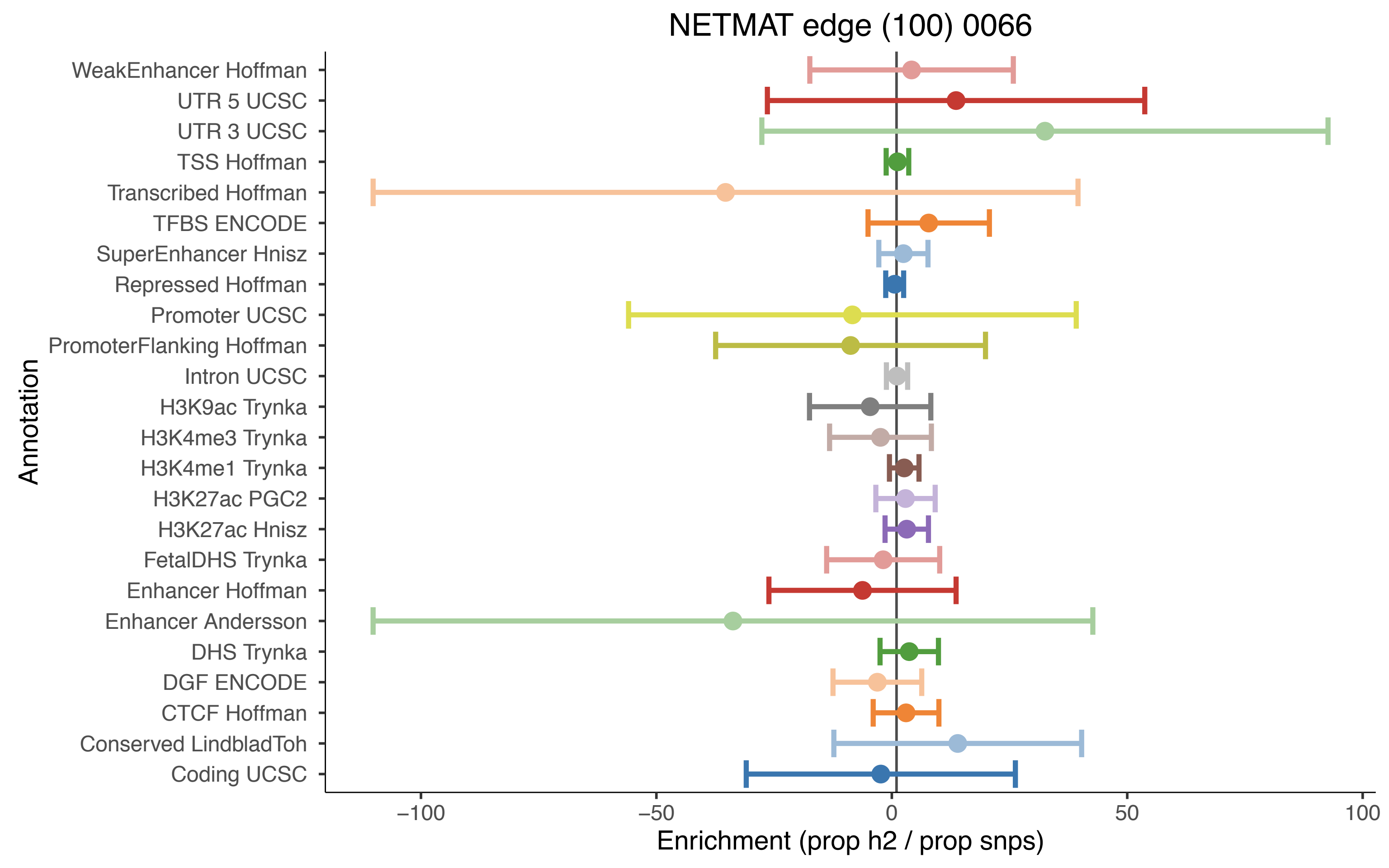

NETMAT edge (100) 0066
Annotation
WeakEnhancer Hoffman
UTR 5 UCSC
UTR 3 UCSC
TSS Hoffman
Transcribed Hoffman
TFBS ENCODE
SuperEnhancer Hnisz
Repressed Hoffman
Promoter UCSC
PromoterFlanking Hoffman
Intron UCSC
H3K9ac Trynka
H3K4me3 Trynka
H3K4me1 Trynka
H3K27ac PGC2
H3K27ac Hnisz
FetalDHS Trynka
Enhancer Hoffman
Enhancer Andersson
DHS Trynka
DGF ENCODE
CTCF Hoffman
Conserved LindbladToh
Coding UCSC
−100
−50
0
50
100
Enrichment (prop h2 / prop snps)

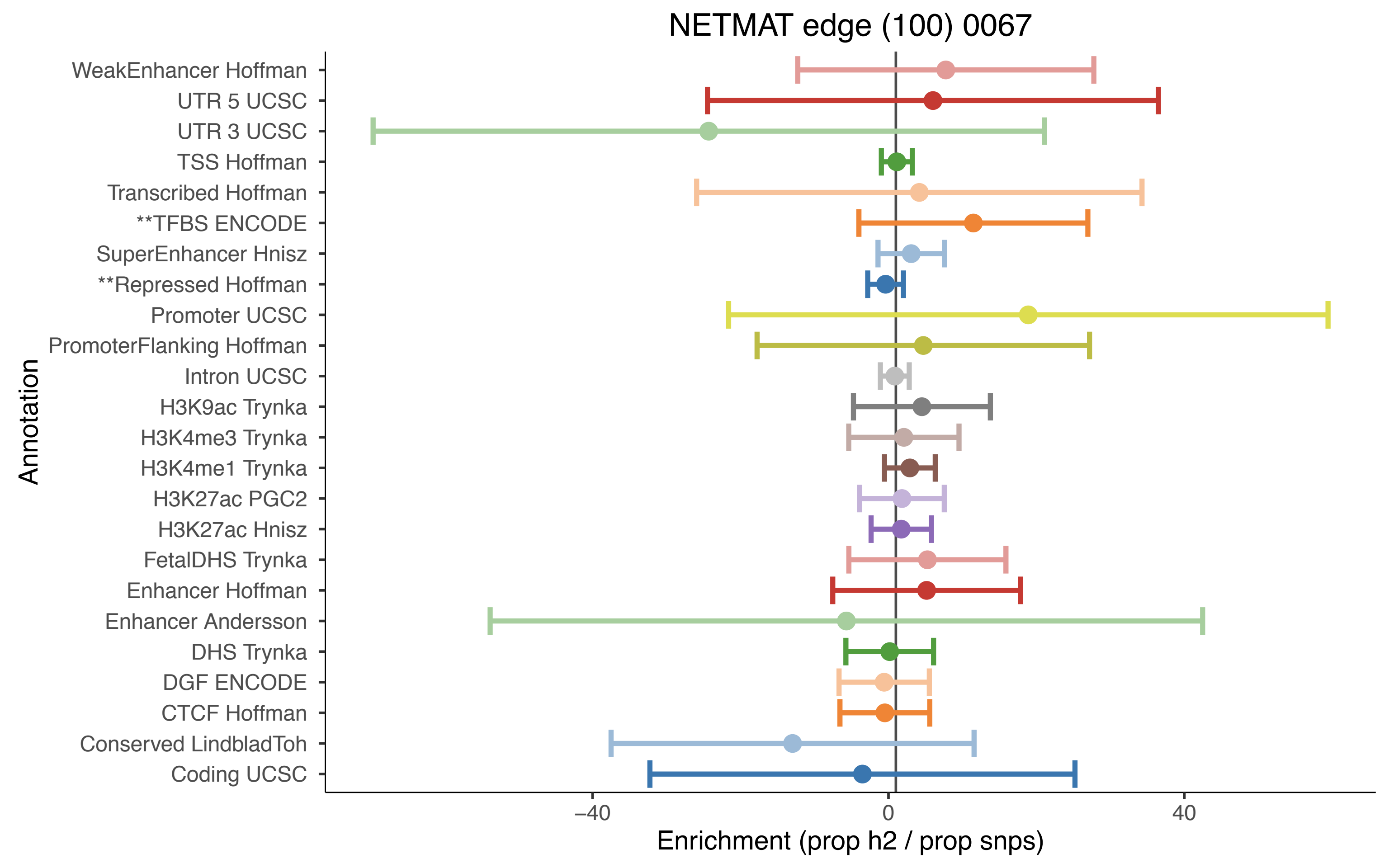
NETMAT edge (100) 0067
Annotation
WeakEnhancer Hoffman
UTR 5 UCSC
UTR 3 UCSC
TSS Hoffman
Transcribed Hoffman
**TFBS ENCODE
SuperEnhancer Hnisz
**Repressed Hoffman
Promoter UCSC
PromoterFlanking Hoffman
Intron UCSC
H3K9ac Trynka
H3K4me3 Trynka
H3K4me1 Trynka
H3K27ac PGC2
H3K27ac Hnisz
FetalDHS Trynka
Enhancer Hoffman
Enhancer Andersson
DHS Trynka
DGF ENCODE
CTCF Hoffman
Conserved LindbladToh
Coding UCSC
−40
0
40
Enrichment (prop h2 / prop snps)

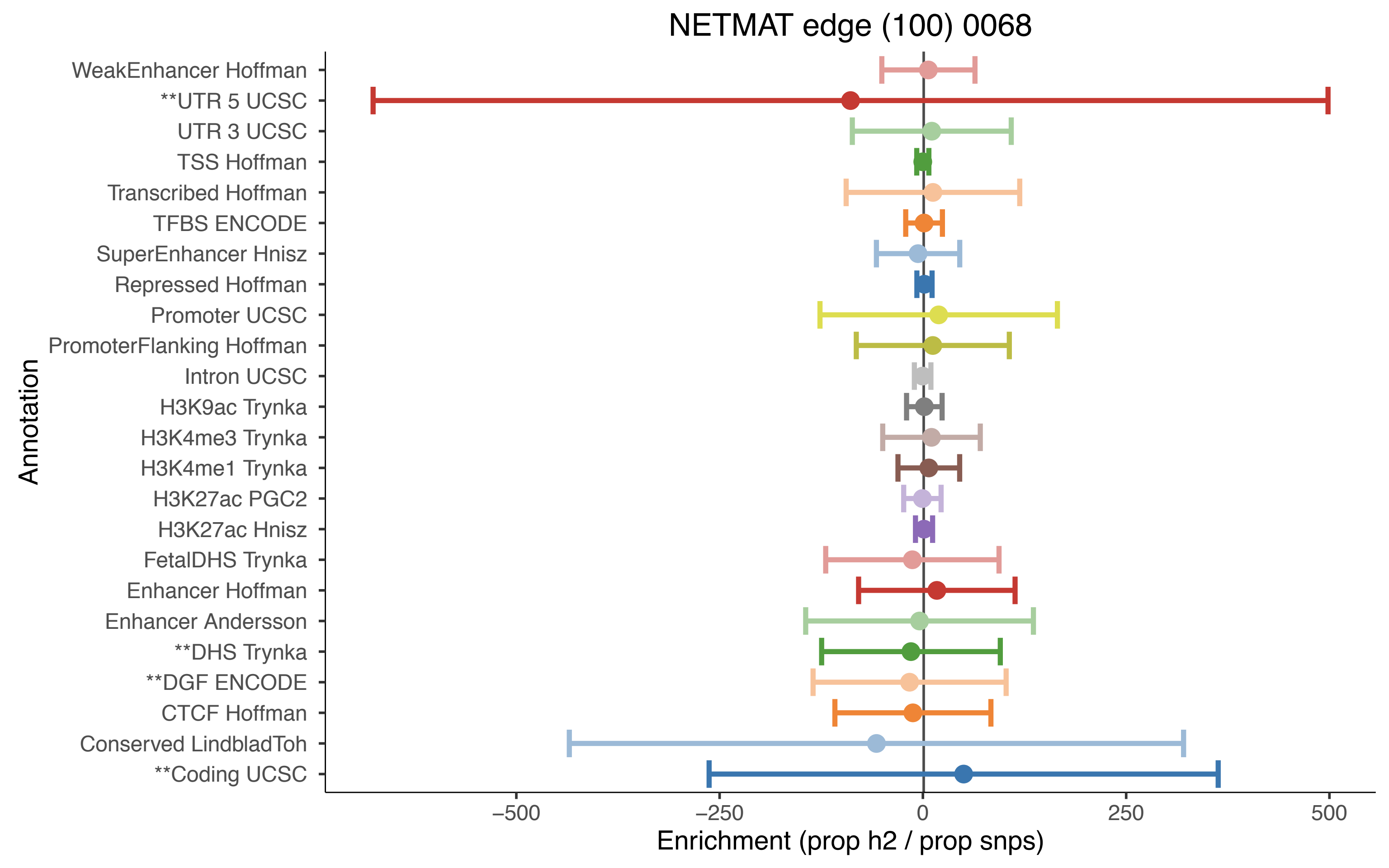
NETMAT edge (100) 0068
WeakEnhancer Hoffman
**UTR 5 UCSC
UTR 3 UCSC
TSS Hoffman
Transcribed Hoffman
TFBS ENCODE
SuperEnhancer Hnisz
Repressed Hoffman
Promoter UCSC
PromoterFlanking Hoffman
Intron UCSC
H3K9ac Trynka
H3K4me3 Trynka
H3K4me1 Trynka
H3K27ac PGC2
H3K27ac Hnisz
FetalDHS Trynka
Enhancer Hoffman
Enhancer Andersson
**DHS Trynka
**DGF ENCODE
CTCF Hoffman
Conserved LindbladToh
**Coding UCSC
Annotation
−500
−250
0
250
500
Enrichment (prop h2 / prop snps)

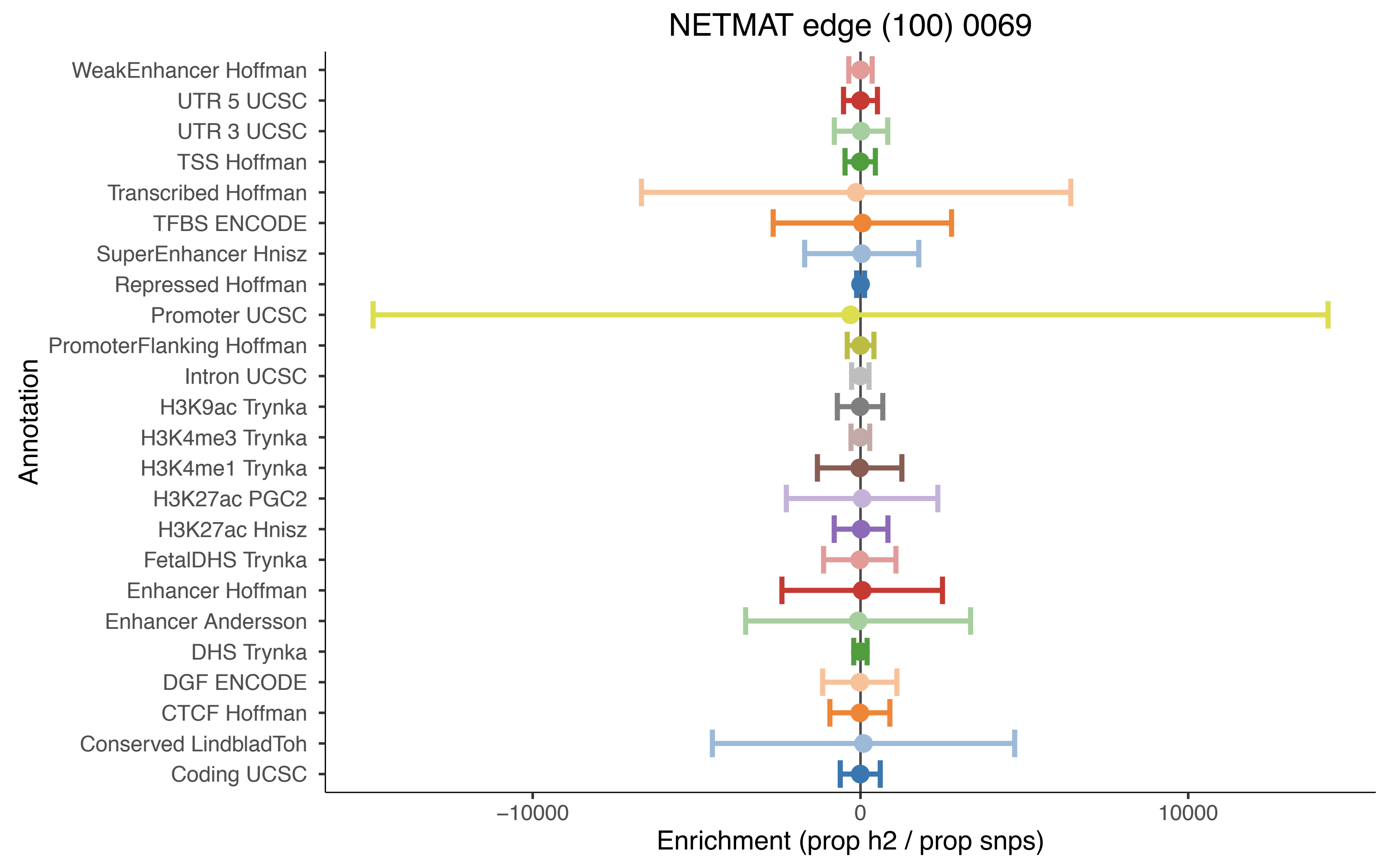
NETMAT edge (100) 0069
WeakEnhancer Hoffman
UTR 5 UCSC
UTR 3 UCSC
TSS Hoffman
Transcribed Hoffman
TFBS ENCODE
SuperEnhancer Hnisz
Repressed Hoffman
Promoter UCSC
PromoterFlanking Hoffman
Intron UCSC
H3K9ac Trynka
H3K4me3 Trynka
H3K4me1 Trynka
H3K27ac PGC2
H3K27ac Hnisz
FetalDHS Trynka
Enhancer Hoffman
Enhancer Andersson
DHS Trynka
DGF ENCODE
CTCF Hoffman
Conserved LindbladToh
Coding UCSC
Annotation
−10000
0
10000
Enrichment (prop h2 / prop snps)

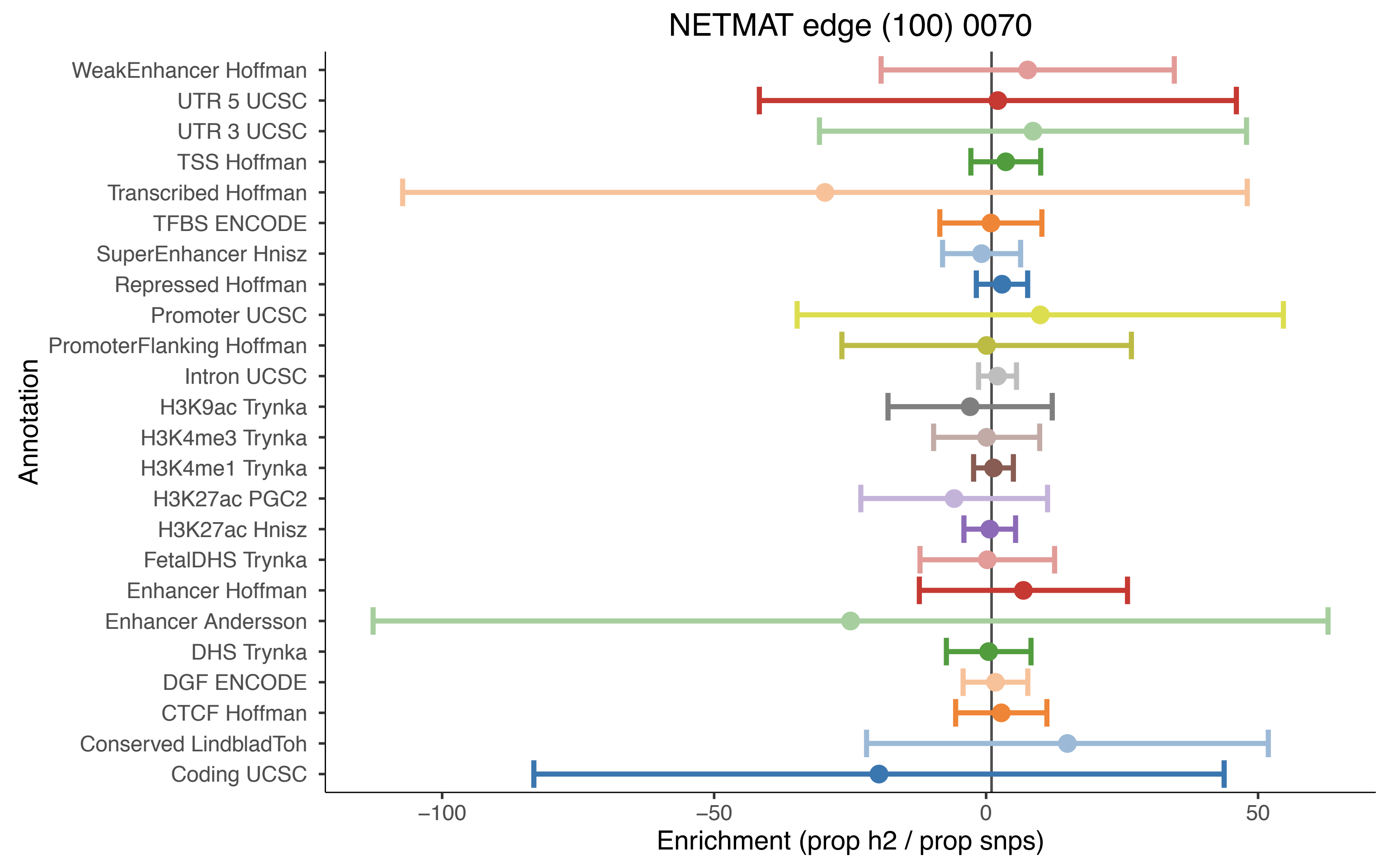

NETMAT edge (100) 0070
Annotation
WeakEnhancer Hoffman
UTR 5 UCSC
UTR 3 UCSC
TSS Hoffman
Transcribed Hoffman
TFBS ENCODE
SuperEnhancer Hnisz
Repressed Hoffman
Promoter UCSC
PromoterFlanking Hoffman
Intron UCSC
H3K9ac Trynka
H3K4me3 Trynka
H3K4me1 Trynka
H3K27ac PGC2
H3K27ac Hnisz
FetalDHS Trynka
Enhancer Hoffman
Enhancer Andersson
DHS Trynka
DGF ENCODE
CTCF Hoffman
Conserved LindbladToh
Coding UCSC
−100
−50
0
50
Enrichment (prop h2 / prop snps)

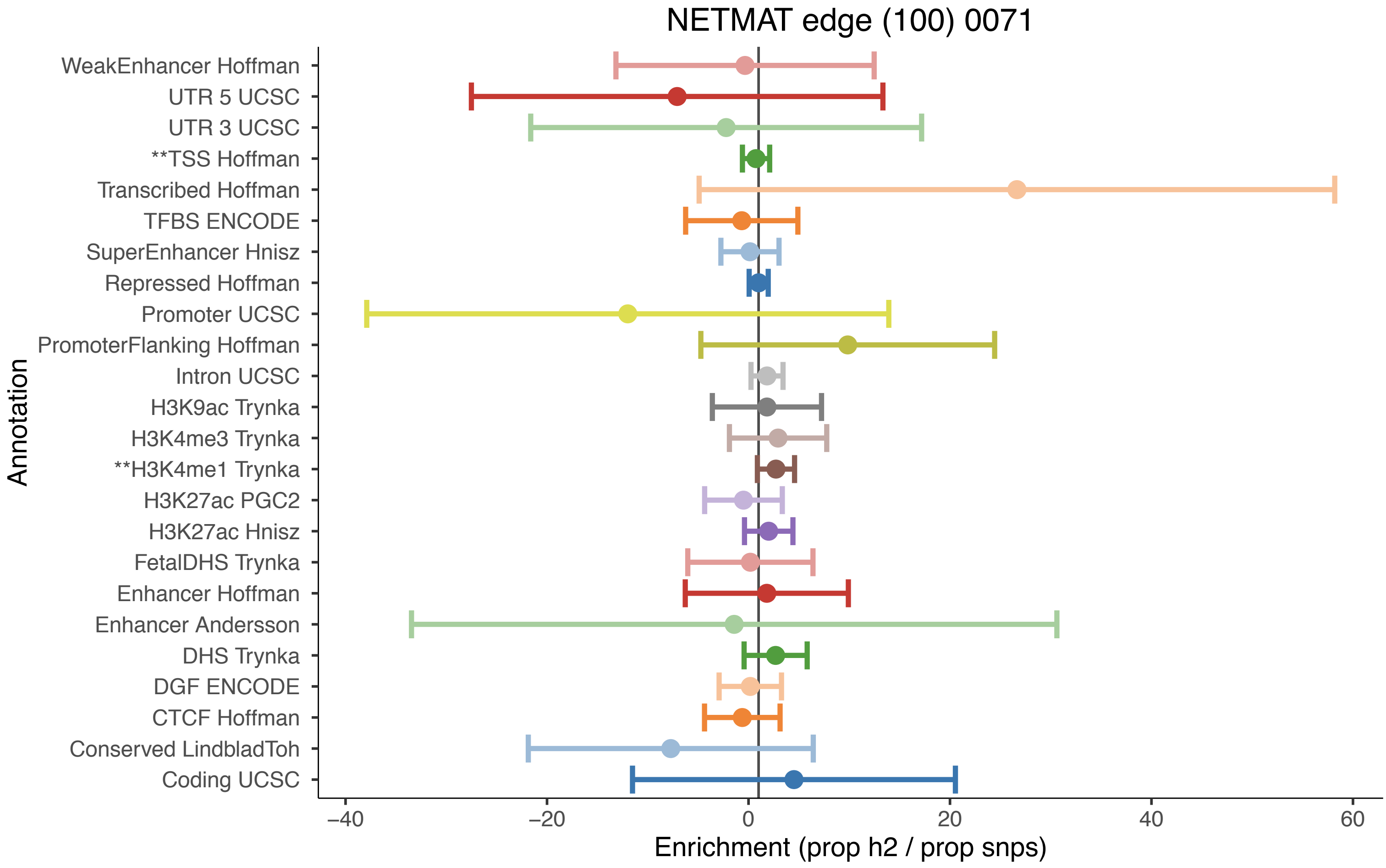
NETMAT edge (100) 0071
WeakEnhancer Hoffman
UTR 5 UCSC
UTR 3 UCSC
**TSS Hoffman
Transcribed Hoffman
TFBS ENCODE
SuperEnhancer Hnisz
Repressed Hoffman
Promoter UCSC
PromoterFlanking Hoffman
Intron UCSC
H3K9ac Trynka
H3K4me3 Trynka
**H3K4me1 Trynka
H3K27ac PGC2
H3K27ac Hnisz
FetalDHS Trynka
Enhancer Hoffman
Enhancer Andersson
DHS Trynka
DGF ENCODE
CTCF Hoffman
Conserved LindbladToh
Coding UCSC
Annotation
−40
−20
0
20
40
60
Enrichment (prop h2 / prop snps)

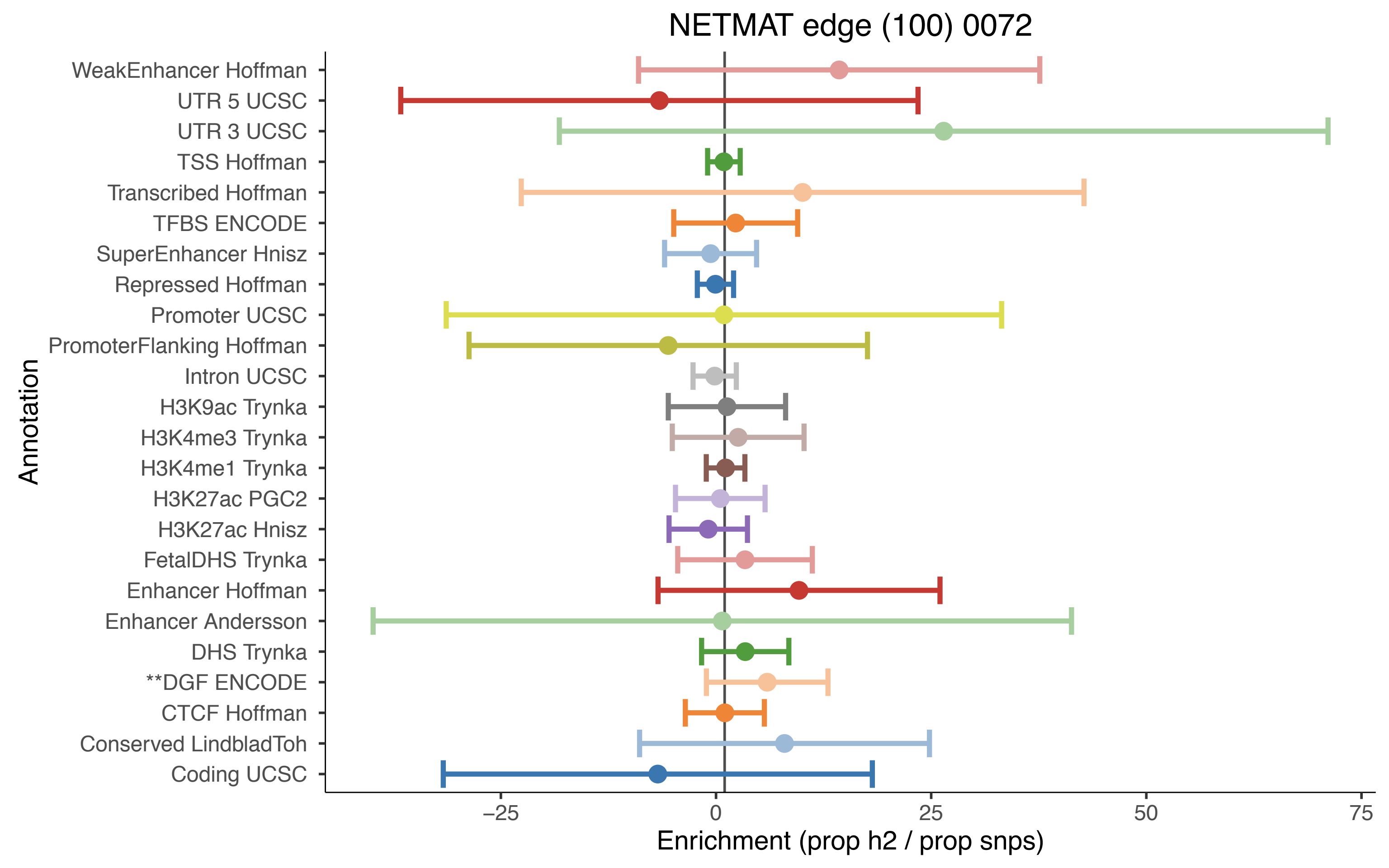
NETMAT edge (100) 0072
Annotation
WeakEnhancer Hoffman
UTR 5 UCSC
UTR 3 UCSC
TSS Hoffman
Transcribed Hoffman
TFBS ENCODE
SuperEnhancer Hnisz
Repressed Hoffman
Promoter UCSC
PromoterFlanking Hoffman
Intron UCSC
H3K9ac Trynka
H3K4me3 Trynka
H3K4me1 Trynka
H3K27ac PGC2
H3K27ac Hnisz
FetalDHS Trynka
Enhancer Hoffman
Enhancer Andersson
DHS Trynka
**DGF ENCODE
CTCF Hoffman
Conserved LindbladToh
Coding UCSC
−25
0
25
50
75
Enrichment (prop h2 / prop snps)

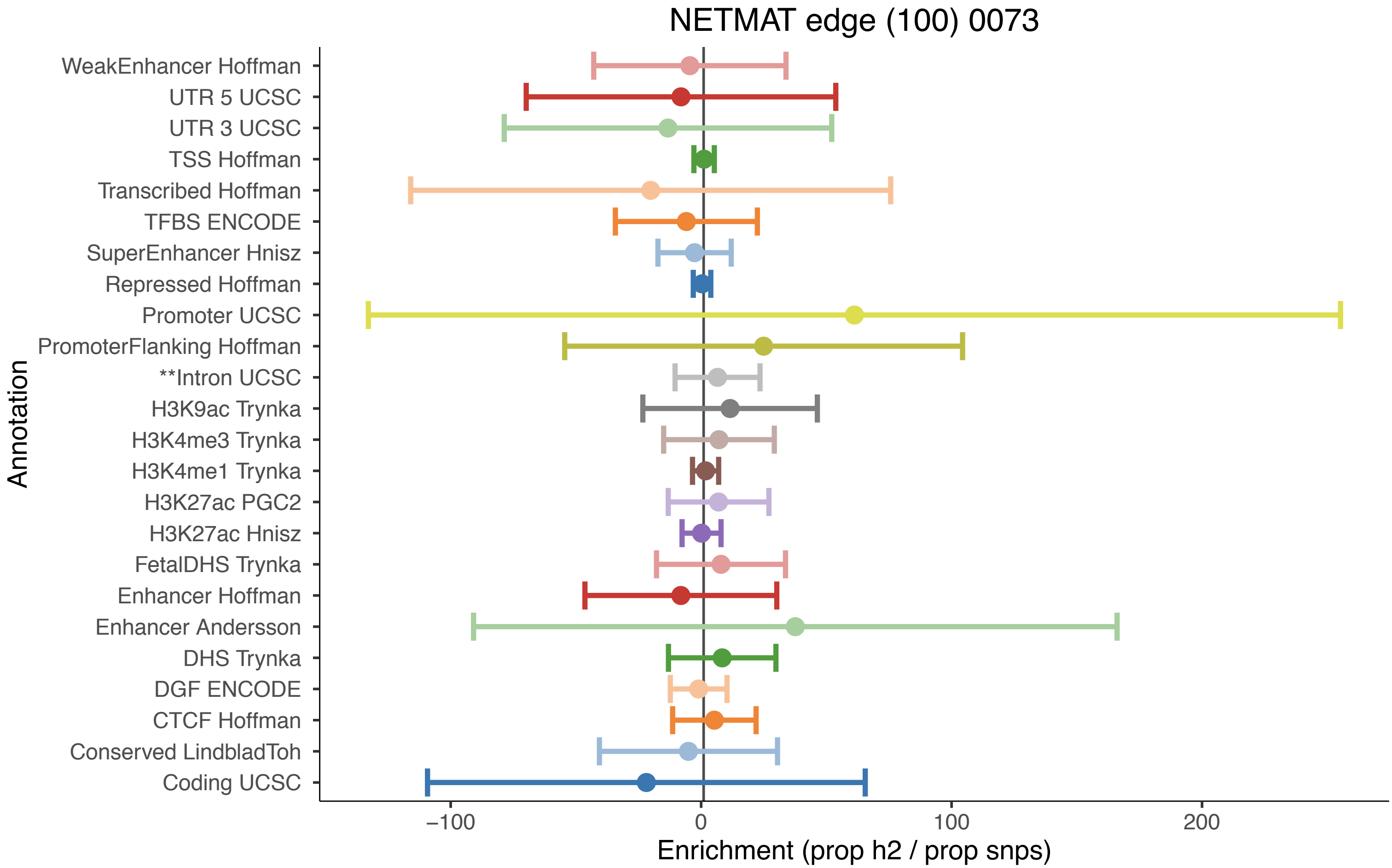

NETMAT edge (100) 0073
WeakEnhancer Hoffman
UTR 5 UCSC
UTR 3 UCSC
TSS Hoffman
Transcribed Hoffman
TFBS ENCODE
SuperEnhancer Hnisz
Repressed Hoffman
Promoter UCSC
PromoterFlanking Hoffman
**Intron UCSC
H3K9ac Trynka
H3K4me3 Trynka
H3K4me1 Trynka
H3K27ac PGC2
H3K27ac Hnisz
FetalDHS Trynka
Enhancer Hoffman
Enhancer Andersson
DHS Trynka
DGF ENCODE
CTCF Hoffman
Conserved LindbladToh
Coding UCSC
Annotation
−100
0
100
200
Enrichment (prop h2 / prop snps)

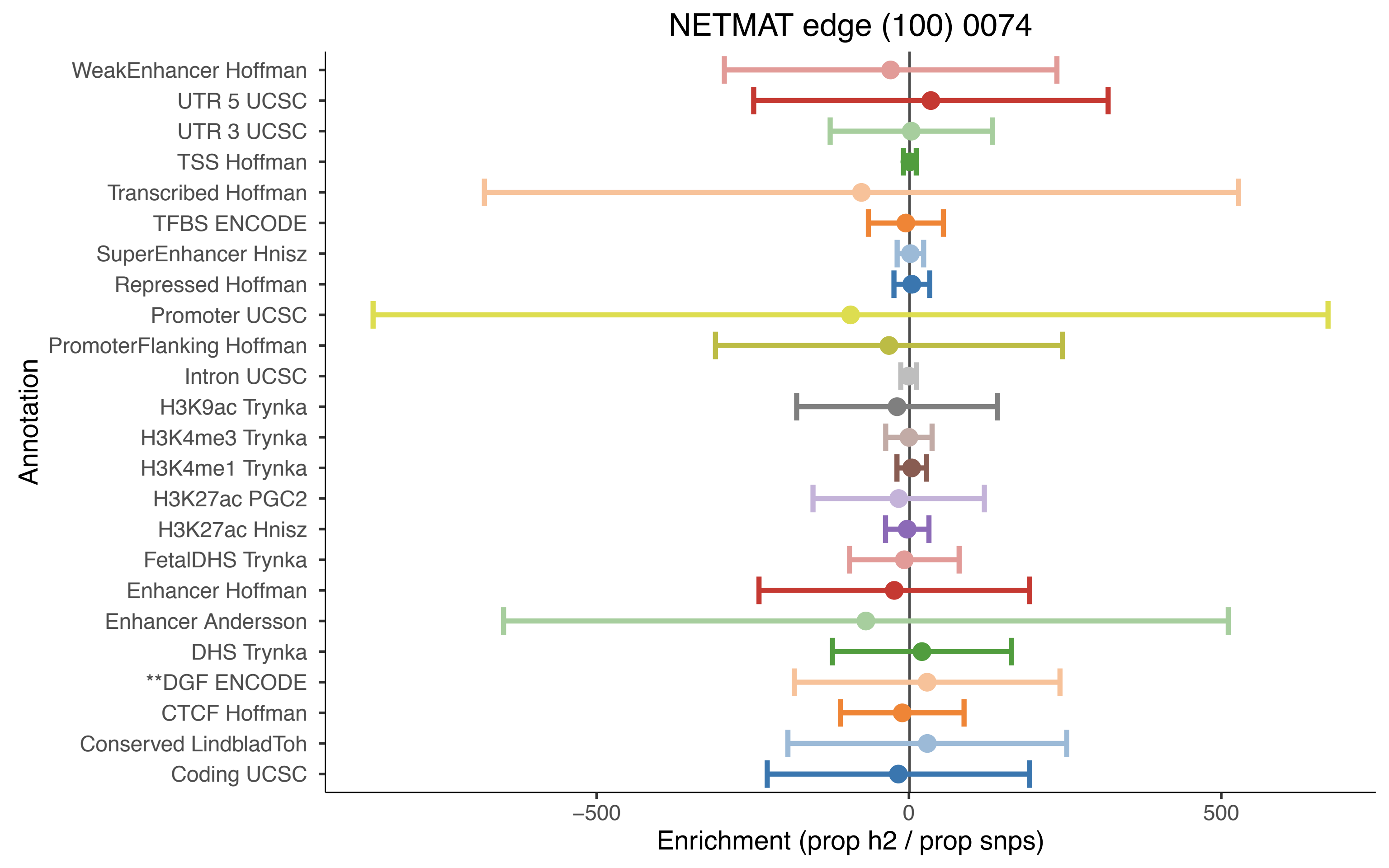
NETMAT edge (100) 0074
WeakEnhancer Hoffman
UTR 5 UCSC
UTR 3 UCSC
TSS Hoffman
Transcribed Hoffman
TFBS ENCODE
SuperEnhancer Hnisz
Repressed Hoffman
Promoter UCSC
PromoterFlanking Hoffman
Intron UCSC
H3K9ac Trynka
H3K4me3 Trynka
H3K4me1 Trynka
H3K27ac PGC2
H3K27ac Hnisz
FetalDHS Trynka
Enhancer Hoffman
Enhancer Andersson
DHS Trynka
**DGF ENCODE
CTCF Hoffman
Conserved LindbladToh
Coding UCSC
Annotation
−500
0
500
Enrichment (prop h2 / prop snps)

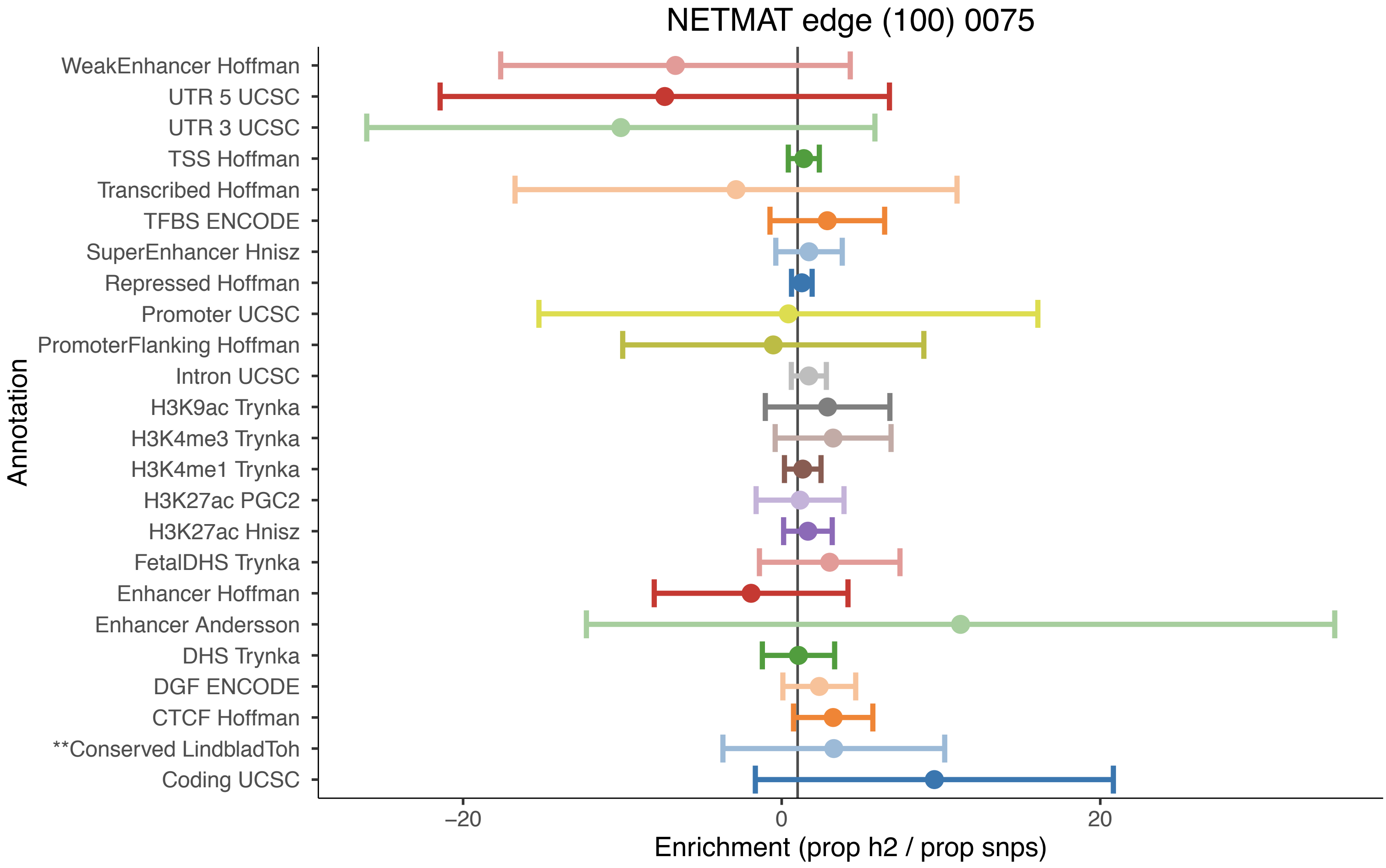
NETMAT edge (100) 0075
WeakEnhancer Hoffman
UTR 5 UCSC
UTR 3 UCSC
TSS Hoffman
Transcribed Hoffman
TFBS ENCODE
SuperEnhancer Hnisz
Repressed Hoffman
Promoter UCSC
PromoterFlanking Hoffman
Intron UCSC
H3K9ac Trynka
H3K4me3 Trynka
H3K4me1 Trynka
H3K27ac PGC2
H3K27ac Hnisz
FetalDHS Trynka
Enhancer Hoffman
Enhancer Andersson
DHS Trynka
DGF ENCODE
CTCF Hoffman
**Conserved LindbladToh
Coding UCSC
Annotation
−20
0
20
Enrichment (prop h2 / prop snps)

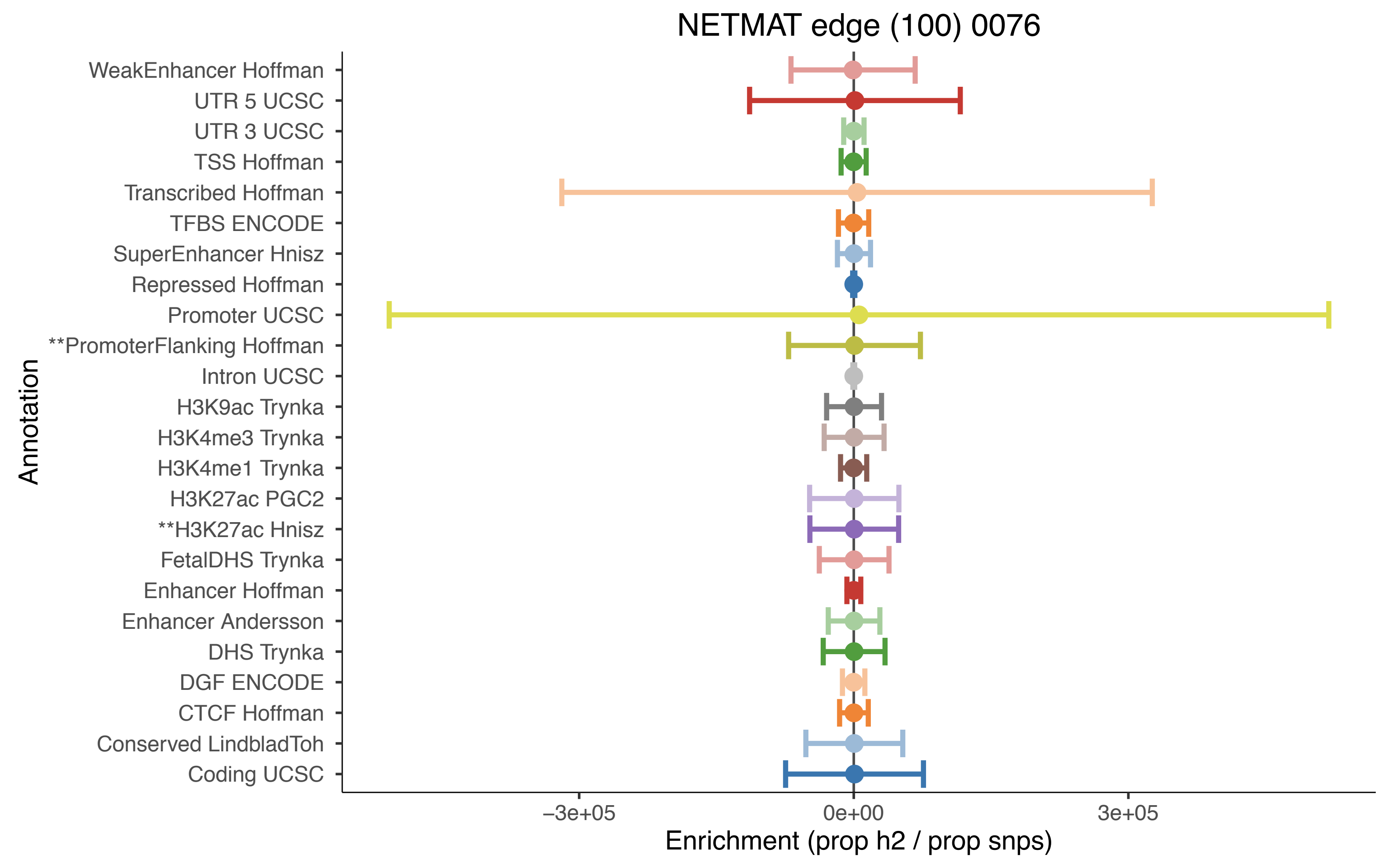

NETMAT edge (100) 0076
WeakEnhancer Hoffman
UTR 5 UCSC
UTR 3 UCSC
TSS Hoffman
Transcribed Hoffman
TFBS ENCODE
SuperEnhancer Hnisz
Repressed Hoffman
Promoter UCSC
**PromoterFlanking Hoffman
Intron UCSC
H3K9ac Trynka
H3K4me3 Trynka
H3K4me1 Trynka
H3K27ac PGC2
**H3K27ac Hnisz
FetalDHS Trynka
Enhancer Hoffman
Enhancer Andersson
DHS Trynka
DGF ENCODE
CTCF Hoffman
Conserved LindbladToh
Coding UCSC
Annotation
−3e+05
0e+00
3e+05
Enrichment (prop h2 / prop snps)

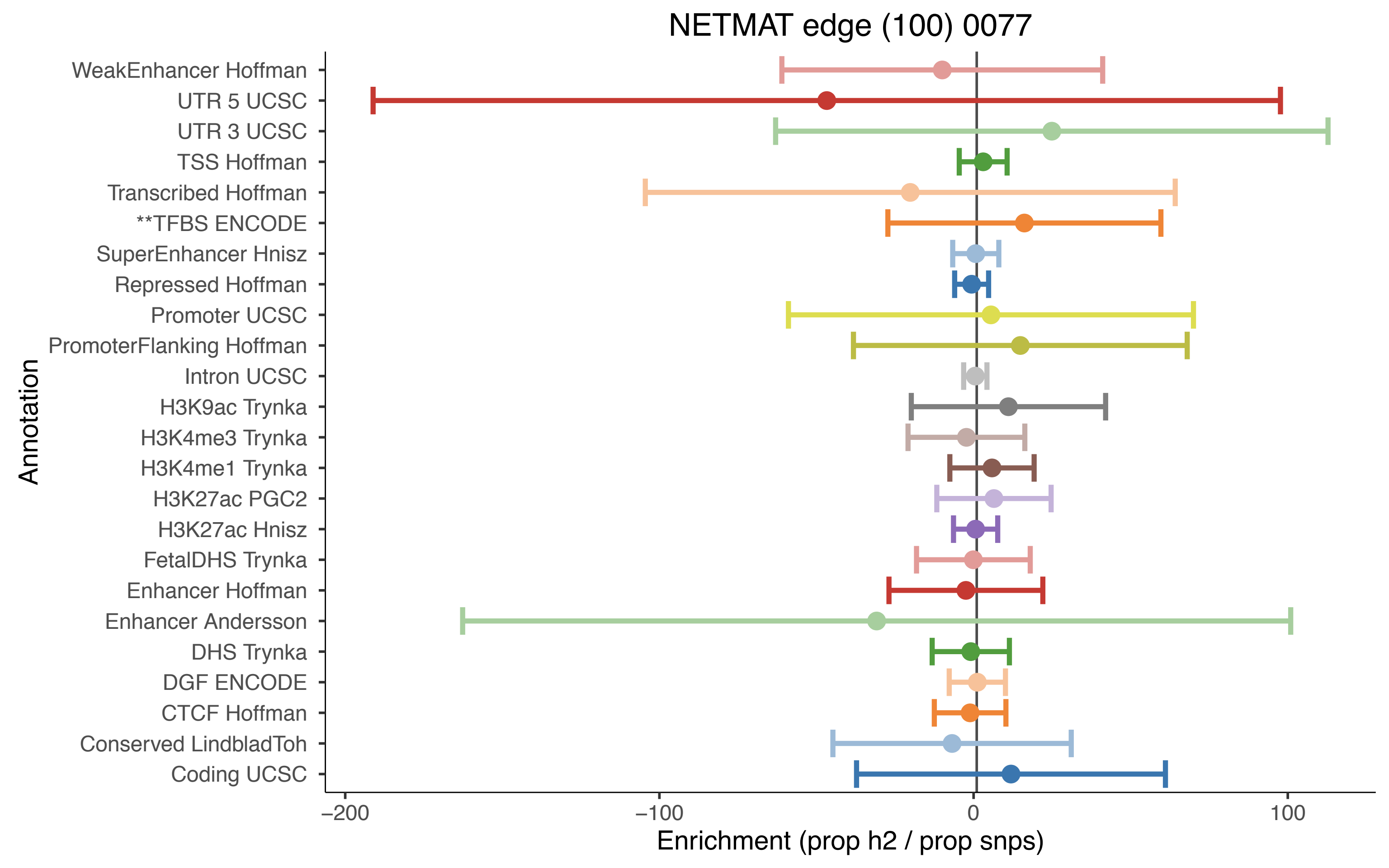

NETMAT edge (100) 0077
WeakEnhancer Hoffman
UTR 5 UCSC
UTR 3 UCSC
TSS Hoffman
Transcribed Hoffman
**TFBS ENCODE
SuperEnhancer Hnisz
Repressed Hoffman
Promoter UCSC
PromoterFlanking Hoffman
Intron UCSC
H3K9ac Trynka
H3K4me3 Trynka
H3K4me1 Trynka
H3K27ac PGC2
H3K27ac Hnisz
FetalDHS Trynka
Enhancer Hoffman
Enhancer Andersson
DHS Trynka
DGF ENCODE
CTCF Hoffman
Conserved LindbladToh
Coding UCSC
Annotation
−200
−100
0
100
Enrichment (prop h2 / prop snps)

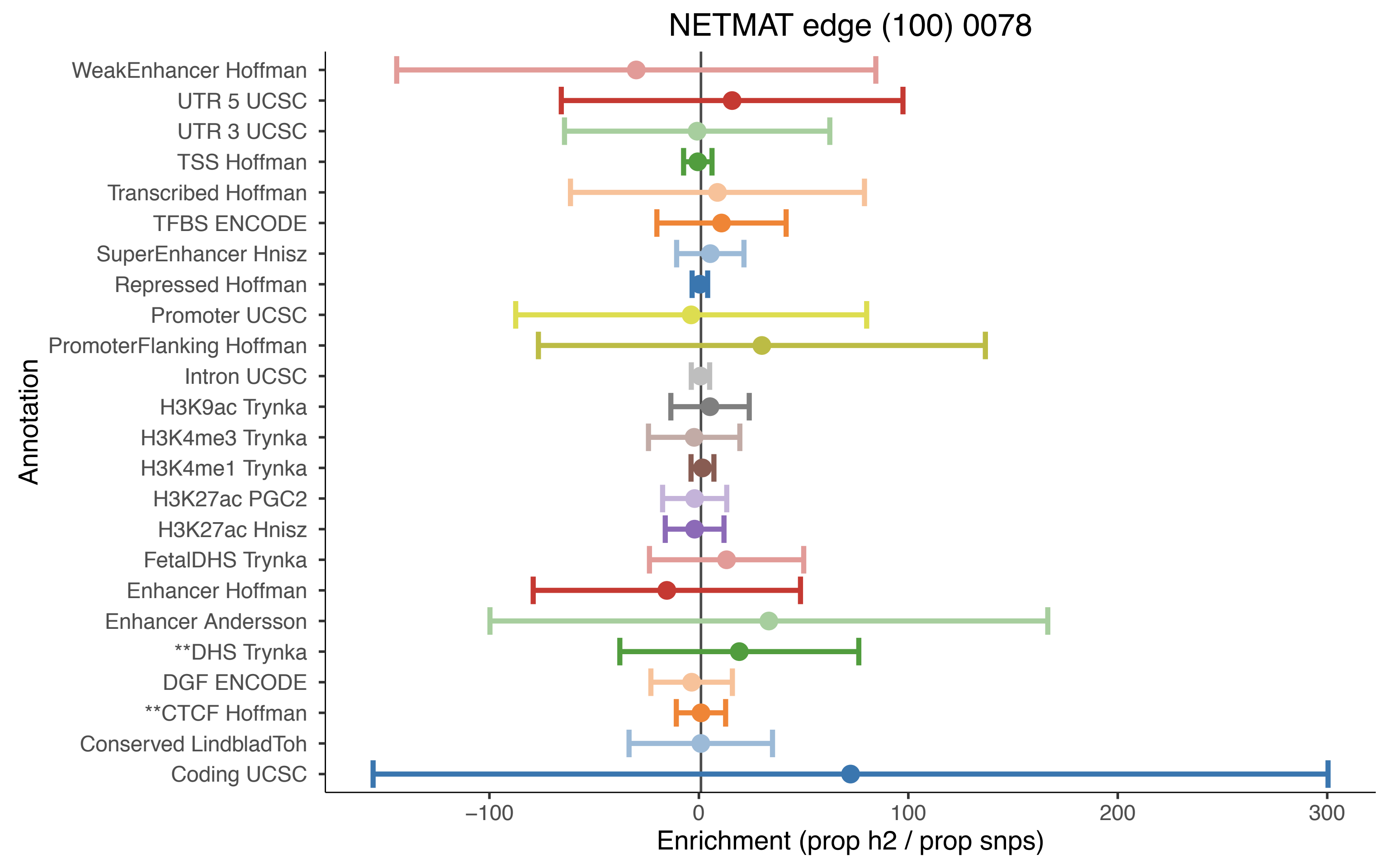
NETMAT edge (100) 0078
WeakEnhancer Hoffman
UTR 5 UCSC
UTR 3 UCSC
TSS Hoffman
Transcribed Hoffman
TFBS ENCODE
SuperEnhancer Hnisz
Repressed Hoffman
Promoter UCSC
PromoterFlanking Hoffman
Intron UCSC
H3K9ac Trynka
H3K4me3 Trynka
H3K4me1 Trynka
H3K27ac PGC2
H3K27ac Hnisz
FetalDHS Trynka
Enhancer Hoffman
Enhancer Andersson
**DHS Trynka
DGF ENCODE
**CTCF Hoffman
Conserved LindbladToh
Coding UCSC
Annotation
−100
0
100
200
300
Enrichment (prop h2 / prop snps)

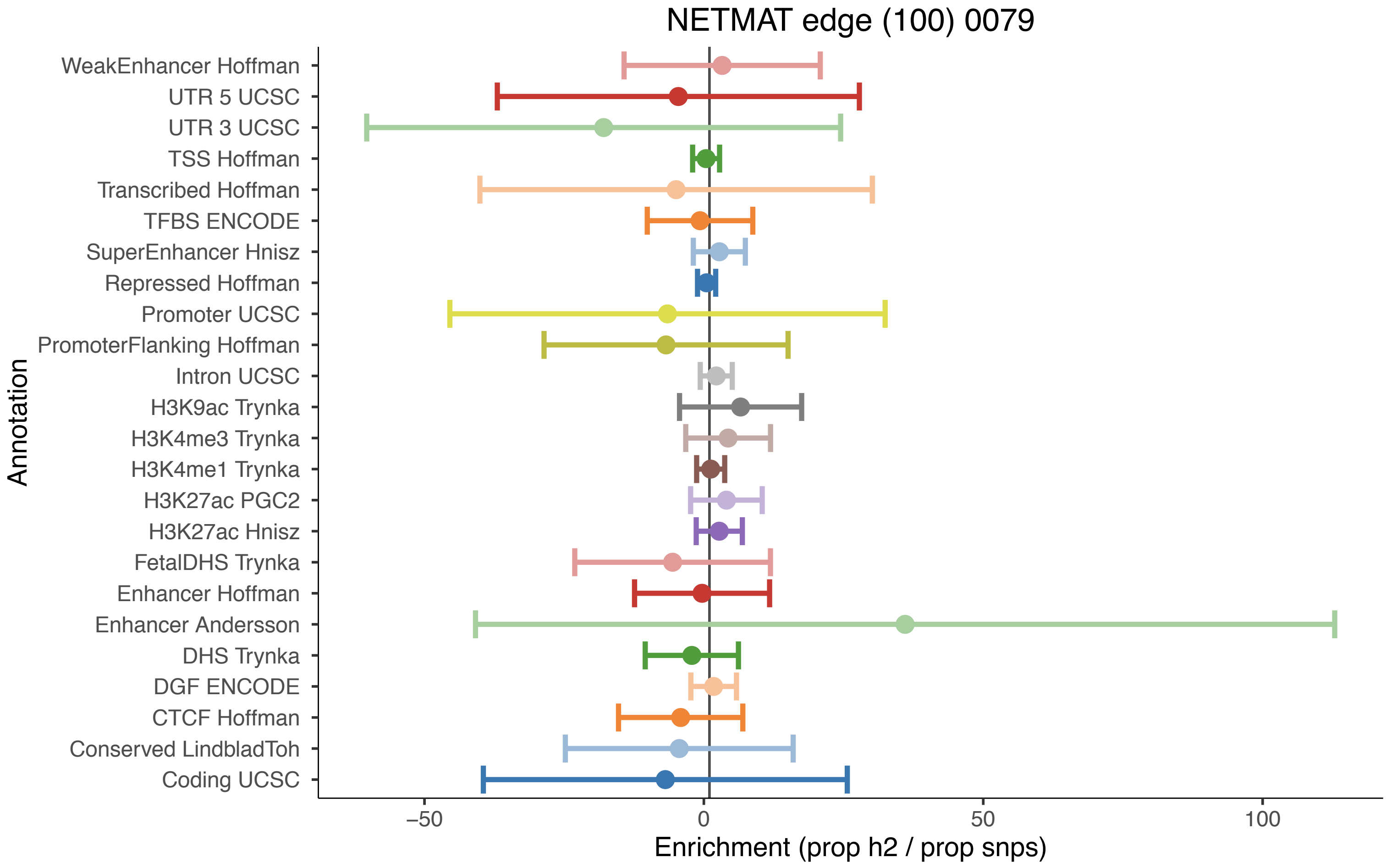

NETMAT edge (100) 0079
WeakEnhancer Hoffman
UTR 5 UCSC
UTR 3 UCSC
TSS Hoffman
Transcribed Hoffman
TFBS ENCODE
SuperEnhancer Hnisz
Repressed Hoffman
Promoter UCSC
PromoterFlanking Hoffman
Intron UCSC
H3K9ac Trynka
H3K4me3 Trynka
H3K4me1 Trynka
H3K27ac PGC2
H3K27ac Hnisz
FetalDHS Trynka
Enhancer Hoffman
Enhancer Andersson
DHS Trynka
DGF ENCODE
CTCF Hoffman
Conserved LindbladToh
Coding UCSC
Annotation
−50
0
50
100
Enrichment (prop h2 / prop snps)

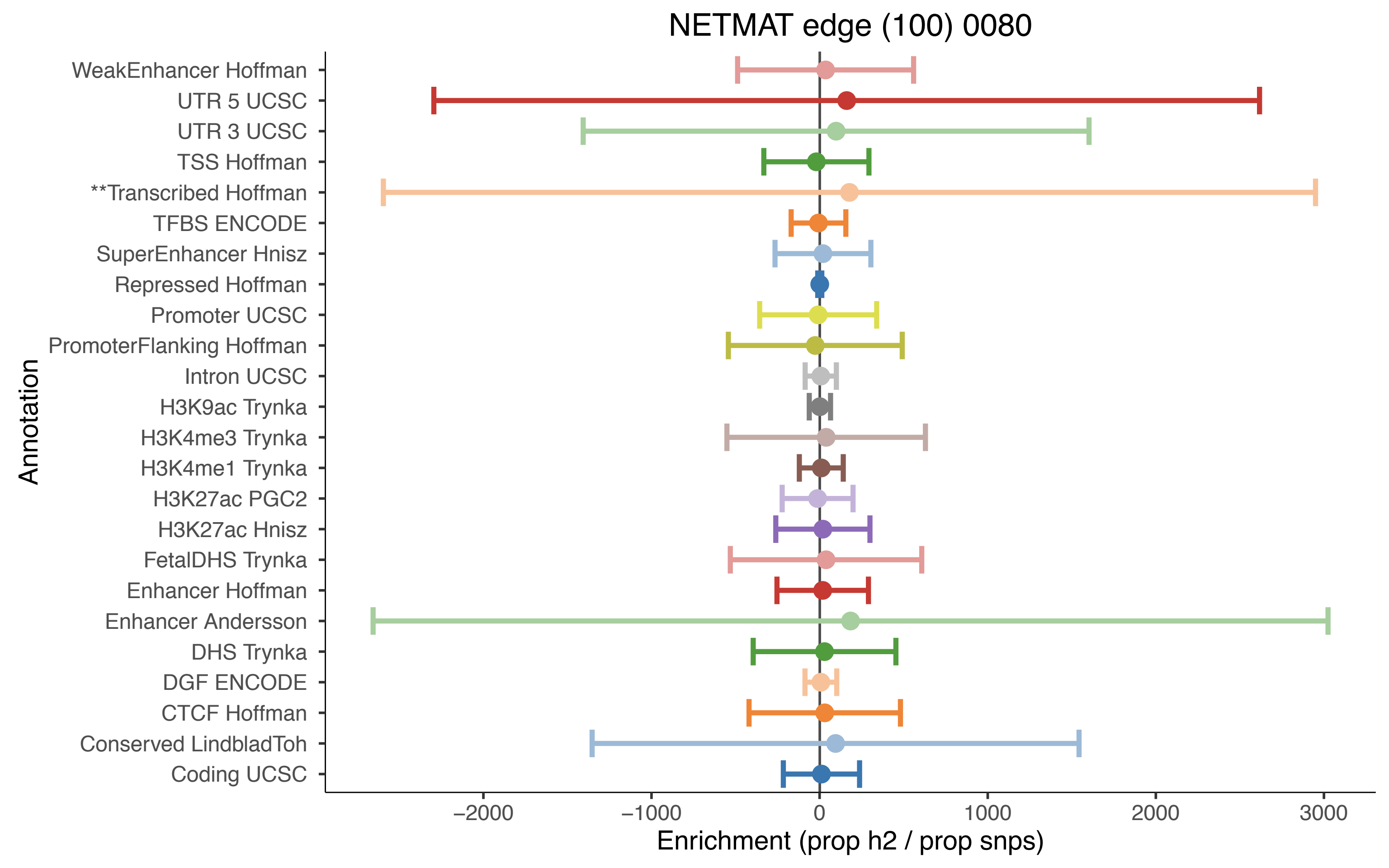
NETMAT edge (100) 0080
WeakEnhancer Hoffman
UTR 5 UCSC
UTR 3 UCSC
TSS Hoffman
**Transcribed Hoffman
TFBS ENCODE
SuperEnhancer Hnisz
Repressed Hoffman
Promoter UCSC
PromoterFlanking Hoffman
Intron UCSC
H3K9ac Trynka
H3K4me3 Trynka
H3K4me1 Trynka
H3K27ac PGC2
H3K27ac Hnisz
FetalDHS Trynka
Enhancer Hoffman
Enhancer Andersson
DHS Trynka
DGF ENCODE
CTCF Hoffman
Conserved LindbladToh
Coding UCSC
Annotation
−2000
−1000
0
1000
2000
3000
Enrichment (prop h2 / prop snps)

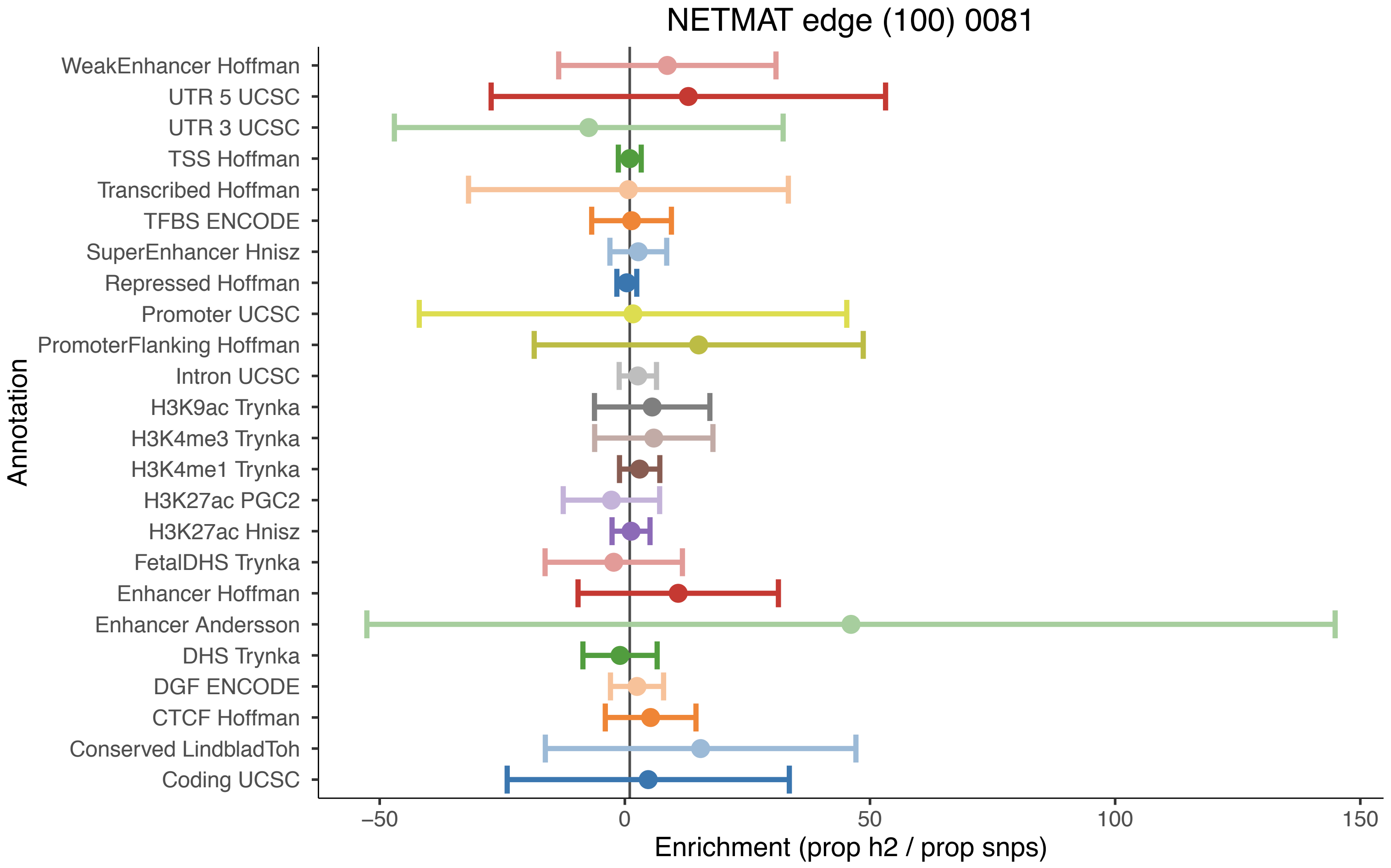
NETMAT edge (100) 0081
WeakEnhancer Hoffman
UTR 5 UCSC
UTR 3 UCSC
TSS Hoffman
Transcribed Hoffman
TFBS ENCODE
SuperEnhancer Hnisz
Repressed Hoffman
Promoter UCSC
PromoterFlanking Hoffman
Intron UCSC
H3K9ac Trynka
H3K4me3 Trynka
H3K4me1 Trynka
H3K27ac PGC2
H3K27ac Hnisz
FetalDHS Trynka
Enhancer Hoffman
Enhancer Andersson
DHS Trynka
DGF ENCODE
CTCF Hoffman
Conserved LindbladToh
Coding UCSC
Annotation
−50
0
50
100
150
Enrichment (prop h2 / prop snps)

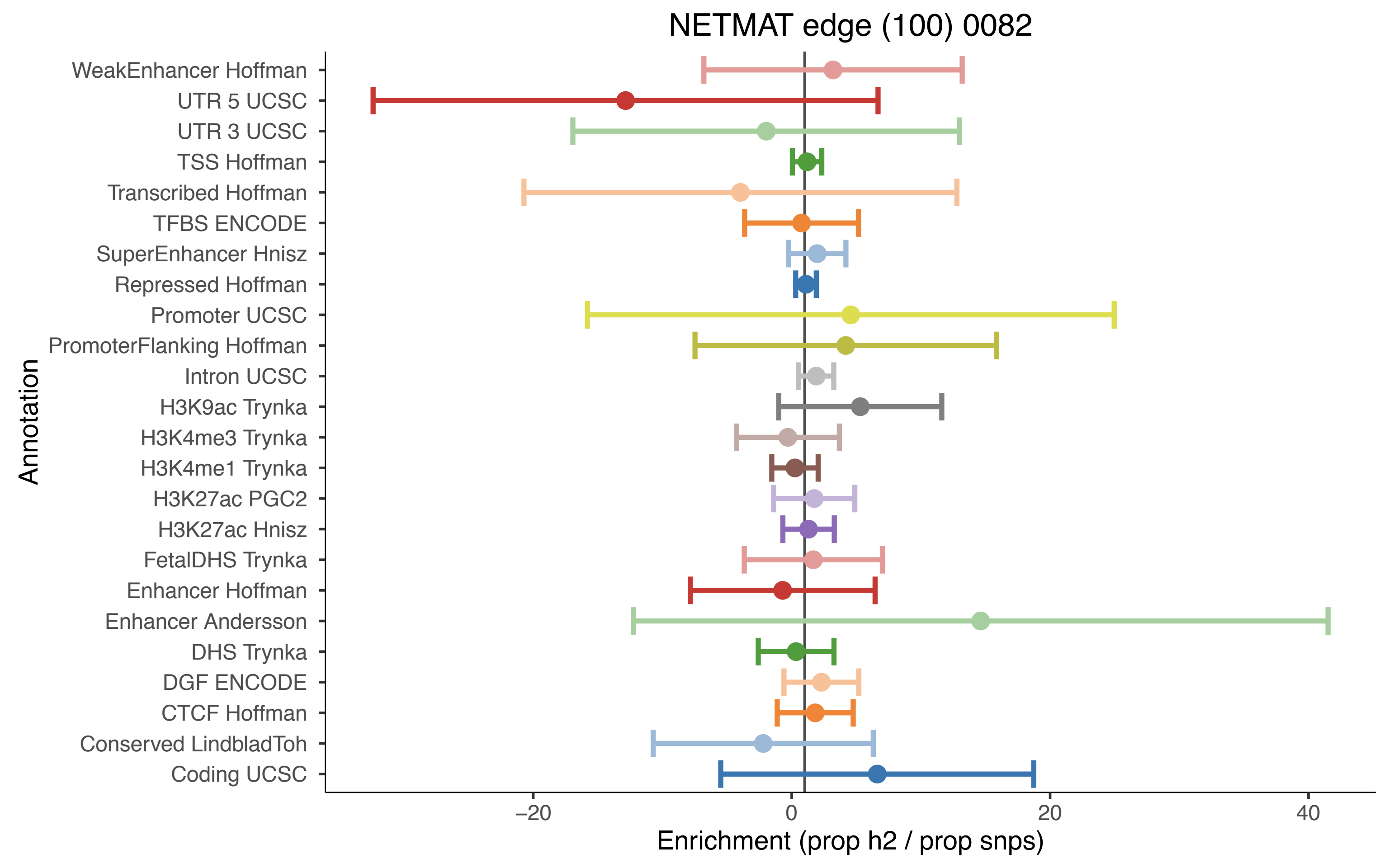
NETMAT edge (100) 0082
WeakEnhancer Hoffman
UTR 5 UCSC
UTR 3 UCSC
TSS Hoffman
Transcribed Hoffman
TFBS ENCODE
SuperEnhancer Hnisz
Repressed Hoffman
Promoter UCSC
PromoterFlanking Hoffman
Intron UCSC
H3K9ac Trynka
H3K4me3 Trynka
H3K4me1 Trynka
H3K27ac PGC2
H3K27ac Hnisz
FetalDHS Trynka
Enhancer Hoffman
Enhancer Andersson
DHS Trynka
DGF ENCODE
CTCF Hoffman
Conserved LindbladToh
Coding UCSC
Annotation
−20
0
20
40
Enrichment (prop h2 / prop snps)

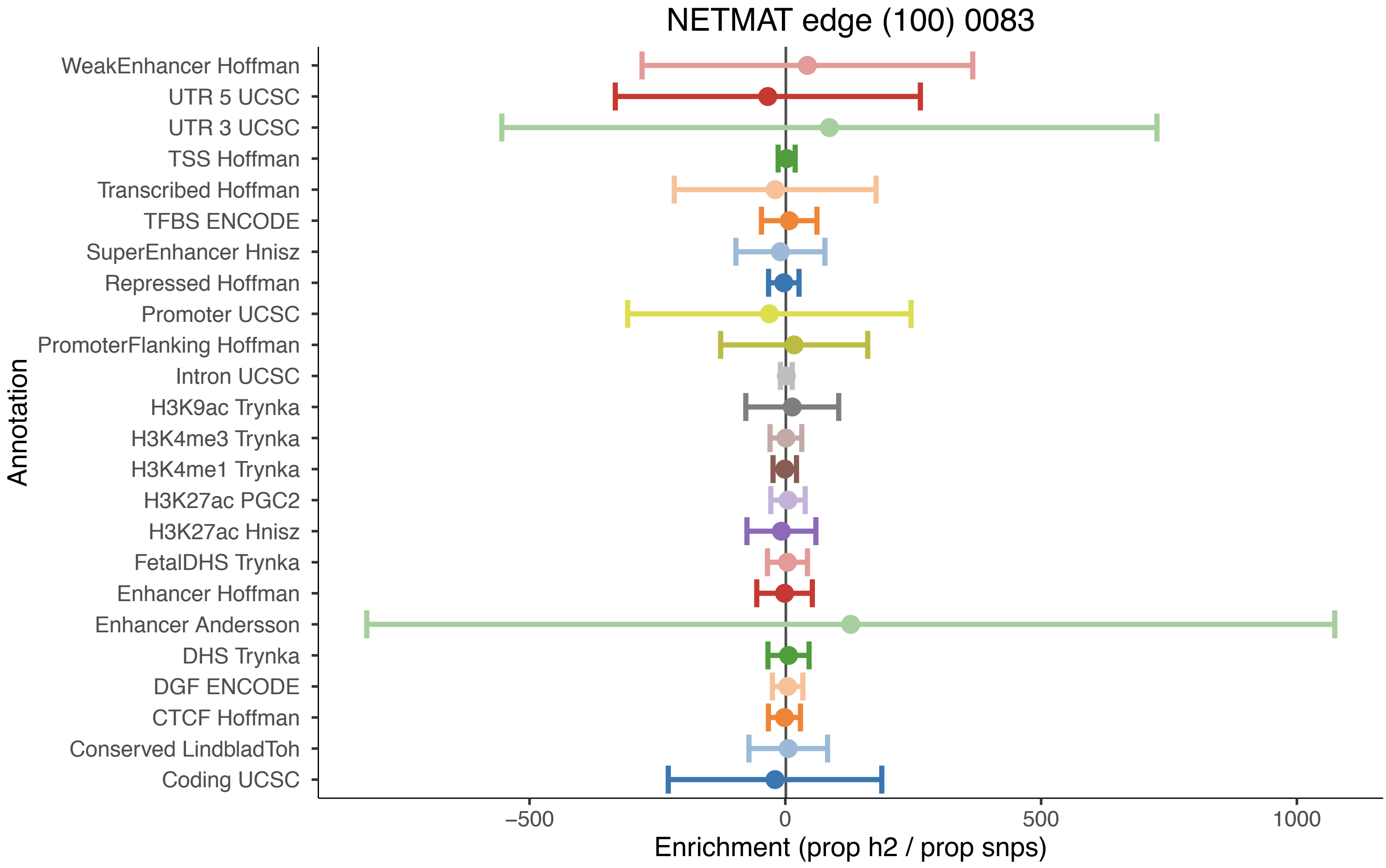

NETMAT edge (100) 0083
WeakEnhancer Hoffman
UTR 5 UCSC
UTR 3 UCSC
TSS Hoffman
Transcribed Hoffman
TFBS ENCODE
SuperEnhancer Hnisz
Repressed Hoffman
Promoter UCSC
PromoterFlanking Hoffman
Intron UCSC
H3K9ac Trynka
H3K4me3 Trynka
H3K4me1 Trynka
H3K27ac PGC2
H3K27ac Hnisz
FetalDHS Trynka
Enhancer Hoffman
Enhancer Andersson
DHS Trynka
DGF ENCODE
CTCF Hoffman
Conserved LindbladToh
Coding UCSC
Annotation
–500
0
500
1000
Enrichment (prop h2 / prop snps)

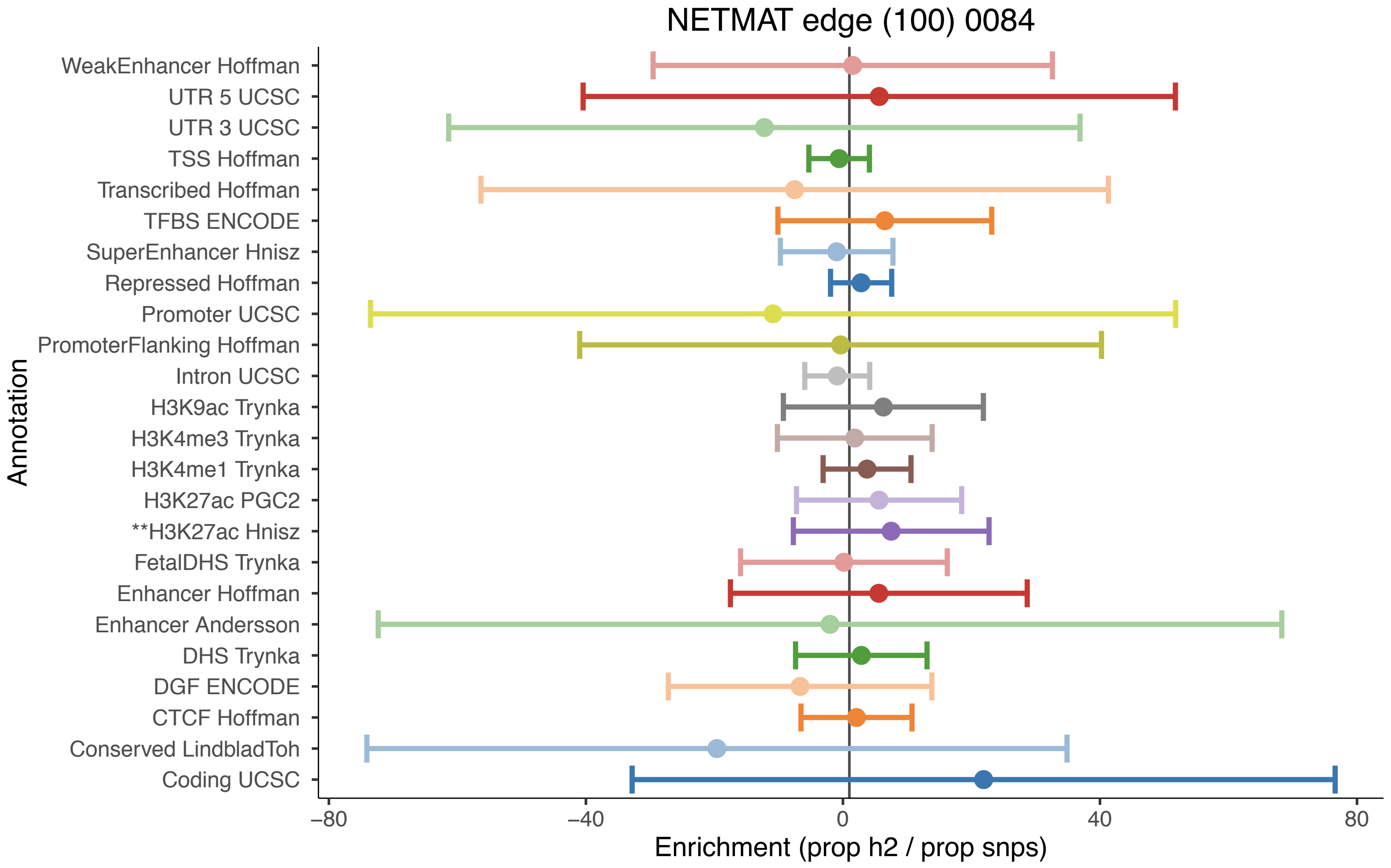

NETMAT edge (100) 0084
WeakEnhancer Hoffman
UTR 5 UCSC
UTR 3 UCSC
TSS Hoffman
Transcribed Hoffman
TFBS ENCODE
SuperEnhancer Hnisz
Repressed Hoffman
Promoter UCSC
PromoterFlanking Hoffman
Intron UCSC
H3K9ac Trynka
H3K4me3 Trynka
H3K4me1 Trynka
H3K27ac PGC2
**H3K27ac Hnisz
FetalDHS Trynka
Enhancer Hoffman
Enhancer Andersson
DHS Trynka
DGF ENCODE
CTCF Hoffman
Conserved LindbladToh
Coding UCSC
Annotation
−80
−40
0
40
80
Enrichment (prop h2 / prop snps)

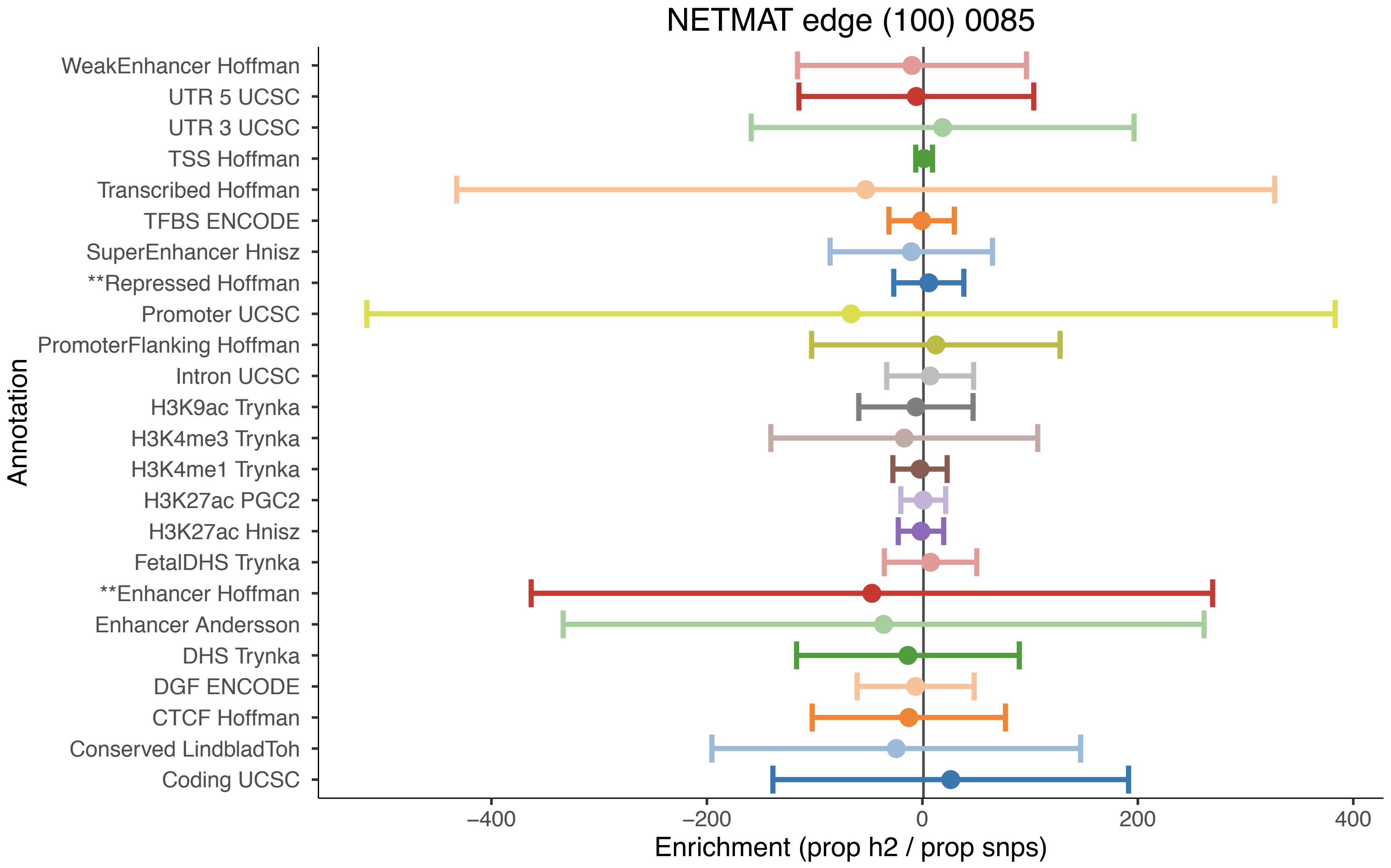

NETMAT edge (100) 0085
WeakEnhancer Hoffman
UTR 5 UCSC
UTR 3 UCSC
TSS Hoffman
Transcribed Hoffman
TFBS ENCODE
SuperEnhancer Hnisz
**Repressed Hoffman
Promoter UCSC
PromoterFlanking Hoffman
Intron UCSC
H3K9ac Trynka
H3K4me3 Trynka
H3K4me1 Trynka
H3K27ac PGC2
H3K27ac Hnisz
FetalDHS Trynka
**Enhancer Hoffman
Enhancer Andersson
DHS Trynka
DGF ENCODE
CTCF Hoffman
Conserved LindbladToh
Coding UCSC
Annotation
−400
−200
0
200
400
Enrichment (prop h2 / prop snps)

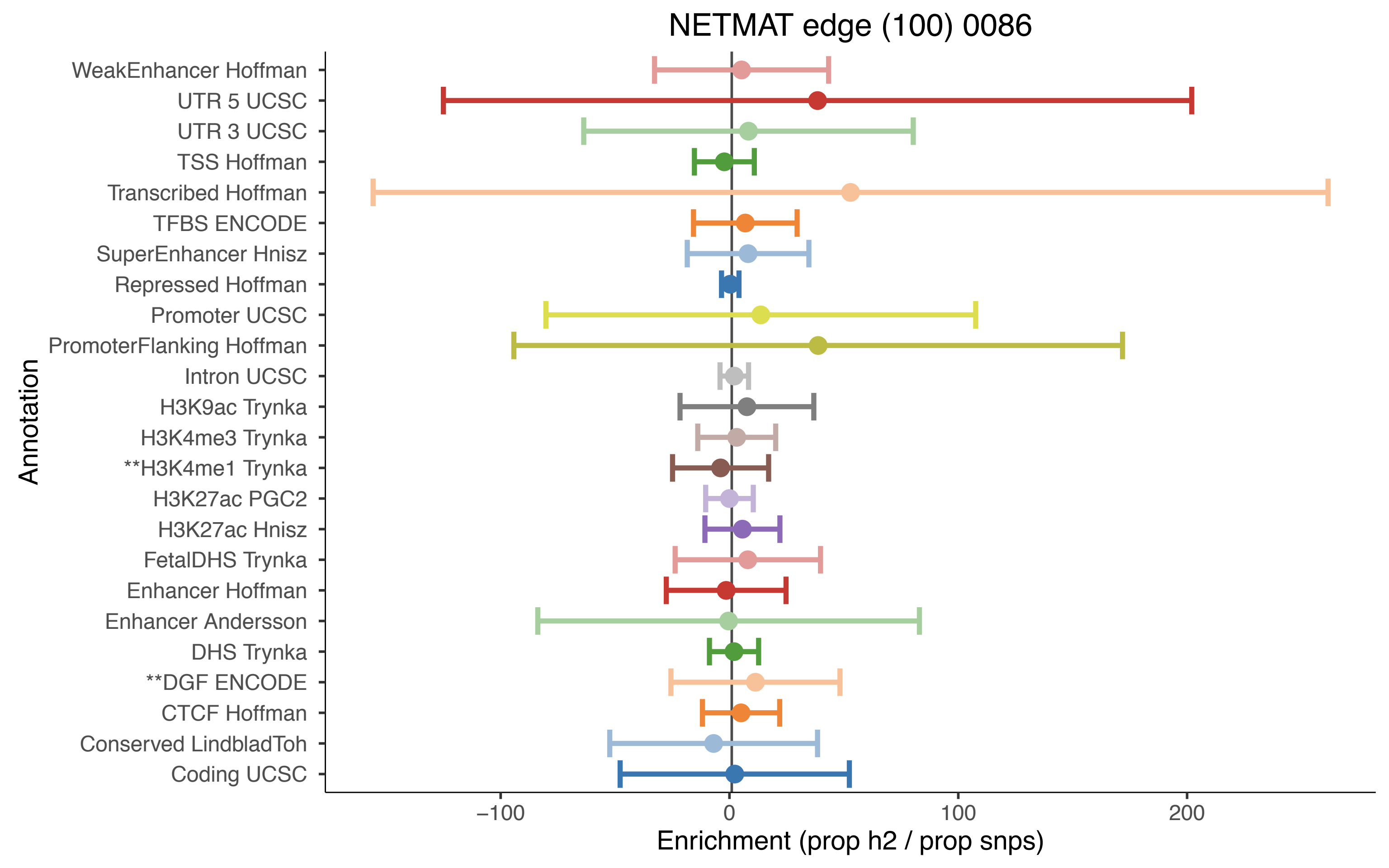

NETMAT edge (100) 0086
Annotation
WeakEnhancer Hoffman
UTR 5 UCSC
UTR 3 UCSC
TSS Hoffman
Transcribed Hoffman
TFBS ENCODE
SuperEnhancer Hnisz
Repressed Hoffman
Promoter UCSC
PromoterFlanking Hoffman
Intron UCSC
H3K9ac Trynka
H3K4me3 Trynka
**H3K4me1 Trynka
H3K27ac PGC2
H3K27ac Hnisz
FetalDHS Trynka
Enhancer Hoffman
Enhancer Andersson
DHS Trynka
**DGF ENCODE
CTCF Hoffman
Conserved LindbladToh
Coding UCSC
−100
0
100
200
Enrichment (prop h2 / prop snps)

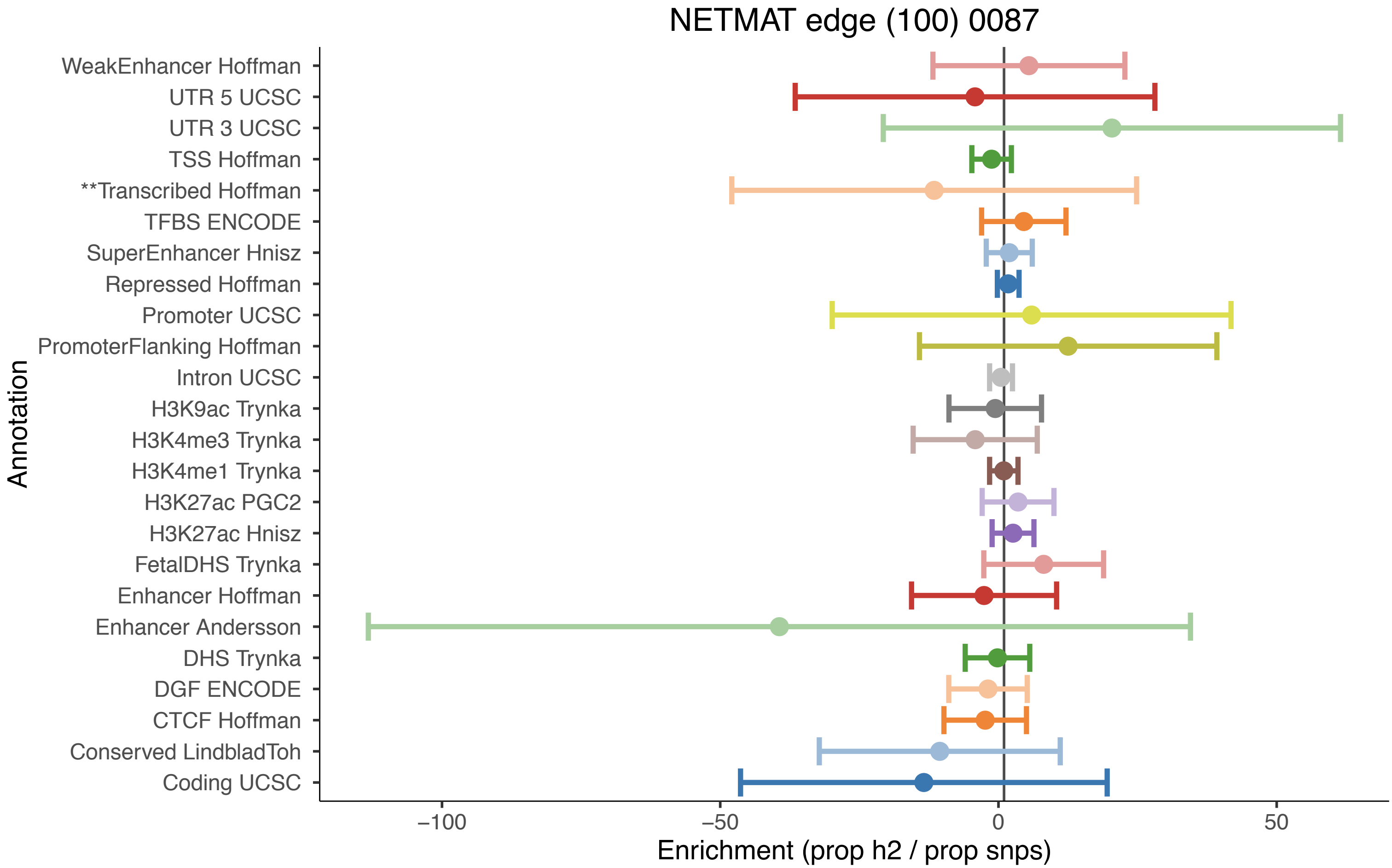

NETMAT edge (100) 0087
WeakEnhancer Hoffman
UTR 5 UCSC
UTR 3 UCSC
TSS Hoffman
**Transcribed Hoffman
TFBS ENCODE
SuperEnhancer Hnisz
Repressed Hoffman
Promoter UCSC
PromoterFlanking Hoffman
Intron UCSC
H3K9ac Trynka
H3K4me3 Trynka
H3K4me1 Trynka
H3K27ac PGC2
H3K27ac Hnisz
FetalDHS Trynka
Enhancer Hoffman
Enhancer Andersson
DHS Trynka
DGF ENCODE
CTCF Hoffman
Conserved LindbladToh
Coding UCSC
Annotation
−100
−50
0
50
Enrichment (prop h2 / prop snps)

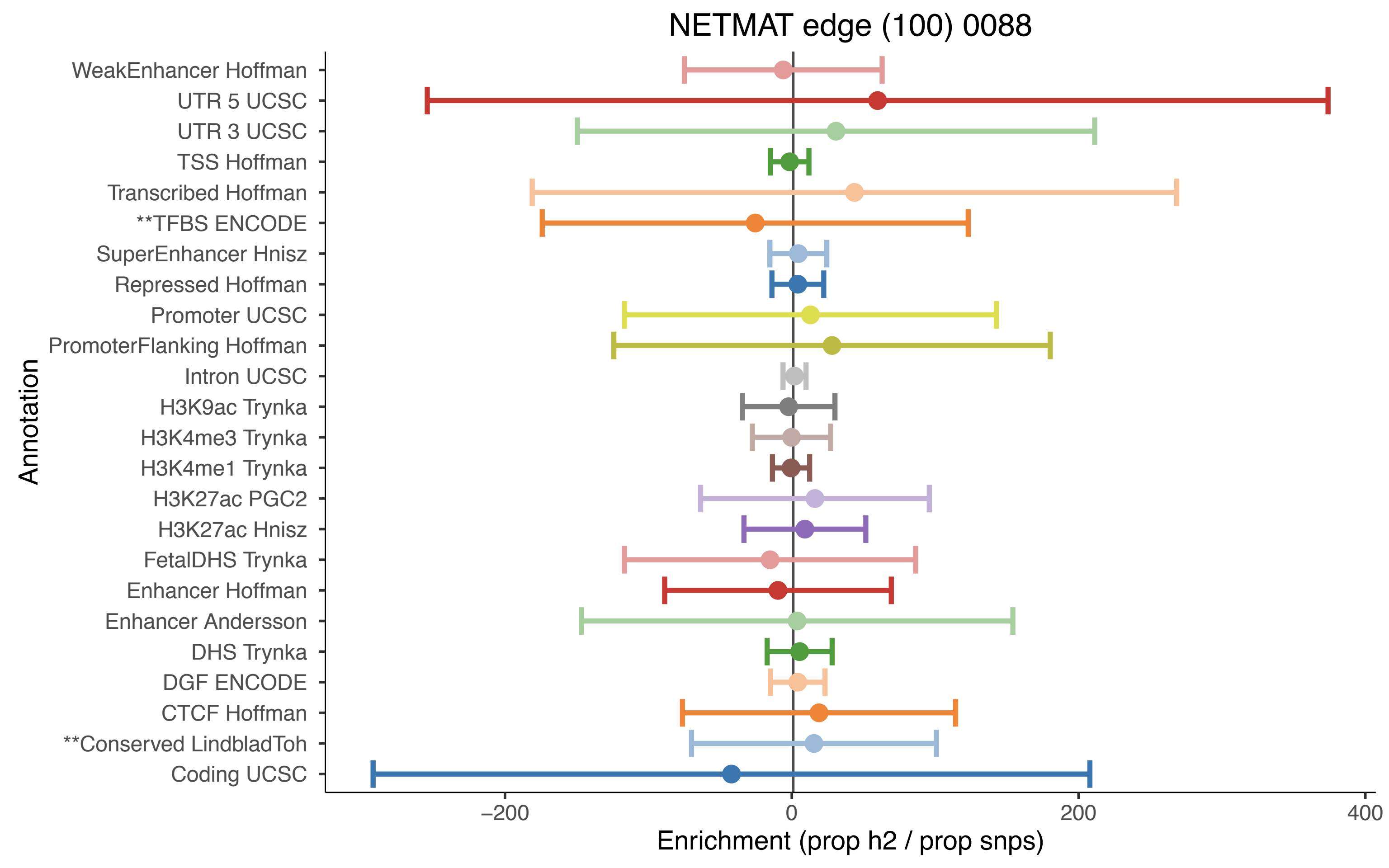

NETMAT edge (100) 0088
Annotation
WeakEnhancer Hoffman
UTR 5 UCSC
UTR 3 UCSC
TSS Hoffman
Transcribed Hoffman
**TFBS ENCODE
SuperEnhancer Hnisz
Repressed Hoffman
Promoter UCSC
PromoterFlanking Hoffman
Intron UCSC
H3K9ac Trynka
H3K4me3 Trynka
H3K4me1 Trynka
H3K27ac PGC2
H3K27ac Hnisz
FetalDHS Trynka
Enhancer Hoffman
Enhancer Andersson
DHS Trynka
DGF ENCODE
CTCF Hoffman
**Conserved LindbladToh
Coding UCSC
−200
0
200
400
Enrichment (prop h2 / prop snps)

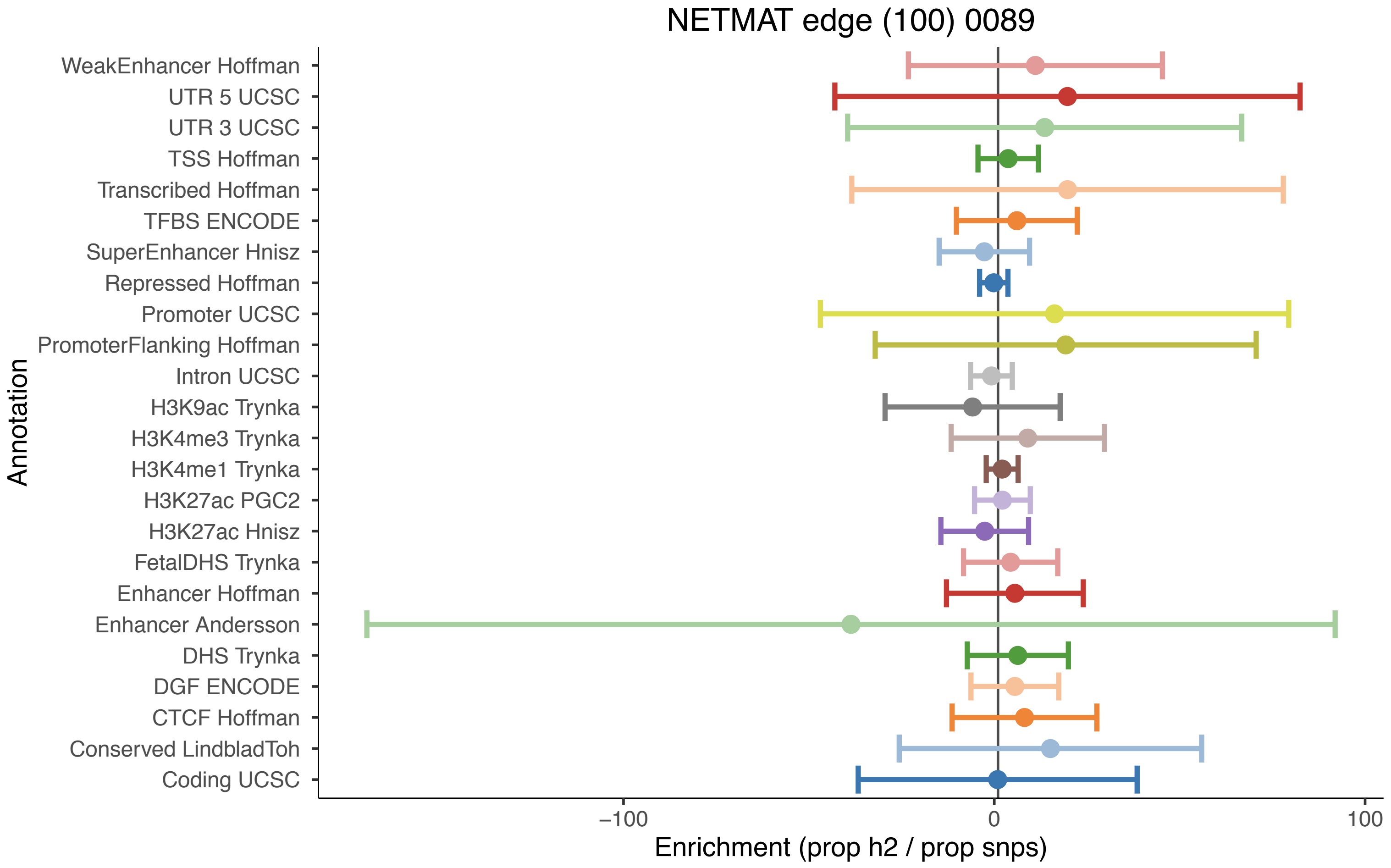

NETMAT edge (100) 0089
Annotation
WeakEnhancer Hoffman
UTR 5 UCSC
UTR 3 UCSC
TSS Hoffman
Transcribed Hoffman
TFBS ENCODE
SuperEnhancer Hnisz
Repressed Hoffman
Promoter UCSC
PromoterFlanking Hoffman
Intron UCSC
H3K9ac Trynka
H3K4me3 Trynka
H3K4me1 Trynka
H3K27ac PGC2
H3K27ac Hnisz
FetalDHS Trynka
Enhancer Hoffman
Enhancer Andersson
DHS Trynka
DGF ENCODE
CTCF Hoffman
Conserved LindbladToh
Coding UCSC
−100
0
100
Enrichment (prop h2 / prop snps)

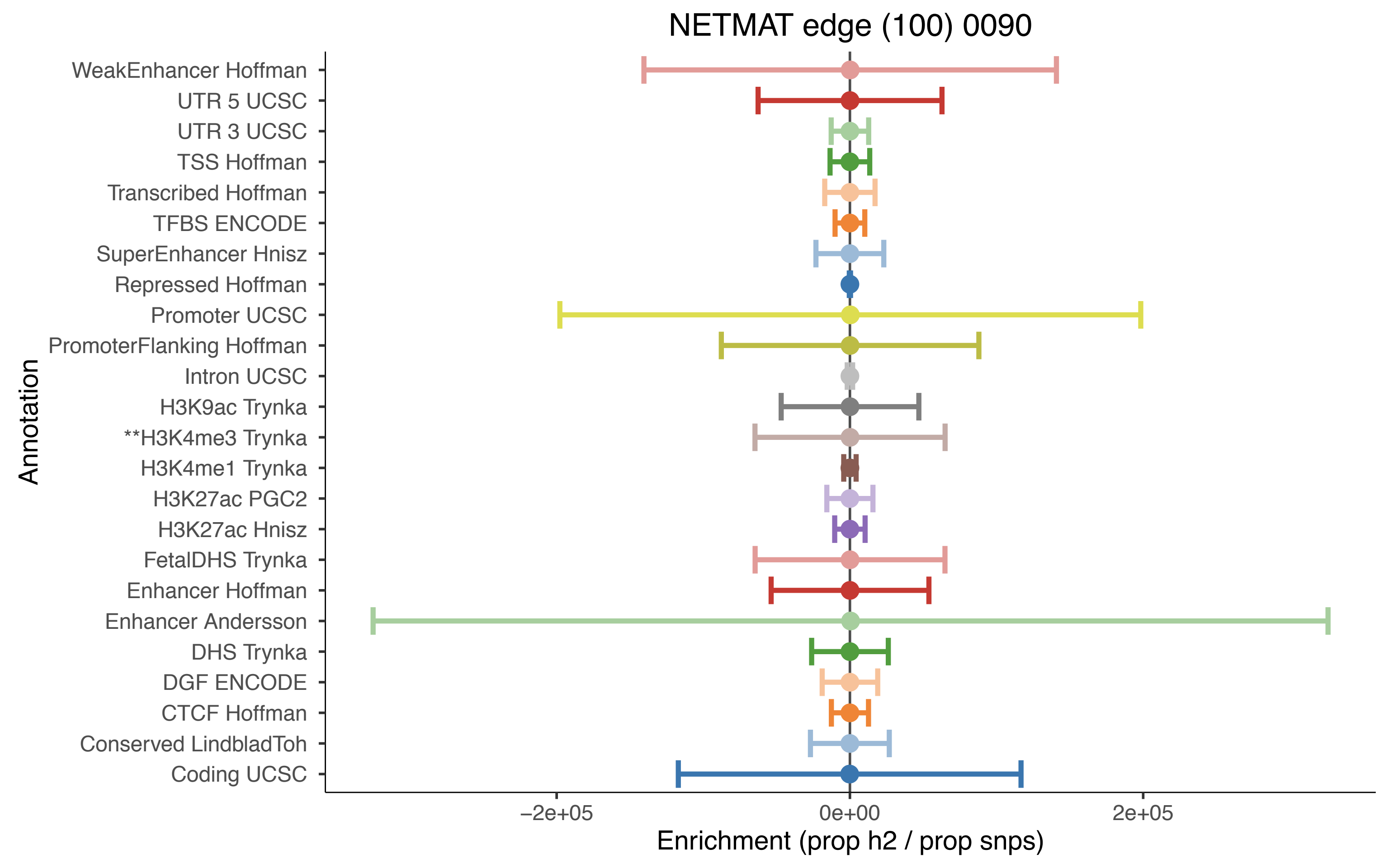
NETMAT edge (100) 0090
WeakEnhancer Hoffman
UTR 5 UCSC
UTR 3 UCSC
TSS Hoffman
Transcribed Hoffman
TFBS ENCODE
SuperEnhancer Hnisz
Repressed Hoffman
Promoter UCSC
PromoterFlanking Hoffman
Intron UCSC
H3K9ac Trynka
**H3K4me3 Trynka
H3K4me1 Trynka
H3K27ac PGC2
H3K27ac Hnisz
FetalDHS Trynka
Enhancer Hoffman
Enhancer Andersson
DHS Trynka
DGF ENCODE
CTCF Hoffman
Conserved LindbladToh
Coding UCSC
Annotation
−2e+05
0e+00
2e+05
Enrichment (prop h2 / prop snps)

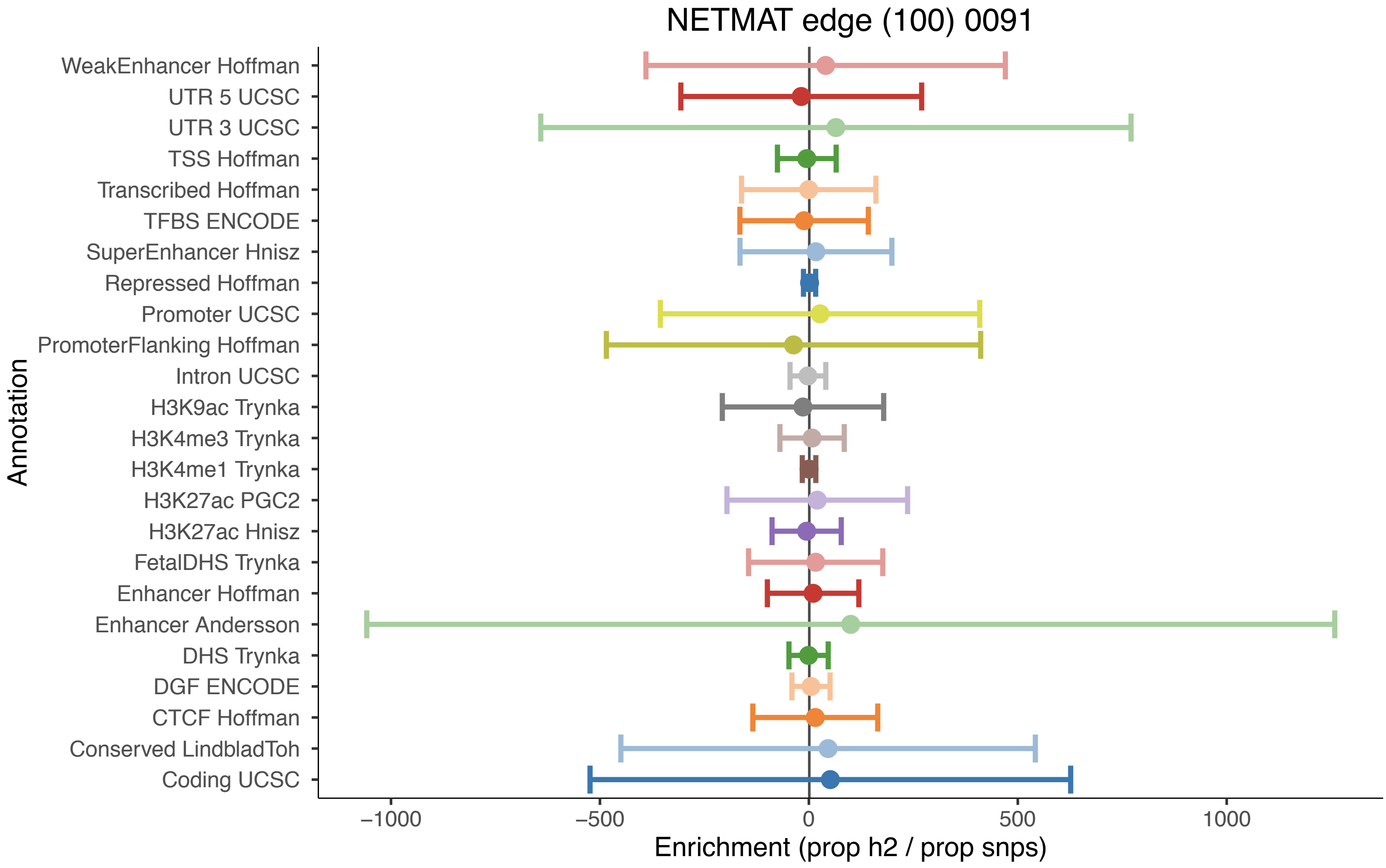

NETMAT edge (100) 0091
WeakEnhancer Hoffman
UTR 5 UCSC
UTR 3 UCSC
TSS Hoffman
Transcribed Hoffman
TFBS ENCODE
SuperEnhancer Hnisz
Repressed Hoffman
Promoter UCSC
PromoterFlanking Hoffman
Intron UCSC
H3K9ac Trynka
H3K4me3 Trynka
H3K4me1 Trynka
H3K27ac PGC2
H3K27ac Hnisz
FetalDHS Trynka
Enhancer Hoffman
Enhancer Andersson
DHS Trynka
DGF ENCODE
CTCF Hoffman
Conserved LindbladToh
Coding UCSC
Annotation
−1000
−500
0
500
1000
Enrichment (prop h2 / prop snps)

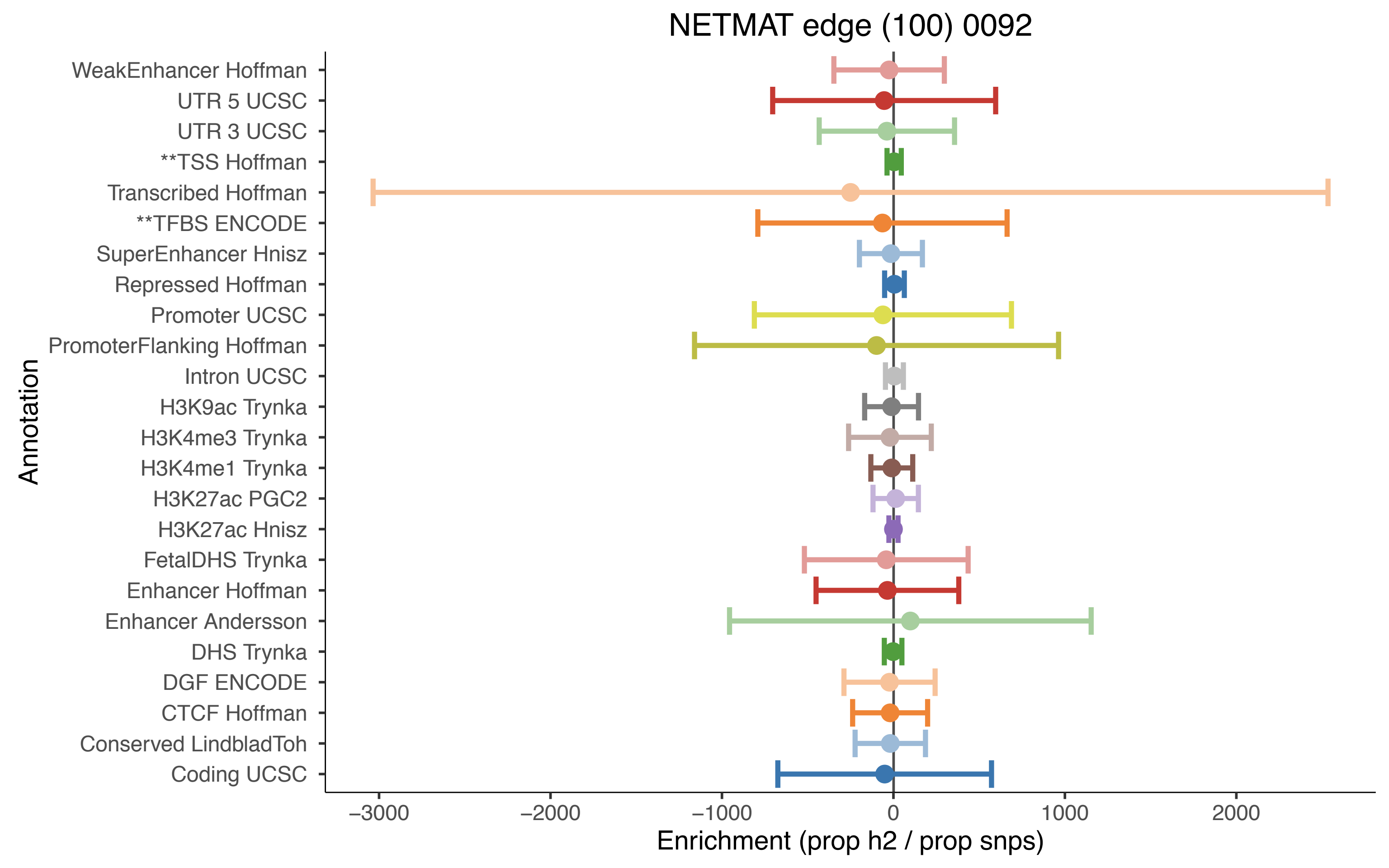
NETMAT edge (100) 0092
WeakEnhancer Hoffman
UTR 5 UCSC
UTR 3 UCSC
**TSS Hoffman
Transcribed Hoffman
**TFBS ENCODE
SuperEnhancer Hnisz
Repressed Hoffman
Promoter UCSC
PromoterFlanking Hoffman
Intron UCSC
H3K9ac Trynka
H3K4me3 Trynka
H3K4me1 Trynka
H3K27ac PGC2
H3K27ac Hnisz
FetalDHS Trynka
Enhancer Hoffman
Enhancer Andersson
DHS Trynka
DGF ENCODE
CTCF Hoffman
Conserved LindbladToh
Coding UCSC
Annotation
−3000
−2000
−1000
0
1000
2000
Enrichment (prop h2 / prop snps)

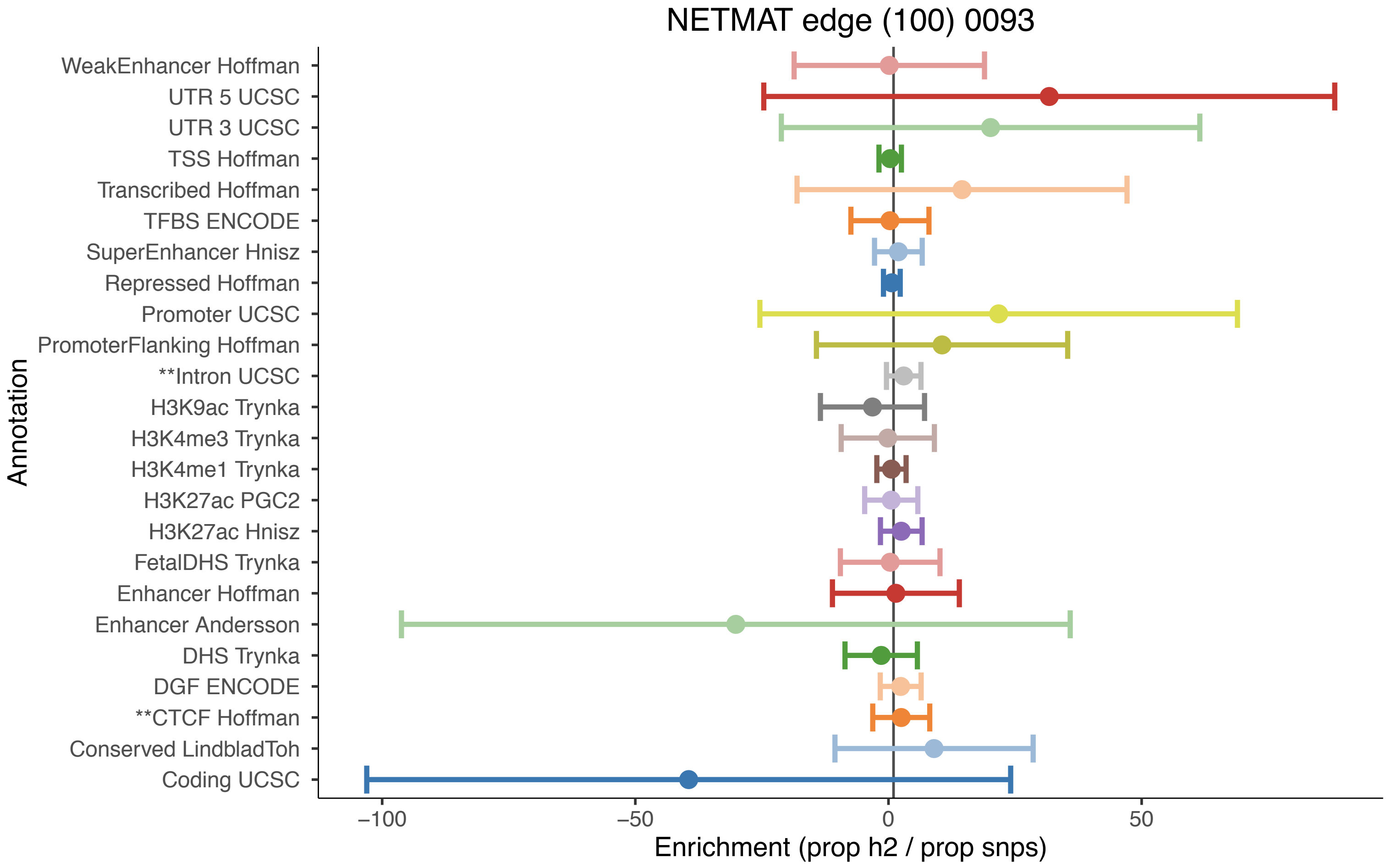
NETMAT edge (100) 0093
WeakEnhancer Hoffman
UTR 5 UCSC
UTR 3 UCSC
TSS Hoffman
Transcribed Hoffman
TFBS ENCODE
SuperEnhancer Hnisz
Repressed Hoffman
Promoter UCSC
PromoterFlanking Hoffman
**Intron UCSC
H3K9ac Trynka
H3K4me3 Trynka
H3K4me1 Trynka
H3K27ac PGC2
H3K27ac Hnisz
FetalDHS Trynka
Enhancer Hoffman
Enhancer Andersson
DHS Trynka
DGF ENCODE
**CTCF Hoffman
Conserved LindbladToh
Coding UCSC
Annotation
−100
−50
0
50
Enrichment (prop h2 / prop snps)

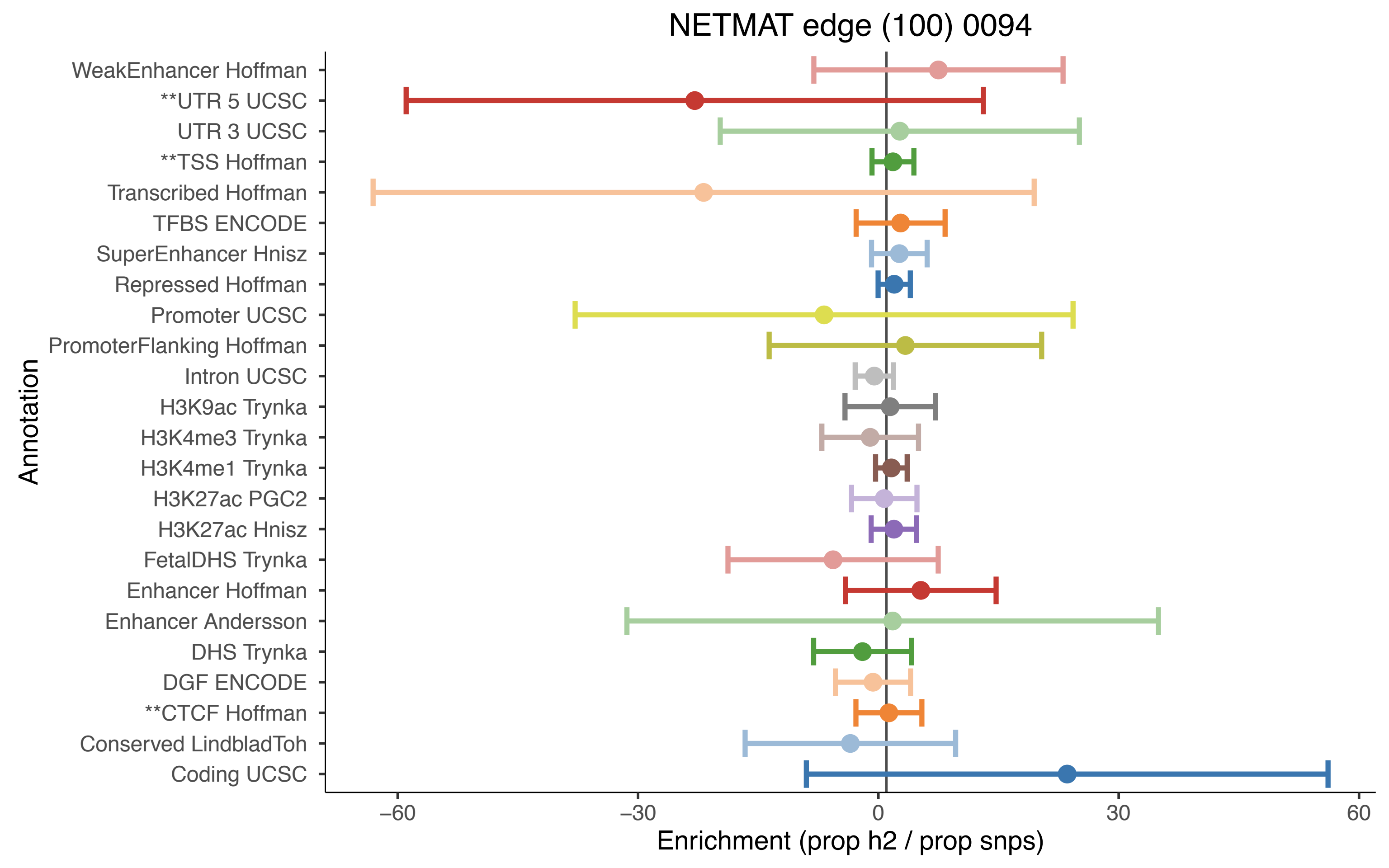

NETMAT edge (100) 0094
WeakEnhancer Hoffman
**UTR 5 UCSC
UTR 3 UCSC
**TSS Hoffman
Transcribed Hoffman
TFBS ENCODE
SuperEnhancer Hnisz
Repressed Hoffman
Promoter UCSC
PromoterFlanking Hoffman
Intron UCSC
H3K9ac Trynka
H3K4me3 Trynka
H3K4me1 Trynka
H3K27ac PGC2
H3K27ac Hnisz
FetalDHS Trynka
Enhancer Hoffman
Enhancer Andersson
DHS Trynka
DGF ENCODE
**CTCF Hoffman
Conserved LindbladToh
Coding UCSC
Annotation
−60
−30
0
30
60
Enrichment (prop h2 / prop snps)

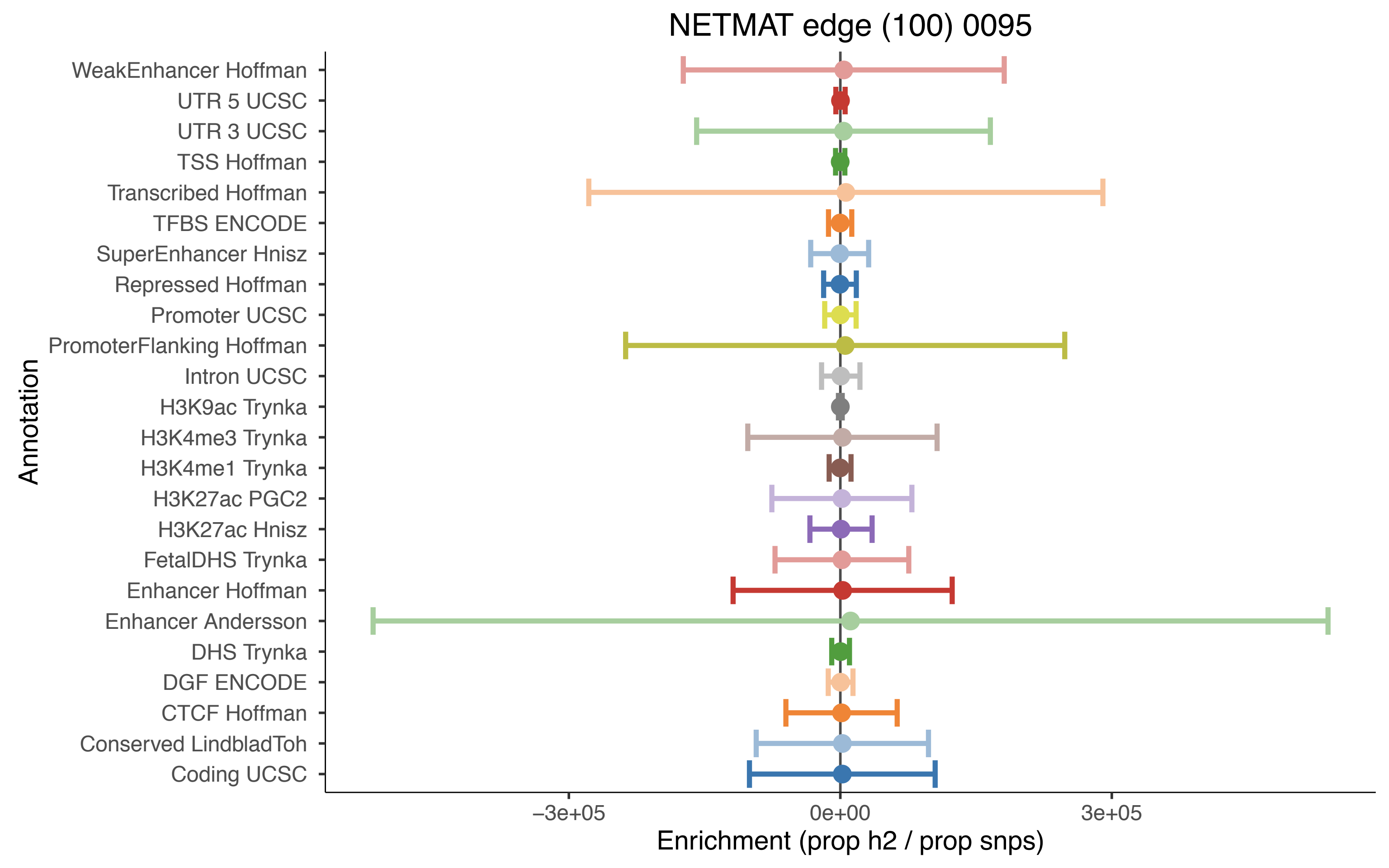
NETMAT edge (100) 0095
WeakEnhancer Hoffman
UTR 5 UCSC
UTR 3 UCSC
TSS Hoffman
Transcribed Hoffman
TFBS ENCODE
SuperEnhancer Hnisz
Repressed Hoffman
Promoter UCSC
PromoterFlanking Hoffman
Intron UCSC
H3K9ac Trynka
H3K4me3 Trynka
H3K4me1 Trynka
H3K27ac PGC2
H3K27ac Hnisz
FetalDHS Trynka
Enhancer Hoffman
Enhancer Andersson
DHS Trynka
DGF ENCODE
CTCF Hoffman
Conserved LindbladToh
Coding UCSC
Annotation
−3e+05
0e+00
3e+05
Enrichment (prop h2 / prop snps)

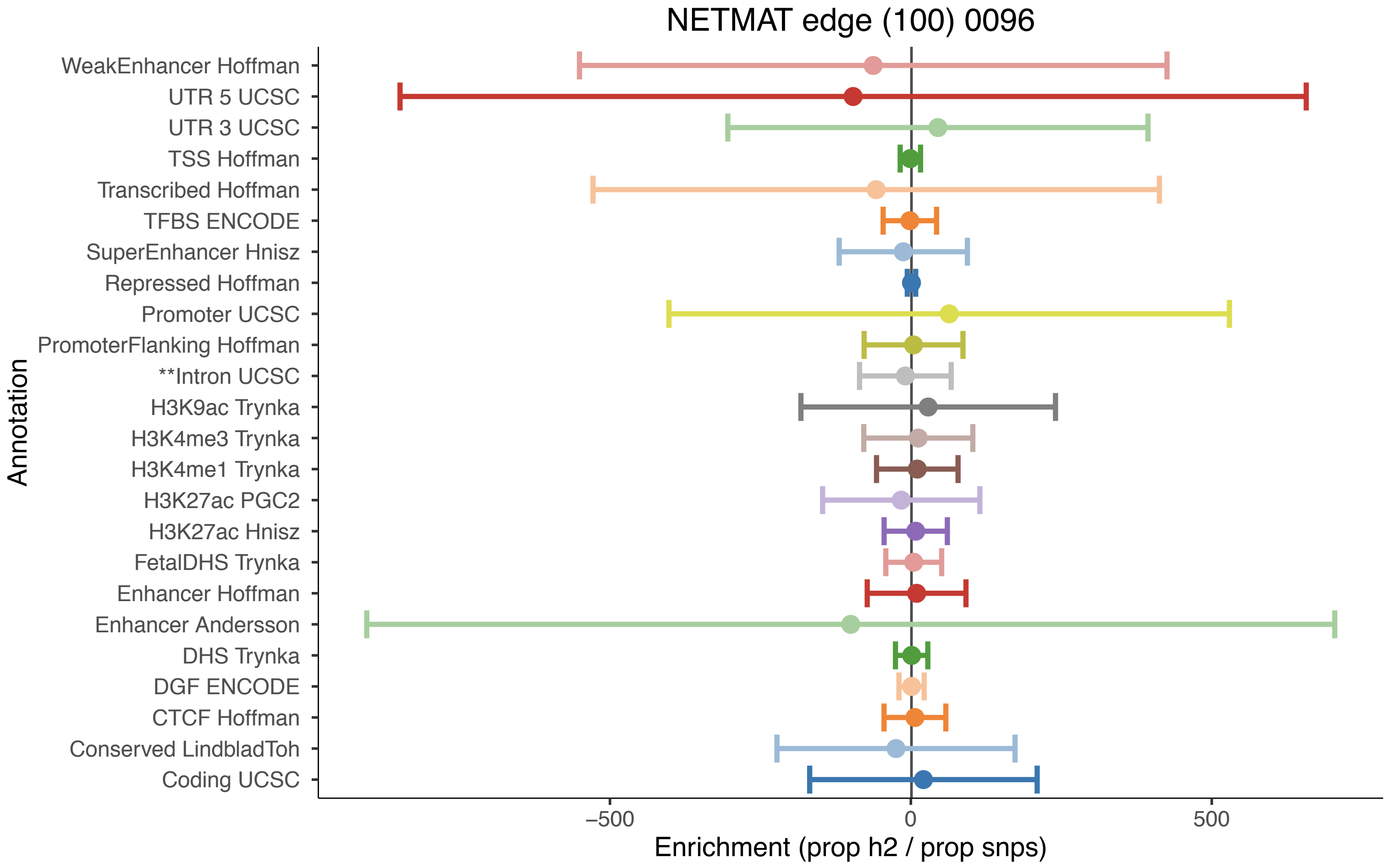

NETMAT edge (100) 0096
WeakEnhancer Hoffman
UTR 5 UCSC
UTR 3 UCSC
TSS Hoffman
Transcribed Hoffman
TFBS ENCODE
SuperEnhancer Hnisz
Repressed Hoffman
Promoter UCSC
PromoterFlanking Hoffman
**Intron UCSC
H3K9ac Trynka
H3K4me3 Trynka
H3K4me1 Trynka
H3K27ac PGC2
H3K27ac Hnisz
FetalDHS Trynka
Enhancer Hoffman
Enhancer Andersson
DHS Trynka
DGF ENCODE
CTCF Hoffman
Conserved LindbladToh
Coding UCSC
Annotation
−500
0
500
Enrichment (prop h2 / prop snps)

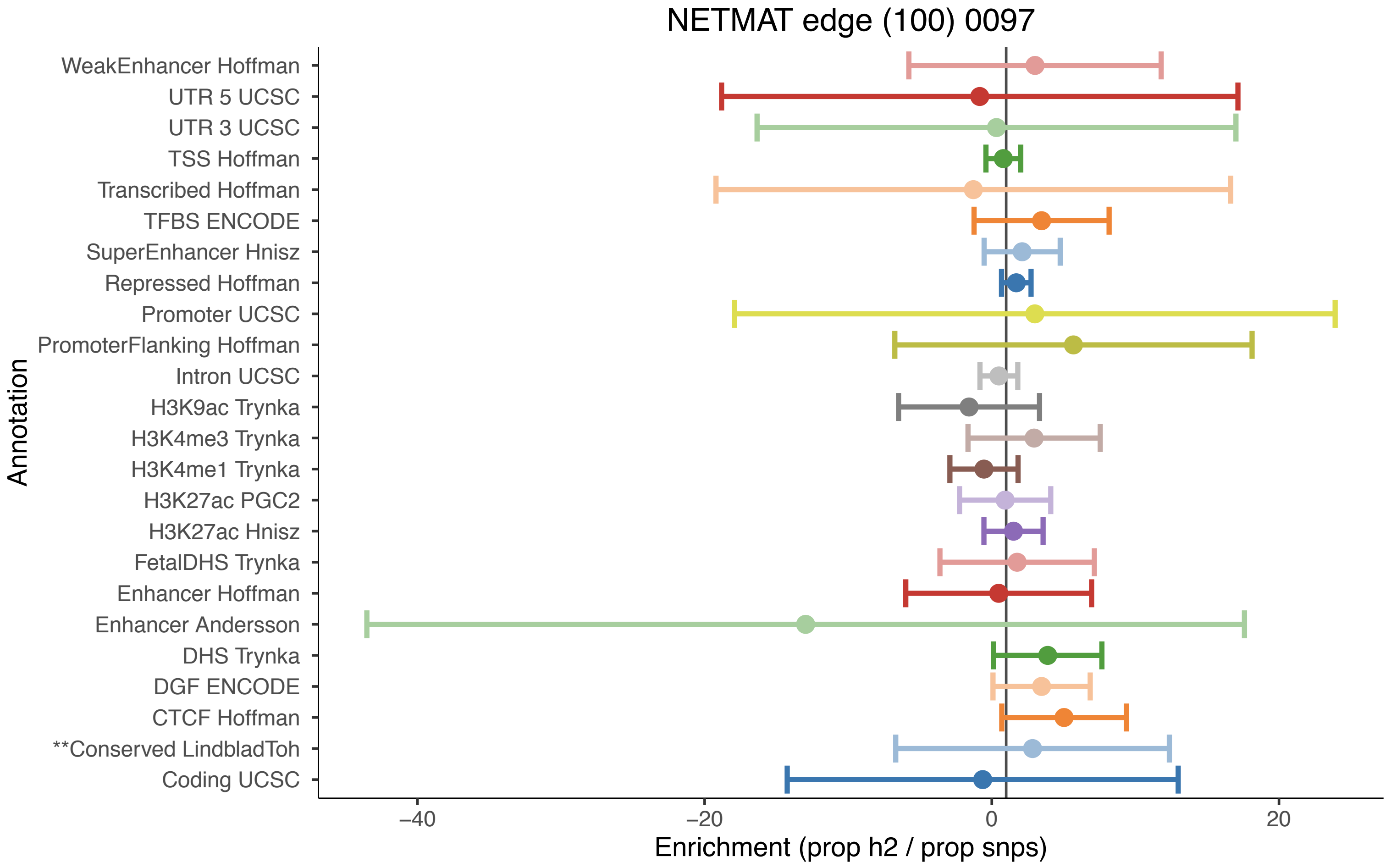
NETMAT edge (100) 0097
WeakEnhancer Hoffman
UTR 5 UCSC
UTR 3 UCSC
TSS Hoffman
Transcribed Hoffman
TFBS ENCODE
SuperEnhancer Hnisz
Repressed Hoffman
Promoter UCSC
PromoterFlanking Hoffman
Intron UCSC
H3K9ac Trynka
H3K4me3 Trynka
H3K4me1 Trynka
H3K27ac PGC2
H3K27ac Hnisz
FetalDHS Trynka
Enhancer Hoffman
Enhancer Andersson
DHS Trynka
DGF ENCODE
CTCF Hoffman
**Conserved LindbladToh
Coding UCSC
Annotation
−40
−20
0
20
Enrichment (prop h2 / prop snps)

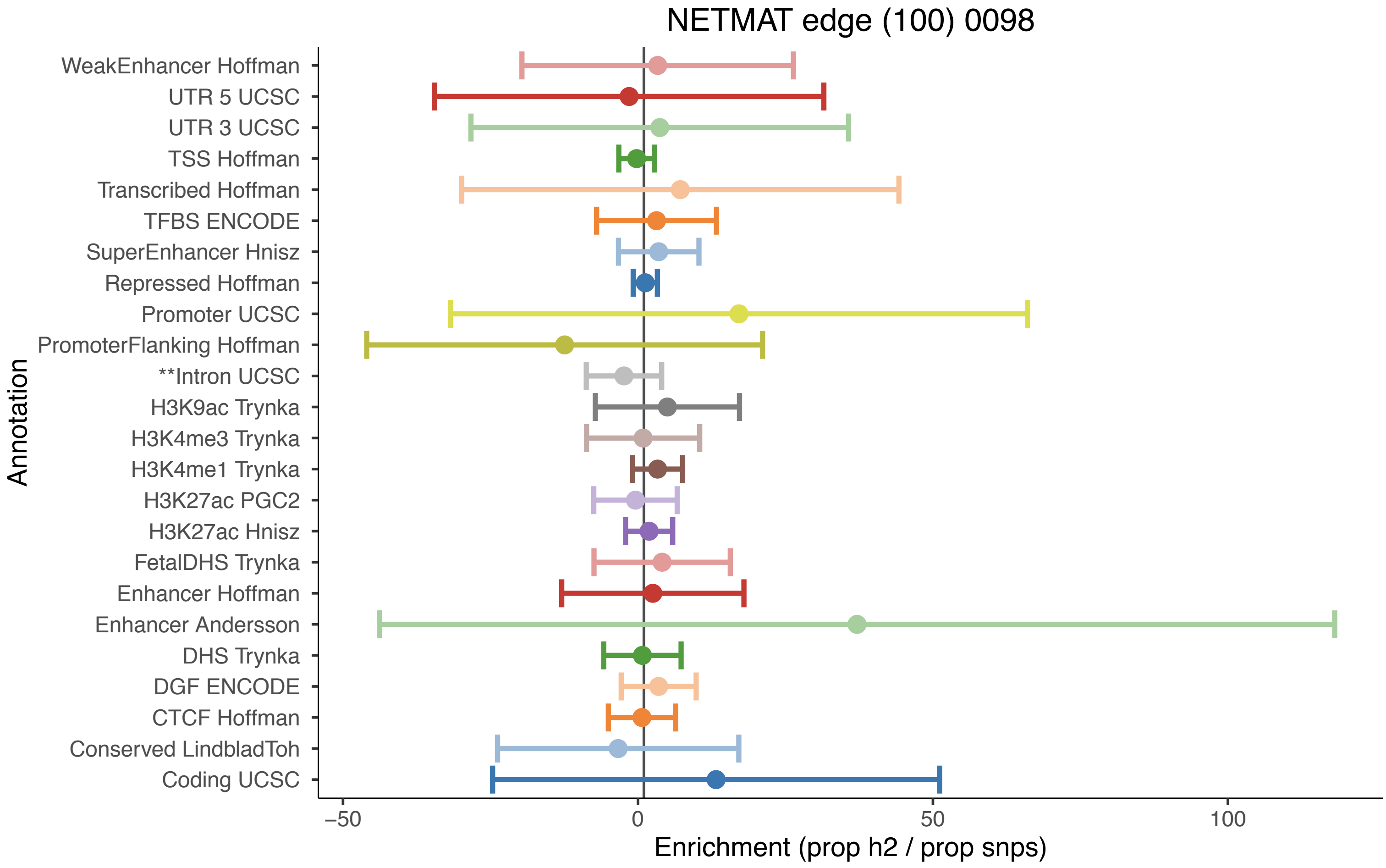
NETMAT edge (100) 0098
WeakEnhancer Hoffman
UTR 5 UCSC
UTR 3 UCSC
TSS Hoffman
Transcribed Hoffman
TFBS ENCODE
SuperEnhancer Hnisz
Repressed Hoffman
Promoter UCSC
PromoterFlanking Hoffman
**Intron UCSC
H3K9ac Trynka
H3K4me3 Trynka
H3K4me1 Trynka
H3K27ac PGC2
H3K27ac Hnisz
FetalDHS Trynka
Enhancer Hoffman
Enhancer Andersson
DHS Trynka
DGF ENCODE
CTCF Hoffman
Conserved LindbladToh
Coding UCSC
Annotation
−50
0
50
100
Enrichment (prop h2 / prop snps)

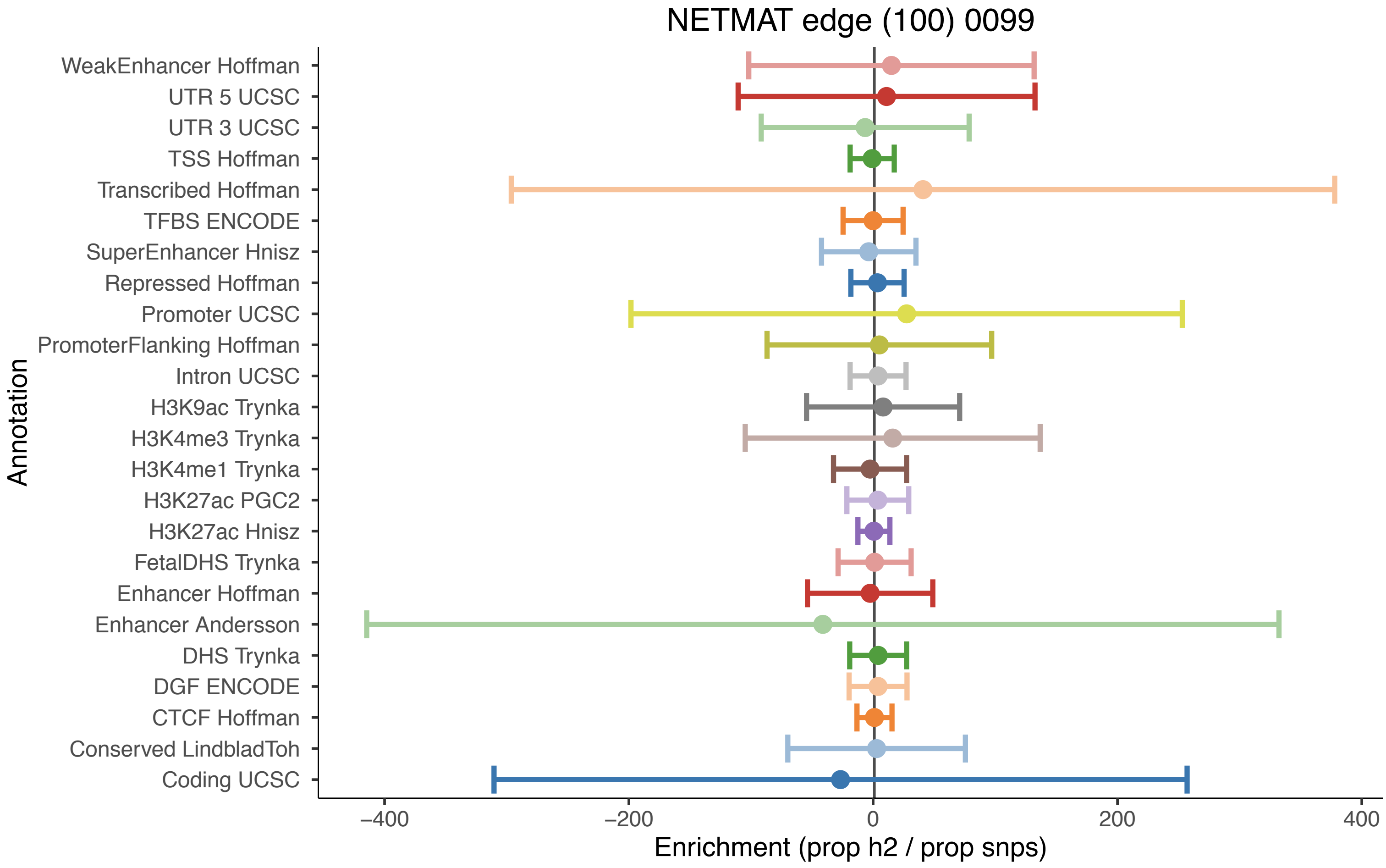

NETMAT edge (100) 0099
Annotation
WeakEnhancer Hoffman
UTR 5 UCSC
UTR 3 UCSC
TSS Hoffman
Transcribed Hoffman
TFBS ENCODE
SuperEnhancer Hnisz
Repressed Hoffman
Promoter UCSC
PromoterFlanking Hoffman
Intron UCSC
H3K9ac Trynka
H3K4me3 Trynka
H3K4me1 Trynka
H3K27ac PGC2
H3K27ac Hnisz
FetalDHS Trynka
Enhancer Hoffman
Enhancer Andersson
DHS Trynka
DGF ENCODE
CTCF Hoffman
Conserved LindbladToh
Coding UCSC
−400
−200
0
200
400
Enrichment (prop h2 / prop snps)

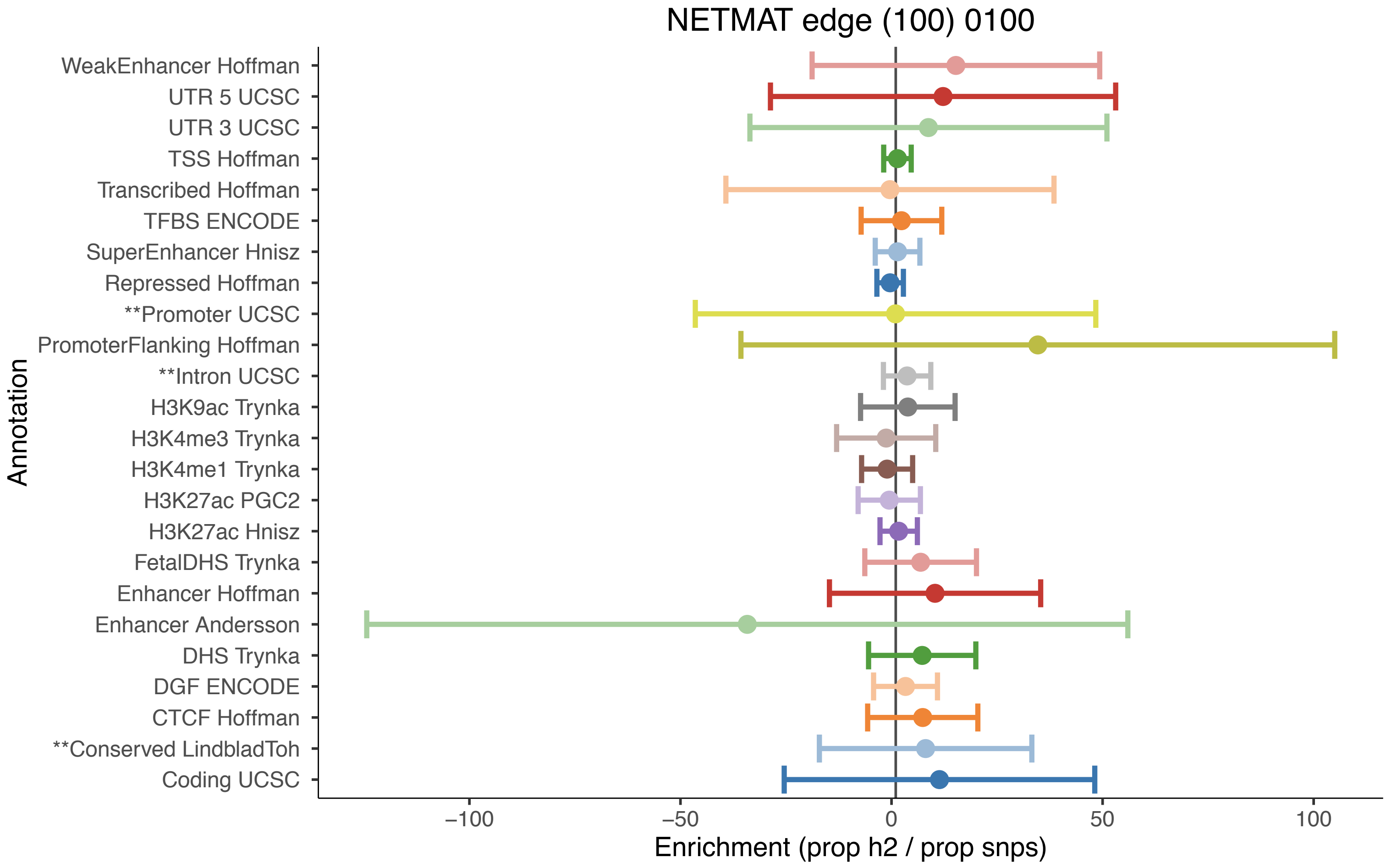

NETMAT edge (100) 0100
Annotation
WeakEnhancer Hoffman
UTR 5 UCSC
UTR 3 UCSC
TSS Hoffman
Transcribed Hoffman
TFBS ENCODE
SuperEnhancer Hnisz
Repressed Hoffman
**Promoter UCSC
PromoterFlanking Hoffman
**Intron UCSC
H3K9ac Trynka
H3K4me3 Trynka
H3K4me1 Trynka
H3K27ac PGC2
H3K27ac Hnisz
FetalDHS Trynka
Enhancer Hoffman
Enhancer Andersson
DHS Trynka
DGF ENCODE
CTCF Hoffman
**Conserved LindbladToh
Coding UCSC
−100
−50
0
50
100
Enrichment (prop h2 / prop snps)

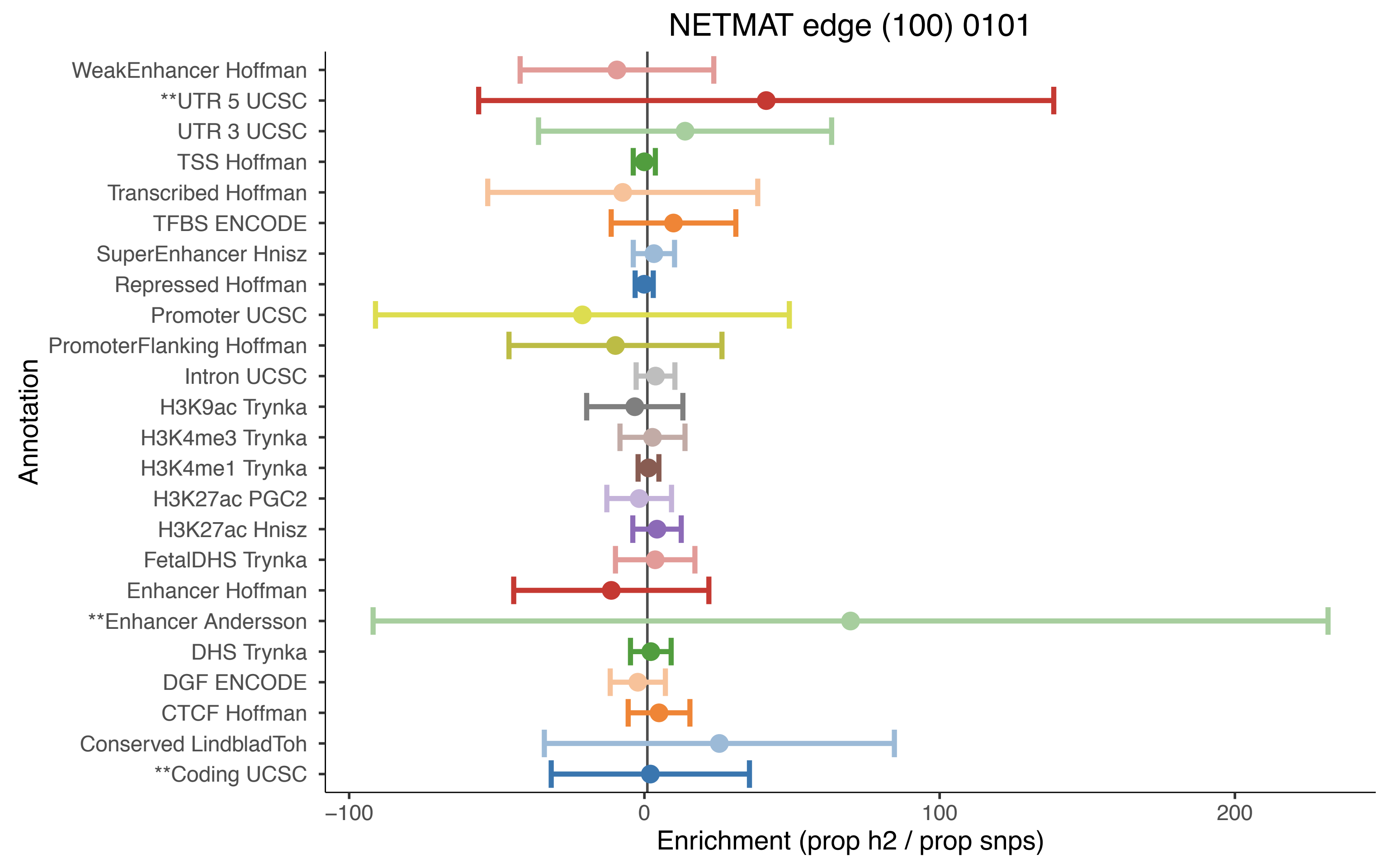
NETMAT edge (100) 0101
Annotation
WeakEnhancer Hoffman
**UTR 5 UCSC
UTR 3 UCSC
TSS Hoffman
Transcribed Hoffman
TFBS ENCODE
SuperEnhancer Hnisz
Repressed Hoffman
Promoter UCSC
PromoterFlanking Hoffman
Intron UCSC
H3K9ac Trynka
H3K4me3 Trynka
H3K4me1 Trynka
H3K27ac PGC2
H3K27ac Hnisz
FetalDHS Trynka
Enhancer Hoffman
**Enhancer Andersson
DHS Trynka
DGF ENCODE
CTCF Hoffman
Conserved LindbladToh
**Coding UCSC
−100
0
100
200
Enrichment (prop h2 / prop snps)

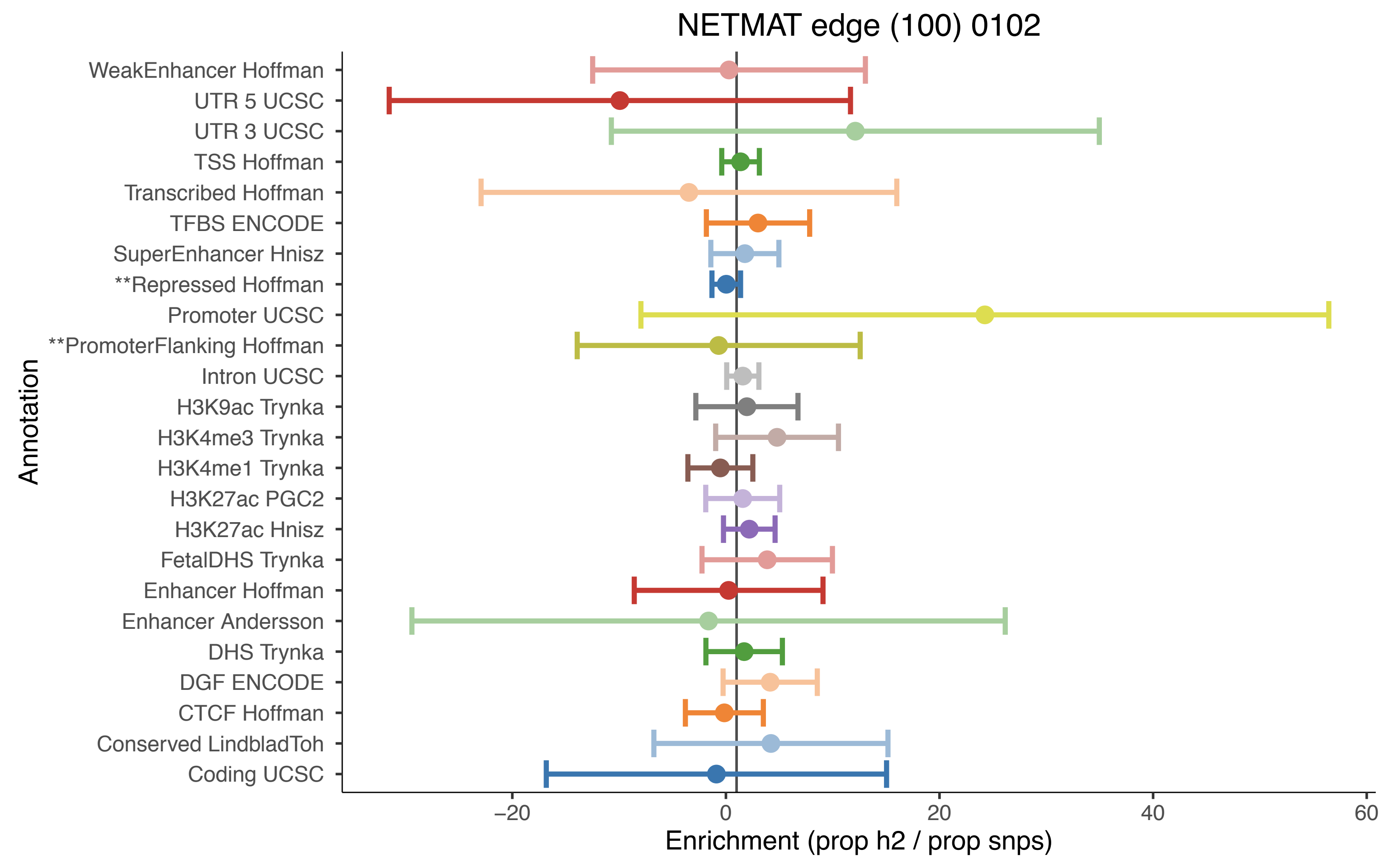
NETMAT edge (100) 0102
WeakEnhancer Hoffman
UTR 5 UCSC
UTR 3 UCSC
TSS Hoffman
Transcribed Hoffman
TFBS ENCODE
SuperEnhancer Hnisz
**Repressed Hoffman
Promoter UCSC
**PromoterFlanking Hoffman
Intron UCSC
H3K9ac Trynka
H3K4me3 Trynka
H3K4me1 Trynka
H3K27ac PGC2
H3K27ac Hnisz
FetalDHS Trynka
Enhancer Hoffman
Enhancer Andersson
DHS Trynka
DGF ENCODE
CTCF Hoffman
Conserved LindbladToh
Coding UCSC
Annotation
−20
0
20
40
60
Enrichment (prop h2 / prop snps)

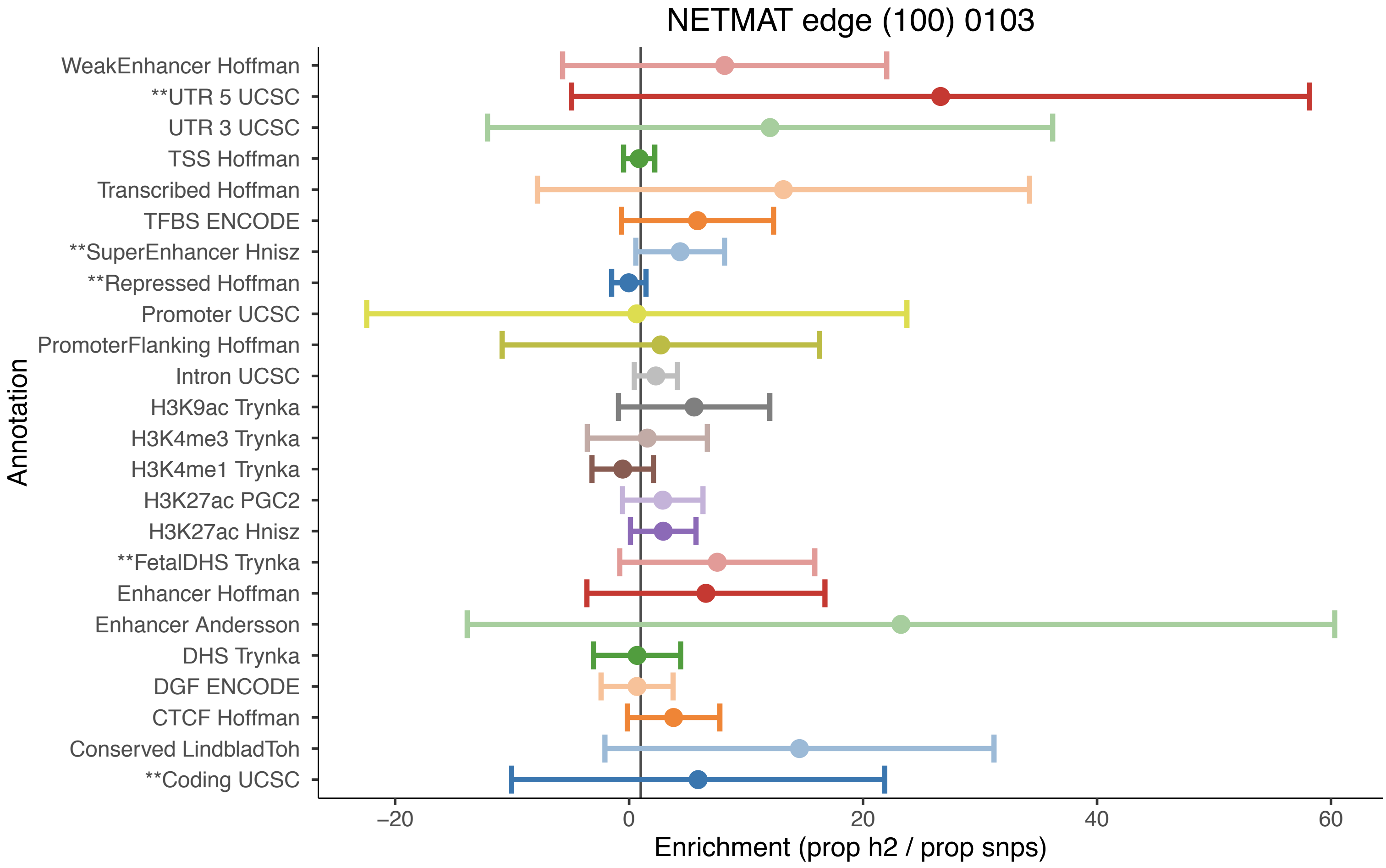
NETMAT edge (100) 0103
WeakEnhancer Hoffman
**UTR 5 UCSC
UTR 3 UCSC
TSS Hoffman
Transcribed Hoffman
TFBS ENCODE
**SuperEnhancer Hnisz
**Repressed Hoffman
Promoter UCSC
PromoterFlanking Hoffman
Intron UCSC
H3K9ac Trynka
H3K4me3 Trynka
H3K4me1 Trynka
H3K27ac PGC2
H3K27ac Hnisz
**FetalDHS Trynka
Enhancer Hoffman
Enhancer Andersson
DHS Trynka
DGF ENCODE
CTCF Hoffman
Conserved LindbladToh
**Coding UCSC
Annotation
−20
0
20
40
60
Enrichment (prop h2 / prop snps)

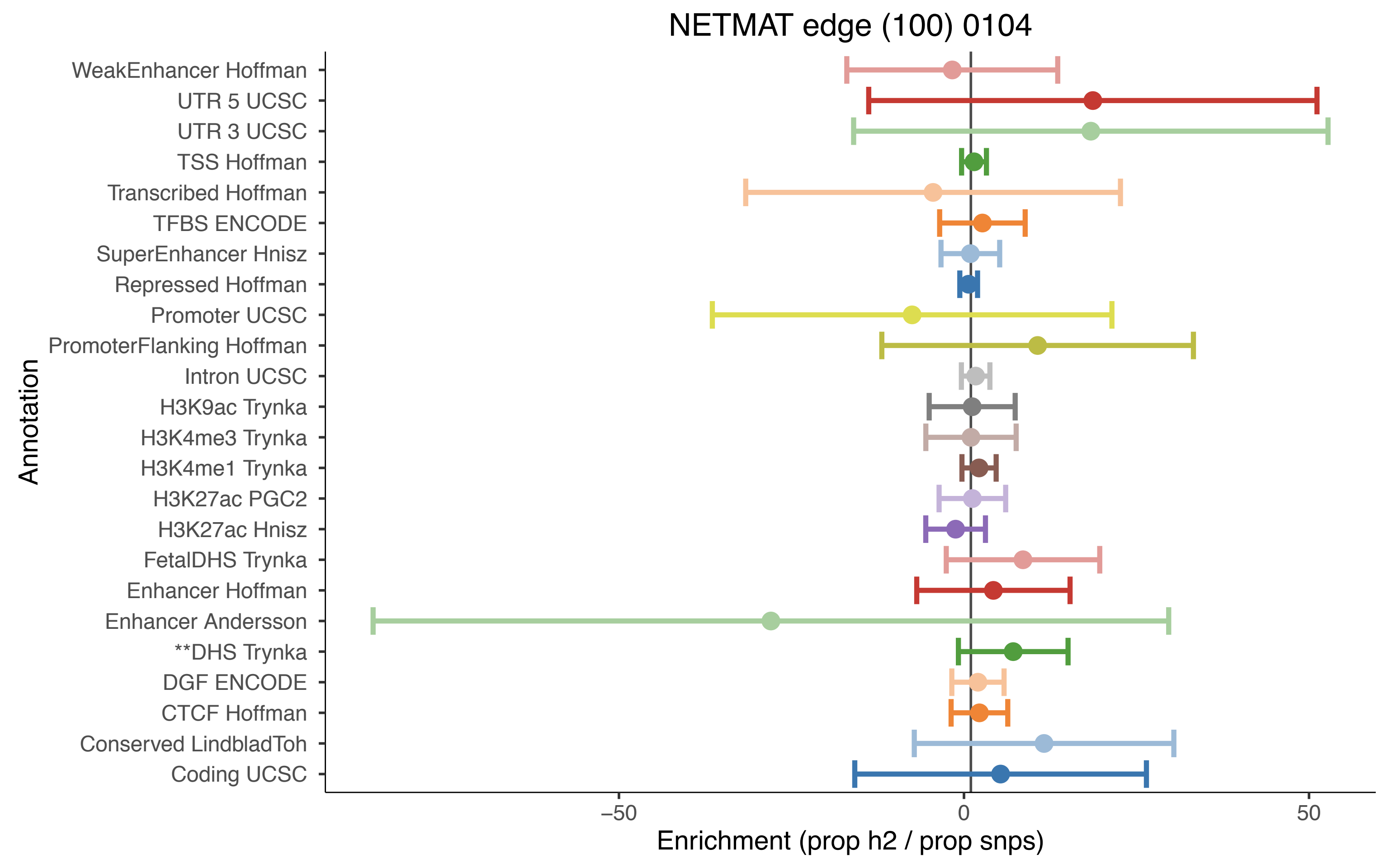

NETMAT edge (100) 0104
WeakEnhancer Hoffman
UTR 5 UCSC
UTR 3 UCSC
TSS Hoffman
Transcribed Hoffman
TFBS ENCODE
SuperEnhancer Hnisz
Repressed Hoffman
Promoter UCSC
PromoterFlanking Hoffman
Intron UCSC
H3K9ac Trynka
H3K4me3 Trynka
H3K4me1 Trynka
H3K27ac PGC2
H3K27ac Hnisz
FetalDHS Trynka
Enhancer Hoffman
Enhancer Andersson
**DHS Trynka
DGF ENCODE
CTCF Hoffman
Conserved LindbladToh
Coding UCSC
Annotation
−50
0
50
Enrichment (prop h2 / prop snps)

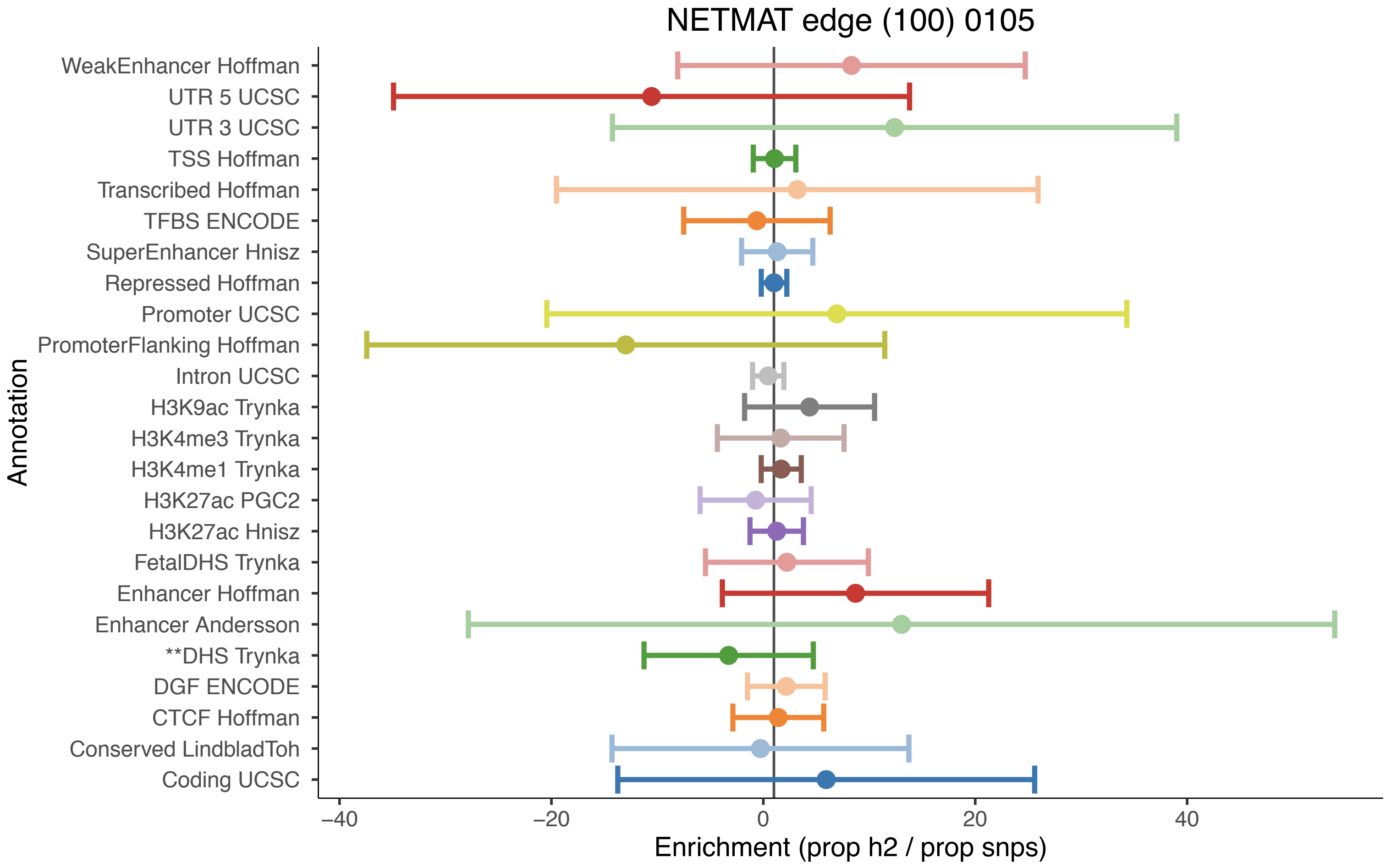

NETMAT edge (100) 0105
WeakEnhancer Hoffman
UTR 5 UCSC
UTR 3 UCSC
TSS Hoffman
Transcribed Hoffman
TFBS ENCODE
SuperEnhancer Hnisz
Repressed Hoffman
Promoter UCSC
PromoterFlanking Hoffman
Intron UCSC
H3K9ac Trynka
H3K4me3 Trynka
H3K4me1 Trynka
H3K27ac PGC2
H3K27ac Hnisz
FetalDHS Trynka
Enhancer Hoffman
Enhancer Andersson
**DHS Trynka
DGF ENCODE
CTCF Hoffman
Conserved LindbladToh
Coding UCSC
Annotation
−40
−20
0
20
40
Enrichment (prop h2 / prop snps)

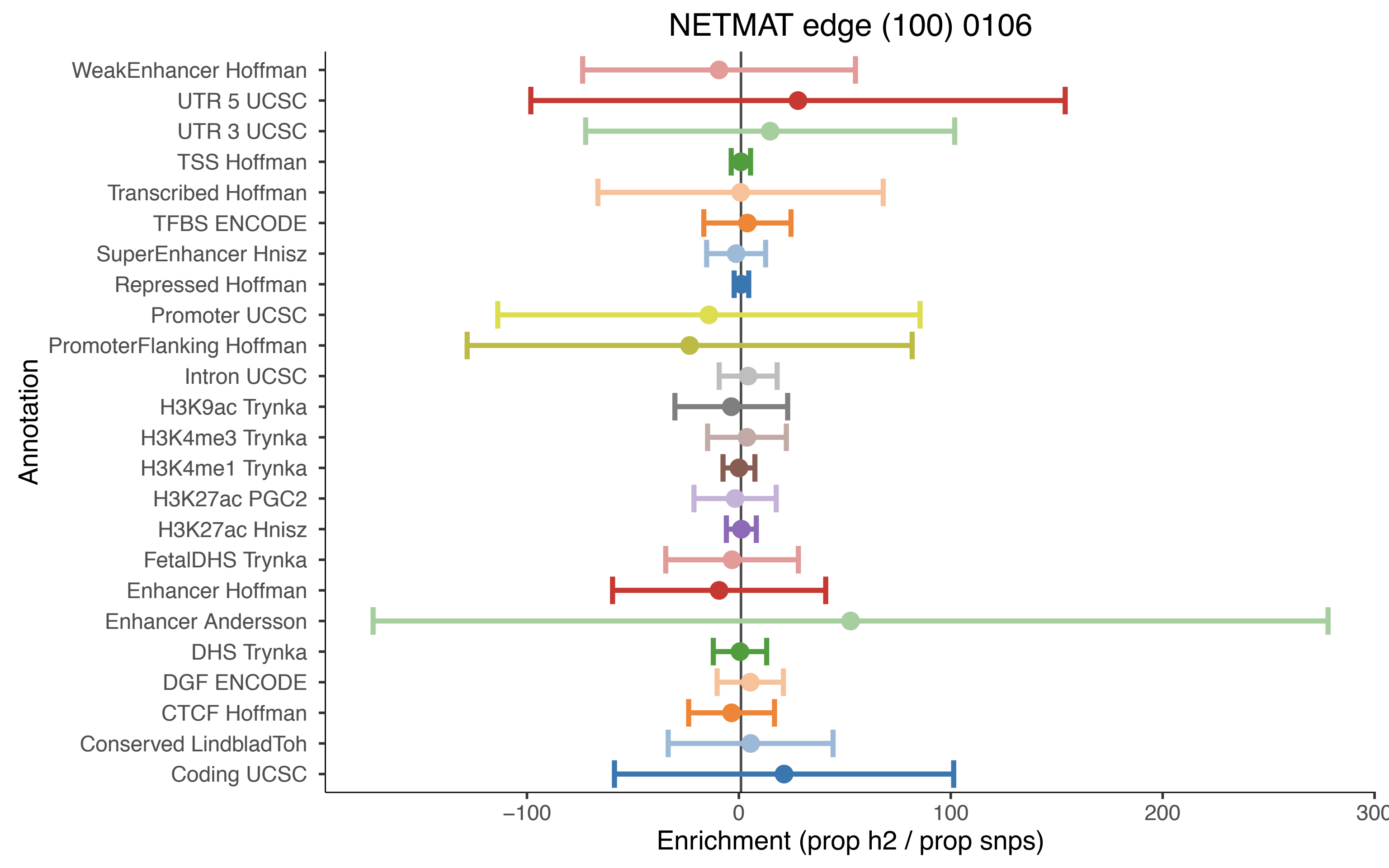

NETMAT edge (100) 0106
WeakEnhancer Hoffman
UTR 5 UCSC
UTR 3 UCSC
TSS Hoffman
Transcribed Hoffman
TFBS ENCODE
SuperEnhancer Hnisz
Repressed Hoffman
Promoter UCSC
PromoterFlanking Hoffman
Intron UCSC
H3K9ac Trynka
H3K4me3 Trynka
H3K4me1 Trynka
H3K27ac PGC2
H3K27ac Hnisz
FetalDHS Trynka
Enhancer Hoffman
Enhancer Andersson
DHS Trynka
DGF ENCODE
CTCF Hoffman
Conserved LindbladToh
Coding UCSC
Annotation
−100
0
100
200
Enrichment (prop h2 / prop snps)

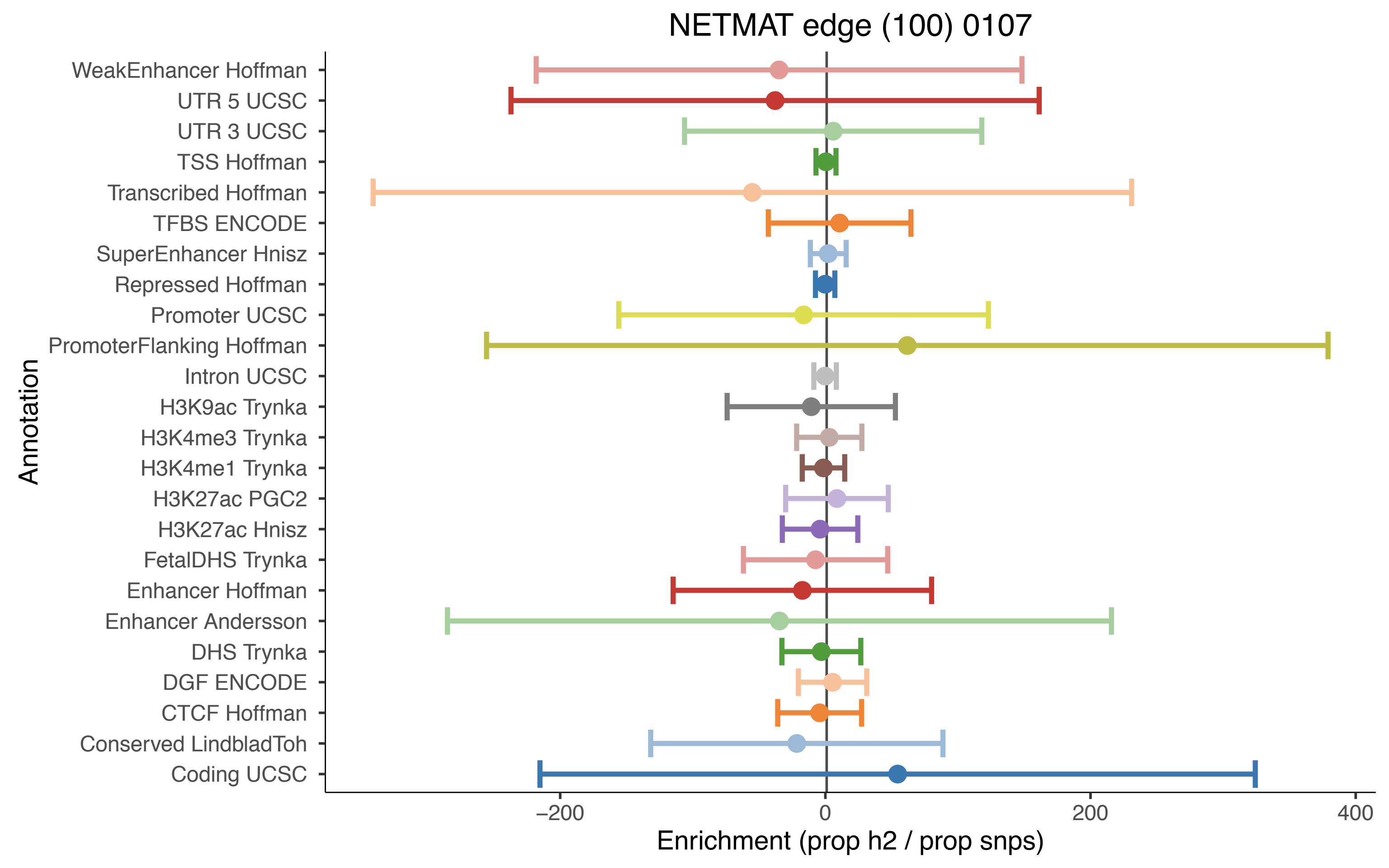

NETMAT edge (100) 0107
WeakEnhancer Hoffman
UTR 5 UCSC
UTR 3 UCSC
TSS Hoffman
Transcribed Hoffman
TFBS ENCODE
SuperEnhancer Hnisz
Repressed Hoffman
Promoter UCSC
PromoterFlanking Hoffman
Intron UCSC
H3K9ac Trynka
H3K4me3 Trynka
H3K4me1 Trynka
H3K27ac PGC2
H3K27ac Hnisz
FetalDHS Trynka
Enhancer Hoffman
Enhancer Andersson
DHS Trynka
DGF ENCODE
CTCF Hoffman
Conserved LindbladToh
Coding UCSC
Annotation
−200
0
200
400
Enrichment (prop h2 / prop snps)

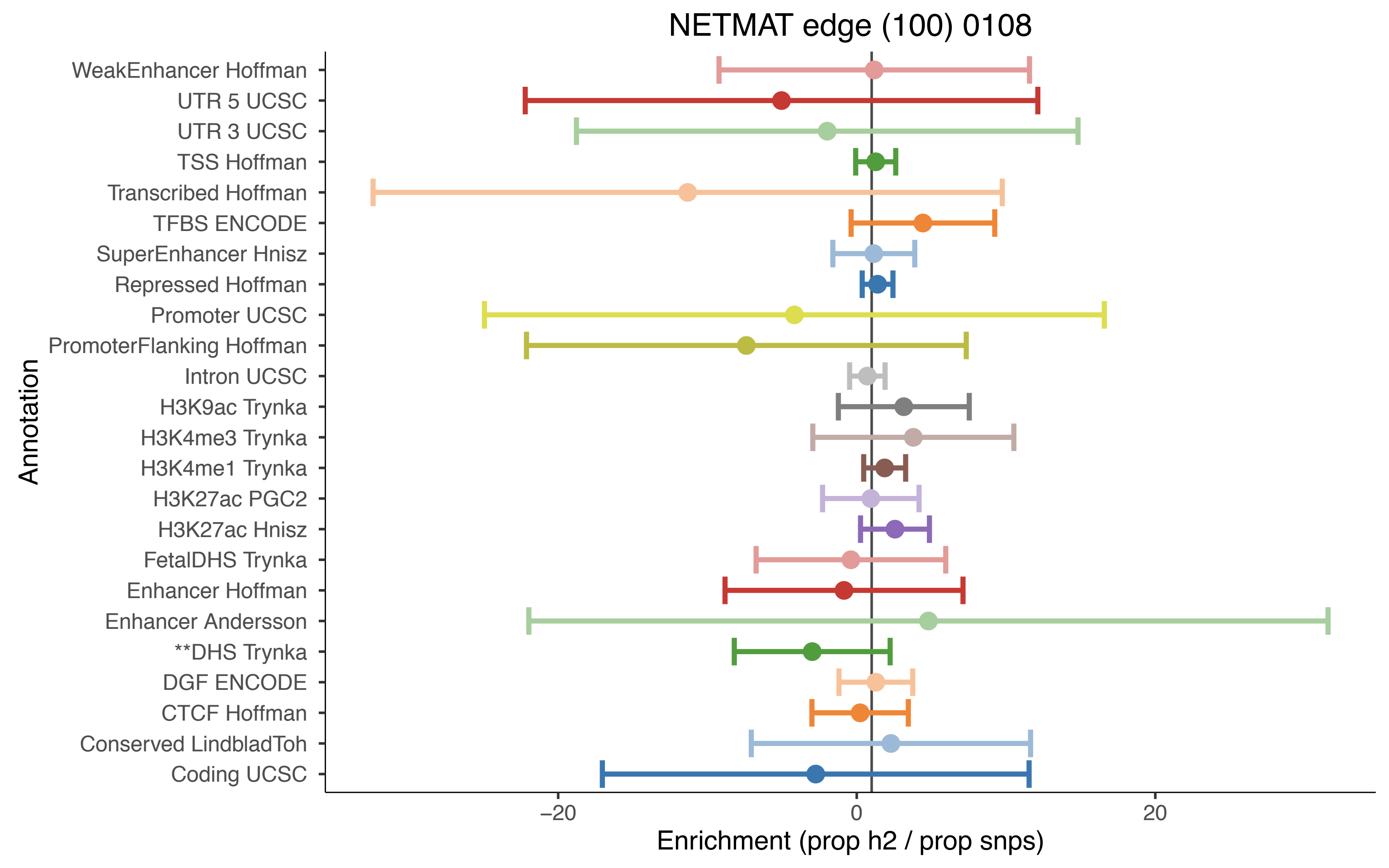
NETMAT edge (100) 0108
WeakEnhancer Hoffman
UTR 5 UCSC
UTR 3 UCSC
TSS Hoffman
Transcribed Hoffman
TFBS ENCODE
SuperEnhancer Hnisz
Repressed Hoffman
Promoter UCSC
PromoterFlanking Hoffman
Intron UCSC
H3K9ac Trynka
H3K4me3 Trynka
H3K4me1 Trynka
H3K27ac PGC2
H3K27ac Hnisz
FetalDHS Trynka
Enhancer Hoffman
Enhancer Andersson
**DHS Trynka
DGF ENCODE
CTCF Hoffman
Conserved LindbladToh
Coding UCSC
Annotation
−20
0
20
Enrichment (prop h2 / prop snps)

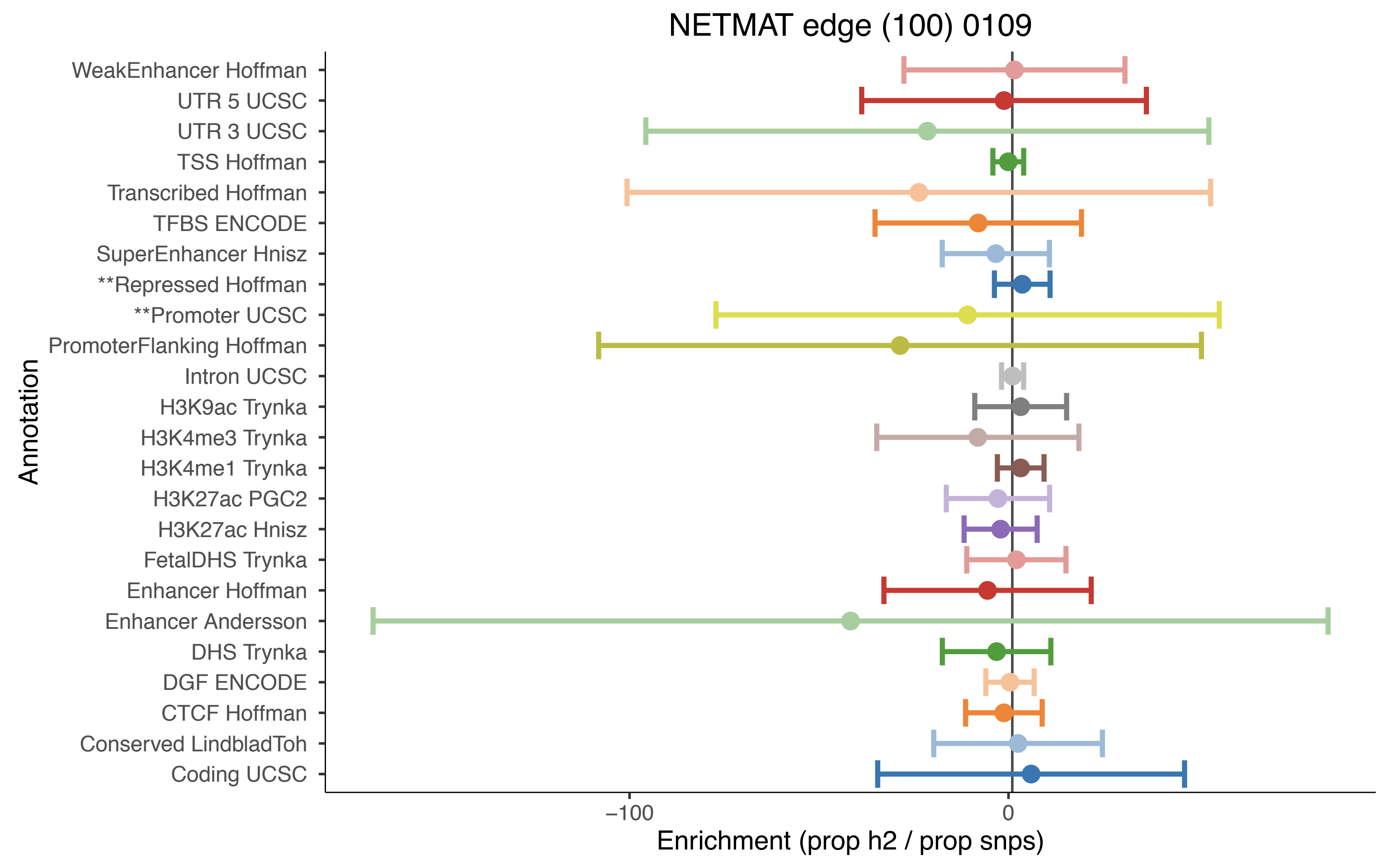
NETMAT edge (100) 0109
Annotation
WeakEnhancer Hoffman
UTR 5 UCSC
UTR 3 UCSC
TSS Hoffman
Transcribed Hoffman
TFBS ENCODE
SuperEnhancer Hnisz
**Repressed Hoffman
**Promoter UCSC
PromoterFlanking Hoffman
Intron UCSC
H3K9ac Trynka
H3K4me3 Trynka
H3K4me1 Trynka
H3K27ac PGC2
H3K27ac Hnisz
FetalDHS Trynka
Enhancer Hoffman
Enhancer Andersson
DHS Trynka
DGF ENCODE
CTCF Hoffman
Conserved LindbladToh
Coding UCSC
−100
0
Enrichment (prop h2 / prop snps)

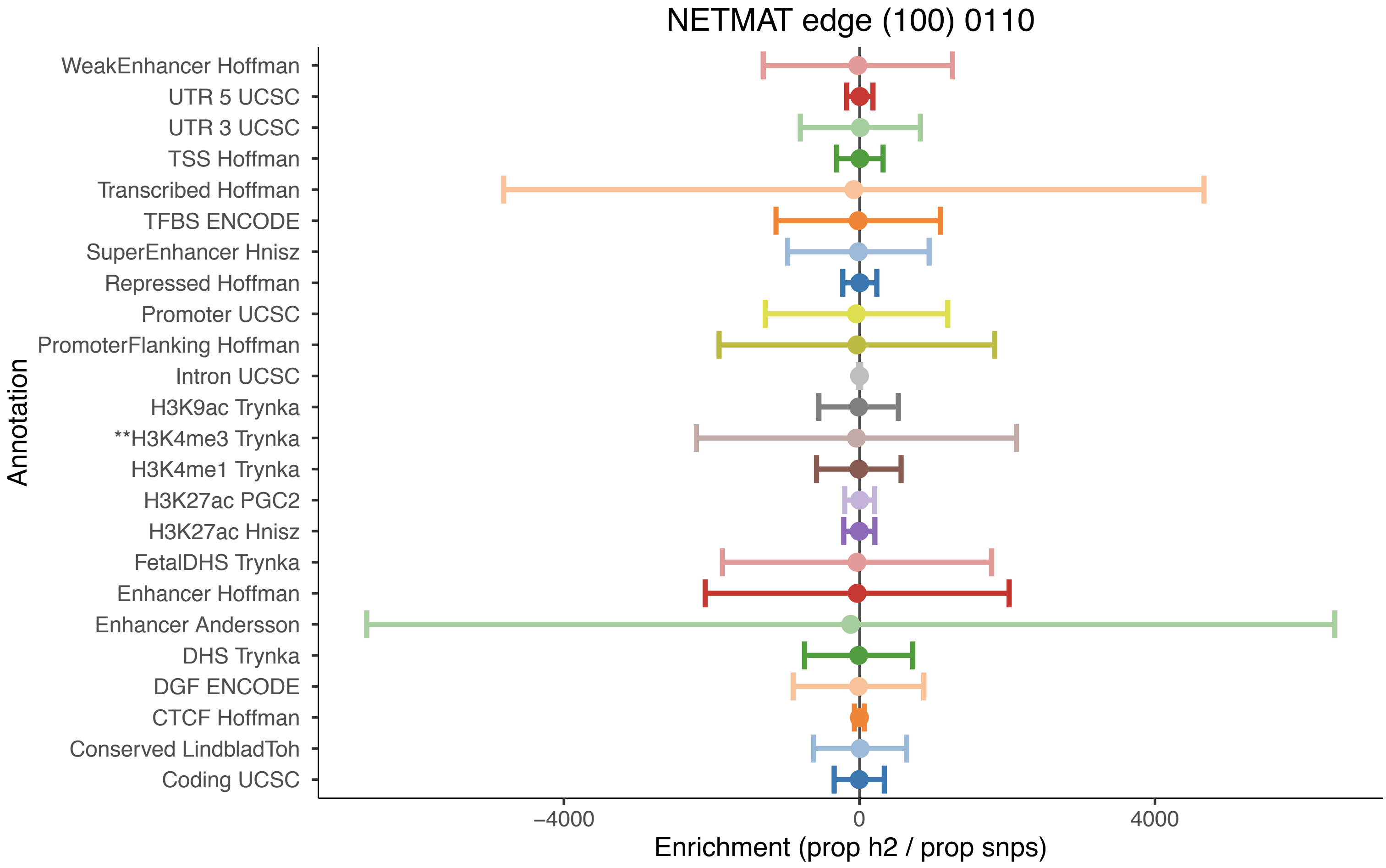

NETMAT edge (100) 0110
WeakEnhancer Hoffman
UTR 5 UCSC
UTR 3 UCSC
TSS Hoffman
Transcribed Hoffman
TFBS ENCODE
SuperEnhancer Hnisz
Repressed Hoffman
Promoter UCSC
PromoterFlanking Hoffman
Intron UCSC
H3K9ac Trynka
**H3K4me3 Trynka
H3K4me1 Trynka
H3K27ac PGC2
H3K27ac Hnisz
FetalDHS Trynka
Enhancer Hoffman
Enhancer Andersson
DHS Trynka
DGF ENCODE
CTCF Hoffman
Conserved LindbladToh
Coding UCSC
Annotation
−4000
0
4000
Enrichment (prop h2 / prop snps)

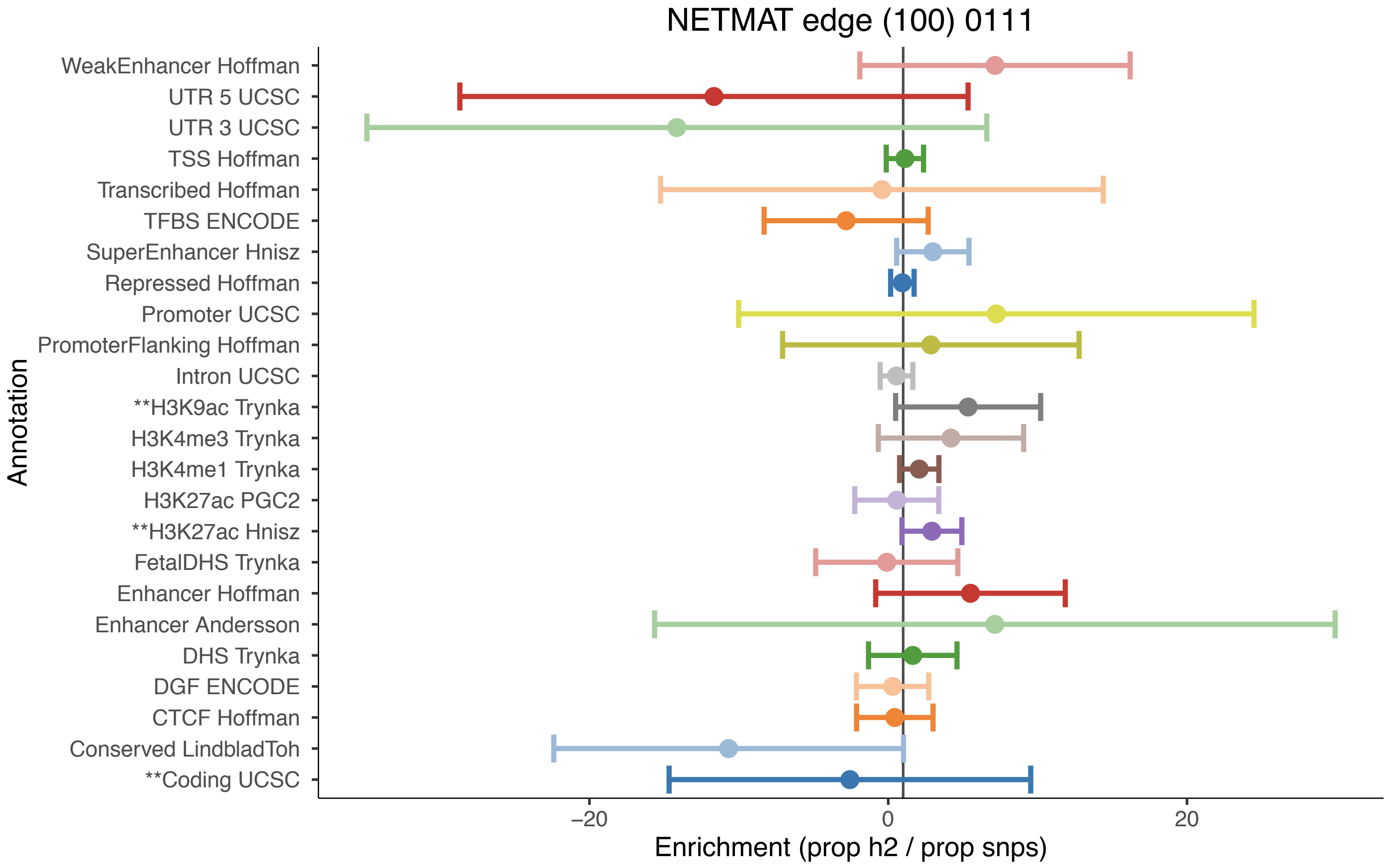

NETMAT edge (100) 0111
WeakEnhancer Hoffman
UTR 5 UCSC
UTR 3 UCSC
TSS Hoffman
Transcribed Hoffman
TFBS ENCODE
SuperEnhancer Hnisz
Repressed Hoffman
Promoter UCSC
PromoterFlanking Hoffman
Intron UCSC
**H3K9ac Trynka
H3K4me3 Trynka
H3K4me1 Trynka
H3K27ac PGC2
**H3K27ac Hnisz
FetalDHS Trynka
Enhancer Hoffman
Enhancer Andersson
DHS Trynka
DGF ENCODE
CTCF Hoffman
Conserved LindbladToh
**Coding UCSC
Annotation
−20
0
20
Enrichment (prop h2 / prop snps)

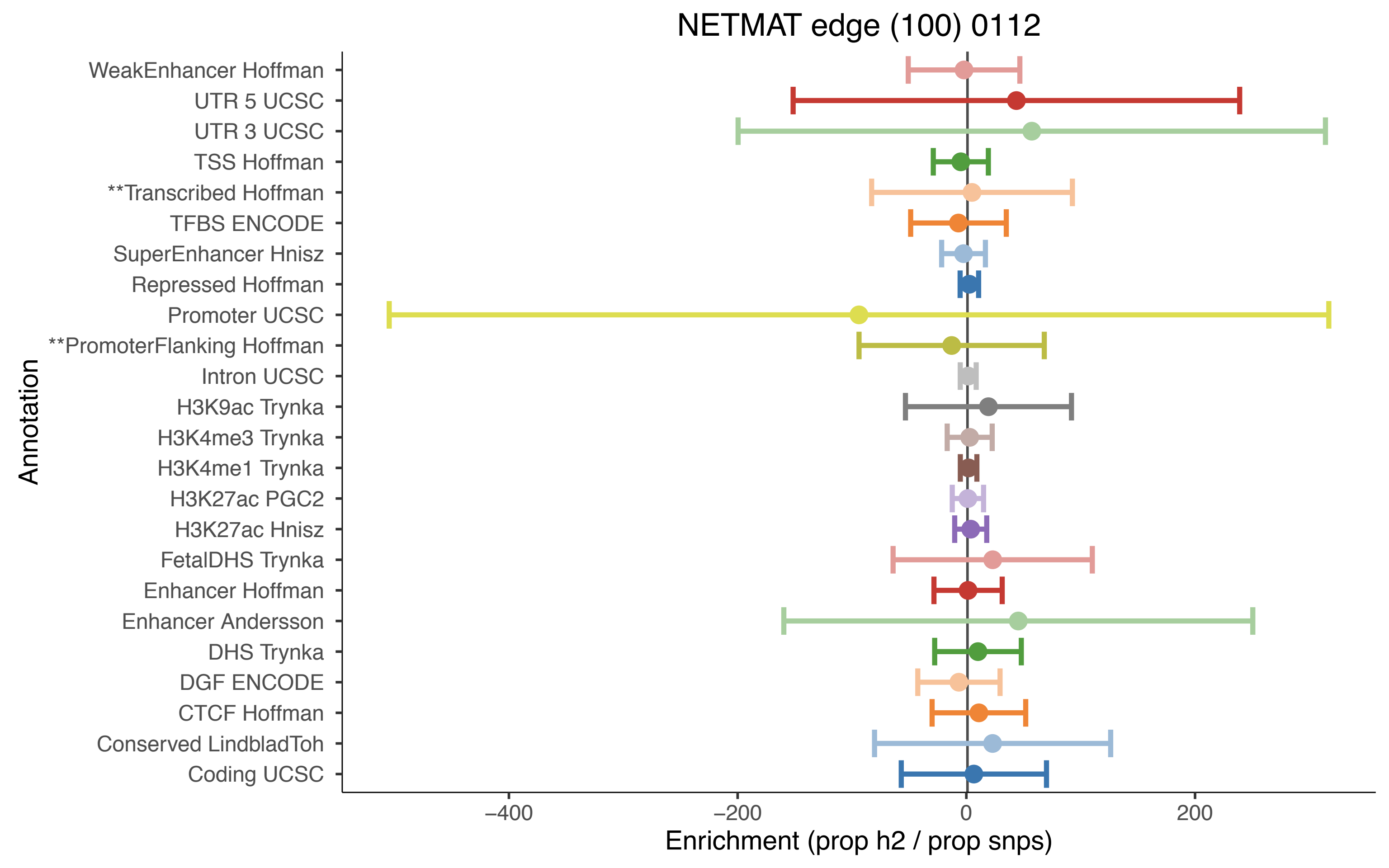
NETMAT edge (100) 0112
WeakEnhancer Hoffman
UTR 5 UCSC
UTR 3 UCSC
TSS Hoffman
**Transcribed Hoffman
TFBS ENCODE
SuperEnhancer Hnisz
Repressed Hoffman
Promoter UCSC
**PromoterFlanking Hoffman
Intron UCSC
H3K9ac Trynka
H3K4me3 Trynka
H3K4me1 Trynka
H3K27ac PGC2
H3K27ac Hnisz
FetalDHS Trynka
Enhancer Hoffman
Enhancer Andersson
DHS Trynka
DGF ENCODE
CTCF Hoffman
Conserved LindbladToh
Coding UCSC
Annotation
−400
−200
0
200
Enrichment (prop h2 / prop snps)

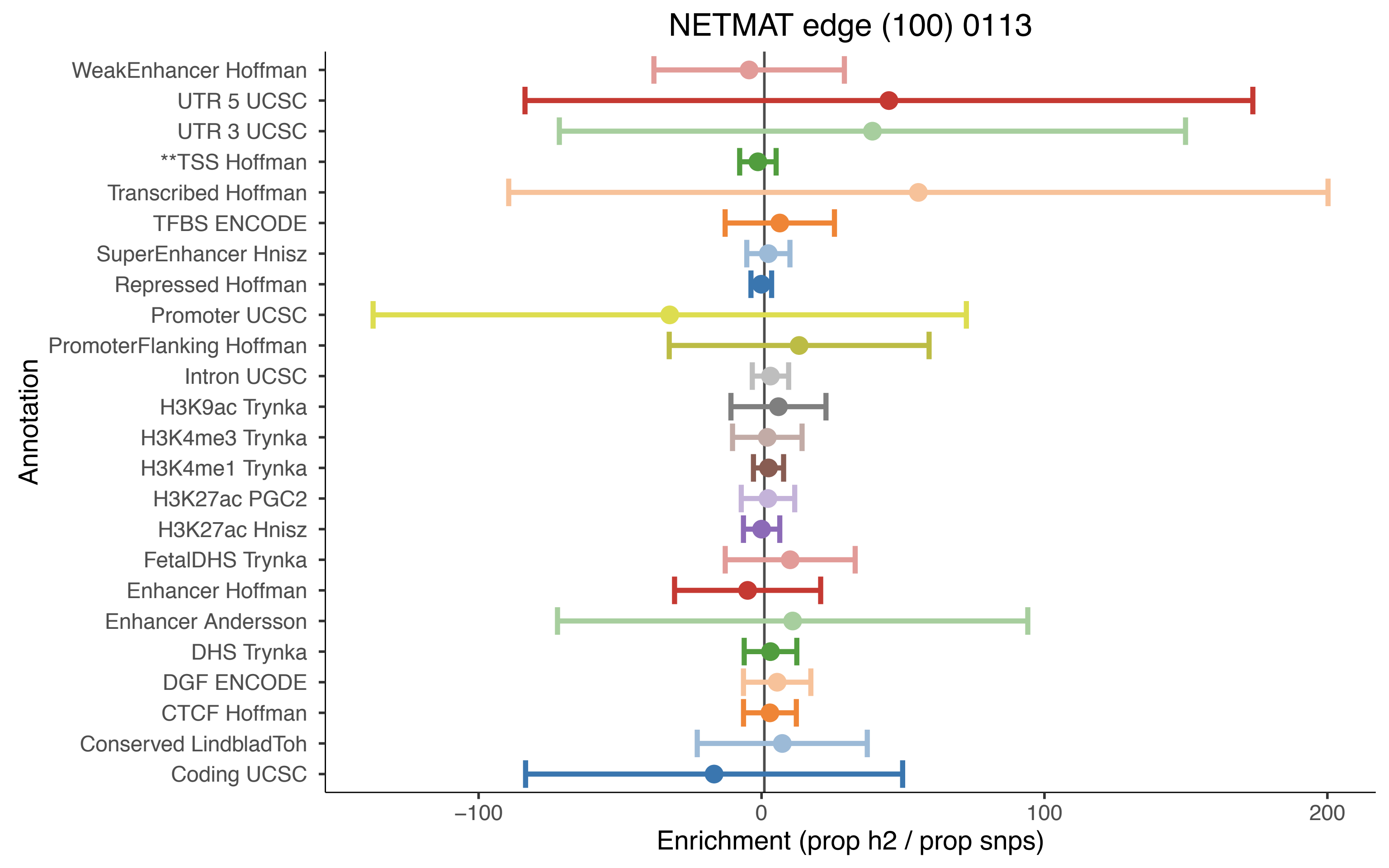
NETMAT edge (100) 0113
WeakEnhancer Hoffman
UTR 5 UCSC
UTR 3 UCSC
**TSS Hoffman
Transcribed Hoffman
TFBS ENCODE
SuperEnhancer Hnisz
Repressed Hoffman
Promoter UCSC
PromoterFlanking Hoffman
Intron UCSC
H3K9ac Trynka
H3K4me3 Trynka
H3K4me1 Trynka
H3K27ac PGC2
H3K27ac Hnisz
FetalDHS Trynka
Enhancer Hoffman
Enhancer Andersson
DHS Trynka
DGF ENCODE
CTCF Hoffman
Conserved LindbladToh
Coding UCSC
Annotation
−100
0
100
200
Enrichment (prop h2 / prop snps)

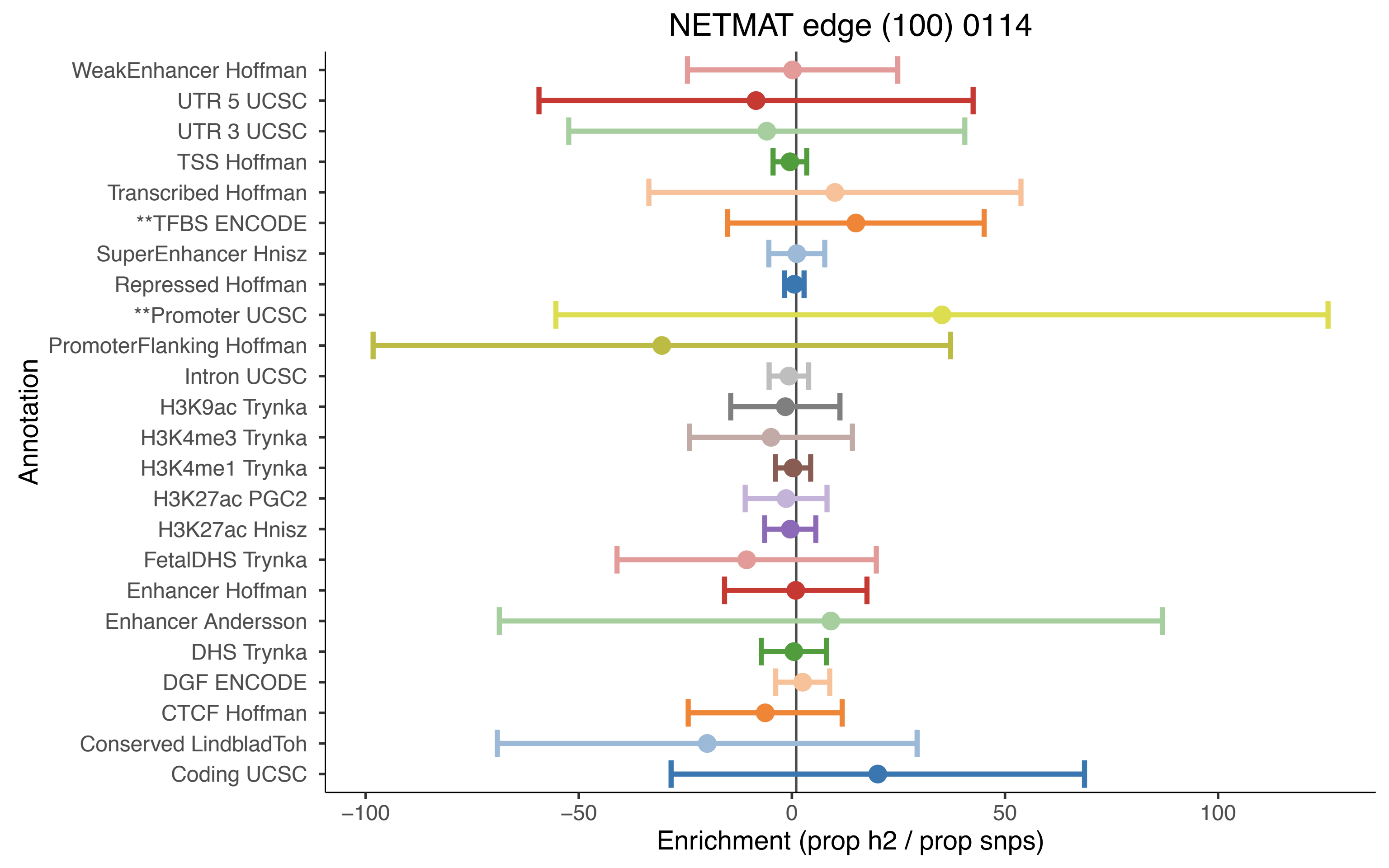

NETMAT edge (100) 0114
WeakEnhancer Hoffman
UTR 5 UCSC
UTR 3 UCSC
TSS Hoffman
Transcribed Hoffman
**TFBS ENCODE
SuperEnhancer Hnisz
Repressed Hoffman
**Promoter UCSC
PromoterFlanking Hoffman
Intron UCSC
H3K9ac Trynka
H3K4me3 Trynka
H3K4me1 Trynka
H3K27ac PGC2
H3K27ac Hnisz
FetalDHS Trynka
Enhancer Hoffman
Enhancer Andersson
DHS Trynka
DGF ENCODE
CTCF Hoffman
Conserved LindbladToh
Coding UCSC
Annotation
−100
−50
0
50
100
Enrichment (prop h2 / prop snps)

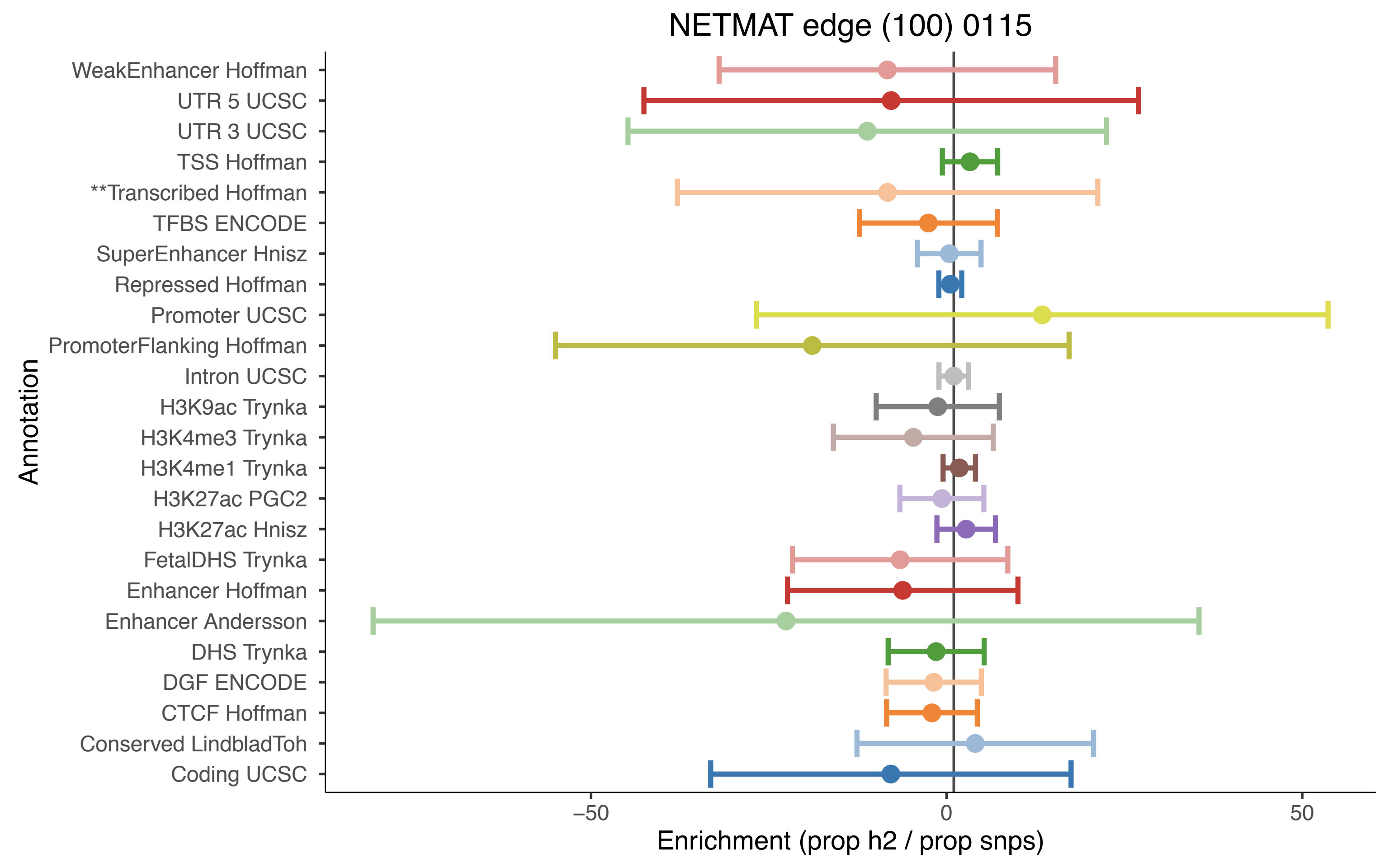
NETMAT edge (100) 0115
WeakEnhancer Hoffman
UTR 5 UCSC
UTR 3 UCSC
TSS Hoffman
**Transcribed Hoffman
TFBS ENCODE
SuperEnhancer Hnisz
Repressed Hoffman
Promoter UCSC
PromoterFlanking Hoffman
Intron UCSC
H3K9ac Trynka
H3K4me3 Trynka
H3K4me1 Trynka
H3K27ac PGC2
H3K27ac Hnisz
FetalDHS Trynka
Enhancer Hoffman
Enhancer Andersson
DHS Trynka
DGF ENCODE
CTCF Hoffman
Conserved LindbladToh
Coding UCSC
Annotation
−50
0
50
Enrichment (prop h2 / prop snps)

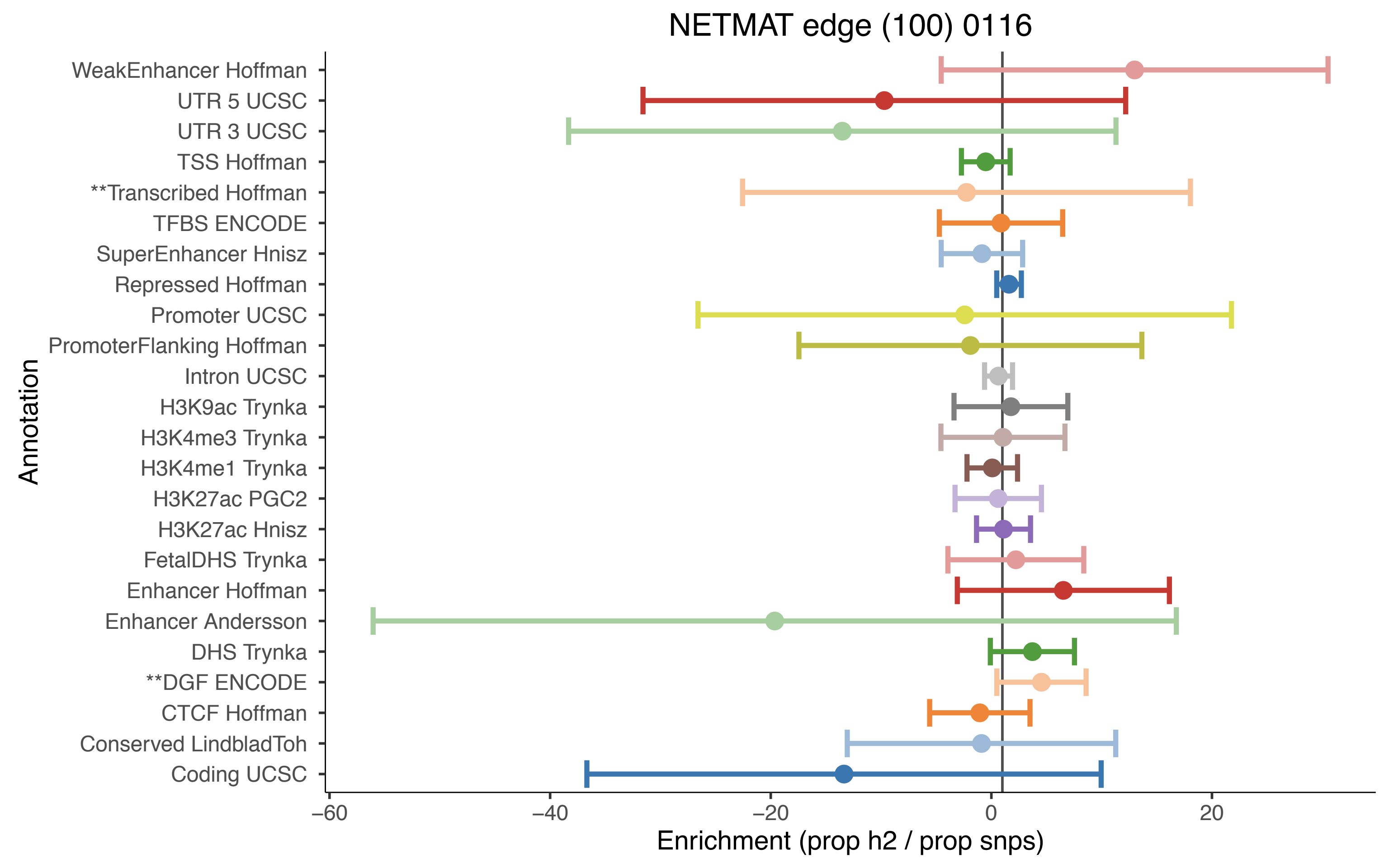
NETMAT edge (100) 0116
WeakEnhancer Hoffman
UTR 5 UCSC
UTR 3 UCSC
TSS Hoffman
**Transcribed Hoffman
TFBS ENCODE
SuperEnhancer Hnisz
Repressed Hoffman
Promoter UCSC
PromoterFlanking Hoffman
Intron UCSC
H3K9ac Trynka
H3K4me3 Trynka
H3K4me1 Trynka
H3K27ac PGC2
H3K27ac Hnisz
FetalDHS Trynka
Enhancer Hoffman
Enhancer Andersson
DHS Trynka
**DGF ENCODE
CTCF Hoffman
Conserved LindbladToh
Coding UCSC
Annotation
−60
−40
−20
0
20
Enrichment (prop h2 / prop snps)

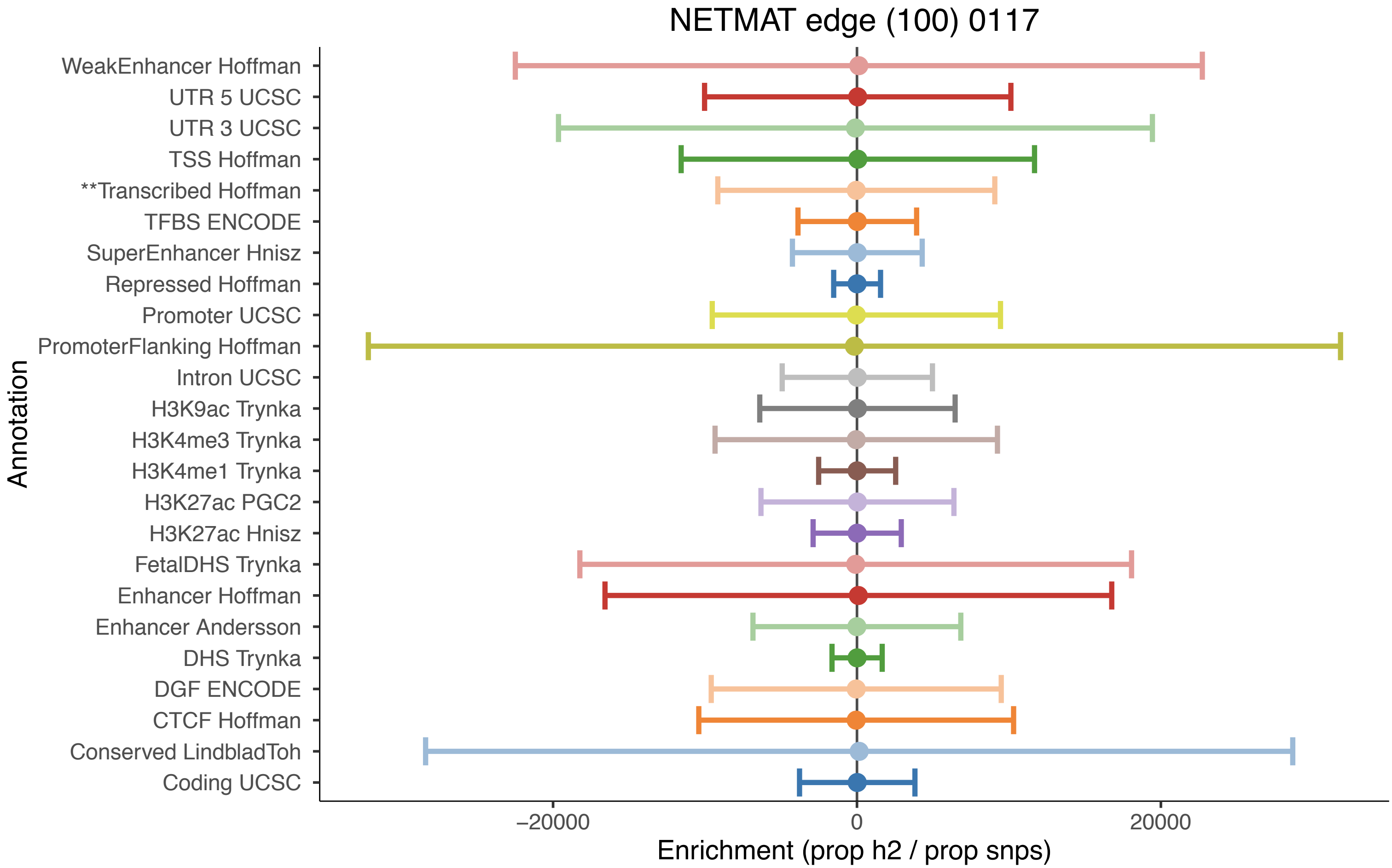

NETMAT edge (100) 0117
WeakEnhancer Hoffman
UTR 5 UCSC
UTR 3 UCSC
TSS Hoffman
**Transcribed Hoffman
TFBS ENCODE
SuperEnhancer Hnisz
Repressed Hoffman
Promoter UCSC
PromoterFlanking Hoffman
Intron UCSC
H3K9ac Trynka
H3K4me3 Trynka
H3K4me1 Trynka
H3K27ac PGC2
H3K27ac Hnisz
FetalDHS Trynka
Enhancer Hoffman
Enhancer Andersson
DHS Trynka
DGF ENCODE
CTCF Hoffman
Conserved LindbladToh
Coding UCSC
Annotation
−20000
0
20000
Enrichment (prop h2 / prop snps)

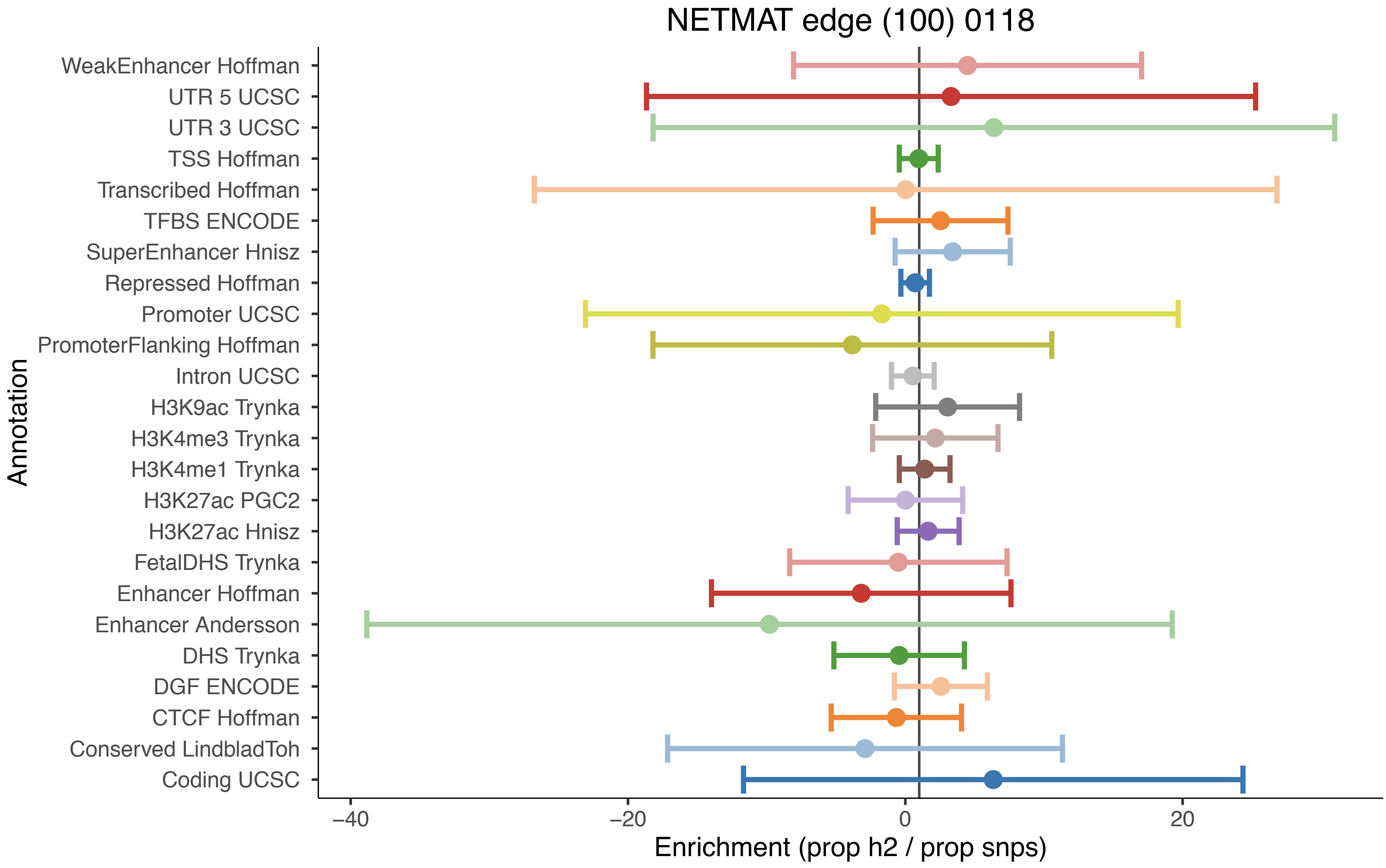
NETMAT edge (100) 0118
WeakEnhancer Hoffman
UTR 5 UCSC
UTR 3 UCSC
TSS Hoffman
Transcribed Hoffman
TFBS ENCODE
SuperEnhancer Hnisz
Repressed Hoffman
Promoter UCSC
PromoterFlanking Hoffman
Intron UCSC
H3K9ac Trynka
H3K4me3 Trynka
H3K4me1 Trynka
H3K27ac PGC2
H3K27ac Hnisz
FetalDHS Trynka
Enhancer Hoffman
Enhancer Andersson
DHS Trynka
DGF ENCODE
CTCF Hoffman
Conserved LindbladToh
Coding UCSC
Annotation
−40
−20
0
20
Enrichment (prop h2 / prop snps)

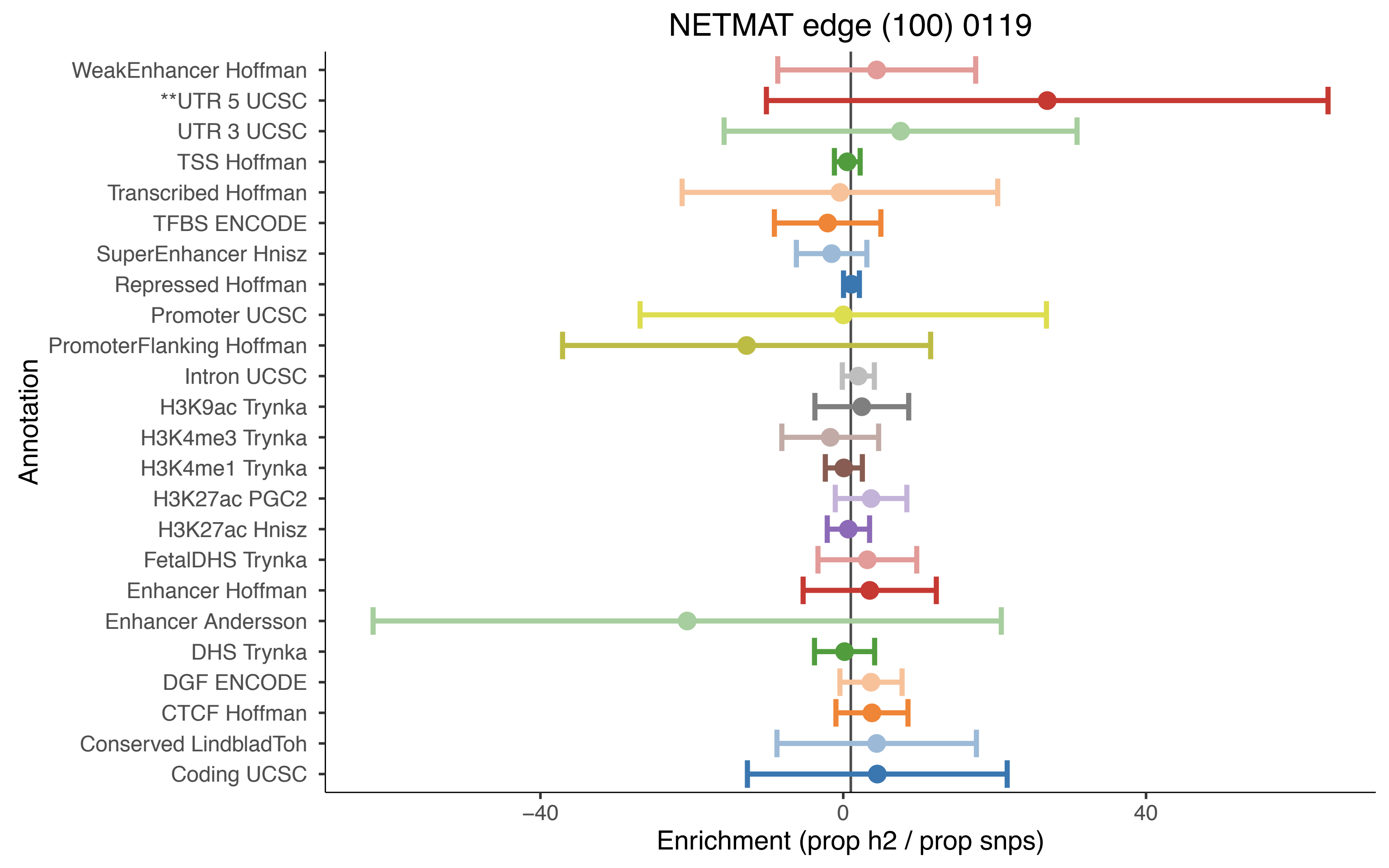

NETMAT edge (100) 0119
WeakEnhancer Hoffman
**UTR 5 UCSC
UTR 3 UCSC
TSS Hoffman
Transcribed Hoffman
TFBS ENCODE
SuperEnhancer Hnisz
Repressed Hoffman
Promoter UCSC
PromoterFlanking Hoffman
Intron UCSC
H3K9ac Trynka
H3K4me3 Trynka
H3K4me1 Trynka
H3K27ac PGC2
H3K27ac Hnisz
FetalDHS Trynka
Enhancer Hoffman
Enhancer Andersson
DHS Trynka
DGF ENCODE
CTCF Hoffman
Conserved LindbladToh
Coding UCSC
Annotation
−40
0
40
Enrichment (prop h2 / prop snps)

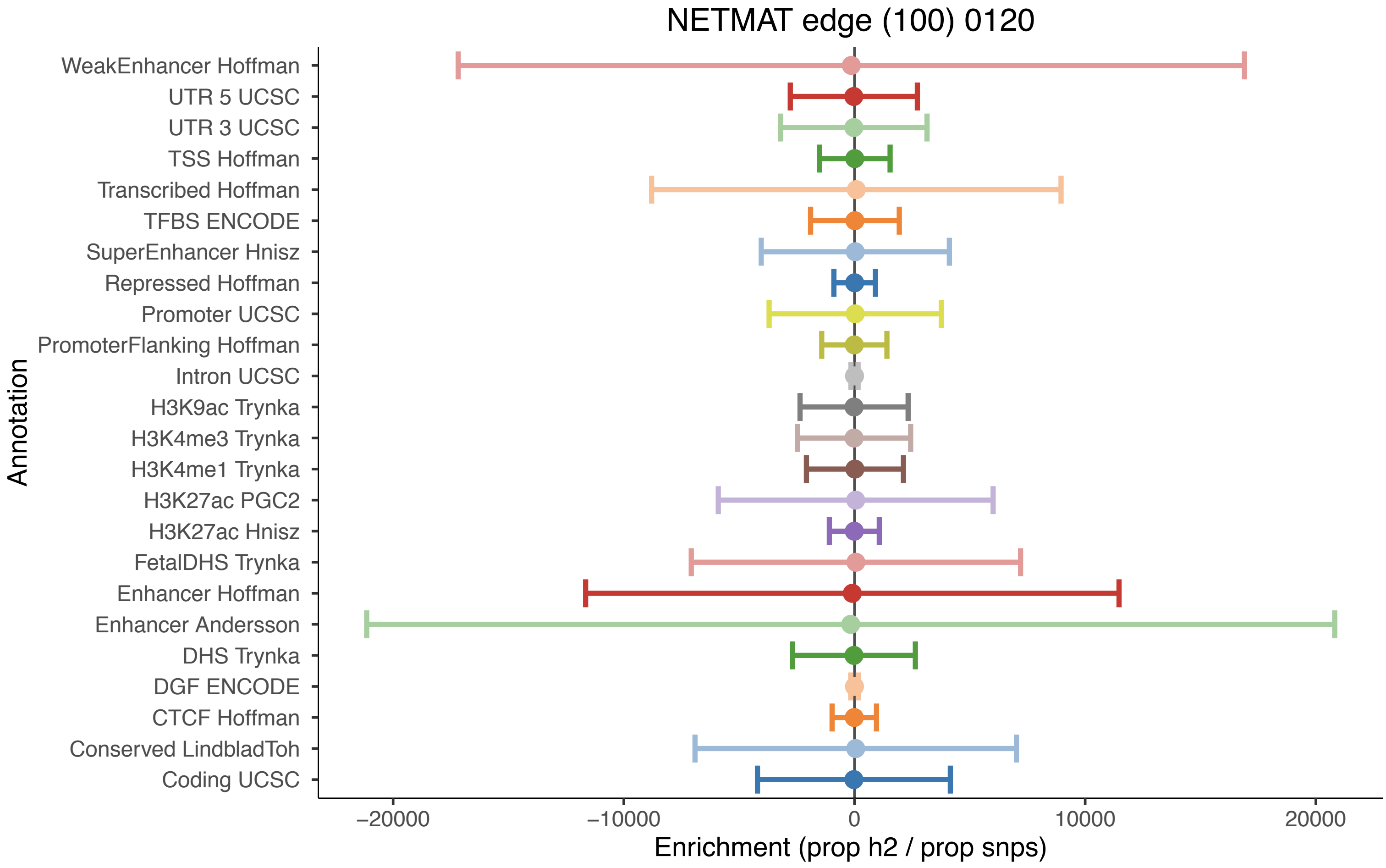

NETMAT edge (100) 0120
WeakEnhancer Hoffman
UTR 5 UCSC
UTR 3 UCSC
TSS Hoffman
Transcribed Hoffman
TFBS ENCODE
SuperEnhancer Hnisz
Repressed Hoffman
Promoter UCSC
PromoterFlanking Hoffman
Intron UCSC
H3K9ac Trynka
H3K4me3 Trynka
H3K4me1 Trynka
H3K27ac PGC2
H3K27ac Hnisz
FetalDHS Trynka
Enhancer Hoffman
Enhancer Andersson
DHS Trynka
DGF ENCODE
CTCF Hoffman
Conserved LindbladToh
Coding UCSC
Annotation
−20000
−10000
0
10000
20000
Enrichment (prop h2 / prop snps)

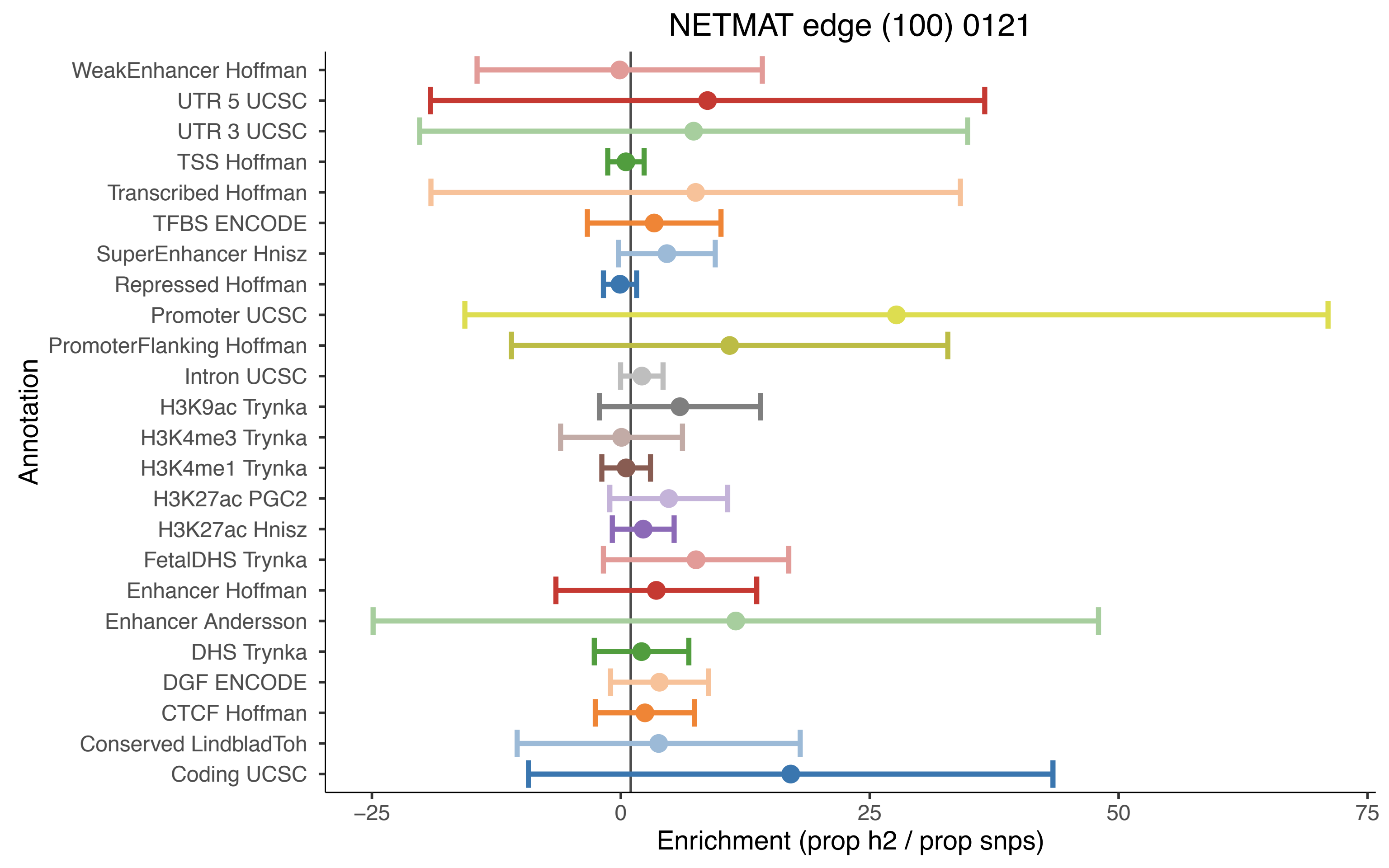
NETMAT edge (100) 0121
Annotation
WeakEnhancer Hoffman
UTR 5 UCSC
UTR 3 UCSC
TSS Hoffman
Transcribed Hoffman
TFBS ENCODE
SuperEnhancer Hnisz
Repressed Hoffman
Promoter UCSC
PromoterFlanking Hoffman
Intron UCSC
H3K9ac Trynka
H3K4me3 Trynka
H3K4me1 Trynka
H3K27ac PGC2
H3K27ac Hnisz
FetalDHS Trynka
Enhancer Hoffman
Enhancer Andersson
DHS Trynka
DGF ENCODE
CTCF Hoffman
Conserved LindbladToh
Coding UCSC
−25
0
25
50
75
Enrichment (prop h2 / prop snps)

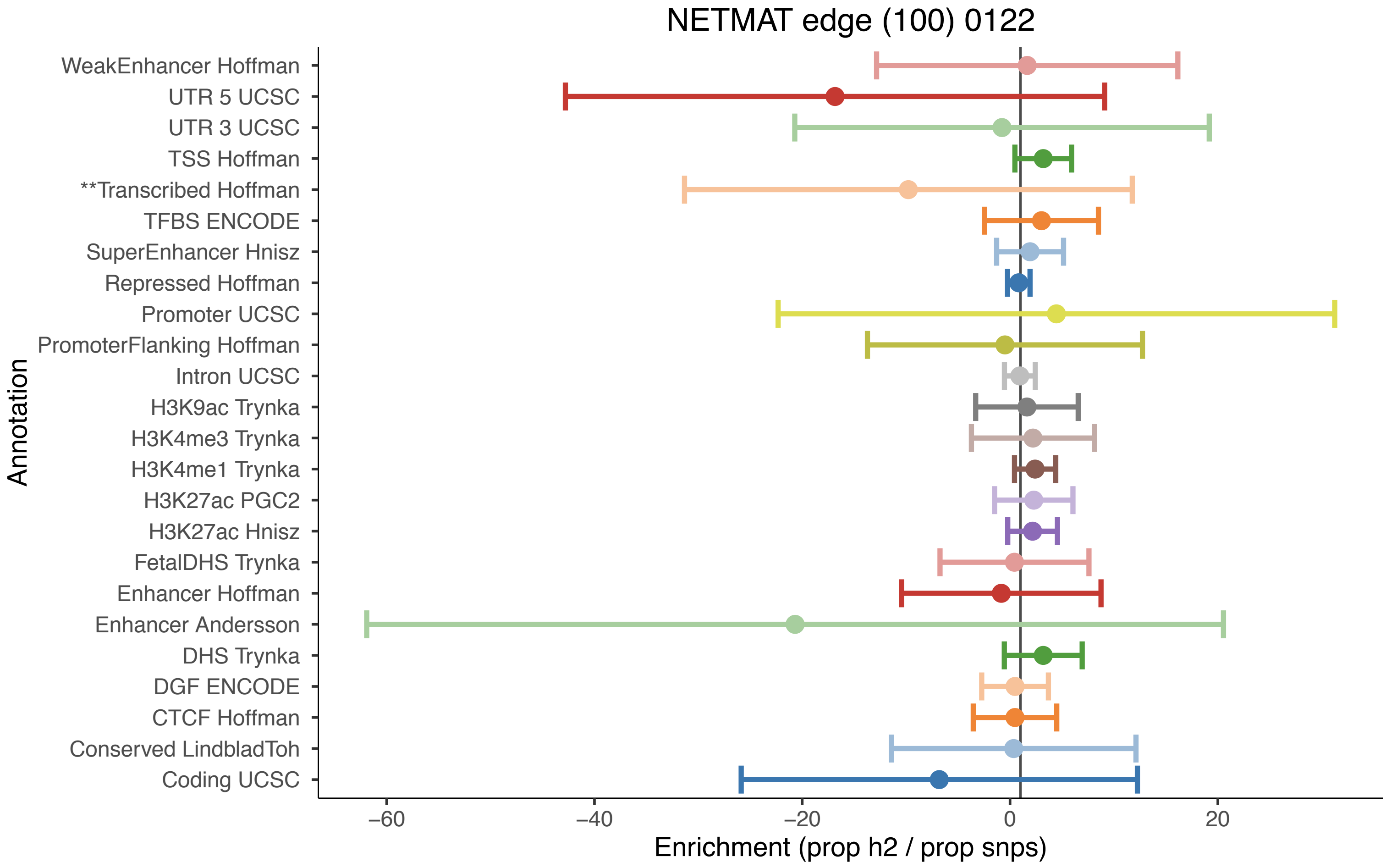

NETMAT edge (100) 0122
WeakEnhancer Hoffman
UTR 5 UCSC
UTR 3 UCSC
TSS Hoffman
**Transcribed Hoffman
TFBS ENCODE
SuperEnhancer Hnisz
Repressed Hoffman
Promoter UCSC
PromoterFlanking Hoffman
Intron UCSC
H3K9ac Trynka
H3K4me3 Trynka
H3K4me1 Trynka
H3K27ac PGC2
H3K27ac Hnisz
FetalDHS Trynka
Enhancer Hoffman
Enhancer Andersson
DHS Trynka
DGF ENCODE
CTCF Hoffman
Conserved LindbladToh
Coding UCSC
Annotation
−60
−40
−20
0
20
Enrichment (prop h2 / prop snps)

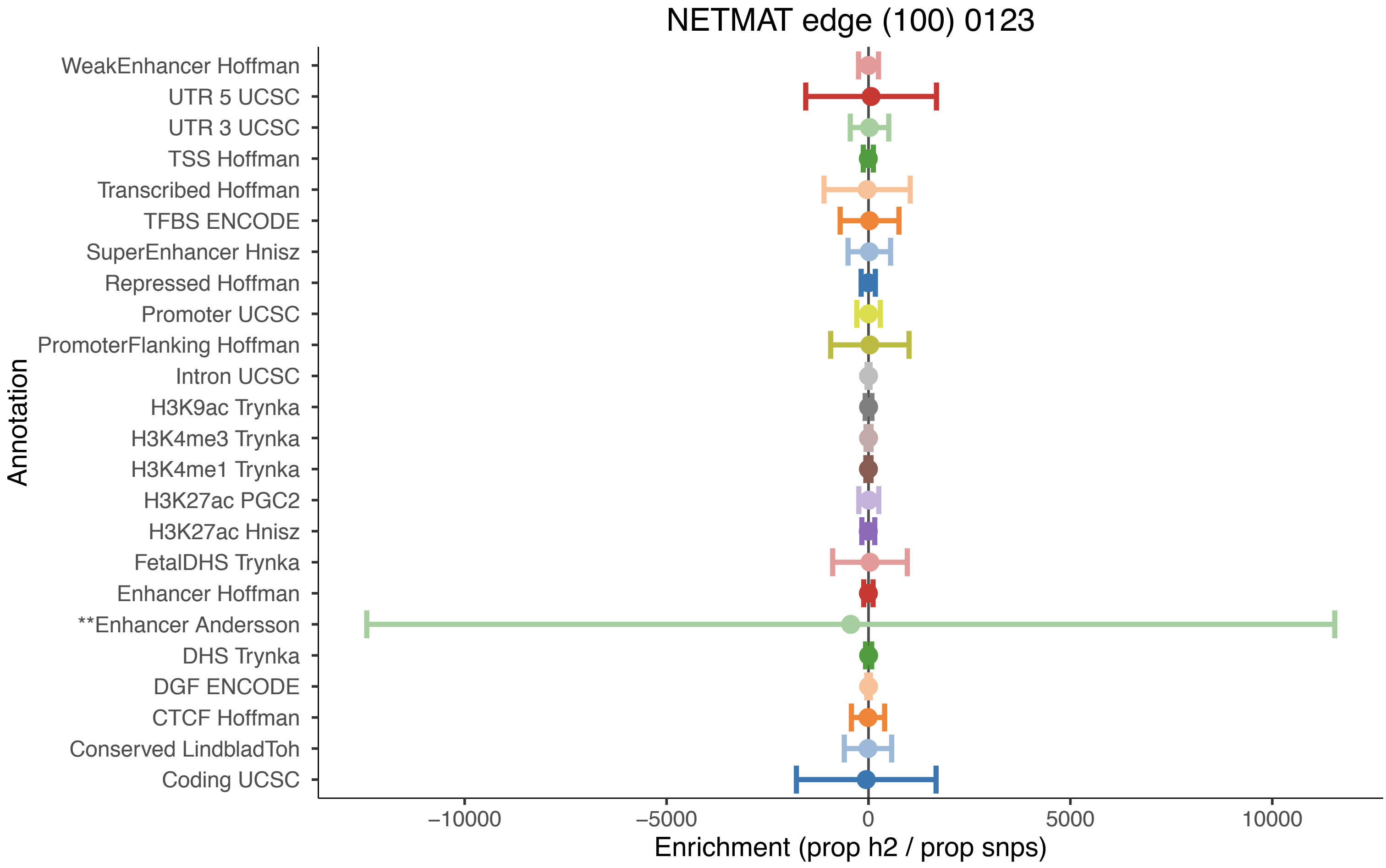
NETMAT edge (100) 0123
WeakEnhancer Hoffman
UTR 5 UCSC
UTR 3 UCSC
TSS Hoffman
Transcribed Hoffman
TFBS ENCODE
SuperEnhancer Hnisz
Repressed Hoffman
Promoter UCSC
PromoterFlanking Hoffman
Intron UCSC
H3K9ac Trynka
H3K4me3 Trynka
H3K4me1 Trynka
H3K27ac PGC2
H3K27ac Hnisz
FetalDHS Trynka
Enhancer Hoffman
**Enhancer Andersson
DHS Trynka
DGF ENCODE
CTCF Hoffman
Conserved LindbladToh
Coding UCSC
Annotation
−10000
−5000
0
5000
10000
Enrichment (prop h2 / prop snps)

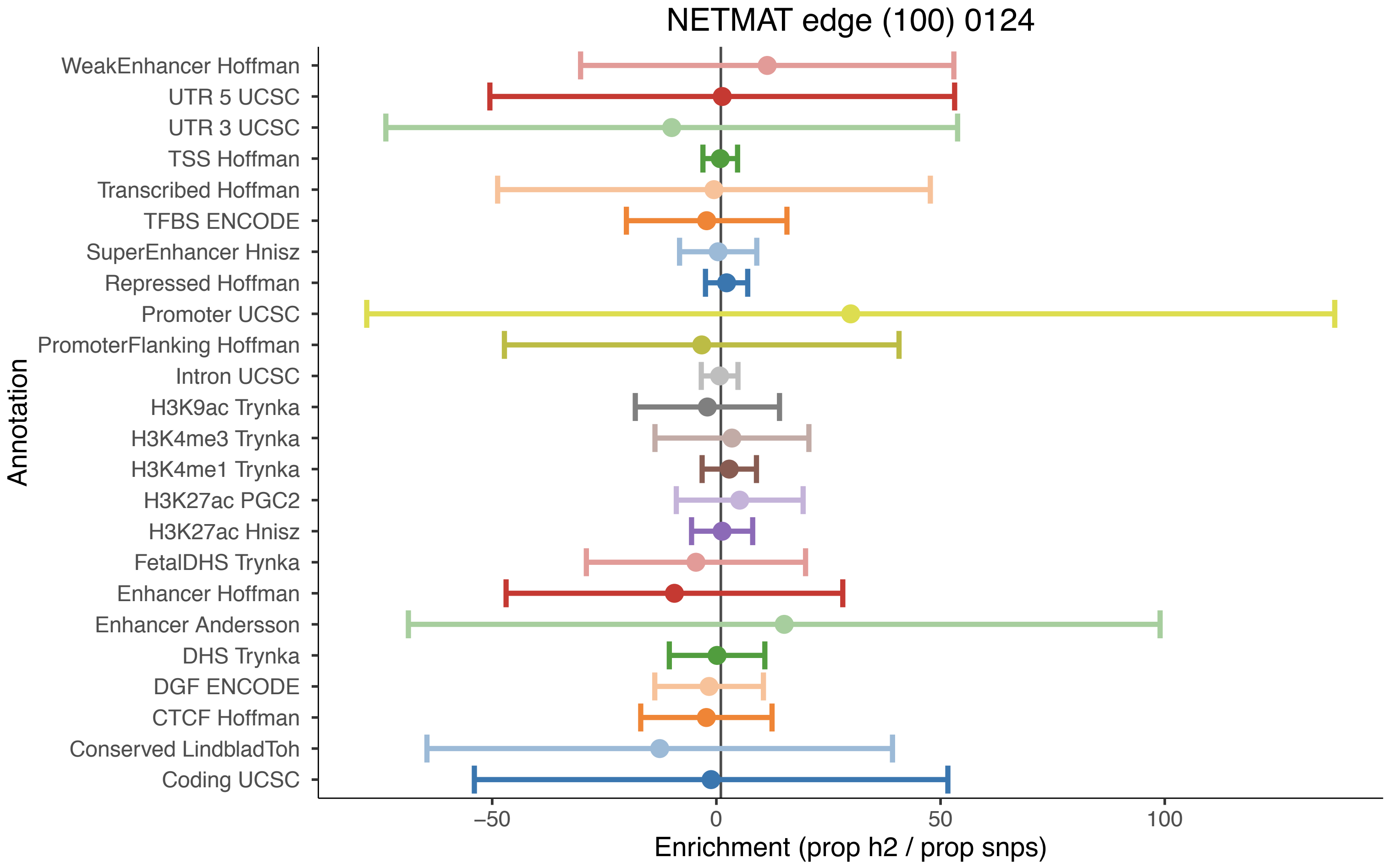
NETMAT edge (100) 0124
WeakEnhancer Hoffman
UTR 5 UCSC
UTR 3 UCSC
TSS Hoffman
Transcribed Hoffman
TFBS ENCODE
SuperEnhancer Hnisz
Repressed Hoffman
Promoter UCSC
PromoterFlanking Hoffman
Intron UCSC
H3K9ac Trynka
H3K4me3 Trynka
H3K4me1 Trynka
H3K27ac PGC2
H3K27ac Hnisz
FetalDHS Trynka
Enhancer Hoffman
Enhancer Andersson
DHS Trynka
DGF ENCODE
CTCF Hoffman
Conserved LindbladToh
Coding UCSC
Annotation
−50
0
50
100
Enrichment (prop h2 / prop snps)

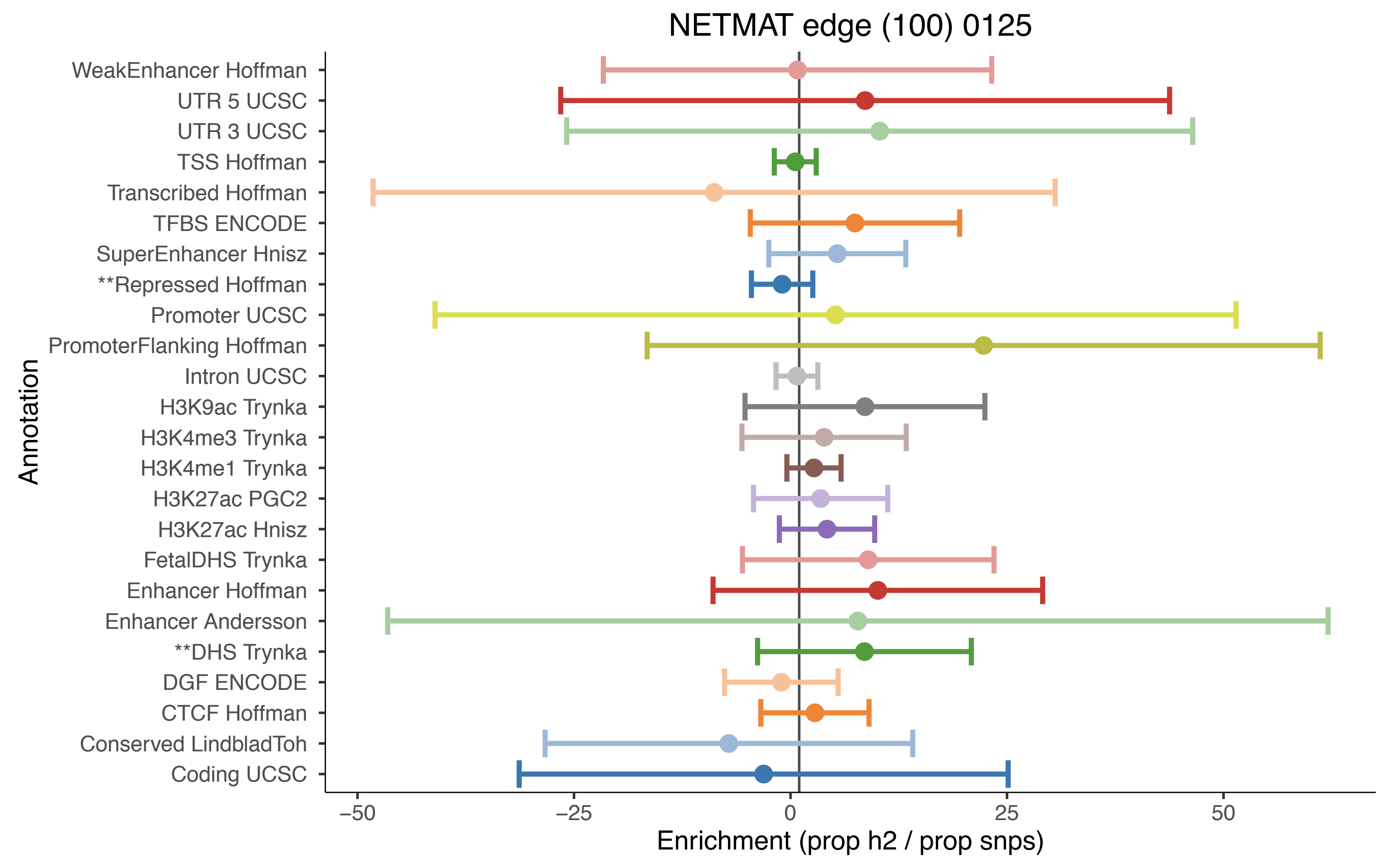
NETMAT edge (100) 0125
Annotation
WeakEnhancer Hoffman
UTR 5 UCSC
UTR 3 UCSC
TSS Hoffman
Transcribed Hoffman
TFBS ENCODE
SuperEnhancer Hnisz
**Repressed Hoffman
Promoter UCSC
PromoterFlanking Hoffman
Intron UCSC
H3K9ac Trynka
H3K4me3 Trynka
H3K4me1 Trynka
H3K27ac PGC2
H3K27ac Hnisz
FetalDHS Trynka
Enhancer Hoffman
Enhancer Andersson
**DHS Trynka
DGF ENCODE
CTCF Hoffman
Conserved LindbladToh
Coding UCSC
−50
−25
0
25
50
Enrichment (prop h2 / prop snps)

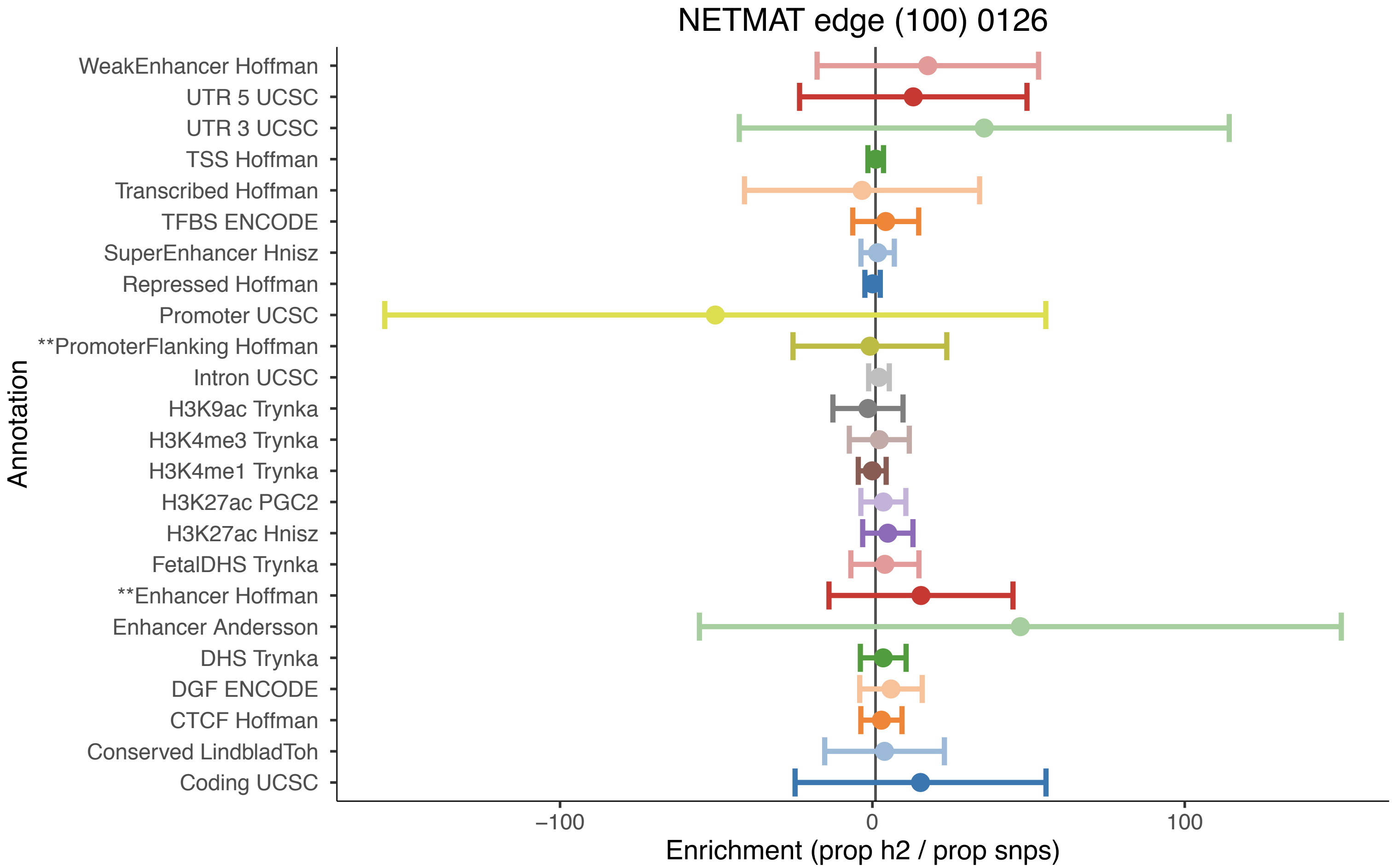
NETMAT edge (100) 0126
WeakEnhancer Hoffman
UTR 5 UCSC
UTR 3 UCSC
TSS Hoffman
Transcribed Hoffman
TFBS ENCODE
SuperEnhancer Hnisz
Repressed Hoffman
Promoter UCSC
**PromoterFlanking Hoffman
Intron UCSC
H3K9ac Trynka
H3K4me3 Trynka
H3K4me1 Trynka
H3K27ac PGC2
H3K27ac Hnisz
FetalDHS Trynka
**Enhancer Hoffman
Enhancer Andersson
DHS Trynka
DGF ENCODE
CTCF Hoffman
Conserved LindbladToh
Coding UCSC
Annotation
−100
0
100
Enrichment (prop h2 / prop snps)

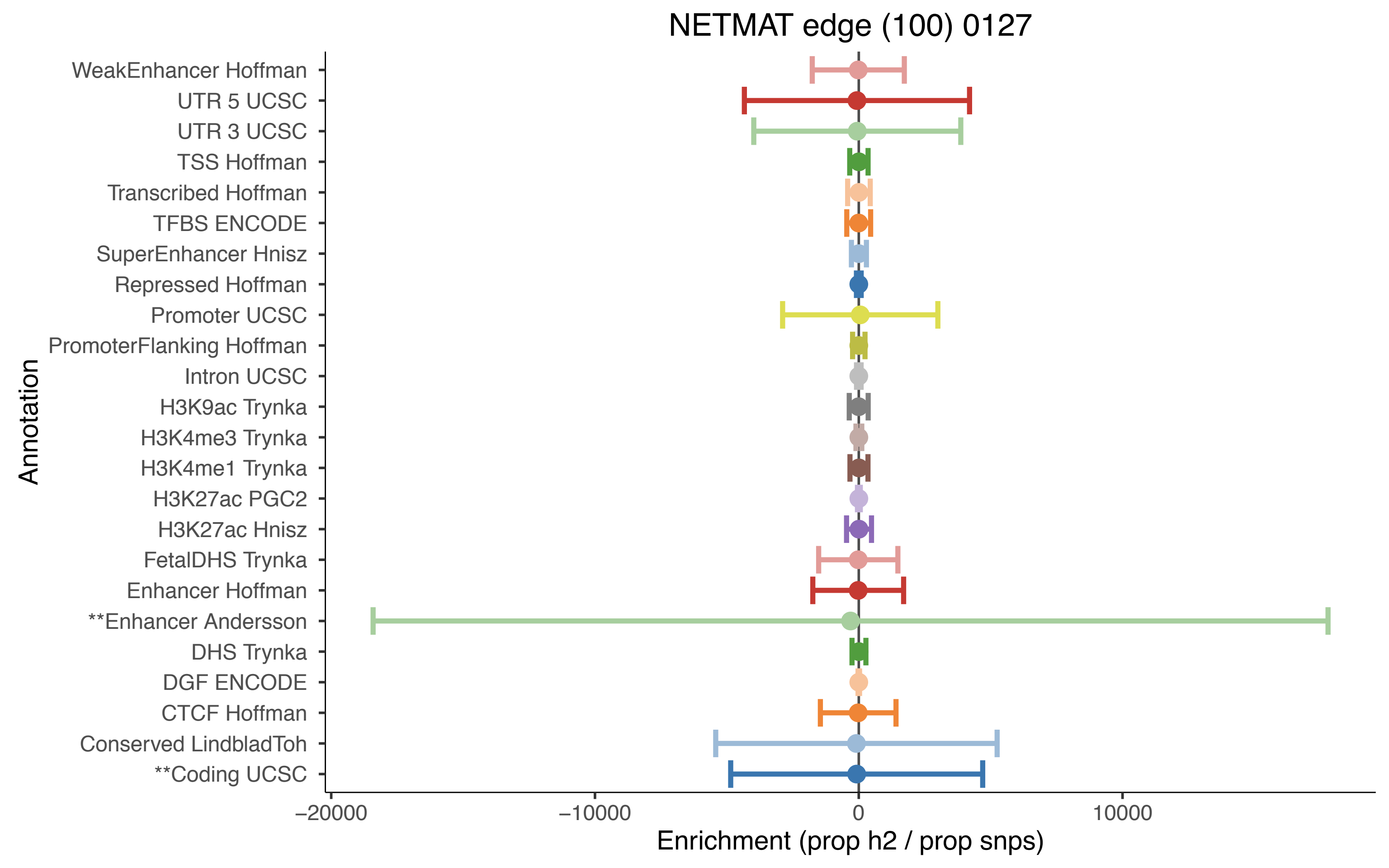
NETMAT edge (100) 0127
Annotation
WeakEnhancer Hoffman
UTR 5 UCSC
UTR 3 UCSC
TSS Hoffman
Transcribed Hoffman
TFBS ENCODE
SuperEnhancer Hnisz
Repressed Hoffman
Promoter UCSC
PromoterFlanking Hoffman
Intron UCSC
H3K9ac Trynka
H3K4me3 Trynka
H3K4me1 Trynka
H3K27ac PGC2
H3K27ac Hnisz
FetalDHS Trynka
Enhancer Hoffman
**Enhancer Andersson
DHS Trynka
DGF ENCODE
CTCF Hoffman
Conserved LindbladToh
**Coding UCSC
−20000
−10000
0
10000
Enrichment (prop h2 / prop snps)

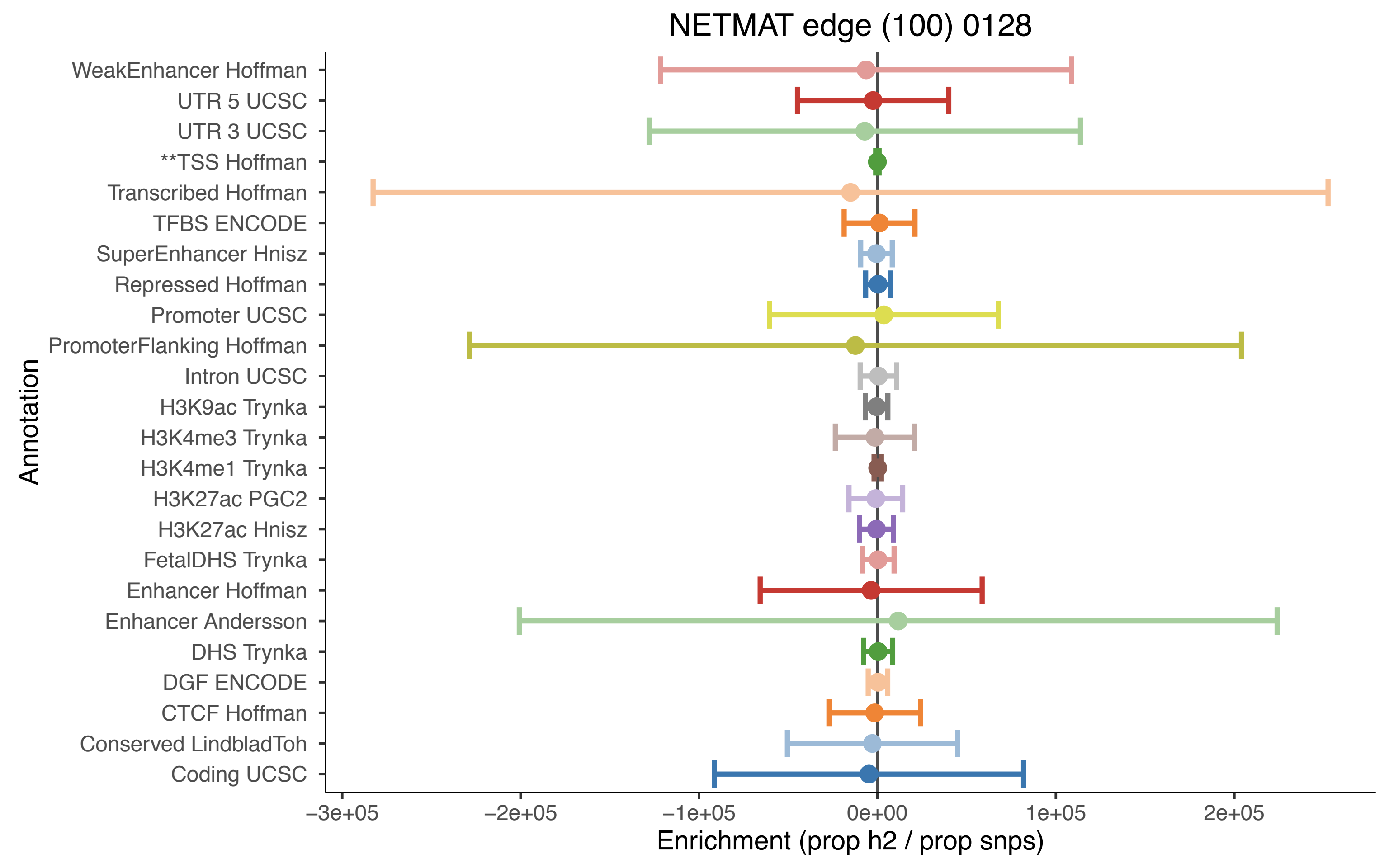

NETMAT edge (100) 0128
WeakEnhancer Hoffman
UTR 5 UCSC
UTR 3 UCSC
**TSS Hoffman
Transcribed Hoffman
TFBS ENCODE
SuperEnhancer Hnisz
Repressed Hoffman
Promoter UCSC
PromoterFlanking Hoffman
Intron UCSC
H3K9ac Trynka
H3K4me3 Trynka
H3K4me1 Trynka
H3K27ac PGC2
H3K27ac Hnisz
FetalDHS Trynka
Enhancer Hoffman
Enhancer Andersson
DHS Trynka
DGF ENCODE
CTCF Hoffman
Conserved LindbladToh
Coding UCSC
Annotation
−3e+05
−2e+05
−1e+05
0e+00
1e+05
2e+05
Enrichment (prop h2 / prop snps)

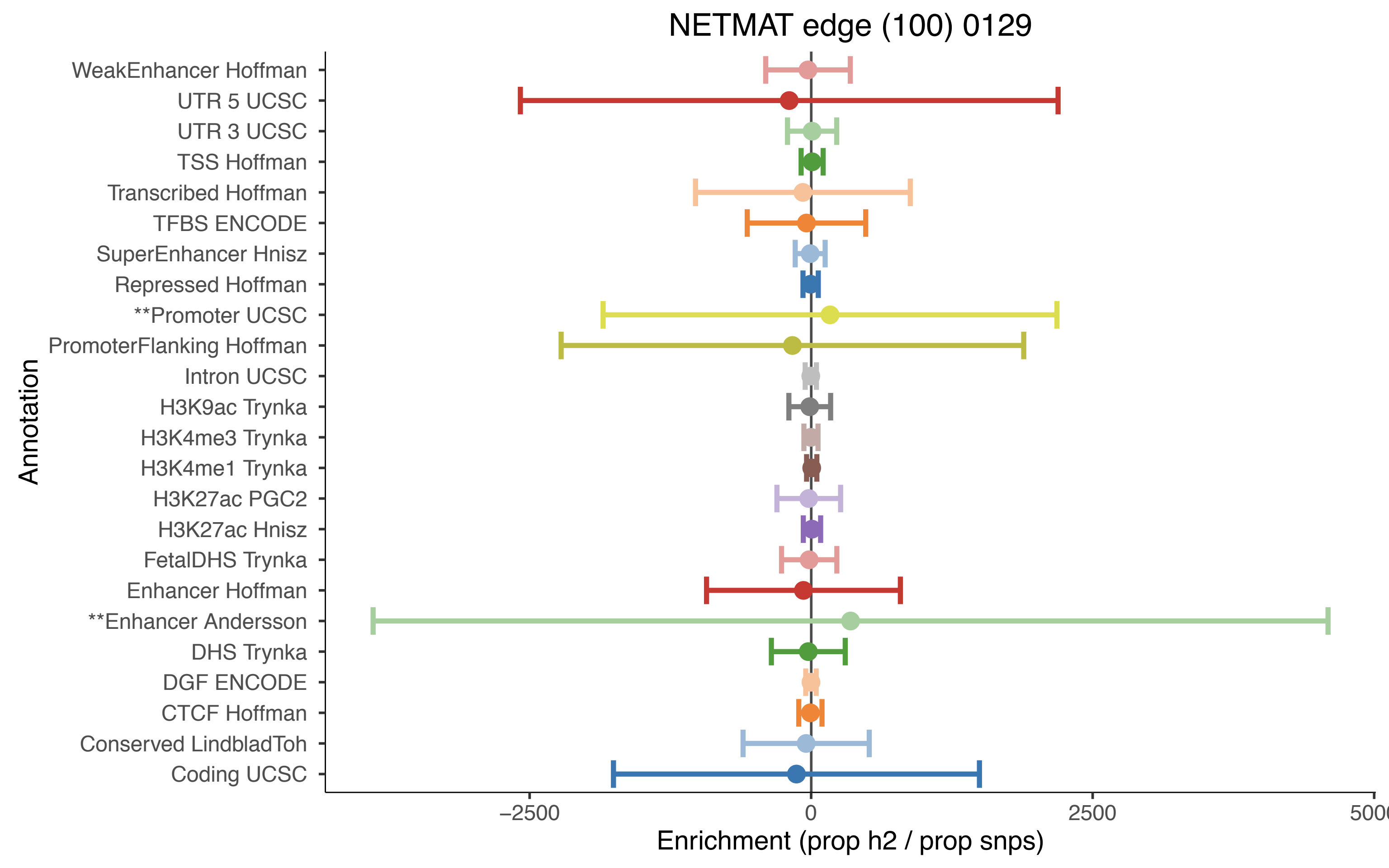
NETMAT edge (100) 0129
WeakEnhancer Hoffman
UTR 5 UCSC
UTR 3 UCSC
TSS Hoffman
Transcribed Hoffman
TFBS ENCODE
SuperEnhancer Hnisz
Repressed Hoffman
**Promoter UCSC
PromoterFlanking Hoffman
Intron UCSC
H3K9ac Trynka
H3K4me3 Trynka
H3K4me1 Trynka
H3K27ac PGC2
H3K27ac Hnisz
FetalDHS Trynka
Enhancer Hoffman
**Enhancer Andersson
DHS Trynka
DGF ENCODE
CTCF Hoffman
Conserved LindbladToh
Coding UCSC
Annotation
−2500
0
2500
Enrichment (prop h2 / prop snps)

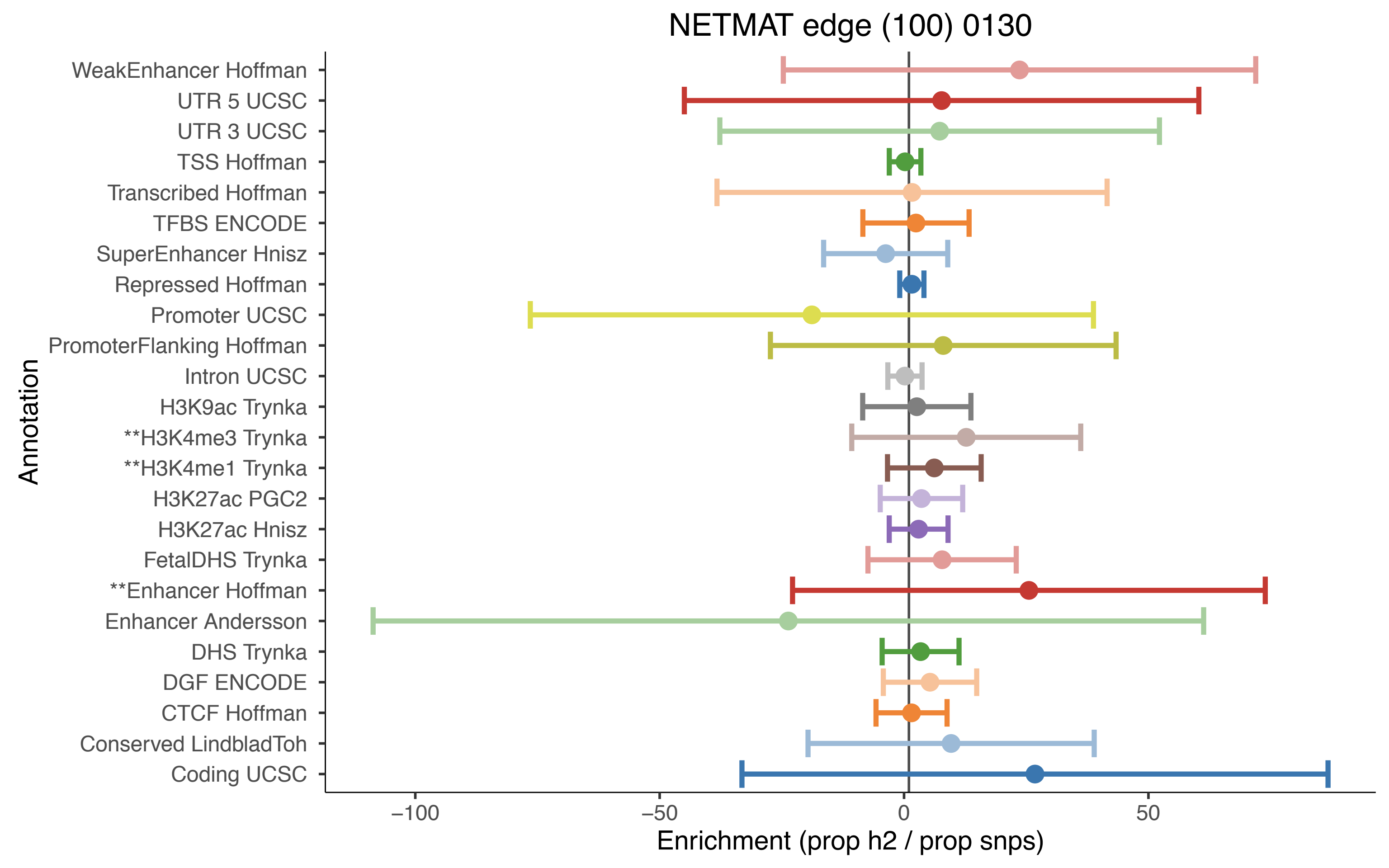
NETMAT edge (100) 0130
WeakEnhancer Hoffman
UTR 5 UCSC
UTR 3 UCSC
TSS Hoffman
Transcribed Hoffman
TFBS ENCODE
SuperEnhancer Hnisz
Repressed Hoffman
Promoter UCSC
PromoterFlanking Hoffman
Intron UCSC
H3K9ac Trynka
**H3K4me3 Trynka
**H3K4me1 Trynka
H3K27ac PGC2
H3K27ac Hnisz
FetalDHS Trynka
**Enhancer Hoffman
Enhancer Andersson
DHS Trynka
DGF ENCODE
CTCF Hoffman
Conserved LindbladToh
Coding UCSC
Annotation
−100
−50
0
50
Enrichment (prop h2 / prop snps)

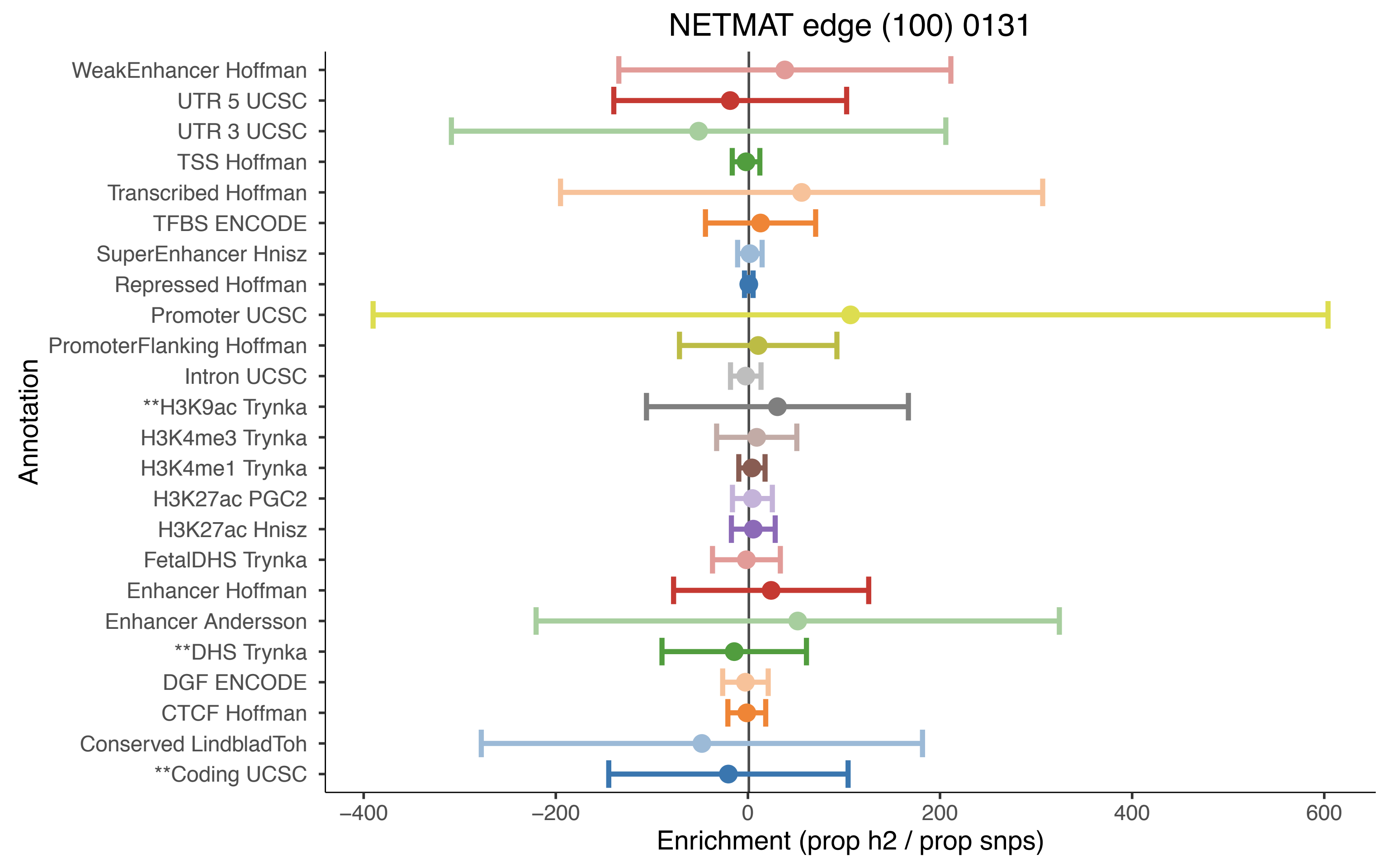
NETMAT edge (100) 0131
WeakEnhancer Hoffman
UTR 5 UCSC
UTR 3 UCSC
TSS Hoffman
Transcribed Hoffman
TFBS ENCODE
SuperEnhancer Hnisz
Repressed Hoffman
Promoter UCSC
PromoterFlanking Hoffman
Intron UCSC
**H3K9ac Trynka
H3K4me3 Trynka
H3K4me1 Trynka
H3K27ac PGC2
H3K27ac Hnisz
FetalDHS Trynka
Enhancer Hoffman
Enhancer Andersson
**DHS Trynka
DGF ENCODE
CTCF Hoffman
Conserved LindbladToh
**Coding UCSC
Annotation
−400
−200
0
200
400
600
Enrichment (prop h2 / prop snps)

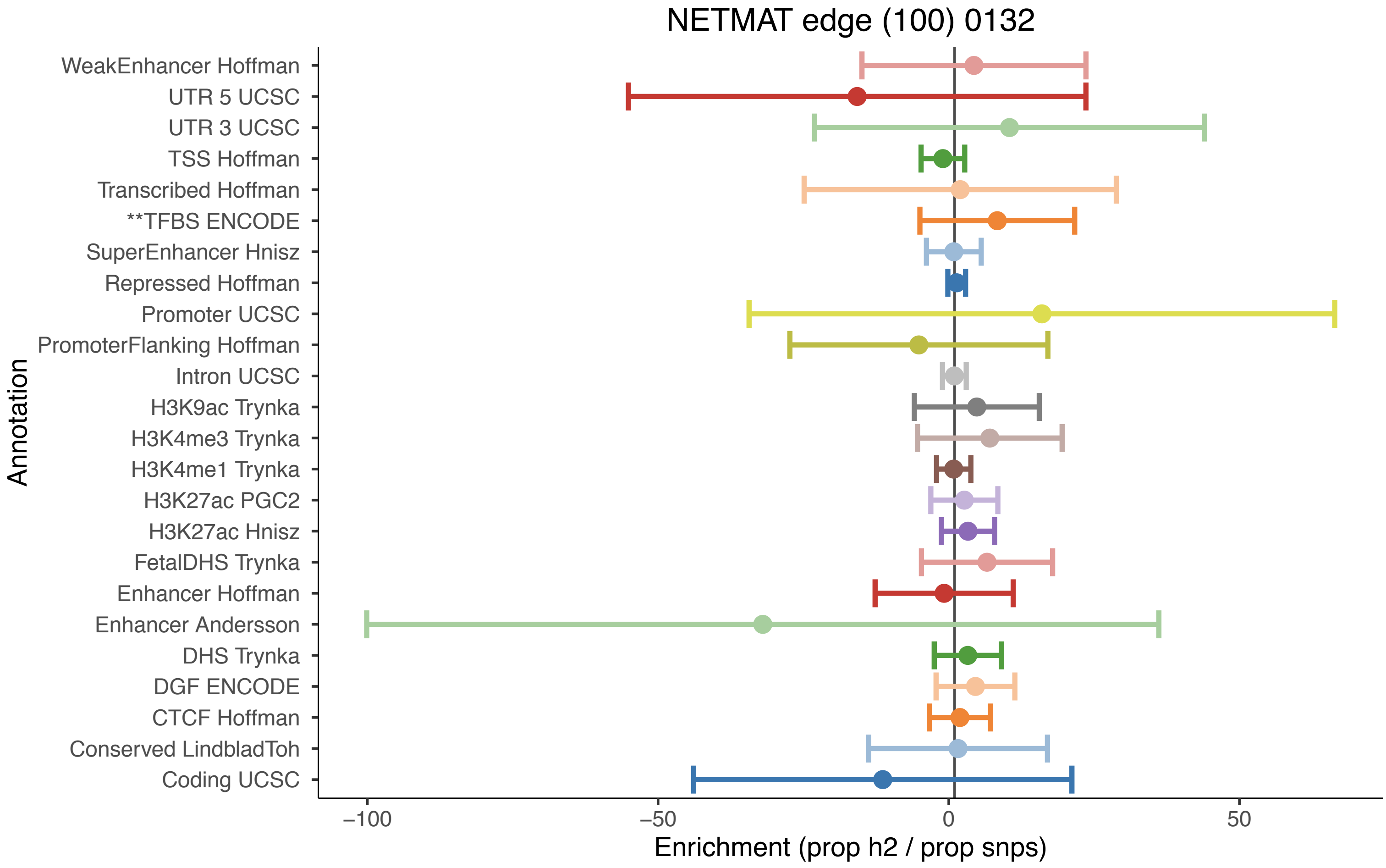

NETMAT edge (100) 0132
WeakEnhancer Hoffman
UTR 5 UCSC
UTR 3 UCSC
TSS Hoffman
Transcribed Hoffman
**TFBS ENCODE
SuperEnhancer Hnisz
Repressed Hoffman
Promoter UCSC
PromoterFlanking Hoffman
Intron UCSC
H3K9ac Trynka
H3K4me3 Trynka
H3K4me1 Trynka
H3K27ac PGC2
H3K27ac Hnisz
FetalDHS Trynka
Enhancer Hoffman
Enhancer Andersson
DHS Trynka
DGF ENCODE
CTCF Hoffman
Conserved LindbladToh
Coding UCSC
Annotation
−100
−50
0
50
Enrichment (prop h2 / prop snps)

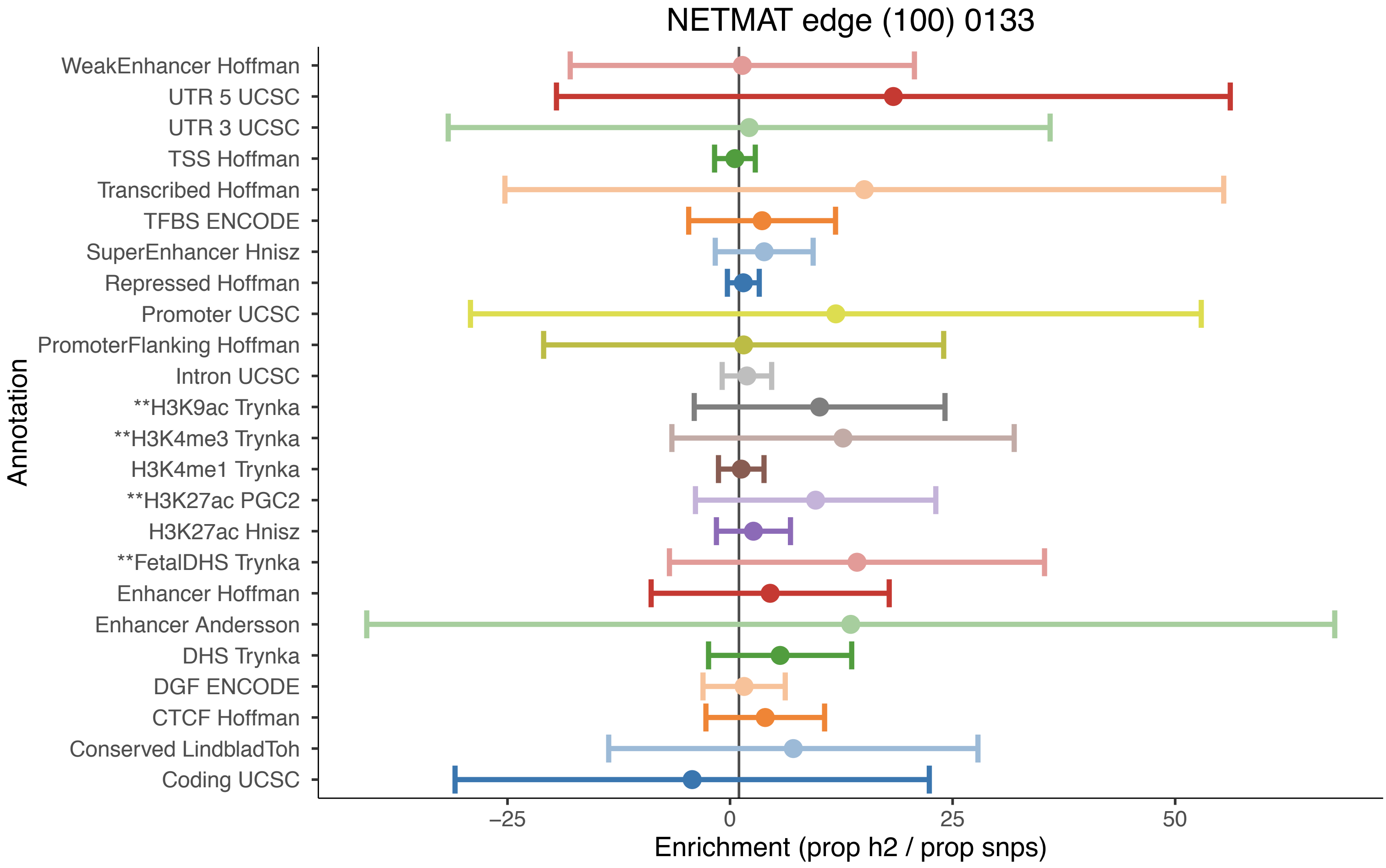
NETMAT edge (100) 0133
WeakEnhancer Hoffman
UTR 5 UCSC
UTR 3 UCSC
TSS Hoffman
Transcribed Hoffman
TFBS ENCODE
SuperEnhancer Hnisz
Repressed Hoffman
Promoter UCSC
PromoterFlanking Hoffman
Intron UCSC
**H3K9ac Trynka
**H3K4me3 Trynka
H3K4me1 Trynka
**H3K27ac PGC2
H3K27ac Hnisz
**FetalDHS Trynka
Enhancer Hoffman
Enhancer Andersson
DHS Trynka
DGF ENCODE
CTCF Hoffman
Conserved LindbladToh
Coding UCSC
Annotation
−25
0
25
50
Enrichment (prop h2 / prop snps)

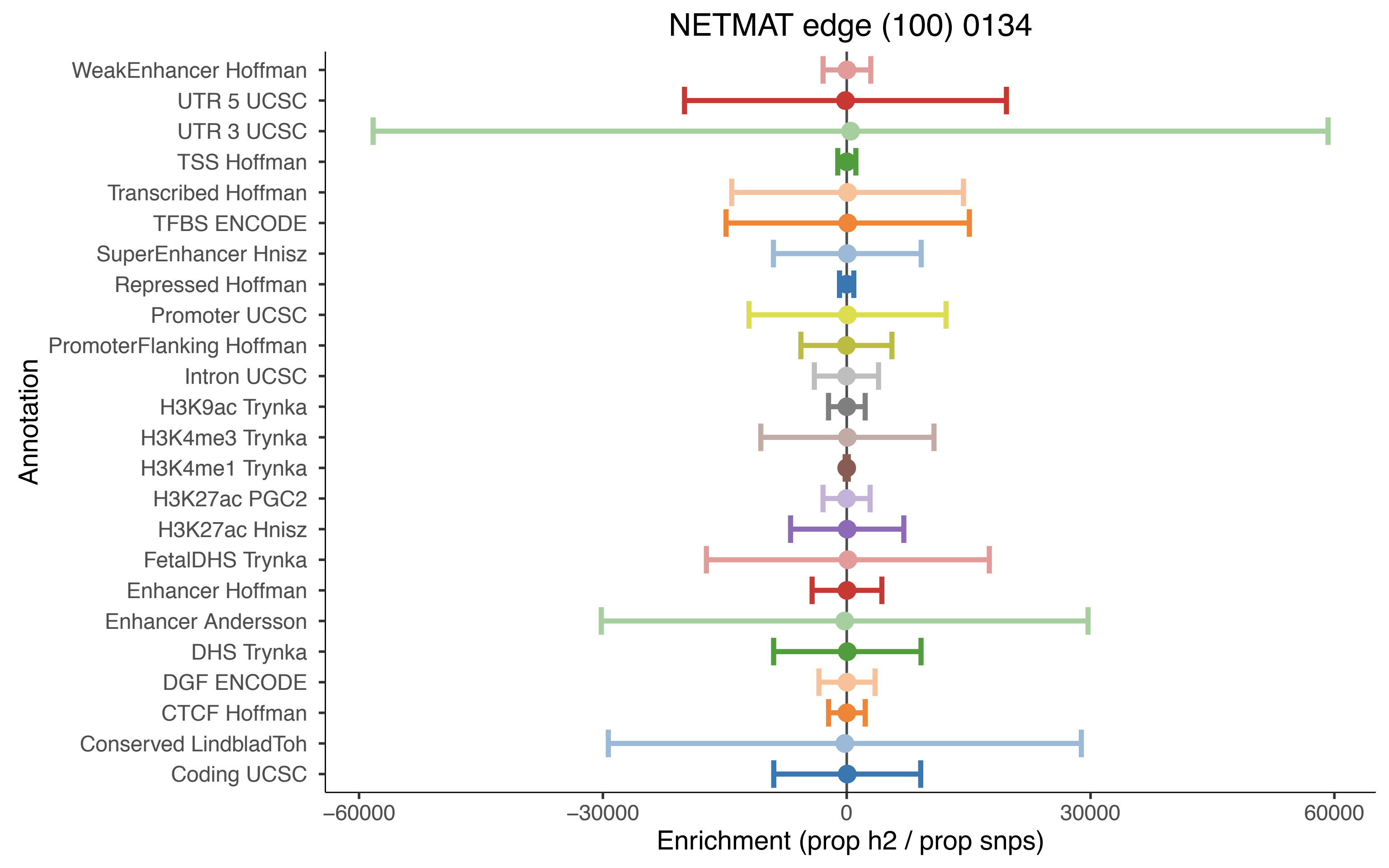
NETMAT edge (100) 0134
Annotation
WeakEnhancer Hoffman
UTR 5 UCSC
UTR 3 UCSC
TSS Hoffman
Transcribed Hoffman
TFBS ENCODE
SuperEnhancer Hnisz
Repressed Hoffman
Promoter UCSC
PromoterFlanking Hoffman
Intron UCSC
H3K9ac Trynka
H3K4me3 Trynka
H3K4me1 Trynka
H3K27ac PGC2
H3K27ac Hnisz
FetalDHS Trynka
Enhancer Hoffman
Enhancer Andersson
DHS Trynka
DGF ENCODE
CTCF Hoffman
Conserved LindbladToh
Coding UCSC
−60000
−30000
0
30000
60000
Enrichment (prop h2 / prop snps)

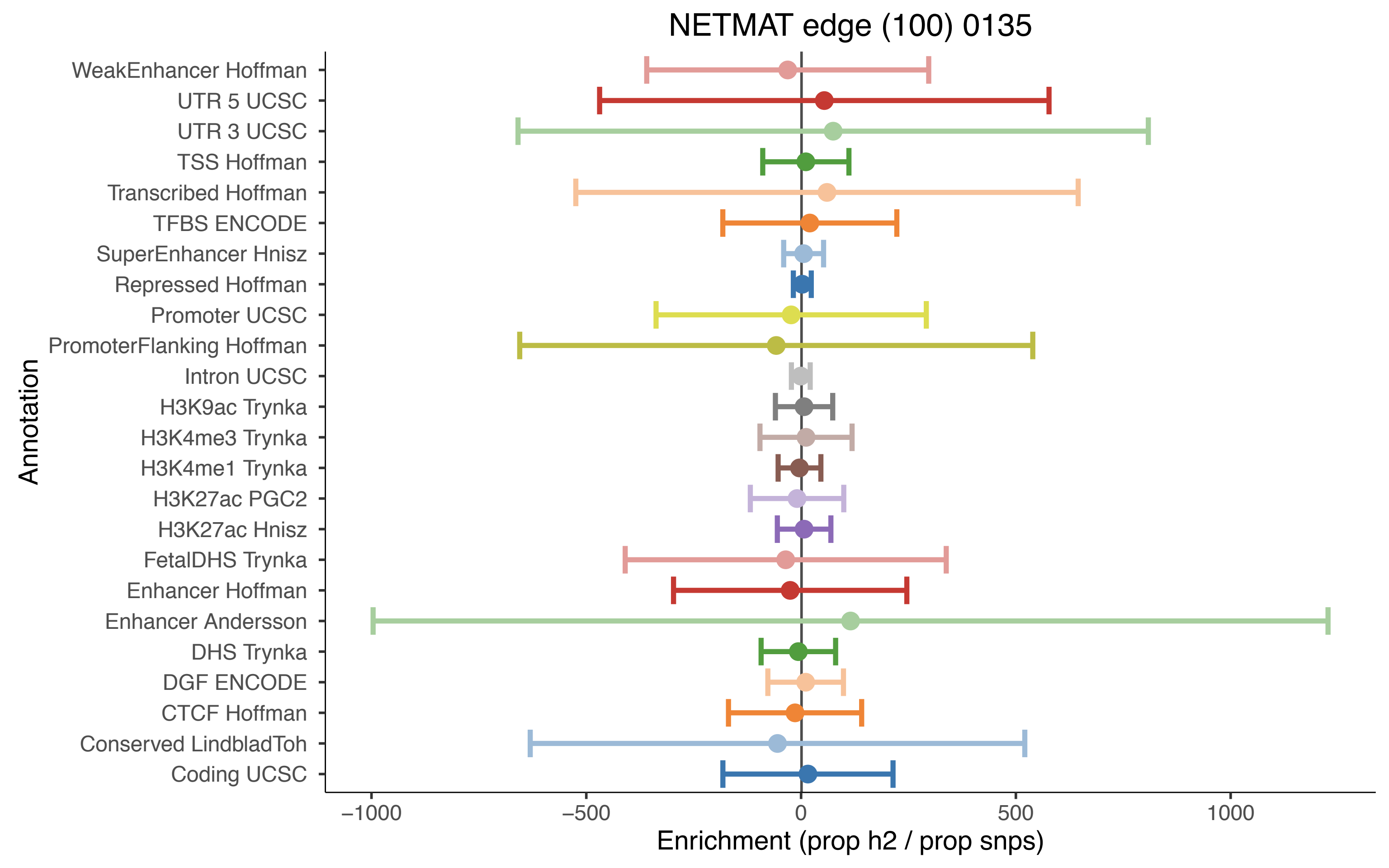
NETMAT edge (100) 0135
WeakEnhancer Hoffman
UTR 5 UCSC
UTR 3 UCSC
TSS Hoffman
Transcribed Hoffman
TFBS ENCODE
SuperEnhancer Hnisz
Repressed Hoffman
Promoter UCSC
PromoterFlanking Hoffman
Intron UCSC
H3K9ac Trynka
H3K4me3 Trynka
H3K4me1 Trynka
H3K27ac PGC2
H3K27ac Hnisz
FetalDHS Trynka
Enhancer Hoffman
Enhancer Andersson
DHS Trynka
DGF ENCODE
CTCF Hoffman
Conserved LindbladToh
Coding UCSC
Annotation
−1000
−500
0
500
1000
Enrichment (prop h2 / prop snps)

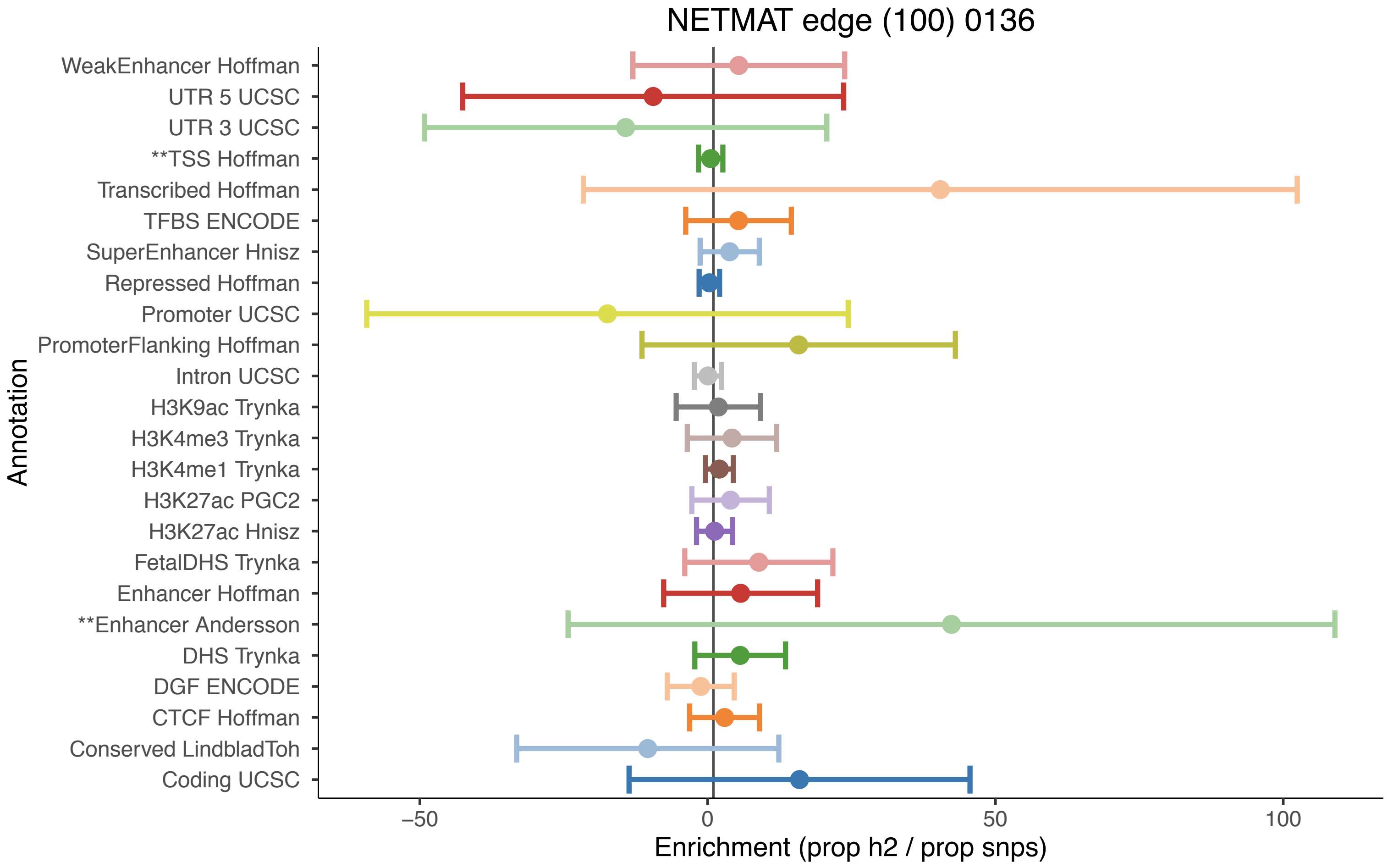
NETMAT edge (100) 0136
WeakEnhancer Hoffman
UTR 5 UCSC
UTR 3 UCSC
**TSS Hoffman
Transcribed Hoffman
TFBS ENCODE
SuperEnhancer Hnisz
Repressed Hoffman
Promoter UCSC
PromoterFlanking Hoffman
Intron UCSC
H3K9ac Trynka
H3K4me3 Trynka
H3K4me1 Trynka
H3K27ac PGC2
H3K27ac Hnisz
FetalDHS Trynka
Enhancer Hoffman
**Enhancer Andersson
DHS Trynka
DGF ENCODE
CTCF Hoffman
Conserved LindbladToh
Coding UCSC
Annotation
−50
0
50
100
Enrichment (prop h2 / prop snps)

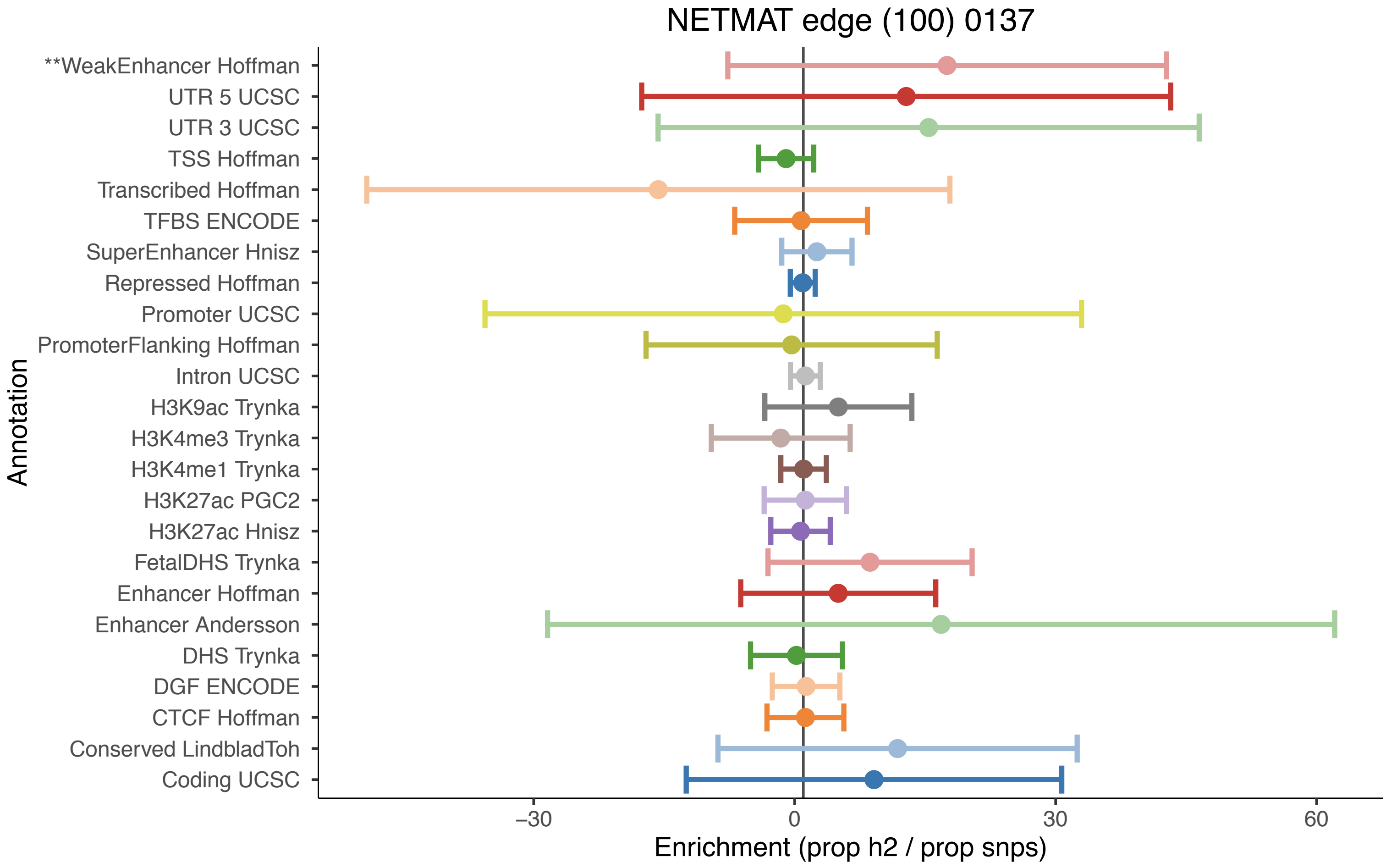

NETMAT edge (100) 0137
**WeakEnhancer Hoffman
UTR 5 UCSC
UTR 3 UCSC
TSS Hoffman
Transcribed Hoffman
TFBS ENCODE
SuperEnhancer Hnisz
Repressed Hoffman
Promoter UCSC
PromoterFlanking Hoffman
Intron UCSC
H3K9ac Trynka
H3K4me3 Trynka
H3K4me1 Trynka
H3K27ac PGC2
H3K27ac Hnisz
FetalDHS Trynka
Enhancer Hoffman
Enhancer Andersson
DHS Trynka
DGF ENCODE
CTCF Hoffman
Conserved LindbladToh
Coding UCSC
Annotation
−30
0
30
60
Enrichment (prop h2 / prop snps)

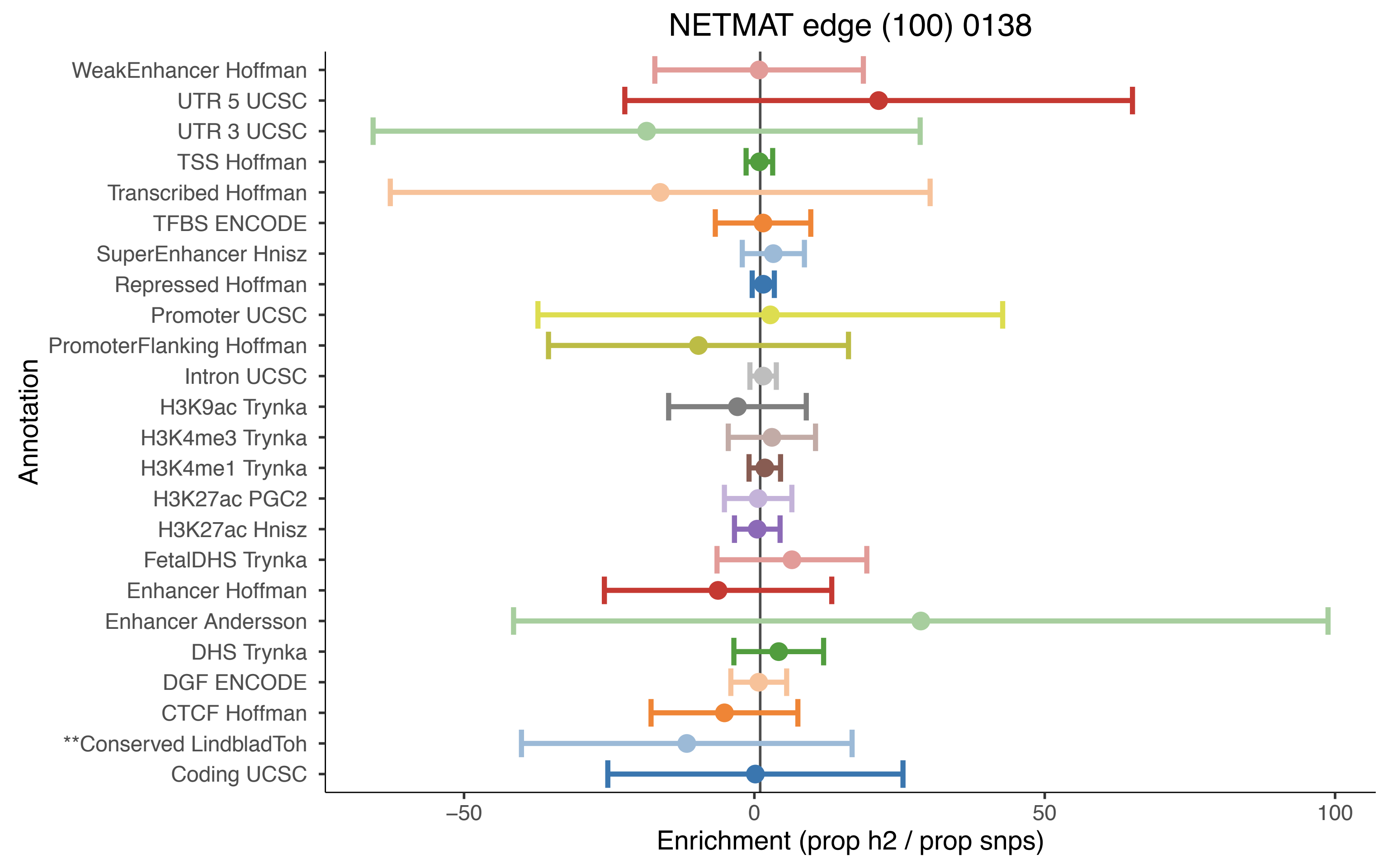
NETMAT edge (100) 0138
WeakEnhancer Hoffman
UTR 5 UCSC
UTR 3 UCSC
TSS Hoffman
Transcribed Hoffman
TFBS ENCODE
SuperEnhancer Hnisz
Repressed Hoffman
Promoter UCSC
PromoterFlanking Hoffman
Intron UCSC
H3K9ac Trynka
H3K4me3 Trynka
H3K4me1 Trynka
H3K27ac PGC2
H3K27ac Hnisz
FetalDHS Trynka
Enhancer Hoffman
Enhancer Andersson
DHS Trynka
DGF ENCODE
CTCF Hoffman
**Conserved LindbladToh
Coding UCSC
Annotation
−50
0
50
100
Enrichment (prop h2 / prop snps)

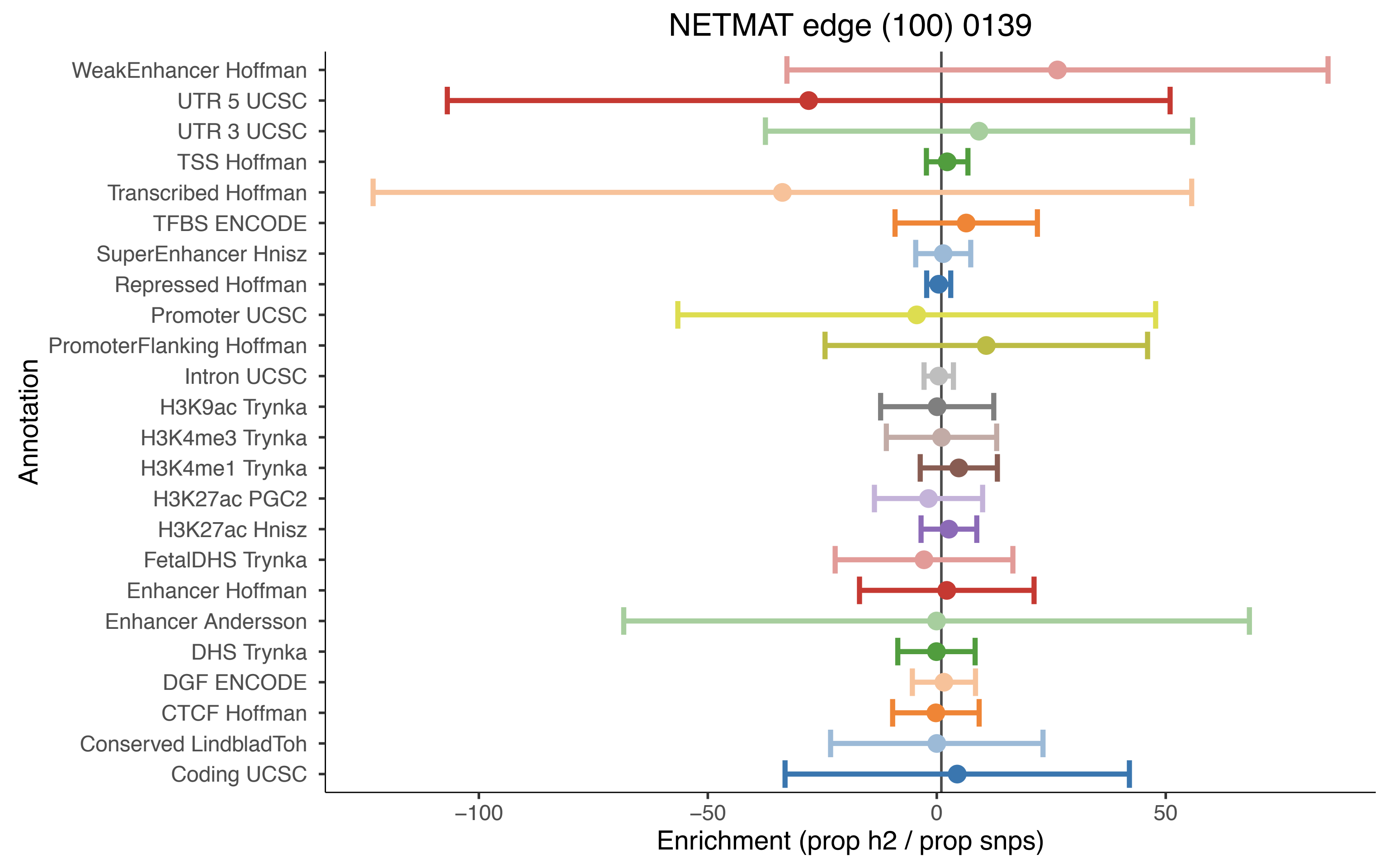
NETMAT edge (100) 0139
WeakEnhancer Hoffman
UTR 5 UCSC
UTR 3 UCSC
TSS Hoffman
Transcribed Hoffman
TFBS ENCODE
SuperEnhancer Hnisz
Repressed Hoffman
Promoter UCSC
PromoterFlanking Hoffman
Intron UCSC
H3K9ac Trynka
H3K4me3 Trynka
H3K4me1 Trynka
H3K27ac PGC2
H3K27ac Hnisz
FetalDHS Trynka
Enhancer Hoffman
Enhancer Andersson
DHS Trynka
DGF ENCODE
CTCF Hoffman
Conserved LindbladToh
Coding UCSC
Annotation
−100
−50
0
50
Enrichment (prop h2 / prop snps)

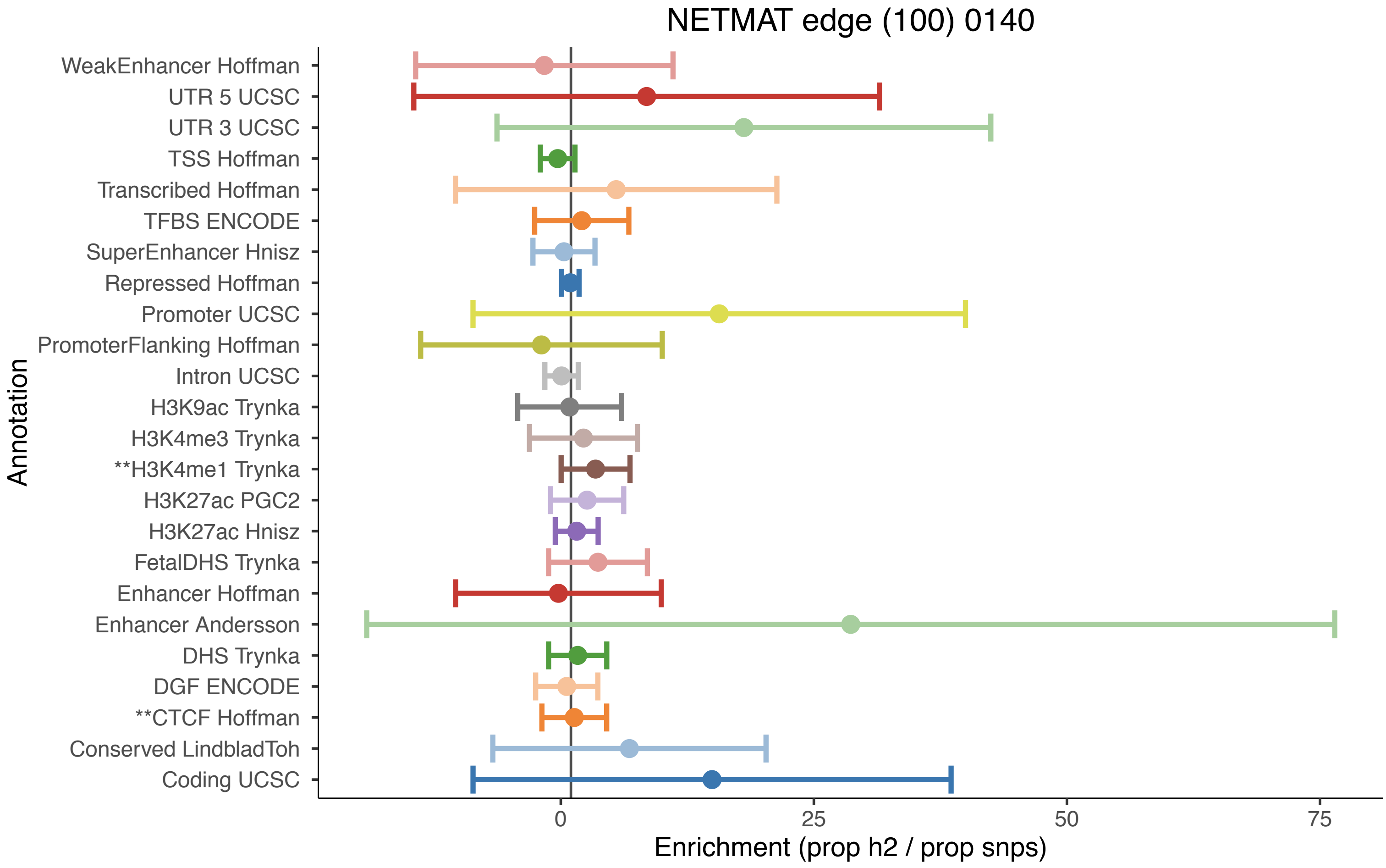
NETMAT edge (100) 0140
WeakEnhancer Hoffman
UTR 5 UCSC
UTR 3 UCSC
TSS Hoffman
Transcribed Hoffman
TFBS ENCODE
SuperEnhancer Hnisz
Repressed Hoffman
Promoter UCSC
PromoterFlanking Hoffman
Intron UCSC
H3K9ac Trynka
H3K4me3 Trynka
**H3K4me1 Trynka
H3K27ac PGC2
H3K27ac Hnisz
FetalDHS Trynka
Enhancer Hoffman
Enhancer Andersson
DHS Trynka
DGF ENCODE
**CTCF Hoffman
Conserved LindbladToh
Coding UCSC
Annotation
0
25
50
75
Enrichment (prop h2 / prop snps)

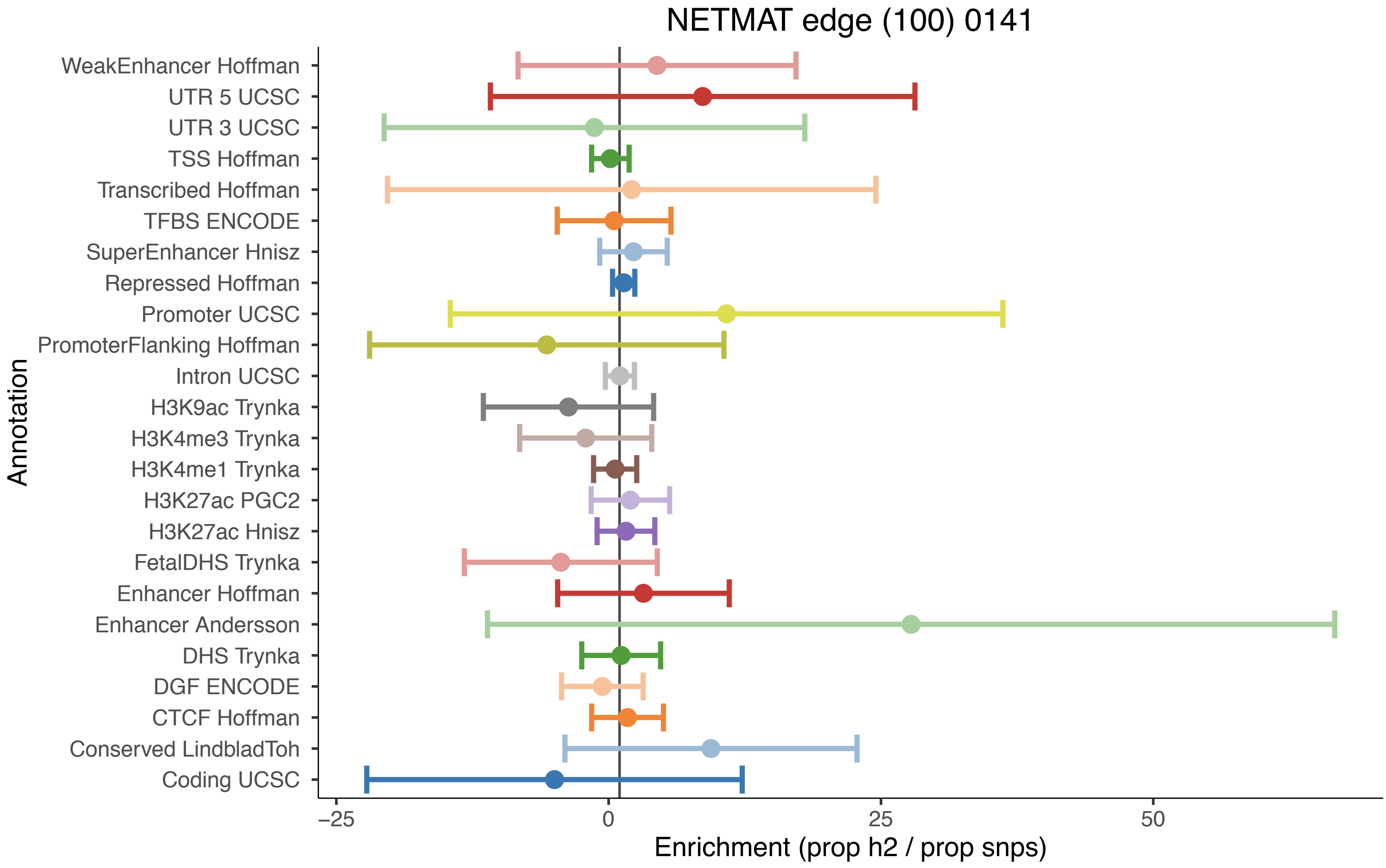
NETMAT edge (100) 0141
WeakEnhancer Hoffman
UTR 5 UCSC
UTR 3 UCSC
TSS Hoffman
Transcribed Hoffman
TFBS ENCODE
SuperEnhancer Hnisz
Repressed Hoffman
Promoter UCSC
PromoterFlanking Hoffman
Intron UCSC
H3K9ac Trynka
H3K4me3 Trynka
H3K4me1 Trynka
H3K27ac PGC2
H3K27ac Hnisz
FetalDHS Trynka
Enhancer Hoffman
Enhancer Andersson
DHS Trynka
DGF ENCODE
CTCF Hoffman
Conserved LindbladToh
Coding UCSC
Annotation
−25
0
25
50
Enrichment (prop h2 / prop snps)

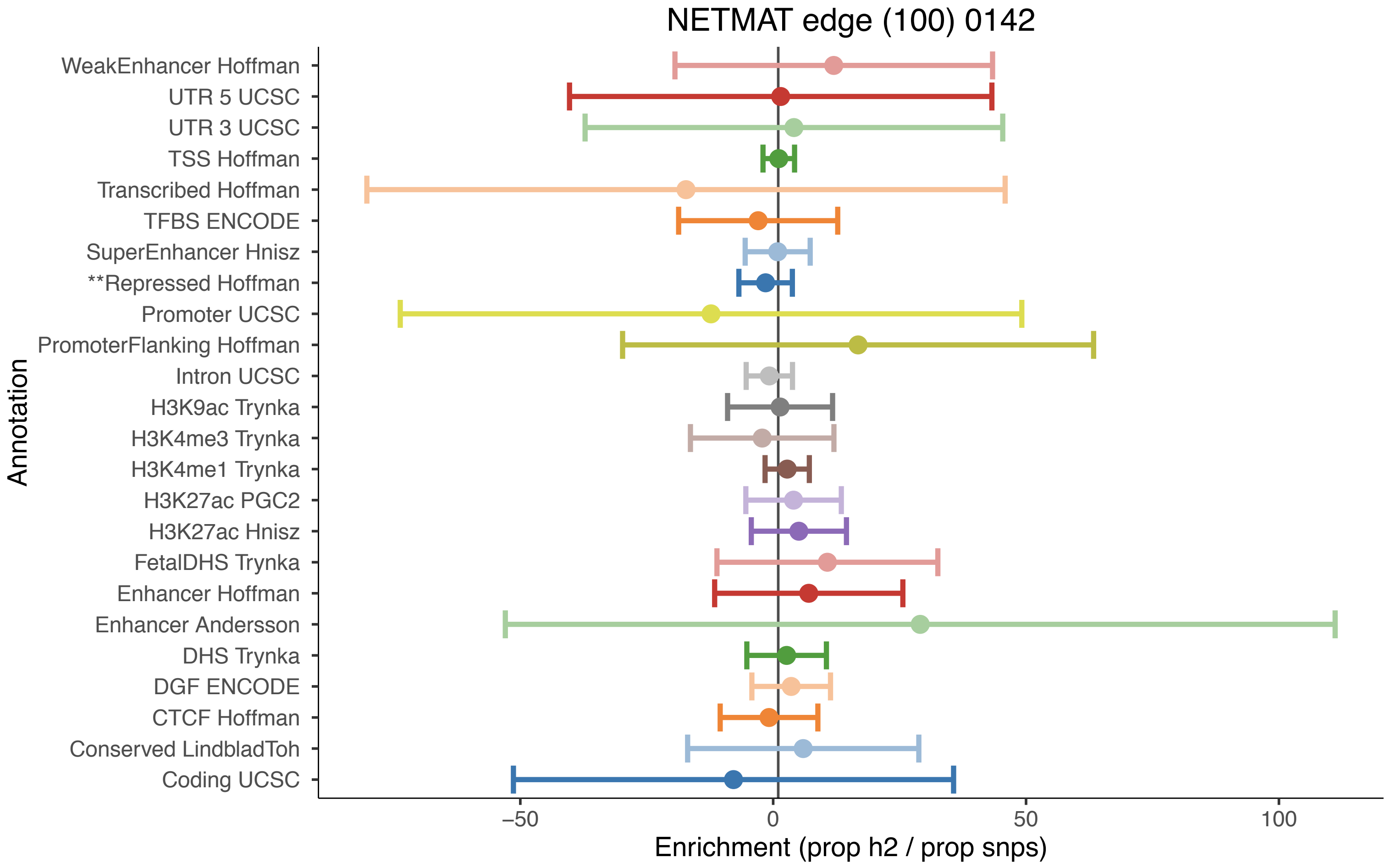
NETMAT edge (100) 0142
WeakEnhancer Hoffman
UTR 5 UCSC
UTR 3 UCSC
TSS Hoffman
Transcribed Hoffman
TFBS ENCODE
SuperEnhancer Hnisz
**Repressed Hoffman
Promoter UCSC
PromoterFlanking Hoffman
Intron UCSC
H3K9ac Trynka
H3K4me3 Trynka
H3K4me1 Trynka
H3K27ac PGC2
H3K27ac Hnisz
FetalDHS Trynka
Enhancer Hoffman
Enhancer Andersson
DHS Trynka
DGF ENCODE
CTCF Hoffman
Conserved LindbladToh
Coding UCSC
Annotation
−50
0
50
100
Enrichment (prop h2 / prop snps)

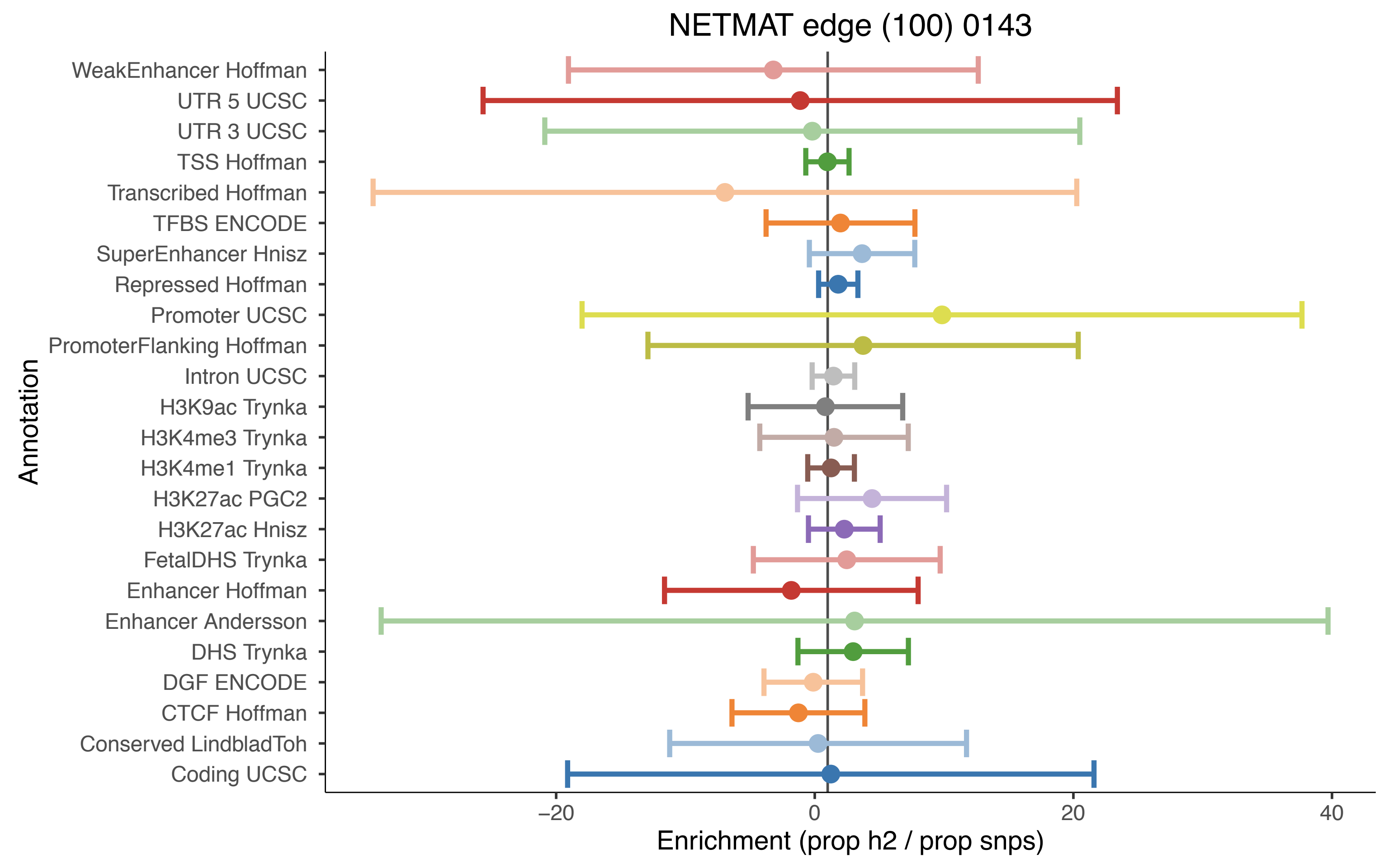
NETMAT edge (100) 0143
Annotation
WeakEnhancer Hoffman
UTR 5 UCSC
UTR 3 UCSC
TSS Hoffman
Transcribed Hoffman
TFBS ENCODE
SuperEnhancer Hnisz
Repressed Hoffman
Promoter UCSC
PromoterFlanking Hoffman
Intron UCSC
H3K9ac Trynka
H3K4me3 Trynka
H3K4me1 Trynka
H3K27ac PGC2
H3K27ac Hnisz
FetalDHS Trynka
Enhancer Hoffman
Enhancer Andersson
DHS Trynka
DGF ENCODE
CTCF Hoffman
Conserved LindbladToh
Coding UCSC
−20
0
20
40
Enrichment (prop h2 / prop snps)

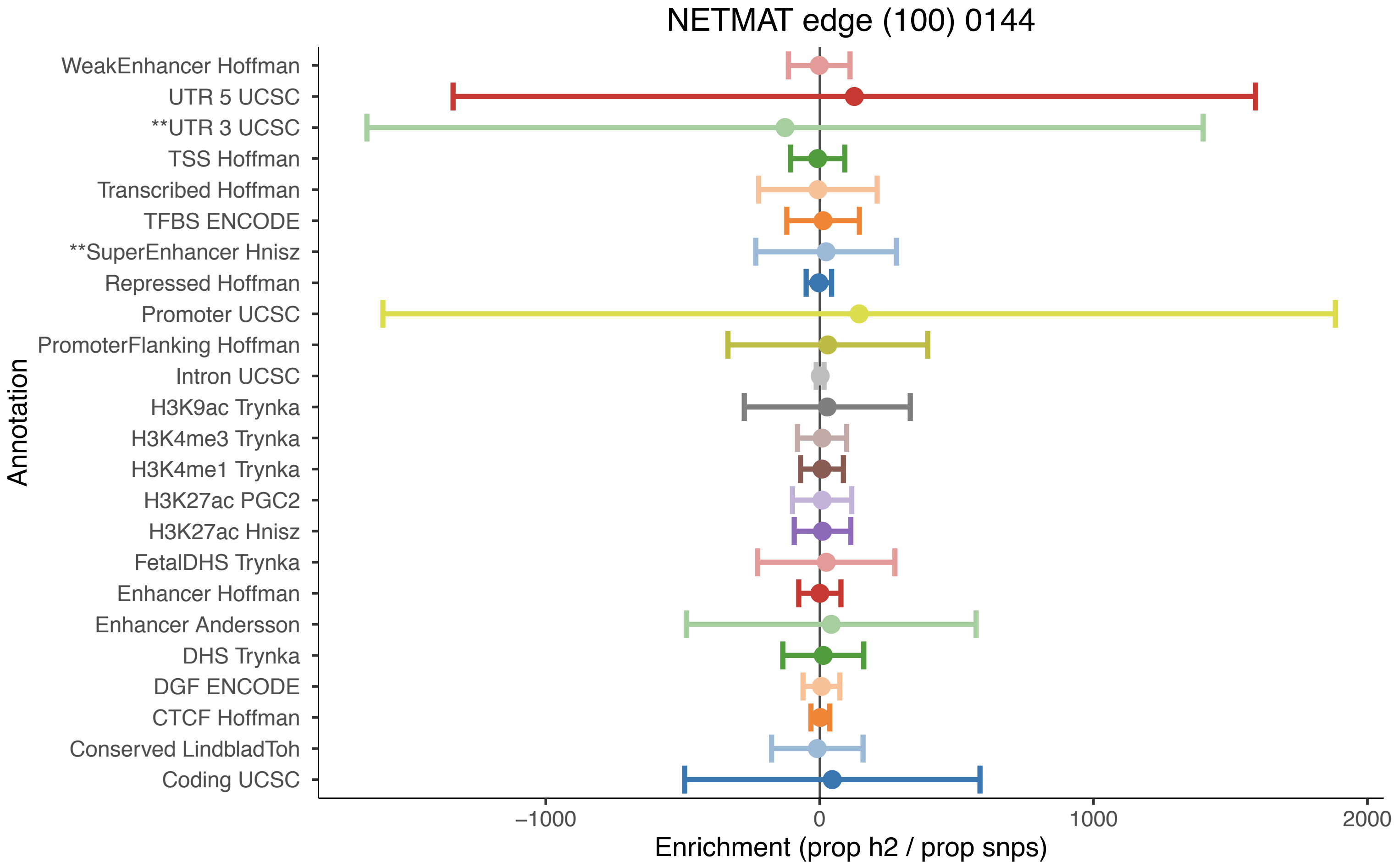
NETMAT edge (100) 0144
WeakEnhancer Hoffman
UTR 5 UCSC
**UTR 3 UCSC
TSS Hoffman
Transcribed Hoffman
TFBS ENCODE
**SuperEnhancer Hnisz
Repressed Hoffman
Promoter UCSC
PromoterFlanking Hoffman
Intron UCSC
H3K9ac Trynka
H3K4me3 Trynka
H3K4me1 Trynka
H3K27ac PGC2
H3K27ac Hnisz
FetalDHS Trynka
Enhancer Hoffman
Enhancer Andersson
DHS Trynka
DGF ENCODE
CTCF Hoffman
Conserved LindbladToh
Coding UCSC
Annotation
−1000
0
1000
2000
Enrichment (prop h2 / prop snps)

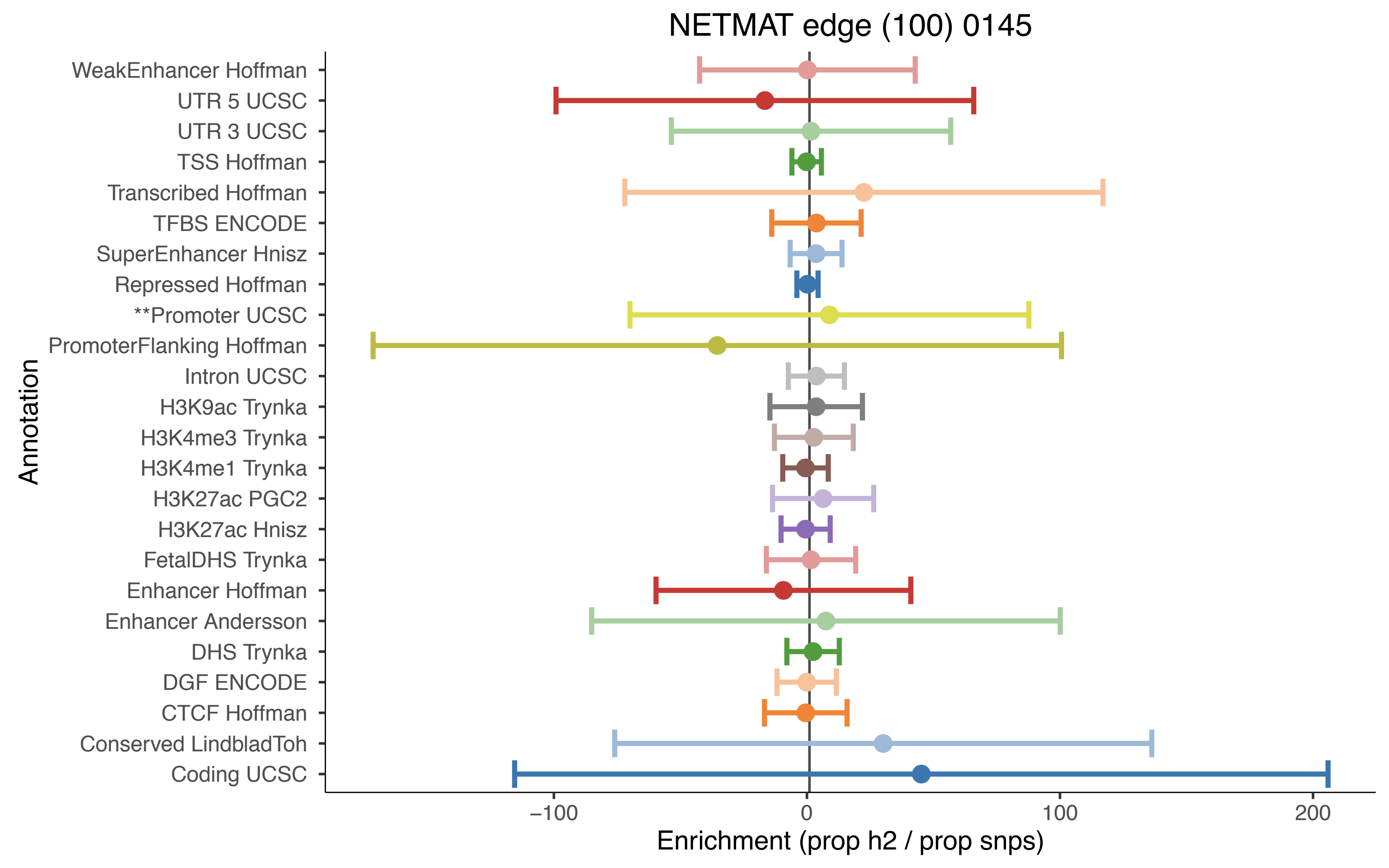

NETMAT edge (100) 0145
WeakEnhancer Hoffman
UTR 5 UCSC
UTR 3 UCSC
TSS Hoffman
Transcribed Hoffman
TFBS ENCODE
SuperEnhancer Hnisz
Repressed Hoffman
**Promoter UCSC
PromoterFlanking Hoffman
Intron UCSC
H3K9ac Trynka
H3K4me3 Trynka
H3K4me1 Trynka
H3K27ac PGC2
H3K27ac Hnisz
FetalDHS Trynka
Enhancer Hoffman
Enhancer Andersson
DHS Trynka
DGF ENCODE
CTCF Hoffman
Conserved LindbladToh
Coding UCSC
Annotation
−100
0
100
200
Enrichment (prop h2 / prop snps)

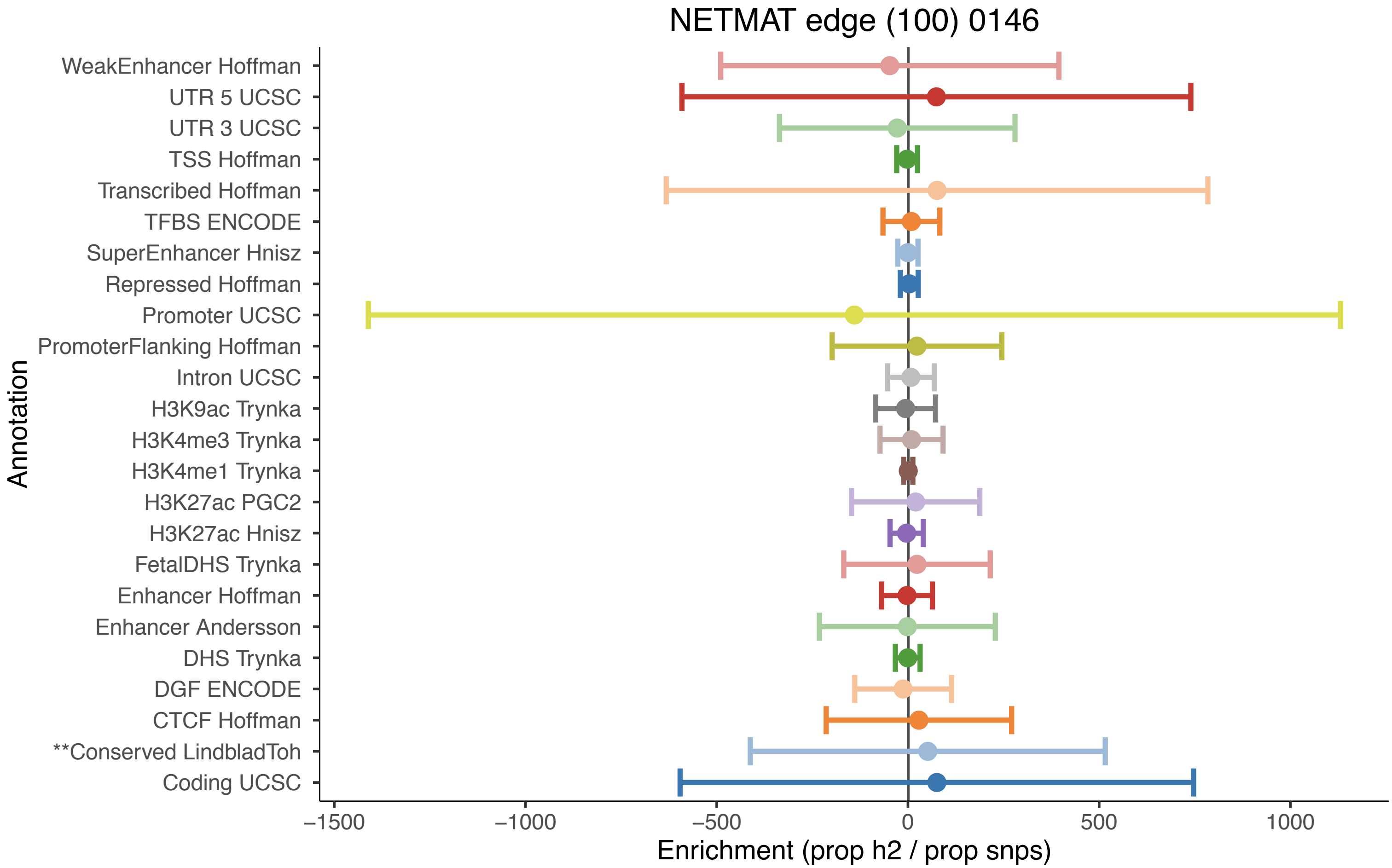
NETMAT edge (100) 0146
WeakEnhancer Hoffman
UTR 5 UCSC
UTR 3 UCSC
TSS Hoffman
Transcribed Hoffman
TFBS ENCODE
SuperEnhancer Hnisz
Repressed Hoffman
Promoter UCSC
PromoterFlanking Hoffman
Intron UCSC
H3K9ac Trynka
H3K4me3 Trynka
H3K4me1 Trynka
H3K27ac PGC2
H3K27ac Hnisz
FetalDHS Trynka
Enhancer Hoffman
Enhancer Andersson
DHS Trynka
DGF ENCODE
CTCF Hoffman
**Conserved LindbladToh
Coding UCSC
Annotation
−1500
−1000
−500
0
500
1000
Enrichment (prop h2 / prop snps)

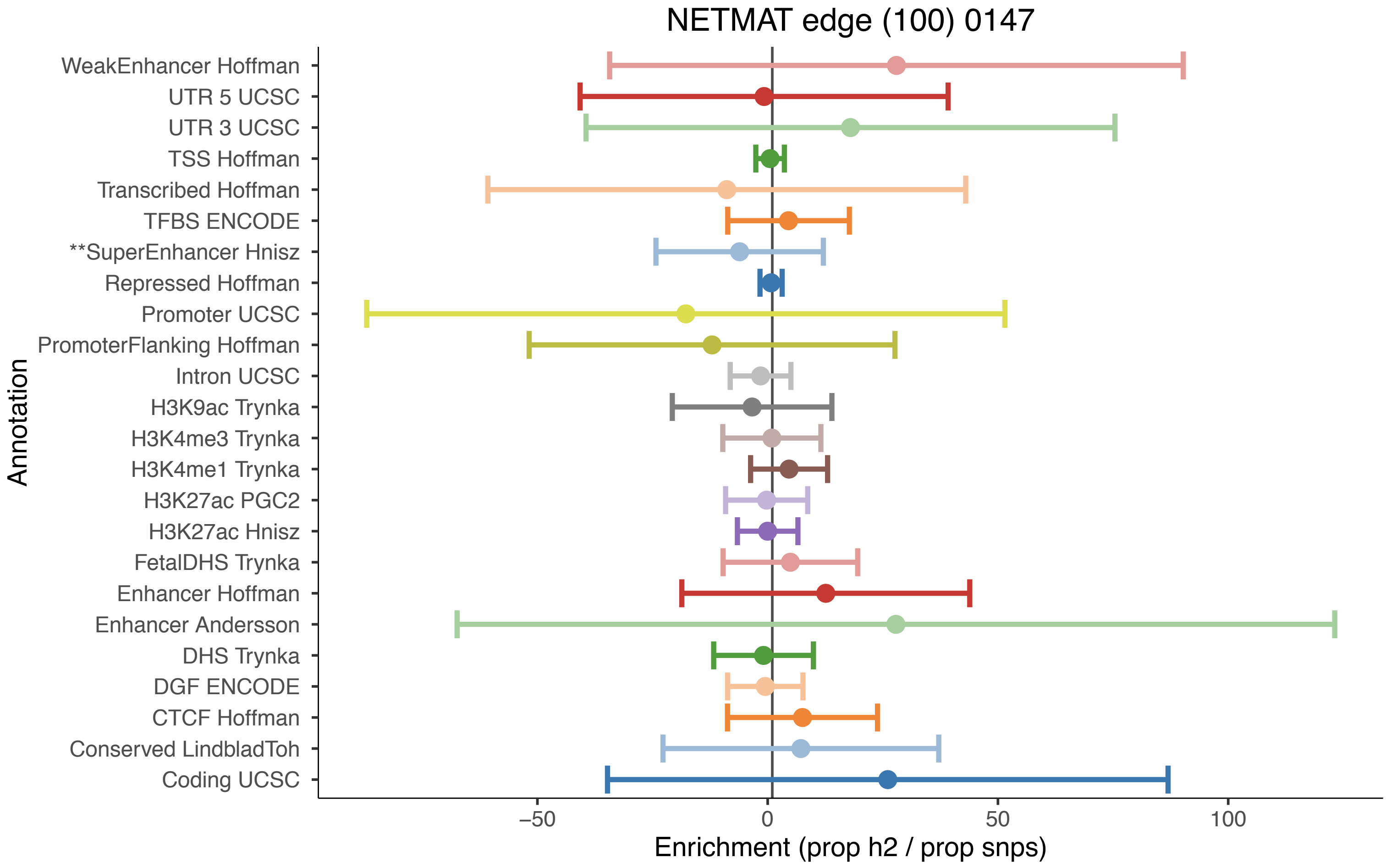
NETMAT edge (100) 0147
WeakEnhancer Hoffman
UTR 5 UCSC
UTR 3 UCSC
TSS Hoffman
Transcribed Hoffman
TFBS ENCODE
**SuperEnhancer Hnisz
Repressed Hoffman
Promoter UCSC
PromoterFlanking Hoffman
Intron UCSC
H3K9ac Trynka
H3K4me3 Trynka
H3K4me1 Trynka
H3K27ac PGC2
H3K27ac Hnisz
FetalDHS Trynka
Enhancer Hoffman
Enhancer Andersson
DHS Trynka
DGF ENCODE
CTCF Hoffman
Conserved LindbladToh
Coding UCSC
Annotation
−50
0
50
100
Enrichment (prop h2 / prop snps)

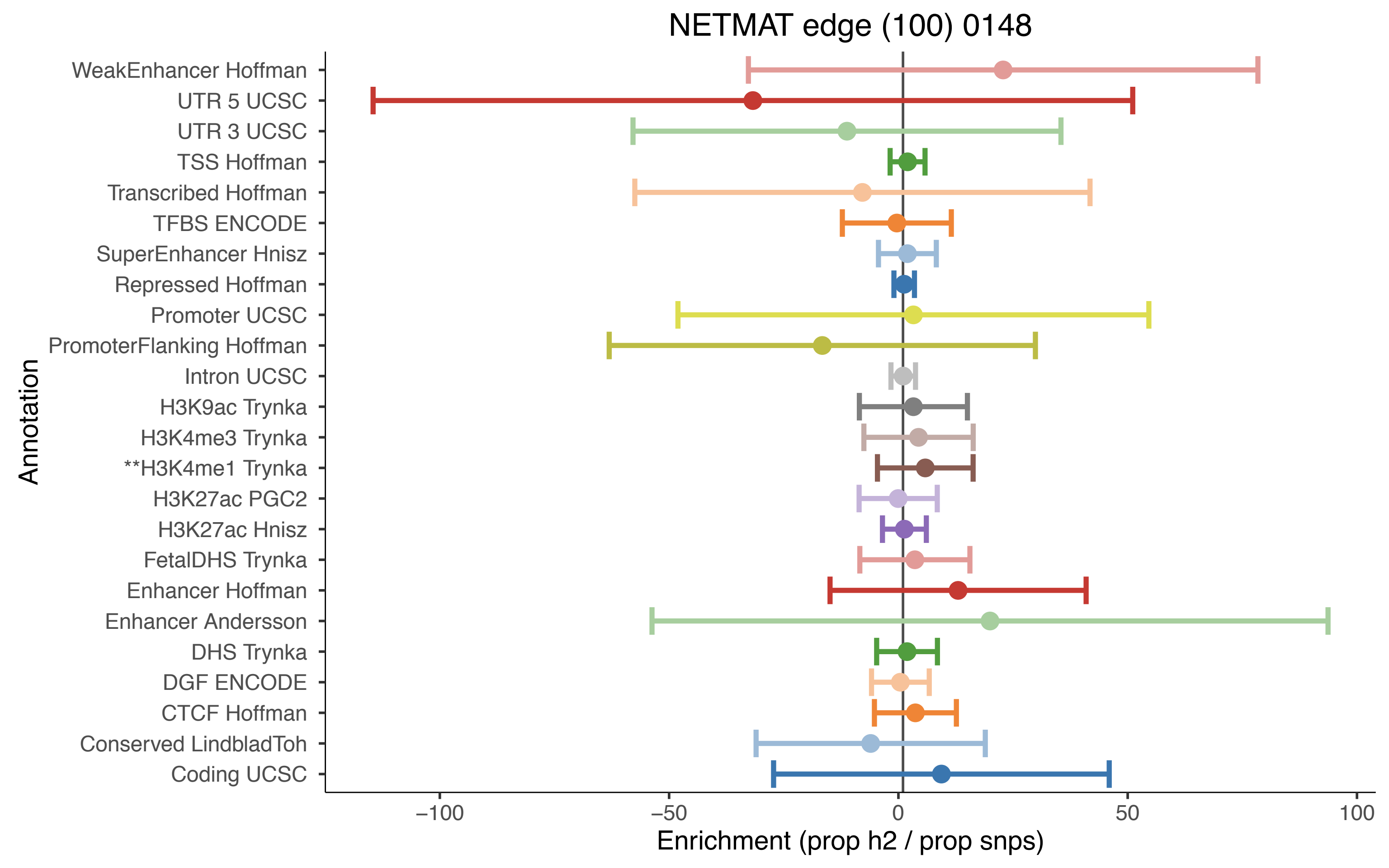

NETMAT edge (100) 0148
Annotation
WeakEnhancer Hoffman
UTR 5 UCSC
UTR 3 UCSC
TSS Hoffman
Transcribed Hoffman
TFBS ENCODE
SuperEnhancer Hnisz
Repressed Hoffman
Promoter UCSC
PromoterFlanking Hoffman
Intron UCSC
H3K9ac Trynka
H3K4me3 Trynka
**H3K4me1 Trynka
H3K27ac PGC2
H3K27ac Hnisz
FetalDHS Trynka
Enhancer Hoffman
Enhancer Andersson
DHS Trynka
DGF ENCODE
CTCF Hoffman
Conserved LindbladToh
Coding UCSC
−100
−50
0
50
100
Enrichment (prop h2 / prop snps)

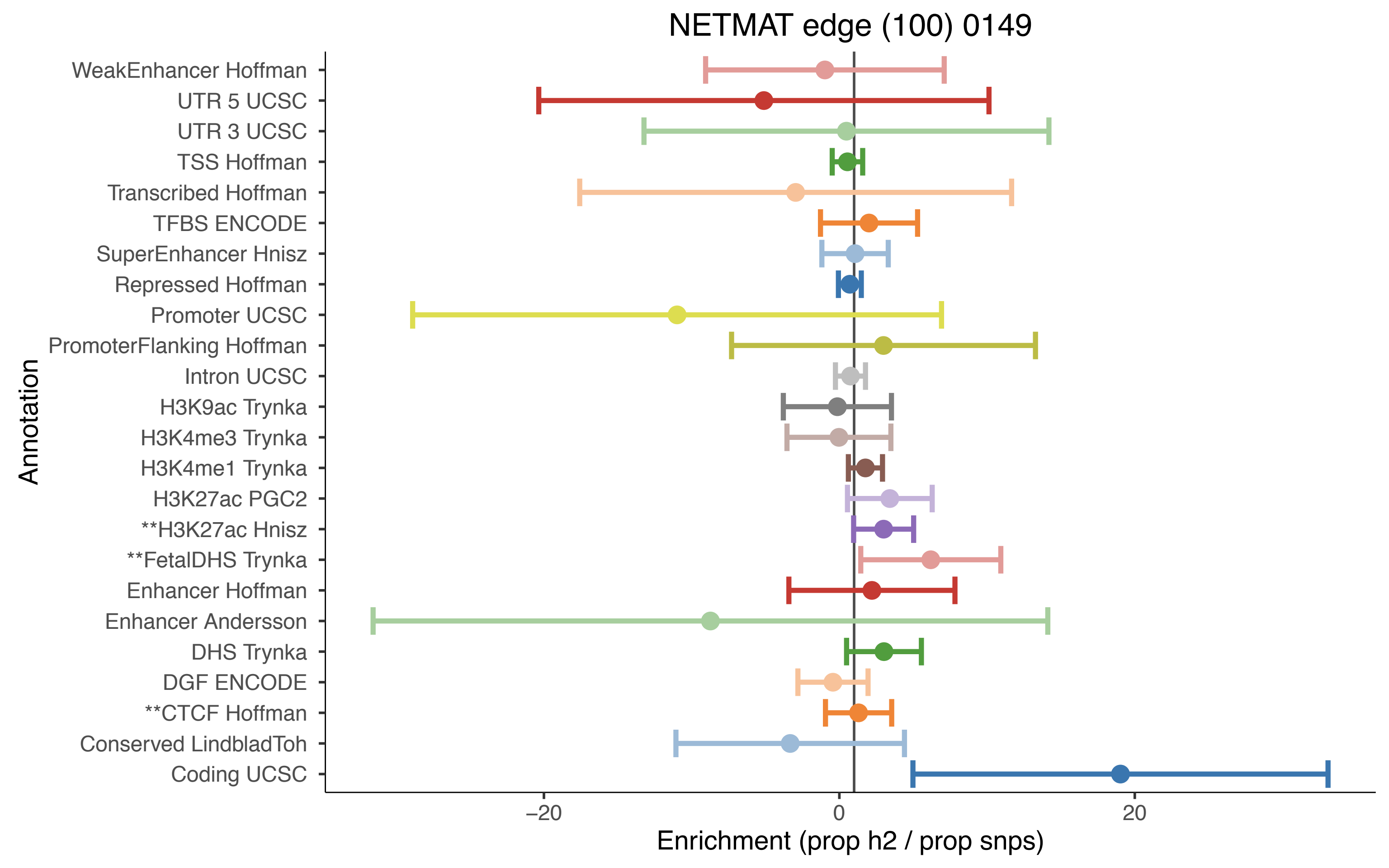
NETMAT edge (100) 0149
WeakEnhancer Hoffman
UTR 5 UCSC
UTR 3 UCSC
TSS Hoffman
Transcribed Hoffman
TFBS ENCODE
SuperEnhancer Hnisz
Repressed Hoffman
Promoter UCSC
PromoterFlanking Hoffman
Intron UCSC
H3K9ac Trynka
H3K4me3 Trynka
H3K4me1 Trynka
H3K27ac PGC2
**H3K27ac Hnisz
**FetalDHS Trynka
Enhancer Hoffman
Enhancer Andersson
DHS Trynka
DGF ENCODE
**CTCF Hoffman
Conserved LindbladToh
Coding UCSC
Annotation
−20
0
20
Enrichment (prop h2 / prop snps)

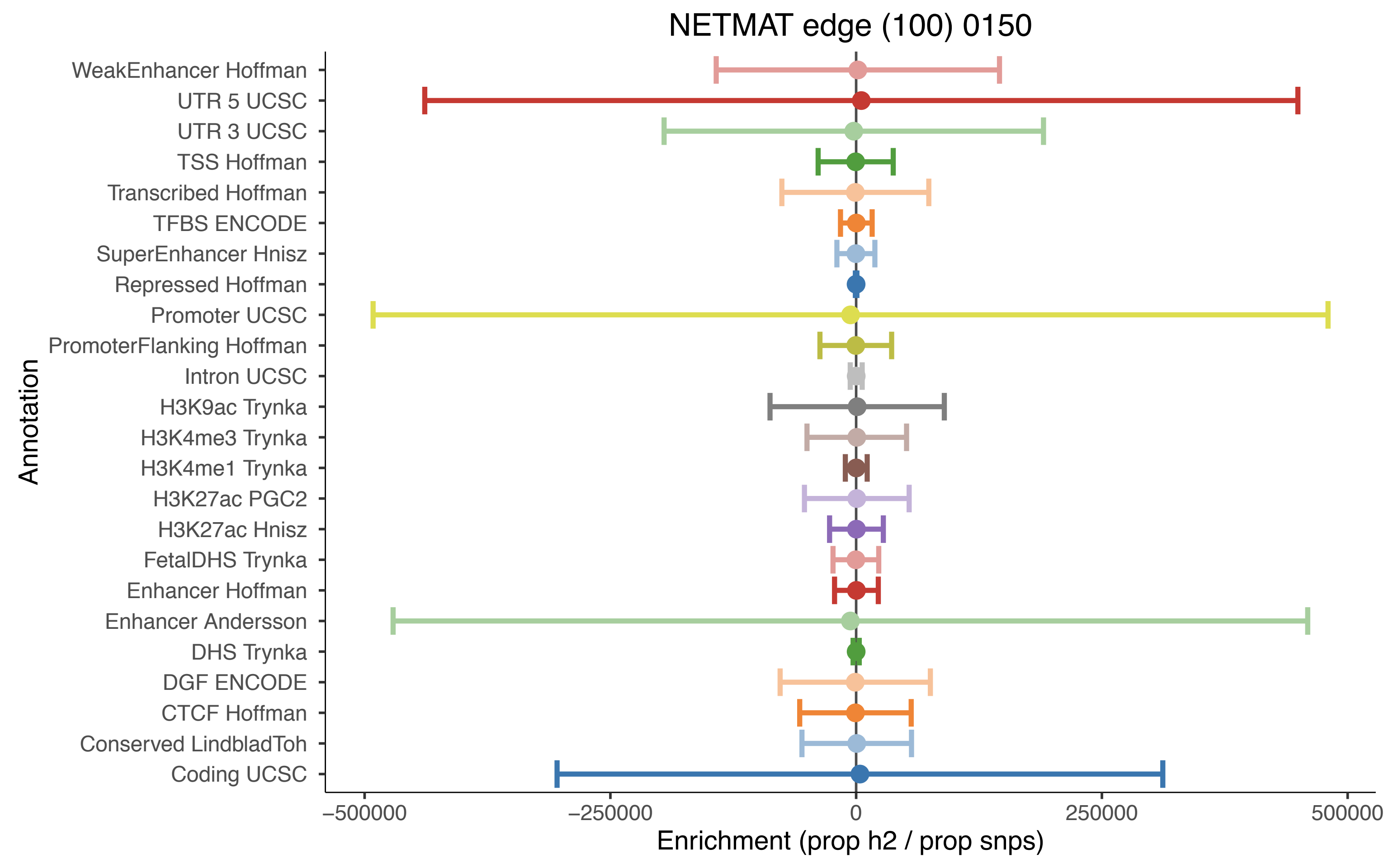

NETMAT edge (100) 0150
WeakEnhancer Hoffman
UTR 5 UCSC
UTR 3 UCSC
TSS Hoffman
Transcribed Hoffman
TFBS ENCODE
SuperEnhancer Hnisz
Repressed Hoffman
Promoter UCSC
PromoterFlanking Hoffman
Intron UCSC
H3K9ac Trynka
H3K4me3 Trynka
H3K4me1 Trynka
H3K27ac PGC2
H3K27ac Hnisz
FetalDHS Trynka
Enhancer Hoffman
Enhancer Andersson
DHS Trynka
DGF ENCODE
CTCF Hoffman
Conserved LindbladToh
Coding UCSC
Annotation
−500000
−250000
0
250000
500000
Enrichment (prop h2 / prop snps)

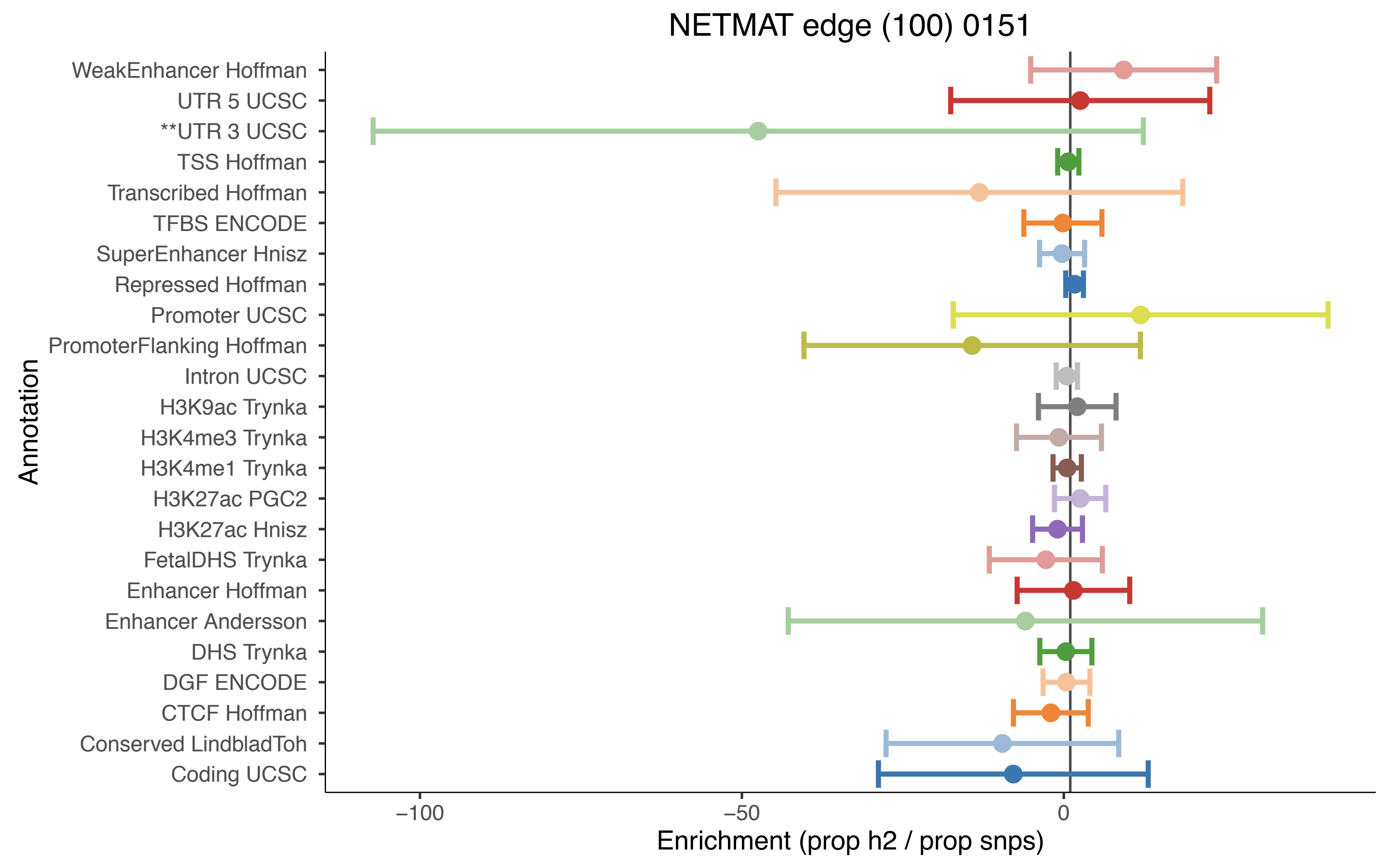
NETMAT edge (100) 0151
WeakEnhancer Hoffman
UTR 5 UCSC
**UTR 3 UCSC
TSS Hoffman
Transcribed Hoffman
TFBS ENCODE
SuperEnhancer Hnisz
Repressed Hoffman
Promoter UCSC
PromoterFlanking Hoffman
Intron UCSC
H3K9ac Trynka
H3K4me3 Trynka
H3K4me1 Trynka
H3K27ac PGC2
H3K27ac Hnisz
FetalDHS Trynka
Enhancer Hoffman
Enhancer Andersson
DHS Trynka
DGF ENCODE
CTCF Hoffman
Conserved LindbladToh
Coding UCSC
Annotation
−100
−50
0
Enrichment (prop h2 / prop snps)

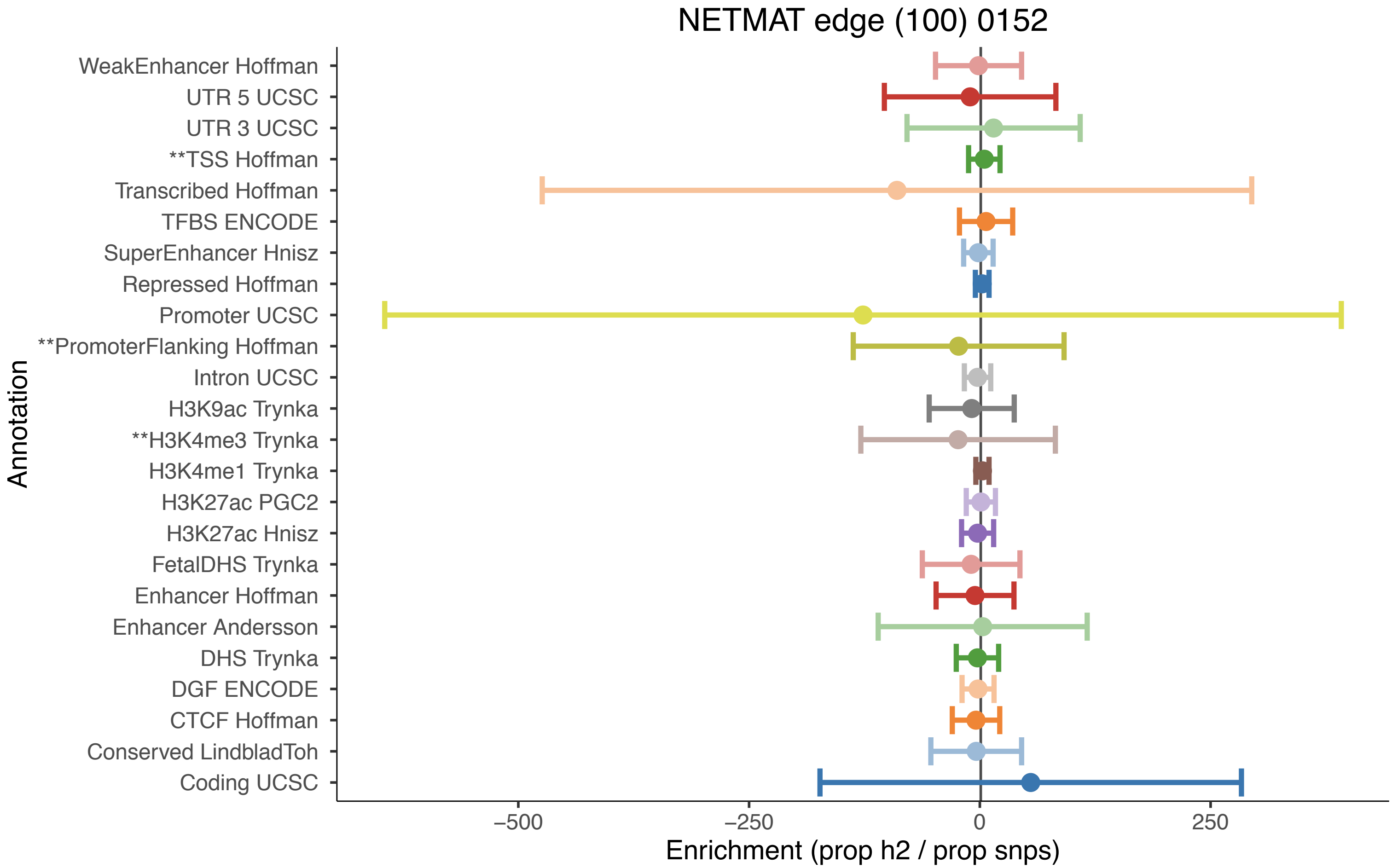
NETMAT edge (100) 0152
WeakEnhancer Hoffman
UTR 5 UCSC
UTR 3 UCSC
**TSS Hoffman
Transcribed Hoffman
TFBS ENCODE
SuperEnhancer Hnisz
Repressed Hoffman
Promoter UCSC
**PromoterFlanking Hoffman
Intron UCSC
H3K9ac Trynka
**H3K4me3 Trynka
H3K4me1 Trynka
H3K27ac PGC2
H3K27ac Hnisz
FetalDHS Trynka
Enhancer Hoffman
Enhancer Andersson
DHS Trynka
DGF ENCODE
CTCF Hoffman
Conserved LindbladToh
Coding UCSC
Annotation
−500
−250
0
250
Enrichment (prop h2 / prop snps)

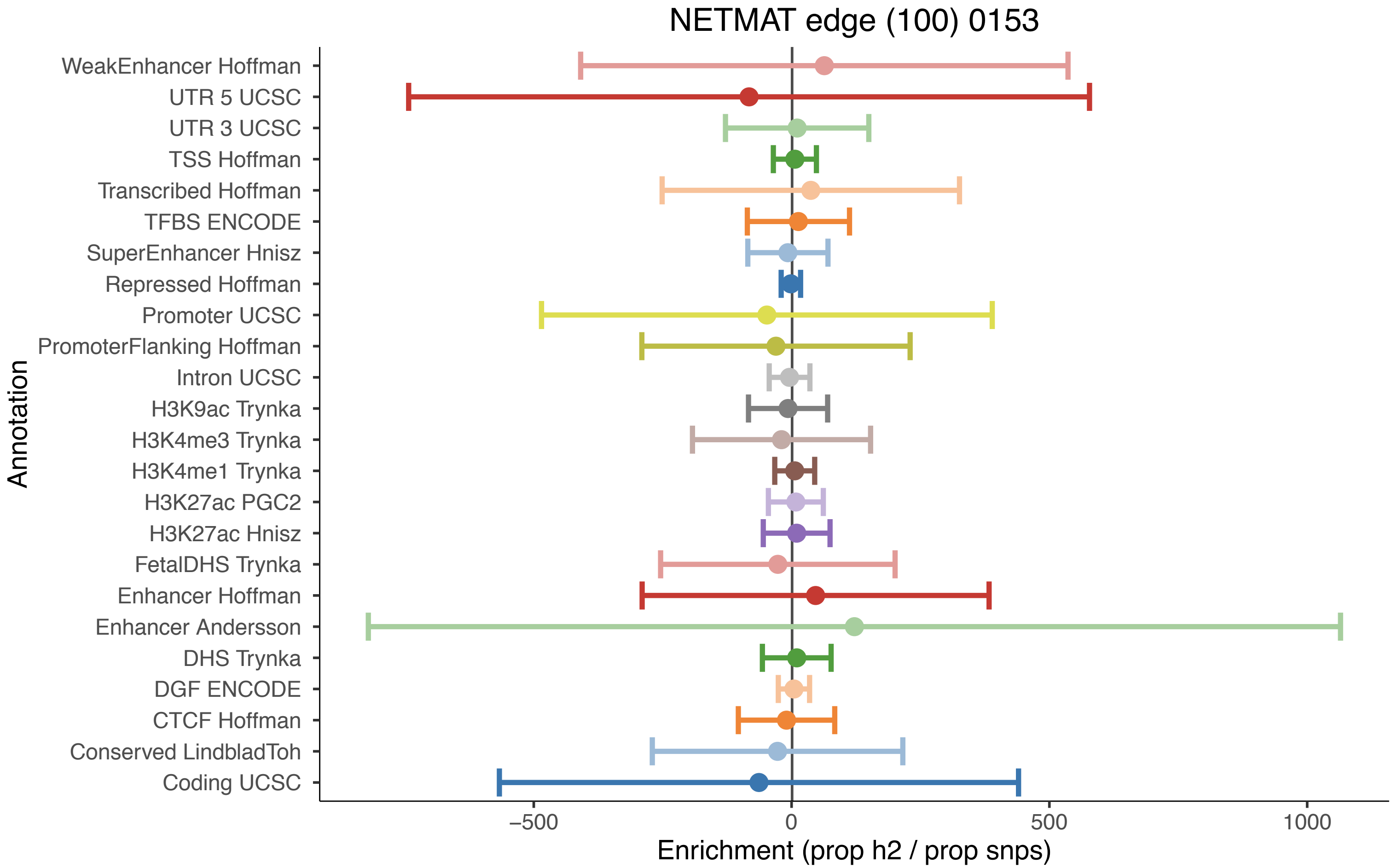

NETMAT edge (100) 0153
WeakEnhancer Hoffman
UTR 5 UCSC
UTR 3 UCSC
TSS Hoffman
Transcribed Hoffman
TFBS ENCODE
SuperEnhancer Hnisz
Repressed Hoffman
Promoter UCSC
PromoterFlanking Hoffman
Intron UCSC
H3K9ac Trynka
H3K4me3 Trynka
H3K4me1 Trynka
H3K27ac PGC2
H3K27ac Hnisz
FetalDHS Trynka
Enhancer Hoffman
Enhancer Andersson
DHS Trynka
DGF ENCODE
CTCF Hoffman
Conserved LindbladToh
Coding UCSC
Annotation
−500
0
500
1000
Enrichment (prop h2 / prop snps)

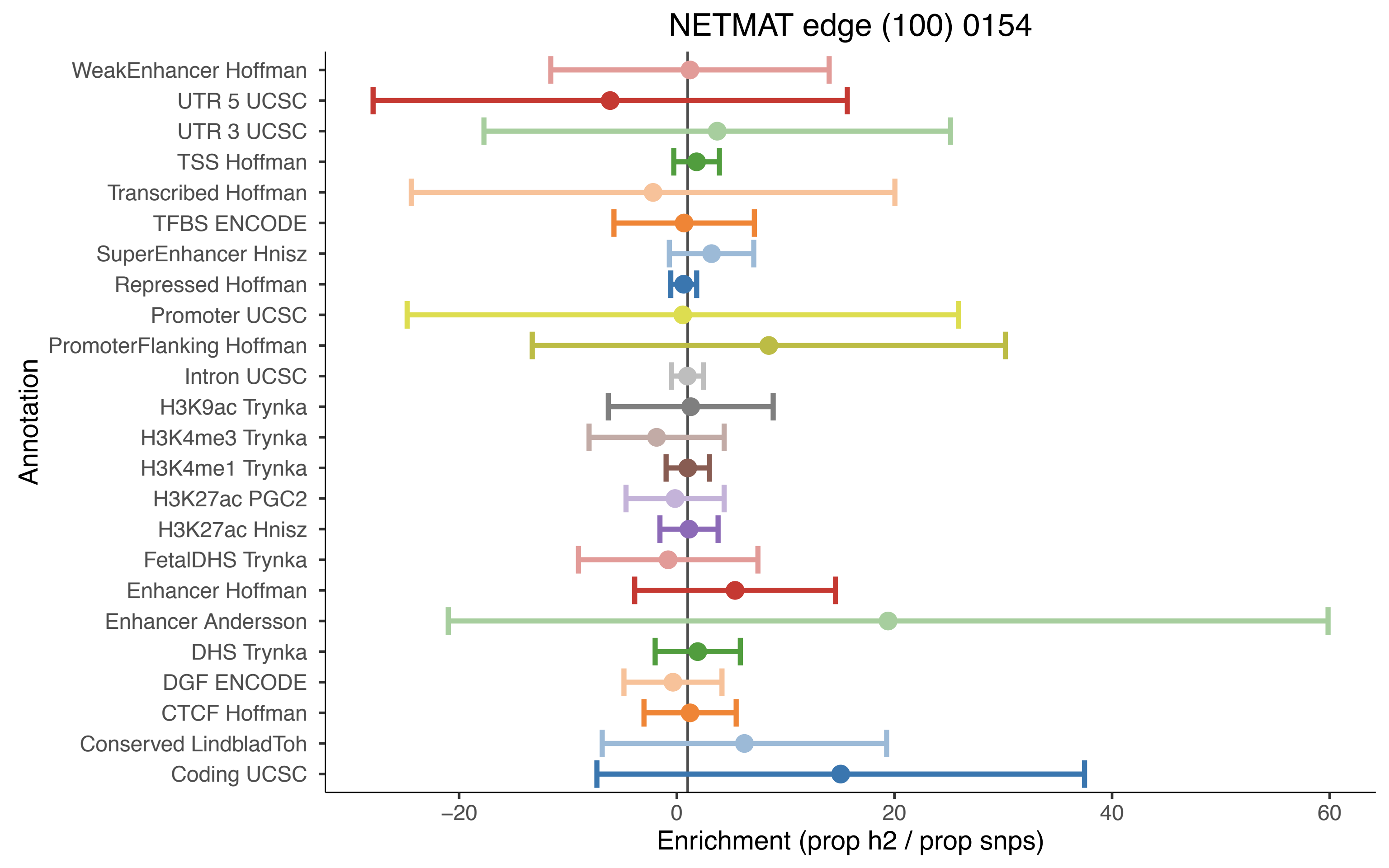
NETMAT edge (100) 0154
WeakEnhancer Hoffman
UTR 5 UCSC
UTR 3 UCSC
TSS Hoffman
Transcribed Hoffman
TFBS ENCODE
SuperEnhancer Hnisz
Repressed Hoffman
Promoter UCSC
PromoterFlanking Hoffman
Intron UCSC
H3K9ac Trynka
H3K4me3 Trynka
H3K4me1 Trynka
H3K27ac PGC2
H3K27ac Hnisz
FetalDHS Trynka
Enhancer Hoffman
Enhancer Andersson
DHS Trynka
DGF ENCODE
CTCF Hoffman
Conserved LindbladToh
Coding UCSC
Annotation
−20
0
20
40
60
Enrichment (prop h2 / prop snps)

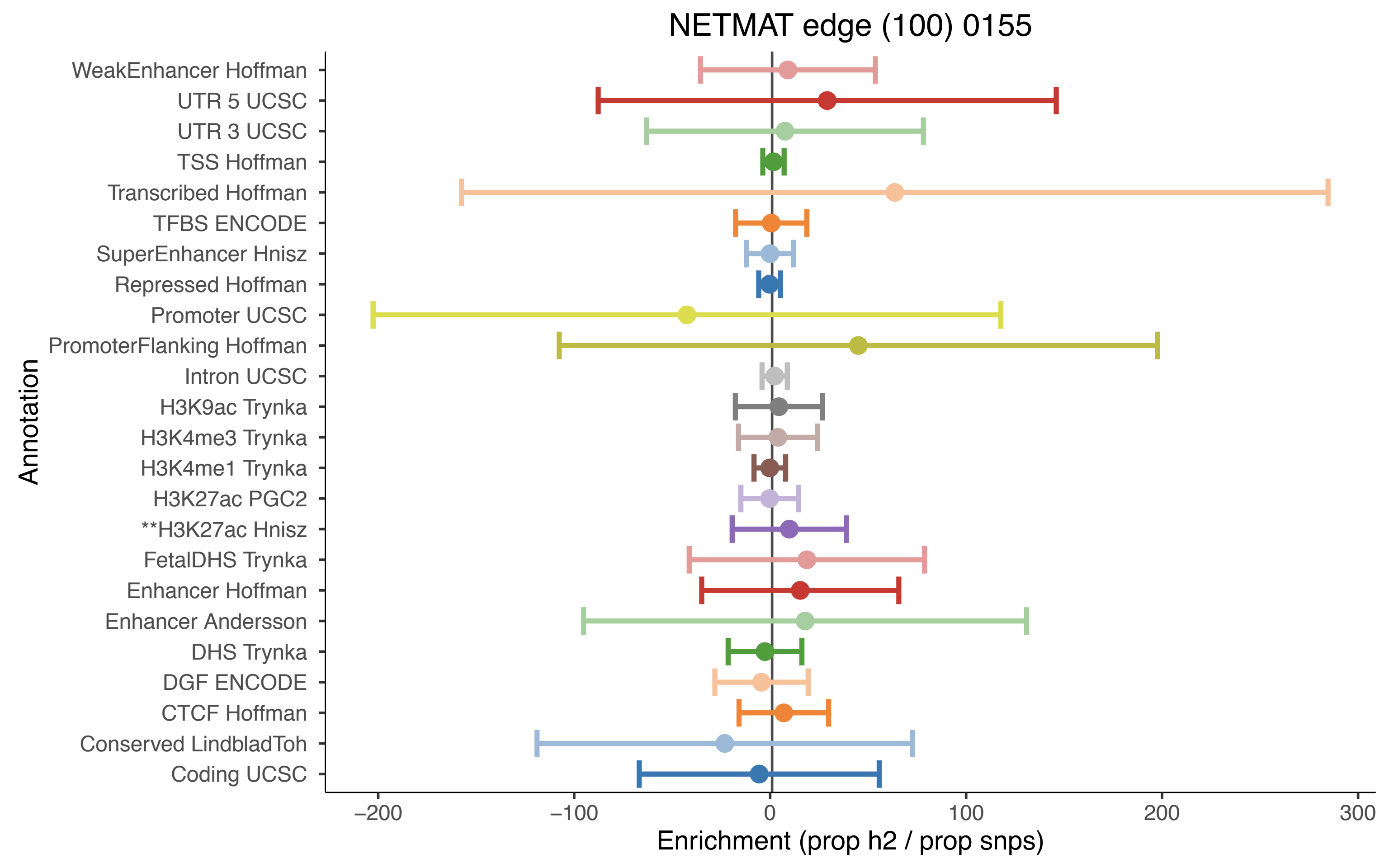

NETMAT edge (100) 0155
WeakEnhancer Hoffman
UTR 5 UCSC
UTR 3 UCSC
TSS Hoffman
Transcribed Hoffman
TFBS ENCODE
SuperEnhancer Hnisz
Repressed Hoffman
Promoter UCSC
PromoterFlanking Hoffman
Intron UCSC
H3K9ac Trynka
H3K4me3 Trynka
H3K4me1 Trynka
H3K27ac PGC2
**H3K27ac Hnisz
FetalDHS Trynka
Enhancer Hoffman
Enhancer Andersson
DHS Trynka
DGF ENCODE
CTCF Hoffman
Conserved LindbladToh
Coding UCSC
Annotation
−200
−100
0
100
200
300
Enrichment (prop h2 / prop snps)

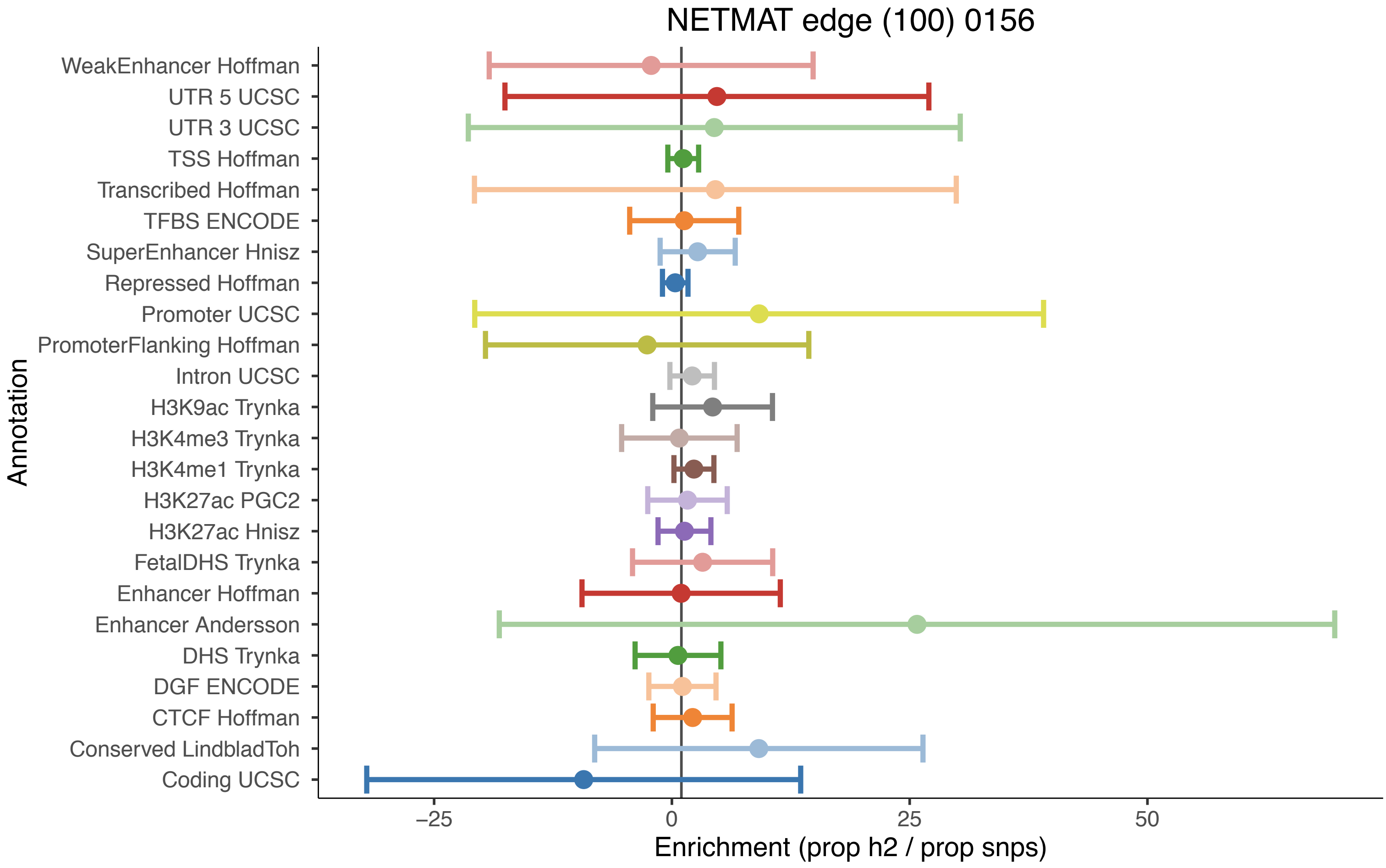
NETMAT edge (100) 0156
WeakEnhancer Hoffman
UTR 5 UCSC
UTR 3 UCSC
TSS Hoffman
Transcribed Hoffman
TFBS ENCODE
SuperEnhancer Hnisz
Repressed Hoffman
Promoter UCSC
PromoterFlanking Hoffman
Intron UCSC
H3K9ac Trynka
H3K4me3 Trynka
H3K4me1 Trynka
H3K27ac PGC2
H3K27ac Hnisz
FetalDHS Trynka
Enhancer Hoffman
Enhancer Andersson
DHS Trynka
DGF ENCODE
CTCF Hoffman
Conserved LindbladToh
Coding UCSC
Annotation
−25
0
25
50
Enrichment (prop h2 / prop snps)

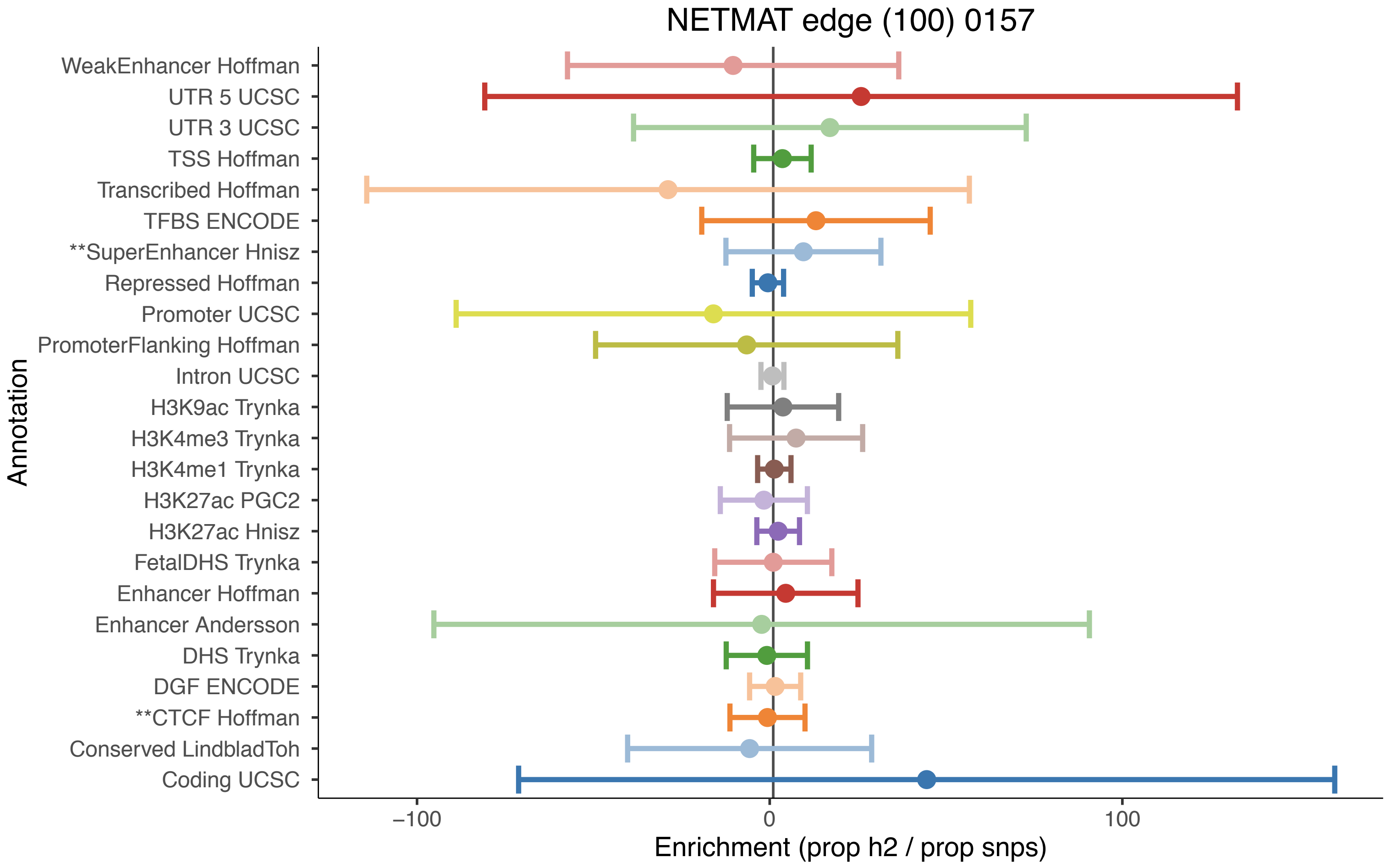
NETMAT edge (100) 0157
WeakEnhancer Hoffman
UTR 5 UCSC
UTR 3 UCSC
TSS Hoffman
Transcribed Hoffman
TFBS ENCODE
**SuperEnhancer Hnisz
Repressed Hoffman
Promoter UCSC
PromoterFlanking Hoffman
Intron UCSC
H3K9ac Trynka
H3K4me3 Trynka
H3K4me1 Trynka
H3K27ac PGC2
H3K27ac Hnisz
FetalDHS Trynka
Enhancer Hoffman
Enhancer Andersson
DHS Trynka
DGF ENCODE
**CTCF Hoffman
Conserved LindbladToh
Coding UCSC
Annotation
−100
0
100
Enrichment (prop h2 / prop snps)

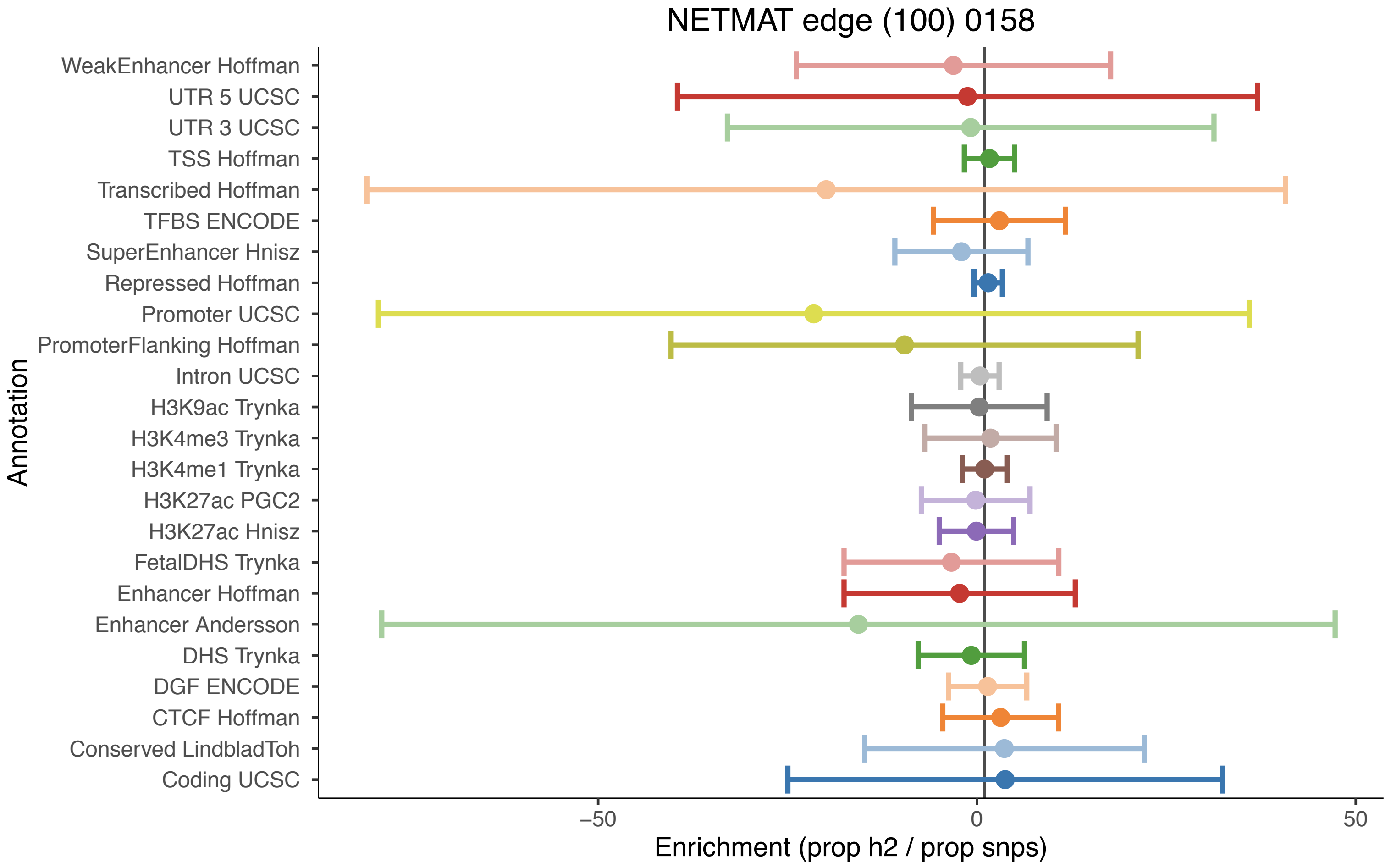
NETMAT edge (100) 0158
WeakEnhancer Hoffman
UTR 5 UCSC
UTR 3 UCSC
TSS Hoffman
Transcribed Hoffman
TFBS ENCODE
SuperEnhancer Hnisz
Repressed Hoffman
Promoter UCSC
PromoterFlanking Hoffman
Intron UCSC
H3K9ac Trynka
H3K4me3 Trynka
H3K4me1 Trynka
H3K27ac PGC2
H3K27ac Hnisz
FetalDHS Trynka
Enhancer Hoffman
Enhancer Andersson
DHS Trynka
DGF ENCODE
CTCF Hoffman
Conserved LindbladToh
Coding UCSC
Annotation
−50
0
50
Enrichment (prop h2 / prop snps)

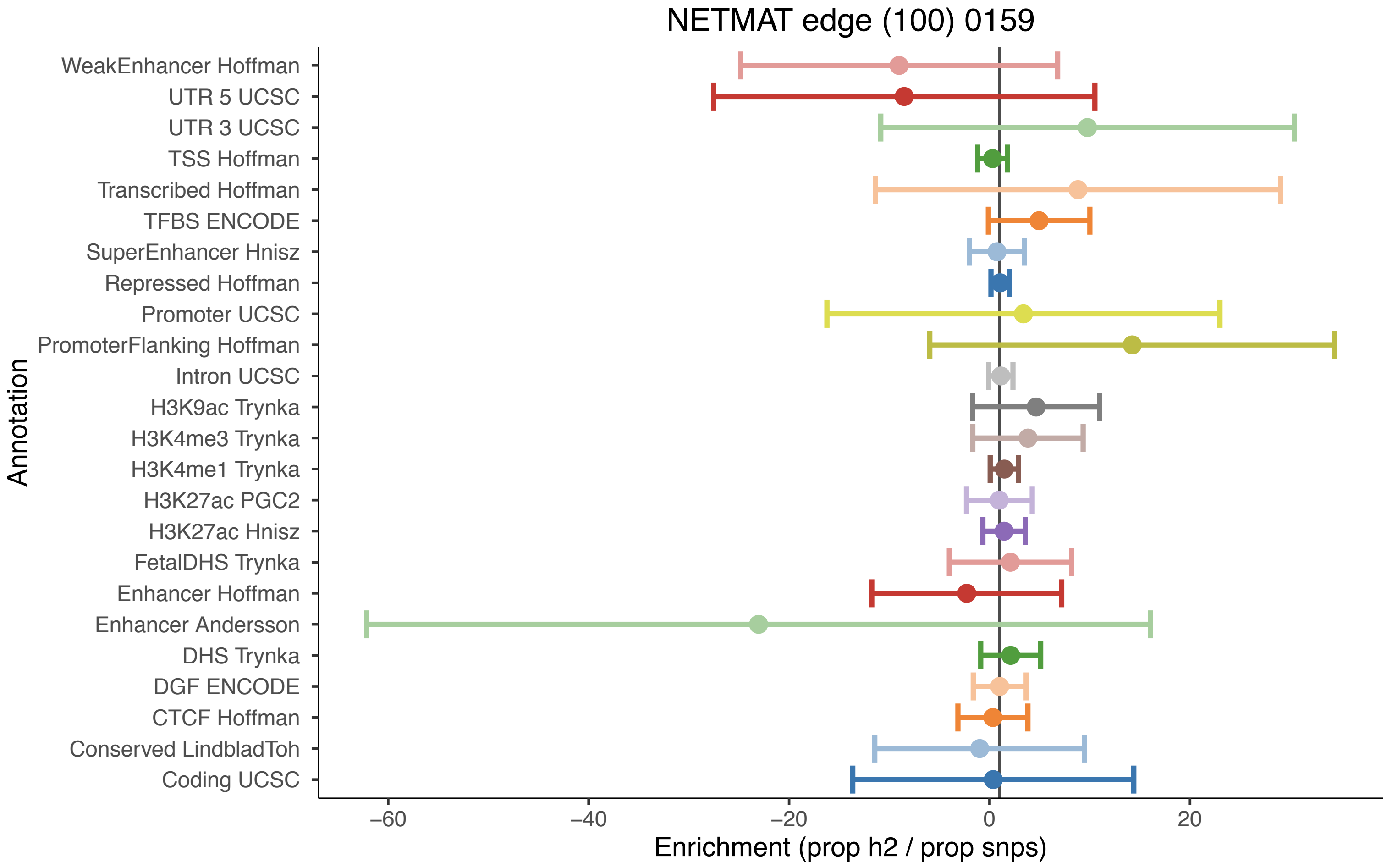

NETMAT edge (100) 0159
WeakEnhancer Hoffman
UTR 5 UCSC
UTR 3 UCSC
TSS Hoffman
Transcribed Hoffman
TFBS ENCODE
SuperEnhancer Hnisz
Repressed Hoffman
Promoter UCSC
PromoterFlanking Hoffman
Intron UCSC
H3K9ac Trynka
H3K4me3 Trynka
H3K4me1 Trynka
H3K27ac PGC2
H3K27ac Hnisz
FetalDHS Trynka
Enhancer Hoffman
Enhancer Andersson
DHS Trynka
DGF ENCODE
CTCF Hoffman
Conserved LindbladToh
Coding UCSC
Annotation
−60
−40
−20
0
20
Enrichment (prop h2 / prop snps)

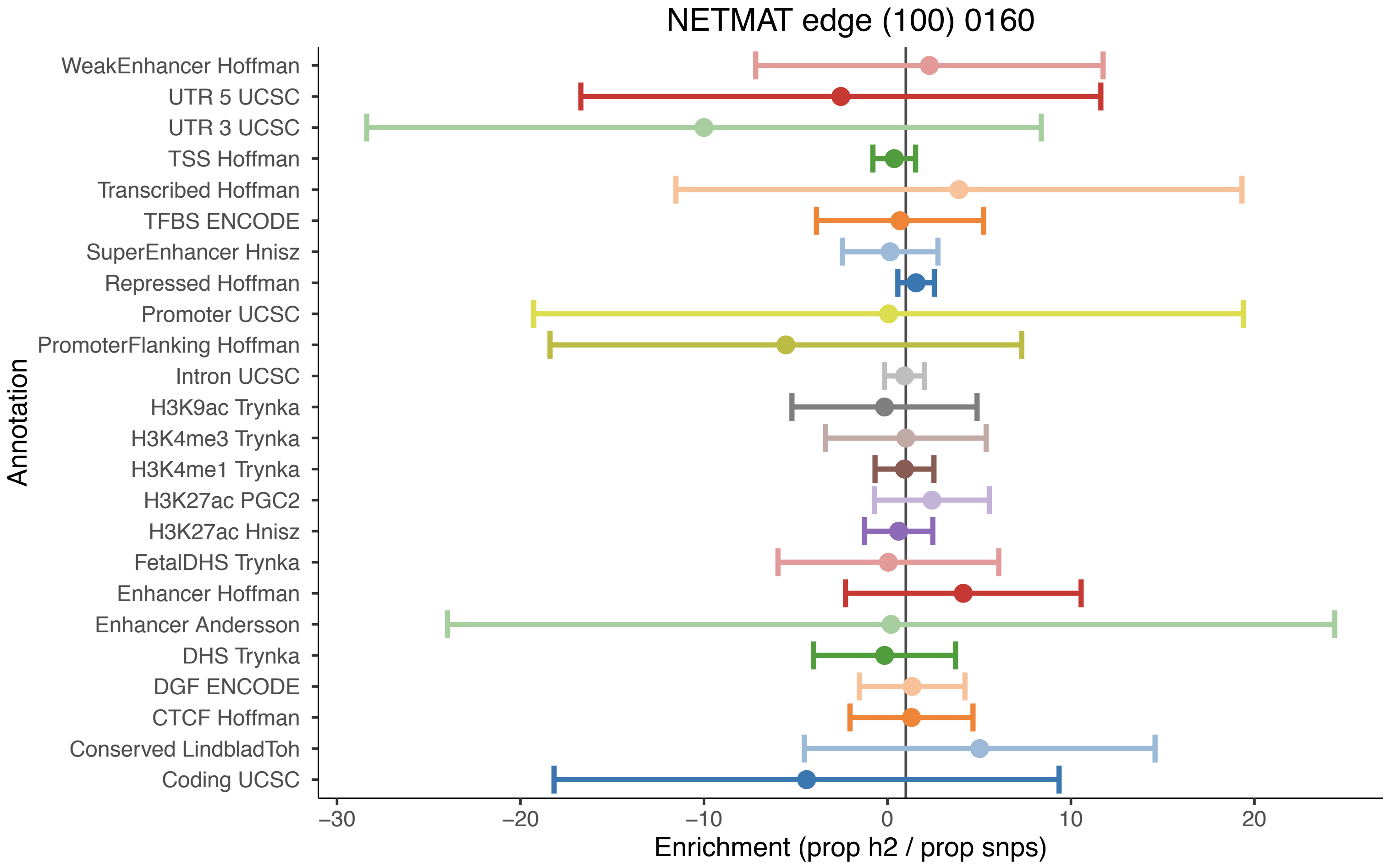
NETMAT edge (100) 0160
WeakEnhancer Hoffman
UTR 5 UCSC
UTR 3 UCSC
TSS Hoffman
Transcribed Hoffman
TFBS ENCODE
SuperEnhancer Hnisz
Repressed Hoffman
Promoter UCSC
PromoterFlanking Hoffman
Intron UCSC
H3K9ac Trynka
H3K4me3 Trynka
H3K4me1 Trynka
H3K27ac PGC2
H3K27ac Hnisz
FetalDHS Trynka
Enhancer Hoffman
Enhancer Andersson
DHS Trynka
DGF ENCODE
CTCF Hoffman
Conserved LindbladToh
Coding UCSC
Annotation
−30
−20
−10
0
10
20
Enrichment (prop h2 / prop snps)

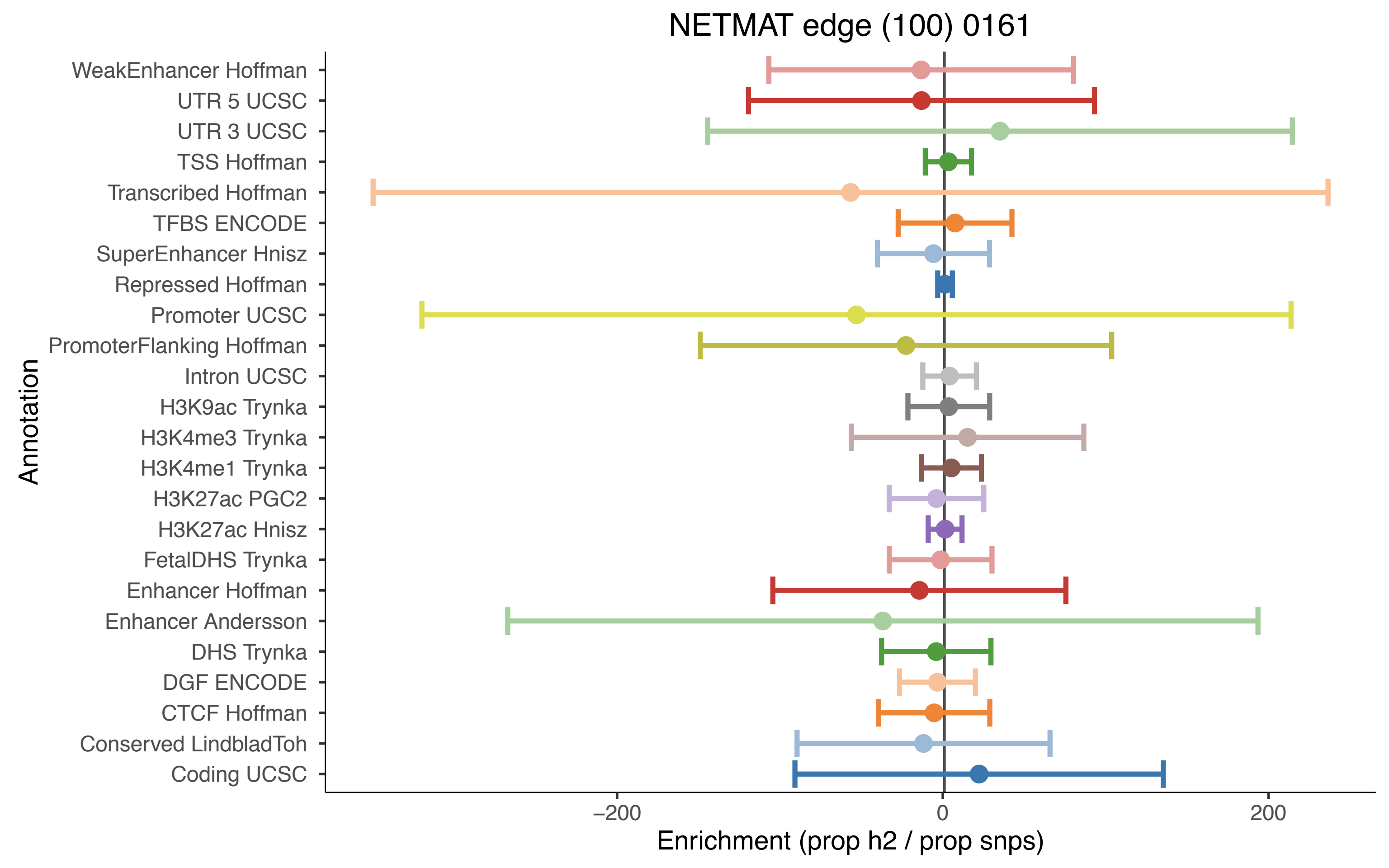

NETMAT edge (100) 0161
WeakEnhancer Hoffman
UTR 5 UCSC
UTR 3 UCSC
TSS Hoffman
Transcribed Hoffman
TFBS ENCODE
SuperEnhancer Hnisz
Repressed Hoffman
Promoter UCSC
PromoterFlanking Hoffman
Intron UCSC
H3K9ac Trynka
H3K4me3 Trynka
H3K4me1 Trynka
H3K27ac PGC2
H3K27ac Hnisz
FetalDHS Trynka
Enhancer Hoffman
Enhancer Andersson
DHS Trynka
DGF ENCODE
CTCF Hoffman
Conserved LindbladToh
Coding UCSC
Annotation
−200
0
200
Enrichment (prop h2 / prop snps)

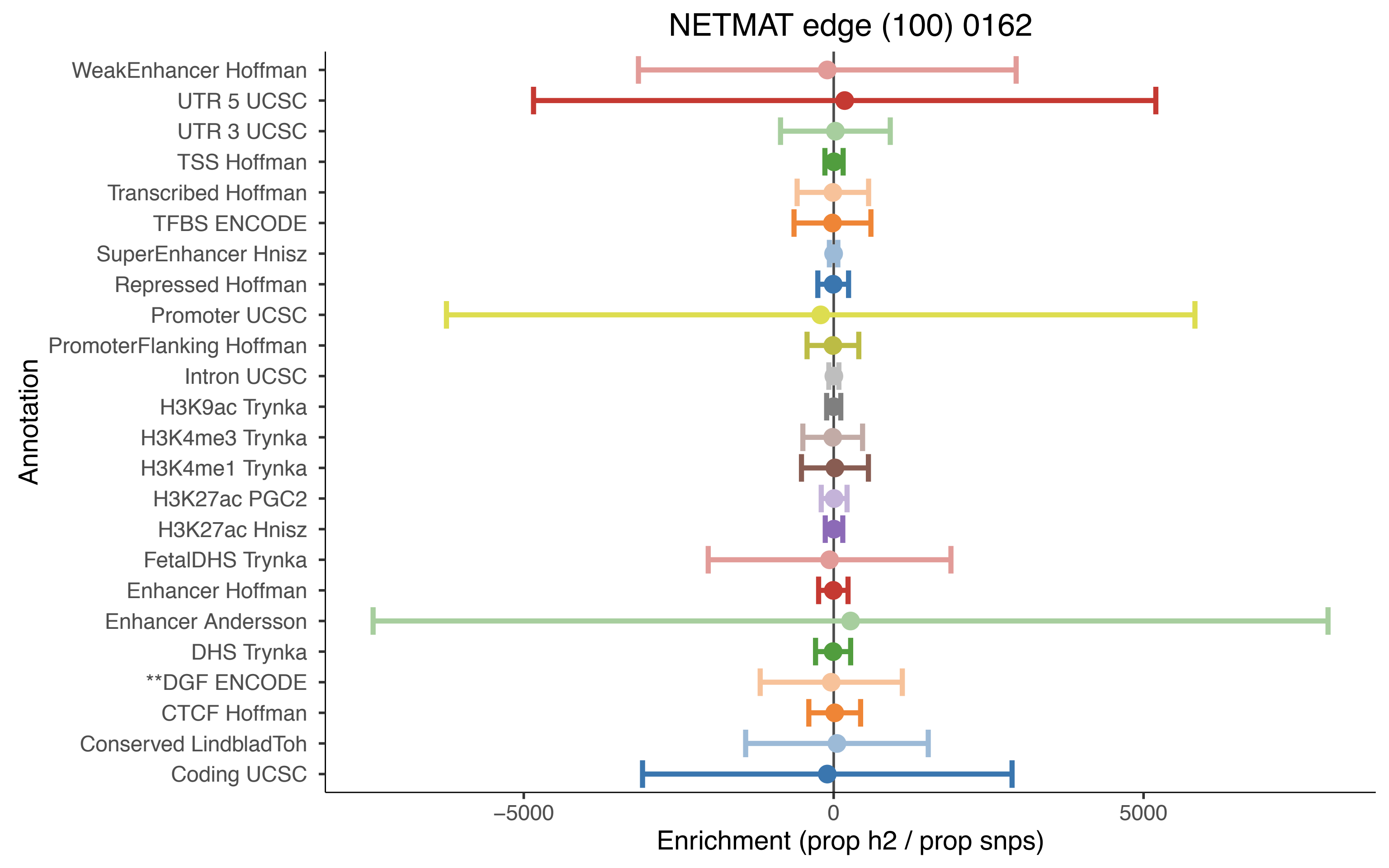
NETMAT edge (100) 0162
WeakEnhancer Hoffman
UTR 5 UCSC
UTR 3 UCSC
TSS Hoffman
Transcribed Hoffman
TFBS ENCODE
SuperEnhancer Hnisz
Repressed Hoffman
Promoter UCSC
PromoterFlanking Hoffman
Intron UCSC
H3K9ac Trynka
H3K4me3 Trynka
H3K4me1 Trynka
H3K27ac PGC2
H3K27ac Hnisz
FetalDHS Trynka
Enhancer Hoffman
Enhancer Andersson
DHS Trynka
**DGF ENCODE
CTCF Hoffman
Conserved LindbladToh
Coding UCSC
Annotation
−5000
0
5000
Enrichment (prop h2 / prop snps)

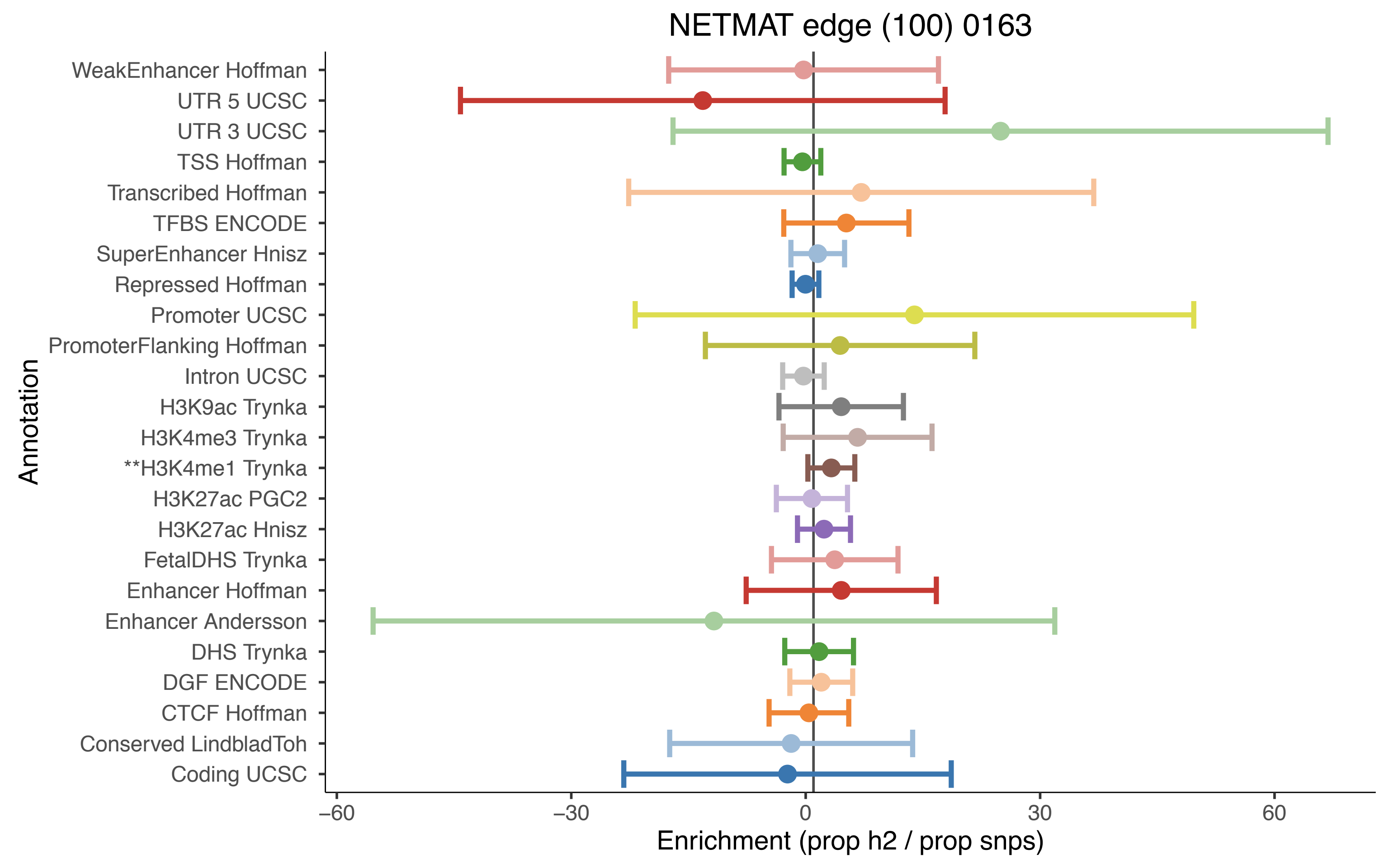
NETMAT edge (100) 0163
WeakEnhancer Hoffman
UTR 5 UCSC
UTR 3 UCSC
TSS Hoffman
Transcribed Hoffman
TFBS ENCODE
SuperEnhancer Hnisz
Repressed Hoffman
Promoter UCSC
PromoterFlanking Hoffman
Intron UCSC
H3K9ac Trynka
H3K4me3 Trynka
**H3K4me1 Trynka
H3K27ac PGC2
H3K27ac Hnisz
FetalDHS Trynka
Enhancer Hoffman
Enhancer Andersson
DHS Trynka
DGF ENCODE
CTCF Hoffman
Conserved LindbladToh
Coding UCSC
Annotation
−60
−30
0
30
60
Enrichment (prop h2 / prop snps)

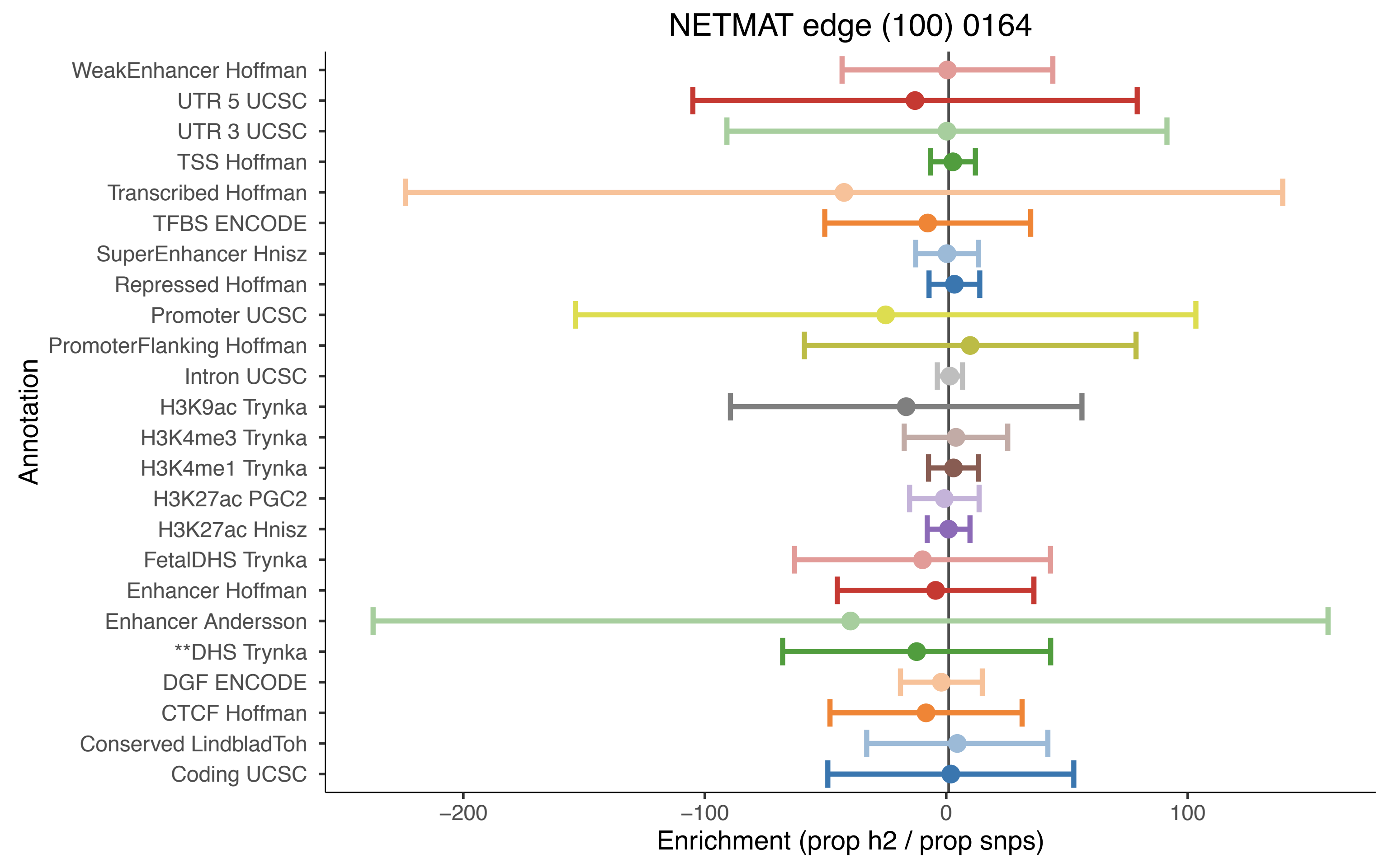
NETMAT edge (100) 0164
Annotation
WeakEnhancer Hoffman
UTR 5 UCSC
UTR 3 UCSC
TSS Hoffman
Transcribed Hoffman
TFBS ENCODE
SuperEnhancer Hnisz
Repressed Hoffman
Promoter UCSC
PromoterFlanking Hoffman
Intron UCSC
H3K9ac Trynka
H3K4me3 Trynka
H3K4me1 Trynka
H3K27ac PGC2
H3K27ac Hnisz
FetalDHS Trynka
Enhancer Hoffman
Enhancer Andersson
**DHS Trynka
DGF ENCODE
CTCF Hoffman
Conserved LindbladToh
Coding UCSC
−200
−100
0
100
Enrichment (prop h2 / prop snps)

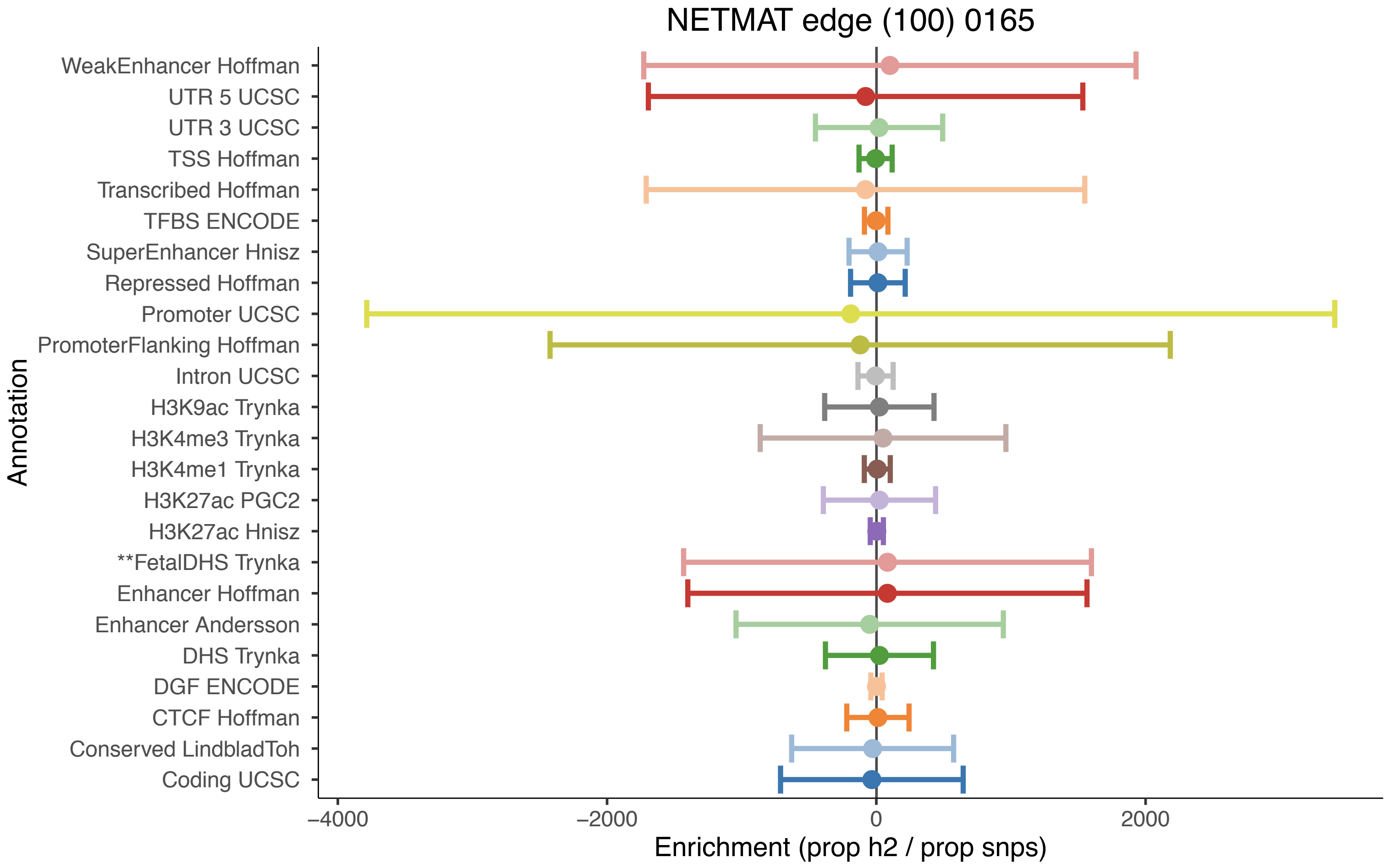
NETMAT edge (100) 0165
WeakEnhancer Hoffman
UTR 5 UCSC
UTR 3 UCSC
TSS Hoffman
Transcribed Hoffman
TFBS ENCODE
SuperEnhancer Hnisz
Repressed Hoffman
Promoter UCSC
PromoterFlanking Hoffman
Intron UCSC
H3K9ac Trynka
H3K4me3 Trynka
H3K4me1 Trynka
H3K27ac PGC2
H3K27ac Hnisz
**FetalDHS Trynka
Enhancer Hoffman
Enhancer Andersson
DHS Trynka
DGF ENCODE
CTCF Hoffman
Conserved LindbladToh
Coding UCSC
Annotation
−4000
−2000
0
2000
Enrichment (prop h2 / prop snps)

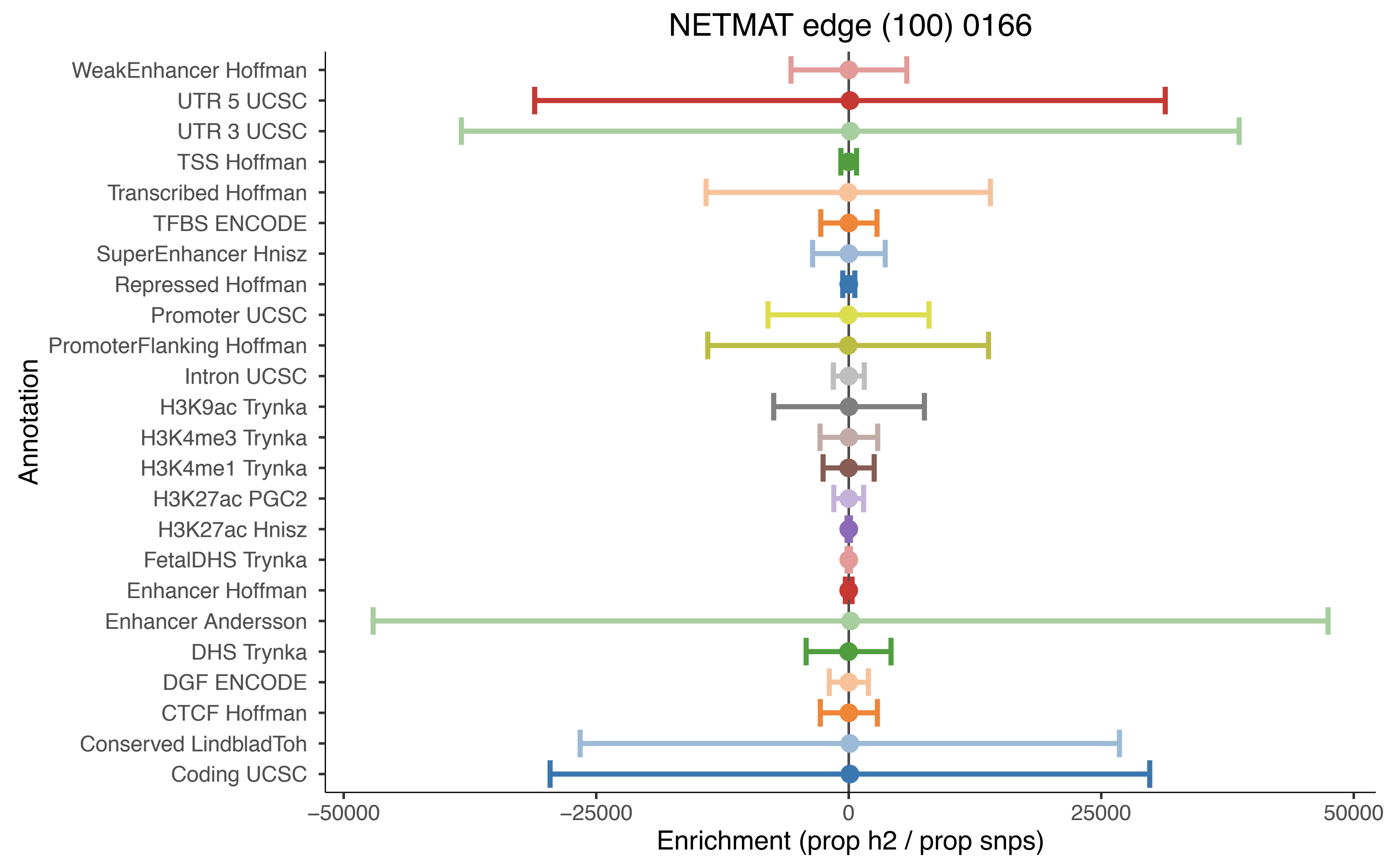
NETMAT edge (100) 0166
Annotation
WeakEnhancer Hoffman
UTR 5 UCSC
UTR 3 UCSC
TSS Hoffman
Transcribed Hoffman
TFBS ENCODE
SuperEnhancer Hnisz
Repressed Hoffman
Promoter UCSC
PromoterFlanking Hoffman
Intron UCSC
H3K9ac Trynka
H3K4me3 Trynka
H3K4me1 Trynka
H3K27ac PGC2
H3K27ac Hnisz
FetalDHS Trynka
Enhancer Hoffman
Enhancer Andersson
DHS Trynka
DGF ENCODE
CTCF Hoffman
Conserved LindbladToh
Coding UCSC
−50000
−25000
0
25000
50000
Enrichment (prop h2 / prop snps)

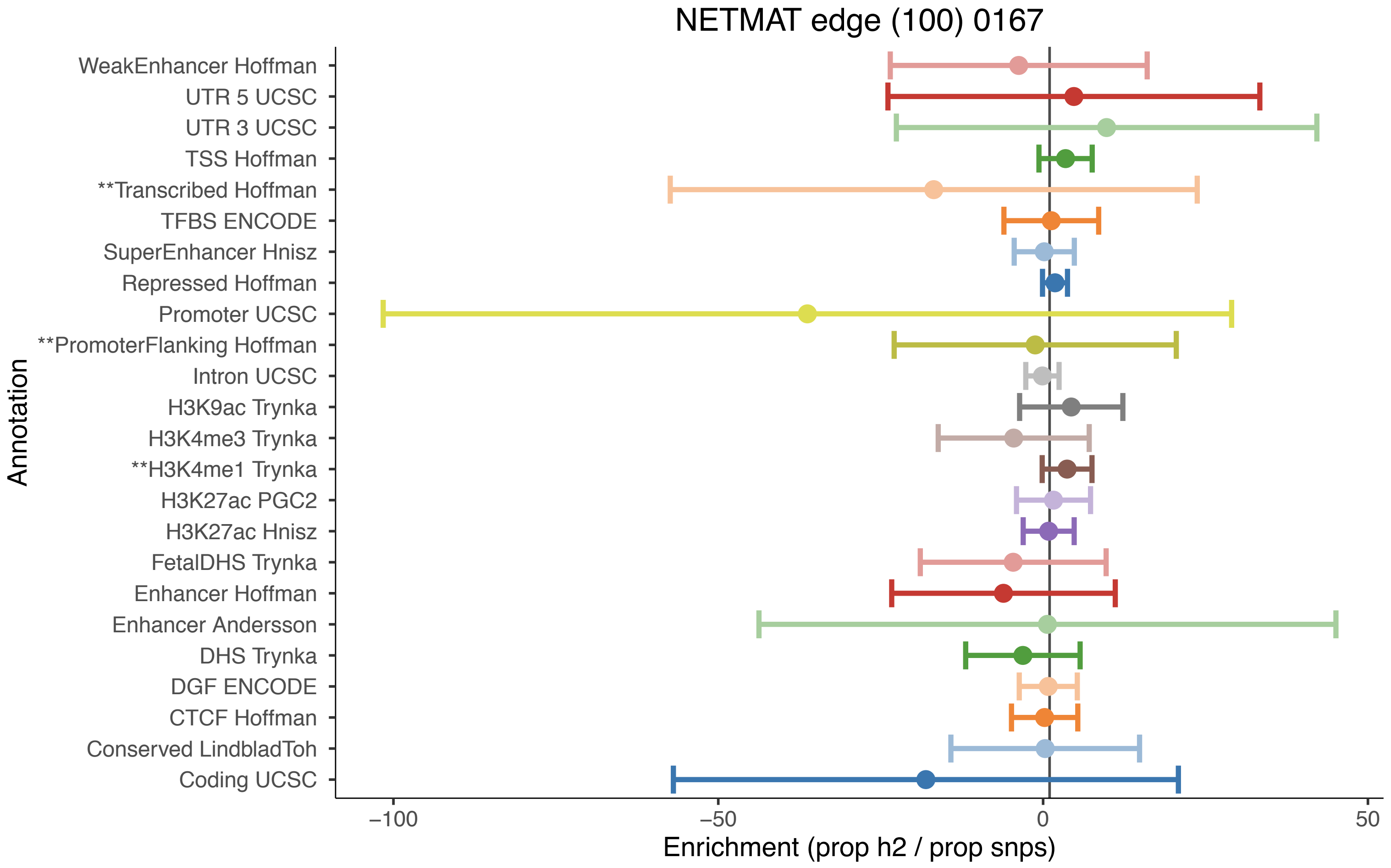

NETMAT edge (100) 0167
WeakEnhancer Hoffman
UTR 5 UCSC
UTR 3 UCSC
TSS Hoffman
**Transcribed Hoffman
TFBS ENCODE
SuperEnhancer Hnisz
Repressed Hoffman
Promoter UCSC
**PromoterFlanking Hoffman
Intron UCSC
H3K9ac Trynka
H3K4me3 Trynka
**H3K4me1 Trynka
H3K27ac PGC2
H3K27ac Hnisz
FetalDHS Trynka
Enhancer Hoffman
Enhancer Andersson
DHS Trynka
DGF ENCODE
CTCF Hoffman
Conserved LindbladToh
Coding UCSC
Annotation
−100
−50
0
50
Enrichment (prop h2 / prop snps)

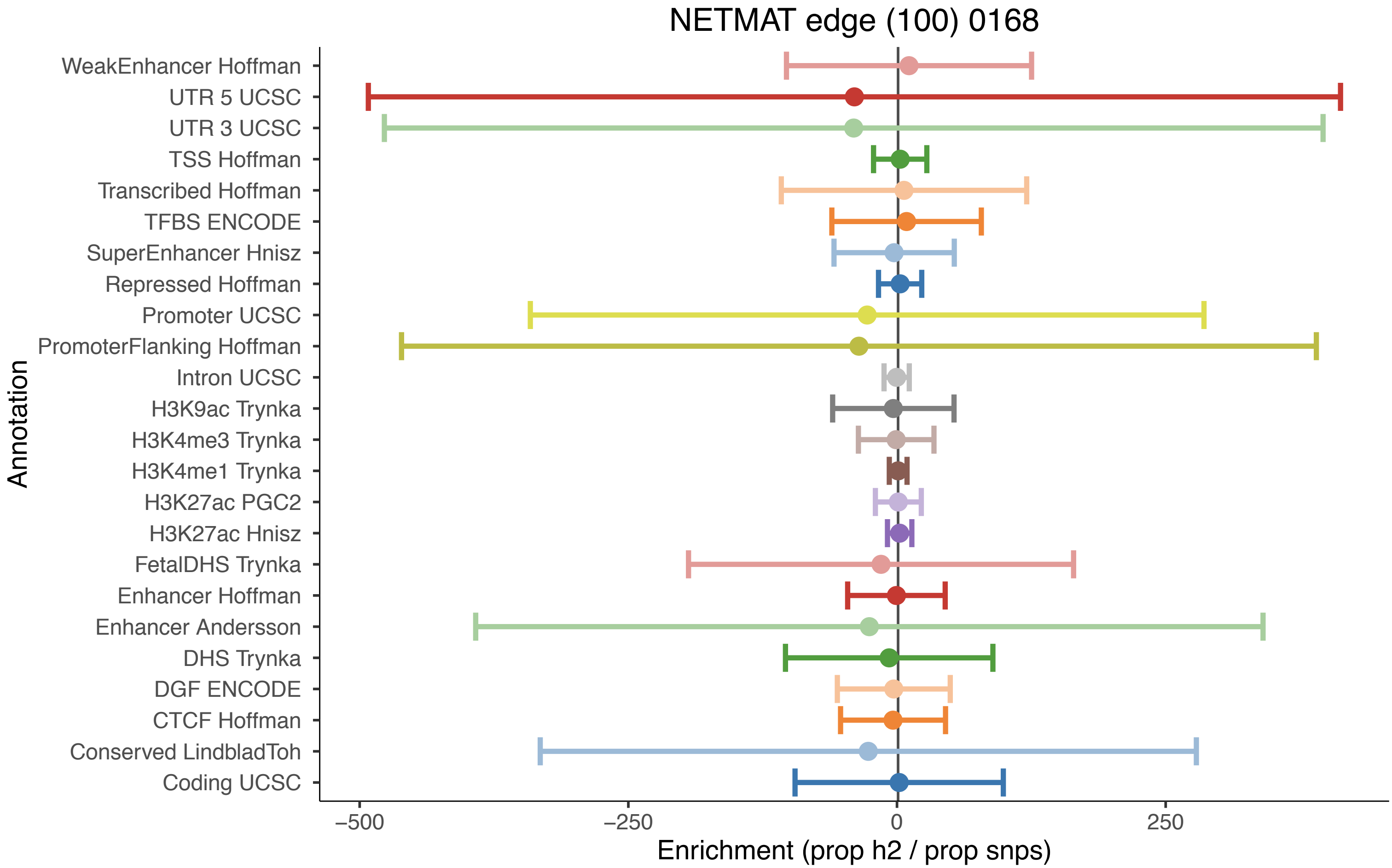

NETMAT edge (100) 0168
WeakEnhancer Hoffman
UTR 5 UCSC
UTR 3 UCSC
TSS Hoffman
Transcribed Hoffman
TFBS ENCODE
SuperEnhancer Hnisz
Repressed Hoffman
Promoter UCSC
PromoterFlanking Hoffman
Intron UCSC
H3K9ac Trynka
H3K4me3 Trynka
H3K4me1 Trynka
H3K27ac PGC2
H3K27ac Hnisz
FetalDHS Trynka
Enhancer Hoffman
Enhancer Andersson
DHS Trynka
DGF ENCODE
CTCF Hoffman
Conserved LindbladToh
Coding UCSC
Annotation
−500
−250
0
250
Enrichment (prop h2 / prop snps)

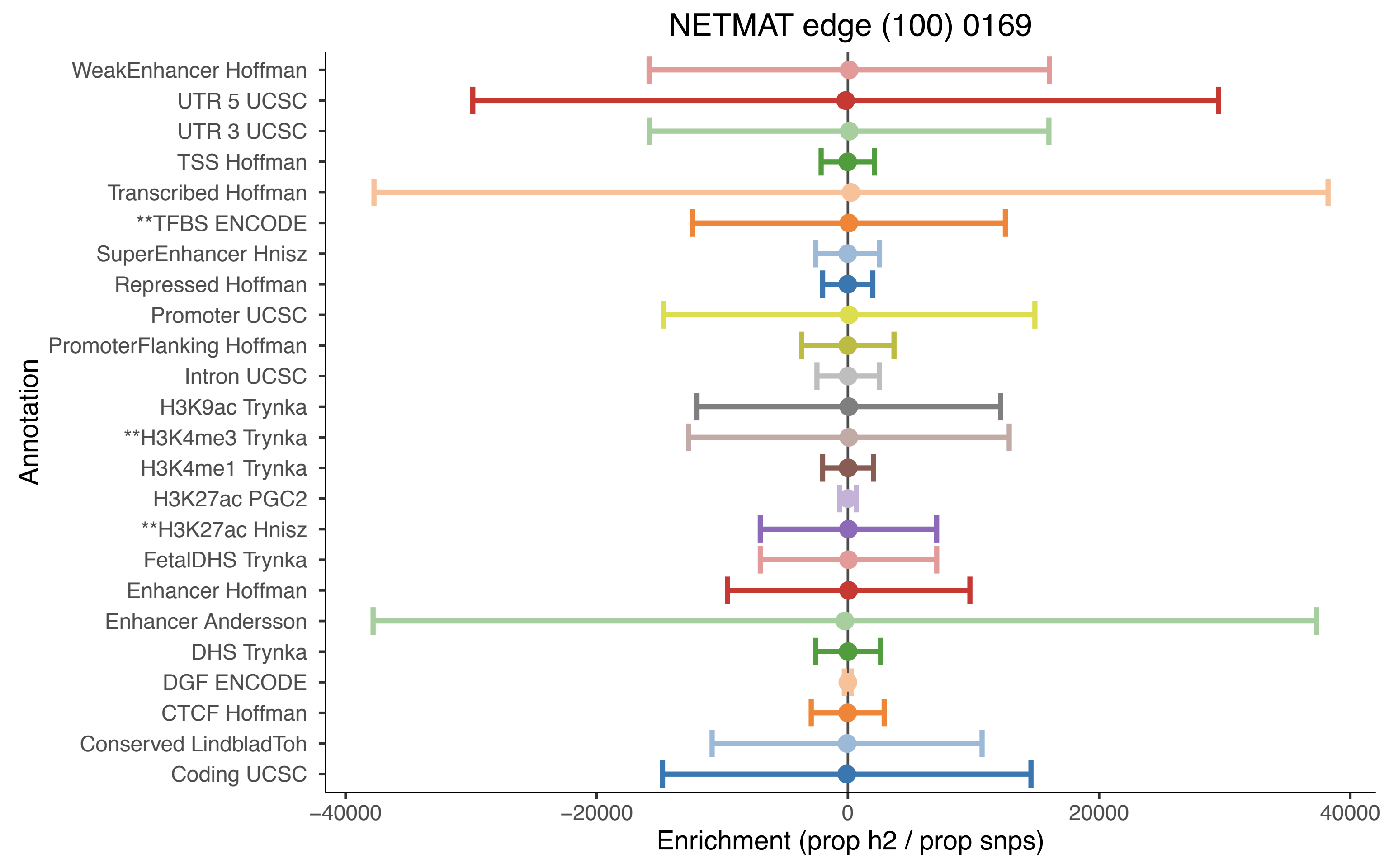
NETMAT edge (100) 0169
WeakEnhancer Hoffman
UTR 5 UCSC
UTR 3 UCSC
TSS Hoffman
Transcribed Hoffman
**TFBS ENCODE
SuperEnhancer Hnisz
Repressed Hoffman
Promoter UCSC
PromoterFlanking Hoffman
Intron UCSC
H3K9ac Trynka
**H3K4me3 Trynka
H3K4me1 Trynka
H3K27ac PGC2
**H3K27ac Hnisz
FetalDHS Trynka
Enhancer Hoffman
Enhancer Andersson
DHS Trynka
DGF ENCODE
CTCF Hoffman
Conserved LindbladToh
Coding UCSC
Annotation
−40000
−20000
0
20000
40000
Enrichment (prop h2 / prop snps)

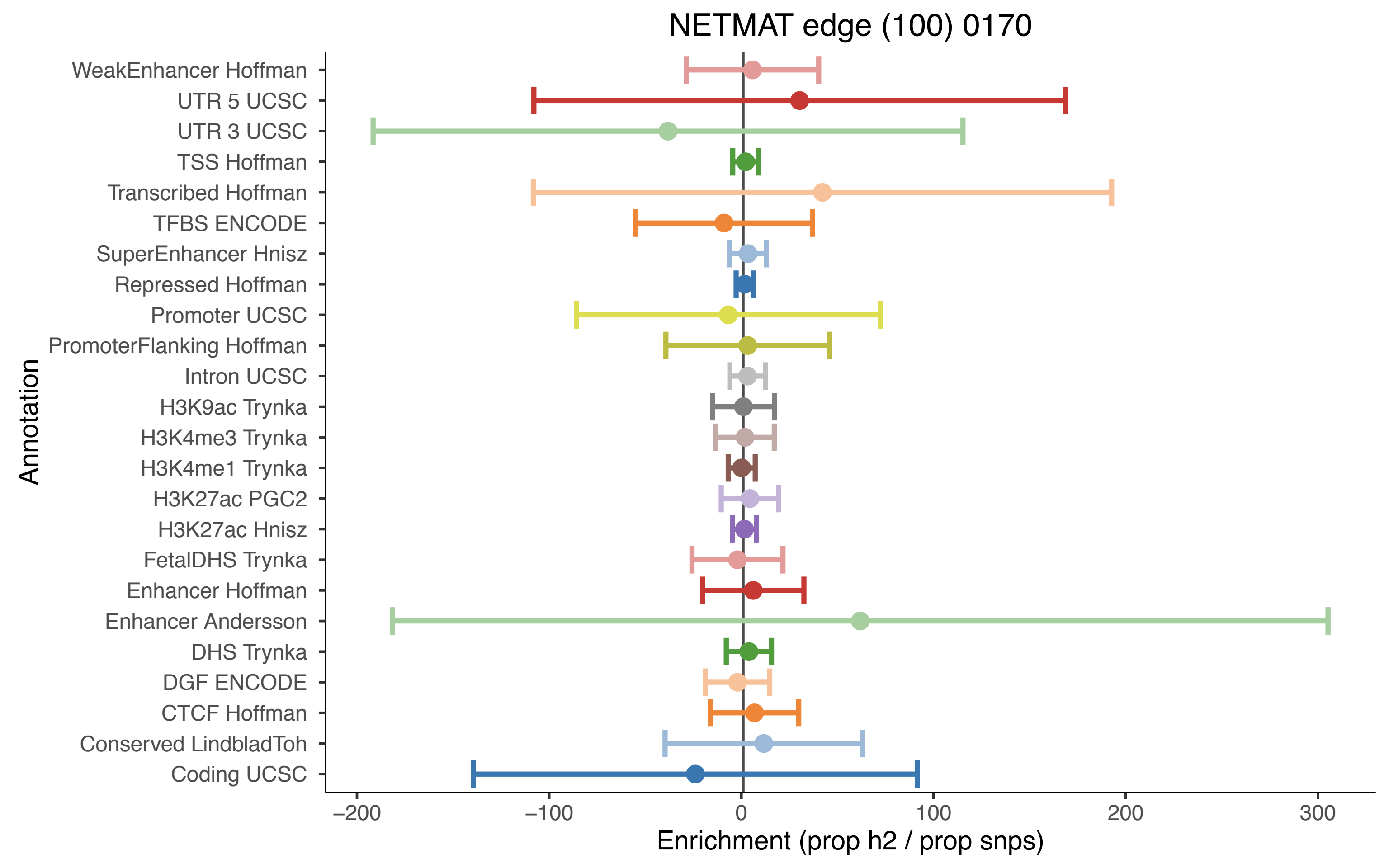
NETMAT edge (100) 0170
WeakEnhancer Hoffman
UTR 5 UCSC
UTR 3 UCSC
TSS Hoffman
Transcribed Hoffman
TFBS ENCODE
SuperEnhancer Hnisz
Repressed Hoffman
Promoter UCSC
PromoterFlanking Hoffman
Intron UCSC
H3K9ac Trynka
H3K4me3 Trynka
H3K4me1 Trynka
H3K27ac PGC2
H3K27ac Hnisz
FetalDHS Trynka
Enhancer Hoffman
Enhancer Andersson
DHS Trynka
DGF ENCODE
CTCF Hoffman
Conserved LindbladToh
Coding UCSC
Annotation
−200
−100
0
100
200
300
Enrichment (prop h2 / prop snps)

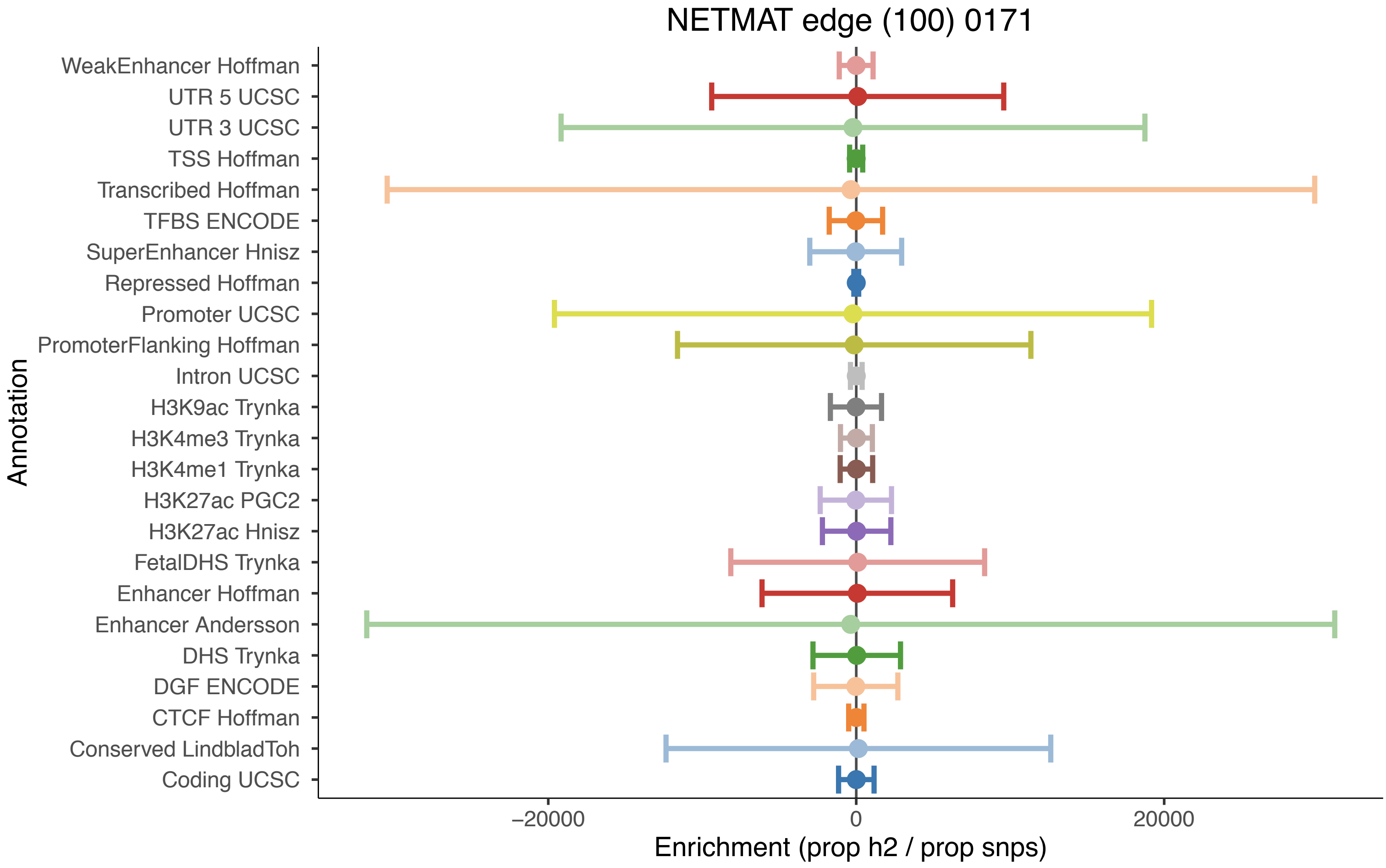
NETMAT edge (100) 0171
WeakEnhancer Hoffman
UTR 5 UCSC
UTR 3 UCSC
TSS Hoffman
Transcribed Hoffman
TFBS ENCODE
SuperEnhancer Hnisz
Repressed Hoffman
Promoter UCSC
PromoterFlanking Hoffman
Intron UCSC
H3K9ac Trynka
H3K4me3 Trynka
H3K4me1 Trynka
H3K27ac PGC2
H3K27ac Hnisz
FetalDHS Trynka
Enhancer Hoffman
Enhancer Andersson
DHS Trynka
DGF ENCODE
CTCF Hoffman
Conserved LindbladToh
Coding UCSC
Annotation
−20000
0
20000
Enrichment (prop h2 / prop snps)

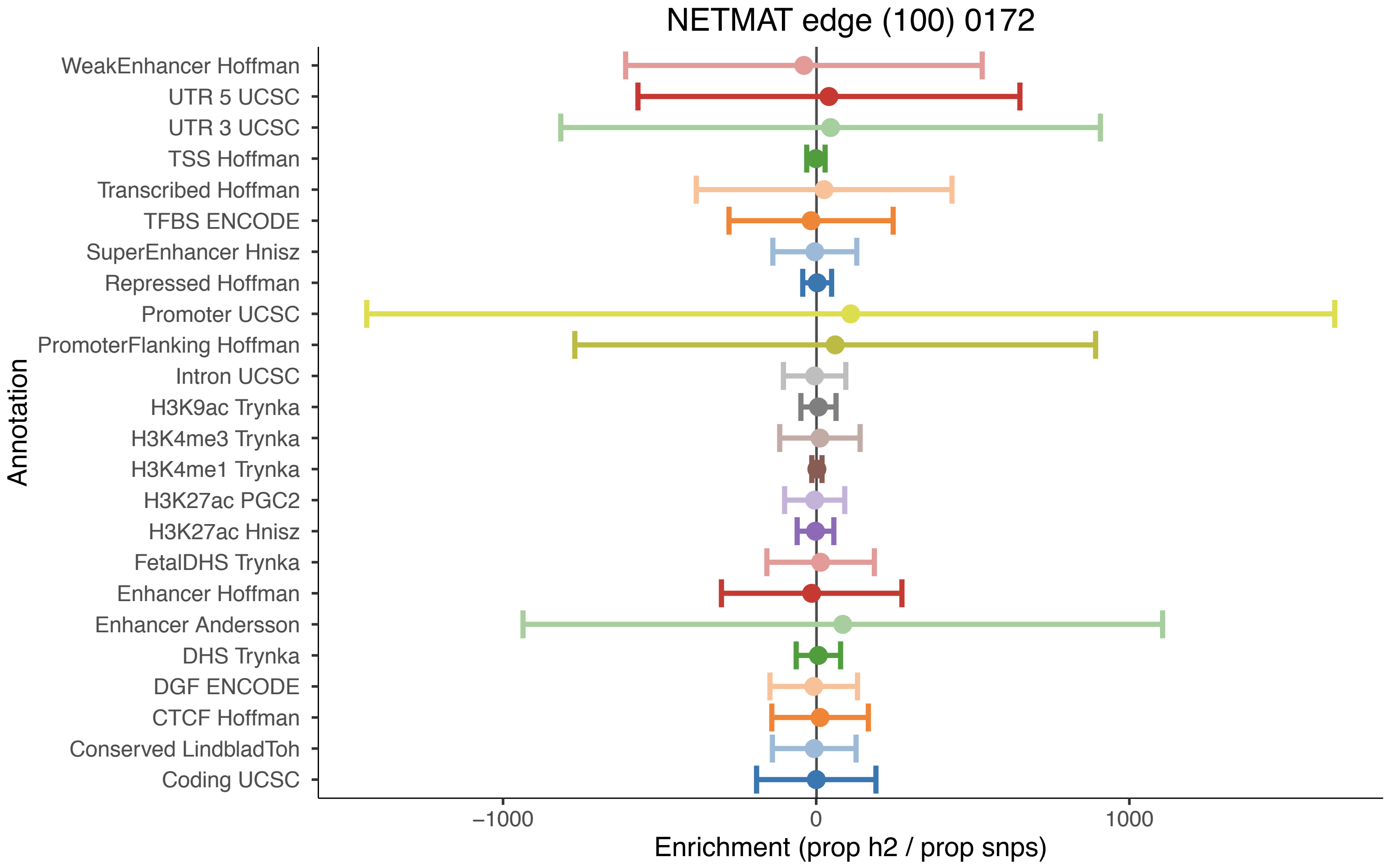
NETMAT edge (100) 0172
WeakEnhancer Hoffman
UTR 5 UCSC
UTR 3 UCSC
TSS Hoffman
Transcribed Hoffman
TFBS ENCODE
SuperEnhancer Hnisz
Repressed Hoffman
Promoter UCSC
PromoterFlanking Hoffman
Intron UCSC
H3K9ac Trynka
H3K4me3 Trynka
H3K4me1 Trynka
H3K27ac PGC2
H3K27ac Hnisz
FetalDHS Trynka
Enhancer Hoffman
Enhancer Andersson
DHS Trynka
DGF ENCODE
CTCF Hoffman
Conserved LindbladToh
Coding UCSC
Annotation
−1000
0
1000
Enrichment (prop h2 / prop snps)

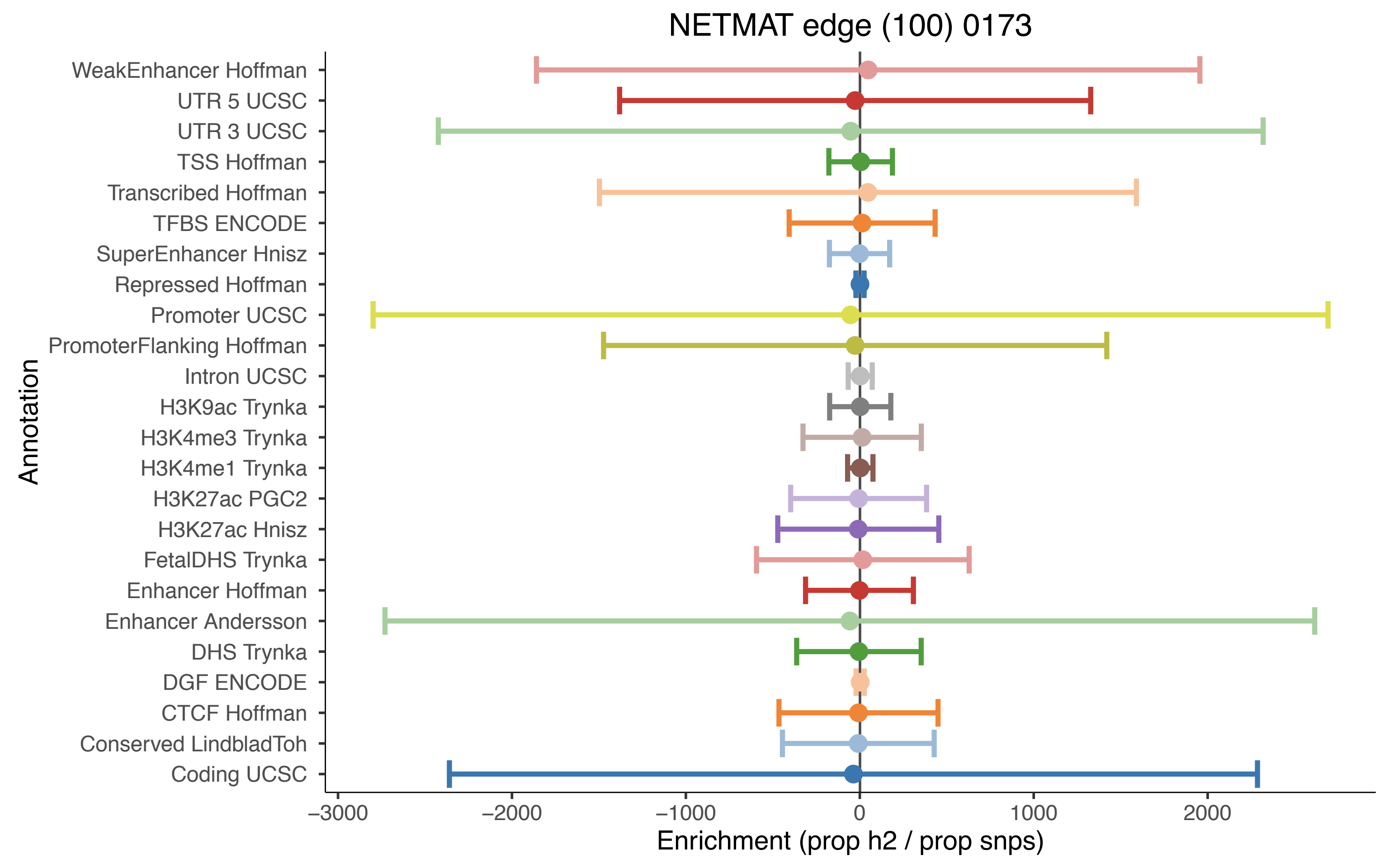

NETMAT edge (100) 0173
Annotation
WeakEnhancer Hoffman
UTR 5 UCSC
UTR 3 UCSC
TSS Hoffman
Transcribed Hoffman
TFBS ENCODE
SuperEnhancer Hnisz
Repressed Hoffman
Promoter UCSC
PromoterFlanking Hoffman
Intron UCSC
H3K9ac Trynka
H3K4me3 Trynka
H3K4me1 Trynka
H3K27ac PGC2
H3K27ac Hnisz
FetalDHS Trynka
Enhancer Hoffman
Enhancer Andersson
DHS Trynka
DGF ENCODE
CTCF Hoffman
Conserved LindbladToh
Coding UCSC
−3000
−2000
−1000
0
1000
2000
Enrichment (prop h2 / prop snps)

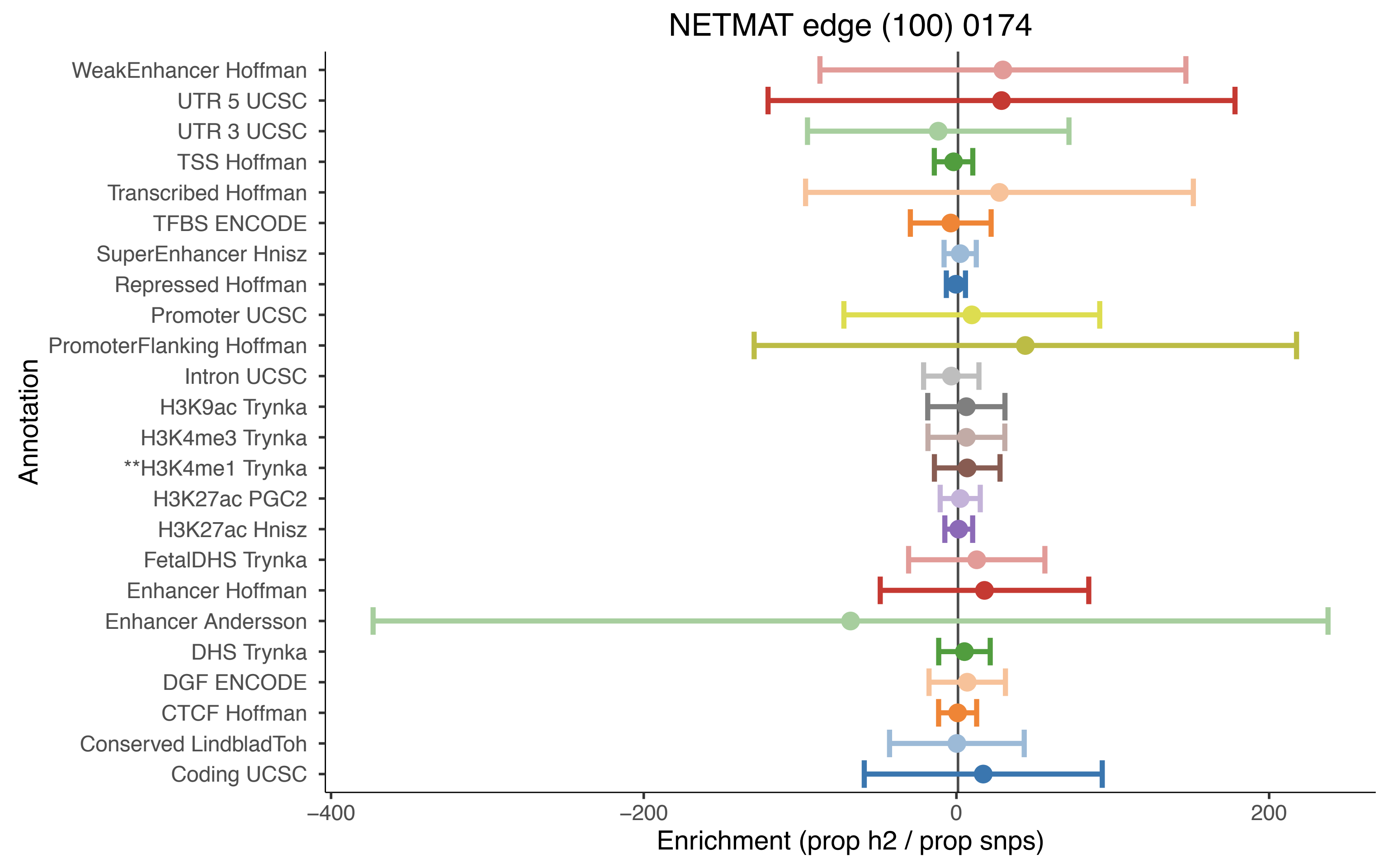
NETMAT edge (100) 0174
WeakEnhancer Hoffman
UTR 5 UCSC
UTR 3 UCSC
TSS Hoffman
Transcribed Hoffman
TFBS ENCODE
SuperEnhancer Hnisz
Repressed Hoffman
Promoter UCSC
PromoterFlanking Hoffman
Intron UCSC
H3K9ac Trynka
H3K4me3 Trynka
**H3K4me1 Trynka
H3K27ac PGC2
H3K27ac Hnisz
FetalDHS Trynka
Enhancer Hoffman
Enhancer Andersson
DHS Trynka
DGF ENCODE
CTCF Hoffman
Conserved LindbladToh
Coding UCSC
Annotation
−400
−200
0
200
Enrichment (prop h2 / prop snps)

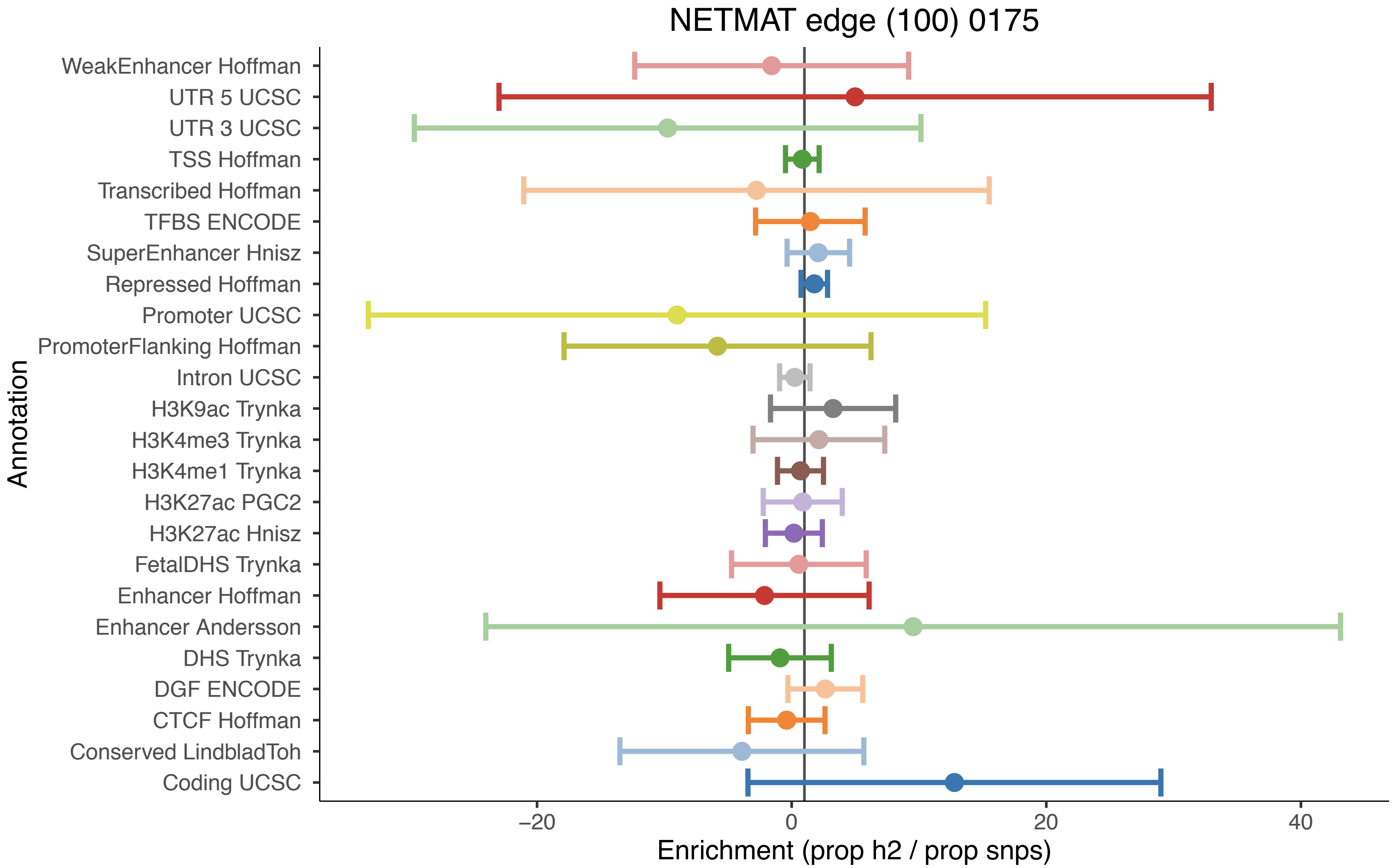

NETMAT edge (100) 0175
WeakEnhancer Hoffman
UTR 5 UCSC
UTR 3 UCSC
TSS Hoffman
Transcribed Hoffman
TFBS ENCODE
SuperEnhancer Hnisz
Repressed Hoffman
Promoter UCSC
PromoterFlanking Hoffman
Intron UCSC
H3K9ac Trynka
H3K4me3 Trynka
H3K4me1 Trynka
H3K27ac PGC2
H3K27ac Hnisz
FetalDHS Trynka
Enhancer Hoffman
Enhancer Andersson
DHS Trynka
DGF ENCODE
CTCF Hoffman
Conserved LindbladToh
Coding UCSC
Annotation
−20
0
20
40
Enrichment (prop h2 / prop snps)

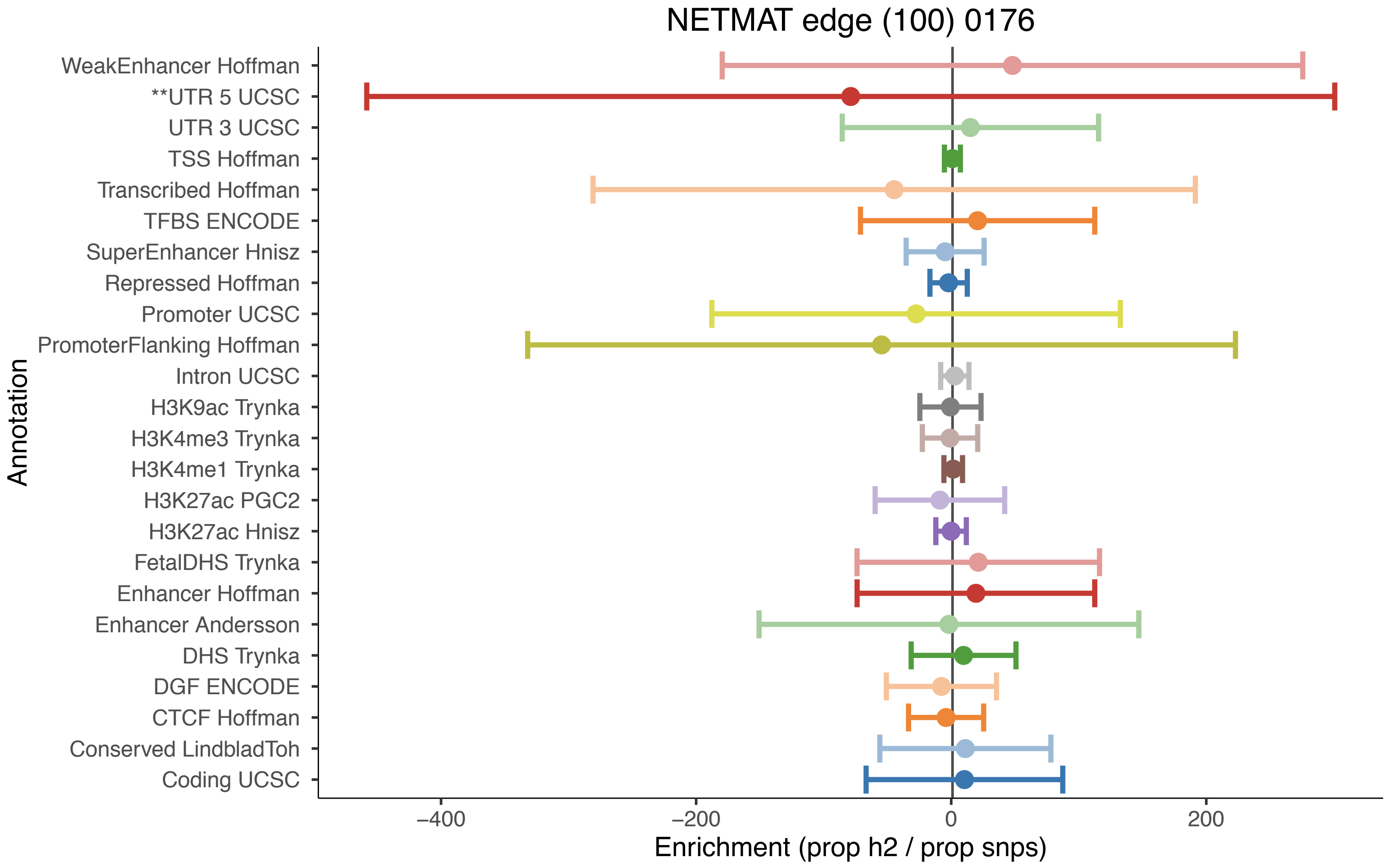
NETMAT edge (100) 0176
WeakEnhancer Hoffman
**UTR 5 UCSC
UTR 3 UCSC
TSS Hoffman
Transcribed Hoffman
TFBS ENCODE
SuperEnhancer Hnisz
Repressed Hoffman
Promoter UCSC
PromoterFlanking Hoffman
Intron UCSC
H3K9ac Trynka
H3K4me3 Trynka
H3K4me1 Trynka
H3K27ac PGC2
H3K27ac Hnisz
FetalDHS Trynka
Enhancer Hoffman
Enhancer Andersson
DHS Trynka
DGF ENCODE
CTCF Hoffman
Conserved LindbladToh
Coding UCSC
Annotation
−400
−200
0
200
Enrichment (prop h2 / prop snps)

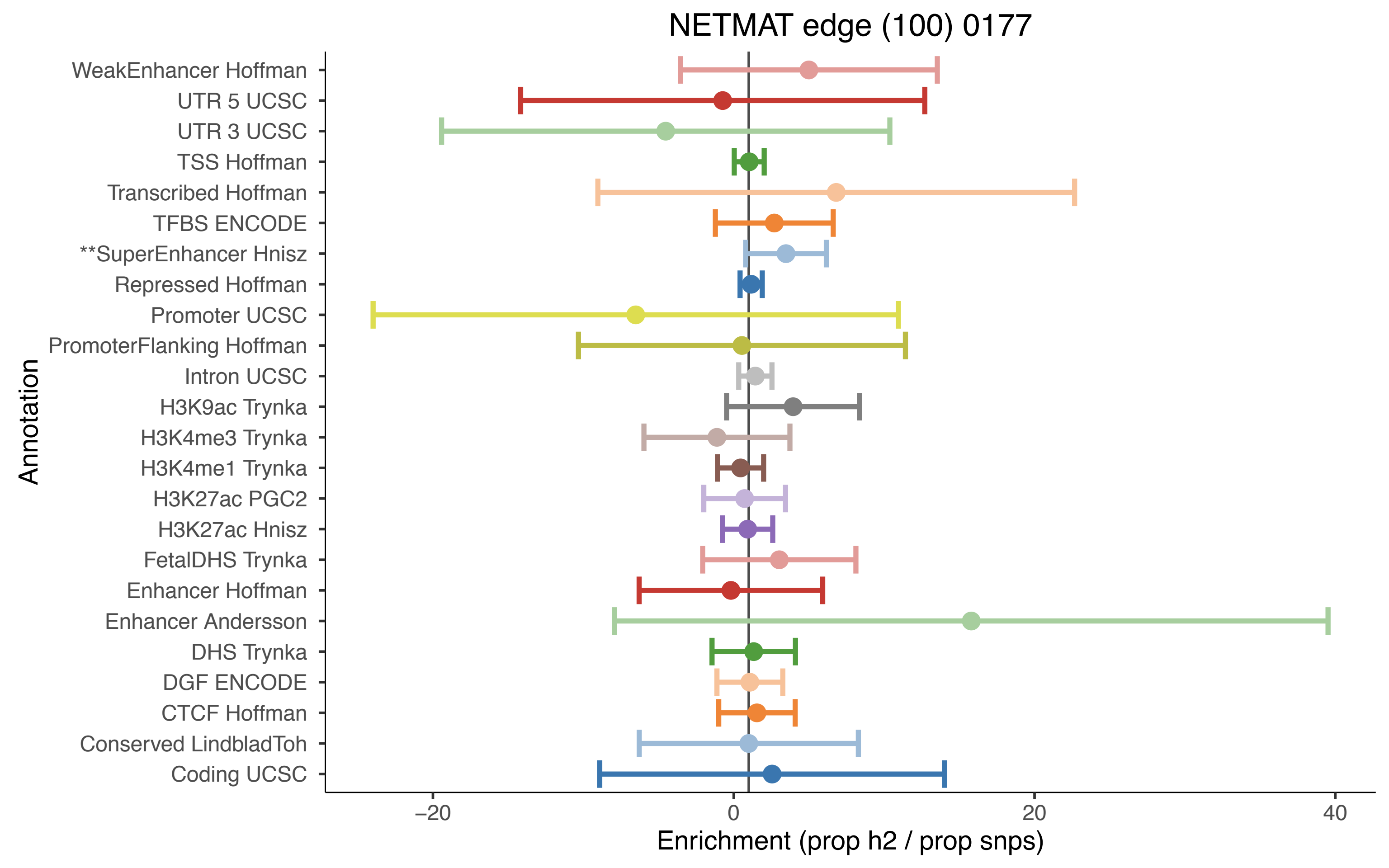

NETMAT edge (100) 0177
WeakEnhancer Hoffman
UTR 5 UCSC
UTR 3 UCSC
TSS Hoffman
Transcribed Hoffman
TFBS ENCODE
**SuperEnhancer Hnisz
Repressed Hoffman
Promoter UCSC
PromoterFlanking Hoffman
Intron UCSC
H3K9ac Trynka
H3K4me3 Trynka
H3K4me1 Trynka
H3K27ac PGC2
H3K27ac Hnisz
FetalDHS Trynka
Enhancer Hoffman
Enhancer Andersson
DHS Trynka
DGF ENCODE
CTCF Hoffman
Conserved LindbladToh
Coding UCSC
Annotation
−20
0
20
40
Enrichment (prop h2 / prop snps)

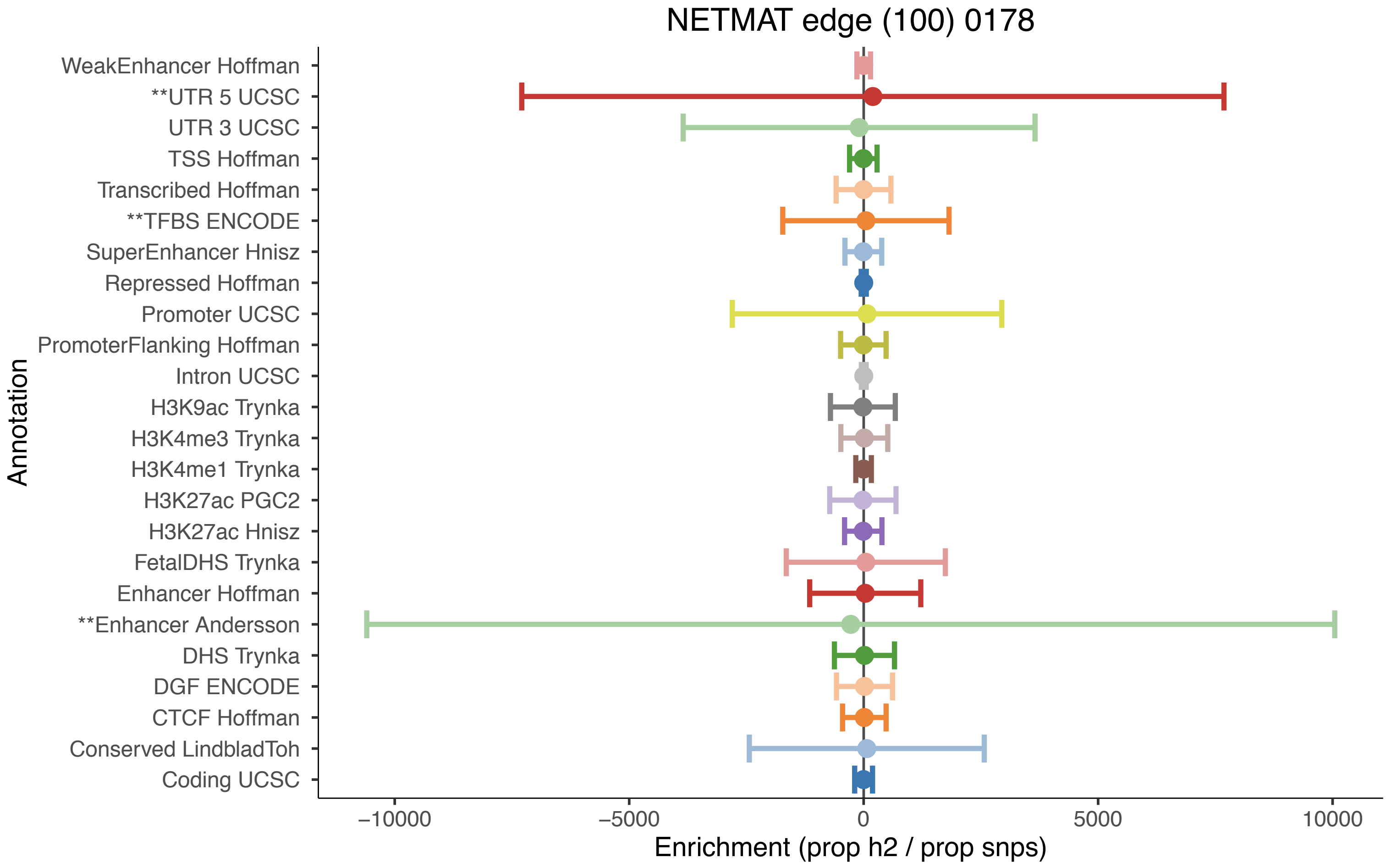
NETMAT edge (100) 0178
WeakEnhancer Hoffman
**UTR 5 UCSC
UTR 3 UCSC
TSS Hoffman
Transcribed Hoffman
**TFBS ENCODE
SuperEnhancer Hnisz
Repressed Hoffman
Promoter UCSC
PromoterFlanking Hoffman
Intron UCSC
H3K9ac Trynka
H3K4me3 Trynka
H3K4me1 Trynka
H3K27ac PGC2
H3K27ac Hnisz
FetalDHS Trynka
Enhancer Hoffman
**Enhancer Andersson
DHS Trynka
DGF ENCODE
CTCF Hoffman
Conserved LindbladToh
Coding UCSC
Annotation
−10000
−5000
0
5000
10000
Enrichment (prop h2 / prop snps)

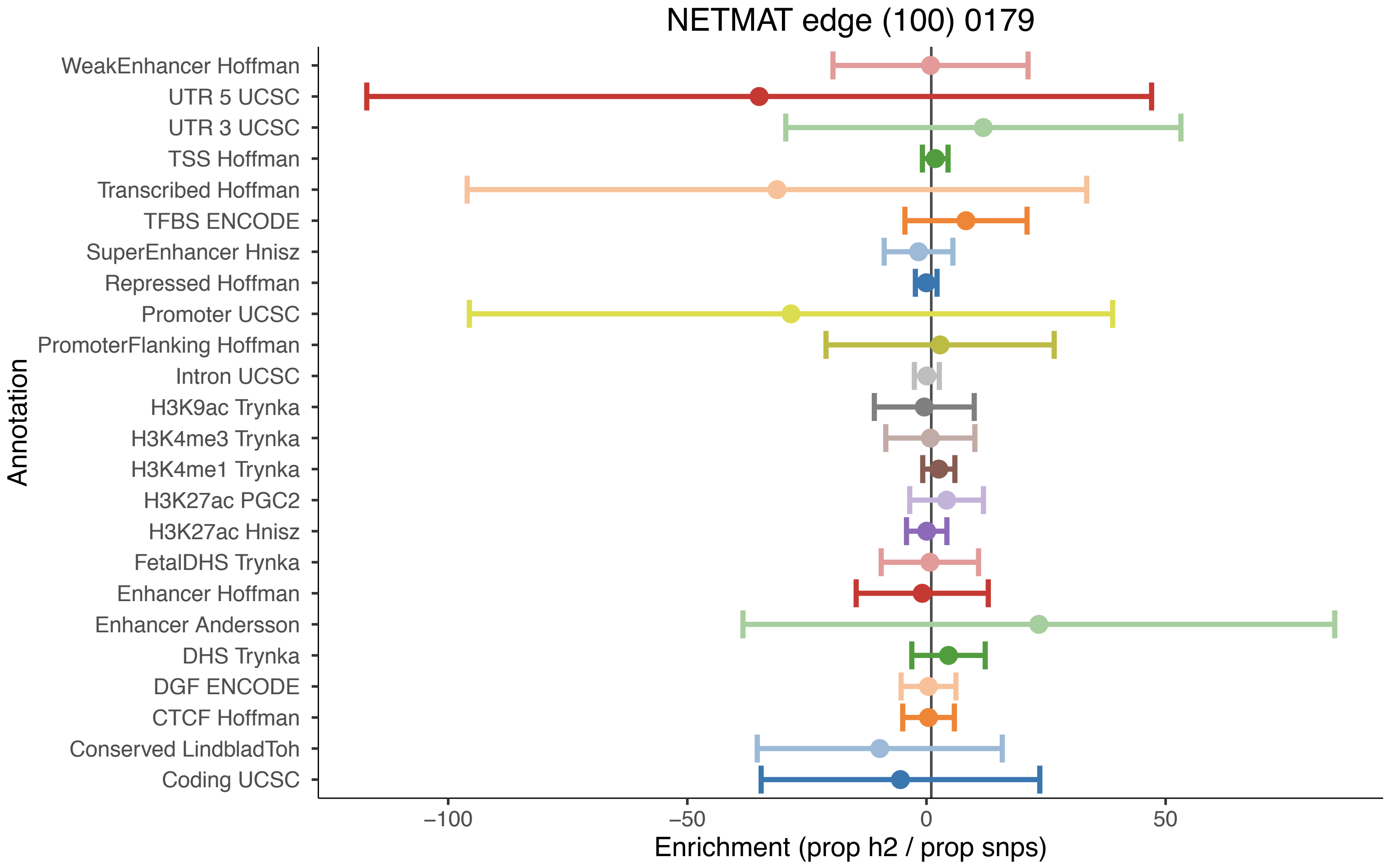

NETMAT edge (100) 0179
WeakEnhancer Hoffman
UTR 5 UCSC
UTR 3 UCSC
TSS Hoffman
Transcribed Hoffman
TFBS ENCODE
SuperEnhancer Hnisz
Repressed Hoffman
Promoter UCSC
PromoterFlanking Hoffman
Intron UCSC
H3K9ac Trynka
H3K4me3 Trynka
H3K4me1 Trynka
H3K27ac PGC2
H3K27ac Hnisz
FetalDHS Trynka
Enhancer Hoffman
Enhancer Andersson
DHS Trynka
DGF ENCODE
CTCF Hoffman
Conserved LindbladToh
Coding UCSC
Annotation
−100
−50
0
50
Enrichment (prop h2 / prop snps)

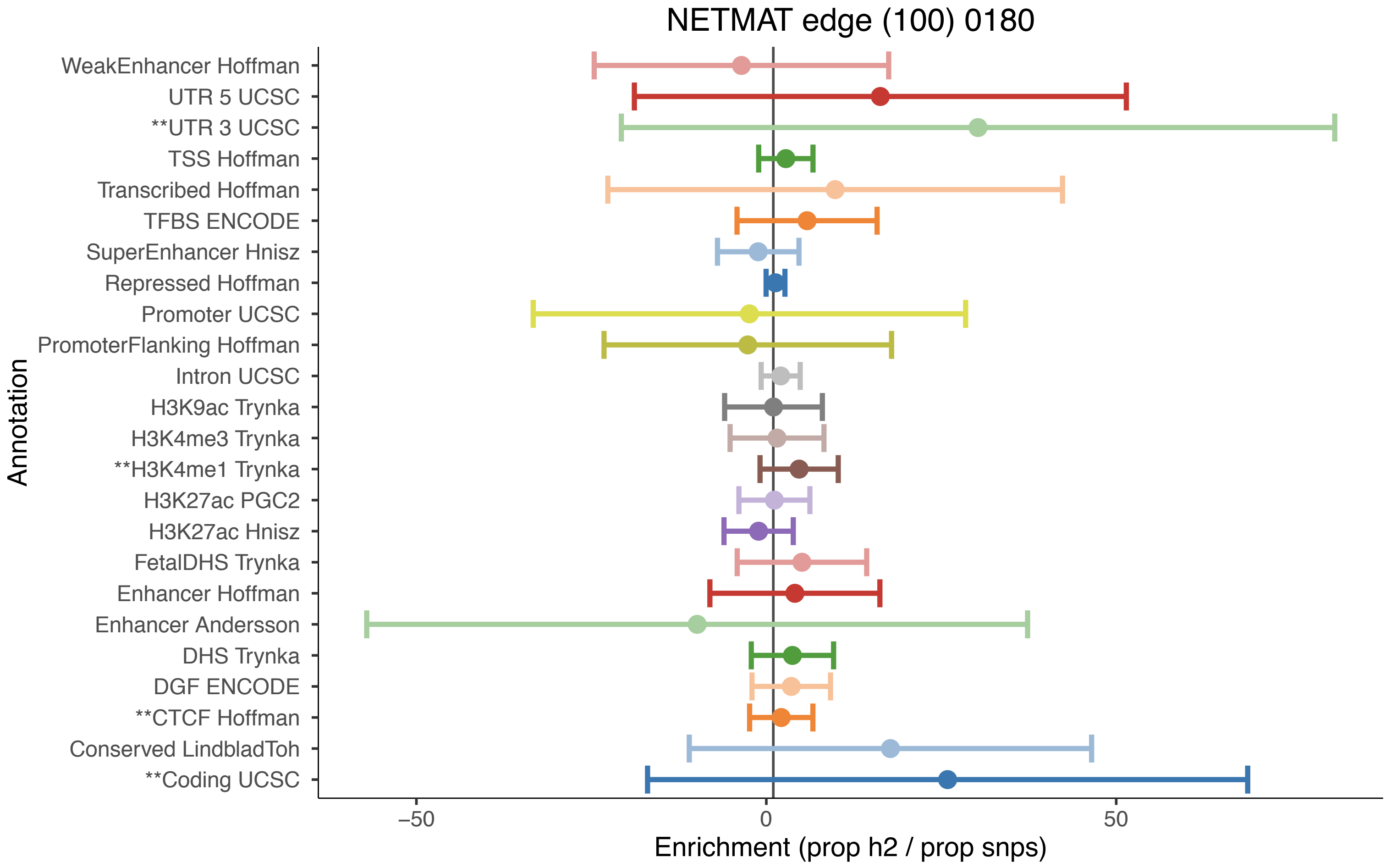
NETMAT edge (100) 0180
WeakEnhancer Hoffman
UTR 5 UCSC
**UTR 3 UCSC
TSS Hoffman
Transcribed Hoffman
TFBS ENCODE
SuperEnhancer Hnisz
Repressed Hoffman
Promoter UCSC
PromoterFlanking Hoffman
Intron UCSC
H3K9ac Trynka
H3K4me3 Trynka
**H3K4me1 Trynka
H3K27ac PGC2
H3K27ac Hnisz
FetalDHS Trynka
Enhancer Hoffman
Enhancer Andersson
DHS Trynka
DGF ENCODE
**CTCF Hoffman
Conserved LindbladToh
**Coding UCSC
Annotation
−50
0
50
Enrichment (prop h2 / prop snps)

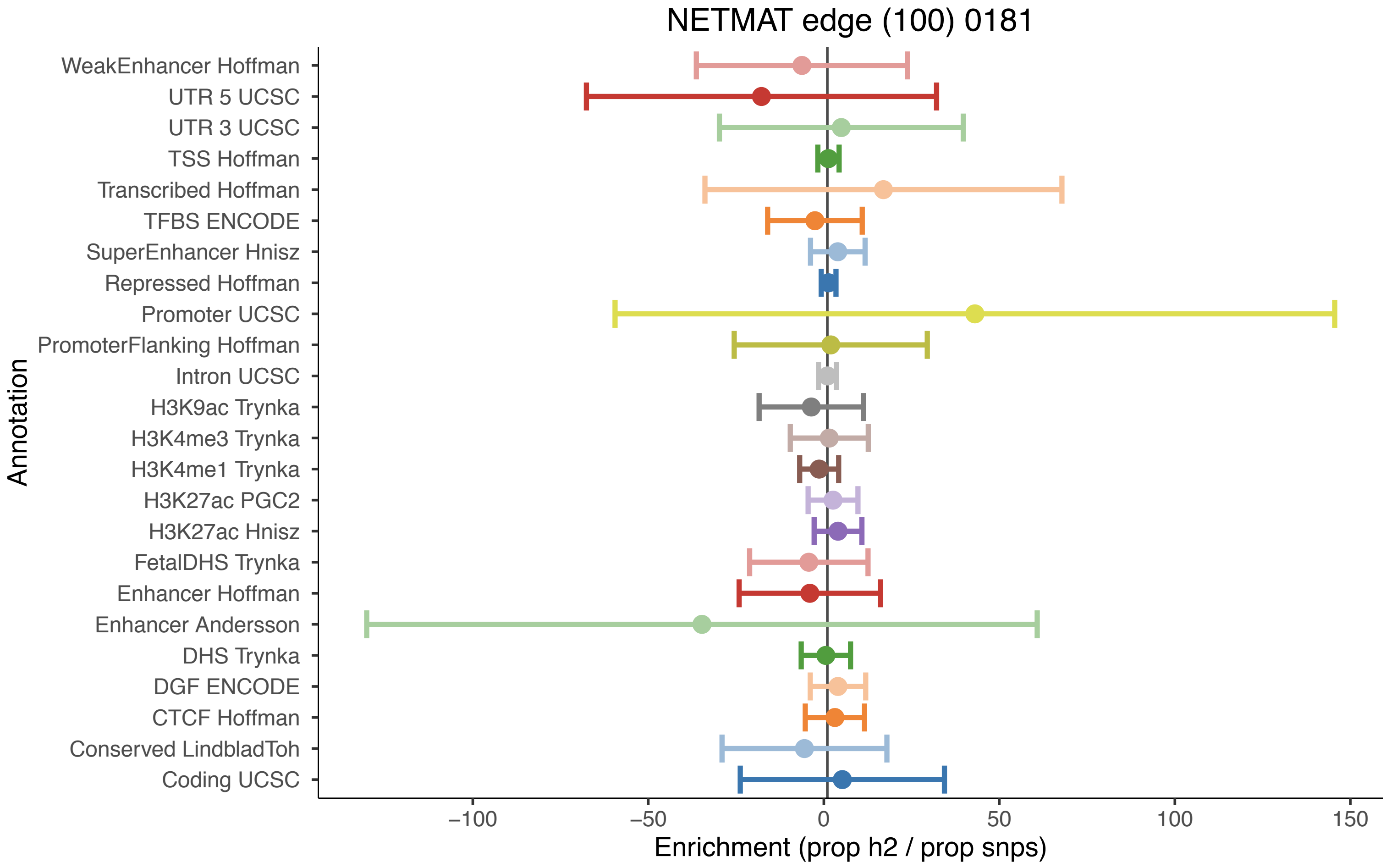
NETMAT edge (100) 0181
Annotation
WeakEnhancer Hoffman
UTR 5 UCSC
UTR 3 UCSC
TSS Hoffman
Transcribed Hoffman
TFBS ENCODE
SuperEnhancer Hnisz
Repressed Hoffman
Promoter UCSC
PromoterFlanking Hoffman
Intron UCSC
H3K9ac Trynka
H3K4me3 Trynka
H3K4me1 Trynka
H3K27ac PGC2
H3K27ac Hnisz
FetalDHS Trynka
Enhancer Hoffman
Enhancer Andersson
DHS Trynka
DGF ENCODE
CTCF Hoffman
Conserved LindbladToh
Coding UCSC
−100
−50
0
50
100
150
Enrichment (prop h2 / prop snps)

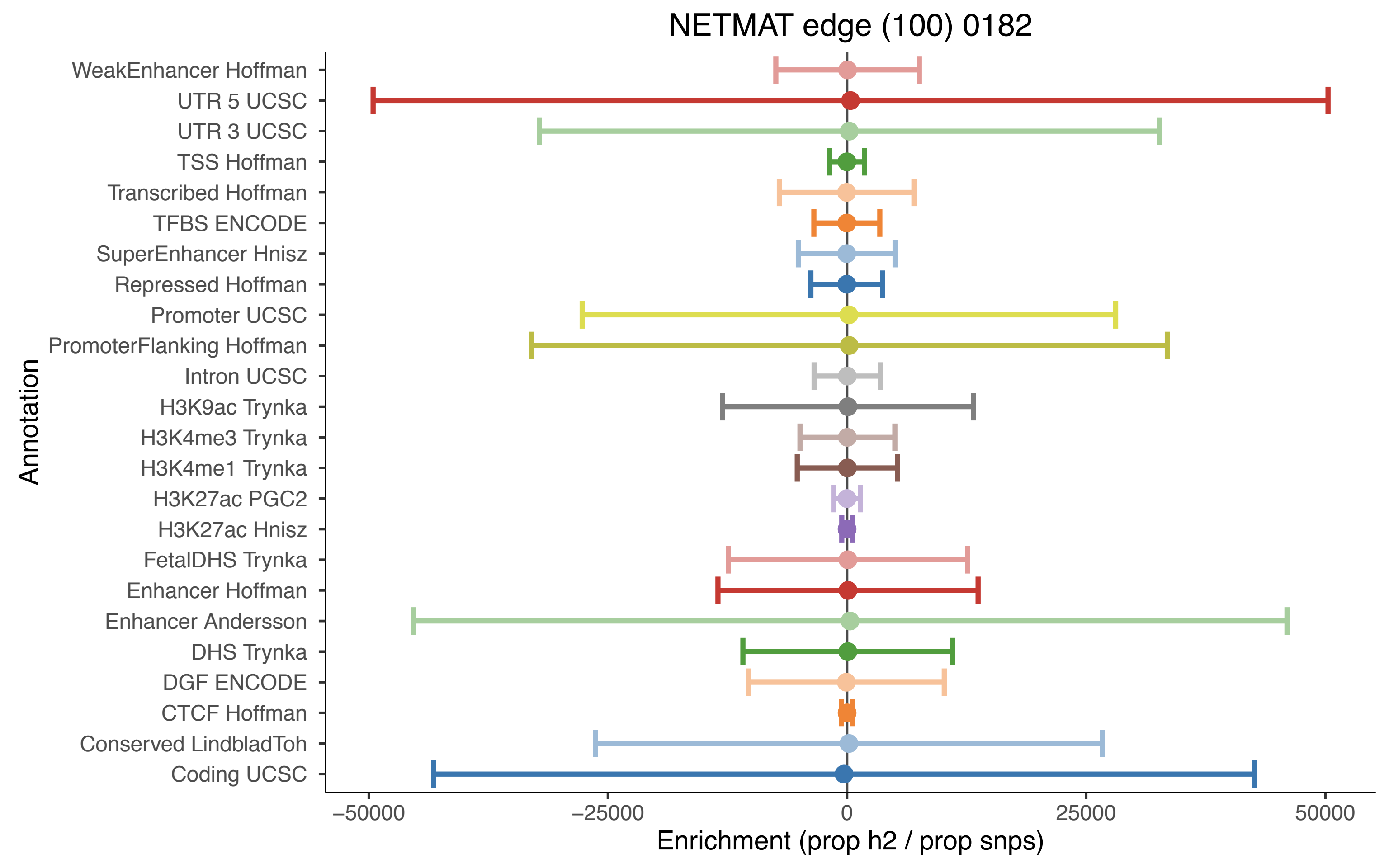
NETMAT edge (100) 0182
Annotation
WeakEnhancer Hoffman
UTR 5 UCSC
UTR 3 UCSC
TSS Hoffman
Transcribed Hoffman
TFBS ENCODE
SuperEnhancer Hnisz
Repressed Hoffman
Promoter UCSC
PromoterFlanking Hoffman
Intron UCSC
H3K9ac Trynka
H3K4me3 Trynka
H3K4me1 Trynka
H3K27ac PGC2
H3K27ac Hnisz
FetalDHS Trynka
Enhancer Hoffman
Enhancer Andersson
DHS Trynka
DGF ENCODE
CTCF Hoffman
Conserved LindbladToh
Coding UCSC
−50000
−25000
0
25000
50000
Enrichment (prop h2 / prop snps)

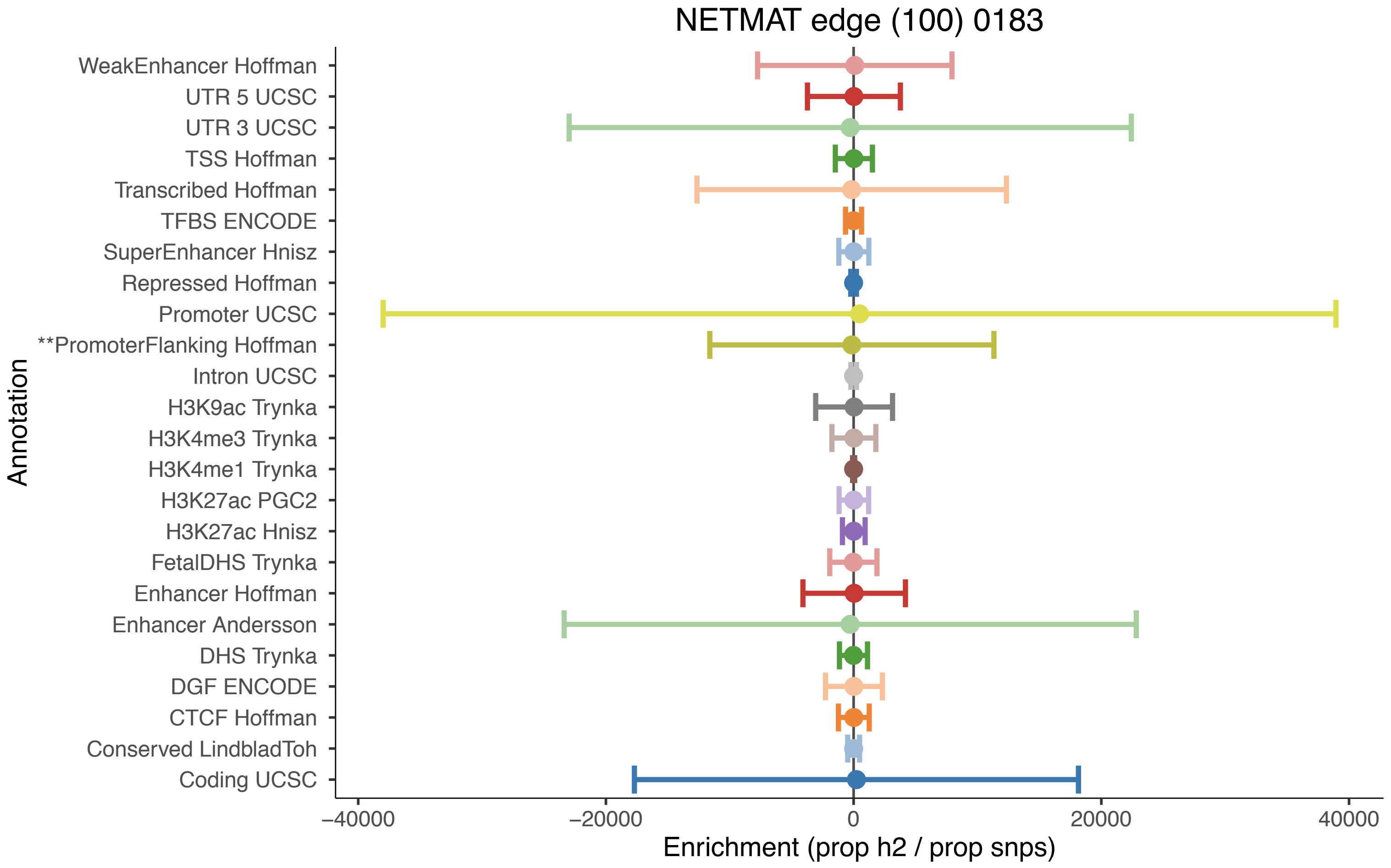
NETMAT edge (100) 0183
WeakEnhancer Hoffman
UTR 5 UCSC
UTR 3 UCSC
TSS Hoffman
Transcribed Hoffman
TFBS ENCODE
SuperEnhancer Hnisz
Repressed Hoffman
Promoter UCSC
**PromoterFlanking Hoffman
Intron UCSC
H3K9ac Trynka
H3K4me3 Trynka
H3K4me1 Trynka
H3K27ac PGC2
H3K27ac Hnisz
FetalDHS Trynka
Enhancer Hoffman
Enhancer Andersson
DHS Trynka
DGF ENCODE
CTCF Hoffman
Conserved LindbladToh
Coding UCSC
Annotation
−40000
−20000
0
20000
40000
Enrichment (prop h2 / prop snps)

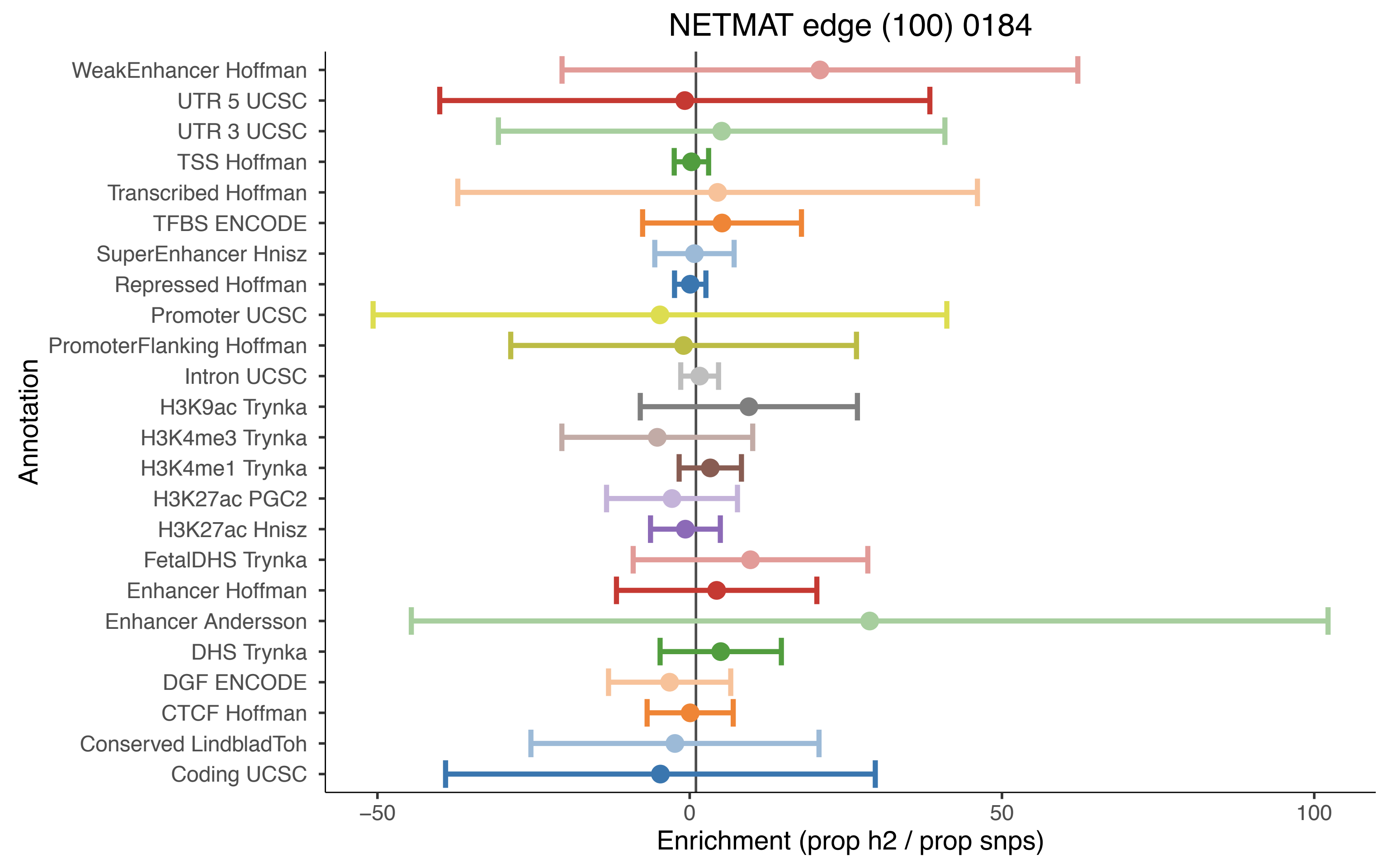

NETMAT edge (100) 0184
WeakEnhancer Hoffman
UTR 5 UCSC
UTR 3 UCSC
TSS Hoffman
Transcribed Hoffman
TFBS ENCODE
SuperEnhancer Hnisz
Repressed Hoffman
Promoter UCSC
PromoterFlanking Hoffman
Intron UCSC
H3K9ac Trynka
H3K4me3 Trynka
H3K4me1 Trynka
H3K27ac PGC2
H3K27ac Hnisz
FetalDHS Trynka
Enhancer Hoffman
Enhancer Andersson
DHS Trynka
DGF ENCODE
CTCF Hoffman
Conserved LindbladToh
Coding UCSC
Annotation
−50
0
50
100
Enrichment (prop h2 / prop snps)

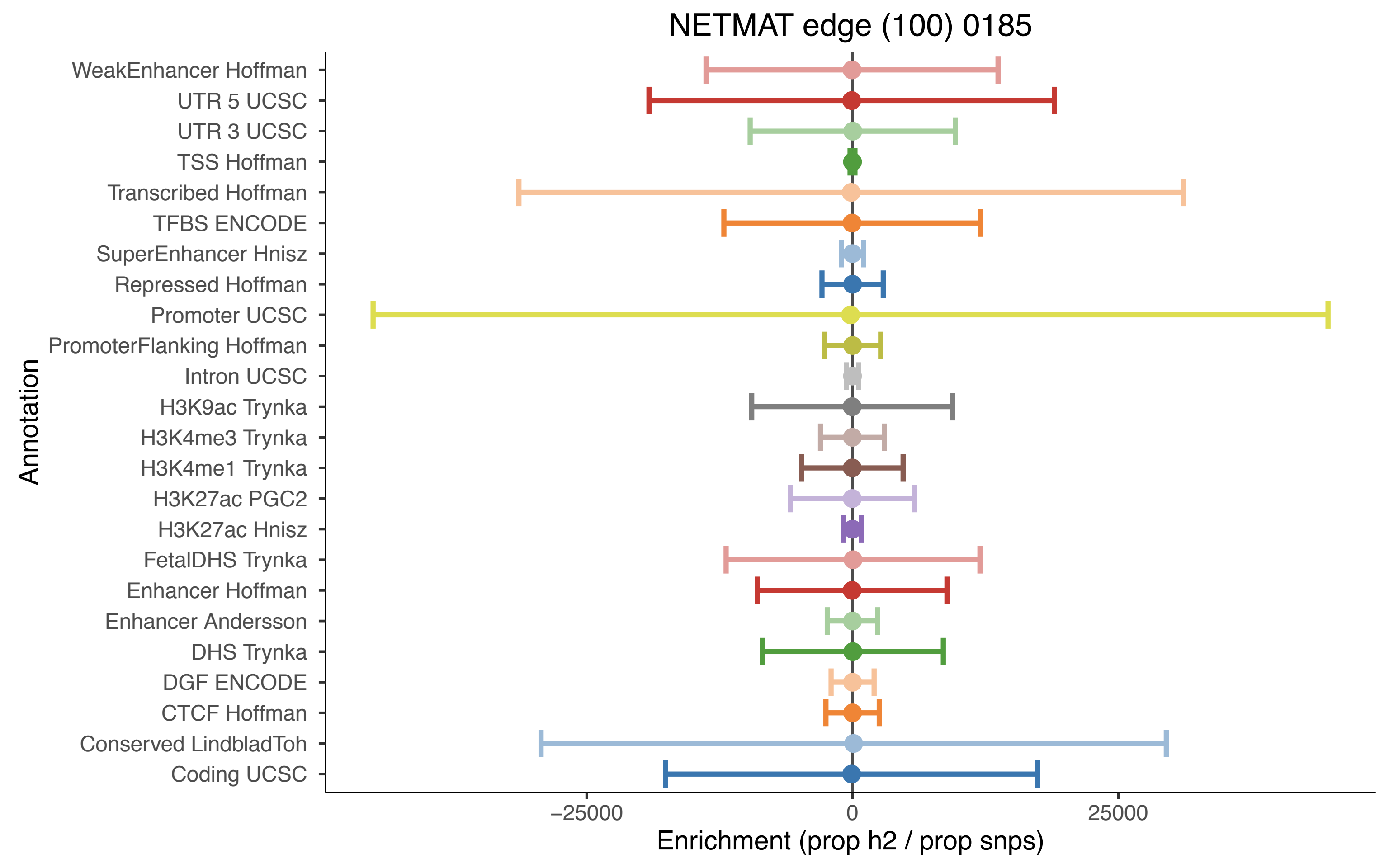

NETMAT edge (100) 0185
WeakEnhancer Hoffman
UTR 5 UCSC
UTR 3 UCSC
TSS Hoffman
Transcribed Hoffman
TFBS ENCODE
SuperEnhancer Hnisz
Repressed Hoffman
Promoter UCSC
PromoterFlanking Hoffman
Intron UCSC
H3K9ac Trynka
H3K4me3 Trynka
H3K4me1 Trynka
H3K27ac PGC2
H3K27ac Hnisz
FetalDHS Trynka
Enhancer Hoffman
Enhancer Andersson
DHS Trynka
DGF ENCODE
CTCF Hoffman
Conserved LindbladToh
Coding UCSC
Annotation
−25000
0
25000
Enrichment (prop h2 / prop snps)

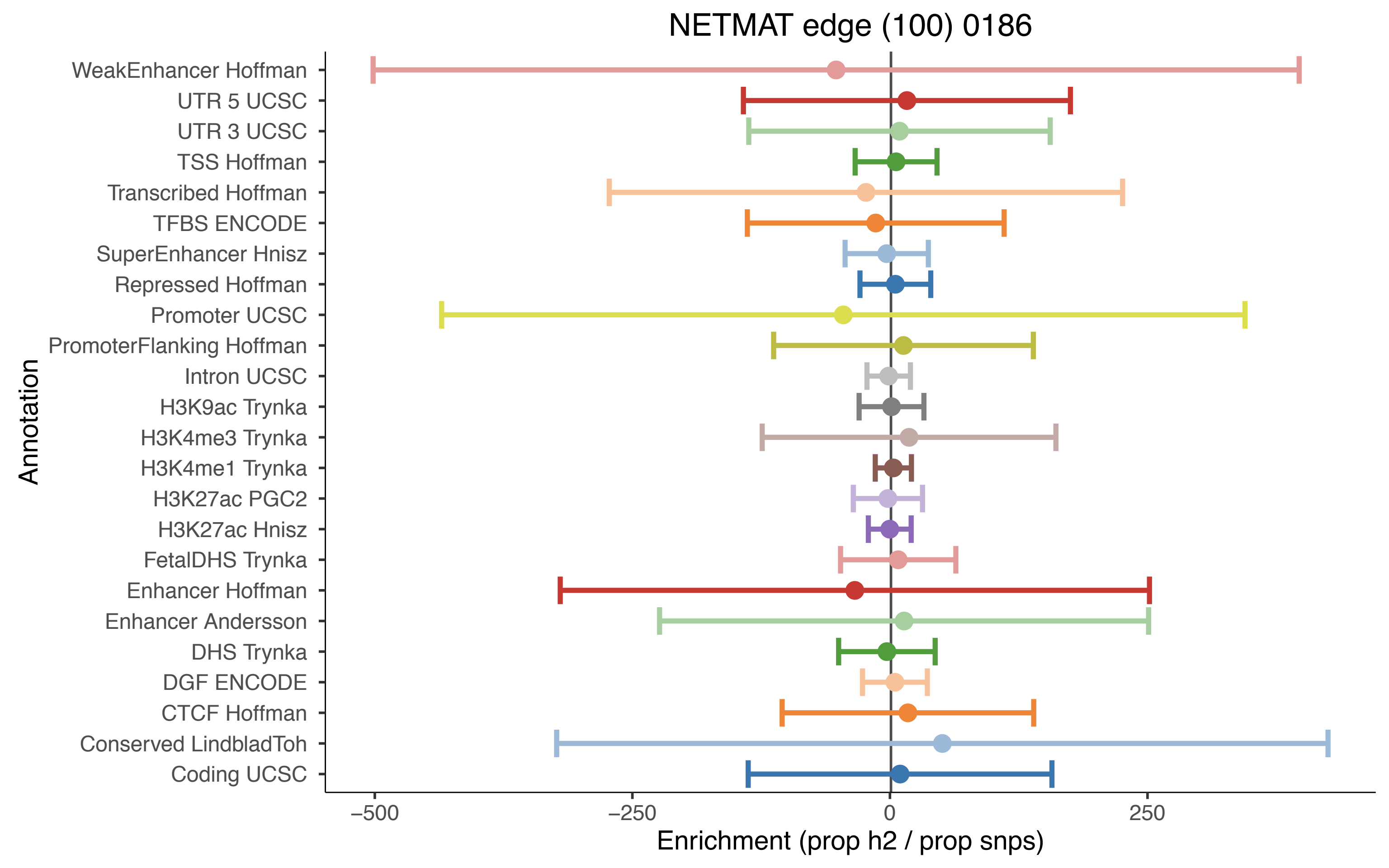

NETMAT edge (100) 0186
Annotation
WeakEnhancer Hoffman
UTR 5 UCSC
UTR 3 UCSC
TSS Hoffman
Transcribed Hoffman
TFBS ENCODE
SuperEnhancer Hnisz
Repressed Hoffman
Promoter UCSC
PromoterFlanking Hoffman
Intron UCSC
H3K9ac Trynka
H3K4me3 Trynka
H3K4me1 Trynka
H3K27ac PGC2
H3K27ac Hnisz
FetalDHS Trynka
Enhancer Hoffman
Enhancer Andersson
DHS Trynka
DGF ENCODE
CTCF Hoffman
Conserved LindbladToh
Coding UCSC
−500
−250
0
250
Enrichment (prop h2 / prop snps)

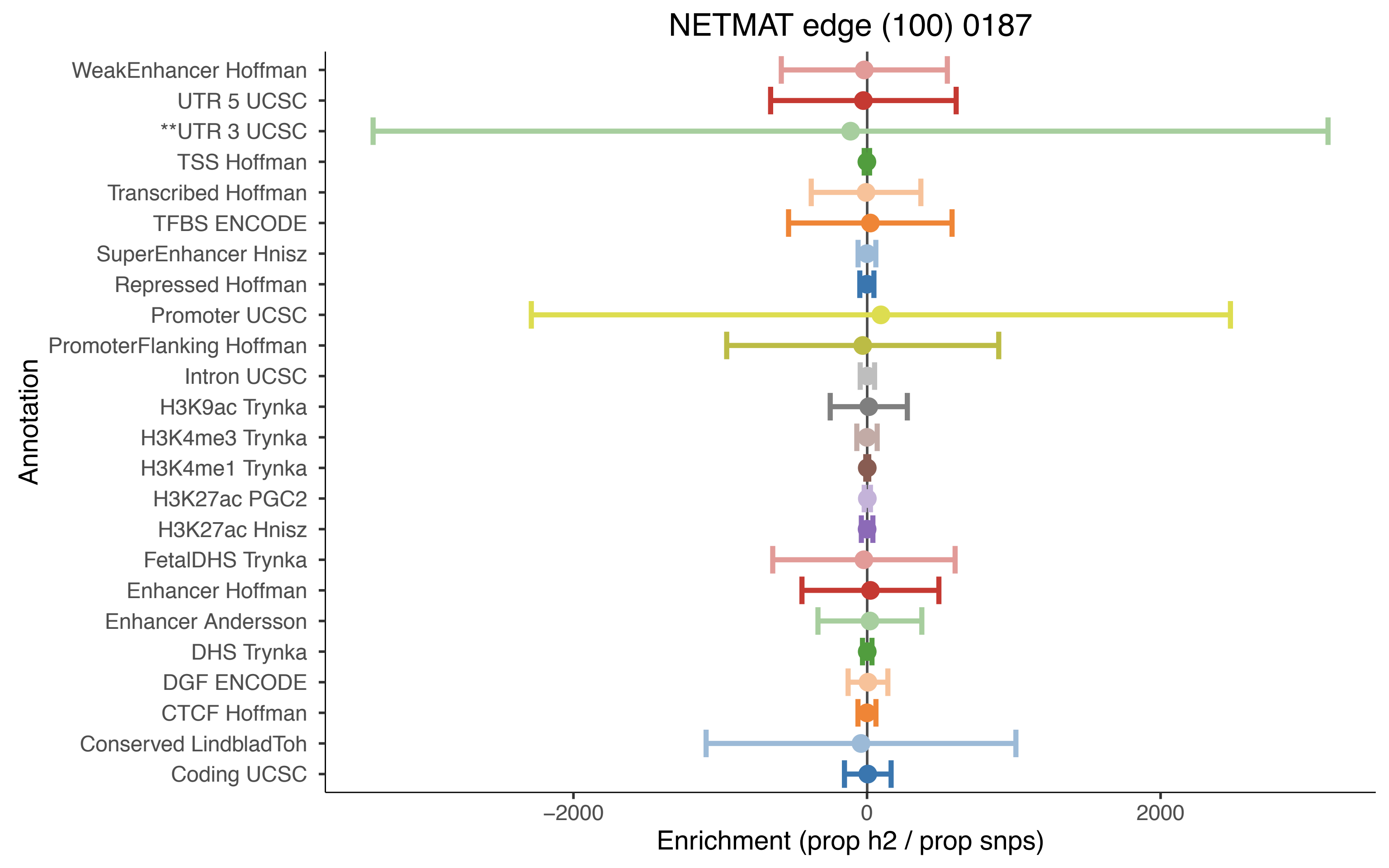

NETMAT edge (100) 0187
WeakEnhancer Hoffman
UTR 5 UCSC
**UTR 3 UCSC
TSS Hoffman
Transcribed Hoffman
TFBS ENCODE
SuperEnhancer Hnisz
Repressed Hoffman
Promoter UCSC
PromoterFlanking Hoffman
Intron UCSC
H3K9ac Trynka
H3K4me3 Trynka
H3K4me1 Trynka
H3K27ac PGC2
H3K27ac Hnisz
FetalDHS Trynka
Enhancer Hoffman
Enhancer Andersson
DHS Trynka
DGF ENCODE
CTCF Hoffman
Conserved LindbladToh
Coding UCSC
Annotation
−2000
0
2000
Enrichment (prop h2 / prop snps)

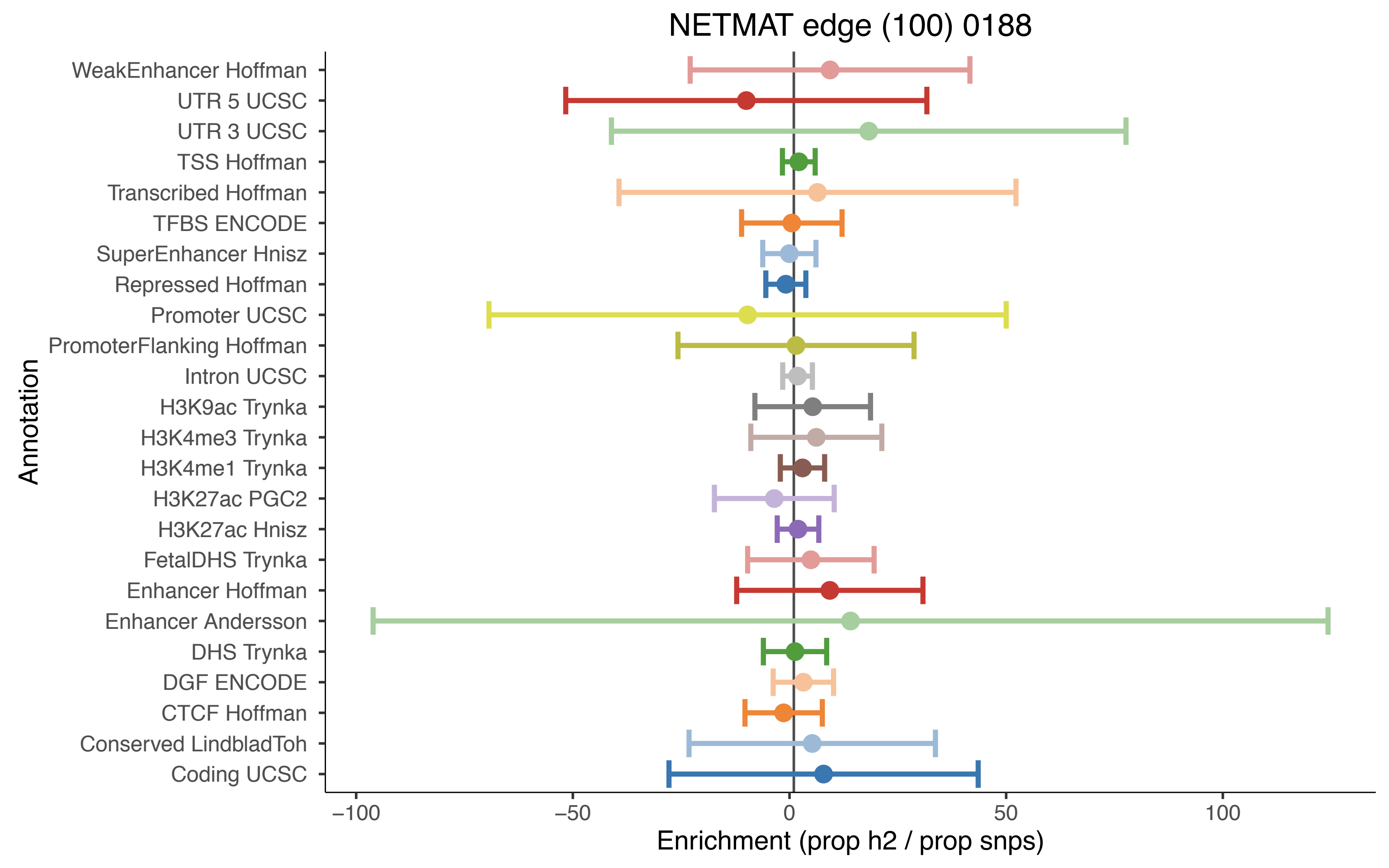
NETMAT edge (100) 0188
WeakEnhancer Hoffman
UTR 5 UCSC
UTR 3 UCSC
TSS Hoffman
Transcribed Hoffman
TFBS ENCODE
SuperEnhancer Hnisz
Repressed Hoffman
Promoter UCSC
PromoterFlanking Hoffman
Intron UCSC
H3K9ac Trynka
H3K4me3 Trynka
H3K4me1 Trynka
H3K27ac PGC2
H3K27ac Hnisz
FetalDHS Trynka
Enhancer Hoffman
Enhancer Andersson
DHS Trynka
DGF ENCODE
CTCF Hoffman
Conserved LindbladToh
Coding UCSC
Annotation
−100
−50
0
50
100
Enrichment (prop h2 / prop snps)

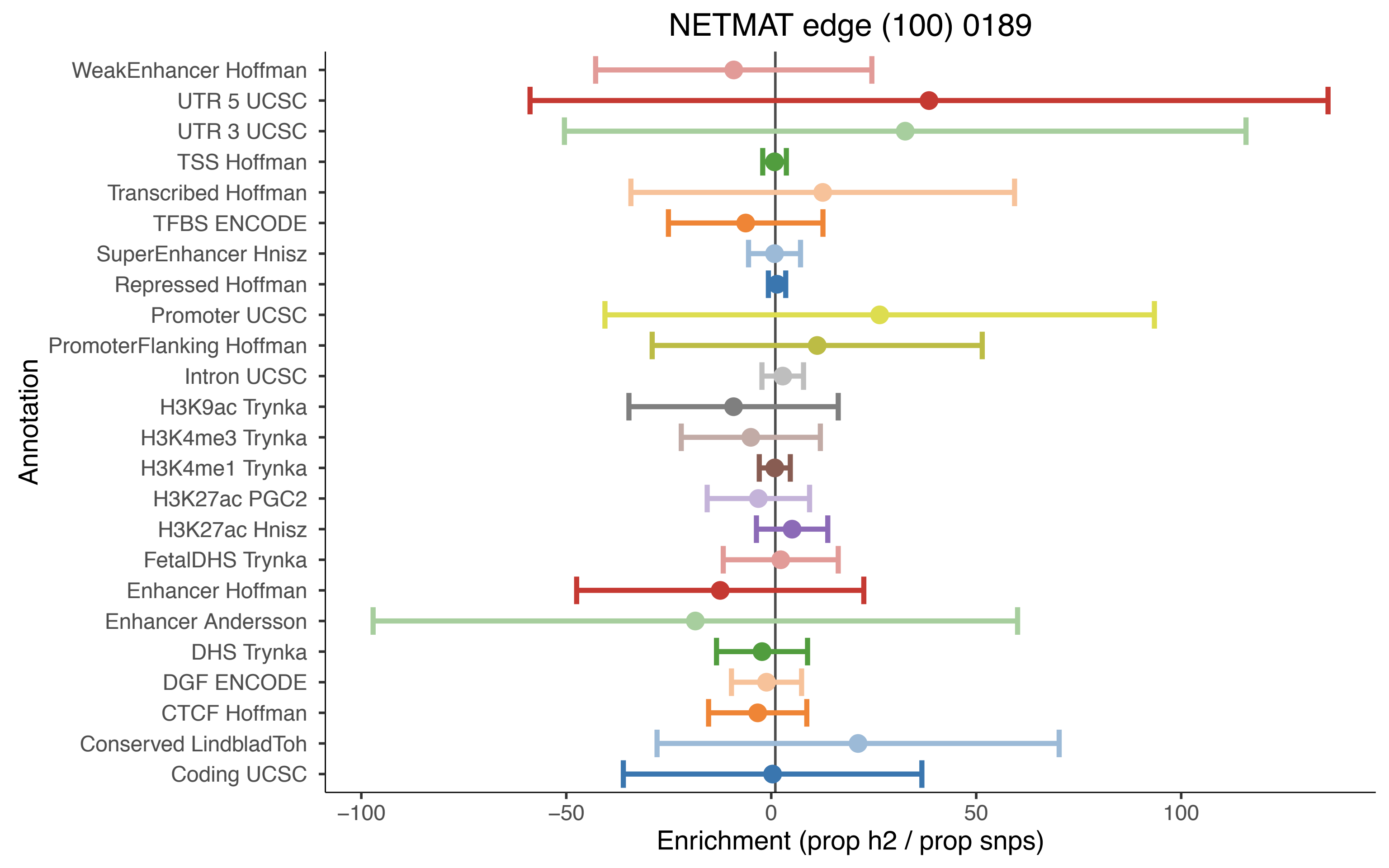
NETMAT edge (100) 0189
Annotation
WeakEnhancer Hoffman
UTR 5 UCSC
UTR 3 UCSC
TSS Hoffman
Transcribed Hoffman
TFBS ENCODE
SuperEnhancer Hnisz
Repressed Hoffman
Promoter UCSC
PromoterFlanking Hoffman
Intron UCSC
H3K9ac Trynka
H3K4me3 Trynka
H3K4me1 Trynka
H3K27ac PGC2
H3K27ac Hnisz
FetalDHS Trynka
Enhancer Hoffman
Enhancer Andersson
DHS Trynka
DGF ENCODE
CTCF Hoffman
Conserved LindbladToh
Coding UCSC
−100
−50
0
50
100
Enrichment (prop h2 / prop snps)

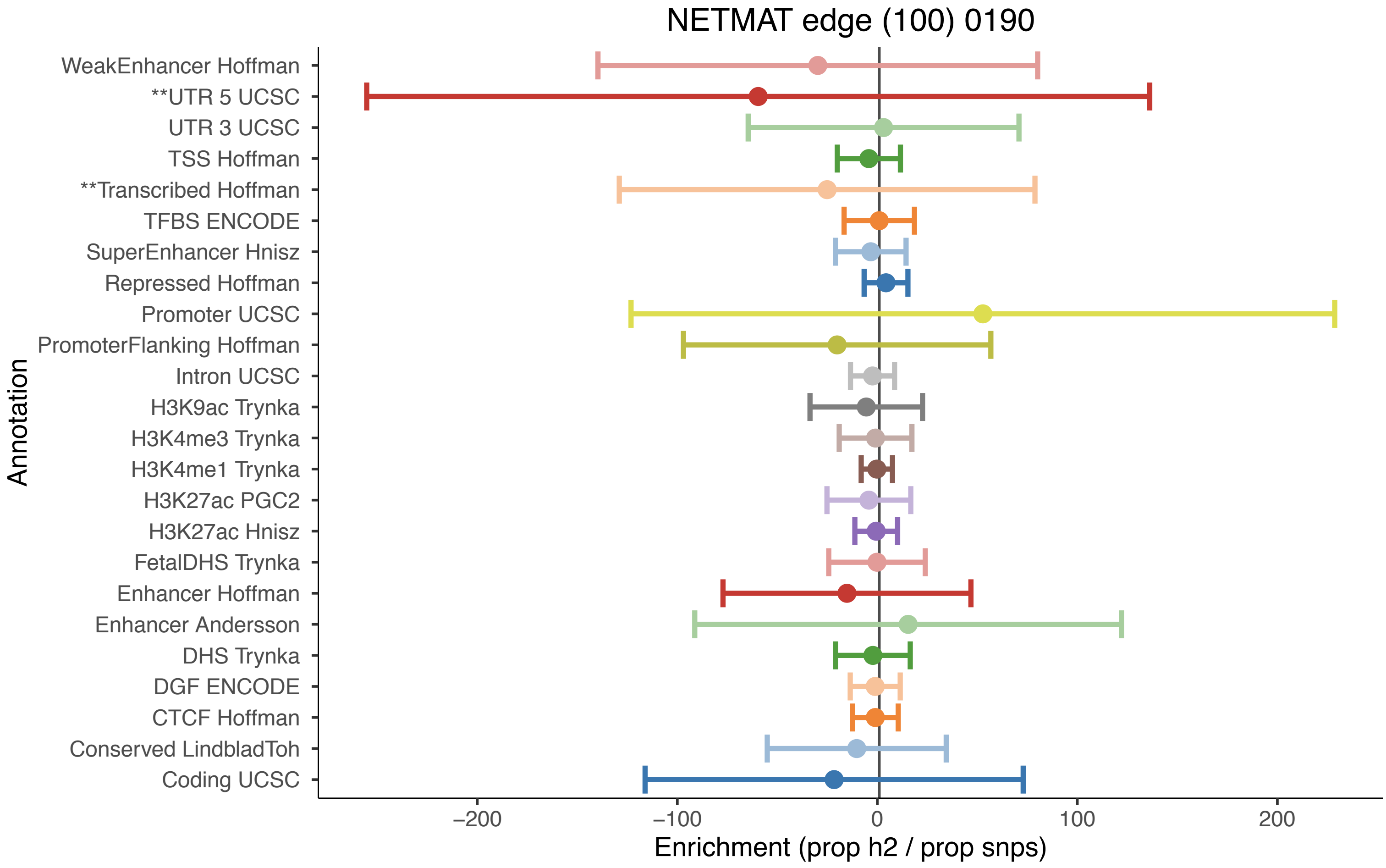
NETMAT edge (100) 0190
WeakEnhancer Hoffman
**UTR 5 UCSC
UTR 3 UCSC
TSS Hoffman
**Transcribed Hoffman
TFBS ENCODE
SuperEnhancer Hnisz
Repressed Hoffman
Promoter UCSC
PromoterFlanking Hoffman
Intron UCSC
H3K9ac Trynka
H3K4me3 Trynka
H3K4me1 Trynka
H3K27ac PGC2
H3K27ac Hnisz
FetalDHS Trynka
Enhancer Hoffman
Enhancer Andersson
DHS Trynka
DGF ENCODE
CTCF Hoffman
Conserved LindbladToh
Coding UCSC
Annotation
−200
−100
0
100
200
Enrichment (prop h2 / prop snps)

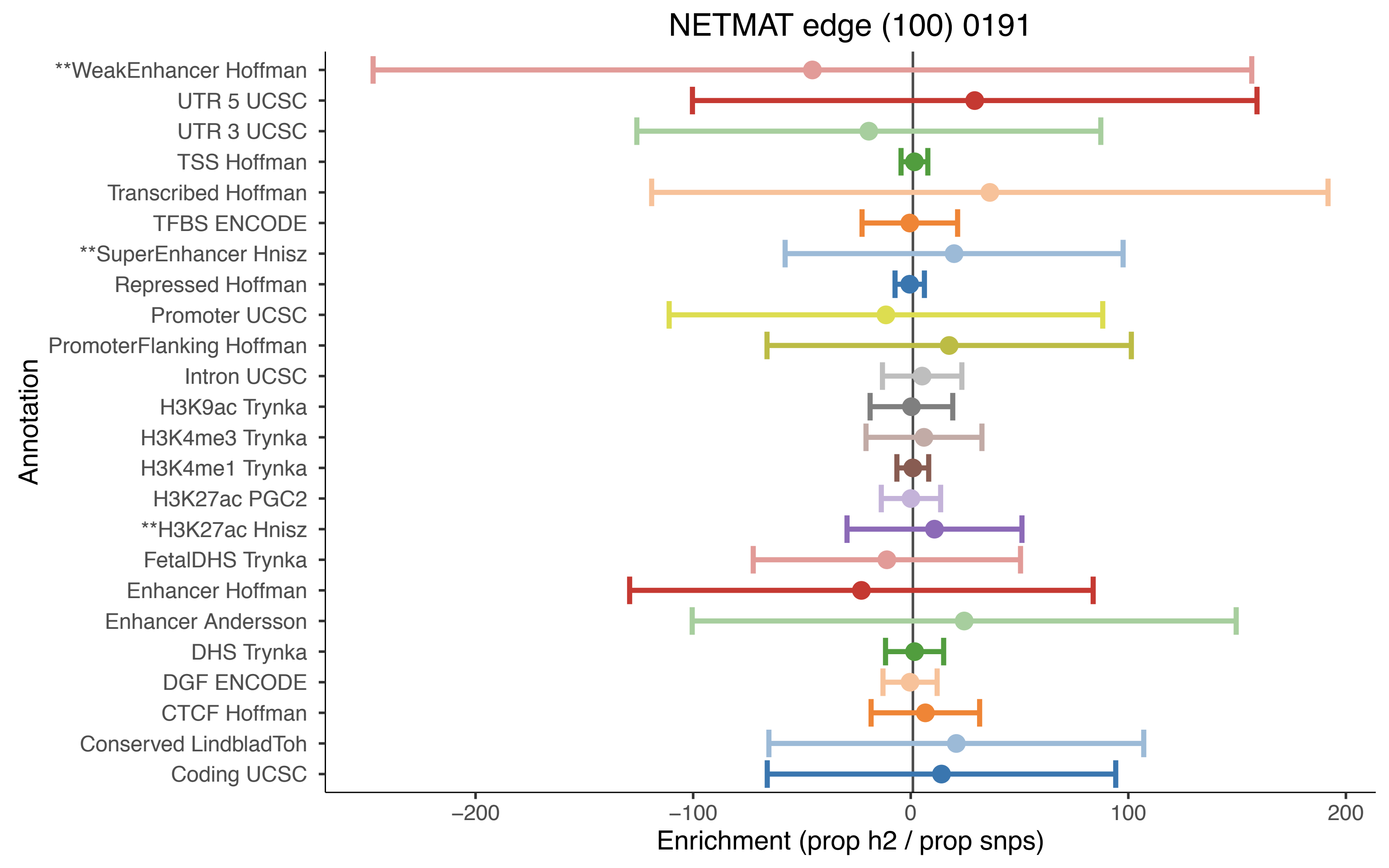
NETMAT edge (100) 0191
**WeakEnhancer Hoffman
UTR 5 UCSC
UTR 3 UCSC
TSS Hoffman
Transcribed Hoffman
TFBS ENCODE
**SuperEnhancer Hnisz
Repressed Hoffman
Promoter UCSC
PromoterFlanking Hoffman
Intron UCSC
H3K9ac Trynka
H3K4me3 Trynka
H3K4me1 Trynka
H3K27ac PGC2
**H3K27ac Hnisz
FetalDHS Trynka
Enhancer Hoffman
Enhancer Andersson
DHS Trynka
DGF ENCODE
CTCF Hoffman
Conserved LindbladToh
Coding UCSC
Annotation
−200
−100
0
100
200
Enrichment (prop h2 / prop snps)

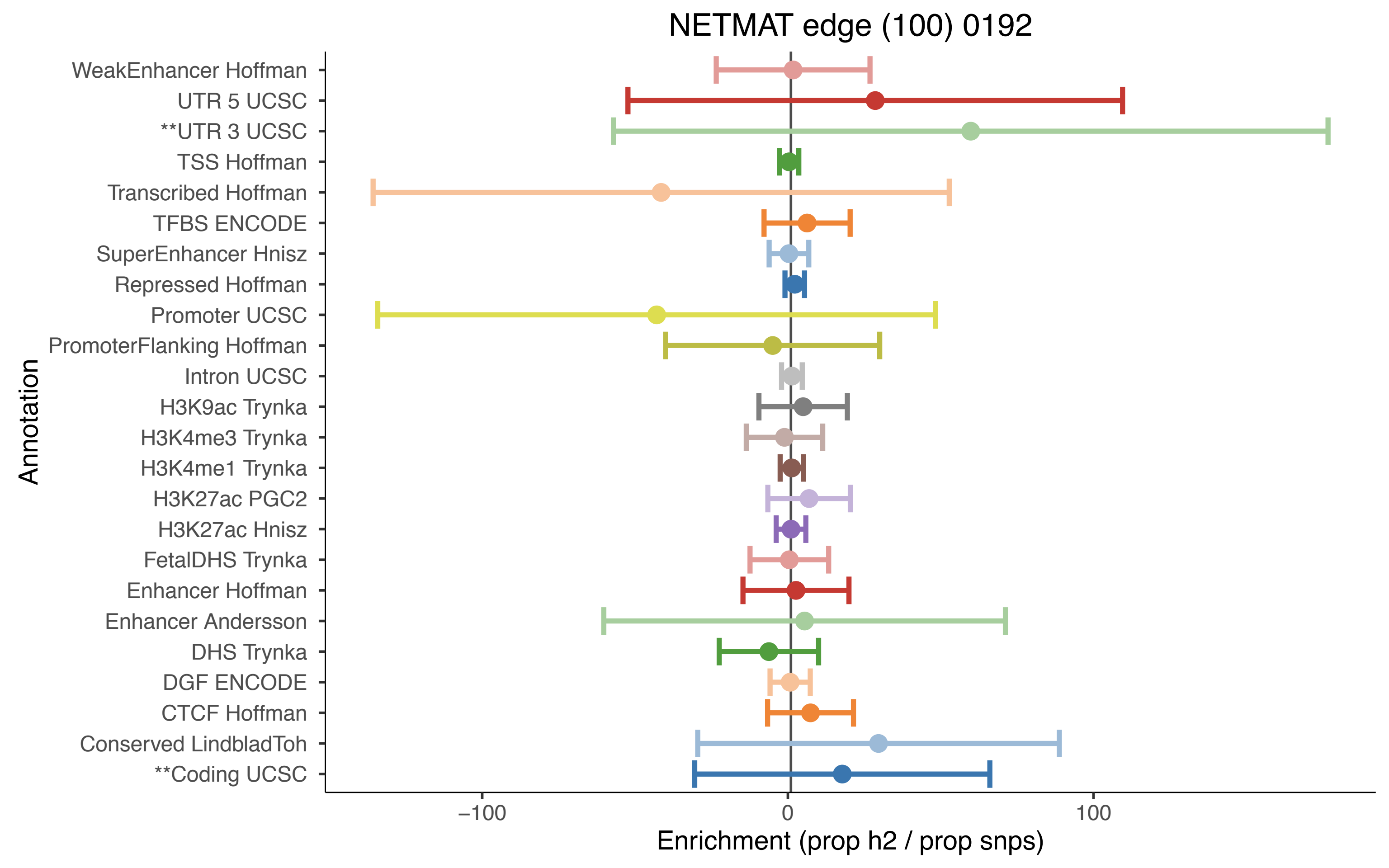
NETMAT edge (100) 0192
WeakEnhancer Hoffman
UTR 5 UCSC
**UTR 3 UCSC
TSS Hoffman
Transcribed Hoffman
TFBS ENCODE
SuperEnhancer Hnisz
Repressed Hoffman
Promoter UCSC
PromoterFlanking Hoffman
Intron UCSC
H3K9ac Trynka
H3K4me3 Trynka
H3K4me1 Trynka
H3K27ac PGC2
H3K27ac Hnisz
FetalDHS Trynka
Enhancer Hoffman
Enhancer Andersson
DHS Trynka
DGF ENCODE
CTCF Hoffman
Conserved LindbladToh
**Coding UCSC
Annotation
−100
0
100
Enrichment (prop h2 / prop snps)

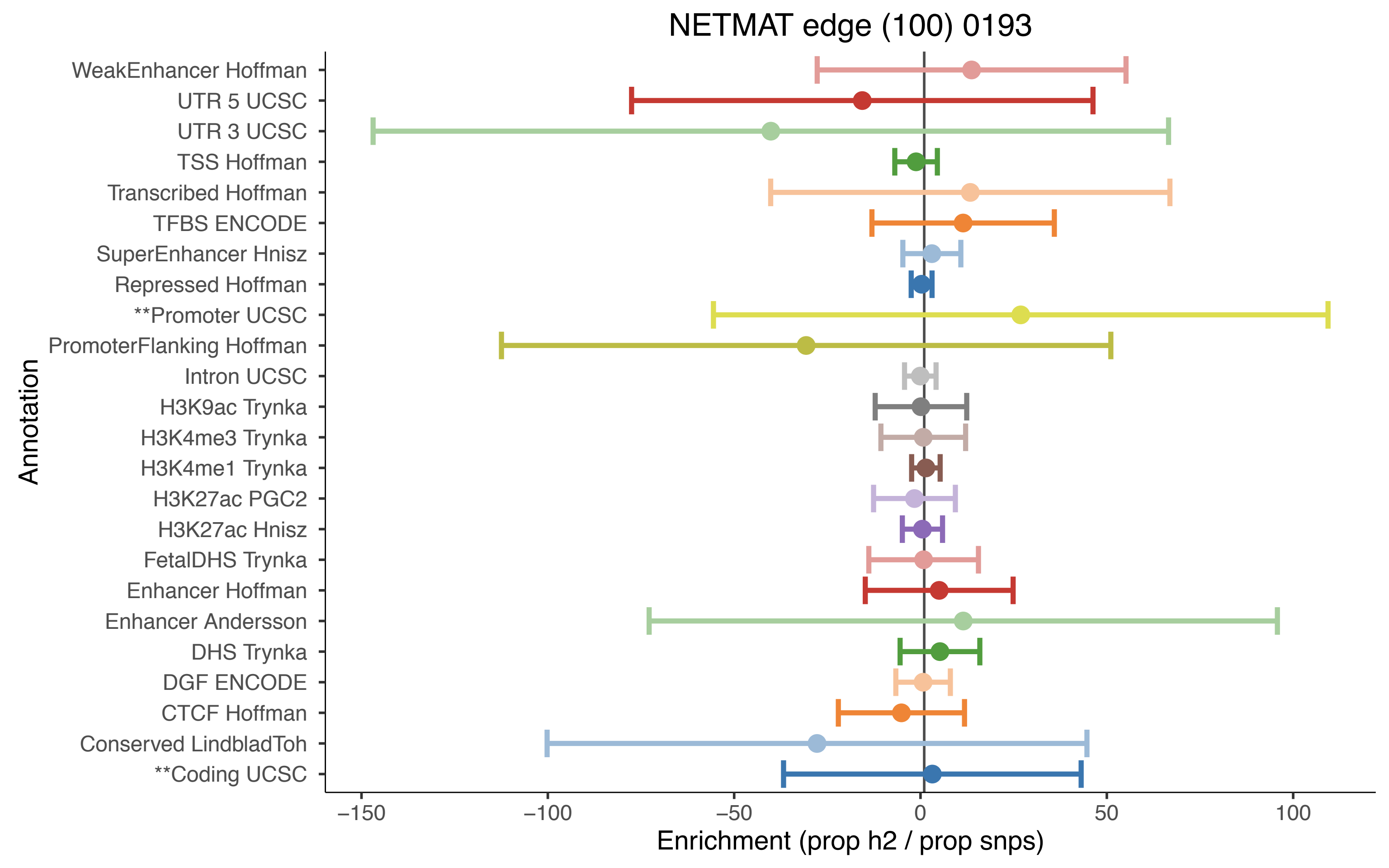
NETMAT edge (100) 0193
WeakEnhancer Hoffman
UTR 5 UCSC
UTR 3 UCSC
TSS Hoffman
Transcribed Hoffman
TFBS ENCODE
SuperEnhancer Hnisz
Repressed Hoffman
**Promoter UCSC
PromoterFlanking Hoffman
Intron UCSC
H3K9ac Trynka
H3K4me3 Trynka
H3K4me1 Trynka
H3K27ac PGC2
H3K27ac Hnisz
FetalDHS Trynka
Enhancer Hoffman
Enhancer Andersson
DHS Trynka
DGF ENCODE
CTCF Hoffman
Conserved LindbladToh
**Coding UCSC
Annotation
−150
−100
−50
0
50
100
Enrichment (prop h2 / prop snps)

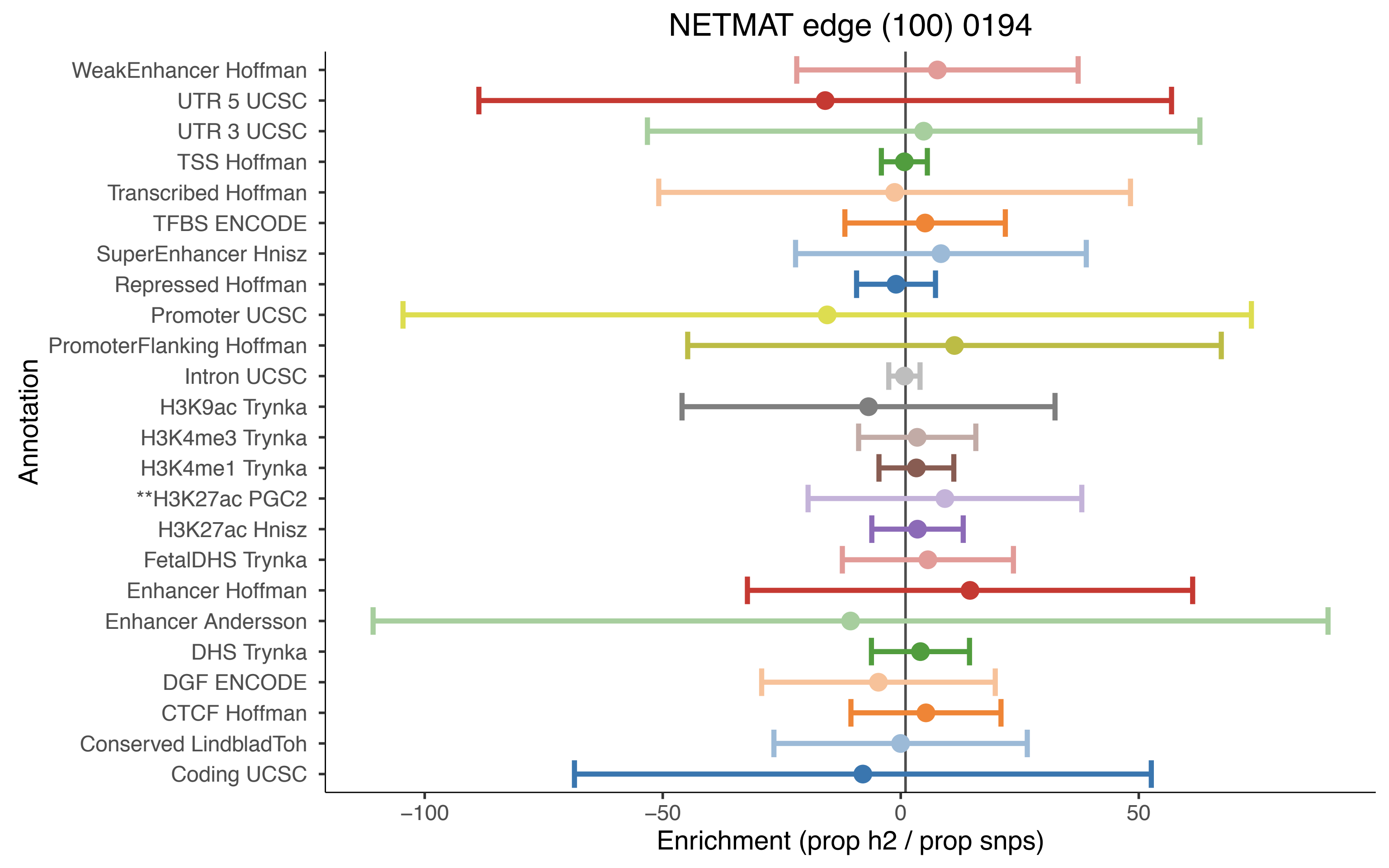
NETMAT edge (100) 0194
WeakEnhancer Hoffman
UTR 5 UCSC
UTR 3 UCSC
TSS Hoffman
Transcribed Hoffman
TFBS ENCODE
SuperEnhancer Hnisz
Repressed Hoffman
Promoter UCSC
PromoterFlanking Hoffman
Intron UCSC
H3K9ac Trynka
H3K4me3 Trynka
H3K4me1 Trynka
**H3K27ac PGC2
H3K27ac Hnisz
FetalDHS Trynka
Enhancer Hoffman
Enhancer Andersson
DHS Trynka
DGF ENCODE
CTCF Hoffman
Conserved LindbladToh
Coding UCSC
Annotation
−100
−50
0
50
Enrichment (prop h2 / prop snps)

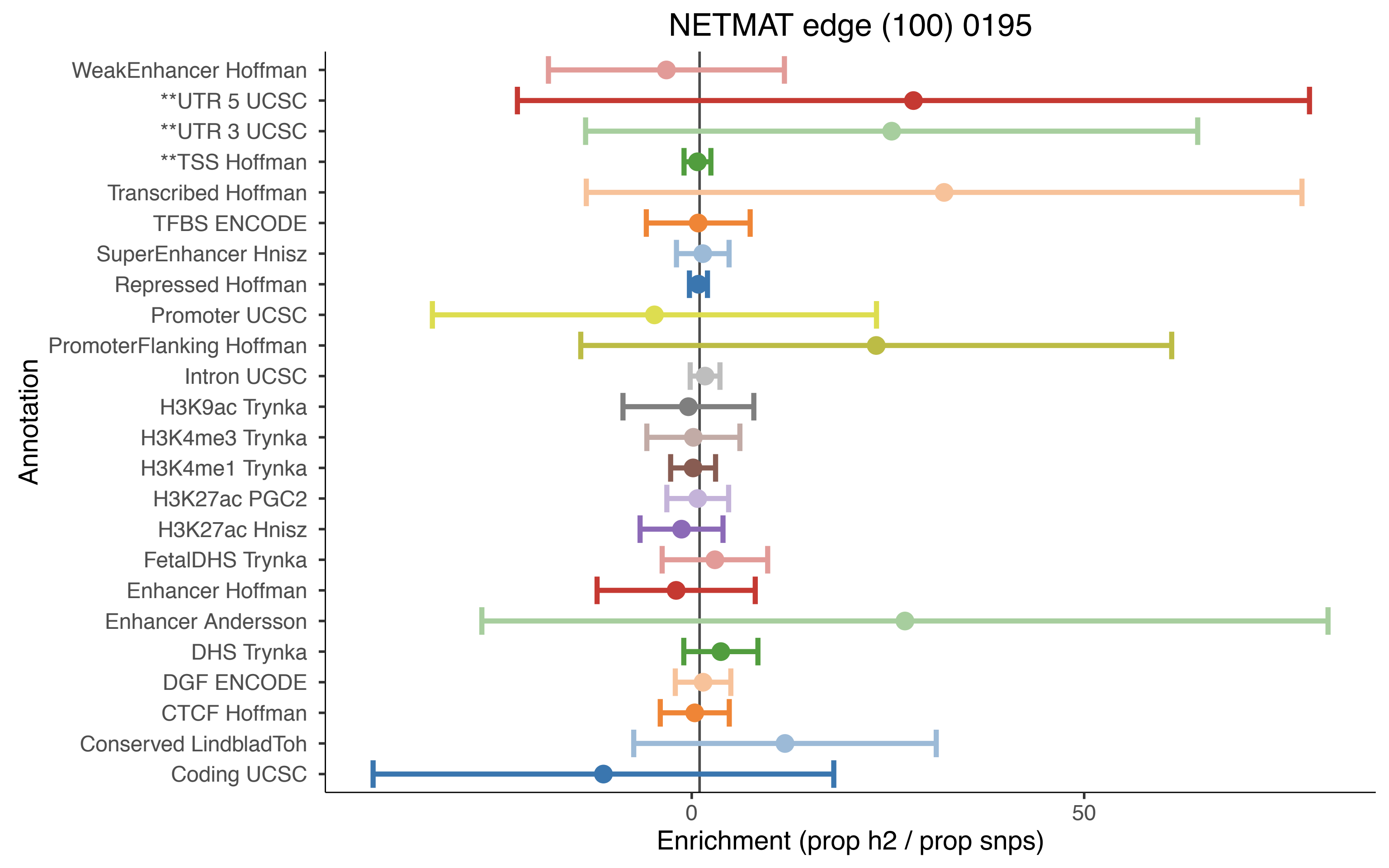
NETMAT edge (100) 0195
WeakEnhancer Hoffman
**UTR 5 UCSC
**UTR 3 UCSC
**TSS Hoffman
Transcribed Hoffman
TFBS ENCODE
SuperEnhancer Hnisz
Repressed Hoffman
Promoter UCSC
PromoterFlanking Hoffman
Intron UCSC
H3K9ac Trynka
H3K4me3 Trynka
H3K4me1 Trynka
H3K27ac PGC2
H3K27ac Hnisz
FetalDHS Trynka
Enhancer Hoffman
Enhancer Andersson
DHS Trynka
DGF ENCODE
CTCF Hoffman
Conserved LindbladToh
Coding UCSC
Annotation
0
50
Enrichment (prop h2 / prop snps)

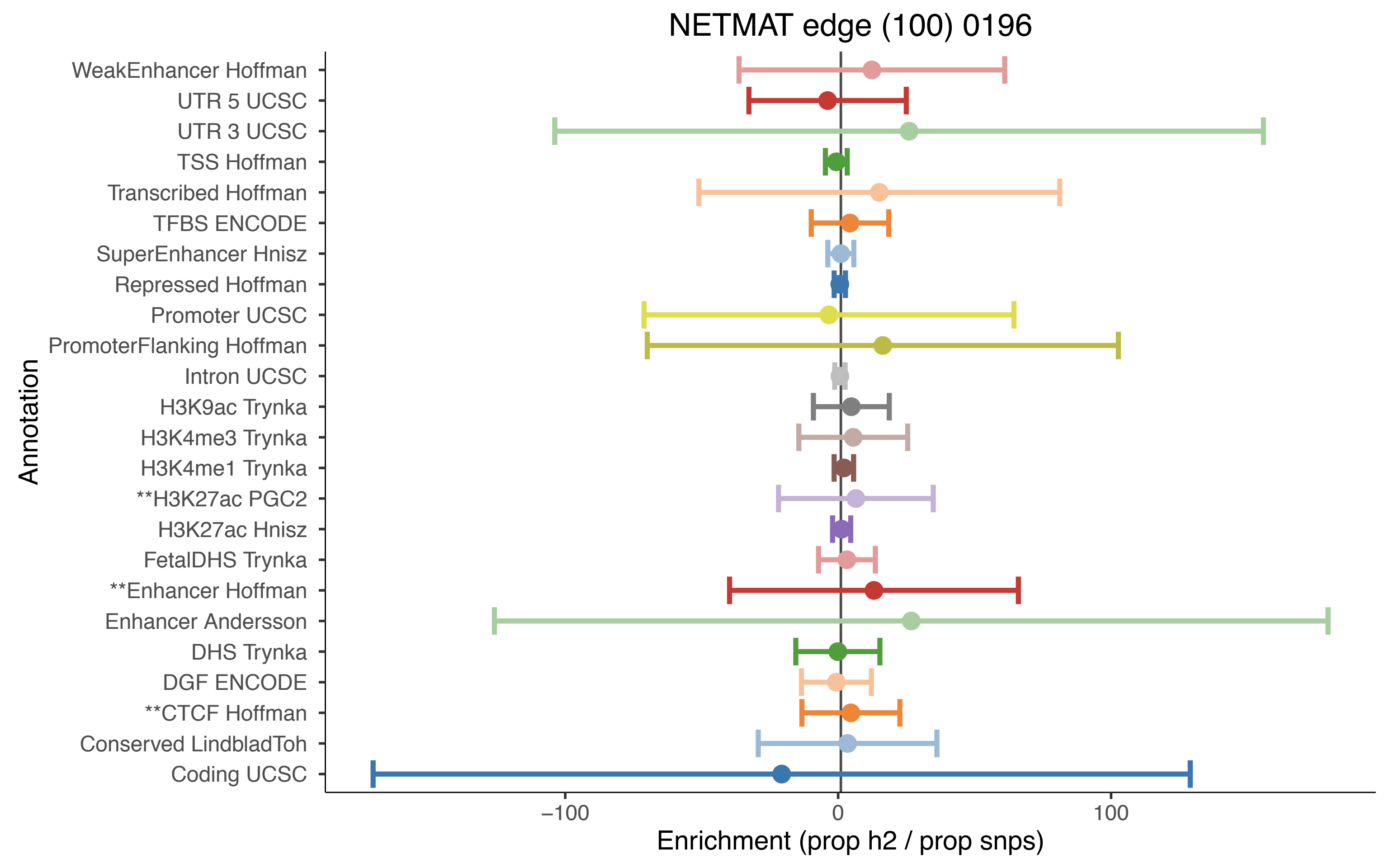

NETMAT edge (100) 0196
WeakEnhancer Hoffman
UTR 5 UCSC
UTR 3 UCSC
TSS Hoffman
Transcribed Hoffman
TFBS ENCODE
SuperEnhancer Hnisz
Repressed Hoffman
Promoter UCSC
PromoterFlanking Hoffman
Intron UCSC
H3K9ac Trynka
H3K4me3 Trynka
H3K4me1 Trynka
**H3K27ac PGC2
H3K27ac Hnisz
FetalDHS Trynka
**Enhancer Hoffman
Enhancer Andersson
DHS Trynka
DGF ENCODE
**CTCF Hoffman
Conserved LindbladToh
Coding UCSC
Annotation
−100
0
100
Enrichment (prop h2 / prop snps)

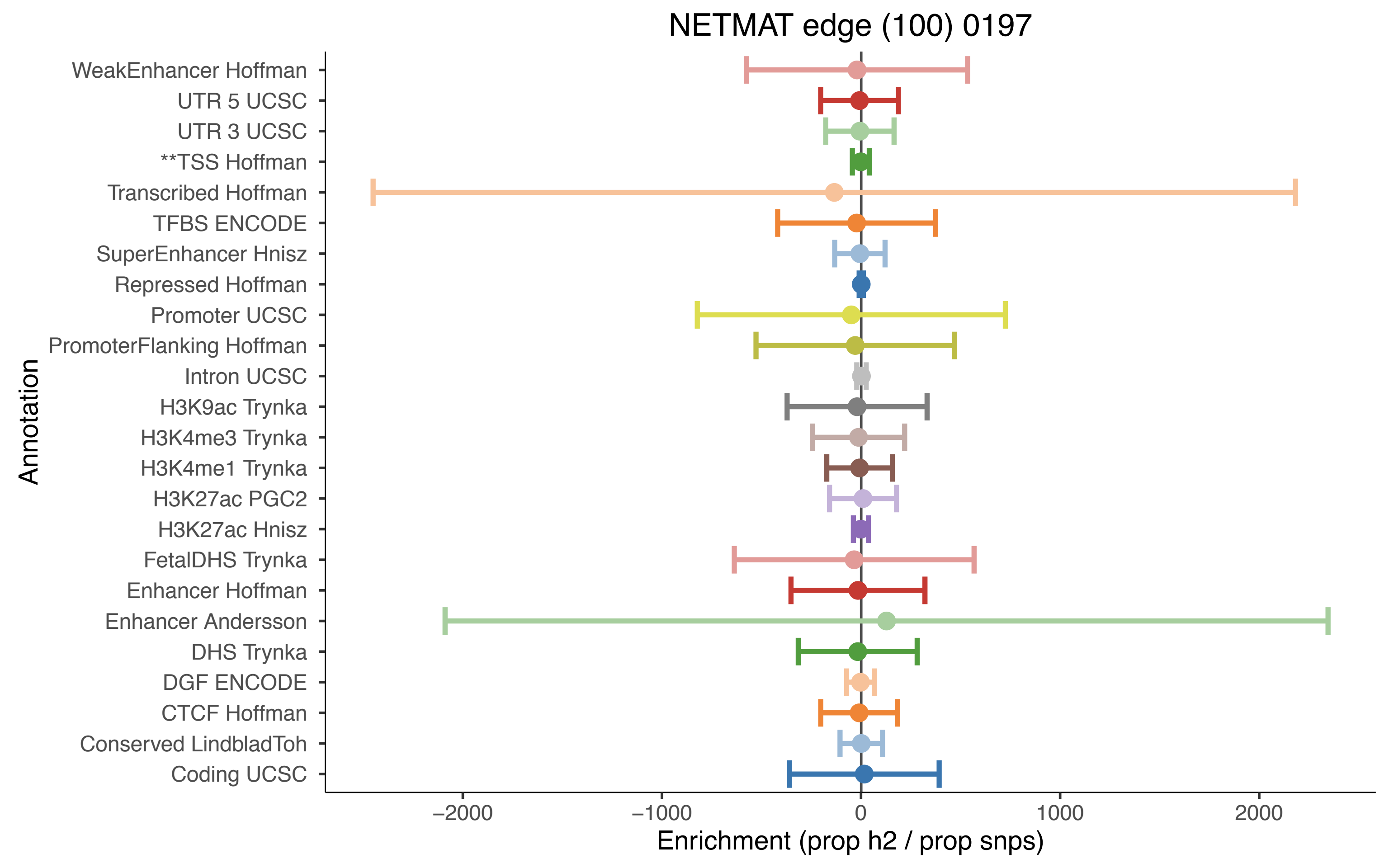

NETMAT edge (100) 0197
Annotation
WeakEnhancer Hoffman
UTR 5 UCSC
UTR 3 UCSC
**TSS Hoffman
Transcribed Hoffman
TFBS ENCODE
SuperEnhancer Hnisz
Repressed Hoffman
Promoter UCSC
PromoterFlanking Hoffman
Intron UCSC
H3K9ac Trynka
H3K4me3 Trynka
H3K4me1 Trynka
H3K27ac PGC2
H3K27ac Hnisz
FetalDHS Trynka
Enhancer Hoffman
Enhancer Andersson
DHS Trynka
DGF ENCODE
CTCF Hoffman
Conserved LindbladToh
Coding UCSC
−2000
−1000
0
1000
2000
Enrichment (prop h2 / prop snps)

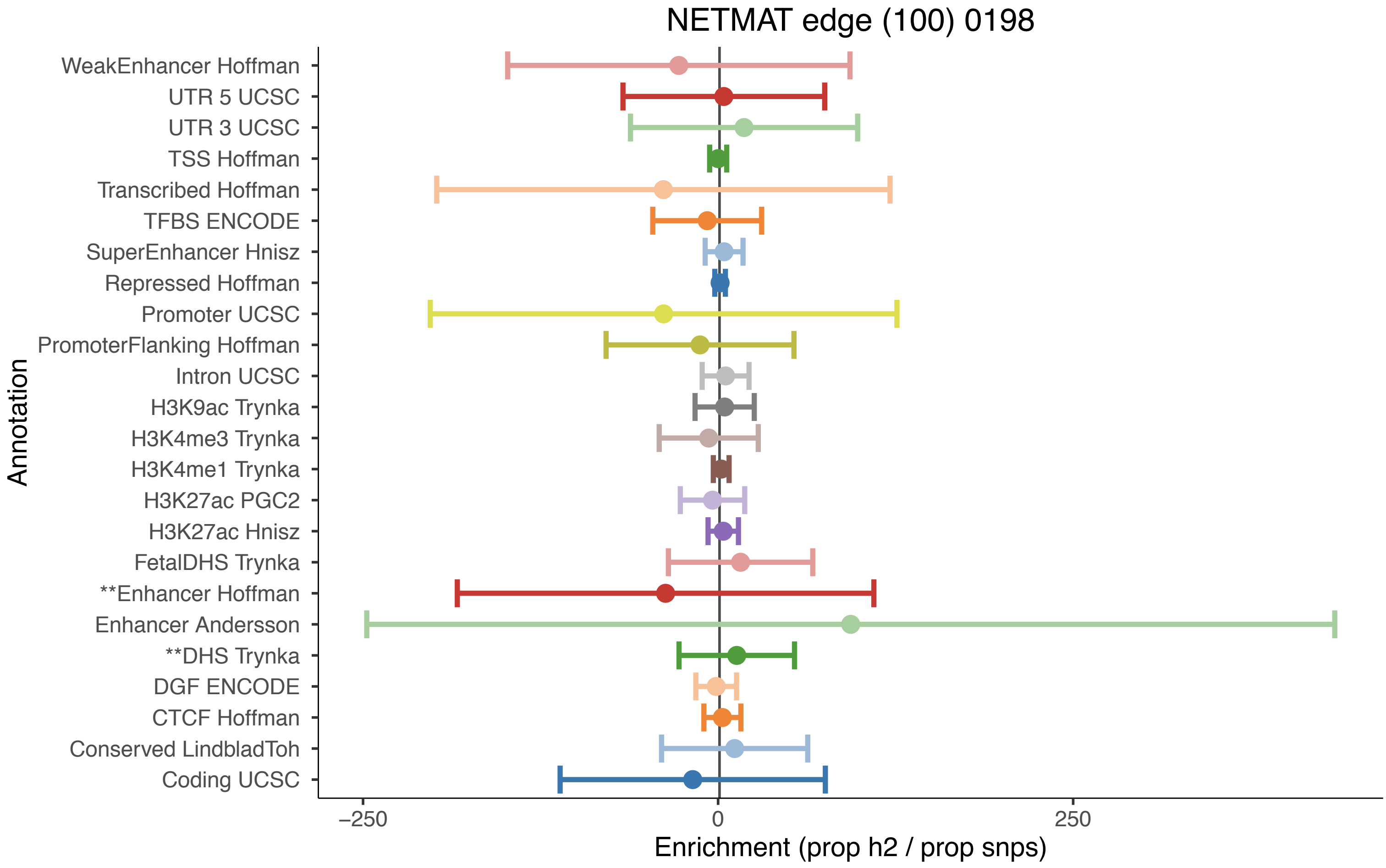

NETMAT edge (100) 0198
WeakEnhancer Hoffman
UTR 5 UCSC
UTR 3 UCSC
TSS Hoffman
Transcribed Hoffman
TFBS ENCODE
SuperEnhancer Hnisz
Repressed Hoffman
Promoter UCSC
PromoterFlanking Hoffman
Intron UCSC
H3K9ac Trynka
H3K4me3 Trynka
H3K4me1 Trynka
H3K27ac PGC2
H3K27ac Hnisz
FetalDHS Trynka
**Enhancer Hoffman
Enhancer Andersson
**DHS Trynka
DGF ENCODE
CTCF Hoffman
Conserved LindbladToh
Coding UCSC
Annotation
−250
0
250
Enrichment (prop h2 / prop snps)

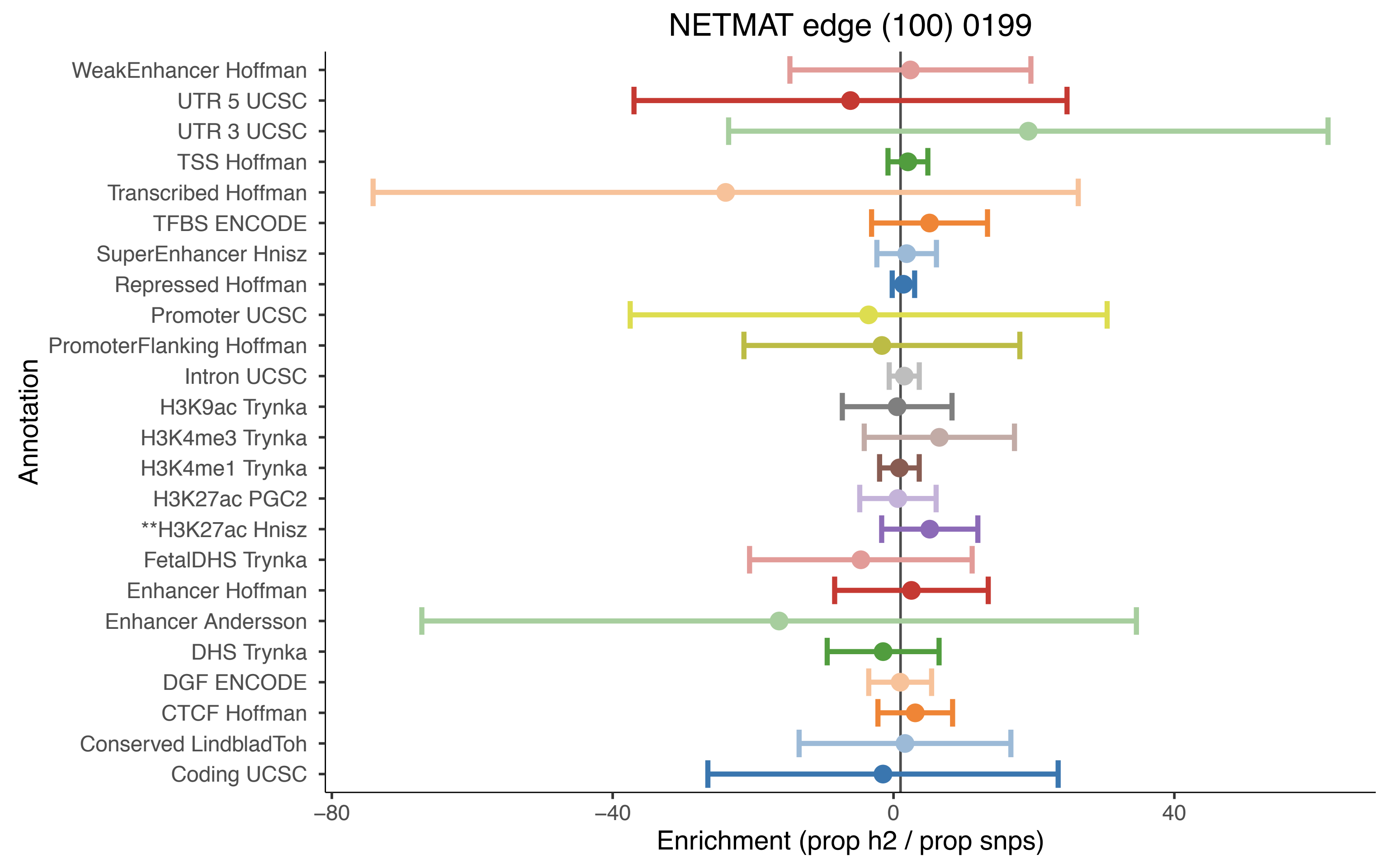

NETMAT edge (100) 0199
WeakEnhancer Hoffman
UTR 5 UCSC
UTR 3 UCSC
TSS Hoffman
Transcribed Hoffman
TFBS ENCODE
SuperEnhancer Hnisz
Repressed Hoffman
Promoter UCSC
PromoterFlanking Hoffman
Intron UCSC
H3K9ac Trynka
H3K4me3 Trynka
H3K4me1 Trynka
H3K27ac PGC2
**H3K27ac Hnisz
FetalDHS Trynka
Enhancer Hoffman
Enhancer Andersson
DHS Trynka
DGF ENCODE
CTCF Hoffman
Conserved LindbladToh
Coding UCSC
Annotation
−80
−40
0
40
Enrichment (prop h2 / prop snps)

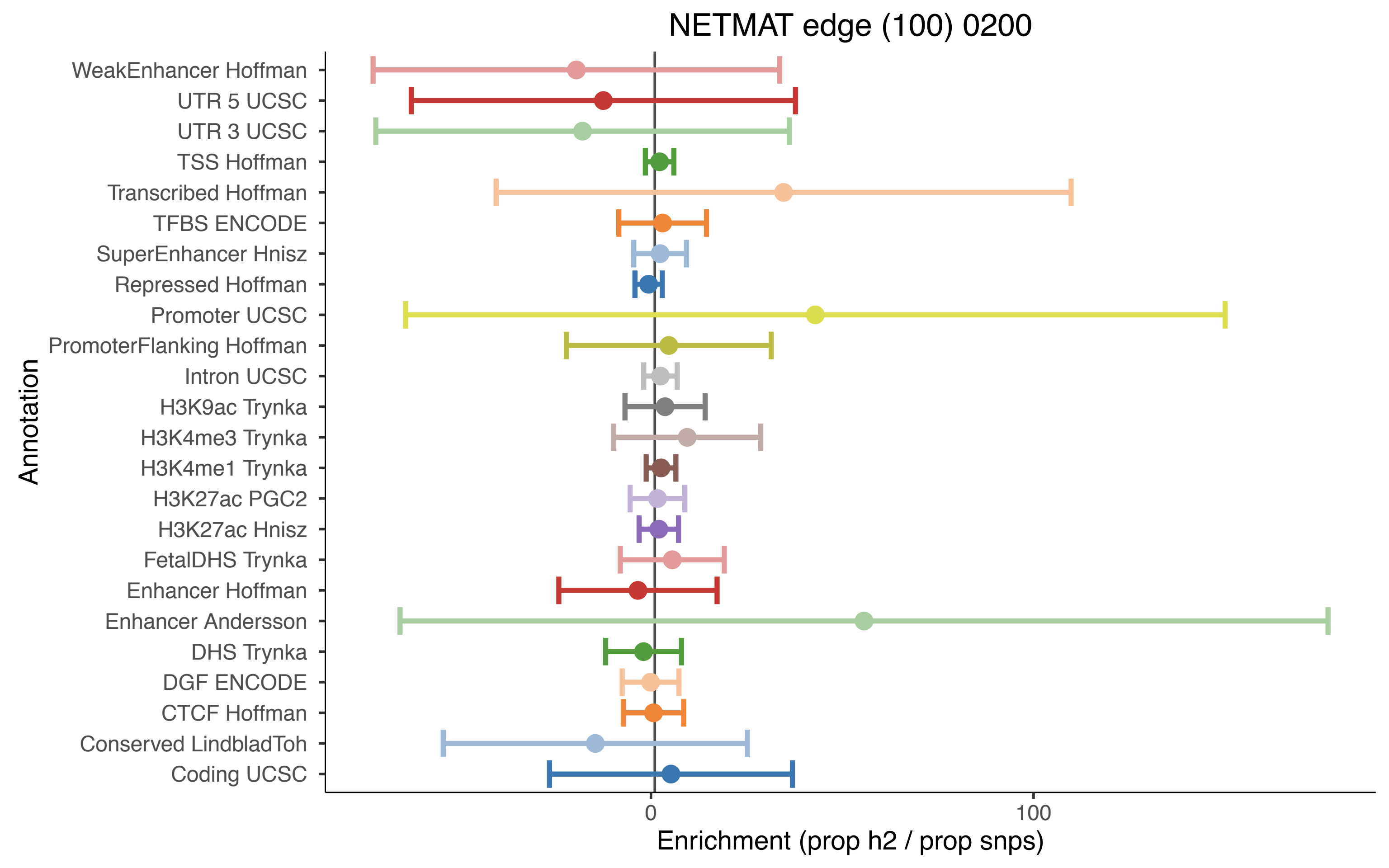
NETMAT edge (100) 0200
WeakEnhancer Hoffman
UTR 5 UCSC
UTR 3 UCSC
TSS Hoffman
Transcribed Hoffman
TFBS ENCODE
SuperEnhancer Hnisz
Repressed Hoffman
Promoter UCSC
PromoterFlanking Hoffman
Intron UCSC
H3K9ac Trynka
H3K4me3 Trynka
H3K4me1 Trynka
H3K27ac PGC2
H3K27ac Hnisz
FetalDHS Trynka
Enhancer Hoffman
Enhancer Andersson
DHS Trynka
DGF ENCODE
CTCF Hoffman
Conserved LindbladToh
Coding UCSC
Annotation
0
100
Enrichment (prop h2 / prop snps)

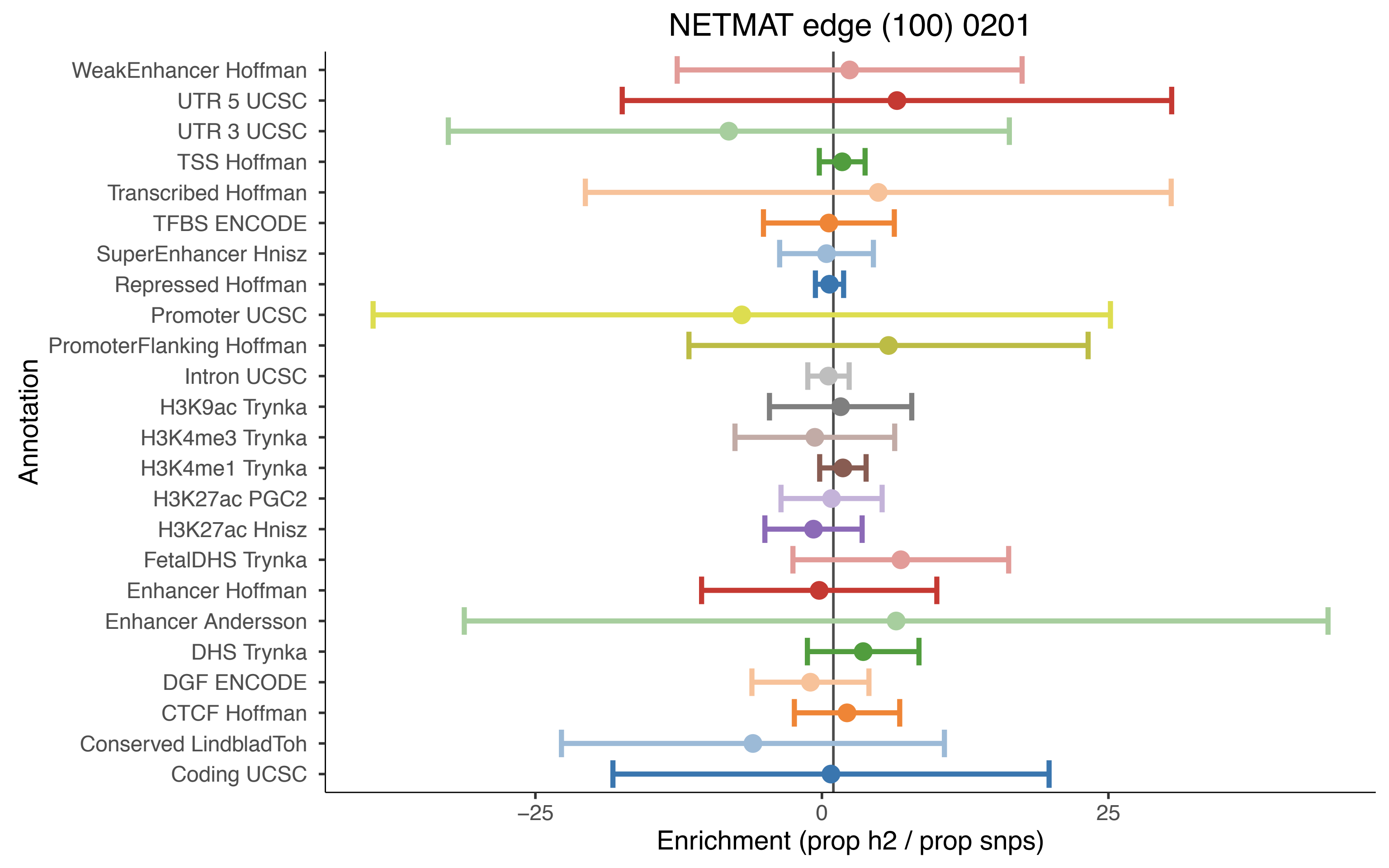
NETMAT edge (100) 0201
WeakEnhancer Hoffman
UTR 5 UCSC
UTR 3 UCSC
TSS Hoffman
Transcribed Hoffman
TFBS ENCODE
SuperEnhancer Hnisz
Repressed Hoffman
Promoter UCSC
PromoterFlanking Hoffman
Intron UCSC
H3K9ac Trynka
H3K4me3 Trynka
H3K4me1 Trynka
H3K27ac PGC2
H3K27ac Hnisz
FetalDHS Trynka
Enhancer Hoffman
Enhancer Andersson
DHS Trynka
DGF ENCODE
CTCF Hoffman
Conserved LindbladToh
Coding UCSC
Annotation
−25
0
25
Enrichment (prop h2 / prop snps)

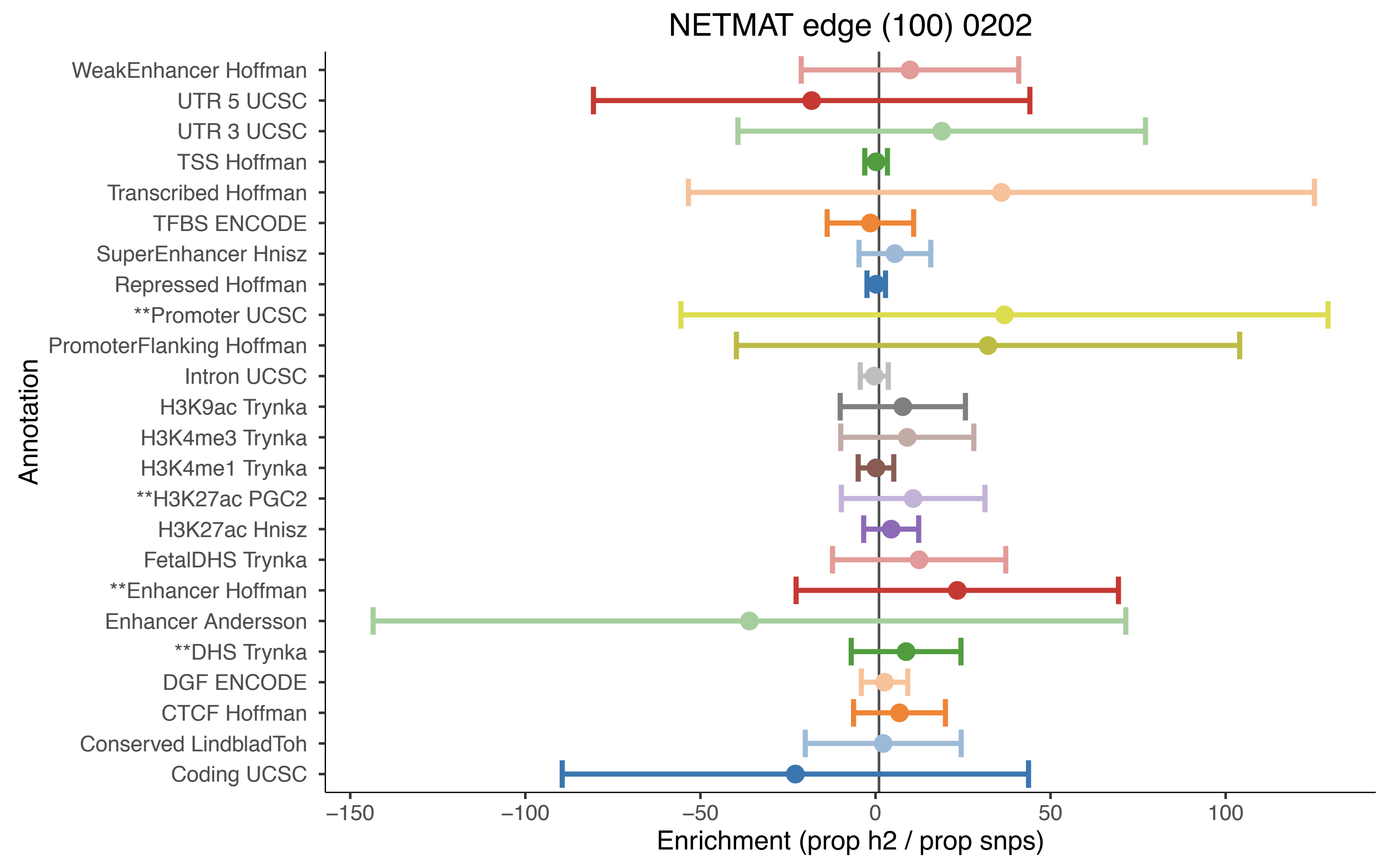
NETMAT edge (100) 0202
WeakEnhancer Hoffman
UTR 5 UCSC
UTR 3 UCSC
TSS Hoffman
Transcribed Hoffman
TFBS ENCODE
SuperEnhancer Hnisz
Repressed Hoffman
**Promoter UCSC
PromoterFlanking Hoffman
Intron UCSC
H3K9ac Trynka
H3K4me3 Trynka
H3K4me1 Trynka
**H3K27ac PGC2
H3K27ac Hnisz
FetalDHS Trynka
**Enhancer Hoffman
Enhancer Andersson
**DHS Trynka
DGF ENCODE
CTCF Hoffman
Conserved LindbladToh
Coding UCSC
Annotation
−150
−100
−50
0
50
100
Enrichment (prop h2 / prop snps)

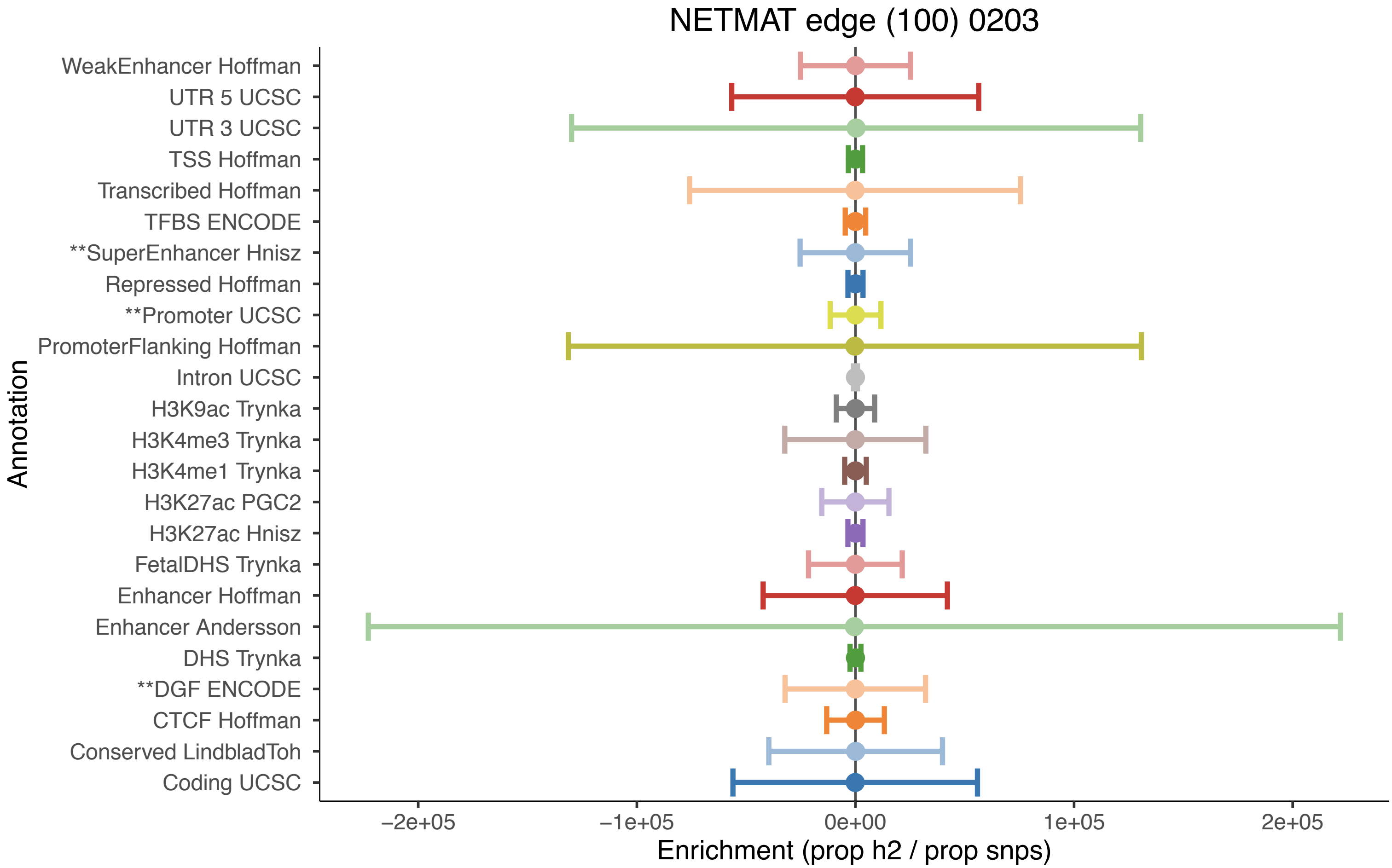
NETMAT edge (100) 0203
WeakEnhancer Hoffman
UTR 5 UCSC
UTR 3 UCSC
TSS Hoffman
Transcribed Hoffman
TFBS ENCODE
**SuperEnhancer Hnisz
Repressed Hoffman
**Promoter UCSC
PromoterFlanking Hoffman
Intron UCSC
H3K9ac Trynka
H3K4me3 Trynka
H3K4me1 Trynka
H3K27ac PGC2
H3K27ac Hnisz
FetalDHS Trynka
Enhancer Hoffman
Enhancer Andersson
DHS Trynka
**DGF ENCODE
CTCF Hoffman
Conserved LindbladToh
Coding UCSC
Annotation
−2e+05
−1e+05
0e+00
1e+05
2e+05
Enrichment (prop h2 / prop snps)

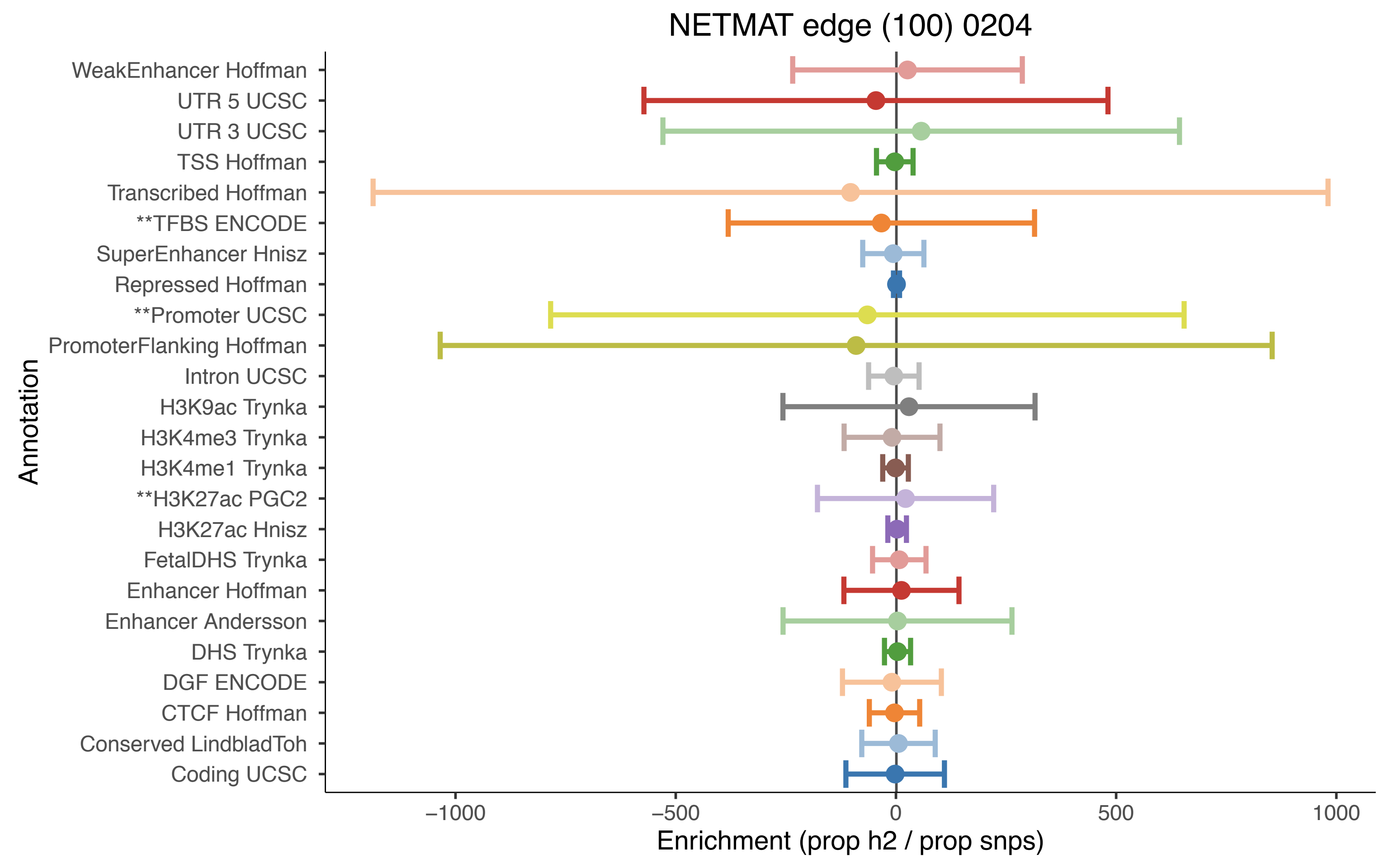

NETMAT edge (100) 0204
WeakEnhancer Hoffman
UTR 5 UCSC
UTR 3 UCSC
TSS Hoffman
Transcribed Hoffman
**TFBS ENCODE
SuperEnhancer Hnisz
Repressed Hoffman
**Promoter UCSC
PromoterFlanking Hoffman
Intron UCSC
H3K9ac Trynka
H3K4me3 Trynka
H3K4me1 Trynka
**H3K27ac PGC2
H3K27ac Hnisz
FetalDHS Trynka
Enhancer Hoffman
Enhancer Andersson
DHS Trynka
DGF ENCODE
CTCF Hoffman
Conserved LindbladToh
Coding UCSC
Annotation
−1000
−500
0
500
1000
Enrichment (prop h2 / prop snps)

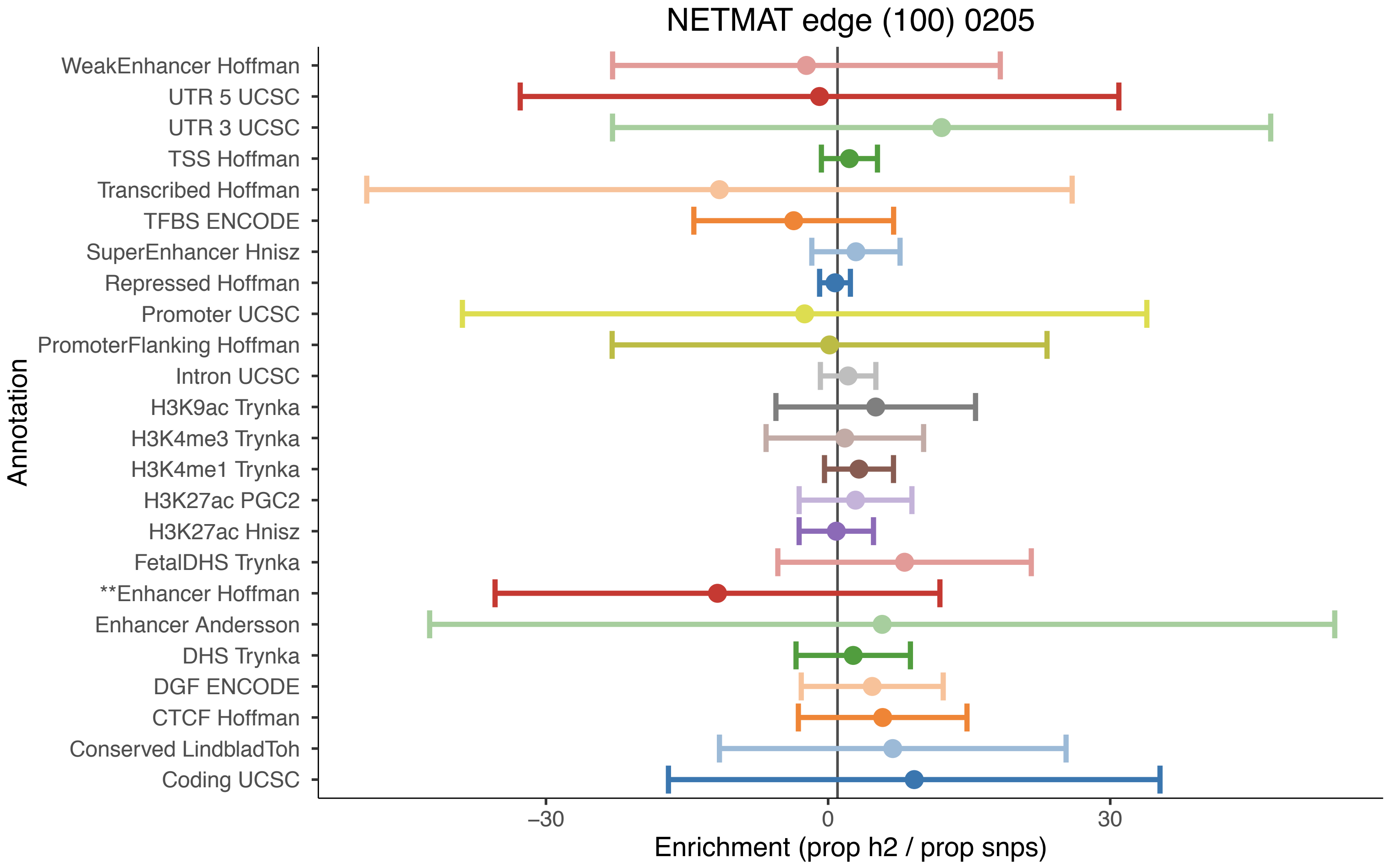
NETMAT edge (100) 0205
WeakEnhancer Hoffman
UTR 5 UCSC
UTR 3 UCSC
TSS Hoffman
Transcribed Hoffman
TFBS ENCODE
SuperEnhancer Hnisz
Repressed Hoffman
Promoter UCSC
PromoterFlanking Hoffman
Intron UCSC
H3K9ac Trynka
H3K4me3 Trynka
H3K4me1 Trynka
H3K27ac PGC2
H3K27ac Hnisz
FetalDHS Trynka
**Enhancer Hoffman
Enhancer Andersson
DHS Trynka
DGF ENCODE
CTCF Hoffman
Conserved LindbladToh
Coding UCSC
Annotation
–30
0
30
Enrichment (prop h2 / prop snps)

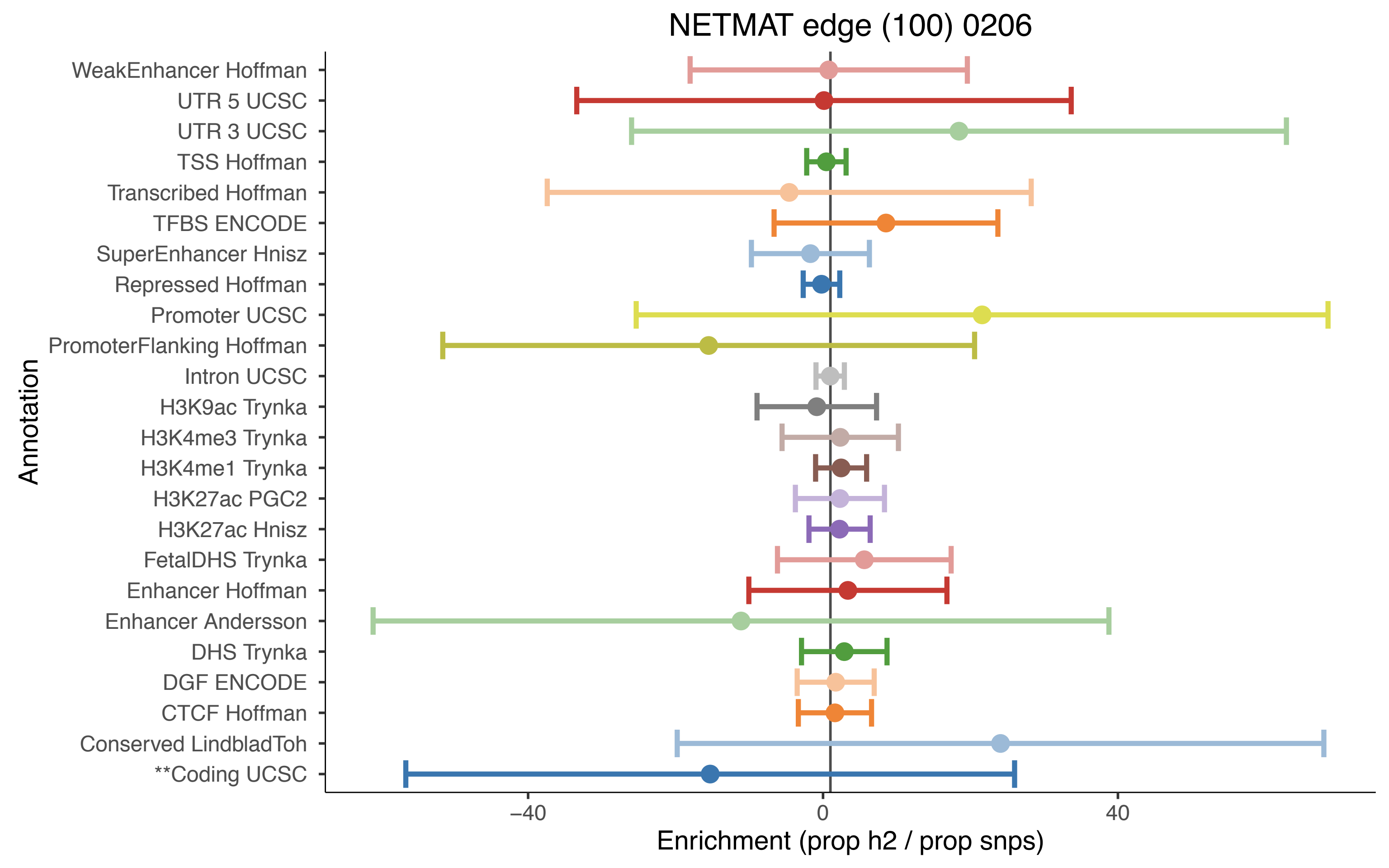

NETMAT edge (100) 0206
WeakEnhancer Hoffman
UTR 5 UCSC
UTR 3 UCSC
TSS Hoffman
Transcribed Hoffman
TFBS ENCODE
SuperEnhancer Hnisz
Repressed Hoffman
Promoter UCSC
PromoterFlanking Hoffman
Intron UCSC
H3K9ac Trynka
H3K4me3 Trynka
H3K4me1 Trynka
H3K27ac PGC2
H3K27ac Hnisz
FetalDHS Trynka
Enhancer Hoffman
Enhancer Andersson
DHS Trynka
DGF ENCODE
CTCF Hoffman
Conserved LindbladToh
**Coding UCSC
Annotation
−40
0
40
Enrichment (prop h2 / prop snps)

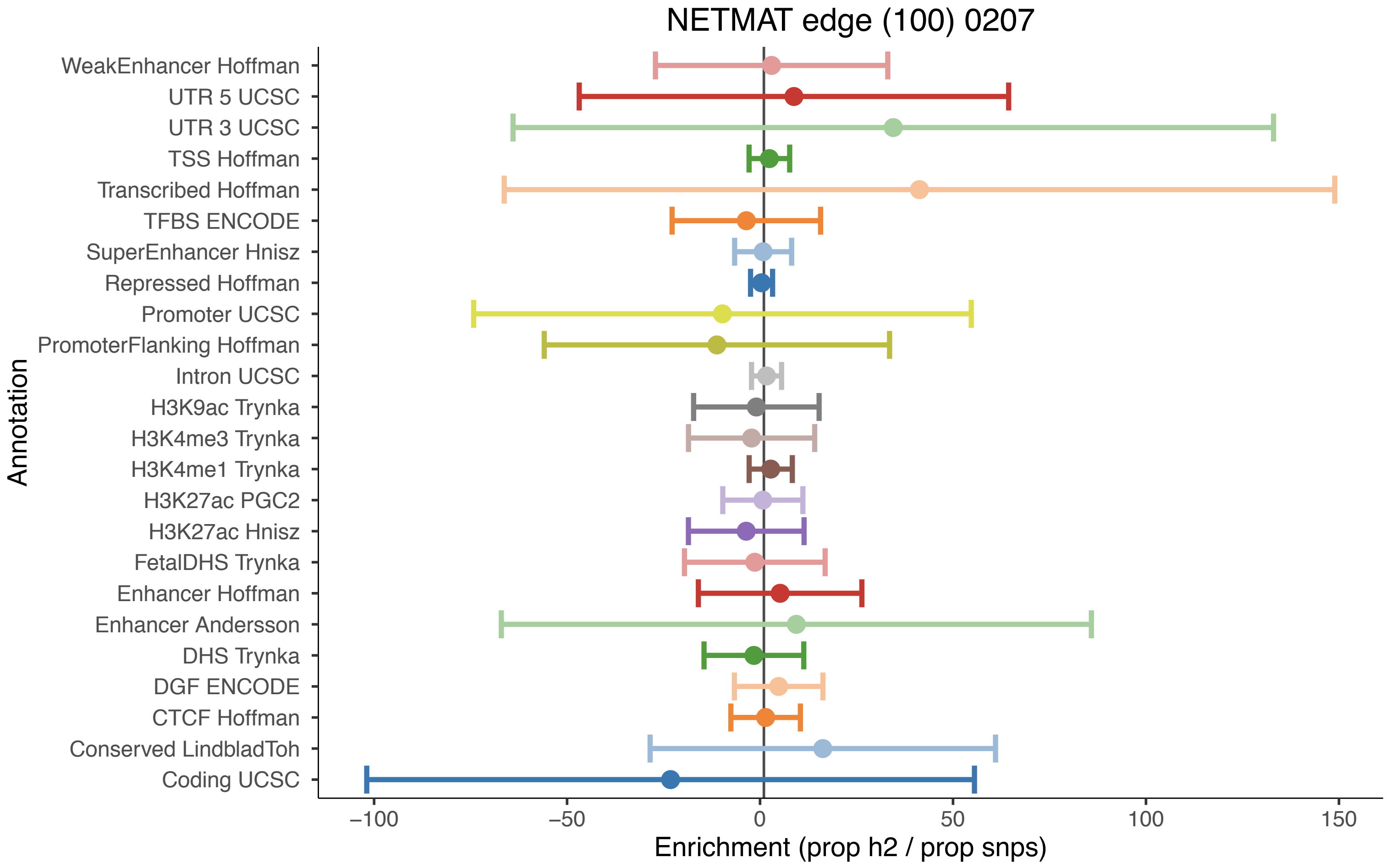

NETMAT edge (100) 0207
WeakEnhancer Hoffman
UTR 5 UCSC
UTR 3 UCSC
TSS Hoffman
Transcribed Hoffman
TFBS ENCODE
SuperEnhancer Hnisz
Repressed Hoffman
Promoter UCSC
PromoterFlanking Hoffman
Intron UCSC
H3K9ac Trynka
H3K4me3 Trynka
H3K4me1 Trynka
H3K27ac PGC2
H3K27ac Hnisz
FetalDHS Trynka
Enhancer Hoffman
Enhancer Andersson
DHS Trynka
DGF ENCODE
CTCF Hoffman
Conserved LindbladToh
Coding UCSC
Annotation
−100
−50
0
50
100
150
Enrichment (prop h2 / prop snps)

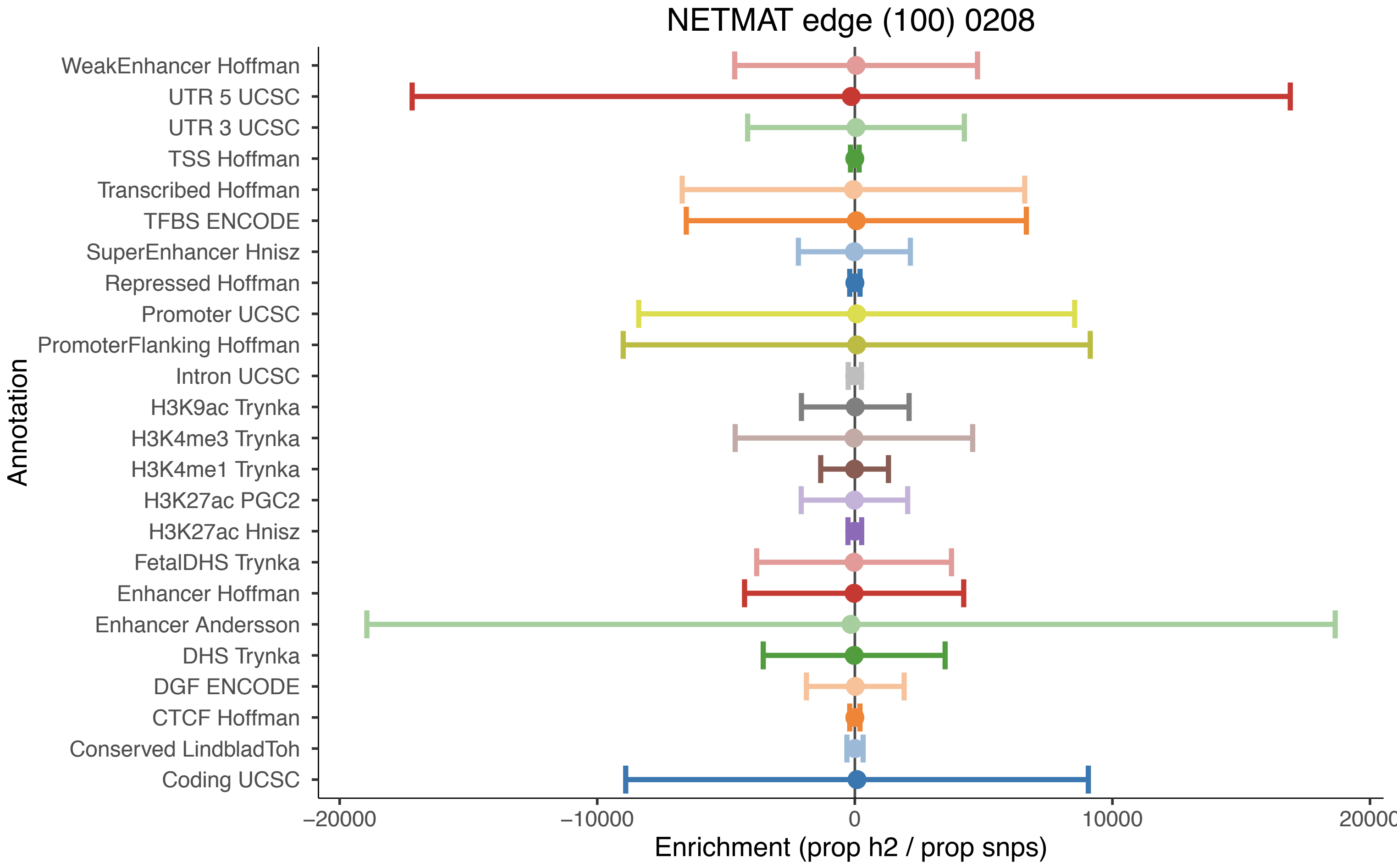
NETMAT edge (100) 0208
Annotation
WeakEnhancer Hoffman
UTR 5 UCSC
UTR 3 UCSC
TSS Hoffman
Transcribed Hoffman
TFBS ENCODE
SuperEnhancer Hnisz
Repressed Hoffman
Promoter UCSC
PromoterFlanking Hoffman
Intron UCSC
H3K9ac Trynka
H3K4me3 Trynka
H3K4me1 Trynka
H3K27ac PGC2
H3K27ac Hnisz
FetalDHS Trynka
Enhancer Hoffman
Enhancer Andersson
DHS Trynka
DGF ENCODE
CTCF Hoffman
Conserved LindbladToh
Coding UCSC
−20000
−10000
0
10000
20000
Enrichment (prop h2 / prop snps)

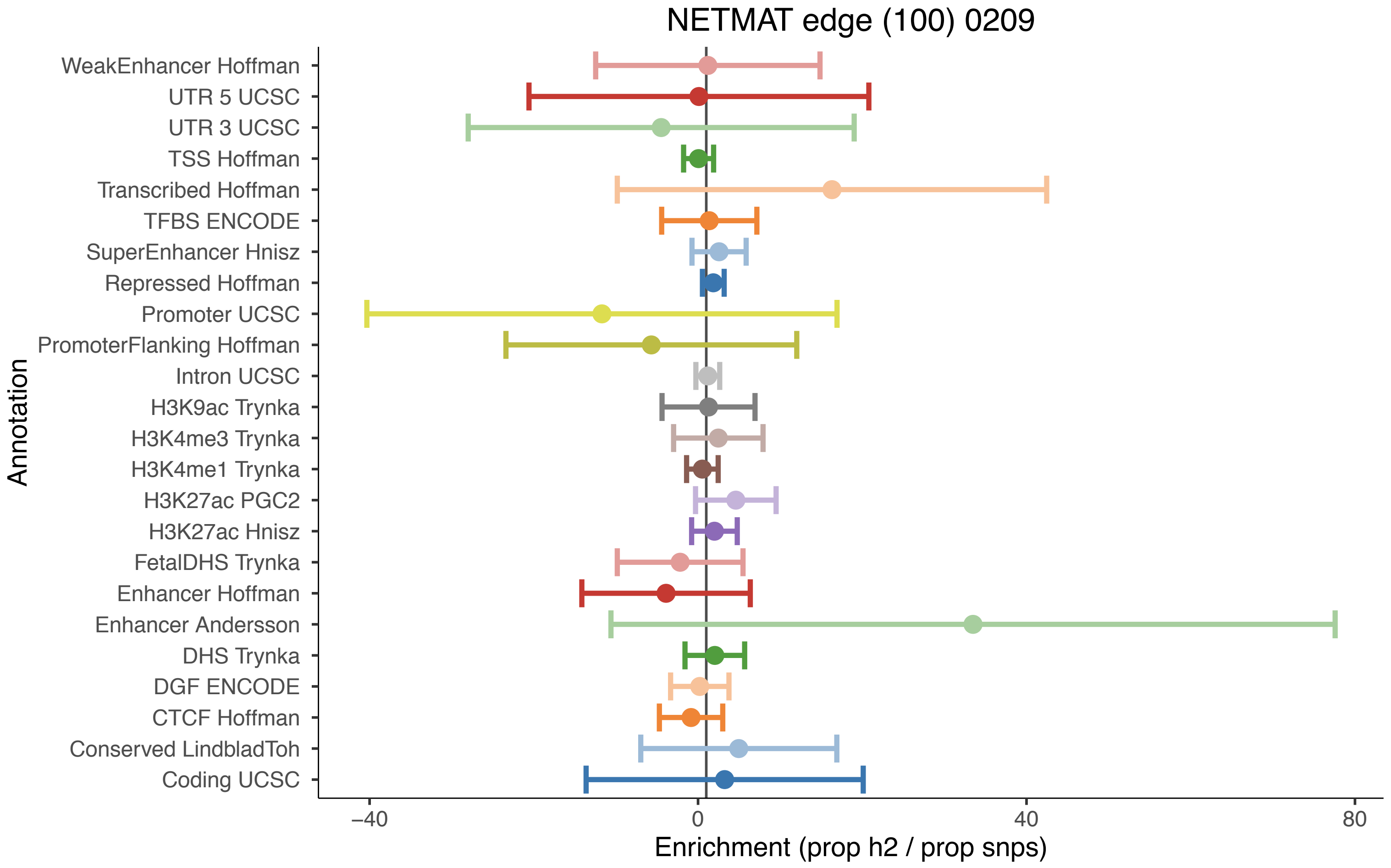

NETMAT edge (100) 0209
WeakEnhancer Hoffman
UTR 5 UCSC
UTR 3 UCSC
TSS Hoffman
Transcribed Hoffman
TFBS ENCODE
SuperEnhancer Hnisz
Repressed Hoffman
Promoter UCSC
PromoterFlanking Hoffman
Intron UCSC
H3K9ac Trynka
H3K4me3 Trynka
H3K4me1 Trynka
H3K27ac PGC2
H3K27ac Hnisz
FetalDHS Trynka
Enhancer Hoffman
Enhancer Andersson
DHS Trynka
DGF ENCODE
CTCF Hoffman
Conserved LindbladToh
Coding UCSC
Annotation
−40
0
40
80
Enrichment (prop h2 / prop snps)

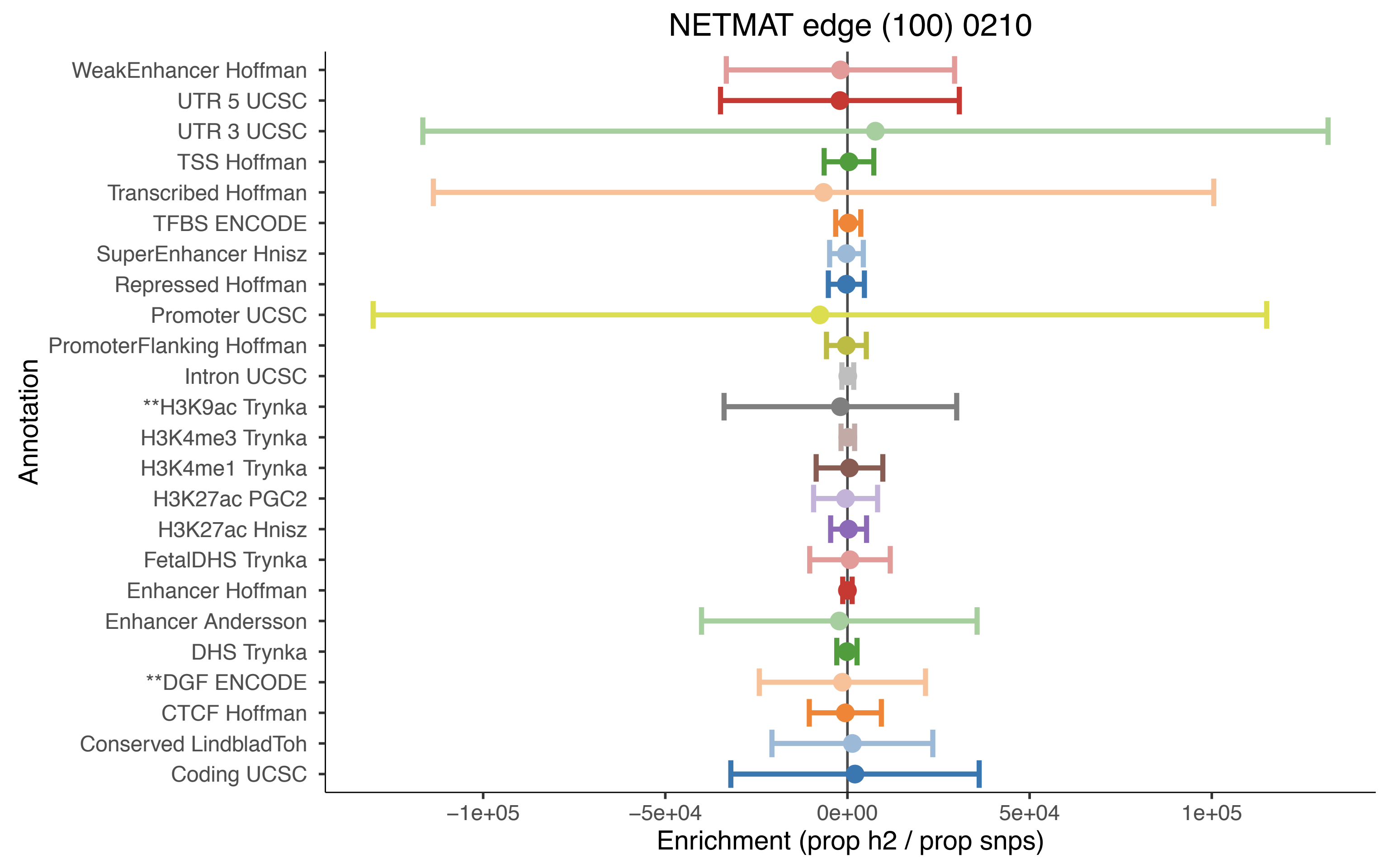
NETMAT edge (100) 0210
Annotation
WeakEnhancer Hoffman
UTR 5 UCSC
UTR 3 UCSC
TSS Hoffman
Transcribed Hoffman
TFBS ENCODE
SuperEnhancer Hnisz
Repressed Hoffman
Promoter UCSC
PromoterFlanking Hoffman
Intron UCSC
**H3K9ac Trynka
H3K4me3 Trynka
H3K4me1 Trynka
H3K27ac PGC2
H3K27ac Hnisz
FetalDHS Trynka
Enhancer Hoffman
Enhancer Andersson
DHS Trynka
**DGF ENCODE
CTCF Hoffman
Conserved LindbladToh
Coding UCSC
−1e+05
−5e+04
0e+00
5e+04
1e+05
Enrichment (prop h2 / prop snps)

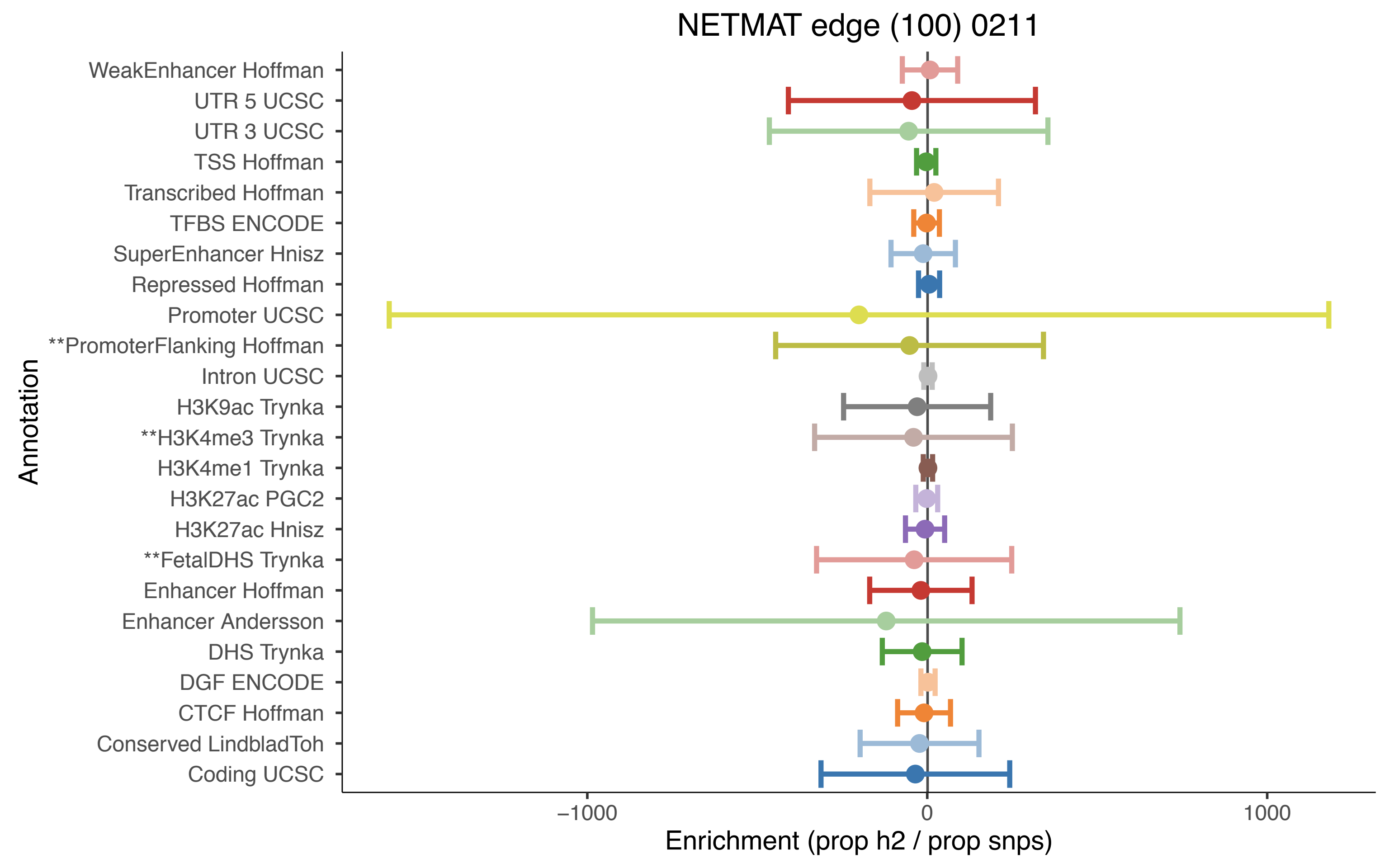
NETMAT edge (100) 0211
WeakEnhancer Hoffman
UTR 5 UCSC
UTR 3 UCSC
TSS Hoffman
Transcribed Hoffman
TFBS ENCODE
SuperEnhancer Hnisz
Repressed Hoffman
Promoter UCSC
**PromoterFlanking Hoffman
Intron UCSC
H3K9ac Trynka
**H3K4me3 Trynka
H3K4me1 Trynka
H3K27ac PGC2
H3K27ac Hnisz
**FetalDHS Trynka
Enhancer Hoffman
Enhancer Andersson
DHS Trynka
DGF ENCODE
CTCF Hoffman
Conserved LindbladToh
Coding UCSC
Annotation
−1000
0
1000
Enrichment (prop h2 / prop snps)

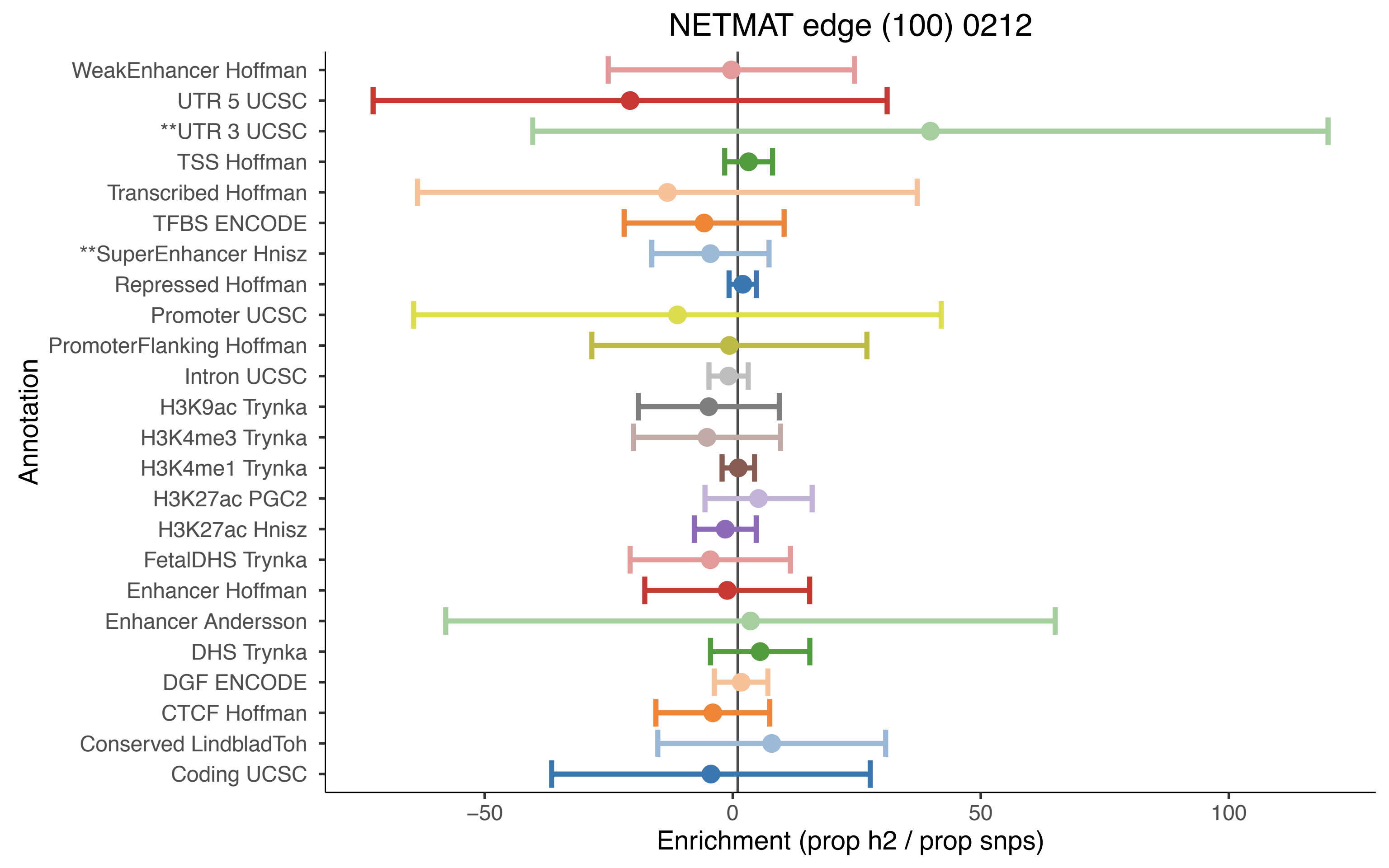
NETMAT edge (100) 0212
WeakEnhancer Hoffman
UTR 5 UCSC
**UTR 3 UCSC
TSS Hoffman
Transcribed Hoffman
TFBS ENCODE
**SuperEnhancer Hnisz
Repressed Hoffman
Promoter UCSC
PromoterFlanking Hoffman
Intron UCSC
H3K9ac Trynka
H3K4me3 Trynka
H3K4me1 Trynka
H3K27ac PGC2
H3K27ac Hnisz
FetalDHS Trynka
Enhancer Hoffman
Enhancer Andersson
DHS Trynka
DGF ENCODE
CTCF Hoffman
Conserved LindbladToh
Coding UCSC
Annotation
−50
0
50
100
Enrichment (prop h2 / prop snps)

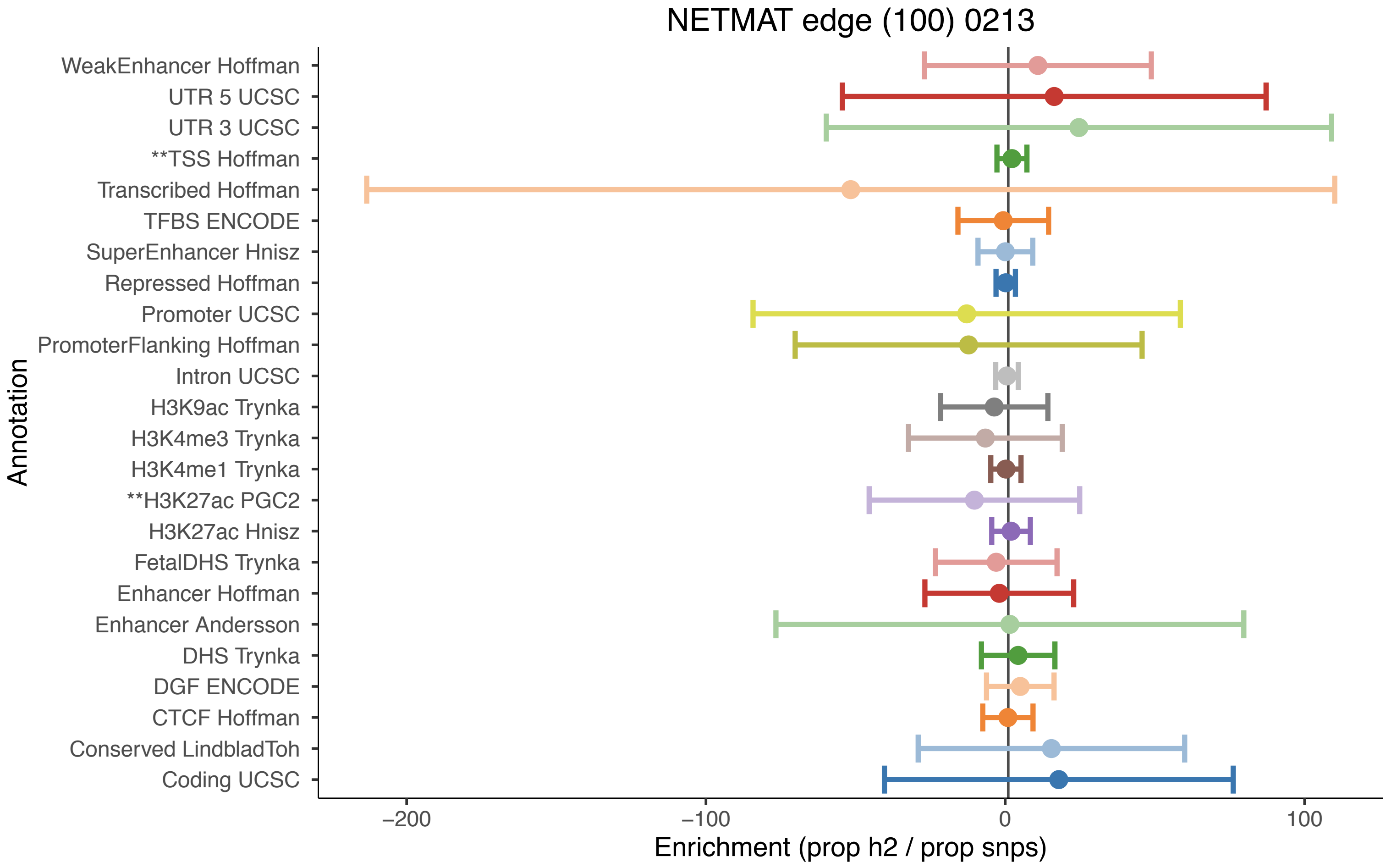
NETMAT edge (100) 0213
WeakEnhancer Hoffman
UTR 5 UCSC
UTR 3 UCSC
**TSS Hoffman
Transcribed Hoffman
TFBS ENCODE
SuperEnhancer Hnisz
Repressed Hoffman
Promoter UCSC
PromoterFlanking Hoffman
Intron UCSC
H3K9ac Trynka
H3K4me3 Trynka
H3K4me1 Trynka
**H3K27ac PGC2
H3K27ac Hnisz
FetalDHS Trynka
Enhancer Hoffman
Enhancer Andersson
DHS Trynka
DGF ENCODE
CTCF Hoffman
Conserved LindbladToh
Coding UCSC
Annotation
−200
−100
0
100
Enrichment (prop h2 / prop snps)

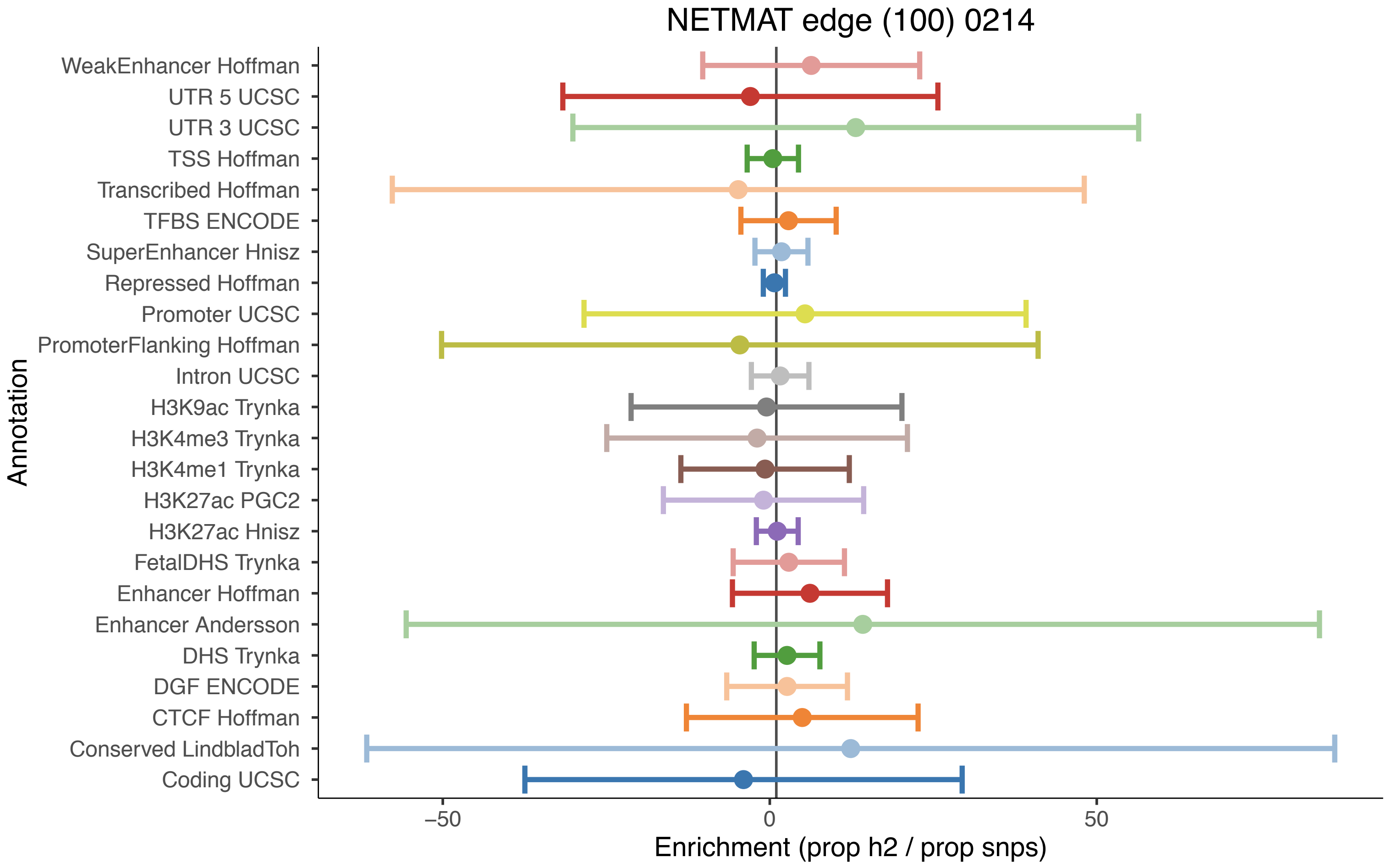
NETMAT edge (100) 0214
WeakEnhancer Hoffman
UTR 5 UCSC
UTR 3 UCSC
TSS Hoffman
Transcribed Hoffman
TFBS ENCODE
SuperEnhancer Hnisz
Repressed Hoffman
Promoter UCSC
PromoterFlanking Hoffman
Intron UCSC
H3K9ac Trynka
H3K4me3 Trynka
H3K4me1 Trynka
H3K27ac PGC2
H3K27ac Hnisz
FetalDHS Trynka
Enhancer Hoffman
Enhancer Andersson
DHS Trynka
DGF ENCODE
CTCF Hoffman
Conserved LindbladToh
Coding UCSC
Annotation
−50
0
50
Enrichment (prop h2 / prop snps)

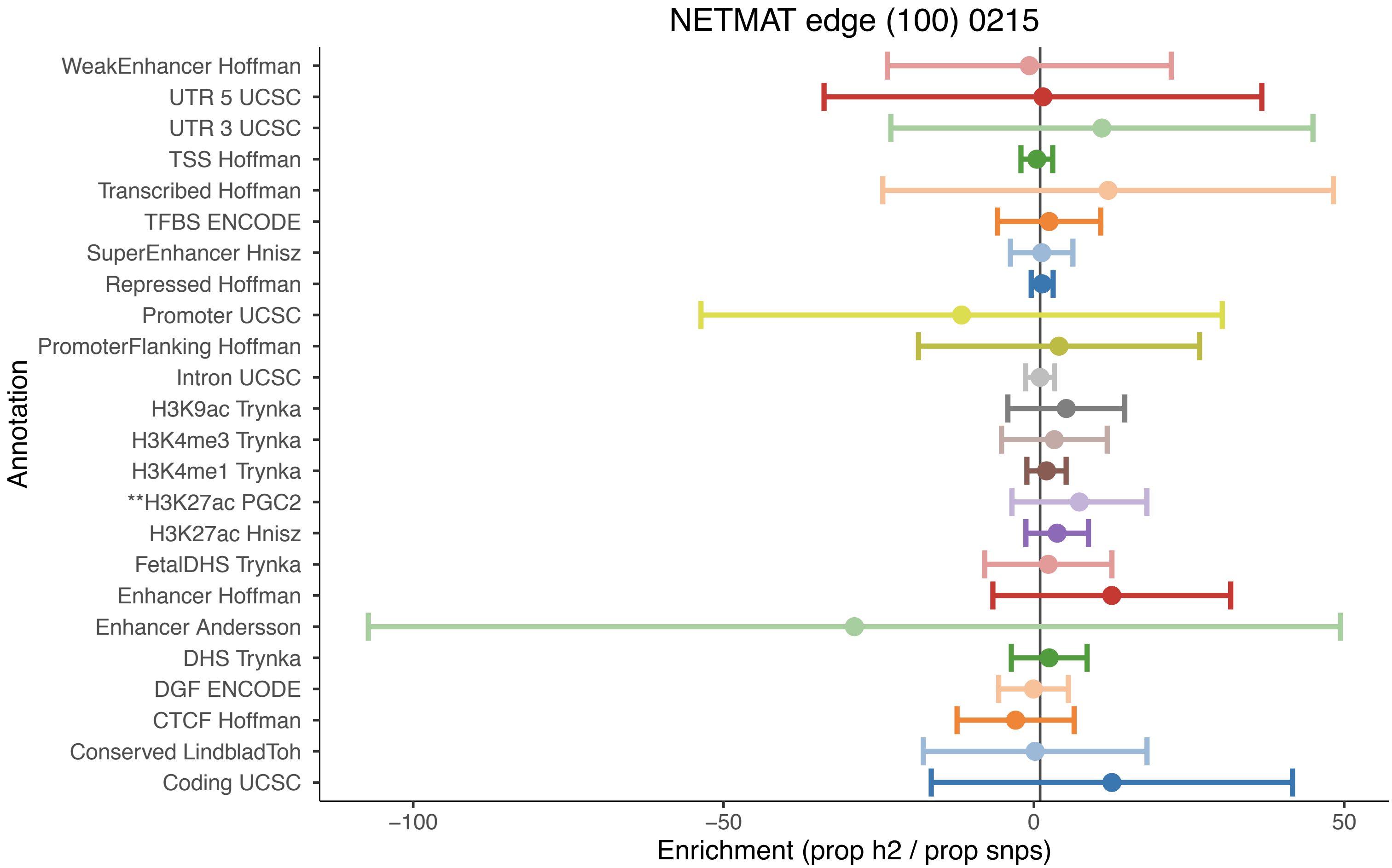
NETMAT edge (100) 0215
Annotation
WeakEnhancer Hoffman
UTR 5 UCSC
UTR 3 UCSC
TSS Hoffman
Transcribed Hoffman
TFBS ENCODE
SuperEnhancer Hnisz
Repressed Hoffman
Promoter UCSC
PromoterFlanking Hoffman
Intron UCSC
H3K9ac Trynka
H3K4me3 Trynka
H3K4me1 Trynka
**H3K27ac PGC2
H3K27ac Hnisz
FetalDHS Trynka
Enhancer Hoffman
Enhancer Andersson
DHS Trynka
DGF ENCODE
CTCF Hoffman
Conserved LindbladToh
Coding UCSC
−100
−50
0
50
Enrichment (prop h2 / prop snps)

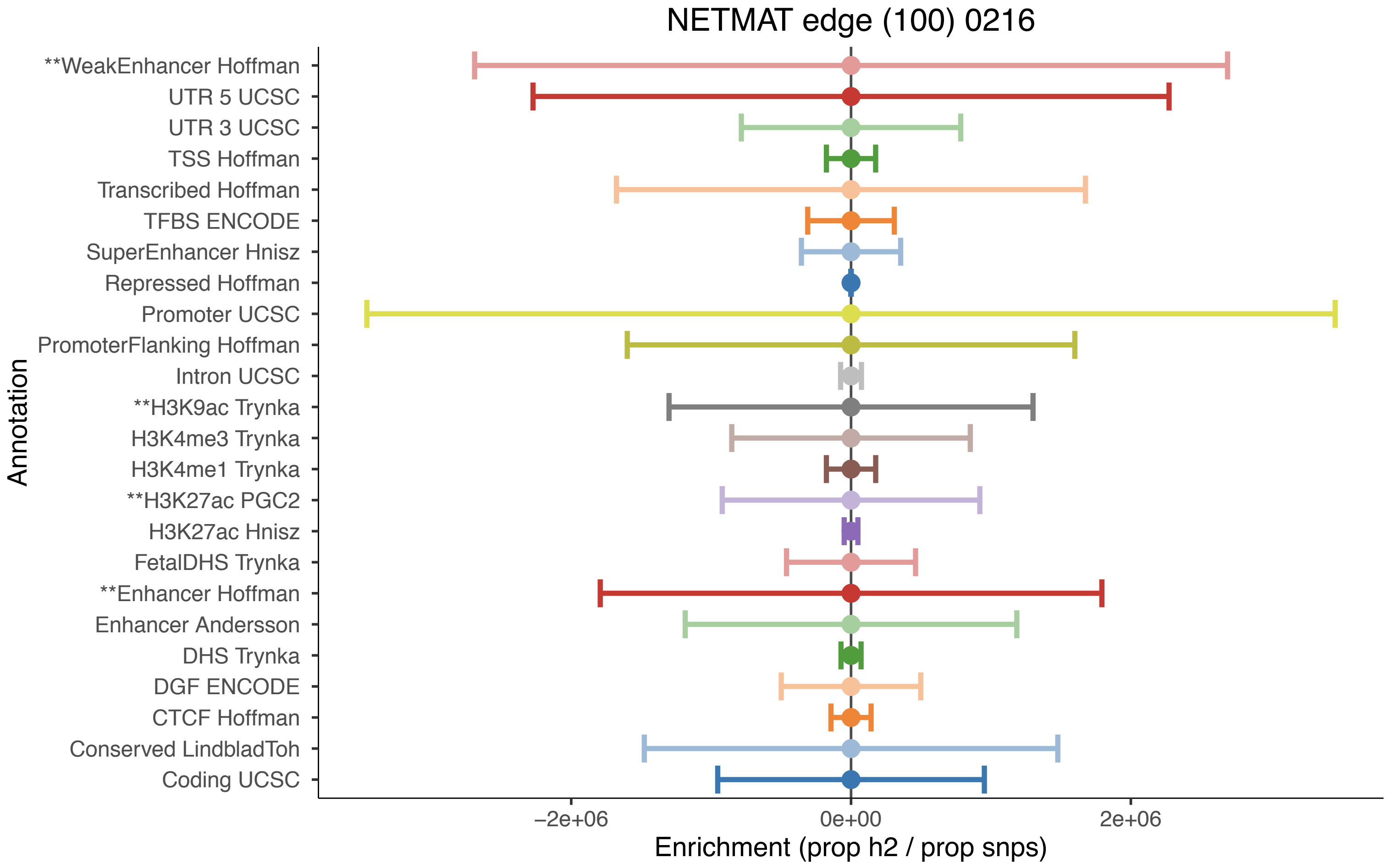

NETMAT edge (100) 0216
**WeakEnhancer Hoffman
UTR 5 UCSC
UTR 3 UCSC
TSS Hoffman
Transcribed Hoffman
TFBS ENCODE
SuperEnhancer Hnisz
Repressed Hoffman
Promoter UCSC
PromoterFlanking Hoffman
Intron UCSC
**H3K9ac Trynka
H3K4me3 Trynka
H3K4me1 Trynka
**H3K27ac PGC2
H3K27ac Hnisz
FetalDHS Trynka
**Enhancer Hoffman
Enhancer Andersson
DHS Trynka
DGF ENCODE
CTCF Hoffman
Conserved LindbladToh
Coding UCSC
Annotation
−2e+06
0e+00
2e+06
Enrichment (prop h2 / prop snps)

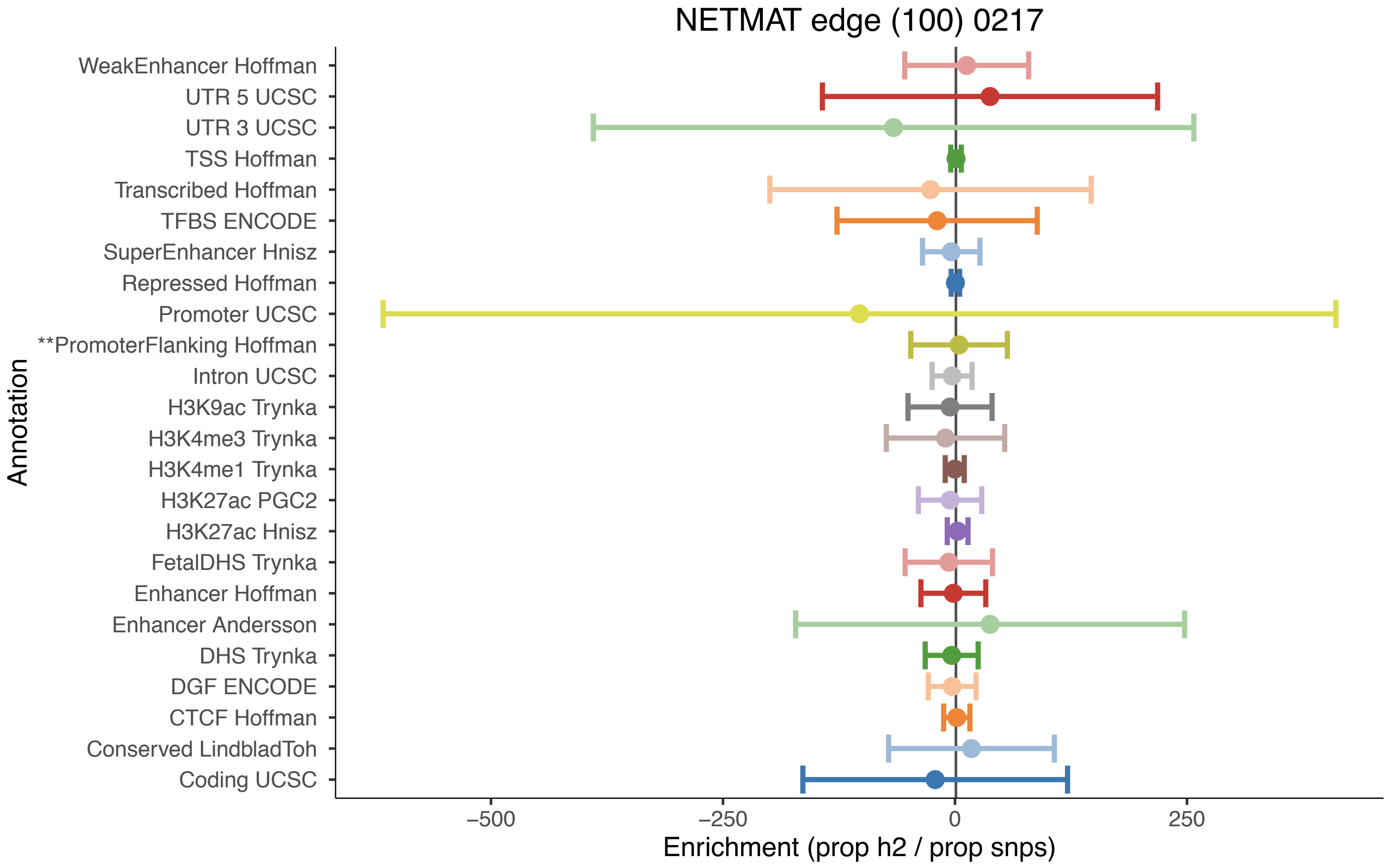
NETMAT edge (100) 0217
WeakEnhancer Hoffman
UTR 5 UCSC
UTR 3 UCSC
TSS Hoffman
Transcribed Hoffman
TFBS ENCODE
SuperEnhancer Hnisz
Repressed Hoffman
Promoter UCSC
**PromoterFlanking Hoffman
Intron UCSC
H3K9ac Trynka
H3K4me3 Trynka
H3K4me1 Trynka
H3K27ac PGC2
H3K27ac Hnisz
FetalDHS Trynka
Enhancer Hoffman
Enhancer Andersson
DHS Trynka
DGF ENCODE
CTCF Hoffman
Conserved LindbladToh
Coding UCSC
Annotation
−500
−250
0
250
Enrichment (prop h2 / prop snps)

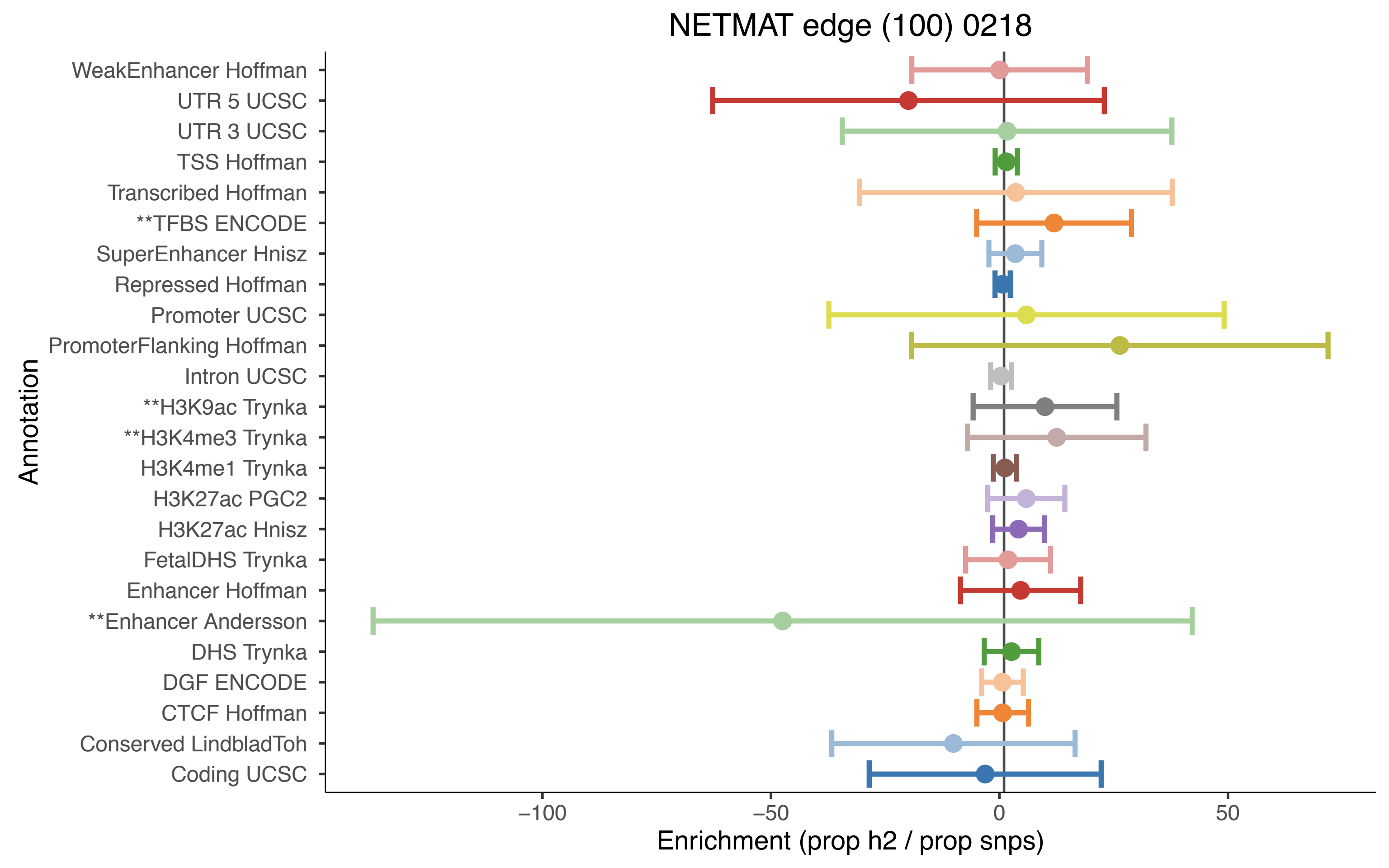

NETMAT edge (100) 0218
WeakEnhancer Hoffman
UTR 5 UCSC
UTR 3 UCSC
TSS Hoffman
Transcribed Hoffman
**TFBS ENCODE
SuperEnhancer Hnisz
Repressed Hoffman
Promoter UCSC
PromoterFlanking Hoffman
Intron UCSC
**H3K9ac Trynka
**H3K4me3 Trynka
H3K4me1 Trynka
H3K27ac PGC2
H3K27ac Hnisz
FetalDHS Trynka
Enhancer Hoffman
**Enhancer Andersson
DHS Trynka
DGF ENCODE
CTCF Hoffman
Conserved LindbladToh
Coding UCSC
Annotation
−100
−50
0
50
Enrichment (prop h2 / prop snps)

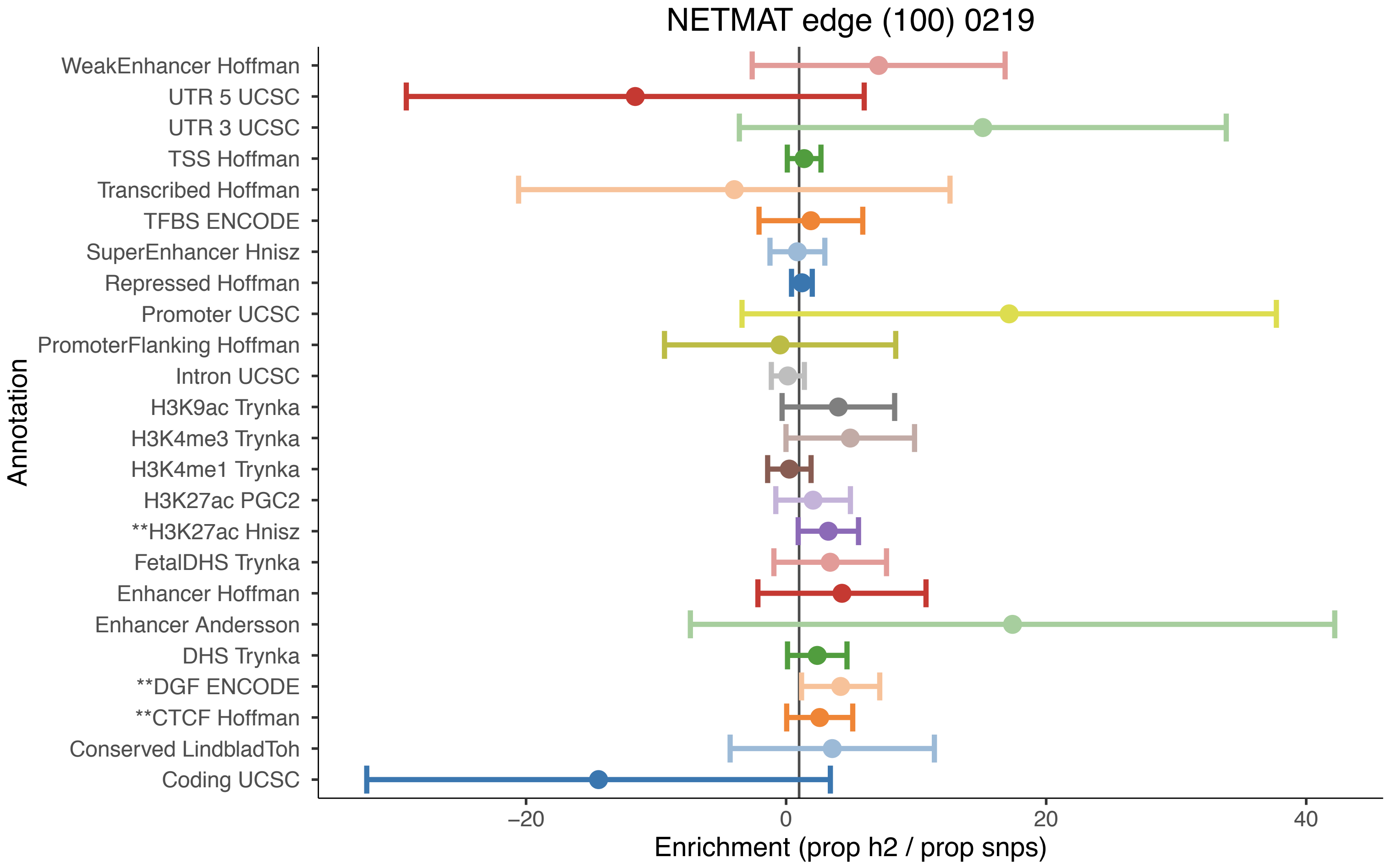
NETMAT edge (100) 0219
WeakEnhancer Hoffman
UTR 5 UCSC
UTR 3 UCSC
TSS Hoffman
Transcribed Hoffman
TFBS ENCODE
SuperEnhancer Hnisz
Repressed Hoffman
Promoter UCSC
PromoterFlanking Hoffman
Intron UCSC
H3K9ac Trynka
H3K4me3 Trynka
H3K4me1 Trynka
H3K27ac PGC2
**H3K27ac Hnisz
FetalDHS Trynka
Enhancer Hoffman
Enhancer Andersson
DHS Trynka
**DGF ENCODE
**CTCF Hoffman
Conserved LindbladToh
Coding UCSC
Annotation
−20
0
20
40
Enrichment (prop h2 / prop snps)

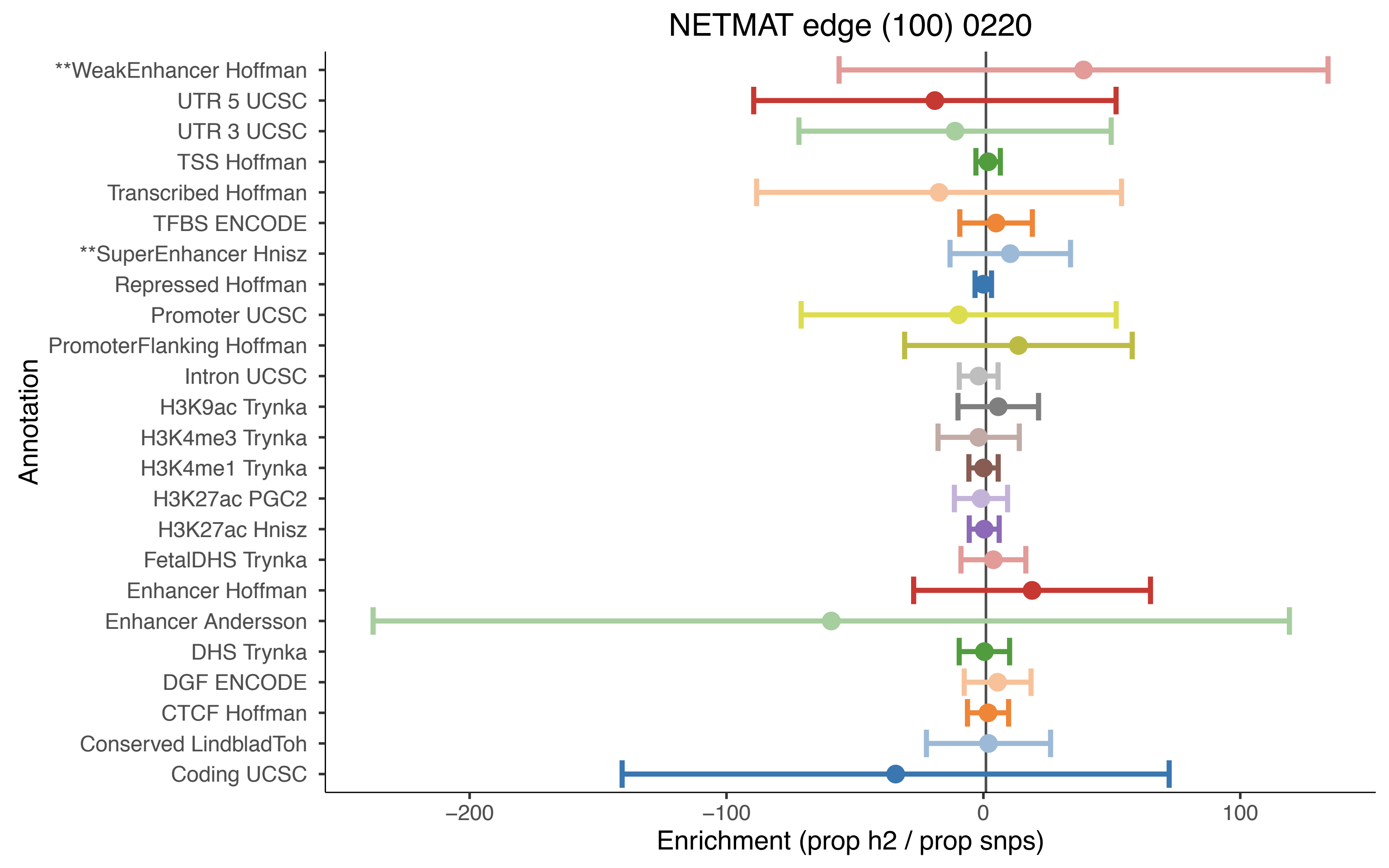

NETMAT edge (100) 0220
**WeakEnhancer Hoffman
UTR 5 UCSC
UTR 3 UCSC
TSS Hoffman
Transcribed Hoffman
TFBS ENCODE
**SuperEnhancer Hnisz
Repressed Hoffman
Promoter UCSC
PromoterFlanking Hoffman
Intron UCSC
H3K9ac Trynka
H3K4me3 Trynka
H3K4me1 Trynka
H3K27ac PGC2
H3K27ac Hnisz
FetalDHS Trynka
Enhancer Hoffman
Enhancer Andersson
DHS Trynka
DGF ENCODE
CTCF Hoffman
Conserved LindbladToh
Coding UCSC
Annotation
−200
−100
0
100
Enrichment (prop h2 / prop snps)

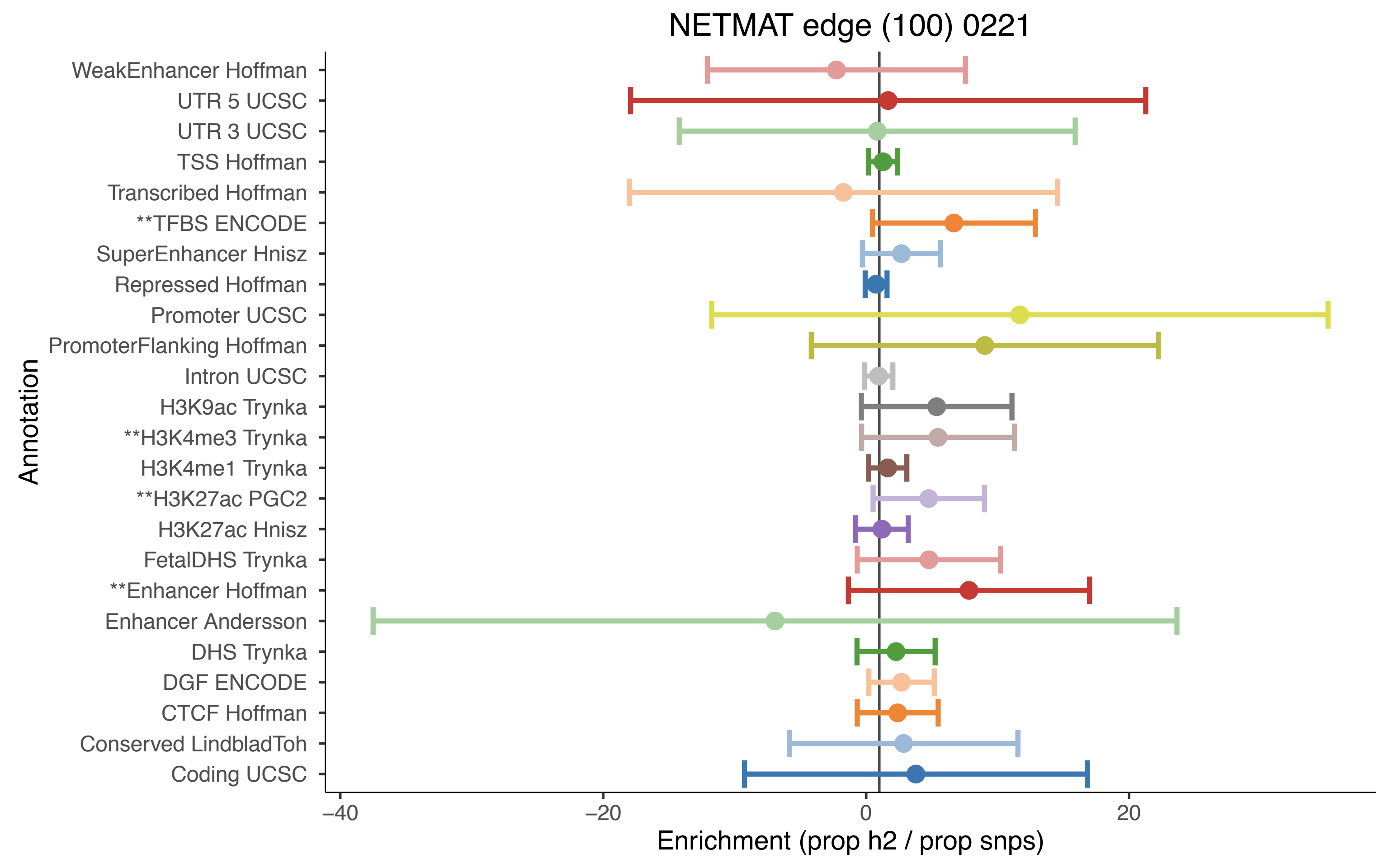

NETMAT edge (100) 0221
WeakEnhancer Hoffman
UTR 5 UCSC
UTR 3 UCSC
TSS Hoffman
Transcribed Hoffman
**TFBS ENCODE
SuperEnhancer Hnisz
Repressed Hoffman
Promoter UCSC
PromoterFlanking Hoffman
Intron UCSC
H3K9ac Trynka
**H3K4me3 Trynka
H3K4me1 Trynka
**H3K27ac PGC2
H3K27ac Hnisz
FetalDHS Trynka
**Enhancer Hoffman
Enhancer Andersson
DHS Trynka
DGF ENCODE
CTCF Hoffman
Conserved LindbladToh
Coding UCSC
Annotation
−40
−20
0
20
Enrichment (prop h2 / prop snps)

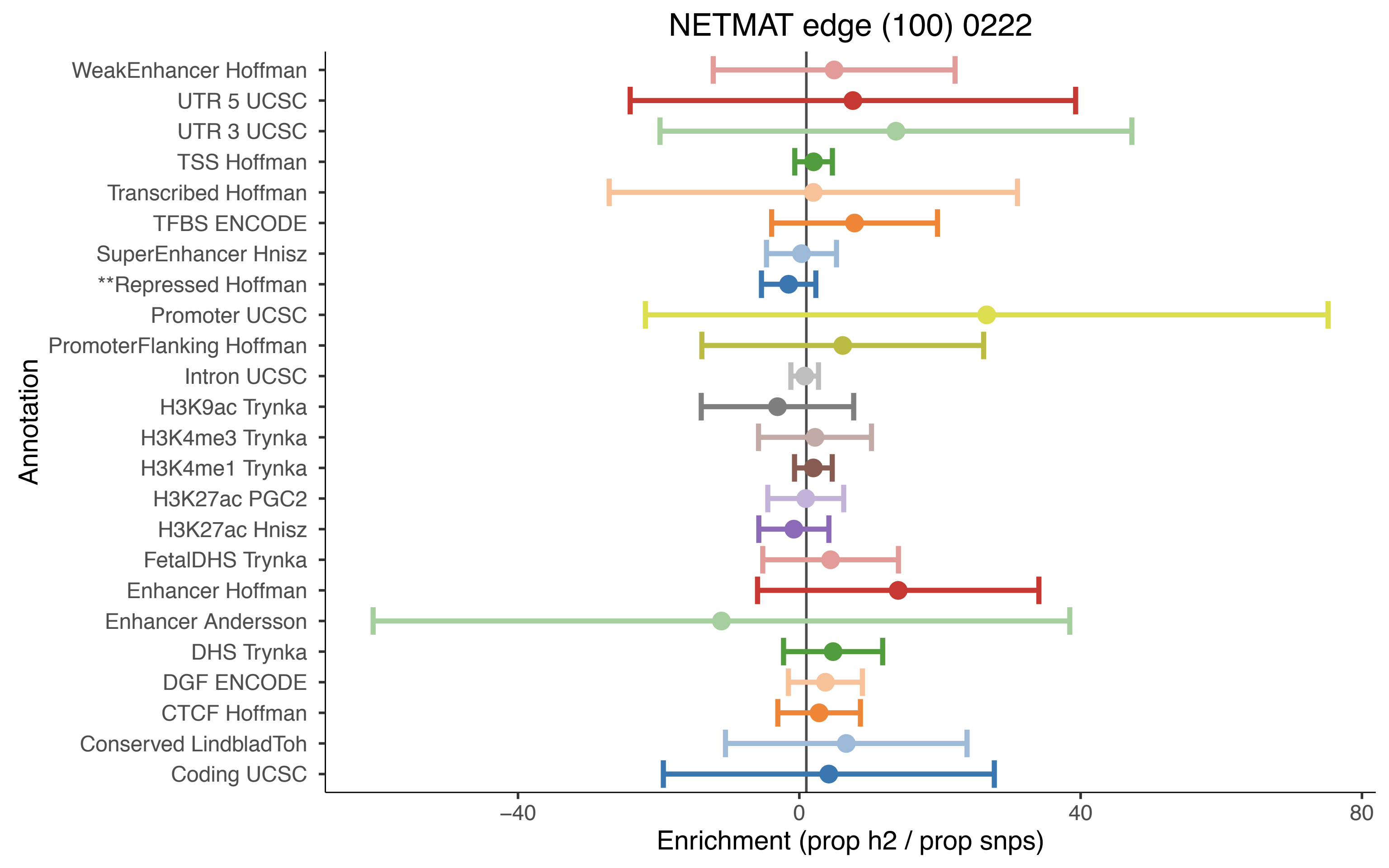
NETMAT edge (100) 0222
WeakEnhancer Hoffman
UTR 5 UCSC
UTR 3 UCSC
TSS Hoffman
Transcribed Hoffman
TFBS ENCODE
SuperEnhancer Hnisz
**Repressed Hoffman
Promoter UCSC
PromoterFlanking Hoffman
Intron UCSC
H3K9ac Trynka
H3K4me3 Trynka
H3K4me1 Trynka
H3K27ac PGC2
H3K27ac Hnisz
FetalDHS Trynka
Enhancer Hoffman
Enhancer Andersson
DHS Trynka
DGF ENCODE
CTCF Hoffman
Conserved LindbladToh
Coding UCSC
Annotation
−40
0
40
80
Enrichment (prop h2 / prop snps)

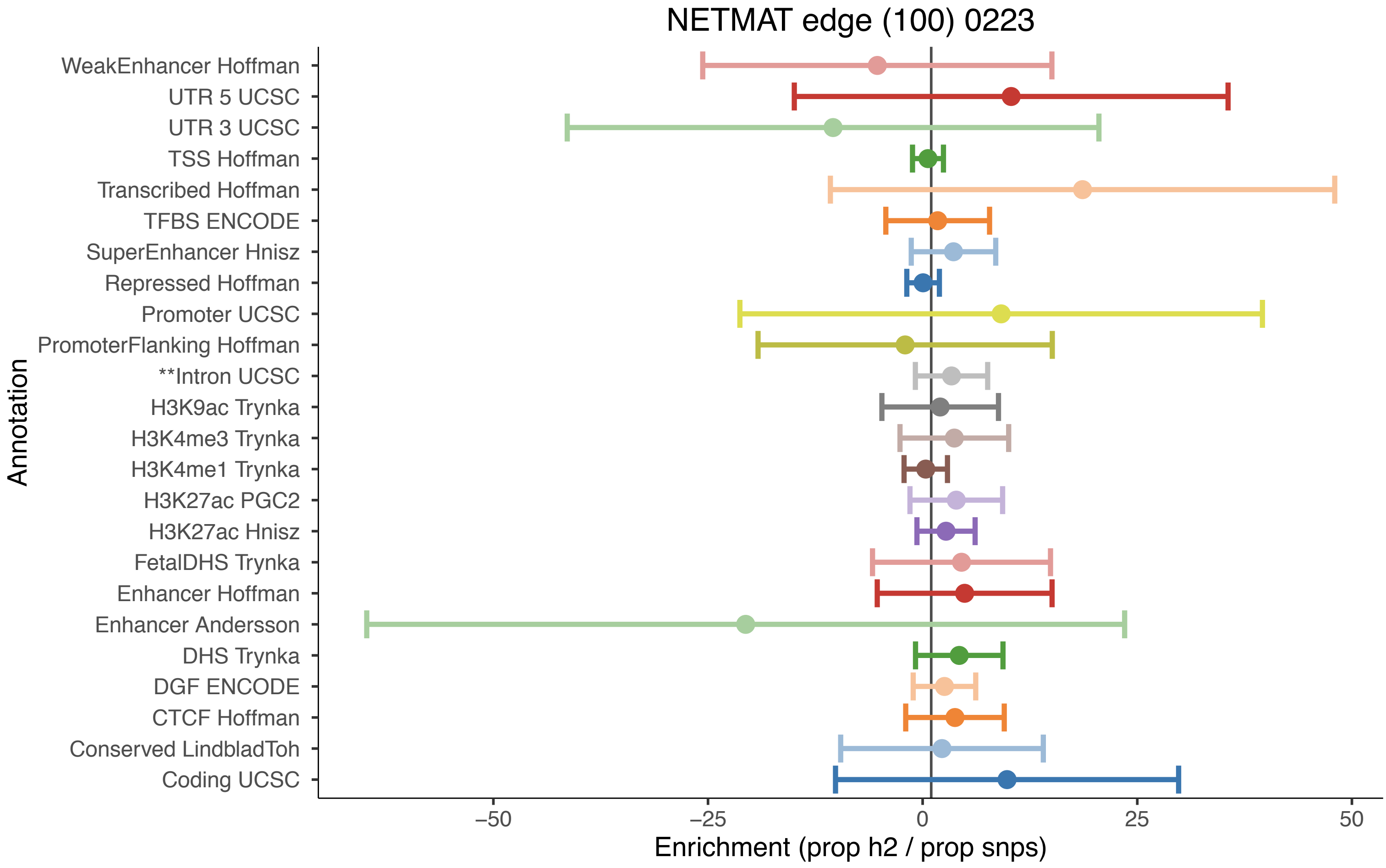

NETMAT edge (100) 0223
WeakEnhancer Hoffman
UTR 5 UCSC
UTR 3 UCSC
TSS Hoffman
Transcribed Hoffman
TFBS ENCODE
SuperEnhancer Hnisz
Repressed Hoffman
Promoter UCSC
PromoterFlanking Hoffman
**Intron UCSC
H3K9ac Trynka
H3K4me3 Trynka
H3K4me1 Trynka
H3K27ac PGC2
H3K27ac Hnisz
FetalDHS Trynka
Enhancer Hoffman
Enhancer Andersson
DHS Trynka
DGF ENCODE
CTCF Hoffman
Conserved LindbladToh
Coding UCSC
Annotation
−50
−25
0
25
50
Enrichment (prop h2 / prop snps)

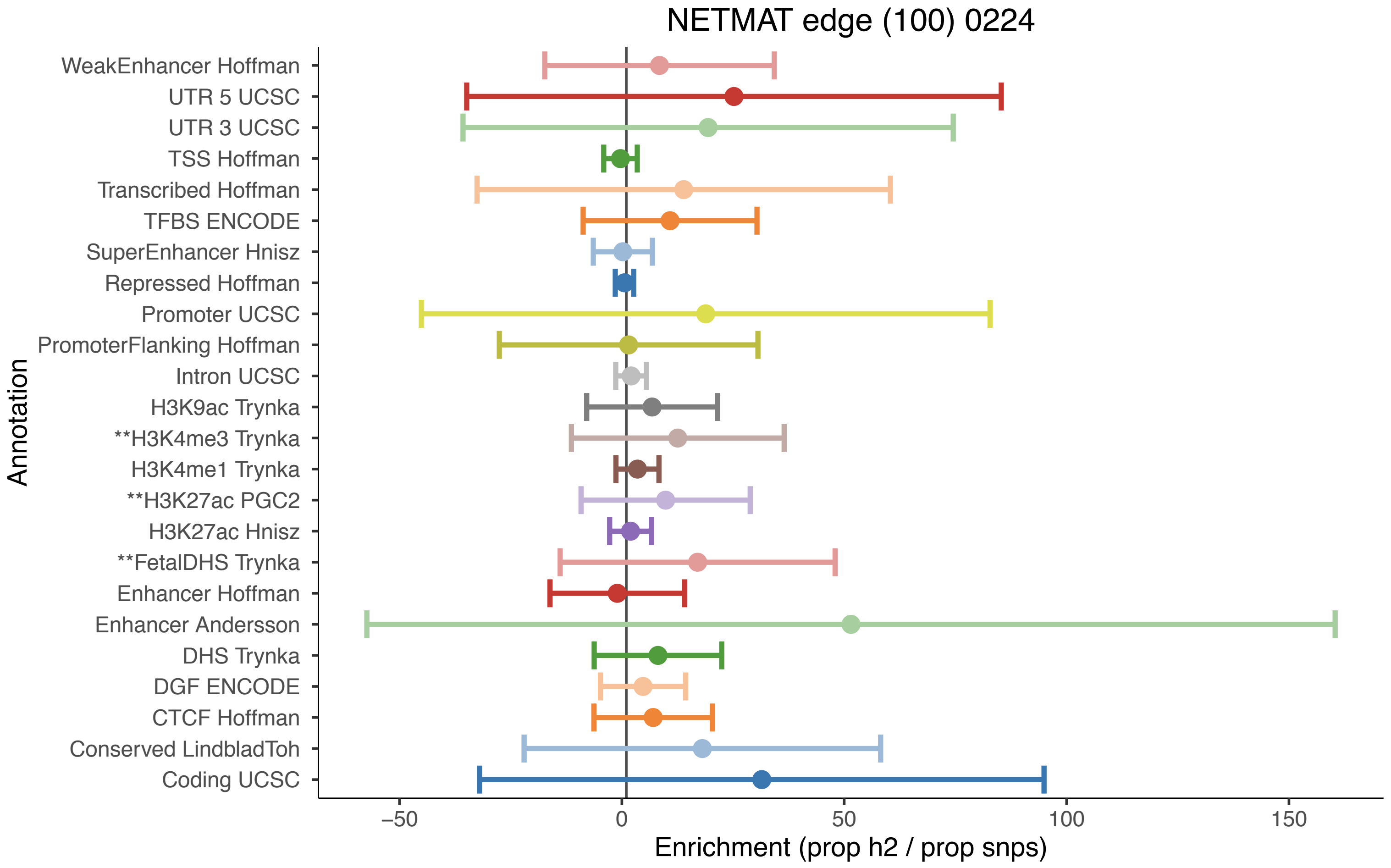

NETMAT edge (100) 0224
Annotation
WeakEnhancer Hoffman
UTR 5 UCSC
UTR 3 UCSC
TSS Hoffman
Transcribed Hoffman
TFBS ENCODE
SuperEnhancer Hnisz
Repressed Hoffman
Promoter UCSC
PromoterFlanking Hoffman
Intron UCSC
H3K9ac Trynka
**H3K4me3 Trynka
H3K4me1 Trynka
**H3K27ac PGC2
H3K27ac Hnisz
**FetalDHS Trynka
Enhancer Hoffman
Enhancer Andersson
DHS Trynka
DGF ENCODE
CTCF Hoffman
Conserved LindbladToh
Coding UCSC
−50
0
50
100
150
Enrichment (prop h2 / prop snps)

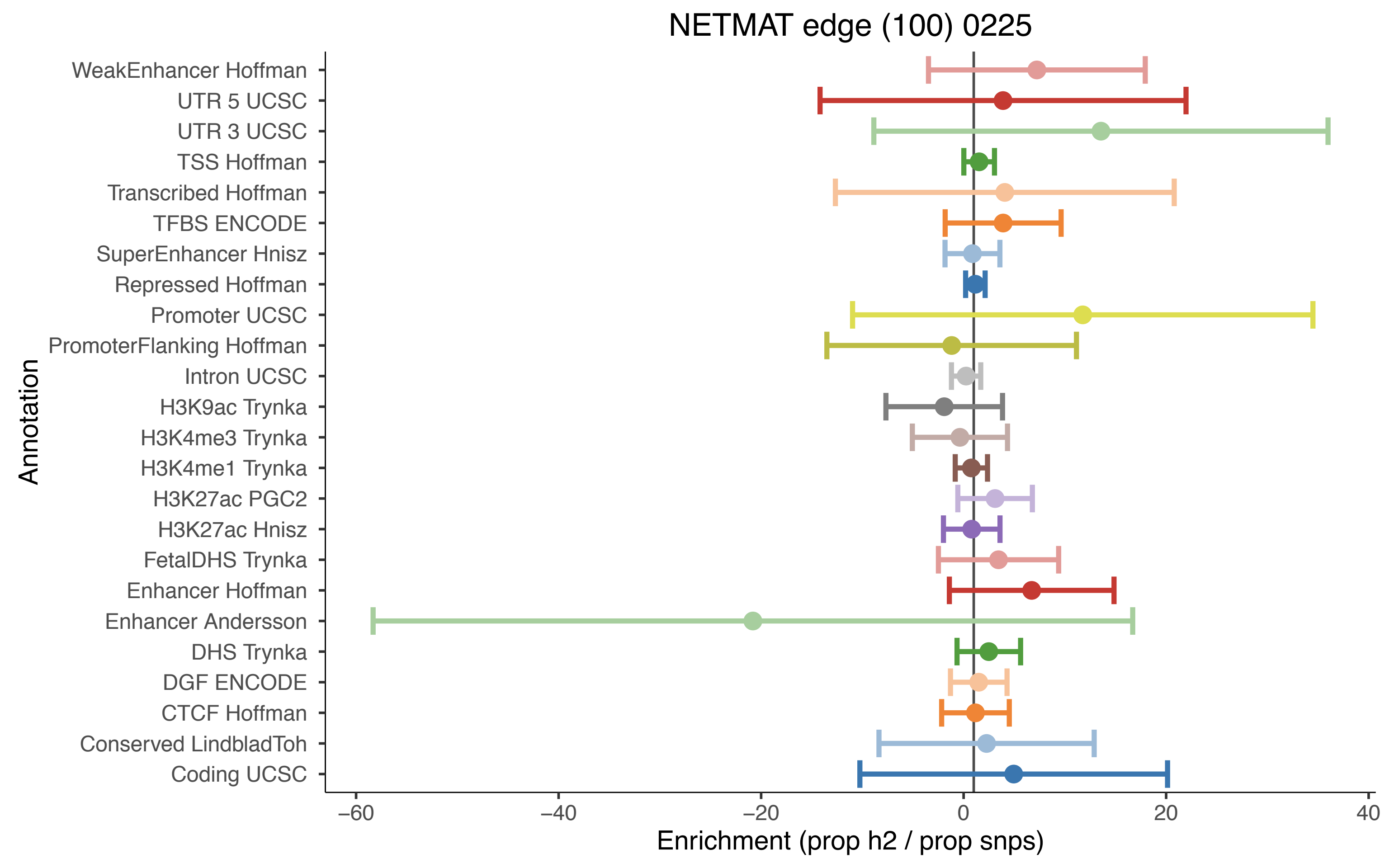
NETMAT edge (100) 0225
WeakEnhancer Hoffman
UTR 5 UCSC
UTR 3 UCSC
TSS Hoffman
Transcribed Hoffman
TFBS ENCODE
SuperEnhancer Hnisz
Repressed Hoffman
Promoter UCSC
PromoterFlanking Hoffman
Intron UCSC
H3K9ac Trynka
H3K4me3 Trynka
H3K4me1 Trynka
H3K27ac PGC2
H3K27ac Hnisz
FetalDHS Trynka
Enhancer Hoffman
Enhancer Andersson
DHS Trynka
DGF ENCODE
CTCF Hoffman
Conserved LindbladToh
Coding UCSC
Annotation
−60
−40
−20
0
20
40
Enrichment (prop h2 / prop snps)

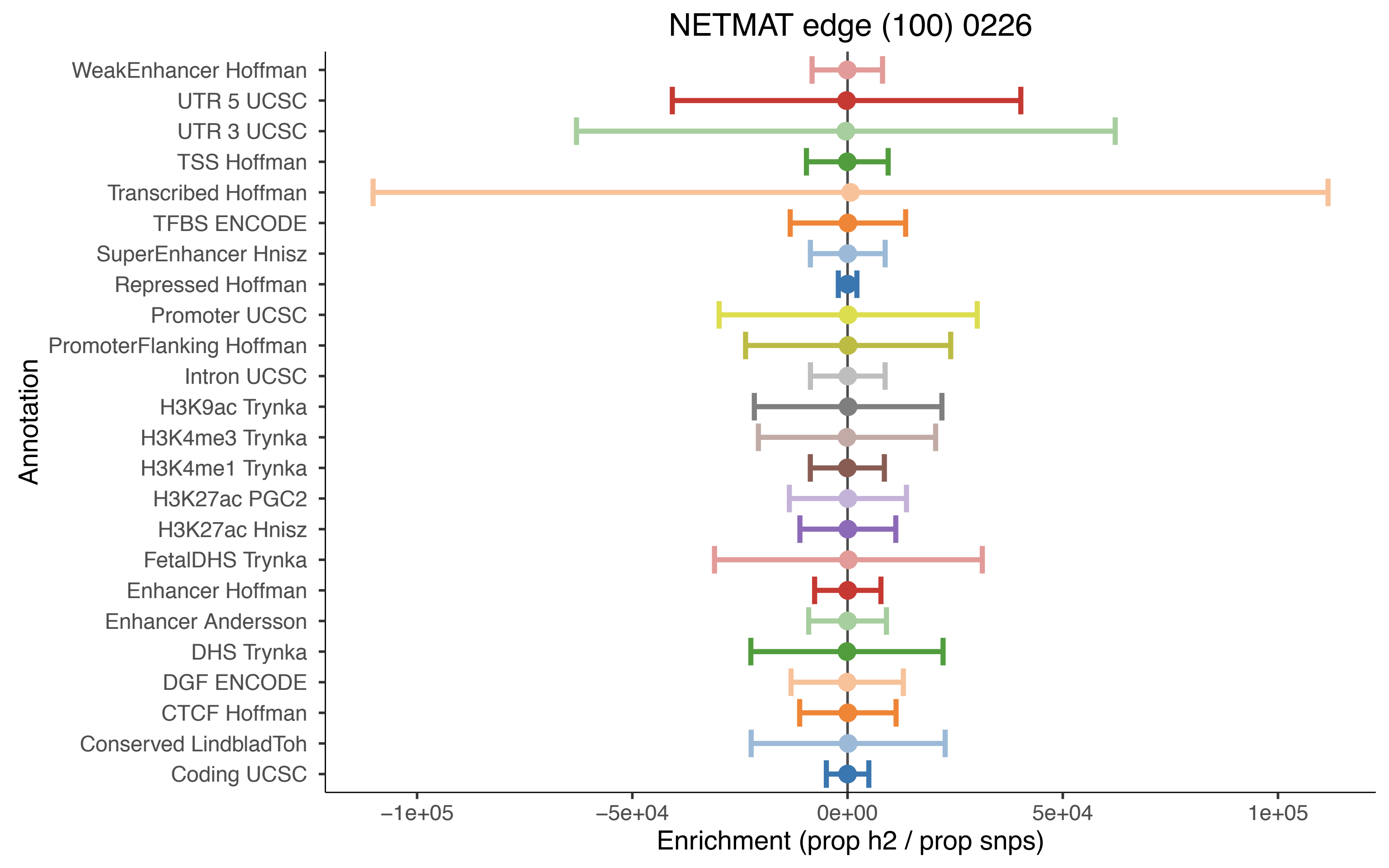

NETMAT edge (100) 0226
WeakEnhancer Hoffman
UTR 5 UCSC
UTR 3 UCSC
TSS Hoffman
Transcribed Hoffman
TFBS ENCODE
SuperEnhancer Hnisz
Repressed Hoffman
Promoter UCSC
PromoterFlanking Hoffman
Intron UCSC
H3K9ac Trynka
H3K4me3 Trynka
H3K4me1 Trynka
H3K27ac PGC2
H3K27ac Hnisz
FetalDHS Trynka
Enhancer Hoffman
Enhancer Andersson
DHS Trynka
DGF ENCODE
CTCF Hoffman
Conserved LindbladToh
Coding UCSC
Annotation
−1e+05
−5e+04
0e+00
5e+04
1e+05
Enrichment (prop h2 / prop snps)

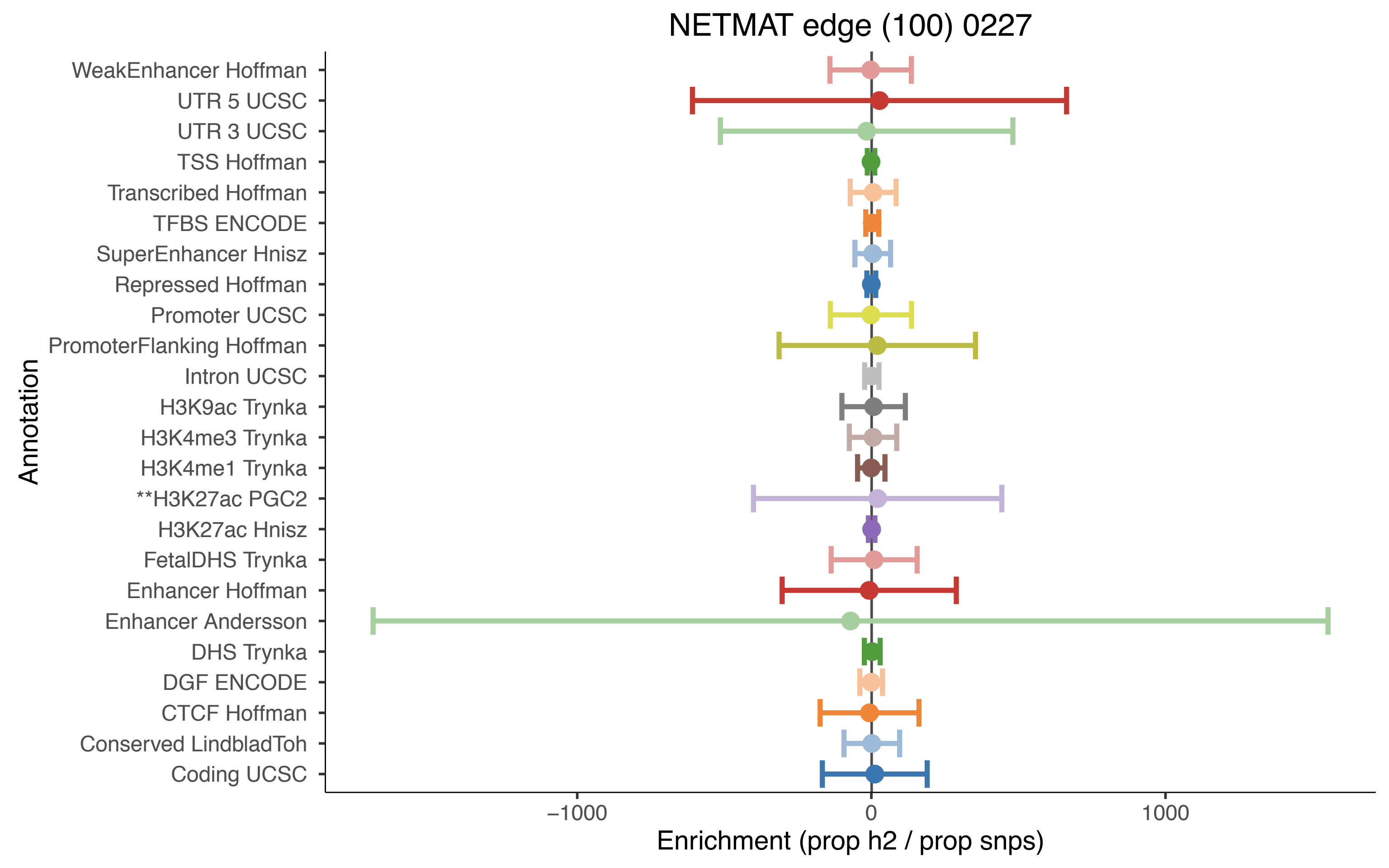
NETMAT edge (100) 0227
Annotation
WeakEnhancer Hoffman
UTR 5 UCSC
UTR 3 UCSC
TSS Hoffman
Transcribed Hoffman
TFBS ENCODE
SuperEnhancer Hnisz
Repressed Hoffman
Promoter UCSC
PromoterFlanking Hoffman
Intron UCSC
H3K9ac Trynka
H3K4me3 Trynka
H3K4me1 Trynka
**H3K27ac PGC2
H3K27ac Hnisz
FetalDHS Trynka
Enhancer Hoffman
Enhancer Andersson
DHS Trynka
DGF ENCODE
CTCF Hoffman
Conserved LindbladToh
Coding UCSC
−1000
0
1000
Enrichment (prop h2 / prop snps)

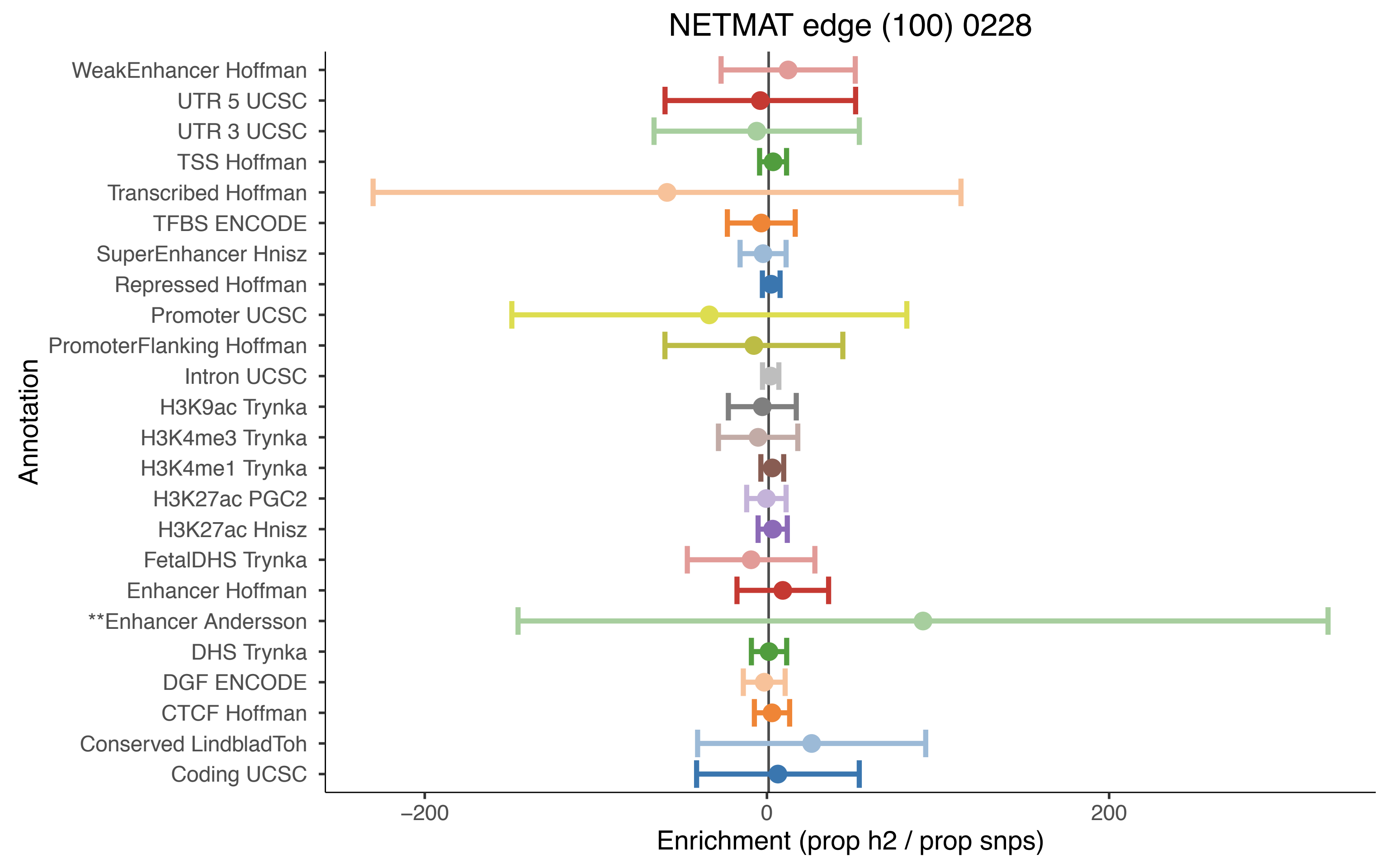
NETMAT edge (100) 0228
WeakEnhancer Hoffman
UTR 5 UCSC
UTR 3 UCSC
TSS Hoffman
Transcribed Hoffman
TFBS ENCODE
SuperEnhancer Hnisz
Repressed Hoffman
Promoter UCSC
PromoterFlanking Hoffman
Intron UCSC
H3K9ac Trynka
H3K4me3 Trynka
H3K4me1 Trynka
H3K27ac PGC2
H3K27ac Hnisz
FetalDHS Trynka
Enhancer Hoffman
**Enhancer Andersson
DHS Trynka
DGF ENCODE
CTCF Hoffman
Conserved LindbladToh
Coding UCSC
Annotation
−200
0
200
Enrichment (prop h2 / prop snps)

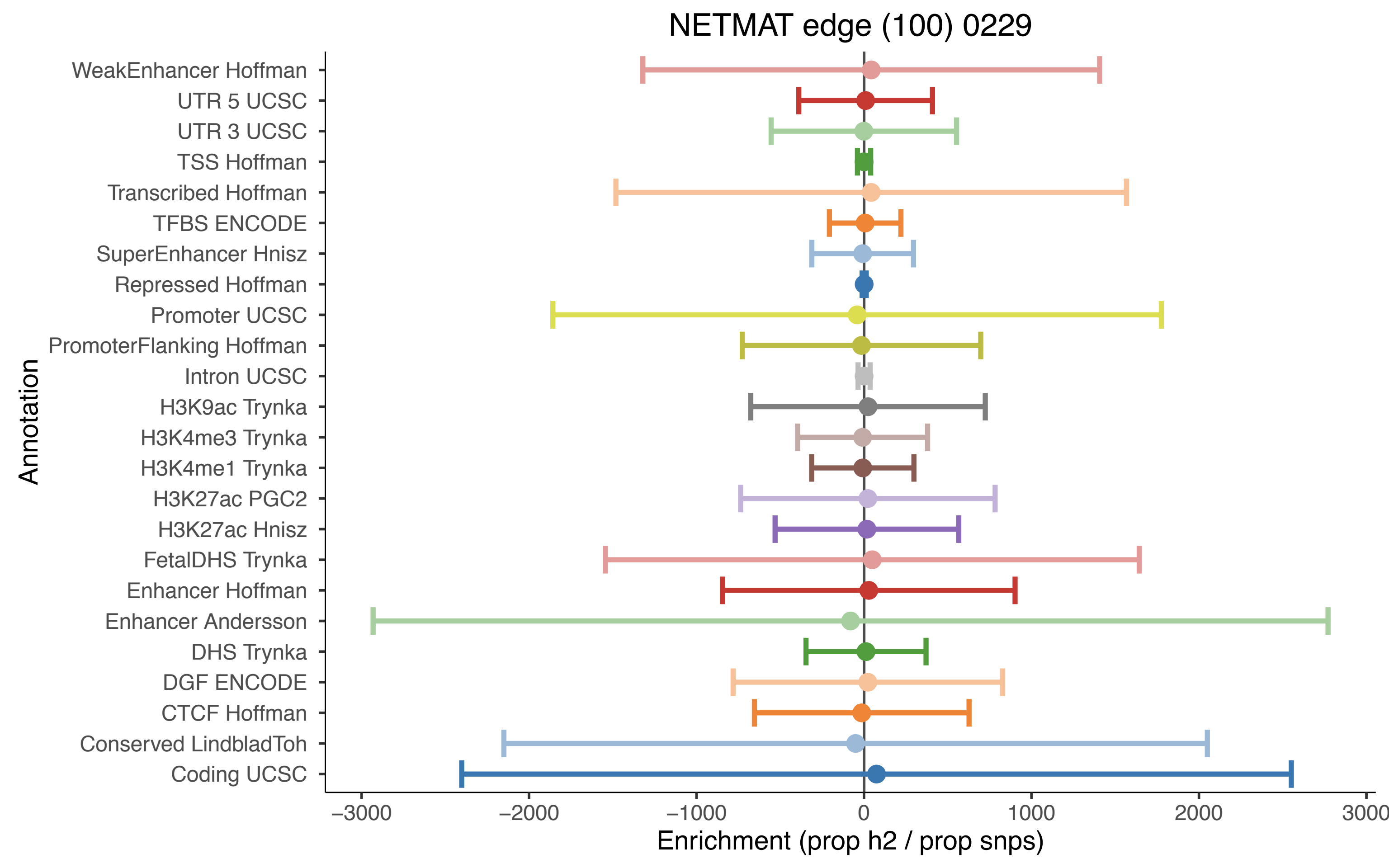
NETMAT edge (100) 0229
WeakEnhancer Hoffman
UTR 5 UCSC
UTR 3 UCSC
TSS Hoffman
Transcribed Hoffman
TFBS ENCODE
SuperEnhancer Hnisz
Repressed Hoffman
Promoter UCSC
PromoterFlanking Hoffman
Intron UCSC
H3K9ac Trynka
H3K4me3 Trynka
H3K4me1 Trynka
H3K27ac PGC2
H3K27ac Hnisz
FetalDHS Trynka
Enhancer Hoffman
Enhancer Andersson
DHS Trynka
DGF ENCODE
CTCF Hoffman
Conserved LindbladToh
Coding UCSC
Annotation
−3000
−2000
−1000
0
1000
2000
3000
Enrichment (prop h2 / prop snps)

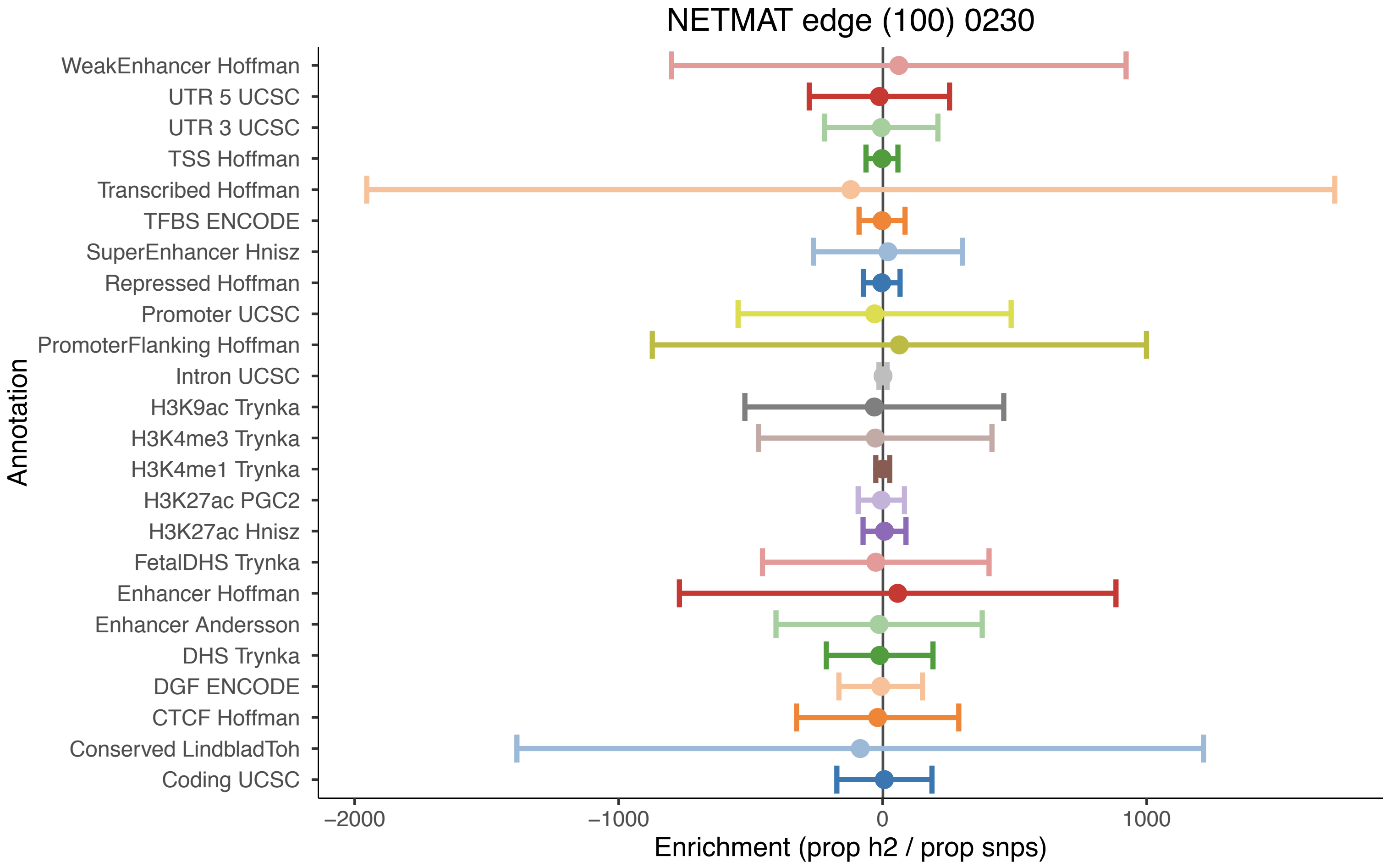
NETMAT edge (100) 0230
Annotation
WeakEnhancer Hoffman
UTR 5 UCSC
UTR 3 UCSC
TSS Hoffman
Transcribed Hoffman
TFBS ENCODE
SuperEnhancer Hnisz
Repressed Hoffman
Promoter UCSC
PromoterFlanking Hoffman
Intron UCSC
H3K9ac Trynka
H3K4me3 Trynka
H3K4me1 Trynka
H3K27ac PGC2
H3K27ac Hnisz
FetalDHS Trynka
Enhancer Hoffman
Enhancer Andersson
DHS Trynka
DGF ENCODE
CTCF Hoffman
Conserved LindbladToh
Coding UCSC
−2000
−1000
0
1000
Enrichment (prop h2 / prop snps)

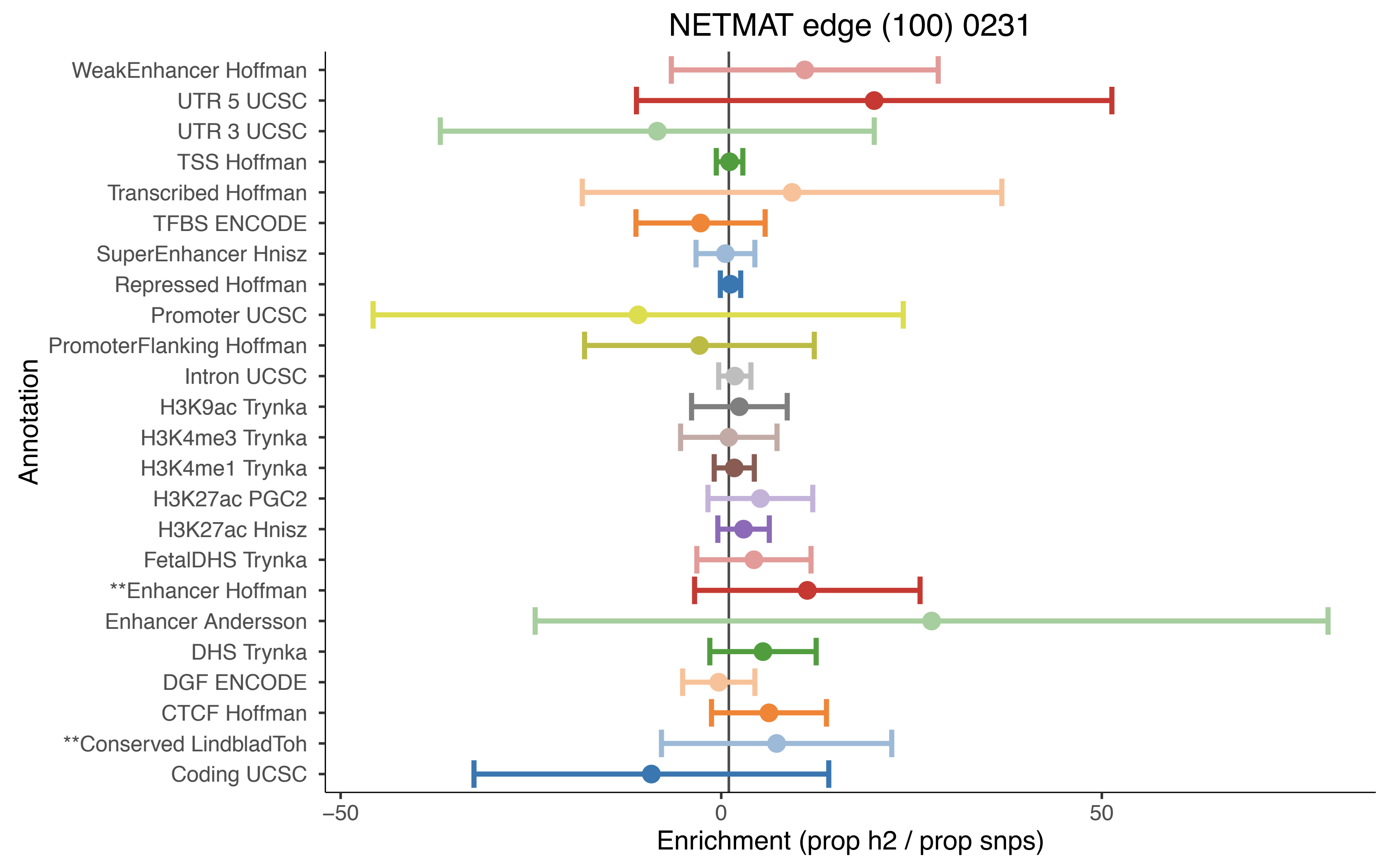

NETMAT edge (100) 0231
Annotation
WeakEnhancer Hoffman
UTR 5 UCSC
UTR 3 UCSC
TSS Hoffman
Transcribed Hoffman
TFBS ENCODE
SuperEnhancer Hnisz
Repressed Hoffman
Promoter UCSC
PromoterFlanking Hoffman
Intron UCSC
H3K9ac Trynka
H3K4me3 Trynka
H3K4me1 Trynka
H3K27ac PGC2
H3K27ac Hnisz
FetalDHS Trynka
**Enhancer Hoffman
Enhancer Andersson
DHS Trynka
DGF ENCODE
CTCF Hoffman
**Conserved LindbladToh
Coding UCSC
−50
0
50
Enrichment (prop h2 / prop snps)

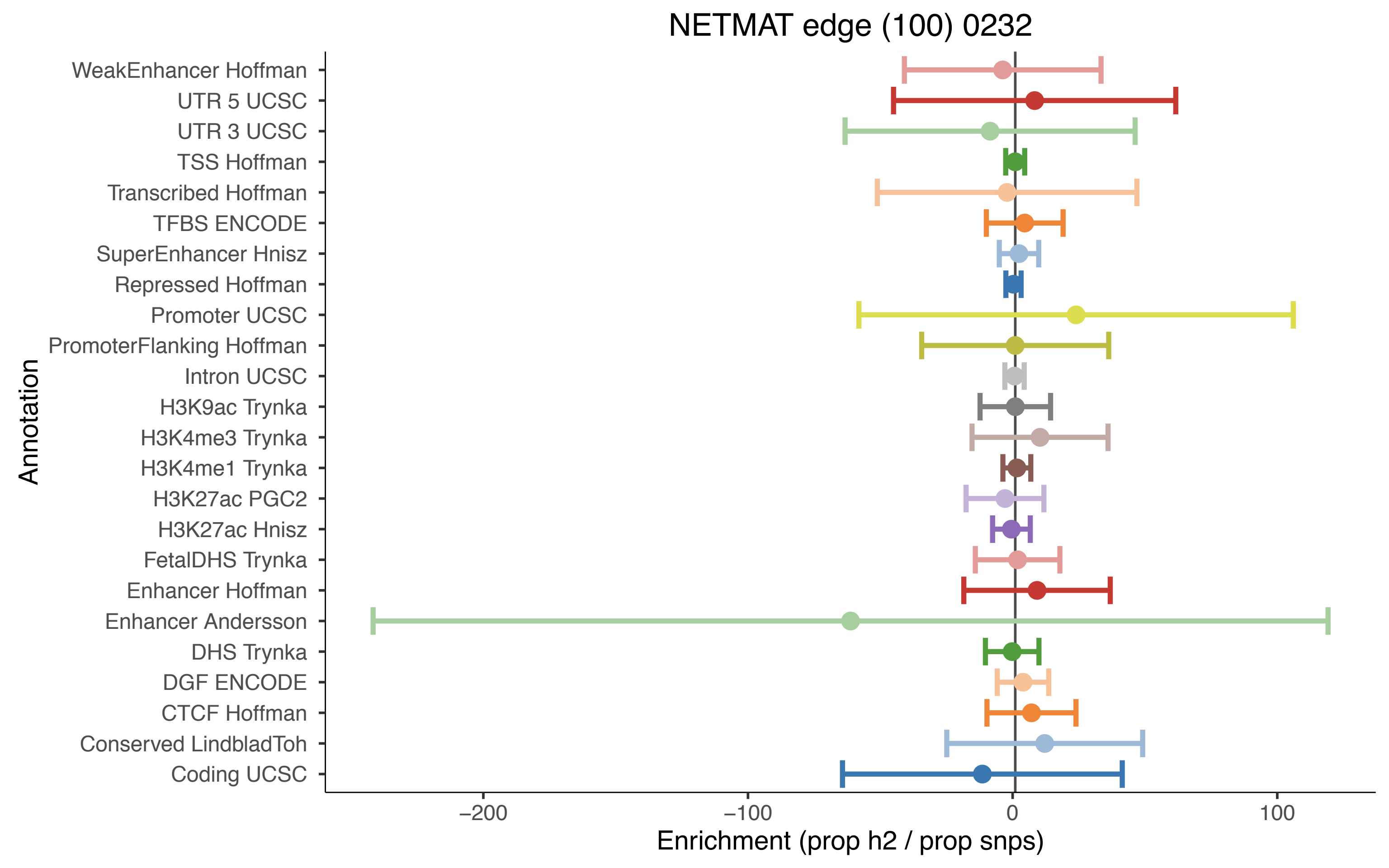

NETMAT edge (100) 0232
WeakEnhancer Hoffman
UTR 5 UCSC
UTR 3 UCSC
TSS Hoffman
Transcribed Hoffman
TFBS ENCODE
SuperEnhancer Hnisz
Repressed Hoffman
Promoter UCSC
PromoterFlanking Hoffman
Intron UCSC
H3K9ac Trynka
H3K4me3 Trynka
H3K4me1 Trynka
H3K27ac PGC2
H3K27ac Hnisz
FetalDHS Trynka
Enhancer Hoffman
Enhancer Andersson
DHS Trynka
DGF ENCODE
CTCF Hoffman
Conserved LindbladToh
Coding UCSC
Annotation
−200
−100
0
100
Enrichment (prop h2 / prop snps)

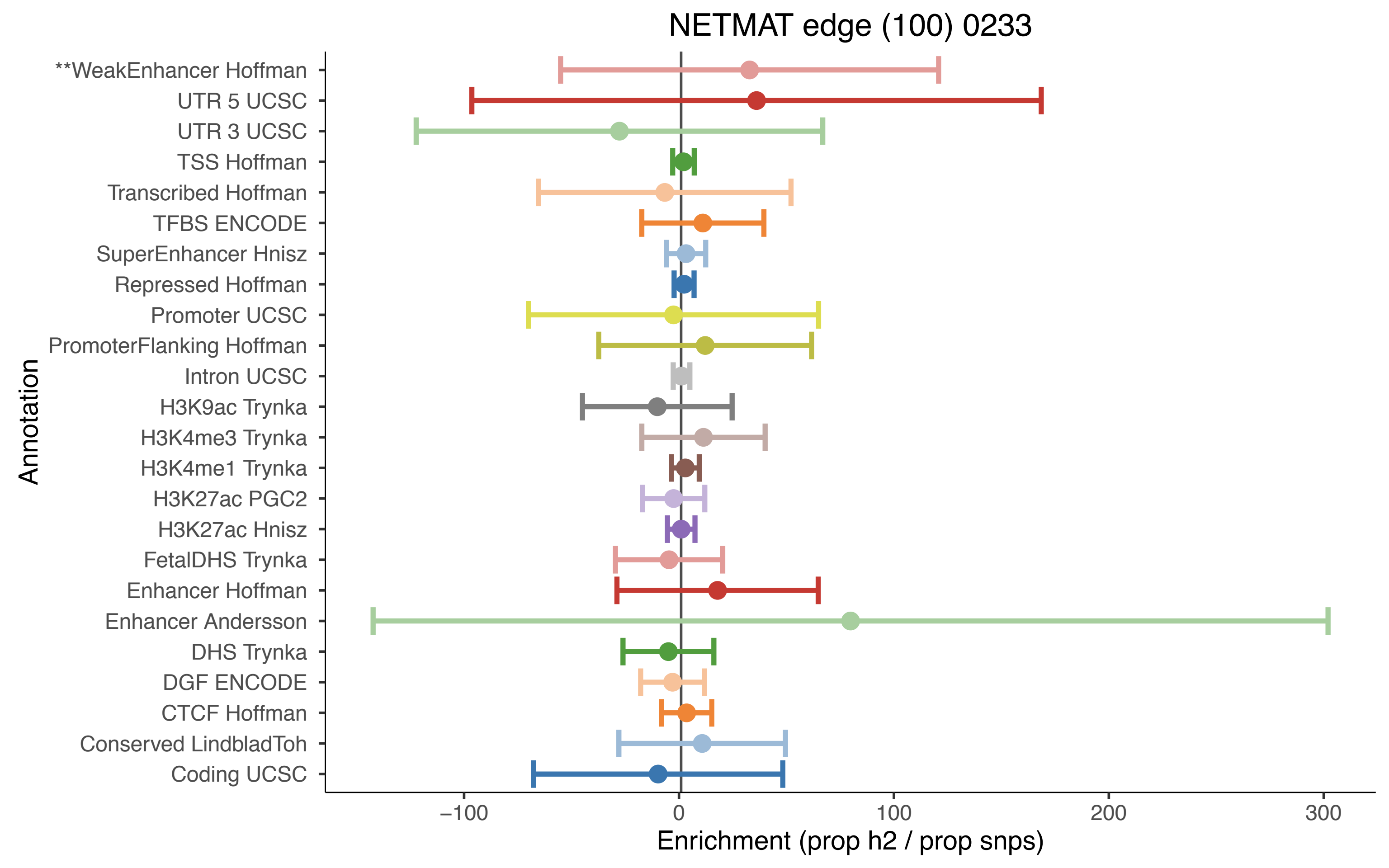
NETMAT edge (100) 0233
Annotation
**WeakEnhancer Hoffman
UTR 5 UCSC
UTR 3 UCSC
TSS Hoffman
Transcribed Hoffman
TFBS ENCODE
SuperEnhancer Hnisz
Repressed Hoffman
Promoter UCSC
PromoterFlanking Hoffman
Intron UCSC
H3K9ac Trynka
H3K4me3 Trynka
H3K4me1 Trynka
H3K27ac PGC2
H3K27ac Hnisz
FetalDHS Trynka
Enhancer Hoffman
Enhancer Andersson
DHS Trynka
DGF ENCODE
CTCF Hoffman
Conserved LindbladToh
Coding UCSC
−100
0
100
200
300
Enrichment (prop h2 / prop snps)

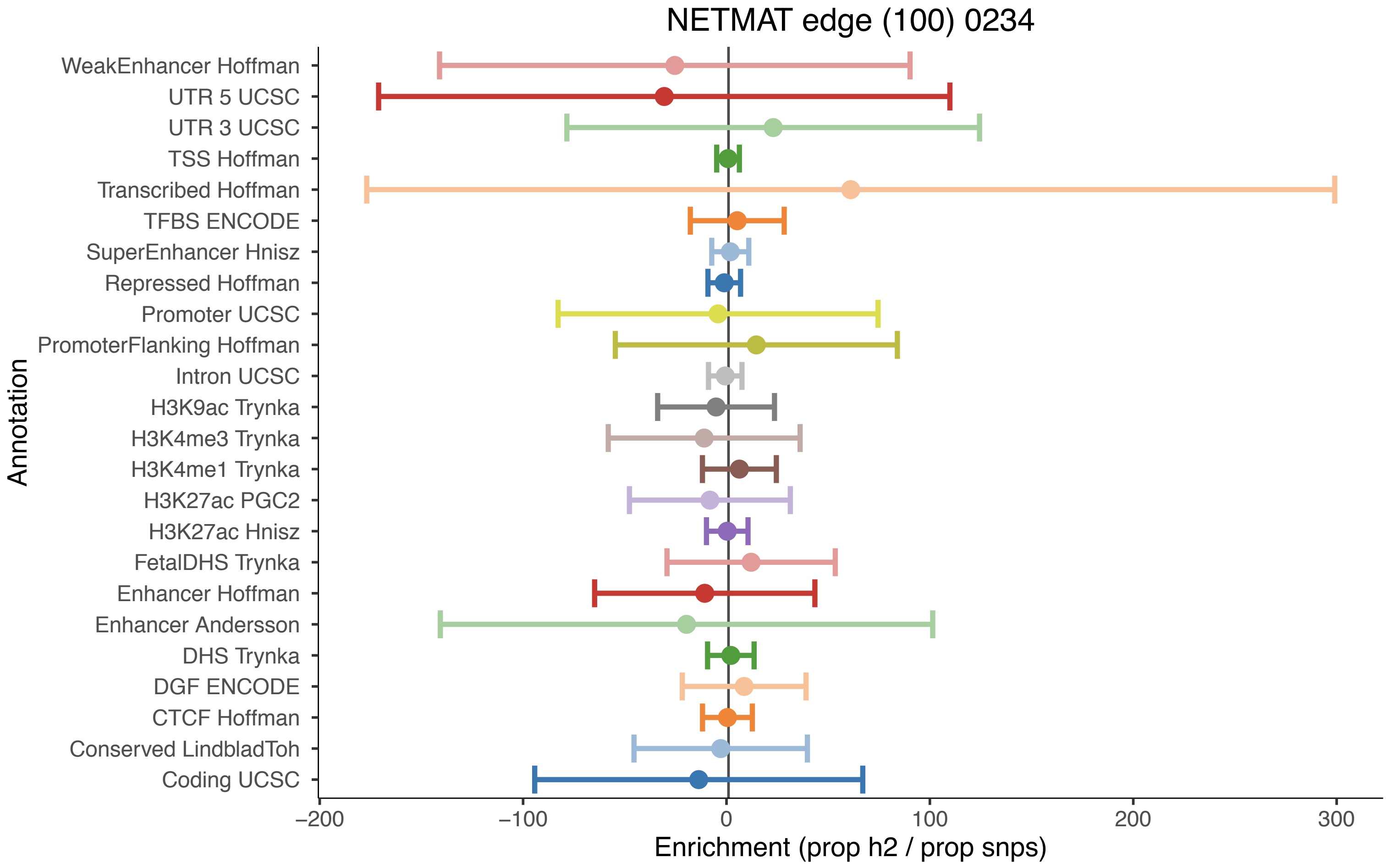
NETMAT edge (100) 0234
WeakEnhancer Hoffman
UTR 5 UCSC
UTR 3 UCSC
TSS Hoffman
Transcribed Hoffman
TFBS ENCODE
SuperEnhancer Hnisz
Repressed Hoffman
Promoter UCSC
PromoterFlanking Hoffman
Intron UCSC
H3K9ac Trynka
H3K4me3 Trynka
H3K4me1 Trynka
H3K27ac PGC2
H3K27ac Hnisz
FetalDHS Trynka
Enhancer Hoffman
Enhancer Andersson
DHS Trynka
DGF ENCODE
CTCF Hoffman
Conserved LindbladToh
Coding UCSC
Annotation
−200
−100
0
100
200
300
Enrichment (prop h2 / prop snps)

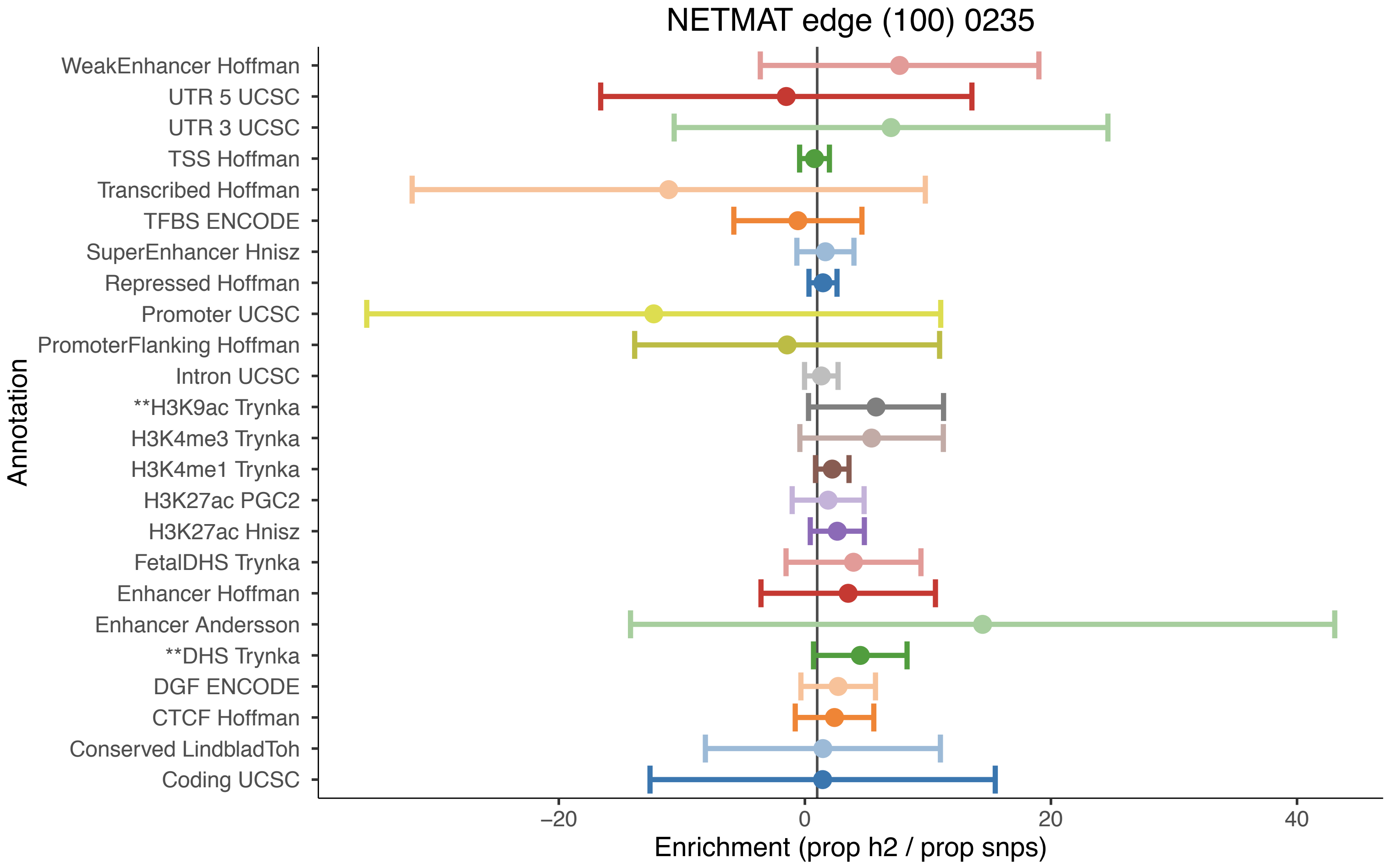

NETMAT edge (100) 0235
WeakEnhancer Hoffman
UTR 5 UCSC
UTR 3 UCSC
TSS Hoffman
Transcribed Hoffman
TFBS ENCODE
SuperEnhancer Hnisz
Repressed Hoffman
Promoter UCSC
PromoterFlanking Hoffman
Intron UCSC
**H3K9ac Trynka
H3K4me3 Trynka
H3K4me1 Trynka
H3K27ac PGC2
H3K27ac Hnisz
FetalDHS Trynka
Enhancer Hoffman
Enhancer Andersson
**DHS Trynka
DGF ENCODE
CTCF Hoffman
Conserved LindbladToh
Coding UCSC
Annotation
−20
0
20
40
Enrichment (prop h2 / prop snps)

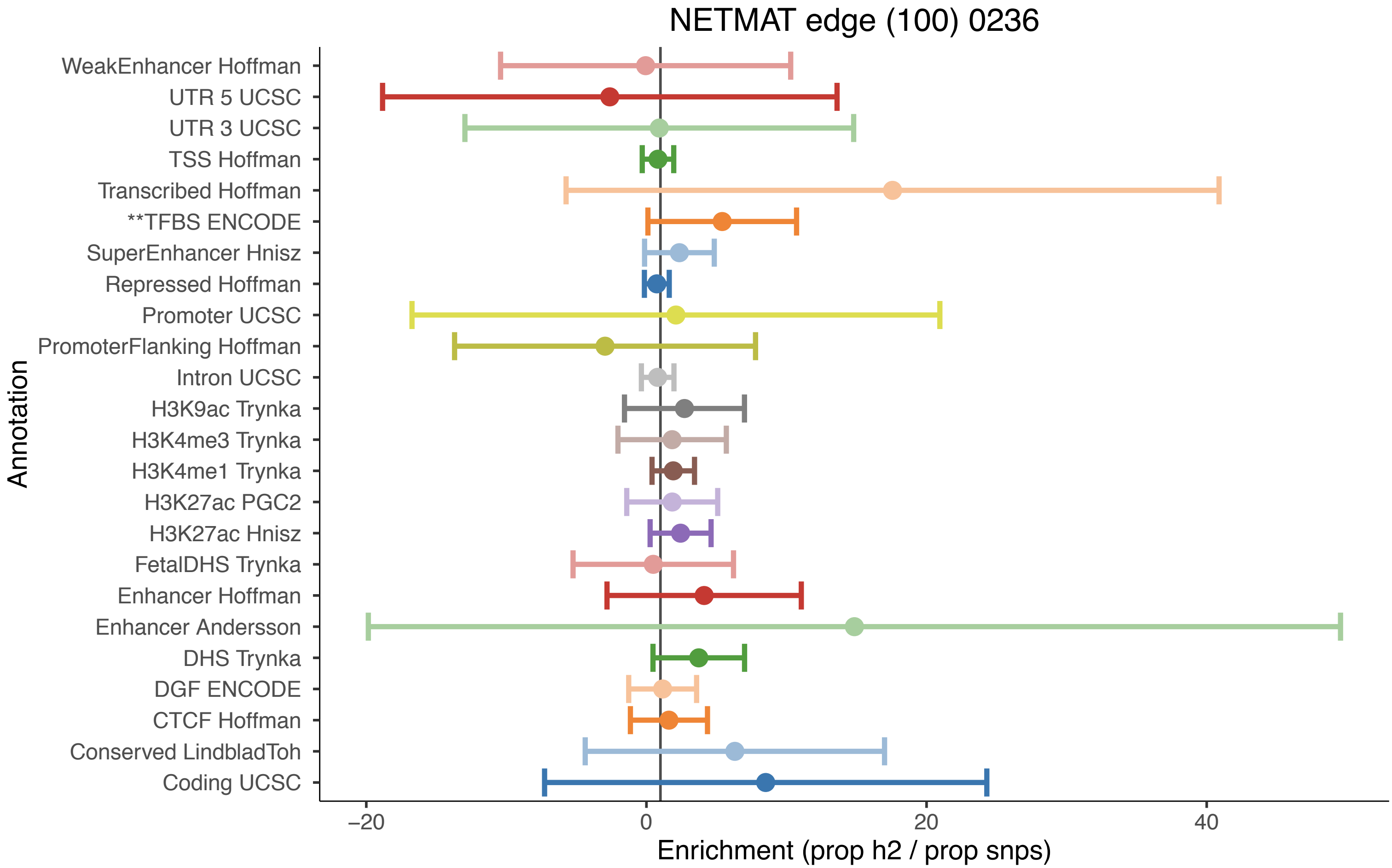
NETMAT edge (100) 0236
WeakEnhancer Hoffman
UTR 5 UCSC
UTR 3 UCSC
TSS Hoffman
Transcribed Hoffman
**TFBS ENCODE
SuperEnhancer Hnisz
Repressed Hoffman
Promoter UCSC
PromoterFlanking Hoffman
Intron UCSC
H3K9ac Trynka
H3K4me3 Trynka
H3K4me1 Trynka
H3K27ac PGC2
H3K27ac Hnisz
FetalDHS Trynka
Enhancer Hoffman
Enhancer Andersson
DHS Trynka
DGF ENCODE
CTCF Hoffman
Conserved LindbladToh
Coding UCSC
Annotation
−20
0
20
40
Enrichment (prop h2 / prop snps)

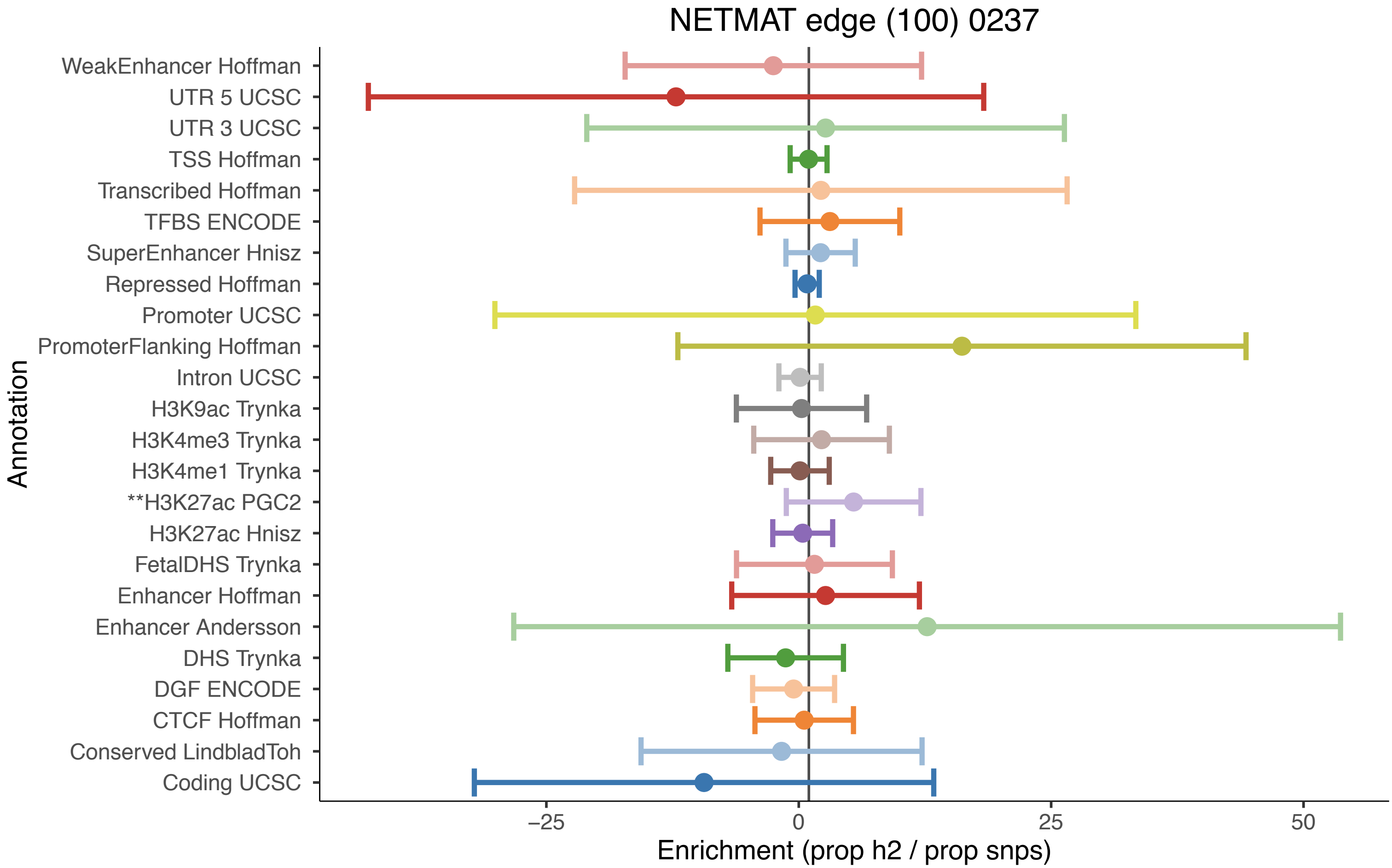
NETMAT edge (100) 0237
WeakEnhancer Hoffman
UTR 5 UCSC
UTR 3 UCSC
TSS Hoffman
Transcribed Hoffman
TFBS ENCODE
SuperEnhancer Hnisz
Repressed Hoffman
Promoter UCSC
PromoterFlanking Hoffman
Intron UCSC
H3K9ac Trynka
H3K4me3 Trynka
H3K4me1 Trynka
**H3K27ac PGC2
H3K27ac Hnisz
FetalDHS Trynka
Enhancer Hoffman
Enhancer Andersson
DHS Trynka
DGF ENCODE
CTCF Hoffman
Conserved LindbladToh
Coding UCSC
Annotation
−25
0
25
50
Enrichment (prop h2 / prop snps)

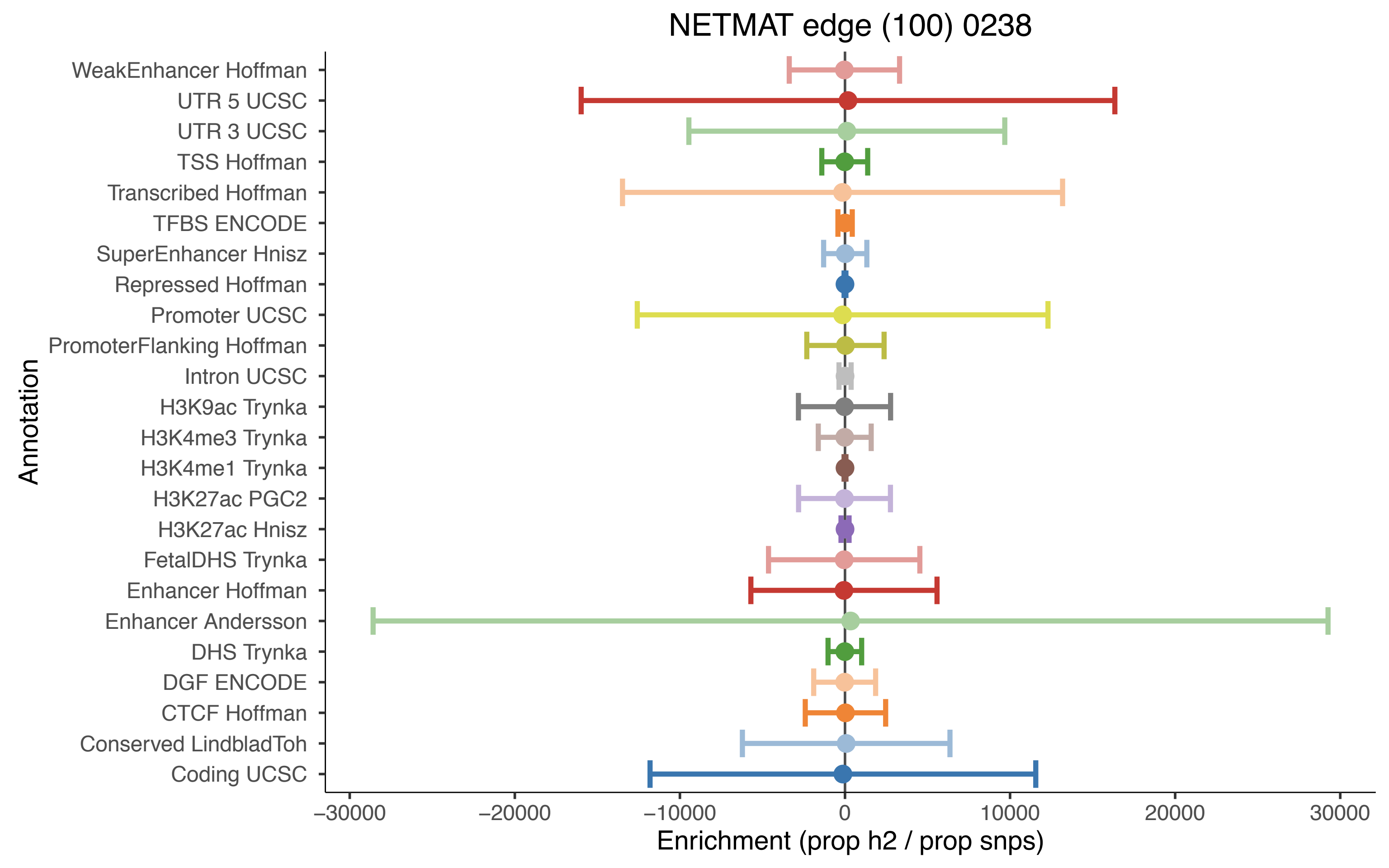
NETMAT edge (100) 0238
WeakEnhancer Hoffman
UTR 5 UCSC
UTR 3 UCSC
TSS Hoffman
Transcribed Hoffman
TFBS ENCODE
SuperEnhancer Hnisz
Repressed Hoffman
Promoter UCSC
PromoterFlanking Hoffman
Intron UCSC
H3K9ac Trynka
H3K4me3 Trynka
H3K4me1 Trynka
H3K27ac PGC2
H3K27ac Hnisz
FetalDHS Trynka
Enhancer Hoffman
Enhancer Andersson
DHS Trynka
DGF ENCODE
CTCF Hoffman
Conserved LindbladToh
Coding UCSC
Annotation
−30000
−20000
−10000
0
10000
20000
30000
Enrichment (prop h2 / prop snps)

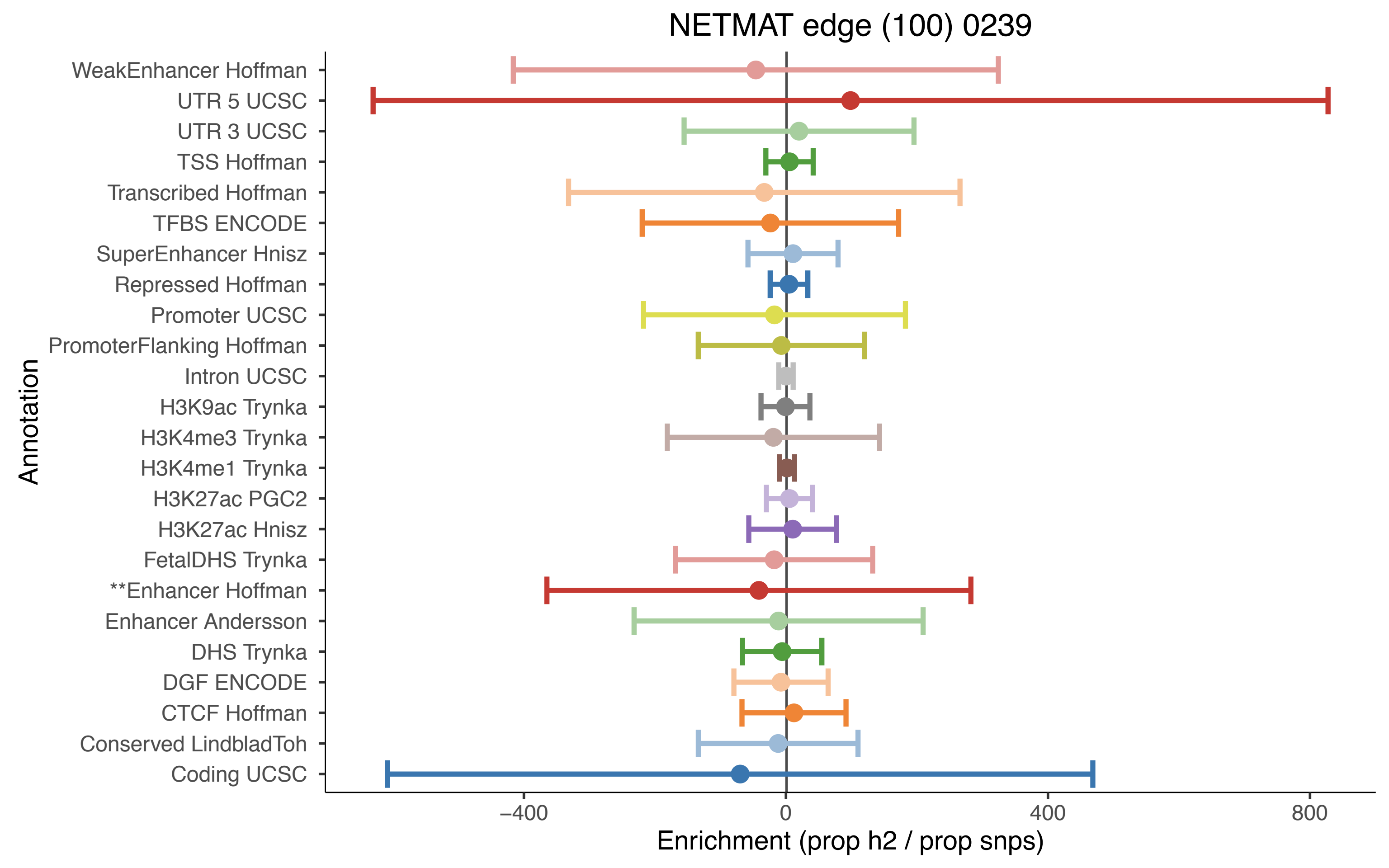

NETMAT edge (100) 0239
Annotation
WeakEnhancer Hoffman
UTR 5 UCSC
UTR 3 UCSC
TSS Hoffman
Transcribed Hoffman
TFBS ENCODE
SuperEnhancer Hnisz
Repressed Hoffman
Promoter UCSC
PromoterFlanking Hoffman
Intron UCSC
H3K9ac Trynka
H3K4me3 Trynka
H3K4me1 Trynka
H3K27ac PGC2
H3K27ac Hnisz
FetalDHS Trynka
**Enhancer Hoffman
Enhancer Andersson
DHS Trynka
DGF ENCODE
CTCF Hoffman
Conserved LindbladToh
Coding UCSC
−400
0
400
800
Enrichment (prop h2 / prop snps)

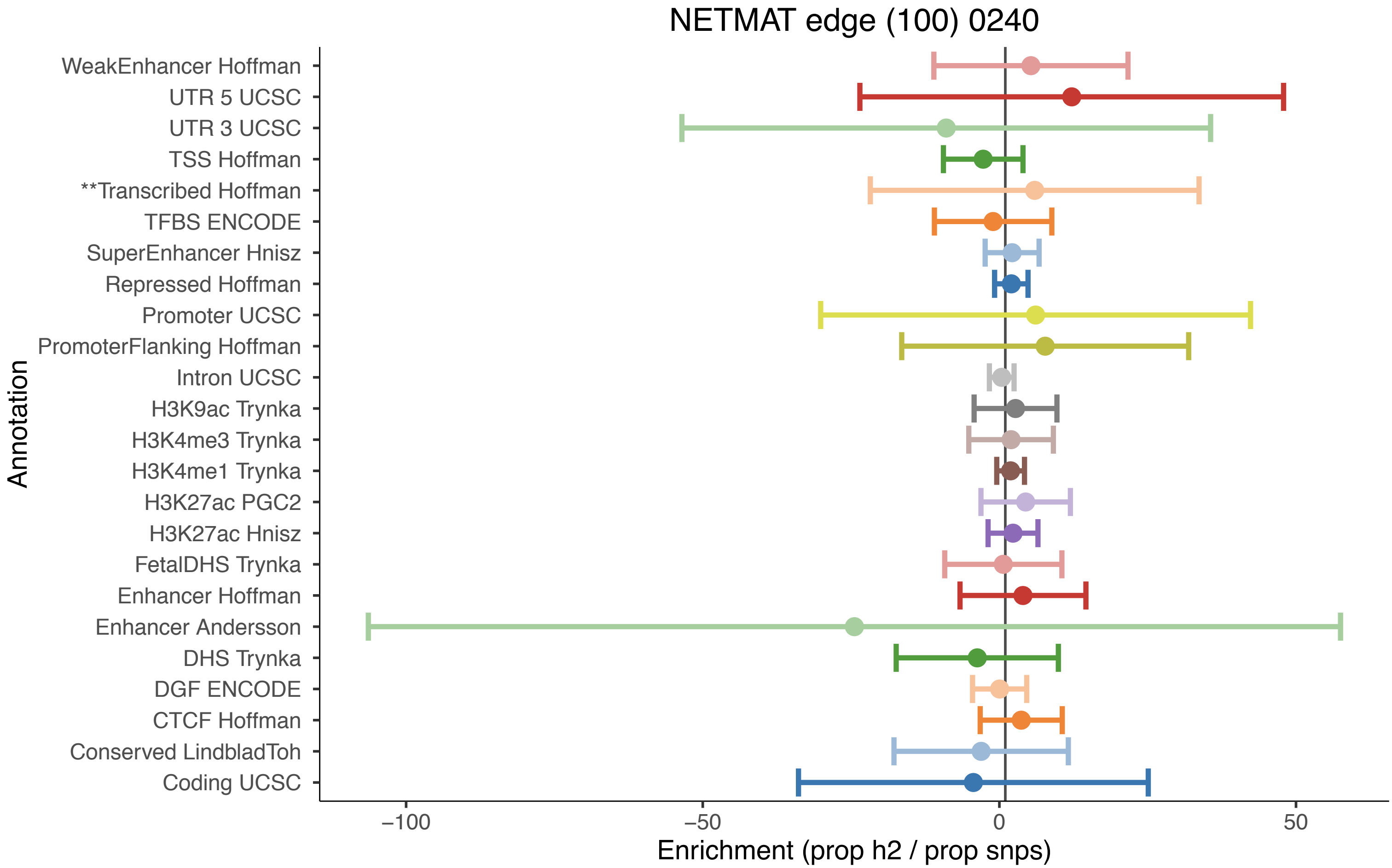

NETMAT edge (100) 0240
Annotation
WeakEnhancer Hoffman
UTR 5 UCSC
UTR 3 UCSC
TSS Hoffman
**Transcribed Hoffman
TFBS ENCODE
SuperEnhancer Hnisz
Repressed Hoffman
Promoter UCSC
PromoterFlanking Hoffman
Intron UCSC
H3K9ac Trynka
H3K4me3 Trynka
H3K4me1 Trynka
H3K27ac PGC2
H3K27ac Hnisz
FetalDHS Trynka
Enhancer Hoffman
Enhancer Andersson
DHS Trynka
DGF ENCODE
CTCF Hoffman
Conserved LindbladToh
Coding UCSC
−100
−50
0
50
Enrichment (prop h2 / prop snps)

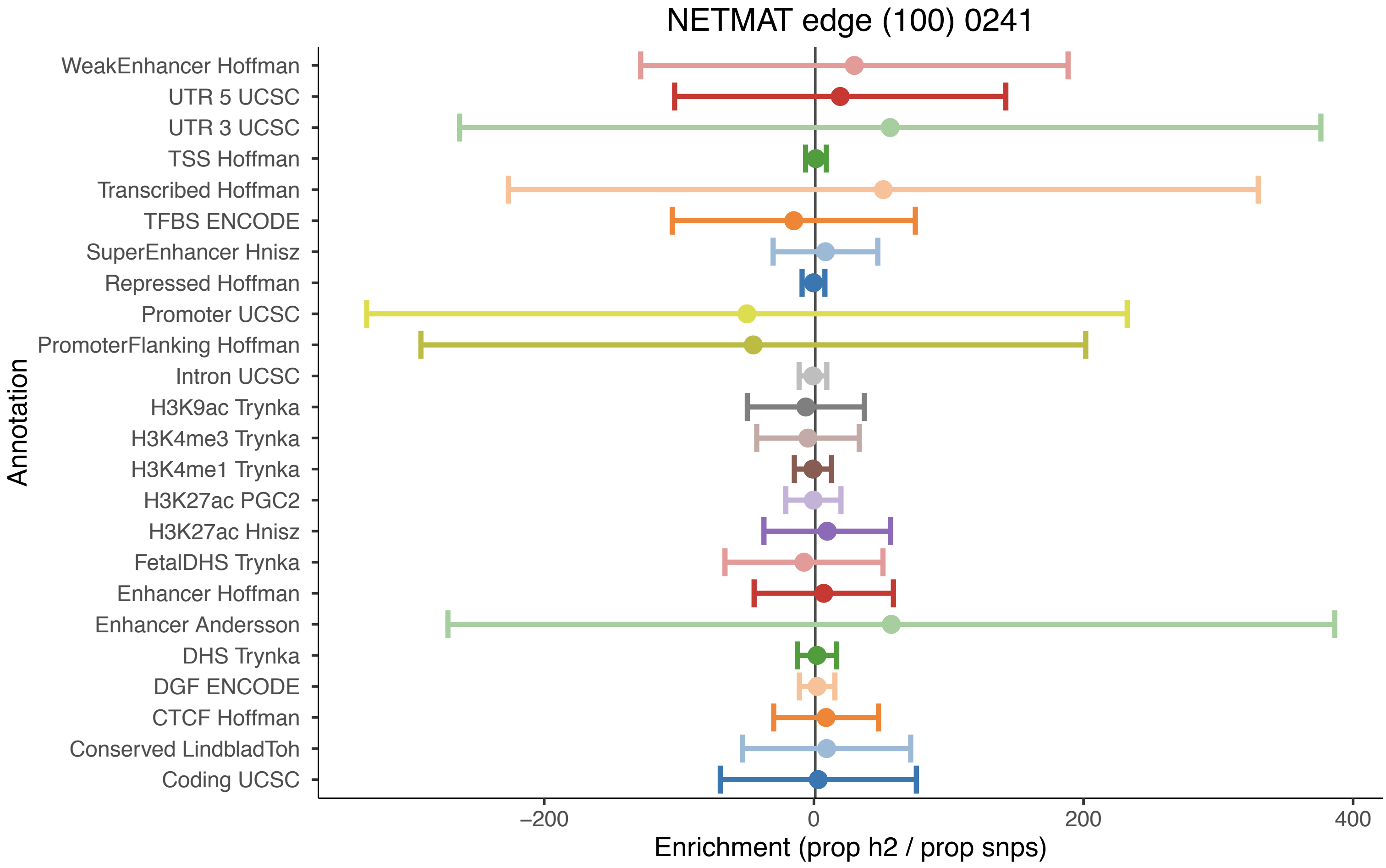
NETMAT edge (100) 0241
Annotation
WeakEnhancer Hoffman
UTR 5 UCSC
UTR 3 UCSC
TSS Hoffman
Transcribed Hoffman
TFBS ENCODE
SuperEnhancer Hnisz
Repressed Hoffman
Promoter UCSC
PromoterFlanking Hoffman
Intron UCSC
H3K9ac Trynka
H3K4me3 Trynka
H3K4me1 Trynka
H3K27ac PGC2
H3K27ac Hnisz
FetalDHS Trynka
Enhancer Hoffman
Enhancer Andersson
DHS Trynka
DGF ENCODE
CTCF Hoffman
Conserved LindbladToh
Coding UCSC
−200
0
200
400
Enrichment (prop h2 / prop snps)

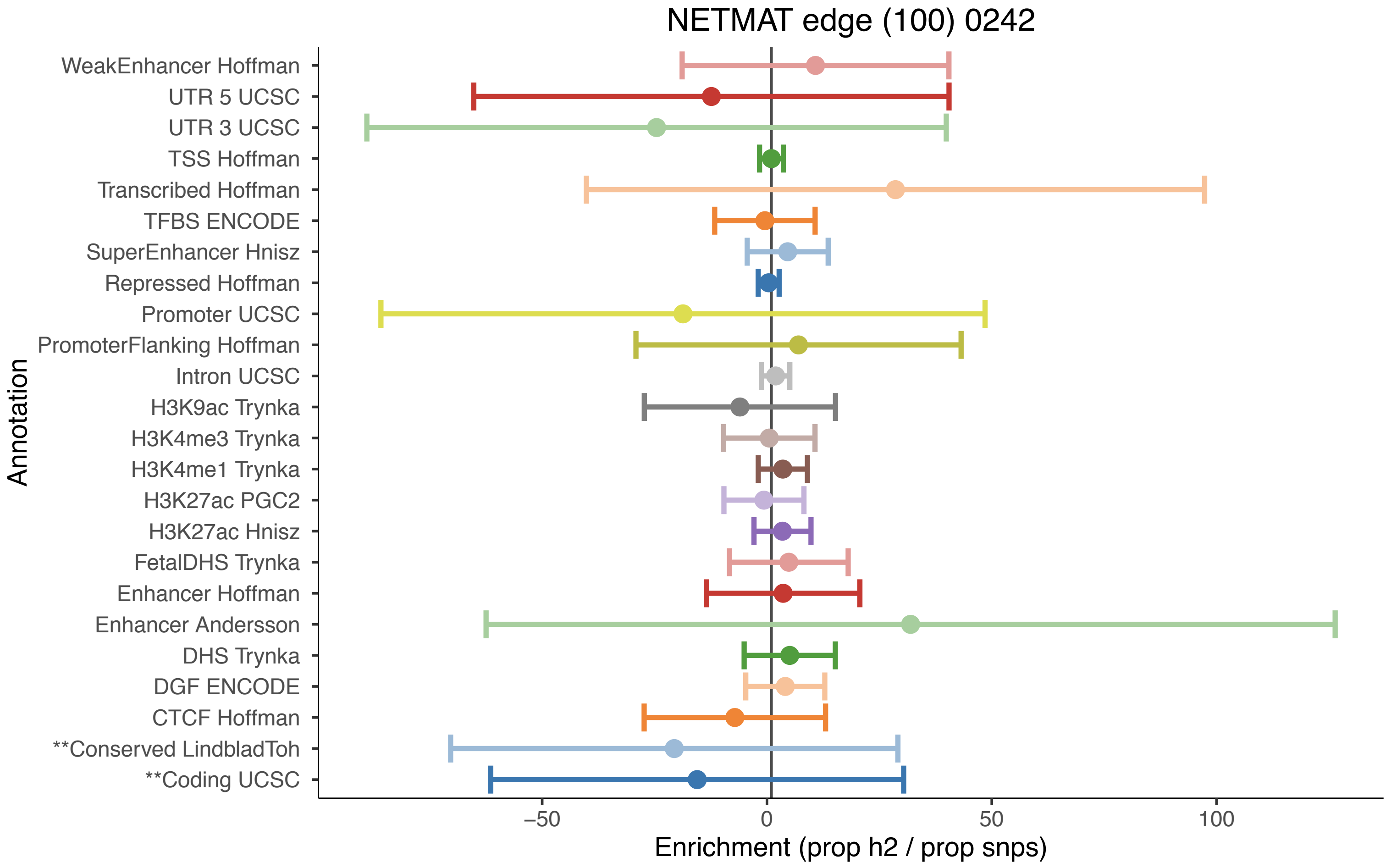
NETMAT edge (100) 0242
WeakEnhancer Hoffman
UTR 5 UCSC
UTR 3 UCSC
TSS Hoffman
Transcribed Hoffman
TFBS ENCODE
SuperEnhancer Hnisz
Repressed Hoffman
Promoter UCSC
PromoterFlanking Hoffman
Intron UCSC
H3K9ac Trynka
H3K4me3 Trynka
H3K4me1 Trynka
H3K27ac PGC2
H3K27ac Hnisz
FetalDHS Trynka
Enhancer Hoffman
Enhancer Andersson
DHS Trynka
DGF ENCODE
CTCF Hoffman
**Conserved LindbladToh
**Coding UCSC
Annotation
−50
0
50
100
Enrichment (prop h2 / prop snps)

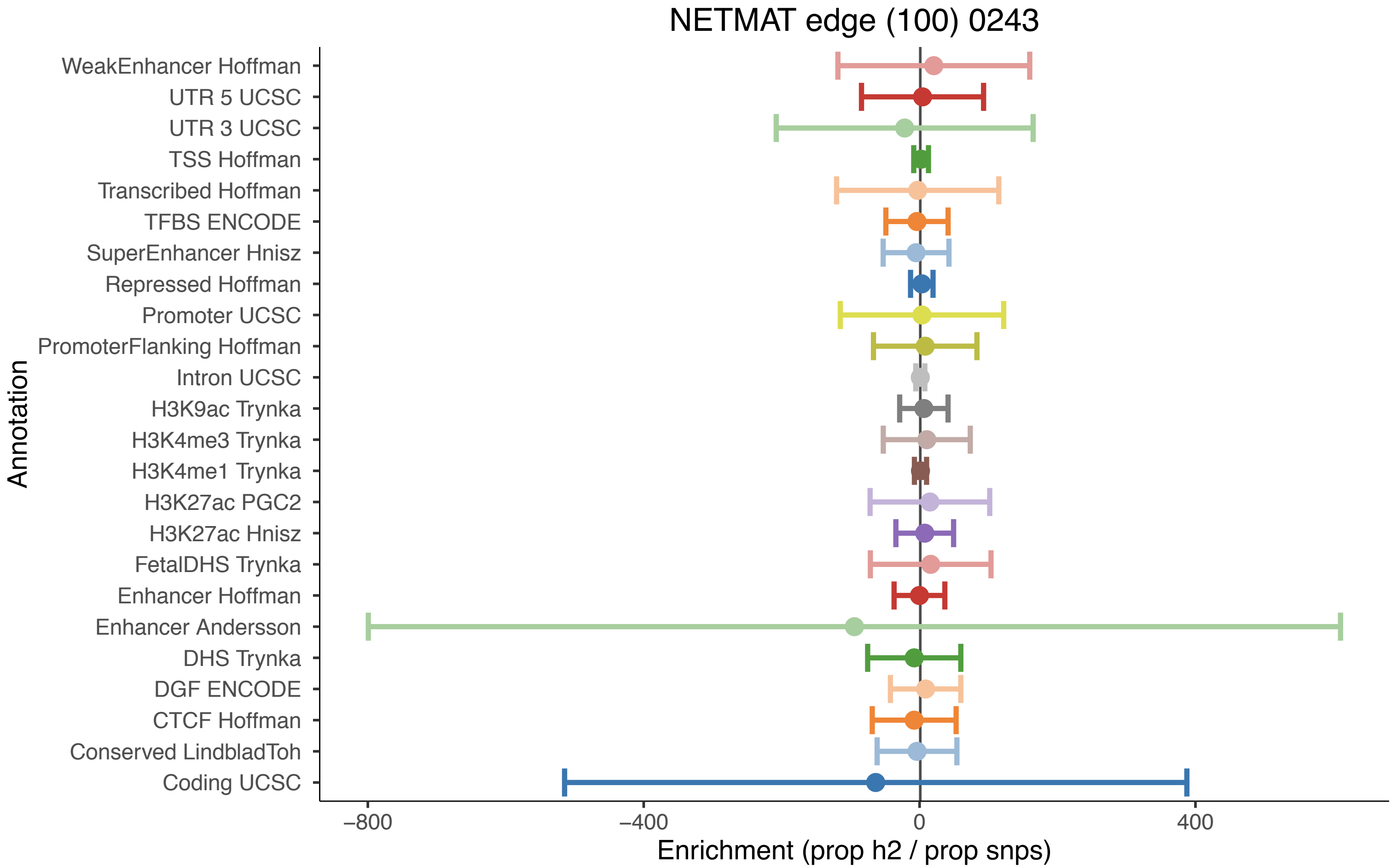

NETMAT edge (100) 0243
WeakEnhancer Hoffman
UTR 5 UCSC
UTR 3 UCSC
TSS Hoffman
Transcribed Hoffman
TFBS ENCODE
SuperEnhancer Hnisz
Repressed Hoffman
Promoter UCSC
PromoterFlanking Hoffman
Intron UCSC
H3K9ac Trynka
H3K4me3 Trynka
H3K4me1 Trynka
H3K27ac PGC2
H3K27ac Hnisz
FetalDHS Trynka
Enhancer Hoffman
Enhancer Andersson
DHS Trynka
DGF ENCODE
CTCF Hoffman
Conserved LindbladToh
Coding UCSC
Annotation
−800
−400
0
400
Enrichment (prop h2 / prop snps)

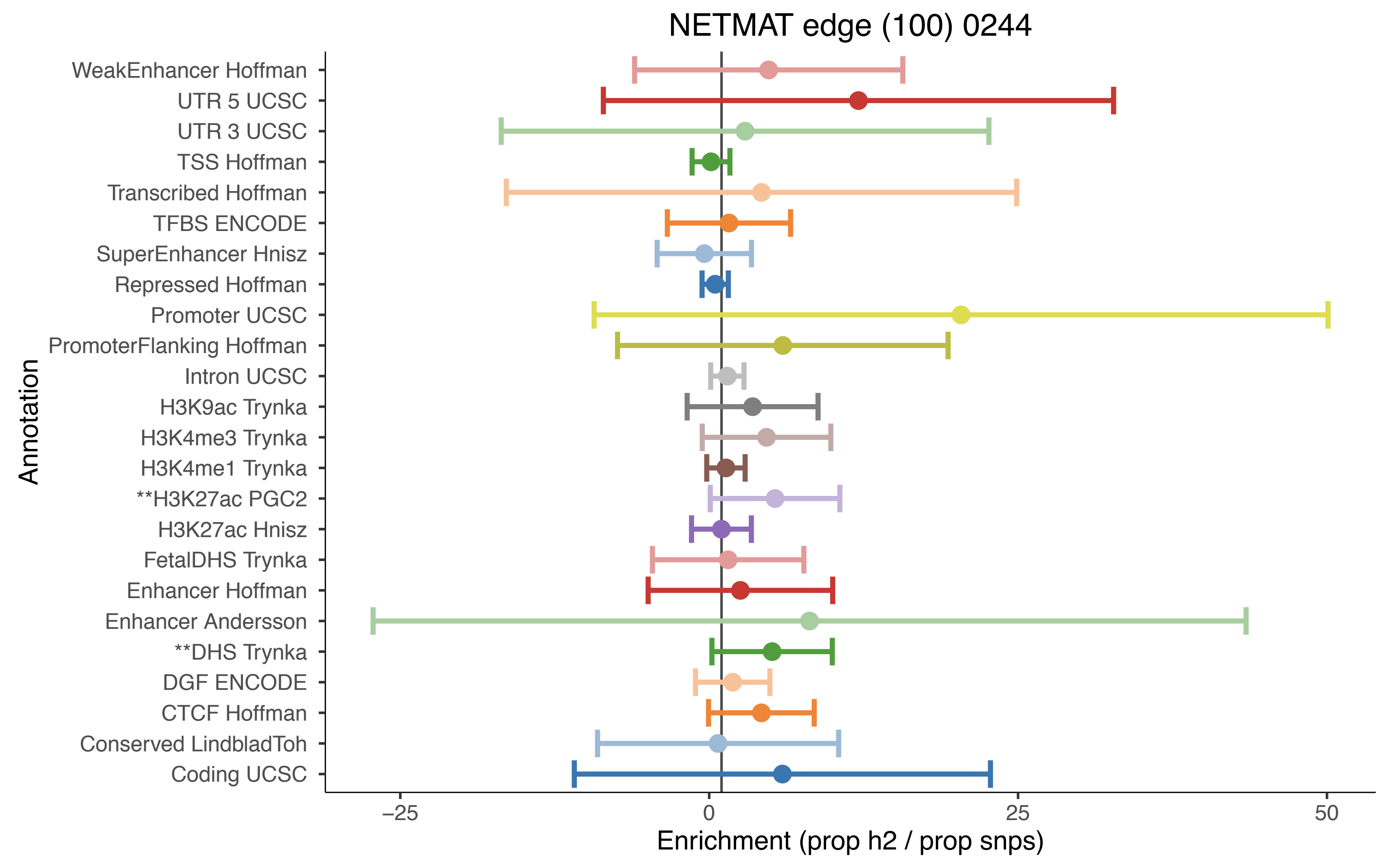
NETMAT edge (100) 0244
WeakEnhancer Hoffman
UTR 5 UCSC
UTR 3 UCSC
TSS Hoffman
Transcribed Hoffman
TFBS ENCODE
SuperEnhancer Hnisz
Repressed Hoffman
Promoter UCSC
PromoterFlanking Hoffman
Intron UCSC
H3K9ac Trynka
H3K4me3 Trynka
H3K4me1 Trynka
**H3K27ac PGC2
H3K27ac Hnisz
FetalDHS Trynka
Enhancer Hoffman
Enhancer Andersson
**DHS Trynka
DGF ENCODE
CTCF Hoffman
Conserved LindbladToh
Coding UCSC
Annotation
−25
0
25
50
Enrichment (prop h2 / prop snps)

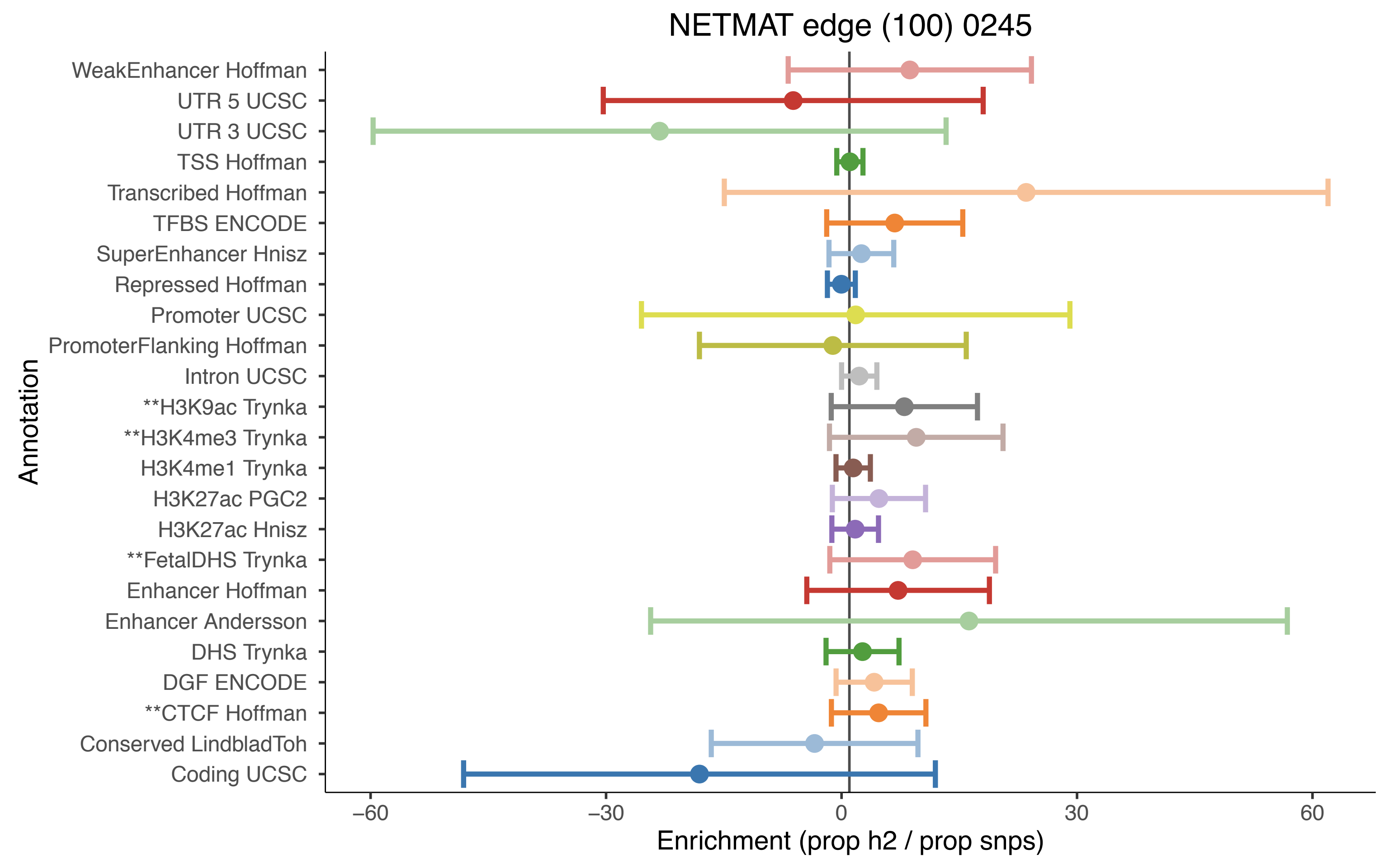
NETMAT edge (100) 0245
WeakEnhancer Hoffman
UTR 5 UCSC
UTR 3 UCSC
TSS Hoffman
Transcribed Hoffman
TFBS ENCODE
SuperEnhancer Hnisz
Repressed Hoffman
Promoter UCSC
PromoterFlanking Hoffman
Intron UCSC
**H3K9ac Trynka
**H3K4me3 Trynka
H3K4me1 Trynka
H3K27ac PGC2
H3K27ac Hnisz
**FetalDHS Trynka
Enhancer Hoffman
Enhancer Andersson
DHS Trynka
DGF ENCODE
**CTCF Hoffman
Conserved LindbladToh
Coding UCSC
Annotation
−60
−30
0
30
60
Enrichment (prop h2 / prop snps)

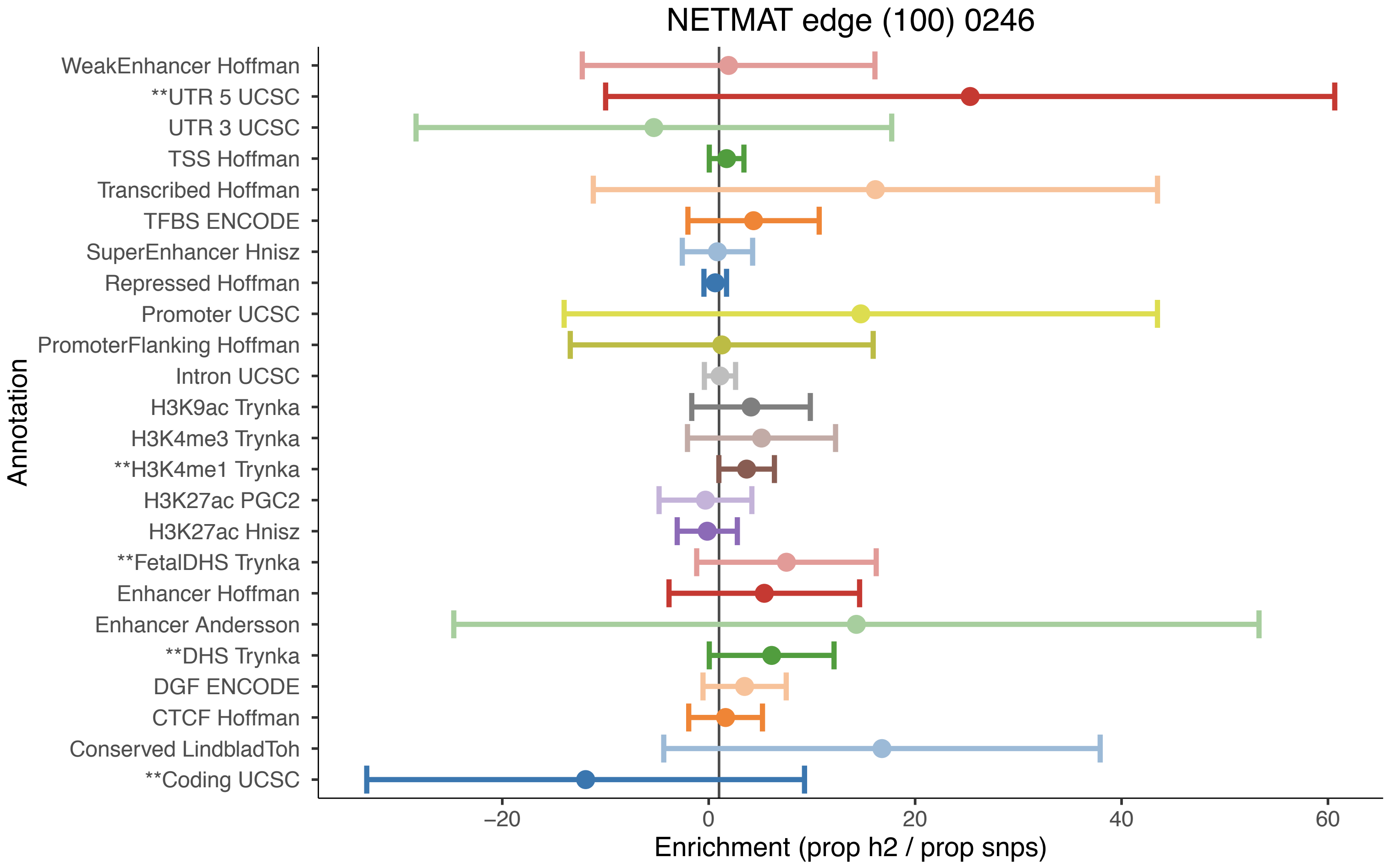

NETMAT edge (100) 0246
WeakEnhancer Hoffman
**UTR 5 UCSC
UTR 3 UCSC
TSS Hoffman
Transcribed Hoffman
TFBS ENCODE
SuperEnhancer Hnisz
Repressed Hoffman
Promoter UCSC
PromoterFlanking Hoffman
Intron UCSC
H3K9ac Trynka
H3K4me3 Trynka
**H3K4me1 Trynka
H3K27ac PGC2
H3K27ac Hnisz
**FetalDHS Trynka
Enhancer Hoffman
Enhancer Andersson
**DHS Trynka
DGF ENCODE
CTCF Hoffman
Conserved LindbladToh
**Coding UCSC
Annotation
−20
0
20
40
60
Enrichment (prop h2 / prop snps)

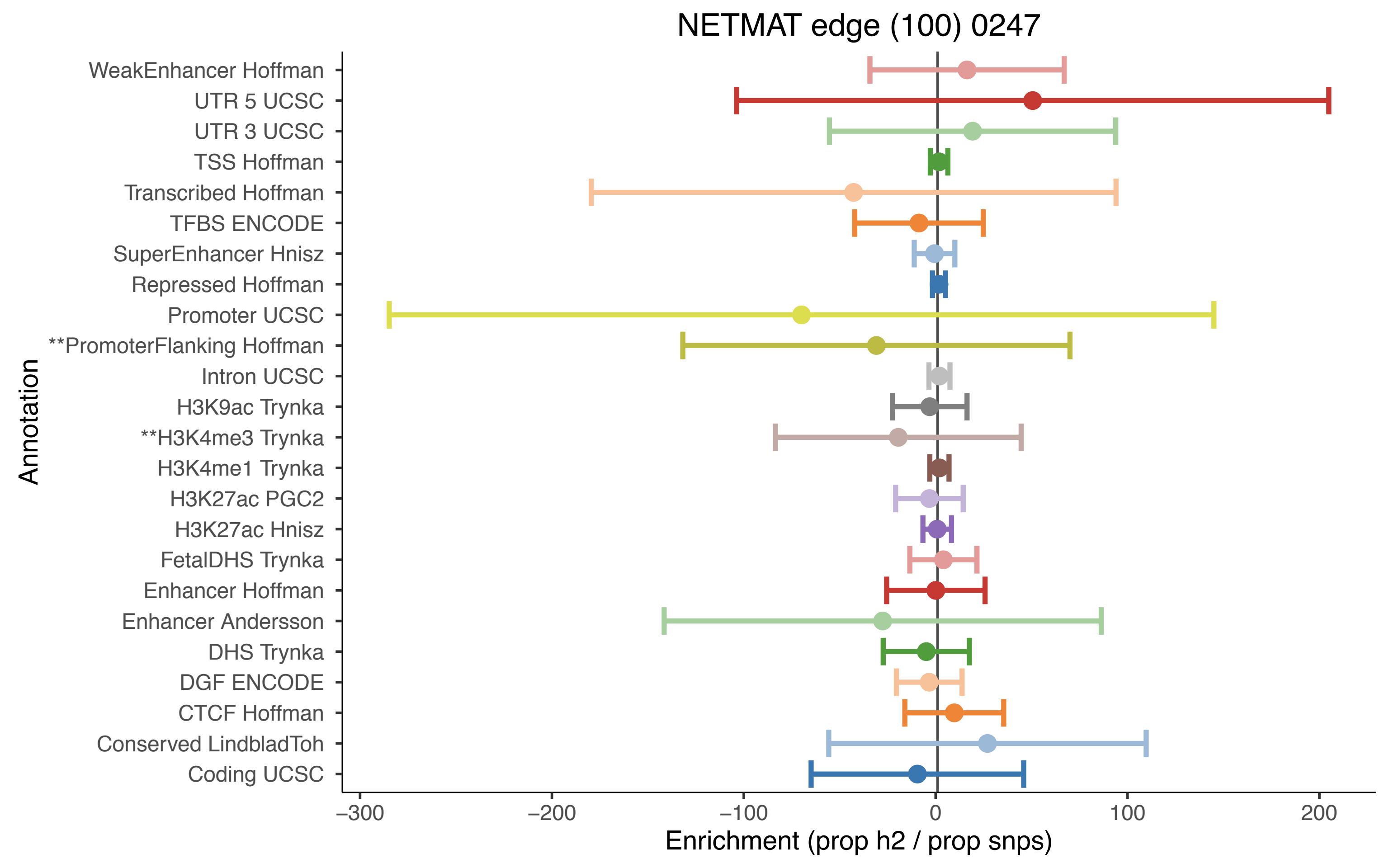
NETMAT edge (100) 0247
WeakEnhancer Hoffman
UTR 5 UCSC
UTR 3 UCSC
TSS Hoffman
Transcribed Hoffman
TFBS ENCODE
SuperEnhancer Hnisz
Repressed Hoffman
Promoter UCSC
**PromoterFlanking Hoffman
Intron UCSC
H3K9ac Trynka
**H3K4me3 Trynka
H3K4me1 Trynka
H3K27ac PGC2
H3K27ac Hnisz
FetalDHS Trynka
Enhancer Hoffman
Enhancer Andersson
DHS Trynka
DGF ENCODE
CTCF Hoffman
Conserved LindbladToh
Coding UCSC
Annotation
−300
−200
−100
0
100
200
Enrichment (prop h2 / prop snps)

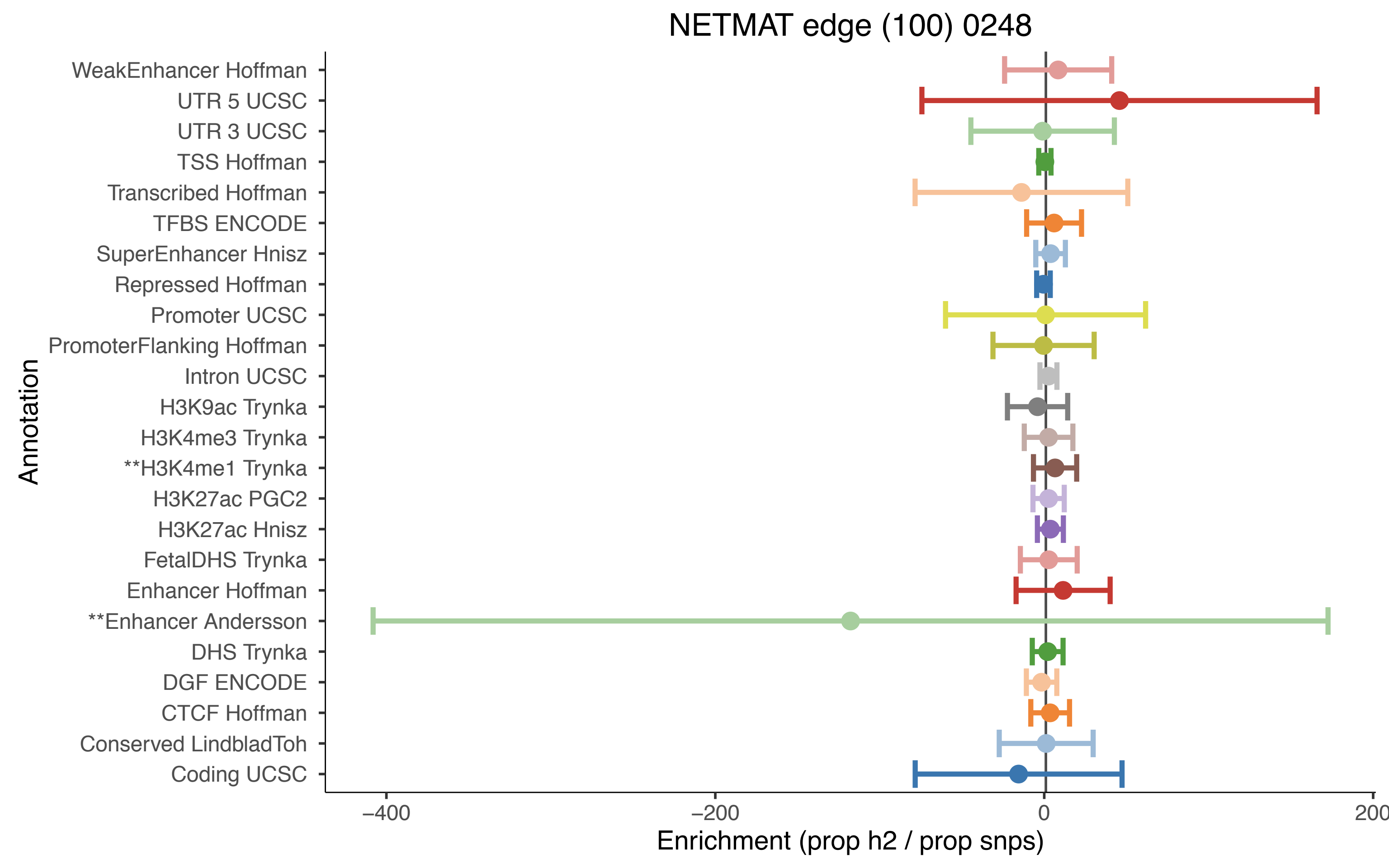

NETMAT edge (100) 0248
WeakEnhancer Hoffman
UTR 5 UCSC
UTR 3 UCSC
TSS Hoffman
Transcribed Hoffman
TFBS ENCODE
SuperEnhancer Hnisz
Repressed Hoffman
Promoter UCSC
PromoterFlanking Hoffman
Intron UCSC
H3K9ac Trynka
H3K4me3 Trynka
**H3K4me1 Trynka
H3K27ac PGC2
H3K27ac Hnisz
FetalDHS Trynka
Enhancer Hoffman
**Enhancer Andersson
DHS Trynka
DGF ENCODE
CTCF Hoffman
Conserved LindbladToh
Coding UCSC
Annotation
−400
−200
0
200
Enrichment (prop h2 / prop snps)

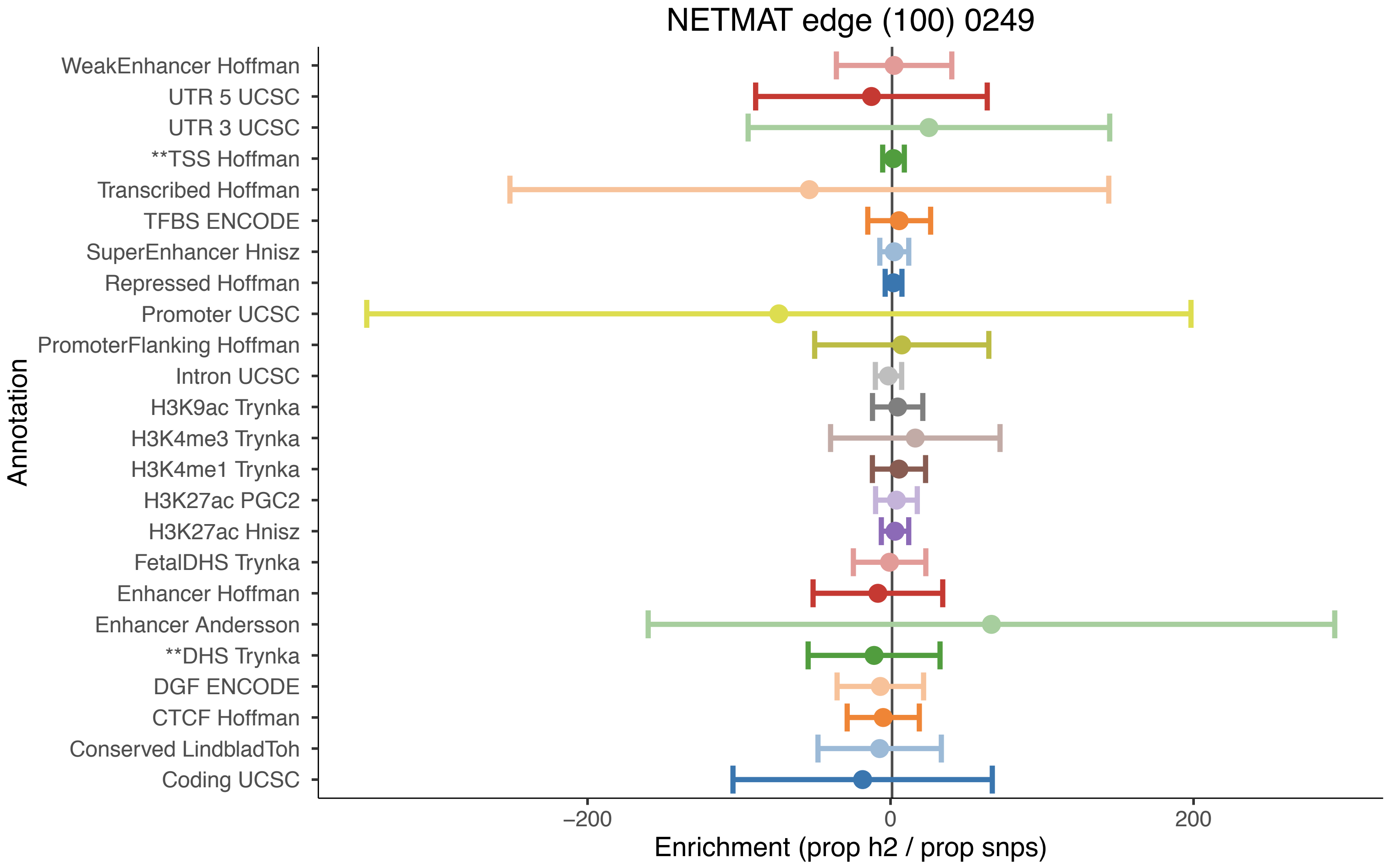
NETMAT edge (100) 0249
WeakEnhancer Hoffman
UTR 5 UCSC
UTR 3 UCSC
**TSS Hoffman
Transcribed Hoffman
TFBS ENCODE
SuperEnhancer Hnisz
Repressed Hoffman
Promoter UCSC
PromoterFlanking Hoffman
Intron UCSC
H3K9ac Trynka
H3K4me3 Trynka
H3K4me1 Trynka
H3K27ac PGC2
H3K27ac Hnisz
FetalDHS Trynka
Enhancer Hoffman
Enhancer Andersson
**DHS Trynka
DGF ENCODE
CTCF Hoffman
Conserved LindbladToh
Coding UCSC
Annotation
−200
0
200
Enrichment (prop h2 / prop snps)

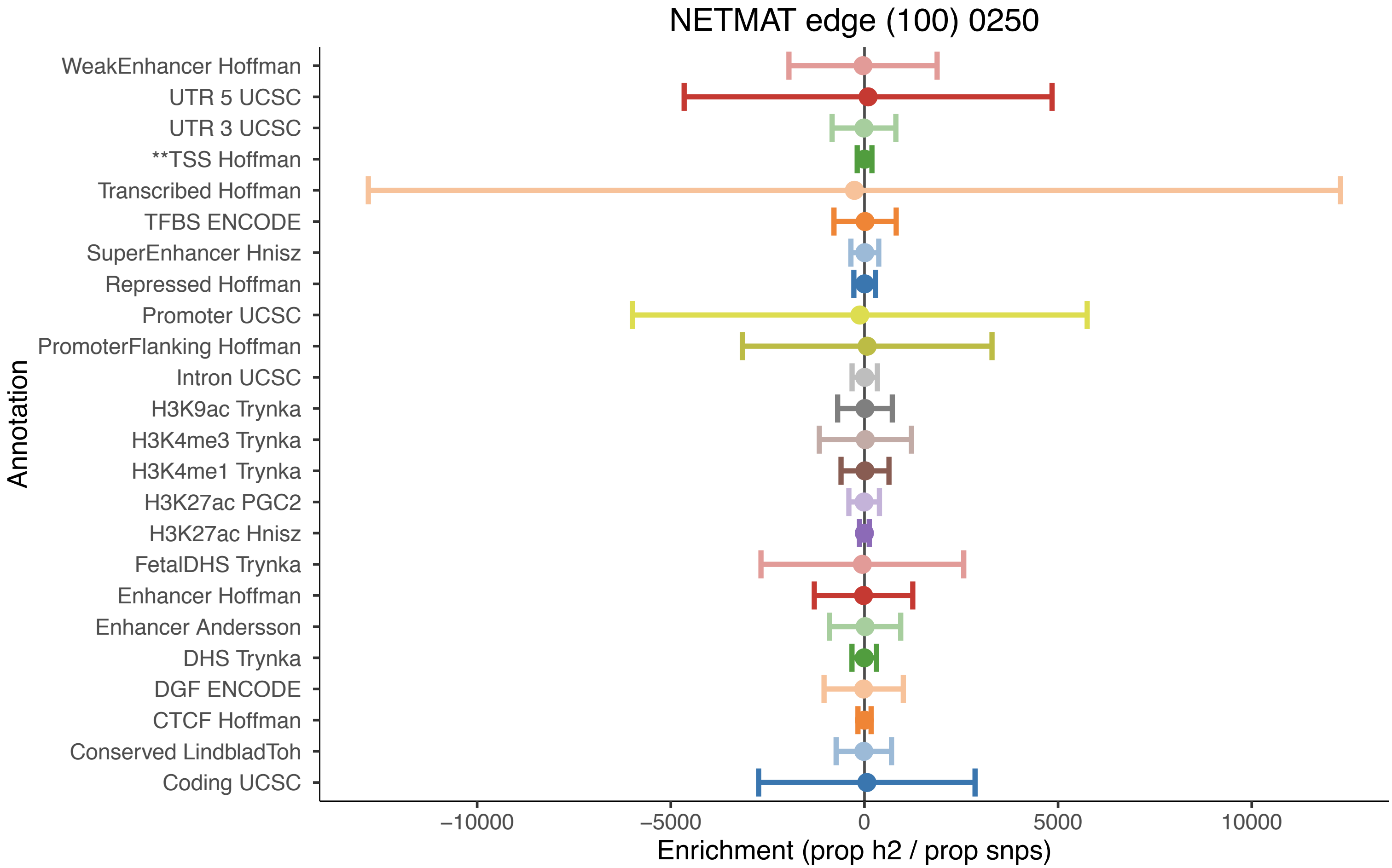

NETMAT edge (100) 0250
WeakEnhancer Hoffman
UTR 5 UCSC
UTR 3 UCSC
**TSS Hoffman
Transcribed Hoffman
TFBS ENCODE
SuperEnhancer Hnisz
Repressed Hoffman
Promoter UCSC
PromoterFlanking Hoffman
Intron UCSC
H3K9ac Trynka
H3K4me3 Trynka
H3K4me1 Trynka
H3K27ac PGC2
H3K27ac Hnisz
FetalDHS Trynka
Enhancer Hoffman
Enhancer Andersson
DHS Trynka
DGF ENCODE
CTCF Hoffman
Conserved LindbladToh
Coding UCSC
Annotation
−10000
−5000
0
5000
10000
Enrichment (prop h2 / prop snps)

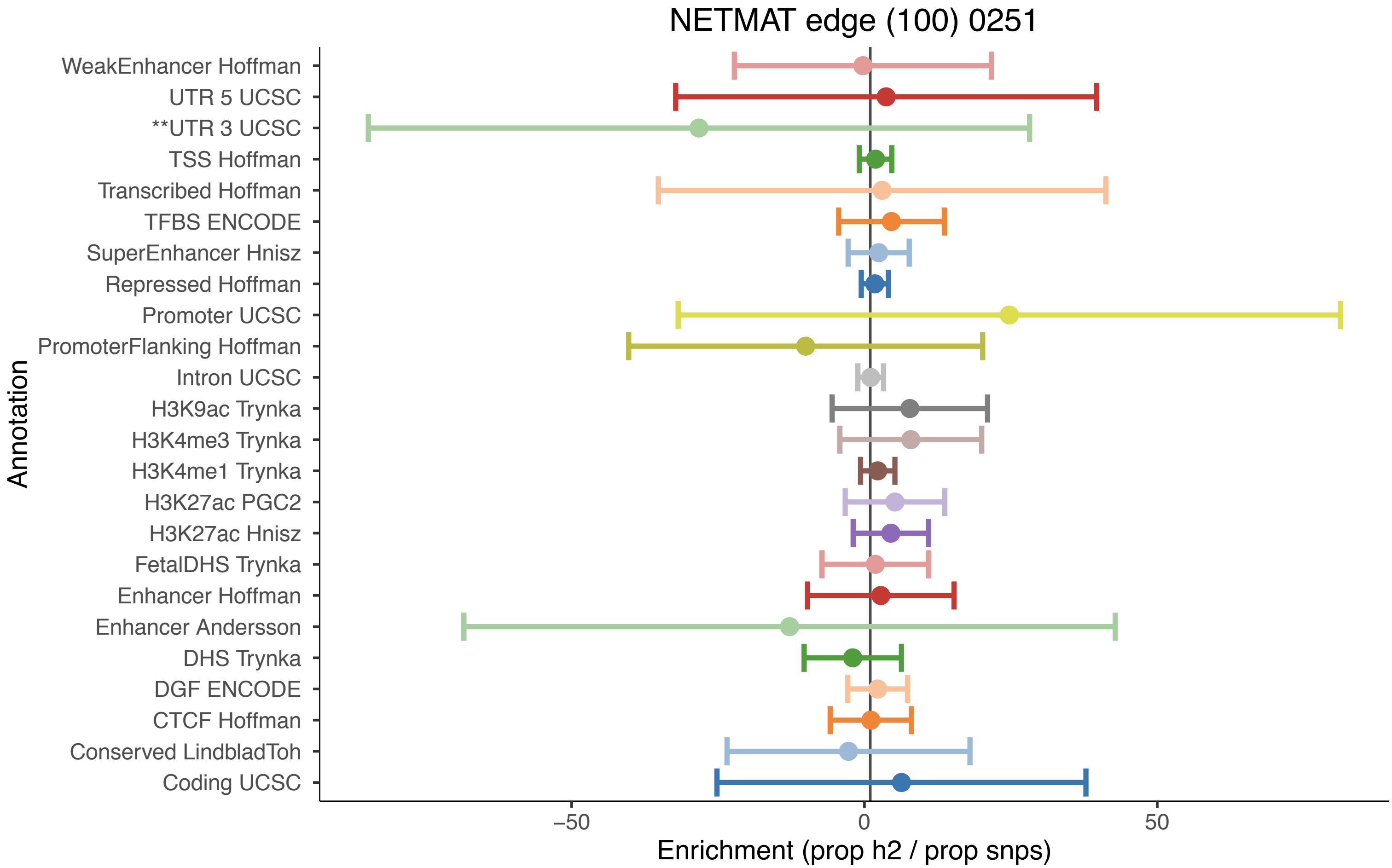

NETMAT edge (100) 0251
WeakEnhancer Hoffman
UTR 5 UCSC
**UTR 3 UCSC
TSS Hoffman
Transcribed Hoffman
TFBS ENCODE
SuperEnhancer Hnisz
Repressed Hoffman
Promoter UCSC
PromoterFlanking Hoffman
Intron UCSC
H3K9ac Trynka
H3K4me3 Trynka
H3K4me1 Trynka
H3K27ac PGC2
H3K27ac Hnisz
FetalDHS Trynka
Enhancer Hoffman
Enhancer Andersson
DHS Trynka
DGF ENCODE
CTCF Hoffman
Conserved LindbladToh
Coding UCSC
Annotation
−50
0
50
Enrichment (prop h2 / prop snps)

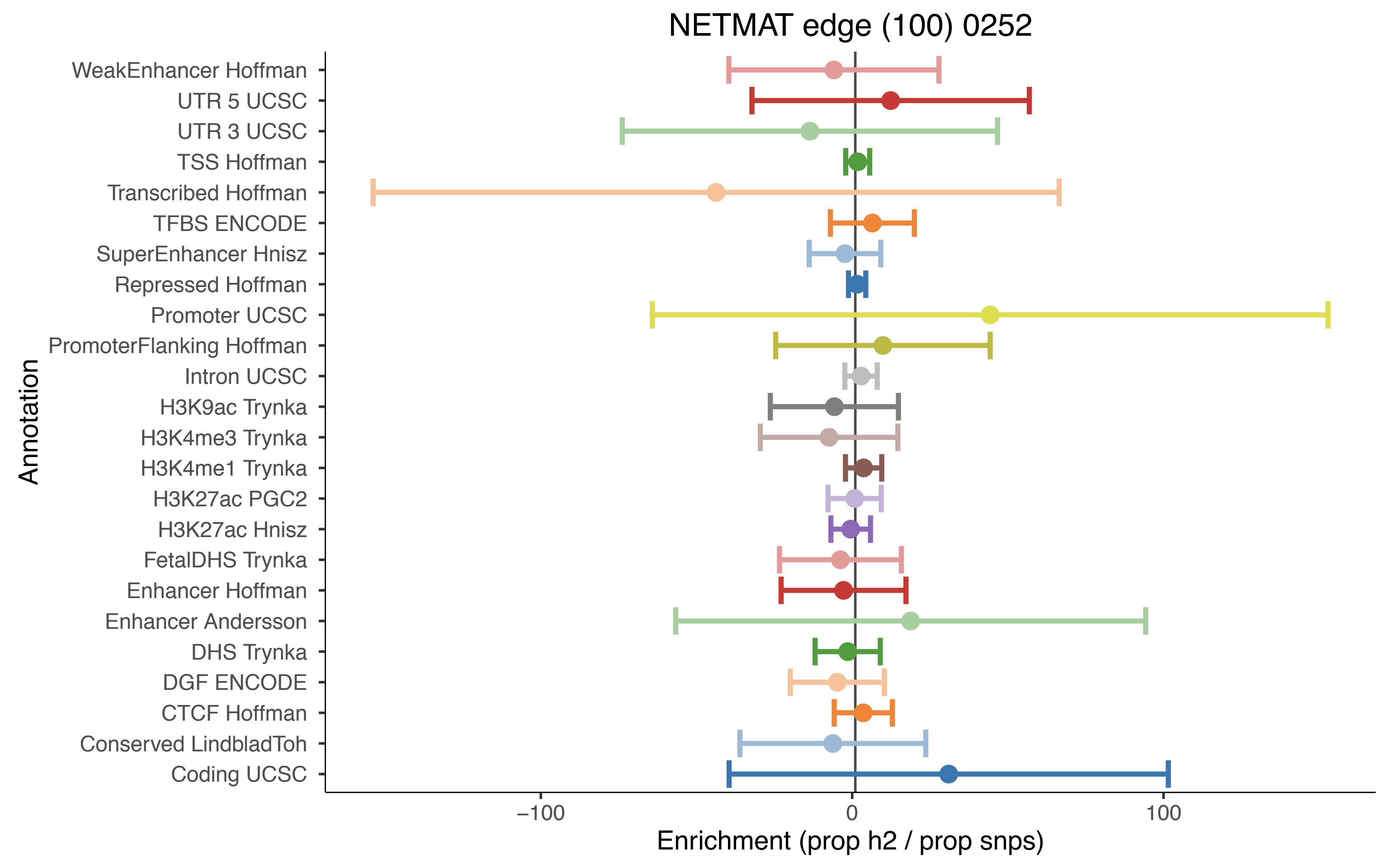
NETMAT edge (100) 0252
Annotation
WeakEnhancer Hoffman
UTR 5 UCSC
UTR 3 UCSC
TSS Hoffman
Transcribed Hoffman
TFBS ENCODE
SuperEnhancer Hnisz
Repressed Hoffman
Promoter UCSC
PromoterFlanking Hoffman
Intron UCSC
H3K9ac Trynka
H3K4me3 Trynka
H3K4me1 Trynka
H3K27ac PGC2
H3K27ac Hnisz
FetalDHS Trynka
Enhancer Hoffman
Enhancer Andersson
DHS Trynka
DGF ENCODE
CTCF Hoffman
Conserved LindbladToh
Coding UCSC
−100
0
100
Enrichment (prop h2 / prop snps)

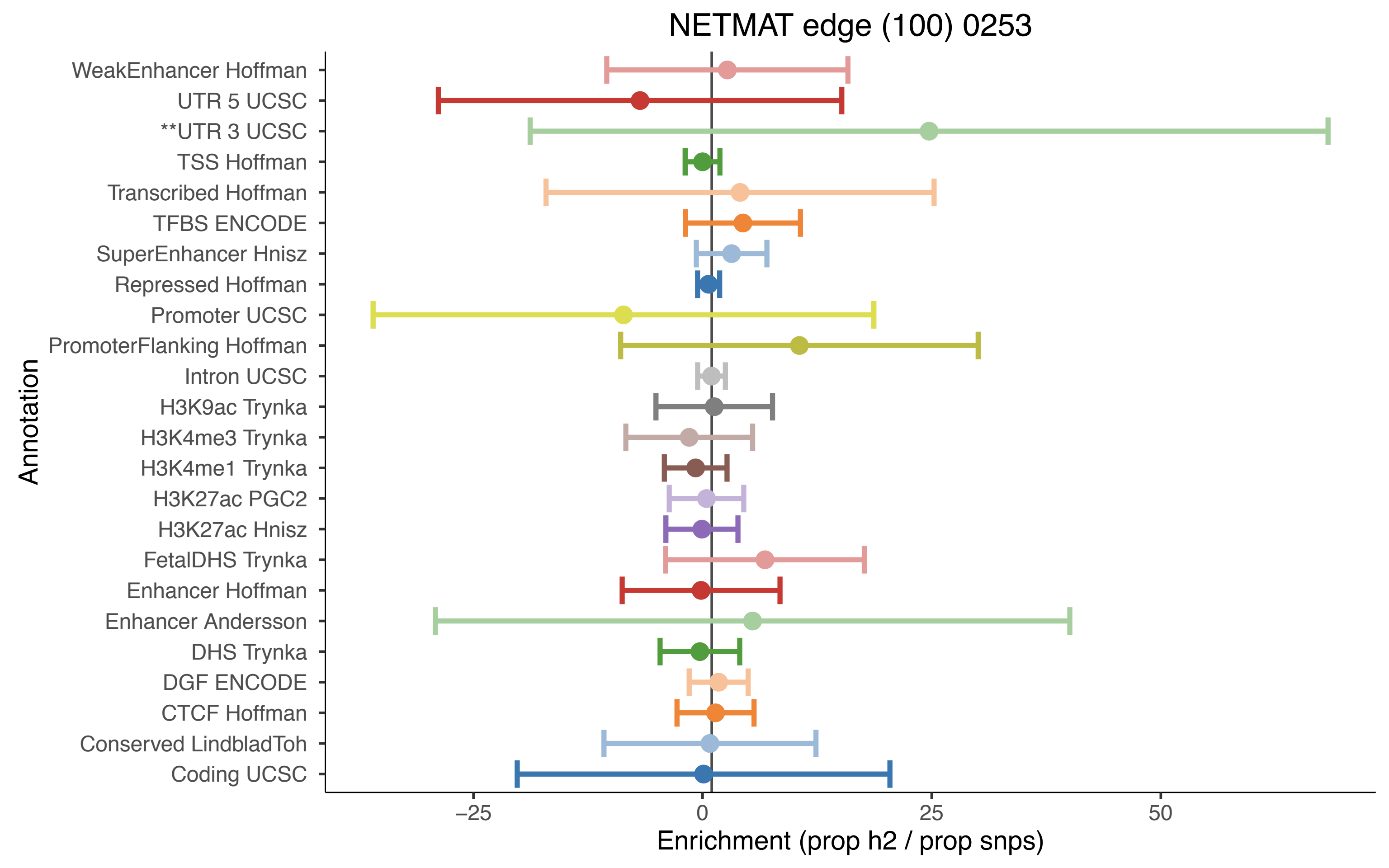
NETMAT edge (100) 0253
WeakEnhancer Hoffman
UTR 5 UCSC
**UTR 3 UCSC
TSS Hoffman
Transcribed Hoffman
TFBS ENCODE
SuperEnhancer Hnisz
Repressed Hoffman
Promoter UCSC
PromoterFlanking Hoffman
Intron UCSC
H3K9ac Trynka
H3K4me3 Trynka
H3K4me1 Trynka
H3K27ac PGC2
H3K27ac Hnisz
FetalDHS Trynka
Enhancer Hoffman
Enhancer Andersson
DHS Trynka
DGF ENCODE
CTCF Hoffman
Conserved LindbladToh
Coding UCSC
Annotation
−25
0
25
50
Enrichment (prop h2 / prop snps)

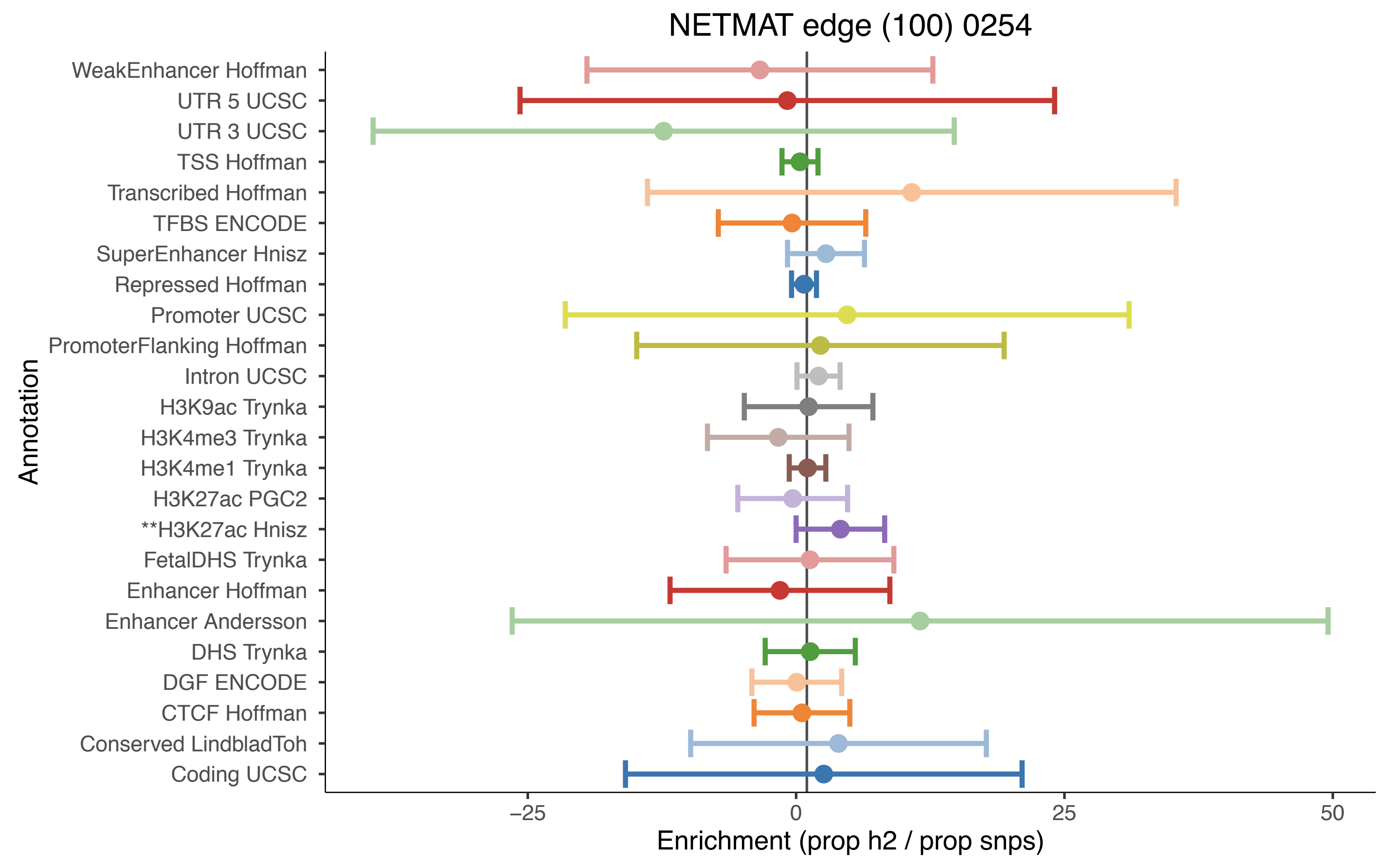

NETMAT edge (100) 0254
WeakEnhancer Hoffman
UTR 5 UCSC
UTR 3 UCSC
TSS Hoffman
Transcribed Hoffman
TFBS ENCODE
SuperEnhancer Hnisz
Repressed Hoffman
Promoter UCSC
PromoterFlanking Hoffman
Intron UCSC
H3K9ac Trynka
H3K4me3 Trynka
H3K4me1 Trynka
H3K27ac PGC2
**H3K27ac Hnisz
FetalDHS Trynka
Enhancer Hoffman
Enhancer Andersson
DHS Trynka
DGF ENCODE
CTCF Hoffman
Conserved LindbladToh
Coding UCSC
Annotation
−25
0
25
50
Enrichment (prop h2 / prop snps)

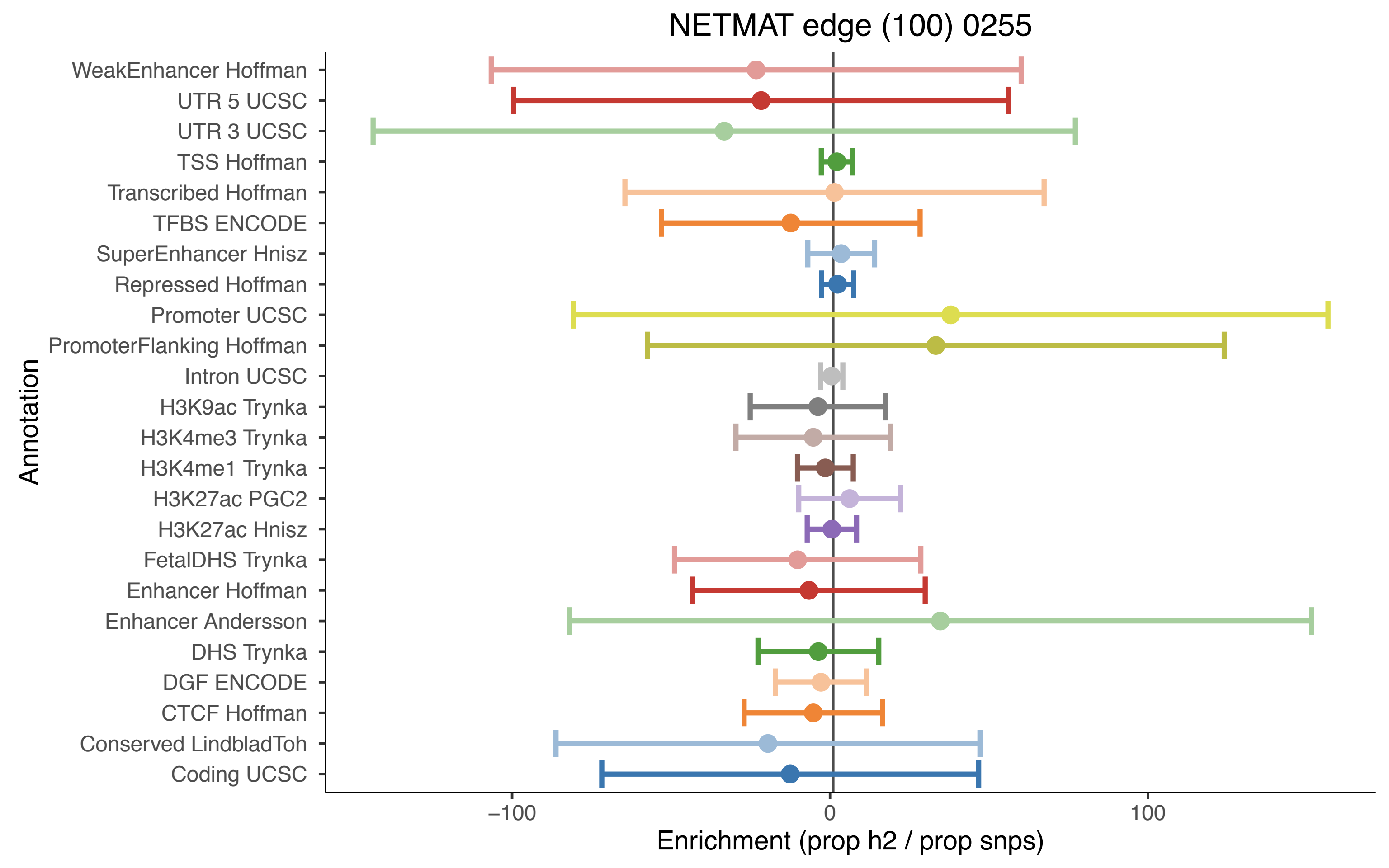

NETMAT edge (100) 0255
WeakEnhancer Hoffman
UTR 5 UCSC
UTR 3 UCSC
TSS Hoffman
Transcribed Hoffman
TFBS ENCODE
SuperEnhancer Hnisz
Repressed Hoffman
Promoter UCSC
PromoterFlanking Hoffman
Intron UCSC
H3K9ac Trynka
H3K4me3 Trynka
H3K4me1 Trynka
H3K27ac PGC2
H3K27ac Hnisz
FetalDHS Trynka
Enhancer Hoffman
Enhancer Andersson
DHS Trynka
DGF ENCODE
CTCF Hoffman
Conserved LindbladToh
Coding UCSC
Annotation
−100
0
100
Enrichment (prop h2 / prop snps)

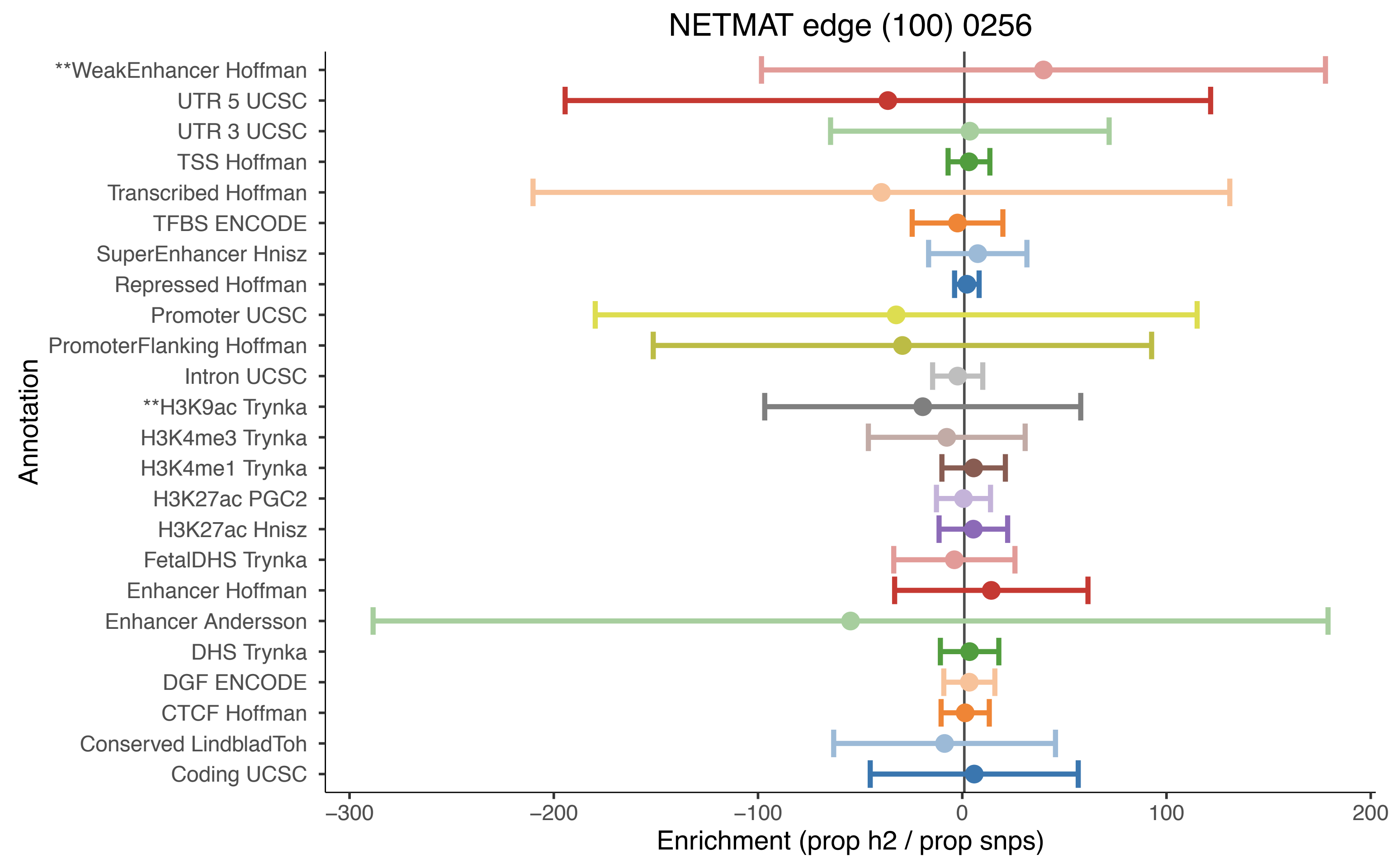
NETMAT edge (100) 0256
**WeakEnhancer Hoffman
UTR 5 UCSC
UTR 3 UCSC
TSS Hoffman
Transcribed Hoffman
TFBS ENCODE
SuperEnhancer Hnisz
Repressed Hoffman
Promoter UCSC
PromoterFlanking Hoffman
Intron UCSC
**H3K9ac Trynka
H3K4me3 Trynka
H3K4me1 Trynka
H3K27ac PGC2
H3K27ac Hnisz
FetalDHS Trynka
Enhancer Hoffman
Enhancer Andersson
DHS Trynka
DGF ENCODE
CTCF Hoffman
Conserved LindbladToh
Coding UCSC
Annotation
−300
−200
−100
0
100
200
Enrichment (prop h2 / prop snps)

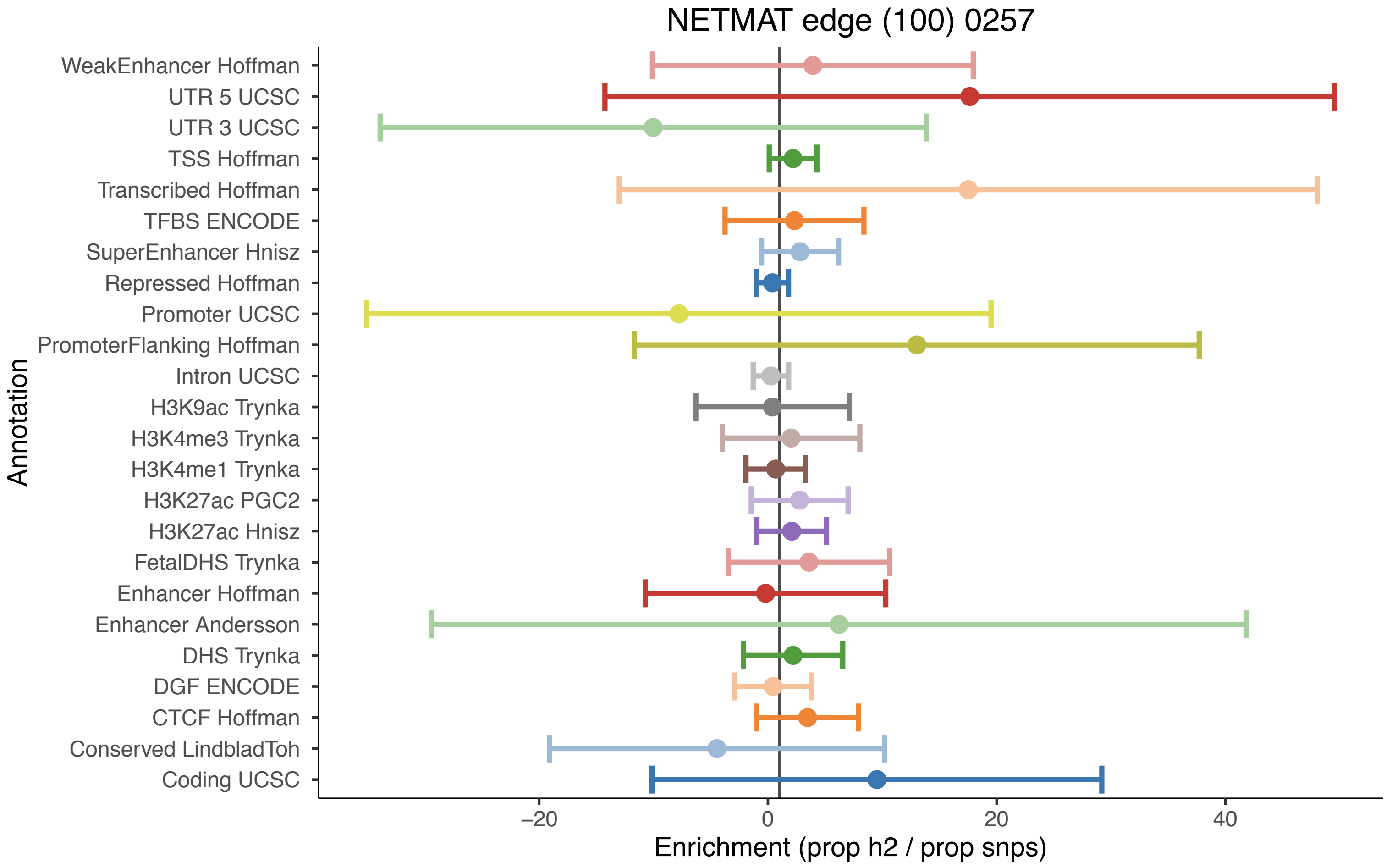
NETMAT edge (100) 0257
WeakEnhancer Hoffman
UTR 5 UCSC
UTR 3 UCSC
TSS Hoffman
Transcribed Hoffman
TFBS ENCODE
SuperEnhancer Hnisz
Repressed Hoffman
Promoter UCSC
PromoterFlanking Hoffman
Intron UCSC
H3K9ac Trynka
H3K4me3 Trynka
H3K4me1 Trynka
H3K27ac PGC2
H3K27ac Hnisz
FetalDHS Trynka
Enhancer Hoffman
Enhancer Andersson
DHS Trynka
DGF ENCODE
CTCF Hoffman
Conserved LindbladToh
Coding UCSC
Annotation
−20
0
20
40
Enrichment (prop h2 / prop snps)

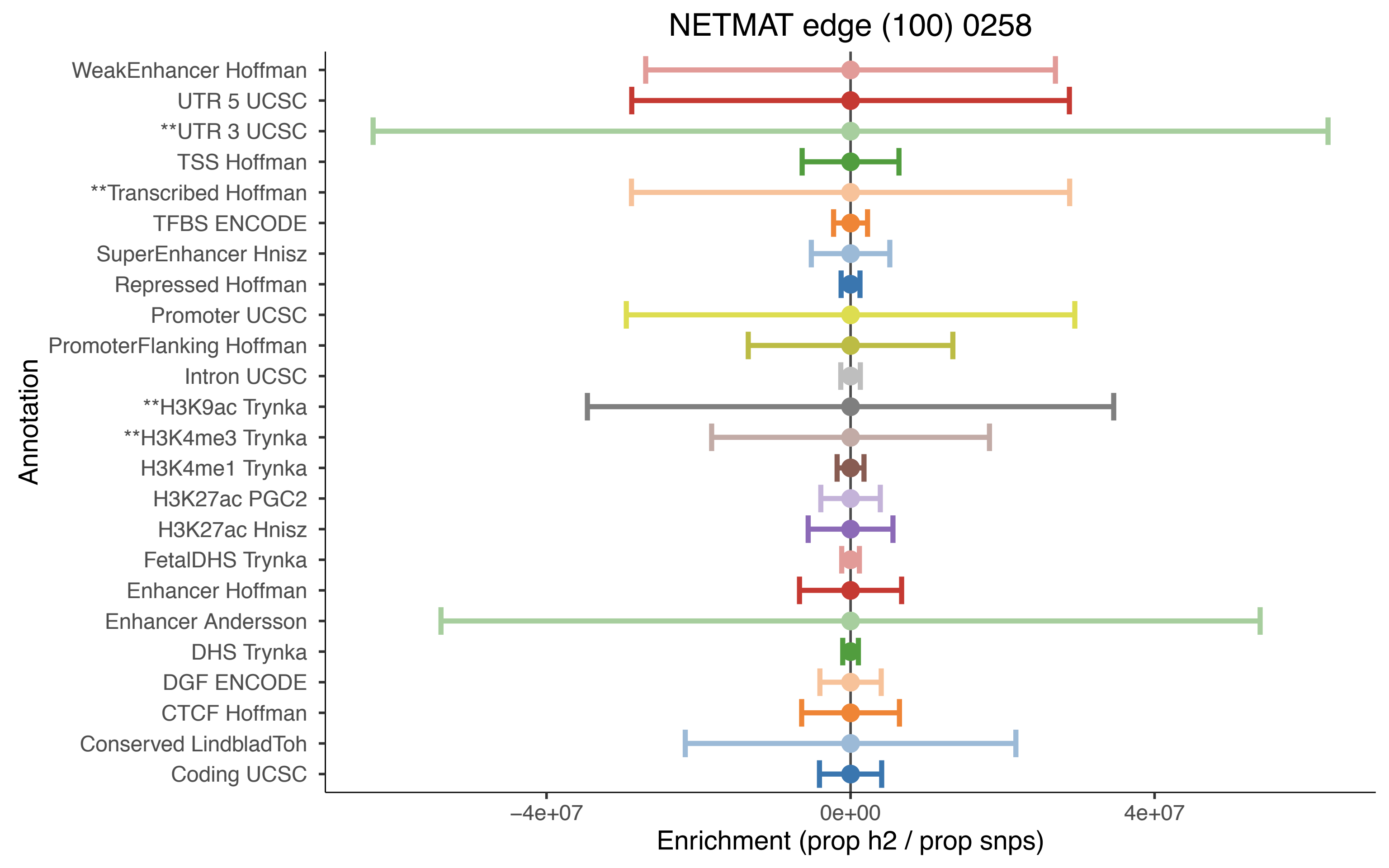
NETMAT edge (100) 0258
WeakEnhancer Hoffman
UTR 5 UCSC
**UTR 3 UCSC
TSS Hoffman
**Transcribed Hoffman
TFBS ENCODE
SuperEnhancer Hnisz
Repressed Hoffman
Promoter UCSC
PromoterFlanking Hoffman
Intron UCSC
**H3K9ac Trynka
**H3K4me3 Trynka
H3K4me1 Trynka
H3K27ac PGC2
H3K27ac Hnisz
FetalDHS Trynka
Enhancer Hoffman
Enhancer Andersson
DHS Trynka
DGF ENCODE
CTCF Hoffman
Conserved LindbladToh
Coding UCSC
Annotation
−4e+07
0e+00
4e+07
Enrichment (prop h2 / prop snps)

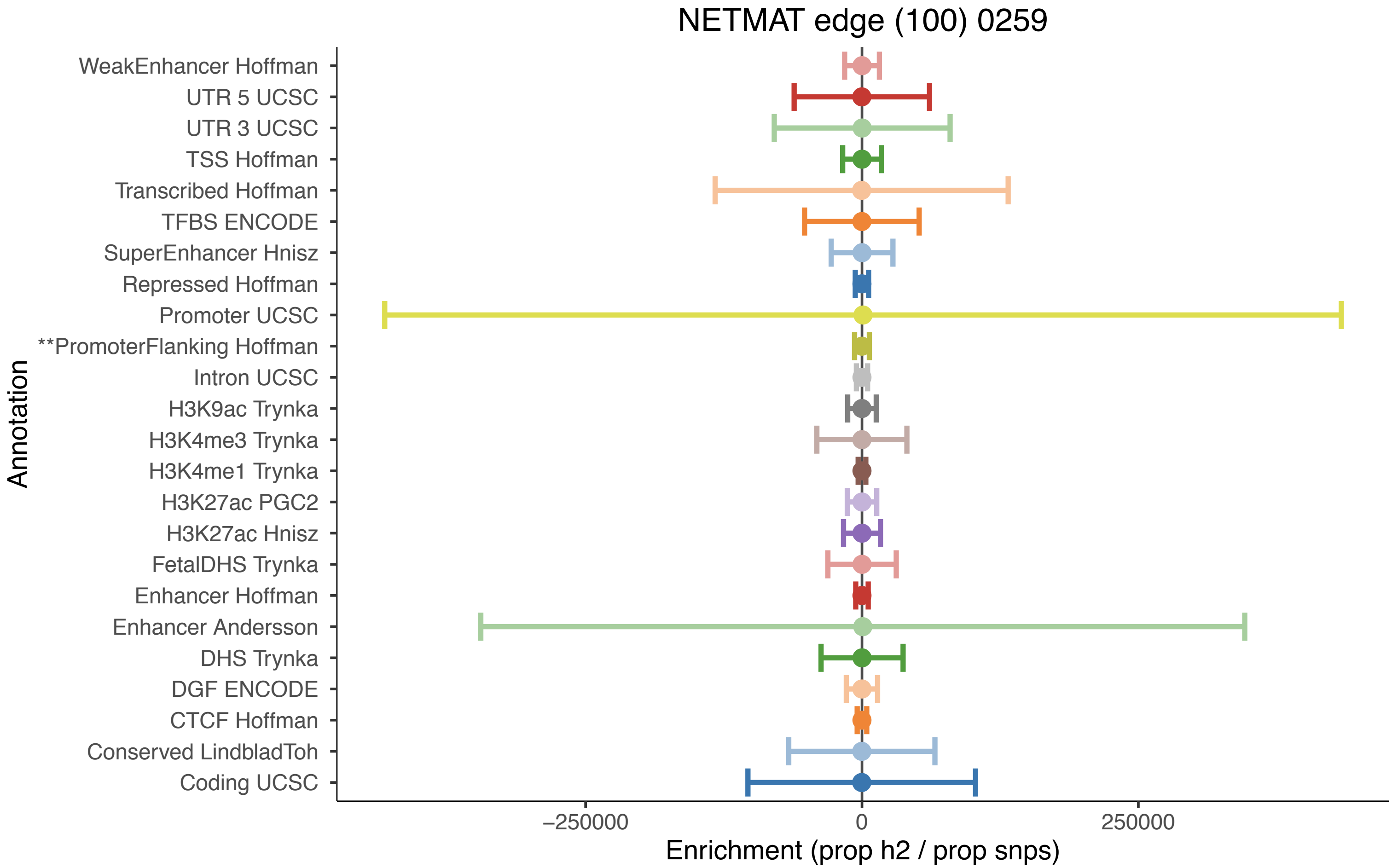
NETMAT edge (100) 0259
WeakEnhancer Hoffman
UTR 5 UCSC
UTR 3 UCSC
TSS Hoffman
Transcribed Hoffman
TFBS ENCODE
SuperEnhancer Hnisz
Repressed Hoffman
Promoter UCSC
**PromoterFlanking Hoffman
Intron UCSC
H3K9ac Trynka
H3K4me3 Trynka
H3K4me1 Trynka
H3K27ac PGC2
H3K27ac Hnisz
FetalDHS Trynka
Enhancer Hoffman
Enhancer Andersson
DHS Trynka
DGF ENCODE
CTCF Hoffman
Conserved LindbladToh
Coding UCSC
Annotation
−250000
0
250000
Enrichment (prop h2 / prop snps)

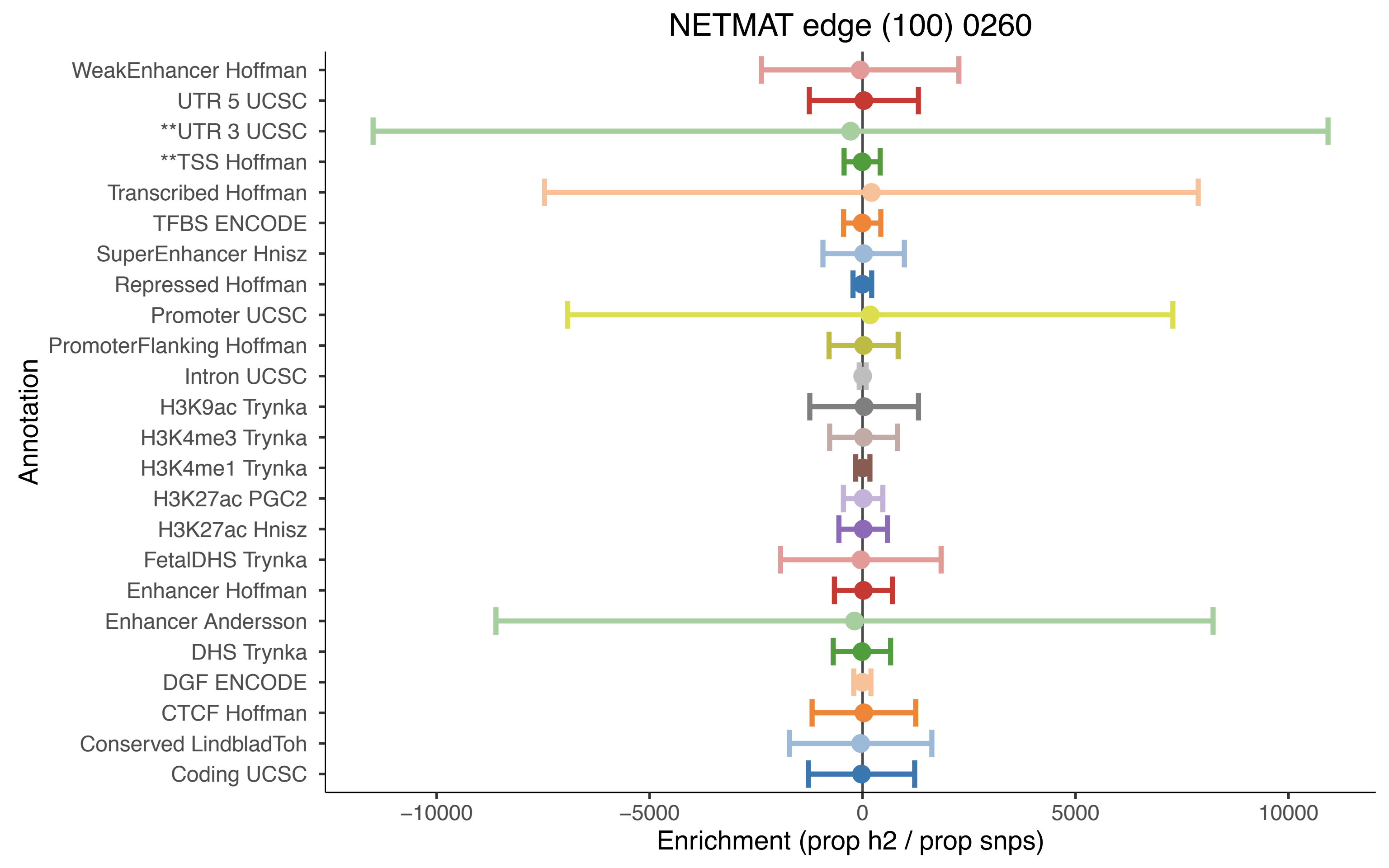

NETMAT edge (100) 0260
WeakEnhancer Hoffman
UTR 5 UCSC
**UTR 3 UCSC
**TSS Hoffman
Transcribed Hoffman
TFBS ENCODE
SuperEnhancer Hnisz
Repressed Hoffman
Promoter UCSC
PromoterFlanking Hoffman
Intron UCSC
H3K9ac Trynka
H3K4me3 Trynka
H3K4me1 Trynka
H3K27ac PGC2
H3K27ac Hnisz
FetalDHS Trynka
Enhancer Hoffman
Enhancer Andersson
DHS Trynka
DGF ENCODE
CTCF Hoffman
Conserved LindbladToh
Coding UCSC
Annotation
−10000
−5000
0
5000
10000
Enrichment (prop h2 / prop snps)

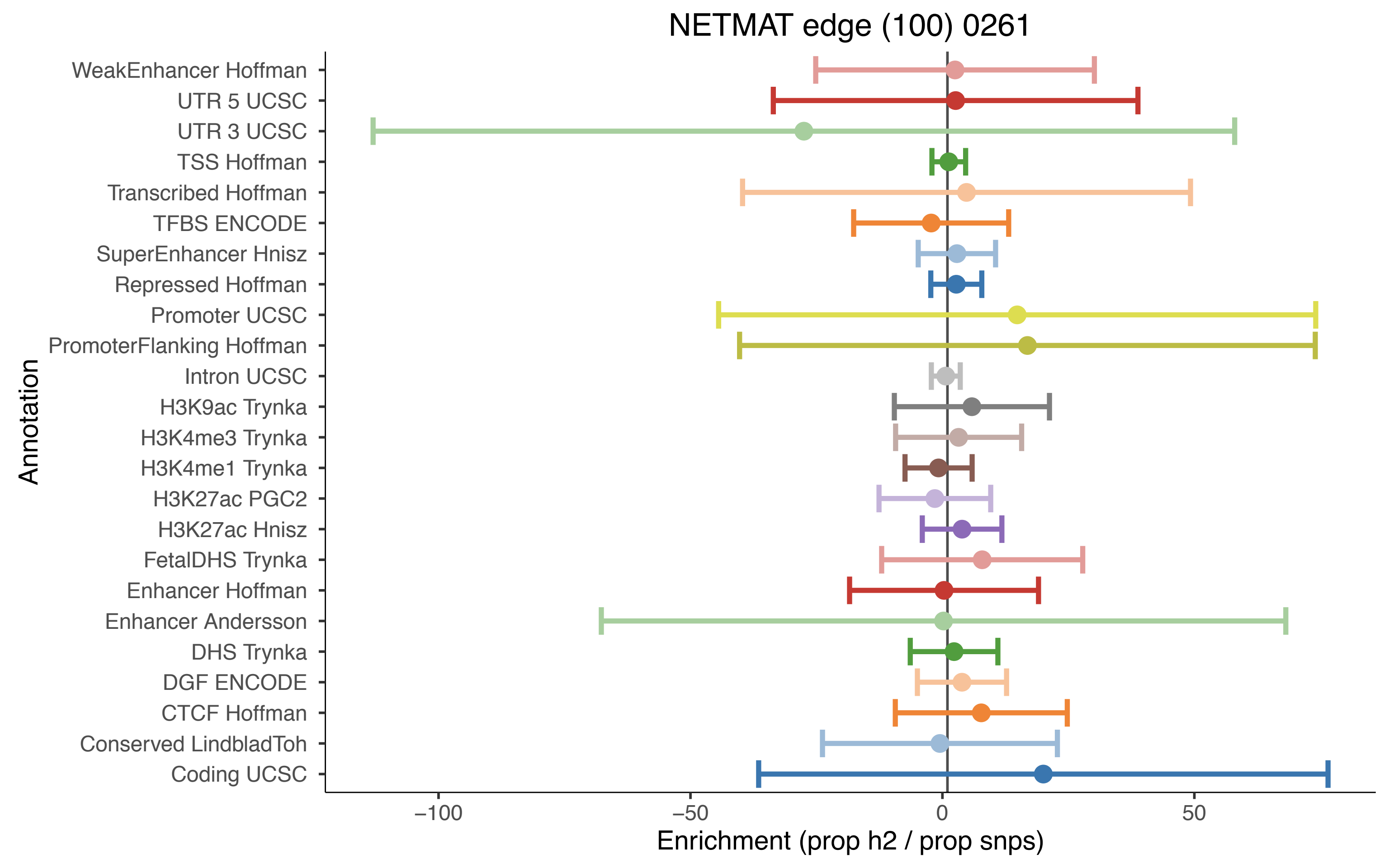
NETMAT edge (100) 0261
WeakEnhancer Hoffman
UTR 5 UCSC
UTR 3 UCSC
TSS Hoffman
Transcribed Hoffman
TFBS ENCODE
SuperEnhancer Hnisz
Repressed Hoffman
Promoter UCSC
PromoterFlanking Hoffman
Intron UCSC
H3K9ac Trynka
H3K4me3 Trynka
H3K4me1 Trynka
H3K27ac PGC2
H3K27ac Hnisz
FetalDHS Trynka
Enhancer Hoffman
Enhancer Andersson
DHS Trynka
DGF ENCODE
CTCF Hoffman
Conserved LindbladToh
Coding UCSC
Annotation
−100
−50
0
50
Enrichment (prop h2 / prop snps)

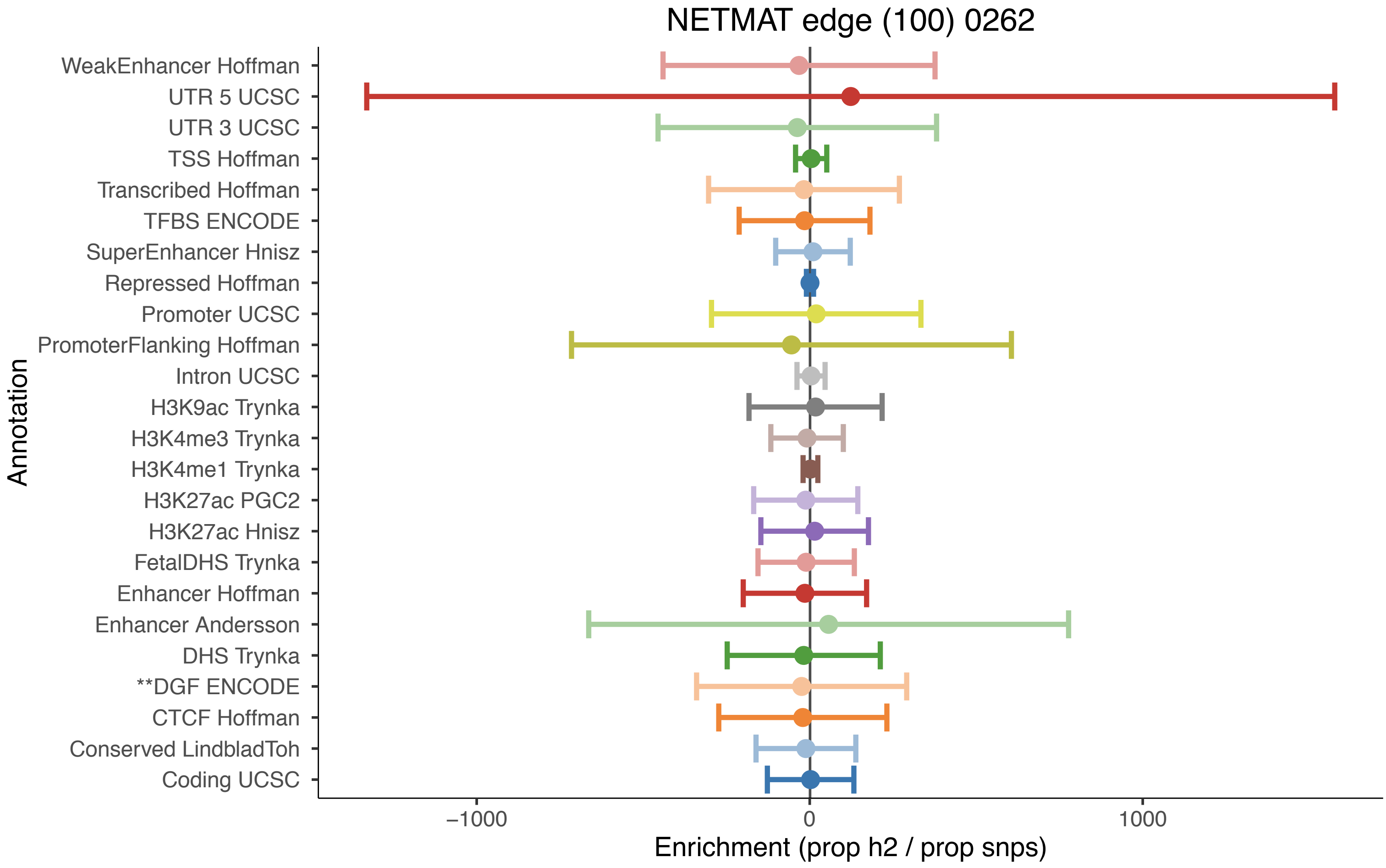
NETMAT edge (100) 0262
Annotation
WeakEnhancer Hoffman
UTR 5 UCSC
UTR 3 UCSC
TSS Hoffman
Transcribed Hoffman
TFBS ENCODE
SuperEnhancer Hnisz
Repressed Hoffman
Promoter UCSC
PromoterFlanking Hoffman
Intron UCSC
H3K9ac Trynka
H3K4me3 Trynka
H3K4me1 Trynka
H3K27ac PGC2
H3K27ac Hnisz
FetalDHS Trynka
Enhancer Hoffman
Enhancer Andersson
DHS Trynka
**DGF ENCODE
CTCF Hoffman
Conserved LindbladToh
Coding UCSC
−1000
0
1000
Enrichment (prop h2 / prop snps)

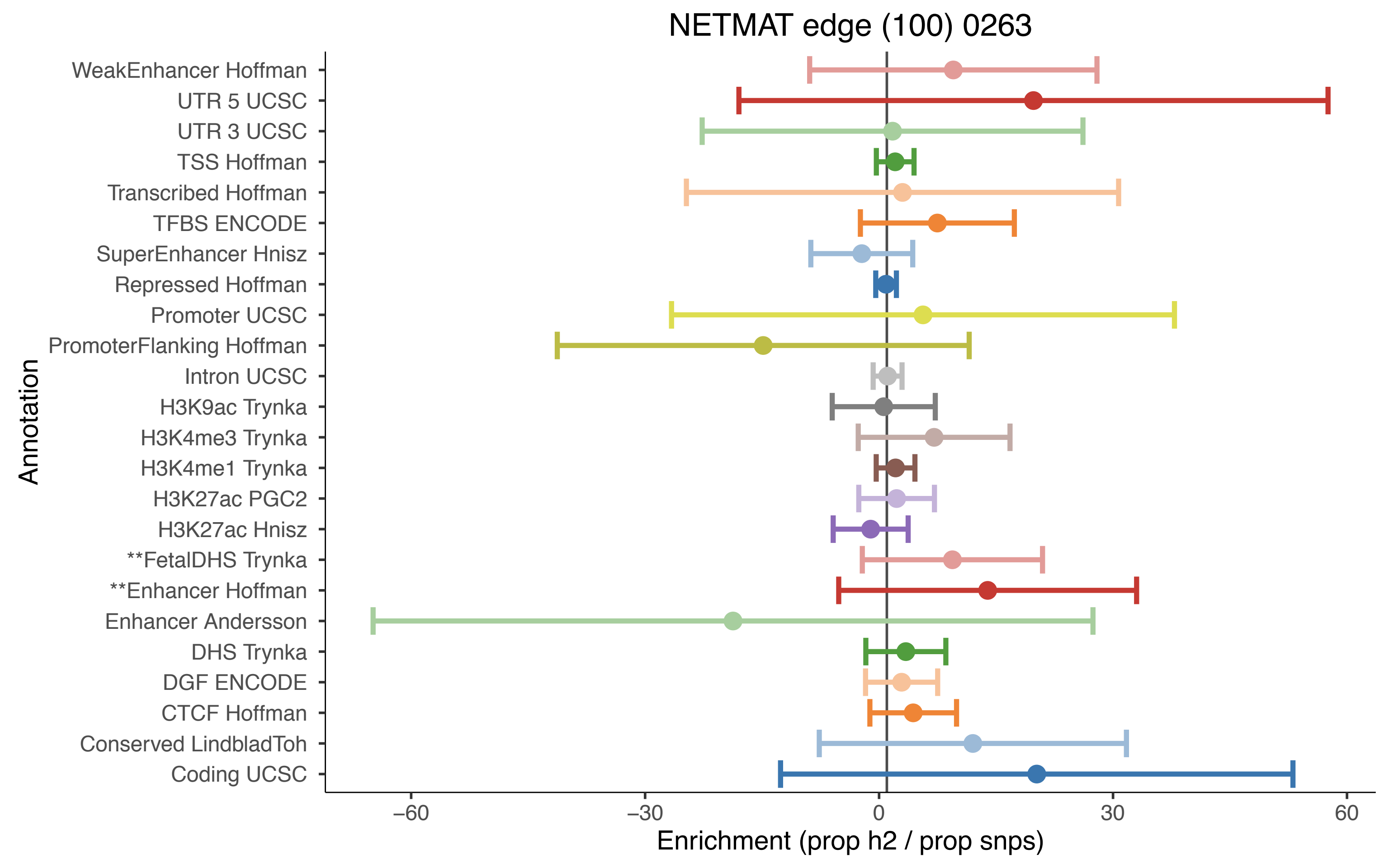
NETMAT edge (100) 0263
WeakEnhancer Hoffman
UTR 5 UCSC
UTR 3 UCSC
TSS Hoffman
Transcribed Hoffman
TFBS ENCODE
SuperEnhancer Hnisz
Repressed Hoffman
Promoter UCSC
PromoterFlanking Hoffman
Intron UCSC
H3K9ac Trynka
H3K4me3 Trynka
H3K4me1 Trynka
H3K27ac PGC2
H3K27ac Hnisz
**FetalDHS Trynka
**Enhancer Hoffman
Enhancer Andersson
DHS Trynka
DGF ENCODE
CTCF Hoffman
Conserved LindbladToh
Coding UCSC
Annotation
−60
−30
0
30
60
Enrichment (prop h2 / prop snps)

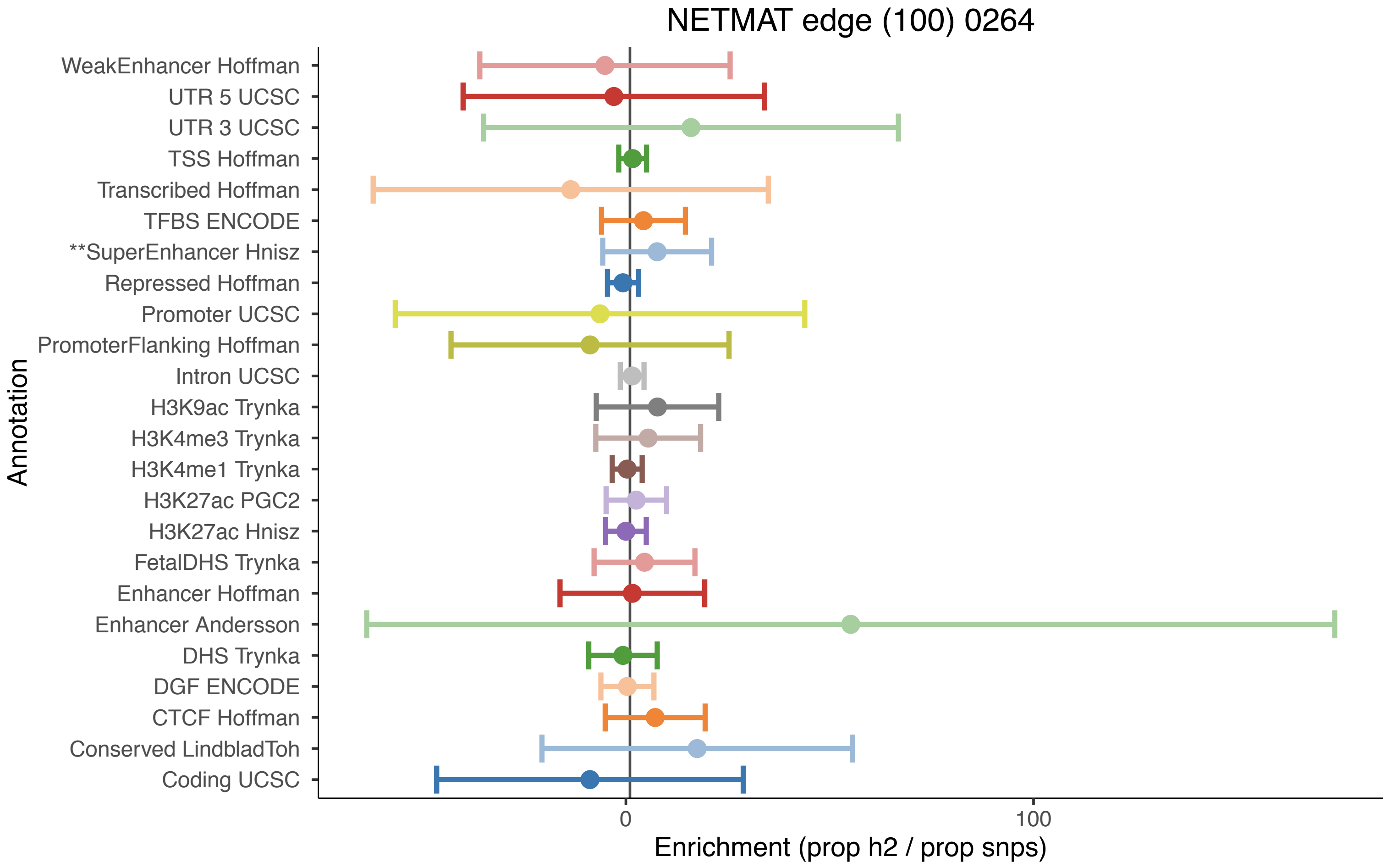

NETMAT edge (100) 0264
WeakEnhancer Hoffman
UTR 5 UCSC
UTR 3 UCSC
TSS Hoffman
Transcribed Hoffman
TFBS ENCODE
**SuperEnhancer Hnisz
Repressed Hoffman
Promoter UCSC
PromoterFlanking Hoffman
Intron UCSC
H3K9ac Trynka
H3K4me3 Trynka
H3K4me1 Trynka
H3K27ac PGC2
H3K27ac Hnisz
FetalDHS Trynka
Enhancer Hoffman
Enhancer Andersson
DHS Trynka
DGF ENCODE
CTCF Hoffman
Conserved LindbladToh
Coding UCSC
Annotation
0
100
Enrichment (prop h2 / prop snps)

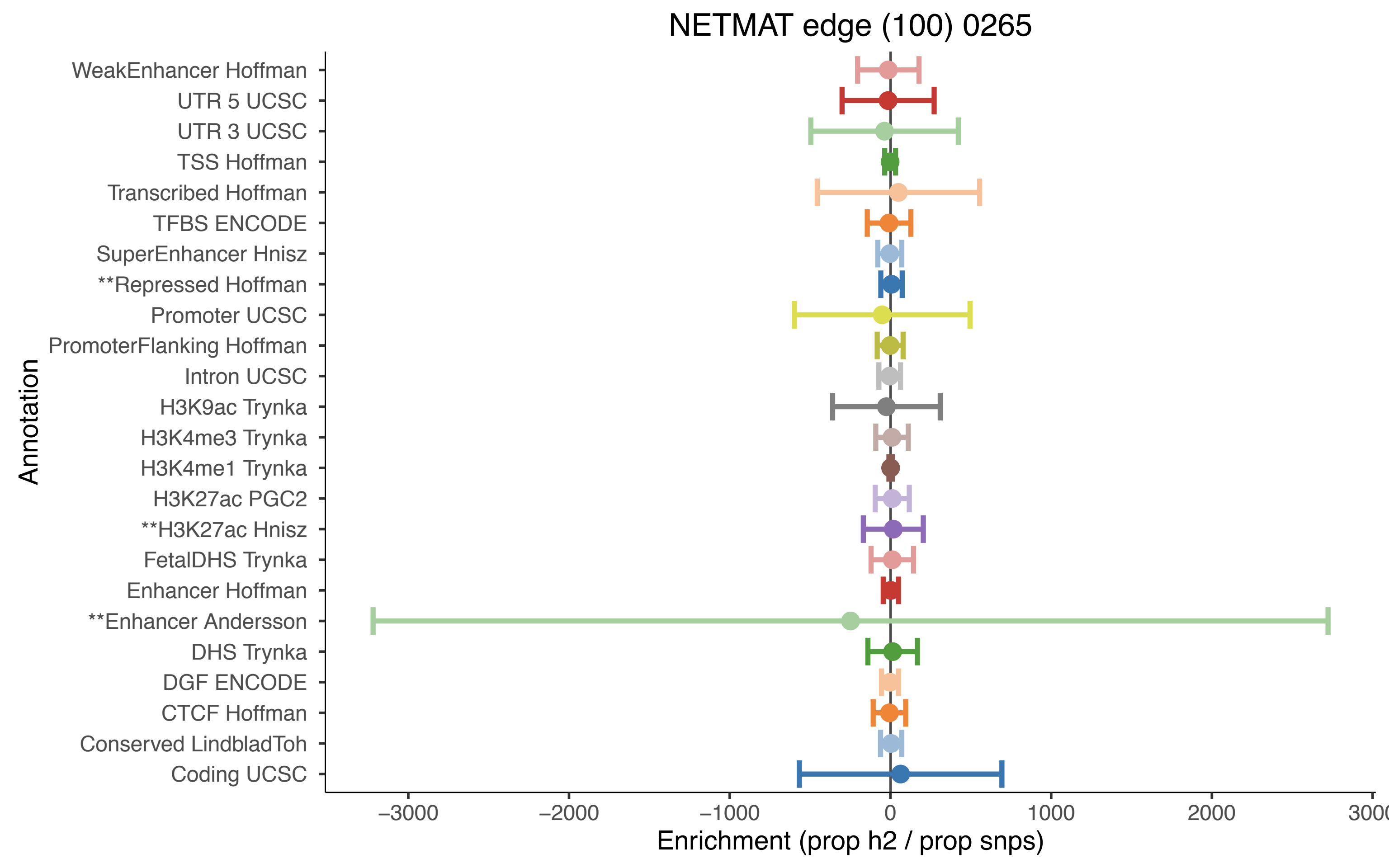

NETMAT edge (100) 0265
WeakEnhancer Hoffman
UTR 5 UCSC
UTR 3 UCSC
TSS Hoffman
Transcribed Hoffman
TFBS ENCODE
SuperEnhancer Hnisz
**Repressed Hoffman
Promoter UCSC
PromoterFlanking Hoffman
Intron UCSC
H3K9ac Trynka
H3K4me3 Trynka
H3K4me1 Trynka
H3K27ac PGC2
**H3K27ac Hnisz
FetalDHS Trynka
Enhancer Hoffman
**Enhancer Andersson
DHS Trynka
DGF ENCODE
CTCF Hoffman
Conserved LindbladToh
Coding UCSC
Annotation
−3000
−2000
−1000
0
1000
2000
Enrichment (prop h2 / prop snps)

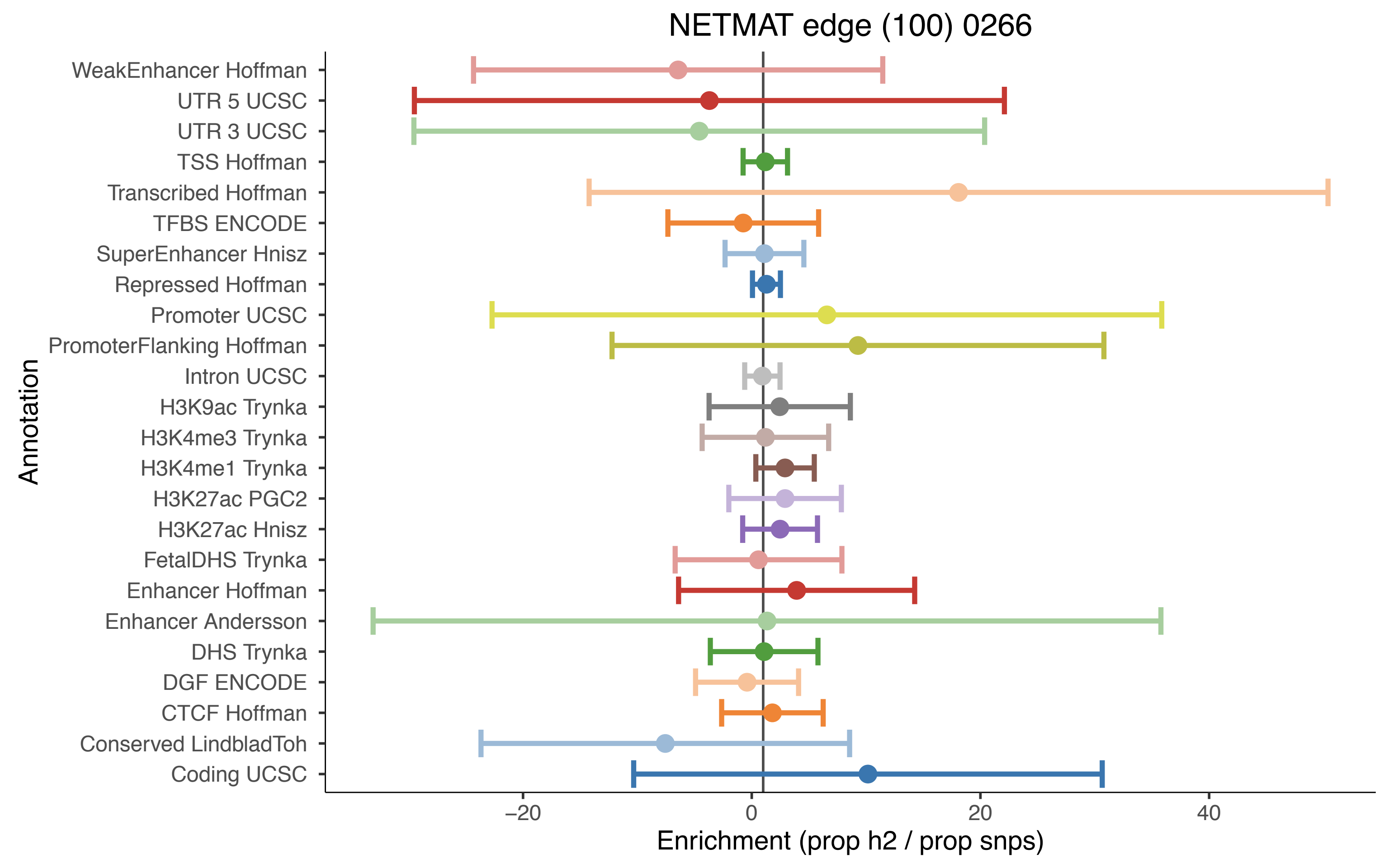
NETMAT edge (100) 0266
WeakEnhancer Hoffman
UTR 5 UCSC
UTR 3 UCSC
TSS Hoffman
Transcribed Hoffman
TFBS ENCODE
SuperEnhancer Hnisz
Repressed Hoffman
Promoter UCSC
PromoterFlanking Hoffman
Intron UCSC
H3K9ac Trynka
H3K4me3 Trynka
H3K4me1 Trynka
H3K27ac PGC2
H3K27ac Hnisz
FetalDHS Trynka
Enhancer Hoffman
Enhancer Andersson
DHS Trynka
DGF ENCODE
CTCF Hoffman
Conserved LindbladToh
Coding UCSC
Annotation
−20
0
20
40
Enrichment (prop h2 / prop snps)

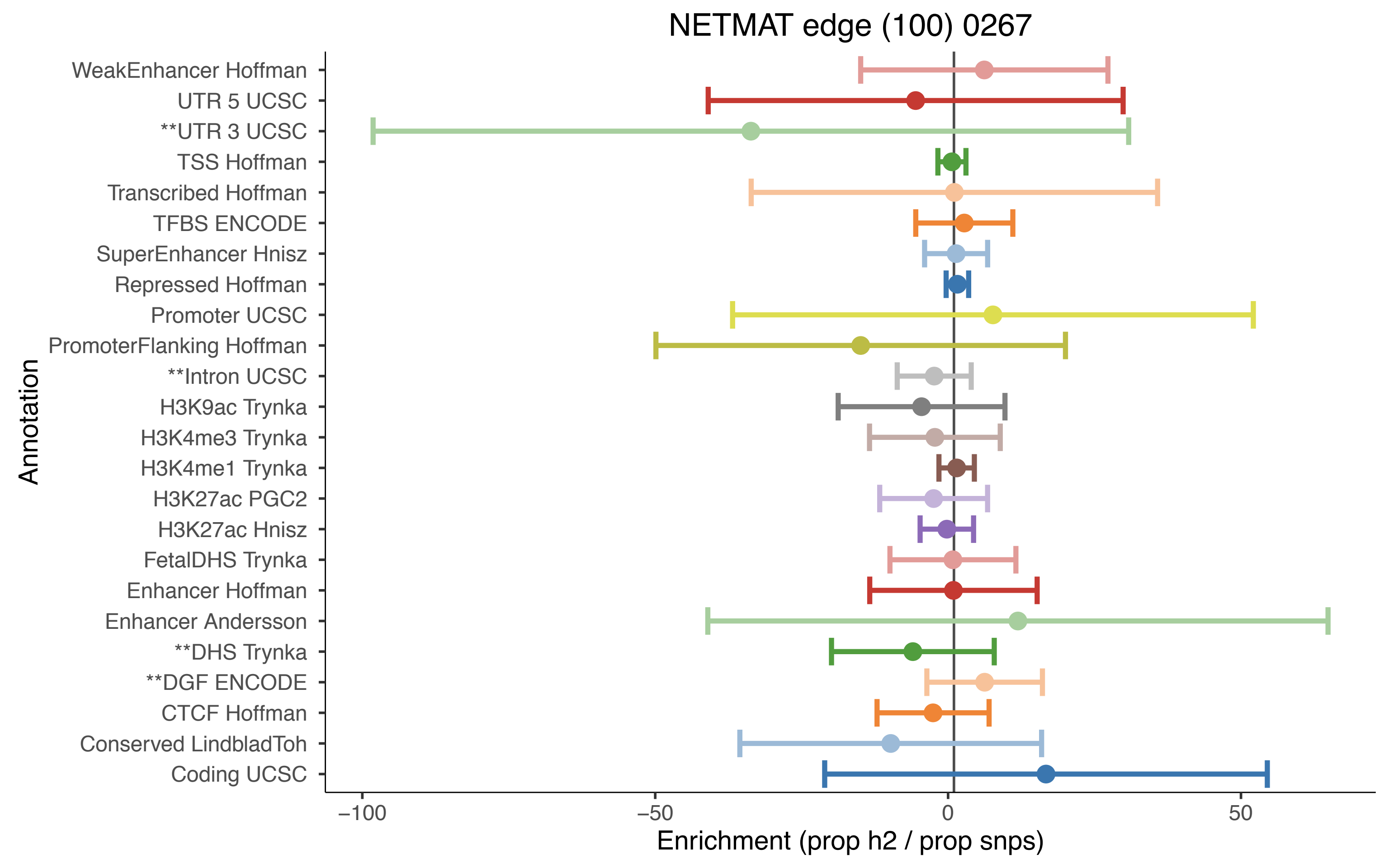

NETMAT edge (100) 0267
WeakEnhancer Hoffman
UTR 5 UCSC
**UTR 3 UCSC
TSS Hoffman
Transcribed Hoffman
TFBS ENCODE
SuperEnhancer Hnisz
Repressed Hoffman
Promoter UCSC
PromoterFlanking Hoffman
**Intron UCSC
H3K9ac Trynka
H3K4me3 Trynka
H3K4me1 Trynka
H3K27ac PGC2
H3K27ac Hnisz
FetalDHS Trynka
Enhancer Hoffman
Enhancer Andersson
**DHS Trynka
**DGF ENCODE
CTCF Hoffman
Conserved LindbladToh
Coding UCSC
Annotation
−100
−50
0
50
Enrichment (prop h2 / prop snps)

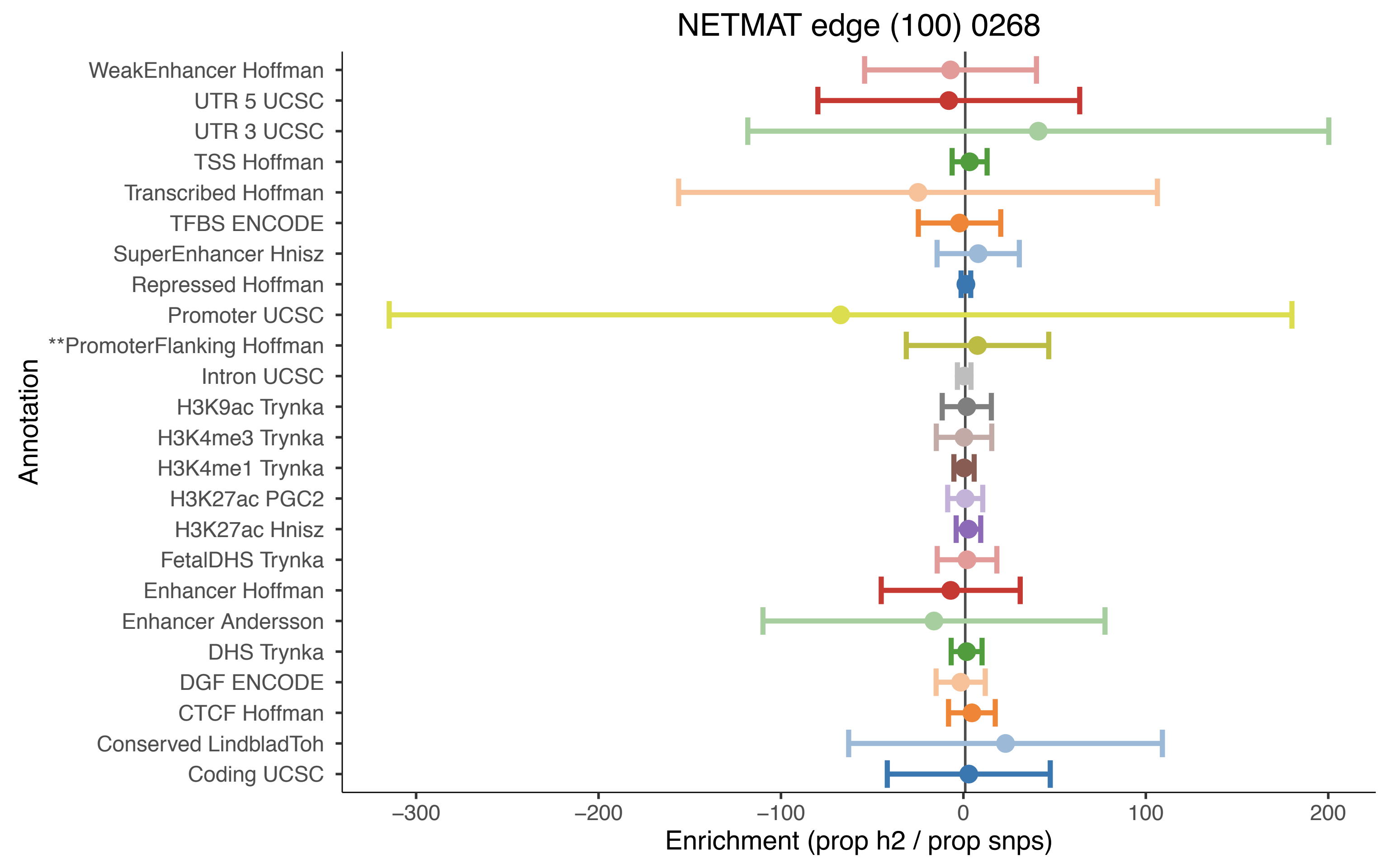

NETMAT edge (100) 0268
WeakEnhancer Hoffman
UTR 5 UCSC
UTR 3 UCSC
TSS Hoffman
Transcribed Hoffman
TFBS ENCODE
SuperEnhancer Hnisz
Repressed Hoffman
Promoter UCSC
**PromoterFlanking Hoffman
Intron UCSC
H3K9ac Trynka
H3K4me3 Trynka
H3K4me1 Trynka
H3K27ac PGC2
H3K27ac Hnisz
FetalDHS Trynka
Enhancer Hoffman
Enhancer Andersson
DHS Trynka
DGF ENCODE
CTCF Hoffman
Conserved LindbladToh
Coding UCSC
Annotation
−300
−200
−100
0
100
200
Enrichment (prop h2 / prop snps)

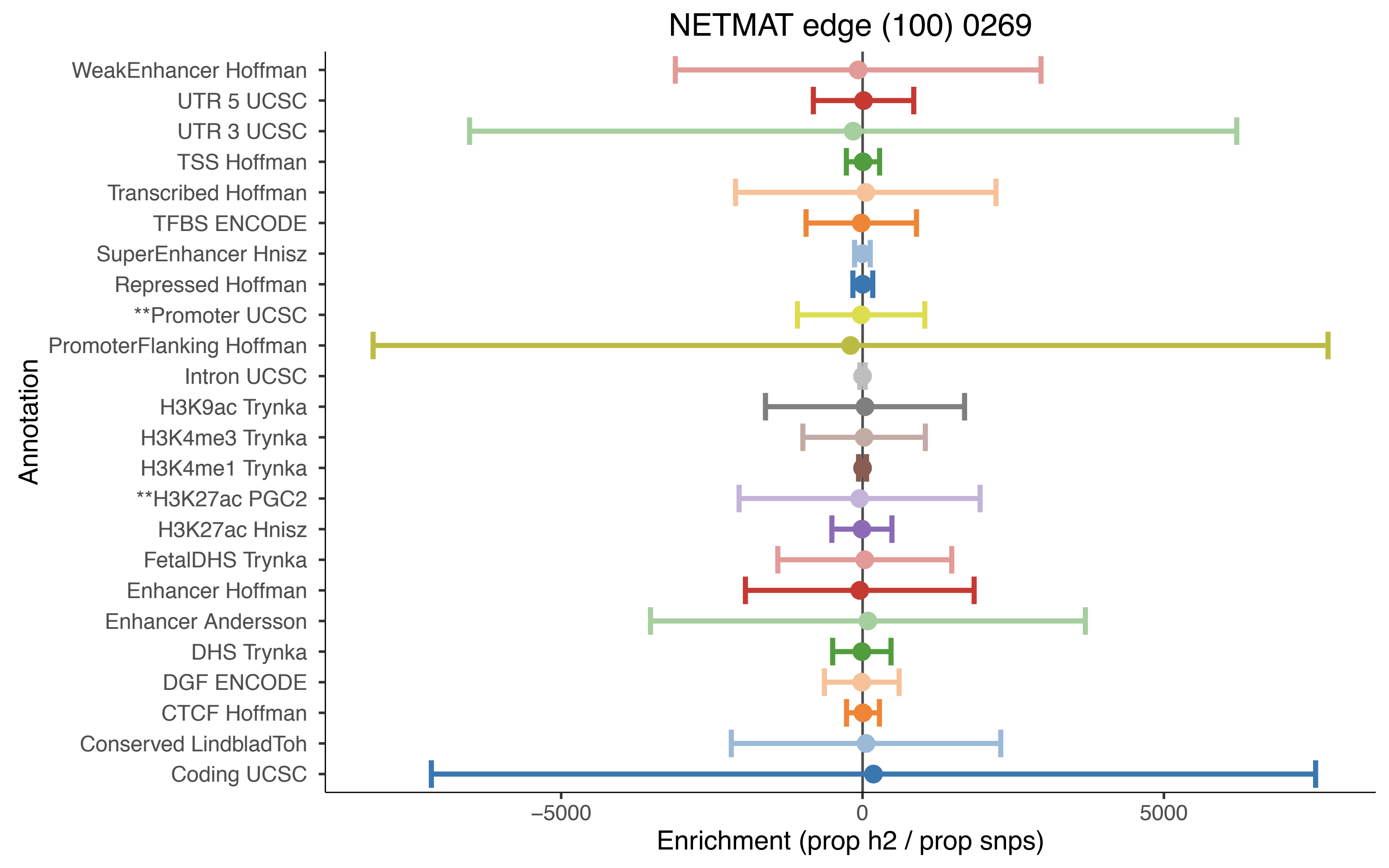
NETMAT edge (100) 0269
WeakEnhancer Hoffman
UTR 5 UCSC
UTR 3 UCSC
TSS Hoffman
Transcribed Hoffman
TFBS ENCODE
SuperEnhancer Hnisz
Repressed Hoffman
**Promoter UCSC
PromoterFlanking Hoffman
Intron UCSC
H3K9ac Trynka
H3K4me3 Trynka
H3K4me1 Trynka
**H3K27ac PGC2
H3K27ac Hnisz
FetalDHS Trynka
Enhancer Hoffman
Enhancer Andersson
DHS Trynka
DGF ENCODE
CTCF Hoffman
Conserved LindbladToh
Coding UCSC
Annotation
−5000
0
5000
Enrichment (prop h2 / prop snps)

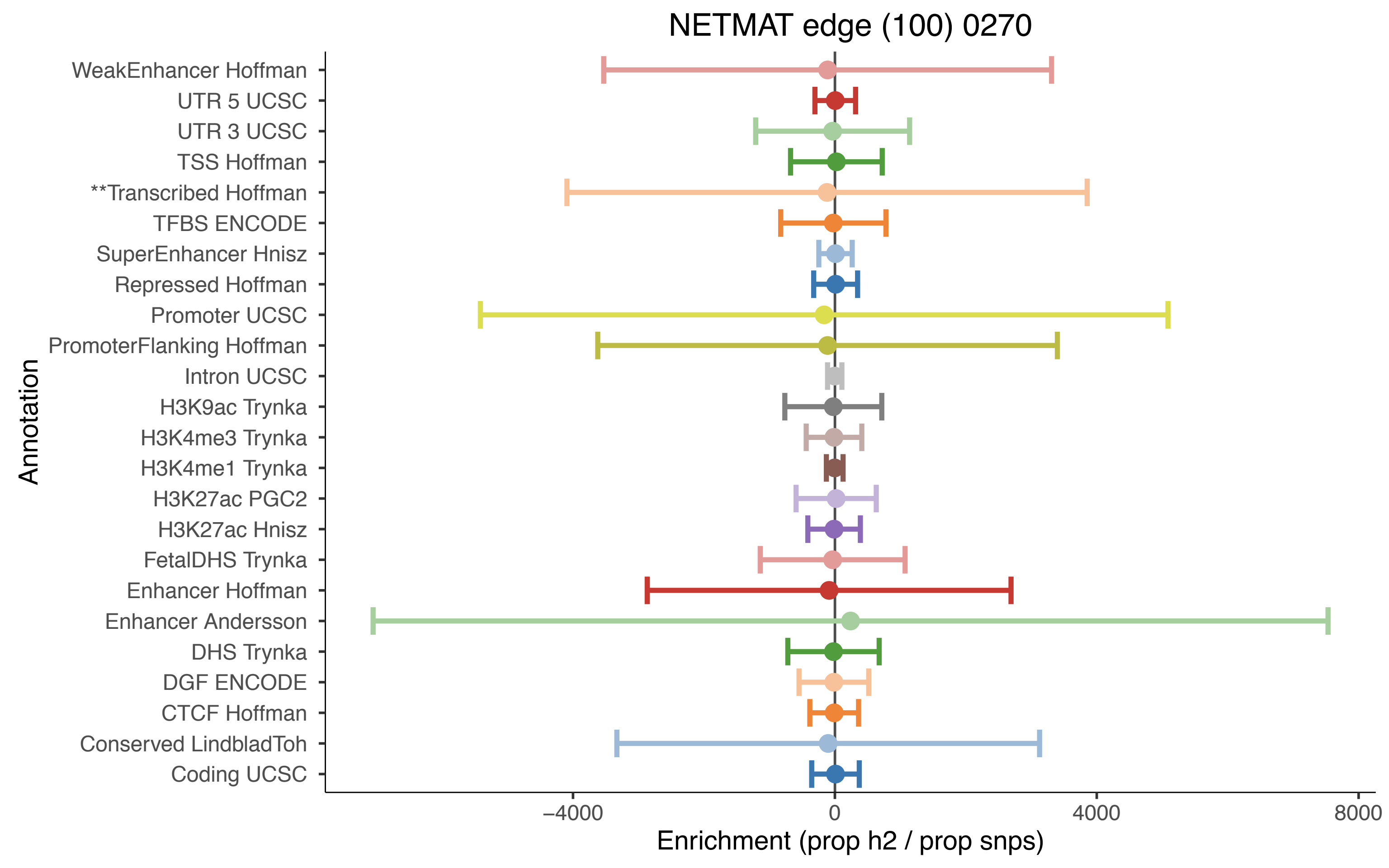

NETMAT edge (100) 0270
WeakEnhancer Hoffman
UTR 5 UCSC
UTR 3 UCSC
TSS Hoffman
**Transcribed Hoffman
TFBS ENCODE
SuperEnhancer Hnisz
Repressed Hoffman
Promoter UCSC
PromoterFlanking Hoffman
Intron UCSC
H3K9ac Trynka
H3K4me3 Trynka
H3K4me1 Trynka
H3K27ac PGC2
H3K27ac Hnisz
FetalDHS Trynka
Enhancer Hoffman
Enhancer Andersson
DHS Trynka
DGF ENCODE
CTCF Hoffman
Conserved LindbladToh
Coding UCSC
Annotation
−4000
0
4000
8000
Enrichment (prop h2 / prop snps)

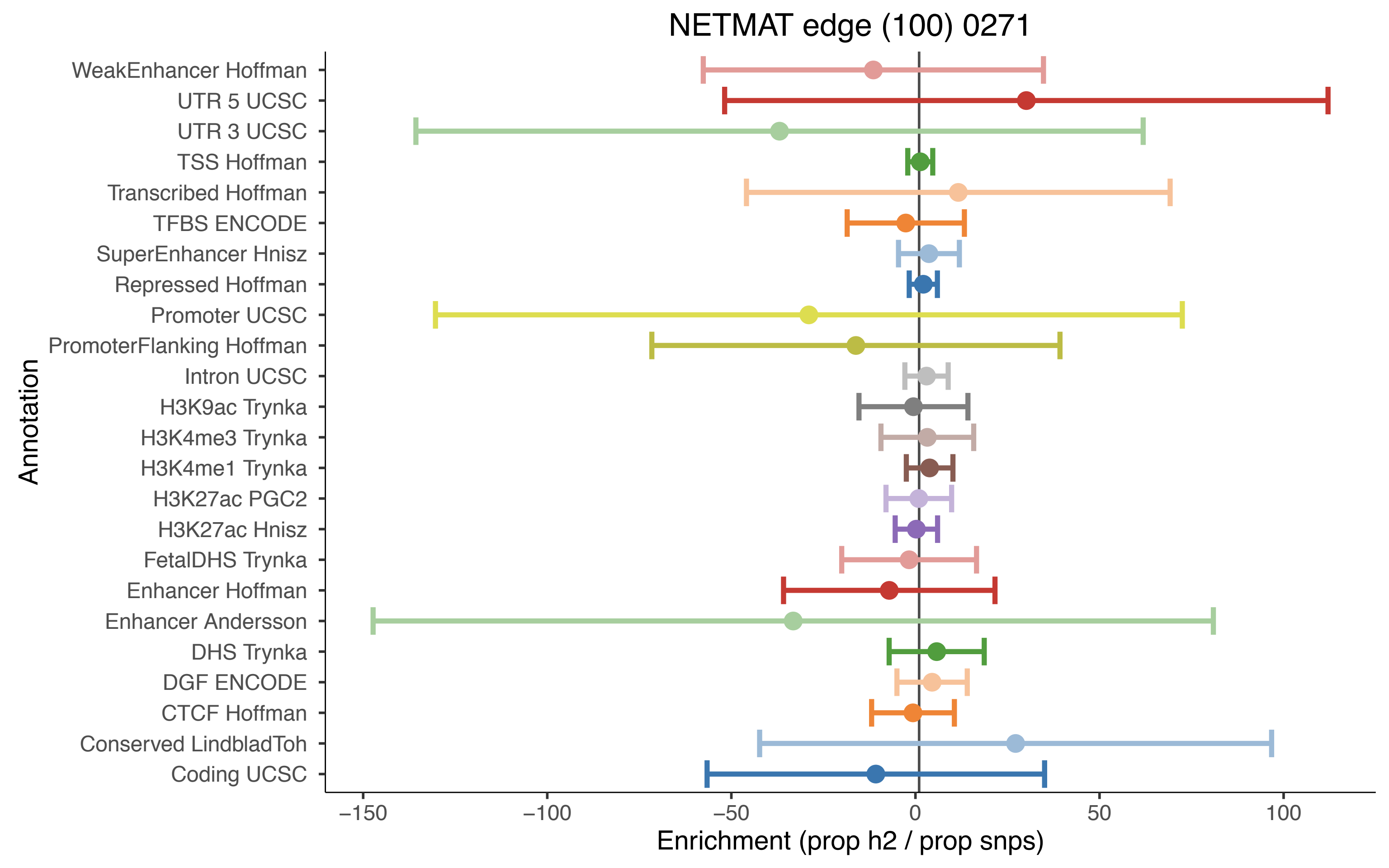

NETMAT edge (100) 0271
Annotation
WeakEnhancer Hoffman
UTR 5 UCSC
UTR 3 UCSC
TSS Hoffman
Transcribed Hoffman
TFBS ENCODE
SuperEnhancer Hnisz
Repressed Hoffman
Promoter UCSC
PromoterFlanking Hoffman
Intron UCSC
H3K9ac Trynka
H3K4me3 Trynka
H3K4me1 Trynka
H3K27ac PGC2
H3K27ac Hnisz
FetalDHS Trynka
Enhancer Hoffman
Enhancer Andersson
DHS Trynka
DGF ENCODE
CTCF Hoffman
Conserved LindbladToh
Coding UCSC
−150
−100
−50
0
50
100
Enrichment (prop h2 / prop snps)

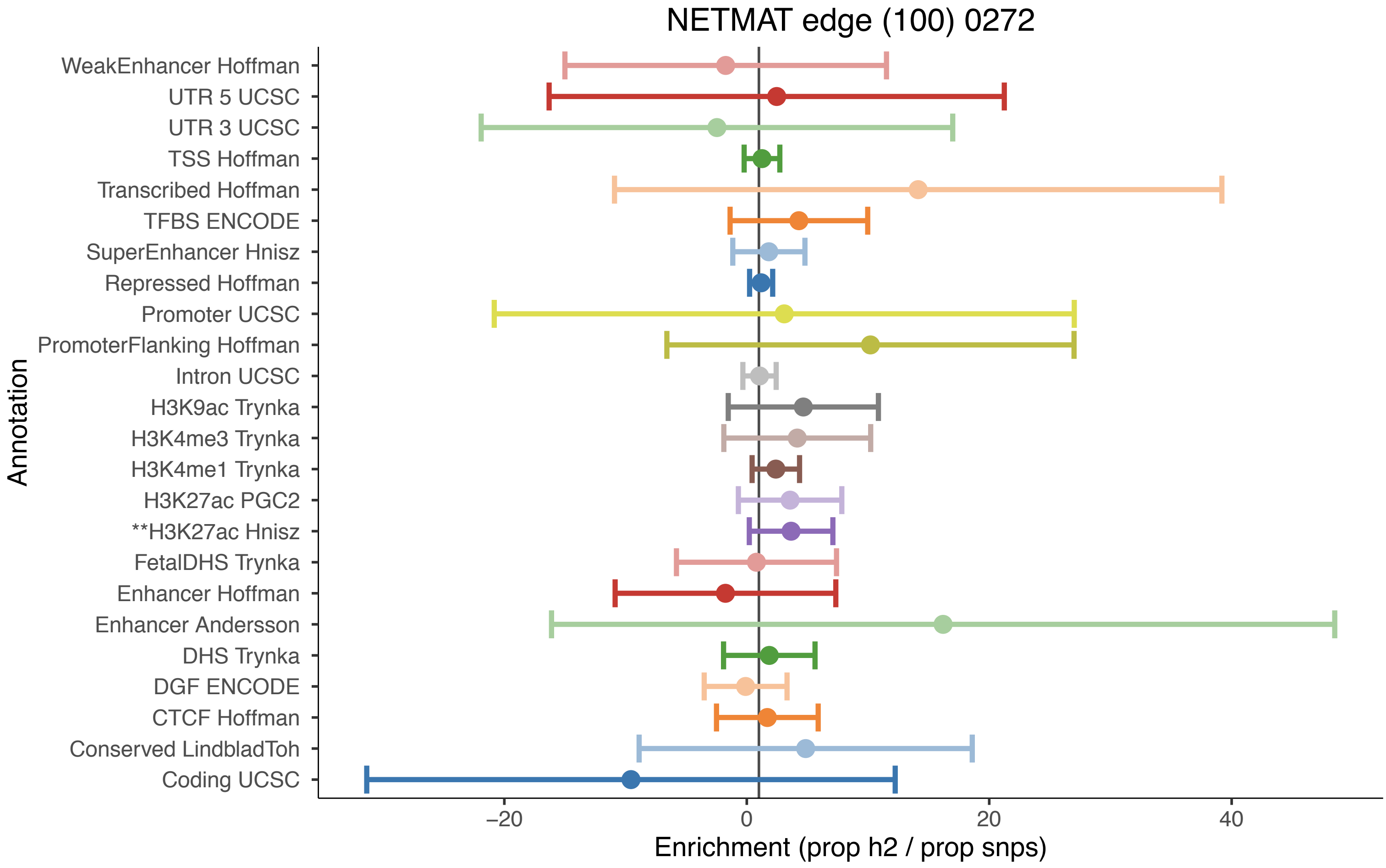
NETMAT edge (100) 0272
Annotation
WeakEnhancer Hoffman
UTR 5 UCSC
UTR 3 UCSC
TSS Hoffman
Transcribed Hoffman
TFBS ENCODE
SuperEnhancer Hnisz
Repressed Hoffman
Promoter UCSC
PromoterFlanking Hoffman
Intron UCSC
H3K9ac Trynka
H3K4me3 Trynka
H3K4me1 Trynka
H3K27ac PGC2
**H3K27ac Hnisz
FetalDHS Trynka
Enhancer Hoffman
Enhancer Andersson
DHS Trynka
DGF ENCODE
CTCF Hoffman
Conserved LindbladToh
Coding UCSC
−20
0
20
40
Enrichment (prop h2 / prop snps)

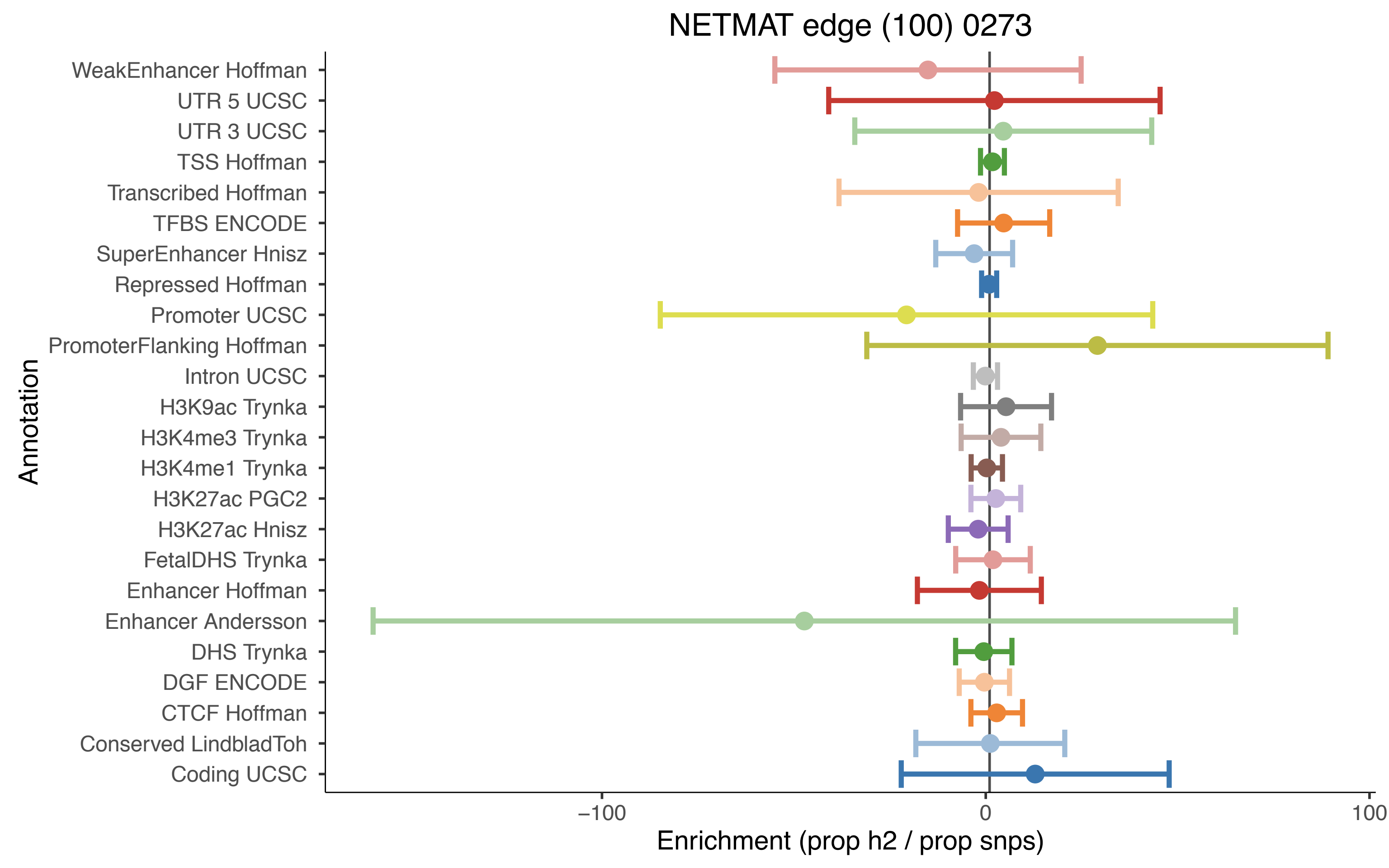

NETMAT edge (100) 0273
WeakEnhancer Hoffman
UTR 5 UCSC
UTR 3 UCSC
TSS Hoffman
Transcribed Hoffman
TFBS ENCODE
SuperEnhancer Hnisz
Repressed Hoffman
Promoter UCSC
PromoterFlanking Hoffman
Intron UCSC
H3K9ac Trynka
H3K4me3 Trynka
H3K4me1 Trynka
H3K27ac PGC2
H3K27ac Hnisz
FetalDHS Trynka
Enhancer Hoffman
Enhancer Andersson
DHS Trynka
DGF ENCODE
CTCF Hoffman
Conserved LindbladToh
Coding UCSC
Annotation
−100
0
100
Enrichment (prop h2 / prop snps)

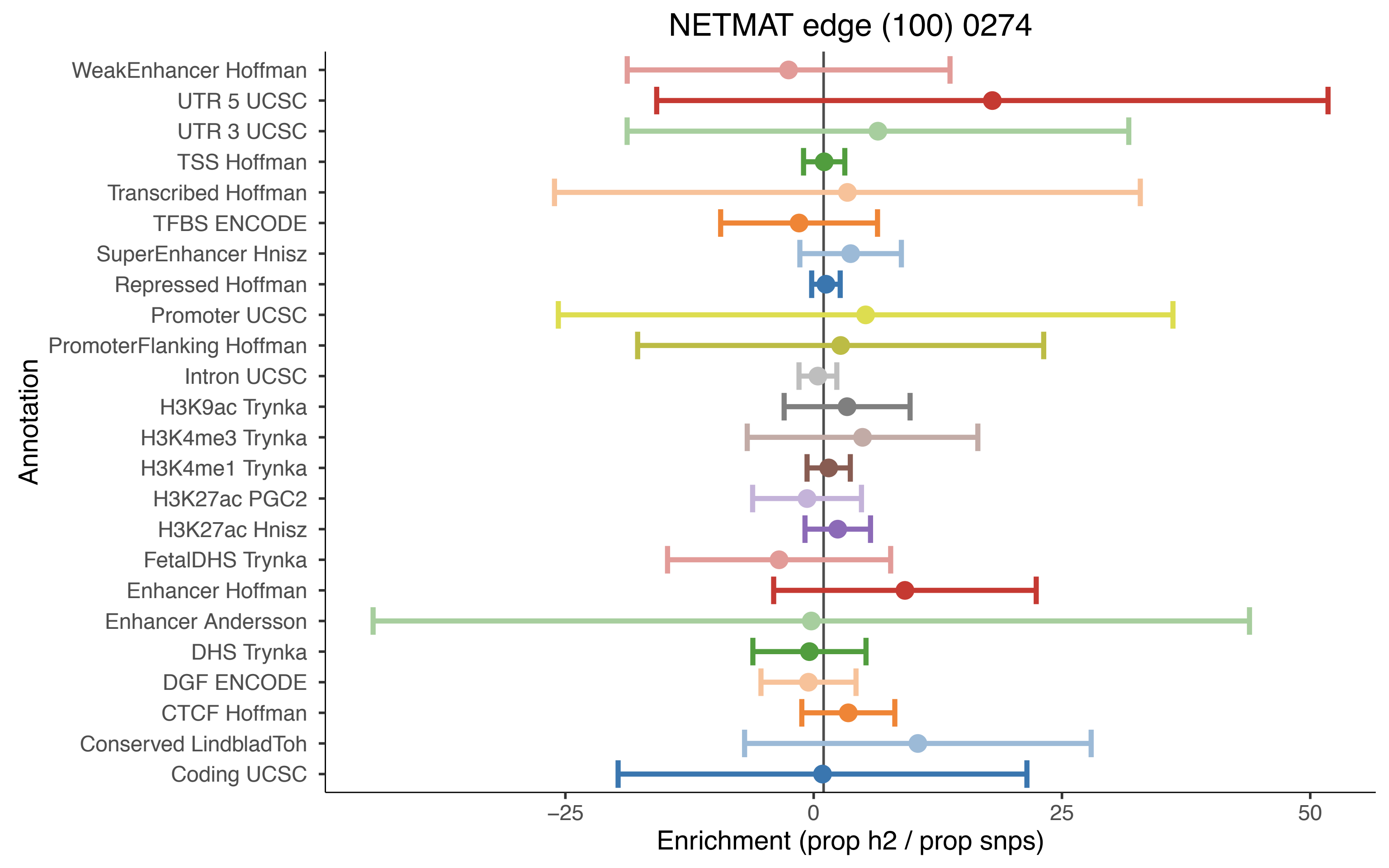
NETMAT edge (100) 0274
Annotation
WeakEnhancer Hoffman
UTR 5 UCSC
UTR 3 UCSC
TSS Hoffman
Transcribed Hoffman
TFBS ENCODE
SuperEnhancer Hnisz
Repressed Hoffman
Promoter UCSC
PromoterFlanking Hoffman
Intron UCSC
H3K9ac Trynka
H3K4me3 Trynka
H3K4me1 Trynka
H3K27ac PGC2
H3K27ac Hnisz
FetalDHS Trynka
Enhancer Hoffman
Enhancer Andersson
DHS Trynka
DGF ENCODE
CTCF Hoffman
Conserved LindbladToh
Coding UCSC
−25
0
25
50
Enrichment (prop h2 / prop snps)

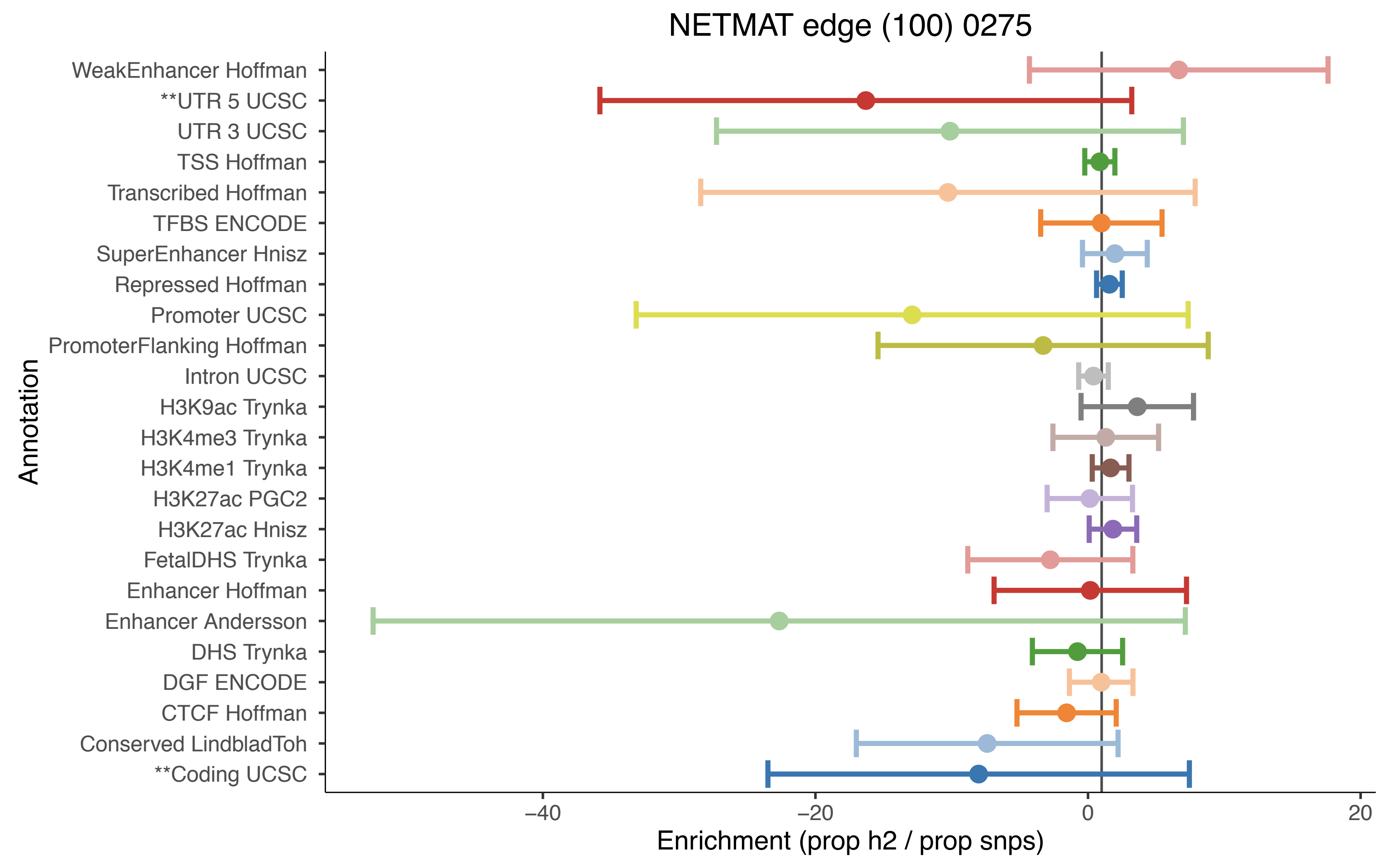
NETMAT edge (100) 0275
WeakEnhancer Hoffman
**UTR 5 UCSC
UTR 3 UCSC
TSS Hoffman
Transcribed Hoffman
TFBS ENCODE
SuperEnhancer Hnisz
Repressed Hoffman
Promoter UCSC
PromoterFlanking Hoffman
Intron UCSC
H3K9ac Trynka
H3K4me3 Trynka
H3K4me1 Trynka
H3K27ac PGC2
H3K27ac Hnisz
FetalDHS Trynka
Enhancer Hoffman
Enhancer Andersson
DHS Trynka
DGF ENCODE
CTCF Hoffman
Conserved LindbladToh
**Coding UCSC
Annotation
−40
−20
0
20
Enrichment (prop h2 / prop snps)

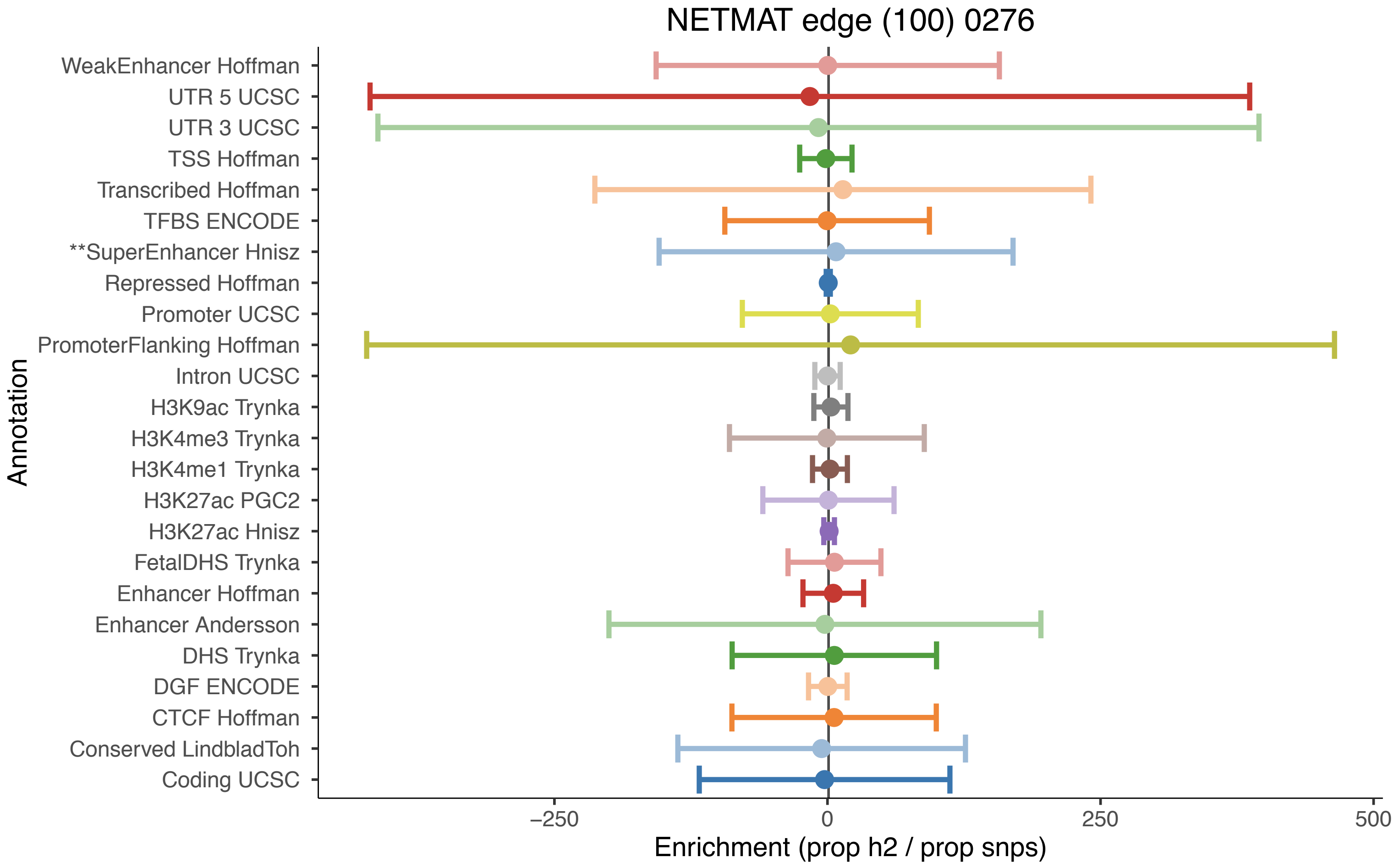

NETMAT edge (100) 0276
WeakEnhancer Hoffman
UTR 5 UCSC
UTR 3 UCSC
TSS Hoffman
Transcribed Hoffman
TFBS ENCODE
**SuperEnhancer Hnisz
Repressed Hoffman
Promoter UCSC
PromoterFlanking Hoffman
Intron UCSC
H3K9ac Trynka
H3K4me3 Trynka
H3K4me1 Trynka
H3K27ac PGC2
H3K27ac Hnisz
FetalDHS Trynka
Enhancer Hoffman
Enhancer Andersson
DHS Trynka
DGF ENCODE
CTCF Hoffman
Conserved LindbladToh
Coding UCSC
Annotation
−250
0
250
500
Enrichment (prop h2 / prop snps)

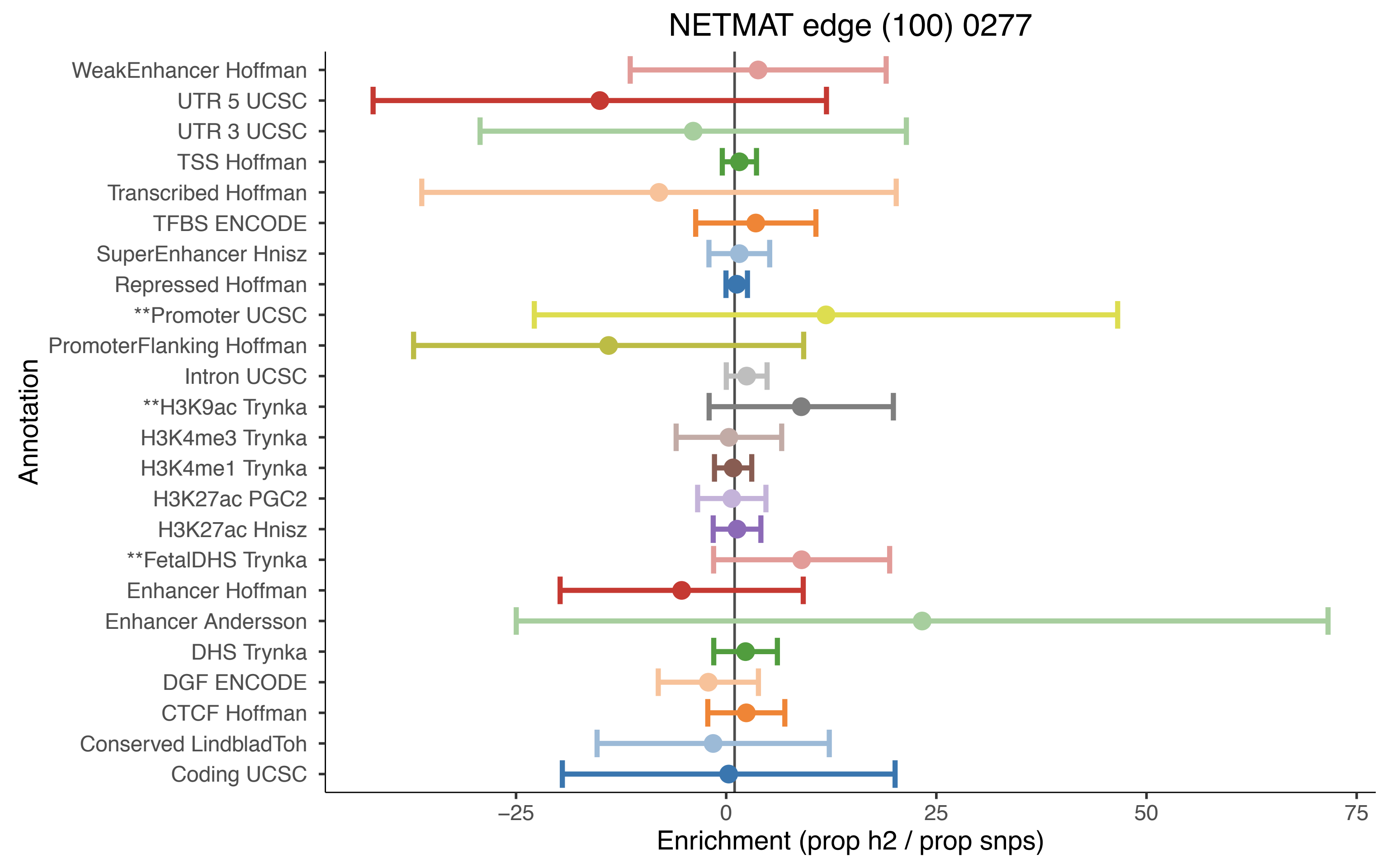

NETMAT edge (100) 0277
WeakEnhancer Hoffman
UTR 5 UCSC
UTR 3 UCSC
TSS Hoffman
Transcribed Hoffman
TFBS ENCODE
SuperEnhancer Hnisz
Repressed Hoffman
**Promoter UCSC
PromoterFlanking Hoffman
Intron UCSC
**H3K9ac Trynka
H3K4me3 Trynka
H3K4me1 Trynka
H3K27ac PGC2
H3K27ac Hnisz
**FetalDHS Trynka
Enhancer Hoffman
Enhancer Andersson
DHS Trynka
DGF ENCODE
CTCF Hoffman
Conserved LindbladToh
Coding UCSC
Annotation
−25
0
25
50
75
Enrichment (prop h2 / prop snps)

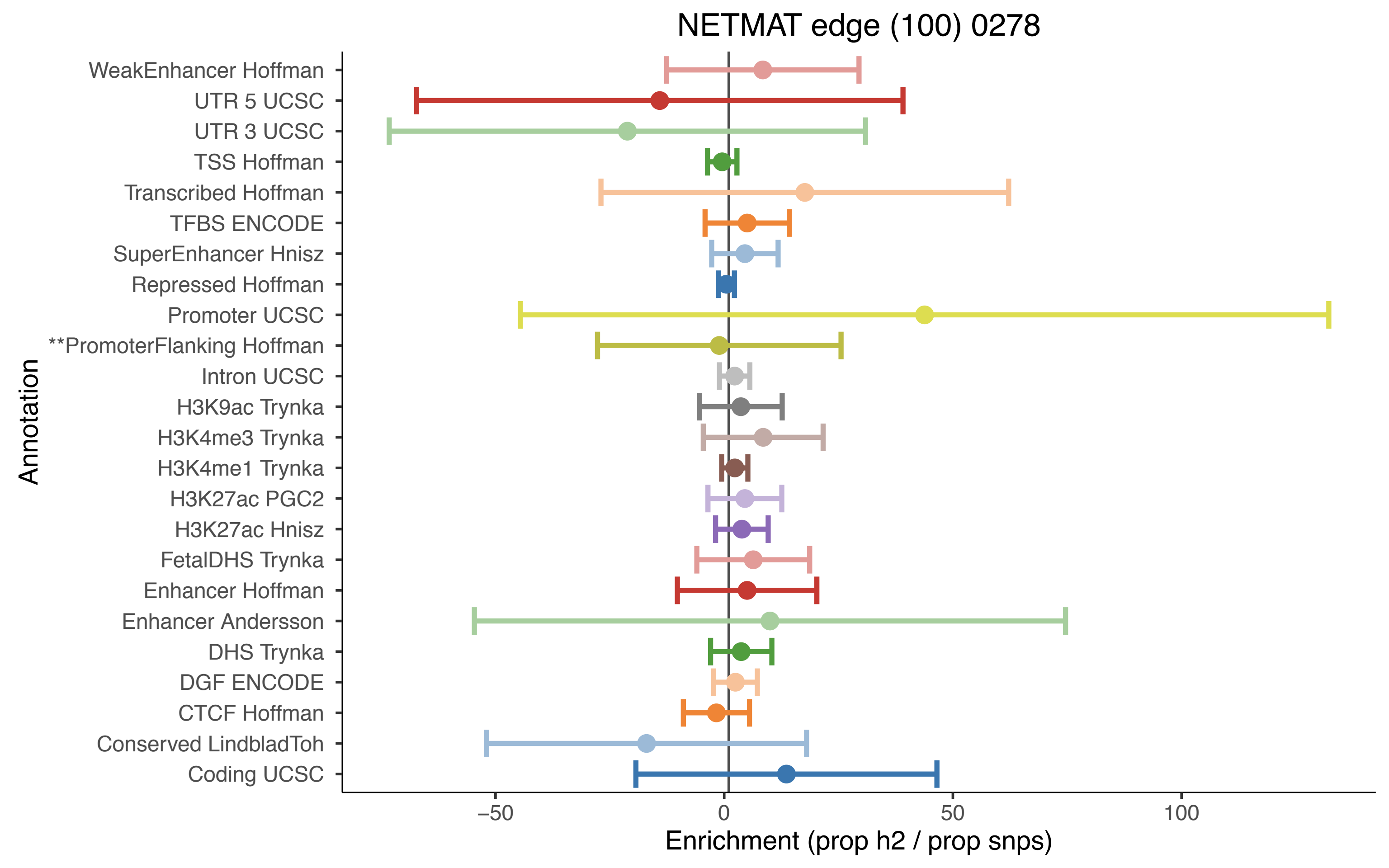

NETMAT edge (100) 0278
WeakEnhancer Hoffman
UTR 5 UCSC
UTR 3 UCSC
TSS Hoffman
Transcribed Hoffman
TFBS ENCODE
SuperEnhancer Hnisz
Repressed Hoffman
Promoter UCSC
**PromoterFlanking Hoffman
Intron UCSC
H3K9ac Trynka
H3K4me3 Trynka
H3K4me1 Trynka
H3K27ac PGC2
H3K27ac Hnisz
FetalDHS Trynka
Enhancer Hoffman
Enhancer Andersson
DHS Trynka
DGF ENCODE
CTCF Hoffman
Conserved LindbladToh
Coding UCSC
Annotation
−50
0
50
100
Enrichment (prop h2 / prop snps)

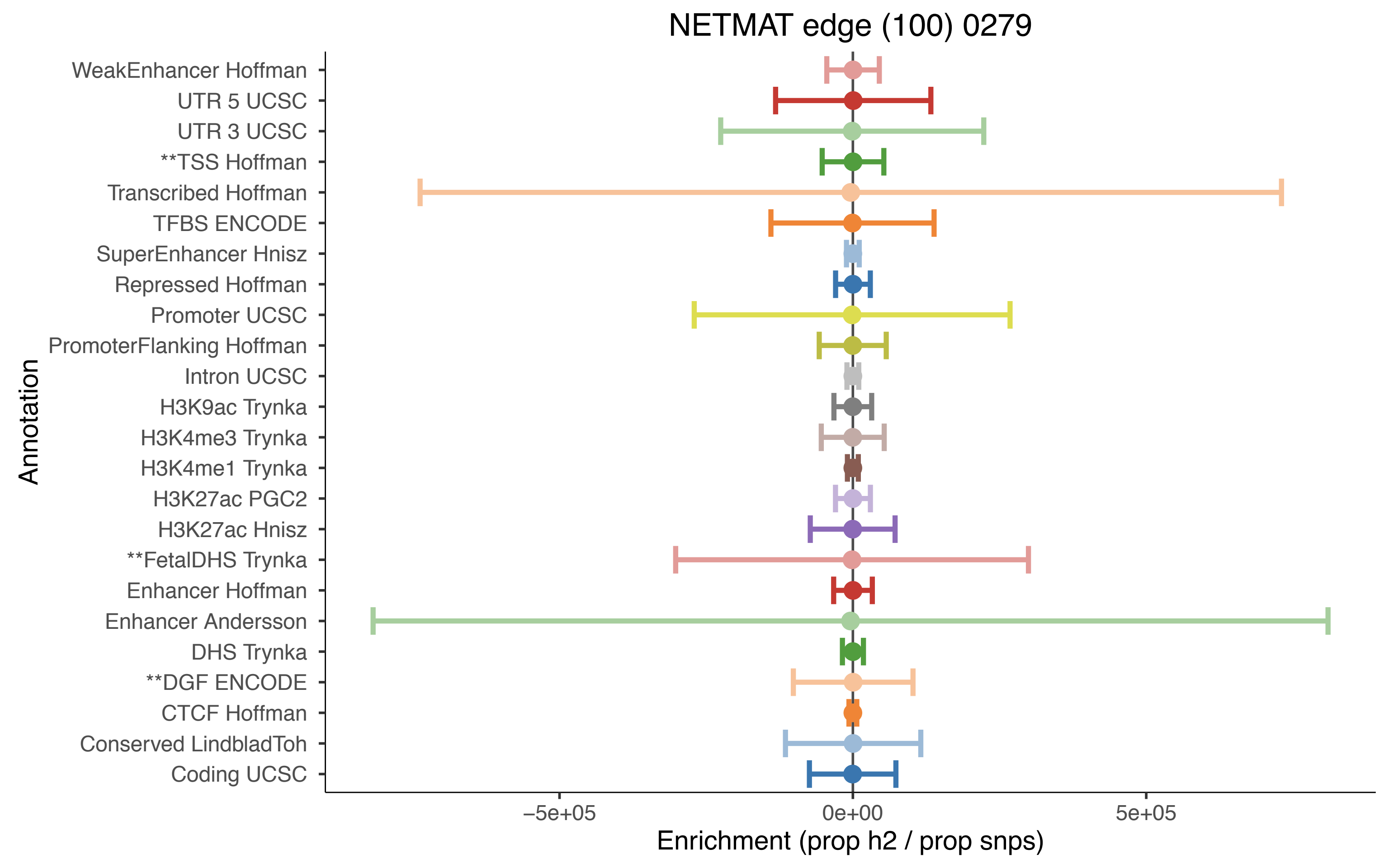
NETMAT edge (100) 0279
WeakEnhancer Hoffman
UTR 5 UCSC
UTR 3 UCSC
**TSS Hoffman
Transcribed Hoffman
TFBS ENCODE
SuperEnhancer Hnisz
Repressed Hoffman
Promoter UCSC
PromoterFlanking Hoffman
Intron UCSC
H3K9ac Trynka
H3K4me3 Trynka
H3K4me1 Trynka
H3K27ac PGC2
H3K27ac Hnisz
**FetalDHS Trynka
Enhancer Hoffman
Enhancer Andersson
DHS Trynka
**DGF ENCODE
CTCF Hoffman
Conserved LindbladToh
Coding UCSC
Annotation
−5e+05
0e+00
5e+05
Enrichment (prop h2 / prop snps)

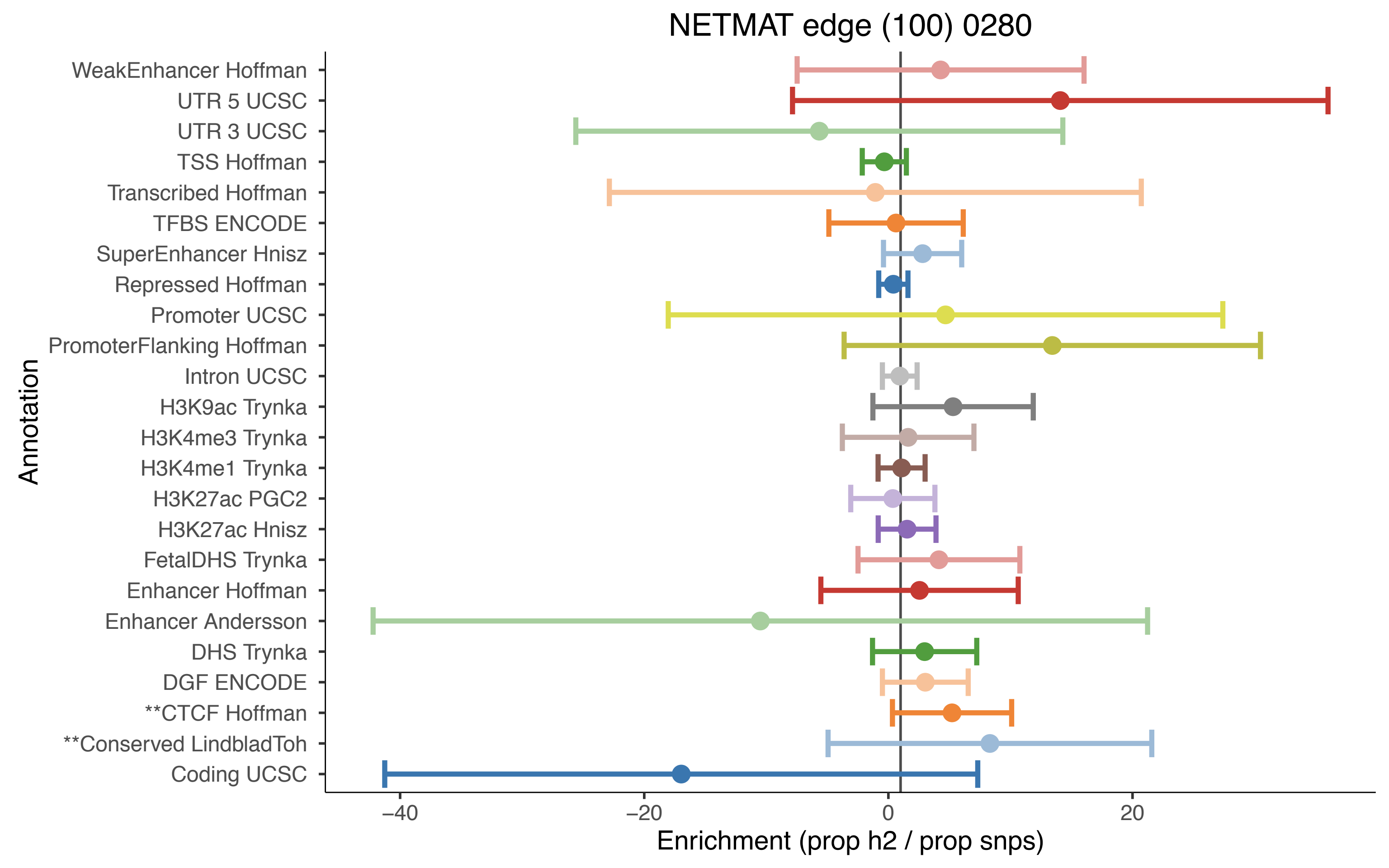
NETMAT edge (100) 0280
WeakEnhancer Hoffman
UTR 5 UCSC
UTR 3 UCSC
TSS Hoffman
Transcribed Hoffman
TFBS ENCODE
SuperEnhancer Hnisz
Repressed Hoffman
Promoter UCSC
PromoterFlanking Hoffman
Intron UCSC
H3K9ac Trynka
H3K4me3 Trynka
H3K4me1 Trynka
H3K27ac PGC2
H3K27ac Hnisz
FetalDHS Trynka
Enhancer Hoffman
Enhancer Andersson
DHS Trynka
DGF ENCODE
**CTCF Hoffman
**Conserved LindbladToh
Coding UCSC
Annotation
−40
−20
0
20
Enrichment (prop h2 / prop snps)

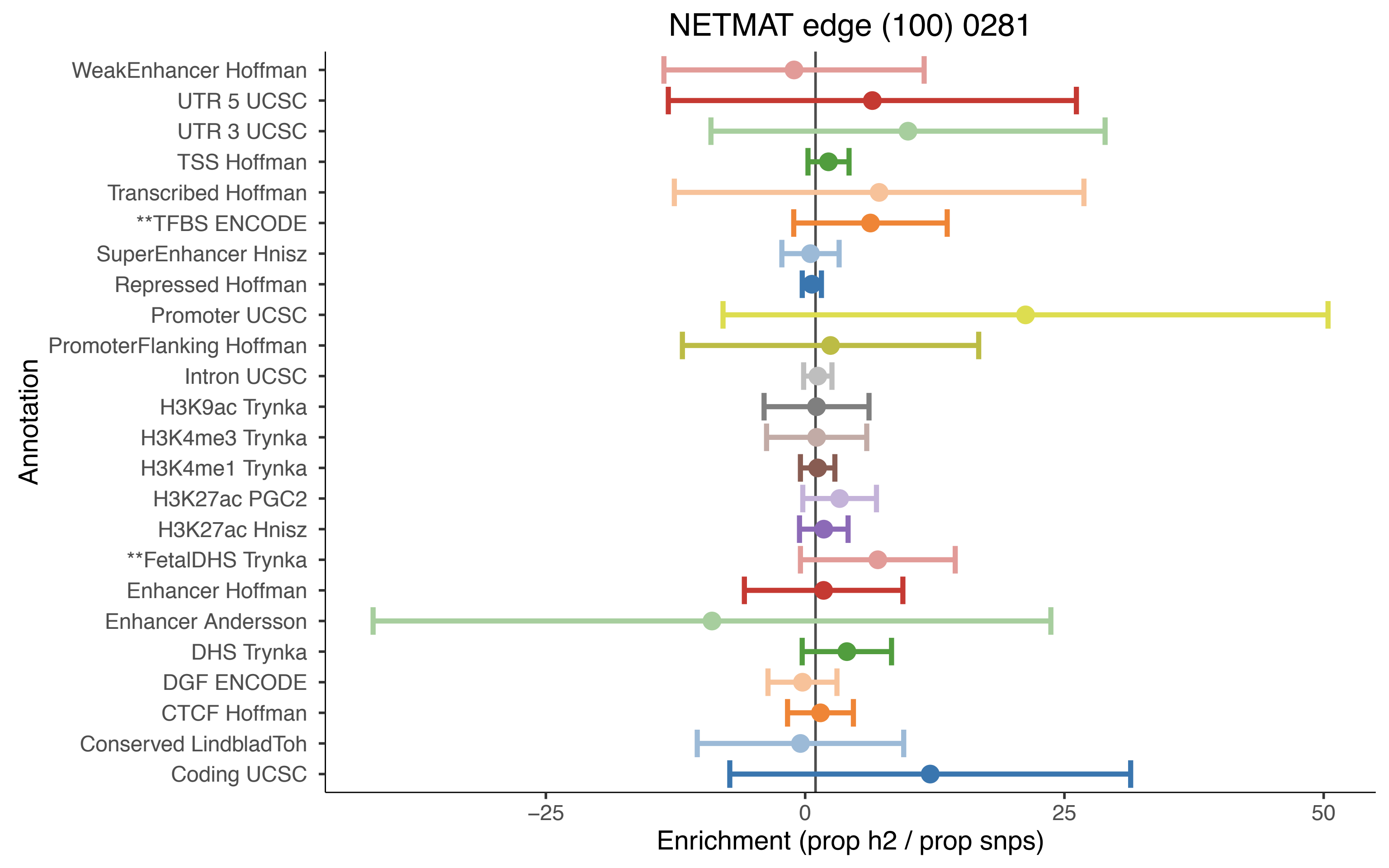
NETMAT edge (100) 0281
WeakEnhancer Hoffman
UTR 5 UCSC
UTR 3 UCSC
TSS Hoffman
Transcribed Hoffman
**TFBS ENCODE
SuperEnhancer Hnisz
Repressed Hoffman
Promoter UCSC
PromoterFlanking Hoffman
Intron UCSC
H3K9ac Trynka
H3K4me3 Trynka
H3K4me1 Trynka
H3K27ac PGC2
H3K27ac Hnisz
**FetalDHS Trynka
Enhancer Hoffman
Enhancer Andersson
DHS Trynka
DGF ENCODE
CTCF Hoffman
Conserved LindbladToh
Coding UCSC
Annotation
−25
0
25
50
Enrichment (prop h2 / prop snps)

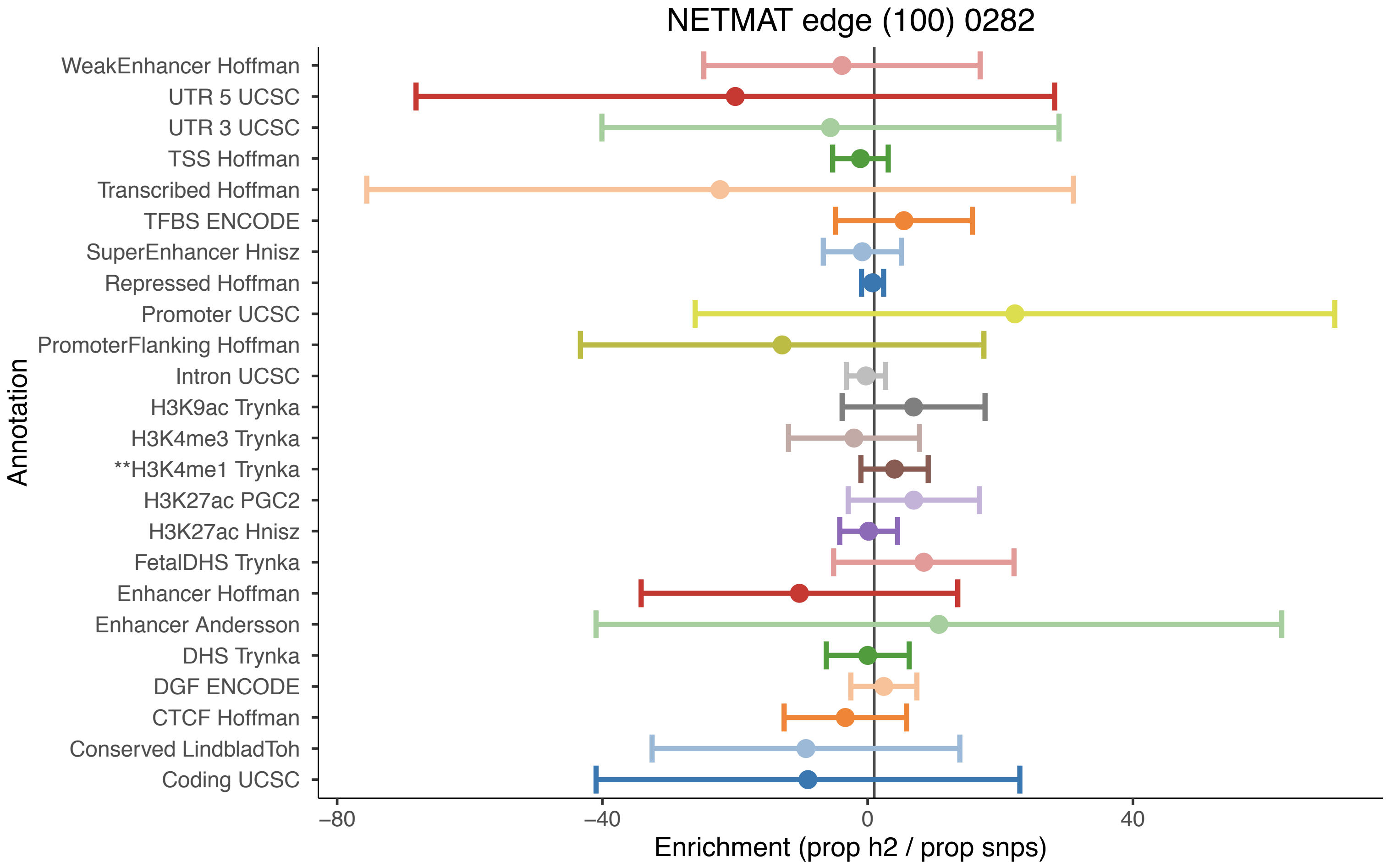
NETMAT edge (100) 0282
WeakEnhancer Hoffman
UTR 5 UCSC
UTR 3 UCSC
TSS Hoffman
Transcribed Hoffman
TFBS ENCODE
SuperEnhancer Hnisz
Repressed Hoffman
Promoter UCSC
PromoterFlanking Hoffman
Intron UCSC
H3K9ac Trynka
H3K4me3 Trynka
**H3K4me1 Trynka
H3K27ac PGC2
H3K27ac Hnisz
FetalDHS Trynka
Enhancer Hoffman
Enhancer Andersson
DHS Trynka
DGF ENCODE
CTCF Hoffman
Conserved LindbladToh
Coding UCSC
Annotation
−80
−40
0
40
Enrichment (prop h2 / prop snps)

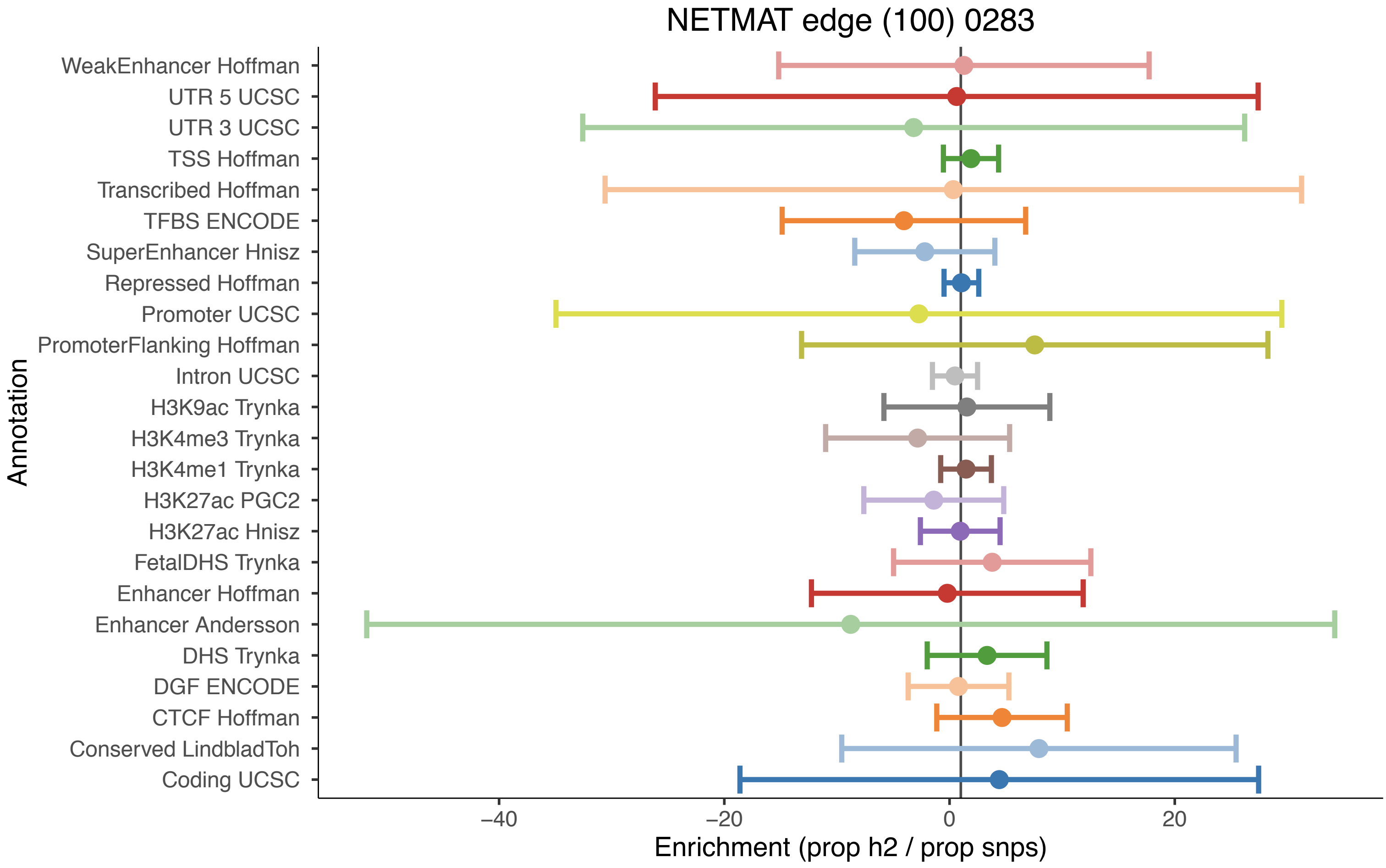

NETMAT edge (100) 0283
WeakEnhancer Hoffman
UTR 5 UCSC
UTR 3 UCSC
TSS Hoffman
Transcribed Hoffman
TFBS ENCODE
SuperEnhancer Hnisz
Repressed Hoffman
Promoter UCSC
PromoterFlanking Hoffman
Intron UCSC
H3K9ac Trynka
H3K4me3 Trynka
H3K4me1 Trynka
H3K27ac PGC2
H3K27ac Hnisz
FetalDHS Trynka
Enhancer Hoffman
Enhancer Andersson
DHS Trynka
DGF ENCODE
CTCF Hoffman
Conserved LindbladToh
Coding UCSC
Annotation
−40
−20
0
20
Enrichment (prop h2 / prop snps)

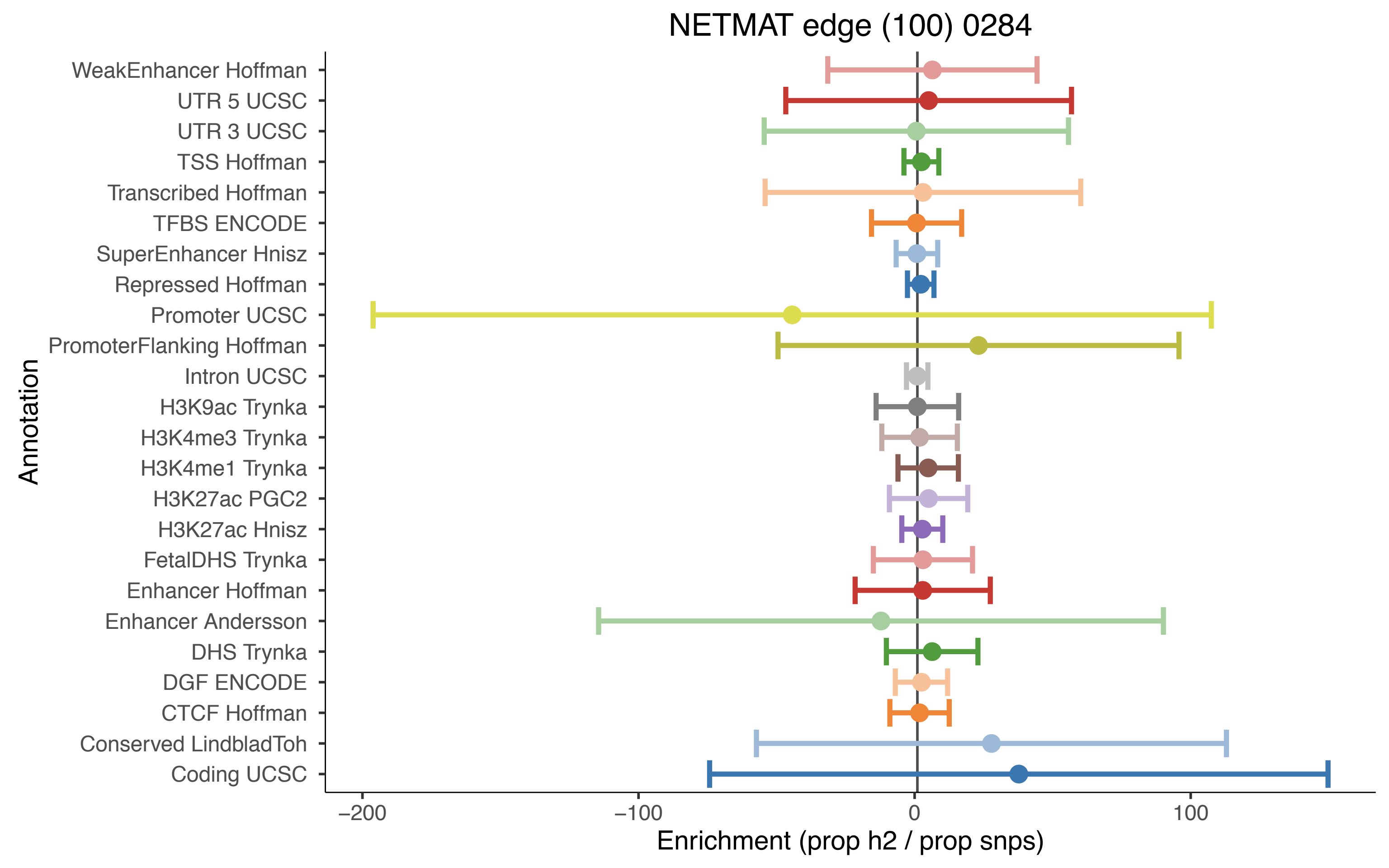

NETMAT edge (100) 0284
Annotation
WeakEnhancer Hoffman
UTR 5 UCSC
UTR 3 UCSC
TSS Hoffman
Transcribed Hoffman
TFBS ENCODE
SuperEnhancer Hnisz
Repressed Hoffman
Promoter UCSC
PromoterFlanking Hoffman
Intron UCSC
H3K9ac Trynka
H3K4me3 Trynka
H3K4me1 Trynka
H3K27ac PGC2
H3K27ac Hnisz
FetalDHS Trynka
Enhancer Hoffman
Enhancer Andersson
DHS Trynka
DGF ENCODE
CTCF Hoffman
Conserved LindbladToh
Coding UCSC
−200
−100
0
100
Enrichment (prop h2 / prop snps)

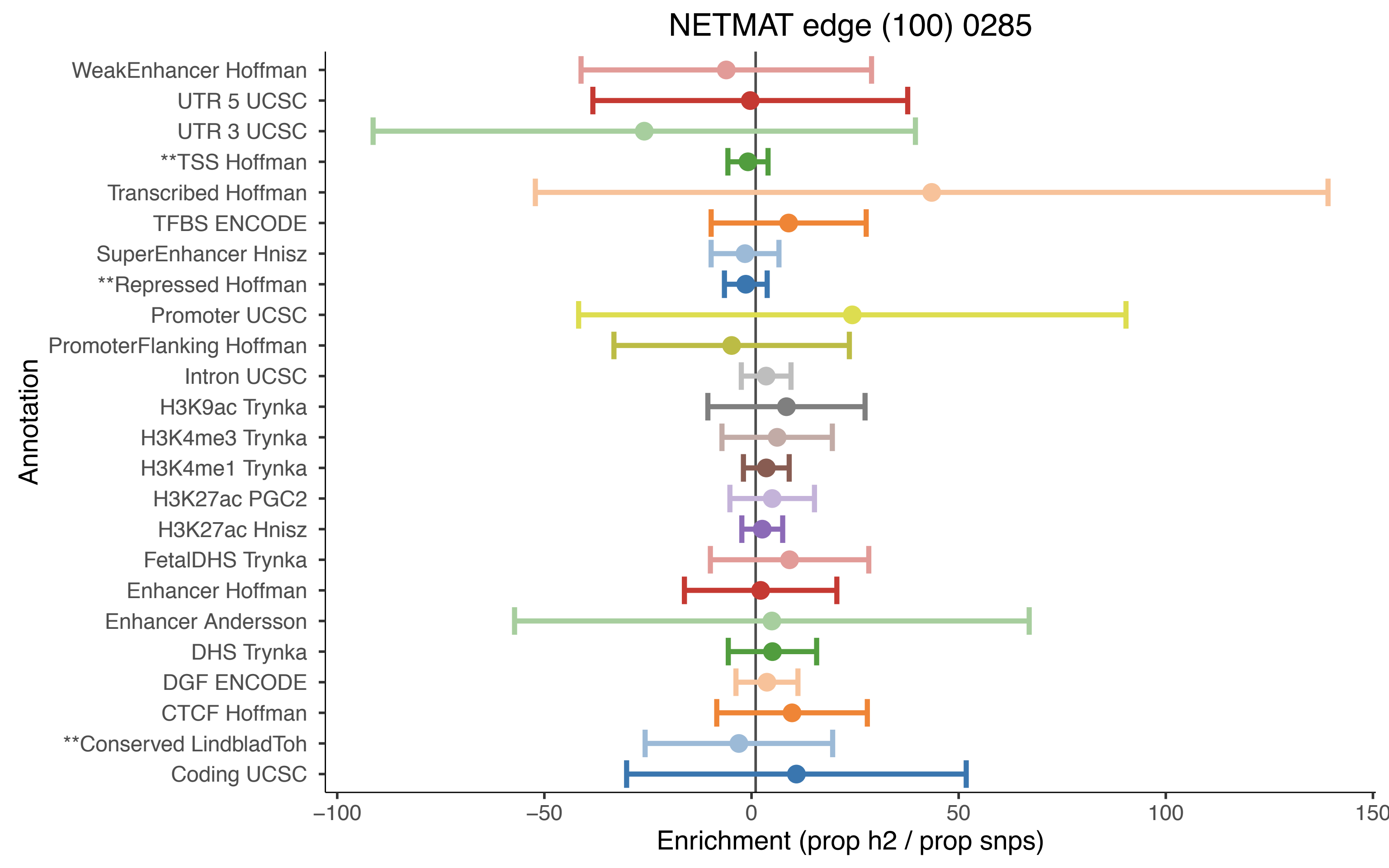
NETMAT edge (100) 0285
Annotation
WeakEnhancer Hoffman
UTR 5 UCSC
UTR 3 UCSC
**TSS Hoffman
Transcribed Hoffman
TFBS ENCODE
SuperEnhancer Hnisz
**Repressed Hoffman
Promoter UCSC
PromoterFlanking Hoffman
Intron UCSC
H3K9ac Trynka
H3K4me3 Trynka
H3K4me1 Trynka
H3K27ac PGC2
H3K27ac Hnisz
FetalDHS Trynka
Enhancer Hoffman
Enhancer Andersson
DHS Trynka
DGF ENCODE
CTCF Hoffman
**Conserved LindbladToh
Coding UCSC
−100
−50
0
50
100
150
Enrichment (prop h2 / prop snps)

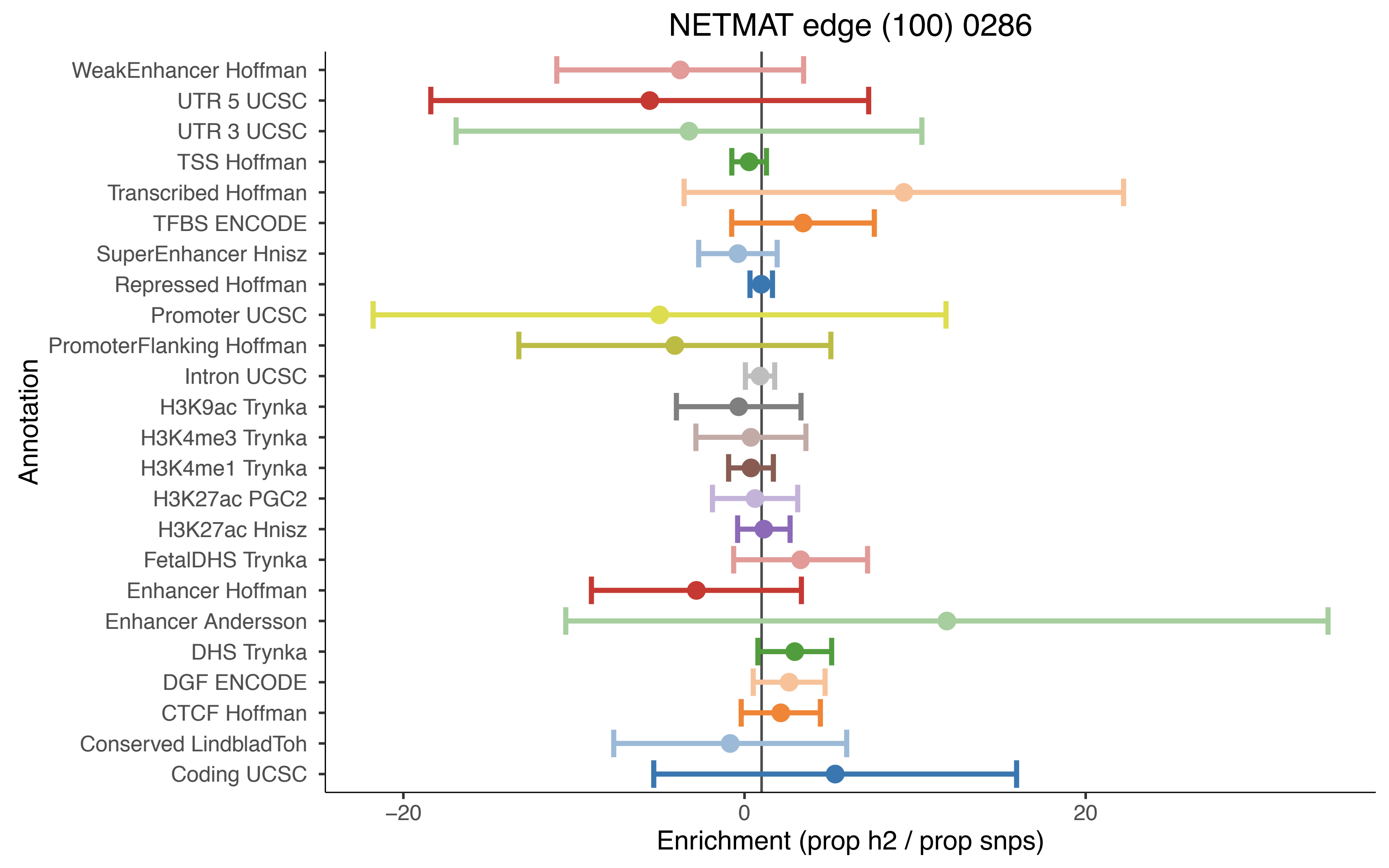
NETMAT edge (100) 0286
WeakEnhancer Hoffman
UTR 5 UCSC
UTR 3 UCSC
TSS Hoffman
Transcribed Hoffman
TFBS ENCODE
SuperEnhancer Hnisz
Repressed Hoffman
Promoter UCSC
PromoterFlanking Hoffman
Intron UCSC
H3K9ac Trynka
H3K4me3 Trynka
H3K4me1 Trynka
H3K27ac PGC2
H3K27ac Hnisz
FetalDHS Trynka
Enhancer Hoffman
Enhancer Andersson
DHS Trynka
DGF ENCODE
CTCF Hoffman
Conserved LindbladToh
Coding UCSC
Annotation
−20
0
20
Enrichment (prop h2 / prop snps)

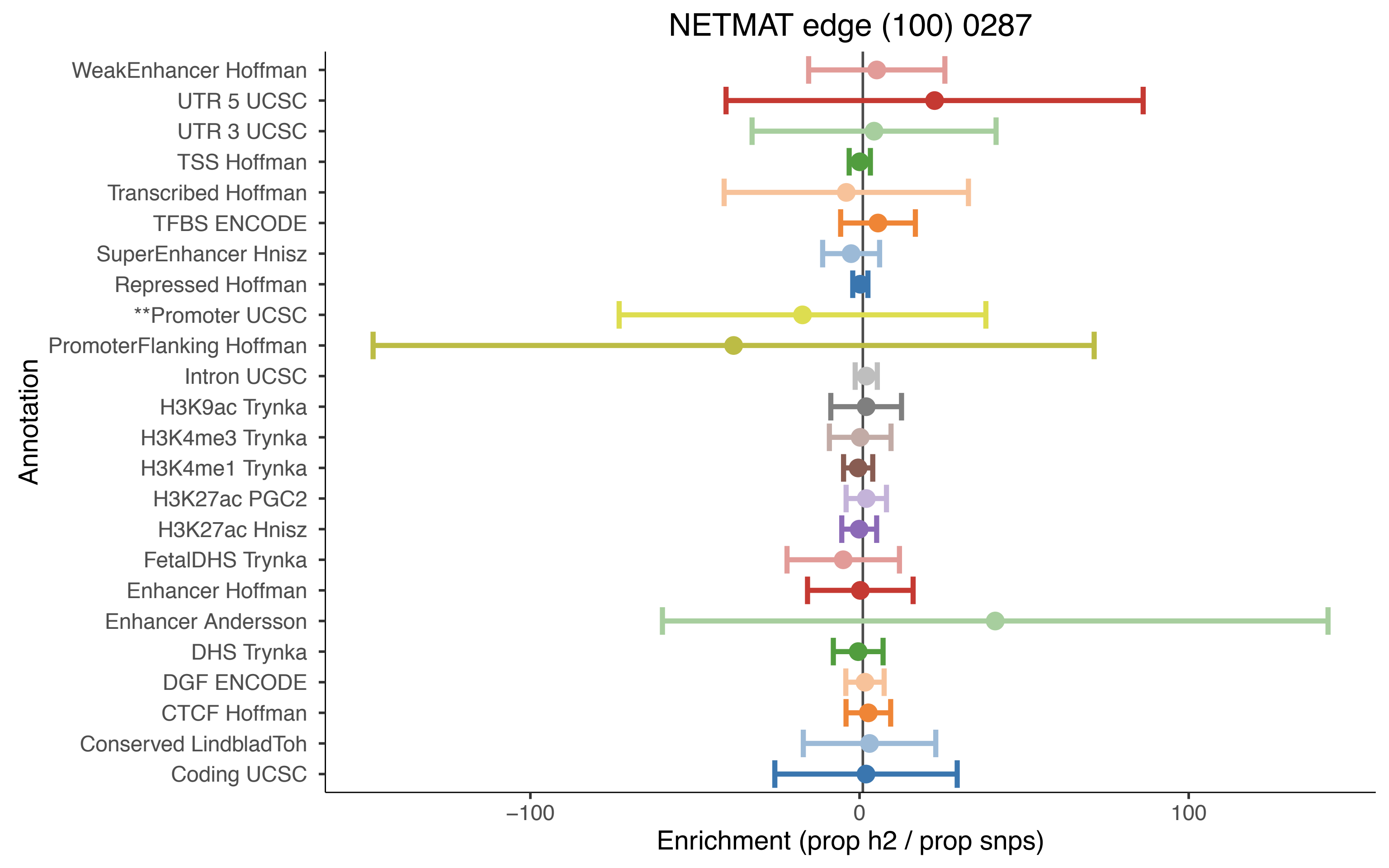

NETMAT edge (100) 0287
WeakEnhancer Hoffman
UTR 5 UCSC
UTR 3 UCSC
TSS Hoffman
Transcribed Hoffman
TFBS ENCODE
SuperEnhancer Hnisz
Repressed Hoffman
**Promoter UCSC
PromoterFlanking Hoffman
Intron UCSC
H3K9ac Trynka
H3K4me3 Trynka
H3K4me1 Trynka
H3K27ac PGC2
H3K27ac Hnisz
FetalDHS Trynka
Enhancer Hoffman
Enhancer Andersson
DHS Trynka
DGF ENCODE
CTCF Hoffman
Conserved LindbladToh
Coding UCSC
Annotation
−100
0
100
Enrichment (prop h2 / prop snps)

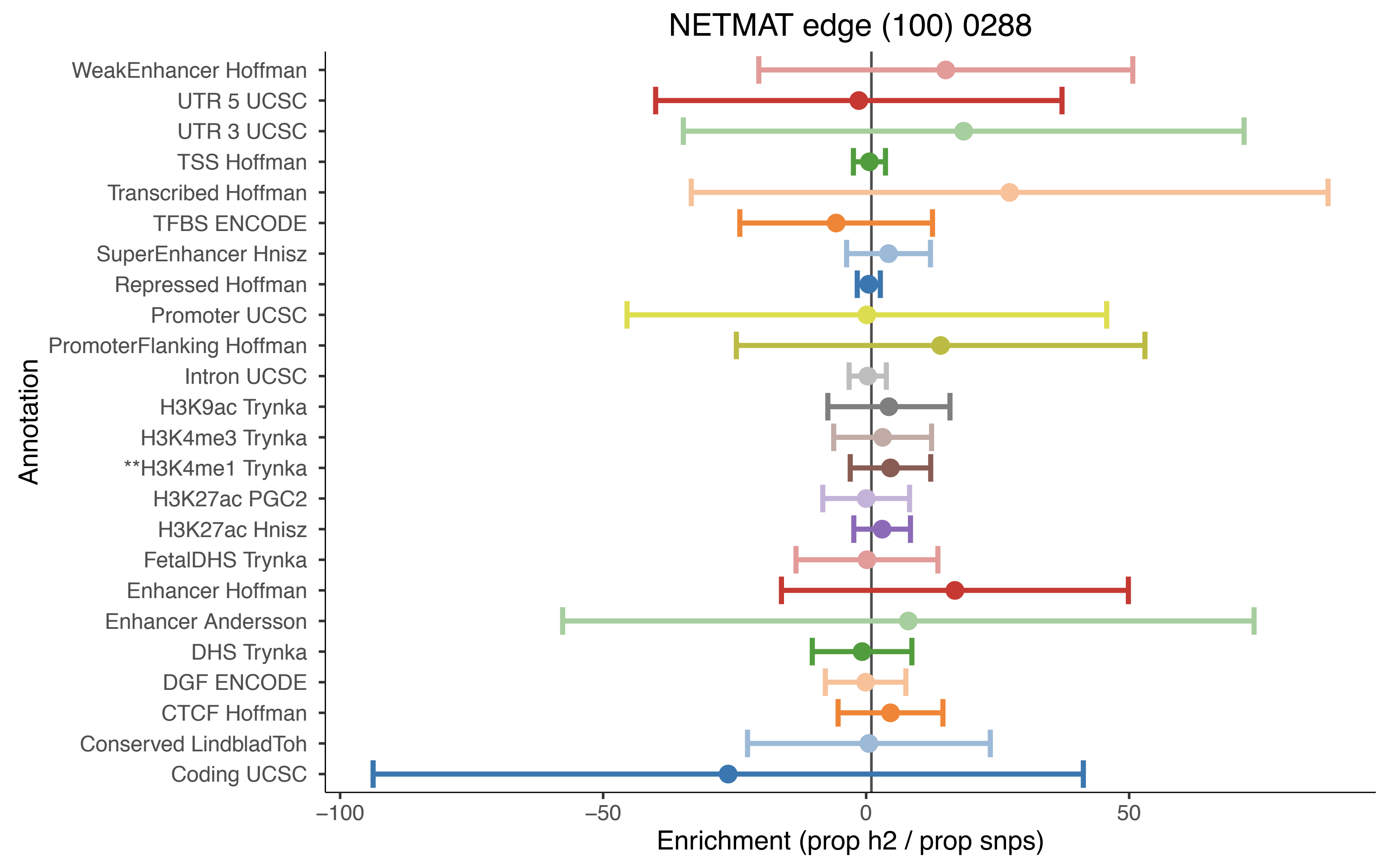
NETMAT edge (100) 0288
WeakEnhancer Hoffman
UTR 5 UCSC
UTR 3 UCSC
TSS Hoffman
Transcribed Hoffman
TFBS ENCODE
SuperEnhancer Hnisz
Repressed Hoffman
Promoter UCSC
PromoterFlanking Hoffman
Intron UCSC
H3K9ac Trynka
H3K4me3 Trynka
**H3K4me1 Trynka
H3K27ac PGC2
H3K27ac Hnisz
FetalDHS Trynka
Enhancer Hoffman
Enhancer Andersson
DHS Trynka
DGF ENCODE
CTCF Hoffman
Conserved LindbladToh
Coding UCSC
Annotation
−100
−50
0
50
Enrichment (prop h2 / prop snps)

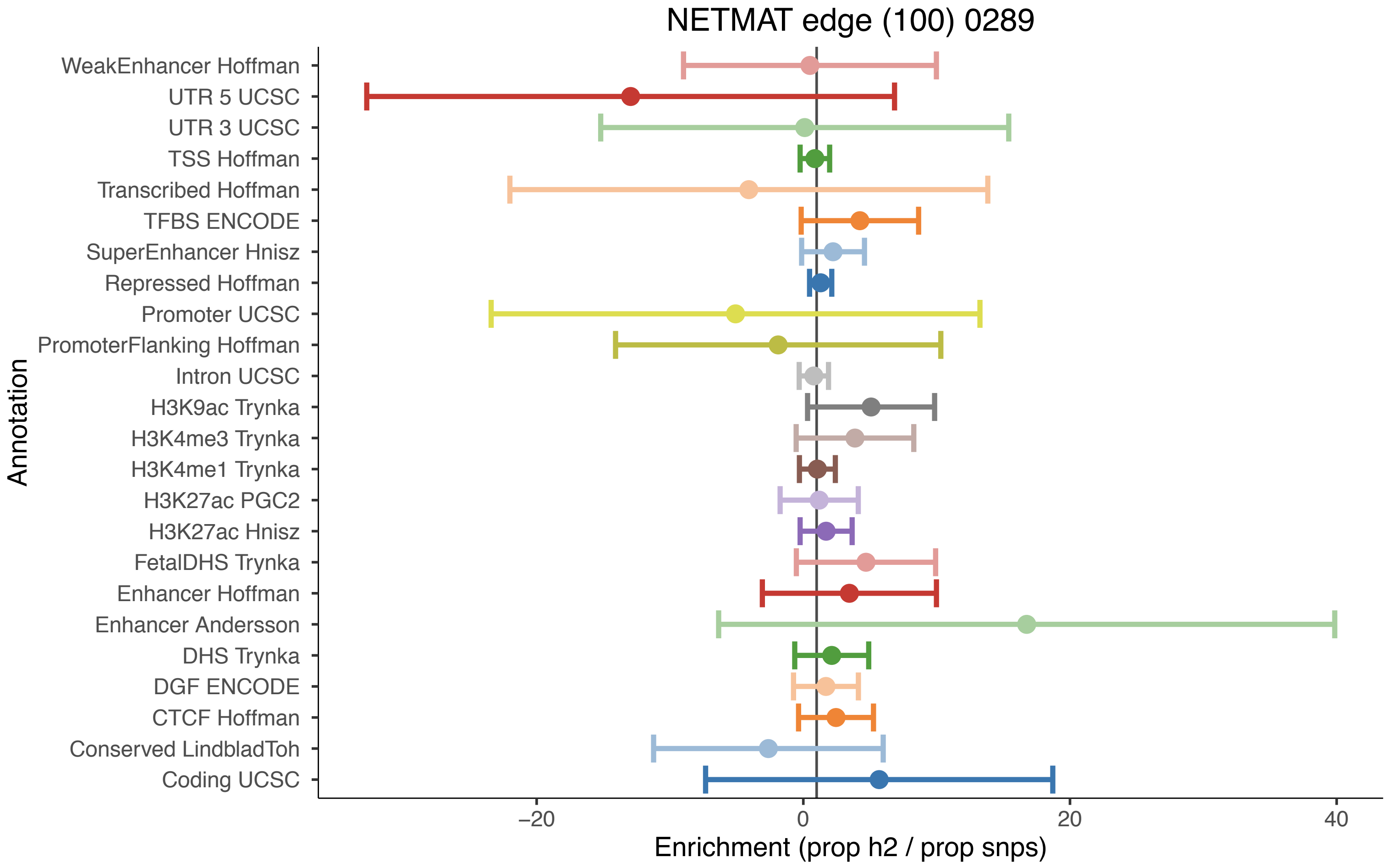

NETMAT edge (100) 0289
WeakEnhancer Hoffman
UTR 5 UCSC
UTR 3 UCSC
TSS Hoffman
Transcribed Hoffman
TFBS ENCODE
SuperEnhancer Hnisz
Repressed Hoffman
Promoter UCSC
PromoterFlanking Hoffman
Intron UCSC
H3K9ac Trynka
H3K4me3 Trynka
H3K4me1 Trynka
H3K27ac PGC2
H3K27ac Hnisz
FetalDHS Trynka
Enhancer Hoffman
Enhancer Andersson
DHS Trynka
DGF ENCODE
CTCF Hoffman
Conserved LindbladToh
Coding UCSC
Annotation
−20
0
20
40
Enrichment (prop h2 / prop snps)

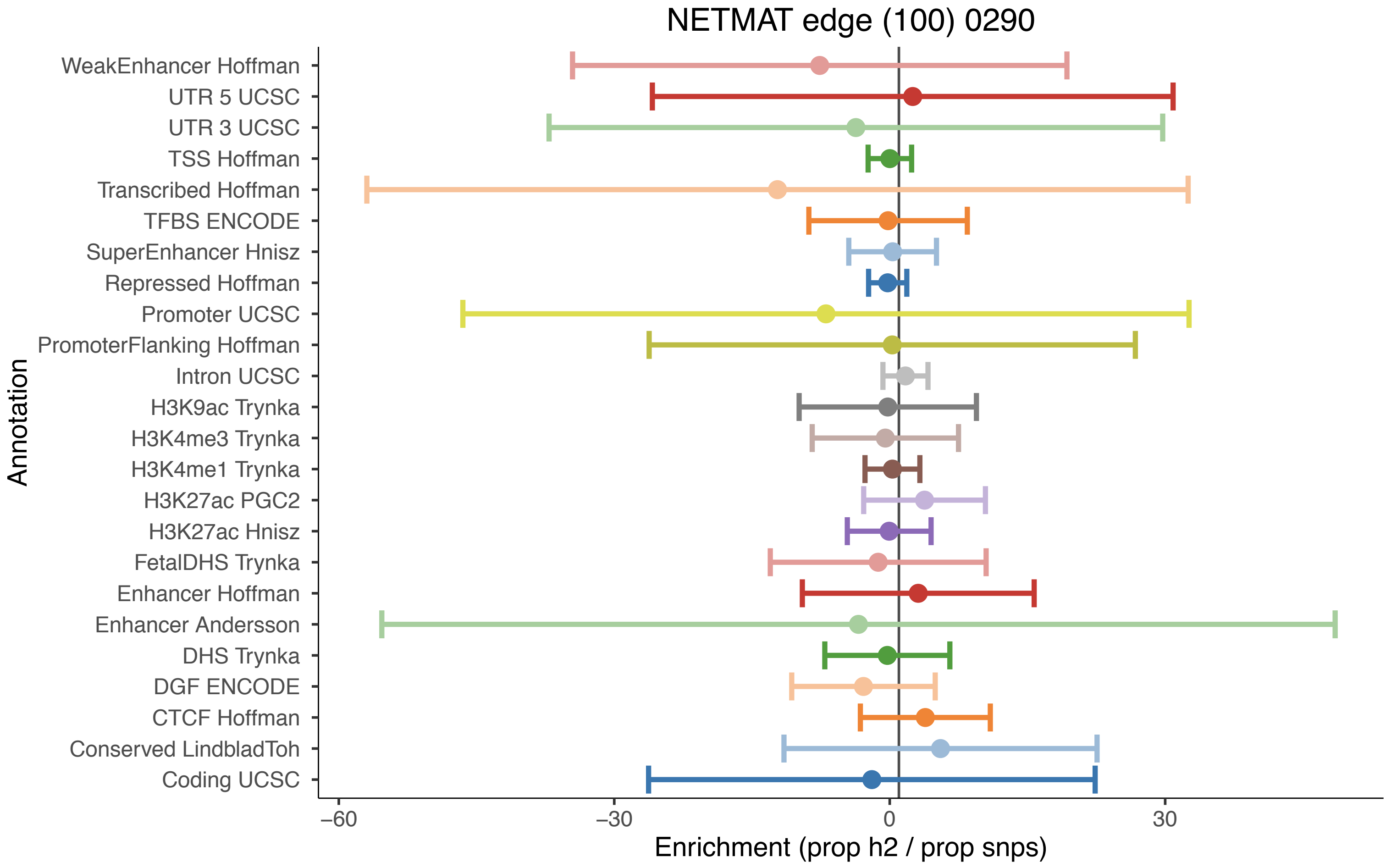
NETMAT edge (100) 0290
WeakEnhancer Hoffman
UTR 5 UCSC
UTR 3 UCSC
TSS Hoffman
Transcribed Hoffman
TFBS ENCODE
SuperEnhancer Hnisz
Repressed Hoffman
Promoter UCSC
PromoterFlanking Hoffman
Intron UCSC
H3K9ac Trynka
H3K4me3 Trynka
H3K4me1 Trynka
H3K27ac PGC2
H3K27ac Hnisz
FetalDHS Trynka
Enhancer Hoffman
Enhancer Andersson
DHS Trynka
DGF ENCODE
CTCF Hoffman
Conserved LindbladToh
Coding UCSC
Annotation
−60
−30
0
30
Enrichment (prop h2 / prop snps)

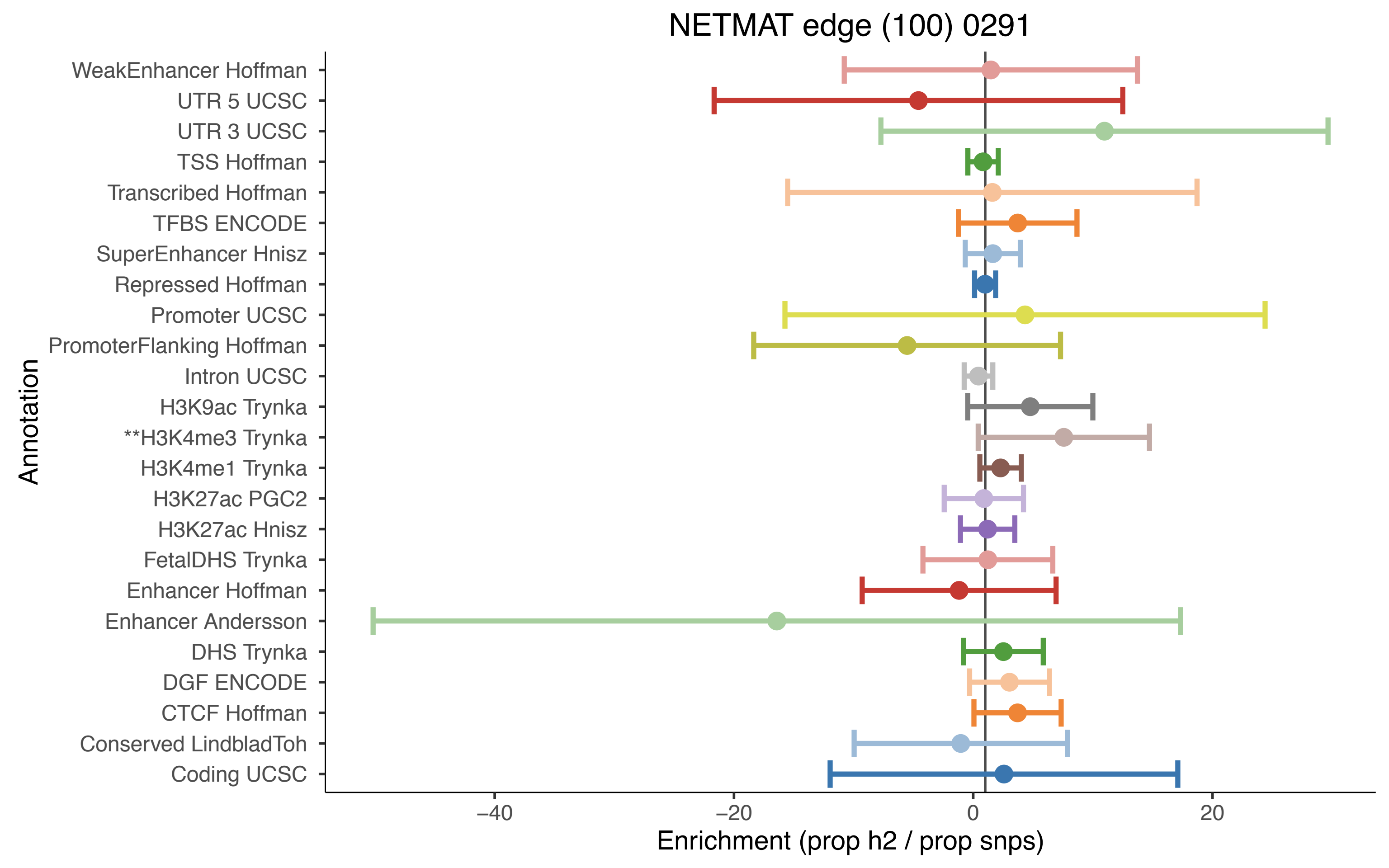

NETMAT edge (100) 0291
WeakEnhancer Hoffman
UTR 5 UCSC
UTR 3 UCSC
TSS Hoffman
Transcribed Hoffman
TFBS ENCODE
SuperEnhancer Hnisz
Repressed Hoffman
Promoter UCSC
PromoterFlanking Hoffman
Intron UCSC
H3K9ac Trynka
**H3K4me3 Trynka
H3K4me1 Trynka
H3K27ac PGC2
H3K27ac Hnisz
FetalDHS Trynka
Enhancer Hoffman
Enhancer Andersson
DHS Trynka
DGF ENCODE
CTCF Hoffman
Conserved LindbladToh
Coding UCSC
Annotation
−40
−20
0
20
Enrichment (prop h2 / prop snps)

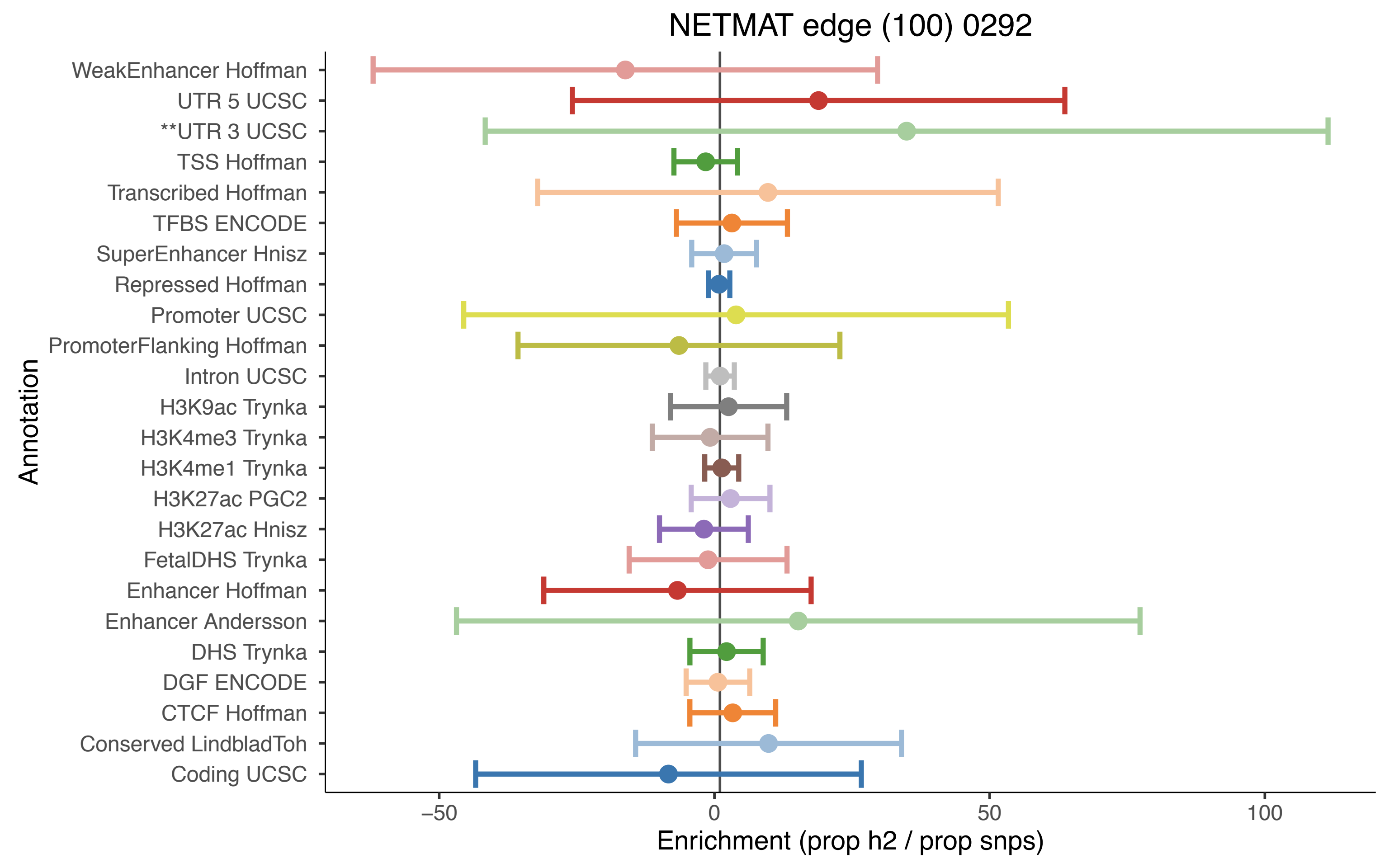
NETMAT edge (100) 0292
WeakEnhancer Hoffman
UTR 5 UCSC
**UTR 3 UCSC
TSS Hoffman
Transcribed Hoffman
TFBS ENCODE
SuperEnhancer Hnisz
Repressed Hoffman
Promoter UCSC
PromoterFlanking Hoffman
Intron UCSC
H3K9ac Trynka
H3K4me3 Trynka
H3K4me1 Trynka
H3K27ac PGC2
H3K27ac Hnisz
FetalDHS Trynka
Enhancer Hoffman
Enhancer Andersson
DHS Trynka
DGF ENCODE
CTCF Hoffman
Conserved LindbladToh
Coding UCSC
Annotation
−50
0
50
100
Enrichment (prop h2 / prop snps)

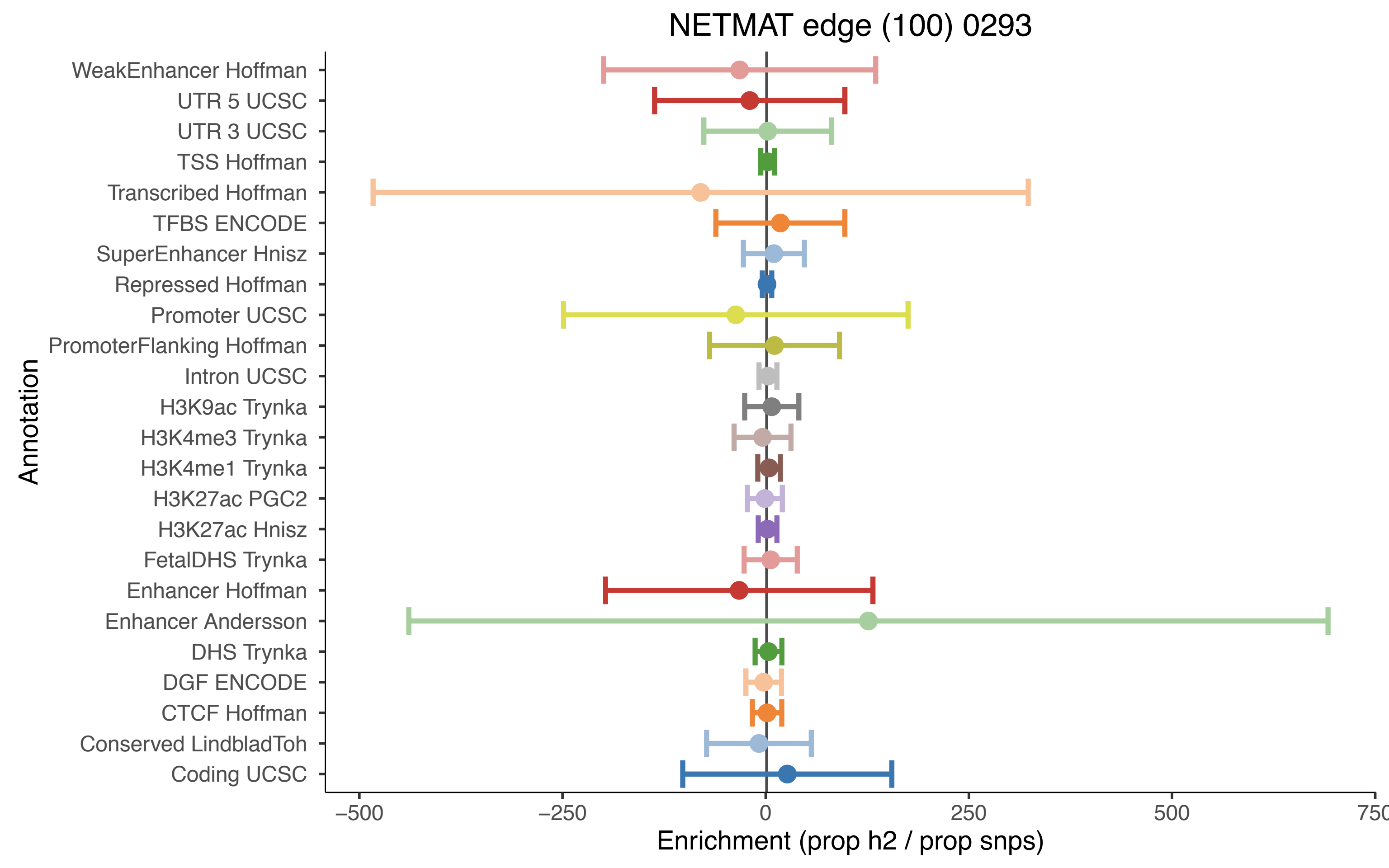

NETMAT edge (100) 0293
WeakEnhancer Hoffman
UTR 5 UCSC
UTR 3 UCSC
TSS Hoffman
Transcribed Hoffman
TFBS ENCODE
SuperEnhancer Hnisz
Repressed Hoffman
Promoter UCSC
PromoterFlanking Hoffman
Intron UCSC
H3K9ac Trynka
H3K4me3 Trynka
H3K4me1 Trynka
H3K27ac PGC2
H3K27ac Hnisz
FetalDHS Trynka
Enhancer Hoffman
Enhancer Andersson
DHS Trynka
DGF ENCODE
CTCF Hoffman
Conserved LindbladToh
Coding UCSC
Annotation
−500
−250
0
250
500
750
Enrichment (prop h2 / prop snps)

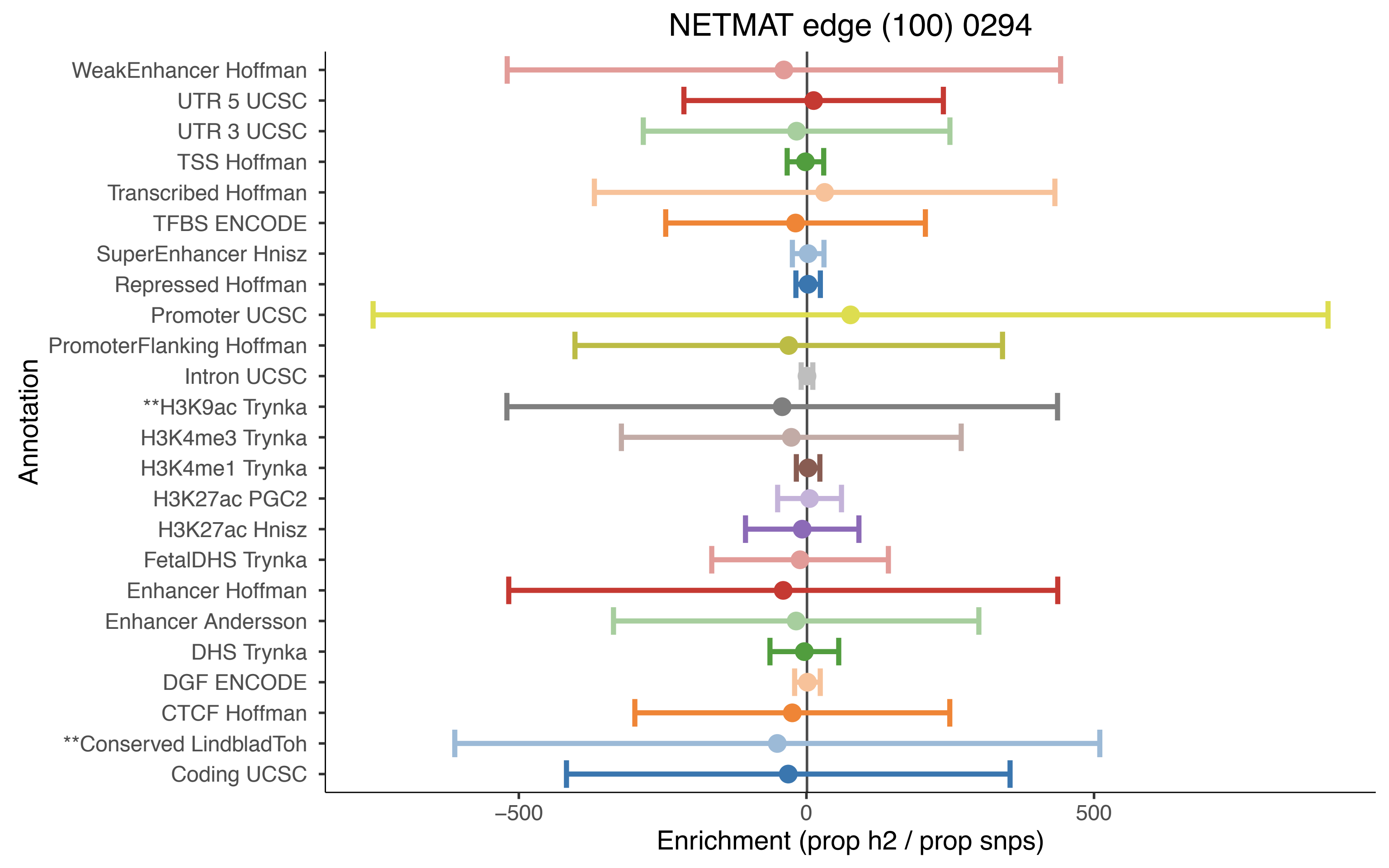
NETMAT edge (100) 0294
WeakEnhancer Hoffman
UTR 5 UCSC
UTR 3 UCSC
TSS Hoffman
Transcribed Hoffman
TFBS ENCODE
SuperEnhancer Hnisz
Repressed Hoffman
Promoter UCSC
PromoterFlanking Hoffman
Intron UCSC
**H3K9ac Trynka
H3K4me3 Trynka
H3K4me1 Trynka
H3K27ac PGC2
H3K27ac Hnisz
FetalDHS Trynka
Enhancer Hoffman
Enhancer Andersson
DHS Trynka
DGF ENCODE
CTCF Hoffman
**Conserved LindbladToh
Coding UCSC
Annotation
−500
0
500
Enrichment (prop h2 / prop snps)

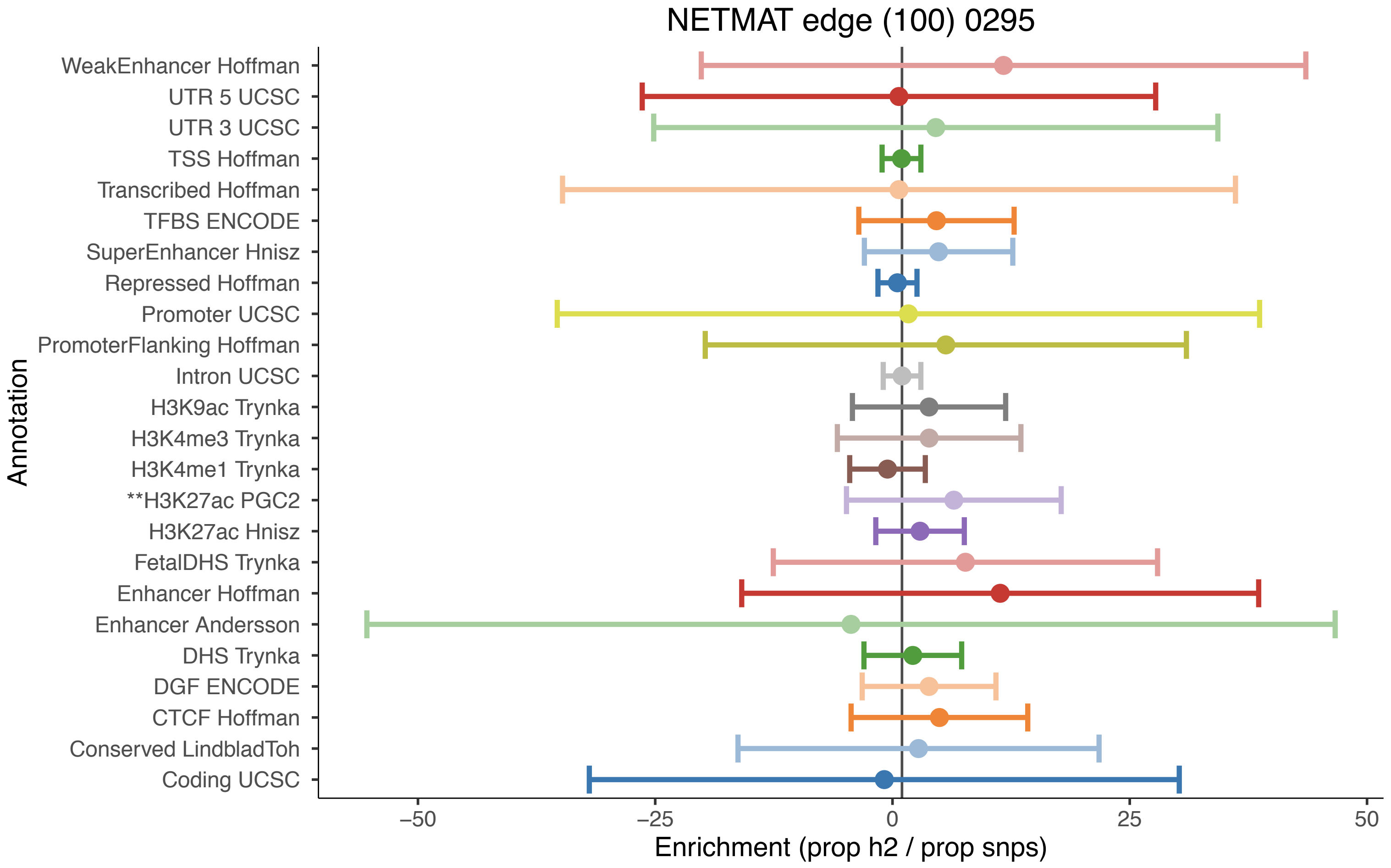

NETMAT edge (100) 0295
Annotation
WeakEnhancer Hoffman
UTR 5 UCSC
UTR 3 UCSC
TSS Hoffman
Transcribed Hoffman
TFBS ENCODE
SuperEnhancer Hnisz
Repressed Hoffman
Promoter UCSC
PromoterFlanking Hoffman
Intron UCSC
H3K9ac Trynka
H3K4me3 Trynka
H3K4me1 Trynka
**H3K27ac PGC2
H3K27ac Hnisz
FetalDHS Trynka
Enhancer Hoffman
Enhancer Andersson
DHS Trynka
DGF ENCODE
CTCF Hoffman
Conserved LindbladToh
Coding UCSC
−50
−25
0
25
50
Enrichment (prop h2 / prop snps)

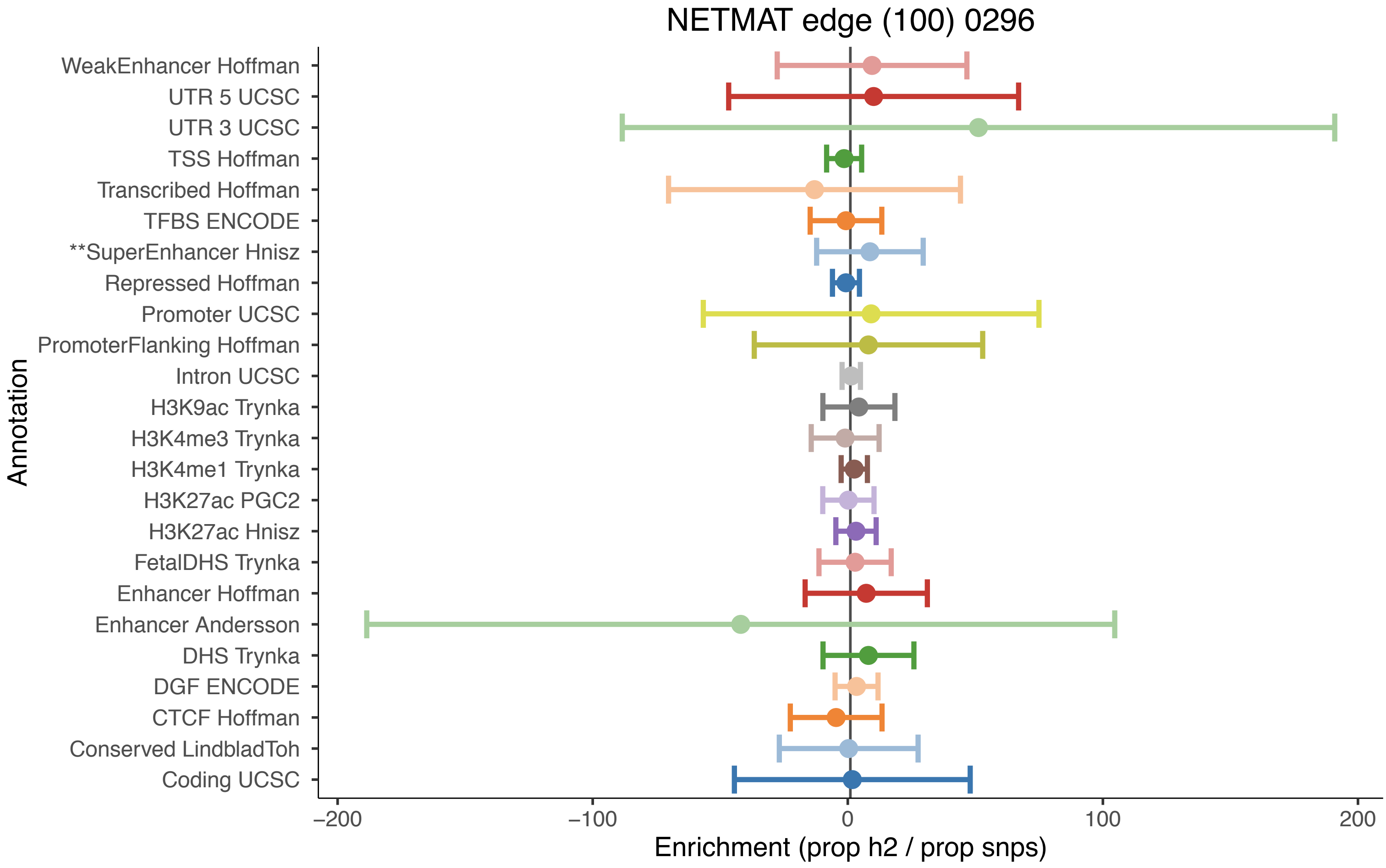
NETMAT edge (100) 0296
WeakEnhancer Hoffman
UTR 5 UCSC
UTR 3 UCSC
TSS Hoffman
Transcribed Hoffman
TFBS ENCODE
**SuperEnhancer Hnisz
Repressed Hoffman
Promoter UCSC
PromoterFlanking Hoffman
Intron UCSC
H3K9ac Trynka
H3K4me3 Trynka
H3K4me1 Trynka
H3K27ac PGC2
H3K27ac Hnisz
FetalDHS Trynka
Enhancer Hoffman
Enhancer Andersson
DHS Trynka
DGF ENCODE
CTCF Hoffman
Conserved LindbladToh
Coding UCSC
Annotation
−200
−100
0
100
200
Enrichment (prop h2 / prop snps)

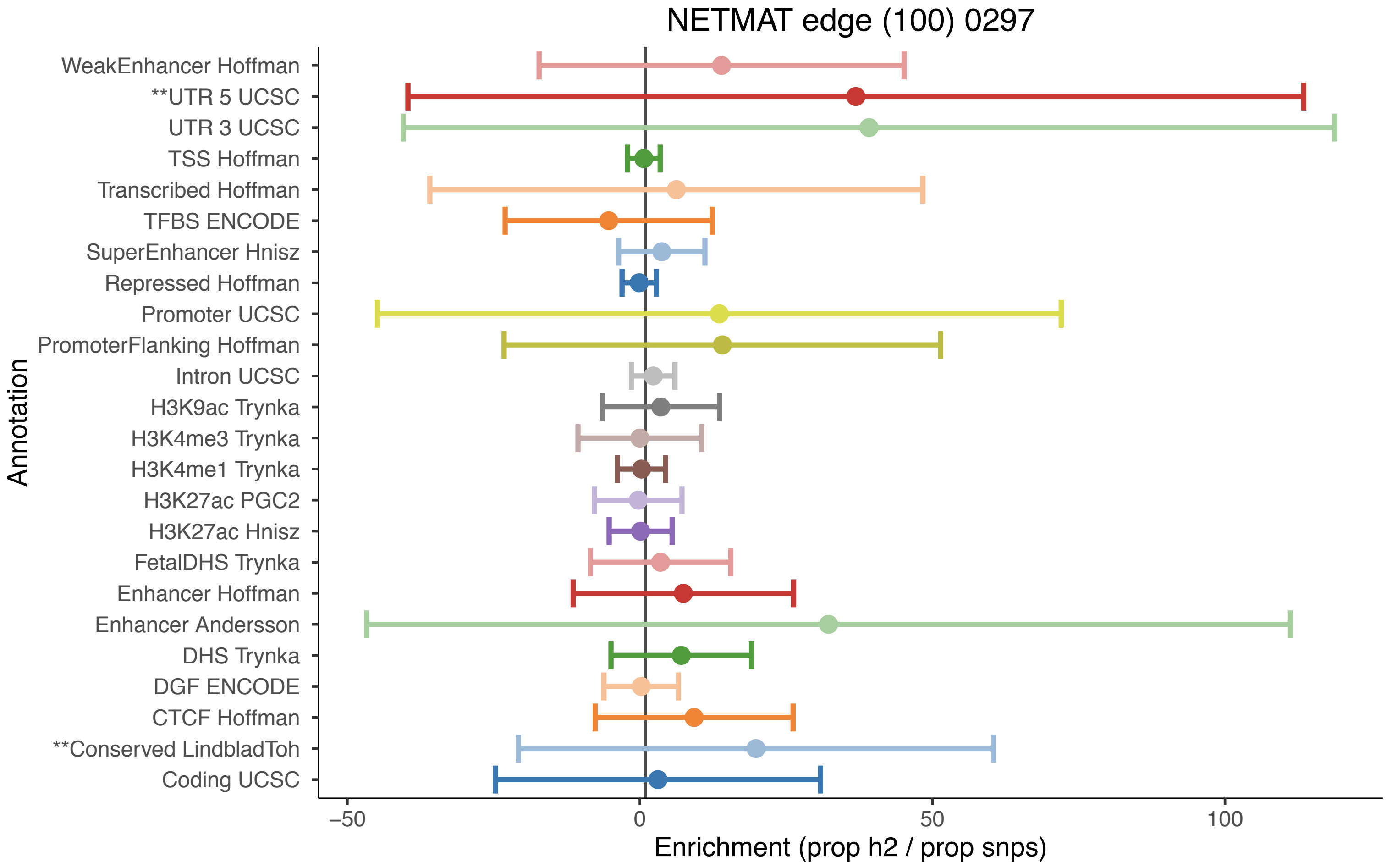

NETMAT edge (100) 0297
WeakEnhancer Hoffman
**UTR 5 UCSC
UTR 3 UCSC
TSS Hoffman
Transcribed Hoffman
TFBS ENCODE
SuperEnhancer Hnisz
Repressed Hoffman
Promoter UCSC
PromoterFlanking Hoffman
Intron UCSC
H3K9ac Trynka
H3K4me3 Trynka
H3K4me1 Trynka
H3K27ac PGC2
H3K27ac Hnisz
FetalDHS Trynka
Enhancer Hoffman
Enhancer Andersson
DHS Trynka
DGF ENCODE
CTCF Hoffman
**Conserved LindbladToh
Coding UCSC
Annotation
−50
0
50
100
Enrichment (prop h2 / prop snps)

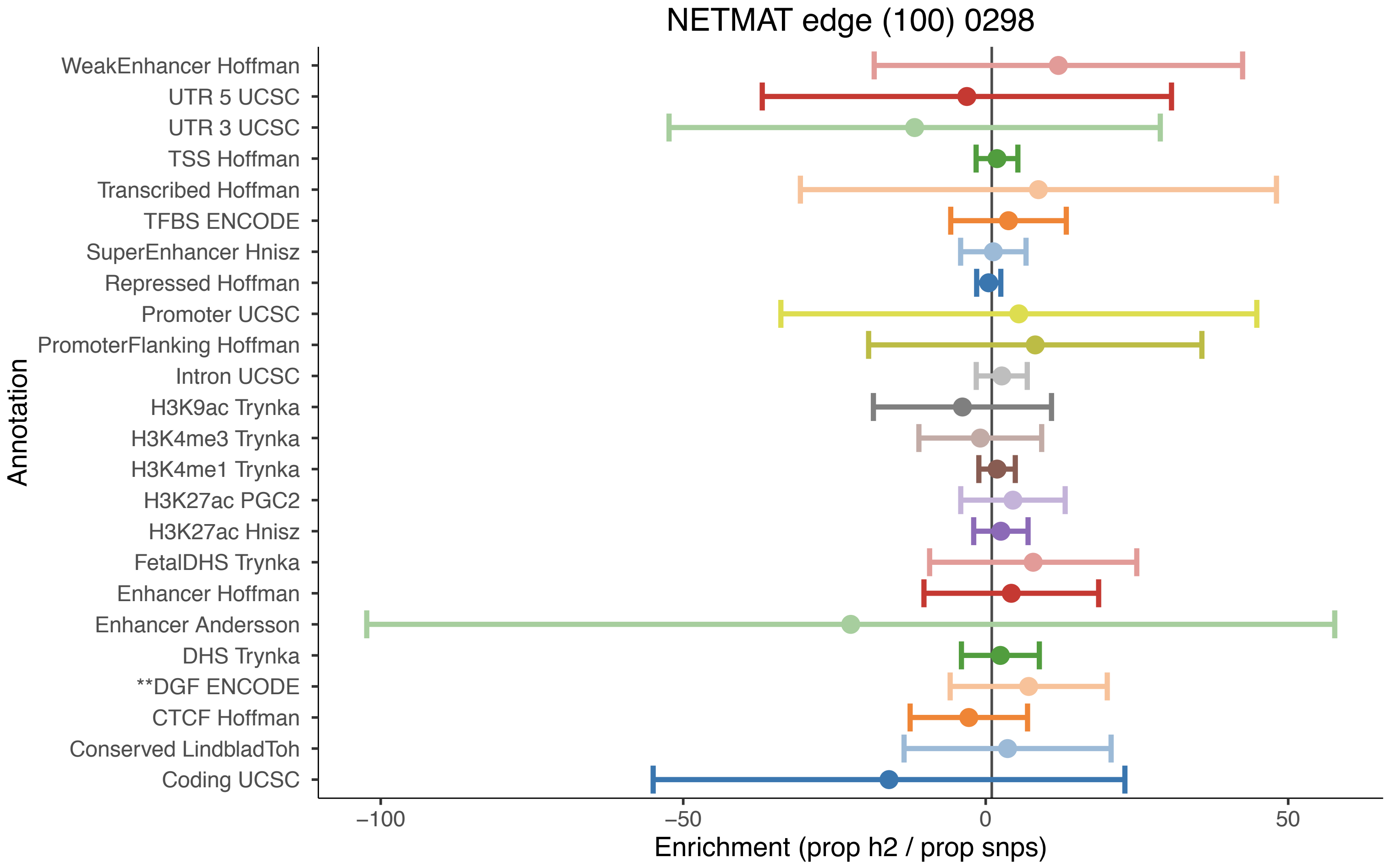
NETMAT edge (100) 0298
WeakEnhancer Hoffman
UTR 5 UCSC
UTR 3 UCSC
TSS Hoffman
Transcribed Hoffman
TFBS ENCODE
SuperEnhancer Hnisz
Repressed Hoffman
Promoter UCSC
PromoterFlanking Hoffman
Intron UCSC
H3K9ac Trynka
H3K4me3 Trynka
H3K4me1 Trynka
H3K27ac PGC2
H3K27ac Hnisz
FetalDHS Trynka
Enhancer Hoffman
Enhancer Andersson
DHS Trynka
**DGF ENCODE
CTCF Hoffman
Conserved LindbladToh
Coding UCSC
Annotation
−100
−50
0
50
Enrichment (prop h2 / prop snps)

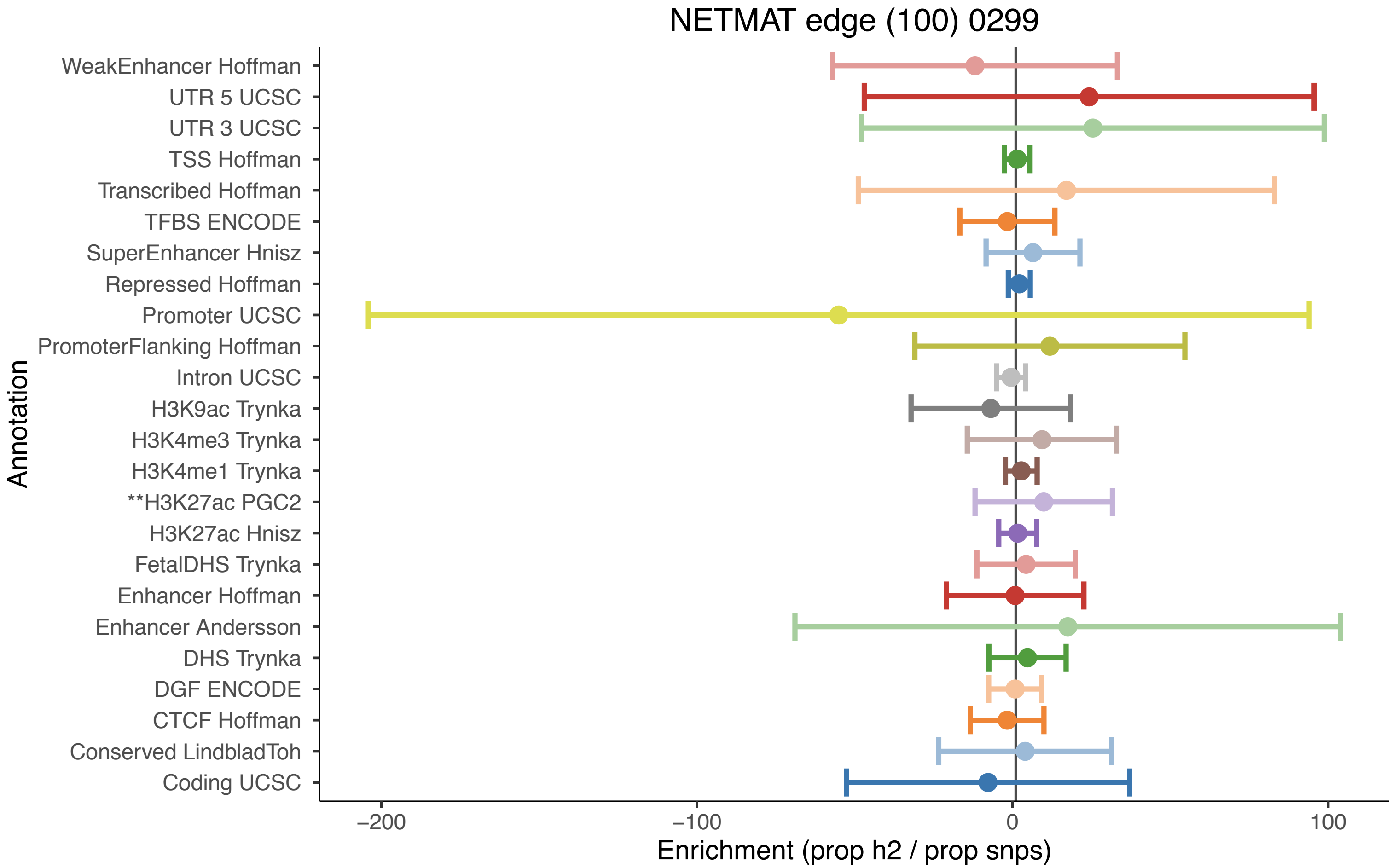
NETMAT edge (100) 0299
WeakEnhancer Hoffman
UTR 5 UCSC
UTR 3 UCSC
TSS Hoffman
Transcribed Hoffman
TFBS ENCODE
SuperEnhancer Hnisz
Repressed Hoffman
Promoter UCSC
PromoterFlanking Hoffman
Intron UCSC
H3K9ac Trynka
H3K4me3 Trynka
H3K4me1 Trynka
**H3K27ac PGC2
H3K27ac Hnisz
FetalDHS Trynka
Enhancer Hoffman
Enhancer Andersson
DHS Trynka
DGF ENCODE
CTCF Hoffman
Conserved LindbladToh
Coding UCSC
Annotation
−200
−100
0
100
Enrichment (prop h2 / prop snps)

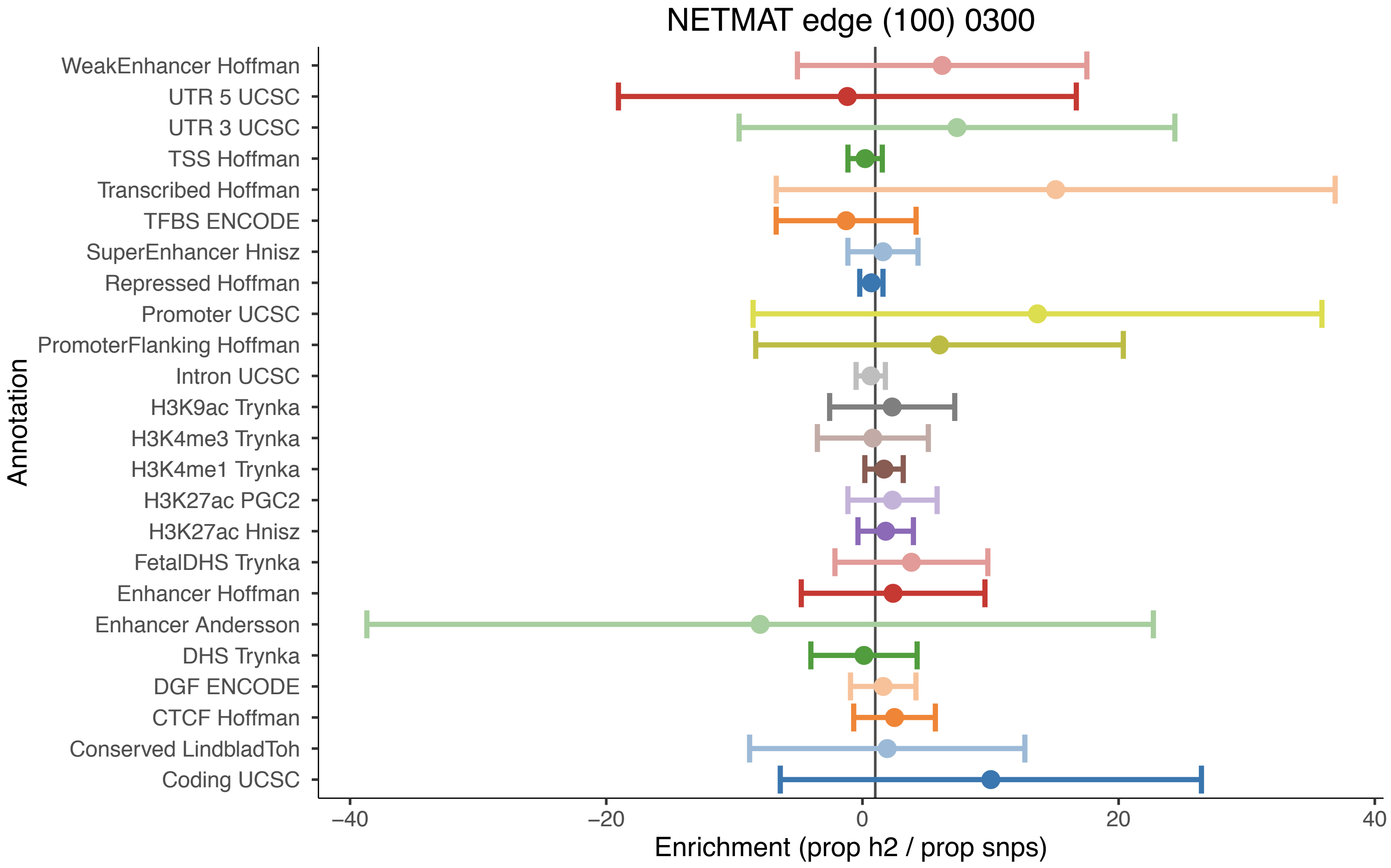

NETMAT edge (100) 0300
Annotation
WeakEnhancer Hoffman
UTR 5 UCSC
UTR 3 UCSC
TSS Hoffman
Transcribed Hoffman
TFBS ENCODE
SuperEnhancer Hnisz
Repressed Hoffman
Promoter UCSC
PromoterFlanking Hoffman
Intron UCSC
H3K9ac Trynka
H3K4me3 Trynka
H3K4me1 Trynka
H3K27ac PGC2
H3K27ac Hnisz
FetalDHS Trynka
Enhancer Hoffman
Enhancer Andersson
DHS Trynka
DGF ENCODE
CTCF Hoffman
Conserved LindbladToh
Coding UCSC
−40
−20
0
20
40
Enrichment (prop h2 / prop snps)

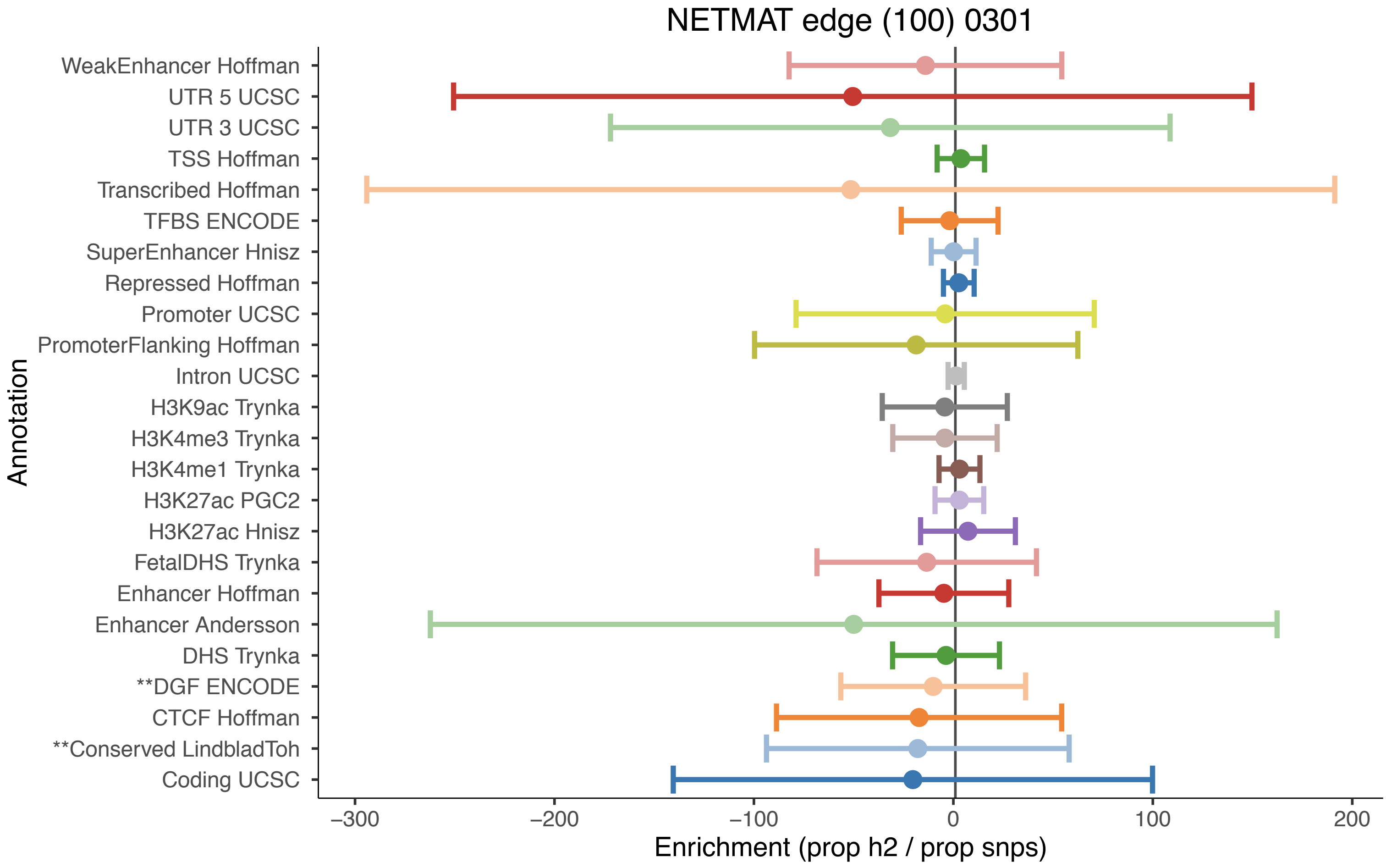

NETMAT edge (100) 0301
Annotation
WeakEnhancer Hoffman
UTR 5 UCSC
UTR 3 UCSC
TSS Hoffman
Transcribed Hoffman
TFBS ENCODE
SuperEnhancer Hnisz
Repressed Hoffman
Promoter UCSC
PromoterFlanking Hoffman
Intron UCSC
H3K9ac Trynka
H3K4me3 Trynka
H3K4me1 Trynka
H3K27ac PGC2
H3K27ac Hnisz
FetalDHS Trynka
Enhancer Hoffman
Enhancer Andersson
DHS Trynka
**DGF ENCODE
CTCF Hoffman
**Conserved LindbladToh
Coding UCSC
−300
−200
−100
0
100
200
Enrichment (prop h2 / prop snps)

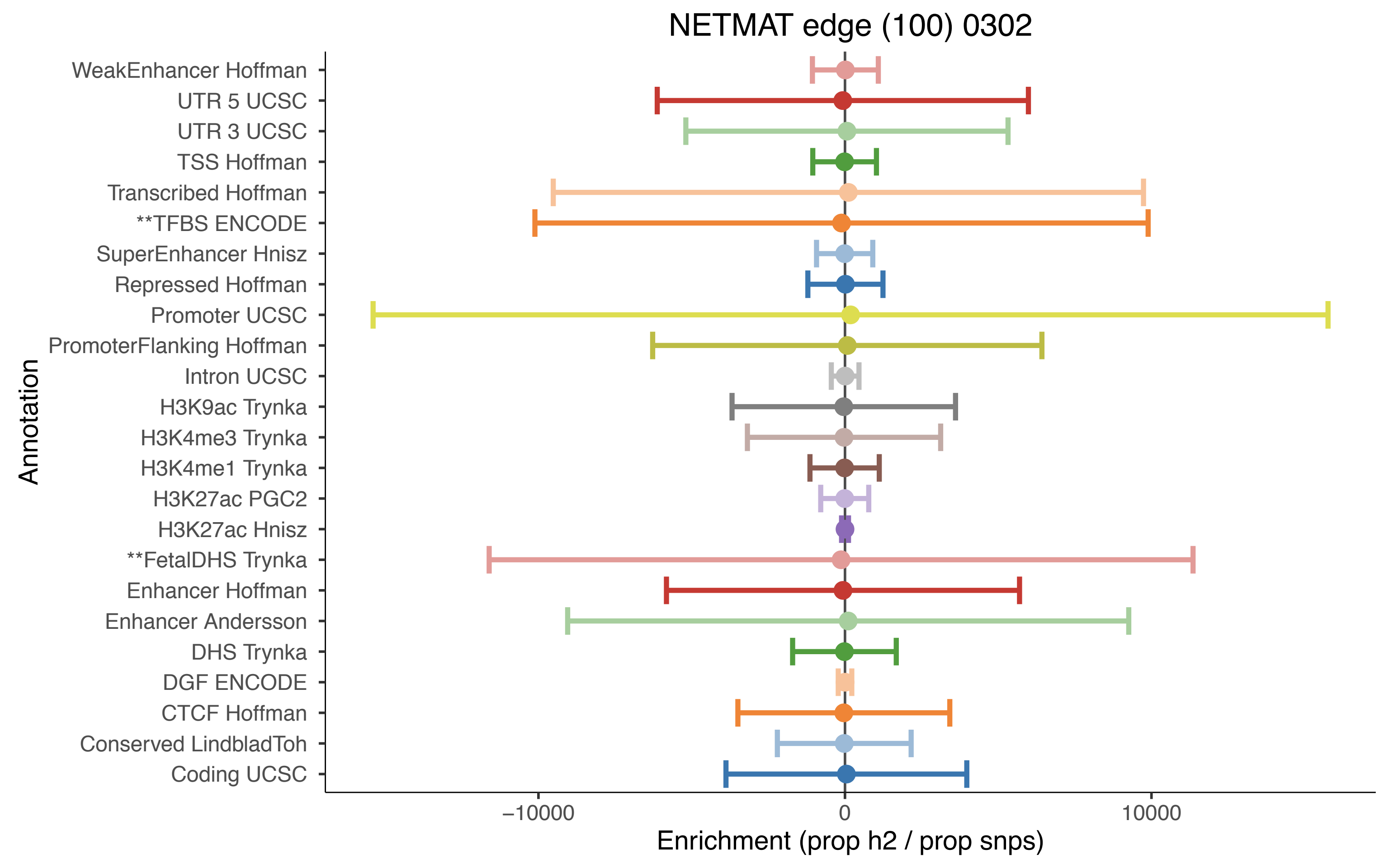
NETMAT edge (100) 0302
WeakEnhancer Hoffman
UTR 5 UCSC
UTR 3 UCSC
TSS Hoffman
Transcribed Hoffman
**TFBS ENCODE
SuperEnhancer Hnisz
Repressed Hoffman
Promoter UCSC
PromoterFlanking Hoffman
Intron UCSC
H3K9ac Trynka
H3K4me3 Trynka
H3K4me1 Trynka
H3K27ac PGC2
H3K27ac Hnisz
**FetalDHS Trynka
Enhancer Hoffman
Enhancer Andersson
DHS Trynka
DGF ENCODE
CTCF Hoffman
Conserved LindbladToh
Coding UCSC
Annotation
−10000
0
10000
Enrichment (prop h2 / prop snps)

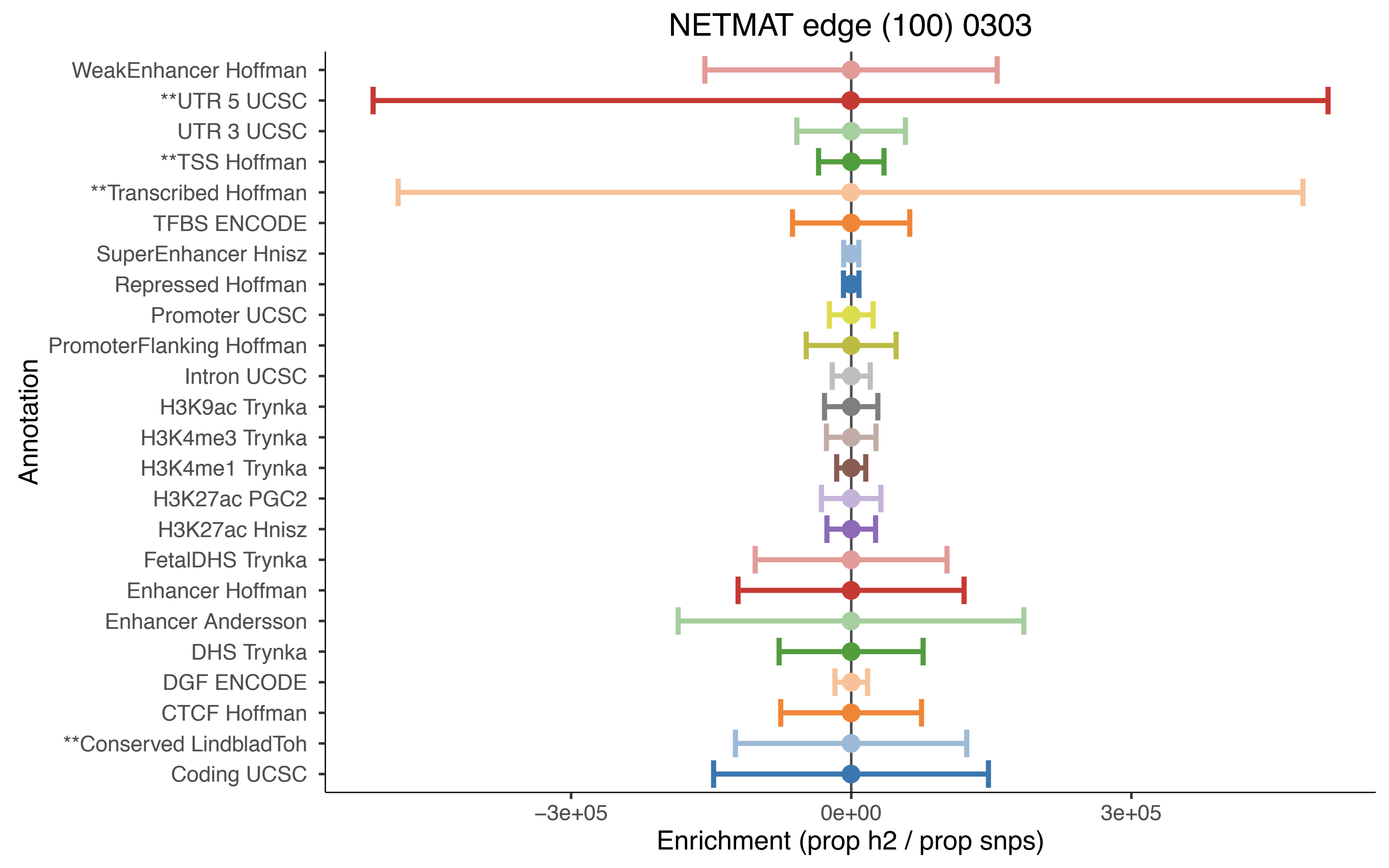

NETMAT edge (100) 0303
WeakEnhancer Hoffman
**UTR 5 UCSC
UTR 3 UCSC
**TSS Hoffman
**Transcribed Hoffman
TFBS ENCODE
SuperEnhancer Hnisz
Repressed Hoffman
Promoter UCSC
PromoterFlanking Hoffman
Intron UCSC
H3K9ac Trynka
H3K4me3 Trynka
H3K4me1 Trynka
H3K27ac PGC2
H3K27ac Hnisz
FetalDHS Trynka
Enhancer Hoffman
Enhancer Andersson
DHS Trynka
DGF ENCODE
CTCF Hoffman
**Conserved LindbladToh
Coding UCSC
Annotation
−3e+05
0e+00
3e+05
Enrichment (prop h2 / prop snps)

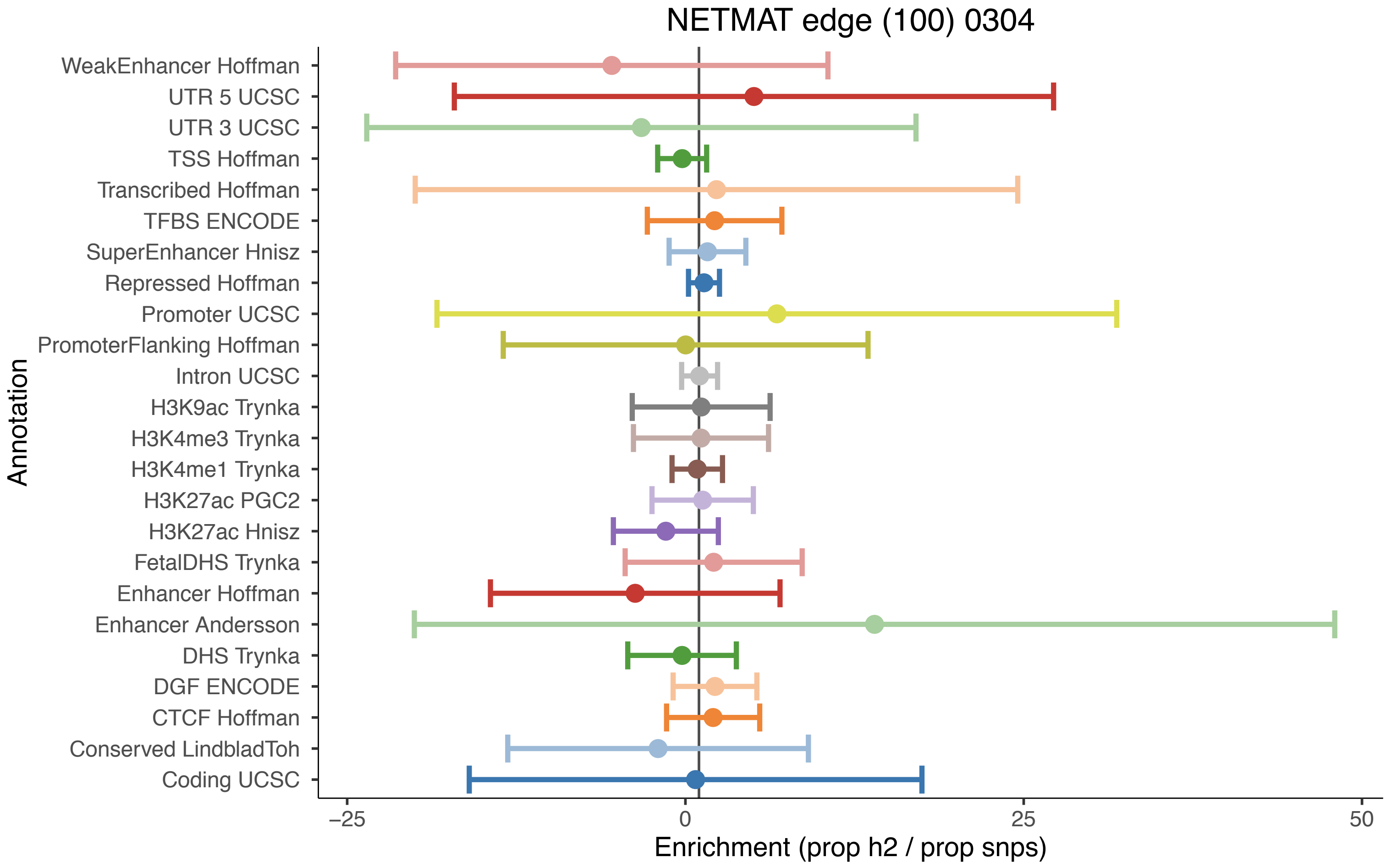
NETMAT edge (100) 0304
WeakEnhancer Hoffman
UTR 5 UCSC
UTR 3 UCSC
TSS Hoffman
Transcribed Hoffman
TFBS ENCODE
SuperEnhancer Hnisz
Repressed Hoffman
Promoter UCSC
PromoterFlanking Hoffman
Intron UCSC
H3K9ac Trynka
H3K4me3 Trynka
H3K4me1 Trynka
H3K27ac PGC2
H3K27ac Hnisz
FetalDHS Trynka
Enhancer Hoffman
Enhancer Andersson
DHS Trynka
DGF ENCODE
CTCF Hoffman
Conserved LindbladToh
Coding UCSC
Annotation
−25
0
25
50
Enrichment (prop h2 / prop snps)

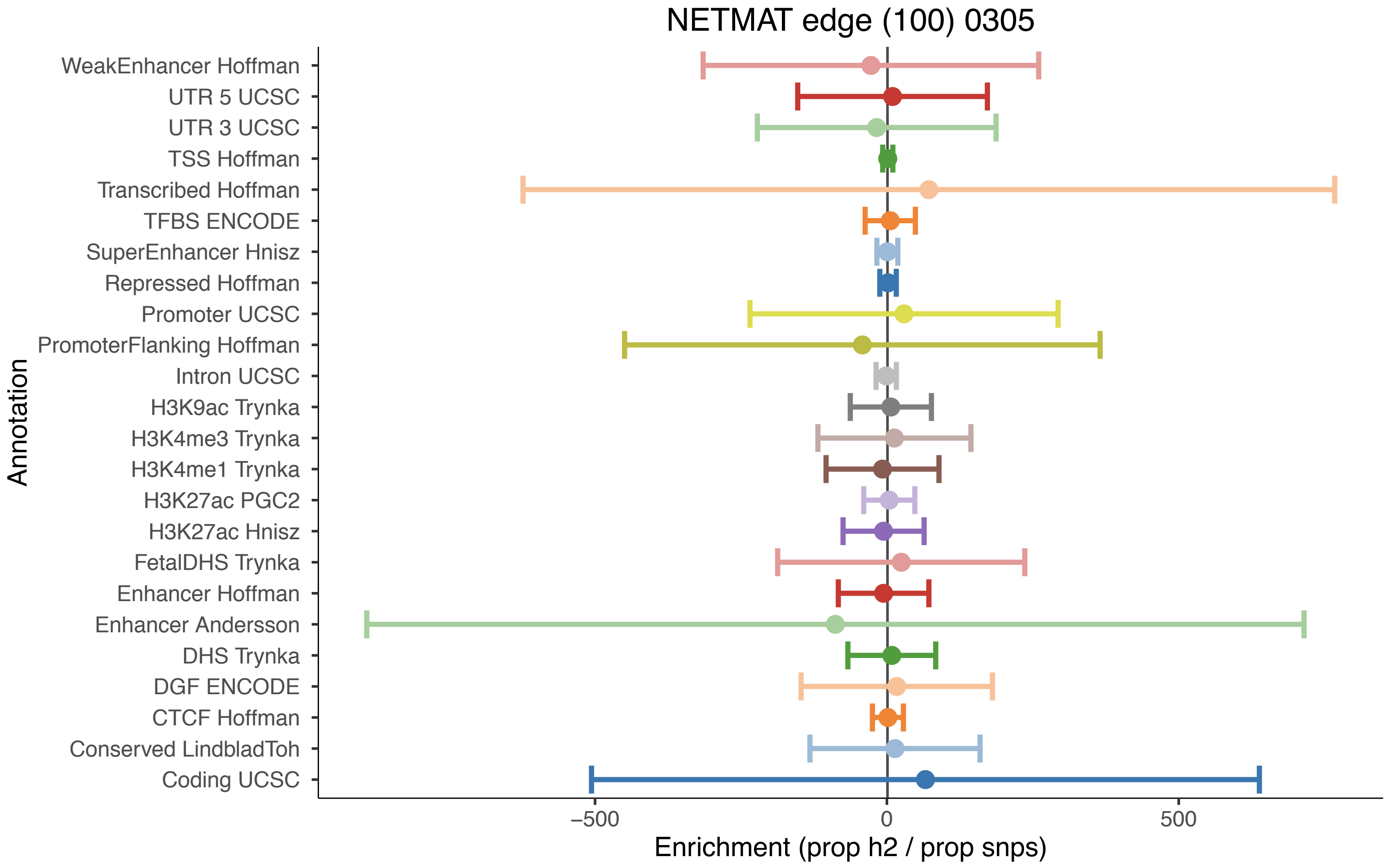
NETMAT edge (100) 0305
WeakEnhancer Hoffman
UTR 5 UCSC
UTR 3 UCSC
TSS Hoffman
Transcribed Hoffman
TFBS ENCODE
SuperEnhancer Hnisz
Repressed Hoffman
Promoter UCSC
PromoterFlanking Hoffman
Intron UCSC
H3K9ac Trynka
H3K4me3 Trynka
H3K4me1 Trynka
H3K27ac PGC2
H3K27ac Hnisz
FetalDHS Trynka
Enhancer Hoffman
Enhancer Andersson
DHS Trynka
DGF ENCODE
CTCF Hoffman
Conserved LindbladToh
Coding UCSC
Annotation
−500
0
500
Enrichment (prop h2 / prop snps)

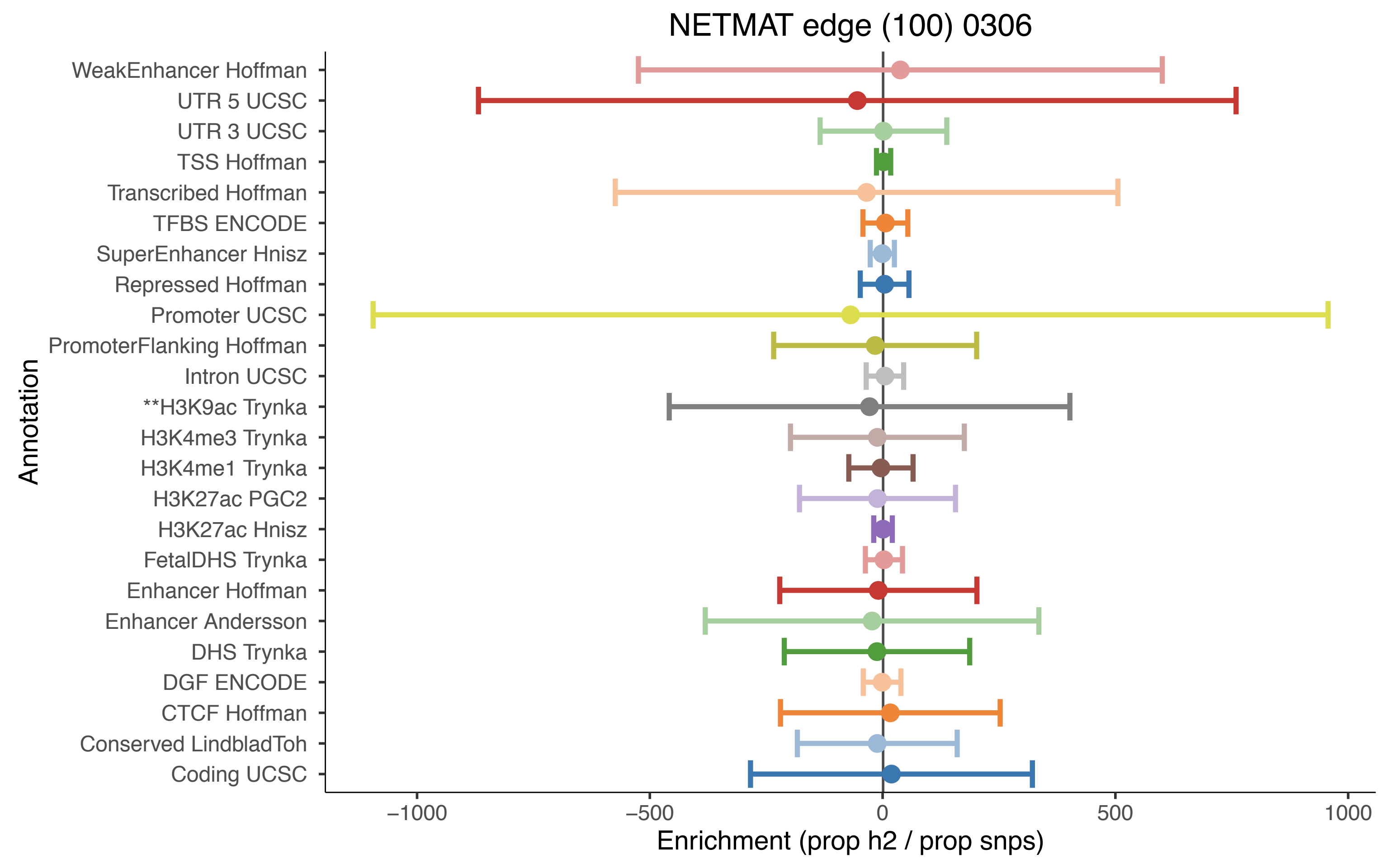

NETMAT edge (100) 0306
WeakEnhancer Hoffman
UTR 5 UCSC
UTR 3 UCSC
TSS Hoffman
Transcribed Hoffman
TFBS ENCODE
SuperEnhancer Hnisz
Repressed Hoffman
Promoter UCSC
PromoterFlanking Hoffman
Intron UCSC
**H3K9ac Trynka
H3K4me3 Trynka
H3K4me1 Trynka
H3K27ac PGC2
H3K27ac Hnisz
FetalDHS Trynka
Enhancer Hoffman
Enhancer Andersson
DHS Trynka
DGF ENCODE
CTCF Hoffman
Conserved LindbladToh
Coding UCSC
Annotation
−1000
−500
0
500
1000
Enrichment (prop h2 / prop snps)

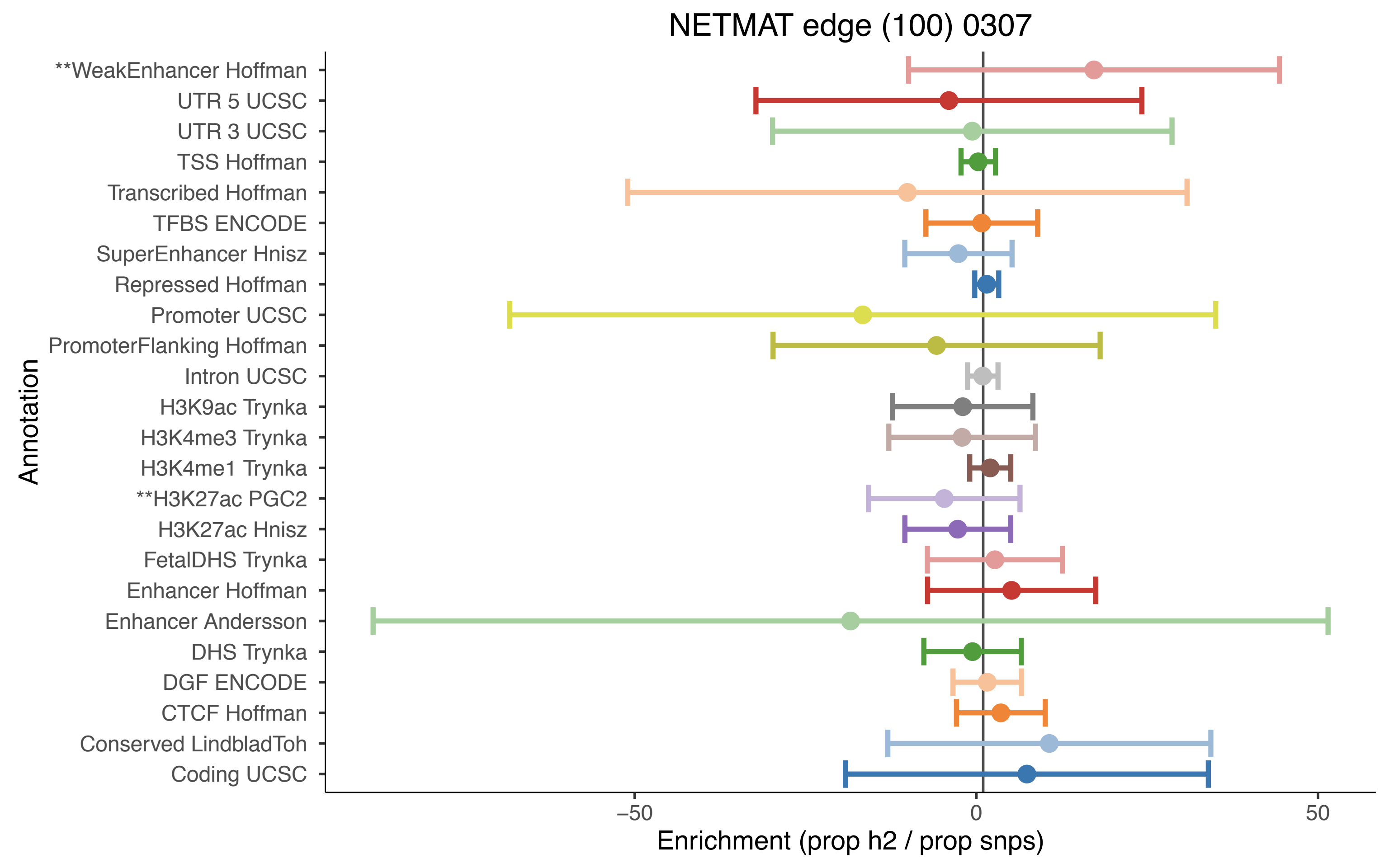
NETMAT edge (100) 0307
**WeakEnhancer Hoffman
UTR 5 UCSC
UTR 3 UCSC
TSS Hoffman
Transcribed Hoffman
TFBS ENCODE
SuperEnhancer Hnisz
Repressed Hoffman
Promoter UCSC
PromoterFlanking Hoffman
Intron UCSC
H3K9ac Trynka
H3K4me3 Trynka
H3K4me1 Trynka
**H3K27ac PGC2
H3K27ac Hnisz
FetalDHS Trynka
Enhancer Hoffman
Enhancer Andersson
DHS Trynka
DGF ENCODE
CTCF Hoffman
Conserved LindbladToh
Coding UCSC
Annotation
−50
0
50
Enrichment (prop h2 / prop snps)

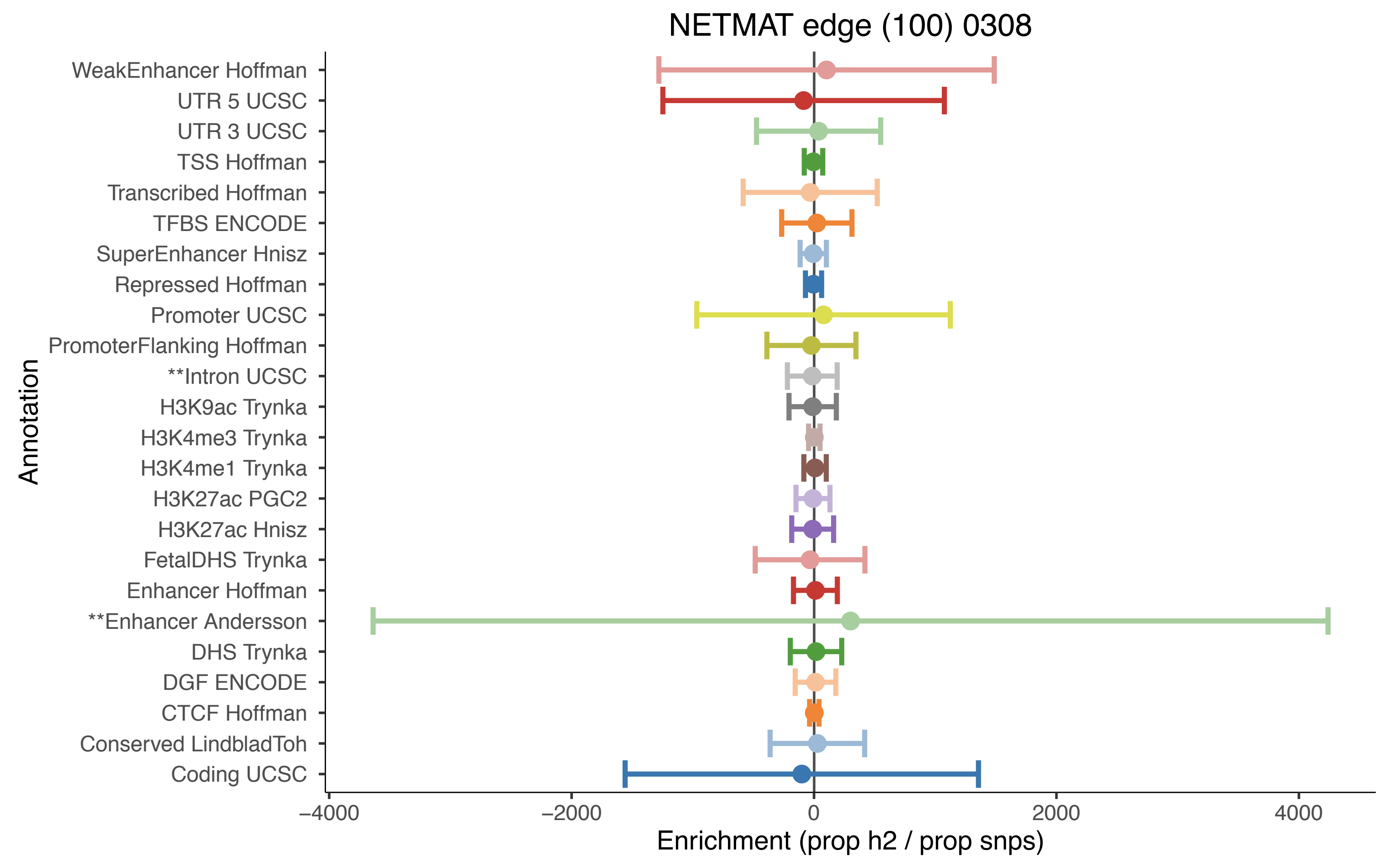
NETMAT edge (100) 0308
WeakEnhancer Hoffman
UTR 5 UCSC
UTR 3 UCSC
TSS Hoffman
Transcribed Hoffman
TFBS ENCODE
SuperEnhancer Hnisz
Repressed Hoffman
Promoter UCSC
PromoterFlanking Hoffman
**Intron UCSC
H3K9ac Trynka
H3K4me3 Trynka
H3K4me1 Trynka
H3K27ac PGC2
H3K27ac Hnisz
FetalDHS Trynka
Enhancer Hoffman
**Enhancer Andersson
DHS Trynka
DGF ENCODE
CTCF Hoffman
Conserved LindbladToh
Coding UCSC
Annotation
−4000
−2000
0
2000
4000
Enrichment (prop h2 / prop snps)

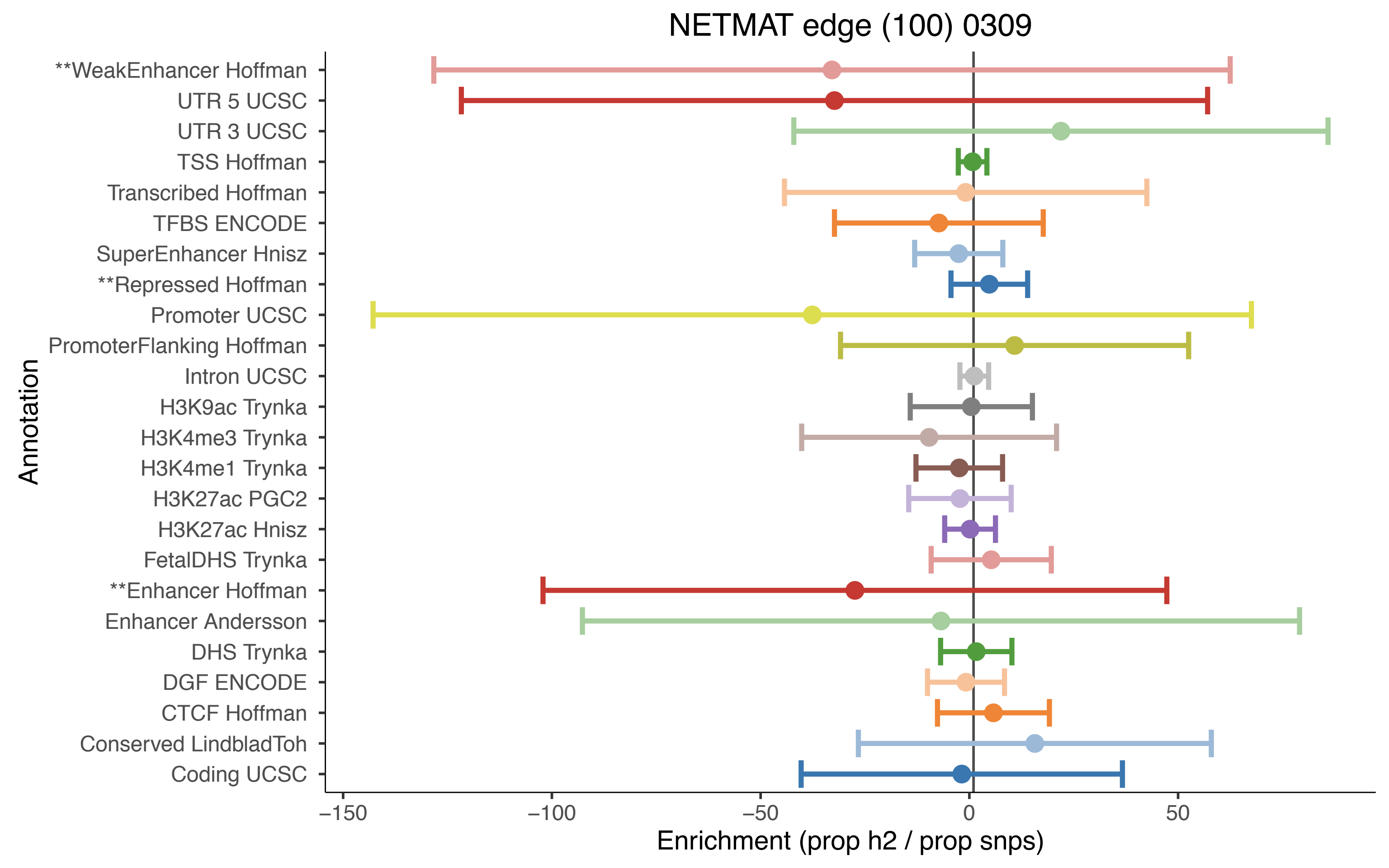

NETMAT edge (100) 0309
**WeakEnhancer Hoffman
UTR 5 UCSC
UTR 3 UCSC
TSS Hoffman
Transcribed Hoffman
TFBS ENCODE
SuperEnhancer Hnisz
**Repressed Hoffman
Promoter UCSC
PromoterFlanking Hoffman
Intron UCSC
H3K9ac Trynka
H3K4me3 Trynka
H3K4me1 Trynka
H3K27ac PGC2
H3K27ac Hnisz
FetalDHS Trynka
**Enhancer Hoffman
Enhancer Andersson
DHS Trynka
DGF ENCODE
CTCF Hoffman
Conserved LindbladToh
Coding UCSC
Annotation
−150
−100
−50
0
50
Enrichment (prop h2 / prop snps)

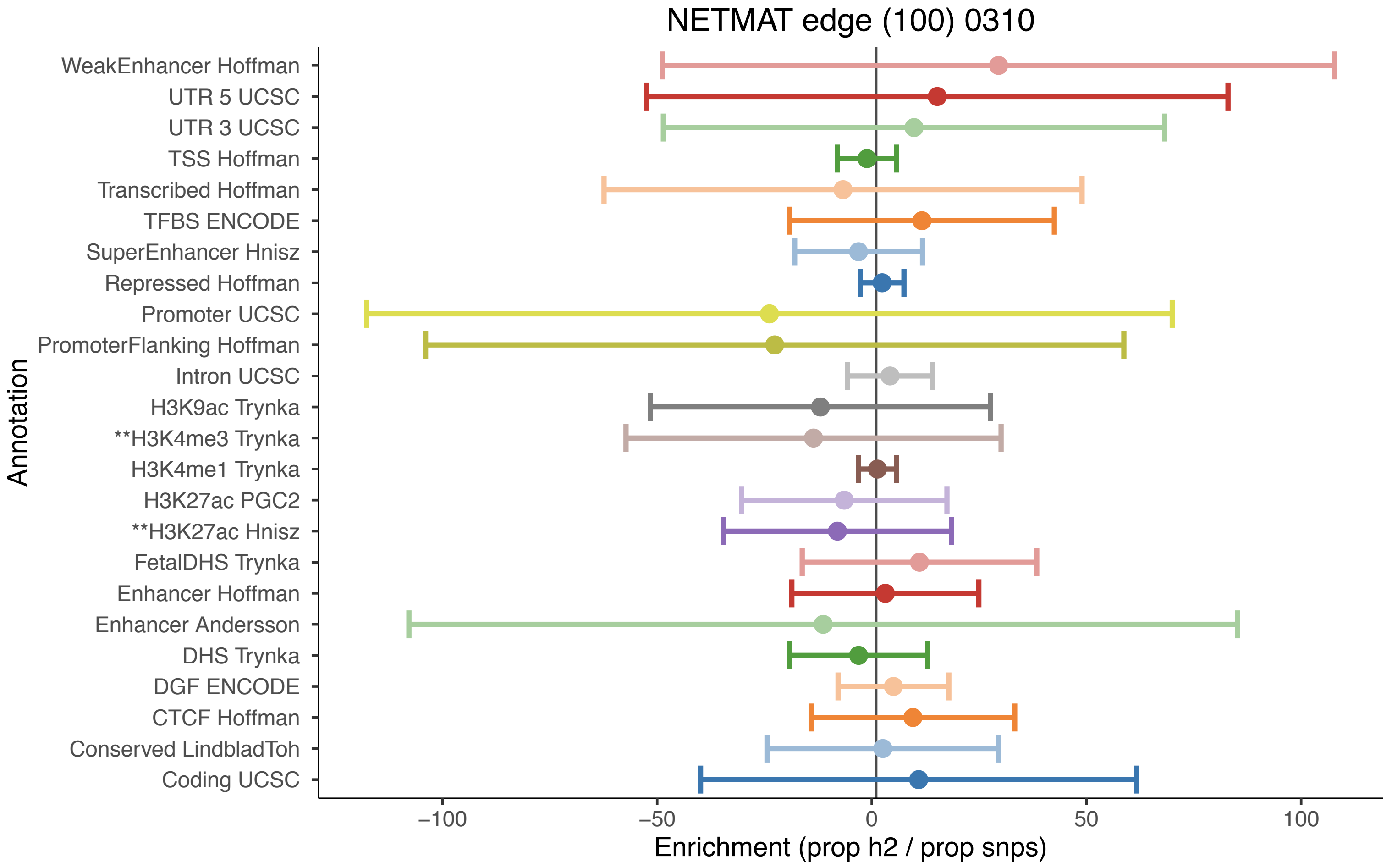
NETMAT edge (100) 0310
WeakEnhancer Hoffman
UTR 5 UCSC
UTR 3 UCSC
TSS Hoffman
Transcribed Hoffman
TFBS ENCODE
SuperEnhancer Hnisz
Repressed Hoffman
Promoter UCSC
PromoterFlanking Hoffman
Intron UCSC
H3K9ac Trynka
**H3K4me3 Trynka
H3K4me1 Trynka
H3K27ac PGC2
**H3K27ac Hnisz
FetalDHS Trynka
Enhancer Hoffman
Enhancer Andersson
DHS Trynka
DGF ENCODE
CTCF Hoffman
Conserved LindbladToh
Coding UCSC
Annotation
−100
−50
0
50
100
Enrichment (prop h2 / prop snps)

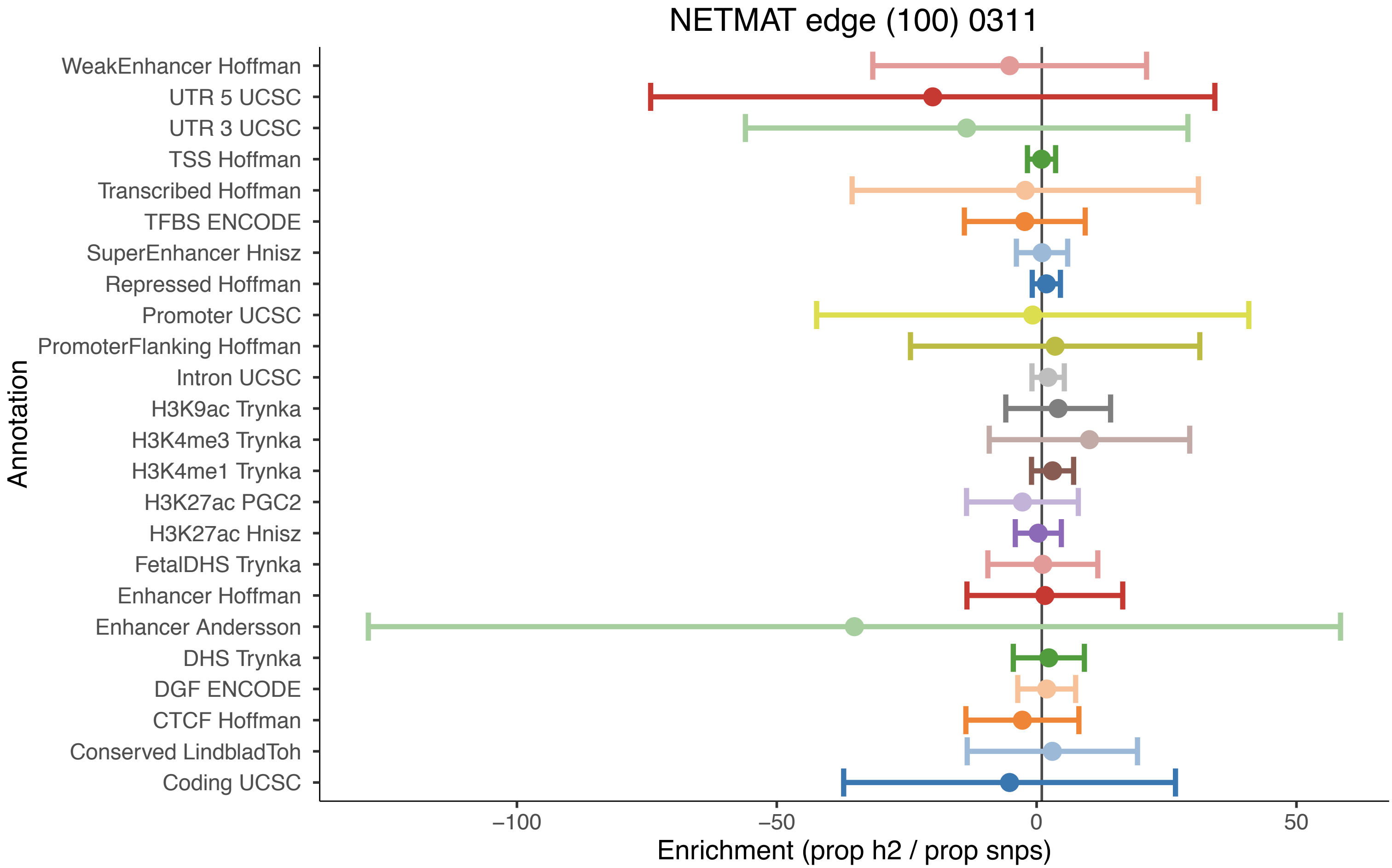

NETMAT edge (100) 0311
WeakEnhancer Hoffman
UTR 5 UCSC
UTR 3 UCSC
TSS Hoffman
Transcribed Hoffman
TFBS ENCODE
SuperEnhancer Hnisz
Repressed Hoffman
Promoter UCSC
PromoterFlanking Hoffman
Intron UCSC
H3K9ac Trynka
H3K4me3 Trynka
H3K4me1 Trynka
H3K27ac PGC2
H3K27ac Hnisz
FetalDHS Trynka
Enhancer Hoffman
Enhancer Andersson
DHS Trynka
DGF ENCODE
CTCF Hoffman
Conserved LindbladToh
Coding UCSC
Annotation
−100
−50
0
50
Enrichment (prop h2 / prop snps)

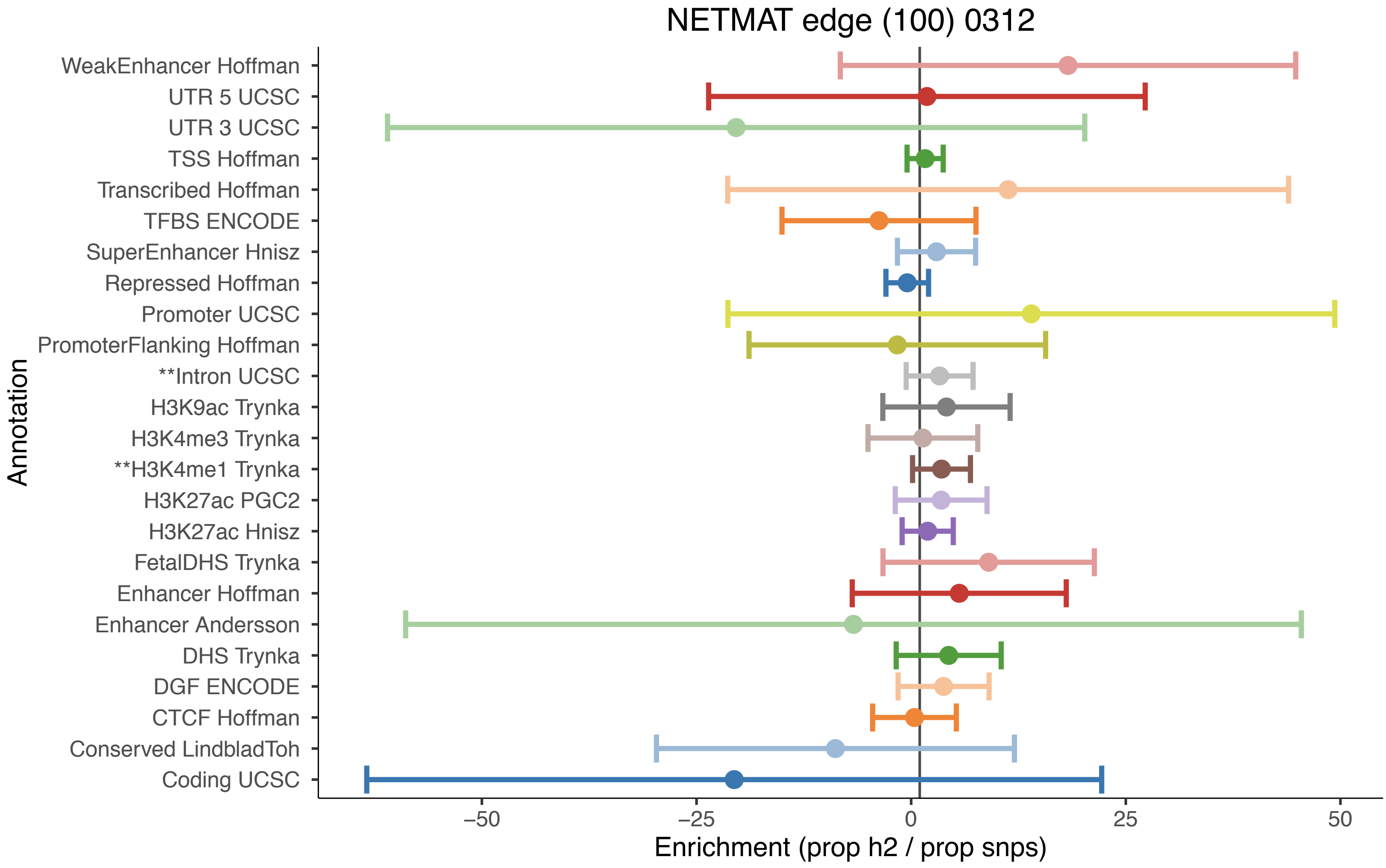

NETMAT edge (100) 0312
WeakEnhancer Hoffman
UTR 5 UCSC
UTR 3 UCSC
TSS Hoffman
Transcribed Hoffman
TFBS ENCODE
SuperEnhancer Hnisz
Repressed Hoffman
Promoter UCSC
PromoterFlanking Hoffman
**Intron UCSC
H3K9ac Trynka
H3K4me3 Trynka
**H3K4me1 Trynka
H3K27ac PGC2
H3K27ac Hnisz
FetalDHS Trynka
Enhancer Hoffman
Enhancer Andersson
DHS Trynka
DGF ENCODE
CTCF Hoffman
Conserved LindbladToh
Coding UCSC
Annotation
−50
−25
0
25
50
Enrichment (prop h2 / prop snps)

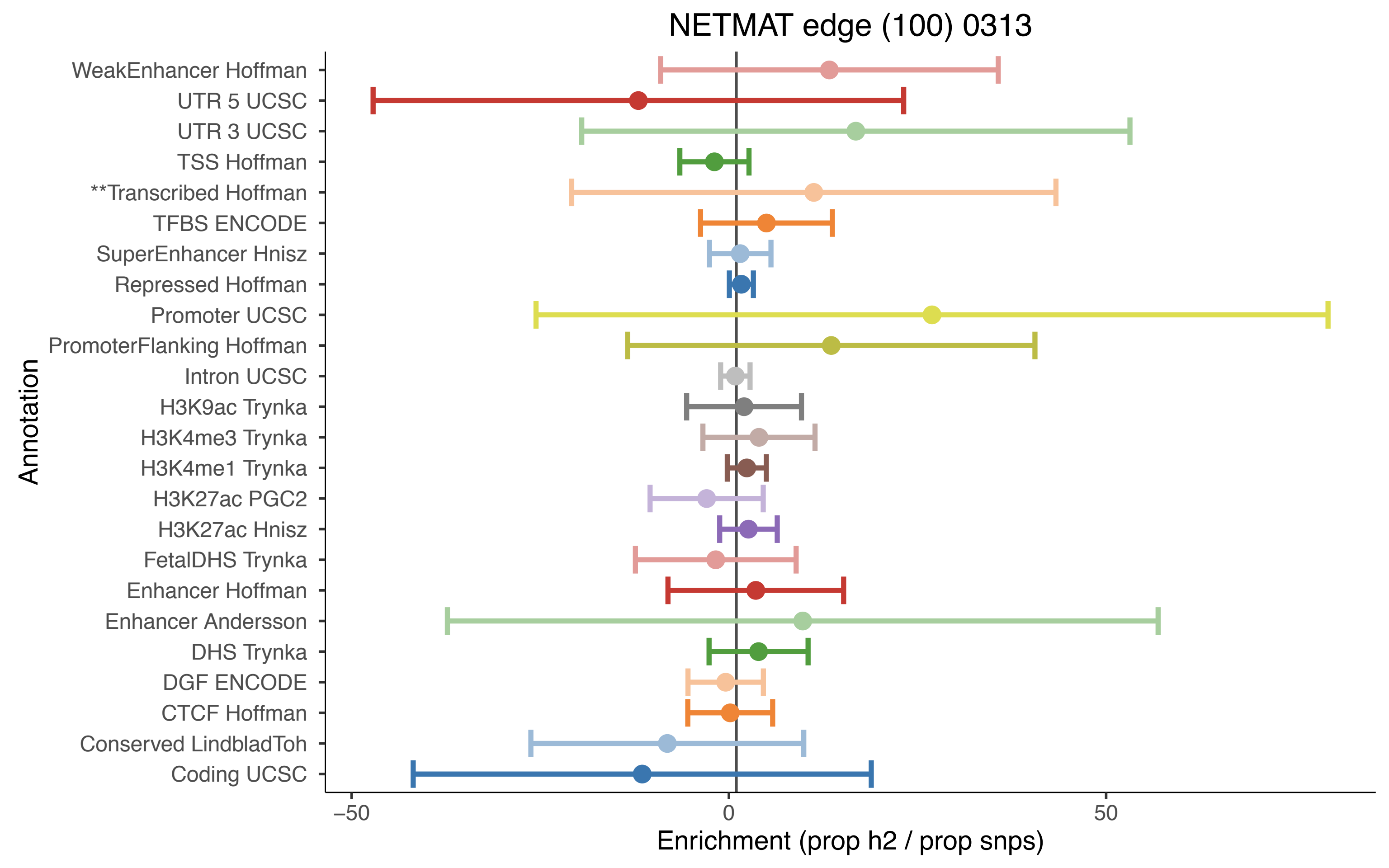
NETMAT edge (100) 0313
WeakEnhancer Hoffman
UTR 5 UCSC
UTR 3 UCSC
TSS Hoffman
**Transcribed Hoffman
TFBS ENCODE
SuperEnhancer Hnisz
Repressed Hoffman
Promoter UCSC
PromoterFlanking Hoffman
Intron UCSC
H3K9ac Trynka
H3K4me3 Trynka
H3K4me1 Trynka
H3K27ac PGC2
H3K27ac Hnisz
FetalDHS Trynka
Enhancer Hoffman
Enhancer Andersson
DHS Trynka
DGF ENCODE
CTCF Hoffman
Conserved LindbladToh
Coding UCSC
Annotation
−50
0
50
Enrichment (prop h2 / prop snps)

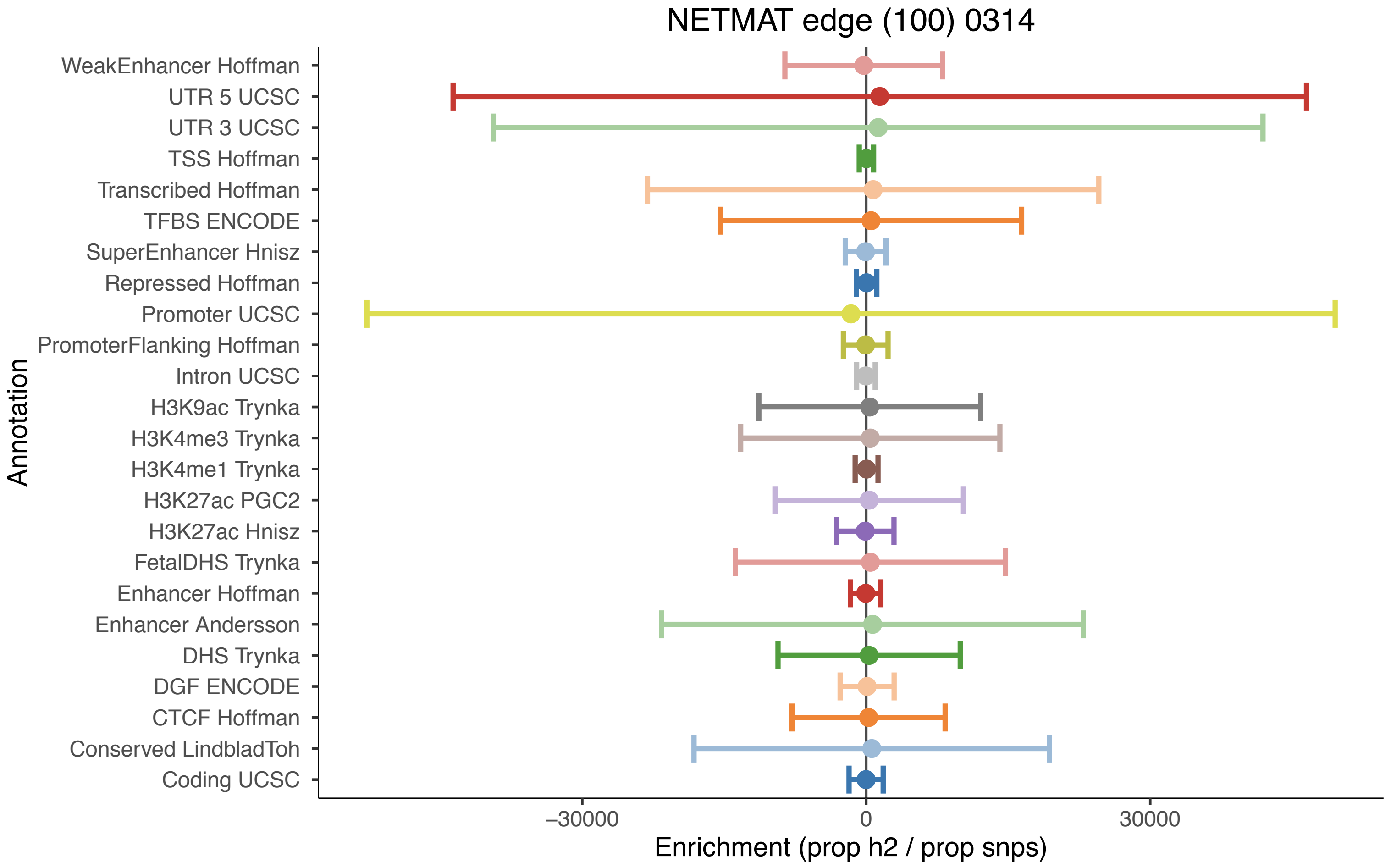
NETMAT edge (100) 0314
WeakEnhancer Hoffman
UTR 5 UCSC
UTR 3 UCSC
TSS Hoffman
Transcribed Hoffman
TFBS ENCODE
SuperEnhancer Hnisz
Repressed Hoffman
Promoter UCSC
PromoterFlanking Hoffman
Intron UCSC
H3K9ac Trynka
H3K4me3 Trynka
H3K4me1 Trynka
H3K27ac PGC2
H3K27ac Hnisz
FetalDHS Trynka
Enhancer Hoffman
Enhancer Andersson
DHS Trynka
DGF ENCODE
CTCF Hoffman
Conserved LindbladToh
Coding UCSC
Annotation
−30000
0
30000
Enrichment (prop h2 / prop snps)

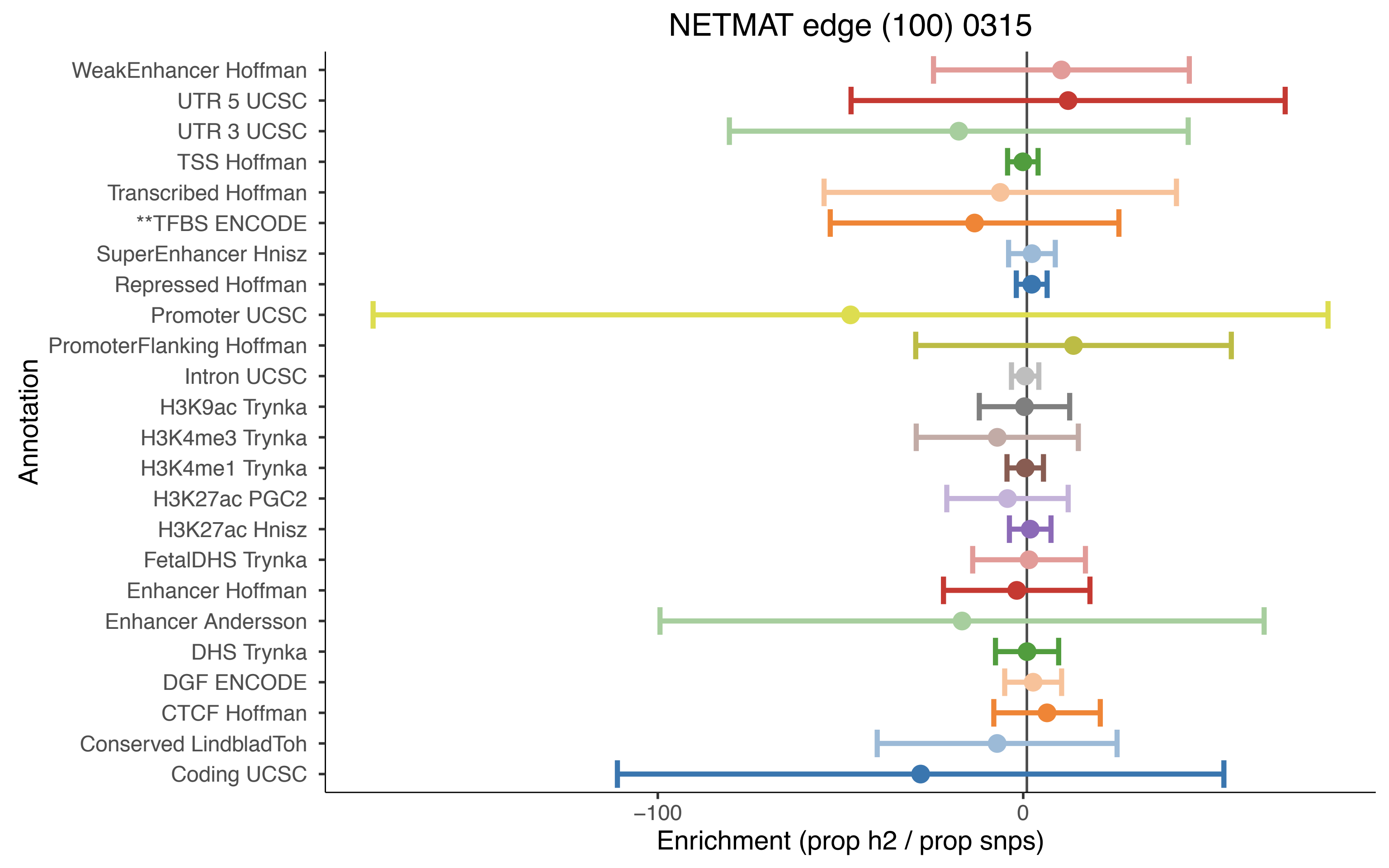
NETMAT edge (100) 0315
WeakEnhancer Hoffman
UTR 5 UCSC
UTR 3 UCSC
TSS Hoffman
Transcribed Hoffman
**TFBS ENCODE
SuperEnhancer Hnisz
Repressed Hoffman
Promoter UCSC
PromoterFlanking Hoffman
Intron UCSC
H3K9ac Trynka
H3K4me3 Trynka
H3K4me1 Trynka
H3K27ac PGC2
H3K27ac Hnisz
FetalDHS Trynka
Enhancer Hoffman
Enhancer Andersson
DHS Trynka
DGF ENCODE
CTCF Hoffman
Conserved LindbladToh
Coding UCSC
Annotation
−100
0
Enrichment (prop h2 / prop snps)

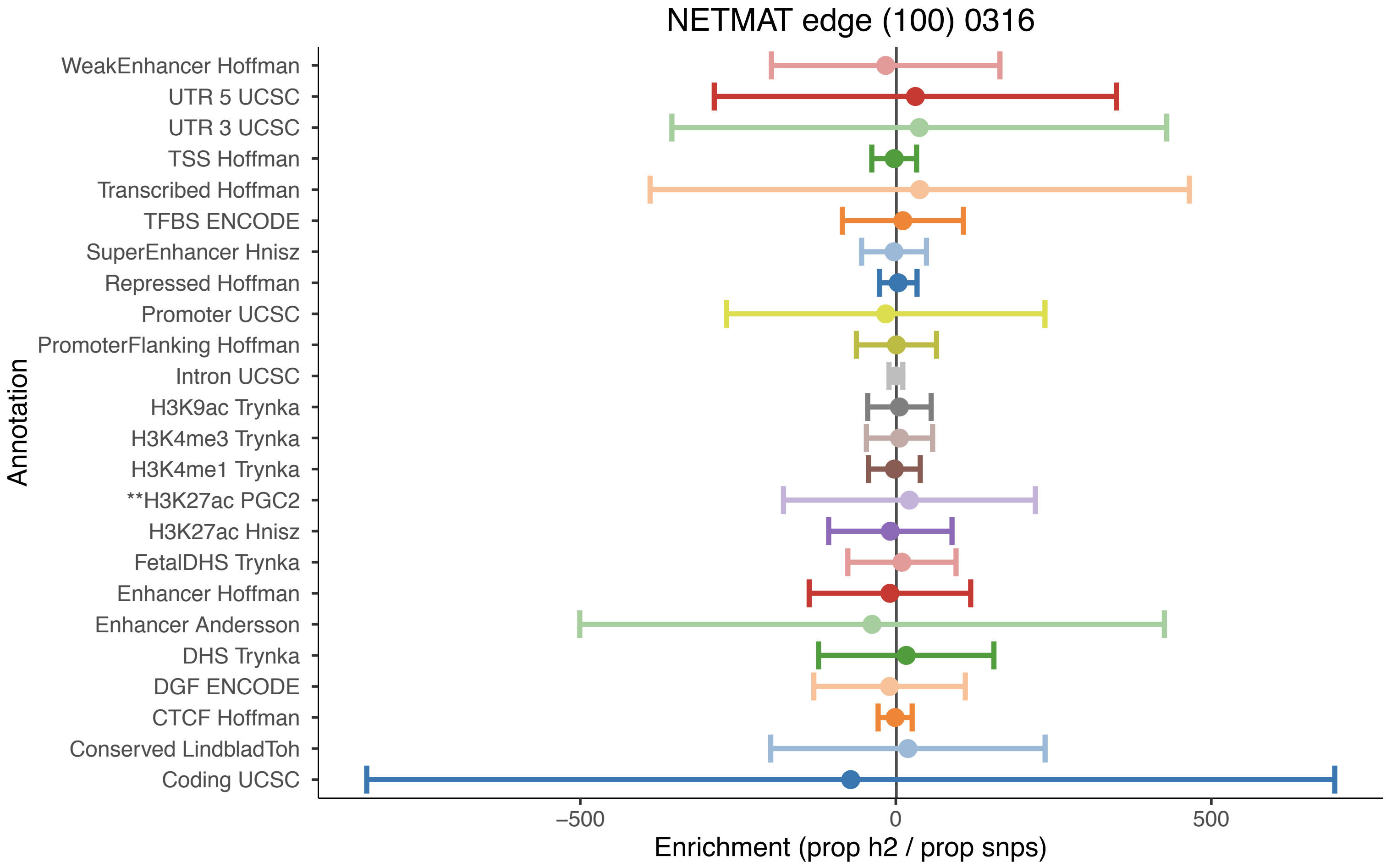

NETMAT edge (100) 0316
Annotation
WeakEnhancer Hoffman
UTR 5 UCSC
UTR 3 UCSC
TSS Hoffman
Transcribed Hoffman
TFBS ENCODE
SuperEnhancer Hnisz
Repressed Hoffman
Promoter UCSC
PromoterFlanking Hoffman
Intron UCSC
H3K9ac Trynka
H3K4me3 Trynka
H3K4me1 Trynka
**H3K27ac PGC2
H3K27ac Hnisz
FetalDHS Trynka
Enhancer Hoffman
Enhancer Andersson
DHS Trynka
DGF ENCODE
CTCF Hoffman
Conserved LindbladToh
Coding UCSC
−500
0
500
Enrichment (prop h2 / prop snps)

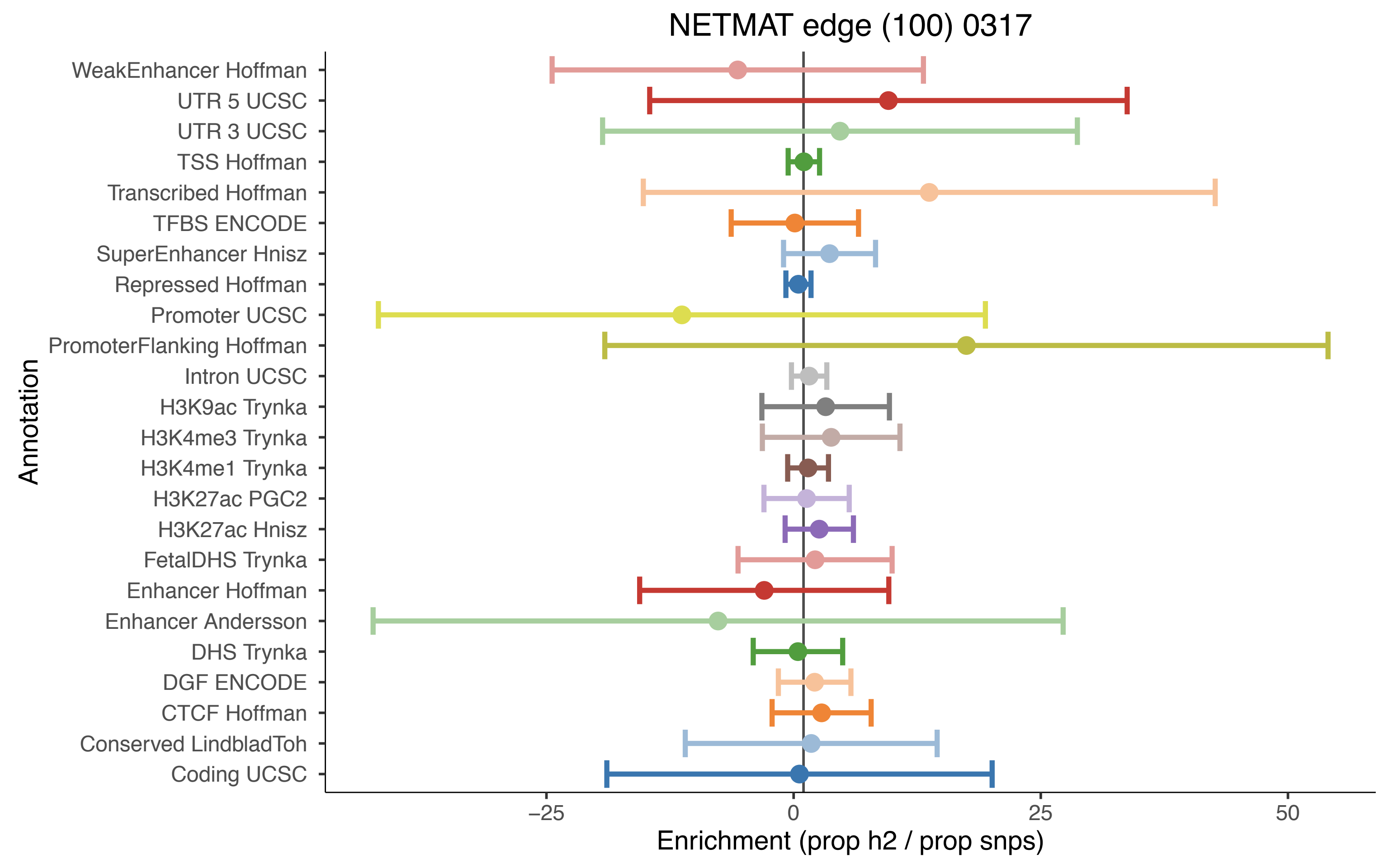
NETMAT edge (100) 0317
WeakEnhancer Hoffman
UTR 5 UCSC
UTR 3 UCSC
TSS Hoffman
Transcribed Hoffman
TFBS ENCODE
SuperEnhancer Hnisz
Repressed Hoffman
Promoter UCSC
PromoterFlanking Hoffman
Intron UCSC
H3K9ac Trynka
H3K4me3 Trynka
H3K4me1 Trynka
H3K27ac PGC2
H3K27ac Hnisz
FetalDHS Trynka
Enhancer Hoffman
Enhancer Andersson
DHS Trynka
DGF ENCODE
CTCF Hoffman
Conserved LindbladToh
Coding UCSC
Annotation
–25
0
25
50
Enrichment (prop h2 / prop snps)

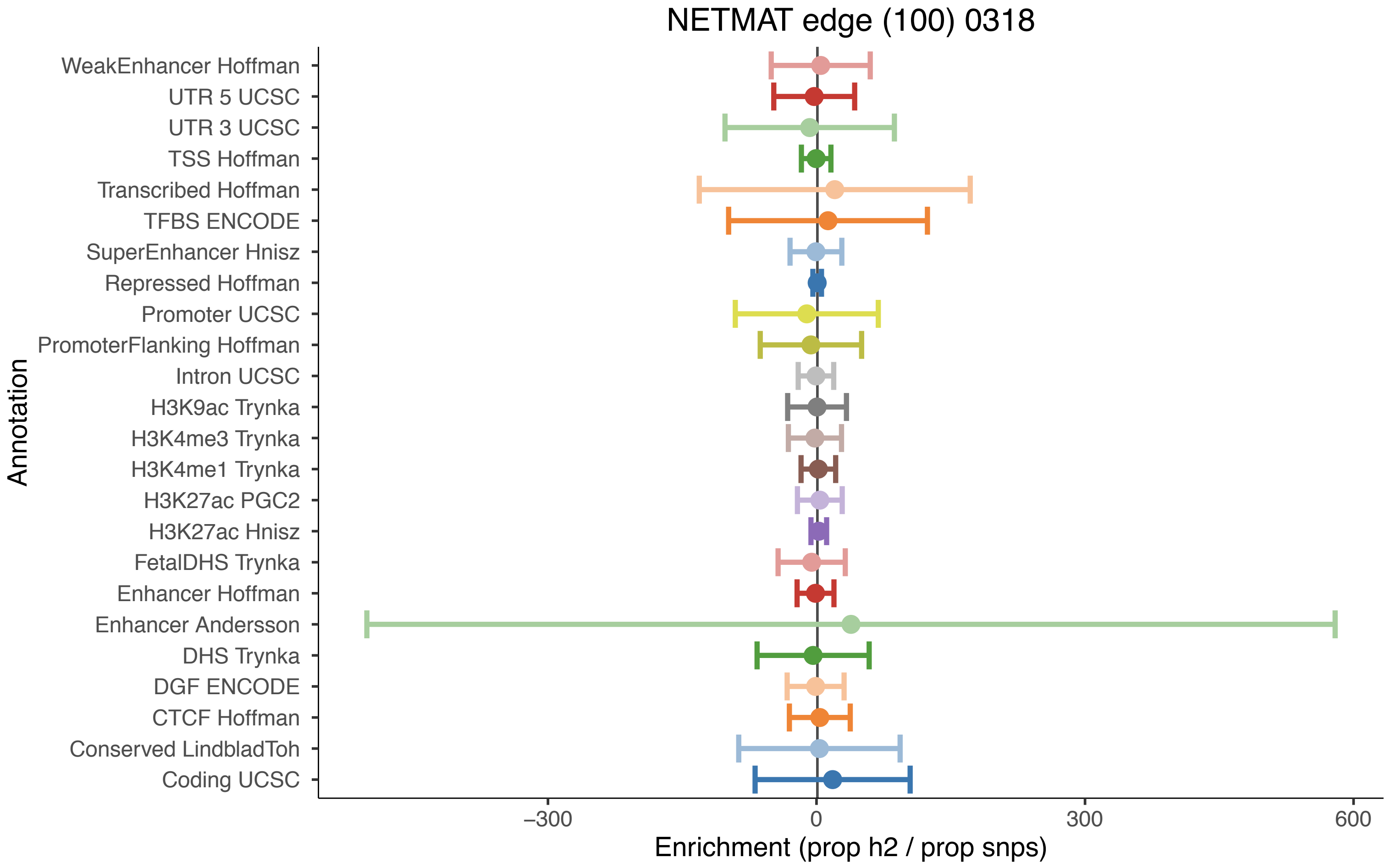

NETMAT edge (100) 0318
WeakEnhancer Hoffman
UTR 5 UCSC
UTR 3 UCSC
TSS Hoffman
Transcribed Hoffman
TFBS ENCODE
SuperEnhancer Hnisz
Repressed Hoffman
Promoter UCSC
PromoterFlanking Hoffman
Intron UCSC
H3K9ac Trynka
H3K4me3 Trynka
H3K4me1 Trynka
H3K27ac PGC2
H3K27ac Hnisz
FetalDHS Trynka
Enhancer Hoffman
Enhancer Andersson
DHS Trynka
DGF ENCODE
CTCF Hoffman
Conserved LindbladToh
Coding UCSC
Annotation
–300
0
300
600
Enrichment (prop h2 / prop snps)

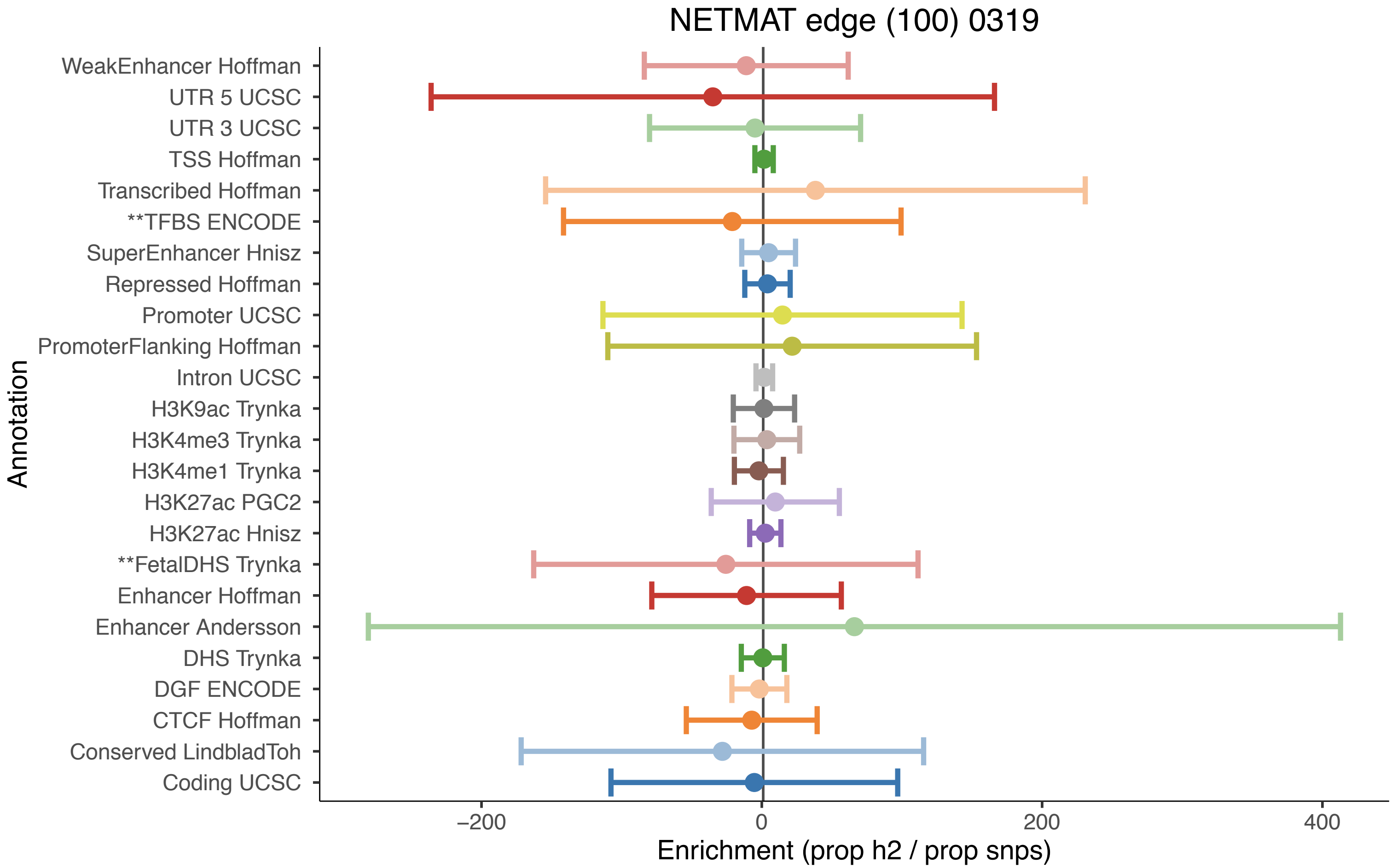

NETMAT edge (100) 0319
WeakEnhancer Hoffman
UTR 5 UCSC
UTR 3 UCSC
TSS Hoffman
Transcribed Hoffman
**TFBS ENCODE
SuperEnhancer Hnisz
Repressed Hoffman
Promoter UCSC
PromoterFlanking Hoffman
Intron UCSC
H3K9ac Trynka
H3K4me3 Trynka
H3K4me1 Trynka
H3K27ac PGC2
H3K27ac Hnisz
**FetalDHS Trynka
Enhancer Hoffman
Enhancer Andersson
DHS Trynka
DGF ENCODE
CTCF Hoffman
Conserved LindbladToh
Coding UCSC
Annotation
−200
0
200
400
Enrichment (prop h2 / prop snps)

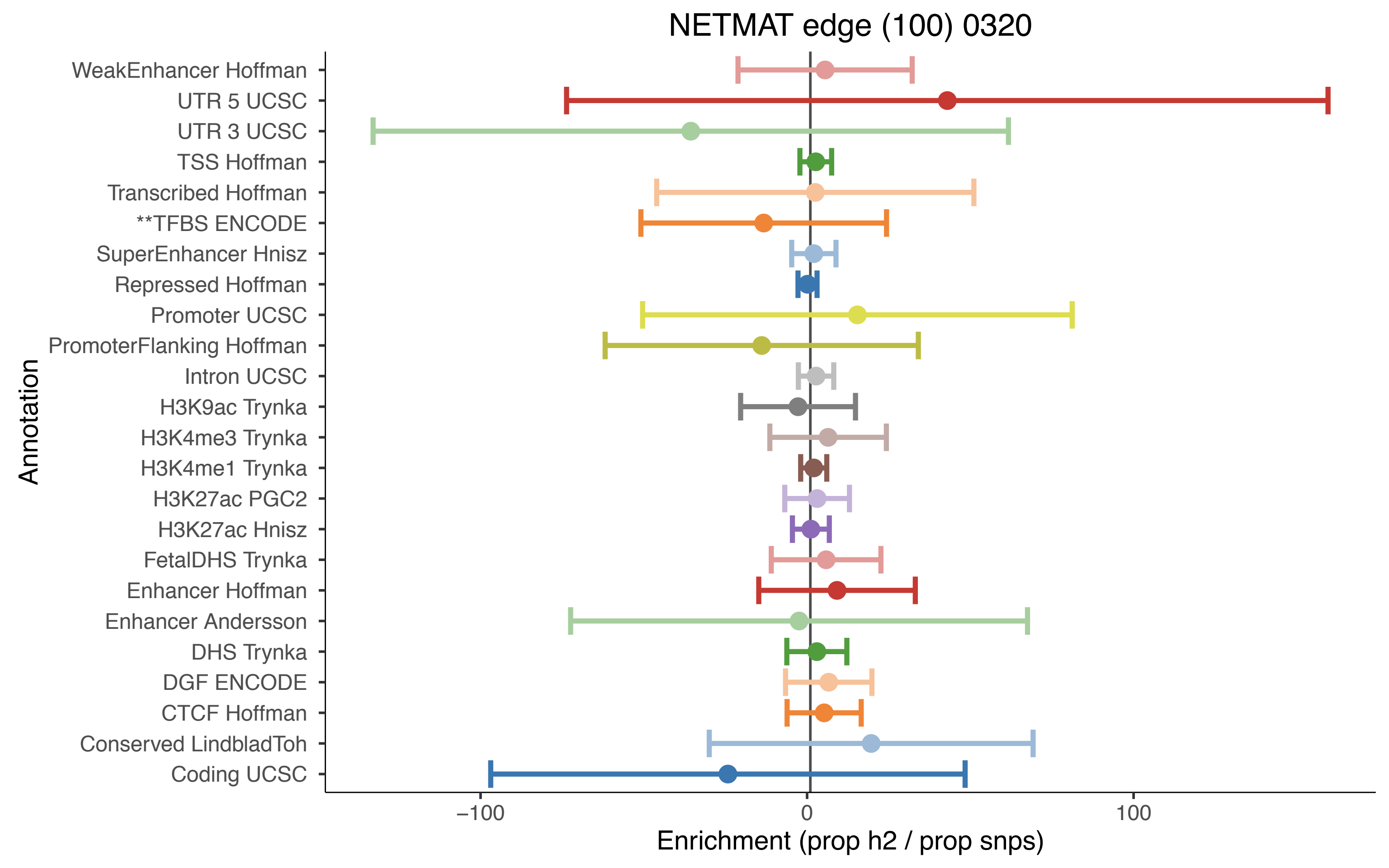

NETMAT edge (100) 0320
WeakEnhancer Hoffman
UTR 5 UCSC
UTR 3 UCSC
TSS Hoffman
Transcribed Hoffman
**TFBS ENCODE
SuperEnhancer Hnisz
Repressed Hoffman
Promoter UCSC
PromoterFlanking Hoffman
Intron UCSC
H3K9ac Trynka
H3K4me3 Trynka
H3K4me1 Trynka
H3K27ac PGC2
H3K27ac Hnisz
FetalDHS Trynka
Enhancer Hoffman
Enhancer Andersson
DHS Trynka
DGF ENCODE
CTCF Hoffman
Conserved LindbladToh
Coding UCSC
Annotation
−100
0
100
Enrichment (prop h2 / prop snps)

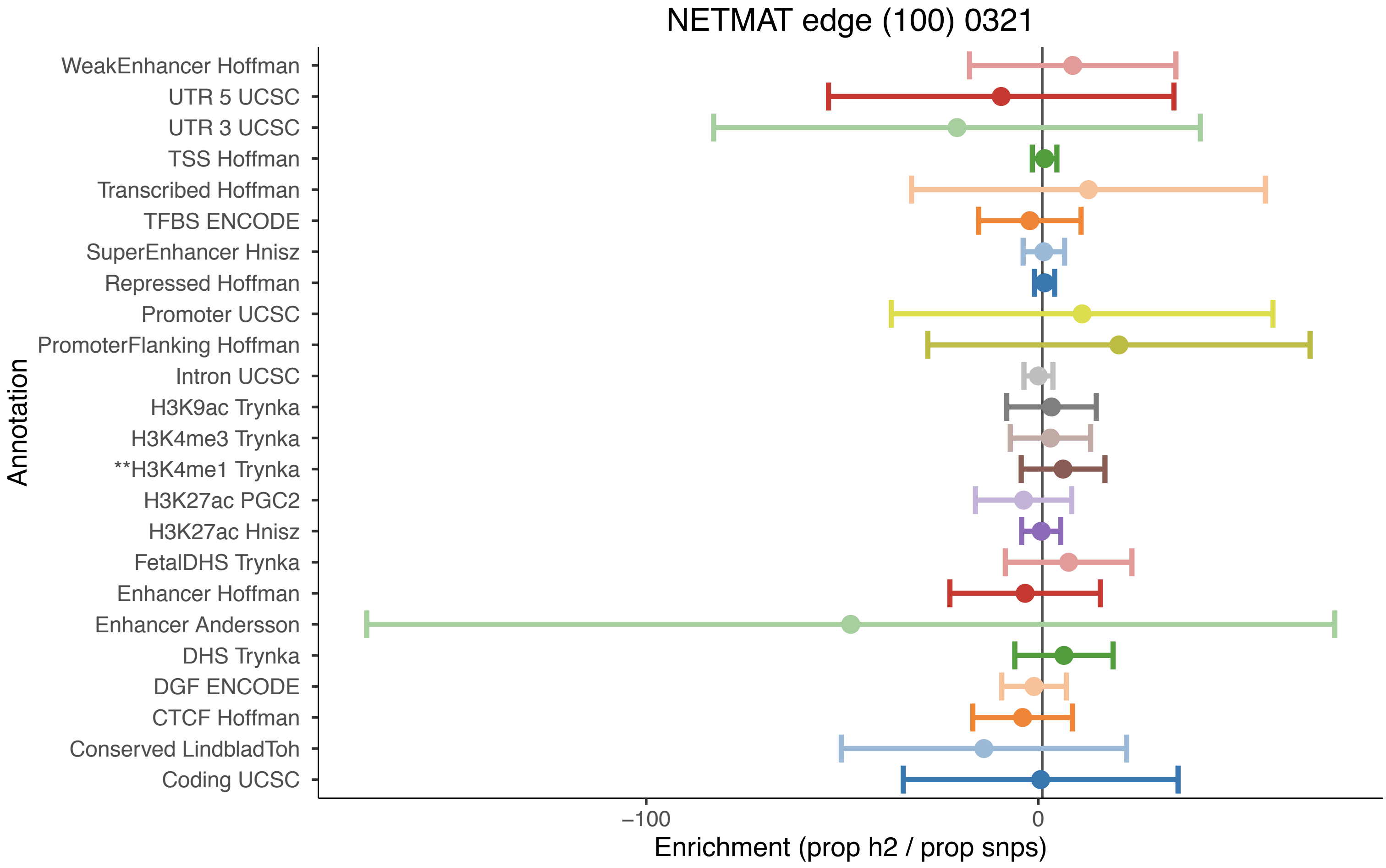

NETMAT edge (100) 0321
WeakEnhancer Hoffman
UTR 5 UCSC
UTR 3 UCSC
TSS Hoffman
Transcribed Hoffman
TFBS ENCODE
SuperEnhancer Hnisz
Repressed Hoffman
Promoter UCSC
PromoterFlanking Hoffman
Intron UCSC
H3K9ac Trynka
H3K4me3 Trynka
**H3K4me1 Trynka
H3K27ac PGC2
H3K27ac Hnisz
FetalDHS Trynka
Enhancer Hoffman
Enhancer Andersson
DHS Trynka
DGF ENCODE
CTCF Hoffman
Conserved LindbladToh
Coding UCSC
Annotation
−100
0
Enrichment (prop h2 / prop snps)

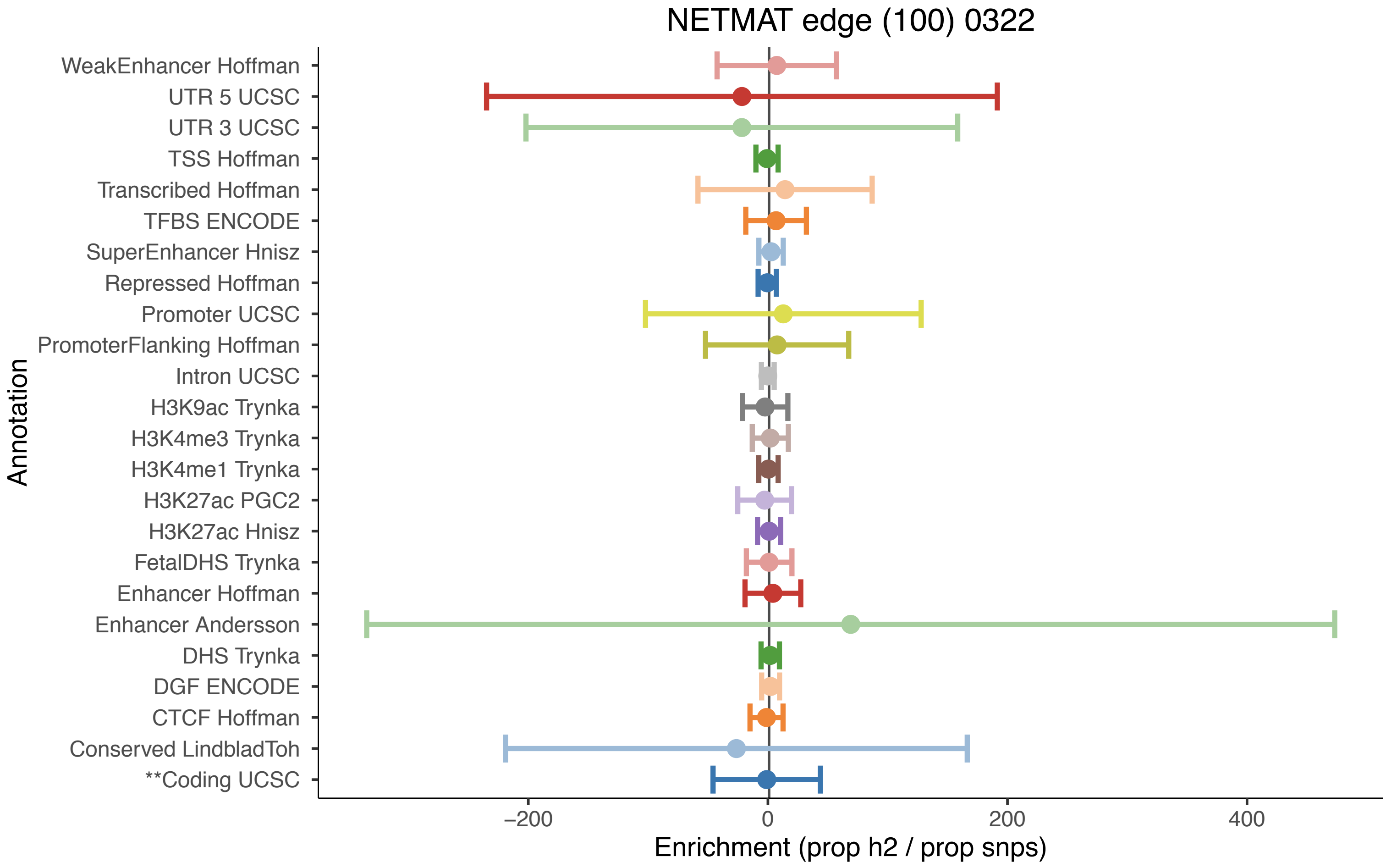
NETMAT edge (100) 0322
WeakEnhancer Hoffman
UTR 5 UCSC
UTR 3 UCSC
TSS Hoffman
Transcribed Hoffman
TFBS ENCODE
SuperEnhancer Hnisz
Repressed Hoffman
Promoter UCSC
PromoterFlanking Hoffman
Intron UCSC
H3K9ac Trynka
H3K4me3 Trynka
H3K4me1 Trynka
H3K27ac PGC2
H3K27ac Hnisz
FetalDHS Trynka
Enhancer Hoffman
Enhancer Andersson
DHS Trynka
DGF ENCODE
CTCF Hoffman
Conserved LindbladToh
**Coding UCSC
Annotation
−200
0
200
400
Enrichment (prop h2 / prop snps)

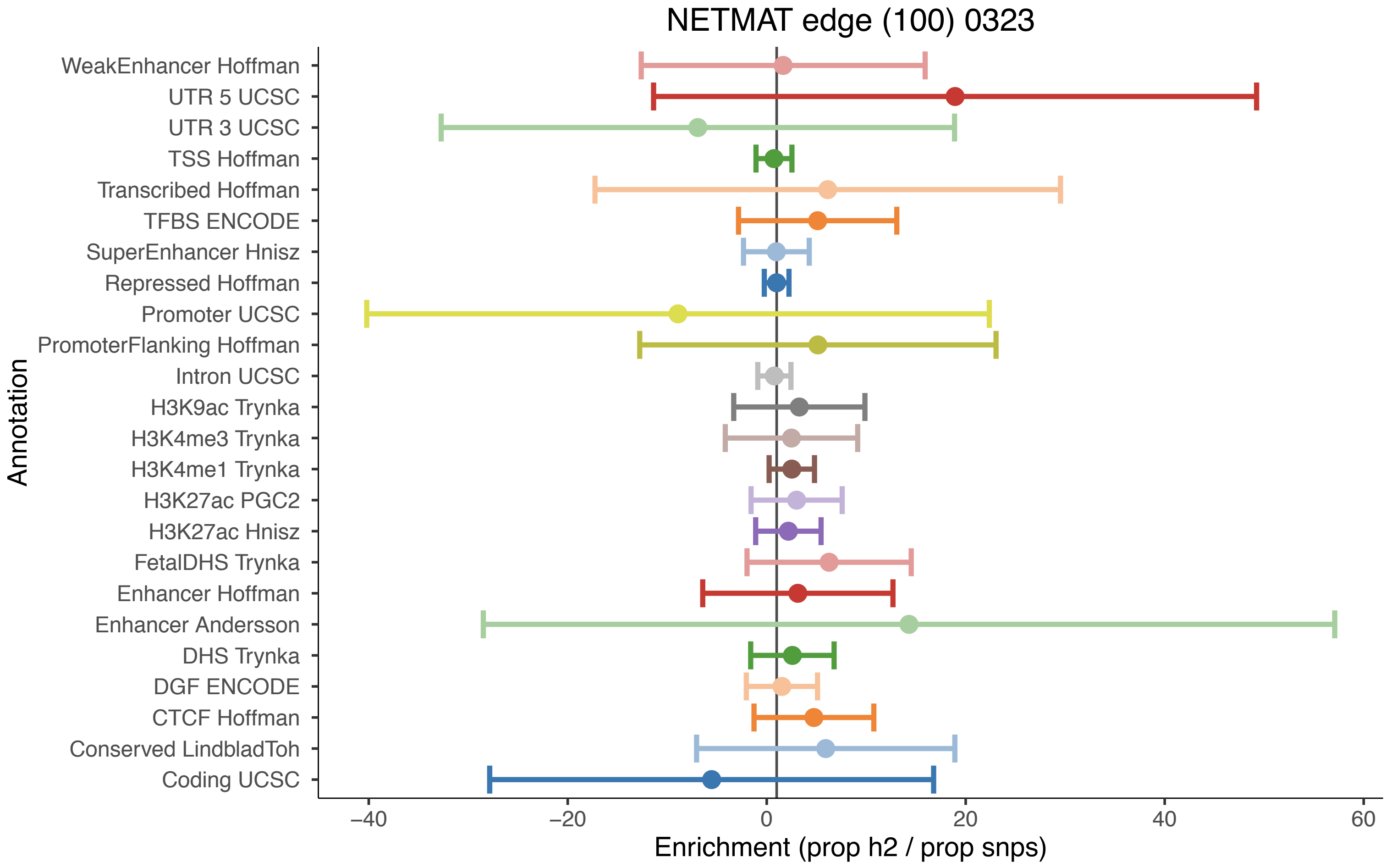

NETMAT edge (100) 0323
WeakEnhancer Hoffman
UTR 5 UCSC
UTR 3 UCSC
TSS Hoffman
Transcribed Hoffman
TFBS ENCODE
SuperEnhancer Hnisz
Repressed Hoffman
Promoter UCSC
PromoterFlanking Hoffman
Intron UCSC
H3K9ac Trynka
H3K4me3 Trynka
H3K4me1 Trynka
H3K27ac PGC2
H3K27ac Hnisz
FetalDHS Trynka
Enhancer Hoffman
Enhancer Andersson
DHS Trynka
DGF ENCODE
CTCF Hoffman
Conserved LindbladToh
Coding UCSC
Annotation
−40
−20
0
20
40
60
Enrichment (prop h2 / prop snps)

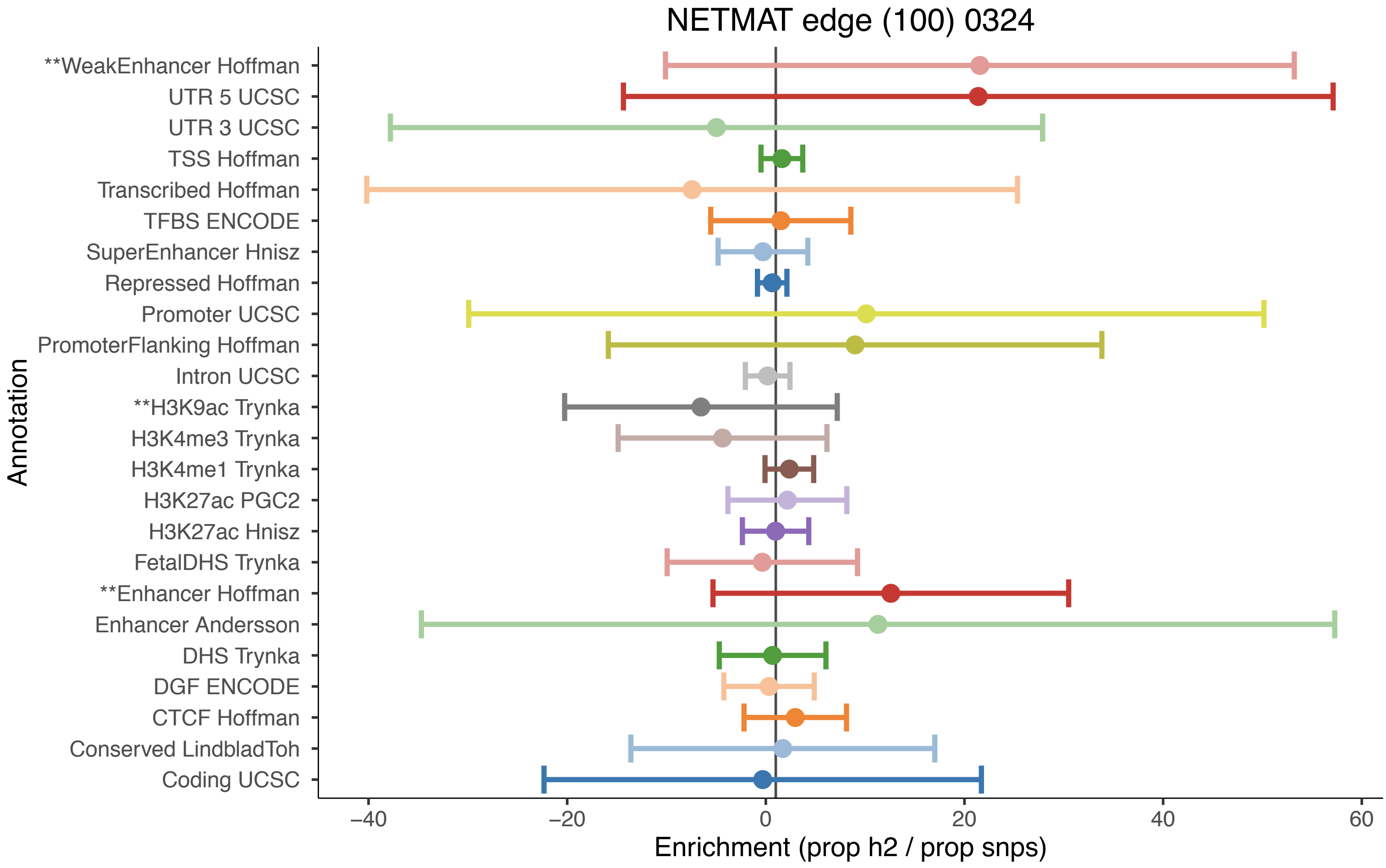
NETMAT edge (100) 0324
**WeakEnhancer Hoffman
UTR 5 UCSC
UTR 3 UCSC
TSS Hoffman
Transcribed Hoffman
TFBS ENCODE
SuperEnhancer Hnisz
Repressed Hoffman
Promoter UCSC
PromoterFlanking Hoffman
Intron UCSC
**H3K9ac Trynka
H3K4me3 Trynka
H3K4me1 Trynka
H3K27ac PGC2
H3K27ac Hnisz
FetalDHS Trynka
**Enhancer Hoffman
Enhancer Andersson
DHS Trynka
DGF ENCODE
CTCF Hoffman
Conserved LindbladToh
Coding UCSC
Annotation
−40
−20
0
20
40
60
Enrichment (prop h2 / prop snps)

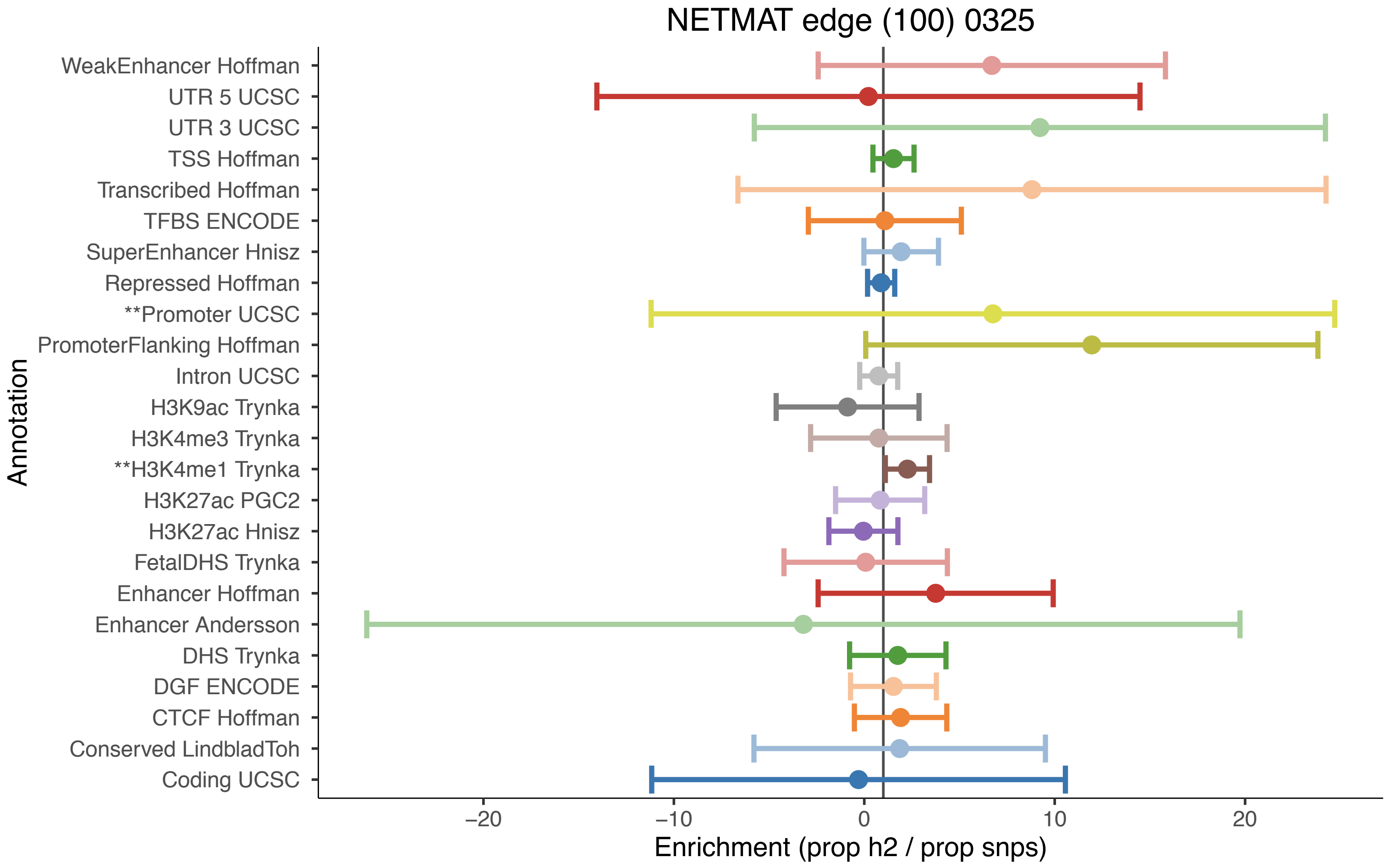
NETMAT edge (100) 0325
WeakEnhancer Hoffman
UTR 5 UCSC
UTR 3 UCSC
TSS Hoffman
Transcribed Hoffman
TFBS ENCODE
SuperEnhancer Hnisz
Repressed Hoffman
**Promoter UCSC
PromoterFlanking Hoffman
Intron UCSC
H3K9ac Trynka
H3K4me3 Trynka
**H3K4me1 Trynka
H3K27ac PGC2
H3K27ac Hnisz
FetalDHS Trynka
Enhancer Hoffman
Enhancer Andersson
DHS Trynka
DGF ENCODE
CTCF Hoffman
Conserved LindbladToh
Coding UCSC
Annotation
−20
−10
0
10
20
Enrichment (prop h2 / prop snps)

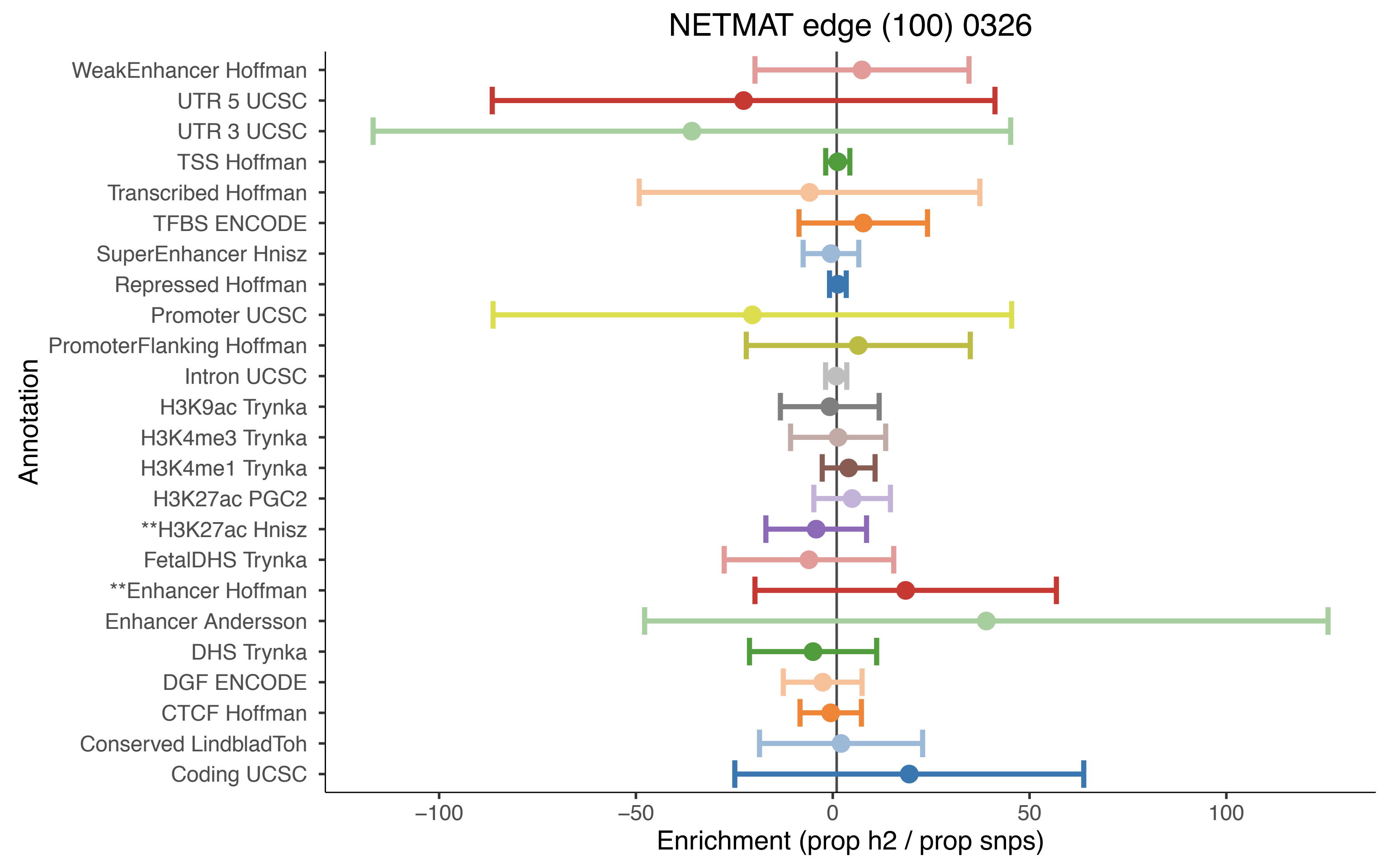

NETMAT edge (100) 0326
WeakEnhancer Hoffman
UTR 5 UCSC
UTR 3 UCSC
TSS Hoffman
Transcribed Hoffman
TFBS ENCODE
SuperEnhancer Hnisz
Repressed Hoffman
Promoter UCSC
PromoterFlanking Hoffman
Intron UCSC
H3K9ac Trynka
H3K4me3 Trynka
H3K4me1 Trynka
H3K27ac PGC2
**H3K27ac Hnisz
FetalDHS Trynka
**Enhancer Hoffman
Enhancer Andersson
DHS Trynka
DGF ENCODE
CTCF Hoffman
Conserved LindbladToh
Coding UCSC
Annotation
−100
−50
0
50
100
Enrichment (prop h2 / prop snps)

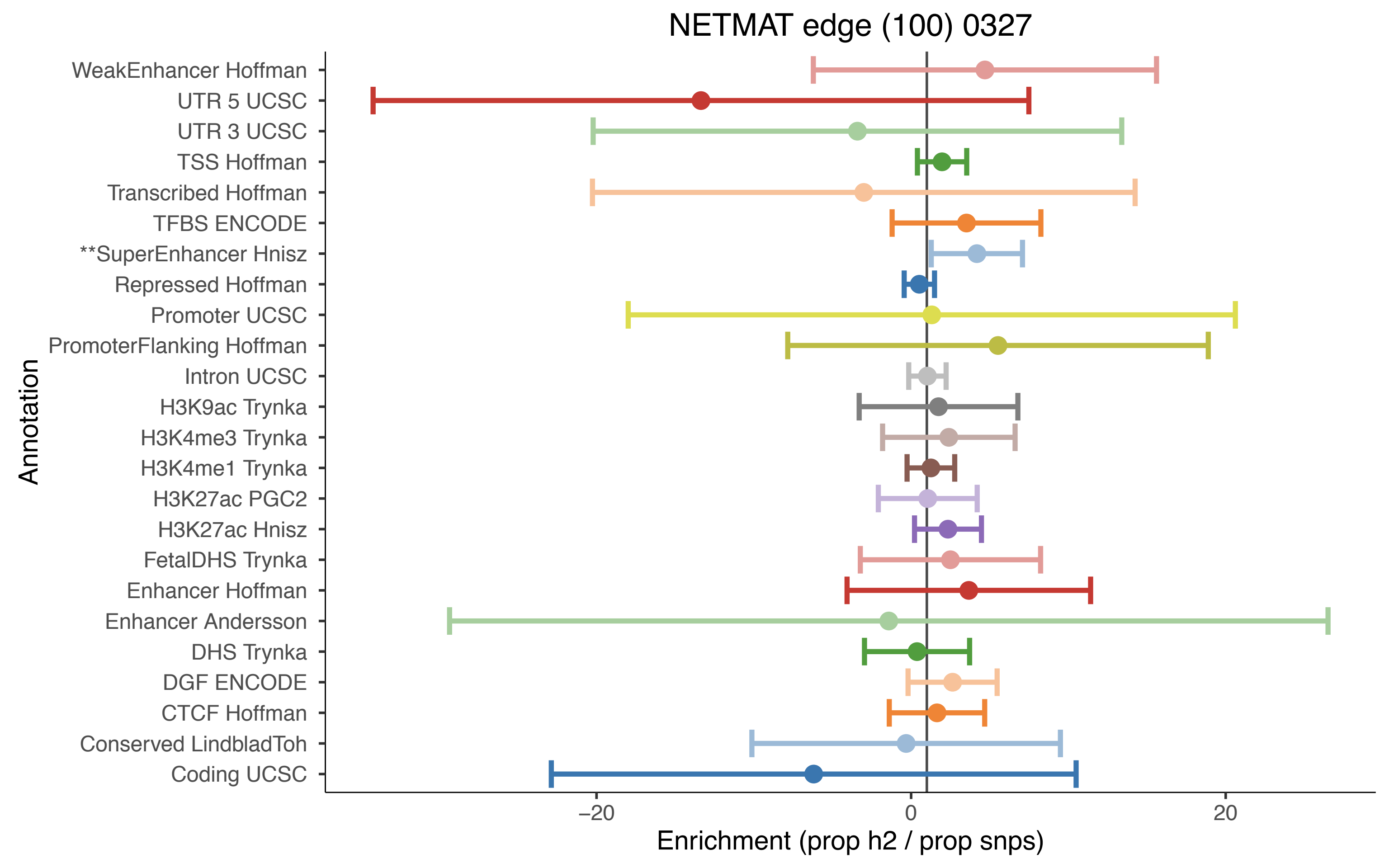
NETMAT edge (100) 0327
WeakEnhancer Hoffman
UTR 5 UCSC
UTR 3 UCSC
TSS Hoffman
Transcribed Hoffman
TFBS ENCODE
**SuperEnhancer Hnisz
Repressed Hoffman
Promoter UCSC
PromoterFlanking Hoffman
Intron UCSC
H3K9ac Trynka
H3K4me3 Trynka
H3K4me1 Trynka
H3K27ac PGC2
H3K27ac Hnisz
FetalDHS Trynka
Enhancer Hoffman
Enhancer Andersson
DHS Trynka
DGF ENCODE
CTCF Hoffman
Conserved LindbladToh
Coding UCSC
Annotation
−20
0
20
Enrichment (prop h2 / prop snps)

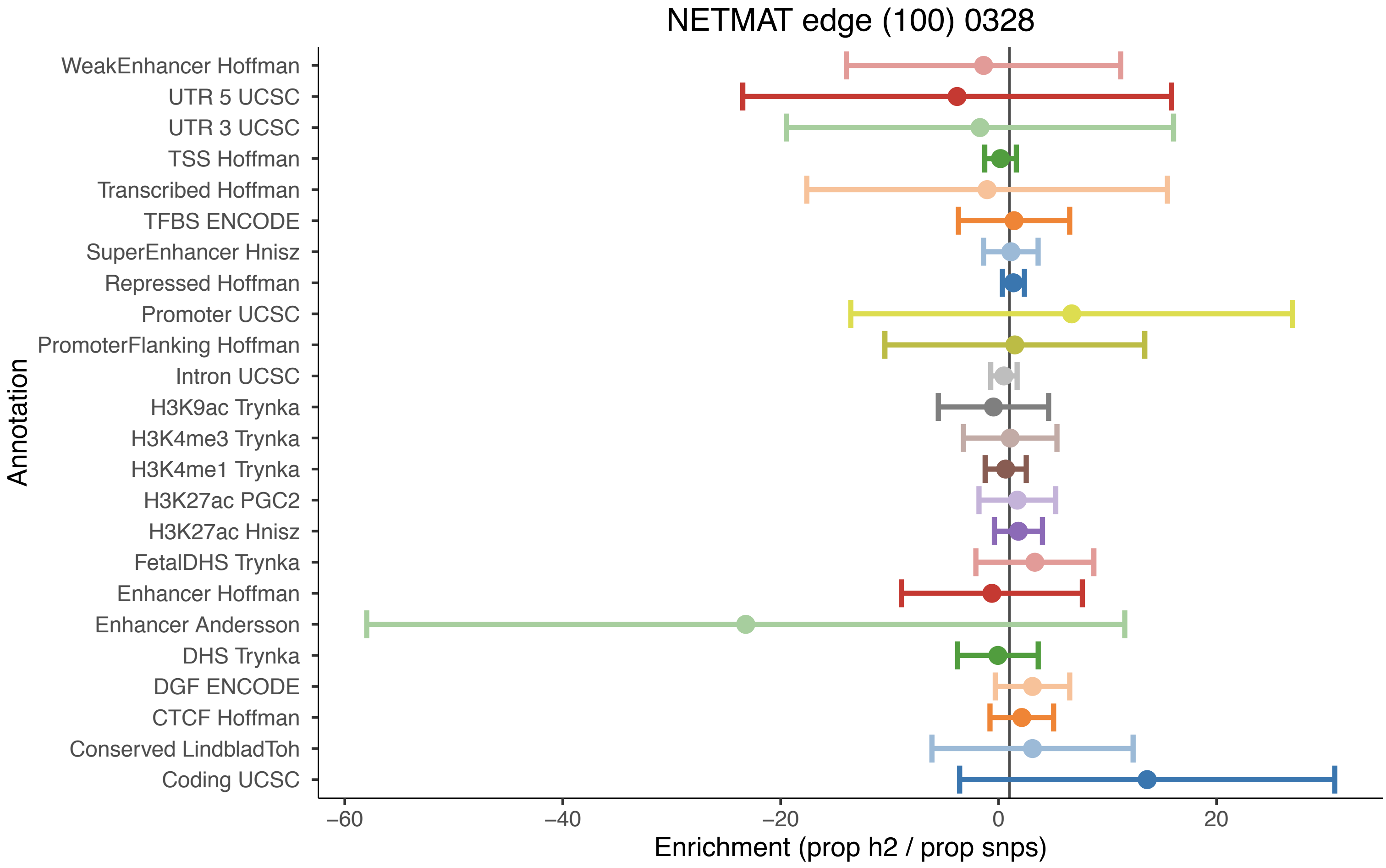
NETMAT edge (100) 0328
Annotation
WeakEnhancer Hoffman
UTR 5 UCSC
UTR 3 UCSC
TSS Hoffman
Transcribed Hoffman
TFBS ENCODE
SuperEnhancer Hnisz
Repressed Hoffman
Promoter UCSC
PromoterFlanking Hoffman
Intron UCSC
H3K9ac Trynka
H3K4me3 Trynka
H3K4me1 Trynka
H3K27ac PGC2
H3K27ac Hnisz
FetalDHS Trynka
Enhancer Hoffman
Enhancer Andersson
DHS Trynka
DGF ENCODE
CTCF Hoffman
Conserved LindbladToh
Coding UCSC
−60
−40
−20
0
20
Enrichment (prop h2 / prop snps)

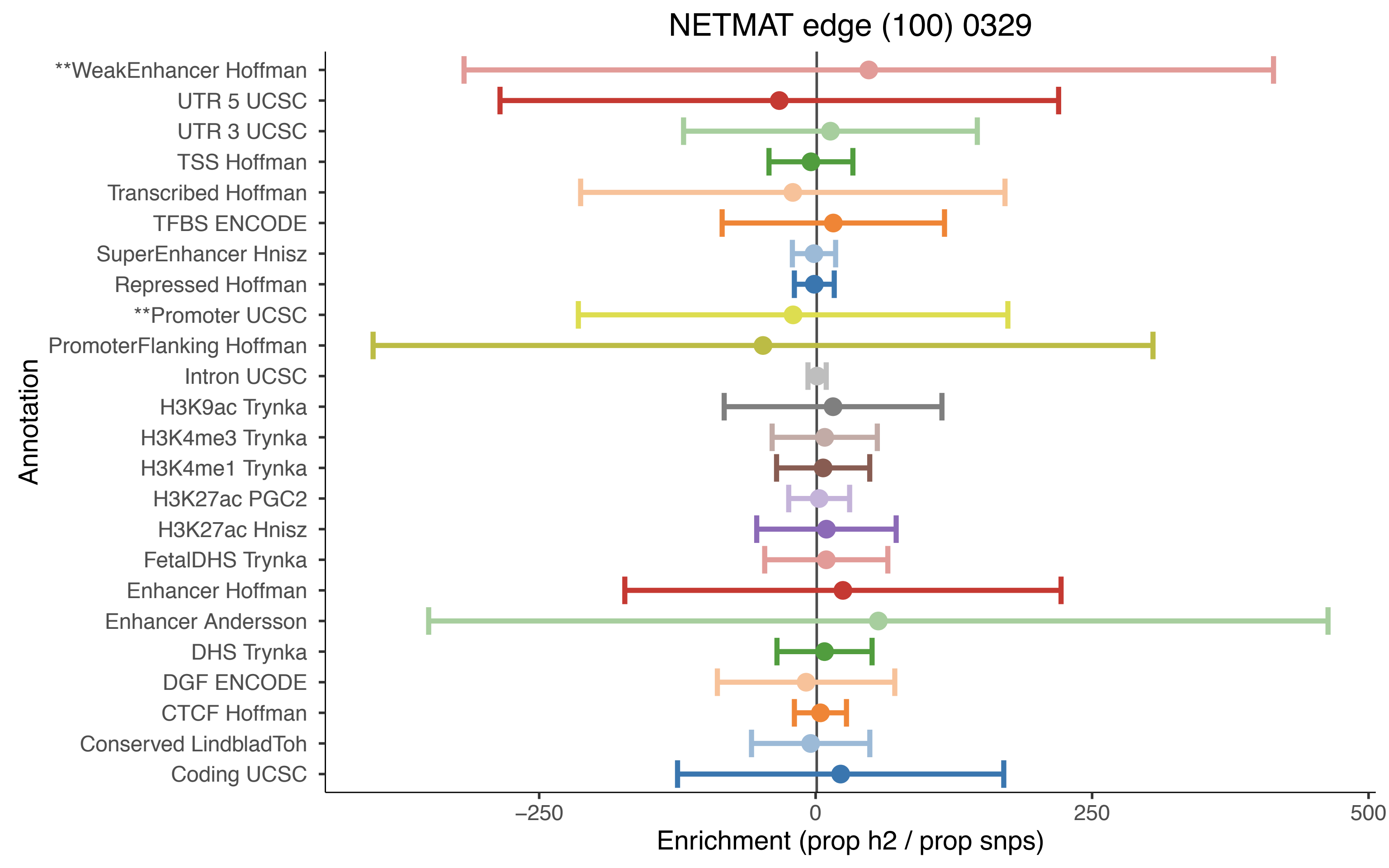

NETMAT edge (100) 0329
**WeakEnhancer Hoffman
UTR 5 UCSC
UTR 3 UCSC
TSS Hoffman
Transcribed Hoffman
TFBS ENCODE
SuperEnhancer Hnisz
Repressed Hoffman
**Promoter UCSC
PromoterFlanking Hoffman
Intron UCSC
H3K9ac Trynka
H3K4me3 Trynka
H3K4me1 Trynka
H3K27ac PGC2
H3K27ac Hnisz
FetalDHS Trynka
Enhancer Hoffman
Enhancer Andersson
DHS Trynka
DGF ENCODE
CTCF Hoffman
Conserved LindbladToh
Coding UCSC
Annotation
−250
0
250
500
Enrichment (prop h2 / prop snps)

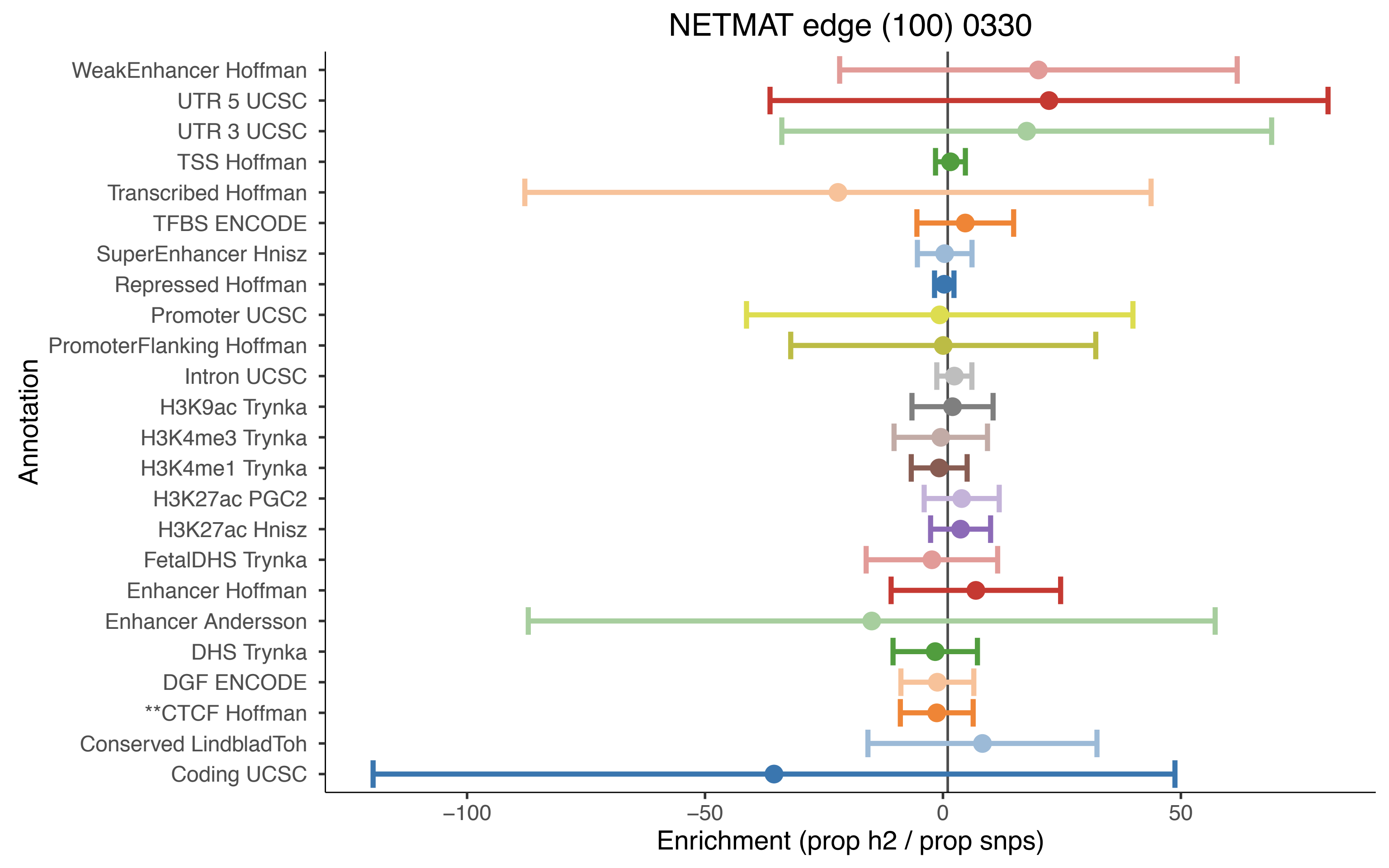

NETMAT edge (100) 0330
WeakEnhancer Hoffman
UTR 5 UCSC
UTR 3 UCSC
TSS Hoffman
Transcribed Hoffman
TFBS ENCODE
SuperEnhancer Hnisz
Repressed Hoffman
Promoter UCSC
PromoterFlanking Hoffman
Intron UCSC
H3K9ac Trynka
H3K4me3 Trynka
H3K4me1 Trynka
H3K27ac PGC2
H3K27ac Hnisz
FetalDHS Trynka
Enhancer Hoffman
Enhancer Andersson
DHS Trynka
DGF ENCODE
**CTCF Hoffman
Conserved LindbladToh
Coding UCSC
Annotation
−100
−50
0
50
Enrichment (prop h2 / prop snps)

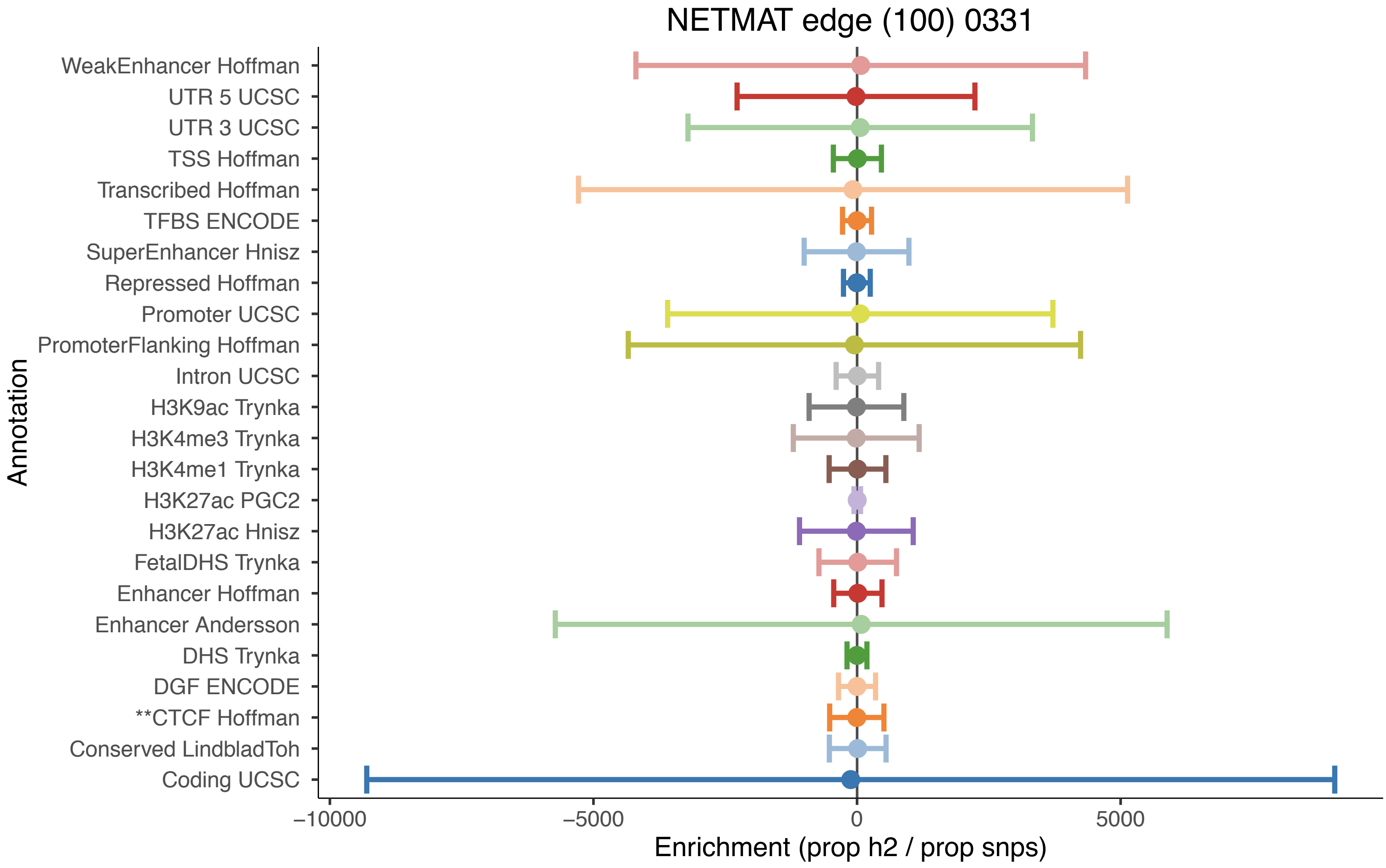
NETMAT edge (100) 0331
WeakEnhancer Hoffman
UTR 5 UCSC
UTR 3 UCSC
TSS Hoffman
Transcribed Hoffman
TFBS ENCODE
SuperEnhancer Hnisz
Repressed Hoffman
Promoter UCSC
PromoterFlanking Hoffman
Intron UCSC
H3K9ac Trynka
H3K4me3 Trynka
H3K4me1 Trynka
H3K27ac PGC2
H3K27ac Hnisz
FetalDHS Trynka
Enhancer Hoffman
Enhancer Andersson
DHS Trynka
DGF ENCODE
**CTCF Hoffman
Conserved LindbladToh
Coding UCSC
Annotation
−10000
−5000
0
5000
Enrichment (prop h2 / prop snps)

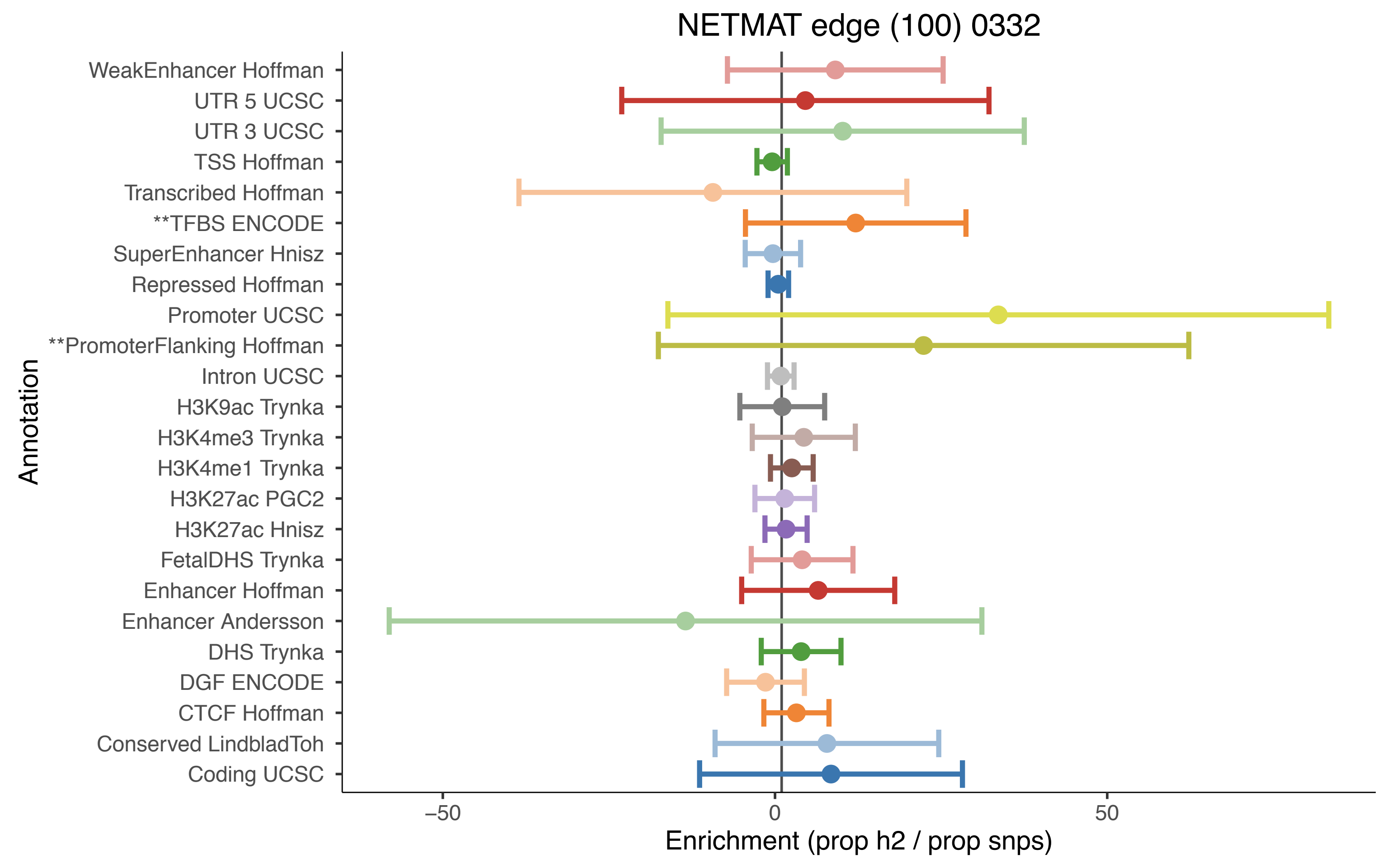
NETMAT edge (100) 0332
WeakEnhancer Hoffman
UTR 5 UCSC
UTR 3 UCSC
TSS Hoffman
Transcribed Hoffman
**TFBS ENCODE
SuperEnhancer Hnisz
Repressed Hoffman
Promoter UCSC
**PromoterFlanking Hoffman
Intron UCSC
H3K9ac Trynka
H3K4me3 Trynka
H3K4me1 Trynka
H3K27ac PGC2
H3K27ac Hnisz
FetalDHS Trynka
Enhancer Hoffman
Enhancer Andersson
DHS Trynka
DGF ENCODE
CTCF Hoffman
Conserved LindbladToh
Coding UCSC
Annotation
−50
0
50
Enrichment (prop h2 / prop snps)

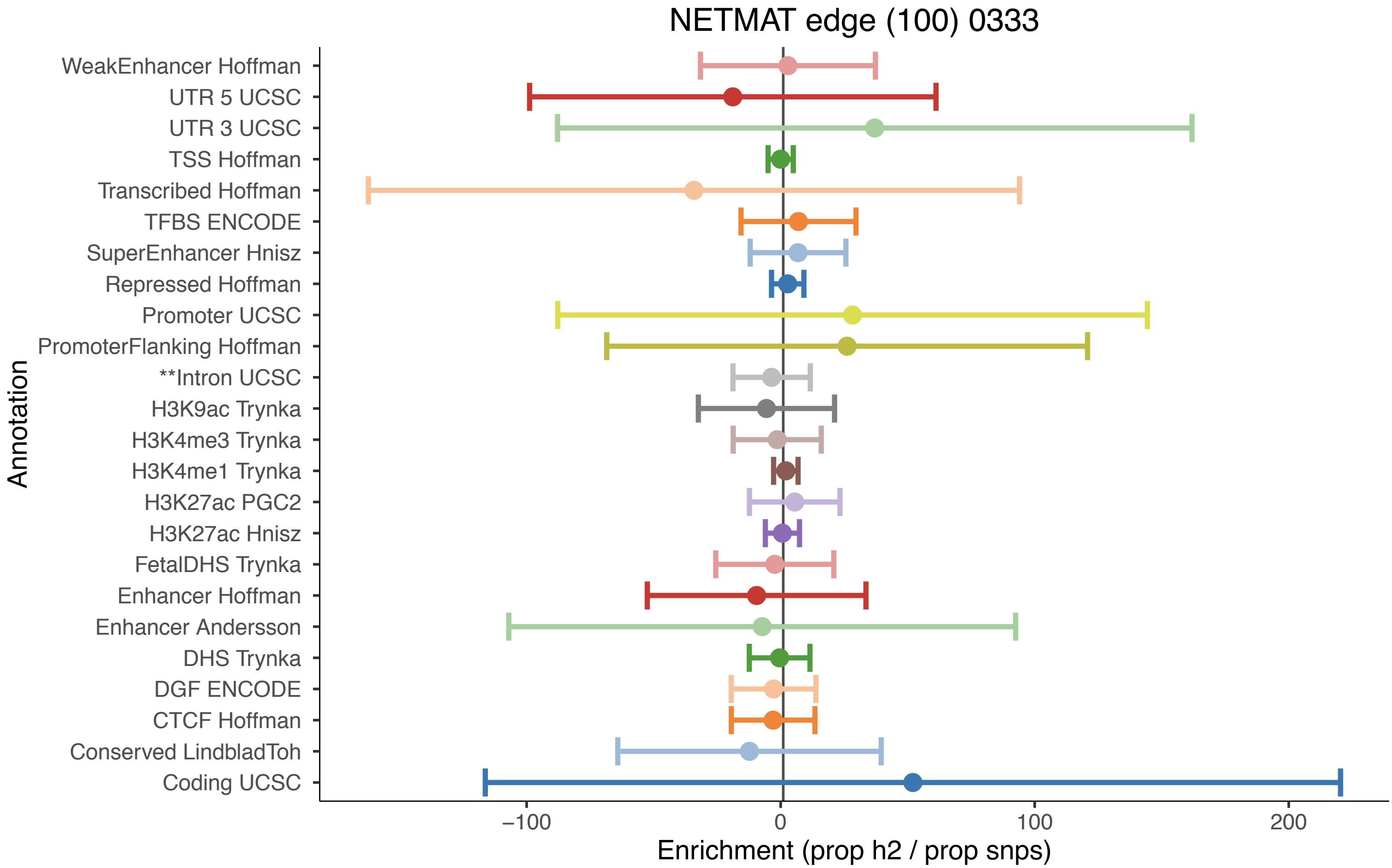
NETMAT edge (100) 0333
Annotation
WeakEnhancer Hoffman
UTR 5 UCSC
UTR 3 UCSC
TSS Hoffman
Transcribed Hoffman
TFBS ENCODE
SuperEnhancer Hnisz
Repressed Hoffman
Promoter UCSC
PromoterFlanking Hoffman
**Intron UCSC
H3K9ac Trynka
H3K4me3 Trynka
H3K4me1 Trynka
H3K27ac PGC2
H3K27ac Hnisz
FetalDHS Trynka
Enhancer Hoffman
Enhancer Andersson
DHS Trynka
DGF ENCODE
CTCF Hoffman
Conserved LindbladToh
Coding UCSC
−100
0
100
200
Enrichment (prop h2 / prop snps)

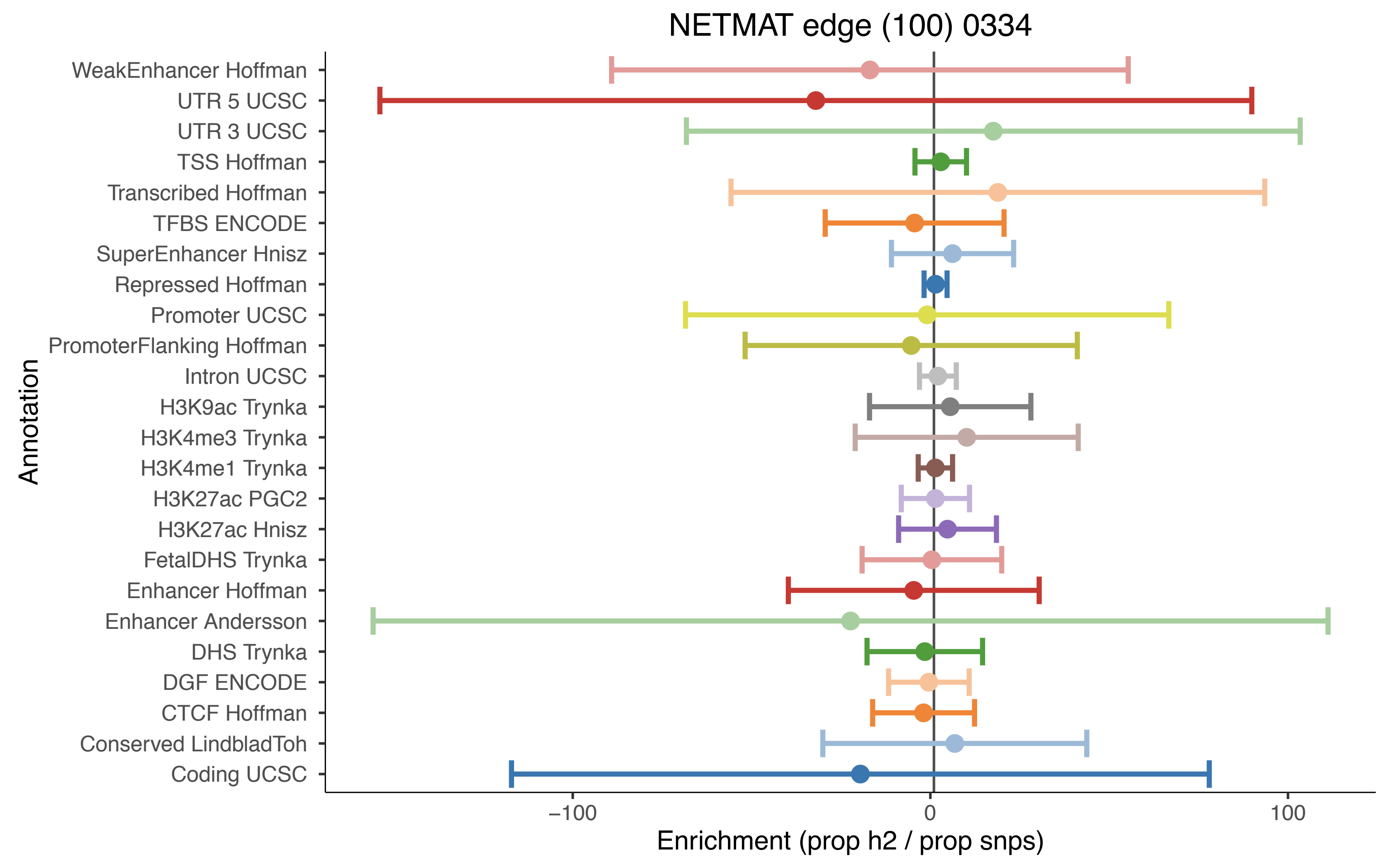
NETMAT edge (100) 0334
WeakEnhancer Hoffman
UTR 5 UCSC
UTR 3 UCSC
TSS Hoffman
Transcribed Hoffman
TFBS ENCODE
SuperEnhancer Hnisz
Repressed Hoffman
Promoter UCSC
PromoterFlanking Hoffman
Intron UCSC
H3K9ac Trynka
H3K4me3 Trynka
H3K4me1 Trynka
H3K27ac PGC2
H3K27ac Hnisz
FetalDHS Trynka
Enhancer Hoffman
Enhancer Andersson
DHS Trynka
DGF ENCODE
CTCF Hoffman
Conserved LindbladToh
Coding UCSC
Annotation
−100
0
100
Enrichment (prop h2 / prop snps)

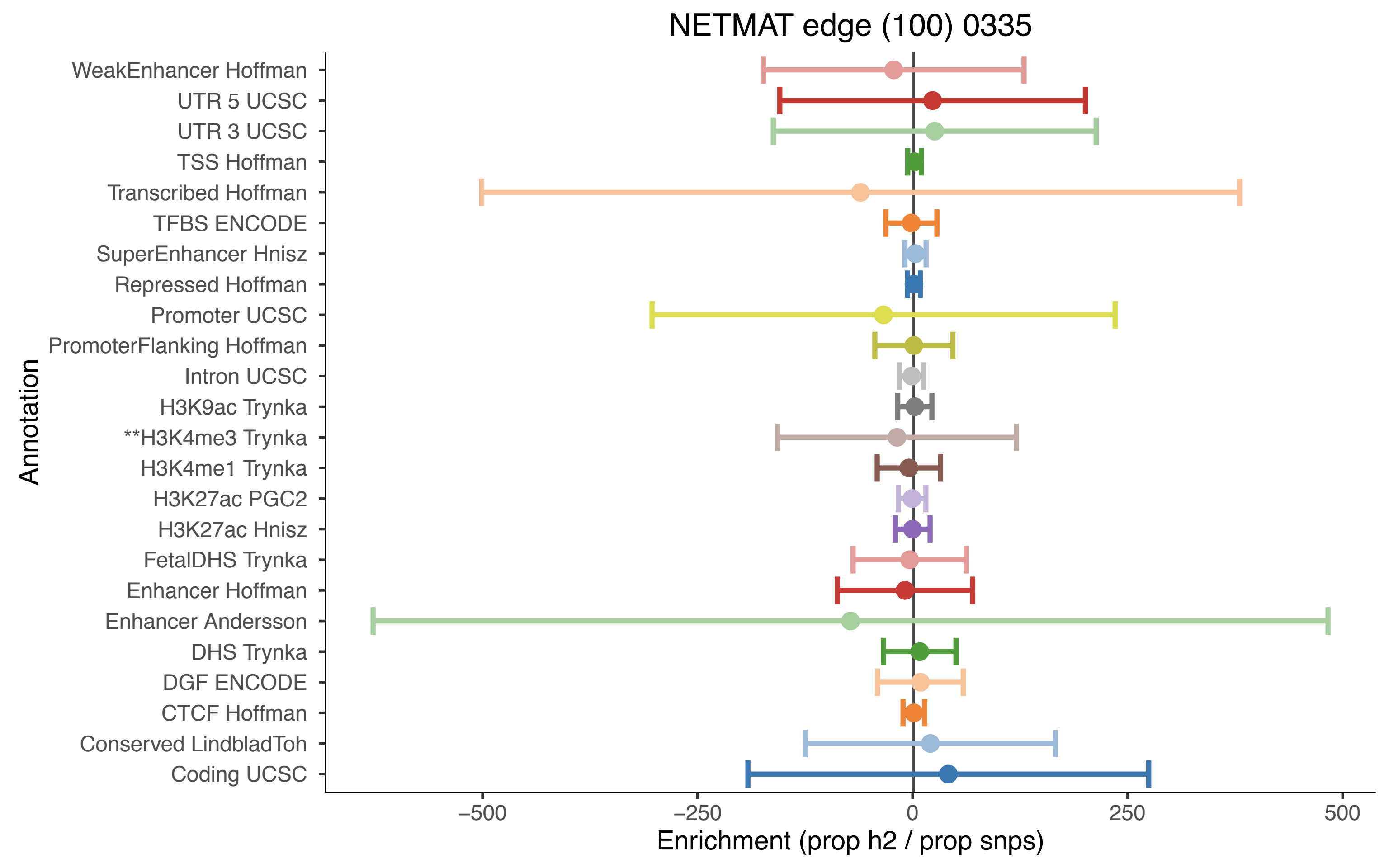
NETMAT edge (100) 0335
WeakEnhancer Hoffman
UTR 5 UCSC
UTR 3 UCSC
TSS Hoffman
Transcribed Hoffman
TFBS ENCODE
SuperEnhancer Hnisz
Repressed Hoffman
Promoter UCSC
PromoterFlanking Hoffman
Intron UCSC
H3K9ac Trynka
**H3K4me3 Trynka
H3K4me1 Trynka
H3K27ac PGC2
H3K27ac Hnisz
FetalDHS Trynka
Enhancer Hoffman
Enhancer Andersson
DHS Trynka
DGF ENCODE
CTCF Hoffman
Conserved LindbladToh
Coding UCSC
Annotation
−500
−250
0
250
500
Enrichment (prop h2 / prop snps)

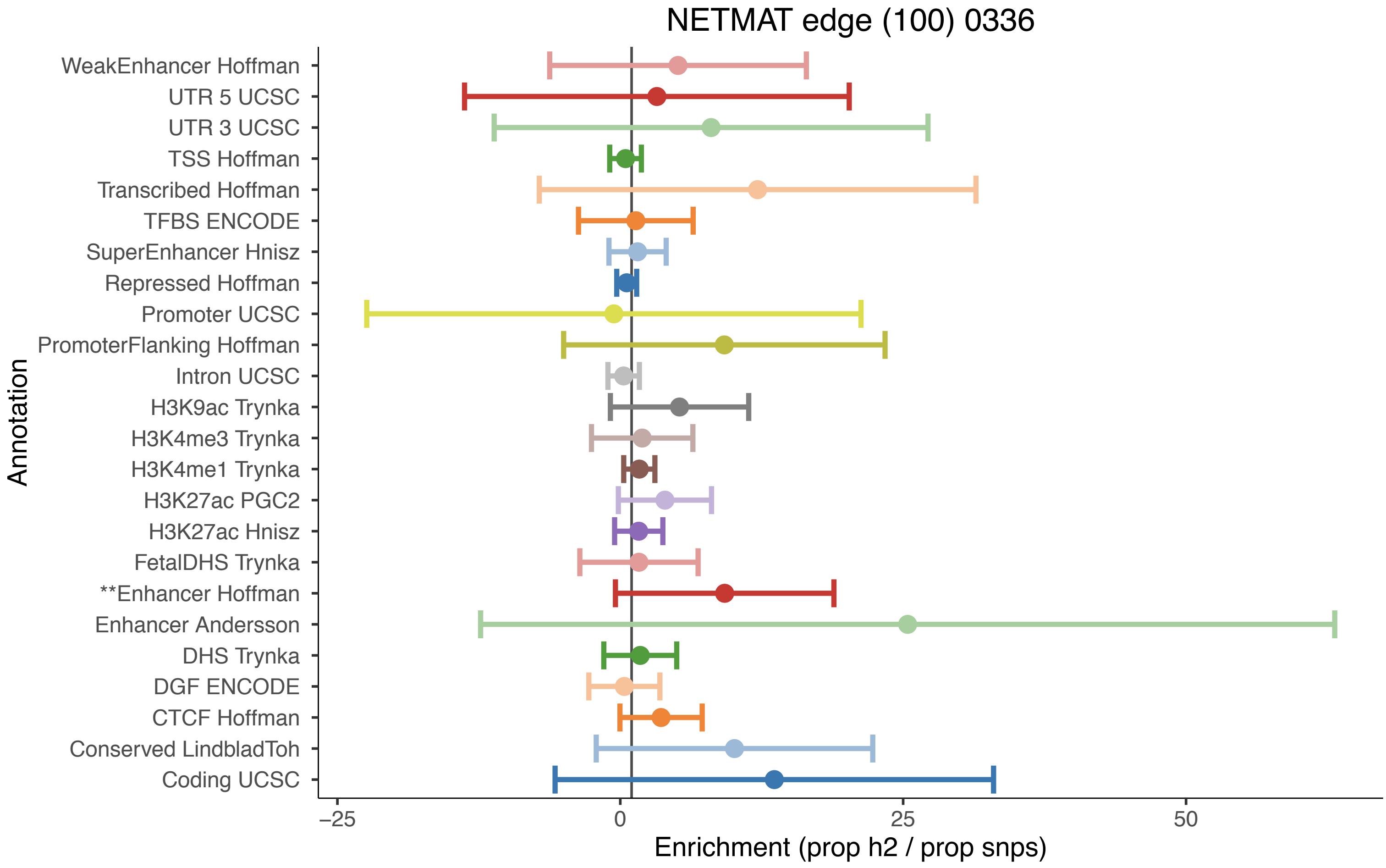
NETMAT edge (100) 0336
WeakEnhancer Hoffman
UTR 5 UCSC
UTR 3 UCSC
TSS Hoffman
Transcribed Hoffman
TFBS ENCODE
SuperEnhancer Hnisz
Repressed Hoffman
Promoter UCSC
PromoterFlanking Hoffman
Intron UCSC
H3K9ac Trynka
H3K4me3 Trynka
H3K4me1 Trynka
H3K27ac PGC2
H3K27ac Hnisz
FetalDHS Trynka
**Enhancer Hoffman
Enhancer Andersson
DHS Trynka
DGF ENCODE
CTCF Hoffman
Conserved LindbladToh
Coding UCSC
Annotation
−25
0
25
50
Enrichment (prop h2 / prop snps)

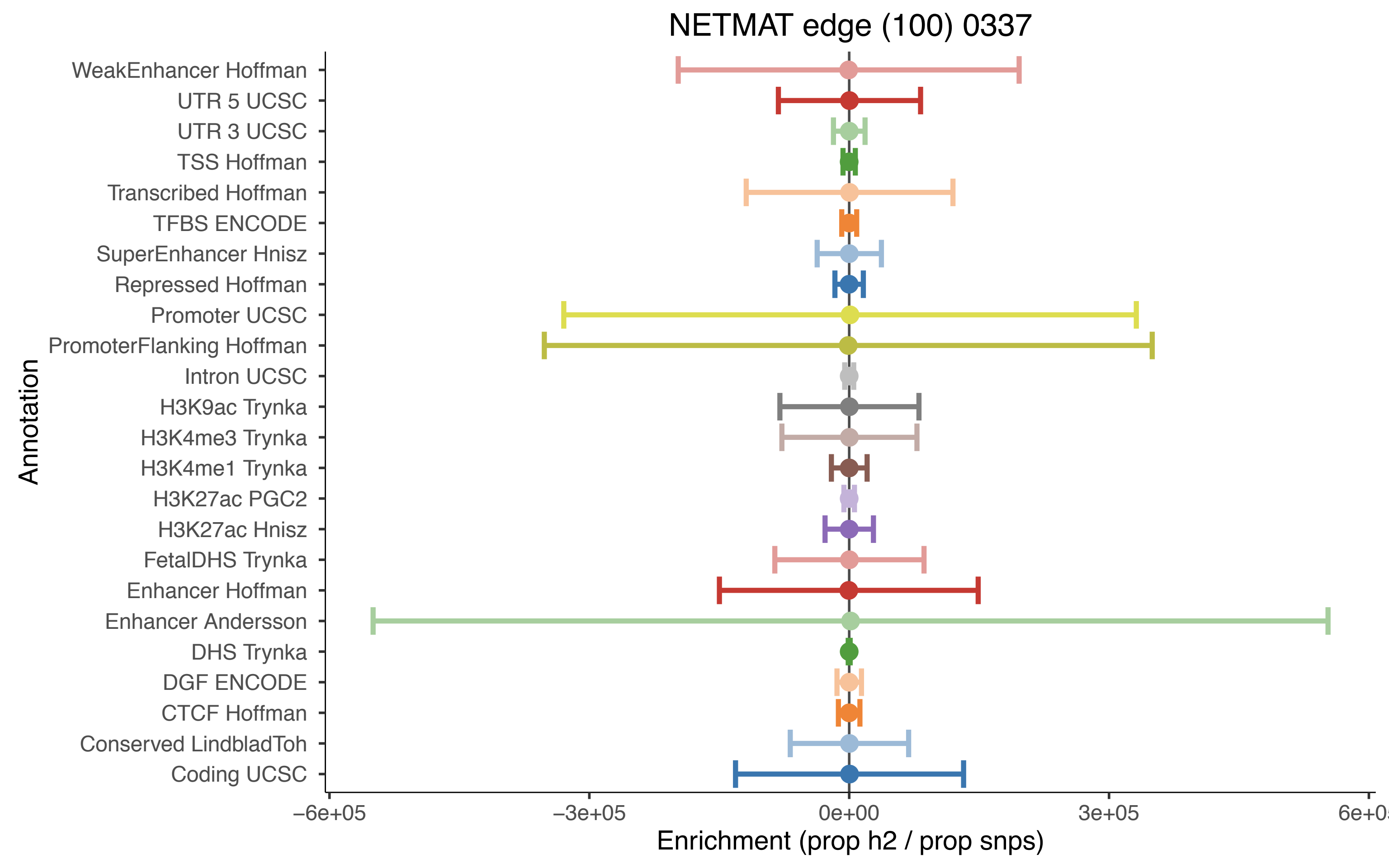
NETMAT edge (100) 0337
Annotation
WeakEnhancer Hoffman
UTR 5 UCSC
UTR 3 UCSC
TSS Hoffman
Transcribed Hoffman
TFBS ENCODE
SuperEnhancer Hnisz
Repressed Hoffman
Promoter UCSC
PromoterFlanking Hoffman
Intron UCSC
H3K9ac Trynka
H3K4me3 Trynka
H3K4me1 Trynka
H3K27ac PGC2
H3K27ac Hnisz
FetalDHS Trynka
Enhancer Hoffman
Enhancer Andersson
DHS Trynka
DGF ENCODE
CTCF Hoffman
Conserved LindbladToh
Coding UCSC
−6e+05
−3e+05
0e+00
3e+05
Enrichment (prop h2 / prop snps)

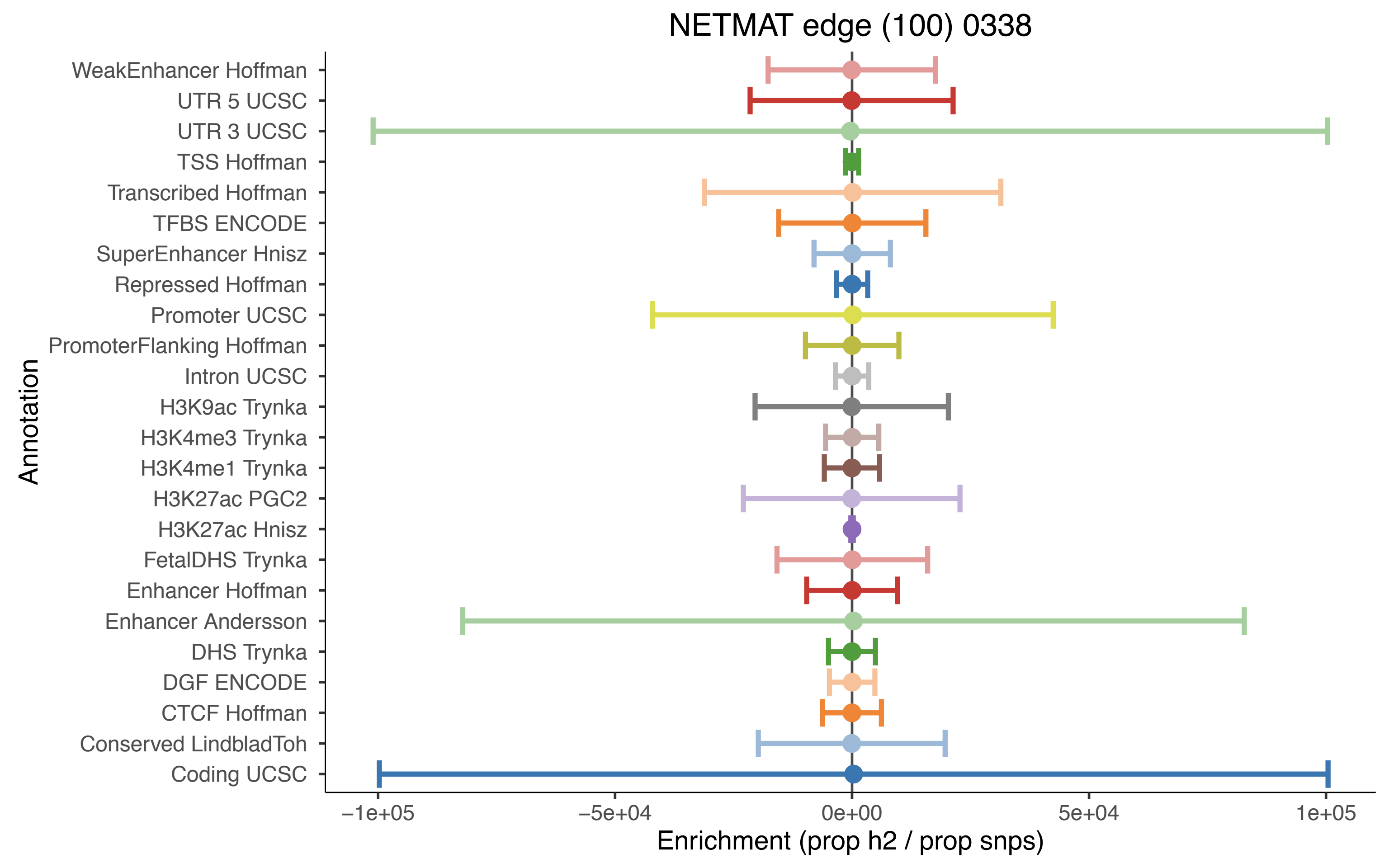

NETMAT edge (100) 0338
Annotation
WeakEnhancer Hoffman
UTR 5 UCSC
UTR 3 UCSC
TSS Hoffman
Transcribed Hoffman
TFBS ENCODE
SuperEnhancer Hnisz
Repressed Hoffman
Promoter UCSC
PromoterFlanking Hoffman
Intron UCSC
H3K9ac Trynka
H3K4me3 Trynka
H3K4me1 Trynka
H3K27ac PGC2
H3K27ac Hnisz
FetalDHS Trynka
Enhancer Hoffman
Enhancer Andersson
DHS Trynka
DGF ENCODE
CTCF Hoffman
Conserved LindbladToh
Coding UCSC
−1e+05
−5e+04
0e+00
5e+04
1e+05
Enrichment (prop h2 / prop snps)

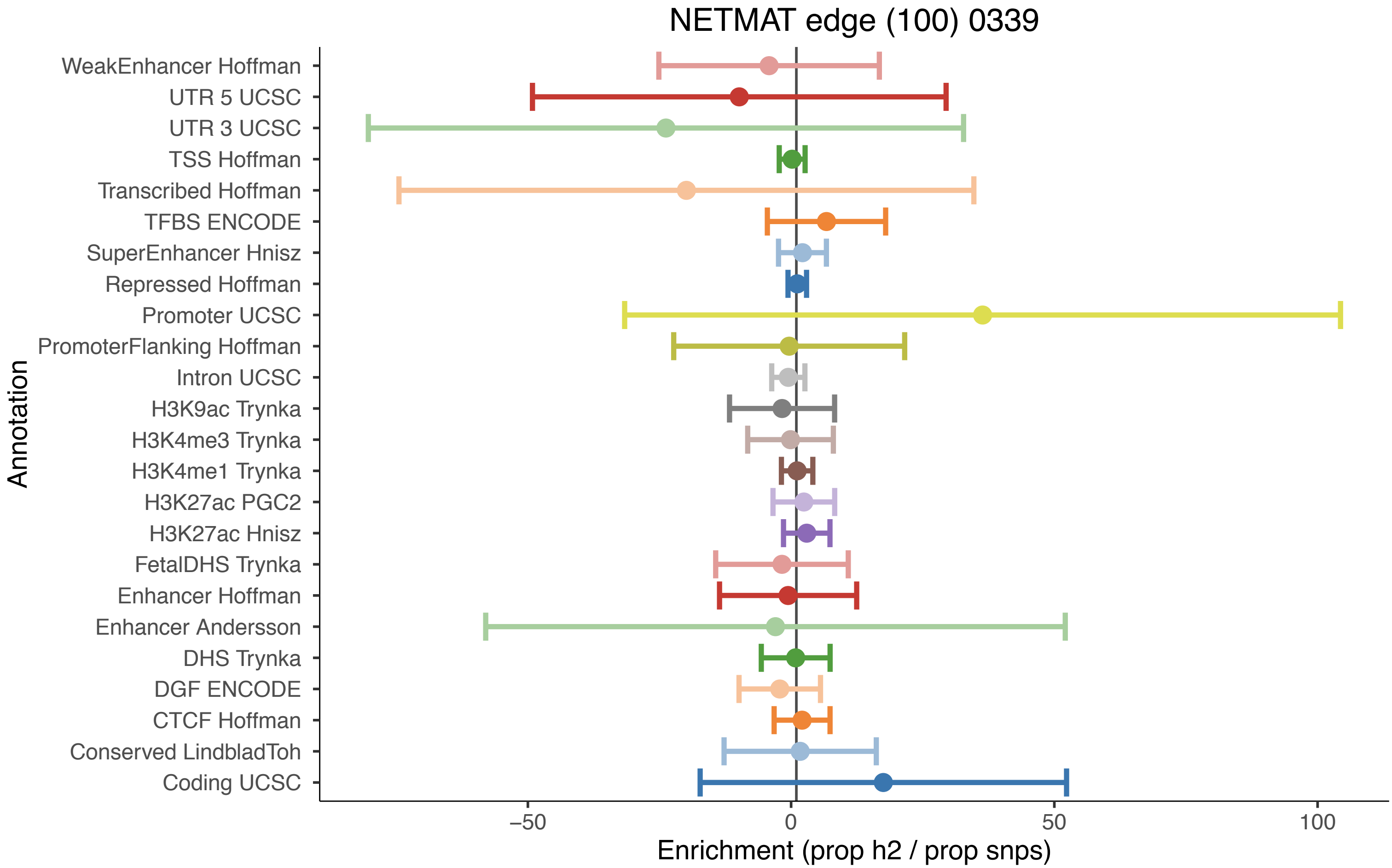

NETMAT edge (100) 0339
WeakEnhancer Hoffman
UTR 5 UCSC
UTR 3 UCSC
TSS Hoffman
Transcribed Hoffman
TFBS ENCODE
SuperEnhancer Hnisz
Repressed Hoffman
Promoter UCSC
PromoterFlanking Hoffman
Intron UCSC
H3K9ac Trynka
H3K4me3 Trynka
H3K4me1 Trynka
H3K27ac PGC2
H3K27ac Hnisz
FetalDHS Trynka
Enhancer Hoffman
Enhancer Andersson
DHS Trynka
DGF ENCODE
CTCF Hoffman
Conserved LindbladToh
Coding UCSC
Annotation
−50
0
50
100
Enrichment (prop h2 / prop snps)

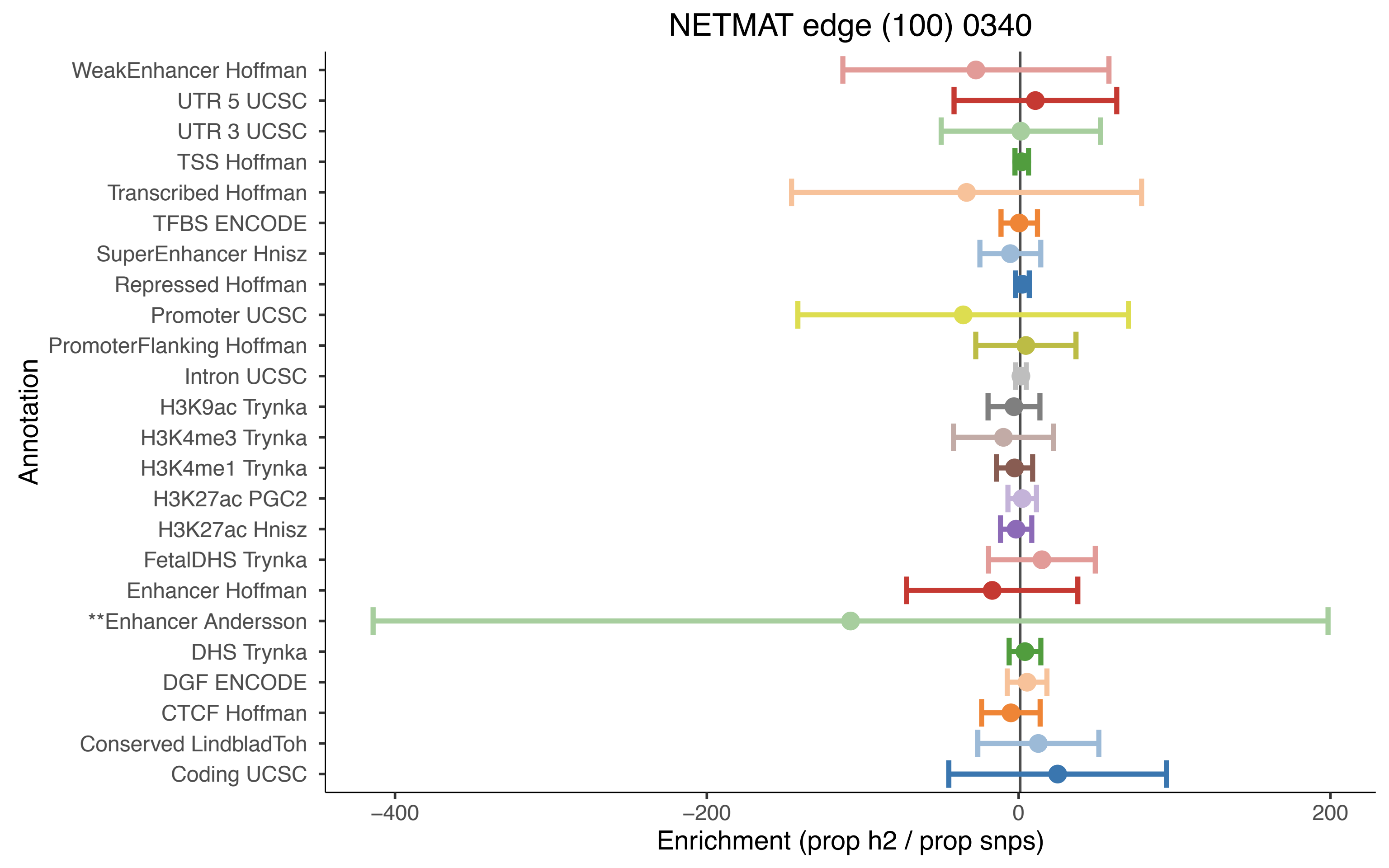
NETMAT edge (100) 0340
WeakEnhancer Hoffman
UTR 5 UCSC
UTR 3 UCSC
TSS Hoffman
Transcribed Hoffman
TFBS ENCODE
SuperEnhancer Hnisz
Repressed Hoffman
Promoter UCSC
PromoterFlanking Hoffman
Intron UCSC
H3K9ac Trynka
H3K4me3 Trynka
H3K4me1 Trynka
H3K27ac PGC2
H3K27ac Hnisz
FetalDHS Trynka
Enhancer Hoffman
**Enhancer Andersson
DHS Trynka
DGF ENCODE
CTCF Hoffman
Conserved LindbladToh
Coding UCSC
Annotation
−400
−200
0
200
Enrichment (prop h2 / prop snps)

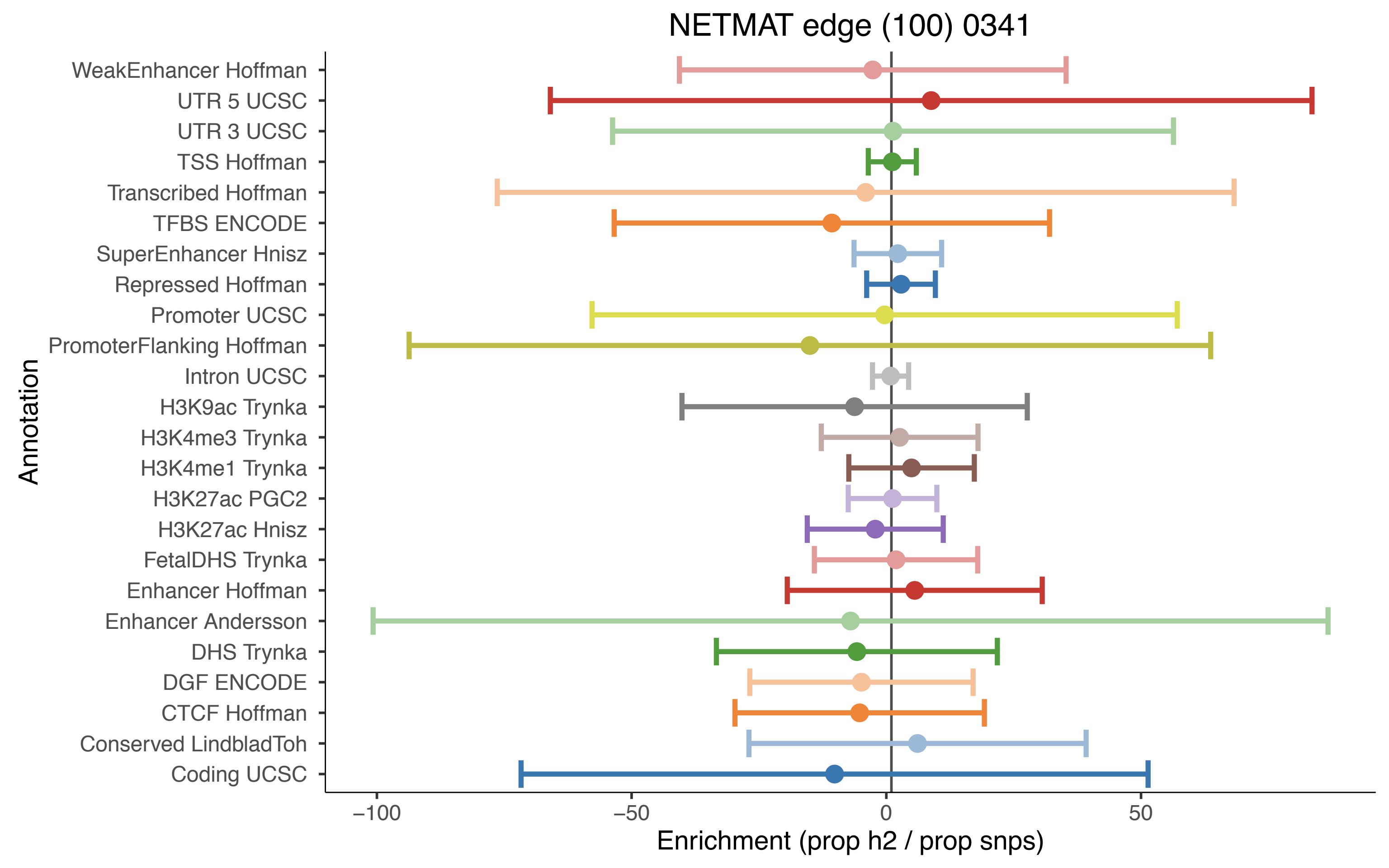
NETMAT edge (100) 0341
WeakEnhancer Hoffman
UTR 5 UCSC
UTR 3 UCSC
TSS Hoffman
Transcribed Hoffman
TFBS ENCODE
SuperEnhancer Hnisz
Repressed Hoffman
Promoter UCSC
PromoterFlanking Hoffman
Intron UCSC
H3K9ac Trynka
H3K4me3 Trynka
H3K4me1 Trynka
H3K27ac PGC2
H3K27ac Hnisz
FetalDHS Trynka
Enhancer Hoffman
Enhancer Andersson
DHS Trynka
DGF ENCODE
CTCF Hoffman
Conserved LindbladToh
Coding UCSC
Annotation
−100
−50
0
50
Enrichment (prop h2 / prop snps)

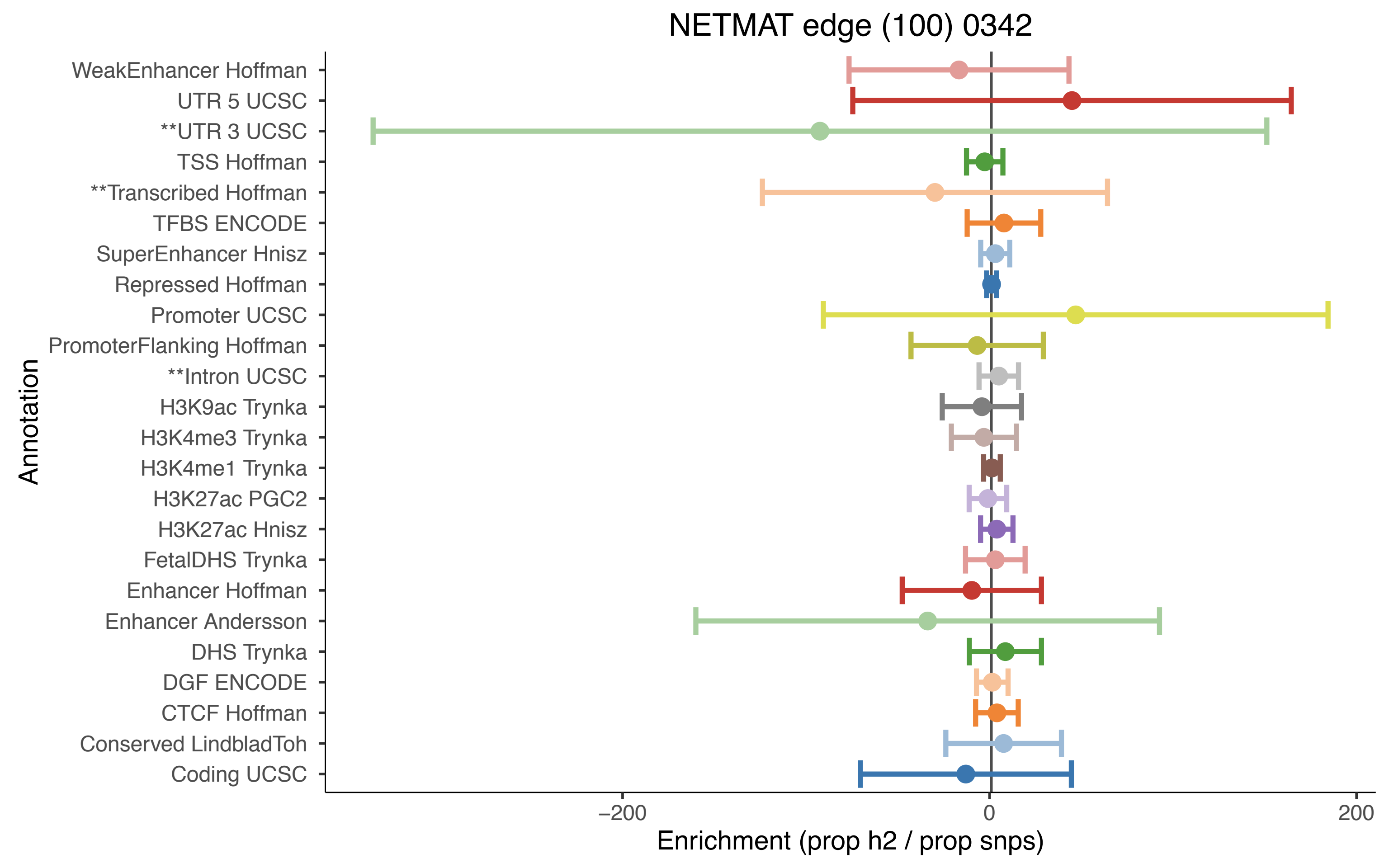

NETMAT edge (100) 0342
WeakEnhancer Hoffman
UTR 5 UCSC
**UTR 3 UCSC
TSS Hoffman
**Transcribed Hoffman
TFBS ENCODE
SuperEnhancer Hnisz
Repressed Hoffman
Promoter UCSC
PromoterFlanking Hoffman
**Intron UCSC
H3K9ac Trynka
H3K4me3 Trynka
H3K4me1 Trynka
H3K27ac PGC2
H3K27ac Hnisz
FetalDHS Trynka
Enhancer Hoffman
Enhancer Andersson
DHS Trynka
DGF ENCODE
CTCF Hoffman
Conserved LindbladToh
Coding UCSC
Annotation
−200
0
200
Enrichment (prop h2 / prop snps)

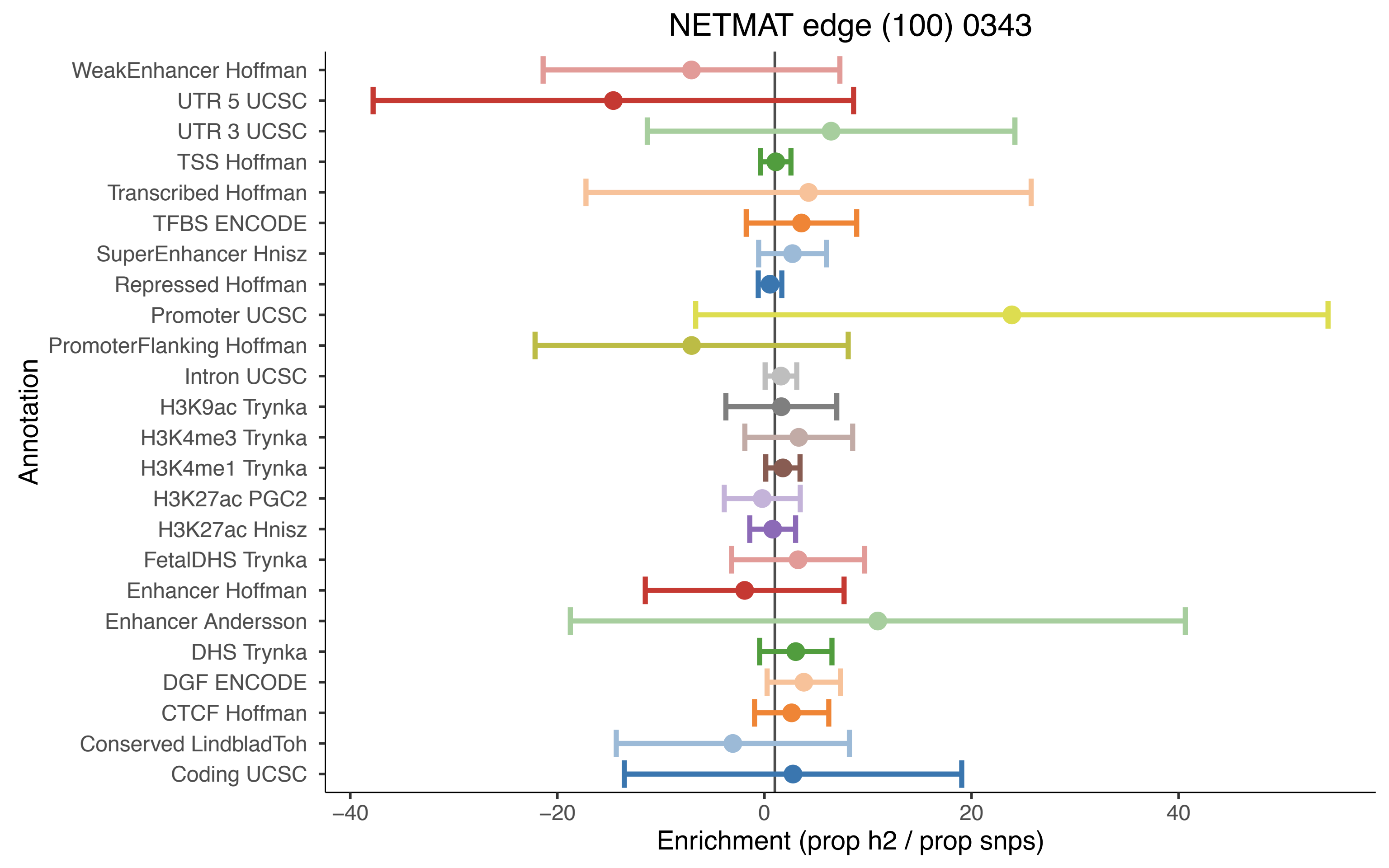
NETMAT edge (100) 0343
WeakEnhancer Hoffman
UTR 5 UCSC
UTR 3 UCSC
TSS Hoffman
Transcribed Hoffman
TFBS ENCODE
SuperEnhancer Hnisz
Repressed Hoffman
Promoter UCSC
PromoterFlanking Hoffman
Intron UCSC
H3K9ac Trynka
H3K4me3 Trynka
H3K4me1 Trynka
H3K27ac PGC2
H3K27ac Hnisz
FetalDHS Trynka
Enhancer Hoffman
Enhancer Andersson
DHS Trynka
DGF ENCODE
CTCF Hoffman
Conserved LindbladToh
Coding UCSC
Annotation
−40
−20
0
20
40
Enrichment (prop h2 / prop snps)

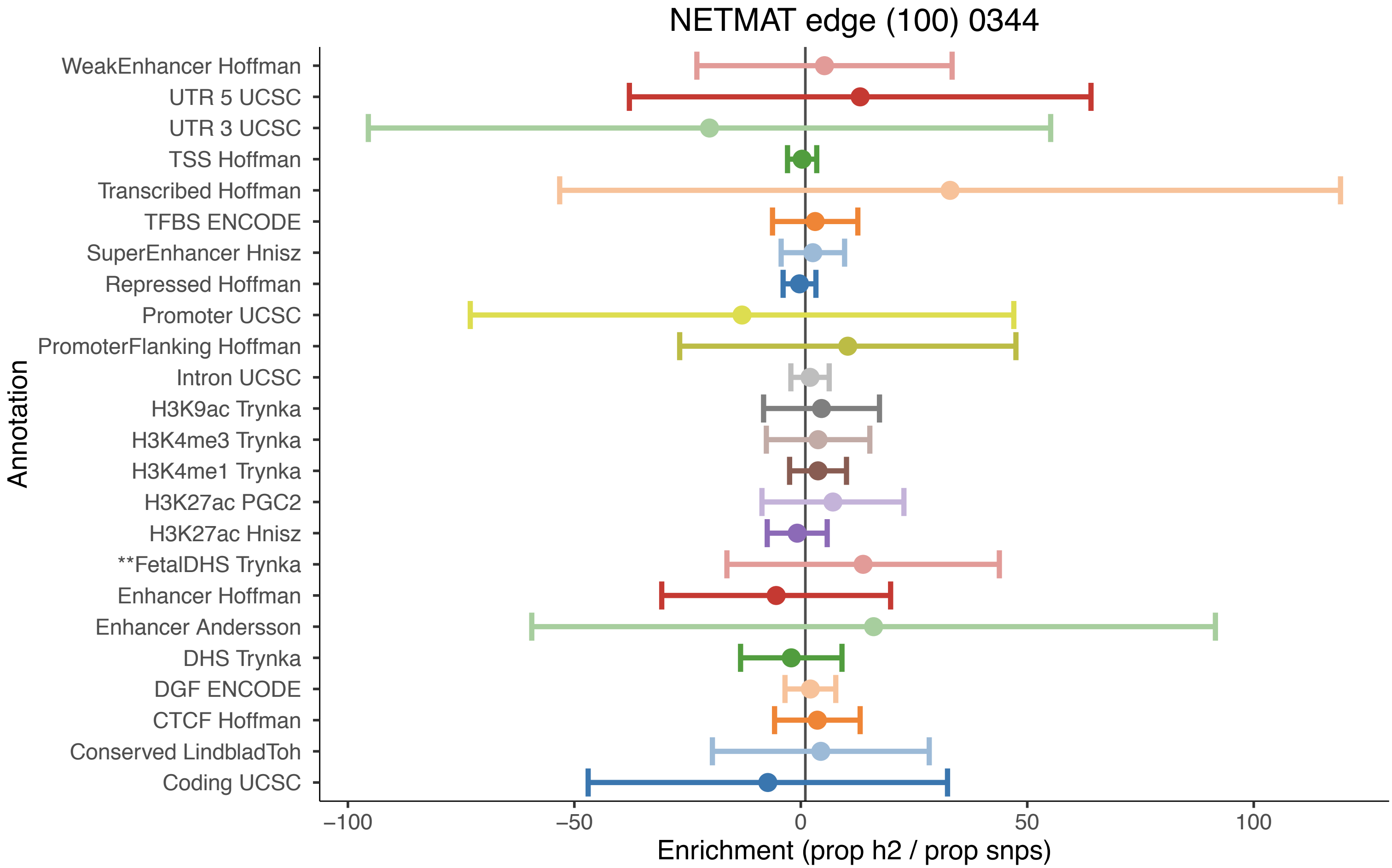

NETMAT edge (100) 0344
Annotation
WeakEnhancer Hoffman
UTR 5 UCSC
UTR 3 UCSC
TSS Hoffman
Transcribed Hoffman
TFBS ENCODE
SuperEnhancer Hnisz
Repressed Hoffman
Promoter UCSC
PromoterFlanking Hoffman
Intron UCSC
H3K9ac Trynka
H3K4me3 Trynka
H3K4me1 Trynka
H3K27ac PGC2
H3K27ac Hnisz
**FetalDHS Trynka
Enhancer Hoffman
Enhancer Andersson
DHS Trynka
DGF ENCODE
CTCF Hoffman
Conserved LindbladToh
Coding UCSC
−100
−50
0
50
100
Enrichment (prop h2 / prop snps)

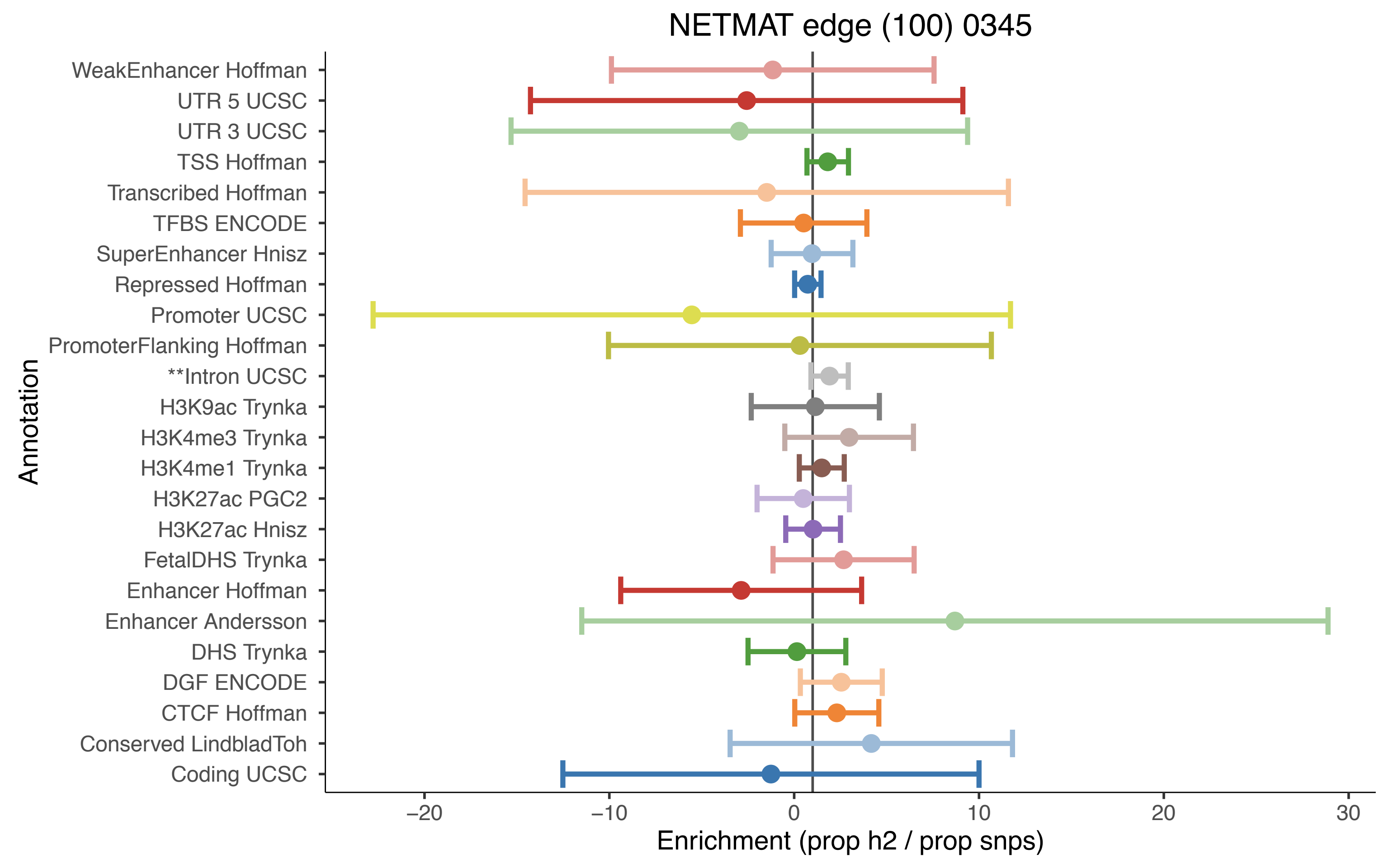
NETMAT edge (100) 0345
WeakEnhancer Hoffman
UTR 5 UCSC
UTR 3 UCSC
TSS Hoffman
Transcribed Hoffman
TFBS ENCODE
SuperEnhancer Hnisz
Repressed Hoffman
Promoter UCSC
PromoterFlanking Hoffman
**Intron UCSC
H3K9ac Trynka
H3K4me3 Trynka
H3K4me1 Trynka
H3K27ac PGC2
H3K27ac Hnisz
FetalDHS Trynka
Enhancer Hoffman
Enhancer Andersson
DHS Trynka
DGF ENCODE
CTCF Hoffman
Conserved LindbladToh
Coding UCSC
Annotation
−20
−10
0
10
20
30
Enrichment (prop h2 / prop snps)

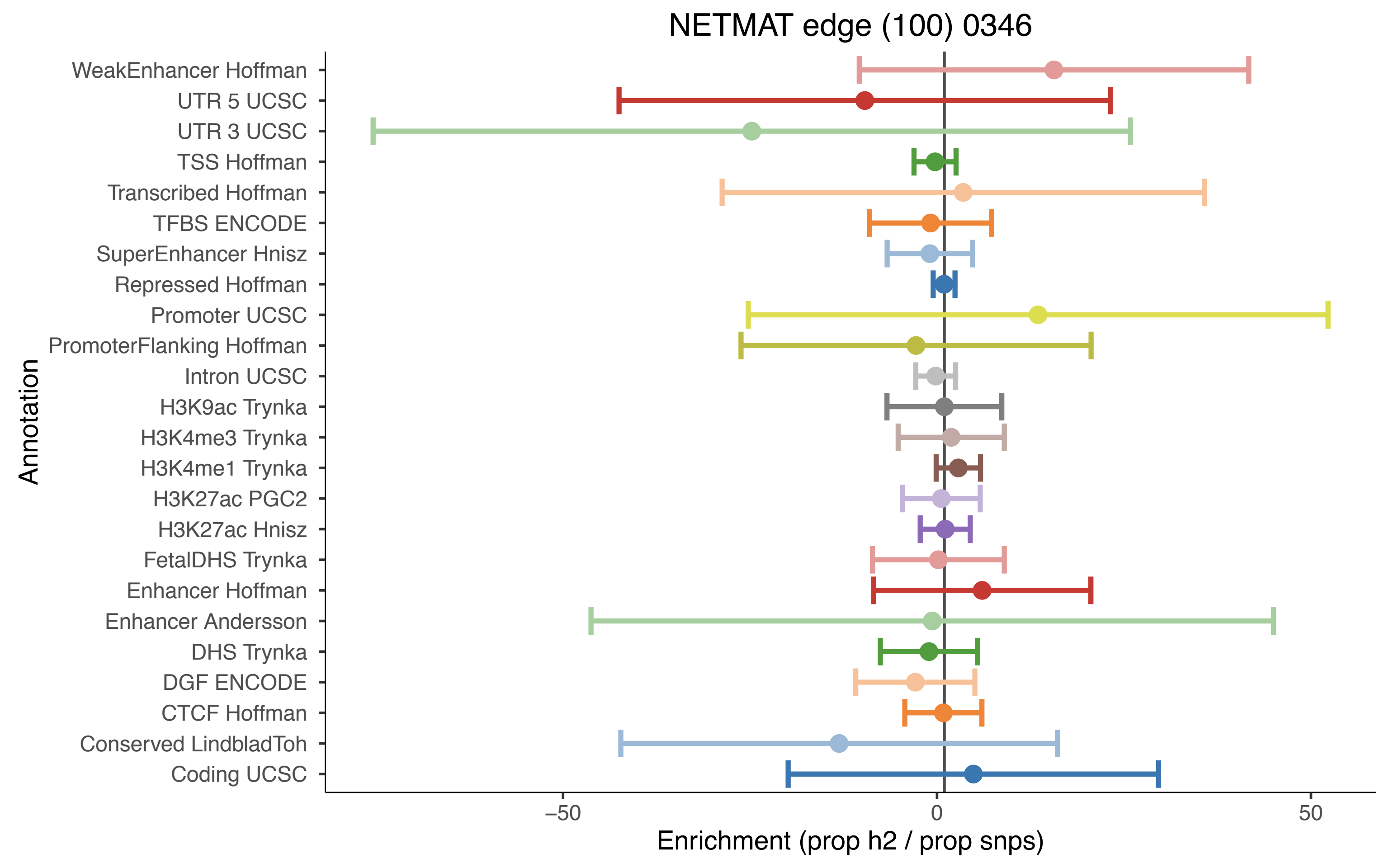
NETMAT edge (100) 0346
Annotation
WeakEnhancer Hoffman
UTR 5 UCSC
UTR 3 UCSC
TSS Hoffman
Transcribed Hoffman
TFBS ENCODE
SuperEnhancer Hnisz
Repressed Hoffman
Promoter UCSC
PromoterFlanking Hoffman
Intron UCSC
H3K9ac Trynka
H3K4me3 Trynka
H3K4me1 Trynka
H3K27ac PGC2
H3K27ac Hnisz
FetalDHS Trynka
Enhancer Hoffman
Enhancer Andersson
DHS Trynka
DGF ENCODE
CTCF Hoffman
Conserved LindbladToh
Coding UCSC
−50
0
50
Enrichment (prop h2 / prop snps)

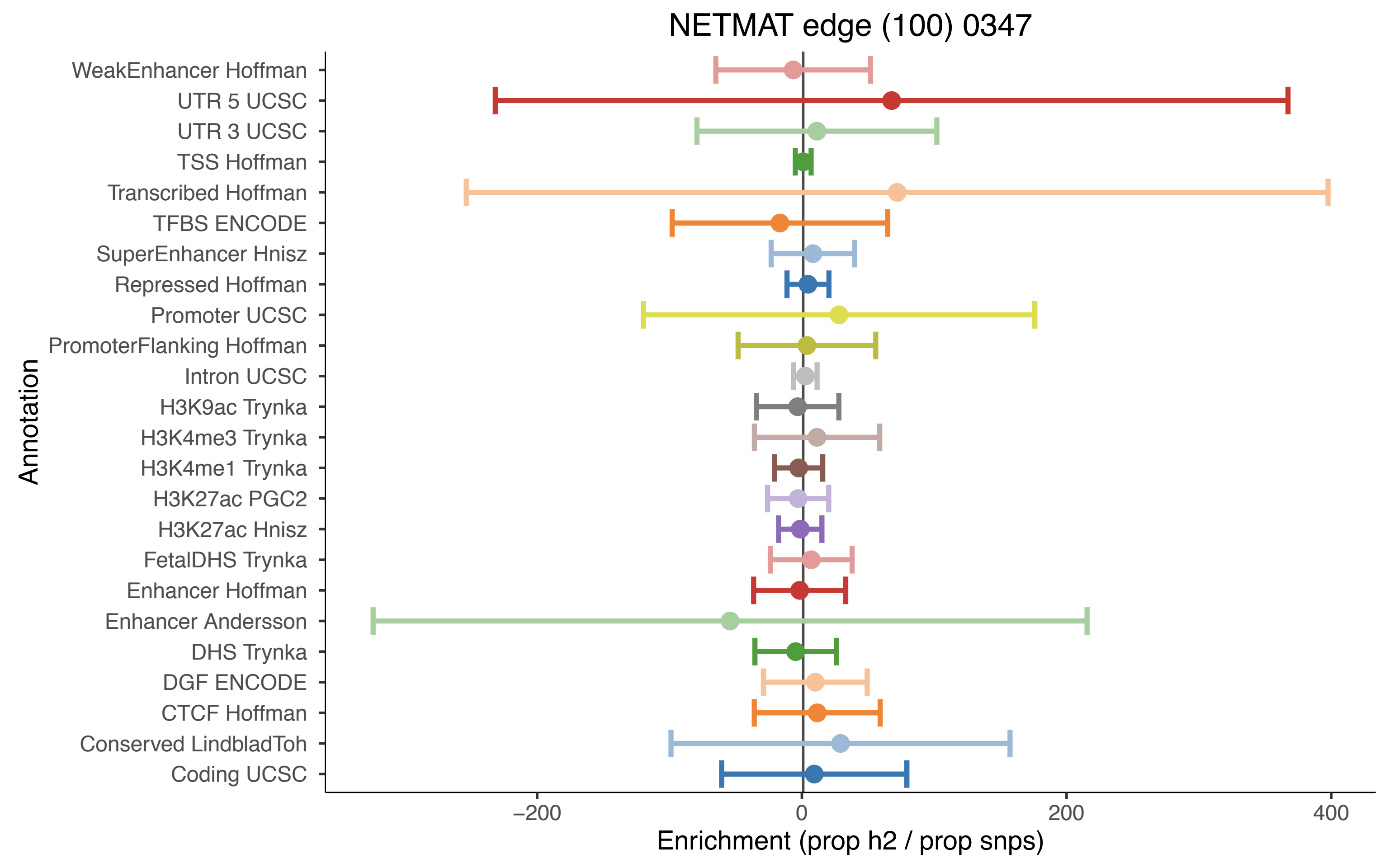
NETMAT edge (100) 0347
WeakEnhancer Hoffman
UTR 5 UCSC
UTR 3 UCSC
TSS Hoffman
Transcribed Hoffman
TFBS ENCODE
SuperEnhancer Hnisz
Repressed Hoffman
Promoter UCSC
PromoterFlanking Hoffman
Intron UCSC
H3K9ac Trynka
H3K4me3 Trynka
H3K4me1 Trynka
H3K27ac PGC2
H3K27ac Hnisz
FetalDHS Trynka
Enhancer Hoffman
Enhancer Andersson
DHS Trynka
DGF ENCODE
CTCF Hoffman
Conserved LindbladToh
Coding UCSC
Annotation
−200
0
200
400
Enrichment (prop h2 / prop snps)

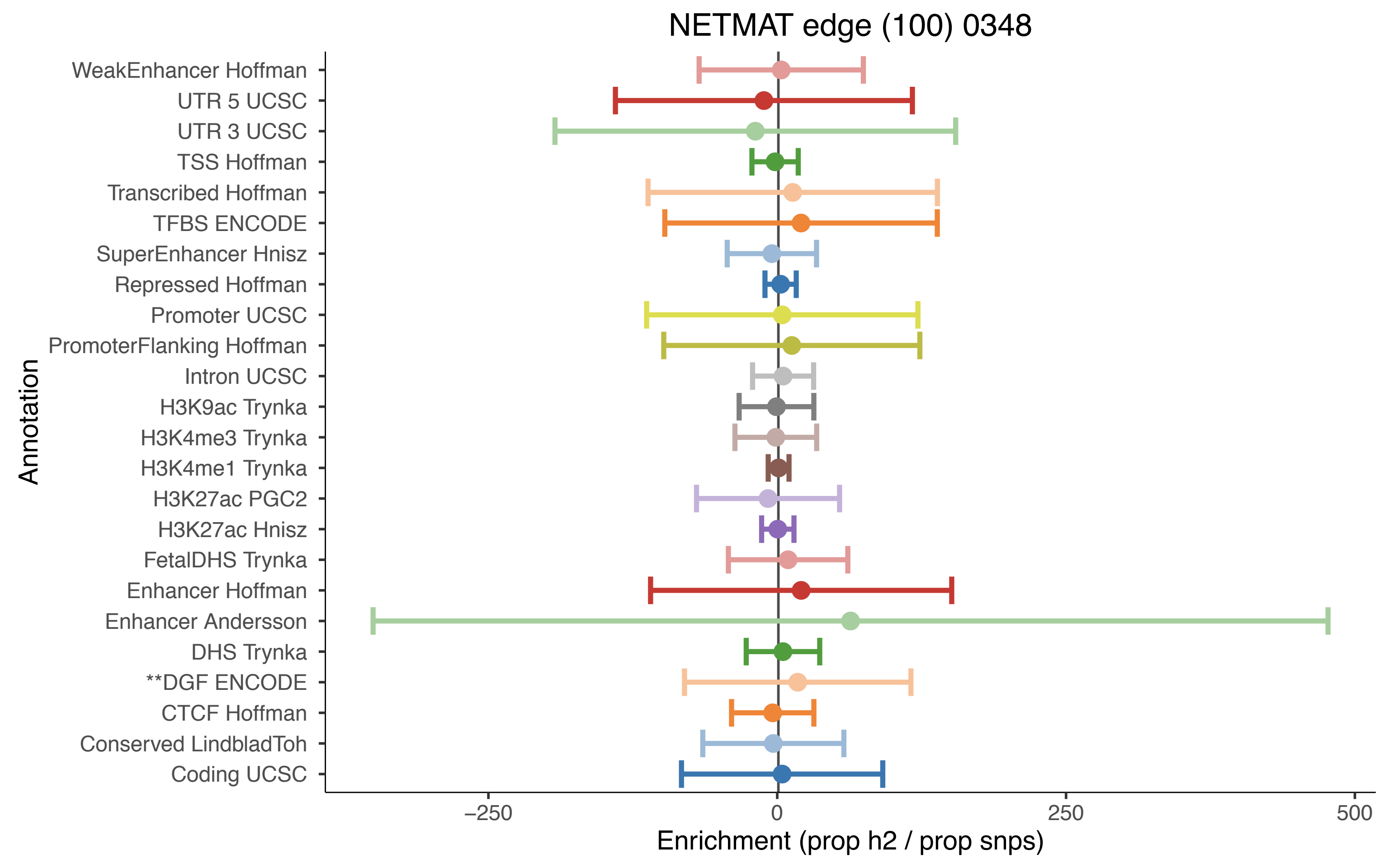

NETMAT edge (100) 0348
WeakEnhancer Hoffman
UTR 5 UCSC
UTR 3 UCSC
TSS Hoffman
Transcribed Hoffman
TFBS ENCODE
SuperEnhancer Hnisz
Repressed Hoffman
Promoter UCSC
PromoterFlanking Hoffman
Intron UCSC
H3K9ac Trynka
H3K4me3 Trynka
H3K4me1 Trynka
H3K27ac PGC2
H3K27ac Hnisz
FetalDHS Trynka
Enhancer Hoffman
Enhancer Andersson
DHS Trynka
**DGF ENCODE
CTCF Hoffman
Conserved LindbladToh
Coding UCSC
Annotation
−250
0
250
500
Enrichment (prop h2 / prop snps)

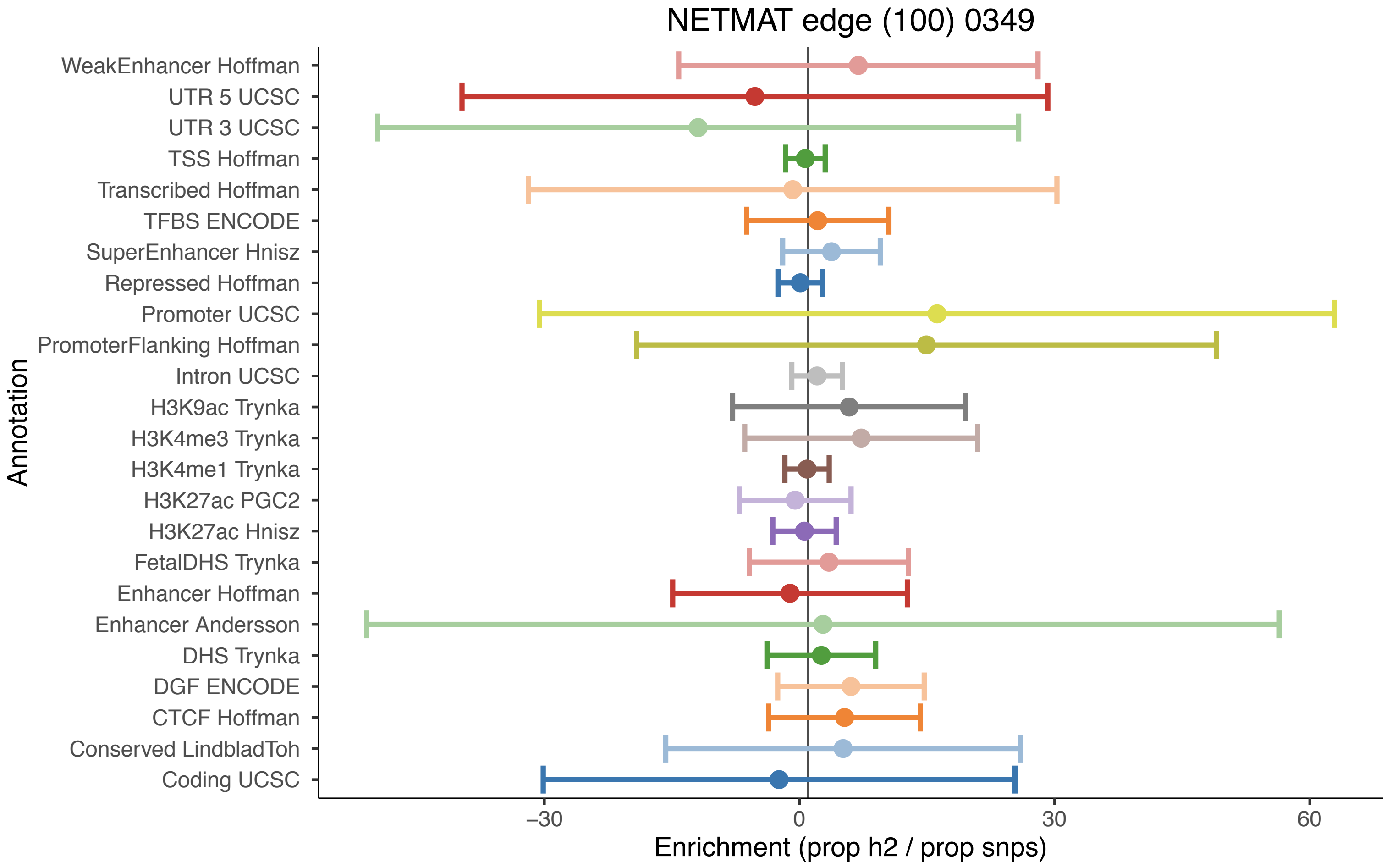
NETMAT edge (100) 0349
WeakEnhancer Hoffman
UTR 5 UCSC
UTR 3 UCSC
TSS Hoffman
Transcribed Hoffman
TFBS ENCODE
SuperEnhancer Hnisz
Repressed Hoffman
Promoter UCSC
PromoterFlanking Hoffman
Intron UCSC
H3K9ac Trynka
H3K4me3 Trynka
H3K4me1 Trynka
H3K27ac PGC2
H3K27ac Hnisz
FetalDHS Trynka
Enhancer Hoffman
Enhancer Andersson
DHS Trynka
DGF ENCODE
CTCF Hoffman
Conserved LindbladToh
Coding UCSC
Annotation
−30
0
30
60
Enrichment (prop h2 / prop snps)

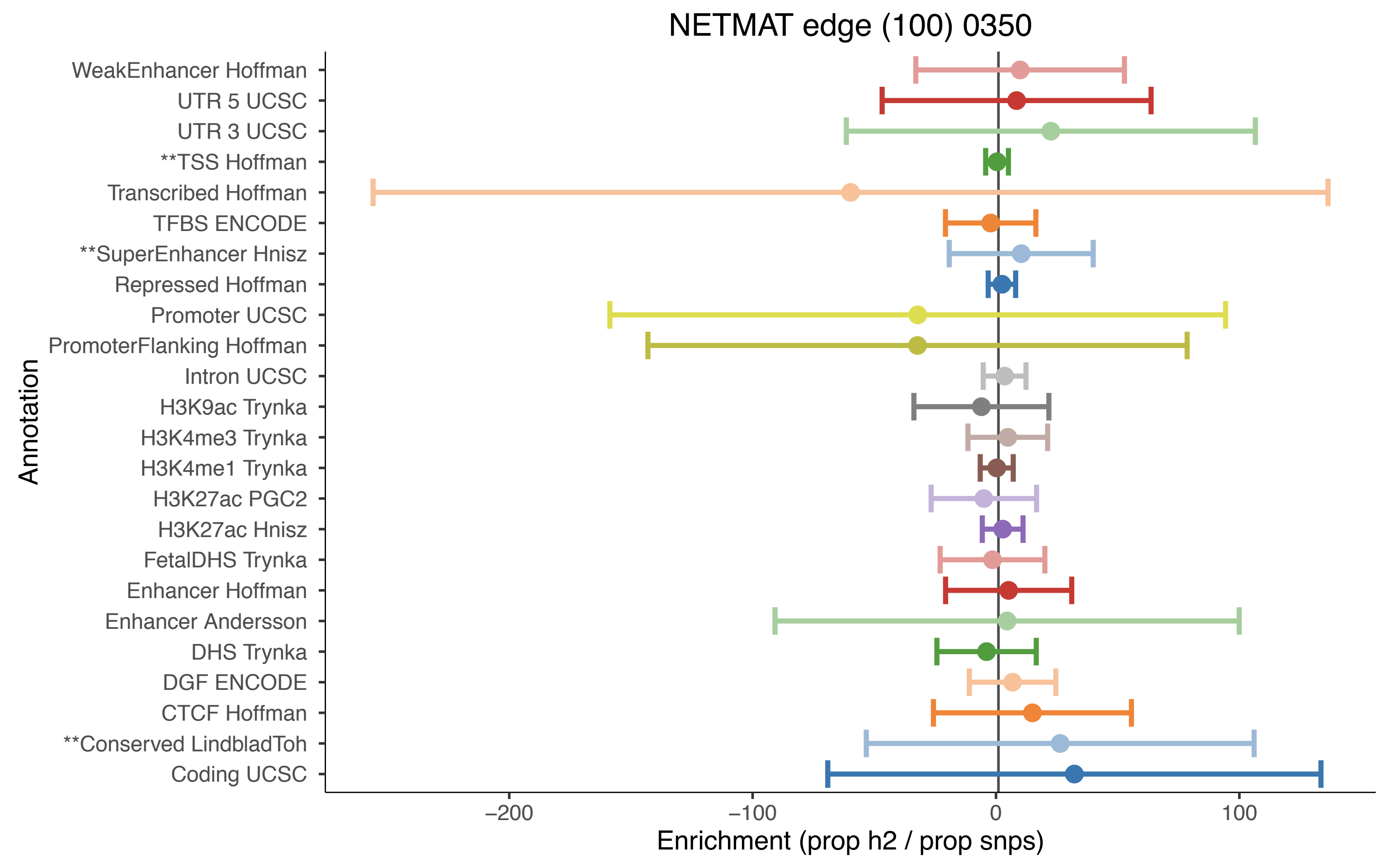
NETMAT edge (100) 0350
WeakEnhancer Hoffman
UTR 5 UCSC
UTR 3 UCSC
**TSS Hoffman
Transcribed Hoffman
TFBS ENCODE
**SuperEnhancer Hnisz
Repressed Hoffman
Promoter UCSC
PromoterFlanking Hoffman
Intron UCSC
H3K9ac Trynka
H3K4me3 Trynka
H3K4me1 Trynka
H3K27ac PGC2
H3K27ac Hnisz
FetalDHS Trynka
Enhancer Hoffman
Enhancer Andersson
DHS Trynka
DGF ENCODE
CTCF Hoffman
**Conserved LindbladToh
Coding UCSC
Annotation
−200
−100
0
100
Enrichment (prop h2 / prop snps)

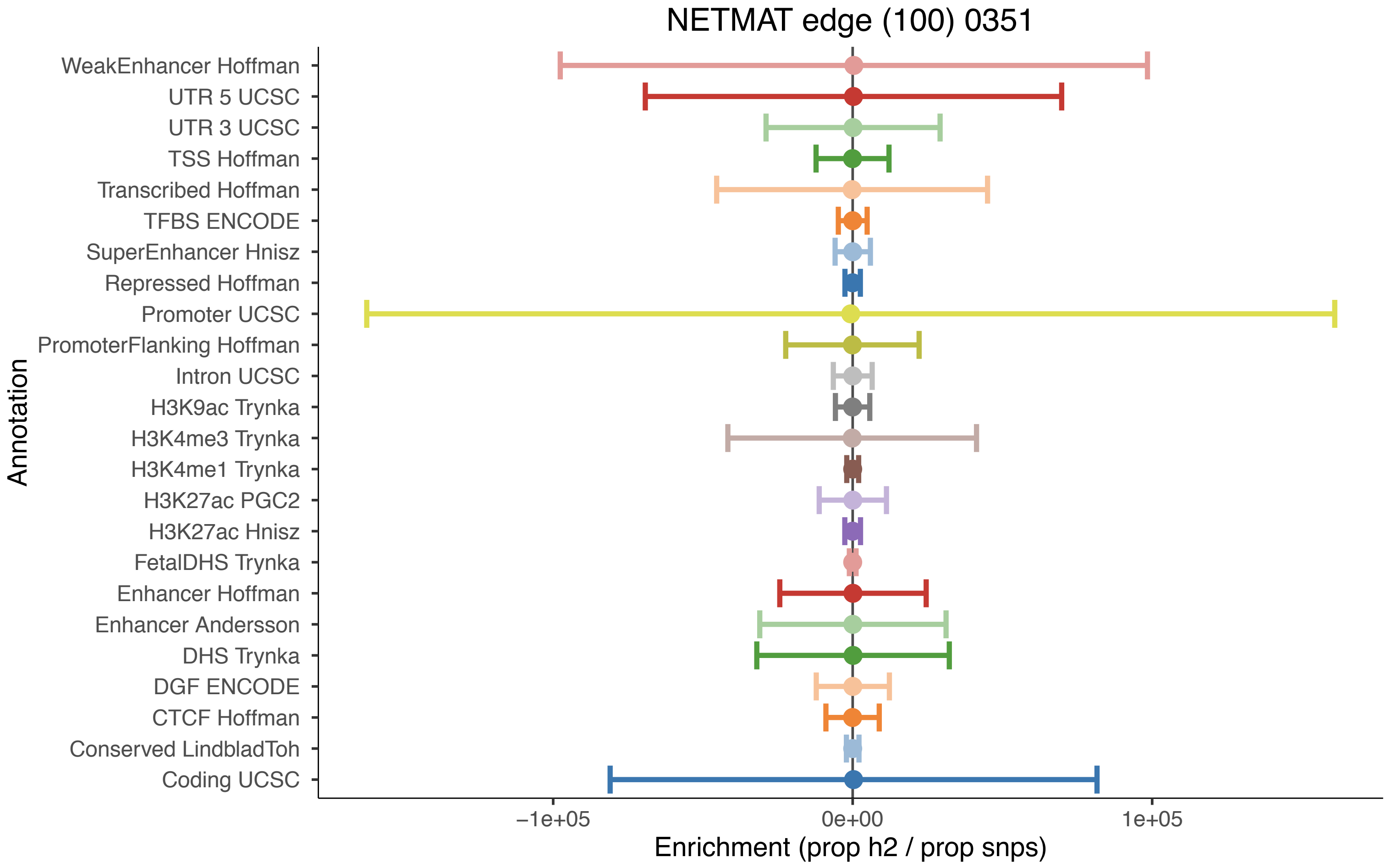

NETMAT edge (100) 0351
Annotation
WeakEnhancer Hoffman
UTR 5 UCSC
UTR 3 UCSC
TSS Hoffman
Transcribed Hoffman
TFBS ENCODE
SuperEnhancer Hnisz
Repressed Hoffman
Promoter UCSC
PromoterFlanking Hoffman
Intron UCSC
H3K9ac Trynka
H3K4me3 Trynka
H3K4me1 Trynka
H3K27ac PGC2
H3K27ac Hnisz
FetalDHS Trynka
Enhancer Hoffman
Enhancer Andersson
DHS Trynka
DGF ENCODE
CTCF Hoffman
Conserved LindbladToh
Coding UCSC
−1e+05
0e+00
1e+05
Enrichment (prop h2 / prop snps)

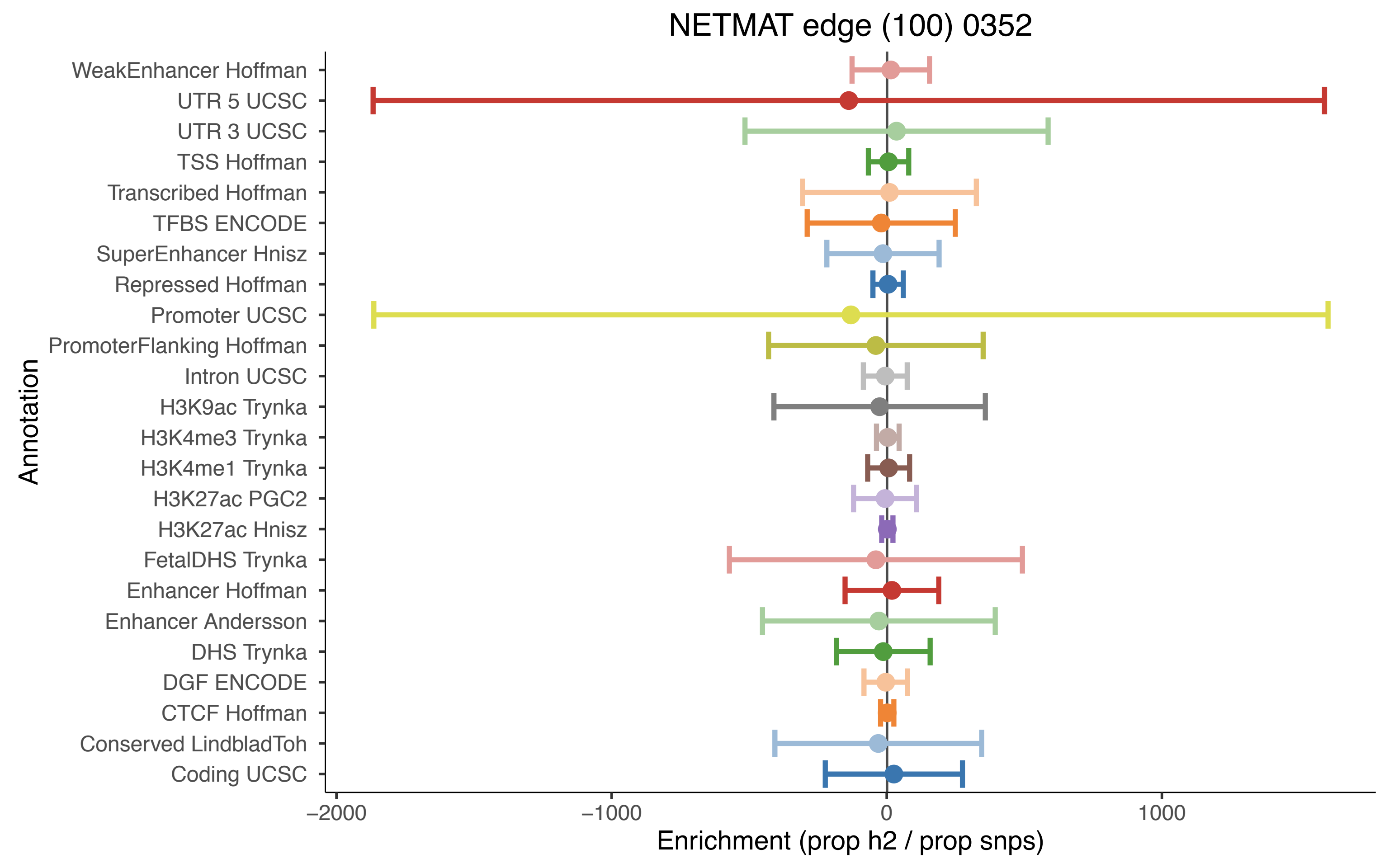

NETMAT edge (100) 0352
Annotation
WeakEnhancer Hoffman
UTR 5 UCSC
UTR 3 UCSC
TSS Hoffman
Transcribed Hoffman
TFBS ENCODE
SuperEnhancer Hnisz
Repressed Hoffman
Promoter UCSC
PromoterFlanking Hoffman
Intron UCSC
H3K9ac Trynka
H3K4me3 Trynka
H3K4me1 Trynka
H3K27ac PGC2
H3K27ac Hnisz
FetalDHS Trynka
Enhancer Hoffman
Enhancer Andersson
DHS Trynka
DGF ENCODE
CTCF Hoffman
Conserved LindbladToh
Coding UCSC
−2000
−1000
0
1000
Enrichment (prop h2 / prop snps)

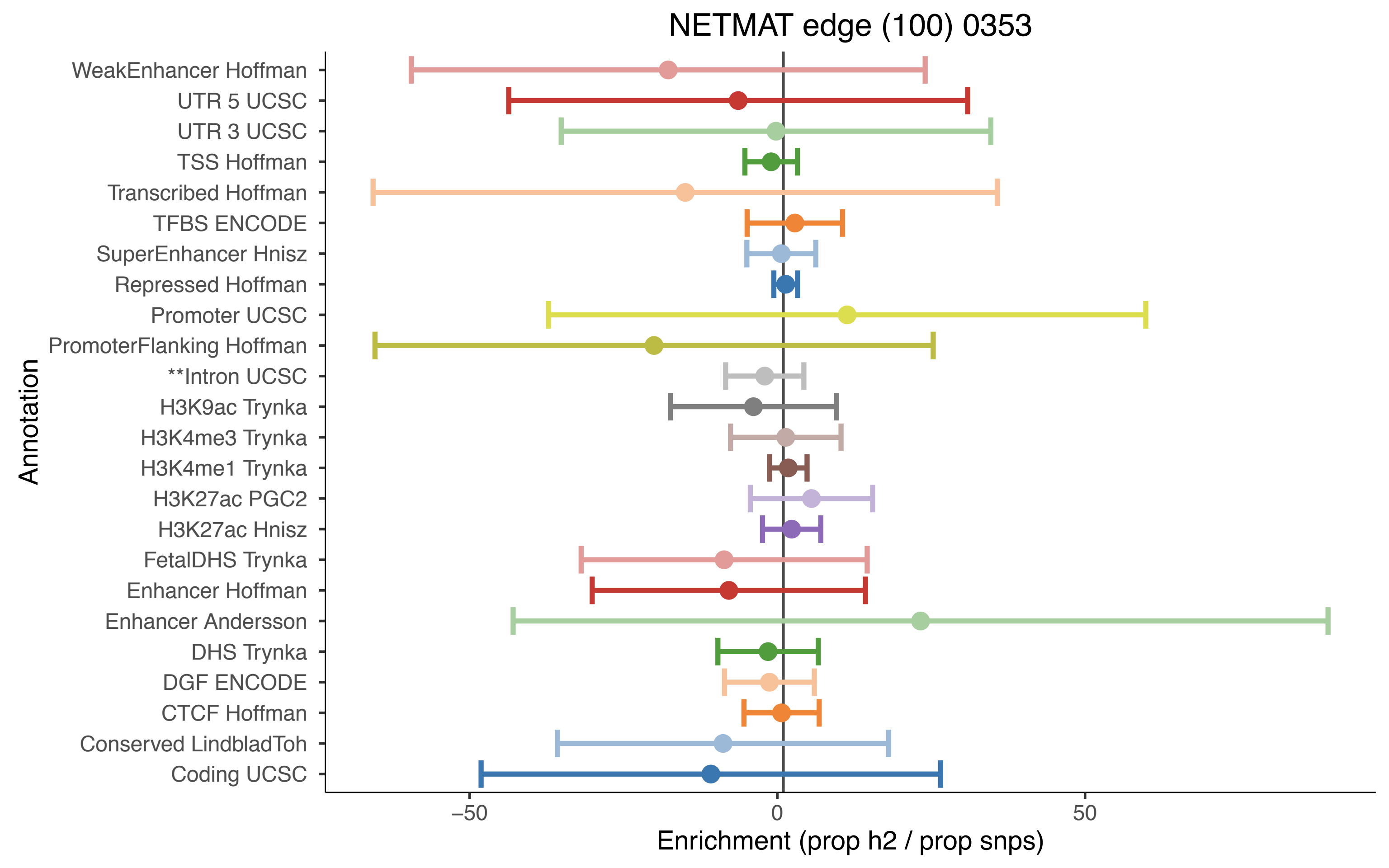
NETMAT edge (100) 0353
WeakEnhancer Hoffman
UTR 5 UCSC
UTR 3 UCSC
TSS Hoffman
Transcribed Hoffman
TFBS ENCODE
SuperEnhancer Hnisz
Repressed Hoffman
Promoter UCSC
PromoterFlanking Hoffman
**Intron UCSC
H3K9ac Trynka
H3K4me3 Trynka
H3K4me1 Trynka
H3K27ac PGC2
H3K27ac Hnisz
FetalDHS Trynka
Enhancer Hoffman
Enhancer Andersson
DHS Trynka
DGF ENCODE
CTCF Hoffman
Conserved LindbladToh
Coding UCSC
Annotation
−50
0
50
Enrichment (prop h2 / prop snps)

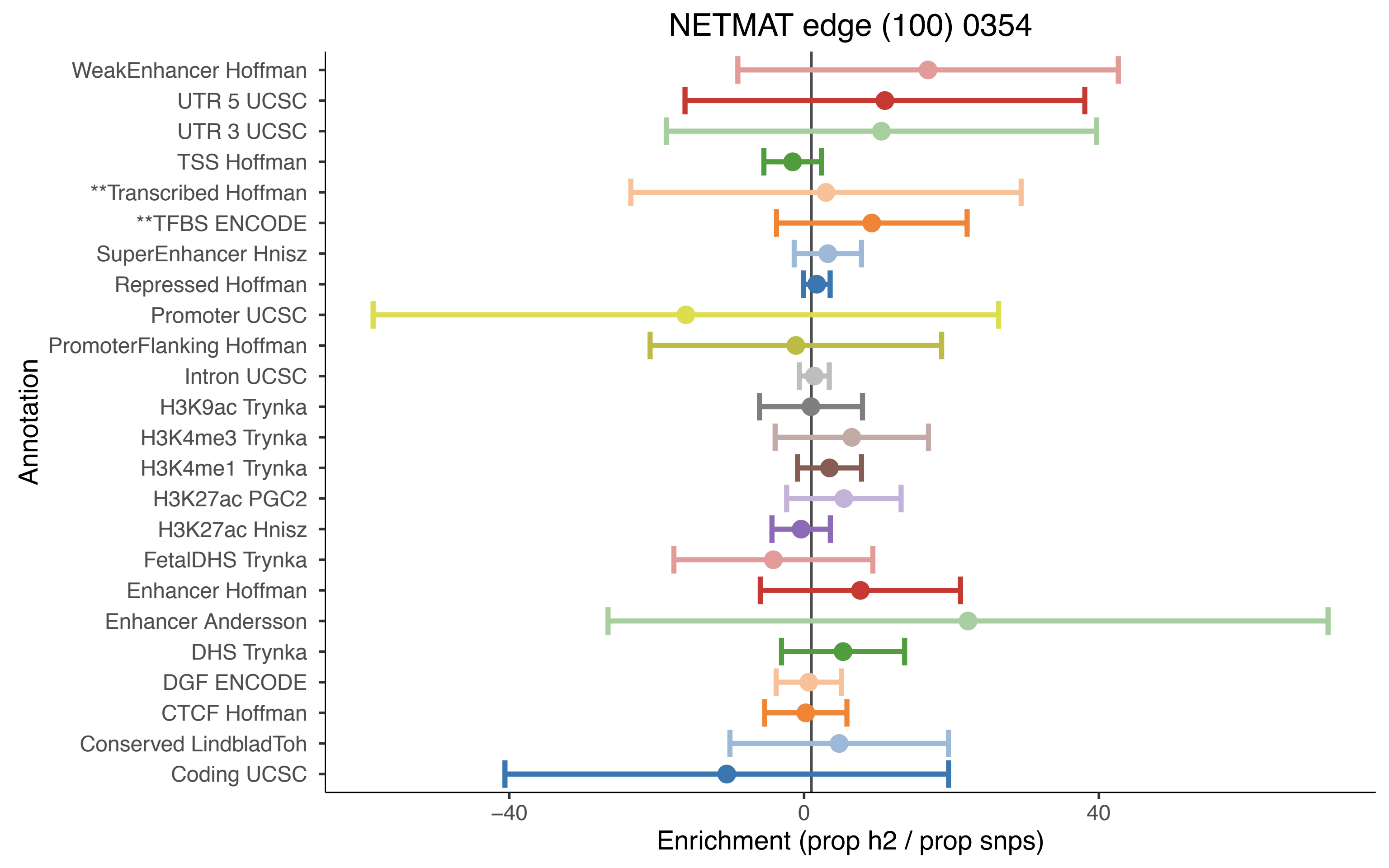

NETMAT edge (100) 0354
WeakEnhancer Hoffman
UTR 5 UCSC
UTR 3 UCSC
TSS Hoffman
**Transcribed Hoffman
**TFBS ENCODE
SuperEnhancer Hnisz
Repressed Hoffman
Promoter UCSC
PromoterFlanking Hoffman
Intron UCSC
H3K9ac Trynka
H3K4me3 Trynka
H3K4me1 Trynka
H3K27ac PGC2
H3K27ac Hnisz
FetalDHS Trynka
Enhancer Hoffman
Enhancer Andersson
DHS Trynka
DGF ENCODE
CTCF Hoffman
Conserved LindbladToh
Coding UCSC
Annotation
−40
0
40
Enrichment (prop h2 / prop snps)

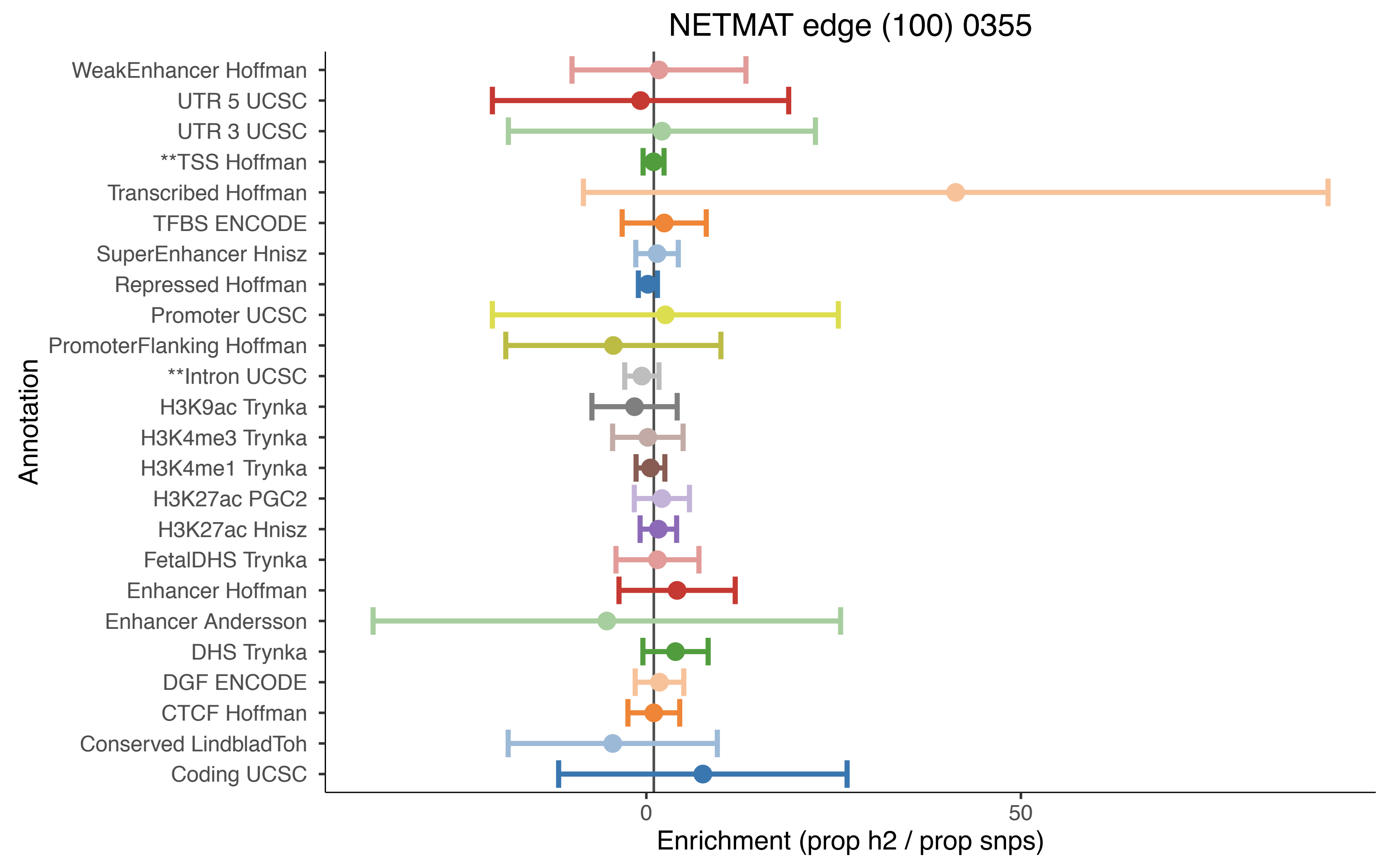

NETMAT edge (100) 0355
WeakEnhancer Hoffman
UTR 5 UCSC
UTR 3 UCSC
**TSS Hoffman
Transcribed Hoffman
TFBS ENCODE
SuperEnhancer Hnisz
Repressed Hoffman
Promoter UCSC
PromoterFlanking Hoffman
**Intron UCSC
H3K9ac Trynka
H3K4me3 Trynka
H3K4me1 Trynka
H3K27ac PGC2
H3K27ac Hnisz
FetalDHS Trynka
Enhancer Hoffman
Enhancer Andersson
DHS Trynka
DGF ENCODE
CTCF Hoffman
Conserved LindbladToh
Coding UCSC
Annotation
0
50
Enrichment (prop h2 / prop snps)

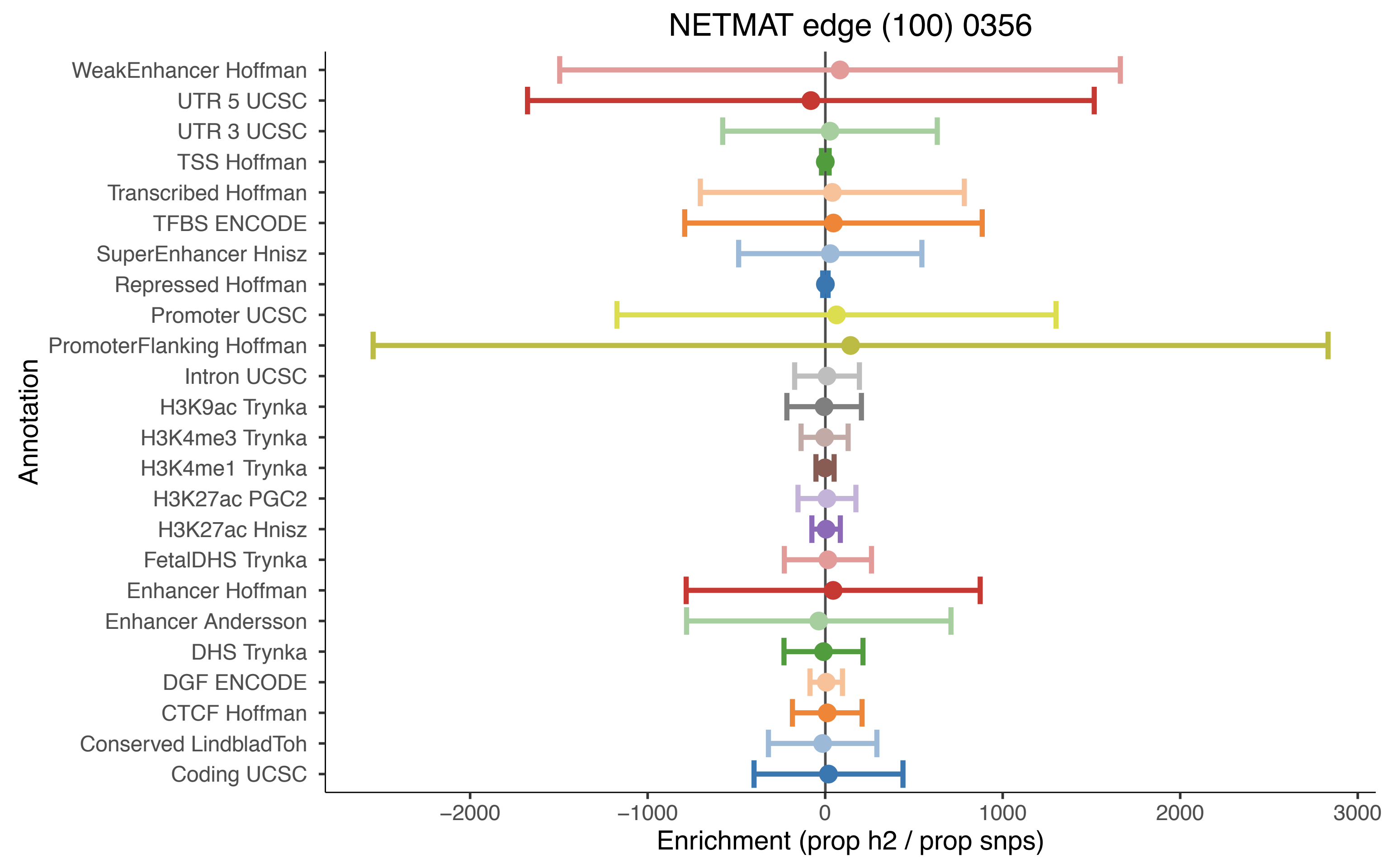
NETMAT edge (100) 0356
WeakEnhancer Hoffman
UTR 5 UCSC
UTR 3 UCSC
TSS Hoffman
Transcribed Hoffman
TFBS ENCODE
SuperEnhancer Hnisz
Repressed Hoffman
Promoter UCSC
PromoterFlanking Hoffman
Intron UCSC
H3K9ac Trynka
H3K4me3 Trynka
H3K4me1 Trynka
H3K27ac PGC2
H3K27ac Hnisz
FetalDHS Trynka
Enhancer Hoffman
Enhancer Andersson
DHS Trynka
DGF ENCODE
CTCF Hoffman
Conserved LindbladToh
Coding UCSC
Annotation
−2000
−1000
0
1000
2000
3000
Enrichment (prop h2 / prop snps)

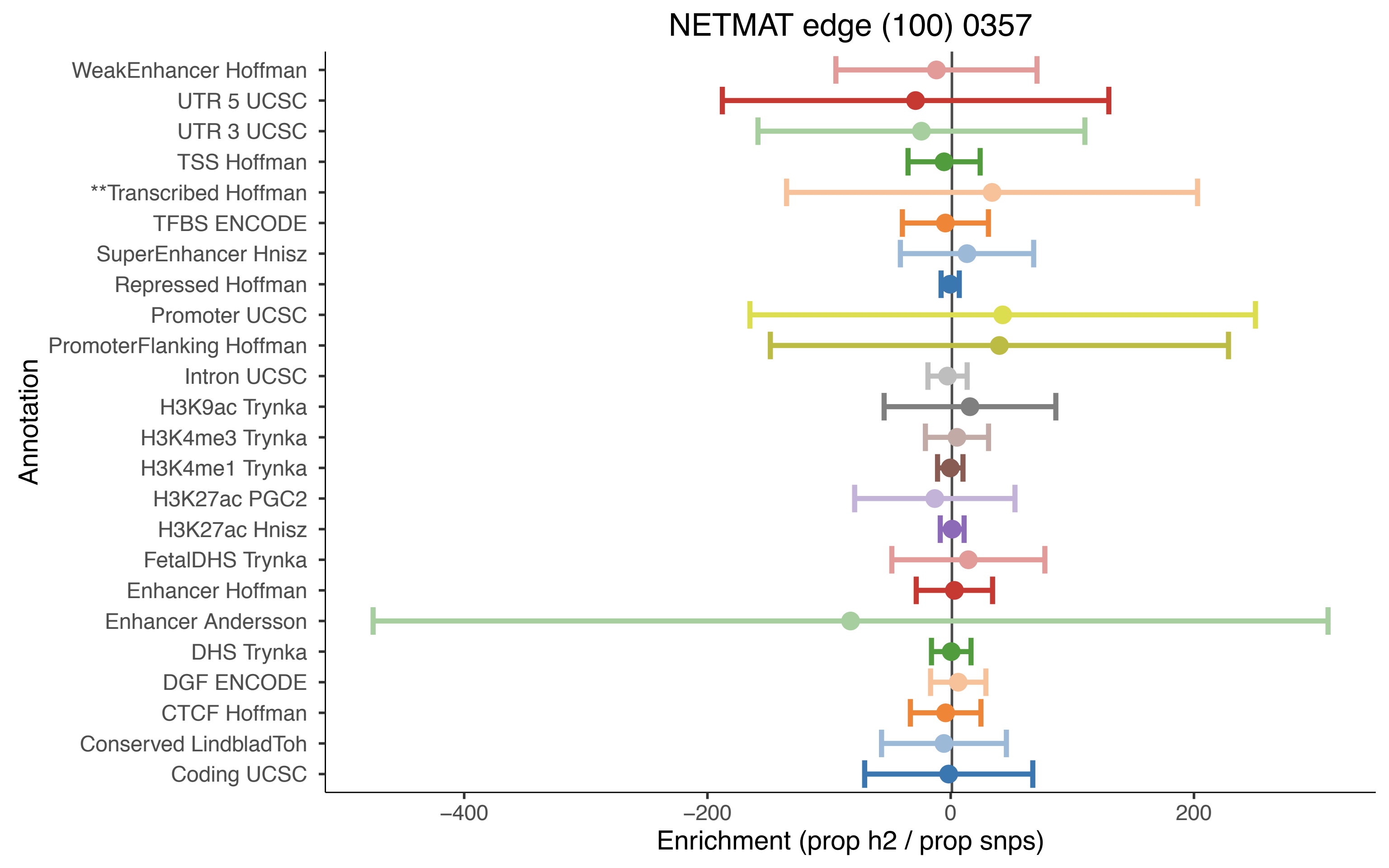

NETMAT edge (100) 0357
WeakEnhancer Hoffman
UTR 5 UCSC
UTR 3 UCSC
TSS Hoffman
**Transcribed Hoffman
TFBS ENCODE
SuperEnhancer Hnisz
Repressed Hoffman
Promoter UCSC
PromoterFlanking Hoffman
Intron UCSC
H3K9ac Trynka
H3K4me3 Trynka
H3K4me1 Trynka
H3K27ac PGC2
H3K27ac Hnisz
FetalDHS Trynka
Enhancer Hoffman
Enhancer Andersson
DHS Trynka
DGF ENCODE
CTCF Hoffman
Conserved LindbladToh
Coding UCSC
Annotation
−400
−200
0
200
Enrichment (prop h2 / prop snps)

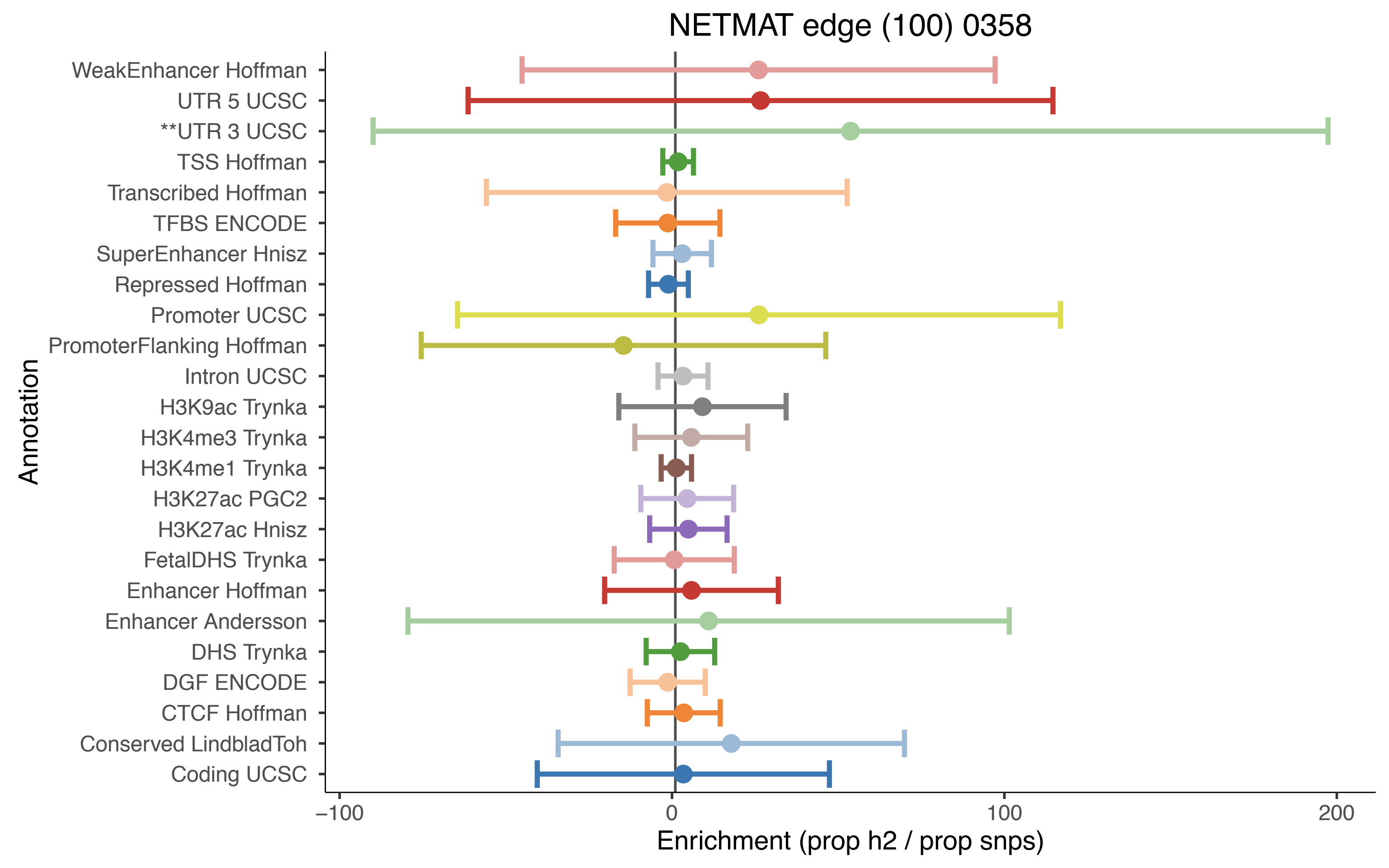
NETMAT edge (100) 0358
WeakEnhancer Hoffman
UTR 5 UCSC
**UTR 3 UCSC
TSS Hoffman
Transcribed Hoffman
TFBS ENCODE
SuperEnhancer Hnisz
Repressed Hoffman
Promoter UCSC
PromoterFlanking Hoffman
Intron UCSC
H3K9ac Trynka
H3K4me3 Trynka
H3K4me1 Trynka
H3K27ac PGC2
H3K27ac Hnisz
FetalDHS Trynka
Enhancer Hoffman
Enhancer Andersson
DHS Trynka
DGF ENCODE
CTCF Hoffman
Conserved LindbladToh
Coding UCSC
Annotation
−100
0
100
200
Enrichment (prop h2 / prop snps)

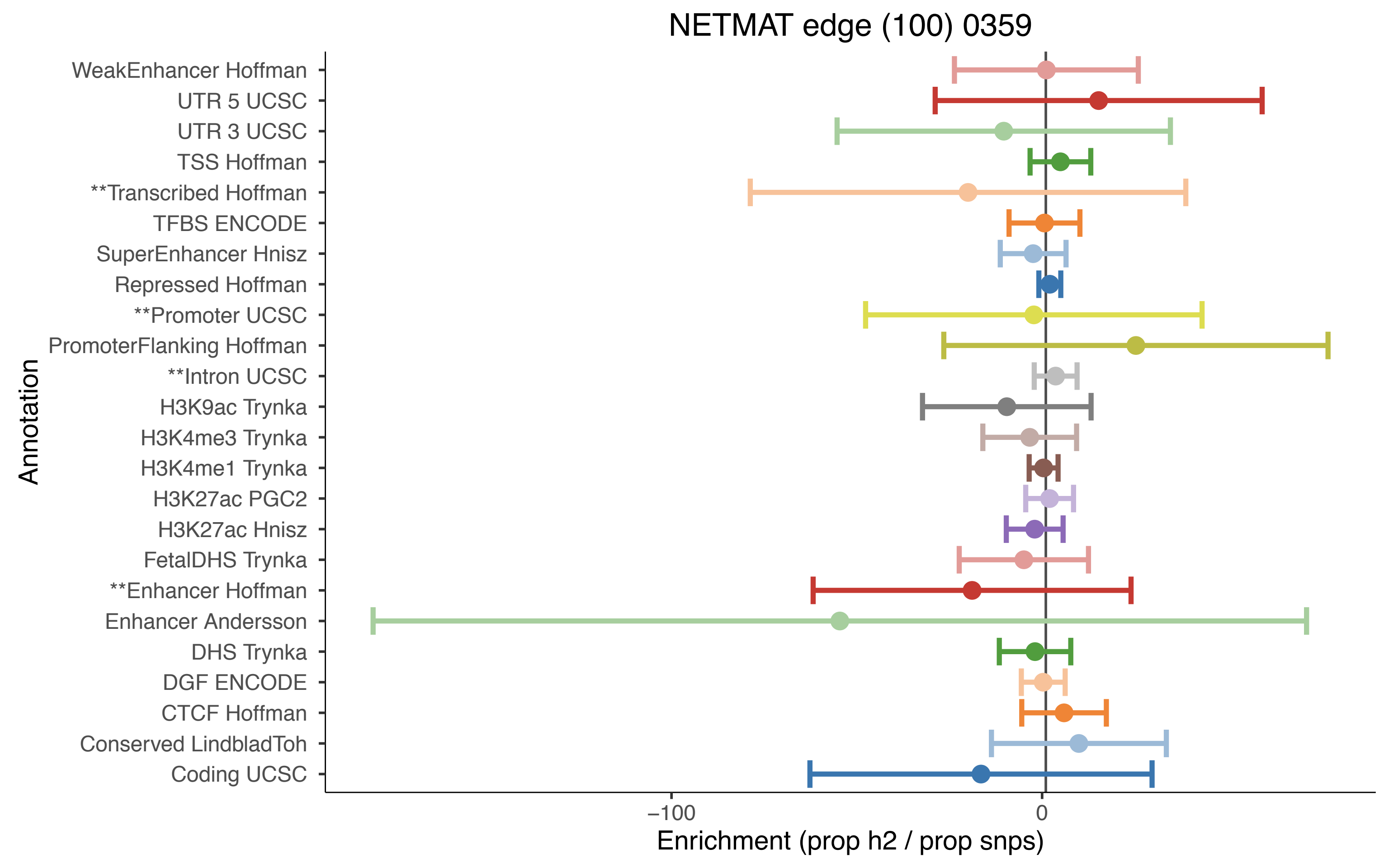

NETMAT edge (100) 0359
WeakEnhancer Hoffman
UTR 5 UCSC
UTR 3 UCSC
TSS Hoffman
**Transcribed Hoffman
TFBS ENCODE
SuperEnhancer Hnisz
Repressed Hoffman
**Promoter UCSC
PromoterFlanking Hoffman
**Intron UCSC
H3K9ac Trynka
H3K4me3 Trynka
H3K4me1 Trynka
H3K27ac PGC2
H3K27ac Hnisz
FetalDHS Trynka
**Enhancer Hoffman
Enhancer Andersson
DHS Trynka
DGF ENCODE
CTCF Hoffman
Conserved LindbladToh
Coding UCSC
Annotation
−100
0
Enrichment (prop h2 / prop snps)

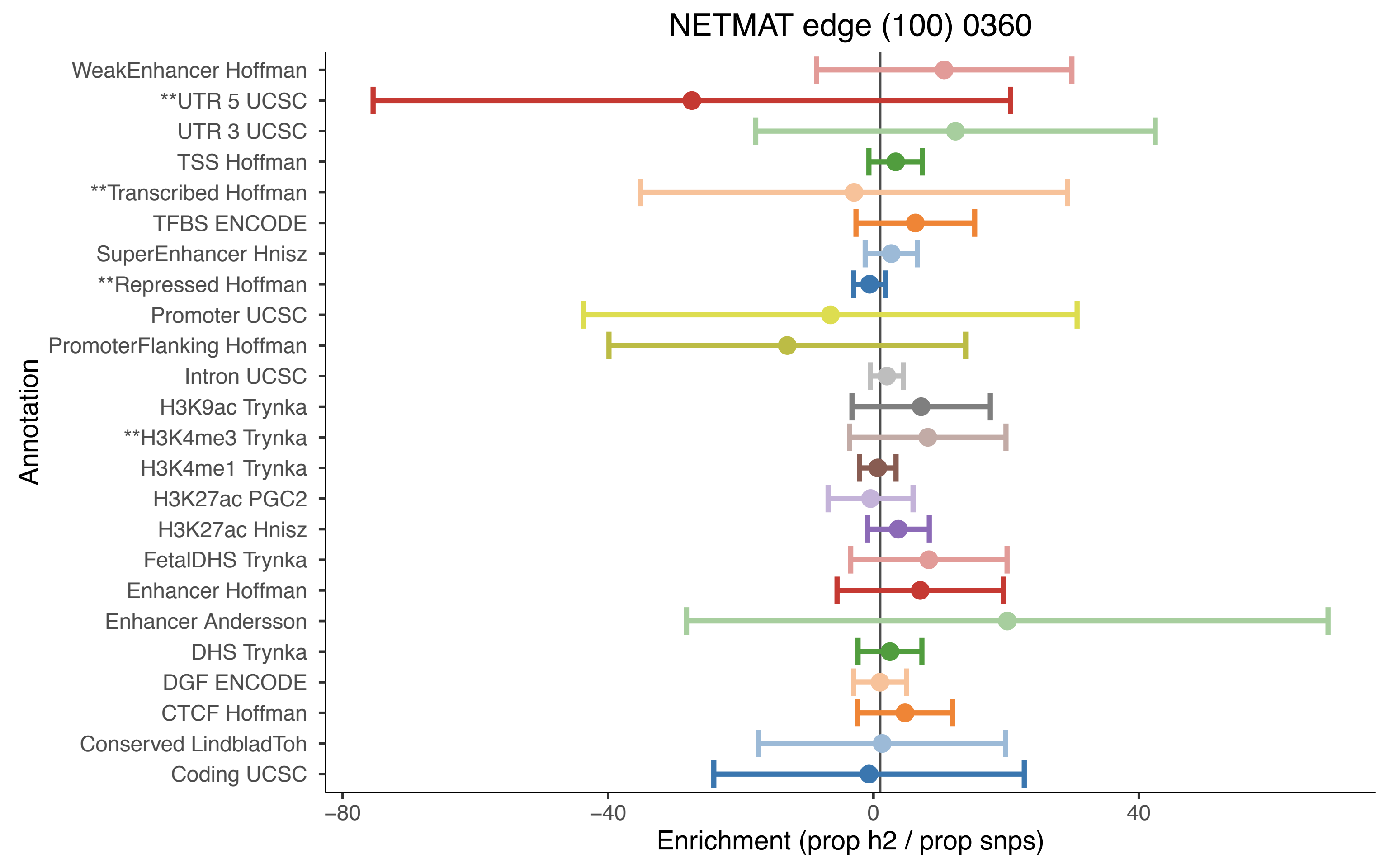

NETMAT edge (100) 0360
WeakEnhancer Hoffman
**UTR 5 UCSC
UTR 3 UCSC
TSS Hoffman
**Transcribed Hoffman
TFBS ENCODE
SuperEnhancer Hnisz
**Repressed Hoffman
Promoter UCSC
PromoterFlanking Hoffman
Intron UCSC
H3K9ac Trynka
**H3K4me3 Trynka
H3K4me1 Trynka
H3K27ac PGC2
H3K27ac Hnisz
FetalDHS Trynka
Enhancer Hoffman
Enhancer Andersson
DHS Trynka
DGF ENCODE
CTCF Hoffman
Conserved LindbladToh
Coding UCSC
Annotation
−80
−40
0
40
Enrichment (prop h2 / prop snps)

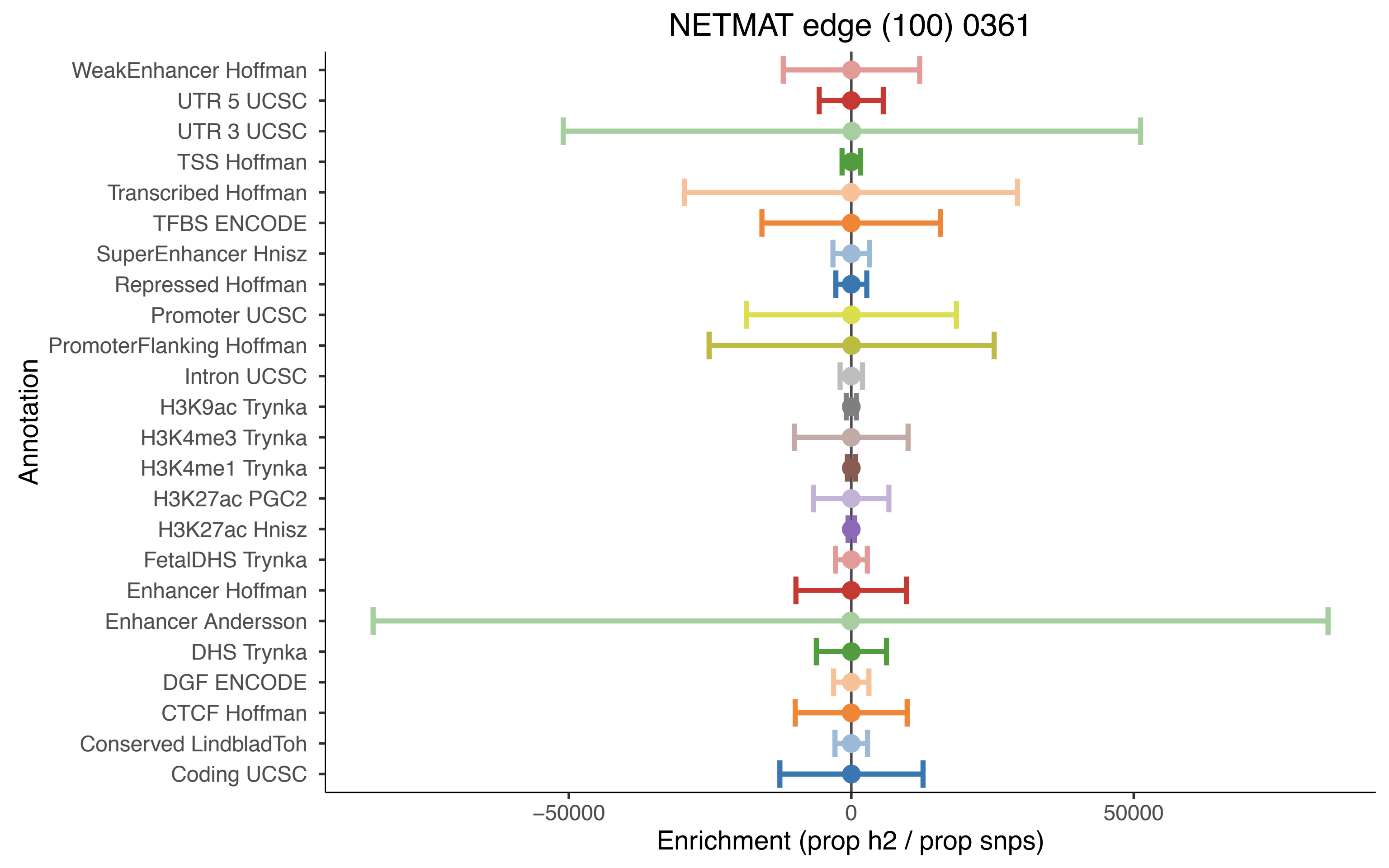
NETMAT edge (100) 0361
WeakEnhancer Hoffman
UTR 5 UCSC
UTR 3 UCSC
TSS Hoffman
Transcribed Hoffman
TFBS ENCODE
SuperEnhancer Hnisz
Repressed Hoffman
Promoter UCSC
PromoterFlanking Hoffman
Intron UCSC
H3K9ac Trynka
H3K4me3 Trynka
H3K4me1 Trynka
H3K27ac PGC2
H3K27ac Hnisz
FetalDHS Trynka
Enhancer Hoffman
Enhancer Andersson
DHS Trynka
DGF ENCODE
CTCF Hoffman
Conserved LindbladToh
Coding UCSC
Annotation
−50000
0
50000
Enrichment (prop h2 / prop snps)

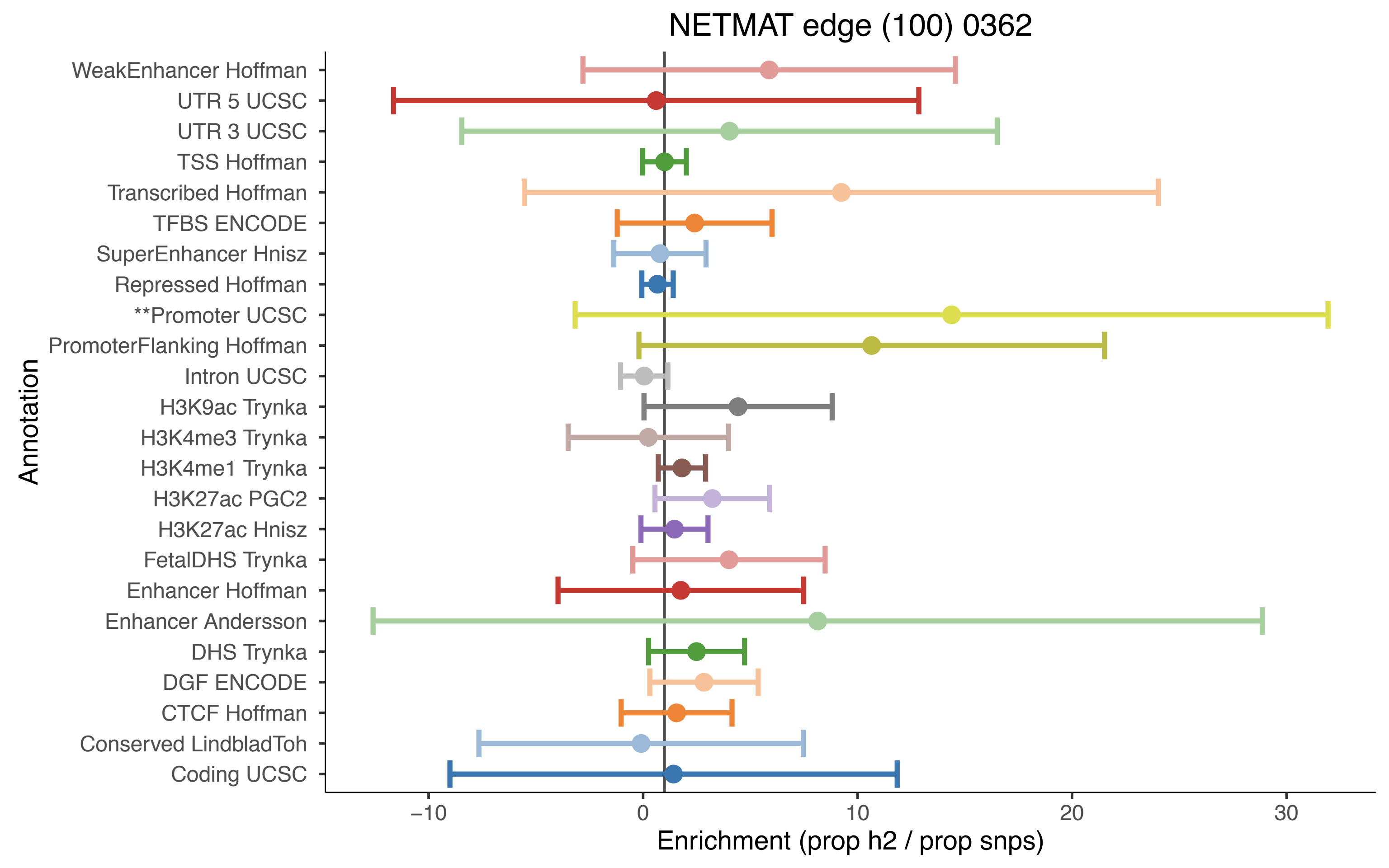

NETMAT edge (100) 0362
WeakEnhancer Hoffman
UTR 5 UCSC
UTR 3 UCSC
TSS Hoffman
Transcribed Hoffman
TFBS ENCODE
SuperEnhancer Hnisz
Repressed Hoffman
**Promoter UCSC
PromoterFlanking Hoffman
Intron UCSC
H3K9ac Trynka
H3K4me3 Trynka
H3K4me1 Trynka
H3K27ac PGC2
H3K27ac Hnisz
FetalDHS Trynka
Enhancer Hoffman
Enhancer Andersson
DHS Trynka
DGF ENCODE
CTCF Hoffman
Conserved LindbladToh
Coding UCSC
Annotation
−10
0
10
20
30
Enrichment (prop h2 / prop snps)

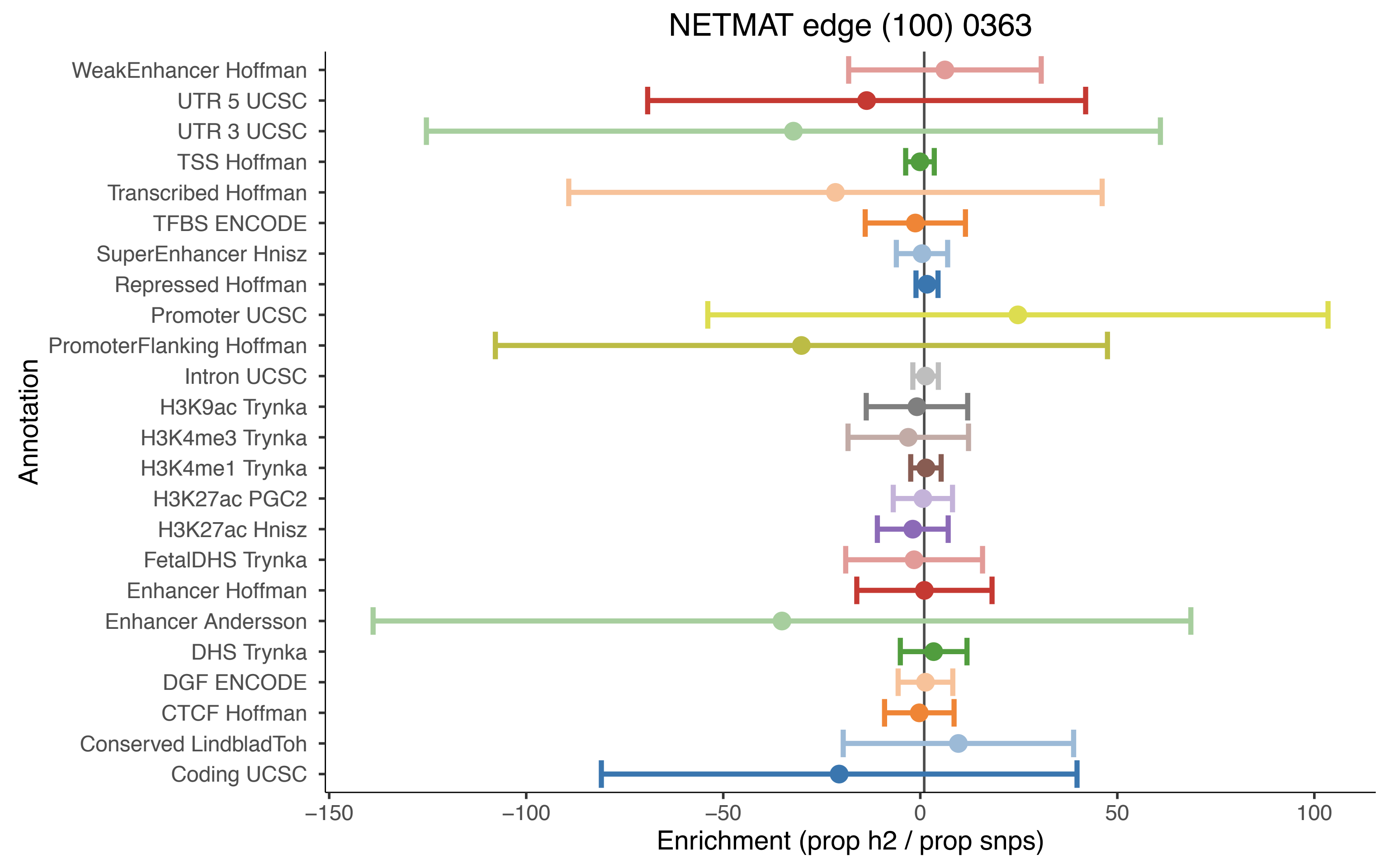

NETMAT edge (100) 0363
WeakEnhancer Hoffman
UTR 5 UCSC
UTR 3 UCSC
TSS Hoffman
Transcribed Hoffman
TFBS ENCODE
SuperEnhancer Hnisz
Repressed Hoffman
Promoter UCSC
PromoterFlanking Hoffman
Intron UCSC
H3K9ac Trynka
H3K4me3 Trynka
H3K4me1 Trynka
H3K27ac PGC2
H3K27ac Hnisz
FetalDHS Trynka
Enhancer Hoffman
Enhancer Andersson
DHS Trynka
DGF ENCODE
CTCF Hoffman
Conserved LindbladToh
Coding UCSC
Annotation
−150
−100
−50
0
50
100
Enrichment (prop h2 / prop snps)

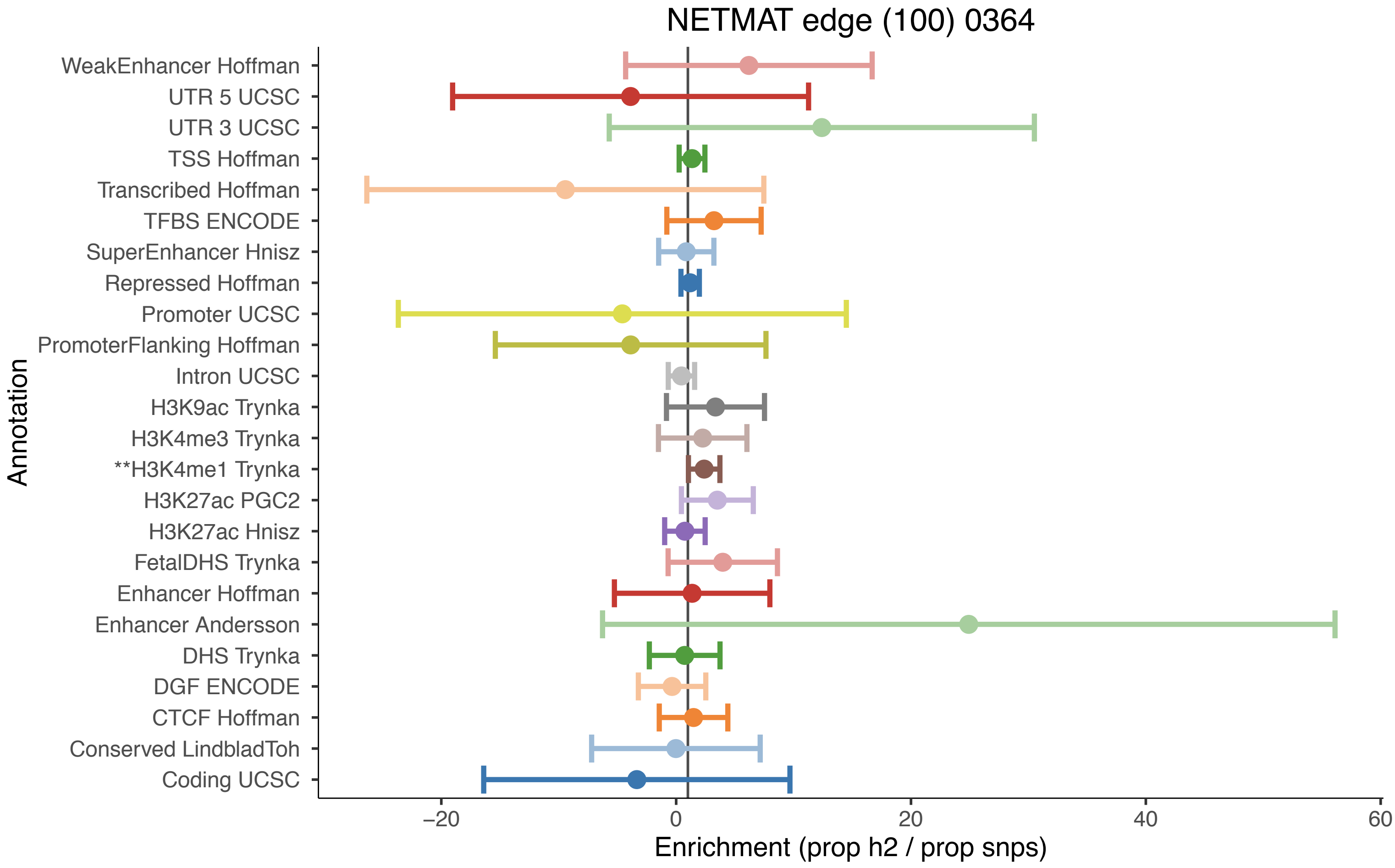
NETMAT edge (100) 0364
WeakEnhancer Hoffman
UTR 5 UCSC
UTR 3 UCSC
TSS Hoffman
Transcribed Hoffman
TFBS ENCODE
SuperEnhancer Hnisz
Repressed Hoffman
Promoter UCSC
PromoterFlanking Hoffman
Intron UCSC
H3K9ac Trynka
H3K4me3 Trynka
**H3K4me1 Trynka
H3K27ac PGC2
H3K27ac Hnisz
FetalDHS Trynka
Enhancer Hoffman
Enhancer Andersson
DHS Trynka
DGF ENCODE
CTCF Hoffman
Conserved LindbladToh
Coding UCSC
Annotation
−20
0
20
40
60
Enrichment (prop h2 / prop snps)

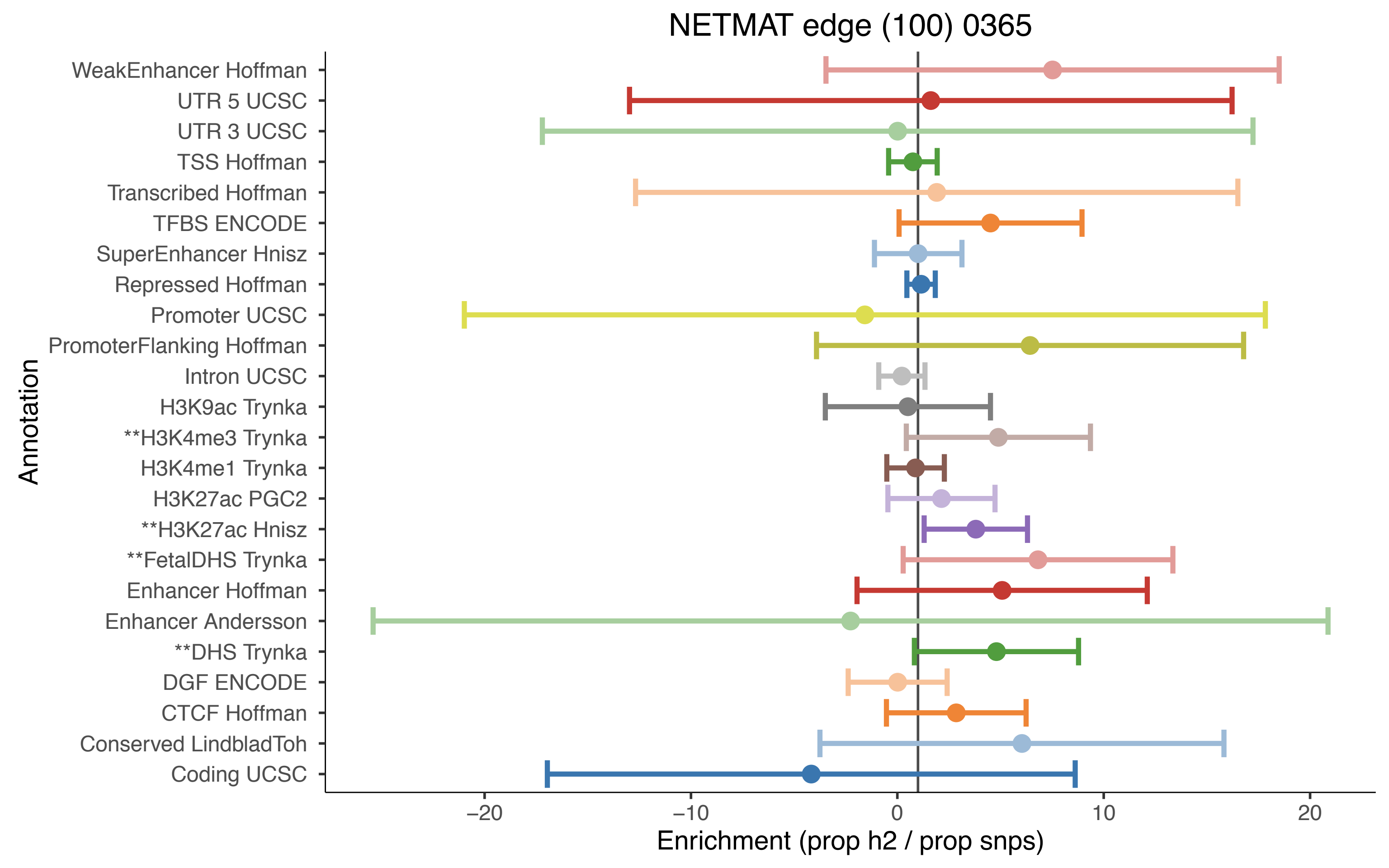

NETMAT edge (100) 0365
WeakEnhancer Hoffman
UTR 5 UCSC
UTR 3 UCSC
TSS Hoffman
Transcribed Hoffman
TFBS ENCODE
SuperEnhancer Hnisz
Repressed Hoffman
Promoter UCSC
PromoterFlanking Hoffman
Intron UCSC
H3K9ac Trynka
**H3K4me3 Trynka
H3K4me1 Trynka
H3K27ac PGC2
**H3K27ac Hnisz
**FetalDHS Trynka
Enhancer Hoffman
Enhancer Andersson
**DHS Trynka
DGF ENCODE
CTCF Hoffman
Conserved LindbladToh
Coding UCSC
Annotation
−20
−10
0
10
20
Enrichment (prop h2 / prop snps)

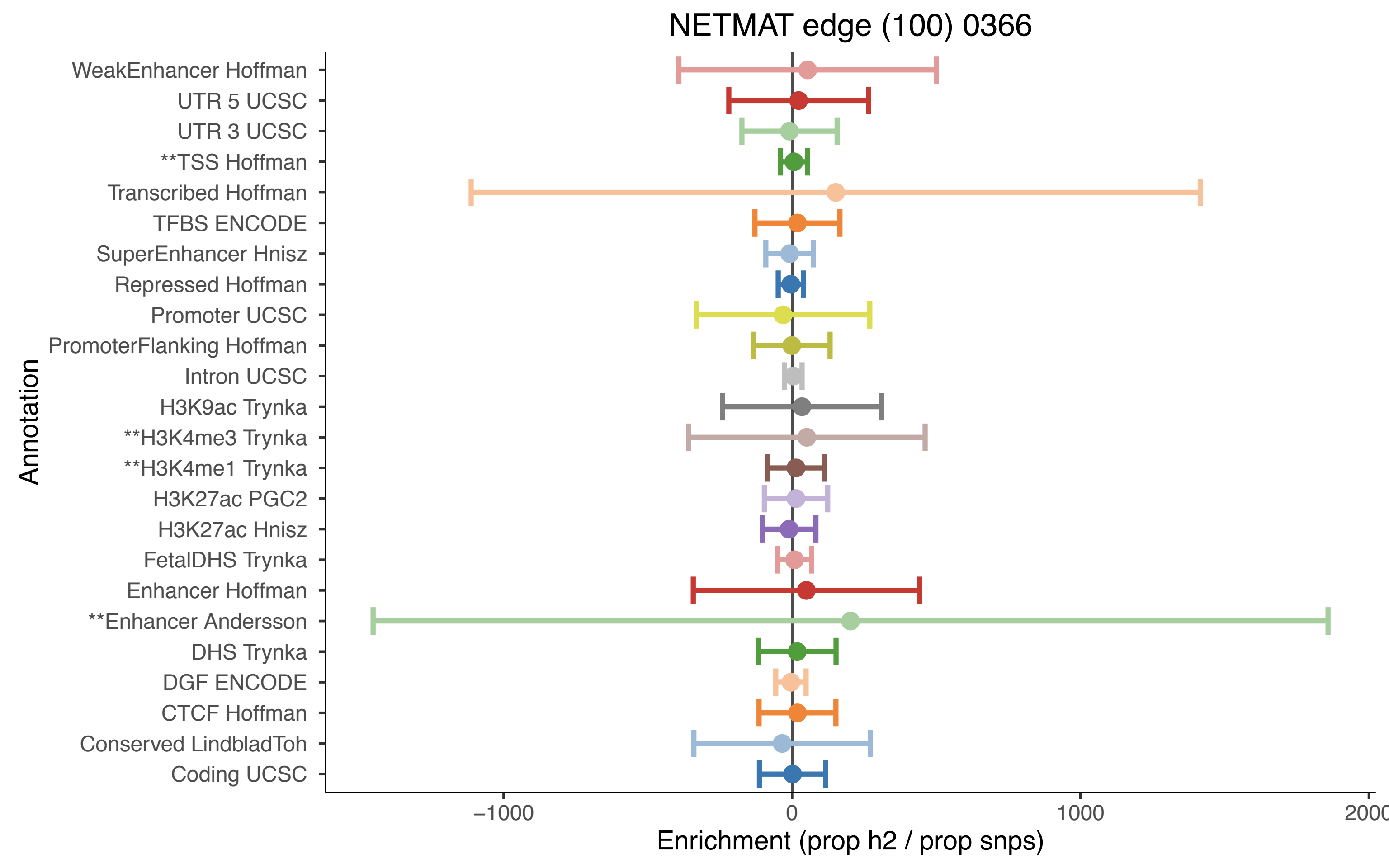
NETMAT edge (100) 0366
WeakEnhancer Hoffman
UTR 5 UCSC
UTR 3 UCSC
**TSS Hoffman
Transcribed Hoffman
TFBS ENCODE
SuperEnhancer Hnisz
Repressed Hoffman
Promoter UCSC
PromoterFlanking Hoffman
Intron UCSC
H3K9ac Trynka
**H3K4me3 Trynka
**H3K4me1 Trynka
H3K27ac PGC2
H3K27ac Hnisz
FetalDHS Trynka
Enhancer Hoffman
**Enhancer Andersson
DHS Trynka
DGF ENCODE
CTCF Hoffman
Conserved LindbladToh
Coding UCSC
Annotation
−1000
0
1000
Enrichment (prop h2 / prop snps)

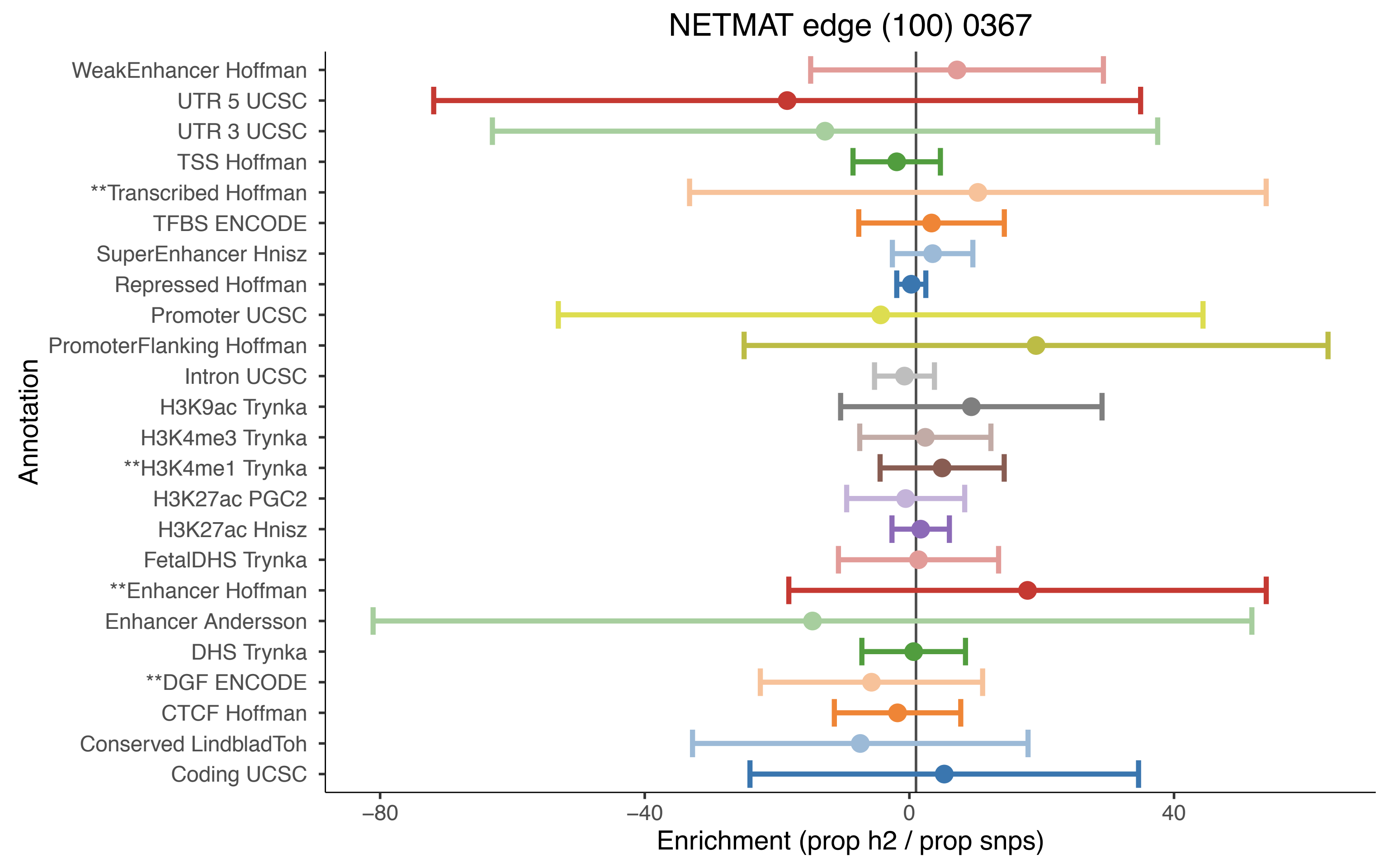
NETMAT edge (100) 0367
WeakEnhancer Hoffman
UTR 5 UCSC
UTR 3 UCSC
TSS Hoffman
**Transcribed Hoffman
TFBS ENCODE
SuperEnhancer Hnisz
Repressed Hoffman
Promoter UCSC
PromoterFlanking Hoffman
Intron UCSC
H3K9ac Trynka
H3K4me3 Trynka
**H3K4me1 Trynka
H3K27ac PGC2
H3K27ac Hnisz
FetalDHS Trynka
**Enhancer Hoffman
Enhancer Andersson
DHS Trynka
**DGF ENCODE
CTCF Hoffman
Conserved LindbladToh
Coding UCSC
Annotation
−80
−40
0
40
Enrichment (prop h2 / prop snps)

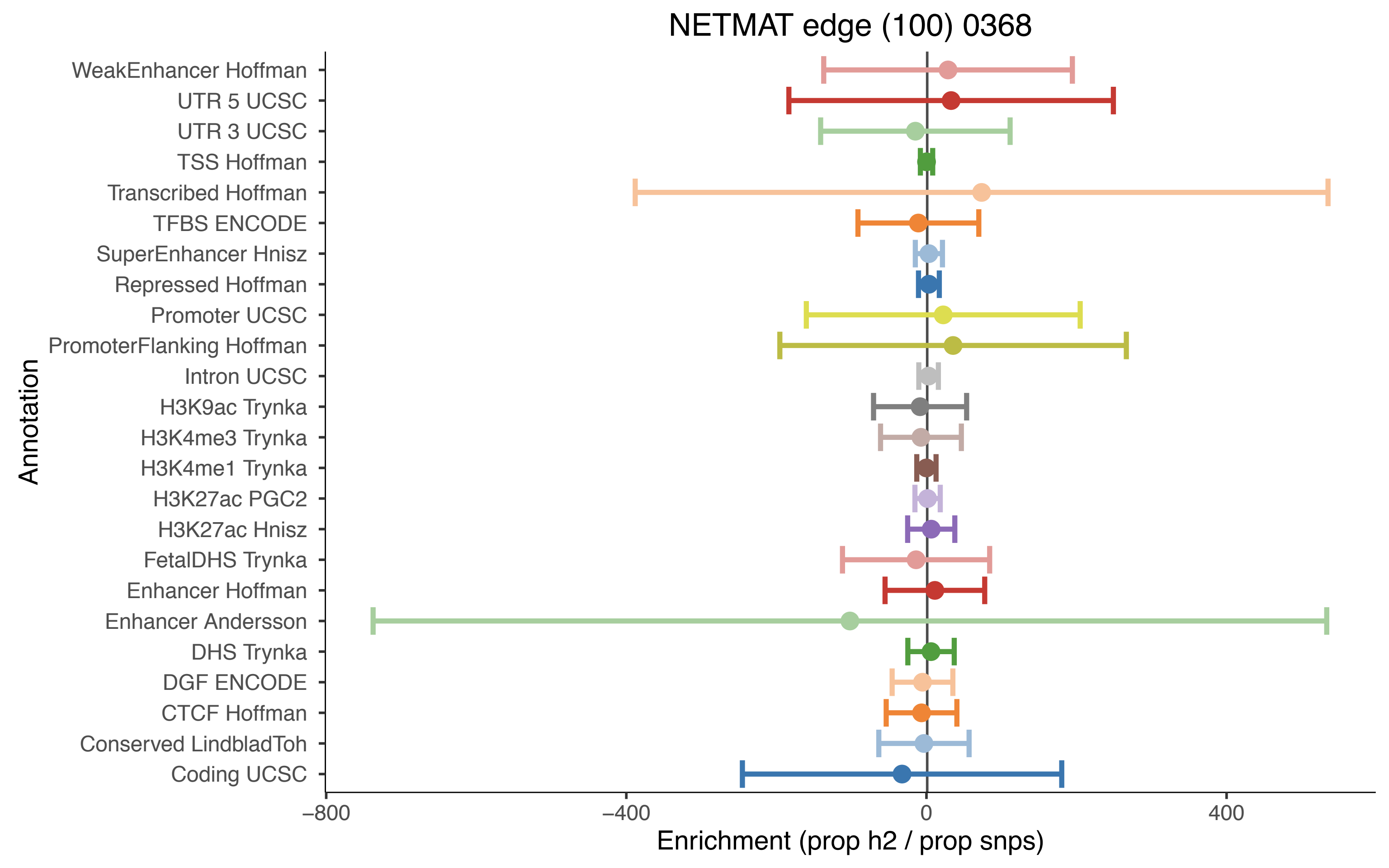

NETMAT edge (100) 0368
Annotation
WeakEnhancer Hoffman
UTR 5 UCSC
UTR 3 UCSC
TSS Hoffman
Transcribed Hoffman
TFBS ENCODE
SuperEnhancer Hnisz
Repressed Hoffman
Promoter UCSC
PromoterFlanking Hoffman
Intron UCSC
H3K9ac Trynka
H3K4me3 Trynka
H3K4me1 Trynka
H3K27ac PGC2
H3K27ac Hnisz
FetalDHS Trynka
Enhancer Hoffman
Enhancer Andersson
DHS Trynka
DGF ENCODE
CTCF Hoffman
Conserved LindbladToh
Coding UCSC
−800
−400
0
400
Enrichment (prop h2 / prop snps)

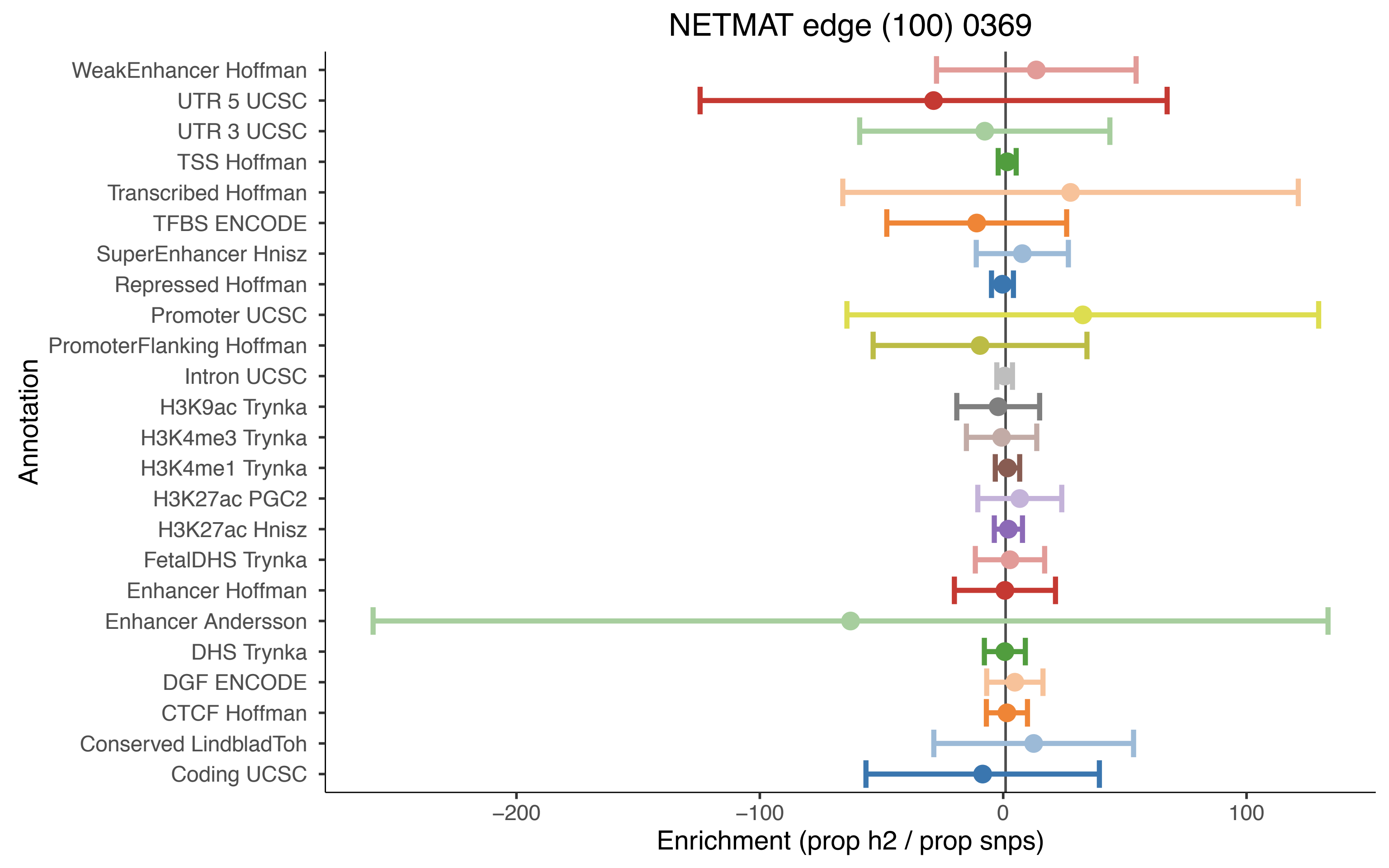

NETMAT edge (100) 0369
WeakEnhancer Hoffman
UTR 5 UCSC
UTR 3 UCSC
TSS Hoffman
Transcribed Hoffman
TFBS ENCODE
SuperEnhancer Hnisz
Repressed Hoffman
Promoter UCSC
PromoterFlanking Hoffman
Intron UCSC
H3K9ac Trynka
H3K4me3 Trynka
H3K4me1 Trynka
H3K27ac PGC2
H3K27ac Hnisz
FetalDHS Trynka
Enhancer Hoffman
Enhancer Andersson
DHS Trynka
DGF ENCODE
CTCF Hoffman
Conserved LindbladToh
Coding UCSC
Annotation
−200
−100
0
100
Enrichment (prop h2 / prop snps)

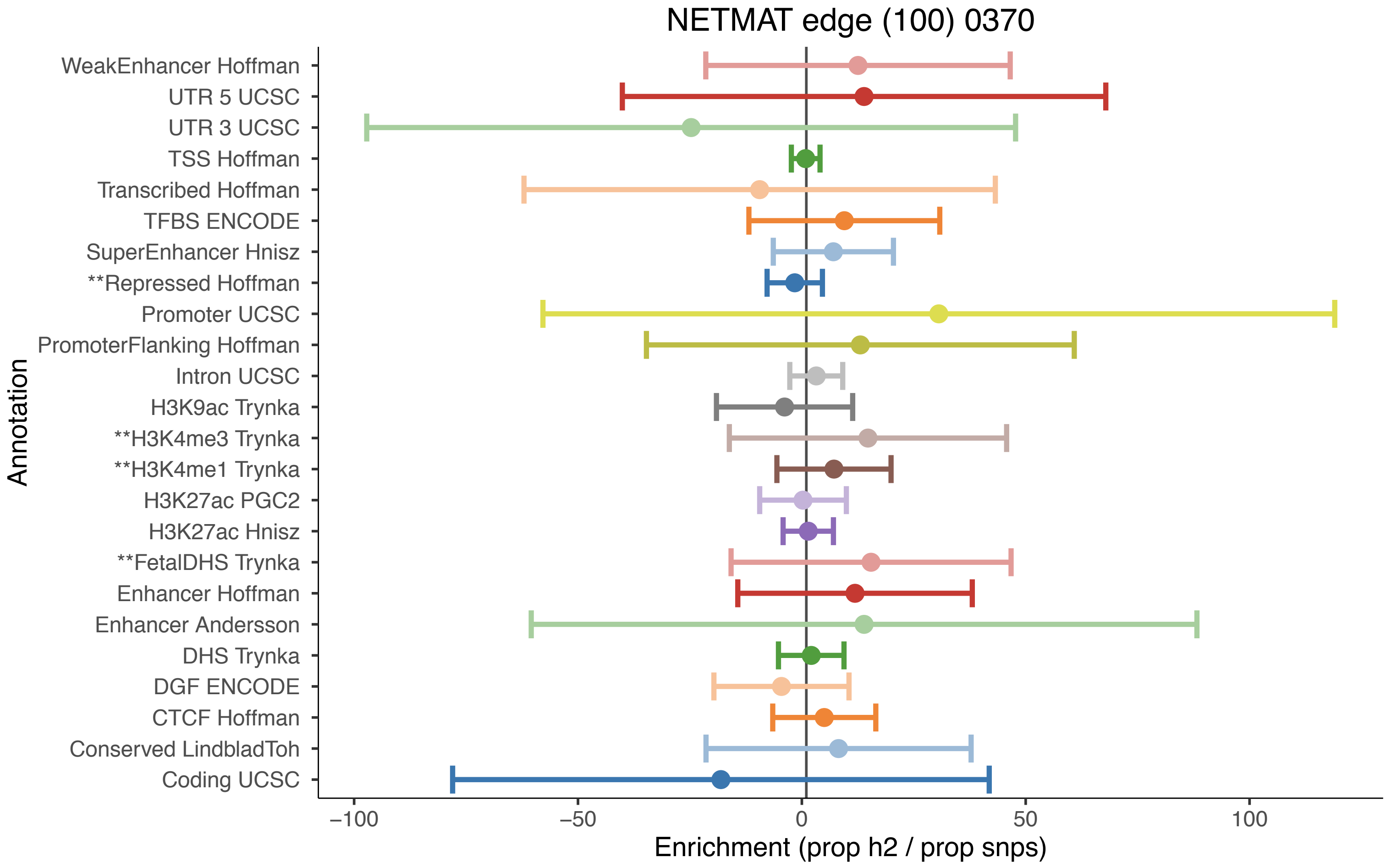

NETMAT edge (100) 0370
WeakEnhancer Hoffman
UTR 5 UCSC
UTR 3 UCSC
TSS Hoffman
Transcribed Hoffman
TFBS ENCODE
SuperEnhancer Hnisz
**Repressed Hoffman
Promoter UCSC
PromoterFlanking Hoffman
Intron UCSC
H3K9ac Trynka
**H3K4me3 Trynka
**H3K4me1 Trynka
H3K27ac PGC2
H3K27ac Hnisz
**FetalDHS Trynka
Enhancer Hoffman
Enhancer Andersson
DHS Trynka
DGF ENCODE
CTCF Hoffman
Conserved LindbladToh
Coding UCSC
Annotation
−100
−50
0
50
100
Enrichment (prop h2 / prop snps)

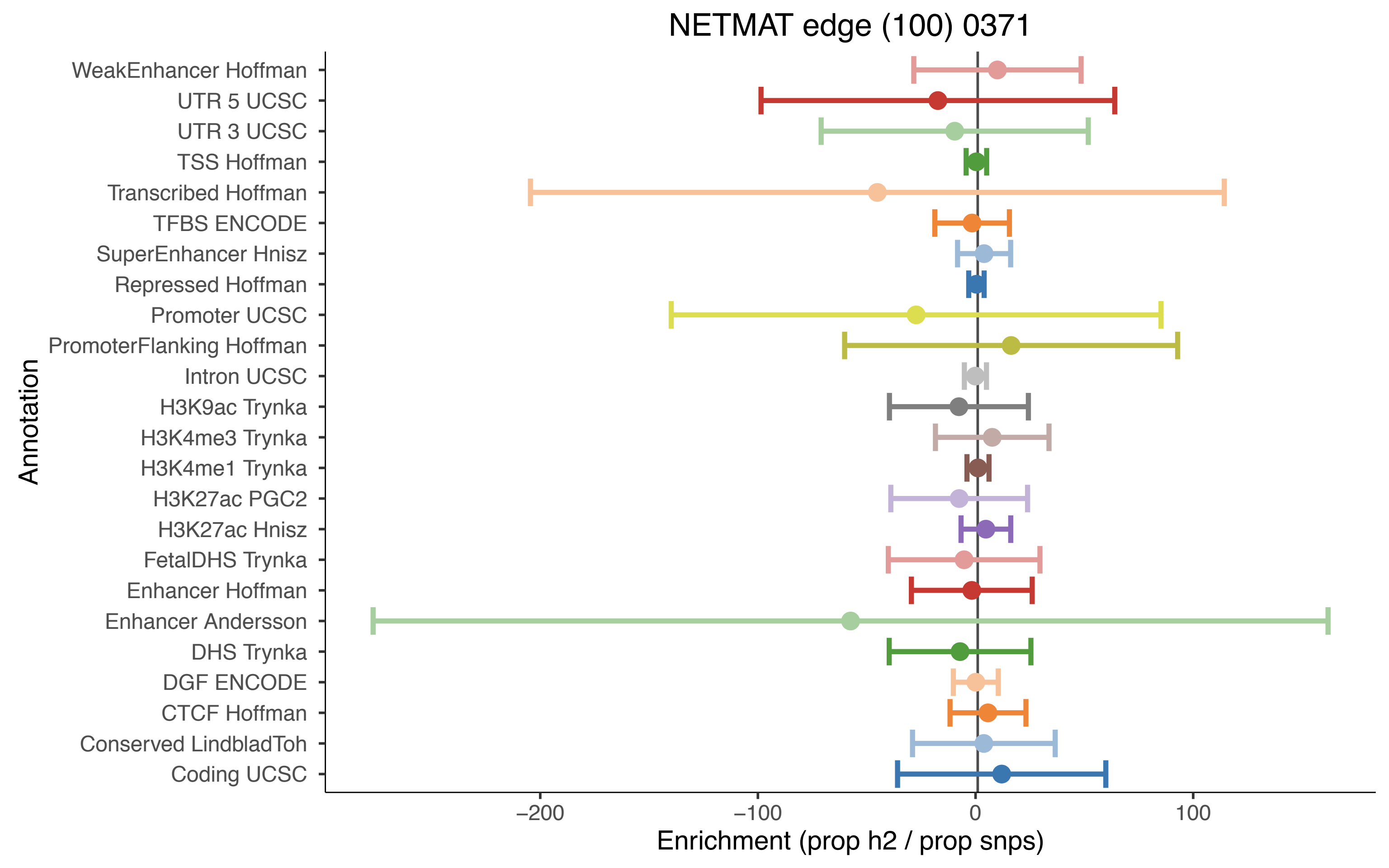
NETMAT edge (100) 0371
Annotation
WeakEnhancer Hoffman
UTR 5 UCSC
UTR 3 UCSC
TSS Hoffman
Transcribed Hoffman
TFBS ENCODE
SuperEnhancer Hnisz
Repressed Hoffman
Promoter UCSC
PromoterFlanking Hoffman
Intron UCSC
H3K9ac Trynka
H3K4me3 Trynka
H3K4me1 Trynka
H3K27ac PGC2
H3K27ac Hnisz
FetalDHS Trynka
Enhancer Hoffman
Enhancer Andersson
DHS Trynka
DGF ENCODE
CTCF Hoffman
Conserved LindbladToh
Coding UCSC
−200
−100
0
100
Enrichment (prop h2 / prop snps)

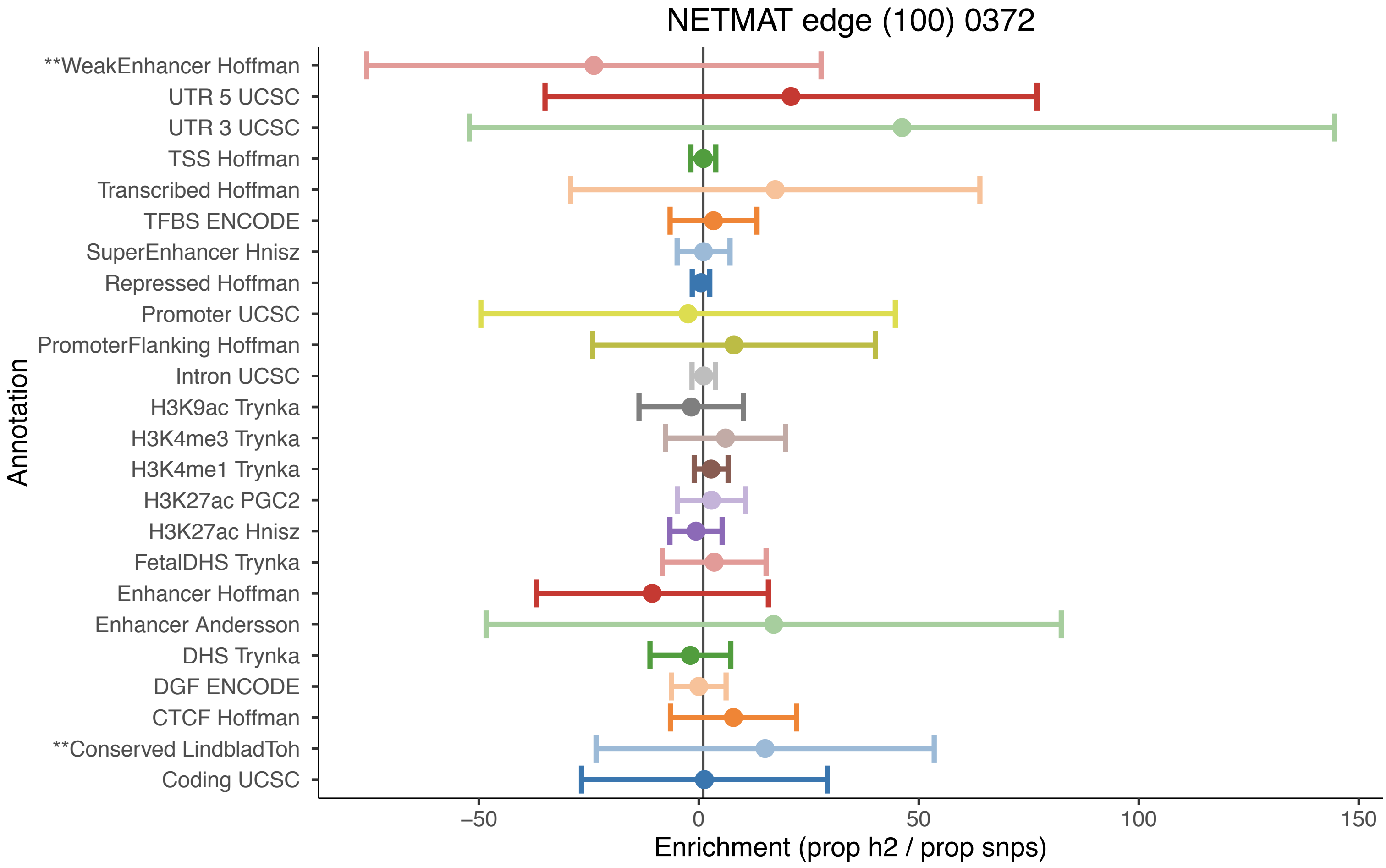

NETMAT edge (100) 0372
**WeakEnhancer Hoffman
UTR 5 UCSC
UTR 3 UCSC
TSS Hoffman
Transcribed Hoffman
TFBS ENCODE
SuperEnhancer Hnisz
Repressed Hoffman
Promoter UCSC
PromoterFlanking Hoffman
Intron UCSC
H3K9ac Trynka
H3K4me3 Trynka
H3K4me1 Trynka
H3K27ac PGC2
H3K27ac Hnisz
FetalDHS Trynka
Enhancer Hoffman
Enhancer Andersson
DHS Trynka
DGF ENCODE
CTCF Hoffman
**Conserved LindbladToh
Coding UCSC
Annotation
−50
0
50
100
150
Enrichment (prop h2 / prop snps)

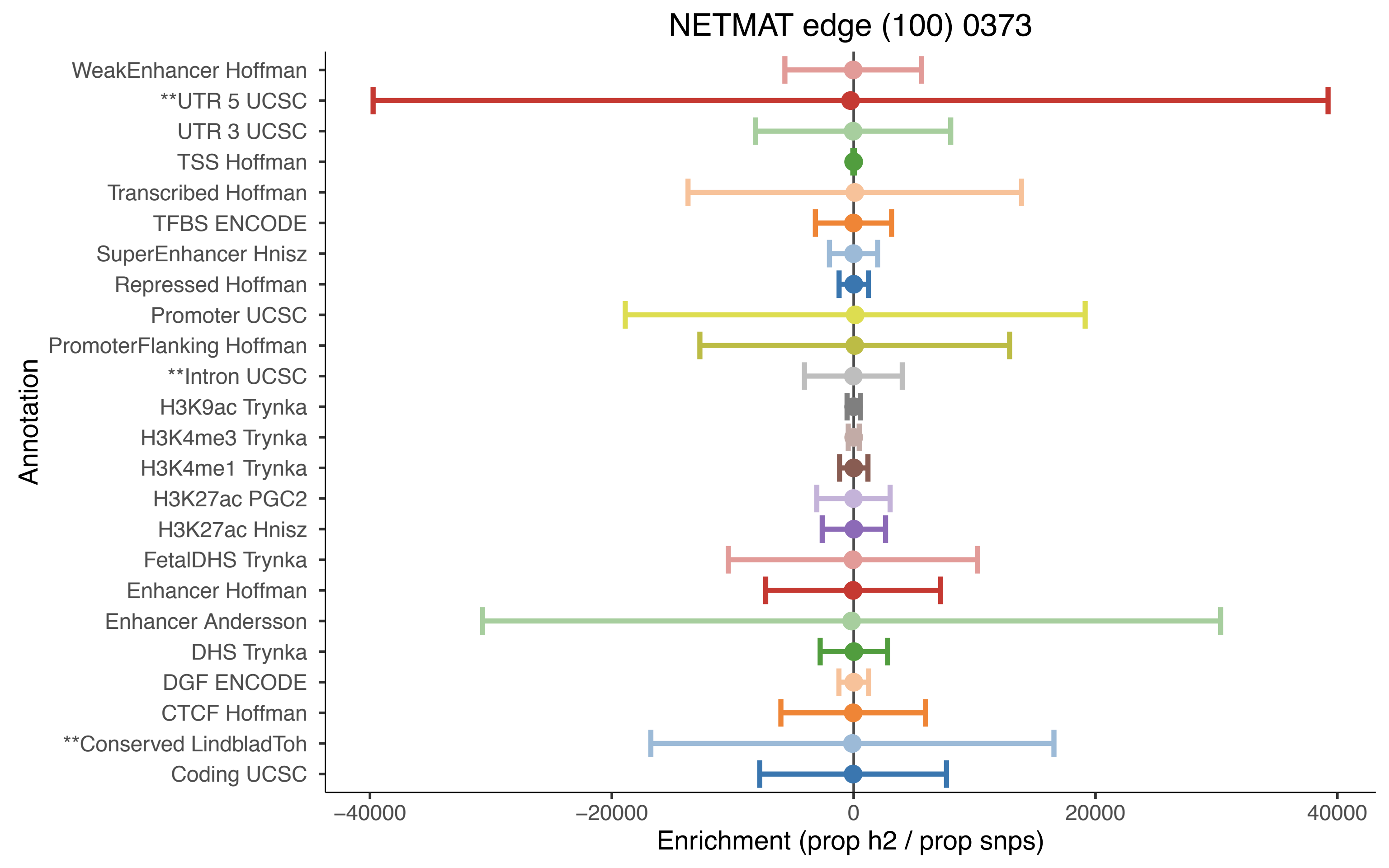

NETMAT edge (100) 0373
WeakEnhancer Hoffman
**UTR 5 UCSC
UTR 3 UCSC
TSS Hoffman
Transcribed Hoffman
TFBS ENCODE
SuperEnhancer Hnisz
Repressed Hoffman
Promoter UCSC
PromoterFlanking Hoffman
**Intron UCSC
H3K9ac Trynka
H3K4me3 Trynka
H3K4me1 Trynka
H3K27ac PGC2
H3K27ac Hnisz
FetalDHS Trynka
Enhancer Hoffman
Enhancer Andersson
DHS Trynka
DGF ENCODE
CTCF Hoffman
**Conserved LindbladToh
Coding UCSC
Annotation
−40000
−20000
0
20000
40000
Enrichment (prop h2 / prop snps)

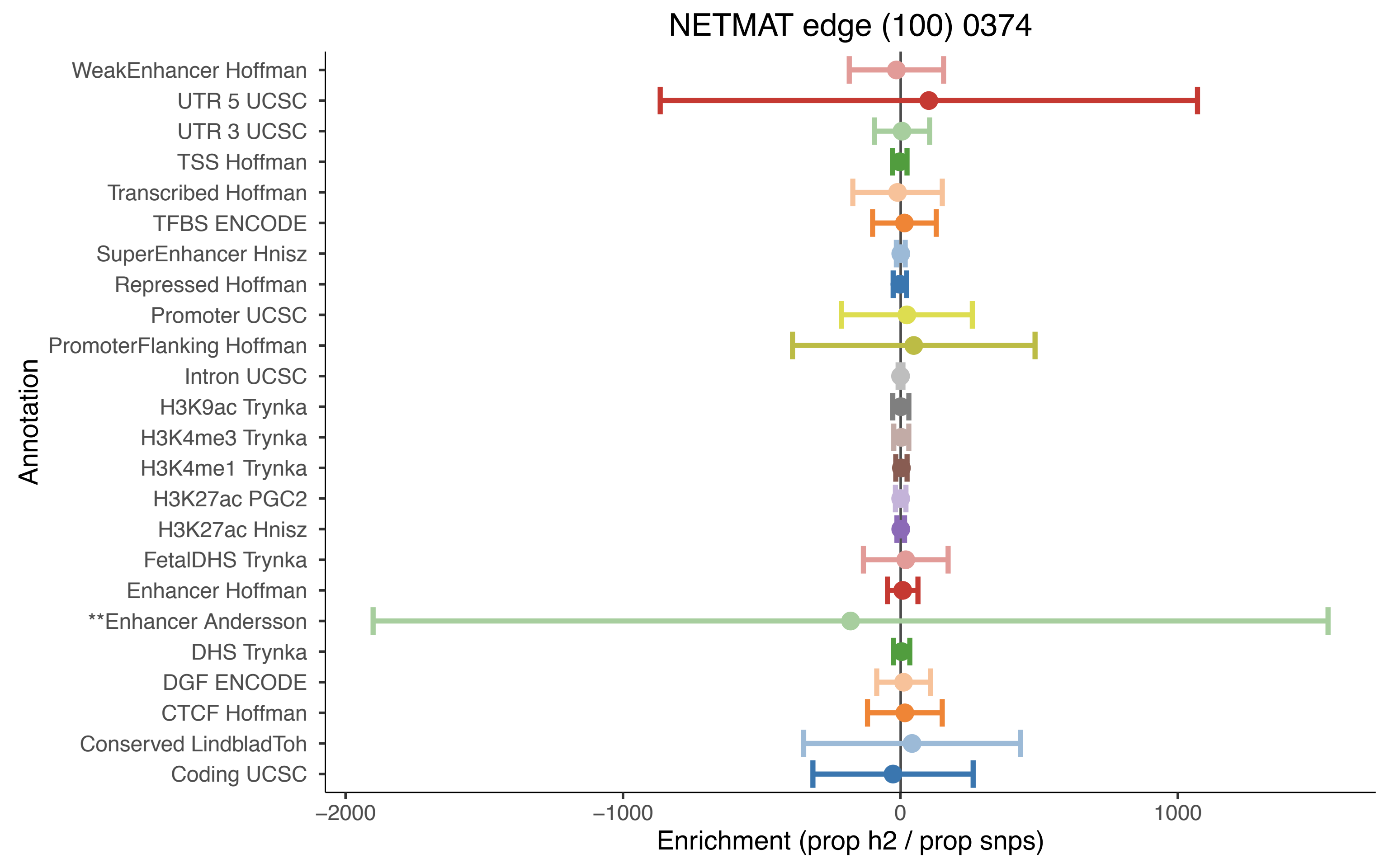

NETMAT edge (100) 0374
Annotation
WeakEnhancer Hoffman
UTR 5 UCSC
UTR 3 UCSC
TSS Hoffman
Transcribed Hoffman
TFBS ENCODE
SuperEnhancer Hnisz
Repressed Hoffman
Promoter UCSC
PromoterFlanking Hoffman
Intron UCSC
H3K9ac Trynka
H3K4me3 Trynka
H3K4me1 Trynka
H3K27ac PGC2
H3K27ac Hnisz
FetalDHS Trynka
Enhancer Hoffman
**Enhancer Andersson
DHS Trynka
DGF ENCODE
CTCF Hoffman
Conserved LindbladToh
Coding UCSC
−2000
−1000
0
1000
Enrichment (prop h2 / prop snps)

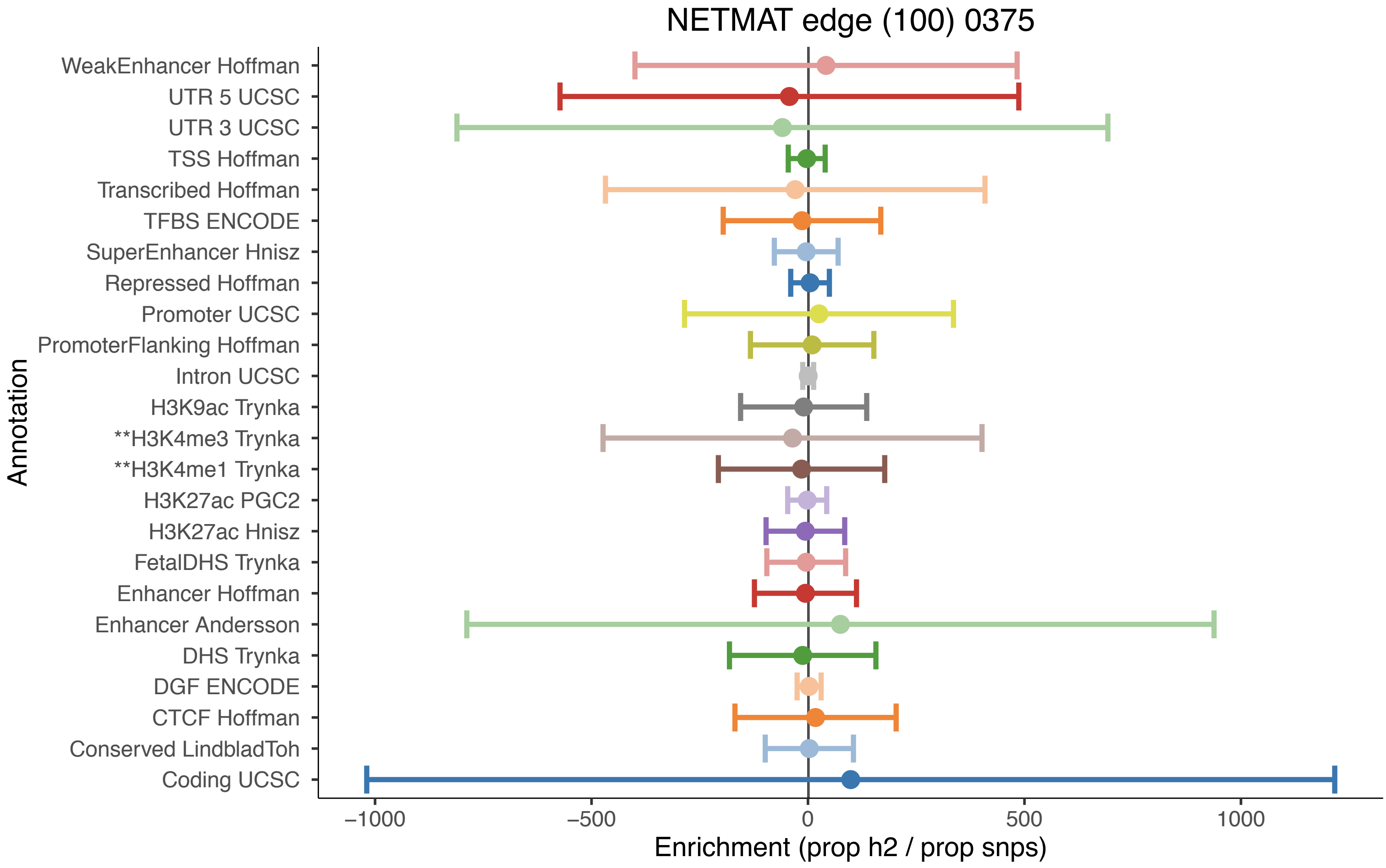
NETMAT edge (100) 0375
WeakEnhancer Hoffman
UTR 5 UCSC
UTR 3 UCSC
TSS Hoffman
Transcribed Hoffman
TFBS ENCODE
SuperEnhancer Hnisz
Repressed Hoffman
Promoter UCSC
PromoterFlanking Hoffman
Intron UCSC
H3K9ac Trynka
**H3K4me3 Trynka
**H3K4me1 Trynka
H3K27ac PGC2
H3K27ac Hnisz
FetalDHS Trynka
Enhancer Hoffman
Enhancer Andersson
DHS Trynka
DGF ENCODE
CTCF Hoffman
Conserved LindbladToh
Coding UCSC
Annotation
−1000
−500
0
500
1000
Enrichment (prop h2 / prop snps)

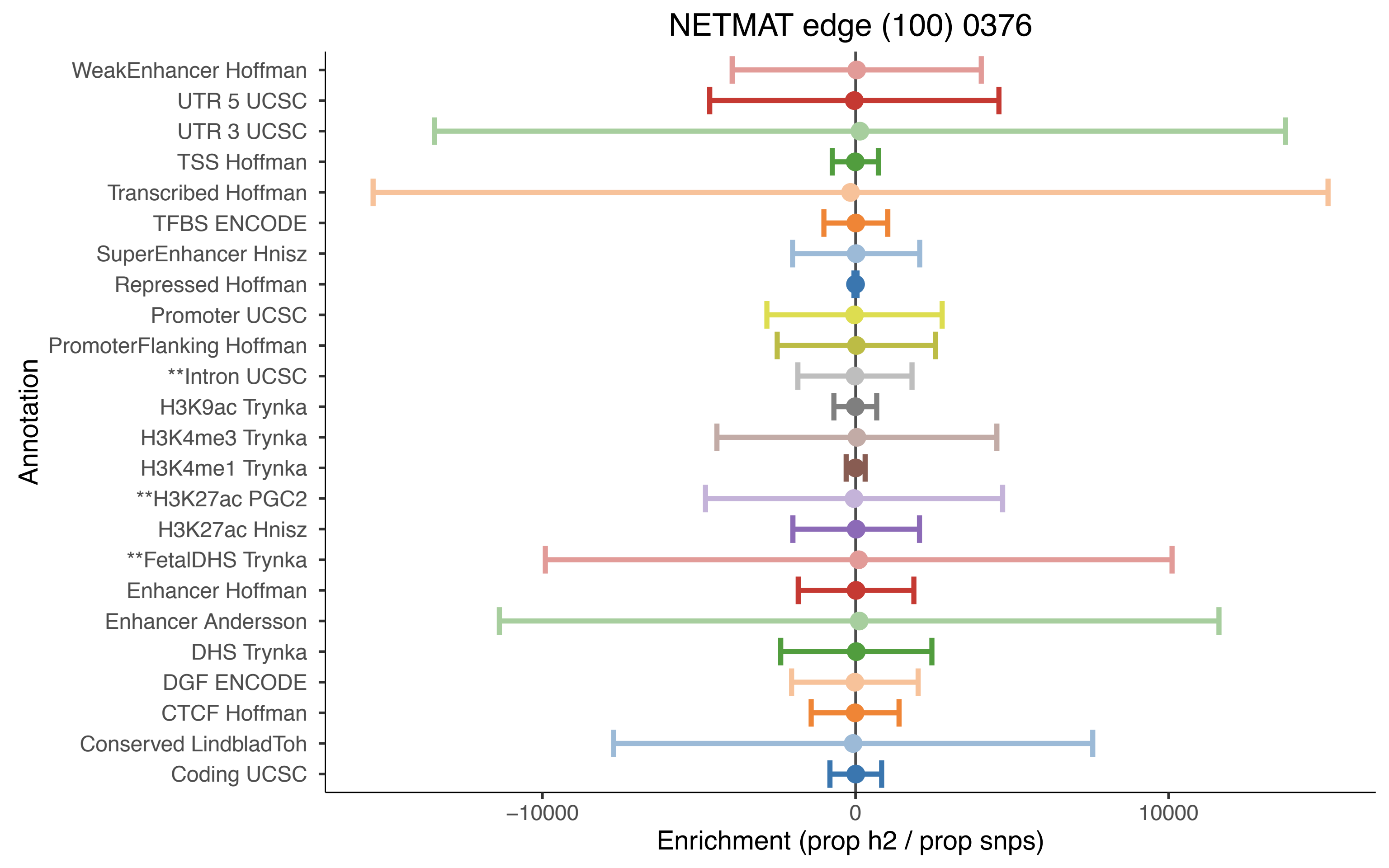

NETMAT edge (100) 0376
WeakEnhancer Hoffman
UTR 5 UCSC
UTR 3 UCSC
TSS Hoffman
Transcribed Hoffman
TFBS ENCODE
SuperEnhancer Hnisz
Repressed Hoffman
Promoter UCSC
PromoterFlanking Hoffman
**Intron UCSC
H3K9ac Trynka
H3K4me3 Trynka
H3K4me1 Trynka
**H3K27ac PGC2
H3K27ac Hnisz
**FetalDHS Trynka
Enhancer Hoffman
Enhancer Andersson
DHS Trynka
DGF ENCODE
CTCF Hoffman
Conserved LindbladToh
Coding UCSC
Annotation
−10000
0
10000
Enrichment (prop h2 / prop snps)

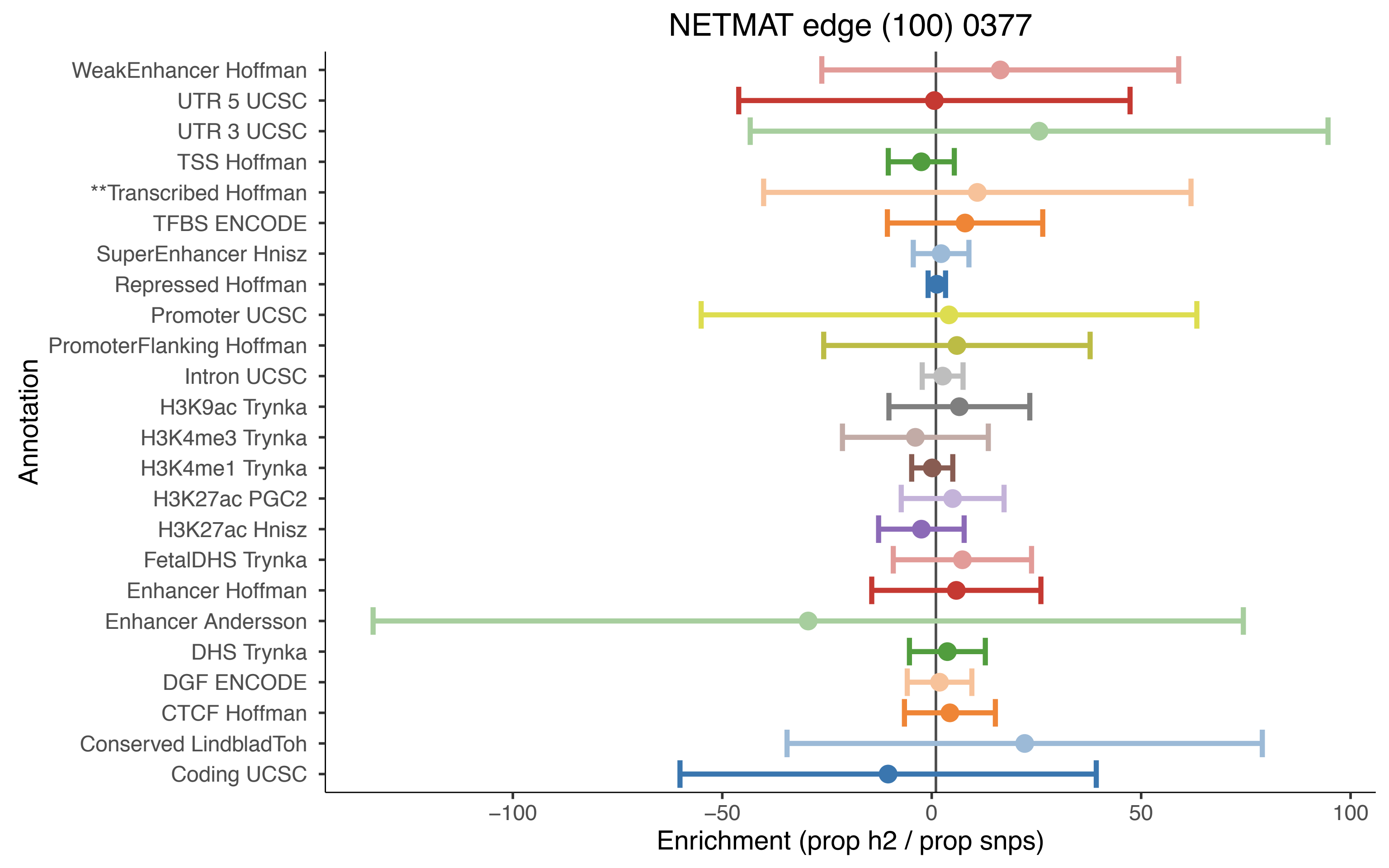
NETMAT edge (100) 0377
WeakEnhancer Hoffman
UTR 5 UCSC
UTR 3 UCSC
TSS Hoffman
**Transcribed Hoffman
TFBS ENCODE
SuperEnhancer Hnisz
Repressed Hoffman
Promoter UCSC
PromoterFlanking Hoffman
Intron UCSC
H3K9ac Trynka
H3K4me3 Trynka
H3K4me1 Trynka
H3K27ac PGC2
H3K27ac Hnisz
FetalDHS Trynka
Enhancer Hoffman
Enhancer Andersson
DHS Trynka
DGF ENCODE
CTCF Hoffman
Conserved LindbladToh
Coding UCSC
Annotation
−100
−50
0
50
100
Enrichment (prop h2 / prop snps)

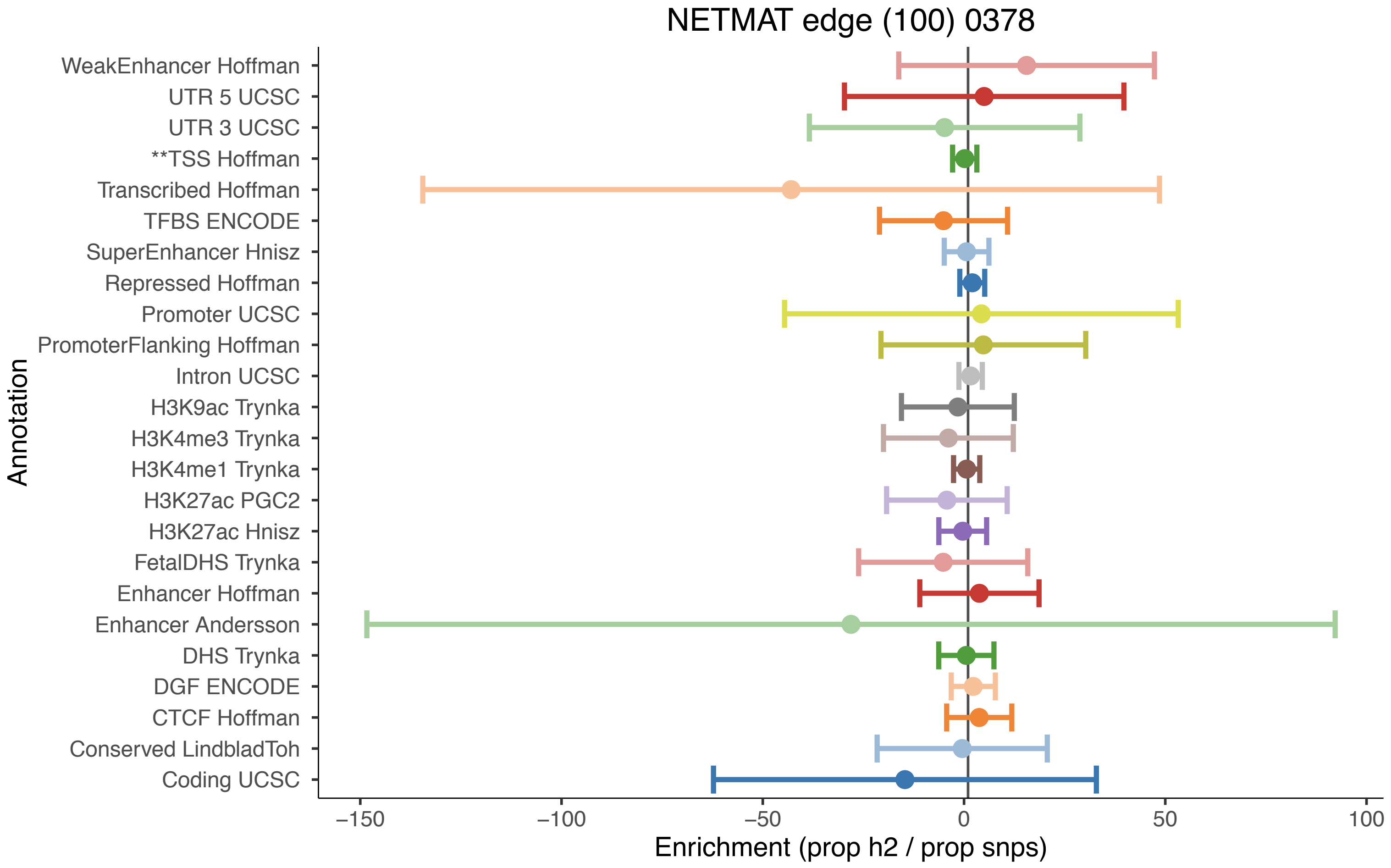

NETMAT edge (100) 0378
WeakEnhancer Hoffman
UTR 5 UCSC
UTR 3 UCSC
**TSS Hoffman
Transcribed Hoffman
TFBS ENCODE
SuperEnhancer Hnisz
Repressed Hoffman
Promoter UCSC
PromoterFlanking Hoffman
Intron UCSC
H3K9ac Trynka
H3K4me3 Trynka
H3K4me1 Trynka
H3K27ac PGC2
H3K27ac Hnisz
FetalDHS Trynka
Enhancer Hoffman
Enhancer Andersson
DHS Trynka
DGF ENCODE
CTCF Hoffman
Conserved LindbladToh
Coding UCSC
Annotation
−150
−100
−50
0
50
100
Enrichment (prop h2 / prop snps)

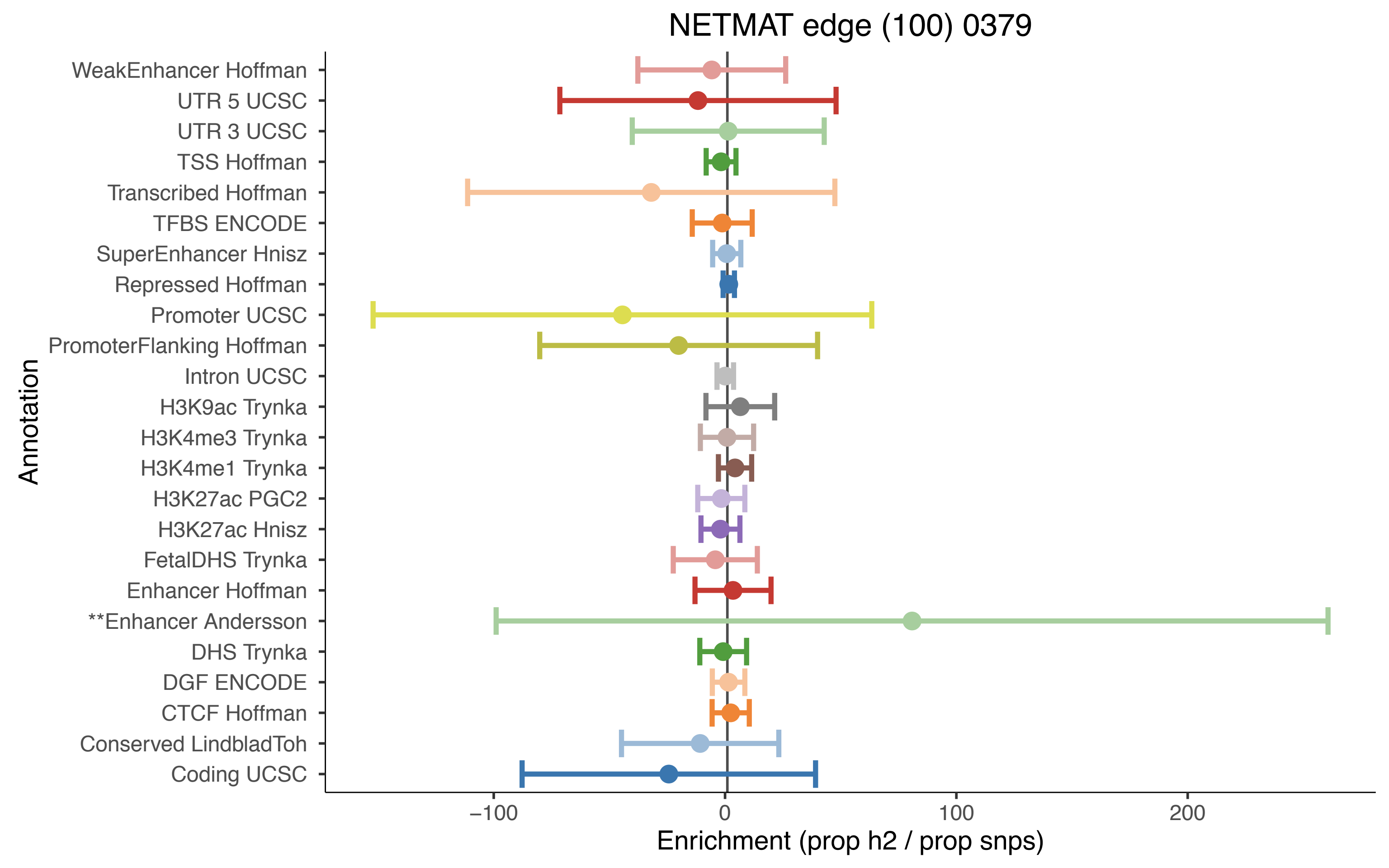
NETMAT edge (100) 0379
WeakEnhancer Hoffman
UTR 5 UCSC
UTR 3 UCSC
TSS Hoffman
Transcribed Hoffman
TFBS ENCODE
SuperEnhancer Hnisz
Repressed Hoffman
Promoter UCSC
PromoterFlanking Hoffman
Intron UCSC
H3K9ac Trynka
H3K4me3 Trynka
H3K4me1 Trynka
H3K27ac PGC2
H3K27ac Hnisz
FetalDHS Trynka
Enhancer Hoffman
**Enhancer Andersson
DHS Trynka
DGF ENCODE
CTCF Hoffman
Conserved LindbladToh
Coding UCSC
Annotation
−100
0
100
200
Enrichment (prop h2 / prop snps)

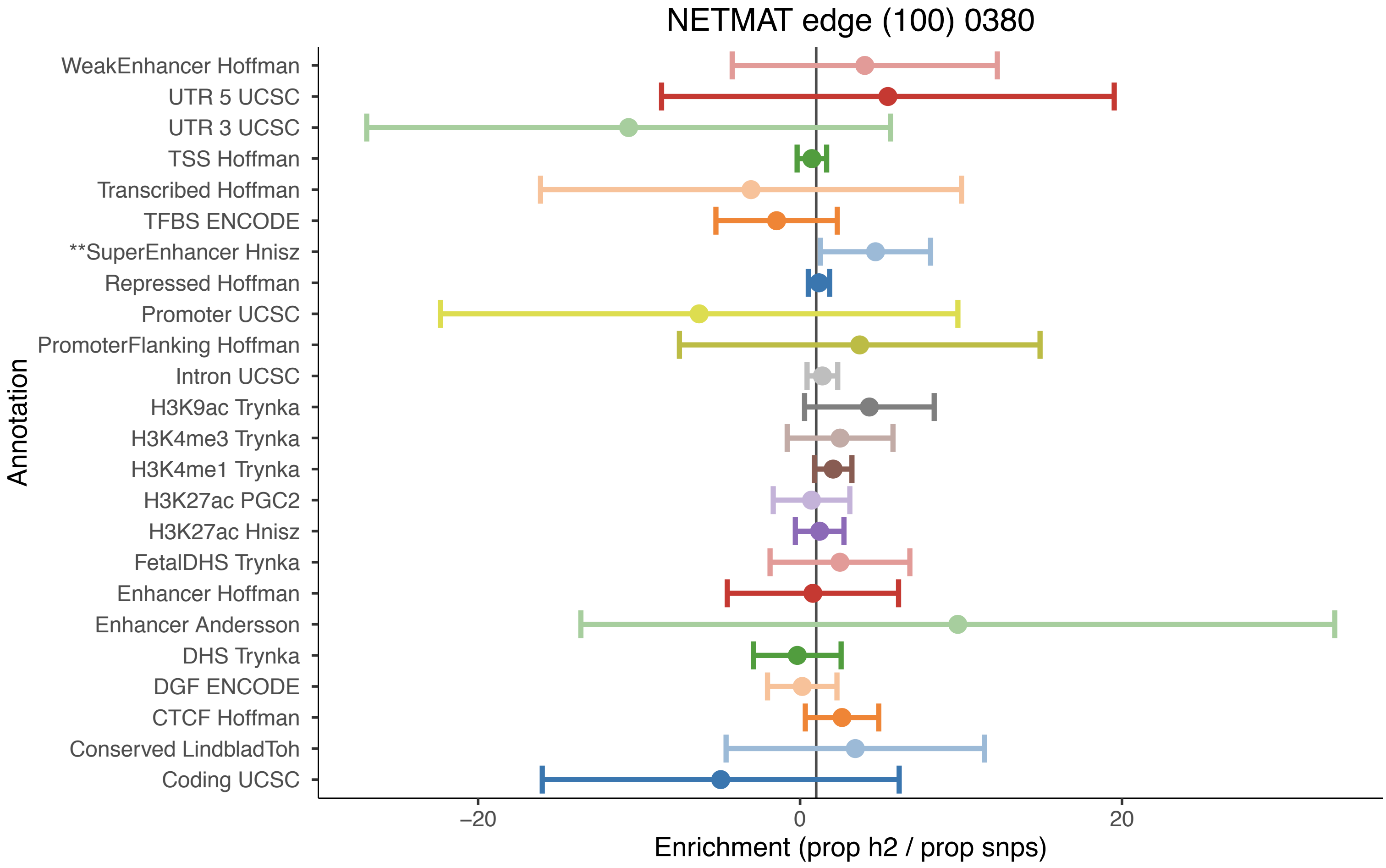
NETMAT edge (100) 0380
Annotation
WeakEnhancer Hoffman
UTR 5 UCSC
UTR 3 UCSC
TSS Hoffman
Transcribed Hoffman
TFBS ENCODE
**SuperEnhancer Hnisz
Repressed Hoffman
Promoter UCSC
PromoterFlanking Hoffman
Intron UCSC
H3K9ac Trynka
H3K4me3 Trynka
H3K4me1 Trynka
H3K27ac PGC2
H3K27ac Hnisz
FetalDHS Trynka
Enhancer Hoffman
Enhancer Andersson
DHS Trynka
DGF ENCODE
CTCF Hoffman
Conserved LindbladToh
Coding UCSC
−20
0
20
Enrichment (prop h2 / prop snps)

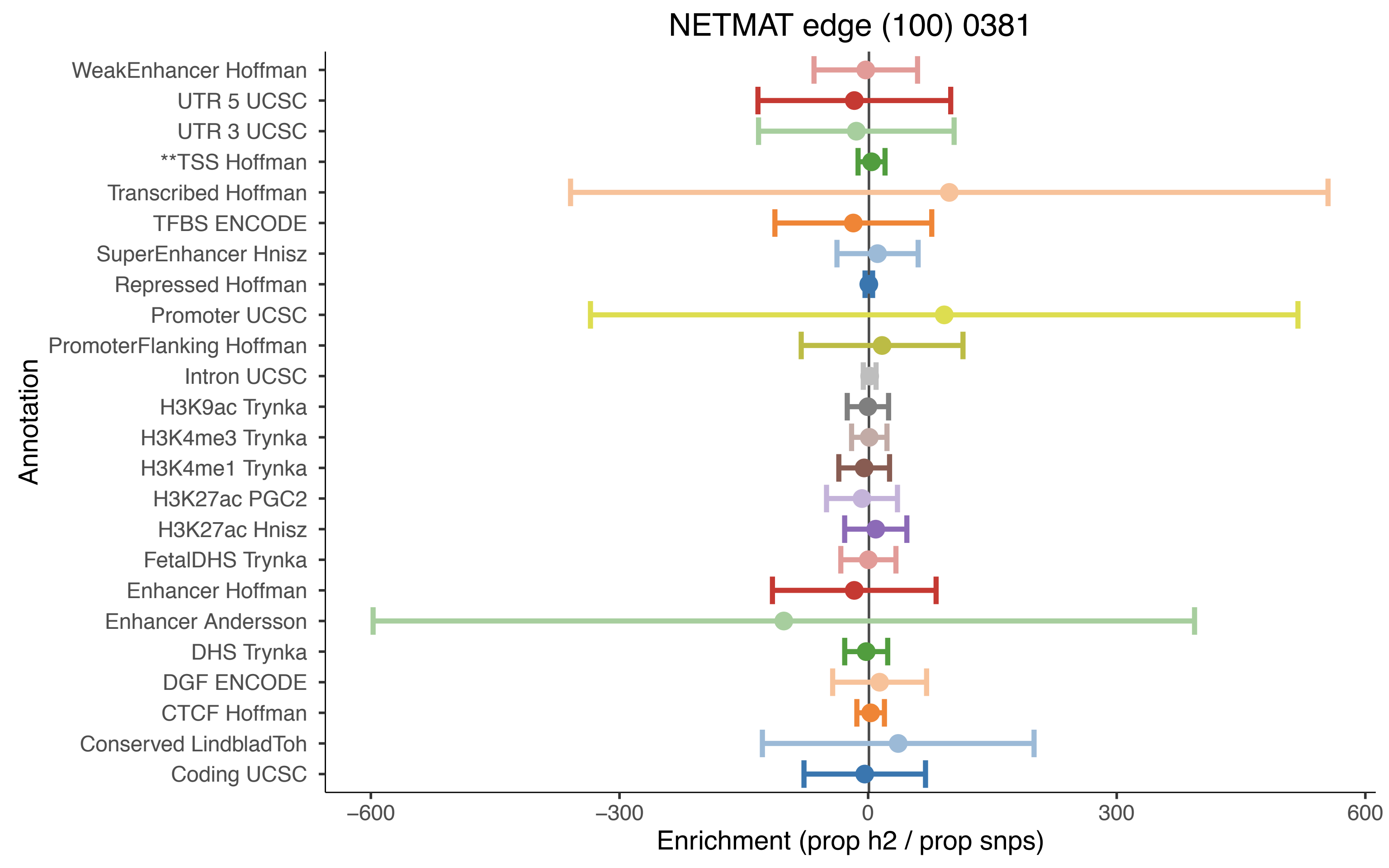

NETMAT edge (100) 0381
WeakEnhancer Hoffman
UTR 5 UCSC
UTR 3 UCSC
**TSS Hoffman
Transcribed Hoffman
TFBS ENCODE
SuperEnhancer Hnisz
Repressed Hoffman
Promoter UCSC
PromoterFlanking Hoffman
Intron UCSC
H3K9ac Trynka
H3K4me3 Trynka
H3K4me1 Trynka
H3K27ac PGC2
H3K27ac Hnisz
FetalDHS Trynka
Enhancer Hoffman
Enhancer Andersson
DHS Trynka
DGF ENCODE
CTCF Hoffman
Conserved LindbladToh
Coding UCSC
Annotation
−600
−300
0
300
600
Enrichment (prop h2 / prop snps)

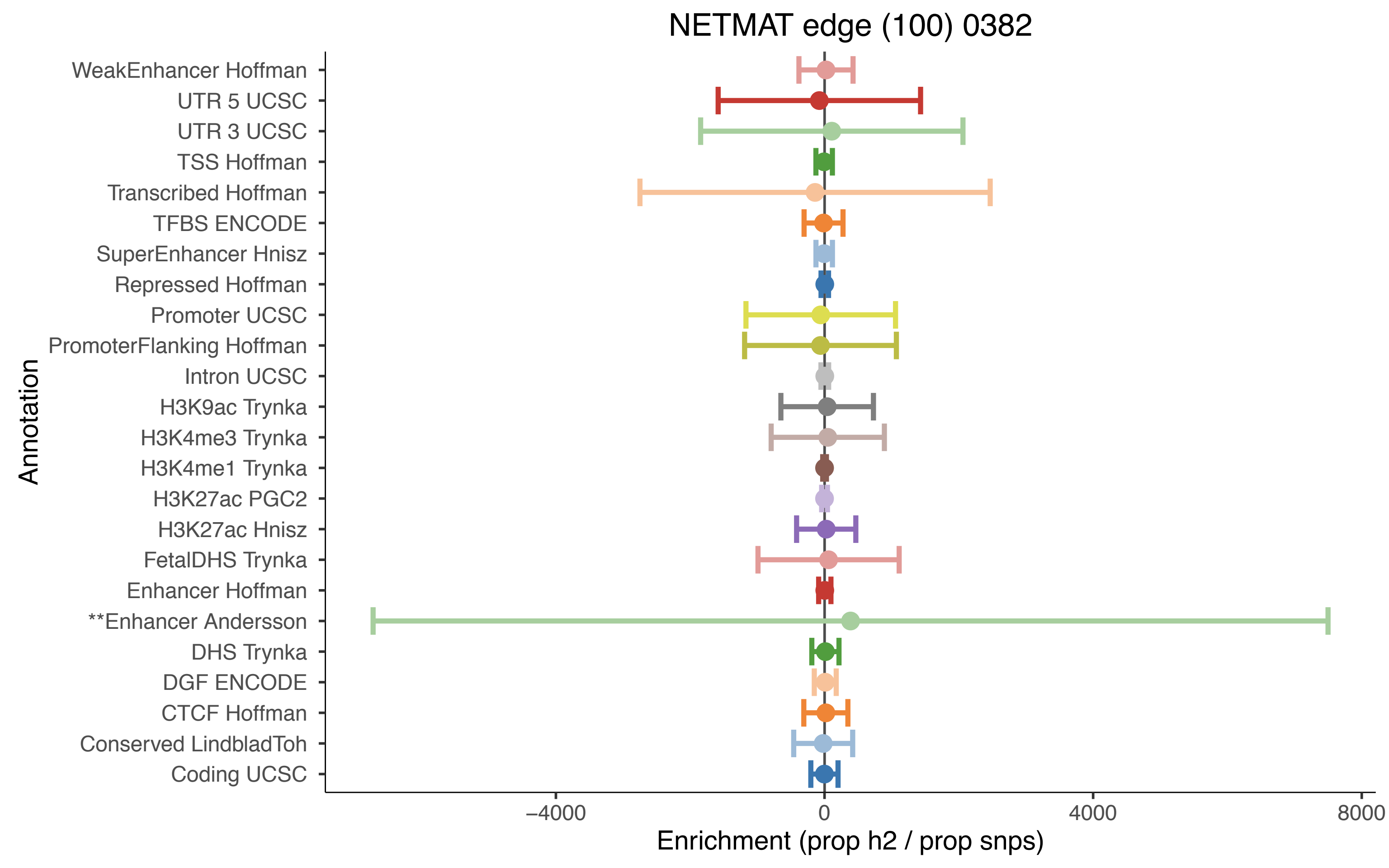

NETMAT edge (100) 0382
Annotation
WeakEnhancer Hoffman
UTR 5 UCSC
UTR 3 UCSC
TSS Hoffman
Transcribed Hoffman
TFBS ENCODE
SuperEnhancer Hnisz
Repressed Hoffman
Promoter UCSC
PromoterFlanking Hoffman
Intron UCSC
H3K9ac Trynka
H3K4me3 Trynka
H3K4me1 Trynka
H3K27ac PGC2
H3K27ac Hnisz
FetalDHS Trynka
Enhancer Hoffman
**Enhancer Andersson
DHS Trynka
DGF ENCODE
CTCF Hoffman
Conserved LindbladToh
Coding UCSC
−4000
0
4000
8000
Enrichment (prop h2 / prop snps)

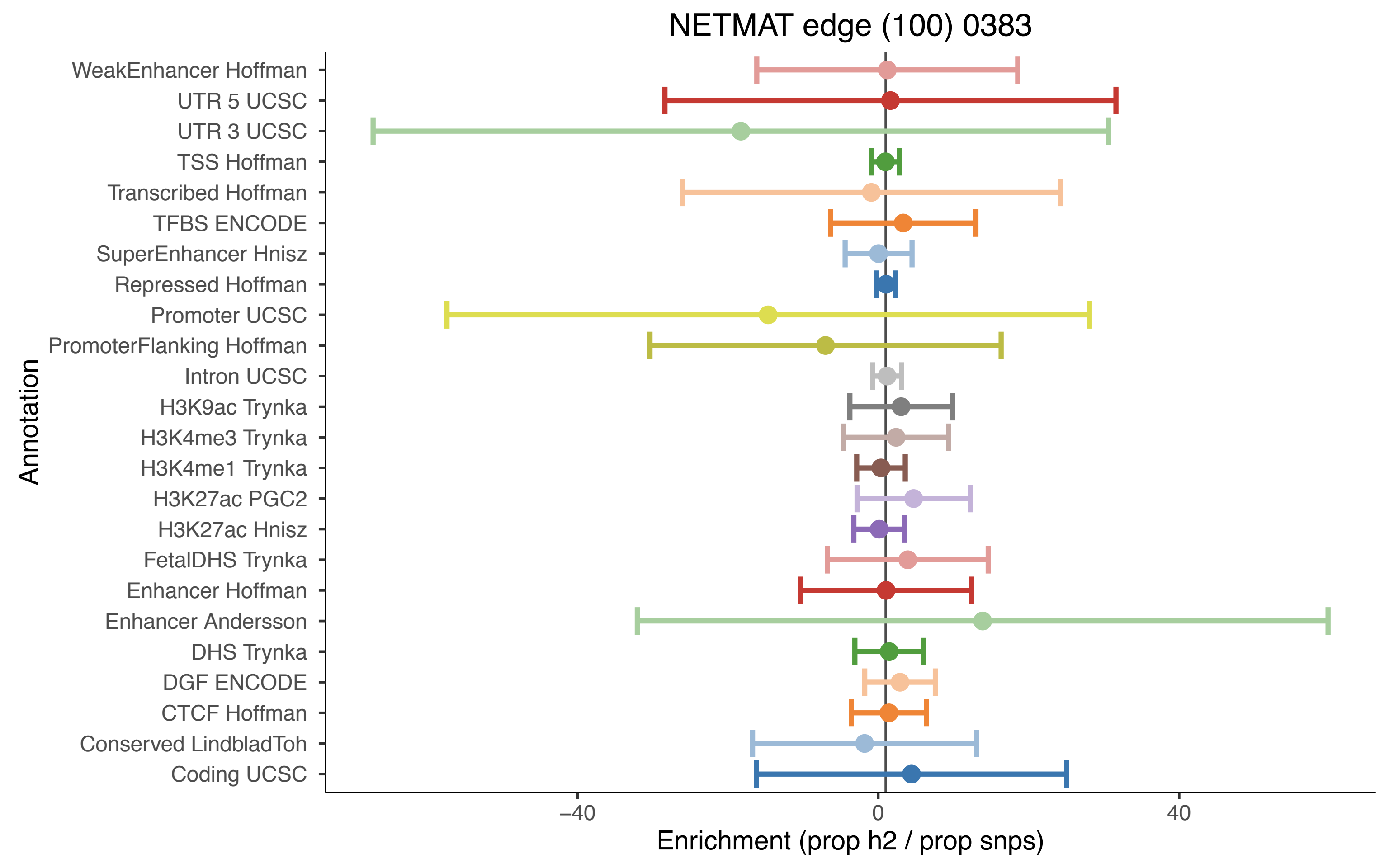
NETMAT edge (100) 0383
WeakEnhancer Hoffman
UTR 5 UCSC
UTR 3 UCSC
TSS Hoffman
Transcribed Hoffman
TFBS ENCODE
SuperEnhancer Hnisz
Repressed Hoffman
Promoter UCSC
PromoterFlanking Hoffman
Intron UCSC
H3K9ac Trynka
H3K4me3 Trynka
H3K4me1 Trynka
H3K27ac PGC2
H3K27ac Hnisz
FetalDHS Trynka
Enhancer Hoffman
Enhancer Andersson
DHS Trynka
DGF ENCODE
CTCF Hoffman
Conserved LindbladToh
Coding UCSC
Annotation
−40
0
40
Enrichment (prop h2 / prop snps)

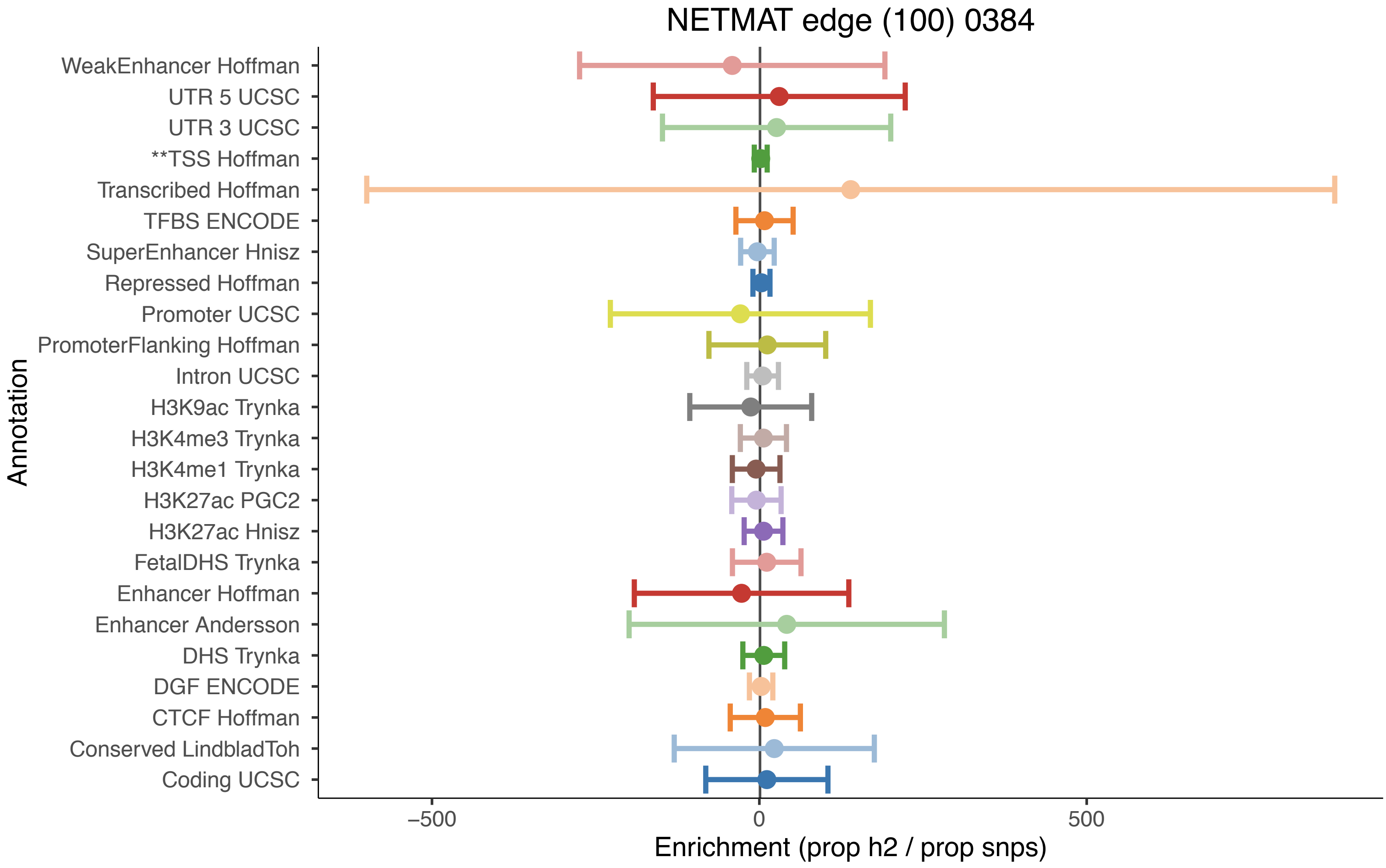
NETMAT edge (100) 0384
WeakEnhancer Hoffman
UTR 5 UCSC
UTR 3 UCSC
**TSS Hoffman
Transcribed Hoffman
TFBS ENCODE
SuperEnhancer Hnisz
Repressed Hoffman
Promoter UCSC
PromoterFlanking Hoffman
Intron UCSC
H3K9ac Trynka
H3K4me3 Trynka
H3K4me1 Trynka
H3K27ac PGC2
H3K27ac Hnisz
FetalDHS Trynka
Enhancer Hoffman
Enhancer Andersson
DHS Trynka
DGF ENCODE
CTCF Hoffman
Conserved LindbladToh
Coding UCSC
Annotation
−500
0
500
Enrichment (prop h2 / prop snps)

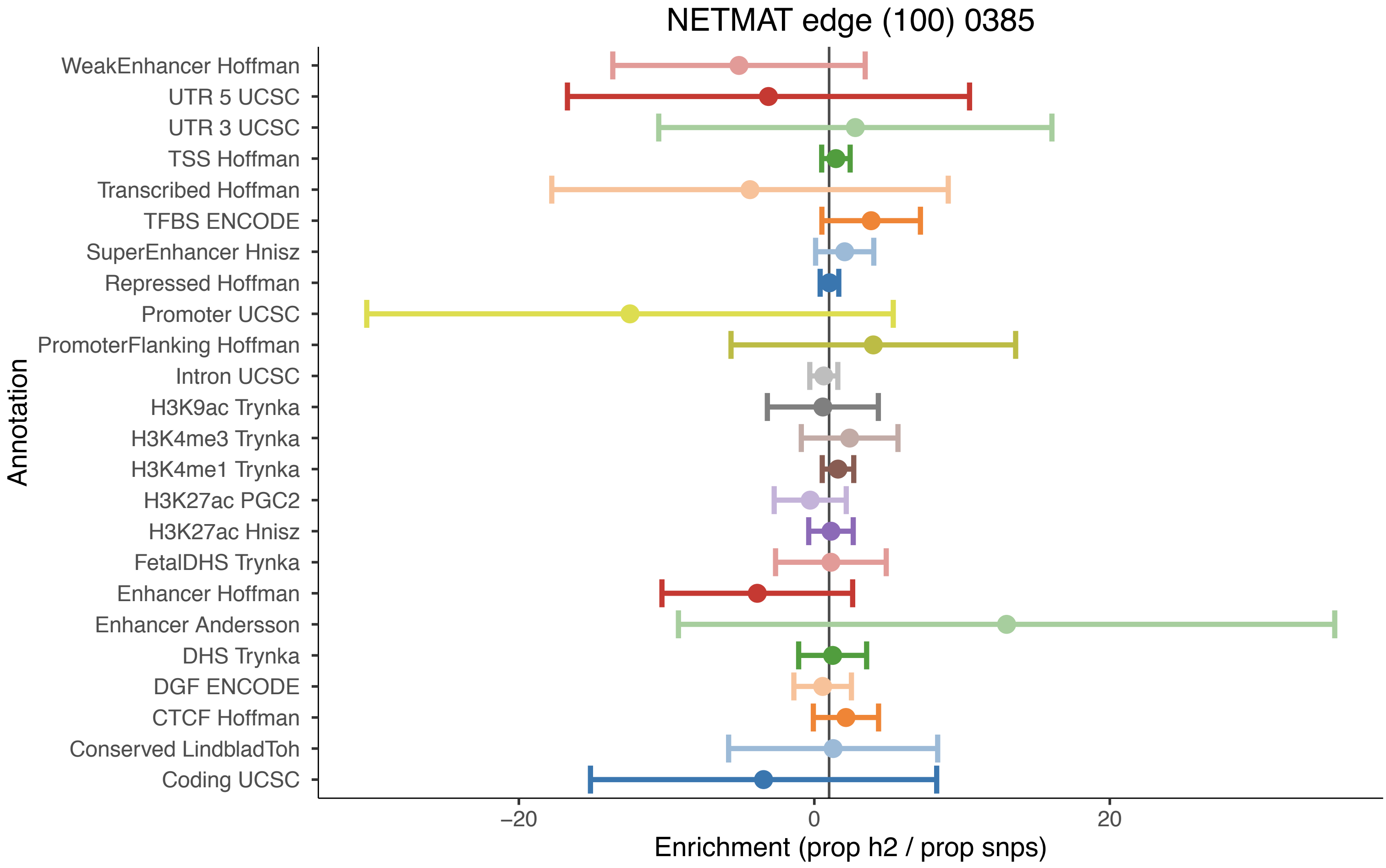

NETMAT edge (100) 0385
Annotation
WeakEnhancer Hoffman
UTR 5 UCSC
UTR 3 UCSC
TSS Hoffman
Transcribed Hoffman
TFBS ENCODE
SuperEnhancer Hnisz
Repressed Hoffman
Promoter UCSC
PromoterFlanking Hoffman
Intron UCSC
H3K9ac Trynka
H3K4me3 Trynka
H3K4me1 Trynka
H3K27ac PGC2
H3K27ac Hnisz
FetalDHS Trynka
Enhancer Hoffman
Enhancer Andersson
DHS Trynka
DGF ENCODE
CTCF Hoffman
Conserved LindbladToh
Coding UCSC
−20
0
20
Enrichment (prop h2 / prop snps)

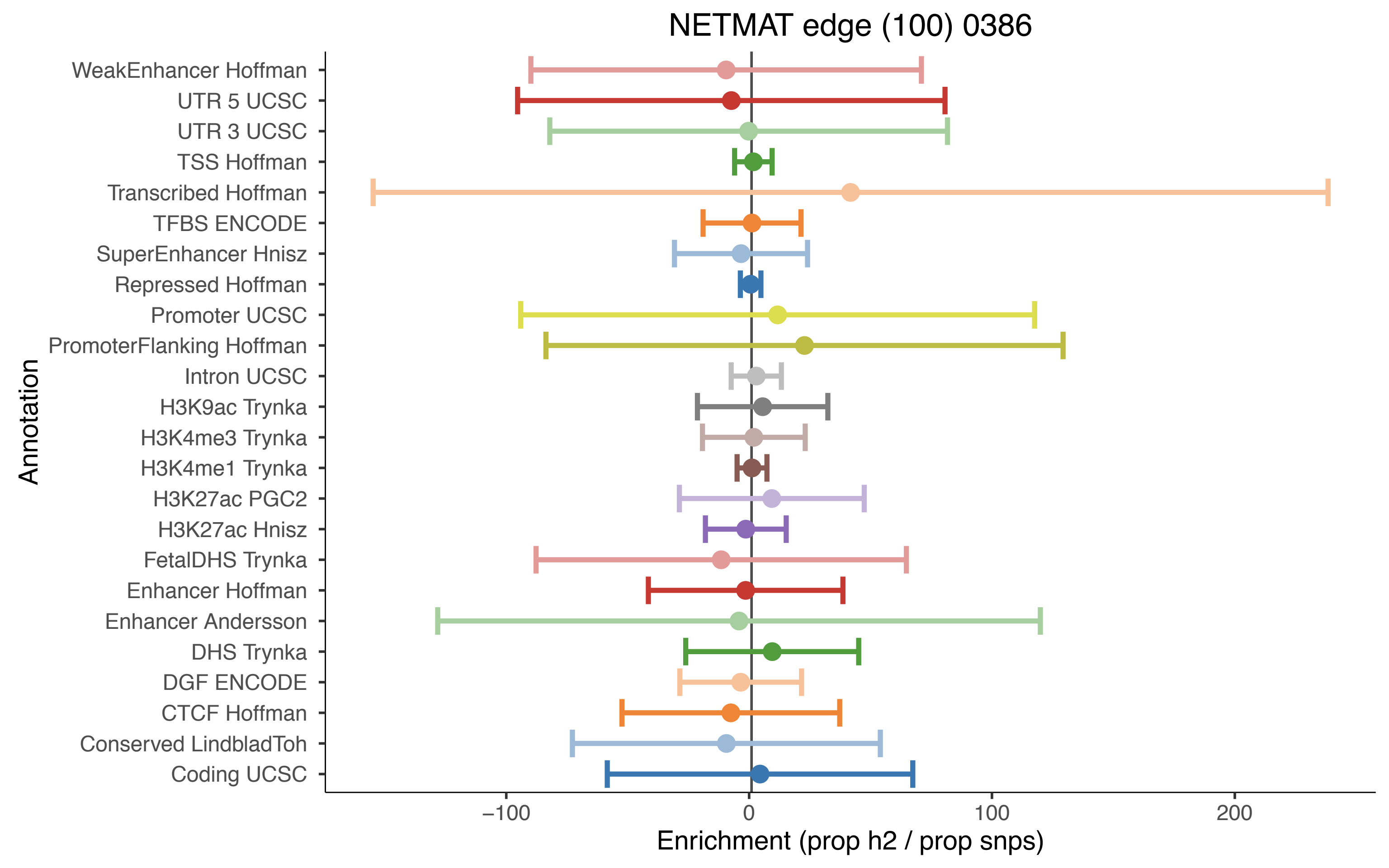
NETMAT edge (100) 0386
Annotation
WeakEnhancer Hoffman
UTR 5 UCSC
UTR 3 UCSC
TSS Hoffman
Transcribed Hoffman
TFBS ENCODE
SuperEnhancer Hnisz
Repressed Hoffman
Promoter UCSC
PromoterFlanking Hoffman
Intron UCSC
H3K9ac Trynka
H3K4me3 Trynka
H3K4me1 Trynka
H3K27ac PGC2
H3K27ac Hnisz
FetalDHS Trynka
Enhancer Hoffman
Enhancer Andersson
DHS Trynka
DGF ENCODE
CTCF Hoffman
Conserved LindbladToh
Coding UCSC
−100
0
100
200
Enrichment (prop h2 / prop snps)

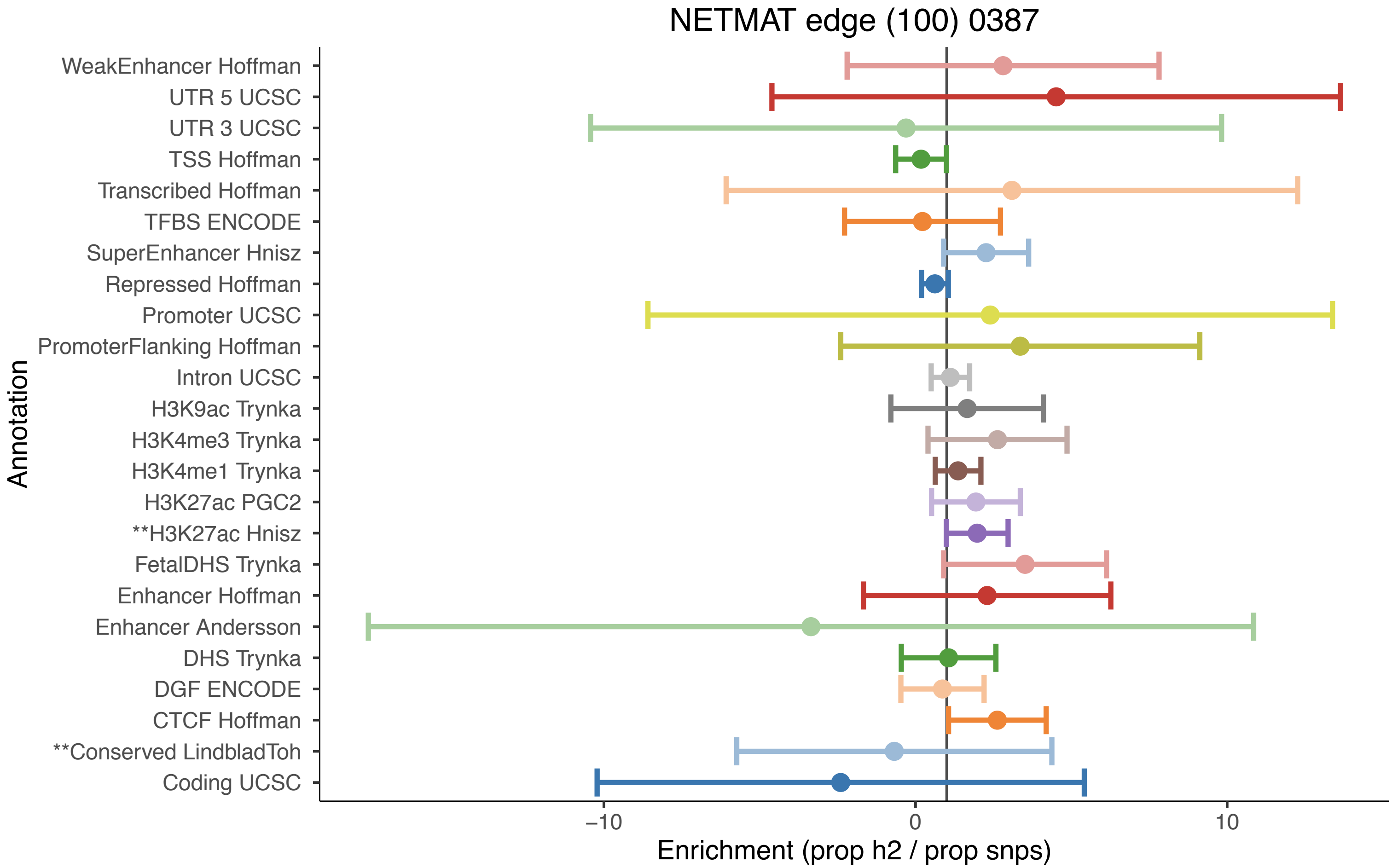
NETMAT edge (100) 0387
WeakEnhancer Hoffman
UTR 5 UCSC
UTR 3 UCSC
TSS Hoffman
Transcribed Hoffman
TFBS ENCODE
SuperEnhancer Hnisz
Repressed Hoffman
Promoter UCSC
PromoterFlanking Hoffman
Intron UCSC
H3K9ac Trynka
H3K4me3 Trynka
H3K4me1 Trynka
H3K27ac PGC2
**H3K27ac Hnisz
FetalDHS Trynka
Enhancer Hoffman
Enhancer Andersson
DHS Trynka
DGF ENCODE
CTCF Hoffman
**Conserved LindbladToh
Coding UCSC
Annotation
−10
0
10
Enrichment (prop h2 / prop snps)

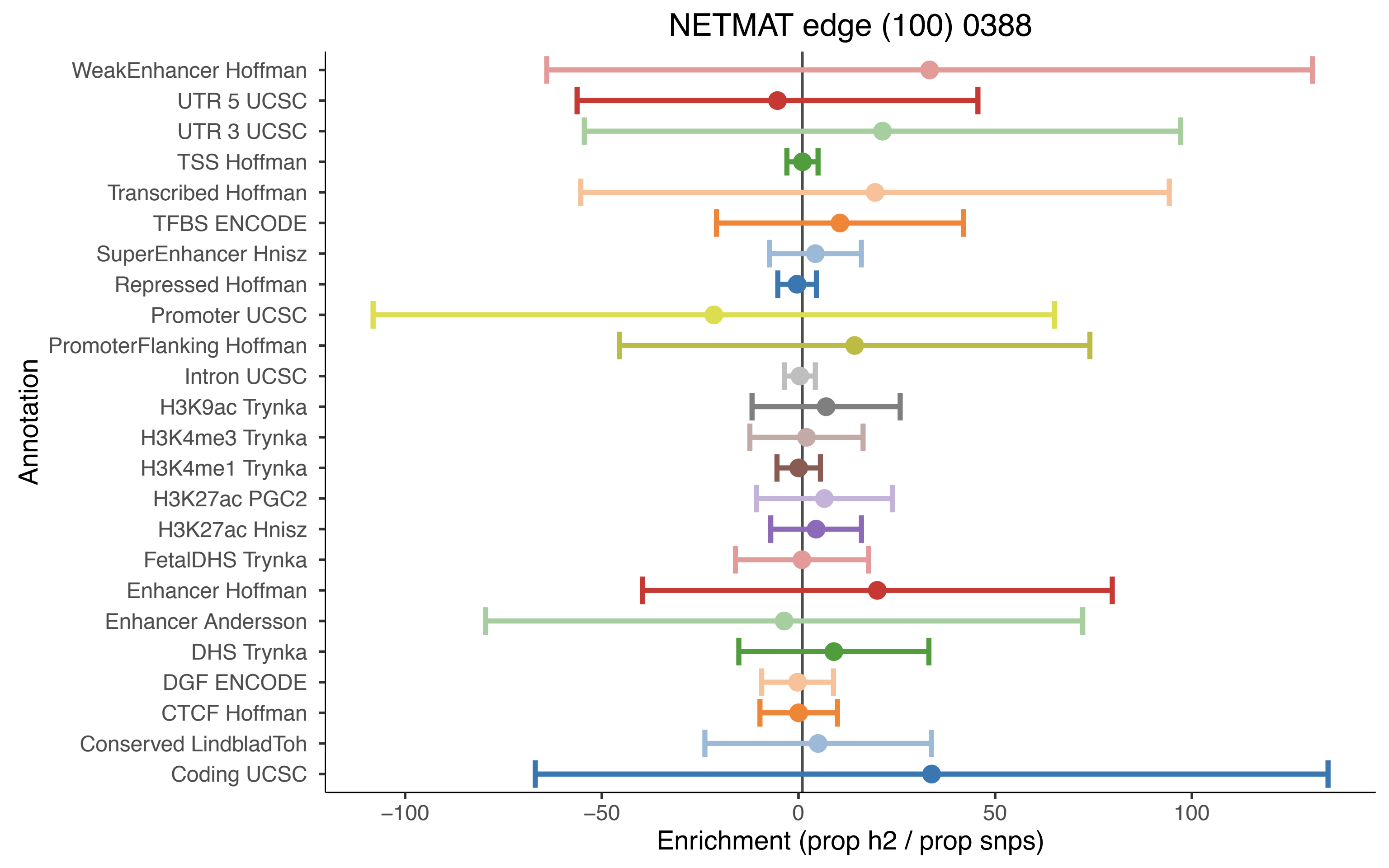

NETMAT edge (100) 0388
WeakEnhancer Hoffman
UTR 5 UCSC
UTR 3 UCSC
TSS Hoffman
Transcribed Hoffman
TFBS ENCODE
SuperEnhancer Hnisz
Repressed Hoffman
Promoter UCSC
PromoterFlanking Hoffman
Intron UCSC
H3K9ac Trynka
H3K4me3 Trynka
H3K4me1 Trynka
H3K27ac PGC2
H3K27ac Hnisz
FetalDHS Trynka
Enhancer Hoffman
Enhancer Andersson
DHS Trynka
DGF ENCODE
CTCF Hoffman
Conserved LindbladToh
Coding UCSC
Annotation
−100
−50
0
50
100
Enrichment (prop h2 / prop snps)

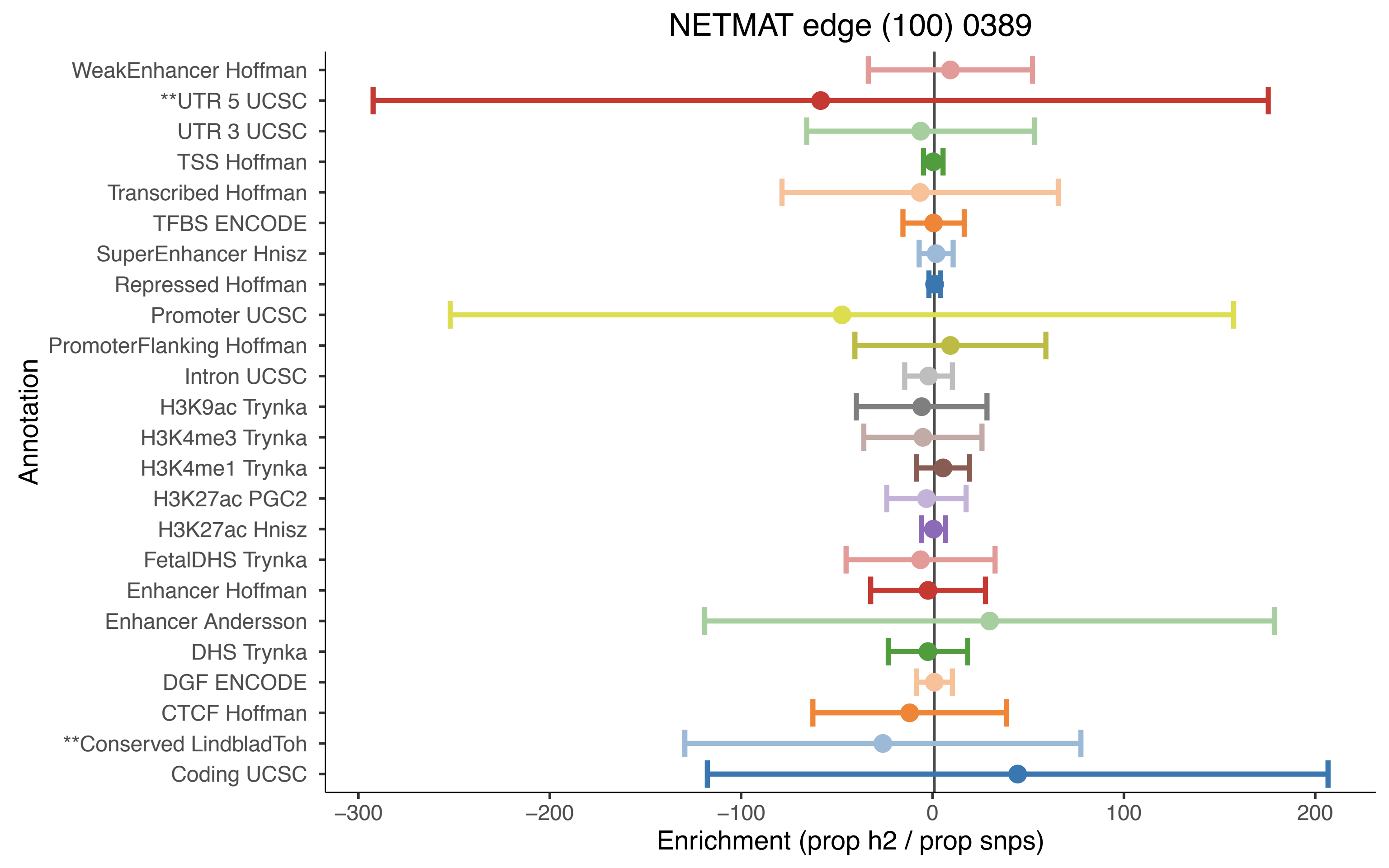

NETMAT edge (100) 0389
WeakEnhancer Hoffman
**UTR 5 UCSC
UTR 3 UCSC
TSS Hoffman
Transcribed Hoffman
TFBS ENCODE
SuperEnhancer Hnisz
Repressed Hoffman
Promoter UCSC
PromoterFlanking Hoffman
Intron UCSC
H3K9ac Trynka
H3K4me3 Trynka
H3K4me1 Trynka
H3K27ac PGC2
H3K27ac Hnisz
FetalDHS Trynka
Enhancer Hoffman
Enhancer Andersson
DHS Trynka
DGF ENCODE
CTCF Hoffman
**Conserved LindbladToh
Coding UCSC
Annotation
−300
−200
−100
0
100
200
Enrichment (prop h2 / prop snps)

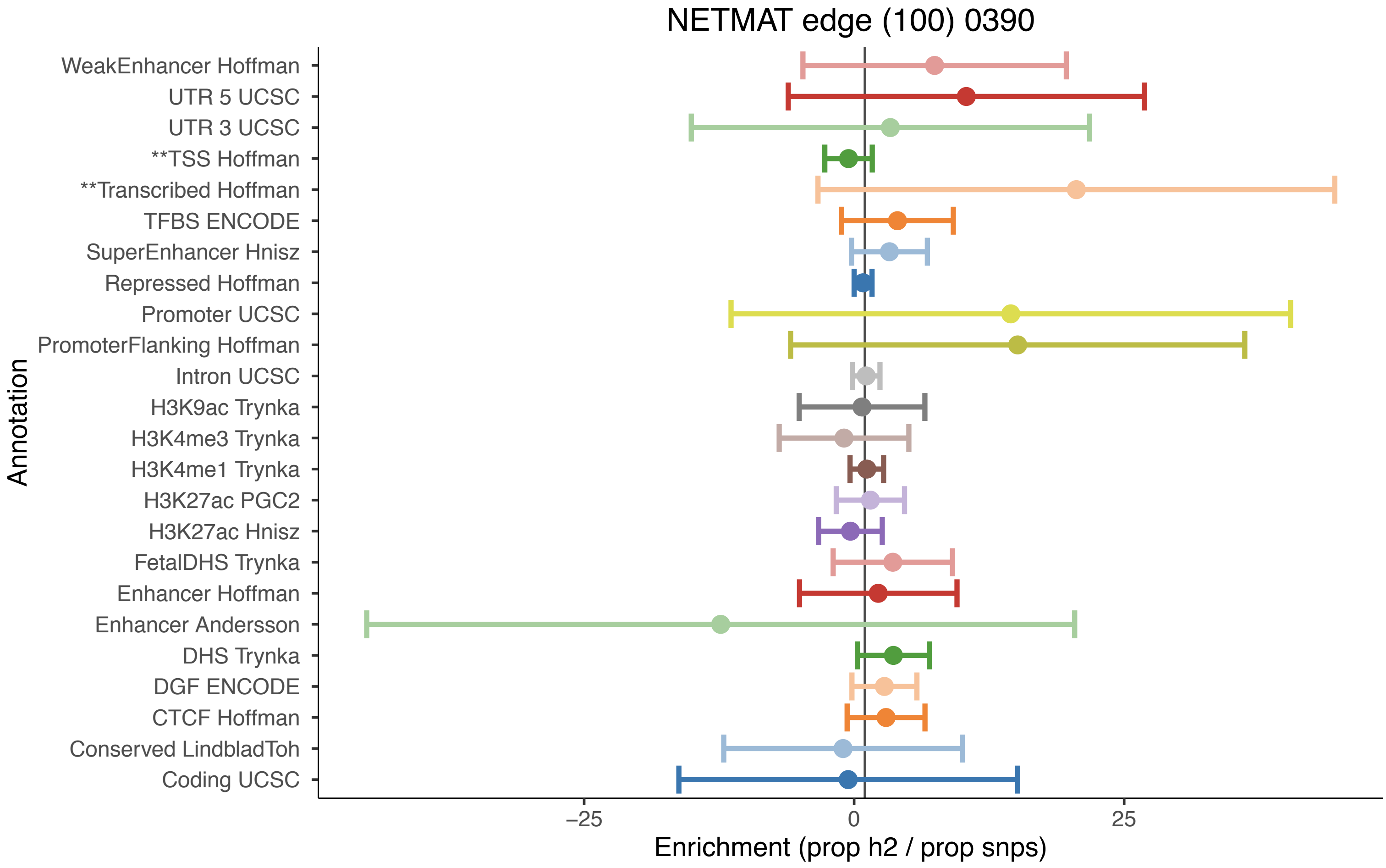

NETMAT edge (100) 0390
WeakEnhancer Hoffman
UTR 5 UCSC
UTR 3 UCSC
**TSS Hoffman
**Transcribed Hoffman
TFBS ENCODE
SuperEnhancer Hnisz
Repressed Hoffman
Promoter UCSC
PromoterFlanking Hoffman
Intron UCSC
H3K9ac Trynka
H3K4me3 Trynka
H3K4me1 Trynka
H3K27ac PGC2
H3K27ac Hnisz
FetalDHS Trynka
Enhancer Hoffman
Enhancer Andersson
DHS Trynka
DGF ENCODE
CTCF Hoffman
Conserved LindbladToh
Coding UCSC
Annotation
−25
0
25
Enrichment (prop h2 / prop snps)

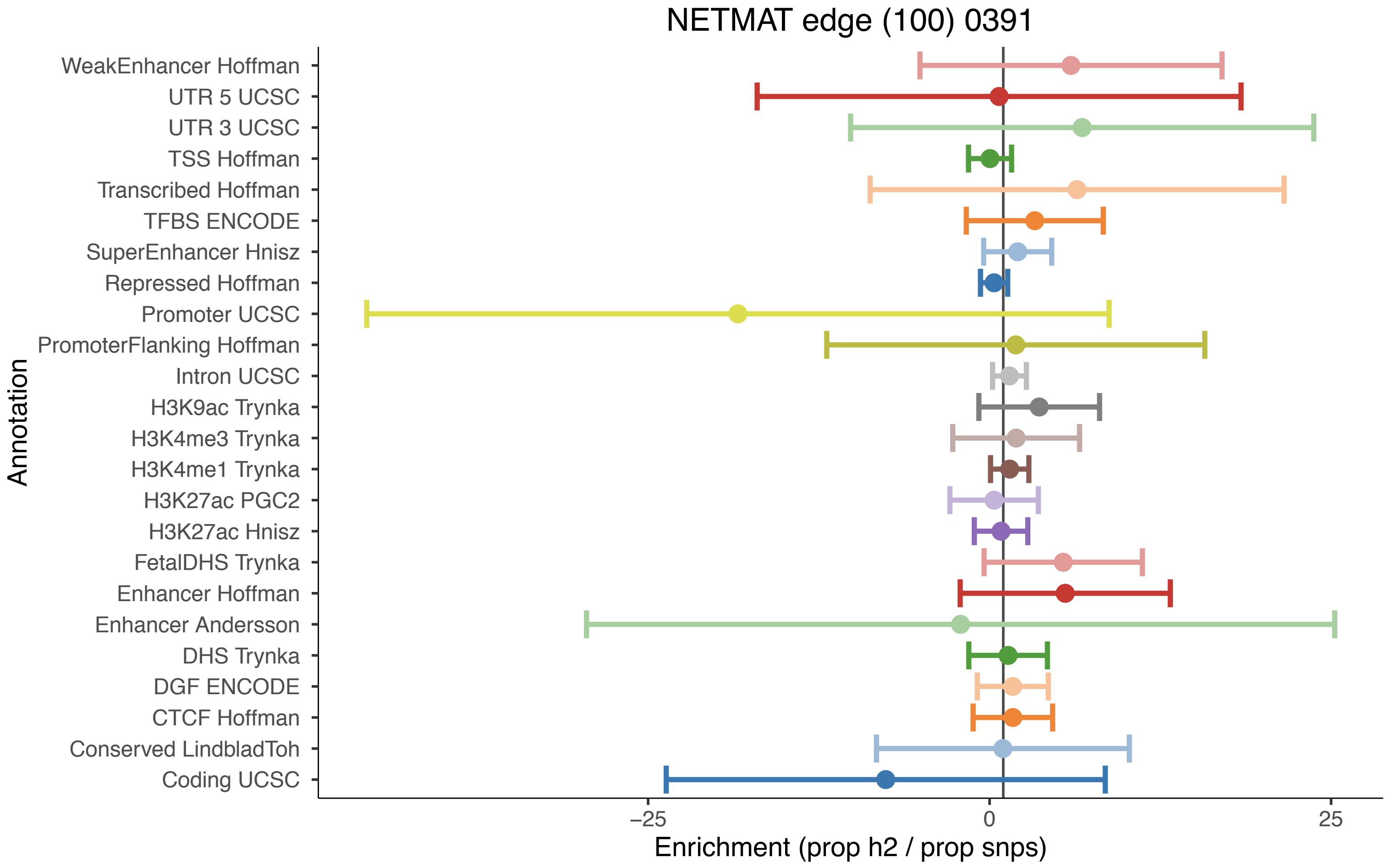

NETMAT edge (100) 0391
WeakEnhancer Hoffman
UTR 5 UCSC
UTR 3 UCSC
TSS Hoffman
Transcribed Hoffman
TFBS ENCODE
SuperEnhancer Hnisz
Repressed Hoffman
Promoter UCSC
PromoterFlanking Hoffman
Intron UCSC
H3K9ac Trynka
H3K4me3 Trynka
H3K4me1 Trynka
H3K27ac PGC2
H3K27ac Hnisz
FetalDHS Trynka
Enhancer Hoffman
Enhancer Andersson
DHS Trynka
DGF ENCODE
CTCF Hoffman
Conserved LindbladToh
Coding UCSC
Annotation
−25
0
25
Enrichment (prop h2 / prop snps)

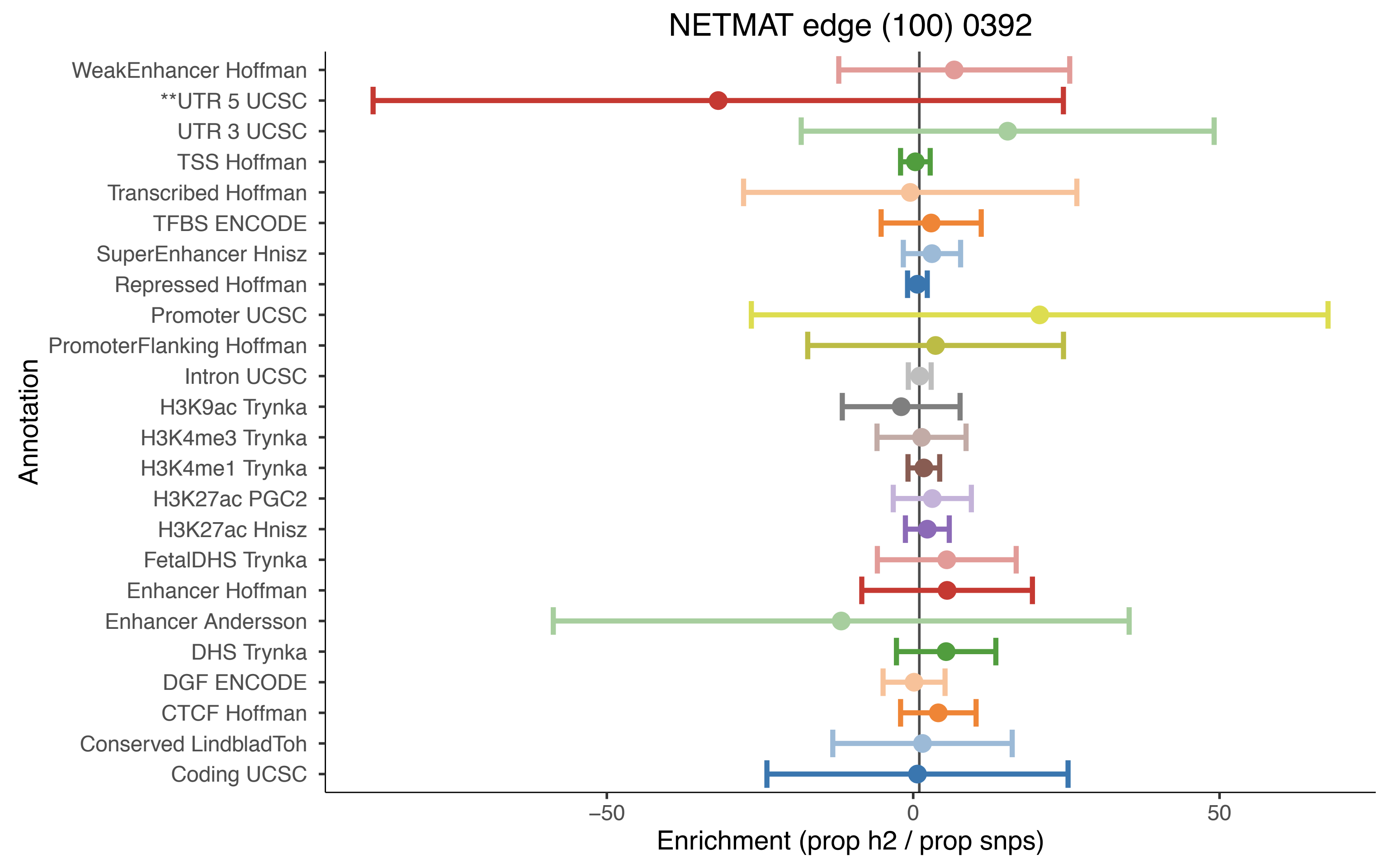

NETMAT edge (100) 0392
Annotation
WeakEnhancer Hoffman
**UTR 5 UCSC
UTR 3 UCSC
TSS Hoffman
Transcribed Hoffman
TFBS ENCODE
SuperEnhancer Hnisz
Repressed Hoffman
Promoter UCSC
PromoterFlanking Hoffman
Intron UCSC
H3K9ac Trynka
H3K4me3 Trynka
H3K4me1 Trynka
H3K27ac PGC2
H3K27ac Hnisz
FetalDHS Trynka
Enhancer Hoffman
Enhancer Andersson
DHS Trynka
DGF ENCODE
CTCF Hoffman
Conserved LindbladToh
Coding UCSC
−50
0
50
Enrichment (prop h2 / prop snps)

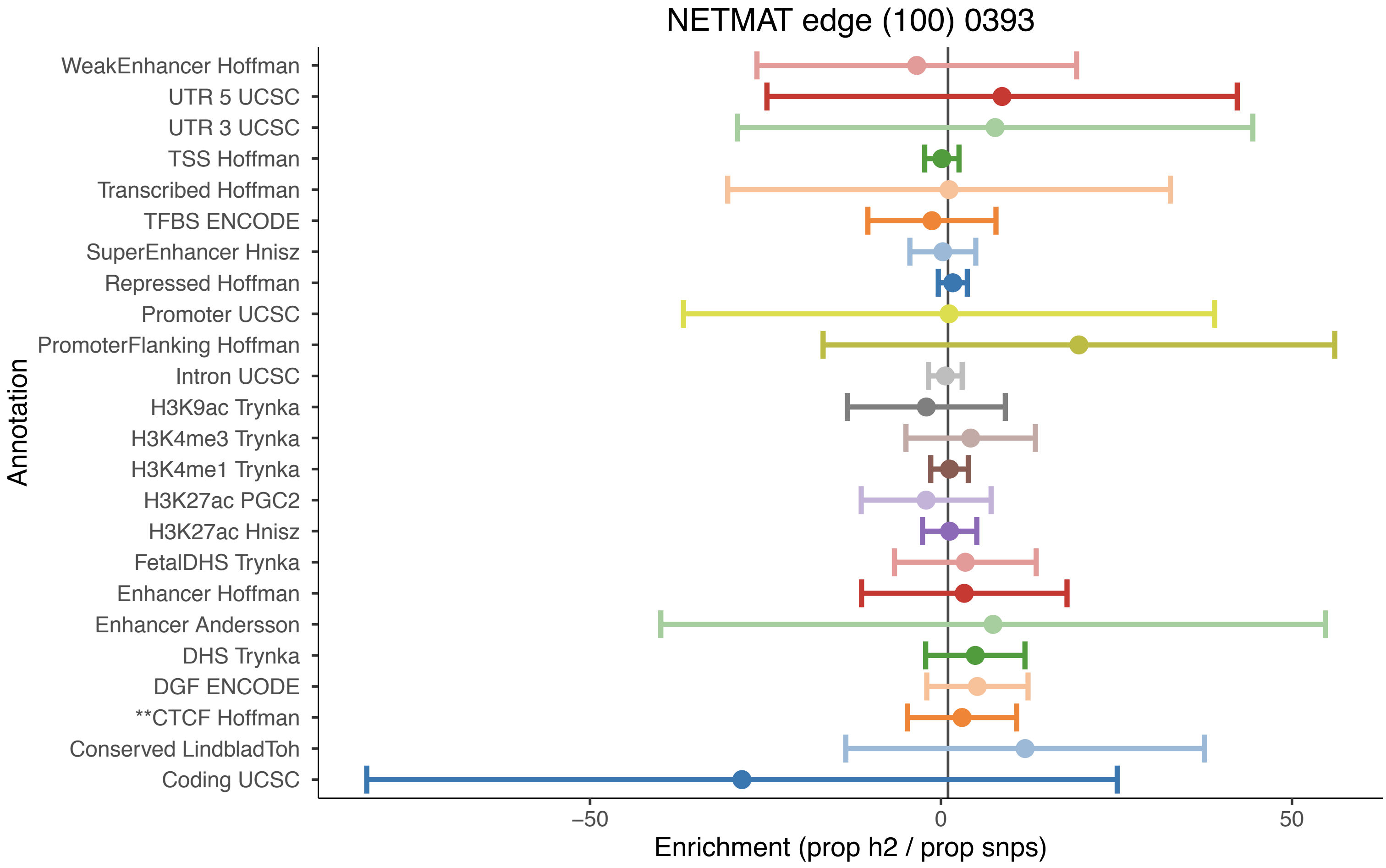

NETMAT edge (100) 0393
Annotation
WeakEnhancer Hoffman
UTR 5 UCSC
UTR 3 UCSC
TSS Hoffman
Transcribed Hoffman
TFBS ENCODE
SuperEnhancer Hnisz
Repressed Hoffman
Promoter UCSC
PromoterFlanking Hoffman
Intron UCSC
H3K9ac Trynka
H3K4me3 Trynka
H3K4me1 Trynka
H3K27ac PGC2
H3K27ac Hnisz
FetalDHS Trynka
Enhancer Hoffman
Enhancer Andersson
DHS Trynka
DGF ENCODE
**CTCF Hoffman
Conserved LindbladToh
Coding UCSC
−50
0
50
Enrichment (prop h2 / prop snps)

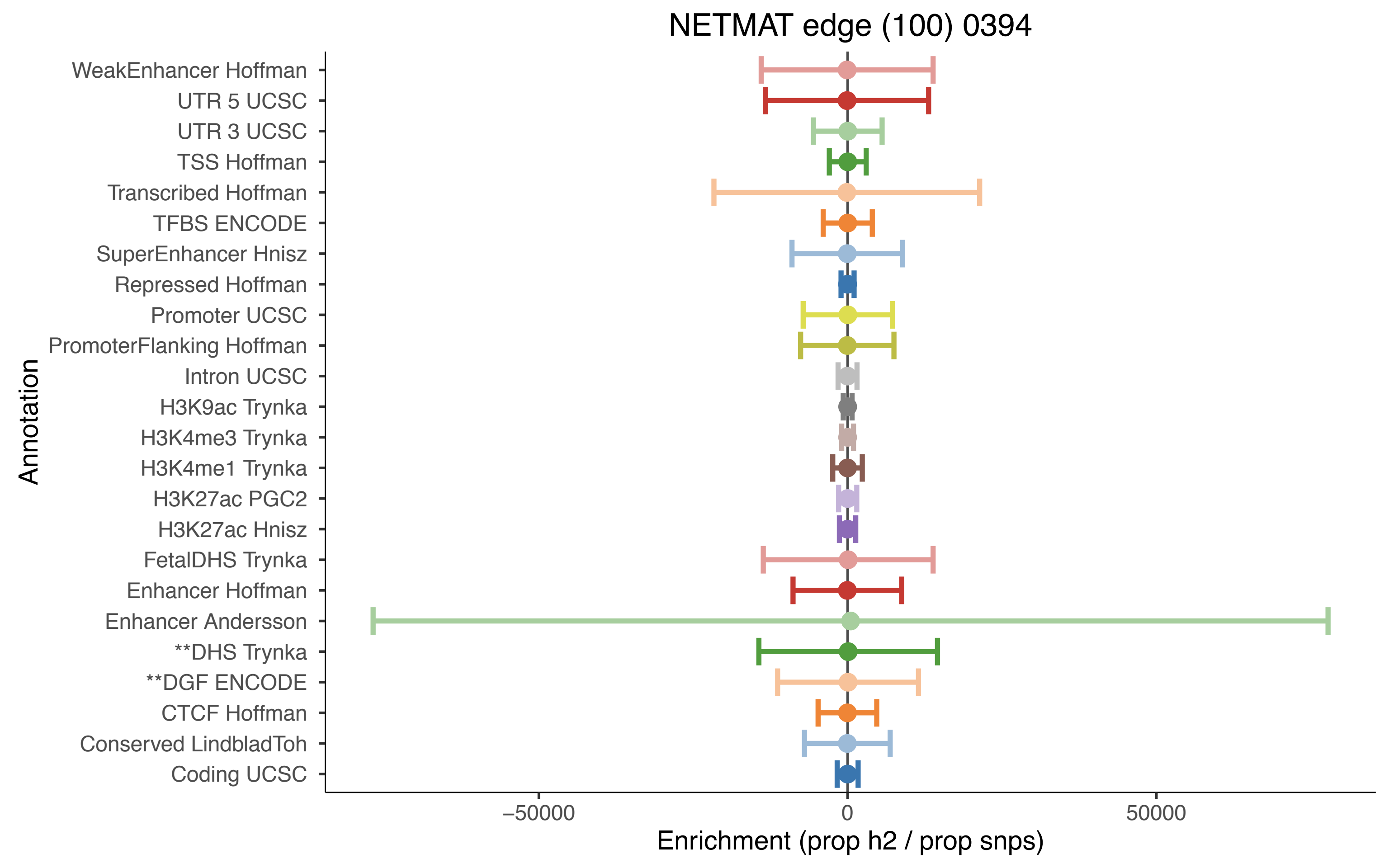
NETMAT edge (100) 0394
Annotation
WeakEnhancer Hoffman
UTR 5 UCSC
UTR 3 UCSC
TSS Hoffman
Transcribed Hoffman
TFBS ENCODE
SuperEnhancer Hnisz
Repressed Hoffman
Promoter UCSC
PromoterFlanking Hoffman
Intron UCSC
H3K9ac Trynka
H3K4me3 Trynka
H3K4me1 Trynka
H3K27ac PGC2
H3K27ac Hnisz
FetalDHS Trynka
Enhancer Hoffman
Enhancer Andersson
**DHS Trynka
**DGF ENCODE
CTCF Hoffman
Conserved LindbladToh
Coding UCSC
−50000
0
50000
Enrichment (prop h2 / prop snps)

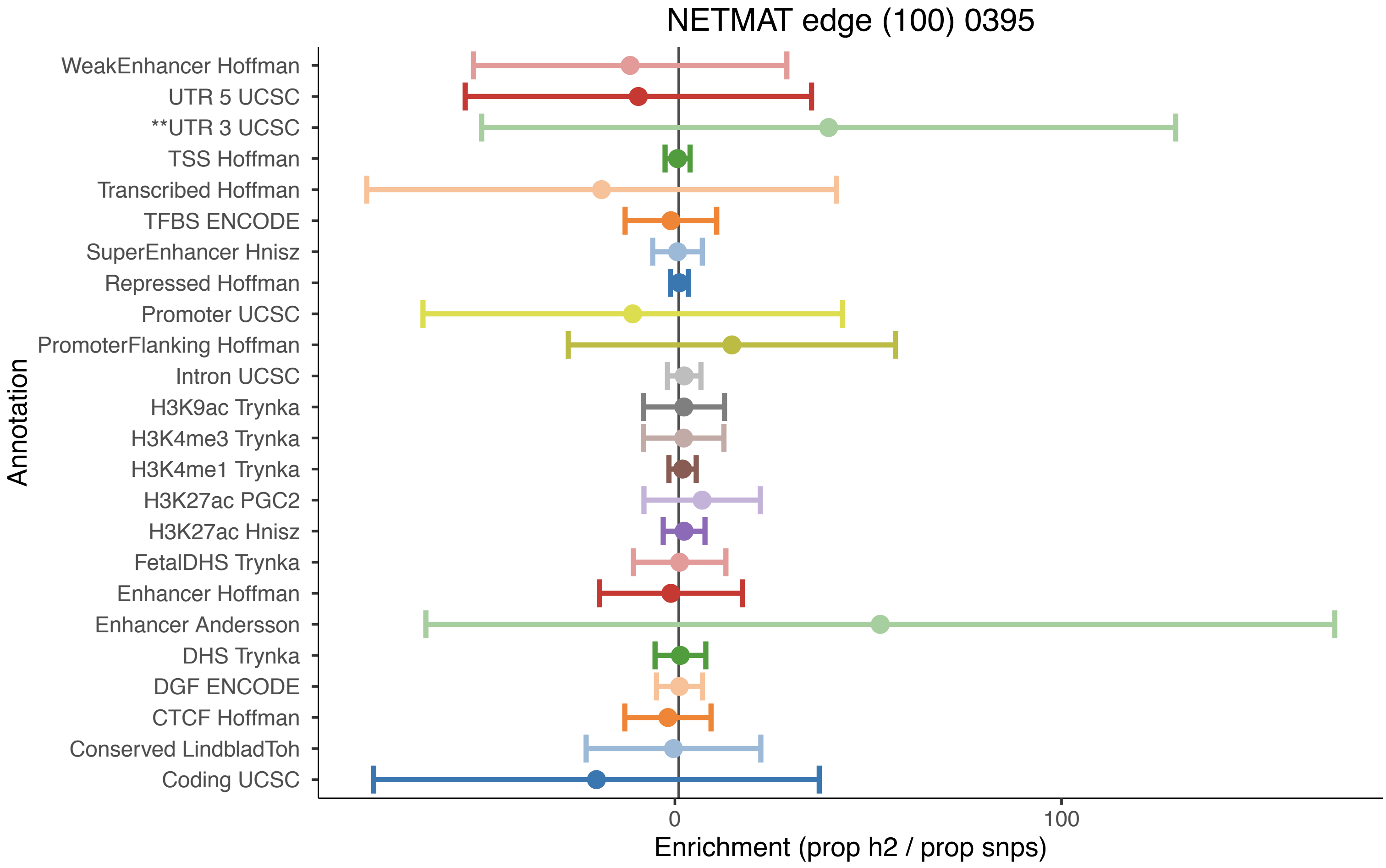

NETMAT edge (100) 0395
WeakEnhancer Hoffman
UTR 5 UCSC
**UTR 3 UCSC
TSS Hoffman
Transcribed Hoffman
TFBS ENCODE
SuperEnhancer Hnisz
Repressed Hoffman
Promoter UCSC
PromoterFlanking Hoffman
Intron UCSC
H3K9ac Trynka
H3K4me3 Trynka
H3K4me1 Trynka
H3K27ac PGC2
H3K27ac Hnisz
FetalDHS Trynka
Enhancer Hoffman
Enhancer Andersson
DHS Trynka
DGF ENCODE
CTCF Hoffman
Conserved LindbladToh
Coding UCSC
Annotation
0
100
Enrichment (prop h2 / prop snps)

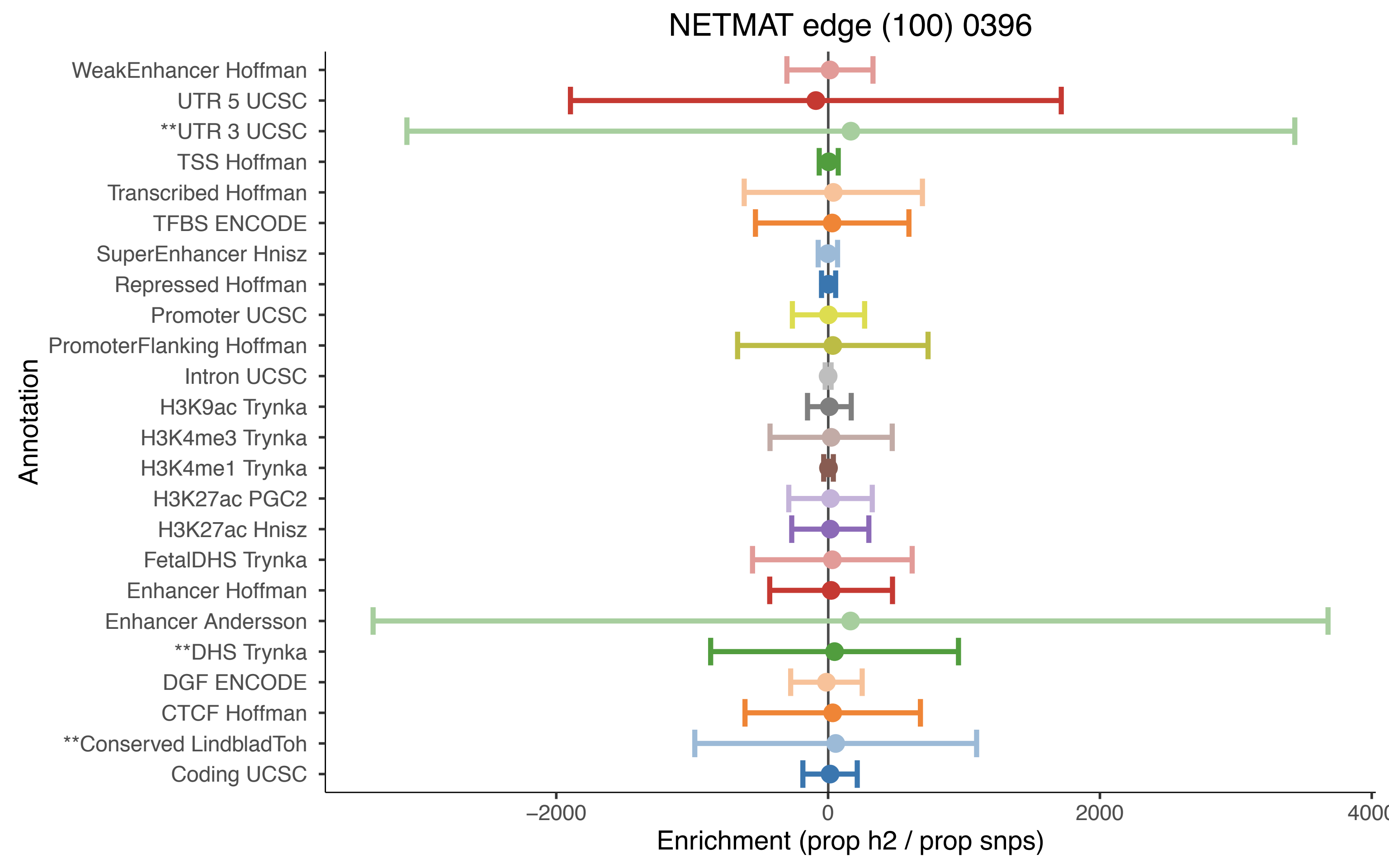

NETMAT edge (100) 0396
WeakEnhancer Hoffman
UTR 5 UCSC
**UTR 3 UCSC
TSS Hoffman
Transcribed Hoffman
TFBS ENCODE
SuperEnhancer Hnisz
Repressed Hoffman
Promoter UCSC
PromoterFlanking Hoffman
Intron UCSC
H3K9ac Trynka
H3K4me3 Trynka
H3K4me1 Trynka
H3K27ac PGC2
H3K27ac Hnisz
FetalDHS Trynka
Enhancer Hoffman
Enhancer Andersson
**DHS Trynka
DGF ENCODE
CTCF Hoffman
**Conserved LindbladToh
Coding UCSC
Annotation
−2000
0
2000
Enrichment (prop h2 / prop snps)

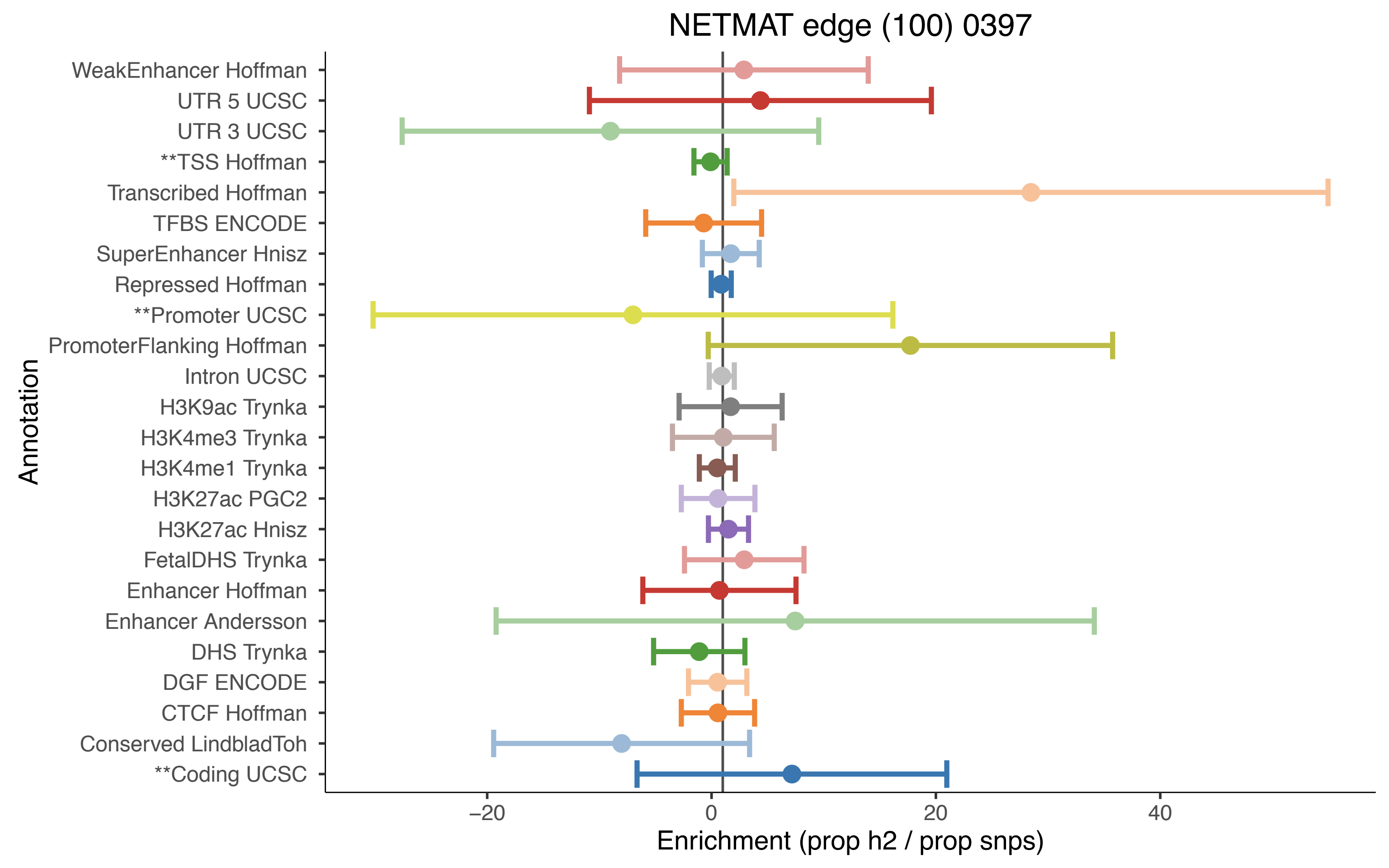

NETMAT edge (100) 0397
WeakEnhancer Hoffman
UTR 5 UCSC
UTR 3 UCSC
**TSS Hoffman
Transcribed Hoffman
TFBS ENCODE
SuperEnhancer Hnisz
Repressed Hoffman
**Promoter UCSC
PromoterFlanking Hoffman
Intron UCSC
H3K9ac Trynka
H3K4me3 Trynka
H3K4me1 Trynka
H3K27ac PGC2
H3K27ac Hnisz
FetalDHS Trynka
Enhancer Hoffman
Enhancer Andersson
DHS Trynka
DGF ENCODE
CTCF Hoffman
Conserved LindbladToh
**Coding UCSC
Annotation
−20
0
20
40
Enrichment (prop h2 / prop snps)

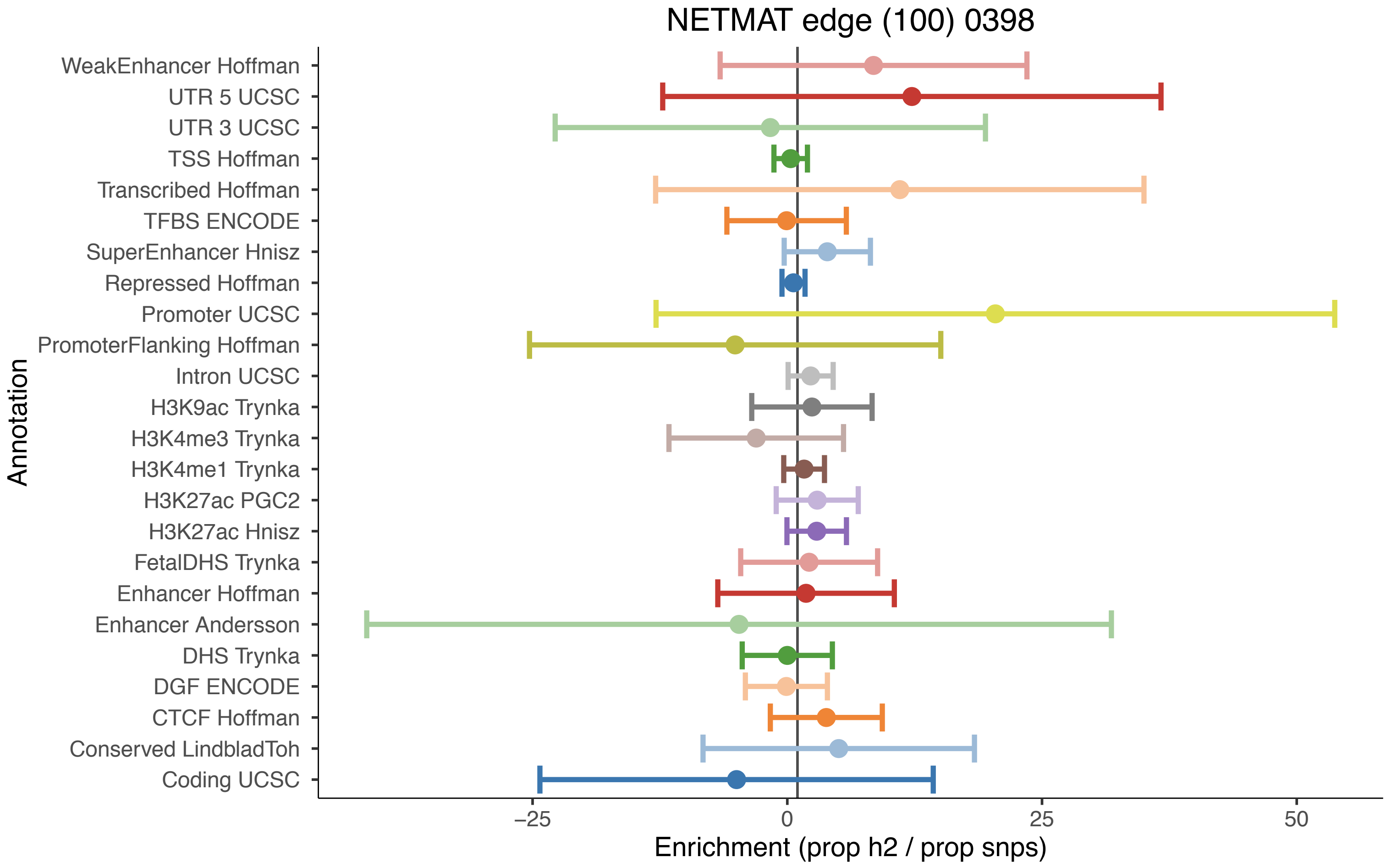
NETMAT edge (100) 0398
WeakEnhancer Hoffman
UTR 5 UCSC
UTR 3 UCSC
TSS Hoffman
Transcribed Hoffman
TFBS ENCODE
SuperEnhancer Hnisz
Repressed Hoffman
Promoter UCSC
PromoterFlanking Hoffman
Intron UCSC
H3K9ac Trynka
H3K4me3 Trynka
H3K4me1 Trynka
H3K27ac PGC2
H3K27ac Hnisz
FetalDHS Trynka
Enhancer Hoffman
Enhancer Andersson
DHS Trynka
DGF ENCODE
CTCF Hoffman
Conserved LindbladToh
Coding UCSC
Annotation
−25
0
25
50
Enrichment (prop h2 / prop snps)

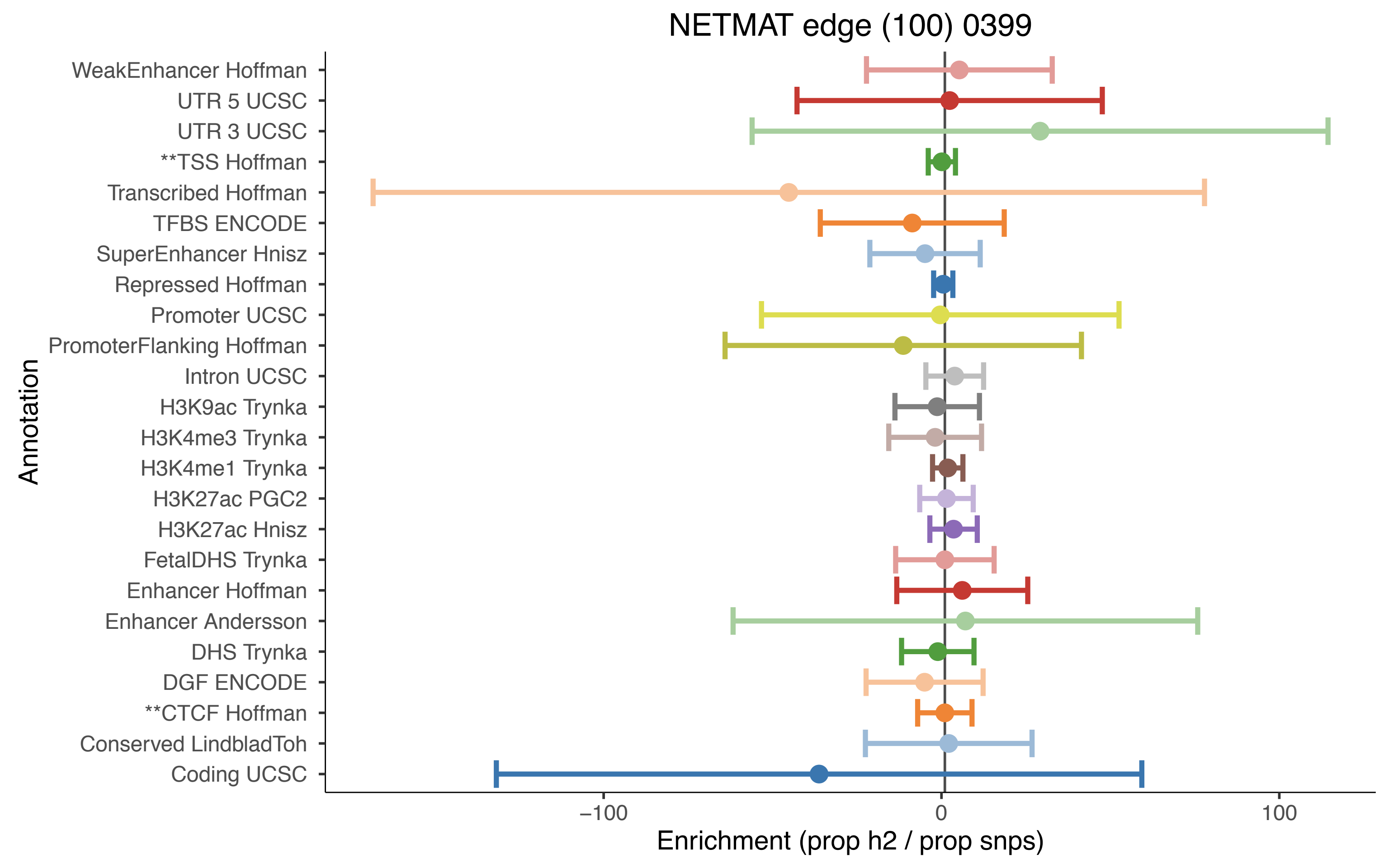
NETMAT edge (100) 0399
Annotation
WeakEnhancer Hoffman
UTR 5 UCSC
UTR 3 UCSC
**TSS Hoffman
Transcribed Hoffman
TFBS ENCODE
SuperEnhancer Hnisz
Repressed Hoffman
Promoter UCSC
PromoterFlanking Hoffman
Intron UCSC
H3K9ac Trynka
H3K4me3 Trynka
H3K4me1 Trynka
H3K27ac PGC2
H3K27ac Hnisz
FetalDHS Trynka
Enhancer Hoffman
Enhancer Andersson
DHS Trynka
DGF ENCODE
**CTCF Hoffman
Conserved LindbladToh
Coding UCSC
−100
0
100
Enrichment (prop h2 / prop snps)

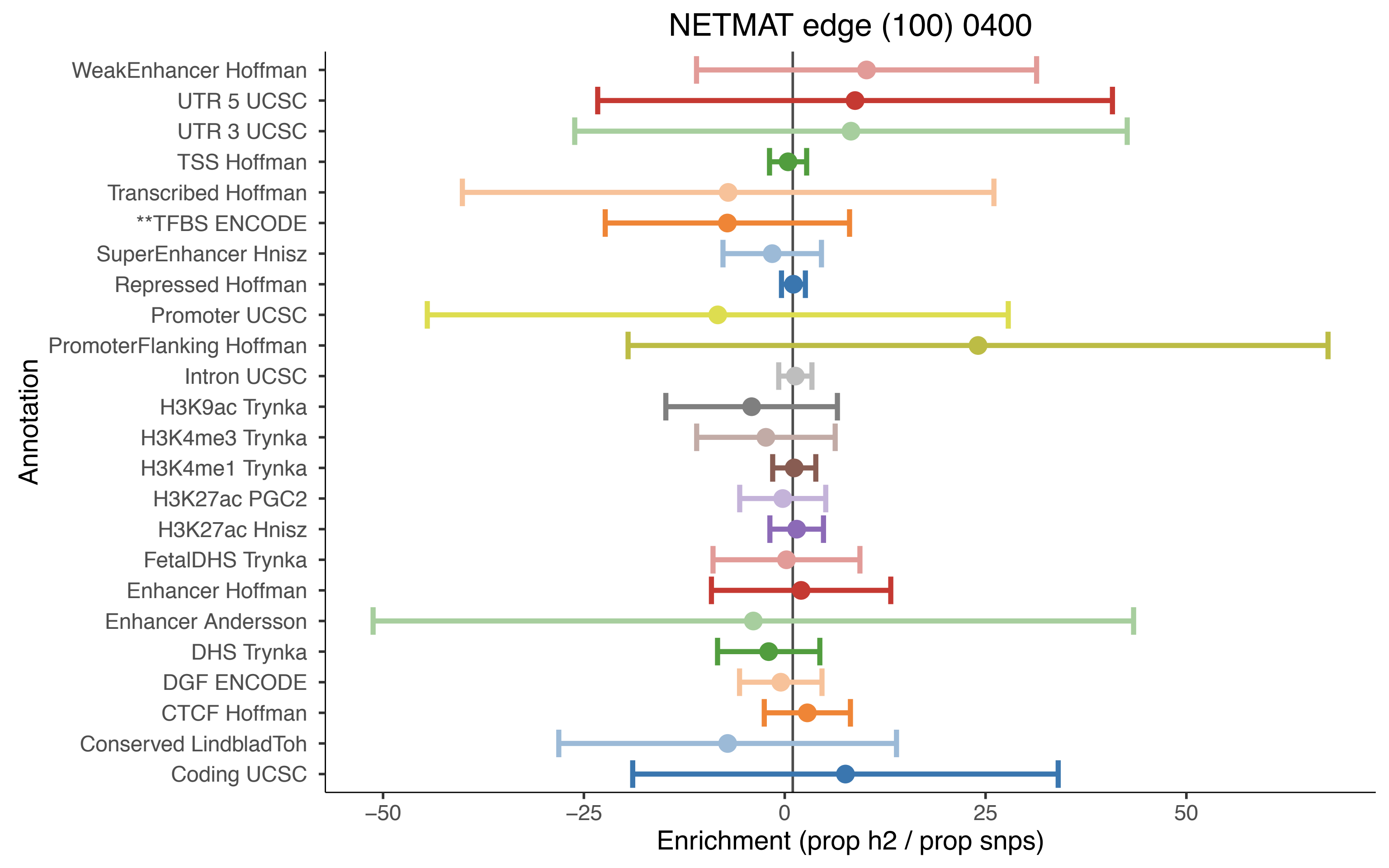
NETMAT edge (100) 0400
WeakEnhancer Hoffman
UTR 5 UCSC
UTR 3 UCSC
TSS Hoffman
Transcribed Hoffman
**TFBS ENCODE
SuperEnhancer Hnisz
Repressed Hoffman
Promoter UCSC
PromoterFlanking Hoffman
Intron UCSC
H3K9ac Trynka
H3K4me3 Trynka
H3K4me1 Trynka
H3K27ac PGC2
H3K27ac Hnisz
FetalDHS Trynka
Enhancer Hoffman
Enhancer Andersson
DHS Trynka
DGF ENCODE
CTCF Hoffman
Conserved LindbladToh
Coding UCSC
Annotation
−50
−25
0
25
50
Enrichment (prop h2 / prop snps)

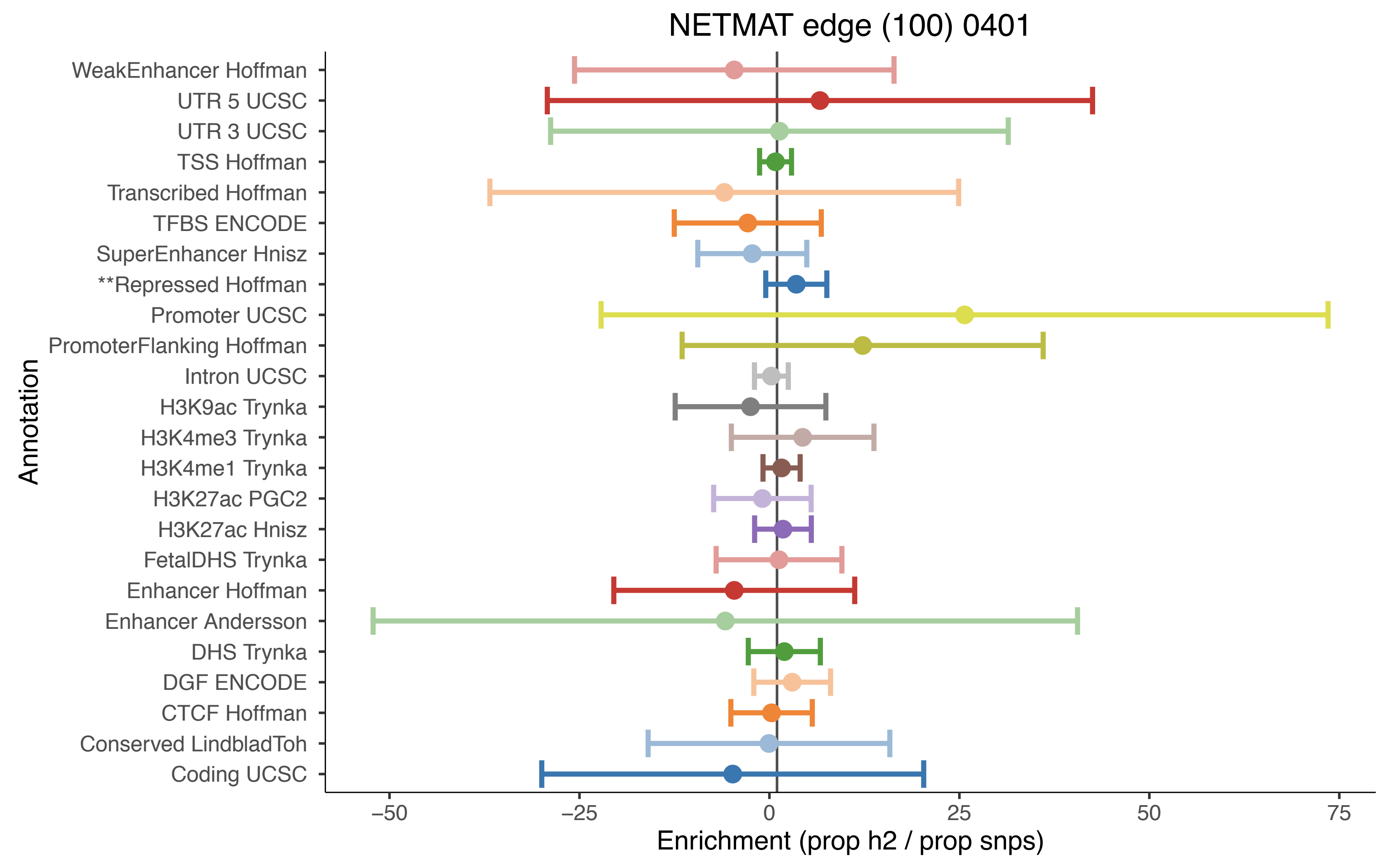

NETMAT edge (100) 0401
WeakEnhancer Hoffman
UTR 5 UCSC
UTR 3 UCSC
TSS Hoffman
Transcribed Hoffman
TFBS ENCODE
SuperEnhancer Hnisz
**Repressed Hoffman
Promoter UCSC
PromoterFlanking Hoffman
Intron UCSC
H3K9ac Trynka
H3K4me3 Trynka
H3K4me1 Trynka
H3K27ac PGC2
H3K27ac Hnisz
FetalDHS Trynka
Enhancer Hoffman
Enhancer Andersson
DHS Trynka
DGF ENCODE
CTCF Hoffman
Conserved LindbladToh
Coding UCSC
Annotation
−50
−25
0
25
50
75
Enrichment (prop h2 / prop snps)

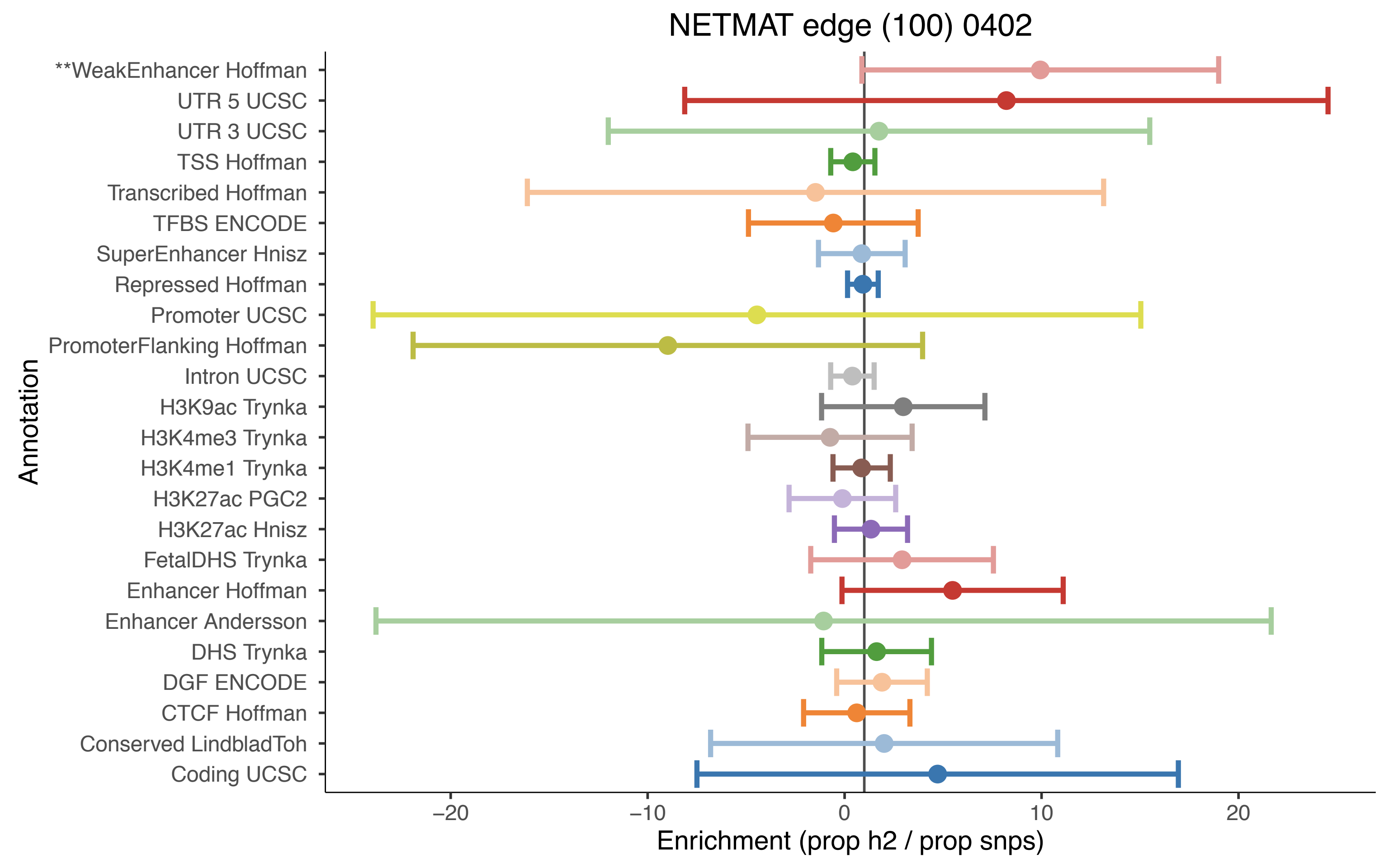
NETMAT edge (100) 0402
**WeakEnhancer Hoffman
UTR 5 UCSC
UTR 3 UCSC
TSS Hoffman
Transcribed Hoffman
TFBS ENCODE
SuperEnhancer Hnisz
Repressed Hoffman
Promoter UCSC
PromoterFlanking Hoffman
Intron UCSC
H3K9ac Trynka
H3K4me3 Trynka
H3K4me1 Trynka
H3K27ac PGC2
H3K27ac Hnisz
FetalDHS Trynka
Enhancer Hoffman
Enhancer Andersson
DHS Trynka
DGF ENCODE
CTCF Hoffman
Conserved LindbladToh
Coding UCSC
Annotation
−20
−10
0
10
20
Enrichment (prop h2 / prop snps)

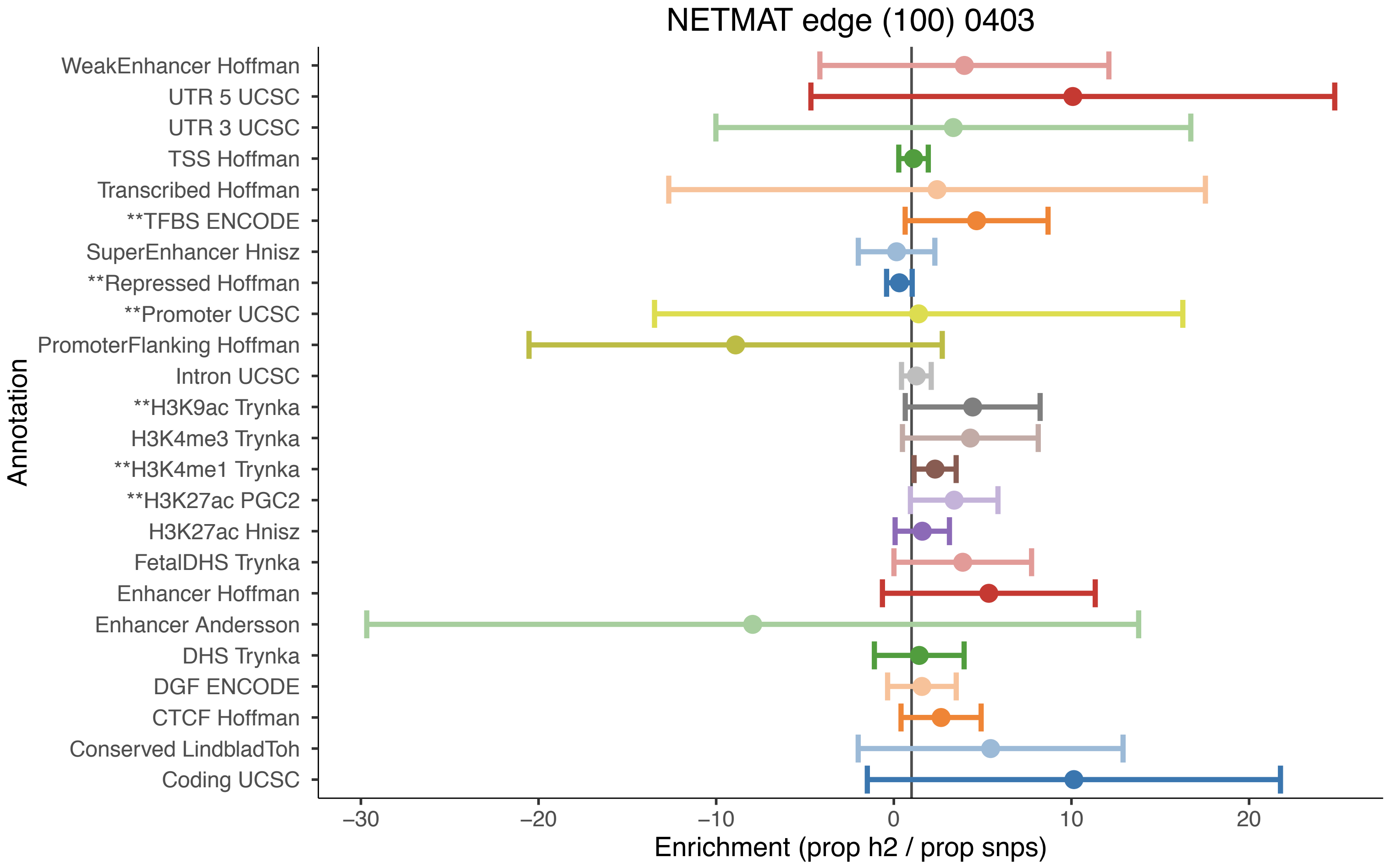

NETMAT edge (100) 0403
WeakEnhancer Hoffman
UTR 5 UCSC
UTR 3 UCSC
TSS Hoffman
Transcribed Hoffman
**TFBS ENCODE
SuperEnhancer Hnisz
**Repressed Hoffman
**Promoter UCSC
PromoterFlanking Hoffman
Intron UCSC
**H3K9ac Trynka
H3K4me3 Trynka
**H3K4me1 Trynka
**H3K27ac PGC2
H3K27ac Hnisz
FetalDHS Trynka
Enhancer Hoffman
Enhancer Andersson
DHS Trynka
DGF ENCODE
CTCF Hoffman
Conserved LindbladToh
Coding UCSC
Annotation
−30
−20
−10
0
10
20
Enrichment (prop h2 / prop snps)

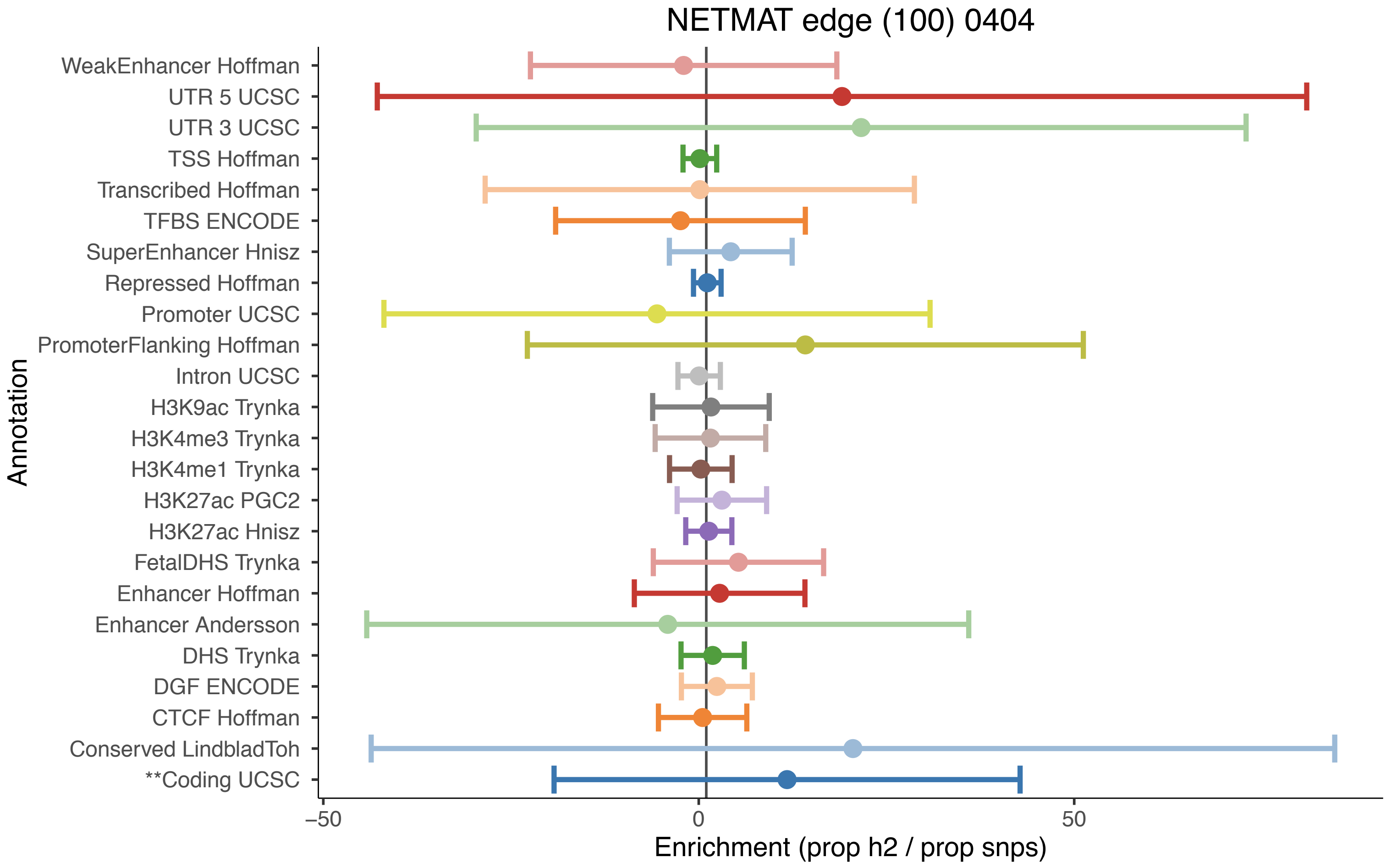

NETMAT edge (100) 0404
WeakEnhancer Hoffman
UTR 5 UCSC
UTR 3 UCSC
TSS Hoffman
Transcribed Hoffman
TFBS ENCODE
SuperEnhancer Hnisz
Repressed Hoffman
Promoter UCSC
PromoterFlanking Hoffman
Intron UCSC
H3K9ac Trynka
H3K4me3 Trynka
H3K4me1 Trynka
H3K27ac PGC2
H3K27ac Hnisz
FetalDHS Trynka
Enhancer Hoffman
Enhancer Andersson
DHS Trynka
DGF ENCODE
CTCF Hoffman
Conserved LindbladToh
**Coding UCSC
Annotation
−50
0
50
Enrichment (prop h2 / prop snps)

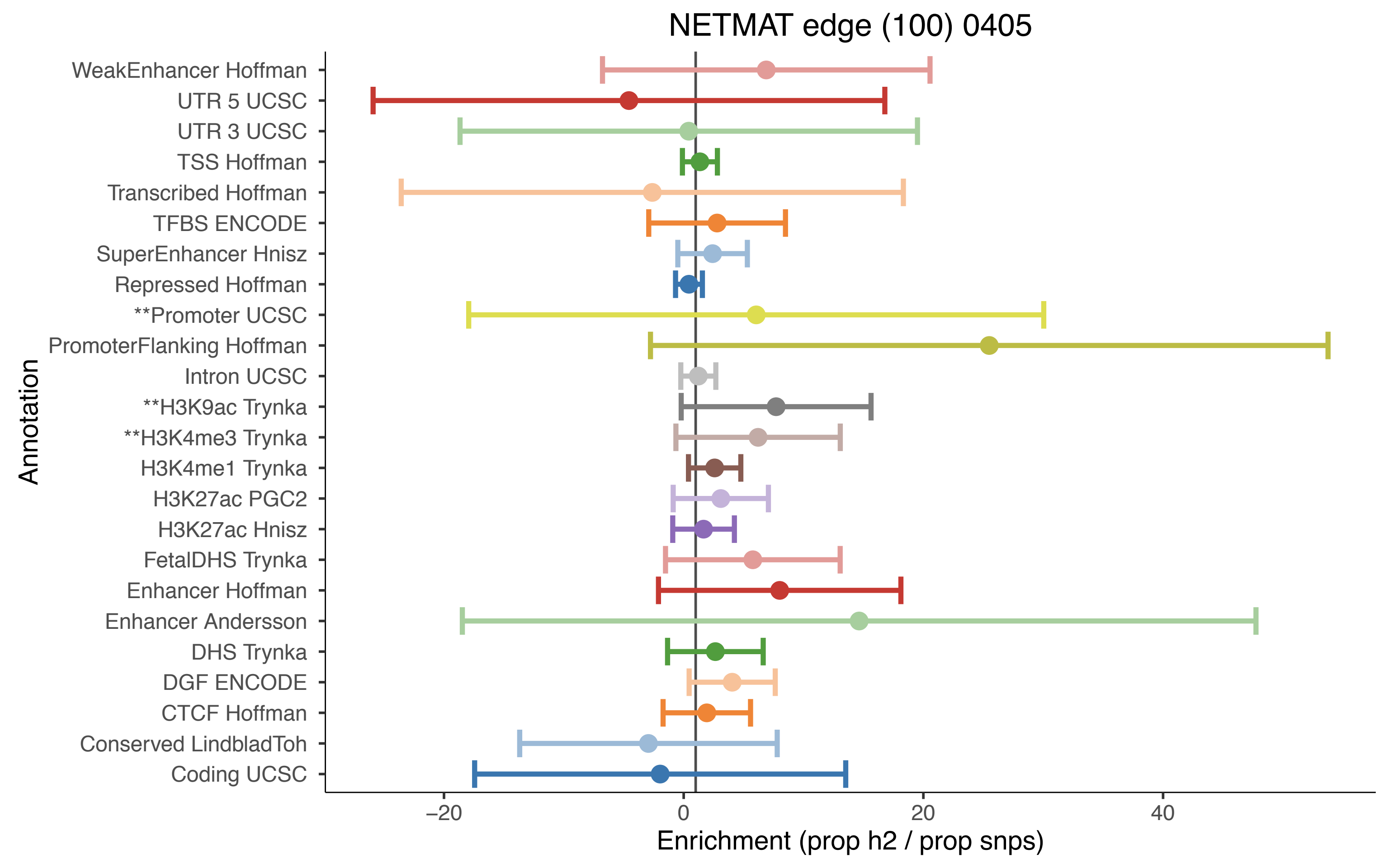

NETMAT edge (100) 0405
WeakEnhancer Hoffman
UTR 5 UCSC
UTR 3 UCSC
TSS Hoffman
Transcribed Hoffman
TFBS ENCODE
SuperEnhancer Hnisz
Repressed Hoffman
**Promoter UCSC
PromoterFlanking Hoffman
Intron UCSC
**H3K9ac Trynka
**H3K4me3 Trynka
H3K4me1 Trynka
H3K27ac PGC2
H3K27ac Hnisz
FetalDHS Trynka
Enhancer Hoffman
Enhancer Andersson
DHS Trynka
DGF ENCODE
CTCF Hoffman
Conserved LindbladToh
Coding UCSC
Annotation
−20
0
20
40
Enrichment (prop h2 / prop snps)

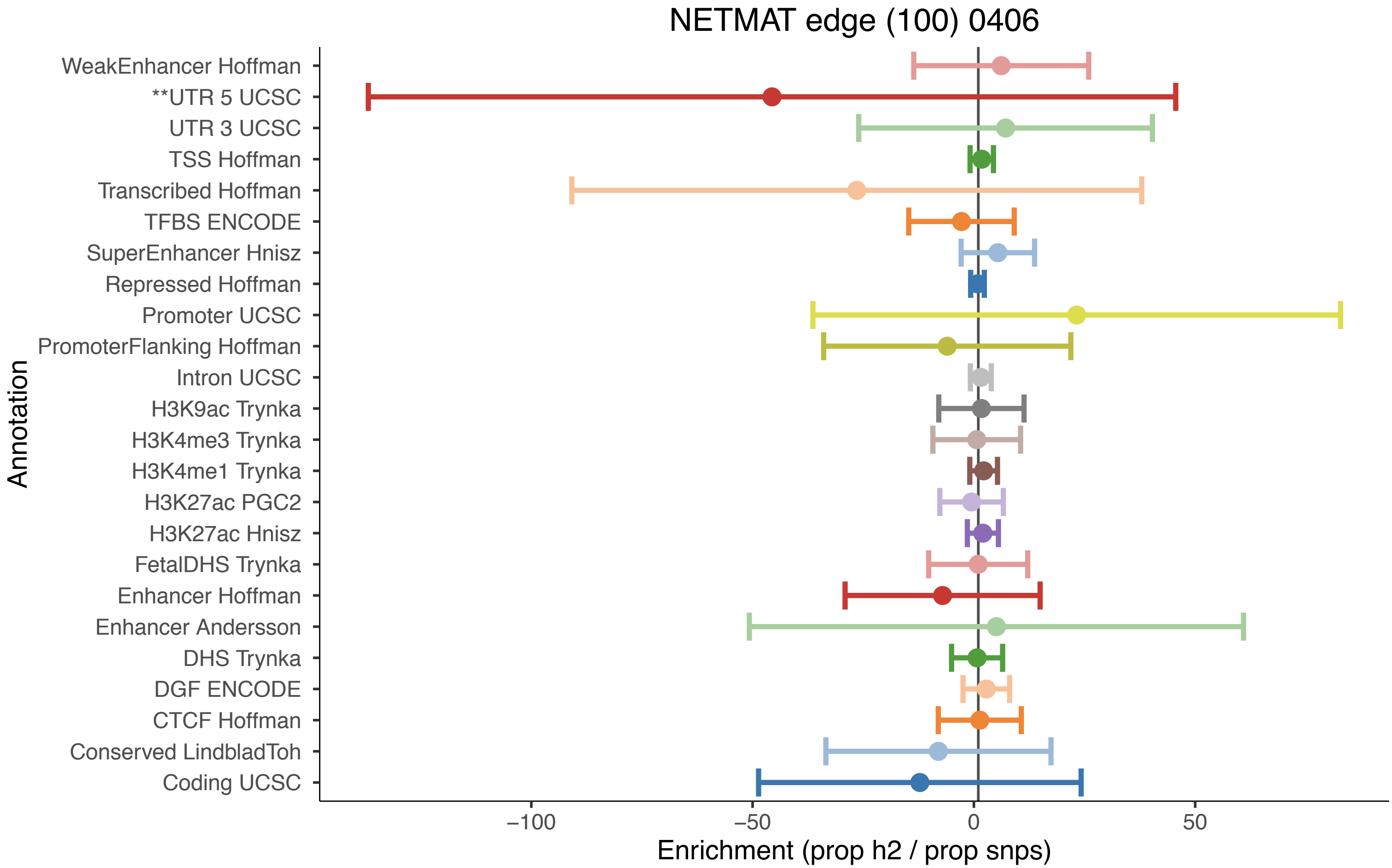

NETMAT edge (100) 0406
WeakEnhancer Hoffman
**UTR 5 UCSC
UTR 3 UCSC
TSS Hoffman
Transcribed Hoffman
TFBS ENCODE
SuperEnhancer Hnisz
Repressed Hoffman
Promoter UCSC
PromoterFlanking Hoffman
Intron UCSC
H3K9ac Trynka
H3K4me3 Trynka
H3K4me1 Trynka
H3K27ac PGC2
H3K27ac Hnisz
FetalDHS Trynka
Enhancer Hoffman
Enhancer Andersson
DHS Trynka
DGF ENCODE
CTCF Hoffman
Conserved LindbladToh
Coding UCSC
Annotation
−100
−50
0
50
Enrichment (prop h2 / prop snps)

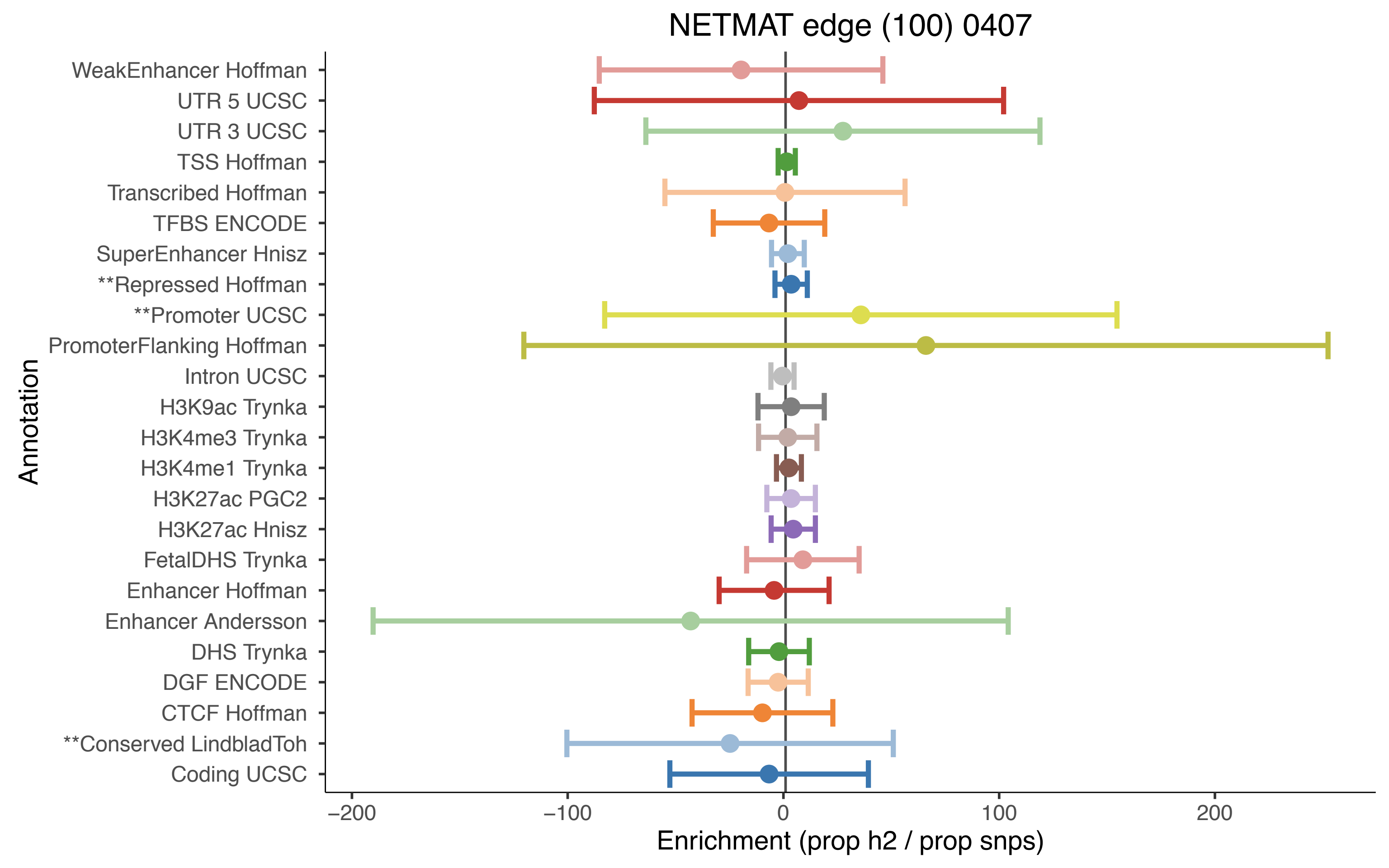

NETMAT edge (100) 0407
WeakEnhancer Hoffman
UTR 5 UCSC
UTR 3 UCSC
TSS Hoffman
Transcribed Hoffman
TFBS ENCODE
SuperEnhancer Hnisz
**Repressed Hoffman
**Promoter UCSC
PromoterFlanking Hoffman
Intron UCSC
H3K9ac Trynka
H3K4me3 Trynka
H3K4me1 Trynka
H3K27ac PGC2
H3K27ac Hnisz
FetalDHS Trynka
Enhancer Hoffman
Enhancer Andersson
DHS Trynka
DGF ENCODE
CTCF Hoffman
**Conserved LindbladToh
Coding UCSC
Annotation
−200
−100
0
100
200
Enrichment (prop h2 / prop snps)

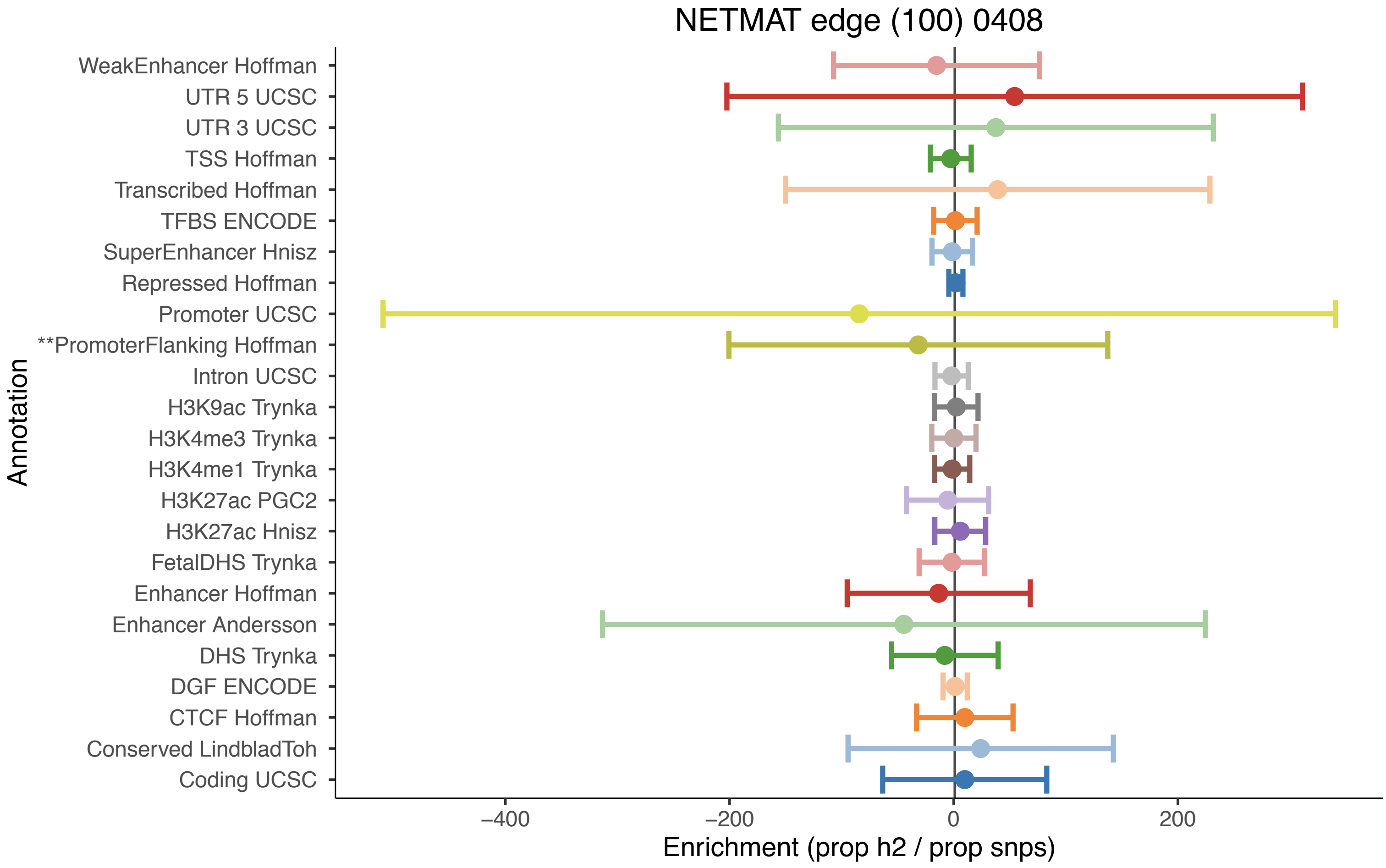
NETMAT edge (100) 0408
WeakEnhancer Hoffman
UTR 5 UCSC
UTR 3 UCSC
TSS Hoffman
Transcribed Hoffman
TFBS ENCODE
SuperEnhancer Hnisz
Repressed Hoffman
Promoter UCSC
**PromoterFlanking Hoffman
Intron UCSC
H3K9ac Trynka
H3K4me3 Trynka
H3K4me1 Trynka
H3K27ac PGC2
H3K27ac Hnisz
FetalDHS Trynka
Enhancer Hoffman
Enhancer Andersson
DHS Trynka
DGF ENCODE
CTCF Hoffman
Conserved LindbladToh
Coding UCSC
Annotation
−400
−200
0
200
Enrichment (prop h2 / prop snps)

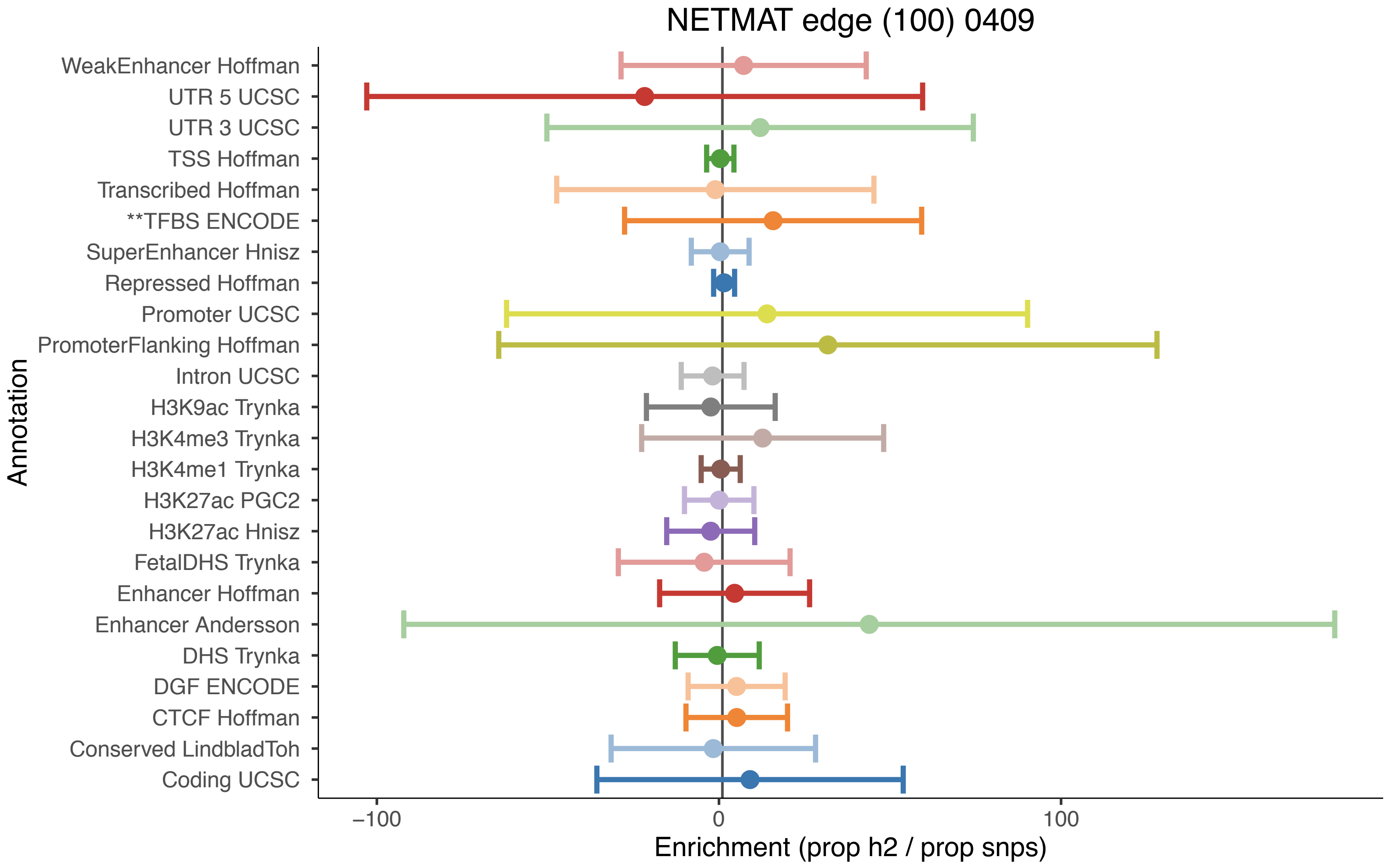
NETMAT edge (100) 0409
WeakEnhancer Hoffman
UTR 5 UCSC
UTR 3 UCSC
TSS Hoffman
Transcribed Hoffman
**TFBS ENCODE
SuperEnhancer Hnisz
Repressed Hoffman
Promoter UCSC
PromoterFlanking Hoffman
Intron UCSC
H3K9ac Trynka
H3K4me3 Trynka
H3K4me1 Trynka
H3K27ac PGC2
H3K27ac Hnisz
FetalDHS Trynka
Enhancer Hoffman
Enhancer Andersson
DHS Trynka
DGF ENCODE
CTCF Hoffman
Conserved LindbladToh
Coding UCSC
Annotation
−100
0
100
Enrichment (prop h2 / prop snps)

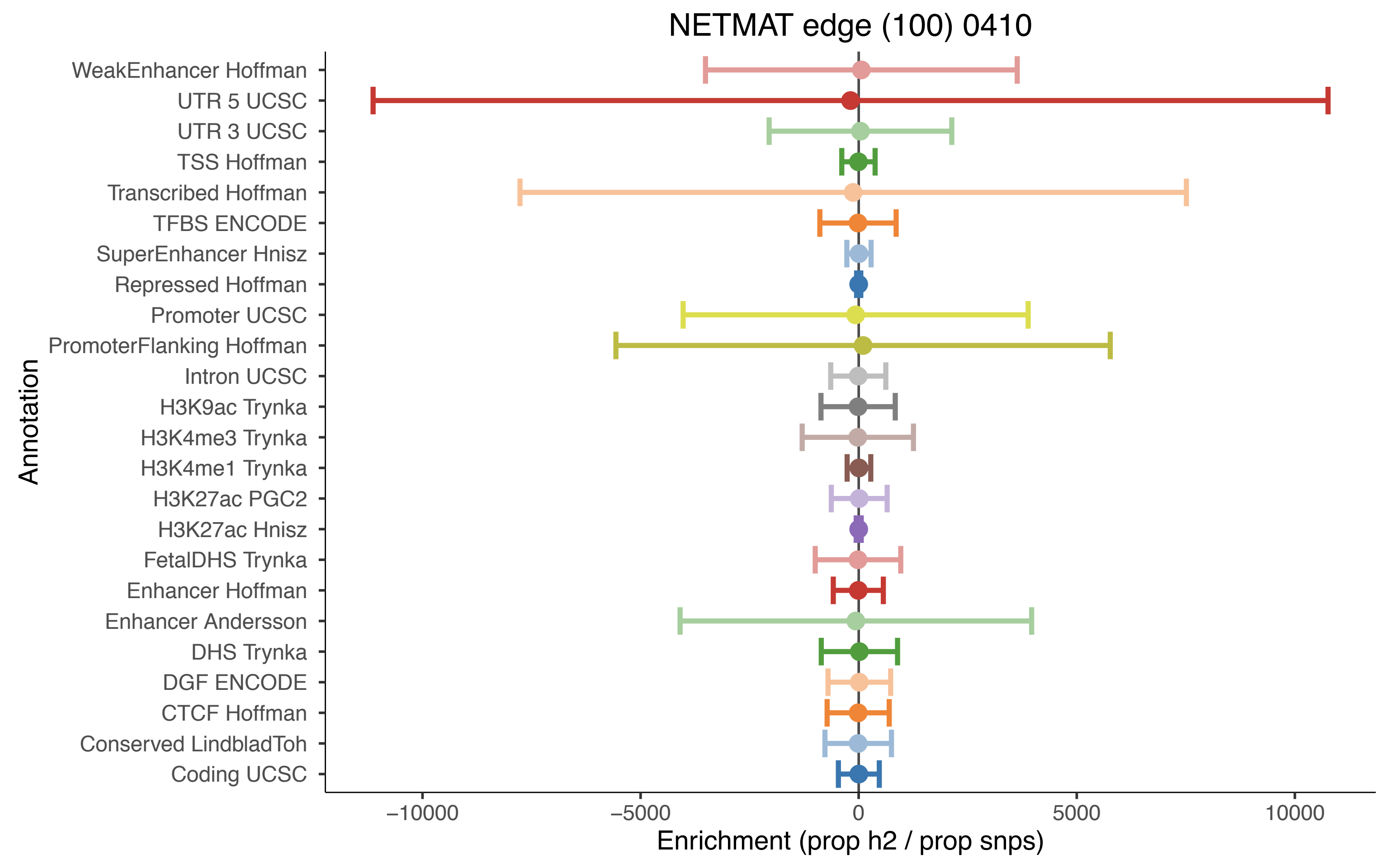
NETMAT edge (100) 0410
WeakEnhancer Hoffman
UTR 5 UCSC
UTR 3 UCSC
TSS Hoffman
Transcribed Hoffman
TFBS ENCODE
SuperEnhancer Hnisz
Repressed Hoffman
Promoter UCSC
PromoterFlanking Hoffman
Intron UCSC
H3K9ac Trynka
H3K4me3 Trynka
H3K4me1 Trynka
H3K27ac PGC2
H3K27ac Hnisz
FetalDHS Trynka
Enhancer Hoffman
Enhancer Andersson
DHS Trynka
DGF ENCODE
CTCF Hoffman
Conserved LindbladToh
Coding UCSC
Annotation
−10000
−5000
0
5000
10000
Enrichment (prop h2 / prop snps)

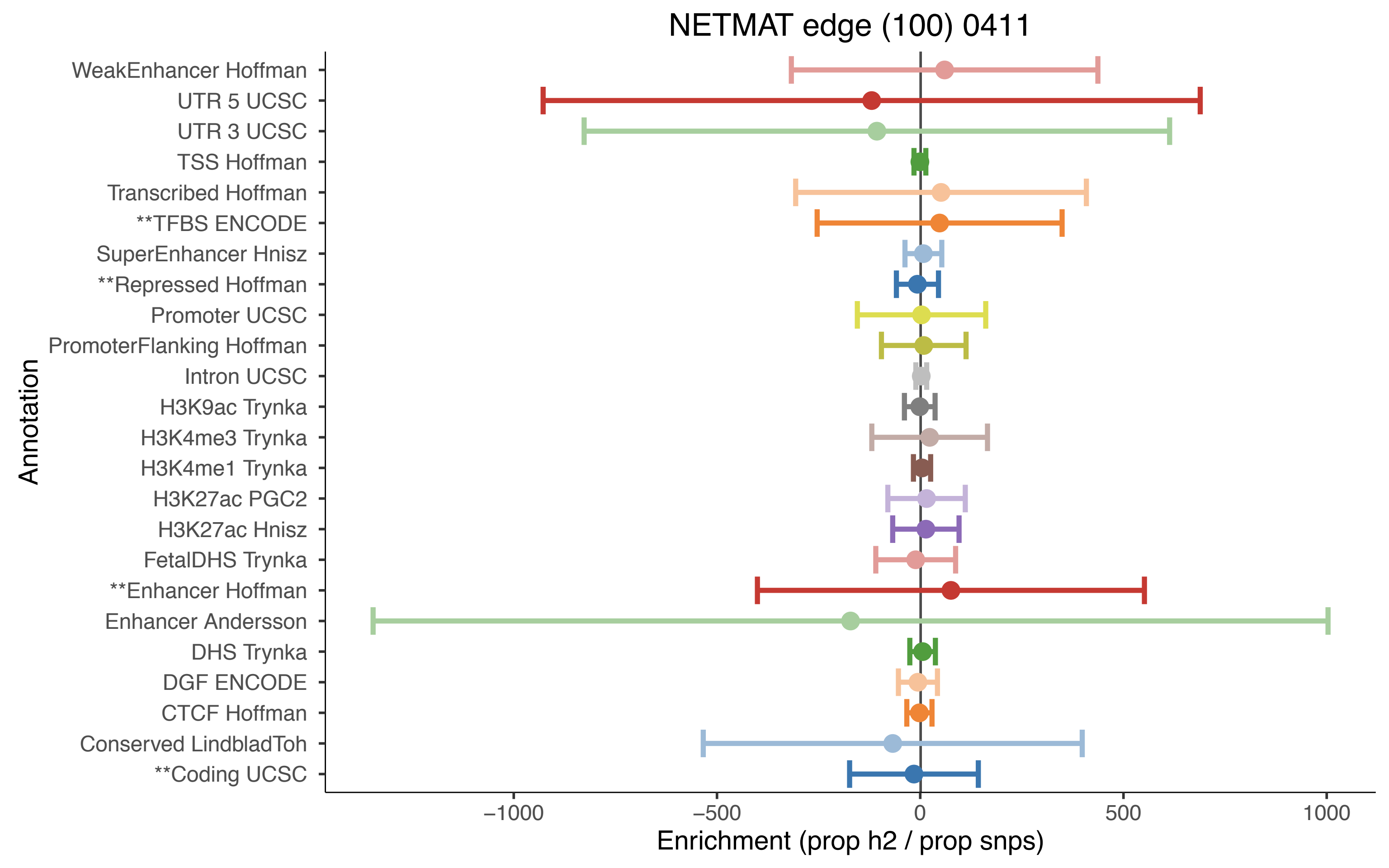
NETMAT edge (100) 0411
WeakEnhancer Hoffman
UTR 5 UCSC
UTR 3 UCSC
TSS Hoffman
Transcribed Hoffman
**TFBS ENCODE
SuperEnhancer Hnisz
**Repressed Hoffman
Promoter UCSC
PromoterFlanking Hoffman
Intron UCSC
H3K9ac Trynka
H3K4me3 Trynka
H3K4me1 Trynka
H3K27ac PGC2
H3K27ac Hnisz
FetalDHS Trynka
**Enhancer Hoffman
Enhancer Andersson
DHS Trynka
DGF ENCODE
CTCF Hoffman
Conserved LindbladToh
**Coding UCSC
Annotation
−1000
−500
0
500
1000
Enrichment (prop h2 / prop snps)

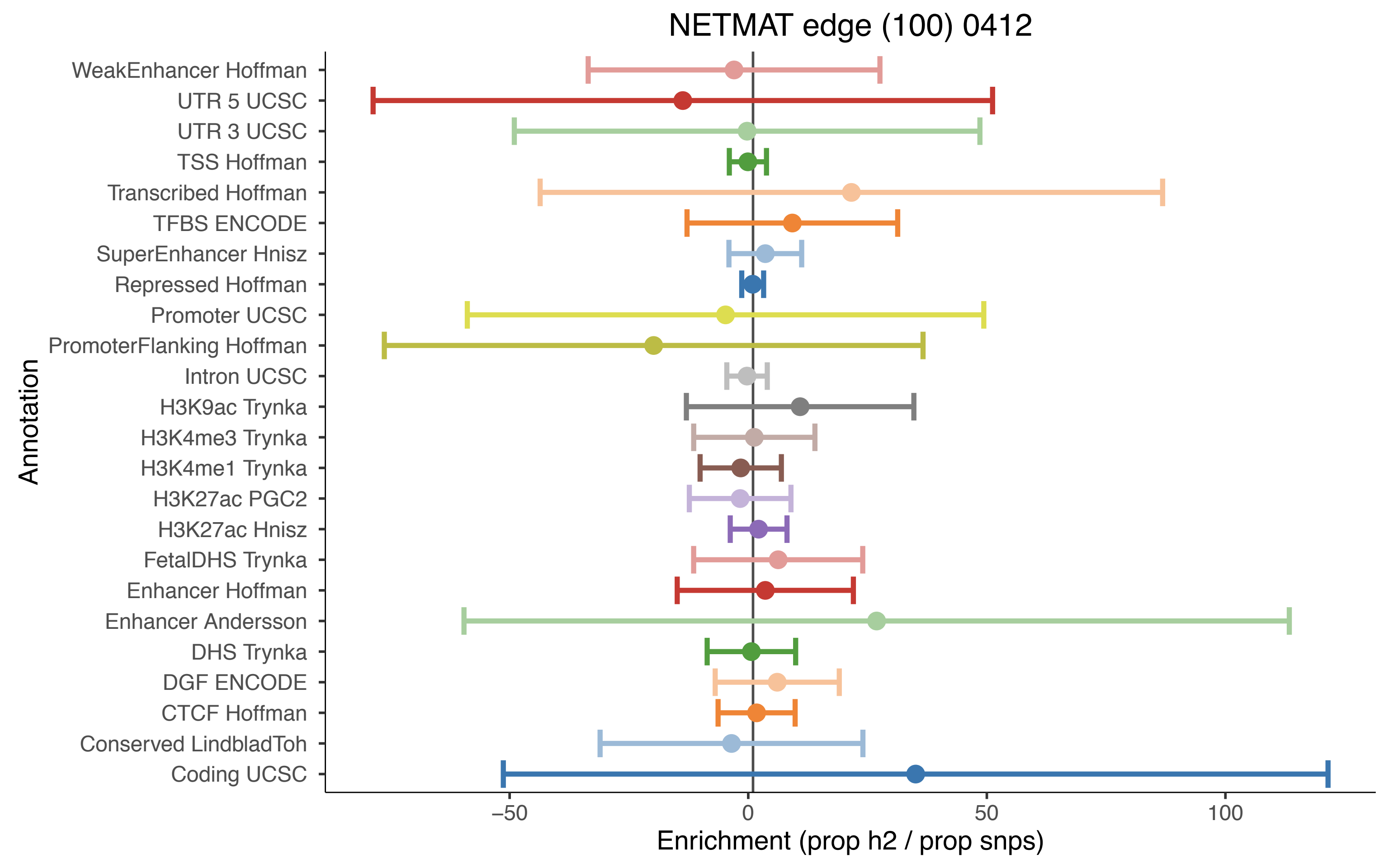
NETMAT edge (100) 0412
WeakEnhancer Hoffman
UTR 5 UCSC
UTR 3 UCSC
TSS Hoffman
Transcribed Hoffman
TFBS ENCODE
SuperEnhancer Hnisz
Repressed Hoffman
Promoter UCSC
PromoterFlanking Hoffman
Intron UCSC
H3K9ac Trynka
H3K4me3 Trynka
H3K4me1 Trynka
H3K27ac PGC2
H3K27ac Hnisz
FetalDHS Trynka
Enhancer Hoffman
Enhancer Andersson
DHS Trynka
DGF ENCODE
CTCF Hoffman
Conserved LindbladToh
Coding UCSC
Annotation
−50
0
50
100
Enrichment (prop h2 / prop snps)

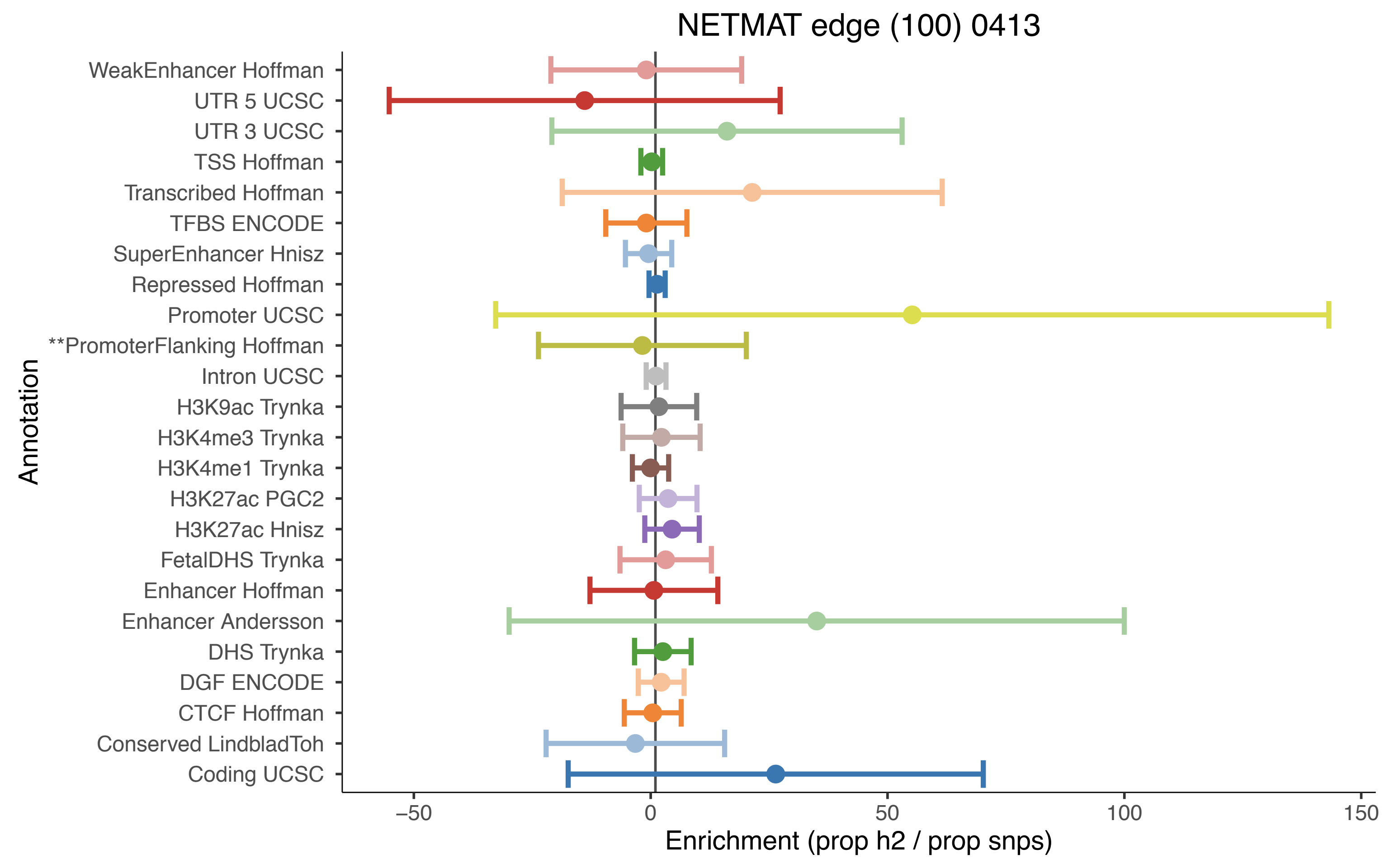
NETMAT edge (100) 0413
WeakEnhancer Hoffman
UTR 5 UCSC
UTR 3 UCSC
TSS Hoffman
Transcribed Hoffman
TFBS ENCODE
SuperEnhancer Hnisz
Repressed Hoffman
Promoter UCSC
**PromoterFlanking Hoffman
Intron UCSC
H3K9ac Trynka
H3K4me3 Trynka
H3K4me1 Trynka
H3K27ac PGC2
H3K27ac Hnisz
FetalDHS Trynka
Enhancer Hoffman
Enhancer Andersson
DHS Trynka
DGF ENCODE
CTCF Hoffman
Conserved LindbladToh
Coding UCSC
Annotation
−50
0
50
100
150
Enrichment (prop h2 / prop snps)

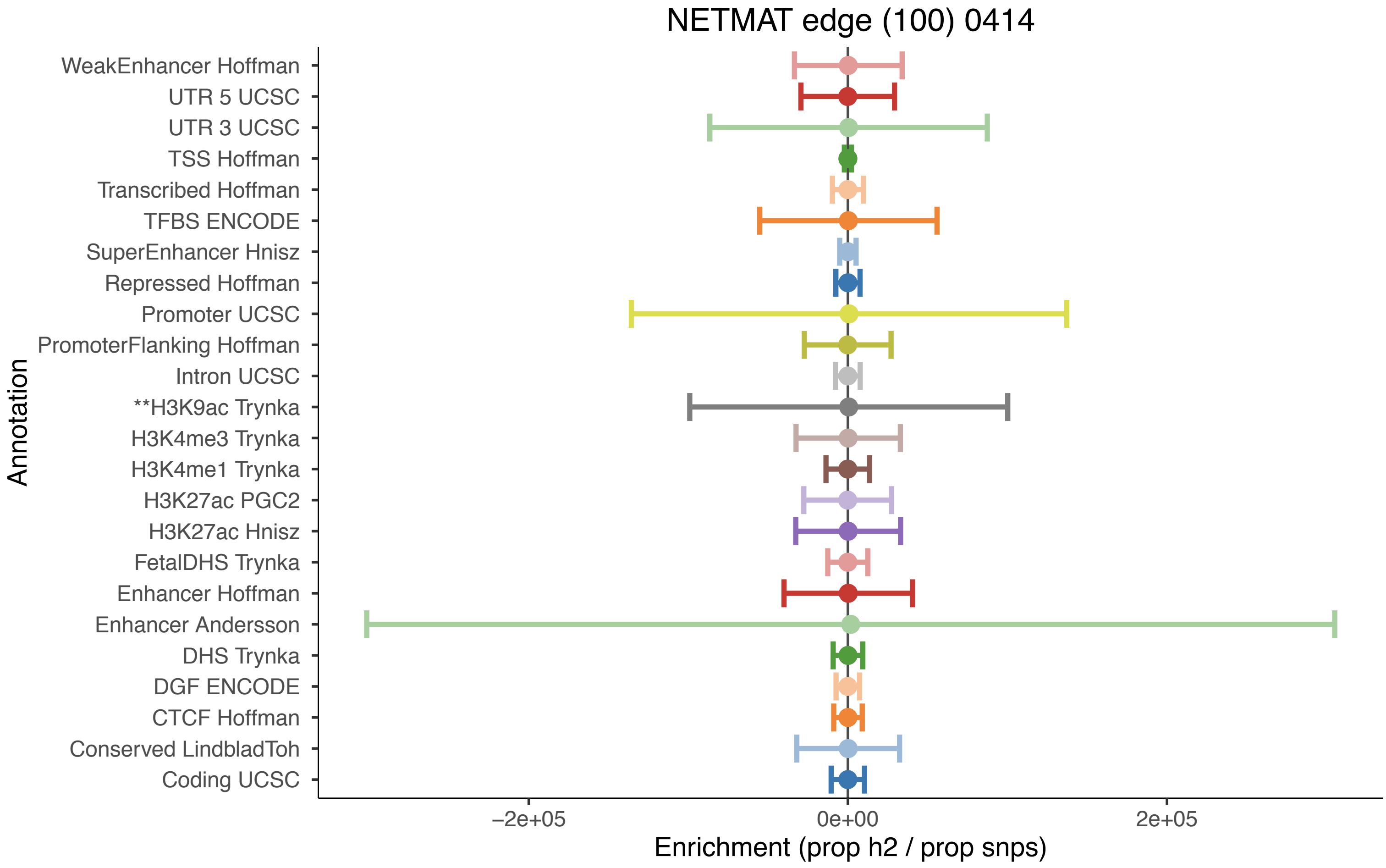

NETMAT edge (100) 0414
Annotation
WeakEnhancer Hoffman
UTR 5 UCSC
UTR 3 UCSC
TSS Hoffman
Transcribed Hoffman
TFBS ENCODE
SuperEnhancer Hnisz
Repressed Hoffman
Promoter UCSC
PromoterFlanking Hoffman
Intron UCSC
**H3K9ac Trynka
H3K4me3 Trynka
H3K4me1 Trynka
H3K27ac PGC2
H3K27ac Hnisz
FetalDHS Trynka
Enhancer Hoffman
Enhancer Andersson
DHS Trynka
DGF ENCODE
CTCF Hoffman
Conserved LindbladToh
Coding UCSC
−2e+05
0e+00
2e+05
Enrichment (prop h2 / prop snps)

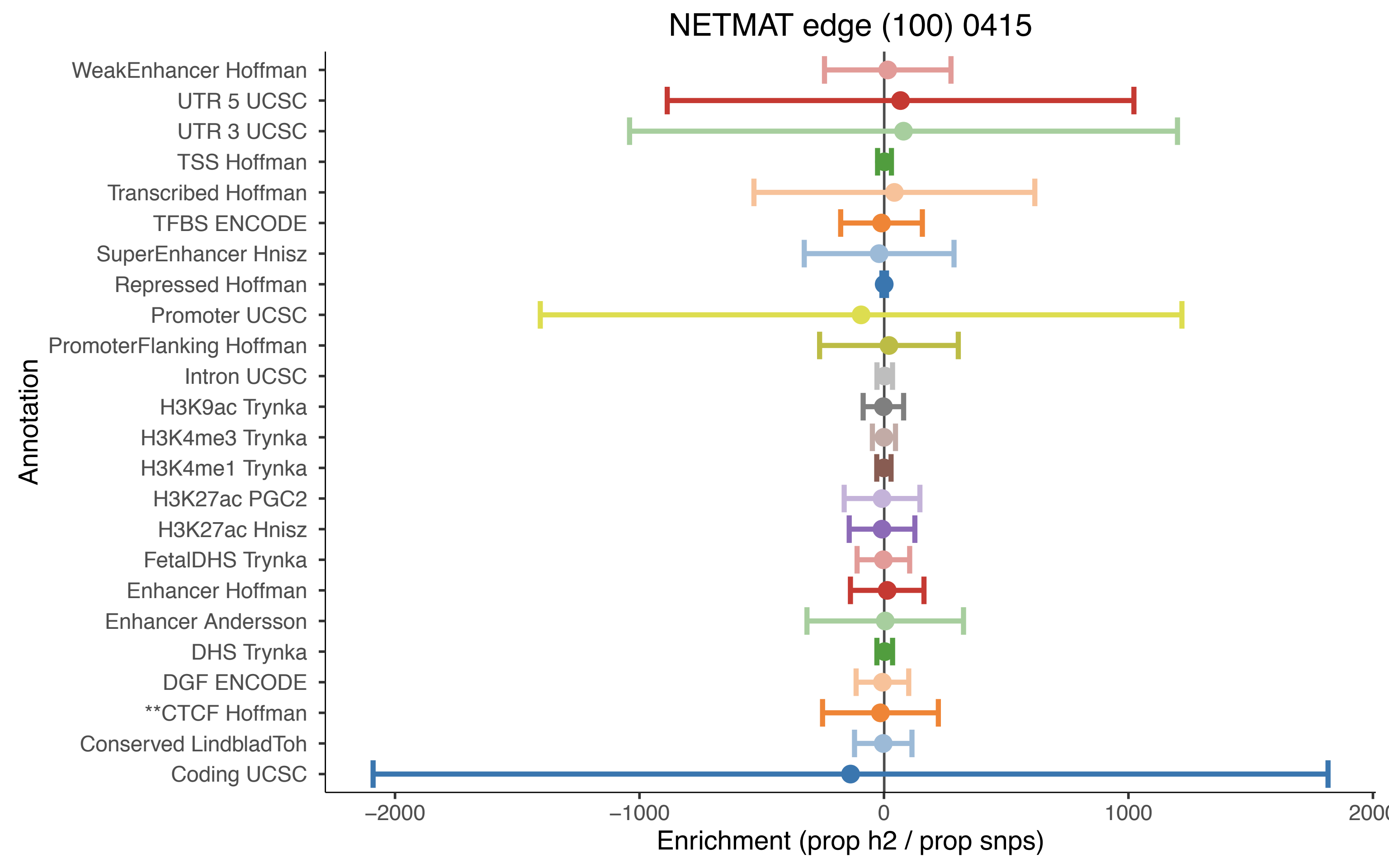

NETMAT edge (100) 0415
Annotation
WeakEnhancer Hoffman
UTR 5 UCSC
UTR 3 UCSC
TSS Hoffman
Transcribed Hoffman
TFBS ENCODE
SuperEnhancer Hnisz
Repressed Hoffman
Promoter UCSC
PromoterFlanking Hoffman
Intron UCSC
H3K9ac Trynka
H3K4me3 Trynka
H3K4me1 Trynka
H3K27ac PGC2
H3K27ac Hnisz
FetalDHS Trynka
Enhancer Hoffman
Enhancer Andersson
DHS Trynka
DGF ENCODE
**CTCF Hoffman
Conserved LindbladToh
Coding UCSC
−2000
−1000
0
1000
Enrichment (prop h2 / prop snps)

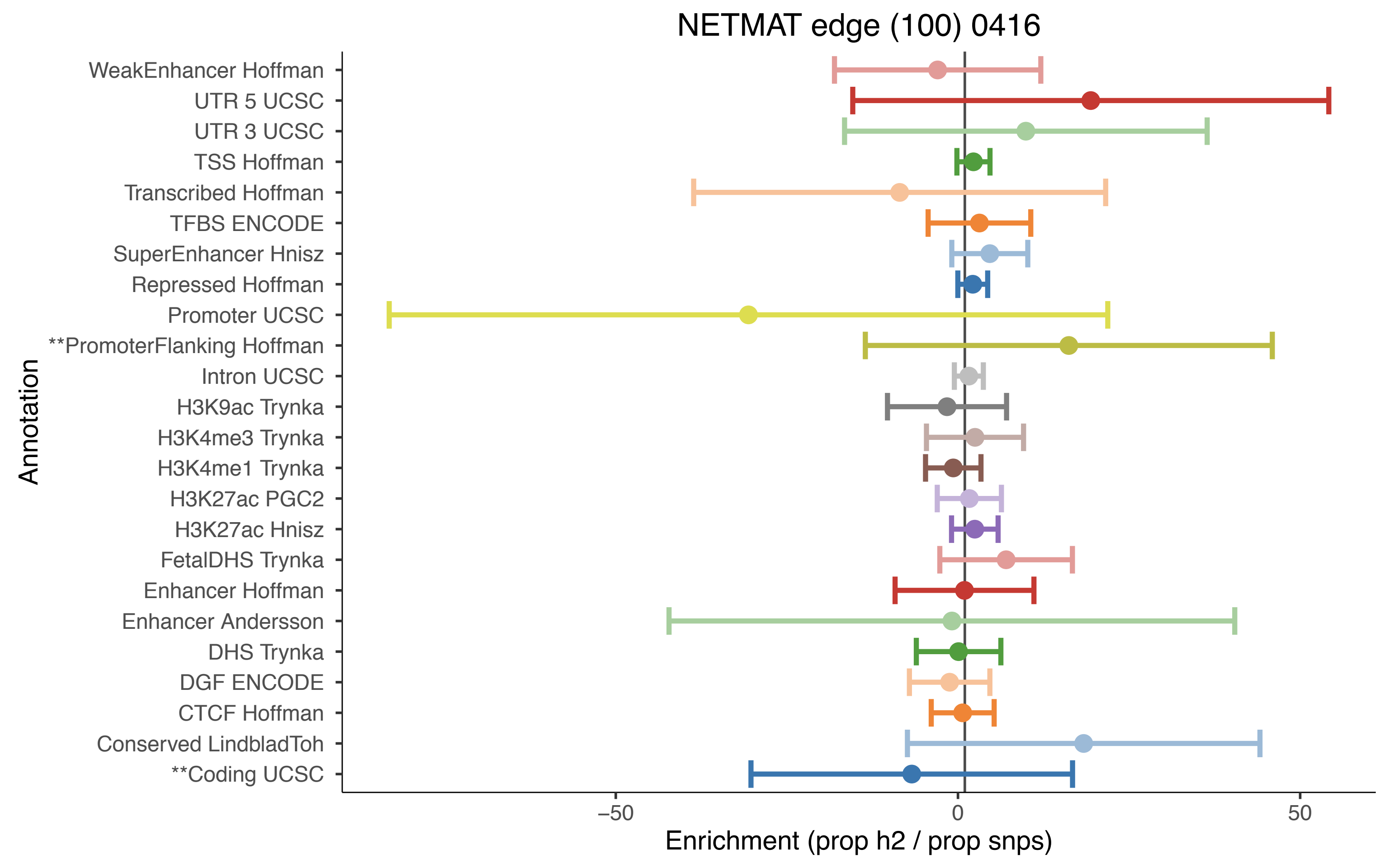
NETMAT edge (100) 0416
WeakEnhancer Hoffman
UTR 5 UCSC
UTR 3 UCSC
TSS Hoffman
Transcribed Hoffman
TFBS ENCODE
SuperEnhancer Hnisz
Repressed Hoffman
Promoter UCSC
**PromoterFlanking Hoffman
Intron UCSC
H3K9ac Trynka
H3K4me3 Trynka
H3K4me1 Trynka
H3K27ac PGC2
H3K27ac Hnisz
FetalDHS Trynka
Enhancer Hoffman
Enhancer Andersson
DHS Trynka
DGF ENCODE
CTCF Hoffman
Conserved LindbladToh
**Coding UCSC
Annotation
−50
0
50
Enrichment (prop h2 / prop snps)

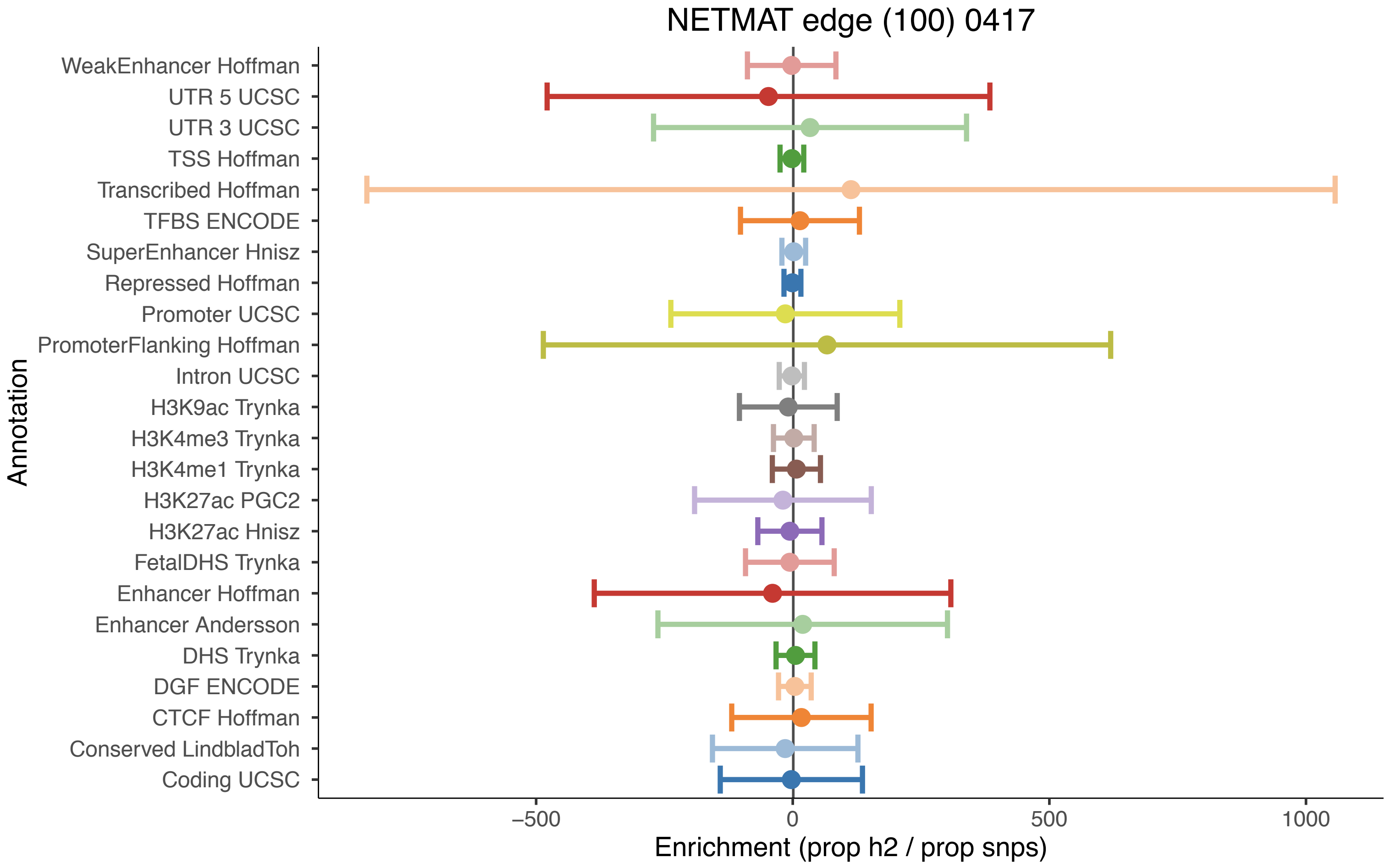

NETMAT edge (100) 0417
WeakEnhancer Hoffman
UTR 5 UCSC
UTR 3 UCSC
TSS Hoffman
Transcribed Hoffman
TFBS ENCODE
SuperEnhancer Hnisz
Repressed Hoffman
Promoter UCSC
PromoterFlanking Hoffman
Intron UCSC
H3K9ac Trynka
H3K4me3 Trynka
H3K4me1 Trynka
H3K27ac PGC2
H3K27ac Hnisz
FetalDHS Trynka
Enhancer Hoffman
Enhancer Andersson
DHS Trynka
DGF ENCODE
CTCF Hoffman
Conserved LindbladToh
Coding UCSC
Annotation
−500
0
500
1000
Enrichment (prop h2 / prop snps)

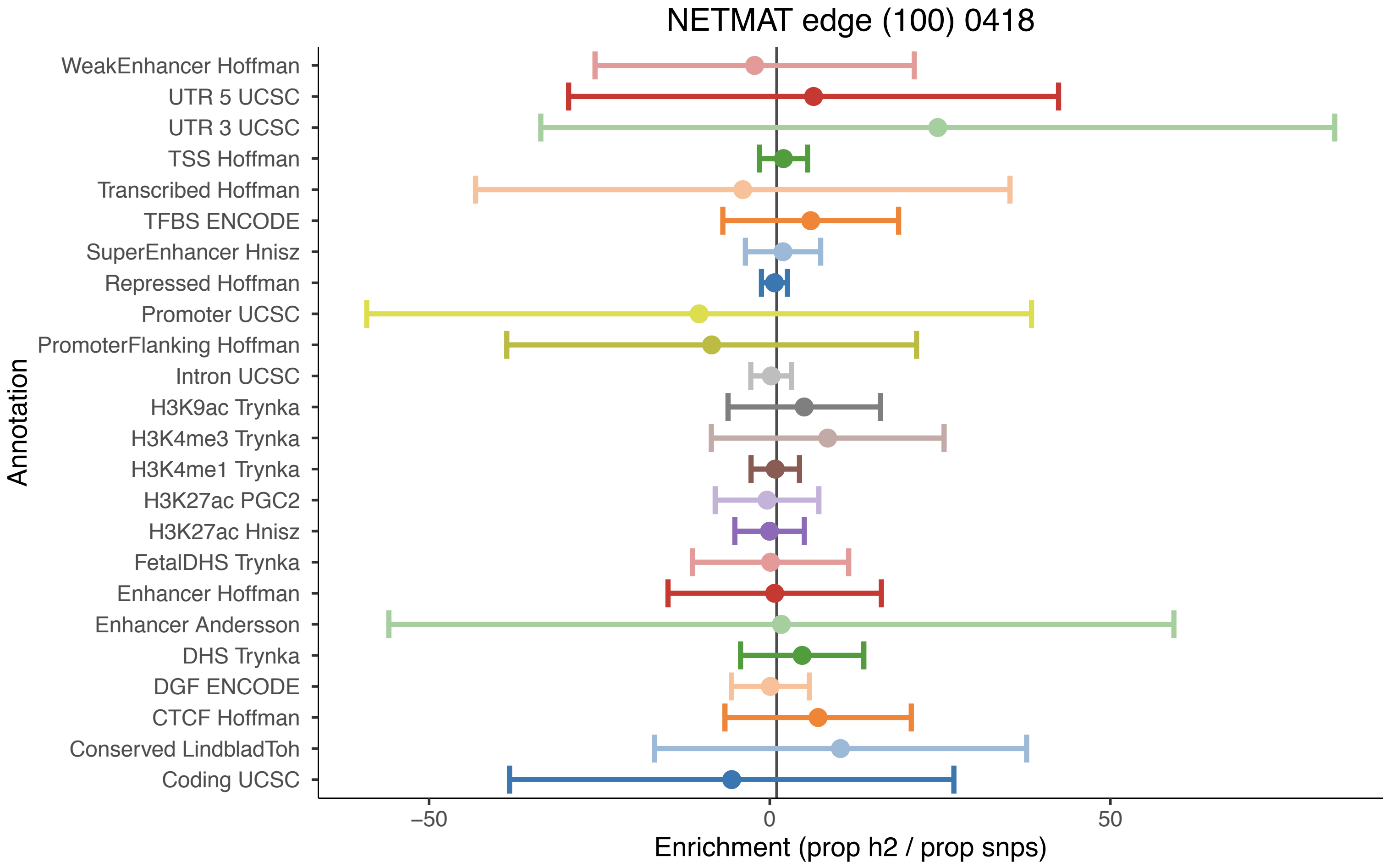

NETMAT edge (100) 0418
WeakEnhancer Hoffman
UTR 5 UCSC
UTR 3 UCSC
TSS Hoffman
Transcribed Hoffman
TFBS ENCODE
SuperEnhancer Hnisz
Repressed Hoffman
Promoter UCSC
PromoterFlanking Hoffman
Intron UCSC
H3K9ac Trynka
H3K4me3 Trynka
H3K4me1 Trynka
H3K27ac PGC2
H3K27ac Hnisz
FetalDHS Trynka
Enhancer Hoffman
Enhancer Andersson
DHS Trynka
DGF ENCODE
CTCF Hoffman
Conserved LindbladToh
Coding UCSC
Annotation
−50
0
50
Enrichment (prop h2 / prop snps)

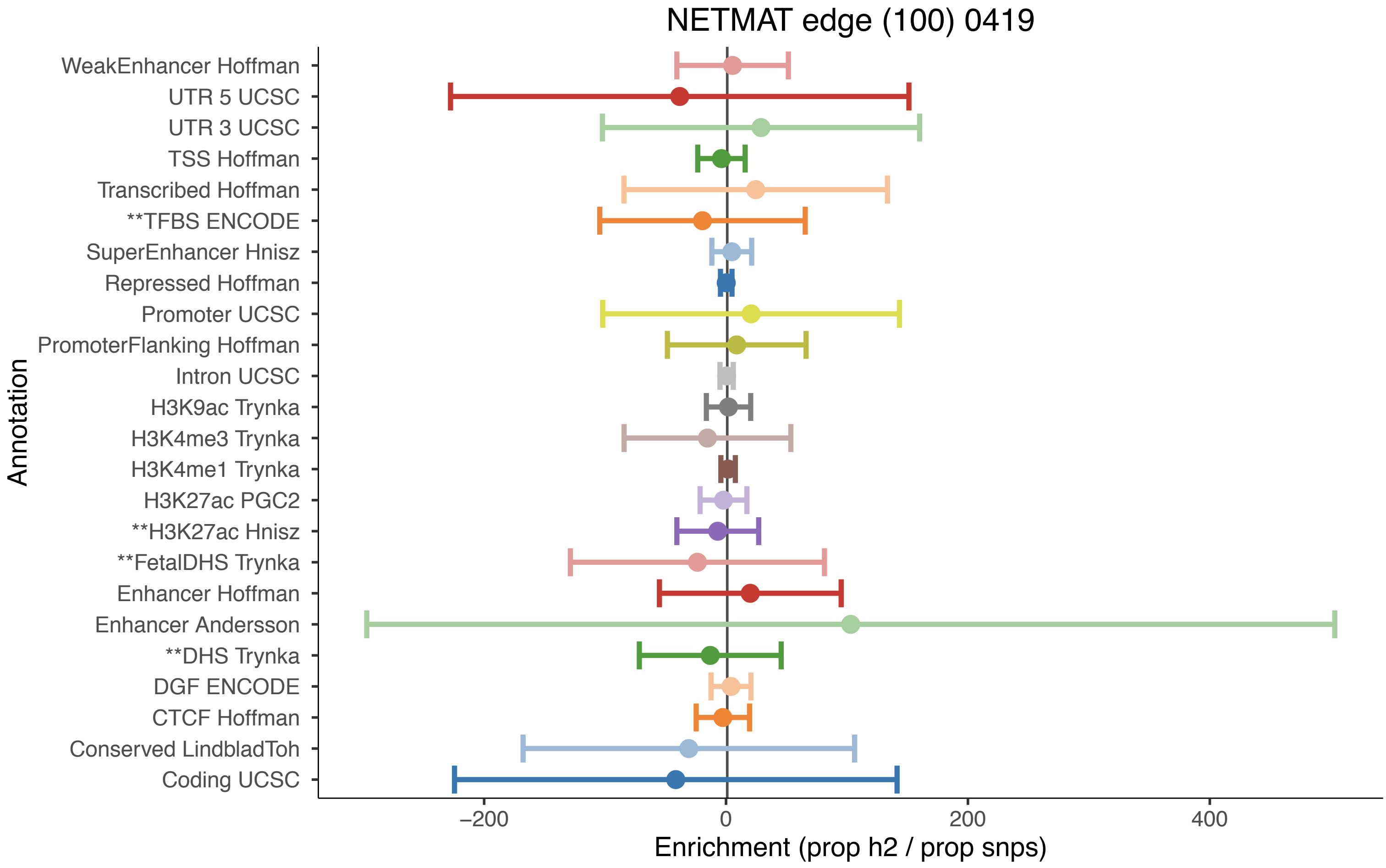

NETMAT edge (100) 0419
Annotation
WeakEnhancer Hoffman
UTR 5 UCSC
UTR 3 UCSC
TSS Hoffman
Transcribed Hoffman
**TFBS ENCODE
SuperEnhancer Hnisz
Repressed Hoffman
Promoter UCSC
PromoterFlanking Hoffman
Intron UCSC
H3K9ac Trynka
H3K4me3 Trynka
H3K4me1 Trynka
H3K27ac PGC2
**H3K27ac Hnisz
**FetalDHS Trynka
Enhancer Hoffman
Enhancer Andersson
**DHS Trynka
DGF ENCODE
CTCF Hoffman
Conserved LindbladToh
Coding UCSC
−200
0
200
400
Enrichment (prop h2 / prop snps)

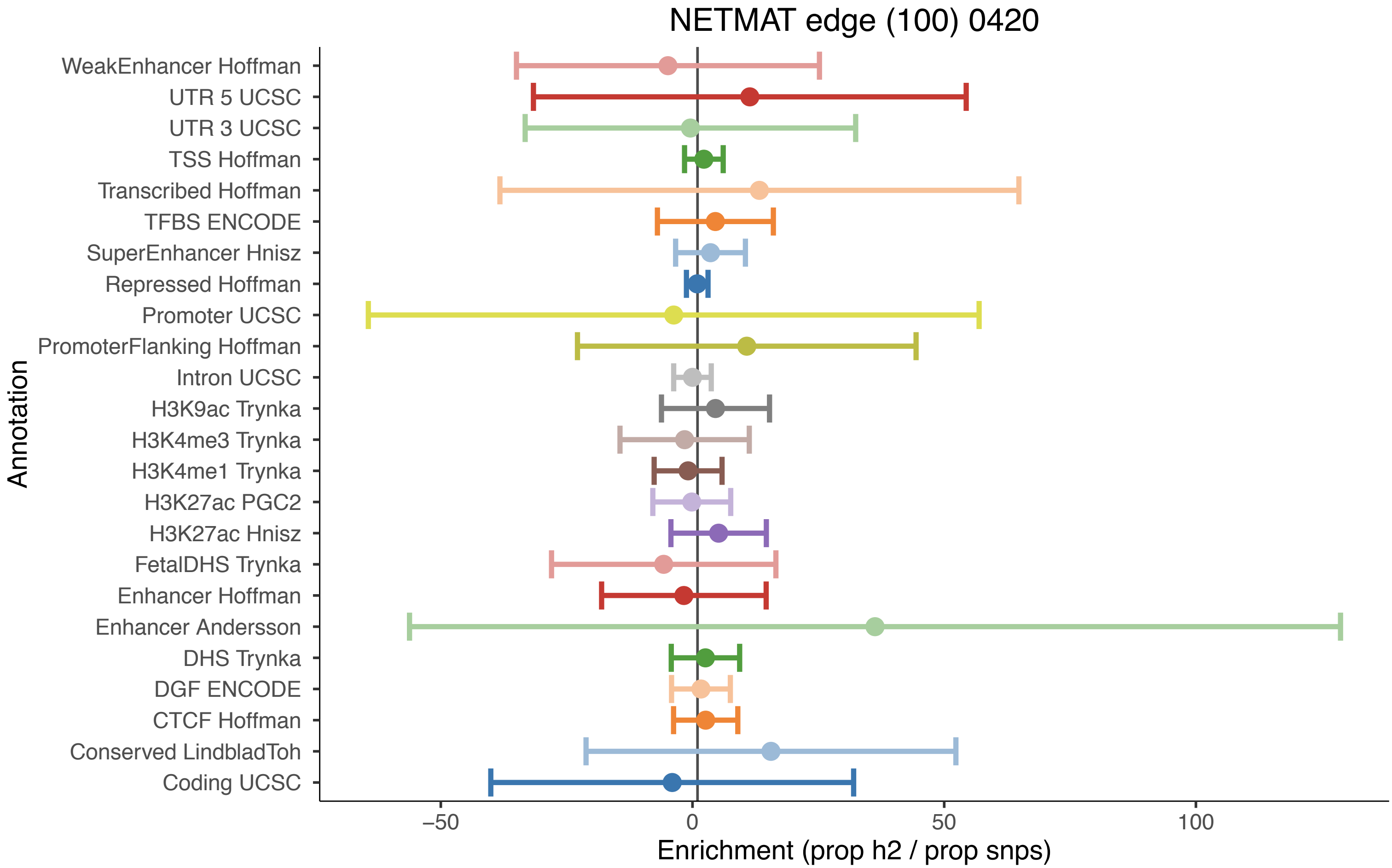
NETMAT edge (100) 0420
WeakEnhancer Hoffman
UTR 5 UCSC
UTR 3 UCSC
TSS Hoffman
Transcribed Hoffman
TFBS ENCODE
SuperEnhancer Hnisz
Repressed Hoffman
Promoter UCSC
PromoterFlanking Hoffman
Intron UCSC
H3K9ac Trynka
H3K4me3 Trynka
H3K4me1 Trynka
H3K27ac PGC2
H3K27ac Hnisz
FetalDHS Trynka
Enhancer Hoffman
Enhancer Andersson
DHS Trynka
DGF ENCODE
CTCF Hoffman
Conserved LindbladToh
Coding UCSC
Annotation
−50
0
50
100
Enrichment (prop h2 / prop snps)

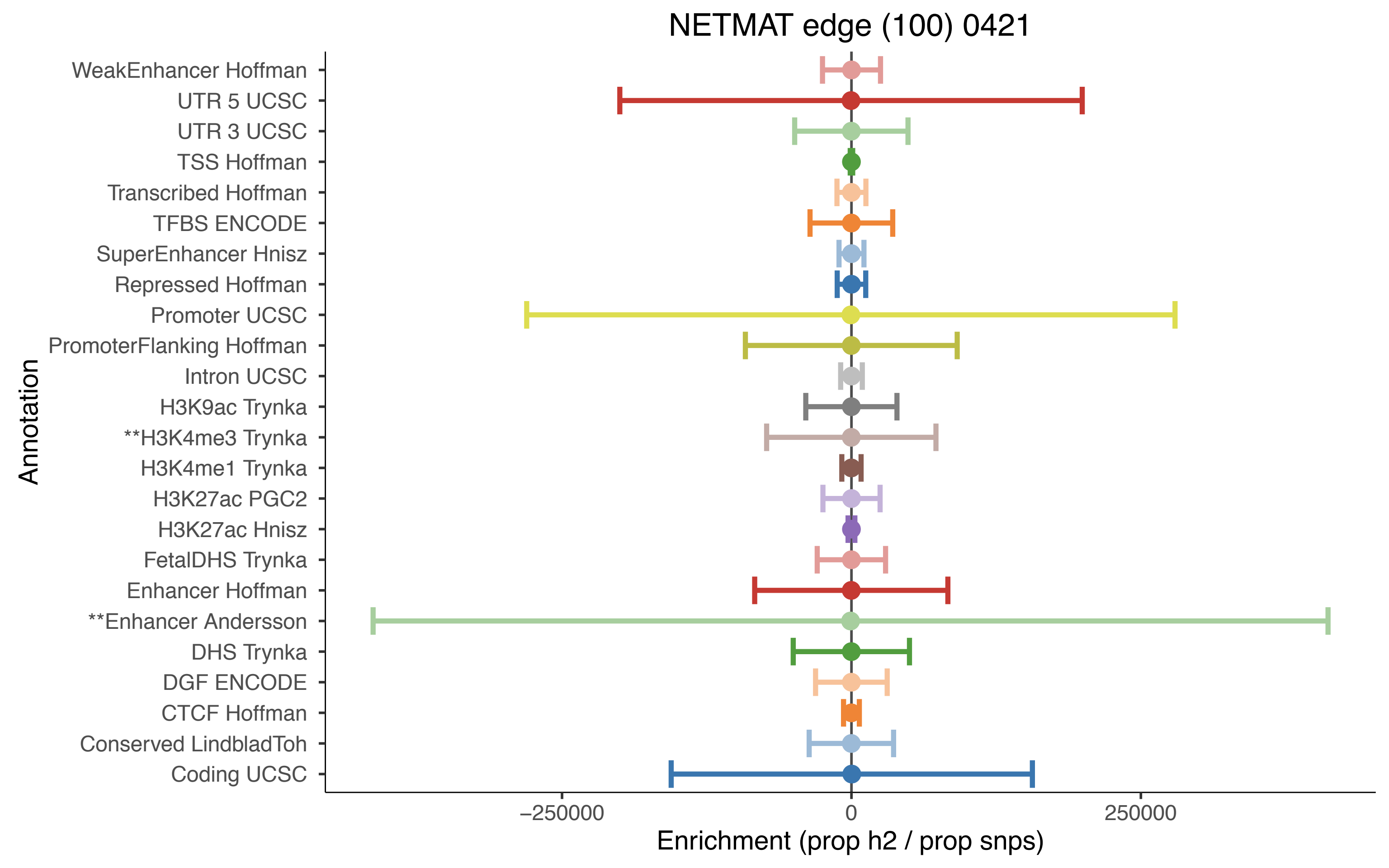
NETMAT edge (100) 0421
Annotation
WeakEnhancer Hoffman
UTR 5 UCSC
UTR 3 UCSC
TSS Hoffman
Transcribed Hoffman
TFBS ENCODE
SuperEnhancer Hnisz
Repressed Hoffman
Promoter UCSC
PromoterFlanking Hoffman
Intron UCSC
H3K9ac Trynka
**H3K4me3 Trynka
H3K4me1 Trynka
H3K27ac PGC2
H3K27ac Hnisz
FetalDHS Trynka
Enhancer Hoffman
**Enhancer Andersson
DHS Trynka
DGF ENCODE
CTCF Hoffman
Conserved LindbladToh
Coding UCSC
−250000
0
250000
Enrichment (prop h2 / prop snps)

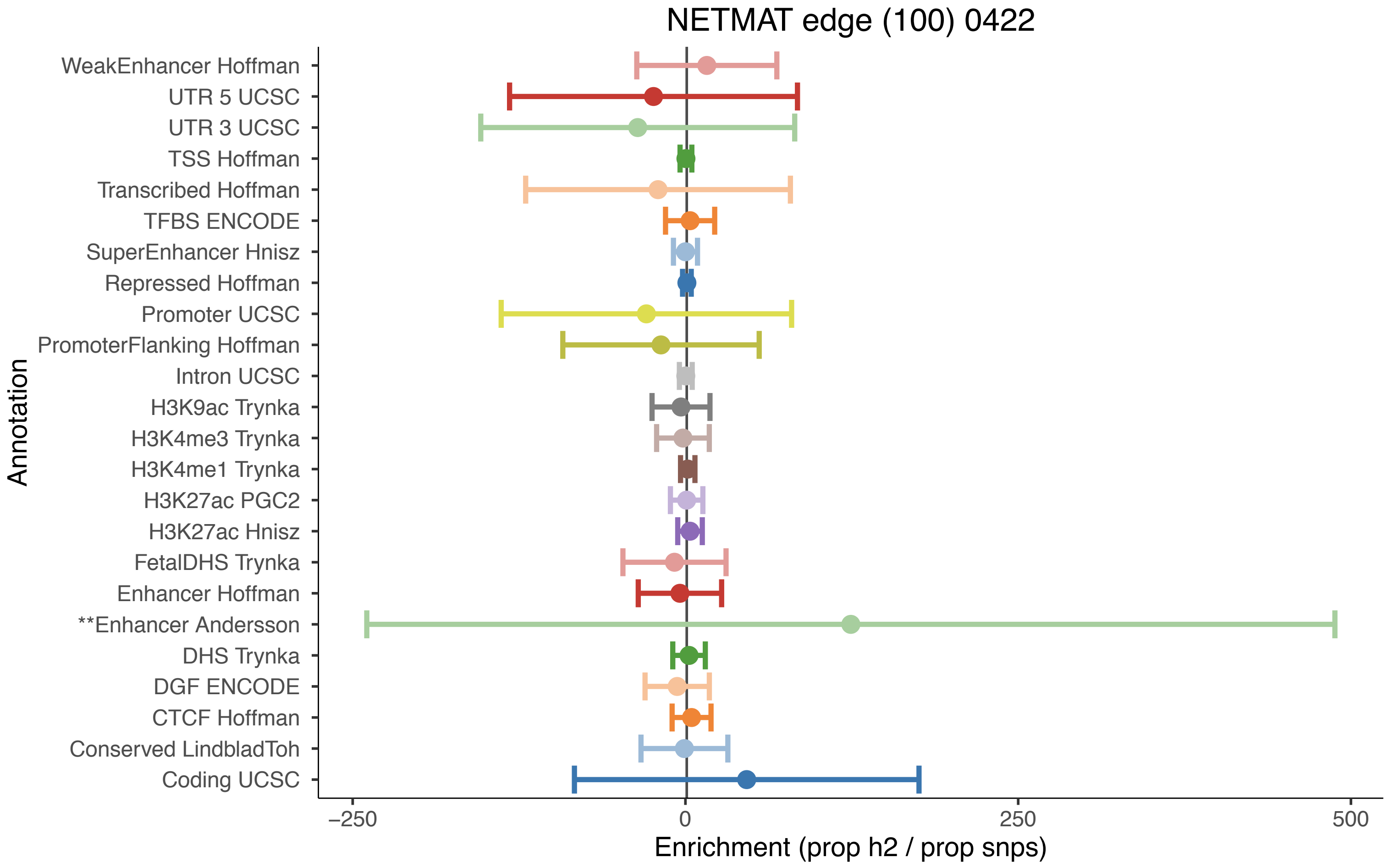
NETMAT edge (100) 0422
WeakEnhancer Hoffman
UTR 5 UCSC
UTR 3 UCSC
TSS Hoffman
Transcribed Hoffman
TFBS ENCODE
SuperEnhancer Hnisz
Repressed Hoffman
Promoter UCSC
PromoterFlanking Hoffman
Intron UCSC
H3K9ac Trynka
H3K4me3 Trynka
H3K4me1 Trynka
H3K27ac PGC2
H3K27ac Hnisz
FetalDHS Trynka
Enhancer Hoffman
**Enhancer Andersson
DHS Trynka
DGF ENCODE
CTCF Hoffman
Conserved LindbladToh
Coding UCSC
Annotation
−250
0
250
500
Enrichment (prop h2 / prop snps)

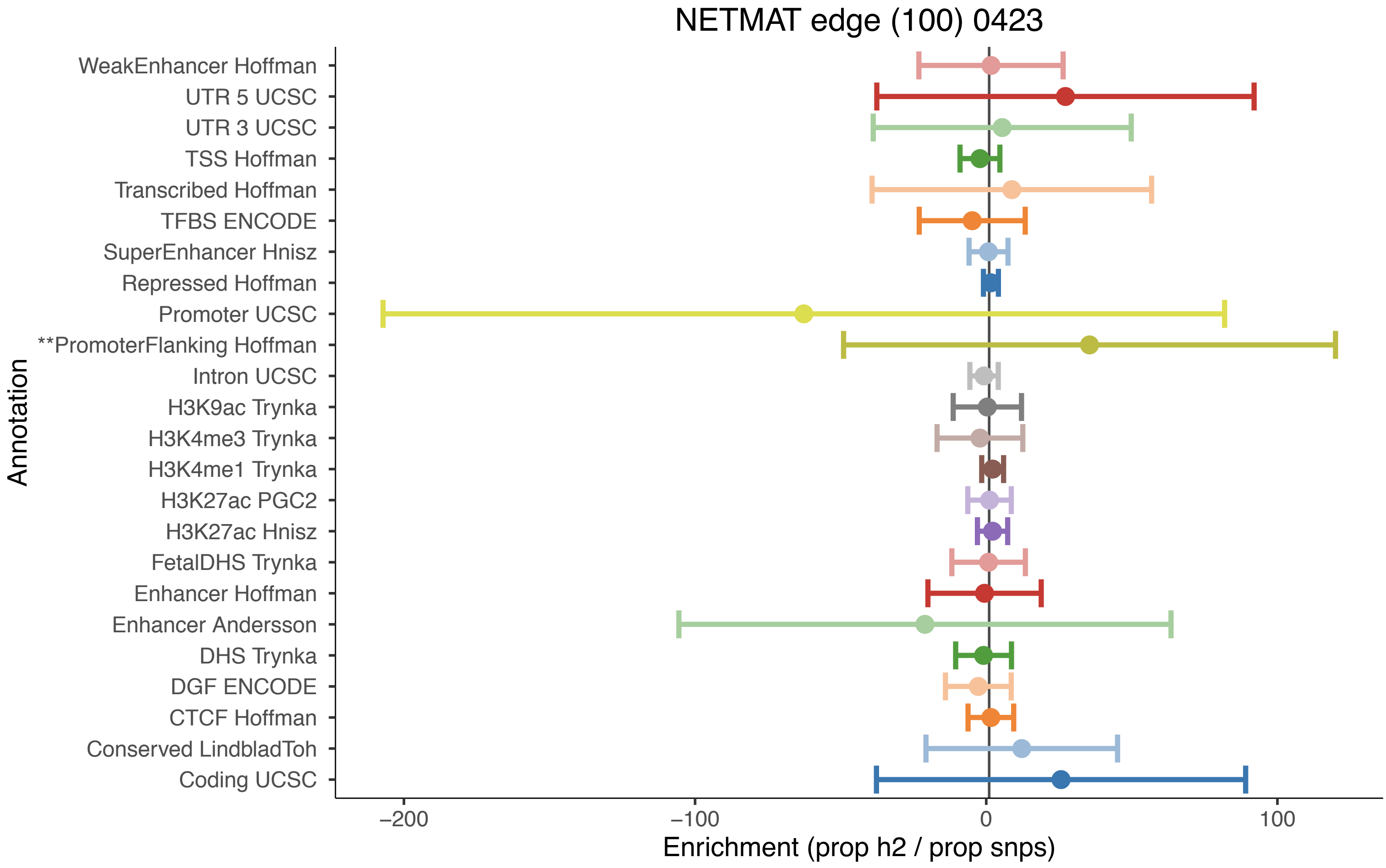
NETMAT edge (100) 0423
WeakEnhancer Hoffman
UTR 5 UCSC
UTR 3 UCSC
TSS Hoffman
Transcribed Hoffman
TFBS ENCODE
SuperEnhancer Hnisz
Repressed Hoffman
Promoter UCSC
**PromoterFlanking Hoffman
Intron UCSC
H3K9ac Trynka
H3K4me3 Trynka
H3K4me1 Trynka
H3K27ac PGC2
H3K27ac Hnisz
FetalDHS Trynka
Enhancer Hoffman
Enhancer Andersson
DHS Trynka
DGF ENCODE
CTCF Hoffman
Conserved LindbladToh
Coding UCSC
Annotation
−200
−100
0
100
Enrichment (prop h2 / prop snps)

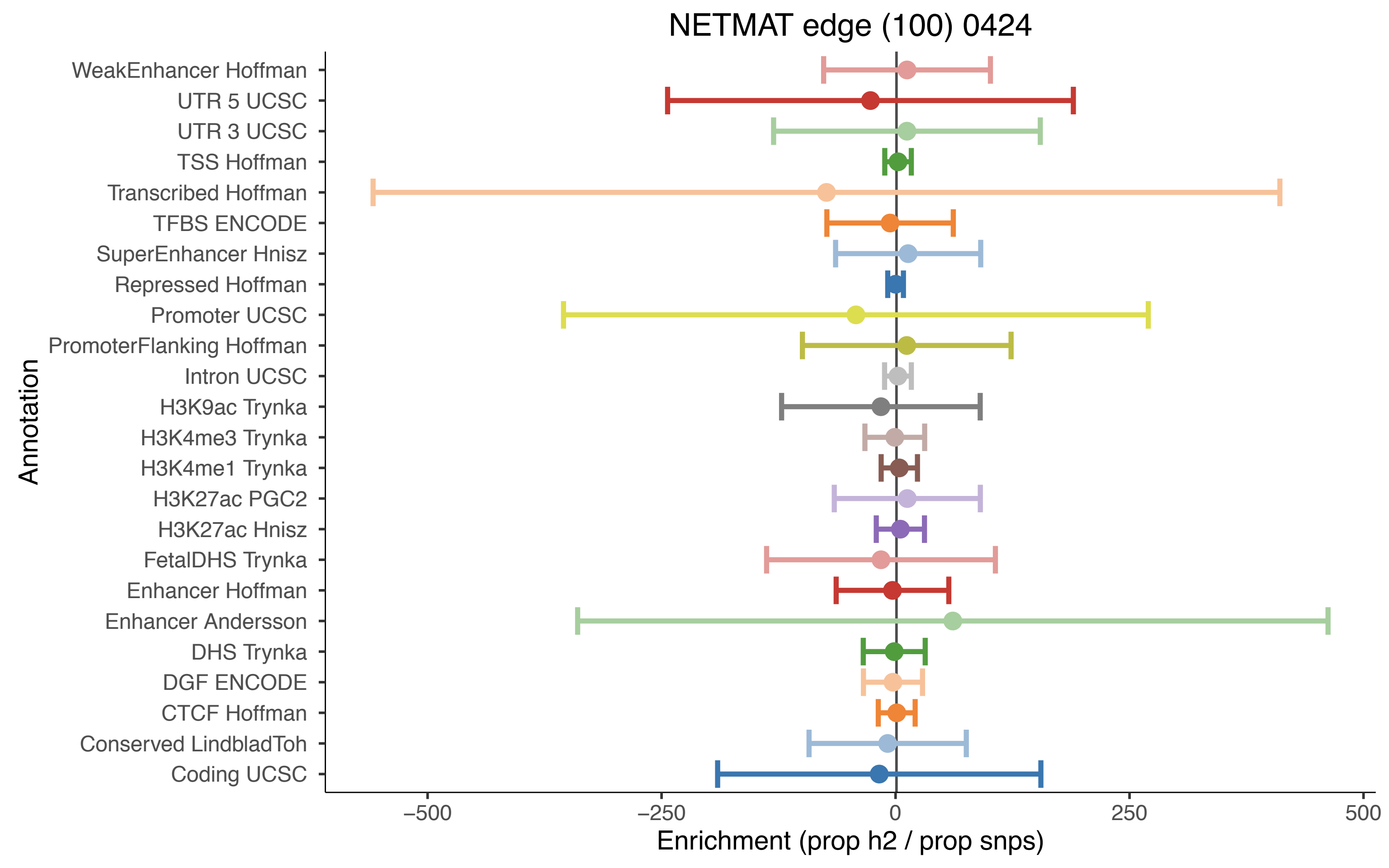

NETMAT edge (100) 0424
WeakEnhancer Hoffman
UTR 5 UCSC
UTR 3 UCSC
TSS Hoffman
Transcribed Hoffman
TFBS ENCODE
SuperEnhancer Hnisz
Repressed Hoffman
Promoter UCSC
PromoterFlanking Hoffman
Intron UCSC
H3K9ac Trynka
H3K4me3 Trynka
H3K4me1 Trynka
H3K27ac PGC2
H3K27ac Hnisz
FetalDHS Trynka
Enhancer Hoffman
Enhancer Andersson
DHS Trynka
DGF ENCODE
CTCF Hoffman
Conserved LindbladToh
Coding UCSC
Annotation
−500
−250
0
250
500
Enrichment (prop h2 / prop snps)

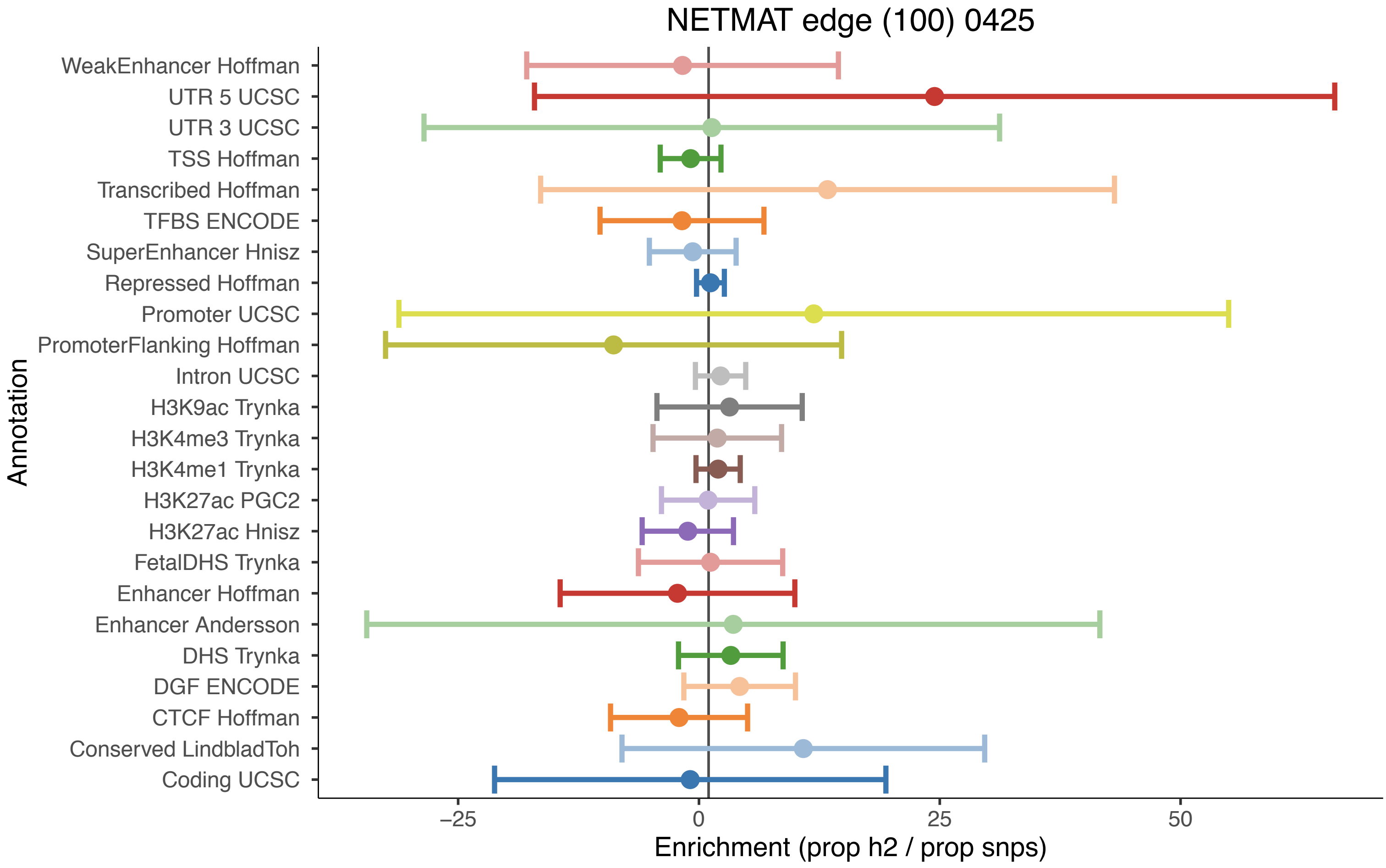

NETMAT edge (100) 0425
WeakEnhancer Hoffman
UTR 5 UCSC
UTR 3 UCSC
TSS Hoffman
Transcribed Hoffman
TFBS ENCODE
SuperEnhancer Hnisz
Repressed Hoffman
Promoter UCSC
PromoterFlanking Hoffman
Intron UCSC
H3K9ac Trynka
H3K4me3 Trynka
H3K4me1 Trynka
H3K27ac PGC2
H3K27ac Hnisz
FetalDHS Trynka
Enhancer Hoffman
Enhancer Andersson
DHS Trynka
DGF ENCODE
CTCF Hoffman
Conserved LindbladToh
Coding UCSC
Annotation
−25
0
25
50
Enrichment (prop h2 / prop snps)

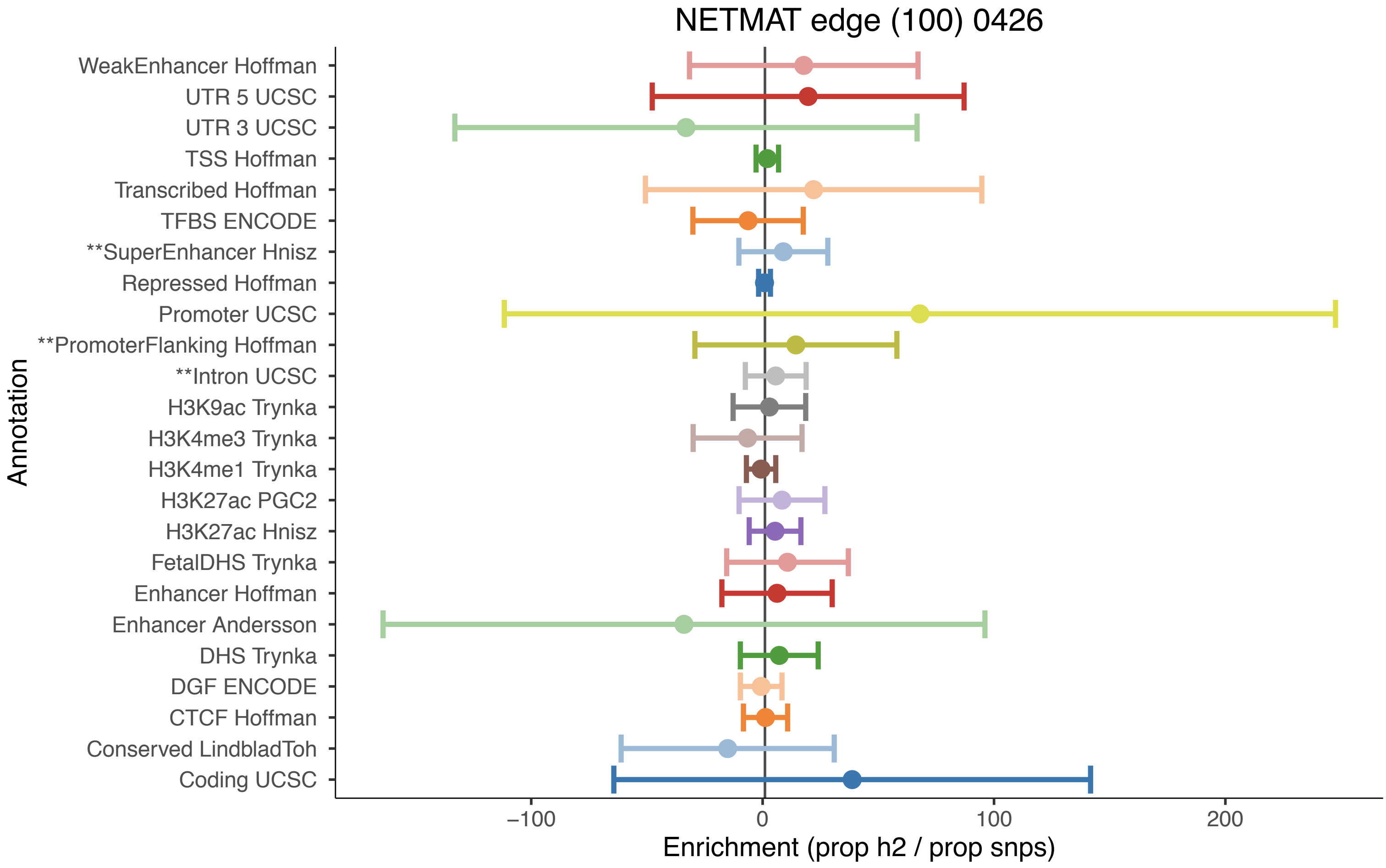
NETMAT edge (100) 0426
WeakEnhancer Hoffman
UTR 5 UCSC
UTR 3 UCSC
TSS Hoffman
Transcribed Hoffman
TFBS ENCODE
**SuperEnhancer Hnisz
Repressed Hoffman
Promoter UCSC
**PromoterFlanking Hoffman
**Intron UCSC
H3K9ac Trynka
H3K4me3 Trynka
H3K4me1 Trynka
H3K27ac PGC2
H3K27ac Hnisz
FetalDHS Trynka
Enhancer Hoffman
Enhancer Andersson
DHS Trynka
DGF ENCODE
CTCF Hoffman
Conserved LindbladToh
Coding UCSC
Annotation
−100
0
100
200
Enrichment (prop h2 / prop snps)

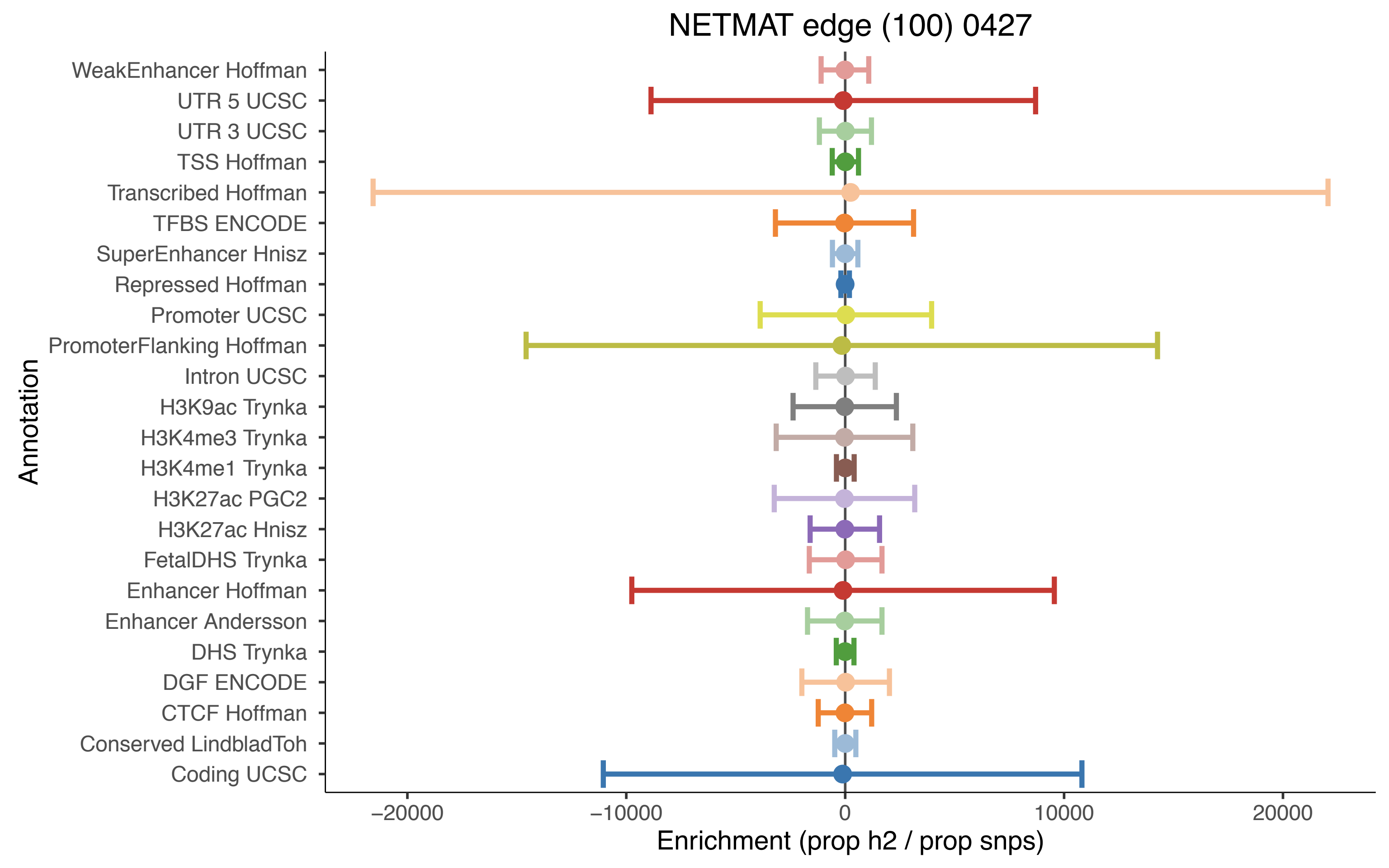
NETMAT edge (100) 0427
WeakEnhancer Hoffman
UTR 5 UCSC
UTR 3 UCSC
TSS Hoffman
Transcribed Hoffman
TFBS ENCODE
SuperEnhancer Hnisz
Repressed Hoffman
Promoter UCSC
PromoterFlanking Hoffman
Intron UCSC
H3K9ac Trynka
H3K4me3 Trynka
H3K4me1 Trynka
H3K27ac PGC2
H3K27ac Hnisz
FetalDHS Trynka
Enhancer Hoffman
Enhancer Andersson
DHS Trynka
DGF ENCODE
CTCF Hoffman
Conserved LindbladToh
Coding UCSC
Annotation
−20000
−10000
0
10000
20000
Enrichment (prop h2 / prop snps)

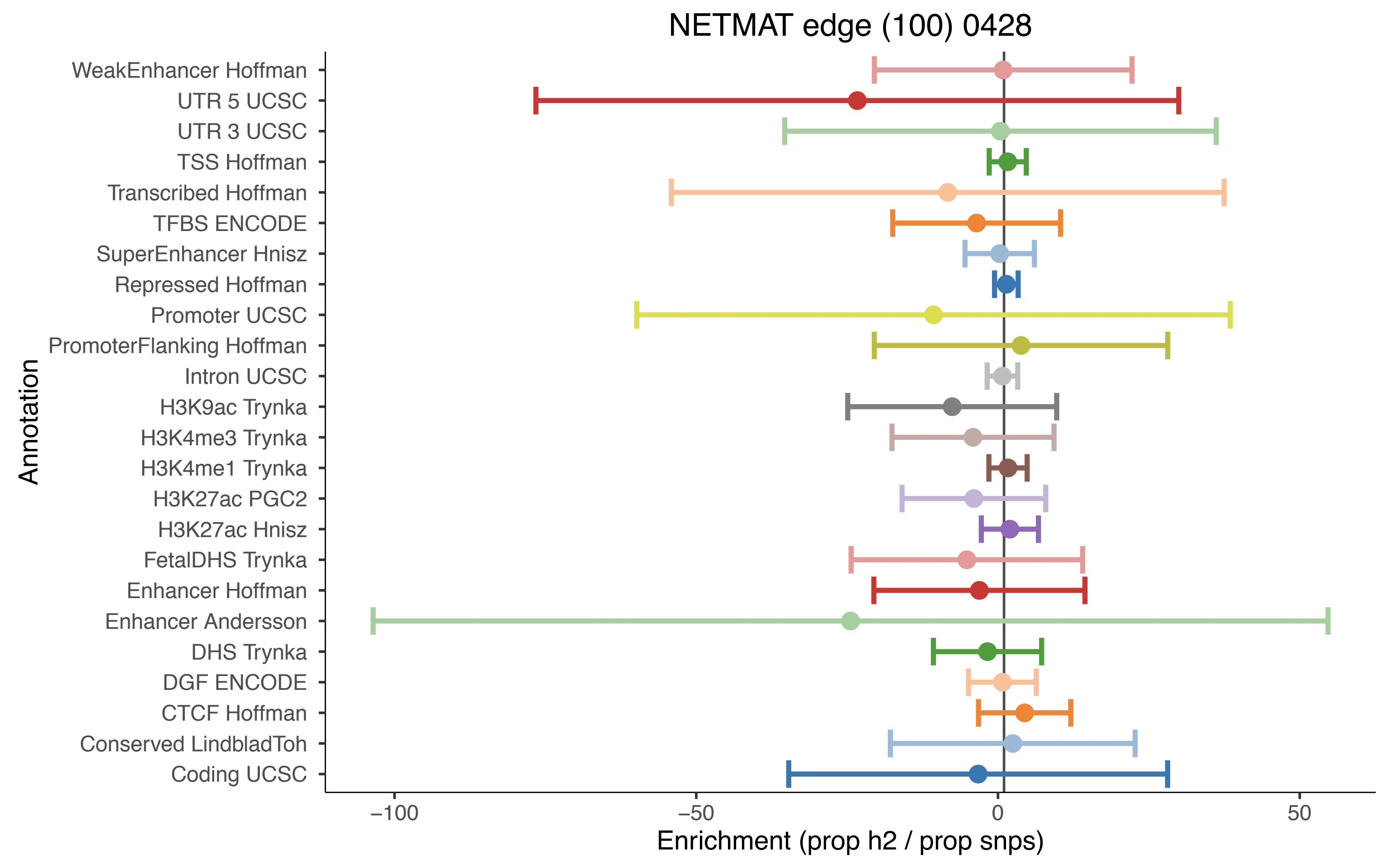
NETMAT edge (100) 0428
Annotation
WeakEnhancer Hoffman
UTR 5 UCSC
UTR 3 UCSC
TSS Hoffman
Transcribed Hoffman
TFBS ENCODE
SuperEnhancer Hnisz
Repressed Hoffman
Promoter UCSC
PromoterFlanking Hoffman
Intron UCSC
H3K9ac Trynka
H3K4me3 Trynka
H3K4me1 Trynka
H3K27ac PGC2
H3K27ac Hnisz
FetalDHS Trynka
Enhancer Hoffman
Enhancer Andersson
DHS Trynka
DGF ENCODE
CTCF Hoffman
Conserved LindbladToh
Coding UCSC
−100
−50
0
50
Enrichment (prop h2 / prop snps)

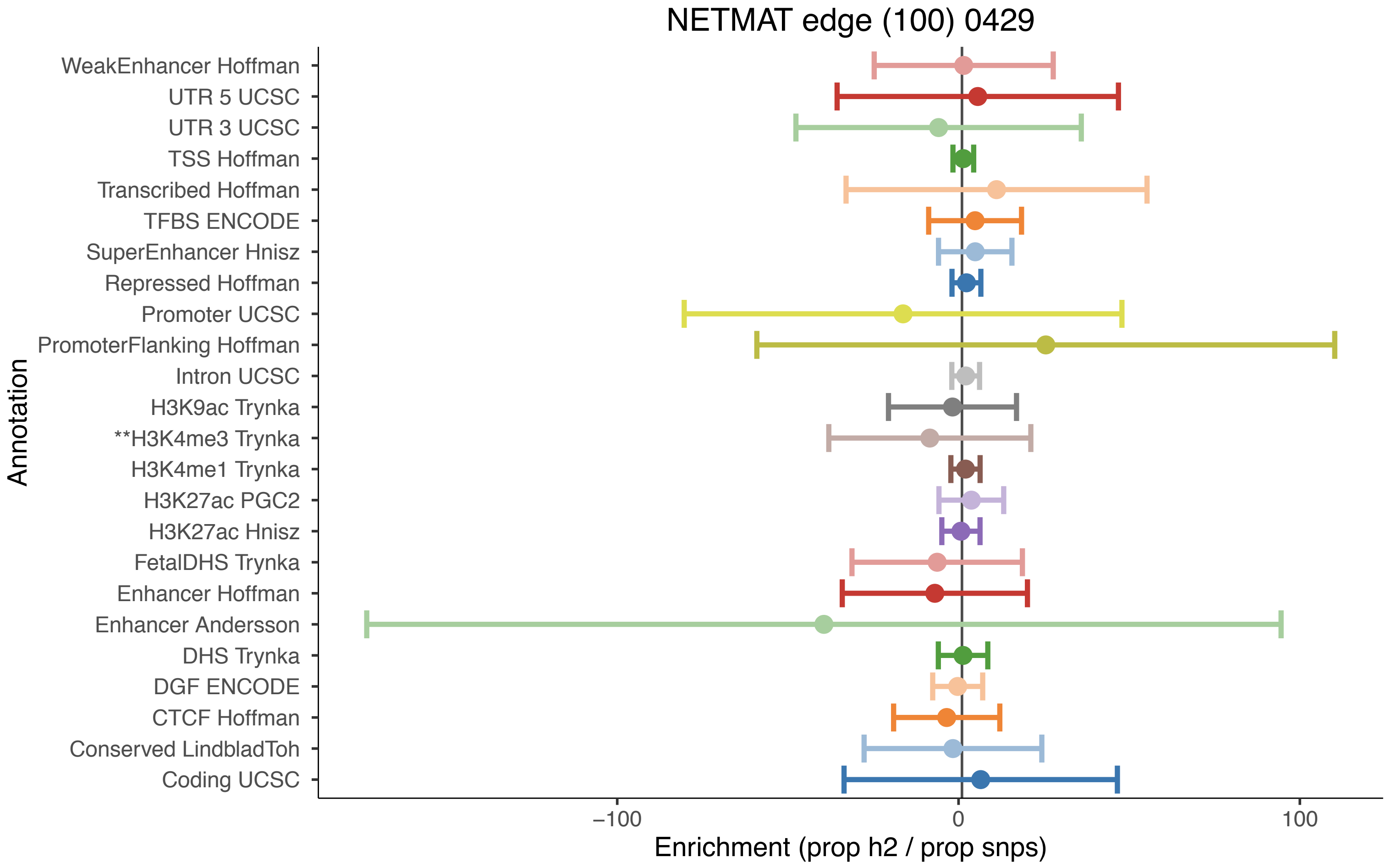

NETMAT edge (100) 0429
WeakEnhancer Hoffman
UTR 5 UCSC
UTR 3 UCSC
TSS Hoffman
Transcribed Hoffman
TFBS ENCODE
SuperEnhancer Hnisz
Repressed Hoffman
Promoter UCSC
PromoterFlanking Hoffman
Intron UCSC
H3K9ac Trynka
**H3K4me3 Trynka
H3K4me1 Trynka
H3K27ac PGC2
H3K27ac Hnisz
FetalDHS Trynka
Enhancer Hoffman
Enhancer Andersson
DHS Trynka
DGF ENCODE
CTCF Hoffman
Conserved LindbladToh
Coding UCSC
Annotation
−100
0
100
Enrichment (prop h2 / prop snps)

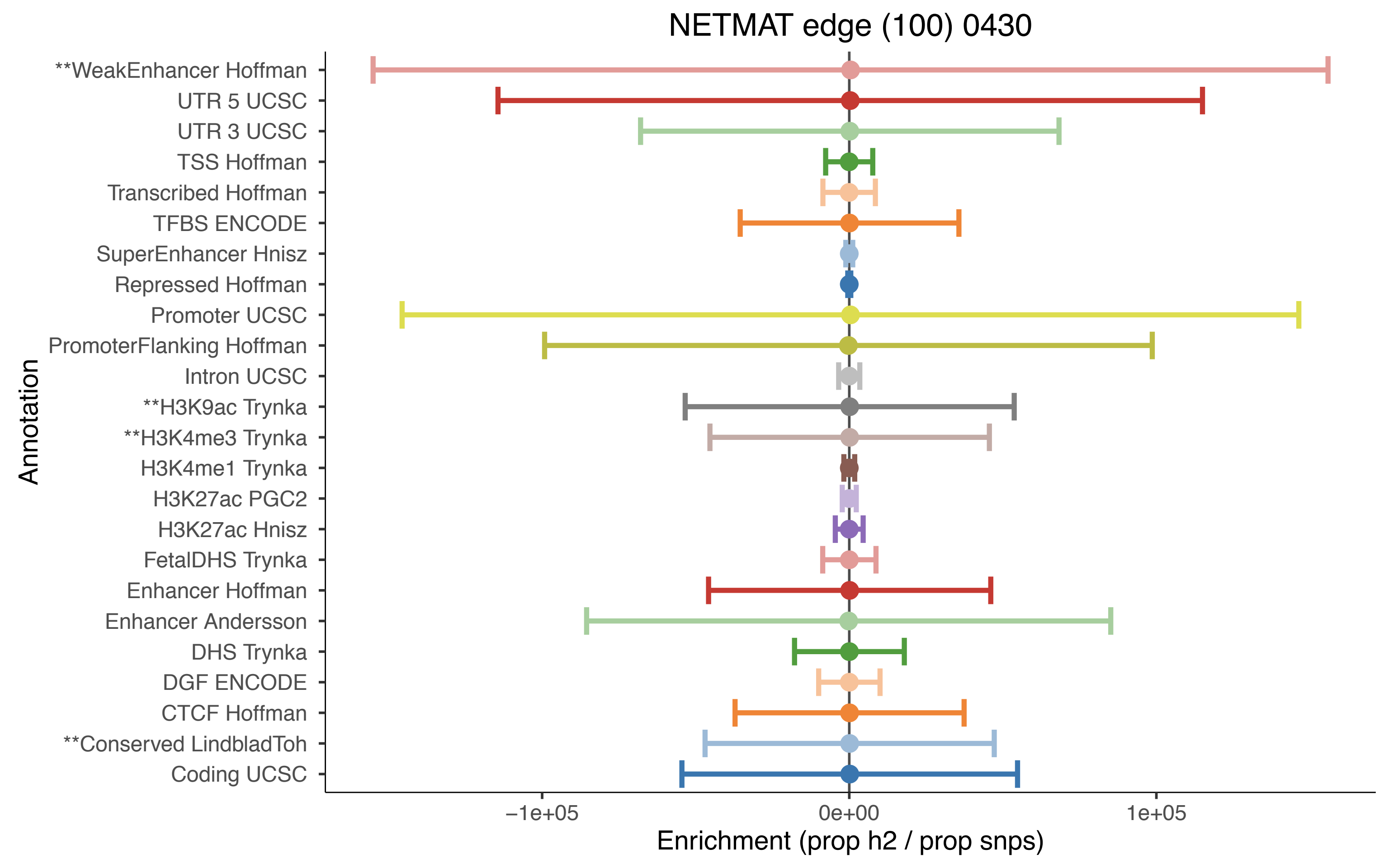

NETMAT edge (100) 0430
**WeakEnhancer Hoffman
UTR 5 UCSC
UTR 3 UCSC
TSS Hoffman
Transcribed Hoffman
TFBS ENCODE
SuperEnhancer Hnisz
Repressed Hoffman
Promoter UCSC
PromoterFlanking Hoffman
Intron UCSC
**H3K9ac Trynka
**H3K4me3 Trynka
H3K4me1 Trynka
H3K27ac PGC2
H3K27ac Hnisz
FetalDHS Trynka
Enhancer Hoffman
Enhancer Andersson
DHS Trynka
DGF ENCODE
CTCF Hoffman
**Conserved LindbladToh
Coding UCSC
Annotation
−1e+05
0e+00
1e+05
Enrichment (prop h2 / prop snps)

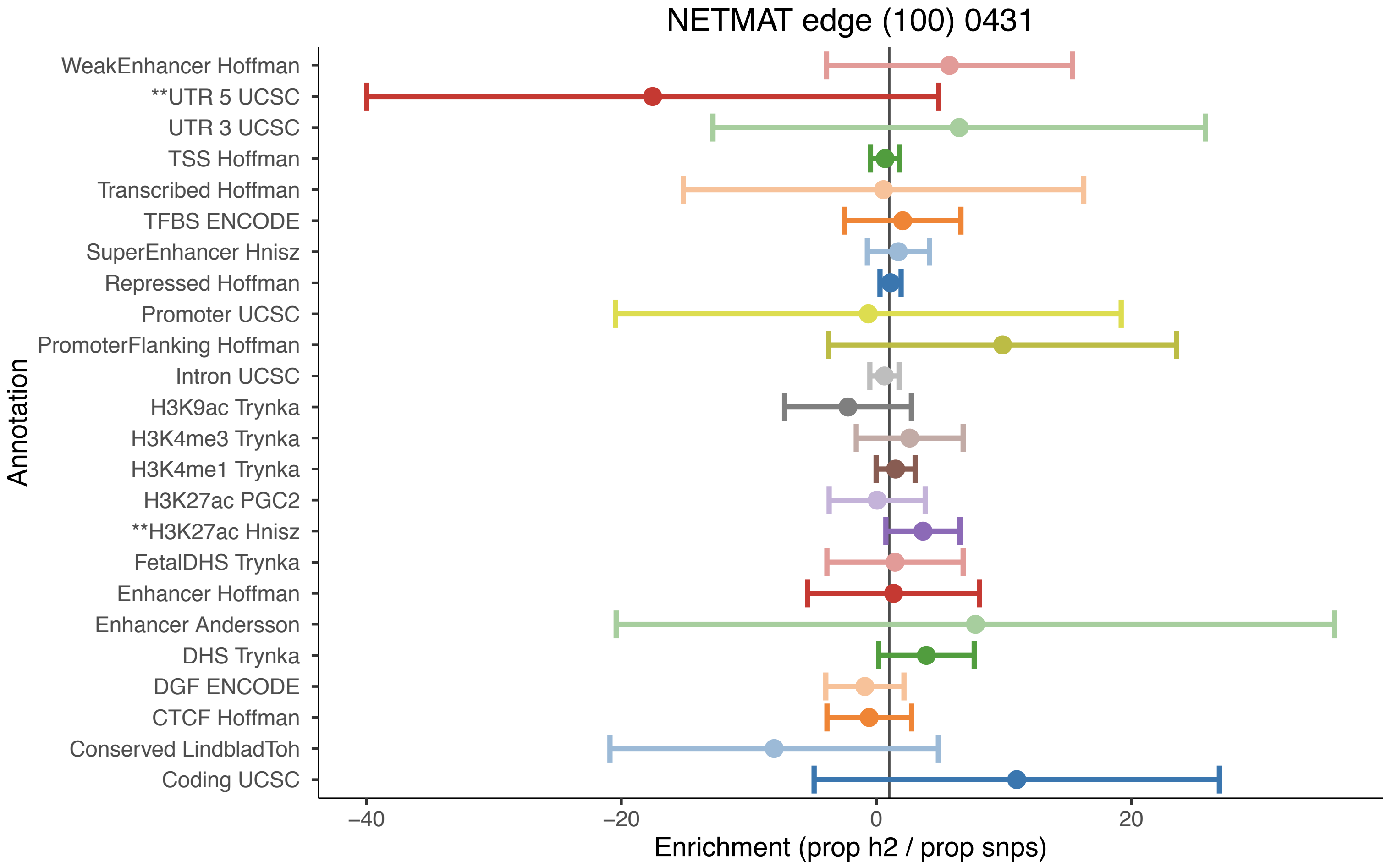

NETMAT edge (100) 0431
WeakEnhancer Hoffman
**UTR 5 UCSC
UTR 3 UCSC
TSS Hoffman
Transcribed Hoffman
TFBS ENCODE
SuperEnhancer Hnisz
Repressed Hoffman
Promoter UCSC
PromoterFlanking Hoffman
Intron UCSC
H3K9ac Trynka
H3K4me3 Trynka
H3K4me1 Trynka
H3K27ac PGC2
**H3K27ac Hnisz
FetalDHS Trynka
Enhancer Hoffman
Enhancer Andersson
DHS Trynka
DGF ENCODE
CTCF Hoffman
Conserved LindbladToh
Coding UCSC
Annotation
−40
−20
0
20
Enrichment (prop h2 / prop snps)

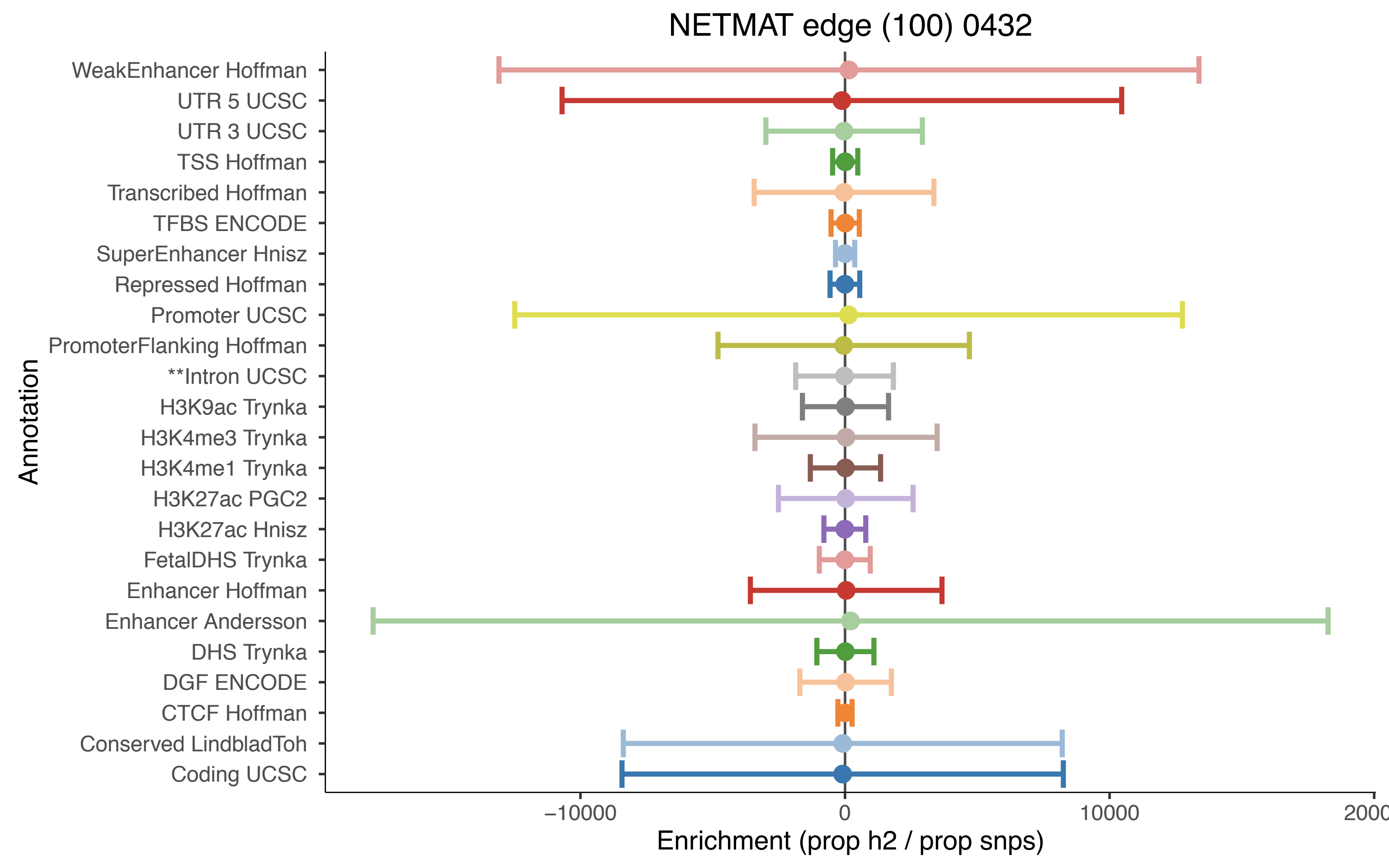

NETMAT edge (100) 0432
WeakEnhancer Hoffman
UTR 5 UCSC
UTR 3 UCSC
TSS Hoffman
Transcribed Hoffman
TFBS ENCODE
SuperEnhancer Hnisz
Repressed Hoffman
Promoter UCSC
PromoterFlanking Hoffman
**Intron UCSC
H3K9ac Trynka
H3K4me3 Trynka
H3K4me1 Trynka
H3K27ac PGC2
H3K27ac Hnisz
FetalDHS Trynka
Enhancer Hoffman
Enhancer Andersson
DHS Trynka
DGF ENCODE
CTCF Hoffman
Conserved LindbladToh
Coding UCSC
Annotation
−10000
0
10000
Enrichment (prop h2 / prop snps)

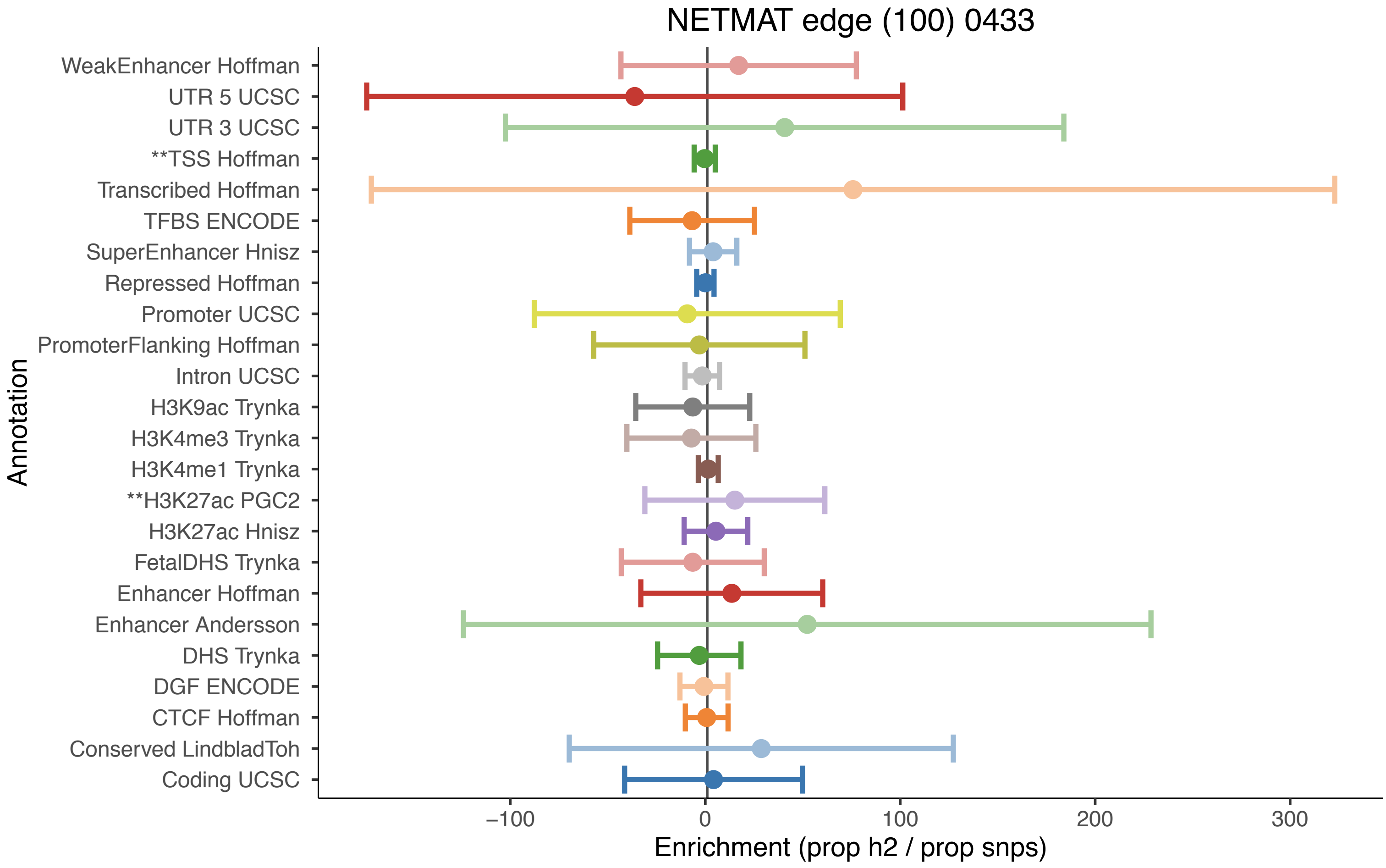

NETMAT edge (100) 0433
WeakEnhancer Hoffman
UTR 5 UCSC
UTR 3 UCSC
**TSS Hoffman
Transcribed Hoffman
TFBS ENCODE
SuperEnhancer Hnisz
Repressed Hoffman
Promoter UCSC
PromoterFlanking Hoffman
Intron UCSC
H3K9ac Trynka
H3K4me3 Trynka
H3K4me1 Trynka
**H3K27ac PGC2
H3K27ac Hnisz
FetalDHS Trynka
Enhancer Hoffman
Enhancer Andersson
DHS Trynka
DGF ENCODE
CTCF Hoffman
Conserved LindbladToh
Coding UCSC
Annotation
−100
0
100
200
300
Enrichment (prop h2 / prop snps)

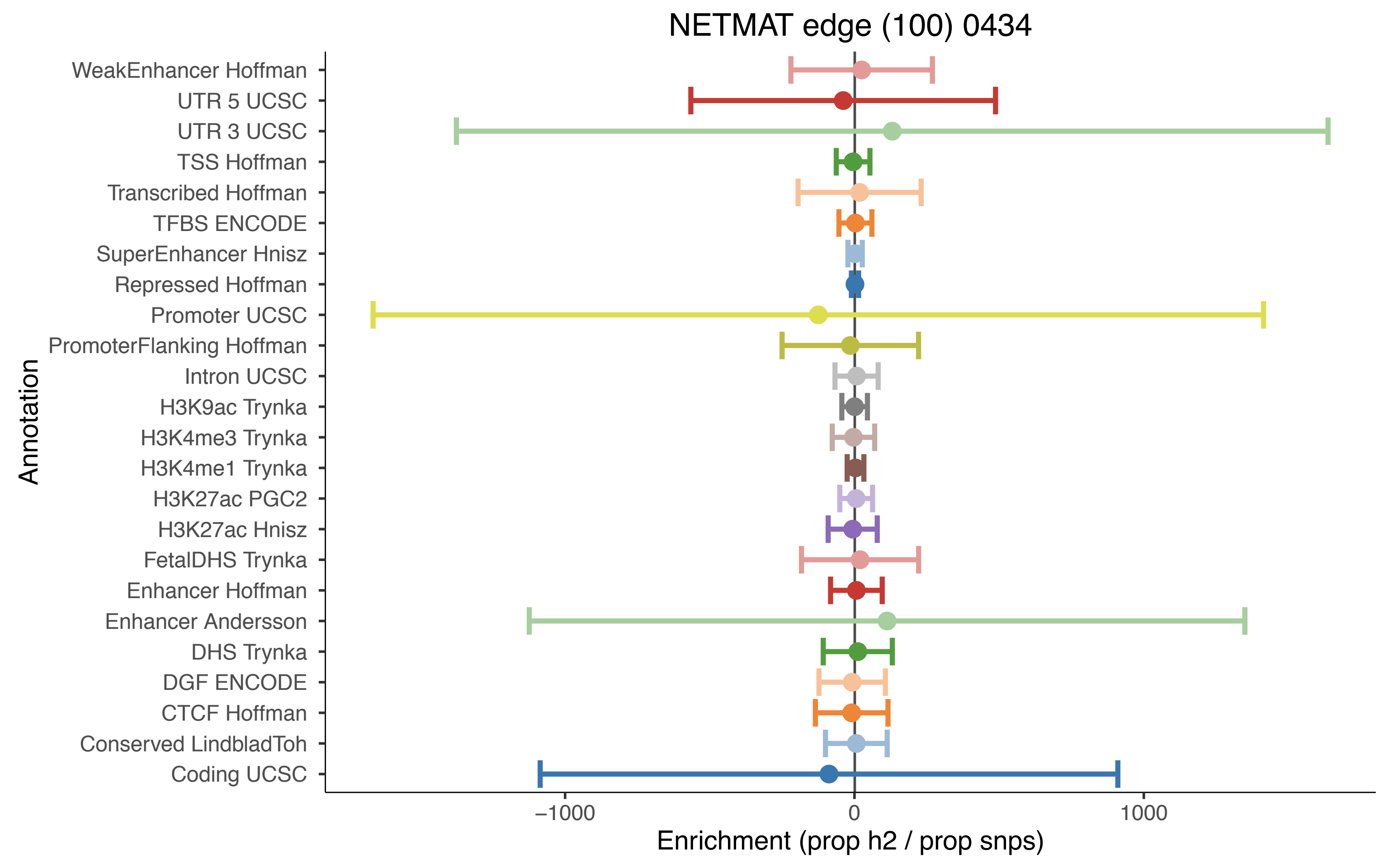

NETMAT edge (100) 0434
WeakEnhancer Hoffman
UTR 5 UCSC
UTR 3 UCSC
TSS Hoffman
Transcribed Hoffman
TFBS ENCODE
SuperEnhancer Hnisz
Repressed Hoffman
Promoter UCSC
PromoterFlanking Hoffman
Intron UCSC
H3K9ac Trynka
H3K4me3 Trynka
H3K4me1 Trynka
H3K27ac PGC2
H3K27ac Hnisz
FetalDHS Trynka
Enhancer Hoffman
Enhancer Andersson
DHS Trynka
DGF ENCODE
CTCF Hoffman
Conserved LindbladToh
Coding UCSC
Annotation
−1000
0
1000
Enrichment (prop h2 / prop snps)

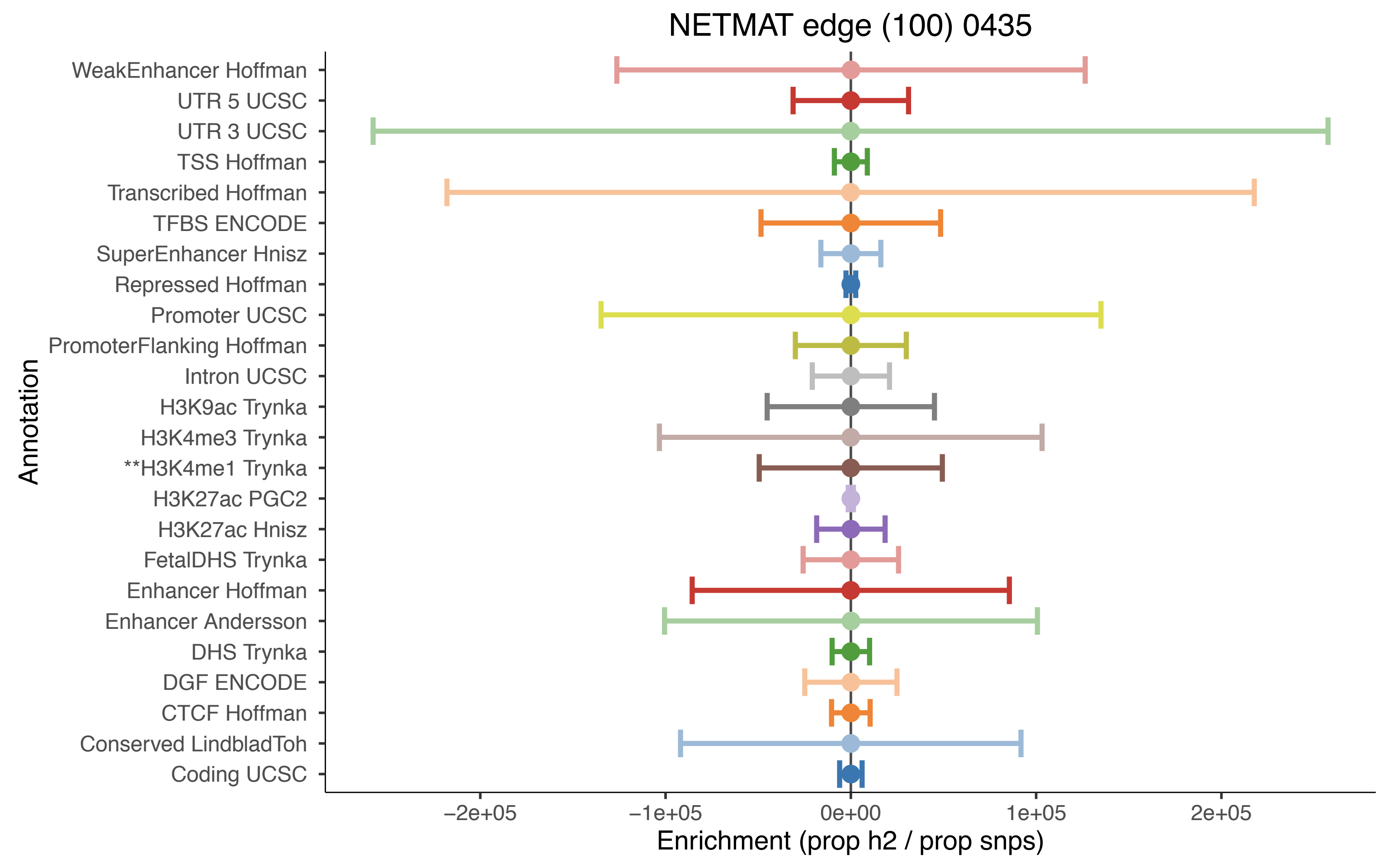
NETMAT edge (100) 0435
WeakEnhancer Hoffman
UTR 5 UCSC
UTR 3 UCSC
TSS Hoffman
Transcribed Hoffman
TFBS ENCODE
SuperEnhancer Hnisz
Repressed Hoffman
Promoter UCSC
PromoterFlanking Hoffman
Intron UCSC
H3K9ac Trynka
H3K4me3 Trynka
**H3K4me1 Trynka
H3K27ac PGC2
H3K27ac Hnisz
FetalDHS Trynka
Enhancer Hoffman
Enhancer Andersson
DHS Trynka
DGF ENCODE
CTCF Hoffman
Conserved LindbladToh
Coding UCSC
Annotation
−2e+05
−1e+05
0e+00
1e+05
2e+05
Enrichment (prop h2 / prop snps)

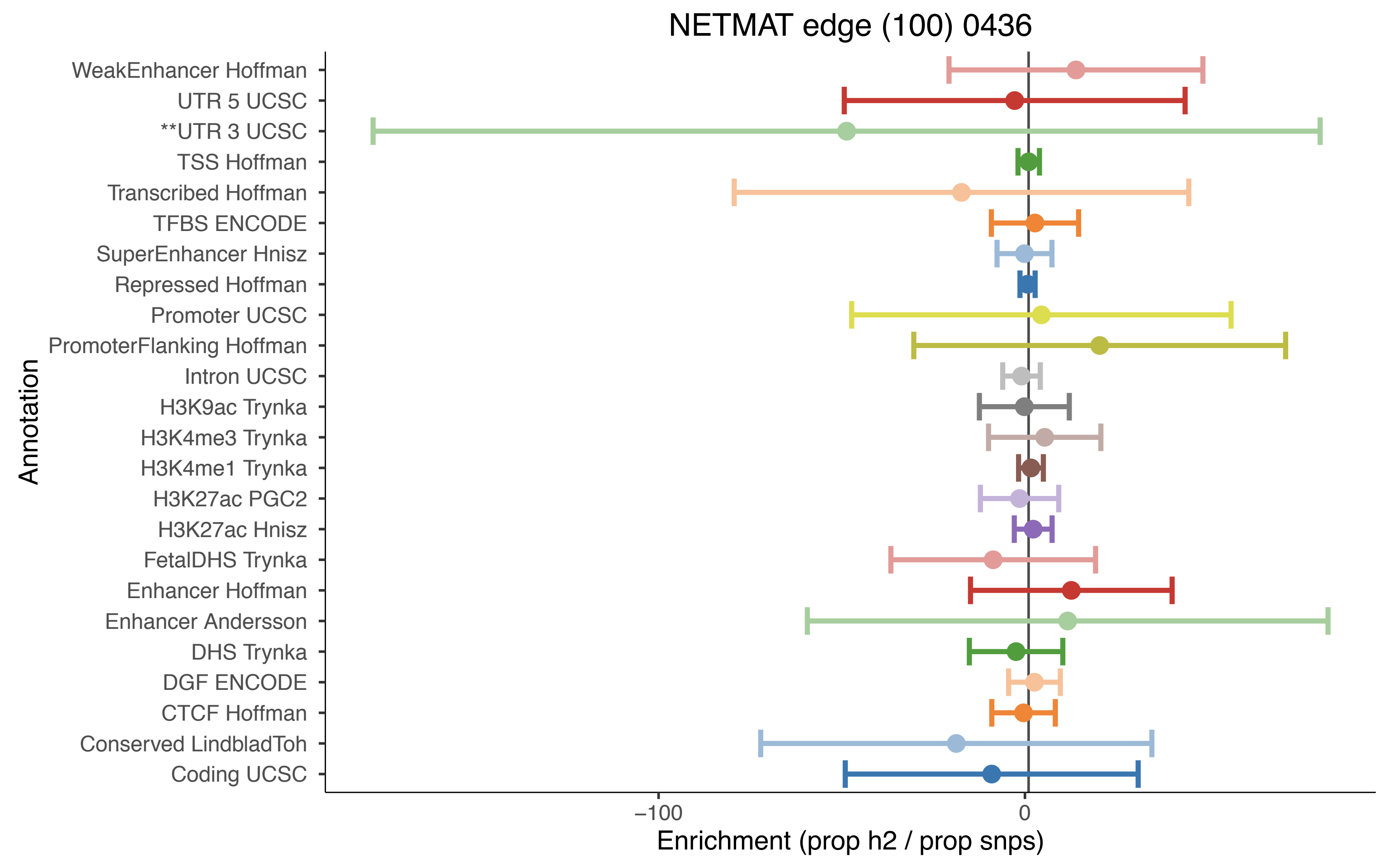
NETMAT edge (100) 0436
WeakEnhancer Hoffman
UTR 5 UCSC
**UTR 3 UCSC
TSS Hoffman
Transcribed Hoffman
TFBS ENCODE
SuperEnhancer Hnisz
Repressed Hoffman
Promoter UCSC
PromoterFlanking Hoffman
Intron UCSC
H3K9ac Trynka
H3K4me3 Trynka
H3K4me1 Trynka
H3K27ac PGC2
H3K27ac Hnisz
FetalDHS Trynka
Enhancer Hoffman
Enhancer Andersson
DHS Trynka
DGF ENCODE
CTCF Hoffman
Conserved LindbladToh
Coding UCSC
Annotation
−100
0
Enrichment (prop h2 / prop snps)

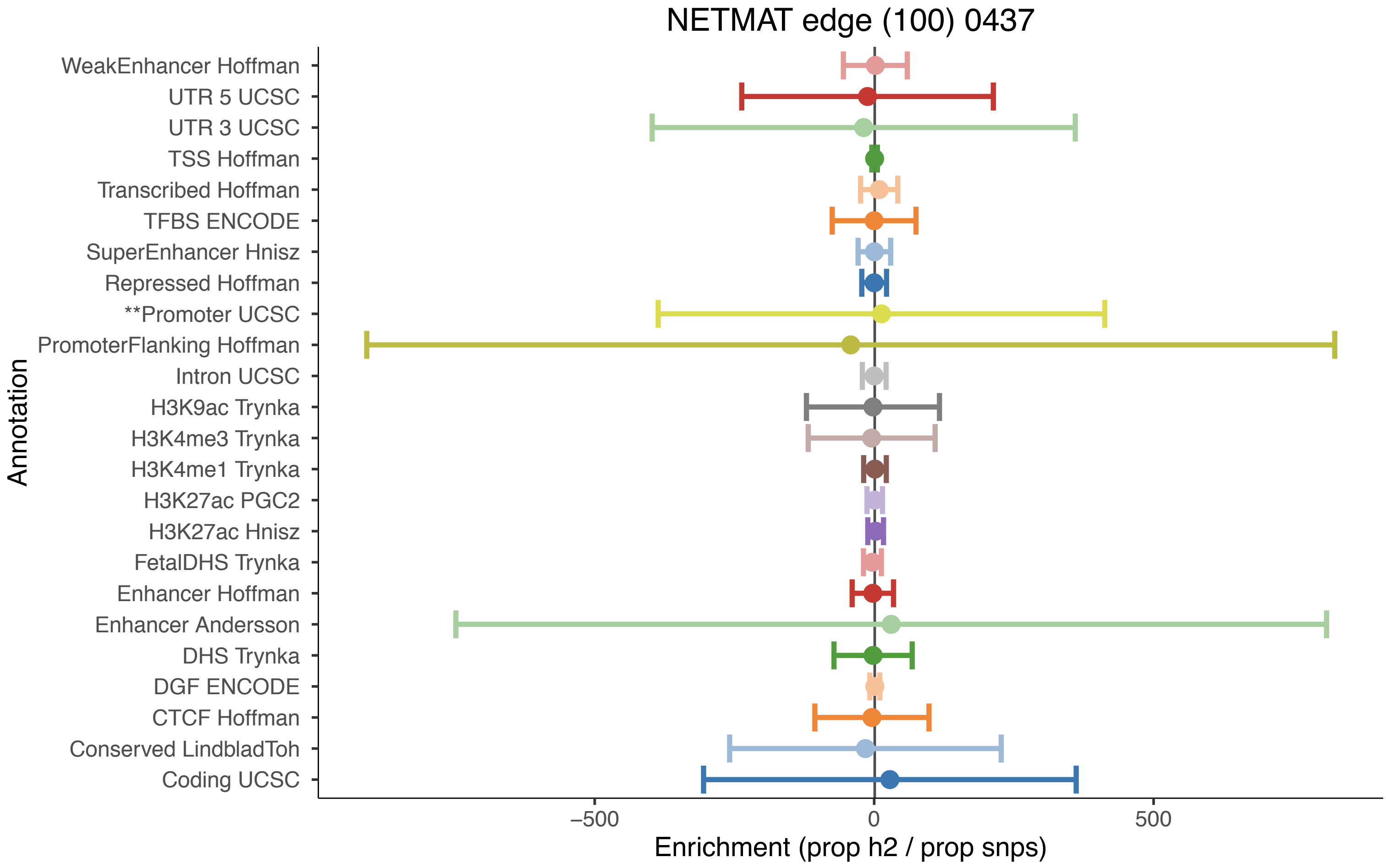
NETMAT edge (100) 0437
WeakEnhancer Hoffman
UTR 5 UCSC
UTR 3 UCSC
TSS Hoffman
Transcribed Hoffman
TFBS ENCODE
SuperEnhancer Hnisz
Repressed Hoffman
**Promoter UCSC
PromoterFlanking Hoffman
Intron UCSC
H3K9ac Trynka
H3K4me3 Trynka
H3K4me1 Trynka
H3K27ac PGC2
H3K27ac Hnisz
FetalDHS Trynka
Enhancer Hoffman
Enhancer Andersson
DHS Trynka
DGF ENCODE
CTCF Hoffman
Conserved LindbladToh
Coding UCSC
Annotation
−500
0
500
Enrichment (prop h2 / prop snps)

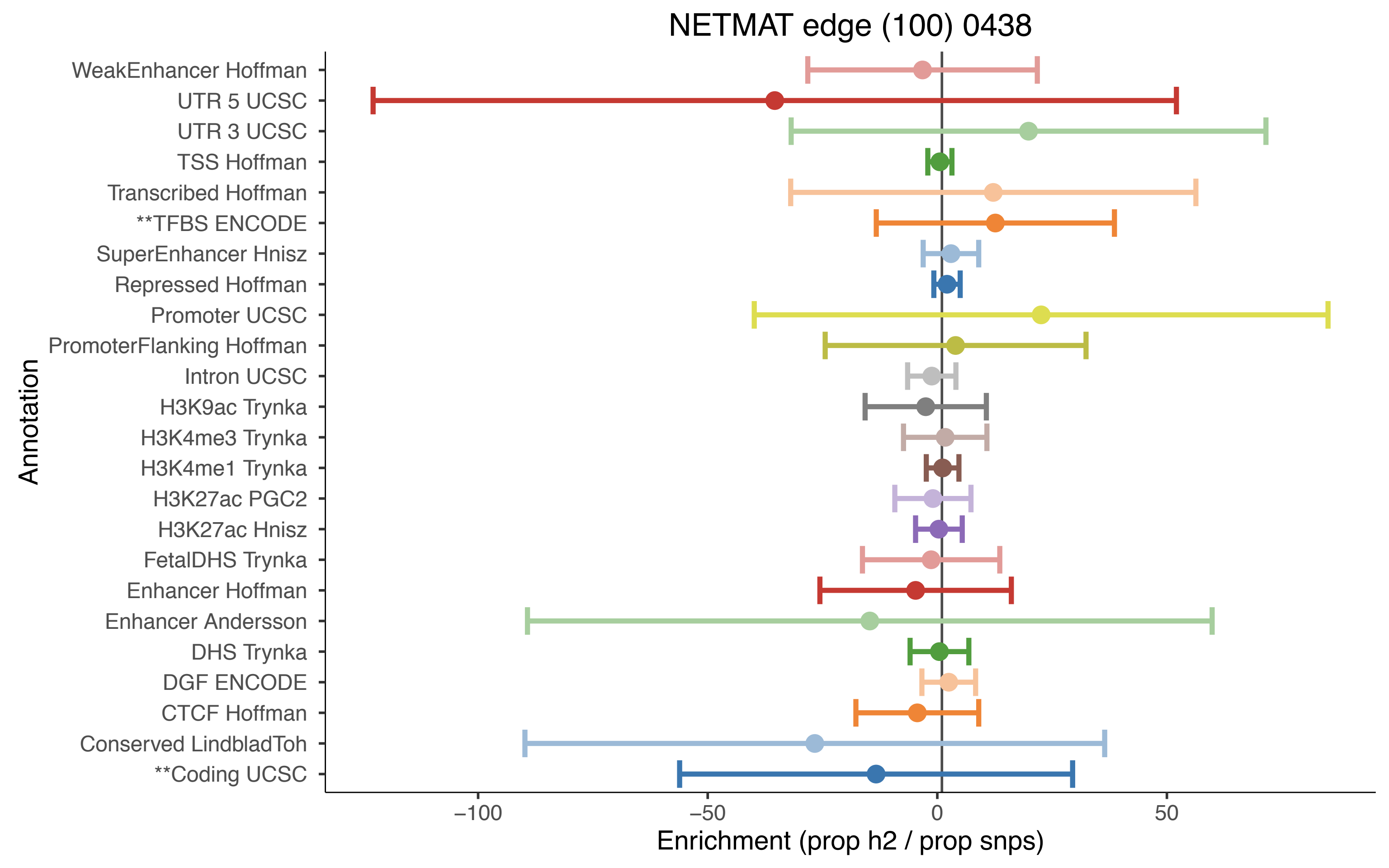
NETMAT edge (100) 0438
WeakEnhancer Hoffman
UTR 5 UCSC
UTR 3 UCSC
TSS Hoffman
Transcribed Hoffman
**TFBS ENCODE
SuperEnhancer Hnisz
Repressed Hoffman
Promoter UCSC
PromoterFlanking Hoffman
Intron UCSC
H3K9ac Trynka
H3K4me3 Trynka
H3K4me1 Trynka
H3K27ac PGC2
H3K27ac Hnisz
FetalDHS Trynka
Enhancer Hoffman
Enhancer Andersson
DHS Trynka
DGF ENCODE
CTCF Hoffman
Conserved LindbladToh
**Coding UCSC
Annotation
−100
−50
0
50
Enrichment (prop h2 / prop snps)

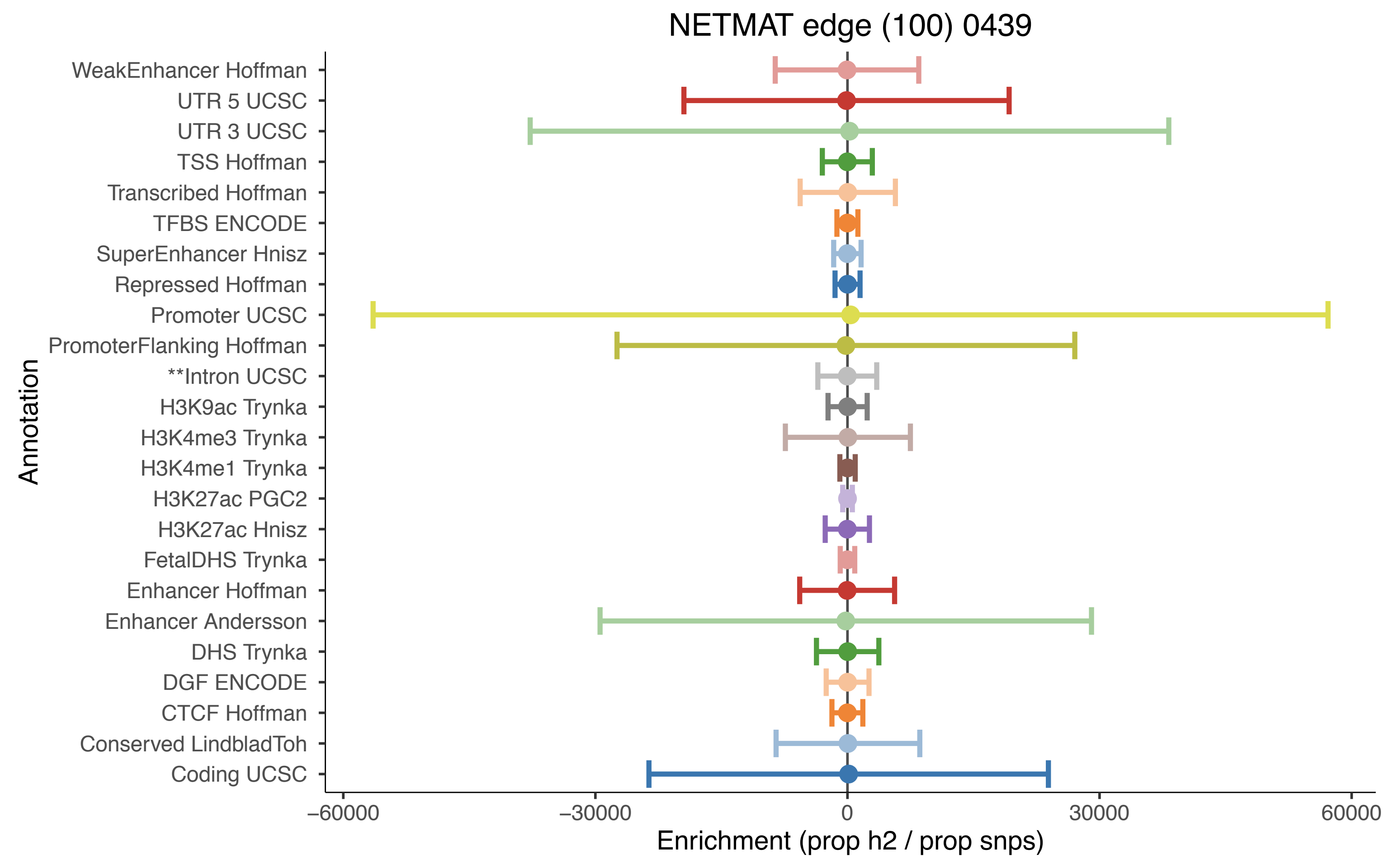
NETMAT edge (100) 0439
WeakEnhancer Hoffman
UTR 5 UCSC
UTR 3 UCSC
TSS Hoffman
Transcribed Hoffman
TFBS ENCODE
SuperEnhancer Hnisz
Repressed Hoffman
Promoter UCSC
PromoterFlanking Hoffman
**Intron UCSC
H3K9ac Trynka
H3K4me3 Trynka
H3K4me1 Trynka
H3K27ac PGC2
H3K27ac Hnisz
FetalDHS Trynka
Enhancer Hoffman
Enhancer Andersson
DHS Trynka
DGF ENCODE
CTCF Hoffman
Conserved LindbladToh
Coding UCSC
Annotation
−60000
−30000
0
30000
60000
Enrichment (prop h2 / prop snps)

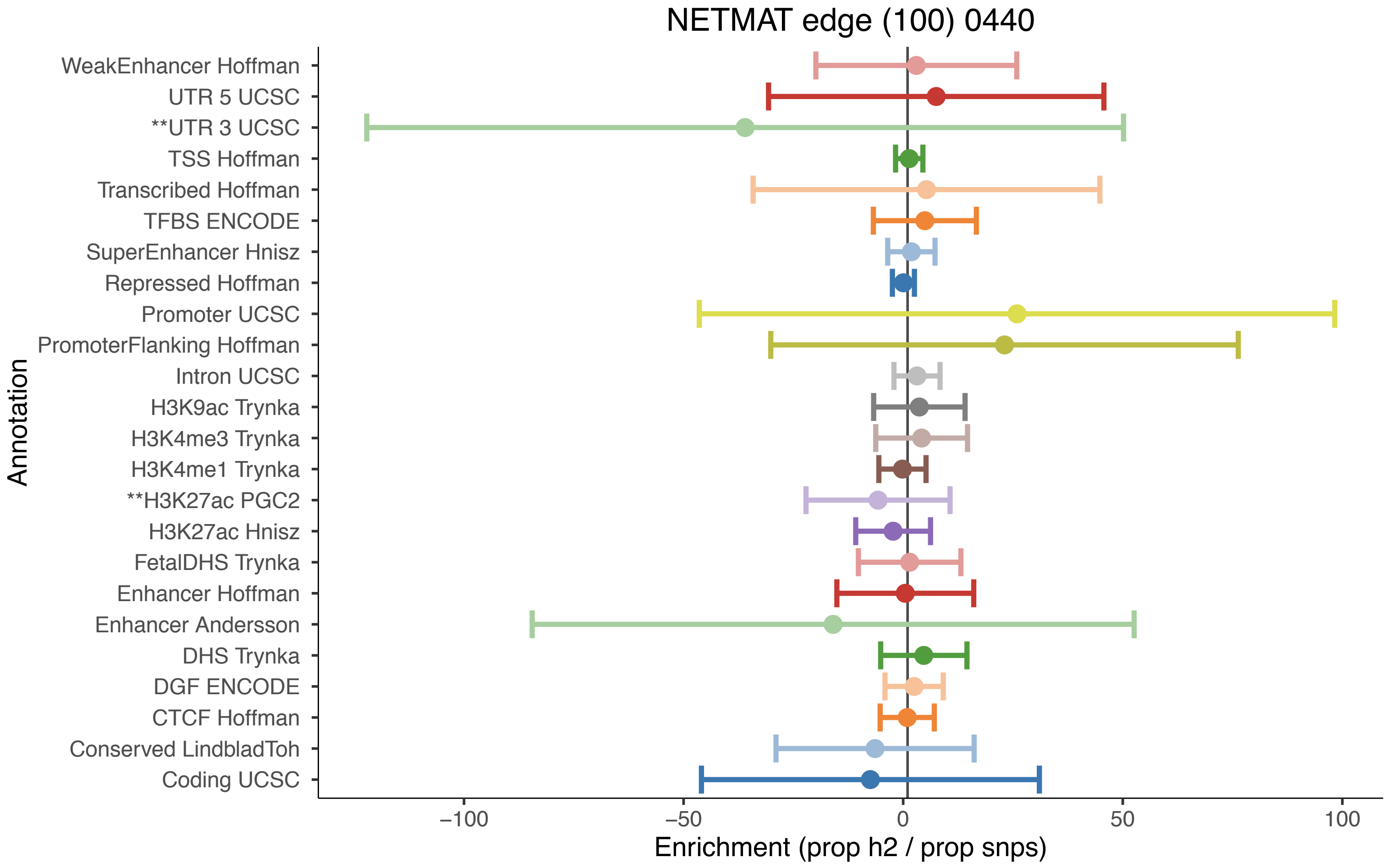

NETMAT edge (100) 0440
WeakEnhancer Hoffman
UTR 5 UCSC
**UTR 3 UCSC
TSS Hoffman
Transcribed Hoffman
TFBS ENCODE
SuperEnhancer Hnisz
Repressed Hoffman
Promoter UCSC
PromoterFlanking Hoffman
Intron UCSC
H3K9ac Trynka
H3K4me3 Trynka
H3K4me1 Trynka
**H3K27ac PGC2
H3K27ac Hnisz
FetalDHS Trynka
Enhancer Hoffman
Enhancer Andersson
DHS Trynka
DGF ENCODE
CTCF Hoffman
Conserved LindbladToh
Coding UCSC
Annotation
−100
−50
0
50
100
Enrichment (prop h2 / prop snps)

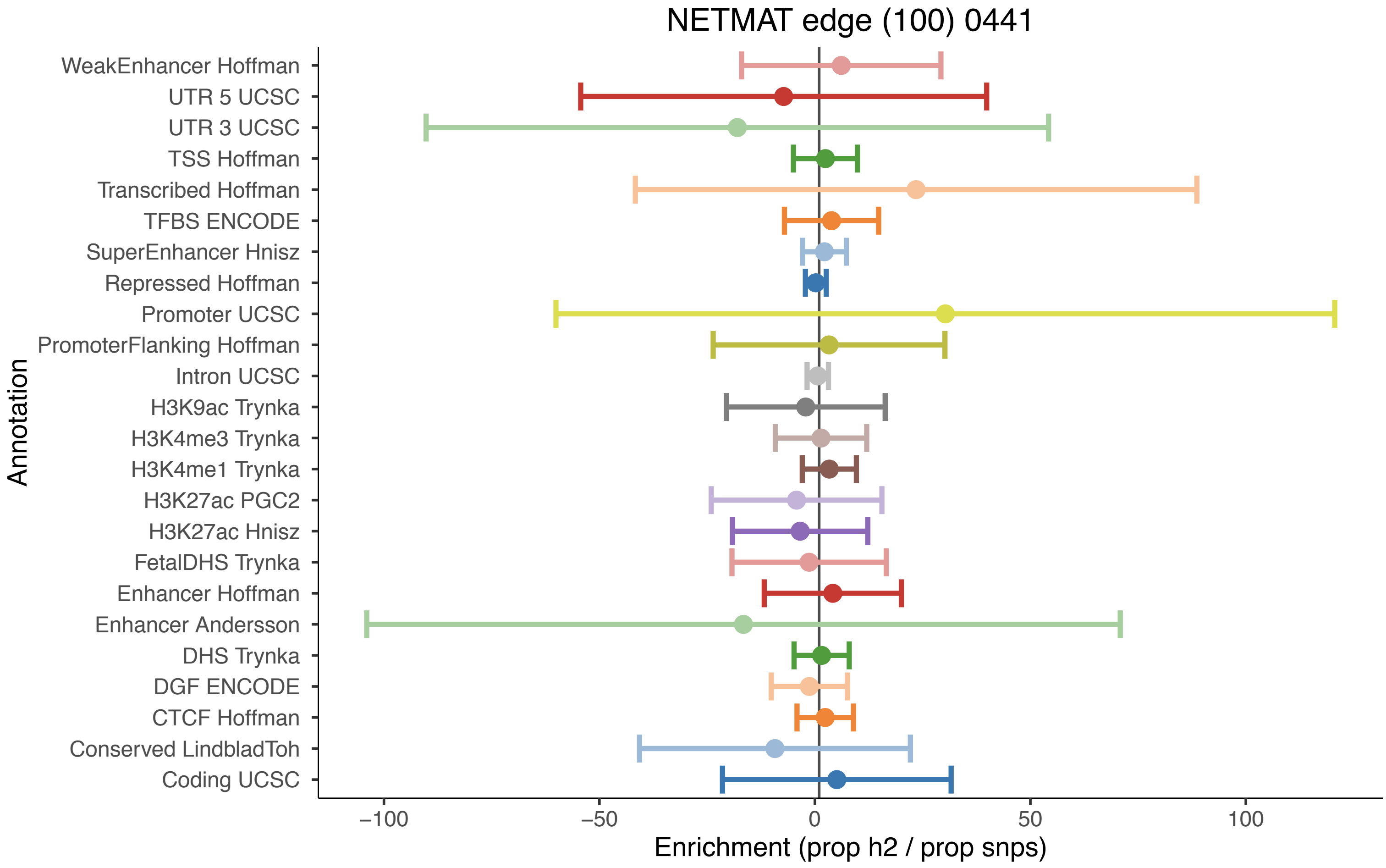
NETMAT edge (100) 0441
WeakEnhancer Hoffman
UTR 5 UCSC
UTR 3 UCSC
TSS Hoffman
Transcribed Hoffman
TFBS ENCODE
SuperEnhancer Hnisz
Repressed Hoffman
Promoter UCSC
PromoterFlanking Hoffman
Intron UCSC
H3K9ac Trynka
H3K4me3 Trynka
H3K4me1 Trynka
H3K27ac PGC2
H3K27ac Hnisz
FetalDHS Trynka
Enhancer Hoffman
Enhancer Andersson
DHS Trynka
DGF ENCODE
CTCF Hoffman
Conserved LindbladToh
Coding UCSC
Annotation
−100
−50
0
50
100
Enrichment (prop h2 / prop snps)

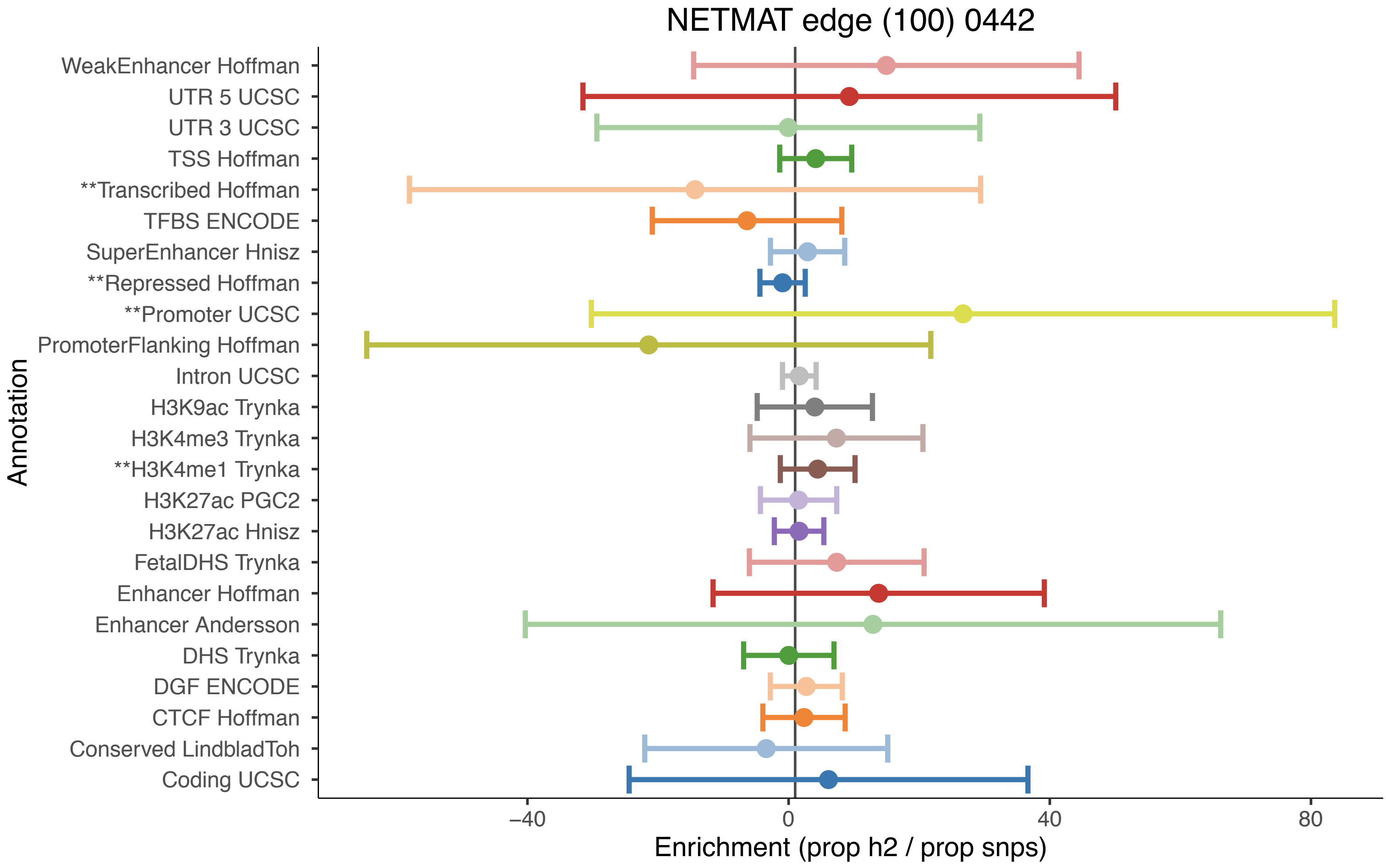

NETMAT edge (100) 0442
WeakEnhancer Hoffman
UTR 5 UCSC
UTR 3 UCSC
TSS Hoffman
**Transcribed Hoffman
TFBS ENCODE
SuperEnhancer Hnisz
**Repressed Hoffman
**Promoter UCSC
PromoterFlanking Hoffman
Intron UCSC
H3K9ac Trynka
H3K4me3 Trynka
**H3K4me1 Trynka
H3K27ac PGC2
H3K27ac Hnisz
FetalDHS Trynka
Enhancer Hoffman
Enhancer Andersson
DHS Trynka
DGF ENCODE
CTCF Hoffman
Conserved LindbladToh
Coding UCSC
Annotation
−40
0
40
80
Enrichment (prop h2 / prop snps)

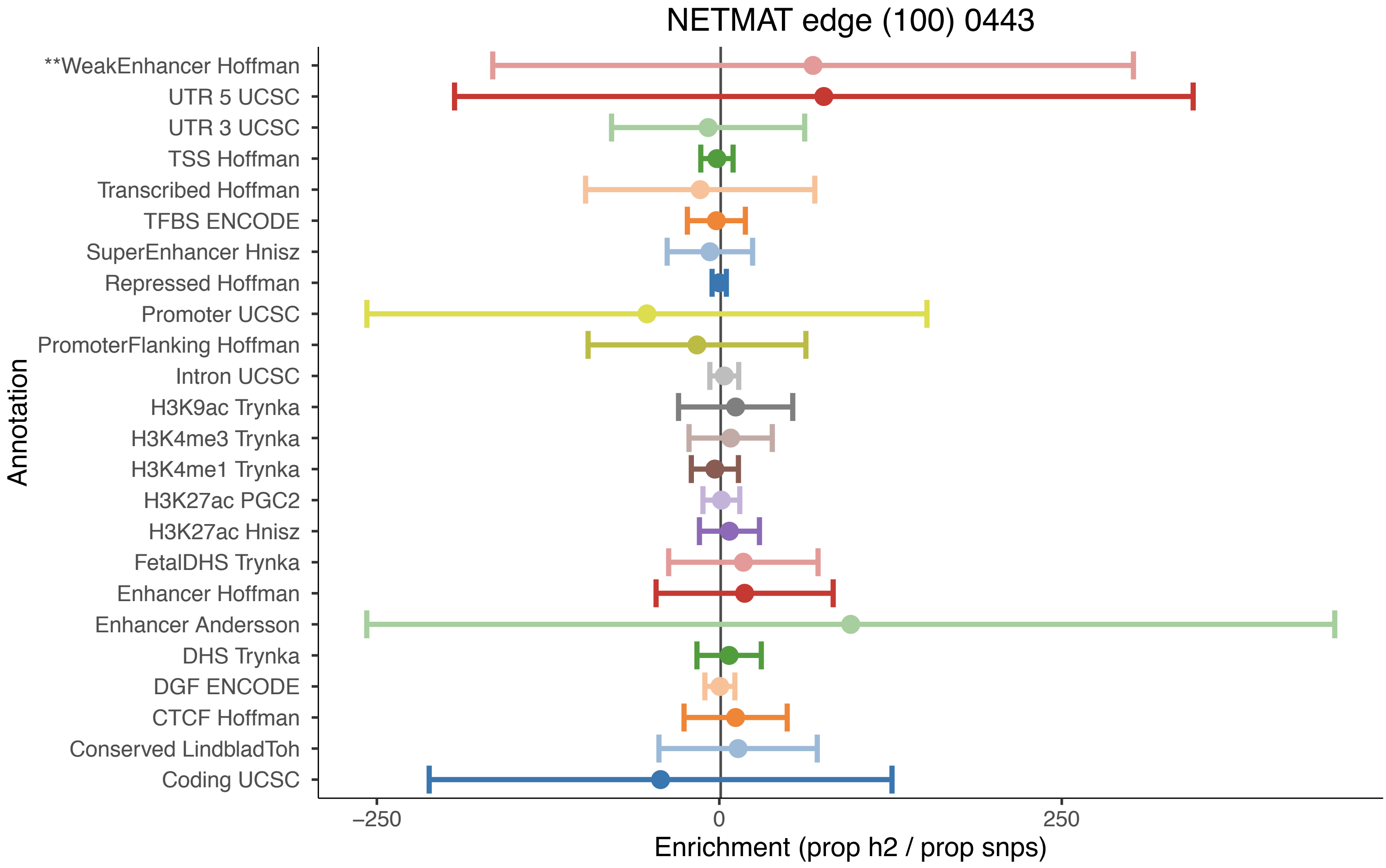

NETMAT edge (100) 0443
**WeakEnhancer Hoffman
UTR 5 UCSC
UTR 3 UCSC
TSS Hoffman
Transcribed Hoffman
TFBS ENCODE
SuperEnhancer Hnisz
Repressed Hoffman
Promoter UCSC
PromoterFlanking Hoffman
Intron UCSC
H3K9ac Trynka
H3K4me3 Trynka
H3K4me1 Trynka
H3K27ac PGC2
H3K27ac Hnisz
FetalDHS Trynka
Enhancer Hoffman
Enhancer Andersson
DHS Trynka
DGF ENCODE
CTCF Hoffman
Conserved LindbladToh
Coding UCSC
Annotation
−250
0
250
Enrichment (prop h2 / prop snps)

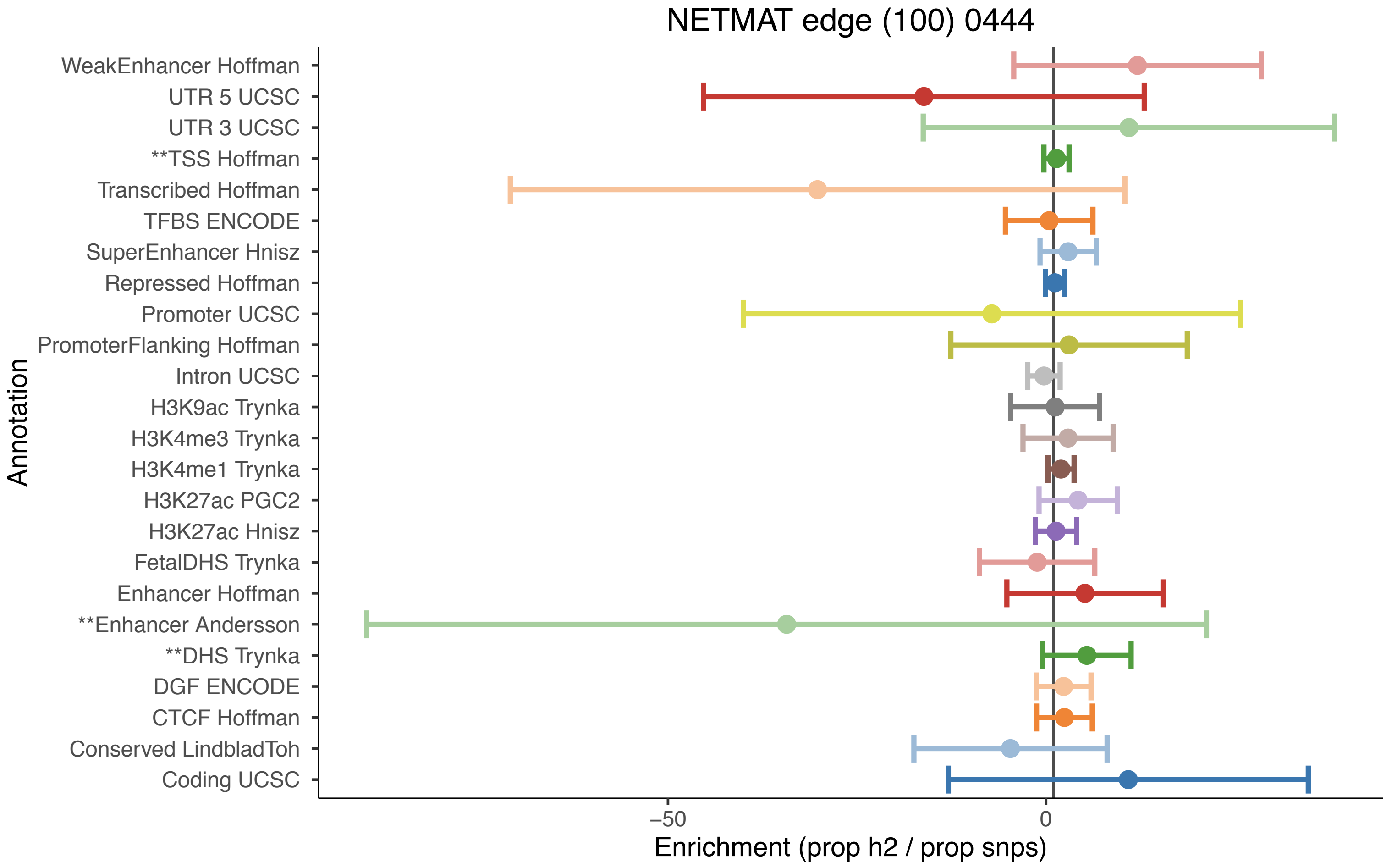

NETMAT edge (100) 0444
WeakEnhancer Hoffman
UTR 5 UCSC
UTR 3 UCSC
**TSS Hoffman
Transcribed Hoffman
TFBS ENCODE
SuperEnhancer Hnisz
Repressed Hoffman
Promoter UCSC
PromoterFlanking Hoffman
Intron UCSC
H3K9ac Trynka
H3K4me3 Trynka
H3K4me1 Trynka
H3K27ac PGC2
H3K27ac Hnisz
FetalDHS Trynka
Enhancer Hoffman
**Enhancer Andersson
**DHS Trynka
DGF ENCODE
CTCF Hoffman
Conserved LindbladToh
Coding UCSC
Annotation
−50
0
Enrichment (prop h2 / prop snps)

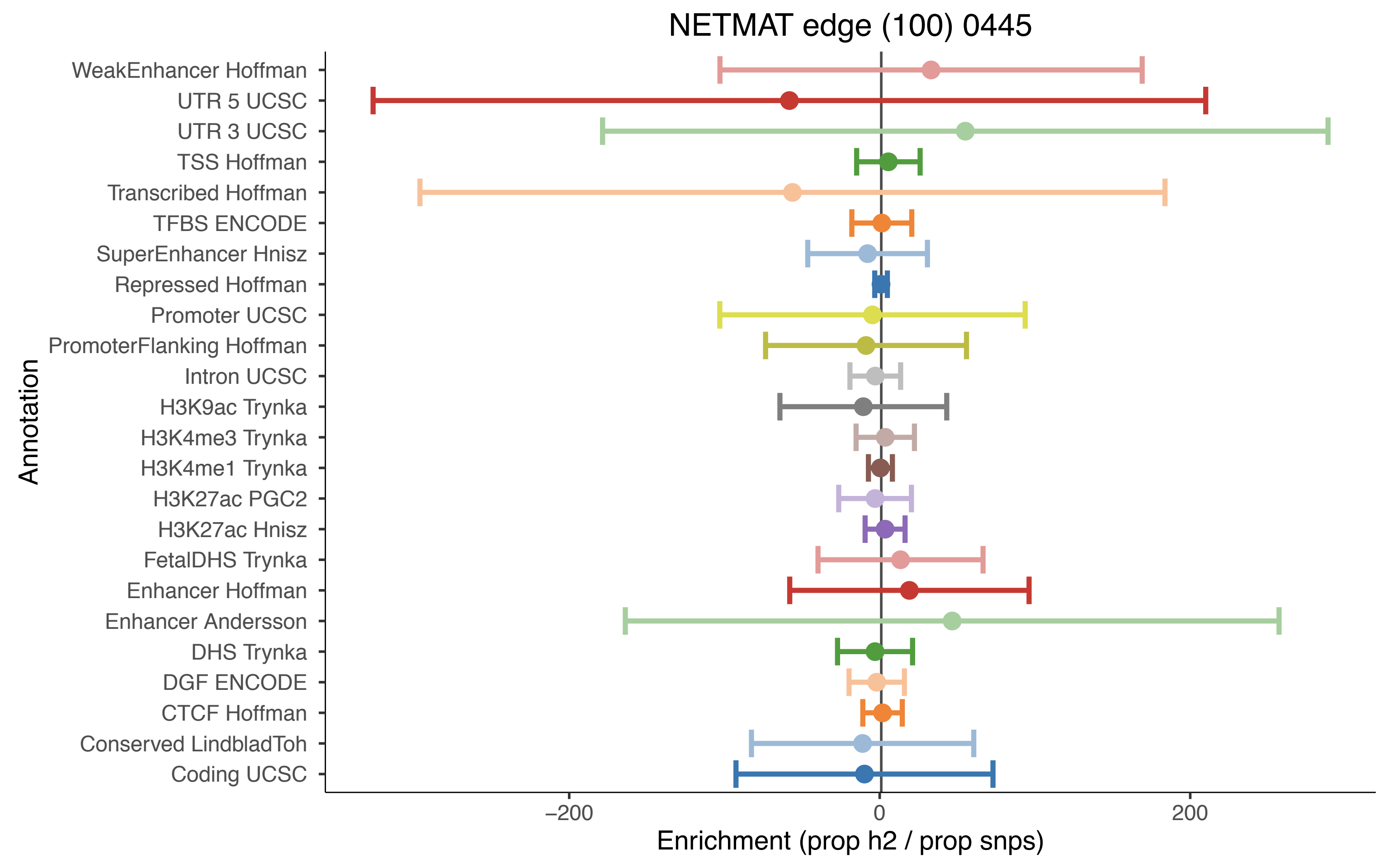
NETMAT edge (100) 0445
WeakEnhancer Hoffman
UTR 5 UCSC
UTR 3 UCSC
TSS Hoffman
Transcribed Hoffman
TFBS ENCODE
SuperEnhancer Hnisz
Repressed Hoffman
Promoter UCSC
PromoterFlanking Hoffman
Intron UCSC
H3K9ac Trynka
H3K4me3 Trynka
H3K4me1 Trynka
H3K27ac PGC2
H3K27ac Hnisz
FetalDHS Trynka
Enhancer Hoffman
Enhancer Andersson
DHS Trynka
DGF ENCODE
CTCF Hoffman
Conserved LindbladToh
Coding UCSC
Annotation
−200
0
200
Enrichment (prop h2 / prop snps)

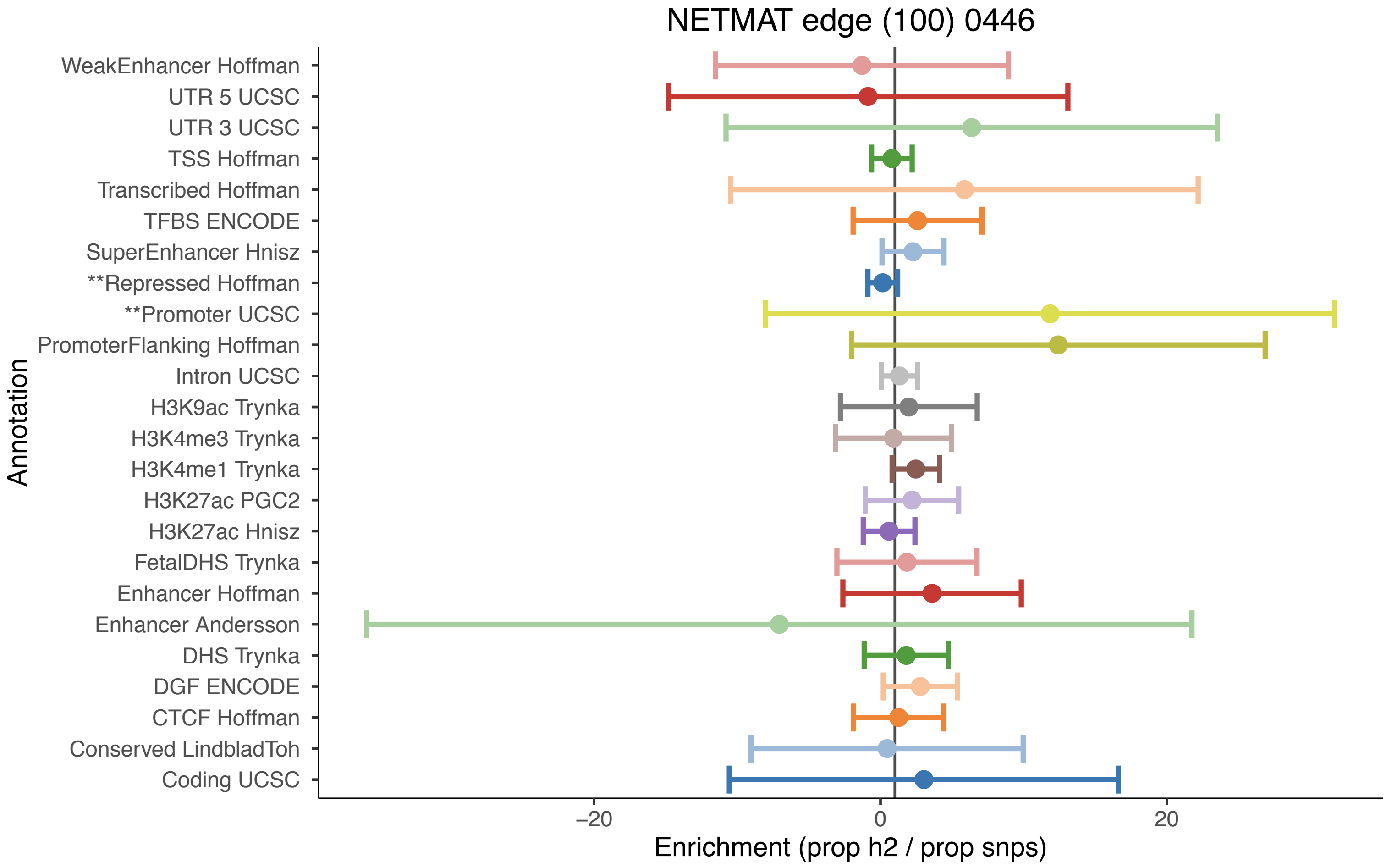
NETMAT edge (100) 0446
WeakEnhancer Hoffman
UTR 5 UCSC
UTR 3 UCSC
TSS Hoffman
Transcribed Hoffman
TFBS ENCODE
SuperEnhancer Hnisz
**Repressed Hoffman
**Promoter UCSC
PromoterFlanking Hoffman
Intron UCSC
H3K9ac Trynka
H3K4me3 Trynka
H3K4me1 Trynka
H3K27ac PGC2
H3K27ac Hnisz
FetalDHS Trynka
Enhancer Hoffman
Enhancer Andersson
DHS Trynka
DGF ENCODE
CTCF Hoffman
Conserved LindbladToh
Coding UCSC
Annotation
−20
0
20
Enrichment (prop h2 / prop snps)

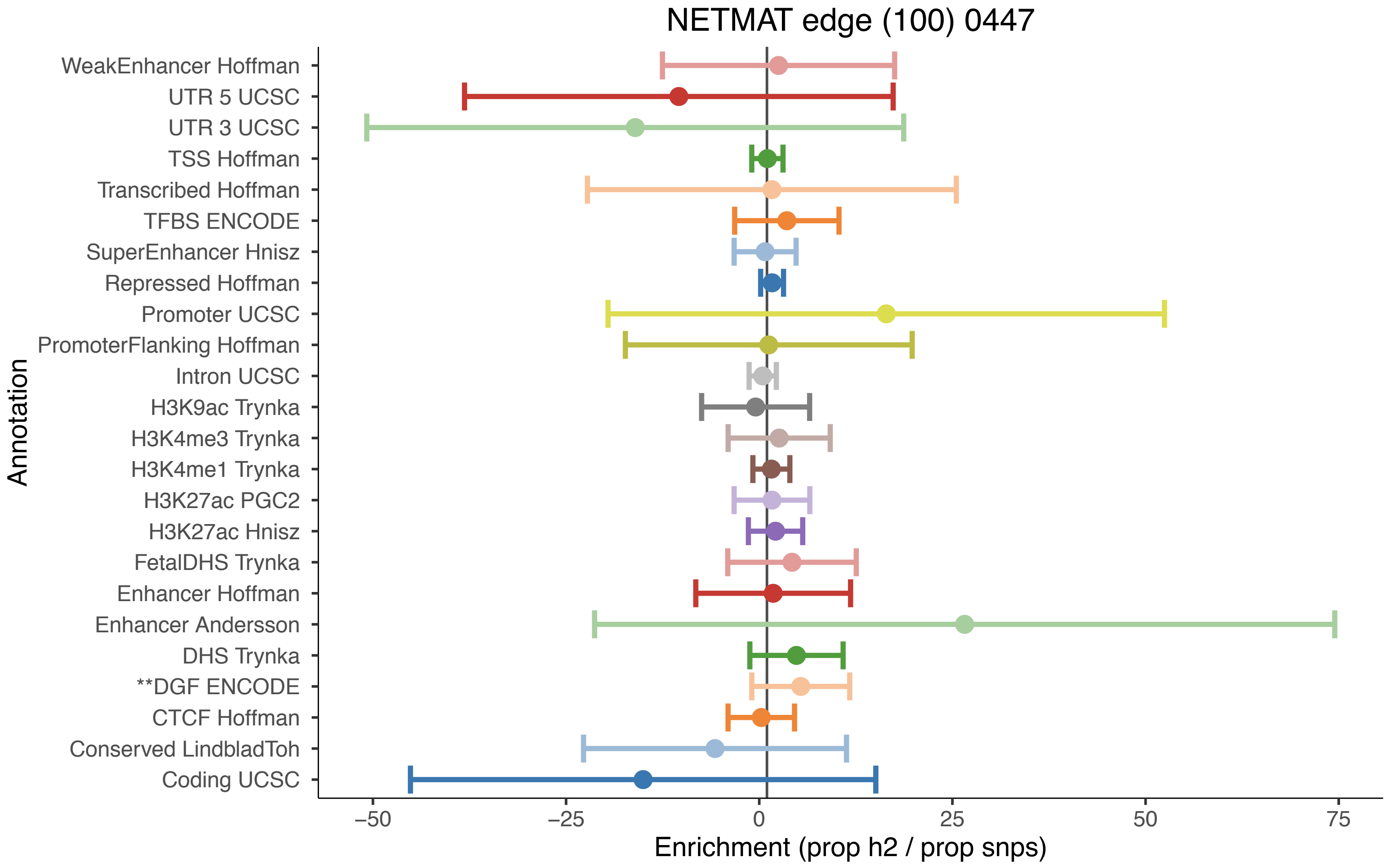

NETMAT edge (100) 0447
WeakEnhancer Hoffman
UTR 5 UCSC
UTR 3 UCSC
TSS Hoffman
Transcribed Hoffman
TFBS ENCODE
SuperEnhancer Hnisz
Repressed Hoffman
Promoter UCSC
PromoterFlanking Hoffman
Intron UCSC
H3K9ac Trynka
H3K4me3 Trynka
H3K4me1 Trynka
H3K27ac PGC2
H3K27ac Hnisz
FetalDHS Trynka
Enhancer Hoffman
Enhancer Andersson
DHS Trynka
**DGF ENCODE
CTCF Hoffman
Conserved LindbladToh
Coding UCSC
Annotation
−50
−25
0
25
50
75
Enrichment (prop h2 / prop snps)

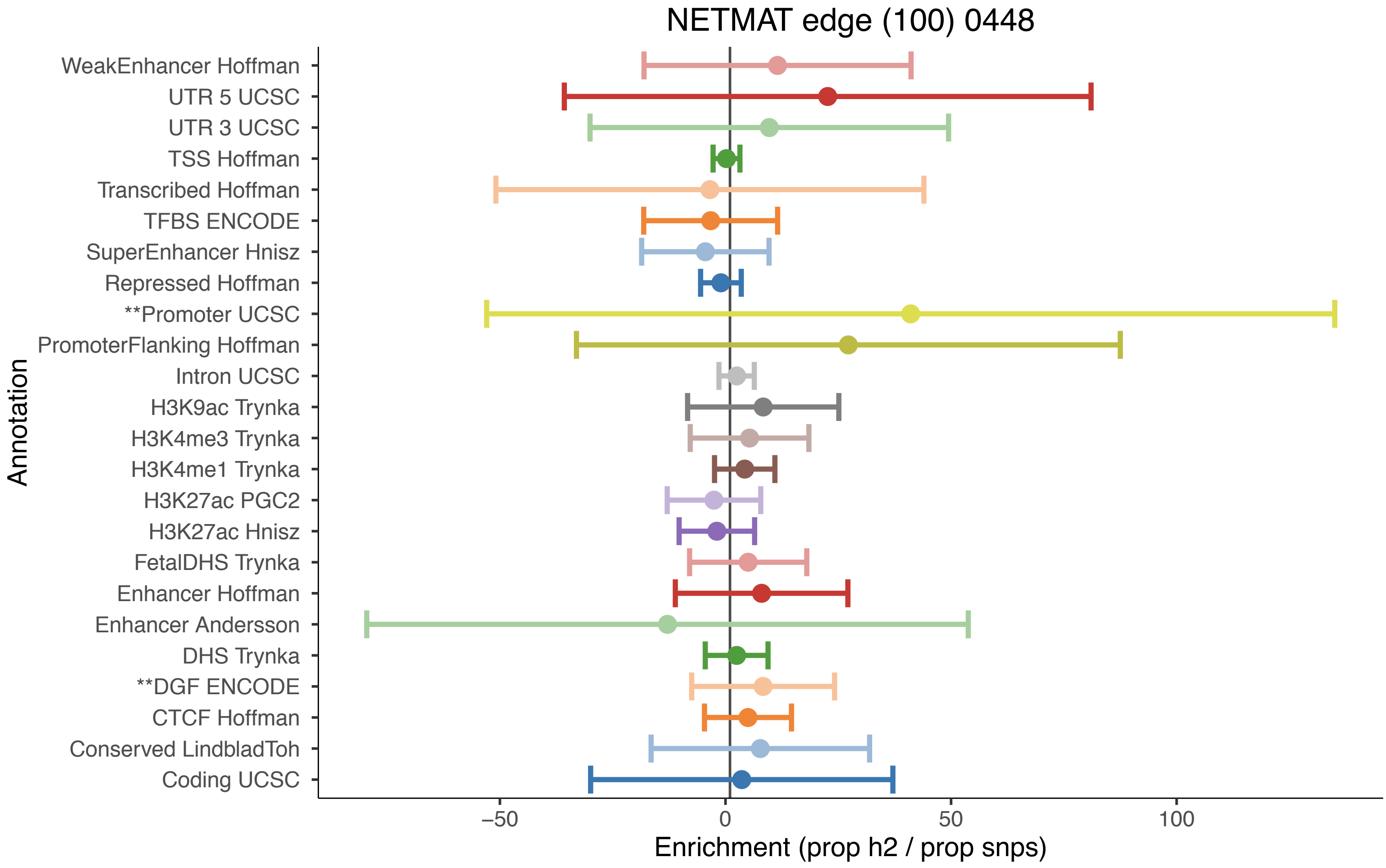

NETMAT edge (100) 0448
WeakEnhancer Hoffman
UTR 5 UCSC
UTR 3 UCSC
TSS Hoffman
Transcribed Hoffman
TFBS ENCODE
SuperEnhancer Hnisz
Repressed Hoffman
**Promoter UCSC
PromoterFlanking Hoffman
Intron UCSC
H3K9ac Trynka
H3K4me3 Trynka
H3K4me1 Trynka
H3K27ac PGC2
H3K27ac Hnisz
FetalDHS Trynka
Enhancer Hoffman
Enhancer Andersson
DHS Trynka
**DGF ENCODE
CTCF Hoffman
Conserved LindbladToh
Coding UCSC
Annotation
−50
0
50
100
Enrichment (prop h2 / prop snps)

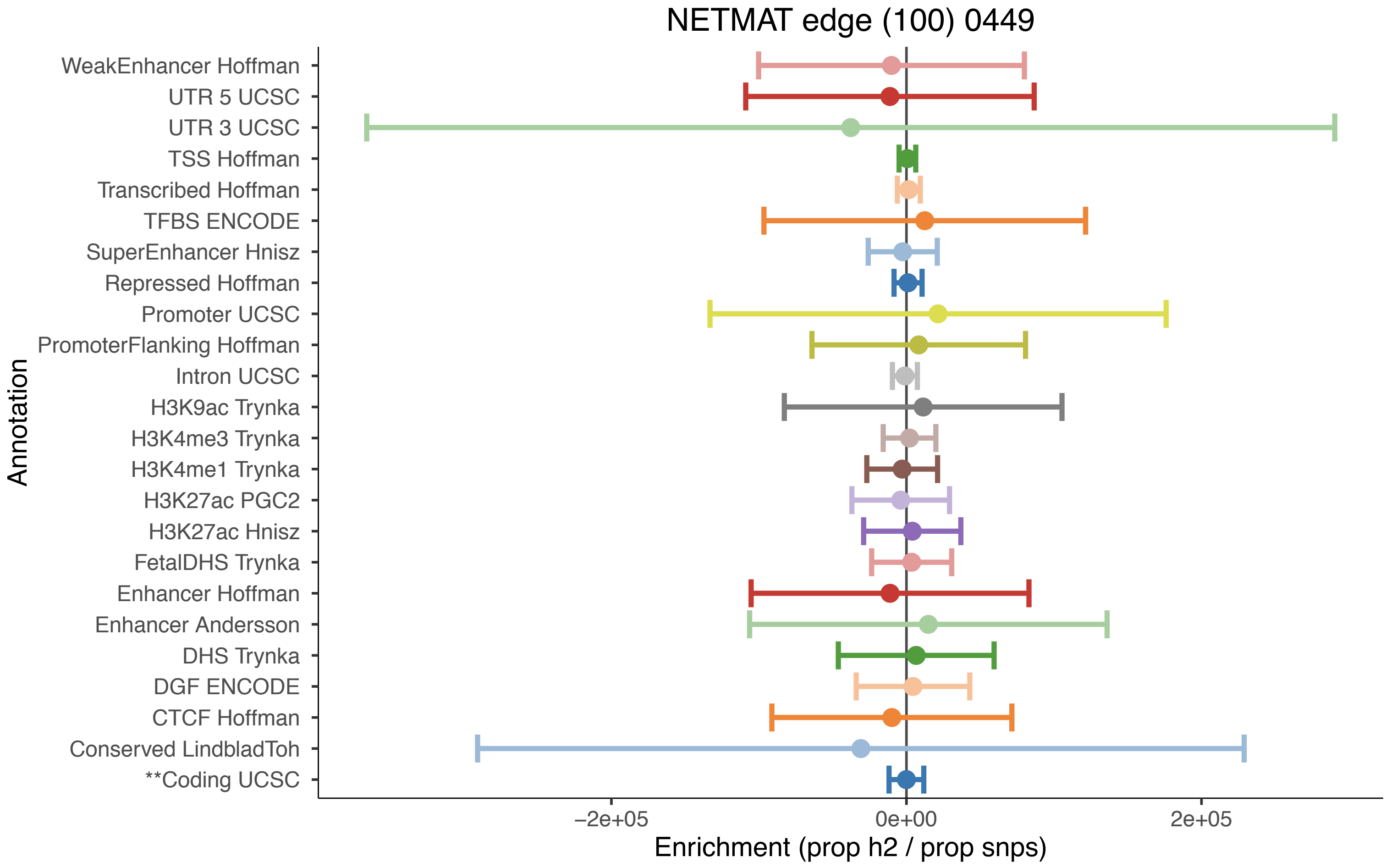

NETMAT edge (100) 0449
Annotation
WeakEnhancer Hoffman
UTR 5 UCSC
UTR 3 UCSC
TSS Hoffman
Transcribed Hoffman
TFBS ENCODE
SuperEnhancer Hnisz
Repressed Hoffman
Promoter UCSC
PromoterFlanking Hoffman
Intron UCSC
H3K9ac Trynka
H3K4me3 Trynka
H3K4me1 Trynka
H3K27ac PGC2
H3K27ac Hnisz
FetalDHS Trynka
Enhancer Hoffman
Enhancer Andersson
DHS Trynka
DGF ENCODE
CTCF Hoffman
Conserved LindbladToh
**Coding UCSC
−2e+05
0e+00
2e+05
Enrichment (prop h2 / prop snps)

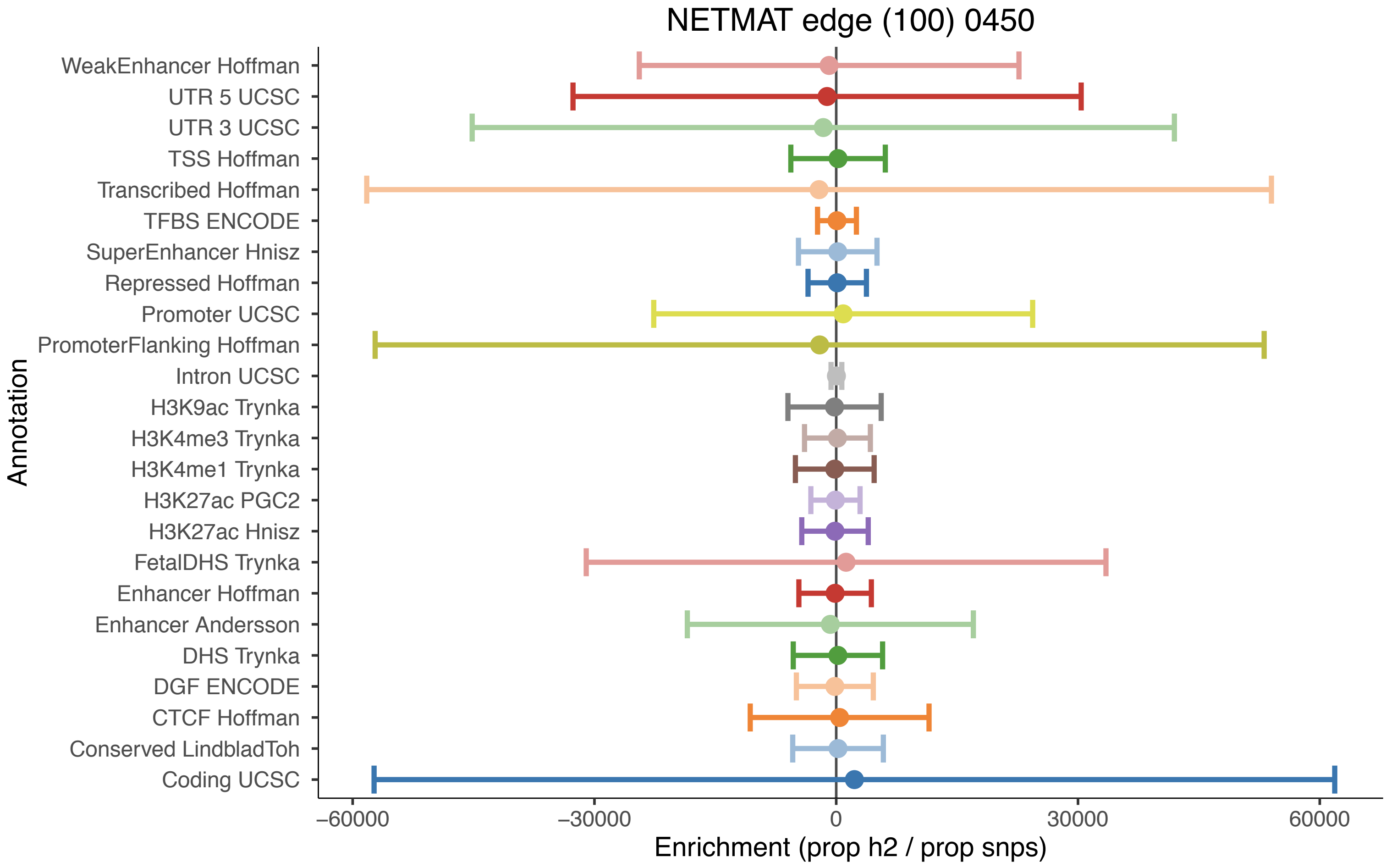
NETMAT edge (100) 0450
Annotation
WeakEnhancer Hoffman
UTR 5 UCSC
UTR 3 UCSC
TSS Hoffman
Transcribed Hoffman
TFBS ENCODE
SuperEnhancer Hnisz
Repressed Hoffman
Promoter UCSC
PromoterFlanking Hoffman
Intron UCSC
H3K9ac Trynka
H3K4me3 Trynka
H3K4me1 Trynka
H3K27ac PGC2
H3K27ac Hnisz
FetalDHS Trynka
Enhancer Hoffman
Enhancer Andersson
DHS Trynka
DGF ENCODE
CTCF Hoffman
Conserved LindbladToh
Coding UCSC
−60000
−30000
0
30000
60000
Enrichment (prop h2 / prop snps)

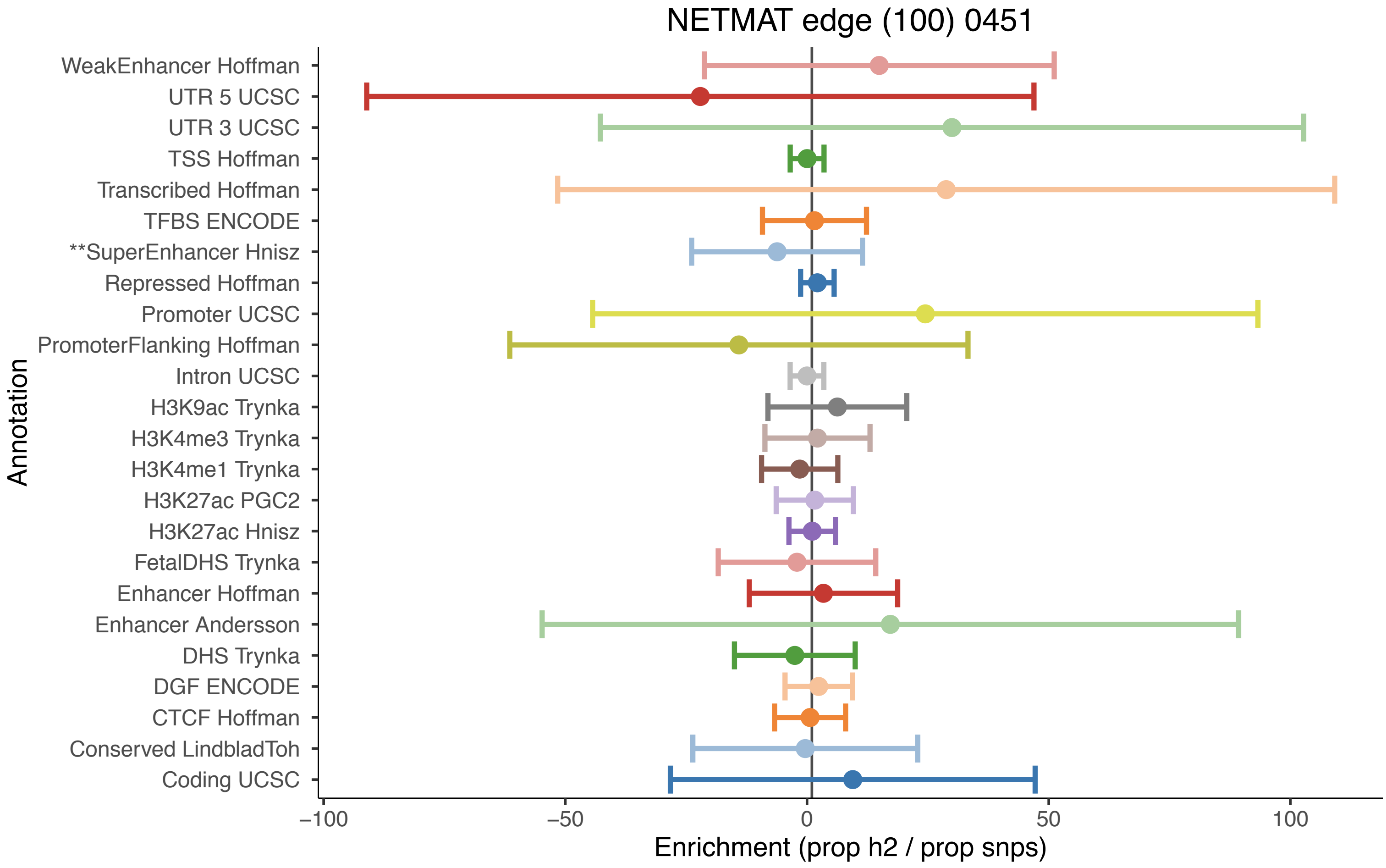

NETMAT edge (100) 0451
WeakEnhancer Hoffman
UTR 5 UCSC
UTR 3 UCSC
TSS Hoffman
Transcribed Hoffman
TFBS ENCODE
**SuperEnhancer Hnisz
Repressed Hoffman
Promoter UCSC
PromoterFlanking Hoffman
Intron UCSC
H3K9ac Trynka
H3K4me3 Trynka
H3K4me1 Trynka
H3K27ac PGC2
H3K27ac Hnisz
FetalDHS Trynka
Enhancer Hoffman
Enhancer Andersson
DHS Trynka
DGF ENCODE
CTCF Hoffman
Conserved LindbladToh
Coding UCSC
Annotation
−100
−50
0
50
100
Enrichment (prop h2 / prop snps)

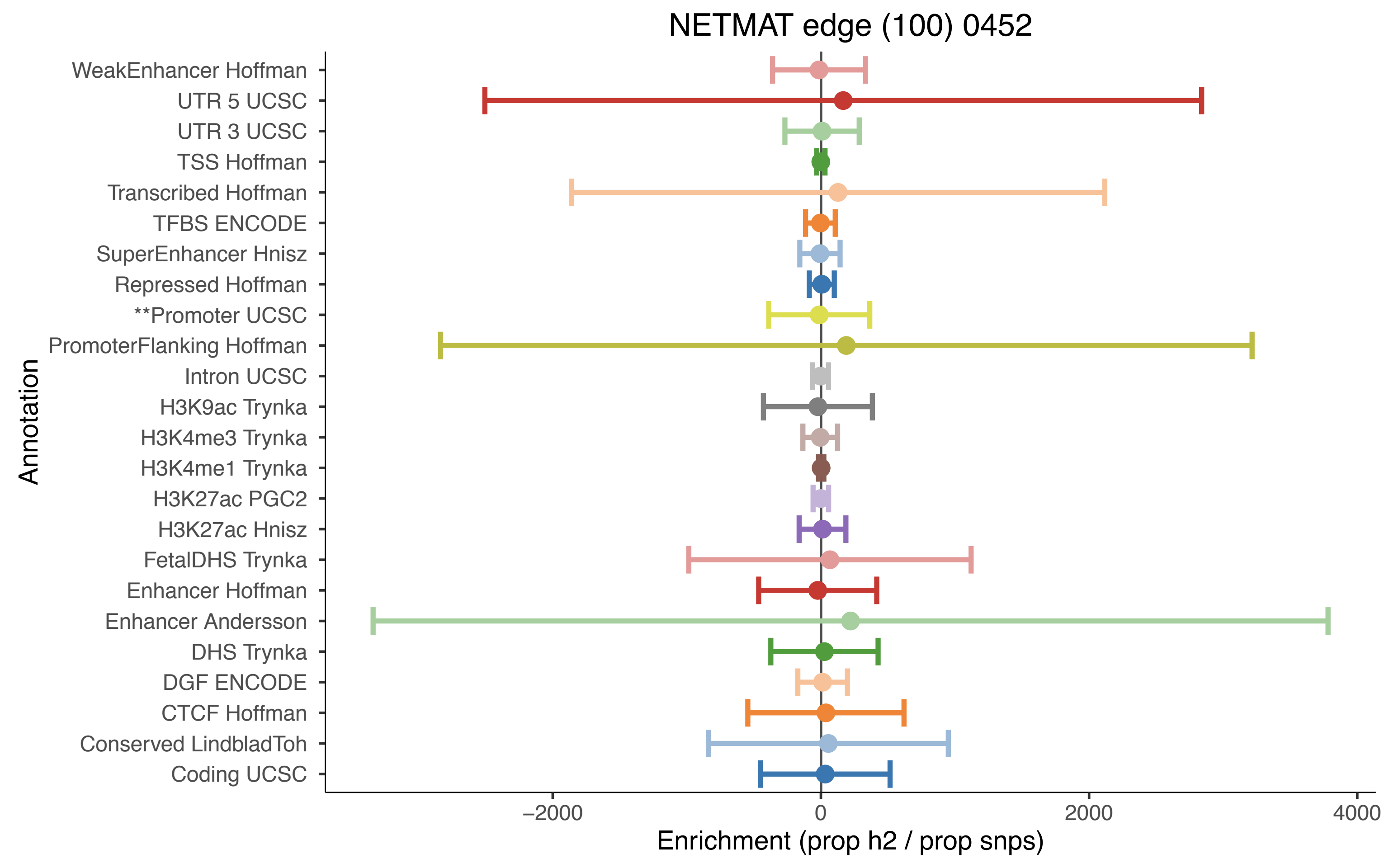
NETMAT edge (100) 0452
WeakEnhancer Hoffman
UTR 5 UCSC
UTR 3 UCSC
TSS Hoffman
Transcribed Hoffman
TFBS ENCODE
SuperEnhancer Hnisz
Repressed Hoffman
**Promoter UCSC
PromoterFlanking Hoffman
Intron UCSC
H3K9ac Trynka
H3K4me3 Trynka
H3K4me1 Trynka
H3K27ac PGC2
H3K27ac Hnisz
FetalDHS Trynka
Enhancer Hoffman
Enhancer Andersson
DHS Trynka
DGF ENCODE
CTCF Hoffman
Conserved LindbladToh
Coding UCSC
Annotation
−2000
0
2000
4000
Enrichment (prop h2 / prop snps)

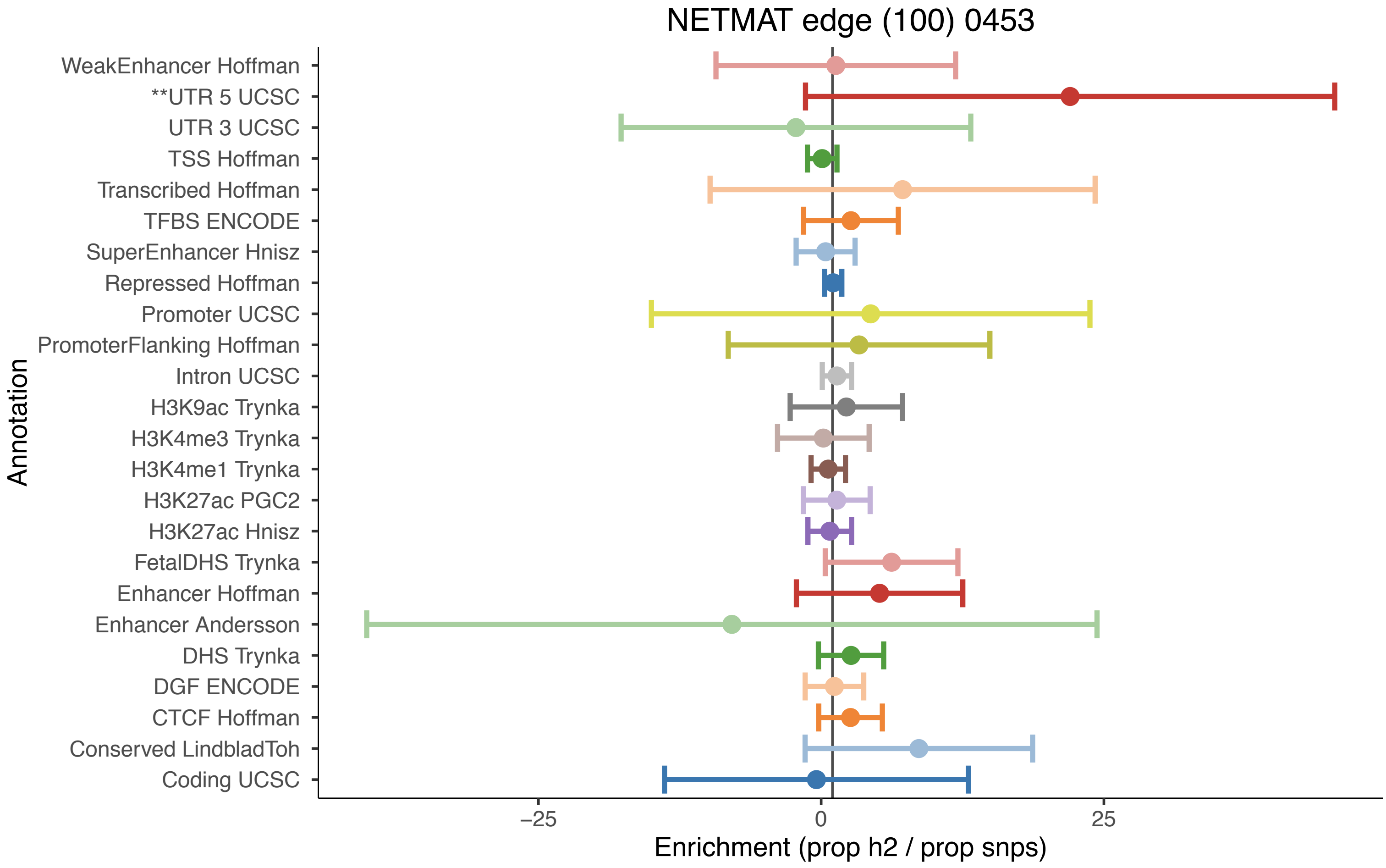

NETMAT edge (100) 0453
WeakEnhancer Hoffman
**UTR 5 UCSC
UTR 3 UCSC
TSS Hoffman
Transcribed Hoffman
TFBS ENCODE
SuperEnhancer Hnisz
Repressed Hoffman
Promoter UCSC
PromoterFlanking Hoffman
Intron UCSC
H3K9ac Trynka
H3K4me3 Trynka
H3K4me1 Trynka
H3K27ac PGC2
H3K27ac Hnisz
FetalDHS Trynka
Enhancer Hoffman
Enhancer Andersson
DHS Trynka
DGF ENCODE
CTCF Hoffman
Conserved LindbladToh
Coding UCSC
Annotation
−25
0
25
Enrichment (prop h2 / prop snps)

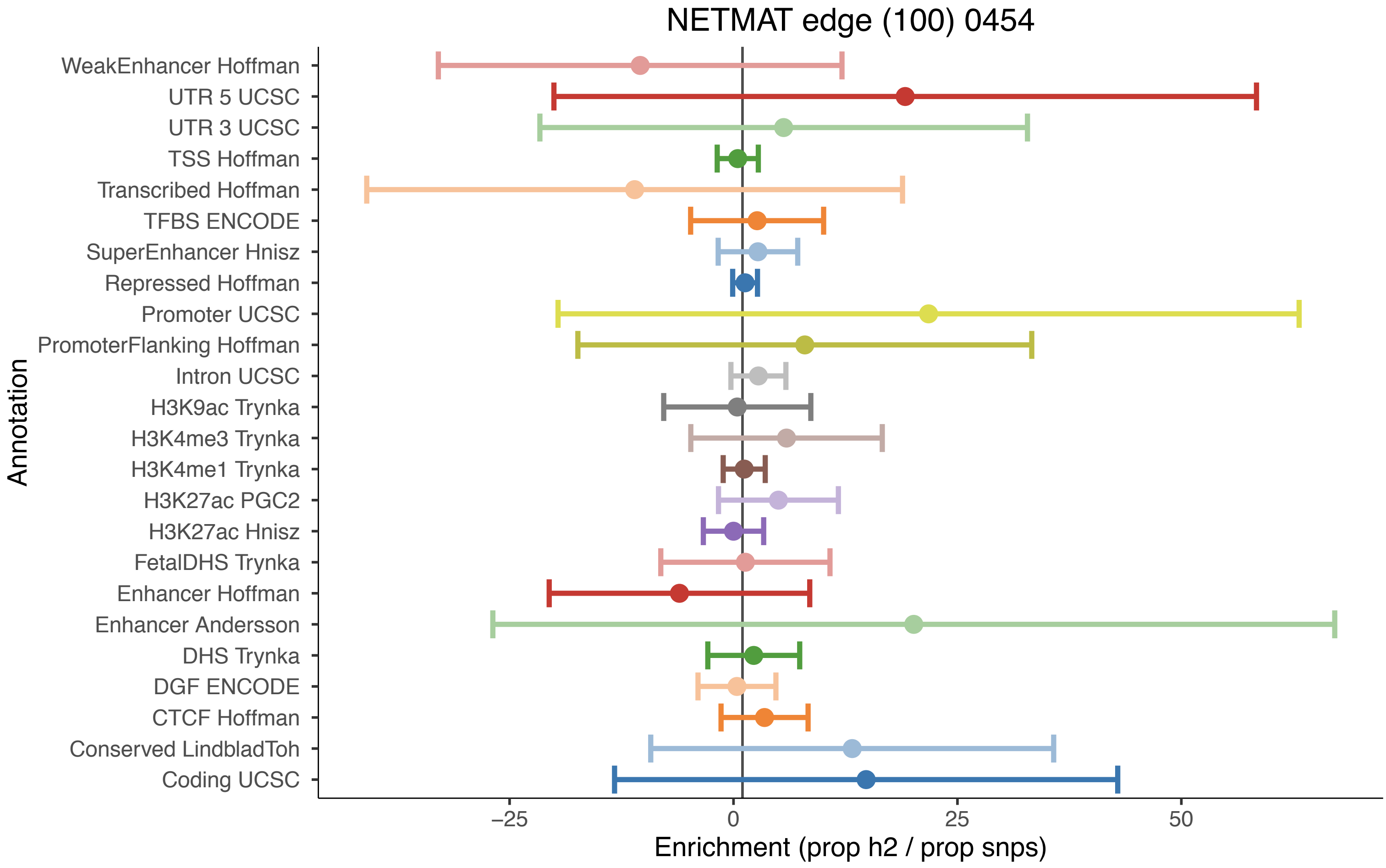

NETMAT edge (100) 0454
WeakEnhancer Hoffman
UTR 5 UCSC
UTR 3 UCSC
TSS Hoffman
Transcribed Hoffman
TFBS ENCODE
SuperEnhancer Hnisz
Repressed Hoffman
Promoter UCSC
PromoterFlanking Hoffman
Intron UCSC
H3K9ac Trynka
H3K4me3 Trynka
H3K4me1 Trynka
H3K27ac PGC2
H3K27ac Hnisz
FetalDHS Trynka
Enhancer Hoffman
Enhancer Andersson
DHS Trynka
DGF ENCODE
CTCF Hoffman
Conserved LindbladToh
Coding UCSC
Annotation
−25
0
25
50
Enrichment (prop h2 / prop snps)

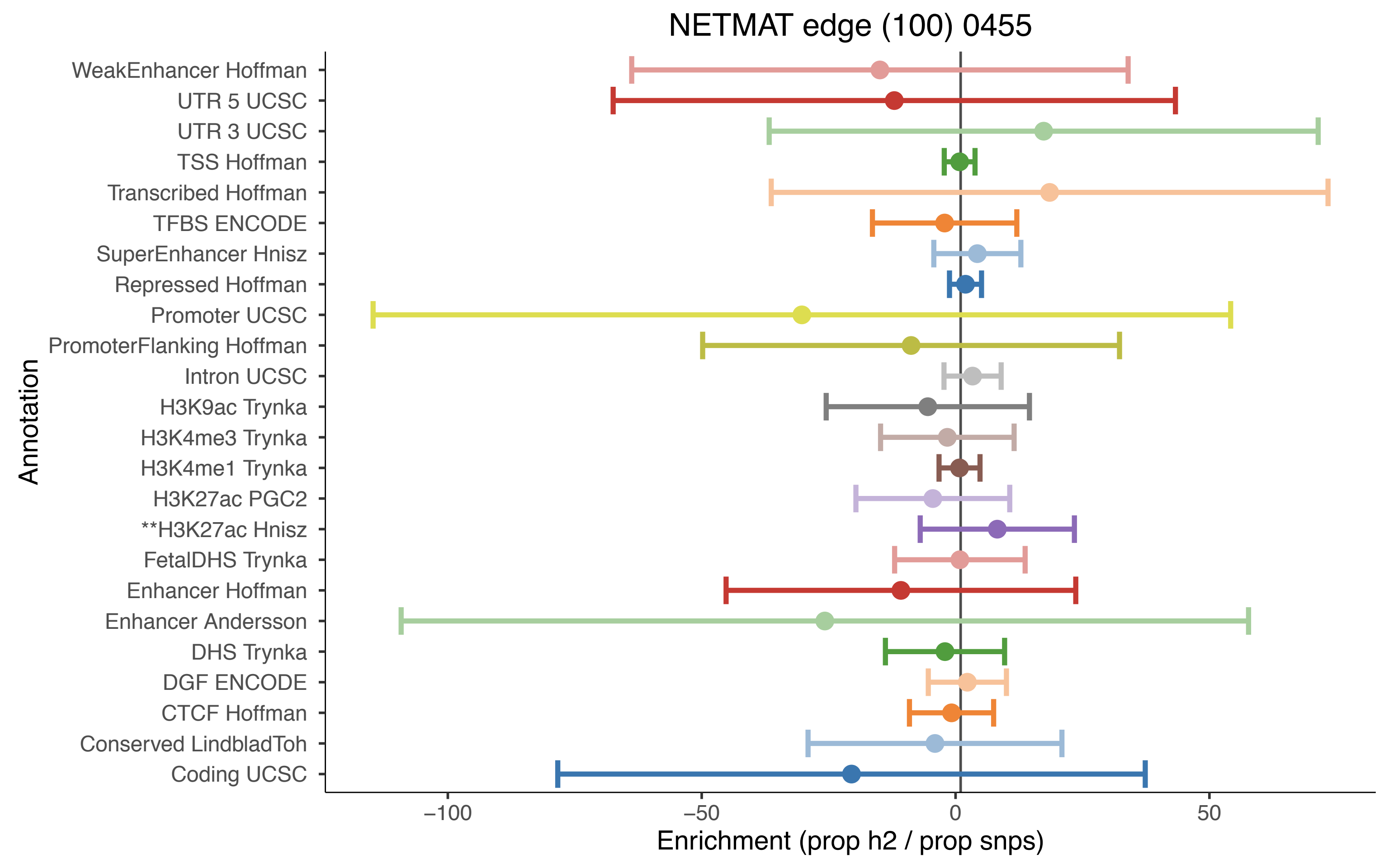
NETMAT edge (100) 0455
WeakEnhancer Hoffman
UTR 5 UCSC
UTR 3 UCSC
TSS Hoffman
Transcribed Hoffman
TFBS ENCODE
SuperEnhancer Hnisz
Repressed Hoffman
Promoter UCSC
PromoterFlanking Hoffman
Intron UCSC
H3K9ac Trynka
H3K4me3 Trynka
H3K4me1 Trynka
H3K27ac PGC2
**H3K27ac Hnisz
FetalDHS Trynka
Enhancer Hoffman
Enhancer Andersson
DHS Trynka
DGF ENCODE
CTCF Hoffman
Conserved LindbladToh
Coding UCSC
Annotation
−100
−50
0
50
Enrichment (prop h2 / prop snps)

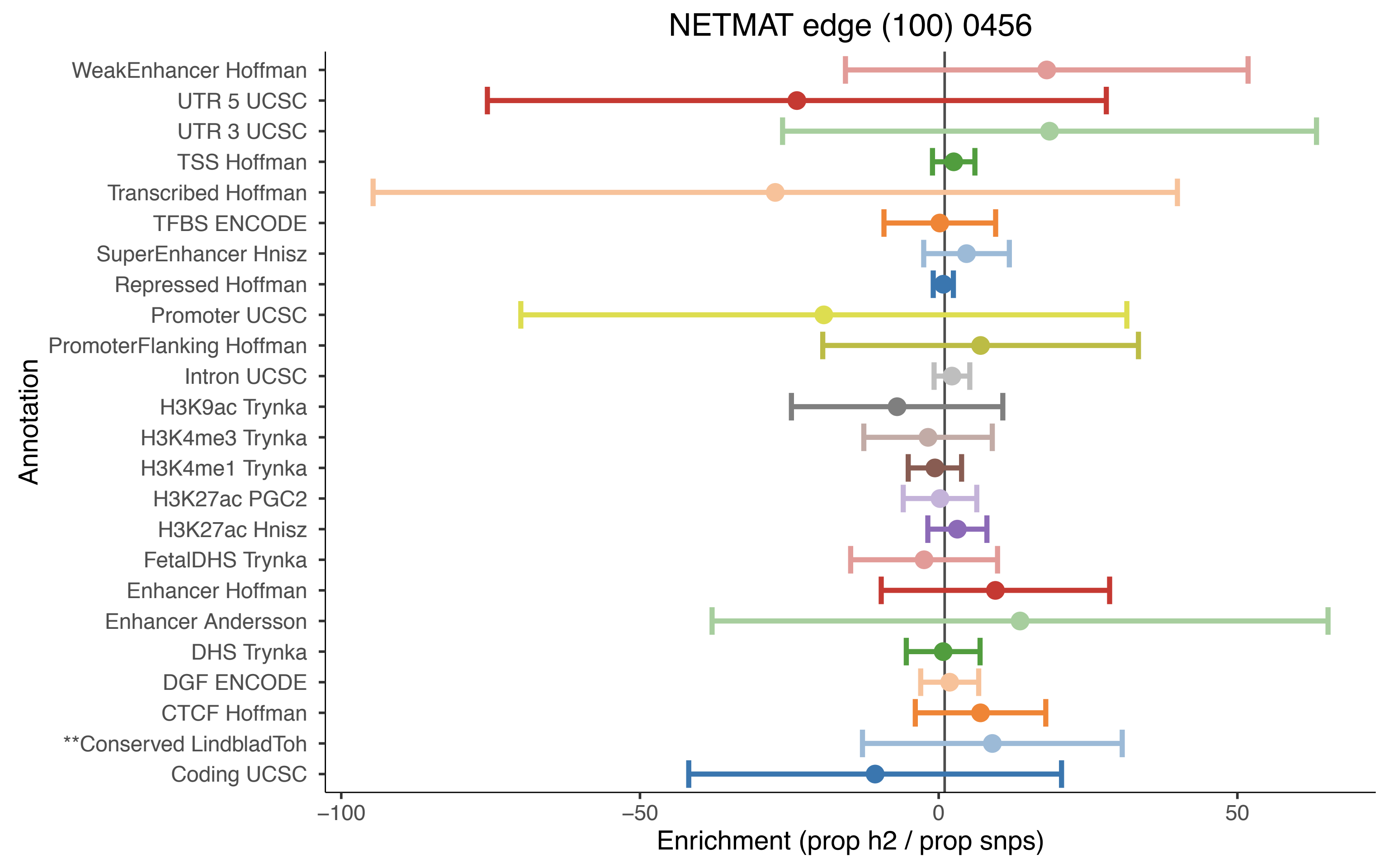
NETMAT edge (100) 0456
WeakEnhancer Hoffman
UTR 5 UCSC
UTR 3 UCSC
TSS Hoffman
Transcribed Hoffman
TFBS ENCODE
SuperEnhancer Hnisz
Repressed Hoffman
Promoter UCSC
PromoterFlanking Hoffman
Intron UCSC
H3K9ac Trynka
H3K4me3 Trynka
H3K4me1 Trynka
H3K27ac PGC2
H3K27ac Hnisz
FetalDHS Trynka
Enhancer Hoffman
Enhancer Andersson
DHS Trynka
DGF ENCODE
CTCF Hoffman
**Conserved LindbladToh
Coding UCSC
Annotation
−100
−50
0
50
Enrichment (prop h2 / prop snps)

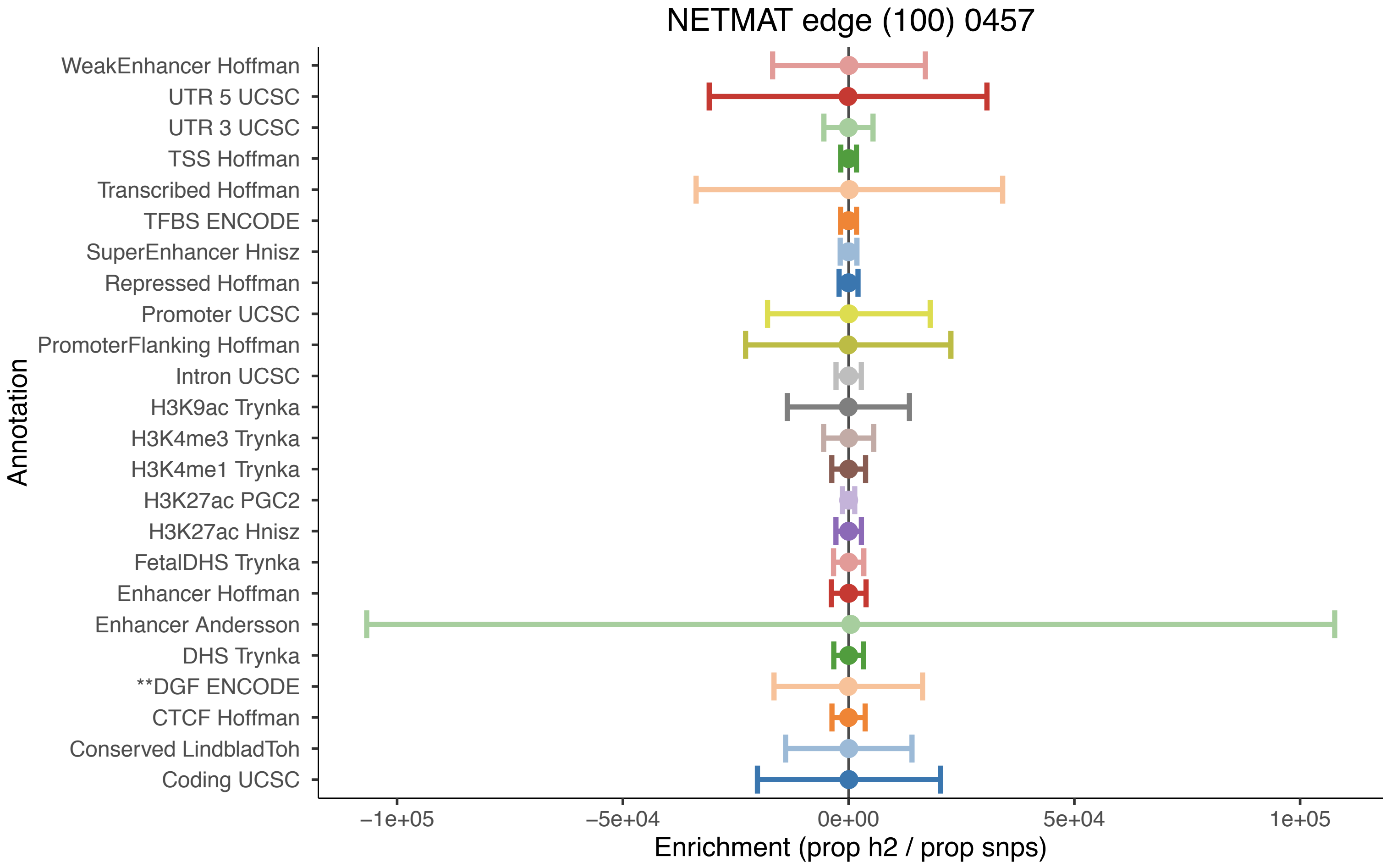
NETMAT edge (100) 0457
WeakEnhancer Hoffman
UTR 5 UCSC
UTR 3 UCSC
TSS Hoffman
Transcribed Hoffman
TFBS ENCODE
SuperEnhancer Hnisz
Repressed Hoffman
Promoter UCSC
PromoterFlanking Hoffman
Intron UCSC
H3K9ac Trynka
H3K4me3 Trynka
H3K4me1 Trynka
H3K27ac PGC2
H3K27ac Hnisz
FetalDHS Trynka
Enhancer Hoffman
Enhancer Andersson
DHS Trynka
**DGF ENCODE
CTCF Hoffman
Conserved LindbladToh
Coding UCSC
Annotation
−1e+05
−5e+04
0e+00
5e+04
1e+05
Enrichment (prop h2 / prop snps)

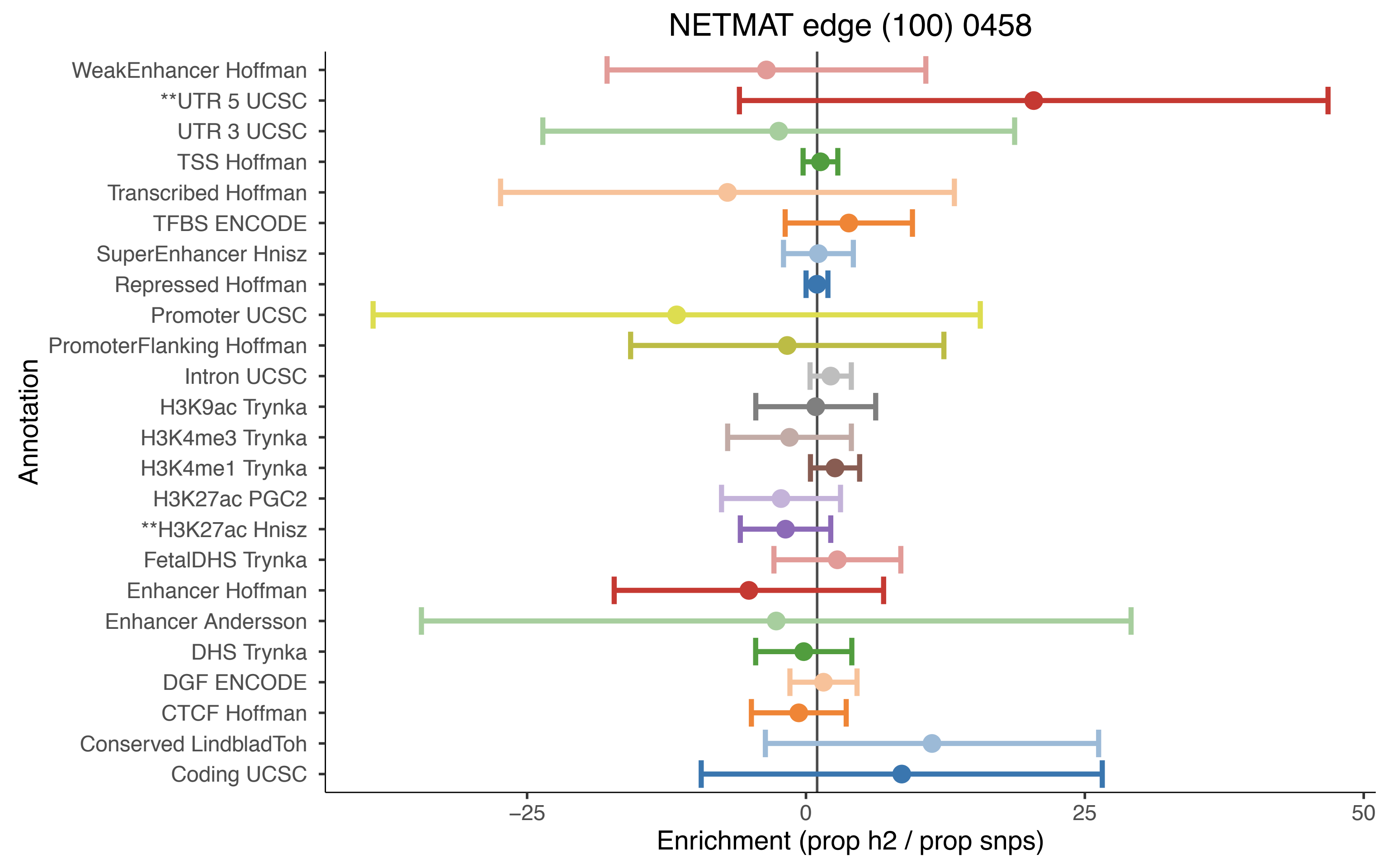
NETMAT edge (100) 0458
WeakEnhancer Hoffman
**UTR 5 UCSC
UTR 3 UCSC
TSS Hoffman
Transcribed Hoffman
TFBS ENCODE
SuperEnhancer Hnisz
Repressed Hoffman
Promoter UCSC
PromoterFlanking Hoffman
Intron UCSC
H3K9ac Trynka
H3K4me3 Trynka
H3K4me1 Trynka
H3K27ac PGC2
**H3K27ac Hnisz
FetalDHS Trynka
Enhancer Hoffman
Enhancer Andersson
DHS Trynka
DGF ENCODE
CTCF Hoffman
Conserved LindbladToh
Coding UCSC
Annotation
−25
0
25
50
Enrichment (prop h2 / prop snps)

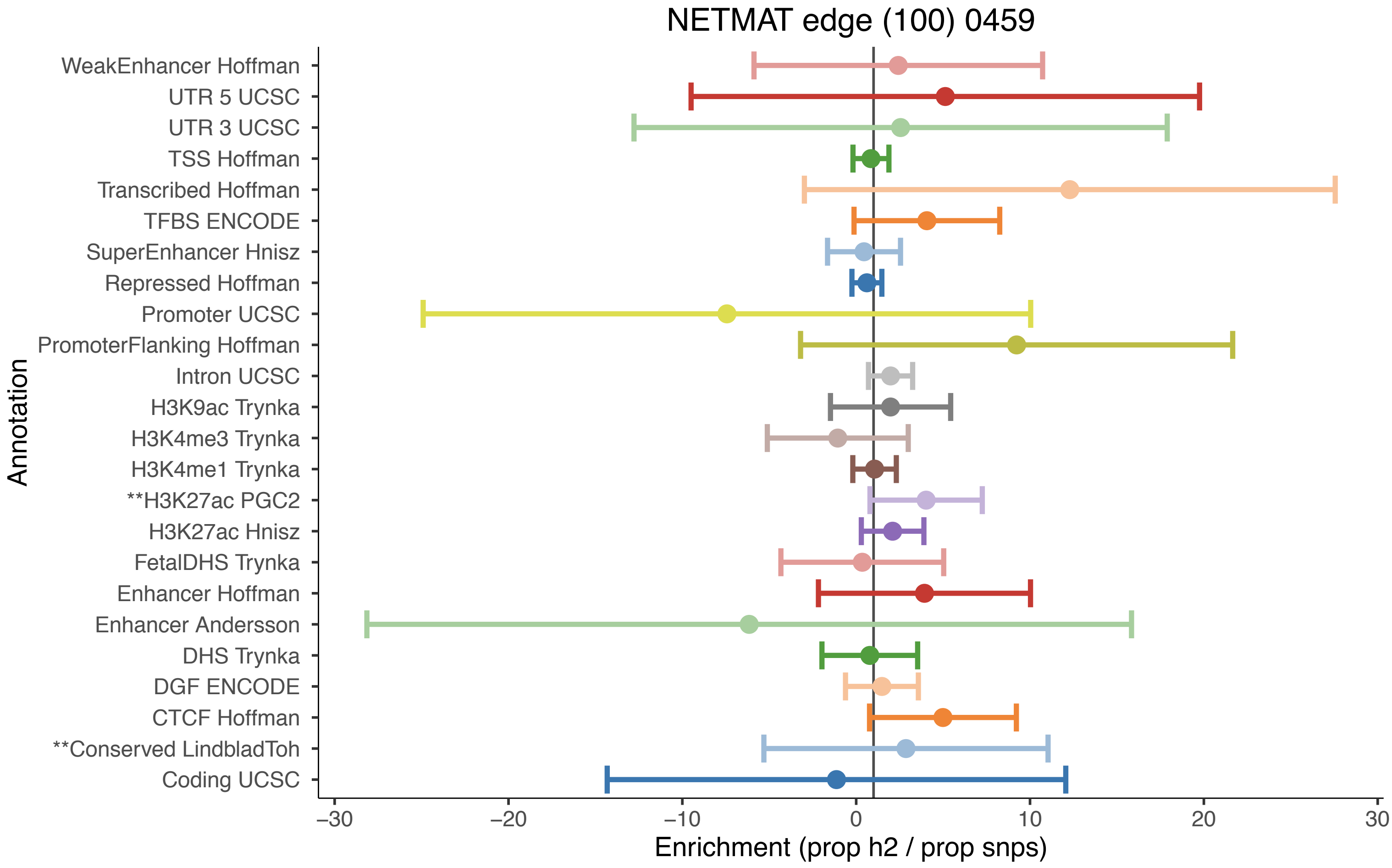
NETMAT edge (100) 0459
WeakEnhancer Hoffman
UTR 5 UCSC
UTR 3 UCSC
TSS Hoffman
Transcribed Hoffman
TFBS ENCODE
SuperEnhancer Hnisz
Repressed Hoffman
Promoter UCSC
PromoterFlanking Hoffman
Intron UCSC
H3K9ac Trynka
H3K4me3 Trynka
H3K4me1 Trynka
**H3K27ac PGC2
H3K27ac Hnisz
FetalDHS Trynka
Enhancer Hoffman
Enhancer Andersson
DHS Trynka
DGF ENCODE
CTCF Hoffman
**Conserved LindbladToh
Coding UCSC
Annotation
−30
−20
−10
0
10
20
30
Enrichment (prop h2 / prop snps)

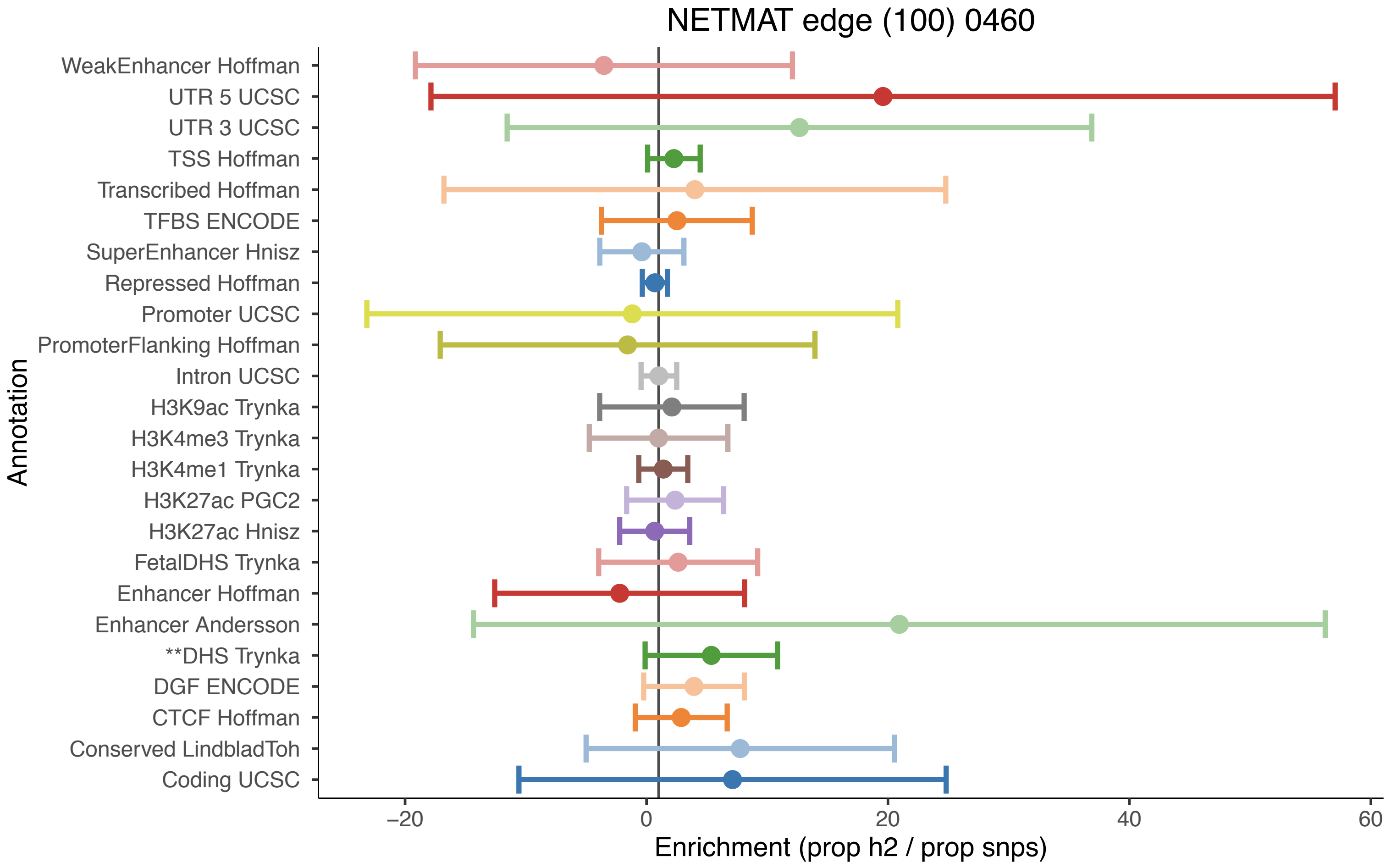
NETMAT edge (100) 0460
WeakEnhancer Hoffman
UTR 5 UCSC
UTR 3 UCSC
TSS Hoffman
Transcribed Hoffman
TFBS ENCODE
SuperEnhancer Hnisz
Repressed Hoffman
Promoter UCSC
PromoterFlanking Hoffman
Intron UCSC
H3K9ac Trynka
H3K4me3 Trynka
H3K4me1 Trynka
H3K27ac PGC2
H3K27ac Hnisz
FetalDHS Trynka
Enhancer Hoffman
Enhancer Andersson
**DHS Trynka
DGF ENCODE
CTCF Hoffman
Conserved LindbladToh
Coding UCSC
Annotation
−20
0
20
40
60
Enrichment (prop h2 / prop snps)

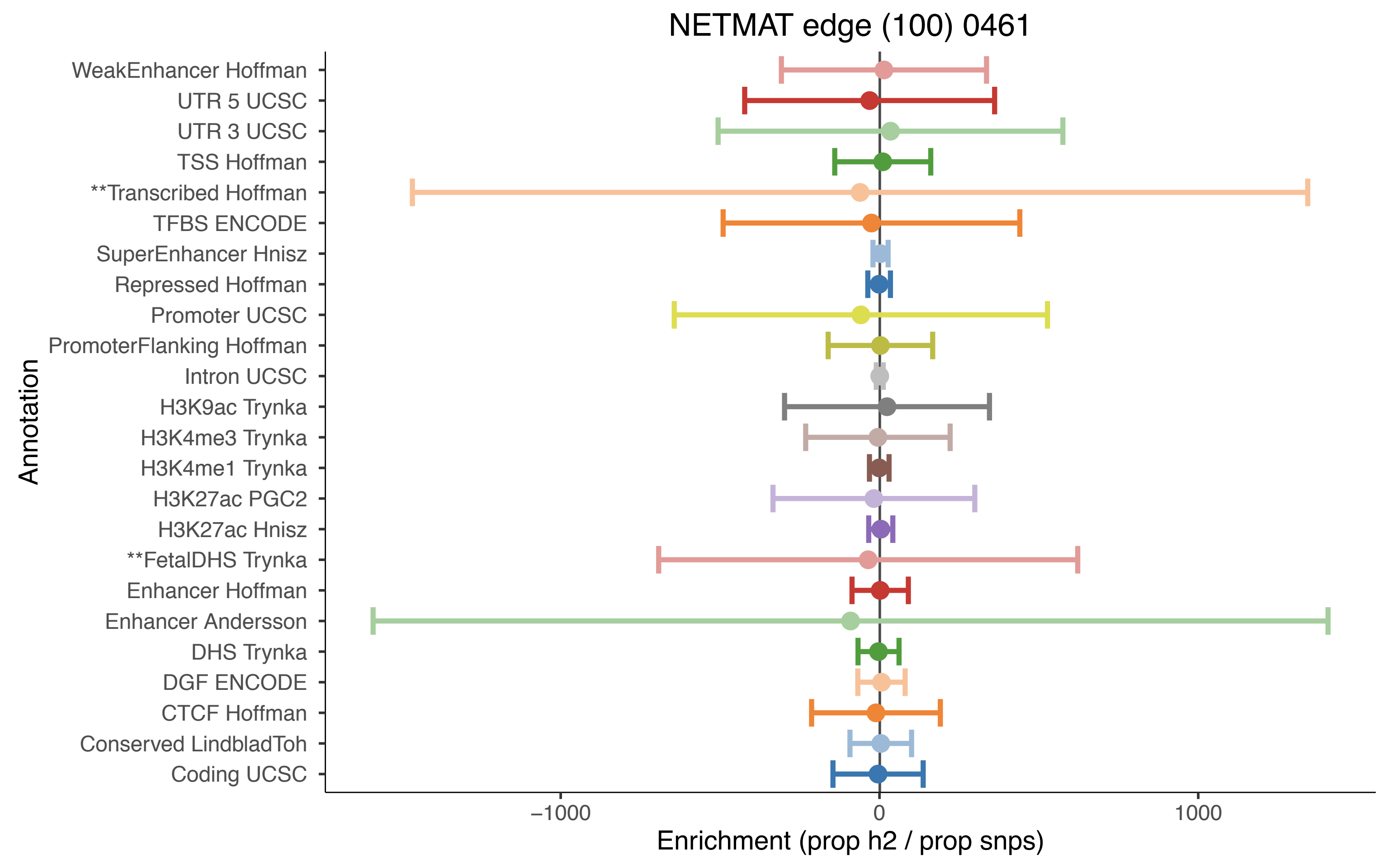

NETMAT edge (100) 0461
WeakEnhancer Hoffman
UTR 5 UCSC
UTR 3 UCSC
TSS Hoffman
**Transcribed Hoffman
TFBS ENCODE
SuperEnhancer Hnisz
Repressed Hoffman
Promoter UCSC
PromoterFlanking Hoffman
Intron UCSC
H3K9ac Trynka
H3K4me3 Trynka
H3K4me1 Trynka
H3K27ac PGC2
H3K27ac Hnisz
**FetalDHS Trynka
Enhancer Hoffman
Enhancer Andersson
DHS Trynka
DGF ENCODE
CTCF Hoffman
Conserved LindbladToh
Coding UCSC
Annotation
−1000
0
1000
Enrichment (prop h2 / prop snps)

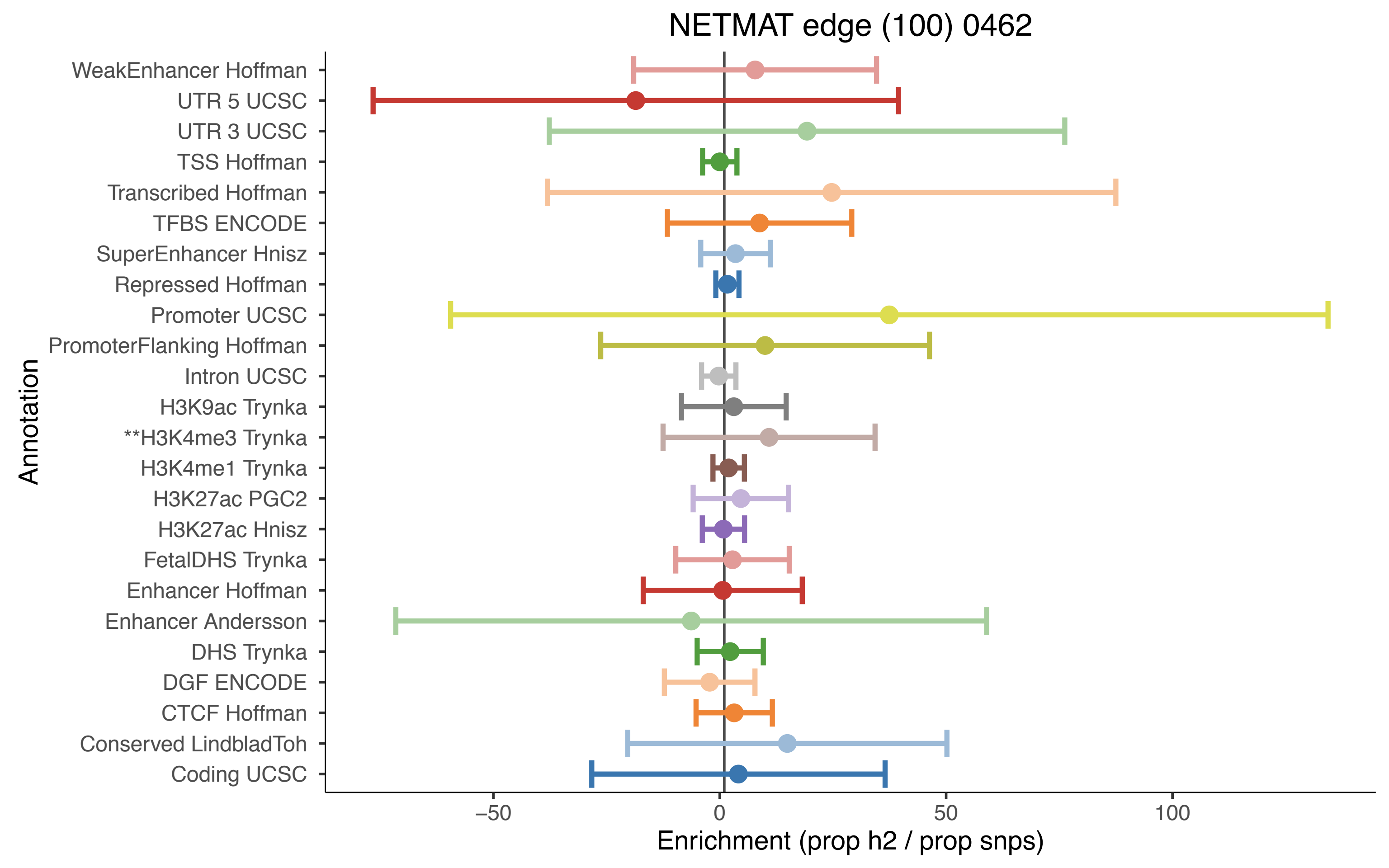
NETMAT edge (100) 0462
Annotation
WeakEnhancer Hoffman
UTR 5 UCSC
UTR 3 UCSC
TSS Hoffman
Transcribed Hoffman
TFBS ENCODE
SuperEnhancer Hnisz
Repressed Hoffman
Promoter UCSC
PromoterFlanking Hoffman
Intron UCSC
H3K9ac Trynka
**H3K4me3 Trynka
H3K4me1 Trynka
H3K27ac PGC2
H3K27ac Hnisz
FetalDHS Trynka
Enhancer Hoffman
Enhancer Andersson
DHS Trynka
DGF ENCODE
CTCF Hoffman
Conserved LindbladToh
Coding UCSC
−50
0
50
100
Enrichment (prop h2 / prop snps)

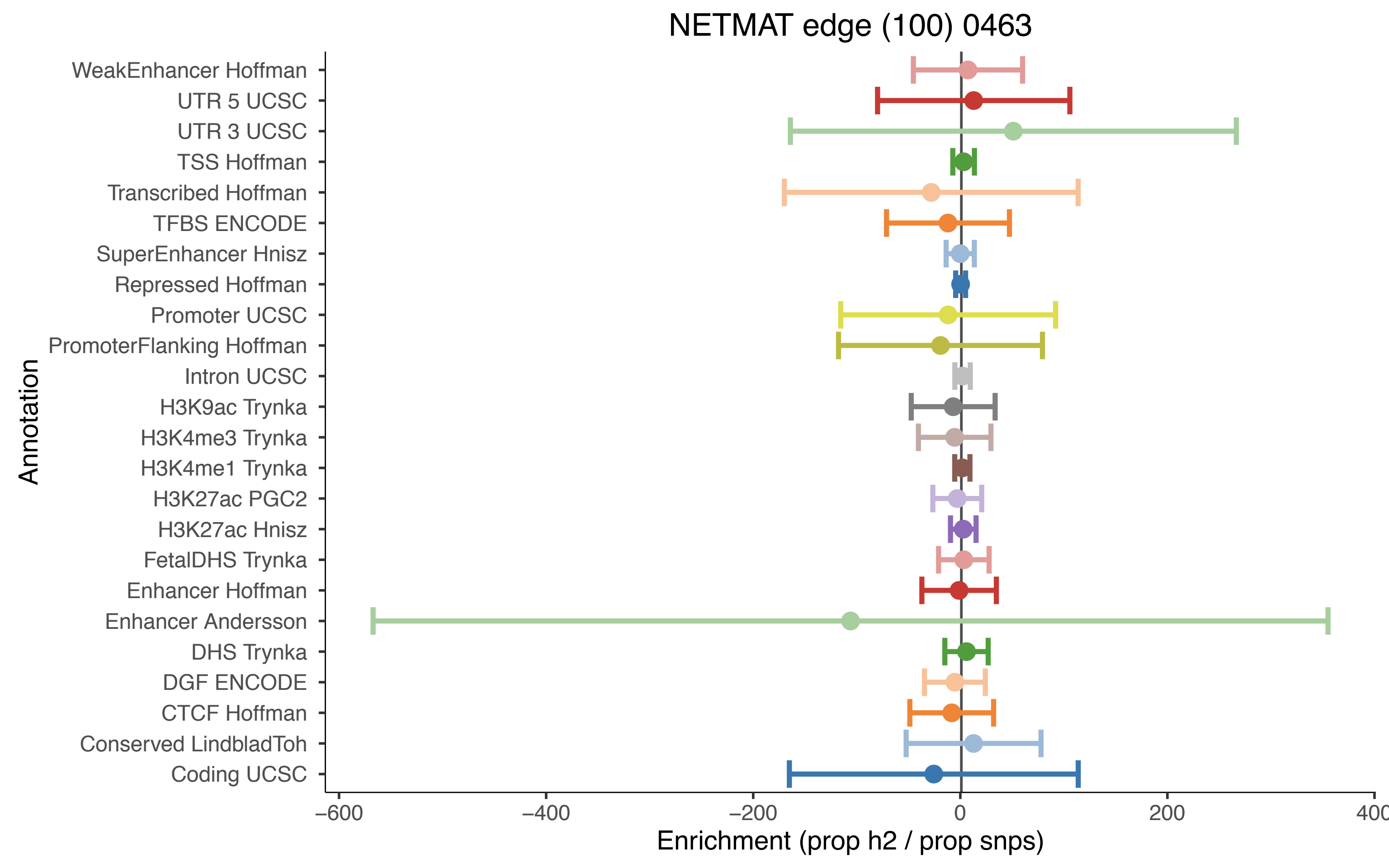

NETMAT edge (100) 0463
WeakEnhancer Hoffman
UTR 5 UCSC
UTR 3 UCSC
TSS Hoffman
Transcribed Hoffman
TFBS ENCODE
SuperEnhancer Hnisz
Repressed Hoffman
Promoter UCSC
PromoterFlanking Hoffman
Intron UCSC
H3K9ac Trynka
H3K4me3 Trynka
H3K4me1 Trynka
H3K27ac PGC2
H3K27ac Hnisz
FetalDHS Trynka
Enhancer Hoffman
Enhancer Andersson
DHS Trynka
DGF ENCODE
CTCF Hoffman
Conserved LindbladToh
Coding UCSC
Annotation
−600
−400
−200
0
200
400
Enrichment (prop h2 / prop snps)

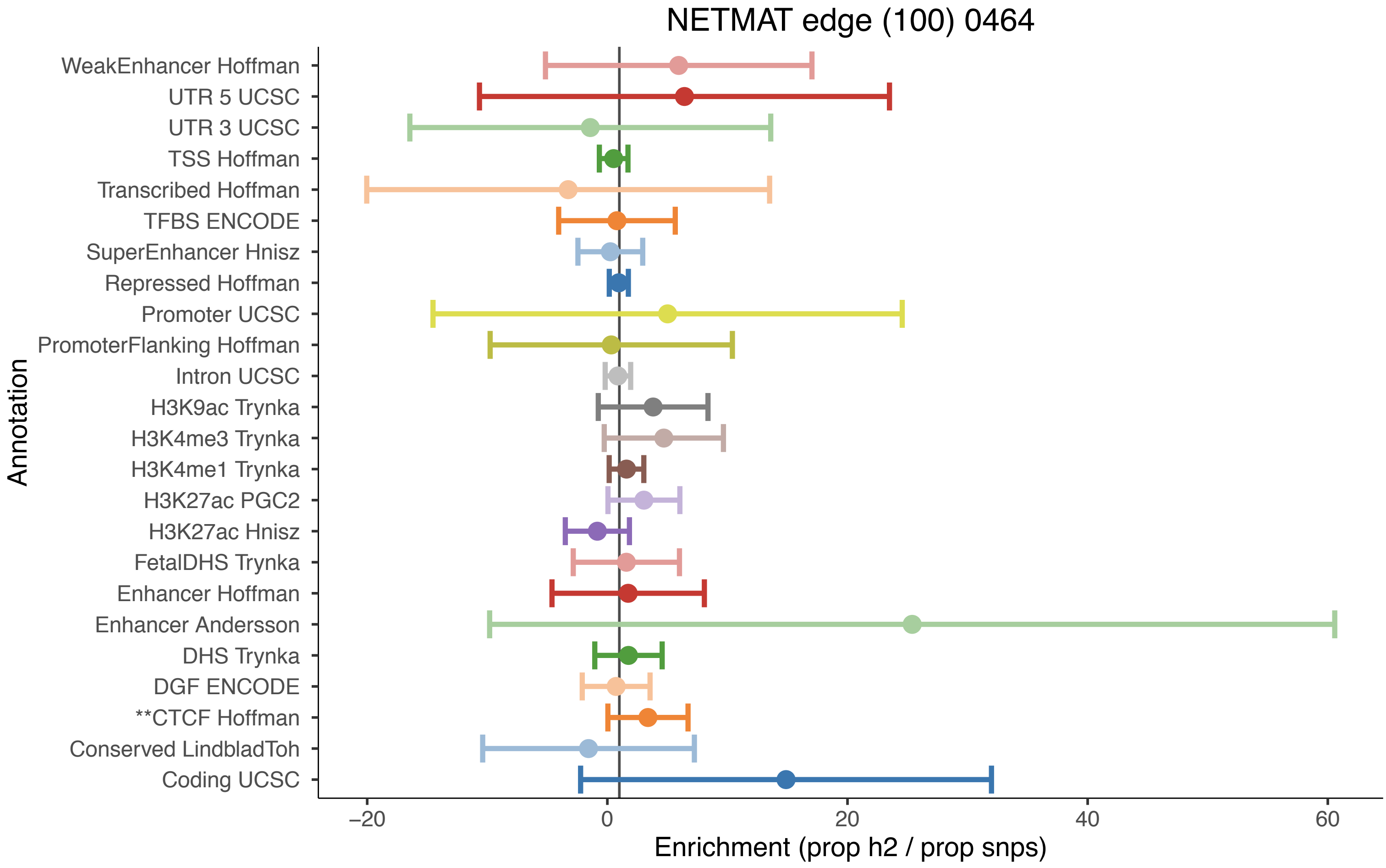

NETMAT edge (100) 0464
WeakEnhancer Hoffman
UTR 5 UCSC
UTR 3 UCSC
TSS Hoffman
Transcribed Hoffman
TFBS ENCODE
SuperEnhancer Hnisz
Repressed Hoffman
Promoter UCSC
PromoterFlanking Hoffman
Intron UCSC
H3K9ac Trynka
H3K4me3 Trynka
H3K4me1 Trynka
H3K27ac PGC2
H3K27ac Hnisz
FetalDHS Trynka
Enhancer Hoffman
Enhancer Andersson
DHS Trynka
DGF ENCODE
**CTCF Hoffman
Conserved LindbladToh
Coding UCSC
Annotation
−20
0
20
40
60
Enrichment (prop h2 / prop snps)

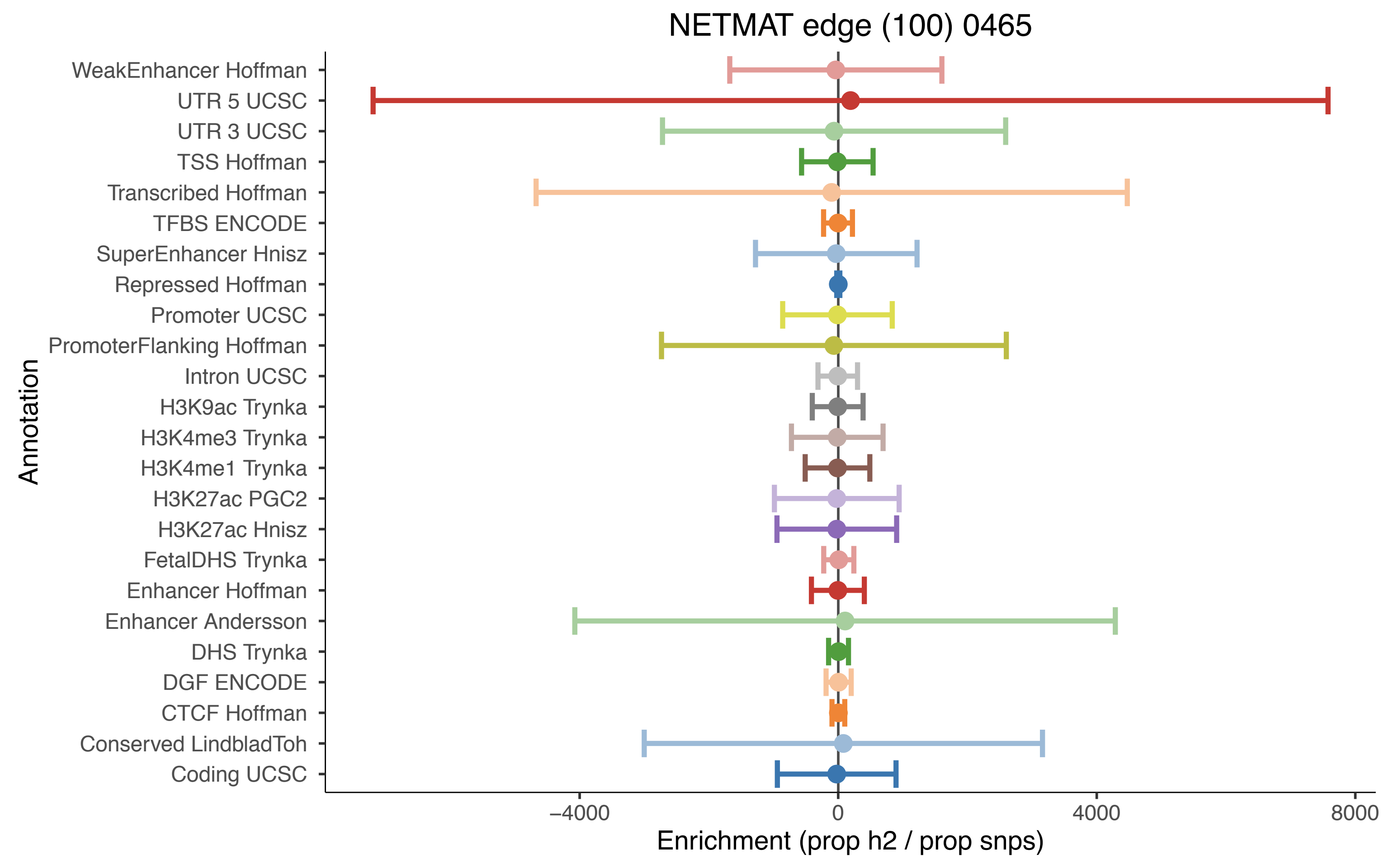

NETMAT edge (100) 0465
Annotation
WeakEnhancer Hoffman
UTR 5 UCSC
UTR 3 UCSC
TSS Hoffman
Transcribed Hoffman
TFBS ENCODE
SuperEnhancer Hnisz
Repressed Hoffman
Promoter UCSC
PromoterFlanking Hoffman
Intron UCSC
H3K9ac Trynka
H3K4me3 Trynka
H3K4me1 Trynka
H3K27ac PGC2
H3K27ac Hnisz
FetalDHS Trynka
Enhancer Hoffman
Enhancer Andersson
DHS Trynka
DGF ENCODE
CTCF Hoffman
Conserved LindbladToh
Coding UCSC
−4000
0
4000
8000
Enrichment (prop h2 / prop snps)

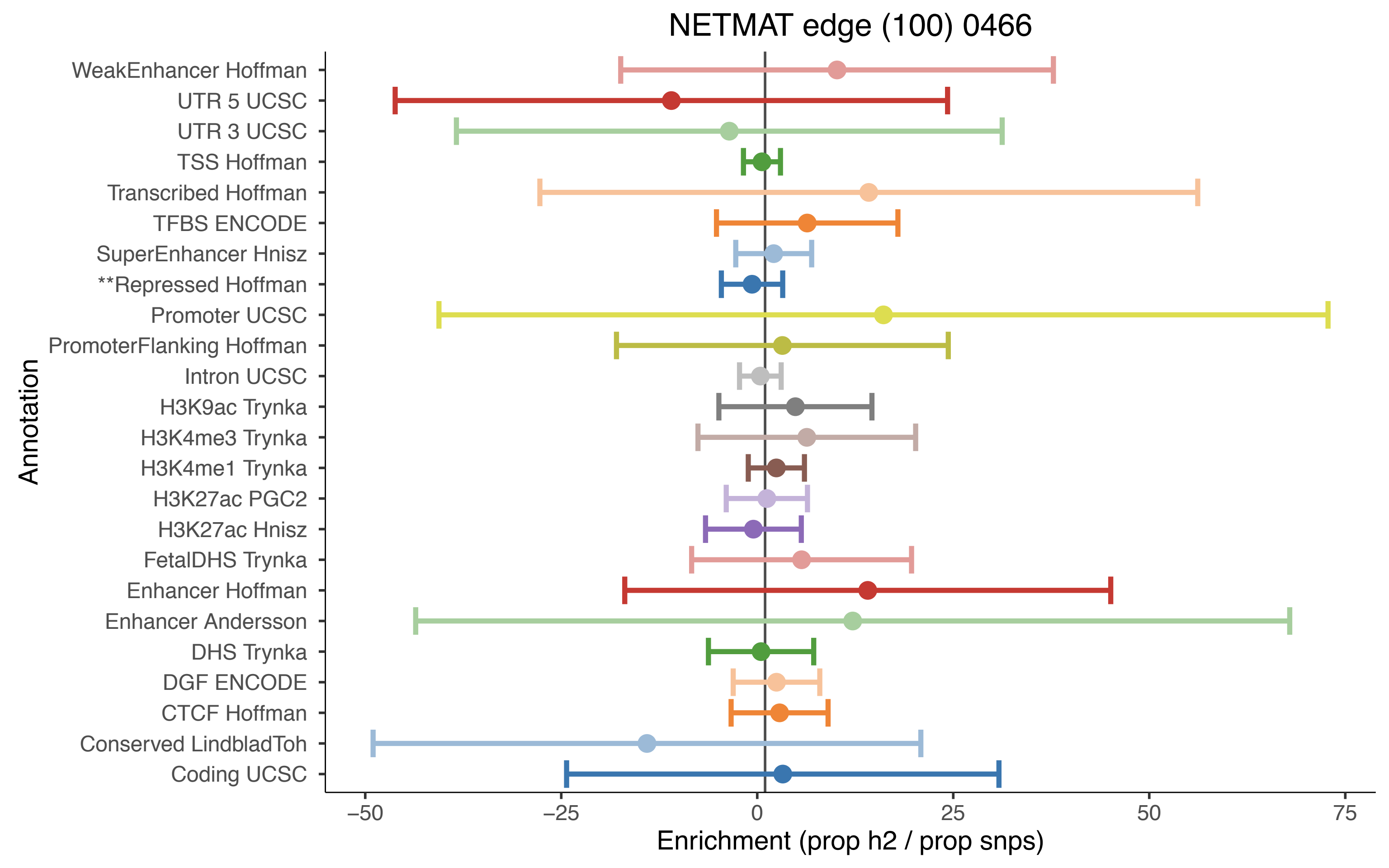

NETMAT edge (100) 0466
WeakEnhancer Hoffman
UTR 5 UCSC
UTR 3 UCSC
TSS Hoffman
Transcribed Hoffman
TFBS ENCODE
SuperEnhancer Hnisz
**Repressed Hoffman
Promoter UCSC
PromoterFlanking Hoffman
Intron UCSC
H3K9ac Trynka
H3K4me3 Trynka
H3K4me1 Trynka
H3K27ac PGC2
H3K27ac Hnisz
FetalDHS Trynka
Enhancer Hoffman
Enhancer Andersson
DHS Trynka
DGF ENCODE
CTCF Hoffman
Conserved LindbladToh
Coding UCSC
Annotation
−50
−25
0
25
50
75
Enrichment (prop h2 / prop snps)

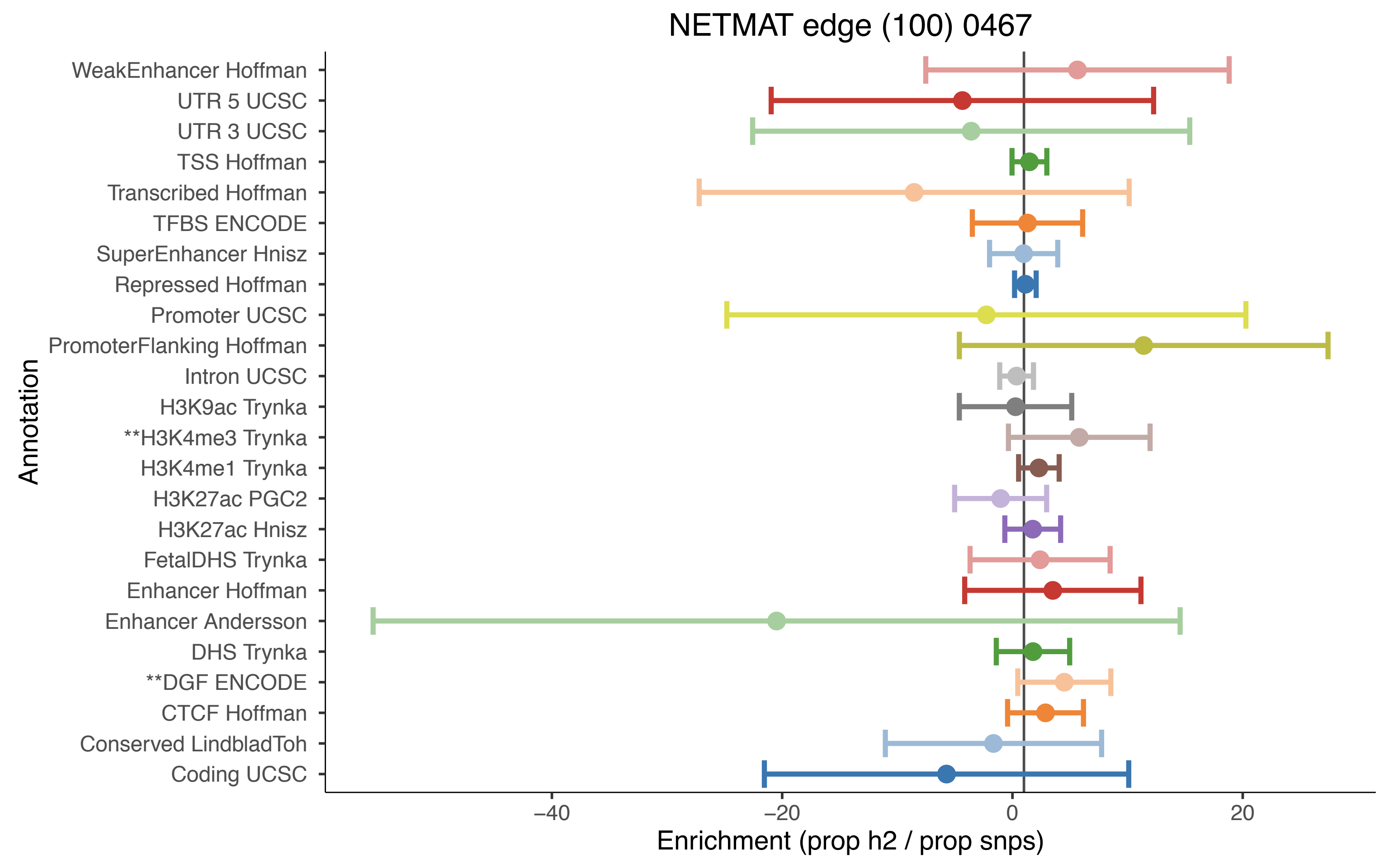
NETMAT edge (100) 0467
WeakEnhancer Hoffman
UTR 5 UCSC
UTR 3 UCSC
TSS Hoffman
Transcribed Hoffman
TFBS ENCODE
SuperEnhancer Hnisz
Repressed Hoffman
Promoter UCSC
PromoterFlanking Hoffman
Intron UCSC
H3K9ac Trynka
**H3K4me3 Trynka
H3K4me1 Trynka
H3K27ac PGC2
H3K27ac Hnisz
FetalDHS Trynka
Enhancer Hoffman
Enhancer Andersson
DHS Trynka
**DGF ENCODE
CTCF Hoffman
Conserved LindbladToh
Coding UCSC
Annotation
−40
−20
0
20
Enrichment (prop h2 / prop snps)

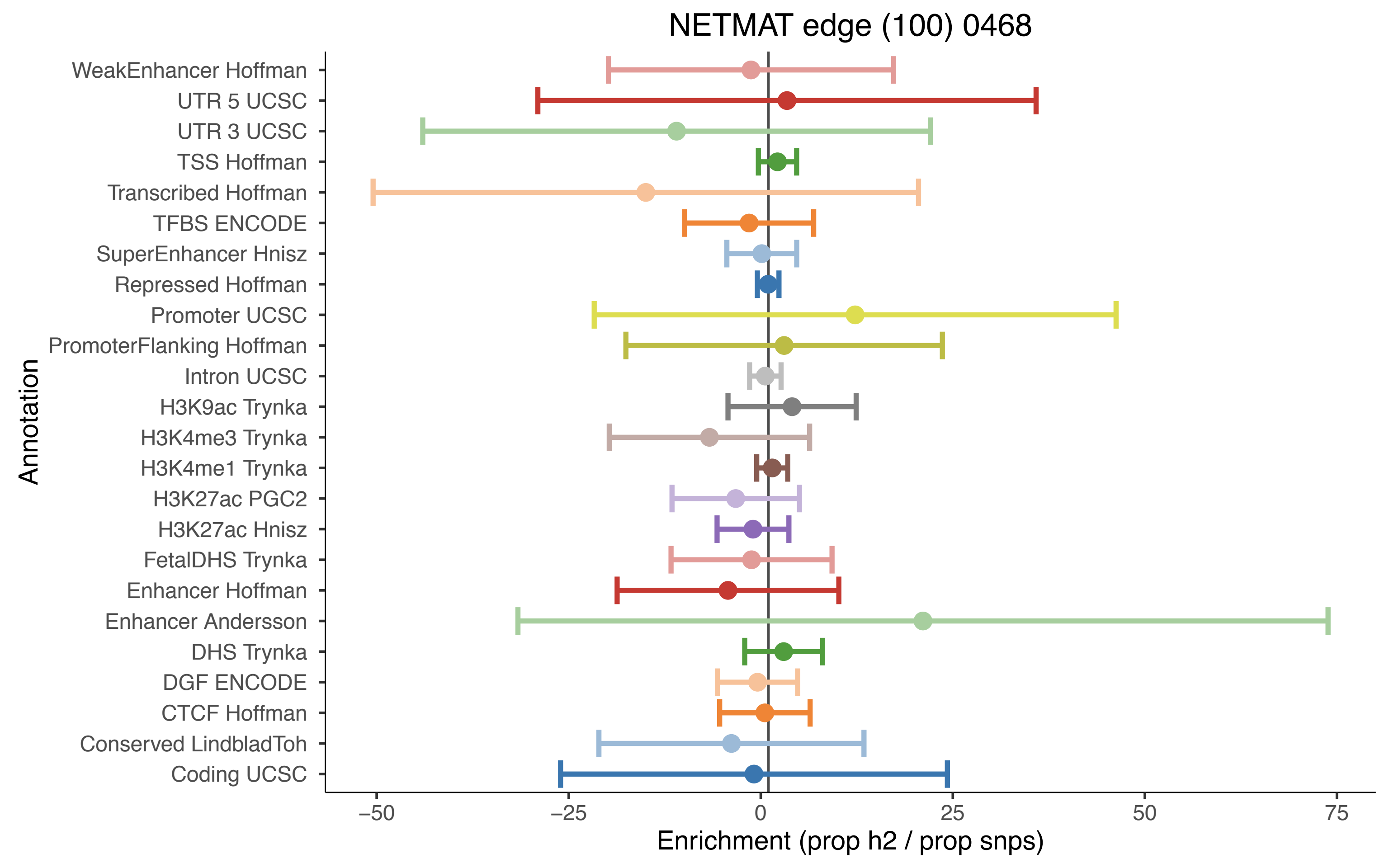
NETMAT edge (100) 0468
WeakEnhancer Hoffman
UTR 5 UCSC
UTR 3 UCSC
TSS Hoffman
Transcribed Hoffman
TFBS ENCODE
SuperEnhancer Hnisz
Repressed Hoffman
Promoter UCSC
PromoterFlanking Hoffman
Intron UCSC
H3K9ac Trynka
H3K4me3 Trynka
H3K4me1 Trynka
H3K27ac PGC2
H3K27ac Hnisz
FetalDHS Trynka
Enhancer Hoffman
Enhancer Andersson
DHS Trynka
DGF ENCODE
CTCF Hoffman
Conserved LindbladToh
Coding UCSC
Annotation
−50
−25
0
25
50
75
Enrichment (prop h2 / prop snps)

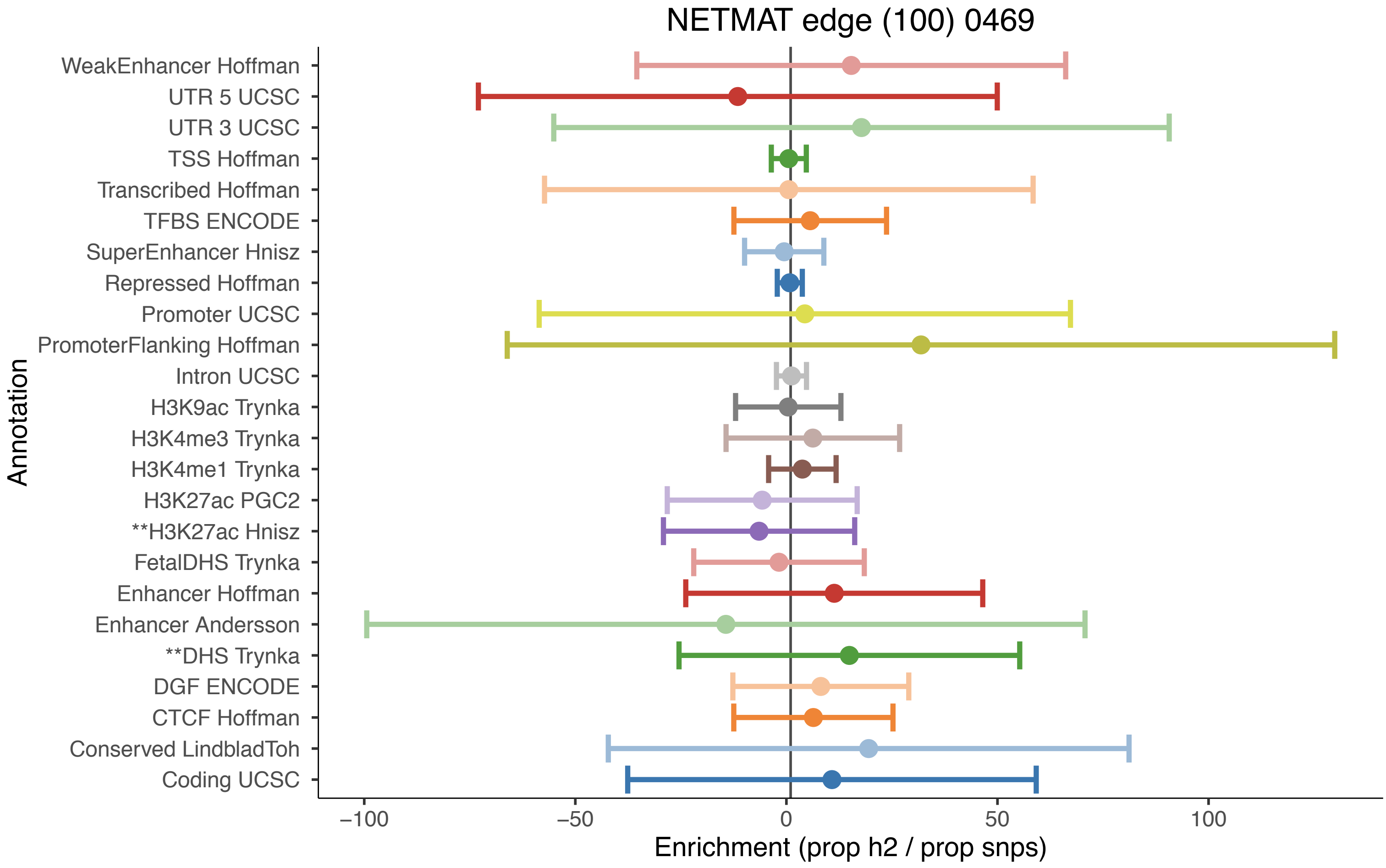

NETMAT edge (100) 0469
Annotation
WeakEnhancer Hoffman
UTR 5 UCSC
UTR 3 UCSC
TSS Hoffman
Transcribed Hoffman
TFBS ENCODE
SuperEnhancer Hnisz
Repressed Hoffman
Promoter UCSC
PromoterFlanking Hoffman
Intron UCSC
H3K9ac Trynka
H3K4me3 Trynka
H3K4me1 Trynka
H3K27ac PGC2
**H3K27ac Hnisz
FetalDHS Trynka
Enhancer Hoffman
Enhancer Andersson
**DHS Trynka
DGF ENCODE
CTCF Hoffman
Conserved LindbladToh
Coding UCSC
−100
−50
0
50
100
Enrichment (prop h2 / prop snps)

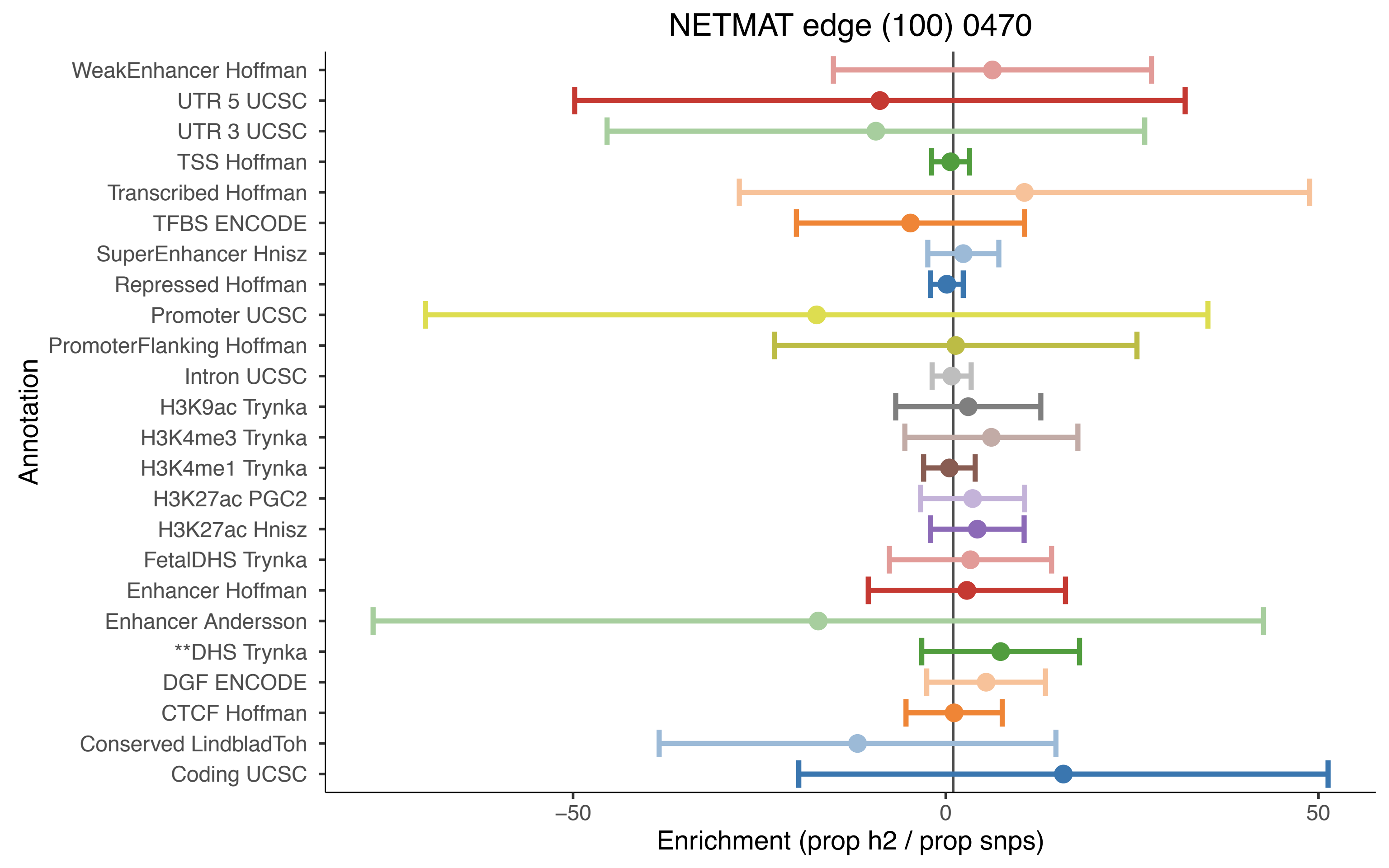
NETMAT edge (100) 0470
WeakEnhancer Hoffman
UTR 5 UCSC
UTR 3 UCSC
TSS Hoffman
Transcribed Hoffman
TFBS ENCODE
SuperEnhancer Hnisz
Repressed Hoffman
Promoter UCSC
PromoterFlanking Hoffman
Intron UCSC
H3K9ac Trynka
H3K4me3 Trynka
H3K4me1 Trynka
H3K27ac PGC2
H3K27ac Hnisz
FetalDHS Trynka
Enhancer Hoffman
Enhancer Andersson
**DHS Trynka
DGF ENCODE
CTCF Hoffman
Conserved LindbladToh
Coding UCSC
Annotation
−50
0
50
Enrichment (prop h2 / prop snps)

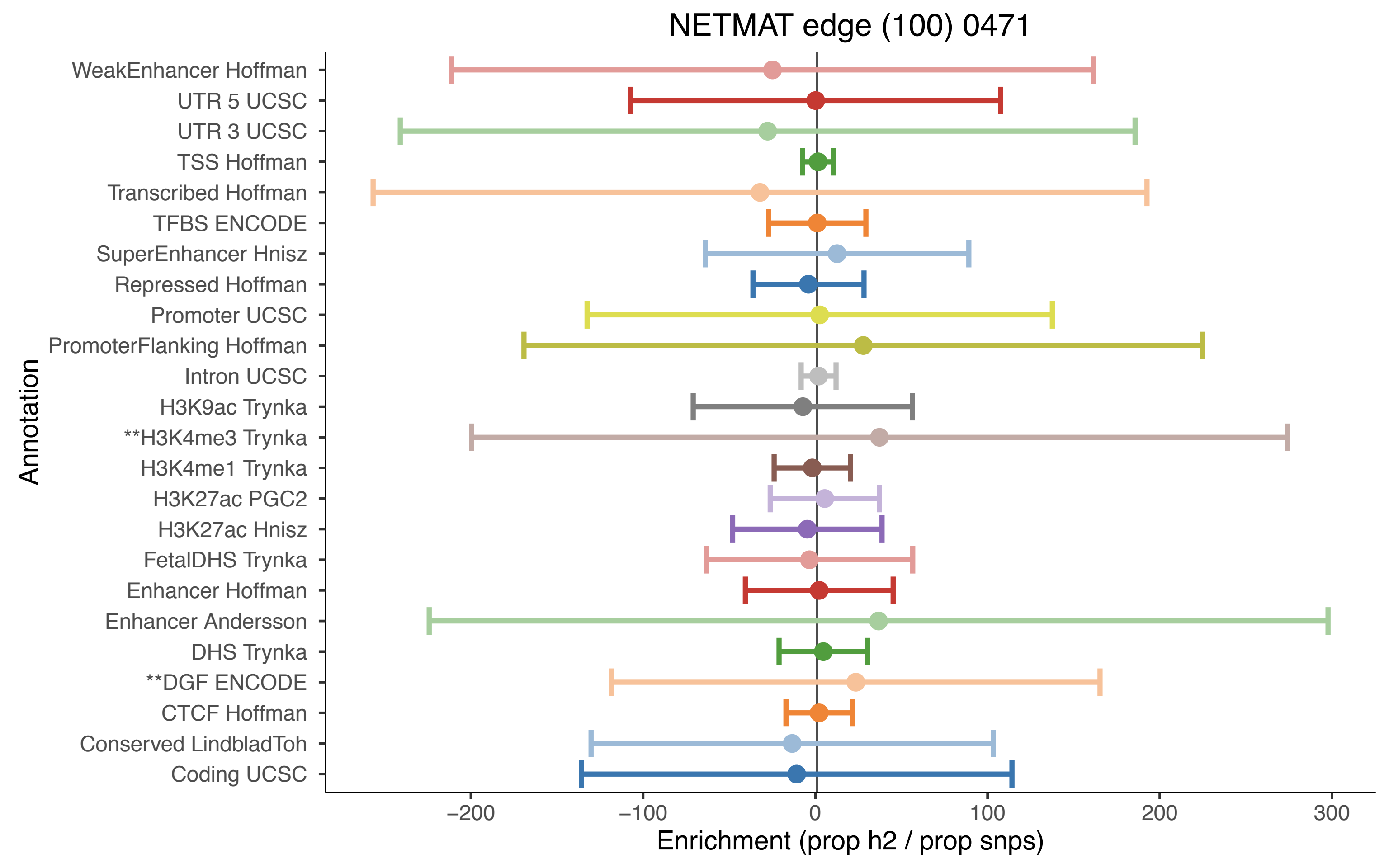

NETMAT edge (100) 0471
Annotation
WeakEnhancer Hoffman
UTR 5 UCSC
UTR 3 UCSC
TSS Hoffman
Transcribed Hoffman
TFBS ENCODE
SuperEnhancer Hnisz
Repressed Hoffman
Promoter UCSC
PromoterFlanking Hoffman
Intron UCSC
H3K9ac Trynka
**H3K4me3 Trynka
H3K4me1 Trynka
H3K27ac PGC2
H3K27ac Hnisz
FetalDHS Trynka
Enhancer Hoffman
Enhancer Andersson
DHS Trynka
**DGF ENCODE
CTCF Hoffman
Conserved LindbladToh
Coding UCSC
−200
−100
0
100
200
300
Enrichment (prop h2 / prop snps)

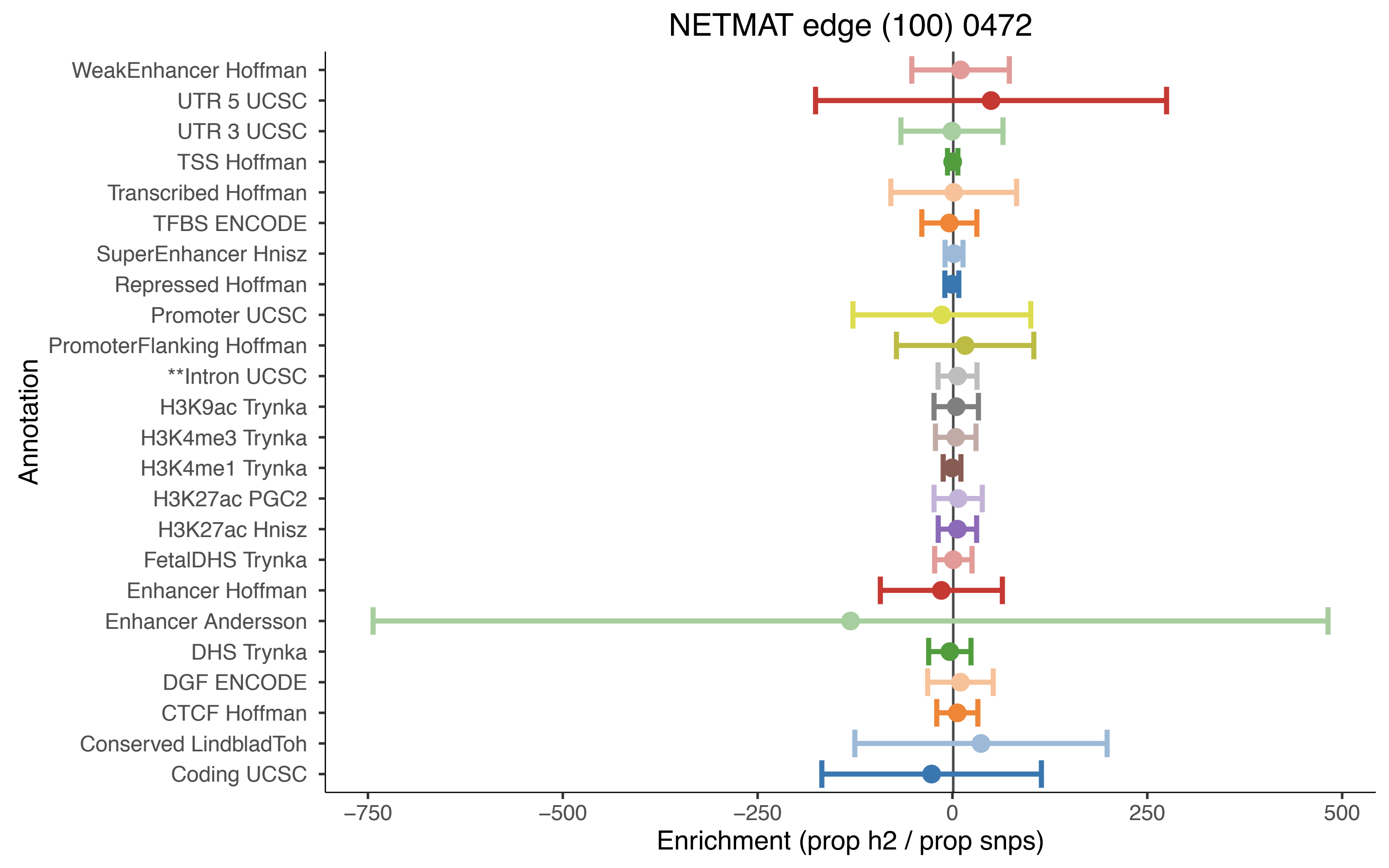
NETMAT edge (100) 0472
WeakEnhancer Hoffman
UTR 5 UCSC
UTR 3 UCSC
TSS Hoffman
Transcribed Hoffman
TFBS ENCODE
SuperEnhancer Hnisz
Repressed Hoffman
Promoter UCSC
PromoterFlanking Hoffman
**Intron UCSC
H3K9ac Trynka
H3K4me3 Trynka
H3K4me1 Trynka
H3K27ac PGC2
H3K27ac Hnisz
FetalDHS Trynka
Enhancer Hoffman
Enhancer Andersson
DHS Trynka
DGF ENCODE
CTCF Hoffman
Conserved LindbladToh
Coding UCSC
Annotation
−750
−500
−250
0
250
500
Enrichment (prop h2 / prop snps)

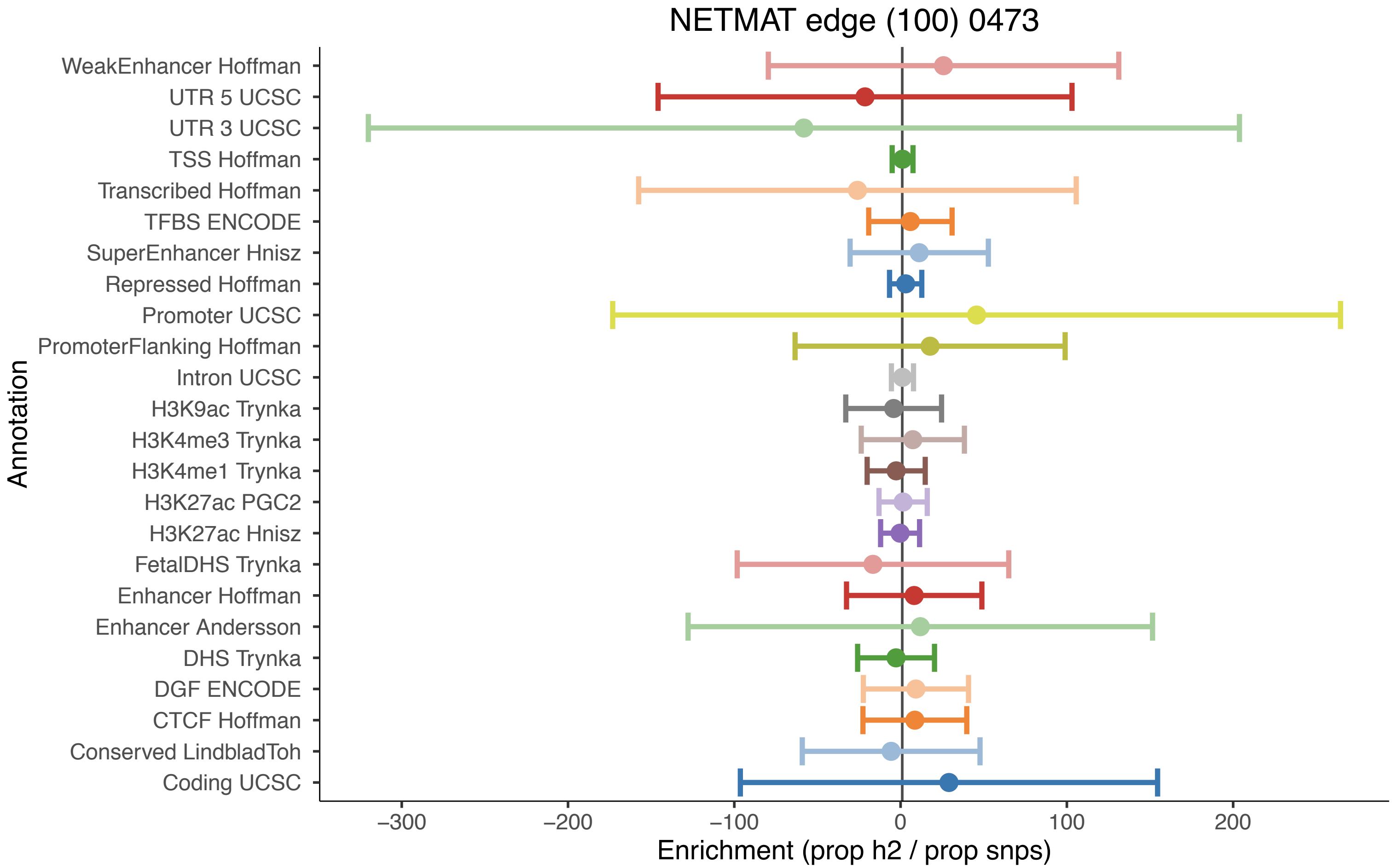
NETMAT edge (100) 0473
Annotation
WeakEnhancer Hoffman
UTR 5 UCSC
UTR 3 UCSC
TSS Hoffman
Transcribed Hoffman
TFBS ENCODE
SuperEnhancer Hnisz
Repressed Hoffman
Promoter UCSC
PromoterFlanking Hoffman
Intron UCSC
H3K9ac Trynka
H3K4me3 Trynka
H3K4me1 Trynka
H3K27ac PGC2
H3K27ac Hnisz
FetalDHS Trynka
Enhancer Hoffman
Enhancer Andersson
DHS Trynka
DGF ENCODE
CTCF Hoffman
Conserved LindbladToh
Coding UCSC
−300
−200
−100
0
100
200
Enrichment (prop h2 / prop snps)

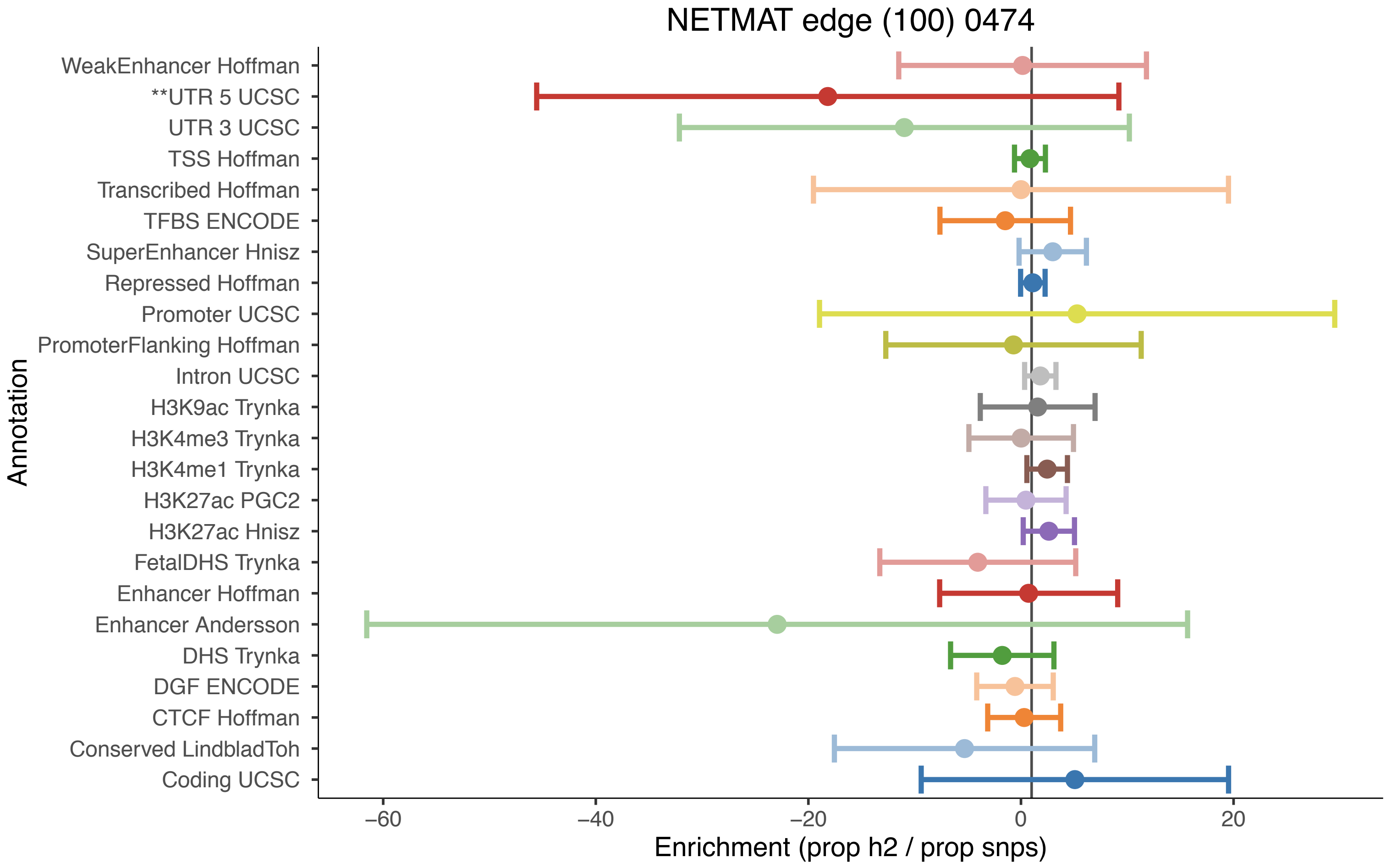

NETMAT edge (100) 0474
WeakEnhancer Hoffman
**UTR 5 UCSC
UTR 3 UCSC
TSS Hoffman
Transcribed Hoffman
TFBS ENCODE
SuperEnhancer Hnisz
Repressed Hoffman
Promoter UCSC
PromoterFlanking Hoffman
Intron UCSC
H3K9ac Trynka
H3K4me3 Trynka
H3K4me1 Trynka
H3K27ac PGC2
H3K27ac Hnisz
FetalDHS Trynka
Enhancer Hoffman
Enhancer Andersson
DHS Trynka
DGF ENCODE
CTCF Hoffman
Conserved LindbladToh
Coding UCSC
Annotation
−60
−40
−20
0
20
Enrichment (prop h2 / prop snps)

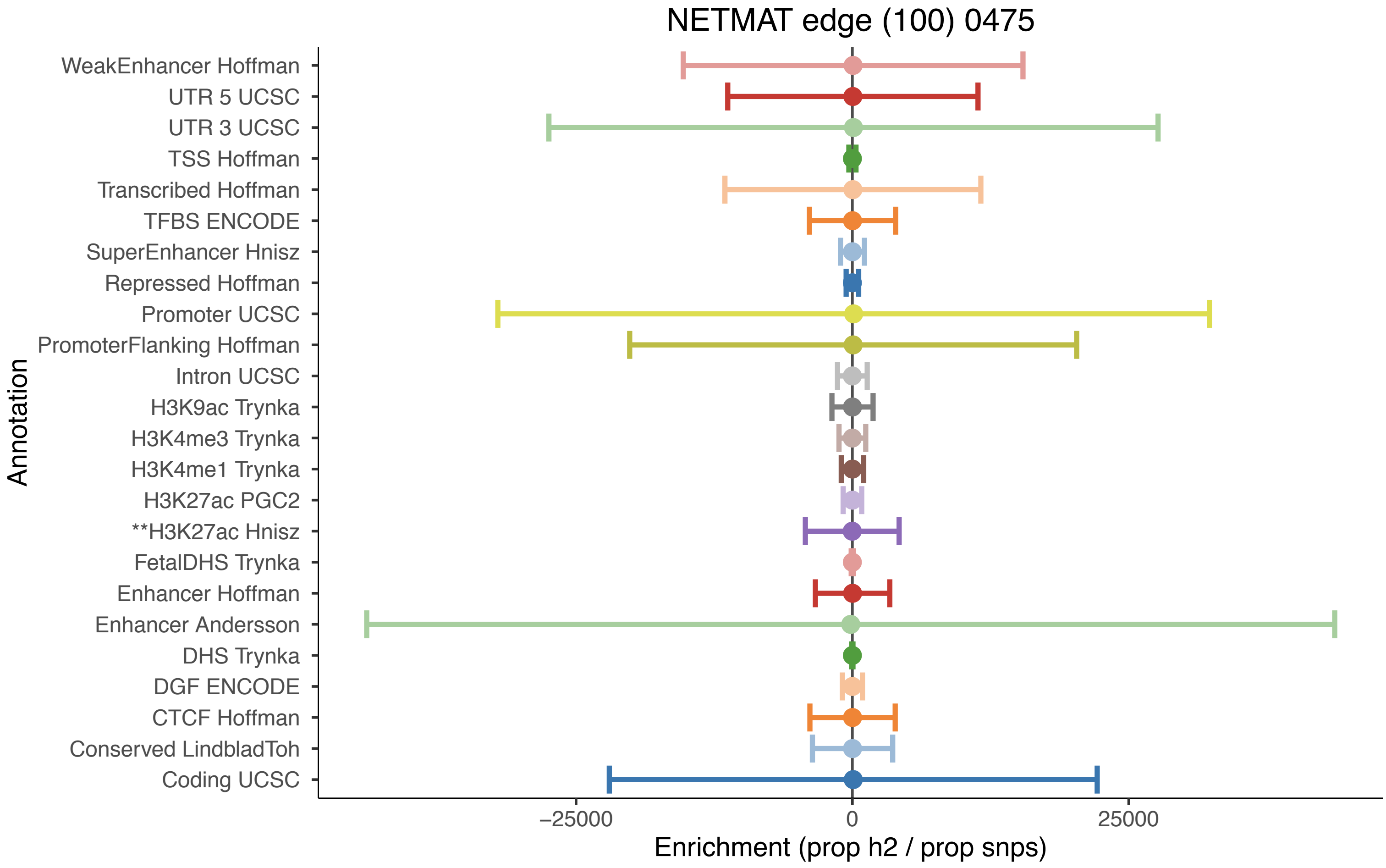
NETMAT edge (100) 0475
WeakEnhancer Hoffman
UTR 5 UCSC
UTR 3 UCSC
TSS Hoffman
Transcribed Hoffman
TFBS ENCODE
SuperEnhancer Hnisz
Repressed Hoffman
Promoter UCSC
PromoterFlanking Hoffman
Intron UCSC
H3K9ac Trynka
H3K4me3 Trynka
H3K4me1 Trynka
H3K27ac PGC2
**H3K27ac Hnisz
FetalDHS Trynka
Enhancer Hoffman
Enhancer Andersson
DHS Trynka
DGF ENCODE
CTCF Hoffman
Conserved LindbladToh
Coding UCSC
Annotation
−25000
0
25000
Enrichment (prop h2 / prop snps)

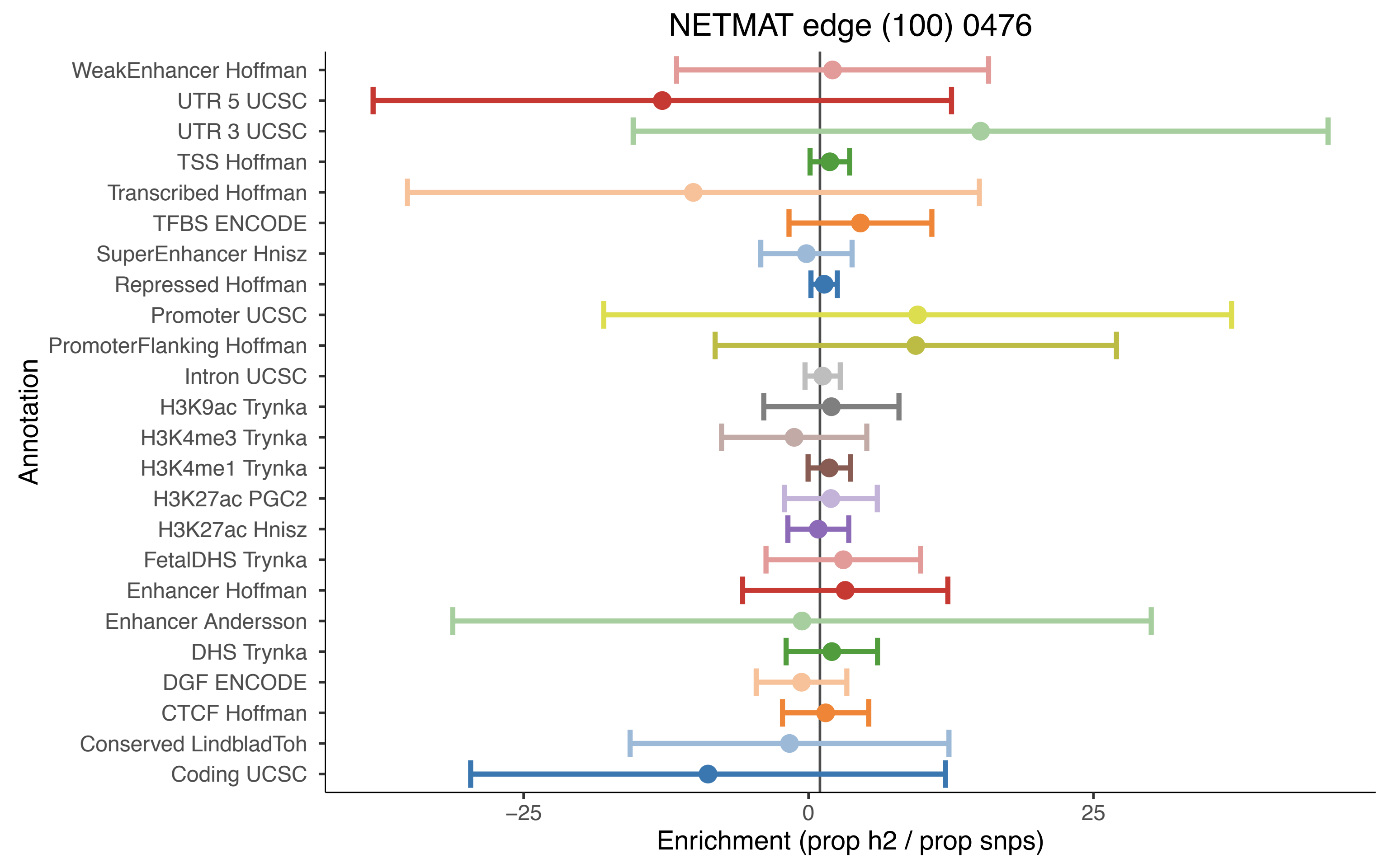

NETMAT edge (100) 0476
Annotation
WeakEnhancer Hoffman
UTR 5 UCSC
UTR 3 UCSC
TSS Hoffman
Transcribed Hoffman
TFBS ENCODE
SuperEnhancer Hnisz
Repressed Hoffman
Promoter UCSC
PromoterFlanking Hoffman
Intron UCSC
H3K9ac Trynka
H3K4me3 Trynka
H3K4me1 Trynka
H3K27ac PGC2
H3K27ac Hnisz
FetalDHS Trynka
Enhancer Hoffman
Enhancer Andersson
DHS Trynka
DGF ENCODE
CTCF Hoffman
Conserved LindbladToh
Coding UCSC
−25
0
25
Enrichment (prop h2 / prop snps)

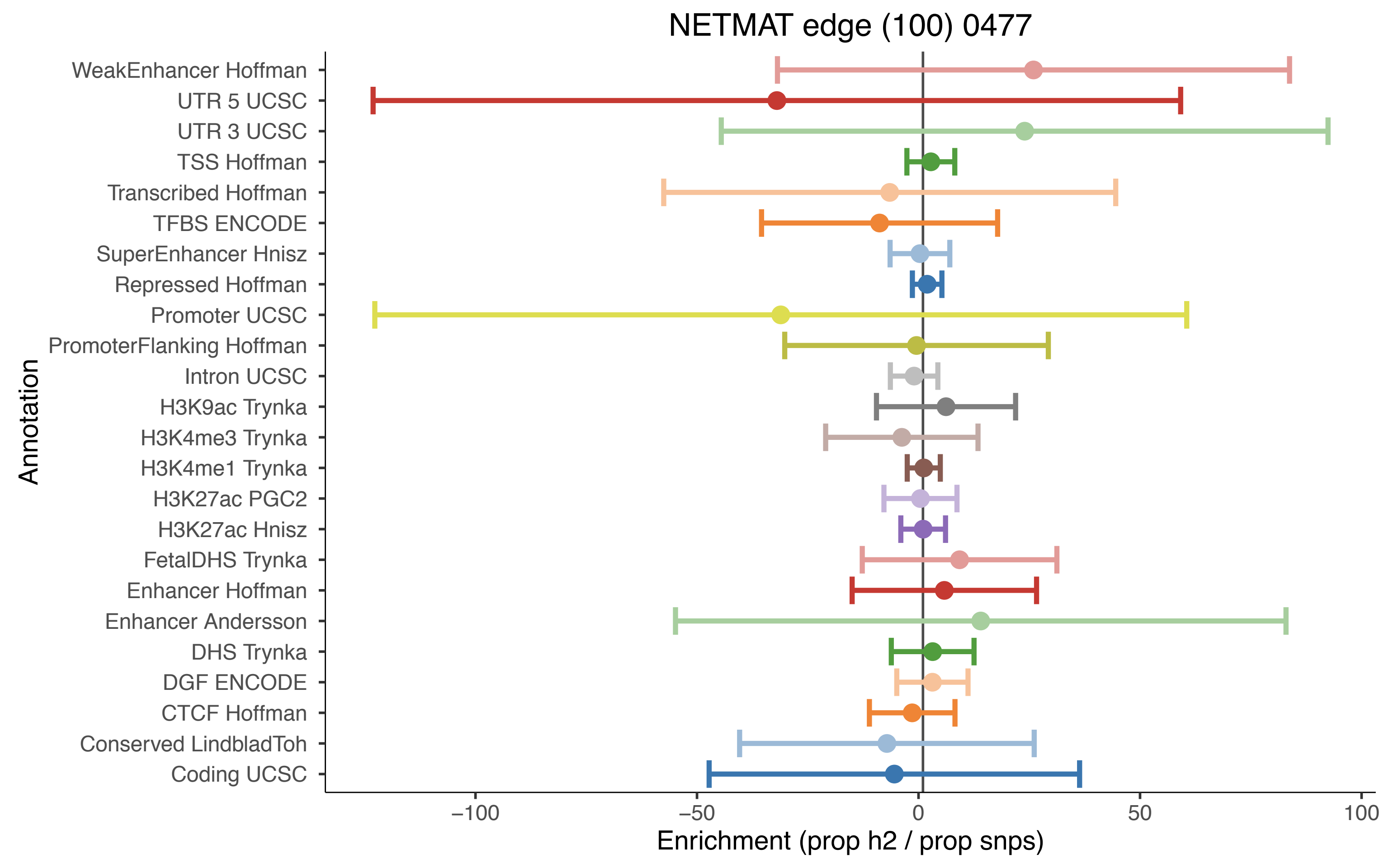
NETMAT edge (100) 0477
WeakEnhancer Hoffman
UTR 5 UCSC
UTR 3 UCSC
TSS Hoffman
Transcribed Hoffman
TFBS ENCODE
SuperEnhancer Hnisz
Repressed Hoffman
Promoter UCSC
PromoterFlanking Hoffman
Intron UCSC
H3K9ac Trynka
H3K4me3 Trynka
H3K4me1 Trynka
H3K27ac PGC2
H3K27ac Hnisz
FetalDHS Trynka
Enhancer Hoffman
Enhancer Andersson
DHS Trynka
DGF ENCODE
CTCF Hoffman
Conserved LindbladToh
Coding UCSC
Annotation
−100
−50
0
50
100
Enrichment (prop h2 / prop snps)

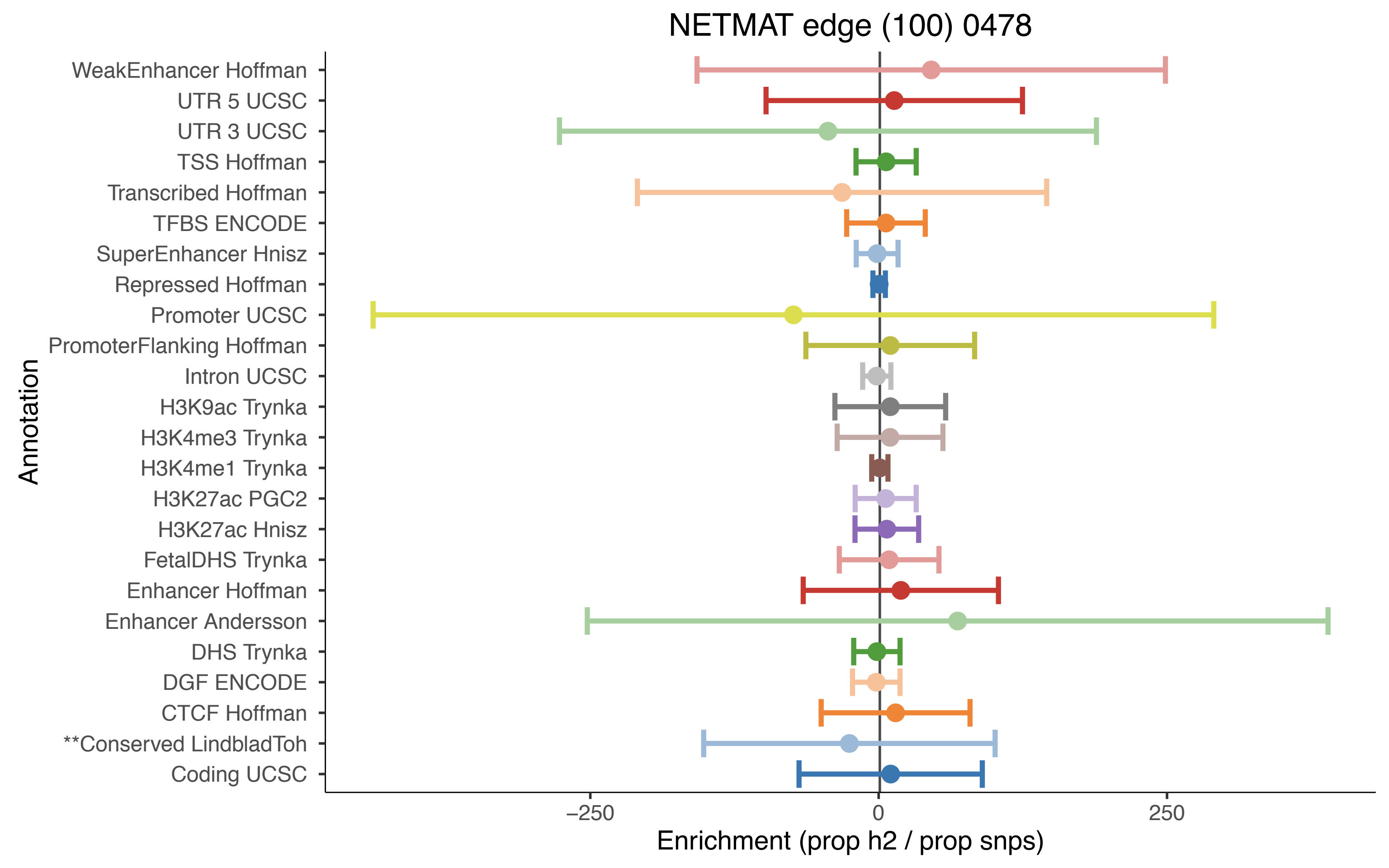

NETMAT edge (100) 0478
WeakEnhancer Hoffman
UTR 5 UCSC
UTR 3 UCSC
TSS Hoffman
Transcribed Hoffman
TFBS ENCODE
SuperEnhancer Hnisz
Repressed Hoffman
Promoter UCSC
PromoterFlanking Hoffman
Intron UCSC
H3K9ac Trynka
H3K4me3 Trynka
H3K4me1 Trynka
H3K27ac PGC2
H3K27ac Hnisz
FetalDHS Trynka
Enhancer Hoffman
Enhancer Andersson
DHS Trynka
DGF ENCODE
CTCF Hoffman
**Conserved LindbladToh
Coding UCSC
Annotation
−250
0
250
Enrichment (prop h2 / prop snps)

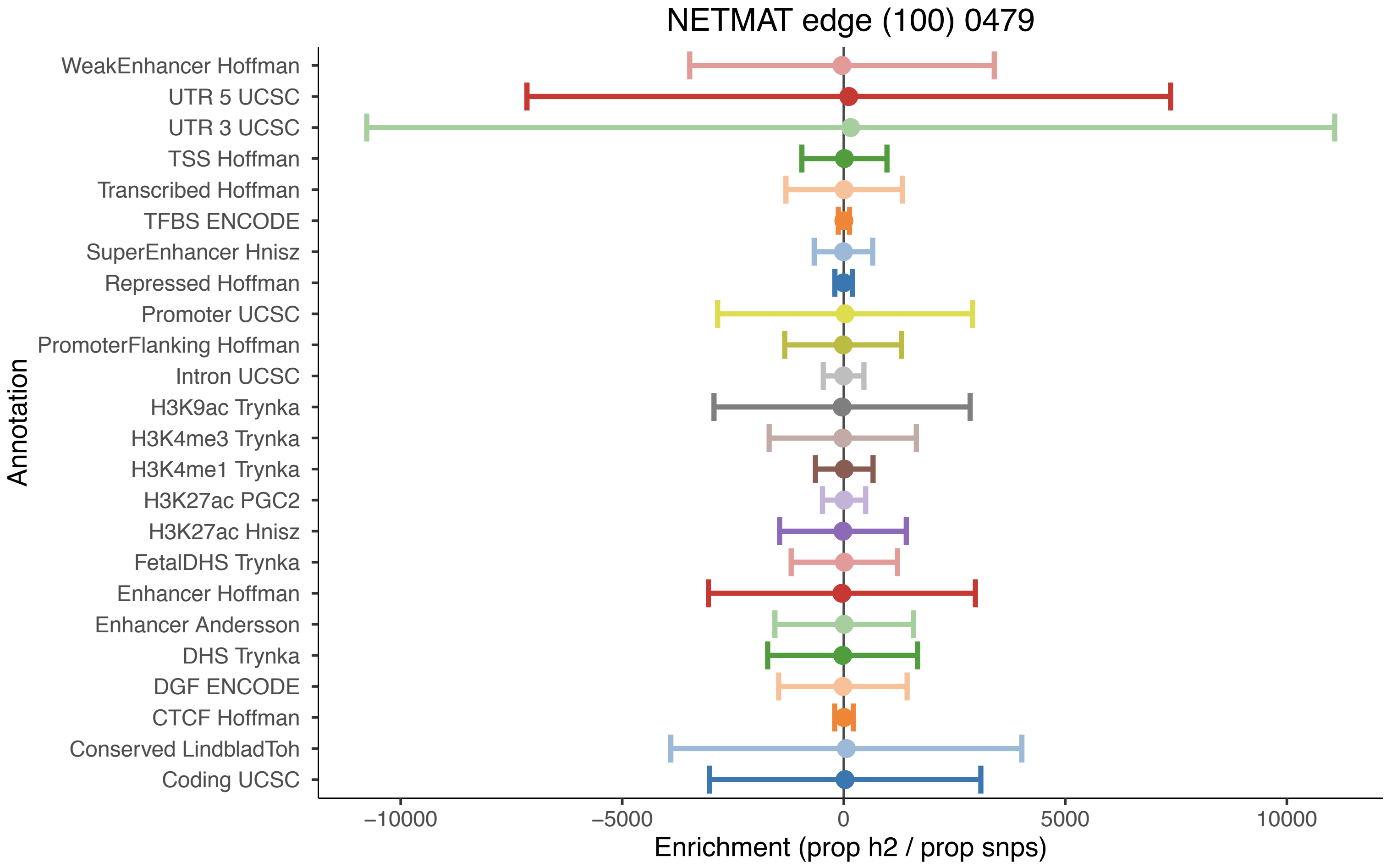
NETMAT edge (100) 0479
WeakEnhancer Hoffman
UTR 5 UCSC
UTR 3 UCSC
TSS Hoffman
Transcribed Hoffman
TFBS ENCODE
SuperEnhancer Hnisz
Repressed Hoffman
Promoter UCSC
PromoterFlanking Hoffman
Intron UCSC
H3K9ac Trynka
H3K4me3 Trynka
H3K4me1 Trynka
H3K27ac PGC2
H3K27ac Hnisz
FetalDHS Trynka
Enhancer Hoffman
Enhancer Andersson
DHS Trynka
DGF ENCODE
CTCF Hoffman
Conserved LindbladToh
Coding UCSC
Annotation
−10000
−5000
0
5000
10000
Enrichment (prop h2 / prop snps)

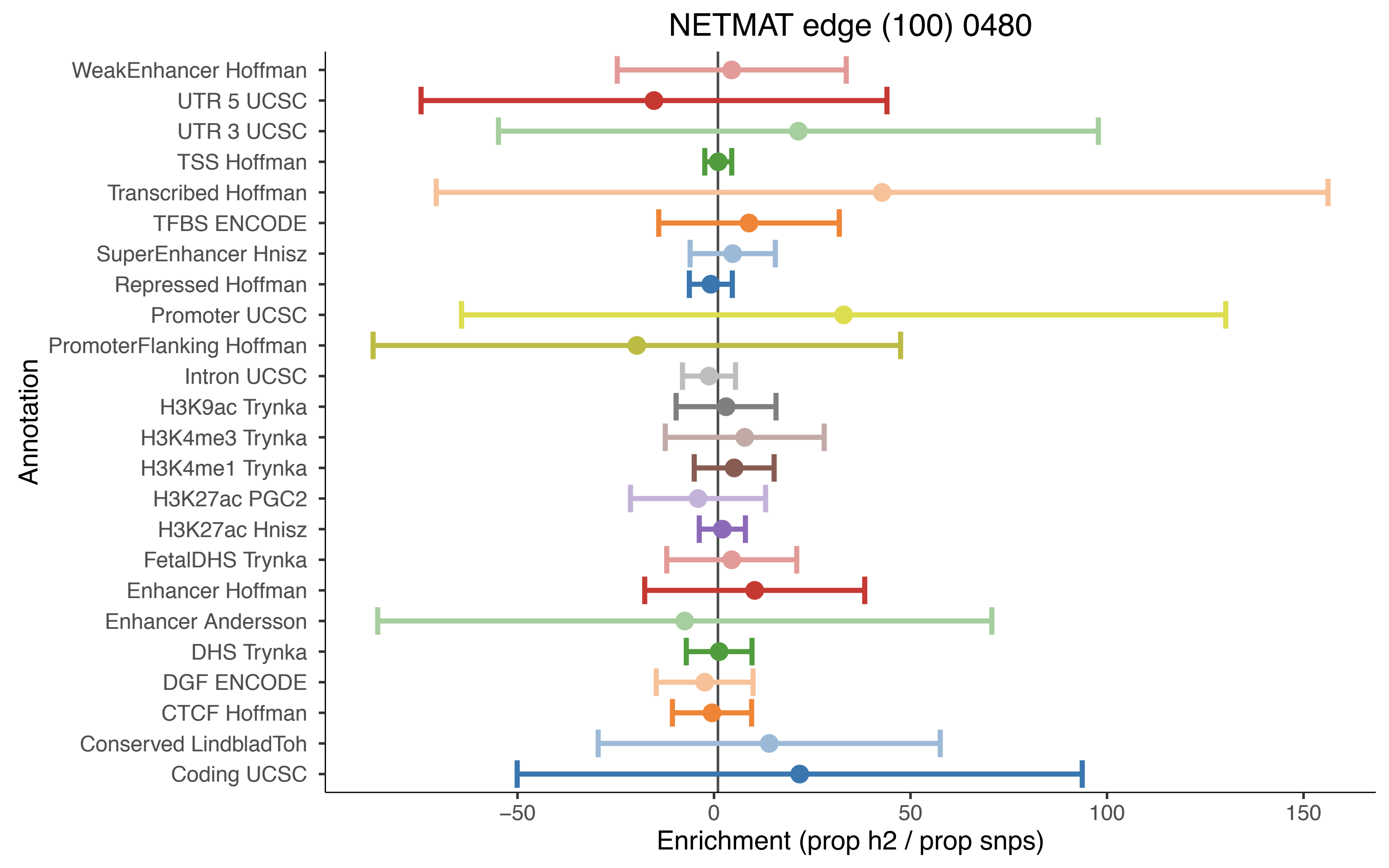
NETMAT edge (100) 0480
Annotation
WeakEnhancer Hoffman
UTR 5 UCSC
UTR 3 UCSC
TSS Hoffman
Transcribed Hoffman
TFBS ENCODE
SuperEnhancer Hnisz
Repressed Hoffman
Promoter UCSC
PromoterFlanking Hoffman
Intron UCSC
H3K9ac Trynka
H3K4me3 Trynka
H3K4me1 Trynka
H3K27ac PGC2
H3K27ac Hnisz
FetalDHS Trynka
Enhancer Hoffman
Enhancer Andersson
DHS Trynka
DGF ENCODE
CTCF Hoffman
Conserved LindbladToh
Coding UCSC
−50
0
50
100
150
Enrichment (prop h2 / prop snps)

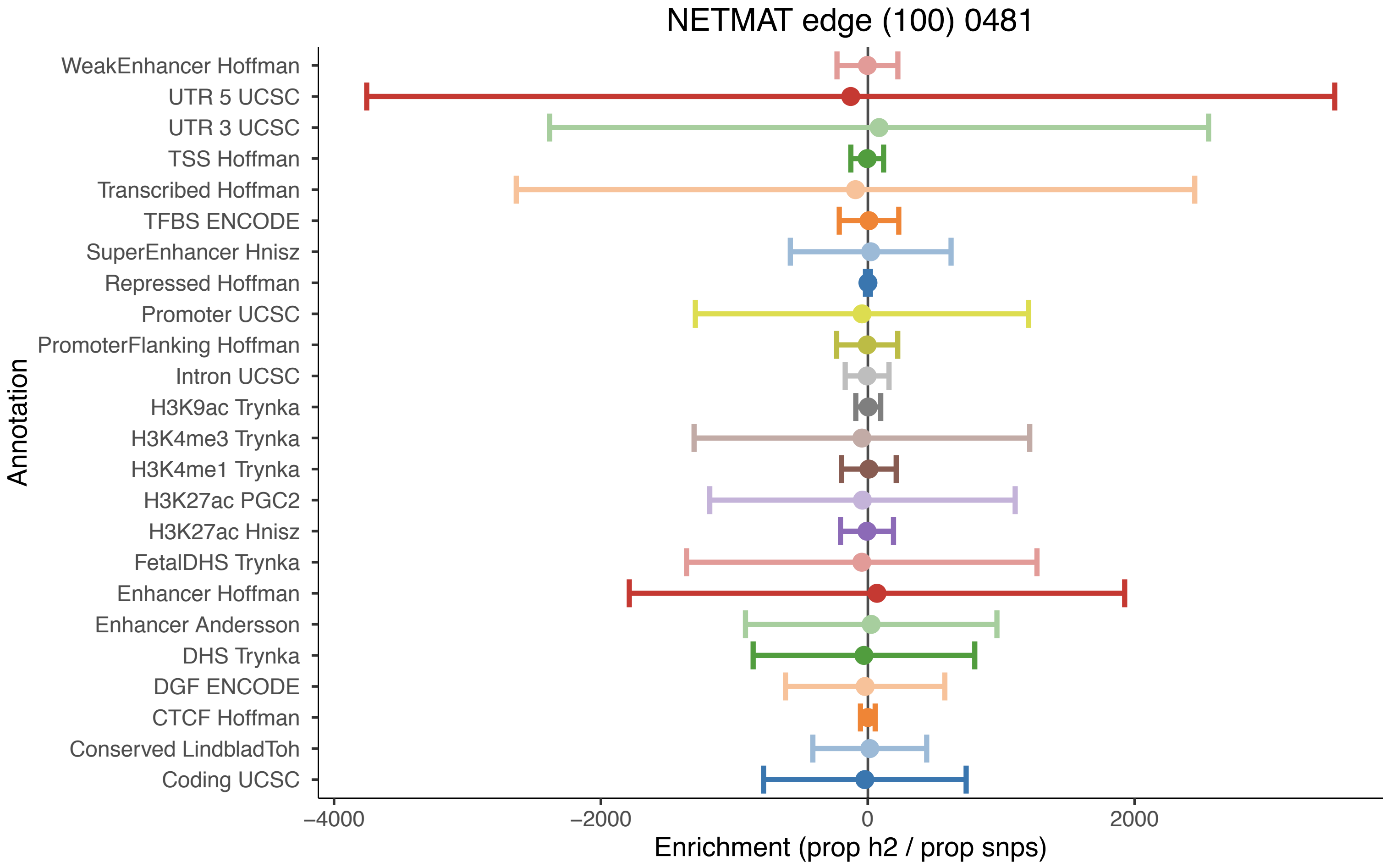

NETMAT edge (100) 0481
Annotation
WeakEnhancer Hoffman
UTR 5 UCSC
UTR 3 UCSC
TSS Hoffman
Transcribed Hoffman
TFBS ENCODE
SuperEnhancer Hnisz
Repressed Hoffman
Promoter UCSC
PromoterFlanking Hoffman
Intron UCSC
H3K9ac Trynka
H3K4me3 Trynka
H3K4me1 Trynka
H3K27ac PGC2
H3K27ac Hnisz
FetalDHS Trynka
Enhancer Hoffman
Enhancer Andersson
DHS Trynka
DGF ENCODE
CTCF Hoffman
Conserved LindbladToh
Coding UCSC
−4000
−2000
0
2000
Enrichment (prop h2 / prop snps)

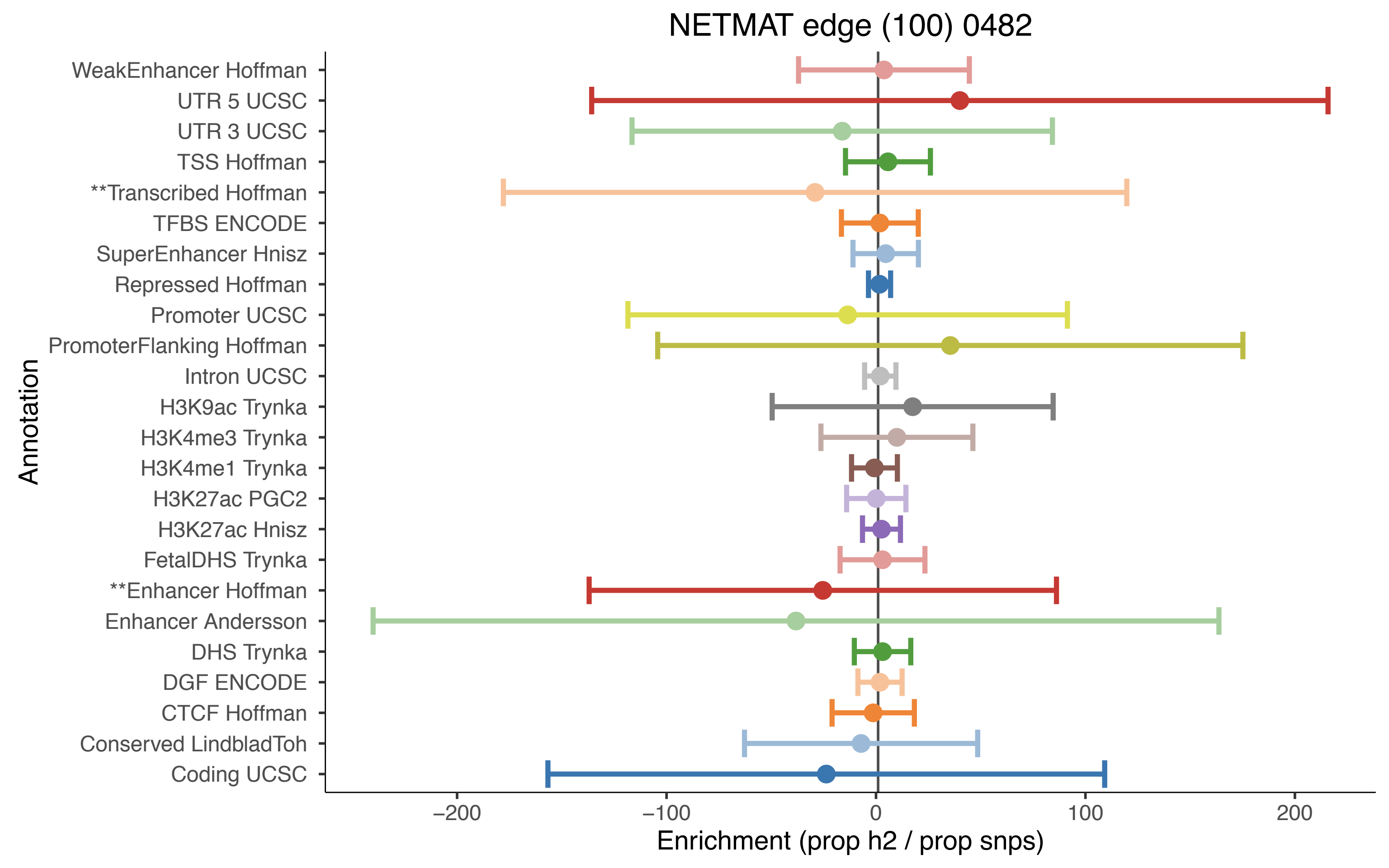

NETMAT edge (100) 0482
WeakEnhancer Hoffman
UTR 5 UCSC
UTR 3 UCSC
TSS Hoffman
**Transcribed Hoffman
TFBS ENCODE
SuperEnhancer Hnisz
Repressed Hoffman
Promoter UCSC
PromoterFlanking Hoffman
Intron UCSC
H3K9ac Trynka
H3K4me3 Trynka
H3K4me1 Trynka
H3K27ac PGC2
H3K27ac Hnisz
FetalDHS Trynka
**Enhancer Hoffman
Enhancer Andersson
DHS Trynka
DGF ENCODE
CTCF Hoffman
Conserved LindbladToh
Coding UCSC
Annotation
−200
−100
0
100
200
Enrichment (prop h2 / prop snps)

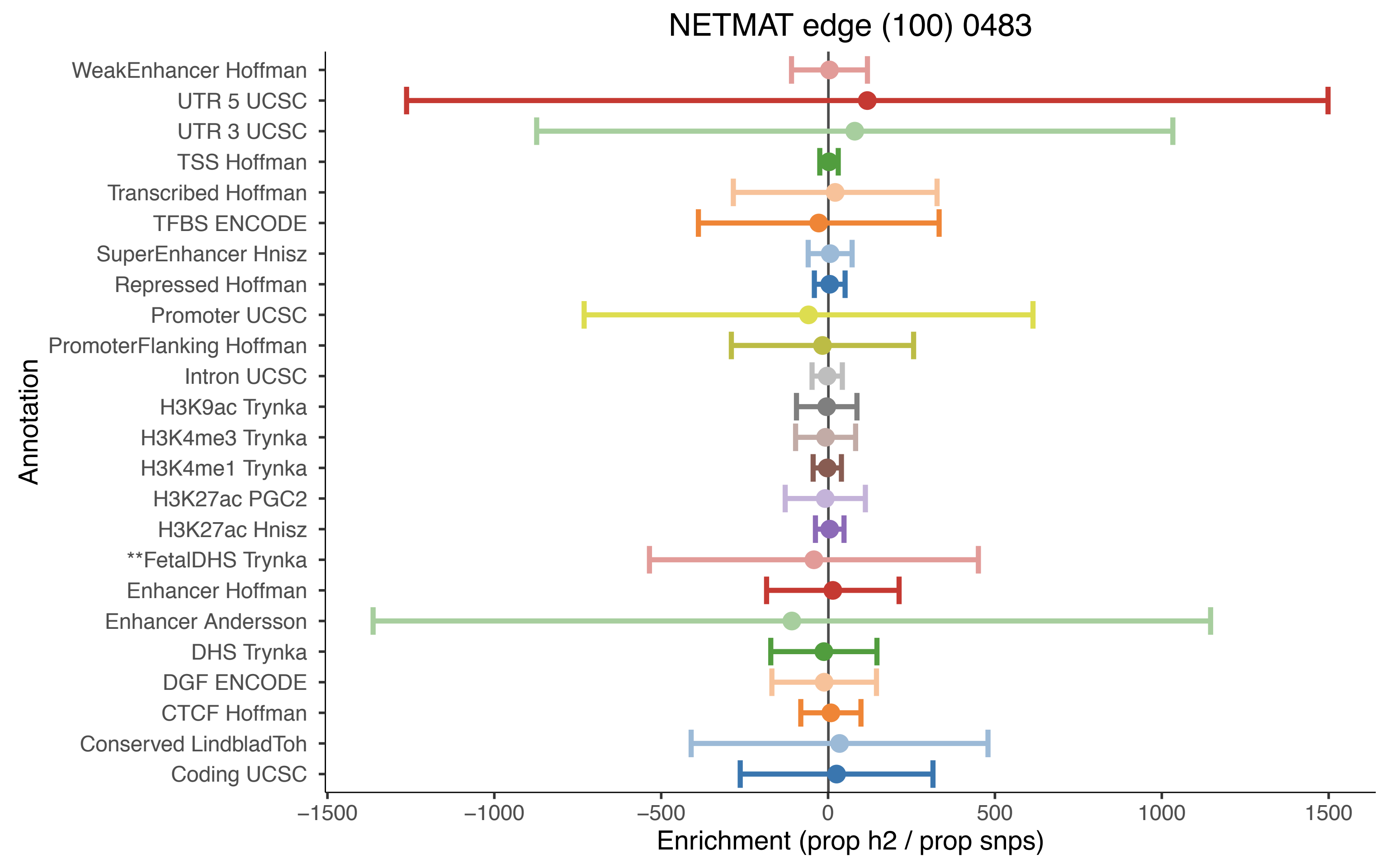

NETMAT edge (100) 0483
WeakEnhancer Hoffman
UTR 5 UCSC
UTR 3 UCSC
TSS Hoffman
Transcribed Hoffman
TFBS ENCODE
SuperEnhancer Hnisz
Repressed Hoffman
Promoter UCSC
PromoterFlanking Hoffman
Intron UCSC
H3K9ac Trynka
H3K4me3 Trynka
H3K4me1 Trynka
H3K27ac PGC2
H3K27ac Hnisz
**FetalDHS Trynka
Enhancer Hoffman
Enhancer Andersson
DHS Trynka
DGF ENCODE
CTCF Hoffman
Conserved LindbladToh
Coding UCSC
Annotation
−1500
−1000
−500
0
500
1000
1500
Enrichment (prop h2 / prop snps)

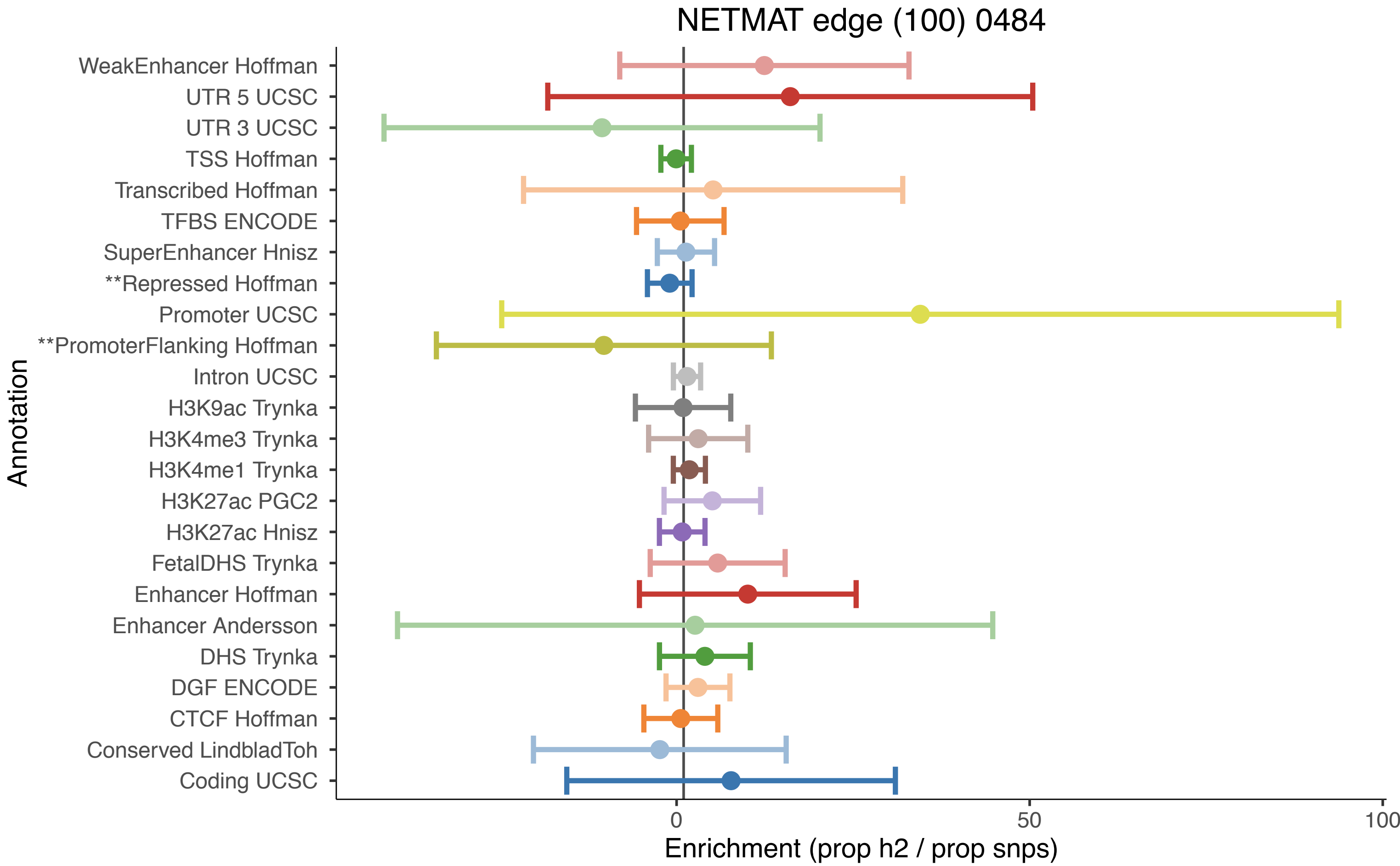

NETMAT edge (100) 0484
WeakEnhancer Hoffman
UTR 5 UCSC
UTR 3 UCSC
TSS Hoffman
Transcribed Hoffman
TFBS ENCODE
SuperEnhancer Hnisz
**Repressed Hoffman
Promoter UCSC
**PromoterFlanking Hoffman
Intron UCSC
H3K9ac Trynka
H3K4me3 Trynka
H3K4me1 Trynka
H3K27ac PGC2
H3K27ac Hnisz
FetalDHS Trynka
Enhancer Hoffman
Enhancer Andersson
DHS Trynka
DGF ENCODE
CTCF Hoffman
Conserved LindbladToh
Coding UCSC
Annotation
0
50
100
Enrichment (prop h2 / prop snps)

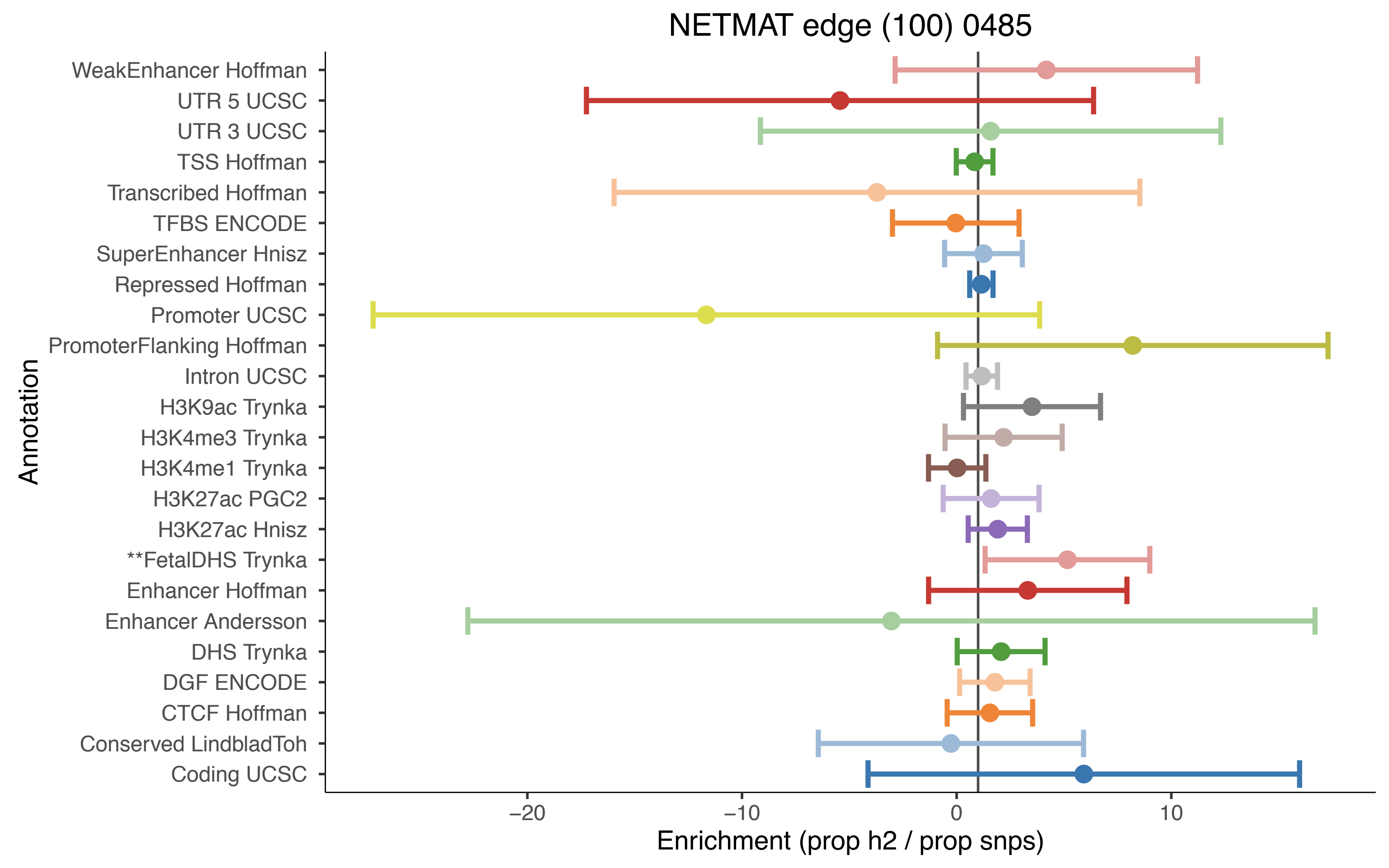

NETMAT edge (100) 0485
Annotation
WeakEnhancer Hoffman
UTR 5 UCSC
UTR 3 UCSC
TSS Hoffman
Transcribed Hoffman
TFBS ENCODE
SuperEnhancer Hnisz
Repressed Hoffman
Promoter UCSC
PromoterFlanking Hoffman
Intron UCSC
H3K9ac Trynka
H3K4me3 Trynka
H3K4me1 Trynka
H3K27ac PGC2
H3K27ac Hnisz
**FetalDHS Trynka
Enhancer Hoffman
Enhancer Andersson
DHS Trynka
DGF ENCODE
CTCF Hoffman
Conserved LindbladToh
Coding UCSC
−20
−10
0
10
Enrichment (prop h2 / prop snps)

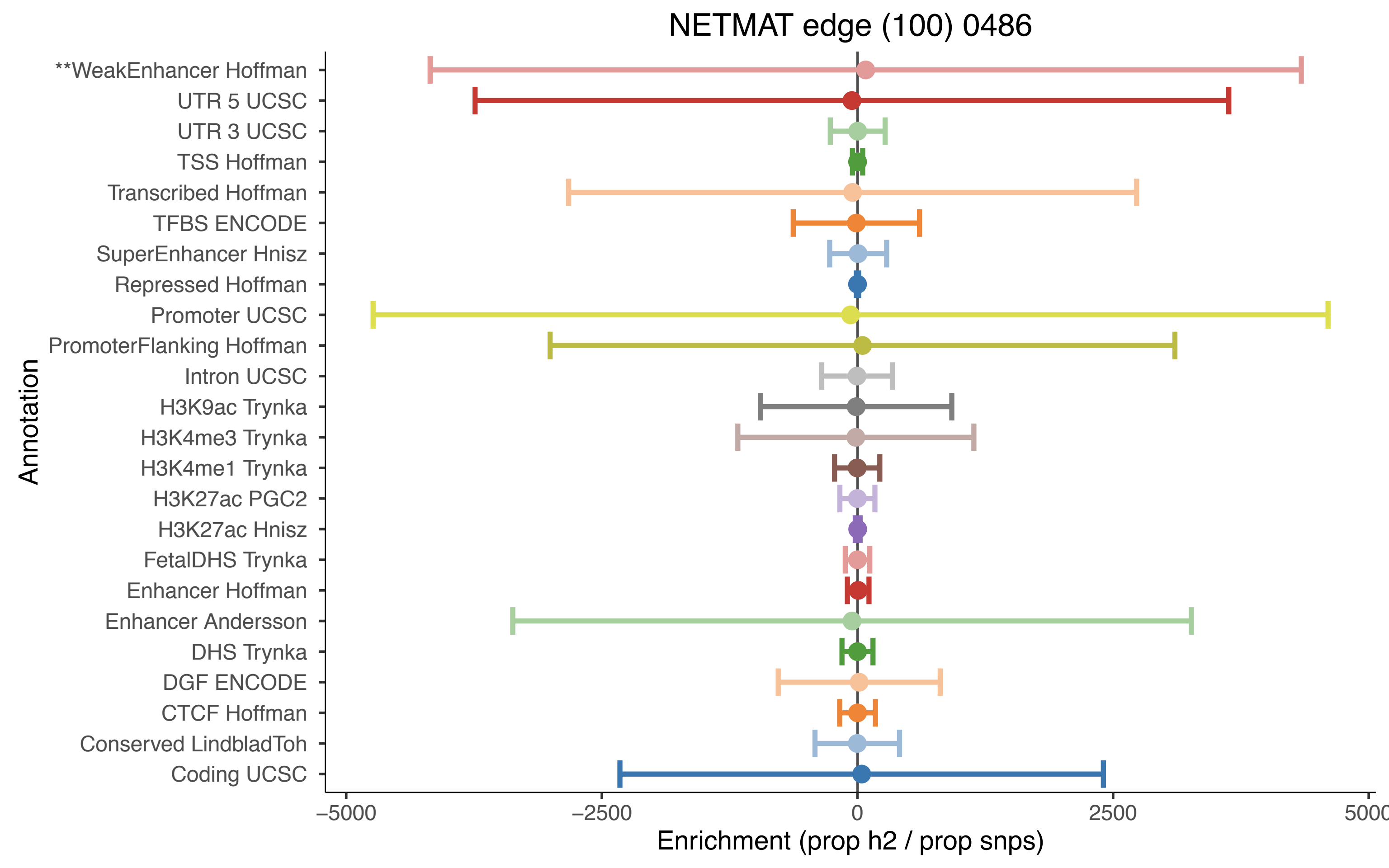

NETMAT edge (100) 0486
**WeakEnhancer Hoffman
UTR 5 UCSC
UTR 3 UCSC
TSS Hoffman
Transcribed Hoffman
TFBS ENCODE
SuperEnhancer Hnisz
Repressed Hoffman
Promoter UCSC
PromoterFlanking Hoffman
Intron UCSC
H3K9ac Trynka
H3K4me3 Trynka
H3K4me1 Trynka
H3K27ac PGC2
H3K27ac Hnisz
FetalDHS Trynka
Enhancer Hoffman
Enhancer Andersson
DHS Trynka
DGF ENCODE
CTCF Hoffman
Conserved LindbladToh
Coding UCSC
Annotation
−5000
−2500
0
2500
5000
Enrichment (prop h2 / prop snps)

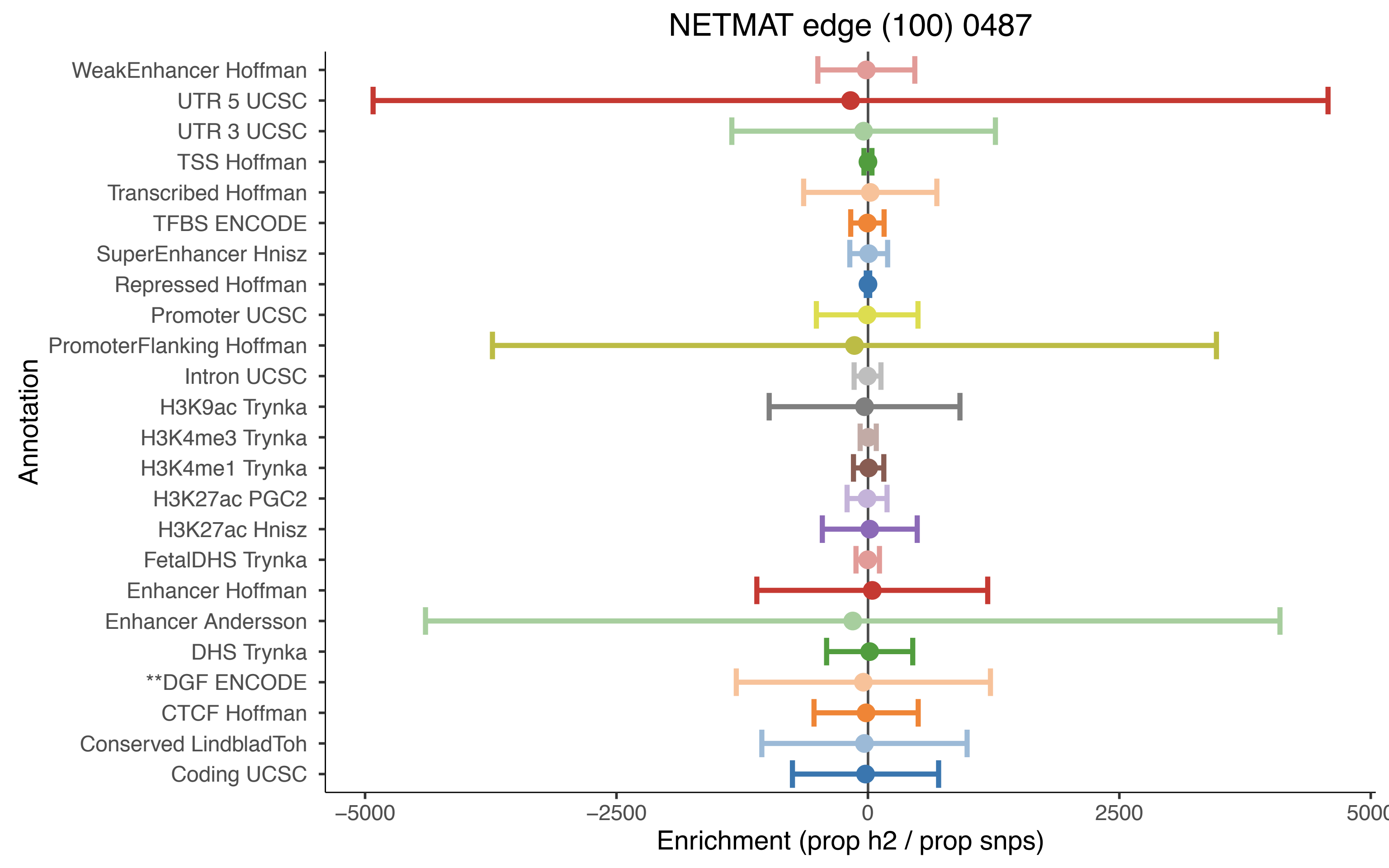
NETMAT edge (100) 0487
WeakEnhancer Hoffman
UTR 5 UCSC
UTR 3 UCSC
TSS Hoffman
Transcribed Hoffman
TFBS ENCODE
SuperEnhancer Hnisz
Repressed Hoffman
Promoter UCSC
PromoterFlanking Hoffman
Intron UCSC
H3K9ac Trynka
H3K4me3 Trynka
H3K4me1 Trynka
H3K27ac PGC2
H3K27ac Hnisz
FetalDHS Trynka
Enhancer Hoffman
Enhancer Andersson
DHS Trynka
**DGF ENCODE
CTCF Hoffman
Conserved LindbladToh
Coding UCSC
Annotation
−5000
−2500
0
2500
5000
Enrichment (prop h2 / prop snps)

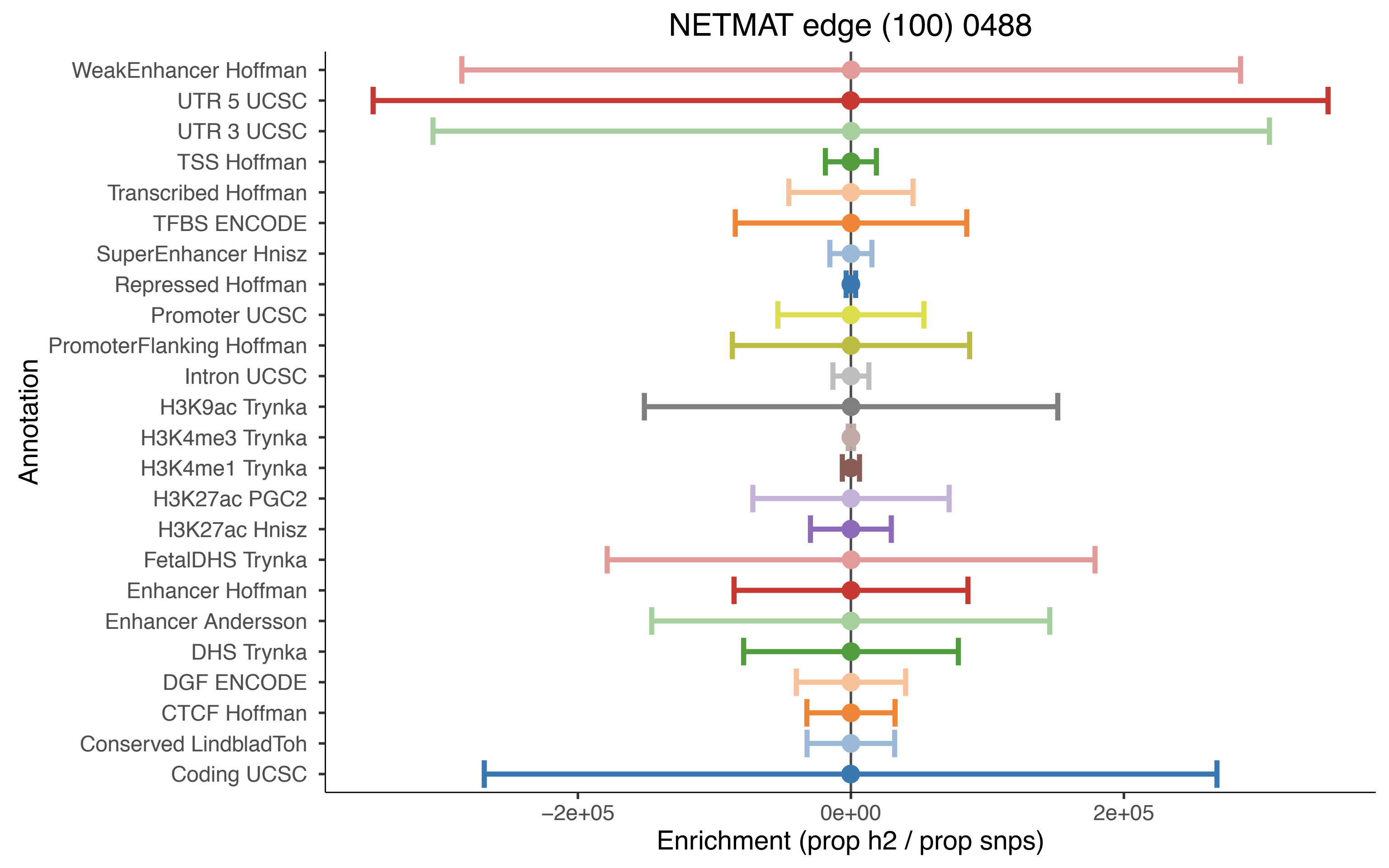
NETMAT edge (100) 0488
WeakEnhancer Hoffman
UTR 5 UCSC
UTR 3 UCSC
TSS Hoffman
Transcribed Hoffman
TFBS ENCODE
SuperEnhancer Hnisz
Repressed Hoffman
Promoter UCSC
PromoterFlanking Hoffman
Intron UCSC
H3K9ac Trynka
H3K4me3 Trynka
H3K4me1 Trynka
H3K27ac PGC2
H3K27ac Hnisz
FetalDHS Trynka
Enhancer Hoffman
Enhancer Andersson
DHS Trynka
DGF ENCODE
CTCF Hoffman
Conserved LindbladToh
Coding UCSC
Annotation
−2e+05
0e+00
2e+05
Enrichment (prop h2 / prop snps)

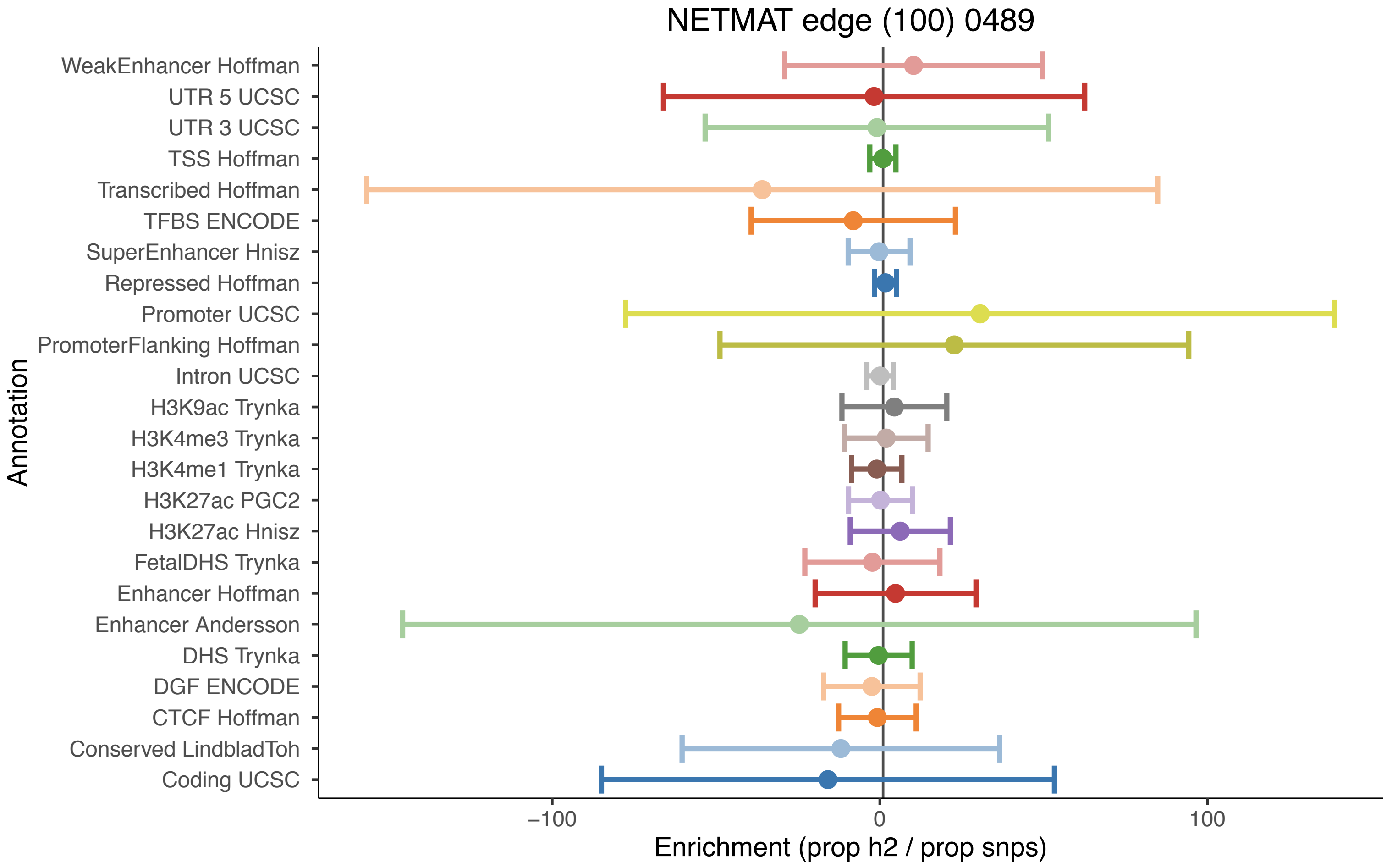

NETMAT edge (100) 0489
WeakEnhancer Hoffman
UTR 5 UCSC
UTR 3 UCSC
TSS Hoffman
Transcribed Hoffman
TFBS ENCODE
SuperEnhancer Hnisz
Repressed Hoffman
Promoter UCSC
PromoterFlanking Hoffman
Intron UCSC
H3K9ac Trynka
H3K4me3 Trynka
H3K4me1 Trynka
H3K27ac PGC2
H3K27ac Hnisz
FetalDHS Trynka
Enhancer Hoffman
Enhancer Andersson
DHS Trynka
DGF ENCODE
CTCF Hoffman
Conserved LindbladToh
Coding UCSC
Annotation
−100
0
100
Enrichment (prop h2 / prop snps)

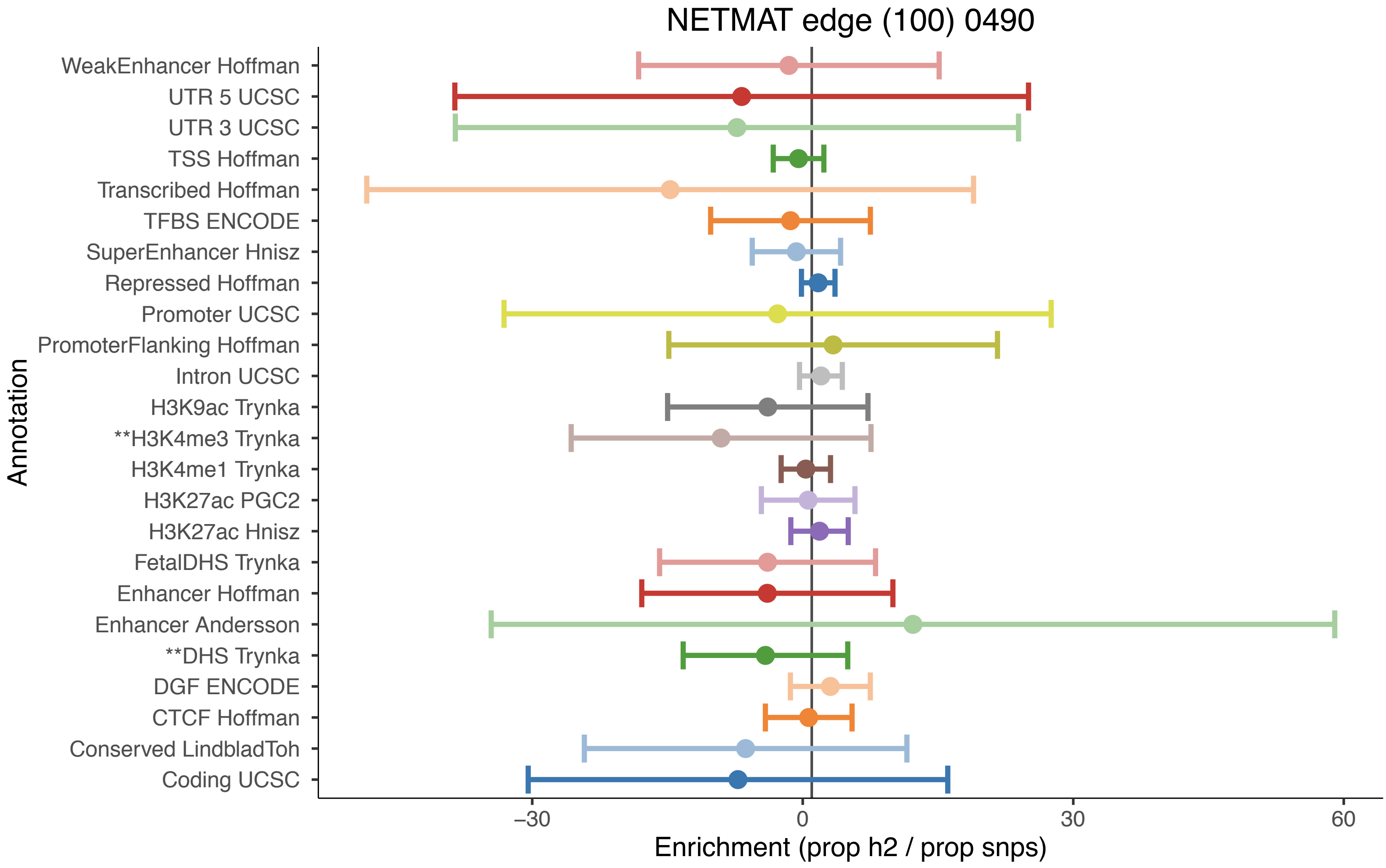

NETMAT edge (100) 0490
Annotation
WeakEnhancer Hoffman
UTR 5 UCSC
UTR 3 UCSC
TSS Hoffman
Transcribed Hoffman
TFBS ENCODE
SuperEnhancer Hnisz
Repressed Hoffman
Promoter UCSC
PromoterFlanking Hoffman
Intron UCSC
H3K9ac Trynka
**H3K4me3 Trynka
H3K4me1 Trynka
H3K27ac PGC2
H3K27ac Hnisz
FetalDHS Trynka
Enhancer Hoffman
Enhancer Andersson
**DHS Trynka
DGF ENCODE
CTCF Hoffman
Conserved LindbladToh
Coding UCSC
−30
0
30
60
Enrichment (prop h2 / prop snps)

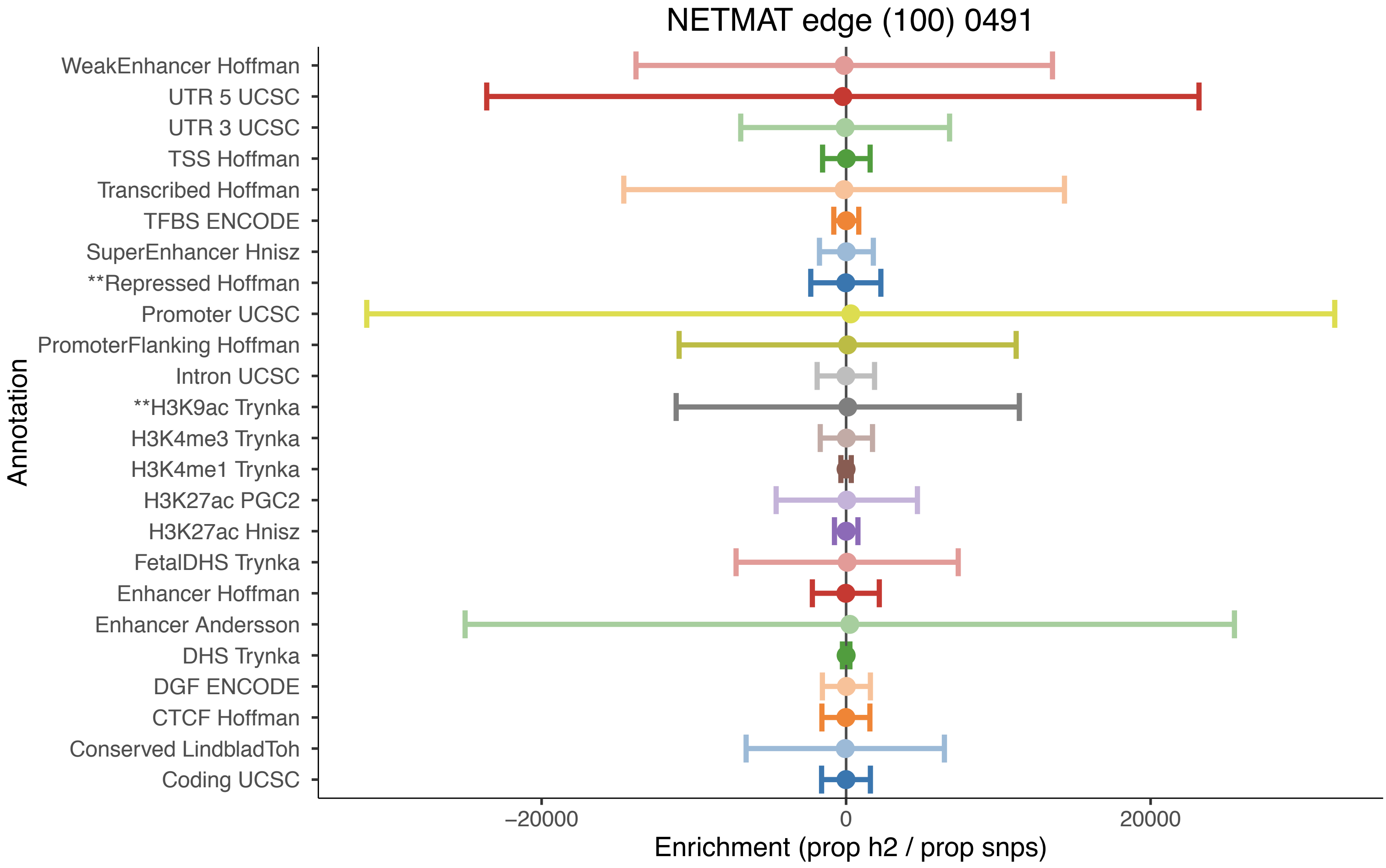
NETMAT edge (100) 0491
WeakEnhancer Hoffman
UTR 5 UCSC
UTR 3 UCSC
TSS Hoffman
Transcribed Hoffman
TFBS ENCODE
SuperEnhancer Hnisz
**Repressed Hoffman
Promoter UCSC
PromoterFlanking Hoffman
Intron UCSC
**H3K9ac Trynka
H3K4me3 Trynka
H3K4me1 Trynka
H3K27ac PGC2
H3K27ac Hnisz
FetalDHS Trynka
Enhancer Hoffman
Enhancer Andersson
DHS Trynka
DGF ENCODE
CTCF Hoffman
Conserved LindbladToh
Coding UCSC
Annotation
−20000
0
20000
Enrichment (prop h2 / prop snps)

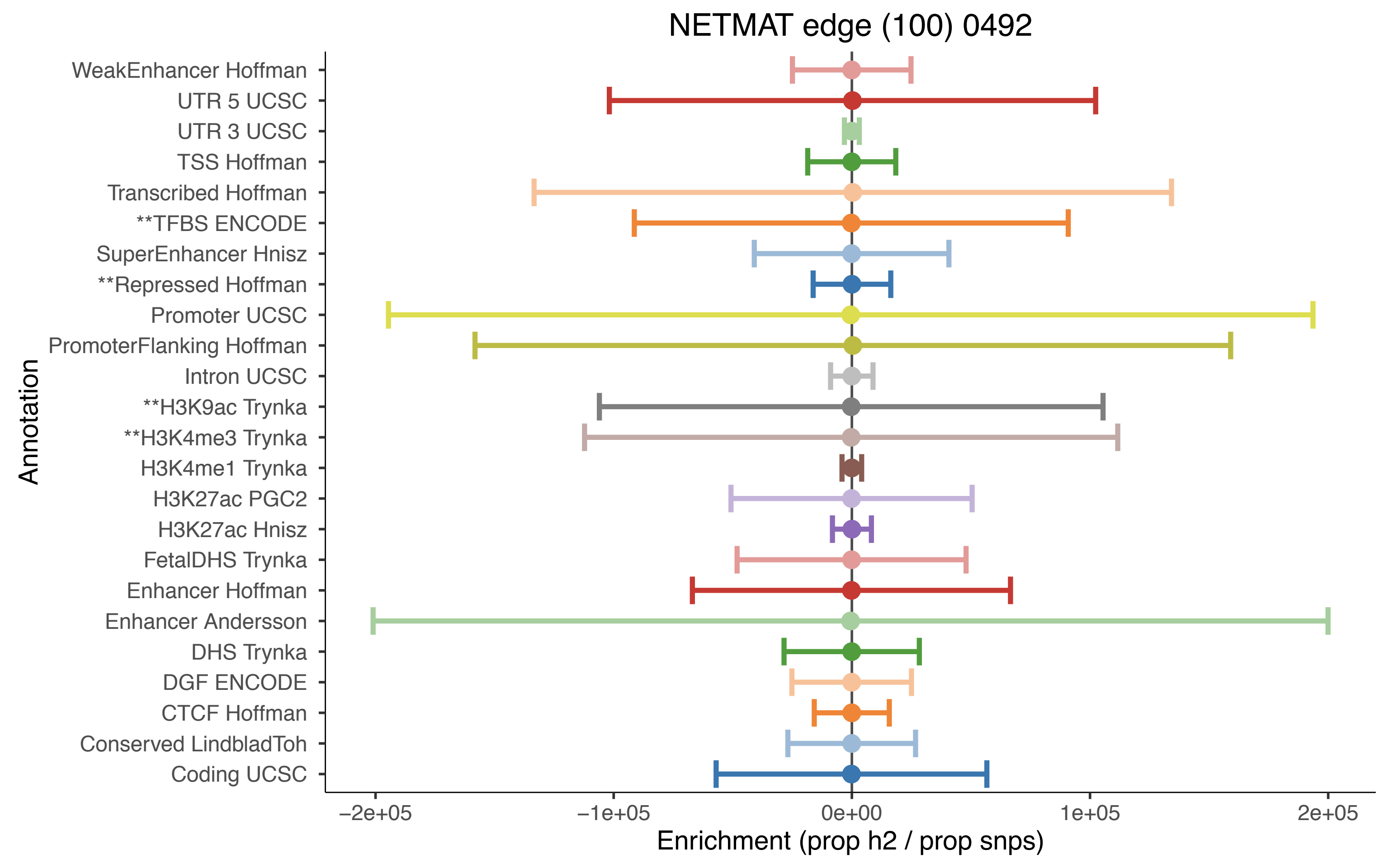

NETMAT edge (100) 0492
WeakEnhancer Hoffman
UTR 5 UCSC
UTR 3 UCSC
TSS Hoffman
Transcribed Hoffman
**TFBS ENCODE
SuperEnhancer Hnisz
**Repressed Hoffman
Promoter UCSC
PromoterFlanking Hoffman
Intron UCSC
**H3K9ac Trynka
**H3K4me3 Trynka
H3K4me1 Trynka
H3K27ac PGC2
H3K27ac Hnisz
FetalDHS Trynka
Enhancer Hoffman
Enhancer Andersson
DHS Trynka
DGF ENCODE
CTCF Hoffman
Conserved LindbladToh
Coding UCSC
Annotation
−2e+05
−1e+05
0e+00
1e+05
2e+05
Enrichment (prop h2 / prop snps)

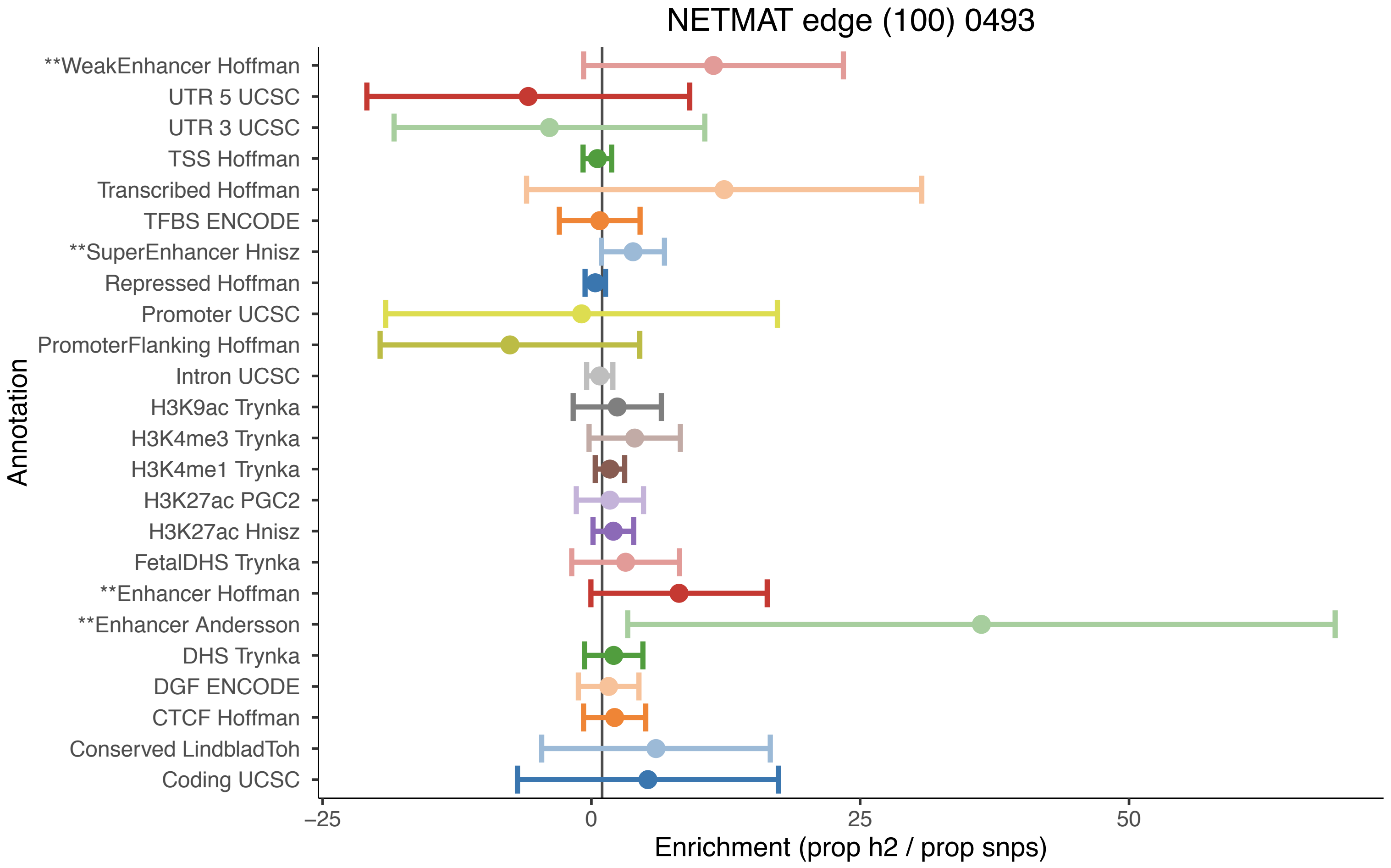

NETMAT edge (100) 0493
**WeakEnhancer Hoffman
UTR 5 UCSC
UTR 3 UCSC
TSS Hoffman
Transcribed Hoffman
TFBS ENCODE
**SuperEnhancer Hnisz
Repressed Hoffman
Promoter UCSC
PromoterFlanking Hoffman
Intron UCSC
H3K9ac Trynka
H3K4me3 Trynka
H3K4me1 Trynka
H3K27ac PGC2
H3K27ac Hnisz
FetalDHS Trynka
**Enhancer Hoffman
**Enhancer Andersson
DHS Trynka
DGF ENCODE
CTCF Hoffman
Conserved LindbladToh
Coding UCSC
Annotation
−25
0
25
50
Enrichment (prop h2 / prop snps)

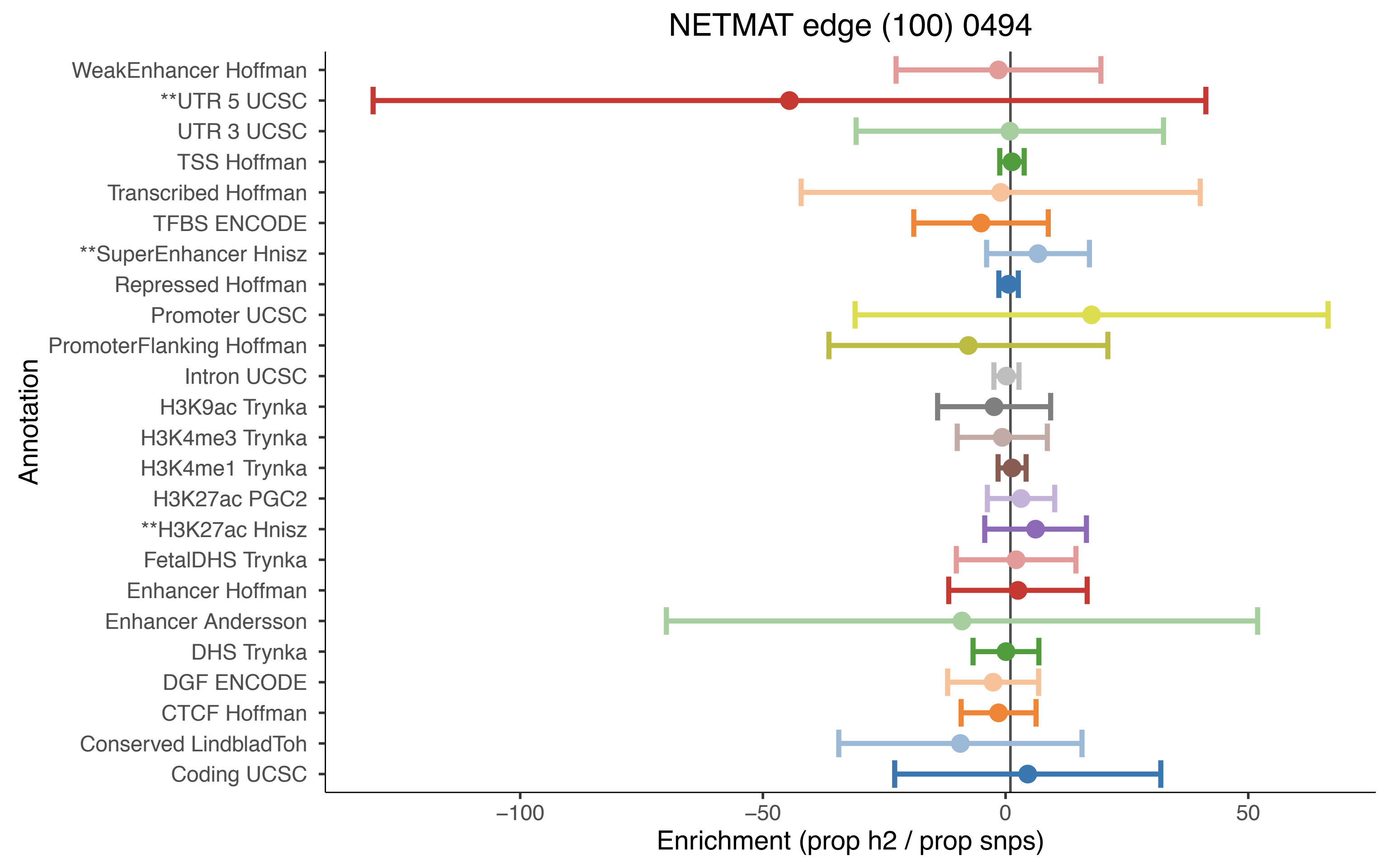
NETMAT edge (100) 0494
WeakEnhancer Hoffman
**UTR 5 UCSC
UTR 3 UCSC
TSS Hoffman
Transcribed Hoffman
TFBS ENCODE
**SuperEnhancer Hnisz
Repressed Hoffman
Promoter UCSC
PromoterFlanking Hoffman
Intron UCSC
H3K9ac Trynka
H3K4me3 Trynka
H3K4me1 Trynka
H3K27ac PGC2
**H3K27ac Hnisz
FetalDHS Trynka
Enhancer Hoffman
Enhancer Andersson
DHS Trynka
DGF ENCODE
CTCF Hoffman
Conserved LindbladToh
Coding UCSC
Annotation
−100
−50
0
50
Enrichment (prop h2 / prop snps)

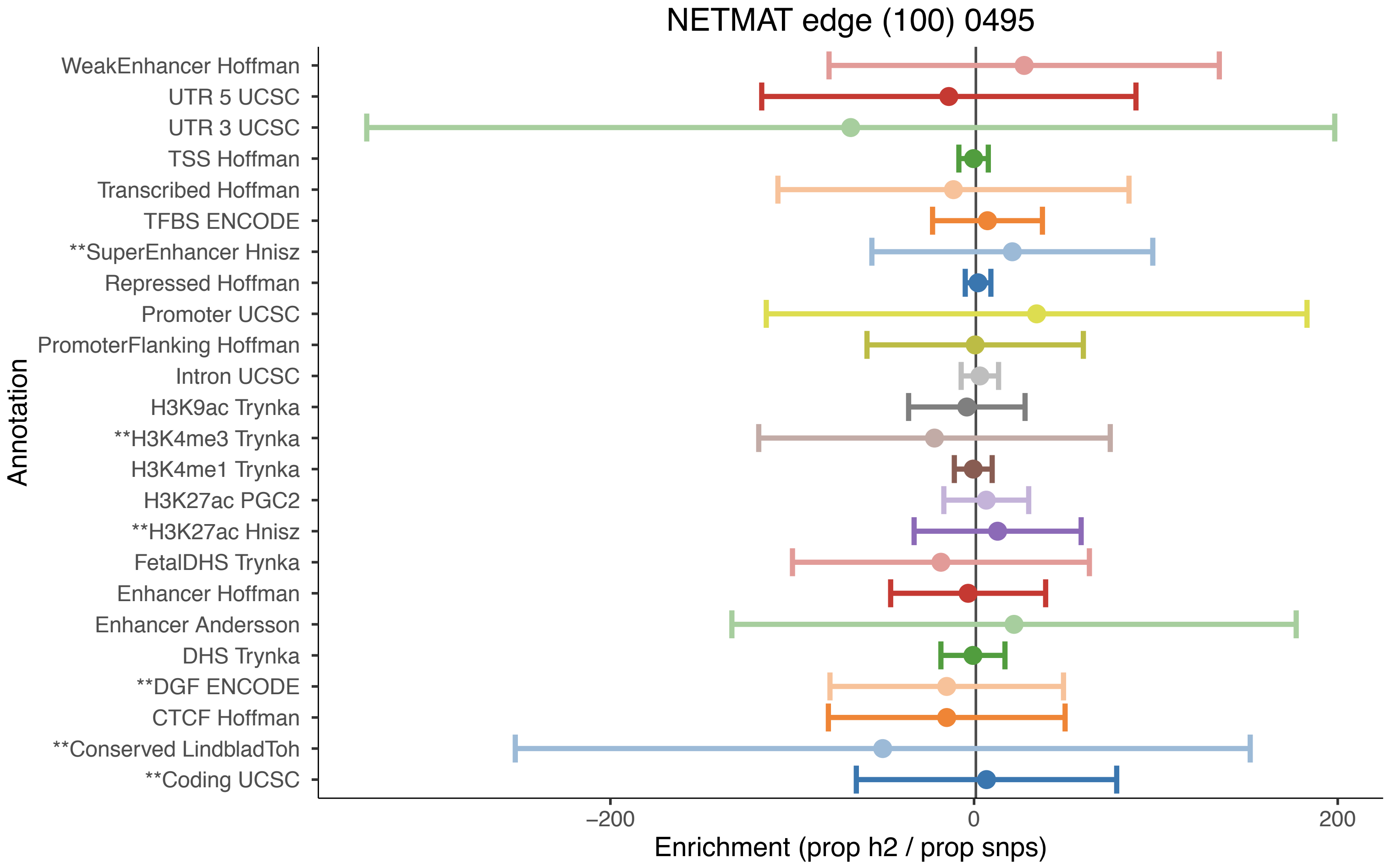
NETMAT edge (100) 0495
WeakEnhancer Hoffman
UTR 5 UCSC
UTR 3 UCSC
TSS Hoffman
Transcribed Hoffman
TFBS ENCODE
**SuperEnhancer Hnisz
Repressed Hoffman
Promoter UCSC
PromoterFlanking Hoffman
Intron UCSC
H3K9ac Trynka
**H3K4me3 Trynka
H3K4me1 Trynka
H3K27ac PGC2
**H3K27ac Hnisz
FetalDHS Trynka
Enhancer Hoffman
Enhancer Andersson
DHS Trynka
**DGF ENCODE
CTCF Hoffman
**Conserved LindbladToh
**Coding UCSC
Annotation
−200
0
200
Enrichment (prop h2 / prop snps)

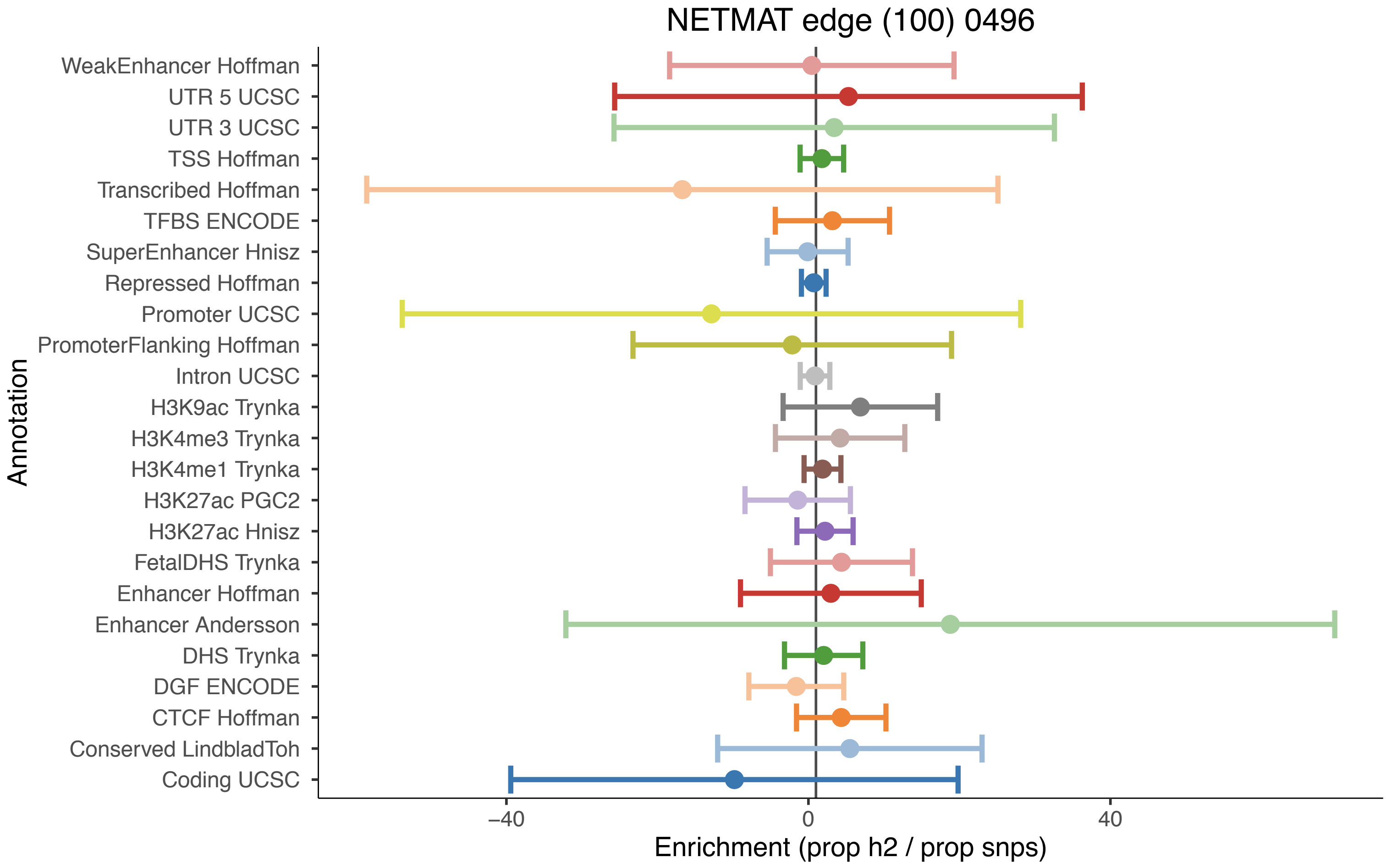

NETMAT edge (100) 0496
WeakEnhancer Hoffman
UTR 5 UCSC
UTR 3 UCSC
TSS Hoffman
Transcribed Hoffman
TFBS ENCODE
SuperEnhancer Hnisz
Repressed Hoffman
Promoter UCSC
PromoterFlanking Hoffman
Intron UCSC
H3K9ac Trynka
H3K4me3 Trynka
H3K4me1 Trynka
H3K27ac PGC2
H3K27ac Hnisz
FetalDHS Trynka
Enhancer Hoffman
Enhancer Andersson
DHS Trynka
DGF ENCODE
CTCF Hoffman
Conserved LindbladToh
Coding UCSC
Annotation
−40
0
40
Enrichment (prop h2 / prop snps)

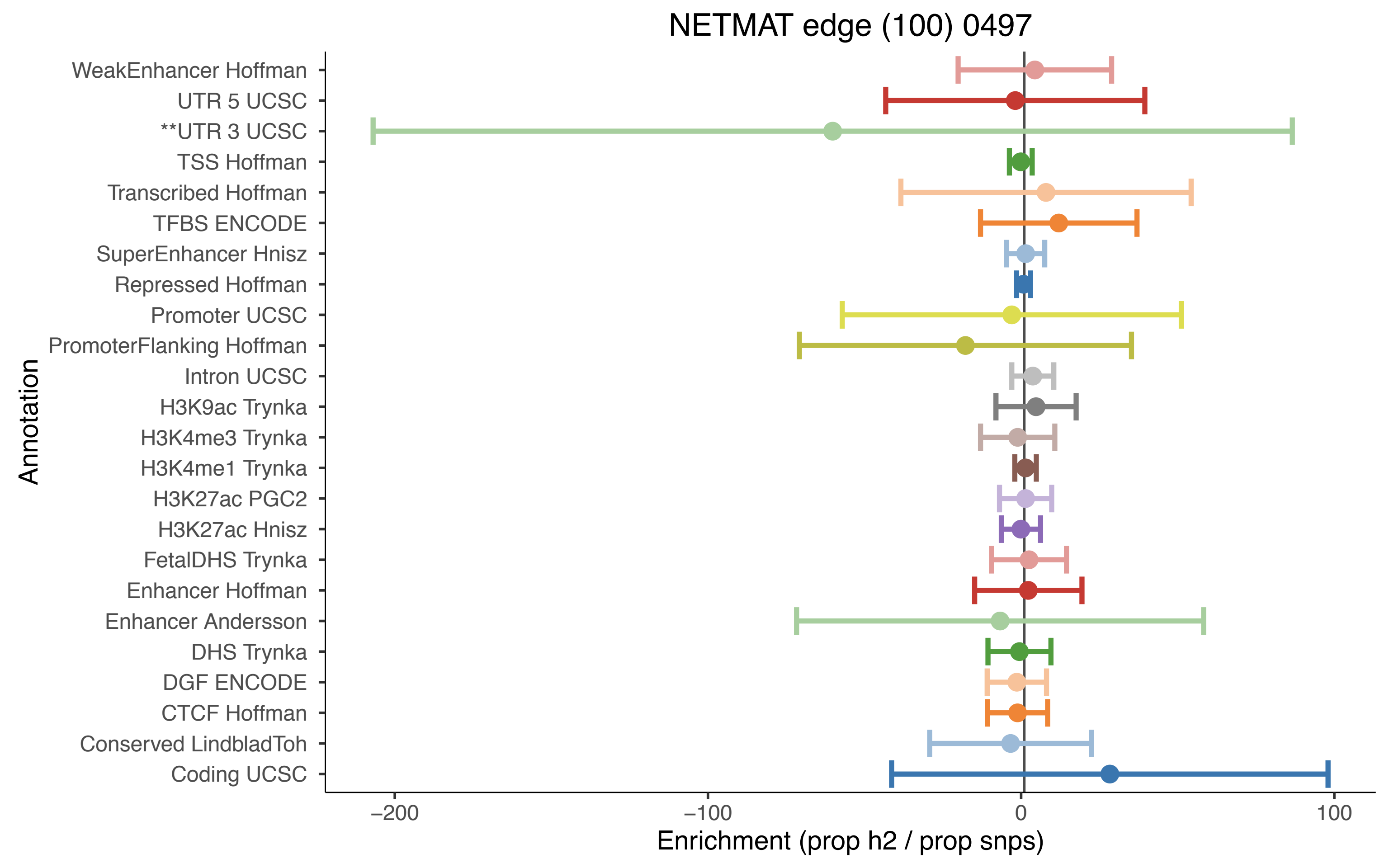
NETMAT edge (100) 0497
Annotation
WeakEnhancer Hoffman
UTR 5 UCSC
**UTR 3 UCSC
TSS Hoffman
Transcribed Hoffman
TFBS ENCODE
SuperEnhancer Hnisz
Repressed Hoffman
Promoter UCSC
PromoterFlanking Hoffman
Intron UCSC
H3K9ac Trynka
H3K4me3 Trynka
H3K4me1 Trynka
H3K27ac PGC2
H3K27ac Hnisz
FetalDHS Trynka
Enhancer Hoffman
Enhancer Andersson
DHS Trynka
DGF ENCODE
CTCF Hoffman
Conserved LindbladToh
Coding UCSC
−200
−100
0
100
Enrichment (prop h2 / prop snps)

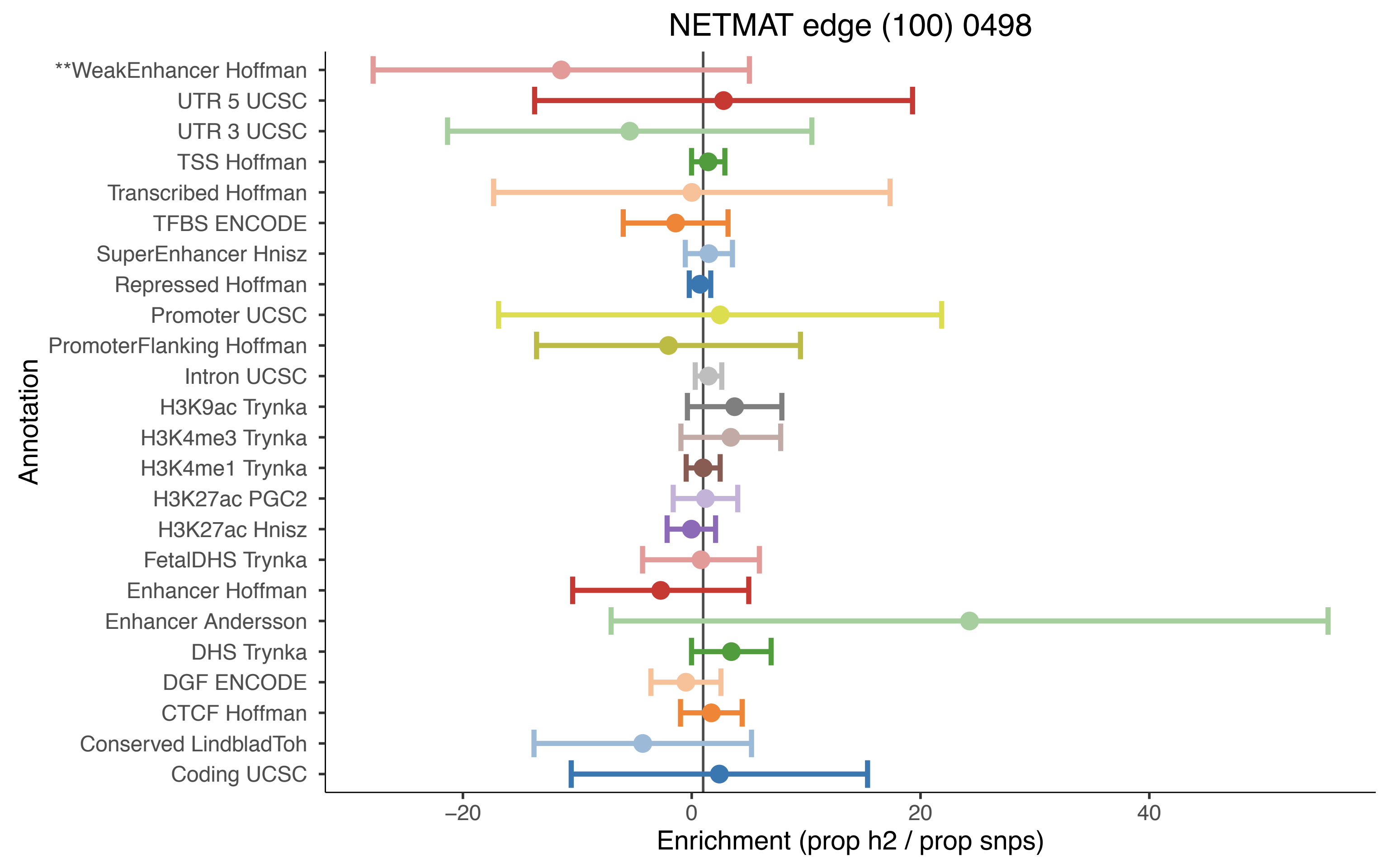

NETMAT edge (100) 0498
Annotation
**WeakEnhancer Hoffman
UTR 5 UCSC
UTR 3 UCSC
TSS Hoffman
Transcribed Hoffman
TFBS ENCODE
SuperEnhancer Hnisz
Repressed Hoffman
Promoter UCSC
PromoterFlanking Hoffman
Intron UCSC
H3K9ac Trynka
H3K4me3 Trynka
H3K4me1 Trynka
H3K27ac PGC2
H3K27ac Hnisz
FetalDHS Trynka
Enhancer Hoffman
Enhancer Andersson
DHS Trynka
DGF ENCODE
CTCF Hoffman
Conserved LindbladToh
Coding UCSC
−20
0
20
40
Enrichment (prop h2 / prop snps)

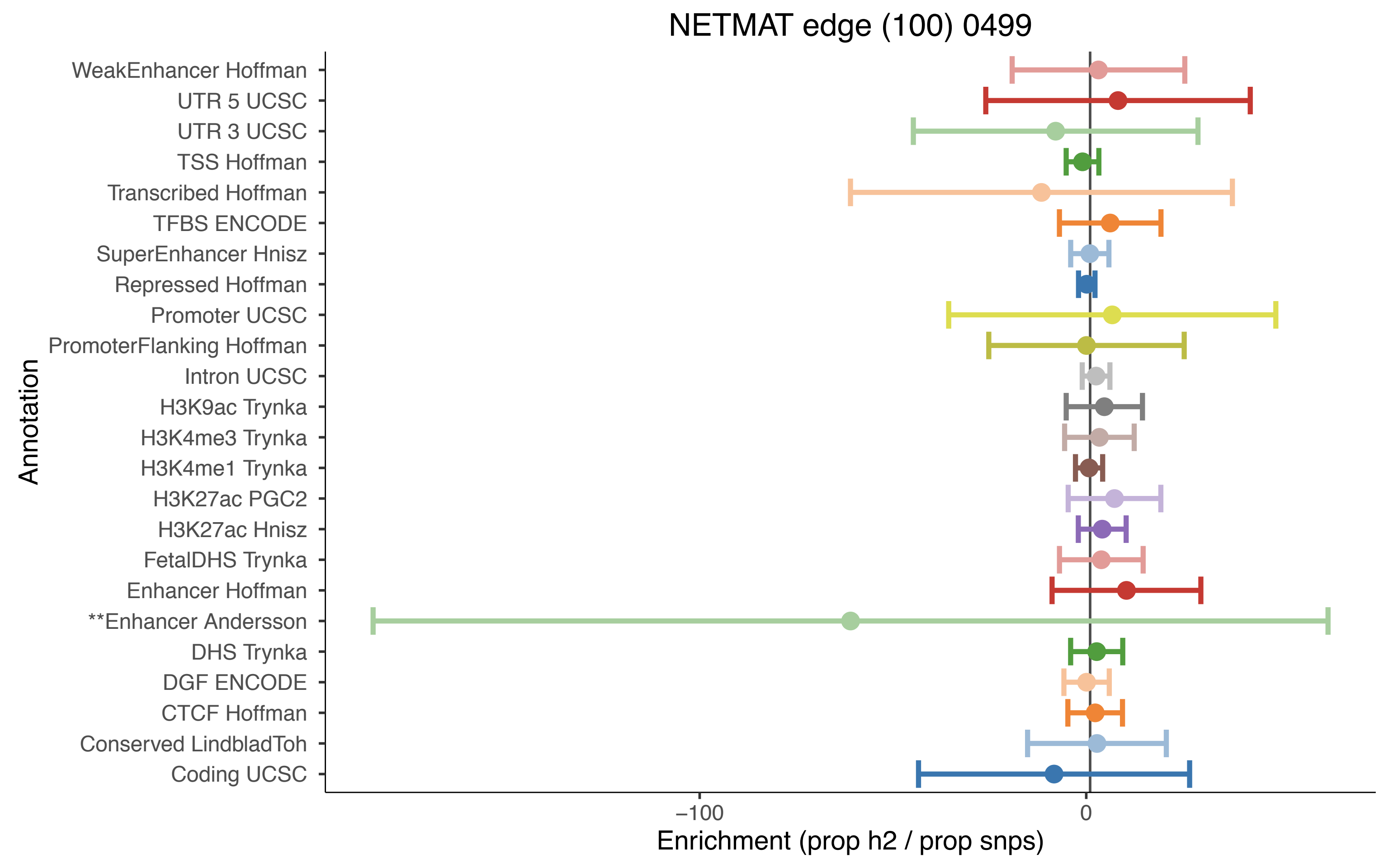

NETMAT edge (100) 0499
WeakEnhancer Hoffman
UTR 5 UCSC
UTR 3 UCSC
TSS Hoffman
Transcribed Hoffman
TFBS ENCODE
SuperEnhancer Hnisz
Repressed Hoffman
Promoter UCSC
PromoterFlanking Hoffman
Intron UCSC
H3K9ac Trynka
H3K4me3 Trynka
H3K4me1 Trynka
H3K27ac PGC2
H3K27ac Hnisz
FetalDHS Trynka
Enhancer Hoffman
**Enhancer Andersson
DHS Trynka
DGF ENCODE
CTCF Hoffman
Conserved LindbladToh
Coding UCSC
Annotation
−100
0
Enrichment (prop h2 / prop snps)

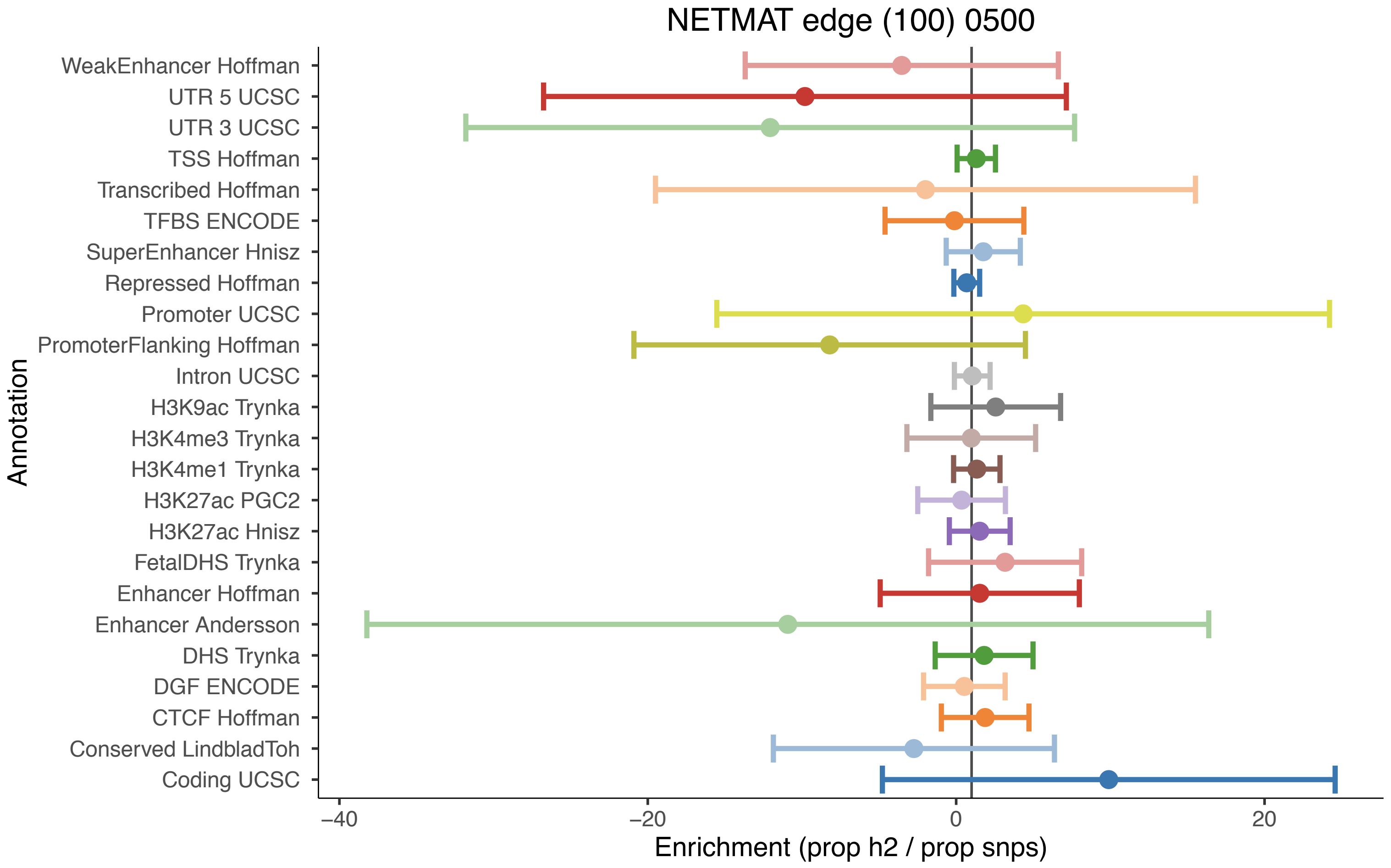
NETMAT edge (100) 0500
WeakEnhancer Hoffman
UTR 5 UCSC
UTR 3 UCSC
TSS Hoffman
Transcribed Hoffman
TFBS ENCODE
SuperEnhancer Hnisz
Repressed Hoffman
Promoter UCSC
PromoterFlanking Hoffman
Intron UCSC
H3K9ac Trynka
H3K4me3 Trynka
H3K4me1 Trynka
H3K27ac PGC2
H3K27ac Hnisz
FetalDHS Trynka
Enhancer Hoffman
Enhancer Andersson
DHS Trynka
DGF ENCODE
CTCF Hoffman
Conserved LindbladToh
Coding UCSC
Annotation
−40
−20
0
20
Enrichment (prop h2 / prop snps)

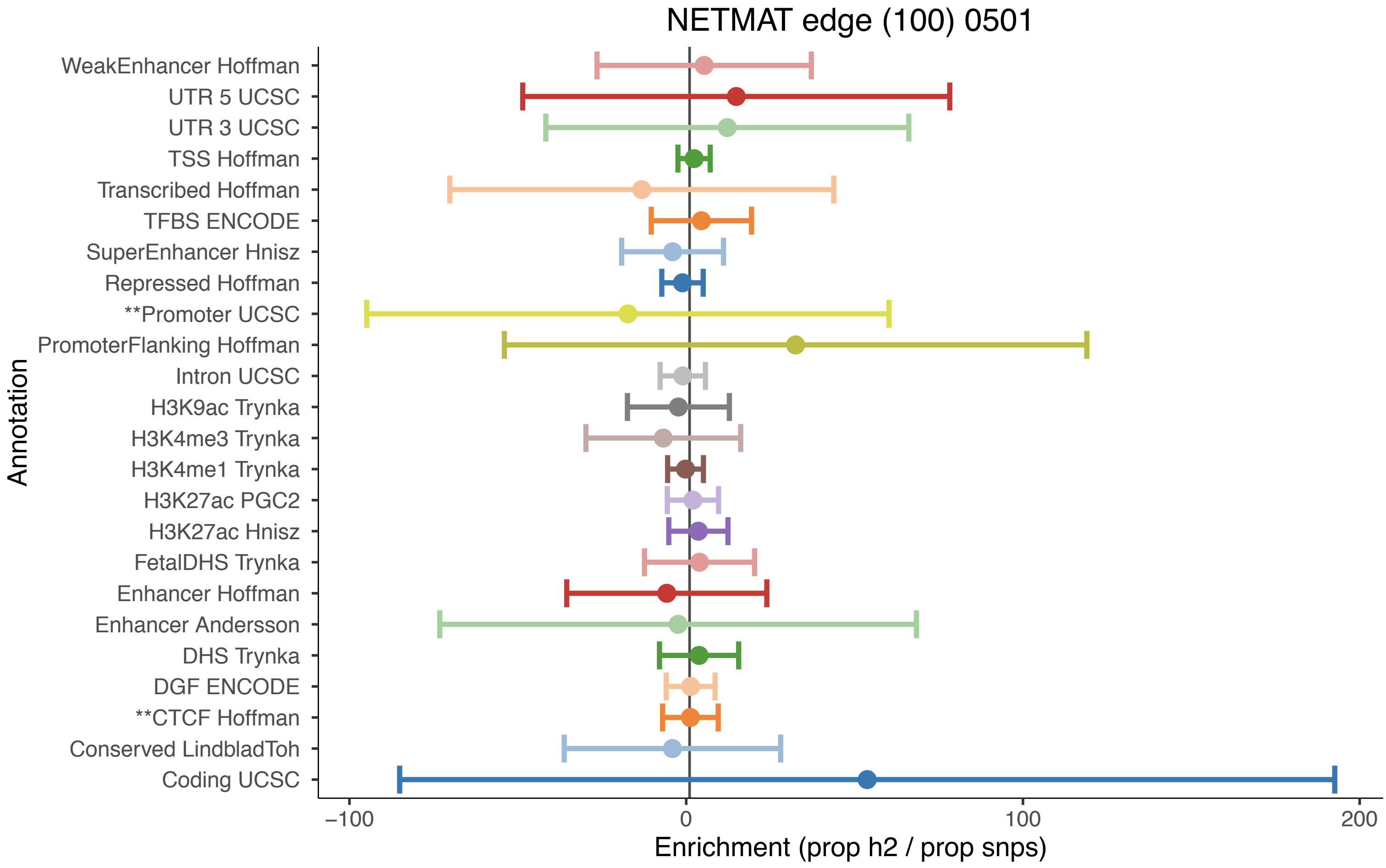

NETMAT edge (100) 0501
WeakEnhancer Hoffman
UTR 5 UCSC
UTR 3 UCSC
TSS Hoffman
Transcribed Hoffman
TFBS ENCODE
SuperEnhancer Hnisz
Repressed Hoffman
**Promoter UCSC
PromoterFlanking Hoffman
Intron UCSC
H3K9ac Trynka
H3K4me3 Trynka
H3K4me1 Trynka
H3K27ac PGC2
H3K27ac Hnisz
FetalDHS Trynka
Enhancer Hoffman
Enhancer Andersson
DHS Trynka
DGF ENCODE
**CTCF Hoffman
Conserved LindbladToh
Coding UCSC
Annotation
−100
0
100
200
Enrichment (prop h2 / prop snps)

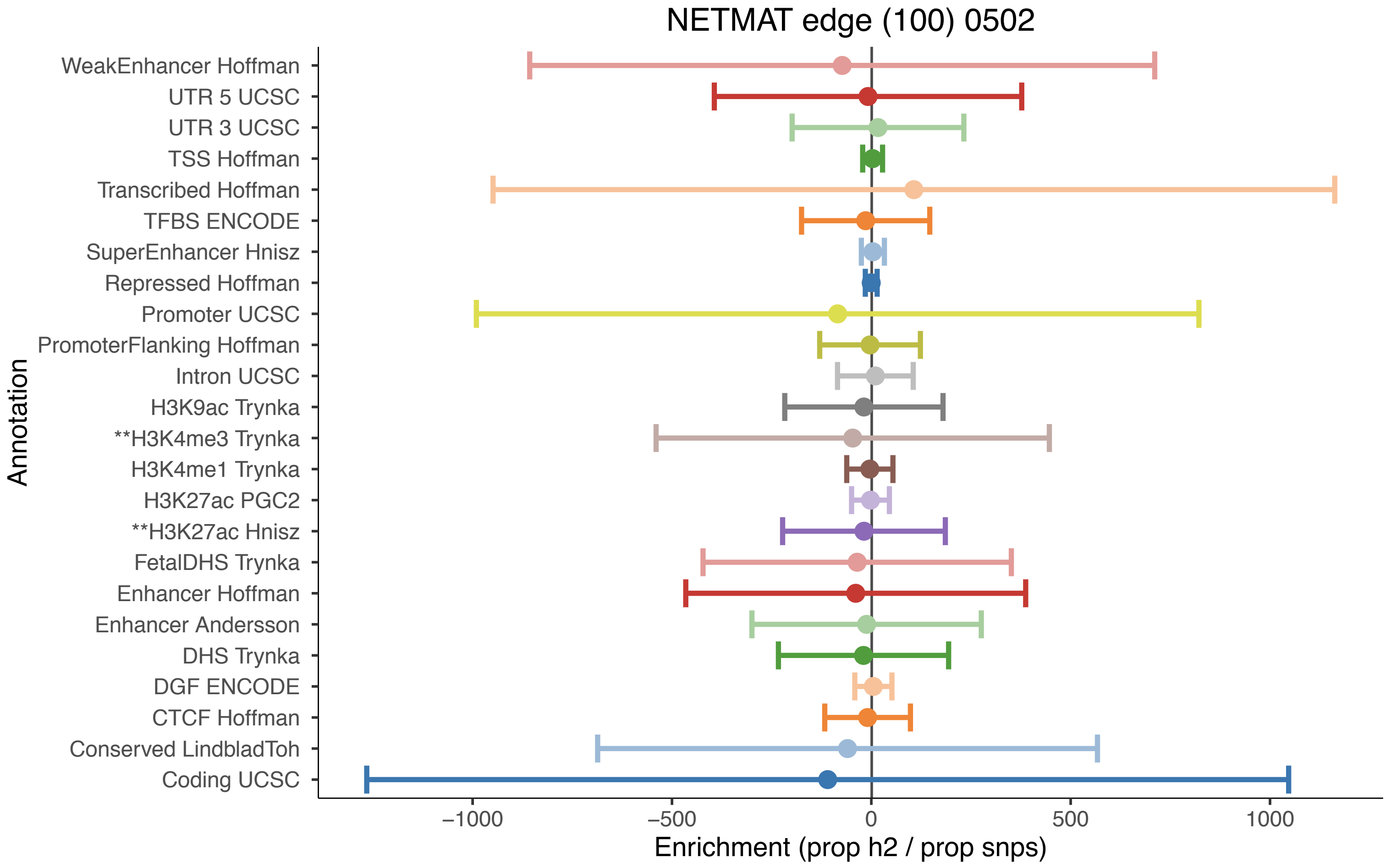
NETMAT edge (100) 0502
WeakEnhancer Hoffman
UTR 5 UCSC
UTR 3 UCSC
TSS Hoffman
Transcribed Hoffman
TFBS ENCODE
SuperEnhancer Hnisz
Repressed Hoffman
Promoter UCSC
PromoterFlanking Hoffman
Intron UCSC
H3K9ac Trynka
**H3K4me3 Trynka
H3K4me1 Trynka
H3K27ac PGC2
**H3K27ac Hnisz
FetalDHS Trynka
Enhancer Hoffman
Enhancer Andersson
DHS Trynka
DGF ENCODE
CTCF Hoffman
Conserved LindbladToh
Coding UCSC
Annotation
−1000
−500
0
500
1000
Enrichment (prop h2 / prop snps)

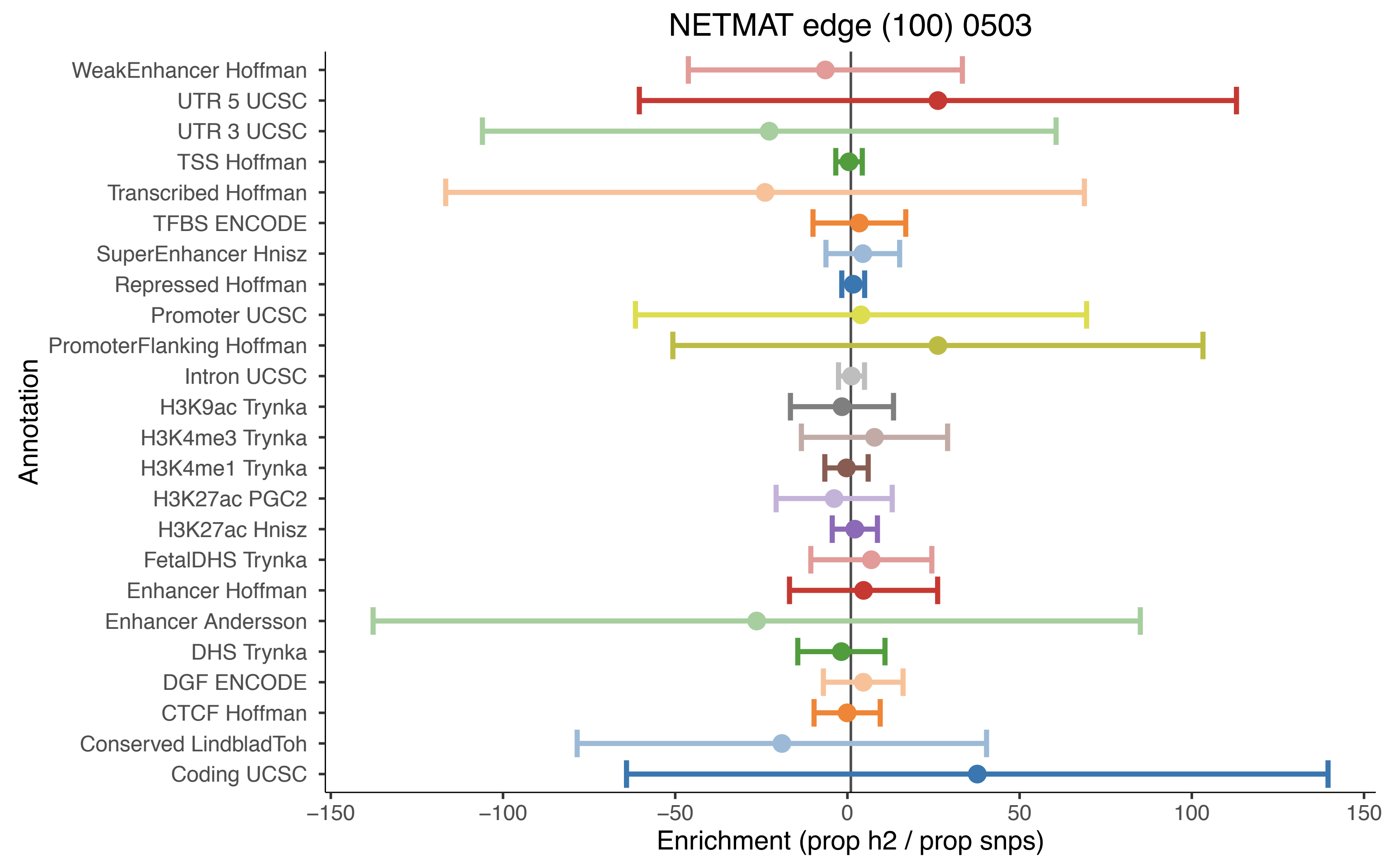

NETMAT edge (100) 0503
WeakEnhancer Hoffman
UTR 5 UCSC
UTR 3 UCSC
TSS Hoffman
Transcribed Hoffman
TFBS ENCODE
SuperEnhancer Hnisz
Repressed Hoffman
Promoter UCSC
PromoterFlanking Hoffman
Intron UCSC
H3K9ac Trynka
H3K4me3 Trynka
H3K4me1 Trynka
H3K27ac PGC2
H3K27ac Hnisz
FetalDHS Trynka
Enhancer Hoffman
Enhancer Andersson
DHS Trynka
DGF ENCODE
CTCF Hoffman
Conserved LindbladToh
Coding UCSC
Annotation
−150
−100
−50
0
50
100
150
Enrichment (prop h2 / prop snps)

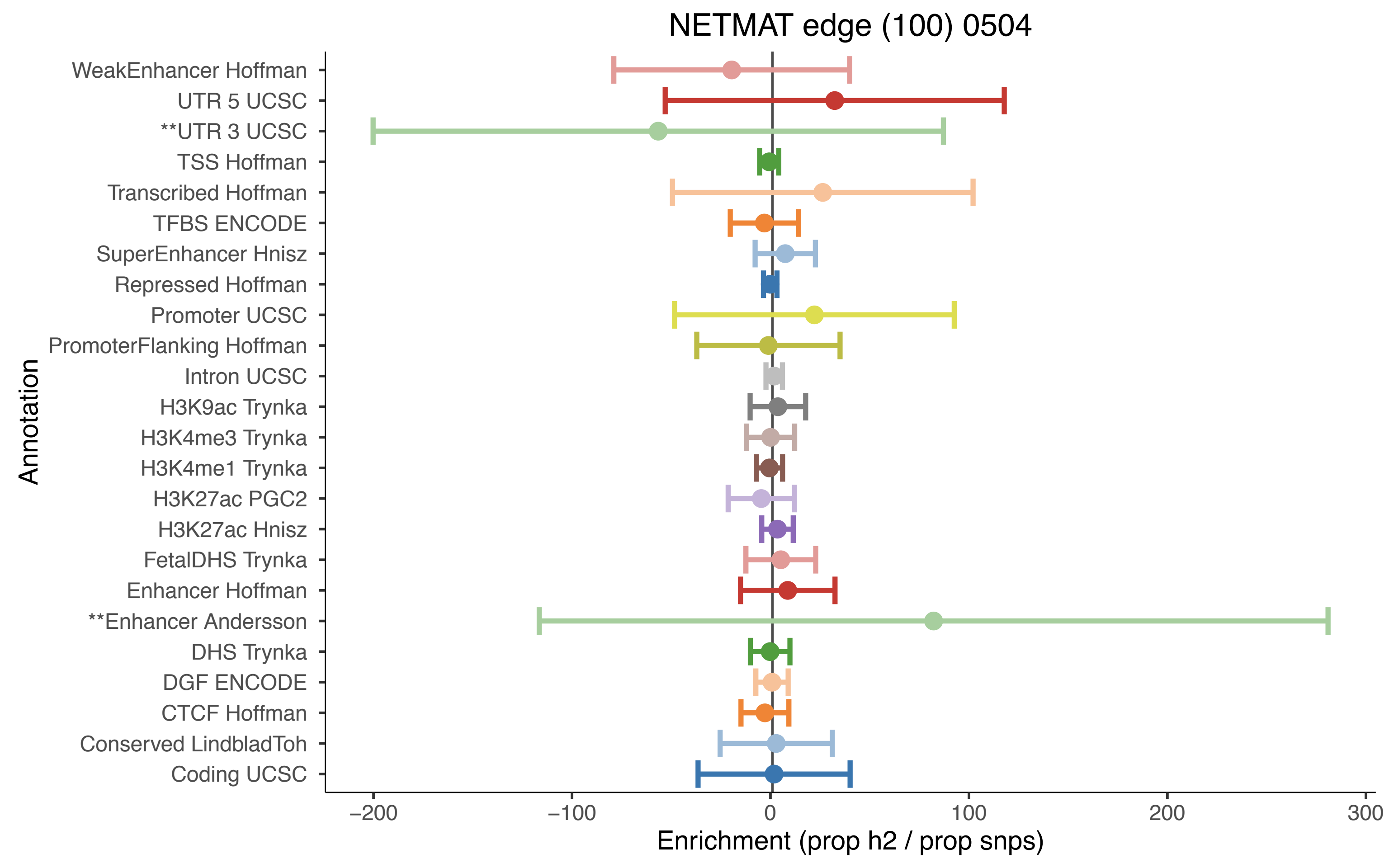
NETMAT edge (100) 0504
WeakEnhancer Hoffman
UTR 5 UCSC
**UTR 3 UCSC
TSS Hoffman
Transcribed Hoffman
TFBS ENCODE
SuperEnhancer Hnisz
Repressed Hoffman
Promoter UCSC
PromoterFlanking Hoffman
Intron UCSC
H3K9ac Trynka
H3K4me3 Trynka
H3K4me1 Trynka
H3K27ac PGC2
H3K27ac Hnisz
FetalDHS Trynka
Enhancer Hoffman
**Enhancer Andersson
DHS Trynka
DGF ENCODE
CTCF Hoffman
Conserved LindbladToh
Coding UCSC
Annotation
−200
−100
0
100
200
300
Enrichment (prop h2 / prop snps)

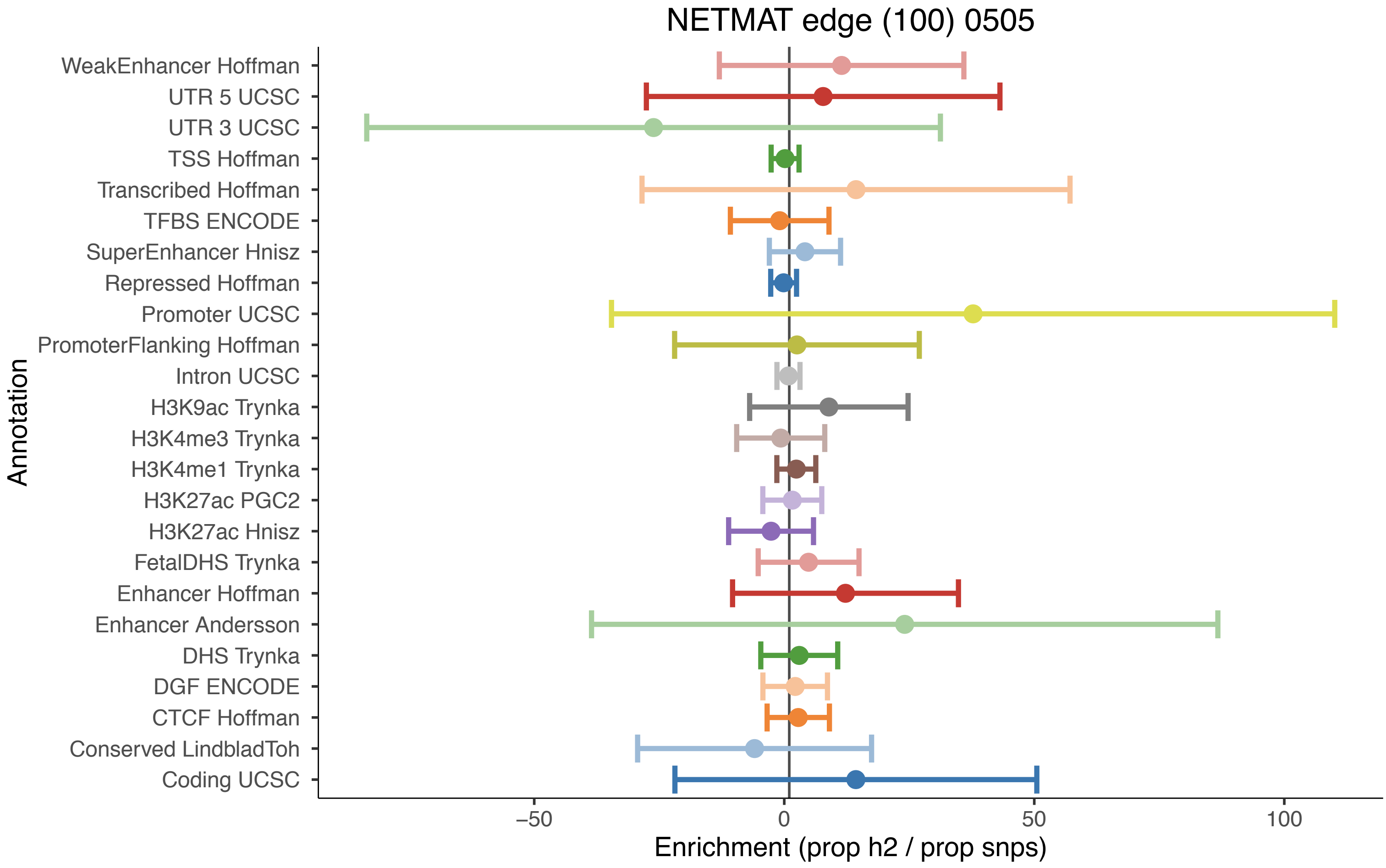

NETMAT edge (100) 0505
WeakEnhancer Hoffman
UTR 5 UCSC
UTR 3 UCSC
TSS Hoffman
Transcribed Hoffman
TFBS ENCODE
SuperEnhancer Hnisz
Repressed Hoffman
Promoter UCSC
PromoterFlanking Hoffman
Intron UCSC
H3K9ac Trynka
H3K4me3 Trynka
H3K4me1 Trynka
H3K27ac PGC2
H3K27ac Hnisz
FetalDHS Trynka
Enhancer Hoffman
Enhancer Andersson
DHS Trynka
DGF ENCODE
CTCF Hoffman
Conserved LindbladToh
Coding UCSC
Annotation
−50
0
50
100
Enrichment (prop h2 / prop snps)

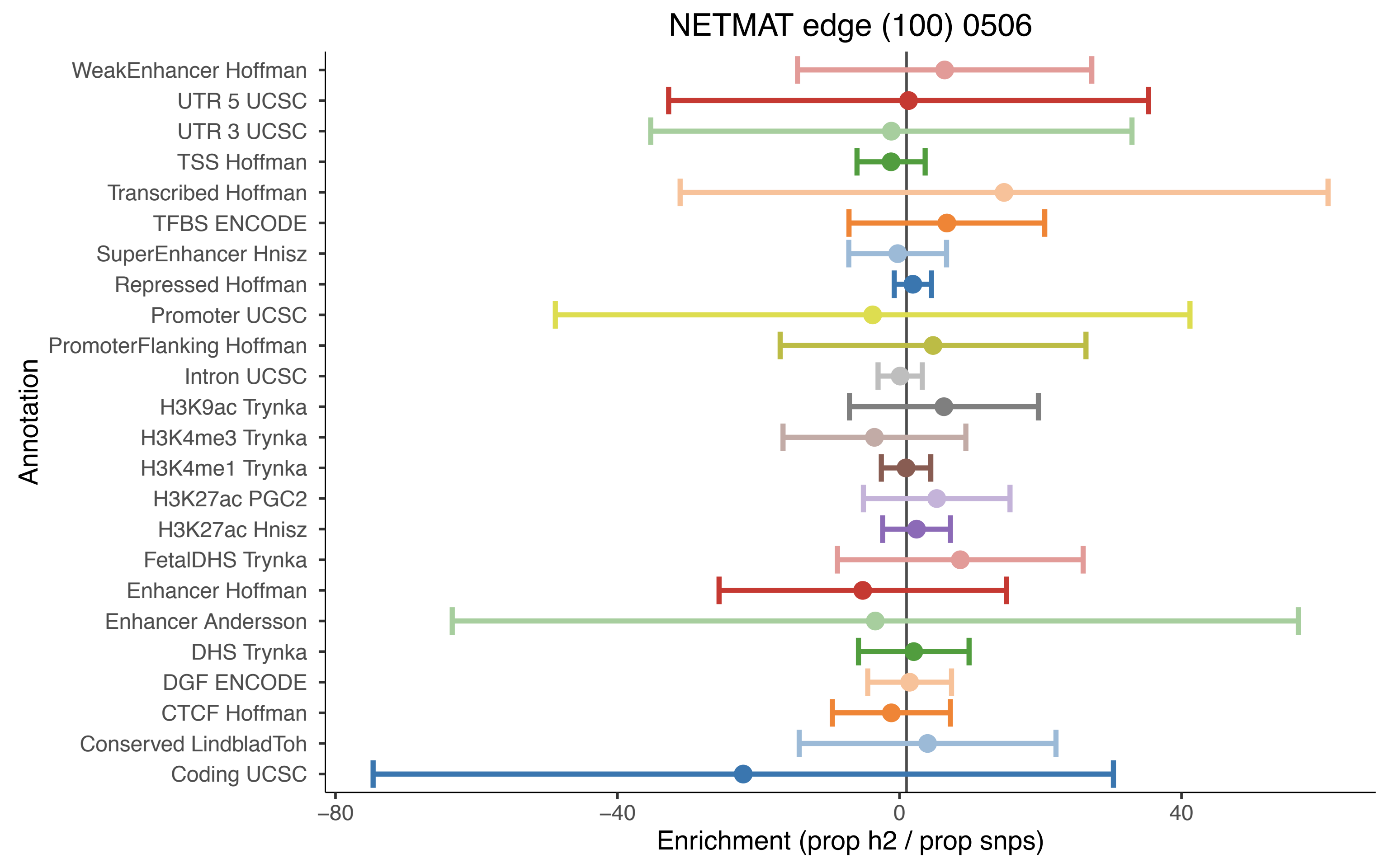
NETMAT edge (100) 0506
WeakEnhancer Hoffman
UTR 5 UCSC
UTR 3 UCSC
TSS Hoffman
Transcribed Hoffman
TFBS ENCODE
SuperEnhancer Hnisz
Repressed Hoffman
Promoter UCSC
PromoterFlanking Hoffman
Intron UCSC
H3K9ac Trynka
H3K4me3 Trynka
H3K4me1 Trynka
H3K27ac PGC2
H3K27ac Hnisz
FetalDHS Trynka
Enhancer Hoffman
Enhancer Andersson
DHS Trynka
DGF ENCODE
CTCF Hoffman
Conserved LindbladToh
Coding UCSC
Annotation
−80
−40
0
40
Enrichment (prop h2 / prop snps)

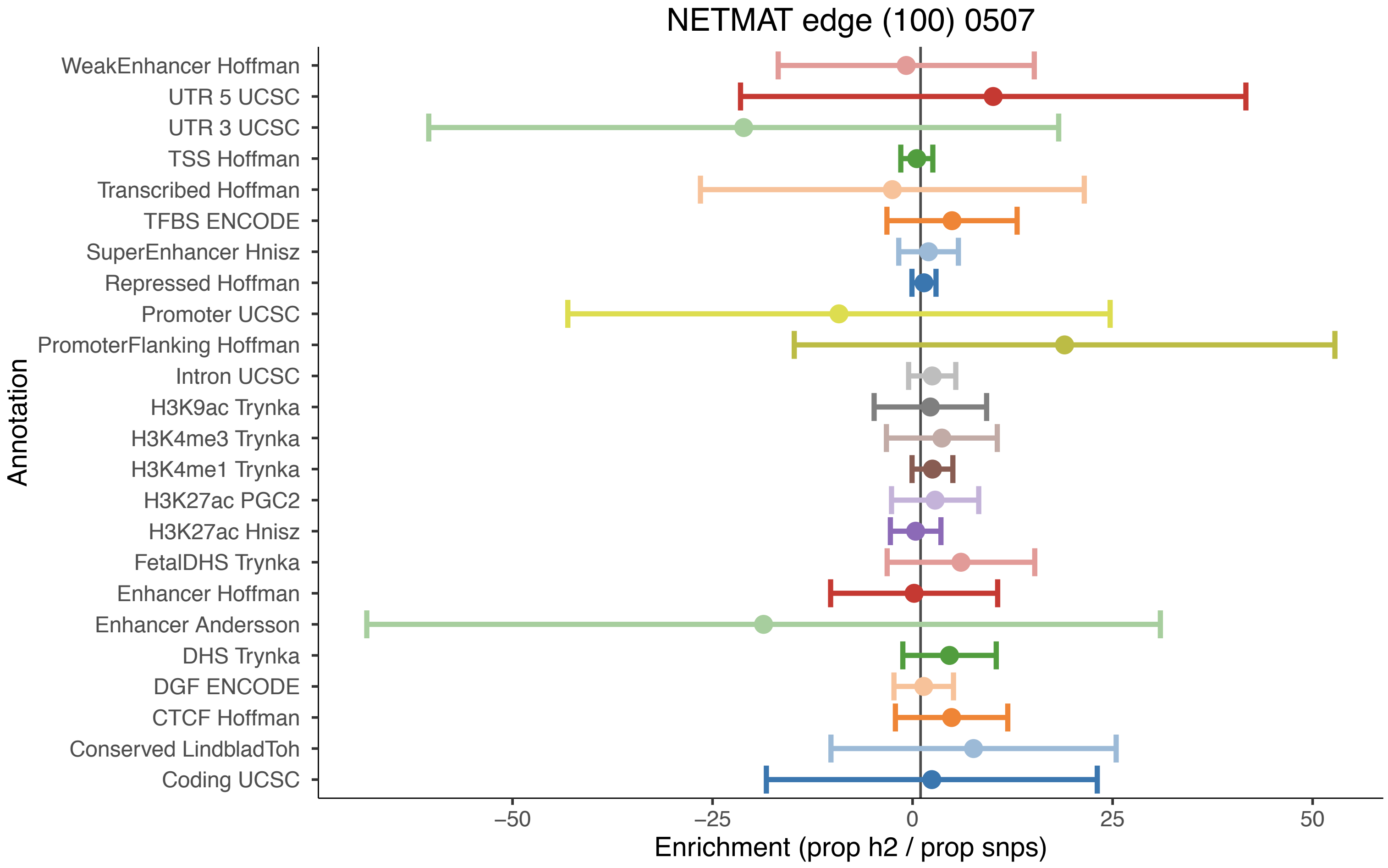

NETMAT edge (100) 0507
WeakEnhancer Hoffman
UTR 5 UCSC
UTR 3 UCSC
TSS Hoffman
Transcribed Hoffman
TFBS ENCODE
SuperEnhancer Hnisz
Repressed Hoffman
Promoter UCSC
PromoterFlanking Hoffman
Intron UCSC
H3K9ac Trynka
H3K4me3 Trynka
H3K4me1 Trynka
H3K27ac PGC2
H3K27ac Hnisz
FetalDHS Trynka
Enhancer Hoffman
Enhancer Andersson
DHS Trynka
DGF ENCODE
CTCF Hoffman
Conserved LindbladToh
Coding UCSC
Annotation
−50
−25
0
25
50
Enrichment (prop h2 / prop snps)

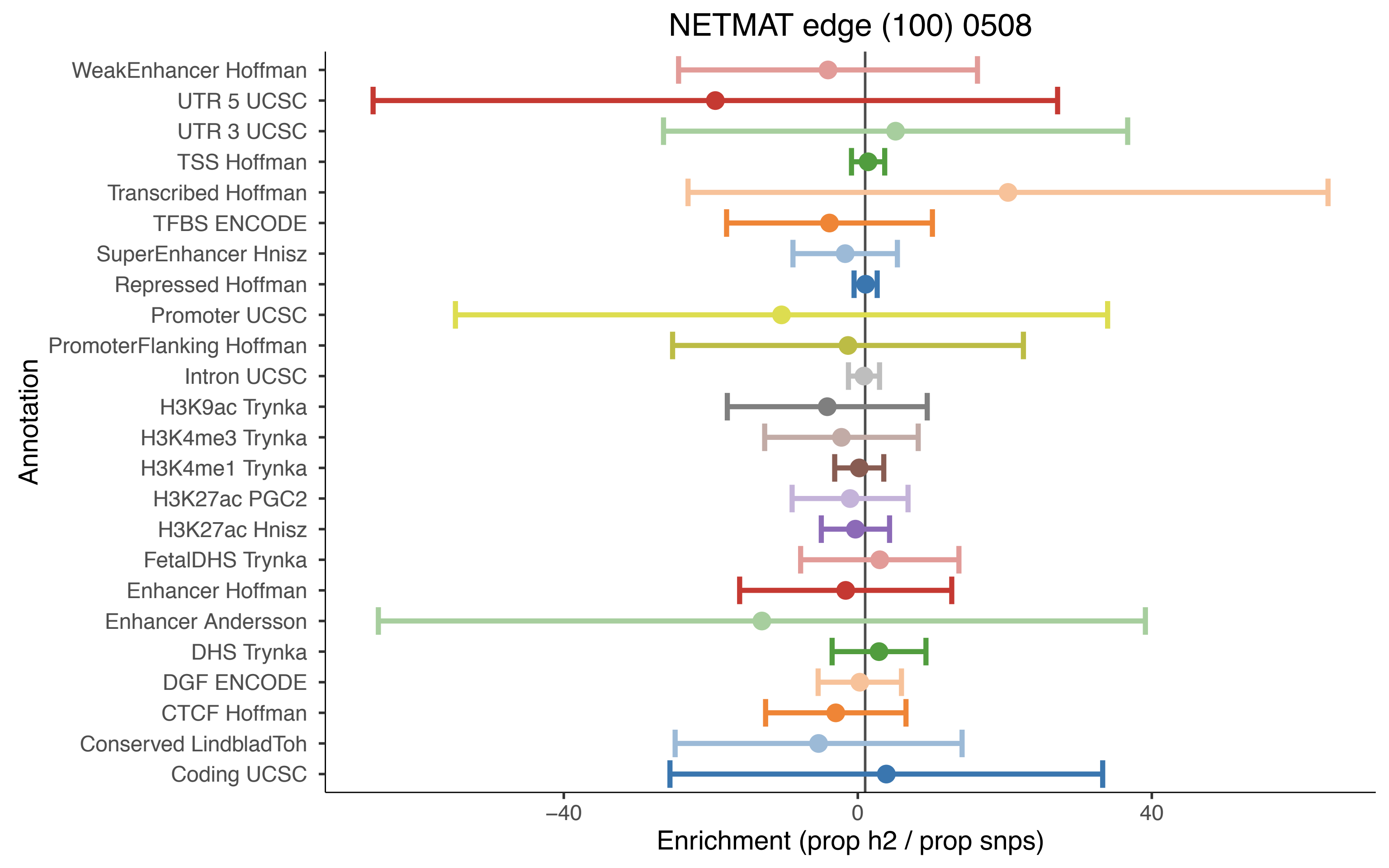
NETMAT edge (100) 0508
WeakEnhancer Hoffman
UTR 5 UCSC
UTR 3 UCSC
TSS Hoffman
Transcribed Hoffman
TFBS ENCODE
SuperEnhancer Hnisz
Repressed Hoffman
Promoter UCSC
PromoterFlanking Hoffman
Intron UCSC
H3K9ac Trynka
H3K4me3 Trynka
H3K4me1 Trynka
H3K27ac PGC2
H3K27ac Hnisz
FetalDHS Trynka
Enhancer Hoffman
Enhancer Andersson
DHS Trynka
DGF ENCODE
CTCF Hoffman
Conserved LindbladToh
Coding UCSC
Annotation
−40
0
40
Enrichment (prop h2 / prop snps)

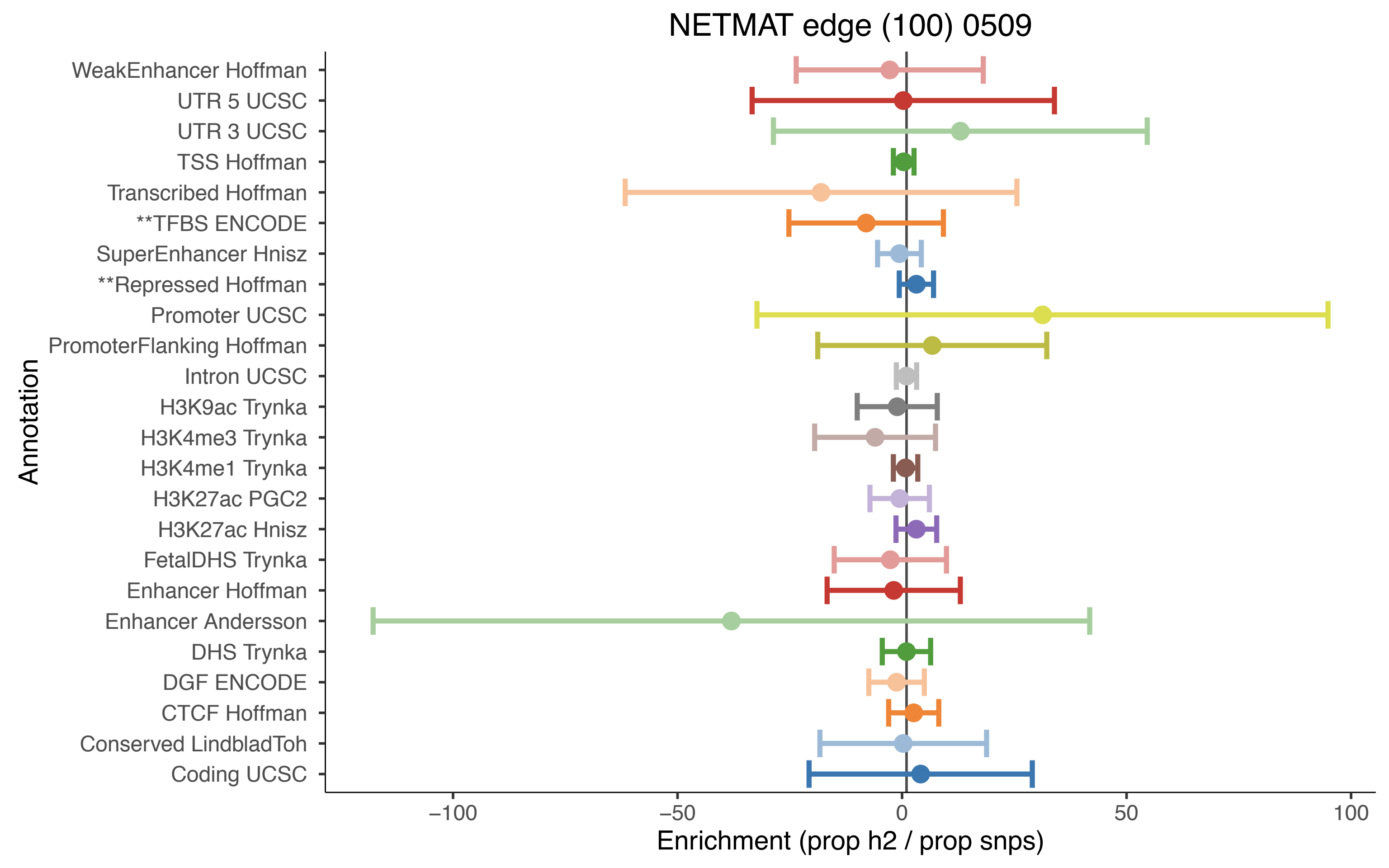
NETMAT edge (100) 0509
Annotation
WeakEnhancer Hoffman
UTR 5 UCSC
UTR 3 UCSC
TSS Hoffman
Transcribed Hoffman
**TFBS ENCODE
SuperEnhancer Hnisz
**Repressed Hoffman
Promoter UCSC
PromoterFlanking Hoffman
Intron UCSC
H3K9ac Trynka
H3K4me3 Trynka
H3K4me1 Trynka
H3K27ac PGC2
H3K27ac Hnisz
FetalDHS Trynka
Enhancer Hoffman
Enhancer Andersson
DHS Trynka
DGF ENCODE
CTCF Hoffman
Conserved LindbladToh
Coding UCSC
−100
−50
0
50
100
Enrichment (prop h2 / prop snps)

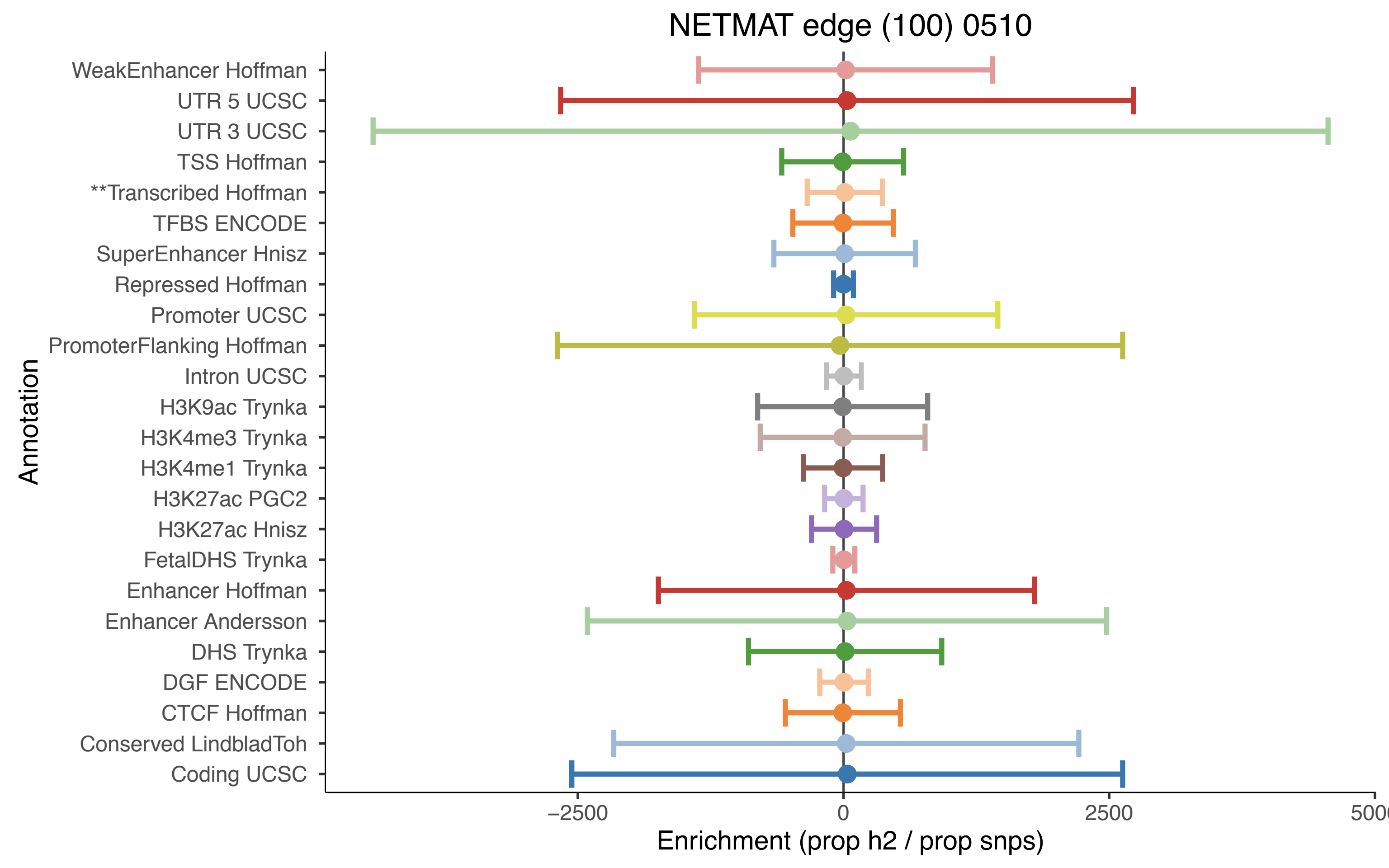

NETMAT edge (100) 0510
WeakEnhancer Hoffman
UTR 5 UCSC
UTR 3 UCSC
TSS Hoffman
**Transcribed Hoffman
TFBS ENCODE
SuperEnhancer Hnisz
Repressed Hoffman
Promoter UCSC
PromoterFlanking Hoffman
Intron UCSC
H3K9ac Trynka
H3K4me3 Trynka
H3K4me1 Trynka
H3K27ac PGC2
H3K27ac Hnisz
FetalDHS Trynka
Enhancer Hoffman
Enhancer Andersson
DHS Trynka
DGF ENCODE
CTCF Hoffman
Conserved LindbladToh
Coding UCSC
Annotation
−2500
0
2500
Enrichment (prop h2 / prop snps)

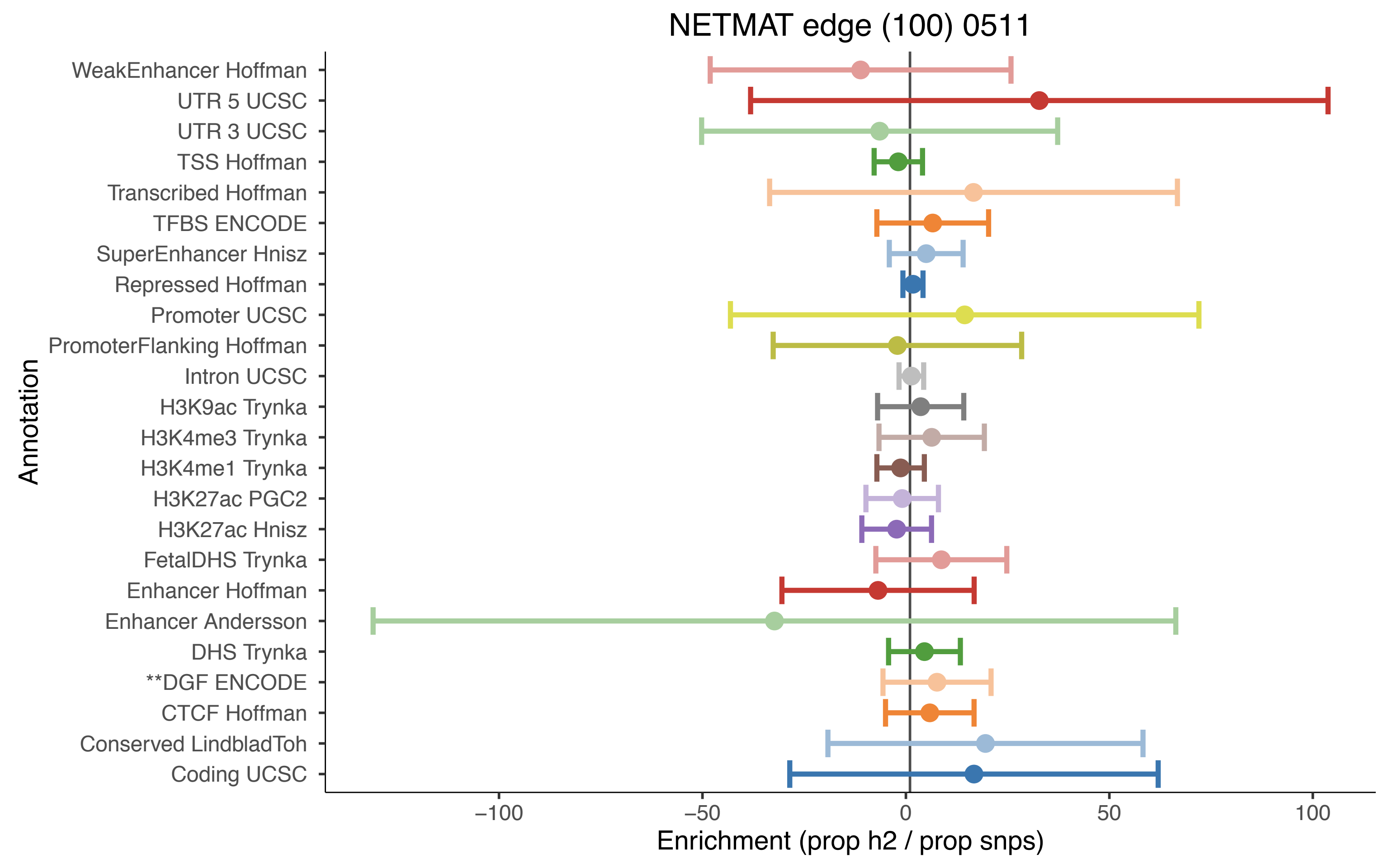
NETMAT edge (100) 0511
Annotation
WeakEnhancer Hoffman
UTR 5 UCSC
UTR 3 UCSC
TSS Hoffman
Transcribed Hoffman
TFBS ENCODE
SuperEnhancer Hnisz
Repressed Hoffman
Promoter UCSC
PromoterFlanking Hoffman
Intron UCSC
H3K9ac Trynka
H3K4me3 Trynka
H3K4me1 Trynka
H3K27ac PGC2
H3K27ac Hnisz
FetalDHS Trynka
Enhancer Hoffman
Enhancer Andersson
DHS Trynka
**DGF ENCODE
CTCF Hoffman
Conserved LindbladToh
Coding UCSC
−100
−50
0
50
100
Enrichment (prop h2 / prop snps)

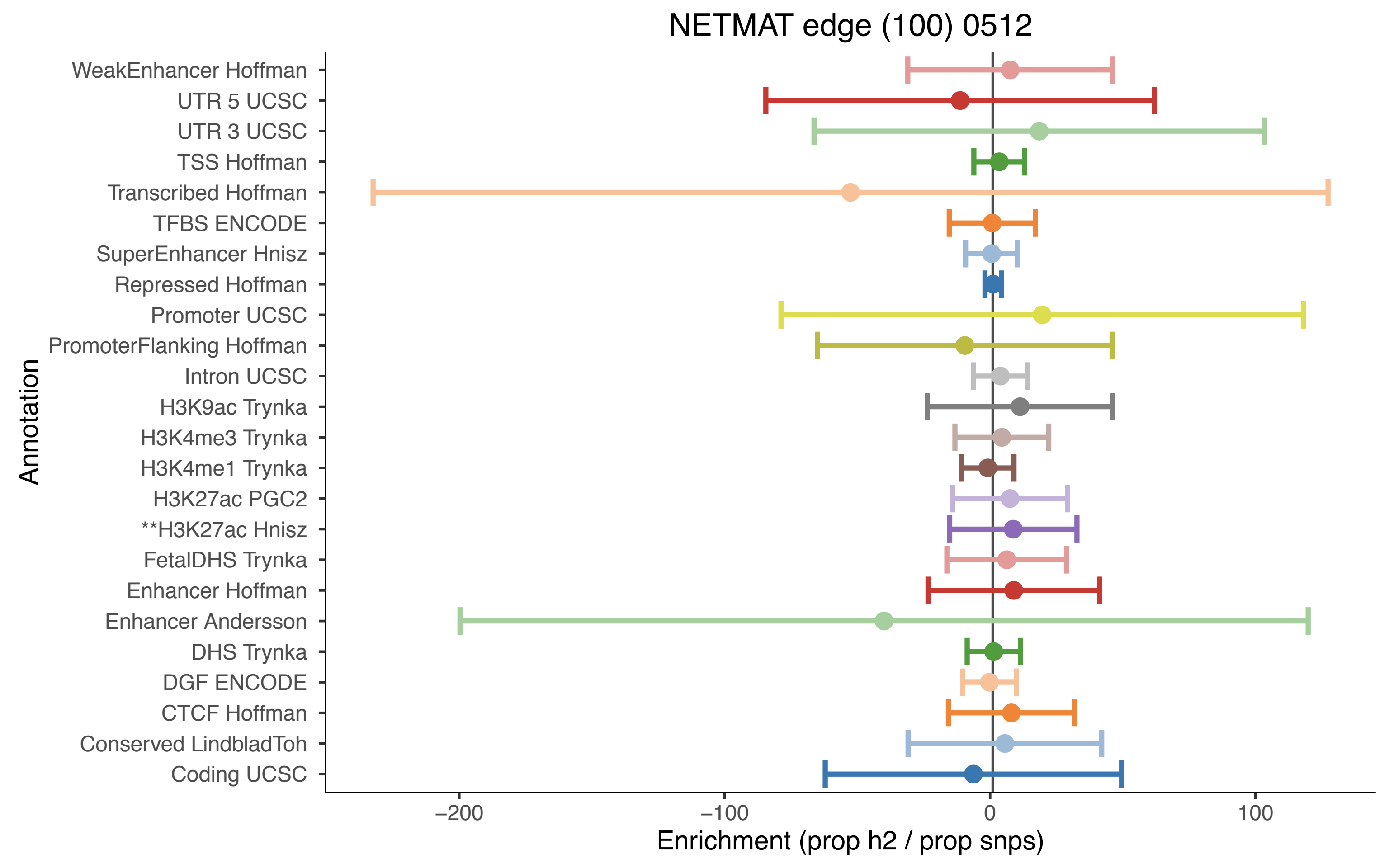

NETMAT edge (100) 0512
WeakEnhancer Hoffman
UTR 5 UCSC
UTR 3 UCSC
TSS Hoffman
Transcribed Hoffman
TFBS ENCODE
SuperEnhancer Hnisz
Repressed Hoffman
Promoter UCSC
PromoterFlanking Hoffman
Intron UCSC
H3K9ac Trynka
H3K4me3 Trynka
H3K4me1 Trynka
H3K27ac PGC2
**H3K27ac Hnisz
FetalDHS Trynka
Enhancer Hoffman
Enhancer Andersson
DHS Trynka
DGF ENCODE
CTCF Hoffman
Conserved LindbladToh
Coding UCSC
Annotation
−200
−100
0
100
Enrichment (prop h2 / prop snps)

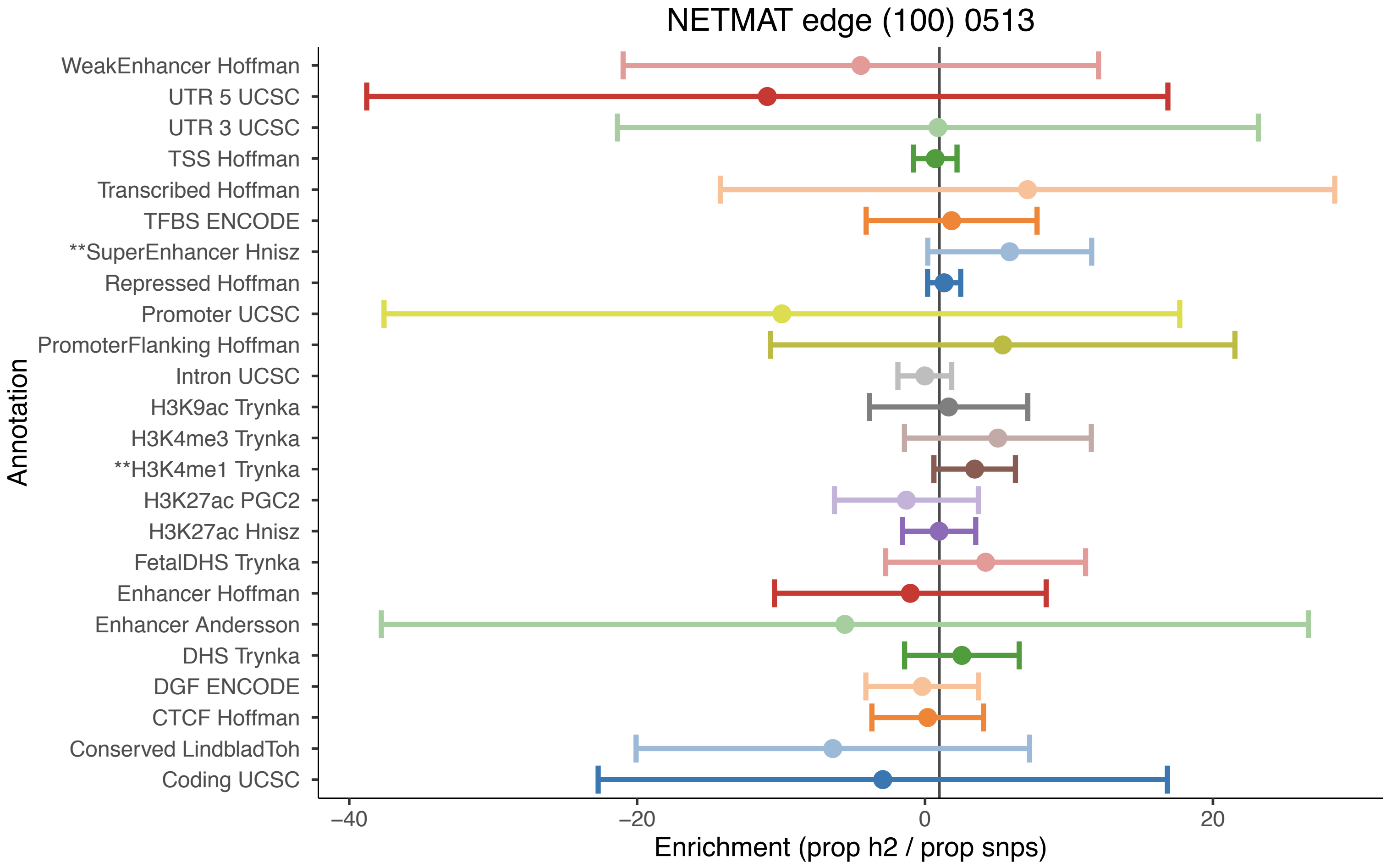
NETMAT edge (100) 0513
WeakEnhancer Hoffman
UTR 5 UCSC
UTR 3 UCSC
TSS Hoffman
Transcribed Hoffman
TFBS ENCODE
**SuperEnhancer Hnisz
Repressed Hoffman
Promoter UCSC
PromoterFlanking Hoffman
Intron UCSC
H3K9ac Trynka
H3K4me3 Trynka
**H3K4me1 Trynka
H3K27ac PGC2
H3K27ac Hnisz
FetalDHS Trynka
Enhancer Hoffman
Enhancer Andersson
DHS Trynka
DGF ENCODE
CTCF Hoffman
Conserved LindbladToh
Coding UCSC
Annotation
−40
−20
0
20
Enrichment (prop h2 / prop snps)

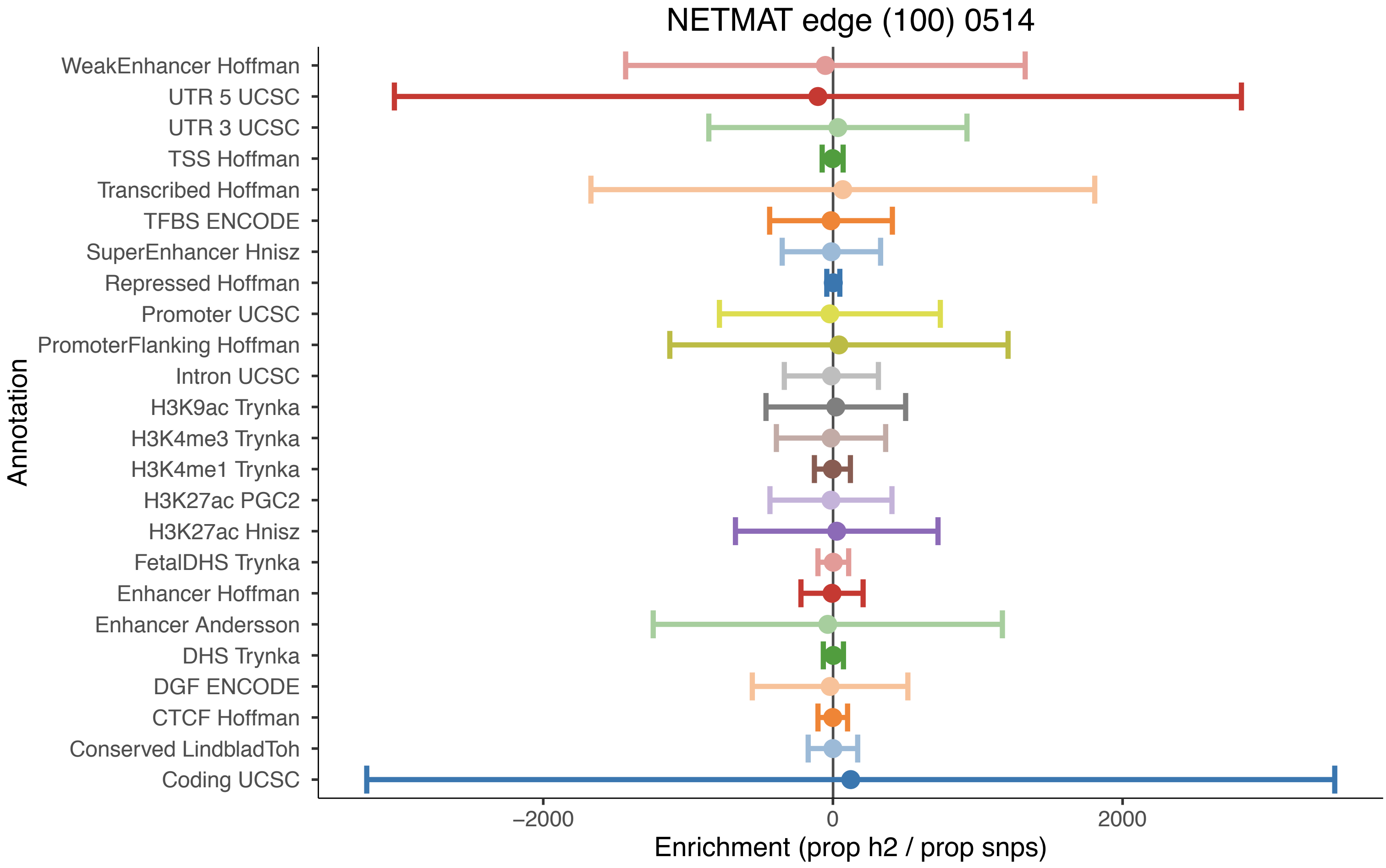
NETMAT edge (100) 0514
Annotation
WeakEnhancer Hoffman
UTR 5 UCSC
UTR 3 UCSC
TSS Hoffman
Transcribed Hoffman
TFBS ENCODE
SuperEnhancer Hnisz
Repressed Hoffman
Promoter UCSC
PromoterFlanking Hoffman
Intron UCSC
H3K9ac Trynka
H3K4me3 Trynka
H3K4me1 Trynka
H3K27ac PGC2
H3K27ac Hnisz
FetalDHS Trynka
Enhancer Hoffman
Enhancer Andersson
DHS Trynka
DGF ENCODE
CTCF Hoffman
Conserved LindbladToh
Coding UCSC
−2000
0
2000
Enrichment (prop h2 / prop snps)

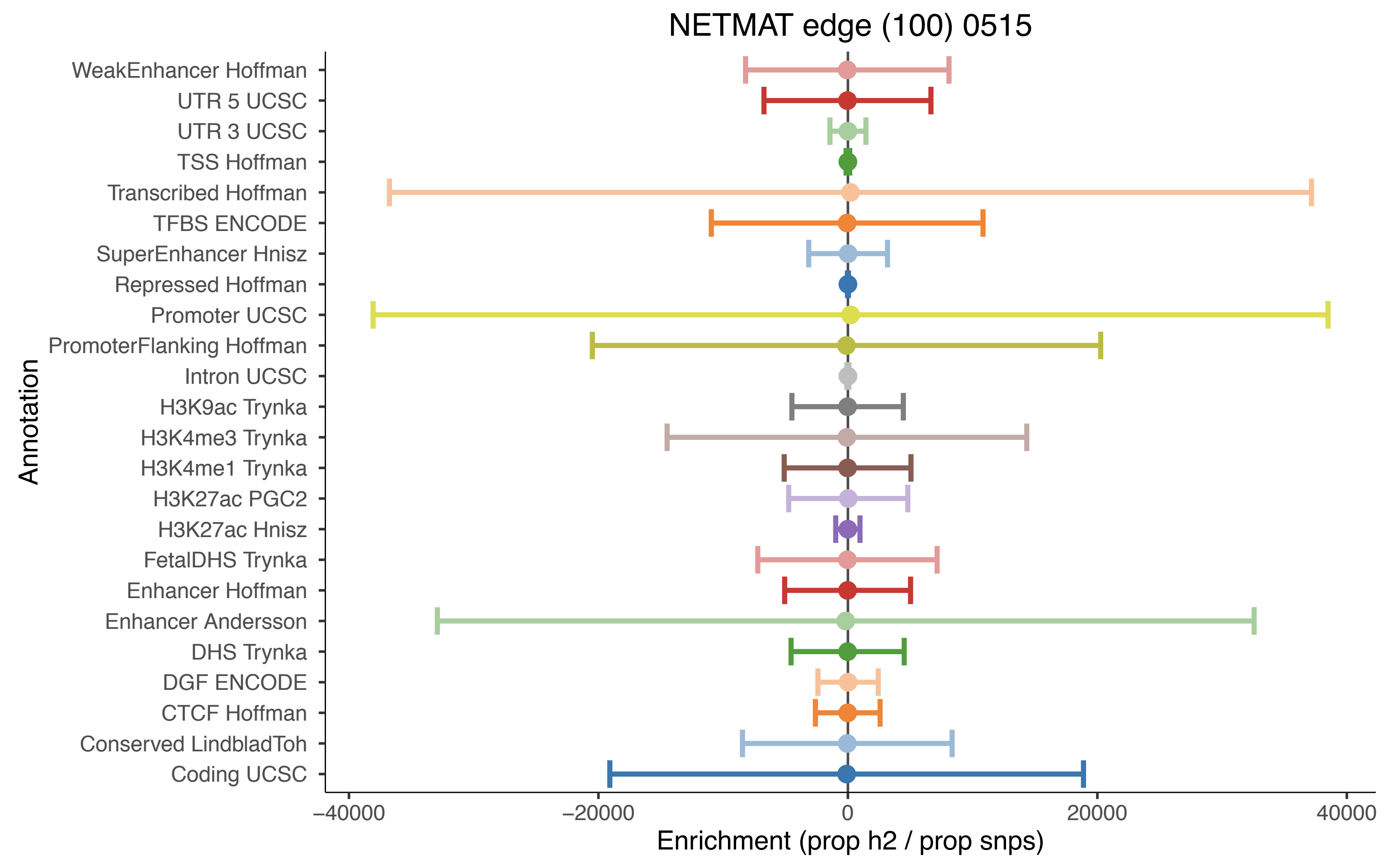

NETMAT edge (100) 0515
Annotation
WeakEnhancer Hoffman
UTR 5 UCSC
UTR 3 UCSC
TSS Hoffman
Transcribed Hoffman
TFBS ENCODE
SuperEnhancer Hnisz
Repressed Hoffman
Promoter UCSC
PromoterFlanking Hoffman
Intron UCSC
H3K9ac Trynka
H3K4me3 Trynka
H3K4me1 Trynka
H3K27ac PGC2
H3K27ac Hnisz
FetalDHS Trynka
Enhancer Hoffman
Enhancer Andersson
DHS Trynka
DGF ENCODE
CTCF Hoffman
Conserved LindbladToh
Coding UCSC
−40000
−20000
0
20000
40000
Enrichment (prop h2 / prop snps)

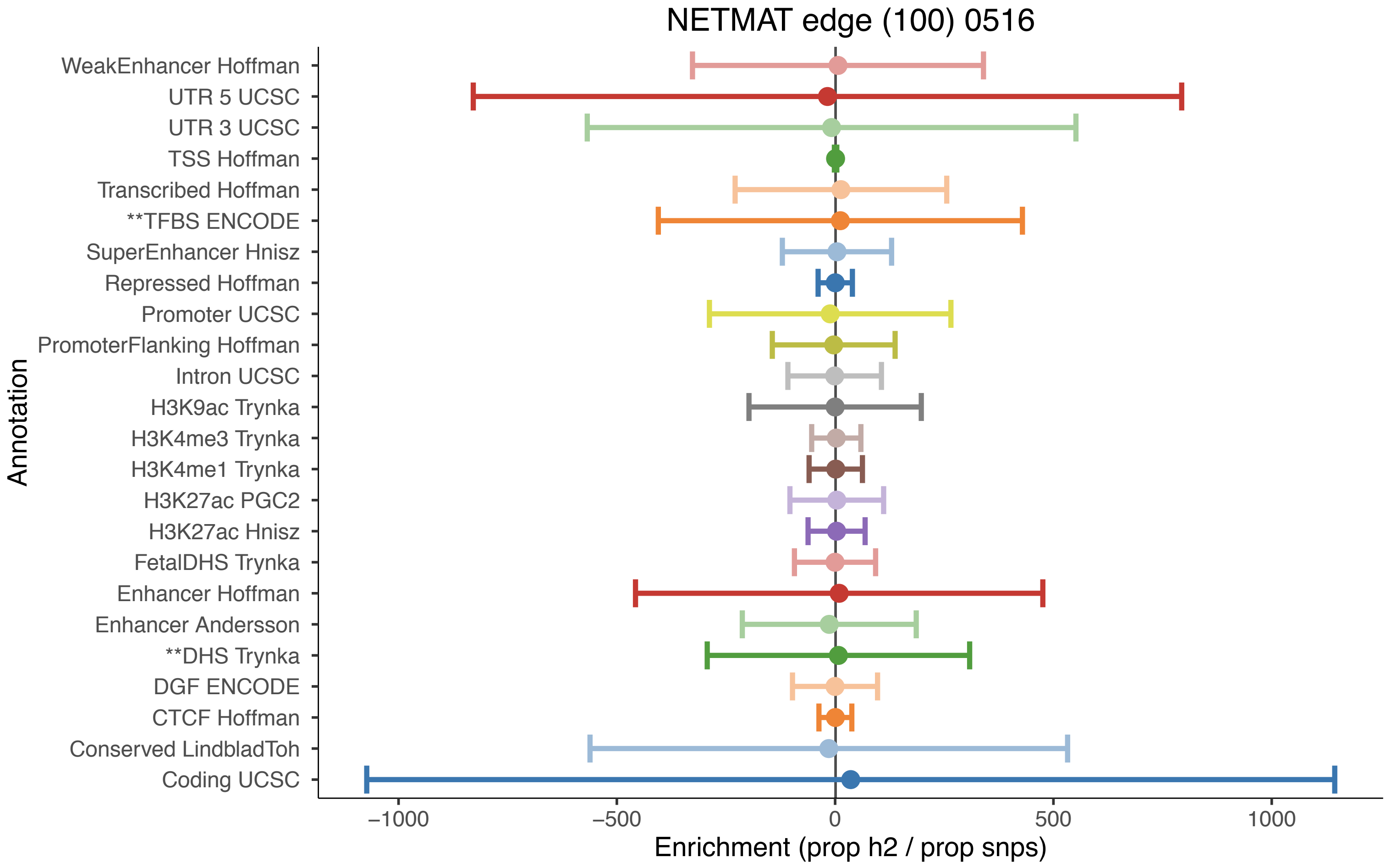

NETMAT edge (100) 0516
WeakEnhancer Hoffman
UTR 5 UCSC
UTR 3 UCSC
TSS Hoffman
Transcribed Hoffman
**TFBS ENCODE
SuperEnhancer Hnisz
Repressed Hoffman
Promoter UCSC
PromoterFlanking Hoffman
Intron UCSC
H3K9ac Trynka
H3K4me3 Trynka
H3K4me1 Trynka
H3K27ac PGC2
H3K27ac Hnisz
FetalDHS Trynka
Enhancer Hoffman
Enhancer Andersson
**DHS Trynka
DGF ENCODE
CTCF Hoffman
Conserved LindbladToh
Coding UCSC
Annotation
−1000
−500
0
500
1000
Enrichment (prop h2 / prop snps)

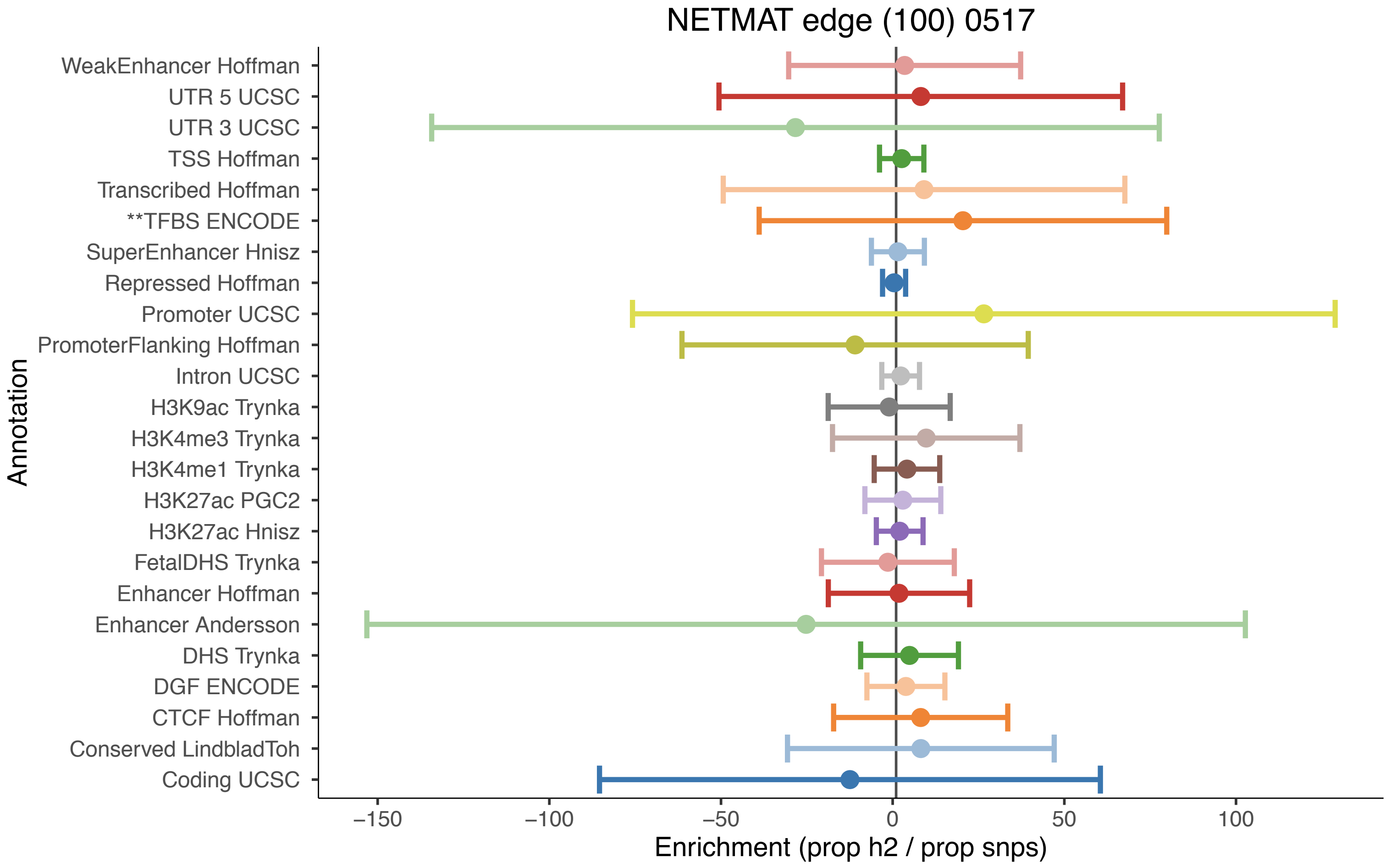

NETMAT edge (100) 0517
WeakEnhancer Hoffman
UTR 5 UCSC
UTR 3 UCSC
TSS Hoffman
Transcribed Hoffman
**TFBS ENCODE
SuperEnhancer Hnisz
Repressed Hoffman
Promoter UCSC
PromoterFlanking Hoffman
Intron UCSC
H3K9ac Trynka
H3K4me3 Trynka
H3K4me1 Trynka
H3K27ac PGC2
H3K27ac Hnisz
FetalDHS Trynka
Enhancer Hoffman
Enhancer Andersson
DHS Trynka
DGF ENCODE
CTCF Hoffman
Conserved LindbladToh
Coding UCSC
Annotation
−150
−100
−50
0
50
100
Enrichment (prop h2 / prop snps)

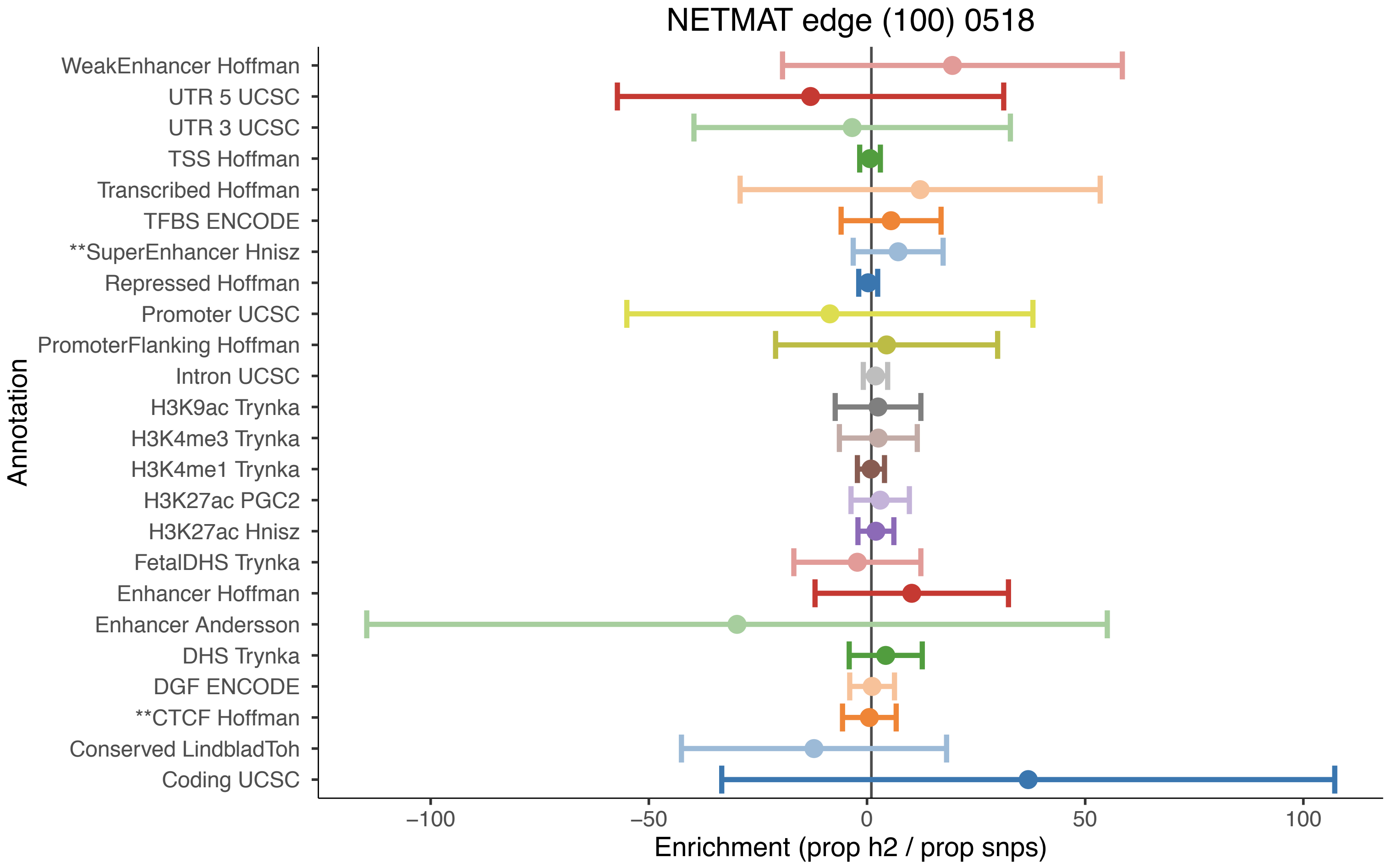
NETMAT edge (100) 0518
WeakEnhancer Hoffman
UTR 5 UCSC
UTR 3 UCSC
TSS Hoffman
Transcribed Hoffman
TFBS ENCODE
**SuperEnhancer Hnisz
Repressed Hoffman
Promoter UCSC
PromoterFlanking Hoffman
Intron UCSC
H3K9ac Trynka
H3K4me3 Trynka
H3K4me1 Trynka
H3K27ac PGC2
H3K27ac Hnisz
FetalDHS Trynka
Enhancer Hoffman
Enhancer Andersson
DHS Trynka
DGF ENCODE
**CTCF Hoffman
Conserved LindbladToh
Coding UCSC
Annotation
−100
−50
0
50
100
Enrichment (prop h2 / prop snps)

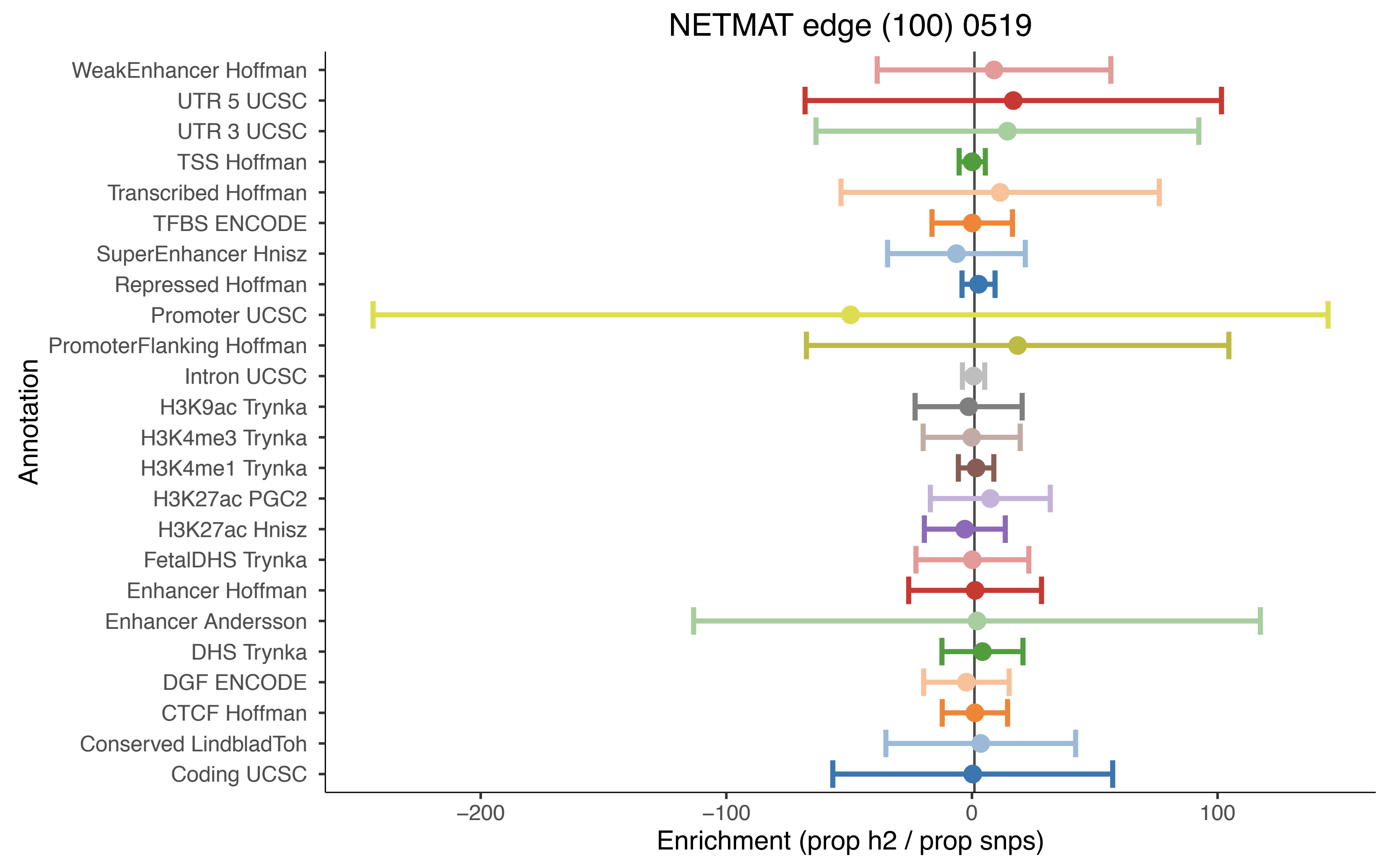
NETMAT edge (100) 0519
Annotation
WeakEnhancer Hoffman
UTR 5 UCSC
UTR 3 UCSC
TSS Hoffman
Transcribed Hoffman
TFBS ENCODE
SuperEnhancer Hnisz
Repressed Hoffman
Promoter UCSC
PromoterFlanking Hoffman
Intron UCSC
H3K9ac Trynka
H3K4me3 Trynka
H3K4me1 Trynka
H3K27ac PGC2
H3K27ac Hnisz
FetalDHS Trynka
Enhancer Hoffman
Enhancer Andersson
DHS Trynka
DGF ENCODE
CTCF Hoffman
Conserved LindbladToh
Coding UCSC
−200
−100
0
100
Enrichment (prop h2 / prop snps)

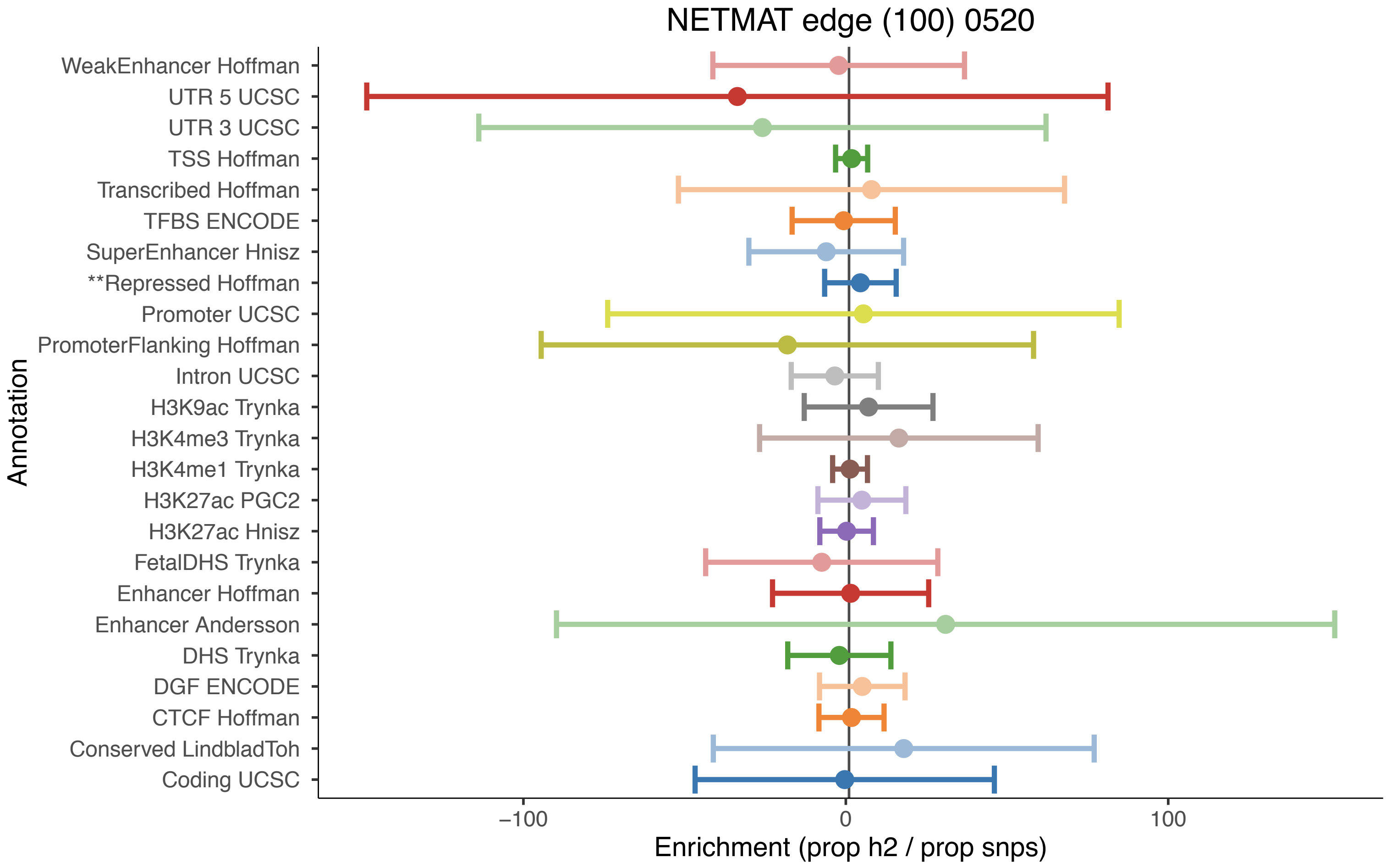
NETMAT edge (100) 0520
WeakEnhancer Hoffman
UTR 5 UCSC
UTR 3 UCSC
TSS Hoffman
Transcribed Hoffman
TFBS ENCODE
SuperEnhancer Hnisz
**Repressed Hoffman
Promoter UCSC
PromoterFlanking Hoffman
Intron UCSC
H3K9ac Trynka
H3K4me3 Trynka
H3K4me1 Trynka
H3K27ac PGC2
H3K27ac Hnisz
FetalDHS Trynka
Enhancer Hoffman
Enhancer Andersson
DHS Trynka
DGF ENCODE
CTCF Hoffman
Conserved LindbladToh
Coding UCSC
Annotation
−100
0
100
Enrichment (prop h2 / prop snps)

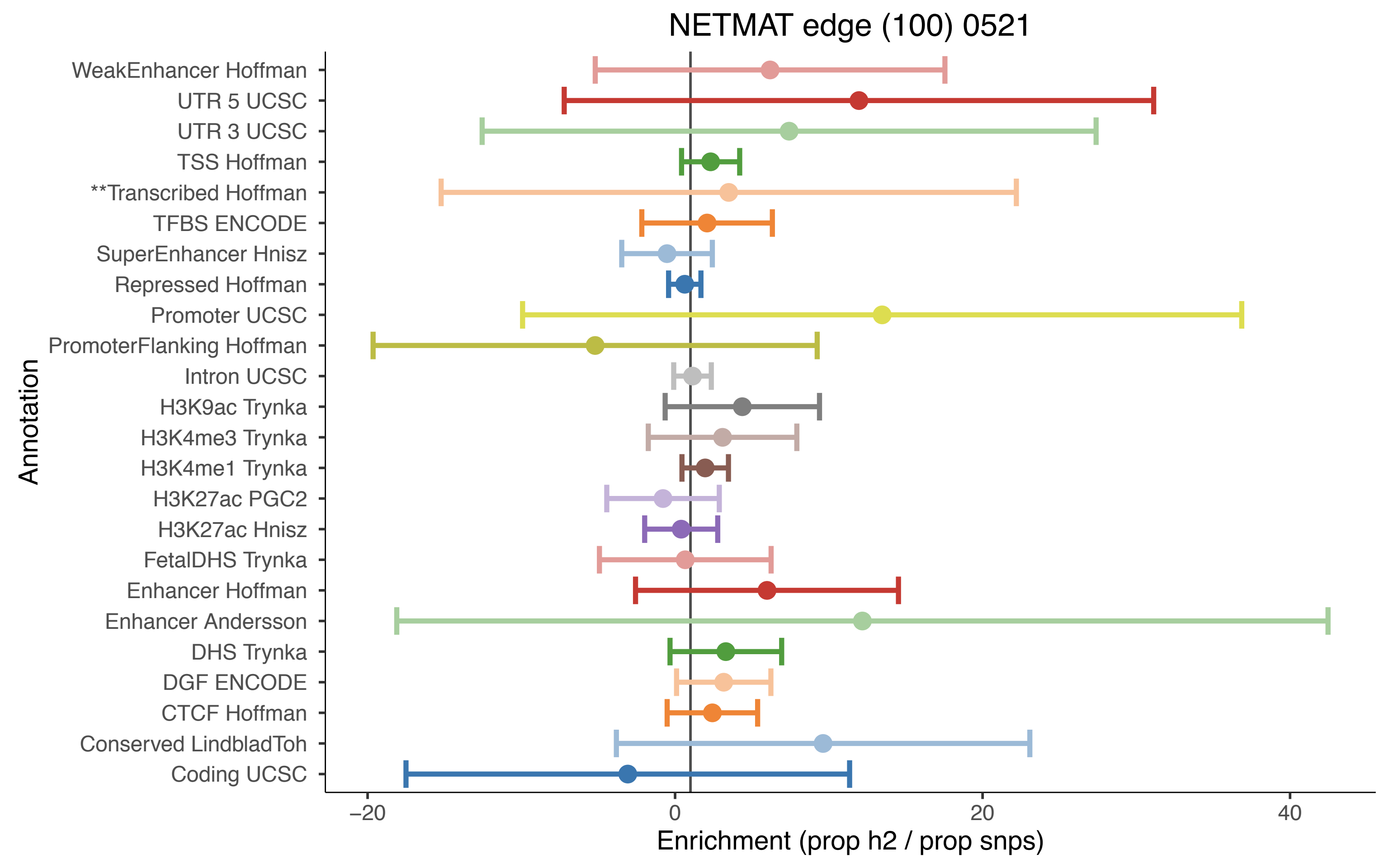
NETMAT edge (100) 0521
WeakEnhancer Hoffman
UTR 5 UCSC
UTR 3 UCSC
TSS Hoffman
**Transcribed Hoffman
TFBS ENCODE
SuperEnhancer Hnisz
Repressed Hoffman
Promoter UCSC
PromoterFlanking Hoffman
Intron UCSC
H3K9ac Trynka
H3K4me3 Trynka
H3K4me1 Trynka
H3K27ac PGC2
H3K27ac Hnisz
FetalDHS Trynka
Enhancer Hoffman
Enhancer Andersson
DHS Trynka
DGF ENCODE
CTCF Hoffman
Conserved LindbladToh
Coding UCSC
Annotation
−20
0
20
40
Enrichment (prop h2 / prop snps)

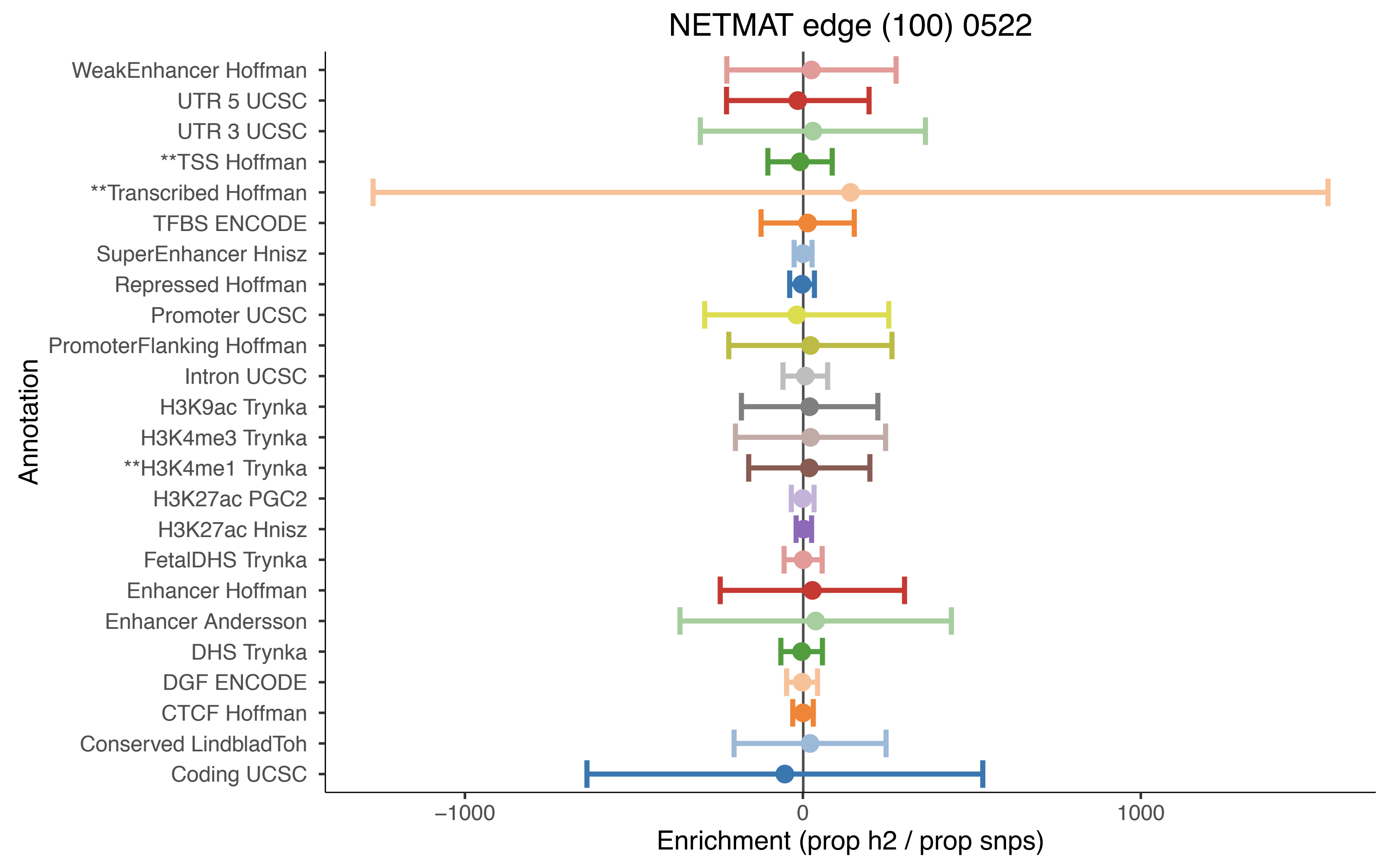
NETMAT edge (100) 0522
WeakEnhancer Hoffman
UTR 5 UCSC
UTR 3 UCSC
**TSS Hoffman
**Transcribed Hoffman
TFBS ENCODE
SuperEnhancer Hnisz
Repressed Hoffman
Promoter UCSC
PromoterFlanking Hoffman
Intron UCSC
H3K9ac Trynka
H3K4me3 Trynka
**H3K4me1 Trynka
H3K27ac PGC2
H3K27ac Hnisz
FetalDHS Trynka
Enhancer Hoffman
Enhancer Andersson
DHS Trynka
DGF ENCODE
CTCF Hoffman
Conserved LindbladToh
Coding UCSC
Annotation
−1000
0
1000
Enrichment (prop h2 / prop snps)

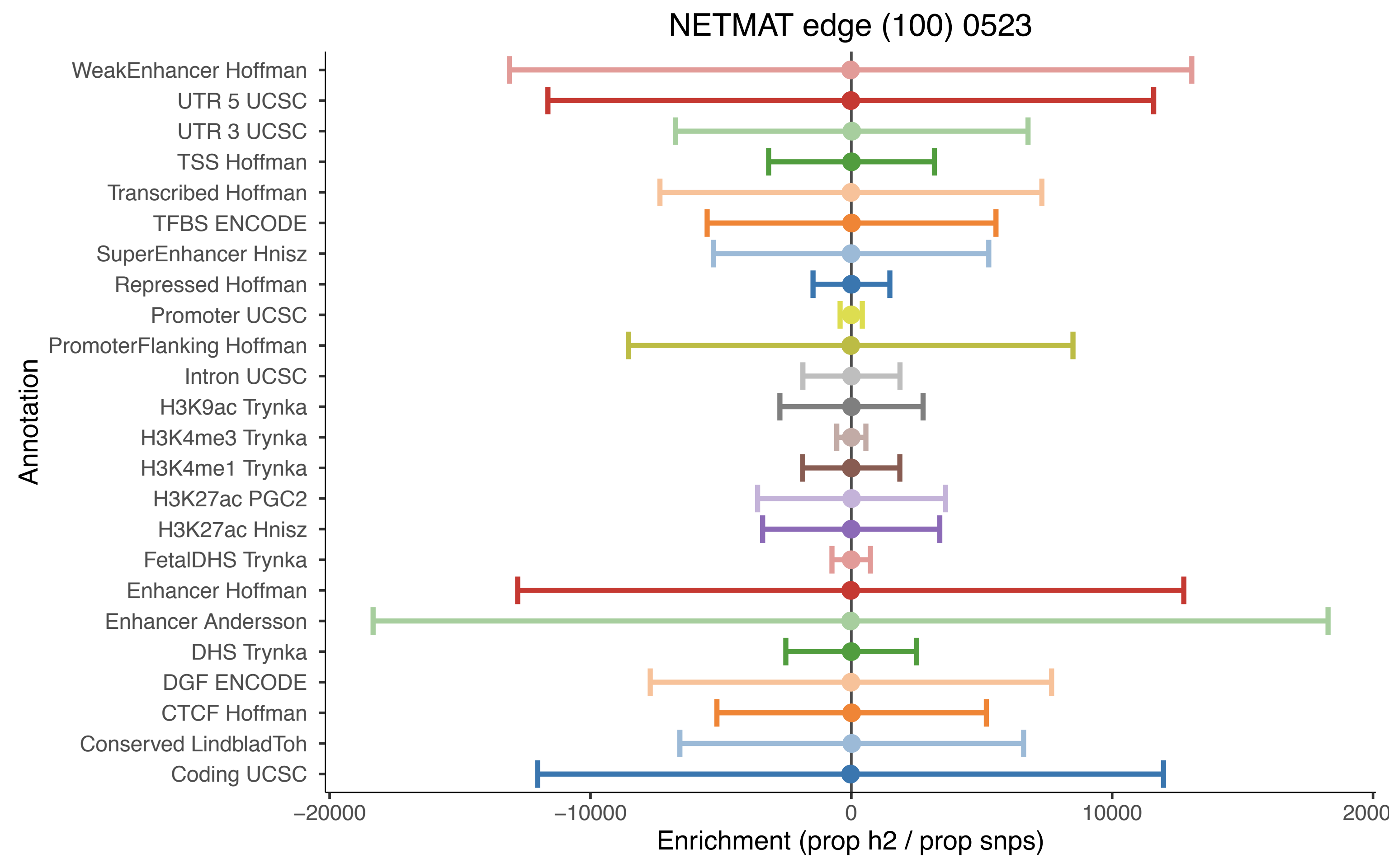

NETMAT edge (100) 0523
Annotation
WeakEnhancer Hoffman
UTR 5 UCSC
UTR 3 UCSC
TSS Hoffman
Transcribed Hoffman
TFBS ENCODE
SuperEnhancer Hnisz
Repressed Hoffman
Promoter UCSC
PromoterFlanking Hoffman
Intron UCSC
H3K9ac Trynka
H3K4me3 Trynka
H3K4me1 Trynka
H3K27ac PGC2
H3K27ac Hnisz
FetalDHS Trynka
Enhancer Hoffman
Enhancer Andersson
DHS Trynka
DGF ENCODE
CTCF Hoffman
Conserved LindbladToh
Coding UCSC
−20000
−10000
0
10000
2000
Enrichment (prop h2 / prop snps)

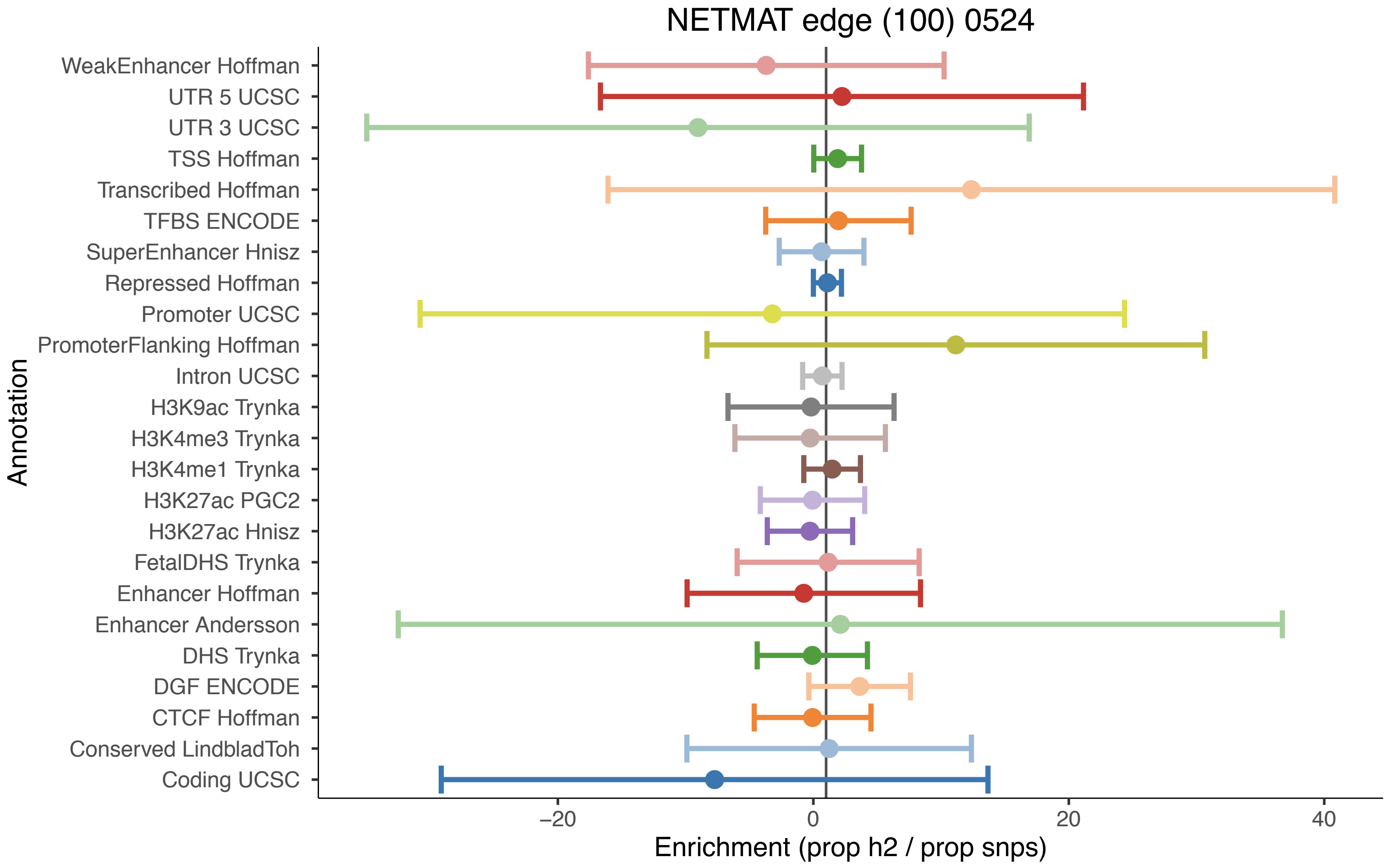

NETMAT edge (100) 0524
Annotation
WeakEnhancer Hoffman
UTR 5 UCSC
UTR 3 UCSC
TSS Hoffman
Transcribed Hoffman
TFBS ENCODE
SuperEnhancer Hnisz
Repressed Hoffman
Promoter UCSC
PromoterFlanking Hoffman
Intron UCSC
H3K9ac Trynka
H3K4me3 Trynka
H3K4me1 Trynka
H3K27ac PGC2
H3K27ac Hnisz
FetalDHS Trynka
Enhancer Hoffman
Enhancer Andersson
DHS Trynka
DGF ENCODE
CTCF Hoffman
Conserved LindbladToh
Coding UCSC
−20
0
20
40
Enrichment (prop h2 / prop snps)

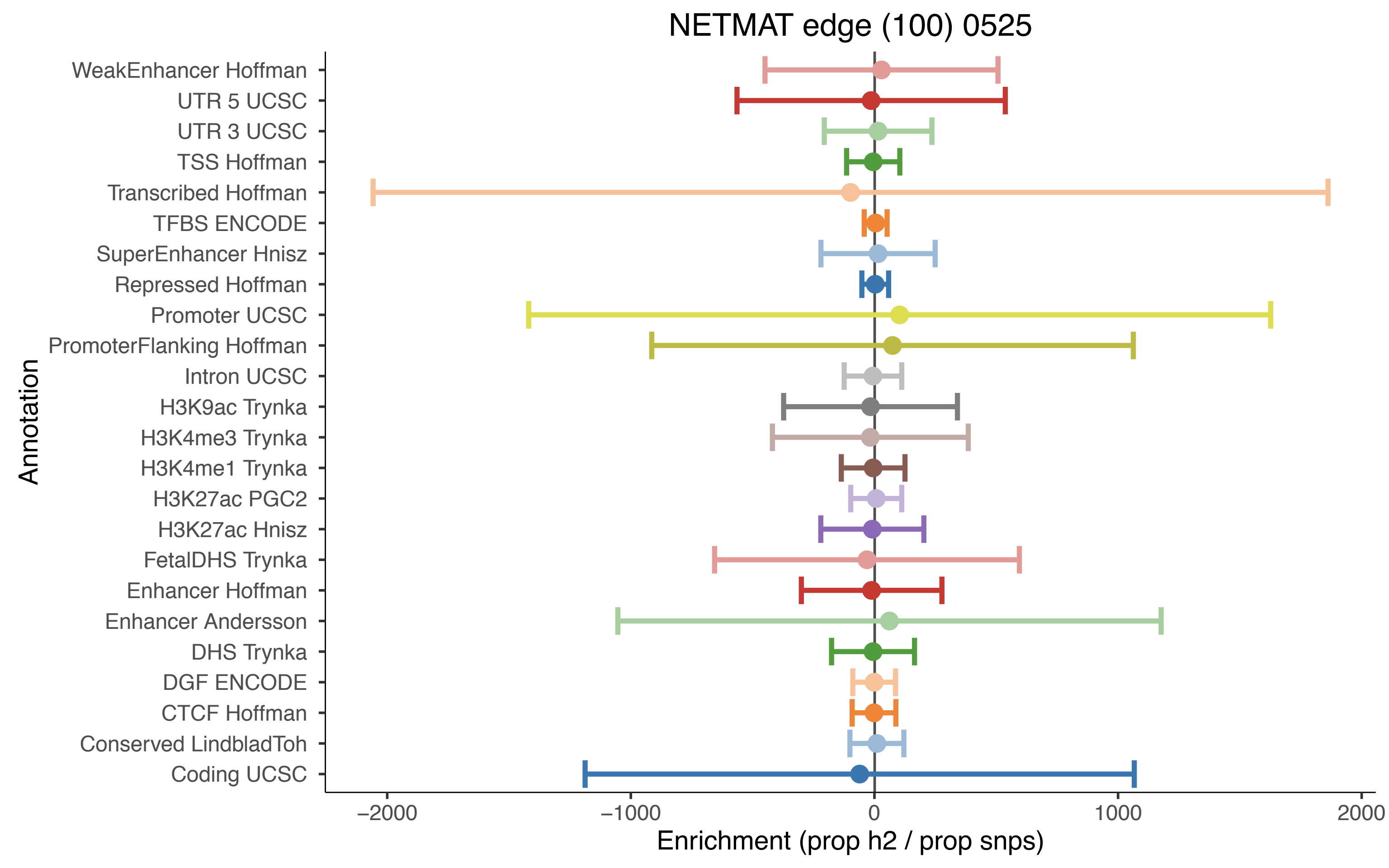

NETMAT edge (100) 0525
Annotation
WeakEnhancer Hoffman
UTR 5 UCSC
UTR 3 UCSC
TSS Hoffman
Transcribed Hoffman
TFBS ENCODE
SuperEnhancer Hnisz
Repressed Hoffman
Promoter UCSC
PromoterFlanking Hoffman
Intron UCSC
H3K9ac Trynka
H3K4me3 Trynka
H3K4me1 Trynka
H3K27ac PGC2
H3K27ac Hnisz
FetalDHS Trynka
Enhancer Hoffman
Enhancer Andersson
DHS Trynka
DGF ENCODE
CTCF Hoffman
Conserved LindbladToh
Coding UCSC
−2000
−1000
0
1000
2000
Enrichment (prop h2 / prop snps)

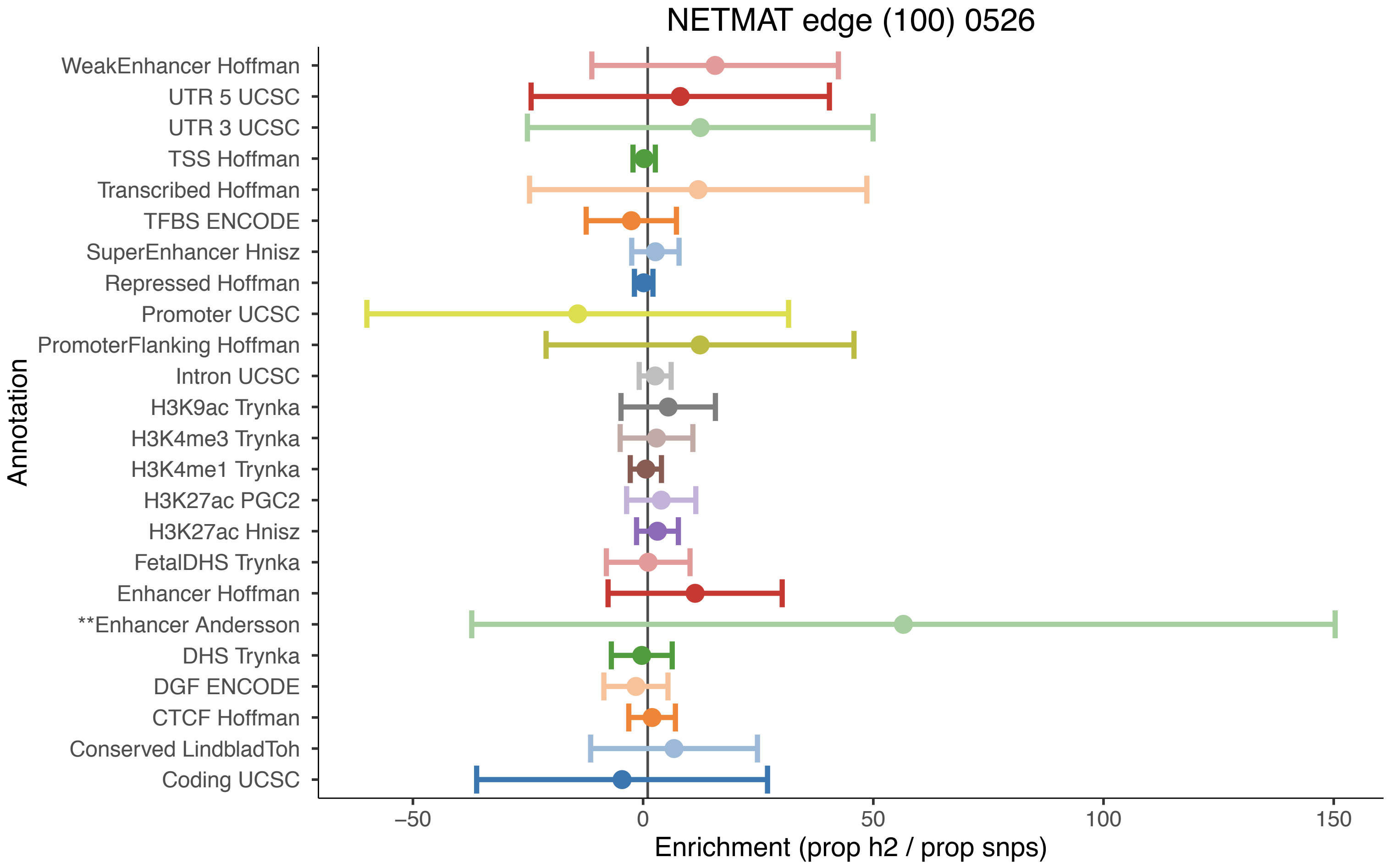
NETMAT edge (100) 0526
Annotation
WeakEnhancer Hoffman
UTR 5 UCSC
UTR 3 UCSC
TSS Hoffman
Transcribed Hoffman
TFBS ENCODE
SuperEnhancer Hnisz
Repressed Hoffman
Promoter UCSC
PromoterFlanking Hoffman
Intron UCSC
H3K9ac Trynka
H3K4me3 Trynka
H3K4me1 Trynka
H3K27ac PGC2
H3K27ac Hnisz
FetalDHS Trynka
Enhancer Hoffman
**Enhancer Andersson
DHS Trynka
DGF ENCODE
CTCF Hoffman
Conserved LindbladToh
Coding UCSC
−50
0
50
100
150
Enrichment (prop h2 / prop snps)

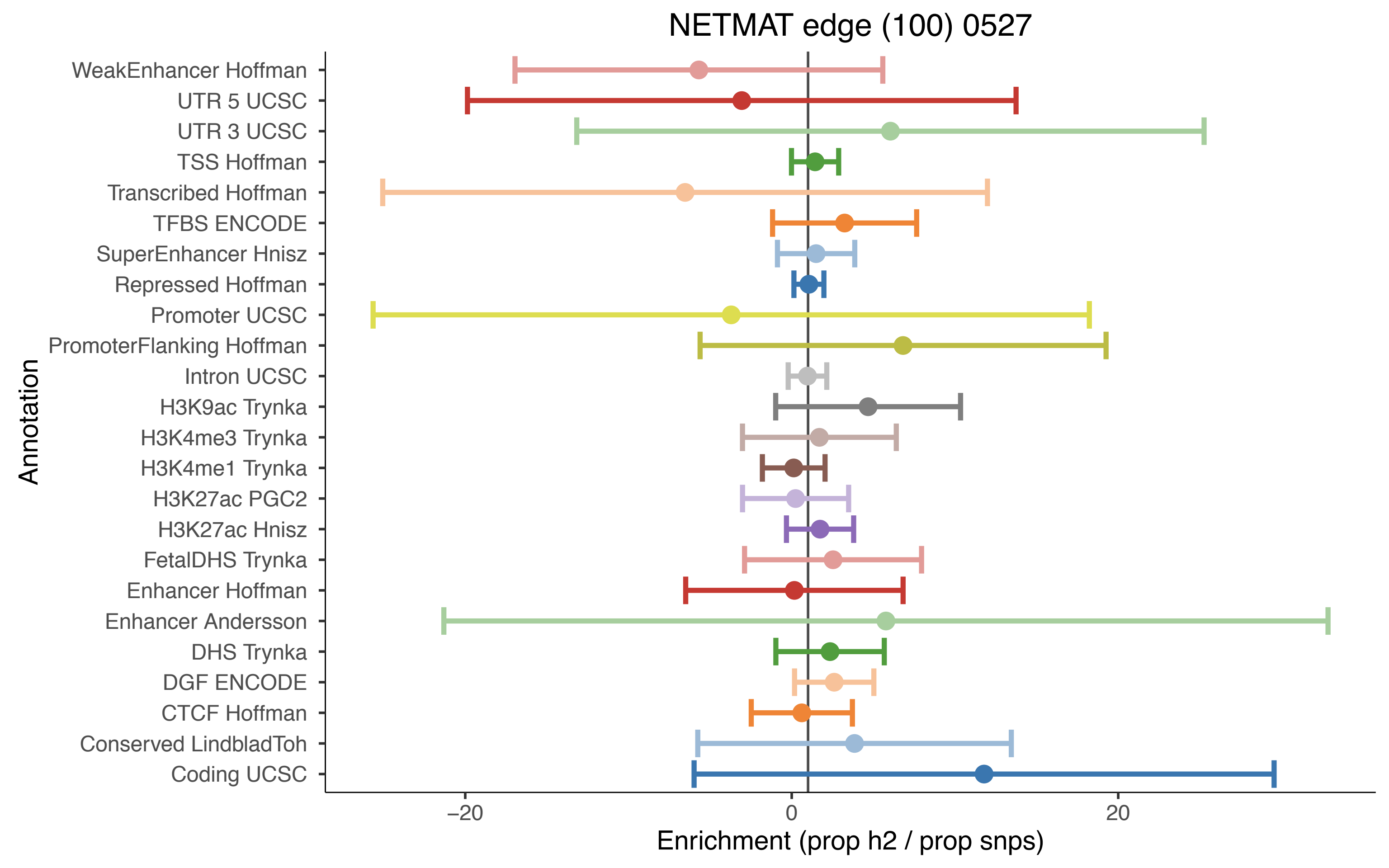

NETMAT edge (100) 0527
WeakEnhancer Hoffman
UTR 5 UCSC
UTR 3 UCSC
TSS Hoffman
Transcribed Hoffman
TFBS ENCODE
SuperEnhancer Hnisz
Repressed Hoffman
Promoter UCSC
PromoterFlanking Hoffman
Intron UCSC
H3K9ac Trynka
H3K4me3 Trynka
H3K4me1 Trynka
H3K27ac PGC2
H3K27ac Hnisz
FetalDHS Trynka
Enhancer Hoffman
Enhancer Andersson
DHS Trynka
DGF ENCODE
CTCF Hoffman
Conserved LindbladToh
Coding UCSC
Annotation
−20
0
20
Enrichment (prop h2 / prop snps)

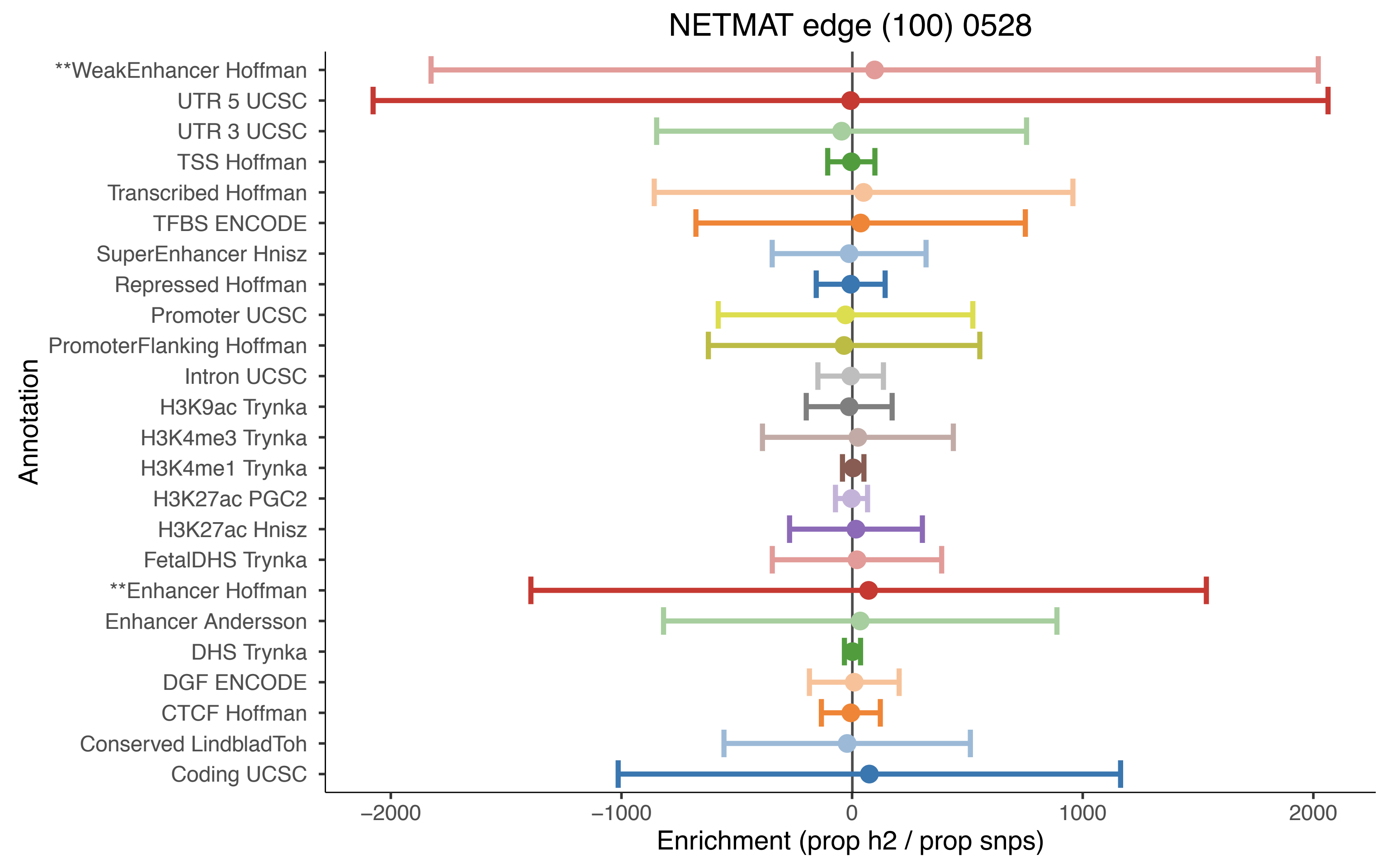
NETMAT edge (100) 0528
**WeakEnhancer Hoffman
UTR 5 UCSC
UTR 3 UCSC
TSS Hoffman
Transcribed Hoffman
TFBS ENCODE
SuperEnhancer Hnisz
Repressed Hoffman
Promoter UCSC
PromoterFlanking Hoffman
Intron UCSC
H3K9ac Trynka
H3K4me3 Trynka
H3K4me1 Trynka
H3K27ac PGC2
H3K27ac Hnisz
FetalDHS Trynka
**Enhancer Hoffman
Enhancer Andersson
DHS Trynka
DGF ENCODE
CTCF Hoffman
Conserved LindbladToh
Coding UCSC
Annotation
−2000
−1000
0
1000
2000
Enrichment (prop h2 / prop snps)

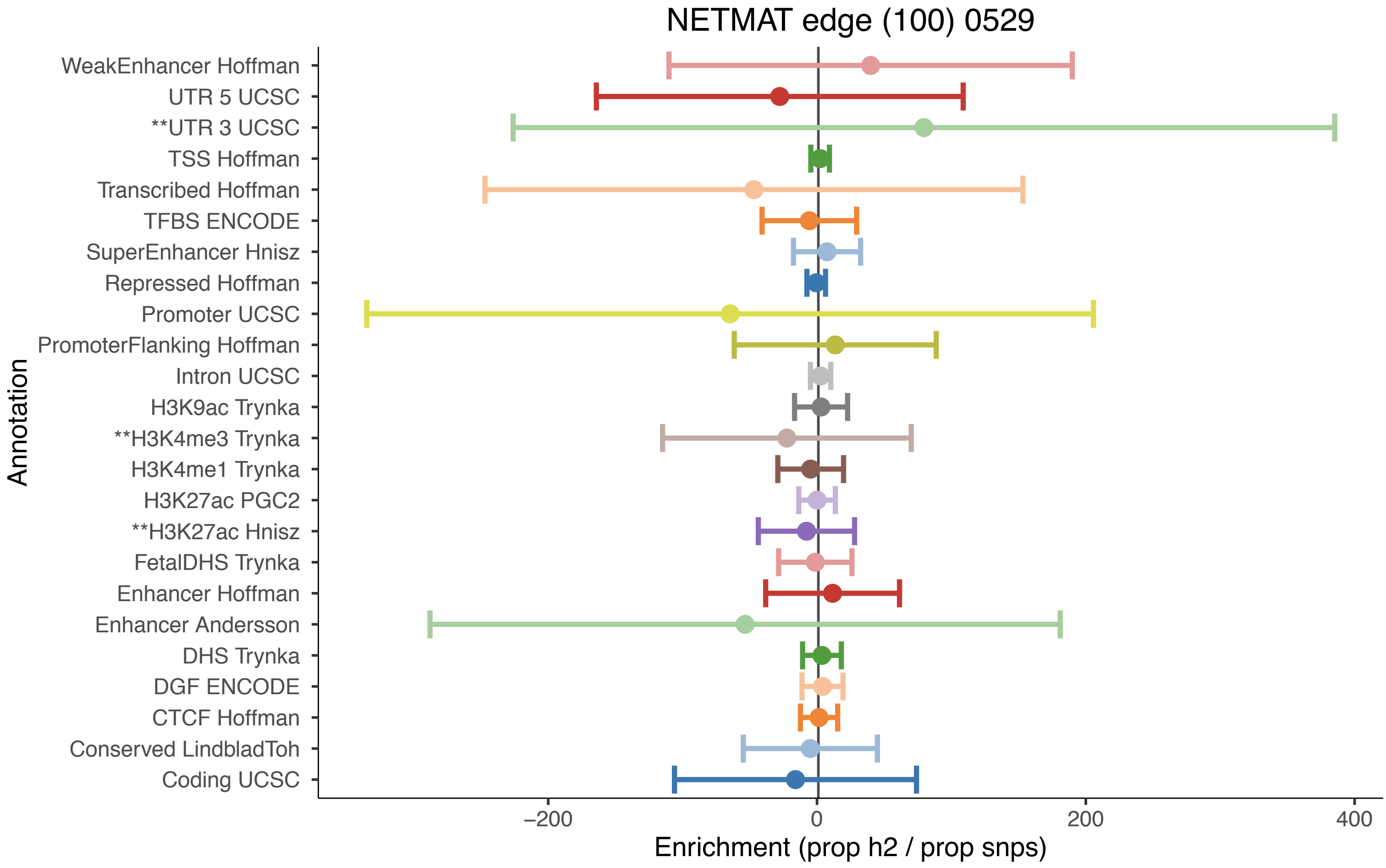
NETMAT edge (100) 0529
WeakEnhancer Hoffman
UTR 5 UCSC
**UTR 3 UCSC
TSS Hoffman
Transcribed Hoffman
TFBS ENCODE
SuperEnhancer Hnisz
Repressed Hoffman
Promoter UCSC
PromoterFlanking Hoffman
Intron UCSC
H3K9ac Trynka
**H3K4me3 Trynka
H3K4me1 Trynka
H3K27ac PGC2
**H3K27ac Hnisz
FetalDHS Trynka
Enhancer Hoffman
Enhancer Andersson
DHS Trynka
DGF ENCODE
CTCF Hoffman
Conserved LindbladToh
Coding UCSC
Annotation
−200
0
200
400
Enrichment (prop h2 / prop snps)

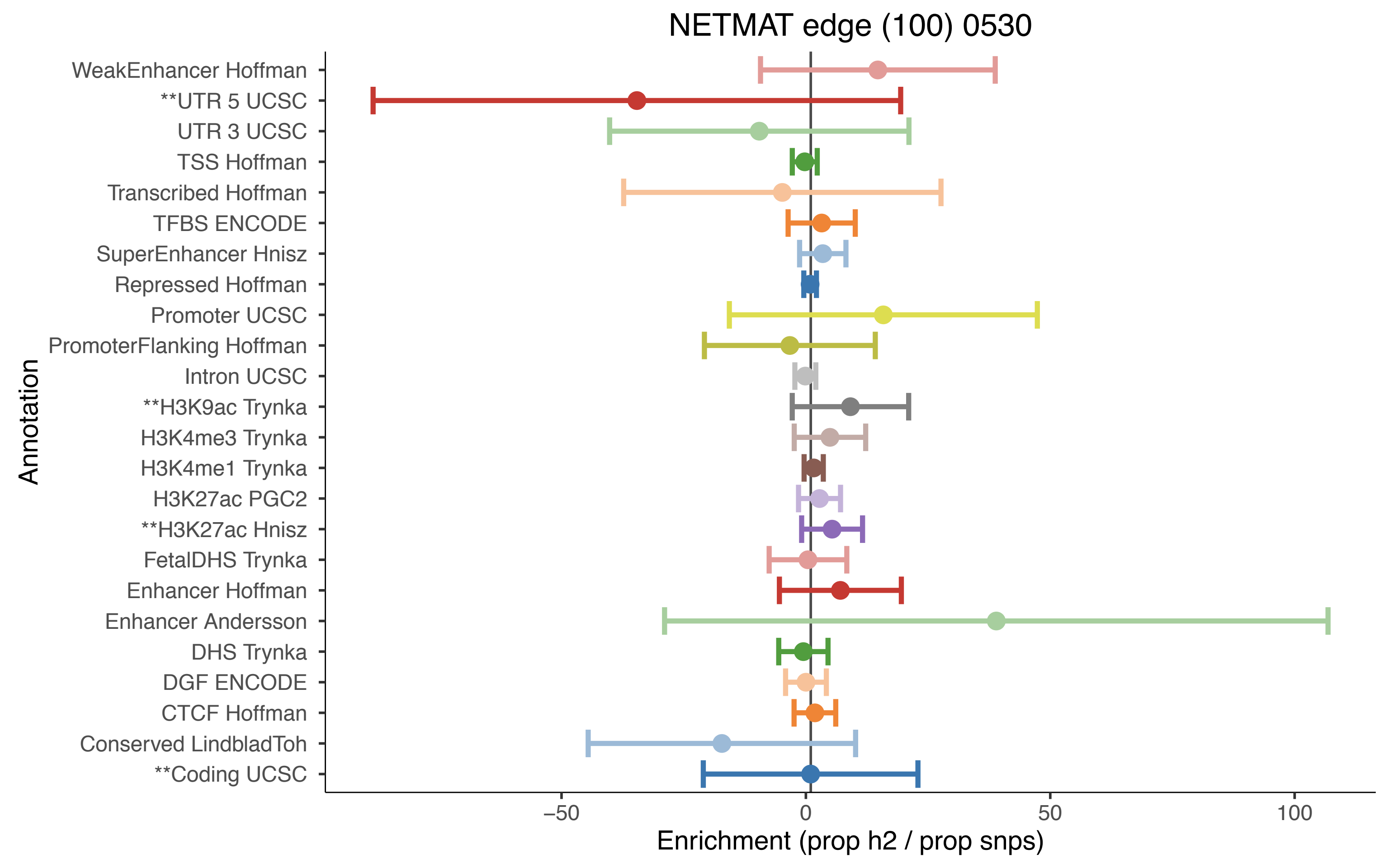
NETMAT edge (100) 0530
WeakEnhancer Hoffman
**UTR 5 UCSC
UTR 3 UCSC
TSS Hoffman
Transcribed Hoffman
TFBS ENCODE
SuperEnhancer Hnisz
Repressed Hoffman
Promoter UCSC
PromoterFlanking Hoffman
Intron UCSC
**H3K9ac Trynka
H3K4me3 Trynka
H3K4me1 Trynka
H3K27ac PGC2
**H3K27ac Hnisz
FetalDHS Trynka
Enhancer Hoffman
Enhancer Andersson
DHS Trynka
DGF ENCODE
CTCF Hoffman
Conserved LindbladToh
**Coding UCSC
Annotation
−50
0
50
100
Enrichment (prop h2 / prop snps)

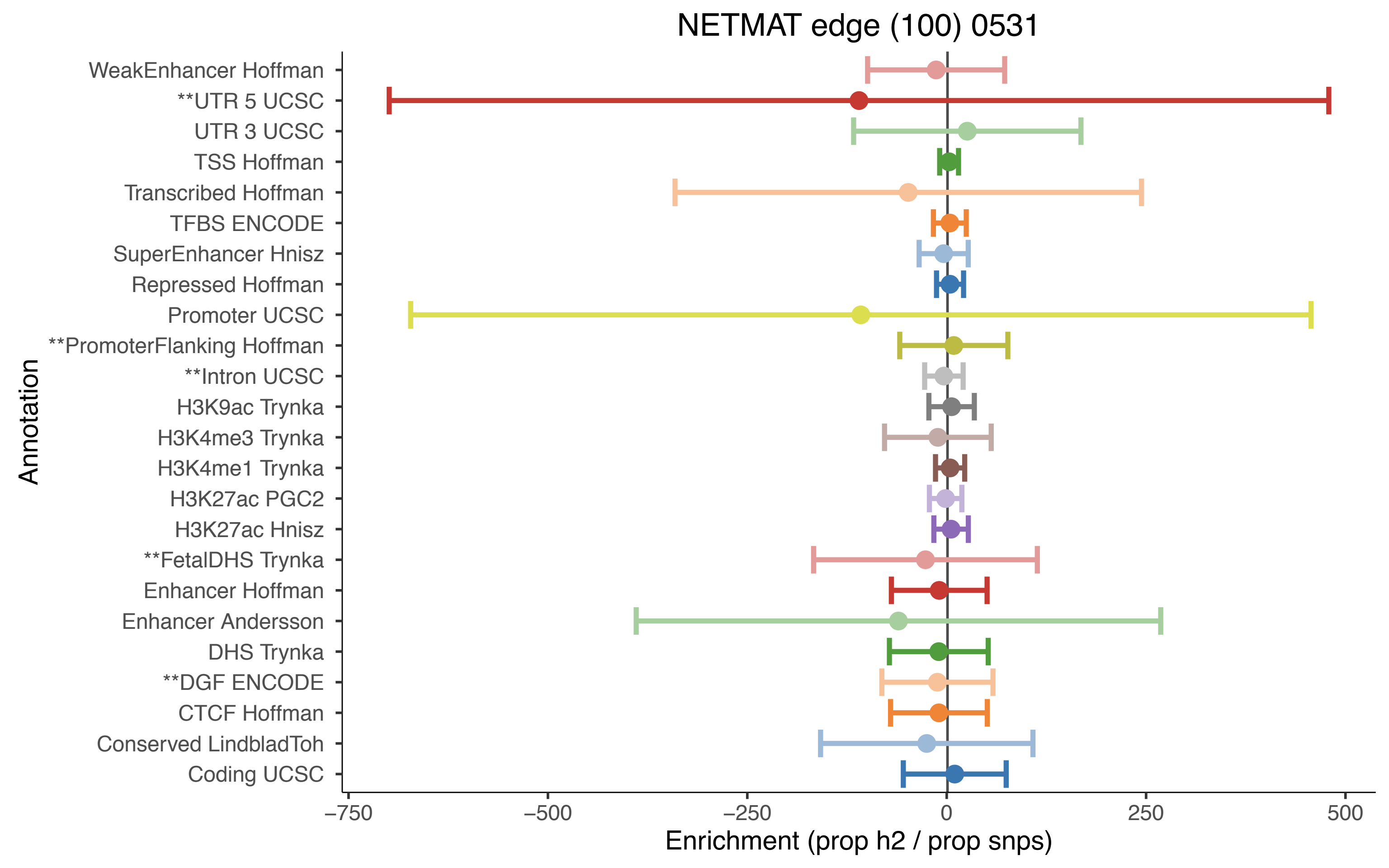

NETMAT edge (100) 0531
WeakEnhancer Hoffman
**UTR 5 UCSC
UTR 3 UCSC
TSS Hoffman
Transcribed Hoffman
TFBS ENCODE
SuperEnhancer Hnisz
Repressed Hoffman
Promoter UCSC
**PromoterFlanking Hoffman
**Intron UCSC
H3K9ac Trynka
H3K4me3 Trynka
H3K4me1 Trynka
H3K27ac PGC2
H3K27ac Hnisz
**FetalDHS Trynka
Enhancer Hoffman
Enhancer Andersson
DHS Trynka
**DGF ENCODE
CTCF Hoffman
Conserved LindbladToh
Coding UCSC
Annotation
−750
−500
−250
0
250
500
Enrichment (prop h2 / prop snps)

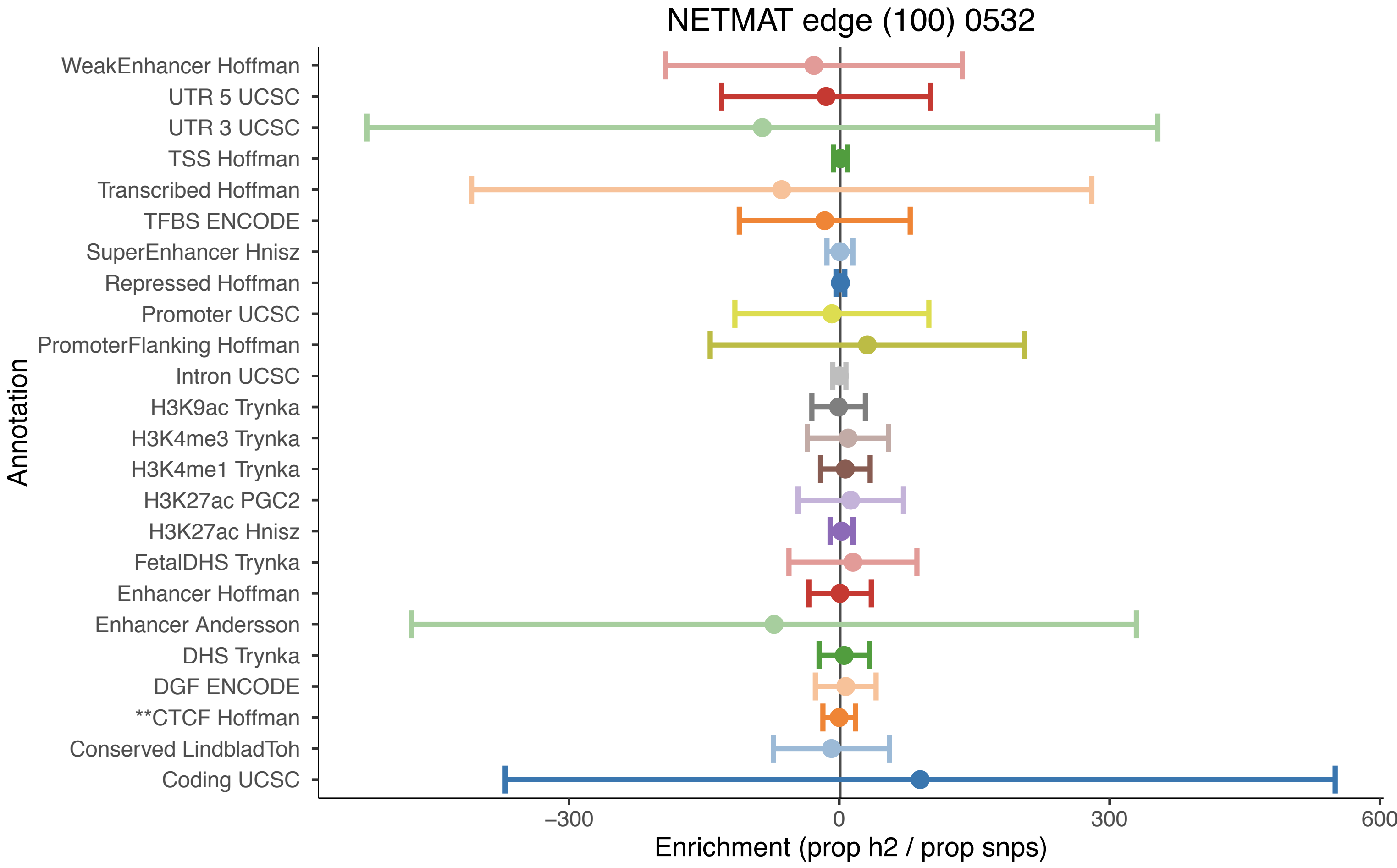
NETMAT edge (100) 0532
Annotation
WeakEnhancer Hoffman
UTR 5 UCSC
UTR 3 UCSC
TSS Hoffman
Transcribed Hoffman
TFBS ENCODE
SuperEnhancer Hnisz
Repressed Hoffman
Promoter UCSC
PromoterFlanking Hoffman
Intron UCSC
H3K9ac Trynka
H3K4me3 Trynka
H3K4me1 Trynka
H3K27ac PGC2
H3K27ac Hnisz
FetalDHS Trynka
Enhancer Hoffman
Enhancer Andersson
DHS Trynka
DGF ENCODE
**CTCF Hoffman
Conserved LindbladToh
Coding UCSC
−300
0
300
600
Enrichment (prop h2 / prop snps)

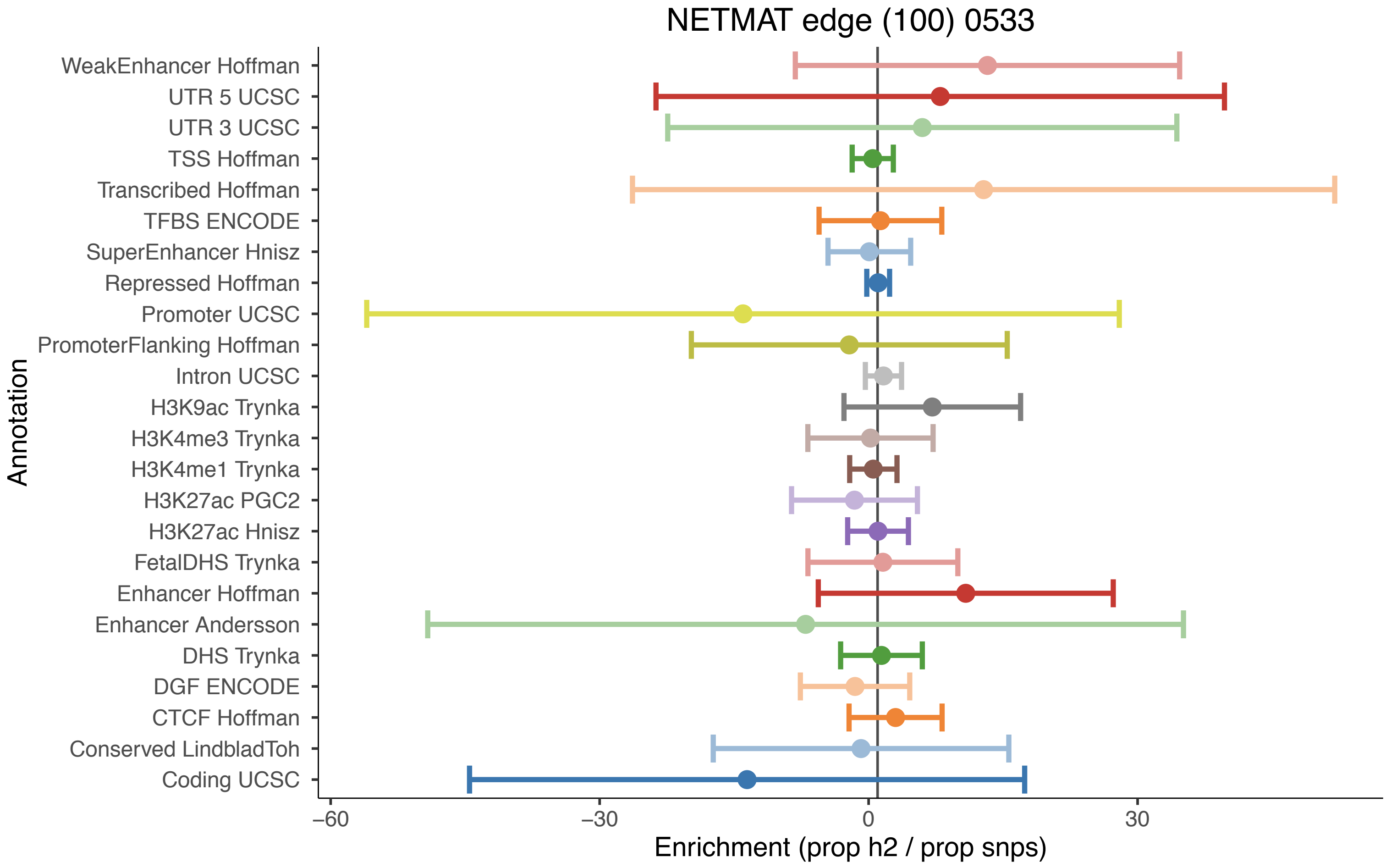

NETMAT edge (100) 0533
WeakEnhancer Hoffman
UTR 5 UCSC
UTR 3 UCSC
TSS Hoffman
Transcribed Hoffman
TFBS ENCODE
SuperEnhancer Hnisz
Repressed Hoffman
Promoter UCSC
PromoterFlanking Hoffman
Intron UCSC
H3K9ac Trynka
H3K4me3 Trynka
H3K4me1 Trynka
H3K27ac PGC2
H3K27ac Hnisz
FetalDHS Trynka
Enhancer Hoffman
Enhancer Andersson
DHS Trynka
DGF ENCODE
CTCF Hoffman
Conserved LindbladToh
Coding UCSC
Annotation
−60
−30
0
30
Enrichment (prop h2 / prop snps)

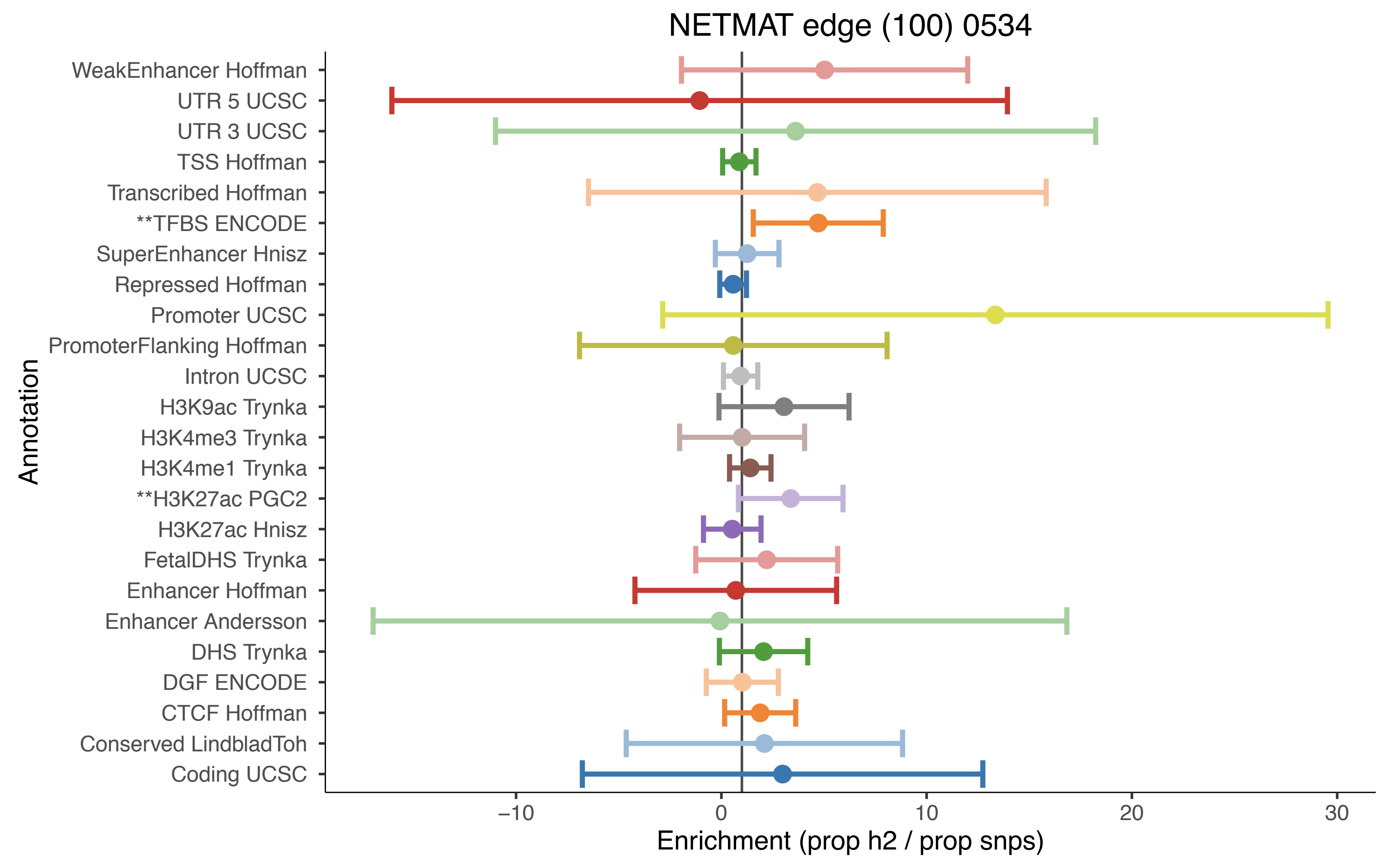

NETMAT edge (100) 0534
WeakEnhancer Hoffman
UTR 5 UCSC
UTR 3 UCSC
TSS Hoffman
Transcribed Hoffman
**TFBS ENCODE
SuperEnhancer Hnisz
Repressed Hoffman
Promoter UCSC
PromoterFlanking Hoffman
Intron UCSC
H3K9ac Trynka
H3K4me3 Trynka
H3K4me1 Trynka
**H3K27ac PGC2
H3K27ac Hnisz
FetalDHS Trynka
Enhancer Hoffman
Enhancer Andersson
DHS Trynka
DGF ENCODE
CTCF Hoffman
Conserved LindbladToh
Coding UCSC
Annotation
−10
0
10
20
30
Enrichment (prop h2 / prop snps)

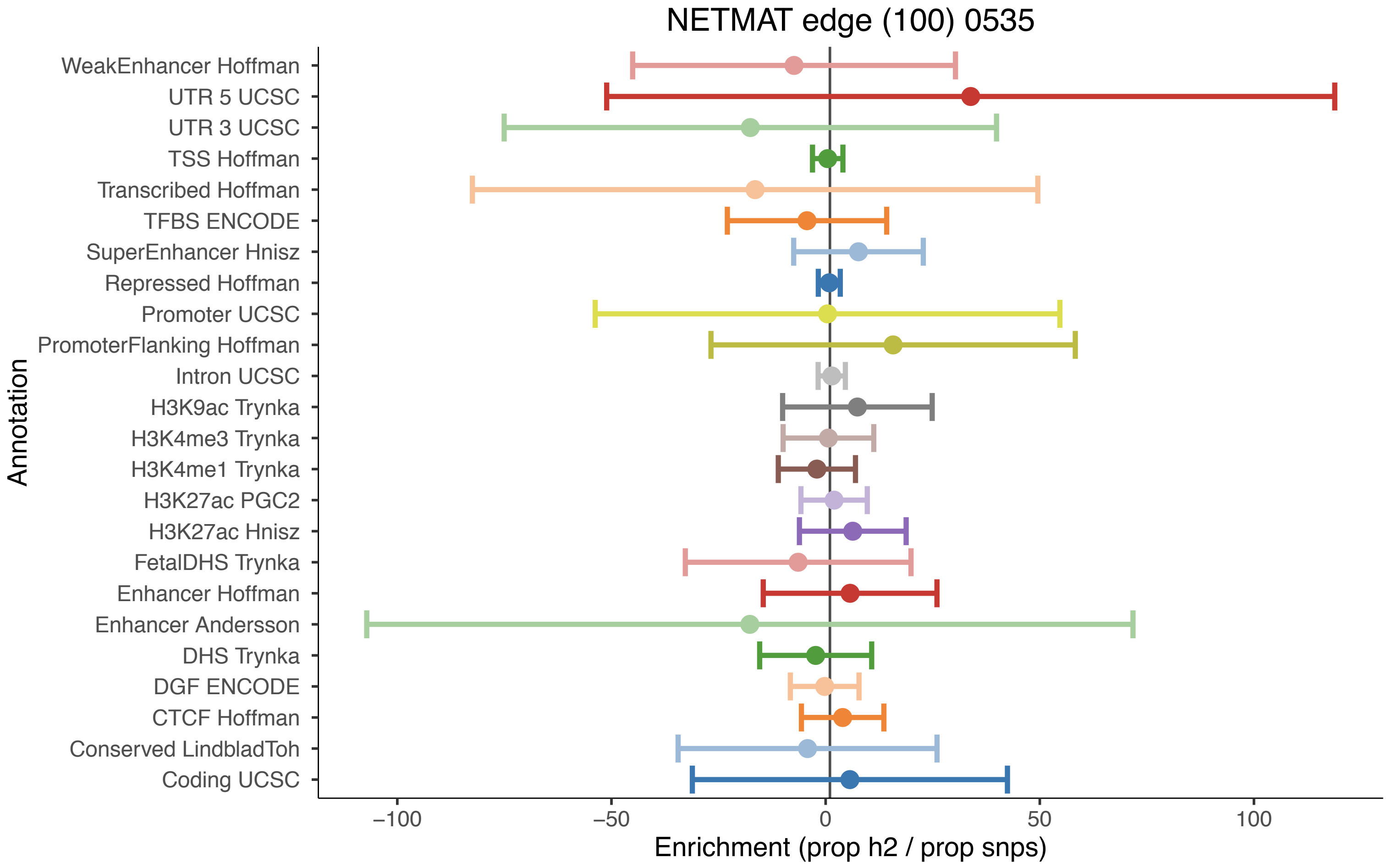
NETMAT edge (100) 0535
WeakEnhancer Hoffman
UTR 5 UCSC
UTR 3 UCSC
TSS Hoffman
Transcribed Hoffman
TFBS ENCODE
SuperEnhancer Hnisz
Repressed Hoffman
Promoter UCSC
PromoterFlanking Hoffman
Intron UCSC
H3K9ac Trynka
H3K4me3 Trynka
H3K4me1 Trynka
H3K27ac PGC2
H3K27ac Hnisz
FetalDHS Trynka
Enhancer Hoffman
Enhancer Andersson
DHS Trynka
DGF ENCODE
CTCF Hoffman
Conserved LindbladToh
Coding UCSC
Annotation
−100
−50
0
50
100
Enrichment (prop h2 / prop snps)

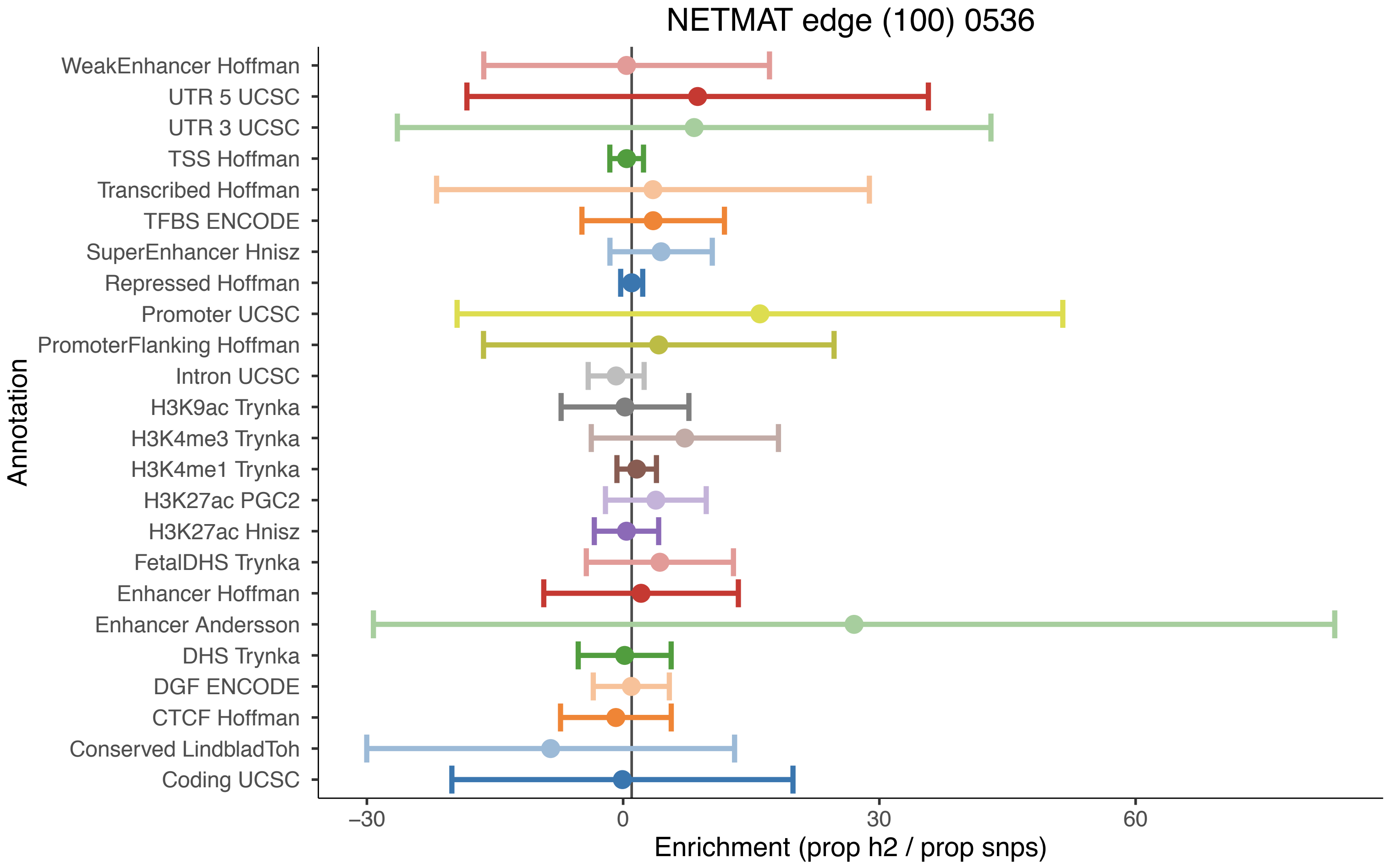

NETMAT edge (100) 0536
WeakEnhancer Hoffman
UTR 5 UCSC
UTR 3 UCSC
TSS Hoffman
Transcribed Hoffman
TFBS ENCODE
SuperEnhancer Hnisz
Repressed Hoffman
Promoter UCSC
PromoterFlanking Hoffman
Intron UCSC
H3K9ac Trynka
H3K4me3 Trynka
H3K4me1 Trynka
H3K27ac PGC2
H3K27ac Hnisz
FetalDHS Trynka
Enhancer Hoffman
Enhancer Andersson
DHS Trynka
DGF ENCODE
CTCF Hoffman
Conserved LindbladToh
Coding UCSC
Annotation
−30
0
30
60
Enrichment (prop h2 / prop snps)

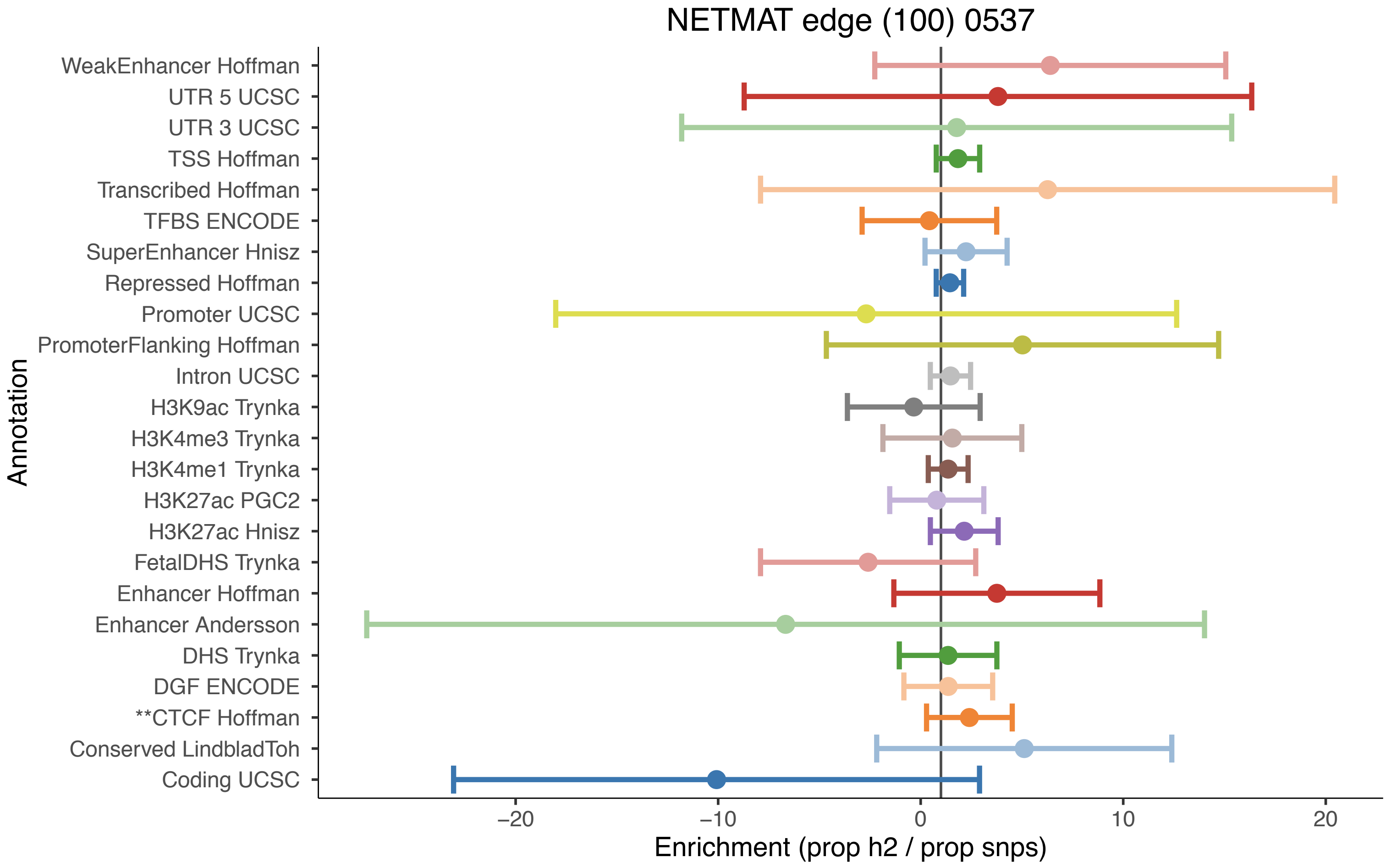

NETMAT edge (100) 0537
WeakEnhancer Hoffman
UTR 5 UCSC
UTR 3 UCSC
TSS Hoffman
Transcribed Hoffman
TFBS ENCODE
SuperEnhancer Hnisz
Repressed Hoffman
Promoter UCSC
PromoterFlanking Hoffman
Intron UCSC
H3K9ac Trynka
H3K4me3 Trynka
H3K4me1 Trynka
H3K27ac PGC2
H3K27ac Hnisz
FetalDHS Trynka
Enhancer Hoffman
Enhancer Andersson
DHS Trynka
DGF ENCODE
**CTCF Hoffman
Conserved LindbladToh
Coding UCSC
Annotation
−20
−10
0
10
20
Enrichment (prop h2 / prop snps)

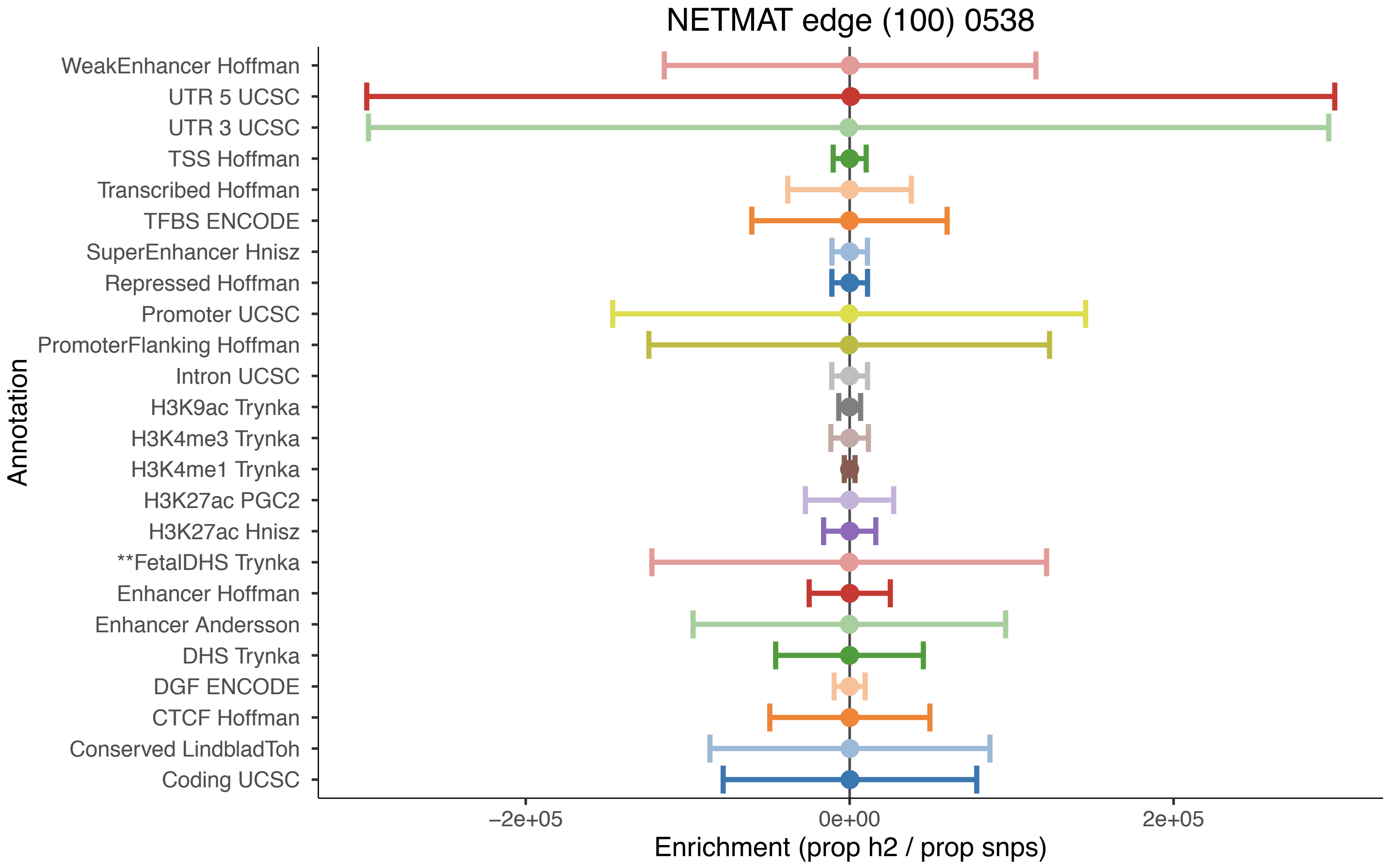
NETMAT edge (100) 0538
WeakEnhancer Hoffman
UTR 5 UCSC
UTR 3 UCSC
TSS Hoffman
Transcribed Hoffman
TFBS ENCODE
SuperEnhancer Hnisz
Repressed Hoffman
Promoter UCSC
PromoterFlanking Hoffman
Intron UCSC
H3K9ac Trynka
H3K4me3 Trynka
H3K4me1 Trynka
H3K27ac PGC2
H3K27ac Hnisz
**FetalDHS Trynka
Enhancer Hoffman
Enhancer Andersson
DHS Trynka
DGF ENCODE
CTCF Hoffman
Conserved LindbladToh
Coding UCSC
Annotation
−2e+05
0e+00
2e+05
Enrichment (prop h2 / prop snps)

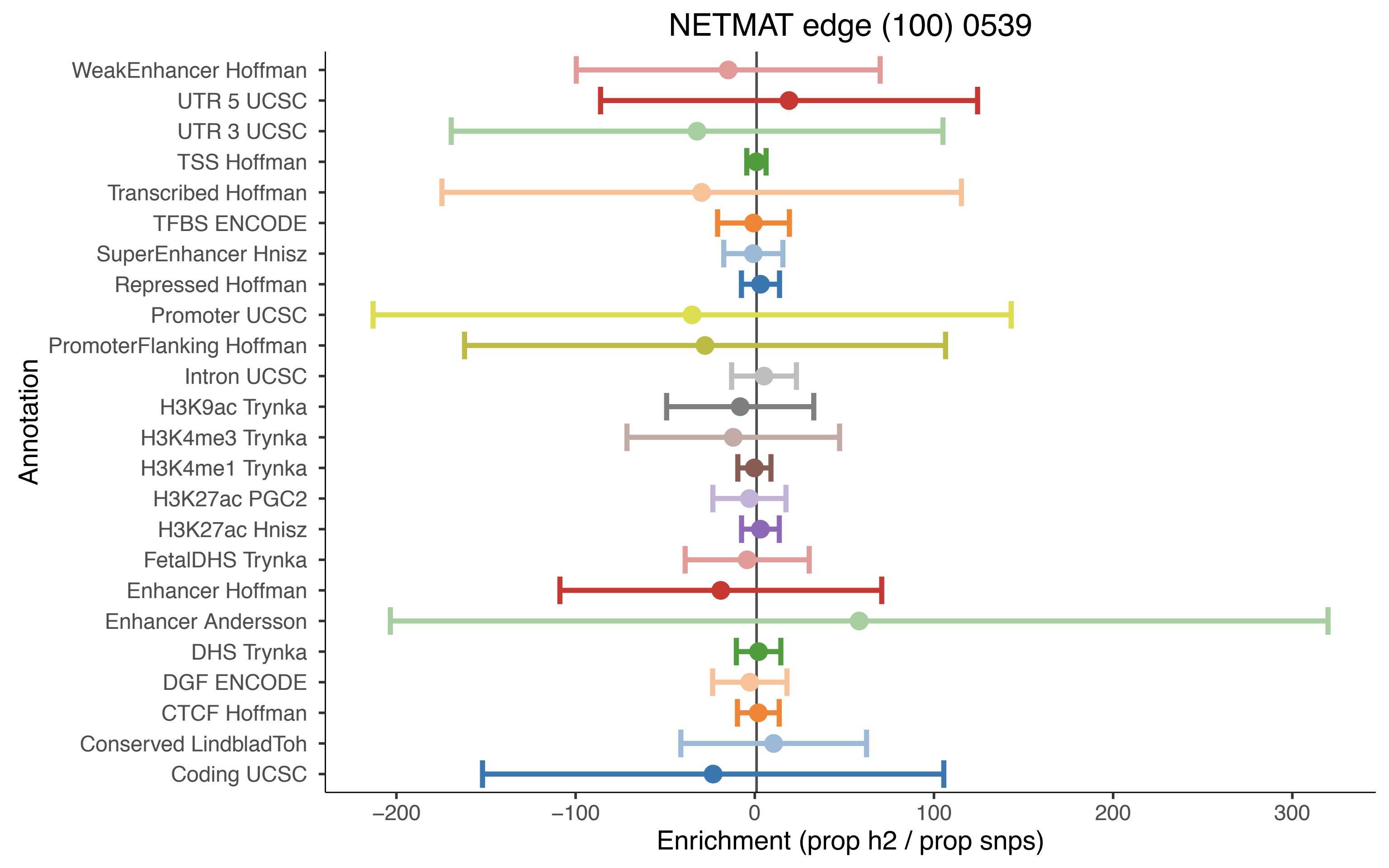
NETMAT edge (100) 0539
WeakEnhancer Hoffman
UTR 5 UCSC
UTR 3 UCSC
TSS Hoffman
Transcribed Hoffman
TFBS ENCODE
SuperEnhancer Hnisz
Repressed Hoffman
Promoter UCSC
PromoterFlanking Hoffman
Intron UCSC
H3K9ac Trynka
H3K4me3 Trynka
H3K4me1 Trynka
H3K27ac PGC2
H3K27ac Hnisz
FetalDHS Trynka
Enhancer Hoffman
Enhancer Andersson
DHS Trynka
DGF ENCODE
CTCF Hoffman
Conserved LindbladToh
Coding UCSC
Annotation
−200
−100
0
100
200
300
Enrichment (prop h2 / prop snps)

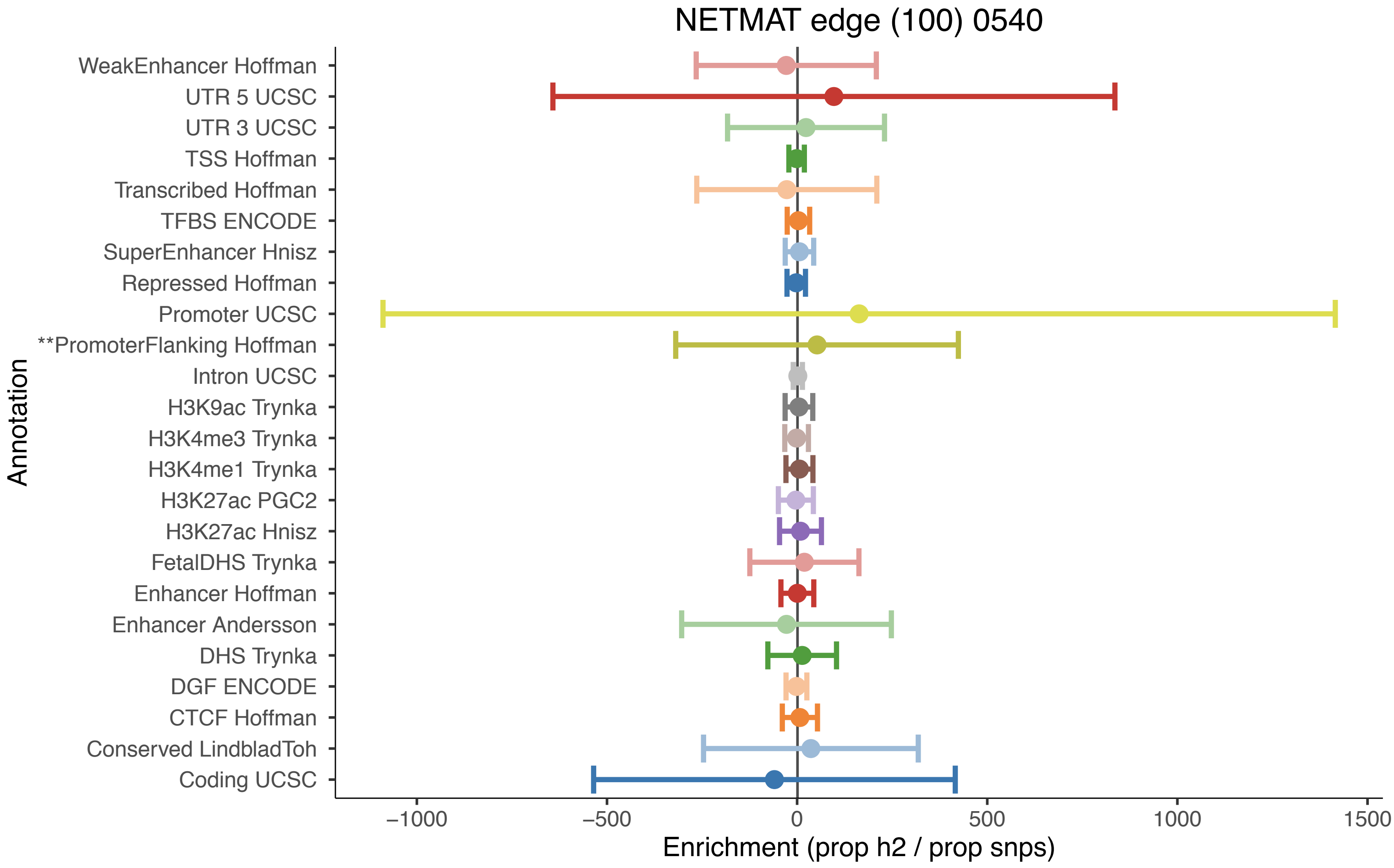

NETMAT edge (100) 0540
WeakEnhancer Hoffman
UTR 5 UCSC
UTR 3 UCSC
TSS Hoffman
Transcribed Hoffman
TFBS ENCODE
SuperEnhancer Hnisz
Repressed Hoffman
Promoter UCSC
**PromoterFlanking Hoffman
Intron UCSC
H3K9ac Trynka
H3K4me3 Trynka
H3K4me1 Trynka
H3K27ac PGC2
H3K27ac Hnisz
FetalDHS Trynka
Enhancer Hoffman
Enhancer Andersson
DHS Trynka
DGF ENCODE
CTCF Hoffman
Conserved LindbladToh
Coding UCSC
Annotation
−1000
−500
0
500
1000
1500
Enrichment (prop h2 / prop snps)

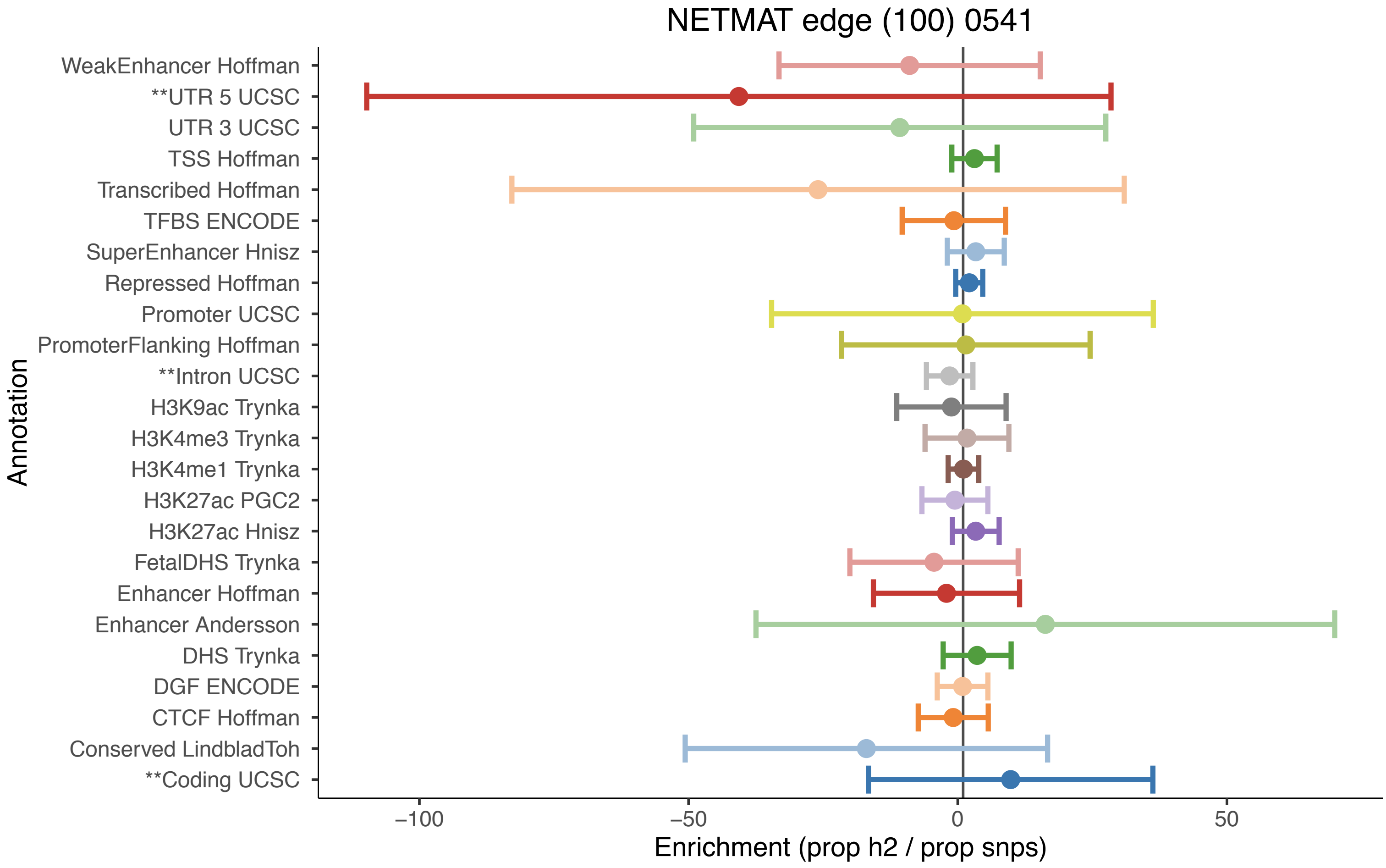
NETMAT edge (100) 0541
WeakEnhancer Hoffman
**UTR 5 UCSC
UTR 3 UCSC
TSS Hoffman
Transcribed Hoffman
TFBS ENCODE
SuperEnhancer Hnisz
Repressed Hoffman
Promoter UCSC
PromoterFlanking Hoffman
**Intron UCSC
H3K9ac Trynka
H3K4me3 Trynka
H3K4me1 Trynka
H3K27ac PGC2
H3K27ac Hnisz
FetalDHS Trynka
Enhancer Hoffman
Enhancer Andersson
DHS Trynka
DGF ENCODE
CTCF Hoffman
Conserved LindbladToh
**Coding UCSC
Annotation
−100
−50
0
50
Enrichment (prop h2 / prop snps)

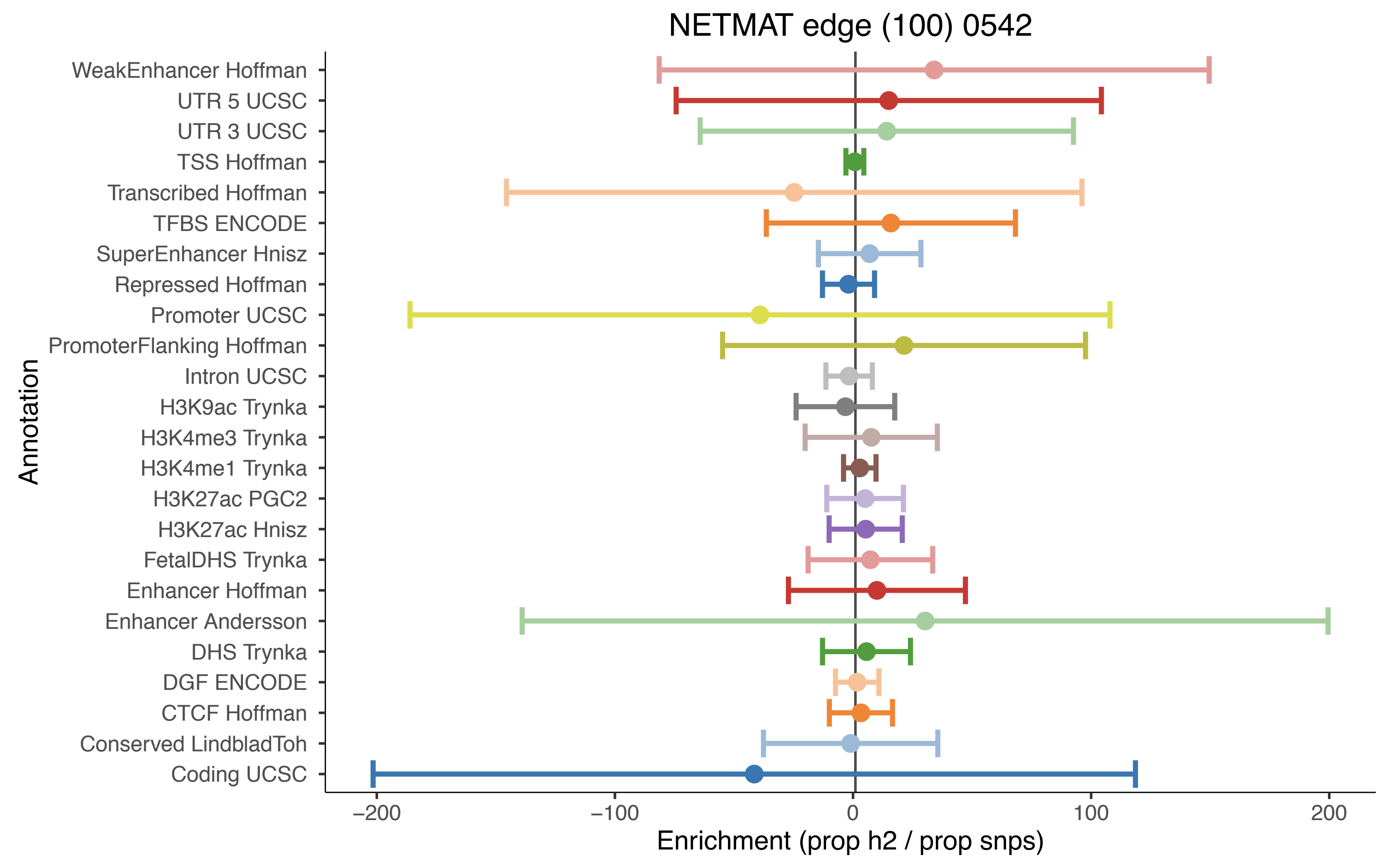

NETMAT edge (100) 0542
Annotation
WeakEnhancer Hoffman
UTR 5 UCSC
UTR 3 UCSC
TSS Hoffman
Transcribed Hoffman
TFBS ENCODE
SuperEnhancer Hnisz
Repressed Hoffman
Promoter UCSC
PromoterFlanking Hoffman
Intron UCSC
H3K9ac Trynka
H3K4me3 Trynka
H3K4me1 Trynka
H3K27ac PGC2
H3K27ac Hnisz
FetalDHS Trynka
Enhancer Hoffman
Enhancer Andersson
DHS Trynka
DGF ENCODE
CTCF Hoffman
Conserved LindbladToh
Coding UCSC
−200
−100
0
100
200
Enrichment (prop h2 / prop snps)

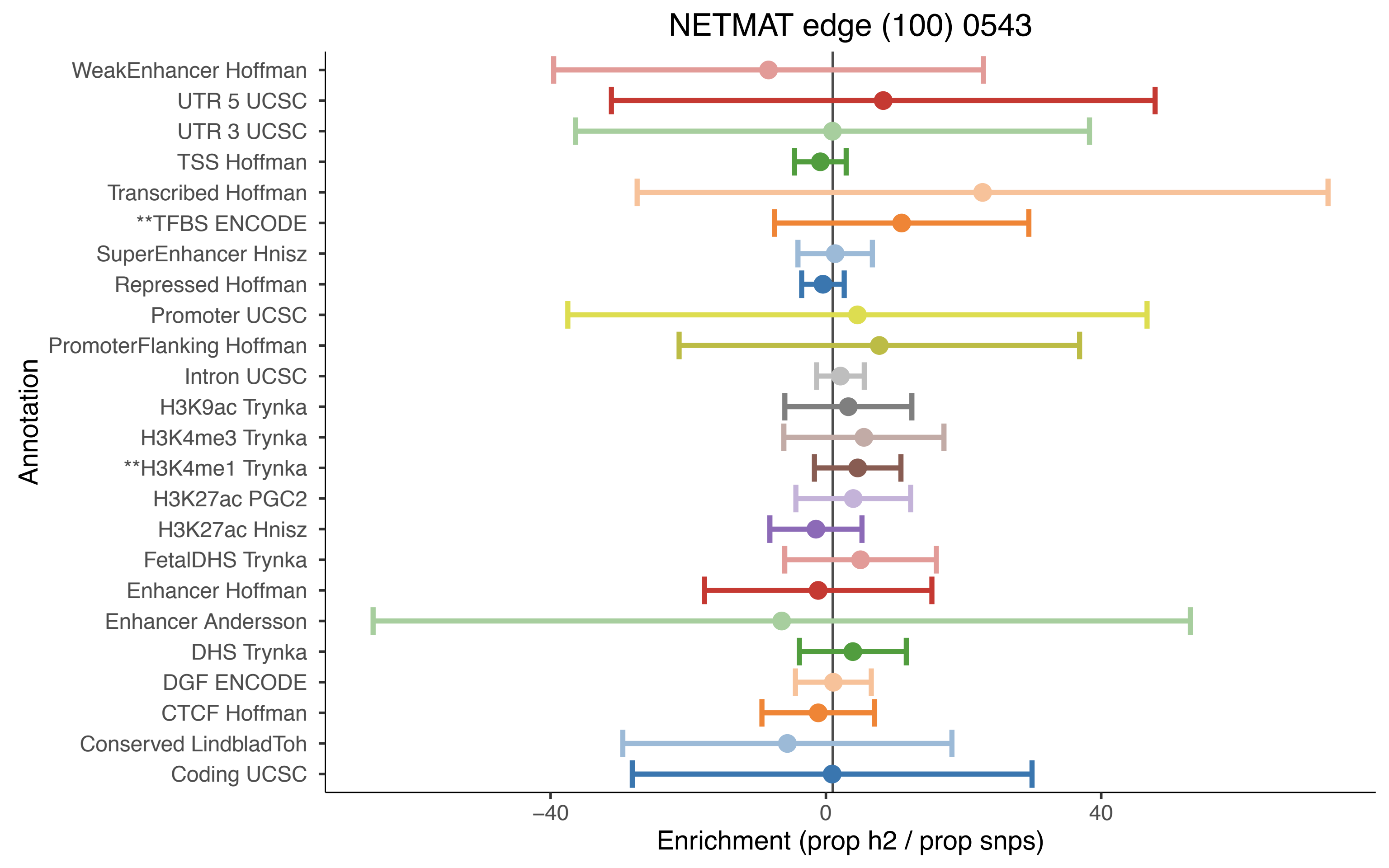

NETMAT edge (100) 0543
WeakEnhancer Hoffman
UTR 5 UCSC
UTR 3 UCSC
TSS Hoffman
Transcribed Hoffman
**TFBS ENCODE
SuperEnhancer Hnisz
Repressed Hoffman
Promoter UCSC
PromoterFlanking Hoffman
Intron UCSC
H3K9ac Trynka
H3K4me3 Trynka
**H3K4me1 Trynka
H3K27ac PGC2
H3K27ac Hnisz
FetalDHS Trynka
Enhancer Hoffman
Enhancer Andersson
DHS Trynka
DGF ENCODE
CTCF Hoffman
Conserved LindbladToh
Coding UCSC
Annotation
−40
0
40
Enrichment (prop h2 / prop snps)

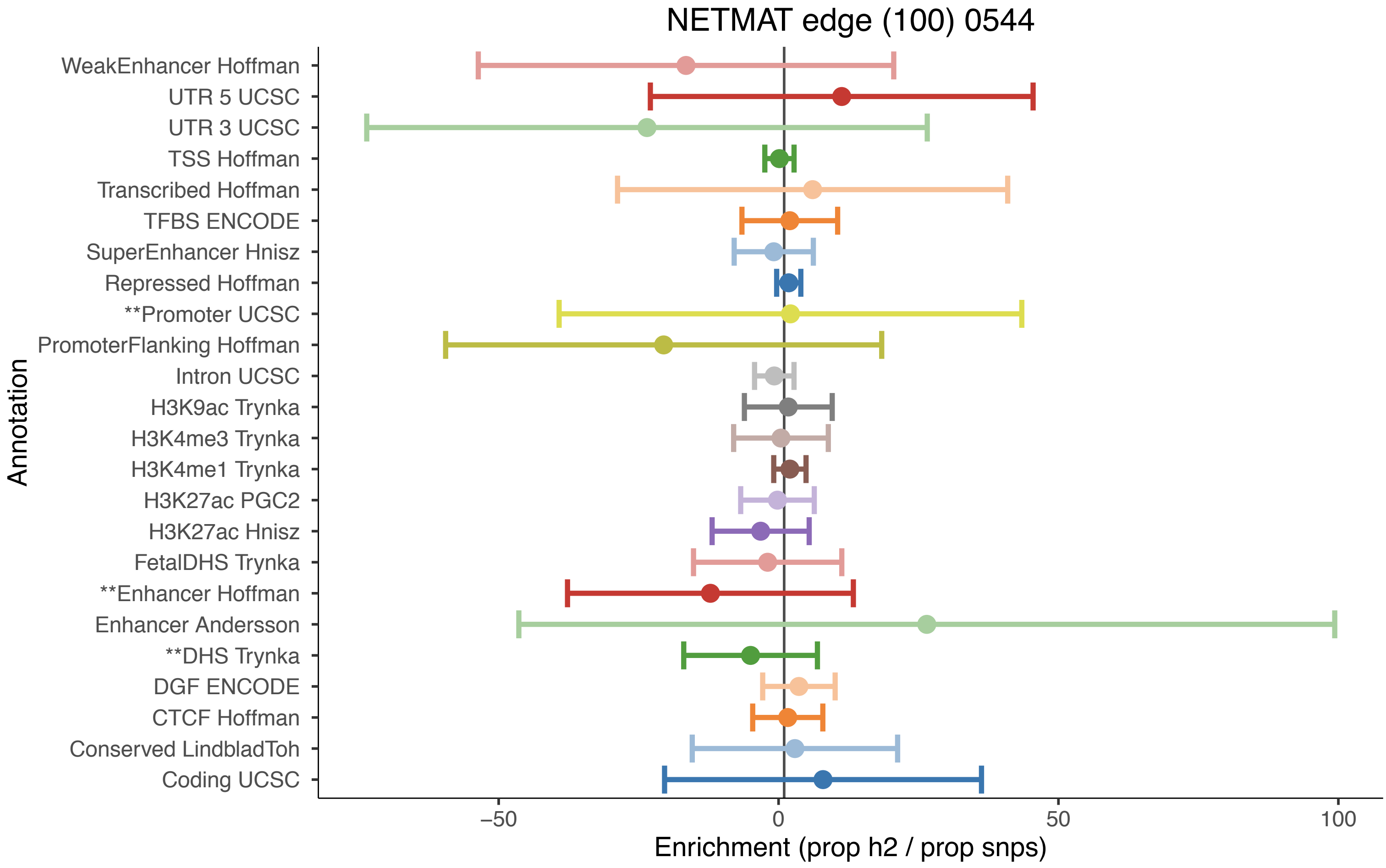
NETMAT edge (100) 0544
Annotation
WeakEnhancer Hoffman
UTR 5 UCSC
UTR 3 UCSC
TSS Hoffman
Transcribed Hoffman
TFBS ENCODE
SuperEnhancer Hnisz
Repressed Hoffman
**Promoter UCSC
PromoterFlanking Hoffman
Intron UCSC
H3K9ac Trynka
H3K4me3 Trynka
H3K4me1 Trynka
H3K27ac PGC2
H3K27ac Hnisz
FetalDHS Trynka
**Enhancer Hoffman
Enhancer Andersson
**DHS Trynka
DGF ENCODE
CTCF Hoffman
Conserved LindbladToh
Coding UCSC
−50
0
50
100
Enrichment (prop h2 / prop snps)

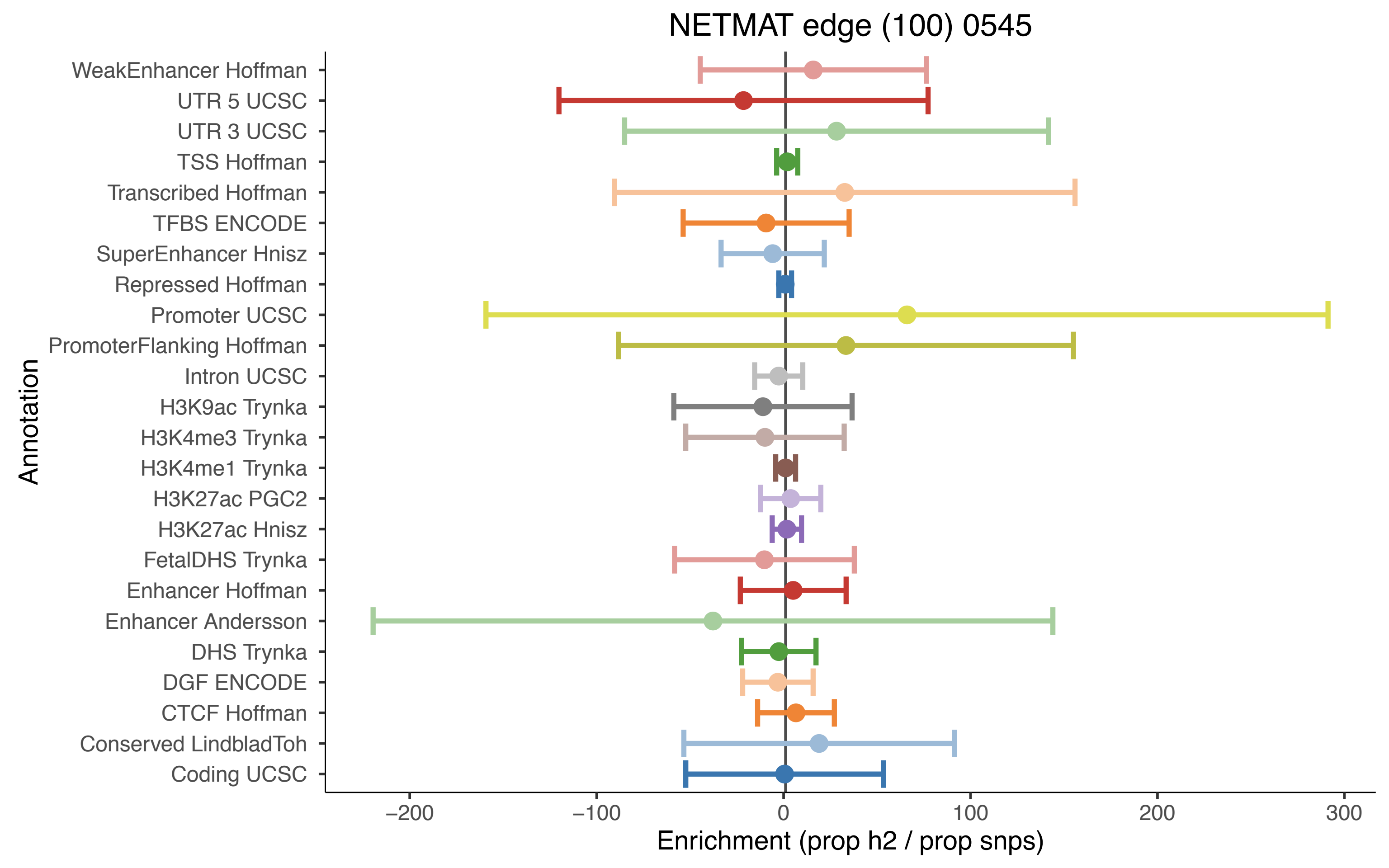

NETMAT edge (100) 0545
WeakEnhancer Hoffman
UTR 5 UCSC
UTR 3 UCSC
TSS Hoffman
Transcribed Hoffman
TFBS ENCODE
SuperEnhancer Hnisz
Repressed Hoffman
Promoter UCSC
PromoterFlanking Hoffman
Intron UCSC
H3K9ac Trynka
H3K4me3 Trynka
H3K4me1 Trynka
H3K27ac PGC2
H3K27ac Hnisz
FetalDHS Trynka
Enhancer Hoffman
Enhancer Andersson
DHS Trynka
DGF ENCODE
CTCF Hoffman
Conserved LindbladToh
Coding UCSC
Annotation
−200
−100
0
100
200
300
Enrichment (prop h2 / prop snps)

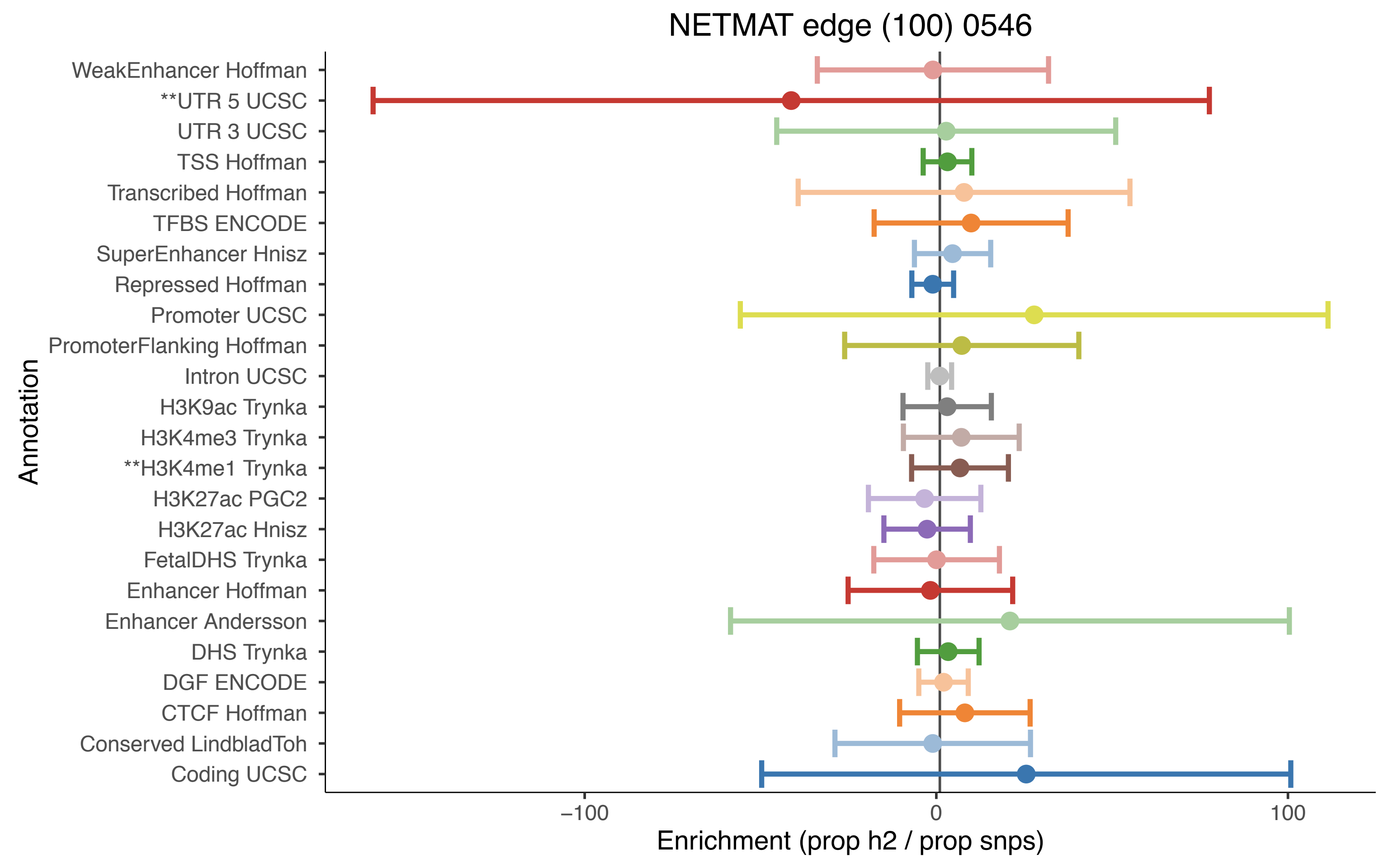
NETMAT edge (100) 0546
WeakEnhancer Hoffman
**UTR 5 UCSC
UTR 3 UCSC
TSS Hoffman
Transcribed Hoffman
TFBS ENCODE
SuperEnhancer Hnisz
Repressed Hoffman
Promoter UCSC
PromoterFlanking Hoffman
Intron UCSC
H3K9ac Trynka
H3K4me3 Trynka
**H3K4me1 Trynka
H3K27ac PGC2
H3K27ac Hnisz
FetalDHS Trynka
Enhancer Hoffman
Enhancer Andersson
DHS Trynka
DGF ENCODE
CTCF Hoffman
Conserved LindbladToh
Coding UCSC
Annotation
−100
0
100
Enrichment (prop h2 / prop snps)

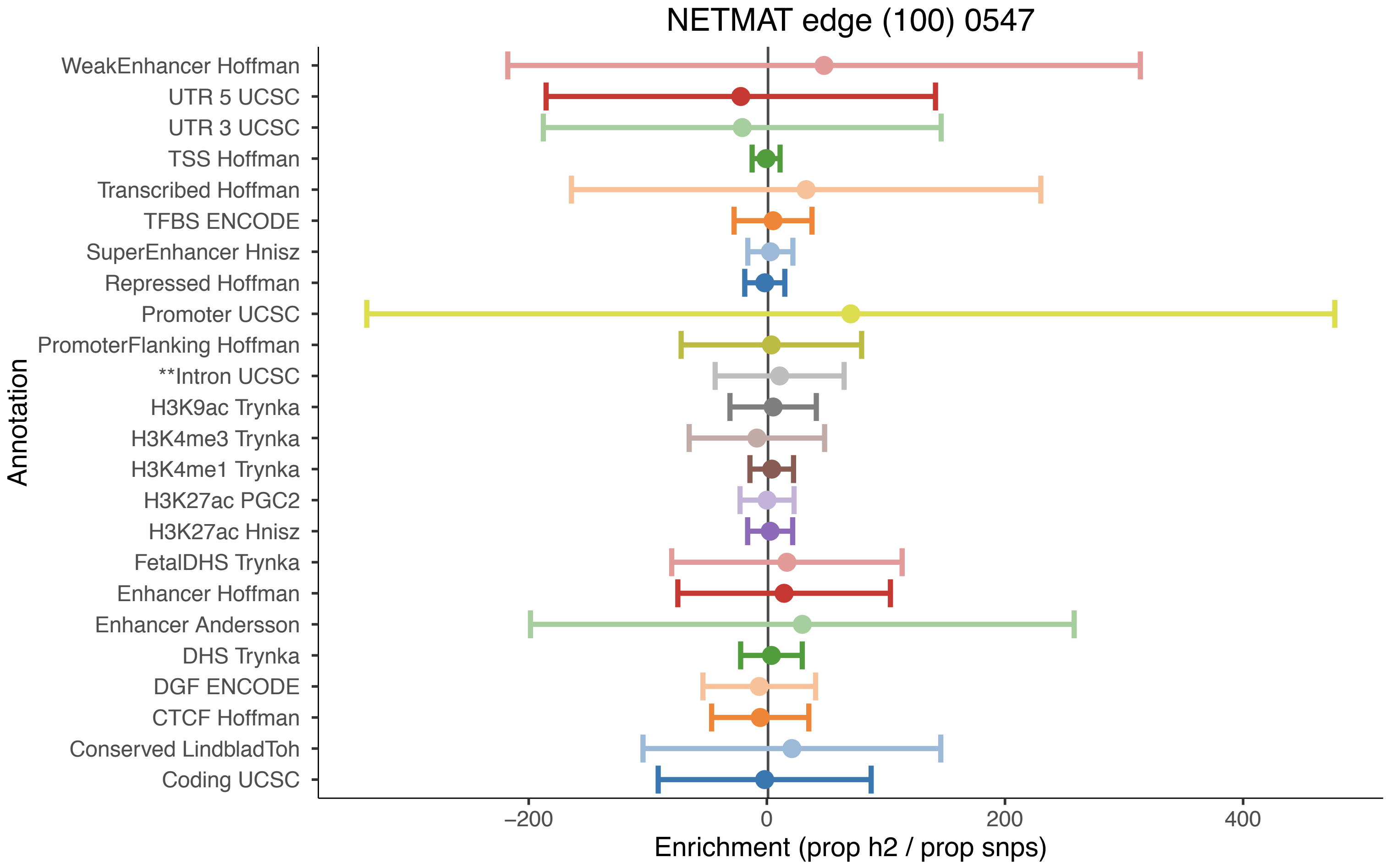

NETMAT edge (100) 0547
WeakEnhancer Hoffman
UTR 5 UCSC
UTR 3 UCSC
TSS Hoffman
Transcribed Hoffman
TFBS ENCODE
SuperEnhancer Hnisz
Repressed Hoffman
Promoter UCSC
PromoterFlanking Hoffman
**Intron UCSC
H3K9ac Trynka
H3K4me3 Trynka
H3K4me1 Trynka
H3K27ac PGC2
H3K27ac Hnisz
FetalDHS Trynka
Enhancer Hoffman
Enhancer Andersson
DHS Trynka
DGF ENCODE
CTCF Hoffman
Conserved LindbladToh
Coding UCSC
Annotation
−200
0
200
400
Enrichment (prop h2 / prop snps)

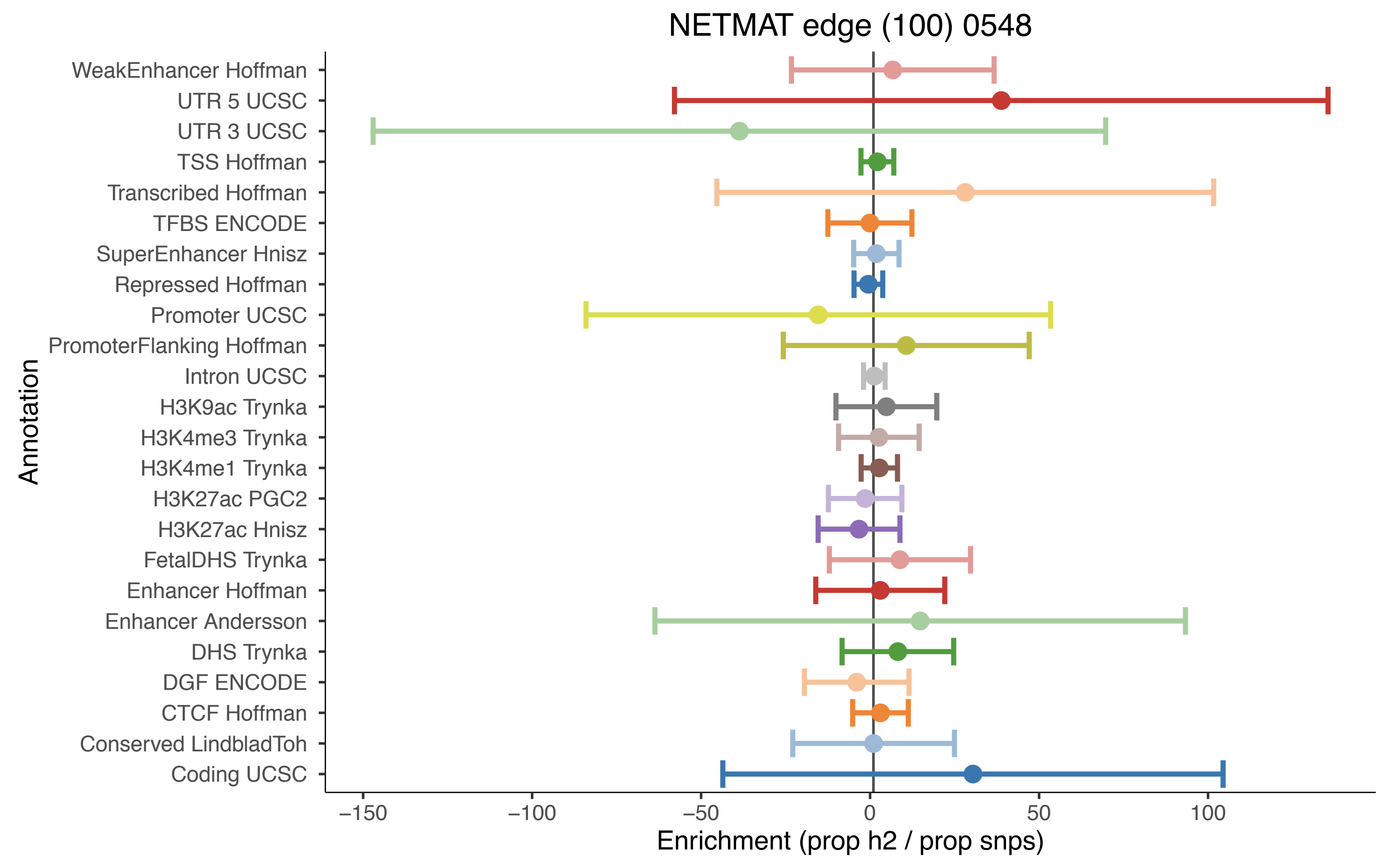

NETMAT edge (100) 0548
WeakEnhancer Hoffman
UTR 5 UCSC
UTR 3 UCSC
TSS Hoffman
Transcribed Hoffman
TFBS ENCODE
SuperEnhancer Hnisz
Repressed Hoffman
Promoter UCSC
PromoterFlanking Hoffman
Intron UCSC
H3K9ac Trynka
H3K4me3 Trynka
H3K4me1 Trynka
H3K27ac PGC2
H3K27ac Hnisz
FetalDHS Trynka
Enhancer Hoffman
Enhancer Andersson
DHS Trynka
DGF ENCODE
CTCF Hoffman
Conserved LindbladToh
Coding UCSC
Annotation
−150
−100
−50
0
50
100
Enrichment (prop h2 / prop snps)

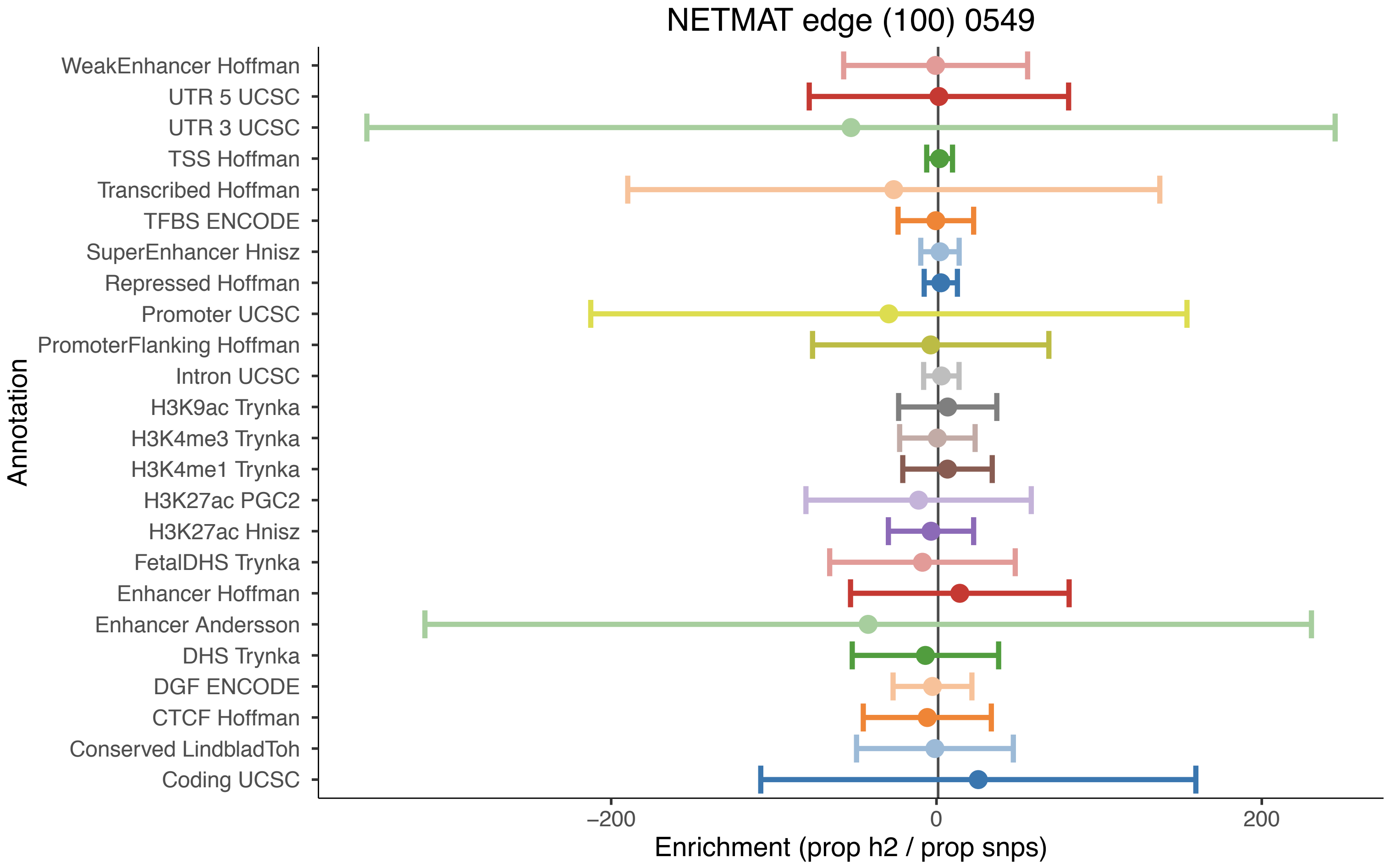
NETMAT edge (100) 0549
WeakEnhancer Hoffman
UTR 5 UCSC
UTR 3 UCSC
TSS Hoffman
Transcribed Hoffman
TFBS ENCODE
SuperEnhancer Hnisz
Repressed Hoffman
Promoter UCSC
PromoterFlanking Hoffman
Intron UCSC
H3K9ac Trynka
H3K4me3 Trynka
H3K4me1 Trynka
H3K27ac PGC2
H3K27ac Hnisz
FetalDHS Trynka
Enhancer Hoffman
Enhancer Andersson
DHS Trynka
DGF ENCODE
CTCF Hoffman
Conserved LindbladToh
Coding UCSC
Annotation
−200
0
200
Enrichment (prop h2 / prop snps)

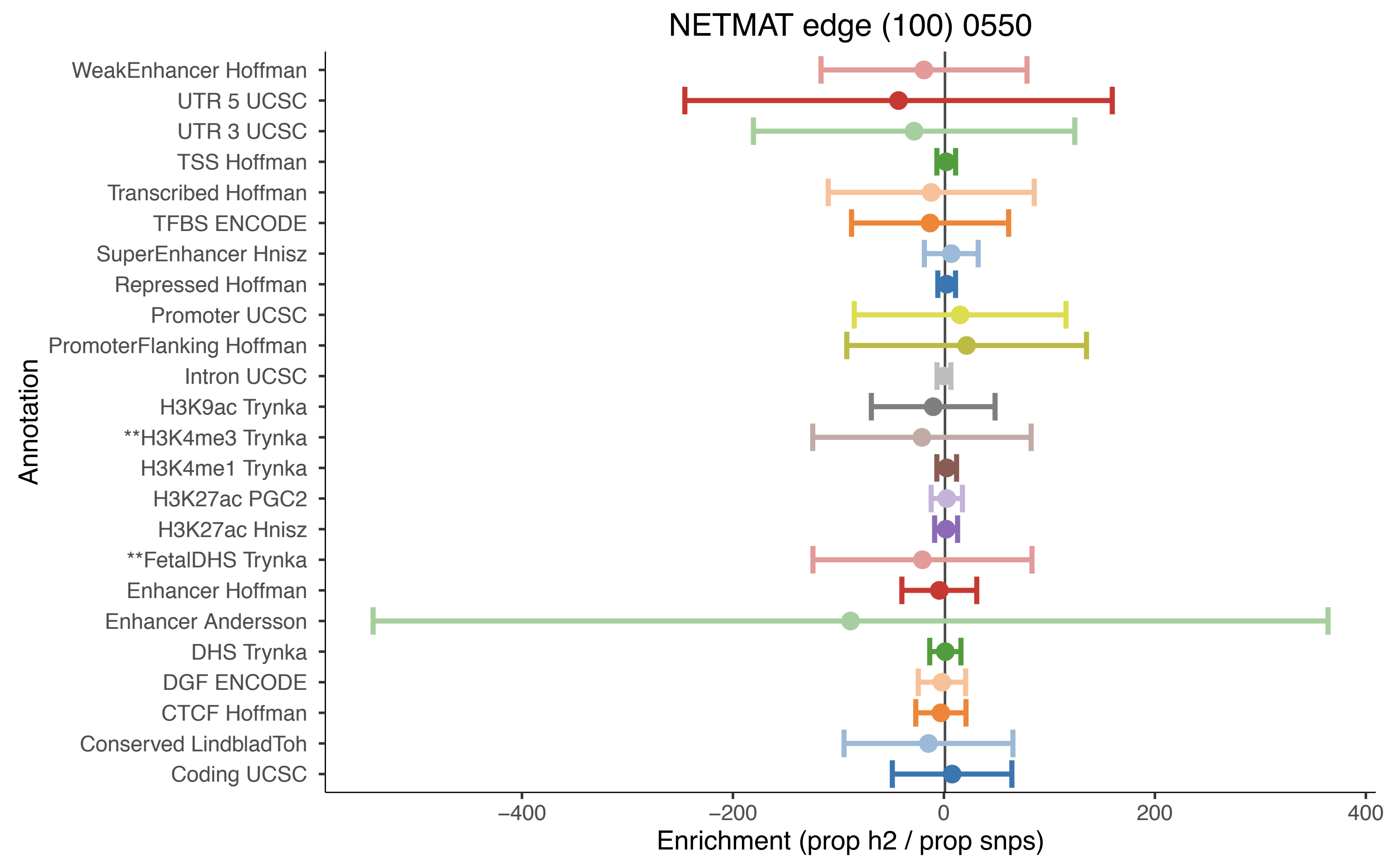
NETMAT edge (100) 0550
WeakEnhancer Hoffman
UTR 5 UCSC
UTR 3 UCSC
TSS Hoffman
Transcribed Hoffman
TFBS ENCODE
SuperEnhancer Hnisz
Repressed Hoffman
Promoter UCSC
PromoterFlanking Hoffman
Intron UCSC
H3K9ac Trynka
**H3K4me3 Trynka
H3K4me1 Trynka
H3K27ac PGC2
H3K27ac Hnisz
**FetalDHS Trynka
Enhancer Hoffman
Enhancer Andersson
DHS Trynka
DGF ENCODE
CTCF Hoffman
Conserved LindbladToh
Coding UCSC
Annotation
−400
−200
0
200
400
Enrichment (prop h2 / prop snps)

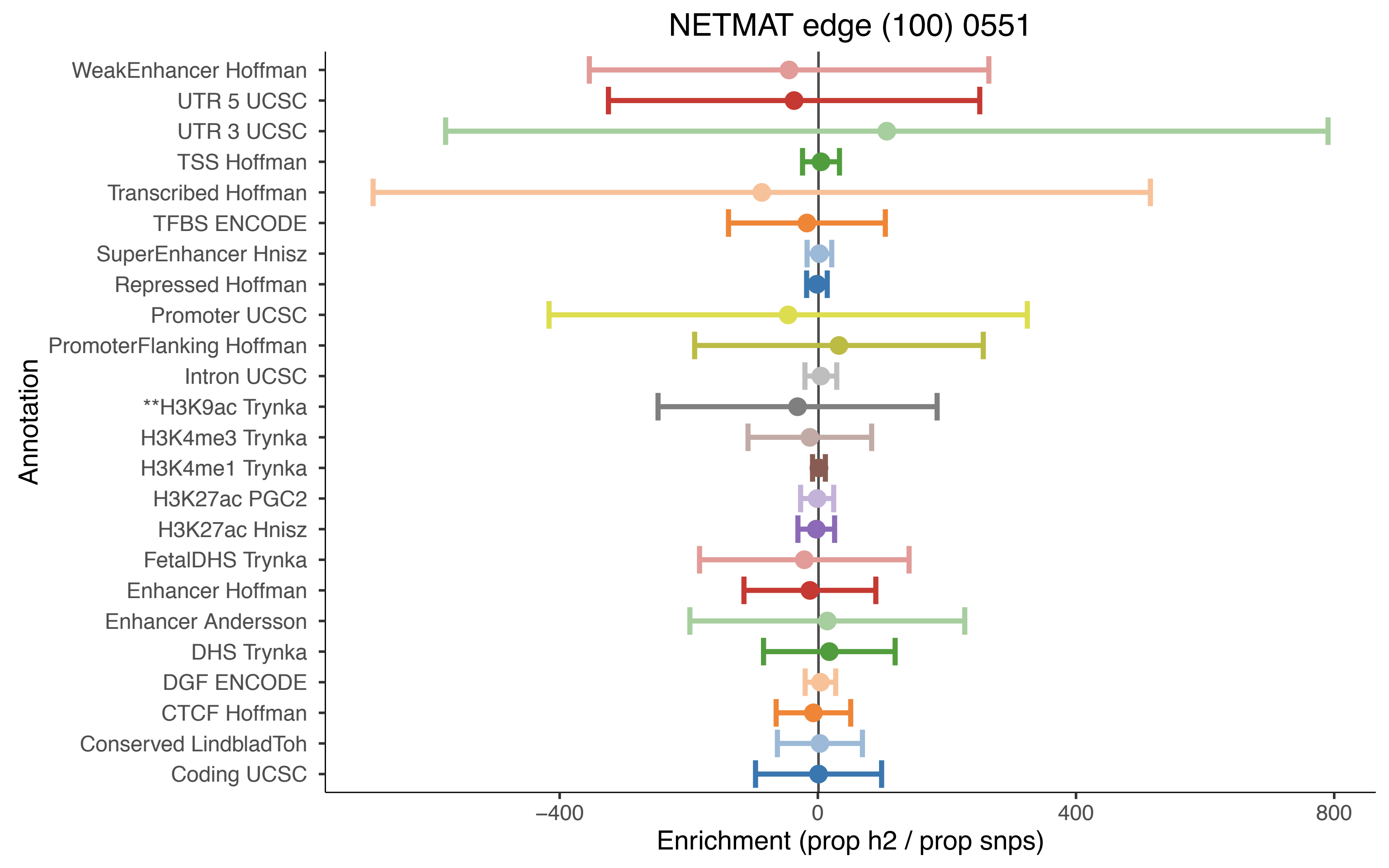

NETMAT edge (100) 0551
WeakEnhancer Hoffman
UTR 5 UCSC
UTR 3 UCSC
TSS Hoffman
Transcribed Hoffman
TFBS ENCODE
SuperEnhancer Hnisz
Repressed Hoffman
Promoter UCSC
PromoterFlanking Hoffman
Intron UCSC
**H3K9ac Trynka
H3K4me3 Trynka
H3K4me1 Trynka
H3K27ac PGC2
H3K27ac Hnisz
FetalDHS Trynka
Enhancer Hoffman
Enhancer Andersson
DHS Trynka
DGF ENCODE
CTCF Hoffman
Conserved LindbladToh
Coding UCSC
Annotation
−400
0
400
800
Enrichment (prop h2 / prop snps)

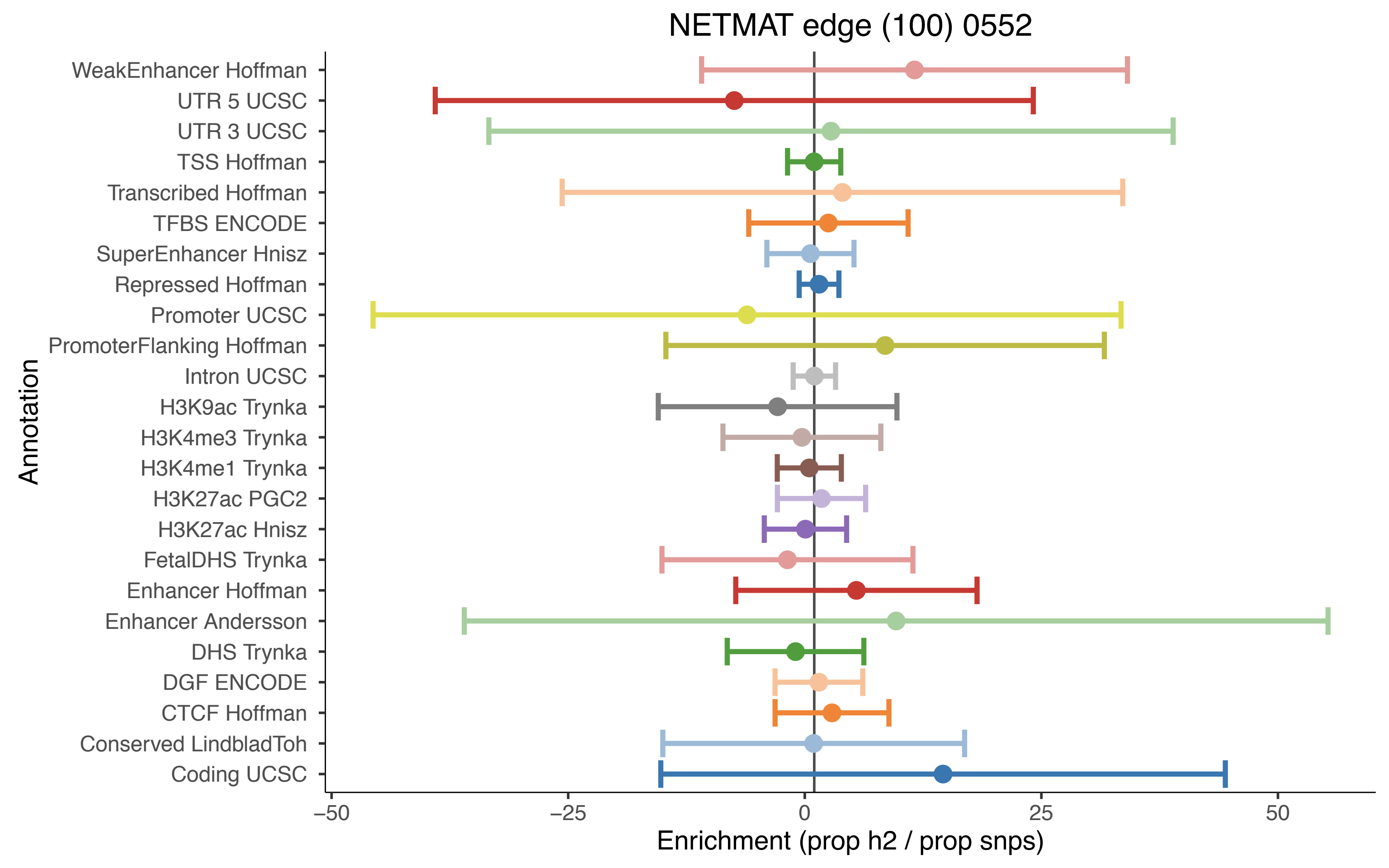

NETMAT edge (100) 0552
WeakEnhancer Hoffman
UTR 5 UCSC
UTR 3 UCSC
TSS Hoffman
Transcribed Hoffman
TFBS ENCODE
SuperEnhancer Hnisz
Repressed Hoffman
Promoter UCSC
PromoterFlanking Hoffman
Intron UCSC
H3K9ac Trynka
H3K4me3 Trynka
H3K4me1 Trynka
H3K27ac PGC2
H3K27ac Hnisz
FetalDHS Trynka
Enhancer Hoffman
Enhancer Andersson
DHS Trynka
DGF ENCODE
CTCF Hoffman
Conserved LindbladToh
Coding UCSC
Annotation
−50
−25
0
25
50
Enrichment (prop h2 / prop snps)

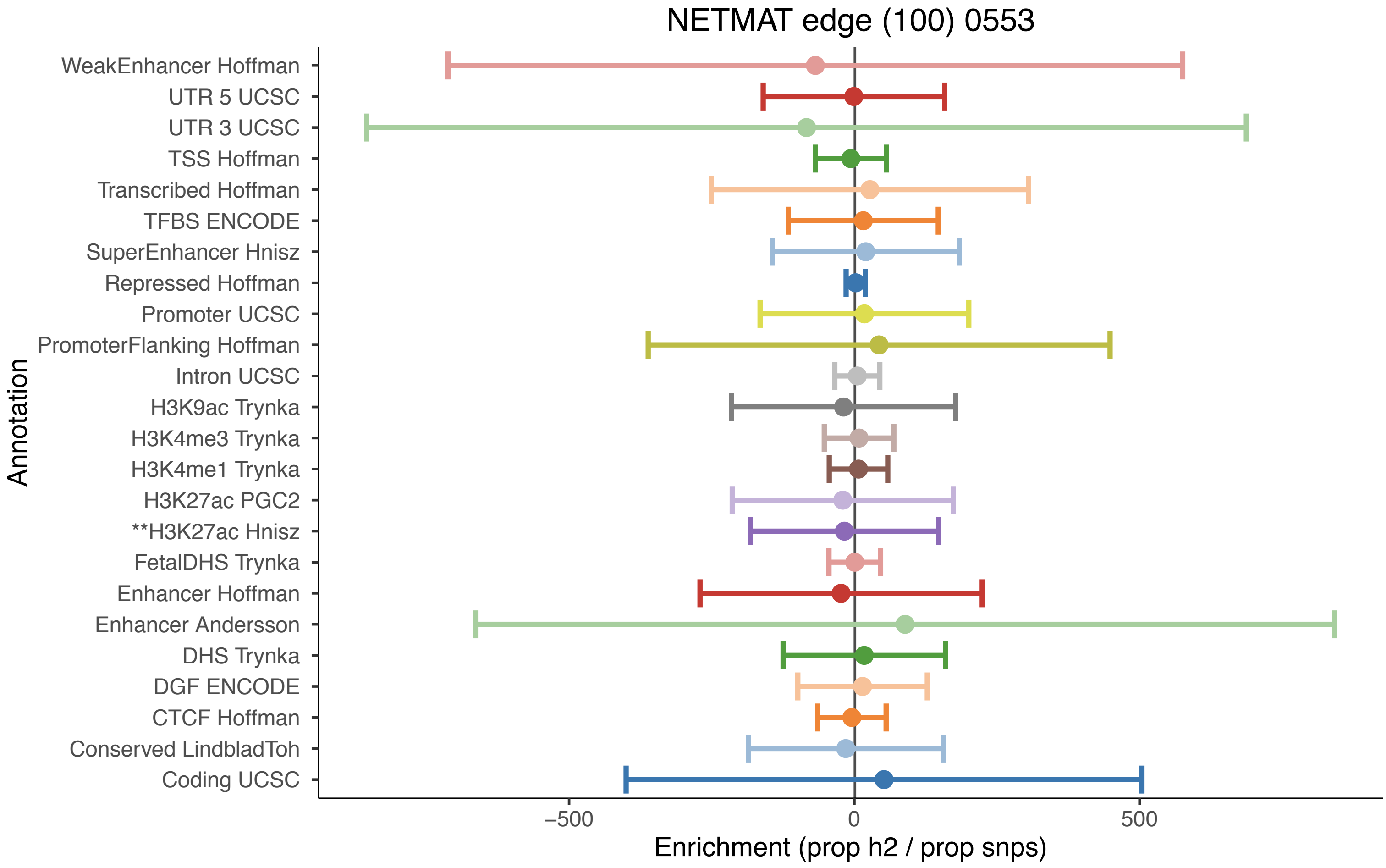
NETMAT edge (100) 0553
WeakEnhancer Hoffman
UTR 5 UCSC
UTR 3 UCSC
TSS Hoffman
Transcribed Hoffman
TFBS ENCODE
SuperEnhancer Hnisz
Repressed Hoffman
Promoter UCSC
PromoterFlanking Hoffman
Intron UCSC
H3K9ac Trynka
H3K4me3 Trynka
H3K4me1 Trynka
H3K27ac PGC2
**H3K27ac Hnisz
FetalDHS Trynka
Enhancer Hoffman
Enhancer Andersson
DHS Trynka
DGF ENCODE
CTCF Hoffman
Conserved LindbladToh
Coding UCSC
Annotation
−500
0
500
Enrichment (prop h2 / prop snps)

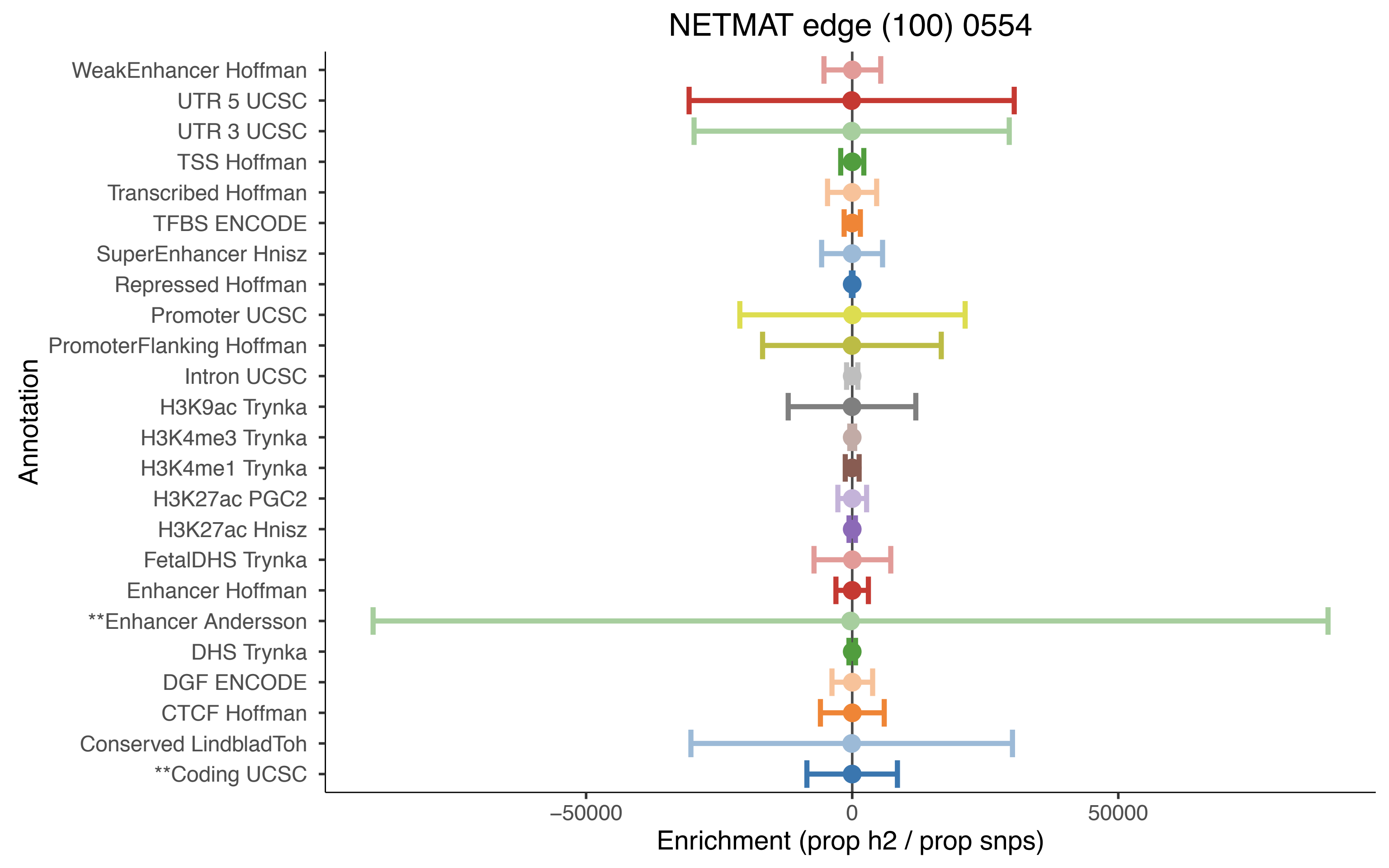
NETMAT edge (100) 0554
WeakEnhancer Hoffman
UTR 5 UCSC
UTR 3 UCSC
TSS Hoffman
Transcribed Hoffman
TFBS ENCODE
SuperEnhancer Hnisz
Repressed Hoffman
Promoter UCSC
PromoterFlanking Hoffman
Intron UCSC
H3K9ac Trynka
H3K4me3 Trynka
H3K4me1 Trynka
H3K27ac PGC2
H3K27ac Hnisz
FetalDHS Trynka
Enhancer Hoffman
**Enhancer Andersson
DHS Trynka
DGF ENCODE
CTCF Hoffman
Conserved LindbladToh
**Coding UCSC
Annotation
−50000
0
50000
Enrichment (prop h2 / prop snps)

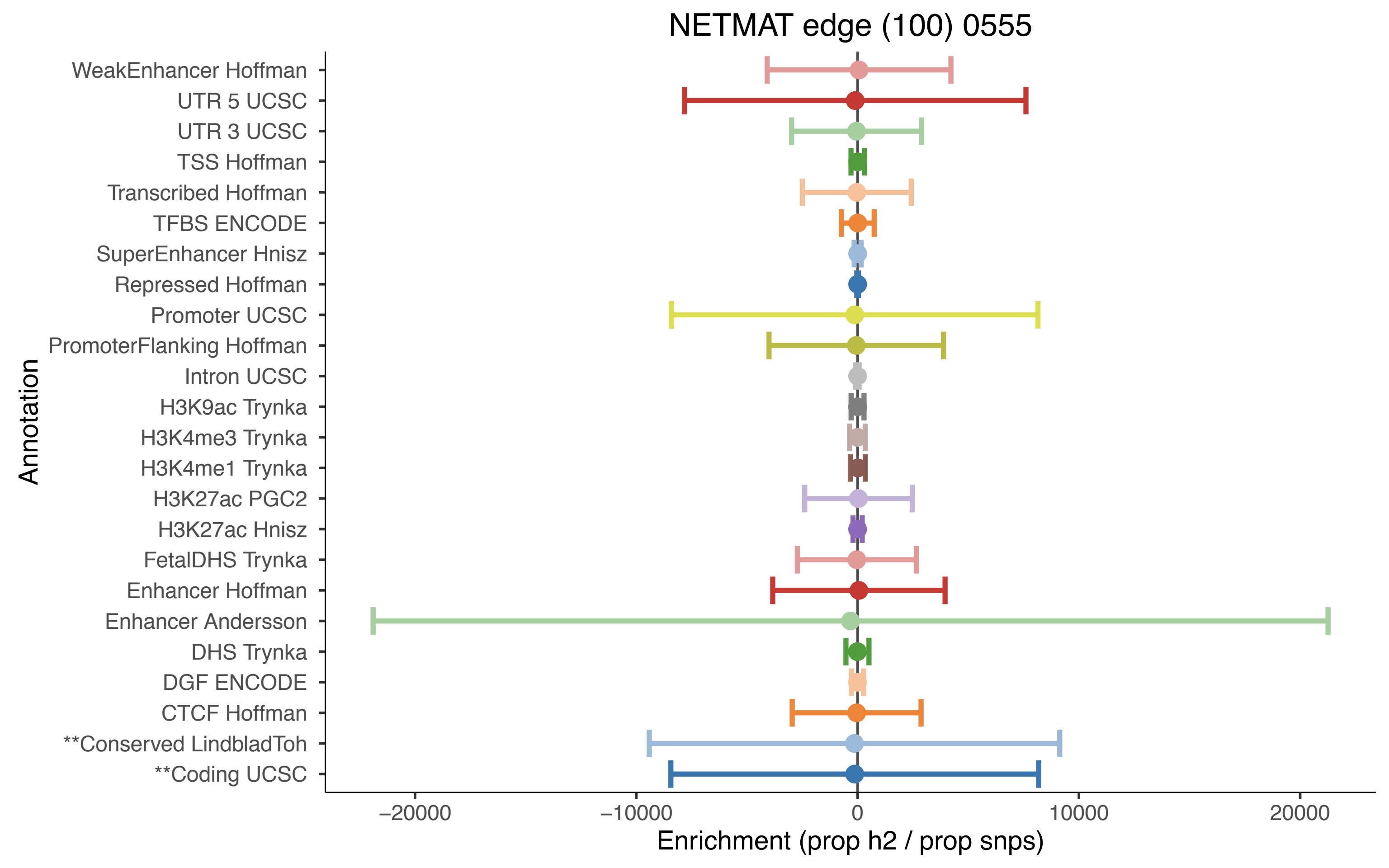

NETMAT edge (100) 0555
WeakEnhancer Hoffman
UTR 5 UCSC
UTR 3 UCSC
TSS Hoffman
Transcribed Hoffman
TFBS ENCODE
SuperEnhancer Hnisz
Repressed Hoffman
Promoter UCSC
PromoterFlanking Hoffman
Intron UCSC
H3K9ac Trynka
H3K4me3 Trynka
H3K4me1 Trynka
H3K27ac PGC2
H3K27ac Hnisz
FetalDHS Trynka
Enhancer Hoffman
Enhancer Andersson
DHS Trynka
DGF ENCODE
CTCF Hoffman
**Conserved LindbladToh
**Coding UCSC
Annotation
−20000
−10000
0
10000
20000
Enrichment (prop h2 / prop snps)

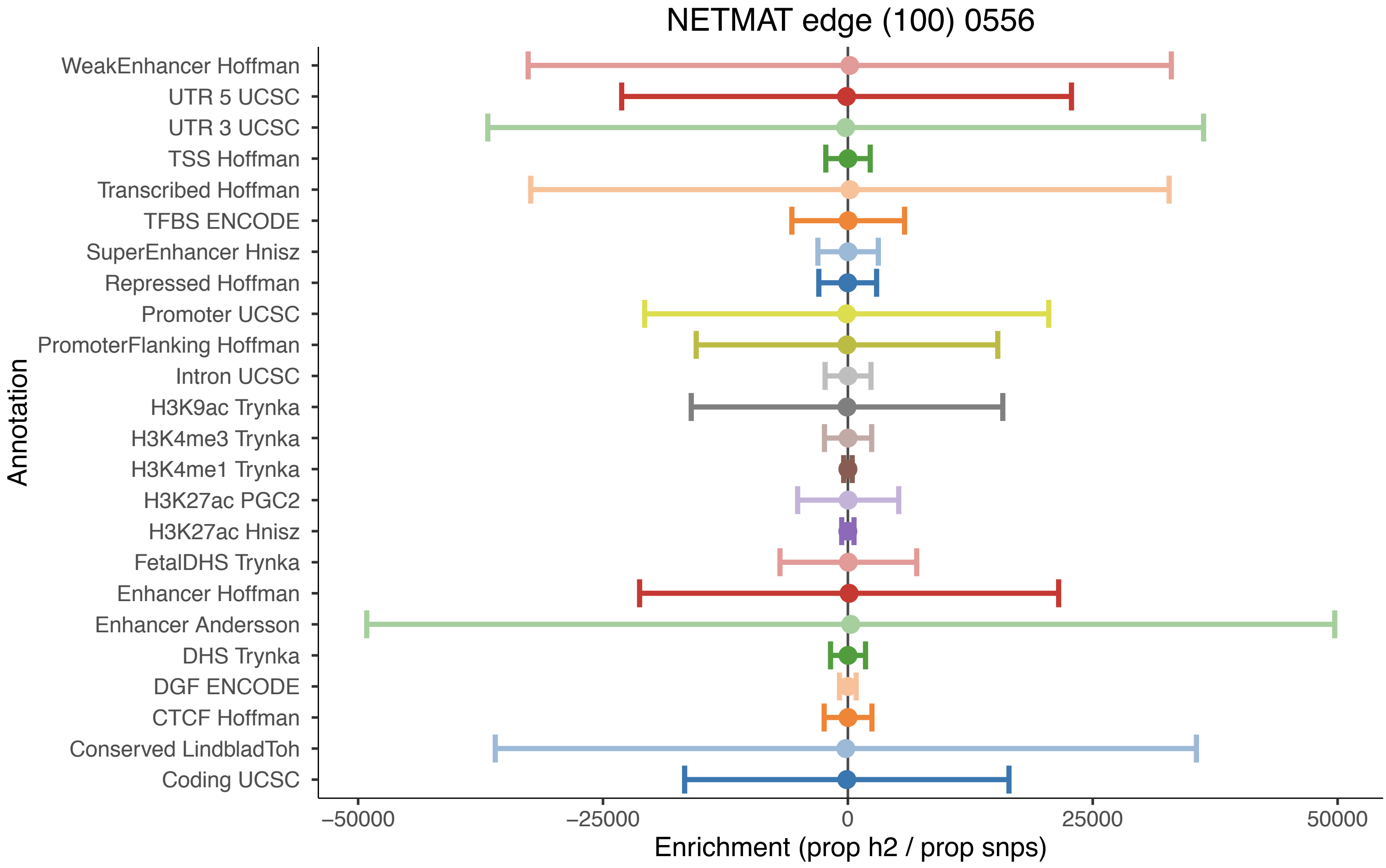
NETMAT edge (100) 0556
WeakEnhancer Hoffman
UTR 5 UCSC
UTR 3 UCSC
TSS Hoffman
Transcribed Hoffman
TFBS ENCODE
SuperEnhancer Hnisz
Repressed Hoffman
Promoter UCSC
PromoterFlanking Hoffman
Intron UCSC
H3K9ac Trynka
H3K4me3 Trynka
H3K4me1 Trynka
H3K27ac PGC2
H3K27ac Hnisz
FetalDHS Trynka
Enhancer Hoffman
Enhancer Andersson
DHS Trynka
DGF ENCODE
CTCF Hoffman
Conserved LindbladToh
Coding UCSC
Annotation
−50000
−25000
0
25000
50000
Enrichment (prop h2 / prop snps)

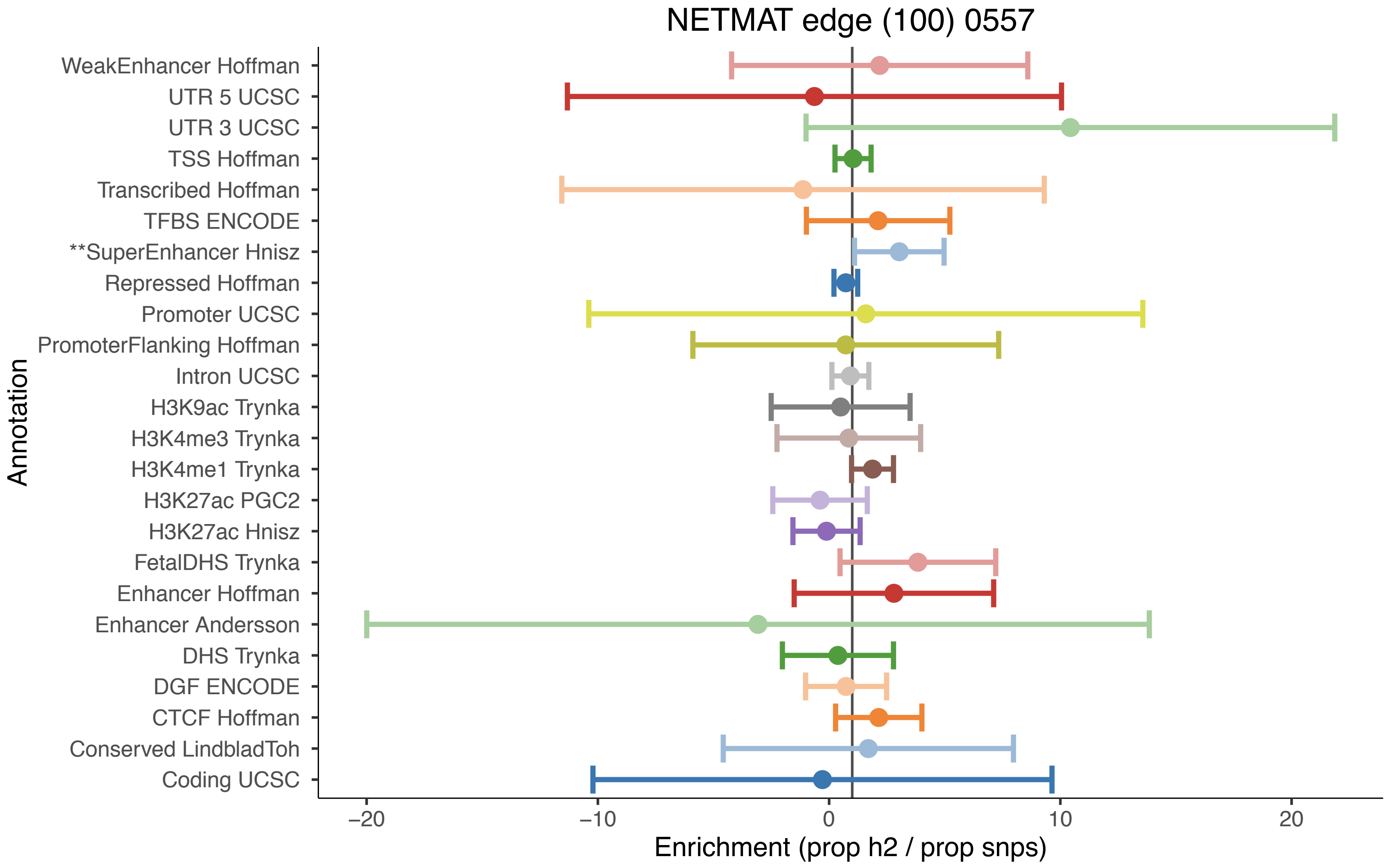

NETMAT edge (100) 0557
WeakEnhancer Hoffman
UTR 5 UCSC
UTR 3 UCSC
TSS Hoffman
Transcribed Hoffman
TFBS ENCODE
**SuperEnhancer Hnisz
Repressed Hoffman
Promoter UCSC
PromoterFlanking Hoffman
Intron UCSC
H3K9ac Trynka
H3K4me3 Trynka
H3K4me1 Trynka
H3K27ac PGC2
H3K27ac Hnisz
FetalDHS Trynka
Enhancer Hoffman
Enhancer Andersson
DHS Trynka
DGF ENCODE
CTCF Hoffman
Conserved LindbladToh
Coding UCSC
Annotation
−20
−10
0
10
20
Enrichment (prop h2 / prop snps)

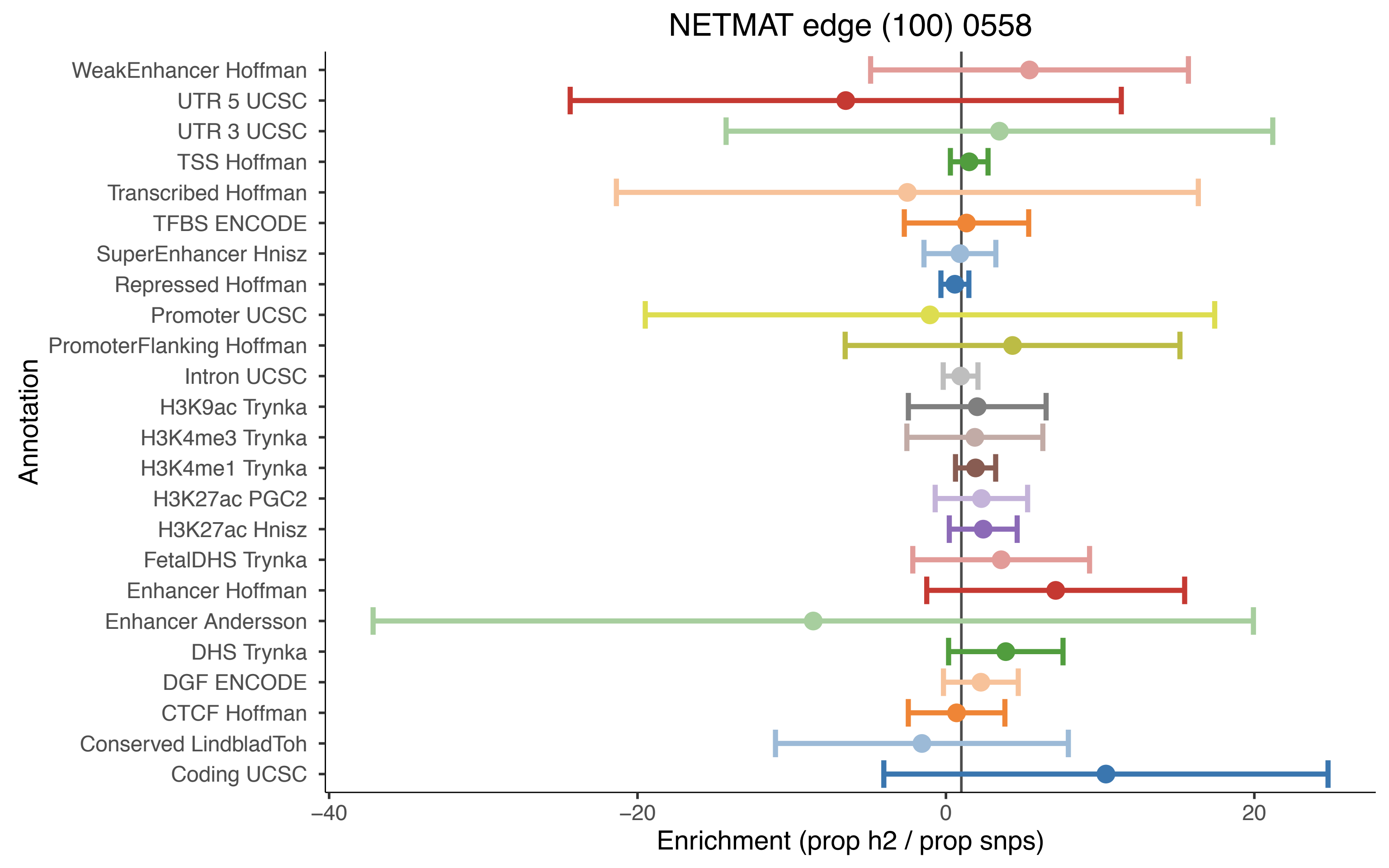

NETMAT edge (100) 0558
WeakEnhancer Hoffman
UTR 5 UCSC
UTR 3 UCSC
TSS Hoffman
Transcribed Hoffman
TFBS ENCODE
SuperEnhancer Hnisz
Repressed Hoffman
Promoter UCSC
PromoterFlanking Hoffman
Intron UCSC
H3K9ac Trynka
H3K4me3 Trynka
H3K4me1 Trynka
H3K27ac PGC2
H3K27ac Hnisz
FetalDHS Trynka
Enhancer Hoffman
Enhancer Andersson
DHS Trynka
DGF ENCODE
CTCF Hoffman
Conserved LindbladToh
Coding UCSC
Annotation
−40
−20
0
20
Enrichment (prop h2 / prop snps)

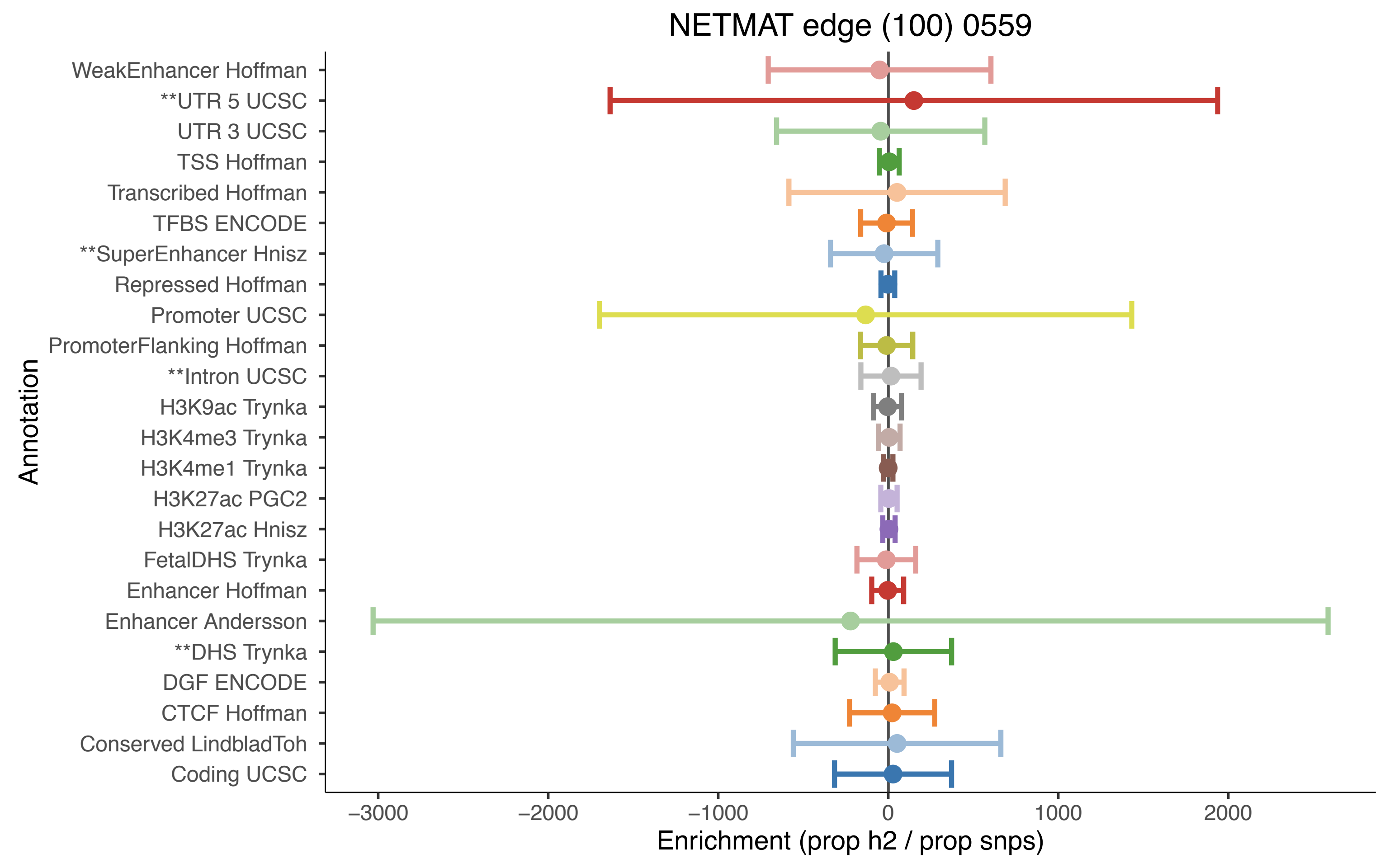
NETMAT edge (100) 0559
WeakEnhancer Hoffman
**UTR 5 UCSC
UTR 3 UCSC
TSS Hoffman
Transcribed Hoffman
TFBS ENCODE
**SuperEnhancer Hnisz
Repressed Hoffman
Promoter UCSC
PromoterFlanking Hoffman
**Intron UCSC
H3K9ac Trynka
H3K4me3 Trynka
H3K4me1 Trynka
H3K27ac PGC2
H3K27ac Hnisz
FetalDHS Trynka
Enhancer Hoffman
Enhancer Andersson
**DHS Trynka
DGF ENCODE
CTCF Hoffman
Conserved LindbladToh
Coding UCSC
Annotation
−3000
−2000
−1000
0
1000
2000
Enrichment (prop h2 / prop snps)

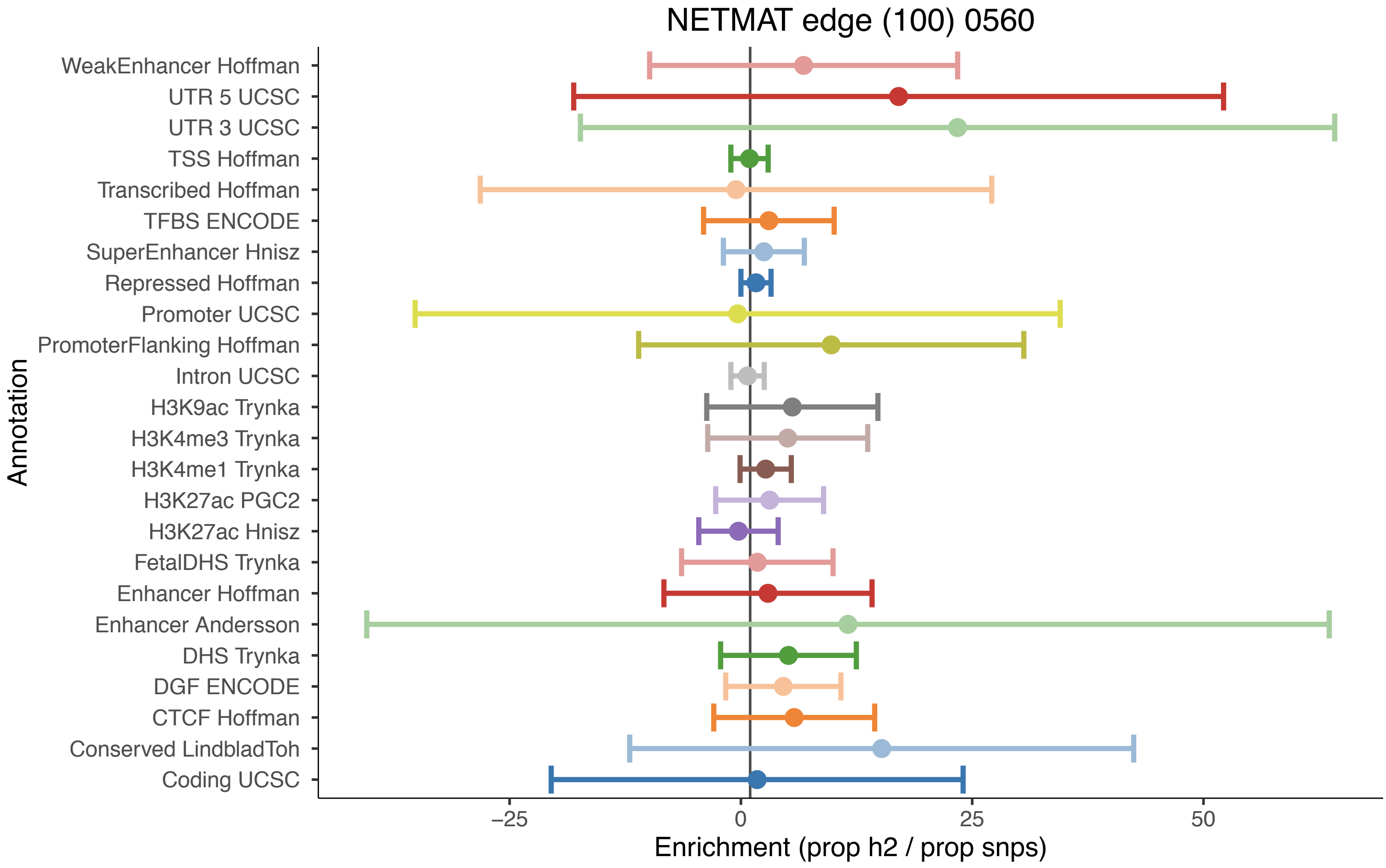

NETMAT edge (100) 0560
Annotation
WeakEnhancer Hoffman
UTR 5 UCSC
UTR 3 UCSC
TSS Hoffman
Transcribed Hoffman
TFBS ENCODE
SuperEnhancer Hnisz
Repressed Hoffman
Promoter UCSC
PromoterFlanking Hoffman
Intron UCSC
H3K9ac Trynka
H3K4me3 Trynka
H3K4me1 Trynka
H3K27ac PGC2
H3K27ac Hnisz
FetalDHS Trynka
Enhancer Hoffman
Enhancer Andersson
DHS Trynka
DGF ENCODE
CTCF Hoffman
Conserved LindbladToh
Coding UCSC
−25
0
25
50
Enrichment (prop h2 / prop snps)

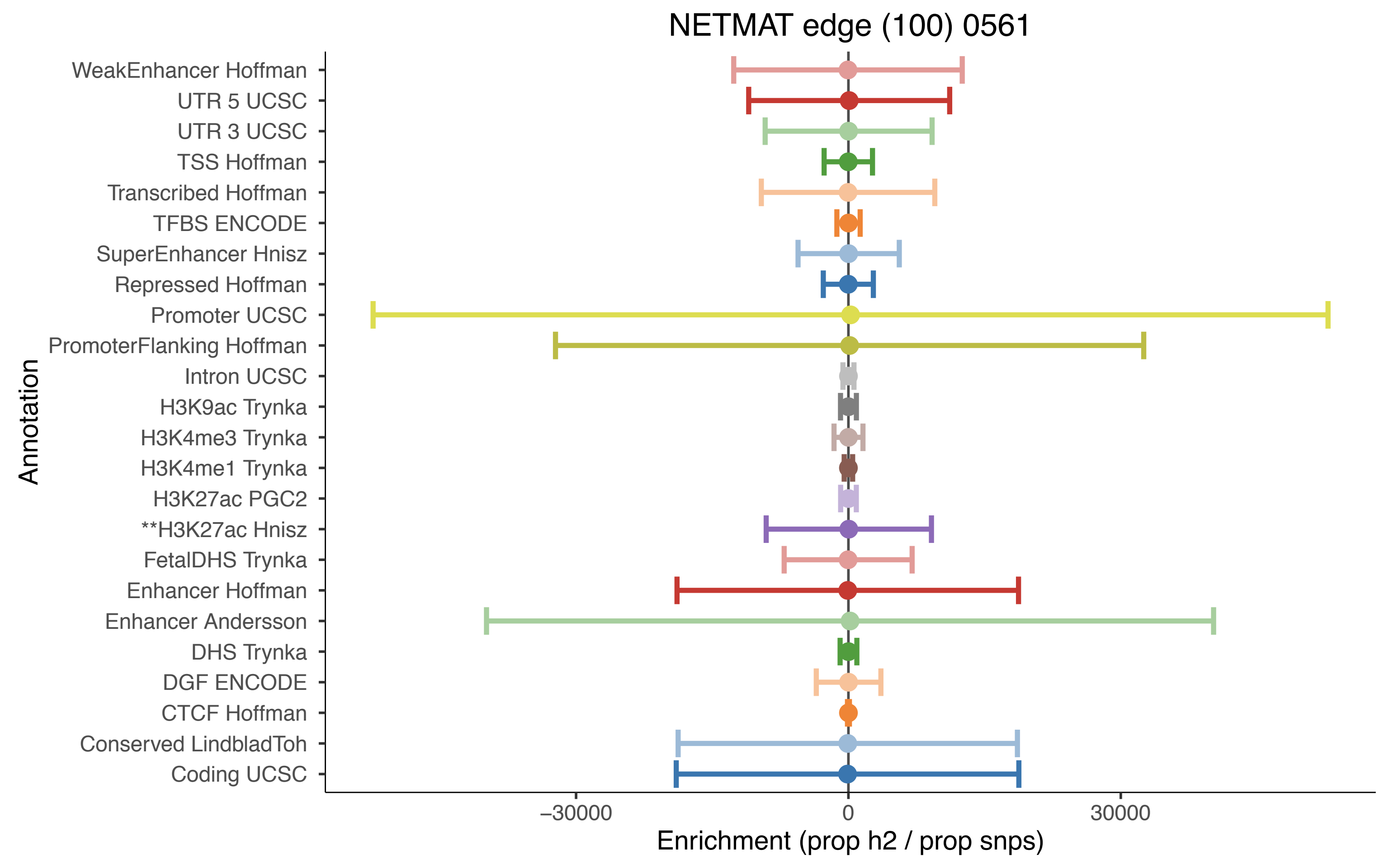
NETMAT edge (100) 0561
Annotation
WeakEnhancer Hoffman
UTR 5 UCSC
UTR 3 UCSC
TSS Hoffman
Transcribed Hoffman
TFBS ENCODE
SuperEnhancer Hnisz
Repressed Hoffman
Promoter UCSC
PromoterFlanking Hoffman
Intron UCSC
H3K9ac Trynka
H3K4me3 Trynka
H3K4me1 Trynka
H3K27ac PGC2
**H3K27ac Hnisz
FetalDHS Trynka
Enhancer Hoffman
Enhancer Andersson
DHS Trynka
DGF ENCODE
CTCF Hoffman
Conserved LindbladToh
Coding UCSC
−30000
0
30000
Enrichment (prop h2 / prop snps)

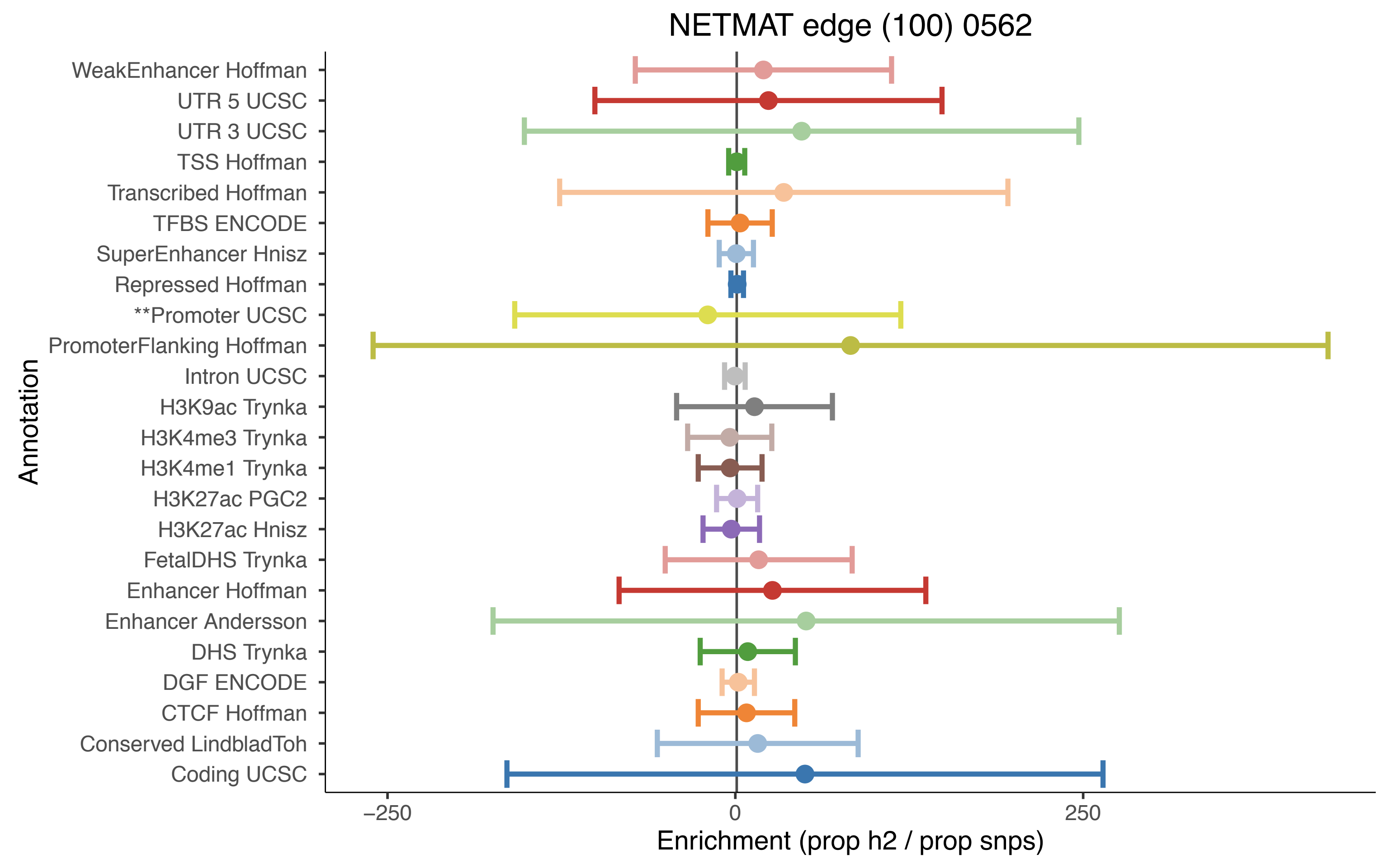
NETMAT edge (100) 0562
WeakEnhancer Hoffman
UTR 5 UCSC
UTR 3 UCSC
TSS Hoffman
Transcribed Hoffman
TFBS ENCODE
SuperEnhancer Hnisz
Repressed Hoffman
**Promoter UCSC
PromoterFlanking Hoffman
Intron UCSC
H3K9ac Trynka
H3K4me3 Trynka
H3K4me1 Trynka
H3K27ac PGC2
H3K27ac Hnisz
FetalDHS Trynka
Enhancer Hoffman
Enhancer Andersson
DHS Trynka
DGF ENCODE
CTCF Hoffman
Conserved LindbladToh
Coding UCSC
Annotation
−250
0
250
Enrichment (prop h2 / prop snps)

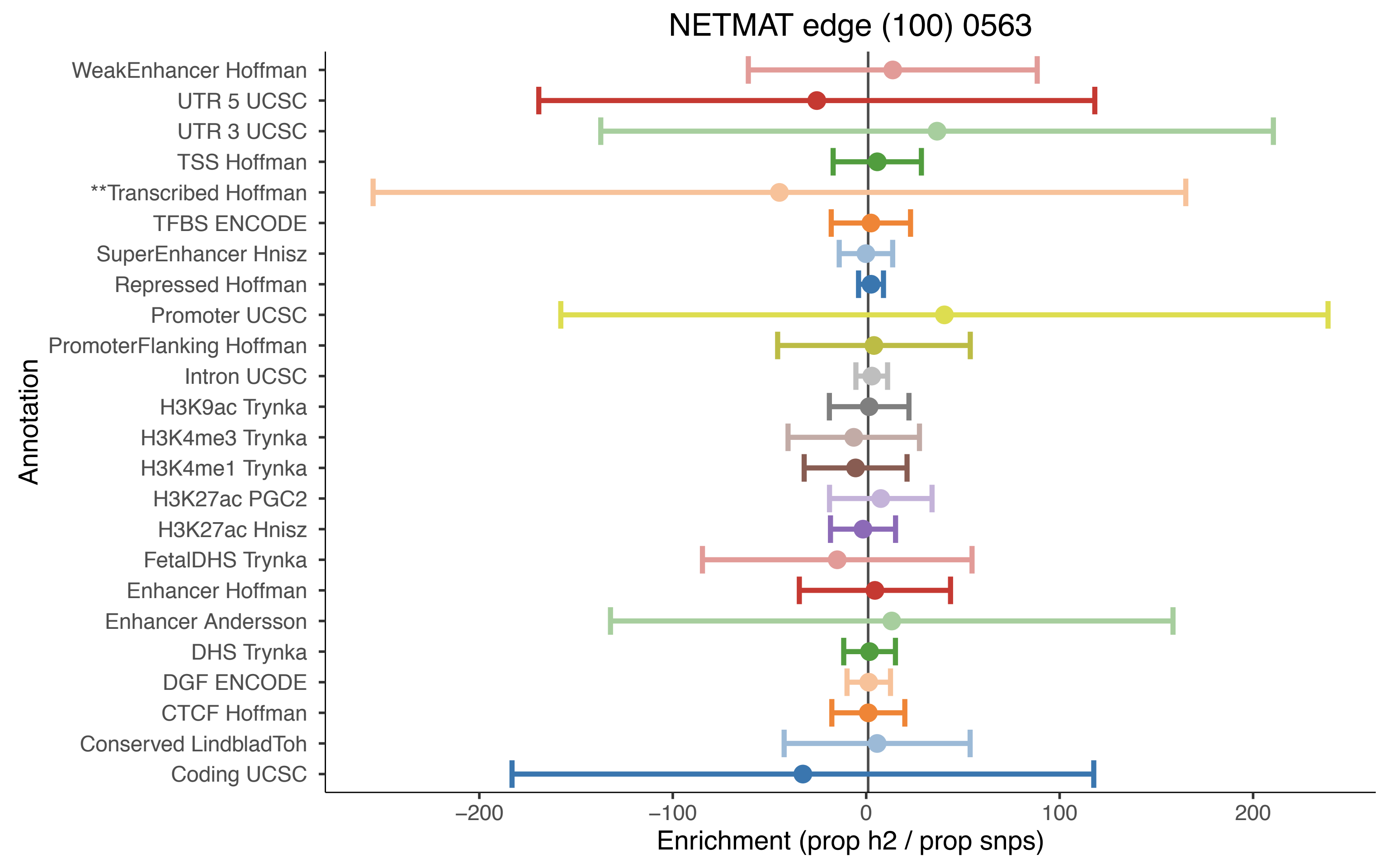

NETMAT edge (100) 0563
WeakEnhancer Hoffman
UTR 5 UCSC
UTR 3 UCSC
TSS Hoffman
**Transcribed Hoffman
TFBS ENCODE
SuperEnhancer Hnisz
Repressed Hoffman
Promoter UCSC
PromoterFlanking Hoffman
Intron UCSC
H3K9ac Trynka
H3K4me3 Trynka
H3K4me1 Trynka
H3K27ac PGC2
H3K27ac Hnisz
FetalDHS Trynka
Enhancer Hoffman
Enhancer Andersson
DHS Trynka
DGF ENCODE
CTCF Hoffman
Conserved LindbladToh
Coding UCSC
Annotation
−200
−100
0
100
200
Enrichment (prop h2 / prop snps)

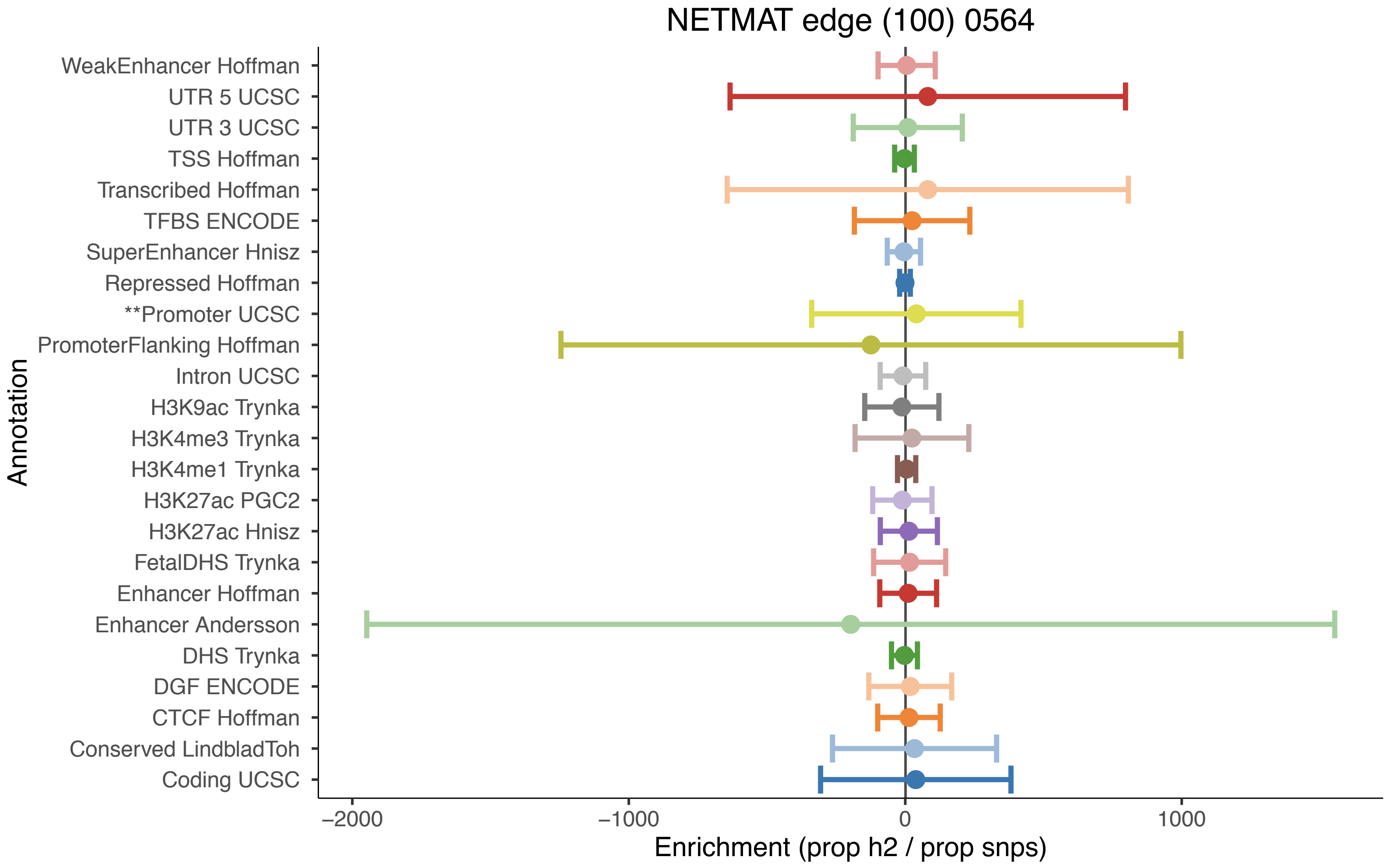

NETMAT edge (100) 0564
WeakEnhancer Hoffman
UTR 5 UCSC
UTR 3 UCSC
TSS Hoffman
Transcribed Hoffman
TFBS ENCODE
SuperEnhancer Hnisz
Repressed Hoffman
**Promoter UCSC
PromoterFlanking Hoffman
Intron UCSC
H3K9ac Trynka
H3K4me3 Trynka
H3K4me1 Trynka
H3K27ac PGC2
H3K27ac Hnisz
FetalDHS Trynka
Enhancer Hoffman
Enhancer Andersson
DHS Trynka
DGF ENCODE
CTCF Hoffman
Conserved LindbladToh
Coding UCSC
Annotation
−2000
−1000
0
1000
Enrichment (prop h2 / prop snps)

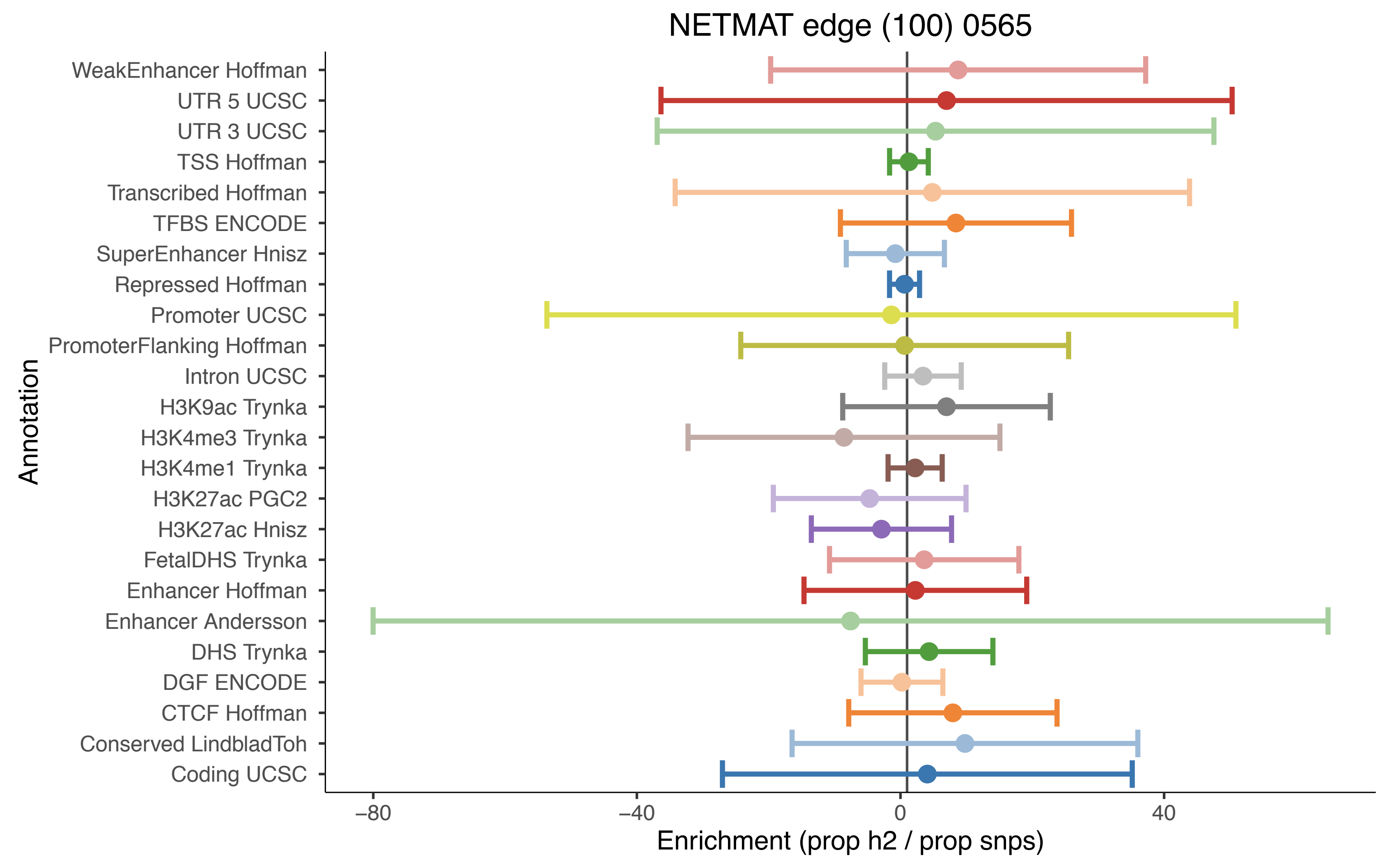

NETMAT edge (100) 0565
WeakEnhancer Hoffman
UTR 5 UCSC
UTR 3 UCSC
TSS Hoffman
Transcribed Hoffman
TFBS ENCODE
SuperEnhancer Hnisz
Repressed Hoffman
Promoter UCSC
PromoterFlanking Hoffman
Intron UCSC
H3K9ac Trynka
H3K4me3 Trynka
H3K4me1 Trynka
H3K27ac PGC2
H3K27ac Hnisz
FetalDHS Trynka
Enhancer Hoffman
Enhancer Andersson
DHS Trynka
DGF ENCODE
CTCF Hoffman
Conserved LindbladToh
Coding UCSC
Annotation
−80
−40
0
40
Enrichment (prop h2 / prop snps)

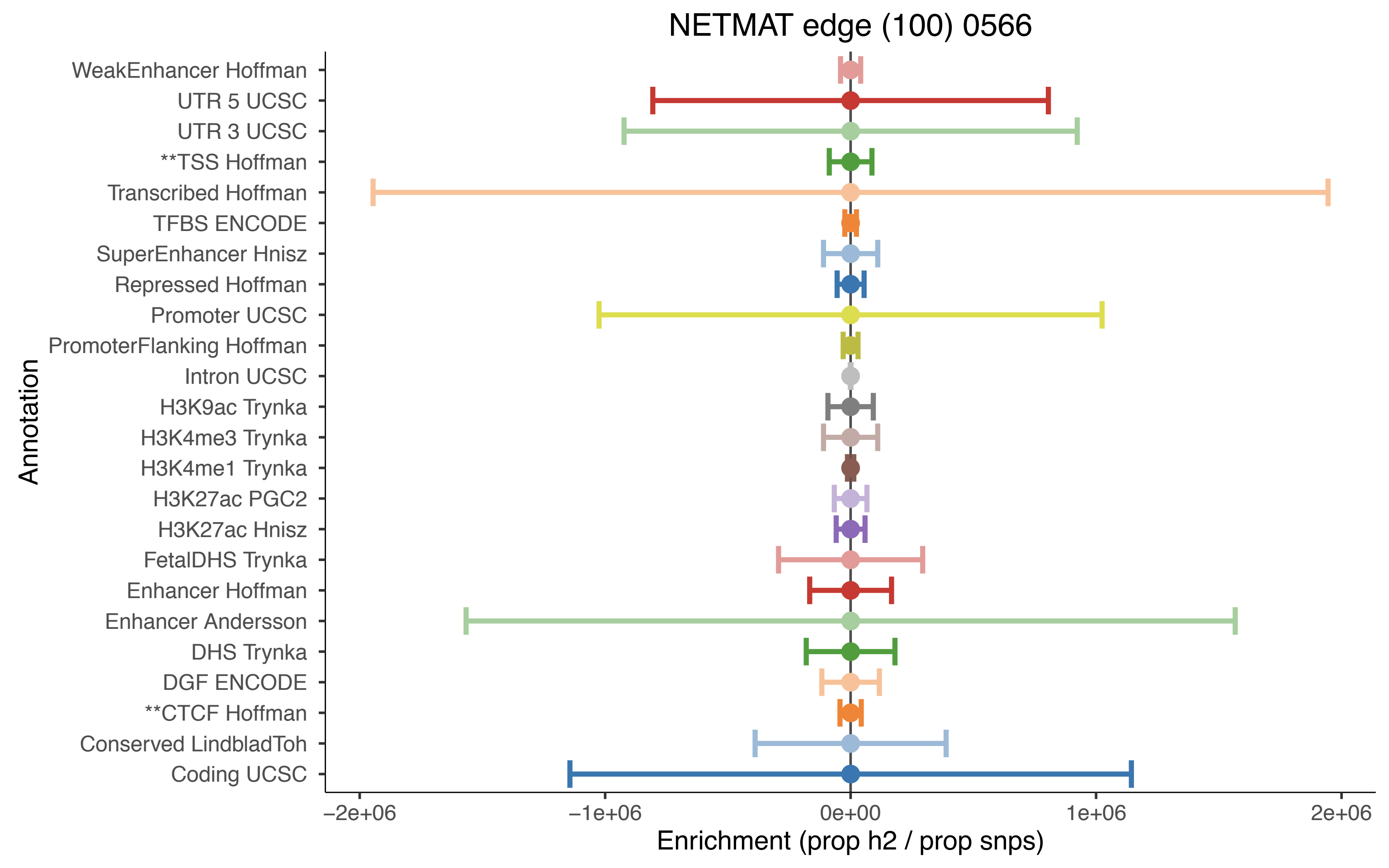

NETMAT edge (100) 0566
Annotation
WeakEnhancer Hoffman
UTR 5 UCSC
UTR 3 UCSC
**TSS Hoffman
Transcribed Hoffman
TFBS ENCODE
SuperEnhancer Hnisz
Repressed Hoffman
Promoter UCSC
PromoterFlanking Hoffman
Intron UCSC
H3K9ac Trynka
H3K4me3 Trynka
H3K4me1 Trynka
H3K27ac PGC2
H3K27ac Hnisz
FetalDHS Trynka
Enhancer Hoffman
Enhancer Andersson
DHS Trynka
DGF ENCODE
**CTCF Hoffman
Conserved LindbladToh
Coding UCSC
−2e+06
−1e+06
0e+00
1e+06
2e+06
Enrichment (prop h2 / prop snps)

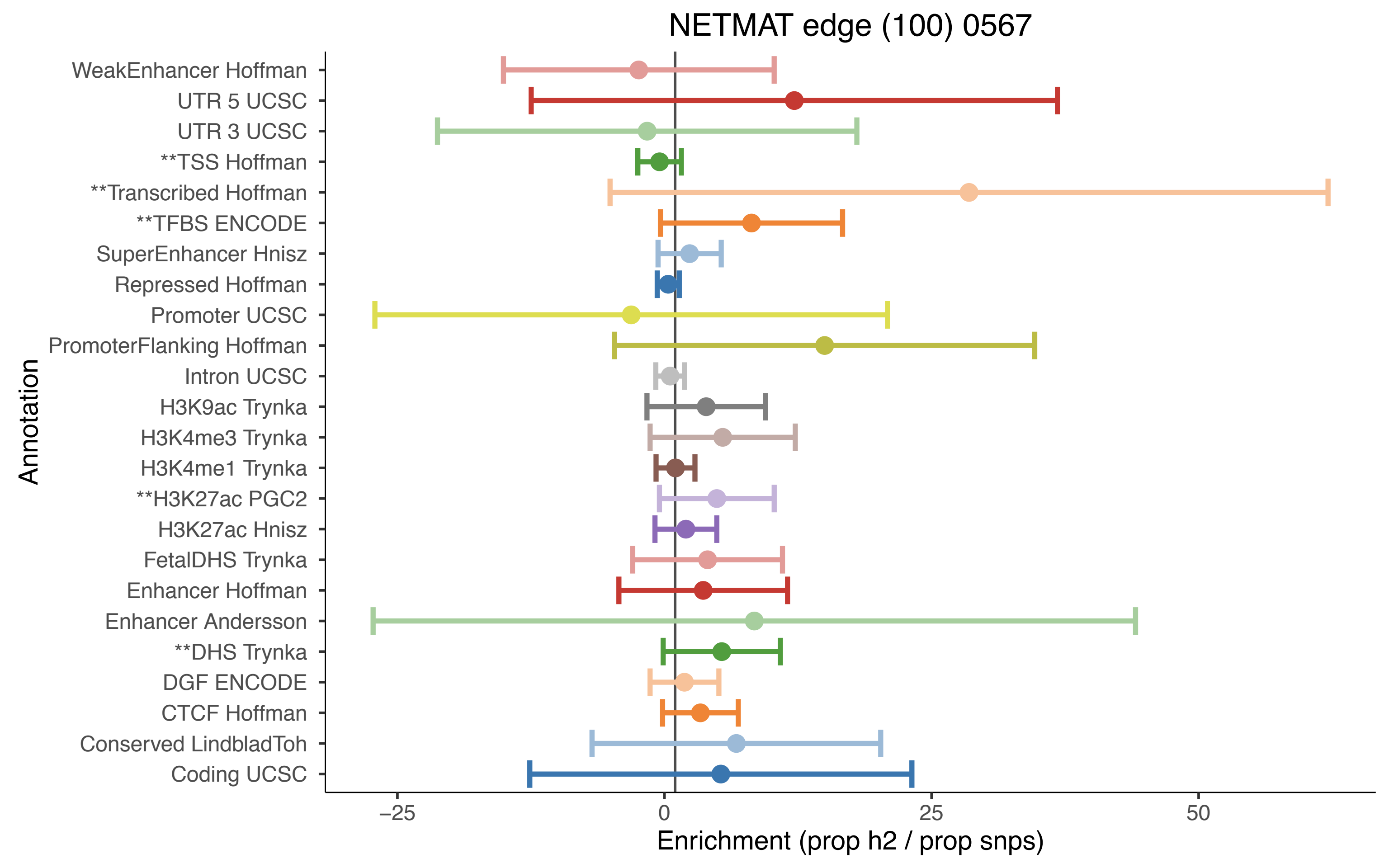
NETMAT edge (100) 0567
WeakEnhancer Hoffman
UTR 5 UCSC
UTR 3 UCSC
**TSS Hoffman
**Transcribed Hoffman
**TFBS ENCODE
SuperEnhancer Hnisz
Repressed Hoffman
Promoter UCSC
PromoterFlanking Hoffman
Intron UCSC
H3K9ac Trynka
H3K4me3 Trynka
H3K4me1 Trynka
**H3K27ac PGC2
H3K27ac Hnisz
FetalDHS Trynka
Enhancer Hoffman
Enhancer Andersson
**DHS Trynka
DGF ENCODE
CTCF Hoffman
Conserved LindbladToh
Coding UCSC
Annotation
−25
0
25
50
Enrichment (prop h2 / prop snps)

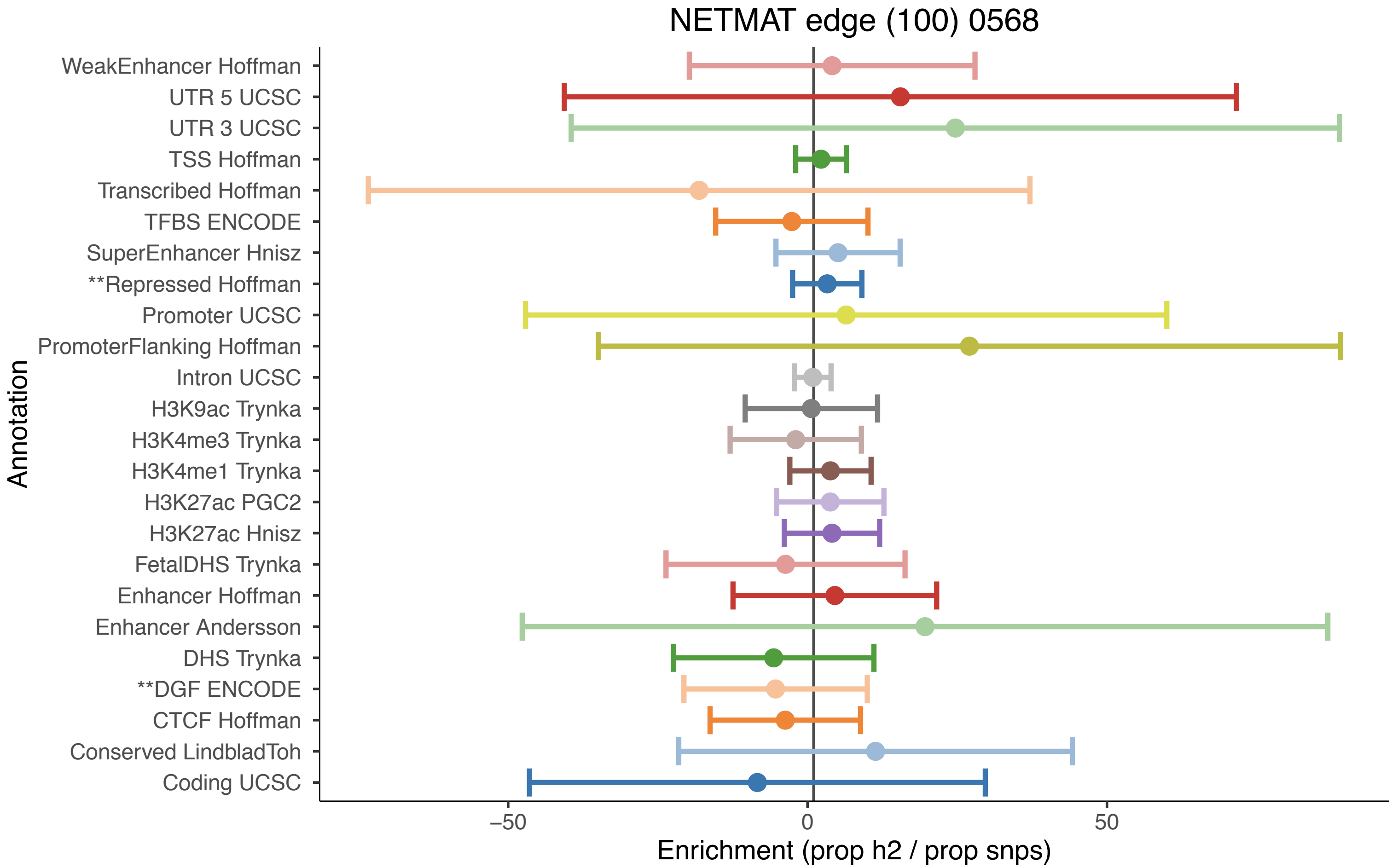

NETMAT edge (100) 0568
WeakEnhancer Hoffman
UTR 5 UCSC
UTR 3 UCSC
TSS Hoffman
Transcribed Hoffman
TFBS ENCODE
SuperEnhancer Hnisz
**Repressed Hoffman
Promoter UCSC
PromoterFlanking Hoffman
Intron UCSC
H3K9ac Trynka
H3K4me3 Trynka
H3K4me1 Trynka
H3K27ac PGC2
H3K27ac Hnisz
FetalDHS Trynka
Enhancer Hoffman
Enhancer Andersson
DHS Trynka
**DGF ENCODE
CTCF Hoffman
Conserved LindbladToh
Coding UCSC
Annotation
−50
0
50
Enrichment (prop h2 / prop snps)

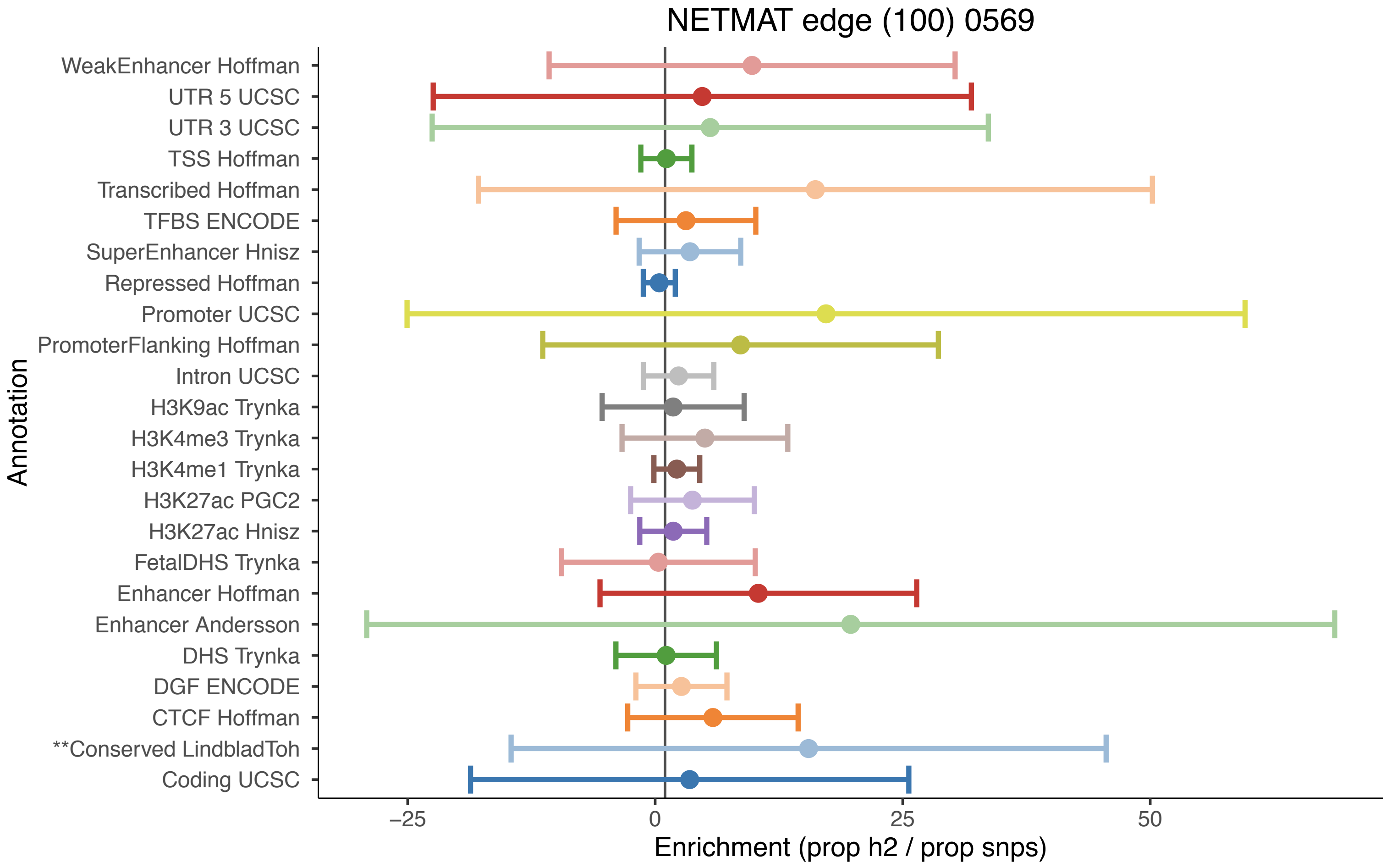
NETMAT edge (100) 0569
WeakEnhancer Hoffman
UTR 5 UCSC
UTR 3 UCSC
TSS Hoffman
Transcribed Hoffman
TFBS ENCODE
SuperEnhancer Hnisz
Repressed Hoffman
Promoter UCSC
PromoterFlanking Hoffman
Intron UCSC
H3K9ac Trynka
H3K4me3 Trynka
H3K4me1 Trynka
H3K27ac PGC2
H3K27ac Hnisz
FetalDHS Trynka
Enhancer Hoffman
Enhancer Andersson
DHS Trynka
DGF ENCODE
CTCF Hoffman
**Conserved LindbladToh
Coding UCSC
Annotation
−25
0
25
50
Enrichment (prop h2 / prop snps)

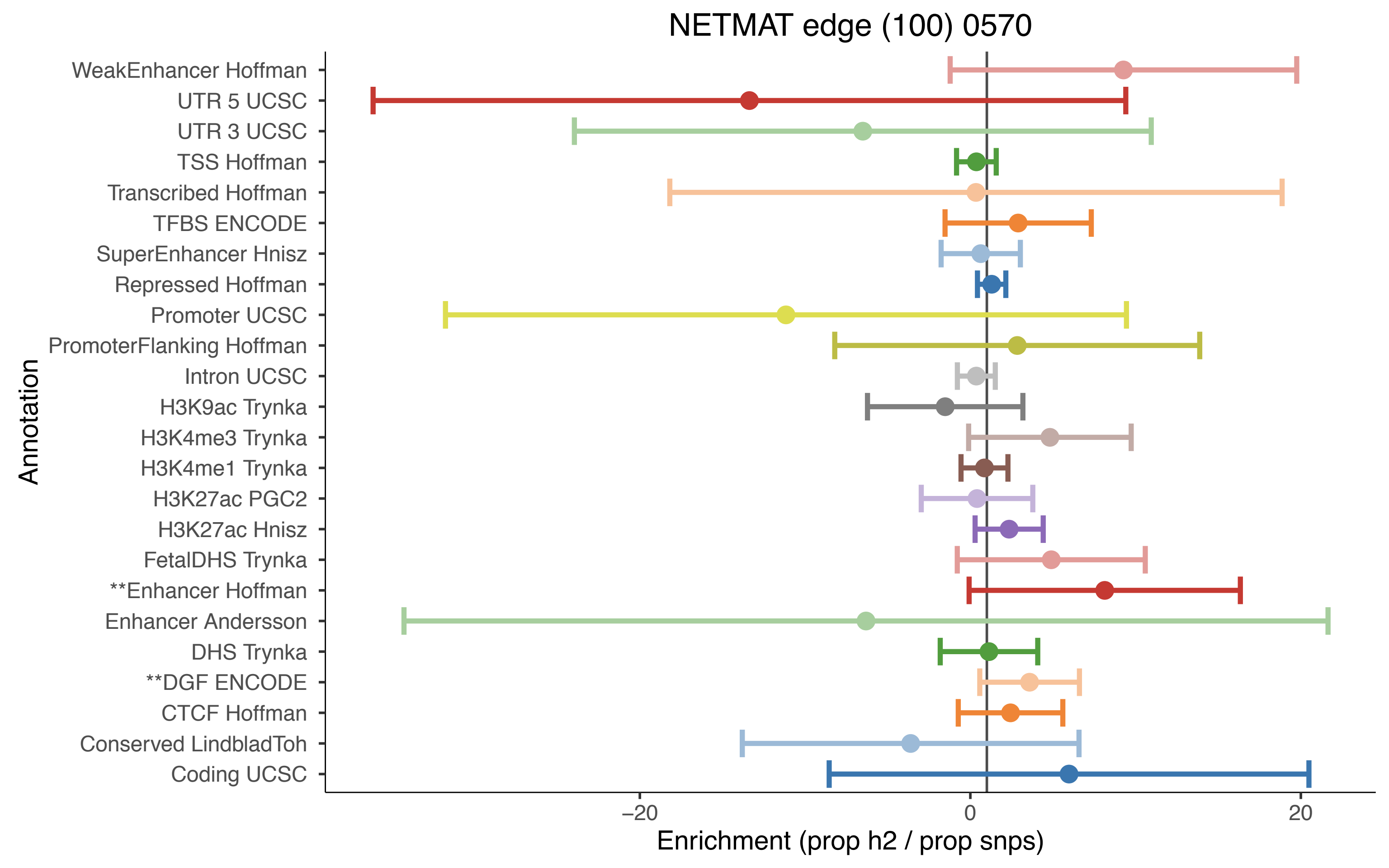
NETMAT edge (100) 0570
WeakEnhancer Hoffman
UTR 5 UCSC
UTR 3 UCSC
TSS Hoffman
Transcribed Hoffman
TFBS ENCODE
SuperEnhancer Hnisz
Repressed Hoffman
Promoter UCSC
PromoterFlanking Hoffman
Intron UCSC
H3K9ac Trynka
H3K4me3 Trynka
H3K4me1 Trynka
H3K27ac PGC2
H3K27ac Hnisz
FetalDHS Trynka
**Enhancer Hoffman
Enhancer Andersson
DHS Trynka
**DGF ENCODE
CTCF Hoffman
Conserved LindbladToh
Coding UCSC
Annotation
−20
0
20
Enrichment (prop h2 / prop snps)

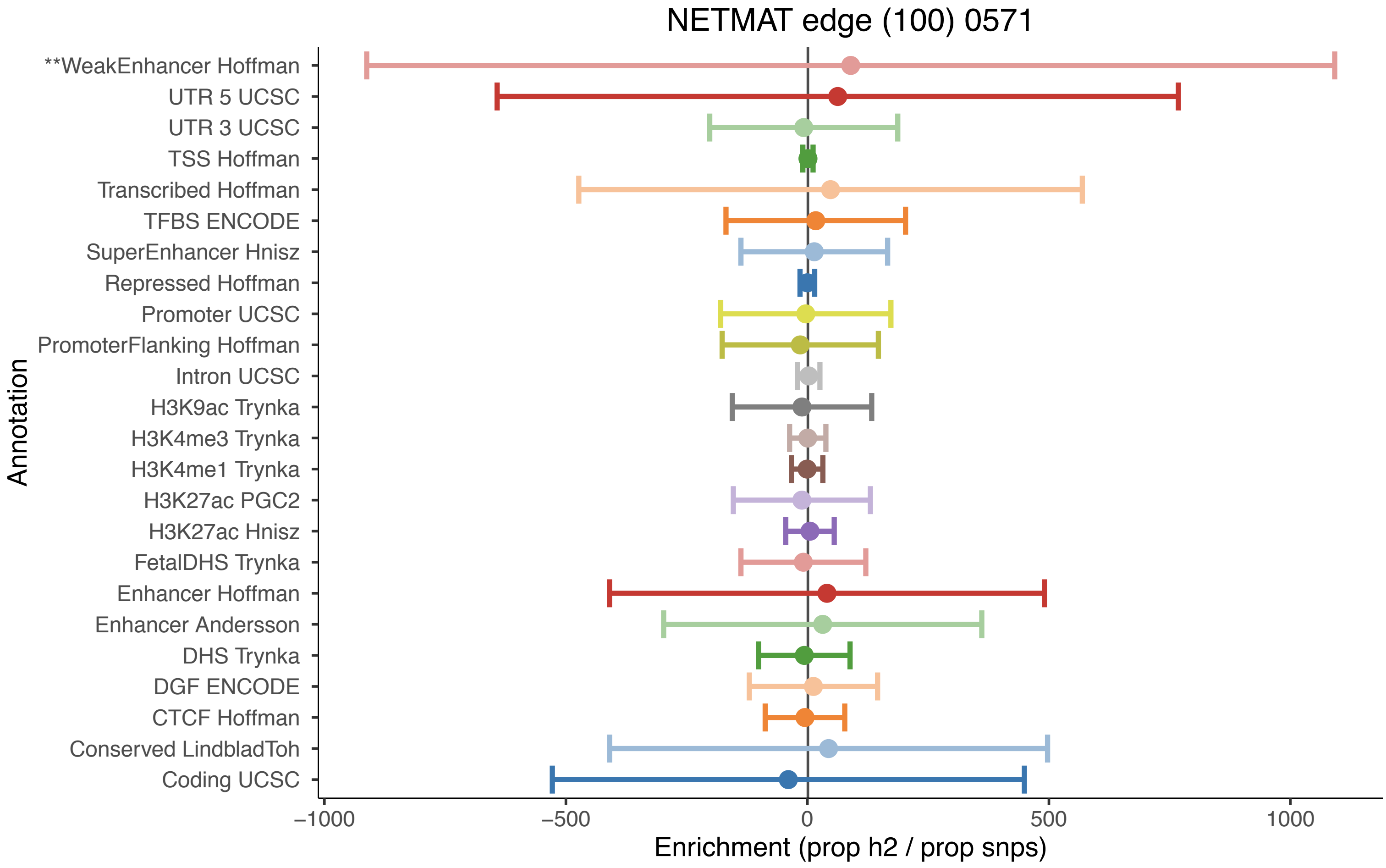

NETMAT edge (100) 0571
**WeakEnhancer Hoffman
UTR 5 UCSC
UTR 3 UCSC
TSS Hoffman
Transcribed Hoffman
TFBS ENCODE
SuperEnhancer Hnisz
Repressed Hoffman
Promoter UCSC
PromoterFlanking Hoffman
Intron UCSC
H3K9ac Trynka
H3K4me3 Trynka
H3K4me1 Trynka
H3K27ac PGC2
H3K27ac Hnisz
FetalDHS Trynka
Enhancer Hoffman
Enhancer Andersson
DHS Trynka
DGF ENCODE
CTCF Hoffman
Conserved LindbladToh
Coding UCSC
Annotation
−1000
−500
0
500
1000
Enrichment (prop h2 / prop snps)

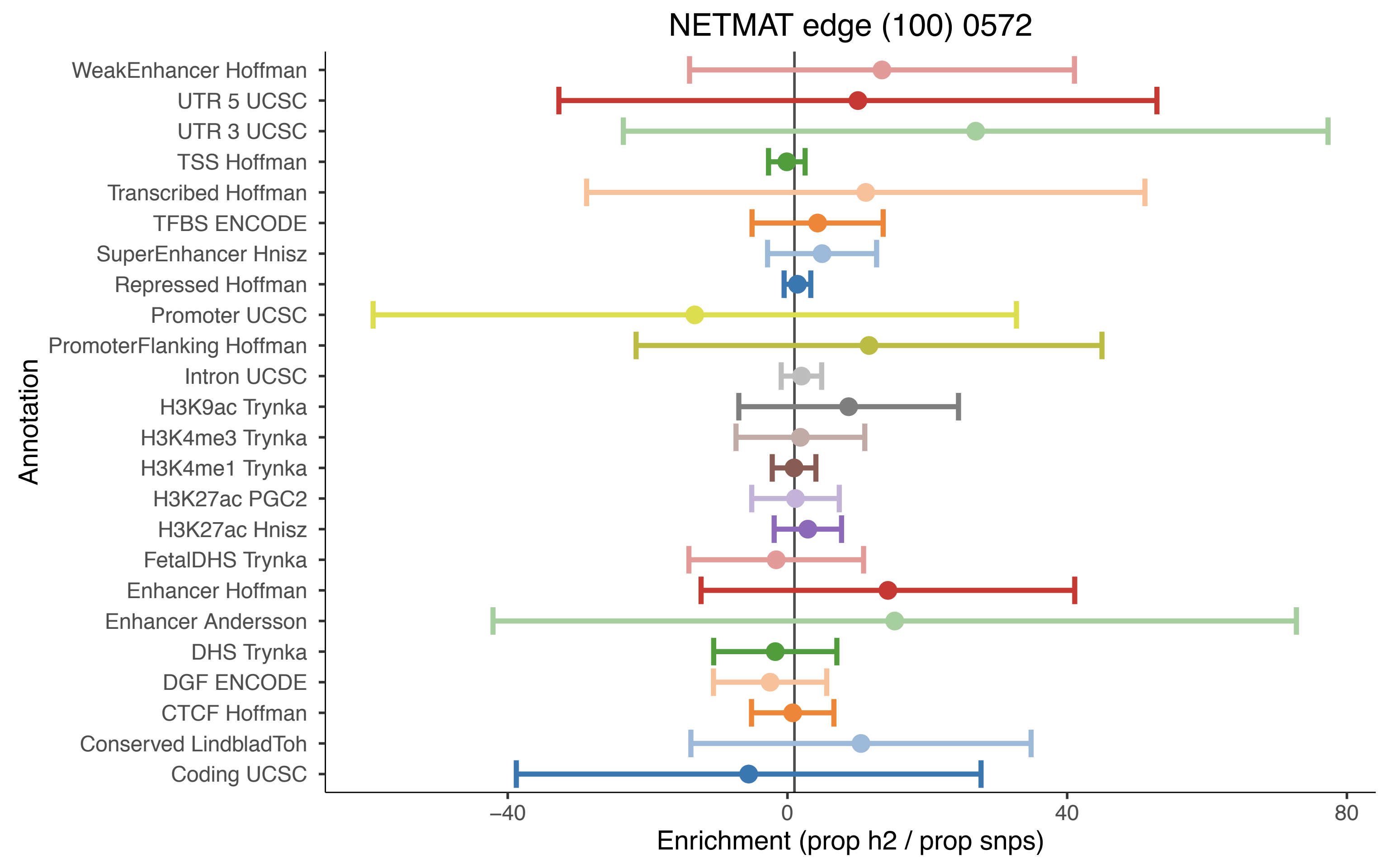

NETMAT edge (100) 0572
WeakEnhancer Hoffman
UTR 5 UCSC
UTR 3 UCSC
TSS Hoffman
Transcribed Hoffman
TFBS ENCODE
SuperEnhancer Hnisz
Repressed Hoffman
Promoter UCSC
PromoterFlanking Hoffman
Intron UCSC
H3K9ac Trynka
H3K4me3 Trynka
H3K4me1 Trynka
H3K27ac PGC2
H3K27ac Hnisz
FetalDHS Trynka
Enhancer Hoffman
Enhancer Andersson
DHS Trynka
DGF ENCODE
CTCF Hoffman
Conserved LindbladToh
Coding UCSC
Annotation
−40
0
40
80
Enrichment (prop h2 / prop snps)

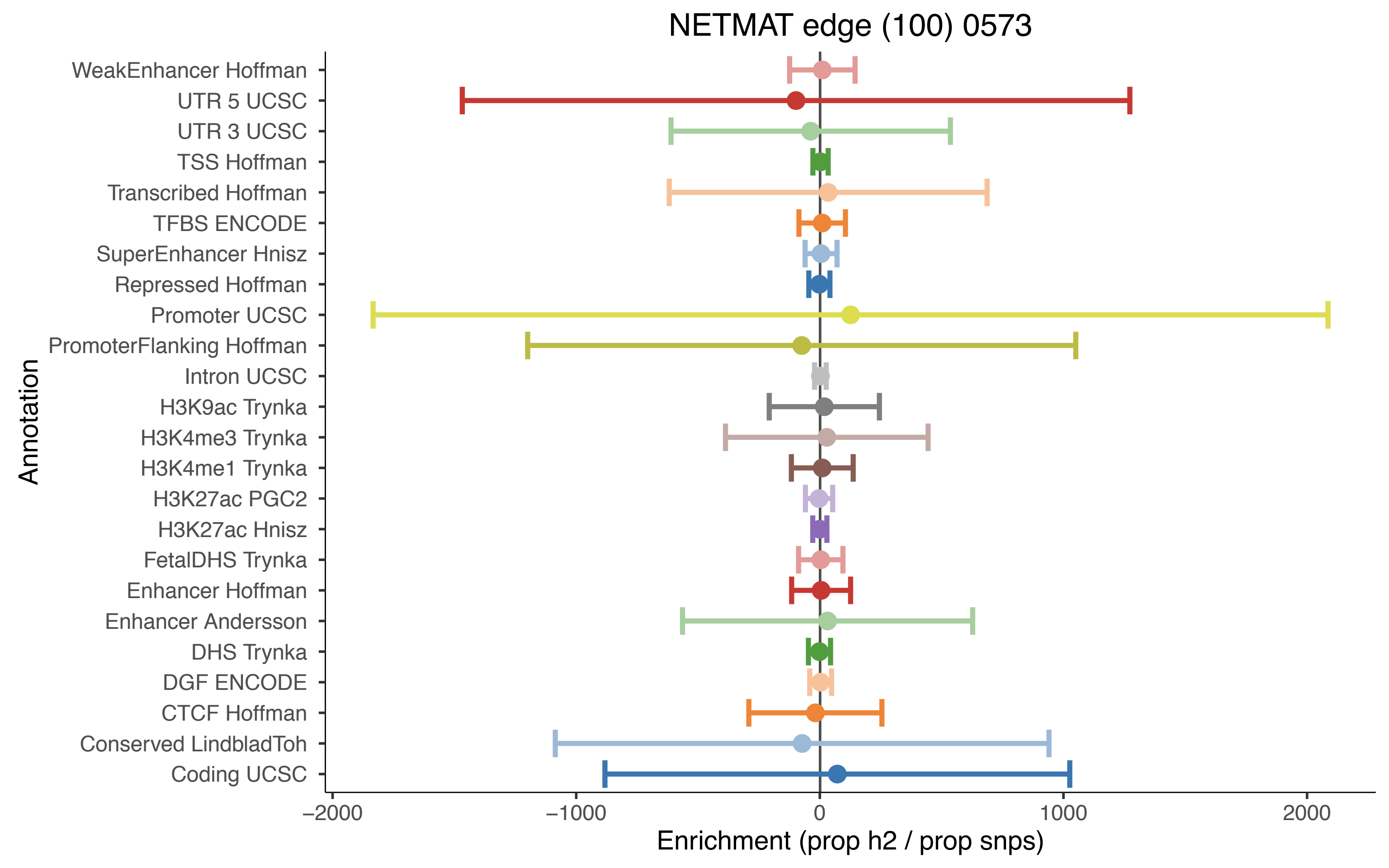

NETMAT edge (100) 0573
Annotation
WeakEnhancer Hoffman
UTR 5 UCSC
UTR 3 UCSC
TSS Hoffman
Transcribed Hoffman
TFBS ENCODE
SuperEnhancer Hnisz
Repressed Hoffman
Promoter UCSC
PromoterFlanking Hoffman
Intron UCSC
H3K9ac Trynka
H3K4me3 Trynka
H3K4me1 Trynka
H3K27ac PGC2
H3K27ac Hnisz
FetalDHS Trynka
Enhancer Hoffman
Enhancer Andersson
DHS Trynka
DGF ENCODE
CTCF Hoffman
Conserved LindbladToh
Coding UCSC
−2000
−1000
0
1000
2000
Enrichment (prop h2 / prop snps)

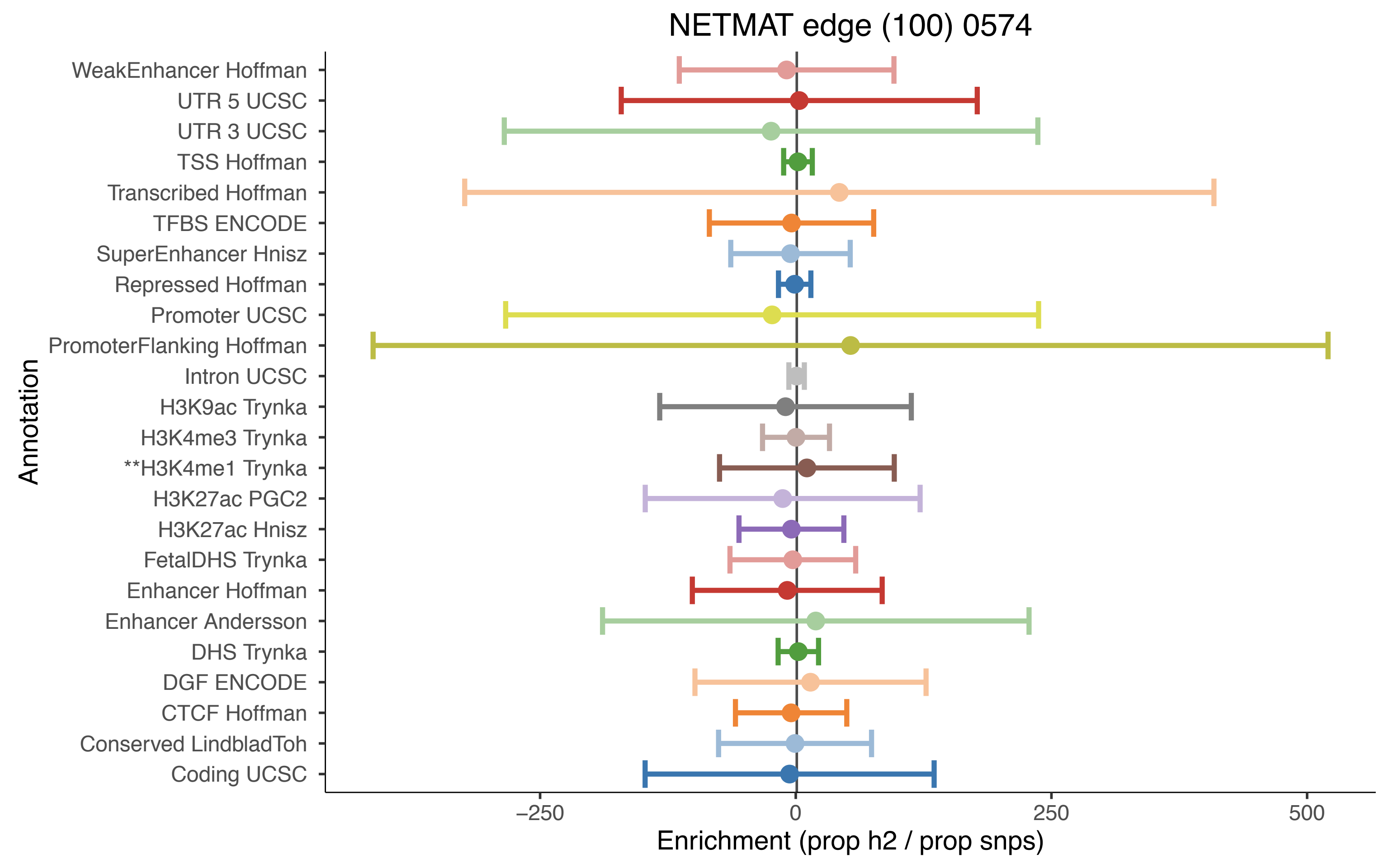
NETMAT edge (100) 0574
WeakEnhancer Hoffman
UTR 5 UCSC
UTR 3 UCSC
TSS Hoffman
Transcribed Hoffman
TFBS ENCODE
SuperEnhancer Hnisz
Repressed Hoffman
Promoter UCSC
PromoterFlanking Hoffman
Intron UCSC
H3K9ac Trynka
H3K4me3 Trynka
**H3K4me1 Trynka
H3K27ac PGC2
H3K27ac Hnisz
FetalDHS Trynka
Enhancer Hoffman
Enhancer Andersson
DHS Trynka
DGF ENCODE
CTCF Hoffman
Conserved LindbladToh
Coding UCSC
Annotation
−250
0
250
500
Enrichment (prop h2 / prop snps)

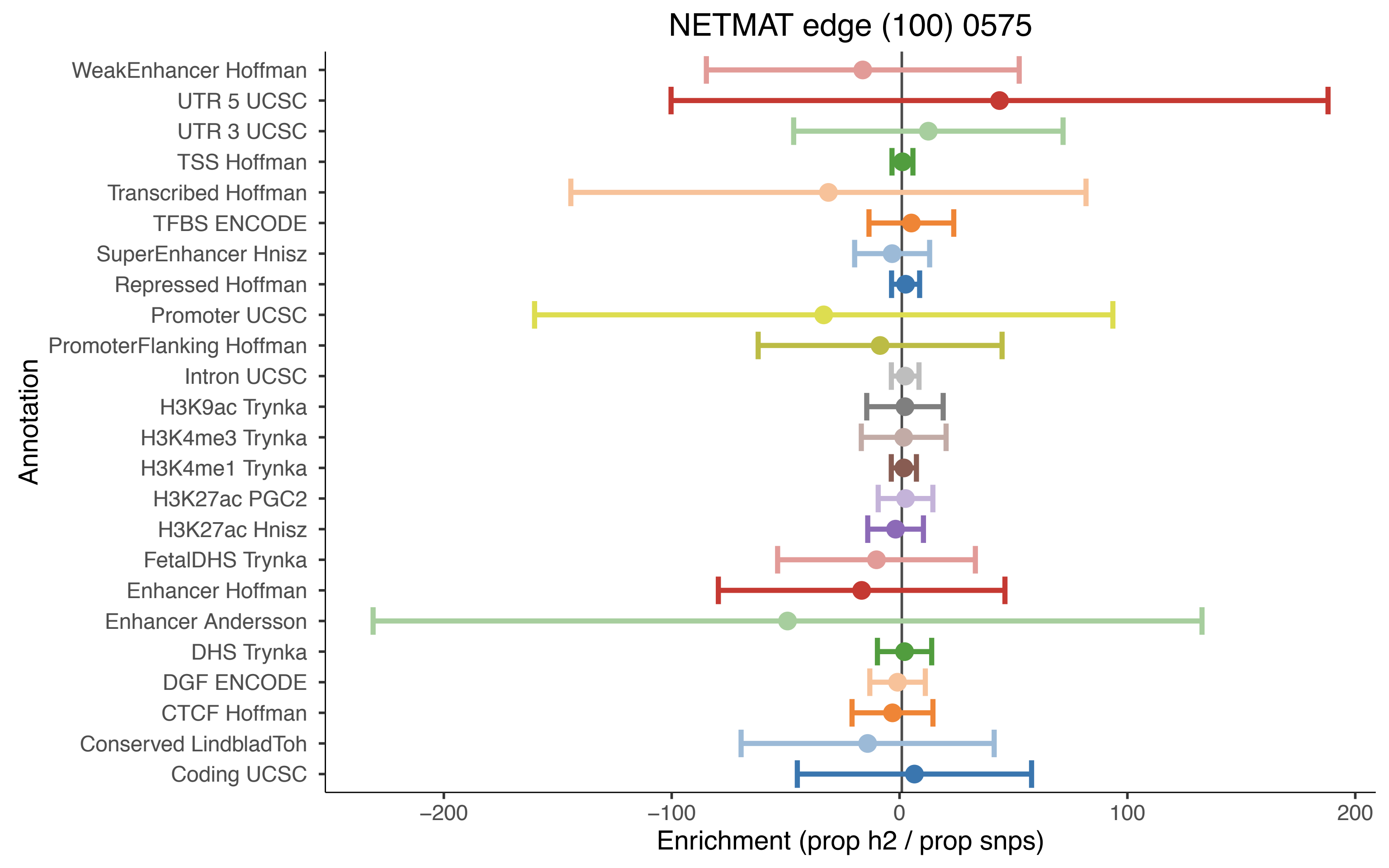
NETMAT edge (100) 0575
WeakEnhancer Hoffman
UTR 5 UCSC
UTR 3 UCSC
TSS Hoffman
Transcribed Hoffman
TFBS ENCODE
SuperEnhancer Hnisz
Repressed Hoffman
Promoter UCSC
PromoterFlanking Hoffman
Intron UCSC
H3K9ac Trynka
H3K4me3 Trynka
H3K4me1 Trynka
H3K27ac PGC2
H3K27ac Hnisz
FetalDHS Trynka
Enhancer Hoffman
Enhancer Andersson
DHS Trynka
DGF ENCODE
CTCF Hoffman
Conserved LindbladToh
Coding UCSC
Annotation
−200
−100
0
100
200
Enrichment (prop h2 / prop snps)

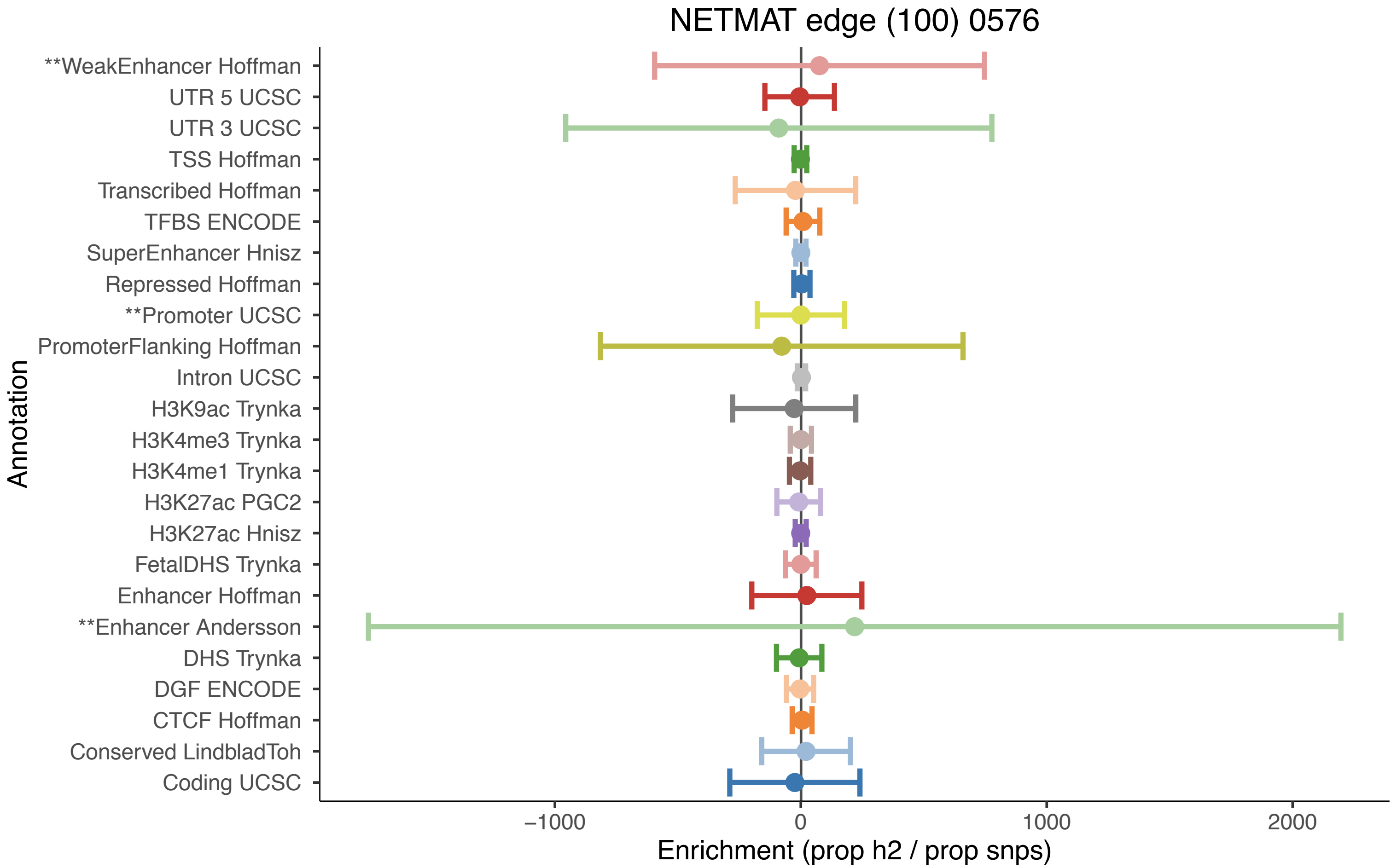
NETMAT edge (100) 0576
**WeakEnhancer Hoffman
UTR 5 UCSC
UTR 3 UCSC
TSS Hoffman
Transcribed Hoffman
TFBS ENCODE
SuperEnhancer Hnisz
Repressed Hoffman
**Promoter UCSC
PromoterFlanking Hoffman
Intron UCSC
H3K9ac Trynka
H3K4me3 Trynka
H3K4me1 Trynka
H3K27ac PGC2
H3K27ac Hnisz
FetalDHS Trynka
Enhancer Hoffman
**Enhancer Andersson
DHS Trynka
DGF ENCODE
CTCF Hoffman
Conserved LindbladToh
Coding UCSC
Annotation
−1000
0
1000
2000
Enrichment (prop h2 / prop snps)

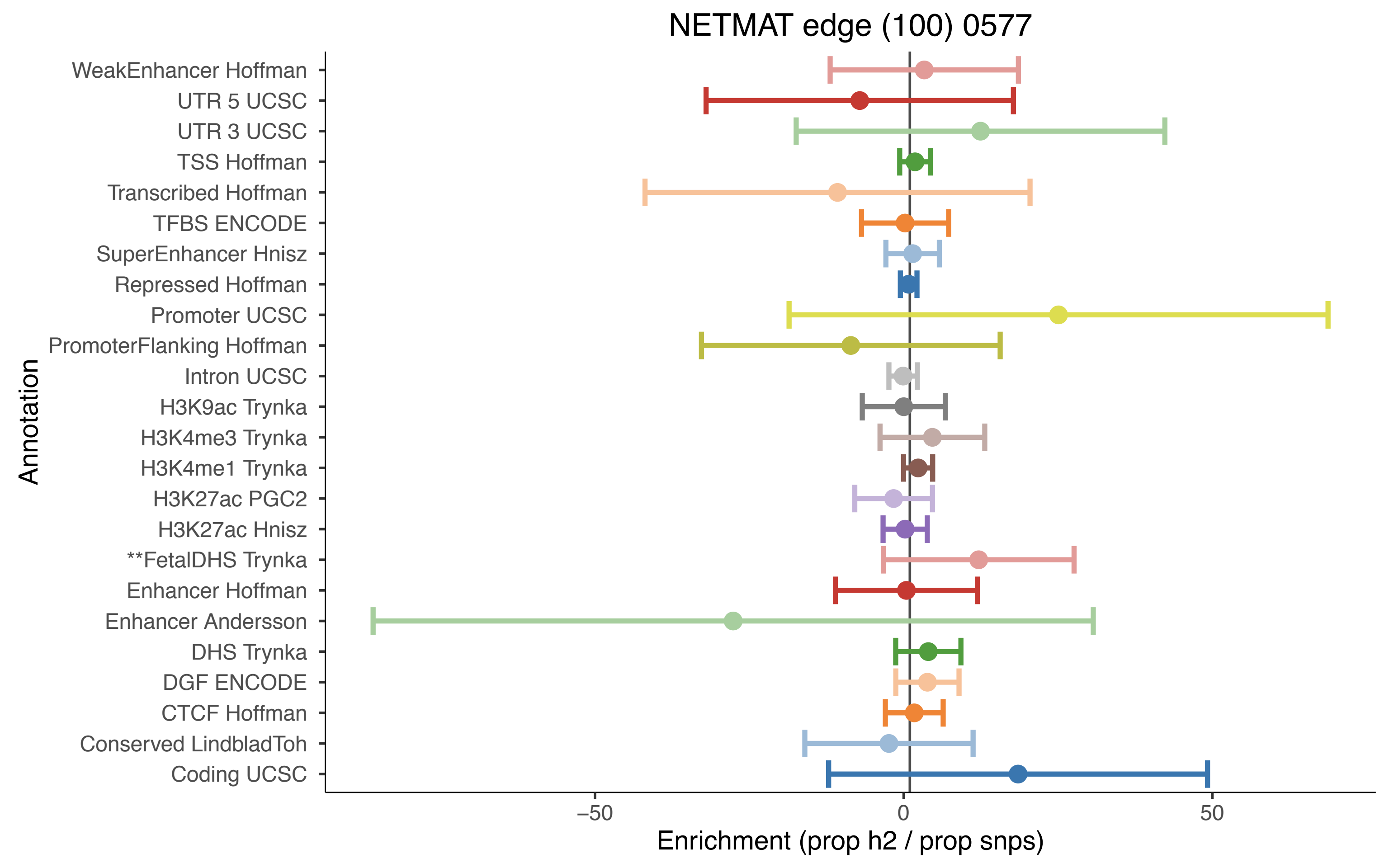
NETMAT edge (100) 0577
Annotation
WeakEnhancer Hoffman
UTR 5 UCSC
UTR 3 UCSC
TSS Hoffman
Transcribed Hoffman
TFBS ENCODE
SuperEnhancer Hnisz
Repressed Hoffman
Promoter UCSC
PromoterFlanking Hoffman
Intron UCSC
H3K9ac Trynka
H3K4me3 Trynka
H3K4me1 Trynka
H3K27ac PGC2
H3K27ac Hnisz
**FetalDHS Trynka
Enhancer Hoffman
Enhancer Andersson
DHS Trynka
DGF ENCODE
CTCF Hoffman
Conserved LindbladToh
Coding UCSC
−50
0
50
Enrichment (prop h2 / prop snps)

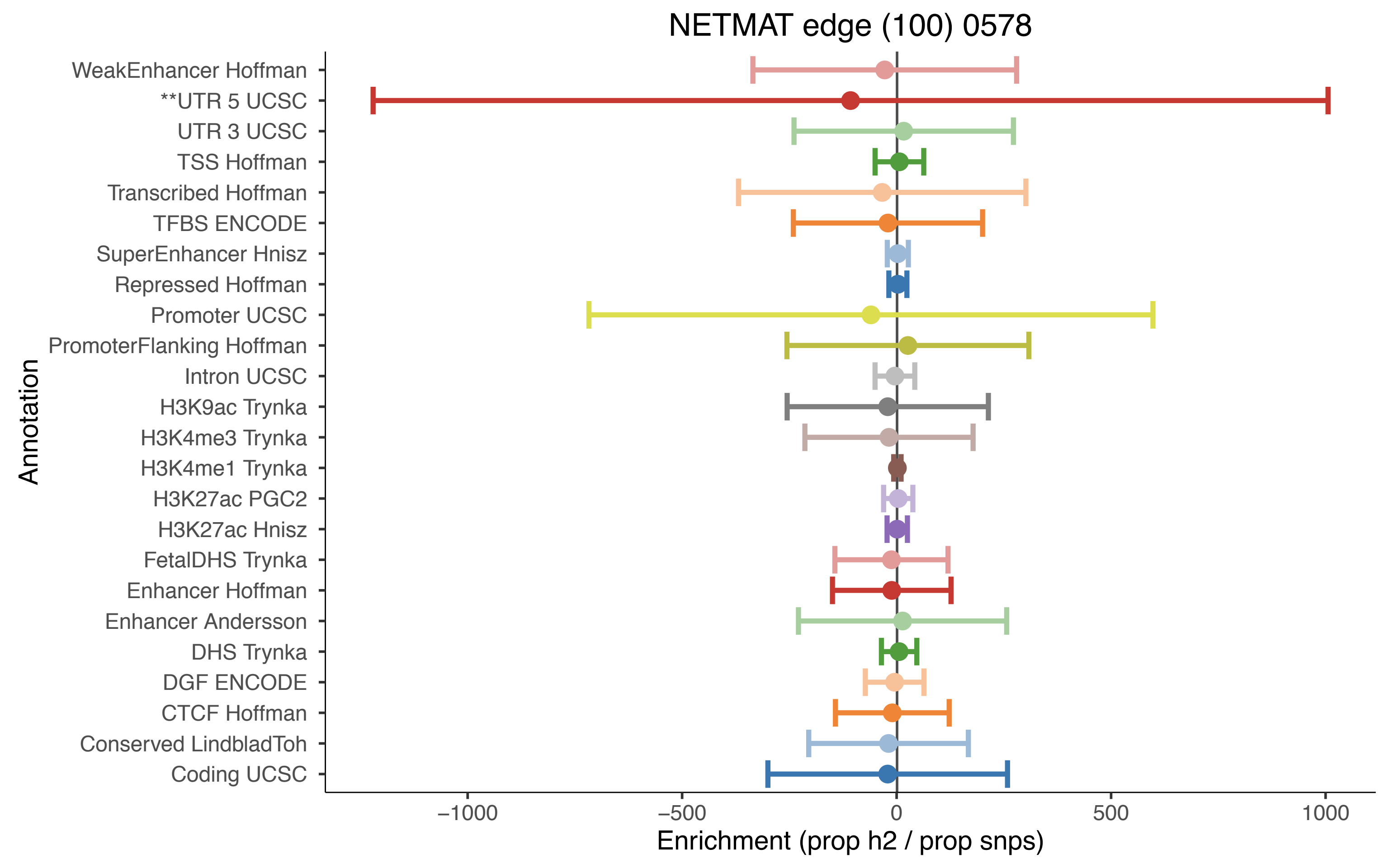

NETMAT edge (100) 0578
Annotation
WeakEnhancer Hoffman
**UTR 5 UCSC
UTR 3 UCSC
TSS Hoffman
Transcribed Hoffman
TFBS ENCODE
SuperEnhancer Hnisz
Repressed Hoffman
Promoter UCSC
PromoterFlanking Hoffman
Intron UCSC
H3K9ac Trynka
H3K4me3 Trynka
H3K4me1 Trynka
H3K27ac PGC2
H3K27ac Hnisz
FetalDHS Trynka
Enhancer Hoffman
Enhancer Andersson
DHS Trynka
DGF ENCODE
CTCF Hoffman
Conserved LindbladToh
Coding UCSC
−1000
−500
0
500
1000
Enrichment (prop h2 / prop snps)

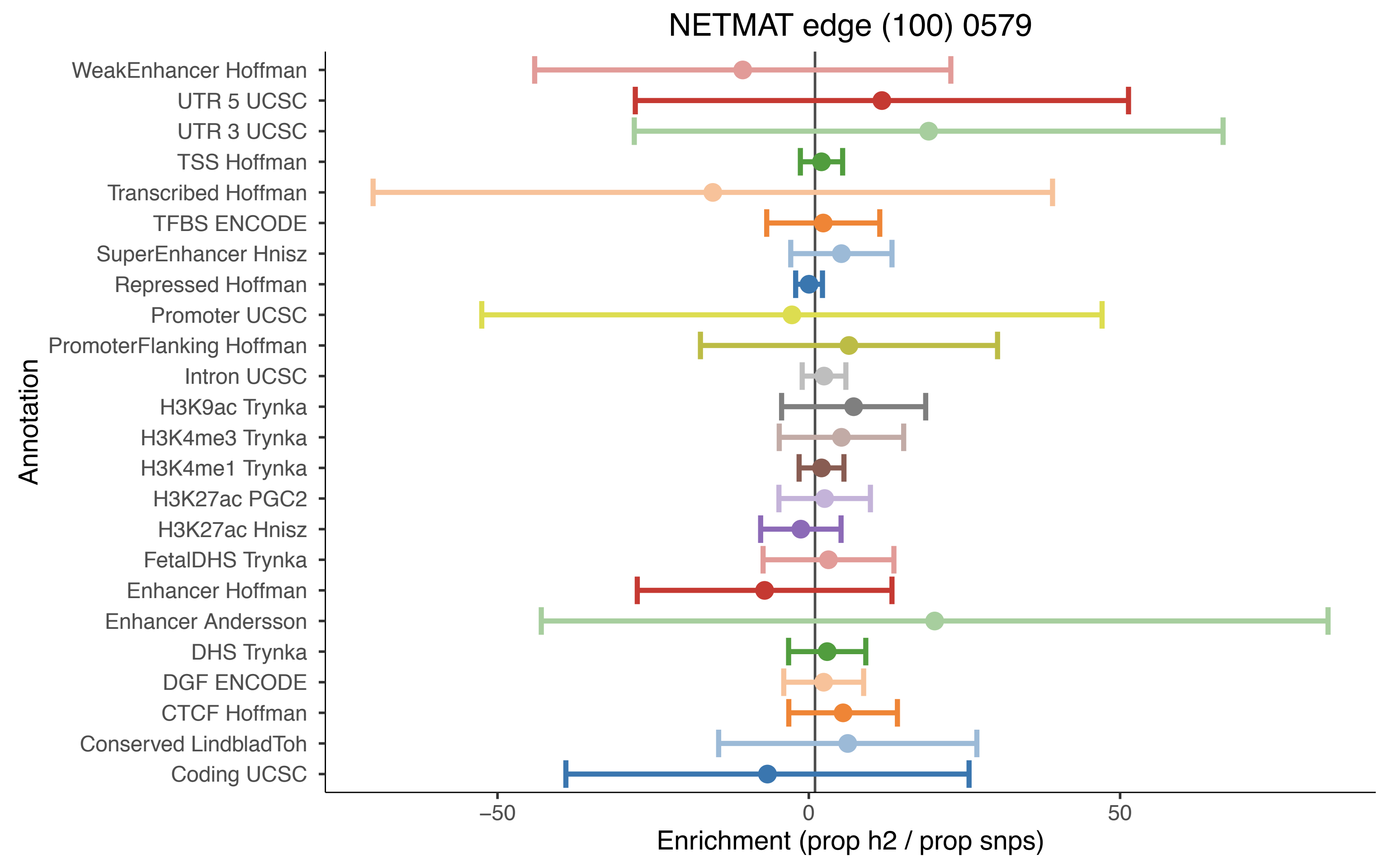
NETMAT edge (100) 0579
Annotation
WeakEnhancer Hoffman
UTR 5 UCSC
UTR 3 UCSC
TSS Hoffman
Transcribed Hoffman
TFBS ENCODE
SuperEnhancer Hnisz
Repressed Hoffman
Promoter UCSC
PromoterFlanking Hoffman
Intron UCSC
H3K9ac Trynka
H3K4me3 Trynka
H3K4me1 Trynka
H3K27ac PGC2
H3K27ac Hnisz
FetalDHS Trynka
Enhancer Hoffman
Enhancer Andersson
DHS Trynka
DGF ENCODE
CTCF Hoffman
Conserved LindbladToh
Coding UCSC
−50
0
50
Enrichment (prop h2 / prop snps)

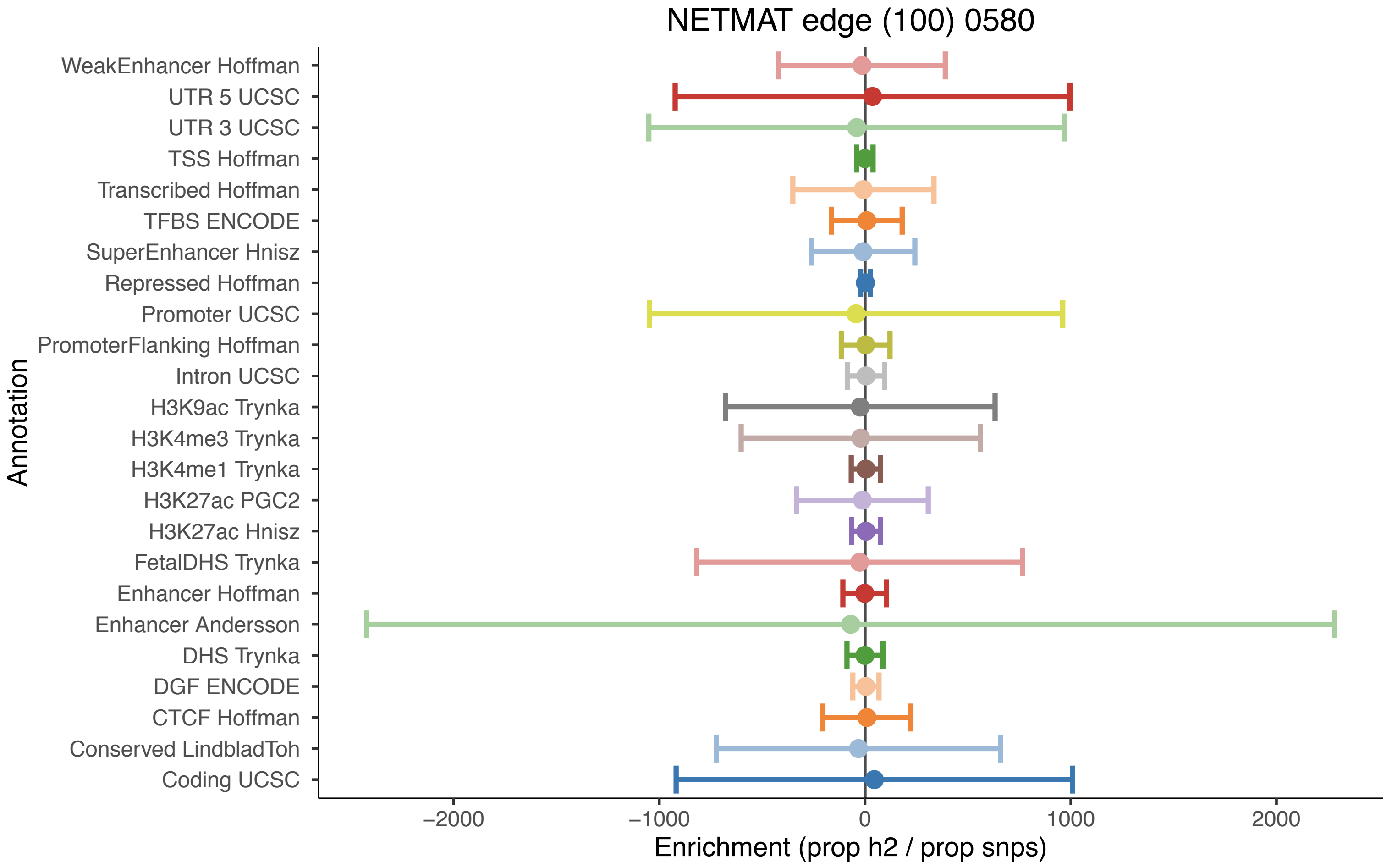

NETMAT edge (100) 0580
WeakEnhancer Hoffman
UTR 5 UCSC
UTR 3 UCSC
TSS Hoffman
Transcribed Hoffman
TFBS ENCODE
SuperEnhancer Hnisz
Repressed Hoffman
Promoter UCSC
PromoterFlanking Hoffman
Intron UCSC
H3K9ac Trynka
H3K4me3 Trynka
H3K4me1 Trynka
H3K27ac PGC2
H3K27ac Hnisz
FetalDHS Trynka
Enhancer Hoffman
Enhancer Andersson
DHS Trynka
DGF ENCODE
CTCF Hoffman
Conserved LindbladToh
Coding UCSC
Annotation
−2000
−1000
0
1000
2000
Enrichment (prop h2 / prop snps)

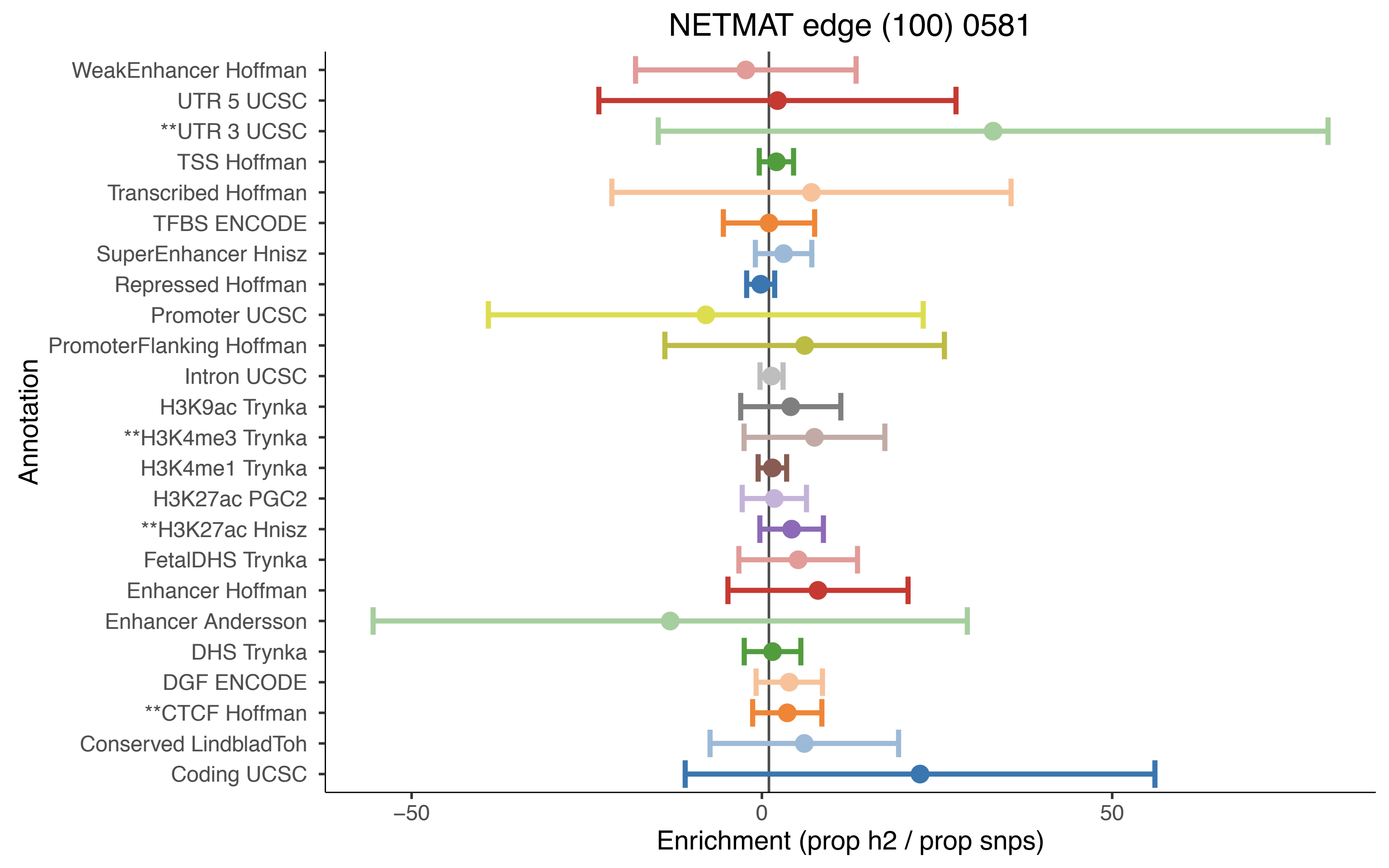

NETMAT edge (100) 0581
Annotation
WeakEnhancer Hoffman
UTR 5 UCSC
**UTR 3 UCSC
TSS Hoffman
Transcribed Hoffman
TFBS ENCODE
SuperEnhancer Hnisz
Repressed Hoffman
Promoter UCSC
PromoterFlanking Hoffman
Intron UCSC
H3K9ac Trynka
**H3K4me3 Trynka
H3K4me1 Trynka
H3K27ac PGC2
**H3K27ac Hnisz
FetalDHS Trynka
Enhancer Hoffman
Enhancer Andersson
DHS Trynka
DGF ENCODE
**CTCF Hoffman
Conserved LindbladToh
Coding UCSC
−50
0
50
Enrichment (prop h2 / prop snps)

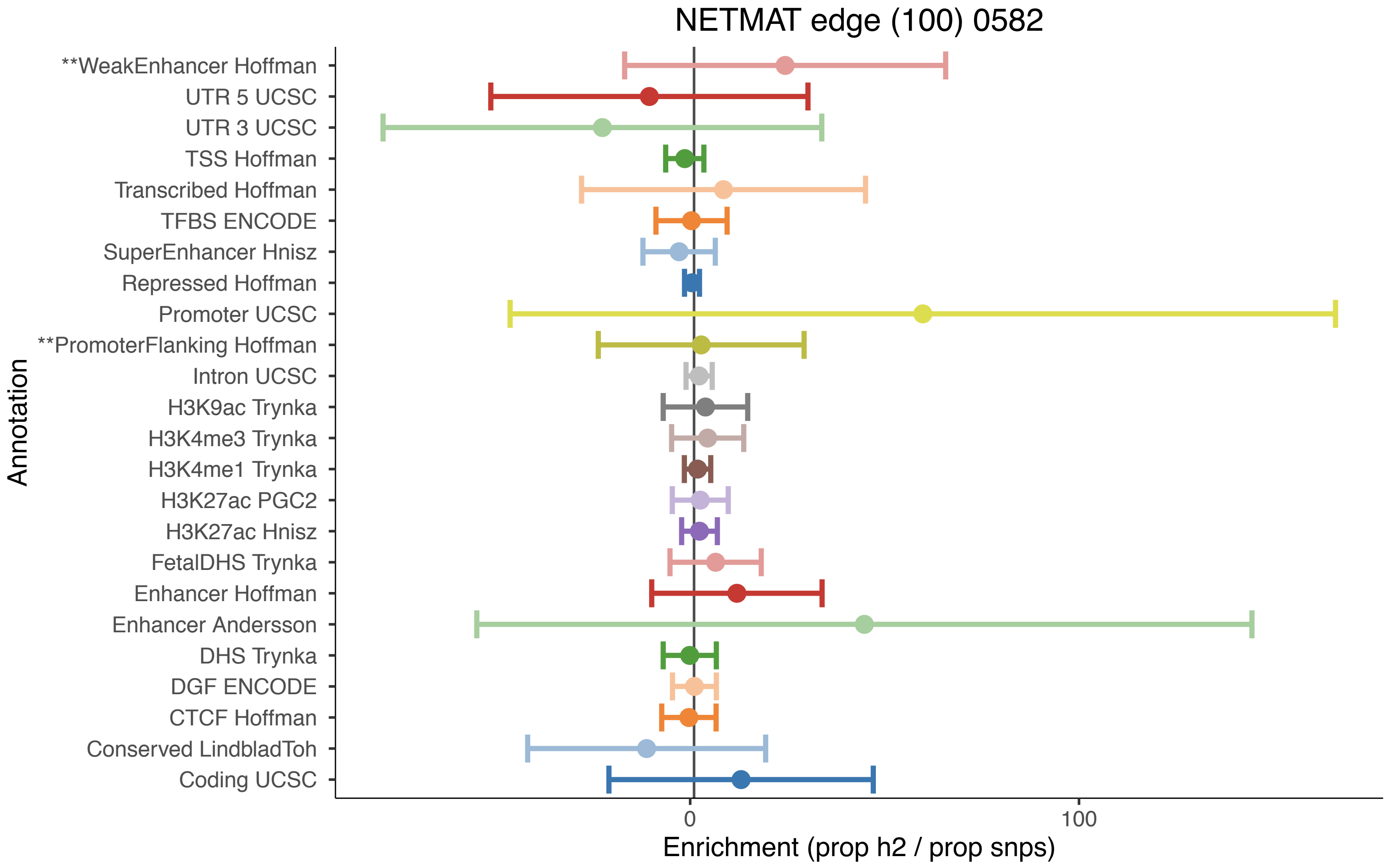

NETMAT edge (100) 0582
**WeakEnhancer Hoffman
UTR 5 UCSC
UTR 3 UCSC
TSS Hoffman
Transcribed Hoffman
TFBS ENCODE
SuperEnhancer Hnisz
Repressed Hoffman
Promoter UCSC
**PromoterFlanking Hoffman
Intron UCSC
H3K9ac Trynka
H3K4me3 Trynka
H3K4me1 Trynka
H3K27ac PGC2
H3K27ac Hnisz
FetalDHS Trynka
Enhancer Hoffman
Enhancer Andersson
DHS Trynka
DGF ENCODE
CTCF Hoffman
Conserved LindbladToh
Coding UCSC
Annotation
0
100
Enrichment (prop h2 / prop snps)

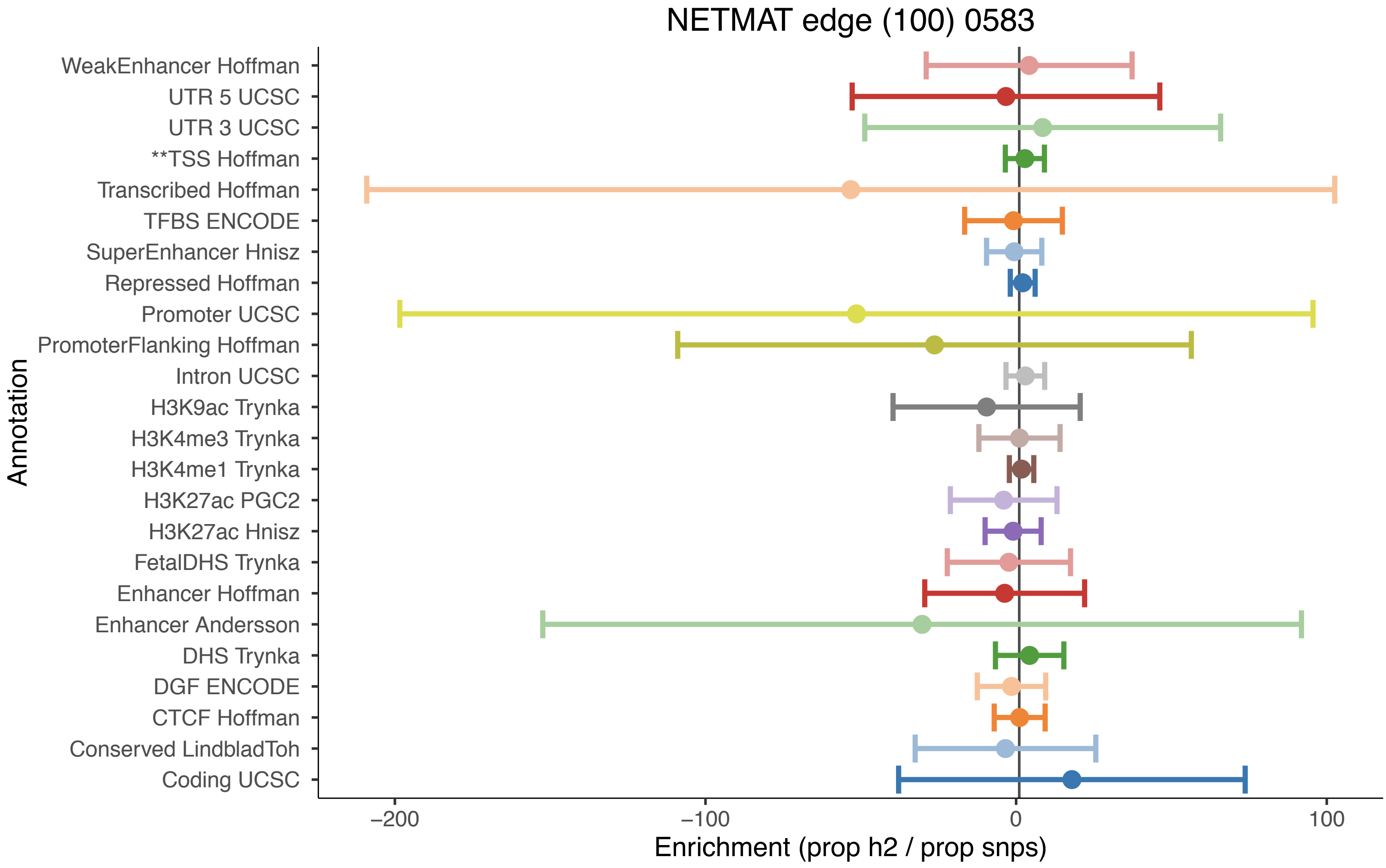

NETMAT edge (100) 0583
WeakEnhancer Hoffman
UTR 5 UCSC
UTR 3 UCSC
**TSS Hoffman
Transcribed Hoffman
TFBS ENCODE
SuperEnhancer Hnisz
Repressed Hoffman
Promoter UCSC
PromoterFlanking Hoffman
Intron UCSC
H3K9ac Trynka
H3K4me3 Trynka
H3K4me1 Trynka
H3K27ac PGC2
H3K27ac Hnisz
FetalDHS Trynka
Enhancer Hoffman
Enhancer Andersson
DHS Trynka
DGF ENCODE
CTCF Hoffman
Conserved LindbladToh
Coding UCSC
Annotation
−200
−100
0
100
Enrichment (prop h2 / prop snps)

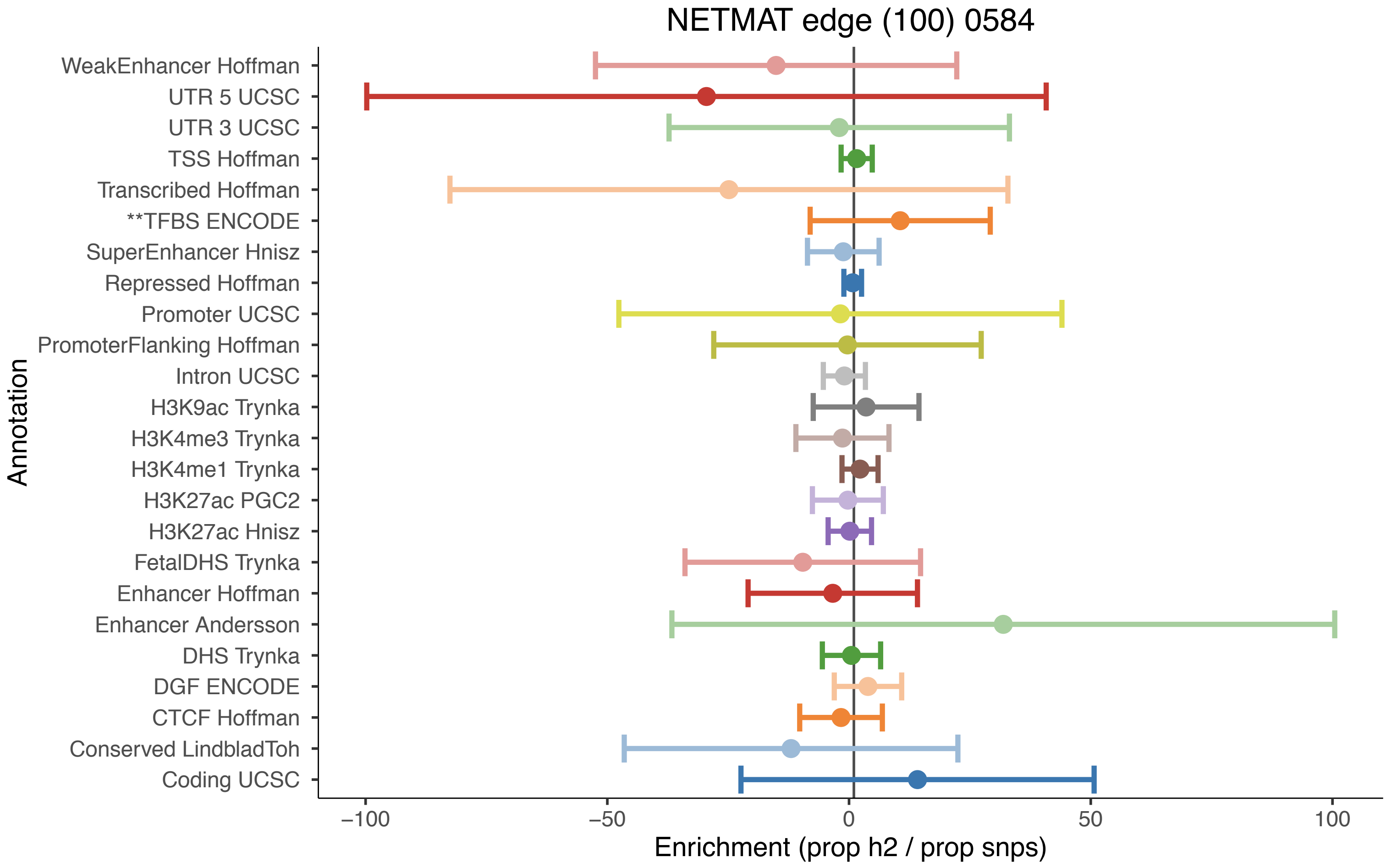
NETMAT edge (100) 0584
WeakEnhancer Hoffman
UTR 5 UCSC
UTR 3 UCSC
TSS Hoffman
Transcribed Hoffman
**TFBS ENCODE
SuperEnhancer Hnisz
Repressed Hoffman
Promoter UCSC
PromoterFlanking Hoffman
Intron UCSC
H3K9ac Trynka
H3K4me3 Trynka
H3K4me1 Trynka
H3K27ac PGC2
H3K27ac Hnisz
FetalDHS Trynka
Enhancer Hoffman
Enhancer Andersson
DHS Trynka
DGF ENCODE
CTCF Hoffman
Conserved LindbladToh
Coding UCSC
Annotation
−100
−50
0
50
100
Enrichment (prop h2 / prop snps)

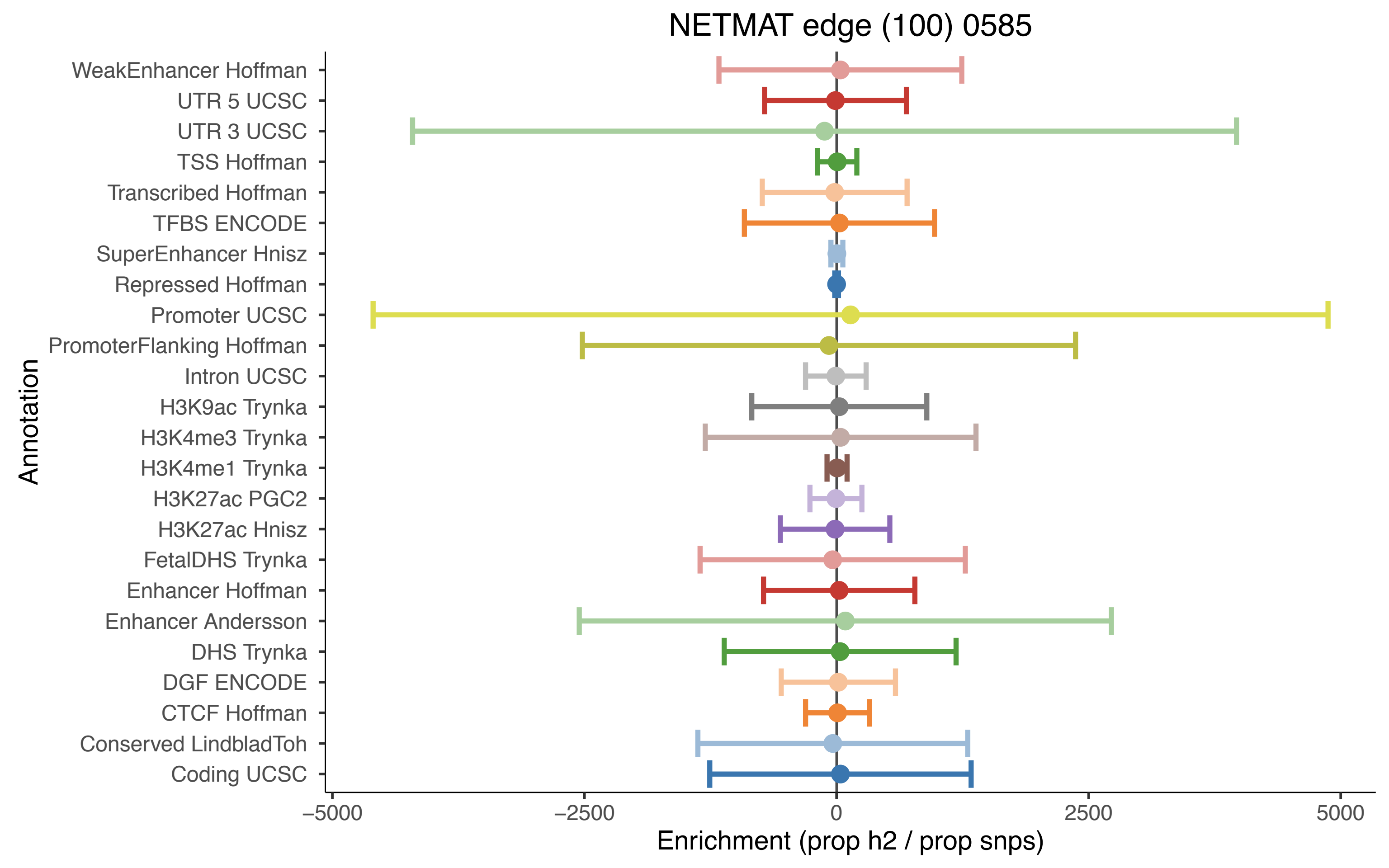

NETMAT edge (100) 0585
Annotation
WeakEnhancer Hoffman
UTR 5 UCSC
UTR 3 UCSC
TSS Hoffman
Transcribed Hoffman
TFBS ENCODE
SuperEnhancer Hnisz
Repressed Hoffman
Promoter UCSC
PromoterFlanking Hoffman
Intron UCSC
H3K9ac Trynka
H3K4me3 Trynka
H3K4me1 Trynka
H3K27ac PGC2
H3K27ac Hnisz
FetalDHS Trynka
Enhancer Hoffman
Enhancer Andersson
DHS Trynka
DGF ENCODE
CTCF Hoffman
Conserved LindbladToh
Coding UCSC
−5000
−2500
0
2500
5000
Enrichment (prop h2 / prop snps)

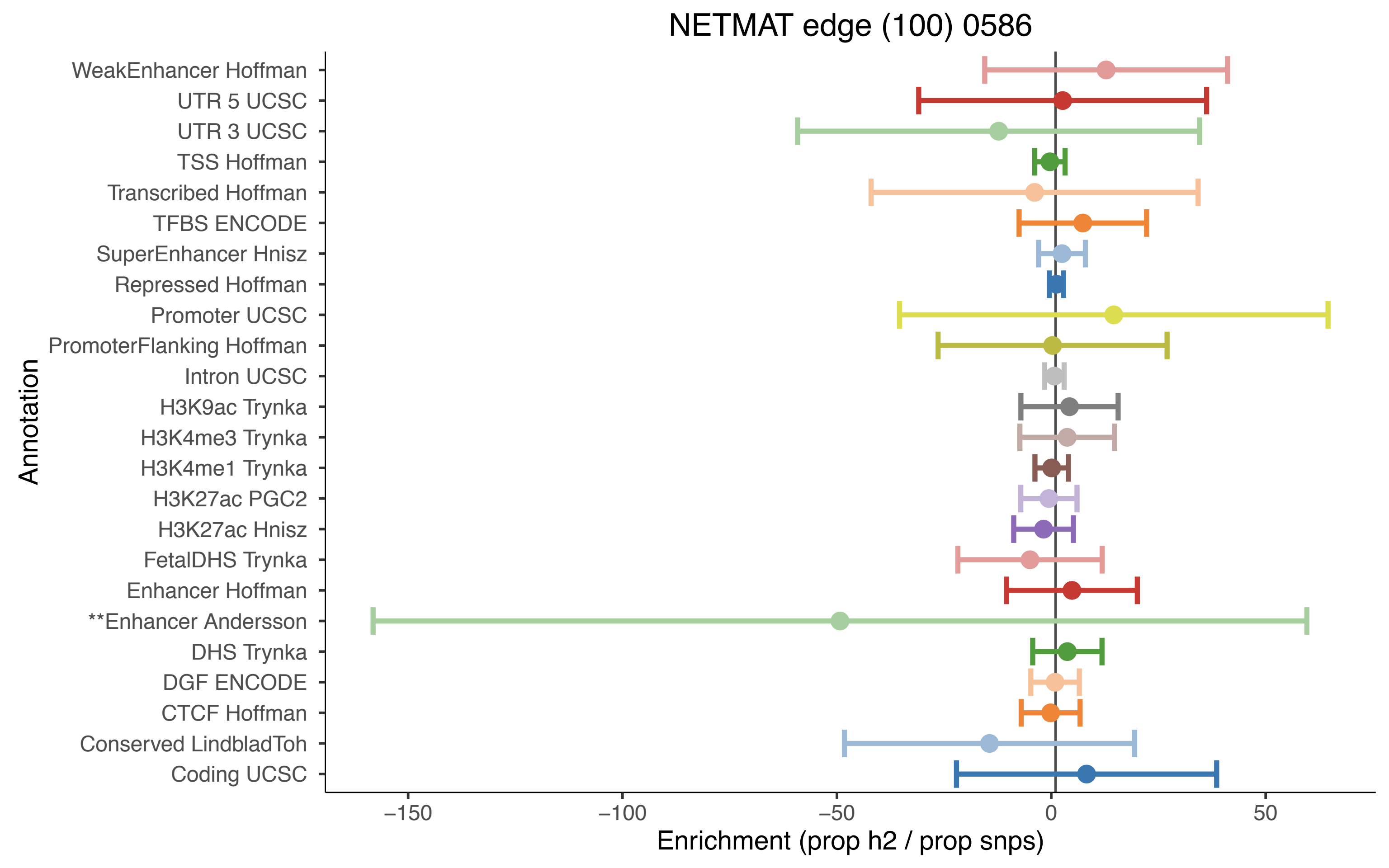
NETMAT edge (100) 0586
WeakEnhancer Hoffman
UTR 5 UCSC
UTR 3 UCSC
TSS Hoffman
Transcribed Hoffman
TFBS ENCODE
SuperEnhancer Hnisz
Repressed Hoffman
Promoter UCSC
PromoterFlanking Hoffman
Intron UCSC
H3K9ac Trynka
H3K4me3 Trynka
H3K4me1 Trynka
H3K27ac PGC2
H3K27ac Hnisz
FetalDHS Trynka
Enhancer Hoffman
**Enhancer Andersson
DHS Trynka
DGF ENCODE
CTCF Hoffman
Conserved LindbladToh
Coding UCSC
Annotation
−150
−100
−50
0
50
Enrichment (prop h2 / prop snps)

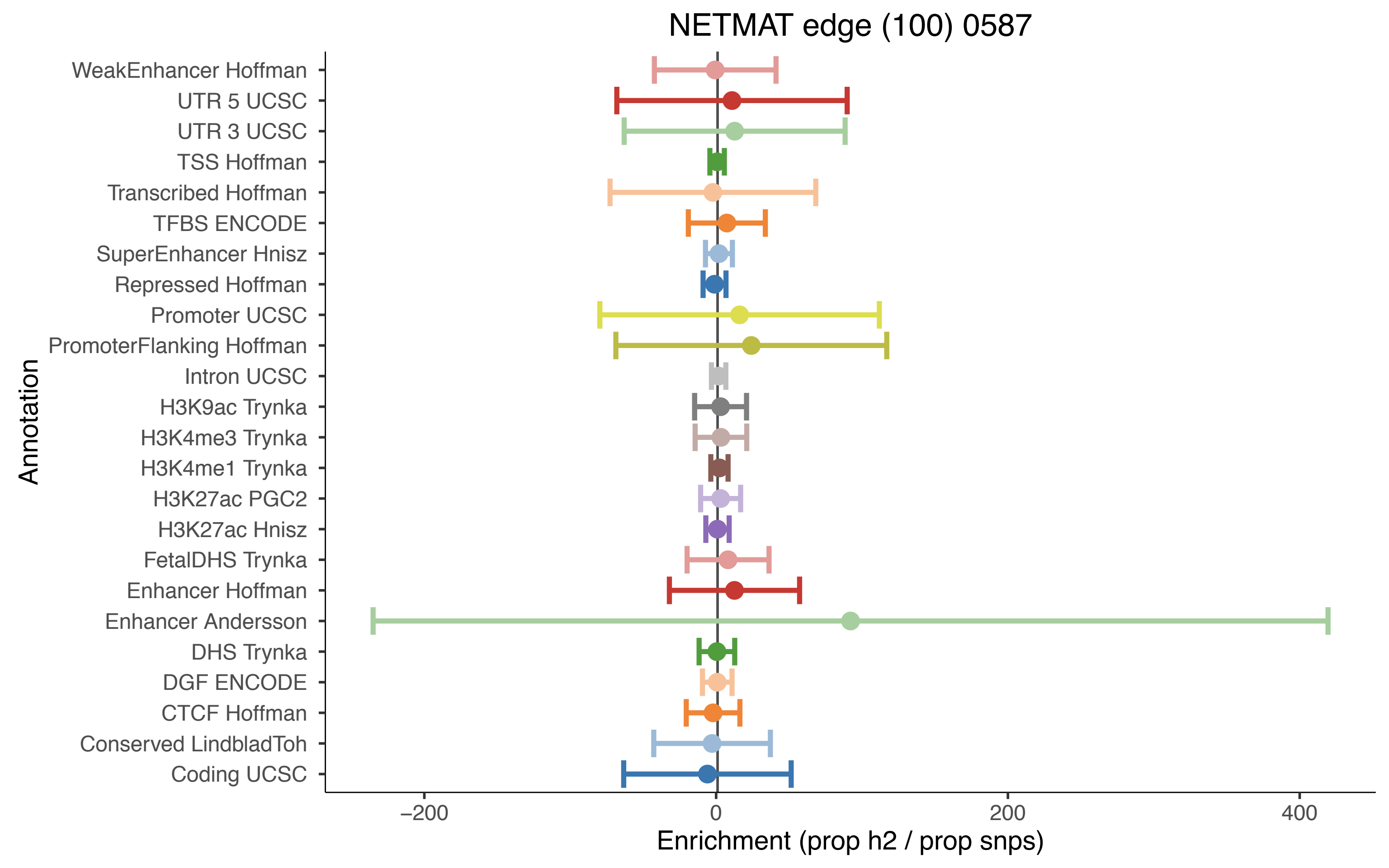
NETMAT edge (100) 0587
Annotation
WeakEnhancer Hoffman
UTR 5 UCSC
UTR 3 UCSC
TSS Hoffman
Transcribed Hoffman
TFBS ENCODE
SuperEnhancer Hnisz
Repressed Hoffman
Promoter UCSC
PromoterFlanking Hoffman
Intron UCSC
H3K9ac Trynka
H3K4me3 Trynka
H3K4me1 Trynka
H3K27ac PGC2
H3K27ac Hnisz
FetalDHS Trynka
Enhancer Hoffman
Enhancer Andersson
DHS Trynka
DGF ENCODE
CTCF Hoffman
Conserved LindbladToh
Coding UCSC
−200
0
200
400
Enrichment (prop h2 / prop snps)

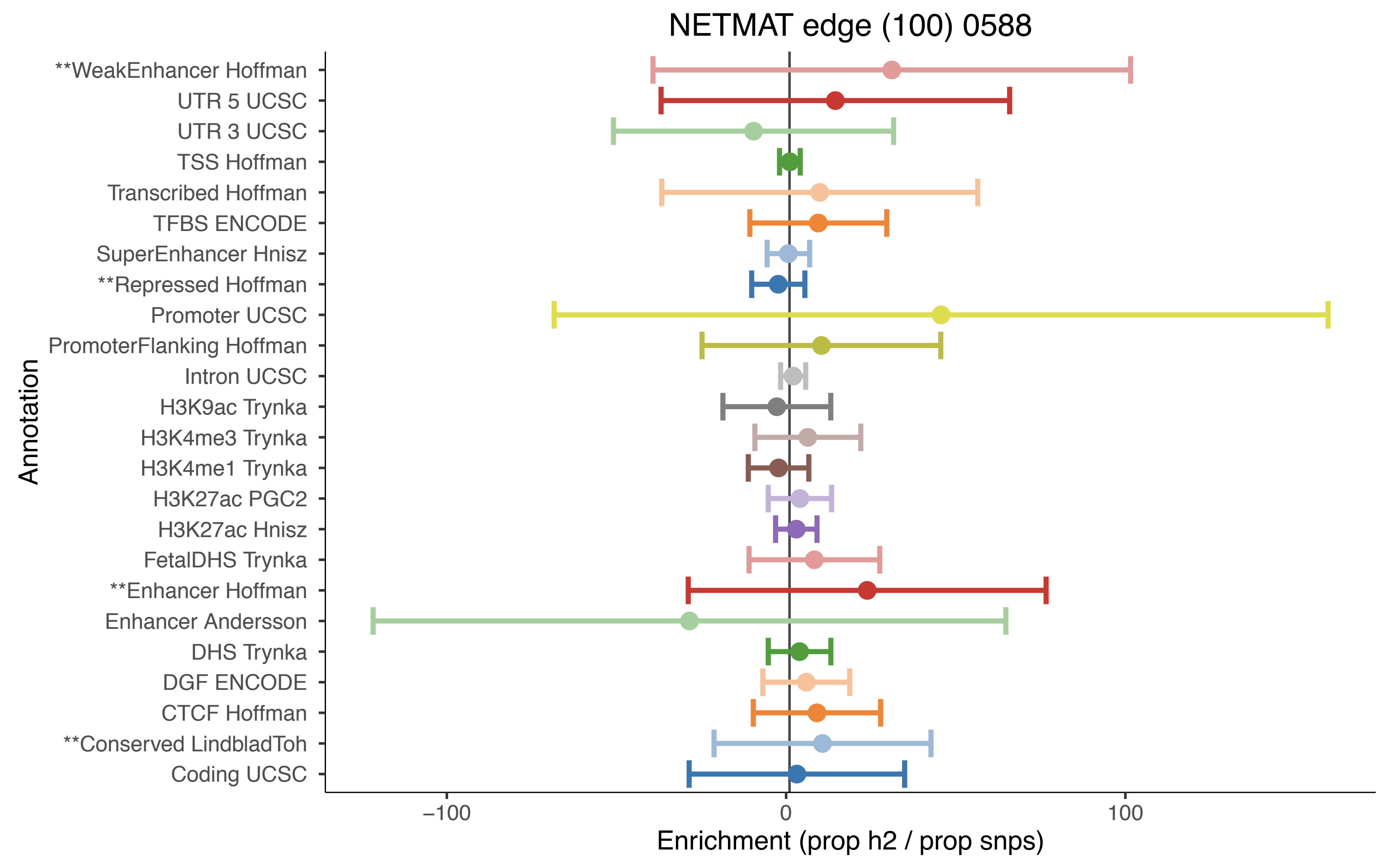
NETMAT edge (100) 0588
**WeakEnhancer Hoffman
UTR 5 UCSC
UTR 3 UCSC
TSS Hoffman
Transcribed Hoffman
TFBS ENCODE
SuperEnhancer Hnisz
**Repressed Hoffman
Promoter UCSC
PromoterFlanking Hoffman
Intron UCSC
H3K9ac Trynka
H3K4me3 Trynka
H3K4me1 Trynka
H3K27ac PGC2
H3K27ac Hnisz
FetalDHS Trynka
**Enhancer Hoffman
Enhancer Andersson
DHS Trynka
DGF ENCODE
CTCF Hoffman
**Conserved LindbladToh
Coding UCSC
Annotation
−100
0
100
Enrichment (prop h2 / prop snps)

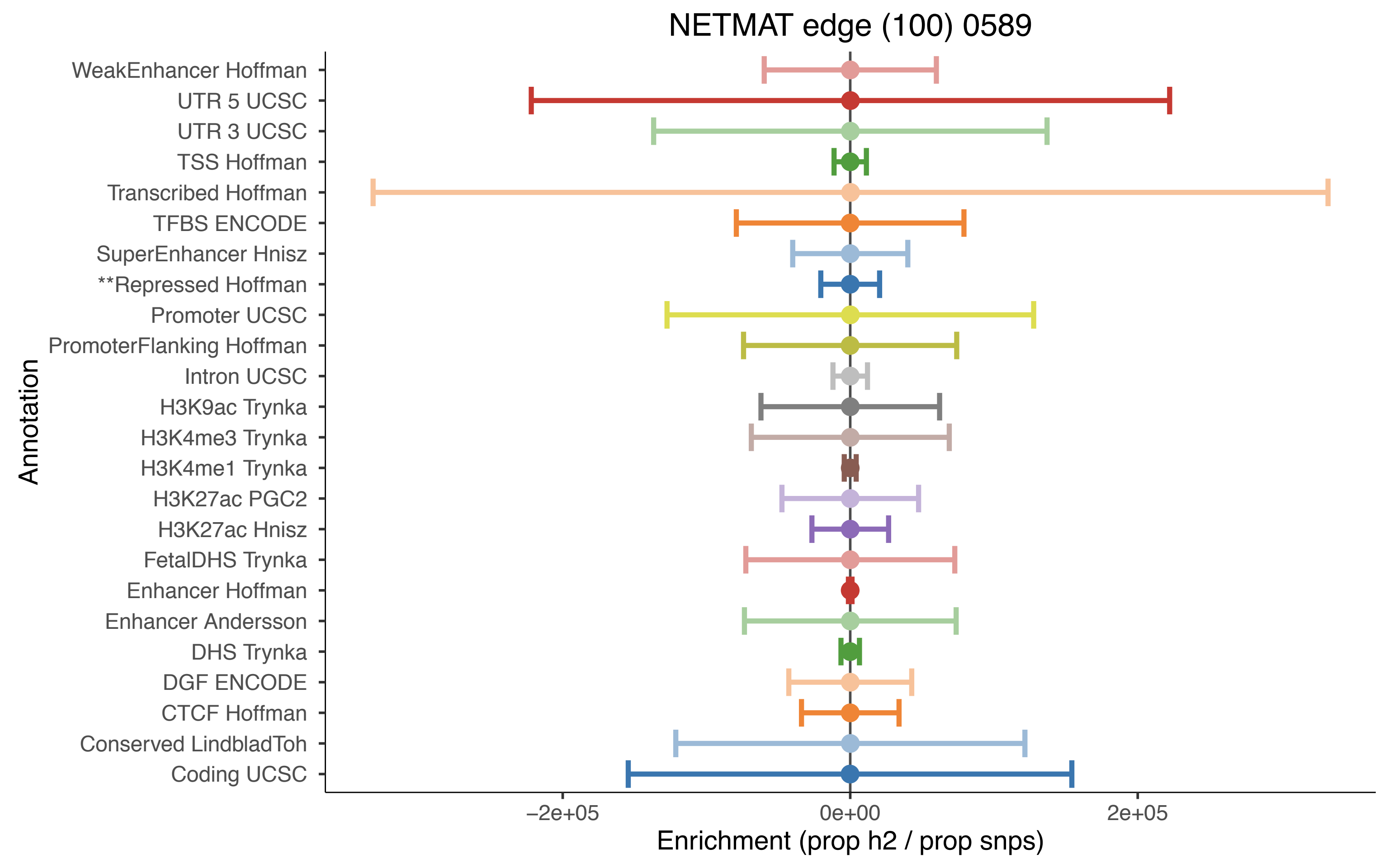
NETMAT edge (100) 0589
WeakEnhancer Hoffman
UTR 5 UCSC
UTR 3 UCSC
TSS Hoffman
Transcribed Hoffman
TFBS ENCODE
SuperEnhancer Hnisz
**Repressed Hoffman
Promoter UCSC
PromoterFlanking Hoffman
Intron UCSC
H3K9ac Trynka
H3K4me3 Trynka
H3K4me1 Trynka
H3K27ac PGC2
H3K27ac Hnisz
FetalDHS Trynka
Enhancer Hoffman
Enhancer Andersson
DHS Trynka
DGF ENCODE
CTCF Hoffman
Conserved LindbladToh
Coding UCSC
Annotation
−2e+05
0e+00
2e+05
Enrichment (prop h2 / prop snps)

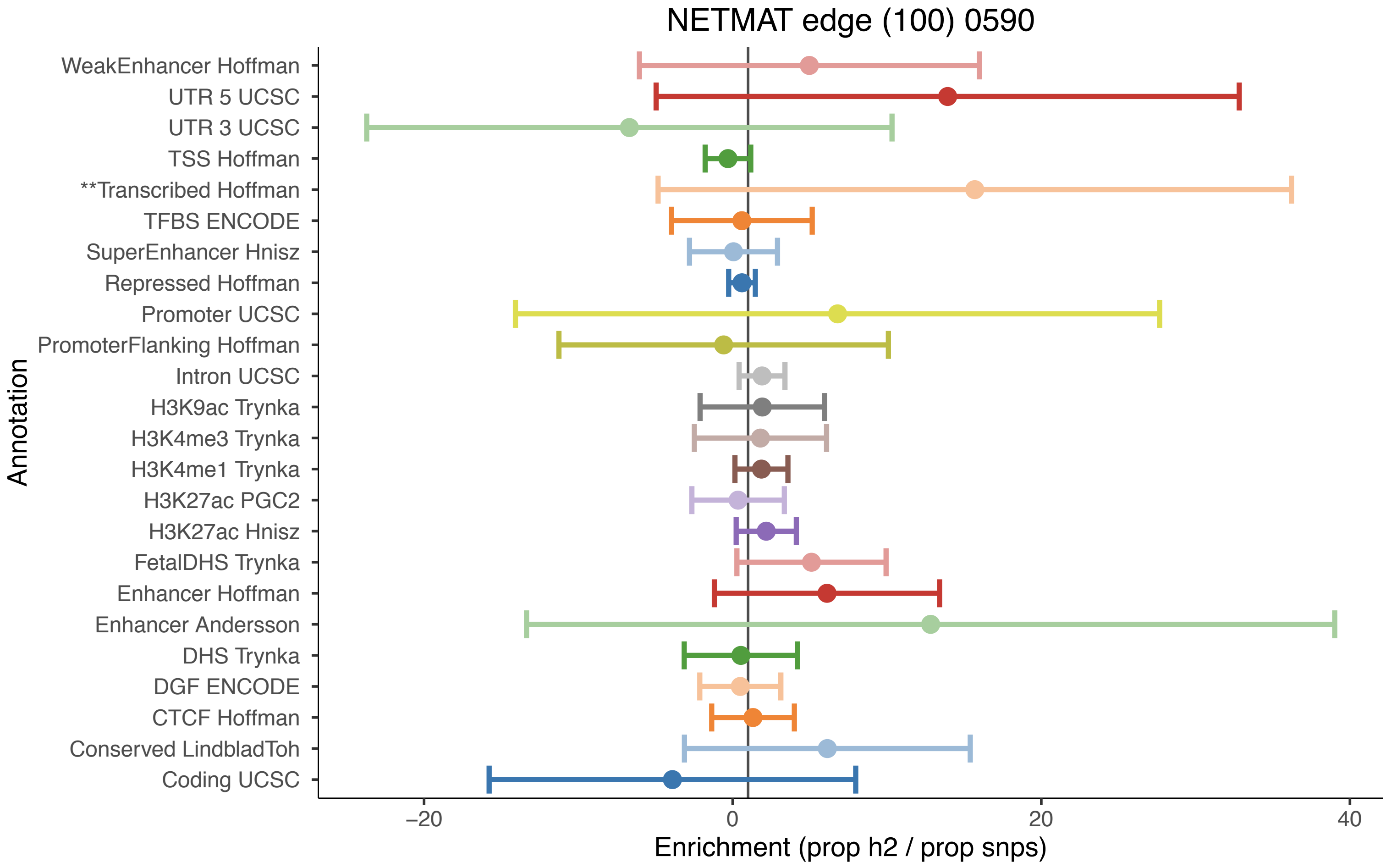

NETMAT edge (100) 0590
WeakEnhancer Hoffman
UTR 5 UCSC
UTR 3 UCSC
TSS Hoffman
**Transcribed Hoffman
TFBS ENCODE
SuperEnhancer Hnisz
Repressed Hoffman
Promoter UCSC
PromoterFlanking Hoffman
Intron UCSC
H3K9ac Trynka
H3K4me3 Trynka
H3K4me1 Trynka
H3K27ac PGC2
H3K27ac Hnisz
FetalDHS Trynka
Enhancer Hoffman
Enhancer Andersson
DHS Trynka
DGF ENCODE
CTCF Hoffman
Conserved LindbladToh
Coding UCSC
Annotation
−20
0
20
40
Enrichment (prop h2 / prop snps)

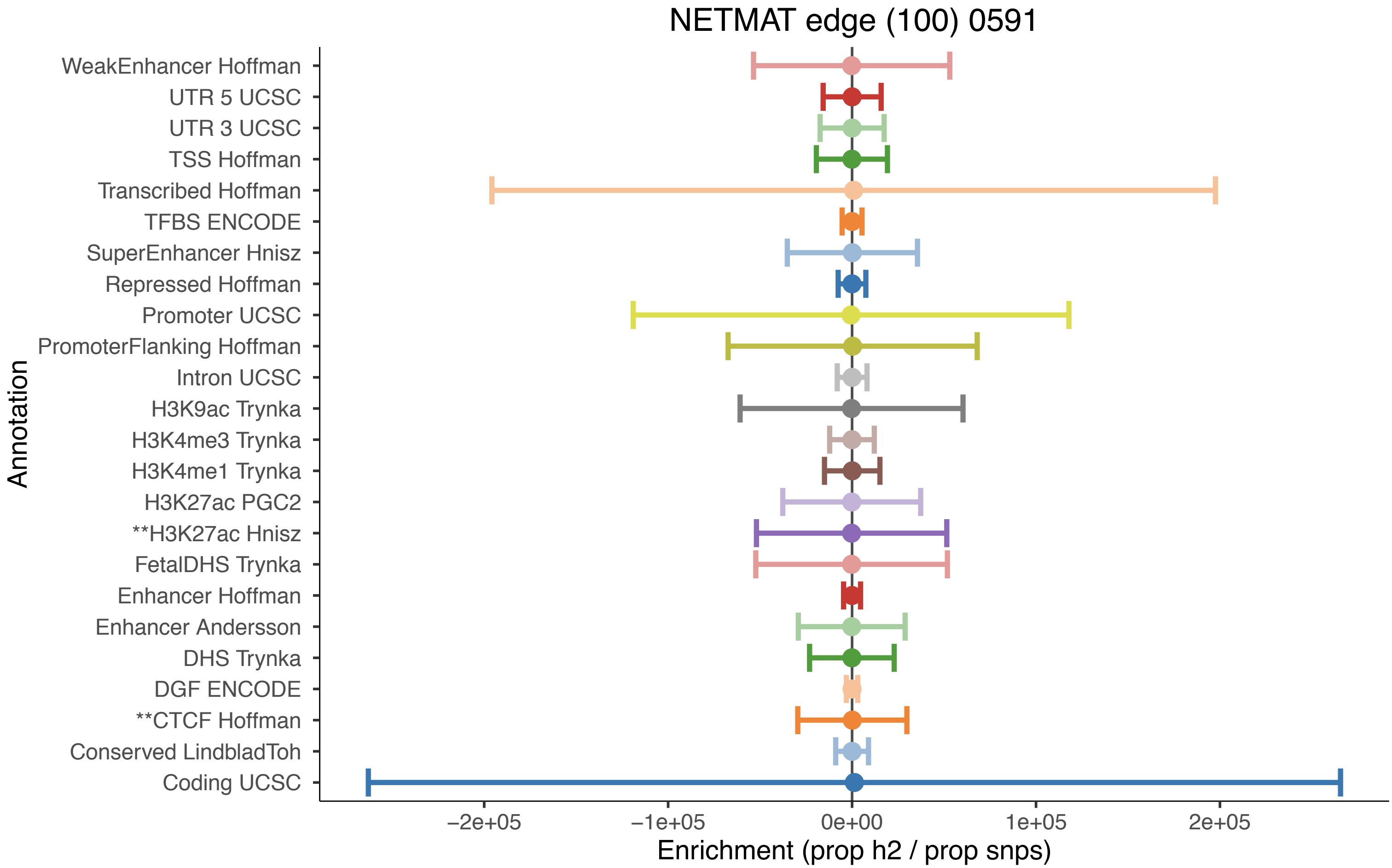

NETMAT edge (100) 0591
WeakEnhancer Hoffman
UTR 5 UCSC
UTR 3 UCSC
TSS Hoffman
Transcribed Hoffman
TFBS ENCODE
SuperEnhancer Hnisz
Repressed Hoffman
Promoter UCSC
PromoterFlanking Hoffman
Intron UCSC
H3K9ac Trynka
H3K4me3 Trynka
H3K4me1 Trynka
H3K27ac PGC2
**H3K27ac Hnisz
FetalDHS Trynka
Enhancer Hoffman
Enhancer Andersson
DHS Trynka
DGF ENCODE
**CTCF Hoffman
Conserved LindbladToh
Coding UCSC
Annotation
−2e+05
−1e+05
0e+00
1e+05
2e+05
Enrichment (prop h2 / prop snps)

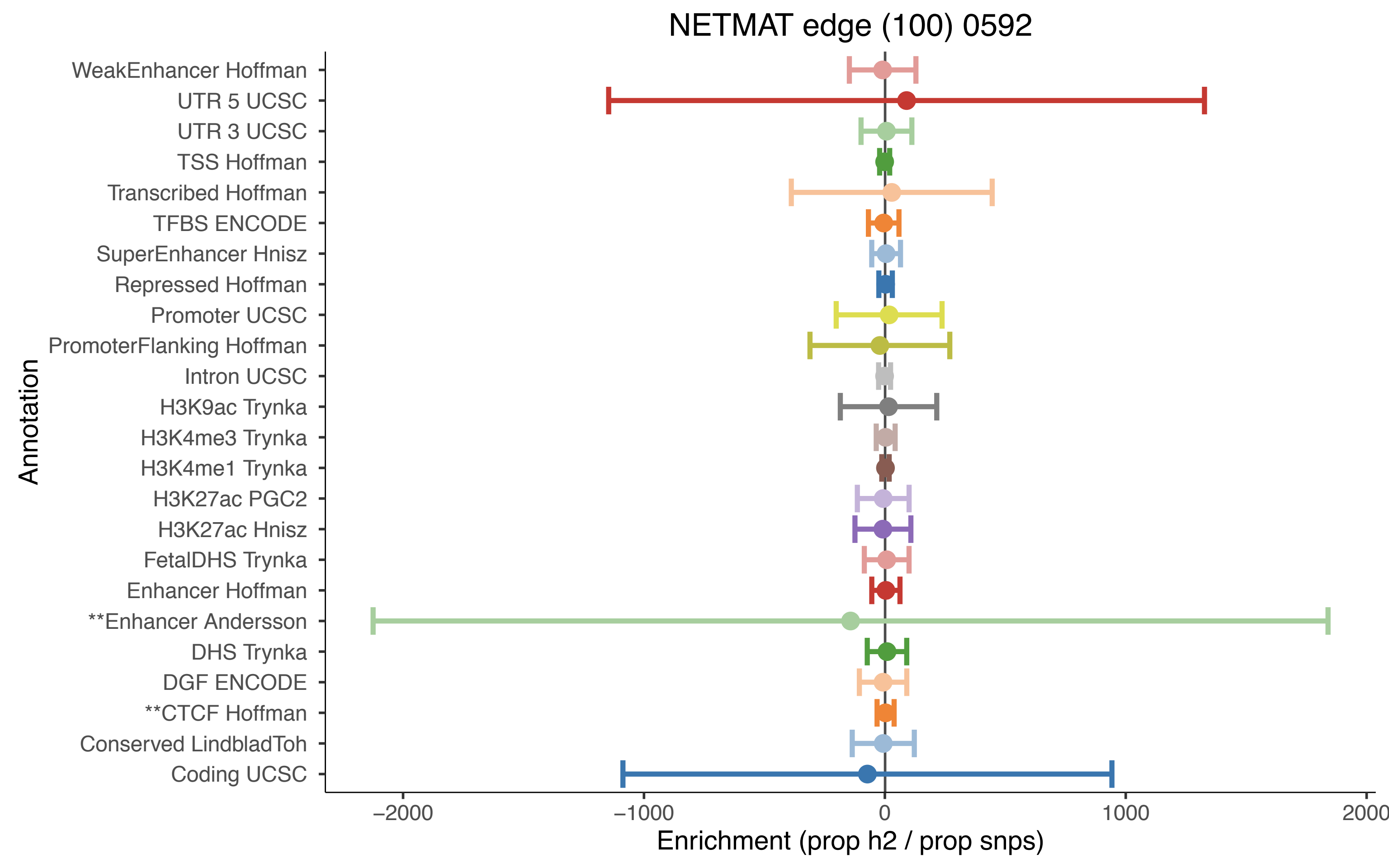
NETMAT edge (100) 0592
WeakEnhancer Hoffman
UTR 5 UCSC
UTR 3 UCSC
TSS Hoffman
Transcribed Hoffman
TFBS ENCODE
SuperEnhancer Hnisz
Repressed Hoffman
Promoter UCSC
PromoterFlanking Hoffman
Intron UCSC
H3K9ac Trynka
H3K4me3 Trynka
H3K4me1 Trynka
H3K27ac PGC2
H3K27ac Hnisz
FetalDHS Trynka
Enhancer Hoffman
**Enhancer Andersson
DHS Trynka
DGF ENCODE
**CTCF Hoffman
Conserved LindbladToh
Coding UCSC
Annotation
−2000
−1000
0
1000
2000
Enrichment (prop h2 / prop snps)

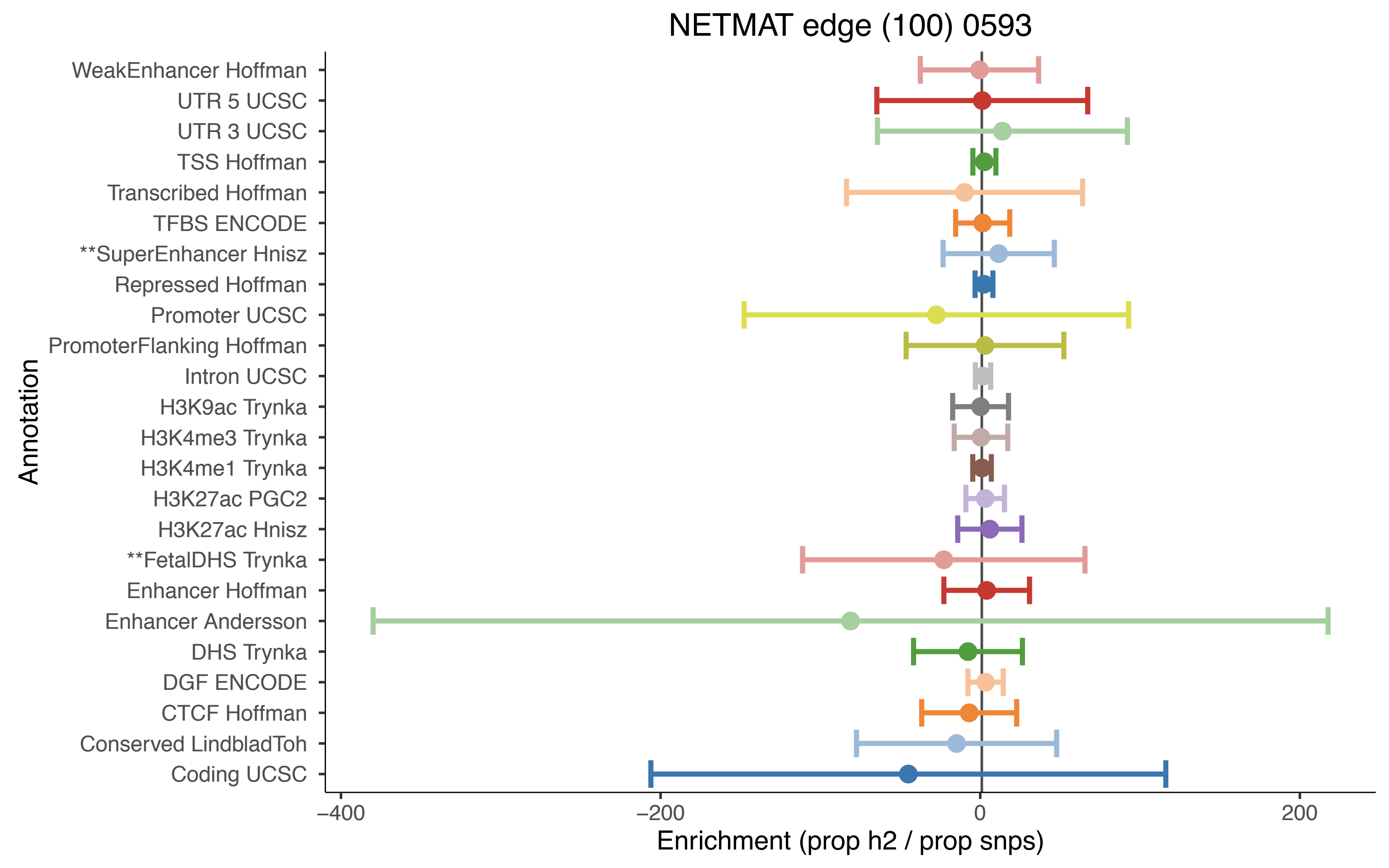
NETMAT edge (100) 0593
WeakEnhancer Hoffman
UTR 5 UCSC
UTR 3 UCSC
TSS Hoffman
Transcribed Hoffman
TFBS ENCODE
**SuperEnhancer Hnisz
Repressed Hoffman
Promoter UCSC
PromoterFlanking Hoffman
Intron UCSC
H3K9ac Trynka
H3K4me3 Trynka
H3K4me1 Trynka
H3K27ac PGC2
H3K27ac Hnisz
**FetalDHS Trynka
Enhancer Hoffman
Enhancer Andersson
DHS Trynka
DGF ENCODE
CTCF Hoffman
Conserved LindbladToh
Coding UCSC
Annotation
−400
−200
0
200
Enrichment (prop h2 / prop snps)

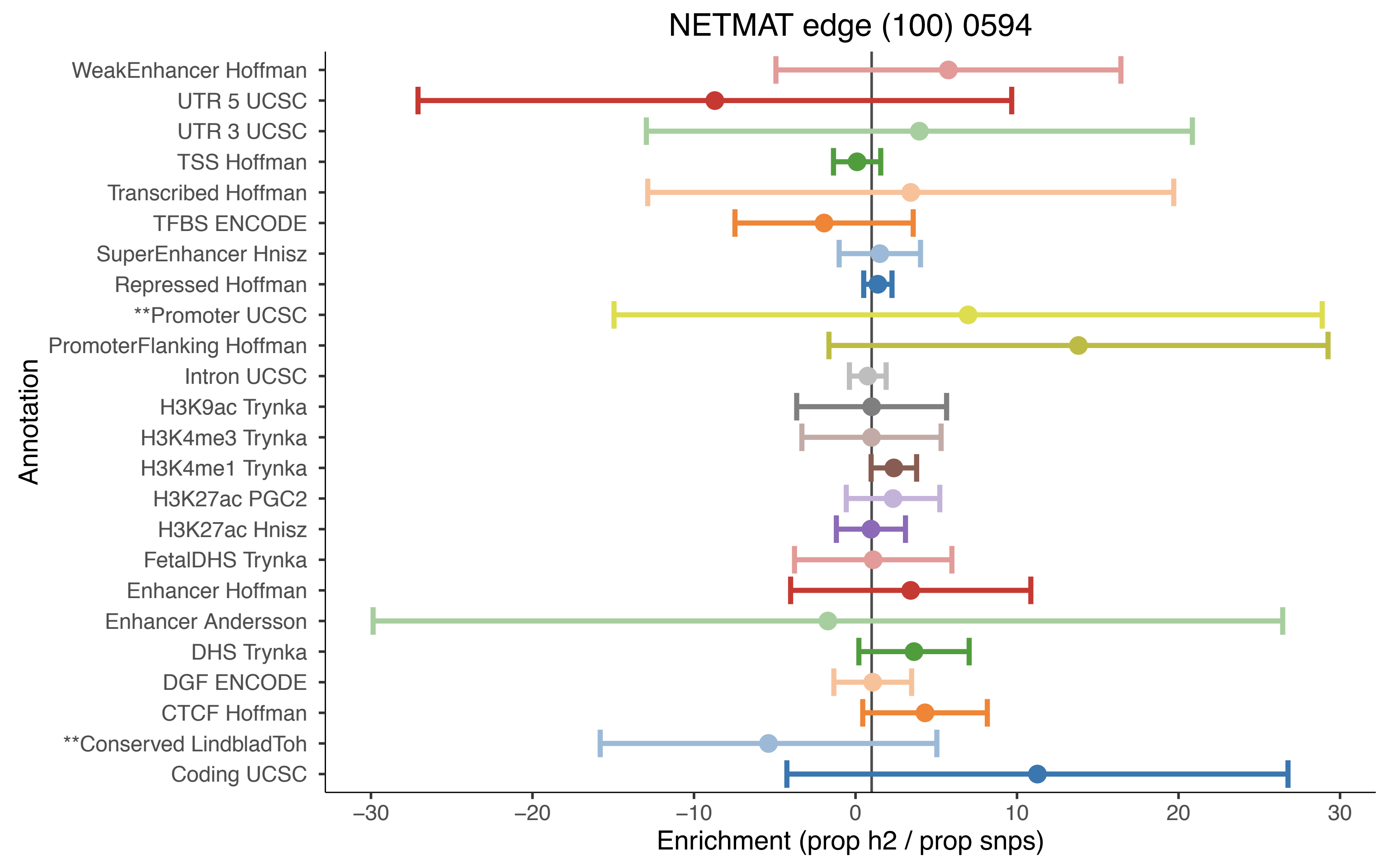
NETMAT edge (100) 0594
WeakEnhancer Hoffman
UTR 5 UCSC
UTR 3 UCSC
TSS Hoffman
Transcribed Hoffman
TFBS ENCODE
SuperEnhancer Hnisz
Repressed Hoffman
**Promoter UCSC
PromoterFlanking Hoffman
Intron UCSC
H3K9ac Trynka
H3K4me3 Trynka
H3K4me1 Trynka
H3K27ac PGC2
H3K27ac Hnisz
FetalDHS Trynka
Enhancer Hoffman
Enhancer Andersson
DHS Trynka
DGF ENCODE
CTCF Hoffman
**Conserved LindbladToh
Coding UCSC
Annotation
−30
−20
−10
0
10
20
30
Enrichment (prop h2 / prop snps)

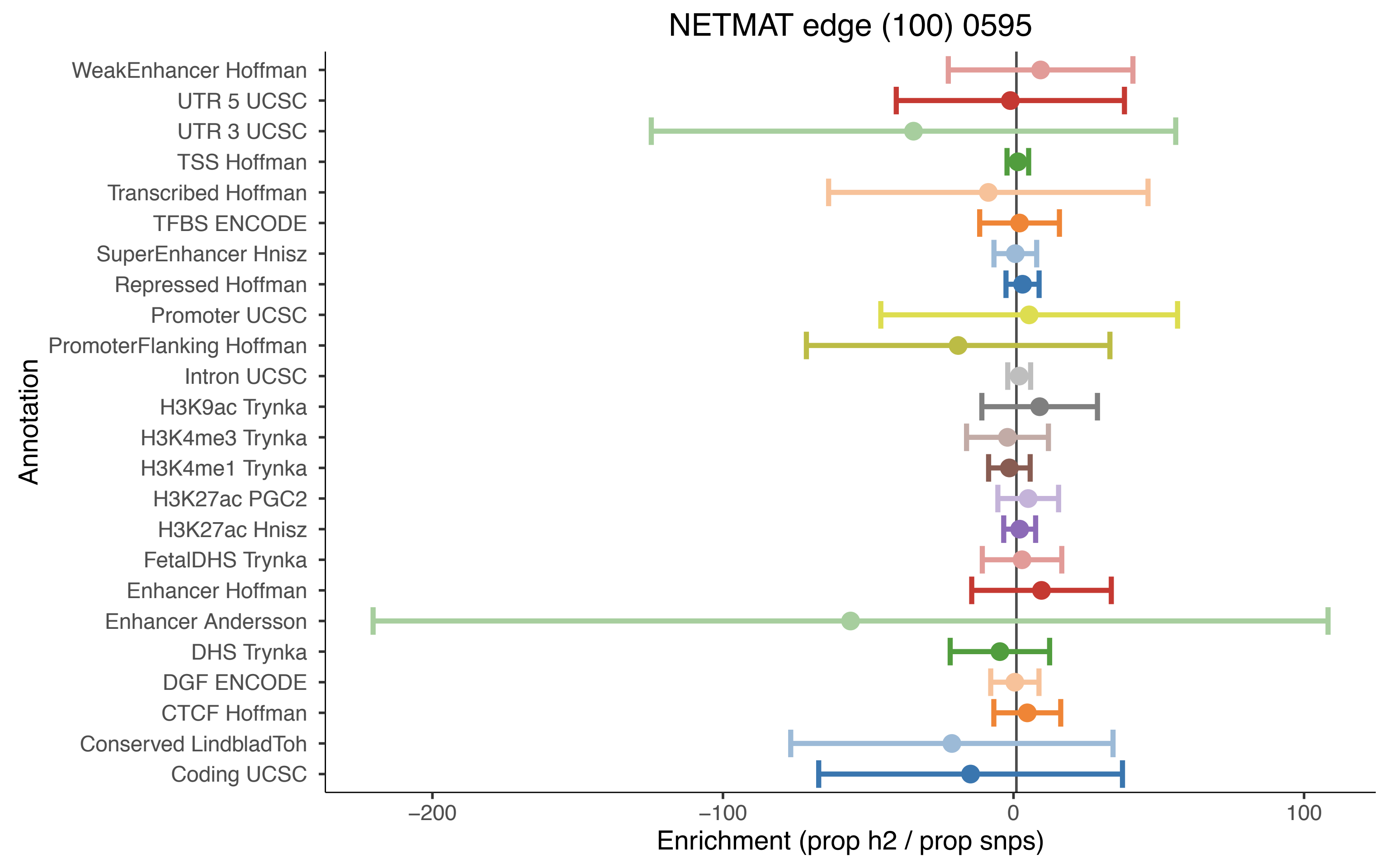
NETMAT edge (100) 0595
WeakEnhancer Hoffman
UTR 5 UCSC
UTR 3 UCSC
TSS Hoffman
Transcribed Hoffman
TFBS ENCODE
SuperEnhancer Hnisz
Repressed Hoffman
Promoter UCSC
PromoterFlanking Hoffman
Intron UCSC
H3K9ac Trynka
H3K4me3 Trynka
H3K4me1 Trynka
H3K27ac PGC2
H3K27ac Hnisz
FetalDHS Trynka
Enhancer Hoffman
Enhancer Andersson
DHS Trynka
DGF ENCODE
CTCF Hoffman
Conserved LindbladToh
Coding UCSC
Annotation
−200
−100
0
100
Enrichment (prop h2 / prop snps)

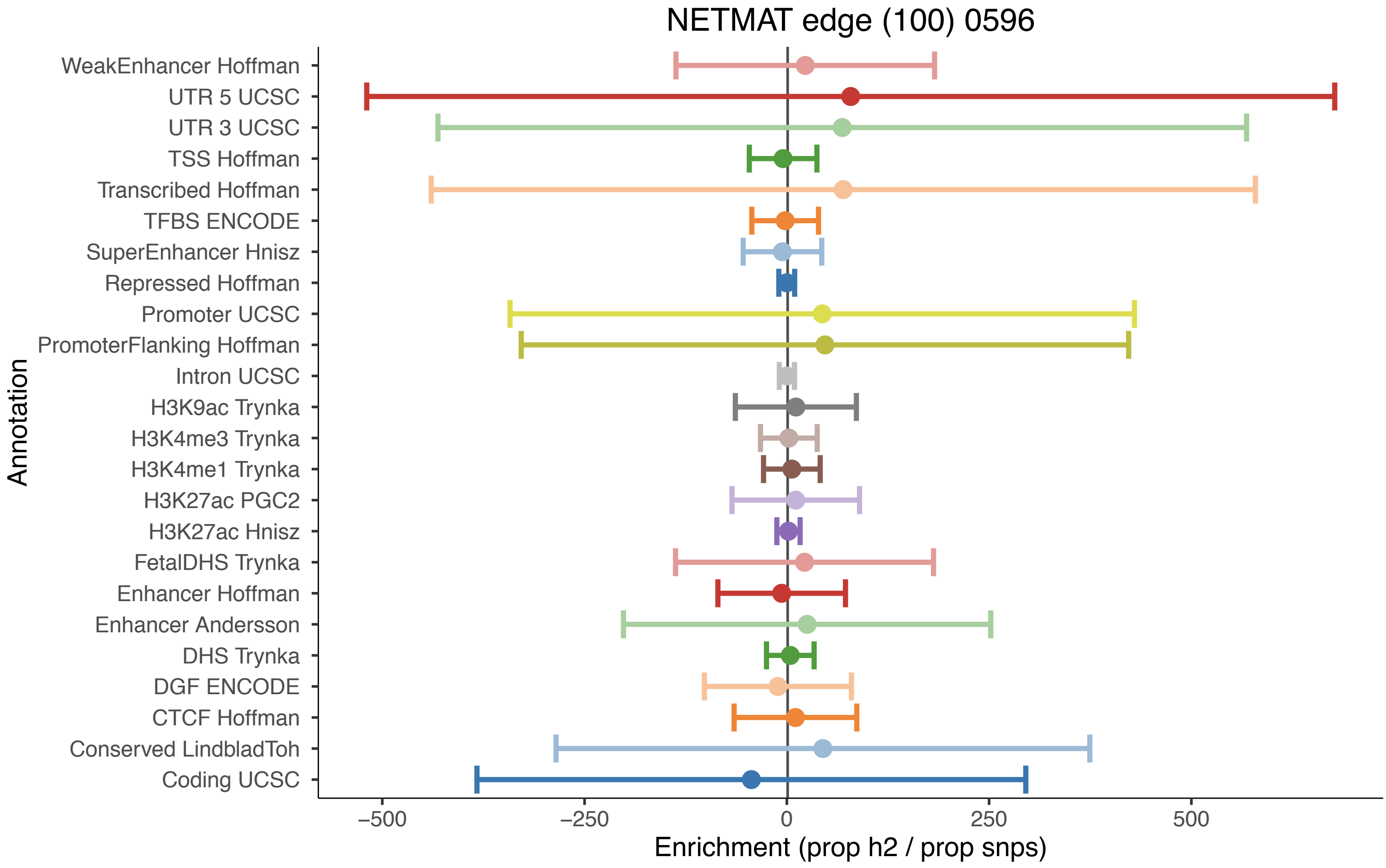

NETMAT edge (100) 0596
Annotation
WeakEnhancer Hoffman
UTR 5 UCSC
UTR 3 UCSC
TSS Hoffman
Transcribed Hoffman
TFBS ENCODE
SuperEnhancer Hnisz
Repressed Hoffman
Promoter UCSC
PromoterFlanking Hoffman
Intron UCSC
H3K9ac Trynka
H3K4me3 Trynka
H3K4me1 Trynka
H3K27ac PGC2
H3K27ac Hnisz
FetalDHS Trynka
Enhancer Hoffman
Enhancer Andersson
DHS Trynka
DGF ENCODE
CTCF Hoffman
Conserved LindbladToh
Coding UCSC
−500
−250
0
250
500
Enrichment (prop h2 / prop snps)

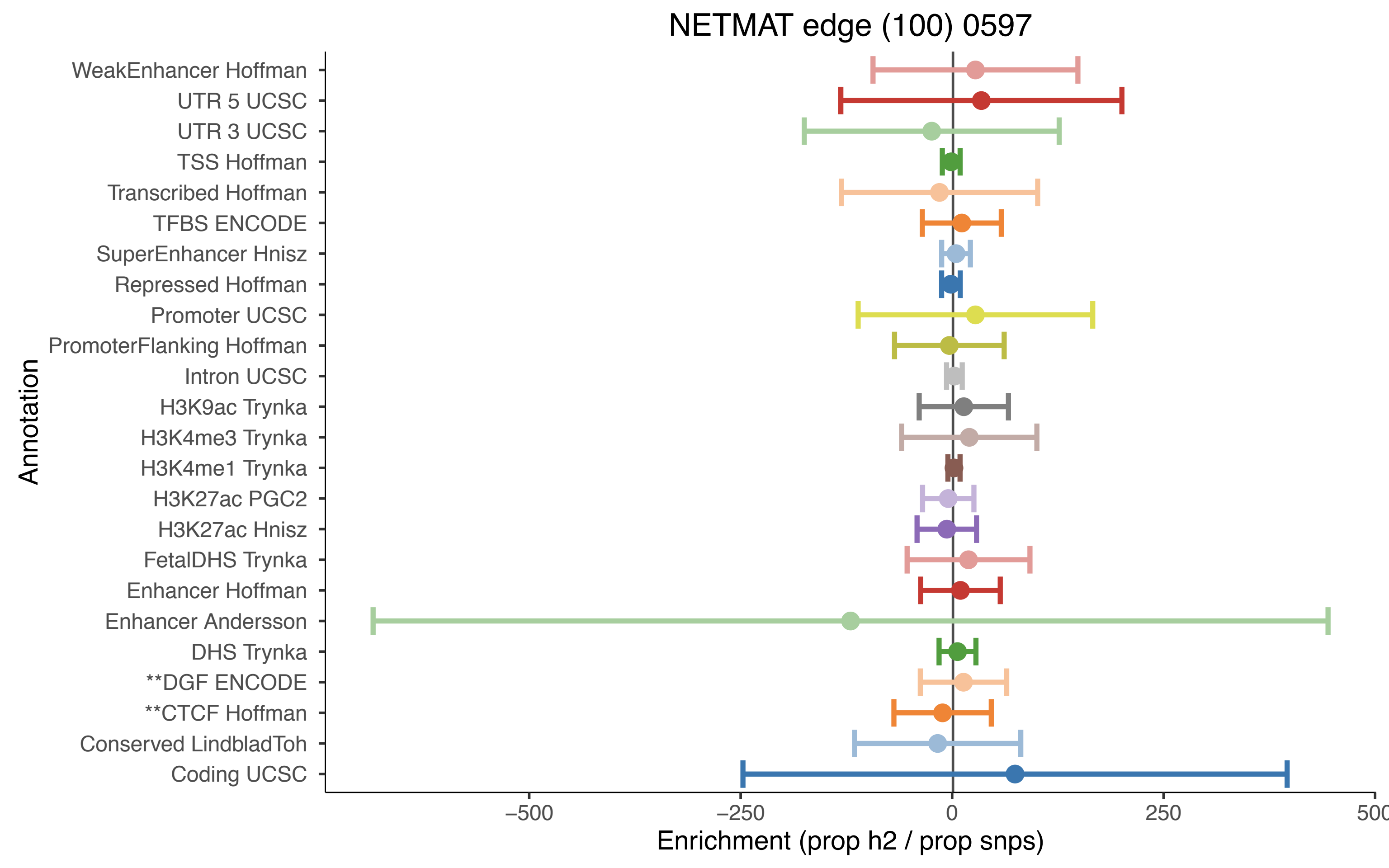
NETMAT edge (100) 0597
WeakEnhancer Hoffman
UTR 5 UCSC
UTR 3 UCSC
TSS Hoffman
Transcribed Hoffman
TFBS ENCODE
SuperEnhancer Hnisz
Repressed Hoffman
Promoter UCSC
PromoterFlanking Hoffman
Intron UCSC
H3K9ac Trynka
H3K4me3 Trynka
H3K4me1 Trynka
H3K27ac PGC2
H3K27ac Hnisz
FetalDHS Trynka
Enhancer Hoffman
Enhancer Andersson
DHS Trynka
**DGF ENCODE
**CTCF Hoffman
Conserved LindbladToh
Coding UCSC
Annotation
−500
−250
0
250
500
Enrichment (prop h2 / prop snps)

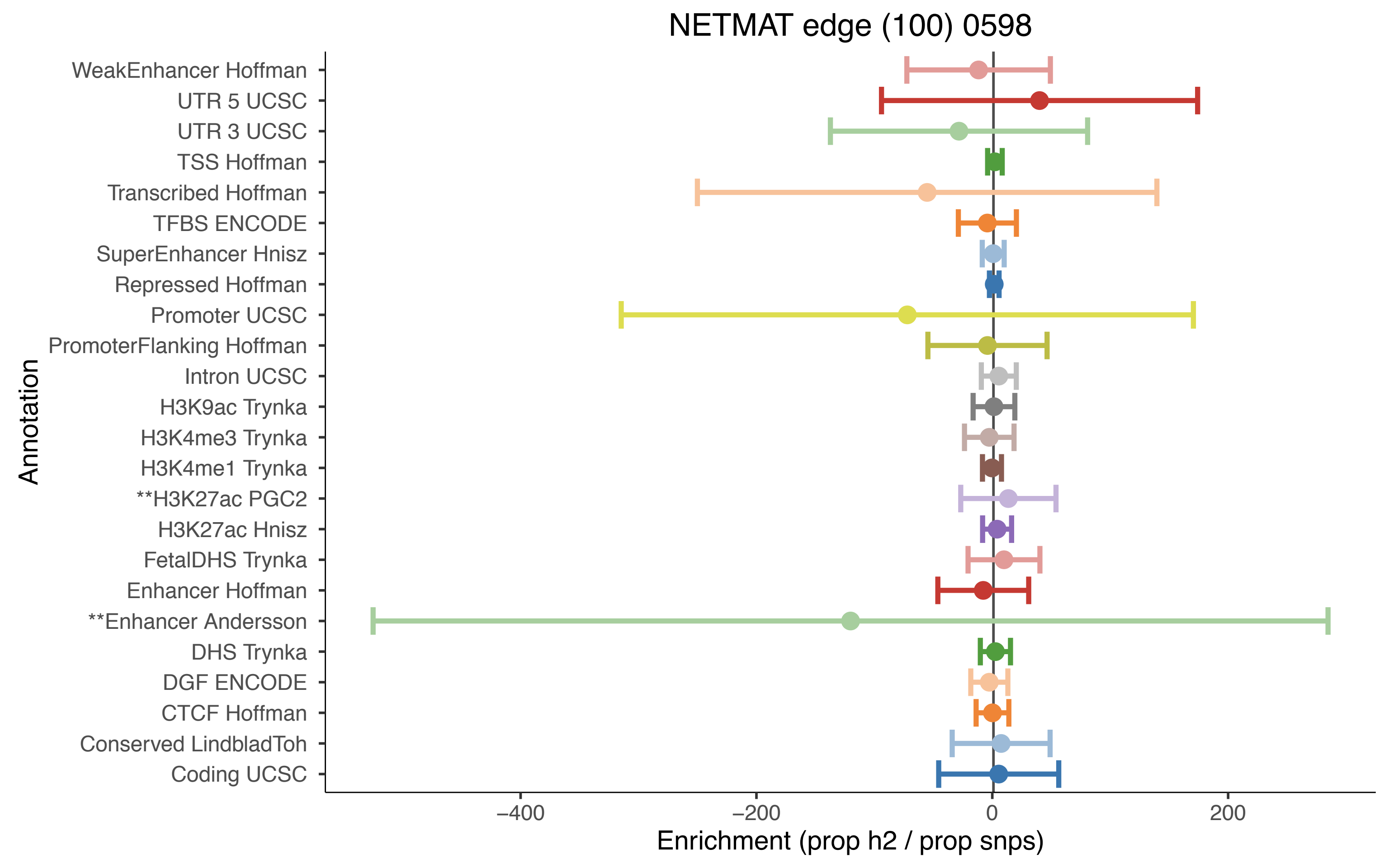

NETMAT edge (100) 0598
Annotation
WeakEnhancer Hoffman
UTR 5 UCSC
UTR 3 UCSC
TSS Hoffman
Transcribed Hoffman
TFBS ENCODE
SuperEnhancer Hnisz
Repressed Hoffman
Promoter UCSC
PromoterFlanking Hoffman
Intron UCSC
H3K9ac Trynka
H3K4me3 Trynka
H3K4me1 Trynka
**H3K27ac PGC2
H3K27ac Hnisz
FetalDHS Trynka
Enhancer Hoffman
**Enhancer Andersson
DHS Trynka
DGF ENCODE
CTCF Hoffman
Conserved LindbladToh
Coding UCSC
−400
−200
0
200
Enrichment (prop h2 / prop snps)

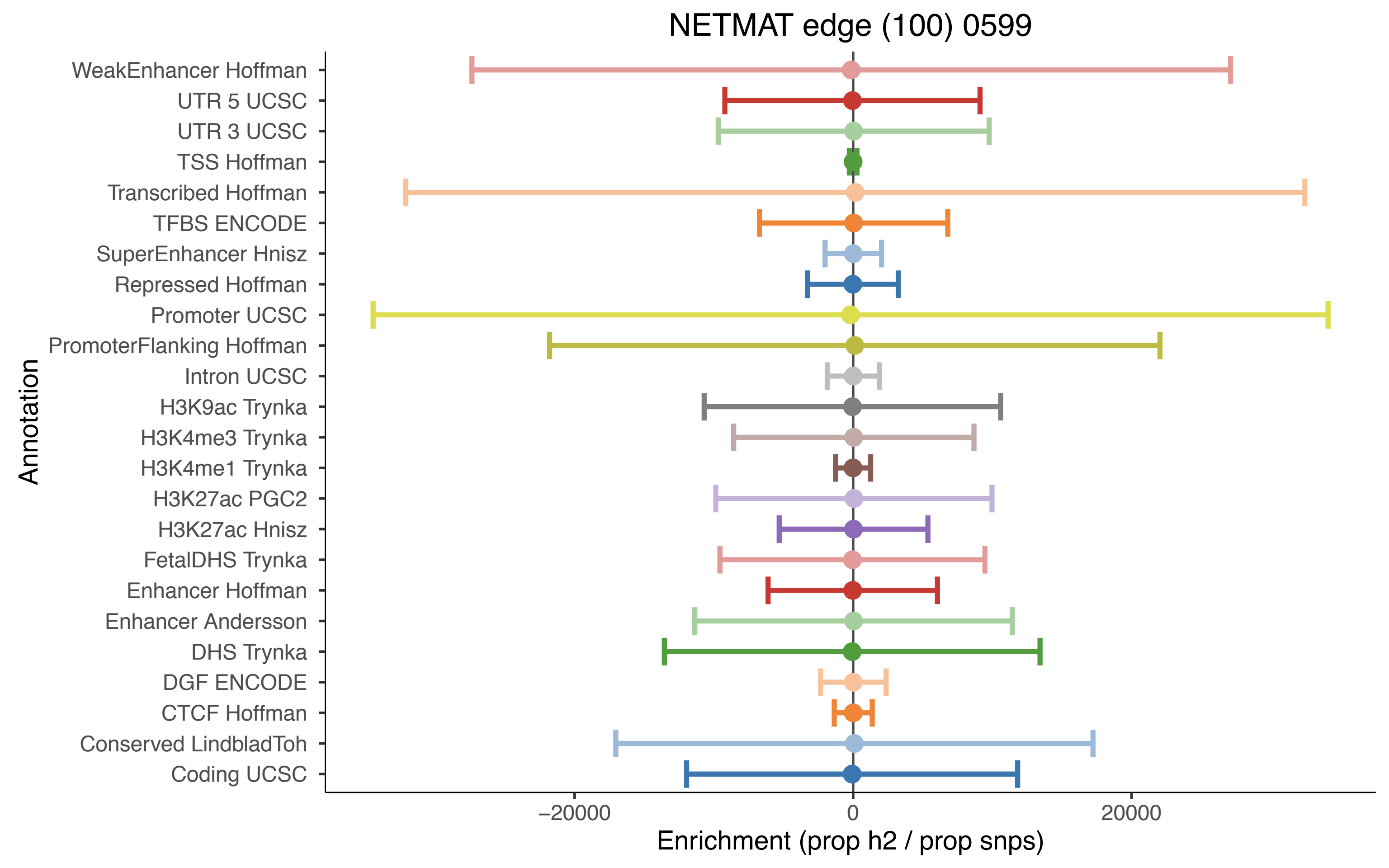

NETMAT edge (100) 0599
WeakEnhancer Hoffman
UTR 5 UCSC
UTR 3 UCSC
TSS Hoffman
Transcribed Hoffman
TFBS ENCODE
SuperEnhancer Hnisz
Repressed Hoffman
Promoter UCSC
PromoterFlanking Hoffman
Intron UCSC
H3K9ac Trynka
H3K4me3 Trynka
H3K4me1 Trynka
H3K27ac PGC2
H3K27ac Hnisz
FetalDHS Trynka
Enhancer Hoffman
Enhancer Andersson
DHS Trynka
DGF ENCODE
CTCF Hoffman
Conserved LindbladToh
Coding UCSC
Annotation
−20000
0
20000
Enrichment (prop h2 / prop snps)

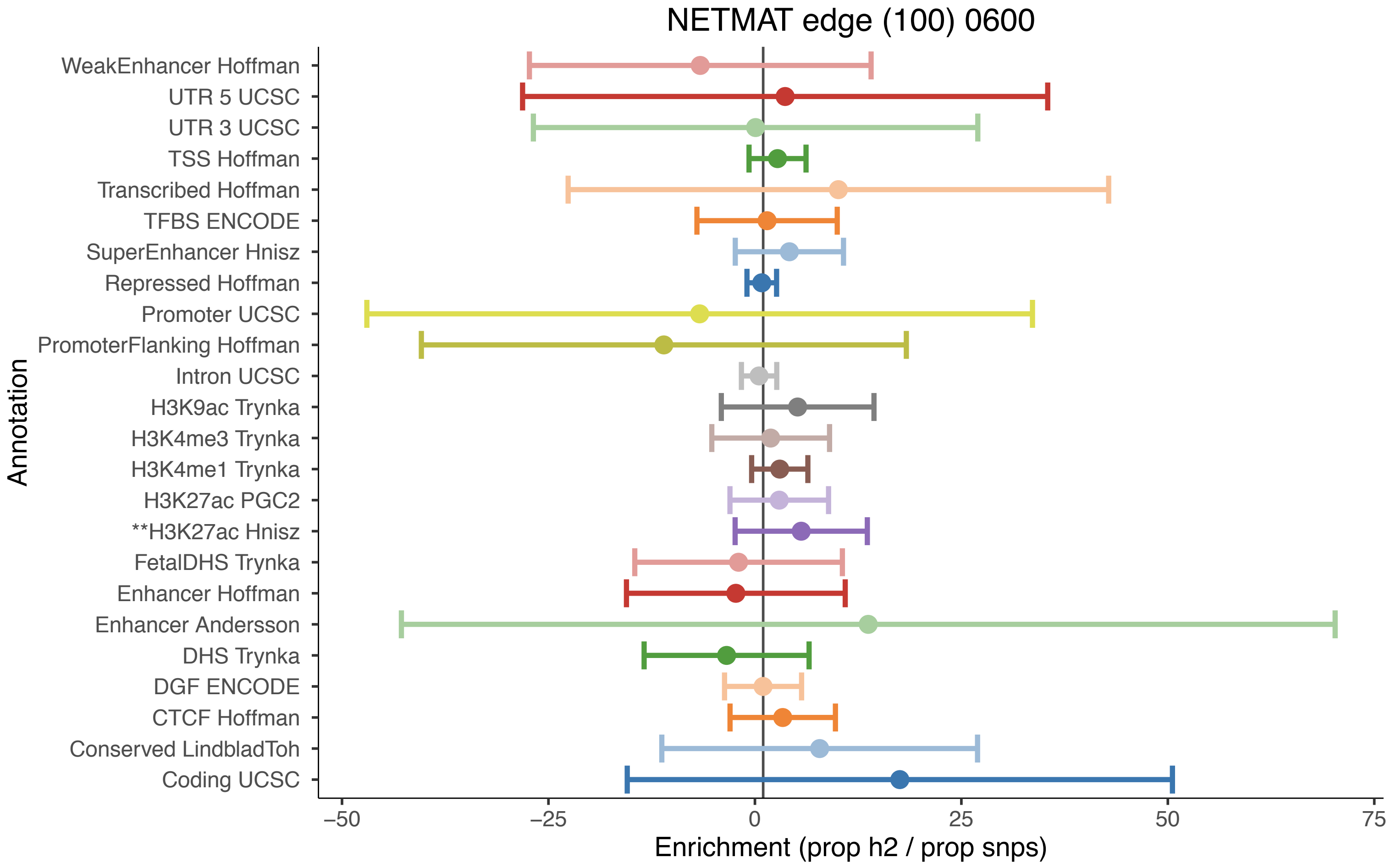

NETMAT edge (100) 0600
WeakEnhancer Hoffman
UTR 5 UCSC
UTR 3 UCSC
TSS Hoffman
Transcribed Hoffman
TFBS ENCODE
SuperEnhancer Hnisz
Repressed Hoffman
Promoter UCSC
PromoterFlanking Hoffman
Intron UCSC
H3K9ac Trynka
H3K4me3 Trynka
H3K4me1 Trynka
H3K27ac PGC2
**H3K27ac Hnisz
FetalDHS Trynka
Enhancer Hoffman
Enhancer Andersson
DHS Trynka
DGF ENCODE
CTCF Hoffman
Conserved LindbladToh
Coding UCSC
Annotation
−50
−25
0
25
50
75
Enrichment (prop h2 / prop snps)

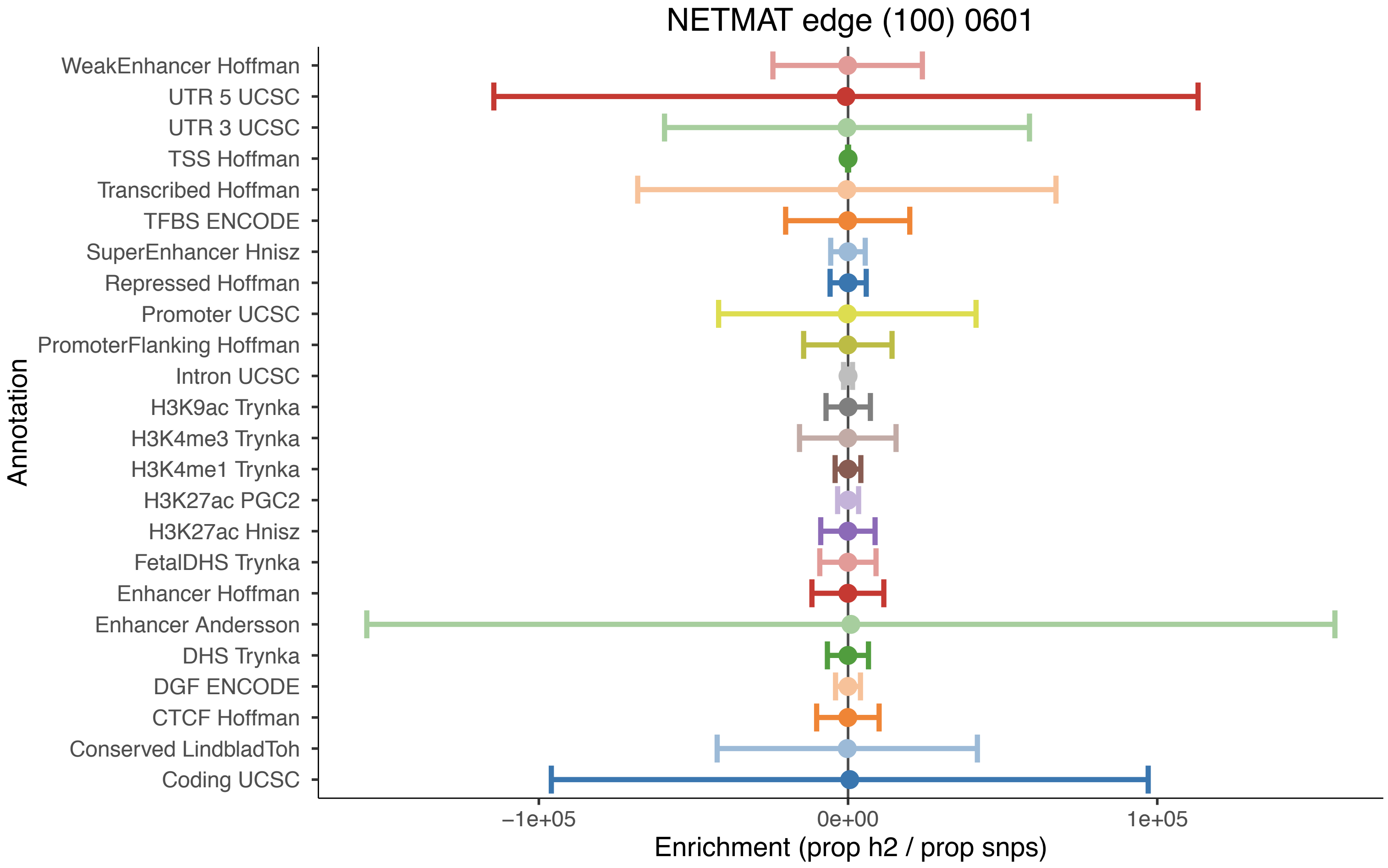
NETMAT edge (100) 0601
WeakEnhancer Hoffman
UTR 5 UCSC
UTR 3 UCSC
TSS Hoffman
Transcribed Hoffman
TFBS ENCODE
SuperEnhancer Hnisz
Repressed Hoffman
Promoter UCSC
PromoterFlanking Hoffman
Intron UCSC
H3K9ac Trynka
H3K4me3 Trynka
H3K4me1 Trynka
H3K27ac PGC2
H3K27ac Hnisz
FetalDHS Trynka
Enhancer Hoffman
Enhancer Andersson
DHS Trynka
DGF ENCODE
CTCF Hoffman
Conserved LindbladToh
Coding UCSC
Annotation
−1e+05
0e+00
1e+05
Enrichment (prop h2 / prop snps)

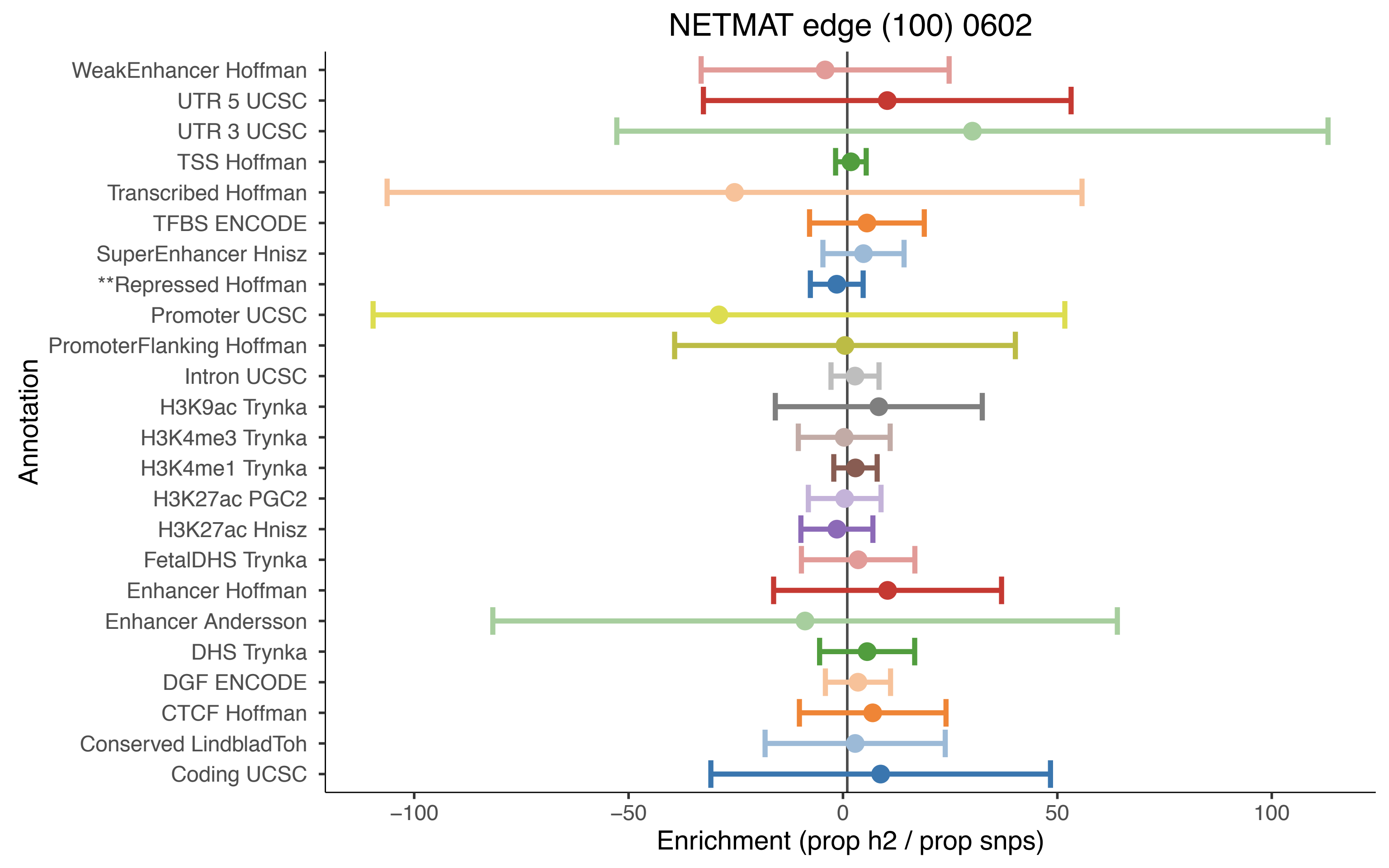
NETMAT edge (100) 0602
WeakEnhancer Hoffman
UTR 5 UCSC
UTR 3 UCSC
TSS Hoffman
Transcribed Hoffman
TFBS ENCODE
SuperEnhancer Hnisz
**Repressed Hoffman
Promoter UCSC
PromoterFlanking Hoffman
Intron UCSC
H3K9ac Trynka
H3K4me3 Trynka
H3K4me1 Trynka
H3K27ac PGC2
H3K27ac Hnisz
FetalDHS Trynka
Enhancer Hoffman
Enhancer Andersson
DHS Trynka
DGF ENCODE
CTCF Hoffman
Conserved LindbladToh
Coding UCSC
Annotation
−100
−50
0
50
100
Enrichment (prop h2 / prop snps)

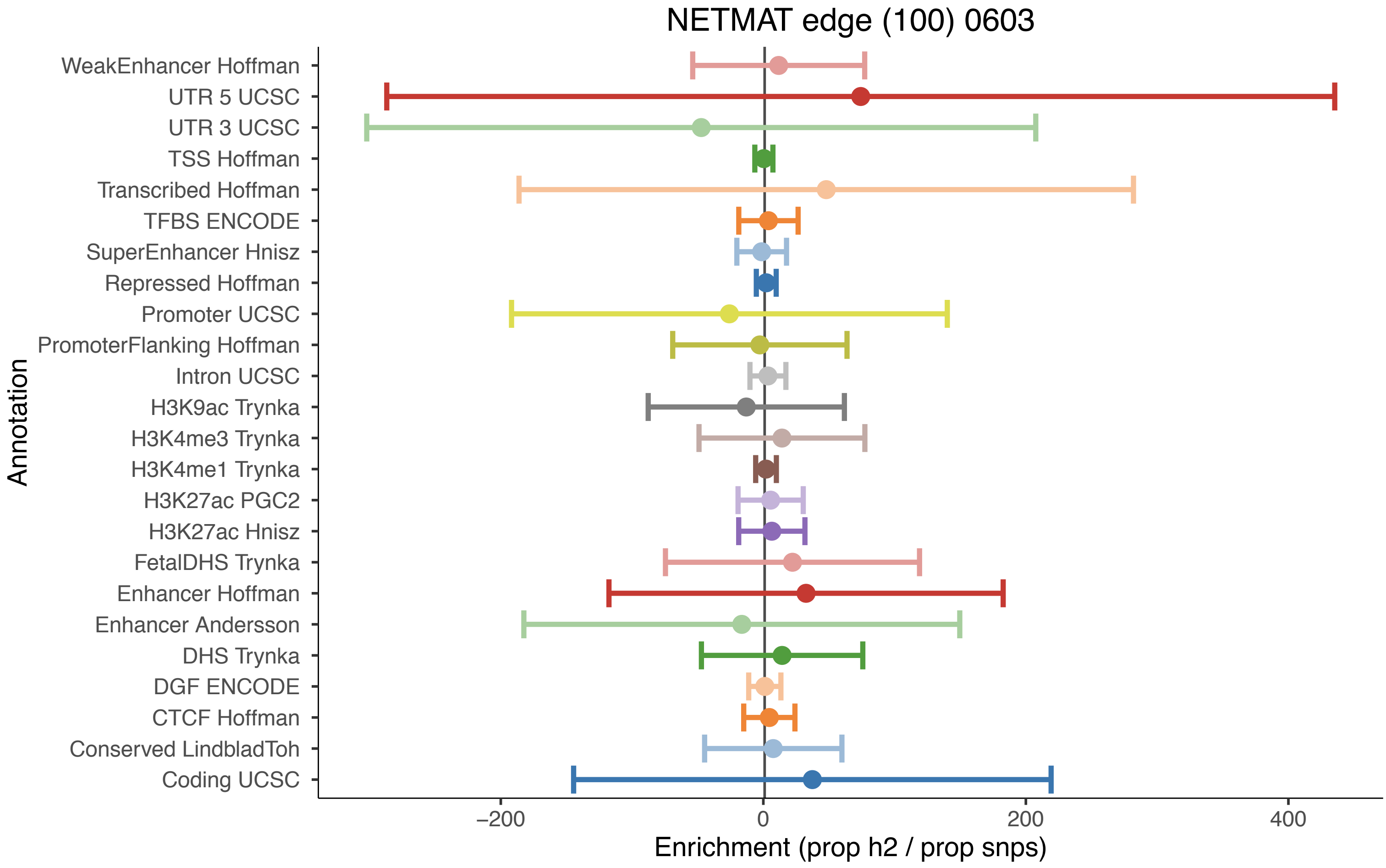
NETMAT edge (100) 0603
WeakEnhancer Hoffman
UTR 5 UCSC
UTR 3 UCSC
TSS Hoffman
Transcribed Hoffman
TFBS ENCODE
SuperEnhancer Hnisz
Repressed Hoffman
Promoter UCSC
PromoterFlanking Hoffman
Intron UCSC
H3K9ac Trynka
H3K4me3 Trynka
H3K4me1 Trynka
H3K27ac PGC2
H3K27ac Hnisz
FetalDHS Trynka
Enhancer Hoffman
Enhancer Andersson
DHS Trynka
DGF ENCODE
CTCF Hoffman
Conserved LindbladToh
Coding UCSC
Annotation
−200
0
200
400
Enrichment (prop h2 / prop snps)

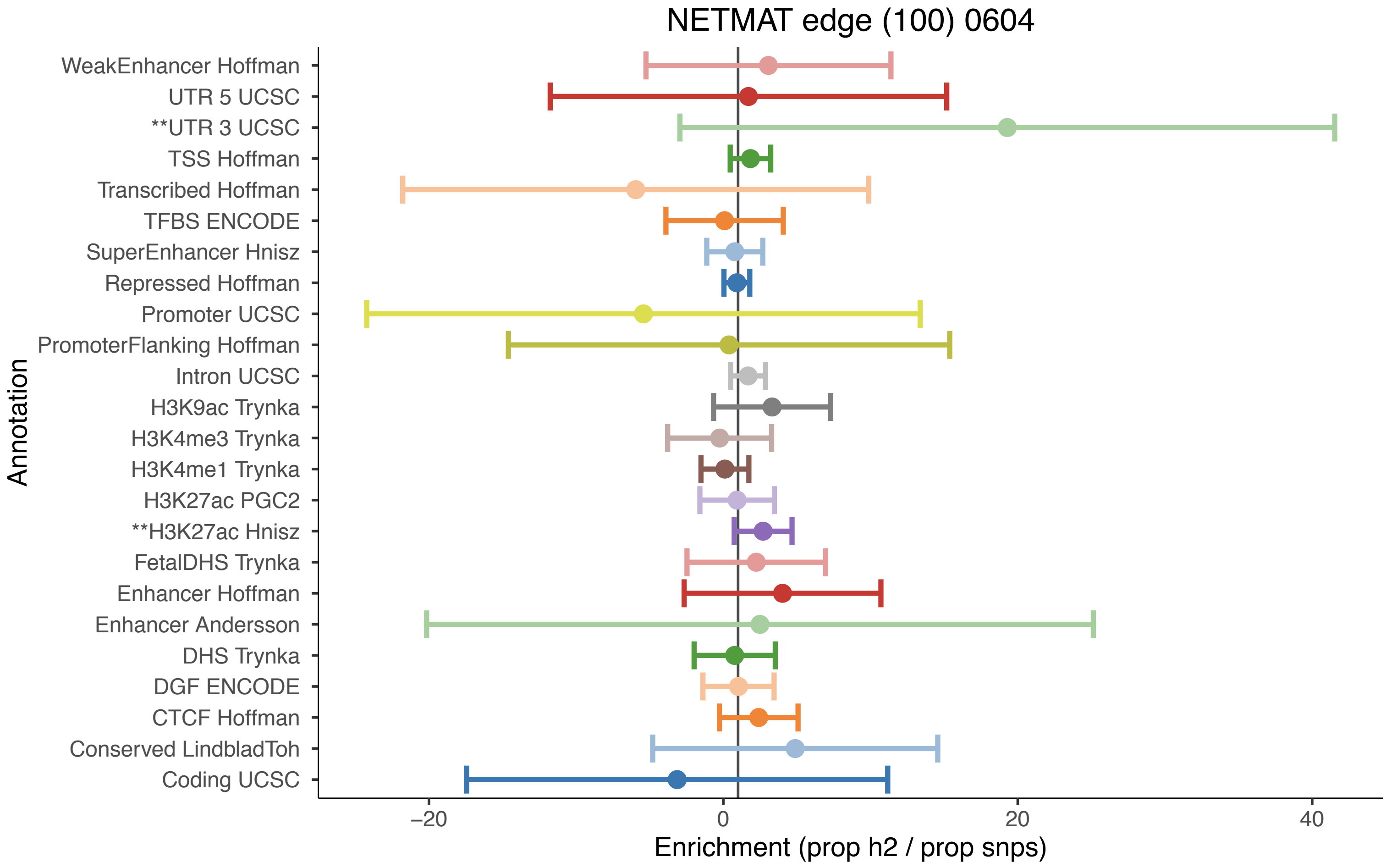

NETMAT edge (100) 0604
WeakEnhancer Hoffman
UTR 5 UCSC
**UTR 3 UCSC
TSS Hoffman
Transcribed Hoffman
TFBS ENCODE
SuperEnhancer Hnisz
Repressed Hoffman
Promoter UCSC
PromoterFlanking Hoffman
Intron UCSC
H3K9ac Trynka
H3K4me3 Trynka
H3K4me1 Trynka
H3K27ac PGC2
**H3K27ac Hnisz
FetalDHS Trynka
Enhancer Hoffman
Enhancer Andersson
DHS Trynka
DGF ENCODE
CTCF Hoffman
Conserved LindbladToh
Coding UCSC
Annotation
−20
0
20
40
Enrichment (prop h2 / prop snps)

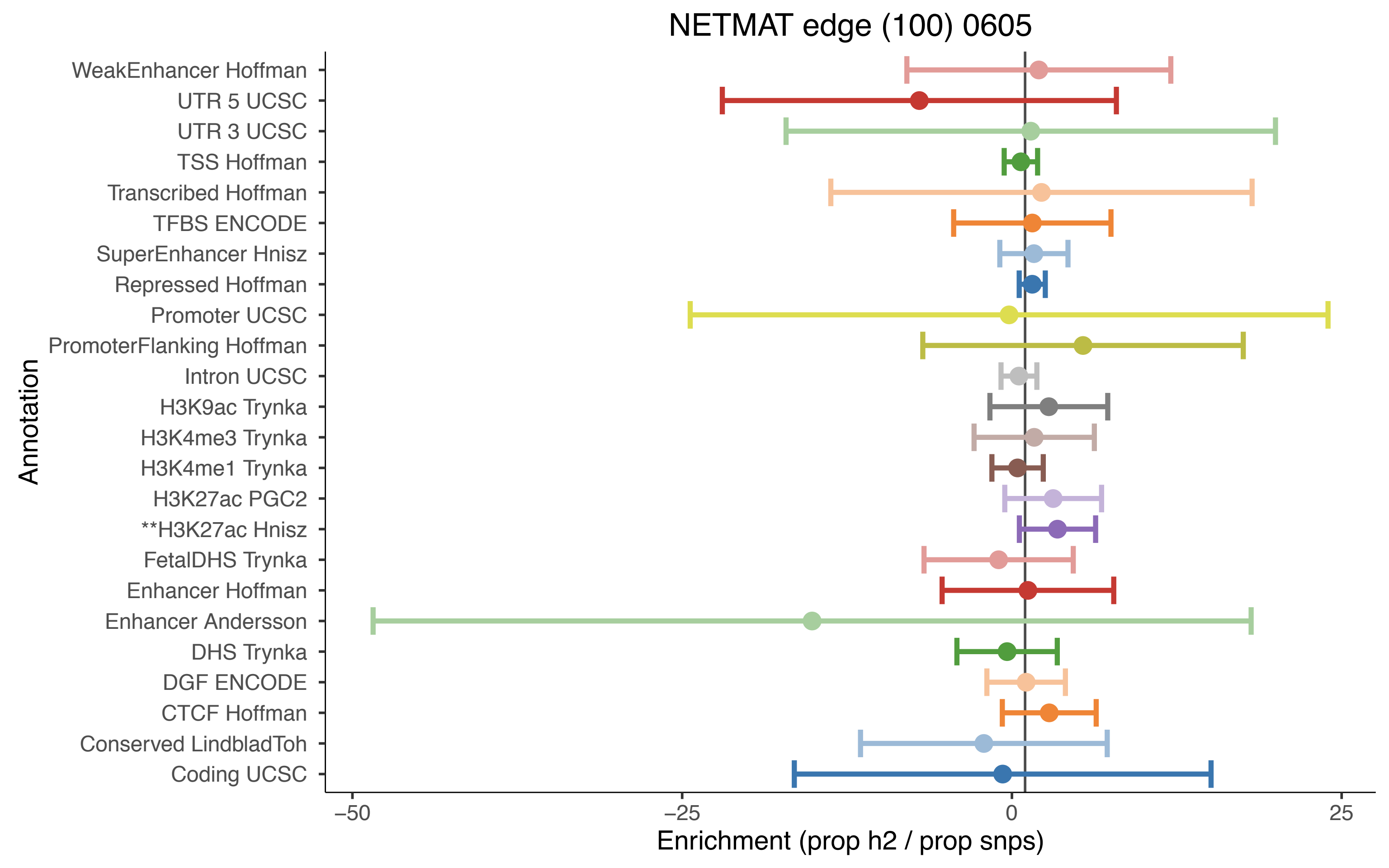
NETMAT edge (100) 0605
WeakEnhancer Hoffman
UTR 5 UCSC
UTR 3 UCSC
TSS Hoffman
Transcribed Hoffman
TFBS ENCODE
SuperEnhancer Hnisz
Repressed Hoffman
Promoter UCSC
PromoterFlanking Hoffman
Intron UCSC
H3K9ac Trynka
H3K4me3 Trynka
H3K4me1 Trynka
H3K27ac PGC2
**H3K27ac Hnisz
FetalDHS Trynka
Enhancer Hoffman
Enhancer Andersson
DHS Trynka
DGF ENCODE
CTCF Hoffman
Conserved LindbladToh
Coding UCSC
Annotation
−50
−25
0
25
Enrichment (prop h2 / prop snps)

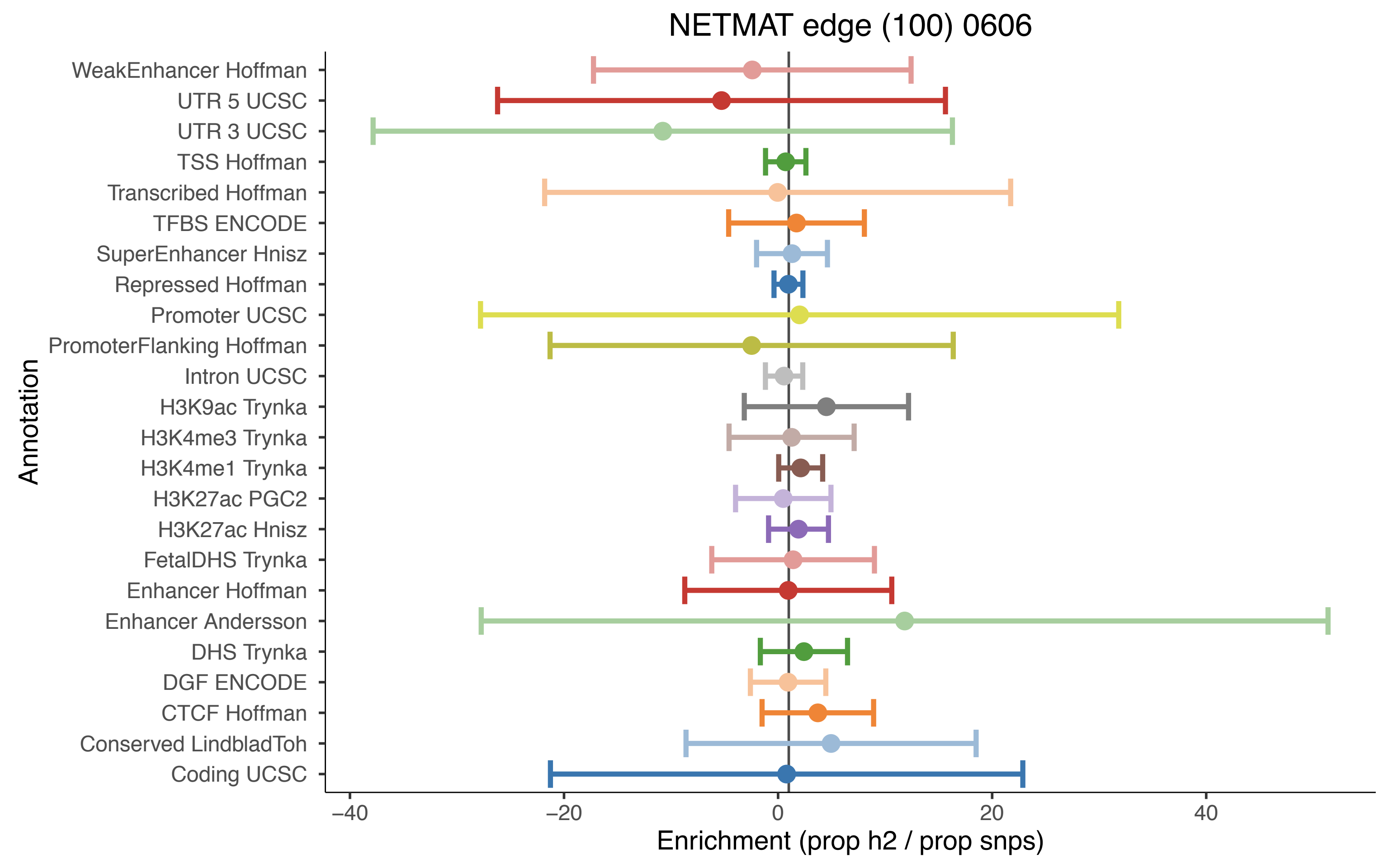
NETMAT edge (100) 0606
Annotation
WeakEnhancer Hoffman
UTR 5 UCSC
UTR 3 UCSC
TSS Hoffman
Transcribed Hoffman
TFBS ENCODE
SuperEnhancer Hnisz
Repressed Hoffman
Promoter UCSC
PromoterFlanking Hoffman
Intron UCSC
H3K9ac Trynka
H3K4me3 Trynka
H3K4me1 Trynka
H3K27ac PGC2
H3K27ac Hnisz
FetalDHS Trynka
Enhancer Hoffman
Enhancer Andersson
DHS Trynka
DGF ENCODE
CTCF Hoffman
Conserved LindbladToh
Coding UCSC
−40
−20
0
20
40
Enrichment (prop h2 / prop snps)

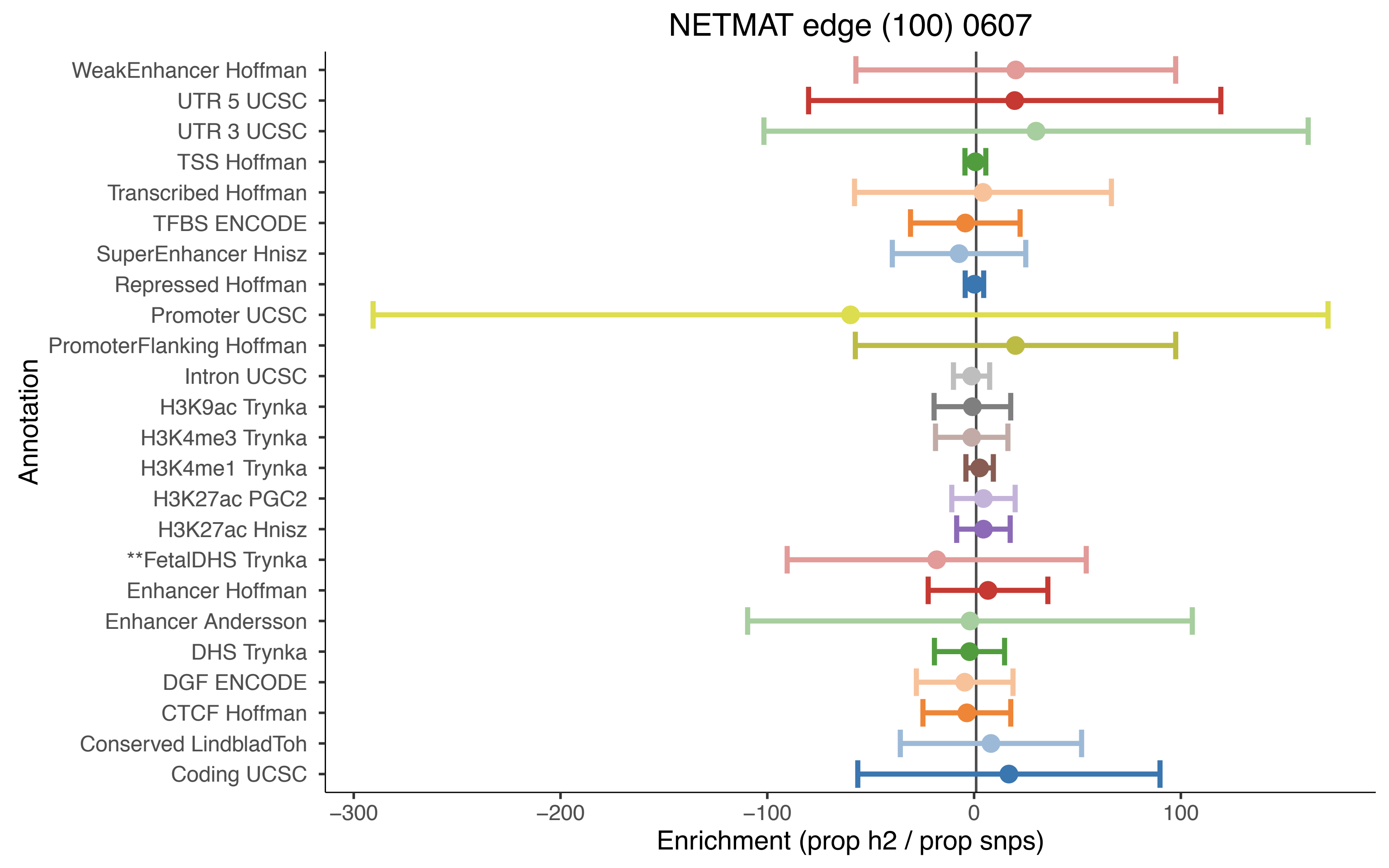

NETMAT edge (100) 0607
WeakEnhancer Hoffman
UTR 5 UCSC
UTR 3 UCSC
TSS Hoffman
Transcribed Hoffman
TFBS ENCODE
SuperEnhancer Hnisz
Repressed Hoffman
Promoter UCSC
PromoterFlanking Hoffman
Intron UCSC
H3K9ac Trynka
H3K4me3 Trynka
H3K4me1 Trynka
H3K27ac PGC2
H3K27ac Hnisz
**FetalDHS Trynka
Enhancer Hoffman
Enhancer Andersson
DHS Trynka
DGF ENCODE
CTCF Hoffman
Conserved LindbladToh
Coding UCSC
Annotation
−300
−200
−100
0
100
Enrichment (prop h2 / prop snps)

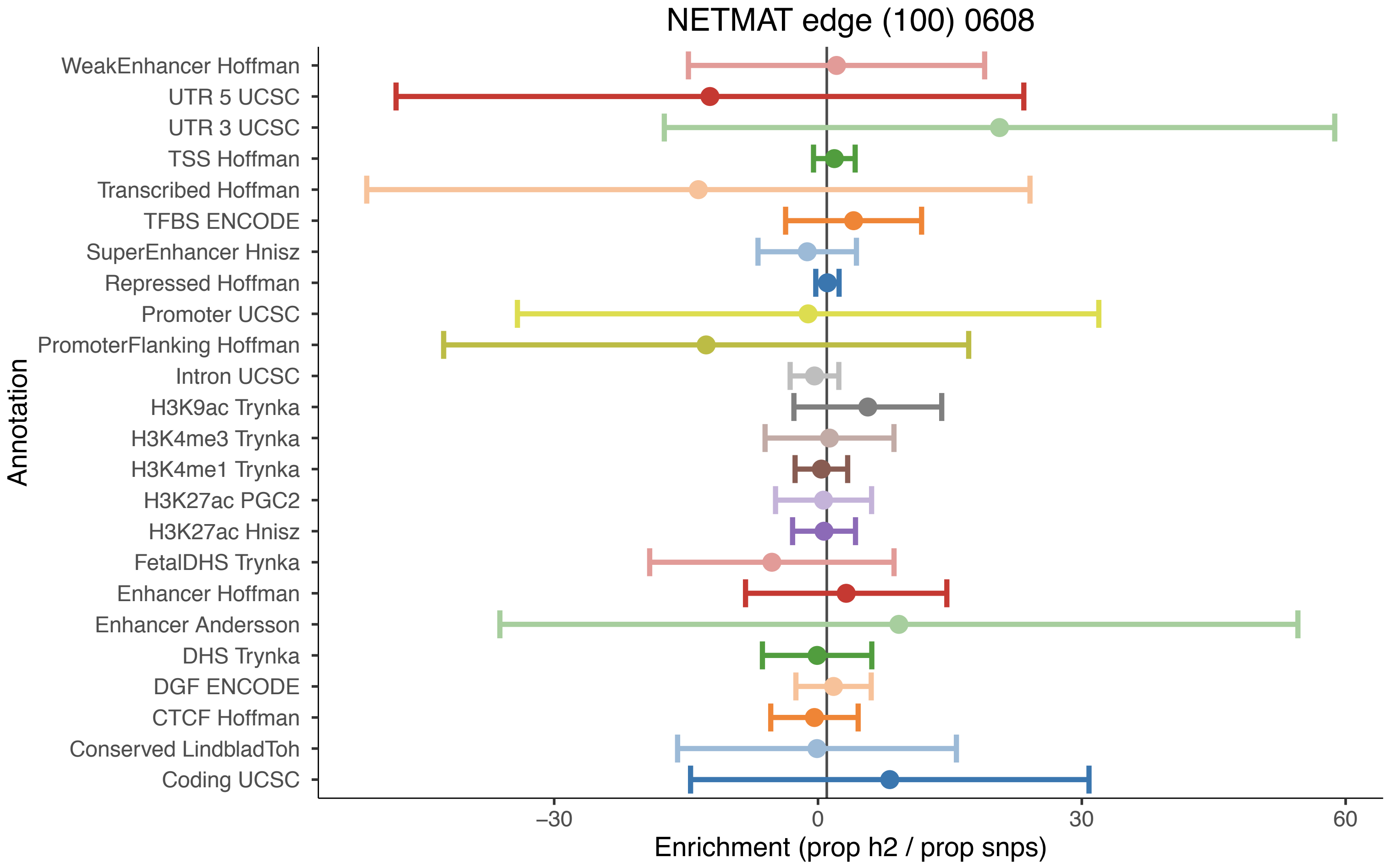
NETMAT edge (100) 0608
Annotation
WeakEnhancer Hoffman
UTR 5 UCSC
UTR 3 UCSC
TSS Hoffman
Transcribed Hoffman
TFBS ENCODE
SuperEnhancer Hnisz
Repressed Hoffman
Promoter UCSC
PromoterFlanking Hoffman
Intron UCSC
H3K9ac Trynka
H3K4me3 Trynka
H3K4me1 Trynka
H3K27ac PGC2
H3K27ac Hnisz
FetalDHS Trynka
Enhancer Hoffman
Enhancer Andersson
DHS Trynka
DGF ENCODE
CTCF Hoffman
Conserved LindbladToh
Coding UCSC
−30
0
30
60
Enrichment (prop h2 / prop snps)

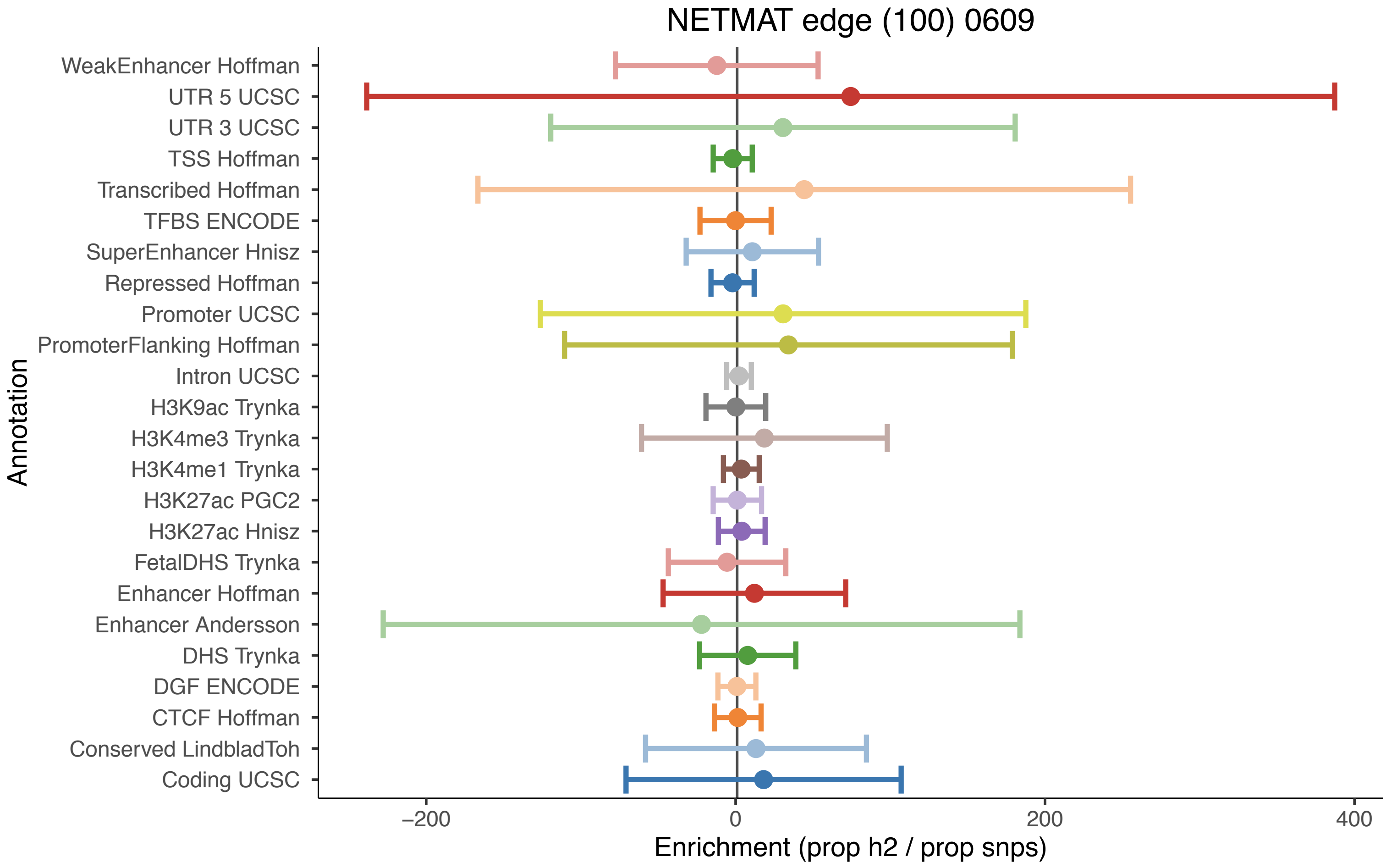
NETMAT edge (100) 0609
Annotation
WeakEnhancer Hoffman
UTR 5 UCSC
UTR 3 UCSC
TSS Hoffman
Transcribed Hoffman
TFBS ENCODE
SuperEnhancer Hnisz
Repressed Hoffman
Promoter UCSC
PromoterFlanking Hoffman
Intron UCSC
H3K9ac Trynka
H3K4me3 Trynka
H3K4me1 Trynka
H3K27ac PGC2
H3K27ac Hnisz
FetalDHS Trynka
Enhancer Hoffman
Enhancer Andersson
DHS Trynka
DGF ENCODE
CTCF Hoffman
Conserved LindbladToh
Coding UCSC
−200
0
200
400
Enrichment (prop h2 / prop snps)

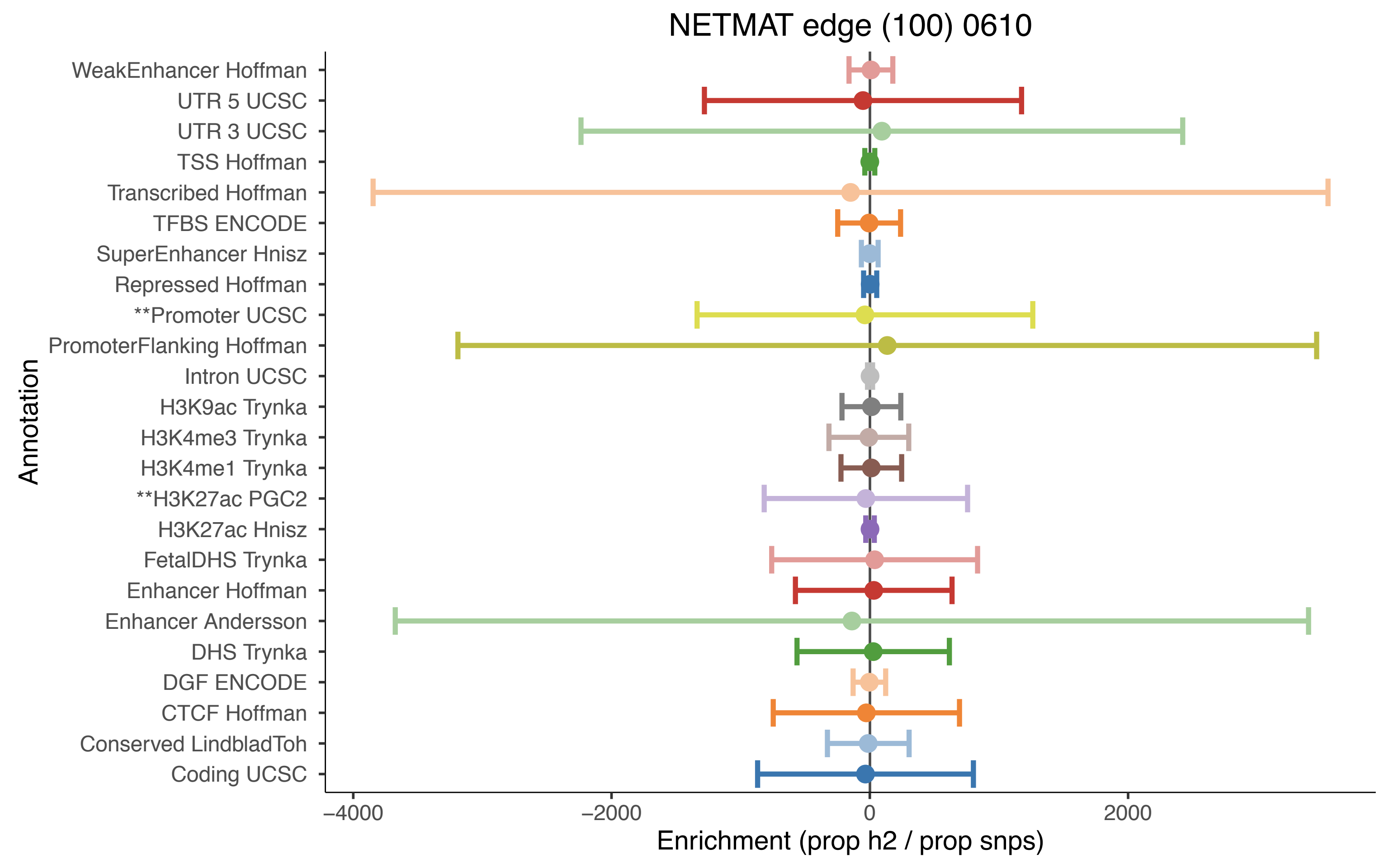
NETMAT edge (100) 0610
WeakEnhancer Hoffman
UTR 5 UCSC
UTR 3 UCSC
TSS Hoffman
Transcribed Hoffman
TFBS ENCODE
SuperEnhancer Hnisz
Repressed Hoffman
**Promoter UCSC
PromoterFlanking Hoffman
Intron UCSC
H3K9ac Trynka
H3K4me3 Trynka
H3K4me1 Trynka
**H3K27ac PGC2
H3K27ac Hnisz
FetalDHS Trynka
Enhancer Hoffman
Enhancer Andersson
DHS Trynka
DGF ENCODE
CTCF Hoffman
Conserved LindbladToh
Coding UCSC
Annotation
−4000
−2000
0
2000
Enrichment (prop h2 / prop snps)

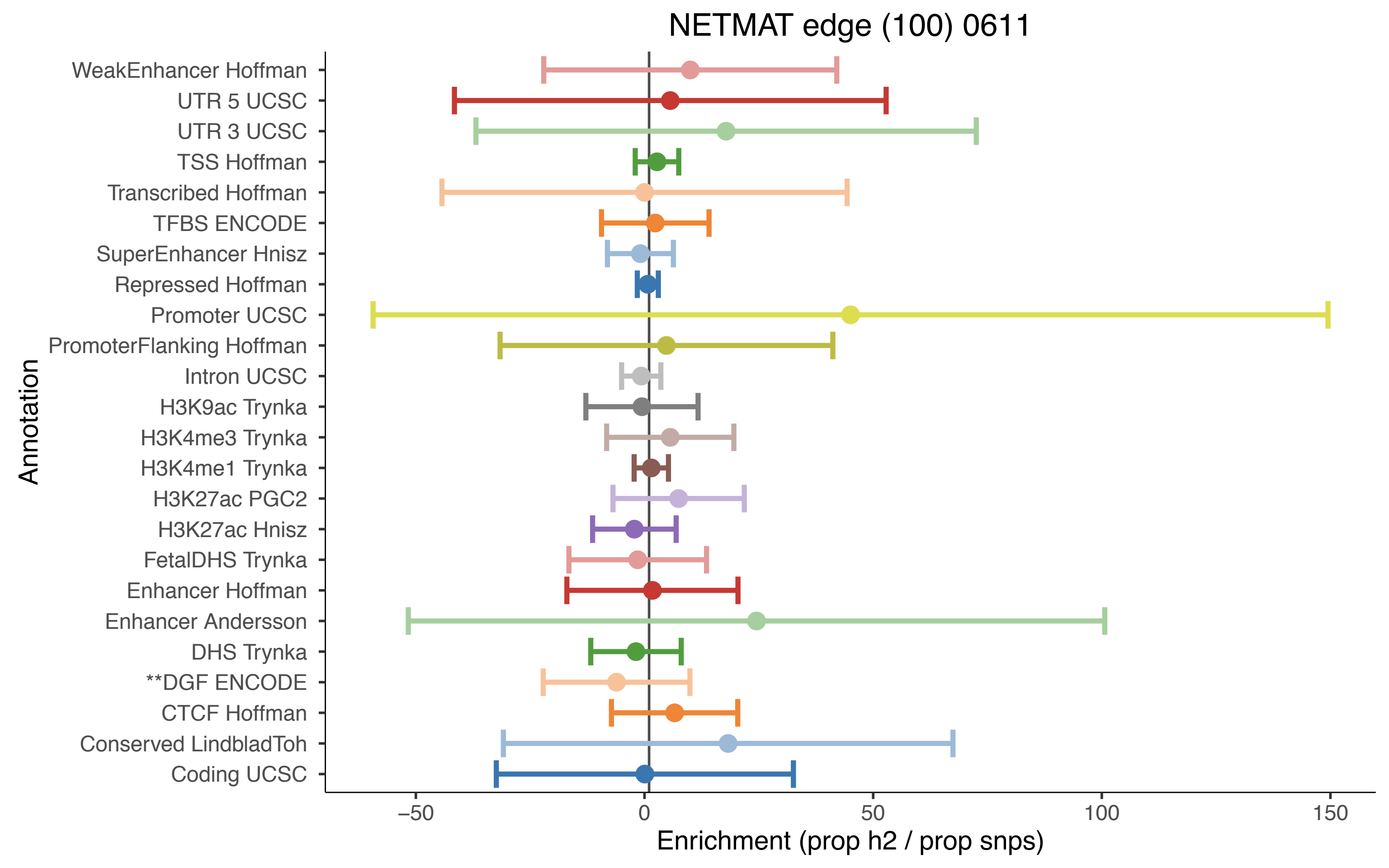

NETMAT edge (100) 0611
Annotation
WeakEnhancer Hoffman
UTR 5 UCSC
UTR 3 UCSC
TSS Hoffman
Transcribed Hoffman
TFBS ENCODE
SuperEnhancer Hnisz
Repressed Hoffman
Promoter UCSC
PromoterFlanking Hoffman
Intron UCSC
H3K9ac Trynka
H3K4me3 Trynka
H3K4me1 Trynka
H3K27ac PGC2
H3K27ac Hnisz
FetalDHS Trynka
Enhancer Hoffman
Enhancer Andersson
DHS Trynka
**DGF ENCODE
CTCF Hoffman
Conserved LindbladToh
Coding UCSC
−50
0
50
100
150
Enrichment (prop h2 / prop snps)

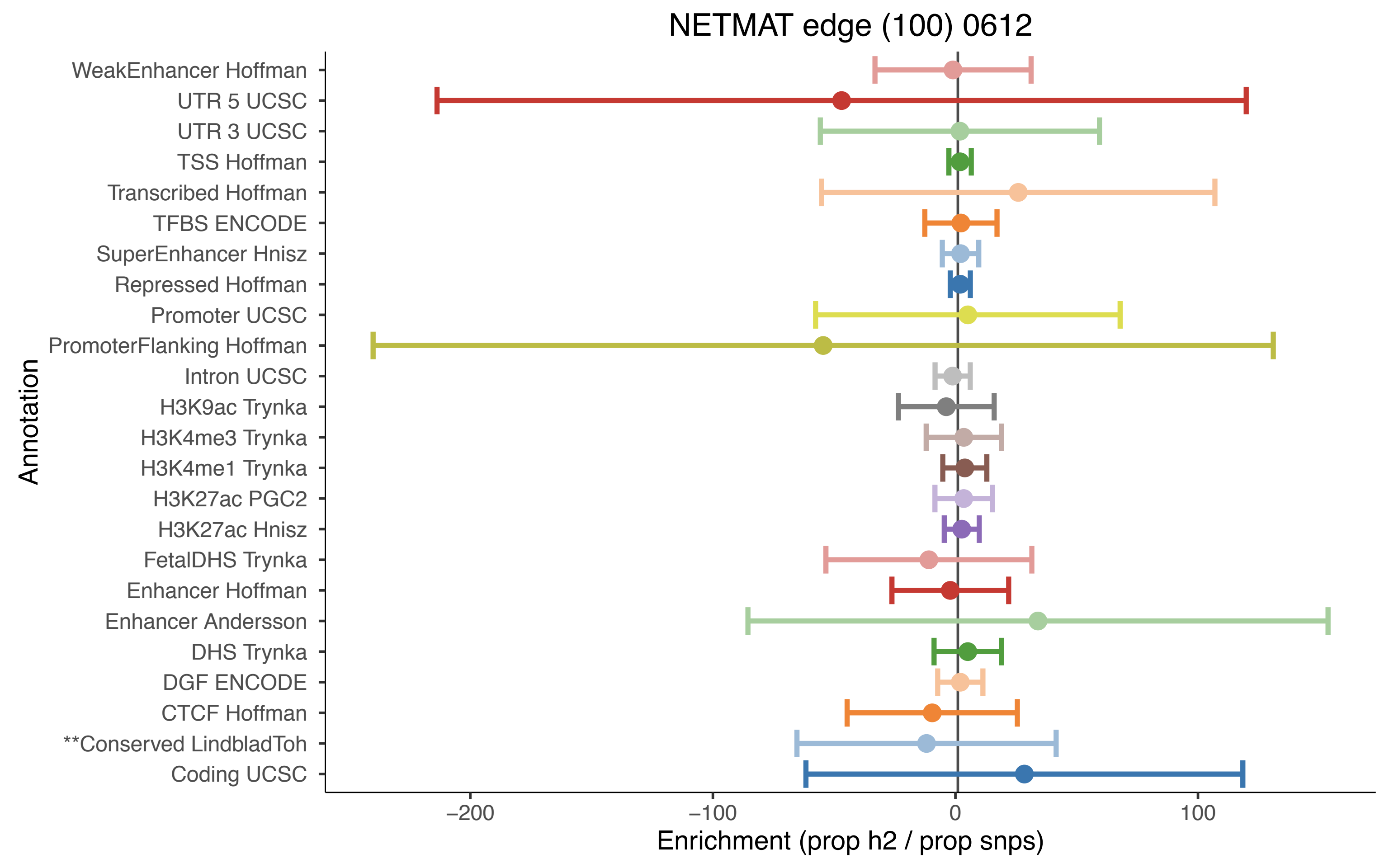
NETMAT edge (100) 0612
WeakEnhancer Hoffman
UTR 5 UCSC
UTR 3 UCSC
TSS Hoffman
Transcribed Hoffman
TFBS ENCODE
SuperEnhancer Hnisz
Repressed Hoffman
Promoter UCSC
PromoterFlanking Hoffman
Intron UCSC
H3K9ac Trynka
H3K4me3 Trynka
H3K4me1 Trynka
H3K27ac PGC2
H3K27ac Hnisz
FetalDHS Trynka
Enhancer Hoffman
Enhancer Andersson
DHS Trynka
DGF ENCODE
CTCF Hoffman
**Conserved LindbladToh
Coding UCSC
Annotation
−200
−100
0
100
Enrichment (prop h2 / prop snps)

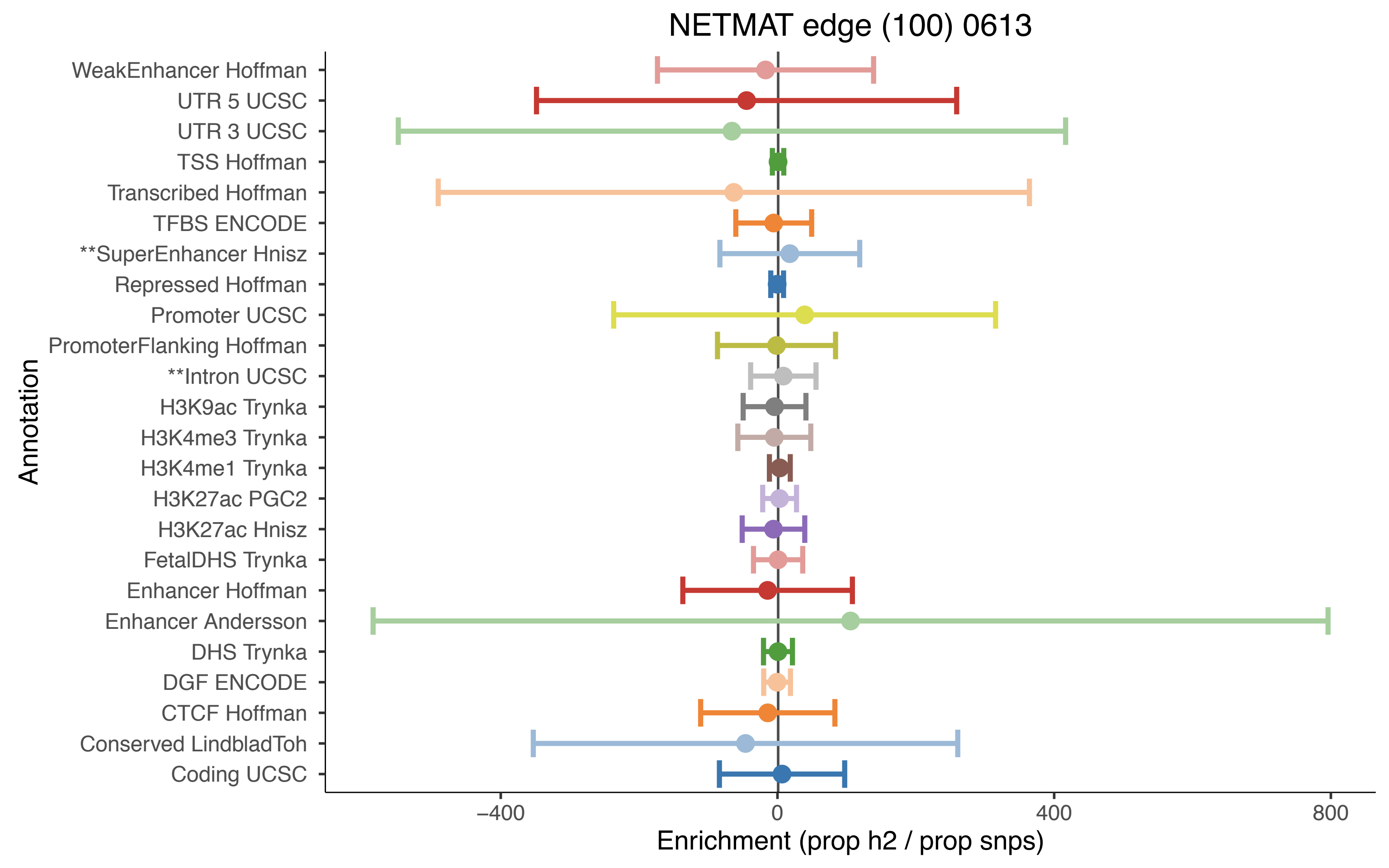
NETMAT edge (100) 0613
WeakEnhancer Hoffman
UTR 5 UCSC
UTR 3 UCSC
TSS Hoffman
Transcribed Hoffman
TFBS ENCODE
**SuperEnhancer Hnisz
Repressed Hoffman
Promoter UCSC
PromoterFlanking Hoffman
**Intron UCSC
H3K9ac Trynka
H3K4me3 Trynka
H3K4me1 Trynka
H3K27ac PGC2
H3K27ac Hnisz
FetalDHS Trynka
Enhancer Hoffman
Enhancer Andersson
DHS Trynka
DGF ENCODE
CTCF Hoffman
Conserved LindbladToh
Coding UCSC
Annotation
−400
0
400
800
Enrichment (prop h2 / prop snps)

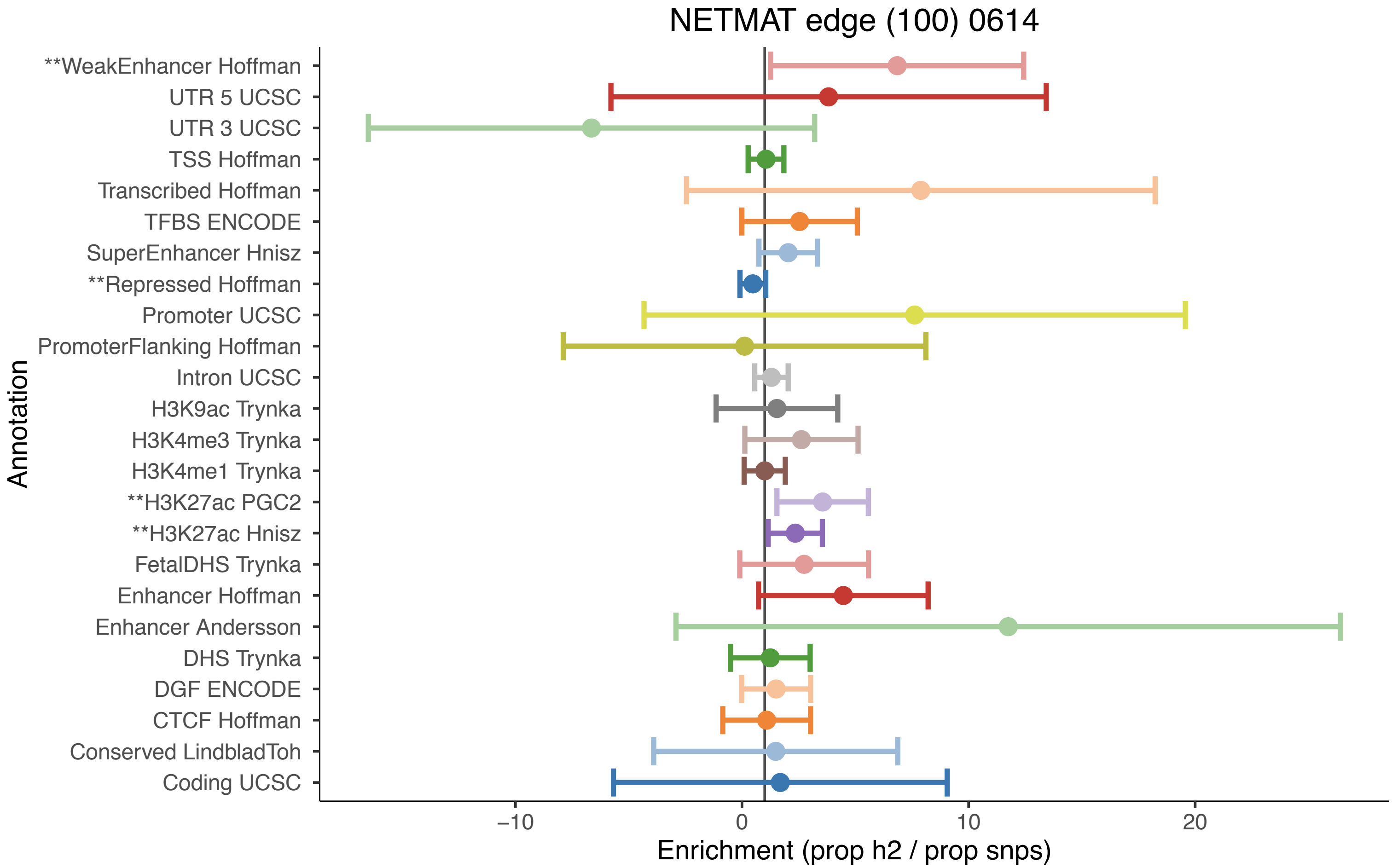
NETMAT edge (100) 0614
**WeakEnhancer Hoffman
UTR 5 UCSC
UTR 3 UCSC
TSS Hoffman
Transcribed Hoffman
TFBS ENCODE
SuperEnhancer Hnisz
**Repressed Hoffman
Promoter UCSC
PromoterFlanking Hoffman
Intron UCSC
H3K9ac Trynka
H3K4me3 Trynka
H3K4me1 Trynka
**H3K27ac PGC2
**H3K27ac Hnisz
FetalDHS Trynka
Enhancer Hoffman
Enhancer Andersson
DHS Trynka
DGF ENCODE
CTCF Hoffman
Conserved LindbladToh
Coding UCSC
Annotation
−10
0
10
20
Enrichment (prop h2 / prop snps)

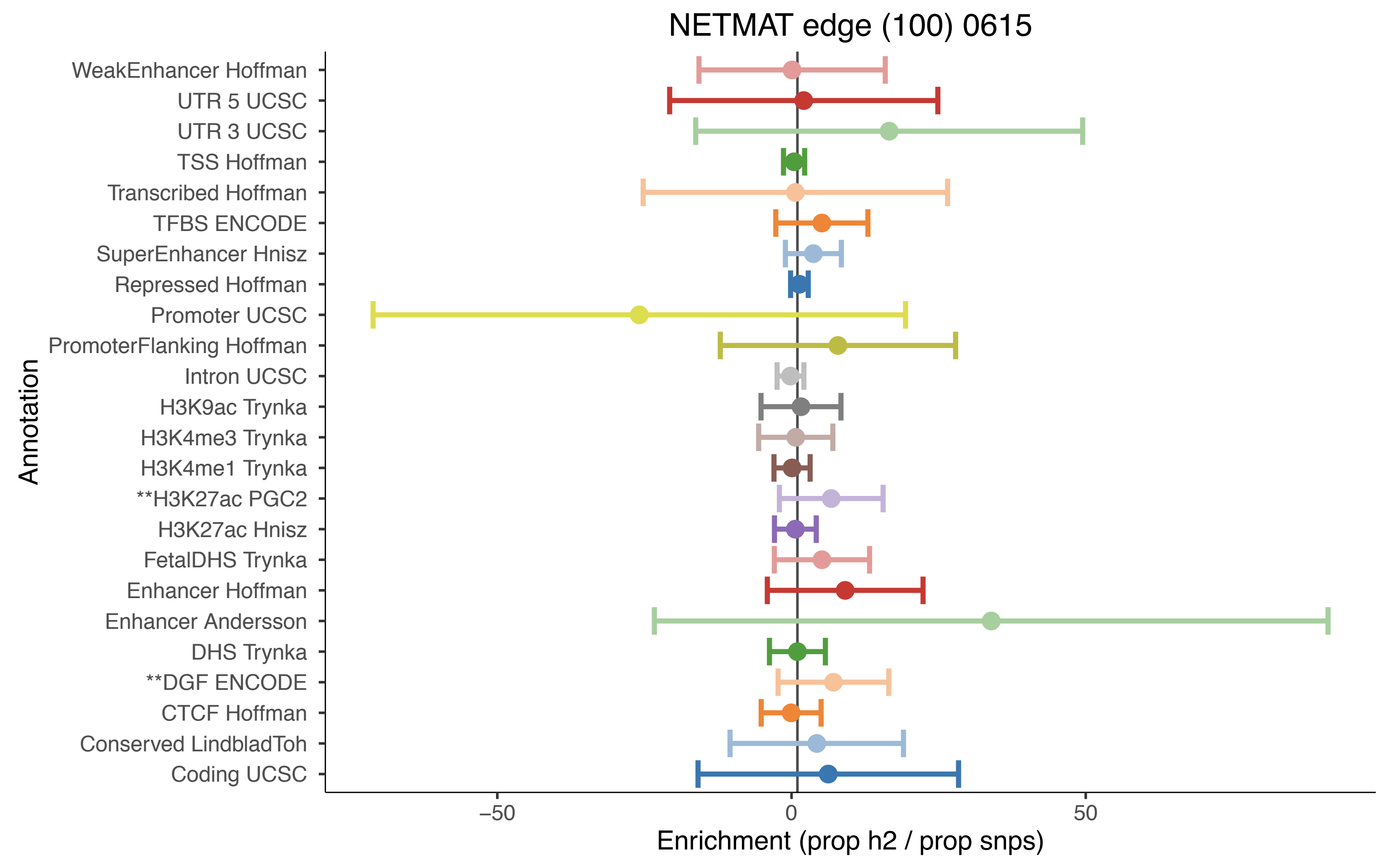

NETMAT edge (100) 0615
WeakEnhancer Hoffman
UTR 5 UCSC
UTR 3 UCSC
TSS Hoffman
Transcribed Hoffman
TFBS ENCODE
SuperEnhancer Hnisz
Repressed Hoffman
Promoter UCSC
PromoterFlanking Hoffman
Intron UCSC
H3K9ac Trynka
H3K4me3 Trynka
H3K4me1 Trynka
**H3K27ac PGC2
H3K27ac Hnisz
FetalDHS Trynka
Enhancer Hoffman
Enhancer Andersson
DHS Trynka
**DGF ENCODE
CTCF Hoffman
Conserved LindbladToh
Coding UCSC
Annotation
−50
0
50
Enrichment (prop h2 / prop snps)

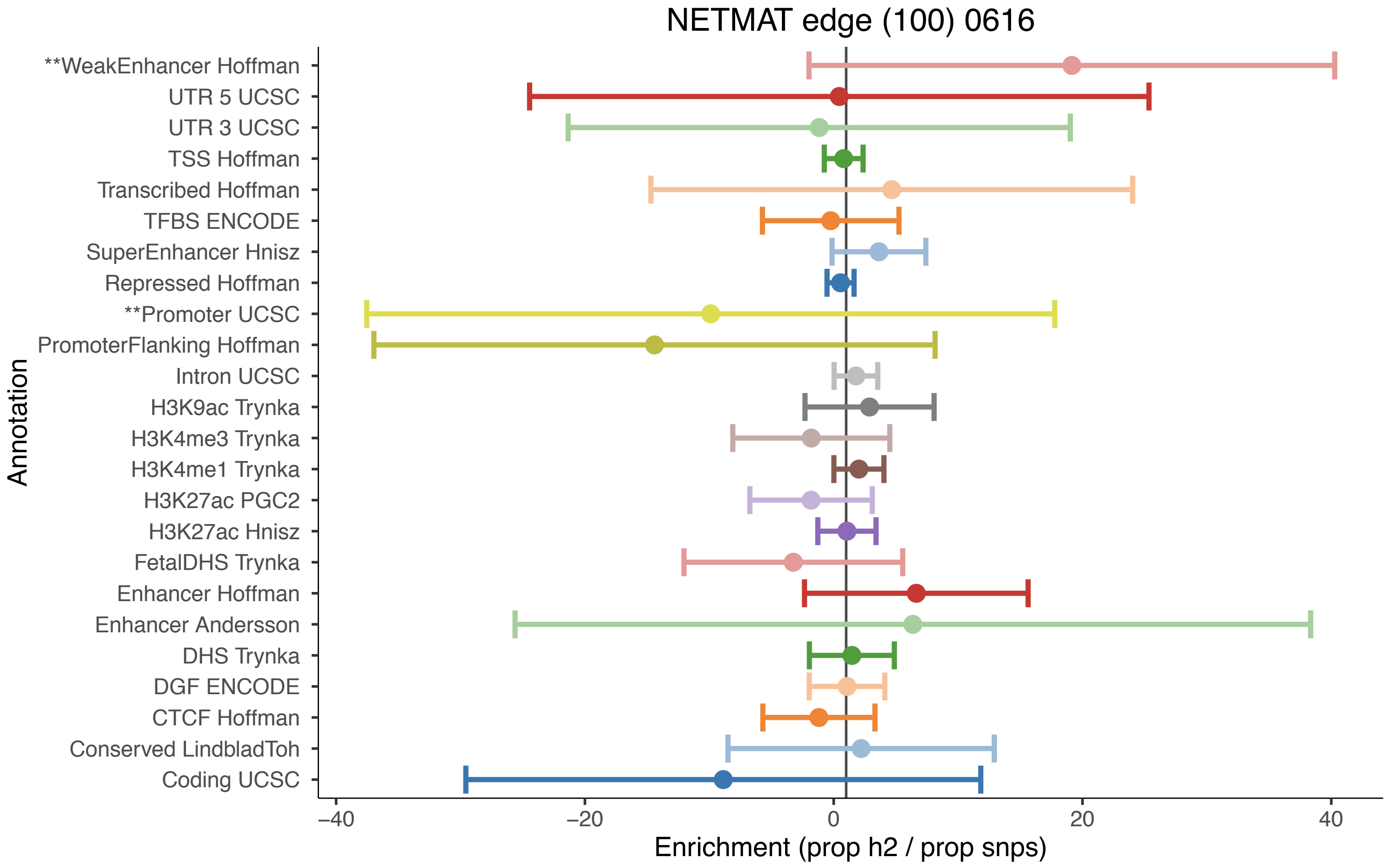
NETMAT edge (100) 0616
**WeakEnhancer Hoffman
UTR 5 UCSC
UTR 3 UCSC
TSS Hoffman
Transcribed Hoffman
TFBS ENCODE
SuperEnhancer Hnisz
Repressed Hoffman
**Promoter UCSC
PromoterFlanking Hoffman
Intron UCSC
H3K9ac Trynka
H3K4me3 Trynka
H3K4me1 Trynka
H3K27ac PGC2
H3K27ac Hnisz
FetalDHS Trynka
Enhancer Hoffman
Enhancer Andersson
DHS Trynka
DGF ENCODE
CTCF Hoffman
Conserved LindbladToh
Coding UCSC
Annotation
−40
−20
0
20
40
Enrichment (prop h2 / prop snps)

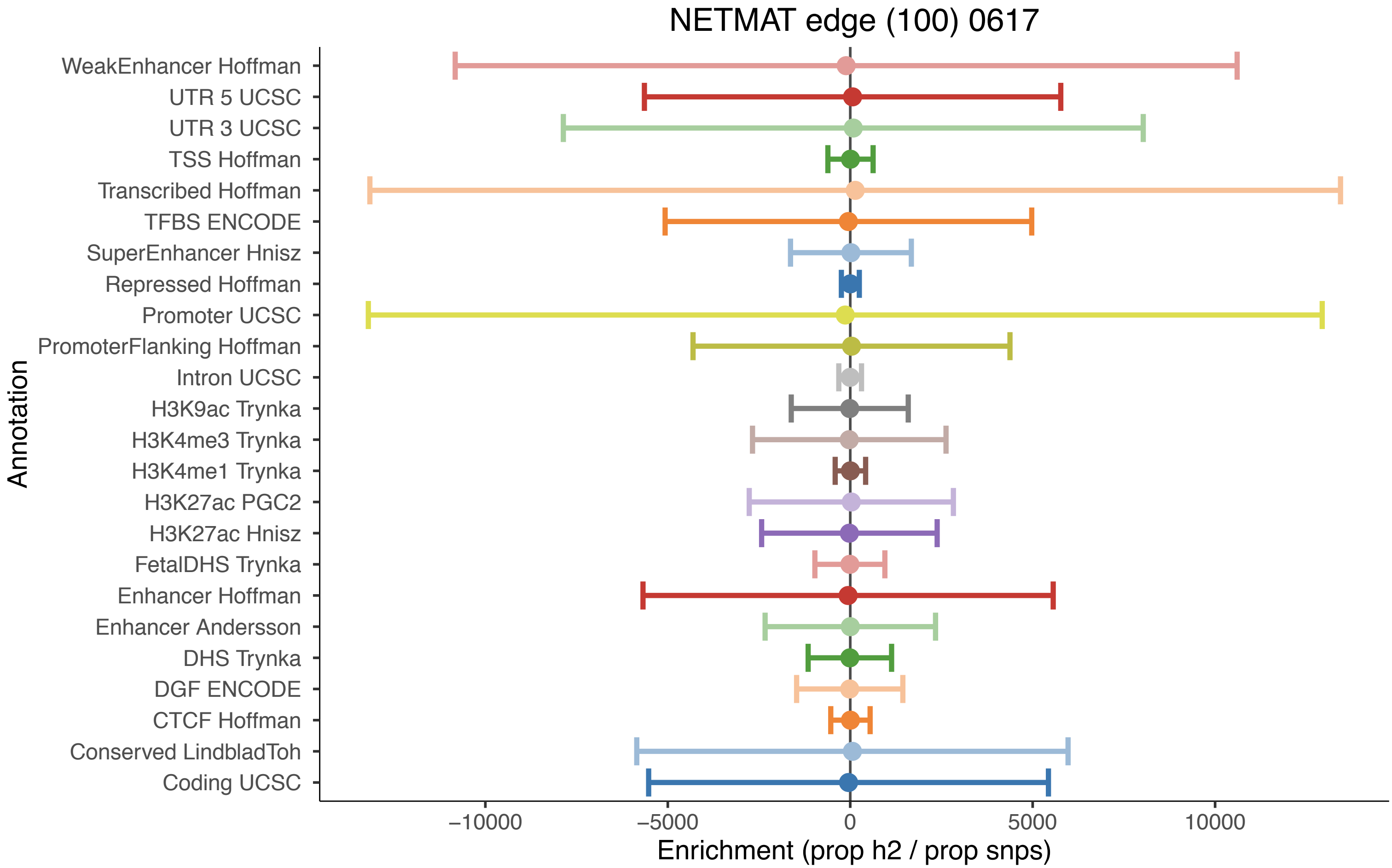
NETMAT edge (100) 0617
WeakEnhancer Hoffman
UTR 5 UCSC
UTR 3 UCSC
TSS Hoffman
Transcribed Hoffman
TFBS ENCODE
SuperEnhancer Hnisz
Repressed Hoffman
Promoter UCSC
PromoterFlanking Hoffman
Intron UCSC
H3K9ac Trynka
H3K4me3 Trynka
H3K4me1 Trynka
H3K27ac PGC2
H3K27ac Hnisz
FetalDHS Trynka
Enhancer Hoffman
Enhancer Andersson
DHS Trynka
DGF ENCODE
CTCF Hoffman
Conserved LindbladToh
Coding UCSC
Annotation
−10000
−5000
0
5000
10000
Enrichment (prop h2 / prop snps)

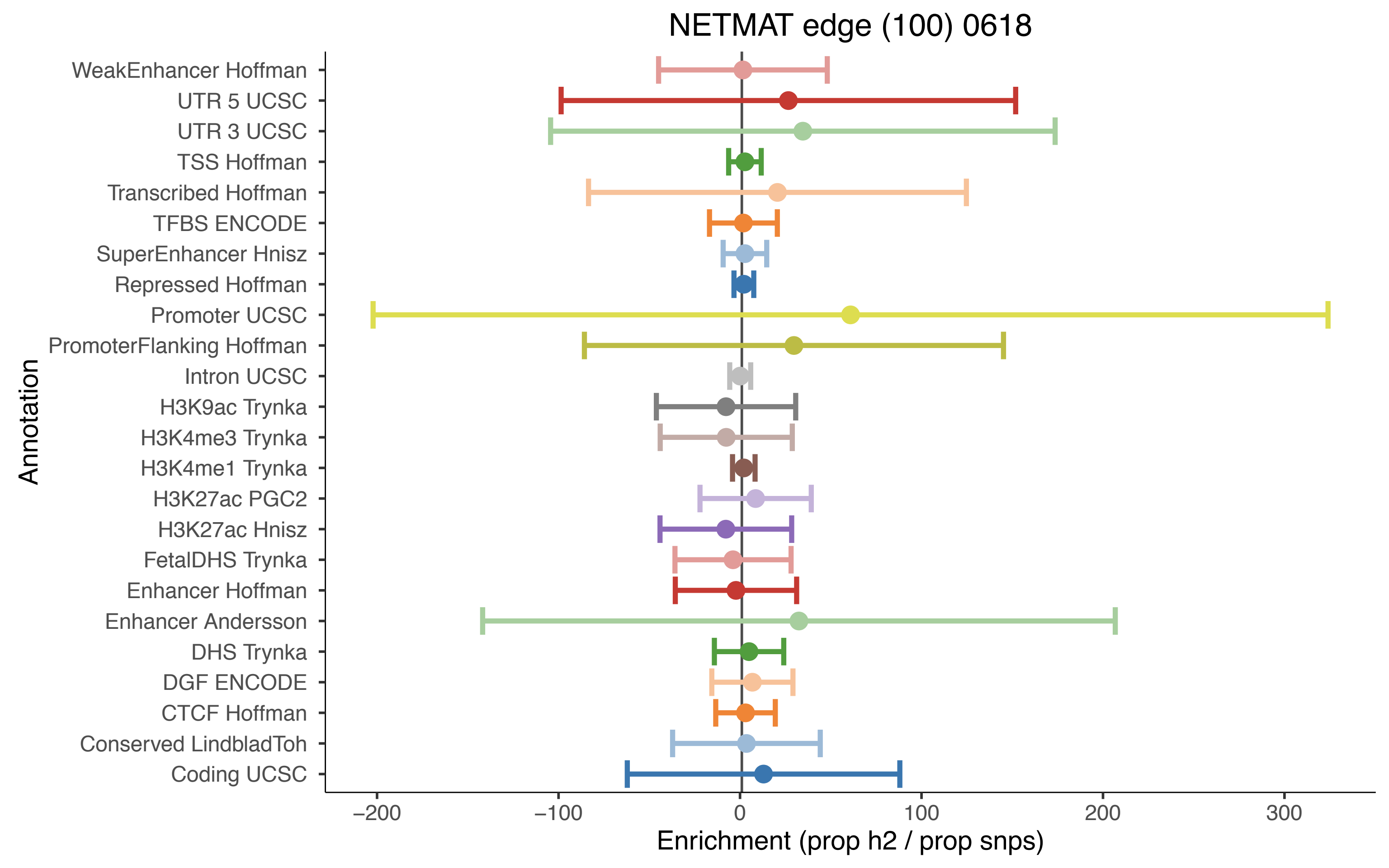

NETMAT edge (100) 0618
WeakEnhancer Hoffman
UTR 5 UCSC
UTR 3 UCSC
TSS Hoffman
Transcribed Hoffman
TFBS ENCODE
SuperEnhancer Hnisz
Repressed Hoffman
Promoter UCSC
PromoterFlanking Hoffman
Intron UCSC
H3K9ac Trynka
H3K4me3 Trynka
H3K4me1 Trynka
H3K27ac PGC2
H3K27ac Hnisz
FetalDHS Trynka
Enhancer Hoffman
Enhancer Andersson
DHS Trynka
DGF ENCODE
CTCF Hoffman
Conserved LindbladToh
Coding UCSC
Annotation
−200
−100
0
100
200
300
Enrichment (prop h2 / prop snps)

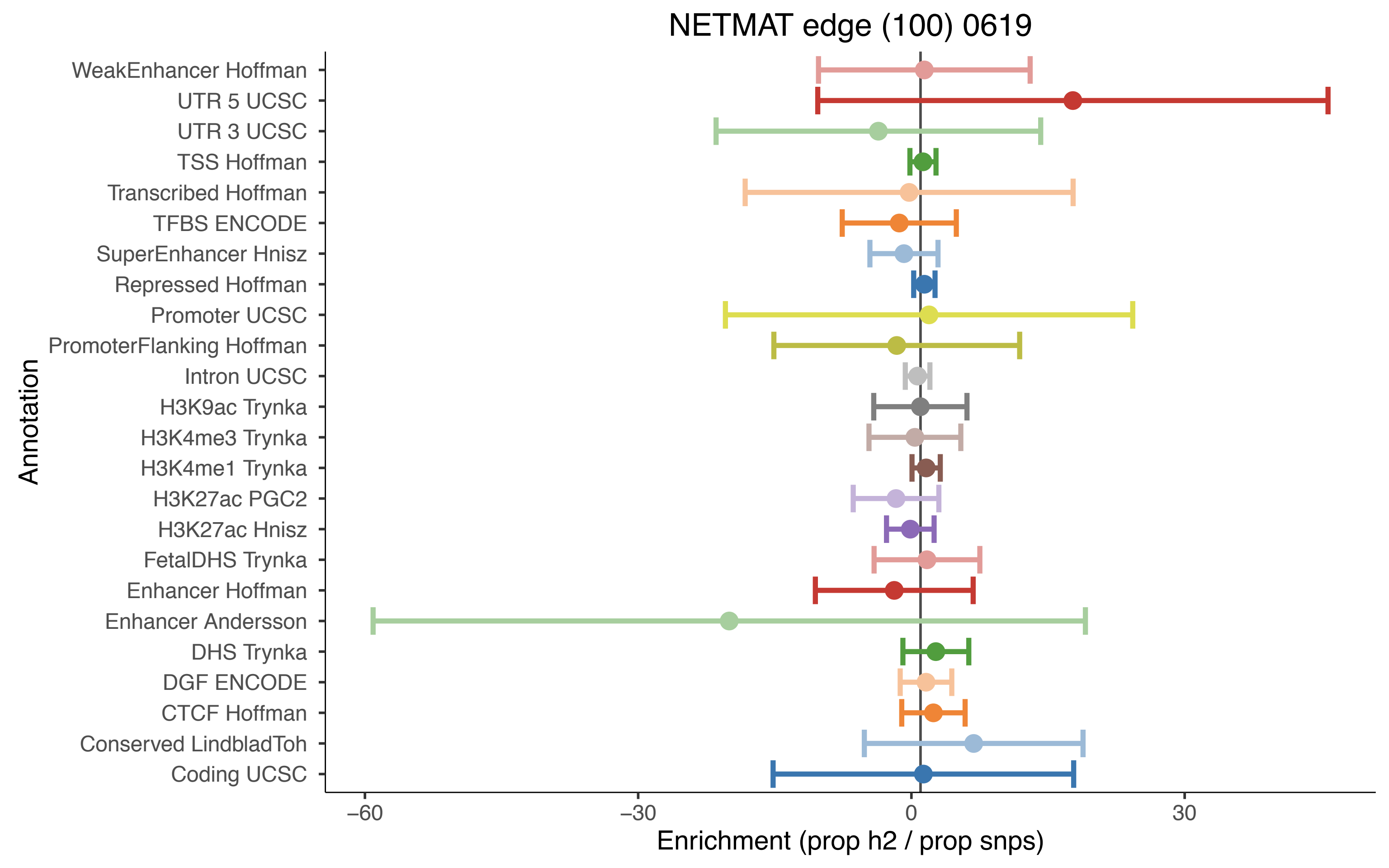
NETMAT edge (100) 0619
WeakEnhancer Hoffman
UTR 5 UCSC
UTR 3 UCSC
TSS Hoffman
Transcribed Hoffman
TFBS ENCODE
SuperEnhancer Hnisz
Repressed Hoffman
Promoter UCSC
PromoterFlanking Hoffman
Intron UCSC
H3K9ac Trynka
H3K4me3 Trynka
H3K4me1 Trynka
H3K27ac PGC2
H3K27ac Hnisz
FetalDHS Trynka
Enhancer Hoffman
Enhancer Andersson
DHS Trynka
DGF ENCODE
CTCF Hoffman
Conserved LindbladToh
Coding UCSC
Annotation
−60
−30
0
30
Enrichment (prop h2 / prop snps)

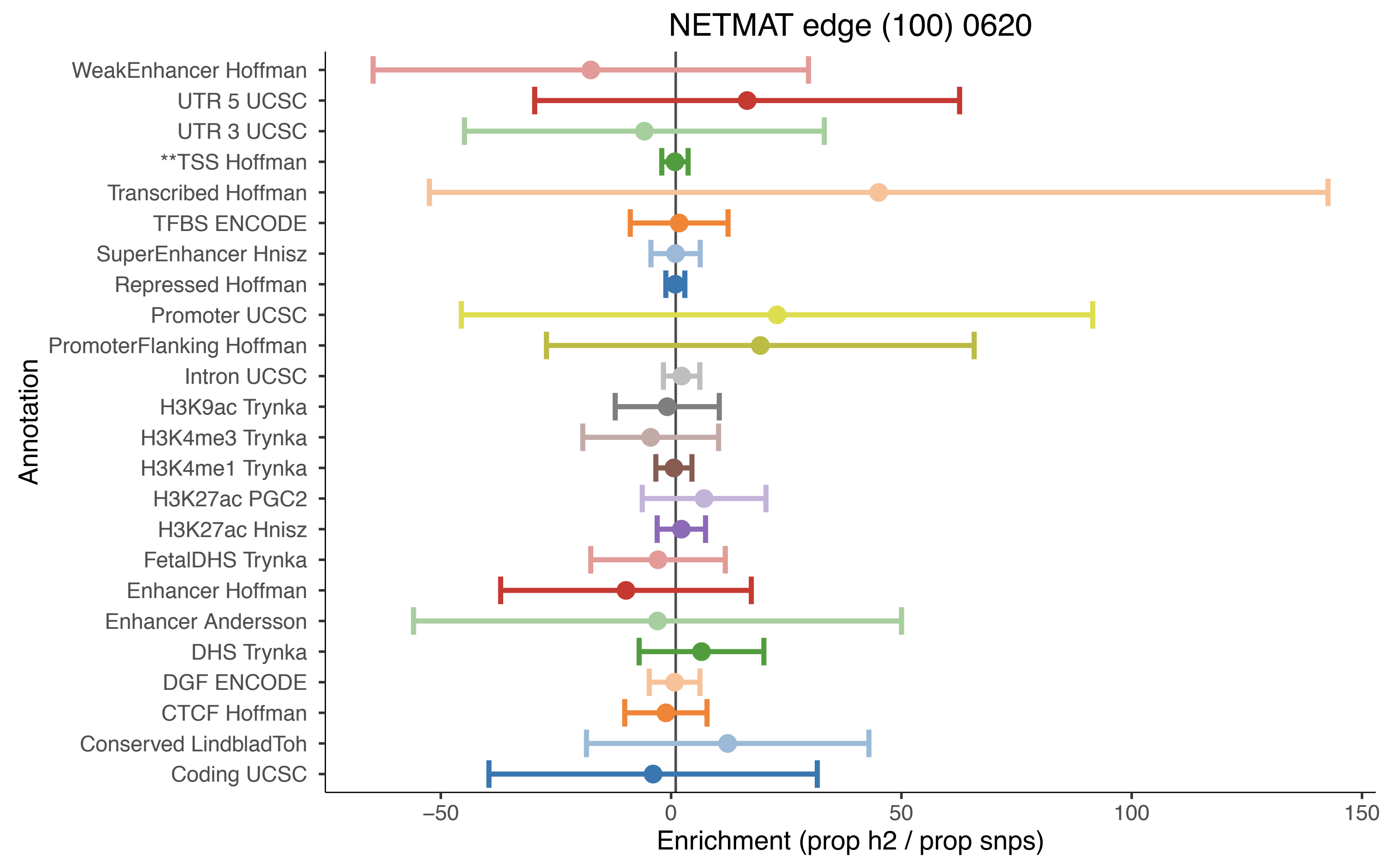
NETMAT edge (100) 0620
WeakEnhancer Hoffman
UTR 5 UCSC
UTR 3 UCSC
**TSS Hoffman
Transcribed Hoffman
TFBS ENCODE
SuperEnhancer Hnisz
Repressed Hoffman
Promoter UCSC
PromoterFlanking Hoffman
Intron UCSC
H3K9ac Trynka
H3K4me3 Trynka
H3K4me1 Trynka
H3K27ac PGC2
H3K27ac Hnisz
FetalDHS Trynka
Enhancer Hoffman
Enhancer Andersson
DHS Trynka
DGF ENCODE
CTCF Hoffman
Conserved LindbladToh
Coding UCSC
Annotation
−50
0
50
100
150
Enrichment (prop h2 / prop snps)

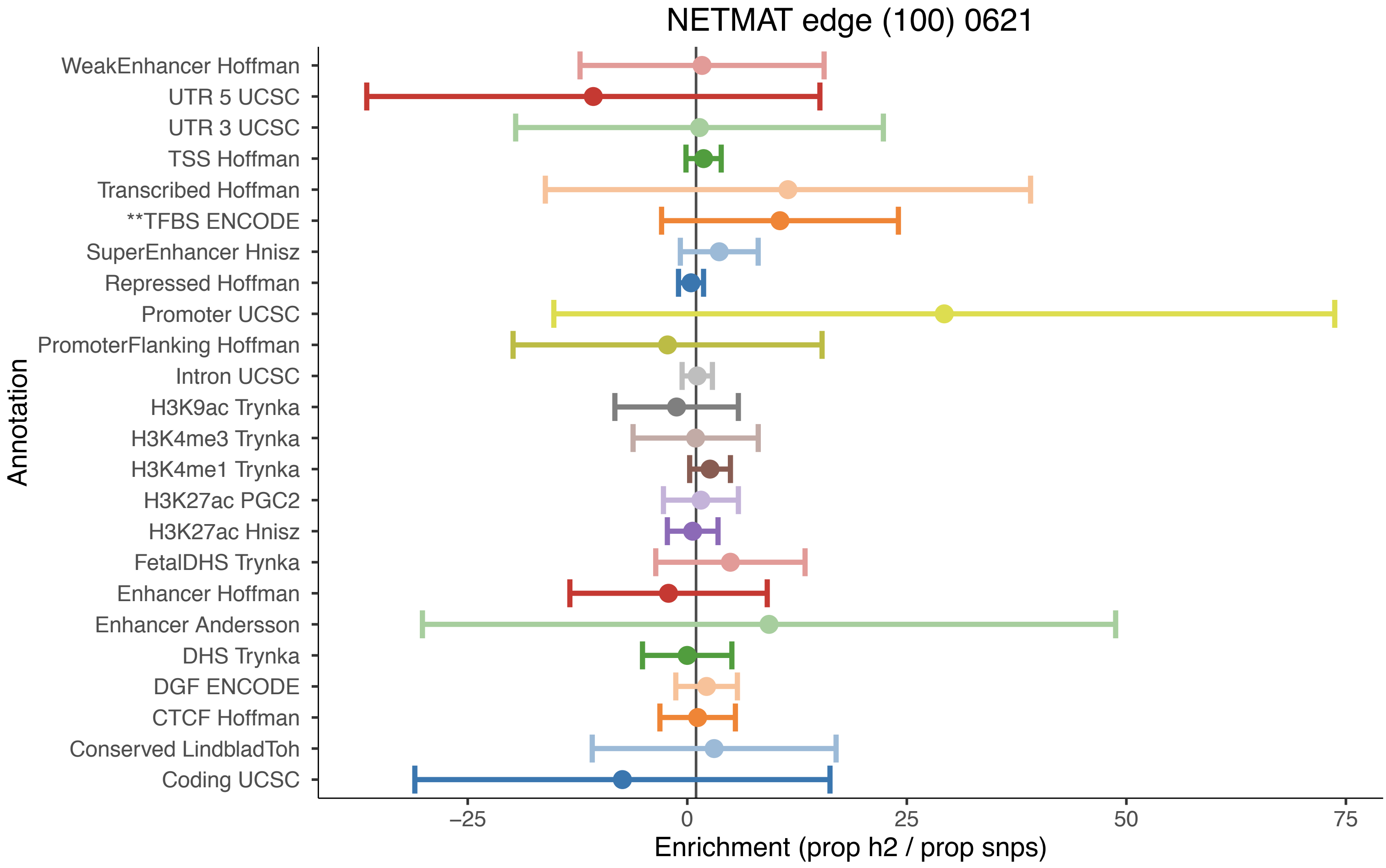

NETMAT edge (100) 0621
WeakEnhancer Hoffman
UTR 5 UCSC
UTR 3 UCSC
TSS Hoffman
Transcribed Hoffman
**TFBS ENCODE
SuperEnhancer Hnisz
Repressed Hoffman
Promoter UCSC
PromoterFlanking Hoffman
Intron UCSC
H3K9ac Trynka
H3K4me3 Trynka
H3K4me1 Trynka
H3K27ac PGC2
H3K27ac Hnisz
FetalDHS Trynka
Enhancer Hoffman
Enhancer Andersson
DHS Trynka
DGF ENCODE
CTCF Hoffman
Conserved LindbladToh
Coding UCSC
Annotation
−25
0
25
50
75
Enrichment (prop h2 / prop snps)

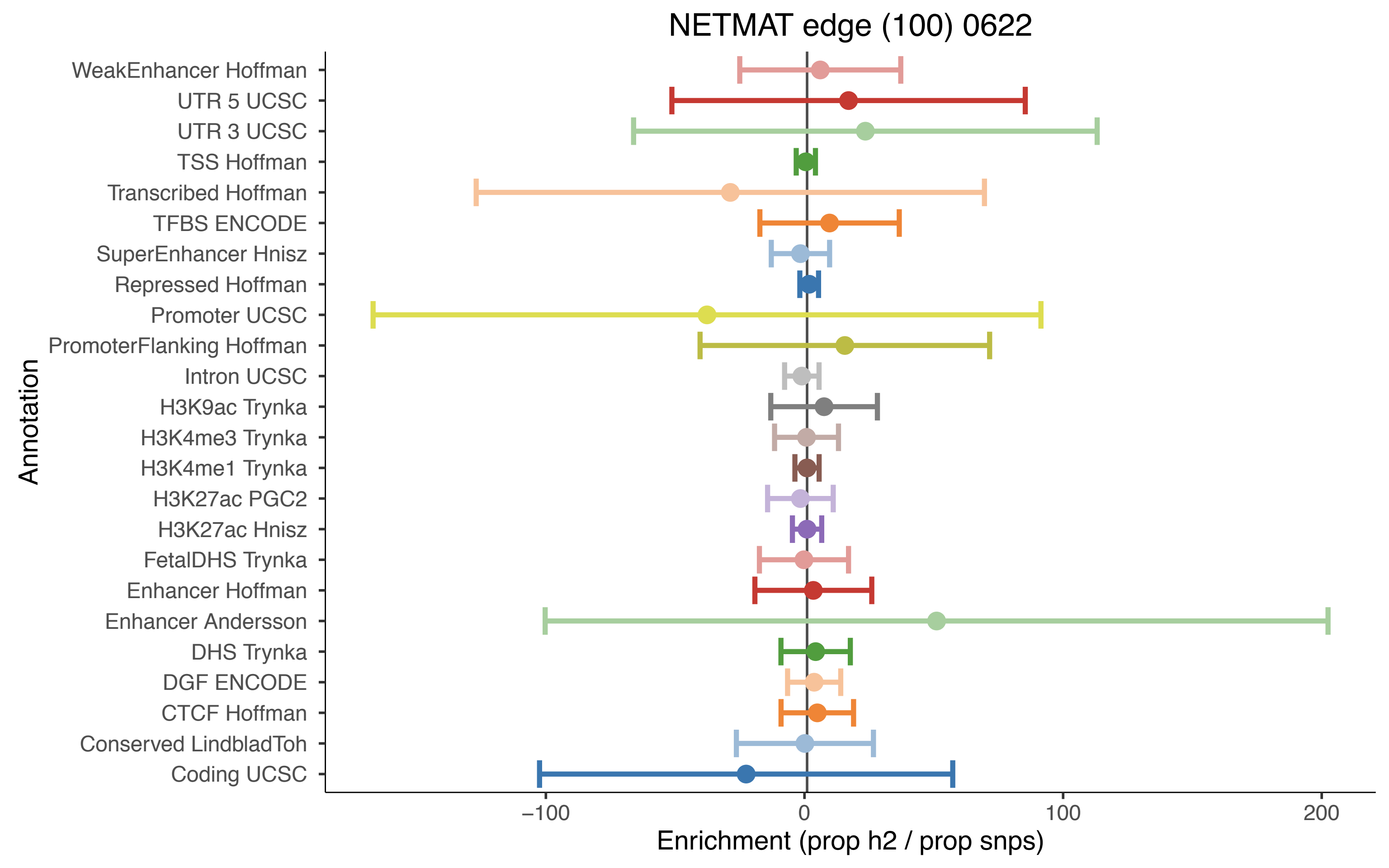

NETMAT edge (100) 0622
WeakEnhancer Hoffman
UTR 5 UCSC
UTR 3 UCSC
TSS Hoffman
Transcribed Hoffman
TFBS ENCODE
SuperEnhancer Hnisz
Repressed Hoffman
Promoter UCSC
PromoterFlanking Hoffman
Intron UCSC
H3K9ac Trynka
H3K4me3 Trynka
H3K4me1 Trynka
H3K27ac PGC2
H3K27ac Hnisz
FetalDHS Trynka
Enhancer Hoffman
Enhancer Andersson
DHS Trynka
DGF ENCODE
CTCF Hoffman
Conserved LindbladToh
Coding UCSC
Annotation
−100
0
100
200
Enrichment (prop h2 / prop snps)

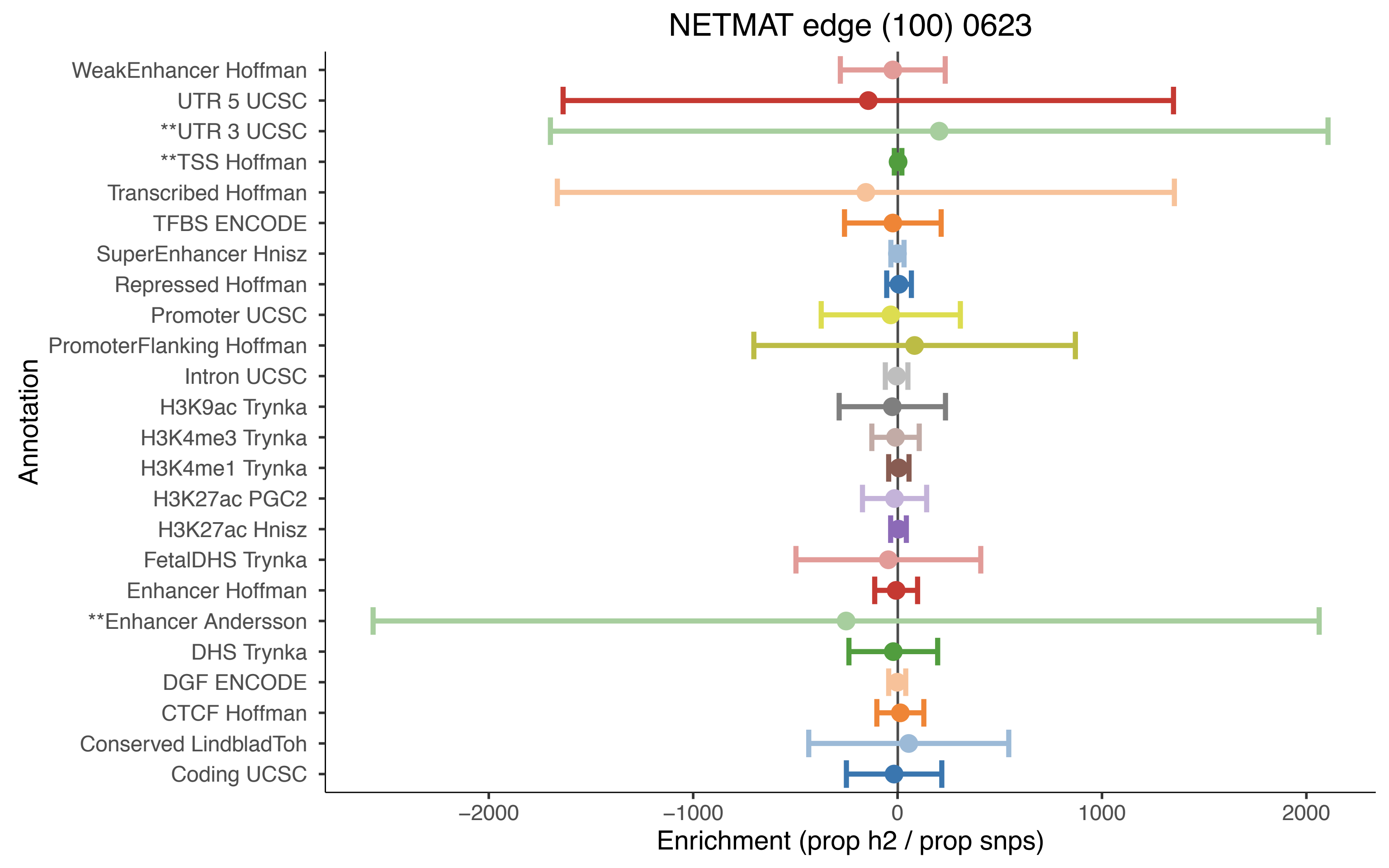

NETMAT edge (100) 0623
WeakEnhancer Hoffman
UTR 5 UCSC
**UTR 3 UCSC
**TSS Hoffman
Transcribed Hoffman
TFBS ENCODE
SuperEnhancer Hnisz
Repressed Hoffman
Promoter UCSC
PromoterFlanking Hoffman
Intron UCSC
H3K9ac Trynka
H3K4me3 Trynka
H3K4me1 Trynka
H3K27ac PGC2
H3K27ac Hnisz
FetalDHS Trynka
Enhancer Hoffman
**Enhancer Andersson
DHS Trynka
DGF ENCODE
CTCF Hoffman
Conserved LindbladToh
Coding UCSC
Annotation
−2000
−1000
0
1000
2000
Enrichment (prop h2 / prop snps)

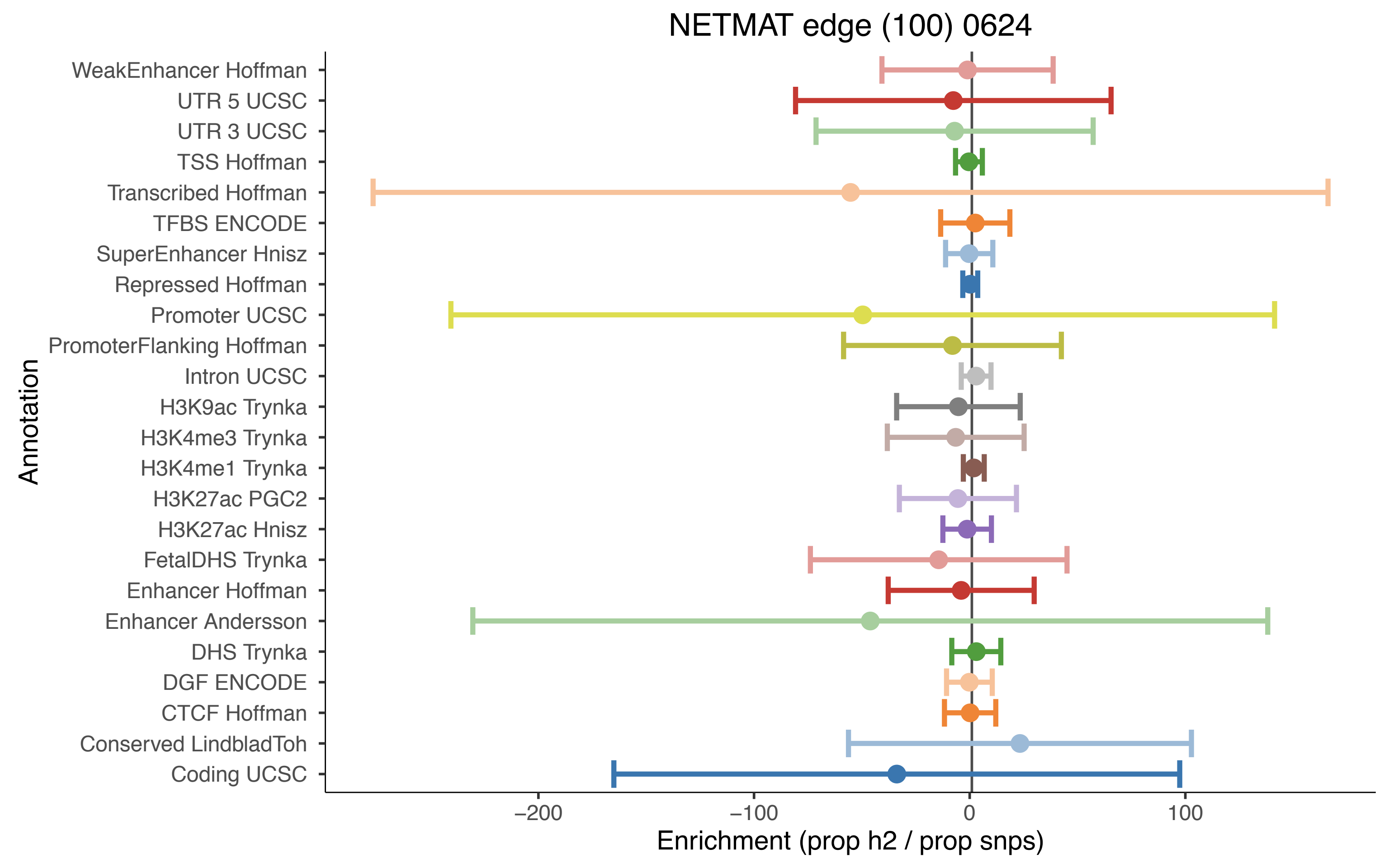
NETMAT edge (100) 0624
Annotation
WeakEnhancer Hoffman
UTR 5 UCSC
UTR 3 UCSC
TSS Hoffman
Transcribed Hoffman
TFBS ENCODE
SuperEnhancer Hnisz
Repressed Hoffman
Promoter UCSC
PromoterFlanking Hoffman
Intron UCSC
H3K9ac Trynka
H3K4me3 Trynka
H3K4me1 Trynka
H3K27ac PGC2
H3K27ac Hnisz
FetalDHS Trynka
Enhancer Hoffman
Enhancer Andersson
DHS Trynka
DGF ENCODE
CTCF Hoffman
Conserved LindbladToh
Coding UCSC
−200
−100
0
100
Enrichment (prop h2 / prop snps)

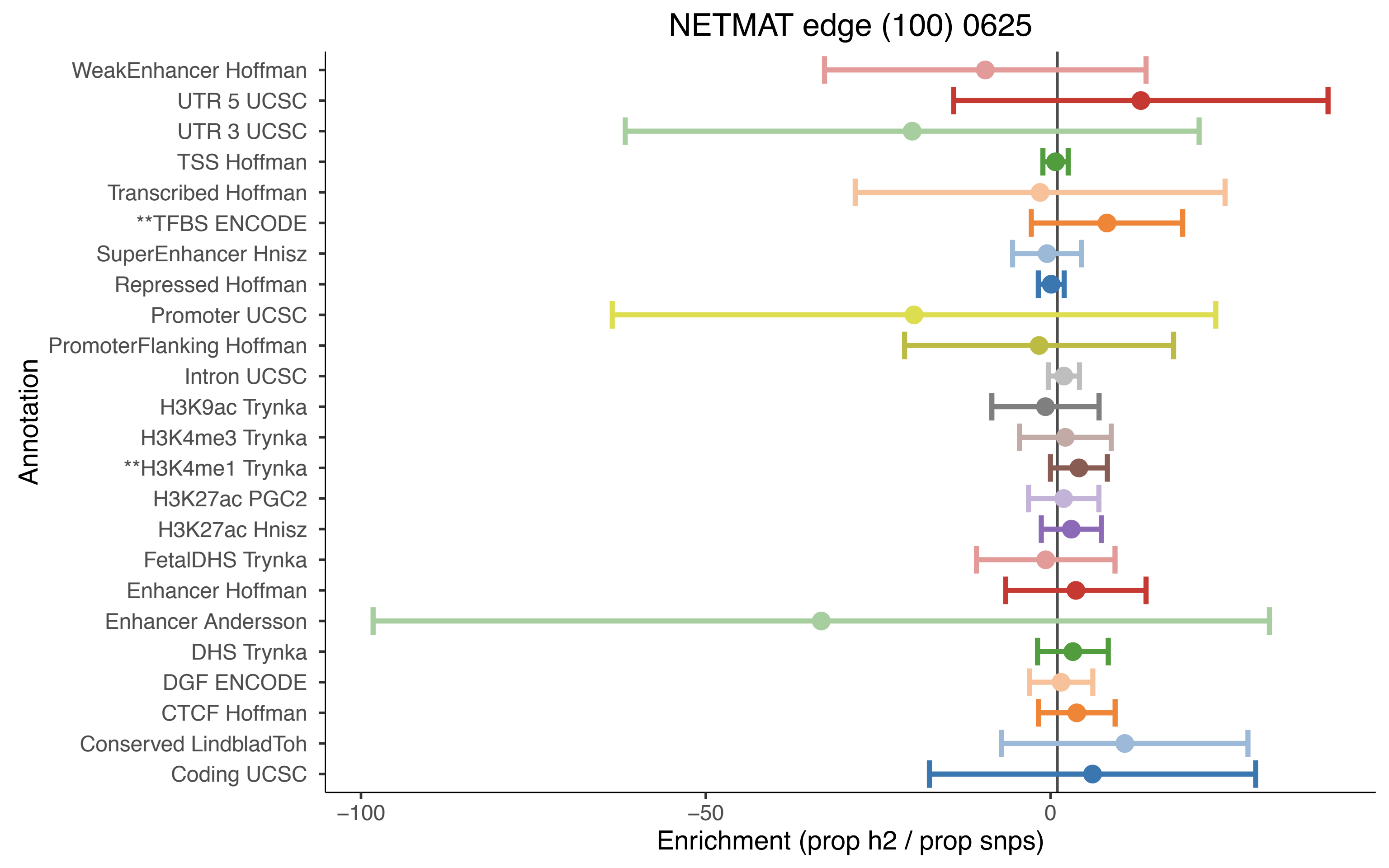

NETMAT edge (100) 0625
WeakEnhancer Hoffman
UTR 5 UCSC
UTR 3 UCSC
TSS Hoffman
Transcribed Hoffman
**TFBS ENCODE
SuperEnhancer Hnisz
Repressed Hoffman
Promoter UCSC
PromoterFlanking Hoffman
Intron UCSC
H3K9ac Trynka
H3K4me3 Trynka
**H3K4me1 Trynka
H3K27ac PGC2
H3K27ac Hnisz
FetalDHS Trynka
Enhancer Hoffman
Enhancer Andersson
DHS Trynka
DGF ENCODE
CTCF Hoffman
Conserved LindbladToh
Coding UCSC
Annotation
−100
−50
0
Enrichment (prop h2 / prop snps)

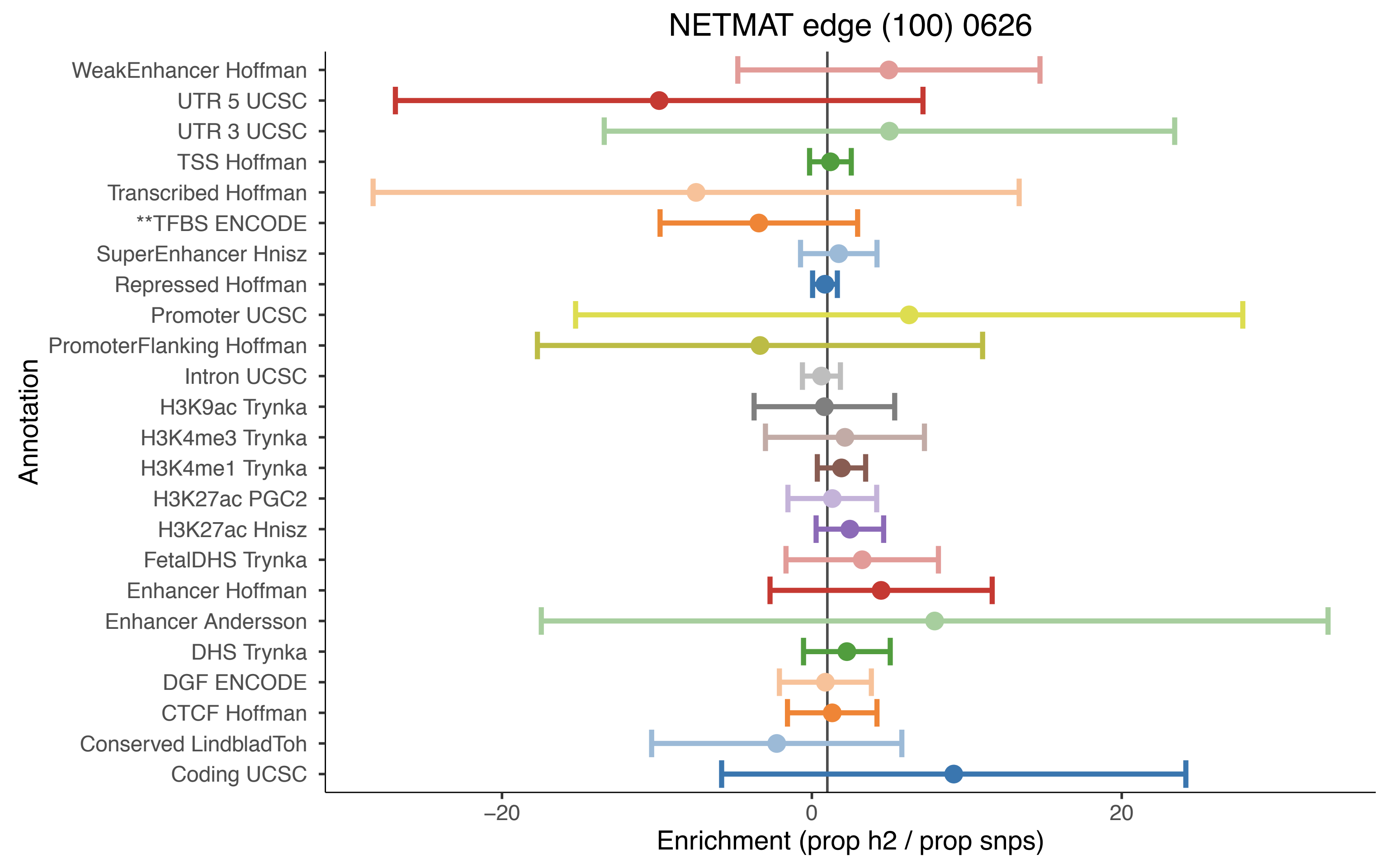

NETMAT edge (100) 0626
WeakEnhancer Hoffman
UTR 5 UCSC
UTR 3 UCSC
TSS Hoffman
Transcribed Hoffman
**TFBS ENCODE
SuperEnhancer Hnisz
Repressed Hoffman
Promoter UCSC
PromoterFlanking Hoffman
Intron UCSC
H3K9ac Trynka
H3K4me3 Trynka
H3K4me1 Trynka
H3K27ac PGC2
H3K27ac Hnisz
FetalDHS Trynka
Enhancer Hoffman
Enhancer Andersson
DHS Trynka
DGF ENCODE
CTCF Hoffman
Conserved LindbladToh
Coding UCSC
Annotation
−20
0
20
Enrichment (prop h2 / prop snps)

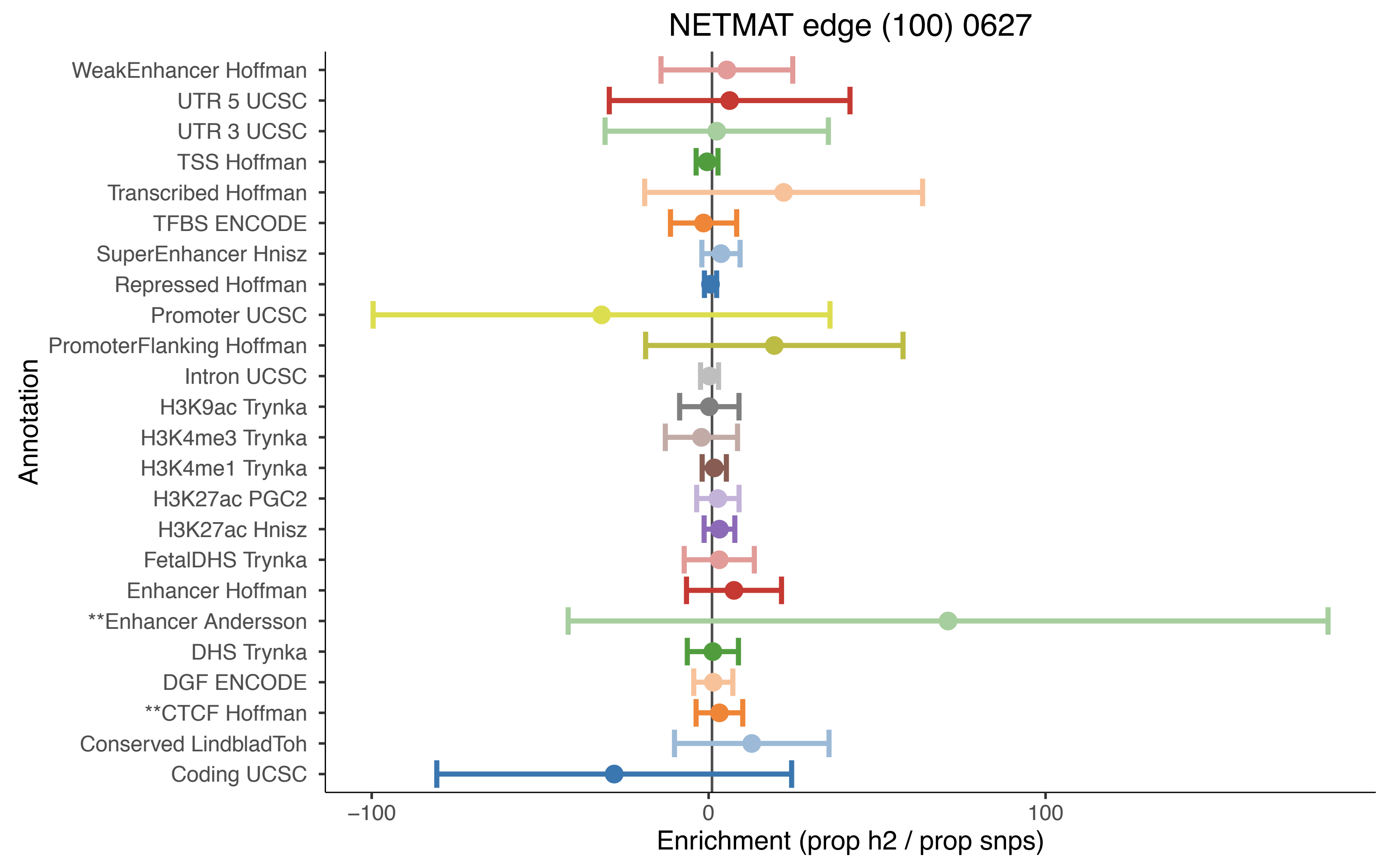
NETMAT edge (100) 0627
WeakEnhancer Hoffman
UTR 5 UCSC
UTR 3 UCSC
TSS Hoffman
Transcribed Hoffman
TFBS ENCODE
SuperEnhancer Hnisz
Repressed Hoffman
Promoter UCSC
PromoterFlanking Hoffman
Intron UCSC
H3K9ac Trynka
H3K4me3 Trynka
H3K4me1 Trynka
H3K27ac PGC2
H3K27ac Hnisz
FetalDHS Trynka
Enhancer Hoffman
**Enhancer Andersson
DHS Trynka
DGF ENCODE
**CTCF Hoffman
Conserved LindbladToh
Coding UCSC
Annotation
−100
0
100
Enrichment (prop h2 / prop snps)

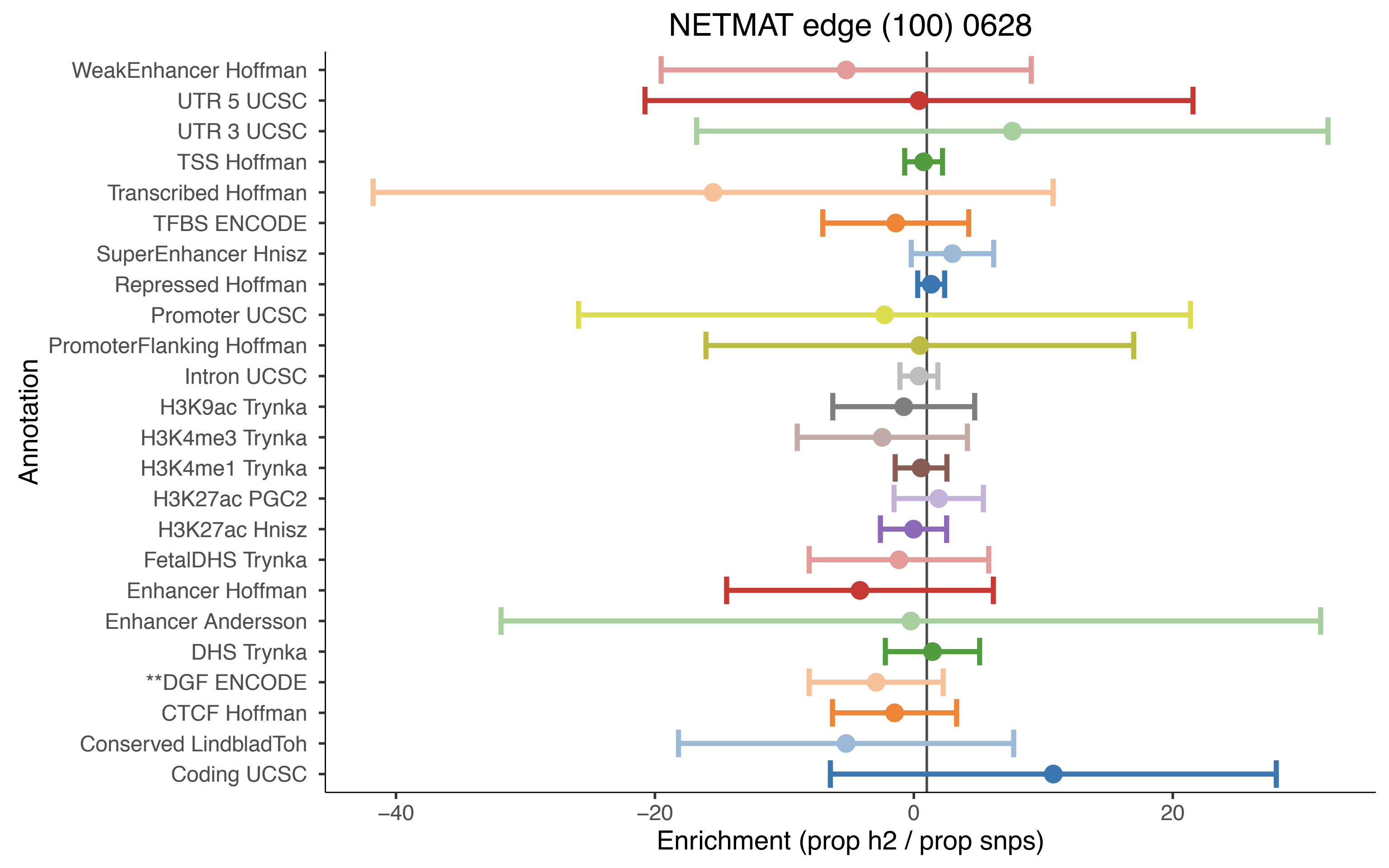
NETMAT edge (100) 0628
WeakEnhancer Hoffman
UTR 5 UCSC
UTR 3 UCSC
TSS Hoffman
Transcribed Hoffman
TFBS ENCODE
SuperEnhancer Hnisz
Repressed Hoffman
Promoter UCSC
PromoterFlanking Hoffman
Intron UCSC
H3K9ac Trynka
H3K4me3 Trynka
H3K4me1 Trynka
H3K27ac PGC2
H3K27ac Hnisz
FetalDHS Trynka
Enhancer Hoffman
Enhancer Andersson
DHS Trynka
**DGF ENCODE
CTCF Hoffman
Conserved LindbladToh
Coding UCSC
Annotation
−40
−20
0
20
Enrichment (prop h2 / prop snps)

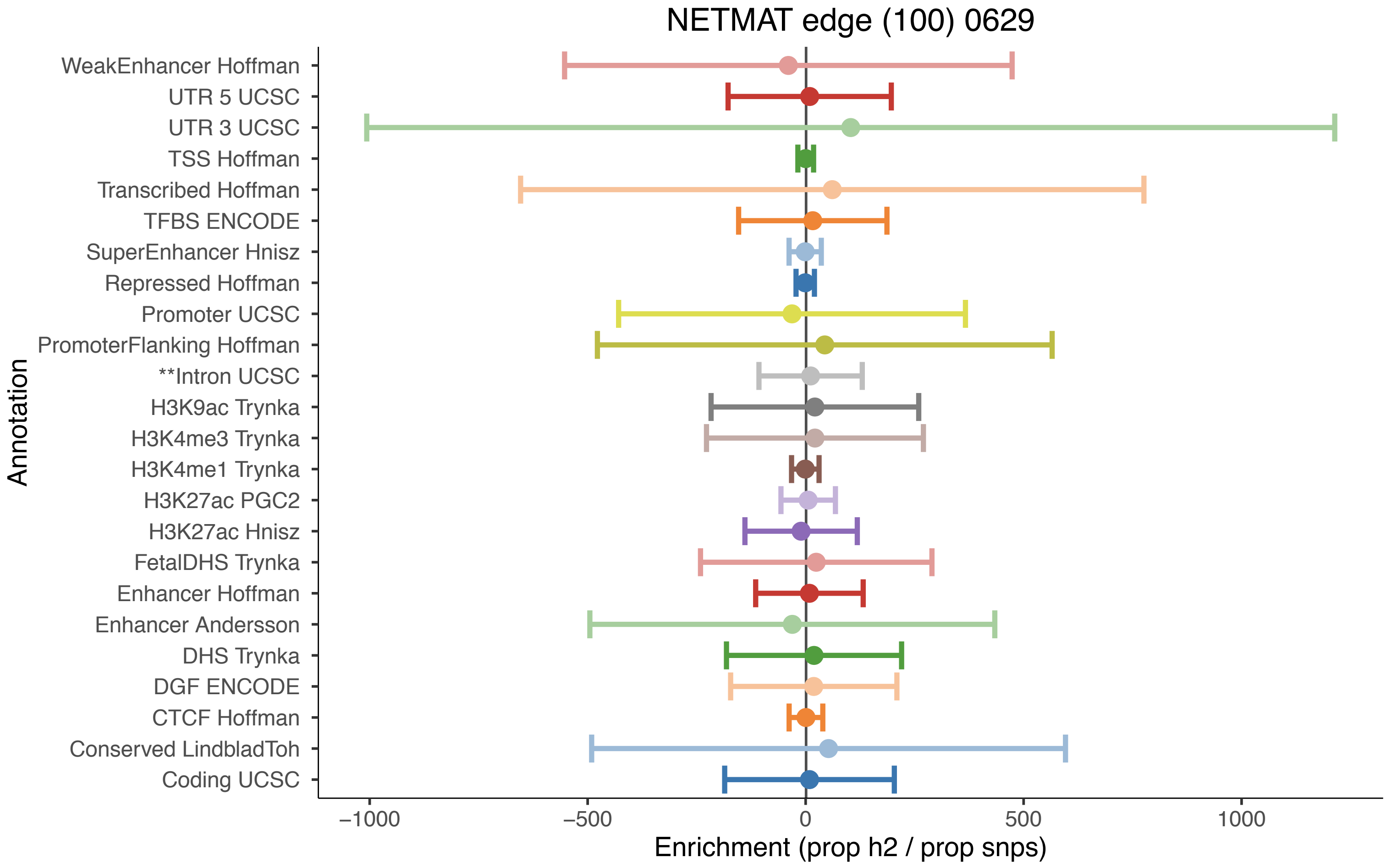
NETMAT edge (100) 0629
WeakEnhancer Hoffman
UTR 5 UCSC
UTR 3 UCSC
TSS Hoffman
Transcribed Hoffman
TFBS ENCODE
SuperEnhancer Hnisz
Repressed Hoffman
Promoter UCSC
PromoterFlanking Hoffman
**Intron UCSC
H3K9ac Trynka
H3K4me3 Trynka
H3K4me1 Trynka
H3K27ac PGC2
H3K27ac Hnisz
FetalDHS Trynka
Enhancer Hoffman
Enhancer Andersson
DHS Trynka
DGF ENCODE
CTCF Hoffman
Conserved LindbladToh
Coding UCSC
Annotation
−1000
−500
0
500
1000
Enrichment (prop h2 / prop snps)

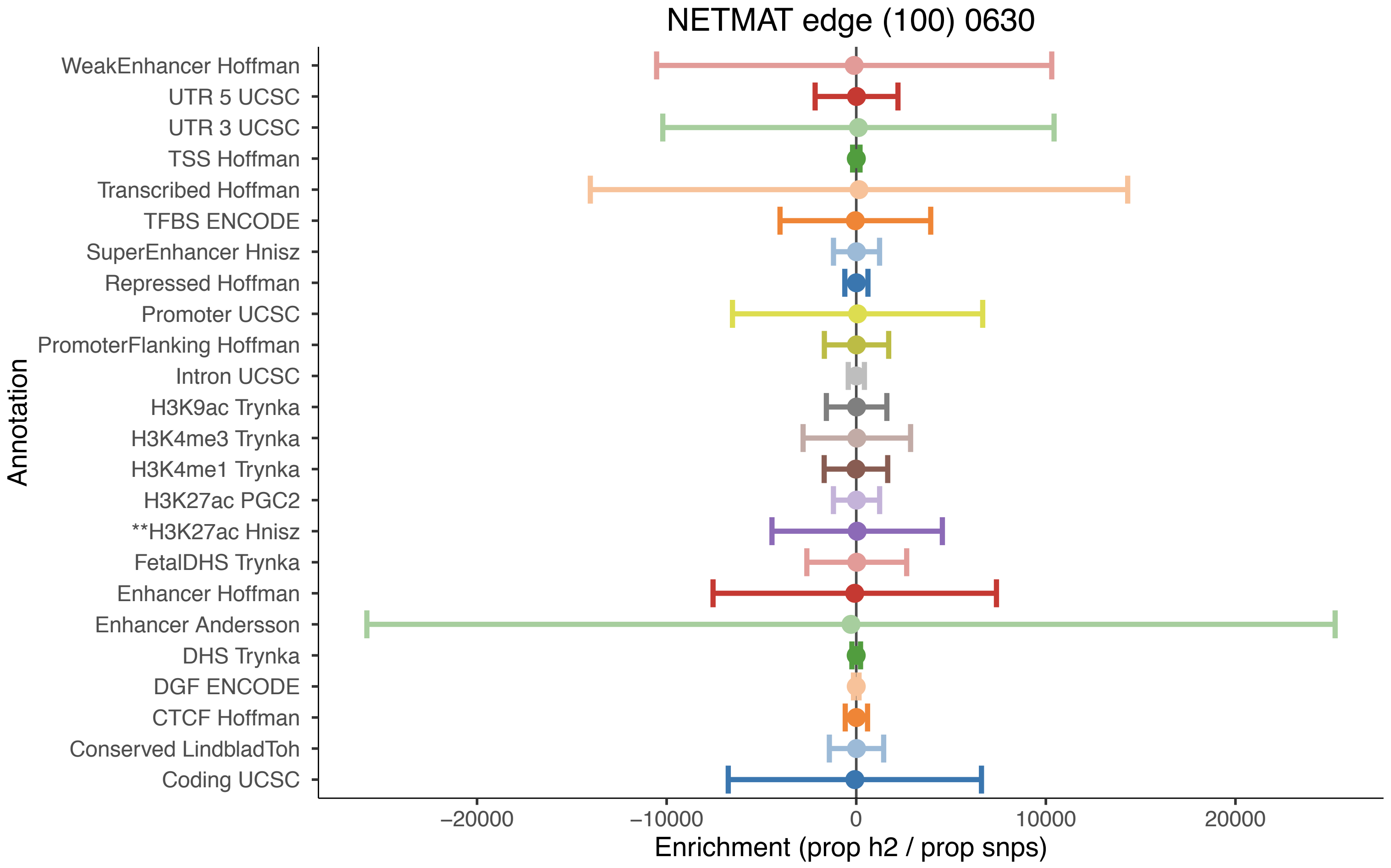

NETMAT edge (100) 0630
WeakEnhancer Hoffman
UTR 5 UCSC
UTR 3 UCSC
TSS Hoffman
Transcribed Hoffman
TFBS ENCODE
SuperEnhancer Hnisz
Repressed Hoffman
Promoter UCSC
PromoterFlanking Hoffman
Intron UCSC
H3K9ac Trynka
H3K4me3 Trynka
H3K4me1 Trynka
H3K27ac PGC2
**H3K27ac Hnisz
FetalDHS Trynka
Enhancer Hoffman
Enhancer Andersson
DHS Trynka
DGF ENCODE
CTCF Hoffman
Conserved LindbladToh
Coding UCSC
Annotation
−20000
−10000
0
10000
20000
Enrichment (prop h2 / prop snps)

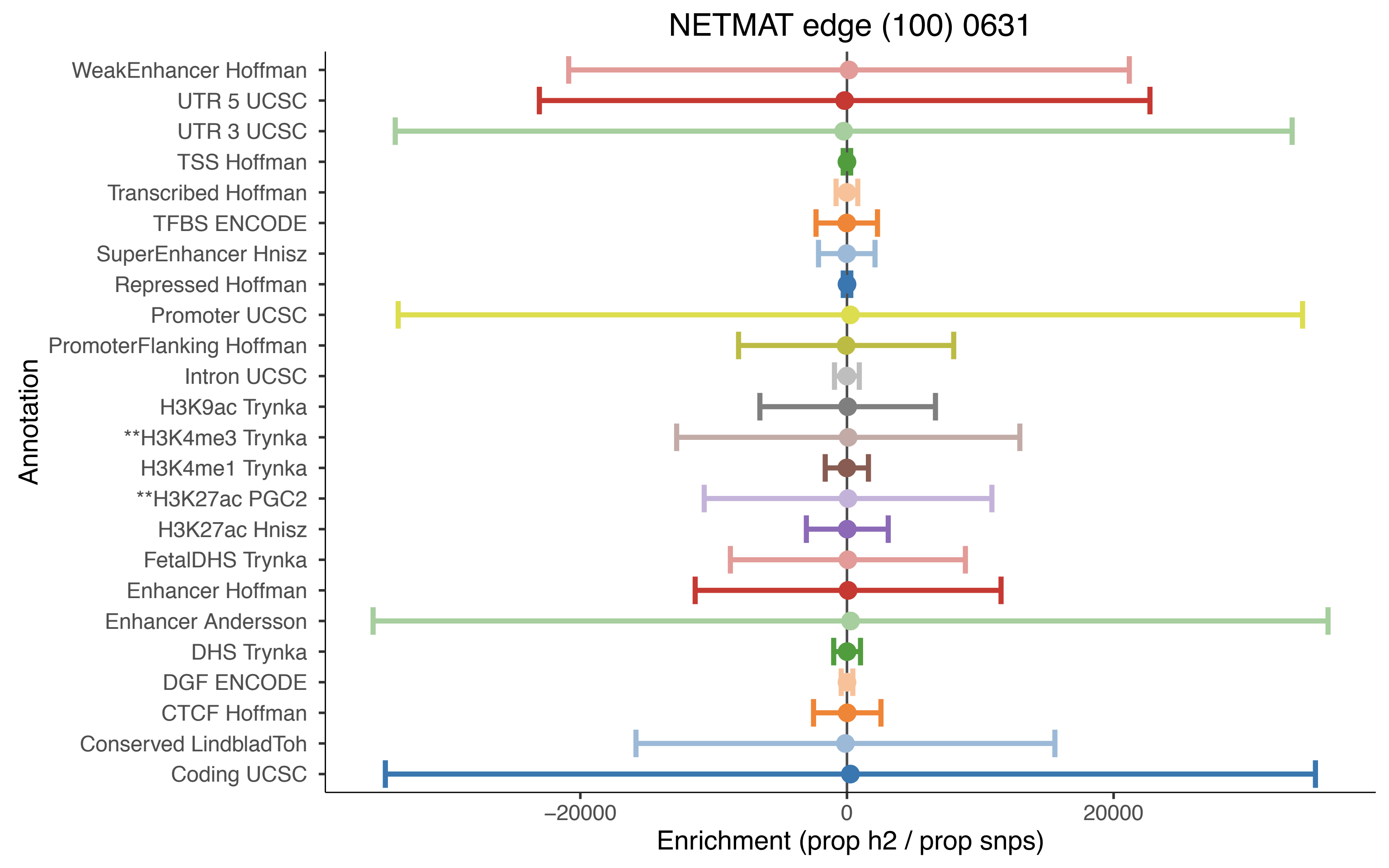
NETMAT edge (100) 0631
WeakEnhancer Hoffman
UTR 5 UCSC
UTR 3 UCSC
TSS Hoffman
Transcribed Hoffman
TFBS ENCODE
SuperEnhancer Hnisz
Repressed Hoffman
Promoter UCSC
PromoterFlanking Hoffman
Intron UCSC
H3K9ac Trynka
**H3K4me3 Trynka
H3K4me1 Trynka
**H3K27ac PGC2
H3K27ac Hnisz
FetalDHS Trynka
Enhancer Hoffman
Enhancer Andersson
DHS Trynka
DGF ENCODE
CTCF Hoffman
Conserved LindbladToh
Coding UCSC
Annotation
−20000
0
20000
Enrichment (prop h2 / prop snps)

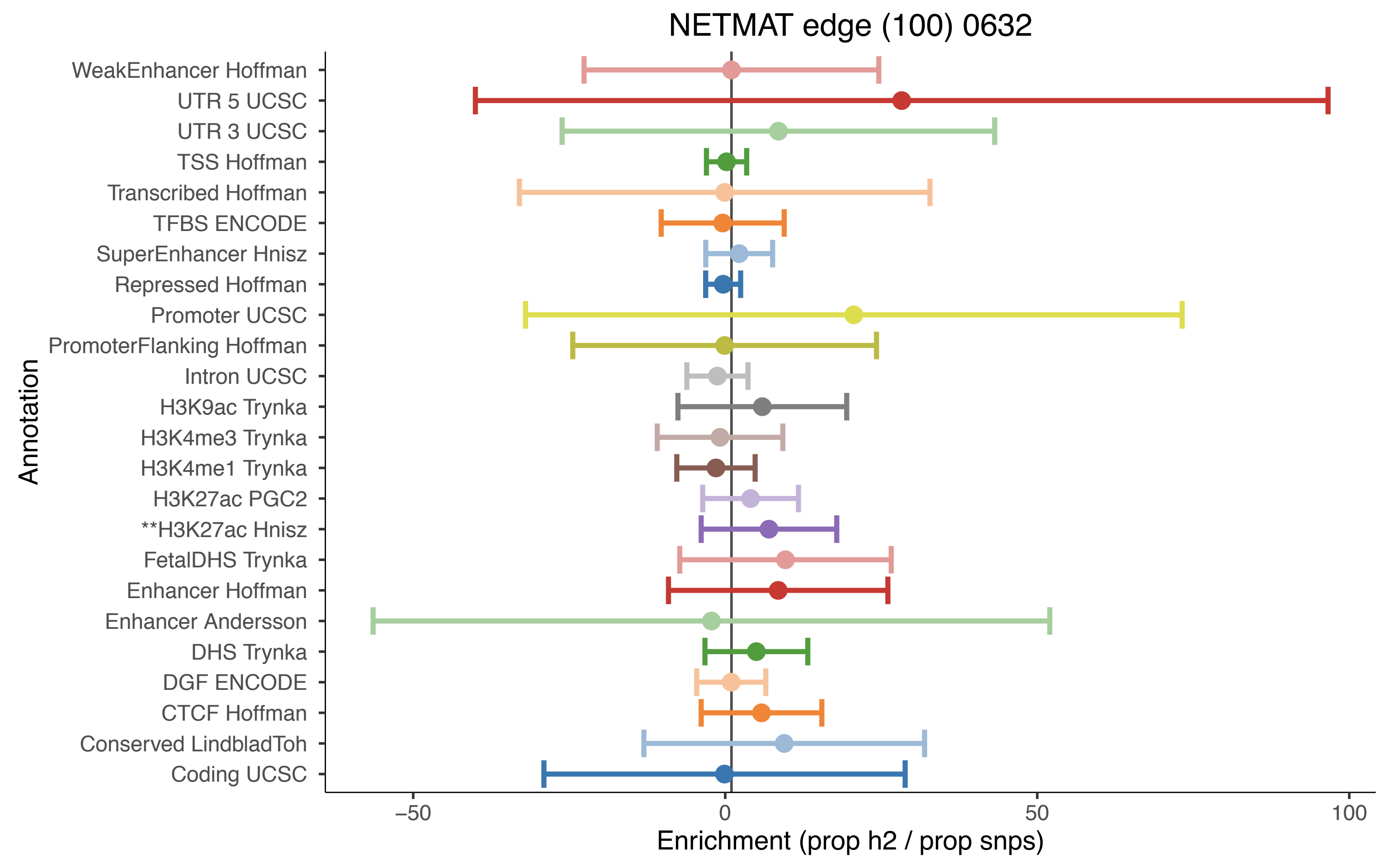

NETMAT edge (100) 0632
WeakEnhancer Hoffman
UTR 5 UCSC
UTR 3 UCSC
TSS Hoffman
Transcribed Hoffman
TFBS ENCODE
SuperEnhancer Hnisz
Repressed Hoffman
Promoter UCSC
PromoterFlanking Hoffman
Intron UCSC
H3K9ac Trynka
H3K4me3 Trynka
H3K4me1 Trynka
H3K27ac PGC2
**H3K27ac Hnisz
FetalDHS Trynka
Enhancer Hoffman
Enhancer Andersson
DHS Trynka
DGF ENCODE
CTCF Hoffman
Conserved LindbladToh
Coding UCSC
Annotation
−50
0
50
100
Enrichment (prop h2 / prop snps)

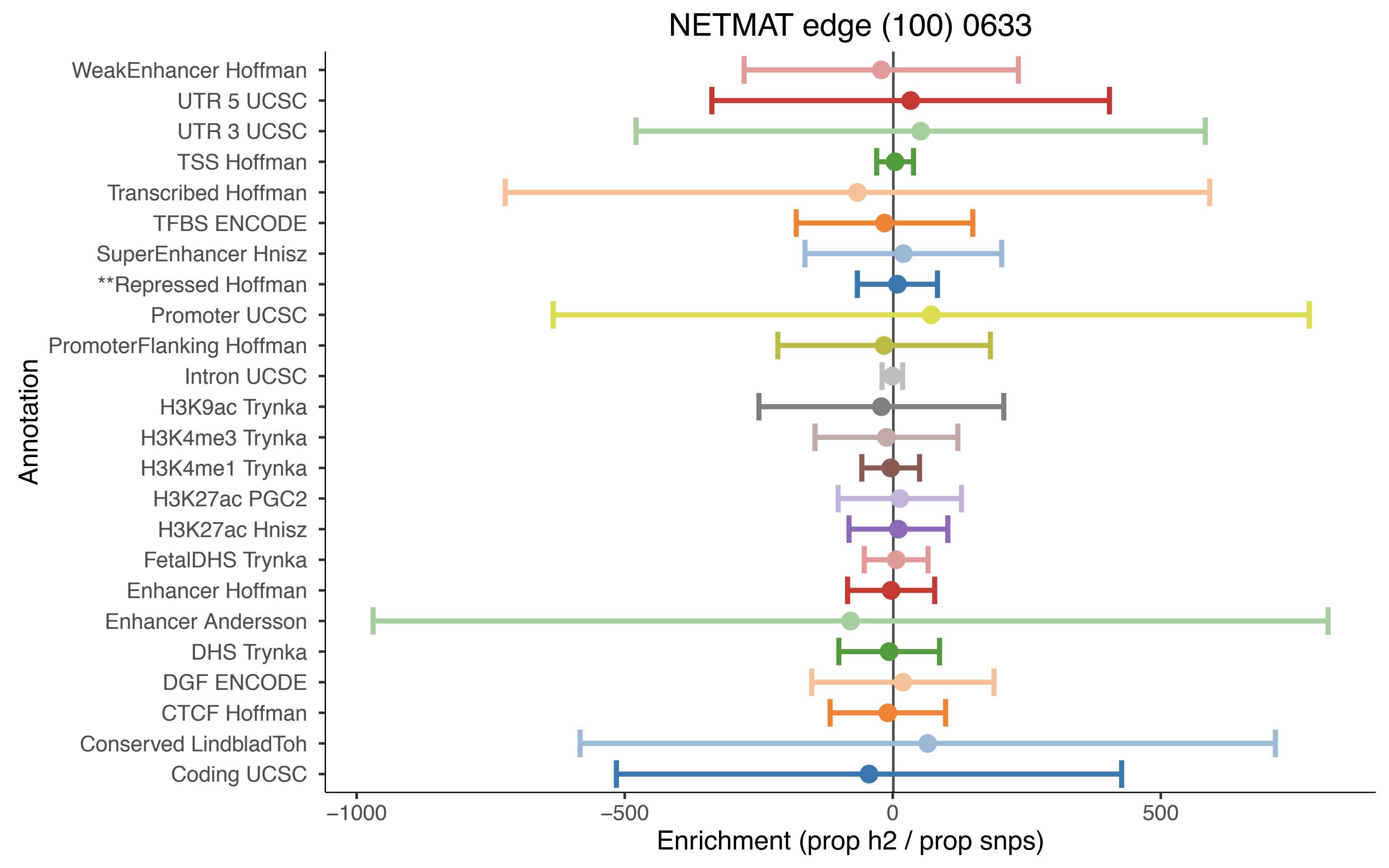
NETMAT edge (100) 0633
WeakEnhancer Hoffman
UTR 5 UCSC
UTR 3 UCSC
TSS Hoffman
Transcribed Hoffman
TFBS ENCODE
SuperEnhancer Hnisz
**Repressed Hoffman
Promoter UCSC
PromoterFlanking Hoffman
Intron UCSC
H3K9ac Trynka
H3K4me3 Trynka
H3K4me1 Trynka
H3K27ac PGC2
H3K27ac Hnisz
FetalDHS Trynka
Enhancer Hoffman
Enhancer Andersson
DHS Trynka
DGF ENCODE
CTCF Hoffman
Conserved LindbladToh
Coding UCSC
Annotation
−1000
−500
0
500
Enrichment (prop h2 / prop snps)

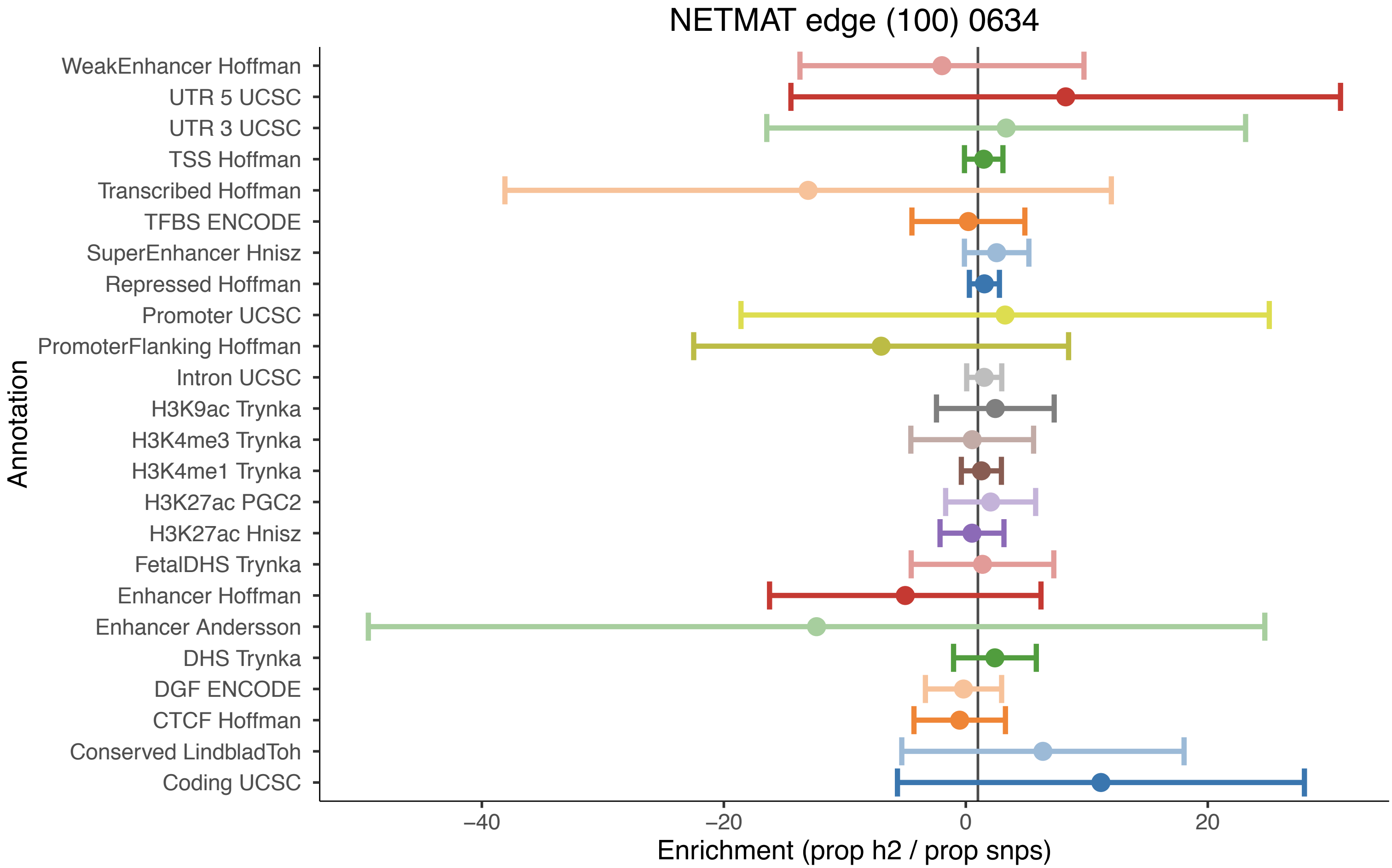
NETMAT edge (100) 0634
WeakEnhancer Hoffman
UTR 5 UCSC
UTR 3 UCSC
TSS Hoffman
Transcribed Hoffman
TFBS ENCODE
SuperEnhancer Hnisz
Repressed Hoffman
Promoter UCSC
PromoterFlanking Hoffman
Intron UCSC
H3K9ac Trynka
H3K4me3 Trynka
H3K4me1 Trynka
H3K27ac PGC2
H3K27ac Hnisz
FetalDHS Trynka
Enhancer Hoffman
Enhancer Andersson
DHS Trynka
DGF ENCODE
CTCF Hoffman
Conserved LindbladToh
Coding UCSC
Annotation
−40
−20
0
20
Enrichment (prop h2 / prop snps)

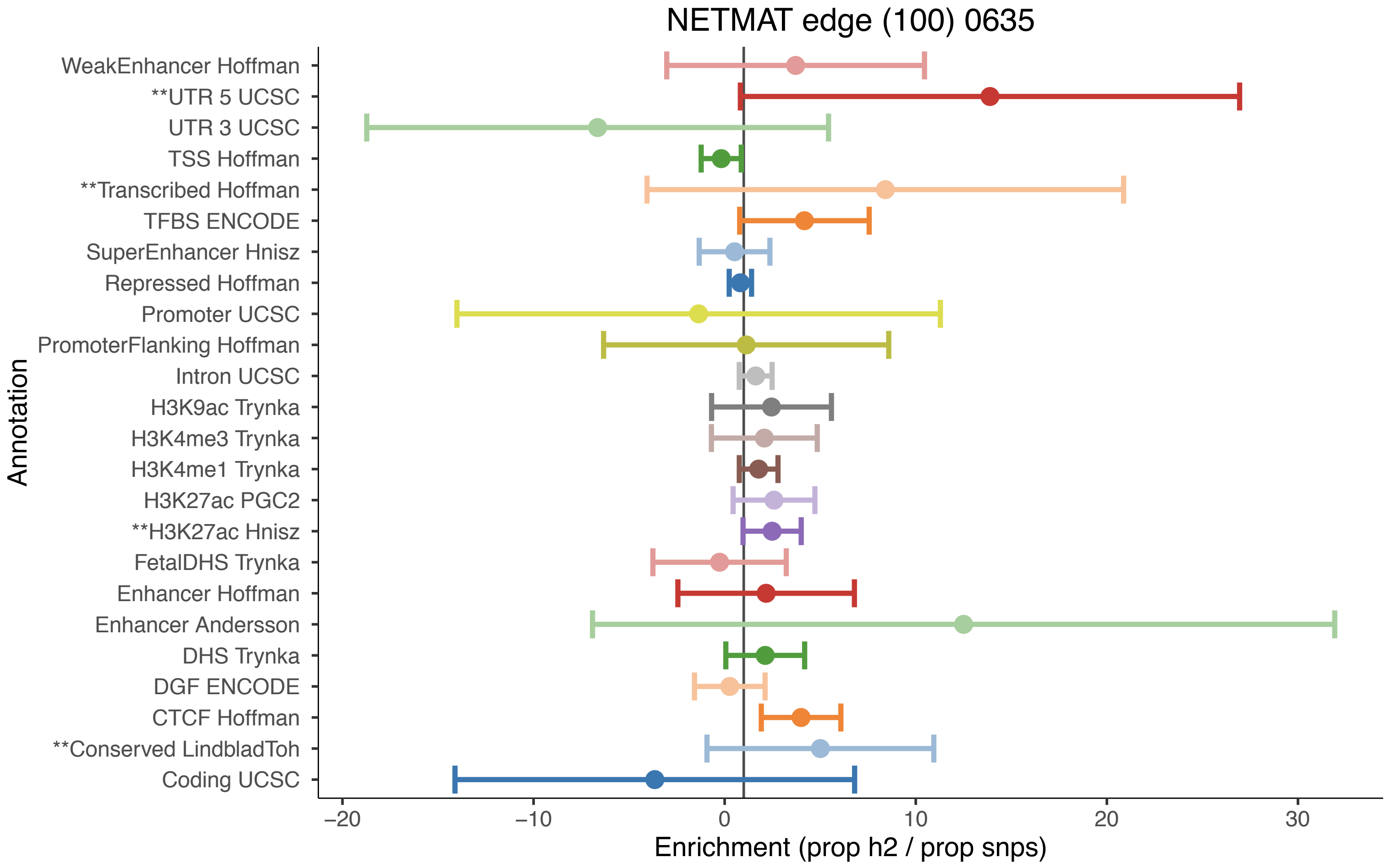

NETMAT edge (100) 0635
WeakEnhancer Hoffman
**UTR 5 UCSC
UTR 3 UCSC
TSS Hoffman
**Transcribed Hoffman
TFBS ENCODE
SuperEnhancer Hnisz
Repressed Hoffman
Promoter UCSC
PromoterFlanking Hoffman
Intron UCSC
H3K9ac Trynka
H3K4me3 Trynka
H3K4me1 Trynka
H3K27ac PGC2
**H3K27ac Hnisz
FetalDHS Trynka
Enhancer Hoffman
Enhancer Andersson
DHS Trynka
DGF ENCODE
CTCF Hoffman
**Conserved LindbladToh
Coding UCSC
Annotation
−20
−10
0
10
20
30
Enrichment (prop h2 / prop snps)

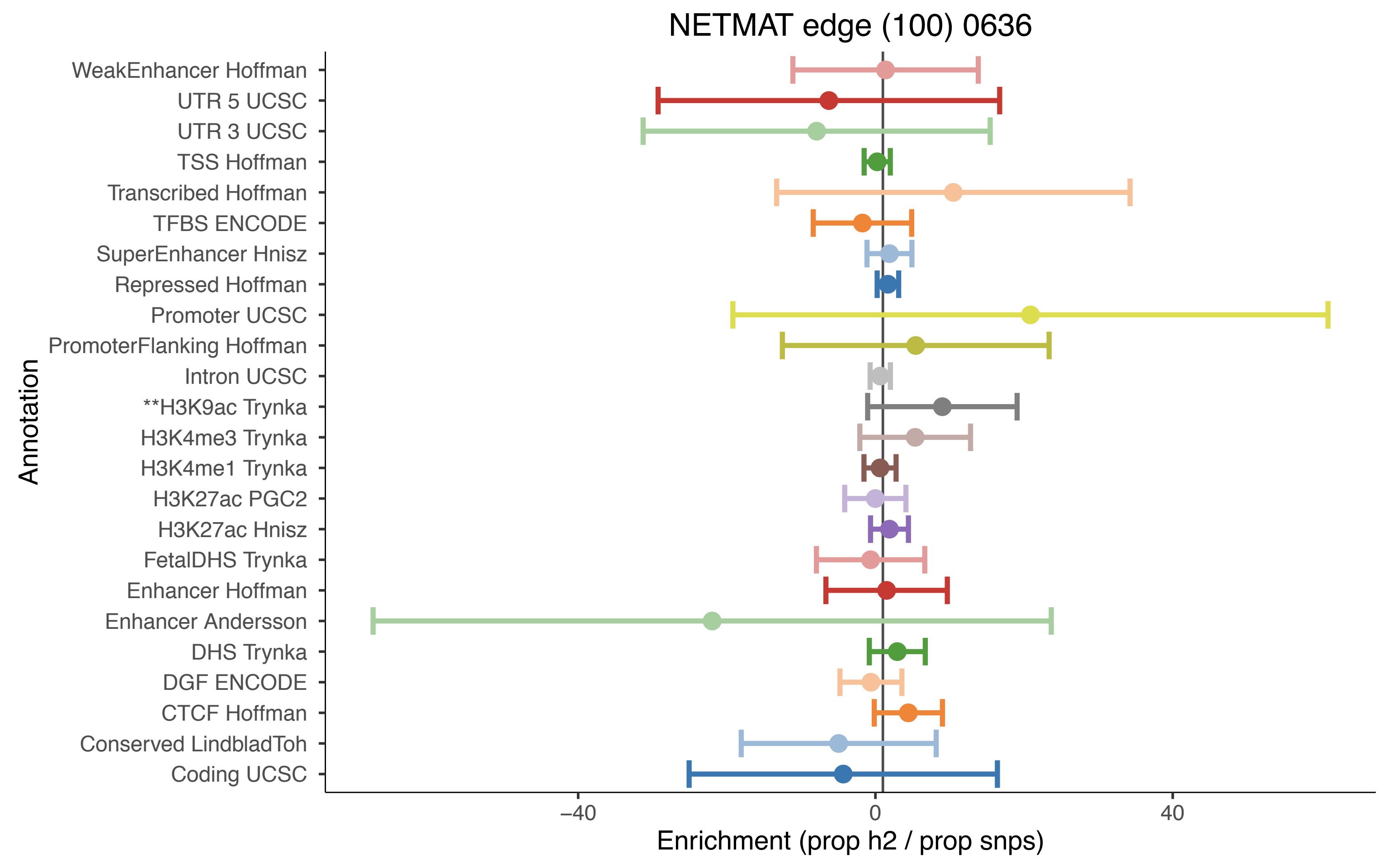

NETMAT edge (100) 0636
WeakEnhancer Hoffman
UTR 5 UCSC
UTR 3 UCSC
TSS Hoffman
Transcribed Hoffman
TFBS ENCODE
SuperEnhancer Hnisz
Repressed Hoffman
Promoter UCSC
PromoterFlanking Hoffman
Intron UCSC
**H3K9ac Trynka
H3K4me3 Trynka
H3K4me1 Trynka
H3K27ac PGC2
H3K27ac Hnisz
FetalDHS Trynka
Enhancer Hoffman
Enhancer Andersson
DHS Trynka
DGF ENCODE
CTCF Hoffman
Conserved LindbladToh
Coding UCSC
Annotation
–40
0
40
Enrichment (prop h2 / prop snps)

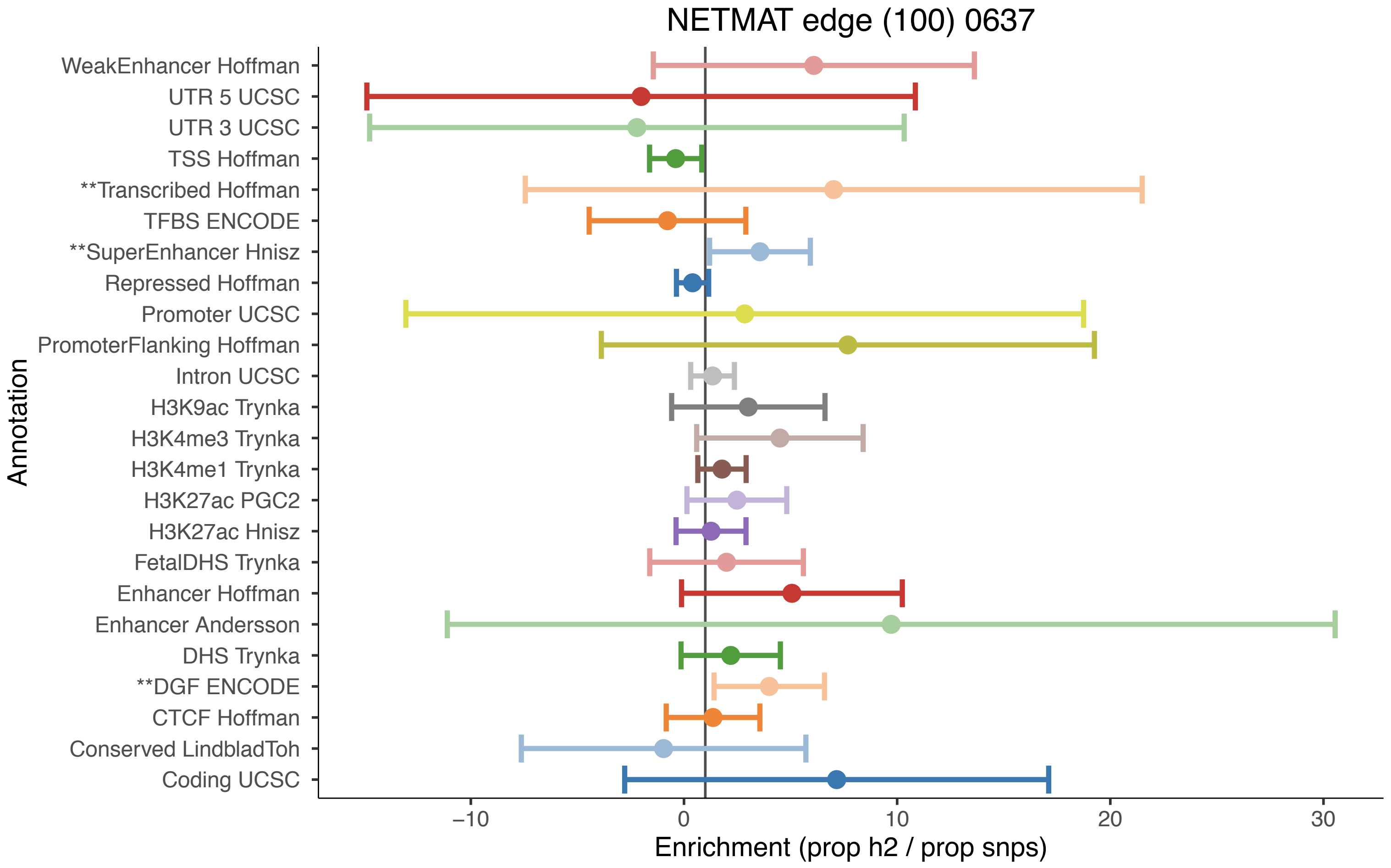

NETMAT edge (100) 0637
WeakEnhancer Hoffman
UTR 5 UCSC
UTR 3 UCSC
TSS Hoffman
**Transcribed Hoffman
TFBS ENCODE
**SuperEnhancer Hnisz
Repressed Hoffman
Promoter UCSC
PromoterFlanking Hoffman
Intron UCSC
H3K9ac Trynka
H3K4me3 Trynka
H3K4me1 Trynka
H3K27ac PGC2
H3K27ac Hnisz
FetalDHS Trynka
Enhancer Hoffman
Enhancer Andersson
DHS Trynka
**DGF ENCODE
CTCF Hoffman
Conserved LindbladToh
Coding UCSC
Annotation
−10
0
10
20
30
Enrichment (prop h2 / prop snps)

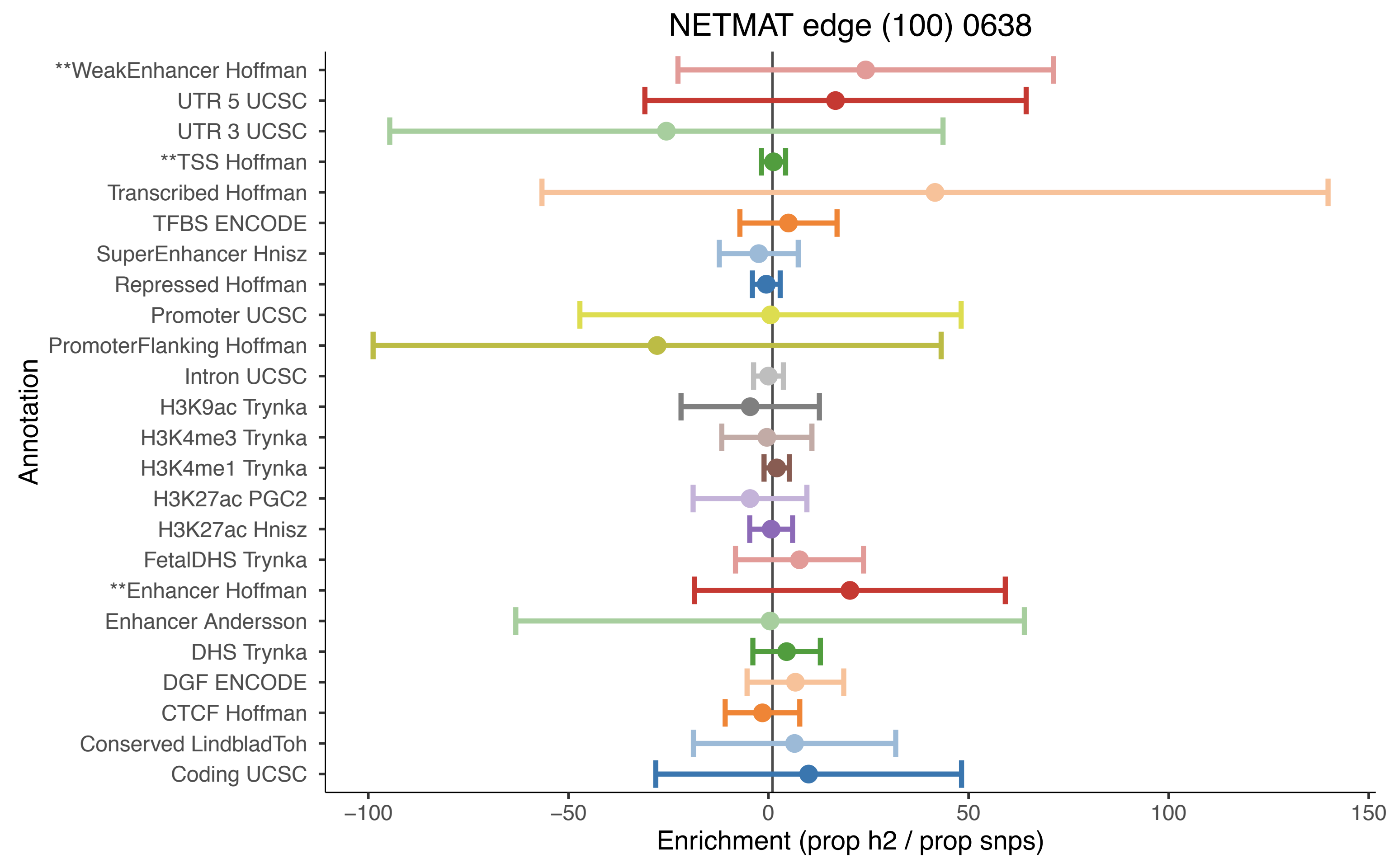
NETMAT edge (100) 0638
**WeakEnhancer Hoffman
UTR 5 UCSC
UTR 3 UCSC
**TSS Hoffman
Transcribed Hoffman
TFBS ENCODE
SuperEnhancer Hnisz
Repressed Hoffman
Promoter UCSC
PromoterFlanking Hoffman
Intron UCSC
H3K9ac Trynka
H3K4me3 Trynka
H3K4me1 Trynka
H3K27ac PGC2
H3K27ac Hnisz
FetalDHS Trynka
**Enhancer Hoffman
Enhancer Andersson
DHS Trynka
DGF ENCODE
CTCF Hoffman
Conserved LindbladToh
Coding UCSC
Annotation
−100
−50
0
50
100
150
Enrichment (prop h2 / prop snps)

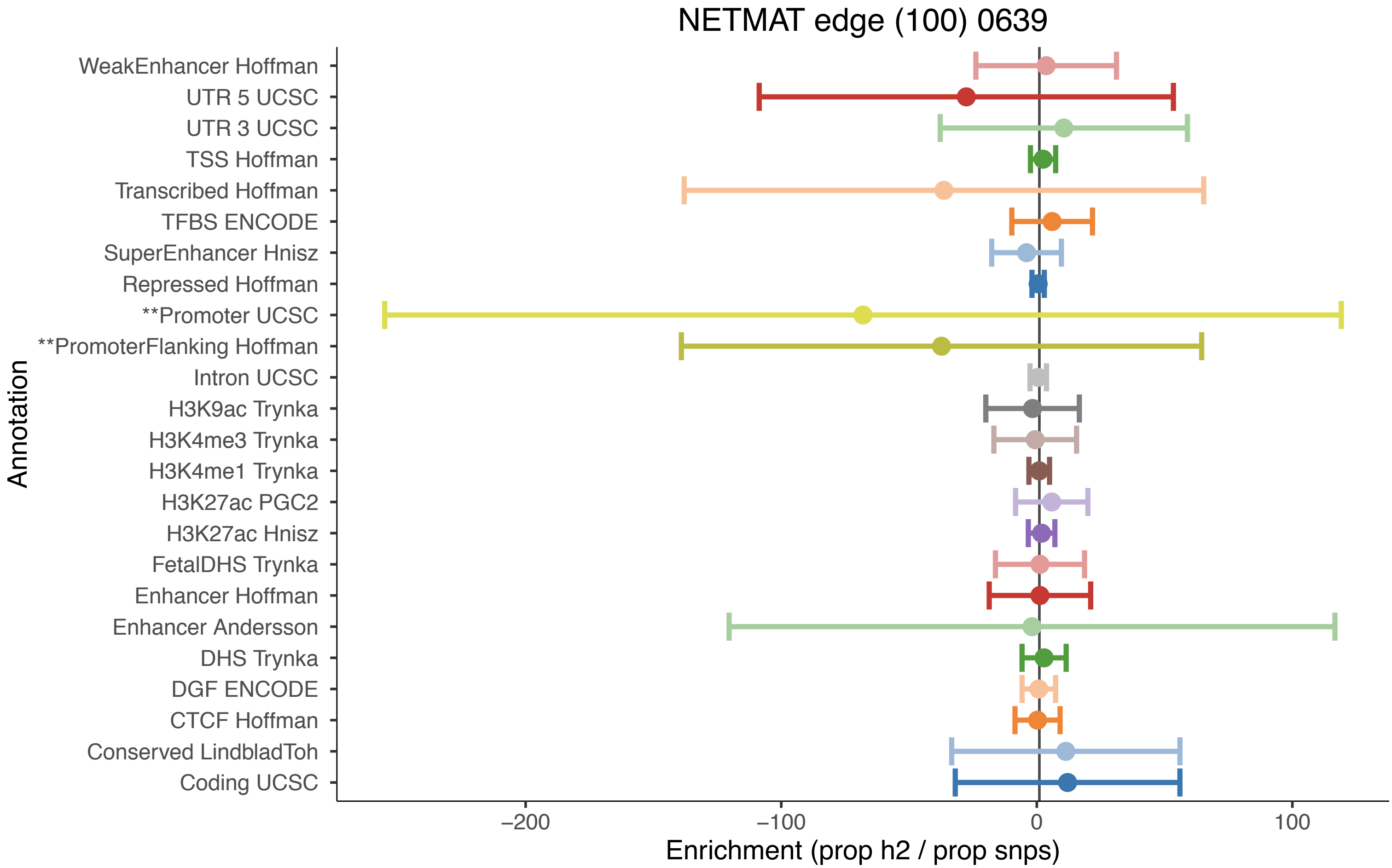

NETMAT edge (100) 0639
WeakEnhancer Hoffman
UTR 5 UCSC
UTR 3 UCSC
TSS Hoffman
Transcribed Hoffman
TFBS ENCODE
SuperEnhancer Hnisz
Repressed Hoffman
**Promoter UCSC
**PromoterFlanking Hoffman
Intron UCSC
H3K9ac Trynka
H3K4me3 Trynka
H3K4me1 Trynka
H3K27ac PGC2
H3K27ac Hnisz
FetalDHS Trynka
Enhancer Hoffman
Enhancer Andersson
DHS Trynka
DGF ENCODE
CTCF Hoffman
Conserved LindbladToh
Coding UCSC
Annotation
−200
−100
0
100
Enrichment (prop h2 / prop snps)

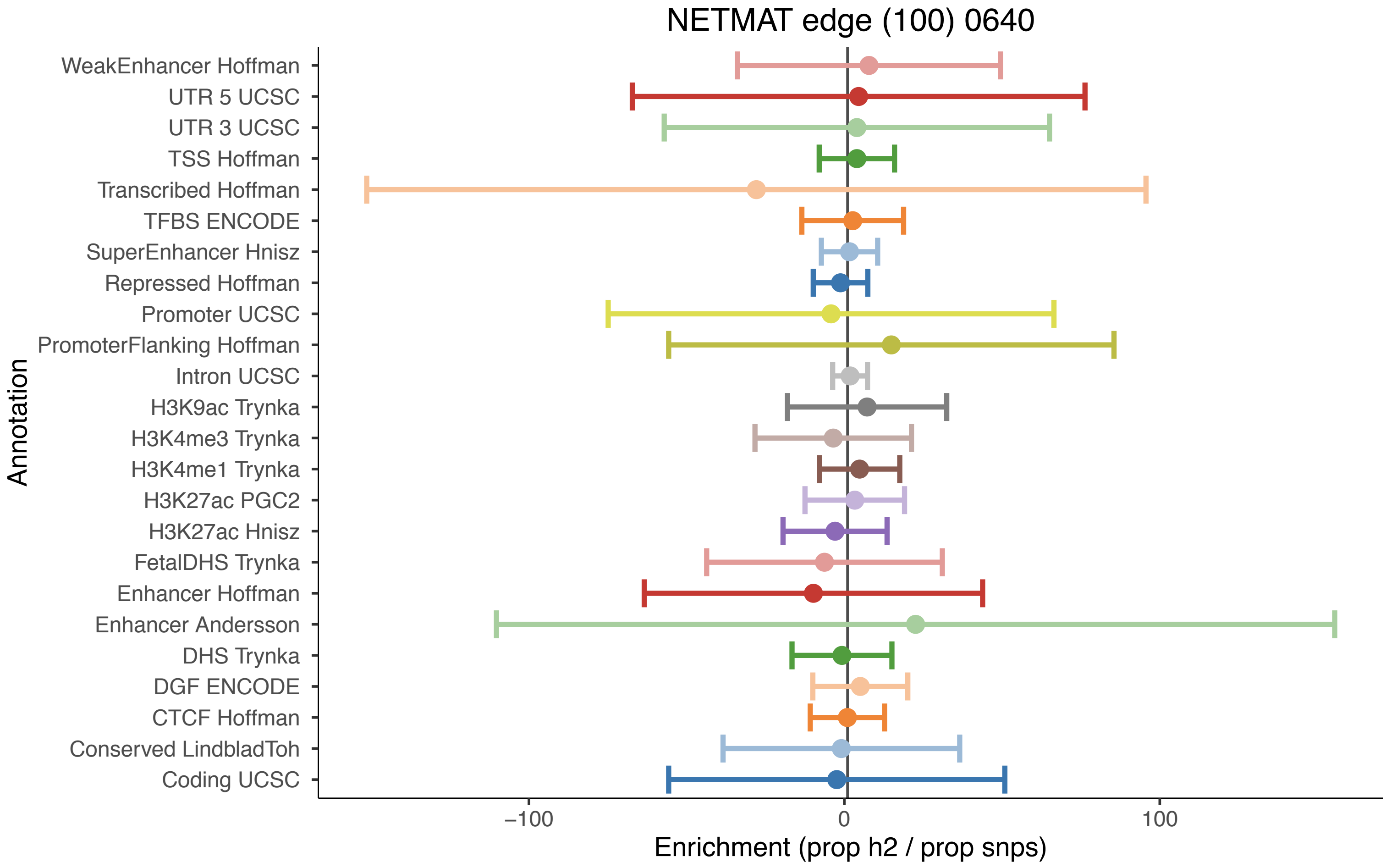

NETMAT edge (100) 0640
WeakEnhancer Hoffman
UTR 5 UCSC
UTR 3 UCSC
TSS Hoffman
Transcribed Hoffman
TFBS ENCODE
SuperEnhancer Hnisz
Repressed Hoffman
Promoter UCSC
PromoterFlanking Hoffman
Intron UCSC
H3K9ac Trynka
H3K4me3 Trynka
H3K4me1 Trynka
H3K27ac PGC2
H3K27ac Hnisz
FetalDHS Trynka
Enhancer Hoffman
Enhancer Andersson
DHS Trynka
DGF ENCODE
CTCF Hoffman
Conserved LindbladToh
Coding UCSC
Annotation
−100
0
100
Enrichment (prop h2 / prop snps)

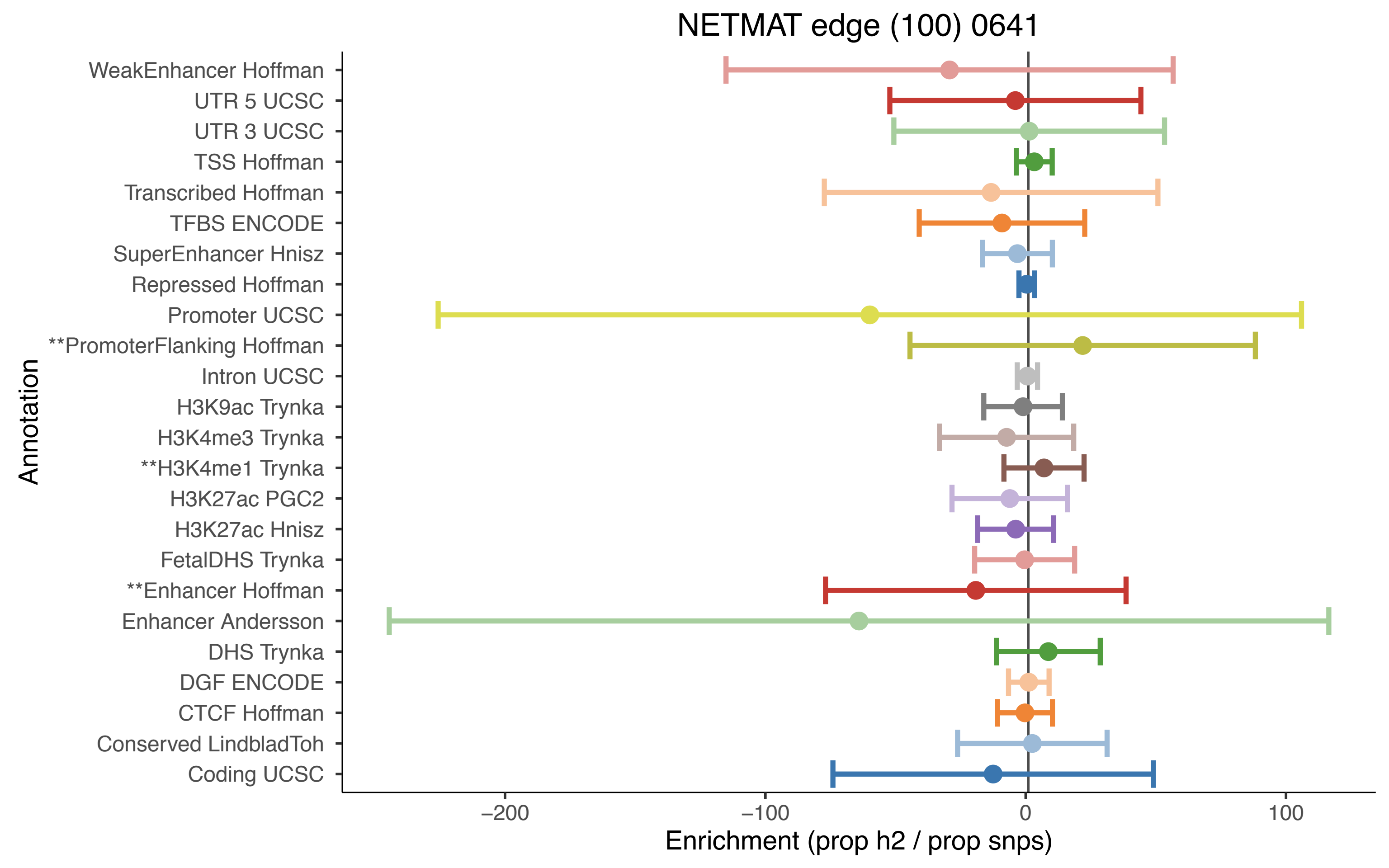
NETMAT edge (100) 0641
WeakEnhancer Hoffman
UTR 5 UCSC
UTR 3 UCSC
TSS Hoffman
Transcribed Hoffman
TFBS ENCODE
SuperEnhancer Hnisz
Repressed Hoffman
Promoter UCSC
**PromoterFlanking Hoffman
Intron UCSC
H3K9ac Trynka
H3K4me3 Trynka
**H3K4me1 Trynka
H3K27ac PGC2
H3K27ac Hnisz
FetalDHS Trynka
**Enhancer Hoffman
Enhancer Andersson
DHS Trynka
DGF ENCODE
CTCF Hoffman
Conserved LindbladToh
Coding UCSC
Annotation
−200
−100
0
100
Enrichment (prop h2 / prop snps)

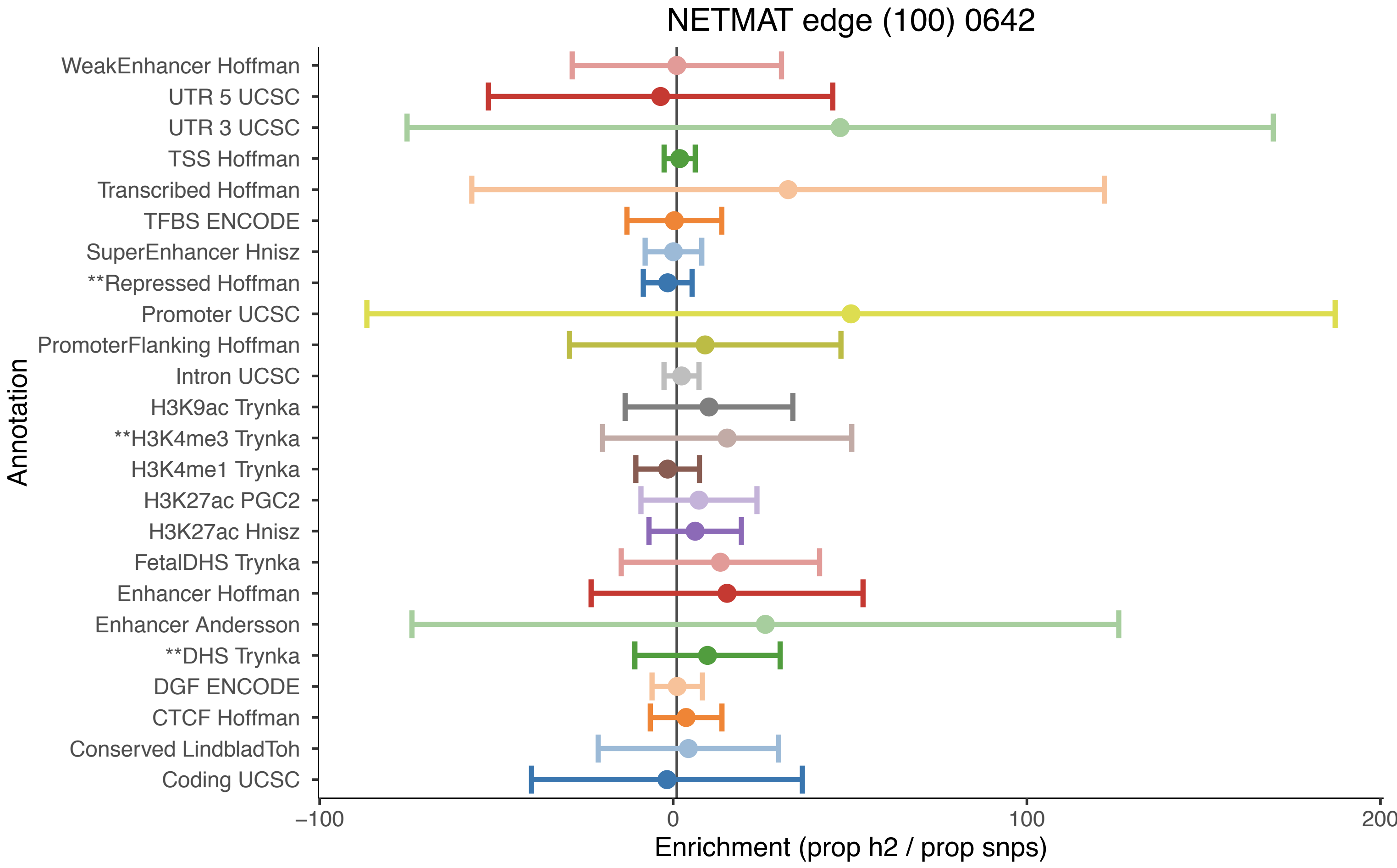

NETMAT edge (100) 0642
WeakEnhancer Hoffman
UTR 5 UCSC
UTR 3 UCSC
TSS Hoffman
Transcribed Hoffman
TFBS ENCODE
SuperEnhancer Hnisz
**Repressed Hoffman
Promoter UCSC
PromoterFlanking Hoffman
Intron UCSC
H3K9ac Trynka
**H3K4me3 Trynka
H3K4me1 Trynka
H3K27ac PGC2
H3K27ac Hnisz
FetalDHS Trynka
Enhancer Hoffman
Enhancer Andersson
**DHS Trynka
DGF ENCODE
CTCF Hoffman
Conserved LindbladToh
Coding UCSC
Annotation
−100
0
100
200
Enrichment (prop h2 / prop snps)

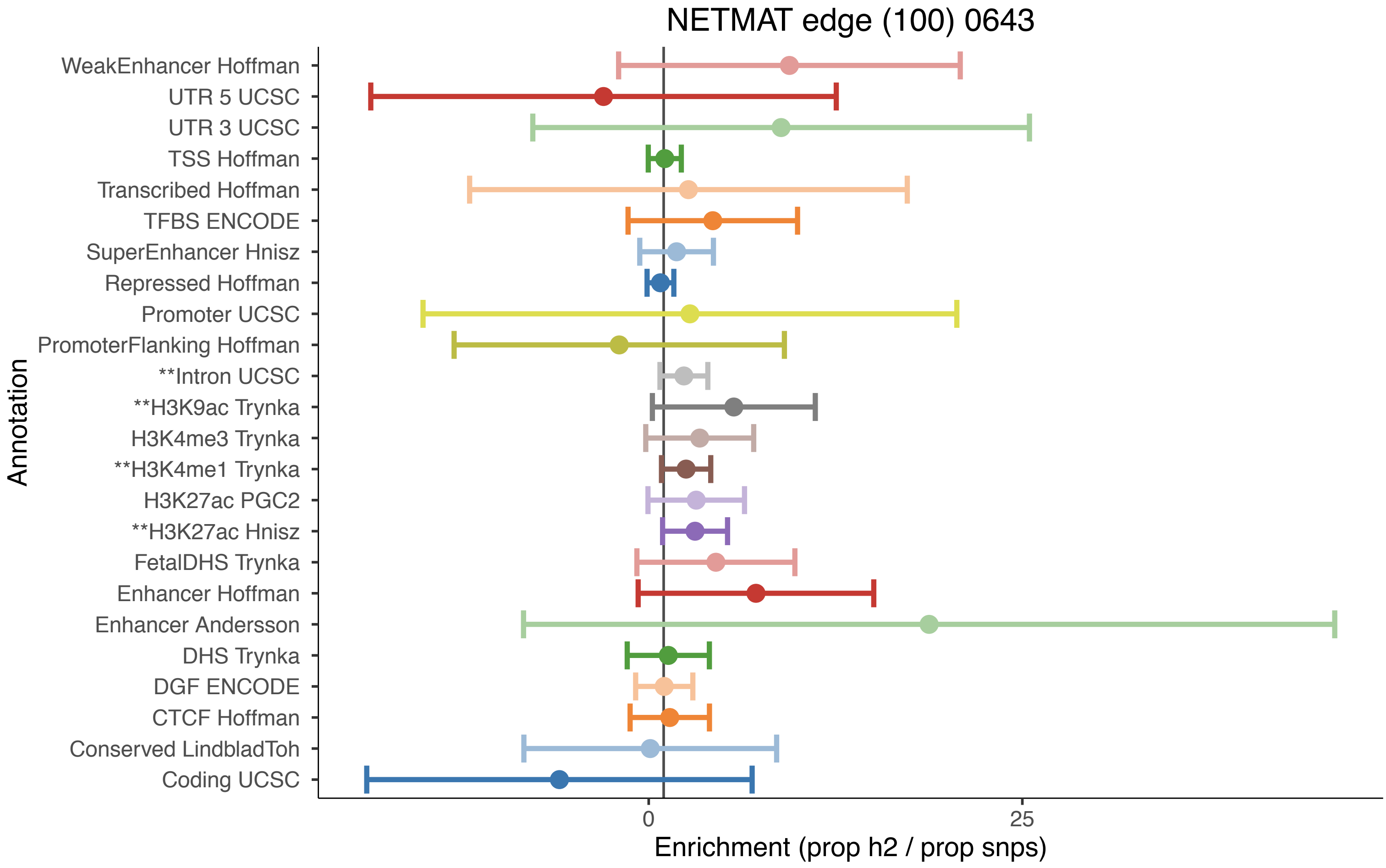
NETMAT edge (100) 0643
Annotation
WeakEnhancer Hoffman
UTR 5 UCSC
UTR 3 UCSC
TSS Hoffman
Transcribed Hoffman
TFBS ENCODE
SuperEnhancer Hnisz
Repressed Hoffman
Promoter UCSC
PromoterFlanking Hoffman
**Intron UCSC
**H3K9ac Trynka
H3K4me3 Trynka
**H3K4me1 Trynka
H3K27ac PGC2
**H3K27ac Hnisz
FetalDHS Trynka
Enhancer Hoffman
Enhancer Andersson
DHS Trynka
DGF ENCODE
CTCF Hoffman
Conserved LindbladToh
Coding UCSC
0
25
Enrichment (prop h2 / prop snps)

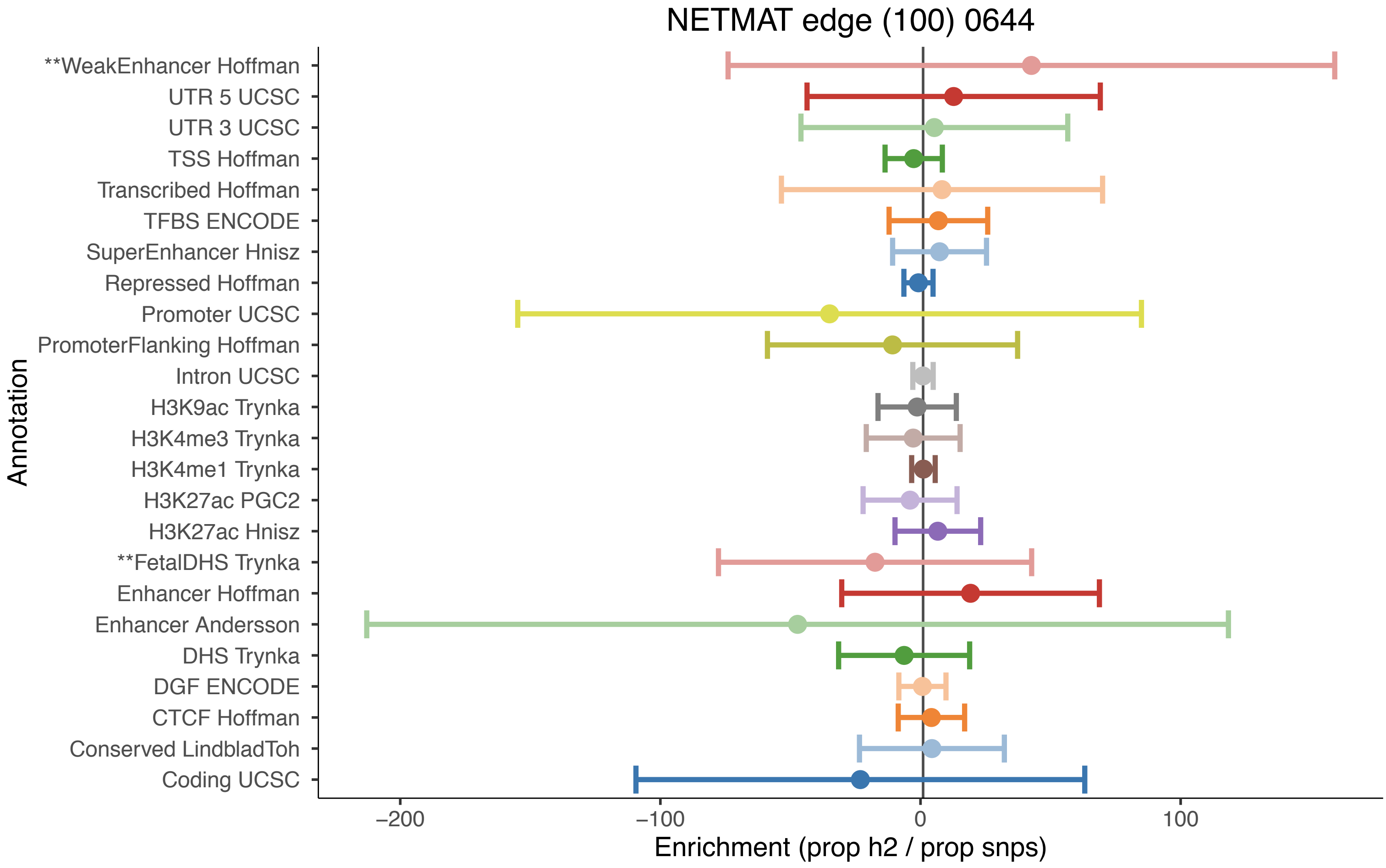
NETMAT edge (100) 0644
**WeakEnhancer Hoffman
UTR 5 UCSC
UTR 3 UCSC
TSS Hoffman
Transcribed Hoffman
TFBS ENCODE
SuperEnhancer Hnisz
Repressed Hoffman
Promoter UCSC
PromoterFlanking Hoffman
Intron UCSC
H3K9ac Trynka
H3K4me3 Trynka
H3K4me1 Trynka
H3K27ac PGC2
H3K27ac Hnisz
**FetalDHS Trynka
Enhancer Hoffman
Enhancer Andersson
DHS Trynka
DGF ENCODE
CTCF Hoffman
Conserved LindbladToh
Coding UCSC
Annotation
−200
−100
0
100
Enrichment (prop h2 / prop snps)

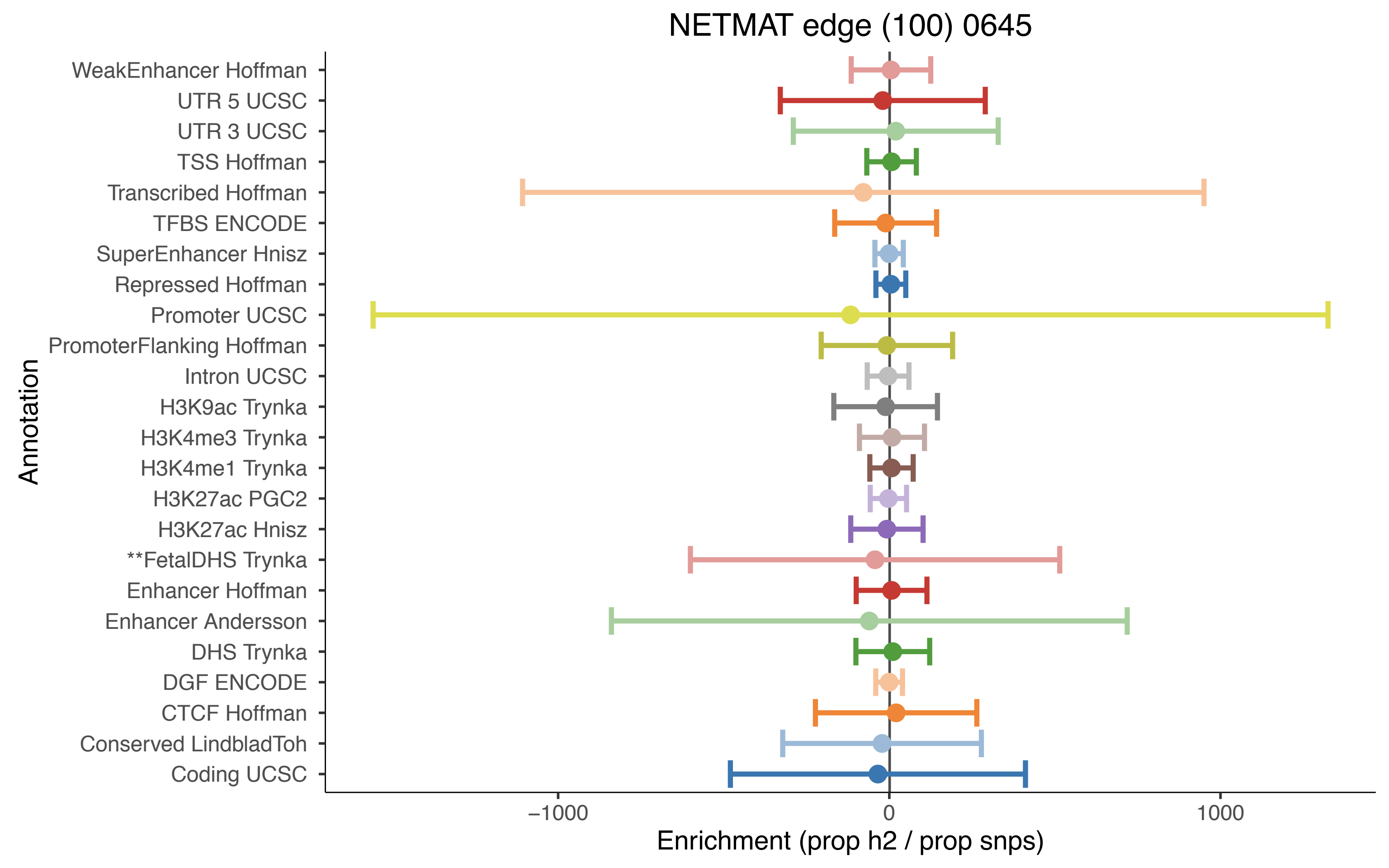

NETMAT edge (100) 0645
WeakEnhancer Hoffman
UTR 5 UCSC
UTR 3 UCSC
TSS Hoffman
Transcribed Hoffman
TFBS ENCODE
SuperEnhancer Hnisz
Repressed Hoffman
Promoter UCSC
PromoterFlanking Hoffman
Intron UCSC
H3K9ac Trynka
H3K4me3 Trynka
H3K4me1 Trynka
H3K27ac PGC2
H3K27ac Hnisz
**FetalDHS Trynka
Enhancer Hoffman
Enhancer Andersson
DHS Trynka
DGF ENCODE
CTCF Hoffman
Conserved LindbladToh
Coding UCSC
Annotation
−1000
0
1000
Enrichment (prop h2 / prop snps)

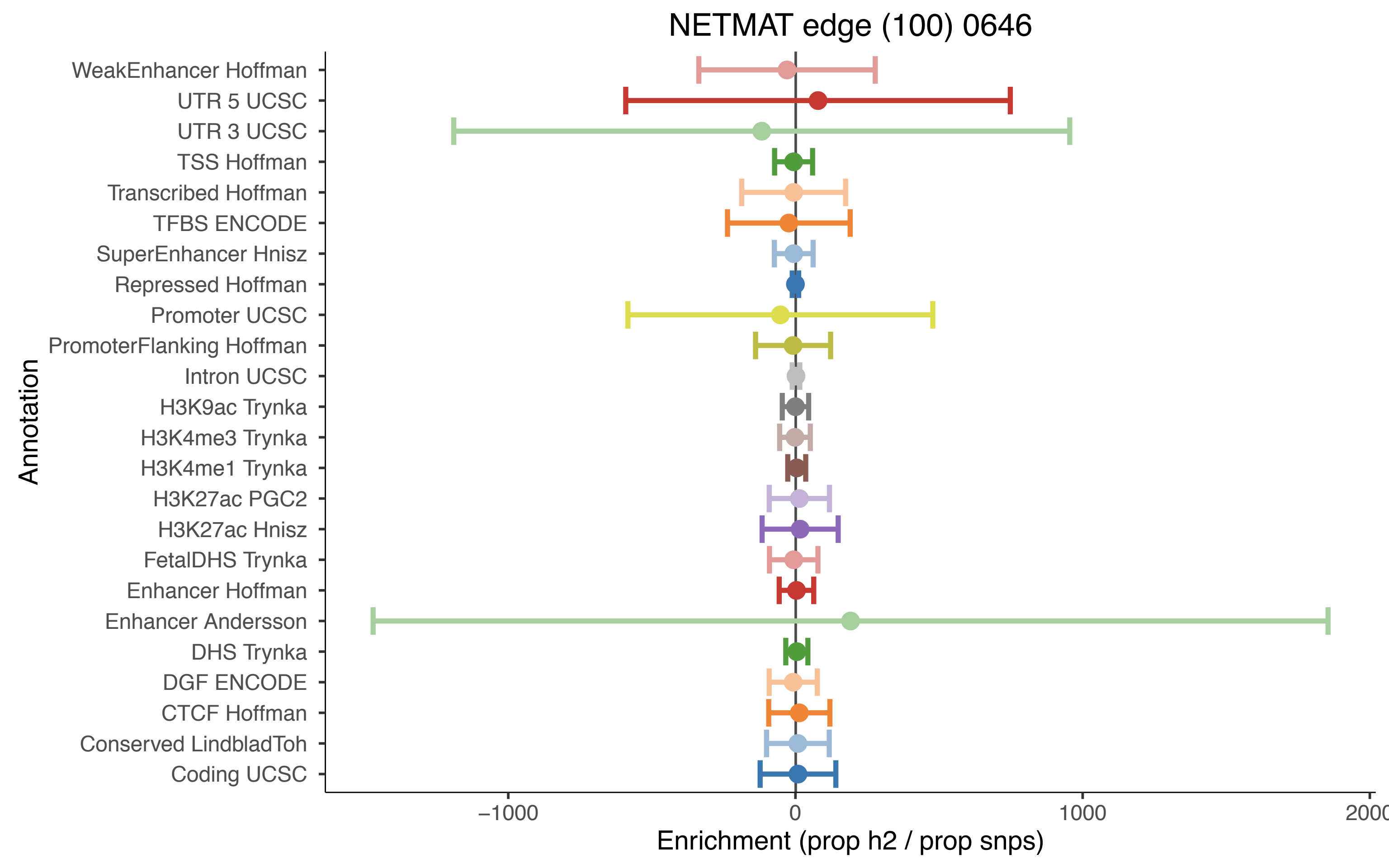
NETMAT edge (100) 0646
WeakEnhancer Hoffman
UTR 5 UCSC
UTR 3 UCSC
TSS Hoffman
Transcribed Hoffman
TFBS ENCODE
SuperEnhancer Hnisz
Repressed Hoffman
Promoter UCSC
PromoterFlanking Hoffman
Intron UCSC
H3K9ac Trynka
H3K4me3 Trynka
H3K4me1 Trynka
H3K27ac PGC2
H3K27ac Hnisz
FetalDHS Trynka
Enhancer Hoffman
Enhancer Andersson
DHS Trynka
DGF ENCODE
CTCF Hoffman
Conserved LindbladToh
Coding UCSC
Annotation
−1000
0
1000
2000
Enrichment (prop h2 / prop snps)

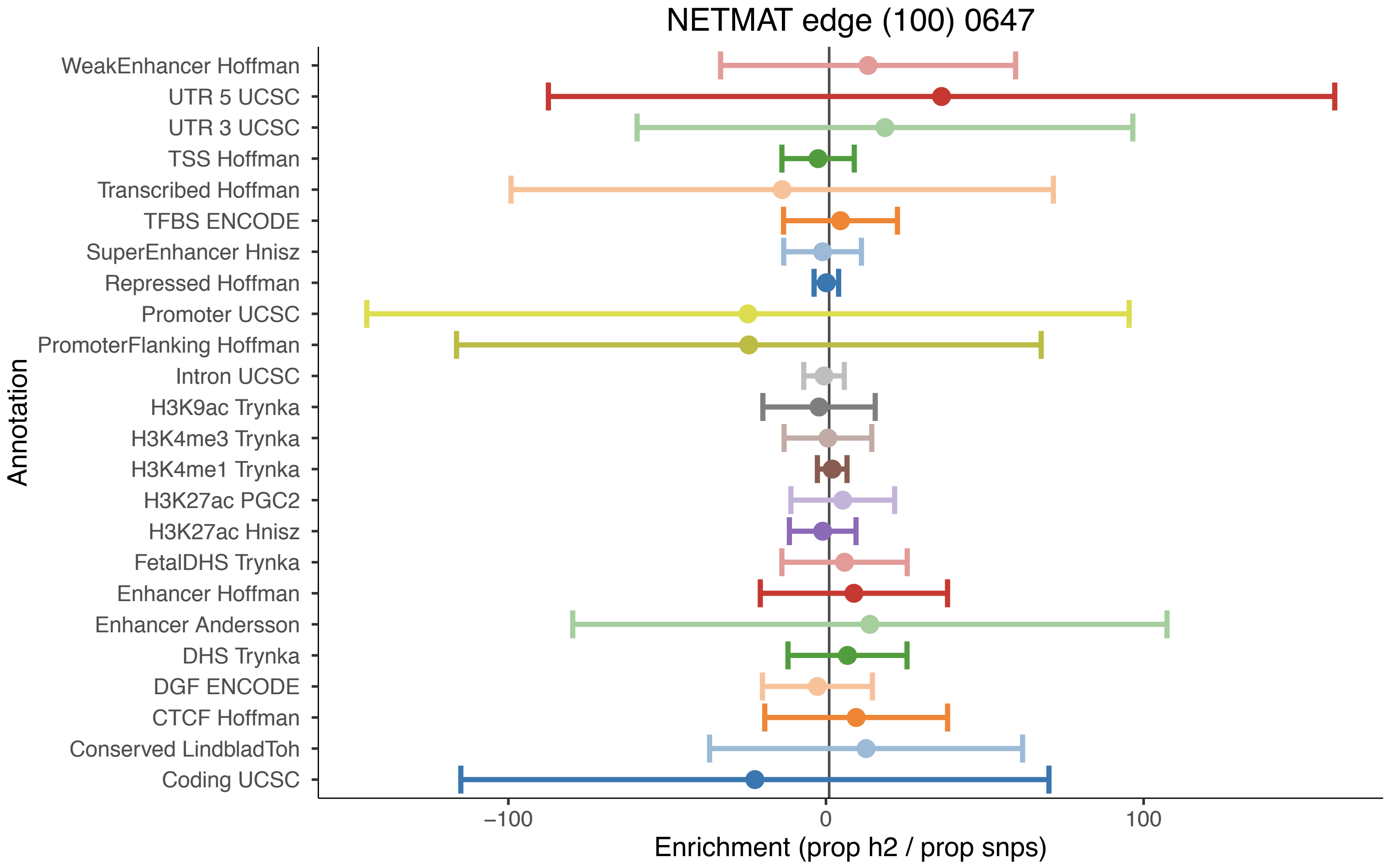

NETMAT edge (100) 0647
WeakEnhancer Hoffman
UTR 5 UCSC
UTR 3 UCSC
TSS Hoffman
Transcribed Hoffman
TFBS ENCODE
SuperEnhancer Hnisz
Repressed Hoffman
Promoter UCSC
PromoterFlanking Hoffman
Intron UCSC
H3K9ac Trynka
H3K4me3 Trynka
H3K4me1 Trynka
H3K27ac PGC2
H3K27ac Hnisz
FetalDHS Trynka
Enhancer Hoffman
Enhancer Andersson
DHS Trynka
DGF ENCODE
CTCF Hoffman
Conserved LindbladToh
Coding UCSC
Annotation
−100
0
100
Enrichment (prop h2 / prop snps)

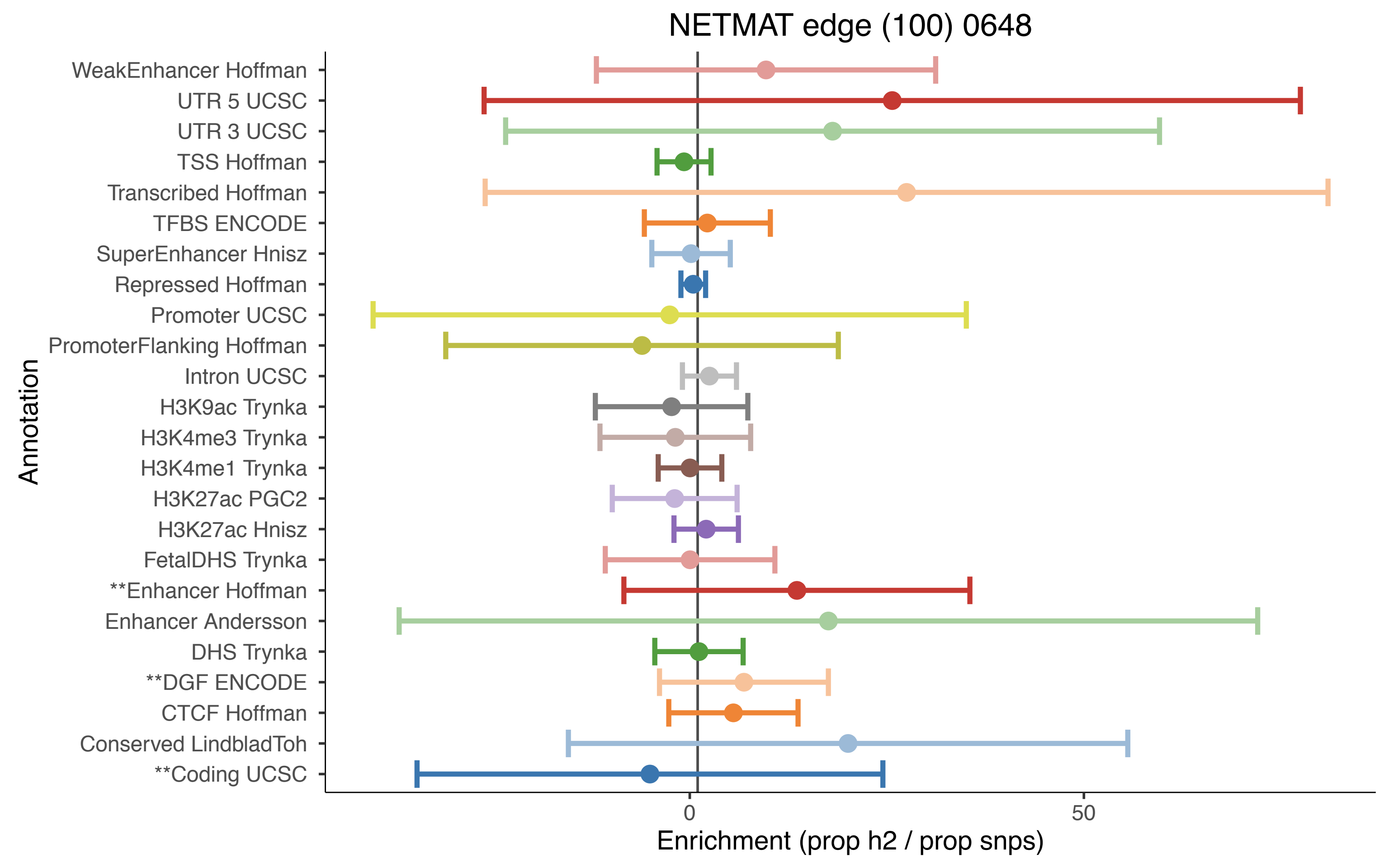

NETMAT edge (100) 0648
Annotation
WeakEnhancer Hoffman
UTR 5 UCSC
UTR 3 UCSC
TSS Hoffman
Transcribed Hoffman
TFBS ENCODE
SuperEnhancer Hnisz
Repressed Hoffman
Promoter UCSC
PromoterFlanking Hoffman
Intron UCSC
H3K9ac Trynka
H3K4me3 Trynka
H3K4me1 Trynka
H3K27ac PGC2
H3K27ac Hnisz
FetalDHS Trynka
**Enhancer Hoffman
Enhancer Andersson
DHS Trynka
**DGF ENCODE
CTCF Hoffman
Conserved LindbladToh
**Coding UCSC
0
50
Enrichment (prop h2 / prop snps)

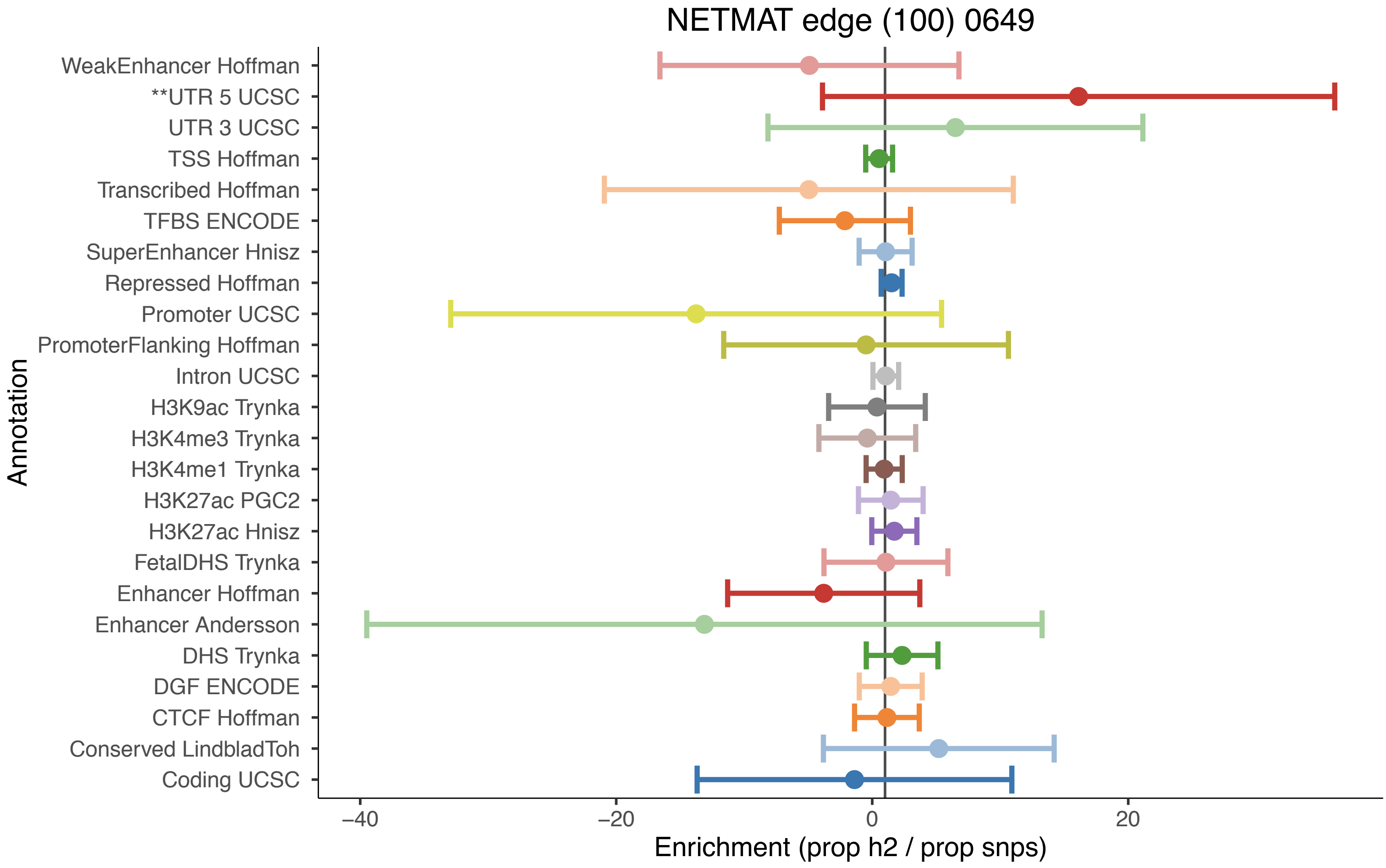
NETMAT edge (100) 0649
WeakEnhancer Hoffman
**UTR 5 UCSC
UTR 3 UCSC
TSS Hoffman
Transcribed Hoffman
TFBS ENCODE
SuperEnhancer Hnisz
Repressed Hoffman
Promoter UCSC
PromoterFlanking Hoffman
Intron UCSC
H3K9ac Trynka
H3K4me3 Trynka
H3K4me1 Trynka
H3K27ac PGC2
H3K27ac Hnisz
FetalDHS Trynka
Enhancer Hoffman
Enhancer Andersson
DHS Trynka
DGF ENCODE
CTCF Hoffman
Conserved LindbladToh
Coding UCSC
Annotation
−40
−20
0
20
Enrichment (prop h2 / prop snps)

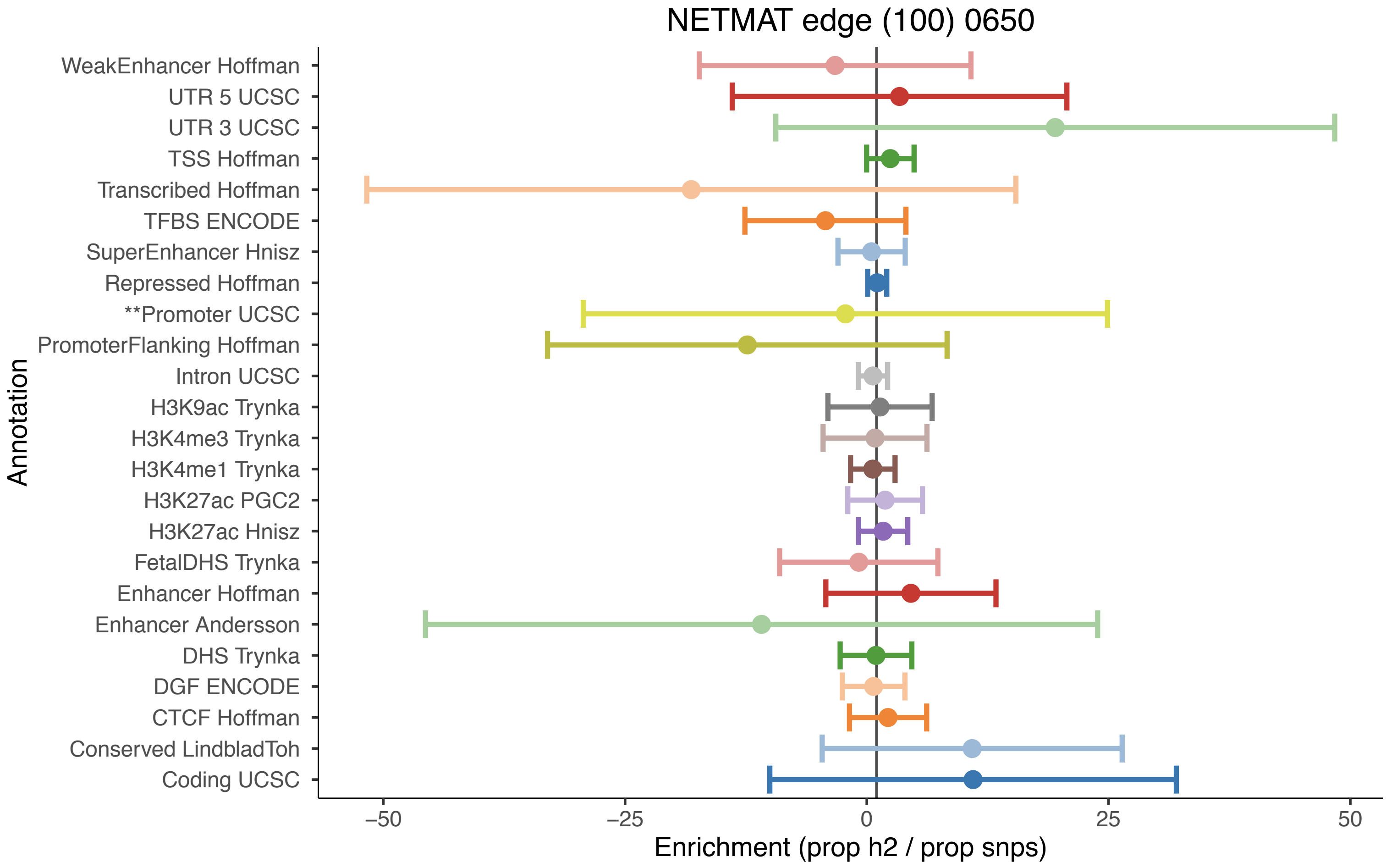
NETMAT edge (100) 0650
Annotation
WeakEnhancer Hoffman
UTR 5 UCSC
UTR 3 UCSC
TSS Hoffman
Transcribed Hoffman
TFBS ENCODE
SuperEnhancer Hnisz
Repressed Hoffman
**Promoter UCSC
PromoterFlanking Hoffman
Intron UCSC
H3K9ac Trynka
H3K4me3 Trynka
H3K4me1 Trynka
H3K27ac PGC2
H3K27ac Hnisz
FetalDHS Trynka
Enhancer Hoffman
Enhancer Andersson
DHS Trynka
DGF ENCODE
CTCF Hoffman
Conserved LindbladToh
Coding UCSC
−50
−25
0
25
50
Enrichment (prop h2 / prop snps)

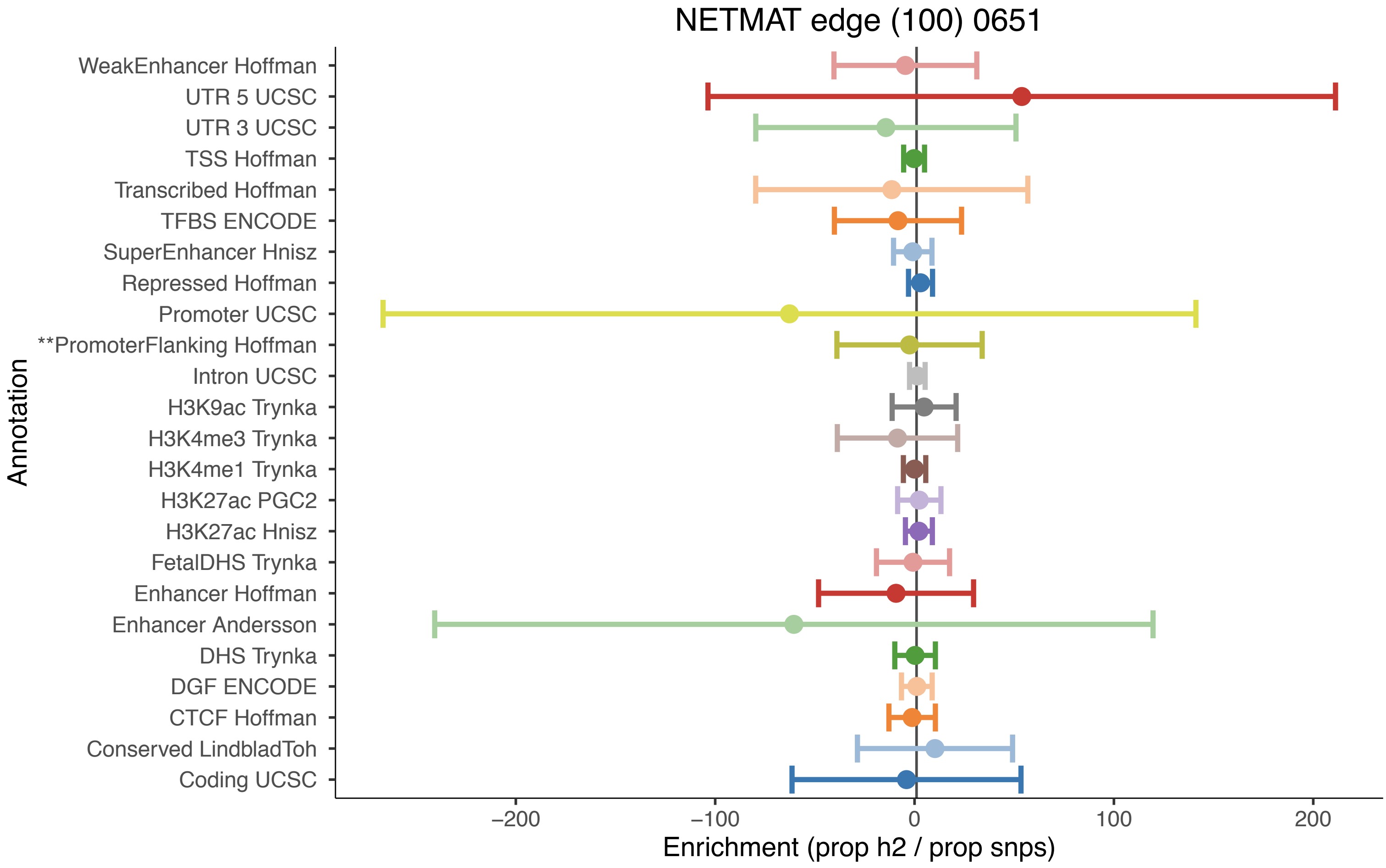
NETMAT edge (100) 0651
WeakEnhancer Hoffman
UTR 5 UCSC
UTR 3 UCSC
TSS Hoffman
Transcribed Hoffman
TFBS ENCODE
SuperEnhancer Hnisz
Repressed Hoffman
Promoter UCSC
**PromoterFlanking Hoffman
Intron UCSC
H3K9ac Trynka
H3K4me3 Trynka
H3K4me1 Trynka
H3K27ac PGC2
H3K27ac Hnisz
FetalDHS Trynka
Enhancer Hoffman
Enhancer Andersson
DHS Trynka
DGF ENCODE
CTCF Hoffman
Conserved LindbladToh
Coding UCSC
Annotation
−200
−100
0
100
200
Enrichment (prop h2 / prop snps)

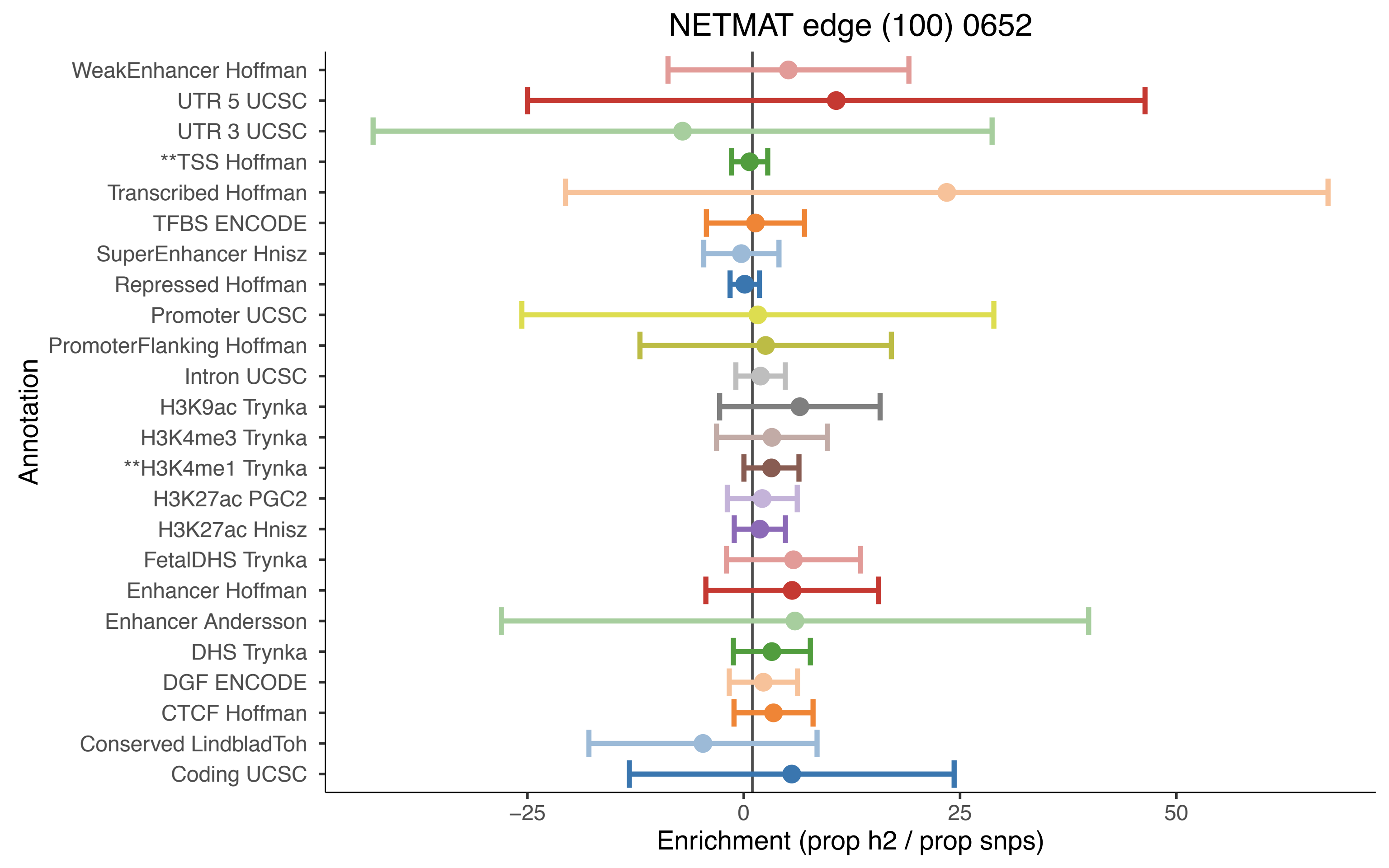
NETMAT edge (100) 0652
WeakEnhancer Hoffman
UTR 5 UCSC
UTR 3 UCSC
**TSS Hoffman
Transcribed Hoffman
TFBS ENCODE
SuperEnhancer Hnisz
Repressed Hoffman
Promoter UCSC
PromoterFlanking Hoffman
Intron UCSC
H3K9ac Trynka
H3K4me3 Trynka
**H3K4me1 Trynka
H3K27ac PGC2
H3K27ac Hnisz
FetalDHS Trynka
Enhancer Hoffman
Enhancer Andersson
DHS Trynka
DGF ENCODE
CTCF Hoffman
Conserved LindbladToh
Coding UCSC
Annotation
−25
0
25
50
Enrichment (prop h2 / prop snps)

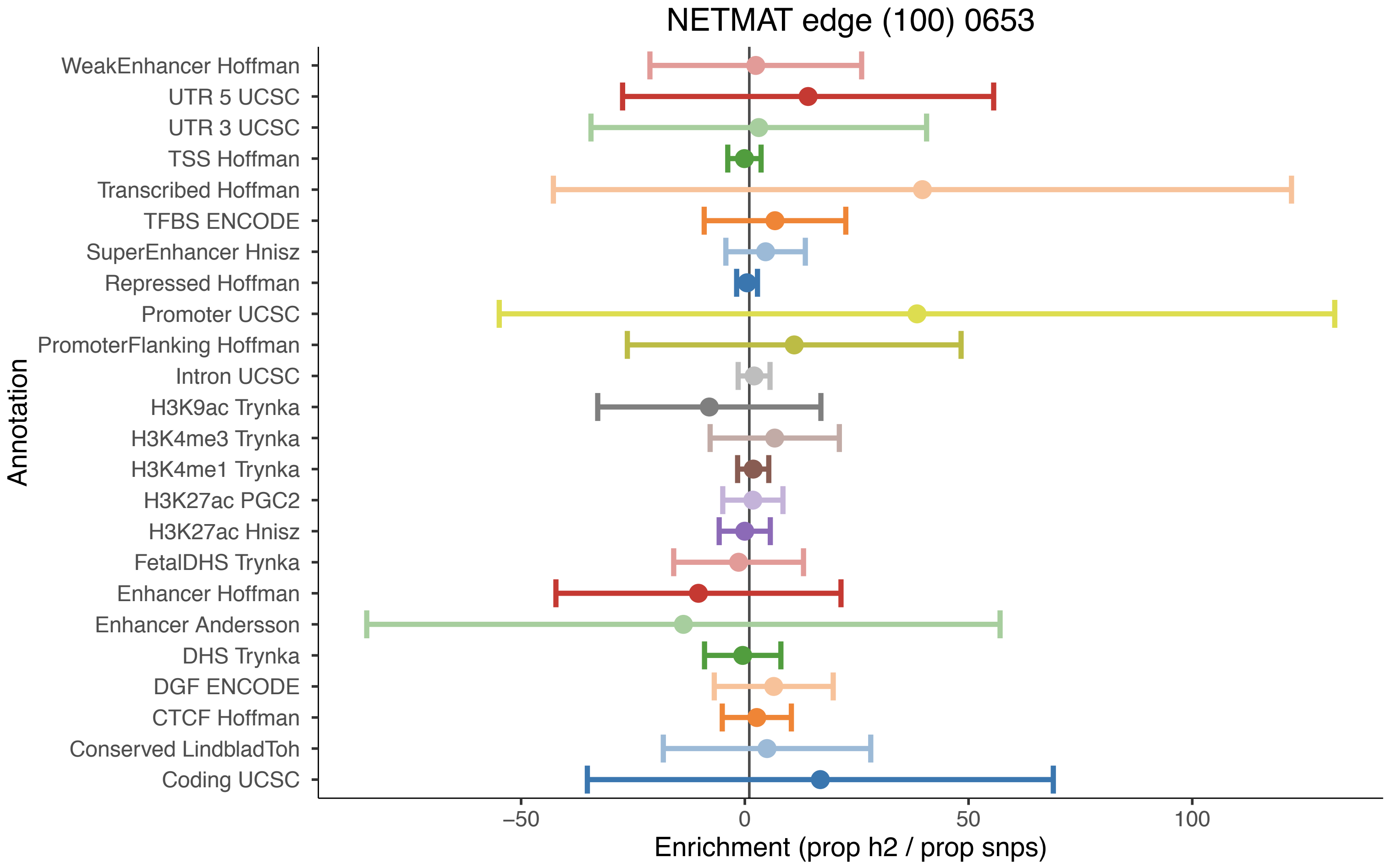
NETMAT edge (100) 0653
Annotation
WeakEnhancer Hoffman
UTR 5 UCSC
UTR 3 UCSC
TSS Hoffman
Transcribed Hoffman
TFBS ENCODE
SuperEnhancer Hnisz
Repressed Hoffman
Promoter UCSC
PromoterFlanking Hoffman
Intron UCSC
H3K9ac Trynka
H3K4me3 Trynka
H3K4me1 Trynka
H3K27ac PGC2
H3K27ac Hnisz
FetalDHS Trynka
Enhancer Hoffman
Enhancer Andersson
DHS Trynka
DGF ENCODE
CTCF Hoffman
Conserved LindbladToh
Coding UCSC
−50
0
50
100
Enrichment (prop h2 / prop snps)

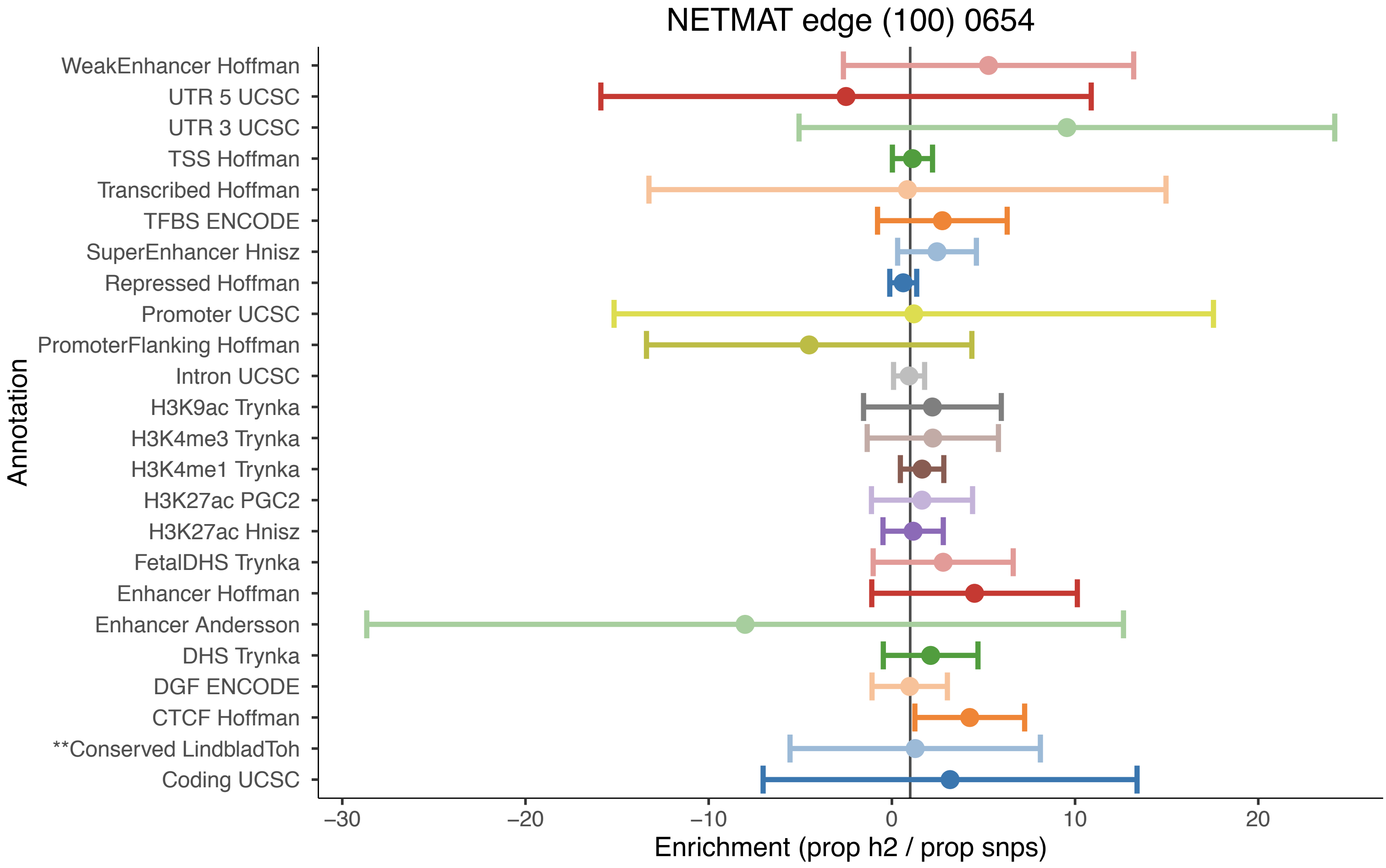

NETMAT edge (100) 0654
WeakEnhancer Hoffman
UTR 5 UCSC
UTR 3 UCSC
TSS Hoffman
Transcribed Hoffman
TFBS ENCODE
SuperEnhancer Hnisz
Repressed Hoffman
Promoter UCSC
PromoterFlanking Hoffman
Intron UCSC
H3K9ac Trynka
H3K4me3 Trynka
H3K4me1 Trynka
H3K27ac PGC2
H3K27ac Hnisz
FetalDHS Trynka
Enhancer Hoffman
Enhancer Andersson
DHS Trynka
DGF ENCODE
CTCF Hoffman
**Conserved LindbladToh
Coding UCSC
Annotation
−30
−20
−10
0
10
20
Enrichment (prop h2 / prop snps)

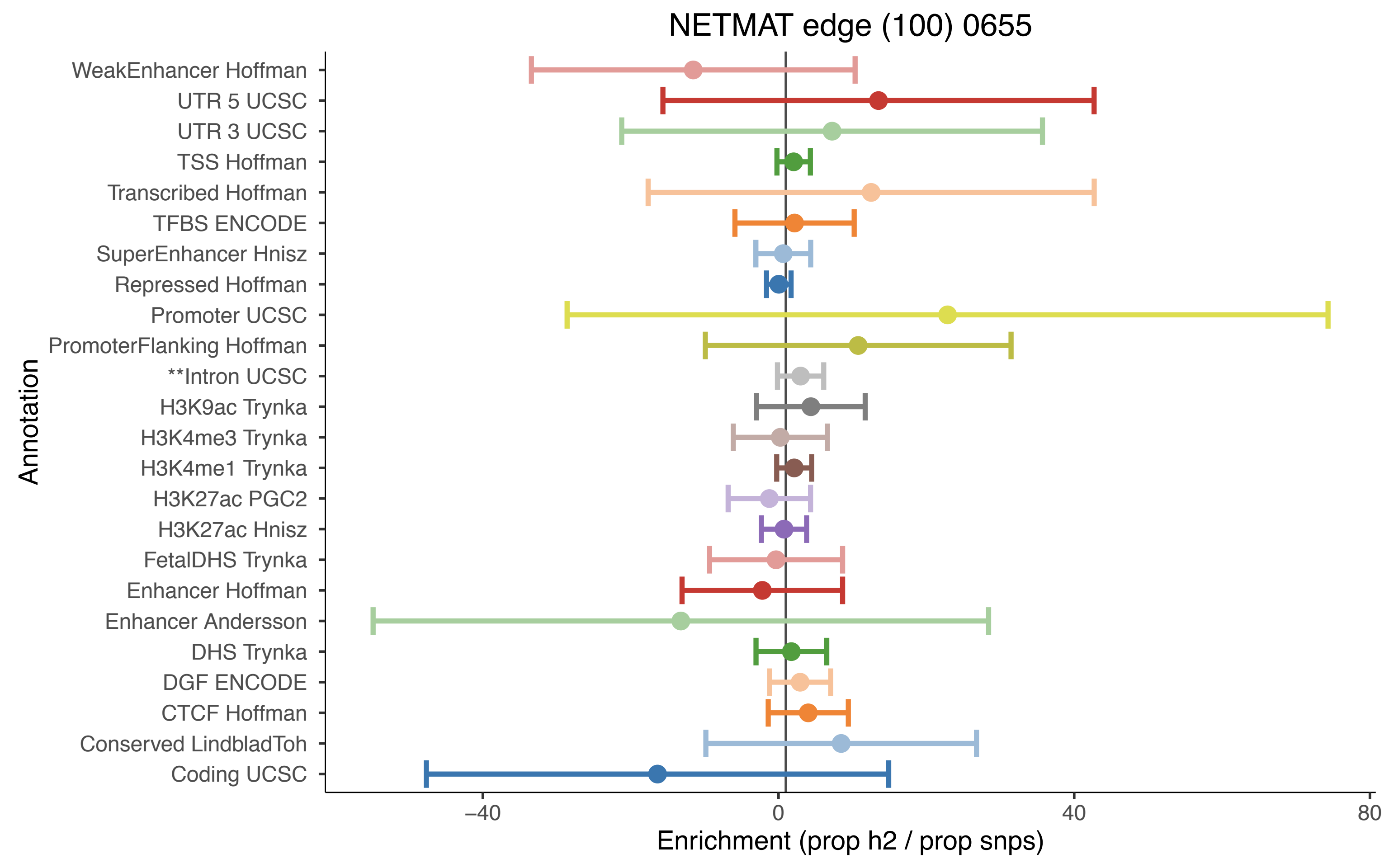

NETMAT edge (100) 0655
WeakEnhancer Hoffman
UTR 5 UCSC
UTR 3 UCSC
TSS Hoffman
Transcribed Hoffman
TFBS ENCODE
SuperEnhancer Hnisz
Repressed Hoffman
Promoter UCSC
PromoterFlanking Hoffman
**Intron UCSC
H3K9ac Trynka
H3K4me3 Trynka
H3K4me1 Trynka
H3K27ac PGC2
H3K27ac Hnisz
FetalDHS Trynka
Enhancer Hoffman
Enhancer Andersson
DHS Trynka
DGF ENCODE
CTCF Hoffman
Conserved LindbladToh
Coding UCSC
Annotation
−40
0
40
80
Enrichment (prop h2 / prop snps)

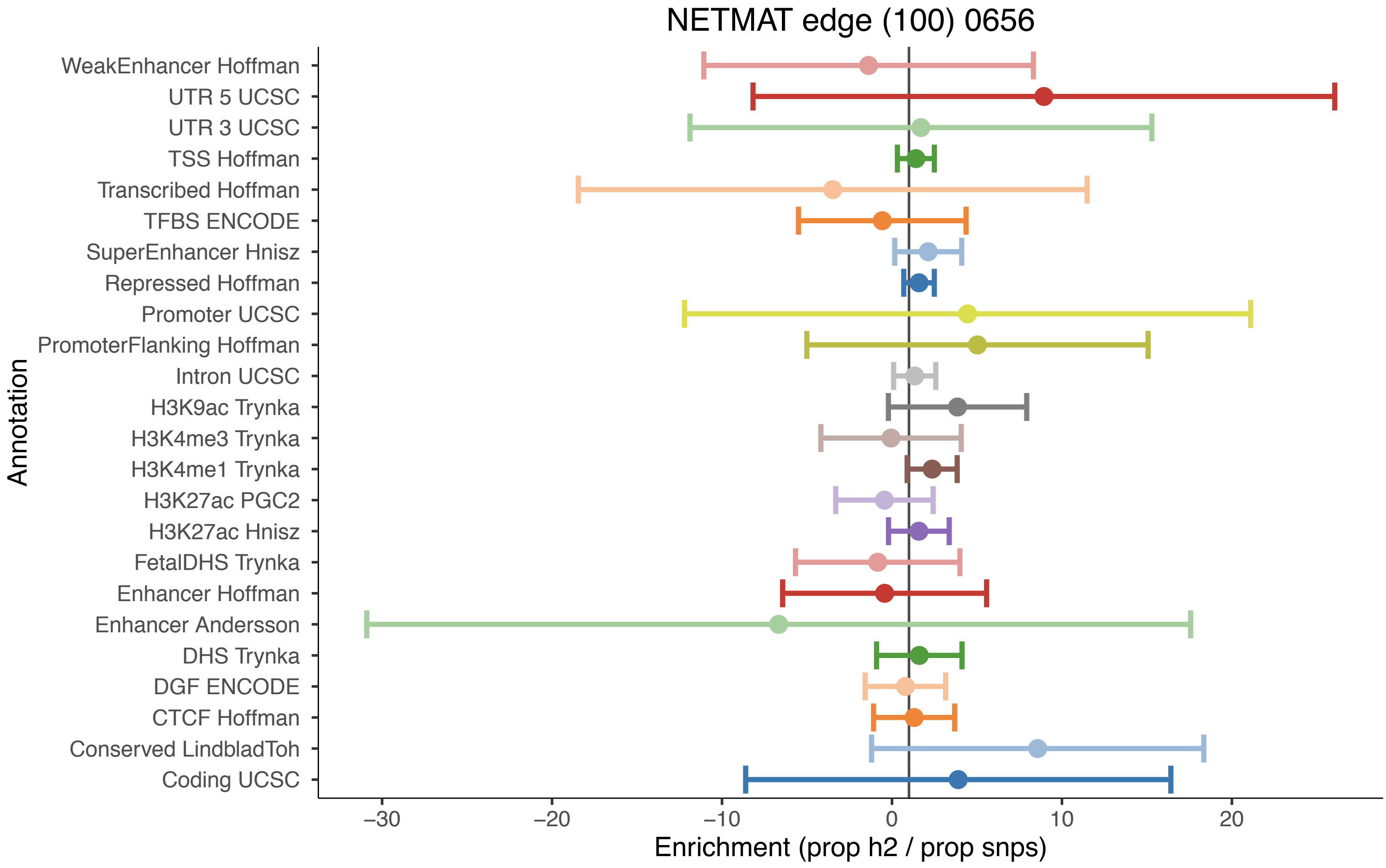
NETMAT edge (100) 0656
WeakEnhancer Hoffman
UTR 5 UCSC
UTR 3 UCSC
TSS Hoffman
Transcribed Hoffman
TFBS ENCODE
SuperEnhancer Hnisz
Repressed Hoffman
Promoter UCSC
PromoterFlanking Hoffman
Intron UCSC
H3K9ac Trynka
H3K4me3 Trynka
H3K4me1 Trynka
H3K27ac PGC2
H3K27ac Hnisz
FetalDHS Trynka
Enhancer Hoffman
Enhancer Andersson
DHS Trynka
DGF ENCODE
CTCF Hoffman
Conserved LindbladToh
Coding UCSC
Annotation
−30
−20
−10
0
10
20
Enrichment (prop h2 / prop snps)

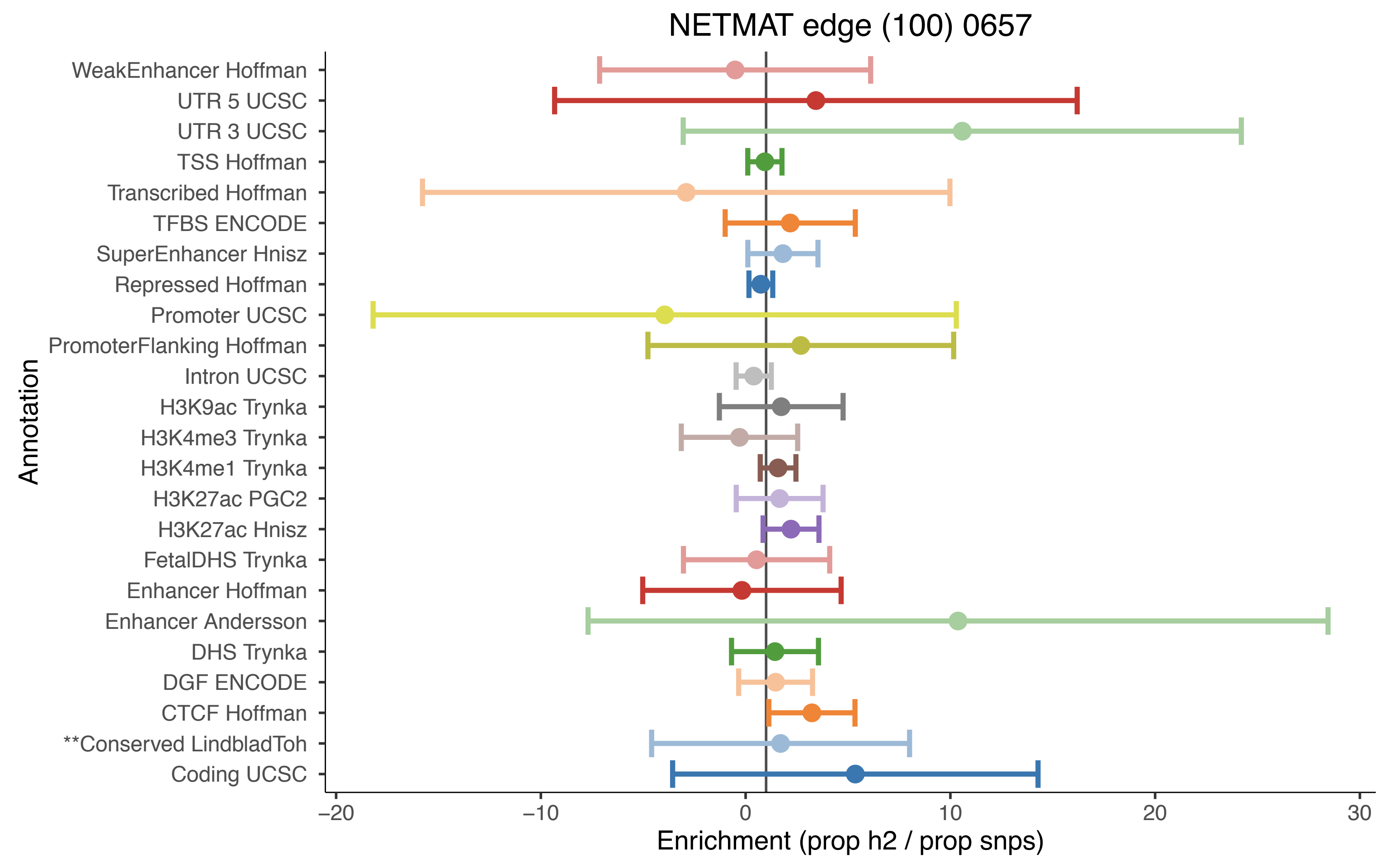
NETMAT edge (100) 0657
WeakEnhancer Hoffman
UTR 5 UCSC
UTR 3 UCSC
TSS Hoffman
Transcribed Hoffman
TFBS ENCODE
SuperEnhancer Hnisz
Repressed Hoffman
Promoter UCSC
PromoterFlanking Hoffman
Intron UCSC
H3K9ac Trynka
H3K4me3 Trynka
H3K4me1 Trynka
H3K27ac PGC2
H3K27ac Hnisz
FetalDHS Trynka
Enhancer Hoffman
Enhancer Andersson
DHS Trynka
DGF ENCODE
CTCF Hoffman
**Conserved LindbladToh
Coding UCSC
Annotation
−20
−10
0
10
20
30
Enrichment (prop h2 / prop snps)

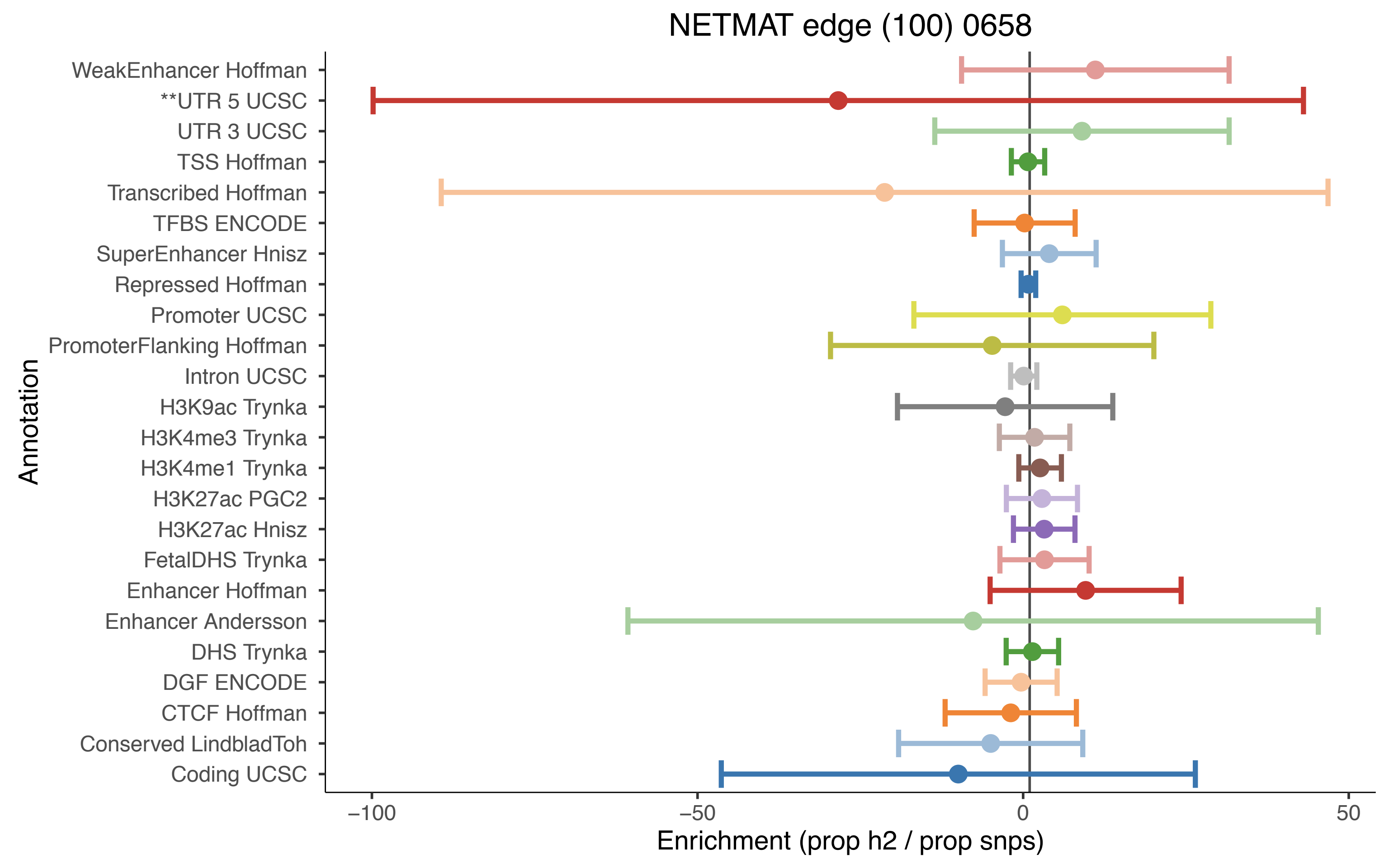

NETMAT edge (100) 0658
WeakEnhancer Hoffman
**UTR 5 UCSC
UTR 3 UCSC
TSS Hoffman
Transcribed Hoffman
TFBS ENCODE
SuperEnhancer Hnisz
Repressed Hoffman
Promoter UCSC
PromoterFlanking Hoffman
Intron UCSC
H3K9ac Trynka
H3K4me3 Trynka
H3K4me1 Trynka
H3K27ac PGC2
H3K27ac Hnisz
FetalDHS Trynka
Enhancer Hoffman
Enhancer Andersson
DHS Trynka
DGF ENCODE
CTCF Hoffman
Conserved LindbladToh
Coding UCSC
Annotation
−100
−50
0
50
Enrichment (prop h2 / prop snps)

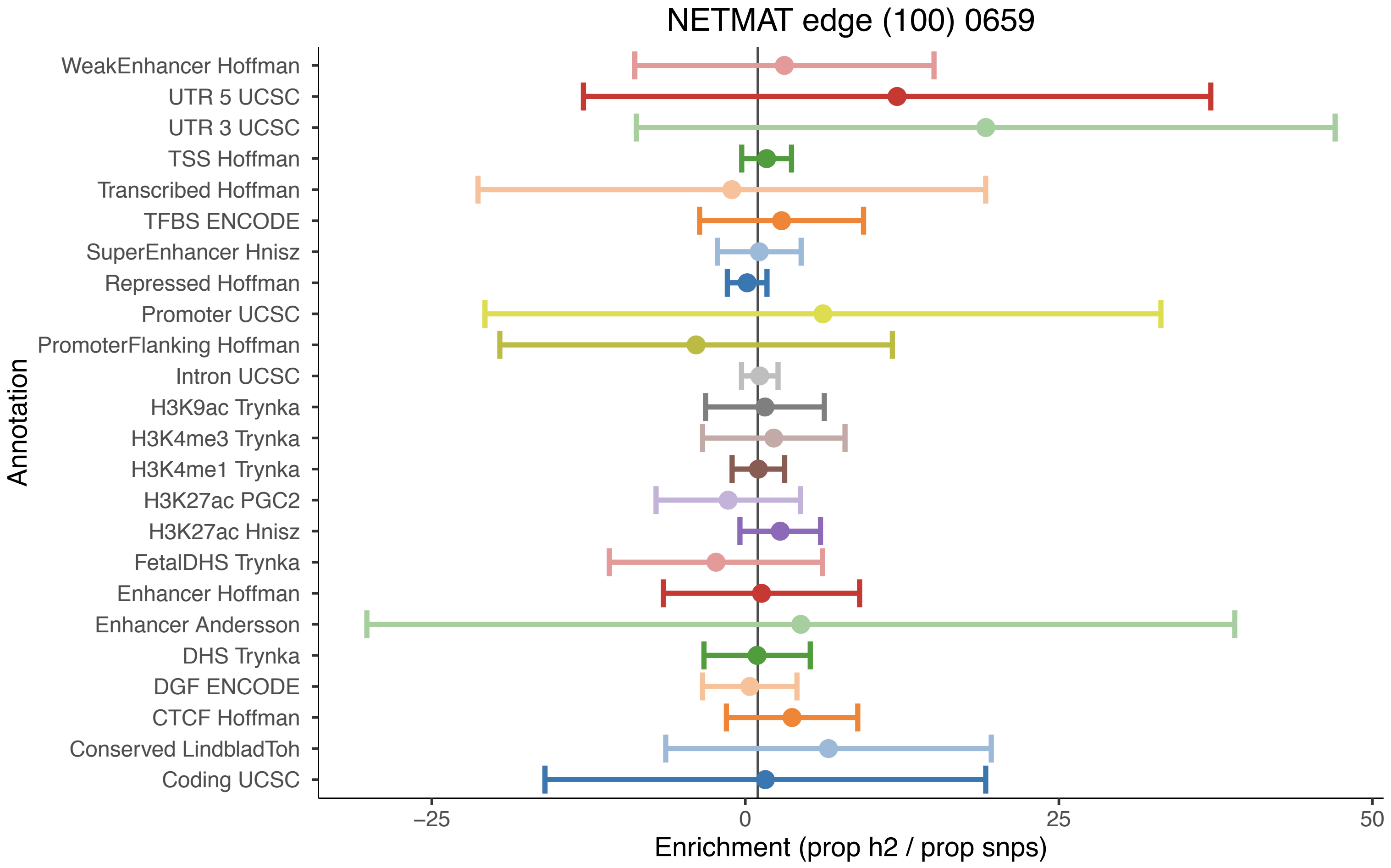

NETMAT edge (100) 0659
Annotation
WeakEnhancer Hoffman
UTR 5 UCSC
UTR 3 UCSC
TSS Hoffman
Transcribed Hoffman
TFBS ENCODE
SuperEnhancer Hnisz
Repressed Hoffman
Promoter UCSC
PromoterFlanking Hoffman
Intron UCSC
H3K9ac Trynka
H3K4me3 Trynka
H3K4me1 Trynka
H3K27ac PGC2
H3K27ac Hnisz
FetalDHS Trynka
Enhancer Hoffman
Enhancer Andersson
DHS Trynka
DGF ENCODE
CTCF Hoffman
Conserved LindbladToh
Coding UCSC
−25
0
25
50
Enrichment (prop h2 / prop snps)

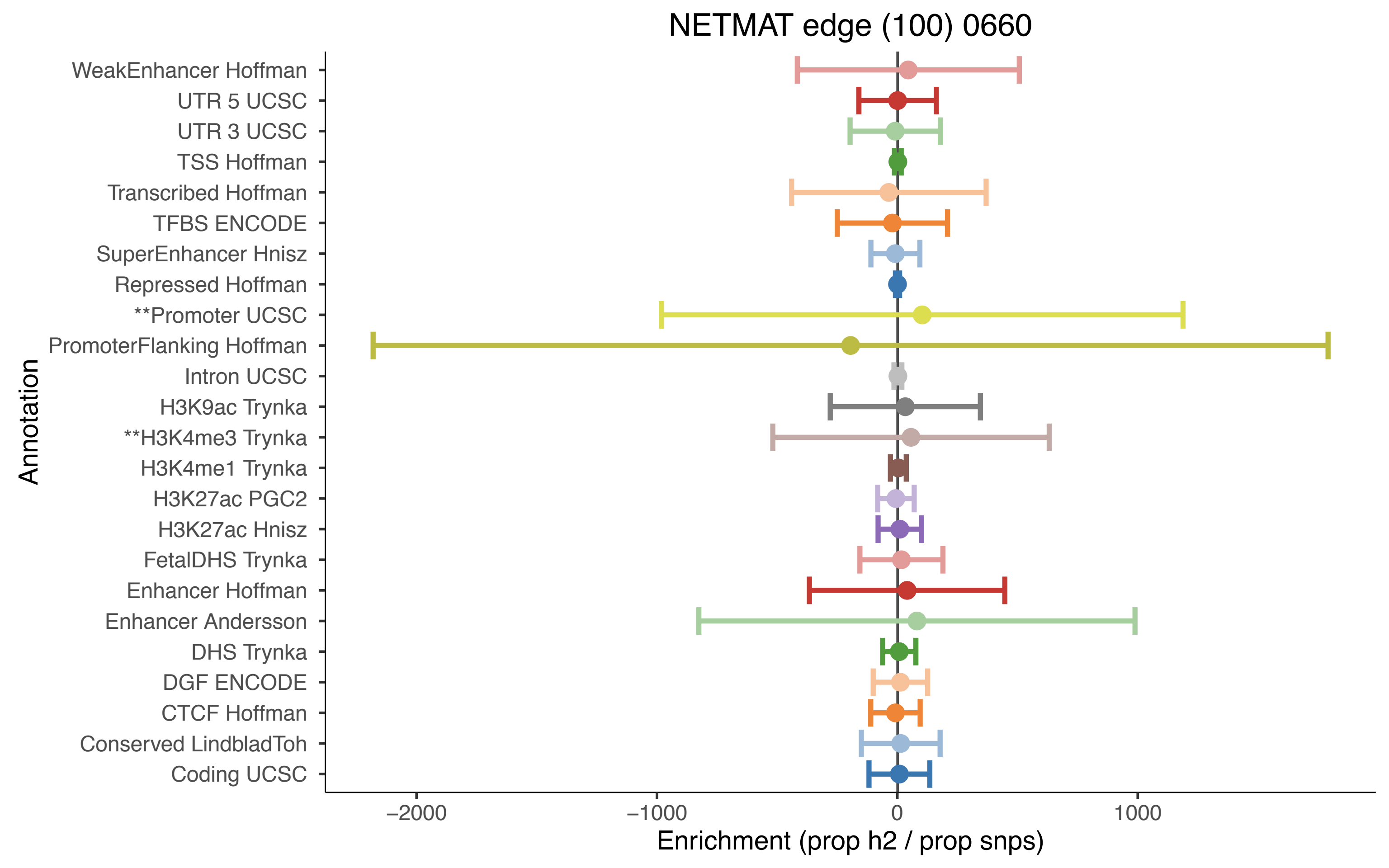

NETMAT edge (100) 0660
WeakEnhancer Hoffman
UTR 5 UCSC
UTR 3 UCSC
TSS Hoffman
Transcribed Hoffman
TFBS ENCODE
SuperEnhancer Hnisz
Repressed Hoffman
**Promoter UCSC
PromoterFlanking Hoffman
Intron UCSC
H3K9ac Trynka
**H3K4me3 Trynka
H3K4me1 Trynka
H3K27ac PGC2
H3K27ac Hnisz
FetalDHS Trynka
Enhancer Hoffman
Enhancer Andersson
DHS Trynka
DGF ENCODE
CTCF Hoffman
Conserved LindbladToh
Coding UCSC
Annotation
−2000
−1000
0
1000
Enrichment (prop h2 / prop snps)

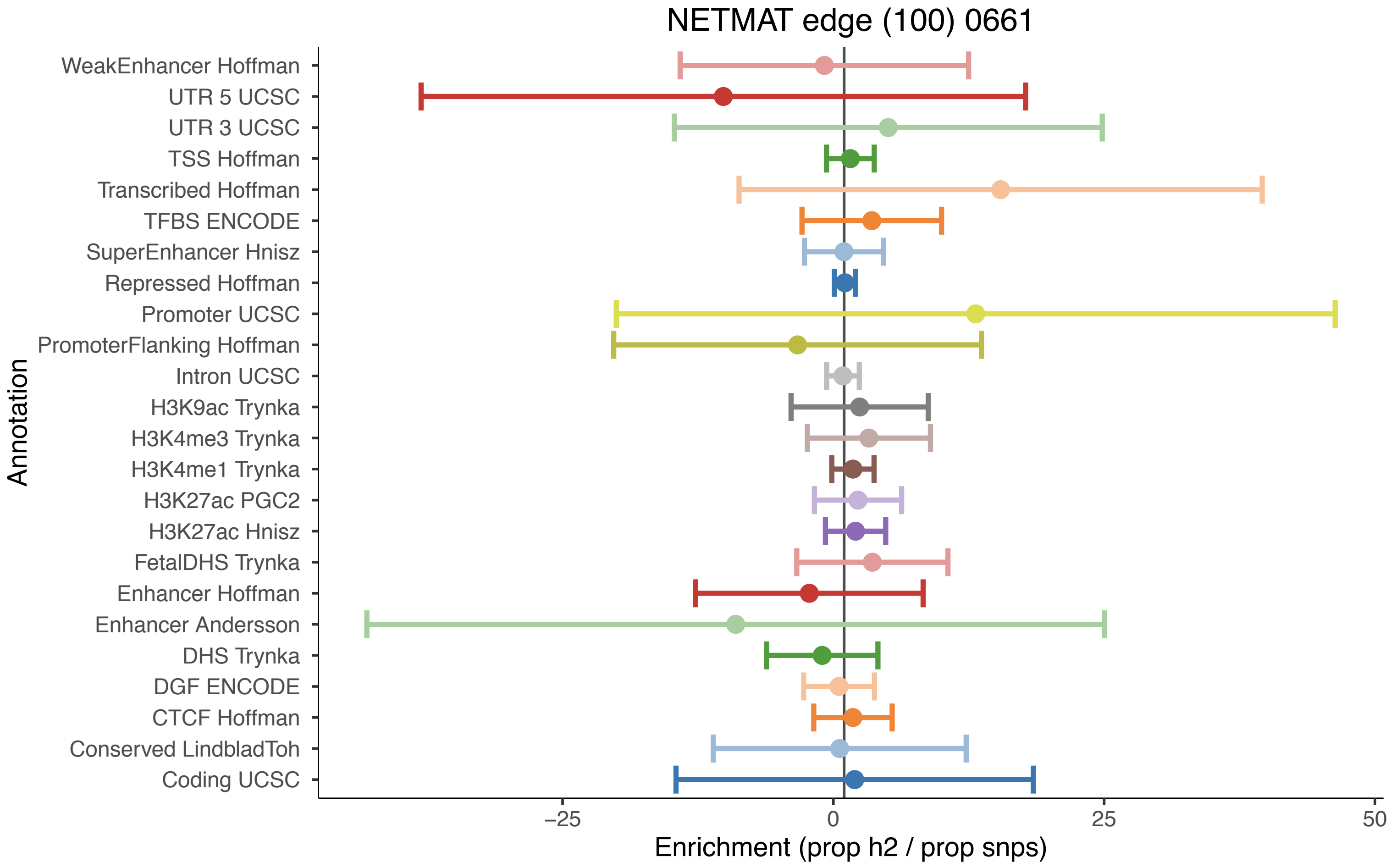

NETMAT edge (100) 0661
WeakEnhancer Hoffman
UTR 5 UCSC
UTR 3 UCSC
TSS Hoffman
Transcribed Hoffman
TFBS ENCODE
SuperEnhancer Hnisz
Repressed Hoffman
Promoter UCSC
PromoterFlanking Hoffman
Intron UCSC
H3K9ac Trynka
H3K4me3 Trynka
H3K4me1 Trynka
H3K27ac PGC2
H3K27ac Hnisz
FetalDHS Trynka
Enhancer Hoffman
Enhancer Andersson
DHS Trynka
DGF ENCODE
CTCF Hoffman
Conserved LindbladToh
Coding UCSC
Annotation
−25
0
25
50
Enrichment (prop h2 / prop snps)

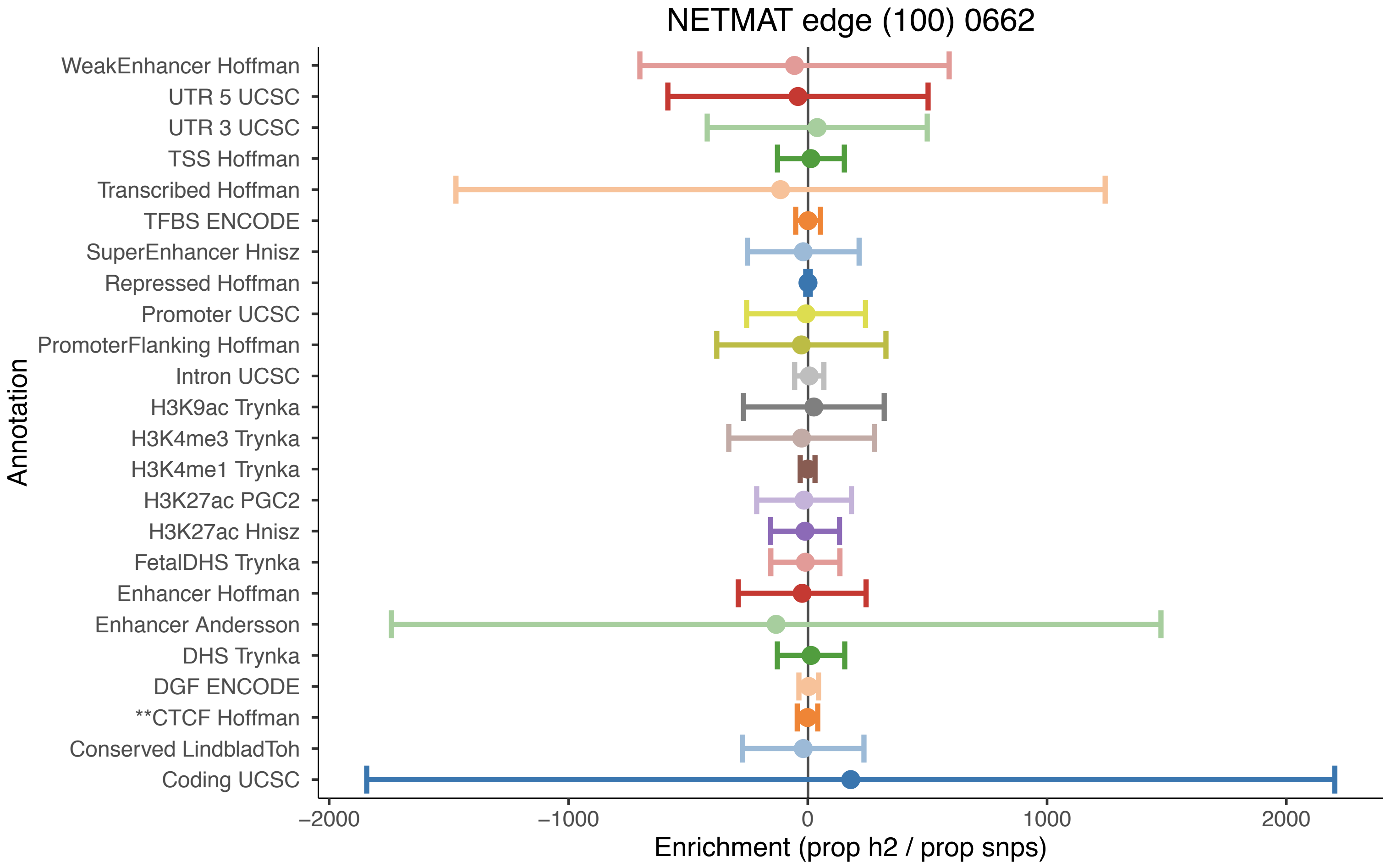
NETMAT edge (100) 0662
WeakEnhancer Hoffman
UTR 5 UCSC
UTR 3 UCSC
TSS Hoffman
Transcribed Hoffman
TFBS ENCODE
SuperEnhancer Hnisz
Repressed Hoffman
Promoter UCSC
PromoterFlanking Hoffman
Intron UCSC
H3K9ac Trynka
H3K4me3 Trynka
H3K4me1 Trynka
H3K27ac PGC2
H3K27ac Hnisz
FetalDHS Trynka
Enhancer Hoffman
Enhancer Andersson
DHS Trynka
DGF ENCODE
**CTCF Hoffman
Conserved LindbladToh
Coding UCSC
Annotation
−2000
−1000
0
1000
2000
Enrichment (prop h2 / prop snps)

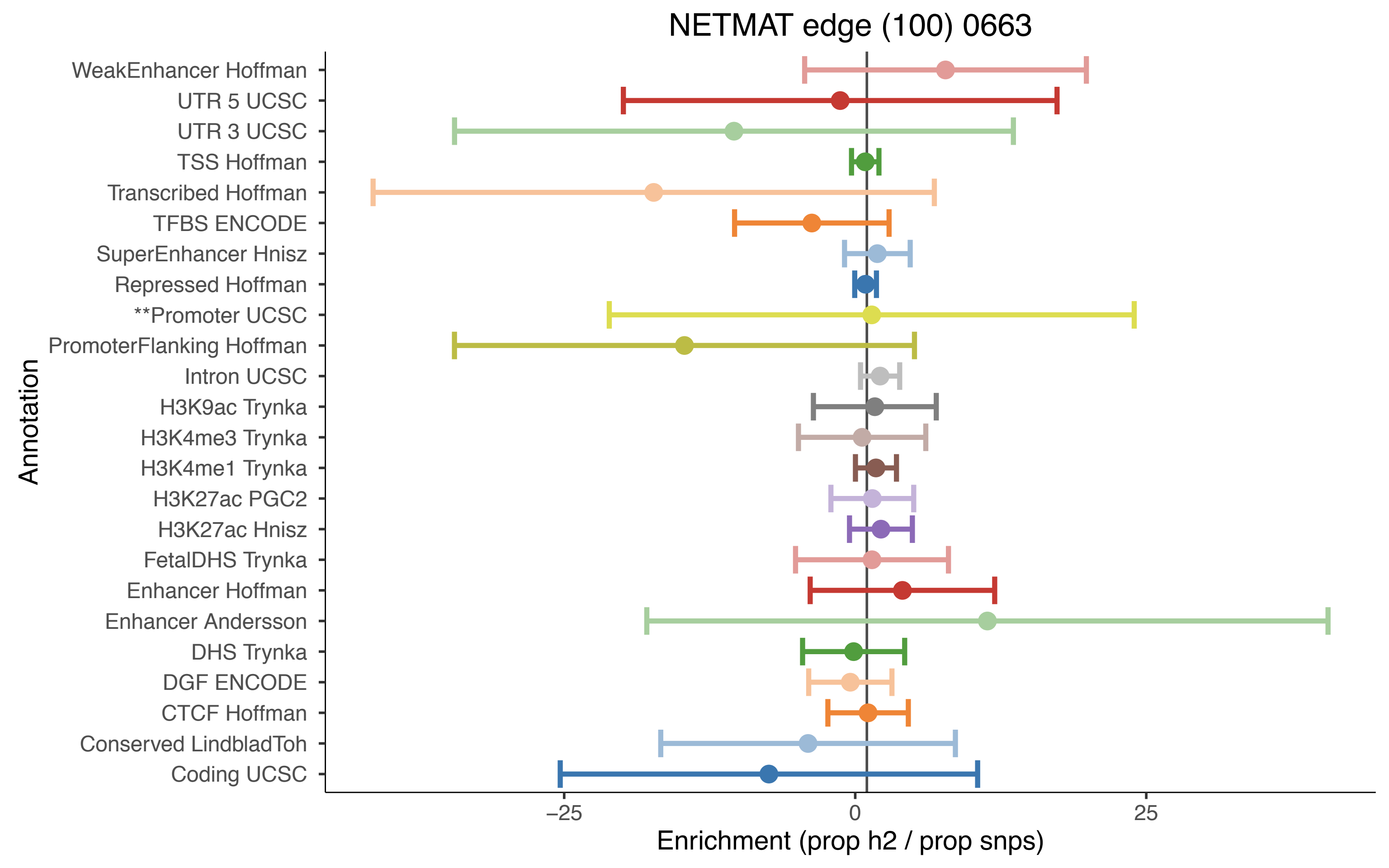

NETMAT edge (100) 0663
WeakEnhancer Hoffman
UTR 5 UCSC
UTR 3 UCSC
TSS Hoffman
Transcribed Hoffman
TFBS ENCODE
SuperEnhancer Hnisz
Repressed Hoffman
**Promoter UCSC
PromoterFlanking Hoffman
Intron UCSC
H3K9ac Trynka
H3K4me3 Trynka
H3K4me1 Trynka
H3K27ac PGC2
H3K27ac Hnisz
FetalDHS Trynka
Enhancer Hoffman
Enhancer Andersson
DHS Trynka
DGF ENCODE
CTCF Hoffman
Conserved LindbladToh
Coding UCSC
Annotation
−25
0
25
Enrichment (prop h2 / prop snps)

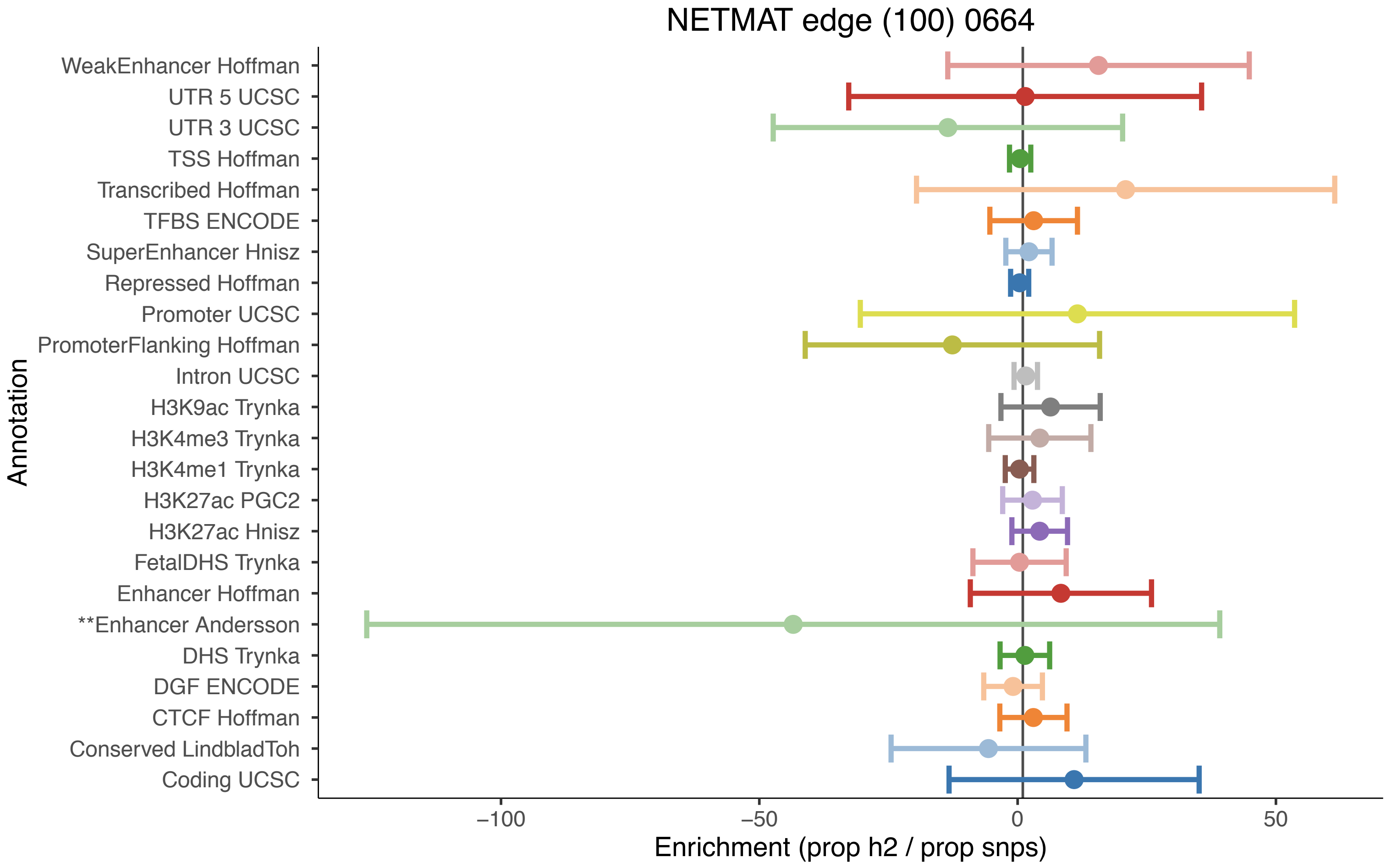
NETMAT edge (100) 0664
WeakEnhancer Hoffman
UTR 5 UCSC
UTR 3 UCSC
TSS Hoffman
Transcribed Hoffman
TFBS ENCODE
SuperEnhancer Hnisz
Repressed Hoffman
Promoter UCSC
PromoterFlanking Hoffman
Intron UCSC
H3K9ac Trynka
H3K4me3 Trynka
H3K4me1 Trynka
H3K27ac PGC2
H3K27ac Hnisz
FetalDHS Trynka
Enhancer Hoffman
**Enhancer Andersson
DHS Trynka
DGF ENCODE
CTCF Hoffman
Conserved LindbladToh
Coding UCSC
Annotation
−100
−50
0
50
Enrichment (prop h2 / prop snps)

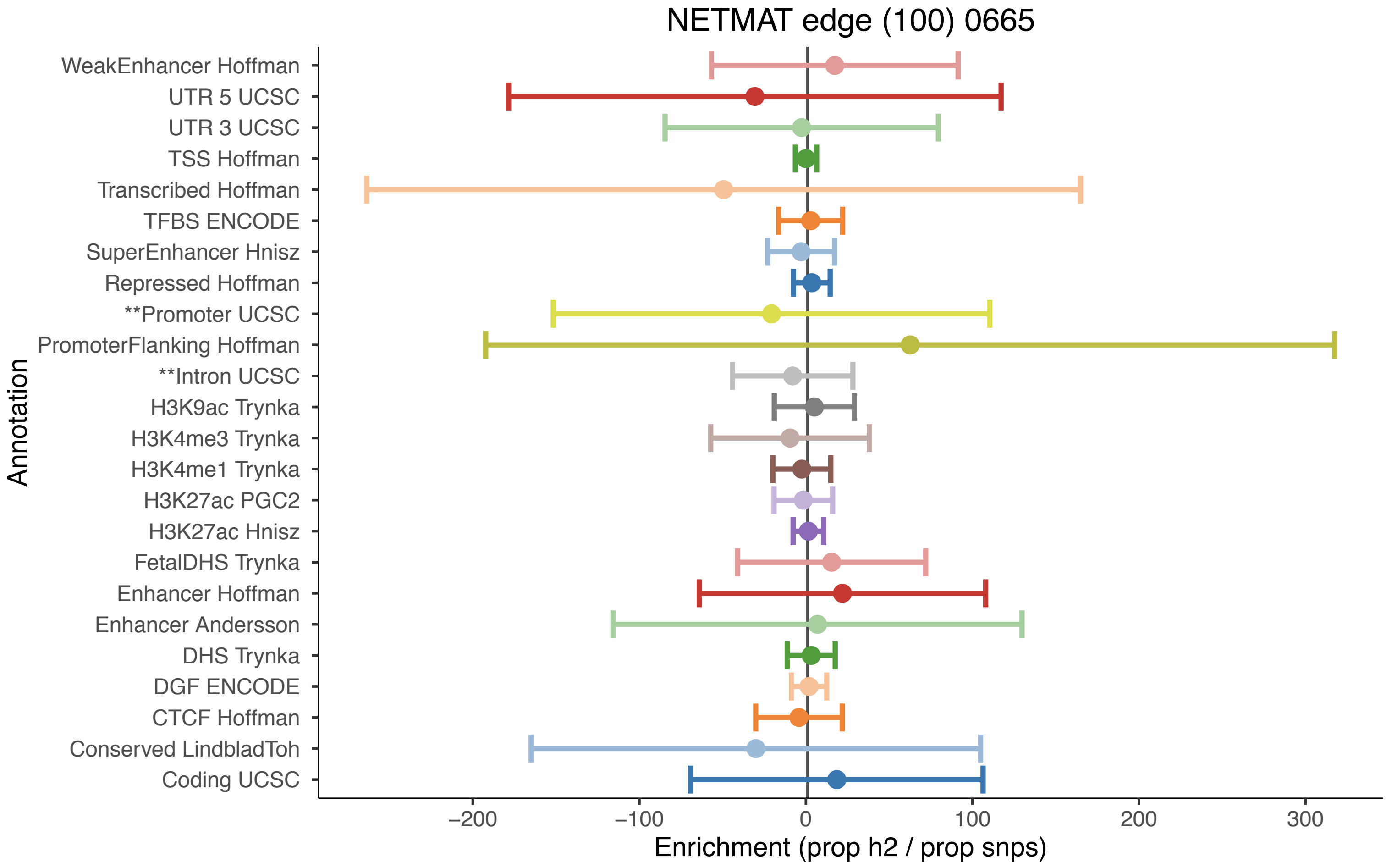
NETMAT edge (100) 0665
WeakEnhancer Hoffman
UTR 5 UCSC
UTR 3 UCSC
TSS Hoffman
Transcribed Hoffman
TFBS ENCODE
SuperEnhancer Hnisz
Repressed Hoffman
**Promoter UCSC
PromoterFlanking Hoffman
**Intron UCSC
H3K9ac Trynka
H3K4me3 Trynka
H3K4me1 Trynka
H3K27ac PGC2
H3K27ac Hnisz
FetalDHS Trynka
Enhancer Hoffman
Enhancer Andersson
DHS Trynka
DGF ENCODE
CTCF Hoffman
Conserved LindbladToh
Coding UCSC
Annotation
−200
−100
0
100
200
300
Enrichment (prop h2 / prop snps)

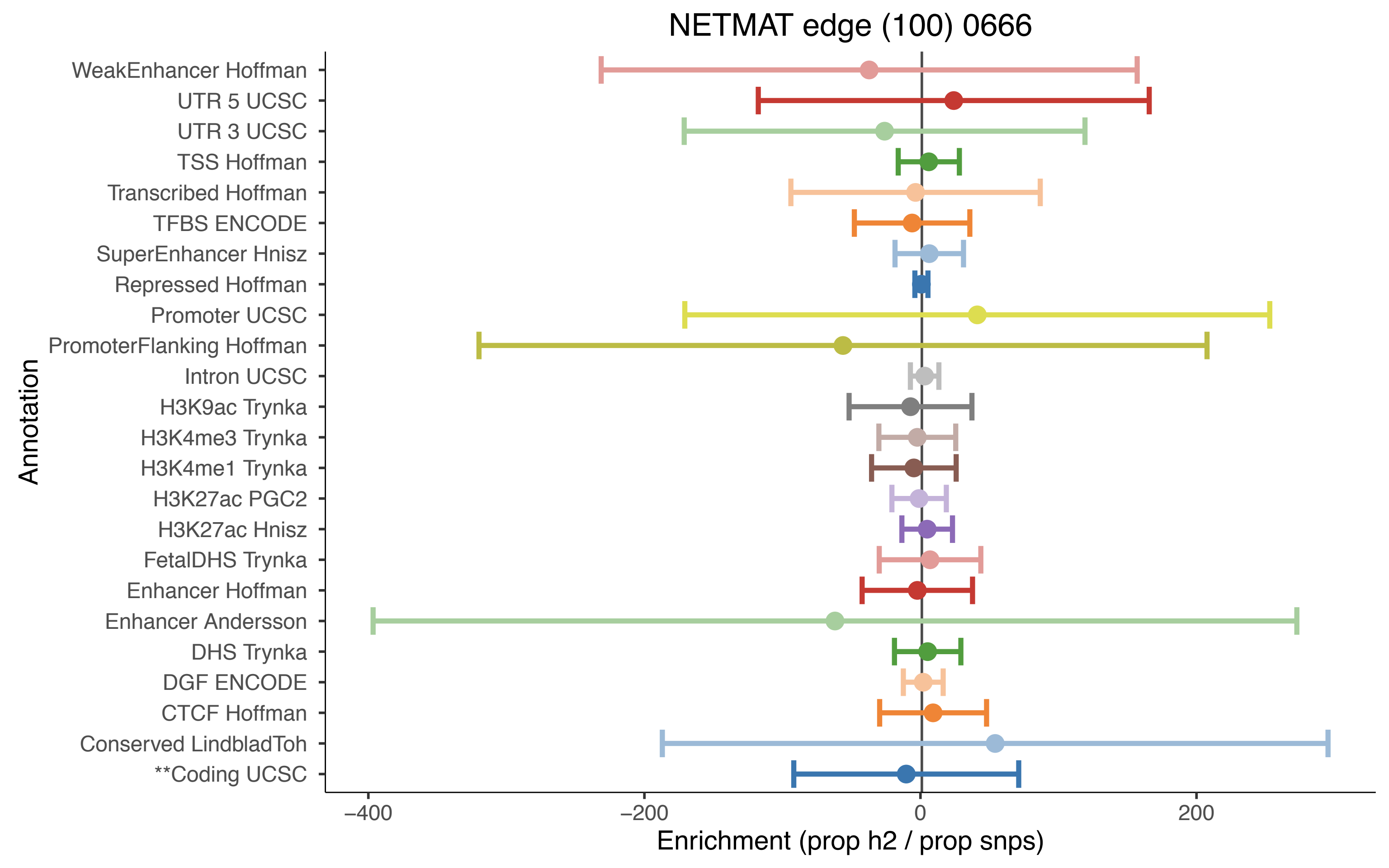

NETMAT edge (100) 0666
WeakEnhancer Hoffman
UTR 5 UCSC
UTR 3 UCSC
TSS Hoffman
Transcribed Hoffman
TFBS ENCODE
SuperEnhancer Hnisz
Repressed Hoffman
Promoter UCSC
PromoterFlanking Hoffman
Intron UCSC
H3K9ac Trynka
H3K4me3 Trynka
H3K4me1 Trynka
H3K27ac PGC2
H3K27ac Hnisz
FetalDHS Trynka
Enhancer Hoffman
Enhancer Andersson
DHS Trynka
DGF ENCODE
CTCF Hoffman
Conserved LindbladToh
**Coding UCSC
Annotation
−400
−200
0
200
Enrichment (prop h2 / prop snps)

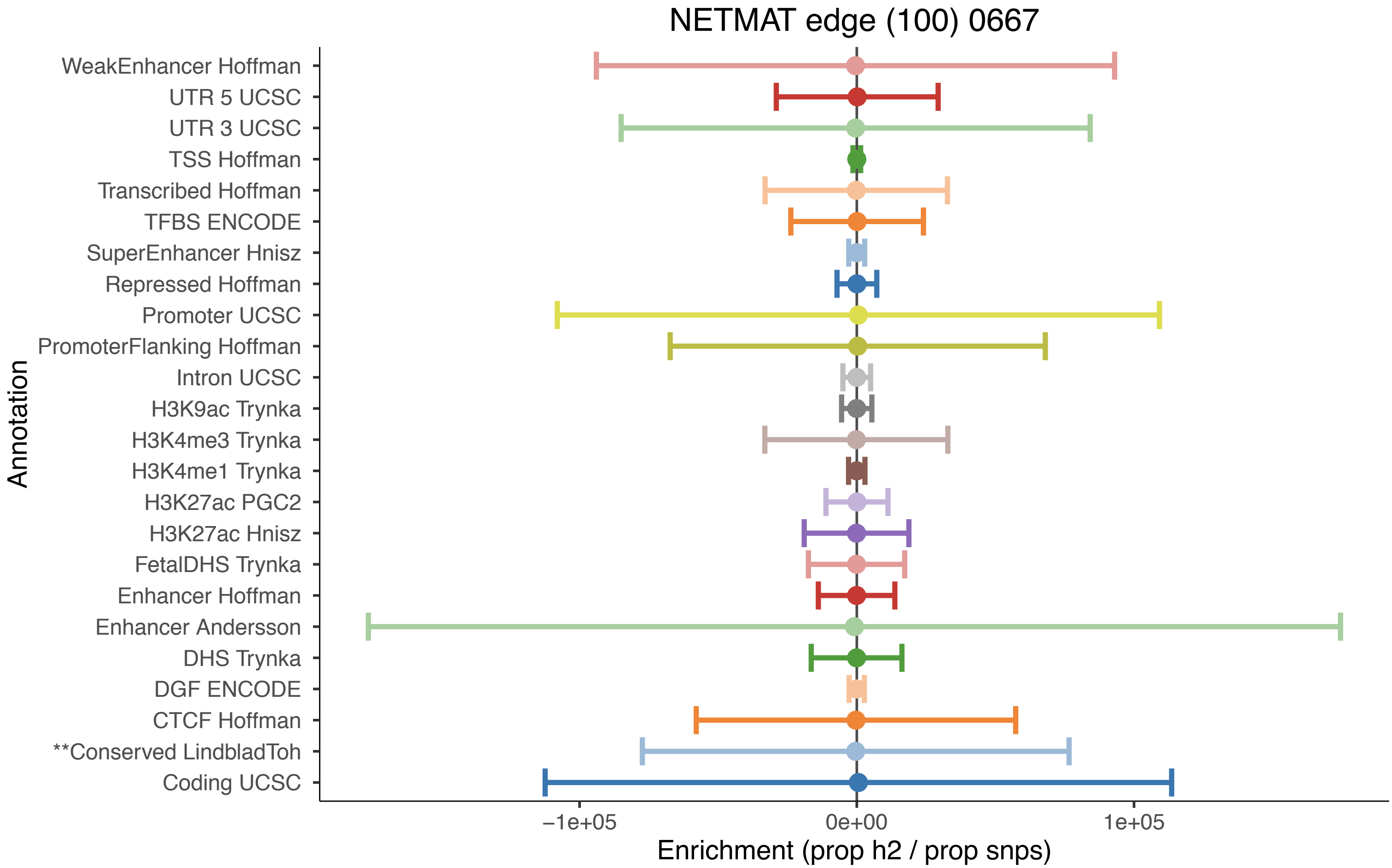
NETMAT edge (100) 0667
Annotation
WeakEnhancer Hoffman
UTR 5 UCSC
UTR 3 UCSC
TSS Hoffman
Transcribed Hoffman
TFBS ENCODE
SuperEnhancer Hnisz
Repressed Hoffman
Promoter UCSC
PromoterFlanking Hoffman
Intron UCSC
H3K9ac Trynka
H3K4me3 Trynka
H3K4me1 Trynka
H3K27ac PGC2
H3K27ac Hnisz
FetalDHS Trynka
Enhancer Hoffman
Enhancer Andersson
DHS Trynka
DGF ENCODE
CTCF Hoffman
**Conserved LindbladToh
Coding UCSC
−1e+05
0e+00
1e+05
Enrichment (prop h2 / prop snps)

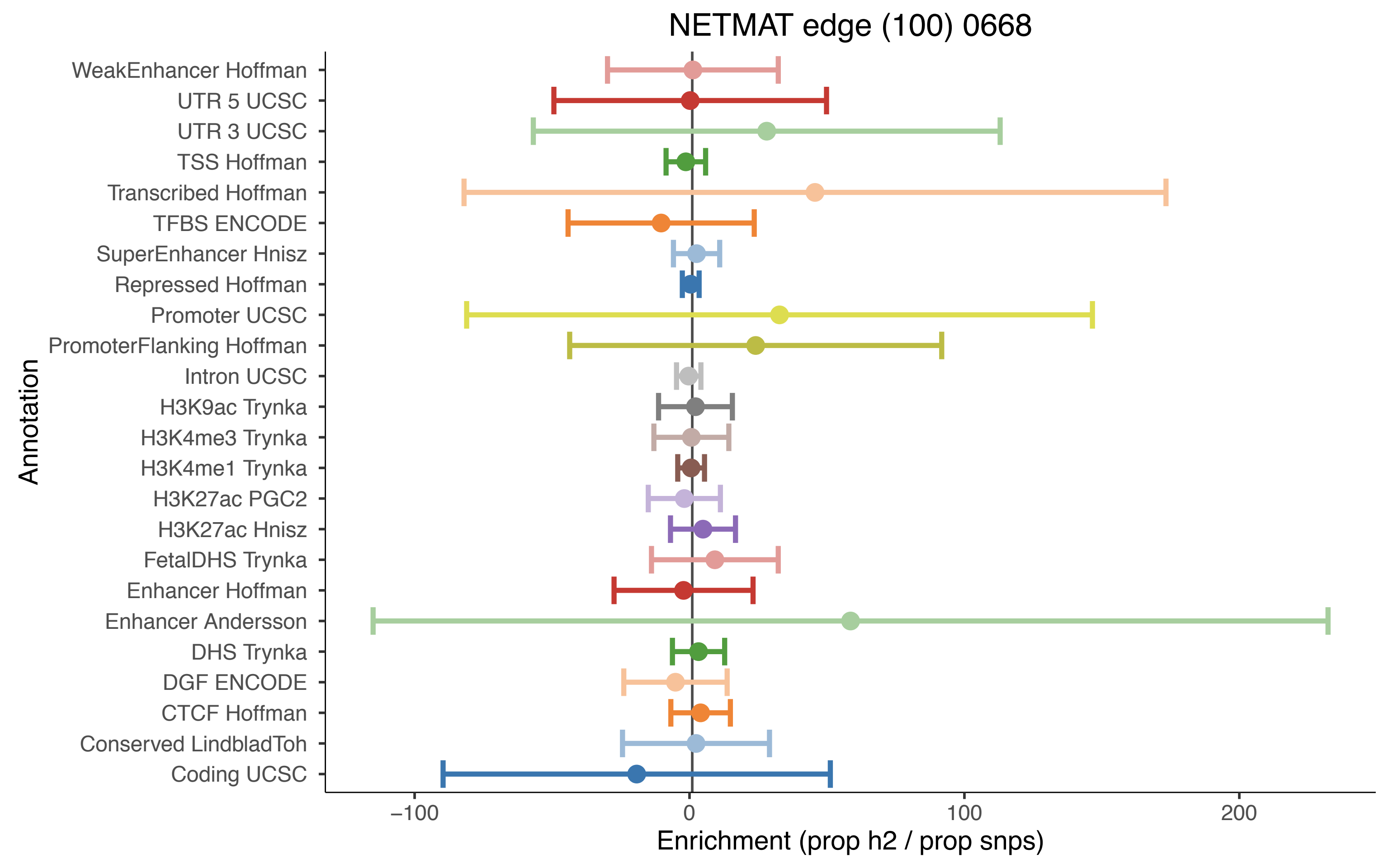
NETMAT edge (100) 0668
WeakEnhancer Hoffman
UTR 5 UCSC
UTR 3 UCSC
TSS Hoffman
Transcribed Hoffman
TFBS ENCODE
SuperEnhancer Hnisz
Repressed Hoffman
Promoter UCSC
PromoterFlanking Hoffman
Intron UCSC
H3K9ac Trynka
H3K4me3 Trynka
H3K4me1 Trynka
H3K27ac PGC2
H3K27ac Hnisz
FetalDHS Trynka
Enhancer Hoffman
Enhancer Andersson
DHS Trynka
DGF ENCODE
CTCF Hoffman
Conserved LindbladToh
Coding UCSC
Annotation
−100
0
100
200
Enrichment (prop h2 / prop snps)

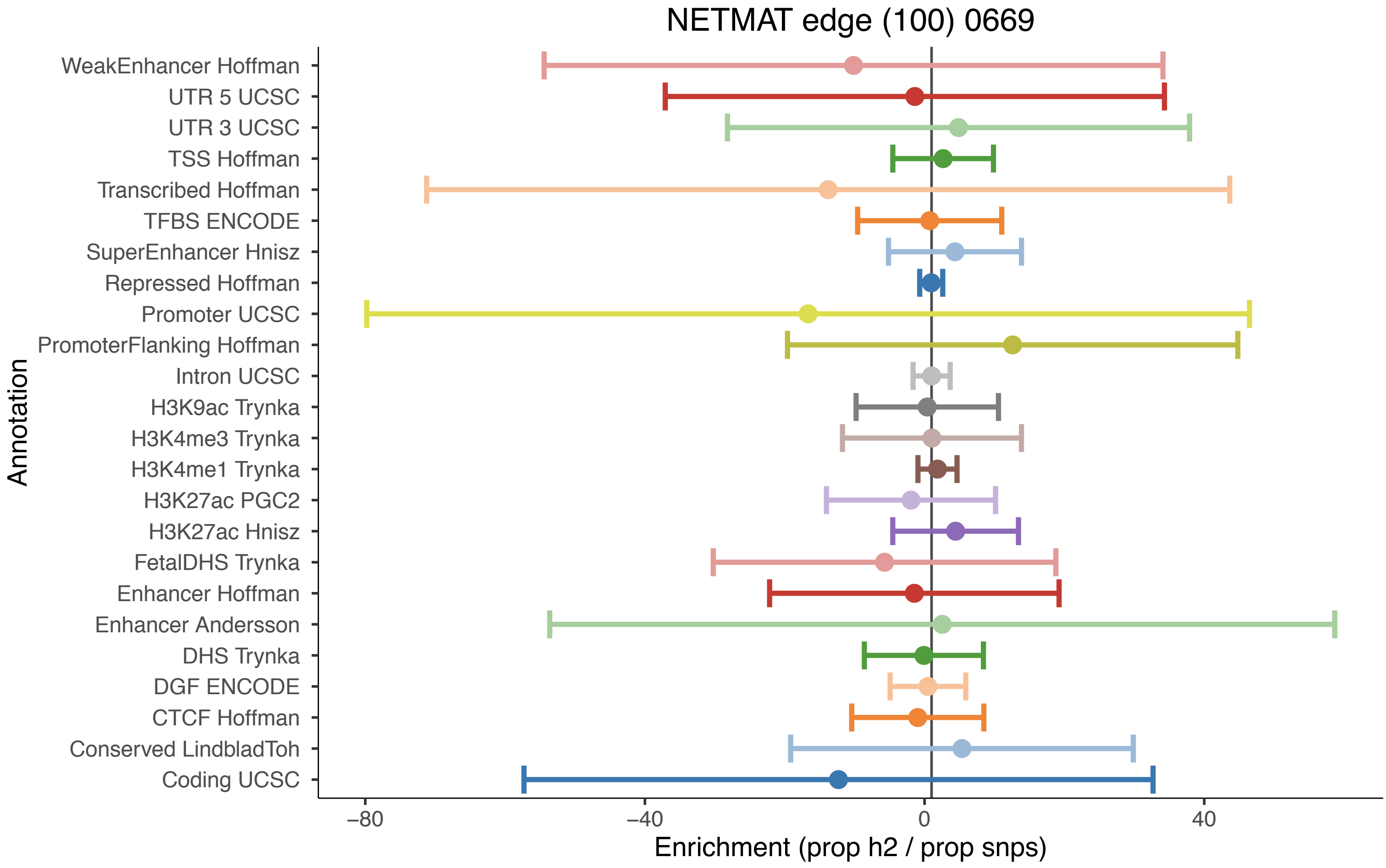
NETMAT edge (100) 0669
WeakEnhancer Hoffman
UTR 5 UCSC
UTR 3 UCSC
TSS Hoffman
Transcribed Hoffman
TFBS ENCODE
SuperEnhancer Hnisz
Repressed Hoffman
Promoter UCSC
PromoterFlanking Hoffman
Intron UCSC
H3K9ac Trynka
H3K4me3 Trynka
H3K4me1 Trynka
H3K27ac PGC2
H3K27ac Hnisz
FetalDHS Trynka
Enhancer Hoffman
Enhancer Andersson
DHS Trynka
DGF ENCODE
CTCF Hoffman
Conserved LindbladToh
Coding UCSC
Annotation
−80
−40
0
40
Enrichment (prop h2 / prop snps)

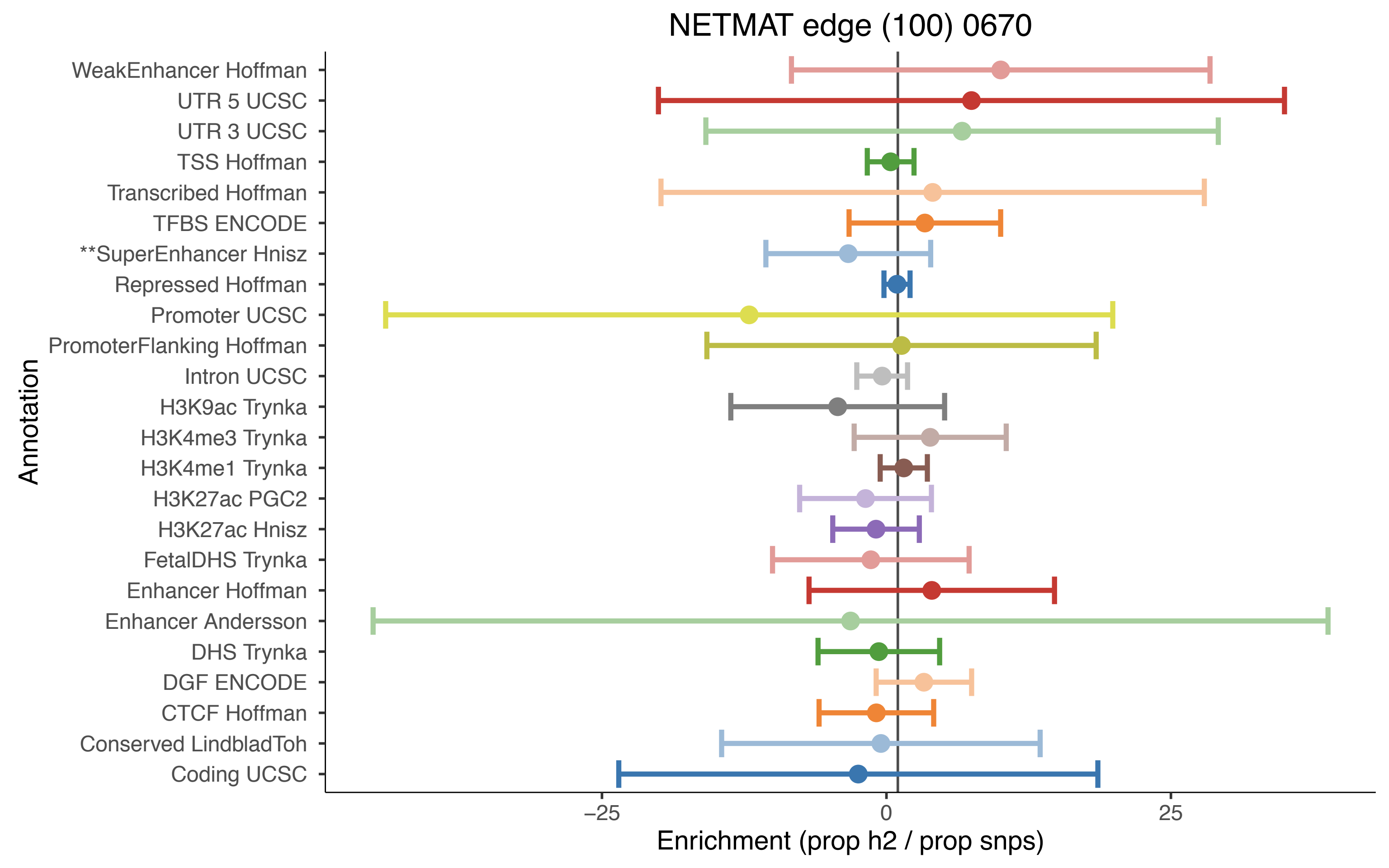
NETMAT edge (100) 0670
WeakEnhancer Hoffman
UTR 5 UCSC
UTR 3 UCSC
TSS Hoffman
Transcribed Hoffman
TFBS ENCODE
**SuperEnhancer Hnisz
Repressed Hoffman
Promoter UCSC
PromoterFlanking Hoffman
Intron UCSC
H3K9ac Trynka
H3K4me3 Trynka
H3K4me1 Trynka
H3K27ac PGC2
H3K27ac Hnisz
FetalDHS Trynka
Enhancer Hoffman
Enhancer Andersson
DHS Trynka
DGF ENCODE
CTCF Hoffman
Conserved LindbladToh
Coding UCSC
Annotation
−25
0
25
Enrichment (prop h2 / prop snps)

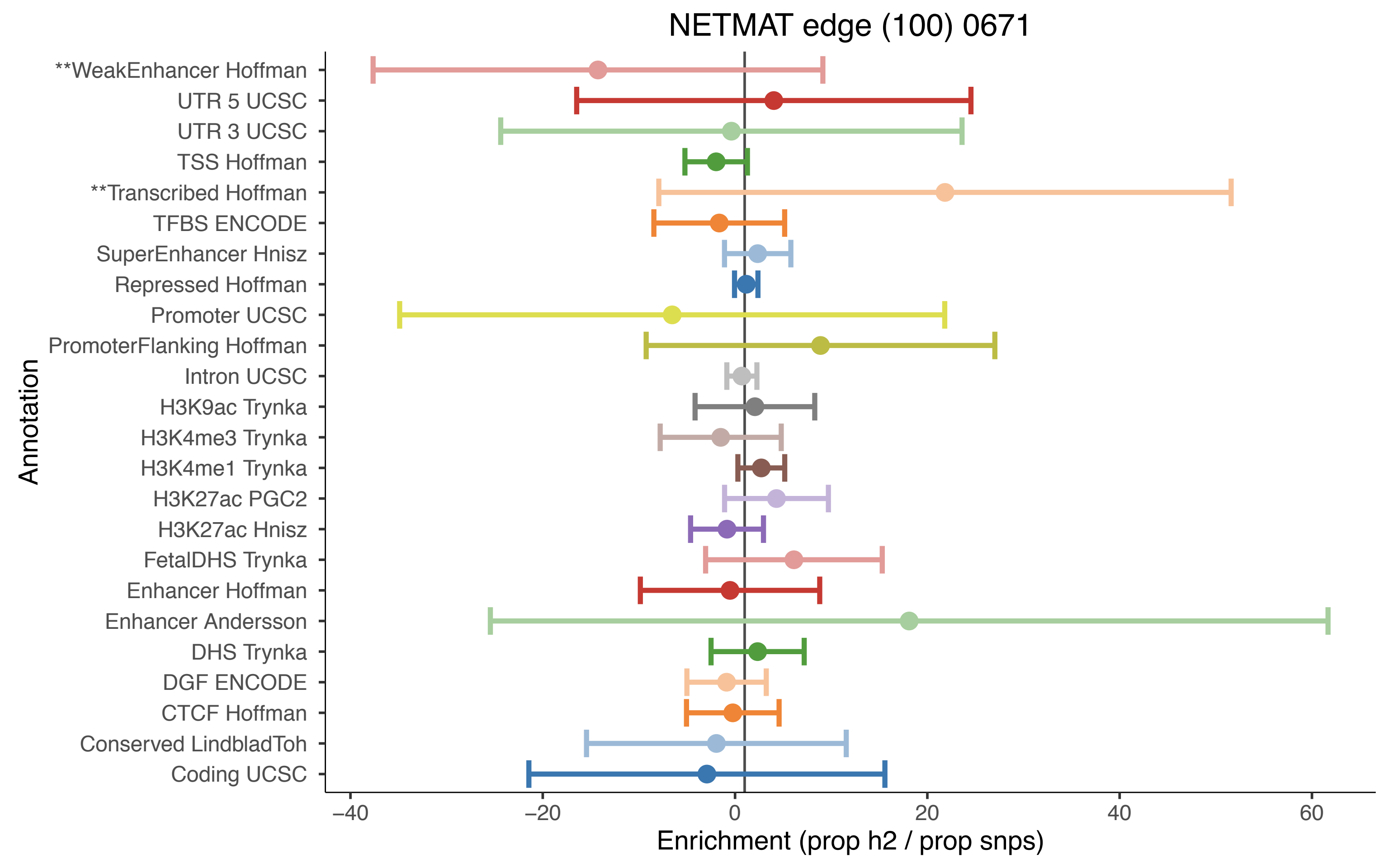
NETMAT edge (100) 0671
**WeakEnhancer Hoffman
UTR 5 UCSC
UTR 3 UCSC
TSS Hoffman
**Transcribed Hoffman
TFBS ENCODE
SuperEnhancer Hnisz
Repressed Hoffman
Promoter UCSC
PromoterFlanking Hoffman
Intron UCSC
H3K9ac Trynka
H3K4me3 Trynka
H3K4me1 Trynka
H3K27ac PGC2
H3K27ac Hnisz
FetalDHS Trynka
Enhancer Hoffman
Enhancer Andersson
DHS Trynka
DGF ENCODE
CTCF Hoffman
Conserved LindbladToh
Coding UCSC
Annotation
−40
−20
0
20
40
60
Enrichment (prop h2 / prop snps)

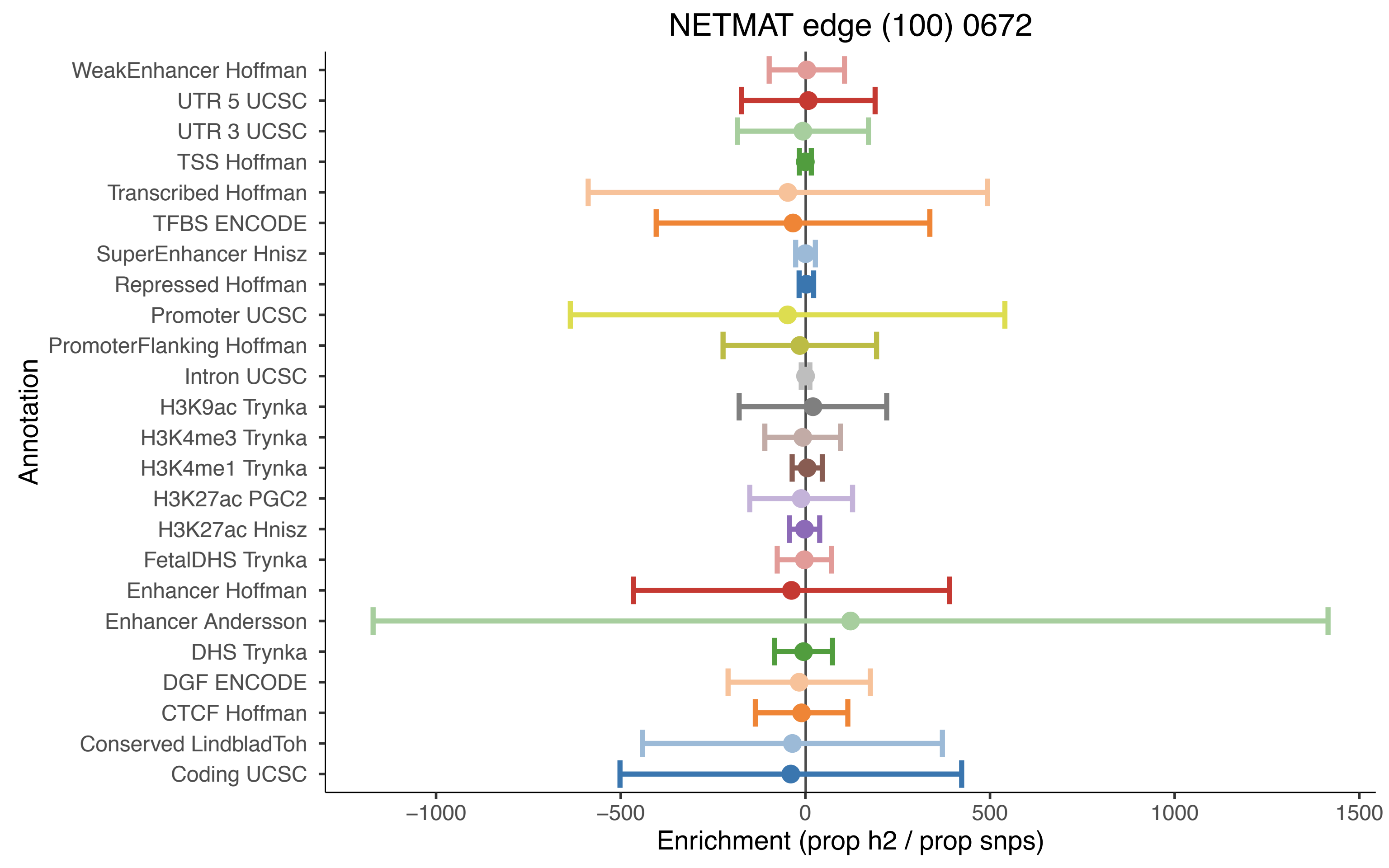
NETMAT edge (100) 0672
WeakEnhancer Hoffman
UTR 5 UCSC
UTR 3 UCSC
TSS Hoffman
Transcribed Hoffman
TFBS ENCODE
SuperEnhancer Hnisz
Repressed Hoffman
Promoter UCSC
PromoterFlanking Hoffman
Intron UCSC
H3K9ac Trynka
H3K4me3 Trynka
H3K4me1 Trynka
H3K27ac PGC2
H3K27ac Hnisz
FetalDHS Trynka
Enhancer Hoffman
Enhancer Andersson
DHS Trynka
DGF ENCODE
CTCF Hoffman
Conserved LindbladToh
Coding UCSC
Annotation
−1000
−500
0
500
1000
1500
Enrichment (prop h2 / prop snps)

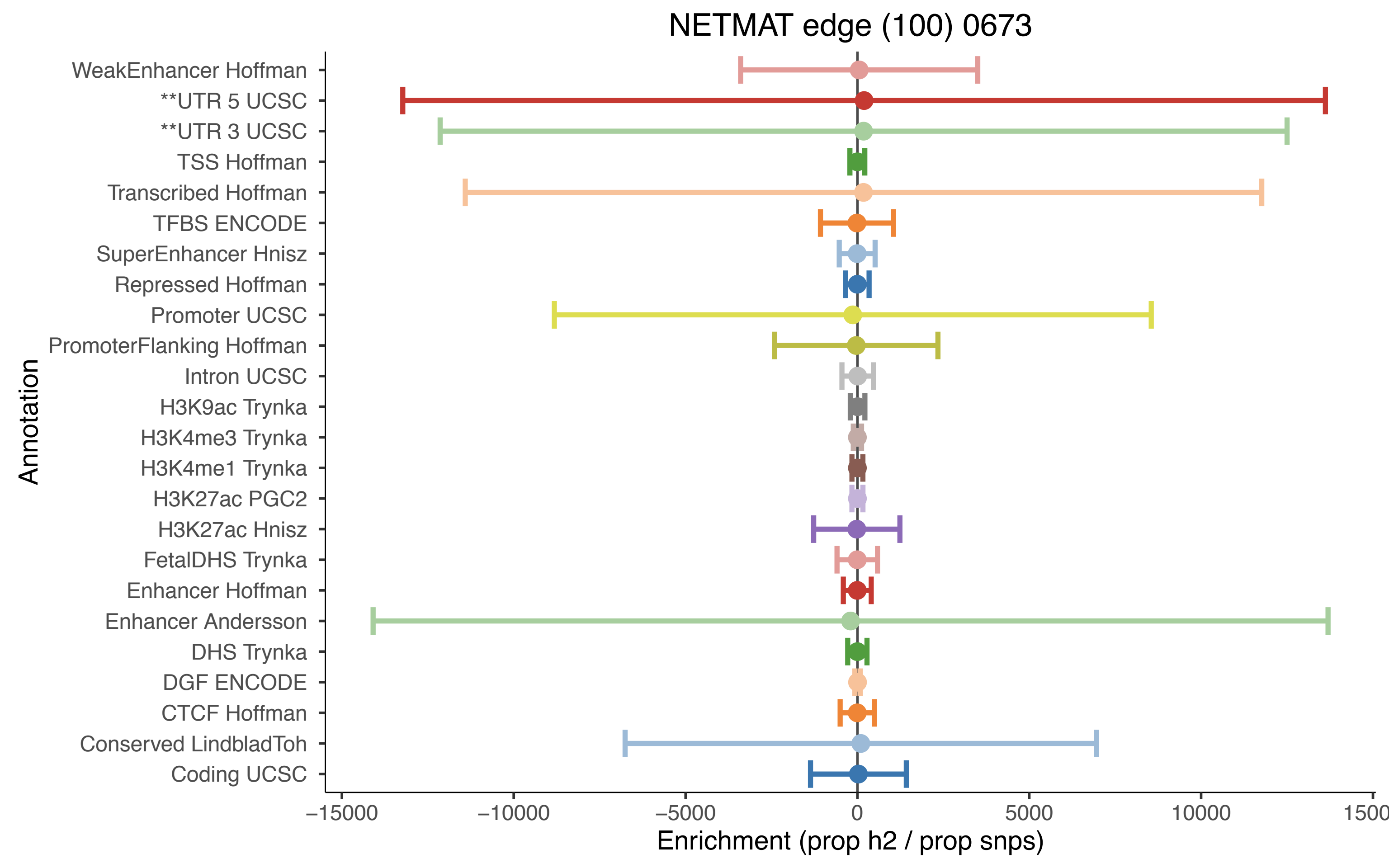

NETMAT edge (100) 0673
WeakEnhancer Hoffman
**UTR 5 UCSC
**UTR 3 UCSC
TSS Hoffman
Transcribed Hoffman
TFBS ENCODE
SuperEnhancer Hnisz
Repressed Hoffman
Promoter UCSC
PromoterFlanking Hoffman
Intron UCSC
H3K9ac Trynka
H3K4me3 Trynka
H3K4me1 Trynka
H3K27ac PGC2
H3K27ac Hnisz
FetalDHS Trynka
Enhancer Hoffman
Enhancer Andersson
DHS Trynka
DGF ENCODE
CTCF Hoffman
Conserved LindbladToh
Coding UCSC
Annotation
−15000
−10000
−5000
0
5000
10000
1500
Enrichment (prop h2 / prop snps)

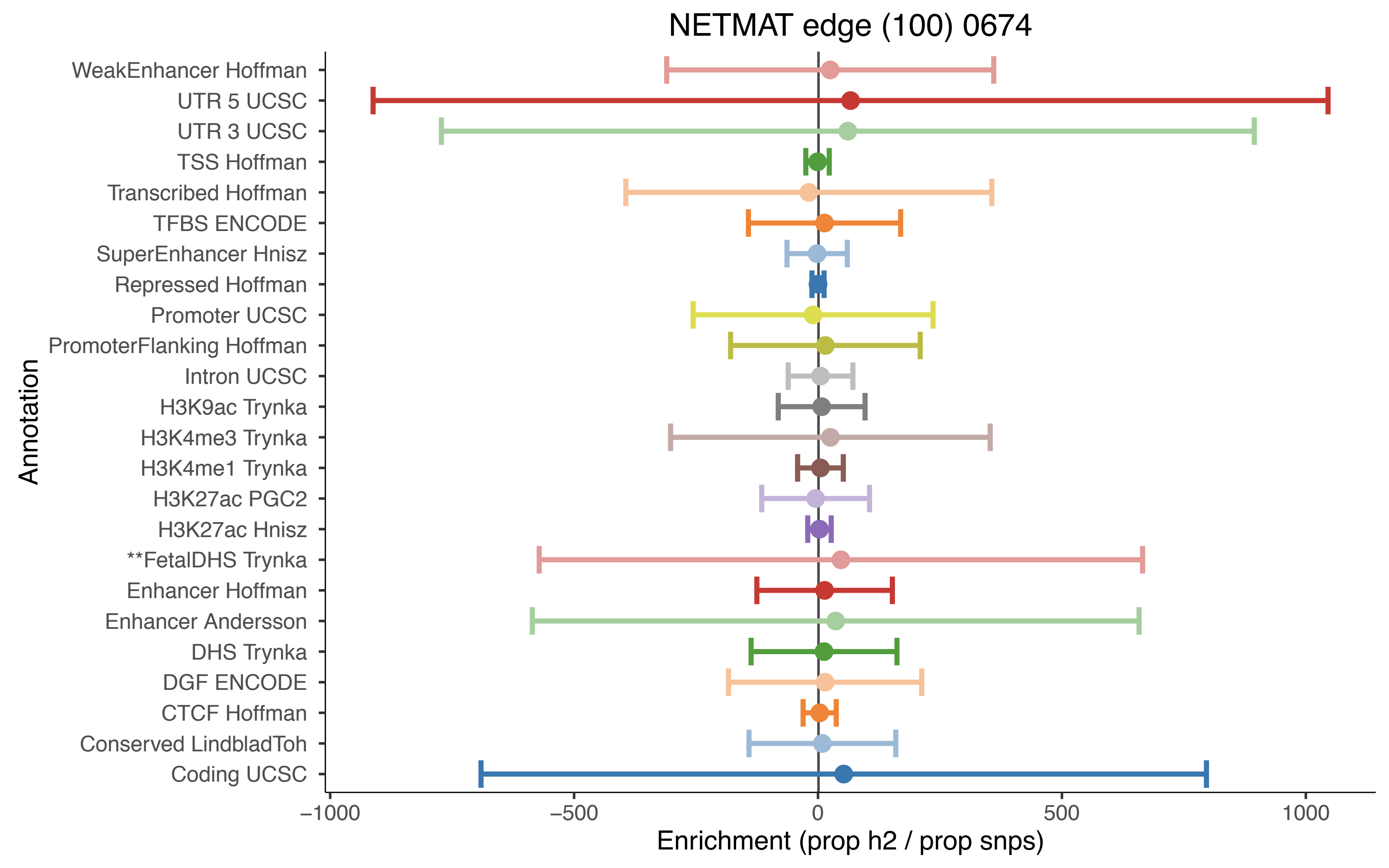

NETMAT edge (100) 0674
WeakEnhancer Hoffman
UTR 5 UCSC
UTR 3 UCSC
TSS Hoffman
Transcribed Hoffman
TFBS ENCODE
SuperEnhancer Hnisz
Repressed Hoffman
Promoter UCSC
PromoterFlanking Hoffman
Intron UCSC
H3K9ac Trynka
H3K4me3 Trynka
H3K4me1 Trynka
H3K27ac PGC2
H3K27ac Hnisz
**FetalDHS Trynka
Enhancer Hoffman
Enhancer Andersson
DHS Trynka
DGF ENCODE
CTCF Hoffman
Conserved LindbladToh
Coding UCSC
Annotation
−1000
−500
0
500
1000
Enrichment (prop h2 / prop snps)

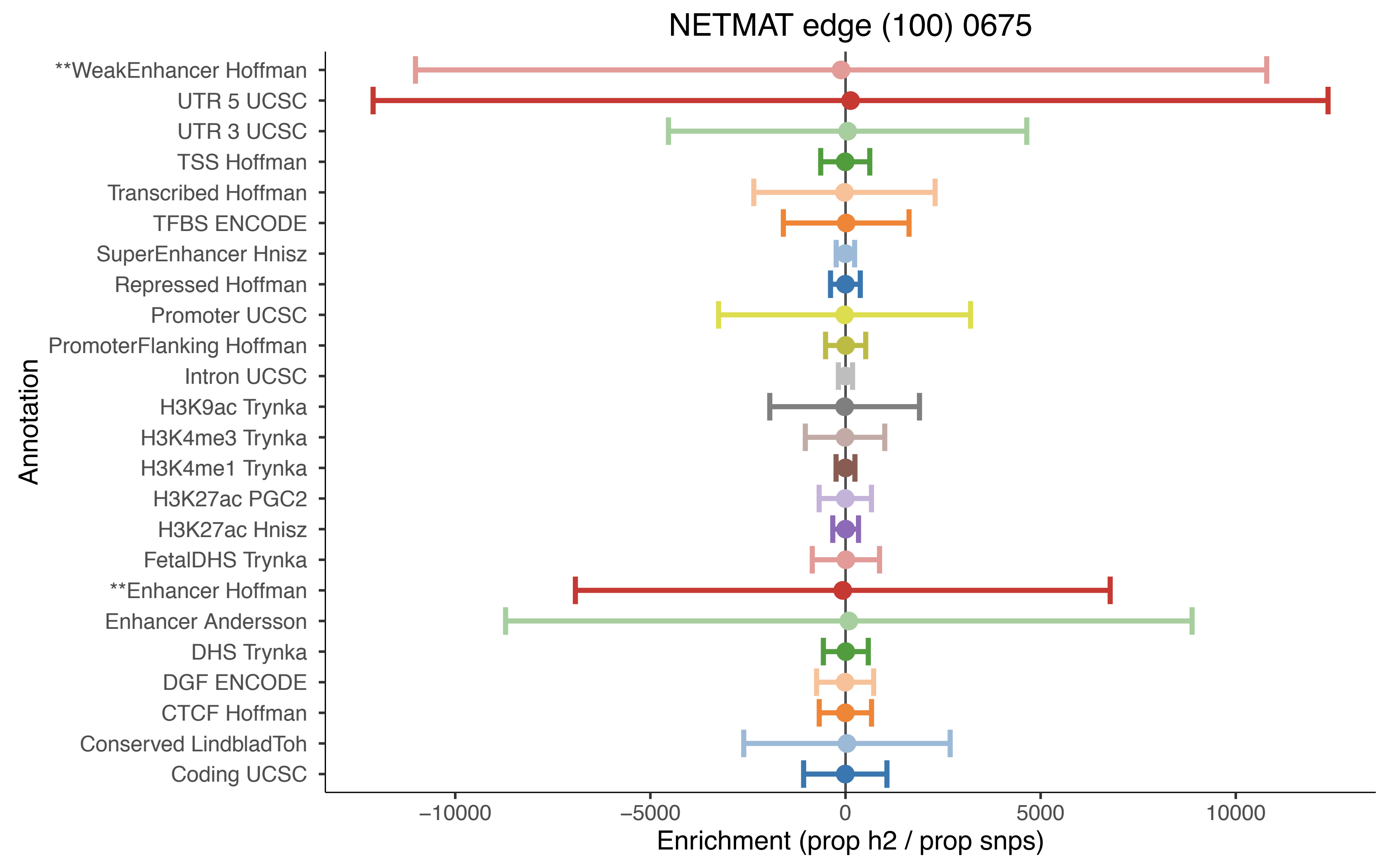

NETMAT edge (100) 0675
**WeakEnhancer Hoffman
UTR 5 UCSC
UTR 3 UCSC
TSS Hoffman
Transcribed Hoffman
TFBS ENCODE
SuperEnhancer Hnisz
Repressed Hoffman
Promoter UCSC
PromoterFlanking Hoffman
Intron UCSC
H3K9ac Trynka
H3K4me3 Trynka
H3K4me1 Trynka
H3K27ac PGC2
H3K27ac Hnisz
FetalDHS Trynka
**Enhancer Hoffman
Enhancer Andersson
DHS Trynka
DGF ENCODE
CTCF Hoffman
Conserved LindbladToh
Coding UCSC
Annotation
−10000
−5000
0
5000
10000
Enrichment (prop h2 / prop snps)

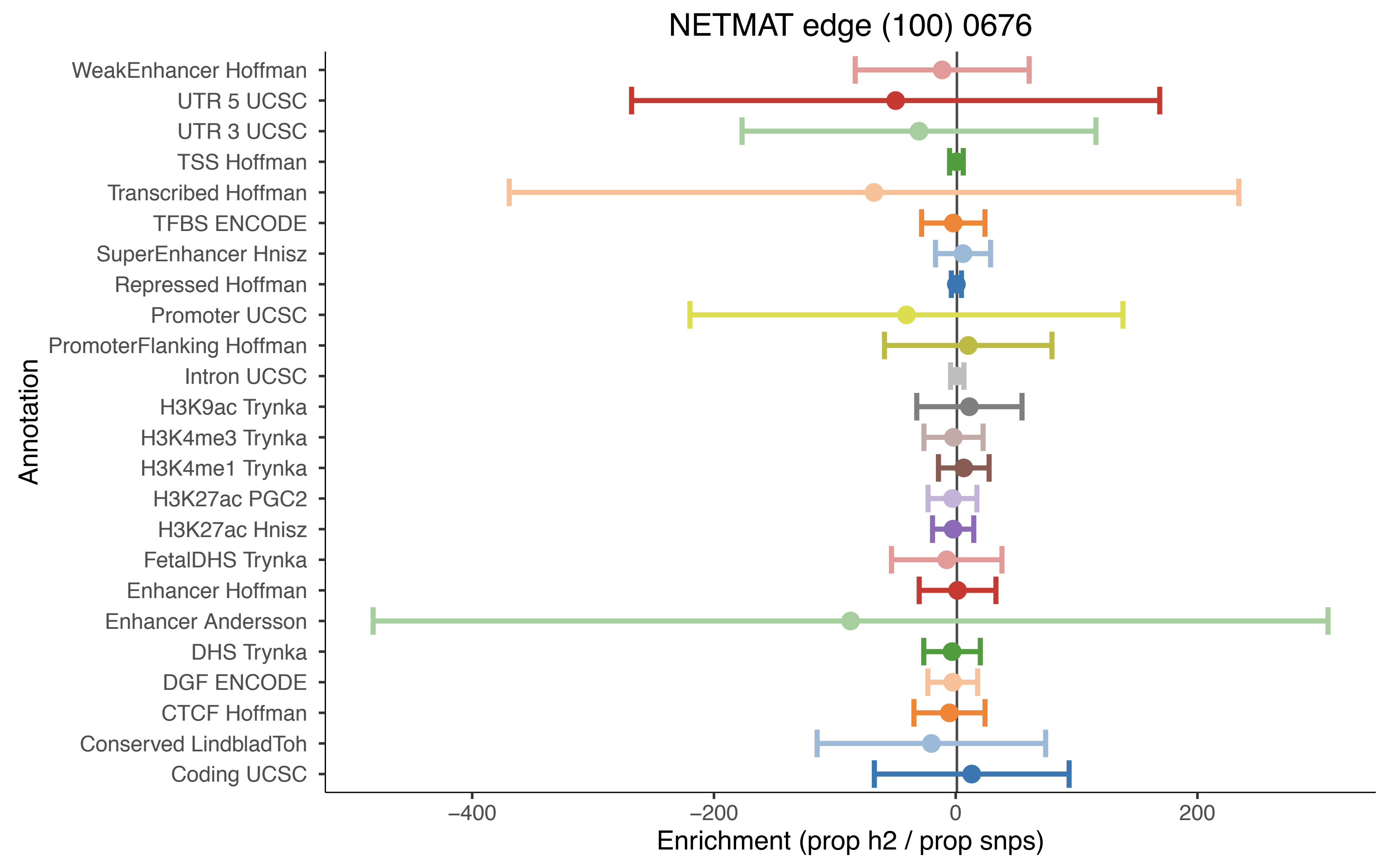

NETMAT edge (100) 0676
WeakEnhancer Hoffman
UTR 5 UCSC
UTR 3 UCSC
TSS Hoffman
Transcribed Hoffman
TFBS ENCODE
SuperEnhancer Hnisz
Repressed Hoffman
Promoter UCSC
PromoterFlanking Hoffman
Intron UCSC
H3K9ac Trynka
H3K4me3 Trynka
H3K4me1 Trynka
H3K27ac PGC2
H3K27ac Hnisz
FetalDHS Trynka
Enhancer Hoffman
Enhancer Andersson
DHS Trynka
DGF ENCODE
CTCF Hoffman
Conserved LindbladToh
Coding UCSC
Annotation
−400
−200
0
200
Enrichment (prop h2 / prop snps)

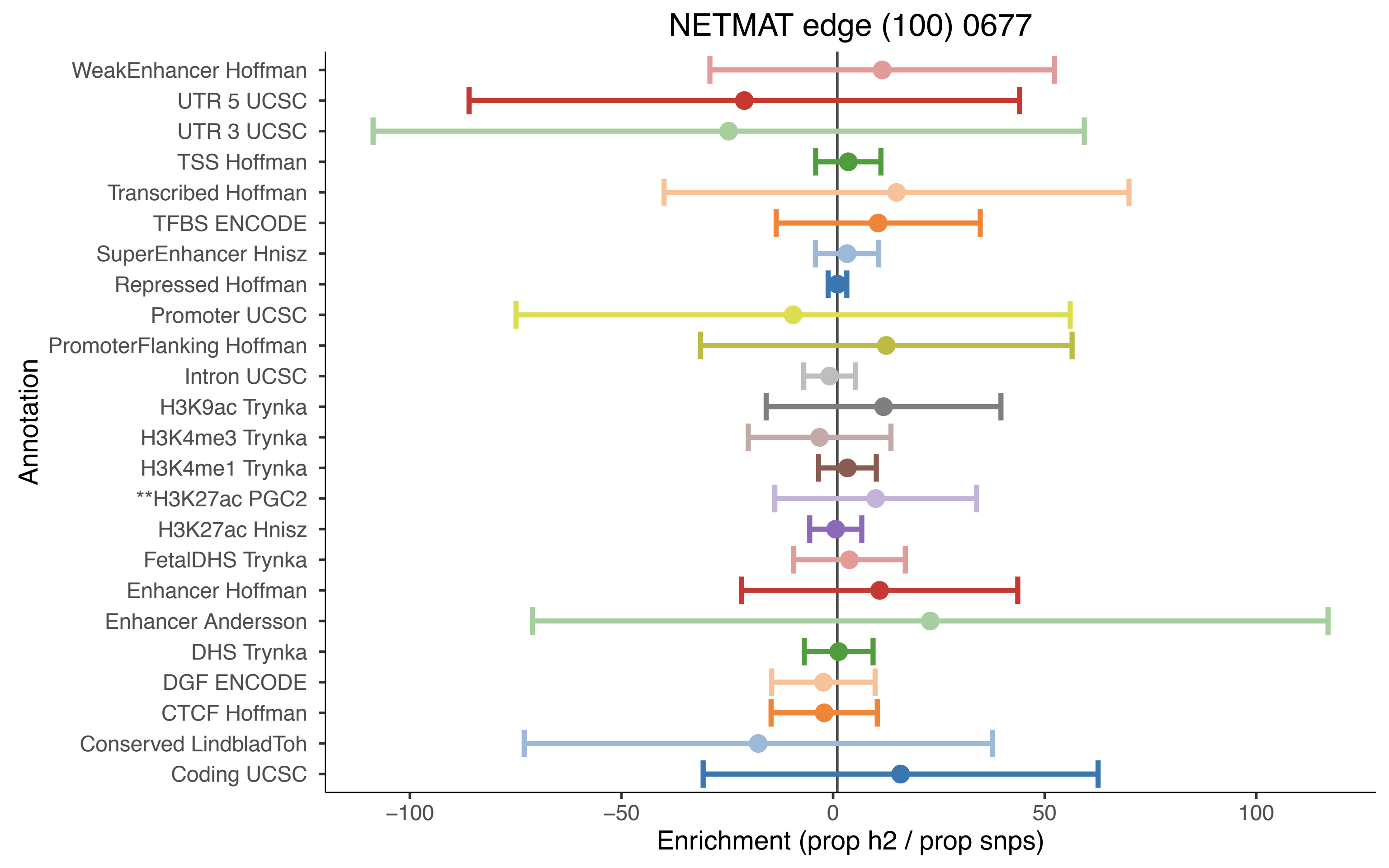
NETMAT edge (100) 0677
Annotation
WeakEnhancer Hoffman
UTR 5 UCSC
UTR 3 UCSC
TSS Hoffman
Transcribed Hoffman
TFBS ENCODE
SuperEnhancer Hnisz
Repressed Hoffman
Promoter UCSC
PromoterFlanking Hoffman
Intron UCSC
H3K9ac Trynka
H3K4me3 Trynka
H3K4me1 Trynka
**H3K27ac PGC2
H3K27ac Hnisz
FetalDHS Trynka
Enhancer Hoffman
Enhancer Andersson
DHS Trynka
DGF ENCODE
CTCF Hoffman
Conserved LindbladToh
Coding UCSC
−100
−50
0
50
100
Enrichment (prop h2 / prop snps)

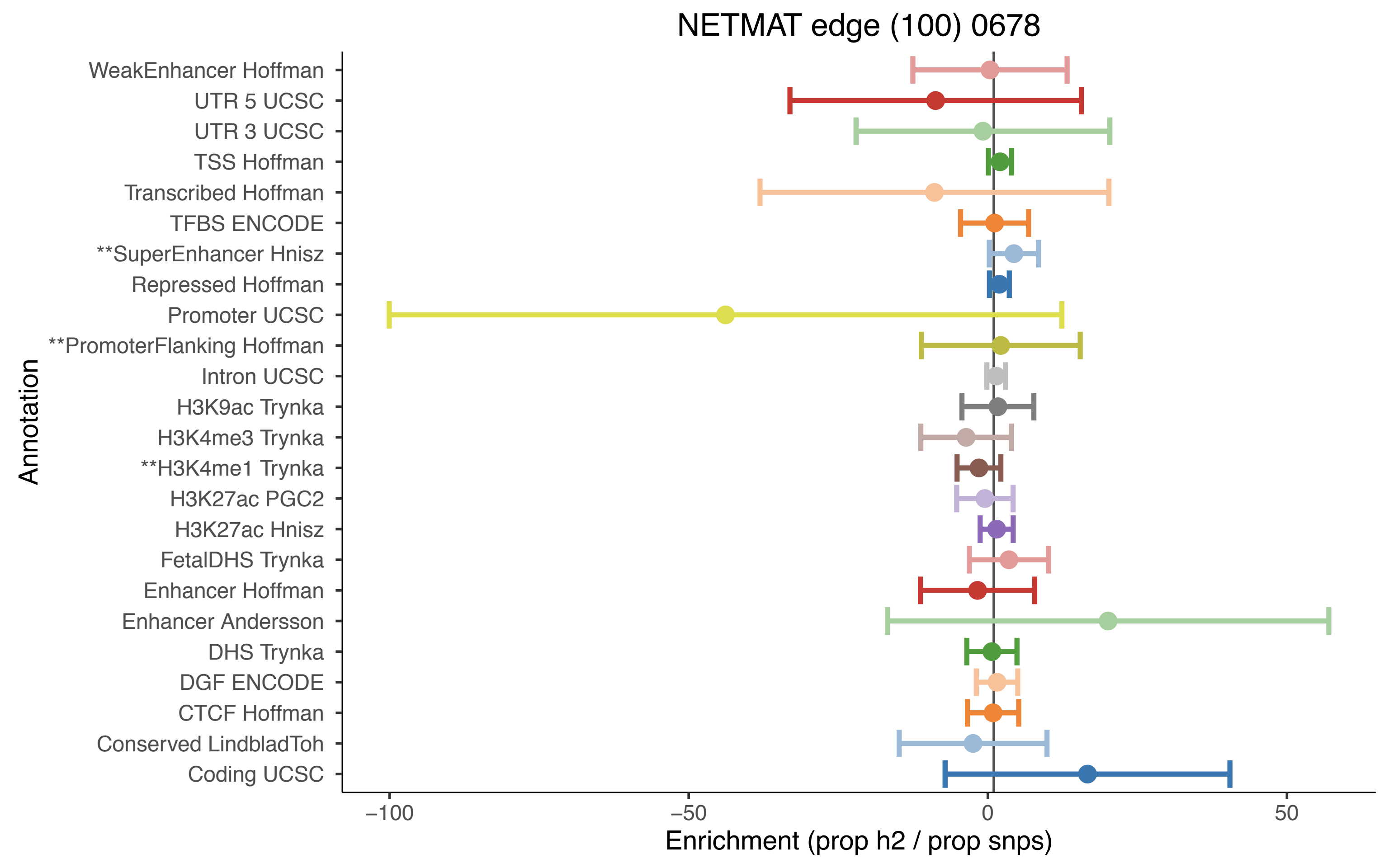
NETMAT edge (100) 0678
Annotation
WeakEnhancer Hoffman
UTR 5 UCSC
UTR 3 UCSC
TSS Hoffman
Transcribed Hoffman
TFBS ENCODE
**SuperEnhancer Hnisz
Repressed Hoffman
Promoter UCSC
**PromoterFlanking Hoffman
Intron UCSC
H3K9ac Trynka
H3K4me3 Trynka
**H3K4me1 Trynka
H3K27ac PGC2
H3K27ac Hnisz
FetalDHS Trynka
Enhancer Hoffman
Enhancer Andersson
DHS Trynka
DGF ENCODE
CTCF Hoffman
Conserved LindbladToh
Coding UCSC
−100
−50
0
50
Enrichment (prop h2 / prop snps)

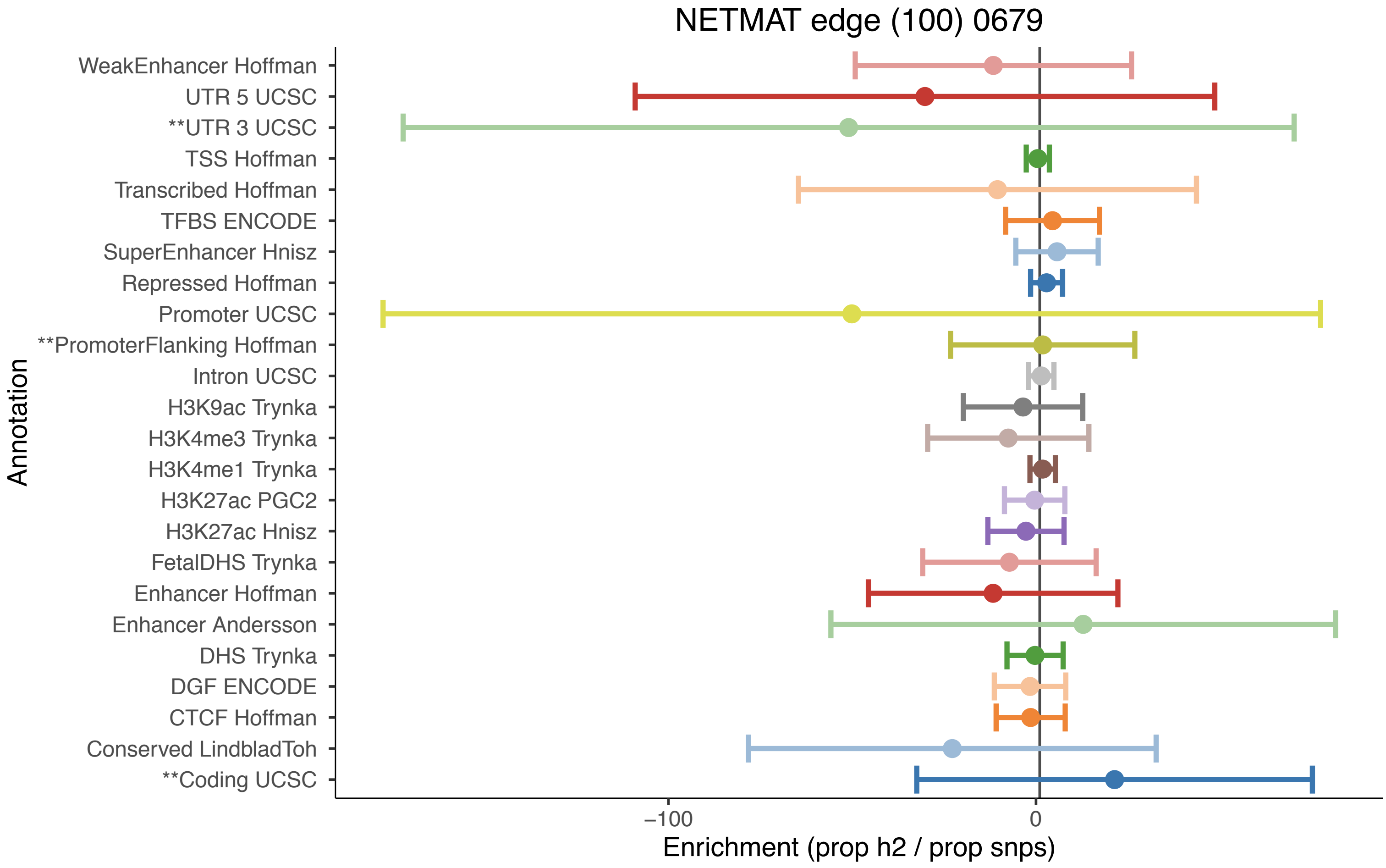

NETMAT edge (100) 0679
WeakEnhancer Hoffman
UTR 5 UCSC
**UTR 3 UCSC
TSS Hoffman
Transcribed Hoffman
TFBS ENCODE
SuperEnhancer Hnisz
Repressed Hoffman
Promoter UCSC
**PromoterFlanking Hoffman
Intron UCSC
H3K9ac Trynka
H3K4me3 Trynka
H3K4me1 Trynka
H3K27ac PGC2
H3K27ac Hnisz
FetalDHS Trynka
Enhancer Hoffman
Enhancer Andersson
DHS Trynka
DGF ENCODE
CTCF Hoffman
Conserved LindbladToh
**Coding UCSC
Annotation
−100
0
Enrichment (prop h2 / prop snps)

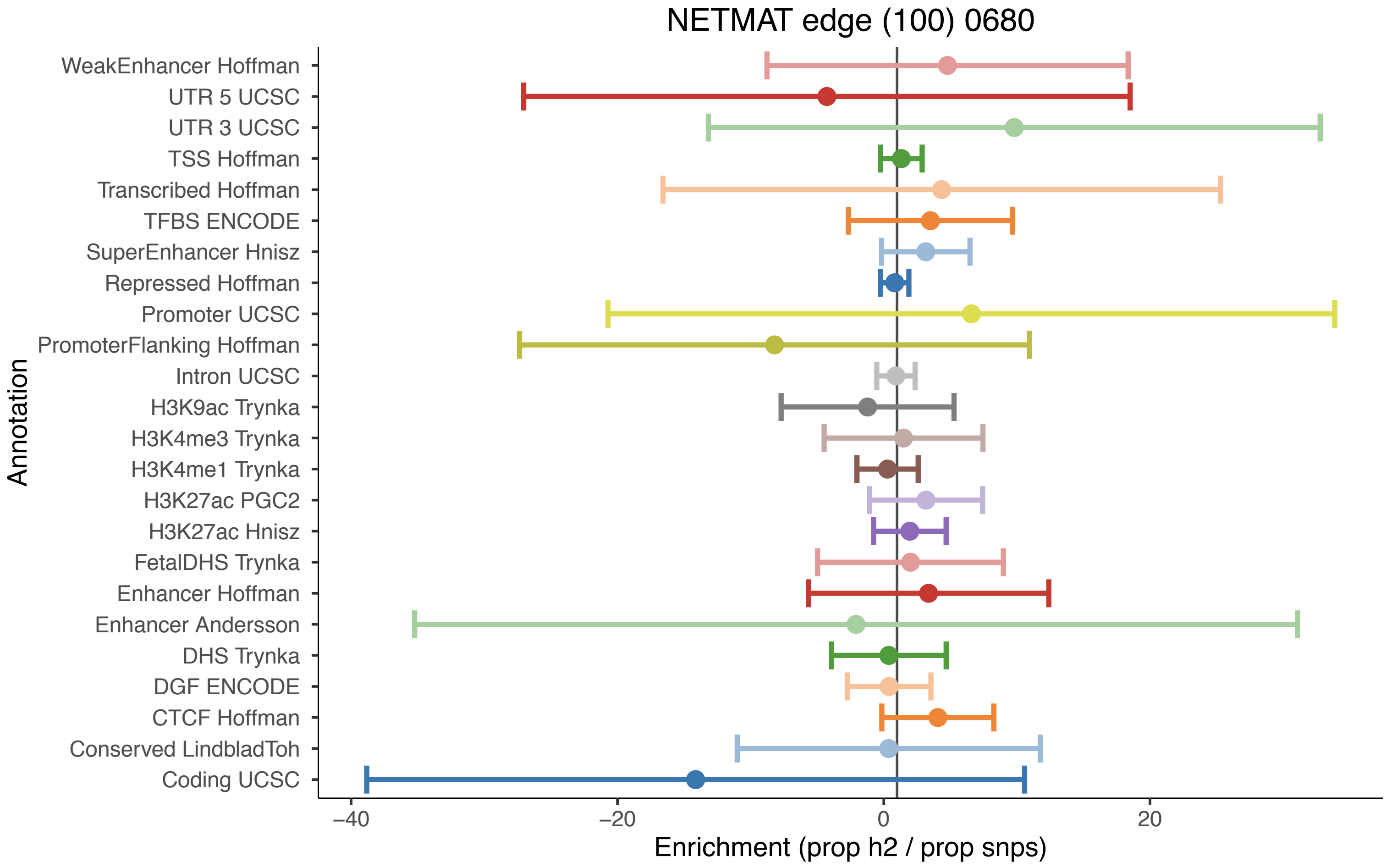

NETMAT edge (100) 0680
Annotation
WeakEnhancer Hoffman
UTR 5 UCSC
UTR 3 UCSC
TSS Hoffman
Transcribed Hoffman
TFBS ENCODE
SuperEnhancer Hnisz
Repressed Hoffman
Promoter UCSC
PromoterFlanking Hoffman
Intron UCSC
H3K9ac Trynka
H3K4me3 Trynka
H3K4me1 Trynka
H3K27ac PGC2
H3K27ac Hnisz
FetalDHS Trynka
Enhancer Hoffman
Enhancer Andersson
DHS Trynka
DGF ENCODE
CTCF Hoffman
Conserved LindbladToh
Coding UCSC
−40
−20
0
20
Enrichment (prop h2 / prop snps)

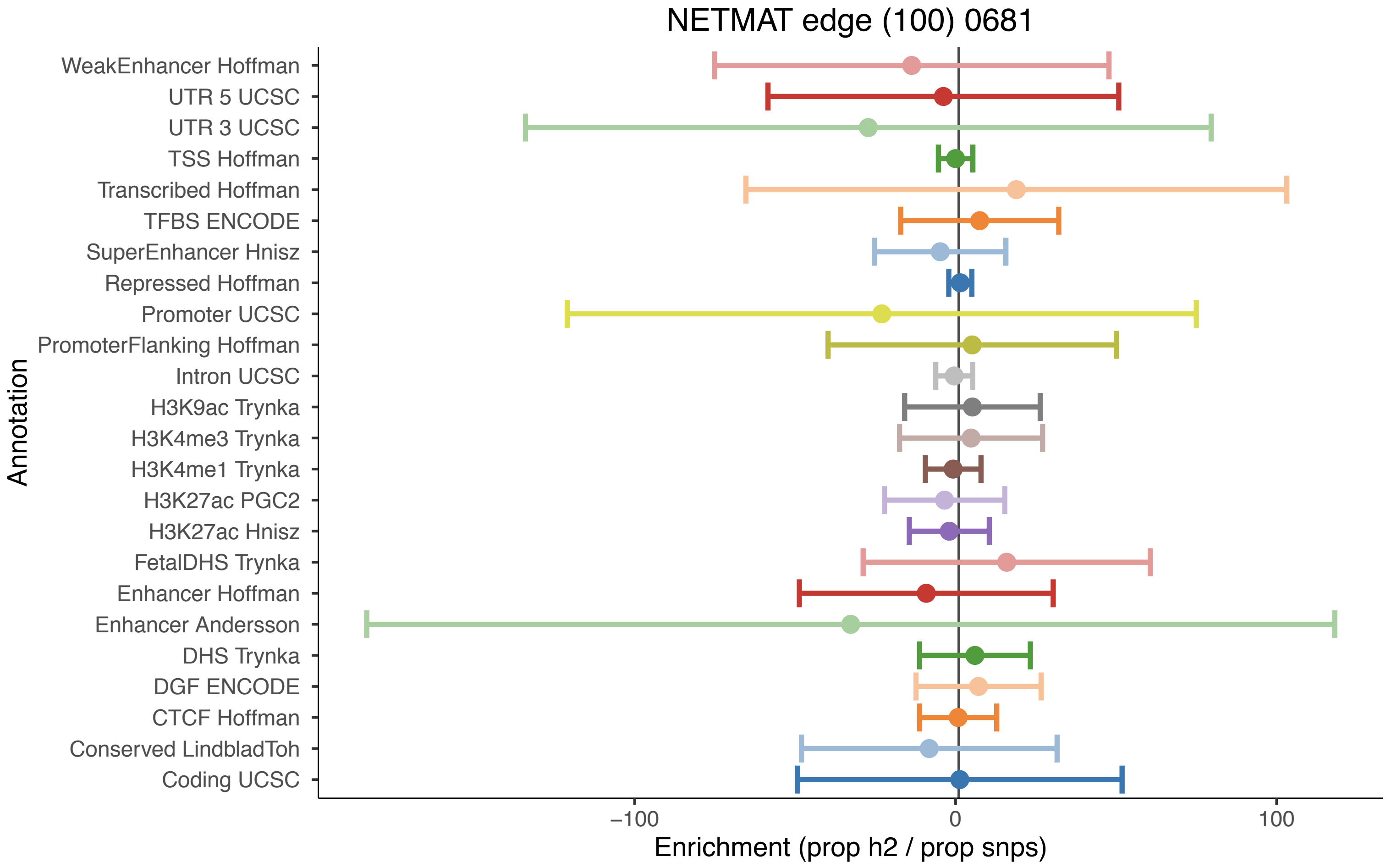
NETMAT edge (100) 0681
WeakEnhancer Hoffman
UTR 5 UCSC
UTR 3 UCSC
TSS Hoffman
Transcribed Hoffman
TFBS ENCODE
SuperEnhancer Hnisz
Repressed Hoffman
Promoter UCSC
PromoterFlanking Hoffman
Intron UCSC
H3K9ac Trynka
H3K4me3 Trynka
H3K4me1 Trynka
H3K27ac PGC2
H3K27ac Hnisz
FetalDHS Trynka
Enhancer Hoffman
Enhancer Andersson
DHS Trynka
DGF ENCODE
CTCF Hoffman
Conserved LindbladToh
Coding UCSC
Annotation
−100
0
100
Enrichment (prop h2 / prop snps)

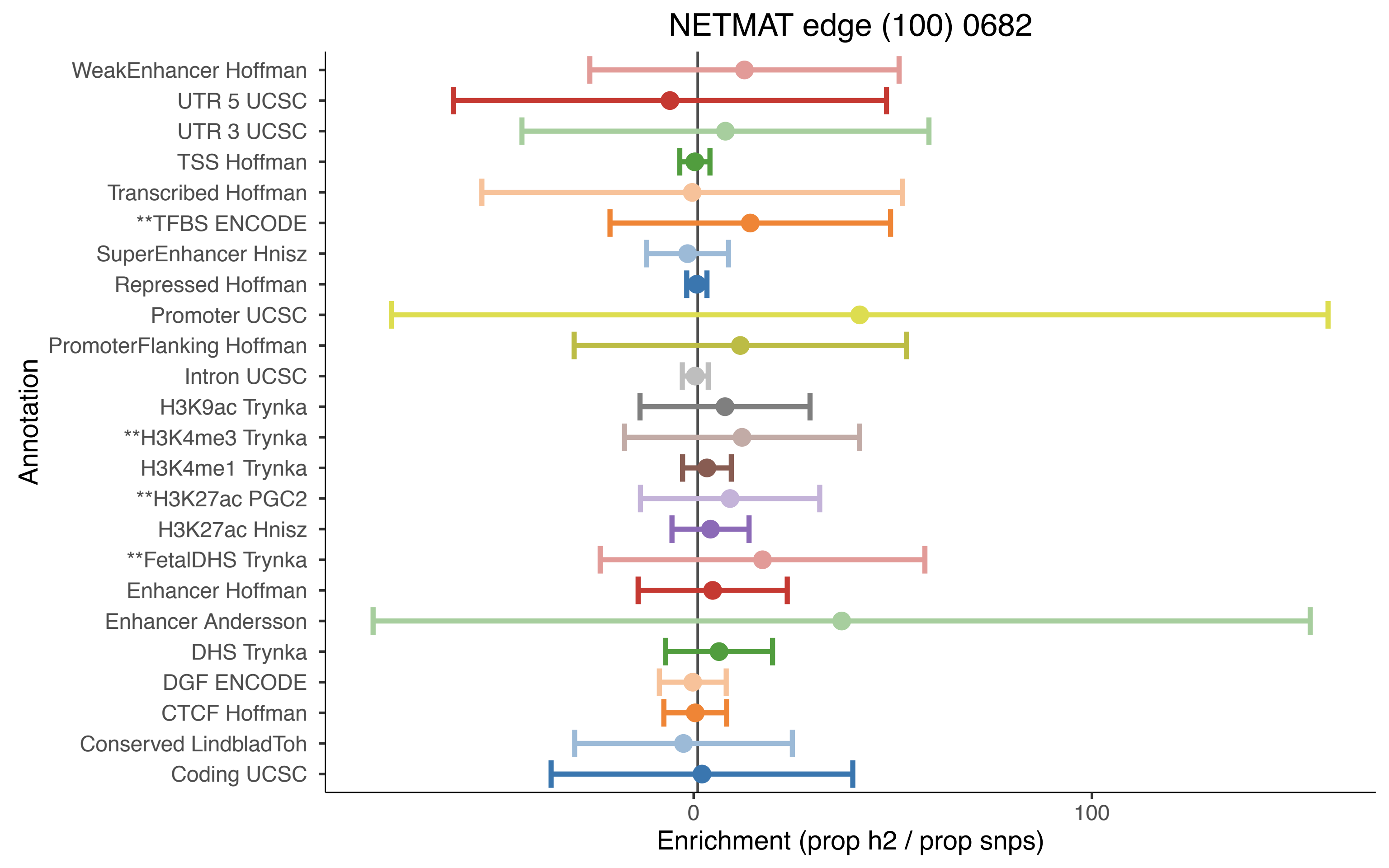
NETMAT edge (100) 0682
WeakEnhancer Hoffman
UTR 5 UCSC
UTR 3 UCSC
TSS Hoffman
Transcribed Hoffman
**TFBS ENCODE
SuperEnhancer Hnisz
Repressed Hoffman
Promoter UCSC
PromoterFlanking Hoffman
Intron UCSC
H3K9ac Trynka
**H3K4me3 Trynka
H3K4me1 Trynka
**H3K27ac PGC2
H3K27ac Hnisz
**FetalDHS Trynka
Enhancer Hoffman
Enhancer Andersson
DHS Trynka
DGF ENCODE
CTCF Hoffman
Conserved LindbladToh
Coding UCSC
Annotation
0
100
Enrichment (prop h2 / prop snps)

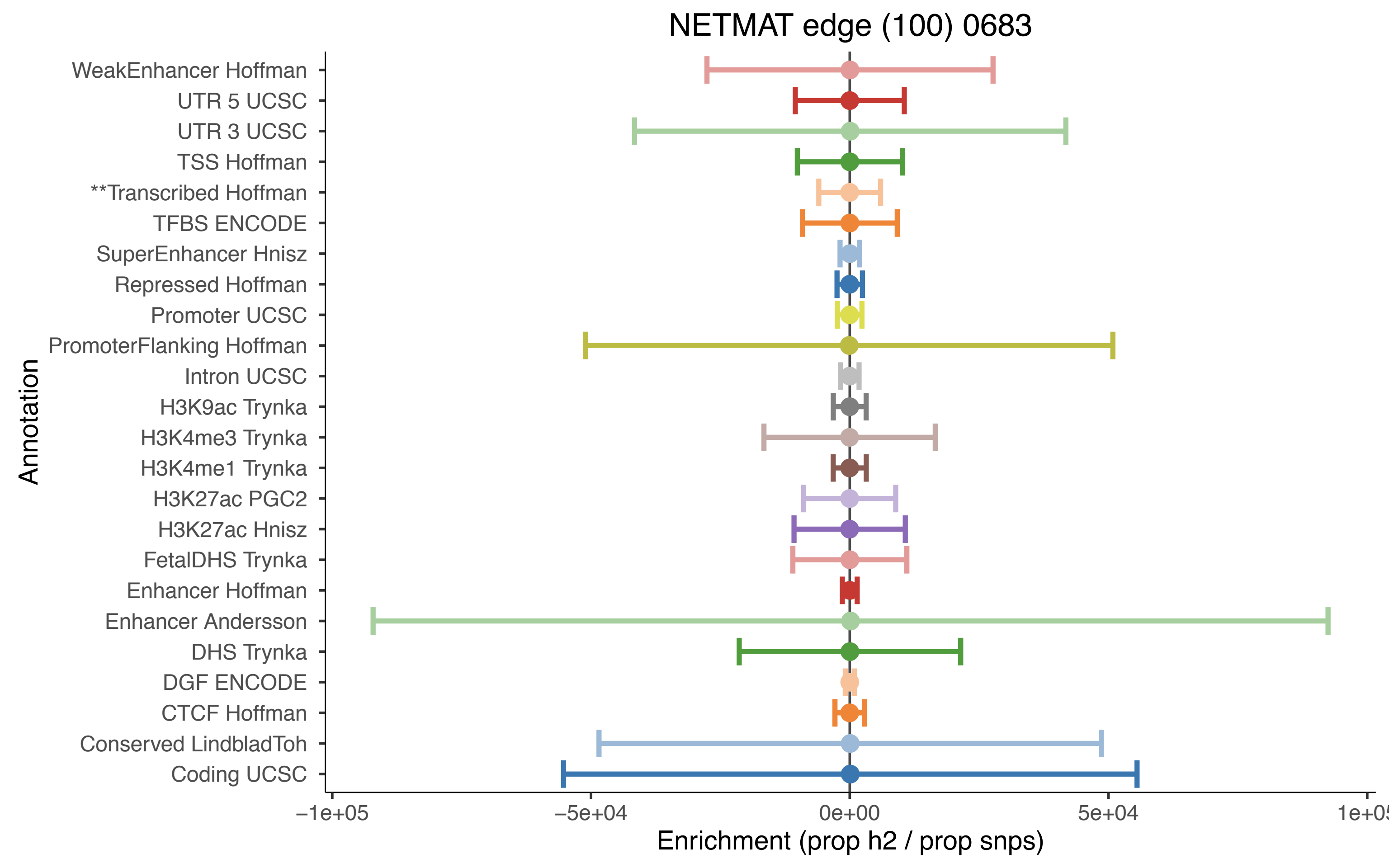

NETMAT edge (100) 0683
WeakEnhancer Hoffman
UTR 5 UCSC
UTR 3 UCSC
TSS Hoffman
**Transcribed Hoffman
TFBS ENCODE
SuperEnhancer Hnisz
Repressed Hoffman
Promoter UCSC
PromoterFlanking Hoffman
Intron UCSC
H3K9ac Trynka
H3K4me3 Trynka
H3K4me1 Trynka
H3K27ac PGC2
H3K27ac Hnisz
FetalDHS Trynka
Enhancer Hoffman
Enhancer Andersson
DHS Trynka
DGF ENCODE
CTCF Hoffman
Conserved LindbladToh
Coding UCSC
Annotation
−1e+05
−5e+04
0e+00
5e+04
Enrichment (prop h2 / prop snps)

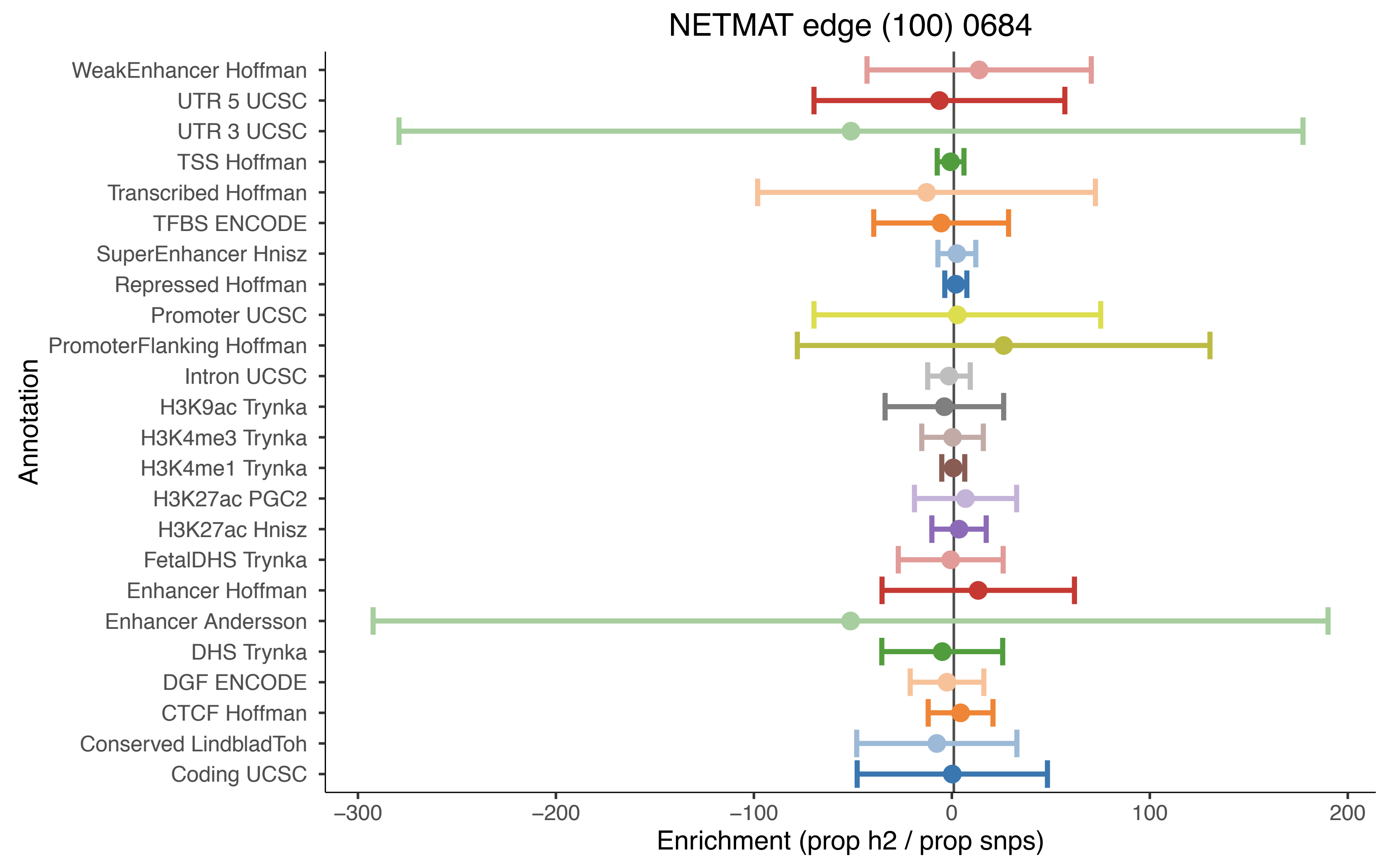
NETMAT edge (100) 0684
Annotation
WeakEnhancer Hoffman
UTR 5 UCSC
UTR 3 UCSC
TSS Hoffman
Transcribed Hoffman
TFBS ENCODE
SuperEnhancer Hnisz
Repressed Hoffman
Promoter UCSC
PromoterFlanking Hoffman
Intron UCSC
H3K9ac Trynka
H3K4me3 Trynka
H3K4me1 Trynka
H3K27ac PGC2
H3K27ac Hnisz
FetalDHS Trynka
Enhancer Hoffman
Enhancer Andersson
DHS Trynka
DGF ENCODE
CTCF Hoffman
Conserved LindbladToh
Coding UCSC
−300
−200
−100
0
100
200
Enrichment (prop h2 / prop snps)

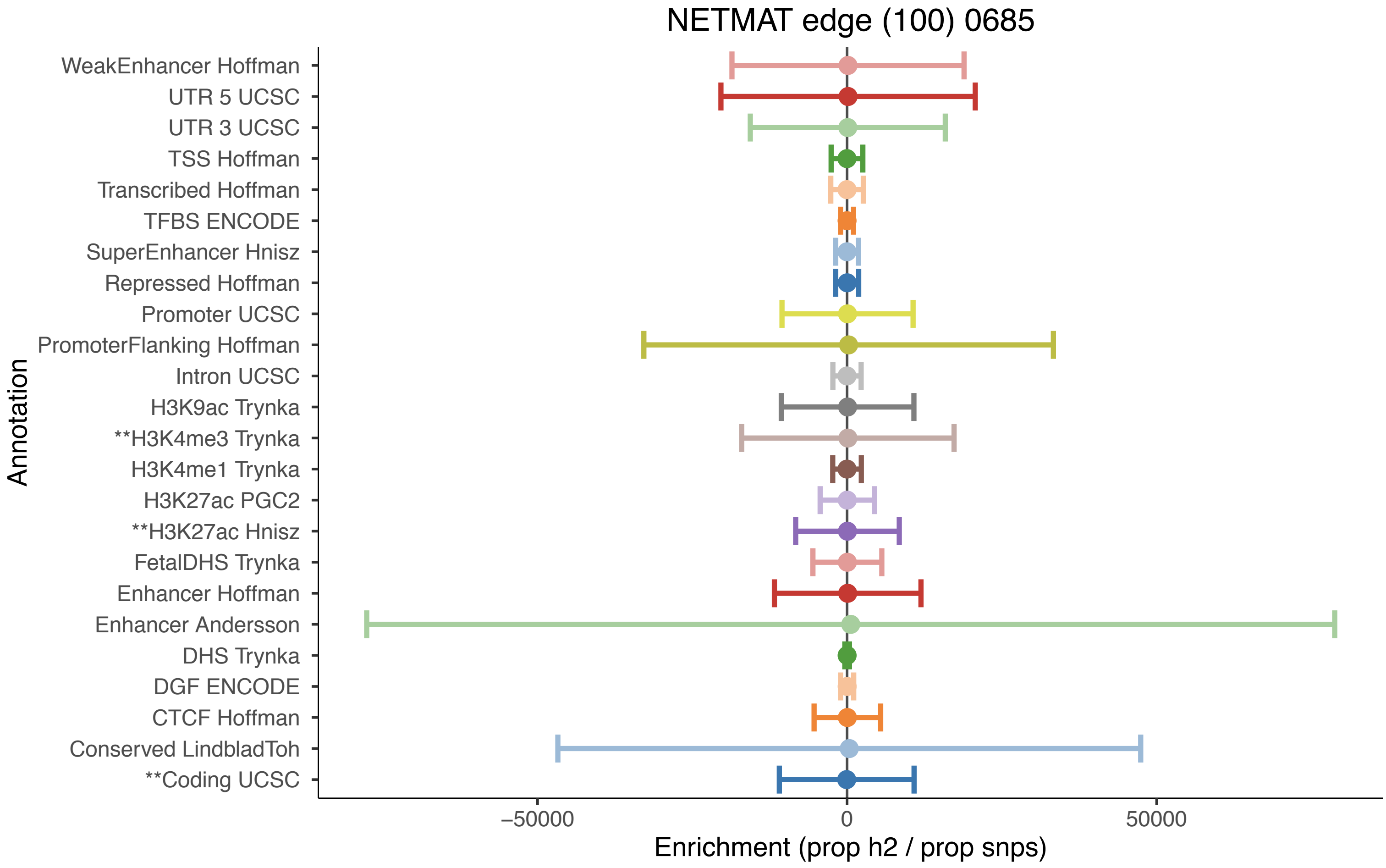
NETMAT edge (100) 0685
Annotation
WeakEnhancer Hoffman
UTR 5 UCSC
UTR 3 UCSC
TSS Hoffman
Transcribed Hoffman
TFBS ENCODE
SuperEnhancer Hnisz
Repressed Hoffman
Promoter UCSC
PromoterFlanking Hoffman
Intron UCSC
H3K9ac Trynka
**H3K4me3 Trynka
H3K4me1 Trynka
H3K27ac PGC2
**H3K27ac Hnisz
FetalDHS Trynka
Enhancer Hoffman
Enhancer Andersson
DHS Trynka
DGF ENCODE
CTCF Hoffman
Conserved LindbladToh
**Coding UCSC
−50000
0
50000
Enrichment (prop h2 / prop snps)

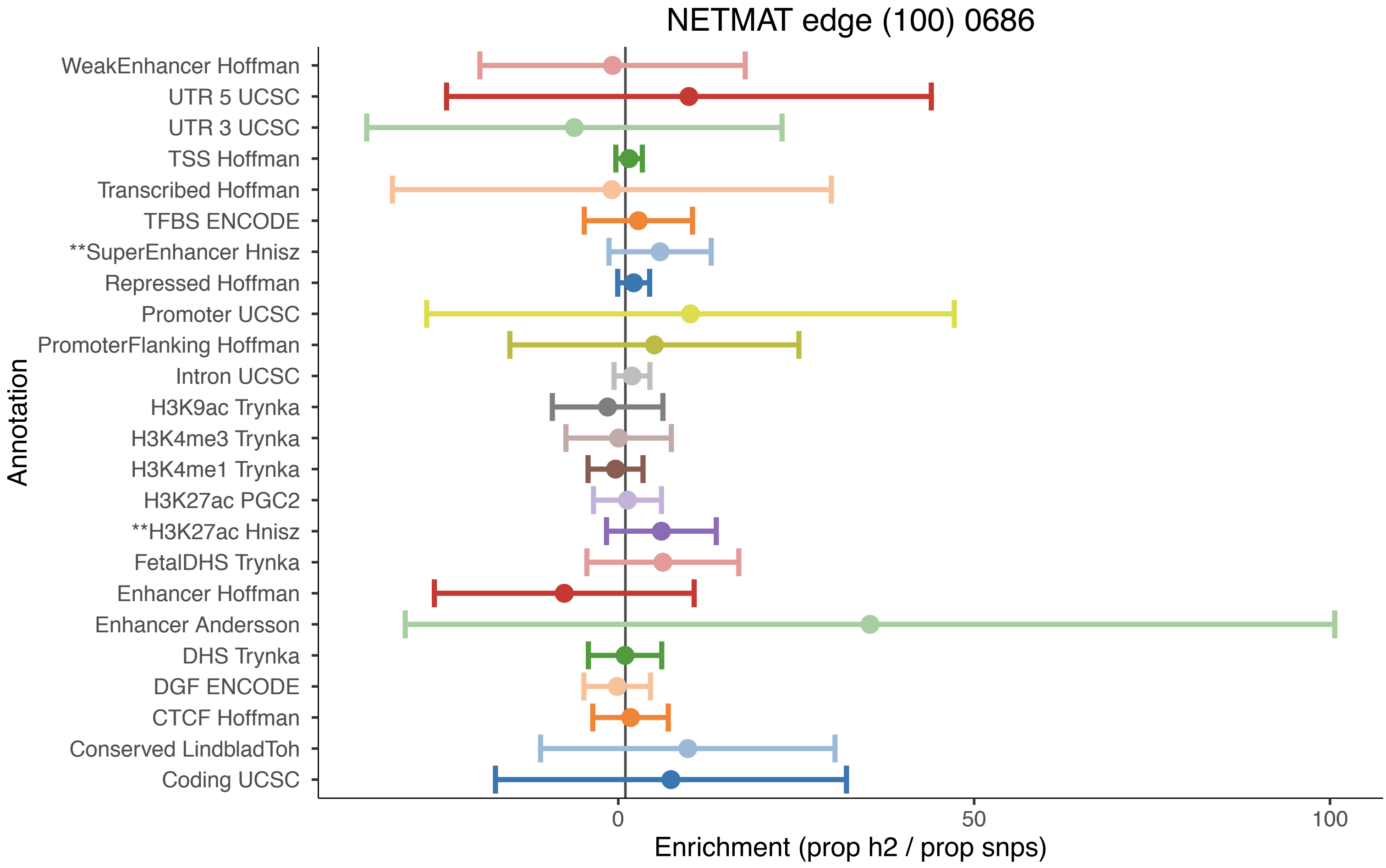
NETMAT edge (100) 0686
WeakEnhancer Hoffman
UTR 5 UCSC
UTR 3 UCSC
TSS Hoffman
Transcribed Hoffman
TFBS ENCODE
**SuperEnhancer Hnisz
Repressed Hoffman
Promoter UCSC
PromoterFlanking Hoffman
Intron UCSC
H3K9ac Trynka
H3K4me3 Trynka
H3K4me1 Trynka
H3K27ac PGC2
**H3K27ac Hnisz
FetalDHS Trynka
Enhancer Hoffman
Enhancer Andersson
DHS Trynka
DGF ENCODE
CTCF Hoffman
Conserved LindbladToh
Coding UCSC
Annotation
0
50
100
Enrichment (prop h2 / prop snps)

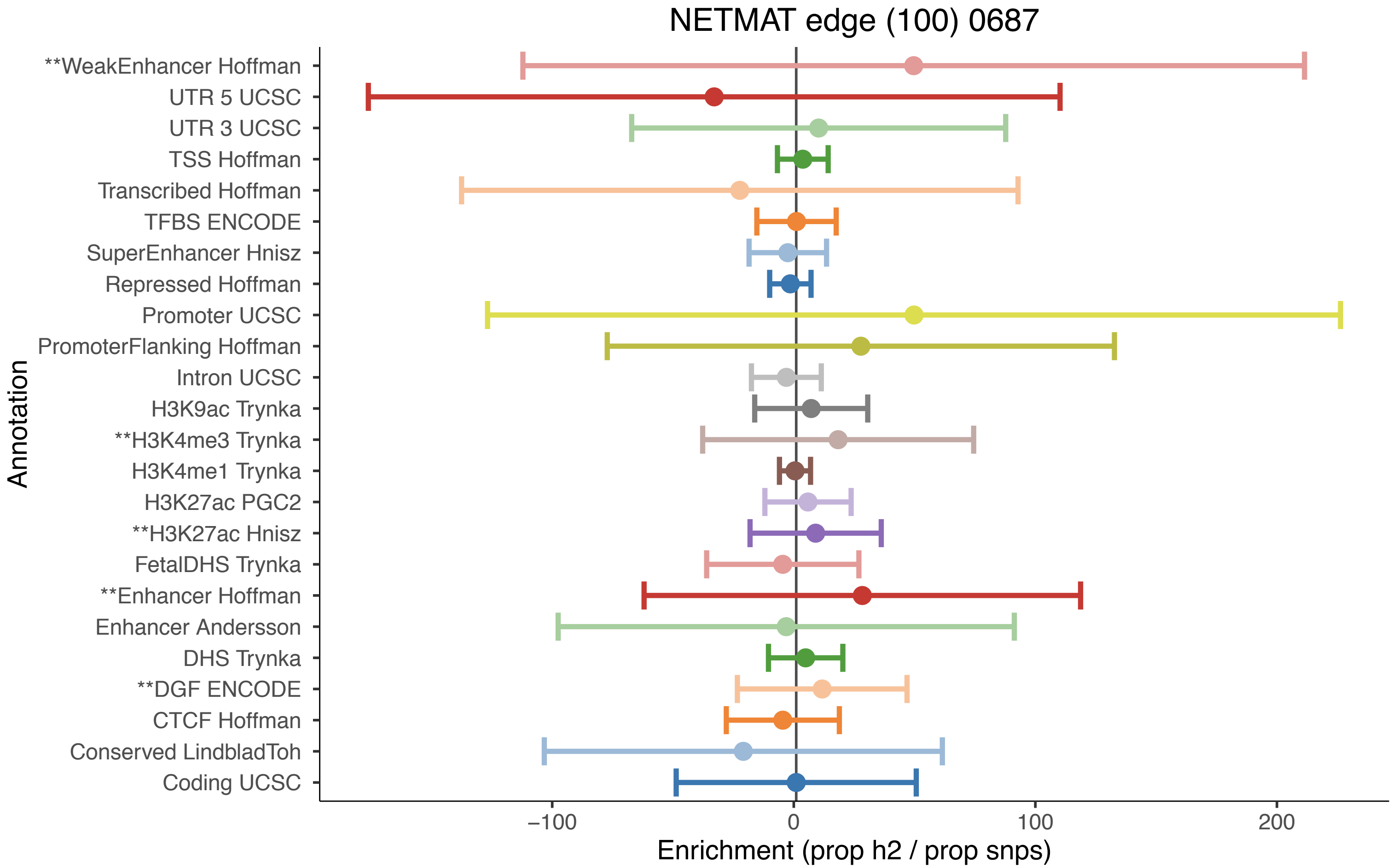
NETMAT edge (100) 0687
**WeakEnhancer Hoffman
UTR 5 UCSC
UTR 3 UCSC
TSS Hoffman
Transcribed Hoffman
TFBS ENCODE
SuperEnhancer Hnisz
Repressed Hoffman
Promoter UCSC
PromoterFlanking Hoffman
Intron UCSC
H3K9ac Trynka
**H3K4me3 Trynka
H3K4me1 Trynka
H3K27ac PGC2
**H3K27ac Hnisz
FetalDHS Trynka
**Enhancer Hoffman
Enhancer Andersson
DHS Trynka
**DGF ENCODE
CTCF Hoffman
Conserved LindbladToh
Coding UCSC
Annotation
−100
0
100
200
Enrichment (prop h2 / prop snps)

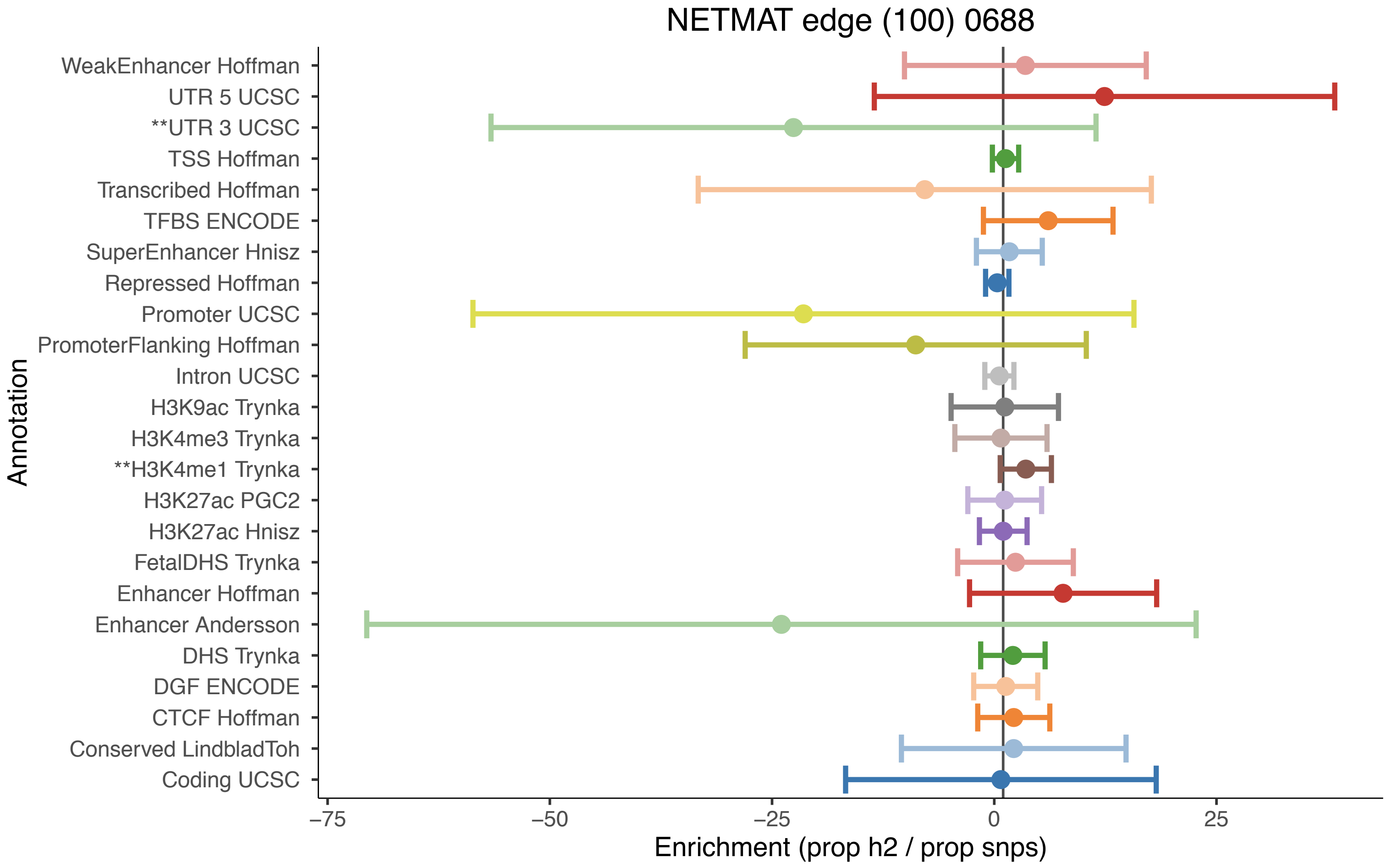
NETMAT edge (100) 0688
WeakEnhancer Hoffman
UTR 5 UCSC
**UTR 3 UCSC
TSS Hoffman
Transcribed Hoffman
TFBS ENCODE
SuperEnhancer Hnisz
Repressed Hoffman
Promoter UCSC
PromoterFlanking Hoffman
Intron UCSC
H3K9ac Trynka
H3K4me3 Trynka
**H3K4me1 Trynka
H3K27ac PGC2
H3K27ac Hnisz
FetalDHS Trynka
Enhancer Hoffman
Enhancer Andersson
DHS Trynka
DGF ENCODE
CTCF Hoffman
Conserved LindbladToh
Coding UCSC
Annotation
−75
−50
−25
0
25
Enrichment (prop h2 / prop snps)

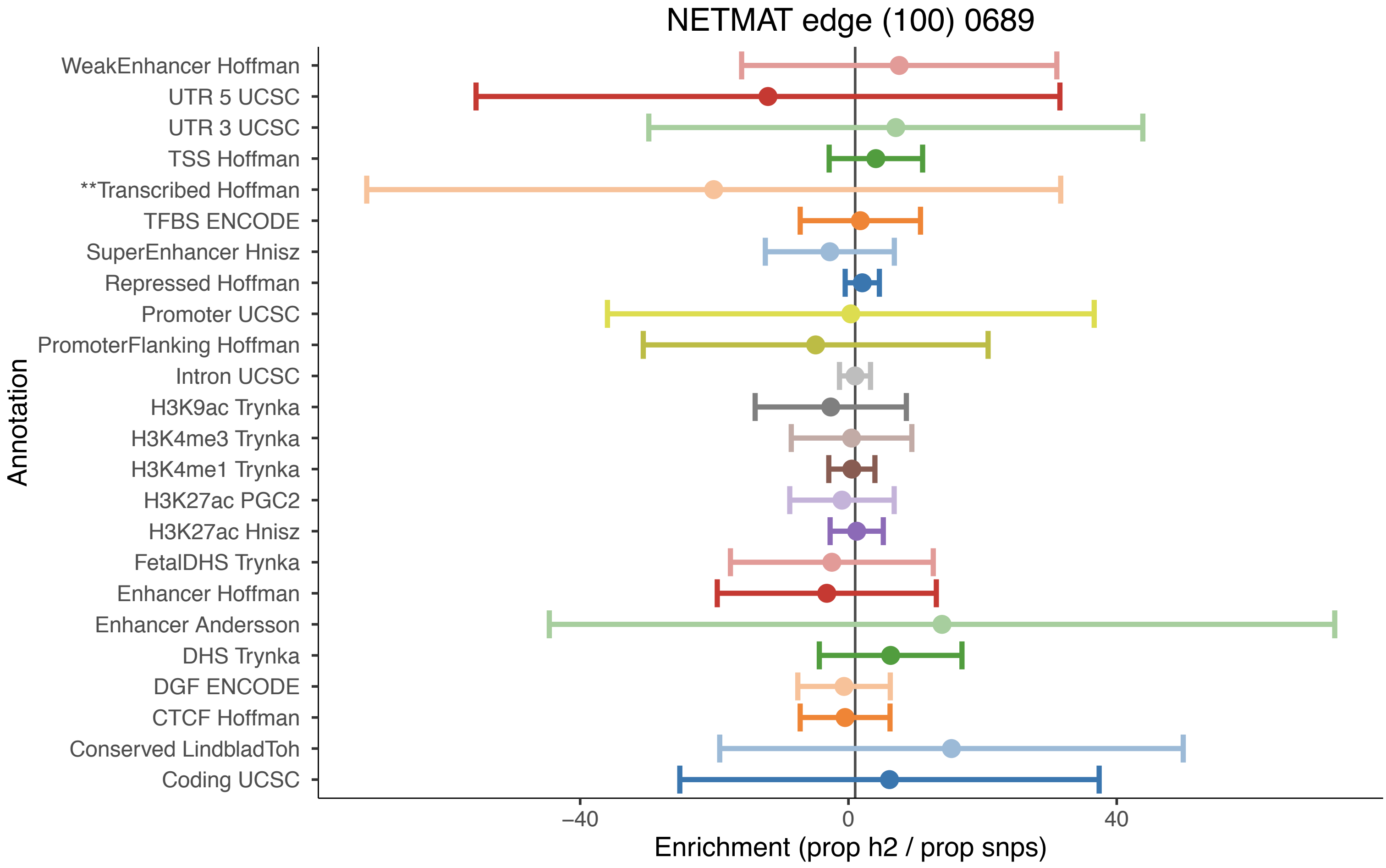
NETMAT edge (100) 0689
WeakEnhancer Hoffman
UTR 5 UCSC
UTR 3 UCSC
TSS Hoffman
**Transcribed Hoffman
TFBS ENCODE
SuperEnhancer Hnisz
Repressed Hoffman
Promoter UCSC
PromoterFlanking Hoffman
Intron UCSC
H3K9ac Trynka
H3K4me3 Trynka
H3K4me1 Trynka
H3K27ac PGC2
H3K27ac Hnisz
FetalDHS Trynka
Enhancer Hoffman
Enhancer Andersson
DHS Trynka
DGF ENCODE
CTCF Hoffman
Conserved LindbladToh
Coding UCSC
Annotation
−40
0
40
Enrichment (prop h2 / prop snps)

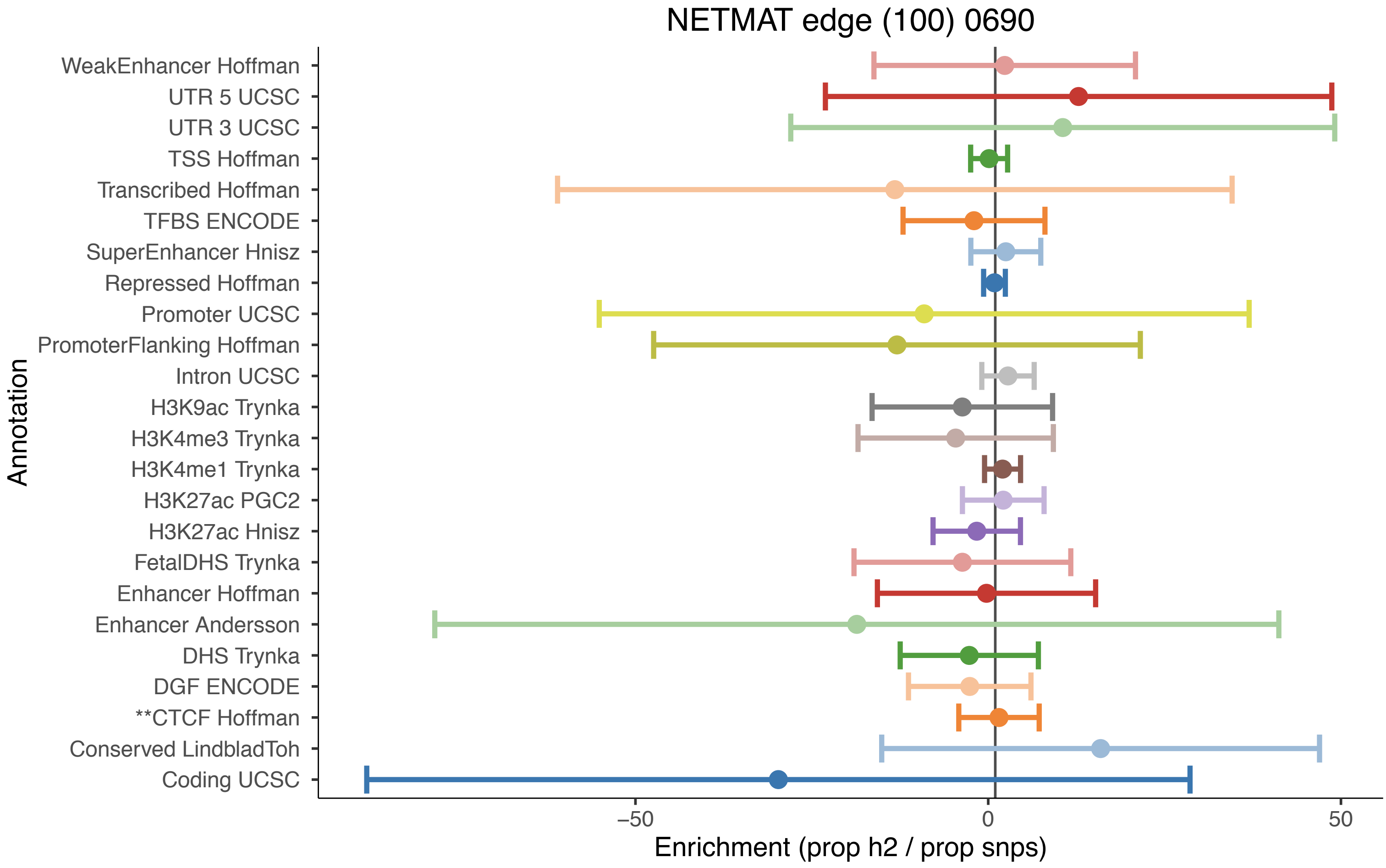

NETMAT edge (100) 0690
WeakEnhancer Hoffman
UTR 5 UCSC
UTR 3 UCSC
TSS Hoffman
Transcribed Hoffman
TFBS ENCODE
SuperEnhancer Hnisz
Repressed Hoffman
Promoter UCSC
PromoterFlanking Hoffman
Intron UCSC
H3K9ac Trynka
H3K4me3 Trynka
H3K4me1 Trynka
H3K27ac PGC2
H3K27ac Hnisz
FetalDHS Trynka
Enhancer Hoffman
Enhancer Andersson
DHS Trynka
DGF ENCODE
**CTCF Hoffman
Conserved LindbladToh
Coding UCSC
Annotation
−50
0
50
Enrichment (prop h2 / prop snps)

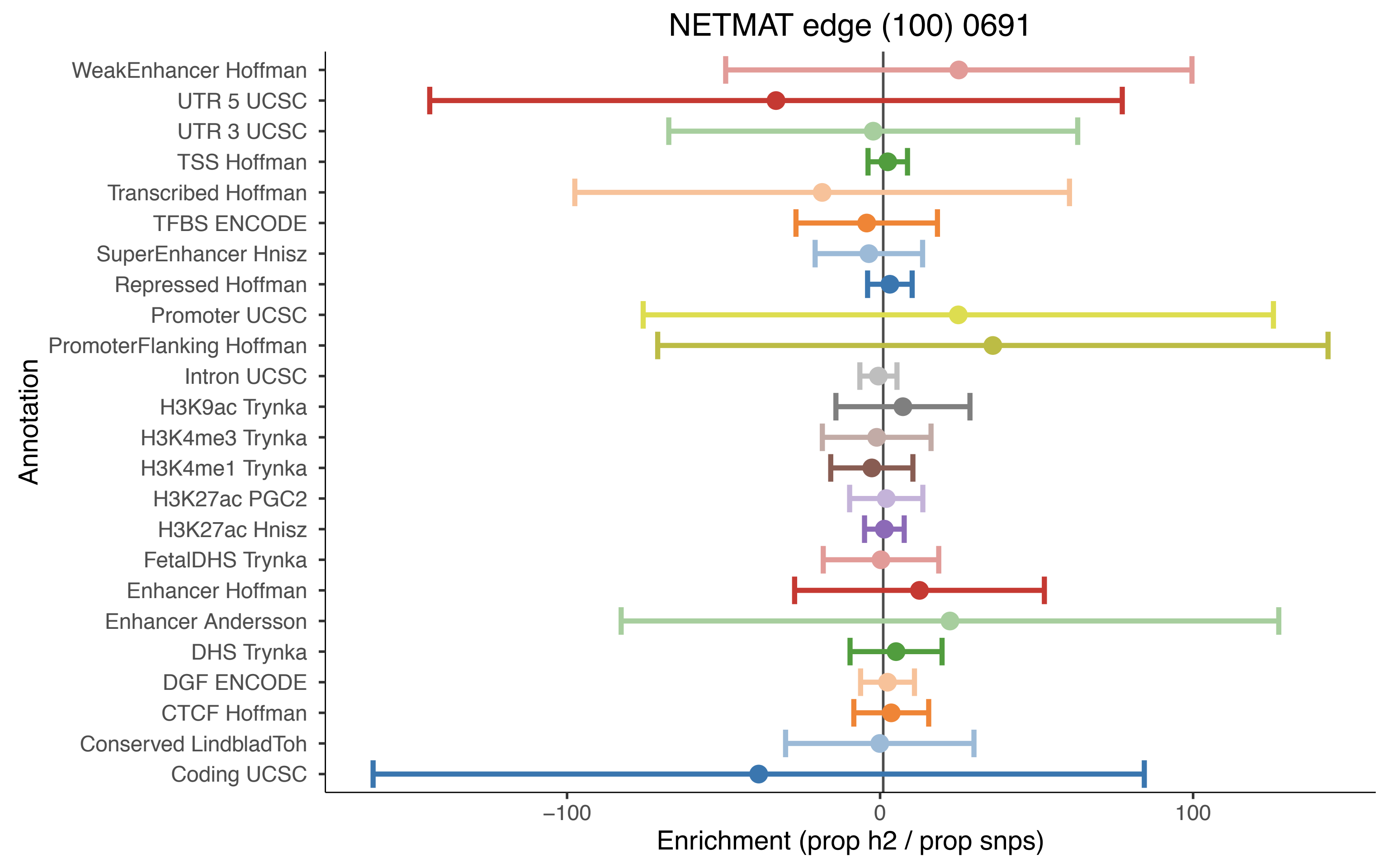

NETMAT edge (100) 0691
Annotation
WeakEnhancer Hoffman
UTR 5 UCSC
UTR 3 UCSC
TSS Hoffman
Transcribed Hoffman
TFBS ENCODE
SuperEnhancer Hnisz
Repressed Hoffman
Promoter UCSC
PromoterFlanking Hoffman
Intron UCSC
H3K9ac Trynka
H3K4me3 Trynka
H3K4me1 Trynka
H3K27ac PGC2
H3K27ac Hnisz
FetalDHS Trynka
Enhancer Hoffman
Enhancer Andersson
DHS Trynka
DGF ENCODE
CTCF Hoffman
Conserved LindbladToh
Coding UCSC
−100
0
100
Enrichment (prop h2 / prop snps)

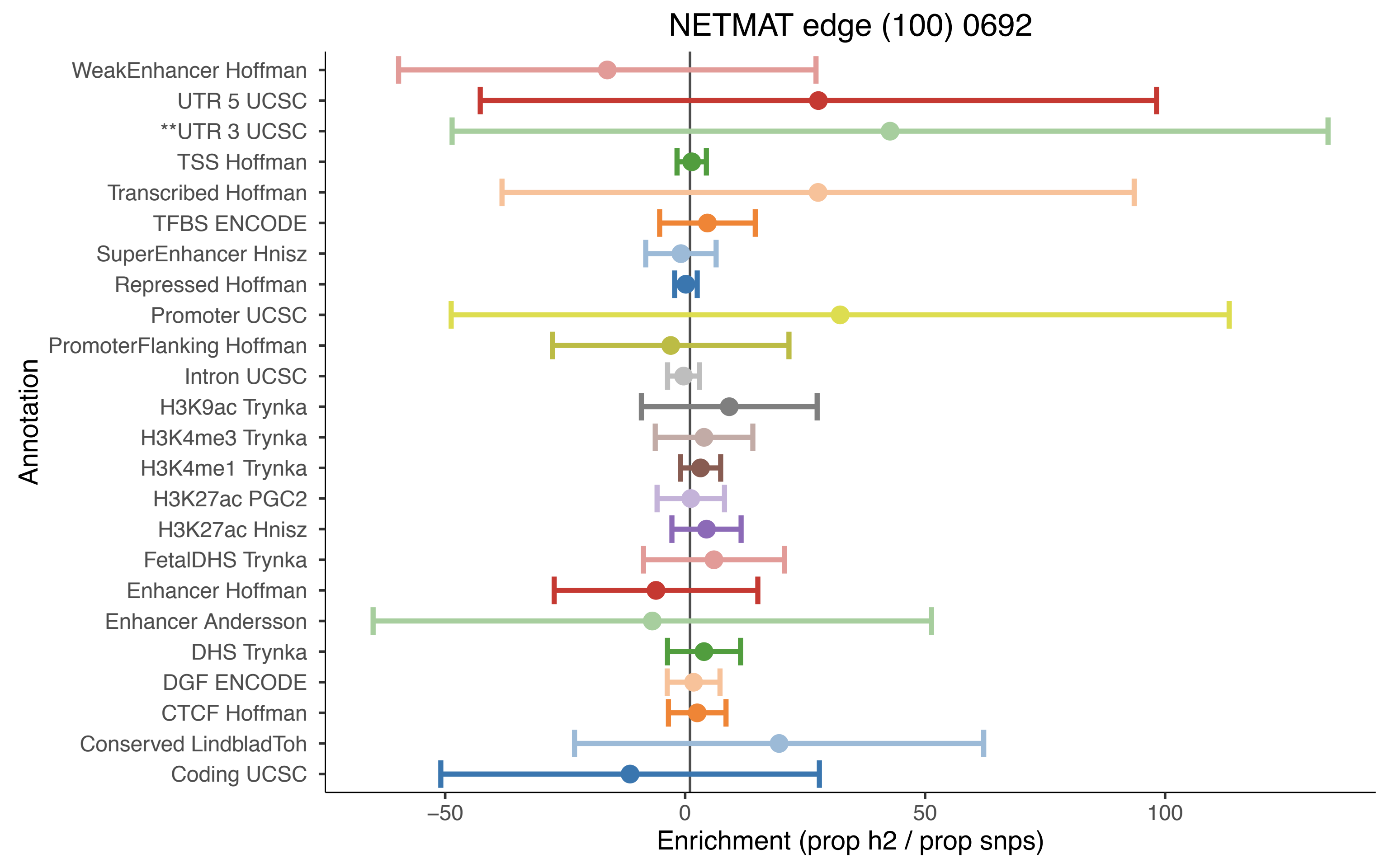

NETMAT edge (100) 0692
WeakEnhancer Hoffman
UTR 5 UCSC
**UTR 3 UCSC
TSS Hoffman
Transcribed Hoffman
TFBS ENCODE
SuperEnhancer Hnisz
Repressed Hoffman
Promoter UCSC
PromoterFlanking Hoffman
Intron UCSC
H3K9ac Trynka
H3K4me3 Trynka
H3K4me1 Trynka
H3K27ac PGC2
H3K27ac Hnisz
FetalDHS Trynka
Enhancer Hoffman
Enhancer Andersson
DHS Trynka
DGF ENCODE
CTCF Hoffman
Conserved LindbladToh
Coding UCSC
Annotation
−50
0
50
100
Enrichment (prop h2 / prop snps)

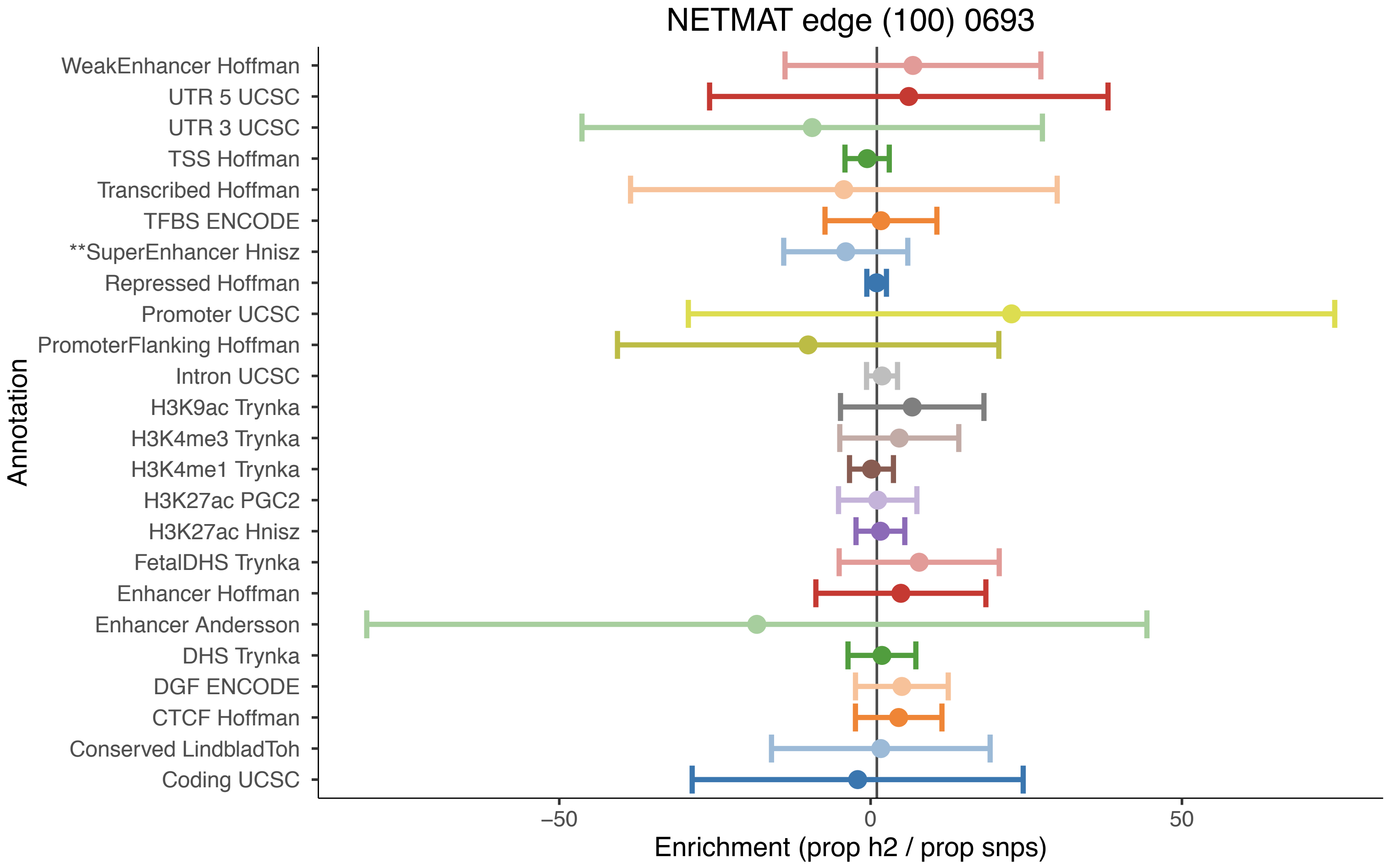

NETMAT edge (100) 0693
WeakEnhancer Hoffman
UTR 5 UCSC
UTR 3 UCSC
TSS Hoffman
Transcribed Hoffman
TFBS ENCODE
**SuperEnhancer Hnisz
Repressed Hoffman
Promoter UCSC
PromoterFlanking Hoffman
Intron UCSC
H3K9ac Trynka
H3K4me3 Trynka
H3K4me1 Trynka
H3K27ac PGC2
H3K27ac Hnisz
FetalDHS Trynka
Enhancer Hoffman
Enhancer Andersson
DHS Trynka
DGF ENCODE
CTCF Hoffman
Conserved LindbladToh
Coding UCSC
Annotation
−50
0
50
Enrichment (prop h2 / prop snps)

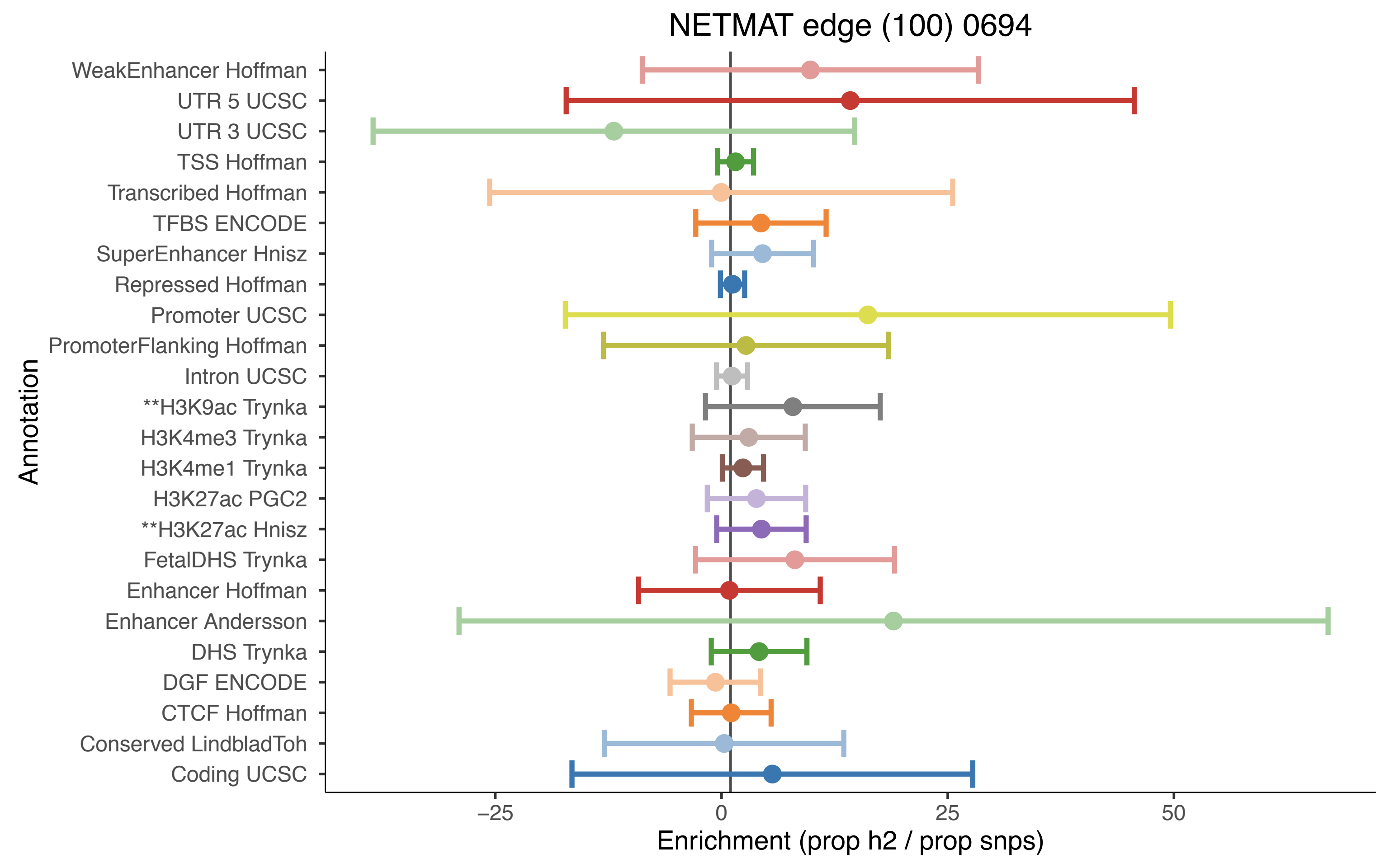
NETMAT edge (100) 0694
WeakEnhancer Hoffman
UTR 5 UCSC
UTR 3 UCSC
TSS Hoffman
Transcribed Hoffman
TFBS ENCODE
SuperEnhancer Hnisz
Repressed Hoffman
Promoter UCSC
PromoterFlanking Hoffman
Intron UCSC
**H3K9ac Trynka
H3K4me3 Trynka
H3K4me1 Trynka
H3K27ac PGC2
**H3K27ac Hnisz
FetalDHS Trynka
Enhancer Hoffman
Enhancer Andersson
DHS Trynka
DGF ENCODE
CTCF Hoffman
Conserved LindbladToh
Coding UCSC
Annotation
−25
0
25
50
Enrichment (prop h2 / prop snps)

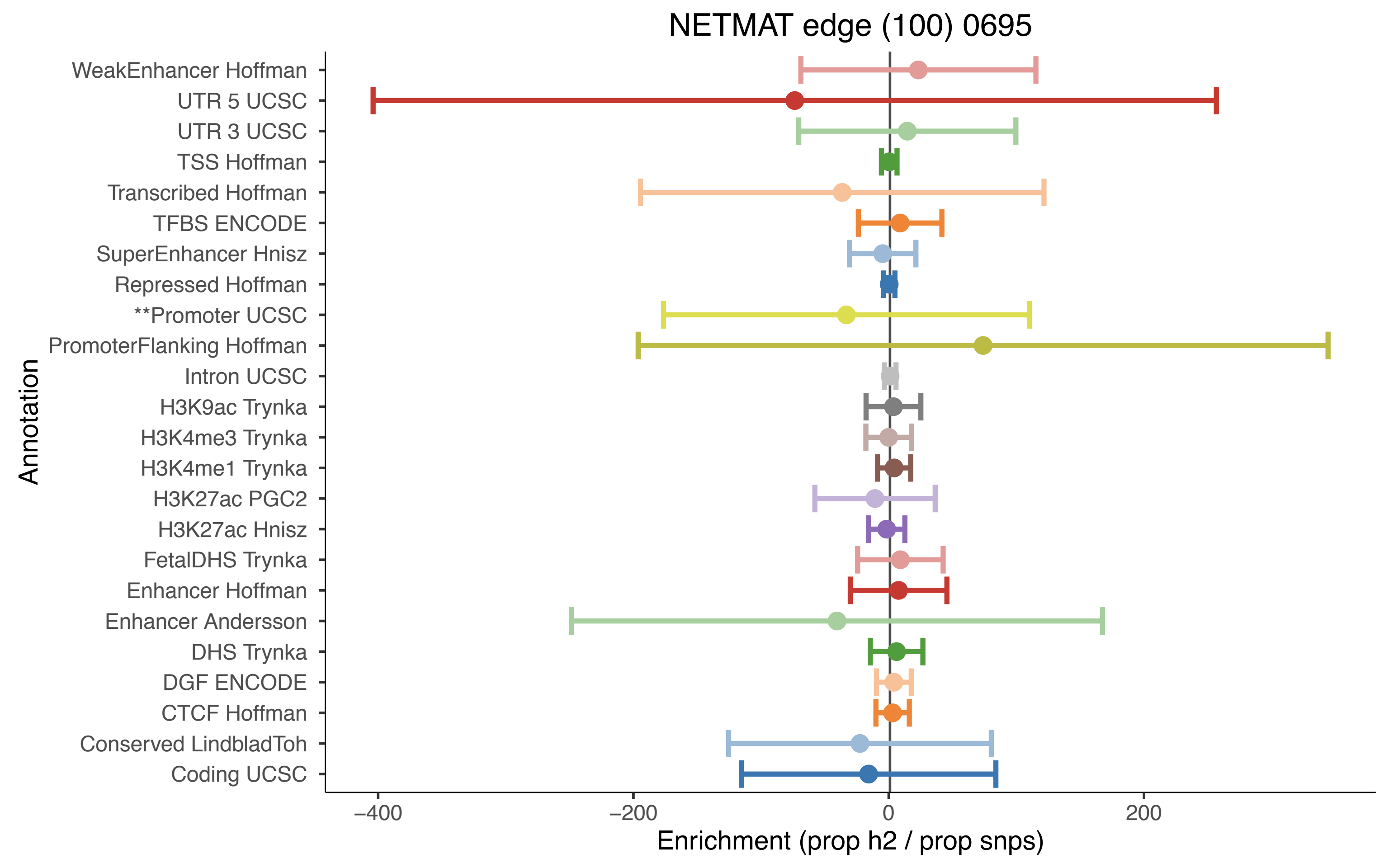
NETMAT edge (100) 0695
WeakEnhancer Hoffman
UTR 5 UCSC
UTR 3 UCSC
TSS Hoffman
Transcribed Hoffman
TFBS ENCODE
SuperEnhancer Hnisz
Repressed Hoffman
**Promoter UCSC
PromoterFlanking Hoffman
Intron UCSC
H3K9ac Trynka
H3K4me3 Trynka
H3K4me1 Trynka
H3K27ac PGC2
H3K27ac Hnisz
FetalDHS Trynka
Enhancer Hoffman
Enhancer Andersson
DHS Trynka
DGF ENCODE
CTCF Hoffman
Conserved LindbladToh
Coding UCSC
Annotation
−400
−200
0
200
Enrichment (prop h2 / prop snps)

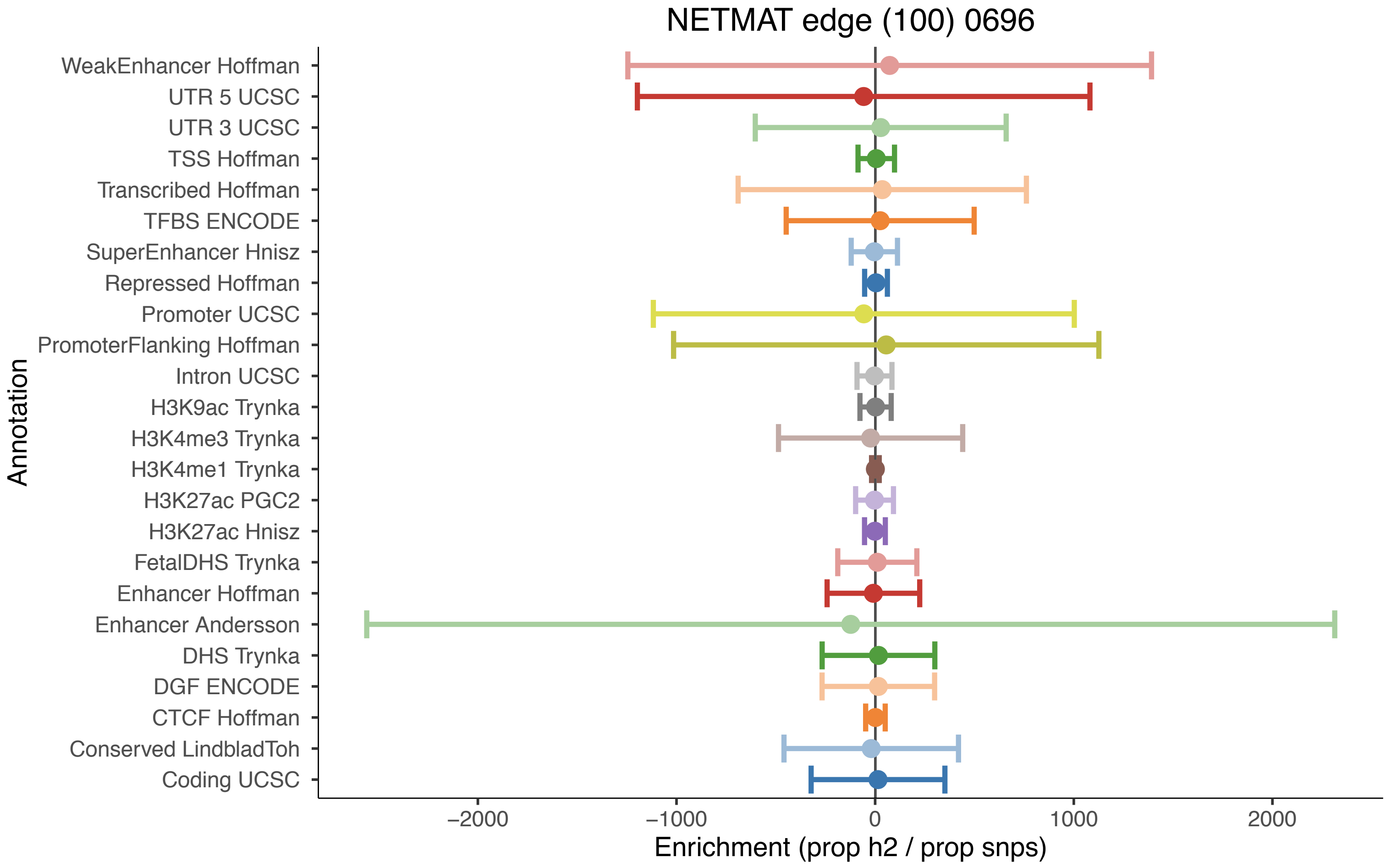
NETMAT edge (100) 0696
Annotation
WeakEnhancer Hoffman
UTR 5 UCSC
UTR 3 UCSC
TSS Hoffman
Transcribed Hoffman
TFBS ENCODE
SuperEnhancer Hnisz
Repressed Hoffman
Promoter UCSC
PromoterFlanking Hoffman
Intron UCSC
H3K9ac Trynka
H3K4me3 Trynka
H3K4me1 Trynka
H3K27ac PGC2
H3K27ac Hnisz
FetalDHS Trynka
Enhancer Hoffman
Enhancer Andersson
DHS Trynka
DGF ENCODE
CTCF Hoffman
Conserved LindbladToh
Coding UCSC
−2000
−1000
0
1000
2000
Enrichment (prop h2 / prop snps)

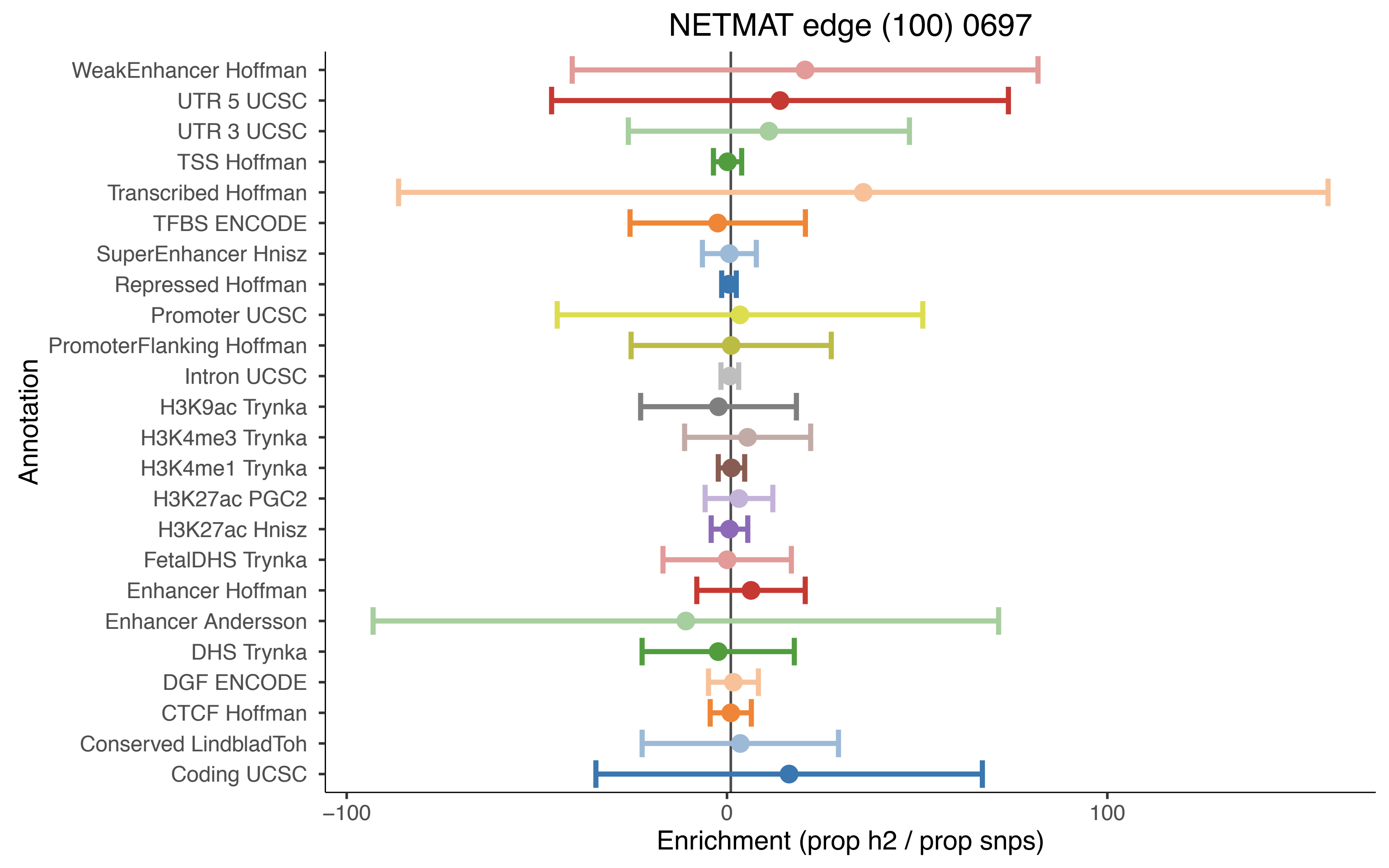
NETMAT edge (100) 0697
WeakEnhancer Hoffman
UTR 5 UCSC
UTR 3 UCSC
TSS Hoffman
Transcribed Hoffman
TFBS ENCODE
SuperEnhancer Hnisz
Repressed Hoffman
Promoter UCSC
PromoterFlanking Hoffman
Intron UCSC
H3K9ac Trynka
H3K4me3 Trynka
H3K4me1 Trynka
H3K27ac PGC2
H3K27ac Hnisz
FetalDHS Trynka
Enhancer Hoffman
Enhancer Andersson
DHS Trynka
DGF ENCODE
CTCF Hoffman
Conserved LindbladToh
Coding UCSC
Annotation
−100
0
100
Enrichment (prop h2 / prop snps)

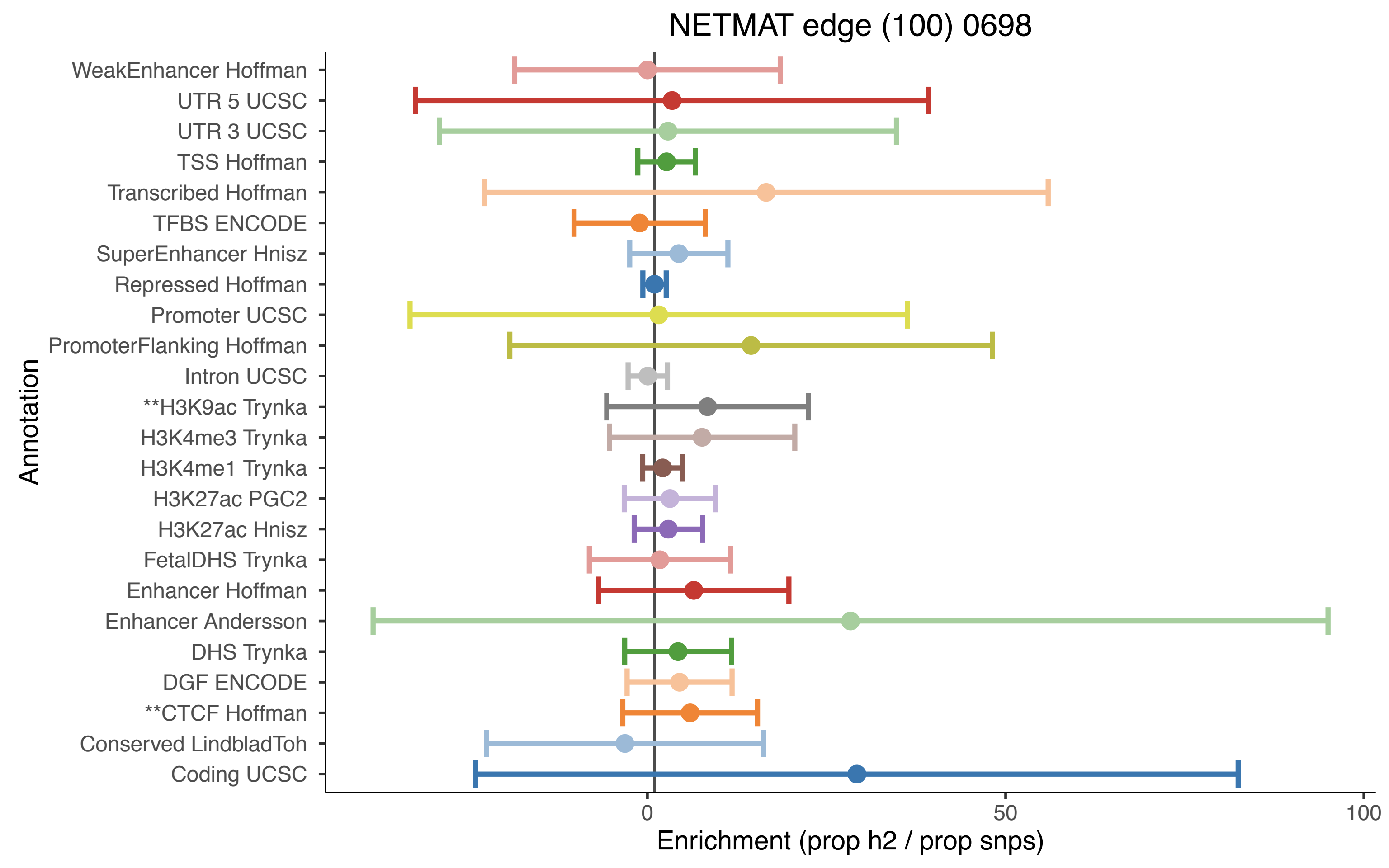
NETMAT edge (100) 0698
WeakEnhancer Hoffman
UTR 5 UCSC
UTR 3 UCSC
TSS Hoffman
Transcribed Hoffman
TFBS ENCODE
SuperEnhancer Hnisz
Repressed Hoffman
Promoter UCSC
PromoterFlanking Hoffman
Intron UCSC
**H3K9ac Trynka
H3K4me3 Trynka
H3K4me1 Trynka
H3K27ac PGC2
H3K27ac Hnisz
FetalDHS Trynka
Enhancer Hoffman
Enhancer Andersson
DHS Trynka
DGF ENCODE
**CTCF Hoffman
Conserved LindbladToh
Coding UCSC
Annotation
0
50
100
Enrichment (prop h2 / prop snps)

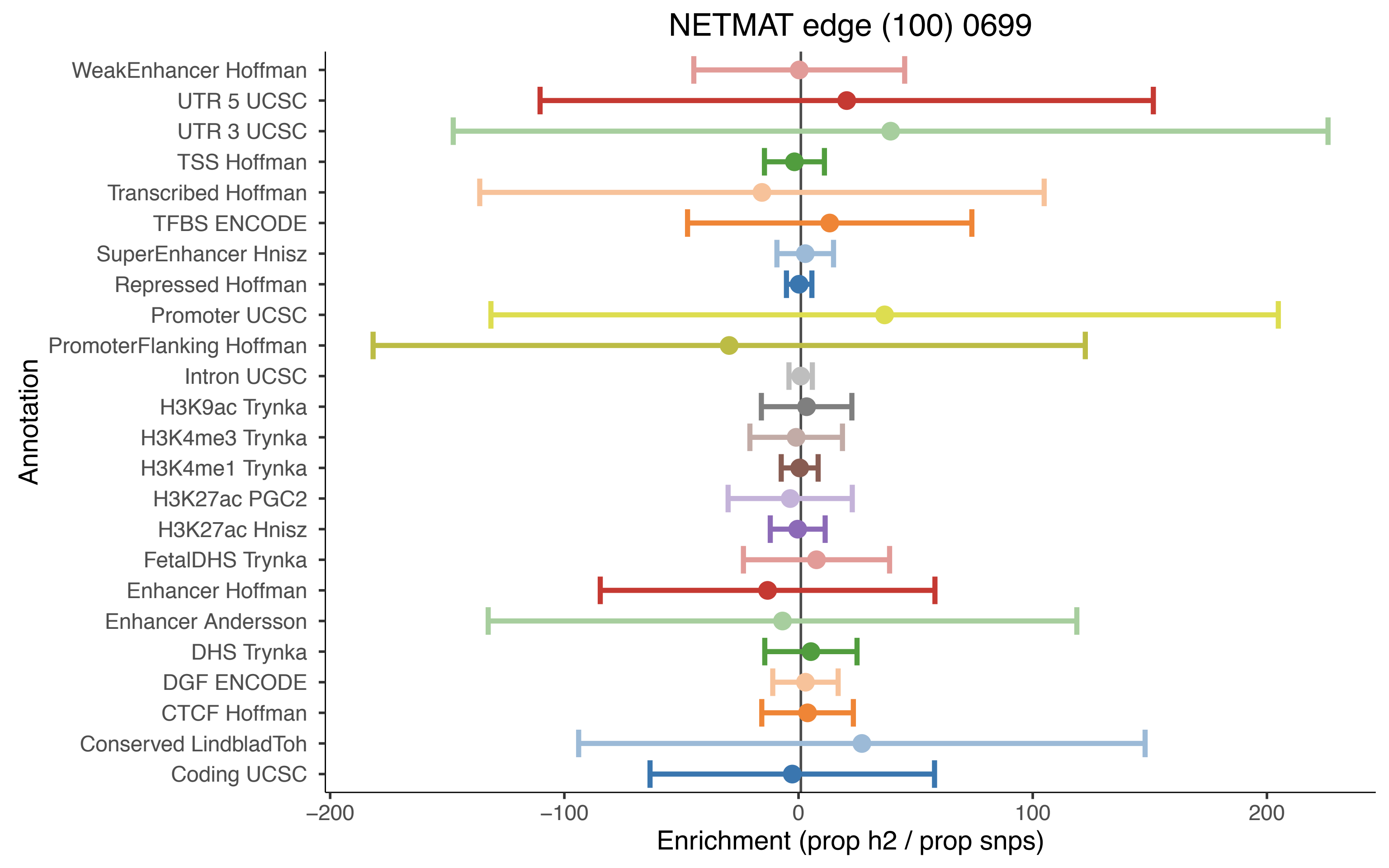
NETMAT edge (100) 0699
WeakEnhancer Hoffman
UTR 5 UCSC
UTR 3 UCSC
TSS Hoffman
Transcribed Hoffman
TFBS ENCODE
SuperEnhancer Hnisz
Repressed Hoffman
Promoter UCSC
PromoterFlanking Hoffman
Intron UCSC
H3K9ac Trynka
H3K4me3 Trynka
H3K4me1 Trynka
H3K27ac PGC2
H3K27ac Hnisz
FetalDHS Trynka
Enhancer Hoffman
Enhancer Andersson
DHS Trynka
DGF ENCODE
CTCF Hoffman
Conserved LindbladToh
Coding UCSC
Annotation
−200
−100
0
100
200
Enrichment (prop h2 / prop snps)

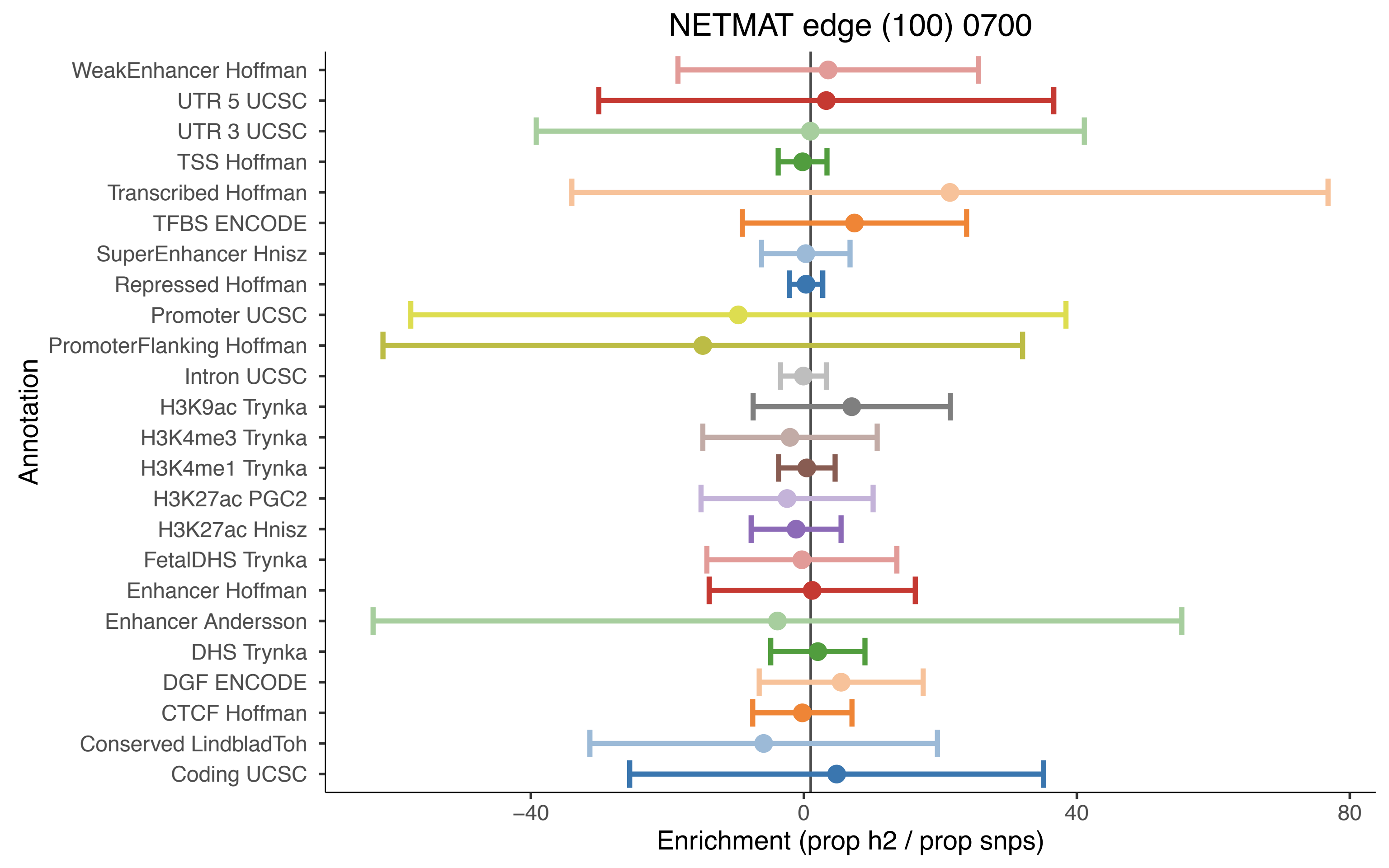

NETMAT edge (100) 0700
Annotation
WeakEnhancer Hoffman
UTR 5 UCSC
UTR 3 UCSC
TSS Hoffman
Transcribed Hoffman
TFBS ENCODE
SuperEnhancer Hnisz
Repressed Hoffman
Promoter UCSC
PromoterFlanking Hoffman
Intron UCSC
H3K9ac Trynka
H3K4me3 Trynka
H3K4me1 Trynka
H3K27ac PGC2
H3K27ac Hnisz
FetalDHS Trynka
Enhancer Hoffman
Enhancer Andersson
DHS Trynka
DGF ENCODE
CTCF Hoffman
Conserved LindbladToh
Coding UCSC
−40
0
40
80
Enrichment (prop h2 / prop snps)

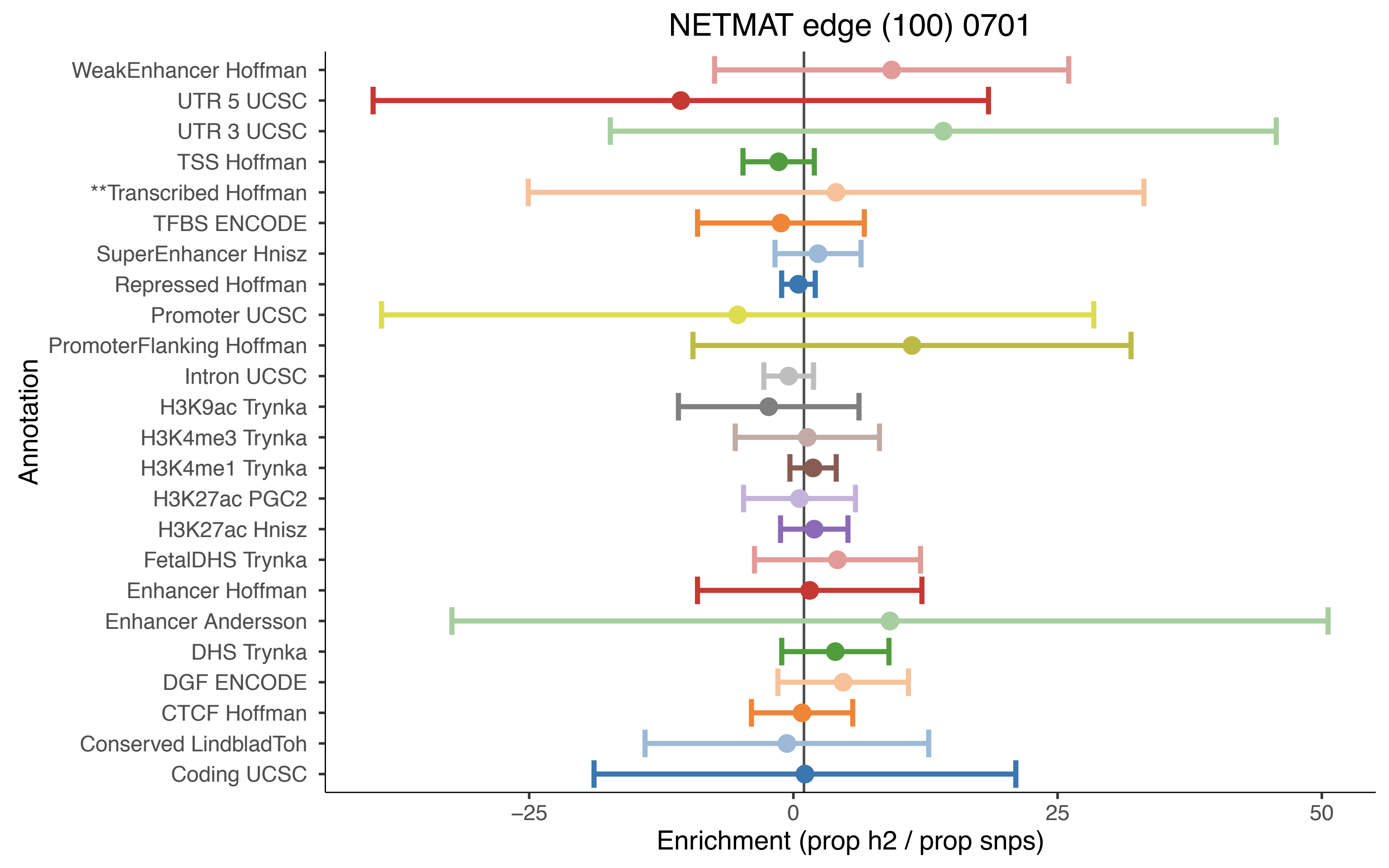
NETMAT edge (100) 0701
WeakEnhancer Hoffman
UTR 5 UCSC
UTR 3 UCSC
TSS Hoffman
**Transcribed Hoffman
TFBS ENCODE
SuperEnhancer Hnisz
Repressed Hoffman
Promoter UCSC
PromoterFlanking Hoffman
Intron UCSC
H3K9ac Trynka
H3K4me3 Trynka
H3K4me1 Trynka
H3K27ac PGC2
H3K27ac Hnisz
FetalDHS Trynka
Enhancer Hoffman
Enhancer Andersson
DHS Trynka
DGF ENCODE
CTCF Hoffman
Conserved LindbladToh
Coding UCSC
Annotation
−25
0
25
50
Enrichment (prop h2 / prop snps)

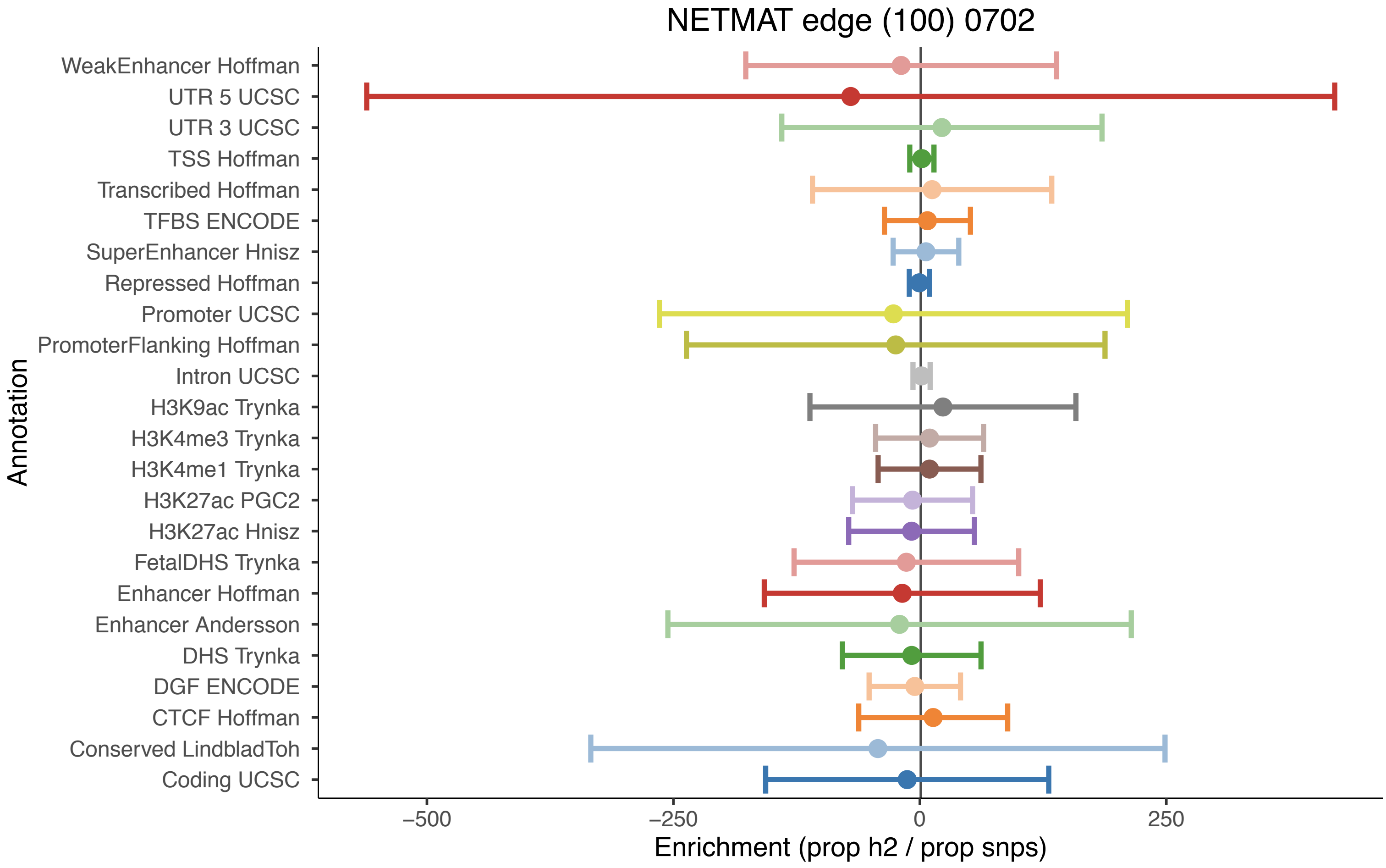
NETMAT edge (100) 0702
Annotation
WeakEnhancer Hoffman
UTR 5 UCSC
UTR 3 UCSC
TSS Hoffman
Transcribed Hoffman
TFBS ENCODE
SuperEnhancer Hnisz
Repressed Hoffman
Promoter UCSC
PromoterFlanking Hoffman
Intron UCSC
H3K9ac Trynka
H3K4me3 Trynka
H3K4me1 Trynka
H3K27ac PGC2
H3K27ac Hnisz
FetalDHS Trynka
Enhancer Hoffman
Enhancer Andersson
DHS Trynka
DGF ENCODE
CTCF Hoffman
Conserved LindbladToh
Coding UCSC
−500
−250
0
250
Enrichment (prop h2 / prop snps)

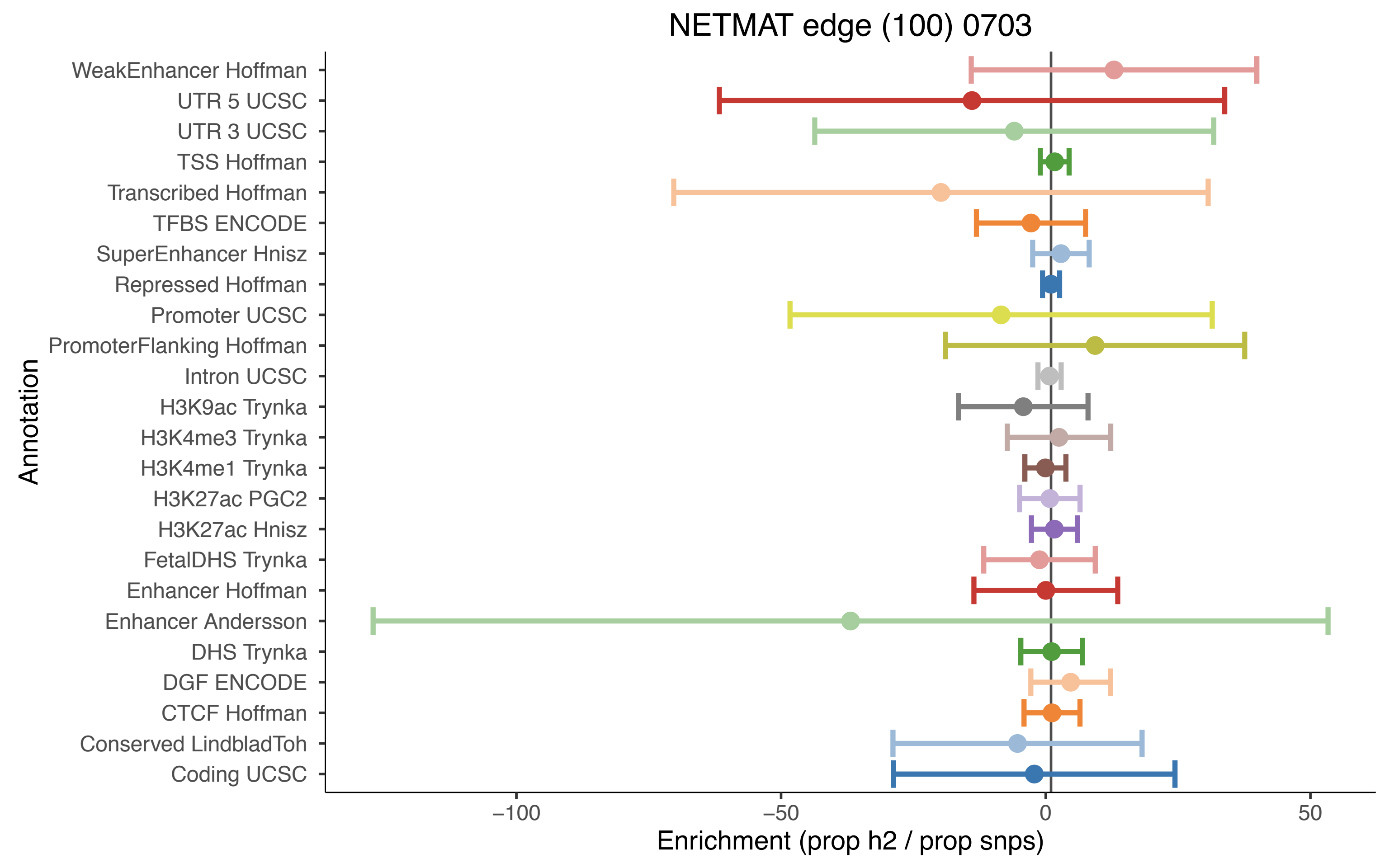
NETMAT edge (100) 0703
WeakEnhancer Hoffman
UTR 5 UCSC
UTR 3 UCSC
TSS Hoffman
Transcribed Hoffman
TFBS ENCODE
SuperEnhancer Hnisz
Repressed Hoffman
Promoter UCSC
PromoterFlanking Hoffman
Intron UCSC
H3K9ac Trynka
H3K4me3 Trynka
H3K4me1 Trynka
H3K27ac PGC2
H3K27ac Hnisz
FetalDHS Trynka
Enhancer Hoffman
Enhancer Andersson
DHS Trynka
DGF ENCODE
CTCF Hoffman
Conserved LindbladToh
Coding UCSC
Annotation
−100
−50
0
50
Enrichment (prop h2 / prop snps)

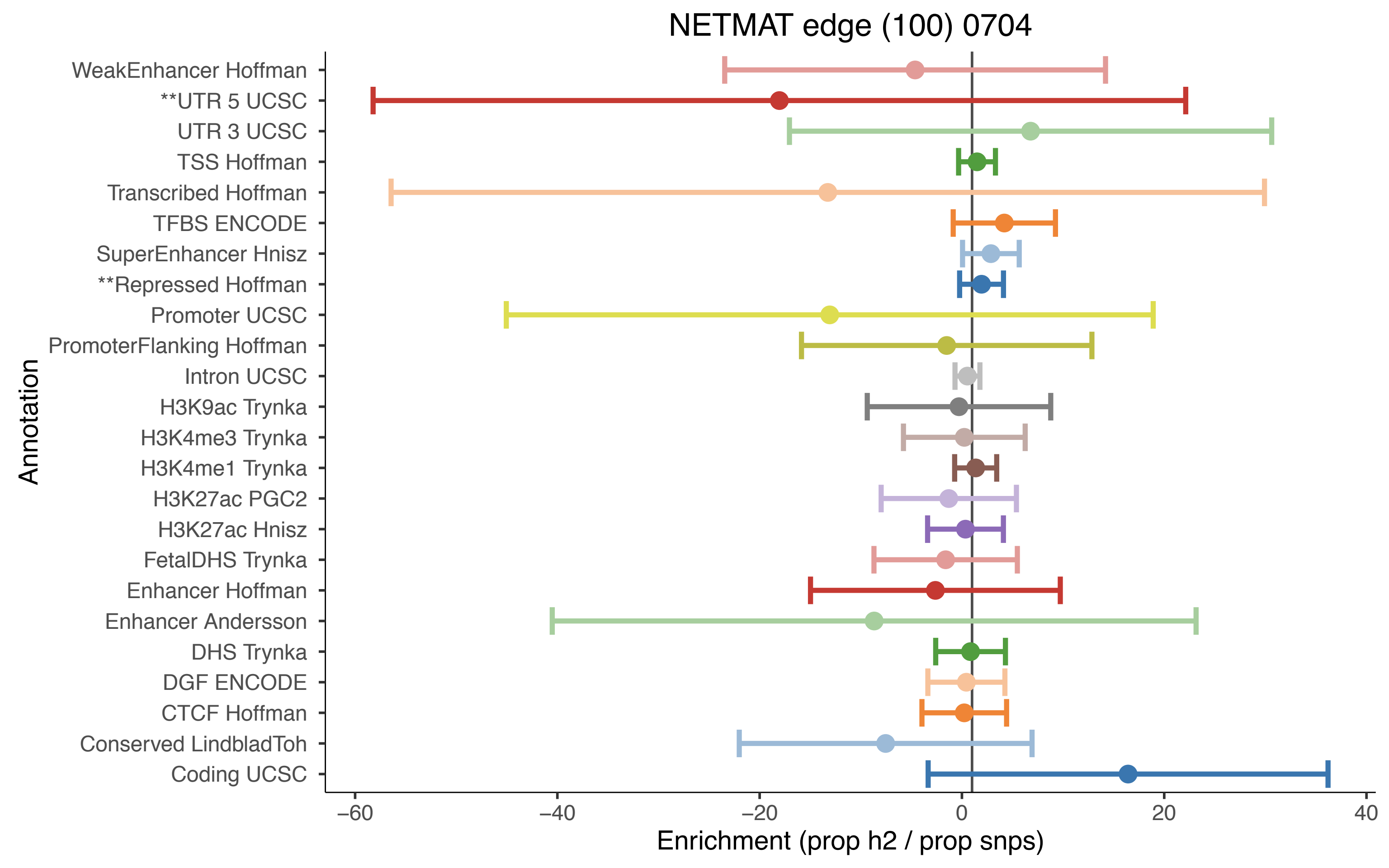
NETMAT edge (100) 0704
WeakEnhancer Hoffman
**UTR 5 UCSC
UTR 3 UCSC
TSS Hoffman
Transcribed Hoffman
TFBS ENCODE
SuperEnhancer Hnisz
**Repressed Hoffman
Promoter UCSC
PromoterFlanking Hoffman
Intron UCSC
H3K9ac Trynka
H3K4me3 Trynka
H3K4me1 Trynka
H3K27ac PGC2
H3K27ac Hnisz
FetalDHS Trynka
Enhancer Hoffman
Enhancer Andersson
DHS Trynka
DGF ENCODE
CTCF Hoffman
Conserved LindbladToh
Coding UCSC
Annotation
−60
−40
−20
0
20
40
Enrichment (prop h2 / prop snps)

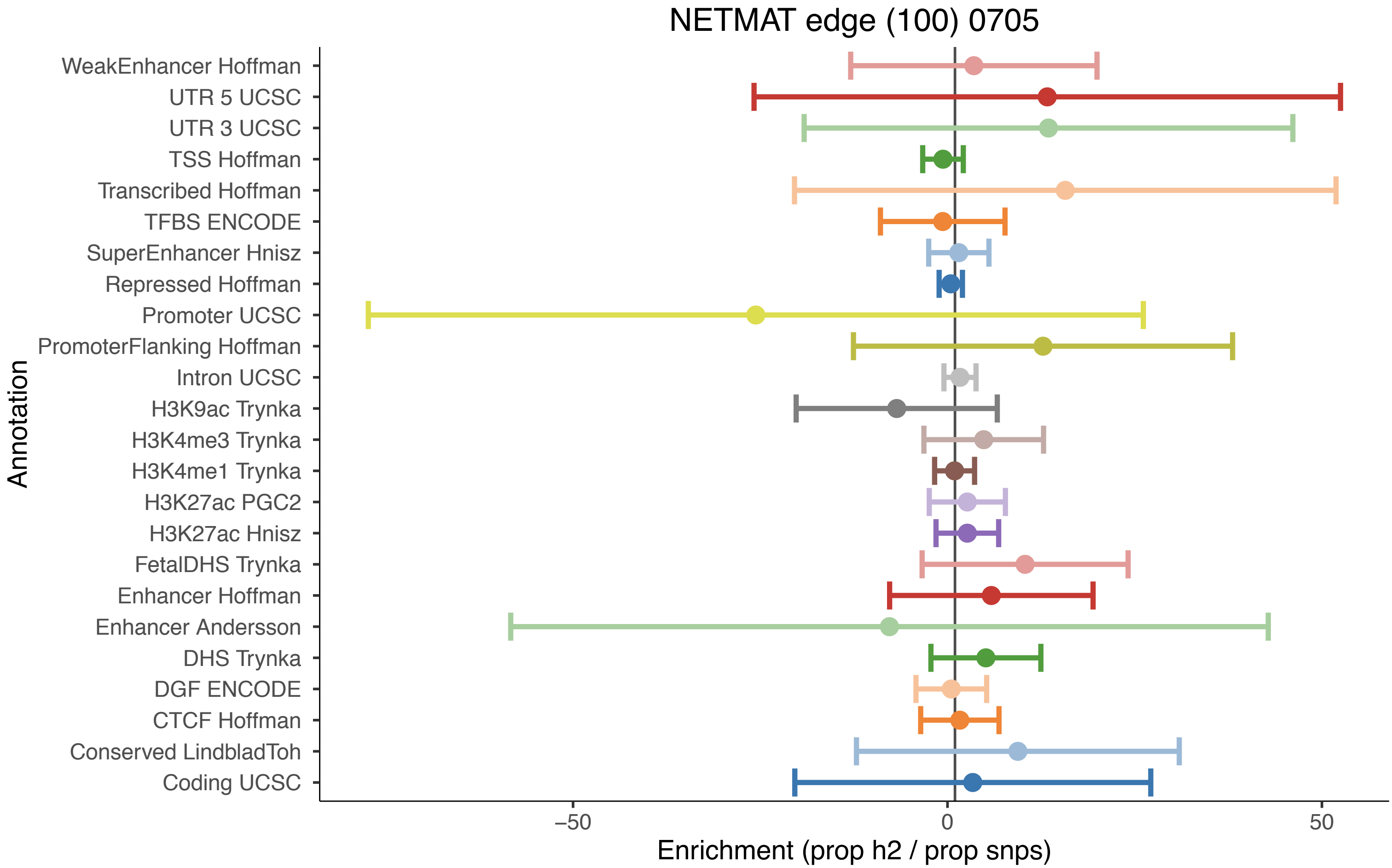
NETMAT edge (100) 0705
WeakEnhancer Hoffman
UTR 5 UCSC
UTR 3 UCSC
TSS Hoffman
Transcribed Hoffman
TFBS ENCODE
SuperEnhancer Hnisz
Repressed Hoffman
Promoter UCSC
PromoterFlanking Hoffman
Intron UCSC
H3K9ac Trynka
H3K4me3 Trynka
H3K4me1 Trynka
H3K27ac PGC2
H3K27ac Hnisz
FetalDHS Trynka
Enhancer Hoffman
Enhancer Andersson
DHS Trynka
DGF ENCODE
CTCF Hoffman
Conserved LindbladToh
Coding UCSC
Annotation
−50
0
50
Enrichment (prop h2 / prop snps)

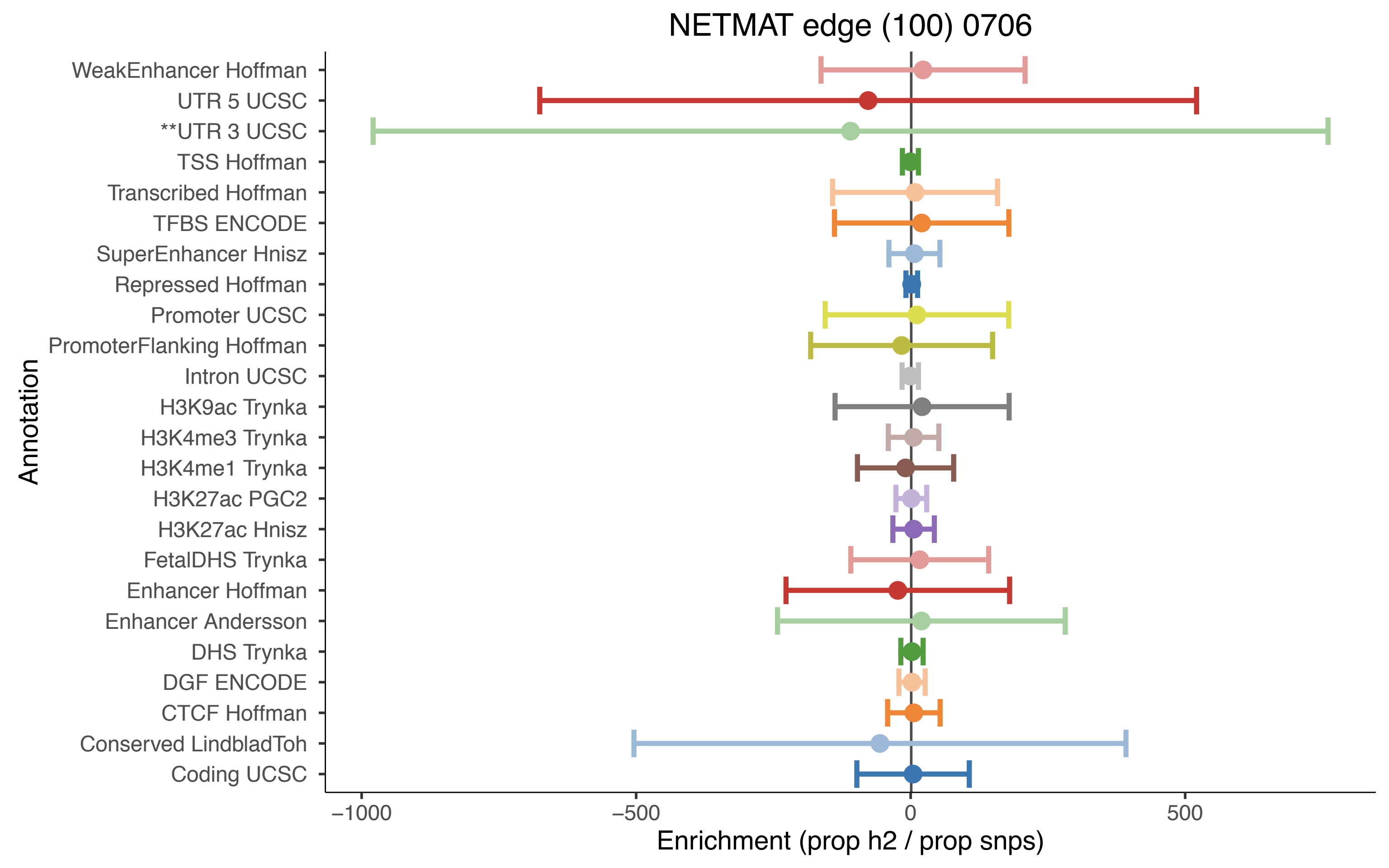

NETMAT edge (100) 0706
WeakEnhancer Hoffman
UTR 5 UCSC
**UTR 3 UCSC
TSS Hoffman
Transcribed Hoffman
TFBS ENCODE
SuperEnhancer Hnisz
Repressed Hoffman
Promoter UCSC
PromoterFlanking Hoffman
Intron UCSC
H3K9ac Trynka
H3K4me3 Trynka
H3K4me1 Trynka
H3K27ac PGC2
H3K27ac Hnisz
FetalDHS Trynka
Enhancer Hoffman
Enhancer Andersson
DHS Trynka
DGF ENCODE
CTCF Hoffman
Conserved LindbladToh
Coding UCSC
Annotation
−1000
−500
0
500
Enrichment (prop h2 / prop snps)

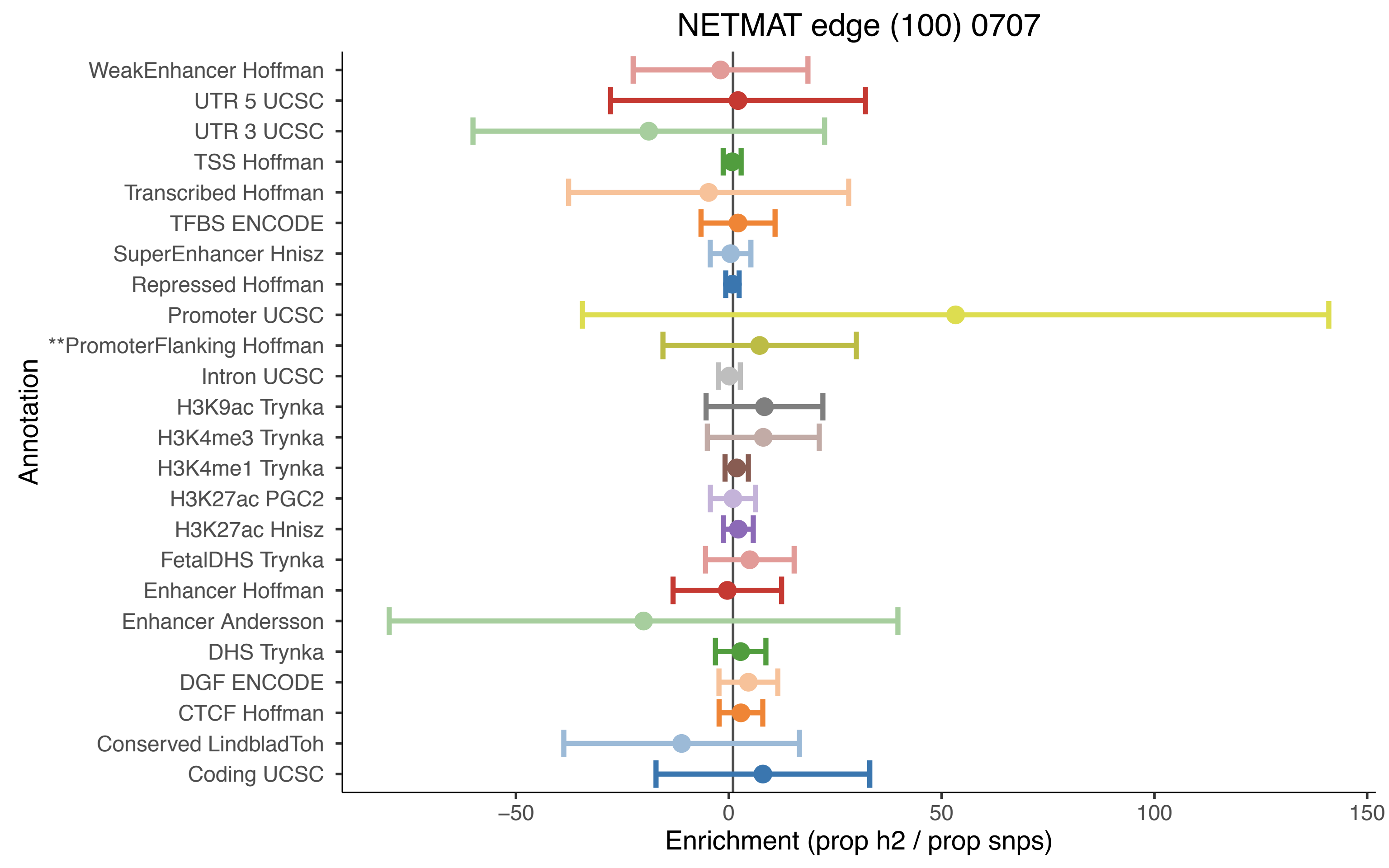

NETMAT edge (100) 0707
WeakEnhancer Hoffman
UTR 5 UCSC
UTR 3 UCSC
TSS Hoffman
Transcribed Hoffman
TFBS ENCODE
SuperEnhancer Hnisz
Repressed Hoffman
Promoter UCSC
**PromoterFlanking Hoffman
Intron UCSC
H3K9ac Trynka
H3K4me3 Trynka
H3K4me1 Trynka
H3K27ac PGC2
H3K27ac Hnisz
FetalDHS Trynka
Enhancer Hoffman
Enhancer Andersson
DHS Trynka
DGF ENCODE
CTCF Hoffman
Conserved LindbladToh
Coding UCSC
Annotation
−50
0
50
100
150
Enrichment (prop h2 / prop snps)

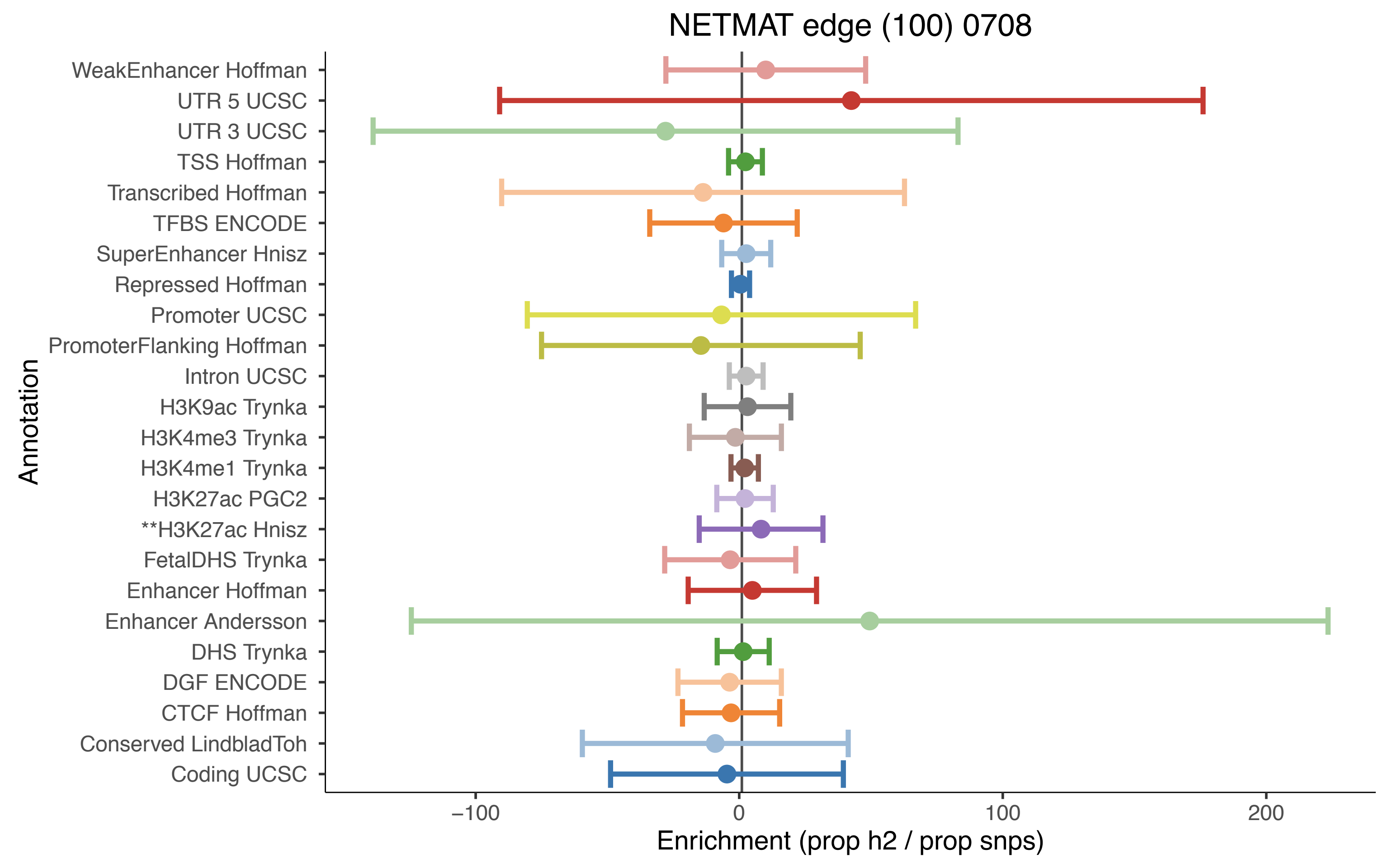
NETMAT edge (100) 0708
WeakEnhancer Hoffman
UTR 5 UCSC
UTR 3 UCSC
TSS Hoffman
Transcribed Hoffman
TFBS ENCODE
SuperEnhancer Hnisz
Repressed Hoffman
Promoter UCSC
PromoterFlanking Hoffman
Intron UCSC
H3K9ac Trynka
H3K4me3 Trynka
H3K4me1 Trynka
H3K27ac PGC2
**H3K27ac Hnisz
FetalDHS Trynka
Enhancer Hoffman
Enhancer Andersson
DHS Trynka
DGF ENCODE
CTCF Hoffman
Conserved LindbladToh
Coding UCSC
Annotation
−100
0
100
200
Enrichment (prop h2 / prop snps)

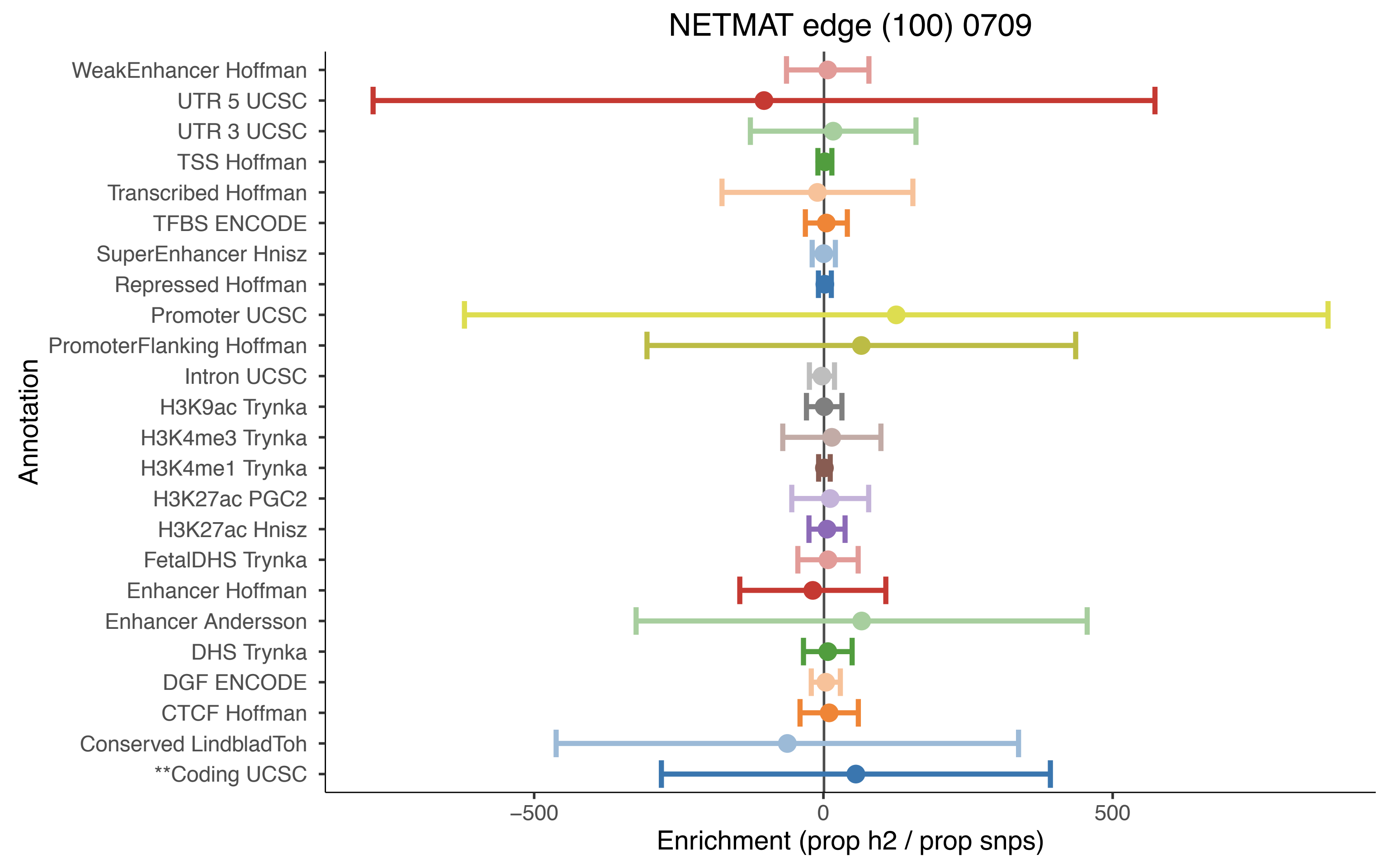
NETMAT edge (100) 0709
WeakEnhancer Hoffman
UTR 5 UCSC
UTR 3 UCSC
TSS Hoffman
Transcribed Hoffman
TFBS ENCODE
SuperEnhancer Hnisz
Repressed Hoffman
Promoter UCSC
PromoterFlanking Hoffman
Intron UCSC
H3K9ac Trynka
H3K4me3 Trynka
H3K4me1 Trynka
H3K27ac PGC2
H3K27ac Hnisz
FetalDHS Trynka
Enhancer Hoffman
Enhancer Andersson
DHS Trynka
DGF ENCODE
CTCF Hoffman
Conserved LindbladToh
**Coding UCSC
Annotation
−500
0
500
Enrichment (prop h2 / prop snps)

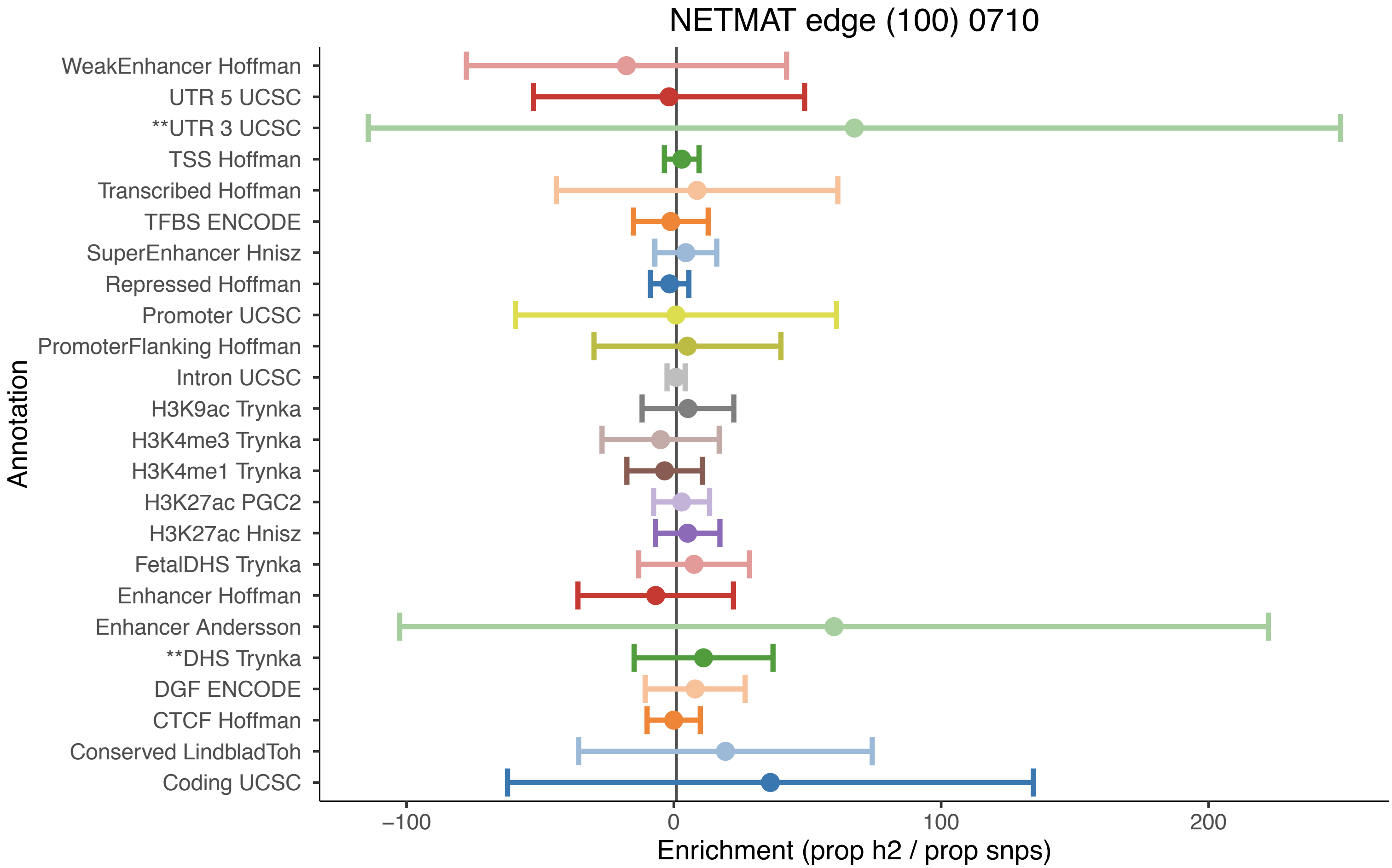
NETMAT edge (100) 0710
WeakEnhancer Hoffman
UTR 5 UCSC
**UTR 3 UCSC
TSS Hoffman
Transcribed Hoffman
TFBS ENCODE
SuperEnhancer Hnisz
Repressed Hoffman
Promoter UCSC
PromoterFlanking Hoffman
Intron UCSC
H3K9ac Trynka
H3K4me3 Trynka
H3K4me1 Trynka
H3K27ac PGC2
H3K27ac Hnisz
FetalDHS Trynka
Enhancer Hoffman
Enhancer Andersson
**DHS Trynka
DGF ENCODE
CTCF Hoffman
Conserved LindbladToh
Coding UCSC
Annotation
−100
0
100
200
Enrichment (prop h2 / prop snps)

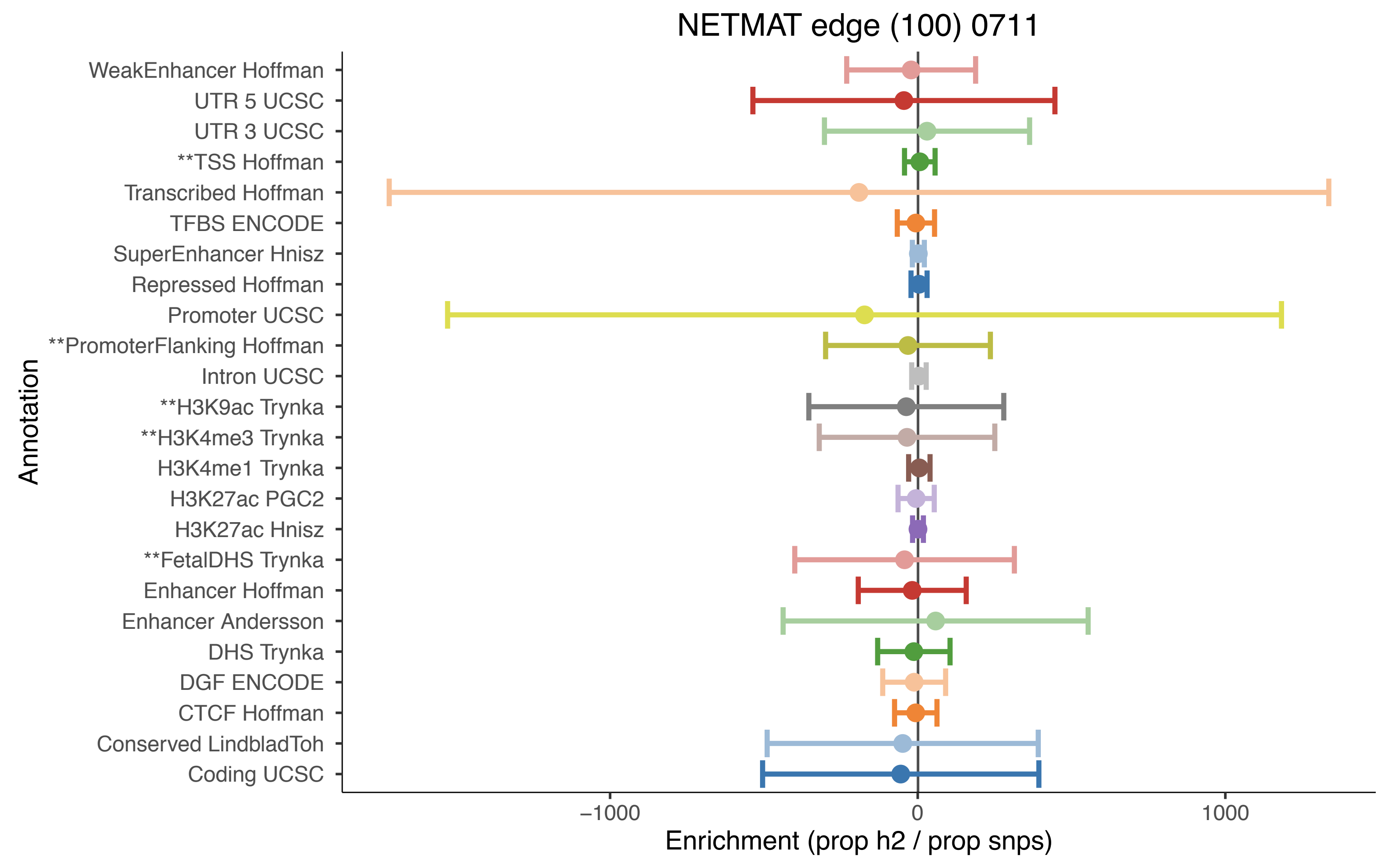
NETMAT edge (100) 0711
WeakEnhancer Hoffman
UTR 5 UCSC
UTR 3 UCSC
**TSS Hoffman
Transcribed Hoffman
TFBS ENCODE
SuperEnhancer Hnisz
Repressed Hoffman
Promoter UCSC
**PromoterFlanking Hoffman
Intron UCSC
**H3K9ac Trynka
**H3K4me3 Trynka
H3K4me1 Trynka
H3K27ac PGC2
H3K27ac Hnisz
**FetalDHS Trynka
Enhancer Hoffman
Enhancer Andersson
DHS Trynka
DGF ENCODE
CTCF Hoffman
Conserved LindbladToh
Coding UCSC
Annotation
−1000
0
1000
Enrichment (prop h2 / prop snps)

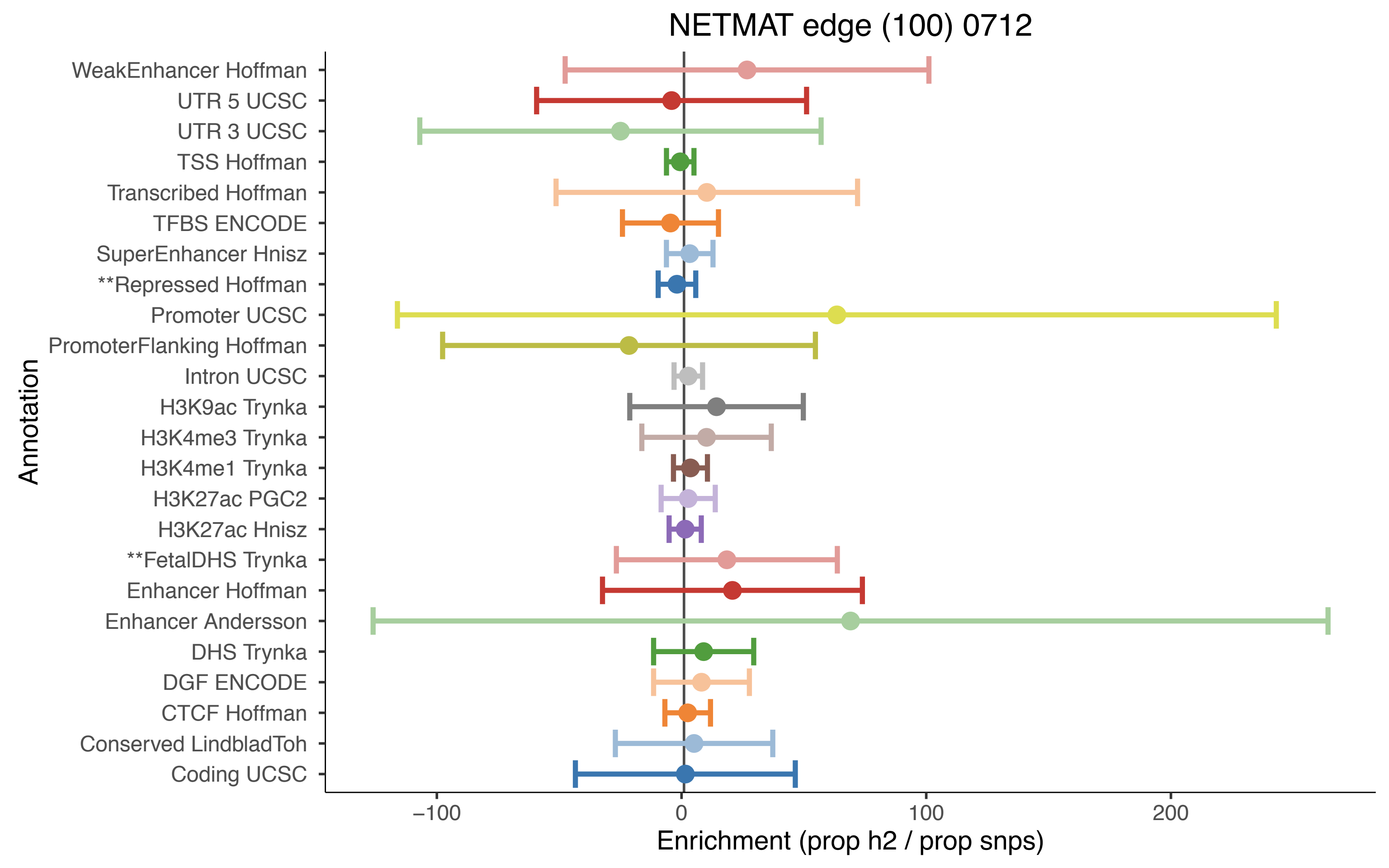
NETMAT edge (100) 0712
WeakEnhancer Hoffman
UTR 5 UCSC
UTR 3 UCSC
TSS Hoffman
Transcribed Hoffman
TFBS ENCODE
SuperEnhancer Hnisz
**Repressed Hoffman
Promoter UCSC
PromoterFlanking Hoffman
Intron UCSC
H3K9ac Trynka
H3K4me3 Trynka
H3K4me1 Trynka
H3K27ac PGC2
H3K27ac Hnisz
**FetalDHS Trynka
Enhancer Hoffman
Enhancer Andersson
DHS Trynka
DGF ENCODE
CTCF Hoffman
Conserved LindbladToh
Coding UCSC
Annotation
−100
0
100
200
Enrichment (prop h2 / prop snps)

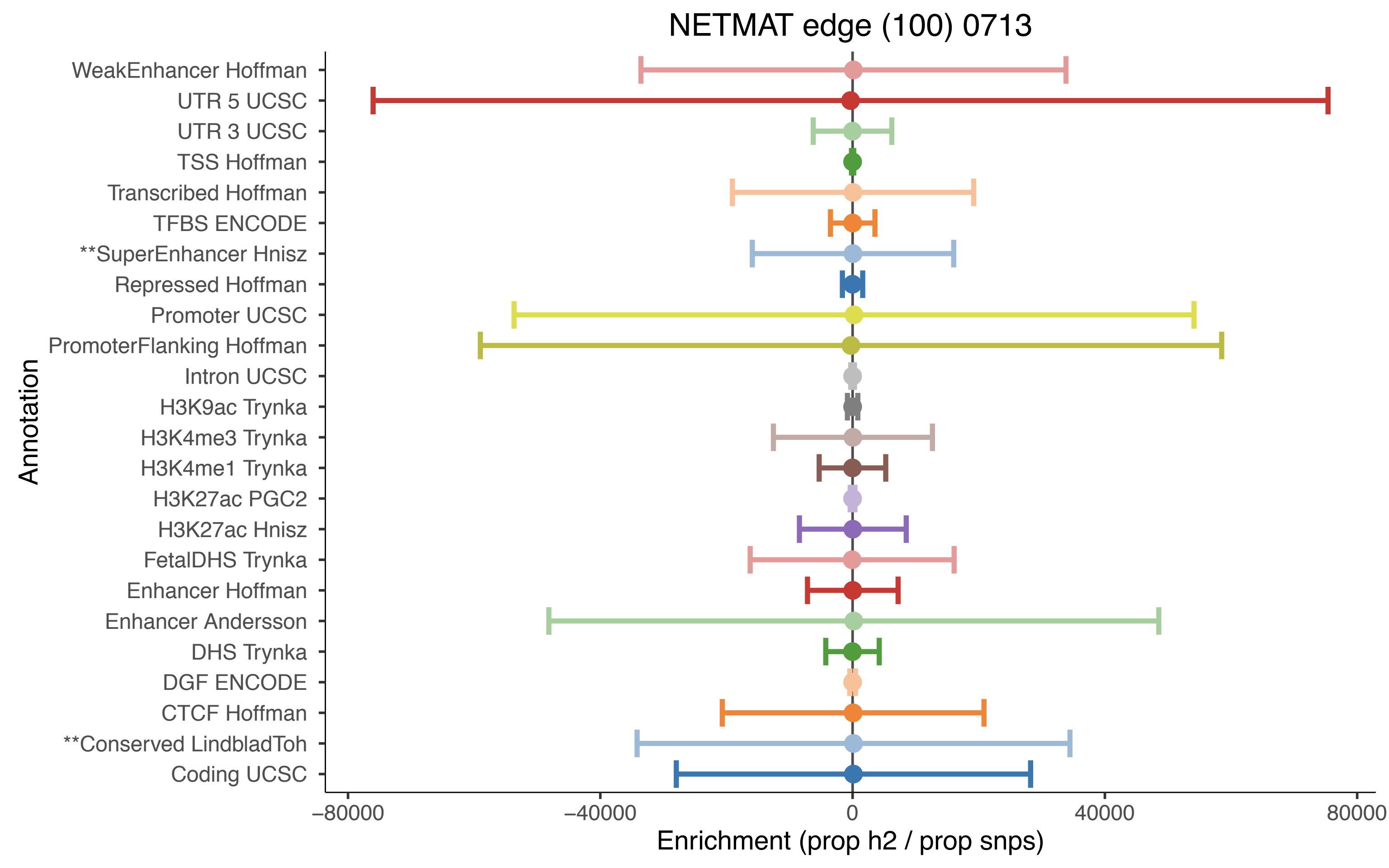

NETMAT edge (100) 0713
WeakEnhancer Hoffman
UTR 5 UCSC
UTR 3 UCSC
TSS Hoffman
Transcribed Hoffman
TFBS ENCODE
**SuperEnhancer Hnisz
Repressed Hoffman
Promoter UCSC
PromoterFlanking Hoffman
Intron UCSC
H3K9ac Trynka
H3K4me3 Trynka
H3K4me1 Trynka
H3K27ac PGC2
H3K27ac Hnisz
FetalDHS Trynka
Enhancer Hoffman
Enhancer Andersson
DHS Trynka
DGF ENCODE
CTCF Hoffman
**Conserved LindbladToh
Coding UCSC
Annotation
−80000
−40000
0
40000
80000
Enrichment (prop h2 / prop snps)

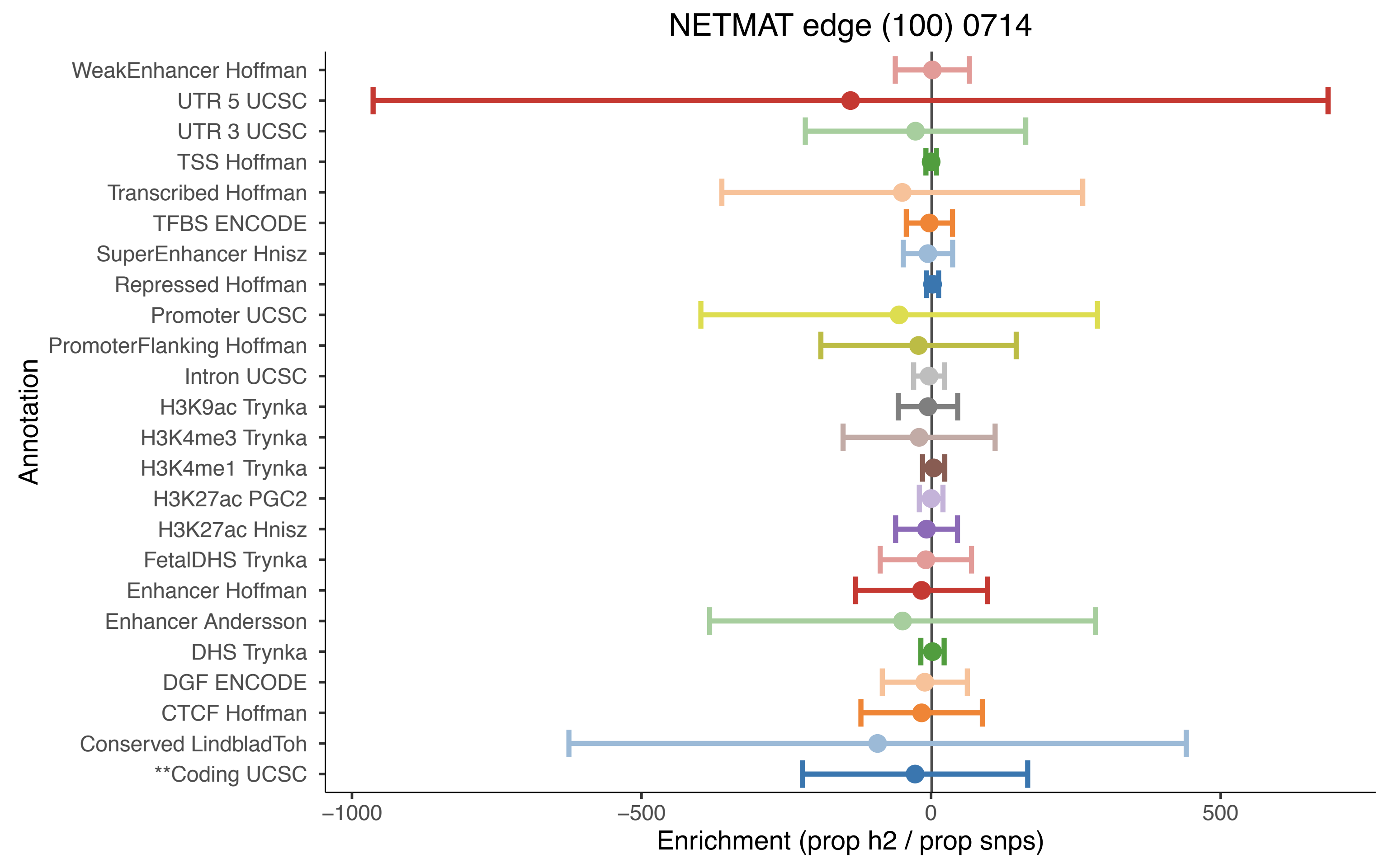
NETMAT edge (100) 0714
Annotation
WeakEnhancer Hoffman
UTR 5 UCSC
UTR 3 UCSC
TSS Hoffman
Transcribed Hoffman
TFBS ENCODE
SuperEnhancer Hnisz
Repressed Hoffman
Promoter UCSC
PromoterFlanking Hoffman
Intron UCSC
H3K9ac Trynka
H3K4me3 Trynka
H3K4me1 Trynka
H3K27ac PGC2
H3K27ac Hnisz
FetalDHS Trynka
Enhancer Hoffman
Enhancer Andersson
DHS Trynka
DGF ENCODE
CTCF Hoffman
Conserved LindbladToh
**Coding UCSC
−1000
−500
0
500
Enrichment (prop h2 / prop snps)

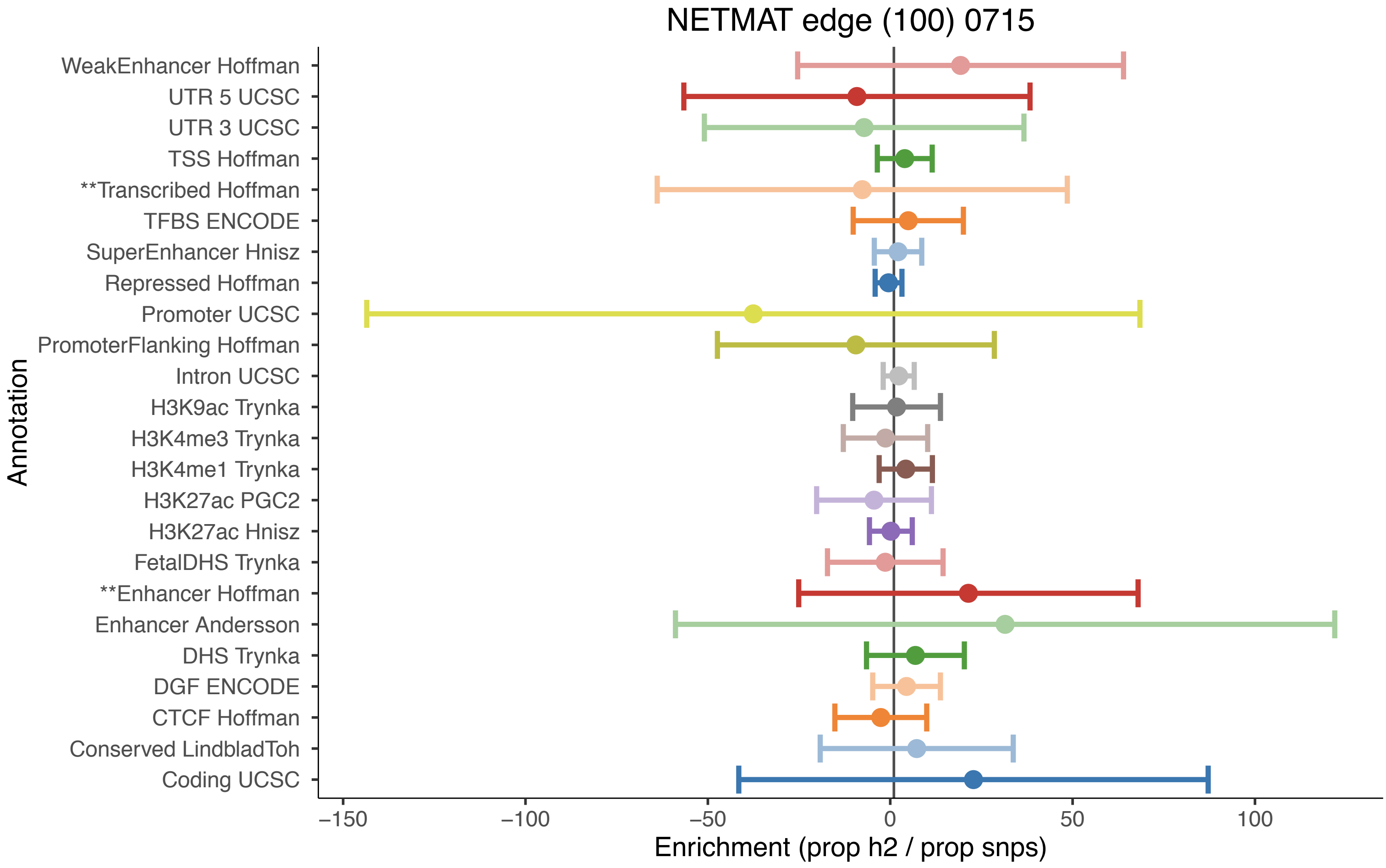

NETMAT edge (100) 0715
WeakEnhancer Hoffman
UTR 5 UCSC
UTR 3 UCSC
TSS Hoffman
**Transcribed Hoffman
TFBS ENCODE
SuperEnhancer Hnisz
Repressed Hoffman
Promoter UCSC
PromoterFlanking Hoffman
Intron UCSC
H3K9ac Trynka
H3K4me3 Trynka
H3K4me1 Trynka
H3K27ac PGC2
H3K27ac Hnisz
FetalDHS Trynka
**Enhancer Hoffman
Enhancer Andersson
DHS Trynka
DGF ENCODE
CTCF Hoffman
Conserved LindbladToh
Coding UCSC
Annotation
−150
−100
−50
0
50
100
Enrichment (prop h2 / prop snps)

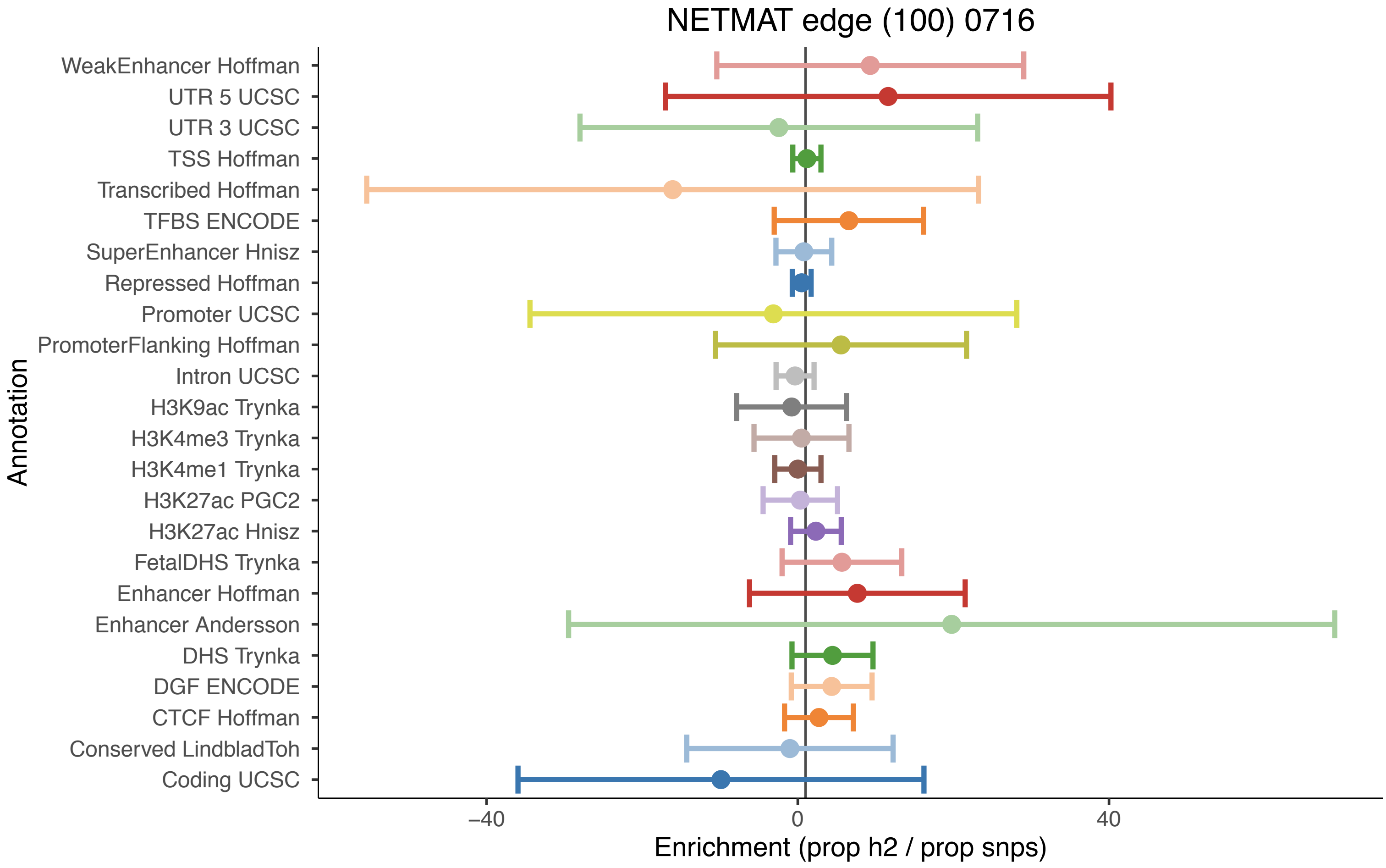

NETMAT edge (100) 0716
WeakEnhancer Hoffman
UTR 5 UCSC
UTR 3 UCSC
TSS Hoffman
Transcribed Hoffman
TFBS ENCODE
SuperEnhancer Hnisz
Repressed Hoffman
Promoter UCSC
PromoterFlanking Hoffman
Intron UCSC
H3K9ac Trynka
H3K4me3 Trynka
H3K4me1 Trynka
H3K27ac PGC2
H3K27ac Hnisz
FetalDHS Trynka
Enhancer Hoffman
Enhancer Andersson
DHS Trynka
DGF ENCODE
CTCF Hoffman
Conserved LindbladToh
Coding UCSC
Annotation
−40
0
40
Enrichment (prop h2 / prop snps)

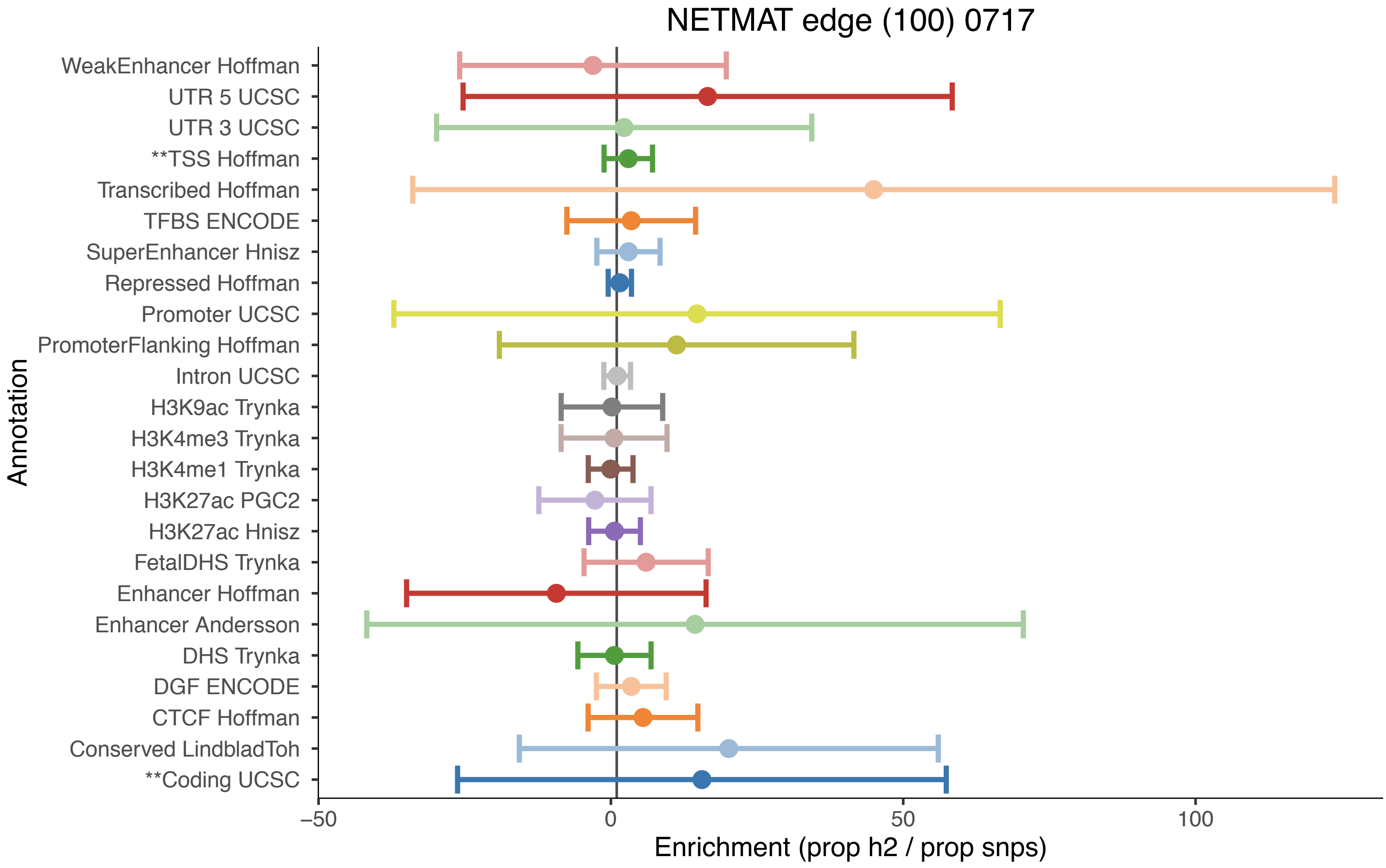

NETMAT edge (100) 0717
WeakEnhancer Hoffman
UTR 5 UCSC
UTR 3 UCSC
**TSS Hoffman
Transcribed Hoffman
TFBS ENCODE
SuperEnhancer Hnisz
Repressed Hoffman
Promoter UCSC
PromoterFlanking Hoffman
Intron UCSC
H3K9ac Trynka
H3K4me3 Trynka
H3K4me1 Trynka
H3K27ac PGC2
H3K27ac Hnisz
FetalDHS Trynka
Enhancer Hoffman
Enhancer Andersson
DHS Trynka
DGF ENCODE
CTCF Hoffman
Conserved LindbladToh
**Coding UCSC
Annotation
−50
0
50
100
Enrichment (prop h2 / prop snps)

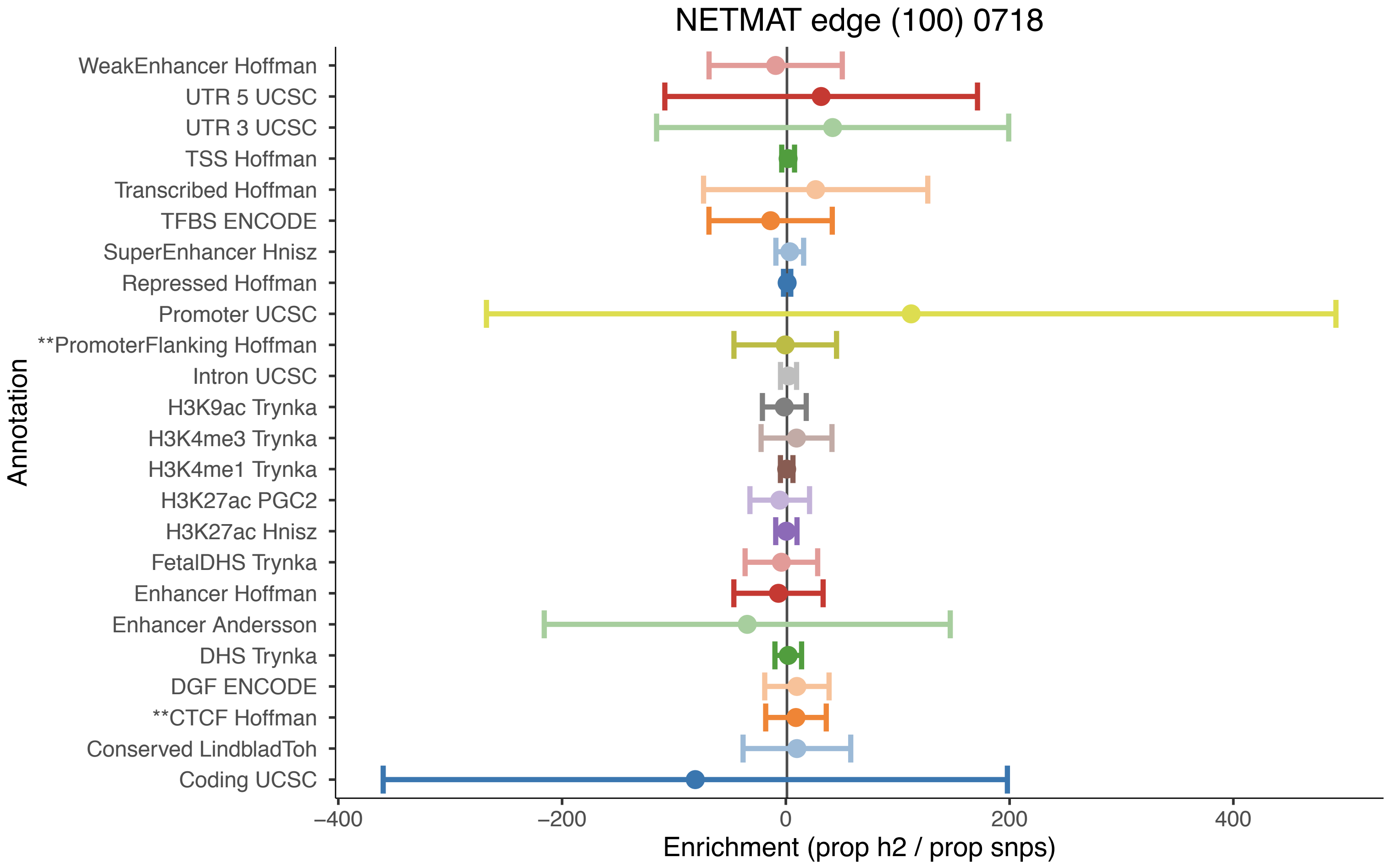

NETMAT edge (100) 0718
WeakEnhancer Hoffman
UTR 5 UCSC
UTR 3 UCSC
TSS Hoffman
Transcribed Hoffman
TFBS ENCODE
SuperEnhancer Hnisz
Repressed Hoffman
Promoter UCSC
**PromoterFlanking Hoffman
Intron UCSC
H3K9ac Trynka
H3K4me3 Trynka
H3K4me1 Trynka
H3K27ac PGC2
H3K27ac Hnisz
FetalDHS Trynka
Enhancer Hoffman
Enhancer Andersson
DHS Trynka
DGF ENCODE
**CTCF Hoffman
Conserved LindbladToh
Coding UCSC
Annotation
−400
−200
0
200
400
Enrichment (prop h2 / prop snps)

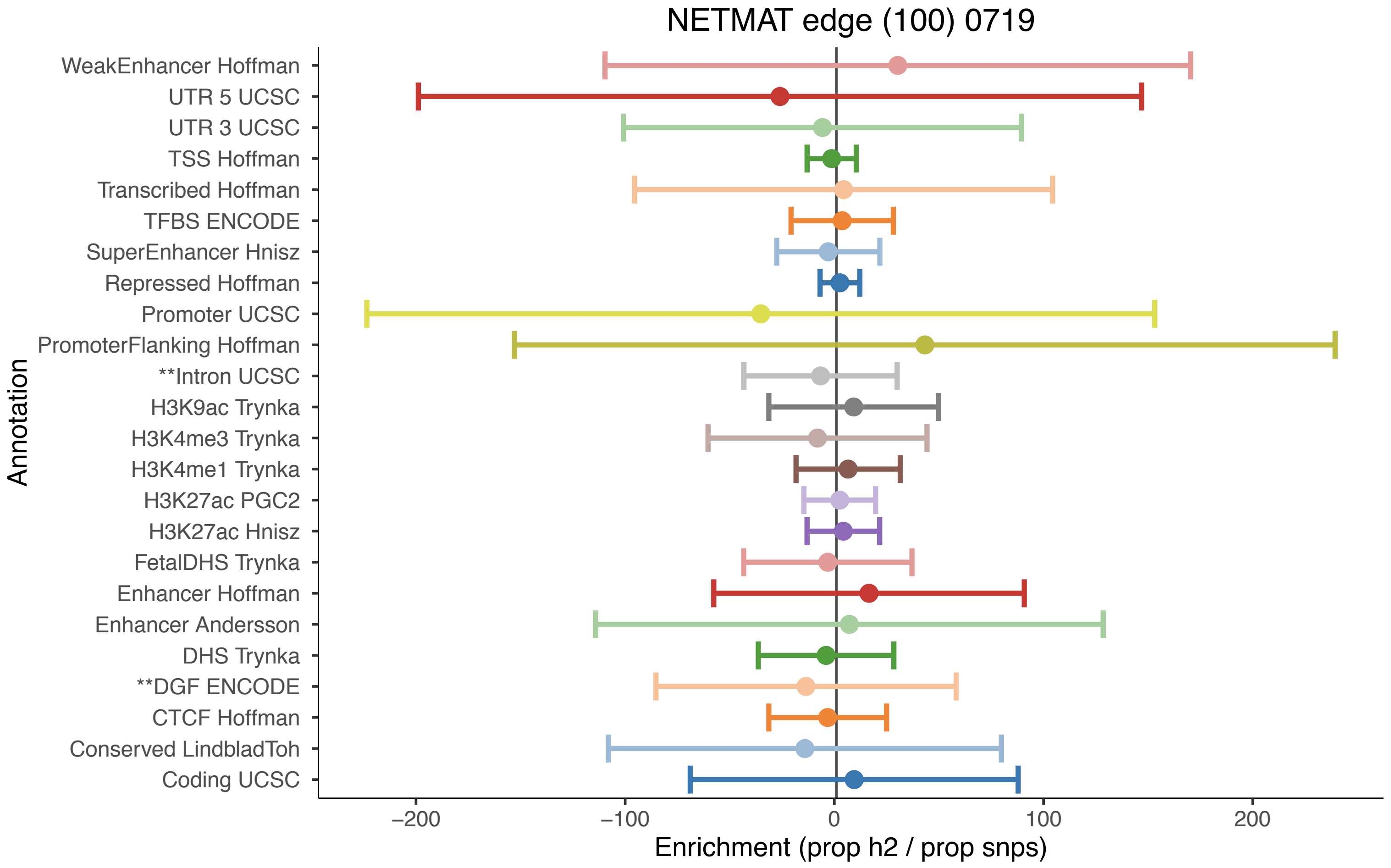

NETMAT edge (100) 0719
WeakEnhancer Hoffman
UTR 5 UCSC
UTR 3 UCSC
TSS Hoffman
Transcribed Hoffman
TFBS ENCODE
SuperEnhancer Hnisz
Repressed Hoffman
Promoter UCSC
PromoterFlanking Hoffman
**Intron UCSC
H3K9ac Trynka
H3K4me3 Trynka
H3K4me1 Trynka
H3K27ac PGC2
H3K27ac Hnisz
FetalDHS Trynka
Enhancer Hoffman
Enhancer Andersson
DHS Trynka
**DGF ENCODE
CTCF Hoffman
Conserved LindbladToh
Coding UCSC
Annotation
−200
−100
0
100
200
Enrichment (prop h2 / prop snps)

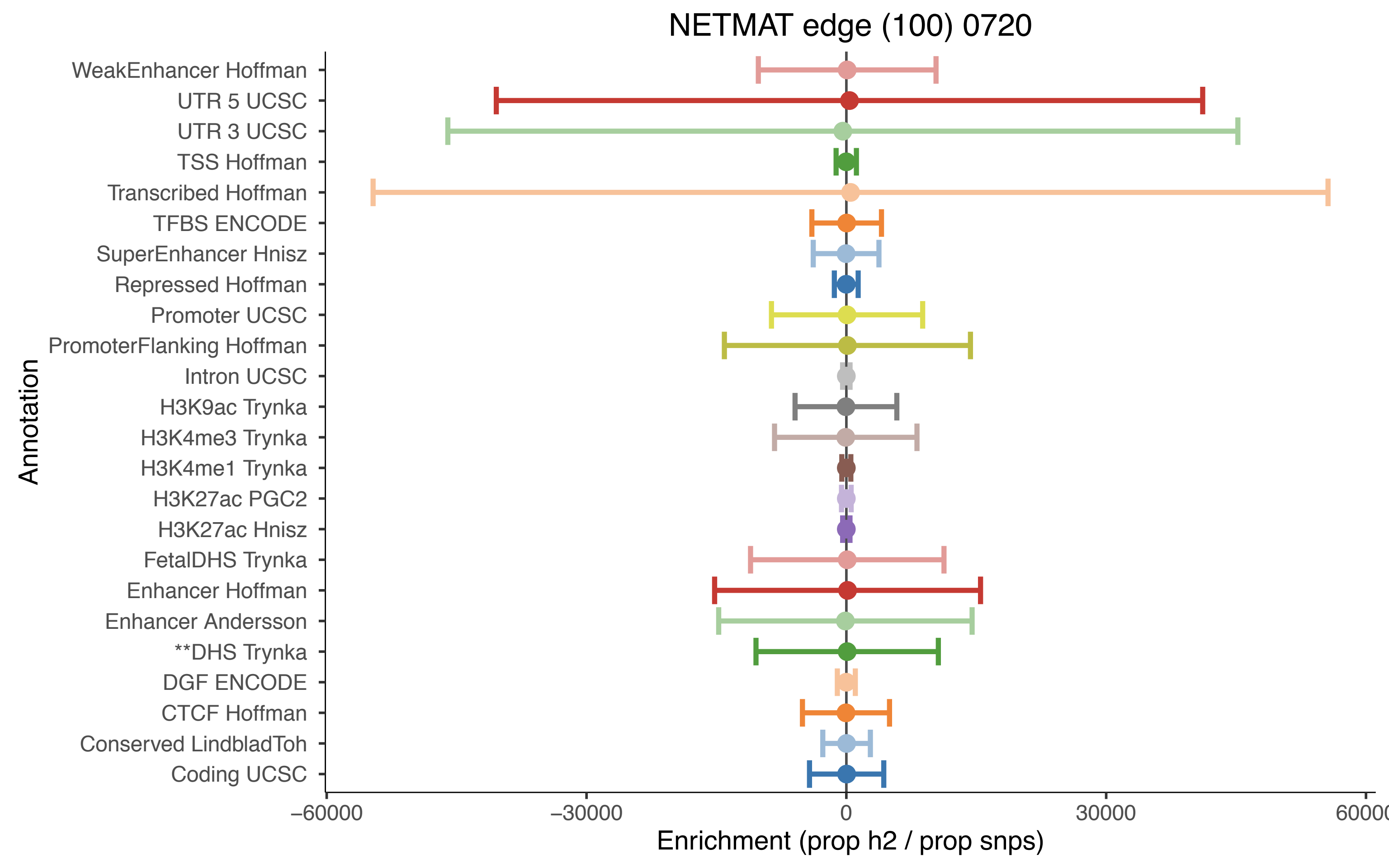

NETMAT edge (100) 0720
WeakEnhancer Hoffman
UTR 5 UCSC
UTR 3 UCSC
TSS Hoffman
Transcribed Hoffman
TFBS ENCODE
SuperEnhancer Hnisz
Repressed Hoffman
Promoter UCSC
PromoterFlanking Hoffman
Intron UCSC
H3K9ac Trynka
H3K4me3 Trynka
H3K4me1 Trynka
H3K27ac PGC2
H3K27ac Hnisz
FetalDHS Trynka
Enhancer Hoffman
Enhancer Andersson
**DHS Trynka
DGF ENCODE
CTCF Hoffman
Conserved LindbladToh
Coding UCSC
Annotation
−60000
−30000
0
30000
Enrichment (prop h2 / prop snps)

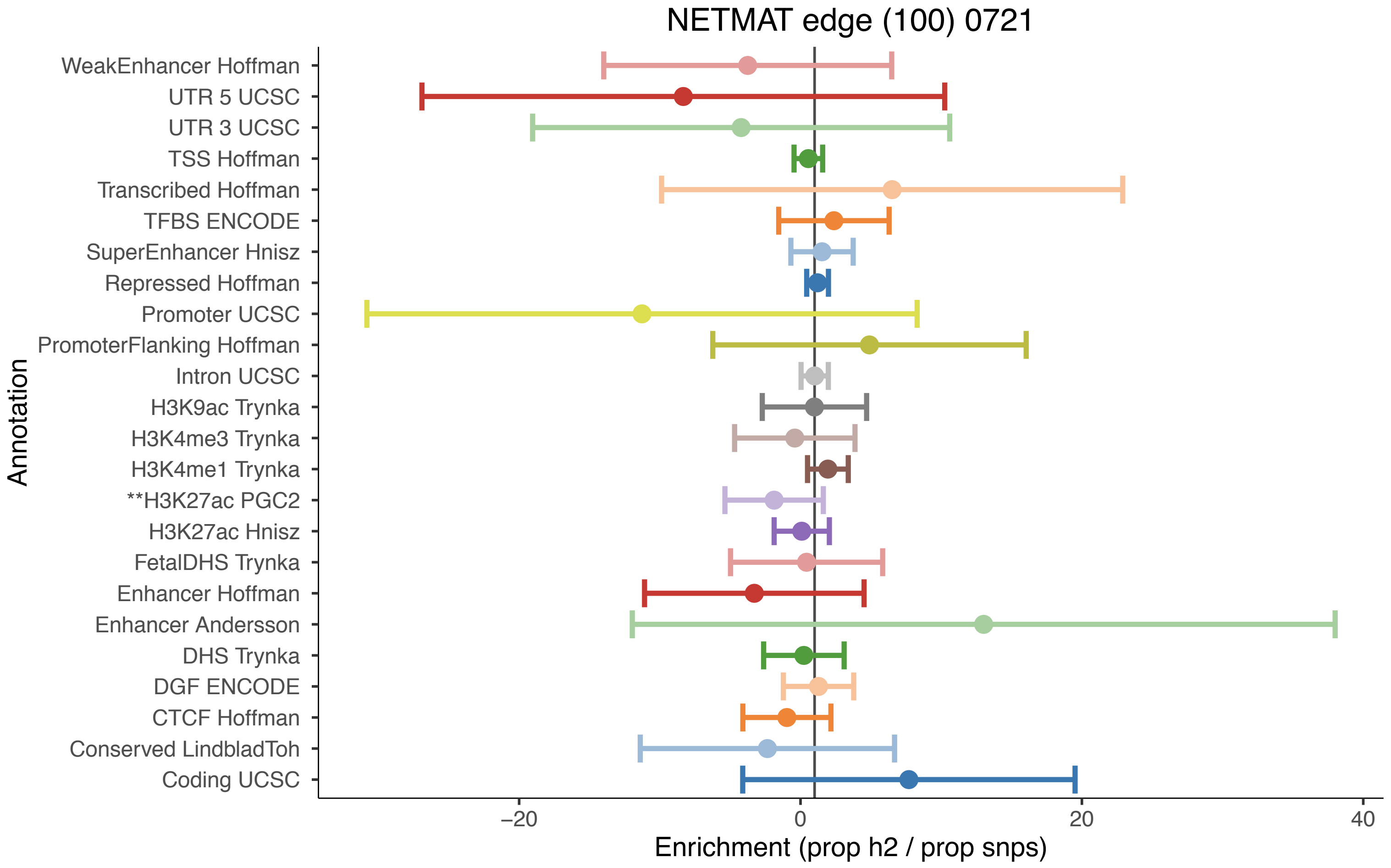
NETMAT edge (100) 0721
WeakEnhancer Hoffman
UTR 5 UCSC
UTR 3 UCSC
TSS Hoffman
Transcribed Hoffman
TFBS ENCODE
SuperEnhancer Hnisz
Repressed Hoffman
Promoter UCSC
PromoterFlanking Hoffman
Intron UCSC
H3K9ac Trynka
H3K4me3 Trynka
H3K4me1 Trynka
**H3K27ac PGC2
H3K27ac Hnisz
FetalDHS Trynka
Enhancer Hoffman
Enhancer Andersson
DHS Trynka
DGF ENCODE
CTCF Hoffman
Conserved LindbladToh
Coding UCSC
Annotation
−20
0
20
40
Enrichment (prop h2 / prop snps)

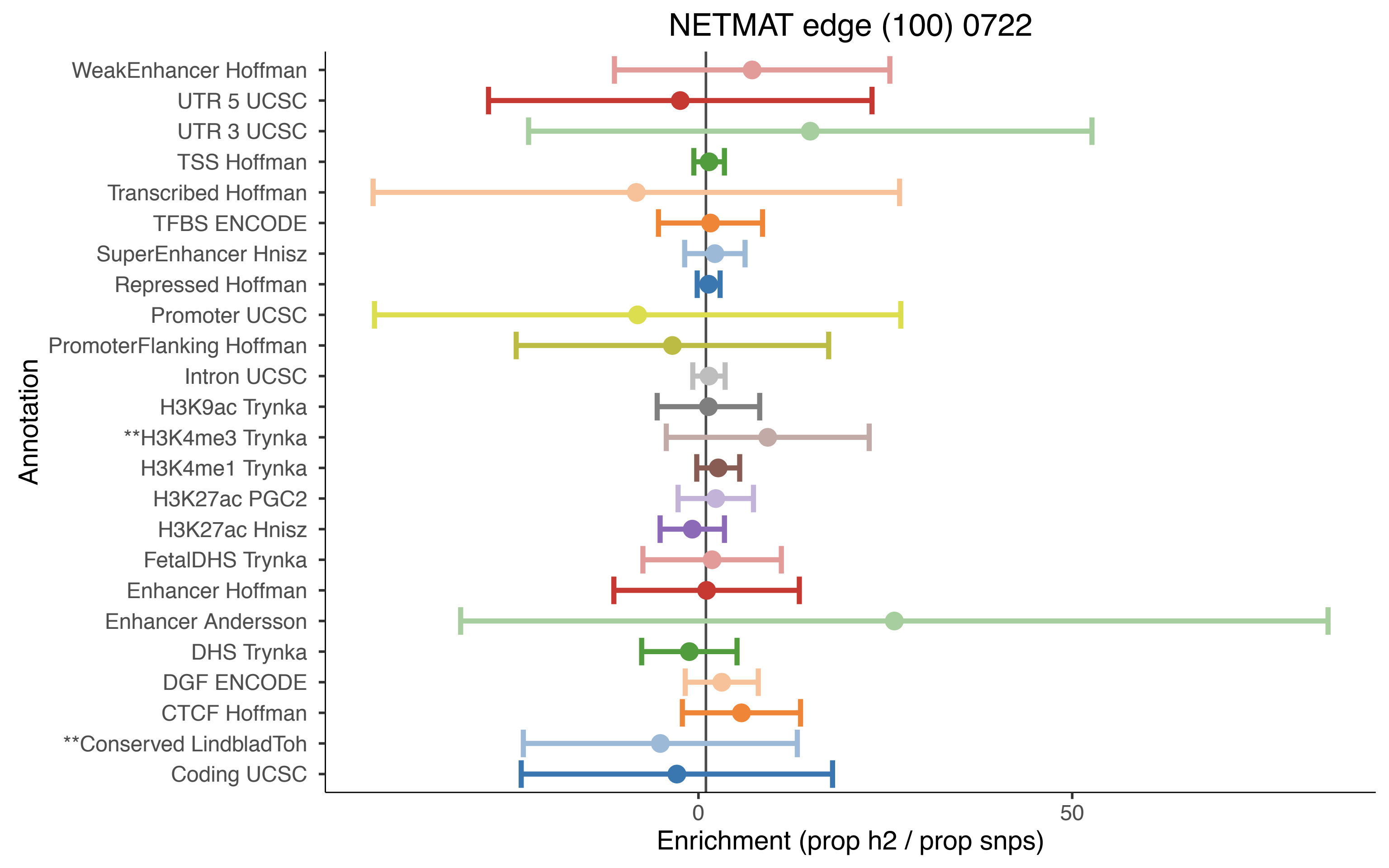

NETMAT edge (100) 0722
WeakEnhancer Hoffman
UTR 5 UCSC
UTR 3 UCSC
TSS Hoffman
Transcribed Hoffman
TFBS ENCODE
SuperEnhancer Hnisz
Repressed Hoffman
Promoter UCSC
PromoterFlanking Hoffman
Intron UCSC
H3K9ac Trynka
**H3K4me3 Trynka
H3K4me1 Trynka
H3K27ac PGC2
H3K27ac Hnisz
FetalDHS Trynka
Enhancer Hoffman
Enhancer Andersson
DHS Trynka
DGF ENCODE
CTCF Hoffman
**Conserved LindbladToh
Coding UCSC
Annotation
0
50
Enrichment (prop h2 / prop snps)

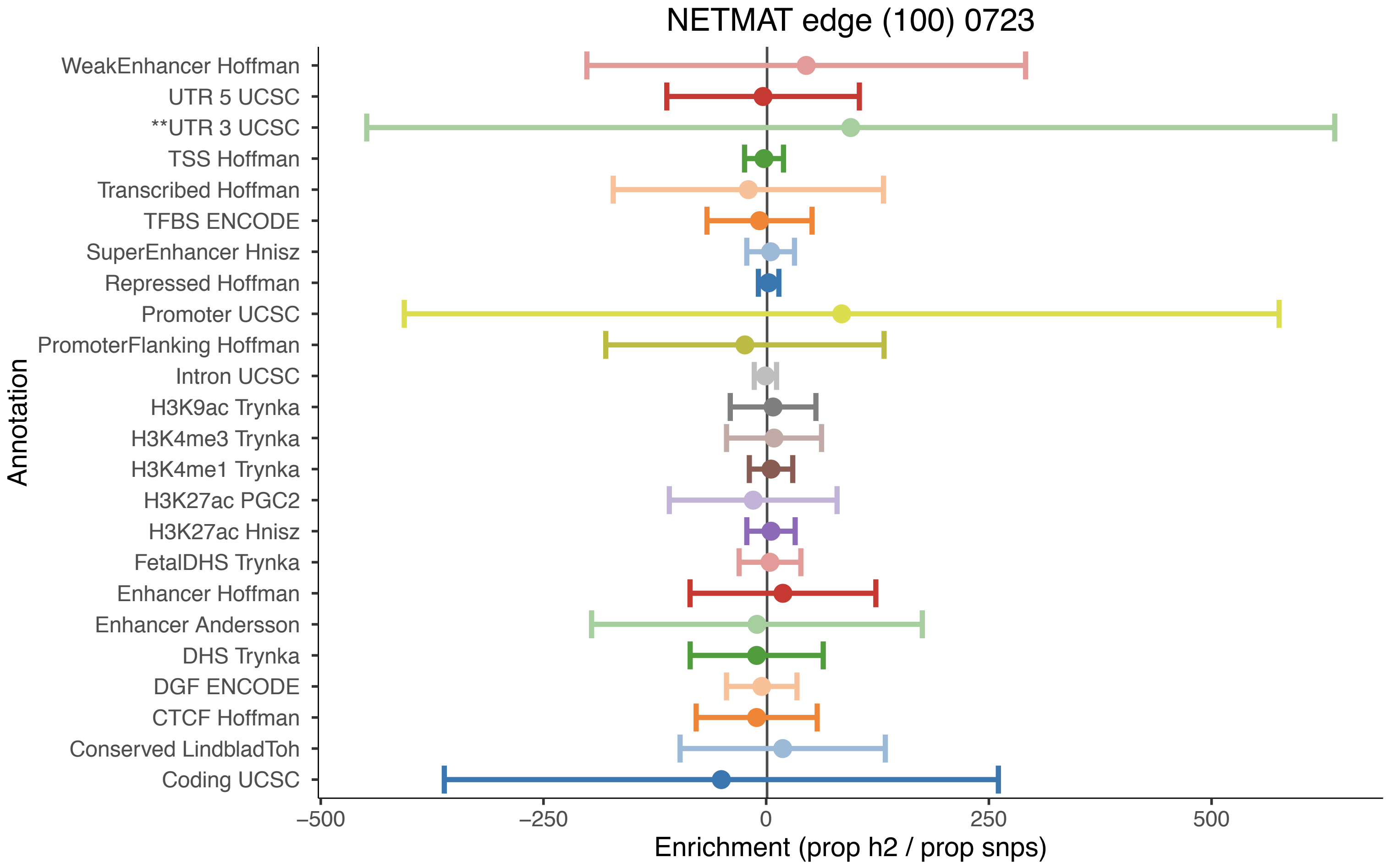
NETMAT edge (100) 0723
WeakEnhancer Hoffman
UTR 5 UCSC
**UTR 3 UCSC
TSS Hoffman
Transcribed Hoffman
TFBS ENCODE
SuperEnhancer Hnisz
Repressed Hoffman
Promoter UCSC
PromoterFlanking Hoffman
Intron UCSC
H3K9ac Trynka
H3K4me3 Trynka
H3K4me1 Trynka
H3K27ac PGC2
H3K27ac Hnisz
FetalDHS Trynka
Enhancer Hoffman
Enhancer Andersson
DHS Trynka
DGF ENCODE
CTCF Hoffman
Conserved LindbladToh
Coding UCSC
Annotation
−500
−250
0
250
500
Enrichment (prop h2 / prop snps)

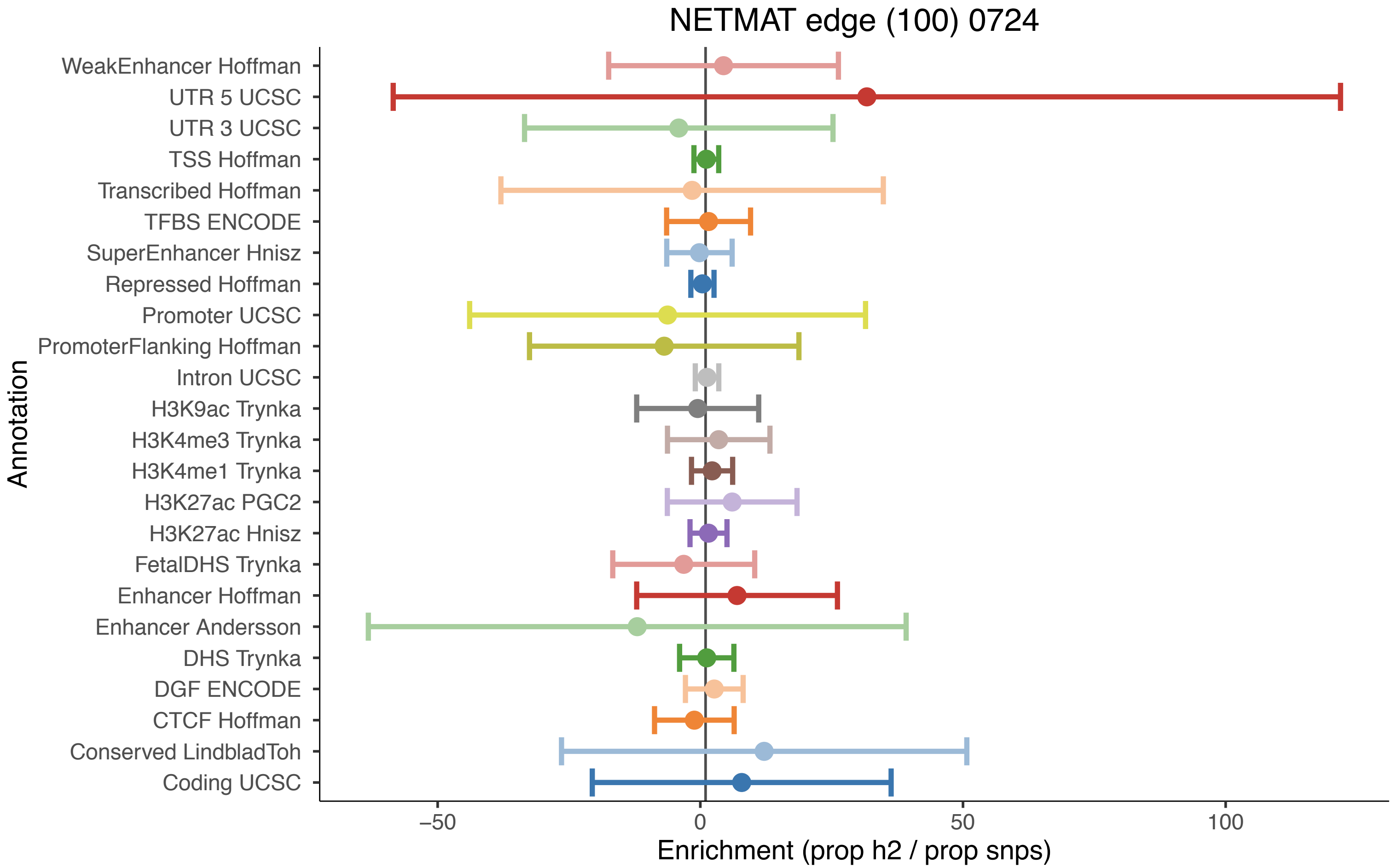
NETMAT edge (100) 0724
WeakEnhancer Hoffman
UTR 5 UCSC
UTR 3 UCSC
TSS Hoffman
Transcribed Hoffman
TFBS ENCODE
SuperEnhancer Hnisz
Repressed Hoffman
Promoter UCSC
PromoterFlanking Hoffman
Intron UCSC
H3K9ac Trynka
H3K4me3 Trynka
H3K4me1 Trynka
H3K27ac PGC2
H3K27ac Hnisz
FetalDHS Trynka
Enhancer Hoffman
Enhancer Andersson
DHS Trynka
DGF ENCODE
CTCF Hoffman
Conserved LindbladToh
Coding UCSC
Annotation
−50
0
50
100
Enrichment (prop h2 / prop snps)

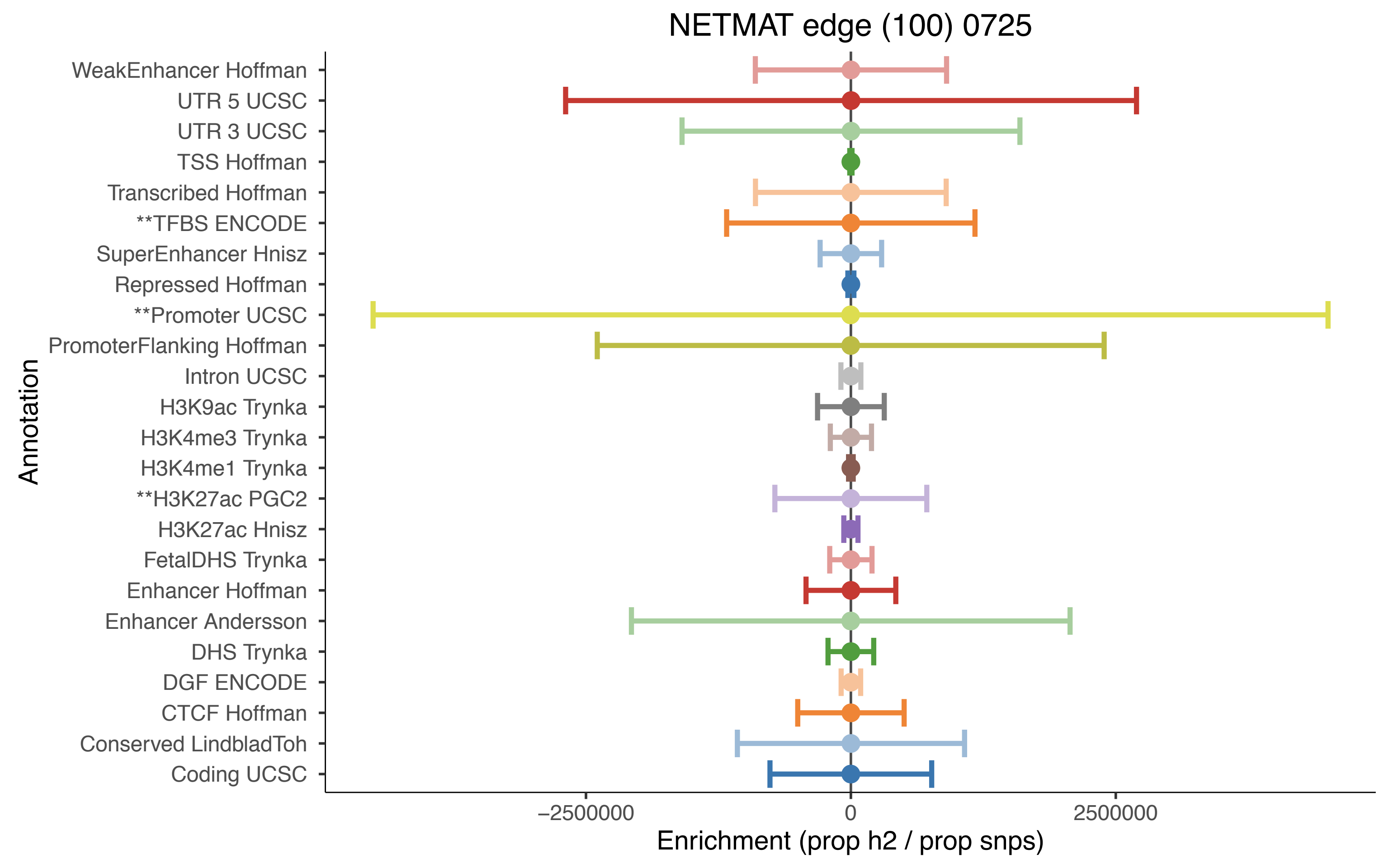
NETMAT edge (100) 0725
WeakEnhancer Hoffman
UTR 5 UCSC
UTR 3 UCSC
TSS Hoffman
Transcribed Hoffman
**TFBS ENCODE
SuperEnhancer Hnisz
Repressed Hoffman
**Promoter UCSC
PromoterFlanking Hoffman
Intron UCSC
H3K9ac Trynka
H3K4me3 Trynka
H3K4me1 Trynka
**H3K27ac PGC2
H3K27ac Hnisz
FetalDHS Trynka
Enhancer Hoffman
Enhancer Andersson
DHS Trynka
DGF ENCODE
CTCF Hoffman
Conserved LindbladToh
Coding UCSC
Annotation
−2500000
0
2500000
Enrichment (prop h2 / prop snps)

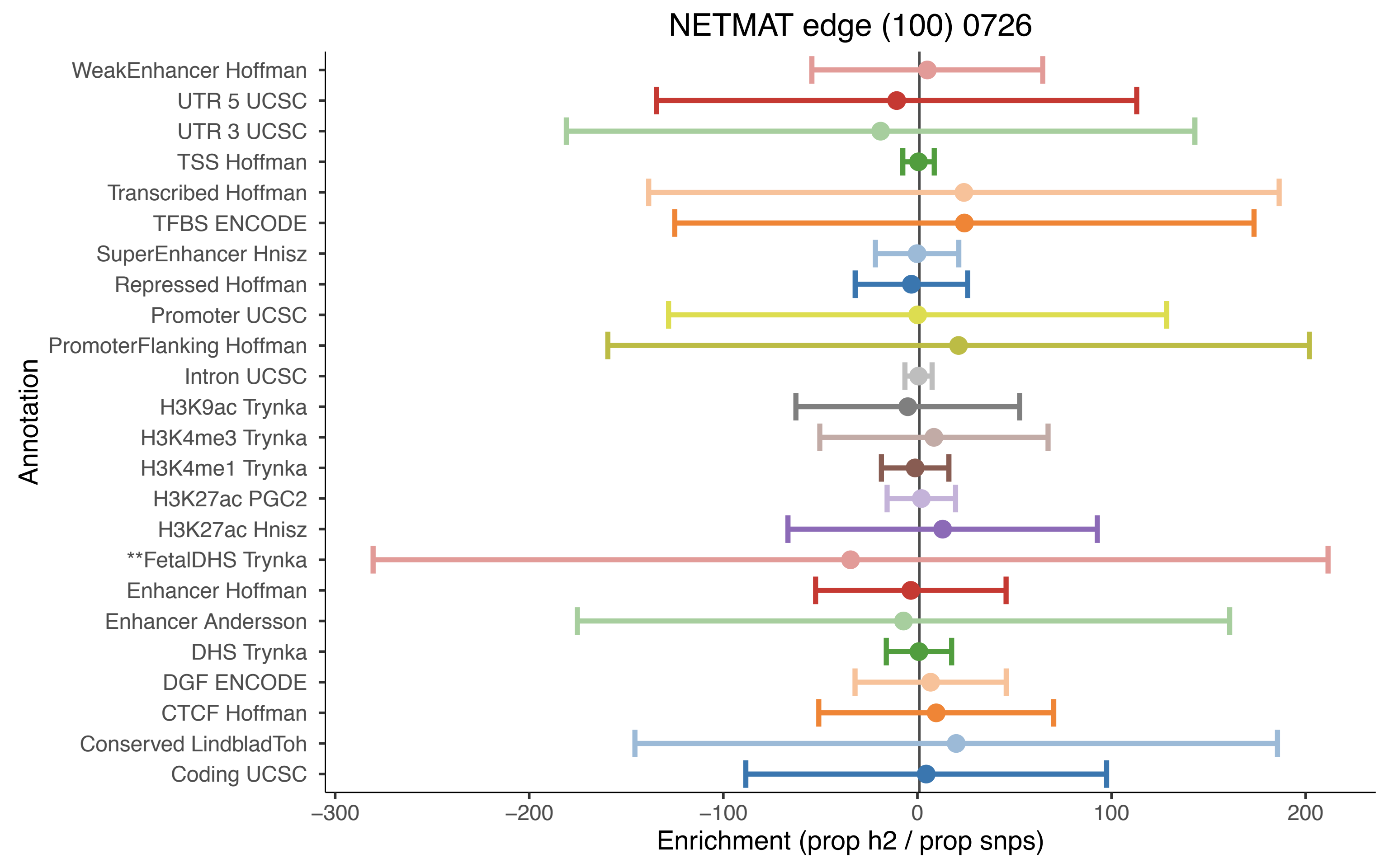
NETMAT edge (100) 0726
Annotation
WeakEnhancer Hoffman
UTR 5 UCSC
UTR 3 UCSC
TSS Hoffman
Transcribed Hoffman
TFBS ENCODE
SuperEnhancer Hnisz
Repressed Hoffman
Promoter UCSC
PromoterFlanking Hoffman
Intron UCSC
H3K9ac Trynka
H3K4me3 Trynka
H3K4me1 Trynka
H3K27ac PGC2
H3K27ac Hnisz
**FetalDHS Trynka
Enhancer Hoffman
Enhancer Andersson
DHS Trynka
DGF ENCODE
CTCF Hoffman
Conserved LindbladToh
Coding UCSC
−300
−200
−100
0
100
200
Enrichment (prop h2 / prop snps)

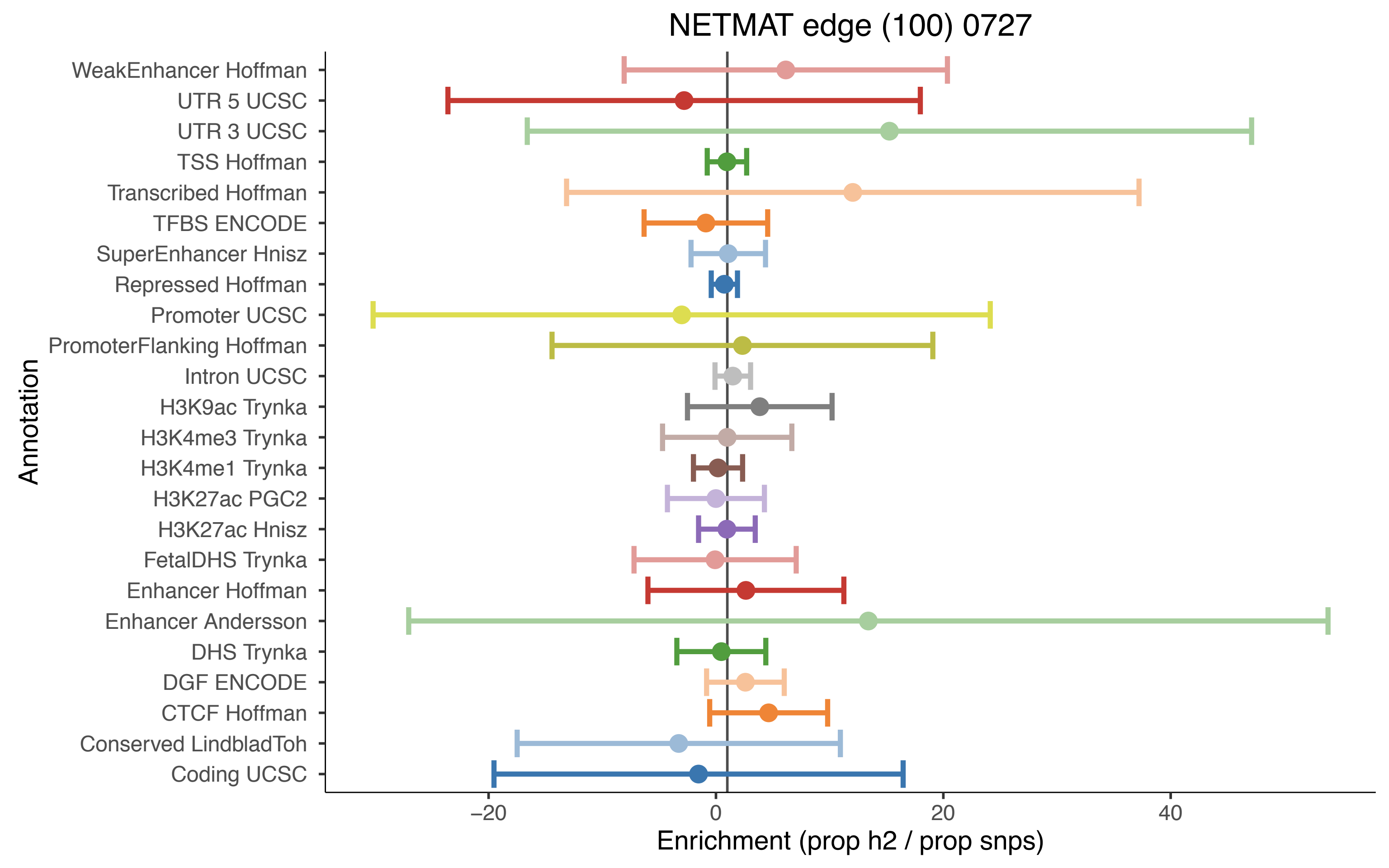
NETMAT edge (100) 0727
WeakEnhancer Hoffman
UTR 5 UCSC
UTR 3 UCSC
TSS Hoffman
Transcribed Hoffman
TFBS ENCODE
SuperEnhancer Hnisz
Repressed Hoffman
Promoter UCSC
PromoterFlanking Hoffman
Intron UCSC
H3K9ac Trynka
H3K4me3 Trynka
H3K4me1 Trynka
H3K27ac PGC2
H3K27ac Hnisz
FetalDHS Trynka
Enhancer Hoffman
Enhancer Andersson
DHS Trynka
DGF ENCODE
CTCF Hoffman
Conserved LindbladToh
Coding UCSC
Annotation
−20
0
20
40
Enrichment (prop h2 / prop snps)

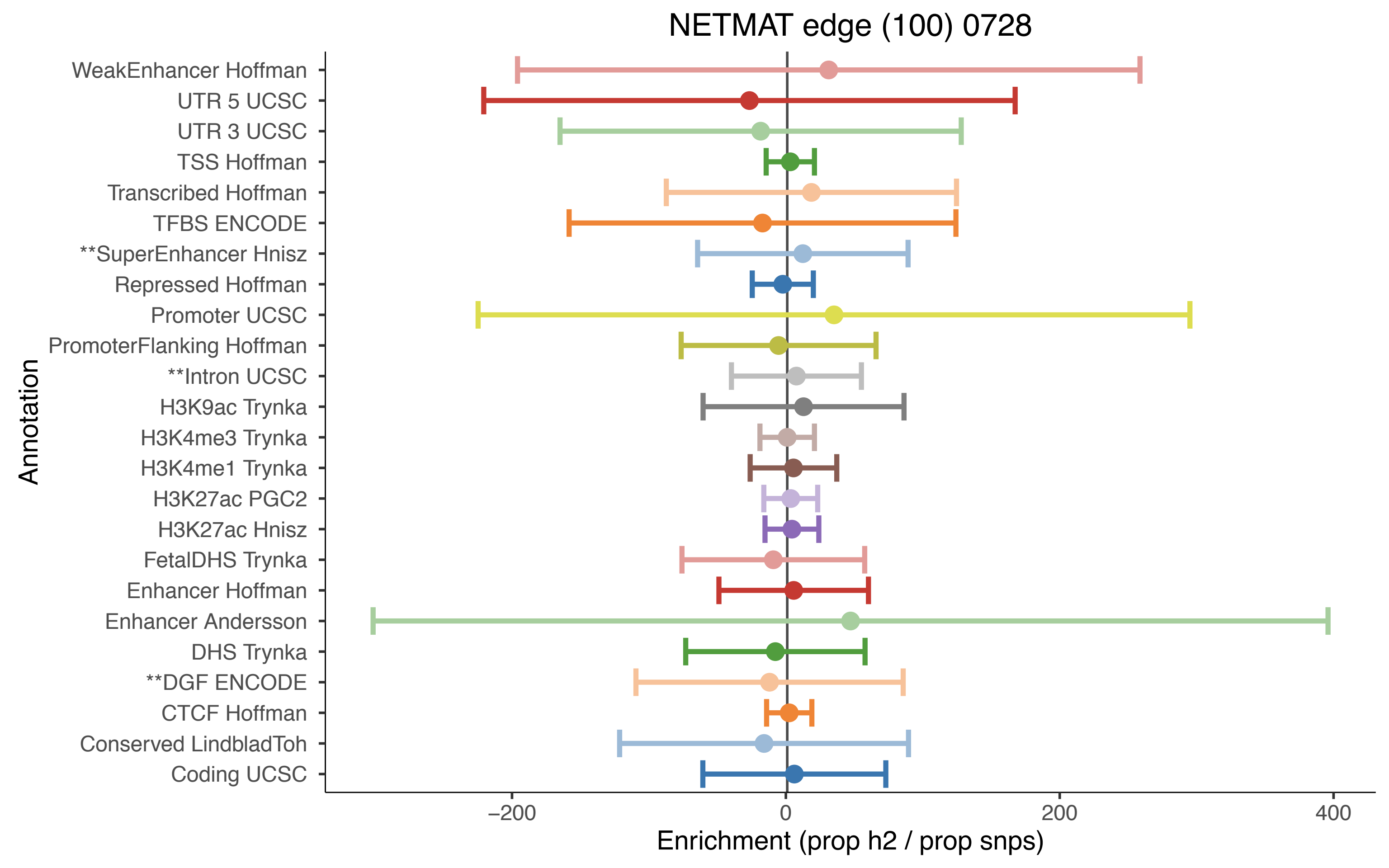
NETMAT edge (100) 0728
WeakEnhancer Hoffman
UTR 5 UCSC
UTR 3 UCSC
TSS Hoffman
Transcribed Hoffman
TFBS ENCODE
**SuperEnhancer Hnisz
Repressed Hoffman
Promoter UCSC
PromoterFlanking Hoffman
**Intron UCSC
H3K9ac Trynka
H3K4me3 Trynka
H3K4me1 Trynka
H3K27ac PGC2
H3K27ac Hnisz
FetalDHS Trynka
Enhancer Hoffman
Enhancer Andersson
DHS Trynka
**DGF ENCODE
CTCF Hoffman
Conserved LindbladToh
Coding UCSC
Annotation
−200
0
200
400
Enrichment (prop h2 / prop snps)

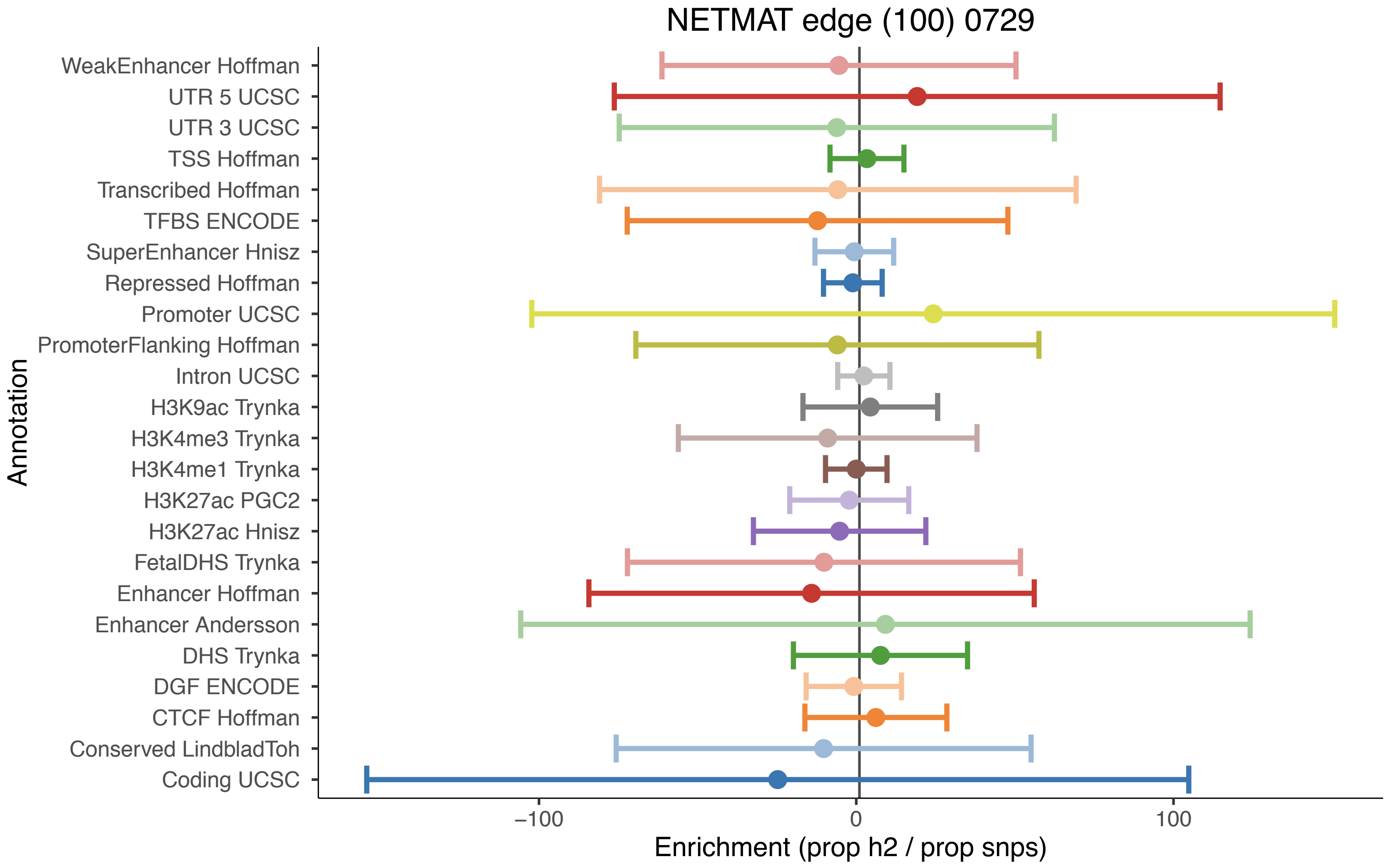
NETMAT edge (100) 0729
WeakEnhancer Hoffman
UTR 5 UCSC
UTR 3 UCSC
TSS Hoffman
Transcribed Hoffman
TFBS ENCODE
SuperEnhancer Hnisz
Repressed Hoffman
Promoter UCSC
PromoterFlanking Hoffman
Intron UCSC
H3K9ac Trynka
H3K4me3 Trynka
H3K4me1 Trynka
H3K27ac PGC2
H3K27ac Hnisz
FetalDHS Trynka
Enhancer Hoffman
Enhancer Andersson
DHS Trynka
DGF ENCODE
CTCF Hoffman
Conserved LindbladToh
Coding UCSC
Annotation
−100
0
100
Enrichment (prop h2 / prop snps)

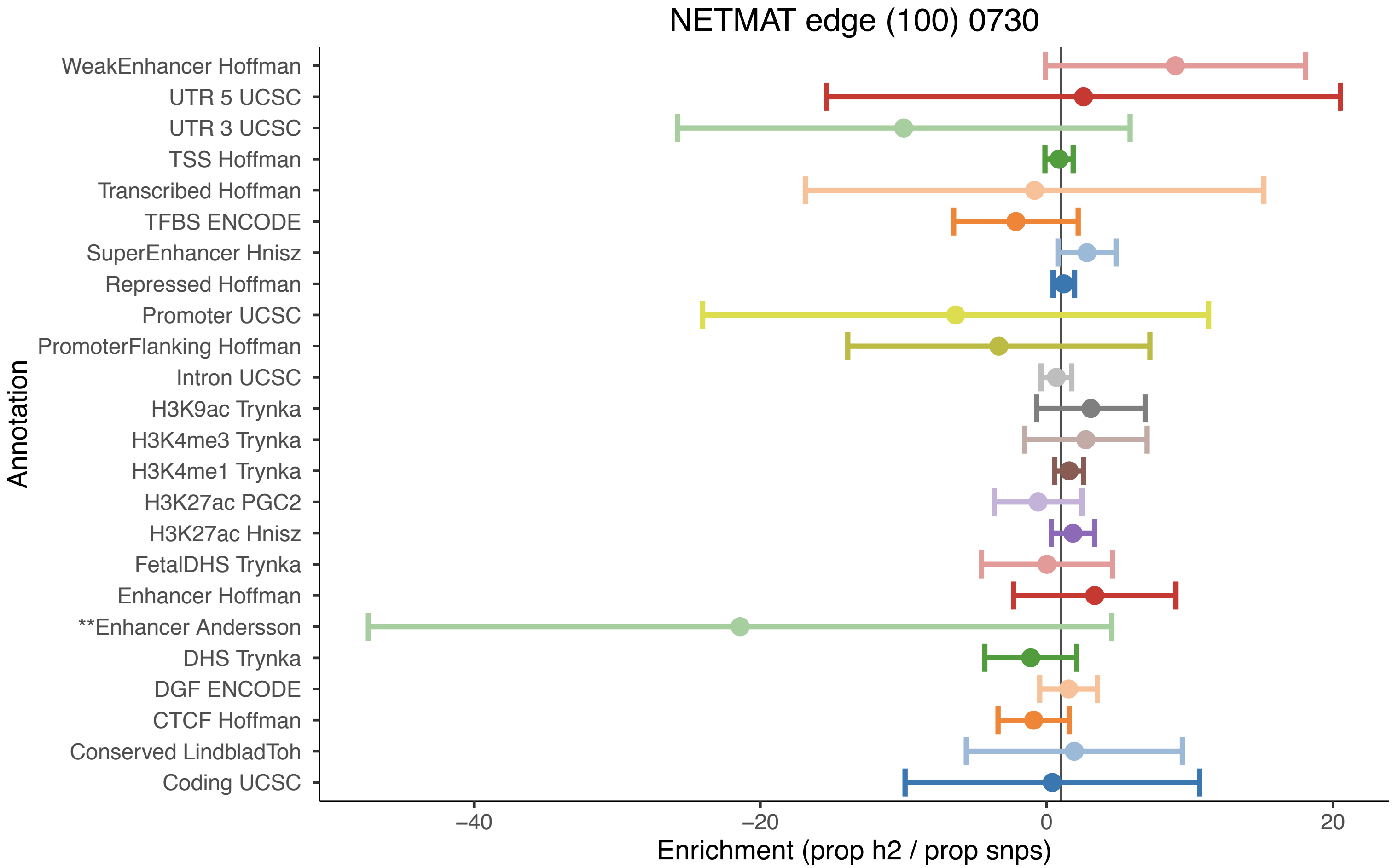
NETMAT edge (100) 0730
WeakEnhancer Hoffman
UTR 5 UCSC
UTR 3 UCSC
TSS Hoffman
Transcribed Hoffman
TFBS ENCODE
SuperEnhancer Hnisz
Repressed Hoffman
Promoter UCSC
PromoterFlanking Hoffman
Intron UCSC
H3K9ac Trynka
H3K4me3 Trynka
H3K4me1 Trynka
H3K27ac PGC2
H3K27ac Hnisz
FetalDHS Trynka
Enhancer Hoffman
**Enhancer Andersson
DHS Trynka
DGF ENCODE
CTCF Hoffman
Conserved LindbladToh
Coding UCSC
Annotation
−40
−20
0
20
Enrichment (prop h2 / prop snps)

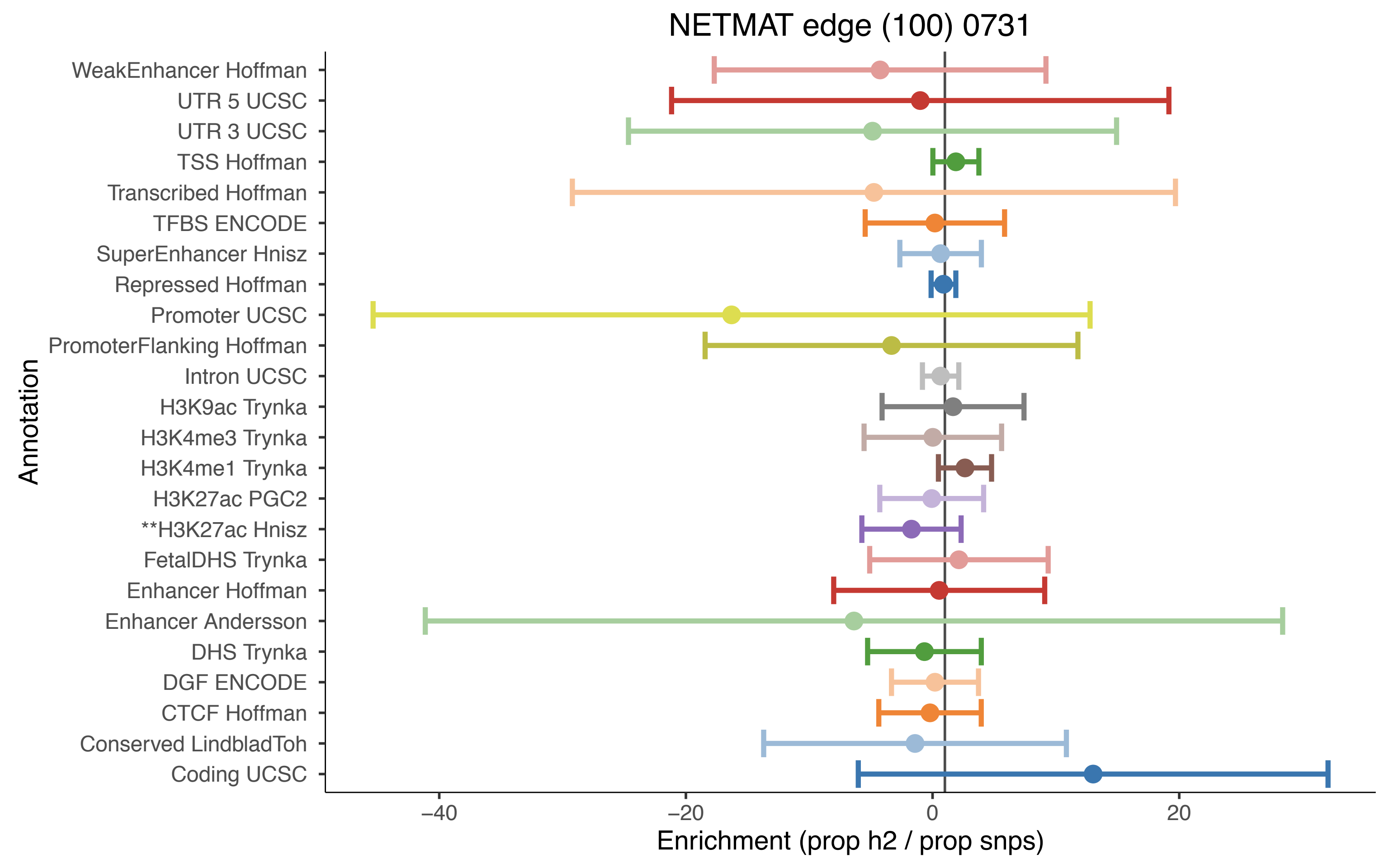
NETMAT edge (100) 0731
WeakEnhancer Hoffman
UTR 5 UCSC
UTR 3 UCSC
TSS Hoffman
Transcribed Hoffman
TFBS ENCODE
SuperEnhancer Hnisz
Repressed Hoffman
Promoter UCSC
PromoterFlanking Hoffman
Intron UCSC
H3K9ac Trynka
H3K4me3 Trynka
H3K4me1 Trynka
H3K27ac PGC2
**H3K27ac Hnisz
FetalDHS Trynka
Enhancer Hoffman
Enhancer Andersson
DHS Trynka
DGF ENCODE
CTCF Hoffman
Conserved LindbladToh
Coding UCSC
Annotation
−40
−20
0
20
Enrichment (prop h2 / prop snps)

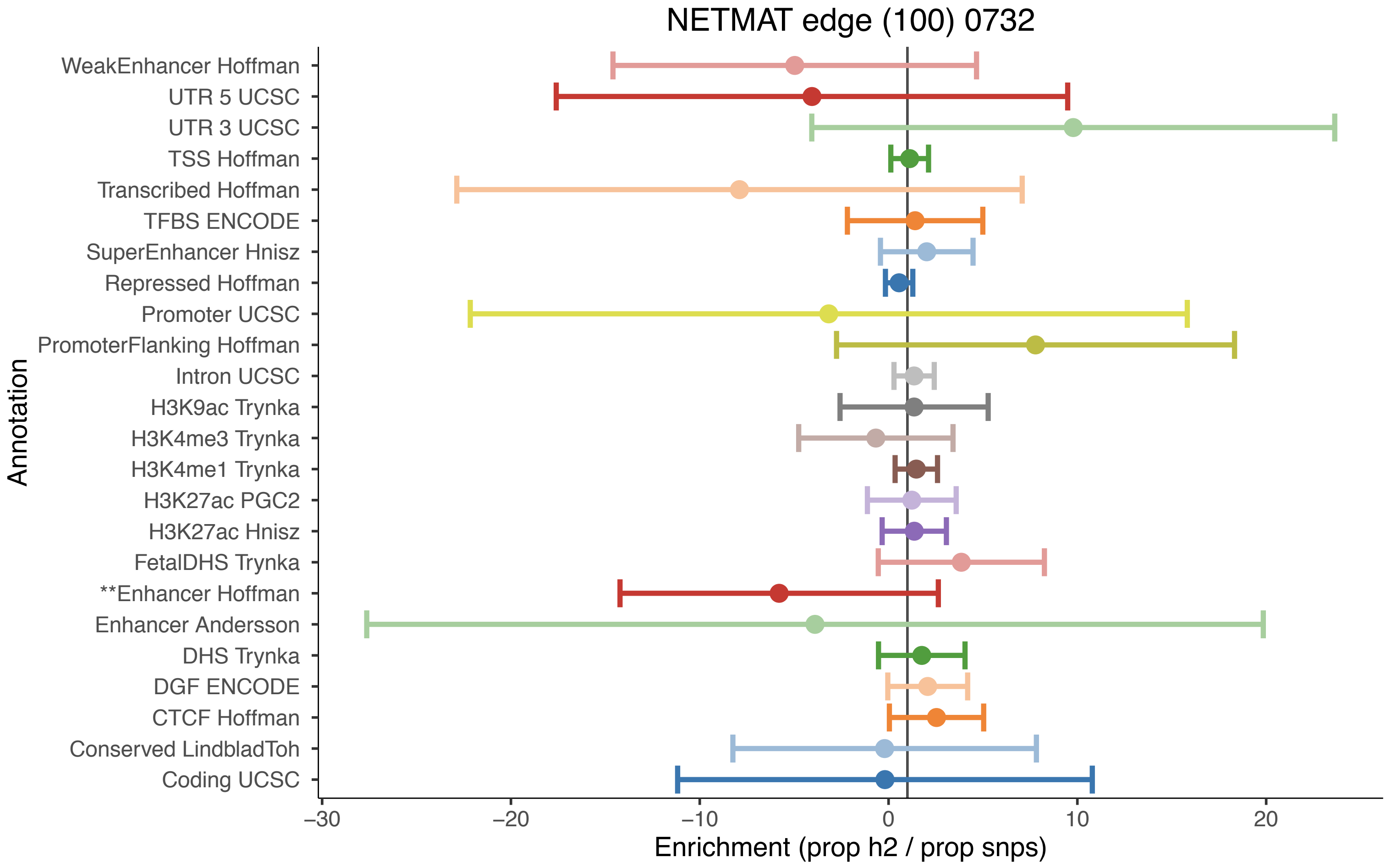
NETMAT edge (100) 0732
WeakEnhancer Hoffman
UTR 5 UCSC
UTR 3 UCSC
TSS Hoffman
Transcribed Hoffman
TFBS ENCODE
SuperEnhancer Hnisz
Repressed Hoffman
Promoter UCSC
PromoterFlanking Hoffman
Intron UCSC
H3K9ac Trynka
H3K4me3 Trynka
H3K4me1 Trynka
H3K27ac PGC2
H3K27ac Hnisz
FetalDHS Trynka
**Enhancer Hoffman
Enhancer Andersson
DHS Trynka
DGF ENCODE
CTCF Hoffman
Conserved LindbladToh
Coding UCSC
Annotation
−30
−20
−10
0
10
20
Enrichment (prop h2 / prop snps)

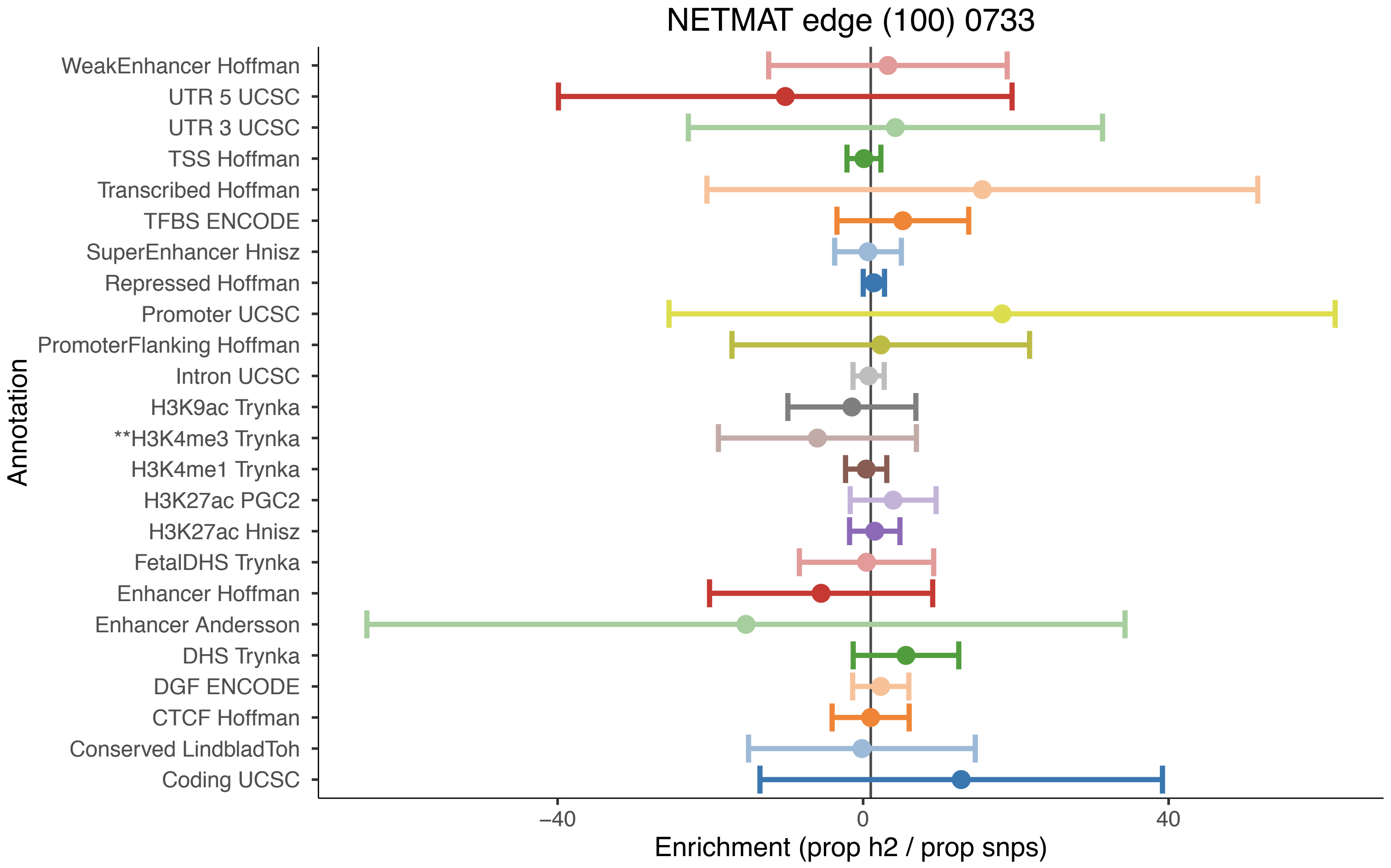

NETMAT edge (100) 0733
WeakEnhancer Hoffman
UTR 5 UCSC
UTR 3 UCSC
TSS Hoffman
Transcribed Hoffman
TFBS ENCODE
SuperEnhancer Hnisz
Repressed Hoffman
Promoter UCSC
PromoterFlanking Hoffman
Intron UCSC
H3K9ac Trynka
**H3K4me3 Trynka
H3K4me1 Trynka
H3K27ac PGC2
H3K27ac Hnisz
FetalDHS Trynka
Enhancer Hoffman
Enhancer Andersson
DHS Trynka
DGF ENCODE
CTCF Hoffman
Conserved LindbladToh
Coding UCSC
Annotation
−40
0
40
Enrichment (prop h2 / prop snps)

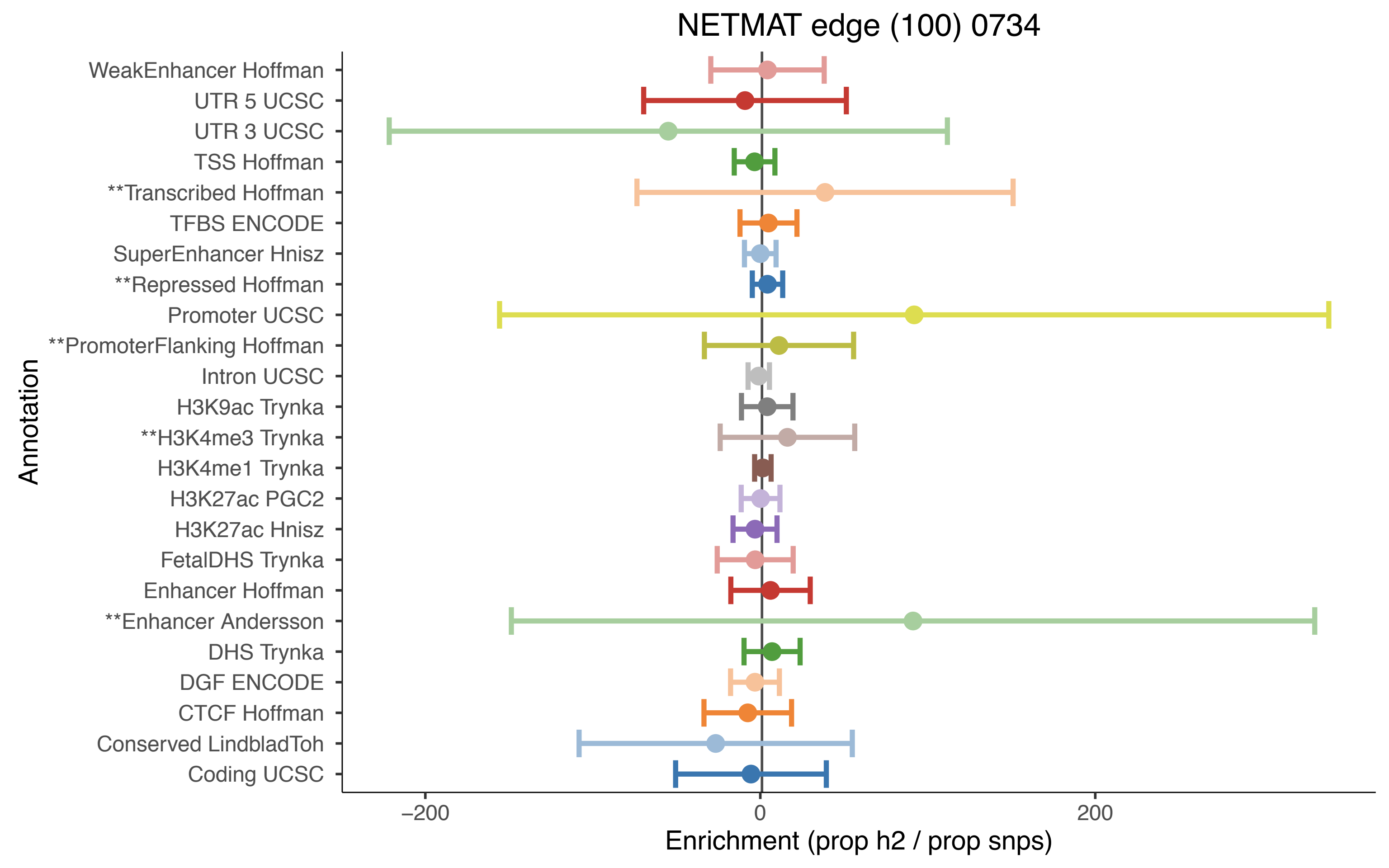
NETMAT edge (100) 0734
WeakEnhancer Hoffman
UTR 5 UCSC
UTR 3 UCSC
TSS Hoffman
**Transcribed Hoffman
TFBS ENCODE
SuperEnhancer Hnisz
**Repressed Hoffman
Promoter UCSC
**PromoterFlanking Hoffman
Intron UCSC
H3K9ac Trynka
**H3K4me3 Trynka
H3K4me1 Trynka
H3K27ac PGC2
H3K27ac Hnisz
FetalDHS Trynka
Enhancer Hoffman
**Enhancer Andersson
DHS Trynka
DGF ENCODE
CTCF Hoffman
Conserved LindbladToh
Coding UCSC
Annotation
−200
0
200
Enrichment (prop h2 / prop snps)

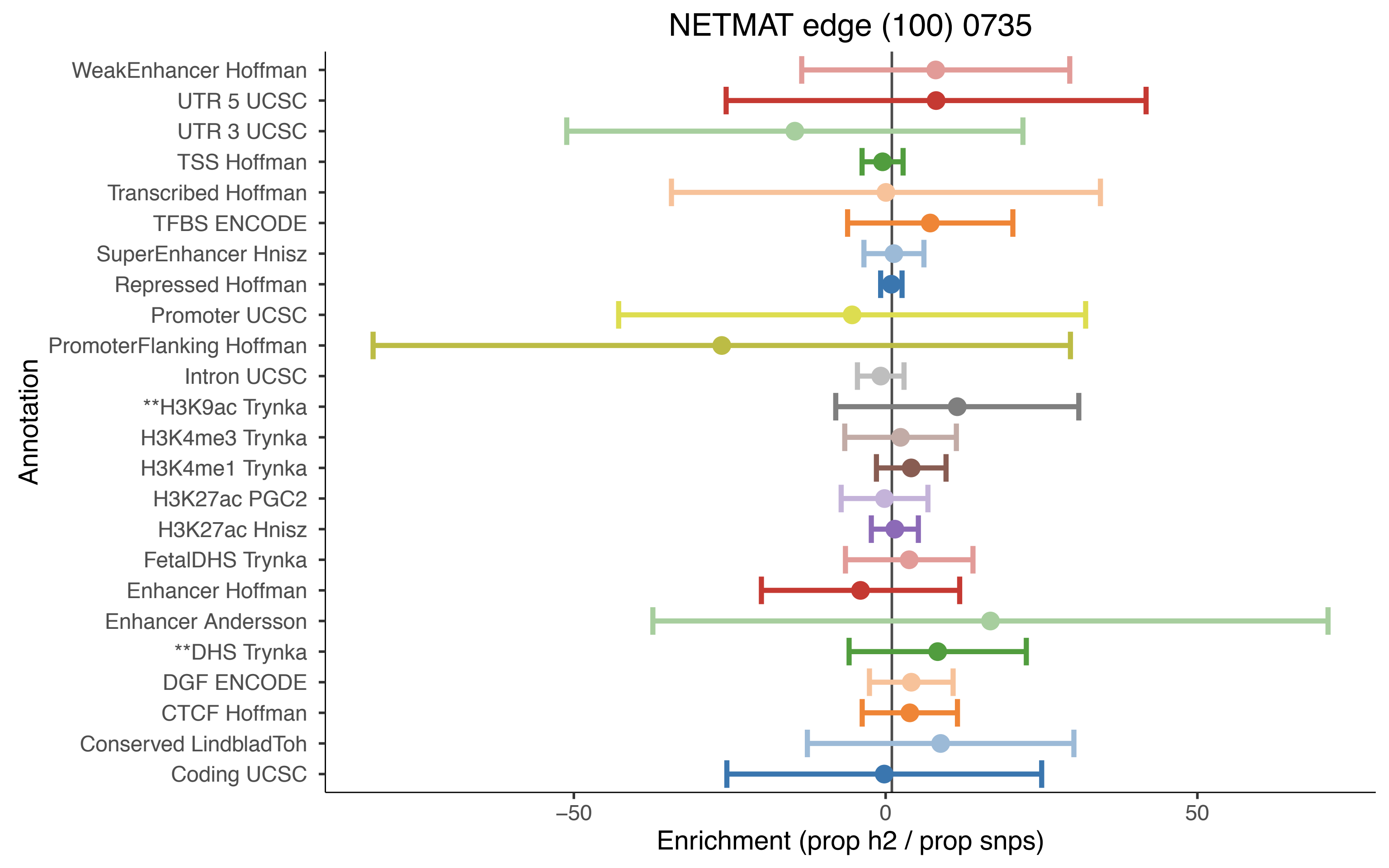
NETMAT edge (100) 0735
WeakEnhancer Hoffman
UTR 5 UCSC
UTR 3 UCSC
TSS Hoffman
Transcribed Hoffman
TFBS ENCODE
SuperEnhancer Hnisz
Repressed Hoffman
Promoter UCSC
PromoterFlanking Hoffman
Intron UCSC
**H3K9ac Trynka
H3K4me3 Trynka
H3K4me1 Trynka
H3K27ac PGC2
H3K27ac Hnisz
FetalDHS Trynka
Enhancer Hoffman
Enhancer Andersson
**DHS Trynka
DGF ENCODE
CTCF Hoffman
Conserved LindbladToh
Coding UCSC
Annotation
−50
0
50
Enrichment (prop h2 / prop snps)

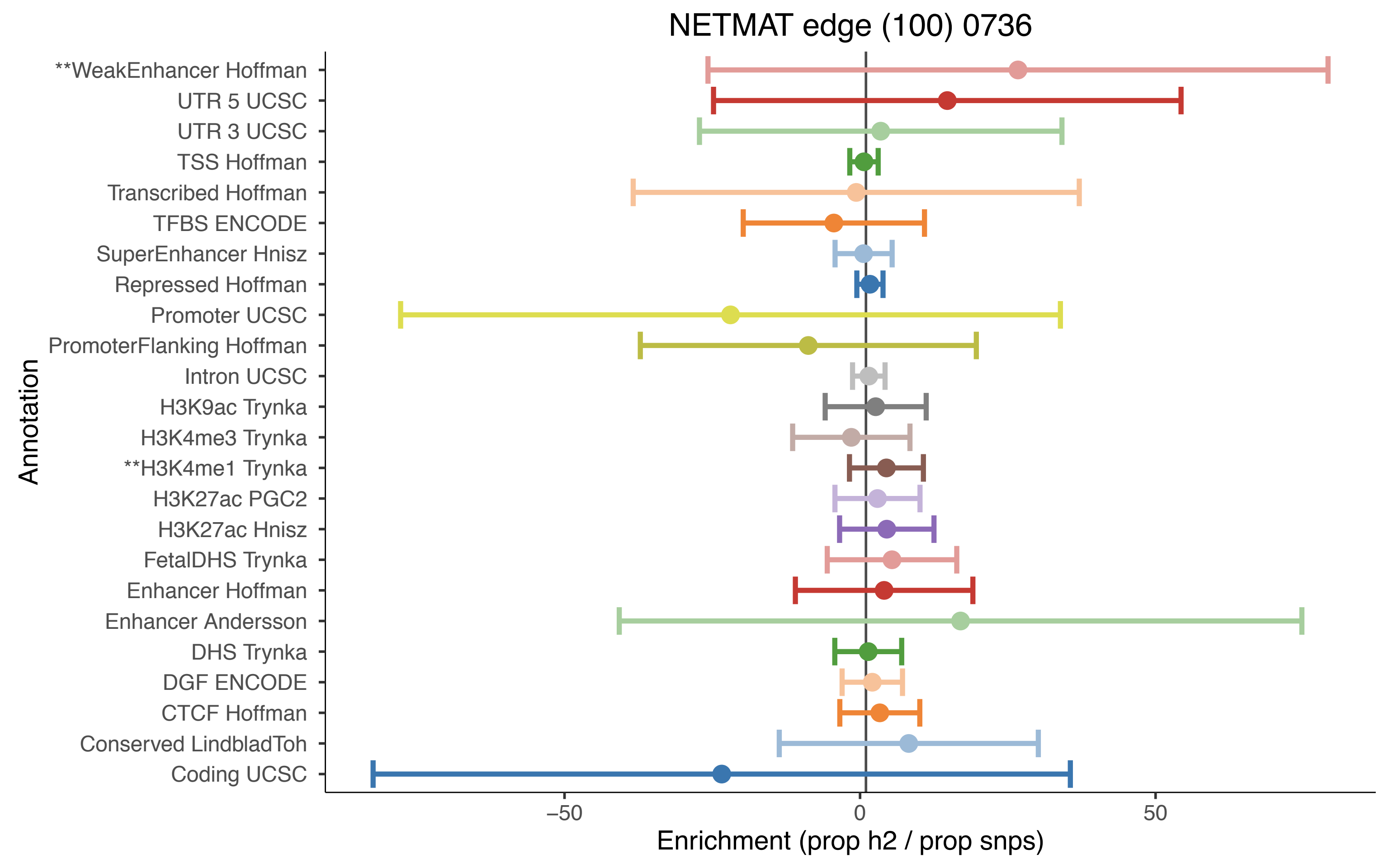

NETMAT edge (100) 0736
**WeakEnhancer Hoffman
UTR 5 UCSC
UTR 3 UCSC
TSS Hoffman
Transcribed Hoffman
TFBS ENCODE
SuperEnhancer Hnisz
Repressed Hoffman
Promoter UCSC
PromoterFlanking Hoffman
Intron UCSC
H3K9ac Trynka
H3K4me3 Trynka
**H3K4me1 Trynka
H3K27ac PGC2
H3K27ac Hnisz
FetalDHS Trynka
Enhancer Hoffman
Enhancer Andersson
DHS Trynka
DGF ENCODE
CTCF Hoffman
Conserved LindbladToh
Coding UCSC
Annotation
−50
0
50
Enrichment (prop h2 / prop snps)

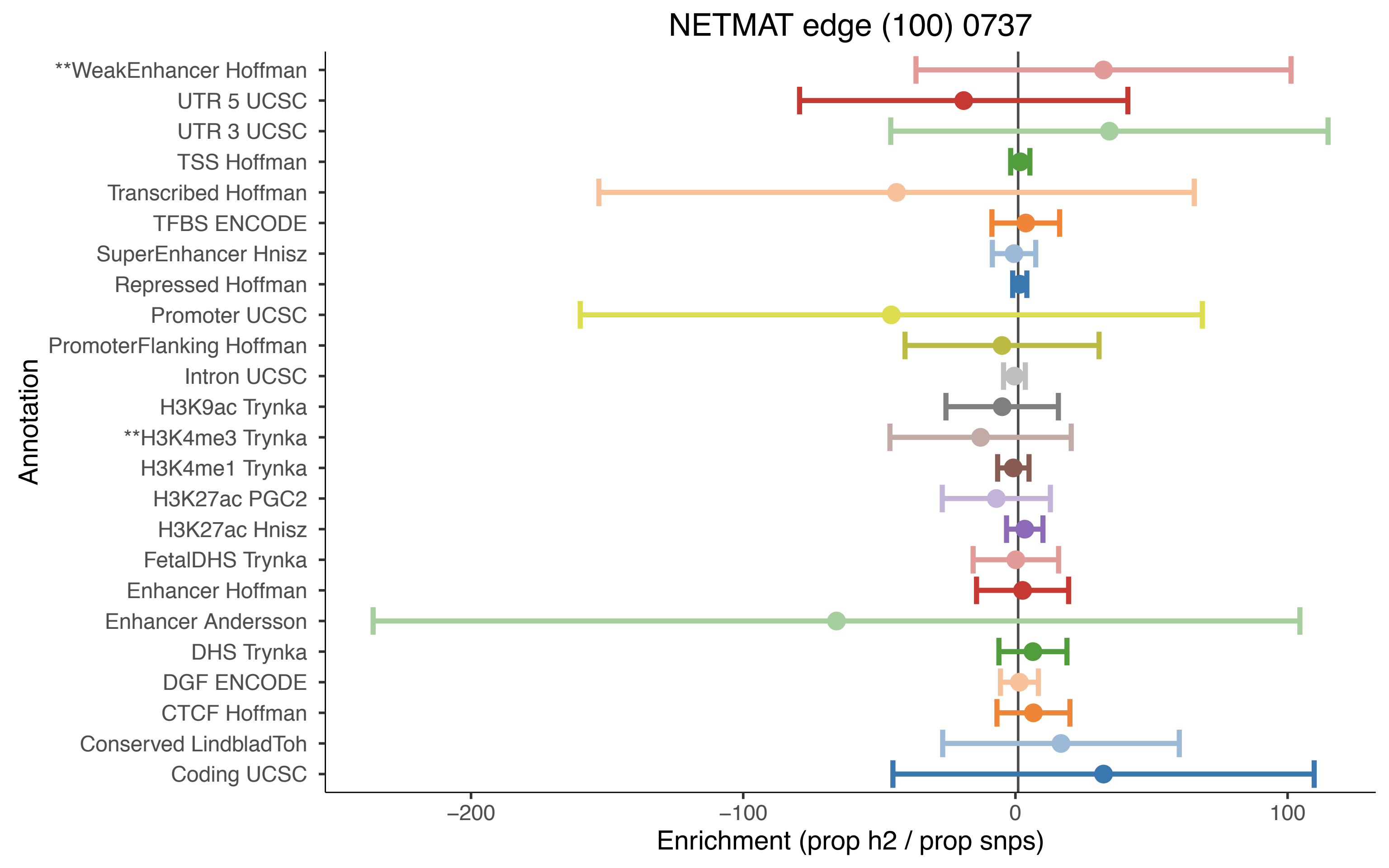

NETMAT edge (100) 0737
**WeakEnhancer Hoffman
UTR 5 UCSC
UTR 3 UCSC
TSS Hoffman
Transcribed Hoffman
TFBS ENCODE
SuperEnhancer Hnisz
Repressed Hoffman
Promoter UCSC
PromoterFlanking Hoffman
Intron UCSC
H3K9ac Trynka
**H3K4me3 Trynka
H3K4me1 Trynka
H3K27ac PGC2
H3K27ac Hnisz
FetalDHS Trynka
Enhancer Hoffman
Enhancer Andersson
DHS Trynka
DGF ENCODE
CTCF Hoffman
Conserved LindbladToh
Coding UCSC
Annotation
−200
−100
0
100
Enrichment (prop h2 / prop snps)

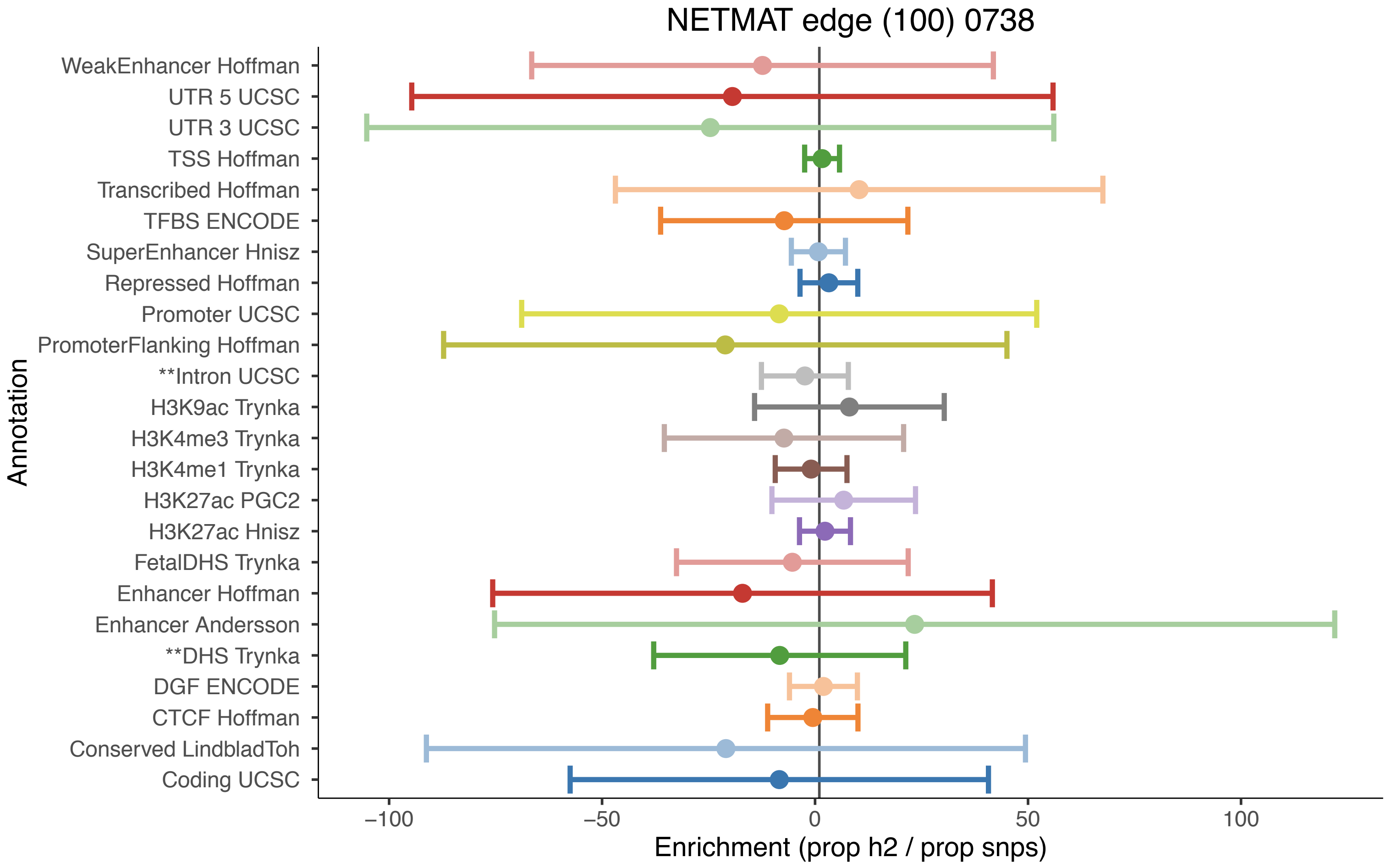
NETMAT edge (100) 0738
WeakEnhancer Hoffman
UTR 5 UCSC
UTR 3 UCSC
TSS Hoffman
Transcribed Hoffman
TFBS ENCODE
SuperEnhancer Hnisz
Repressed Hoffman
Promoter UCSC
PromoterFlanking Hoffman
**Intron UCSC
H3K9ac Trynka
H3K4me3 Trynka
H3K4me1 Trynka
H3K27ac PGC2
H3K27ac Hnisz
FetalDHS Trynka
Enhancer Hoffman
Enhancer Andersson
**DHS Trynka
DGF ENCODE
CTCF Hoffman
Conserved LindbladToh
Coding UCSC
Annotation
−100
−50
0
50
100
Enrichment (prop h2 / prop snps)

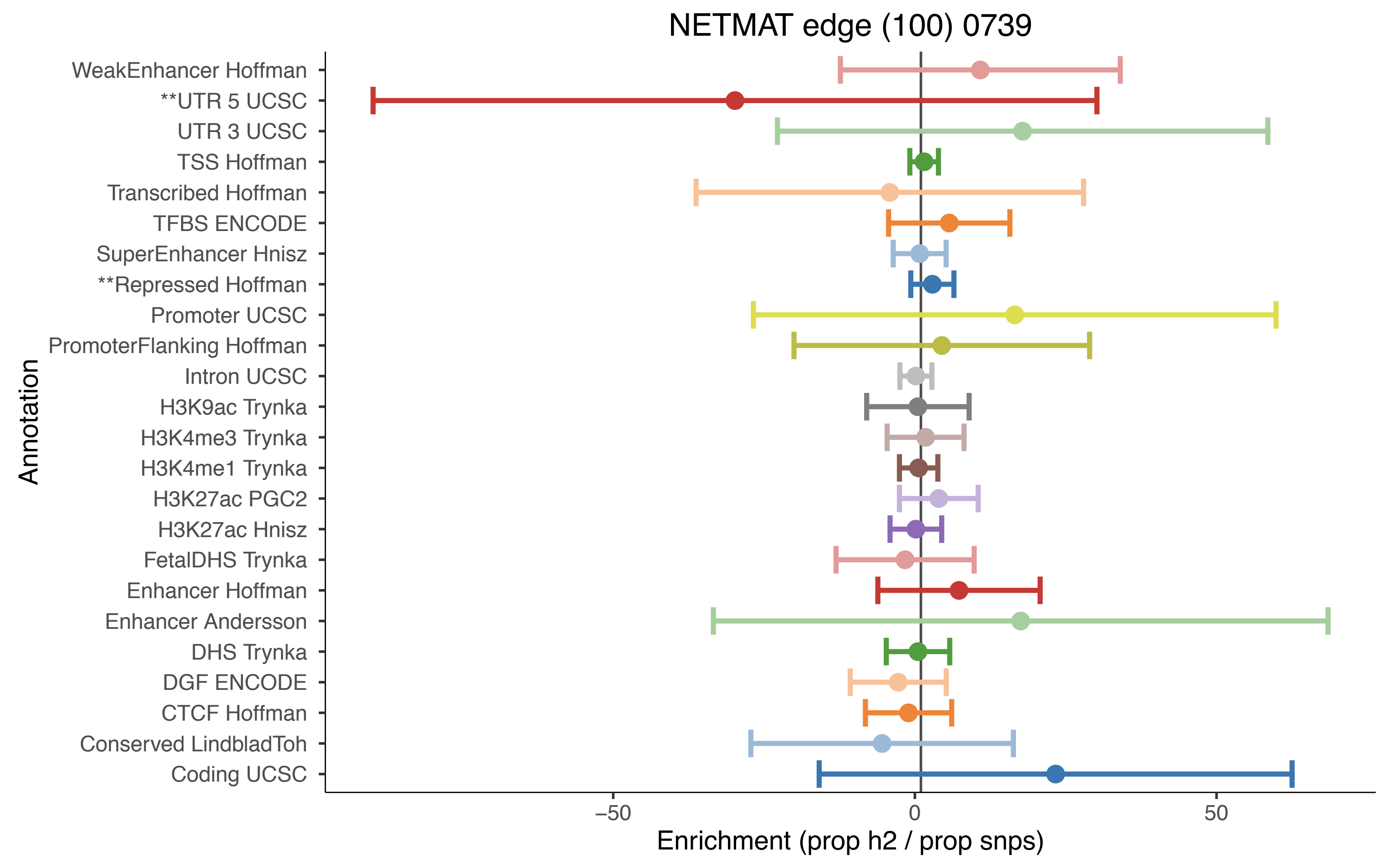
NETMAT edge (100) 0739
WeakEnhancer Hoffman
**UTR 5 UCSC
UTR 3 UCSC
TSS Hoffman
Transcribed Hoffman
TFBS ENCODE
SuperEnhancer Hnisz
**Repressed Hoffman
Promoter UCSC
PromoterFlanking Hoffman
Intron UCSC
H3K9ac Trynka
H3K4me3 Trynka
H3K4me1 Trynka
H3K27ac PGC2
H3K27ac Hnisz
FetalDHS Trynka
Enhancer Hoffman
Enhancer Andersson
DHS Trynka
DGF ENCODE
CTCF Hoffman
Conserved LindbladToh
Coding UCSC
Annotation
−50
0
50
Enrichment (prop h2 / prop snps)

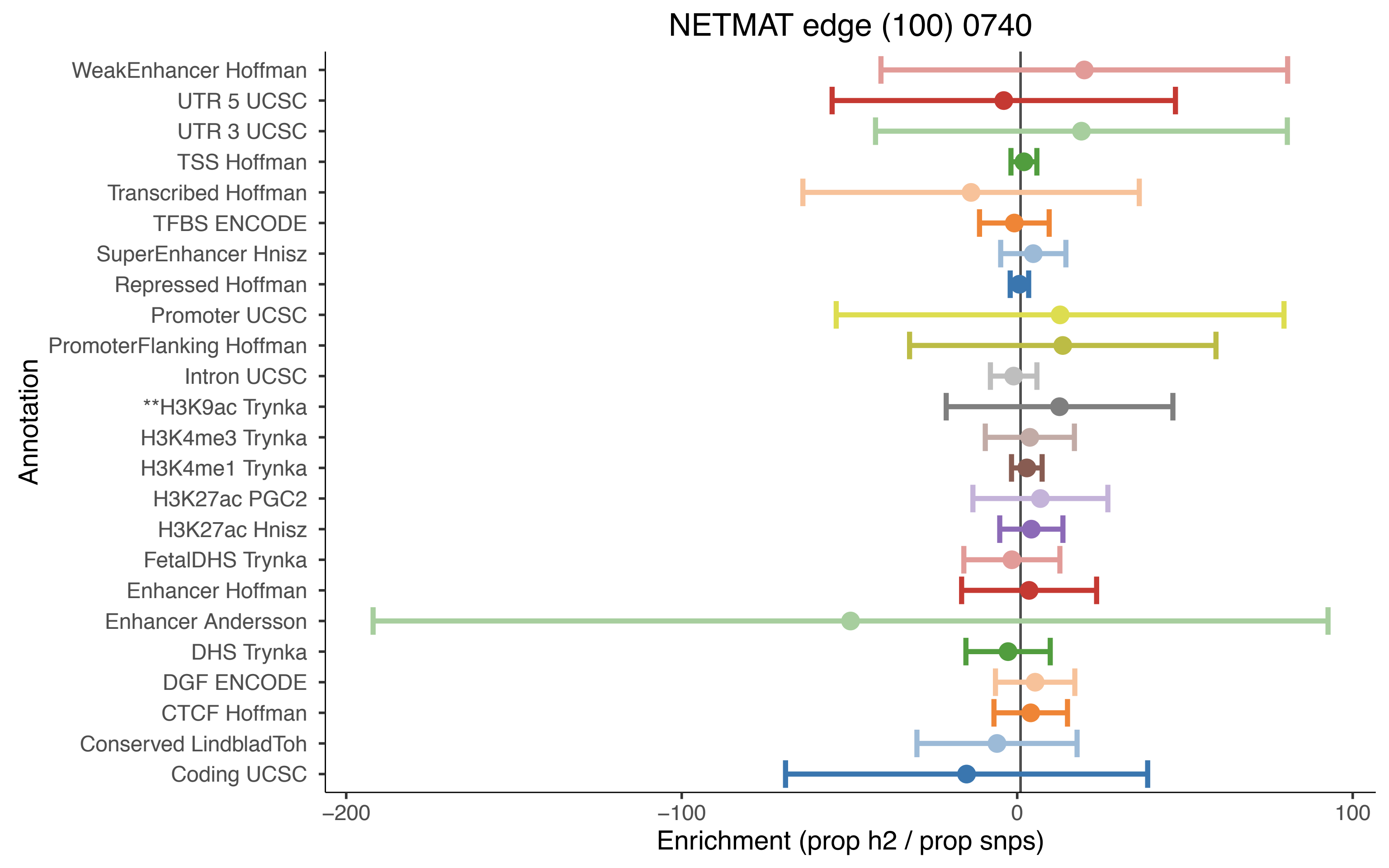
NETMAT edge (100) 0740
WeakEnhancer Hoffman
UTR 5 UCSC
UTR 3 UCSC
TSS Hoffman
Transcribed Hoffman
TFBS ENCODE
SuperEnhancer Hnisz
Repressed Hoffman
Promoter UCSC
PromoterFlanking Hoffman
Intron UCSC
**H3K9ac Trynka
H3K4me3 Trynka
H3K4me1 Trynka
H3K27ac PGC2
H3K27ac Hnisz
FetalDHS Trynka
Enhancer Hoffman
Enhancer Andersson
DHS Trynka
DGF ENCODE
CTCF Hoffman
Conserved LindbladToh
Coding UCSC
Annotation
−200
−100
0
100
Enrichment (prop h2 / prop snps)

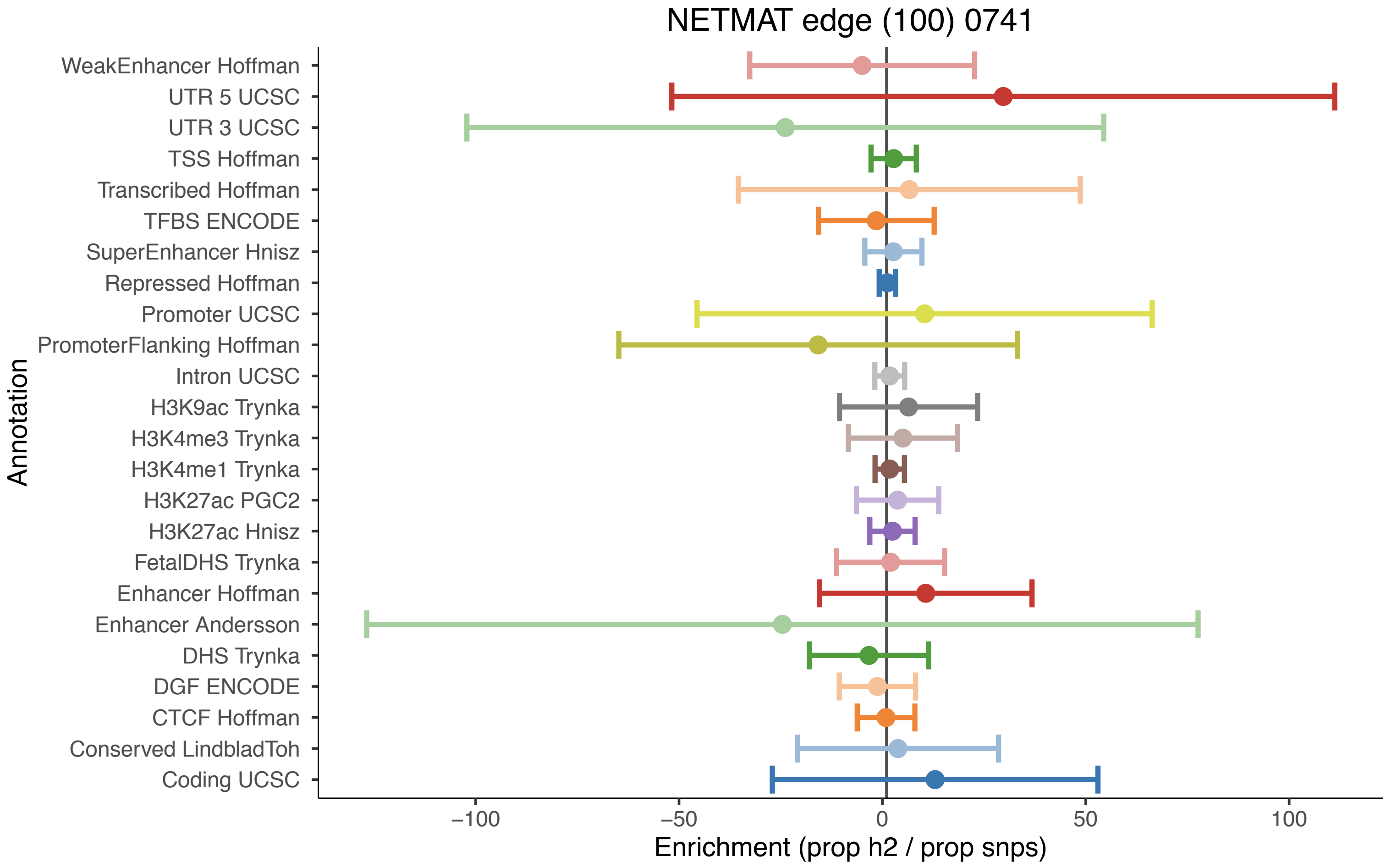

NETMAT edge (100) 0741
WeakEnhancer Hoffman
UTR 5 UCSC
UTR 3 UCSC
TSS Hoffman
Transcribed Hoffman
TFBS ENCODE
SuperEnhancer Hnisz
Repressed Hoffman
Promoter UCSC
PromoterFlanking Hoffman
Intron UCSC
H3K9ac Trynka
H3K4me3 Trynka
H3K4me1 Trynka
H3K27ac PGC2
H3K27ac Hnisz
FetalDHS Trynka
Enhancer Hoffman
Enhancer Andersson
DHS Trynka
DGF ENCODE
CTCF Hoffman
Conserved LindbladToh
Coding UCSC
Annotation
−100
−50
0
50
100
Enrichment (prop h2 / prop snps)

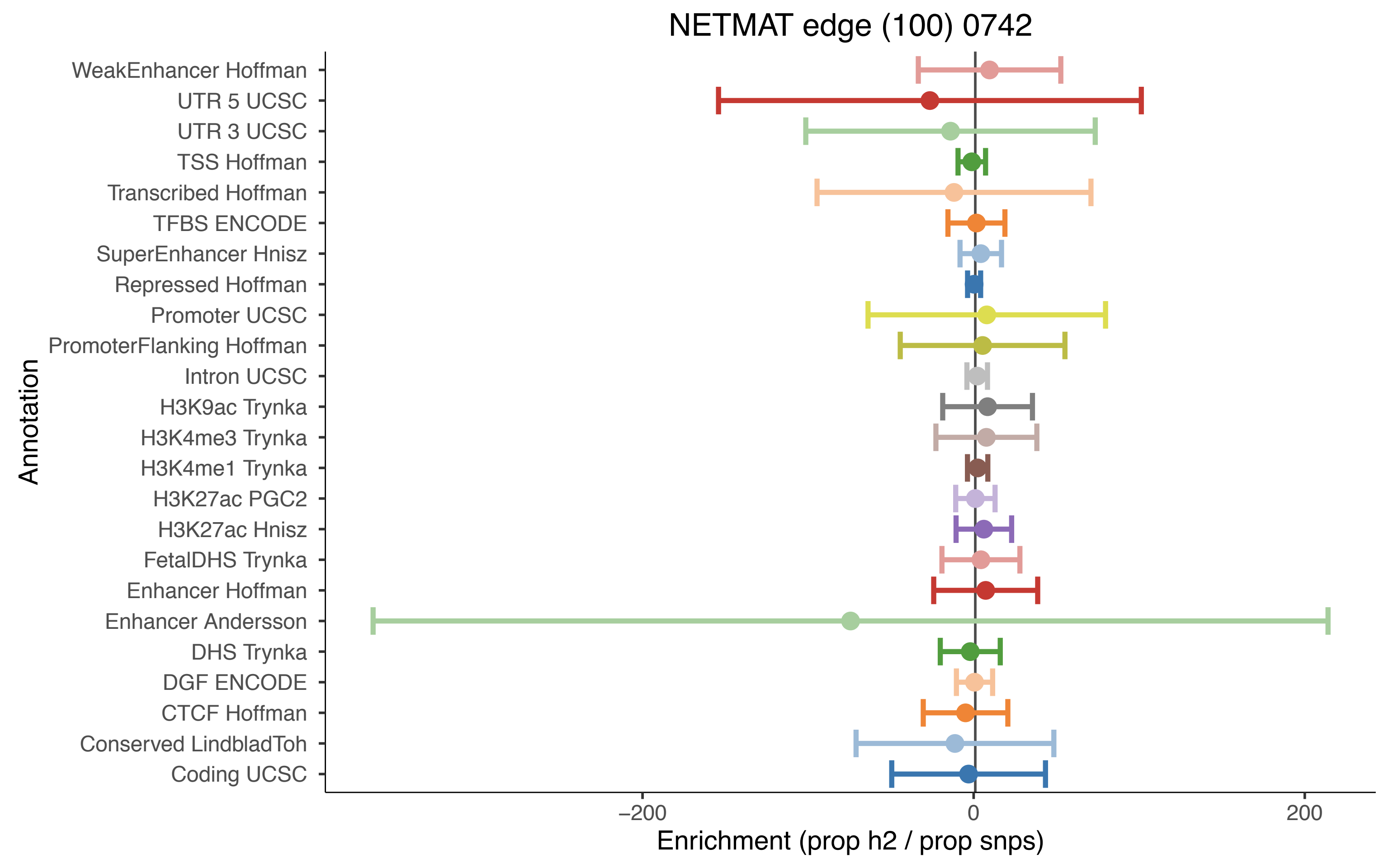

NETMAT edge (100) 0742
WeakEnhancer Hoffman
UTR 5 UCSC
UTR 3 UCSC
TSS Hoffman
Transcribed Hoffman
TFBS ENCODE
SuperEnhancer Hnisz
Repressed Hoffman
Promoter UCSC
PromoterFlanking Hoffman
Intron UCSC
H3K9ac Trynka
H3K4me3 Trynka
H3K4me1 Trynka
H3K27ac PGC2
H3K27ac Hnisz
FetalDHS Trynka
Enhancer Hoffman
Enhancer Andersson
DHS Trynka
DGF ENCODE
CTCF Hoffman
Conserved LindbladToh
Coding UCSC
Annotation
−200
0
200
Enrichment (prop h2 / prop snps)

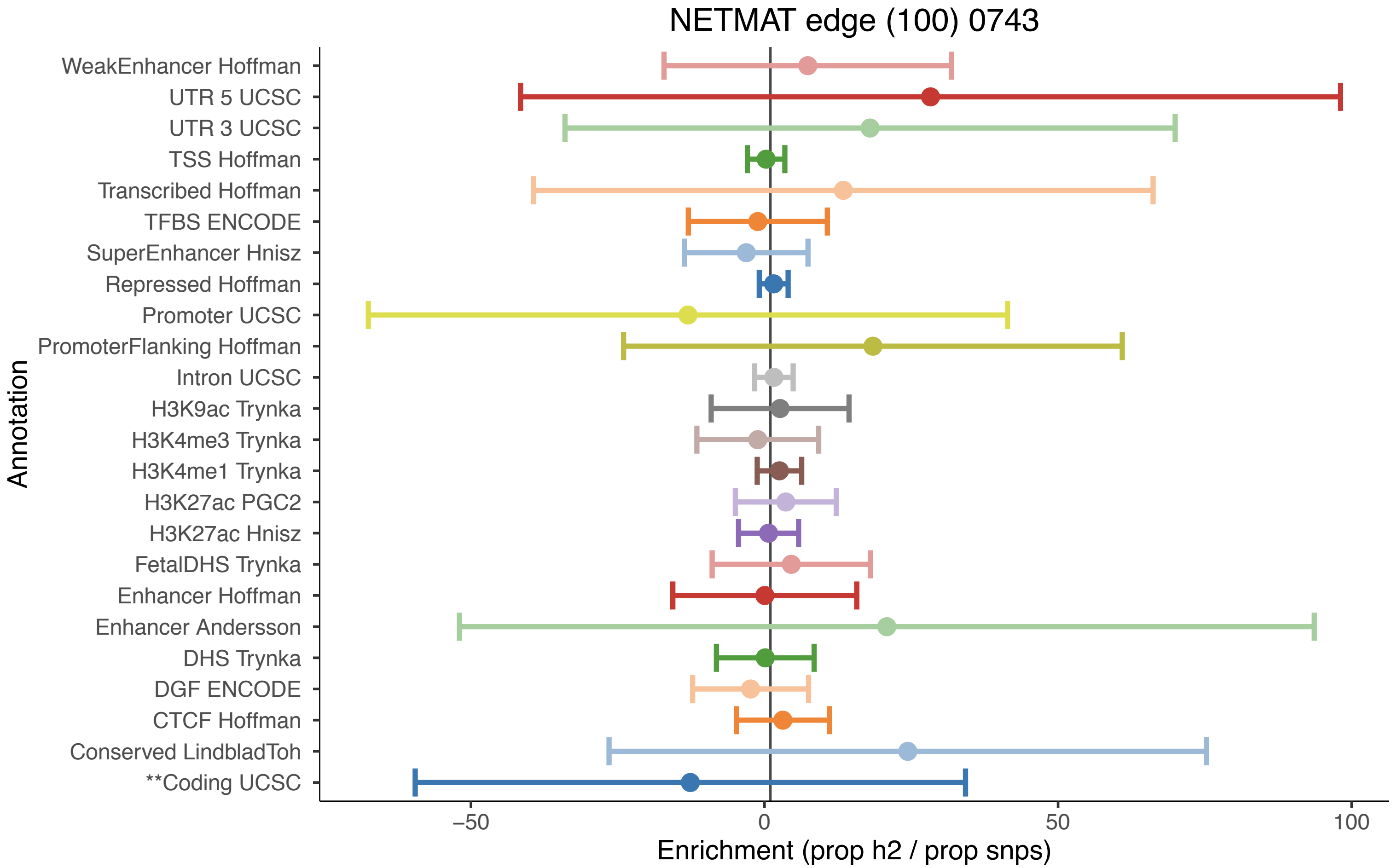
NETMAT edge (100) 0743
Annotation
WeakEnhancer Hoffman
UTR 5 UCSC
UTR 3 UCSC
TSS Hoffman
Transcribed Hoffman
TFBS ENCODE
SuperEnhancer Hnisz
Repressed Hoffman
Promoter UCSC
PromoterFlanking Hoffman
Intron UCSC
H3K9ac Trynka
H3K4me3 Trynka
H3K4me1 Trynka
H3K27ac PGC2
H3K27ac Hnisz
FetalDHS Trynka
Enhancer Hoffman
Enhancer Andersson
DHS Trynka
DGF ENCODE
CTCF Hoffman
Conserved LindbladToh
**Coding UCSC
−50
0
50
100
Enrichment (prop h2 / prop snps)

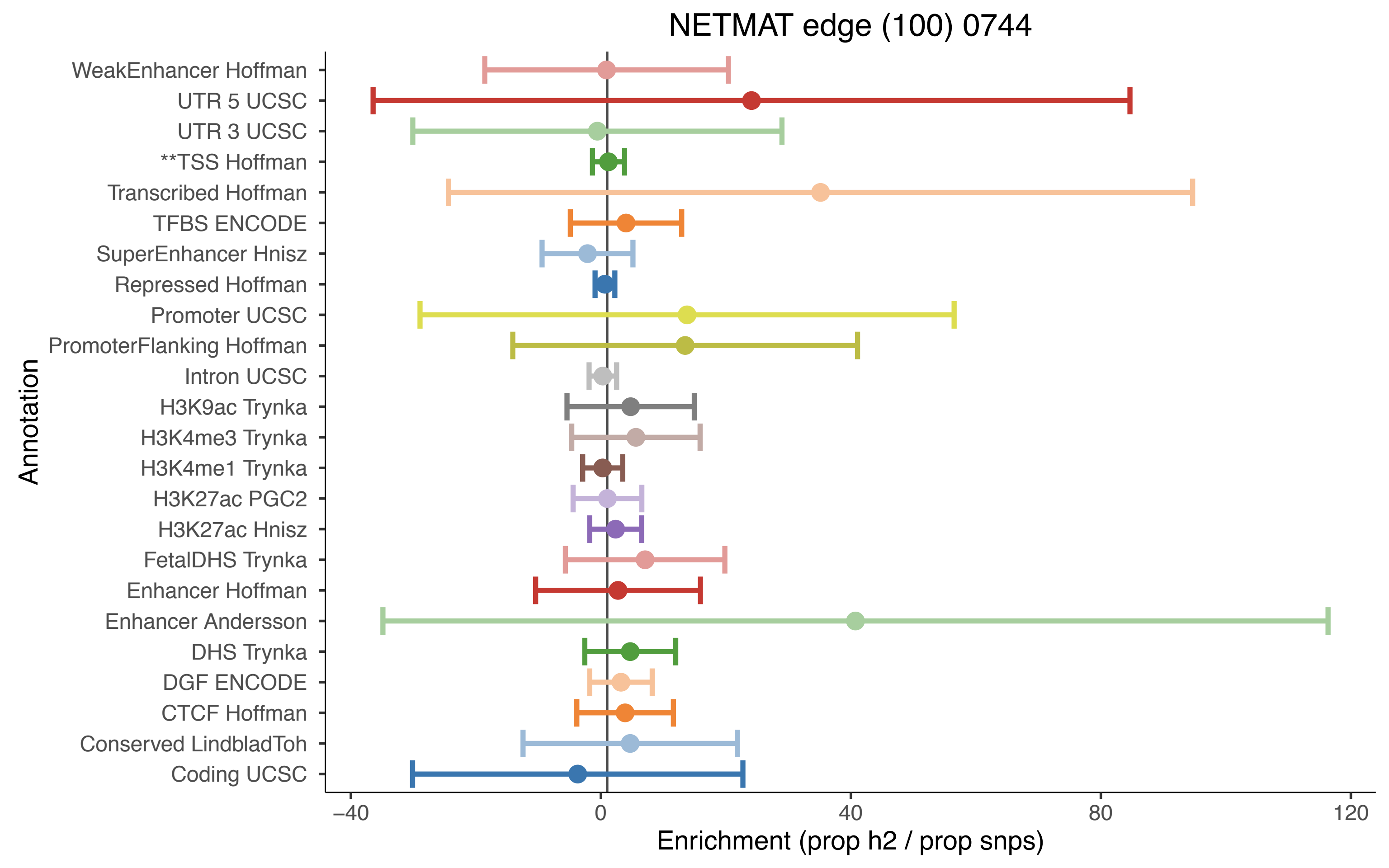
NETMAT edge (100) 0744
WeakEnhancer Hoffman
UTR 5 UCSC
UTR 3 UCSC
**TSS Hoffman
Transcribed Hoffman
TFBS ENCODE
SuperEnhancer Hnisz
Repressed Hoffman
Promoter UCSC
PromoterFlanking Hoffman
Intron UCSC
H3K9ac Trynka
H3K4me3 Trynka
H3K4me1 Trynka
H3K27ac PGC2
H3K27ac Hnisz
FetalDHS Trynka
Enhancer Hoffman
Enhancer Andersson
DHS Trynka
DGF ENCODE
CTCF Hoffman
Conserved LindbladToh
Coding UCSC
Annotation
−40
0
40
80
120
Enrichment (prop h2 / prop snps)

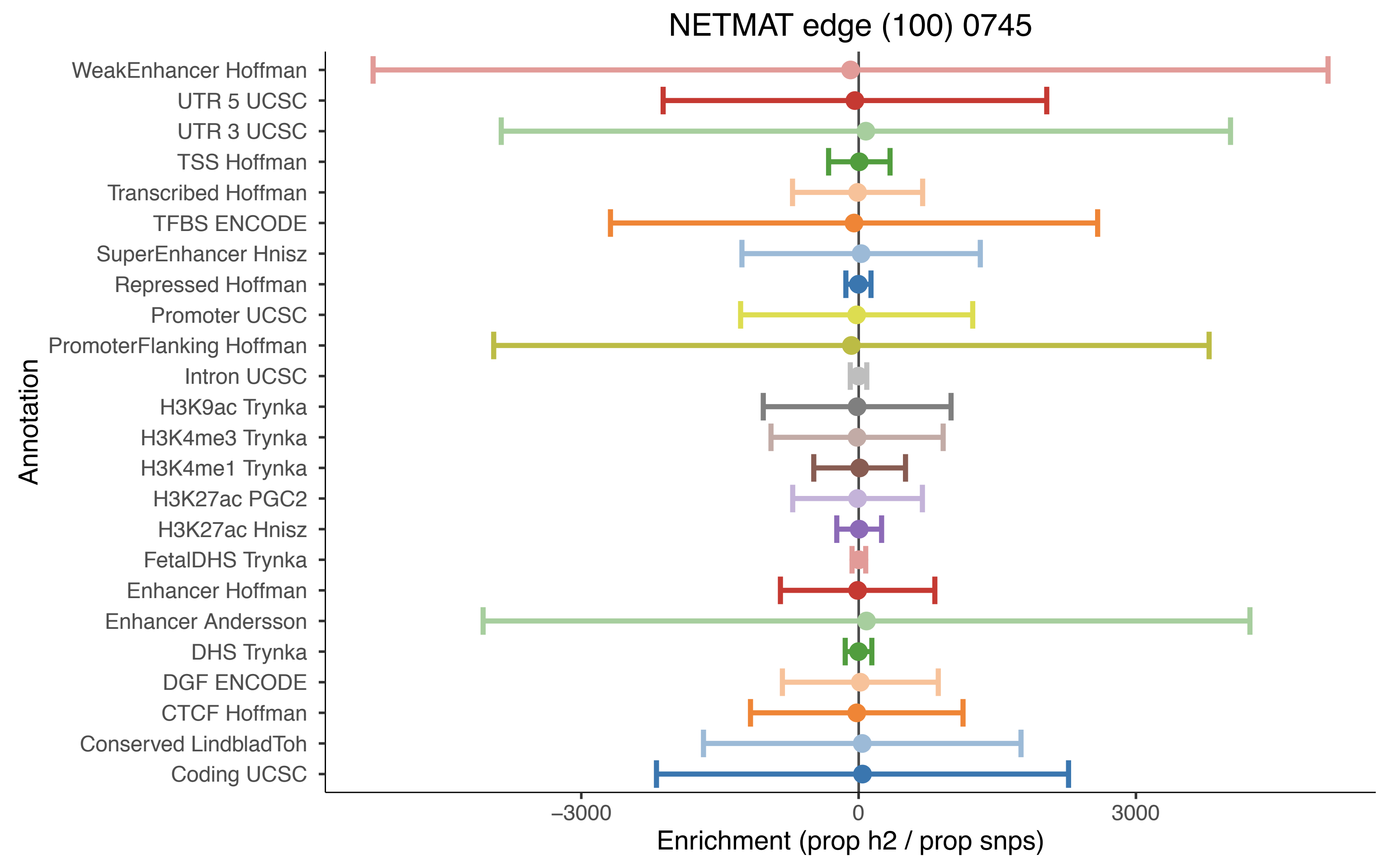

NETMAT edge (100) 0745
WeakEnhancer Hoffman
UTR 5 UCSC
UTR 3 UCSC
TSS Hoffman
Transcribed Hoffman
TFBS ENCODE
SuperEnhancer Hnisz
Repressed Hoffman
Promoter UCSC
PromoterFlanking Hoffman
Intron UCSC
H3K9ac Trynka
H3K4me3 Trynka
H3K4me1 Trynka
H3K27ac PGC2
H3K27ac Hnisz
FetalDHS Trynka
Enhancer Hoffman
Enhancer Andersson
DHS Trynka
DGF ENCODE
CTCF Hoffman
Conserved LindbladToh
Coding UCSC
Annotation
−3000
0
3000
Enrichment (prop h2 / prop snps)

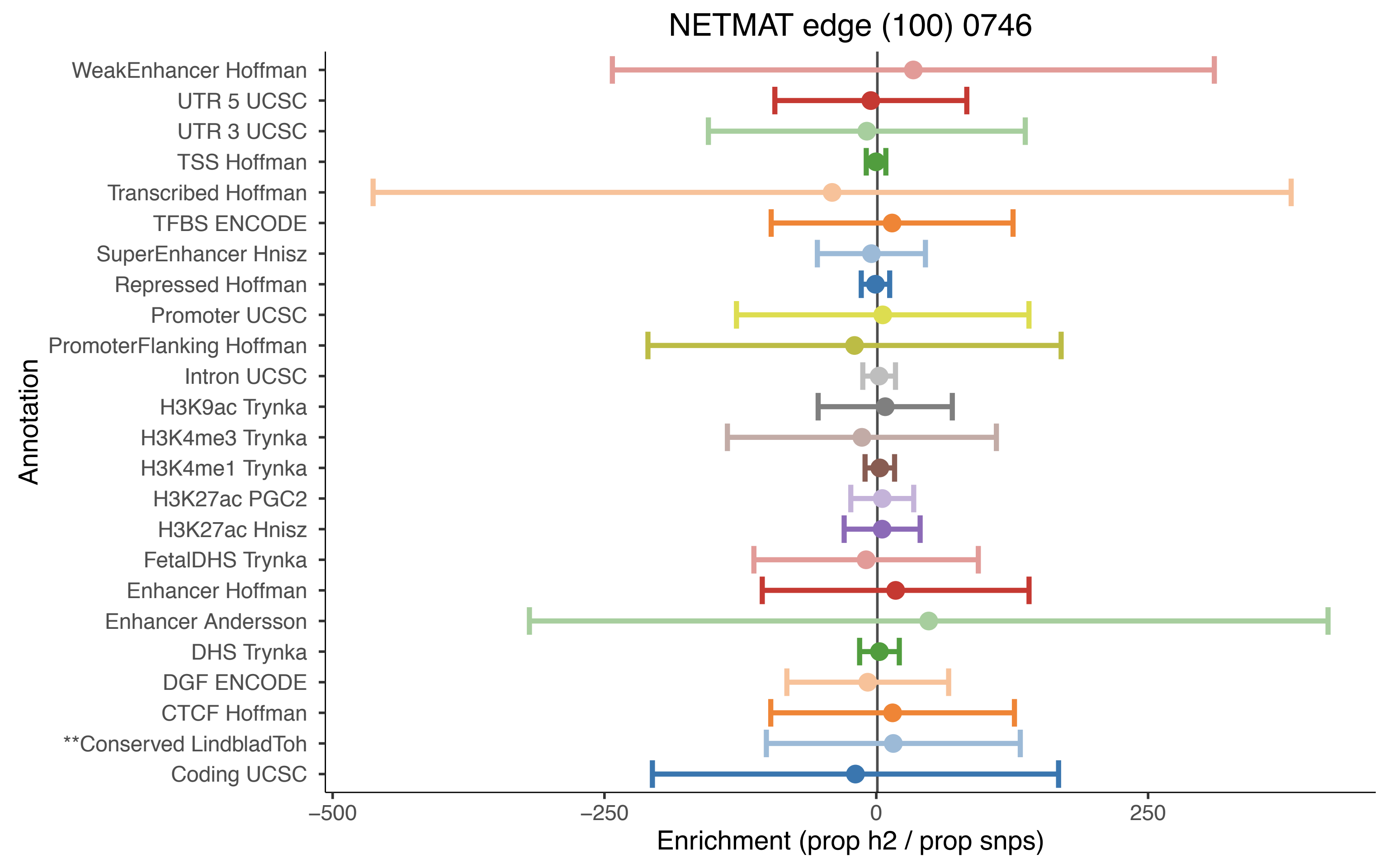
NETMAT edge (100) 0746
Annotation
WeakEnhancer Hoffman
UTR 5 UCSC
UTR 3 UCSC
TSS Hoffman
Transcribed Hoffman
TFBS ENCODE
SuperEnhancer Hnisz
Repressed Hoffman
Promoter UCSC
PromoterFlanking Hoffman
Intron UCSC
H3K9ac Trynka
H3K4me3 Trynka
H3K4me1 Trynka
H3K27ac PGC2
H3K27ac Hnisz
FetalDHS Trynka
Enhancer Hoffman
Enhancer Andersson
DHS Trynka
DGF ENCODE
CTCF Hoffman
**Conserved LindbladToh
Coding UCSC
−500
−250
0
250
Enrichment (prop h2 / prop snps)

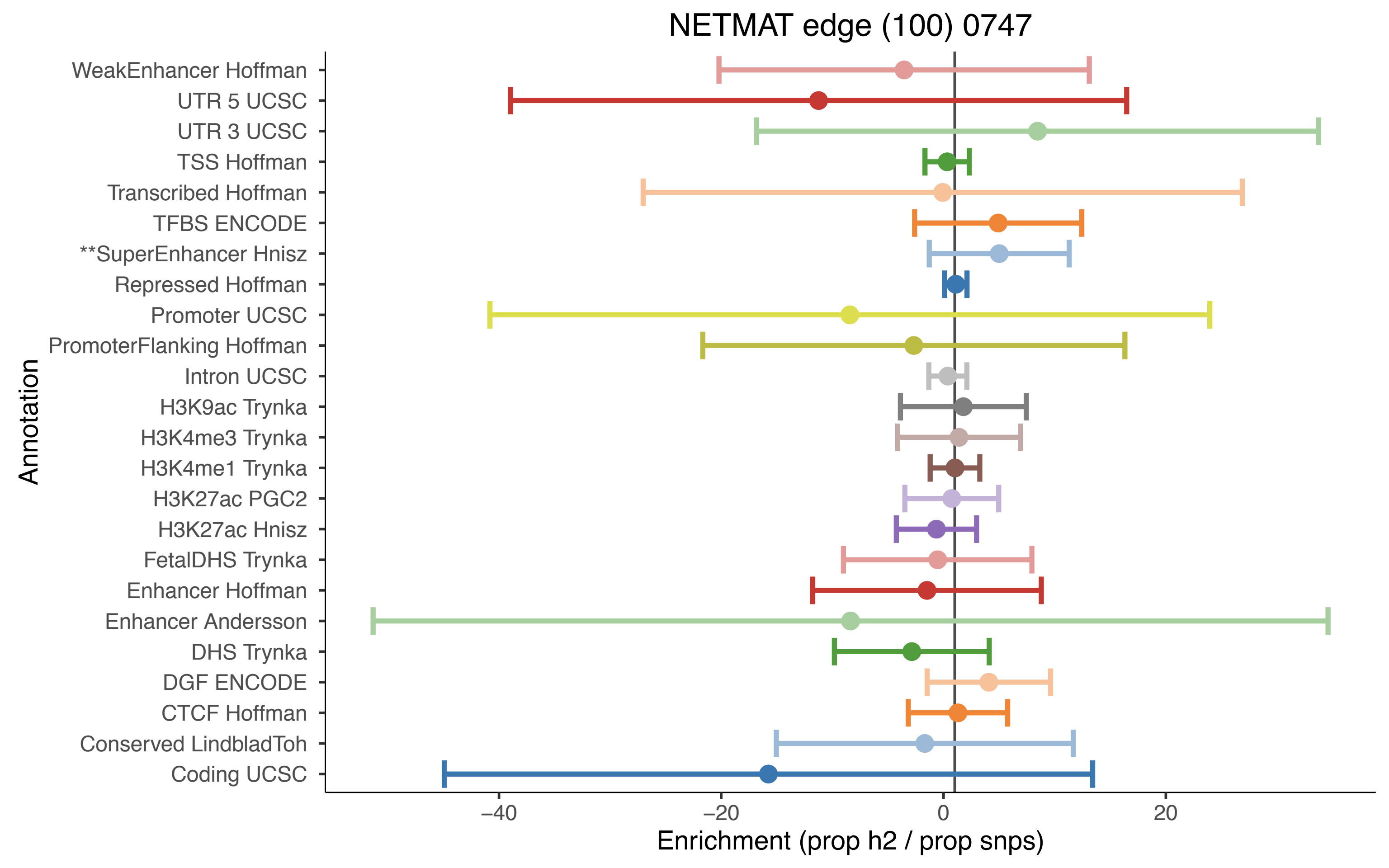
NETMAT edge (100) 0747
WeakEnhancer Hoffman
UTR 5 UCSC
UTR 3 UCSC
TSS Hoffman
Transcribed Hoffman
TFBS ENCODE
**SuperEnhancer Hnisz
Repressed Hoffman
Promoter UCSC
PromoterFlanking Hoffman
Intron UCSC
H3K9ac Trynka
H3K4me3 Trynka
H3K4me1 Trynka
H3K27ac PGC2
H3K27ac Hnisz
FetalDHS Trynka
Enhancer Hoffman
Enhancer Andersson
DHS Trynka
DGF ENCODE
CTCF Hoffman
Conserved LindbladToh
Coding UCSC
Annotation
−40
−20
0
20
Enrichment (prop h2 / prop snps)

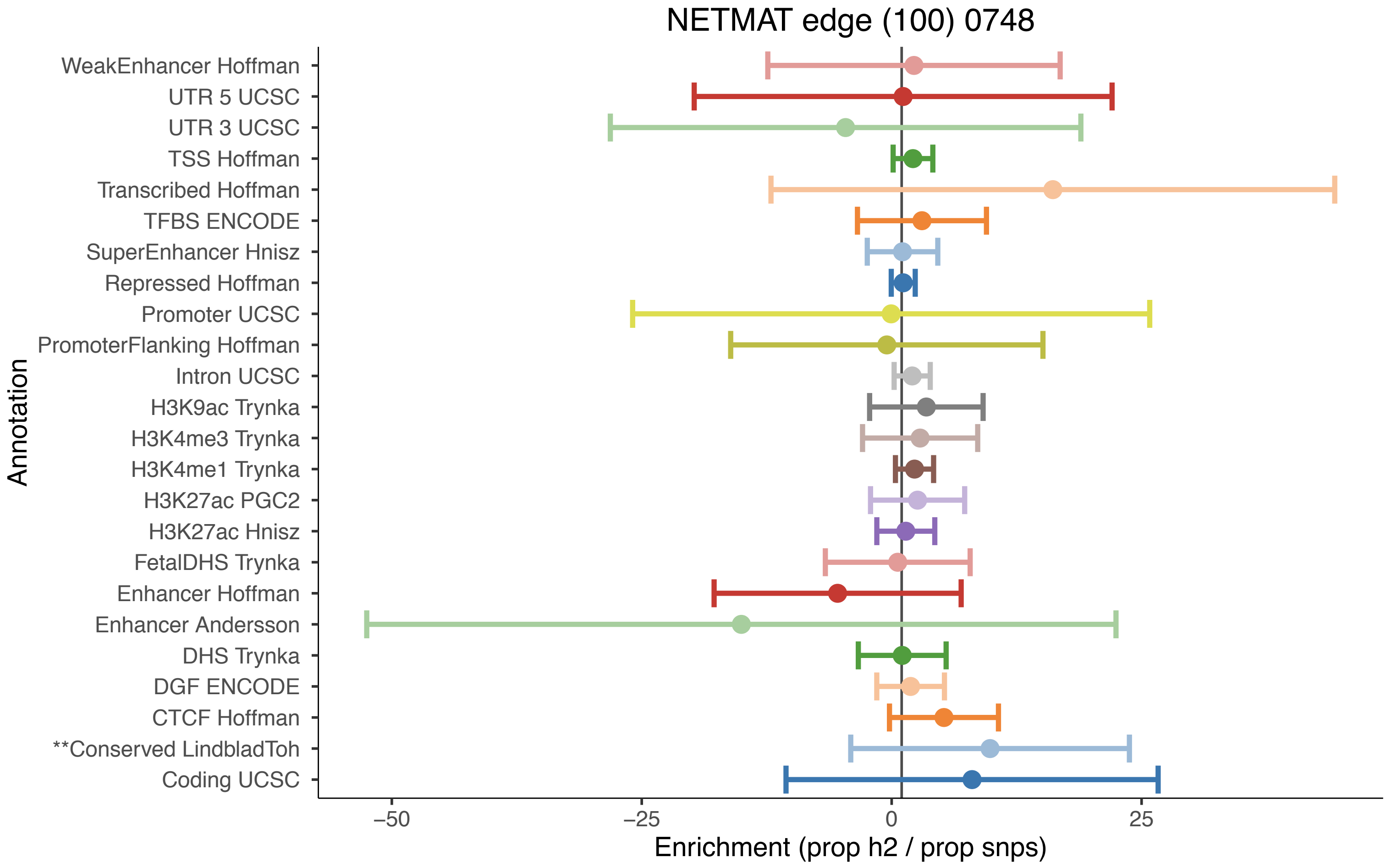
NETMAT edge (100) 0748
Annotation
WeakEnhancer Hoffman
UTR 5 UCSC
UTR 3 UCSC
TSS Hoffman
Transcribed Hoffman
TFBS ENCODE
SuperEnhancer Hnisz
Repressed Hoffman
Promoter UCSC
PromoterFlanking Hoffman
Intron UCSC
H3K9ac Trynka
H3K4me3 Trynka
H3K4me1 Trynka
H3K27ac PGC2
H3K27ac Hnisz
FetalDHS Trynka
Enhancer Hoffman
Enhancer Andersson
DHS Trynka
DGF ENCODE
CTCF Hoffman
**Conserved LindbladToh
Coding UCSC
−50
−25
0
25
Enrichment (prop h2 / prop snps)

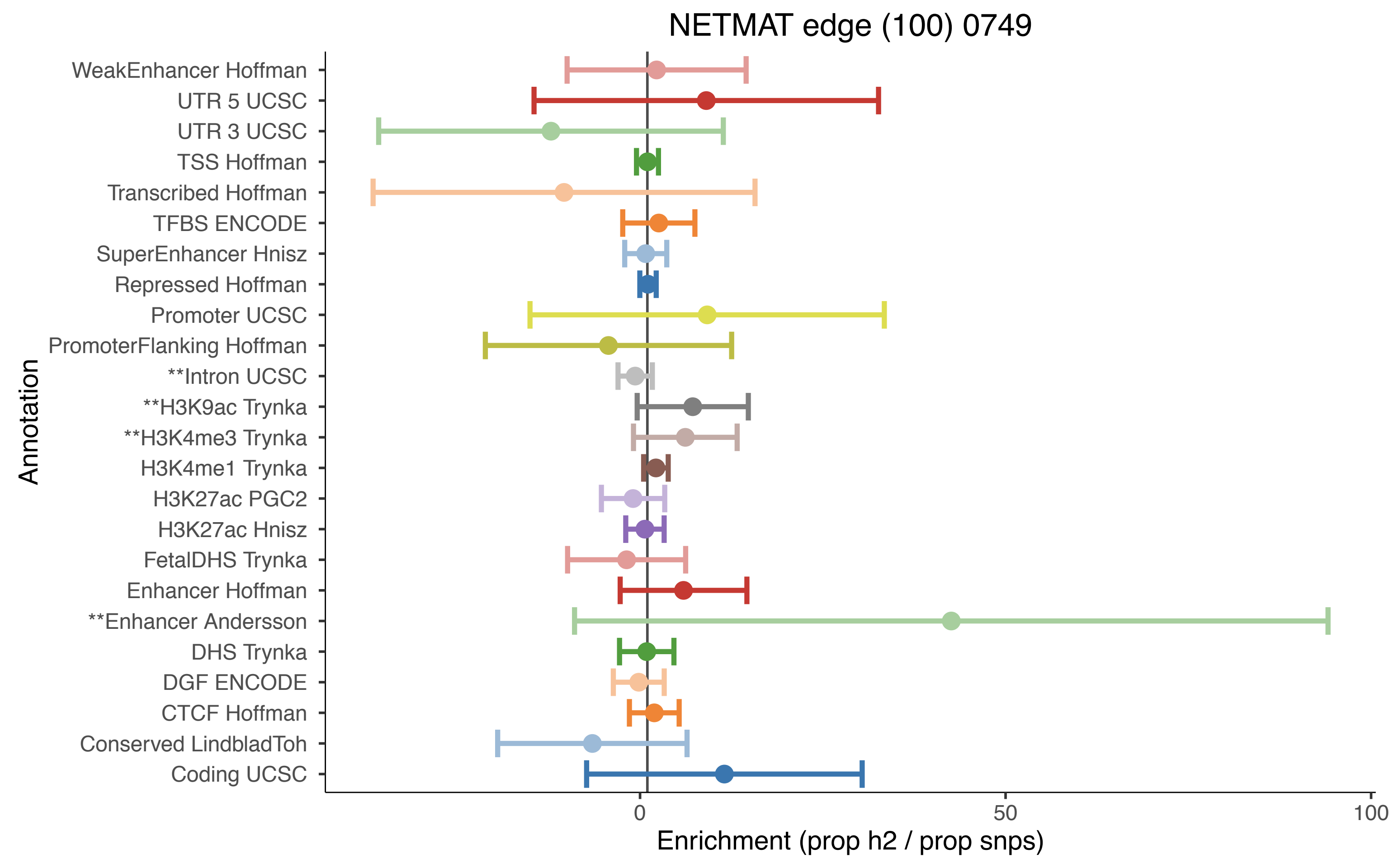

NETMAT edge (100) 0749
Annotation
WeakEnhancer Hoffman
UTR 5 UCSC
UTR 3 UCSC
TSS Hoffman
Transcribed Hoffman
TFBS ENCODE
SuperEnhancer Hnisz
Repressed Hoffman
Promoter UCSC
PromoterFlanking Hoffman
**Intron UCSC
**H3K9ac Trynka
**H3K4me3 Trynka
H3K4me1 Trynka
H3K27ac PGC2
H3K27ac Hnisz
FetalDHS Trynka
Enhancer Hoffman
**Enhancer Andersson
DHS Trynka
DGF ENCODE
CTCF Hoffman
Conserved LindbladToh
Coding UCSC
0
50
100
Enrichment (prop h2 / prop snps)

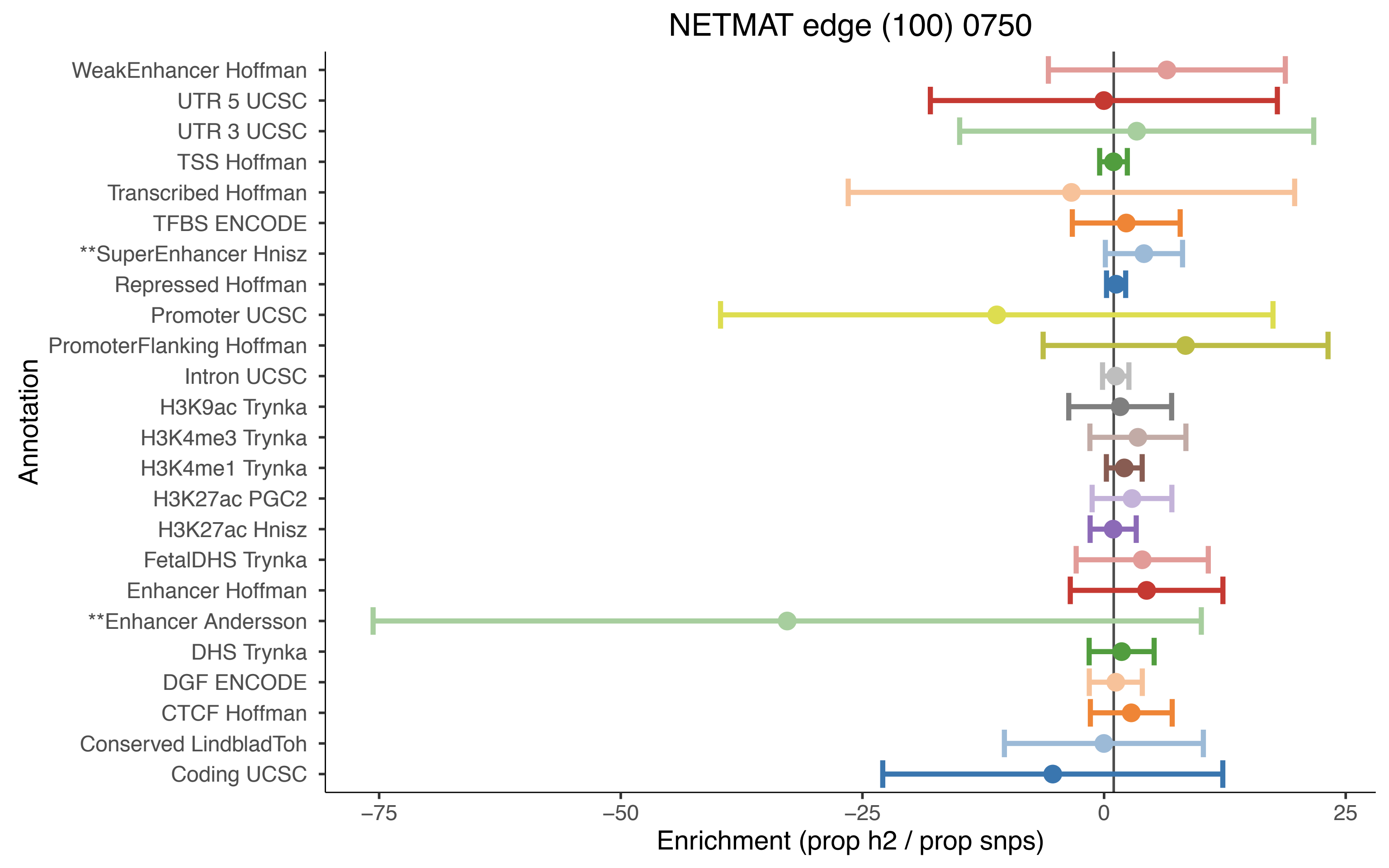
NETMAT edge (100) 0750
WeakEnhancer Hoffman
UTR 5 UCSC
UTR 3 UCSC
TSS Hoffman
Transcribed Hoffman
TFBS ENCODE
**SuperEnhancer Hnisz
Repressed Hoffman
Promoter UCSC
PromoterFlanking Hoffman
Intron UCSC
H3K9ac Trynka
H3K4me3 Trynka
H3K4me1 Trynka
H3K27ac PGC2
H3K27ac Hnisz
FetalDHS Trynka
Enhancer Hoffman
**Enhancer Andersson
DHS Trynka
DGF ENCODE
CTCF Hoffman
Conserved LindbladToh
Coding UCSC
Annotation
−75
−50
−25
0
25
Enrichment (prop h2 / prop snps)

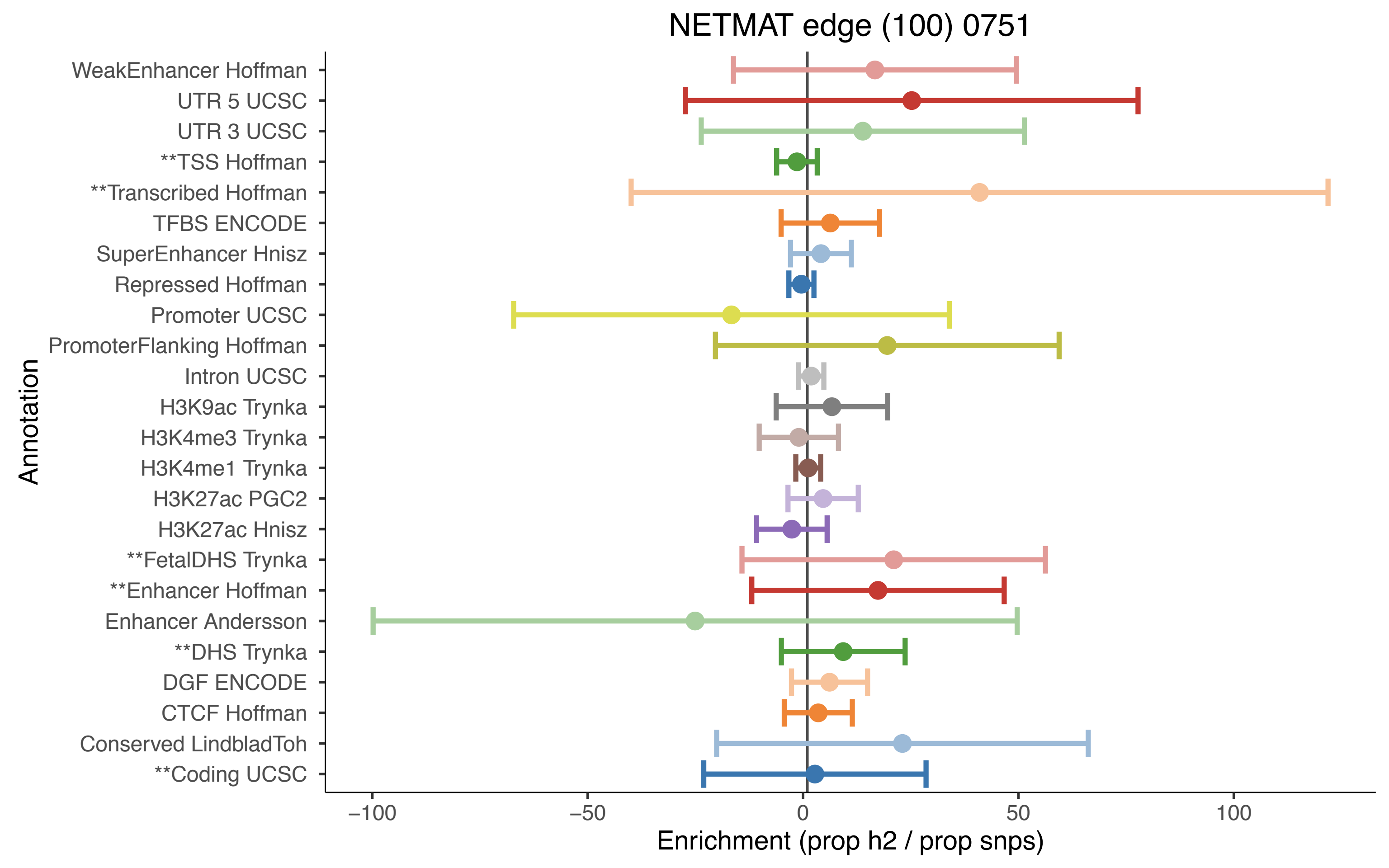
NETMAT edge (100) 0751
WeakEnhancer Hoffman
UTR 5 UCSC
UTR 3 UCSC
**TSS Hoffman
**Transcribed Hoffman
TFBS ENCODE
SuperEnhancer Hnisz
Repressed Hoffman
Promoter UCSC
PromoterFlanking Hoffman
Intron UCSC
H3K9ac Trynka
H3K4me3 Trynka
H3K4me1 Trynka
H3K27ac PGC2
H3K27ac Hnisz
**FetalDHS Trynka
**Enhancer Hoffman
Enhancer Andersson
**DHS Trynka
DGF ENCODE
CTCF Hoffman
Conserved LindbladToh
**Coding UCSC
Annotation
−100
−50
0
50
100
Enrichment (prop h2 / prop snps)

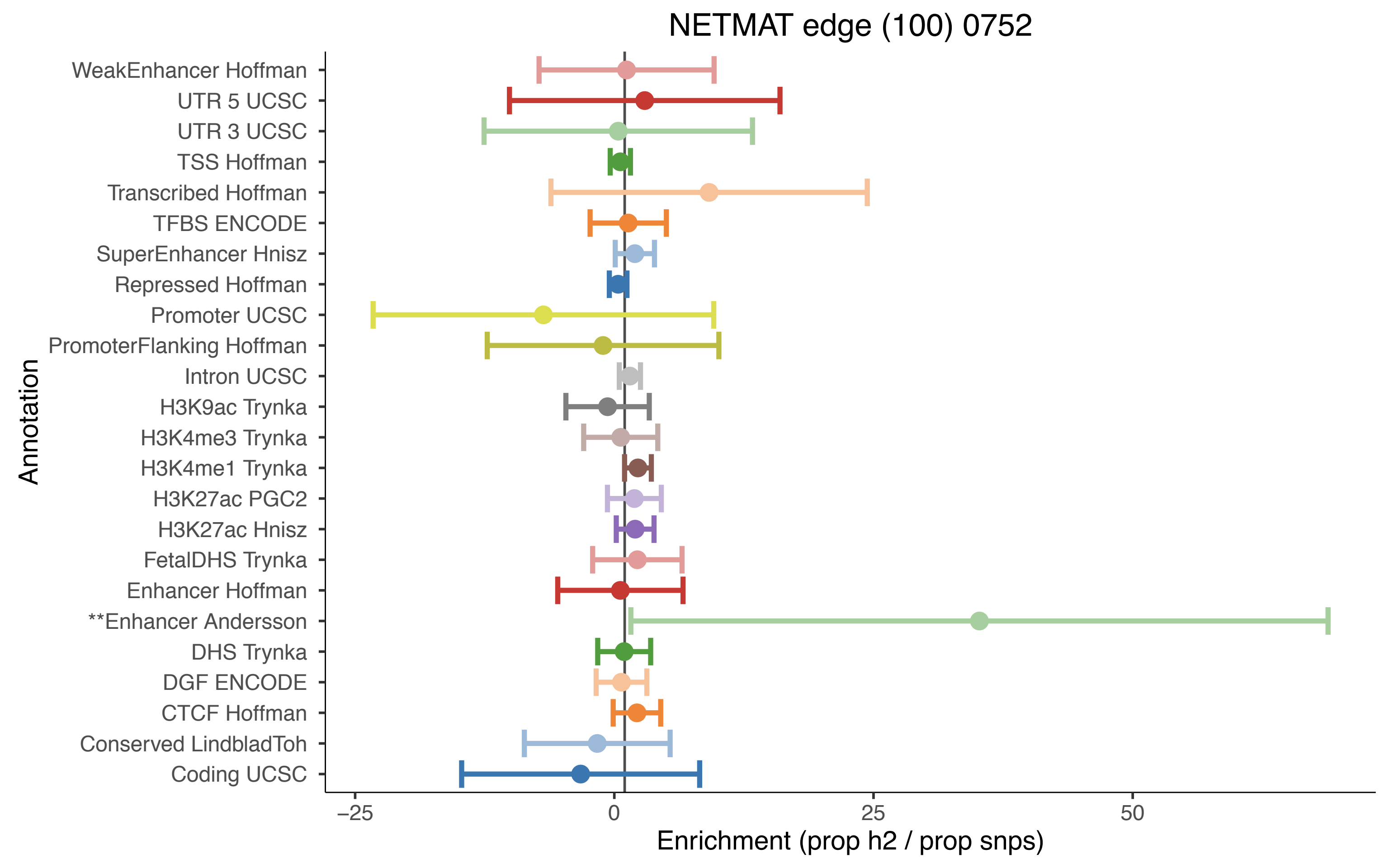
NETMAT edge (100) 0752
Annotation
WeakEnhancer Hoffman
UTR 5 UCSC
UTR 3 UCSC
TSS Hoffman
Transcribed Hoffman
TFBS ENCODE
SuperEnhancer Hnisz
Repressed Hoffman
Promoter UCSC
PromoterFlanking Hoffman
Intron UCSC
H3K9ac Trynka
H3K4me3 Trynka
H3K4me1 Trynka
H3K27ac PGC2
H3K27ac Hnisz
FetalDHS Trynka
Enhancer Hoffman
**Enhancer Andersson
DHS Trynka
DGF ENCODE
CTCF Hoffman
Conserved LindbladToh
Coding UCSC
−25
0
25
50
Enrichment (prop h2 / prop snps)

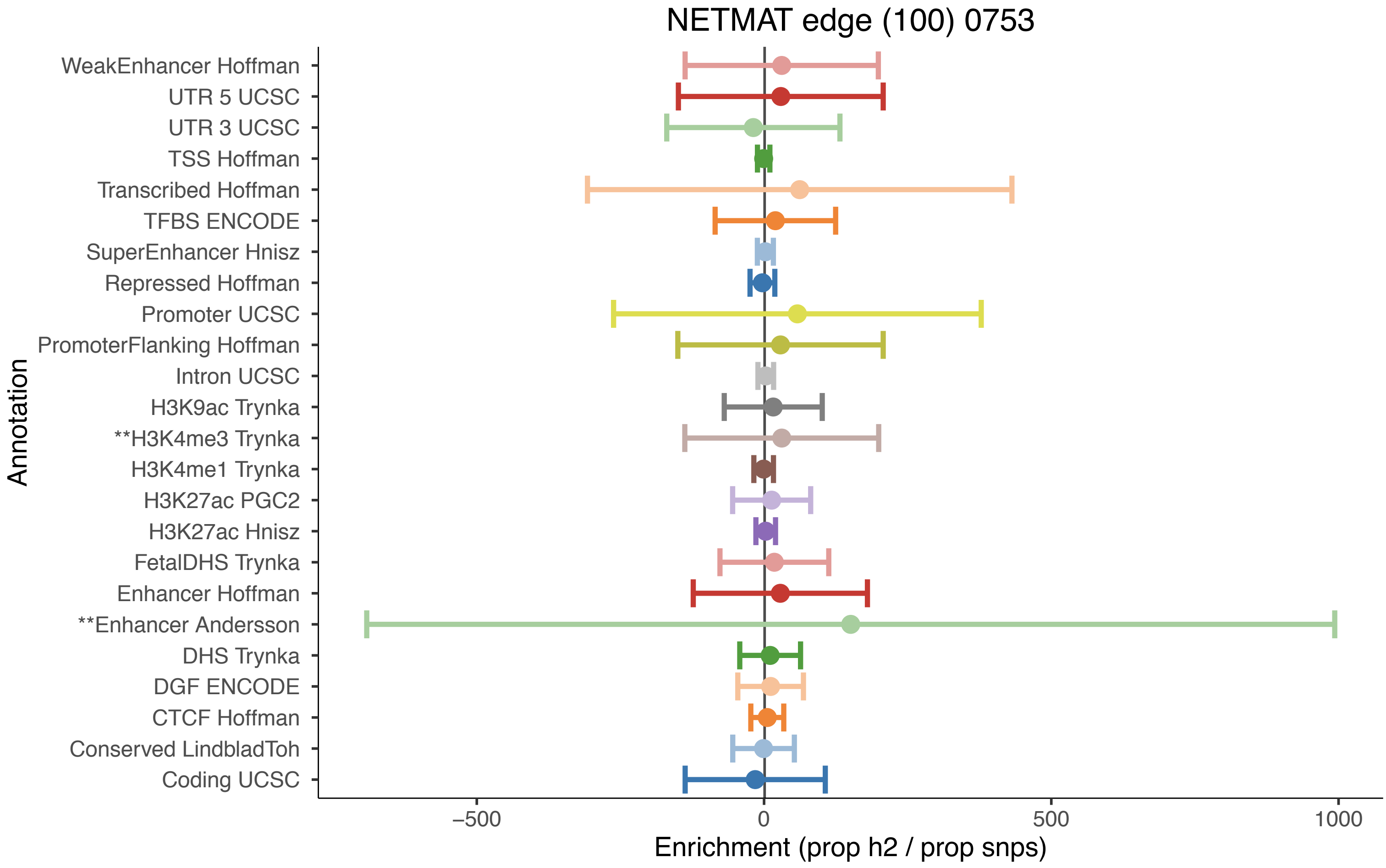
NETMAT edge (100) 0753
Annotation
WeakEnhancer Hoffman
UTR 5 UCSC
UTR 3 UCSC
TSS Hoffman
Transcribed Hoffman
TFBS ENCODE
SuperEnhancer Hnisz
Repressed Hoffman
Promoter UCSC
PromoterFlanking Hoffman
Intron UCSC
H3K9ac Trynka
**H3K4me3 Trynka
H3K4me1 Trynka
H3K27ac PGC2
H3K27ac Hnisz
FetalDHS Trynka
Enhancer Hoffman
**Enhancer Andersson
DHS Trynka
DGF ENCODE
CTCF Hoffman
Conserved LindbladToh
Coding UCSC
−500
0
500
1000
Enrichment (prop h2 / prop snps)

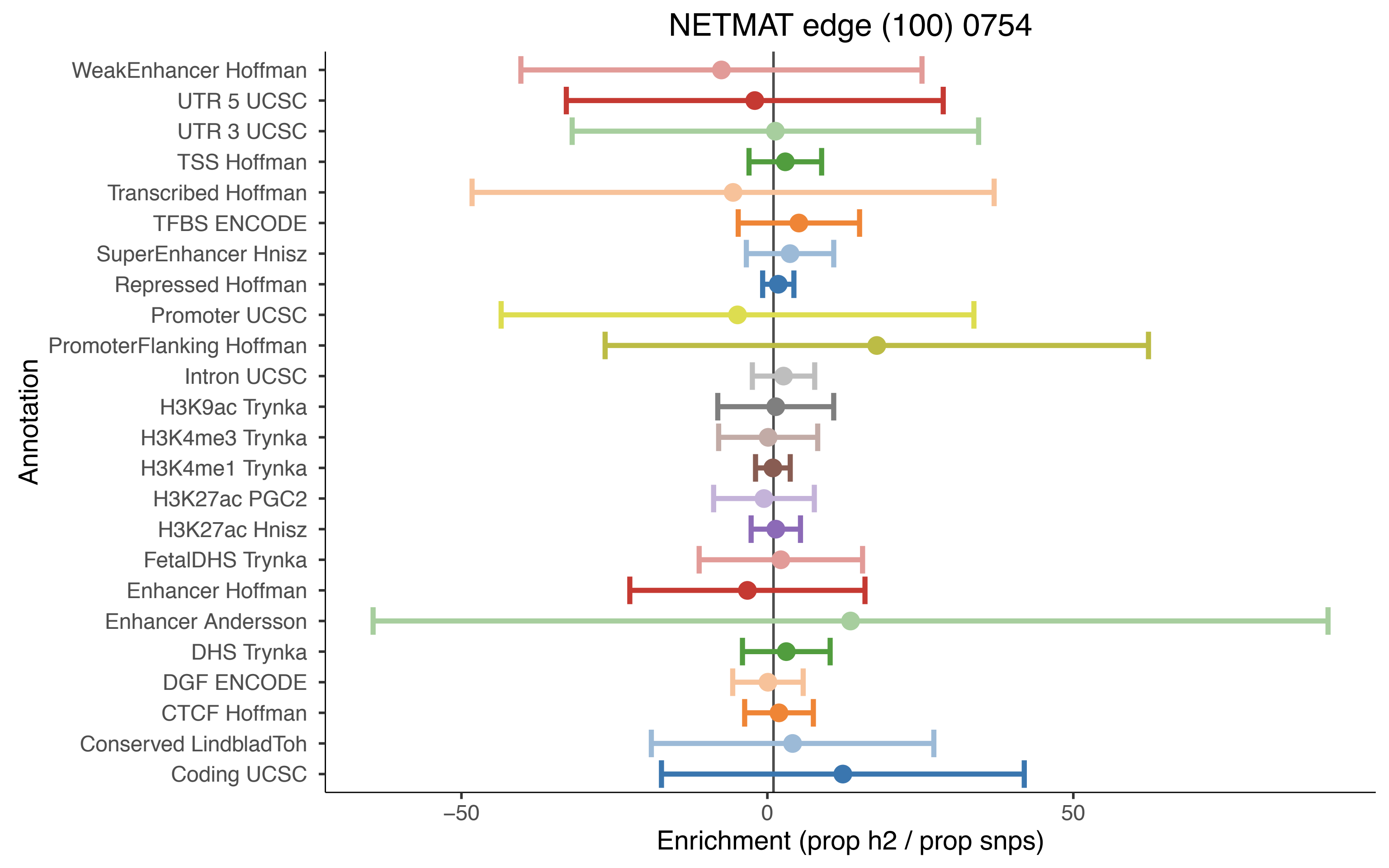
NETMAT edge (100) 0754
Annotation
WeakEnhancer Hoffman
UTR 5 UCSC
UTR 3 UCSC
TSS Hoffman
Transcribed Hoffman
TFBS ENCODE
SuperEnhancer Hnisz
Repressed Hoffman
Promoter UCSC
PromoterFlanking Hoffman
Intron UCSC
H3K9ac Trynka
H3K4me3 Trynka
H3K4me1 Trynka
H3K27ac PGC2
H3K27ac Hnisz
FetalDHS Trynka
Enhancer Hoffman
Enhancer Andersson
DHS Trynka
DGF ENCODE
CTCF Hoffman
Conserved LindbladToh
Coding UCSC
−50
0
50
Enrichment (prop h2 / prop snps)

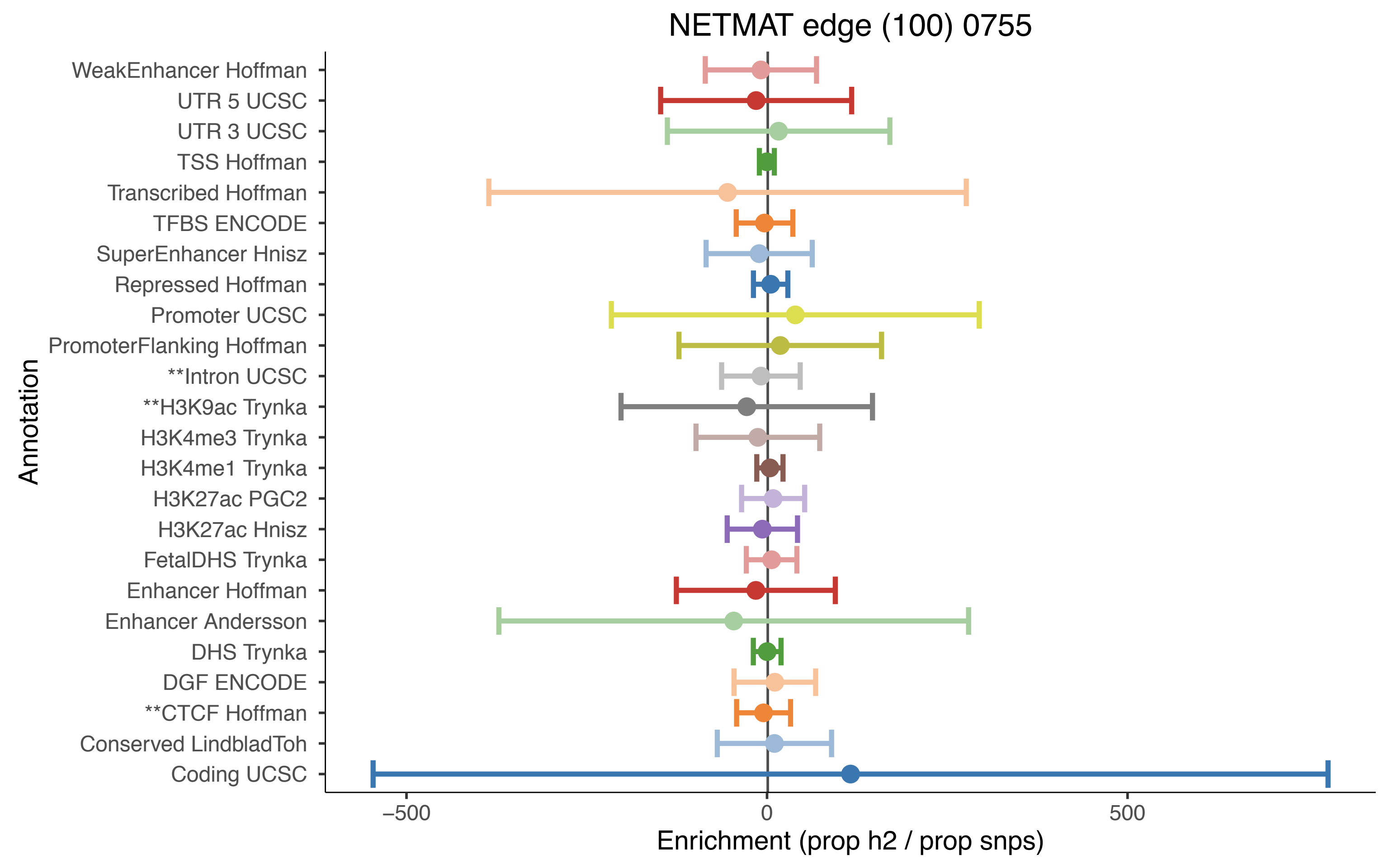
NETMAT edge (100) 0755
WeakEnhancer Hoffman
UTR 5 UCSC
UTR 3 UCSC
TSS Hoffman
Transcribed Hoffman
TFBS ENCODE
SuperEnhancer Hnisz
Repressed Hoffman
Promoter UCSC
PromoterFlanking Hoffman
**Intron UCSC
**H3K9ac Trynka
H3K4me3 Trynka
H3K4me1 Trynka
H3K27ac PGC2
H3K27ac Hnisz
FetalDHS Trynka
Enhancer Hoffman
Enhancer Andersson
DHS Trynka
DGF ENCODE
**CTCF Hoffman
Conserved LindbladToh
Coding UCSC
Annotation
−500
0
500
Enrichment (prop h2 / prop snps)

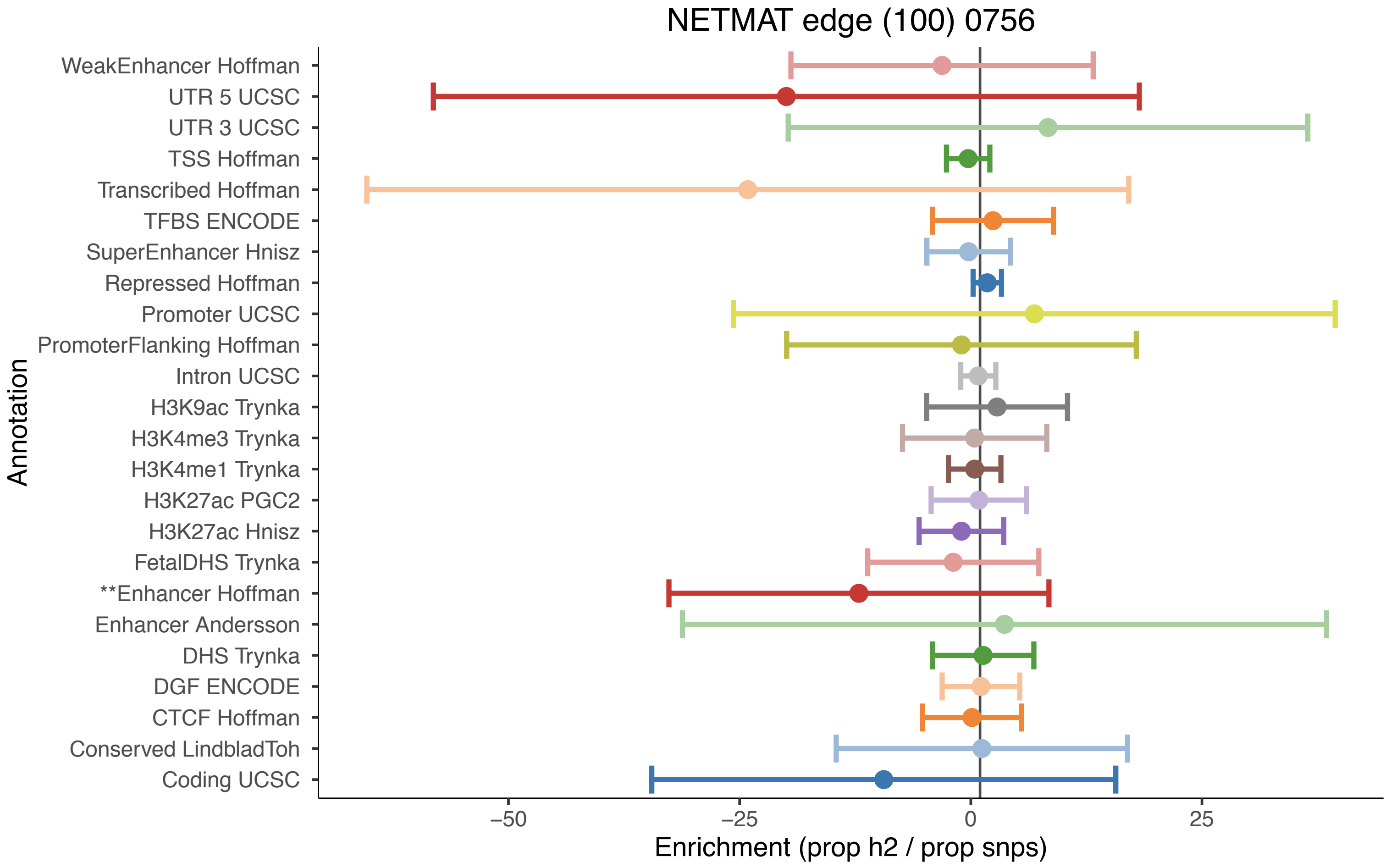

NETMAT edge (100) 0756
Annotation
WeakEnhancer Hoffman
UTR 5 UCSC
UTR 3 UCSC
TSS Hoffman
Transcribed Hoffman
TFBS ENCODE
SuperEnhancer Hnisz
Repressed Hoffman
Promoter UCSC
PromoterFlanking Hoffman
Intron UCSC
H3K9ac Trynka
H3K4me3 Trynka
H3K4me1 Trynka
H3K27ac PGC2
H3K27ac Hnisz
FetalDHS Trynka
**Enhancer Hoffman
Enhancer Andersson
DHS Trynka
DGF ENCODE
CTCF Hoffman
Conserved LindbladToh
Coding UCSC
−50
−25
0
25
Enrichment (prop h2 / prop snps)

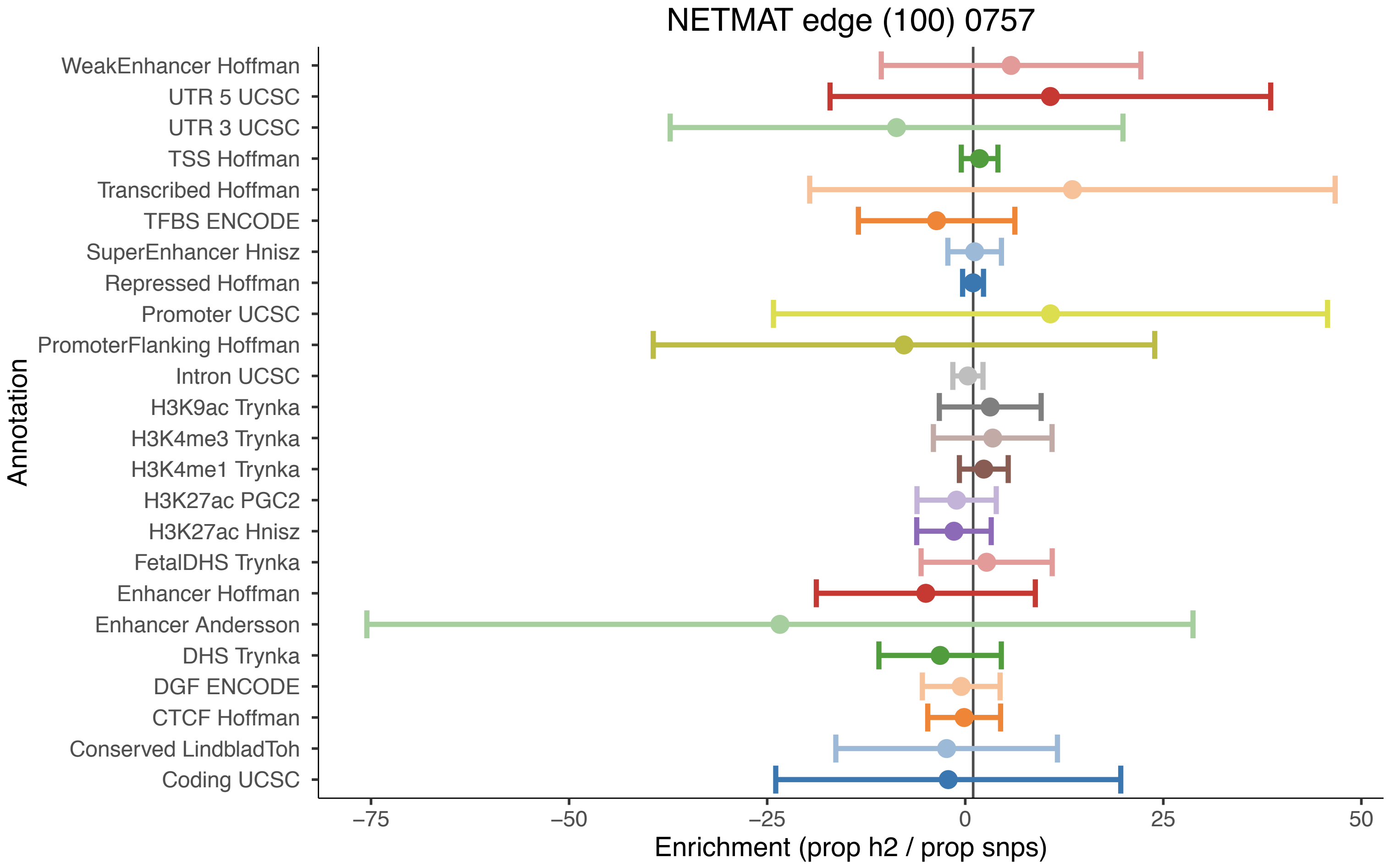
NETMAT edge (100) 0757
WeakEnhancer Hoffman
UTR 5 UCSC
UTR 3 UCSC
TSS Hoffman
Transcribed Hoffman
TFBS ENCODE
SuperEnhancer Hnisz
Repressed Hoffman
Promoter UCSC
PromoterFlanking Hoffman
Intron UCSC
H3K9ac Trynka
H3K4me3 Trynka
H3K4me1 Trynka
H3K27ac PGC2
H3K27ac Hnisz
FetalDHS Trynka
Enhancer Hoffman
Enhancer Andersson
DHS Trynka
DGF ENCODE
CTCF Hoffman
Conserved LindbladToh
Coding UCSC
Annotation
−75
−50
−25
0
25
50
Enrichment (prop h2 / prop snps)

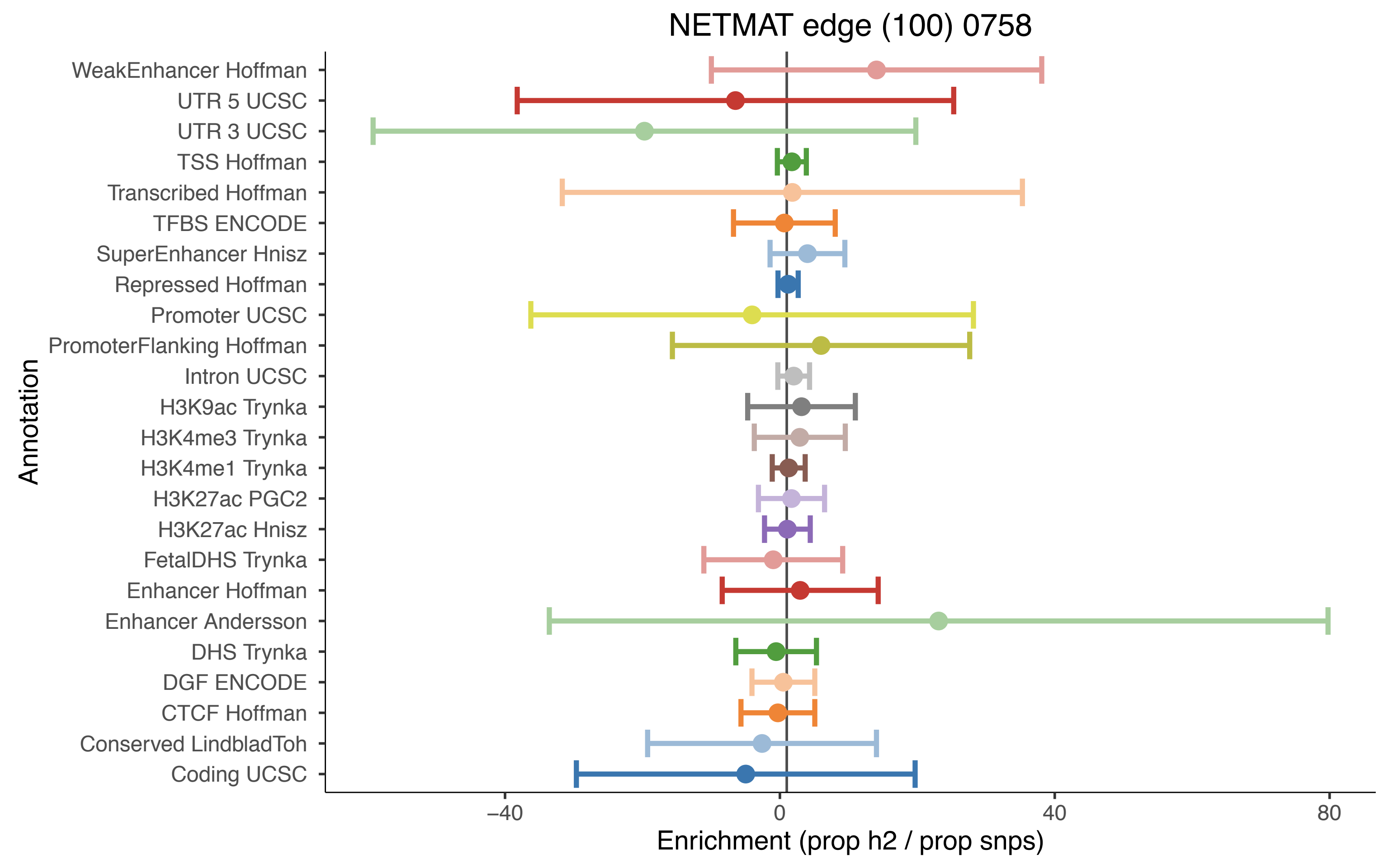
NETMAT edge (100) 0758
WeakEnhancer Hoffman
UTR 5 UCSC
UTR 3 UCSC
TSS Hoffman
Transcribed Hoffman
TFBS ENCODE
SuperEnhancer Hnisz
Repressed Hoffman
Promoter UCSC
PromoterFlanking Hoffman
Intron UCSC
H3K9ac Trynka
H3K4me3 Trynka
H3K4me1 Trynka
H3K27ac PGC2
H3K27ac Hnisz
FetalDHS Trynka
Enhancer Hoffman
Enhancer Andersson
DHS Trynka
DGF ENCODE
CTCF Hoffman
Conserved LindbladToh
Coding UCSC
Annotation
−40
0
40
80
Enrichment (prop h2 / prop snps)

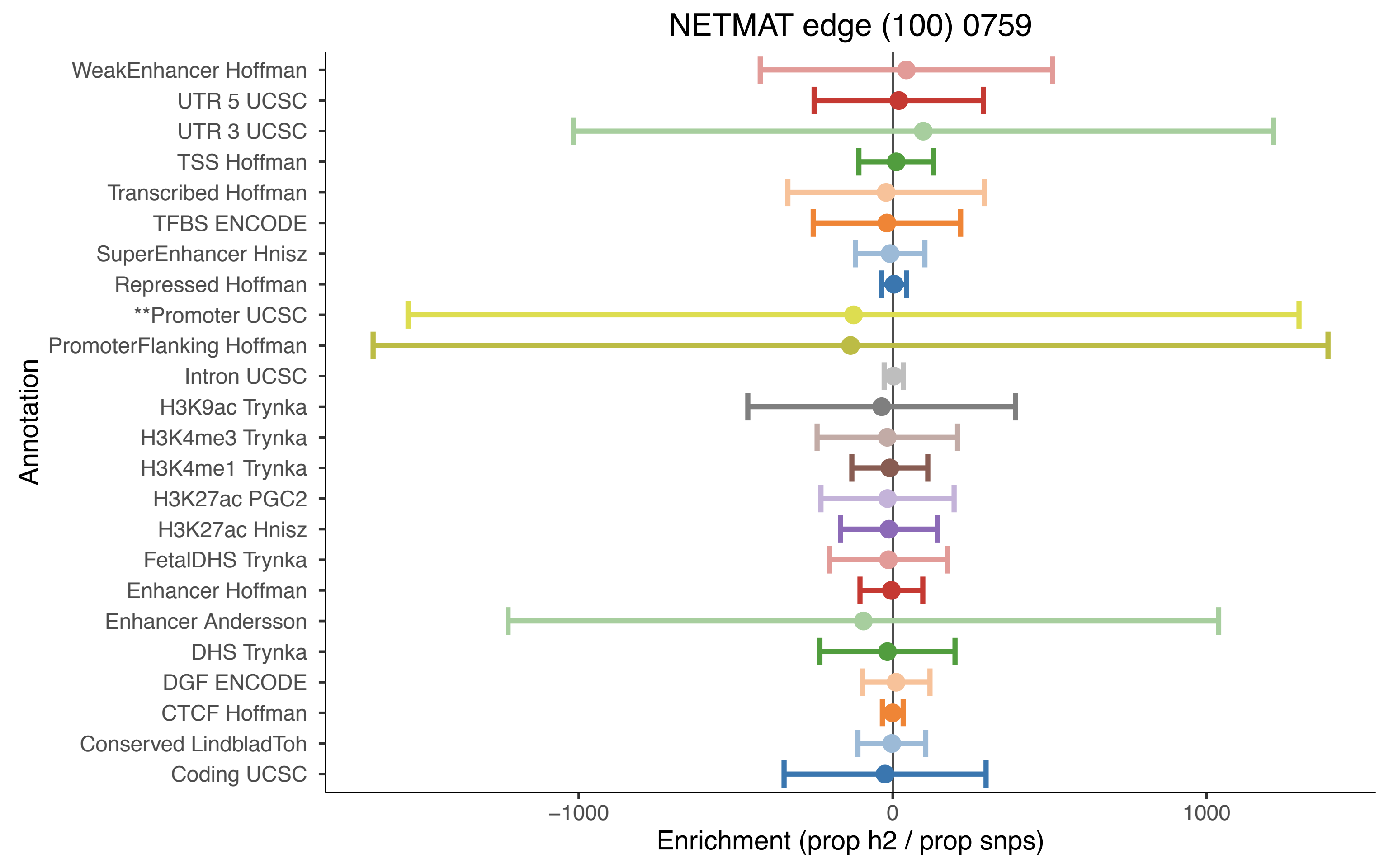
NETMAT edge (100) 0759
WeakEnhancer Hoffman
UTR 5 UCSC
UTR 3 UCSC
TSS Hoffman
Transcribed Hoffman
TFBS ENCODE
SuperEnhancer Hnisz
Repressed Hoffman
**Promoter UCSC
PromoterFlanking Hoffman
Intron UCSC
H3K9ac Trynka
H3K4me3 Trynka
H3K4me1 Trynka
H3K27ac PGC2
H3K27ac Hnisz
FetalDHS Trynka
Enhancer Hoffman
Enhancer Andersson
DHS Trynka
DGF ENCODE
CTCF Hoffman
Conserved LindbladToh
Coding UCSC
Annotation
−1000
0
1000
Enrichment (prop h2 / prop snps)

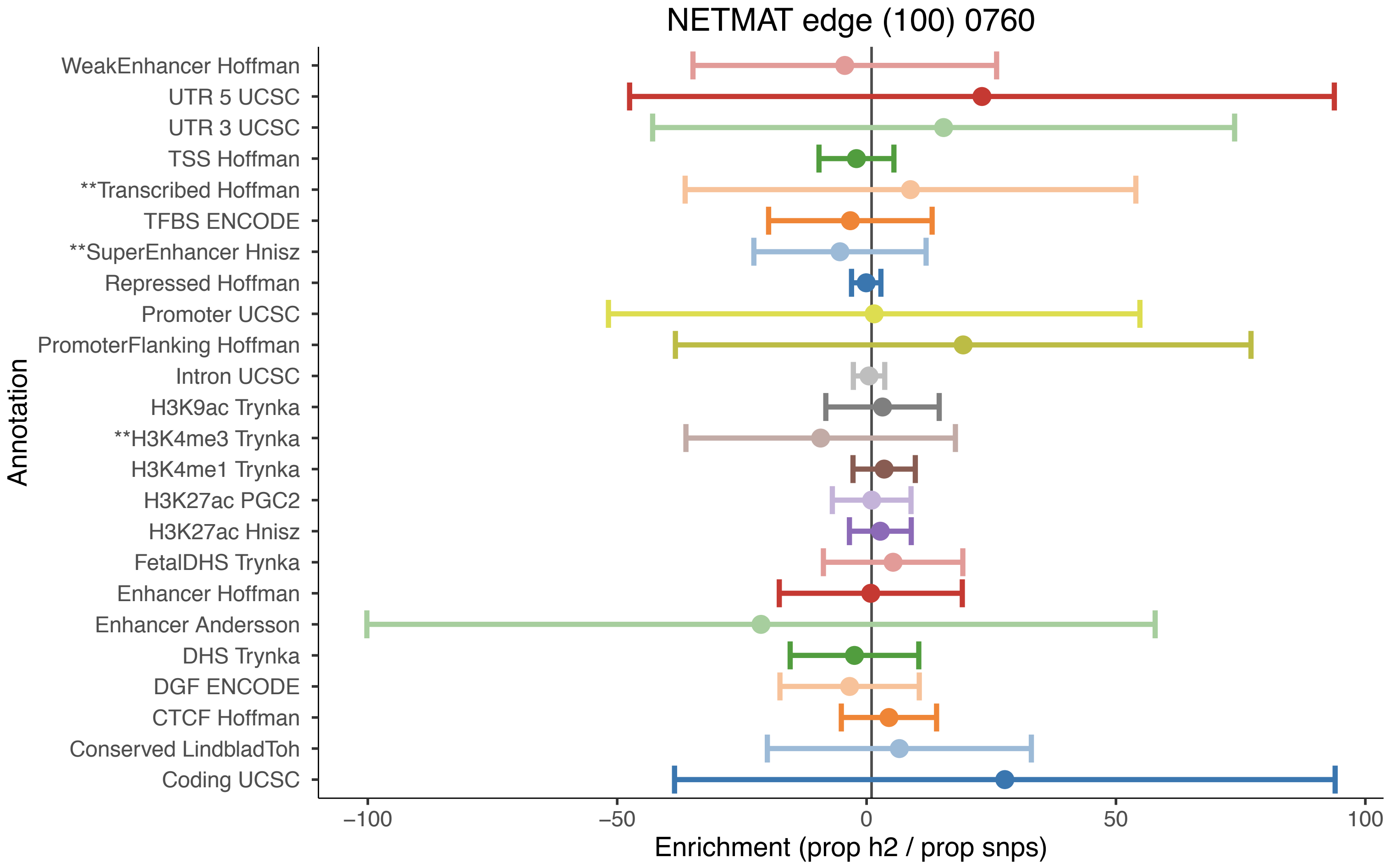

NETMAT edge (100) 0760
WeakEnhancer Hoffman
UTR 5 UCSC
UTR 3 UCSC
TSS Hoffman
**Transcribed Hoffman
TFBS ENCODE
**SuperEnhancer Hnisz
Repressed Hoffman
Promoter UCSC
PromoterFlanking Hoffman
Intron UCSC
H3K9ac Trynka
**H3K4me3 Trynka
H3K4me1 Trynka
H3K27ac PGC2
H3K27ac Hnisz
FetalDHS Trynka
Enhancer Hoffman
Enhancer Andersson
DHS Trynka
DGF ENCODE
CTCF Hoffman
Conserved LindbladToh
Coding UCSC
Annotation
−100
−50
0
50
100
Enrichment (prop h2 / prop snps)

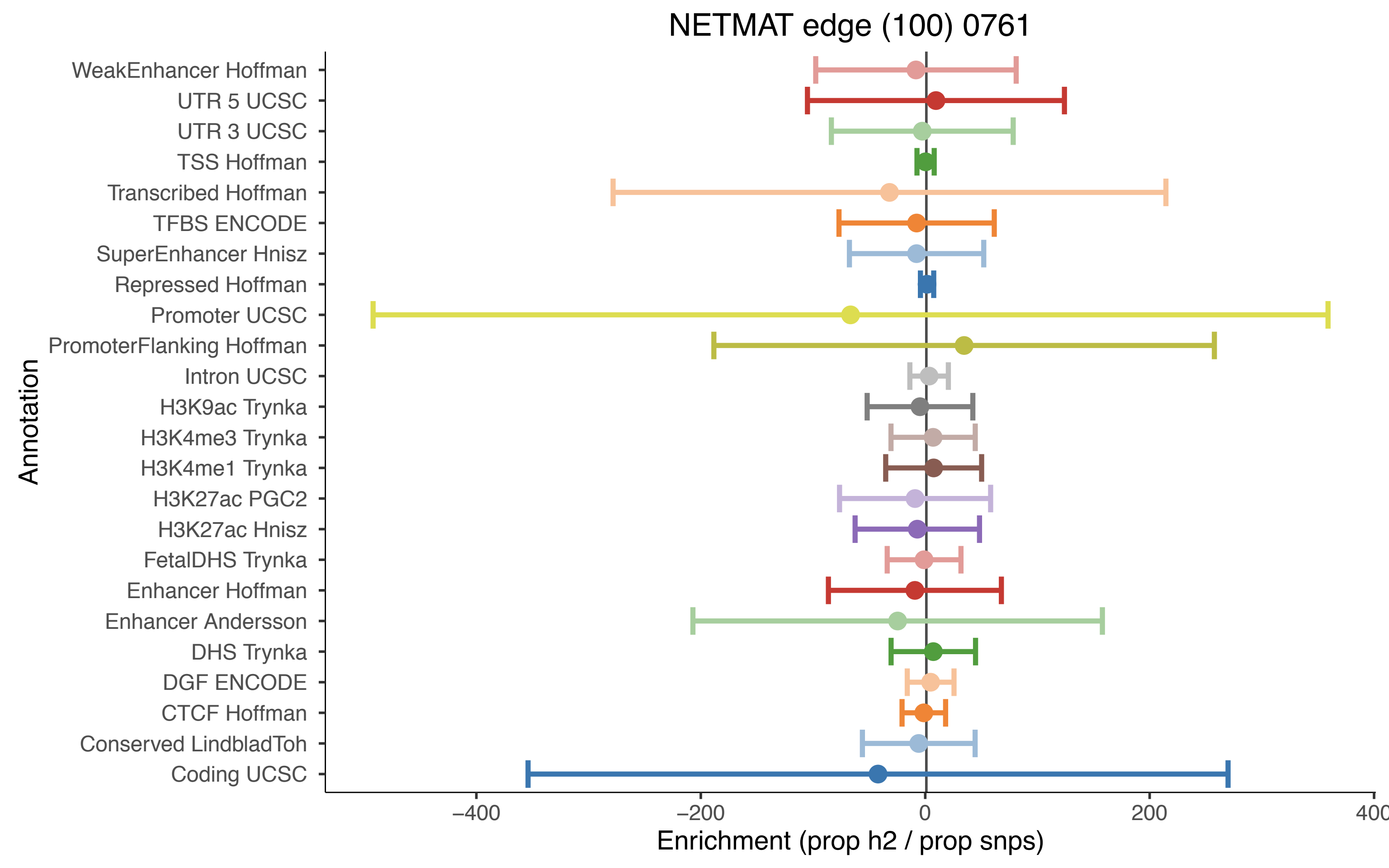

NETMAT edge (100) 0761
Annotation
WeakEnhancer Hoffman
UTR 5 UCSC
UTR 3 UCSC
TSS Hoffman
Transcribed Hoffman
TFBS ENCODE
SuperEnhancer Hnisz
Repressed Hoffman
Promoter UCSC
PromoterFlanking Hoffman
Intron UCSC
H3K9ac Trynka
H3K4me3 Trynka
H3K4me1 Trynka
H3K27ac PGC2
H3K27ac Hnisz
FetalDHS Trynka
Enhancer Hoffman
Enhancer Andersson
DHS Trynka
DGF ENCODE
CTCF Hoffman
Conserved LindbladToh
Coding UCSC
−400
−200
0
200
400
Enrichment (prop h2 / prop snps)

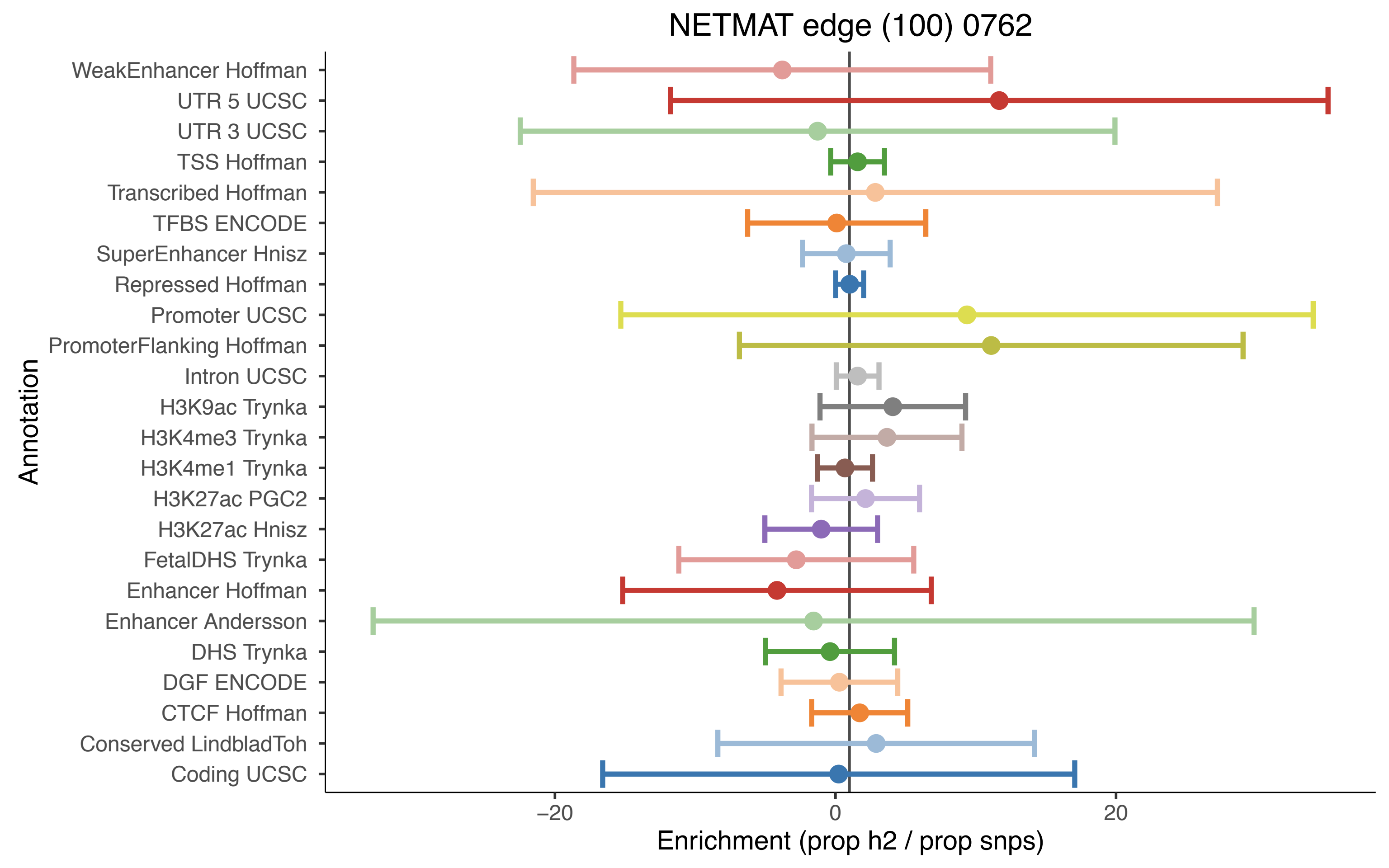
NETMAT edge (100) 0762
WeakEnhancer Hoffman
UTR 5 UCSC
UTR 3 UCSC
TSS Hoffman
Transcribed Hoffman
TFBS ENCODE
SuperEnhancer Hnisz
Repressed Hoffman
Promoter UCSC
PromoterFlanking Hoffman
Intron UCSC
H3K9ac Trynka
H3K4me3 Trynka
H3K4me1 Trynka
H3K27ac PGC2
H3K27ac Hnisz
FetalDHS Trynka
Enhancer Hoffman
Enhancer Andersson
DHS Trynka
DGF ENCODE
CTCF Hoffman
Conserved LindbladToh
Coding UCSC
Annotation
−20
0
20
Enrichment (prop h2 / prop snps)

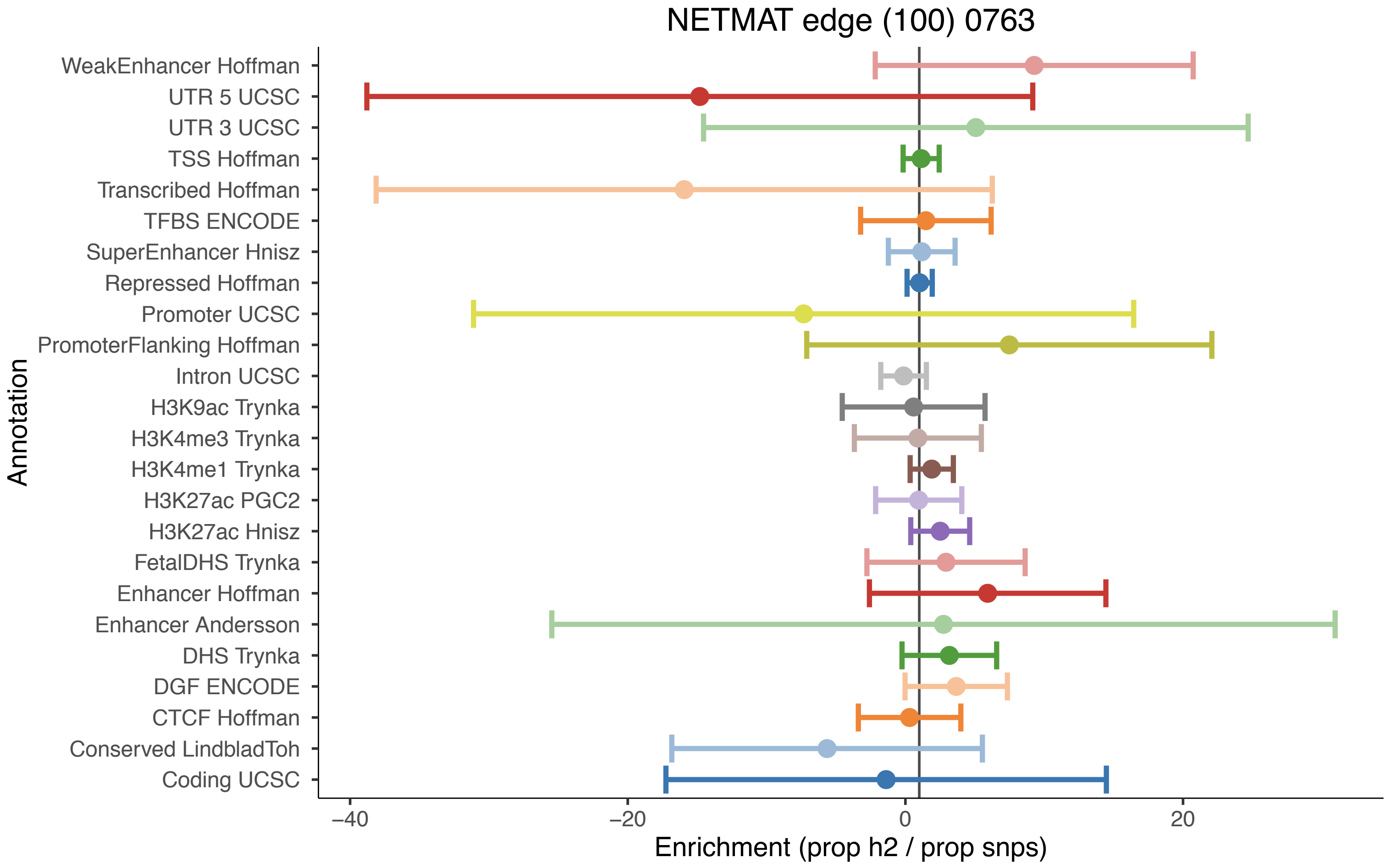
NETMAT edge (100) 0763
WeakEnhancer Hoffman
UTR 5 UCSC
UTR 3 UCSC
TSS Hoffman
Transcribed Hoffman
TFBS ENCODE
SuperEnhancer Hnisz
Repressed Hoffman
Promoter UCSC
PromoterFlanking Hoffman
Intron UCSC
H3K9ac Trynka
H3K4me3 Trynka
H3K4me1 Trynka
H3K27ac PGC2
H3K27ac Hnisz
FetalDHS Trynka
Enhancer Hoffman
Enhancer Andersson
DHS Trynka
DGF ENCODE
CTCF Hoffman
Conserved LindbladToh
Coding UCSC
Annotation
−40
−20
0
20
Enrichment (prop h2 / prop snps)

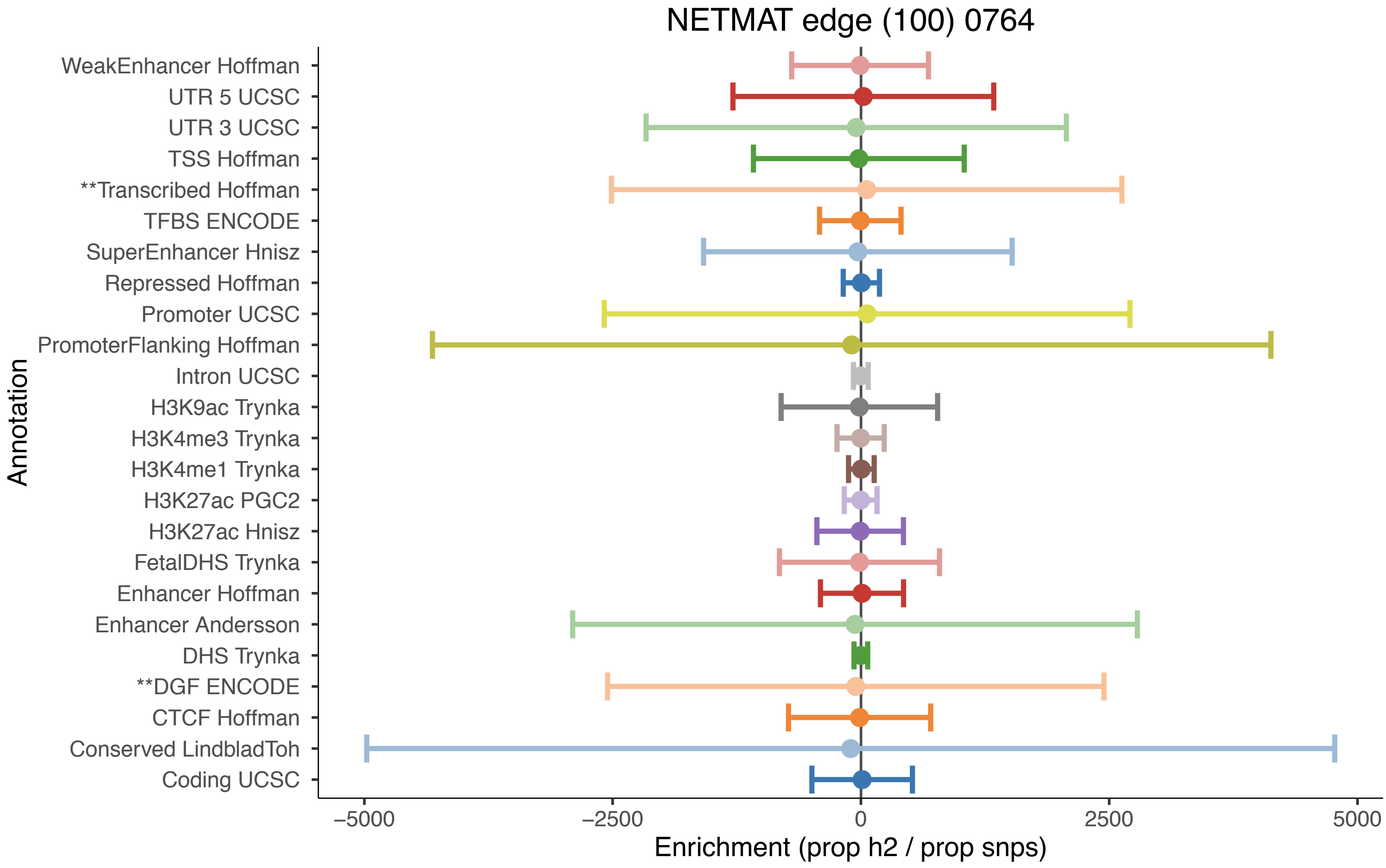
NETMAT edge (100) 0764
WeakEnhancer Hoffman
UTR 5 UCSC
UTR 3 UCSC
TSS Hoffman
**Transcribed Hoffman
TFBS ENCODE
SuperEnhancer Hnisz
Repressed Hoffman
Promoter UCSC
PromoterFlanking Hoffman
Intron UCSC
H3K9ac Trynka
H3K4me3 Trynka
H3K4me1 Trynka
H3K27ac PGC2
H3K27ac Hnisz
FetalDHS Trynka
Enhancer Hoffman
Enhancer Andersson
DHS Trynka
**DGF ENCODE
CTCF Hoffman
Conserved LindbladToh
Coding UCSC
Annotation
−5000
−2500
0
2500
5000
Enrichment (prop h2 / prop snps)

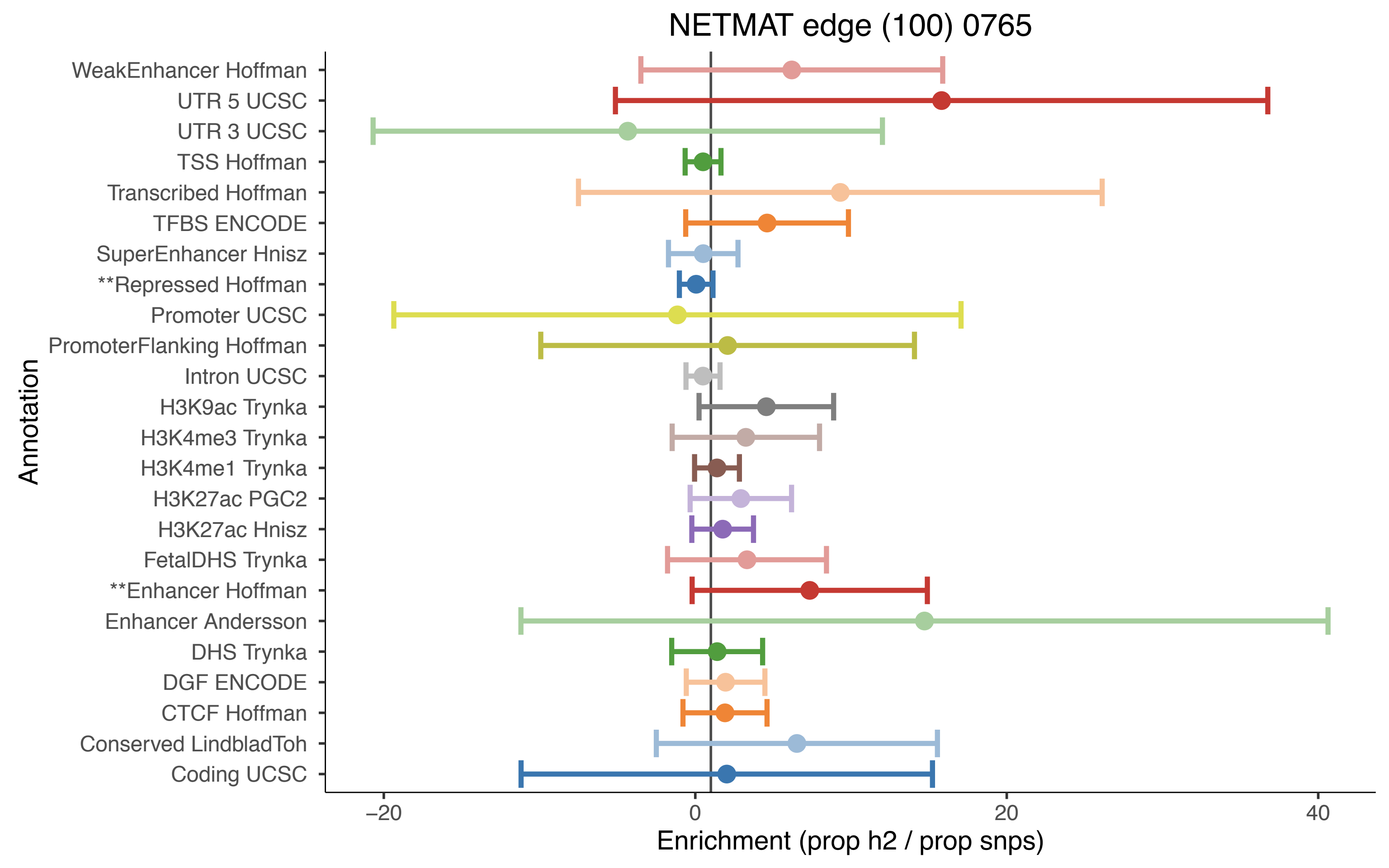

NETMAT edge (100) 0765
WeakEnhancer Hoffman
UTR 5 UCSC
UTR 3 UCSC
TSS Hoffman
Transcribed Hoffman
TFBS ENCODE
SuperEnhancer Hnisz
**Repressed Hoffman
Promoter UCSC
PromoterFlanking Hoffman
Intron UCSC
H3K9ac Trynka
H3K4me3 Trynka
H3K4me1 Trynka
H3K27ac PGC2
H3K27ac Hnisz
FetalDHS Trynka
**Enhancer Hoffman
Enhancer Andersson
DHS Trynka
DGF ENCODE
CTCF Hoffman
Conserved LindbladToh
Coding UCSC
Annotation
−20
0
20
40
Enrichment (prop h2 / prop snps)

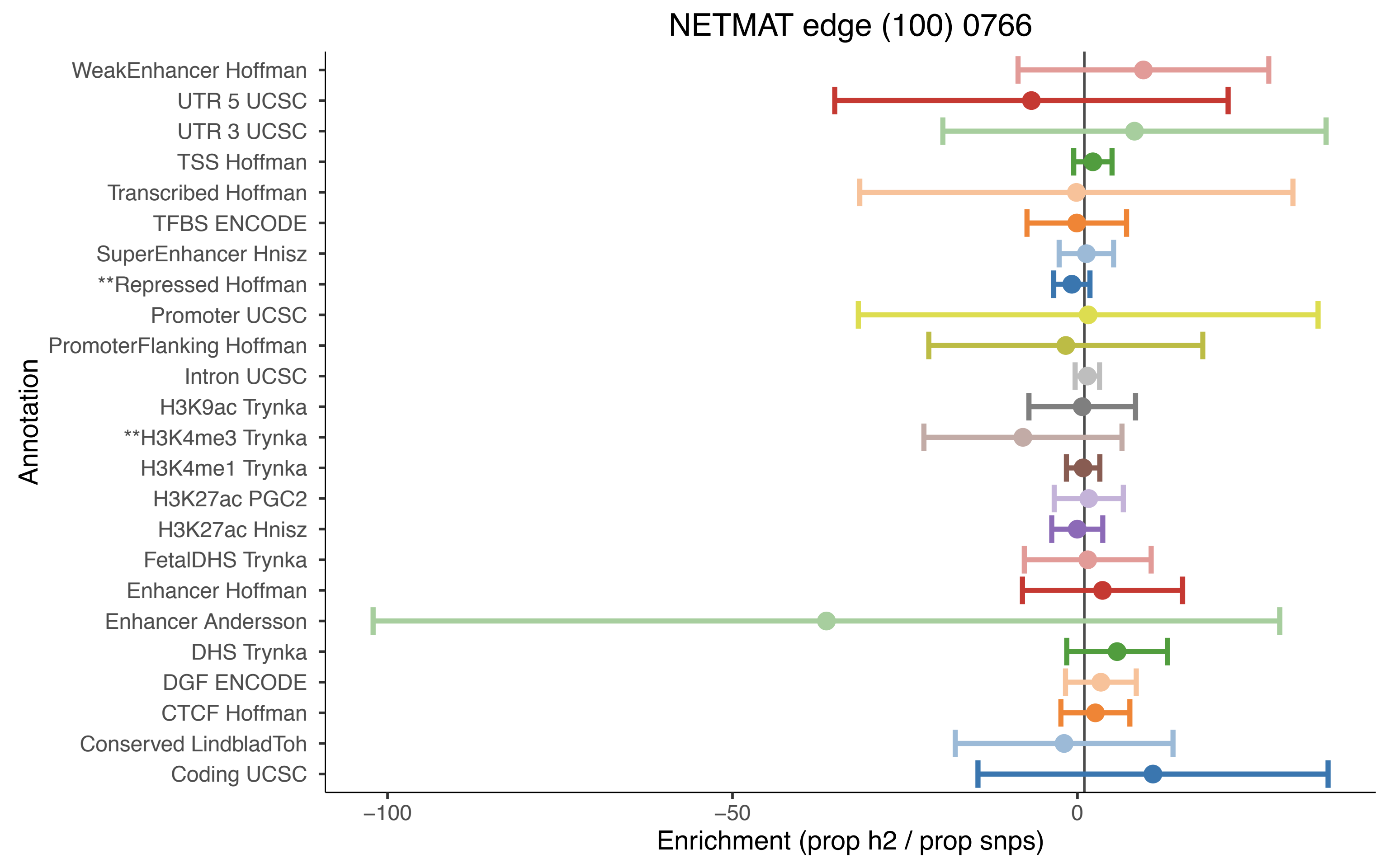
NETMAT edge (100) 0766
Annotation
WeakEnhancer Hoffman
UTR 5 UCSC
UTR 3 UCSC
TSS Hoffman
Transcribed Hoffman
TFBS ENCODE
SuperEnhancer Hnisz
**Repressed Hoffman
Promoter UCSC
PromoterFlanking Hoffman
Intron UCSC
H3K9ac Trynka
**H3K4me3 Trynka
H3K4me1 Trynka
H3K27ac PGC2
H3K27ac Hnisz
FetalDHS Trynka
Enhancer Hoffman
Enhancer Andersson
DHS Trynka
DGF ENCODE
CTCF Hoffman
Conserved LindbladToh
Coding UCSC
−100
−50
0
Enrichment (prop h2 / prop snps)

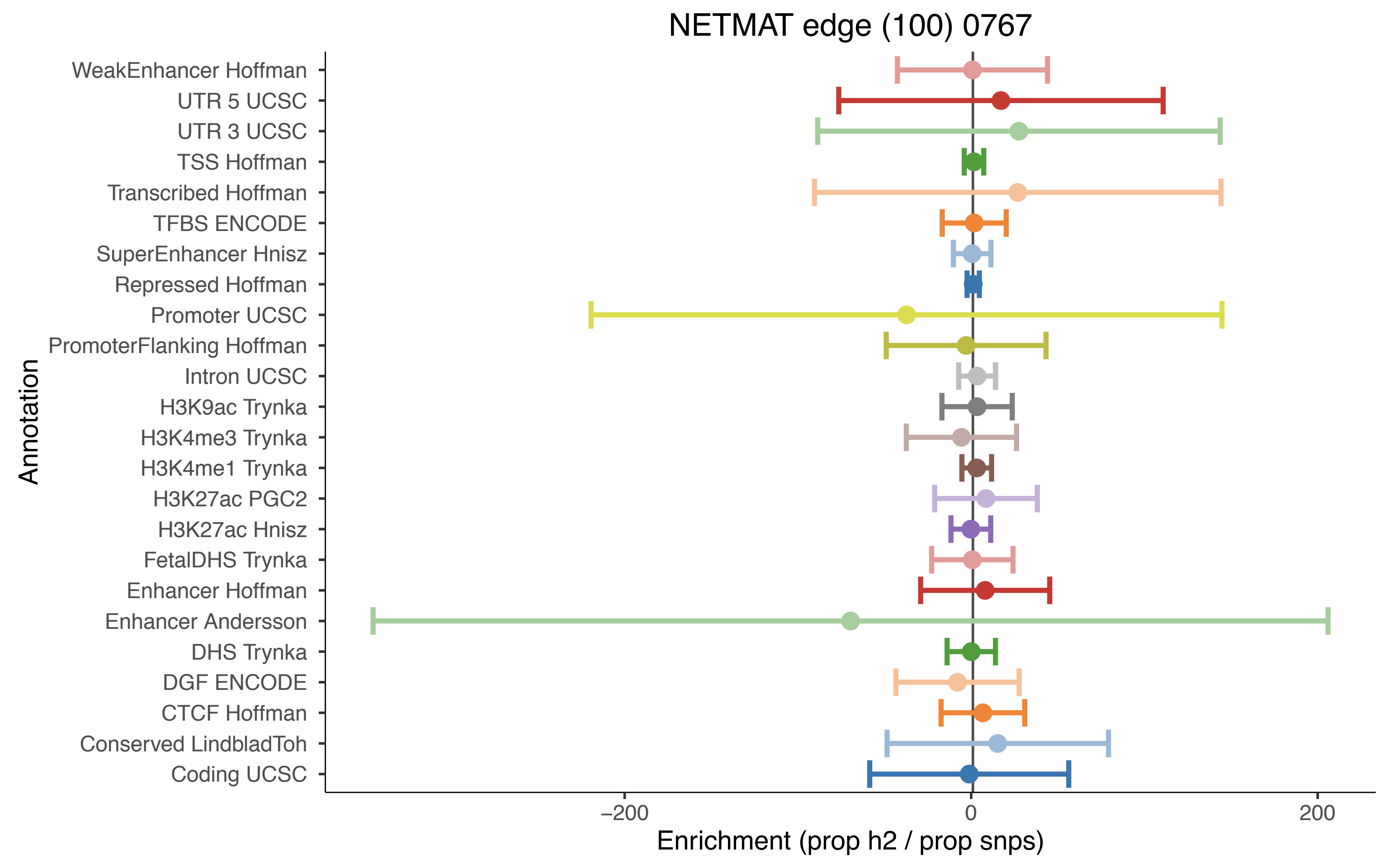
NETMAT edge (100) 0767
WeakEnhancer Hoffman
UTR 5 UCSC
UTR 3 UCSC
TSS Hoffman
Transcribed Hoffman
TFBS ENCODE
SuperEnhancer Hnisz
Repressed Hoffman
Promoter UCSC
PromoterFlanking Hoffman
Intron UCSC
H3K9ac Trynka
H3K4me3 Trynka
H3K4me1 Trynka
H3K27ac PGC2
H3K27ac Hnisz
FetalDHS Trynka
Enhancer Hoffman
Enhancer Andersson
DHS Trynka
DGF ENCODE
CTCF Hoffman
Conserved LindbladToh
Coding UCSC
Annotation
−200
0
200
Enrichment (prop h2 / prop snps)

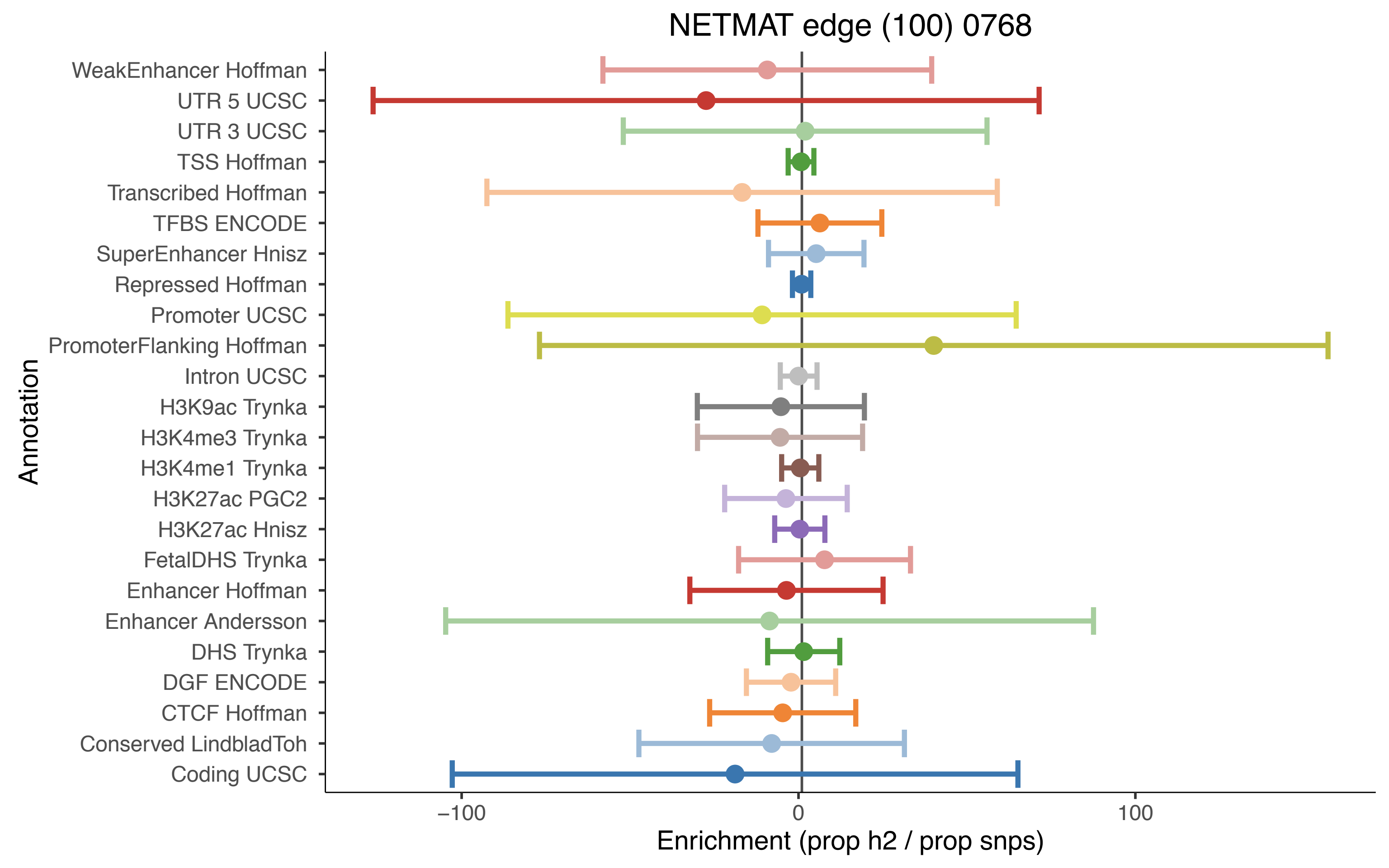

NETMAT edge (100) 0768
WeakEnhancer Hoffman
UTR 5 UCSC
UTR 3 UCSC
TSS Hoffman
Transcribed Hoffman
TFBS ENCODE
SuperEnhancer Hnisz
Repressed Hoffman
Promoter UCSC
PromoterFlanking Hoffman
Intron UCSC
H3K9ac Trynka
H3K4me3 Trynka
H3K4me1 Trynka
H3K27ac PGC2
H3K27ac Hnisz
FetalDHS Trynka
Enhancer Hoffman
Enhancer Andersson
DHS Trynka
DGF ENCODE
CTCF Hoffman
Conserved LindbladToh
Coding UCSC
Annotation
−100
0
100
Enrichment (prop h2 / prop snps)

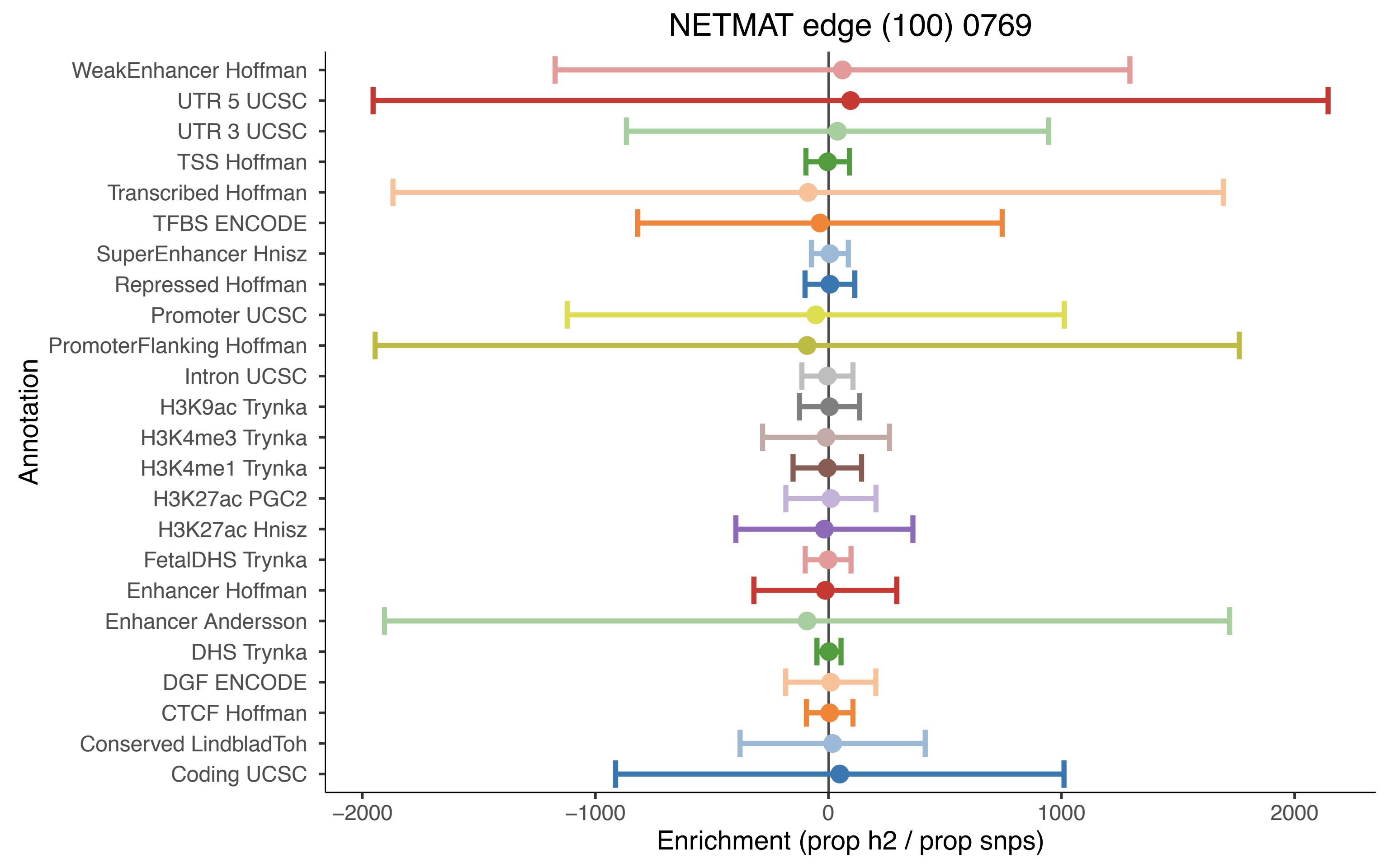

NETMAT edge (100) 0769
WeakEnhancer Hoffman
UTR 5 UCSC
UTR 3 UCSC
TSS Hoffman
Transcribed Hoffman
TFBS ENCODE
SuperEnhancer Hnisz
Repressed Hoffman
Promoter UCSC
PromoterFlanking Hoffman
Intron UCSC
H3K9ac Trynka
H3K4me3 Trynka
H3K4me1 Trynka
H3K27ac PGC2
H3K27ac Hnisz
FetalDHS Trynka
Enhancer Hoffman
Enhancer Andersson
DHS Trynka
DGF ENCODE
CTCF Hoffman
Conserved LindbladToh
Coding UCSC
Annotation
−2000
−1000
0
1000
2000
Enrichment (prop h2 / prop snps)

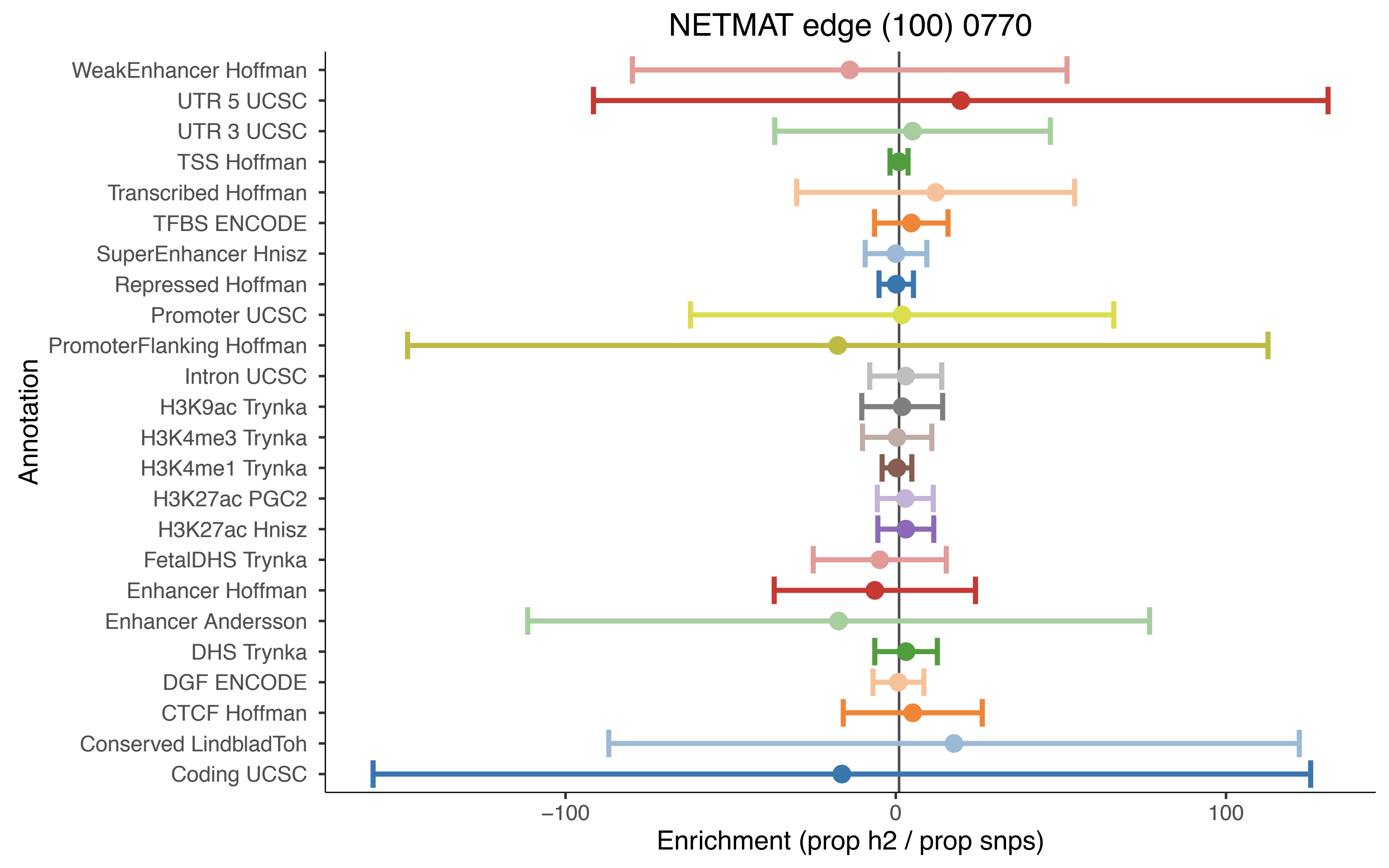
NETMAT edge (100) 0770
WeakEnhancer Hoffman
UTR 5 UCSC
UTR 3 UCSC
TSS Hoffman
Transcribed Hoffman
TFBS ENCODE
SuperEnhancer Hnisz
Repressed Hoffman
Promoter UCSC
PromoterFlanking Hoffman
Intron UCSC
H3K9ac Trynka
H3K4me3 Trynka
H3K4me1 Trynka
H3K27ac PGC2
H3K27ac Hnisz
FetalDHS Trynka
Enhancer Hoffman
Enhancer Andersson
DHS Trynka
DGF ENCODE
CTCF Hoffman
Conserved LindbladToh
Coding UCSC
Annotation
−100
0
100
Enrichment (prop h2 / prop snps)

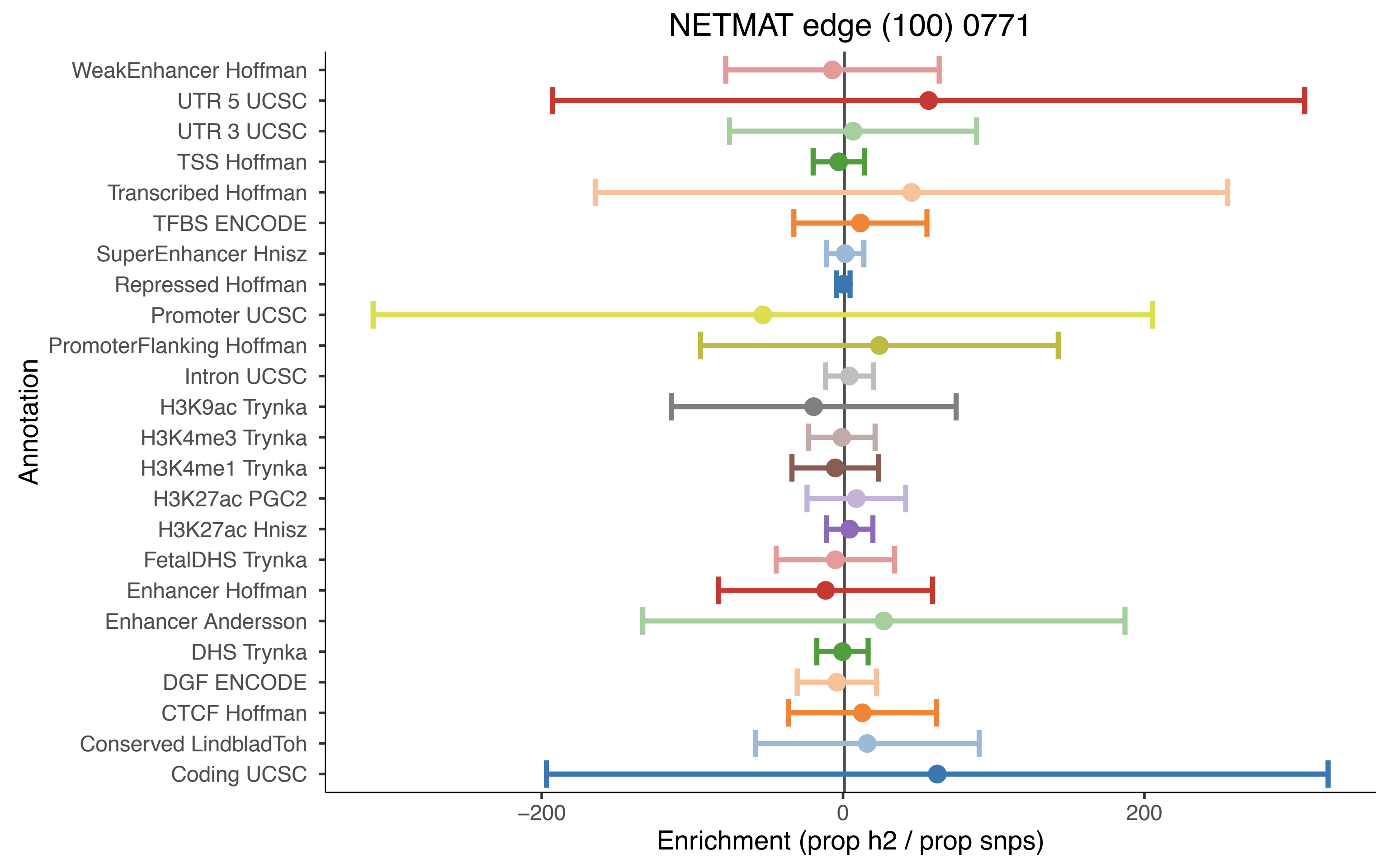
NETMAT edge (100) 0771
WeakEnhancer Hoffman
UTR 5 UCSC
UTR 3 UCSC
TSS Hoffman
Transcribed Hoffman
TFBS ENCODE
SuperEnhancer Hnisz
Repressed Hoffman
Promoter UCSC
PromoterFlanking Hoffman
Intron UCSC
H3K9ac Trynka
H3K4me3 Trynka
H3K4me1 Trynka
H3K27ac PGC2
H3K27ac Hnisz
FetalDHS Trynka
Enhancer Hoffman
Enhancer Andersson
DHS Trynka
DGF ENCODE
CTCF Hoffman
Conserved LindbladToh
Coding UCSC
Annotation
−200
0
200
Enrichment (prop h2 / prop snps)

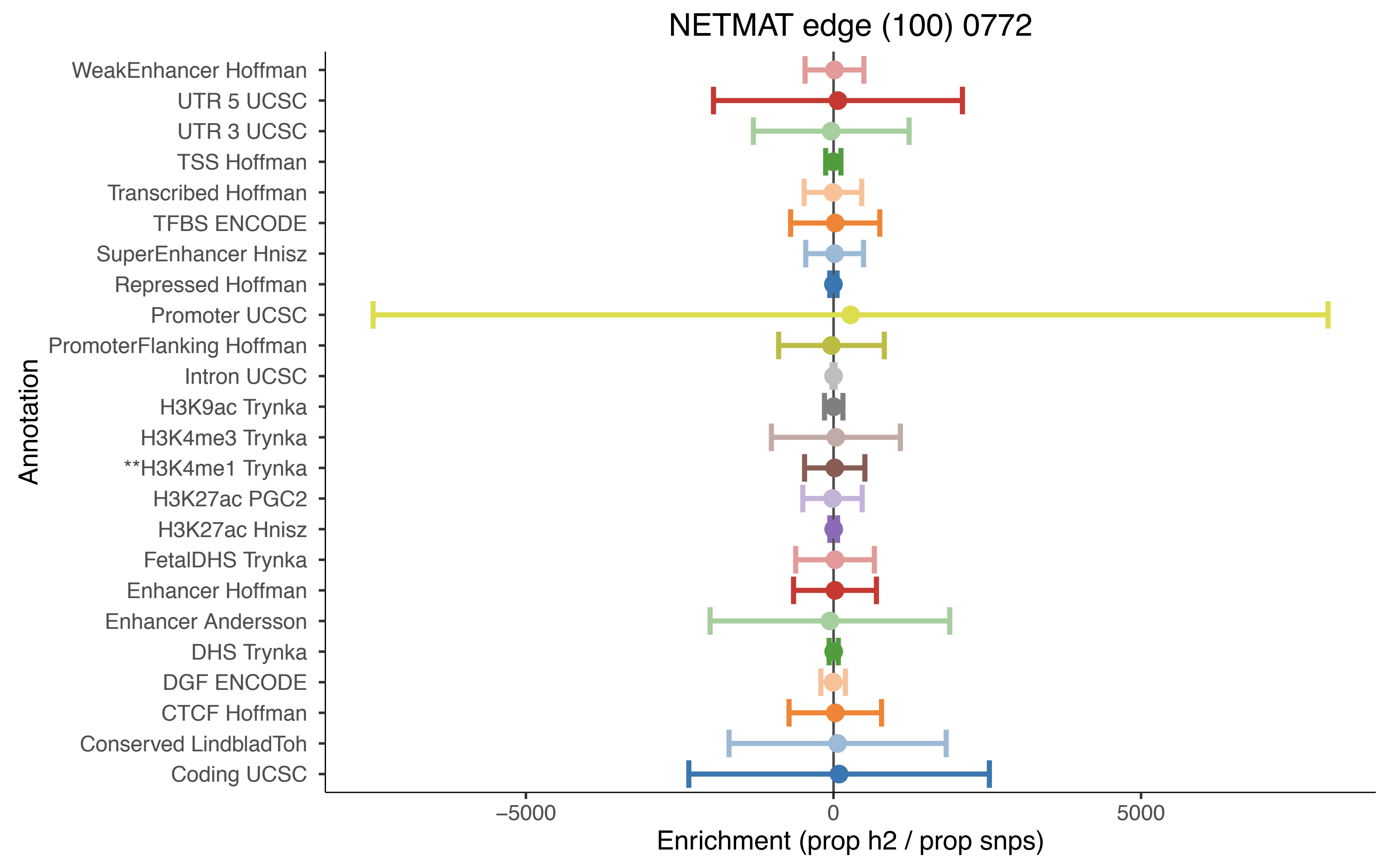

NETMAT edge (100) 0772
WeakEnhancer Hoffman
UTR 5 UCSC
UTR 3 UCSC
TSS Hoffman
Transcribed Hoffman
TFBS ENCODE
SuperEnhancer Hnisz
Repressed Hoffman
Promoter UCSC
PromoterFlanking Hoffman
Intron UCSC
H3K9ac Trynka
H3K4me3 Trynka
**H3K4me1 Trynka
H3K27ac PGC2
H3K27ac Hnisz
FetalDHS Trynka
Enhancer Hoffman
Enhancer Andersson
DHS Trynka
DGF ENCODE
CTCF Hoffman
Conserved LindbladToh
Coding UCSC
Annotation
−5000
0
5000
Enrichment (prop h2 / prop snps)

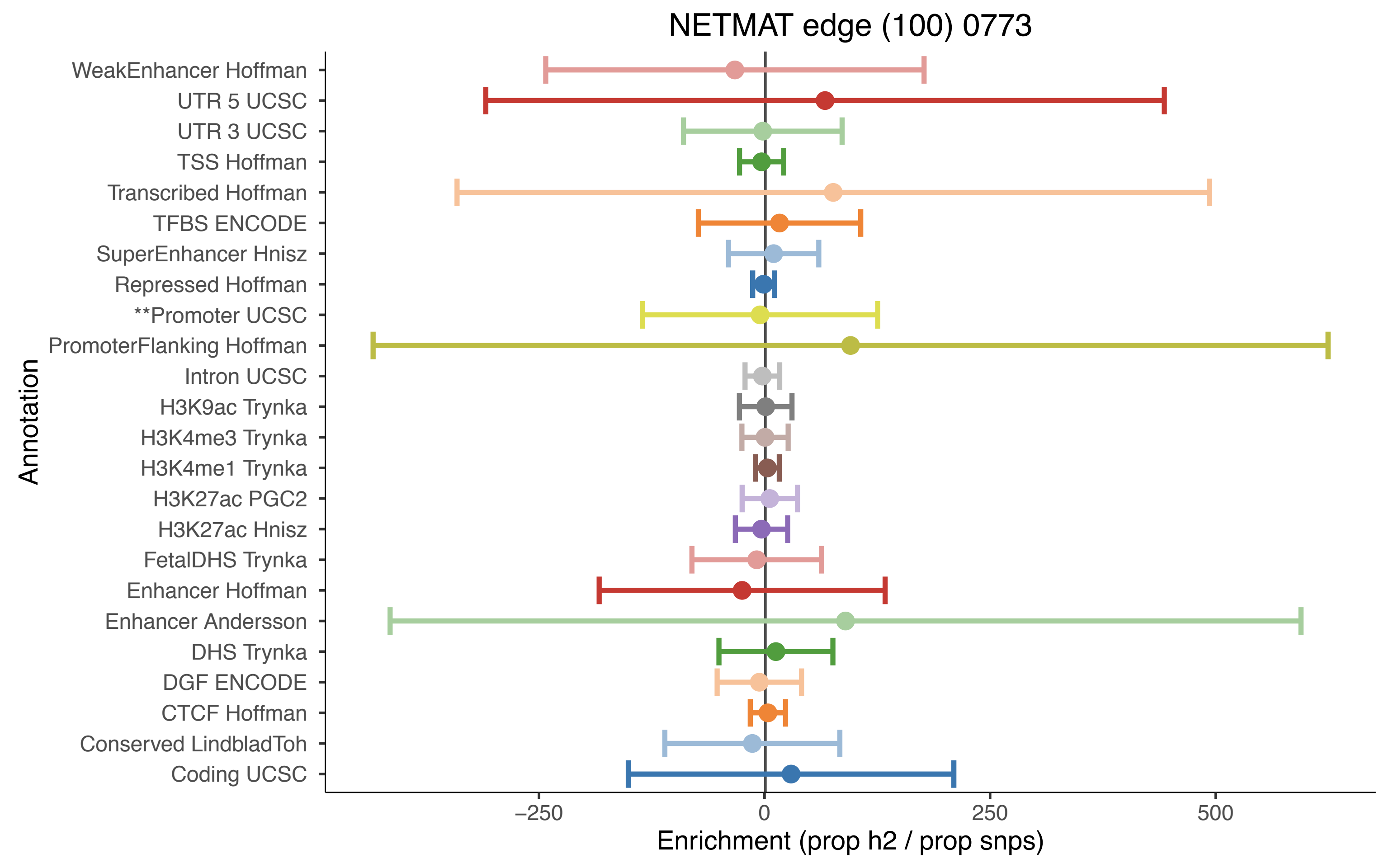
NETMAT edge (100) 0773
WeakEnhancer Hoffman
UTR 5 UCSC
UTR 3 UCSC
TSS Hoffman
Transcribed Hoffman
TFBS ENCODE
SuperEnhancer Hnisz
Repressed Hoffman
**Promoter UCSC
PromoterFlanking Hoffman
Intron UCSC
H3K9ac Trynka
H3K4me3 Trynka
H3K4me1 Trynka
H3K27ac PGC2
H3K27ac Hnisz
FetalDHS Trynka
Enhancer Hoffman
Enhancer Andersson
DHS Trynka
DGF ENCODE
CTCF Hoffman
Conserved LindbladToh
Coding UCSC
Annotation
−250
0
250
500
Enrichment (prop h2 / prop snps)

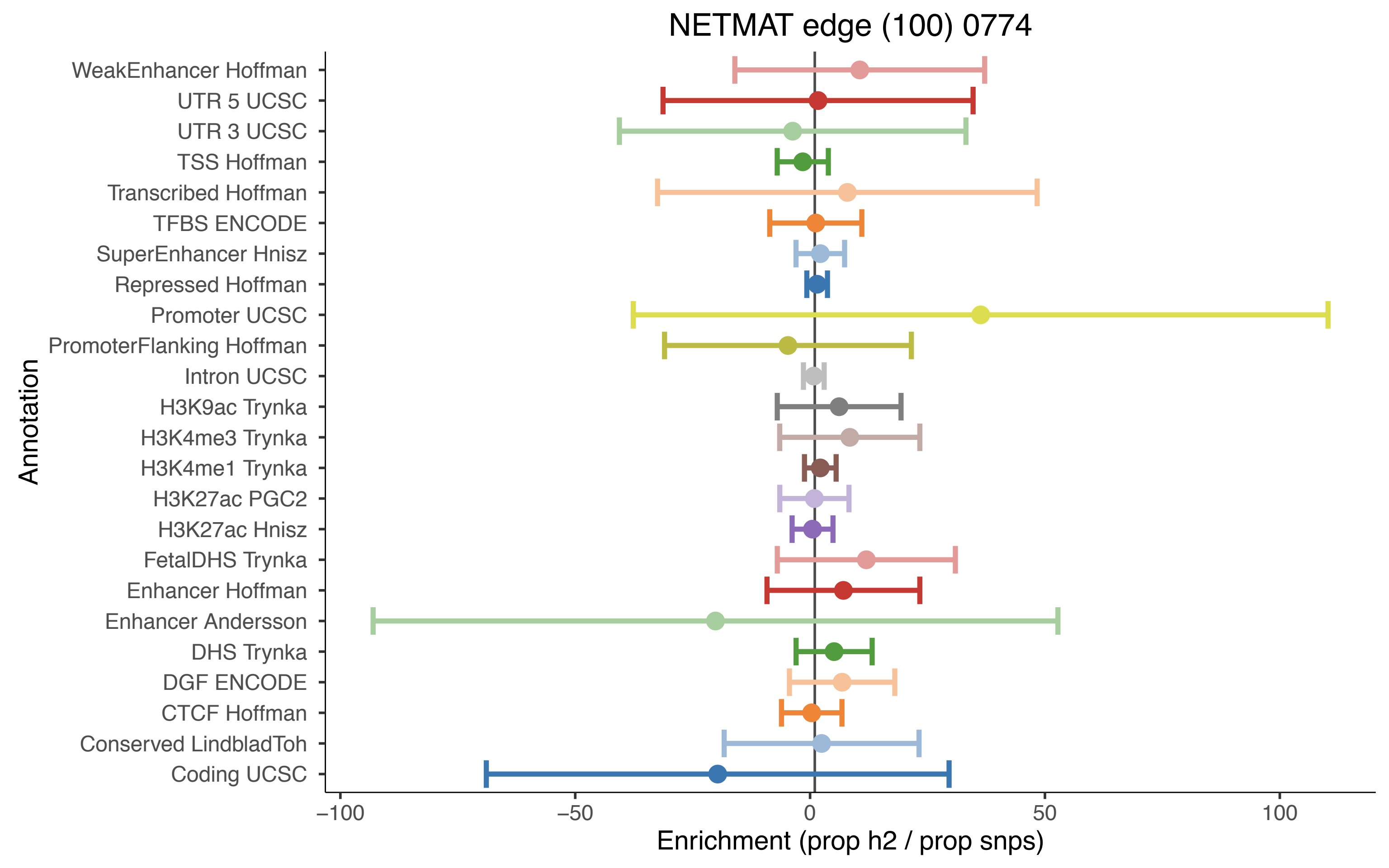
NETMAT edge (100) 0774
Annotation
WeakEnhancer Hoffman
UTR 5 UCSC
UTR 3 UCSC
TSS Hoffman
Transcribed Hoffman
TFBS ENCODE
SuperEnhancer Hnisz
Repressed Hoffman
Promoter UCSC
PromoterFlanking Hoffman
Intron UCSC
H3K9ac Trynka
H3K4me3 Trynka
H3K4me1 Trynka
H3K27ac PGC2
H3K27ac Hnisz
FetalDHS Trynka
Enhancer Hoffman
Enhancer Andersson
DHS Trynka
DGF ENCODE
CTCF Hoffman
Conserved LindbladToh
Coding UCSC
−100
−50
0
50
100
Enrichment (prop h2 / prop snps)

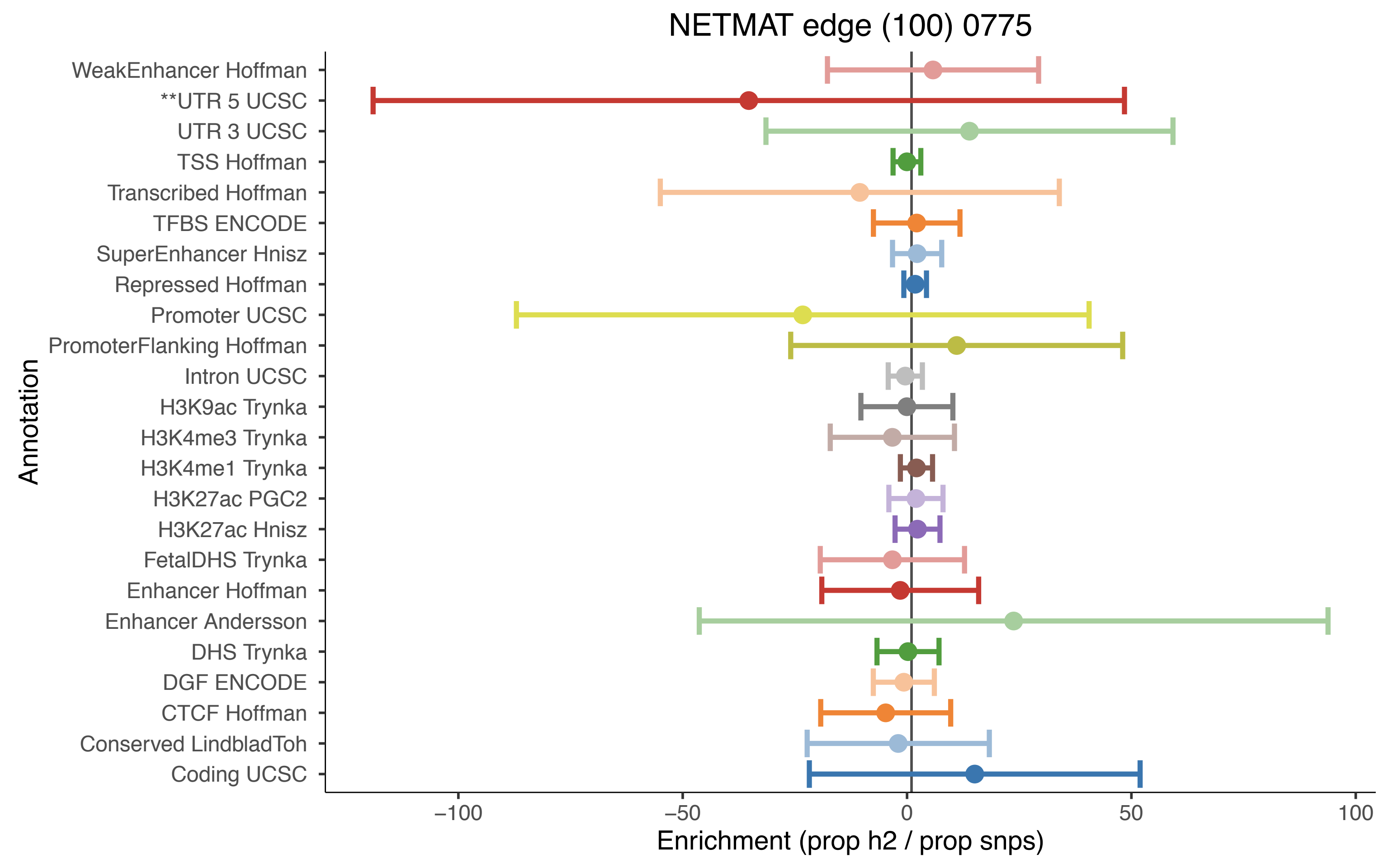

NETMAT edge (100) 0775
WeakEnhancer Hoffman
**UTR 5 UCSC
UTR 3 UCSC
TSS Hoffman
Transcribed Hoffman
TFBS ENCODE
SuperEnhancer Hnisz
Repressed Hoffman
Promoter UCSC
PromoterFlanking Hoffman
Intron UCSC
H3K9ac Trynka
H3K4me3 Trynka
H3K4me1 Trynka
H3K27ac PGC2
H3K27ac Hnisz
FetalDHS Trynka
Enhancer Hoffman
Enhancer Andersson
DHS Trynka
DGF ENCODE
CTCF Hoffman
Conserved LindbladToh
Coding UCSC
Annotation
−100
−50
0
50
100
Enrichment (prop h2 / prop snps)

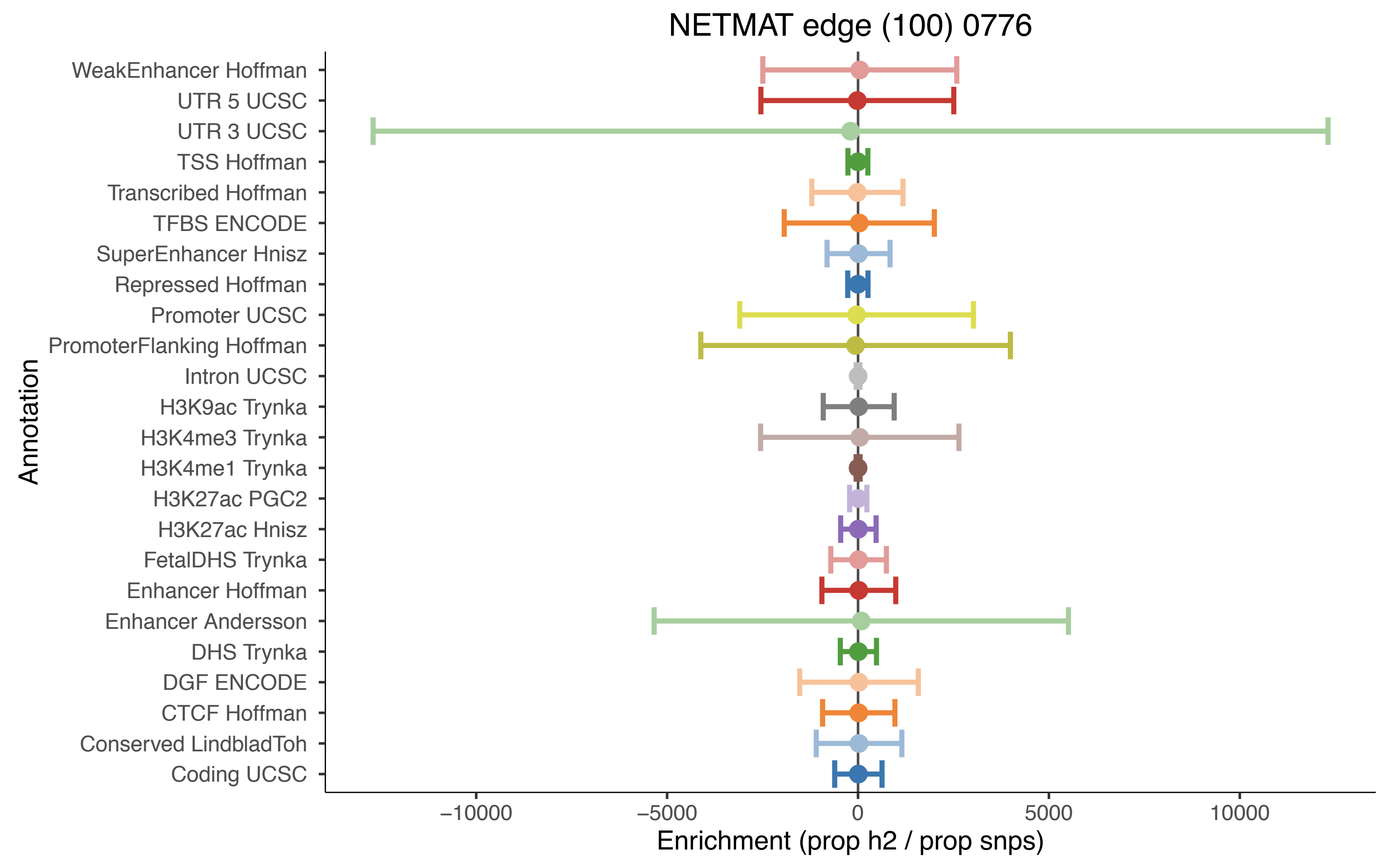

NETMAT edge (100) 0776
WeakEnhancer Hoffman
UTR 5 UCSC
UTR 3 UCSC
TSS Hoffman
Transcribed Hoffman
TFBS ENCODE
SuperEnhancer Hnisz
Repressed Hoffman
Promoter UCSC
PromoterFlanking Hoffman
Intron UCSC
H3K9ac Trynka
H3K4me3 Trynka
H3K4me1 Trynka
H3K27ac PGC2
H3K27ac Hnisz
FetalDHS Trynka
Enhancer Hoffman
Enhancer Andersson
DHS Trynka
DGF ENCODE
CTCF Hoffman
Conserved LindbladToh
Coding UCSC
Annotation
−10000
−5000
0
5000
10000
Enrichment (prop h2 / prop snps)

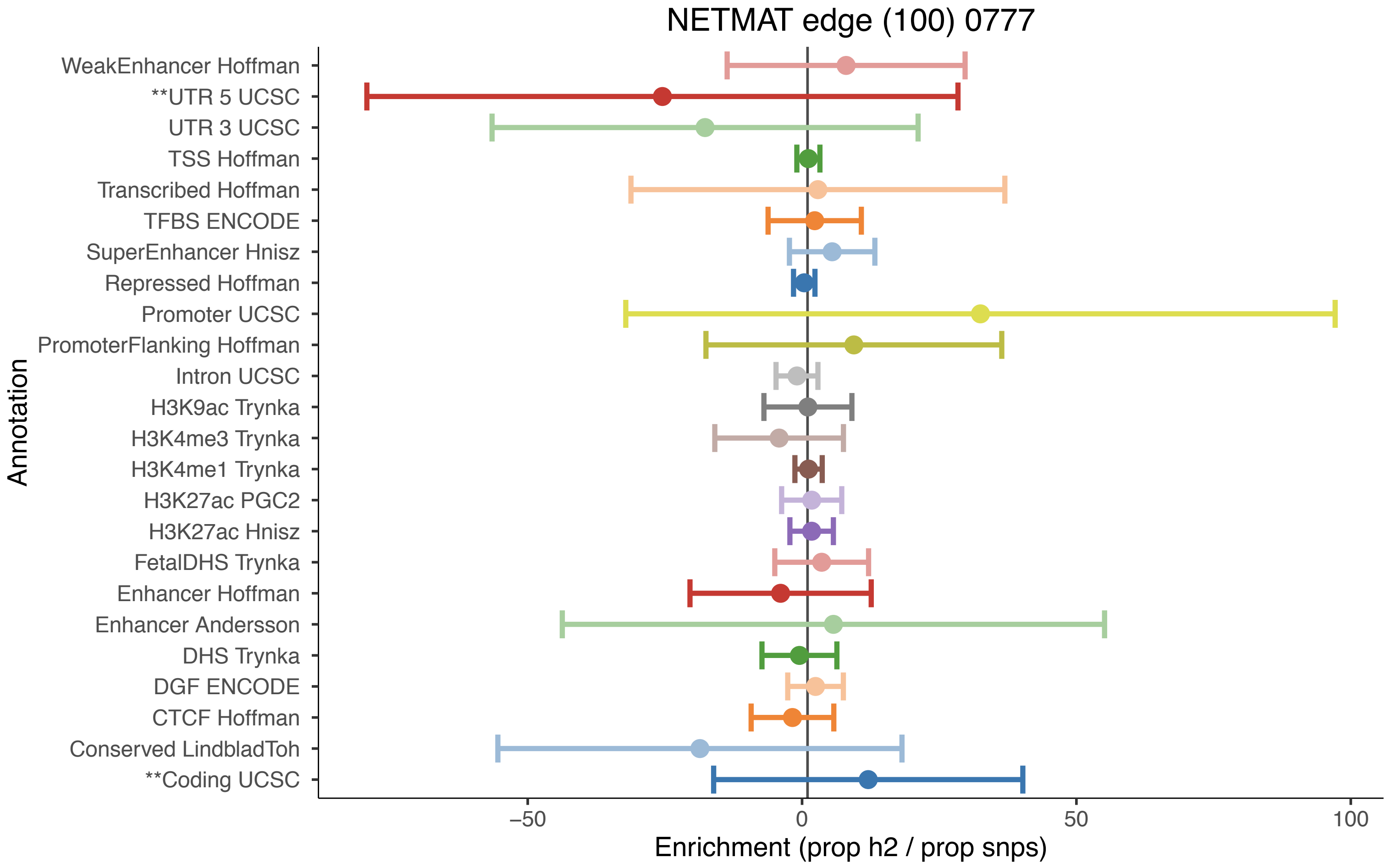
NETMAT edge (100) 0777
WeakEnhancer Hoffman
**UTR 5 UCSC
UTR 3 UCSC
TSS Hoffman
Transcribed Hoffman
TFBS ENCODE
SuperEnhancer Hnisz
Repressed Hoffman
Promoter UCSC
PromoterFlanking Hoffman
Intron UCSC
H3K9ac Trynka
H3K4me3 Trynka
H3K4me1 Trynka
H3K27ac PGC2
H3K27ac Hnisz
FetalDHS Trynka
Enhancer Hoffman
Enhancer Andersson
DHS Trynka
DGF ENCODE
CTCF Hoffman
Conserved LindbladToh
**Coding UCSC
Annotation
−50
0
50
100
Enrichment (prop h2 / prop snps)

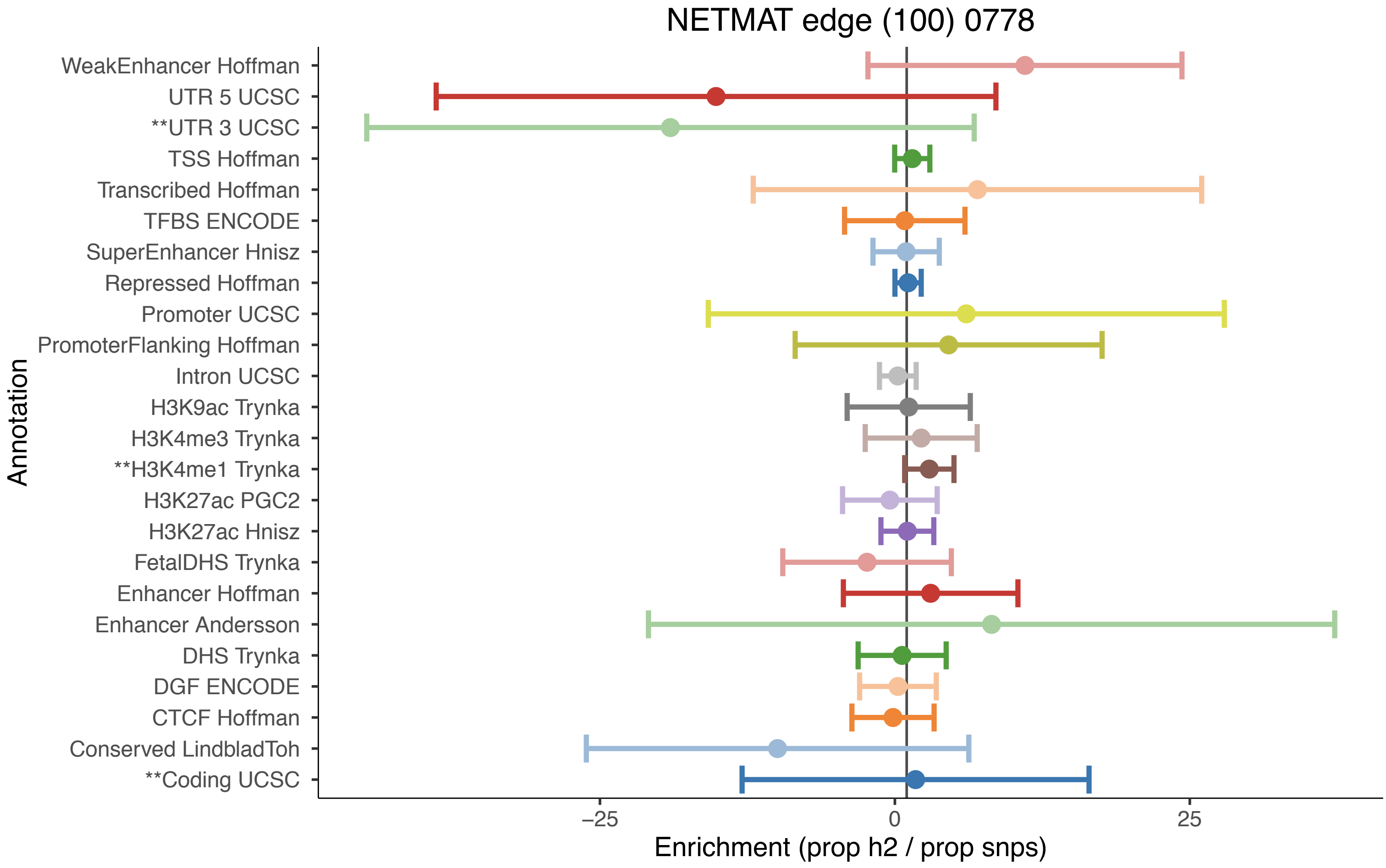
NETMAT edge (100) 0778
WeakEnhancer Hoffman
UTR 5 UCSC
**UTR 3 UCSC
TSS Hoffman
Transcribed Hoffman
TFBS ENCODE
SuperEnhancer Hnisz
Repressed Hoffman
Promoter UCSC
PromoterFlanking Hoffman
Intron UCSC
H3K9ac Trynka
H3K4me3 Trynka
**H3K4me1 Trynka
H3K27ac PGC2
H3K27ac Hnisz
FetalDHS Trynka
Enhancer Hoffman
Enhancer Andersson
DHS Trynka
DGF ENCODE
CTCF Hoffman
Conserved LindbladToh
**Coding UCSC
Annotation
−25
0
25
Enrichment (prop h2 / prop snps)

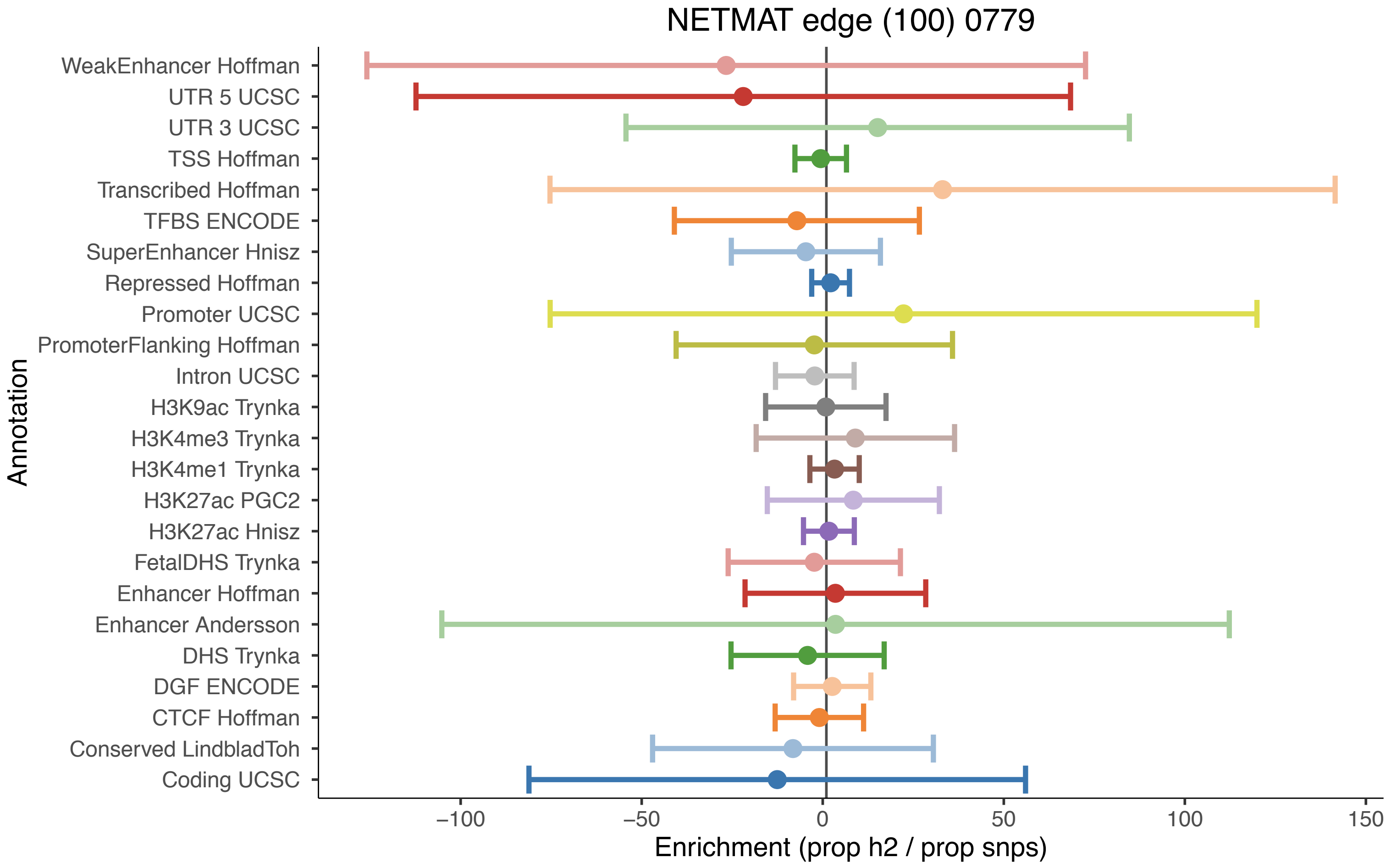

NETMAT edge (100) 0779
WeakEnhancer Hoffman
UTR 5 UCSC
UTR 3 UCSC
TSS Hoffman
Transcribed Hoffman
TFBS ENCODE
SuperEnhancer Hnisz
Repressed Hoffman
Promoter UCSC
PromoterFlanking Hoffman
Intron UCSC
H3K9ac Trynka
H3K4me3 Trynka
H3K4me1 Trynka
H3K27ac PGC2
H3K27ac Hnisz
FetalDHS Trynka
Enhancer Hoffman
Enhancer Andersson
DHS Trynka
DGF ENCODE
CTCF Hoffman
Conserved LindbladToh
Coding UCSC
Annotation
−100
−50
0
50
100
150
Enrichment (prop h2 / prop snps)

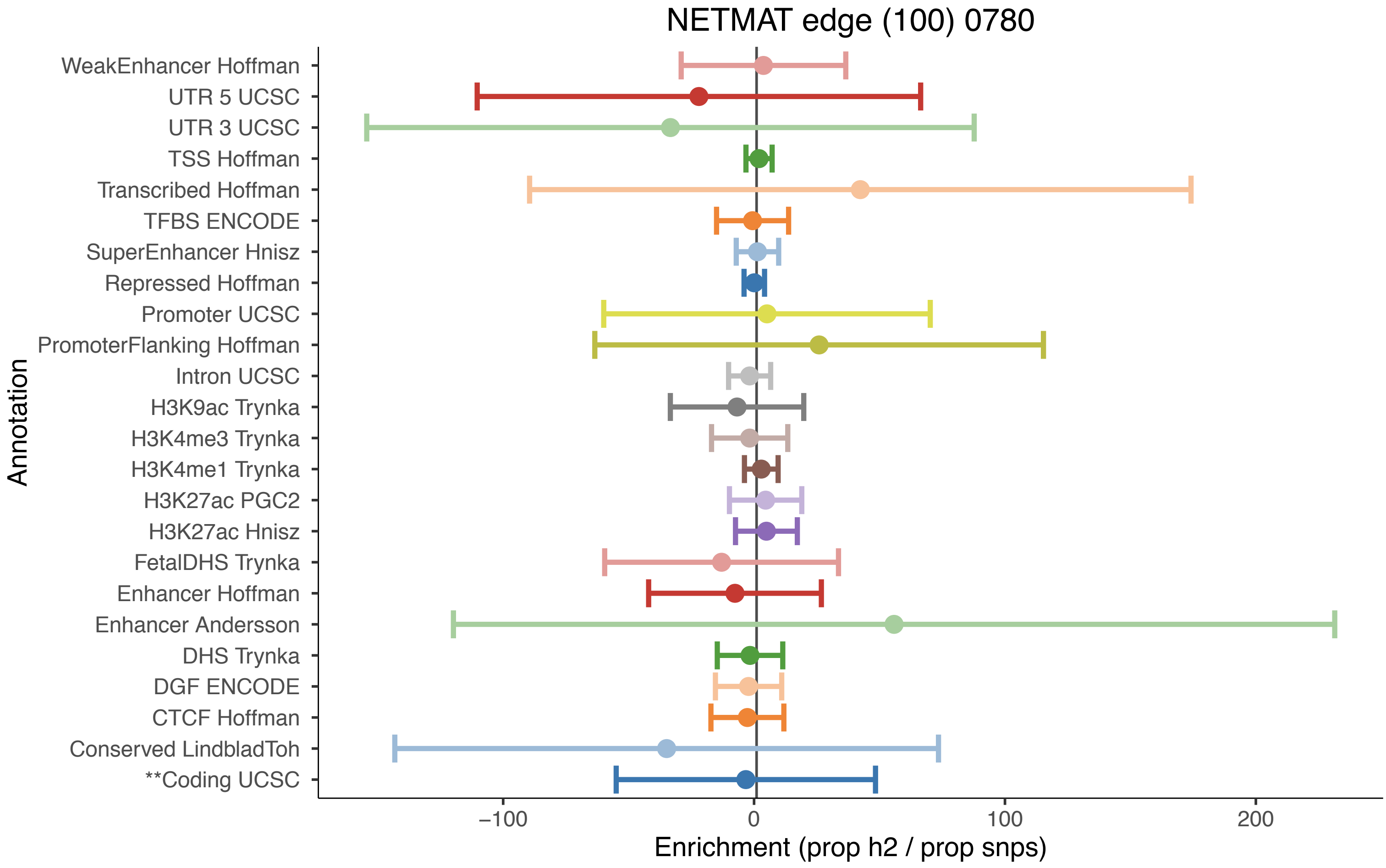
NETMAT edge (100) 0780
WeakEnhancer Hoffman
UTR 5 UCSC
UTR 3 UCSC
TSS Hoffman
Transcribed Hoffman
TFBS ENCODE
SuperEnhancer Hnisz
Repressed Hoffman
Promoter UCSC
PromoterFlanking Hoffman
Intron UCSC
H3K9ac Trynka
H3K4me3 Trynka
H3K4me1 Trynka
H3K27ac PGC2
H3K27ac Hnisz
FetalDHS Trynka
Enhancer Hoffman
Enhancer Andersson
DHS Trynka
DGF ENCODE
CTCF Hoffman
Conserved LindbladToh
**Coding UCSC
Annotation
−100
0
100
200
Enrichment (prop h2 / prop snps)

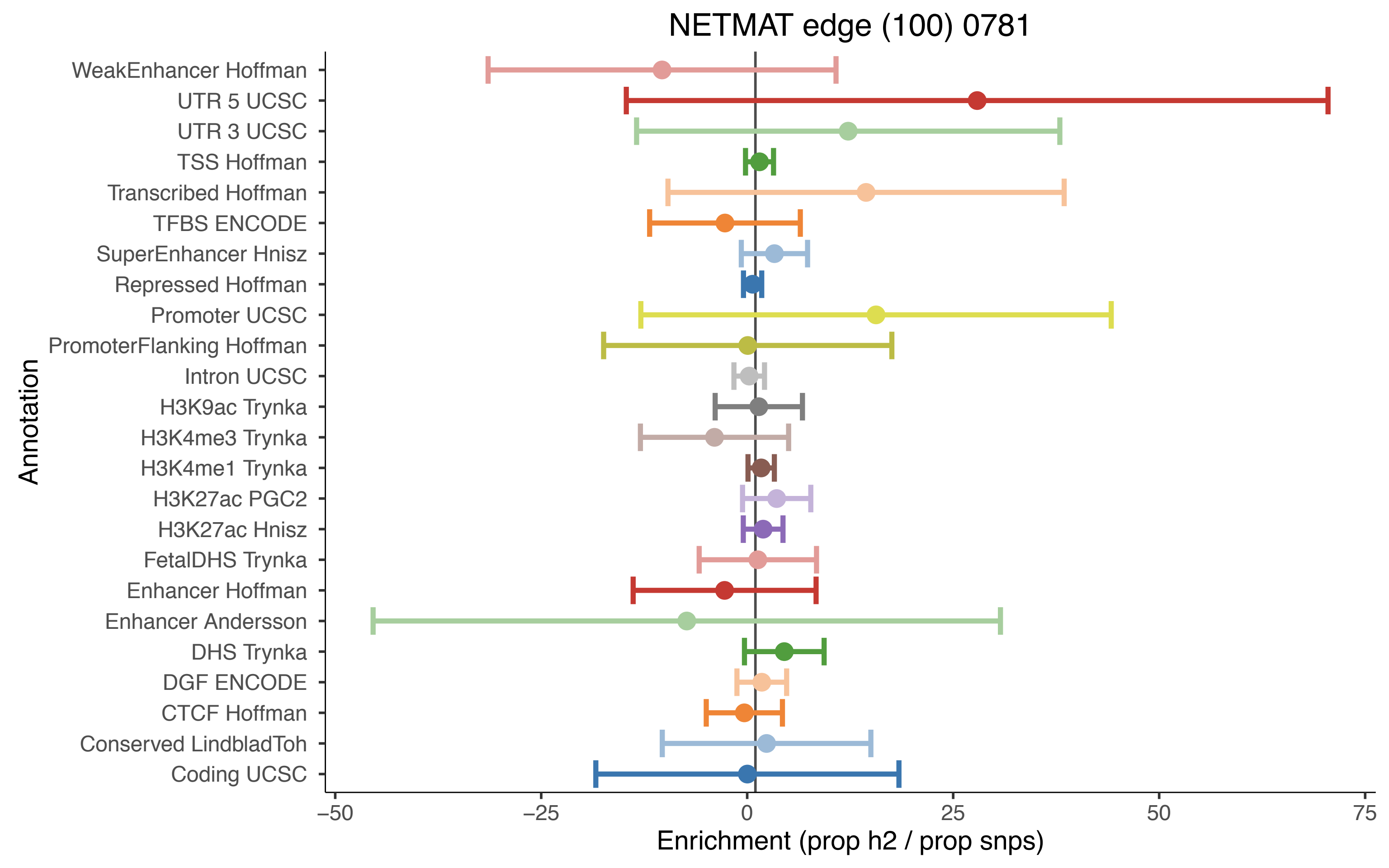
NETMAT edge (100) 0781
Annotation
WeakEnhancer Hoffman
UTR 5 UCSC
UTR 3 UCSC
TSS Hoffman
Transcribed Hoffman
TFBS ENCODE
SuperEnhancer Hnisz
Repressed Hoffman
Promoter UCSC
PromoterFlanking Hoffman
Intron UCSC
H3K9ac Trynka
H3K4me3 Trynka
H3K4me1 Trynka
H3K27ac PGC2
H3K27ac Hnisz
FetalDHS Trynka
Enhancer Hoffman
Enhancer Andersson
DHS Trynka
DGF ENCODE
CTCF Hoffman
Conserved LindbladToh
Coding UCSC
−50
−25
0
25
50
75
Enrichment (prop h2 / prop snps)

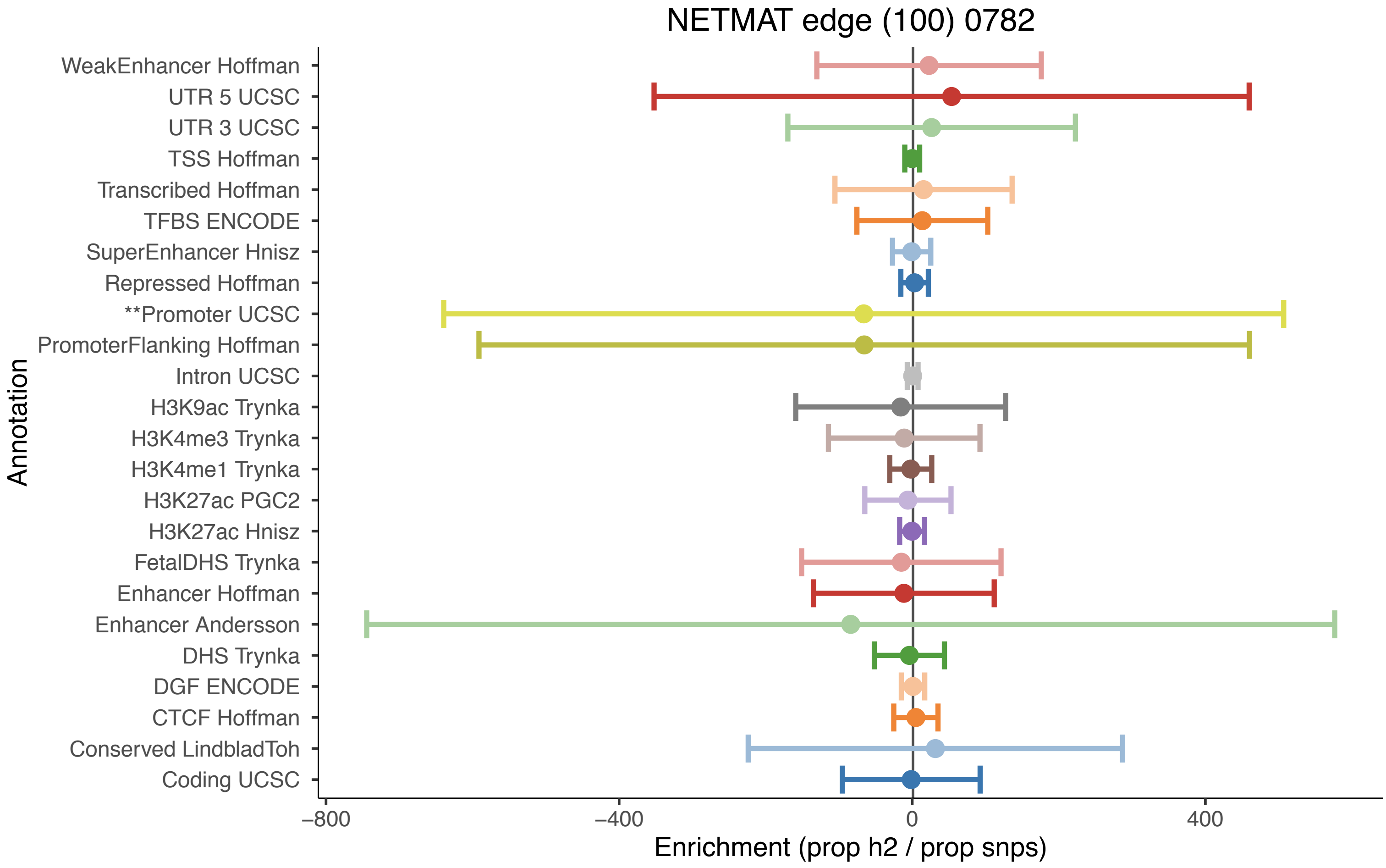

NETMAT edge (100) 0782
WeakEnhancer Hoffman
UTR 5 UCSC
UTR 3 UCSC
TSS Hoffman
Transcribed Hoffman
TFBS ENCODE
SuperEnhancer Hnisz
Repressed Hoffman
**Promoter UCSC
PromoterFlanking Hoffman
Intron UCSC
H3K9ac Trynka
H3K4me3 Trynka
H3K4me1 Trynka
H3K27ac PGC2
H3K27ac Hnisz
FetalDHS Trynka
Enhancer Hoffman
Enhancer Andersson
DHS Trynka
DGF ENCODE
CTCF Hoffman
Conserved LindbladToh
Coding UCSC
Annotation
−800
−400
0
400
Enrichment (prop h2 / prop snps)

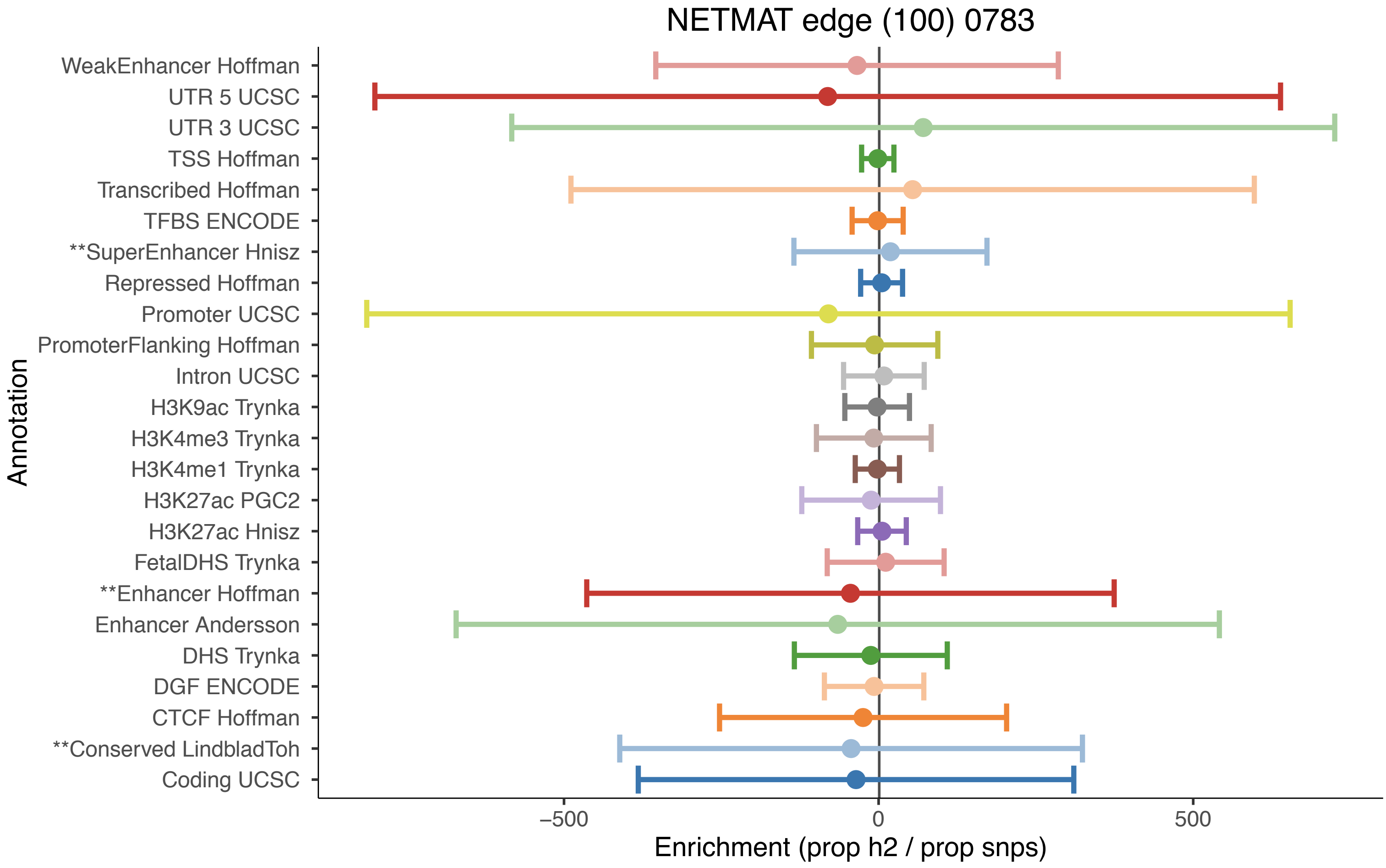
NETMAT edge (100) 0783
WeakEnhancer Hoffman
UTR 5 UCSC
UTR 3 UCSC
TSS Hoffman
Transcribed Hoffman
TFBS ENCODE
**SuperEnhancer Hnisz
Repressed Hoffman
Promoter UCSC
PromoterFlanking Hoffman
Intron UCSC
H3K9ac Trynka
H3K4me3 Trynka
H3K4me1 Trynka
H3K27ac PGC2
H3K27ac Hnisz
FetalDHS Trynka
**Enhancer Hoffman
Enhancer Andersson
DHS Trynka
DGF ENCODE
CTCF Hoffman
**Conserved LindbladToh
Coding UCSC
Annotation
−500
0
500
Enrichment (prop h2 / prop snps)

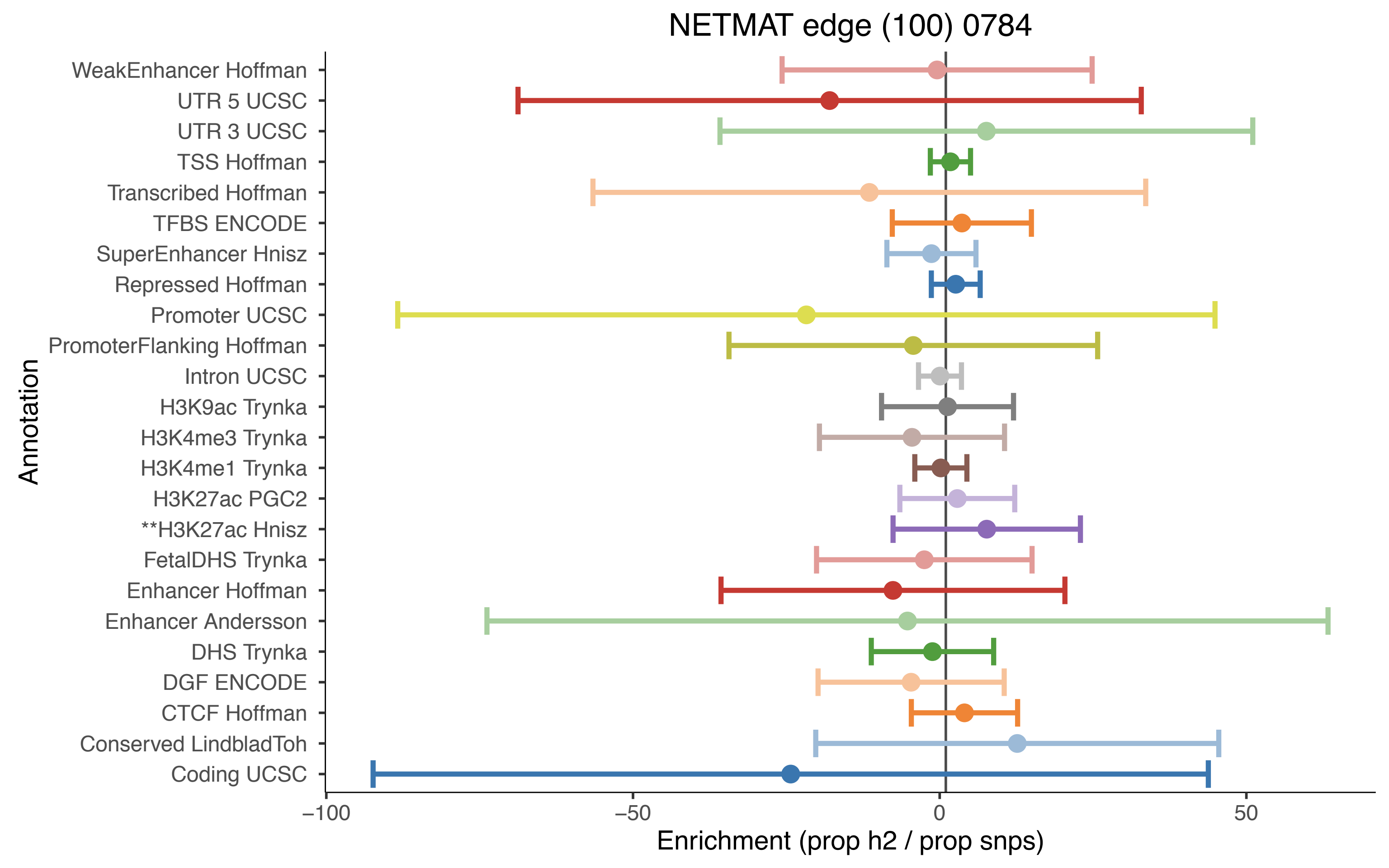
NETMAT edge (100) 0784
WeakEnhancer Hoffman
UTR 5 UCSC
UTR 3 UCSC
TSS Hoffman
Transcribed Hoffman
TFBS ENCODE
SuperEnhancer Hnisz
Repressed Hoffman
Promoter UCSC
PromoterFlanking Hoffman
Intron UCSC
H3K9ac Trynka
H3K4me3 Trynka
H3K4me1 Trynka
H3K27ac PGC2
**H3K27ac Hnisz
FetalDHS Trynka
Enhancer Hoffman
Enhancer Andersson
DHS Trynka
DGF ENCODE
CTCF Hoffman
Conserved LindbladToh
Coding UCSC
Annotation
−100
−50
0
50
Enrichment (prop h2 / prop snps)

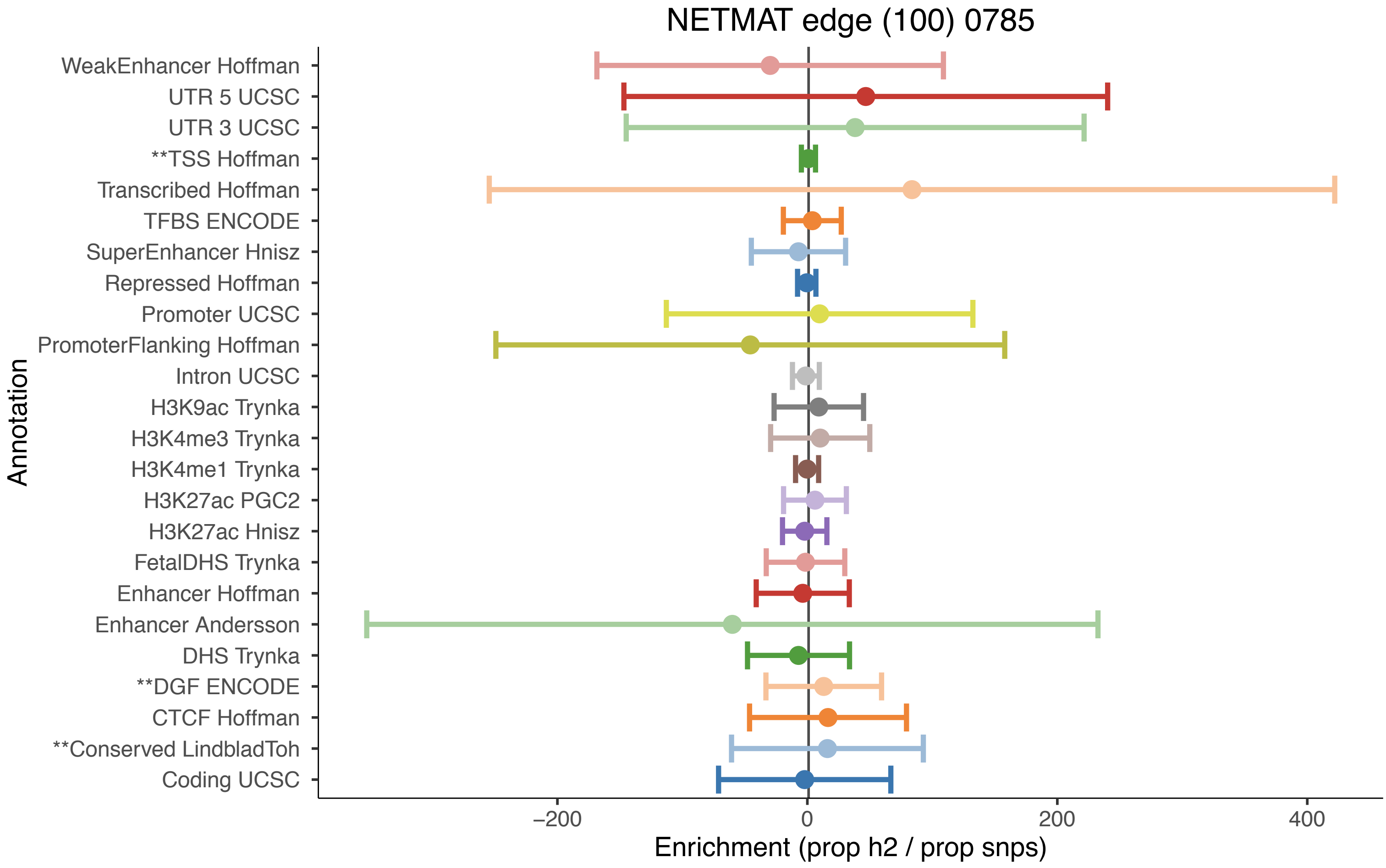
NETMAT edge (100) 0785
WeakEnhancer Hoffman
UTR 5 UCSC
UTR 3 UCSC
**TSS Hoffman
Transcribed Hoffman
TFBS ENCODE
SuperEnhancer Hnisz
Repressed Hoffman
Promoter UCSC
PromoterFlanking Hoffman
Intron UCSC
H3K9ac Trynka
H3K4me3 Trynka
H3K4me1 Trynka
H3K27ac PGC2
H3K27ac Hnisz
FetalDHS Trynka
Enhancer Hoffman
Enhancer Andersson
DHS Trynka
**DGF ENCODE
CTCF Hoffman
**Conserved LindbladToh
Coding UCSC
Annotation
−200
0
200
400
Enrichment (prop h2 / prop snps)

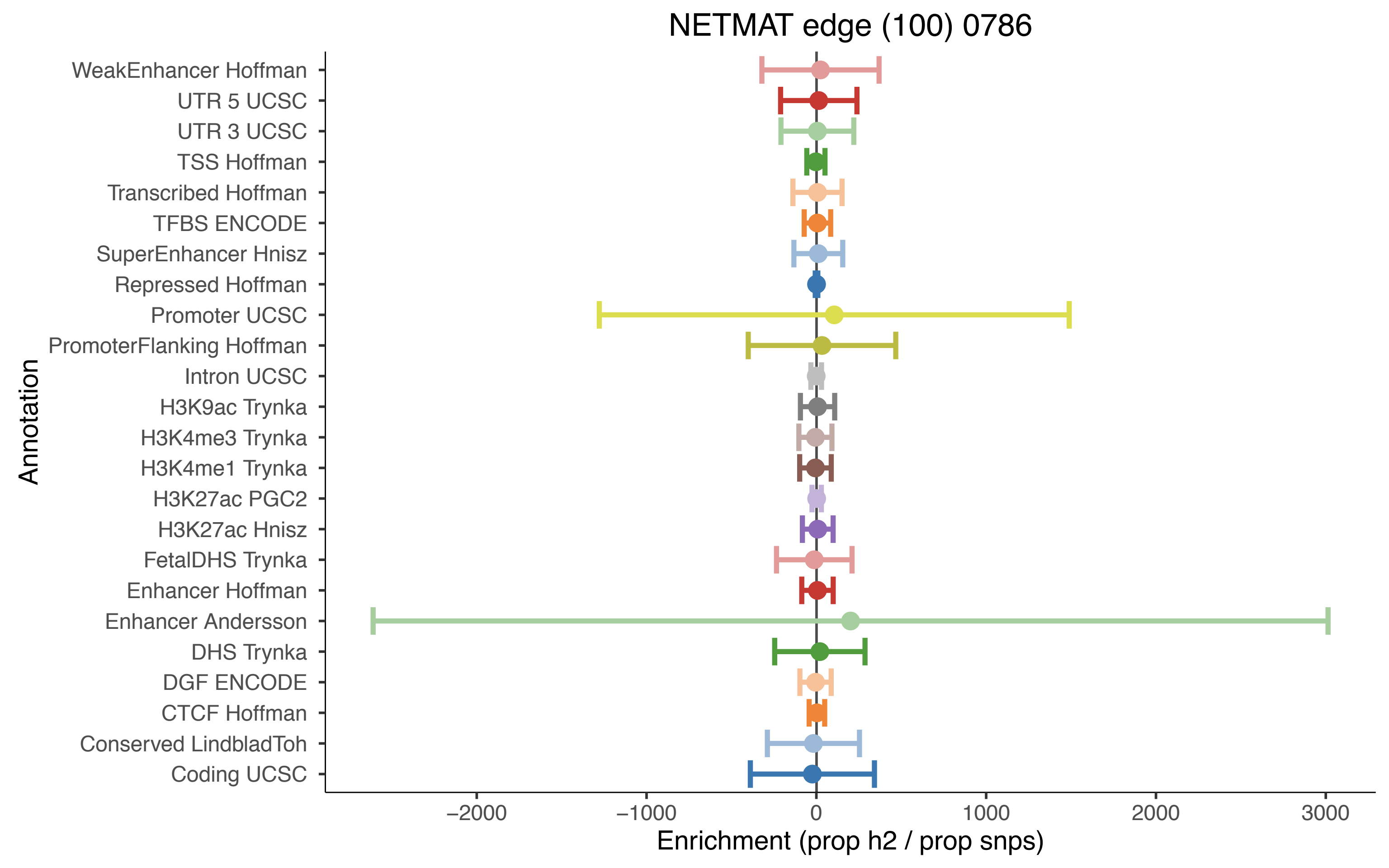
NETMAT edge (100) 0786
WeakEnhancer Hoffman
UTR 5 UCSC
UTR 3 UCSC
TSS Hoffman
Transcribed Hoffman
TFBS ENCODE
SuperEnhancer Hnisz
Repressed Hoffman
Promoter UCSC
PromoterFlanking Hoffman
Intron UCSC
H3K9ac Trynka
H3K4me3 Trynka
H3K4me1 Trynka
H3K27ac PGC2
H3K27ac Hnisz
FetalDHS Trynka
Enhancer Hoffman
Enhancer Andersson
DHS Trynka
DGF ENCODE
CTCF Hoffman
Conserved LindbladToh
Coding UCSC
Annotation
−2000
−1000
0
1000
2000
3000
Enrichment (prop h2 / prop snps)

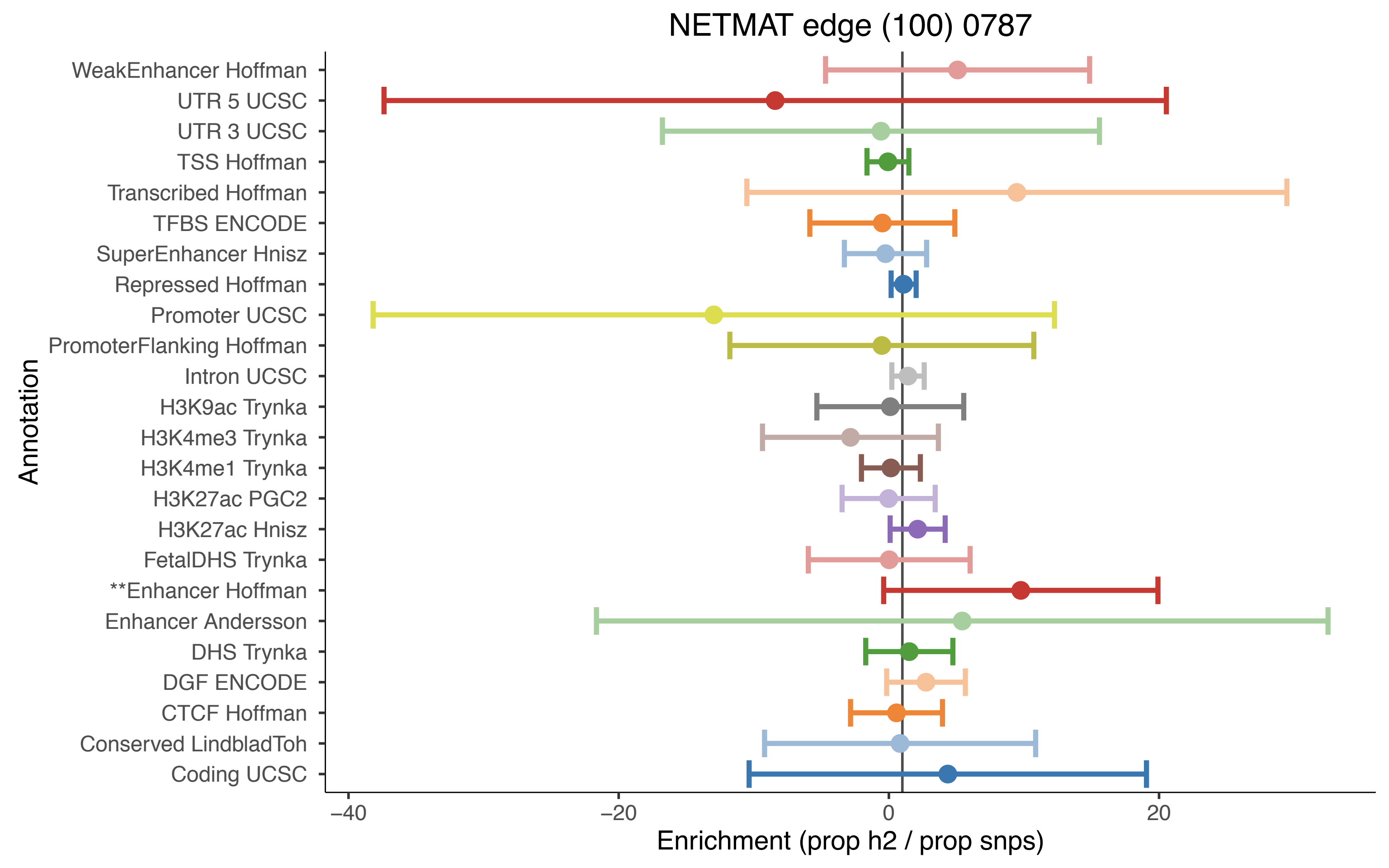
NETMAT edge (100) 0787
WeakEnhancer Hoffman
UTR 5 UCSC
UTR 3 UCSC
TSS Hoffman
Transcribed Hoffman
TFBS ENCODE
SuperEnhancer Hnisz
Repressed Hoffman
Promoter UCSC
PromoterFlanking Hoffman
Intron UCSC
H3K9ac Trynka
H3K4me3 Trynka
H3K4me1 Trynka
H3K27ac PGC2
H3K27ac Hnisz
FetalDHS Trynka
**Enhancer Hoffman
Enhancer Andersson
DHS Trynka
DGF ENCODE
CTCF Hoffman
Conserved LindbladToh
Coding UCSC
Annotation
−40
−20
0
20
Enrichment (prop h2 / prop snps)

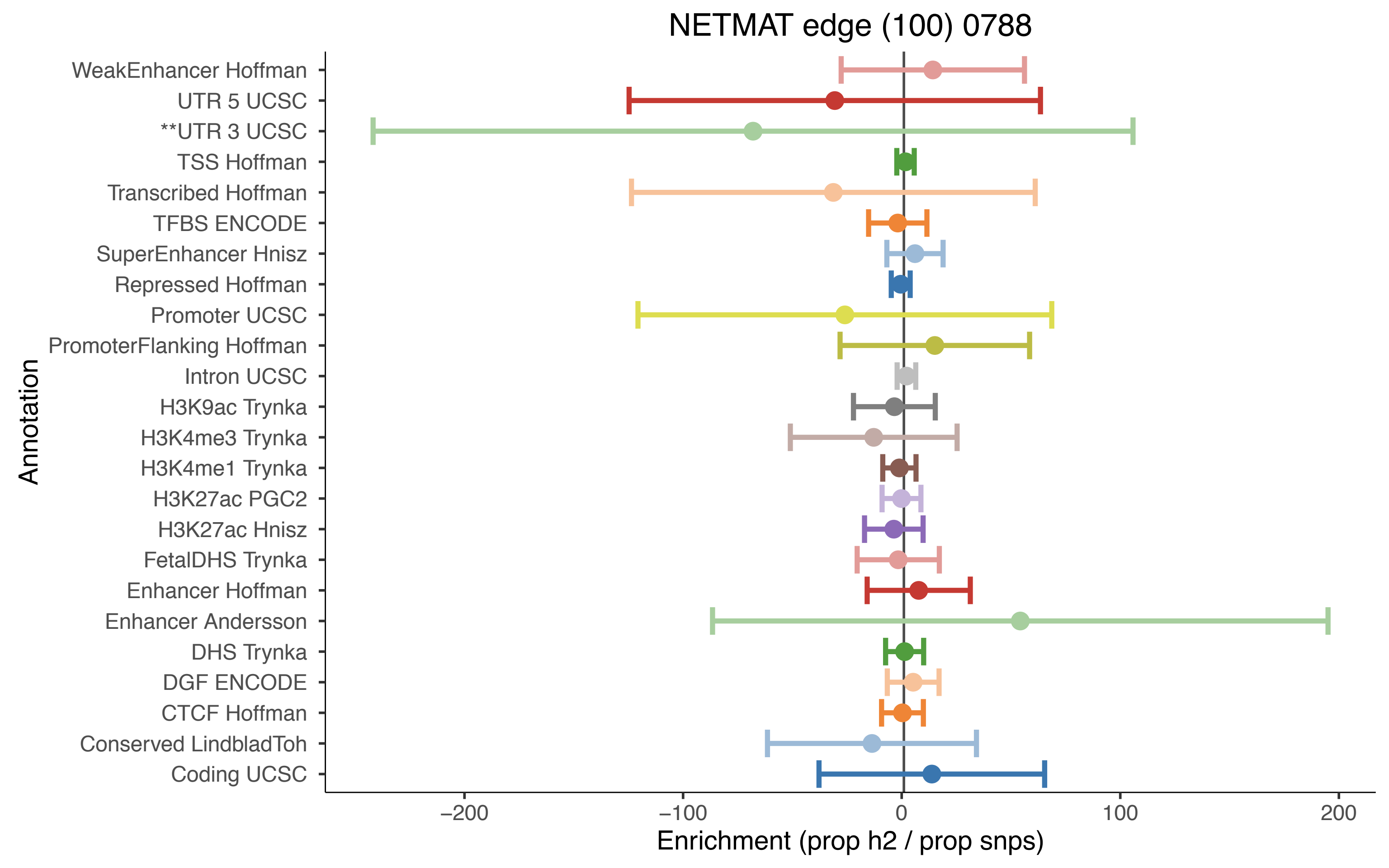

NETMAT edge (100) 0788
WeakEnhancer Hoffman
UTR 5 UCSC
**UTR 3 UCSC
TSS Hoffman
Transcribed Hoffman
TFBS ENCODE
SuperEnhancer Hnisz
Repressed Hoffman
Promoter UCSC
PromoterFlanking Hoffman
Intron UCSC
H3K9ac Trynka
H3K4me3 Trynka
H3K4me1 Trynka
H3K27ac PGC2
H3K27ac Hnisz
FetalDHS Trynka
Enhancer Hoffman
Enhancer Andersson
DHS Trynka
DGF ENCODE
CTCF Hoffman
Conserved LindbladToh
Coding UCSC
Annotation
−200
−100
0
100
200
Enrichment (prop h2 / prop snps)

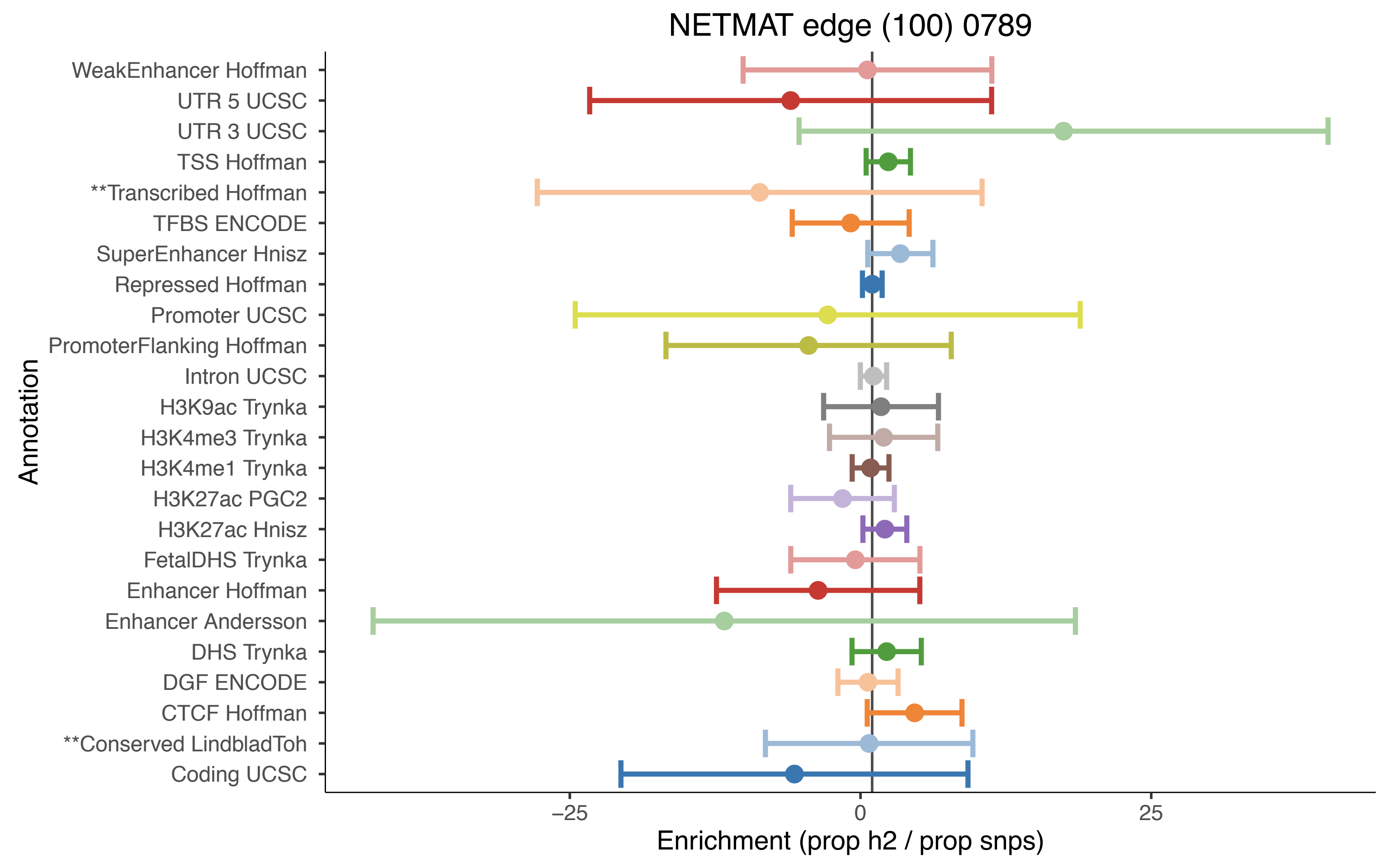

NETMAT edge (100) 0789
WeakEnhancer Hoffman
UTR 5 UCSC
UTR 3 UCSC
TSS Hoffman
**Transcribed Hoffman
TFBS ENCODE
SuperEnhancer Hnisz
Repressed Hoffman
Promoter UCSC
PromoterFlanking Hoffman
Intron UCSC
H3K9ac Trynka
H3K4me3 Trynka
H3K4me1 Trynka
H3K27ac PGC2
H3K27ac Hnisz
FetalDHS Trynka
Enhancer Hoffman
Enhancer Andersson
DHS Trynka
DGF ENCODE
CTCF Hoffman
**Conserved LindbladToh
Coding UCSC
Annotation
−25
0
25
Enrichment (prop h2 / prop snps)

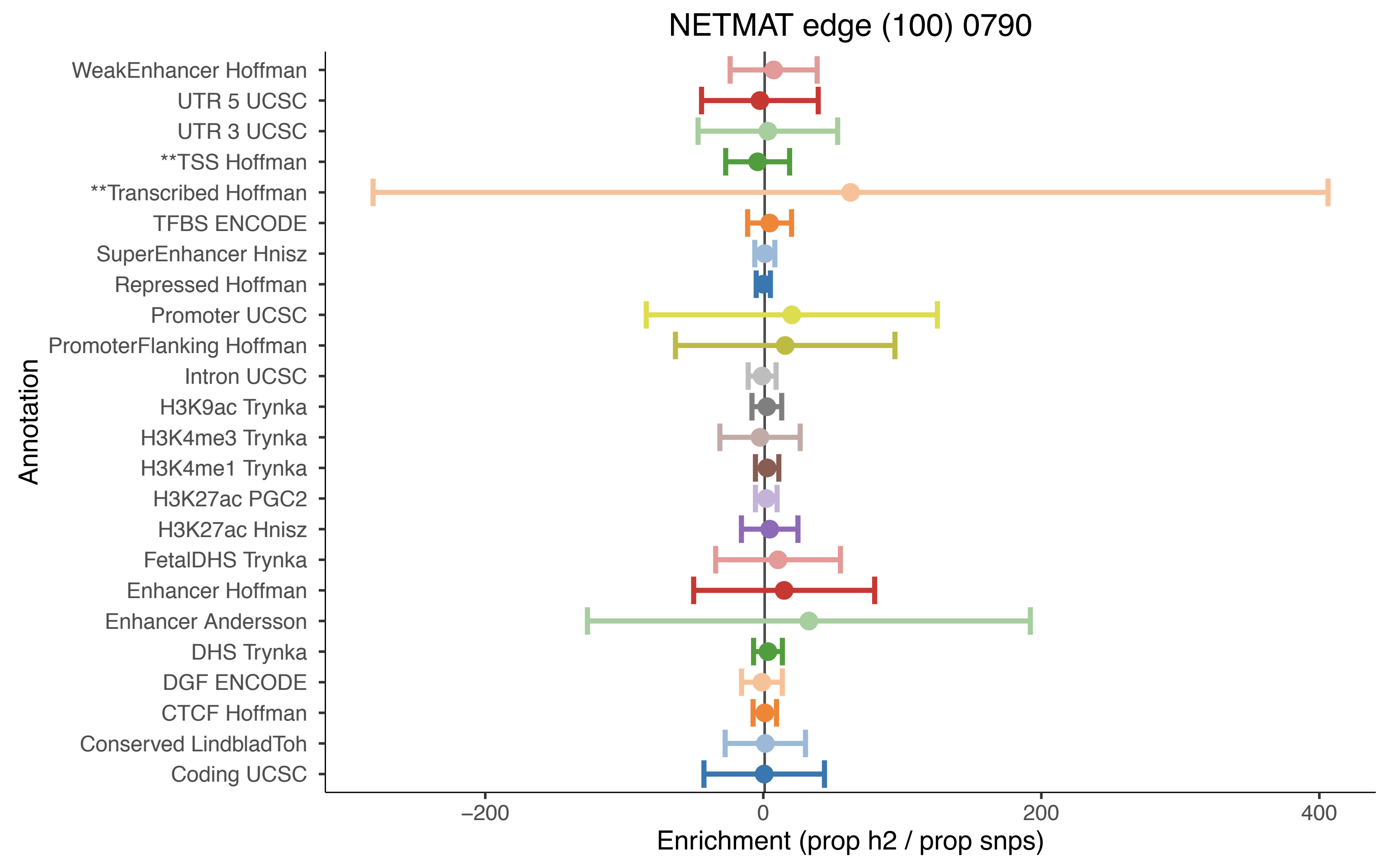

NETMAT edge (100) 0790
WeakEnhancer Hoffman
UTR 5 UCSC
UTR 3 UCSC
**TSS Hoffman
**Transcribed Hoffman
TFBS ENCODE
SuperEnhancer Hnisz
Repressed Hoffman
Promoter UCSC
PromoterFlanking Hoffman
Intron UCSC
H3K9ac Trynka
H3K4me3 Trynka
H3K4me1 Trynka
H3K27ac PGC2
H3K27ac Hnisz
FetalDHS Trynka
Enhancer Hoffman
Enhancer Andersson
DHS Trynka
DGF ENCODE
CTCF Hoffman
Conserved LindbladToh
Coding UCSC
Annotation
−200
0
200
400
Enrichment (prop h2 / prop snps)

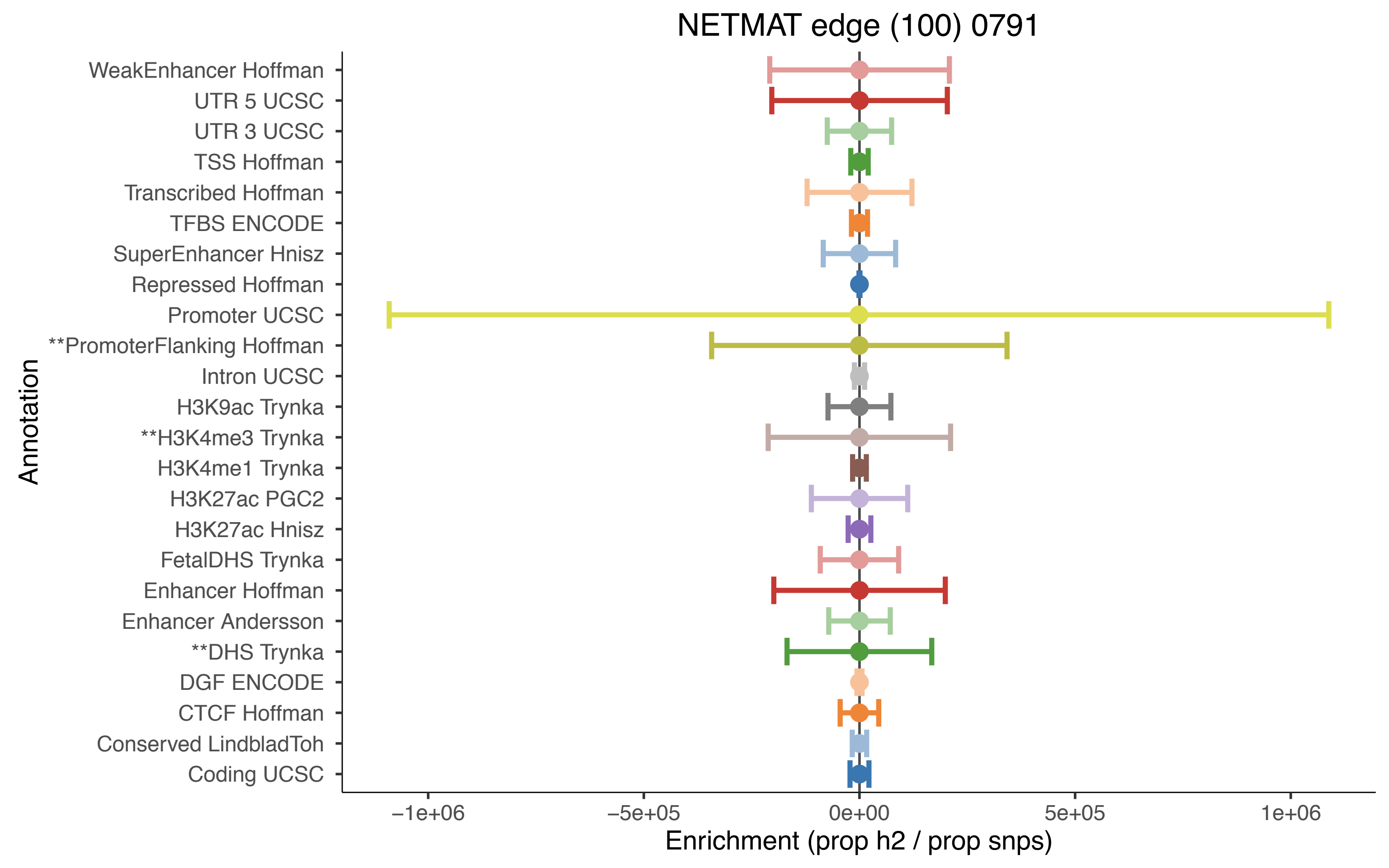
NETMAT edge (100) 0791
Annotation
WeakEnhancer Hoffman
UTR 5 UCSC
UTR 3 UCSC
TSS Hoffman
Transcribed Hoffman
TFBS ENCODE
SuperEnhancer Hnisz
Repressed Hoffman
Promoter UCSC
**PromoterFlanking Hoffman
Intron UCSC
H3K9ac Trynka
**H3K4me3 Trynka
H3K4me1 Trynka
H3K27ac PGC2
H3K27ac Hnisz
FetalDHS Trynka
Enhancer Hoffman
Enhancer Andersson
**DHS Trynka
DGF ENCODE
CTCF Hoffman
Conserved LindbladToh
Coding UCSC
−1e+06
−5e+05
0e+00
5e+05
1e+06
Enrichment (prop h2 / prop snps)

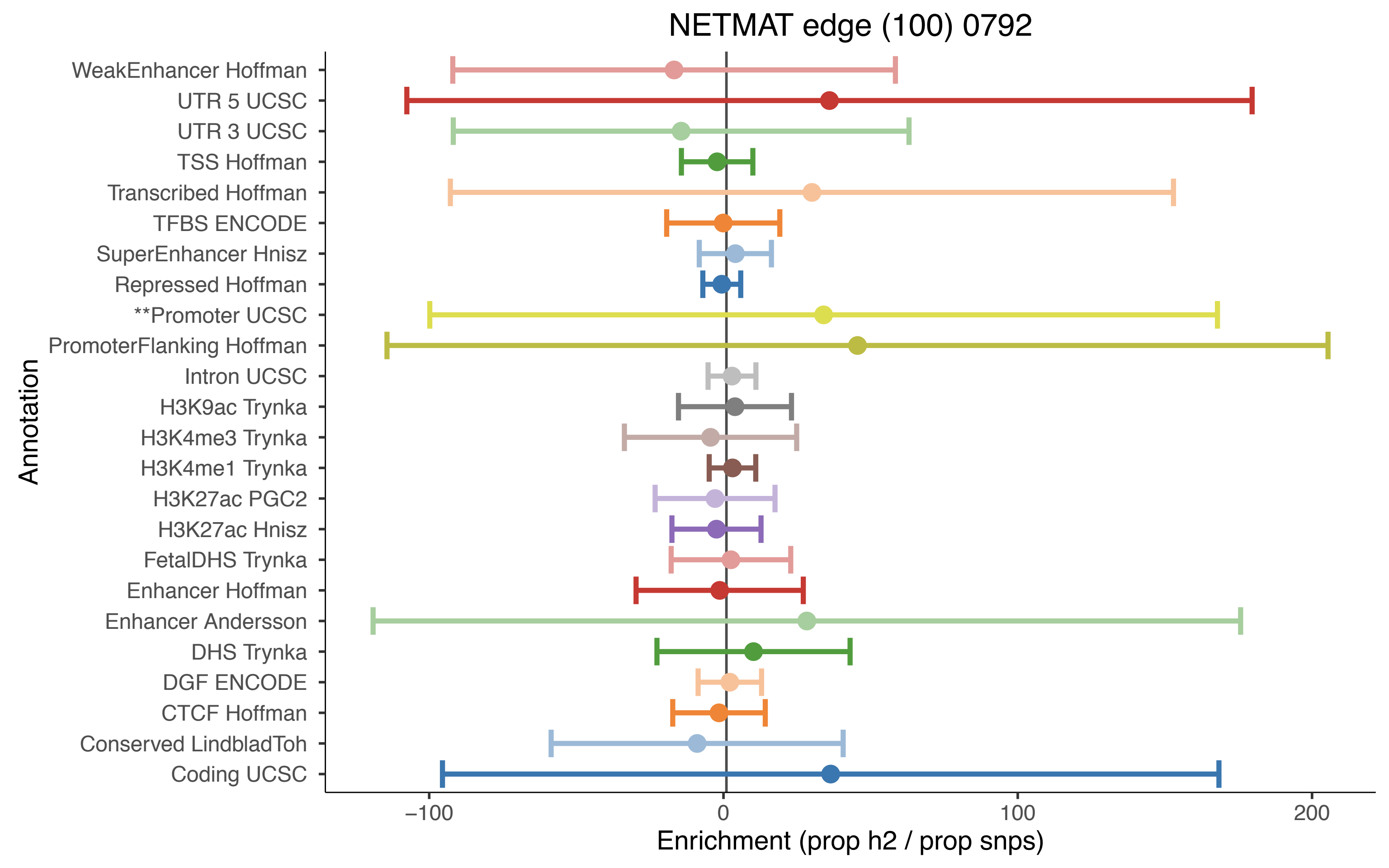
NETMAT edge (100) 0792
WeakEnhancer Hoffman
UTR 5 UCSC
UTR 3 UCSC
TSS Hoffman
Transcribed Hoffman
TFBS ENCODE
SuperEnhancer Hnisz
Repressed Hoffman
**Promoter UCSC
PromoterFlanking Hoffman
Intron UCSC
H3K9ac Trynka
H3K4me3 Trynka
H3K4me1 Trynka
H3K27ac PGC2
H3K27ac Hnisz
FetalDHS Trynka
Enhancer Hoffman
Enhancer Andersson
DHS Trynka
DGF ENCODE
CTCF Hoffman
Conserved LindbladToh
Coding UCSC
Annotation
−100
0
100
200
Enrichment (prop h2 / prop snps)

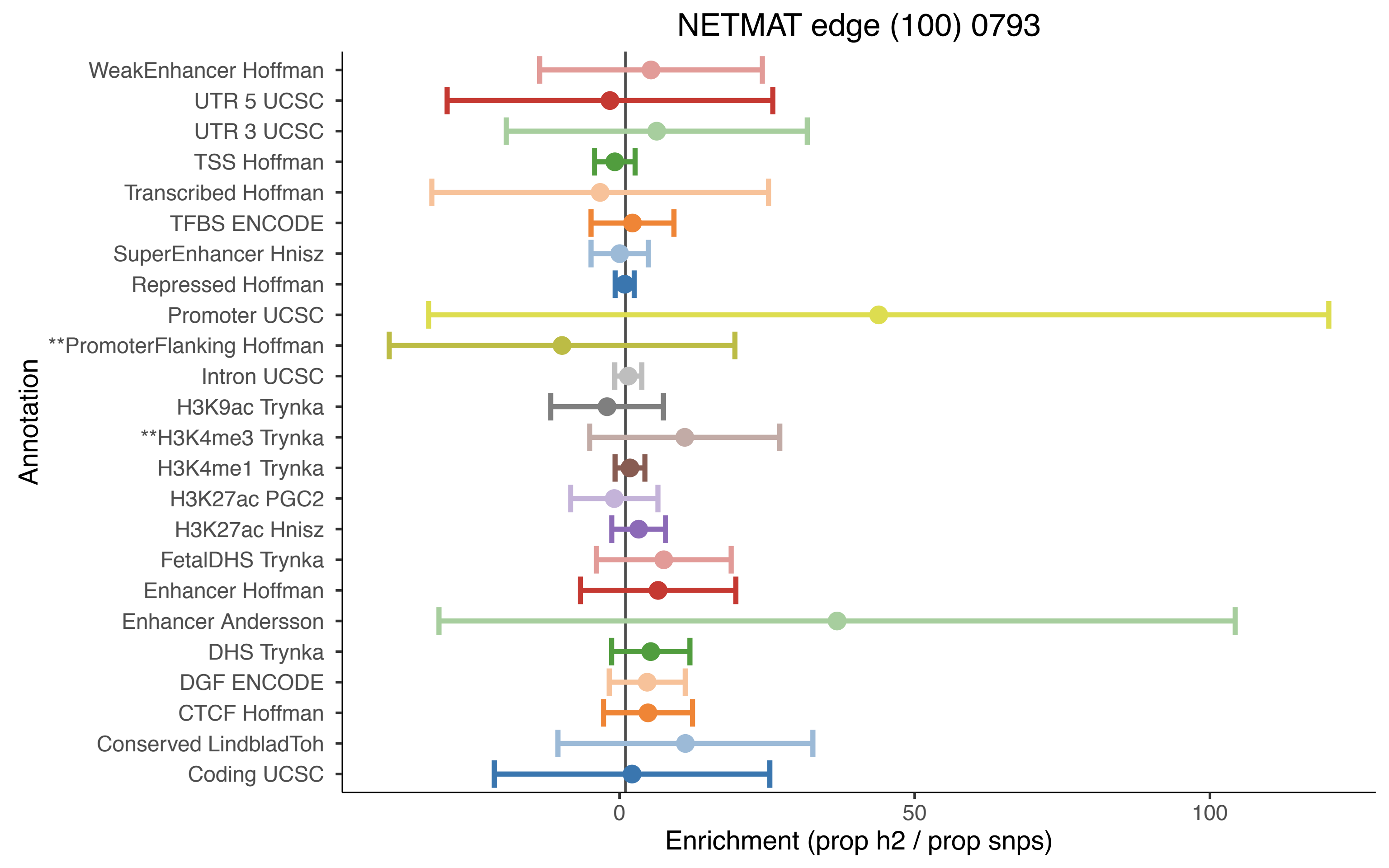
NETMAT edge (100) 0793
WeakEnhancer Hoffman
UTR 5 UCSC
UTR 3 UCSC
TSS Hoffman
Transcribed Hoffman
TFBS ENCODE
SuperEnhancer Hnisz
Repressed Hoffman
Promoter UCSC
**PromoterFlanking Hoffman
Intron UCSC
H3K9ac Trynka
**H3K4me3 Trynka
H3K4me1 Trynka
H3K27ac PGC2
H3K27ac Hnisz
FetalDHS Trynka
Enhancer Hoffman
Enhancer Andersson
DHS Trynka
DGF ENCODE
CTCF Hoffman
Conserved LindbladToh
Coding UCSC
Annotation
0
50
100
Enrichment (prop h2 / prop snps)

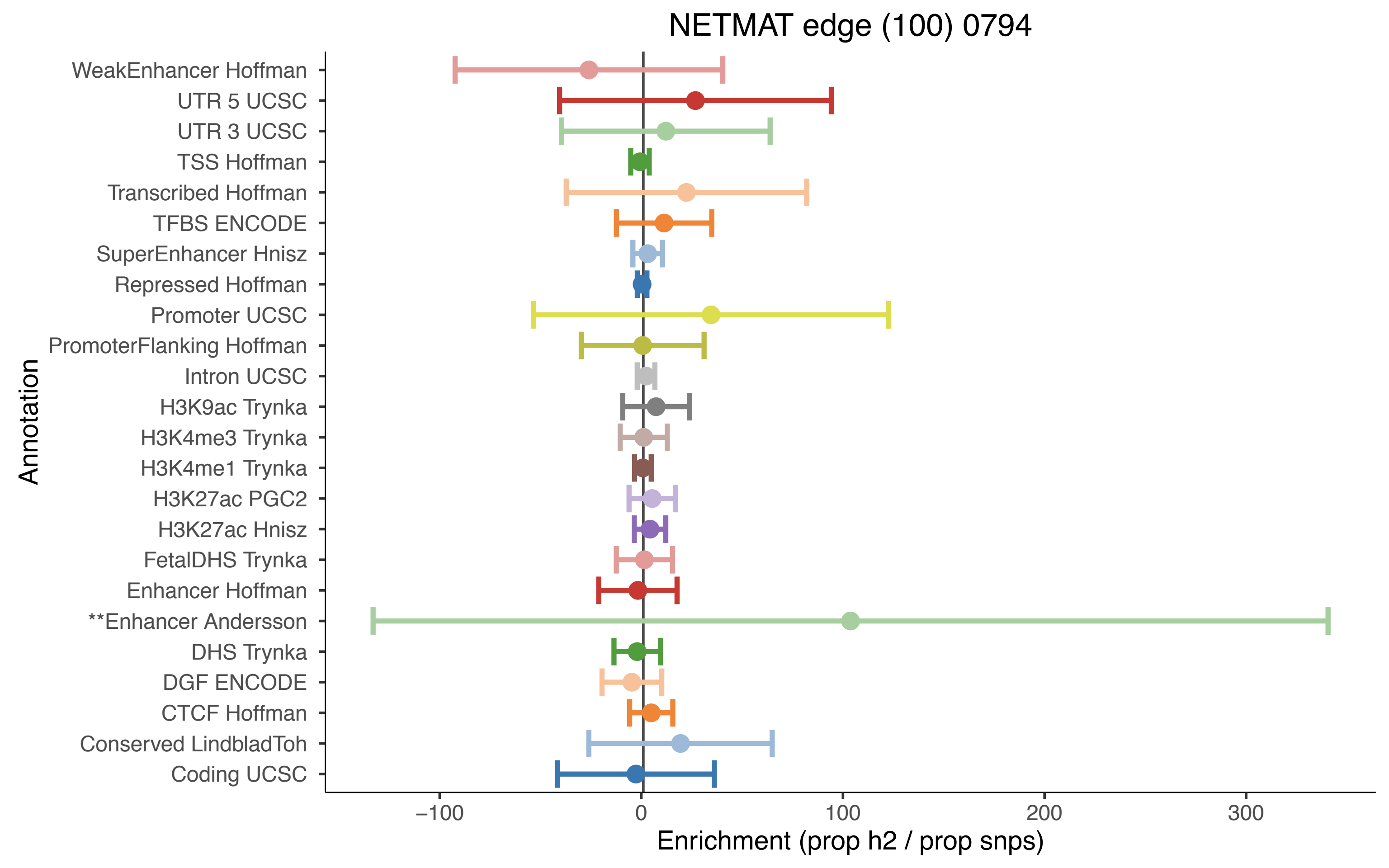
NETMAT edge (100) 0794
Annotation
WeakEnhancer Hoffman
UTR 5 UCSC
UTR 3 UCSC
TSS Hoffman
Transcribed Hoffman
TFBS ENCODE
SuperEnhancer Hnisz
Repressed Hoffman
Promoter UCSC
PromoterFlanking Hoffman
Intron UCSC
H3K9ac Trynka
H3K4me3 Trynka
H3K4me1 Trynka
H3K27ac PGC2
H3K27ac Hnisz
FetalDHS Trynka
Enhancer Hoffman
**Enhancer Andersson
DHS Trynka
DGF ENCODE
CTCF Hoffman
Conserved LindbladToh
Coding UCSC
−100
0
100
200
300
Enrichment (prop h2 / prop snps)

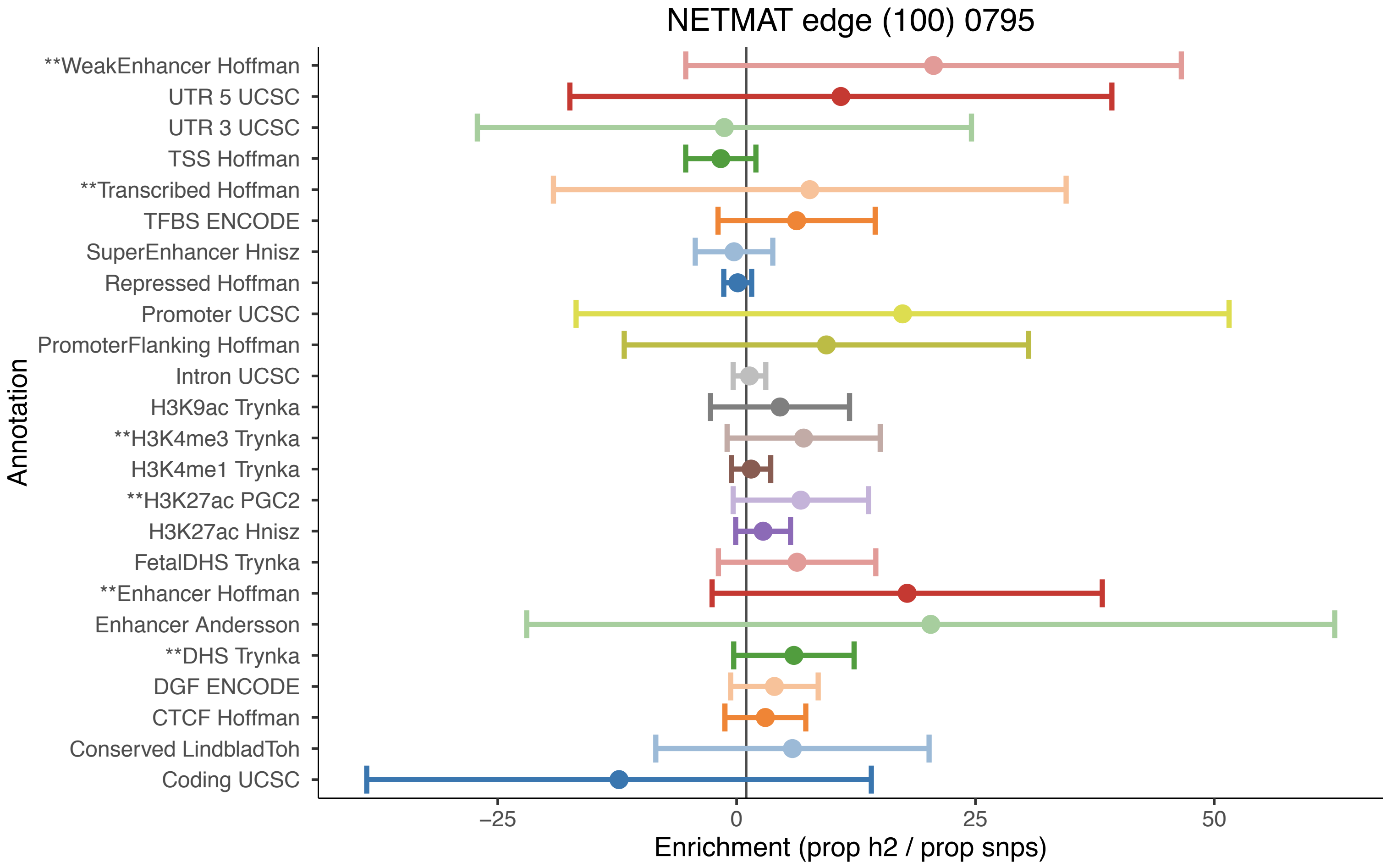
NETMAT edge (100) 0795
**WeakEnhancer Hoffman
UTR 5 UCSC
UTR 3 UCSC
TSS Hoffman
**Transcribed Hoffman
TFBS ENCODE
SuperEnhancer Hnisz
Repressed Hoffman
Promoter UCSC
PromoterFlanking Hoffman
Intron UCSC
H3K9ac Trynka
**H3K4me3 Trynka
H3K4me1 Trynka
**H3K27ac PGC2
H3K27ac Hnisz
FetalDHS Trynka
**Enhancer Hoffman
Enhancer Andersson
**DHS Trynka
DGF ENCODE
CTCF Hoffman
Conserved LindbladToh
Coding UCSC
Annotation
−25
0
25
50
Enrichment (prop h2 / prop snps)

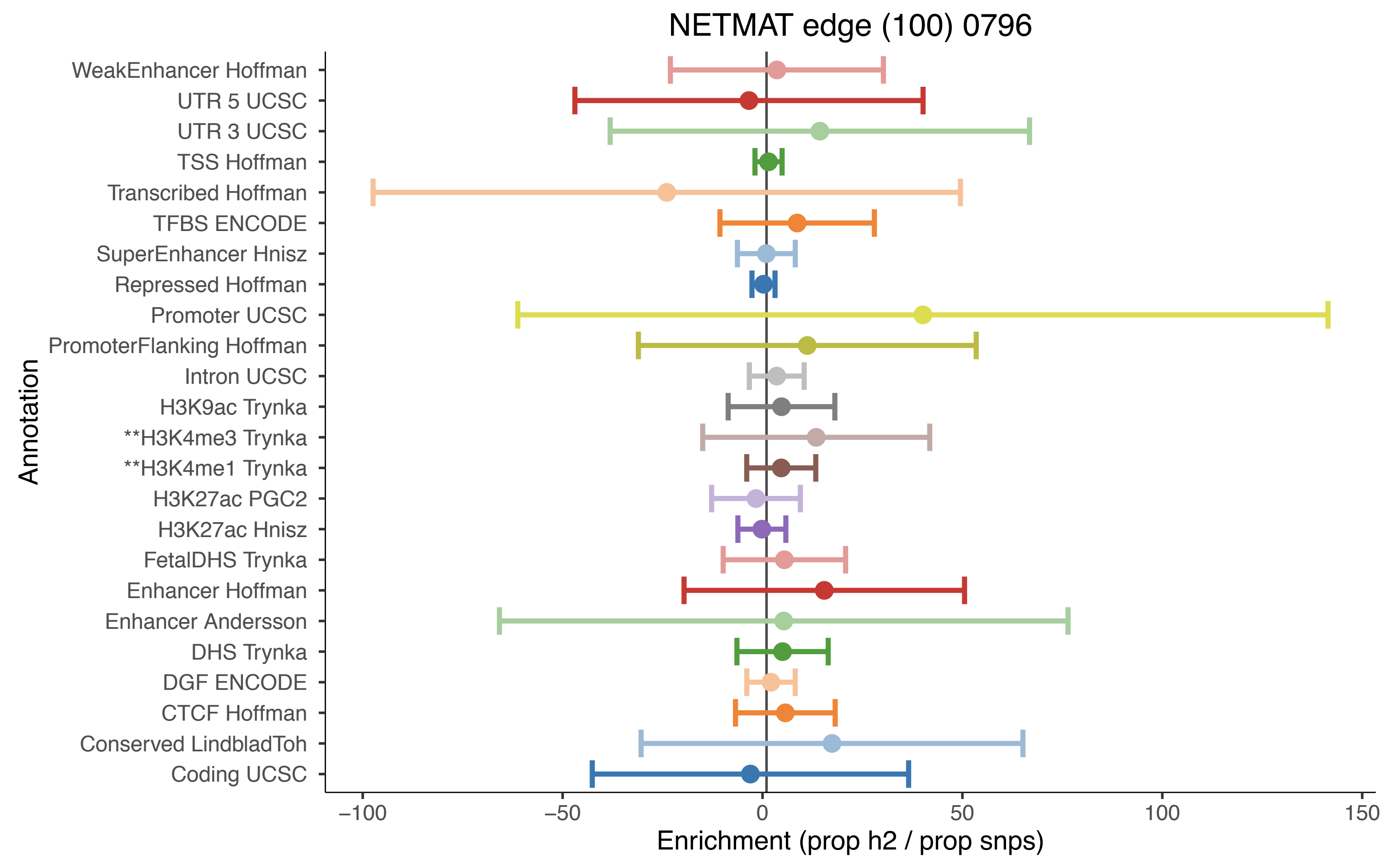
NETMAT edge (100) 0796
WeakEnhancer Hoffman
UTR 5 UCSC
UTR 3 UCSC
TSS Hoffman
Transcribed Hoffman
TFBS ENCODE
SuperEnhancer Hnisz
Repressed Hoffman
Promoter UCSC
PromoterFlanking Hoffman
Intron UCSC
H3K9ac Trynka
**H3K4me3 Trynka
**H3K4me1 Trynka
H3K27ac PGC2
H3K27ac Hnisz
FetalDHS Trynka
Enhancer Hoffman
Enhancer Andersson
DHS Trynka
DGF ENCODE
CTCF Hoffman
Conserved LindbladToh
Coding UCSC
Annotation
−100
−50
0
50
100
150
Enrichment (prop h2 / prop snps)

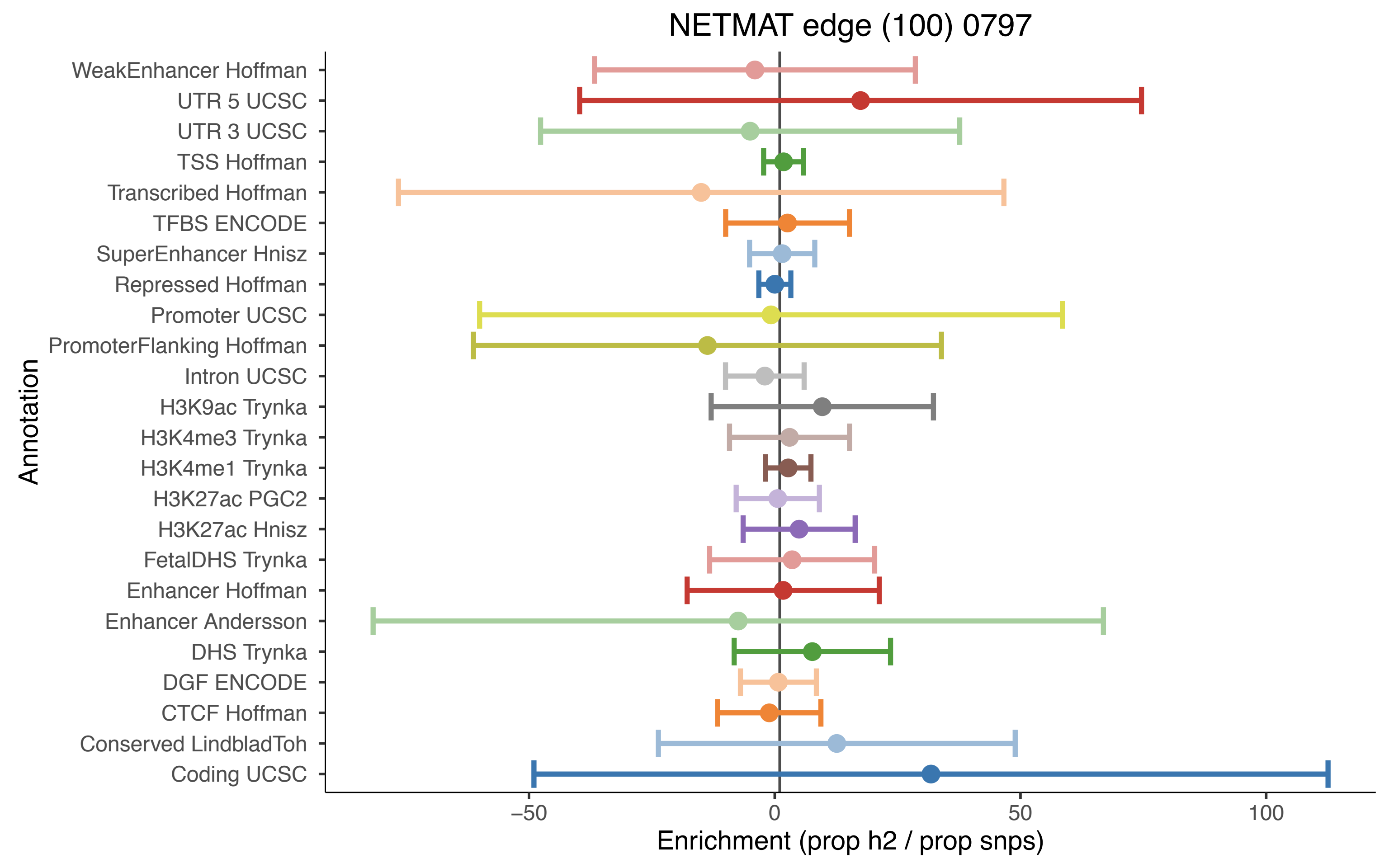
NETMAT edge (100) 0797
WeakEnhancer Hoffman
UTR 5 UCSC
UTR 3 UCSC
TSS Hoffman
Transcribed Hoffman
TFBS ENCODE
SuperEnhancer Hnisz
Repressed Hoffman
Promoter UCSC
PromoterFlanking Hoffman
Intron UCSC
H3K9ac Trynka
H3K4me3 Trynka
H3K4me1 Trynka
H3K27ac PGC2
H3K27ac Hnisz
FetalDHS Trynka
Enhancer Hoffman
Enhancer Andersson
DHS Trynka
DGF ENCODE
CTCF Hoffman
Conserved LindbladToh
Coding UCSC
Annotation
−50
0
50
100
Enrichment (prop h2 / prop snps)

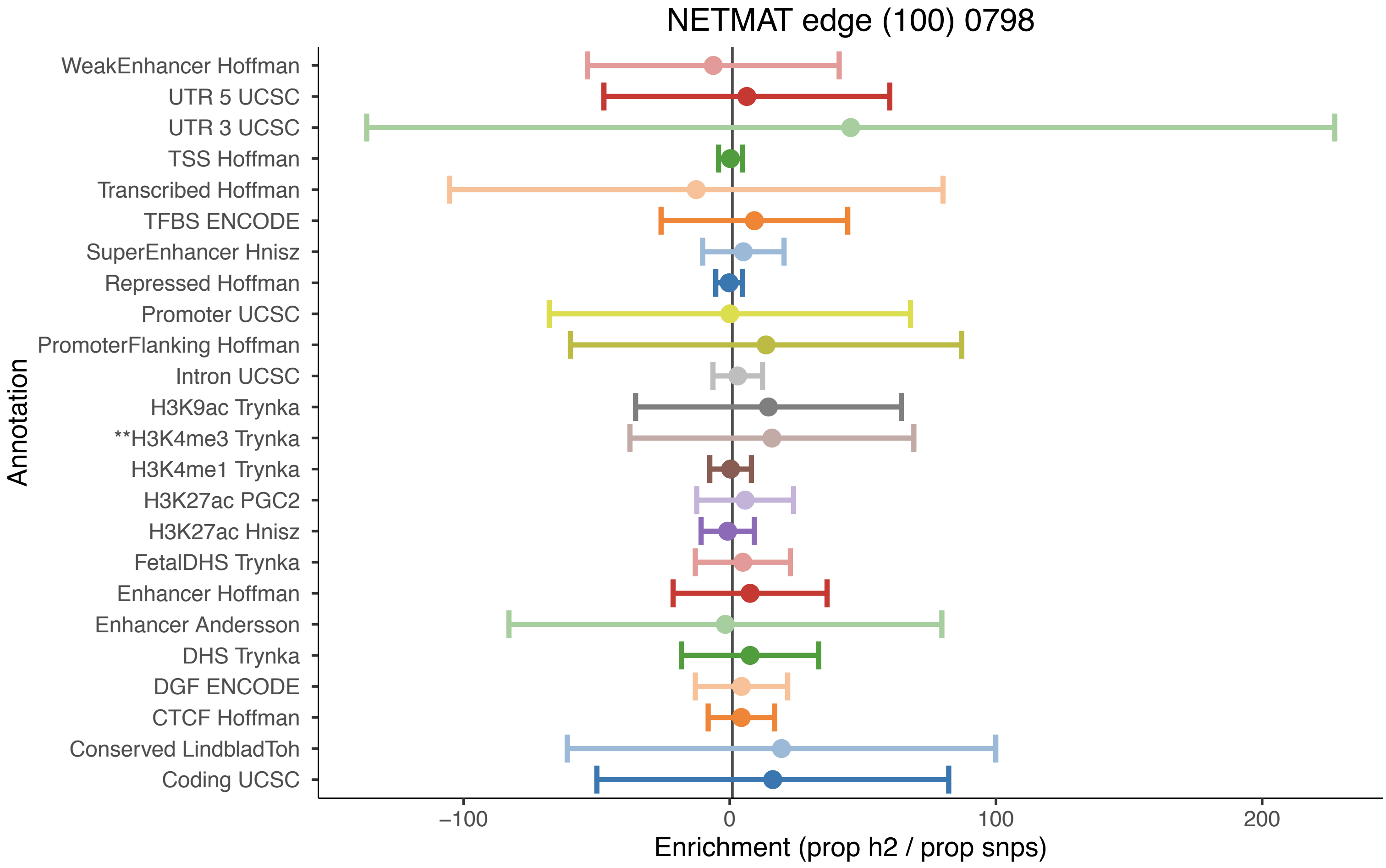
NETMAT edge (100) 0798
Annotation
WeakEnhancer Hoffman
UTR 5 UCSC
UTR 3 UCSC
TSS Hoffman
Transcribed Hoffman
TFBS ENCODE
SuperEnhancer Hnisz
Repressed Hoffman
Promoter UCSC
PromoterFlanking Hoffman
Intron UCSC
H3K9ac Trynka
**H3K4me3 Trynka
H3K4me1 Trynka
H3K27ac PGC2
H3K27ac Hnisz
FetalDHS Trynka
Enhancer Hoffman
Enhancer Andersson
DHS Trynka
DGF ENCODE
CTCF Hoffman
Conserved LindbladToh
Coding UCSC
−100
0
100
200
Enrichment (prop h2 / prop snps)

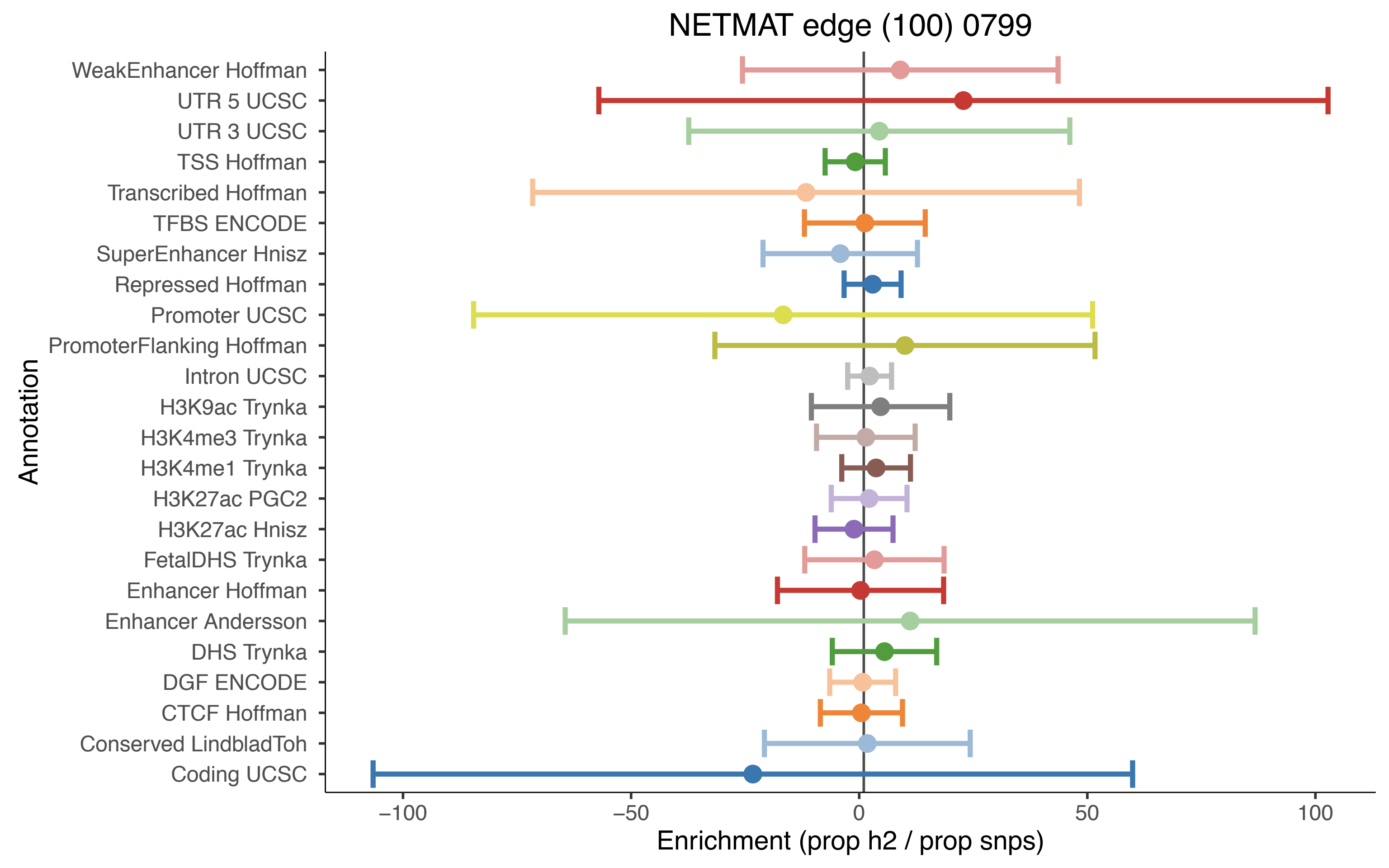
NETMAT edge (100) 0799
WeakEnhancer Hoffman
UTR 5 UCSC
UTR 3 UCSC
TSS Hoffman
Transcribed Hoffman
TFBS ENCODE
SuperEnhancer Hnisz
Repressed Hoffman
Promoter UCSC
PromoterFlanking Hoffman
Intron UCSC
H3K9ac Trynka
H3K4me3 Trynka
H3K4me1 Trynka
H3K27ac PGC2
H3K27ac Hnisz
FetalDHS Trynka
Enhancer Hoffman
Enhancer Andersson
DHS Trynka
DGF ENCODE
CTCF Hoffman
Conserved LindbladToh
Coding UCSC
Annotation
−100
−50
0
50
100
Enrichment (prop h2 / prop snps)

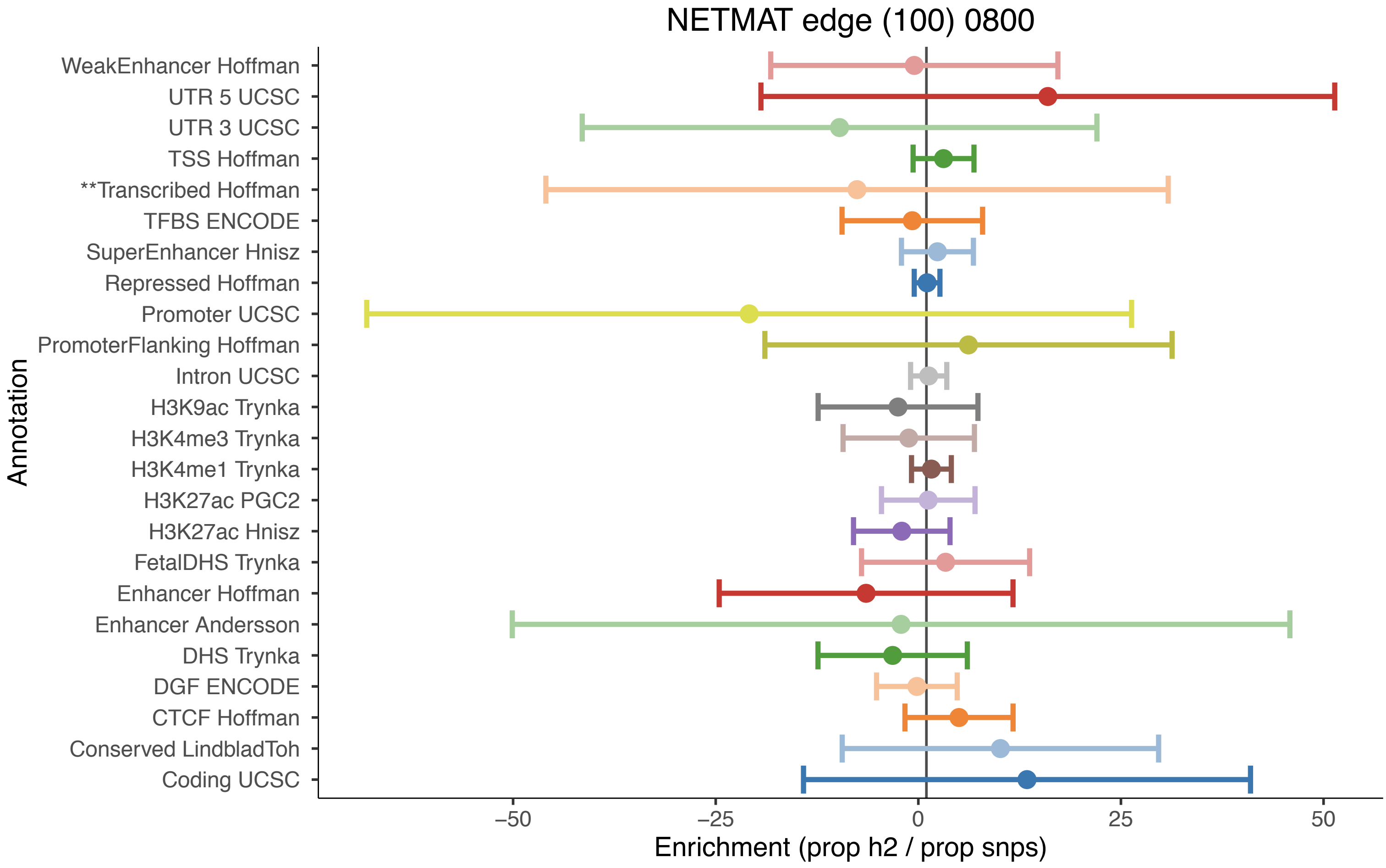

NETMAT edge (100) 0800
Annotation
WeakEnhancer Hoffman
UTR 5 UCSC
UTR 3 UCSC
TSS Hoffman
**Transcribed Hoffman
TFBS ENCODE
SuperEnhancer Hnisz
Repressed Hoffman
Promoter UCSC
PromoterFlanking Hoffman
Intron UCSC
H3K9ac Trynka
H3K4me3 Trynka
H3K4me1 Trynka
H3K27ac PGC2
H3K27ac Hnisz
FetalDHS Trynka
Enhancer Hoffman
Enhancer Andersson
DHS Trynka
DGF ENCODE
CTCF Hoffman
Conserved LindbladToh
Coding UCSC
−50
−25
0
25
50
Enrichment (prop h2 / prop snps)

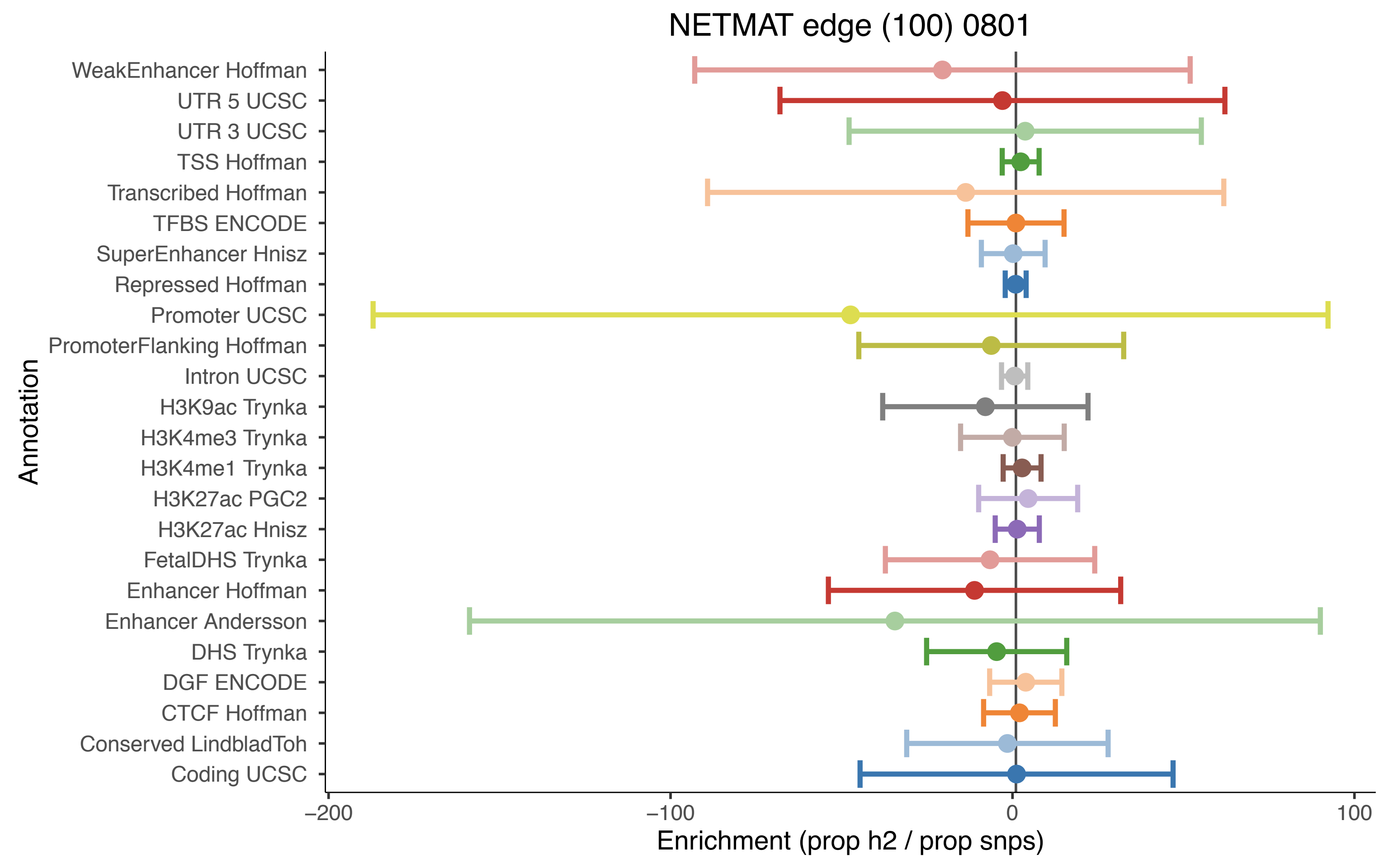
NETMAT edge (100) 0801
Annotation
WeakEnhancer Hoffman
UTR 5 UCSC
UTR 3 UCSC
TSS Hoffman
Transcribed Hoffman
TFBS ENCODE
SuperEnhancer Hnisz
Repressed Hoffman
Promoter UCSC
PromoterFlanking Hoffman
Intron UCSC
H3K9ac Trynka
H3K4me3 Trynka
H3K4me1 Trynka
H3K27ac PGC2
H3K27ac Hnisz
FetalDHS Trynka
Enhancer Hoffman
Enhancer Andersson
DHS Trynka
DGF ENCODE
CTCF Hoffman
Conserved LindbladToh
Coding UCSC
−200
−100
0
100
Enrichment (prop h2 / prop snps)

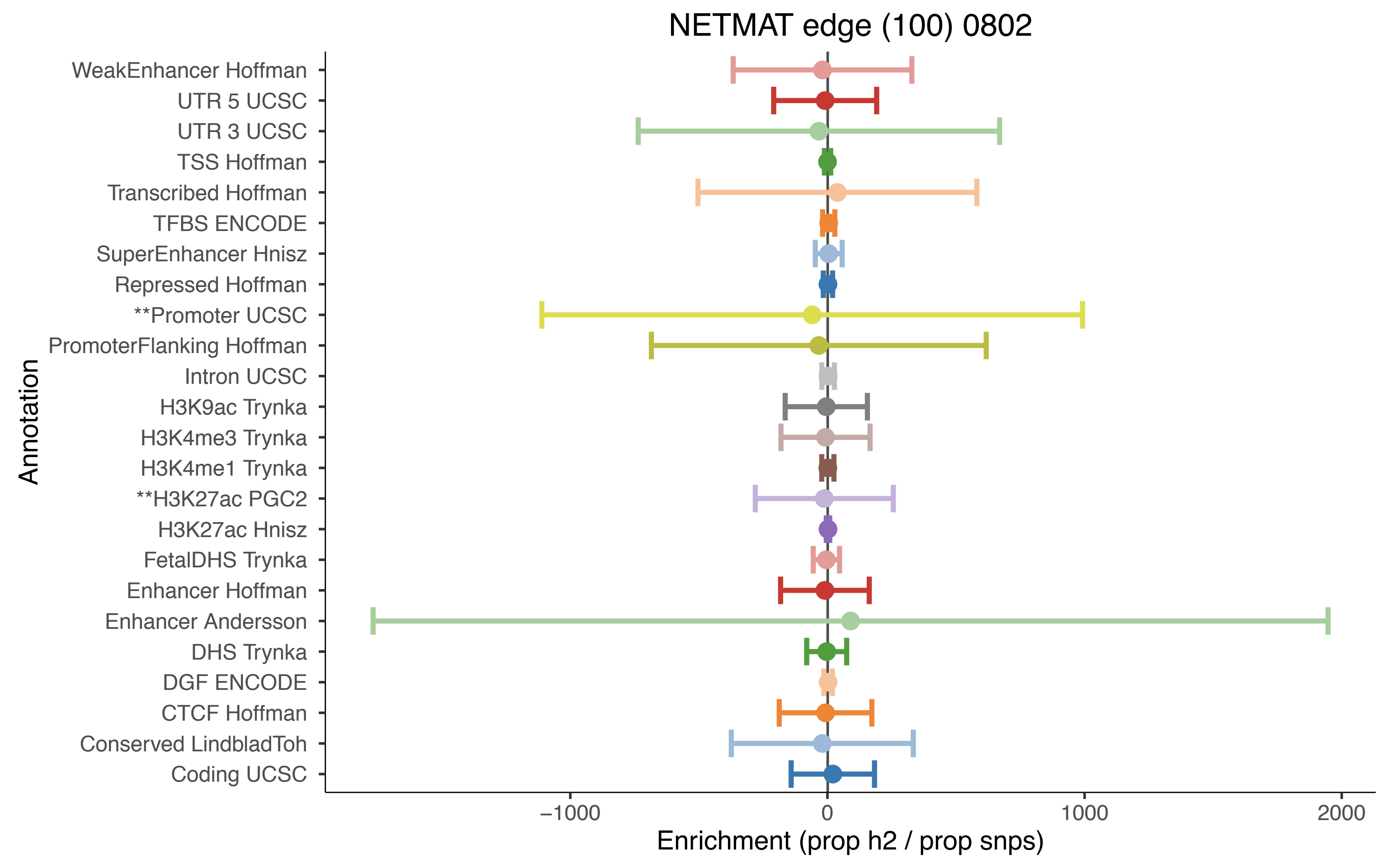
NETMAT edge (100) 0802
Annotation
WeakEnhancer Hoffman
UTR 5 UCSC
UTR 3 UCSC
TSS Hoffman
Transcribed Hoffman
TFBS ENCODE
SuperEnhancer Hnisz
Repressed Hoffman
**Promoter UCSC
PromoterFlanking Hoffman
Intron UCSC
H3K9ac Trynka
H3K4me3 Trynka
H3K4me1 Trynka
**H3K27ac PGC2
H3K27ac Hnisz
FetalDHS Trynka
Enhancer Hoffman
Enhancer Andersson
DHS Trynka
DGF ENCODE
CTCF Hoffman
Conserved LindbladToh
Coding UCSC
−1000
0
1000
2000
Enrichment (prop h2 / prop snps)

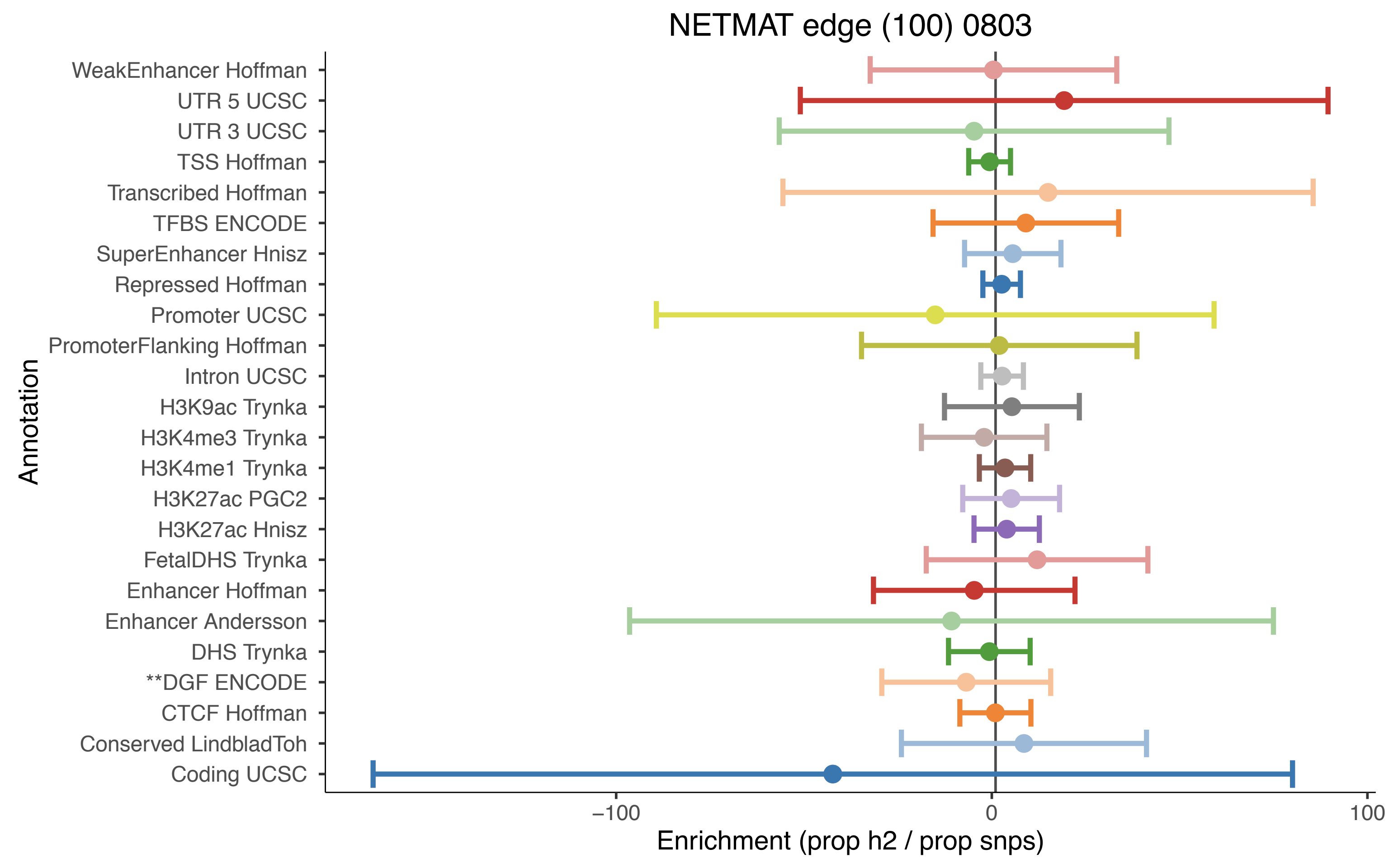
NETMAT edge (100) 0803
WeakEnhancer Hoffman
UTR 5 UCSC
UTR 3 UCSC
TSS Hoffman
Transcribed Hoffman
TFBS ENCODE
SuperEnhancer Hnisz
Repressed Hoffman
Promoter UCSC
PromoterFlanking Hoffman
Intron UCSC
H3K9ac Trynka
H3K4me3 Trynka
H3K4me1 Trynka
H3K27ac PGC2
H3K27ac Hnisz
FetalDHS Trynka
Enhancer Hoffman
Enhancer Andersson
DHS Trynka
**DGF ENCODE
CTCF Hoffman
Conserved LindbladToh
Coding UCSC
Annotation
−100
0
100
Enrichment (prop h2 / prop snps)

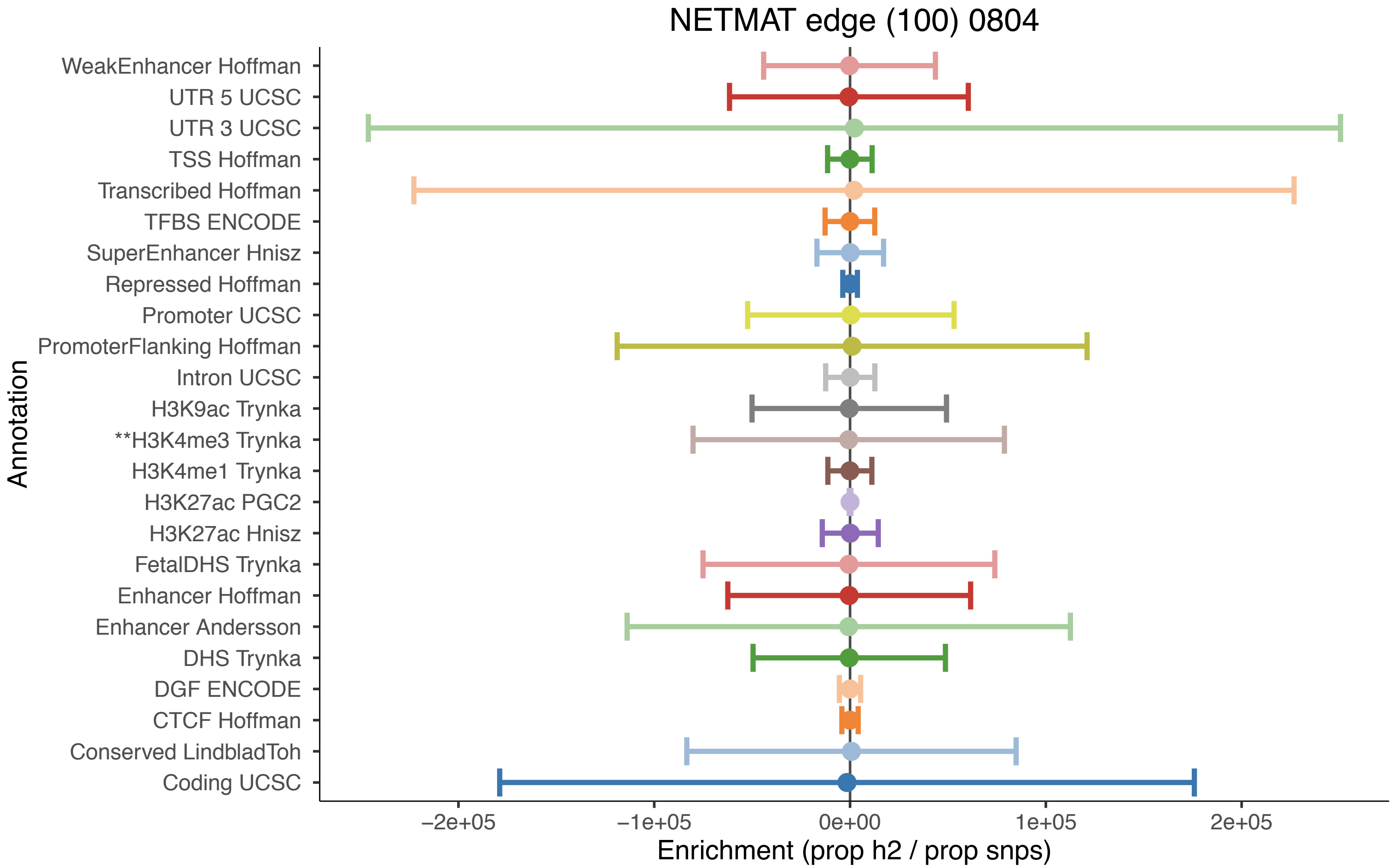
NETMAT edge (100) 0804
Annotation
WeakEnhancer Hoffman
UTR 5 UCSC
UTR 3 UCSC
TSS Hoffman
Transcribed Hoffman
TFBS ENCODE
SuperEnhancer Hnisz
Repressed Hoffman
Promoter UCSC
PromoterFlanking Hoffman
Intron UCSC
H3K9ac Trynka
**H3K4me3 Trynka
H3K4me1 Trynka
H3K27ac PGC2
H3K27ac Hnisz
FetalDHS Trynka
Enhancer Hoffman
Enhancer Andersson
DHS Trynka
DGF ENCODE
CTCF Hoffman
Conserved LindbladToh
Coding UCSC
−2e+05
−1e+05
0e+00
1e+05
2e+05
Enrichment (prop h2 / prop snps)

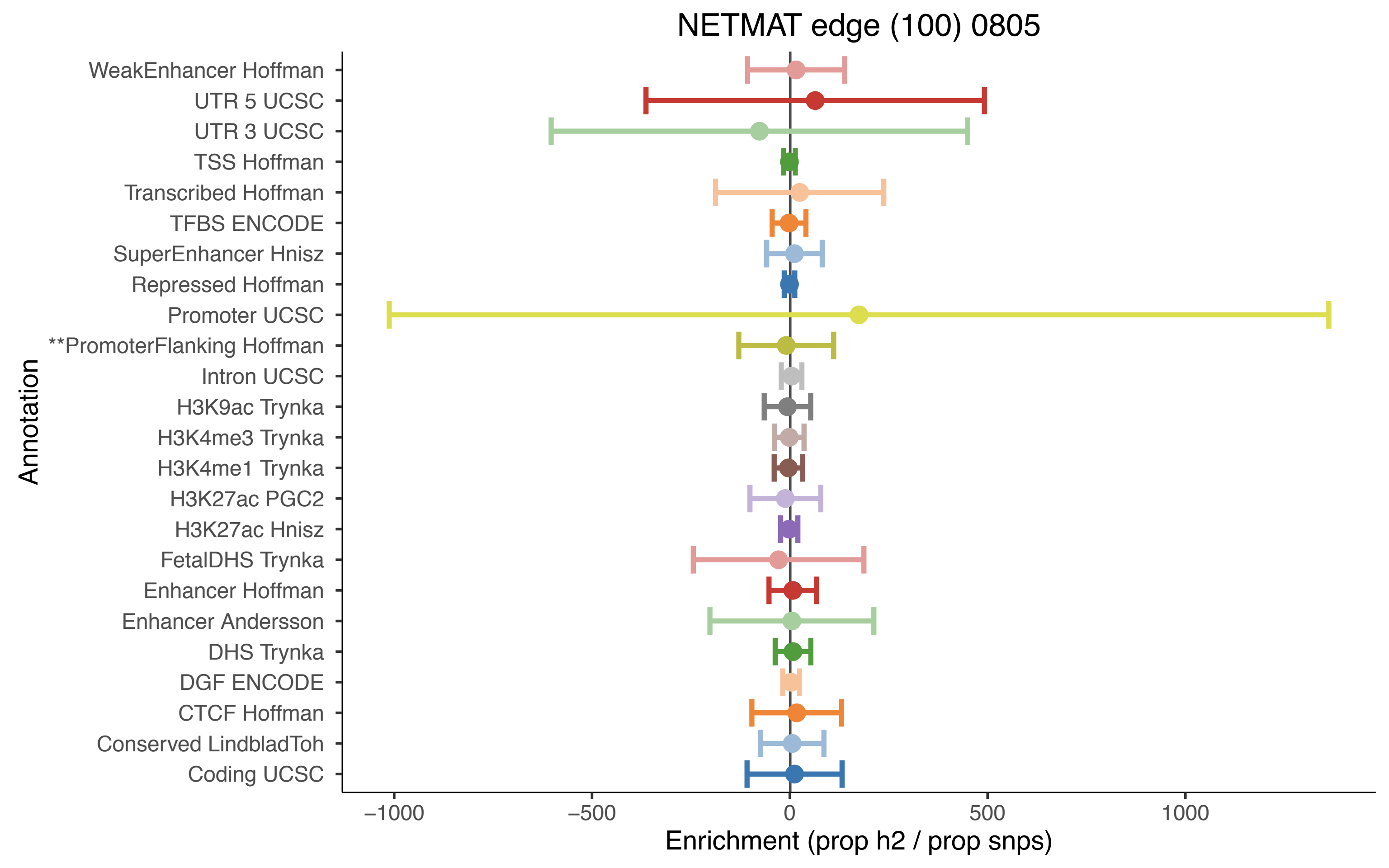
NETMAT edge (100) 0805
WeakEnhancer Hoffman
UTR 5 UCSC
UTR 3 UCSC
TSS Hoffman
Transcribed Hoffman
TFBS ENCODE
SuperEnhancer Hnisz
Repressed Hoffman
Promoter UCSC
**PromoterFlanking Hoffman
Intron UCSC
H3K9ac Trynka
H3K4me3 Trynka
H3K4me1 Trynka
H3K27ac PGC2
H3K27ac Hnisz
FetalDHS Trynka
Enhancer Hoffman
Enhancer Andersson
DHS Trynka
DGF ENCODE
CTCF Hoffman
Conserved LindbladToh
Coding UCSC
Annotation
−1000
−500
0
500
1000
Enrichment (prop h2 / prop snps)

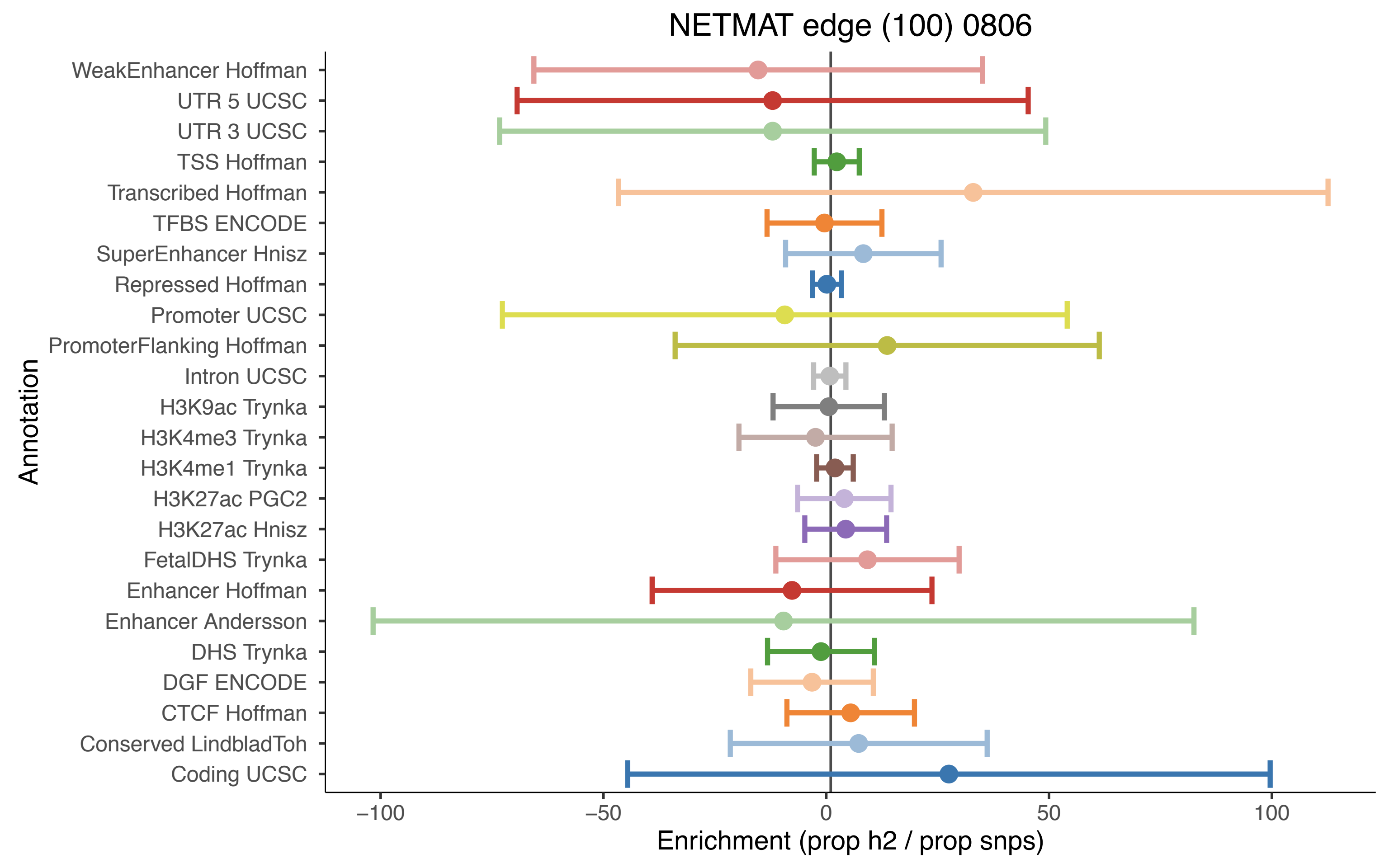
NETMAT edge (100) 0806
WeakEnhancer Hoffman
UTR 5 UCSC
UTR 3 UCSC
TSS Hoffman
Transcribed Hoffman
TFBS ENCODE
SuperEnhancer Hnisz
Repressed Hoffman
Promoter UCSC
PromoterFlanking Hoffman
Intron UCSC
H3K9ac Trynka
H3K4me3 Trynka
H3K4me1 Trynka
H3K27ac PGC2
H3K27ac Hnisz
FetalDHS Trynka
Enhancer Hoffman
Enhancer Andersson
DHS Trynka
DGF ENCODE
CTCF Hoffman
Conserved LindbladToh
Coding UCSC
Annotation
−100
−50
0
50
100
Enrichment (prop h2 / prop snps)

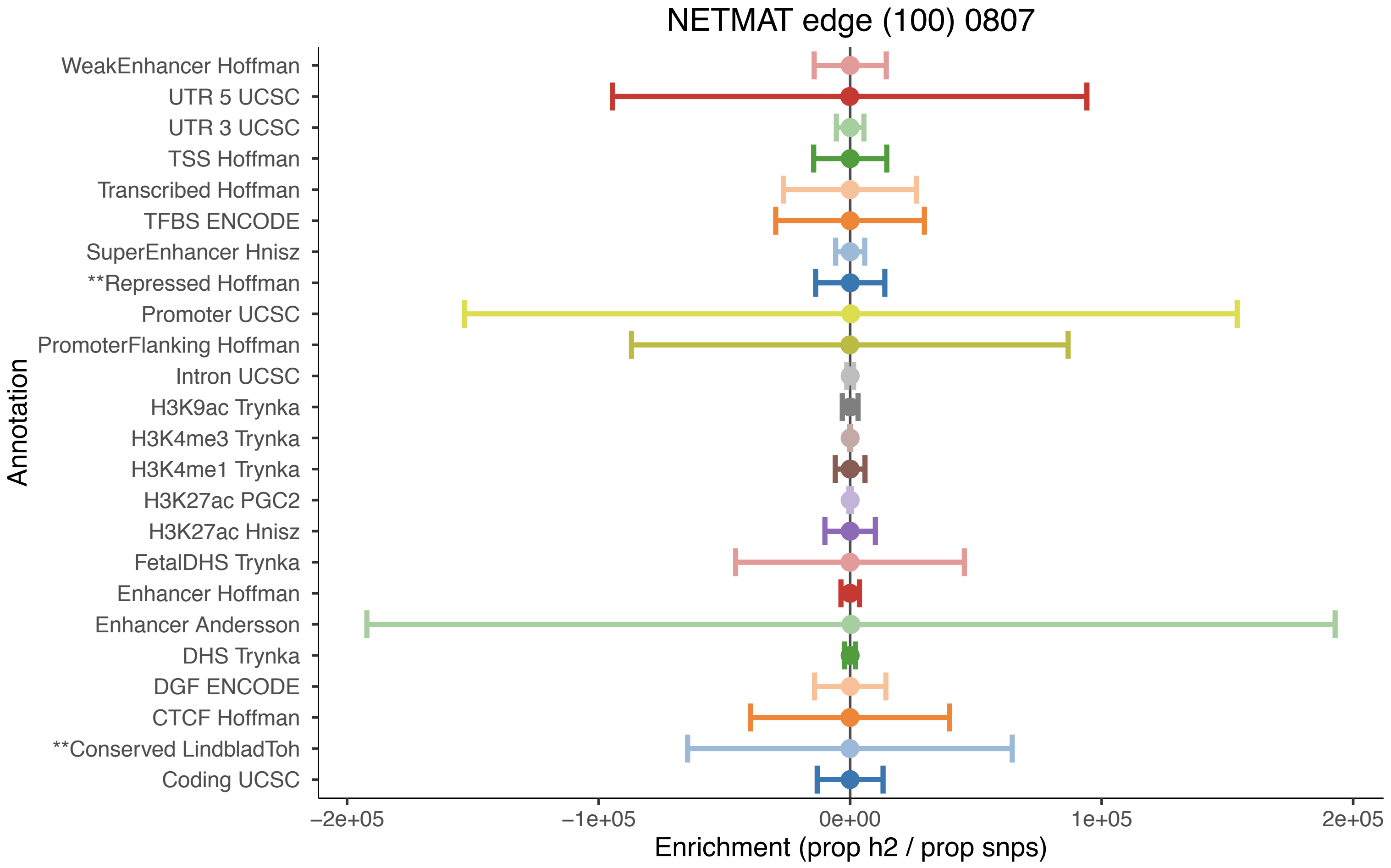
NETMAT edge (100) 0807
WeakEnhancer Hoffman
UTR 5 UCSC
UTR 3 UCSC
TSS Hoffman
Transcribed Hoffman
TFBS ENCODE
SuperEnhancer Hnisz
**Repressed Hoffman
Promoter UCSC
PromoterFlanking Hoffman
Intron UCSC
H3K9ac Trynka
H3K4me3 Trynka
H3K4me1 Trynka
H3K27ac PGC2
H3K27ac Hnisz
FetalDHS Trynka
Enhancer Hoffman
Enhancer Andersson
DHS Trynka
DGF ENCODE
CTCF Hoffman
**Conserved LindbladToh
Coding UCSC
Annotation
−2e+05
−1e+05
0e+00
1e+05
2e+05
Enrichment (prop h2 / prop snps)

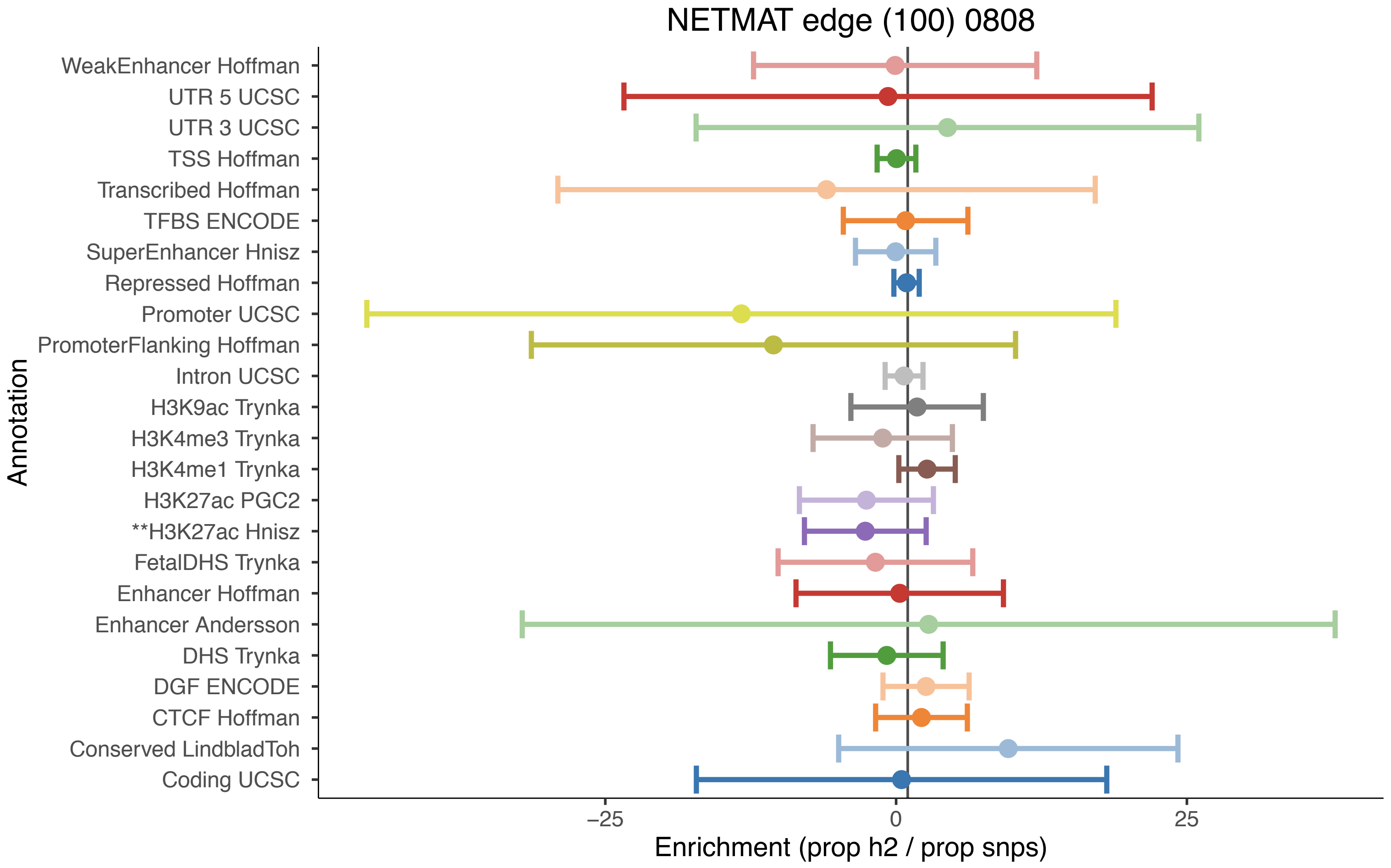
NETMAT edge (100) 0808
WeakEnhancer Hoffman
UTR 5 UCSC
UTR 3 UCSC
TSS Hoffman
Transcribed Hoffman
TFBS ENCODE
SuperEnhancer Hnisz
Repressed Hoffman
Promoter UCSC
PromoterFlanking Hoffman
Intron UCSC
H3K9ac Trynka
H3K4me3 Trynka
H3K4me1 Trynka
H3K27ac PGC2
**H3K27ac Hnisz
FetalDHS Trynka
Enhancer Hoffman
Enhancer Andersson
DHS Trynka
DGF ENCODE
CTCF Hoffman
Conserved LindbladToh
Coding UCSC
Annotation
−25
0
25
Enrichment (prop h2 / prop snps)

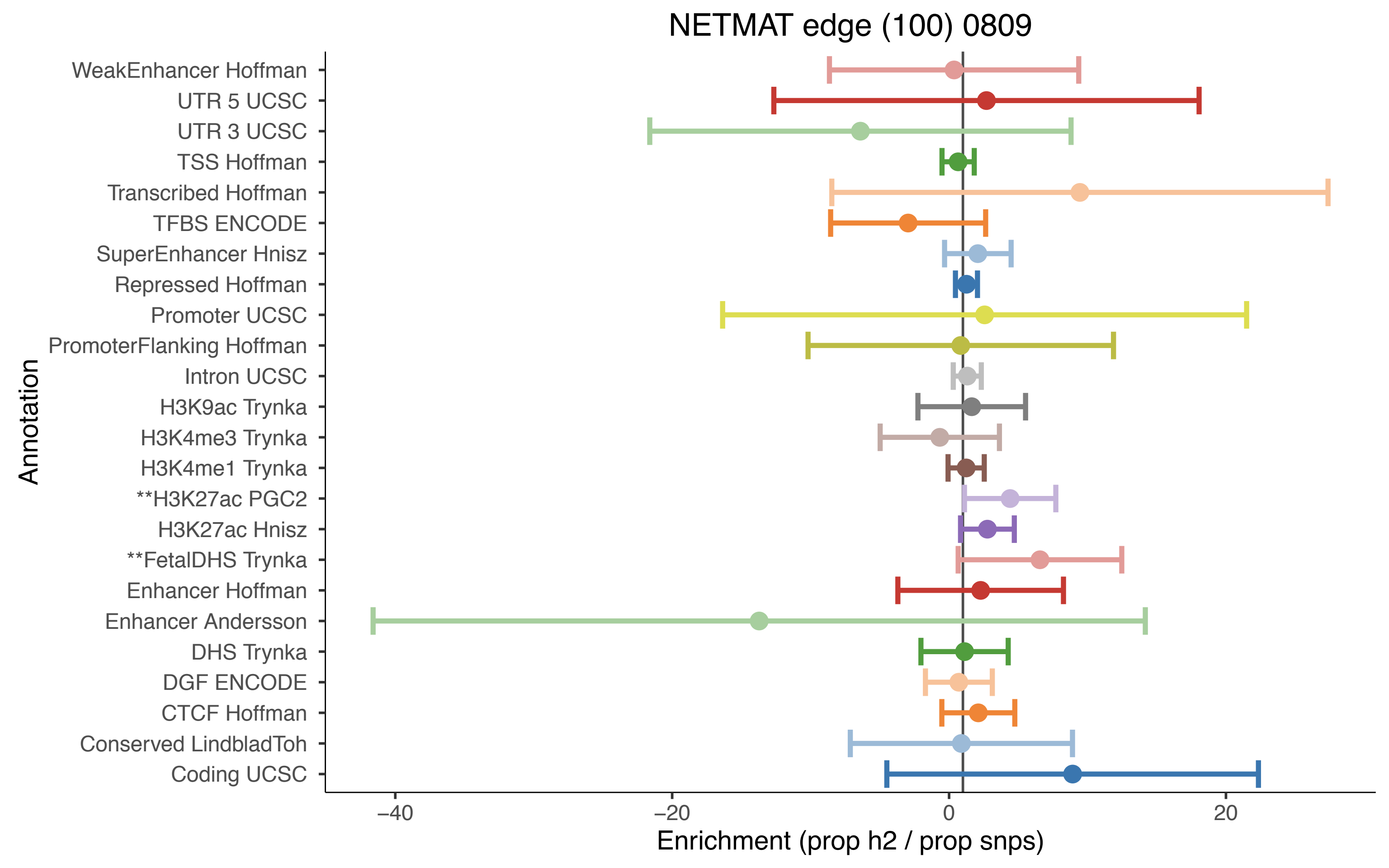
NETMAT edge (100) 0809
WeakEnhancer Hoffman
UTR 5 UCSC
UTR 3 UCSC
TSS Hoffman
Transcribed Hoffman
TFBS ENCODE
SuperEnhancer Hnisz
Repressed Hoffman
Promoter UCSC
PromoterFlanking Hoffman
Intron UCSC
H3K9ac Trynka
H3K4me3 Trynka
H3K4me1 Trynka
**H3K27ac PGC2
H3K27ac Hnisz
**FetalDHS Trynka
Enhancer Hoffman
Enhancer Andersson
DHS Trynka
DGF ENCODE
CTCF Hoffman
Conserved LindbladToh
Coding UCSC
Annotation
−40
−20
0
20
Enrichment (prop h2 / prop snps)

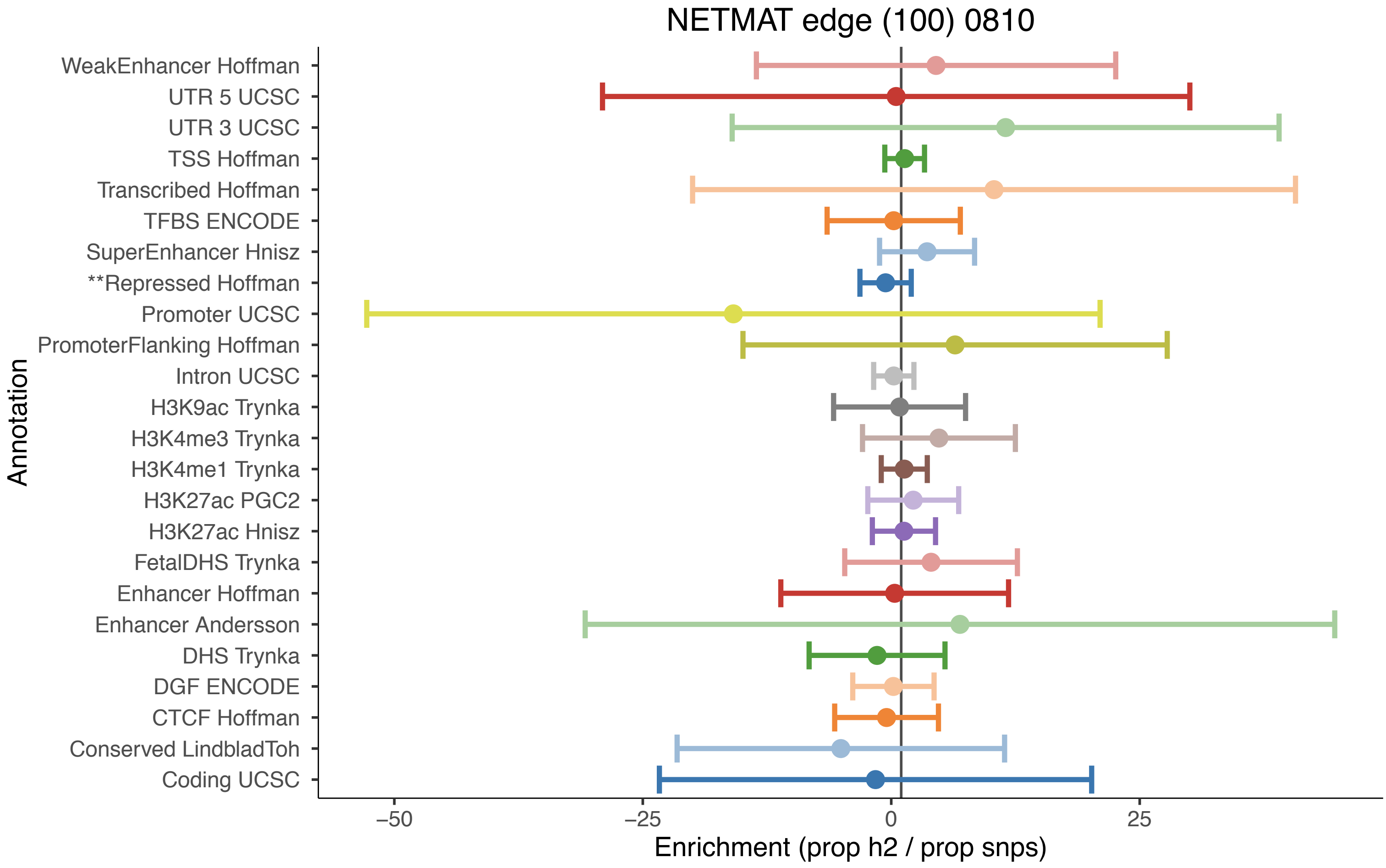
NETMAT edge (100) 0810
Annotation
WeakEnhancer Hoffman
UTR 5 UCSC
UTR 3 UCSC
TSS Hoffman
Transcribed Hoffman
TFBS ENCODE
SuperEnhancer Hnisz
**Repressed Hoffman
Promoter UCSC
PromoterFlanking Hoffman
Intron UCSC
H3K9ac Trynka
H3K4me3 Trynka
H3K4me1 Trynka
H3K27ac PGC2
H3K27ac Hnisz
FetalDHS Trynka
Enhancer Hoffman
Enhancer Andersson
DHS Trynka
DGF ENCODE
CTCF Hoffman
Conserved LindbladToh
Coding UCSC
−50
−25
0
25
Enrichment (prop h2 / prop snps)

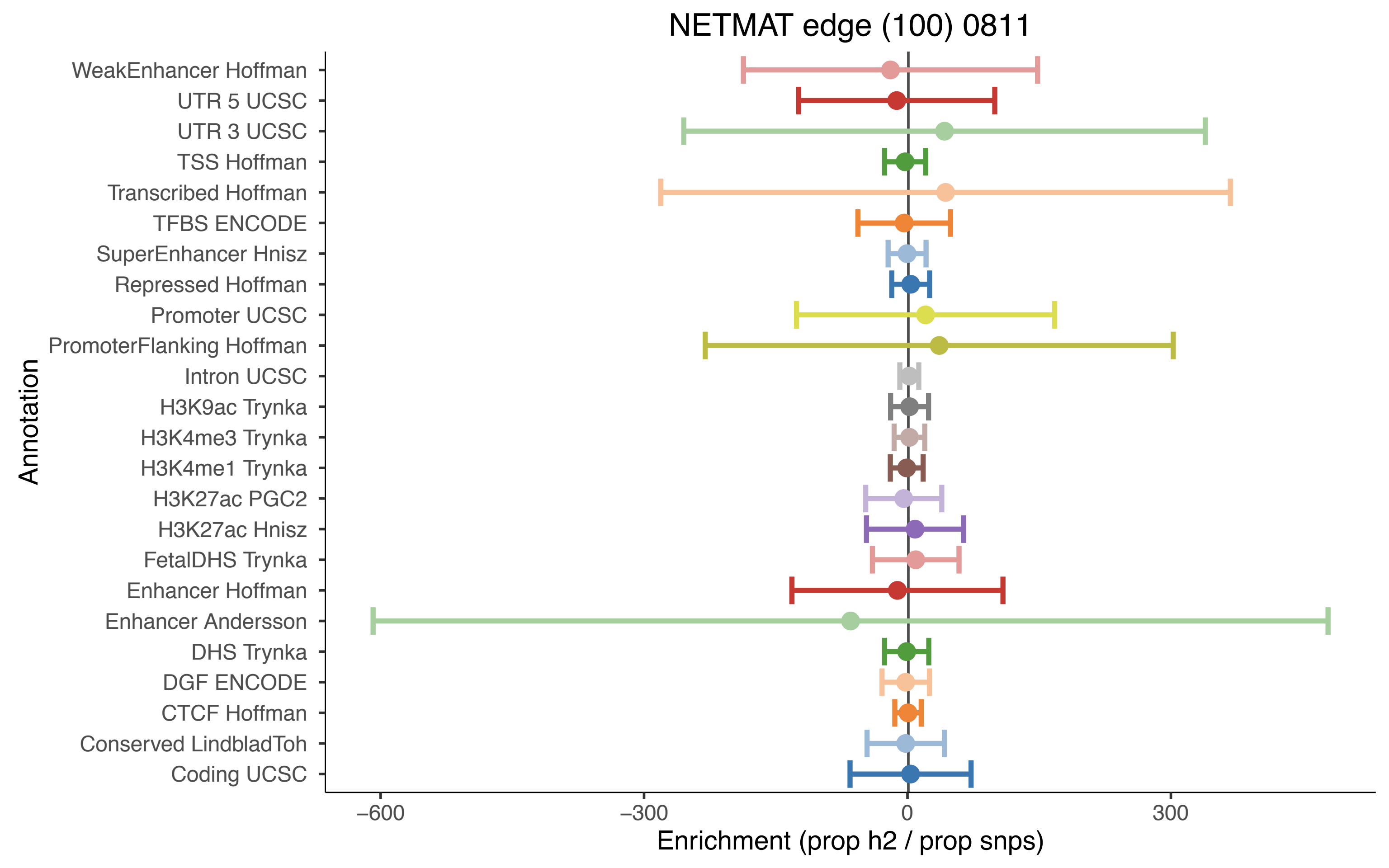
NETMAT edge (100) 0811
Annotation
WeakEnhancer Hoffman
UTR 5 UCSC
UTR 3 UCSC
TSS Hoffman
Transcribed Hoffman
TFBS ENCODE
SuperEnhancer Hnisz
Repressed Hoffman
Promoter UCSC
PromoterFlanking Hoffman
Intron UCSC
H3K9ac Trynka
H3K4me3 Trynka
H3K4me1 Trynka
H3K27ac PGC2
H3K27ac Hnisz
FetalDHS Trynka
Enhancer Hoffman
Enhancer Andersson
DHS Trynka
DGF ENCODE
CTCF Hoffman
Conserved LindbladToh
Coding UCSC
−600
−300
0
300
Enrichment (prop h2 / prop snps)

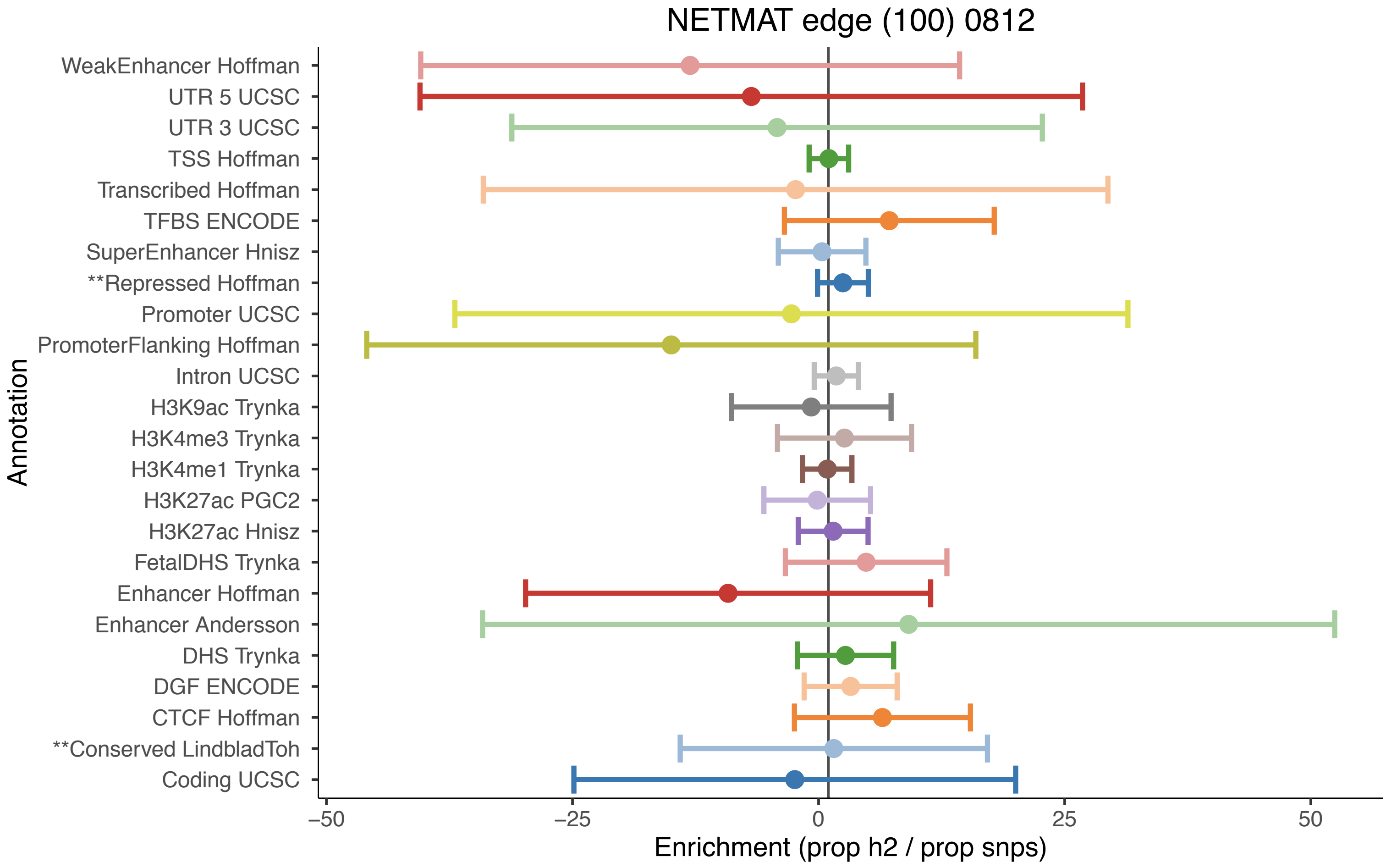
NETMAT edge (100) 0812
WeakEnhancer Hoffman
UTR 5 UCSC
UTR 3 UCSC
TSS Hoffman
Transcribed Hoffman
TFBS ENCODE
SuperEnhancer Hnisz
**Repressed Hoffman
Promoter UCSC
PromoterFlanking Hoffman
Intron UCSC
H3K9ac Trynka
H3K4me3 Trynka
H3K4me1 Trynka
H3K27ac PGC2
H3K27ac Hnisz
FetalDHS Trynka
Enhancer Hoffman
Enhancer Andersson
DHS Trynka
DGF ENCODE
CTCF Hoffman
**Conserved LindbladToh
Coding UCSC
Annotation
−50
−25
0
25
50
Enrichment (prop h2 / prop snps)

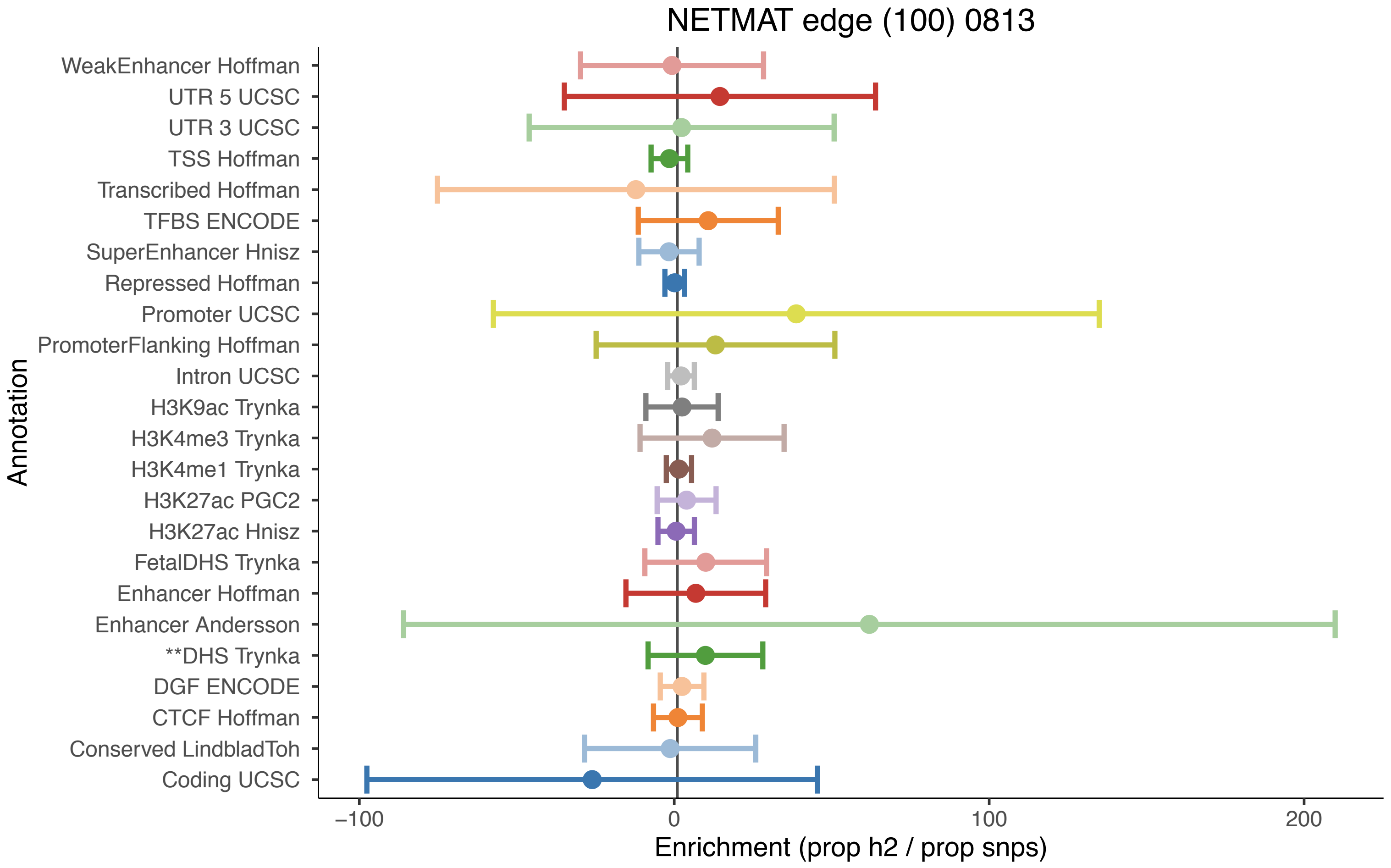
NETMAT edge (100) 0813
Annotation
WeakEnhancer Hoffman
UTR 5 UCSC
UTR 3 UCSC
TSS Hoffman
Transcribed Hoffman
TFBS ENCODE
SuperEnhancer Hnisz
Repressed Hoffman
Promoter UCSC
PromoterFlanking Hoffman
Intron UCSC
H3K9ac Trynka
H3K4me3 Trynka
H3K4me1 Trynka
H3K27ac PGC2
H3K27ac Hnisz
FetalDHS Trynka
Enhancer Hoffman
Enhancer Andersson
**DHS Trynka
DGF ENCODE
CTCF Hoffman
Conserved LindbladToh
Coding UCSC
−100
0
100
200
Enrichment (prop h2 / prop snps)

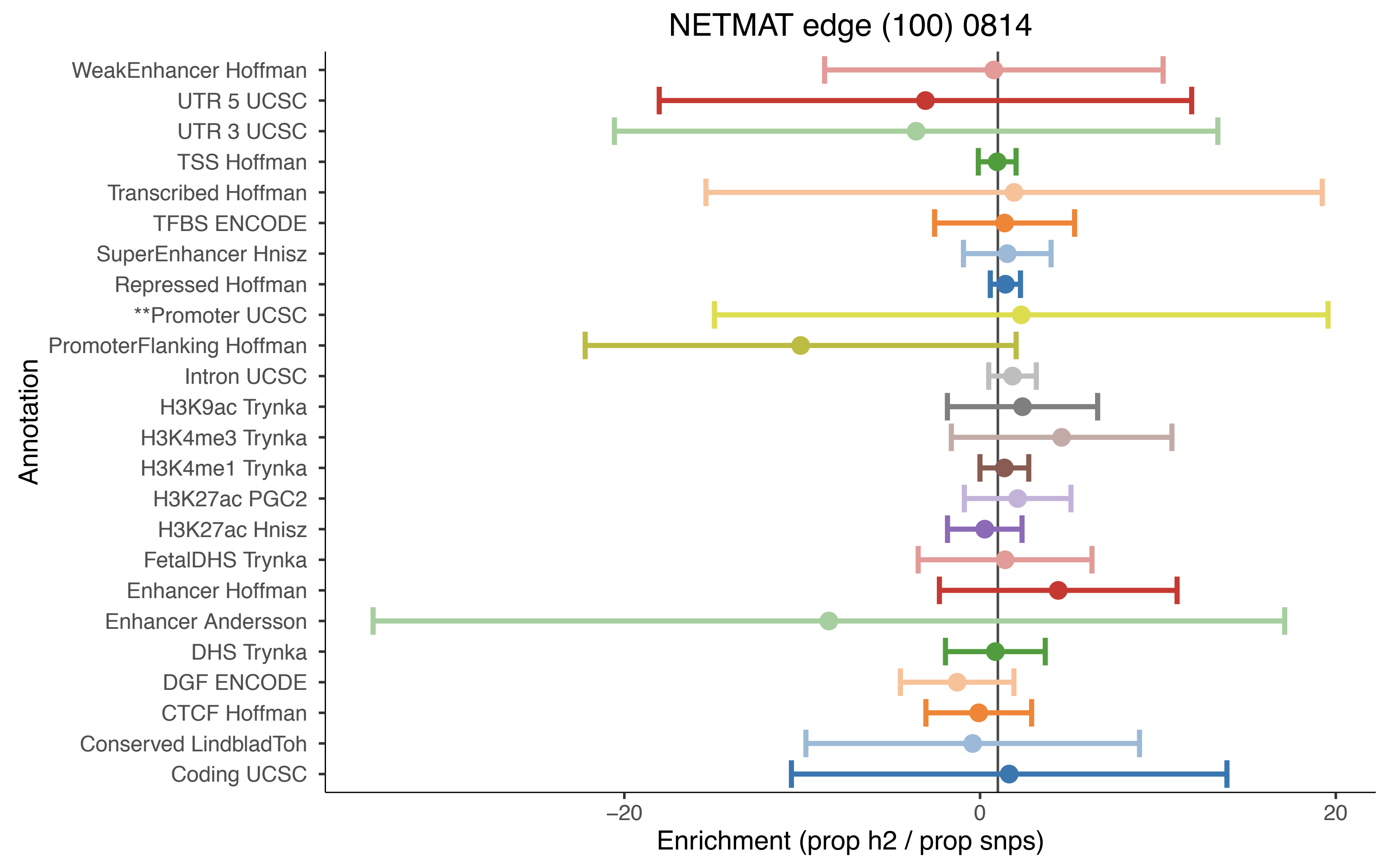
NETMAT edge (100) 0814
WeakEnhancer Hoffman
UTR 5 UCSC
UTR 3 UCSC
TSS Hoffman
Transcribed Hoffman
TFBS ENCODE
SuperEnhancer Hnisz
Repressed Hoffman
**Promoter UCSC
PromoterFlanking Hoffman
Intron UCSC
H3K9ac Trynka
H3K4me3 Trynka
H3K4me1 Trynka
H3K27ac PGC2
H3K27ac Hnisz
FetalDHS Trynka
Enhancer Hoffman
Enhancer Andersson
DHS Trynka
DGF ENCODE
CTCF Hoffman
Conserved LindbladToh
Coding UCSC
Annotation
−20
0
20
Enrichment (prop h2 / prop snps)

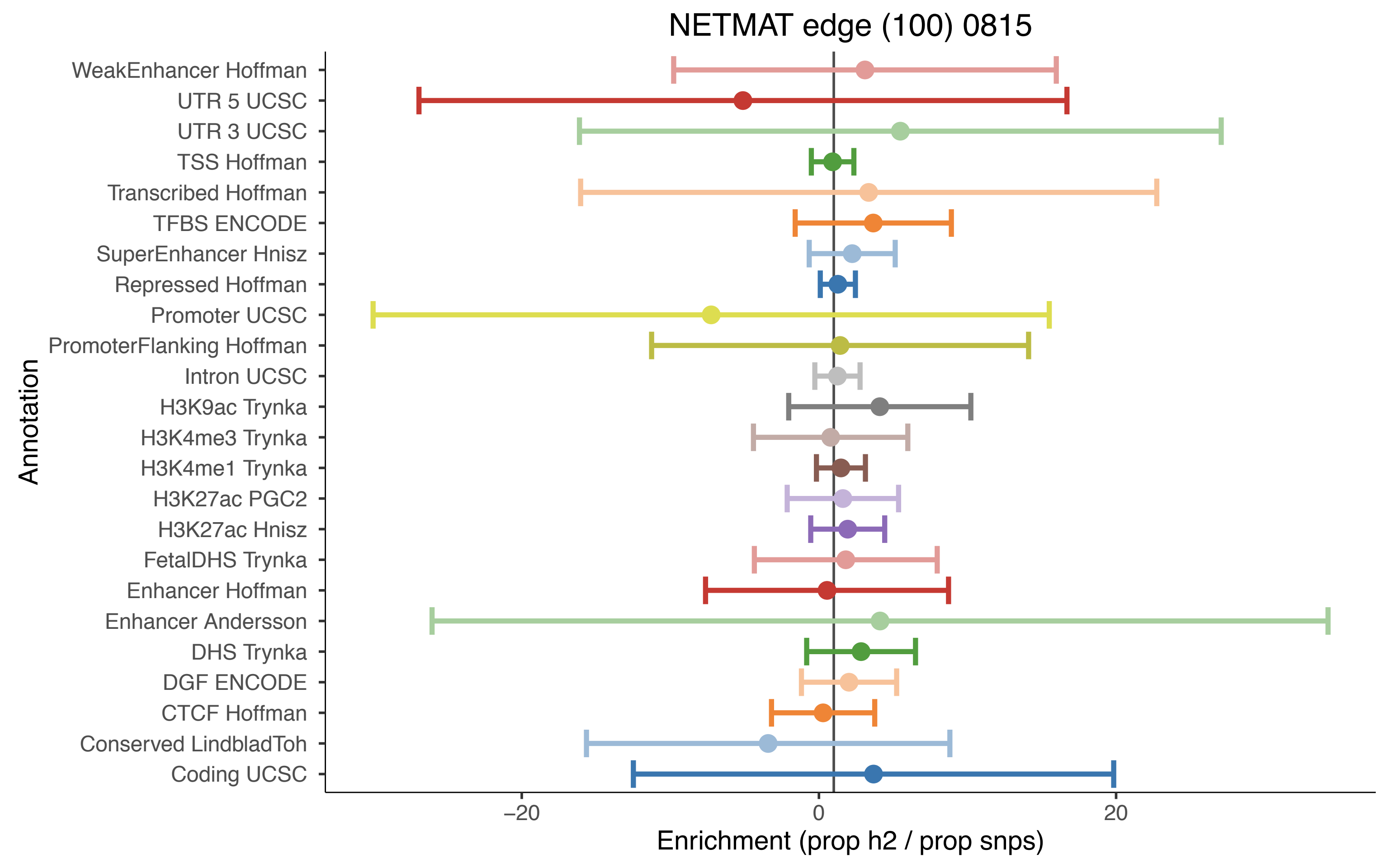
NETMAT edge (100) 0815
WeakEnhancer Hoffman
UTR 5 UCSC
UTR 3 UCSC
TSS Hoffman
Transcribed Hoffman
TFBS ENCODE
SuperEnhancer Hnisz
Repressed Hoffman
Promoter UCSC
PromoterFlanking Hoffman
Intron UCSC
H3K9ac Trynka
H3K4me3 Trynka
H3K4me1 Trynka
H3K27ac PGC2
H3K27ac Hnisz
FetalDHS Trynka
Enhancer Hoffman
Enhancer Andersson
DHS Trynka
DGF ENCODE
CTCF Hoffman
Conserved LindbladToh
Coding UCSC
Annotation
−20
0
20
Enrichment (prop h2 / prop snps)

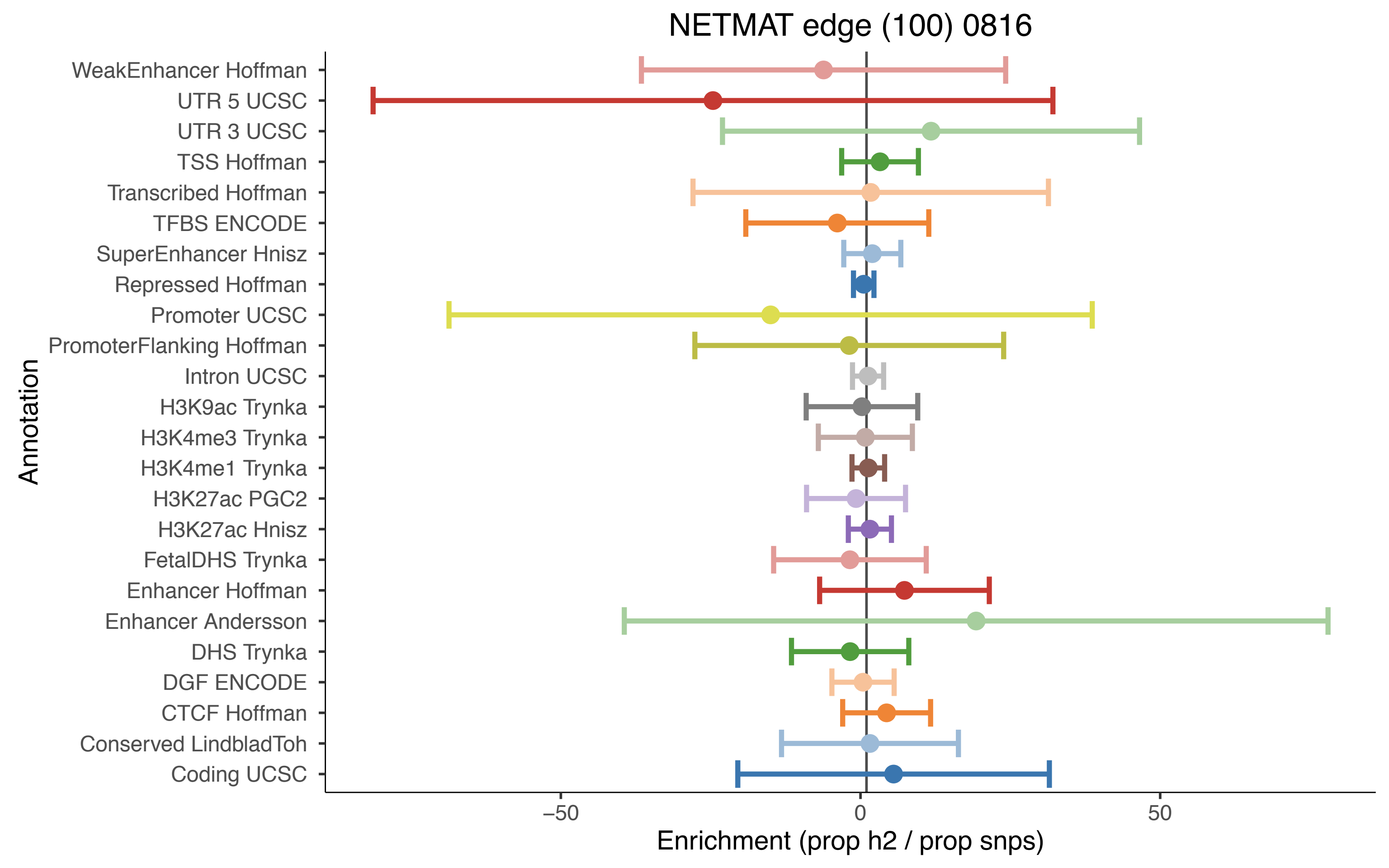
NETMAT edge (100) 0816
WeakEnhancer Hoffman
UTR 5 UCSC
UTR 3 UCSC
TSS Hoffman
Transcribed Hoffman
TFBS ENCODE
SuperEnhancer Hnisz
Repressed Hoffman
Promoter UCSC
PromoterFlanking Hoffman
Intron UCSC
H3K9ac Trynka
H3K4me3 Trynka
H3K4me1 Trynka
H3K27ac PGC2
H3K27ac Hnisz
FetalDHS Trynka
Enhancer Hoffman
Enhancer Andersson
DHS Trynka
DGF ENCODE
CTCF Hoffman
Conserved LindbladToh
Coding UCSC
Annotation
−50
0
50
Enrichment (prop h2 / prop snps)

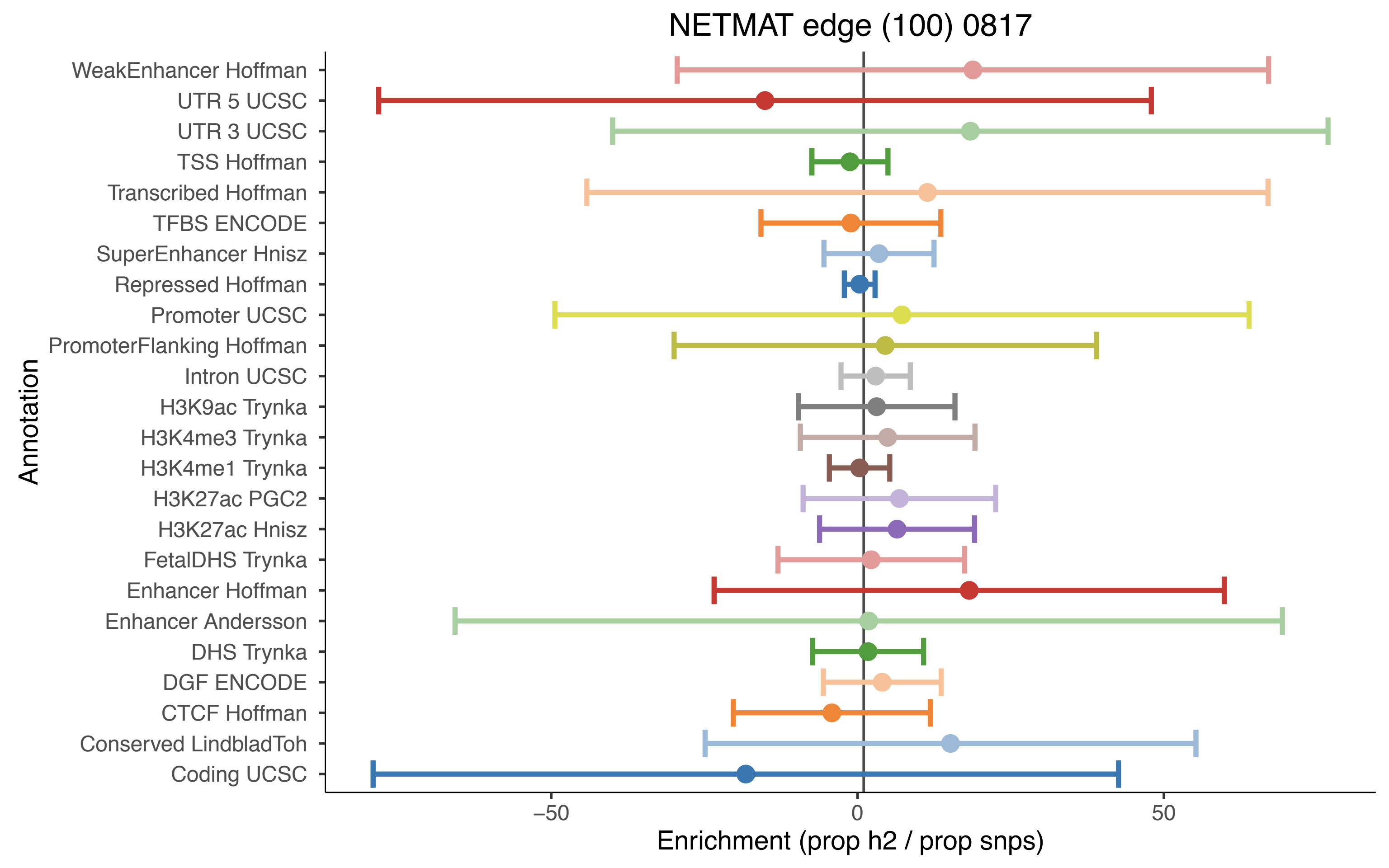
NETMAT edge (100) 0817
WeakEnhancer Hoffman
UTR 5 UCSC
UTR 3 UCSC
TSS Hoffman
Transcribed Hoffman
TFBS ENCODE
SuperEnhancer Hnisz
Repressed Hoffman
Promoter UCSC
PromoterFlanking Hoffman
Intron UCSC
H3K9ac Trynka
H3K4me3 Trynka
H3K4me1 Trynka
H3K27ac PGC2
H3K27ac Hnisz
FetalDHS Trynka
Enhancer Hoffman
Enhancer Andersson
DHS Trynka
DGF ENCODE
CTCF Hoffman
Conserved LindbladToh
Coding UCSC
Annotation
−50
0
50
Enrichment (prop h2 / prop snps)

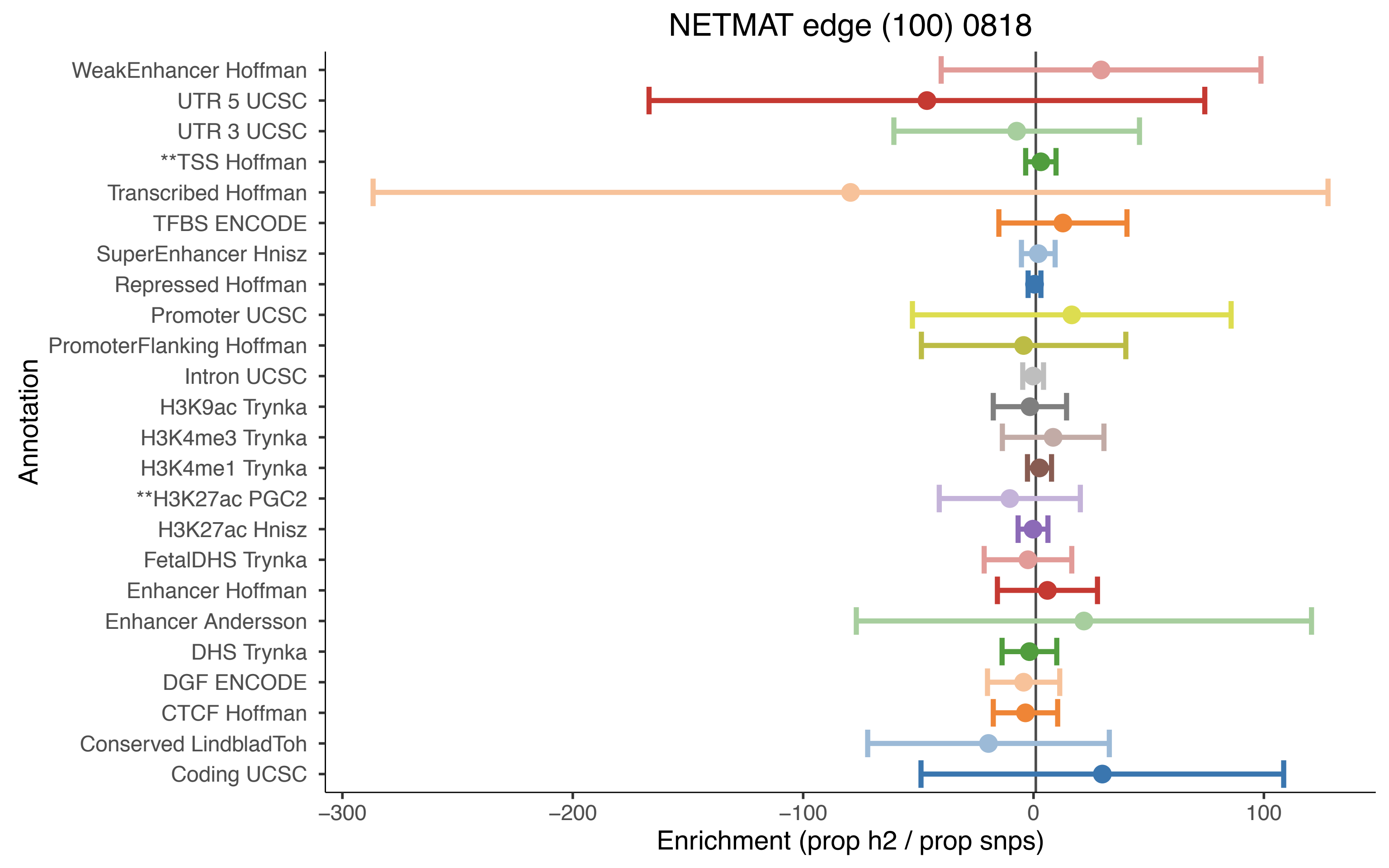

NETMAT edge (100) 0818
WeakEnhancer Hoffman
UTR 5 UCSC
UTR 3 UCSC
**TSS Hoffman
Transcribed Hoffman
TFBS ENCODE
SuperEnhancer Hnisz
Repressed Hoffman
Promoter UCSC
PromoterFlanking Hoffman
Intron UCSC
H3K9ac Trynka
H3K4me3 Trynka
H3K4me1 Trynka
**H3K27ac PGC2
H3K27ac Hnisz
FetalDHS Trynka
Enhancer Hoffman
Enhancer Andersson
DHS Trynka
DGF ENCODE
CTCF Hoffman
Conserved LindbladToh
Coding UCSC
Annotation
−300
−200
−100
0
100
Enrichment (prop h2 / prop snps)

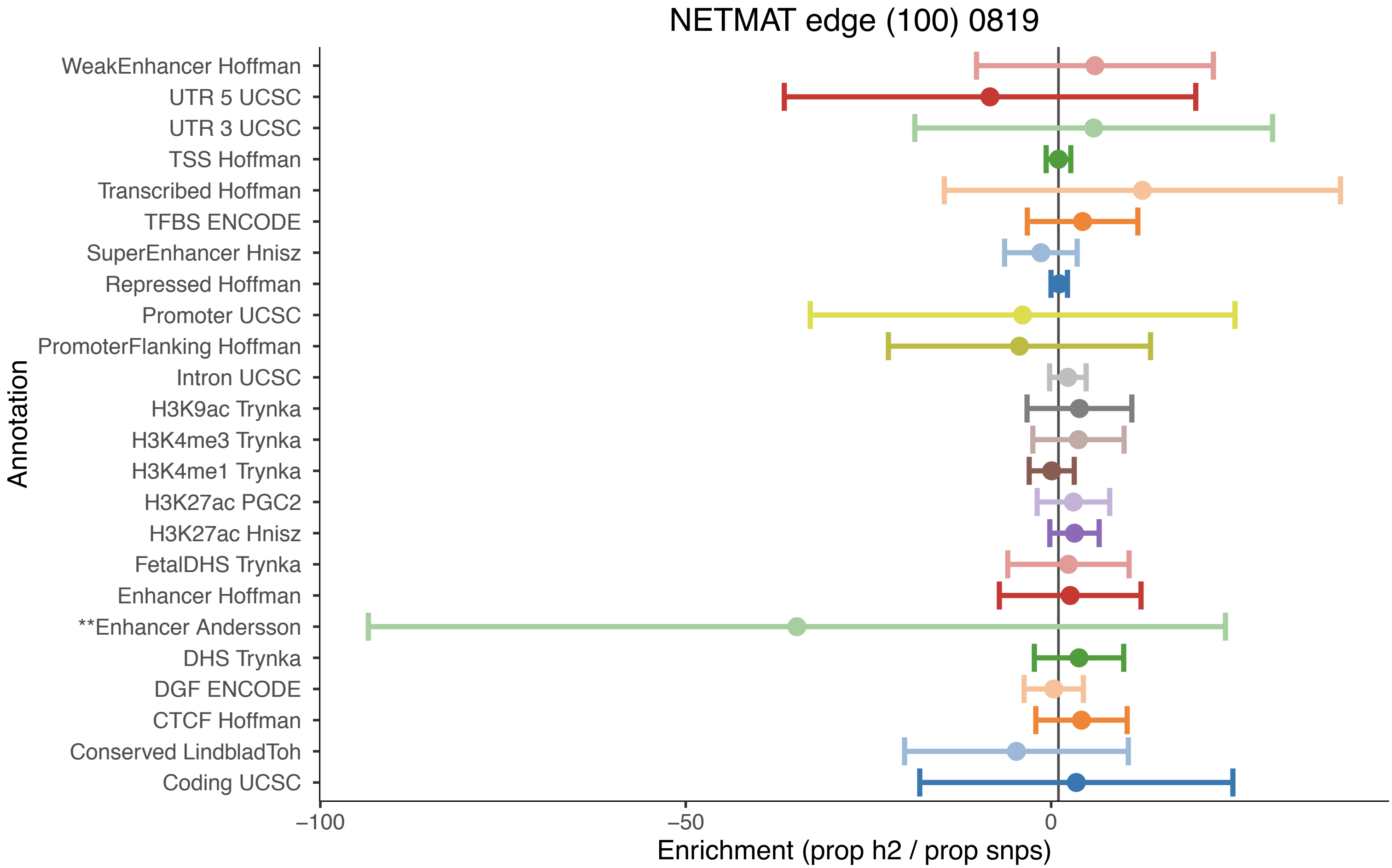

NETMAT edge (100) 0819
WeakEnhancer Hoffman
UTR 5 UCSC
UTR 3 UCSC
TSS Hoffman
Transcribed Hoffman
TFBS ENCODE
SuperEnhancer Hnisz
Repressed Hoffman
Promoter UCSC
PromoterFlanking Hoffman
Intron UCSC
H3K9ac Trynka
H3K4me3 Trynka
H3K4me1 Trynka
H3K27ac PGC2
H3K27ac Hnisz
FetalDHS Trynka
Enhancer Hoffman
**Enhancer Andersson
DHS Trynka
DGF ENCODE
CTCF Hoffman
Conserved LindbladToh
Coding UCSC
Annotation
−100
−50
0
Enrichment (prop h2 / prop snps)

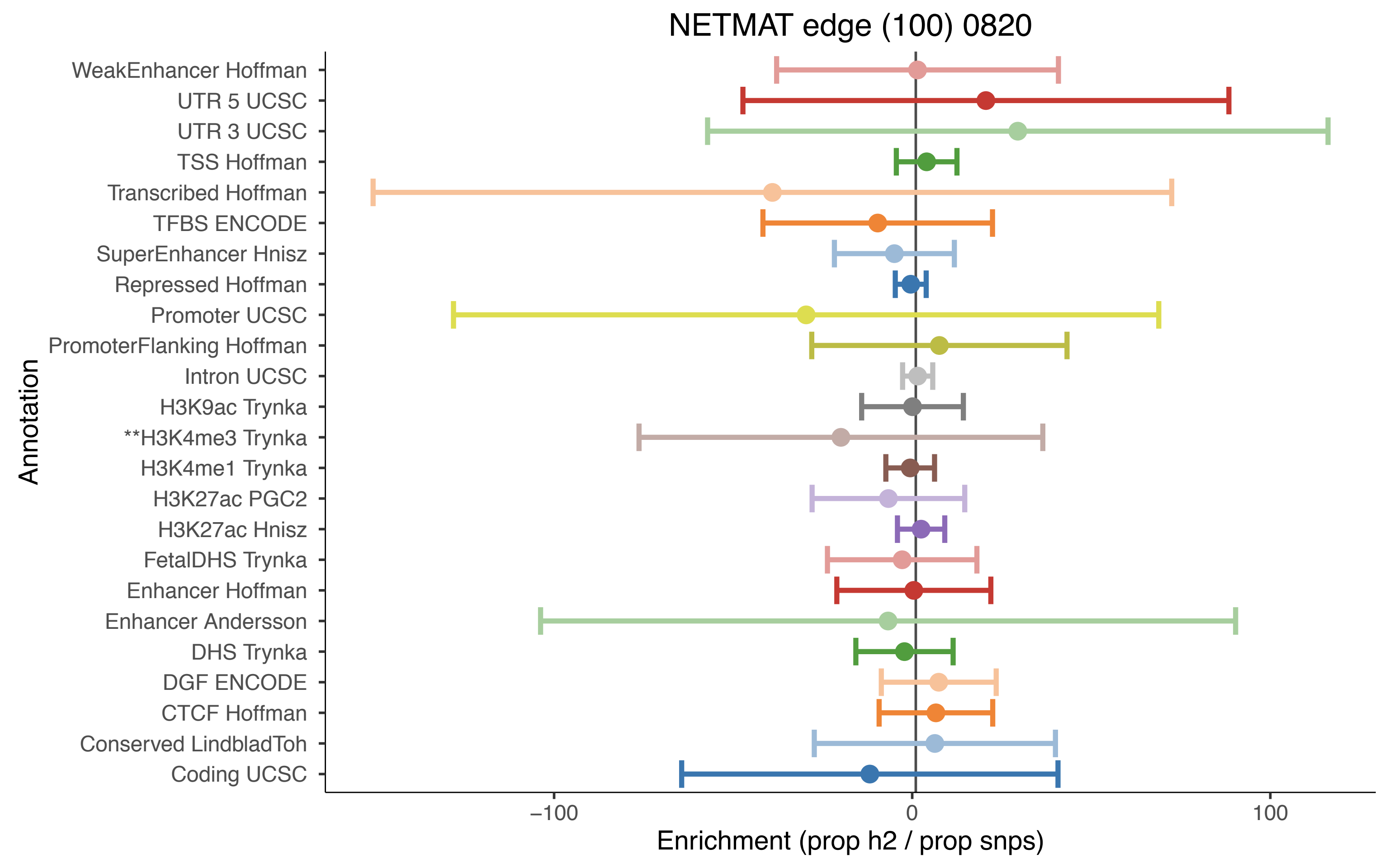
NETMAT edge (100) 0820
WeakEnhancer Hoffman
UTR 5 UCSC
UTR 3 UCSC
TSS Hoffman
Transcribed Hoffman
TFBS ENCODE
SuperEnhancer Hnisz
Repressed Hoffman
Promoter UCSC
PromoterFlanking Hoffman
Intron UCSC
H3K9ac Trynka
**H3K4me3 Trynka
H3K4me1 Trynka
H3K27ac PGC2
H3K27ac Hnisz
FetalDHS Trynka
Enhancer Hoffman
Enhancer Andersson
DHS Trynka
DGF ENCODE
CTCF Hoffman
Conserved LindbladToh
Coding UCSC
Annotation
−100
0
100
Enrichment (prop h2 / prop snps)

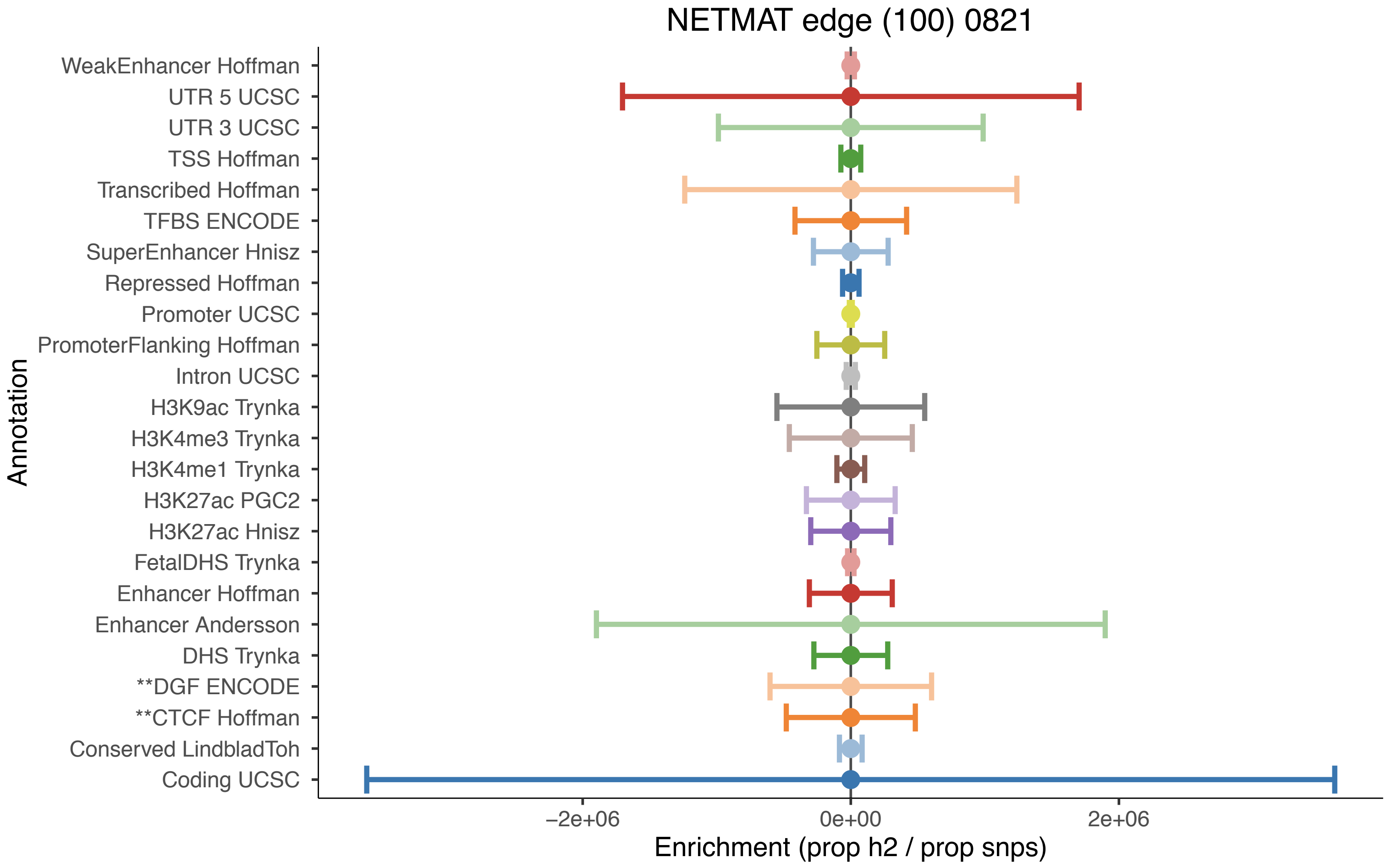

NETMAT edge (100) 0821
WeakEnhancer Hoffman
UTR 5 UCSC
UTR 3 UCSC
TSS Hoffman
Transcribed Hoffman
TFBS ENCODE
SuperEnhancer Hnisz
Repressed Hoffman
Promoter UCSC
PromoterFlanking Hoffman
Intron UCSC
H3K9ac Trynka
H3K4me3 Trynka
H3K4me1 Trynka
H3K27ac PGC2
H3K27ac Hnisz
FetalDHS Trynka
Enhancer Hoffman
Enhancer Andersson
DHS Trynka
**DGF ENCODE
**CTCF Hoffman
Conserved LindbladToh
Coding UCSC
Annotation
−2e+06
0e+00
2e+06
Enrichment (prop h2 / prop snps)

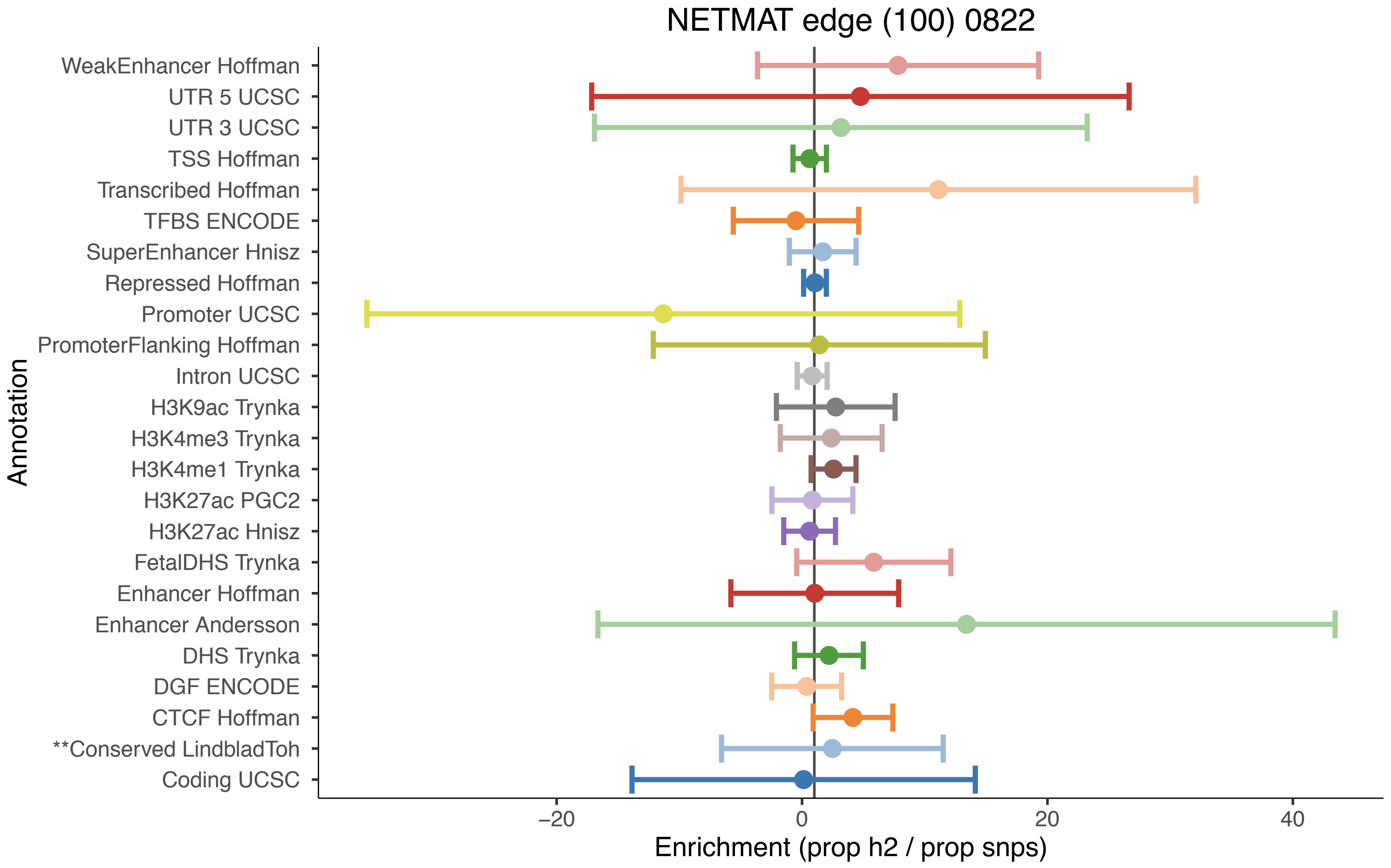
NETMAT edge (100) 0822
WeakEnhancer Hoffman
UTR 5 UCSC
UTR 3 UCSC
TSS Hoffman
Transcribed Hoffman
TFBS ENCODE
SuperEnhancer Hnisz
Repressed Hoffman
Promoter UCSC
PromoterFlanking Hoffman
Intron UCSC
H3K9ac Trynka
H3K4me3 Trynka
H3K4me1 Trynka
H3K27ac PGC2
H3K27ac Hnisz
FetalDHS Trynka
Enhancer Hoffman
Enhancer Andersson
DHS Trynka
DGF ENCODE
CTCF Hoffman
**Conserved LindbladToh
Coding UCSC
Annotation
−20
0
20
40
Enrichment (prop h2 / prop snps)

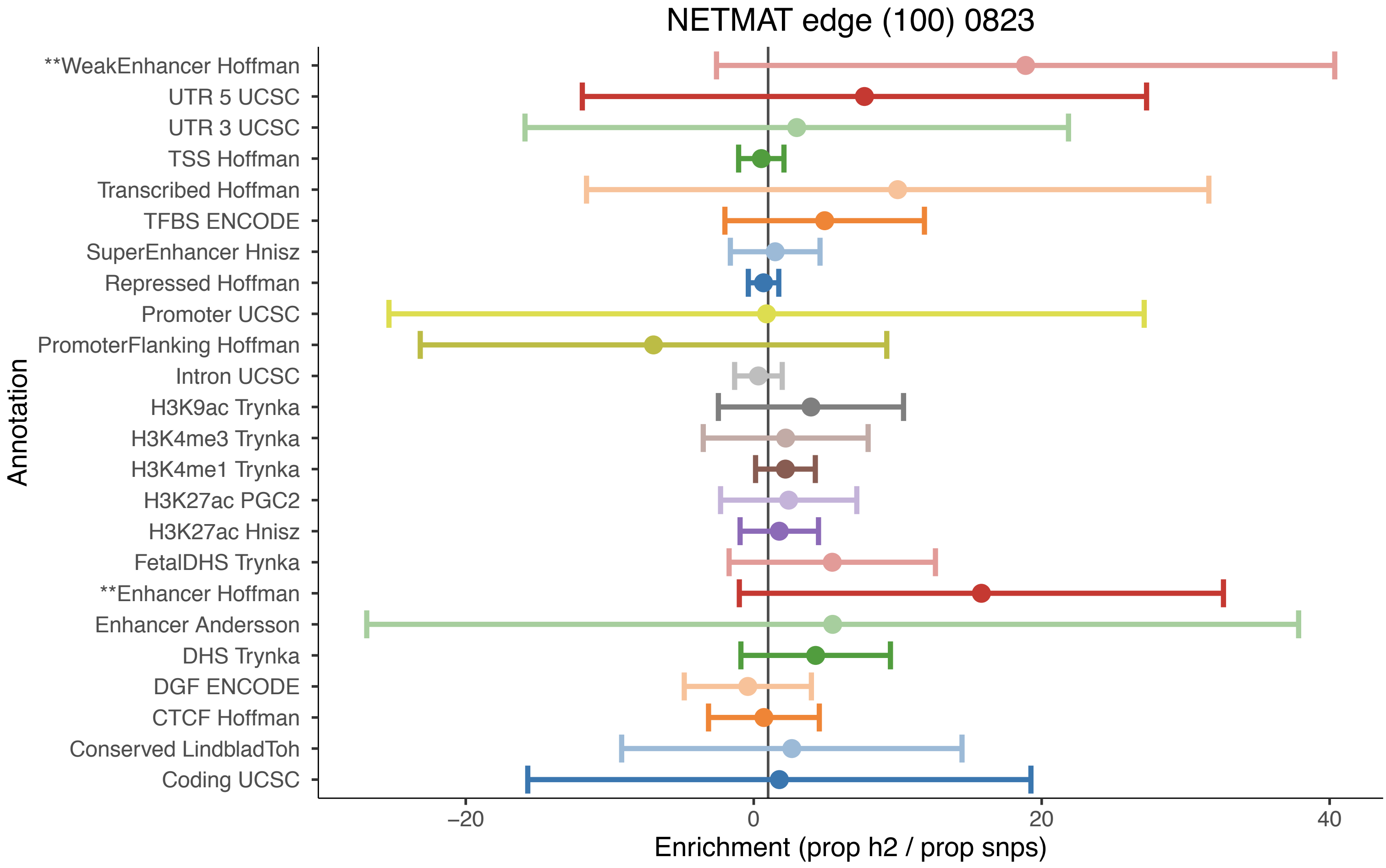
NETMAT edge (100) 0823
**WeakEnhancer Hoffman
UTR 5 UCSC
UTR 3 UCSC
TSS Hoffman
Transcribed Hoffman
TFBS ENCODE
SuperEnhancer Hnisz
Repressed Hoffman
Promoter UCSC
PromoterFlanking Hoffman
Intron UCSC
H3K9ac Trynka
H3K4me3 Trynka
H3K4me1 Trynka
H3K27ac PGC2
H3K27ac Hnisz
FetalDHS Trynka
**Enhancer Hoffman
Enhancer Andersson
DHS Trynka
DGF ENCODE
CTCF Hoffman
Conserved LindbladToh
Coding UCSC
Annotation
−20
0
20
40
Enrichment (prop h2 / prop snps)

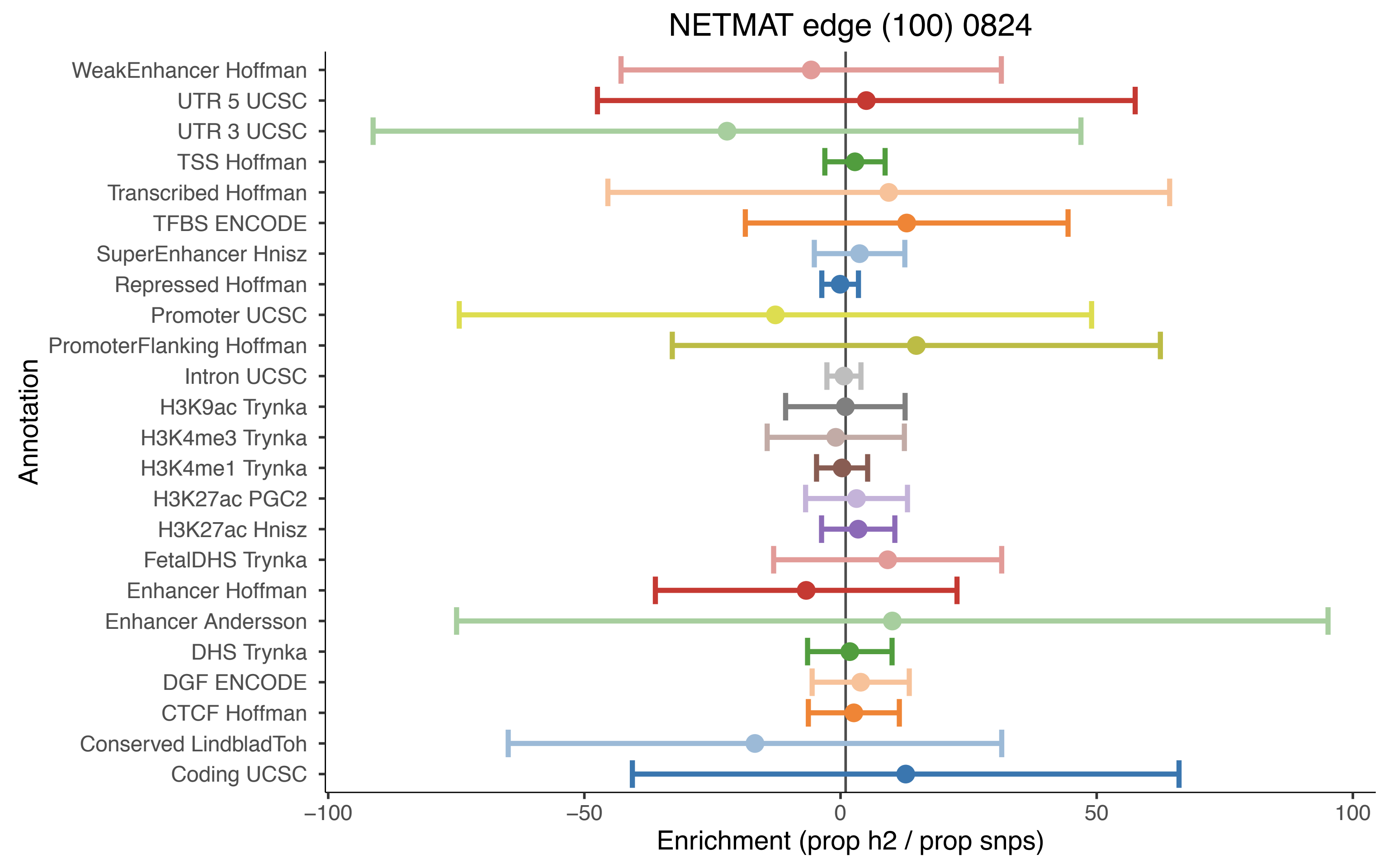

NETMAT edge (100) 0824
Annotation
WeakEnhancer Hoffman
UTR 5 UCSC
UTR 3 UCSC
TSS Hoffman
Transcribed Hoffman
TFBS ENCODE
SuperEnhancer Hnisz
Repressed Hoffman
Promoter UCSC
PromoterFlanking Hoffman
Intron UCSC
H3K9ac Trynka
H3K4me3 Trynka
H3K4me1 Trynka
H3K27ac PGC2
H3K27ac Hnisz
FetalDHS Trynka
Enhancer Hoffman
Enhancer Andersson
DHS Trynka
DGF ENCODE
CTCF Hoffman
Conserved LindbladToh
Coding UCSC
−100
−50
0
50
100
Enrichment (prop h2 / prop snps)

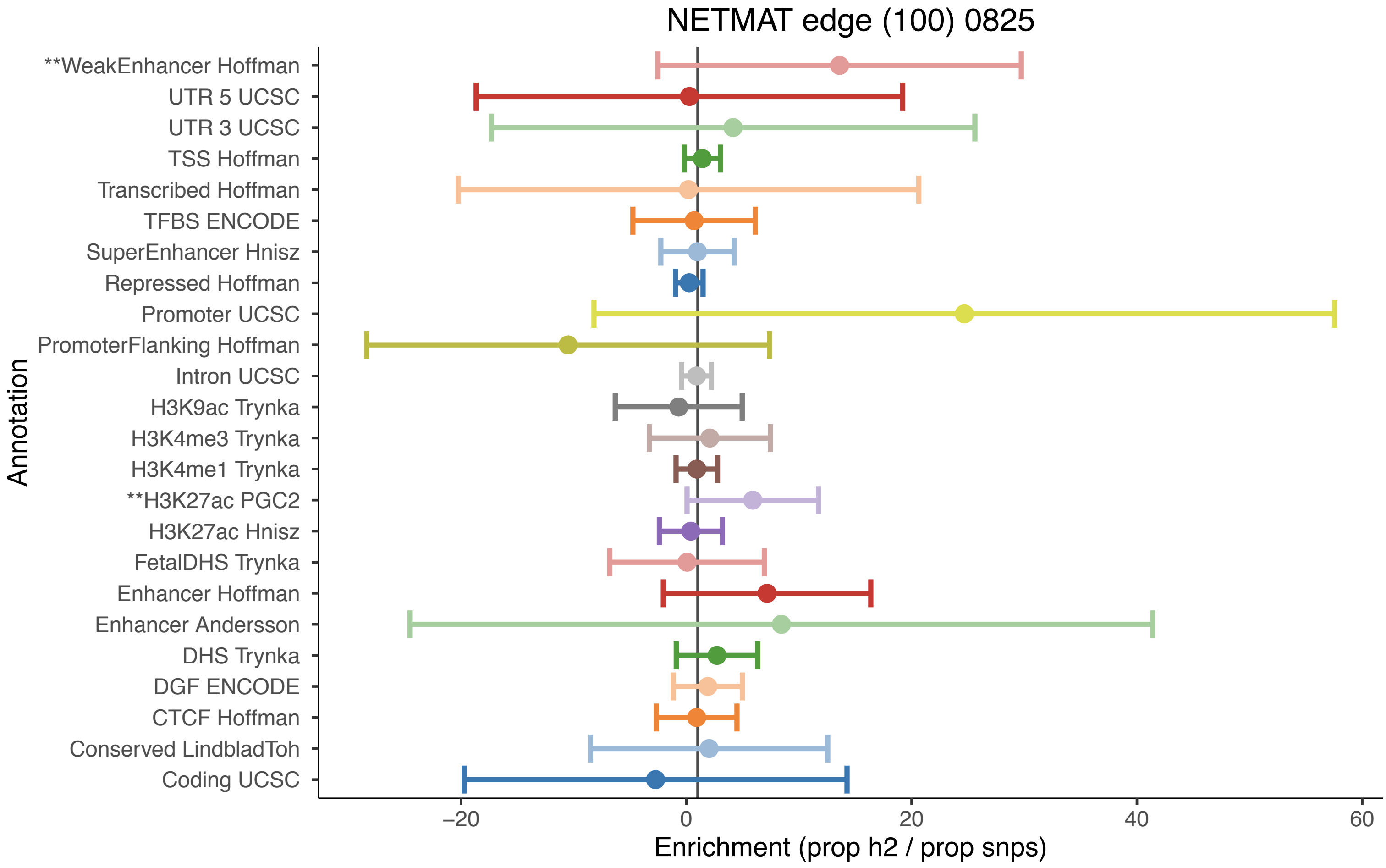

NETMAT edge (100) 0825
**WeakEnhancer Hoffman
UTR 5 UCSC
UTR 3 UCSC
TSS Hoffman
Transcribed Hoffman
TFBS ENCODE
SuperEnhancer Hnisz
Repressed Hoffman
Promoter UCSC
PromoterFlanking Hoffman
Intron UCSC
H3K9ac Trynka
H3K4me3 Trynka
H3K4me1 Trynka
**H3K27ac PGC2
H3K27ac Hnisz
FetalDHS Trynka
Enhancer Hoffman
Enhancer Andersson
DHS Trynka
DGF ENCODE
CTCF Hoffman
Conserved LindbladToh
Coding UCSC
Annotation
−20
0
20
40
60
Enrichment (prop h2 / prop snps)

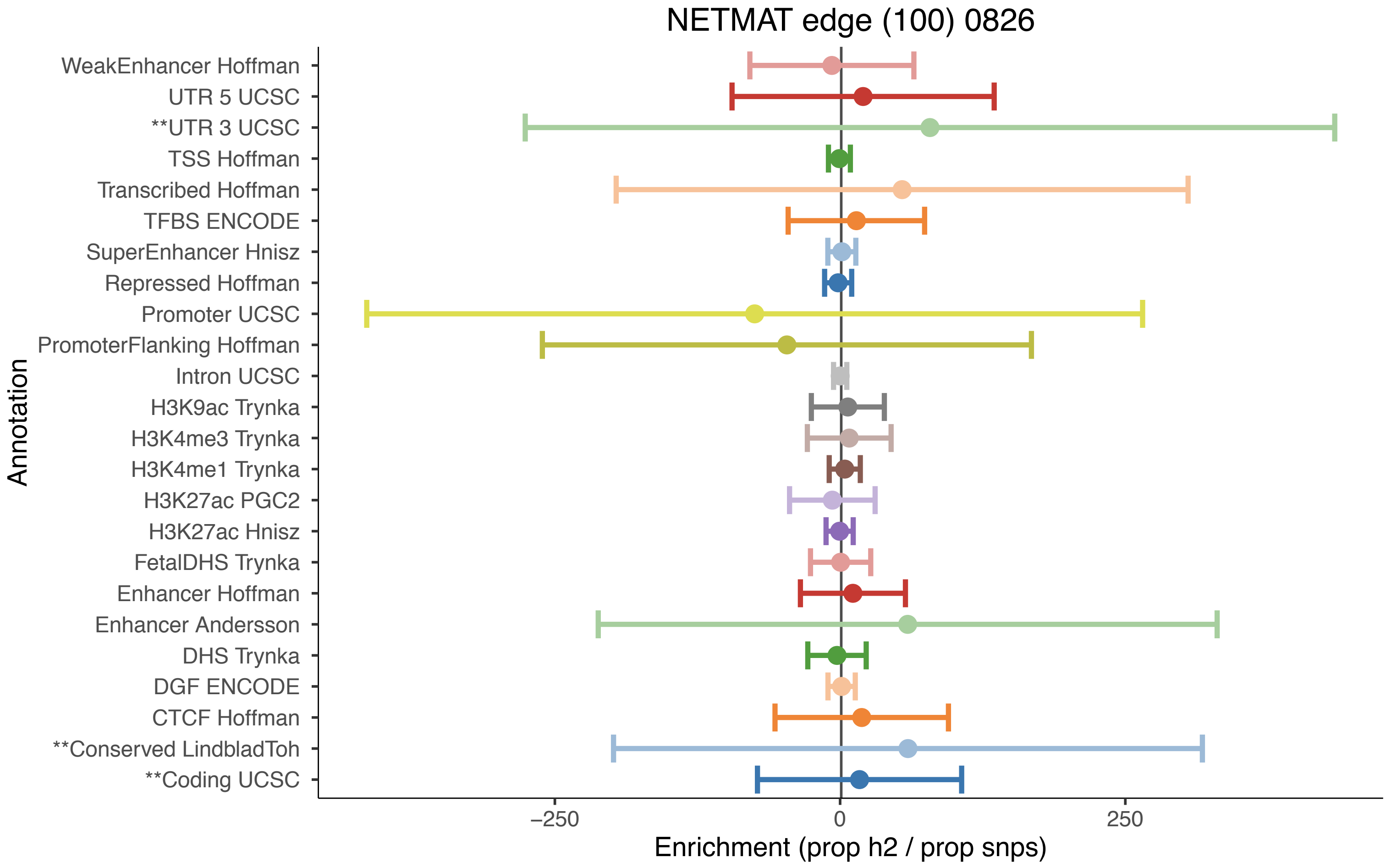

NETMAT edge (100) 0826
WeakEnhancer Hoffman
UTR 5 UCSC
**UTR 3 UCSC
TSS Hoffman
Transcribed Hoffman
TFBS ENCODE
SuperEnhancer Hnisz
Repressed Hoffman
Promoter UCSC
PromoterFlanking Hoffman
Intron UCSC
H3K9ac Trynka
H3K4me3 Trynka
H3K4me1 Trynka
H3K27ac PGC2
H3K27ac Hnisz
FetalDHS Trynka
Enhancer Hoffman
Enhancer Andersson
DHS Trynka
DGF ENCODE
CTCF Hoffman
**Conserved LindbladToh
**Coding UCSC
Annotation
−250
0
250
Enrichment (prop h2 / prop snps)

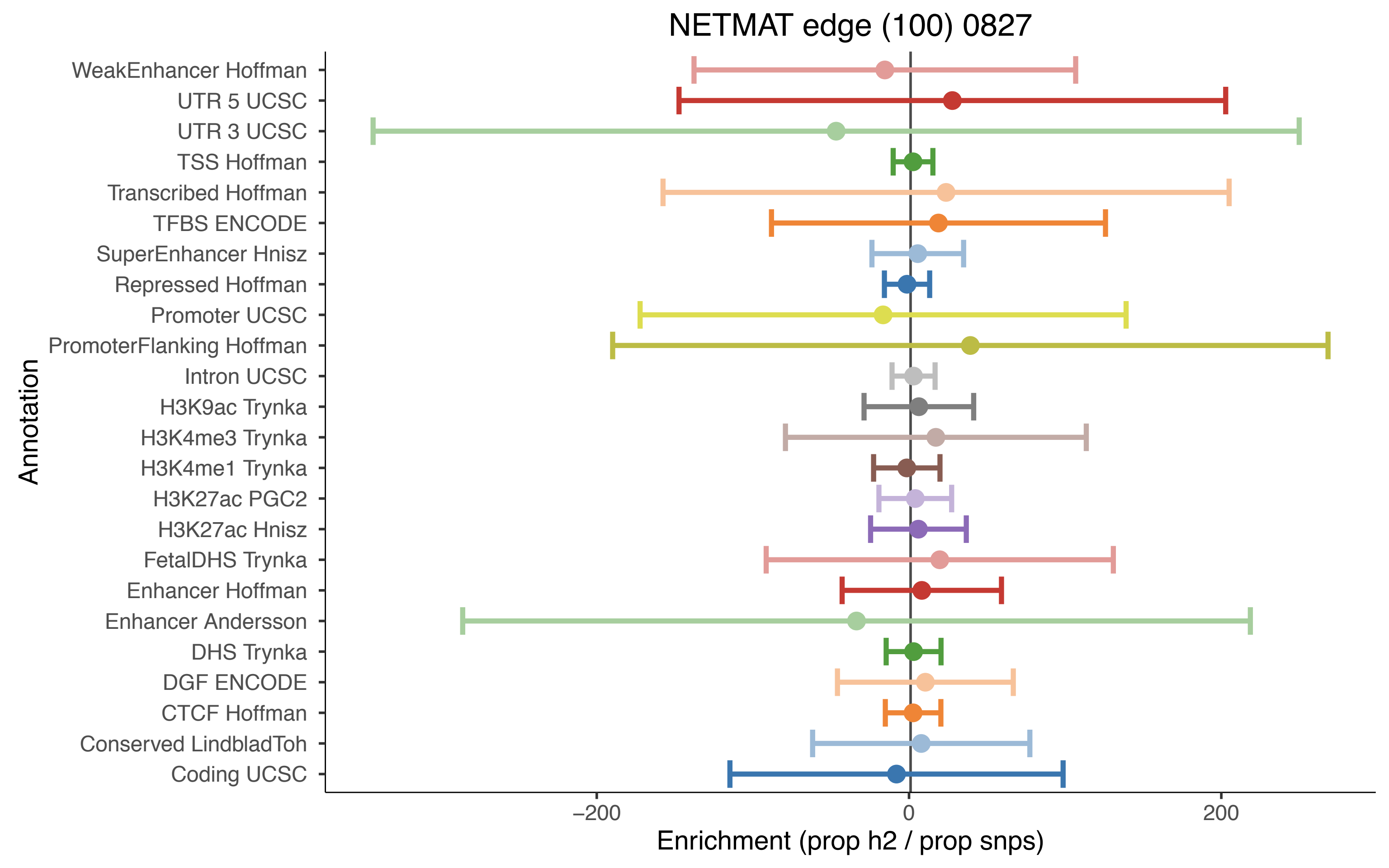
NETMAT edge (100) 0827
Annotation
WeakEnhancer Hoffman
UTR 5 UCSC
UTR 3 UCSC
TSS Hoffman
Transcribed Hoffman
TFBS ENCODE
SuperEnhancer Hnisz
Repressed Hoffman
Promoter UCSC
PromoterFlanking Hoffman
Intron UCSC
H3K9ac Trynka
H3K4me3 Trynka
H3K4me1 Trynka
H3K27ac PGC2
H3K27ac Hnisz
FetalDHS Trynka
Enhancer Hoffman
Enhancer Andersson
DHS Trynka
DGF ENCODE
CTCF Hoffman
Conserved LindbladToh
Coding UCSC
−200
0
200
Enrichment (prop h2 / prop snps)

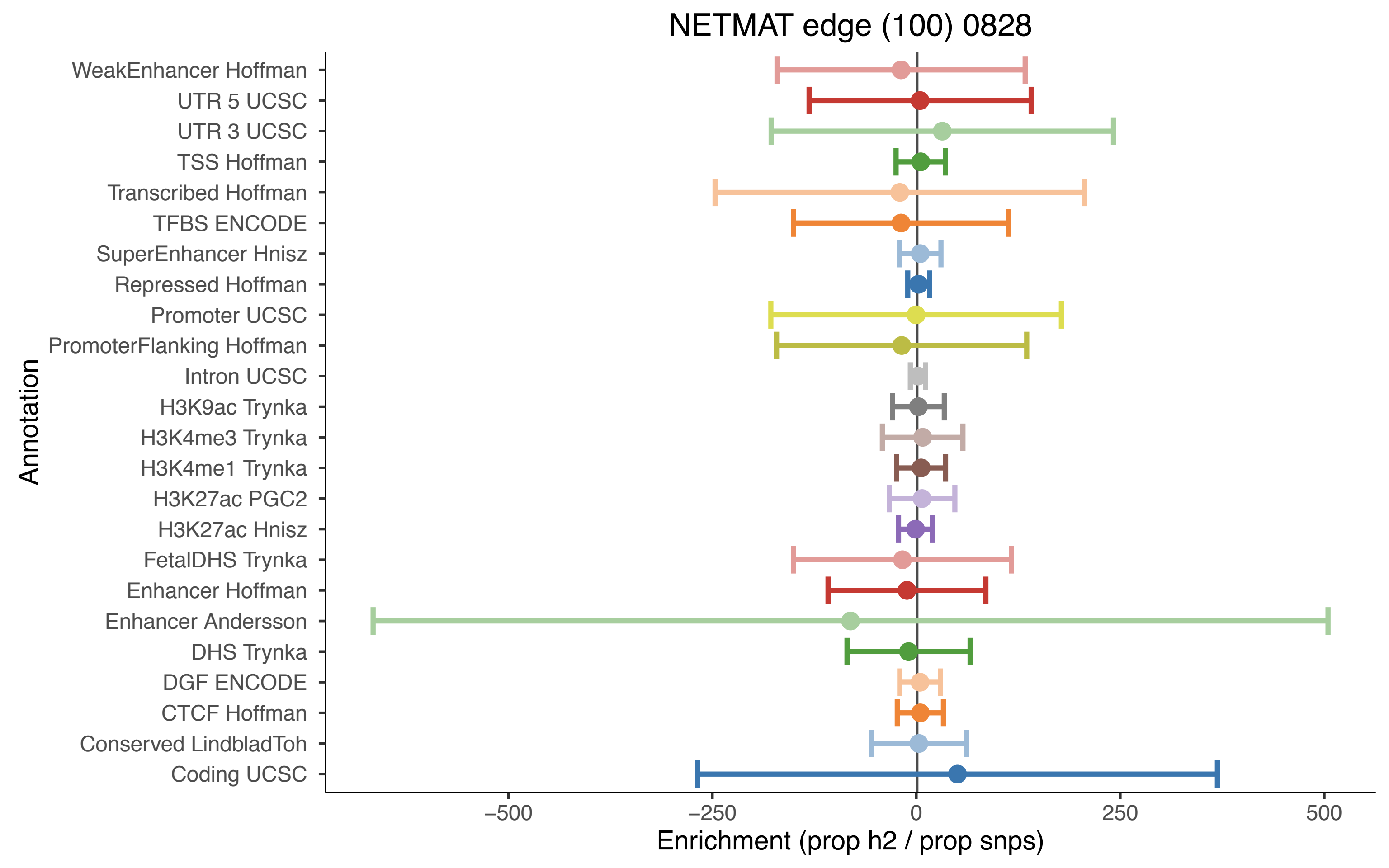
NETMAT edge (100) 0828
WeakEnhancer Hoffman
UTR 5 UCSC
UTR 3 UCSC
TSS Hoffman
Transcribed Hoffman
TFBS ENCODE
SuperEnhancer Hnisz
Repressed Hoffman
Promoter UCSC
PromoterFlanking Hoffman
Intron UCSC
H3K9ac Trynka
H3K4me3 Trynka
H3K4me1 Trynka
H3K27ac PGC2
H3K27ac Hnisz
FetalDHS Trynka
Enhancer Hoffman
Enhancer Andersson
DHS Trynka
DGF ENCODE
CTCF Hoffman
Conserved LindbladToh
Coding UCSC
Annotation
−500
−250
0
250
500
Enrichment (prop h2 / prop snps)

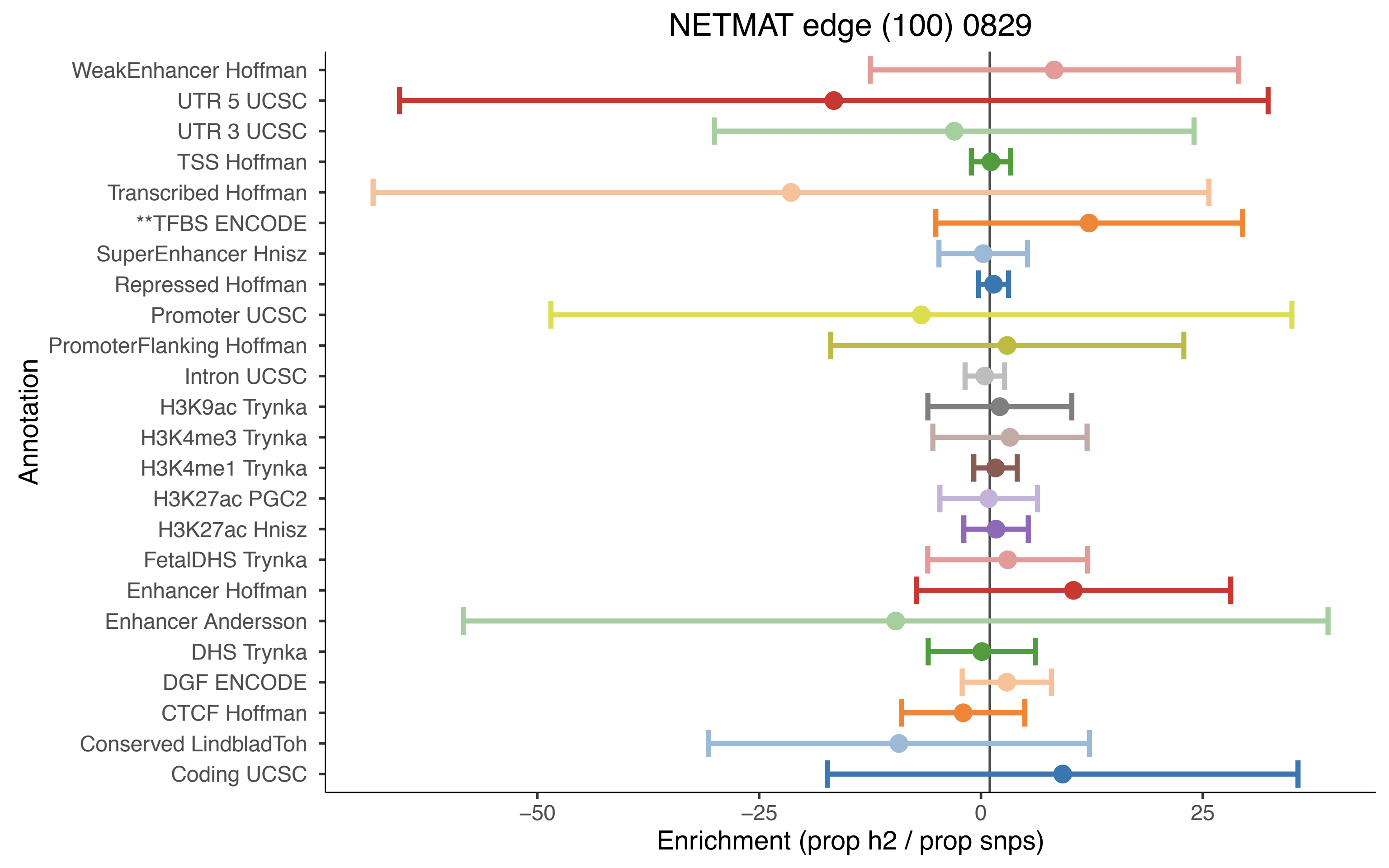

NETMAT edge (100) 0829
WeakEnhancer Hoffman
UTR 5 UCSC
UTR 3 UCSC
TSS Hoffman
Transcribed Hoffman
**TFBS ENCODE
SuperEnhancer Hnisz
Repressed Hoffman
Promoter UCSC
PromoterFlanking Hoffman
Intron UCSC
H3K9ac Trynka
H3K4me3 Trynka
H3K4me1 Trynka
H3K27ac PGC2
H3K27ac Hnisz
FetalDHS Trynka
Enhancer Hoffman
Enhancer Andersson
DHS Trynka
DGF ENCODE
CTCF Hoffman
Conserved LindbladToh
Coding UCSC
Annotation
−50
−25
0
25
Enrichment (prop h2 / prop snps)

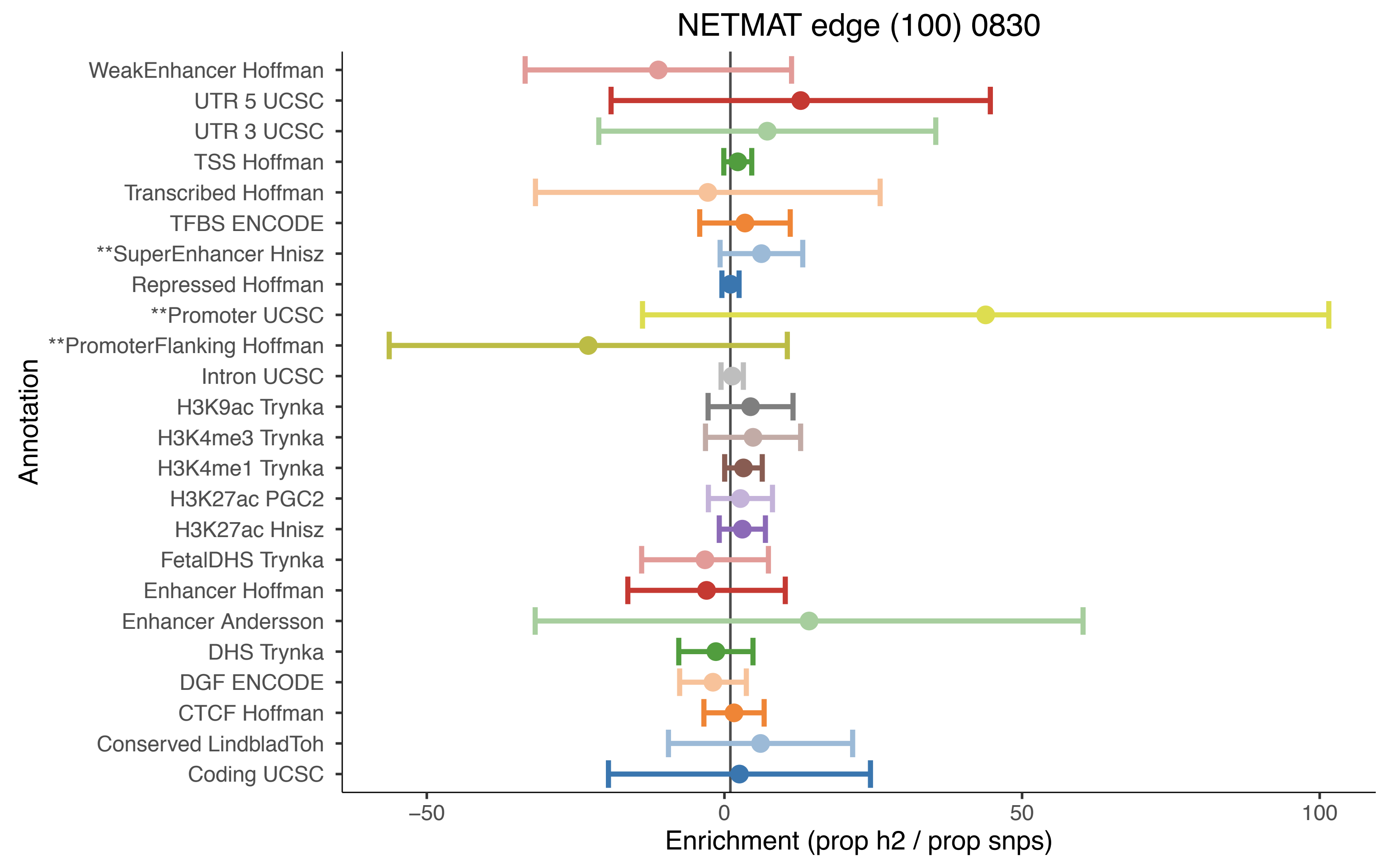
NETMAT edge (100) 0830
WeakEnhancer Hoffman
UTR 5 UCSC
UTR 3 UCSC
TSS Hoffman
Transcribed Hoffman
TFBS ENCODE
**SuperEnhancer Hnisz
Repressed Hoffman
**Promoter UCSC
**PromoterFlanking Hoffman
Intron UCSC
H3K9ac Trynka
H3K4me3 Trynka
H3K4me1 Trynka
H3K27ac PGC2
H3K27ac Hnisz
FetalDHS Trynka
Enhancer Hoffman
Enhancer Andersson
DHS Trynka
DGF ENCODE
CTCF Hoffman
Conserved LindbladToh
Coding UCSC
Annotation
−50
0
50
100
Enrichment (prop h2 / prop snps)

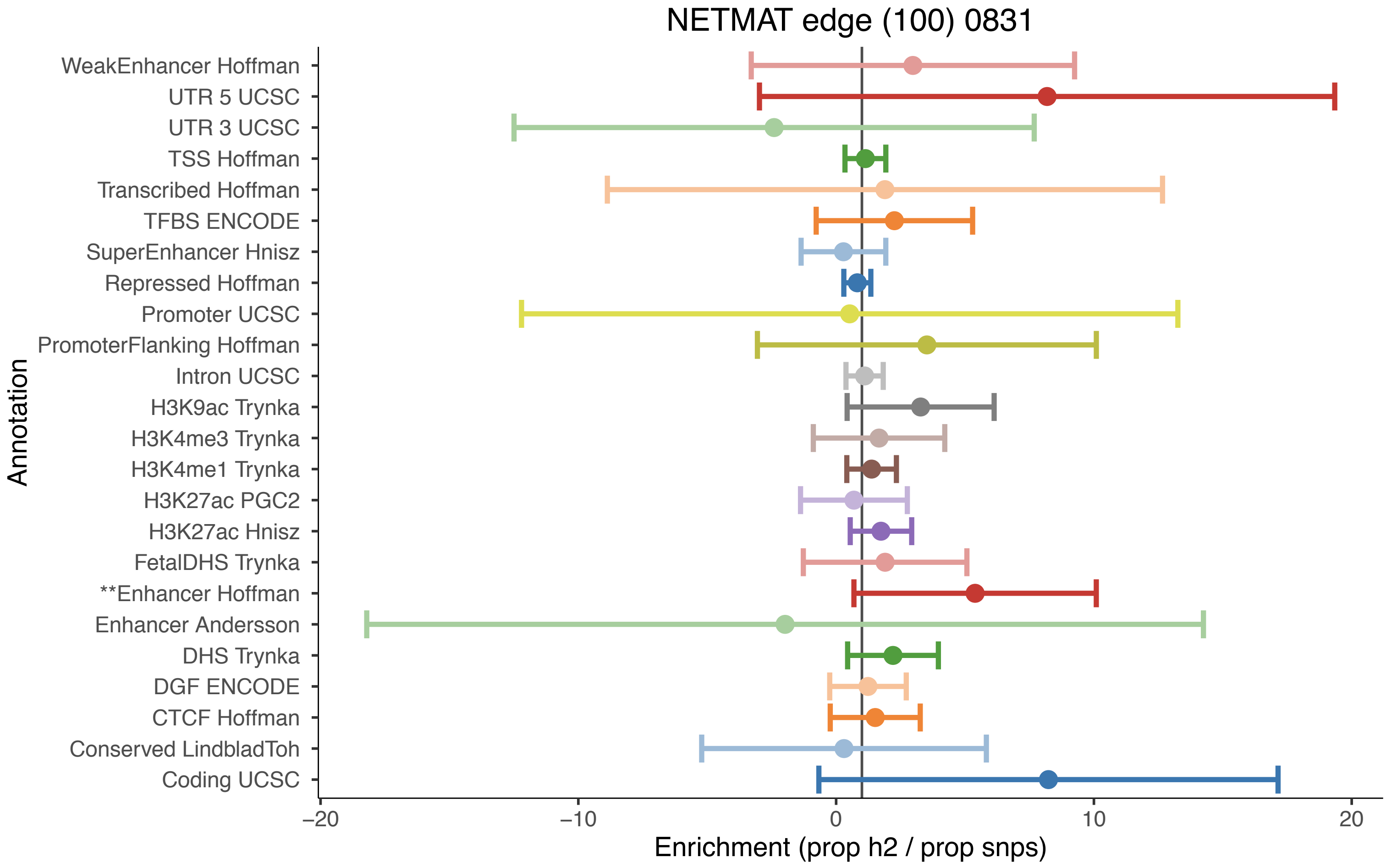
NETMAT edge (100) 0831
WeakEnhancer Hoffman
UTR 5 UCSC
UTR 3 UCSC
TSS Hoffman
Transcribed Hoffman
TFBS ENCODE
SuperEnhancer Hnisz
Repressed Hoffman
Promoter UCSC
PromoterFlanking Hoffman
Intron UCSC
H3K9ac Trynka
H3K4me3 Trynka
H3K4me1 Trynka
H3K27ac PGC2
H3K27ac Hnisz
FetalDHS Trynka
**Enhancer Hoffman
Enhancer Andersson
DHS Trynka
DGF ENCODE
CTCF Hoffman
Conserved LindbladToh
Coding UCSC
Annotation
−20
−10
0
10
20
Enrichment (prop h2 / prop snps)

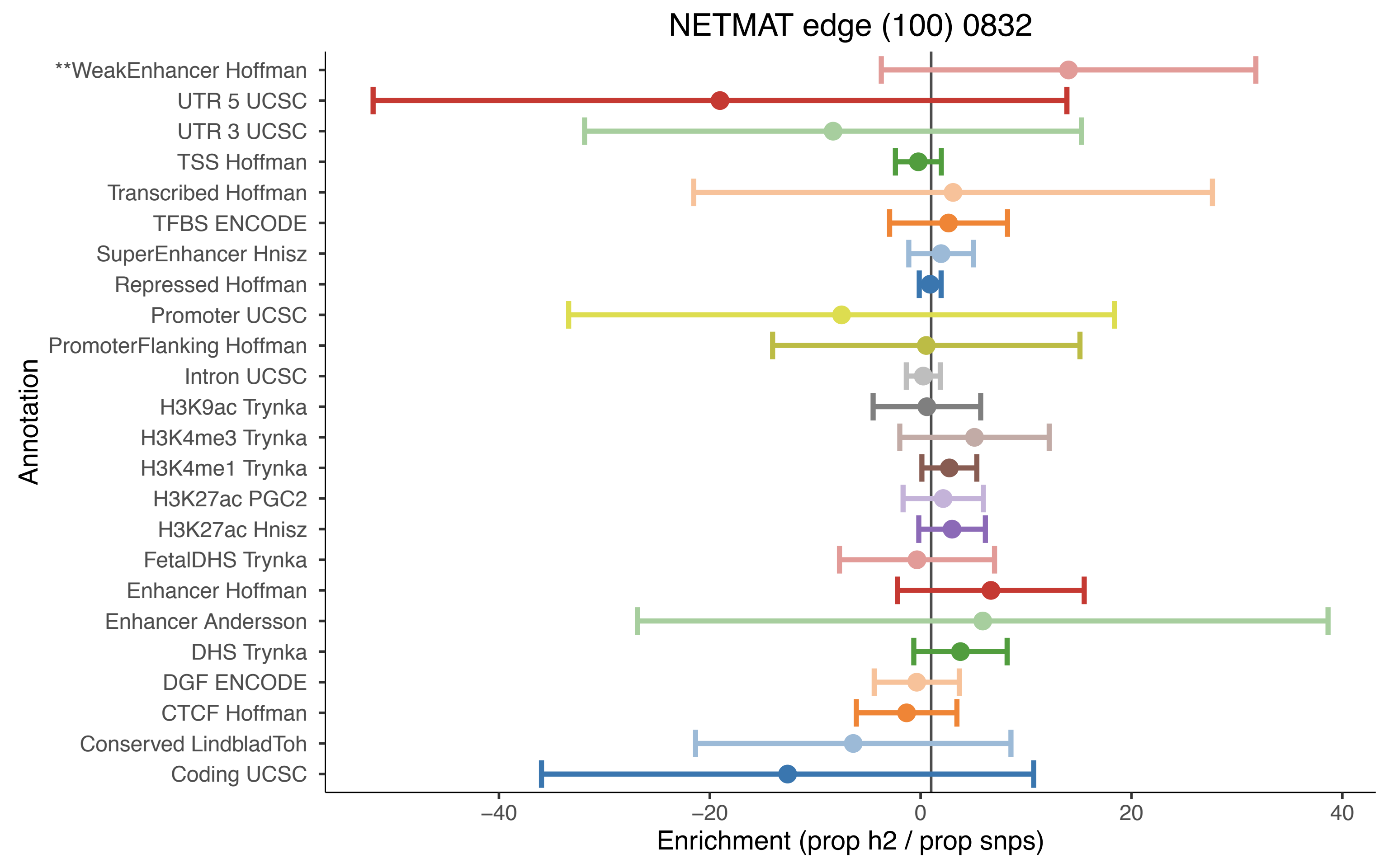

NETMAT edge (100) 0832
**WeakEnhancer Hoffman
UTR 5 UCSC
UTR 3 UCSC
TSS Hoffman
Transcribed Hoffman
TFBS ENCODE
SuperEnhancer Hnisz
Repressed Hoffman
Promoter UCSC
PromoterFlanking Hoffman
Intron UCSC
H3K9ac Trynka
H3K4me3 Trynka
H3K4me1 Trynka
H3K27ac PGC2
H3K27ac Hnisz
FetalDHS Trynka
Enhancer Hoffman
Enhancer Andersson
DHS Trynka
DGF ENCODE
CTCF Hoffman
Conserved LindbladToh
Coding UCSC
Annotation
−40
−20
0
20
40
Enrichment (prop h2 / prop snps)

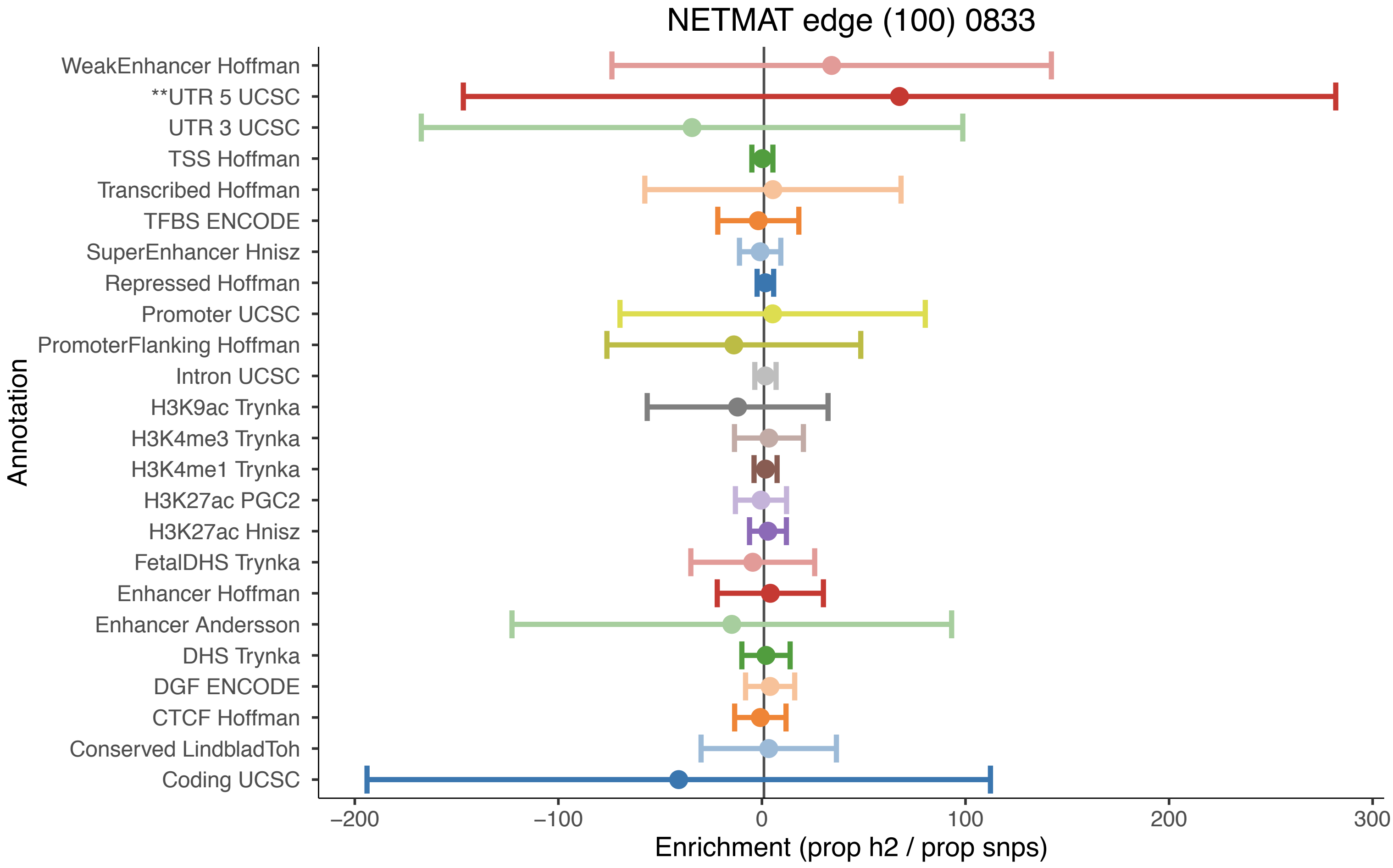
NETMAT edge (100) 0833
WeakEnhancer Hoffman
**UTR 5 UCSC
UTR 3 UCSC
TSS Hoffman
Transcribed Hoffman
TFBS ENCODE
SuperEnhancer Hnisz
Repressed Hoffman
Promoter UCSC
PromoterFlanking Hoffman
Intron UCSC
H3K9ac Trynka
H3K4me3 Trynka
H3K4me1 Trynka
H3K27ac PGC2
H3K27ac Hnisz
FetalDHS Trynka
Enhancer Hoffman
Enhancer Andersson
DHS Trynka
DGF ENCODE
CTCF Hoffman
Conserved LindbladToh
Coding UCSC
Annotation
−200
−100
0
100
200
300
Enrichment (prop h2 / prop snps)

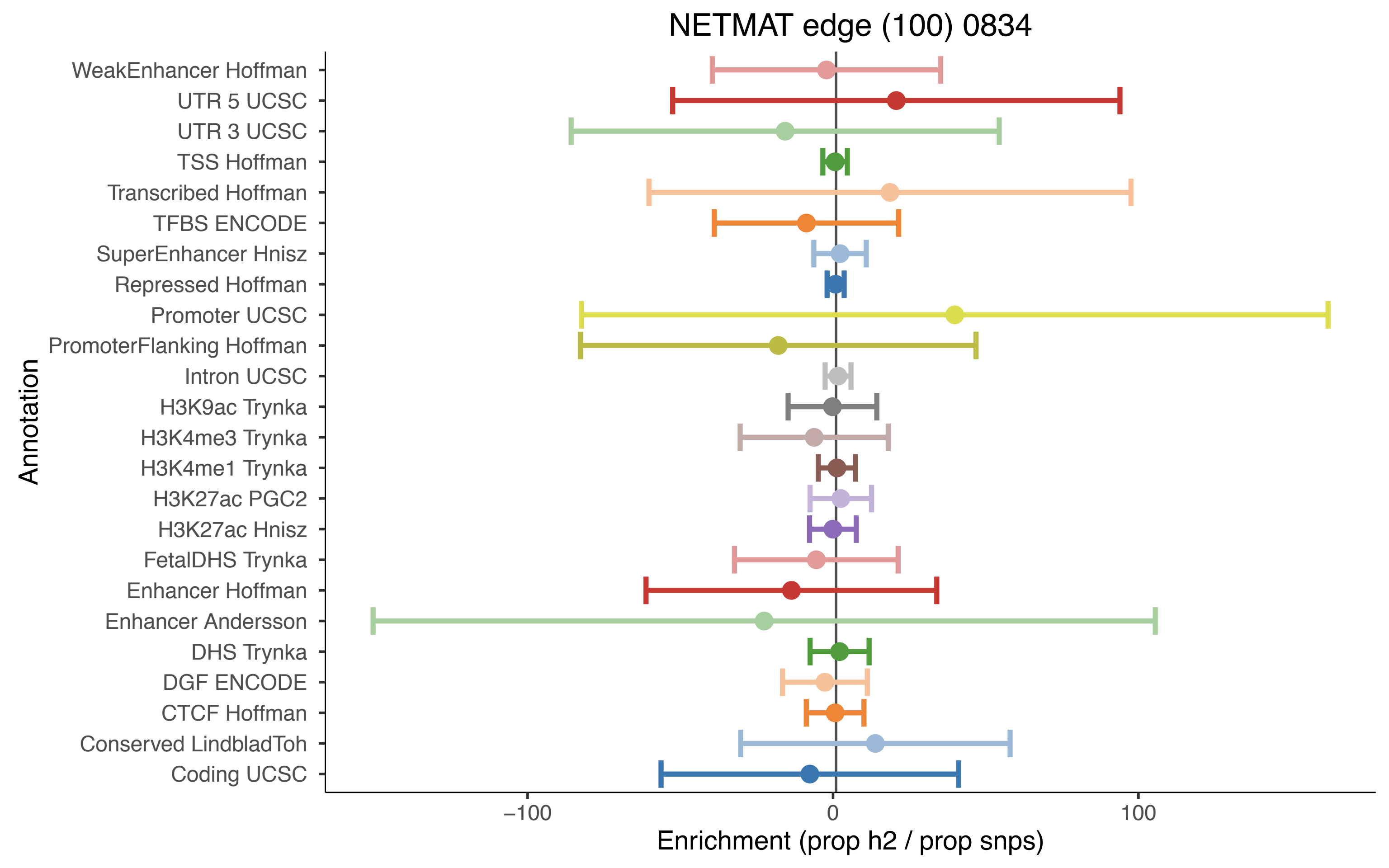

NETMAT edge (100) 0834
WeakEnhancer Hoffman
UTR 5 UCSC
UTR 3 UCSC
TSS Hoffman
Transcribed Hoffman
TFBS ENCODE
SuperEnhancer Hnisz
Repressed Hoffman
Promoter UCSC
PromoterFlanking Hoffman
Intron UCSC
H3K9ac Trynka
H3K4me3 Trynka
H3K4me1 Trynka
H3K27ac PGC2
H3K27ac Hnisz
FetalDHS Trynka
Enhancer Hoffman
Enhancer Andersson
DHS Trynka
DGF ENCODE
CTCF Hoffman
Conserved LindbladToh
Coding UCSC
Annotation
−100
0
100
Enrichment (prop h2 / prop snps)

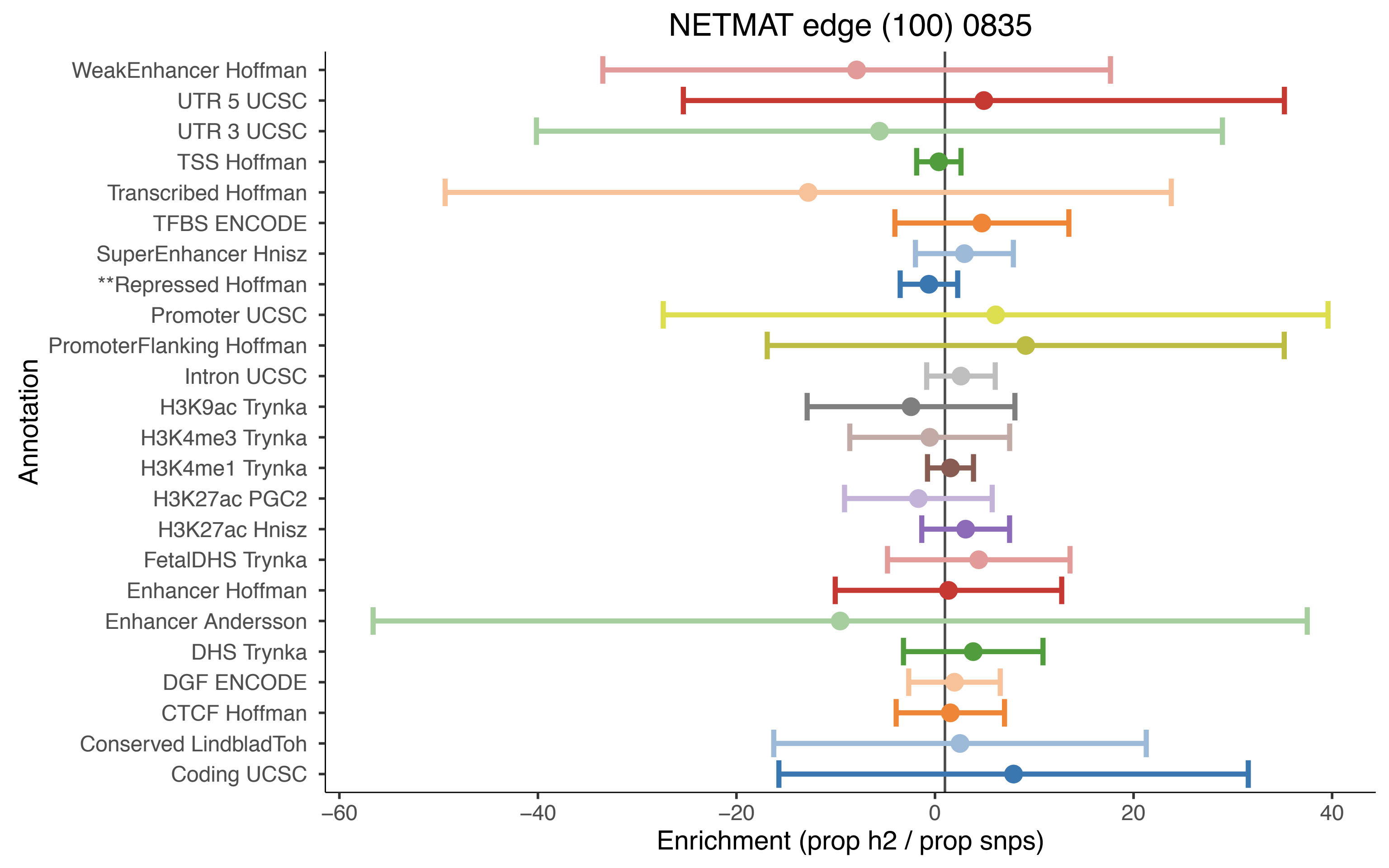
NETMAT edge (100) 0835
WeakEnhancer Hoffman
UTR 5 UCSC
UTR 3 UCSC
TSS Hoffman
Transcribed Hoffman
TFBS ENCODE
SuperEnhancer Hnisz
**Repressed Hoffman
Promoter UCSC
PromoterFlanking Hoffman
Intron UCSC
H3K9ac Trynka
H3K4me3 Trynka
H3K4me1 Trynka
H3K27ac PGC2
H3K27ac Hnisz
FetalDHS Trynka
Enhancer Hoffman
Enhancer Andersson
DHS Trynka
DGF ENCODE
CTCF Hoffman
Conserved LindbladToh
Coding UCSC
Annotation
−60
−40
−20
0
20
40
Enrichment (prop h2 / prop snps)

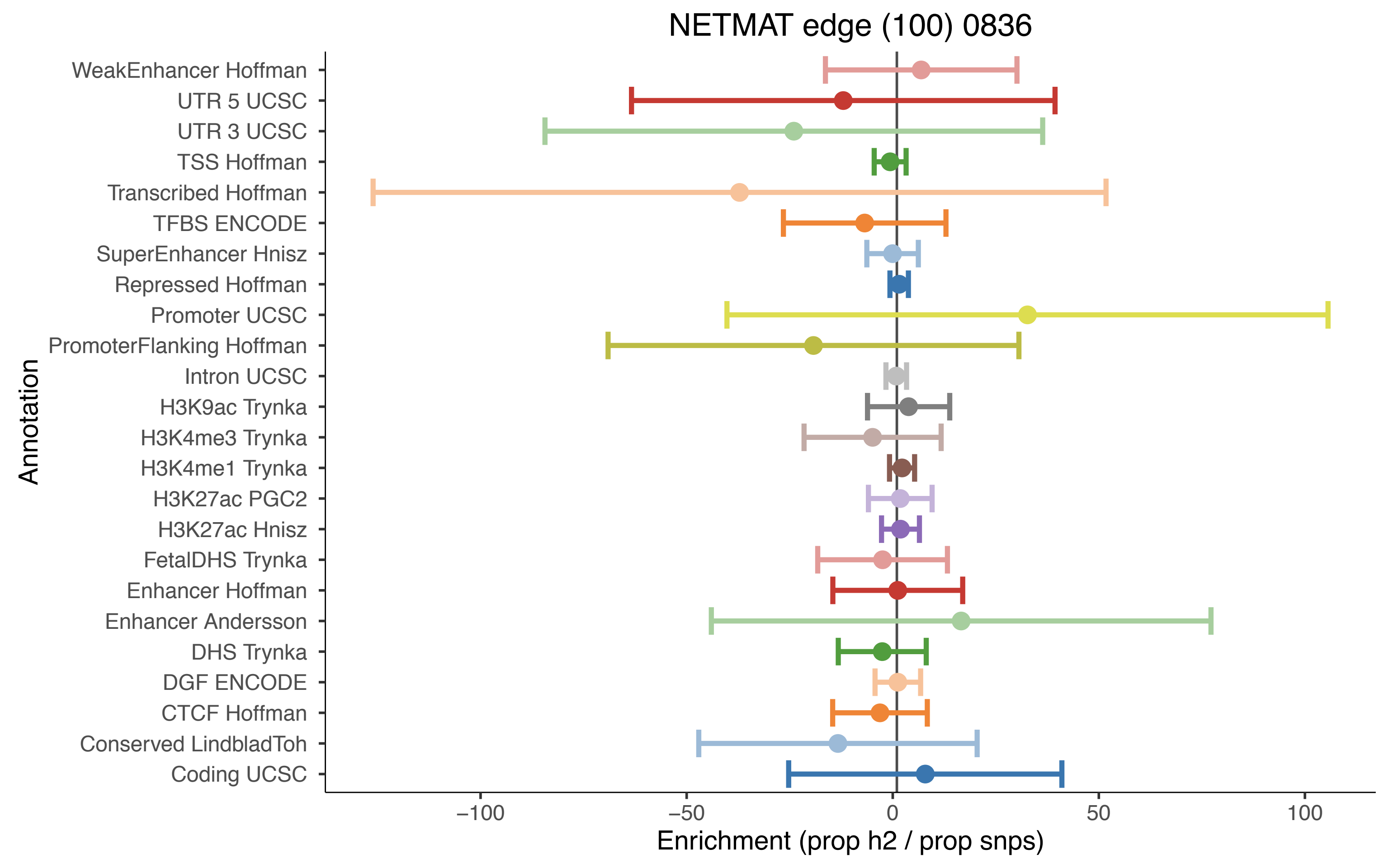

NETMAT edge (100) 0836
WeakEnhancer Hoffman
UTR 5 UCSC
UTR 3 UCSC
TSS Hoffman
Transcribed Hoffman
TFBS ENCODE
SuperEnhancer Hnisz
Repressed Hoffman
Promoter UCSC
PromoterFlanking Hoffman
Intron UCSC
H3K9ac Trynka
H3K4me3 Trynka
H3K4me1 Trynka
H3K27ac PGC2
H3K27ac Hnisz
FetalDHS Trynka
Enhancer Hoffman
Enhancer Andersson
DHS Trynka
DGF ENCODE
CTCF Hoffman
Conserved LindbladToh
Coding UCSC
Annotation
−100
−50
0
50
100
Enrichment (prop h2 / prop snps)

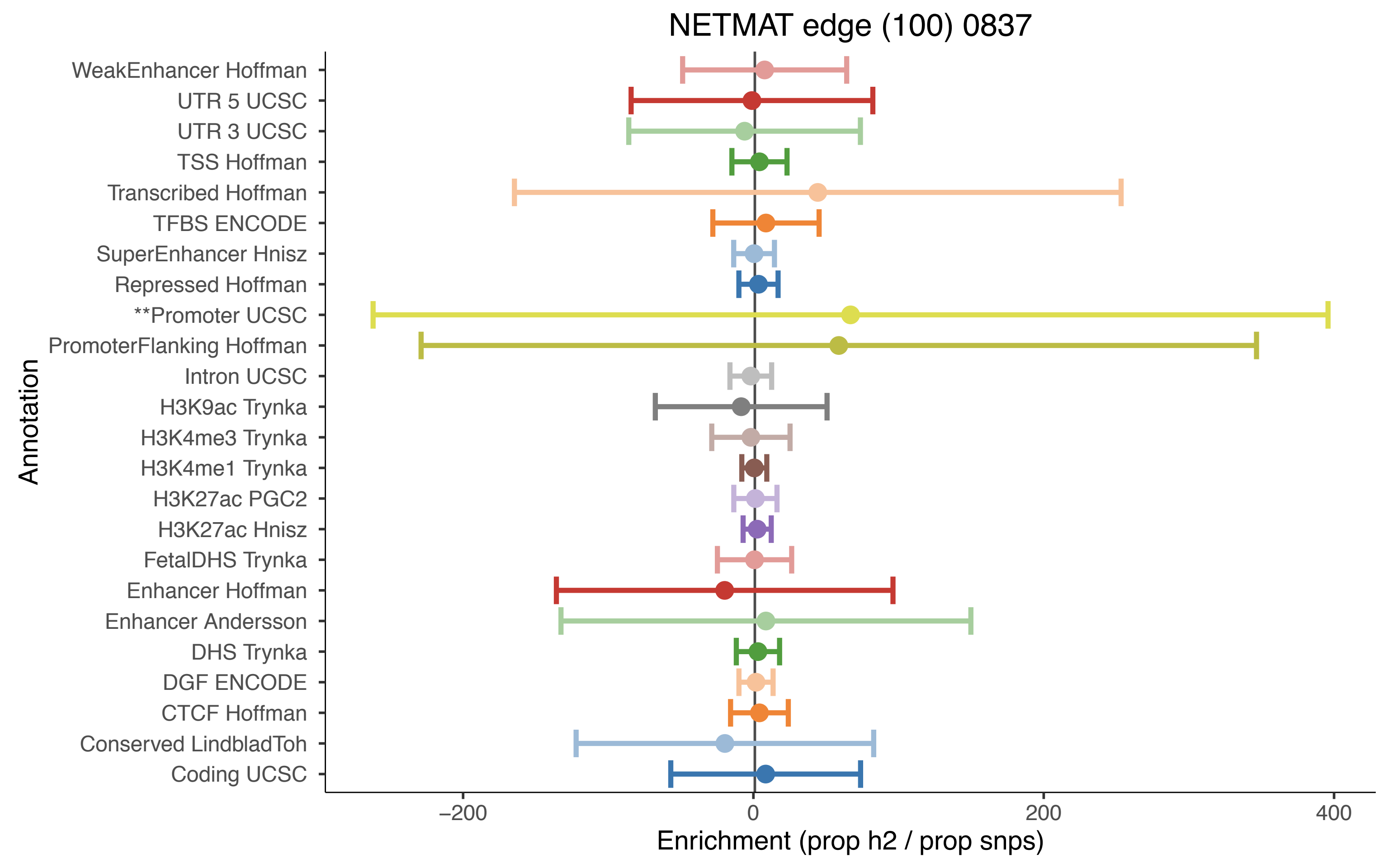
NETMAT edge (100) 0837
WeakEnhancer Hoffman
UTR 5 UCSC
UTR 3 UCSC
TSS Hoffman
Transcribed Hoffman
TFBS ENCODE
SuperEnhancer Hnisz
Repressed Hoffman
**Promoter UCSC
PromoterFlanking Hoffman
Intron UCSC
H3K9ac Trynka
H3K4me3 Trynka
H3K4me1 Trynka
H3K27ac PGC2
H3K27ac Hnisz
FetalDHS Trynka
Enhancer Hoffman
Enhancer Andersson
DHS Trynka
DGF ENCODE
CTCF Hoffman
Conserved LindbladToh
Coding UCSC
Annotation
−200
0
200
400
Enrichment (prop h2 / prop snps)

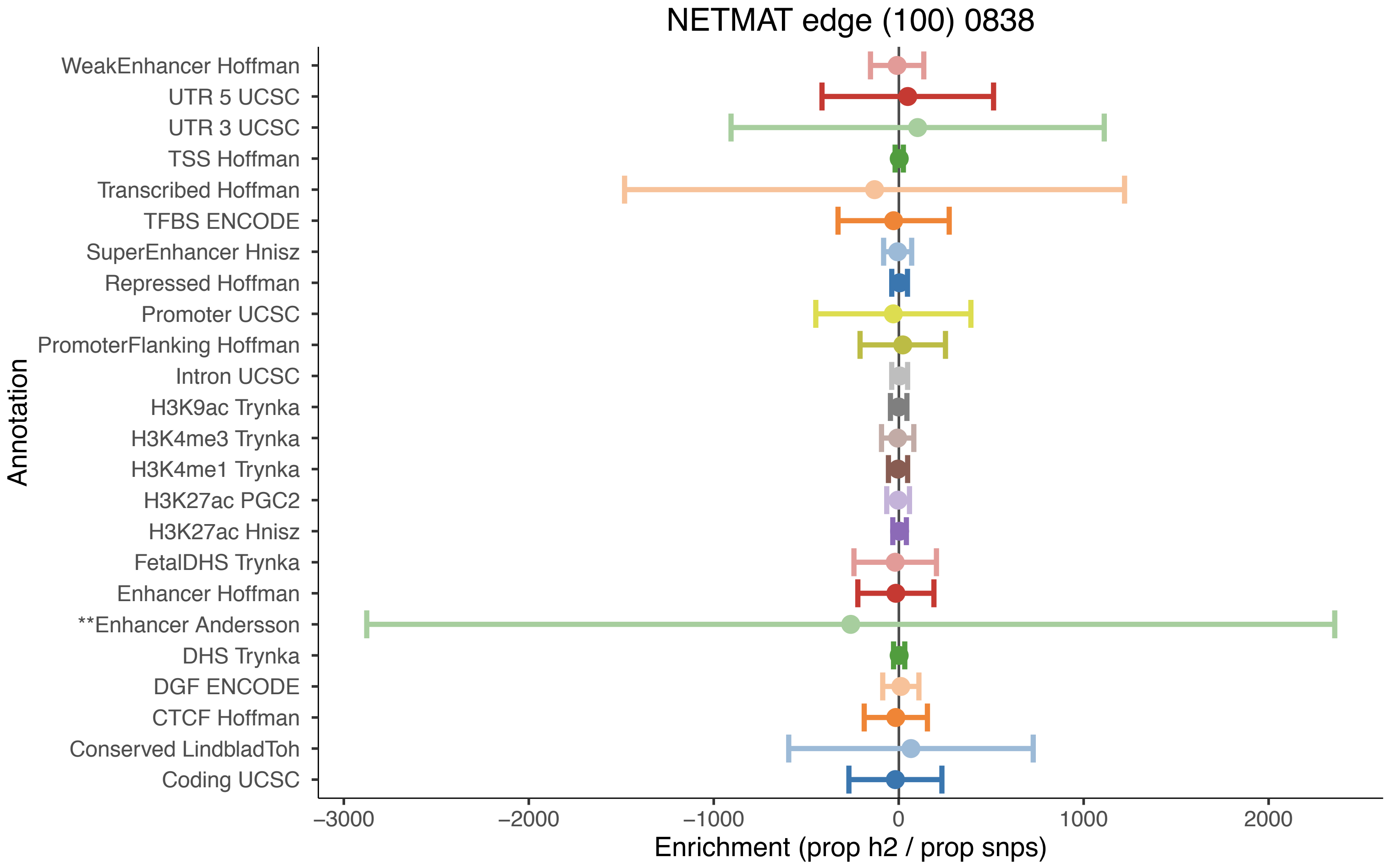
NETMAT edge (100) 0838
Annotation
WeakEnhancer Hoffman
UTR 5 UCSC
UTR 3 UCSC
TSS Hoffman
Transcribed Hoffman
TFBS ENCODE
SuperEnhancer Hnisz
Repressed Hoffman
Promoter UCSC
PromoterFlanking Hoffman
Intron UCSC
H3K9ac Trynka
H3K4me3 Trynka
H3K4me1 Trynka
H3K27ac PGC2
H3K27ac Hnisz
FetalDHS Trynka
Enhancer Hoffman
**Enhancer Andersson
DHS Trynka
DGF ENCODE
CTCF Hoffman
Conserved LindbladToh
Coding UCSC
−3000
−2000
−1000
0
1000
2000
Enrichment (prop h2 / prop snps)

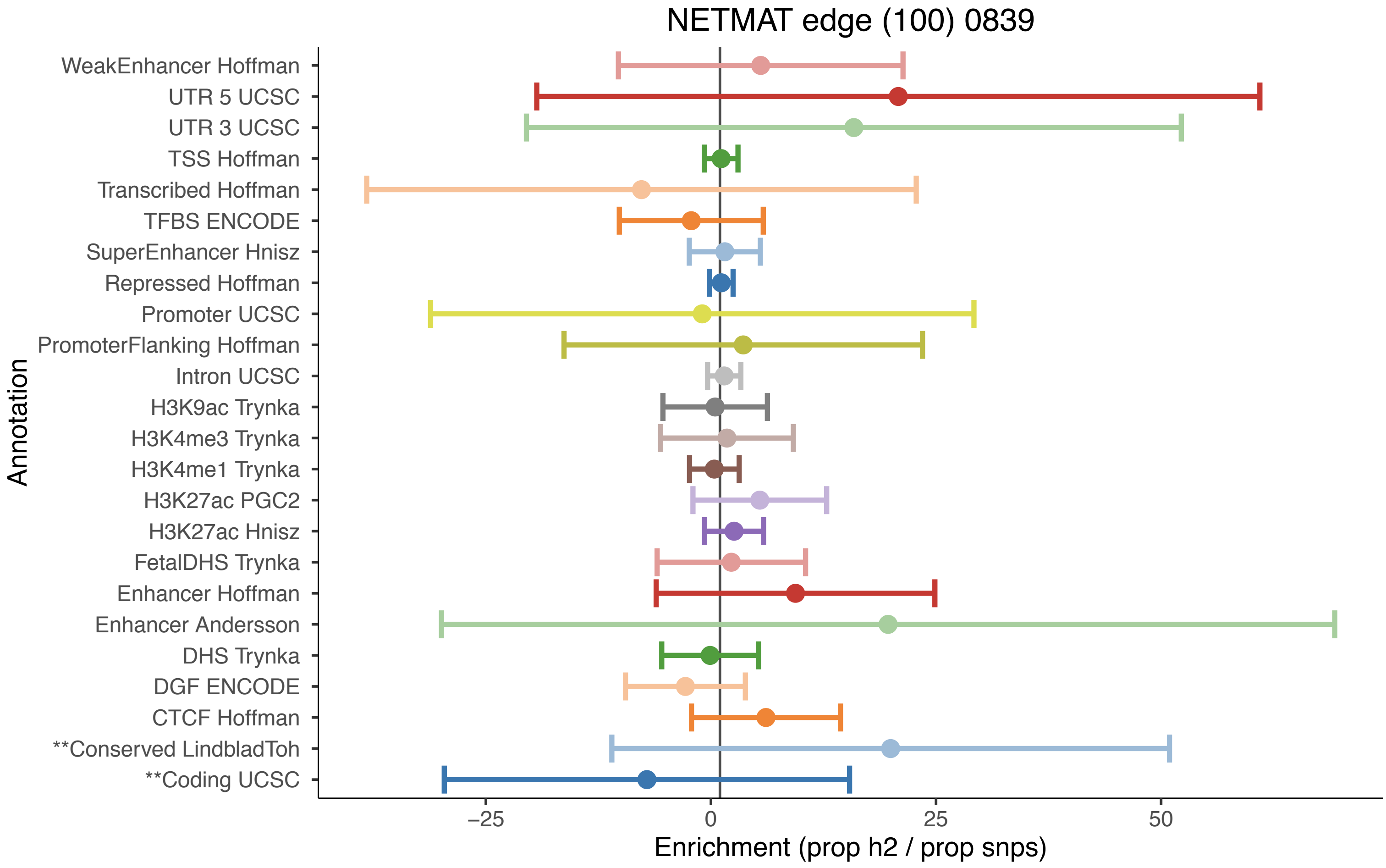
NETMAT edge (100) 0839
Annotation
WeakEnhancer Hoffman
UTR 5 UCSC
UTR 3 UCSC
TSS Hoffman
Transcribed Hoffman
TFBS ENCODE
SuperEnhancer Hnisz
Repressed Hoffman
Promoter UCSC
PromoterFlanking Hoffman
Intron UCSC
H3K9ac Trynka
H3K4me3 Trynka
H3K4me1 Trynka
H3K27ac PGC2
H3K27ac Hnisz
FetalDHS Trynka
Enhancer Hoffman
Enhancer Andersson
DHS Trynka
DGF ENCODE
CTCF Hoffman
**Conserved LindbladToh
**Coding UCSC
−25
0
25
50
Enrichment (prop h2 / prop snps)

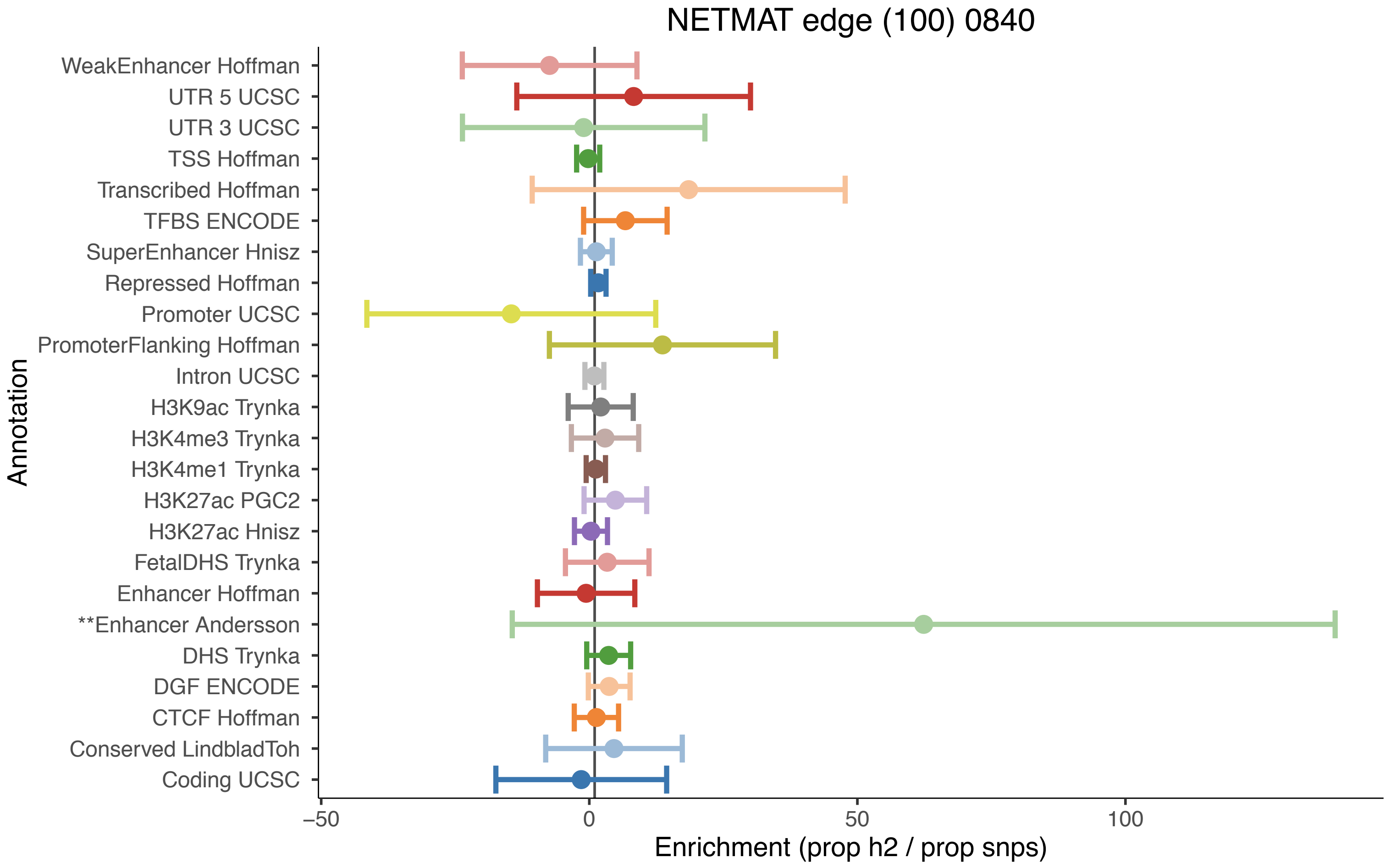
NETMAT edge (100) 0840
Annotation
WeakEnhancer Hoffman
UTR 5 UCSC
UTR 3 UCSC
TSS Hoffman
Transcribed Hoffman
TFBS ENCODE
SuperEnhancer Hnisz
Repressed Hoffman
Promoter UCSC
PromoterFlanking Hoffman
Intron UCSC
H3K9ac Trynka
H3K4me3 Trynka
H3K4me1 Trynka
H3K27ac PGC2
H3K27ac Hnisz
FetalDHS Trynka
Enhancer Hoffman
**Enhancer Andersson
DHS Trynka
DGF ENCODE
CTCF Hoffman
Conserved LindbladToh
Coding UCSC
−50
0
50
100
Enrichment (prop h2 / prop snps)

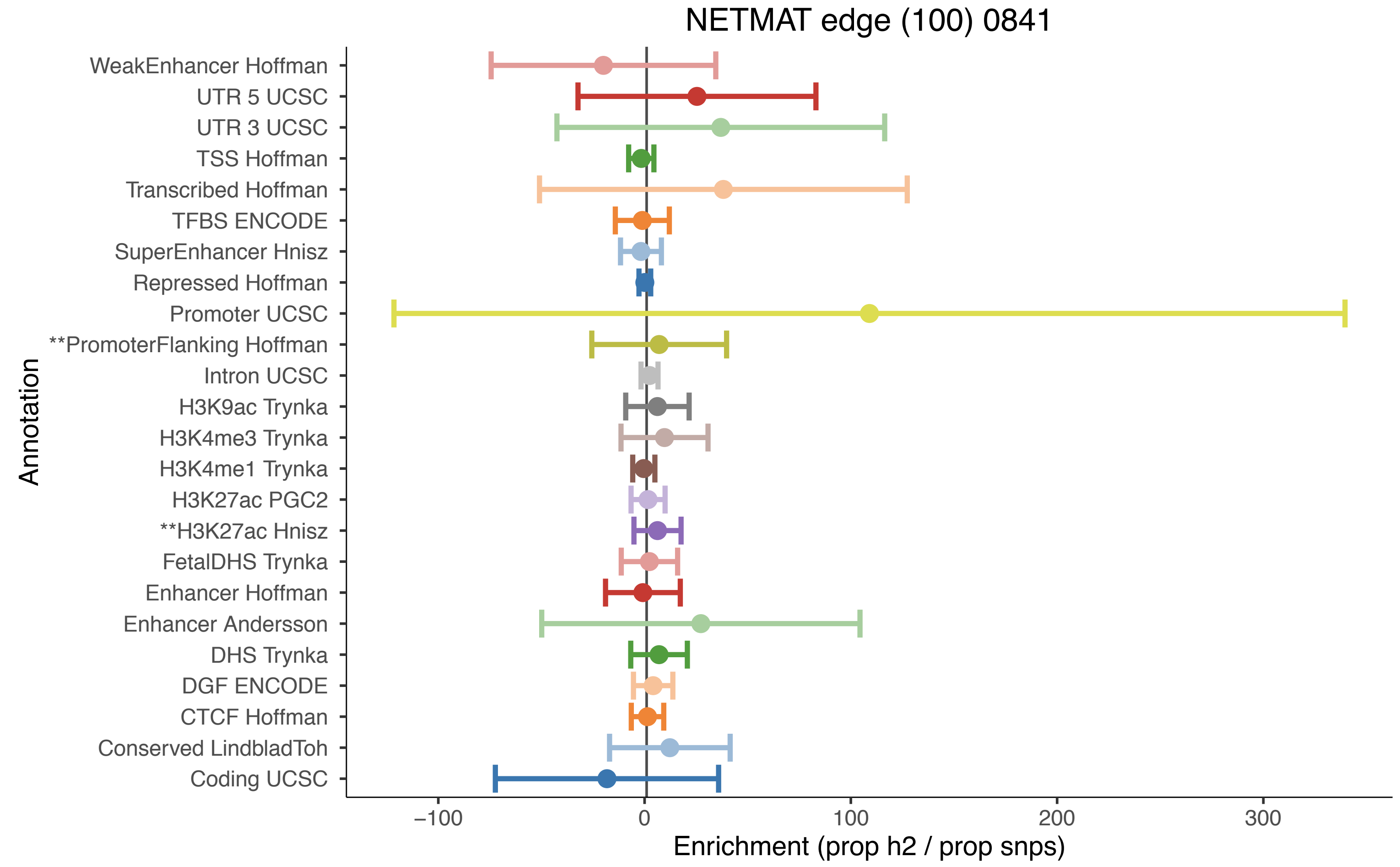
NETMAT edge (100) 0841
WeakEnhancer Hoffman
UTR 5 UCSC
UTR 3 UCSC
TSS Hoffman
Transcribed Hoffman
TFBS ENCODE
SuperEnhancer Hnisz
Repressed Hoffman
Promoter UCSC
**PromoterFlanking Hoffman
Intron UCSC
H3K9ac Trynka
H3K4me3 Trynka
H3K4me1 Trynka
H3K27ac PGC2
**H3K27ac Hnisz
FetalDHS Trynka
Enhancer Hoffman
Enhancer Andersson
DHS Trynka
DGF ENCODE
CTCF Hoffman
Conserved LindbladToh
Coding UCSC
Annotation
−100
0
100
200
300
Enrichment (prop h2 / prop snps)

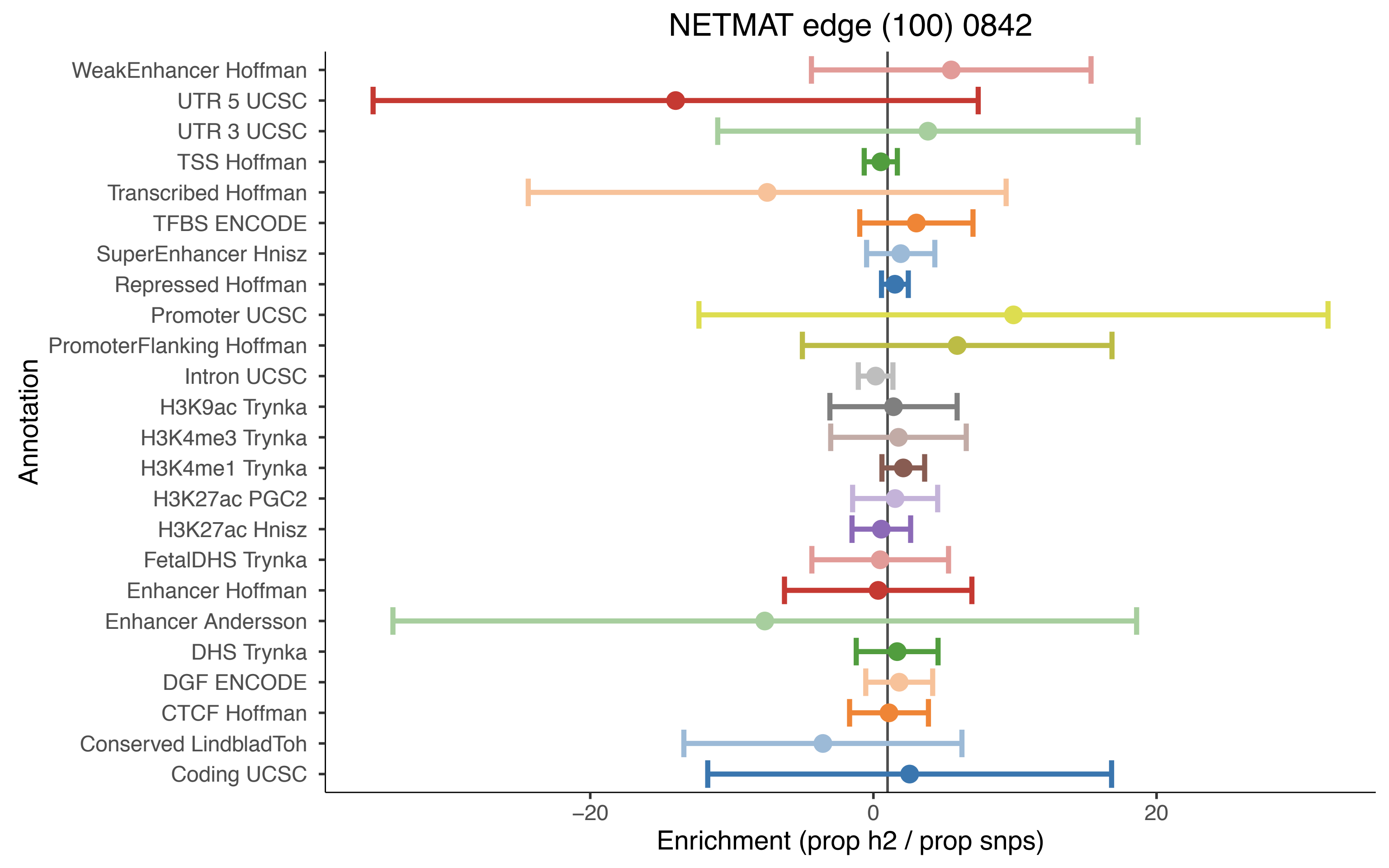

NETMAT edge (100) 0842
Annotation
WeakEnhancer Hoffman
UTR 5 UCSC
UTR 3 UCSC
TSS Hoffman
Transcribed Hoffman
TFBS ENCODE
SuperEnhancer Hnisz
Repressed Hoffman
Promoter UCSC
PromoterFlanking Hoffman
Intron UCSC
H3K9ac Trynka
H3K4me3 Trynka
H3K4me1 Trynka
H3K27ac PGC2
H3K27ac Hnisz
FetalDHS Trynka
Enhancer Hoffman
Enhancer Andersson
DHS Trynka
DGF ENCODE
CTCF Hoffman
Conserved LindbladToh
Coding UCSC
−20
0
20
Enrichment (prop h2 / prop snps)

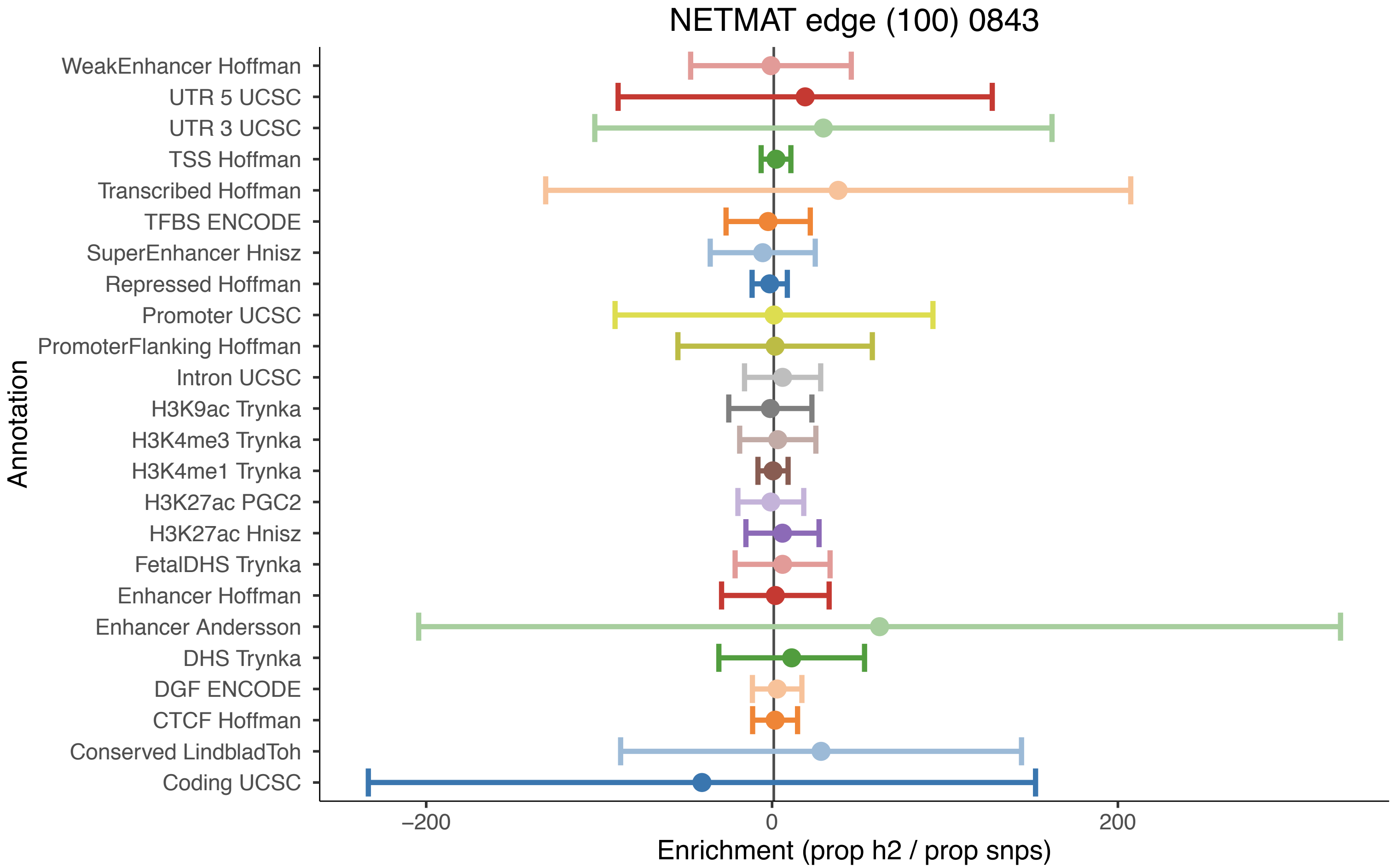
NETMAT edge (100) 0843
WeakEnhancer Hoffman
UTR 5 UCSC
UTR 3 UCSC
TSS Hoffman
Transcribed Hoffman
TFBS ENCODE
SuperEnhancer Hnisz
Repressed Hoffman
Promoter UCSC
PromoterFlanking Hoffman
Intron UCSC
H3K9ac Trynka
H3K4me3 Trynka
H3K4me1 Trynka
H3K27ac PGC2
H3K27ac Hnisz
FetalDHS Trynka
Enhancer Hoffman
Enhancer Andersson
DHS Trynka
DGF ENCODE
CTCF Hoffman
Conserved LindbladToh
Coding UCSC
Annotation
−200
0
200
Enrichment (prop h2 / prop snps)

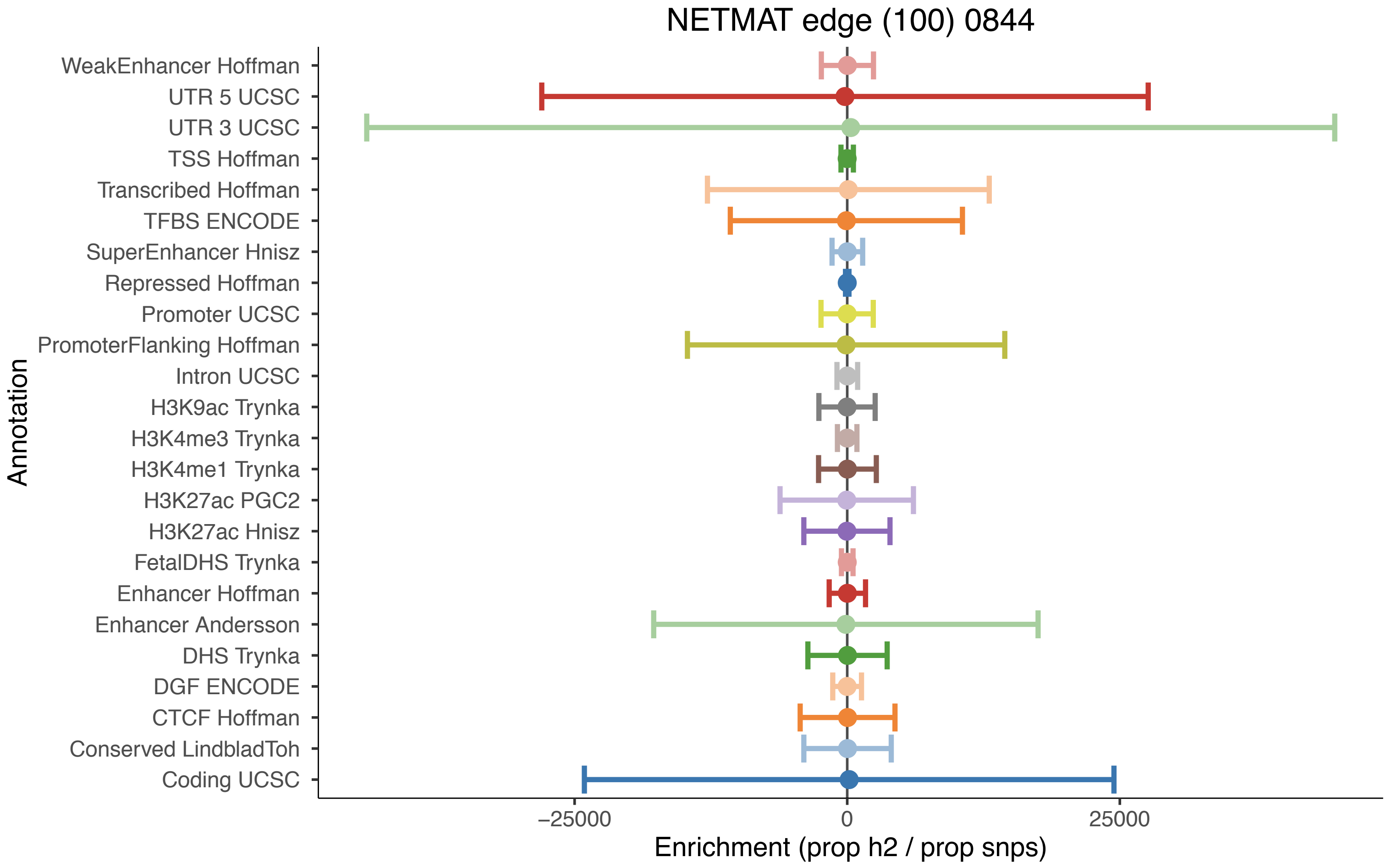

NETMAT edge (100) 0844
WeakEnhancer Hoffman
UTR 5 UCSC
UTR 3 UCSC
TSS Hoffman
Transcribed Hoffman
TFBS ENCODE
SuperEnhancer Hnisz
Repressed Hoffman
Promoter UCSC
PromoterFlanking Hoffman
Intron UCSC
H3K9ac Trynka
H3K4me3 Trynka
H3K4me1 Trynka
H3K27ac PGC2
H3K27ac Hnisz
FetalDHS Trynka
Enhancer Hoffman
Enhancer Andersson
DHS Trynka
DGF ENCODE
CTCF Hoffman
Conserved LindbladToh
Coding UCSC
Annotation
−25000
0
25000
Enrichment (prop h2 / prop snps)

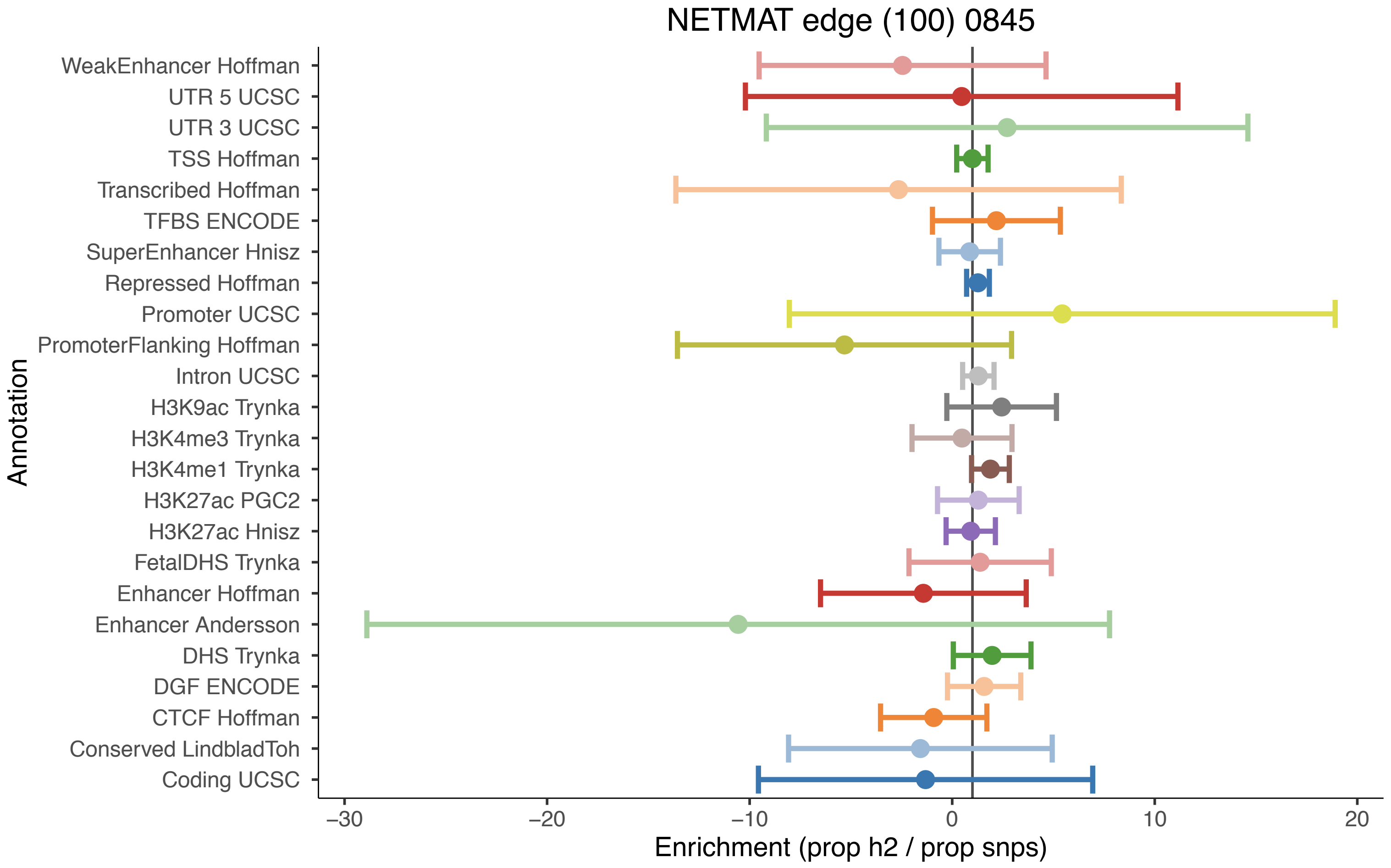
NETMAT edge (100) 0845
WeakEnhancer Hoffman
UTR 5 UCSC
UTR 3 UCSC
TSS Hoffman
Transcribed Hoffman
TFBS ENCODE
SuperEnhancer Hnisz
Repressed Hoffman
Promoter UCSC
PromoterFlanking Hoffman
Intron UCSC
H3K9ac Trynka
H3K4me3 Trynka
H3K4me1 Trynka
H3K27ac PGC2
H3K27ac Hnisz
FetalDHS Trynka
Enhancer Hoffman
Enhancer Andersson
DHS Trynka
DGF ENCODE
CTCF Hoffman
Conserved LindbladToh
Coding UCSC
Annotation
−30
−20
−10
0
10
20
Enrichment (prop h2 / prop snps)

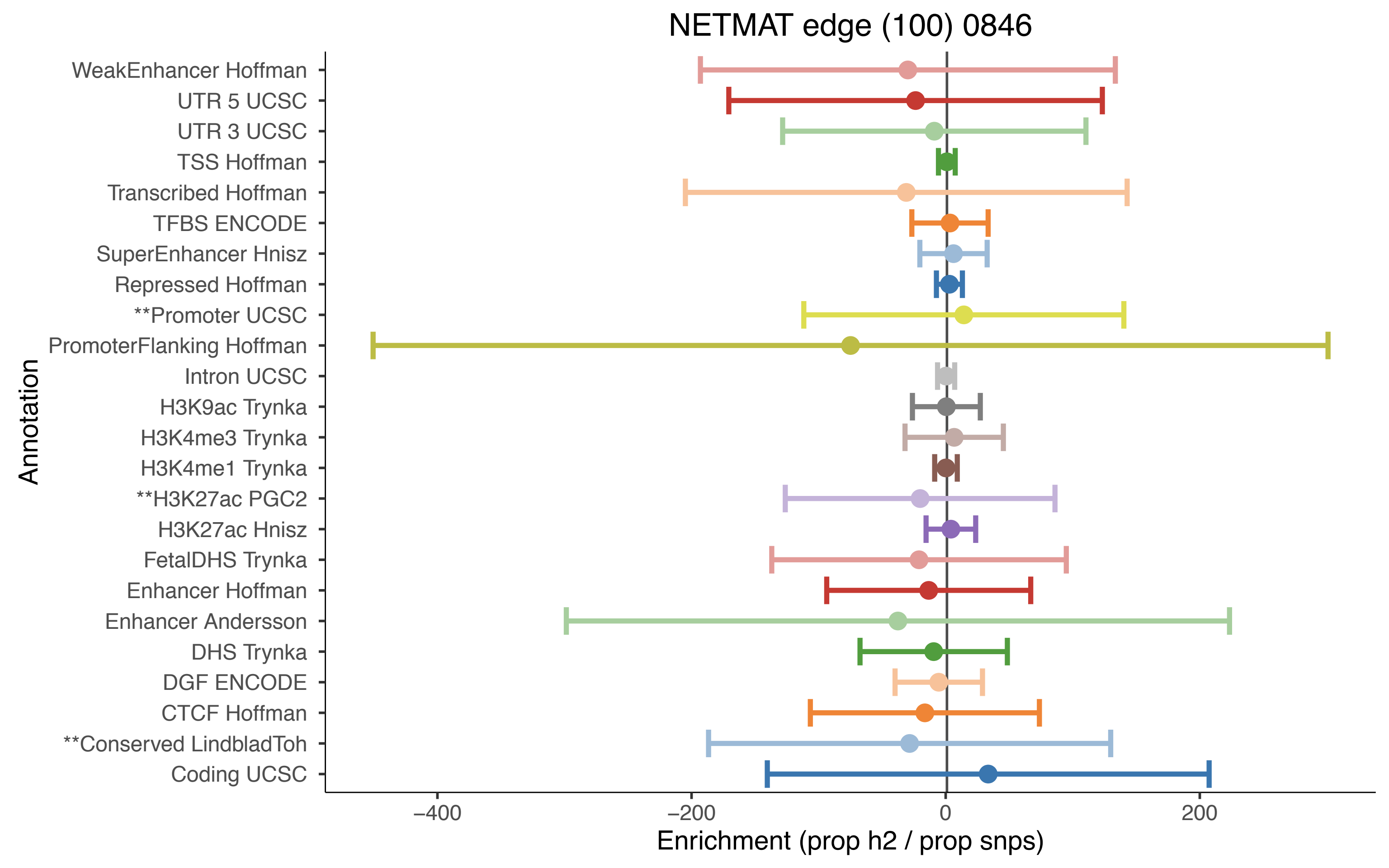

NETMAT edge (100) 0846
WeakEnhancer Hoffman
UTR 5 UCSC
UTR 3 UCSC
TSS Hoffman
Transcribed Hoffman
TFBS ENCODE
SuperEnhancer Hnisz
Repressed Hoffman
**Promoter UCSC
PromoterFlanking Hoffman
Intron UCSC
H3K9ac Trynka
H3K4me3 Trynka
H3K4me1 Trynka
**H3K27ac PGC2
H3K27ac Hnisz
FetalDHS Trynka
Enhancer Hoffman
Enhancer Andersson
DHS Trynka
DGF ENCODE
CTCF Hoffman
**Conserved LindbladToh
Coding UCSC
Annotation
−400
−200
0
200
Enrichment (prop h2 / prop snps)

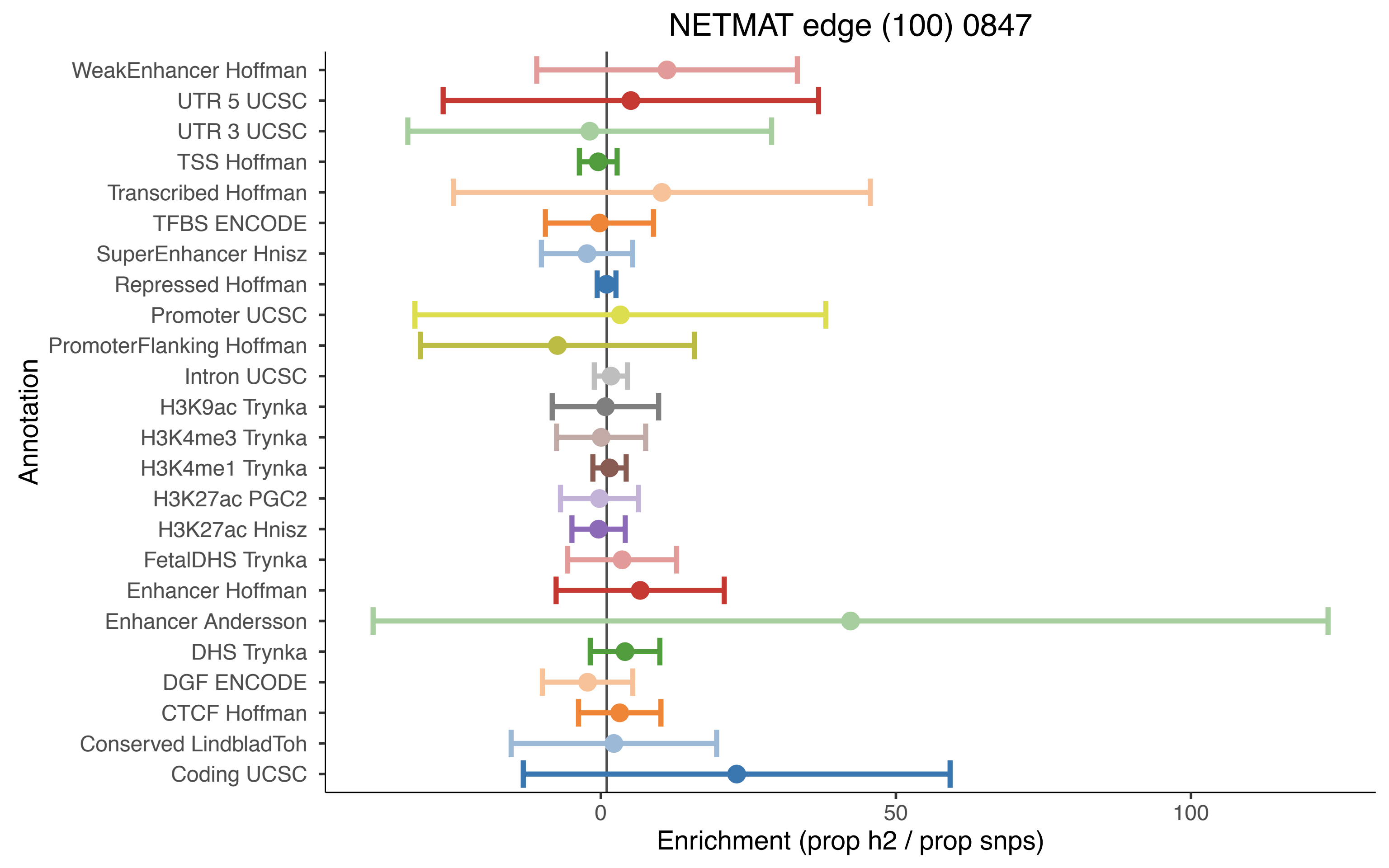

NETMAT edge (100) 0847
Annotation
WeakEnhancer Hoffman
UTR 5 UCSC
UTR 3 UCSC
TSS Hoffman
Transcribed Hoffman
TFBS ENCODE
SuperEnhancer Hnisz
Repressed Hoffman
Promoter UCSC
PromoterFlanking Hoffman
Intron UCSC
H3K9ac Trynka
H3K4me3 Trynka
H3K4me1 Trynka
H3K27ac PGC2
H3K27ac Hnisz
FetalDHS Trynka
Enhancer Hoffman
Enhancer Andersson
DHS Trynka
DGF ENCODE
CTCF Hoffman
Conserved LindbladToh
Coding UCSC
0
50
100
Enrichment (prop h2 / prop snps)

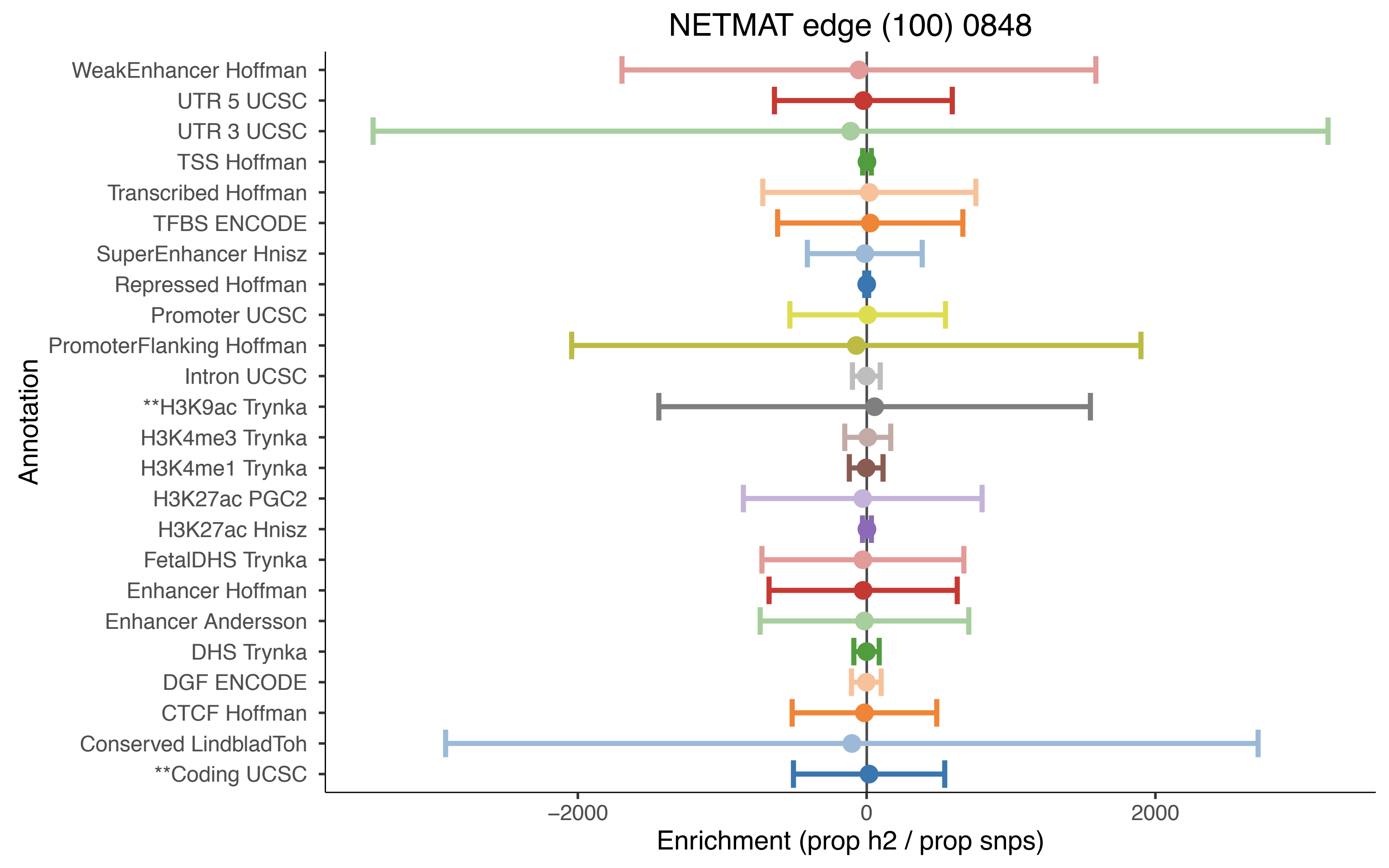
NETMAT edge (100) 0848
Annotation
WeakEnhancer Hoffman
UTR 5 UCSC
UTR 3 UCSC
TSS Hoffman
Transcribed Hoffman
TFBS ENCODE
SuperEnhancer Hnisz
Repressed Hoffman
Promoter UCSC
PromoterFlanking Hoffman
Intron UCSC
**H3K9ac Trynka
H3K4me3 Trynka
H3K4me1 Trynka
H3K27ac PGC2
H3K27ac Hnisz
FetalDHS Trynka
Enhancer Hoffman
Enhancer Andersson
DHS Trynka
DGF ENCODE
CTCF Hoffman
Conserved LindbladToh
**Coding UCSC
−2000
0
2000
Enrichment (prop h2 / prop snps)

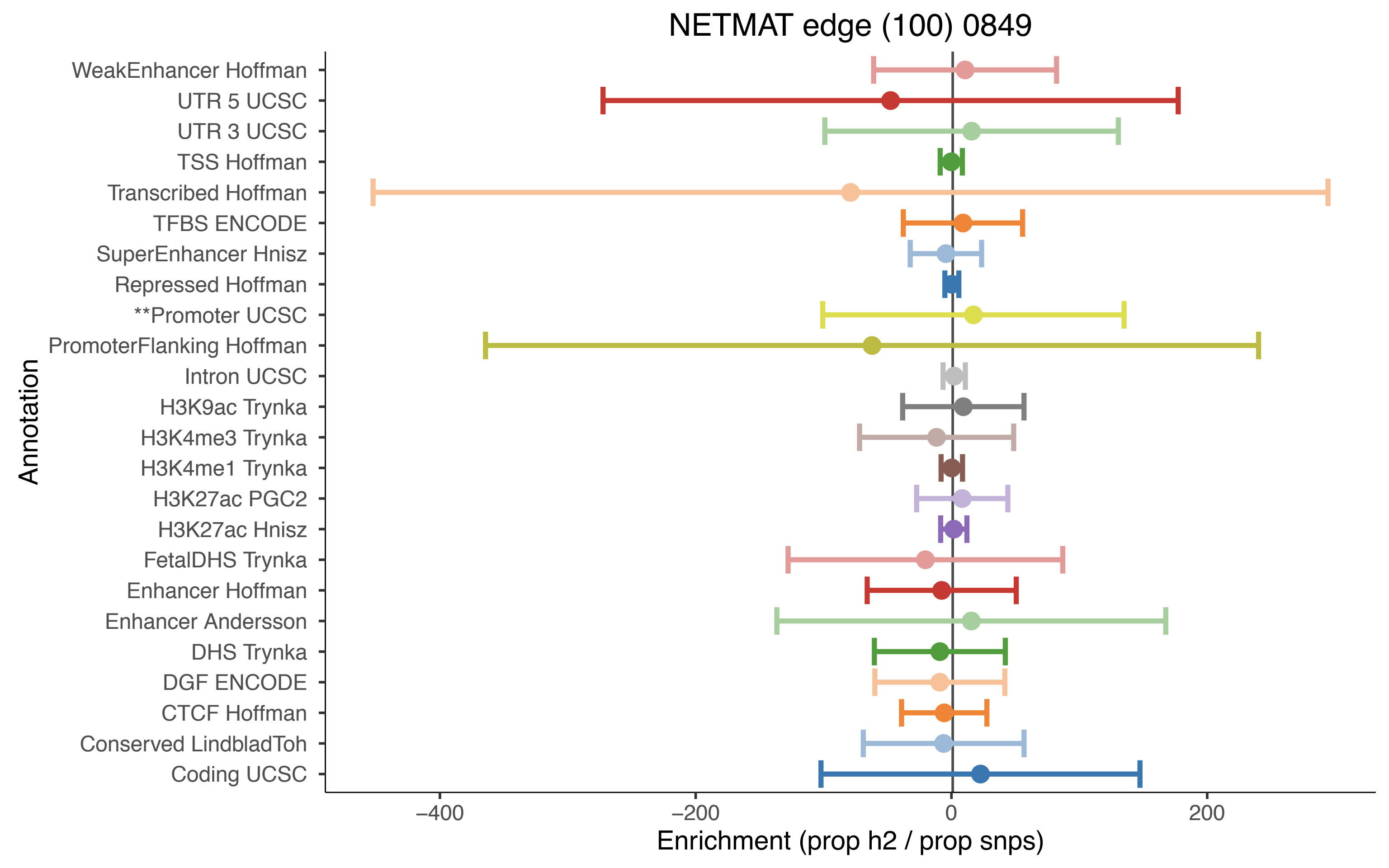

NETMAT edge (100) 0849
WeakEnhancer Hoffman
UTR 5 UCSC
UTR 3 UCSC
TSS Hoffman
Transcribed Hoffman
TFBS ENCODE
SuperEnhancer Hnisz
Repressed Hoffman
**Promoter UCSC
PromoterFlanking Hoffman
Intron UCSC
H3K9ac Trynka
H3K4me3 Trynka
H3K4me1 Trynka
H3K27ac PGC2
H3K27ac Hnisz
FetalDHS Trynka
Enhancer Hoffman
Enhancer Andersson
DHS Trynka
DGF ENCODE
CTCF Hoffman
Conserved LindbladToh
Coding UCSC
Annotation
−400
−200
0
200
Enrichment (prop h2 / prop snps)

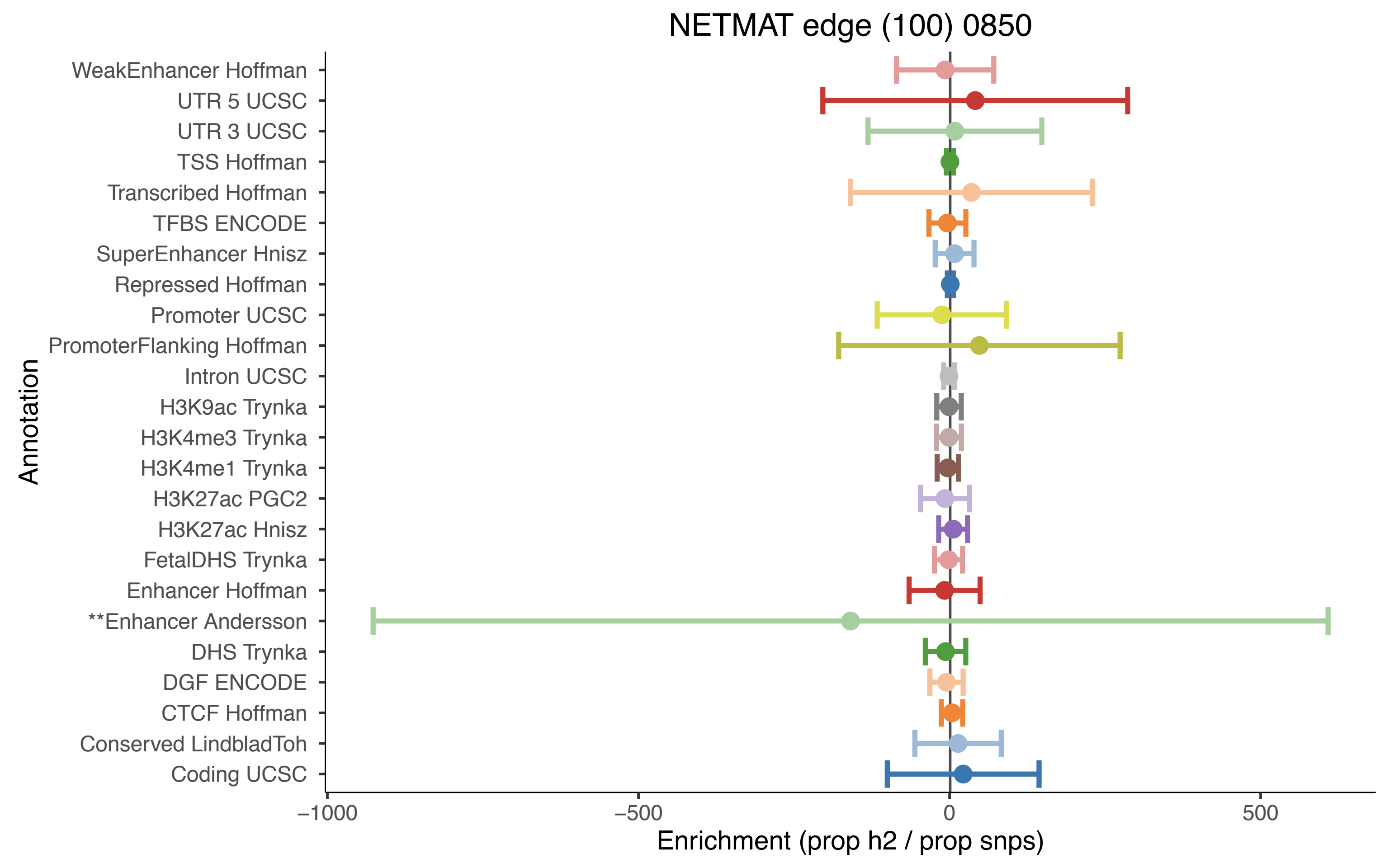

NETMAT edge (100) 0850
Annotation
WeakEnhancer Hoffman
UTR 5 UCSC
UTR 3 UCSC
TSS Hoffman
Transcribed Hoffman
TFBS ENCODE
SuperEnhancer Hnisz
Repressed Hoffman
Promoter UCSC
PromoterFlanking Hoffman
Intron UCSC
H3K9ac Trynka
H3K4me3 Trynka
H3K4me1 Trynka
H3K27ac PGC2
H3K27ac Hnisz
FetalDHS Trynka
Enhancer Hoffman
**Enhancer Andersson
DHS Trynka
DGF ENCODE
CTCF Hoffman
Conserved LindbladToh
Coding UCSC
−1000
−500
0
500
Enrichment (prop h2 / prop snps)

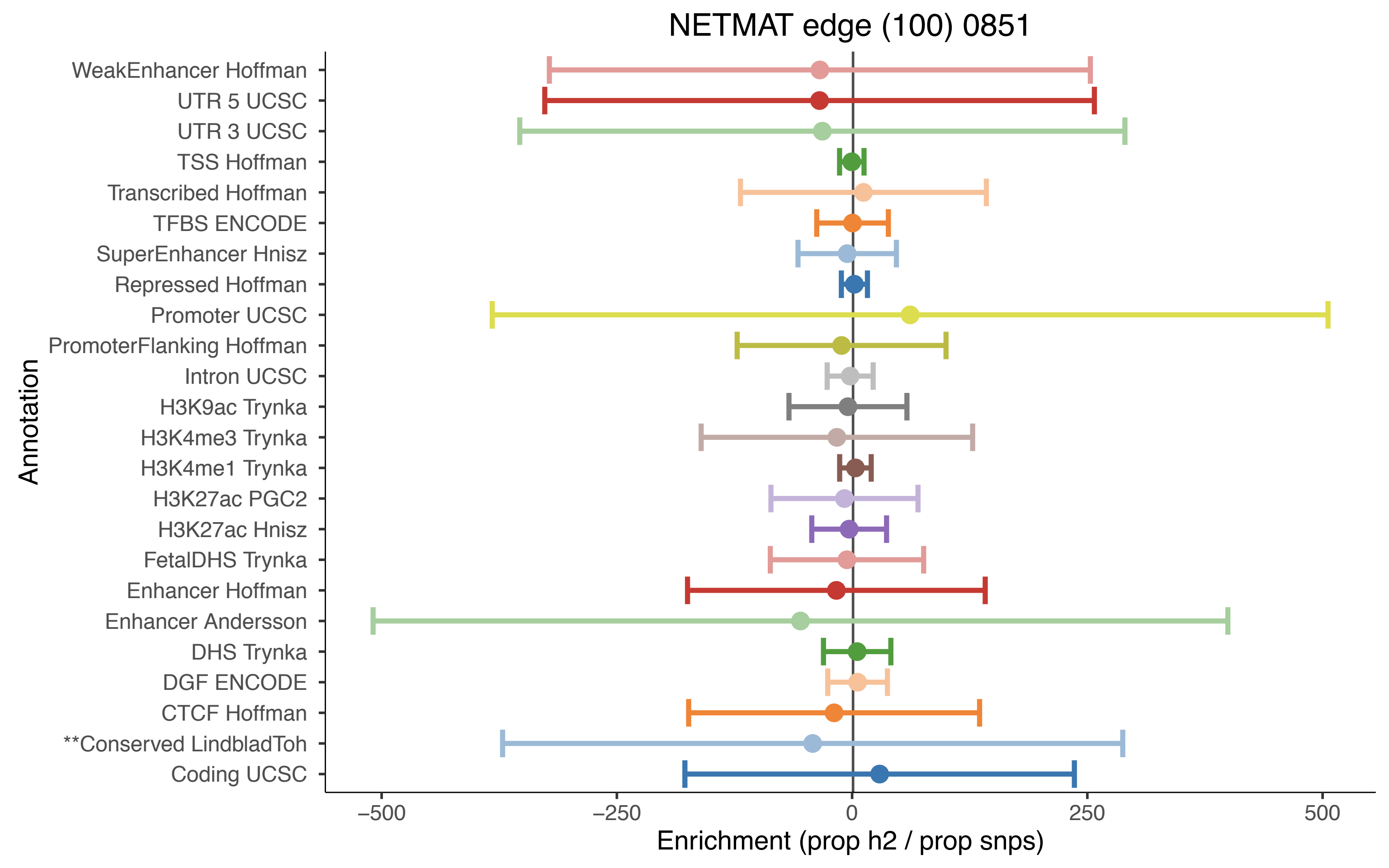
NETMAT edge (100) 0851
WeakEnhancer Hoffman
UTR 5 UCSC
UTR 3 UCSC
TSS Hoffman
Transcribed Hoffman
TFBS ENCODE
SuperEnhancer Hnisz
Repressed Hoffman
Promoter UCSC
PromoterFlanking Hoffman
Intron UCSC
H3K9ac Trynka
H3K4me3 Trynka
H3K4me1 Trynka
H3K27ac PGC2
H3K27ac Hnisz
FetalDHS Trynka
Enhancer Hoffman
Enhancer Andersson
DHS Trynka
DGF ENCODE
CTCF Hoffman
**Conserved LindbladToh
Coding UCSC
Annotation
−500
−250
0
250
500
Enrichment (prop h2 / prop snps)

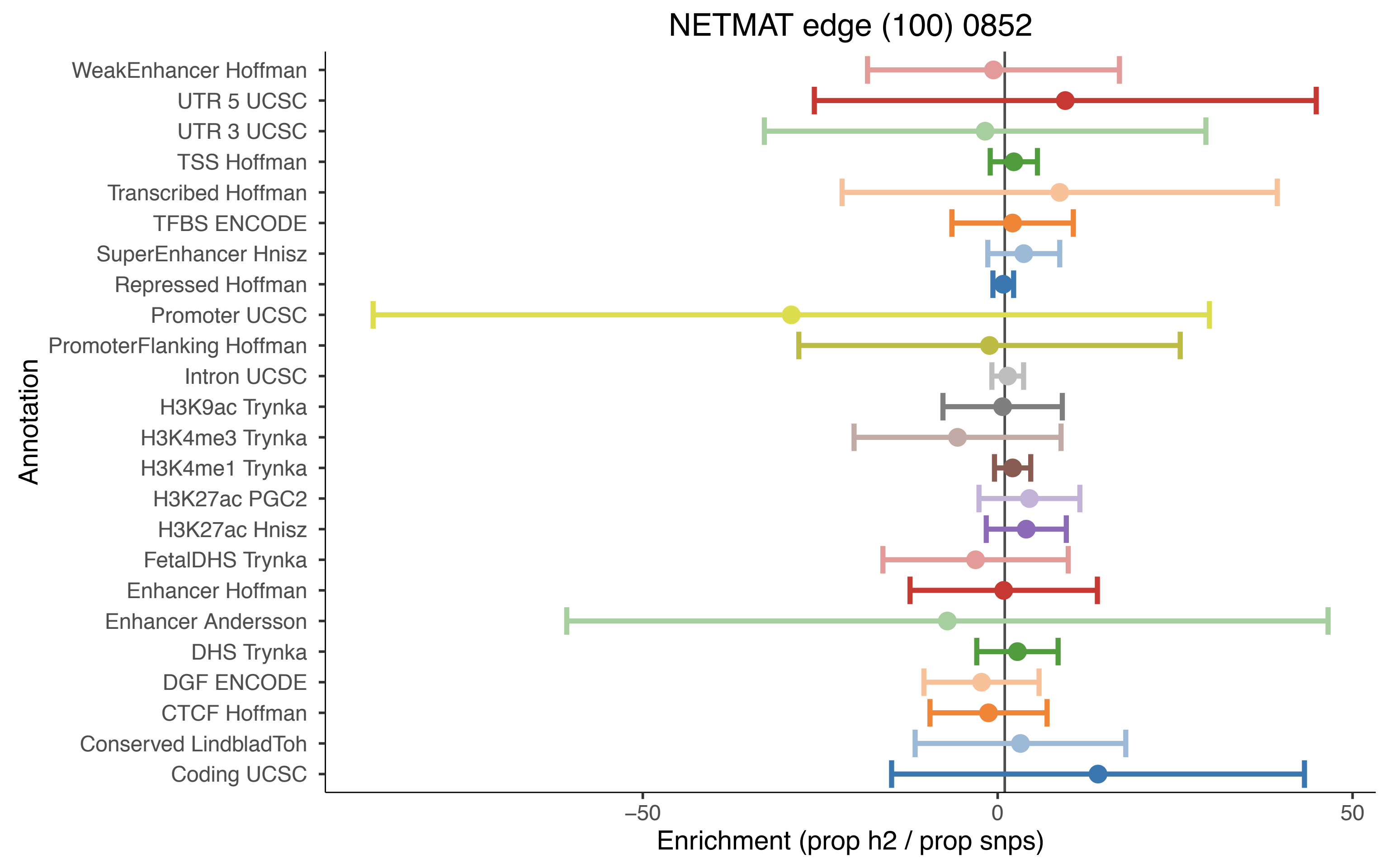
NETMAT edge (100) 0852
WeakEnhancer Hoffman
UTR 5 UCSC
UTR 3 UCSC
TSS Hoffman
Transcribed Hoffman
TFBS ENCODE
SuperEnhancer Hnisz
Repressed Hoffman
Promoter UCSC
PromoterFlanking Hoffman
Intron UCSC
H3K9ac Trynka
H3K4me3 Trynka
H3K4me1 Trynka
H3K27ac PGC2
H3K27ac Hnisz
FetalDHS Trynka
Enhancer Hoffman
Enhancer Andersson
DHS Trynka
DGF ENCODE
CTCF Hoffman
Conserved LindbladToh
Coding UCSC
Annotation
−50
0
50
Enrichment (prop h2 / prop snps)

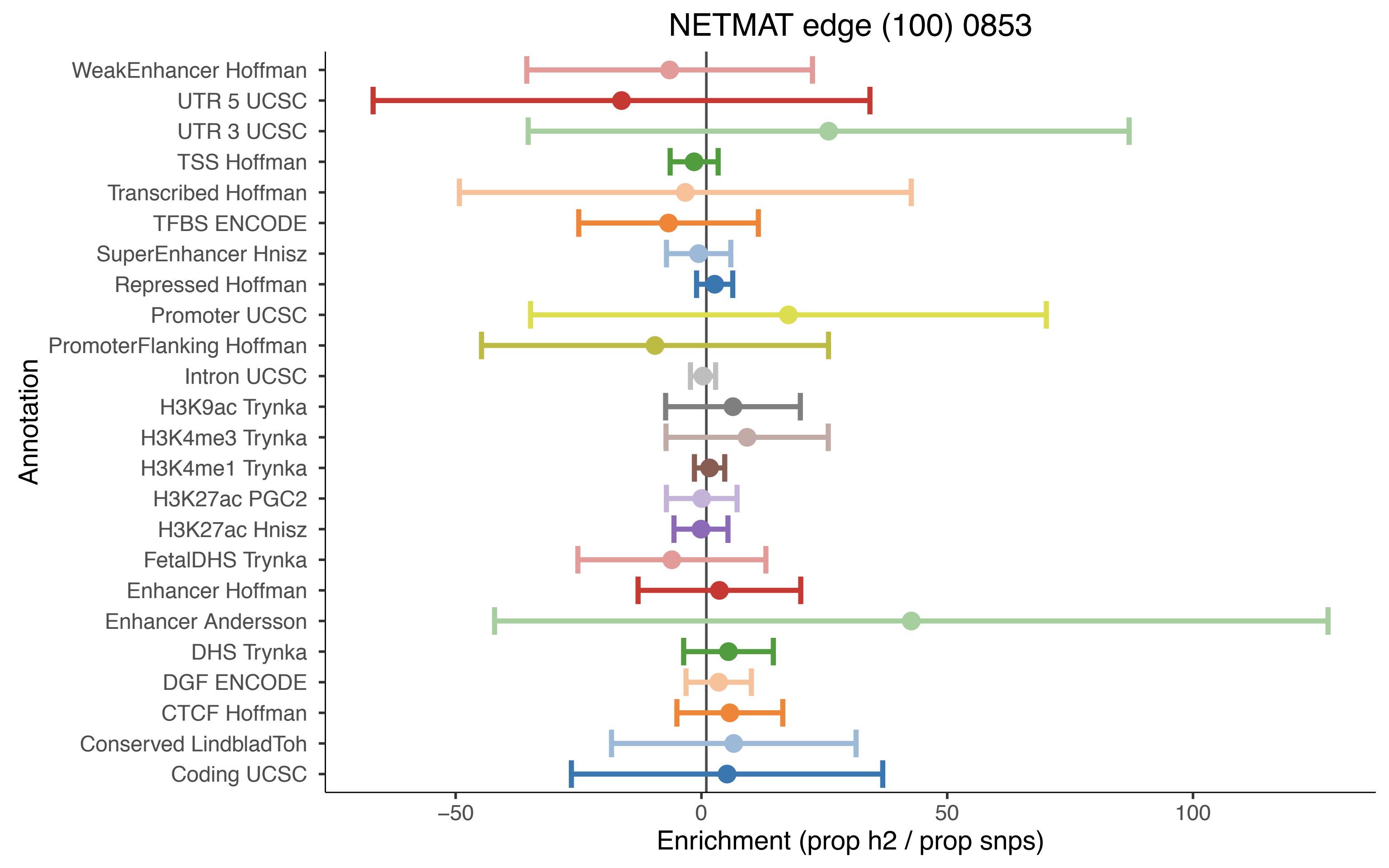
NETMAT edge (100) 0853
Annotation
WeakEnhancer Hoffman
UTR 5 UCSC
UTR 3 UCSC
TSS Hoffman
Transcribed Hoffman
TFBS ENCODE
SuperEnhancer Hnisz
Repressed Hoffman
Promoter UCSC
PromoterFlanking Hoffman
Intron UCSC
H3K9ac Trynka
H3K4me3 Trynka
H3K4me1 Trynka
H3K27ac PGC2
H3K27ac Hnisz
FetalDHS Trynka
Enhancer Hoffman
Enhancer Andersson
DHS Trynka
DGF ENCODE
CTCF Hoffman
Conserved LindbladToh
Coding UCSC
−50
0
50
100
Enrichment (prop h2 / prop snps)

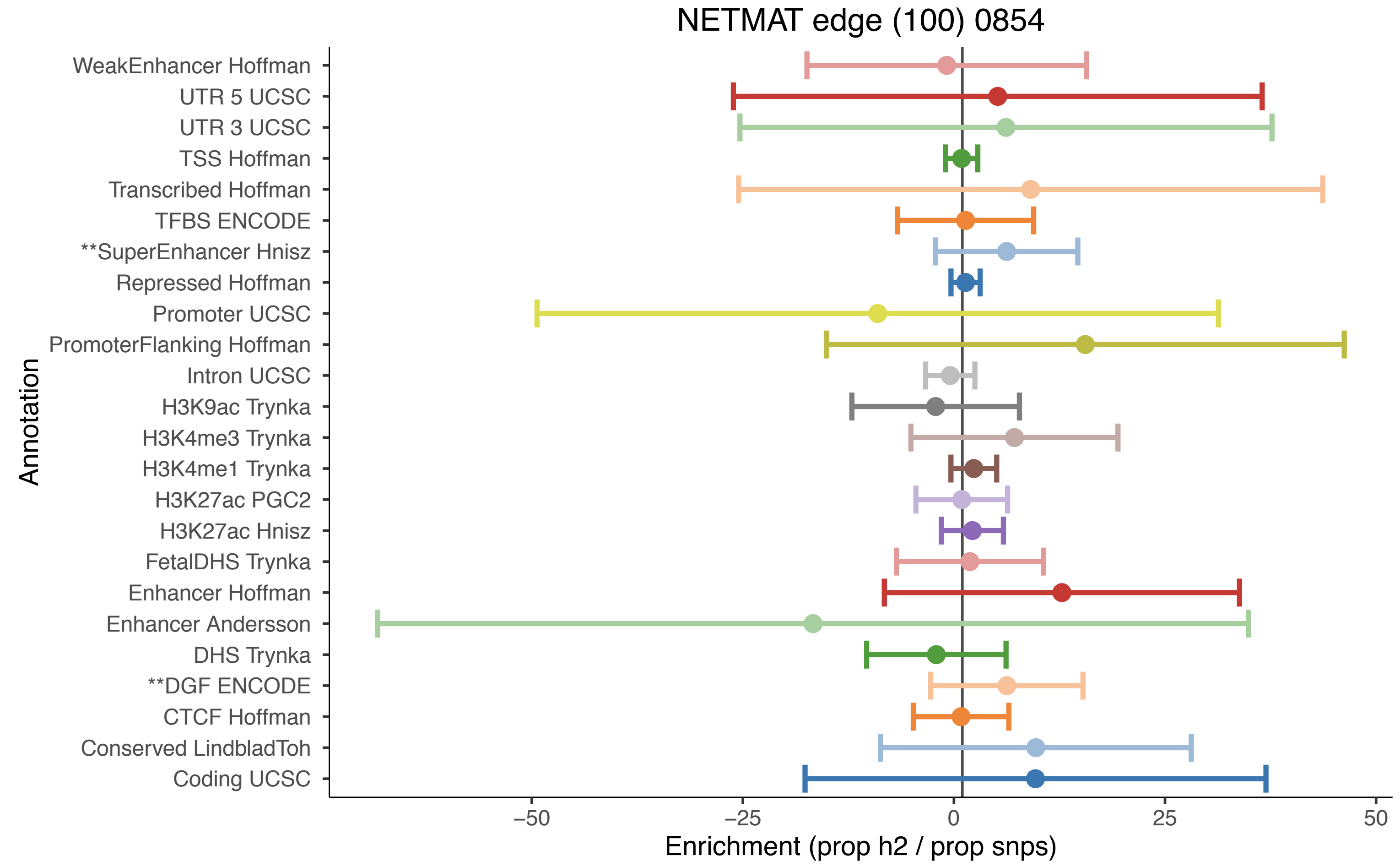

NETMAT edge (100) 0854
WeakEnhancer Hoffman
UTR 5 UCSC
UTR 3 UCSC
TSS Hoffman
Transcribed Hoffman
TFBS ENCODE
**SuperEnhancer Hnisz
Repressed Hoffman
Promoter UCSC
PromoterFlanking Hoffman
Intron UCSC
H3K9ac Trynka
H3K4me3 Trynka
H3K4me1 Trynka
H3K27ac PGC2
H3K27ac Hnisz
FetalDHS Trynka
Enhancer Hoffman
Enhancer Andersson
DHS Trynka
**DGF ENCODE
CTCF Hoffman
Conserved LindbladToh
Coding UCSC
Annotation
−50
−25
0
25
50
Enrichment (prop h2 / prop snps)

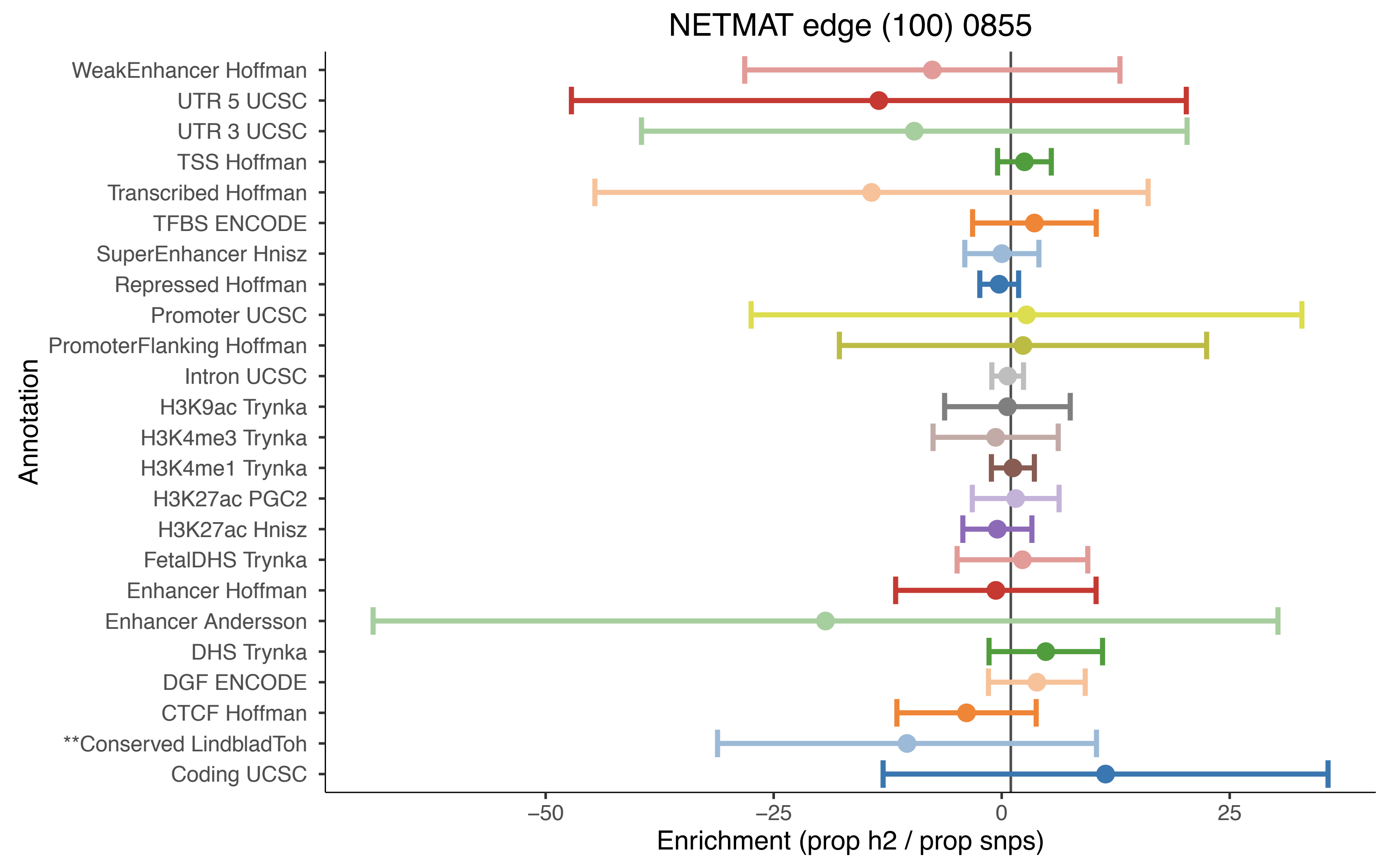

NETMAT edge (100) 0855
WeakEnhancer Hoffman
UTR 5 UCSC
UTR 3 UCSC
TSS Hoffman
Transcribed Hoffman
TFBS ENCODE
SuperEnhancer Hnisz
Repressed Hoffman
Promoter UCSC
PromoterFlanking Hoffman
Intron UCSC
H3K9ac Trynka
H3K4me3 Trynka
H3K4me1 Trynka
H3K27ac PGC2
H3K27ac Hnisz
FetalDHS Trynka
Enhancer Hoffman
Enhancer Andersson
DHS Trynka
DGF ENCODE
CTCF Hoffman
**Conserved LindbladToh
Coding UCSC
Annotation
−50
−25
0
25
Enrichment (prop h2 / prop snps)

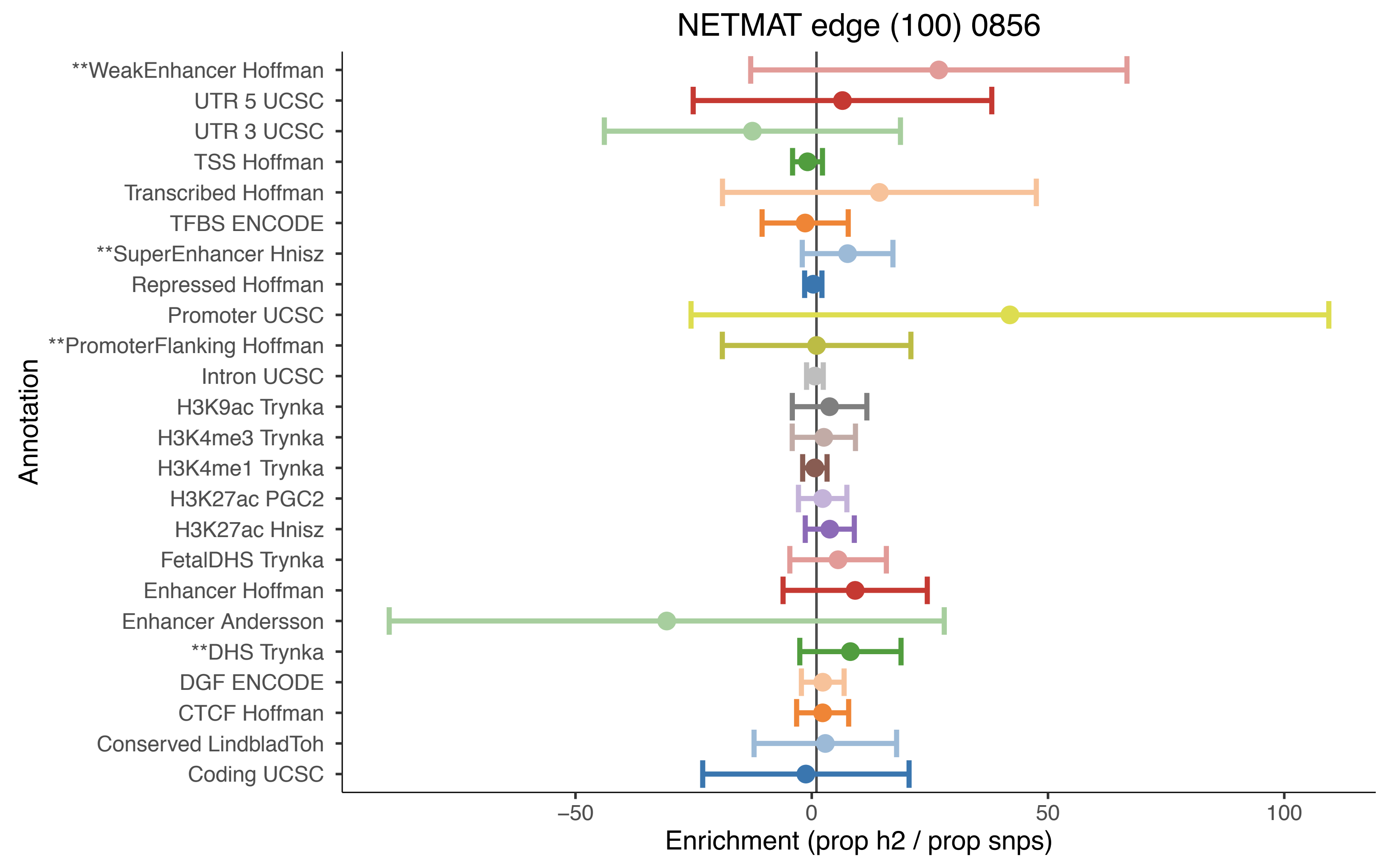
NETMAT edge (100) 0856
**WeakEnhancer Hoffman
UTR 5 UCSC
UTR 3 UCSC
TSS Hoffman
Transcribed Hoffman
TFBS ENCODE
**SuperEnhancer Hnisz
Repressed Hoffman
Promoter UCSC
**PromoterFlanking Hoffman
Intron UCSC
H3K9ac Trynka
H3K4me3 Trynka
H3K4me1 Trynka
H3K27ac PGC2
H3K27ac Hnisz
FetalDHS Trynka
Enhancer Hoffman
Enhancer Andersson
**DHS Trynka
DGF ENCODE
CTCF Hoffman
Conserved LindbladToh
Coding UCSC
Annotation
−50
0
50
100
Enrichment (prop h2 / prop snps)

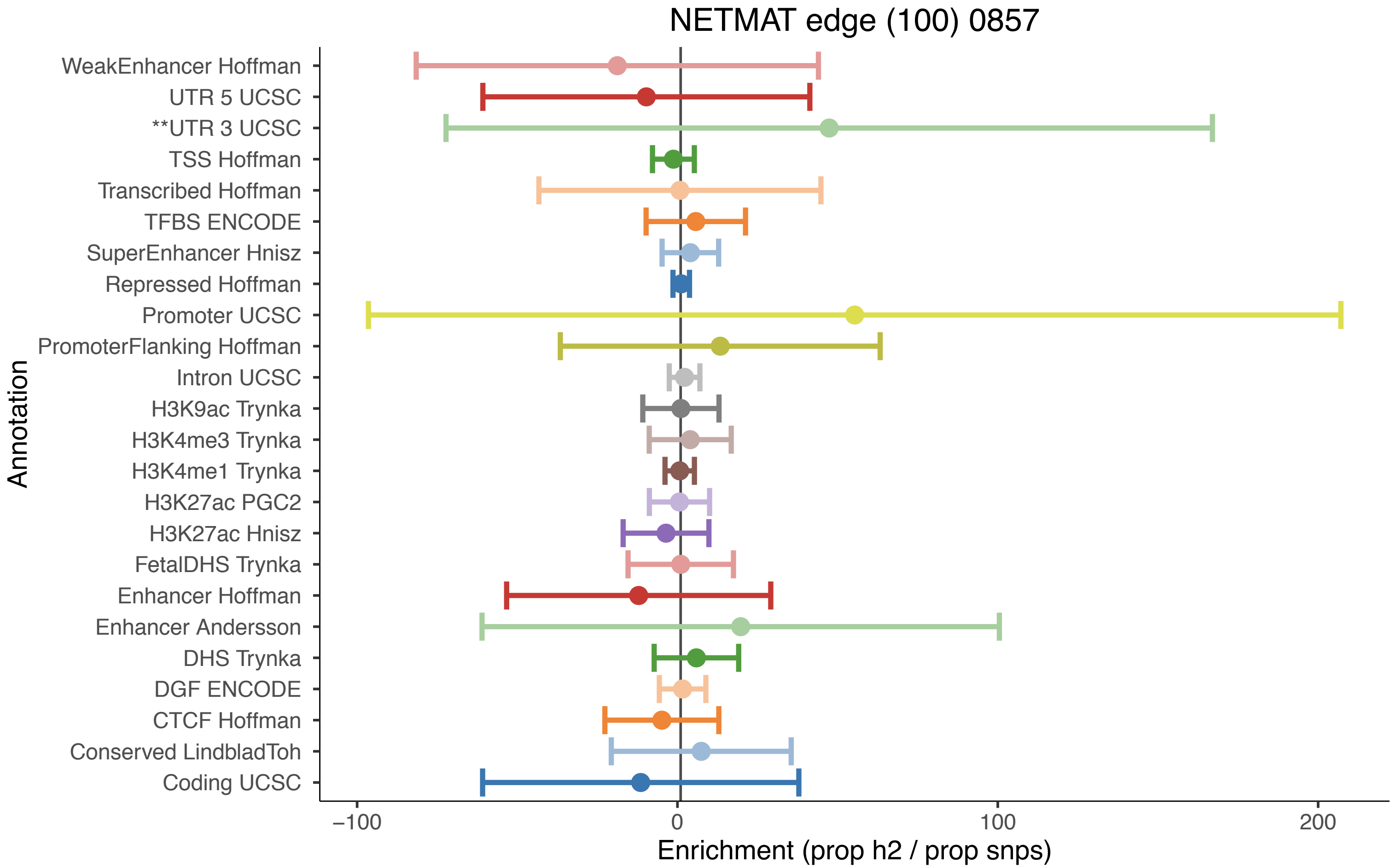

NETMAT edge (100) 0857
WeakEnhancer Hoffman
UTR 5 UCSC
**UTR 3 UCSC
TSS Hoffman
Transcribed Hoffman
TFBS ENCODE
SuperEnhancer Hnisz
Repressed Hoffman
Promoter UCSC
PromoterFlanking Hoffman
Intron UCSC
H3K9ac Trynka
H3K4me3 Trynka
H3K4me1 Trynka
H3K27ac PGC2
H3K27ac Hnisz
FetalDHS Trynka
Enhancer Hoffman
Enhancer Andersson
DHS Trynka
DGF ENCODE
CTCF Hoffman
Conserved LindbladToh
Coding UCSC
Annotation
−100
0
100
200
Enrichment (prop h2 / prop snps)

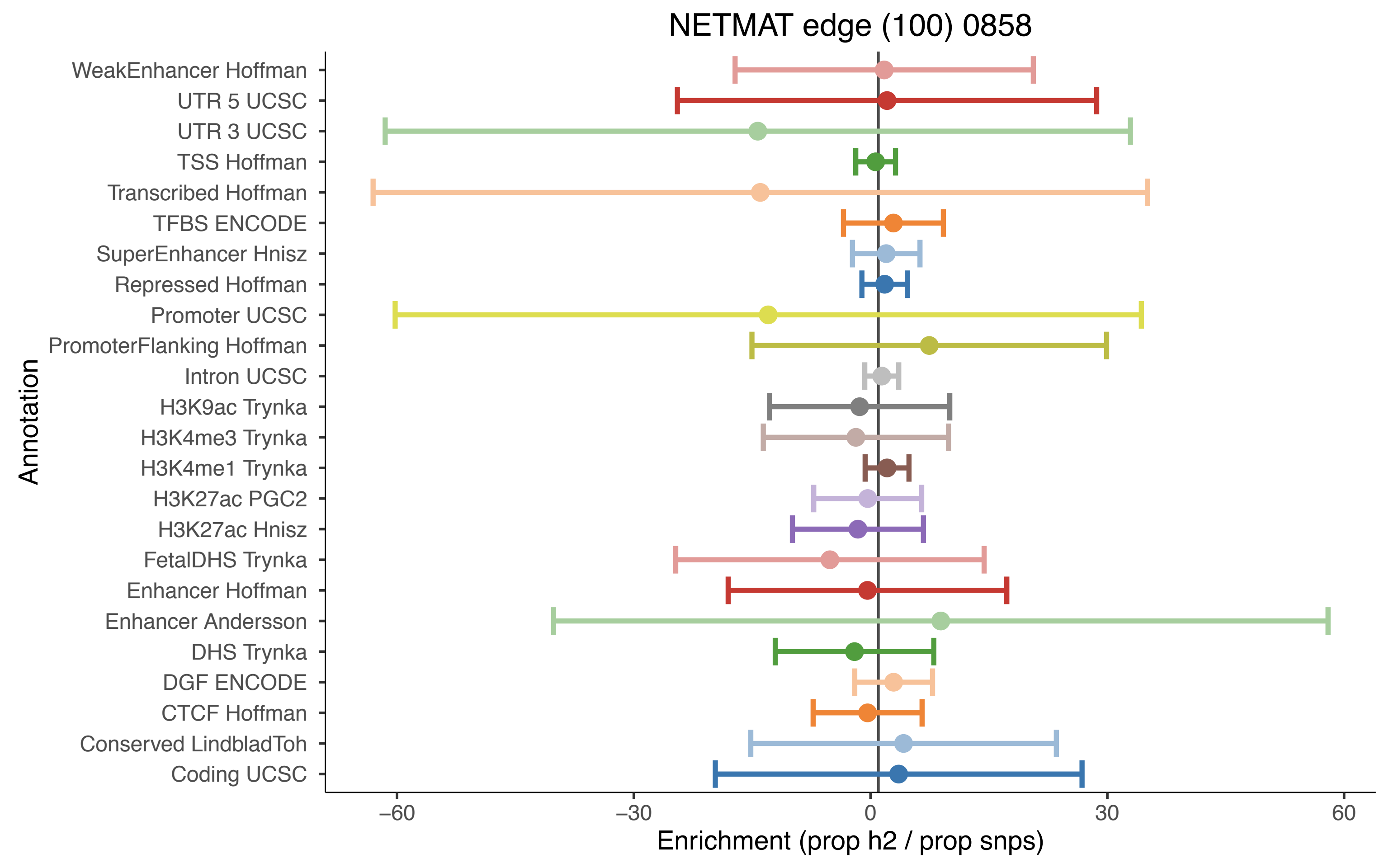
NETMAT edge (100) 0858
Annotation
WeakEnhancer Hoffman
UTR 5 UCSC
UTR 3 UCSC
TSS Hoffman
Transcribed Hoffman
TFBS ENCODE
SuperEnhancer Hnisz
Repressed Hoffman
Promoter UCSC
PromoterFlanking Hoffman
Intron UCSC
H3K9ac Trynka
H3K4me3 Trynka
H3K4me1 Trynka
H3K27ac PGC2
H3K27ac Hnisz
FetalDHS Trynka
Enhancer Hoffman
Enhancer Andersson
DHS Trynka
DGF ENCODE
CTCF Hoffman
Conserved LindbladToh
Coding UCSC
−60
−30
0
30
60
Enrichment (prop h2 / prop snps)

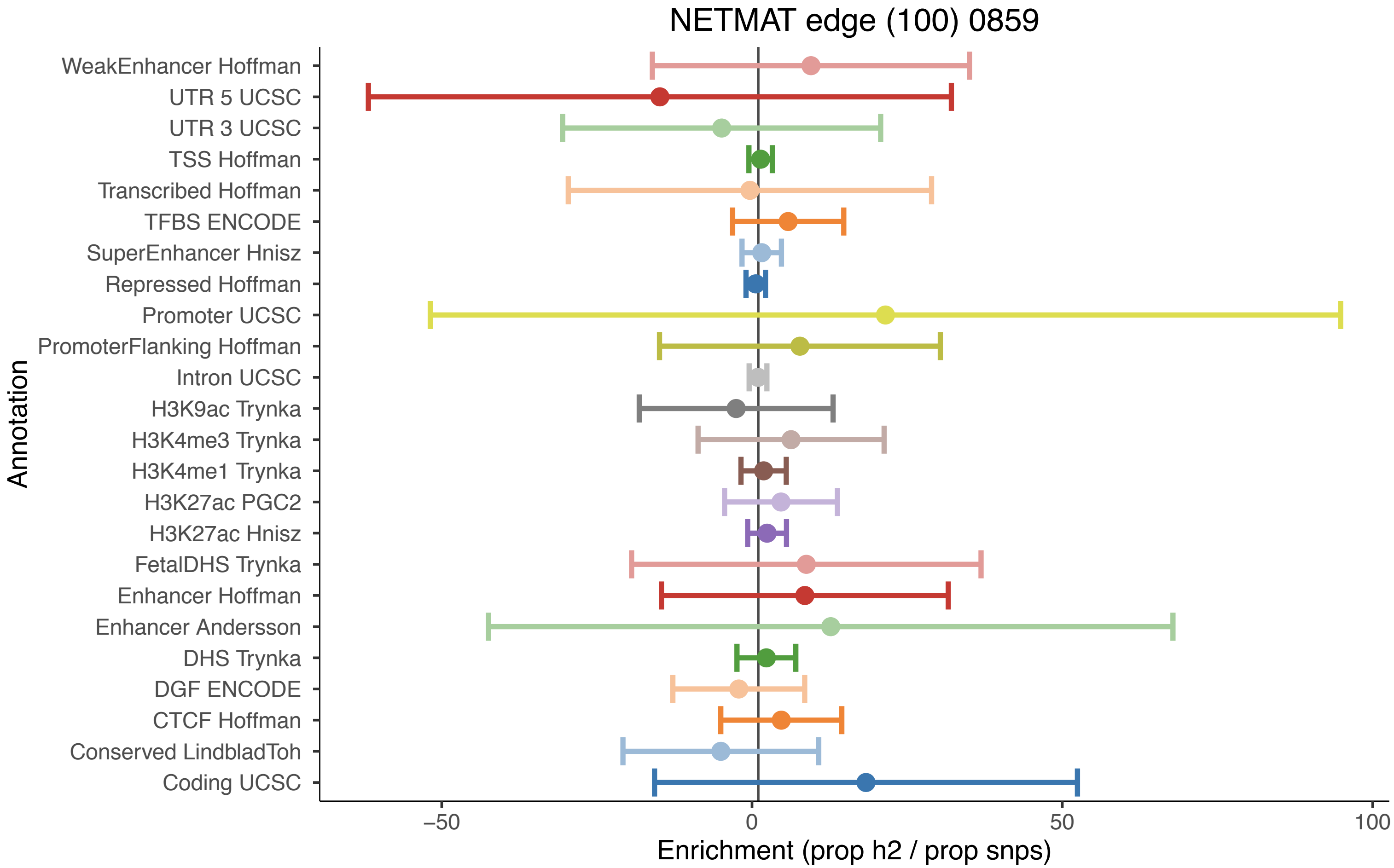

NETMAT edge (100) 0859
WeakEnhancer Hoffman
UTR 5 UCSC
UTR 3 UCSC
TSS Hoffman
Transcribed Hoffman
TFBS ENCODE
SuperEnhancer Hnisz
Repressed Hoffman
Promoter UCSC
PromoterFlanking Hoffman
Intron UCSC
H3K9ac Trynka
H3K4me3 Trynka
H3K4me1 Trynka
H3K27ac PGC2
H3K27ac Hnisz
FetalDHS Trynka
Enhancer Hoffman
Enhancer Andersson
DHS Trynka
DGF ENCODE
CTCF Hoffman
Conserved LindbladToh
Coding UCSC
Annotation
−50
0
50
100
Enrichment (prop h2 / prop snps)

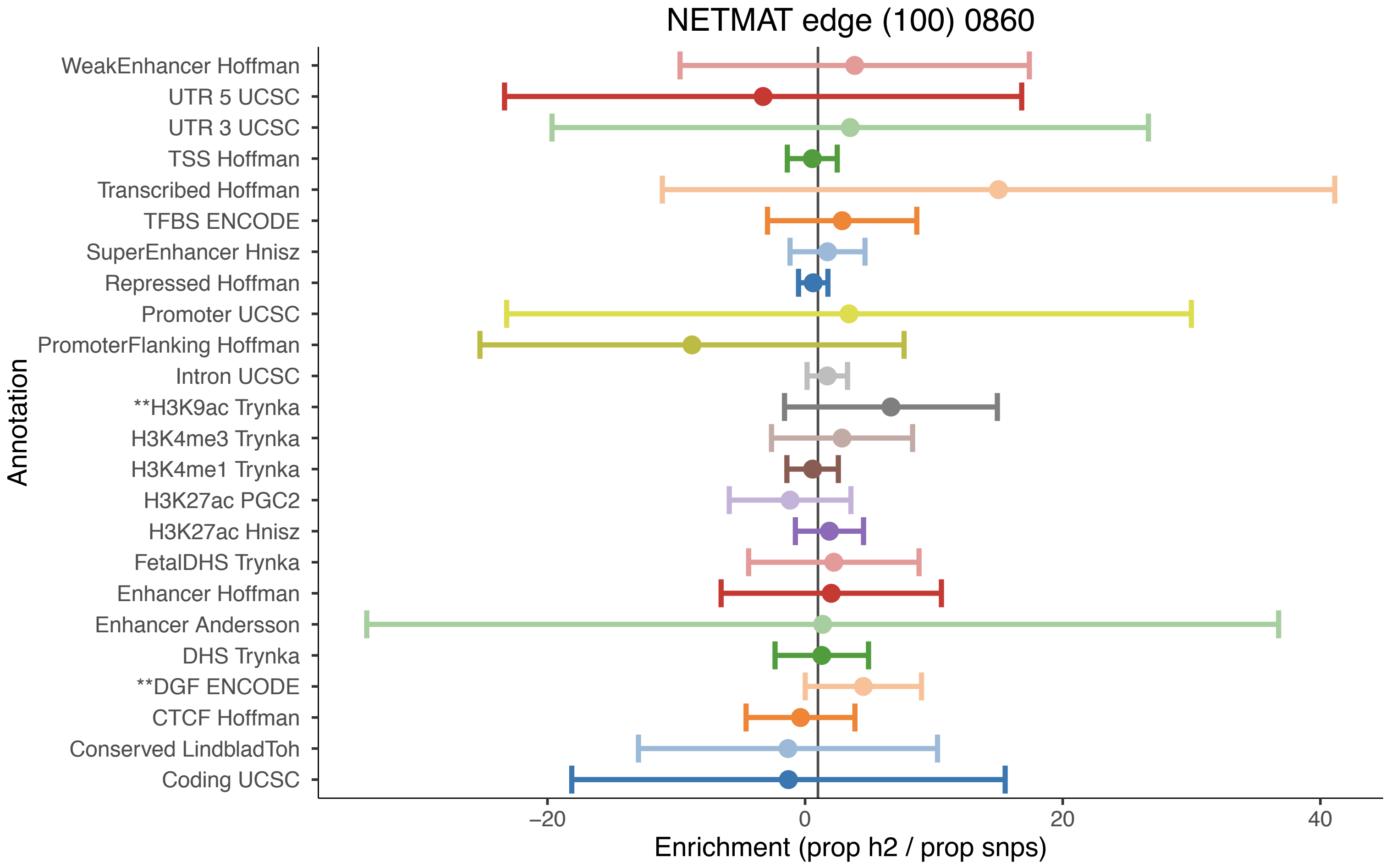
NETMAT edge (100) 0860
WeakEnhancer Hoffman
UTR 5 UCSC
UTR 3 UCSC
TSS Hoffman
Transcribed Hoffman
TFBS ENCODE
SuperEnhancer Hnisz
Repressed Hoffman
Promoter UCSC
PromoterFlanking Hoffman
Intron UCSC
**H3K9ac Trynka
H3K4me3 Trynka
H3K4me1 Trynka
H3K27ac PGC2
H3K27ac Hnisz
FetalDHS Trynka
Enhancer Hoffman
Enhancer Andersson
DHS Trynka
**DGF ENCODE
CTCF Hoffman
Conserved LindbladToh
Coding UCSC
Annotation
−20
0
20
40
Enrichment (prop h2 / prop snps)

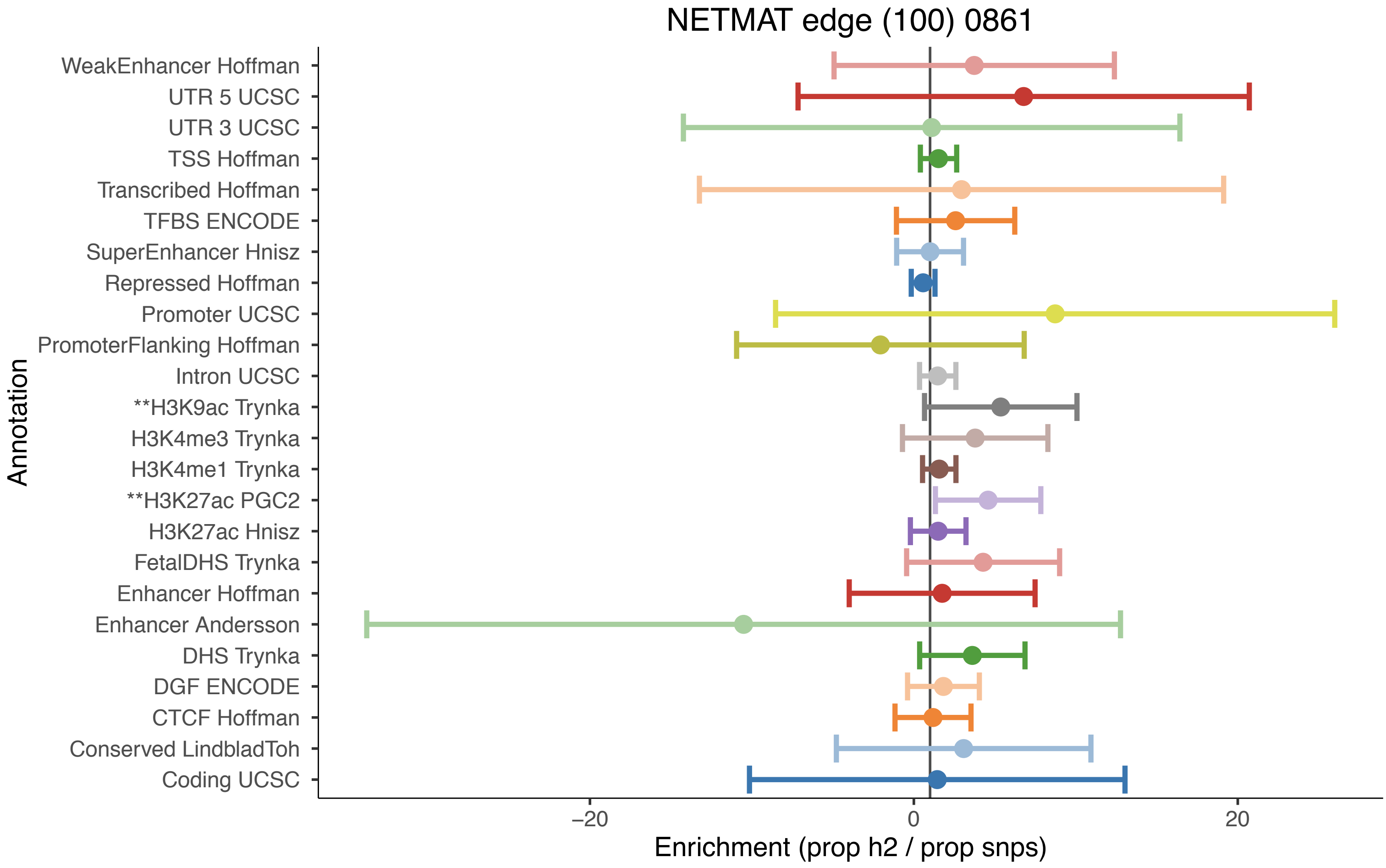

NETMAT edge (100) 0861
WeakEnhancer Hoffman
UTR 5 UCSC
UTR 3 UCSC
TSS Hoffman
Transcribed Hoffman
TFBS ENCODE
SuperEnhancer Hnisz
Repressed Hoffman
Promoter UCSC
PromoterFlanking Hoffman
Intron UCSC
**H3K9ac Trynka
H3K4me3 Trynka
H3K4me1 Trynka
**H3K27ac PGC2
H3K27ac Hnisz
FetalDHS Trynka
Enhancer Hoffman
Enhancer Andersson
DHS Trynka
DGF ENCODE
CTCF Hoffman
Conserved LindbladToh
Coding UCSC
Annotation
−20
0
20
Enrichment (prop h2 / prop snps)

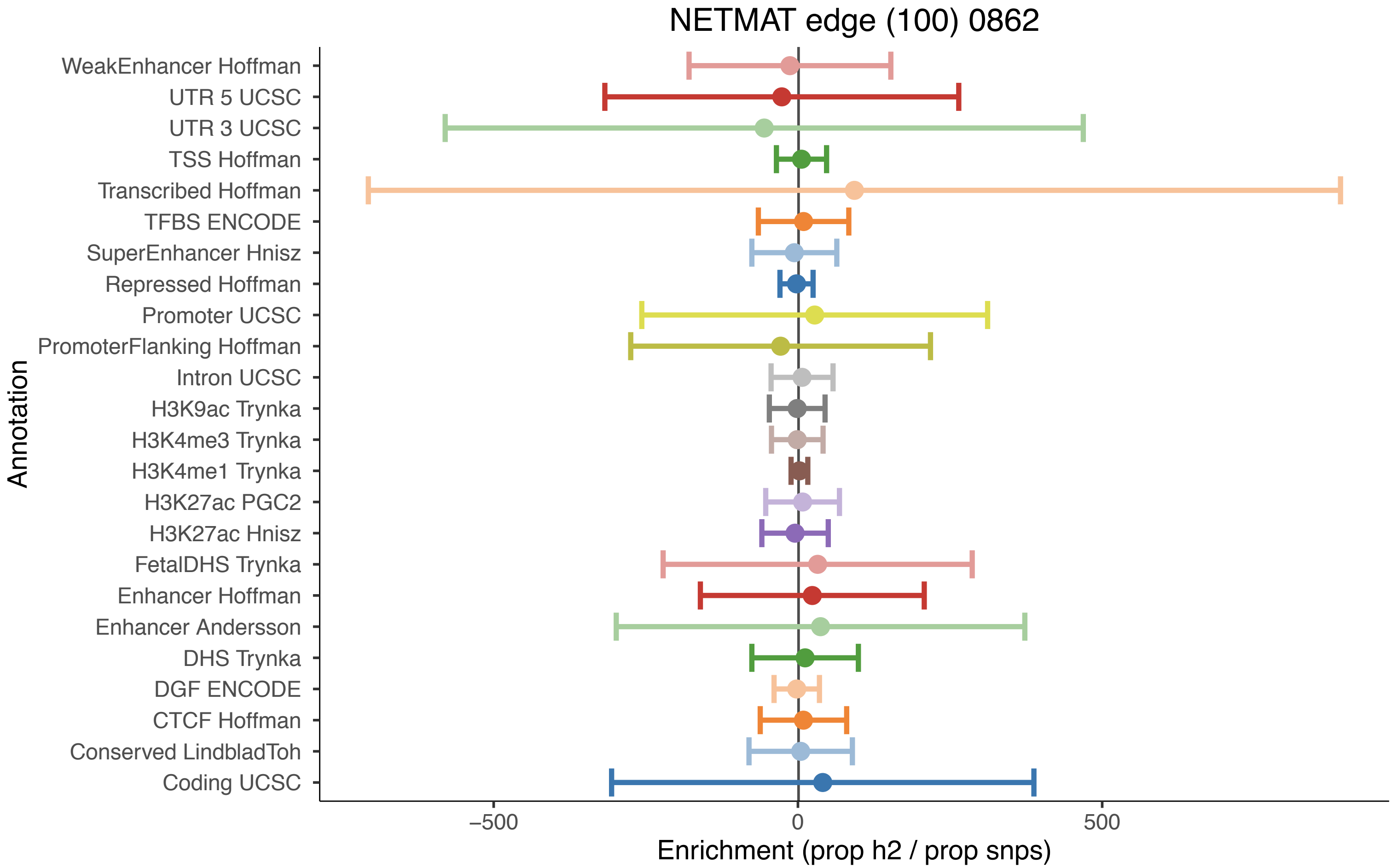
NETMAT edge (100) 0862
Annotation
WeakEnhancer Hoffman
UTR 5 UCSC
UTR 3 UCSC
TSS Hoffman
Transcribed Hoffman
TFBS ENCODE
SuperEnhancer Hnisz
Repressed Hoffman
Promoter UCSC
PromoterFlanking Hoffman
Intron UCSC
H3K9ac Trynka
H3K4me3 Trynka
H3K4me1 Trynka
H3K27ac PGC2
H3K27ac Hnisz
FetalDHS Trynka
Enhancer Hoffman
Enhancer Andersson
DHS Trynka
DGF ENCODE
CTCF Hoffman
Conserved LindbladToh
Coding UCSC
−500
0
500
Enrichment (prop h2 / prop snps)

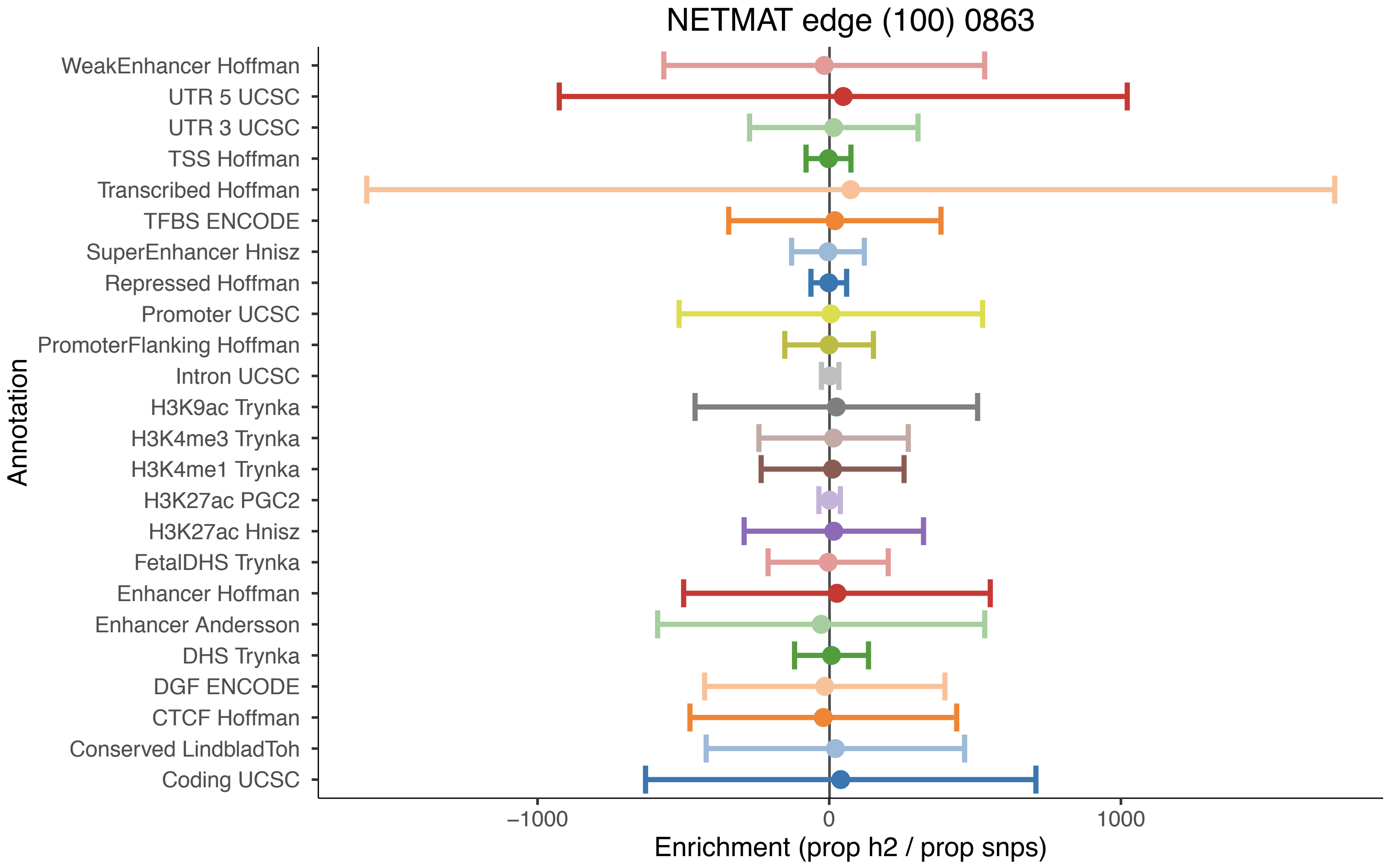
NETMAT edge (100) 0863
WeakEnhancer Hoffman
UTR 5 UCSC
UTR 3 UCSC
TSS Hoffman
Transcribed Hoffman
TFBS ENCODE
SuperEnhancer Hnisz
Repressed Hoffman
Promoter UCSC
PromoterFlanking Hoffman
Intron UCSC
H3K9ac Trynka
H3K4me3 Trynka
H3K4me1 Trynka
H3K27ac PGC2
H3K27ac Hnisz
FetalDHS Trynka
Enhancer Hoffman
Enhancer Andersson
DHS Trynka
DGF ENCODE
CTCF Hoffman
Conserved LindbladToh
Coding UCSC
Annotation
−1000
0
1000
Enrichment (prop h2 / prop snps)

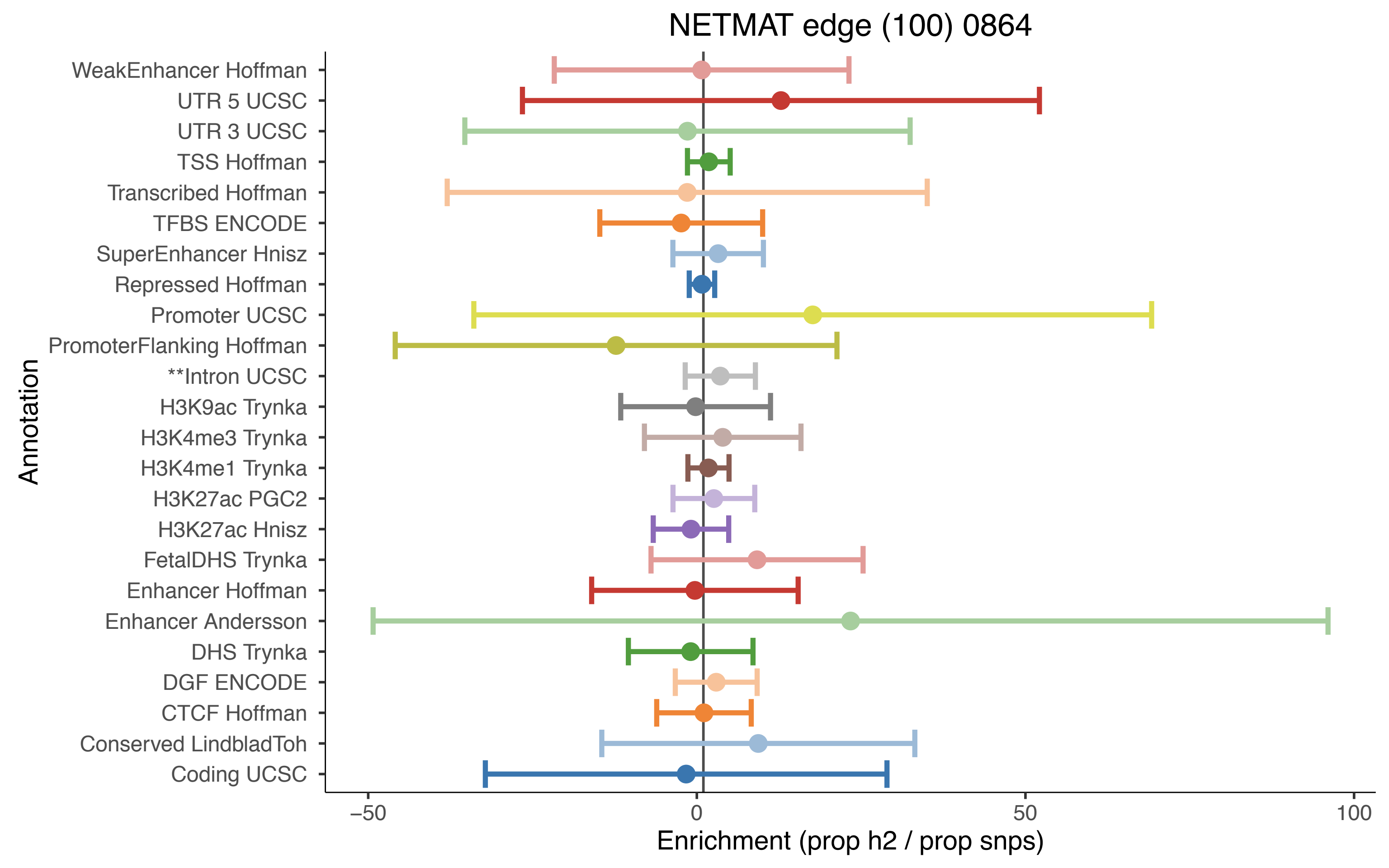

NETMAT edge (100) 0864
Annotation
WeakEnhancer Hoffman
UTR 5 UCSC
UTR 3 UCSC
TSS Hoffman
Transcribed Hoffman
TFBS ENCODE
SuperEnhancer Hnisz
Repressed Hoffman
Promoter UCSC
PromoterFlanking Hoffman
**Intron UCSC
H3K9ac Trynka
H3K4me3 Trynka
H3K4me1 Trynka
H3K27ac PGC2
H3K27ac Hnisz
FetalDHS Trynka
Enhancer Hoffman
Enhancer Andersson
DHS Trynka
DGF ENCODE
CTCF Hoffman
Conserved LindbladToh
Coding UCSC
−50
0
50
100
Enrichment (prop h2 / prop snps)

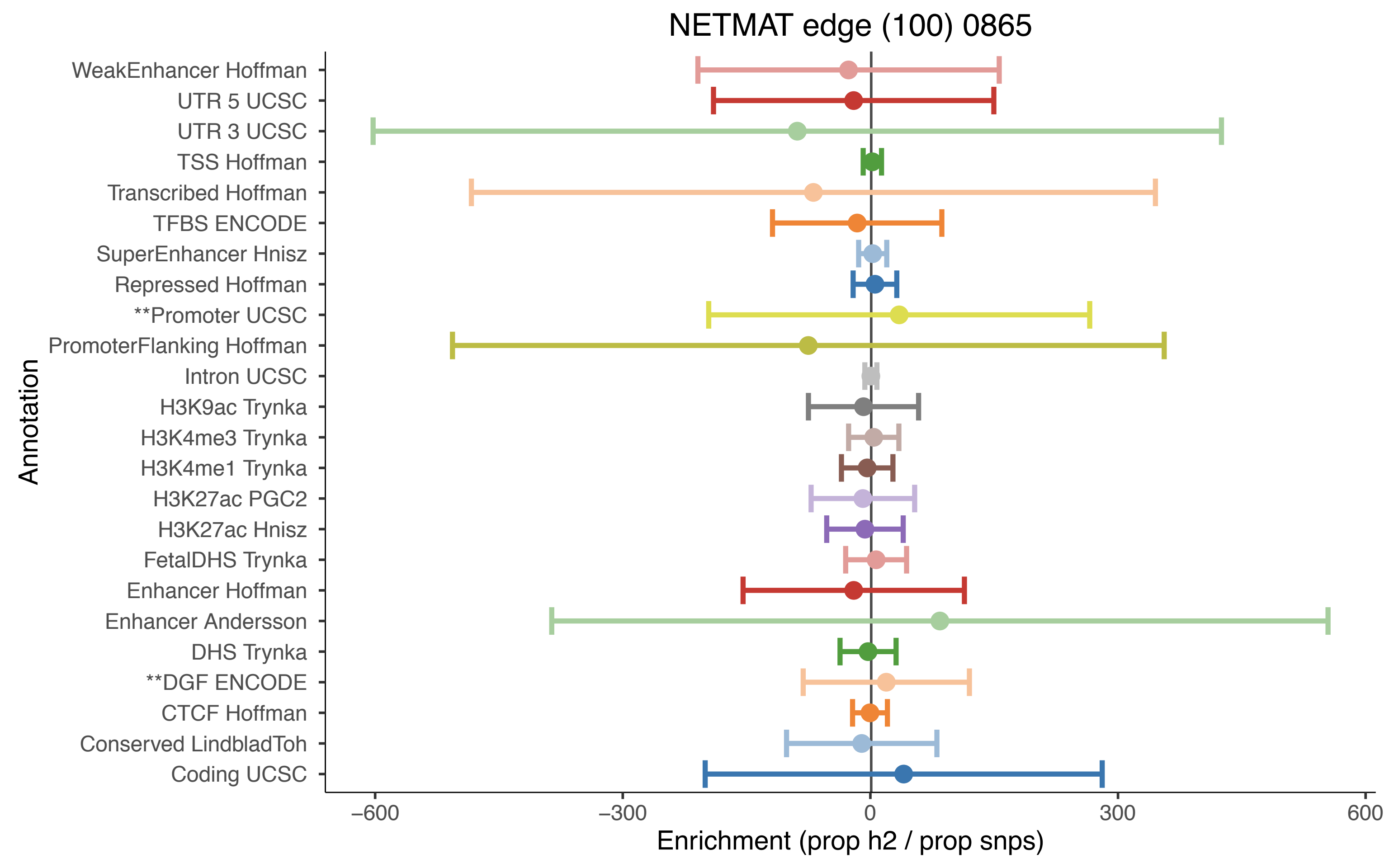
NETMAT edge (100) 0865
WeakEnhancer Hoffman
UTR 5 UCSC
UTR 3 UCSC
TSS Hoffman
Transcribed Hoffman
TFBS ENCODE
SuperEnhancer Hnisz
Repressed Hoffman
**Promoter UCSC
PromoterFlanking Hoffman
Intron UCSC
H3K9ac Trynka
H3K4me3 Trynka
H3K4me1 Trynka
H3K27ac PGC2
H3K27ac Hnisz
FetalDHS Trynka
Enhancer Hoffman
Enhancer Andersson
DHS Trynka
**DGF ENCODE
CTCF Hoffman
Conserved LindbladToh
Coding UCSC
Annotation
−600
−300
0
300
600
Enrichment (prop h2 / prop snps)

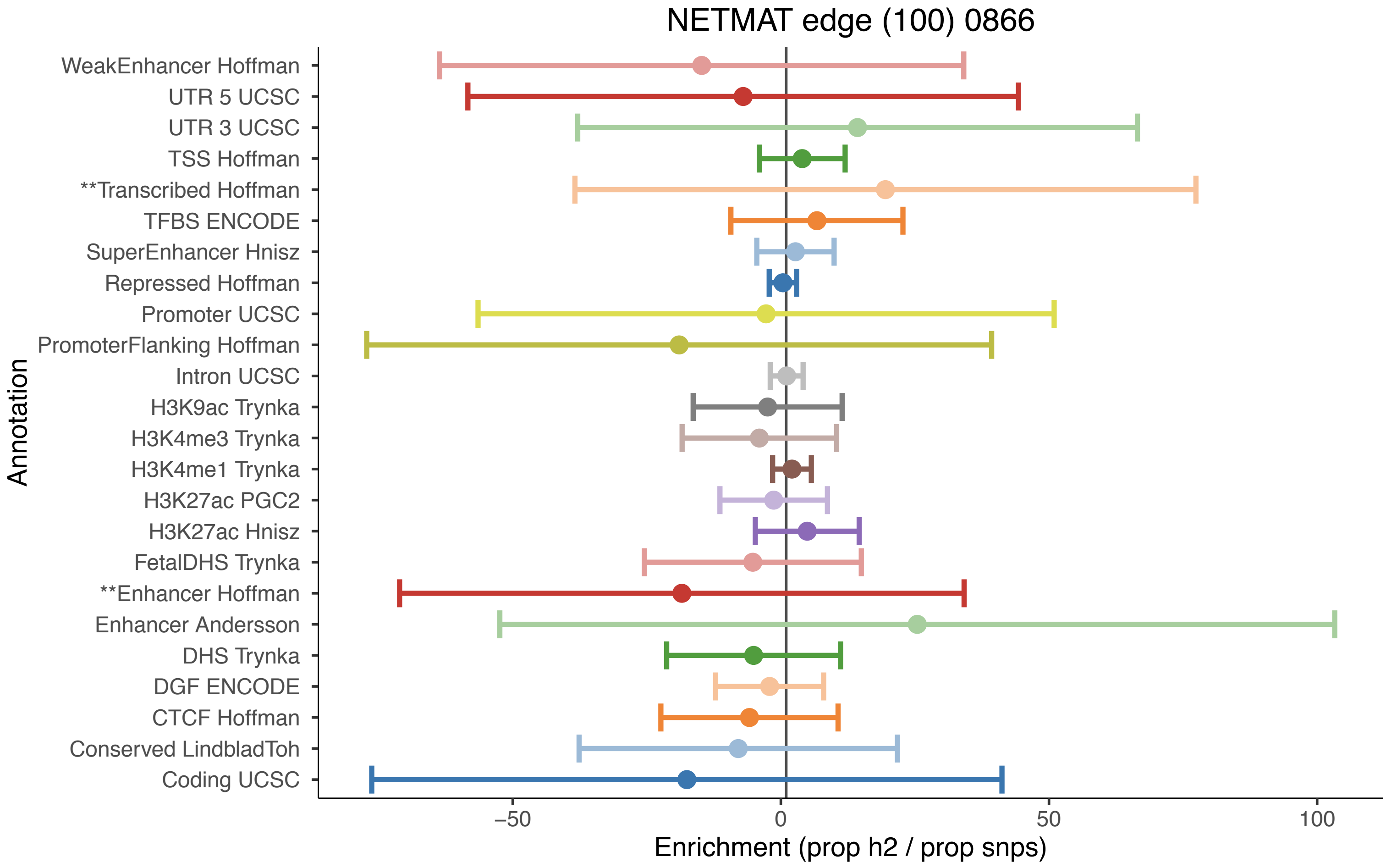
NETMAT edge (100) 0866
WeakEnhancer Hoffman
UTR 5 UCSC
UTR 3 UCSC
TSS Hoffman
**Transcribed Hoffman
TFBS ENCODE
SuperEnhancer Hnisz
Repressed Hoffman
Promoter UCSC
PromoterFlanking Hoffman
Intron UCSC
H3K9ac Trynka
H3K4me3 Trynka
H3K4me1 Trynka
H3K27ac PGC2
H3K27ac Hnisz
FetalDHS Trynka
**Enhancer Hoffman
Enhancer Andersson
DHS Trynka
DGF ENCODE
CTCF Hoffman
Conserved LindbladToh
Coding UCSC
Annotation
−50
0
50
100
Enrichment (prop h2 / prop snps)

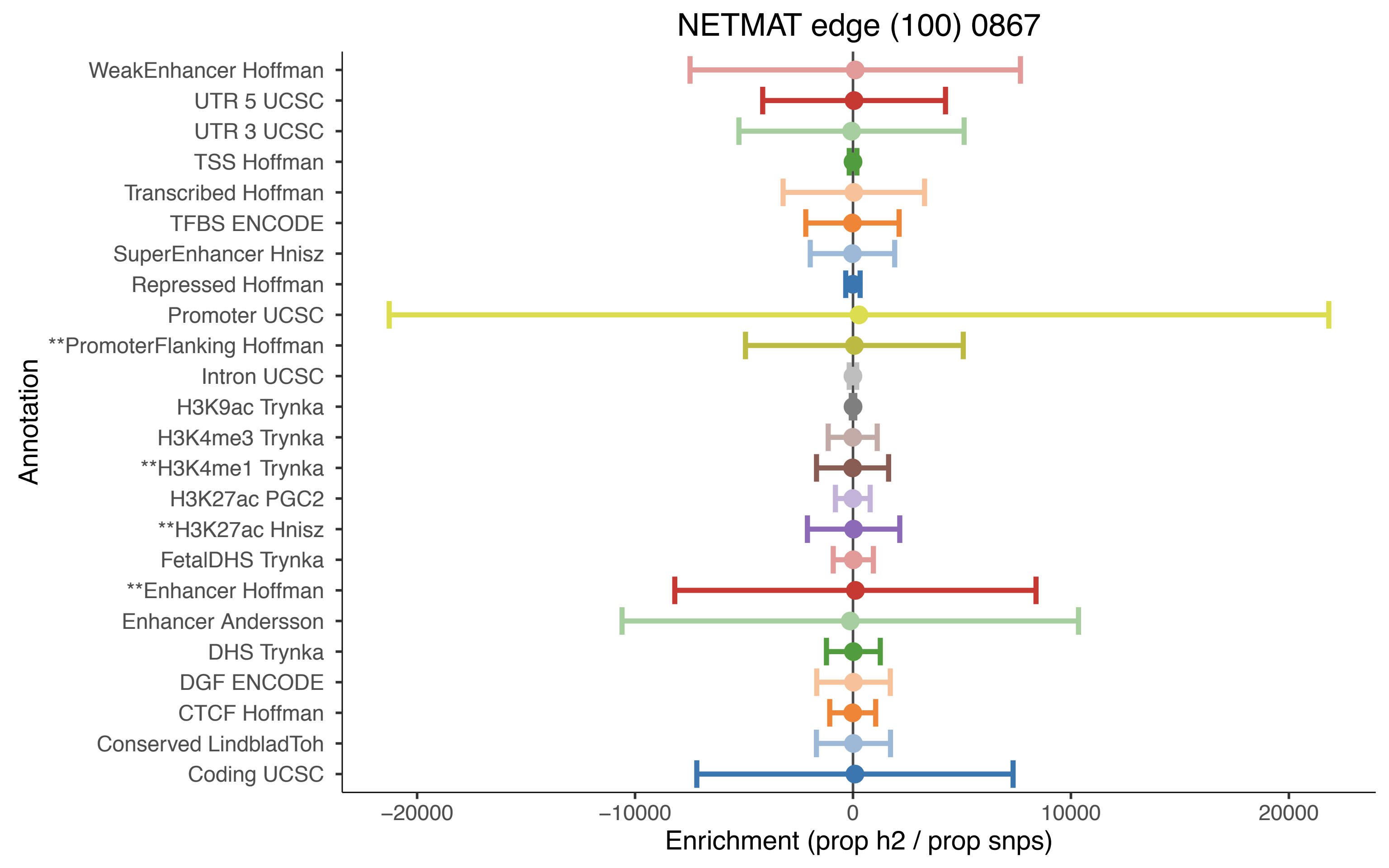
NETMAT edge (100) 0867
Annotation
WeakEnhancer Hoffman
UTR 5 UCSC
UTR 3 UCSC
TSS Hoffman
Transcribed Hoffman
TFBS ENCODE
SuperEnhancer Hnisz
Repressed Hoffman
Promoter UCSC
**PromoterFlanking Hoffman
Intron UCSC
H3K9ac Trynka
H3K4me3 Trynka
**H3K4me1 Trynka
H3K27ac PGC2
**H3K27ac Hnisz
FetalDHS Trynka
**Enhancer Hoffman
Enhancer Andersson
DHS Trynka
DGF ENCODE
CTCF Hoffman
Conserved LindbladToh
Coding UCSC
−20000
−10000
0
10000
20000
Enrichment (prop h2 / prop snps)

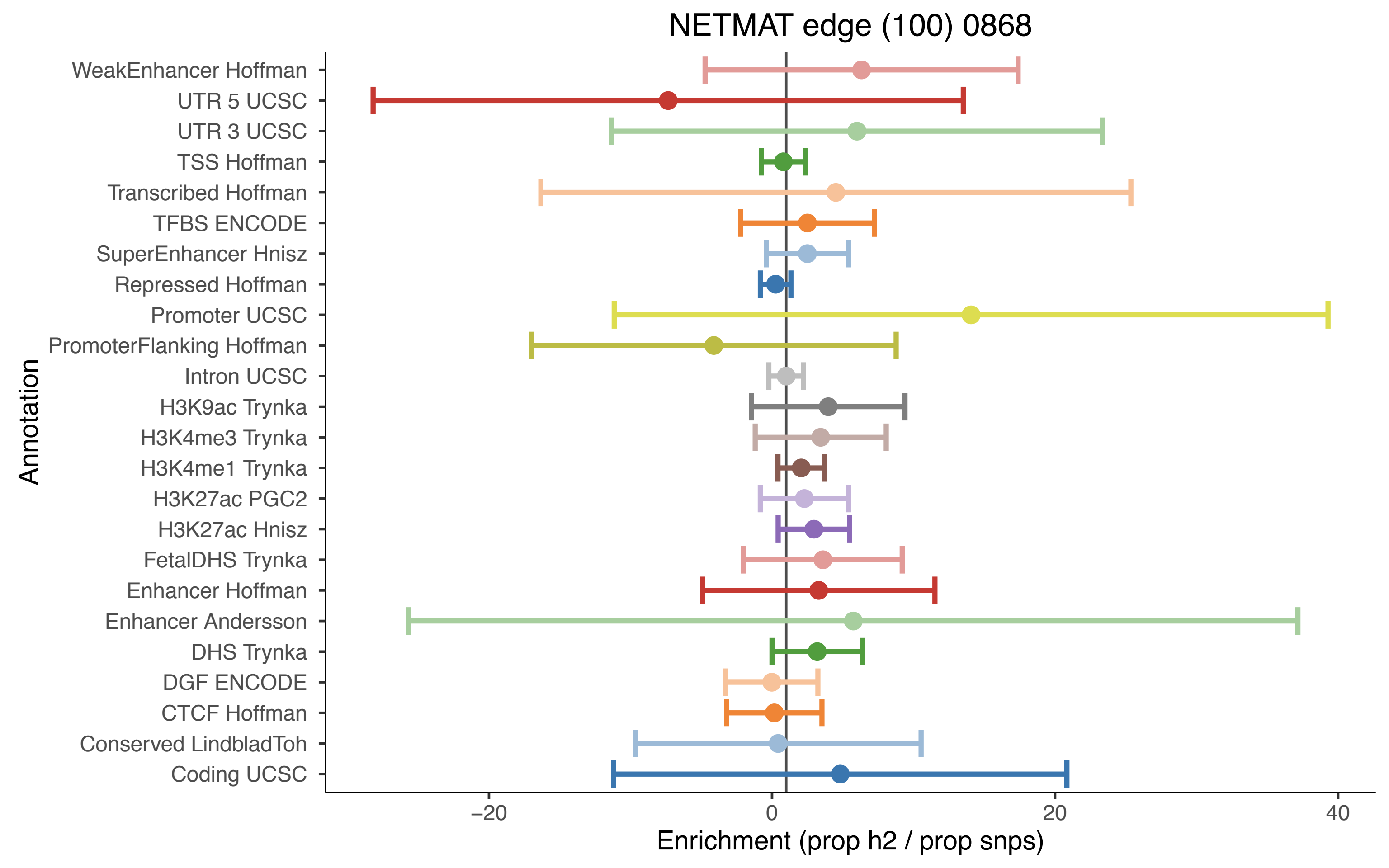

NETMAT edge (100) 0868
WeakEnhancer Hoffman
UTR 5 UCSC
UTR 3 UCSC
TSS Hoffman
Transcribed Hoffman
TFBS ENCODE
SuperEnhancer Hnisz
Repressed Hoffman
Promoter UCSC
PromoterFlanking Hoffman
Intron UCSC
H3K9ac Trynka
H3K4me3 Trynka
H3K4me1 Trynka
H3K27ac PGC2
H3K27ac Hnisz
FetalDHS Trynka
Enhancer Hoffman
Enhancer Andersson
DHS Trynka
DGF ENCODE
CTCF Hoffman
Conserved LindbladToh
Coding UCSC
Annotation
−20
0
20
40
Enrichment (prop h2 / prop snps)

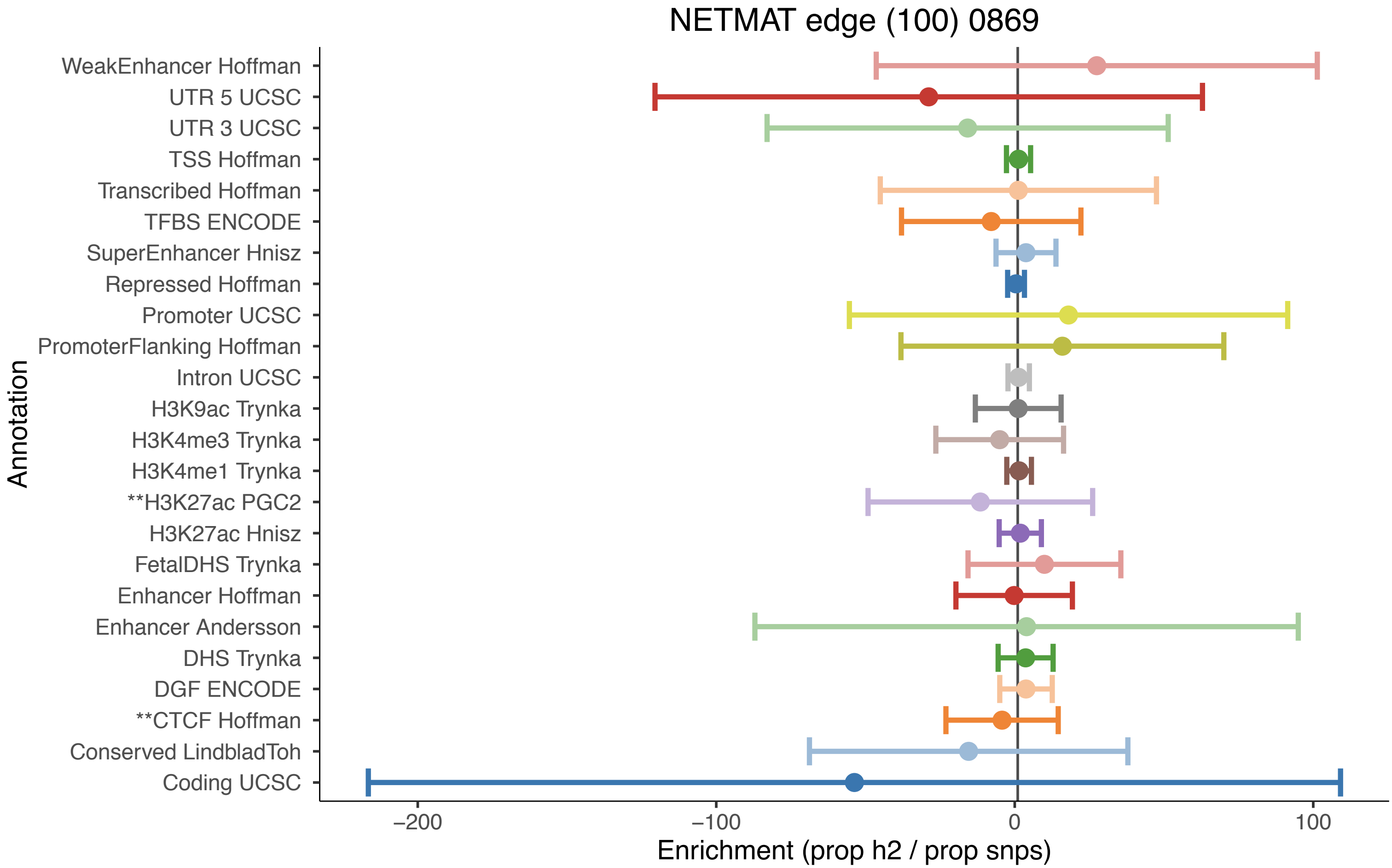

NETMAT edge (100) 0869
WeakEnhancer Hoffman
UTR 5 UCSC
UTR 3 UCSC
TSS Hoffman
Transcribed Hoffman
TFBS ENCODE
SuperEnhancer Hnisz
Repressed Hoffman
Promoter UCSC
PromoterFlanking Hoffman
Intron UCSC
H3K9ac Trynka
H3K4me3 Trynka
H3K4me1 Trynka
**H3K27ac PGC2
H3K27ac Hnisz
FetalDHS Trynka
Enhancer Hoffman
Enhancer Andersson
DHS Trynka
DGF ENCODE
**CTCF Hoffman
Conserved LindbladToh
Coding UCSC
Annotation
−200
−100
0
100
Enrichment (prop h2 / prop snps)

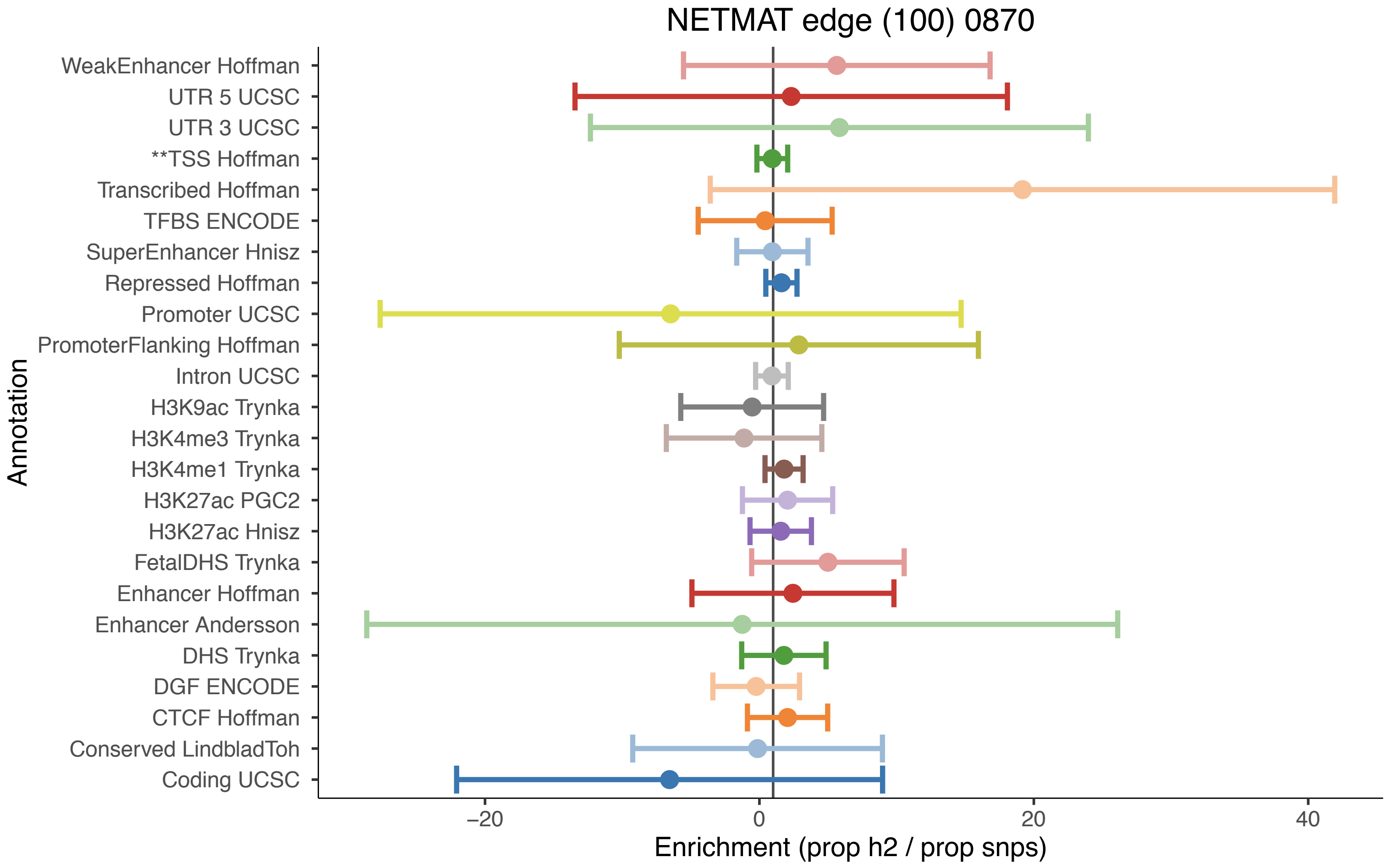
NETMAT edge (100) 0870
WeakEnhancer Hoffman
UTR 5 UCSC
UTR 3 UCSC
**TSS Hoffman
Transcribed Hoffman
TFBS ENCODE
SuperEnhancer Hnisz
Repressed Hoffman
Promoter UCSC
PromoterFlanking Hoffman
Intron UCSC
H3K9ac Trynka
H3K4me3 Trynka
H3K4me1 Trynka
H3K27ac PGC2
H3K27ac Hnisz
FetalDHS Trynka
Enhancer Hoffman
Enhancer Andersson
DHS Trynka
DGF ENCODE
CTCF Hoffman
Conserved LindbladToh
Coding UCSC
Annotation
−20
0
20
40
Enrichment (prop h2 / prop snps)

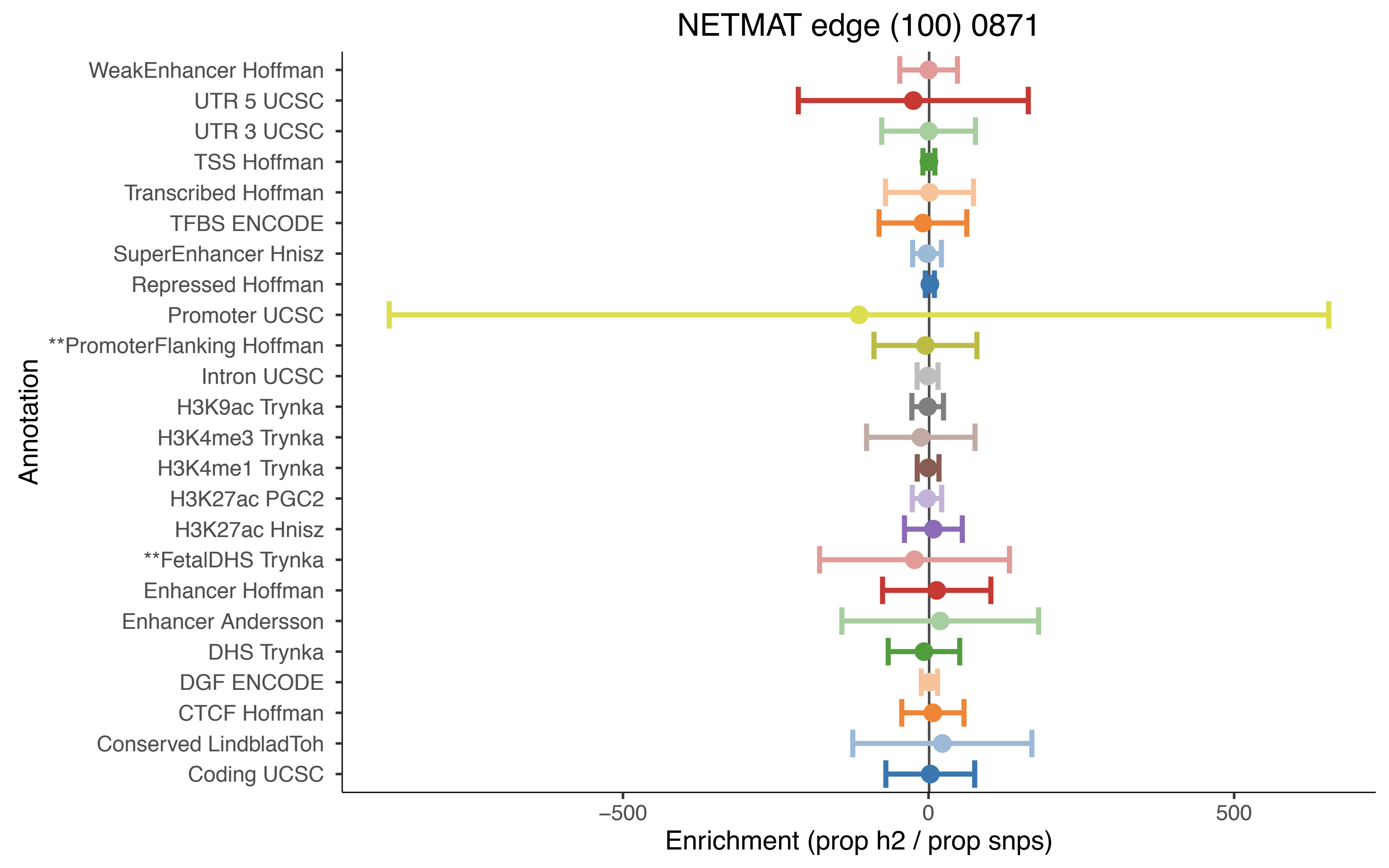
NETMAT edge (100) 0871
WeakEnhancer Hoffman
UTR 5 UCSC
UTR 3 UCSC
TSS Hoffman
Transcribed Hoffman
TFBS ENCODE
SuperEnhancer Hnisz
Repressed Hoffman
Promoter UCSC
**PromoterFlanking Hoffman
Intron UCSC
H3K9ac Trynka
H3K4me3 Trynka
H3K4me1 Trynka
H3K27ac PGC2
H3K27ac Hnisz
**FetalDHS Trynka
Enhancer Hoffman
Enhancer Andersson
DHS Trynka
DGF ENCODE
CTCF Hoffman
Conserved LindbladToh
Coding UCSC
Annotation
−500
0
500
Enrichment (prop h2 / prop snps)

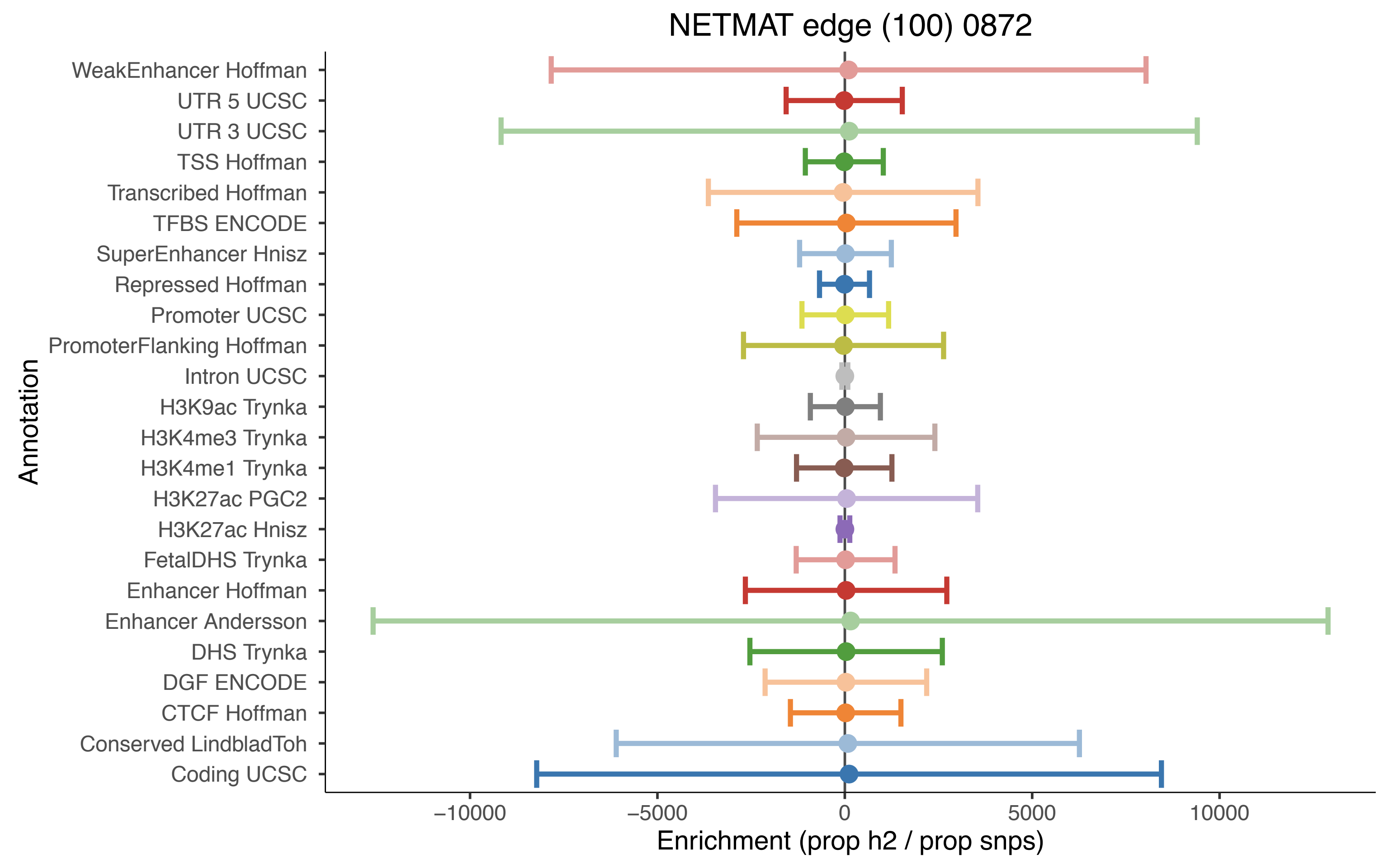
NETMAT edge (100) 0872
WeakEnhancer Hoffman
UTR 5 UCSC
UTR 3 UCSC
TSS Hoffman
Transcribed Hoffman
TFBS ENCODE
SuperEnhancer Hnisz
Repressed Hoffman
Promoter UCSC
PromoterFlanking Hoffman
Intron UCSC
H3K9ac Trynka
H3K4me3 Trynka
H3K4me1 Trynka
H3K27ac PGC2
H3K27ac Hnisz
FetalDHS Trynka
Enhancer Hoffman
Enhancer Andersson
DHS Trynka
DGF ENCODE
CTCF Hoffman
Conserved LindbladToh
Coding UCSC
Annotation
−10000
−5000
0
5000
10000
Enrichment (prop h2 / prop snps)

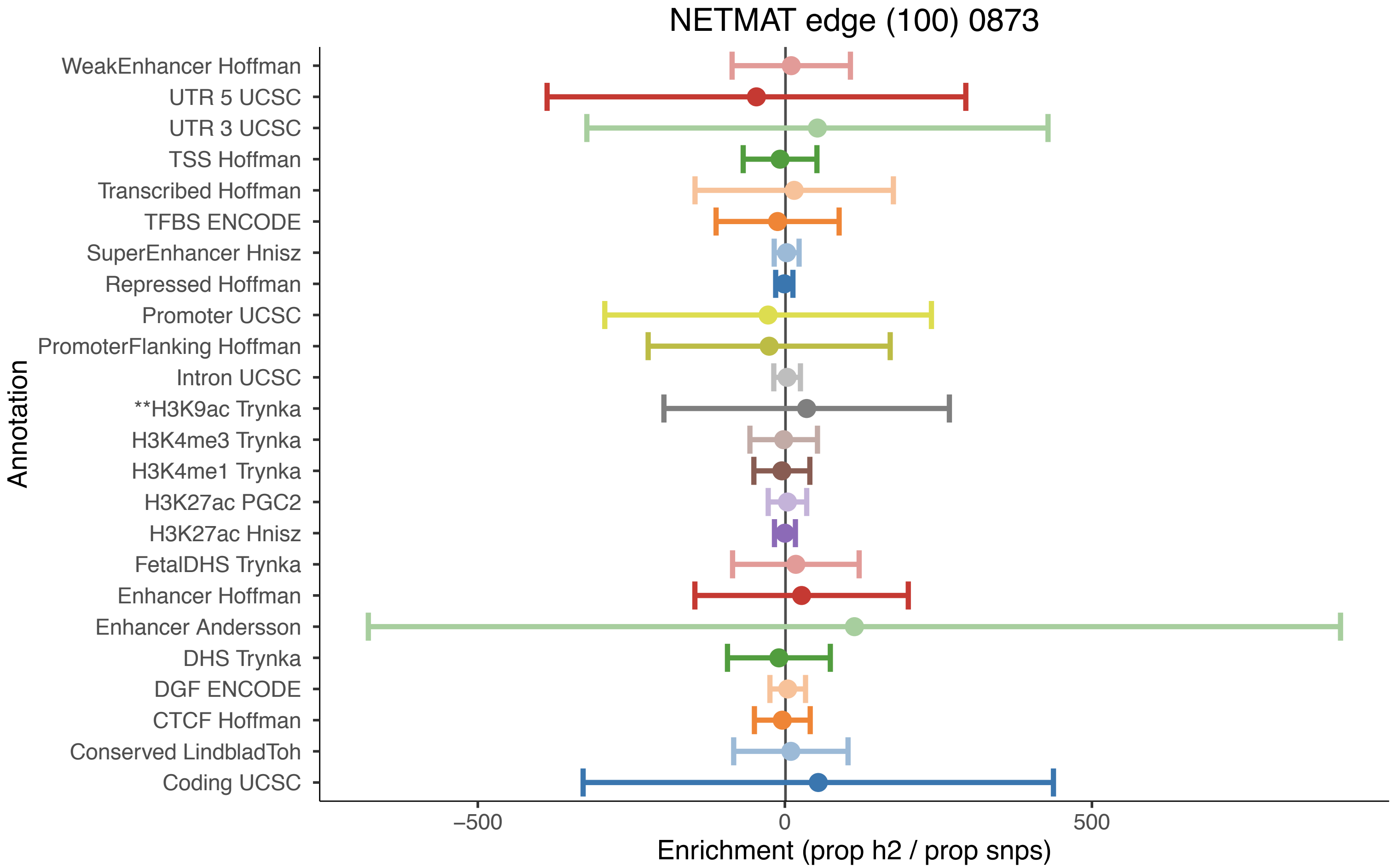
NETMAT edge (100) 0873
WeakEnhancer Hoffman
UTR 5 UCSC
UTR 3 UCSC
TSS Hoffman
Transcribed Hoffman
TFBS ENCODE
SuperEnhancer Hnisz
Repressed Hoffman
Promoter UCSC
PromoterFlanking Hoffman
Intron UCSC
**H3K9ac Trynka
H3K4me3 Trynka
H3K4me1 Trynka
H3K27ac PGC2
H3K27ac Hnisz
FetalDHS Trynka
Enhancer Hoffman
Enhancer Andersson
DHS Trynka
DGF ENCODE
CTCF Hoffman
Conserved LindbladToh
Coding UCSC
Annotation
−500
0
500
Enrichment (prop h2 / prop snps)

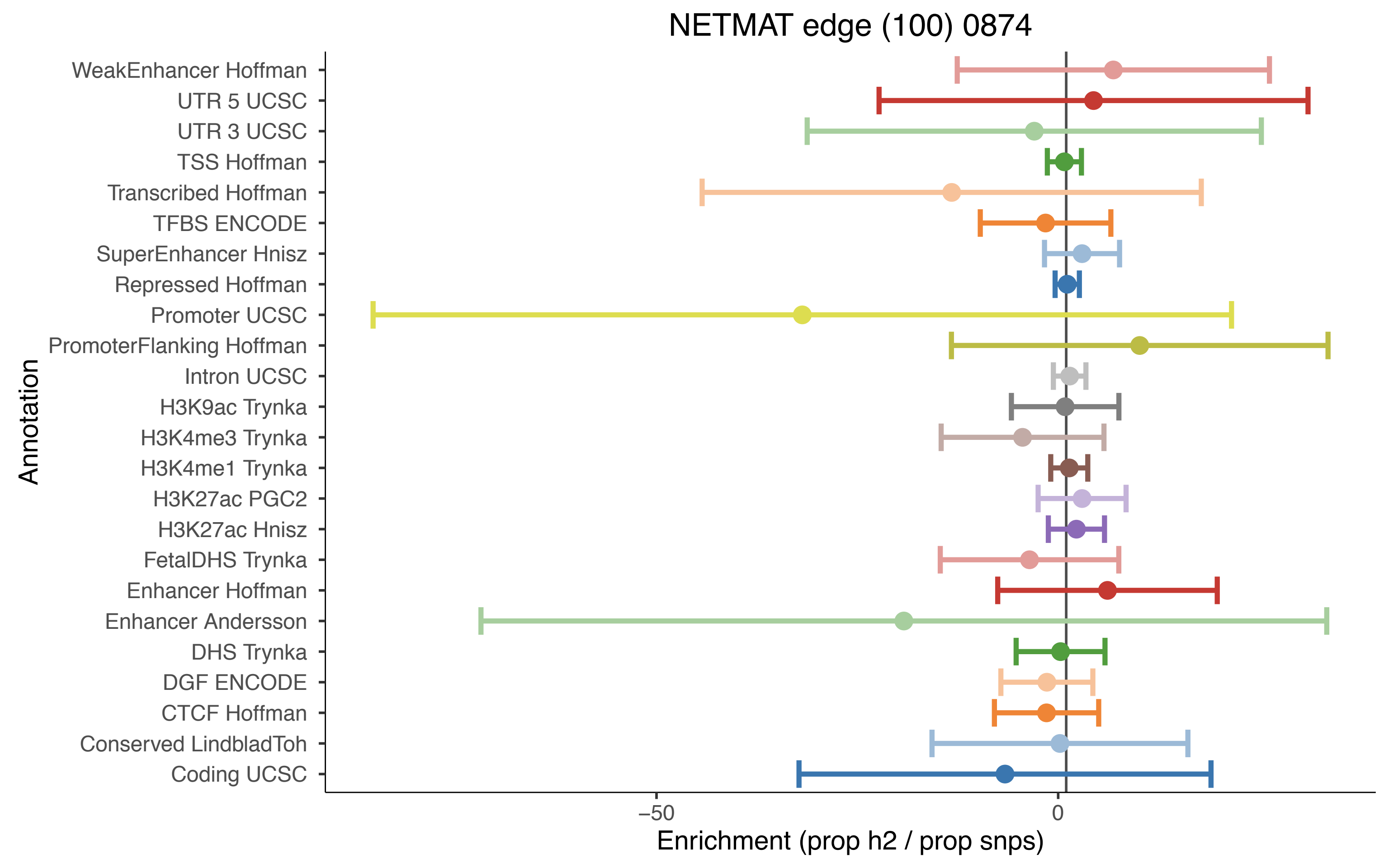
NETMAT edge (100) 0874
WeakEnhancer Hoffman
UTR 5 UCSC
UTR 3 UCSC
TSS Hoffman
Transcribed Hoffman
TFBS ENCODE
SuperEnhancer Hnisz
Repressed Hoffman
Promoter UCSC
PromoterFlanking Hoffman
Intron UCSC
H3K9ac Trynka
H3K4me3 Trynka
H3K4me1 Trynka
H3K27ac PGC2
H3K27ac Hnisz
FetalDHS Trynka
Enhancer Hoffman
Enhancer Andersson
DHS Trynka
DGF ENCODE
CTCF Hoffman
Conserved LindbladToh
Coding UCSC
Annotation
−50
0
Enrichment (prop h2 / prop snps)

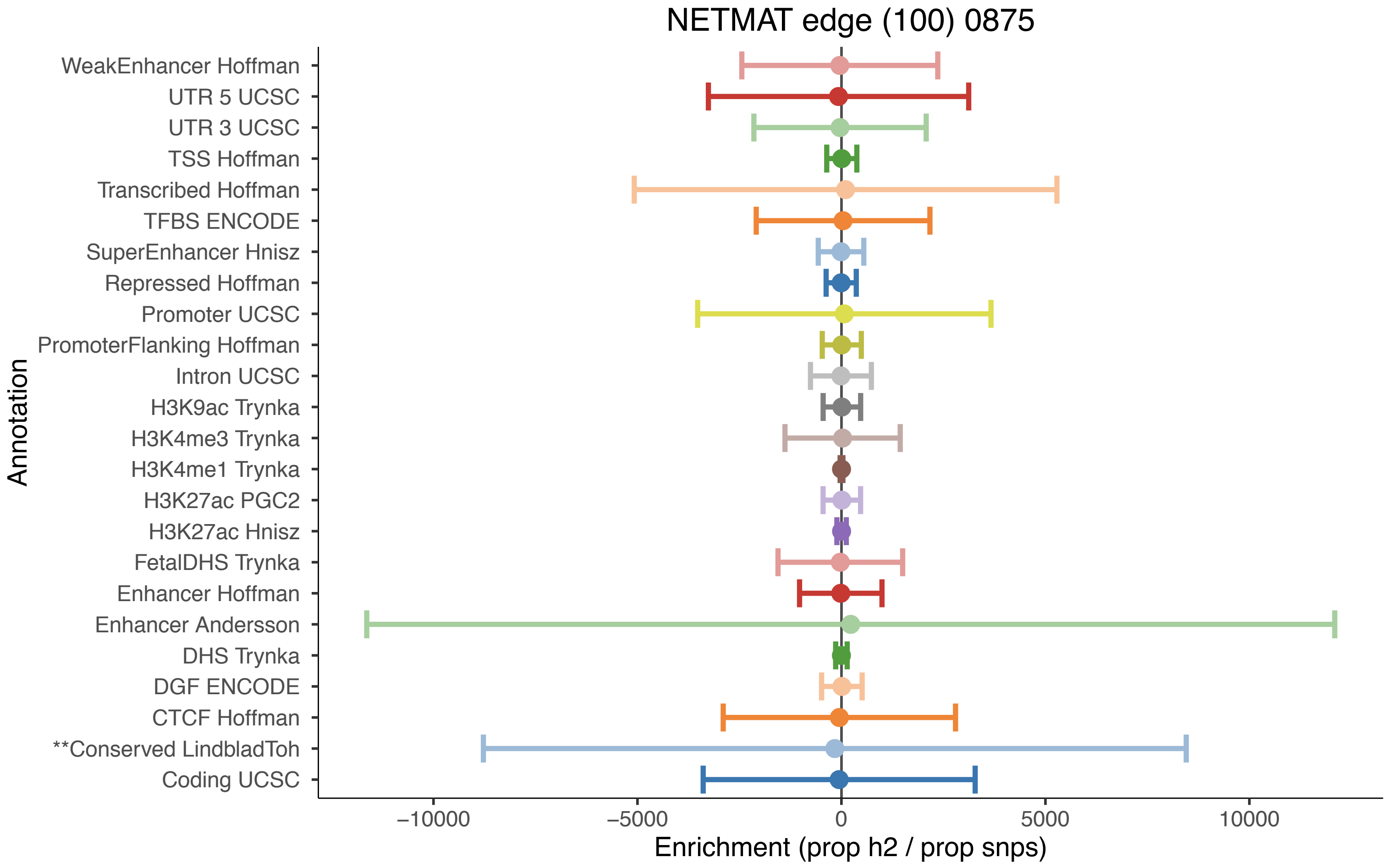
NETMAT edge (100) 0875
WeakEnhancer Hoffman
UTR 5 UCSC
UTR 3 UCSC
TSS Hoffman
Transcribed Hoffman
TFBS ENCODE
SuperEnhancer Hnisz
Repressed Hoffman
Promoter UCSC
PromoterFlanking Hoffman
Intron UCSC
H3K9ac Trynka
H3K4me3 Trynka
H3K4me1 Trynka
H3K27ac PGC2
H3K27ac Hnisz
FetalDHS Trynka
Enhancer Hoffman
Enhancer Andersson
DHS Trynka
DGF ENCODE
CTCF Hoffman
**Conserved LindbladToh
Coding UCSC
Annotation
−10000
−5000
0
5000
10000
Enrichment (prop h2 / prop snps)

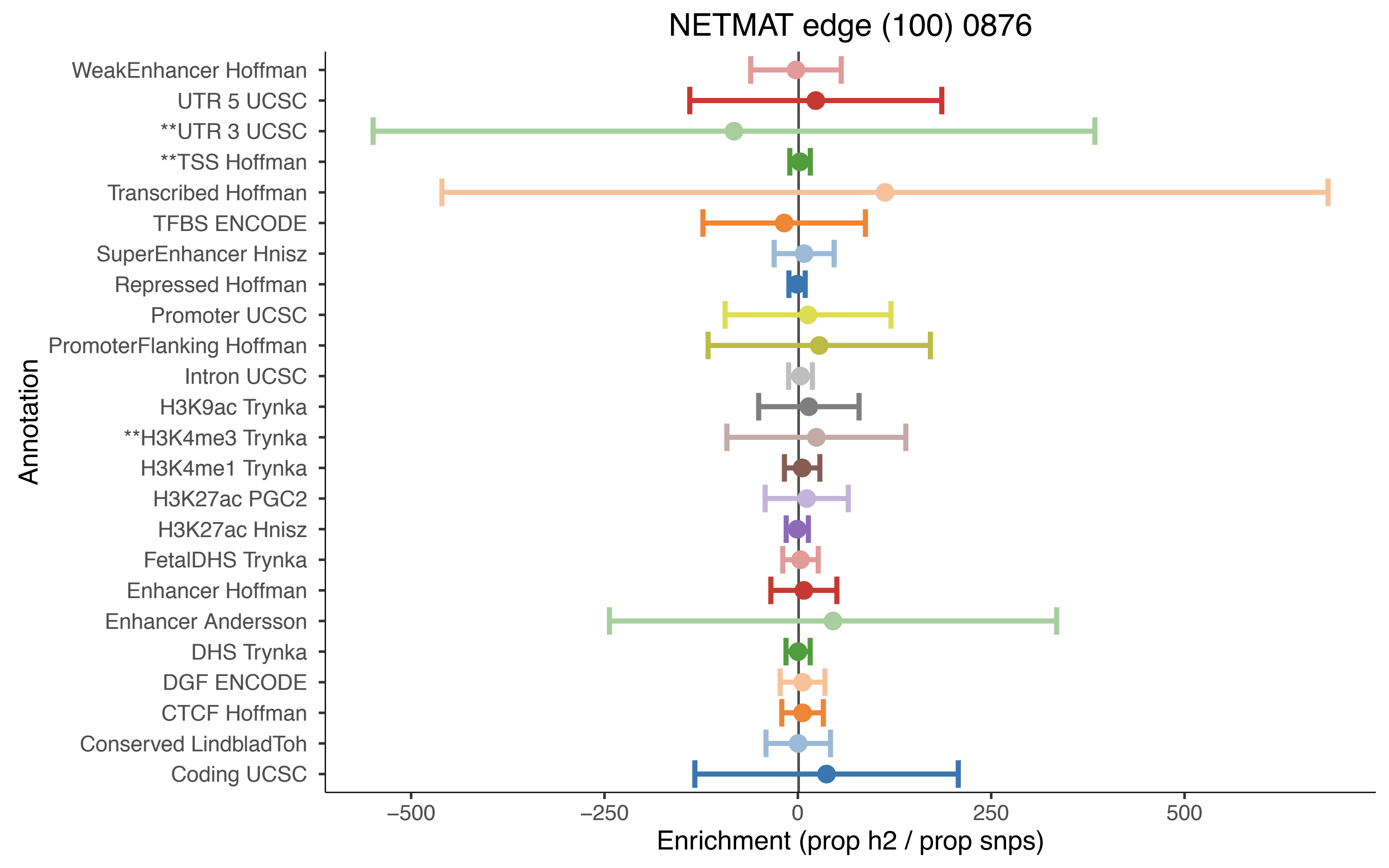
NETMAT edge (100) 0876
WeakEnhancer Hoffman
UTR 5 UCSC
**UTR 3 UCSC
**TSS Hoffman
Transcribed Hoffman
TFBS ENCODE
SuperEnhancer Hnisz
Repressed Hoffman
Promoter UCSC
PromoterFlanking Hoffman
Intron UCSC
H3K9ac Trynka
**H3K4me3 Trynka
H3K4me1 Trynka
H3K27ac PGC2
H3K27ac Hnisz
FetalDHS Trynka
Enhancer Hoffman
Enhancer Andersson
DHS Trynka
DGF ENCODE
CTCF Hoffman
Conserved LindbladToh
Coding UCSC
Annotation
−500
−250
0
250
500
Enrichment (prop h2 / prop snps)

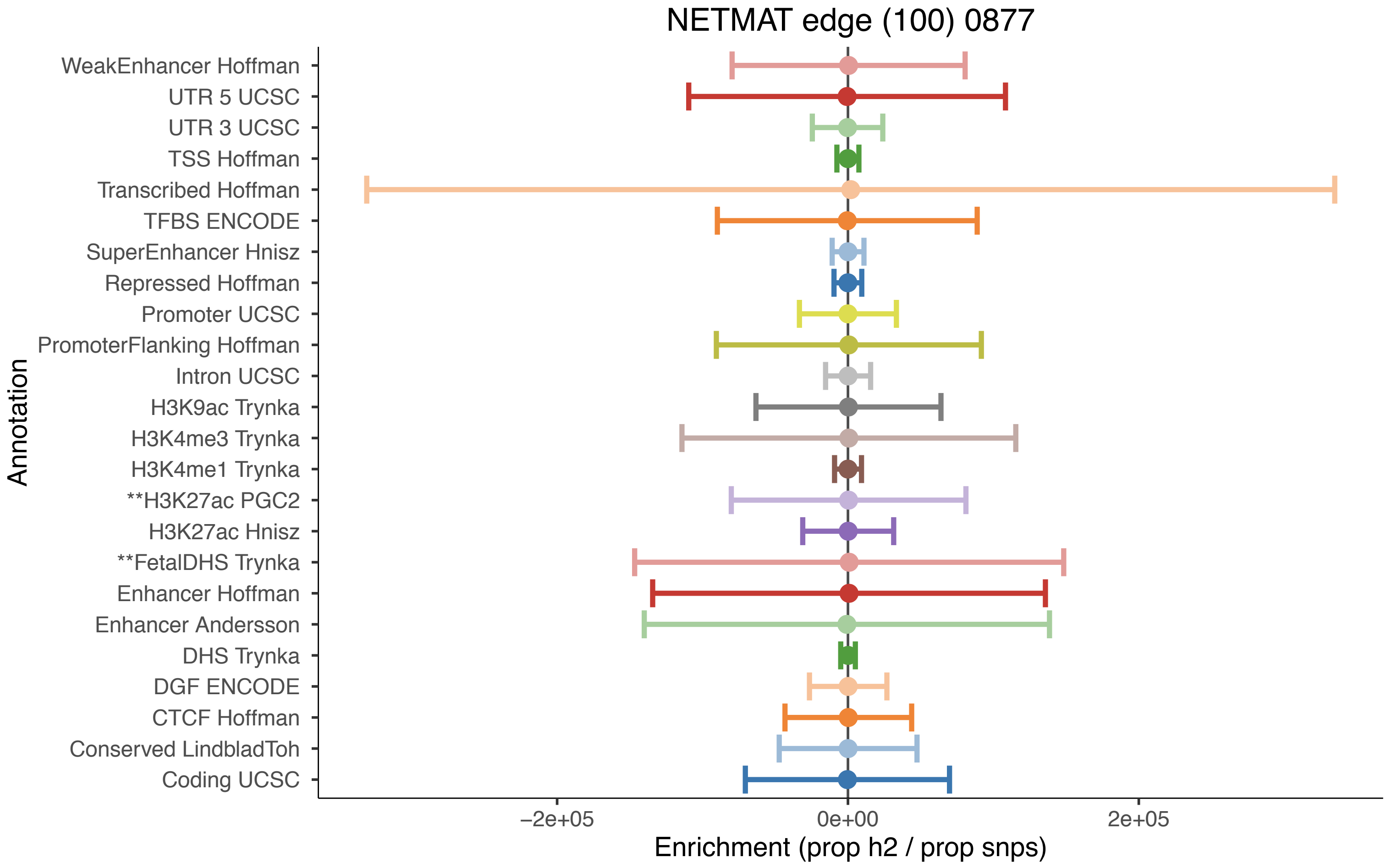

NETMAT edge (100) 0877
WeakEnhancer Hoffman
UTR 5 UCSC
UTR 3 UCSC
TSS Hoffman
Transcribed Hoffman
TFBS ENCODE
SuperEnhancer Hnisz
Repressed Hoffman
Promoter UCSC
PromoterFlanking Hoffman
Intron UCSC
H3K9ac Trynka
H3K4me3 Trynka
H3K4me1 Trynka
**H3K27ac PGC2
H3K27ac Hnisz
**FetalDHS Trynka
Enhancer Hoffman
Enhancer Andersson
DHS Trynka
DGF ENCODE
CTCF Hoffman
Conserved LindbladToh
Coding UCSC
Annotation
−2e+05
0e+00
2e+05
Enrichment (prop h2 / prop snps)

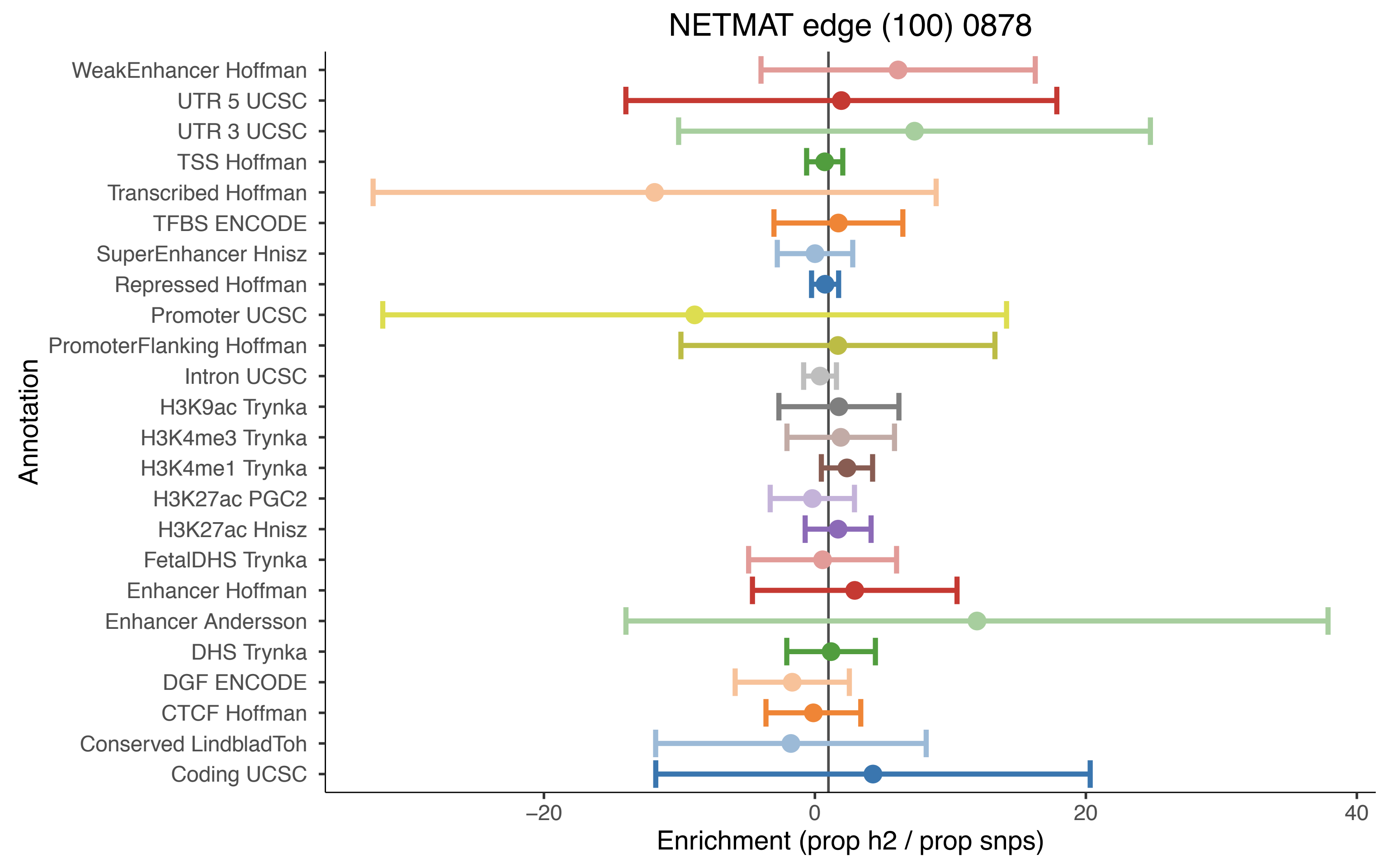
NETMAT edge (100) 0878
WeakEnhancer Hoffman
UTR 5 UCSC
UTR 3 UCSC
TSS Hoffman
Transcribed Hoffman
TFBS ENCODE
SuperEnhancer Hnisz
Repressed Hoffman
Promoter UCSC
PromoterFlanking Hoffman
Intron UCSC
H3K9ac Trynka
H3K4me3 Trynka
H3K4me1 Trynka
H3K27ac PGC2
H3K27ac Hnisz
FetalDHS Trynka
Enhancer Hoffman
Enhancer Andersson
DHS Trynka
DGF ENCODE
CTCF Hoffman
Conserved LindbladToh
Coding UCSC
Annotation
−20
0
20
40
Enrichment (prop h2 / prop snps)

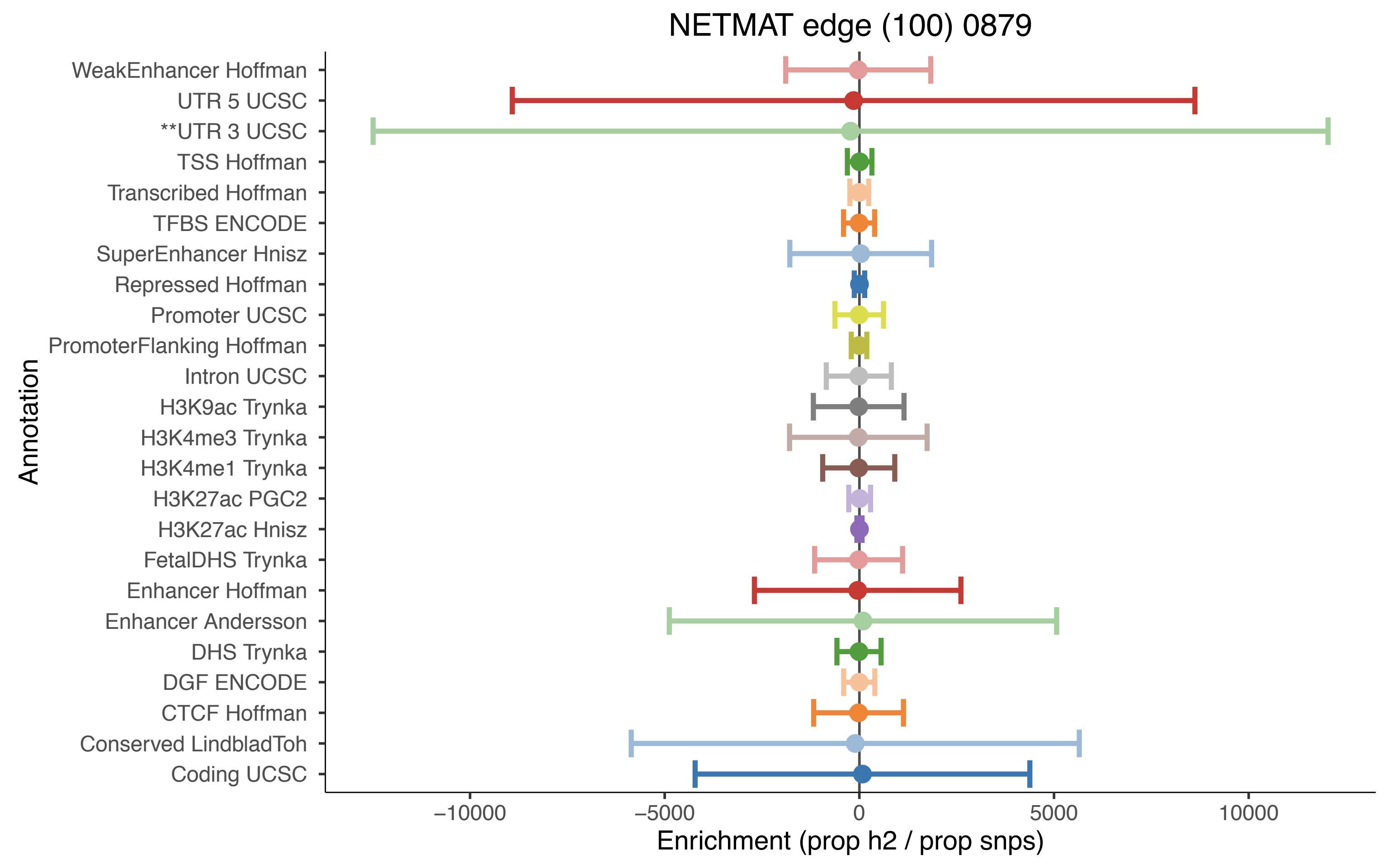

NETMAT edge (100) 0879
WeakEnhancer Hoffman
UTR 5 UCSC
**UTR 3 UCSC
TSS Hoffman
Transcribed Hoffman
TFBS ENCODE
SuperEnhancer Hnisz
Repressed Hoffman
Promoter UCSC
PromoterFlanking Hoffman
Intron UCSC
H3K9ac Trynka
H3K4me3 Trynka
H3K4me1 Trynka
H3K27ac PGC2
H3K27ac Hnisz
FetalDHS Trynka
Enhancer Hoffman
Enhancer Andersson
DHS Trynka
DGF ENCODE
CTCF Hoffman
Conserved LindbladToh
Coding UCSC
Annotation
−10000
−5000
0
5000
10000
Enrichment (prop h2 / prop snps)

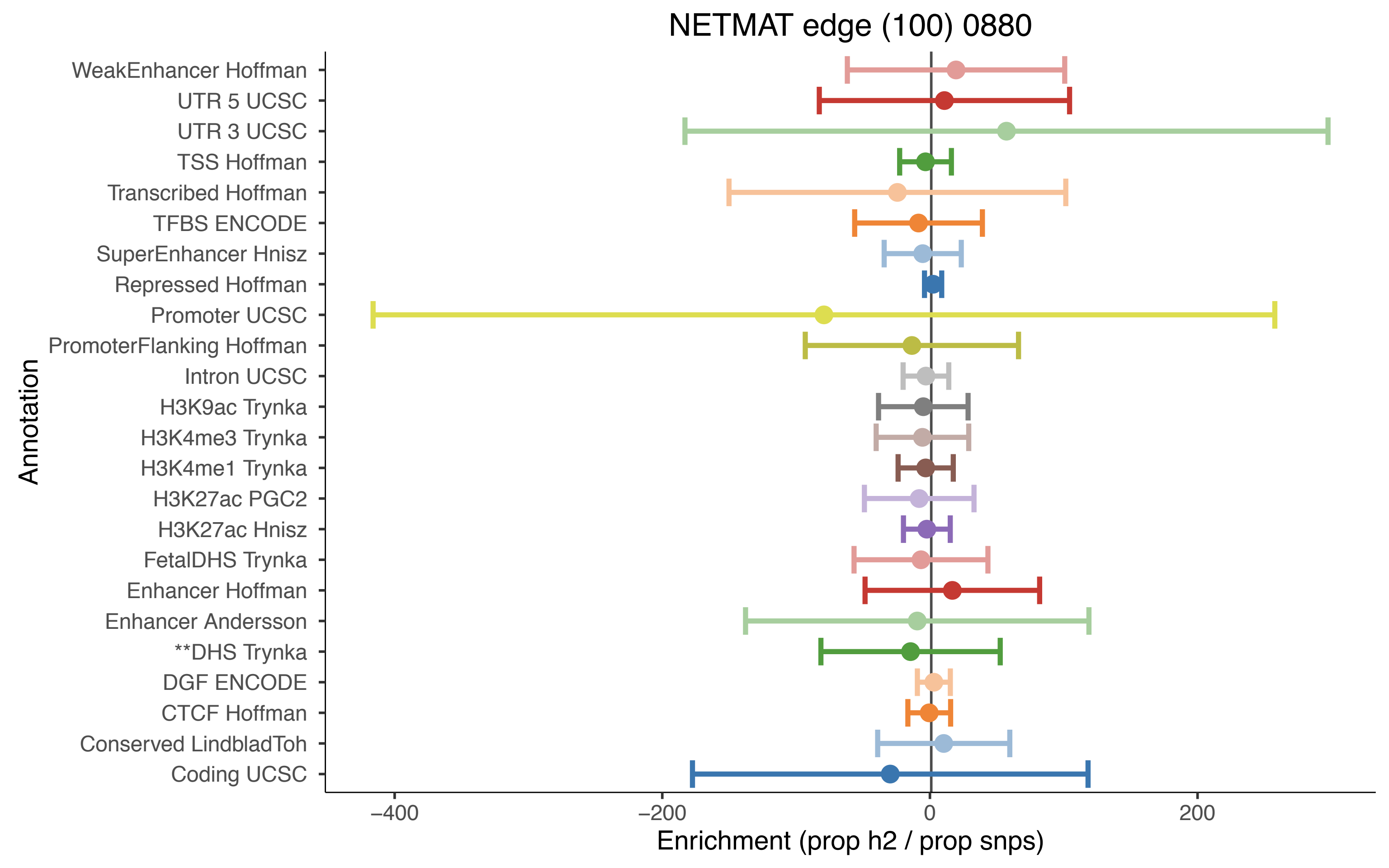
NETMAT edge (100) 0880
WeakEnhancer Hoffman
UTR 5 UCSC
UTR 3 UCSC
TSS Hoffman
Transcribed Hoffman
TFBS ENCODE
SuperEnhancer Hnisz
Repressed Hoffman
Promoter UCSC
PromoterFlanking Hoffman
Intron UCSC
H3K9ac Trynka
H3K4me3 Trynka
H3K4me1 Trynka
H3K27ac PGC2
H3K27ac Hnisz
FetalDHS Trynka
Enhancer Hoffman
Enhancer Andersson
**DHS Trynka
DGF ENCODE
CTCF Hoffman
Conserved LindbladToh
Coding UCSC
Annotation
−400
−200
0
200
Enrichment (prop h2 / prop snps)

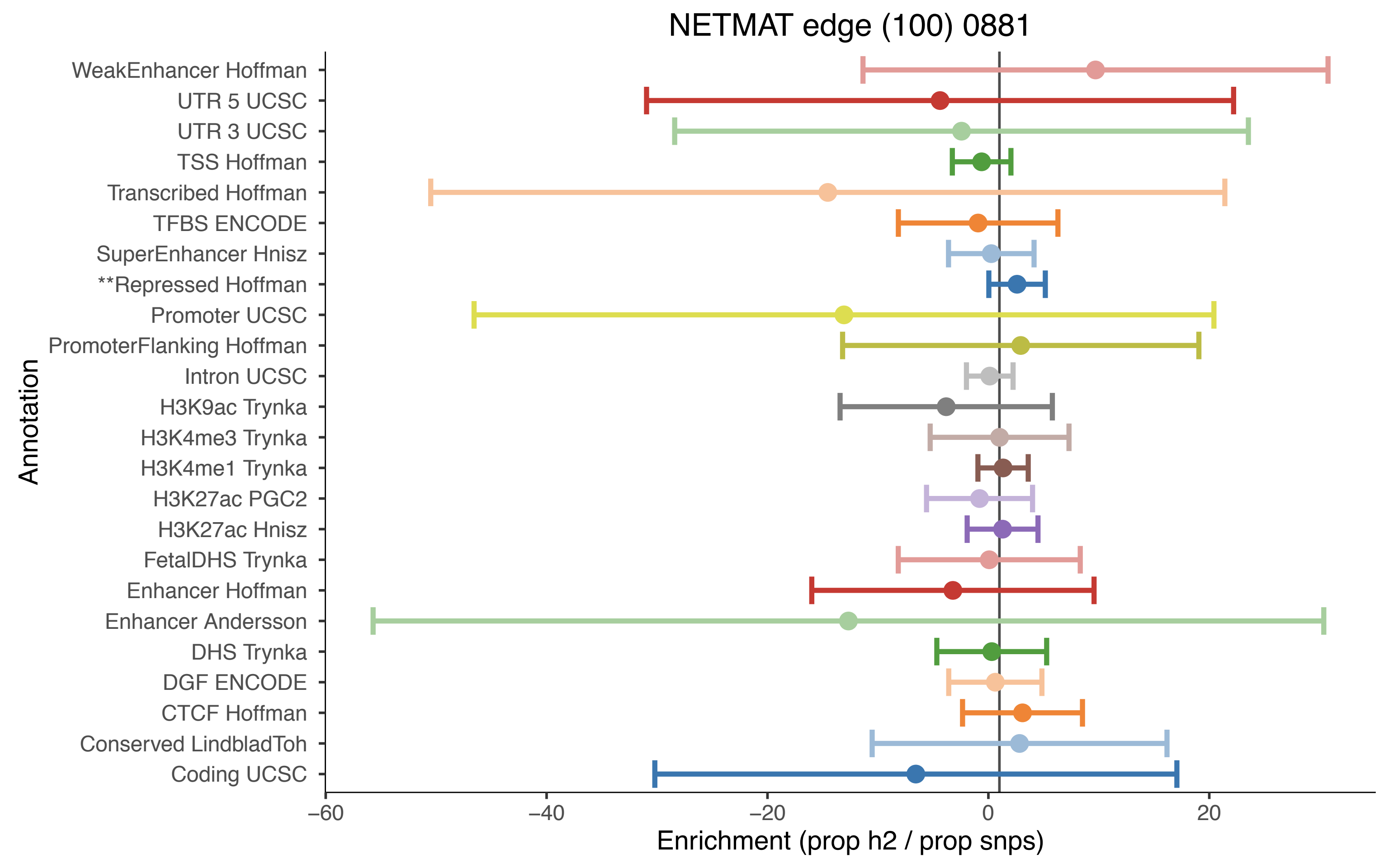

NETMAT edge (100) 0881
WeakEnhancer Hoffman
UTR 5 UCSC
UTR 3 UCSC
TSS Hoffman
Transcribed Hoffman
TFBS ENCODE
SuperEnhancer Hnisz
**Repressed Hoffman
Promoter UCSC
PromoterFlanking Hoffman
Intron UCSC
H3K9ac Trynka
H3K4me3 Trynka
H3K4me1 Trynka
H3K27ac PGC2
H3K27ac Hnisz
FetalDHS Trynka
Enhancer Hoffman
Enhancer Andersson
DHS Trynka
DGF ENCODE
CTCF Hoffman
Conserved LindbladToh
Coding UCSC
Annotation
−60
−40
−20
0
20
Enrichment (prop h2 / prop snps)

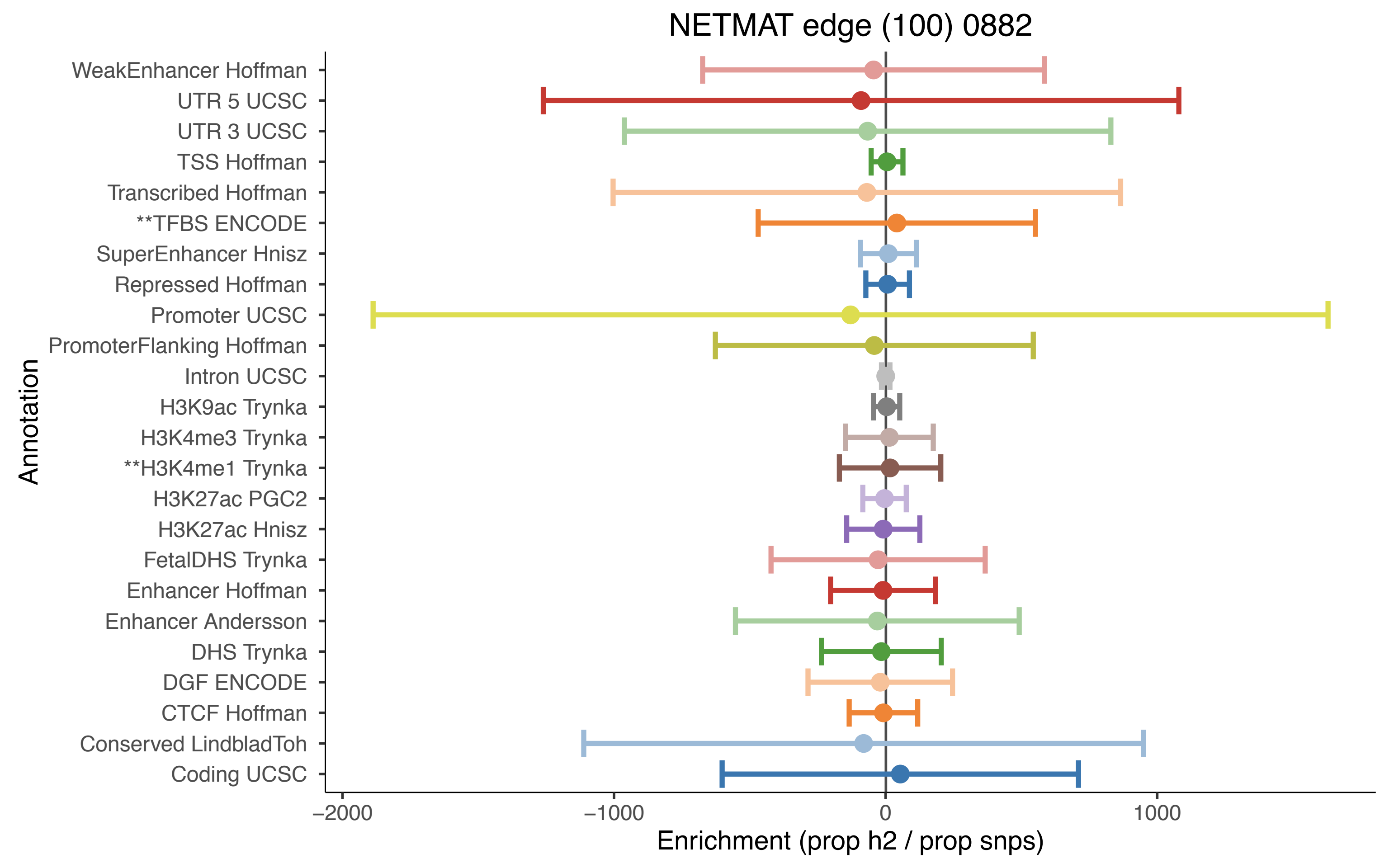
NETMAT edge (100) 0882
Annotation
WeakEnhancer Hoffman
UTR 5 UCSC
UTR 3 UCSC
TSS Hoffman
Transcribed Hoffman
**TFBS ENCODE
SuperEnhancer Hnisz
Repressed Hoffman
Promoter UCSC
PromoterFlanking Hoffman
Intron UCSC
H3K9ac Trynka
H3K4me3 Trynka
**H3K4me1 Trynka
H3K27ac PGC2
H3K27ac Hnisz
FetalDHS Trynka
Enhancer Hoffman
Enhancer Andersson
DHS Trynka
DGF ENCODE
CTCF Hoffman
Conserved LindbladToh
Coding UCSC
−2000
−1000
0
1000
Enrichment (prop h2 / prop snps)

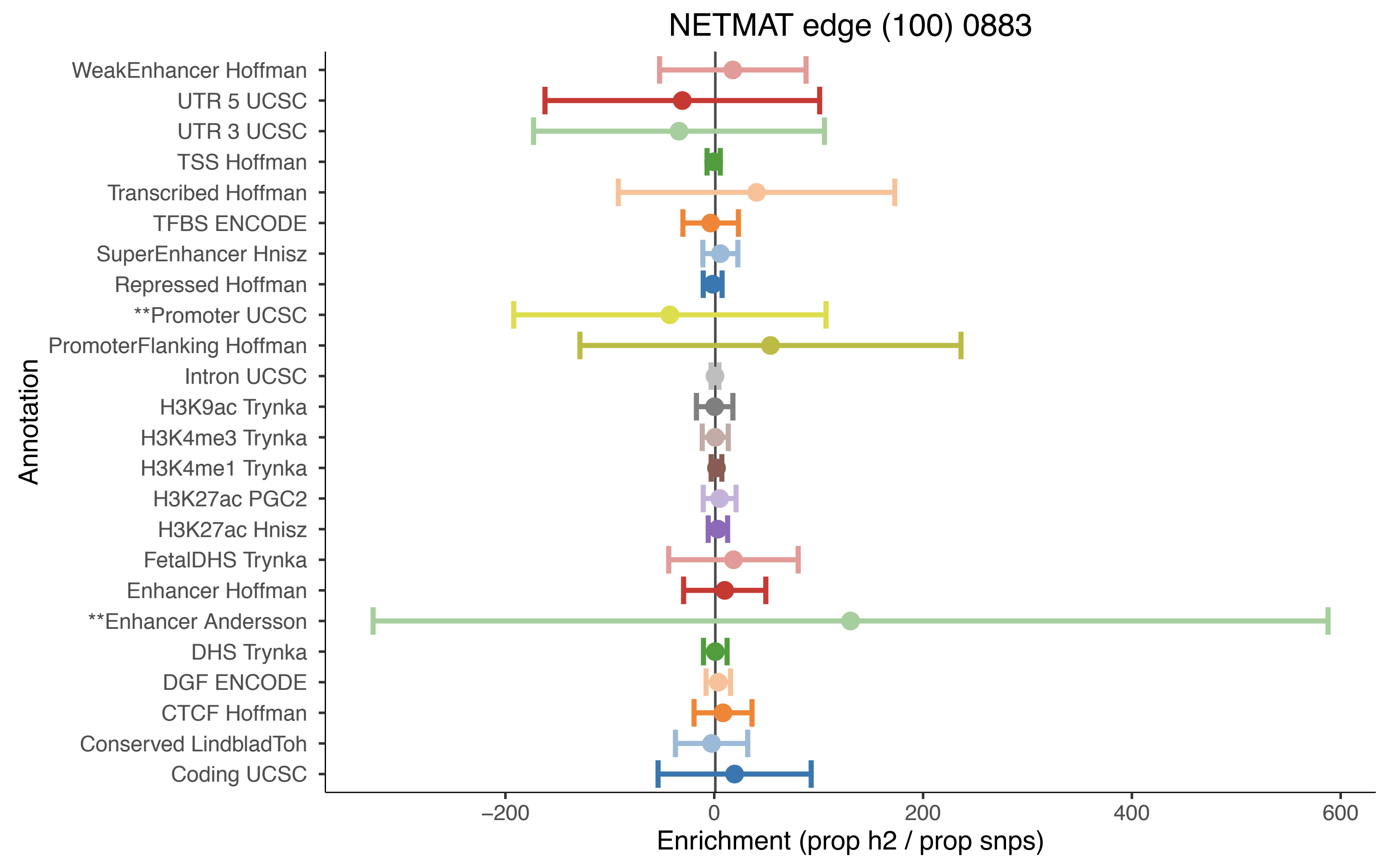
NETMAT edge (100) 0883
WeakEnhancer Hoffman
UTR 5 UCSC
UTR 3 UCSC
TSS Hoffman
Transcribed Hoffman
TFBS ENCODE
SuperEnhancer Hnisz
Repressed Hoffman
**Promoter UCSC
PromoterFlanking Hoffman
Intron UCSC
H3K9ac Trynka
H3K4me3 Trynka
H3K4me1 Trynka
H3K27ac PGC2
H3K27ac Hnisz
FetalDHS Trynka
Enhancer Hoffman
**Enhancer Andersson
DHS Trynka
DGF ENCODE
CTCF Hoffman
Conserved LindbladToh
Coding UCSC
Annotation
−200
0
200
400
600
Enrichment (prop h2 / prop snps)

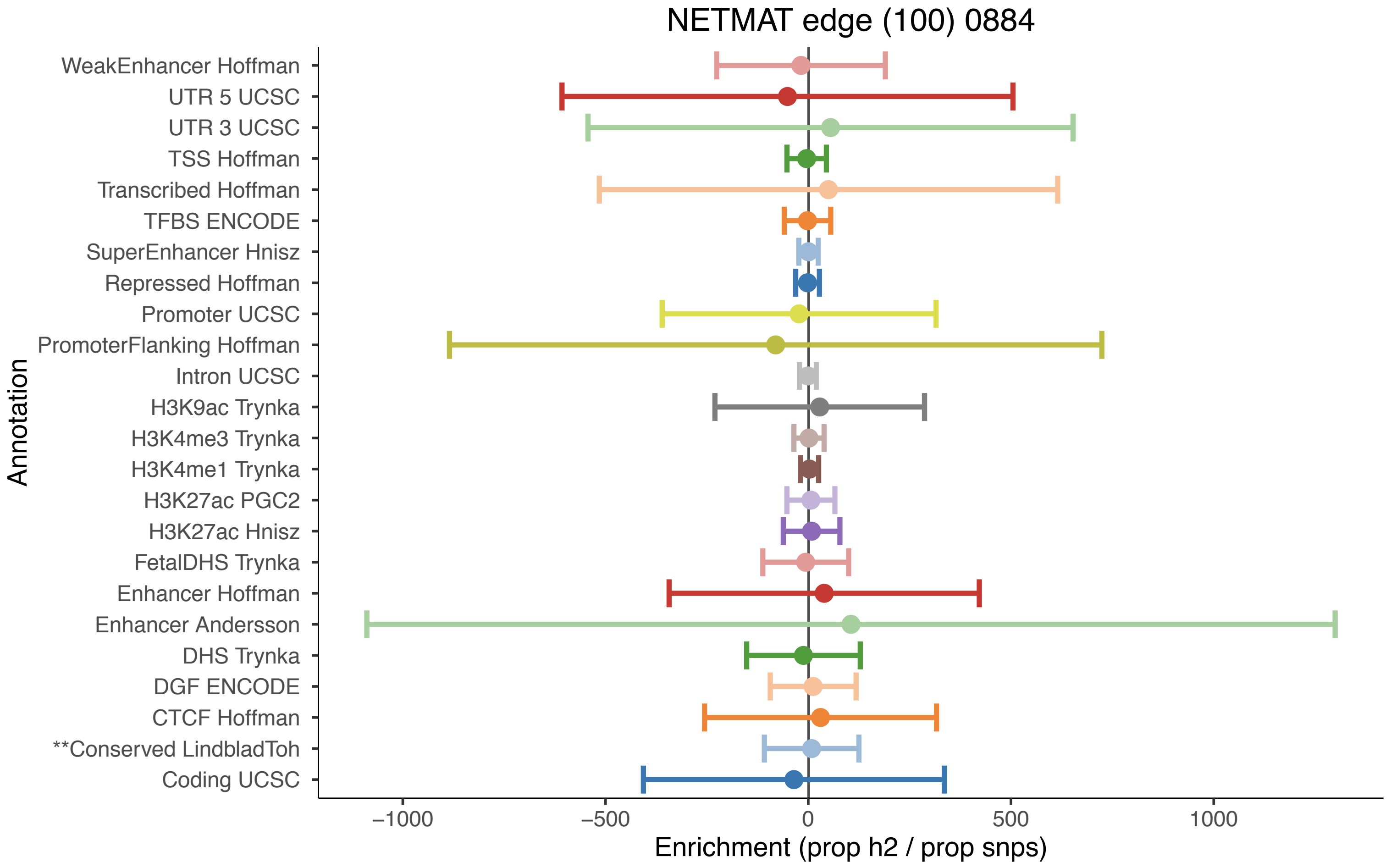
NETMAT edge (100) 0884
WeakEnhancer Hoffman
UTR 5 UCSC
UTR 3 UCSC
TSS Hoffman
Transcribed Hoffman
TFBS ENCODE
SuperEnhancer Hnisz
Repressed Hoffman
Promoter UCSC
PromoterFlanking Hoffman
Intron UCSC
H3K9ac Trynka
H3K4me3 Trynka
H3K4me1 Trynka
H3K27ac PGC2
H3K27ac Hnisz
FetalDHS Trynka
Enhancer Hoffman
Enhancer Andersson
DHS Trynka
DGF ENCODE
CTCF Hoffman
**Conserved LindbladToh
Coding UCSC
Annotation
−1000
−500
0
500
1000
Enrichment (prop h2 / prop snps)

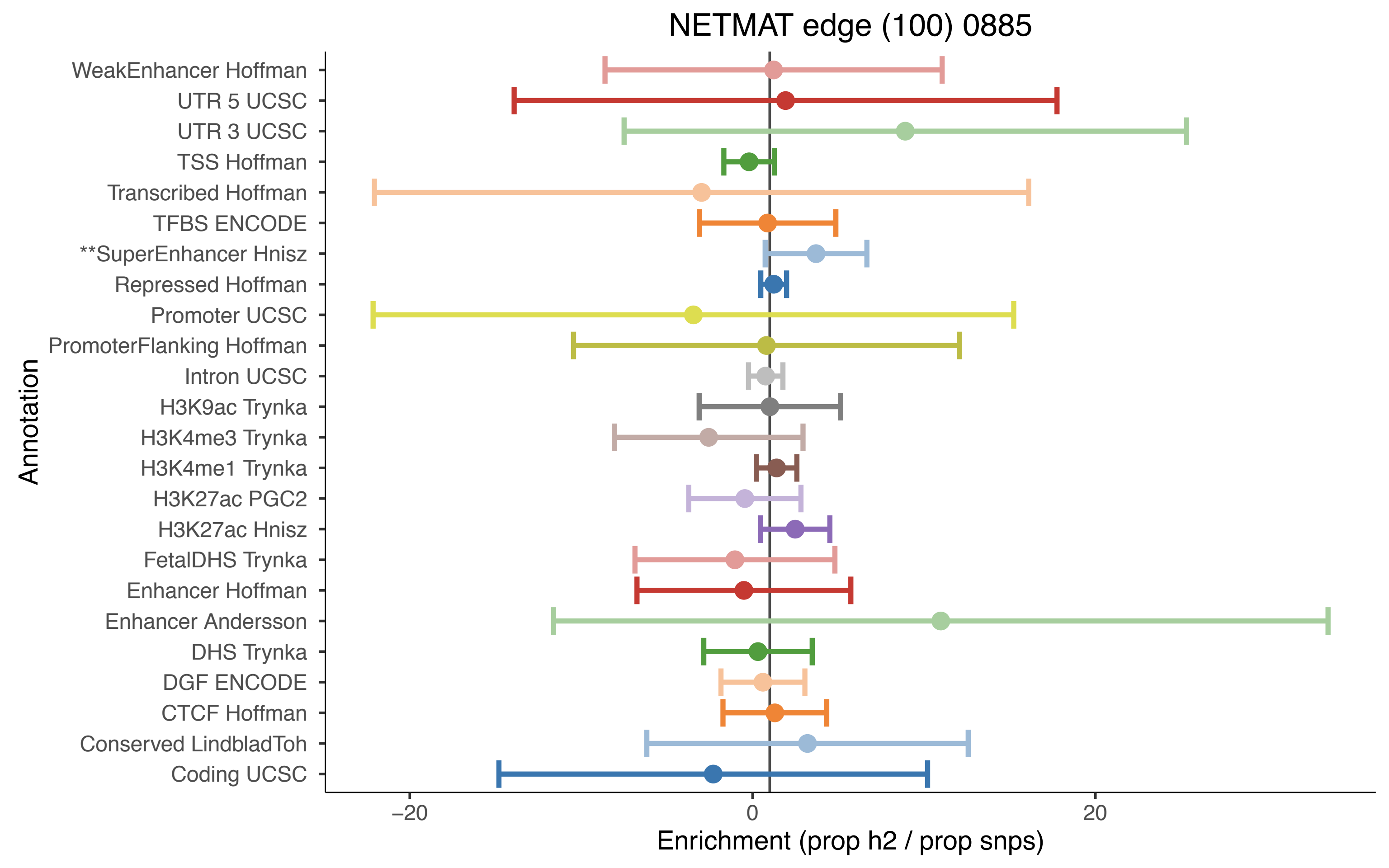
NETMAT edge (100) 0885
WeakEnhancer Hoffman
UTR 5 UCSC
UTR 3 UCSC
TSS Hoffman
Transcribed Hoffman
TFBS ENCODE
**SuperEnhancer Hnisz
Repressed Hoffman
Promoter UCSC
PromoterFlanking Hoffman
Intron UCSC
H3K9ac Trynka
H3K4me3 Trynka
H3K4me1 Trynka
H3K27ac PGC2
H3K27ac Hnisz
FetalDHS Trynka
Enhancer Hoffman
Enhancer Andersson
DHS Trynka
DGF ENCODE
CTCF Hoffman
Conserved LindbladToh
Coding UCSC
Annotation
−20
0
20
Enrichment (prop h2 / prop snps)

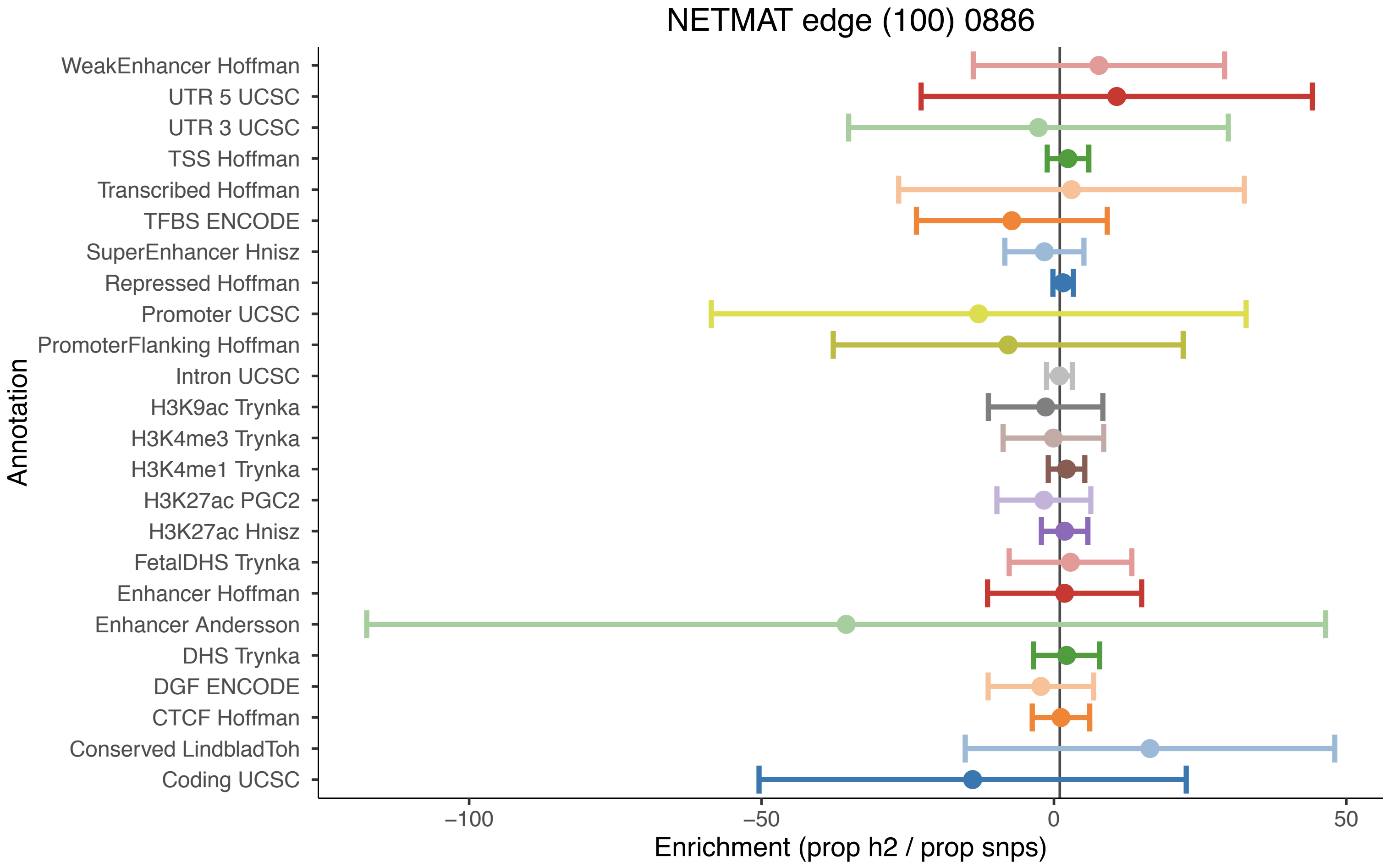
NETMAT edge (100) 0886
WeakEnhancer Hoffman
UTR 5 UCSC
UTR 3 UCSC
TSS Hoffman
Transcribed Hoffman
TFBS ENCODE
SuperEnhancer Hnisz
Repressed Hoffman
Promoter UCSC
PromoterFlanking Hoffman
Intron UCSC
H3K9ac Trynka
H3K4me3 Trynka
H3K4me1 Trynka
H3K27ac PGC2
H3K27ac Hnisz
FetalDHS Trynka
Enhancer Hoffman
Enhancer Andersson
DHS Trynka
DGF ENCODE
CTCF Hoffman
Conserved LindbladToh
Coding UCSC
Annotation
−100
−50
0
50
Enrichment (prop h2 / prop snps)

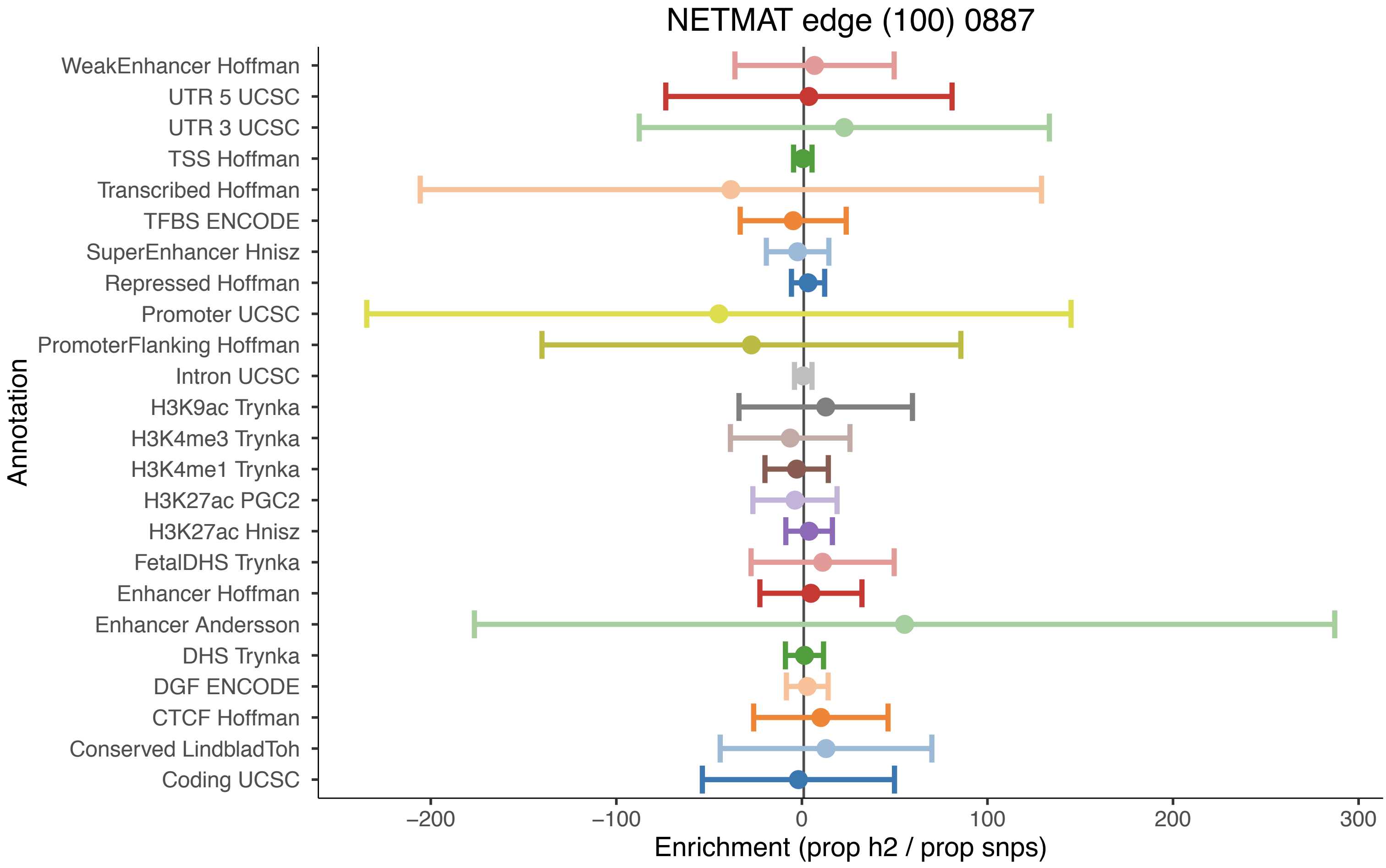

NETMAT edge (100) 0887
WeakEnhancer Hoffman
UTR 5 UCSC
UTR 3 UCSC
TSS Hoffman
Transcribed Hoffman
TFBS ENCODE
SuperEnhancer Hnisz
Repressed Hoffman
Promoter UCSC
PromoterFlanking Hoffman
Intron UCSC
H3K9ac Trynka
H3K4me3 Trynka
H3K4me1 Trynka
H3K27ac PGC2
H3K27ac Hnisz
FetalDHS Trynka
Enhancer Hoffman
Enhancer Andersson
DHS Trynka
DGF ENCODE
CTCF Hoffman
Conserved LindbladToh
Coding UCSC
Annotation
−200
−100
0
100
200
300
Enrichment (prop h2 / prop snps)

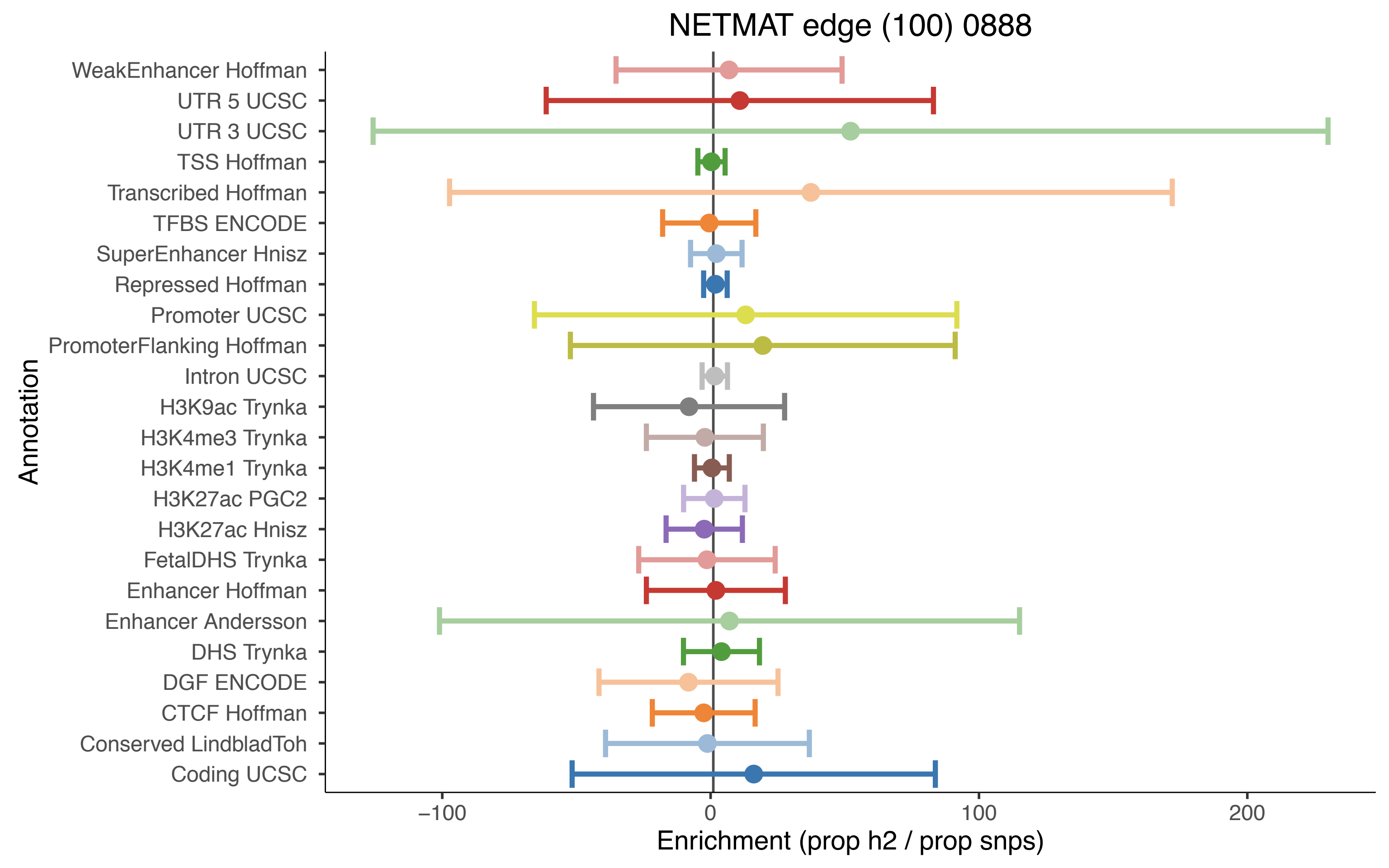

NETMAT edge (100) 0888
Annotation
WeakEnhancer Hoffman
UTR 5 UCSC
UTR 3 UCSC
TSS Hoffman
Transcribed Hoffman
TFBS ENCODE
SuperEnhancer Hnisz
Repressed Hoffman
Promoter UCSC
PromoterFlanking Hoffman
Intron UCSC
H3K9ac Trynka
H3K4me3 Trynka
H3K4me1 Trynka
H3K27ac PGC2
H3K27ac Hnisz
FetalDHS Trynka
Enhancer Hoffman
Enhancer Andersson
DHS Trynka
DGF ENCODE
CTCF Hoffman
Conserved LindbladToh
Coding UCSC
−100
0
100
200
Enrichment (prop h2 / prop snps)

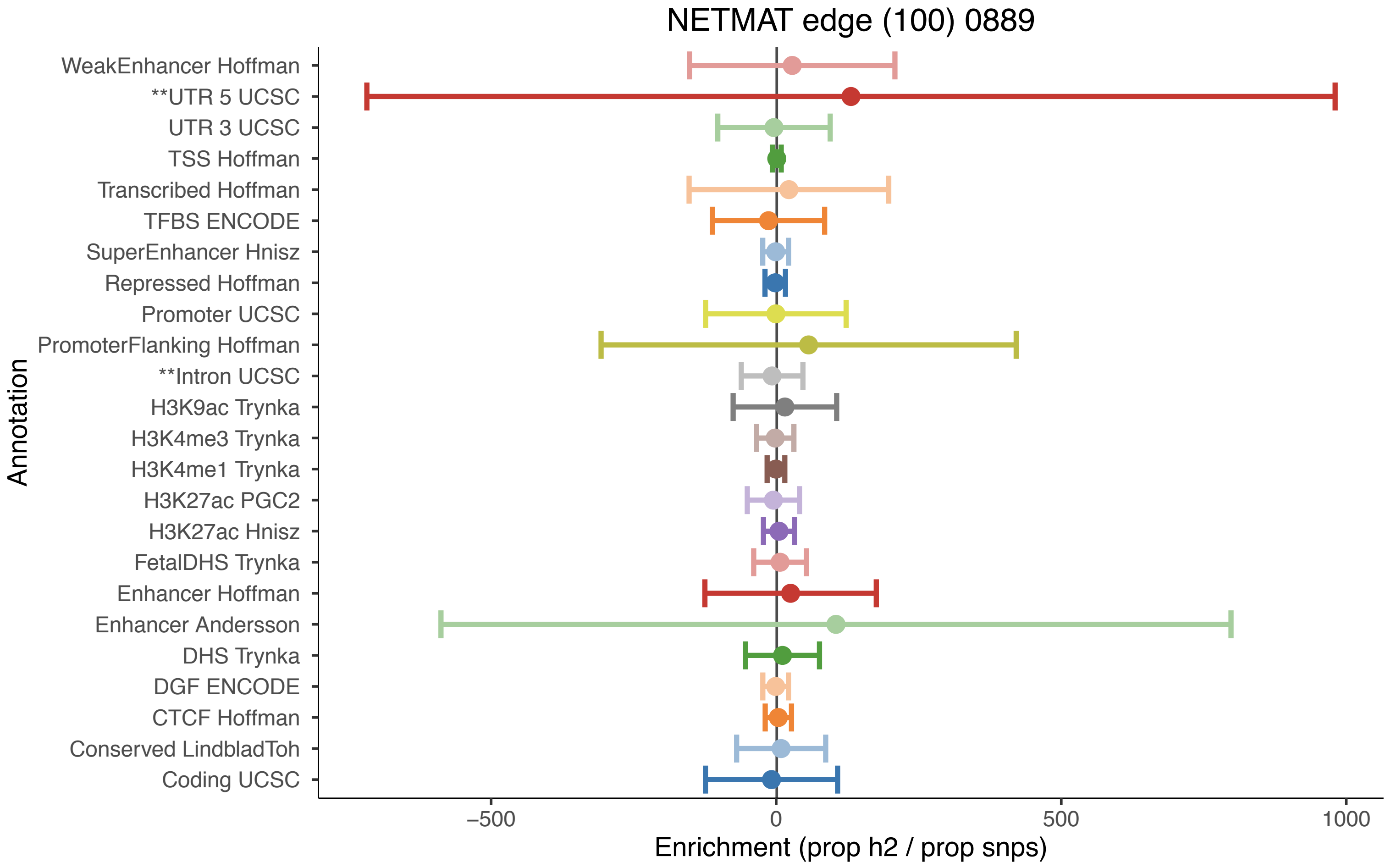

NETMAT edge (100) 0889
WeakEnhancer Hoffman
**UTR 5 UCSC
UTR 3 UCSC
TSS Hoffman
Transcribed Hoffman
TFBS ENCODE
SuperEnhancer Hnisz
Repressed Hoffman
Promoter UCSC
PromoterFlanking Hoffman
**Intron UCSC
H3K9ac Trynka
H3K4me3 Trynka
H3K4me1 Trynka
H3K27ac PGC2
H3K27ac Hnisz
FetalDHS Trynka
Enhancer Hoffman
Enhancer Andersson
DHS Trynka
DGF ENCODE
CTCF Hoffman
Conserved LindbladToh
Coding UCSC
Annotation
−500
0
500
1000
Enrichment (prop h2 / prop snps)

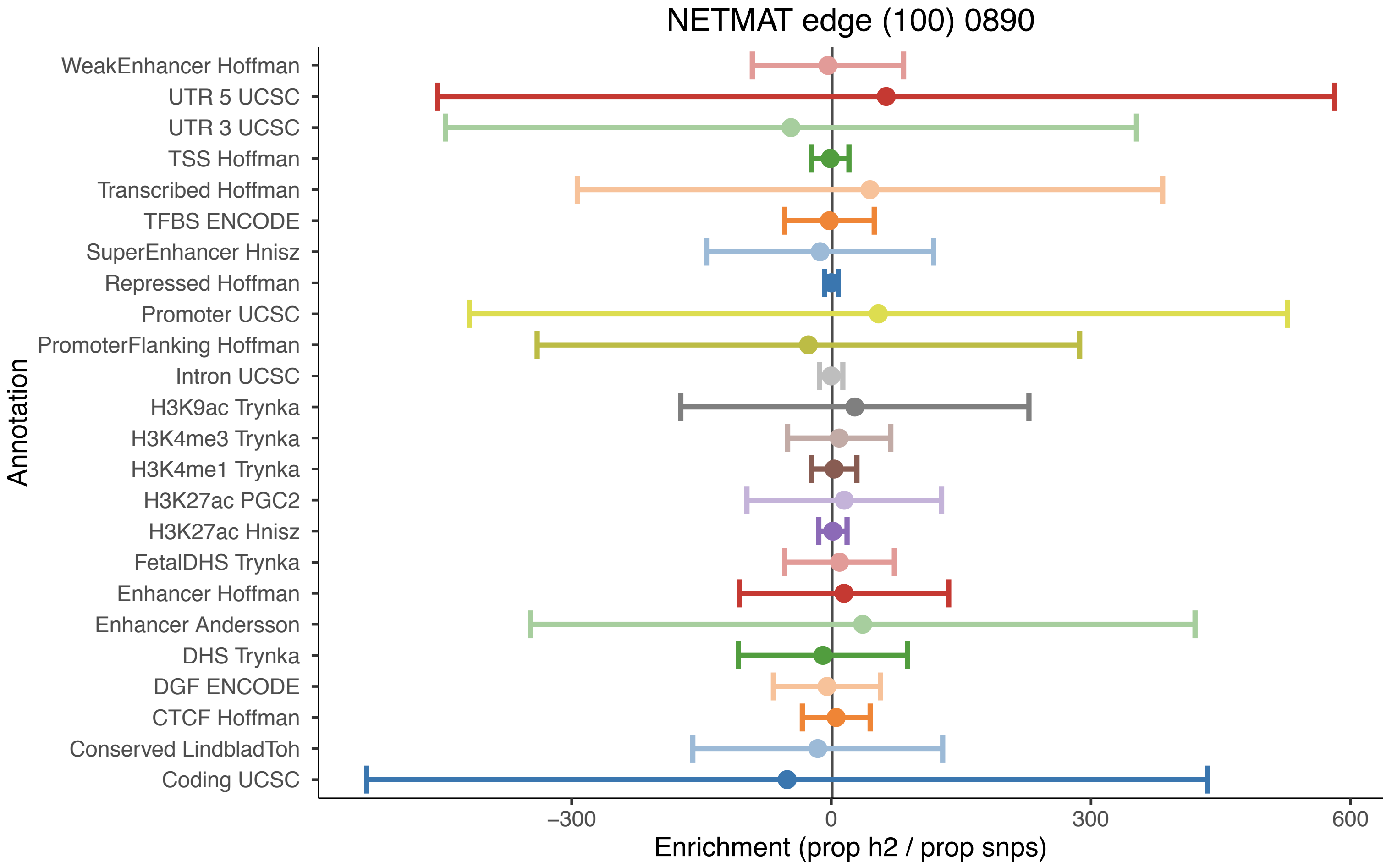

NETMAT edge (100) 0890
WeakEnhancer Hoffman
UTR 5 UCSC
UTR 3 UCSC
TSS Hoffman
Transcribed Hoffman
TFBS ENCODE
SuperEnhancer Hnisz
Repressed Hoffman
Promoter UCSC
PromoterFlanking Hoffman
Intron UCSC
H3K9ac Trynka
H3K4me3 Trynka
H3K4me1 Trynka
H3K27ac PGC2
H3K27ac Hnisz
FetalDHS Trynka
Enhancer Hoffman
Enhancer Andersson
DHS Trynka
DGF ENCODE
CTCF Hoffman
Conserved LindbladToh
Coding UCSC
Annotation
−300
0
300
600
Enrichment (prop h2 / prop snps)

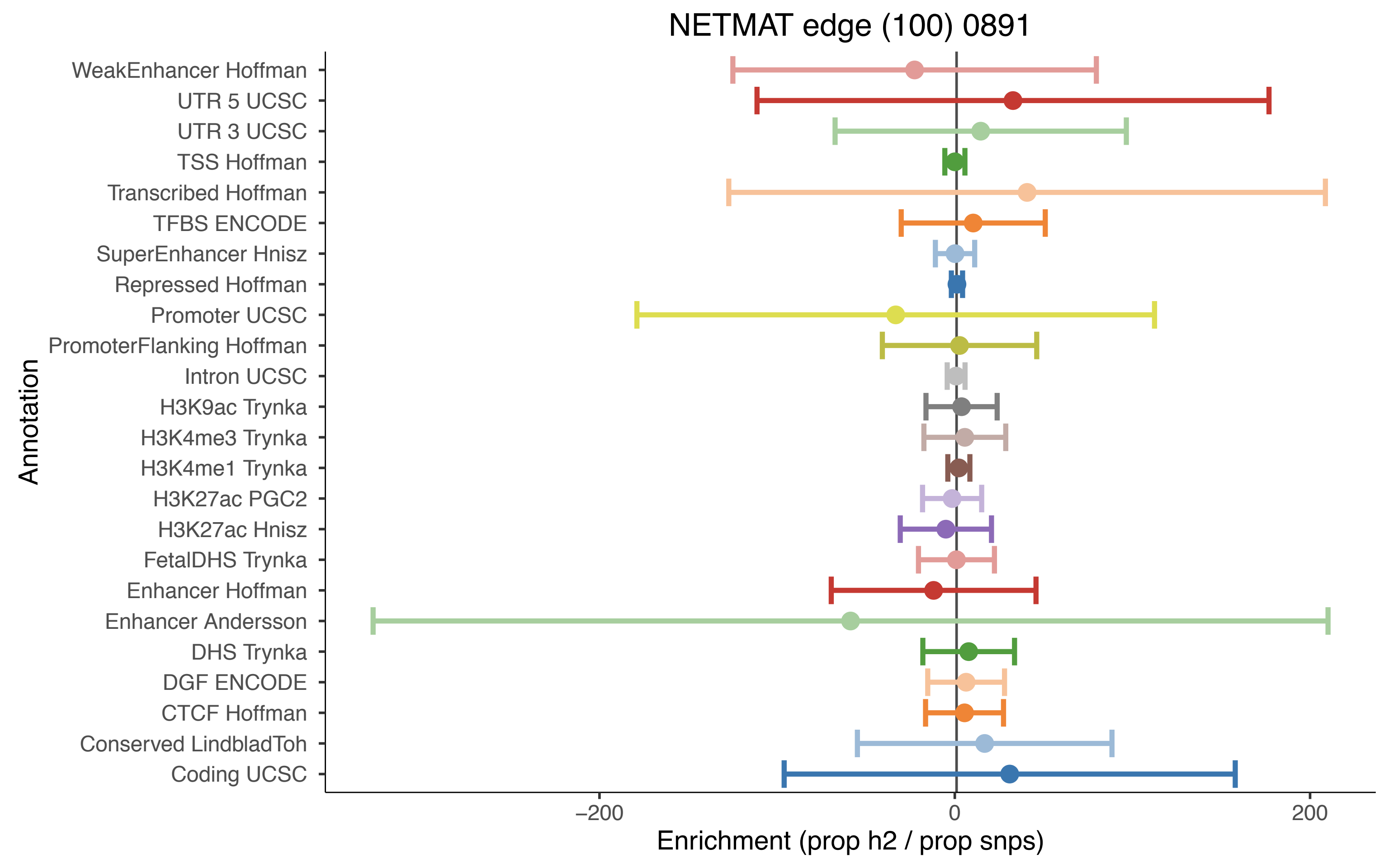
NETMAT edge (100) 0891
WeakEnhancer Hoffman
UTR 5 UCSC
UTR 3 UCSC
TSS Hoffman
Transcribed Hoffman
TFBS ENCODE
SuperEnhancer Hnisz
Repressed Hoffman
Promoter UCSC
PromoterFlanking Hoffman
Intron UCSC
H3K9ac Trynka
H3K4me3 Trynka
H3K4me1 Trynka
H3K27ac PGC2
H3K27ac Hnisz
FetalDHS Trynka
Enhancer Hoffman
Enhancer Andersson
DHS Trynka
DGF ENCODE
CTCF Hoffman
Conserved LindbladToh
Coding UCSC
Annotation
−200
0
200
Enrichment (prop h2 / prop snps)

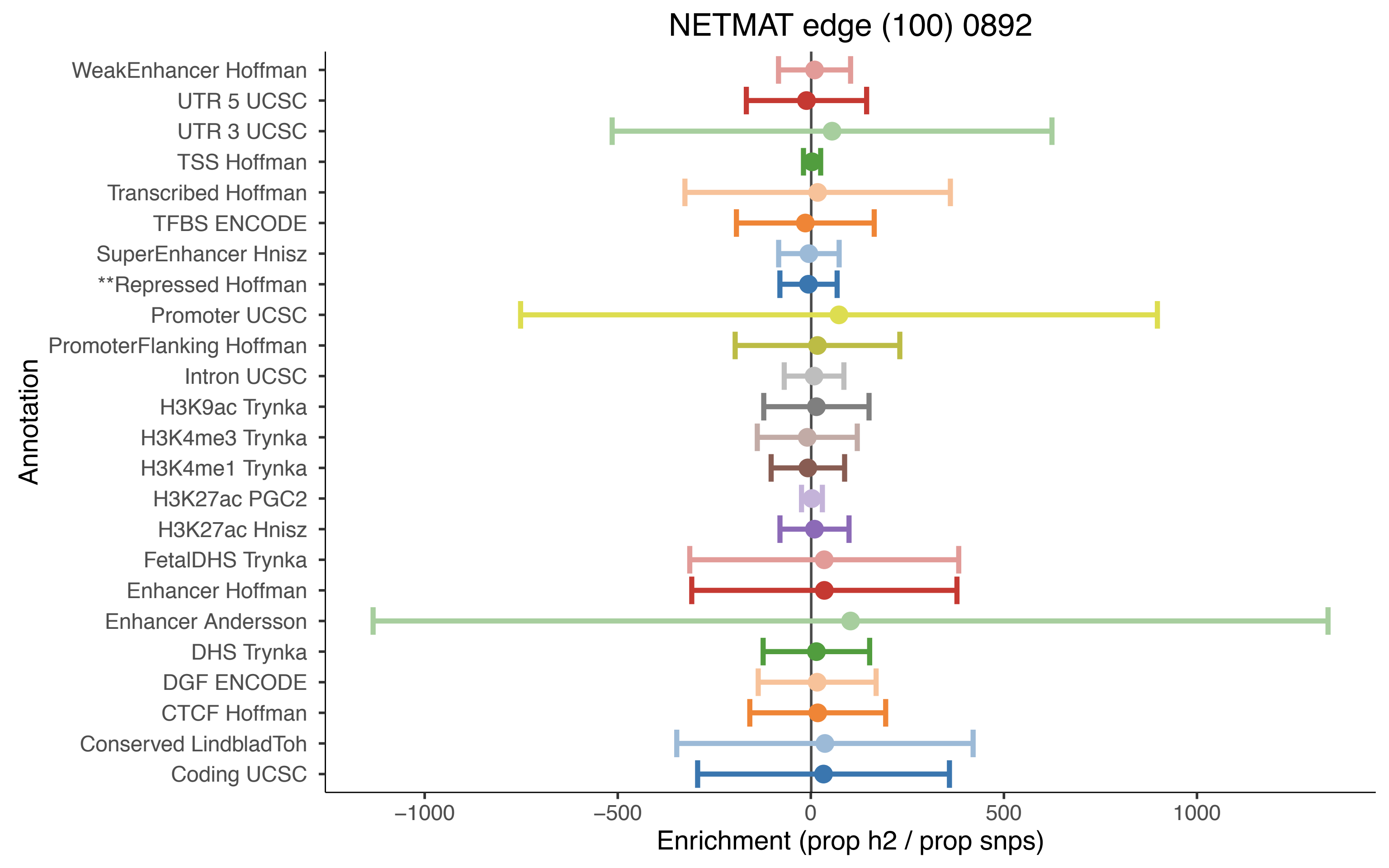

NETMAT edge (100) 0892
WeakEnhancer Hoffman
UTR 5 UCSC
UTR 3 UCSC
TSS Hoffman
Transcribed Hoffman
TFBS ENCODE
SuperEnhancer Hnisz
**Repressed Hoffman
Promoter UCSC
PromoterFlanking Hoffman
Intron UCSC
H3K9ac Trynka
H3K4me3 Trynka
H3K4me1 Trynka
H3K27ac PGC2
H3K27ac Hnisz
FetalDHS Trynka
Enhancer Hoffman
Enhancer Andersson
DHS Trynka
DGF ENCODE
CTCF Hoffman
Conserved LindbladToh
Coding UCSC
Annotation
−1000
−500
0
500
1000
Enrichment (prop h2 / prop snps)

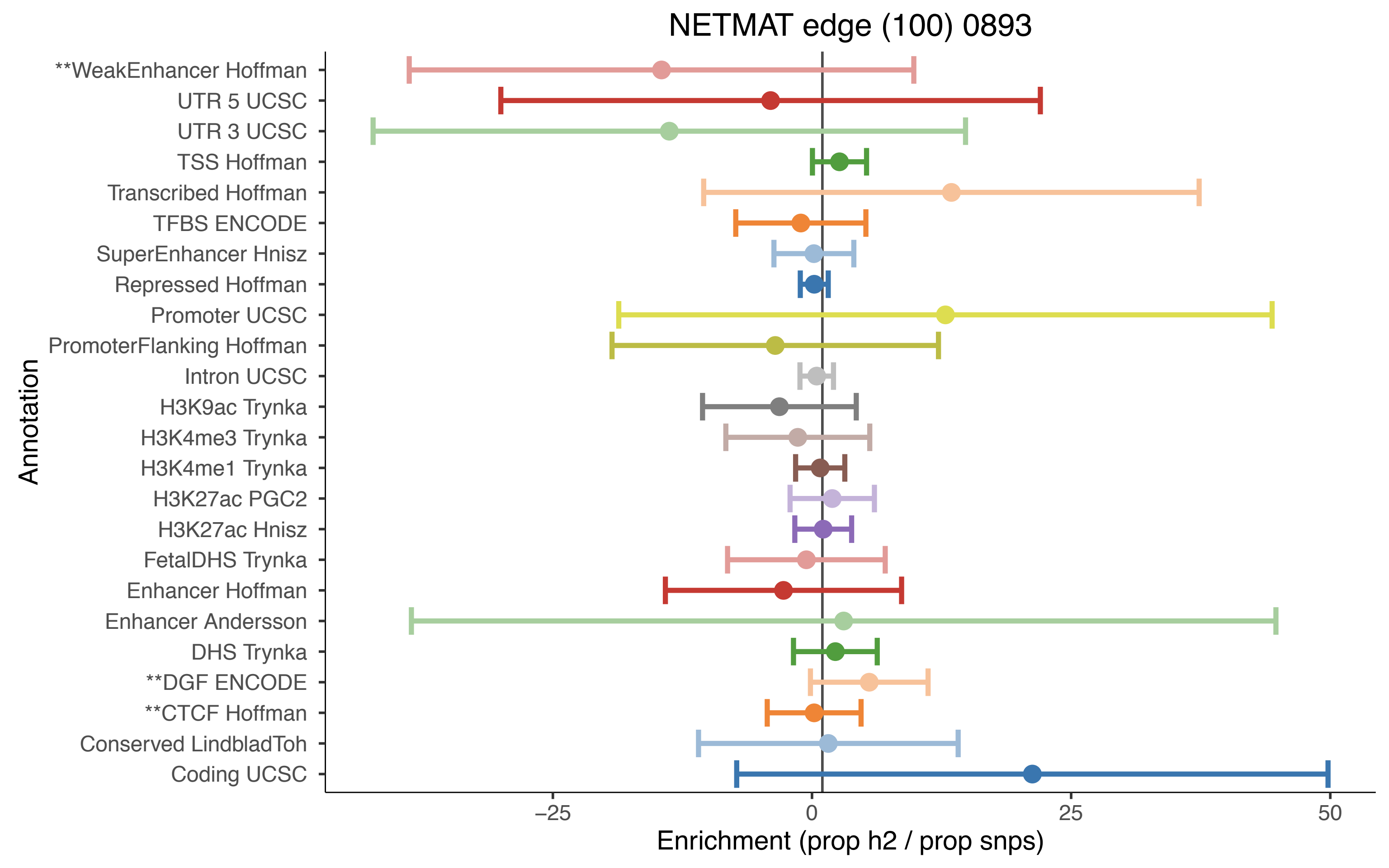
NETMAT edge (100) 0893
**WeakEnhancer Hoffman
UTR 5 UCSC
UTR 3 UCSC
TSS Hoffman
Transcribed Hoffman
TFBS ENCODE
SuperEnhancer Hnisz
Repressed Hoffman
Promoter UCSC
PromoterFlanking Hoffman
Intron UCSC
H3K9ac Trynka
H3K4me3 Trynka
H3K4me1 Trynka
H3K27ac PGC2
H3K27ac Hnisz
FetalDHS Trynka
Enhancer Hoffman
Enhancer Andersson
DHS Trynka
**DGF ENCODE
**CTCF Hoffman
Conserved LindbladToh
Coding UCSC
Annotation
−25
0
25
50
Enrichment (prop h2 / prop snps)

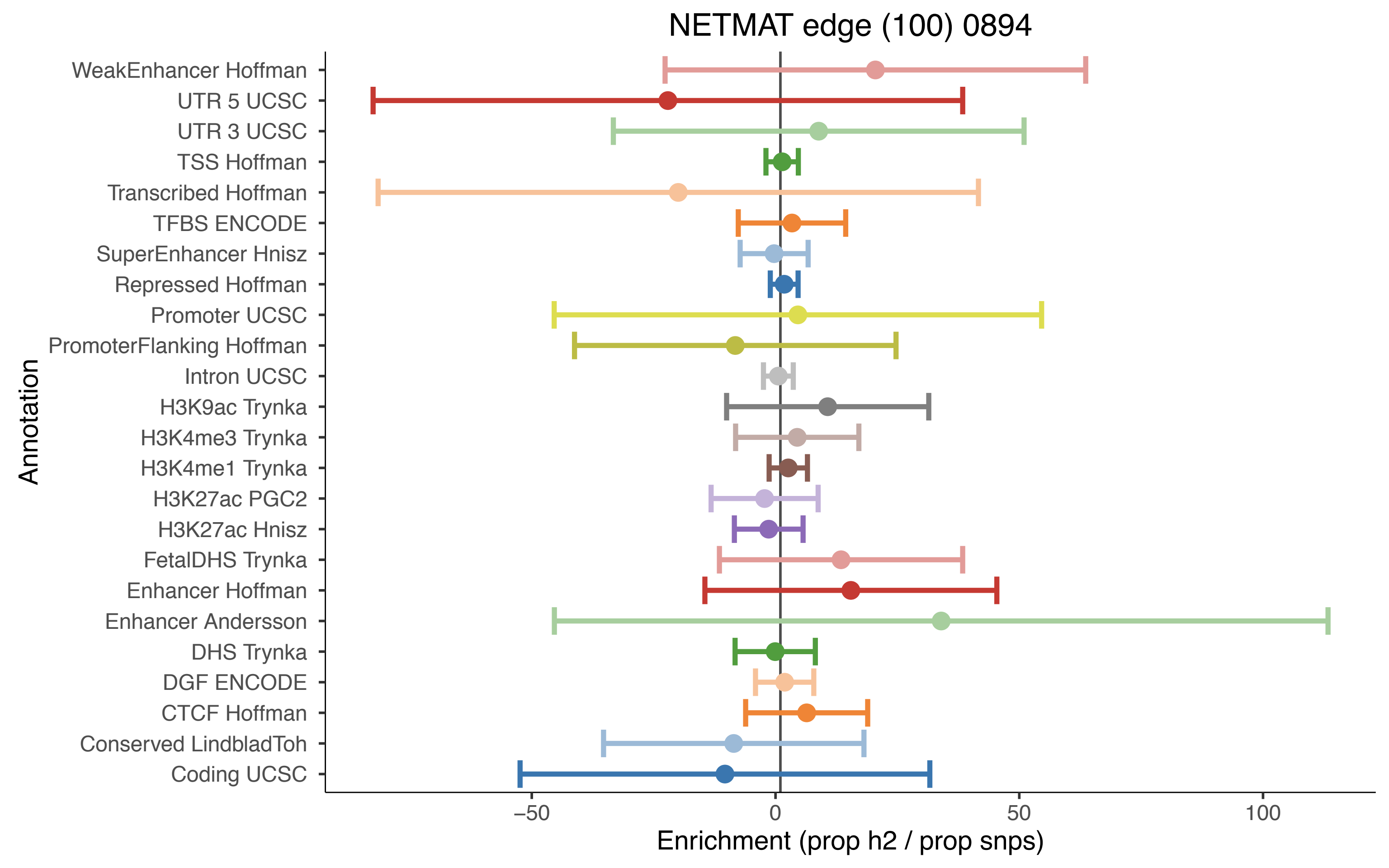
NETMAT edge (100) 0894
WeakEnhancer Hoffman
UTR 5 UCSC
UTR 3 UCSC
TSS Hoffman
Transcribed Hoffman
TFBS ENCODE
SuperEnhancer Hnisz
Repressed Hoffman
Promoter UCSC
PromoterFlanking Hoffman
Intron UCSC
H3K9ac Trynka
H3K4me3 Trynka
H3K4me1 Trynka
H3K27ac PGC2
H3K27ac Hnisz
FetalDHS Trynka
Enhancer Hoffman
Enhancer Andersson
DHS Trynka
DGF ENCODE
CTCF Hoffman
Conserved LindbladToh
Coding UCSC
Annotation
−50
0
50
100
Enrichment (prop h2 / prop snps)

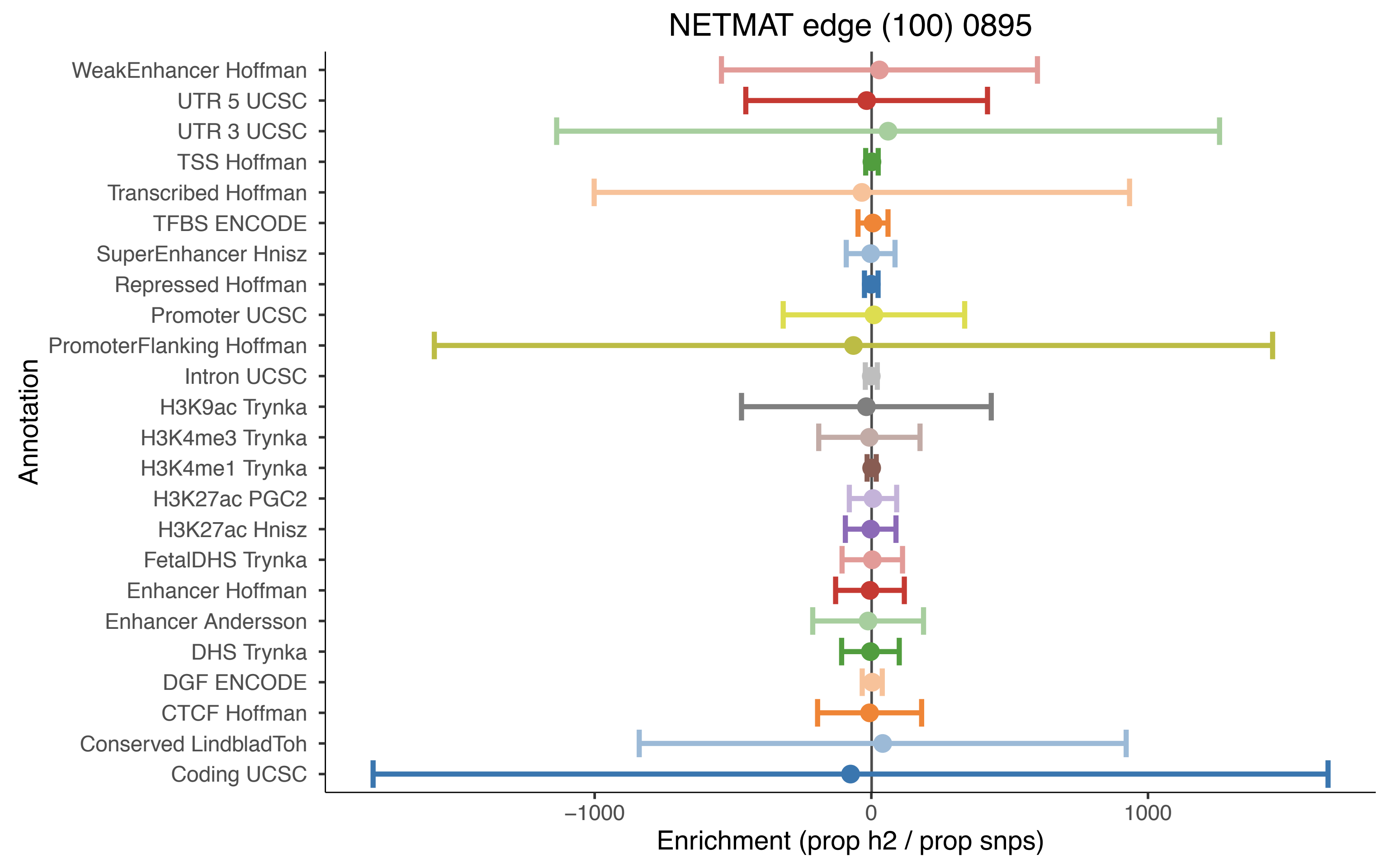
NETMAT edge (100) 0895
Annotation
WeakEnhancer Hoffman
UTR 5 UCSC
UTR 3 UCSC
TSS Hoffman
Transcribed Hoffman
TFBS ENCODE
SuperEnhancer Hnisz
Repressed Hoffman
Promoter UCSC
PromoterFlanking Hoffman
Intron UCSC
H3K9ac Trynka
H3K4me3 Trynka
H3K4me1 Trynka
H3K27ac PGC2
H3K27ac Hnisz
FetalDHS Trynka
Enhancer Hoffman
Enhancer Andersson
DHS Trynka
DGF ENCODE
CTCF Hoffman
Conserved LindbladToh
Coding UCSC
−1000
0
1000
Enrichment (prop h2 / prop snps)

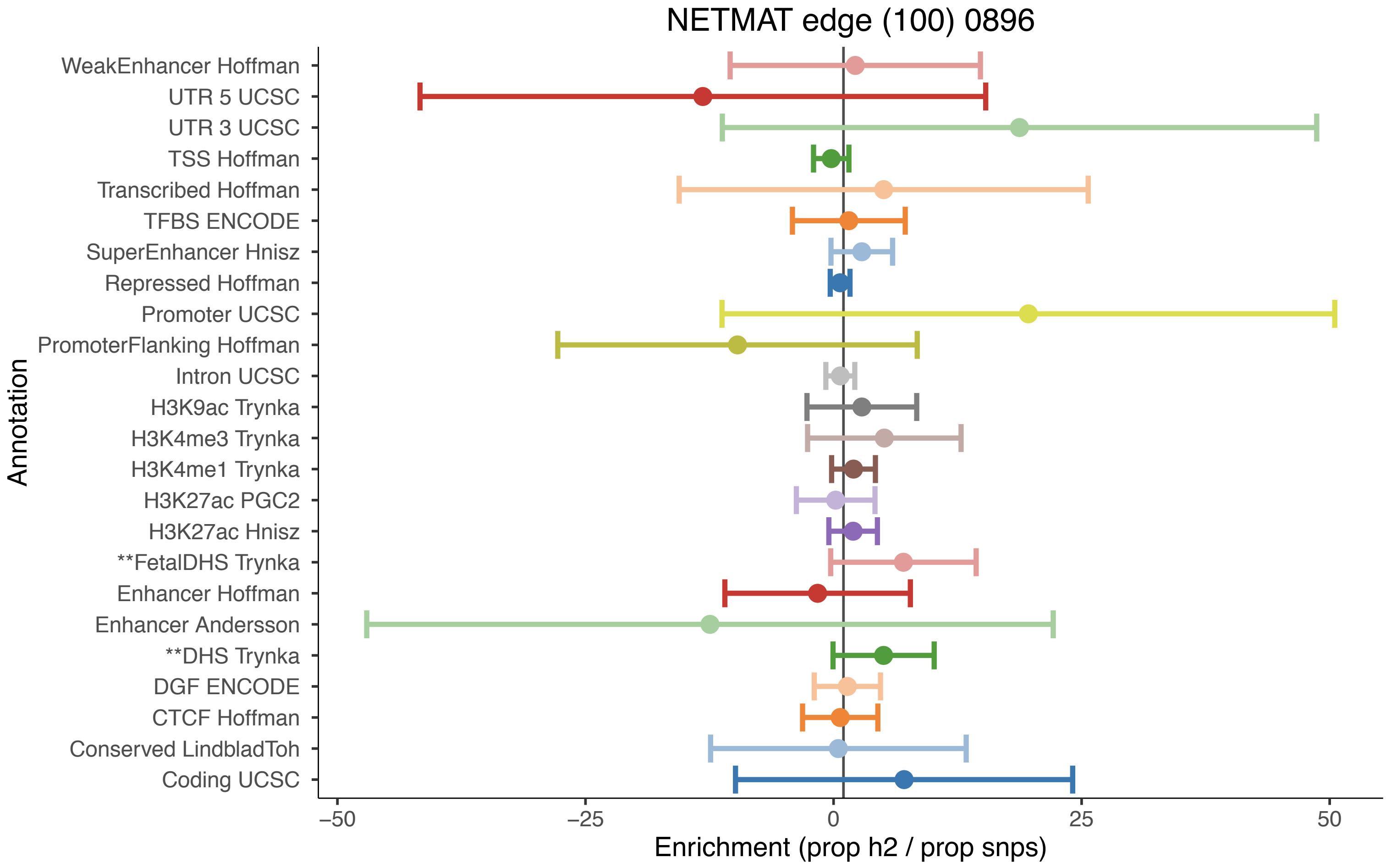
NETMAT edge (100) 0896
WeakEnhancer Hoffman
UTR 5 UCSC
UTR 3 UCSC
TSS Hoffman
Transcribed Hoffman
TFBS ENCODE
SuperEnhancer Hnisz
Repressed Hoffman
Promoter UCSC
PromoterFlanking Hoffman
Intron UCSC
H3K9ac Trynka
H3K4me3 Trynka
H3K4me1 Trynka
H3K27ac PGC2
H3K27ac Hnisz
**FetalDHS Trynka
Enhancer Hoffman
Enhancer Andersson
**DHS Trynka
DGF ENCODE
CTCF Hoffman
Conserved LindbladToh
Coding UCSC
Annotation
−50
−25
0
25
50
Enrichment (prop h2 / prop snps)

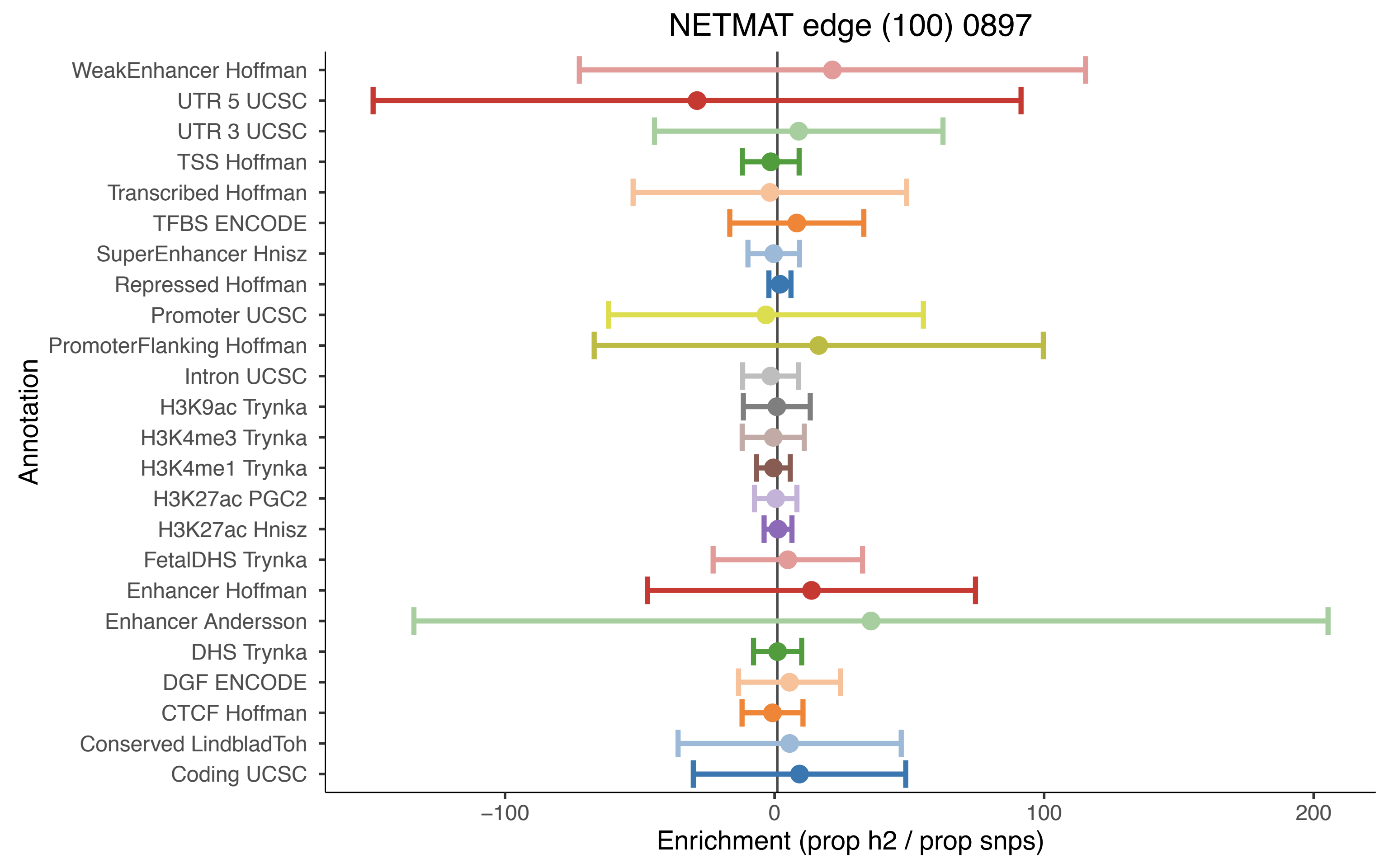
NETMAT edge (100) 0897
Annotation
WeakEnhancer Hoffman
UTR 5 UCSC
UTR 3 UCSC
TSS Hoffman
Transcribed Hoffman
TFBS ENCODE
SuperEnhancer Hnisz
Repressed Hoffman
Promoter UCSC
PromoterFlanking Hoffman
Intron UCSC
H3K9ac Trynka
H3K4me3 Trynka
H3K4me1 Trynka
H3K27ac PGC2
H3K27ac Hnisz
FetalDHS Trynka
Enhancer Hoffman
Enhancer Andersson
DHS Trynka
DGF ENCODE
CTCF Hoffman
Conserved LindbladToh
Coding UCSC
−100
0
100
200
Enrichment (prop h2 / prop snps)

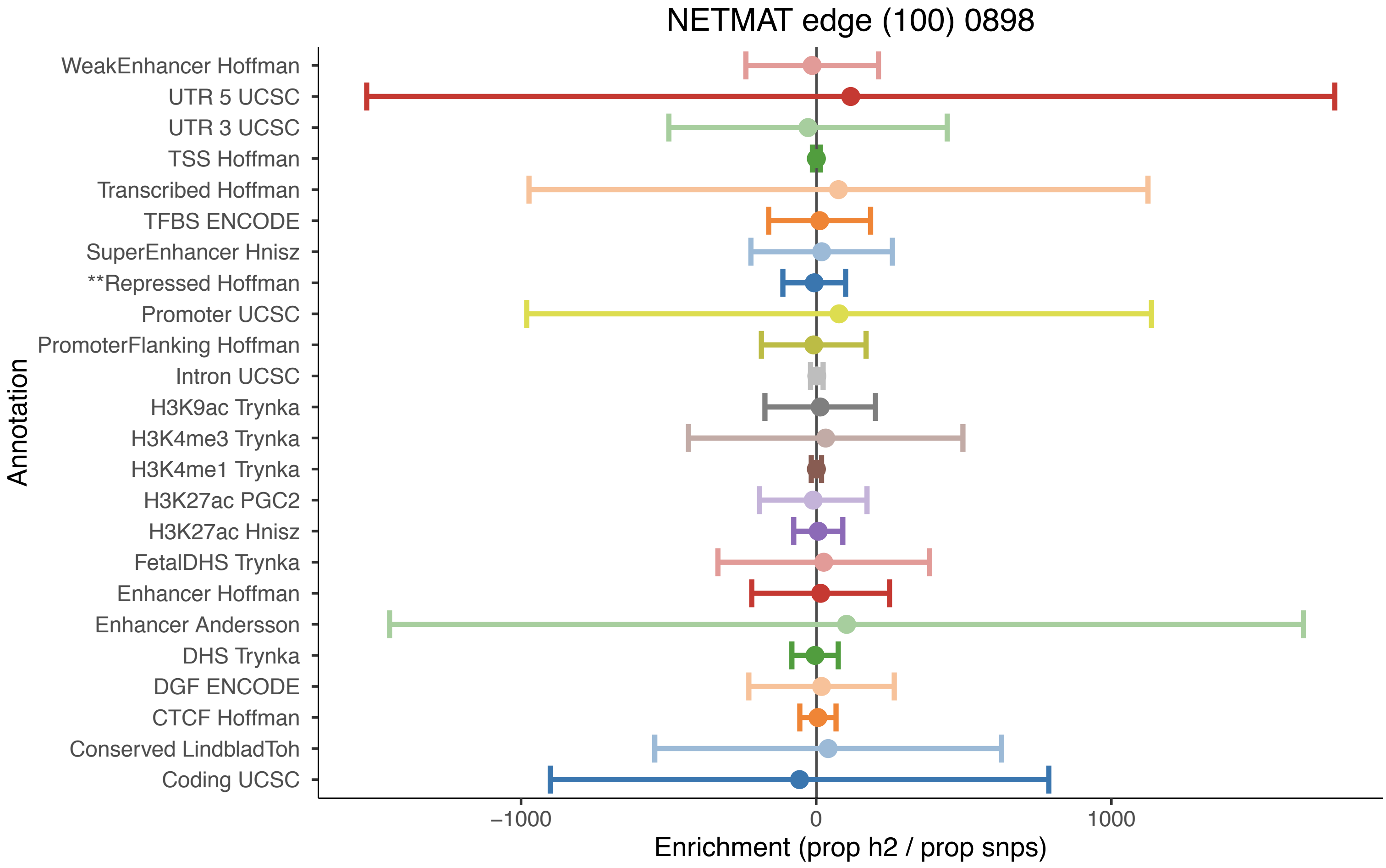
NETMAT edge (100) 0898
WeakEnhancer Hoffman
UTR 5 UCSC
UTR 3 UCSC
TSS Hoffman
Transcribed Hoffman
TFBS ENCODE
SuperEnhancer Hnisz
**Repressed Hoffman
Promoter UCSC
PromoterFlanking Hoffman
Intron UCSC
H3K9ac Trynka
H3K4me3 Trynka
H3K4me1 Trynka
H3K27ac PGC2
H3K27ac Hnisz
FetalDHS Trynka
Enhancer Hoffman
Enhancer Andersson
DHS Trynka
DGF ENCODE
CTCF Hoffman
Conserved LindbladToh
Coding UCSC
Annotation
−1000
0
1000
Enrichment (prop h2 / prop snps)

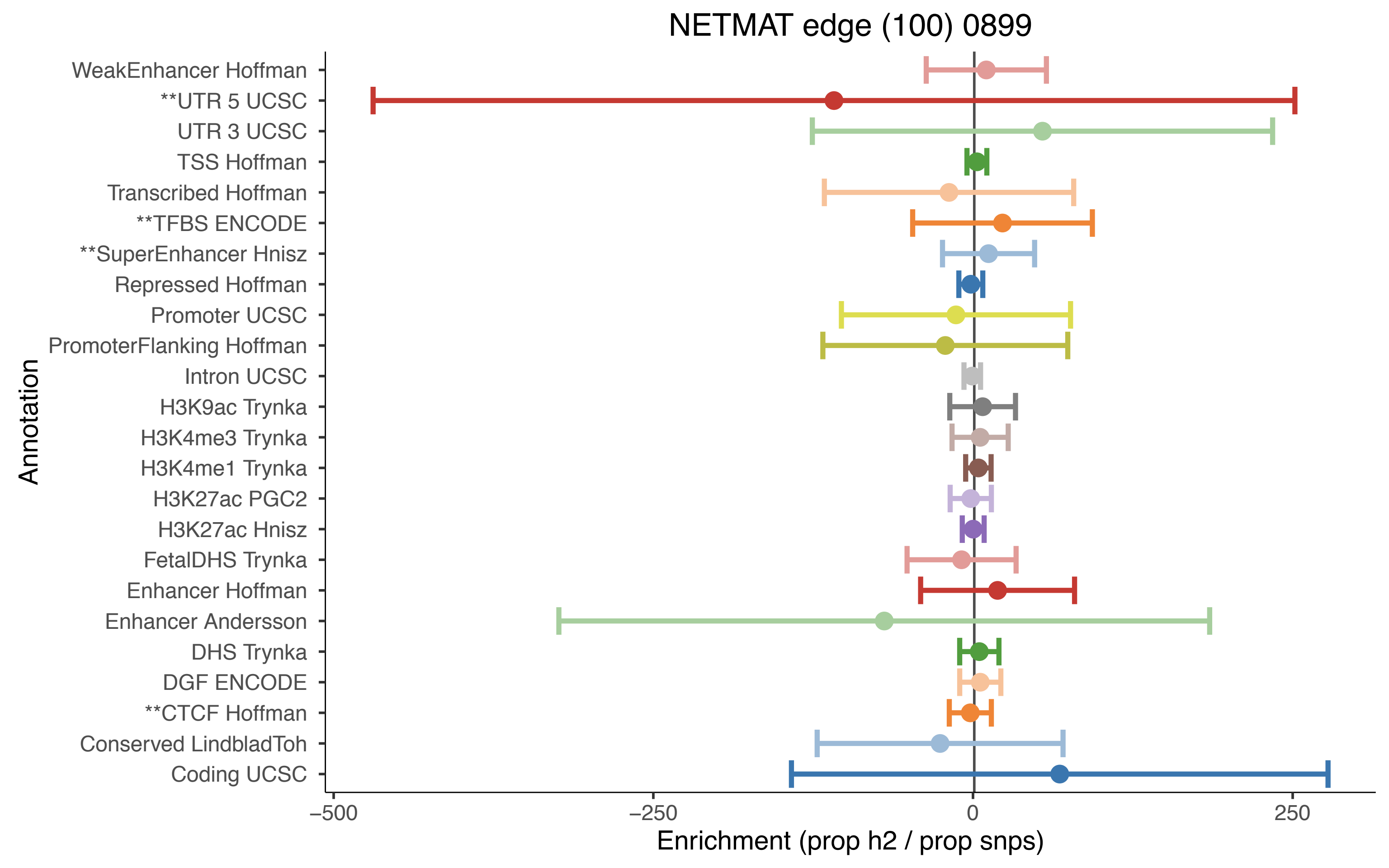
NETMAT edge (100) 0899
WeakEnhancer Hoffman
**UTR 5 UCSC
UTR 3 UCSC
TSS Hoffman
Transcribed Hoffman
**TFBS ENCODE
**SuperEnhancer Hnisz
Repressed Hoffman
Promoter UCSC
PromoterFlanking Hoffman
Intron UCSC
H3K9ac Trynka
H3K4me3 Trynka
H3K4me1 Trynka
H3K27ac PGC2
H3K27ac Hnisz
FetalDHS Trynka
Enhancer Hoffman
Enhancer Andersson
DHS Trynka
DGF ENCODE
**CTCF Hoffman
Conserved LindbladToh
Coding UCSC
Annotation
−500
−250
0
250
Enrichment (prop h2 / prop snps)

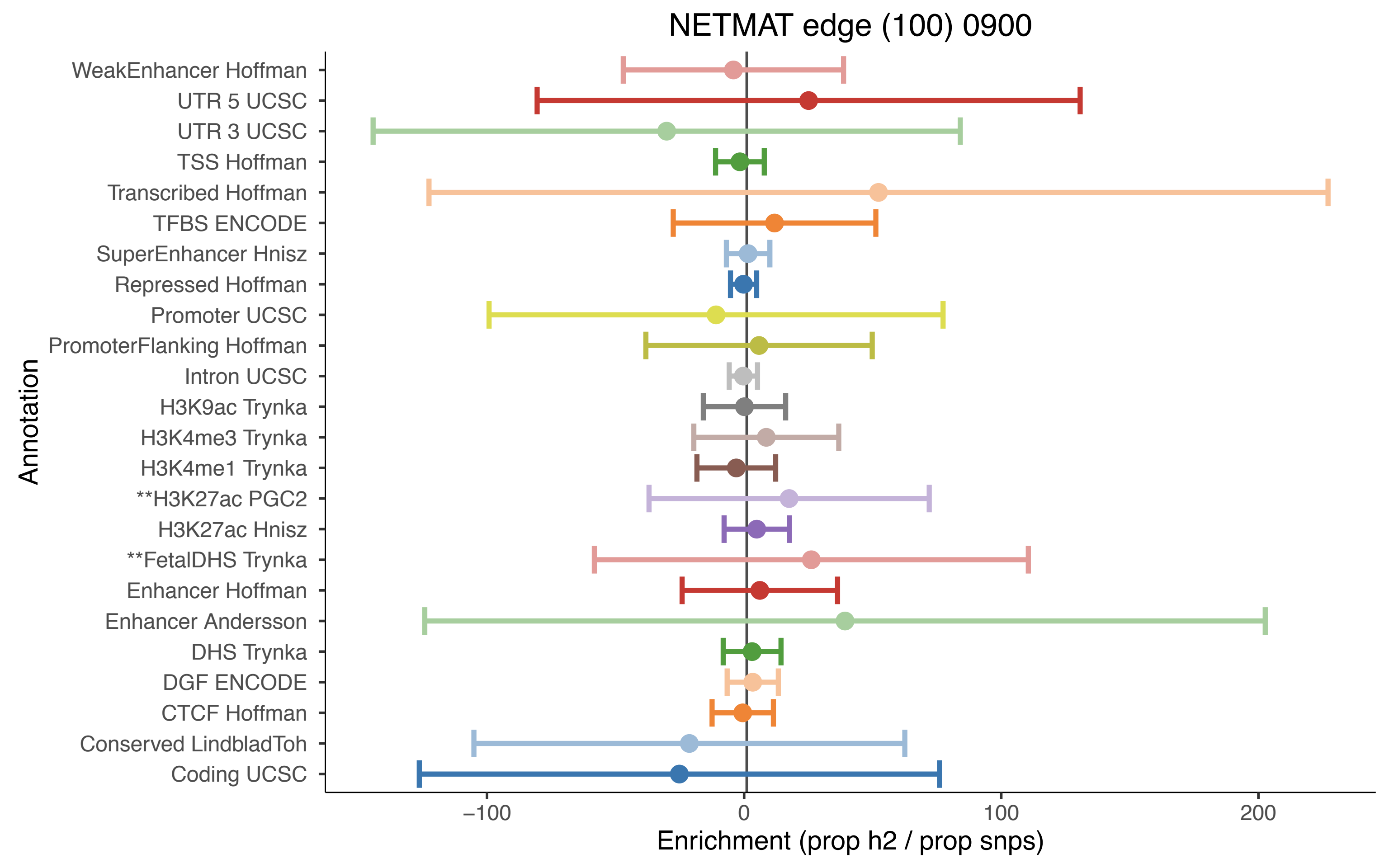

NETMAT edge (100) 0900
WeakEnhancer Hoffman
UTR 5 UCSC
UTR 3 UCSC
TSS Hoffman
Transcribed Hoffman
TFBS ENCODE
SuperEnhancer Hnisz
Repressed Hoffman
Promoter UCSC
PromoterFlanking Hoffman
Intron UCSC
H3K9ac Trynka
H3K4me3 Trynka
H3K4me1 Trynka
**H3K27ac PGC2
H3K27ac Hnisz
**FetalDHS Trynka
Enhancer Hoffman
Enhancer Andersson
DHS Trynka
DGF ENCODE
CTCF Hoffman
Conserved LindbladToh
Coding UCSC
Annotation
−100
0
100
200
Enrichment (prop h2 / prop snps)

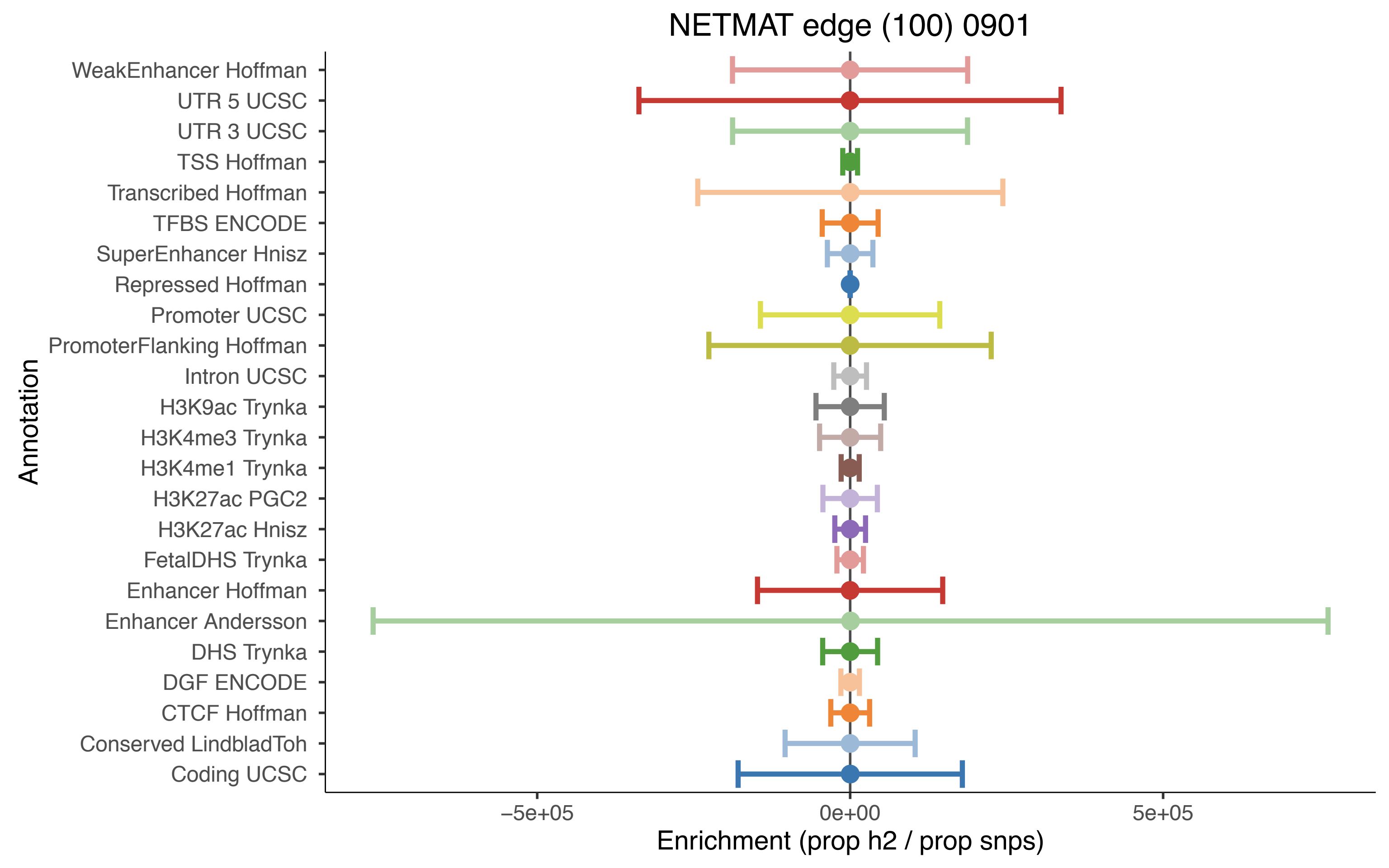
NETMAT edge (100) 0901
WeakEnhancer Hoffman
UTR 5 UCSC
UTR 3 UCSC
TSS Hoffman
Transcribed Hoffman
TFBS ENCODE
SuperEnhancer Hnisz
Repressed Hoffman
Promoter UCSC
PromoterFlanking Hoffman
Intron UCSC
H3K9ac Trynka
H3K4me3 Trynka
H3K4me1 Trynka
H3K27ac PGC2
H3K27ac Hnisz
FetalDHS Trynka
Enhancer Hoffman
Enhancer Andersson
DHS Trynka
DGF ENCODE
CTCF Hoffman
Conserved LindbladToh
Coding UCSC
Annotation
−5e+05
0e+00
5e+05
Enrichment (prop h2 / prop snps)

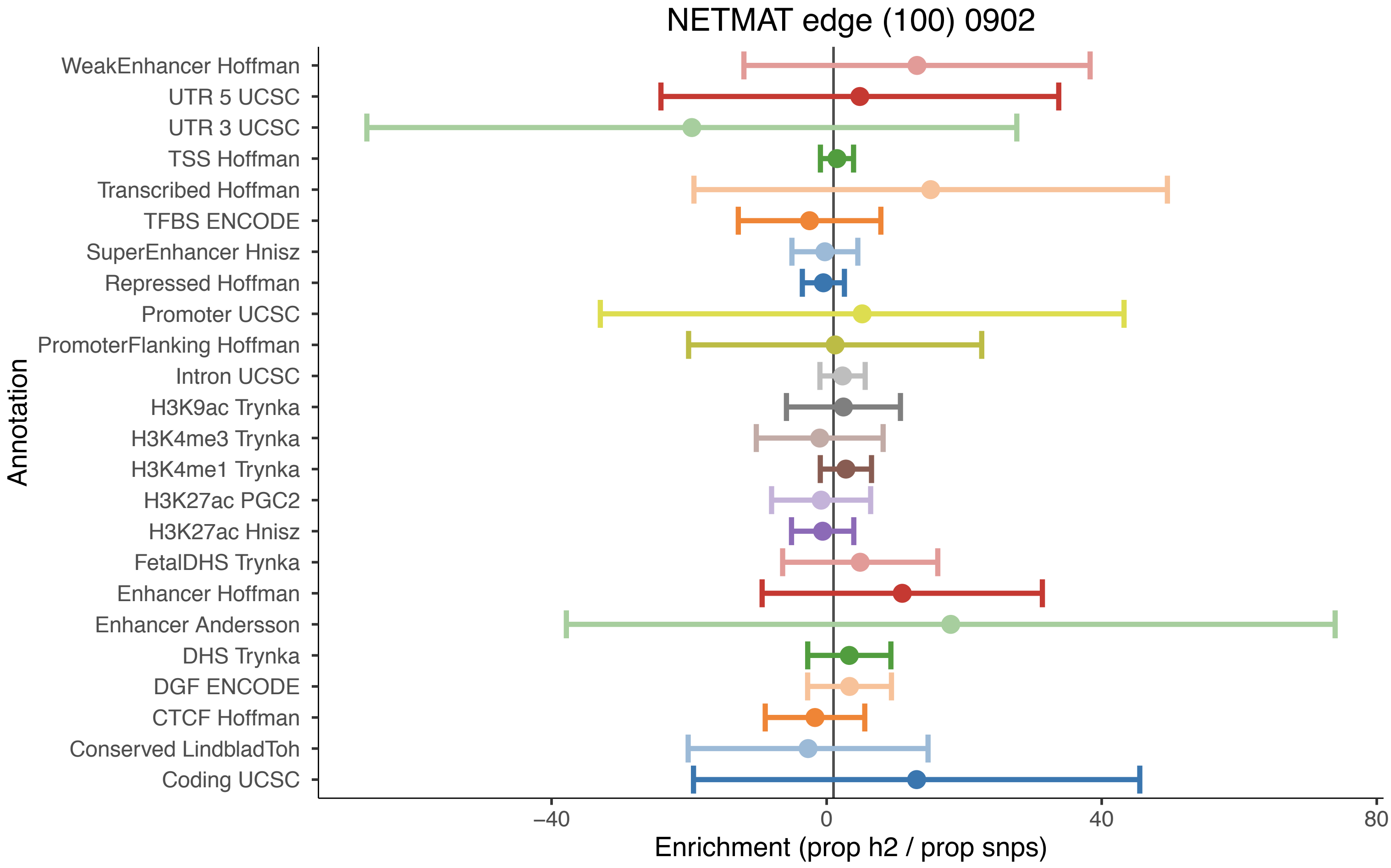
NETMAT edge (100) 0902
Annotation
WeakEnhancer Hoffman
UTR 5 UCSC
UTR 3 UCSC
TSS Hoffman
Transcribed Hoffman
TFBS ENCODE
SuperEnhancer Hnisz
Repressed Hoffman
Promoter UCSC
PromoterFlanking Hoffman
Intron UCSC
H3K9ac Trynka
H3K4me3 Trynka
H3K4me1 Trynka
H3K27ac PGC2
H3K27ac Hnisz
FetalDHS Trynka
Enhancer Hoffman
Enhancer Andersson
DHS Trynka
DGF ENCODE
CTCF Hoffman
Conserved LindbladToh
Coding UCSC
−40
0
40
80
Enrichment (prop h2 / prop snps)

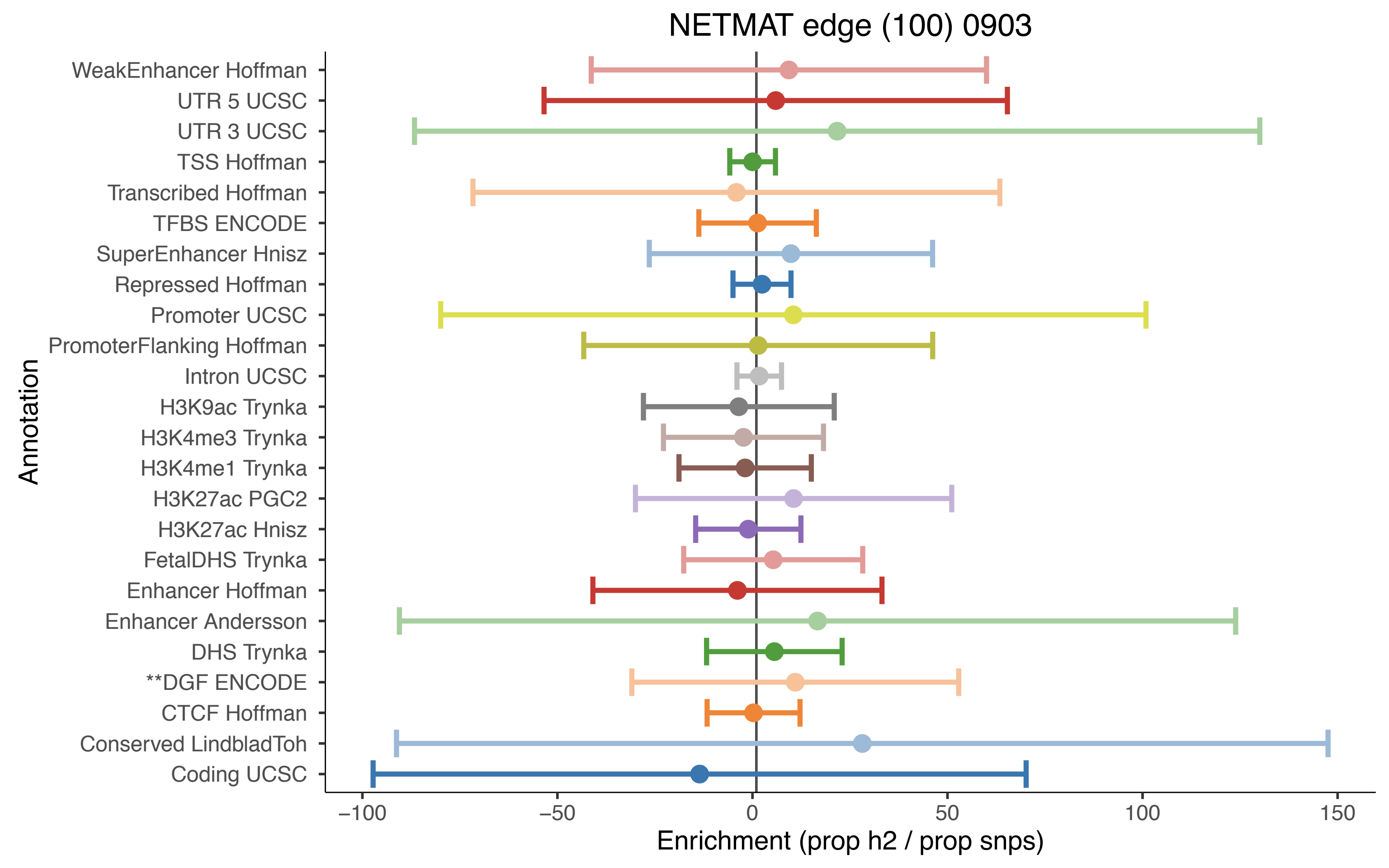
NETMAT edge (100) 0903
WeakEnhancer Hoffman
UTR 5 UCSC
UTR 3 UCSC
TSS Hoffman
Transcribed Hoffman
TFBS ENCODE
SuperEnhancer Hnisz
Repressed Hoffman
Promoter UCSC
PromoterFlanking Hoffman
Intron UCSC
H3K9ac Trynka
H3K4me3 Trynka
H3K4me1 Trynka
H3K27ac PGC2
H3K27ac Hnisz
FetalDHS Trynka
Enhancer Hoffman
Enhancer Andersson
DHS Trynka
**DGF ENCODE
CTCF Hoffman
Conserved LindbladToh
Coding UCSC
Annotation
−100
−50
0
50
100
150
Enrichment (prop h2 / prop snps)

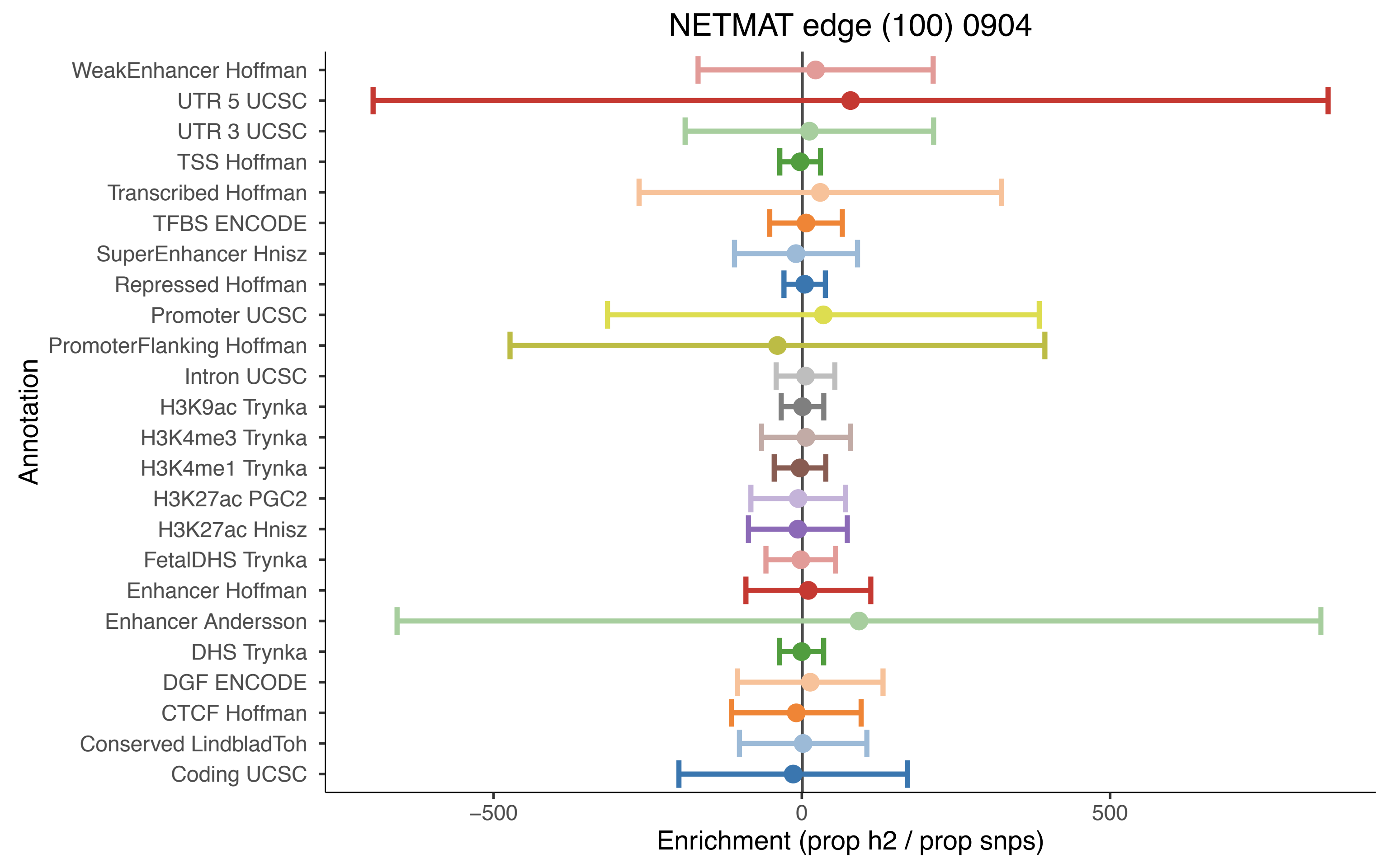
NETMAT edge (100) 0904
WeakEnhancer Hoffman
UTR 5 UCSC
UTR 3 UCSC
TSS Hoffman
Transcribed Hoffman
TFBS ENCODE
SuperEnhancer Hnisz
Repressed Hoffman
Promoter UCSC
PromoterFlanking Hoffman
Intron UCSC
H3K9ac Trynka
H3K4me3 Trynka
H3K4me1 Trynka
H3K27ac PGC2
H3K27ac Hnisz
FetalDHS Trynka
Enhancer Hoffman
Enhancer Andersson
DHS Trynka
DGF ENCODE
CTCF Hoffman
Conserved LindbladToh
Coding UCSC
Annotation
−500
0
500
Enrichment (prop h2 / prop snps)

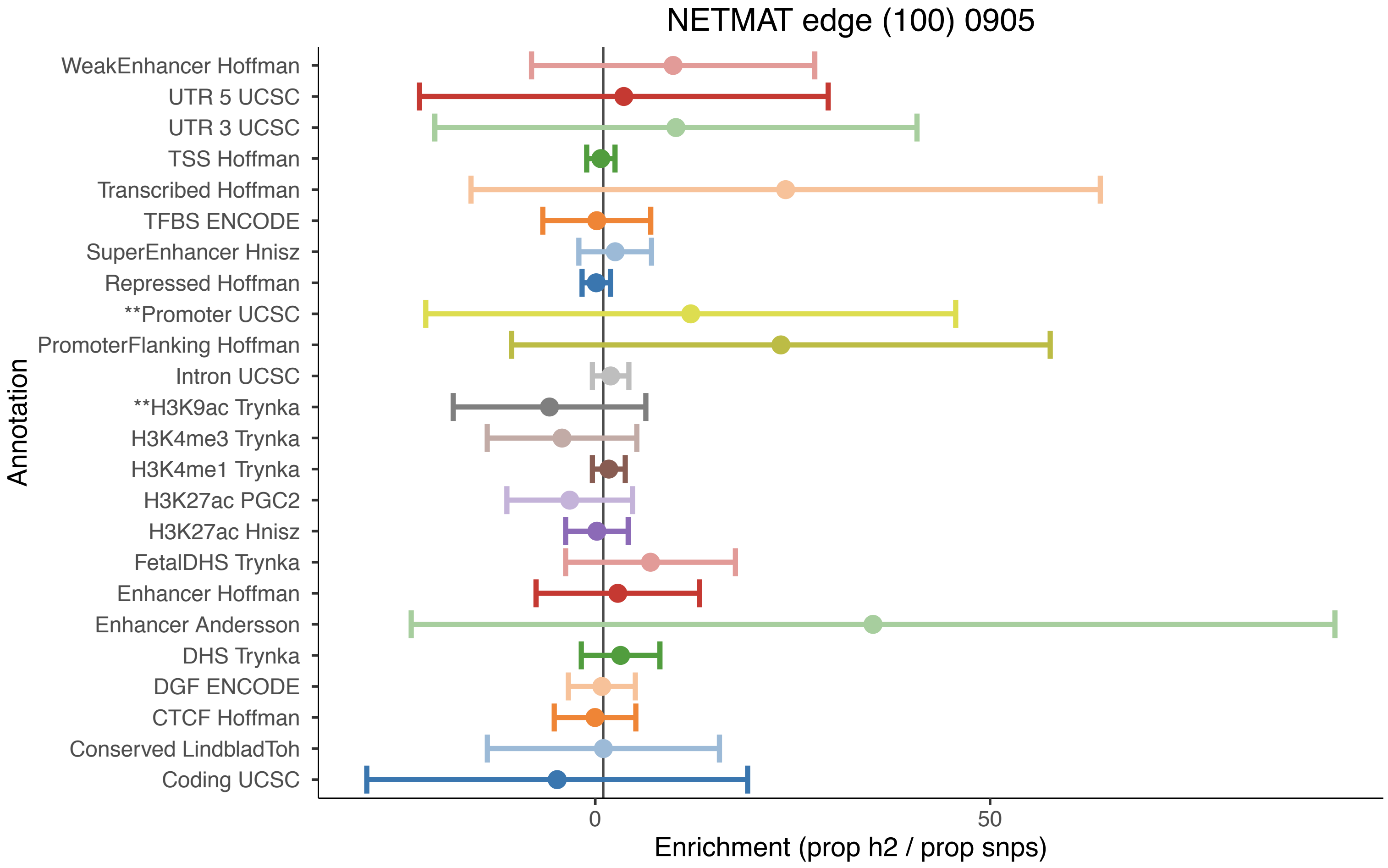

NETMAT edge (100) 0905
WeakEnhancer Hoffman
UTR 5 UCSC
UTR 3 UCSC
TSS Hoffman
Transcribed Hoffman
TFBS ENCODE
SuperEnhancer Hnisz
Repressed Hoffman
**Promoter UCSC
PromoterFlanking Hoffman
Intron UCSC
**H3K9ac Trynka
H3K4me3 Trynka
H3K4me1 Trynka
H3K27ac PGC2
H3K27ac Hnisz
FetalDHS Trynka
Enhancer Hoffman
Enhancer Andersson
DHS Trynka
DGF ENCODE
CTCF Hoffman
Conserved LindbladToh
Coding UCSC
Annotation
0
50
Enrichment (prop h2 / prop snps)

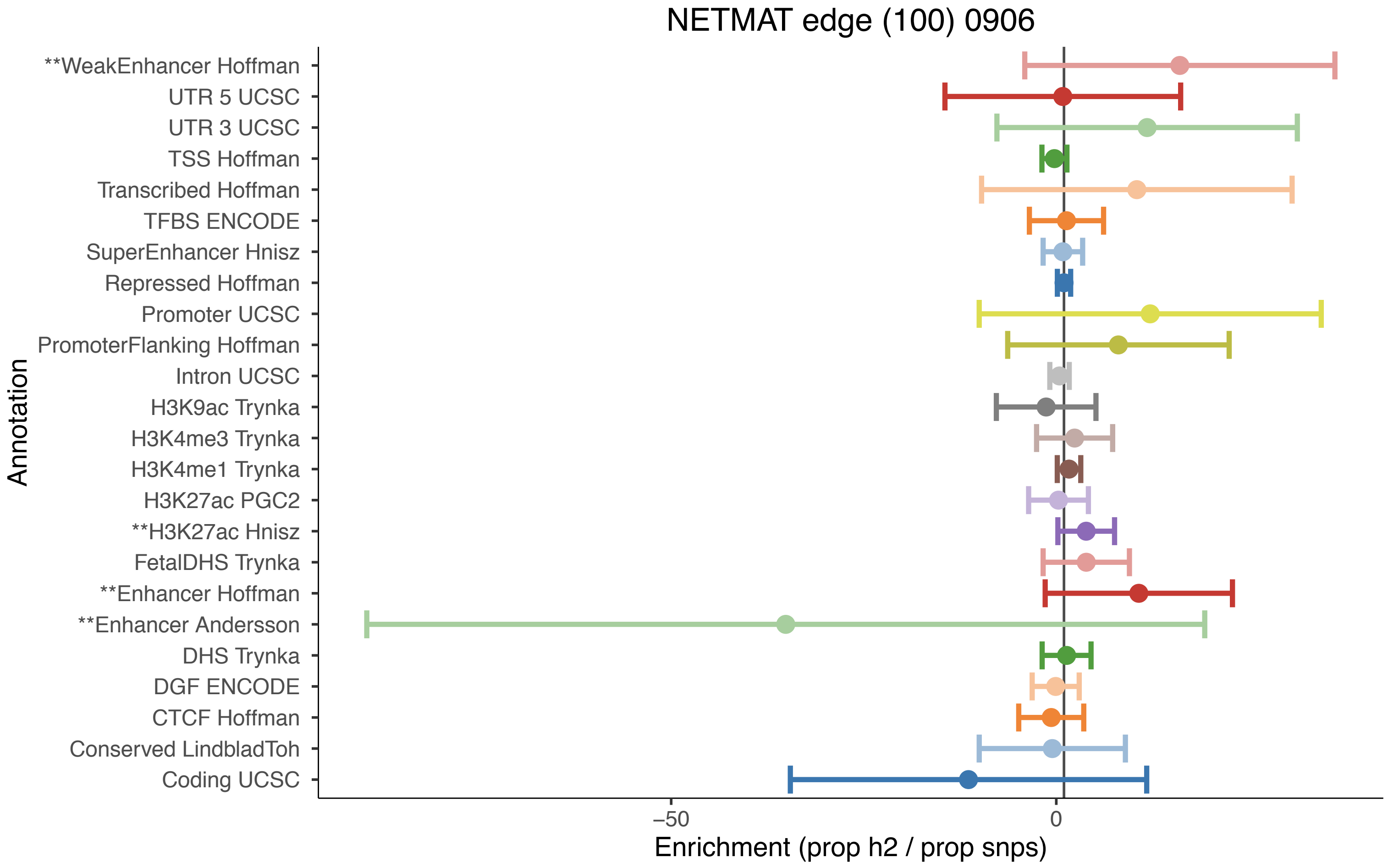
NETMAT edge (100) 0906
**WeakEnhancer Hoffman
UTR 5 UCSC
UTR 3 UCSC
TSS Hoffman
Transcribed Hoffman
TFBS ENCODE
SuperEnhancer Hnisz
Repressed Hoffman
Promoter UCSC
PromoterFlanking Hoffman
Intron UCSC
H3K9ac Trynka
H3K4me3 Trynka
H3K4me1 Trynka
H3K27ac PGC2
**H3K27ac Hnisz
FetalDHS Trynka
**Enhancer Hoffman
**Enhancer Andersson
DHS Trynka
DGF ENCODE
CTCF Hoffman
Conserved LindbladToh
Coding UCSC
Annotation
−50
0
Enrichment (prop h2 / prop snps)

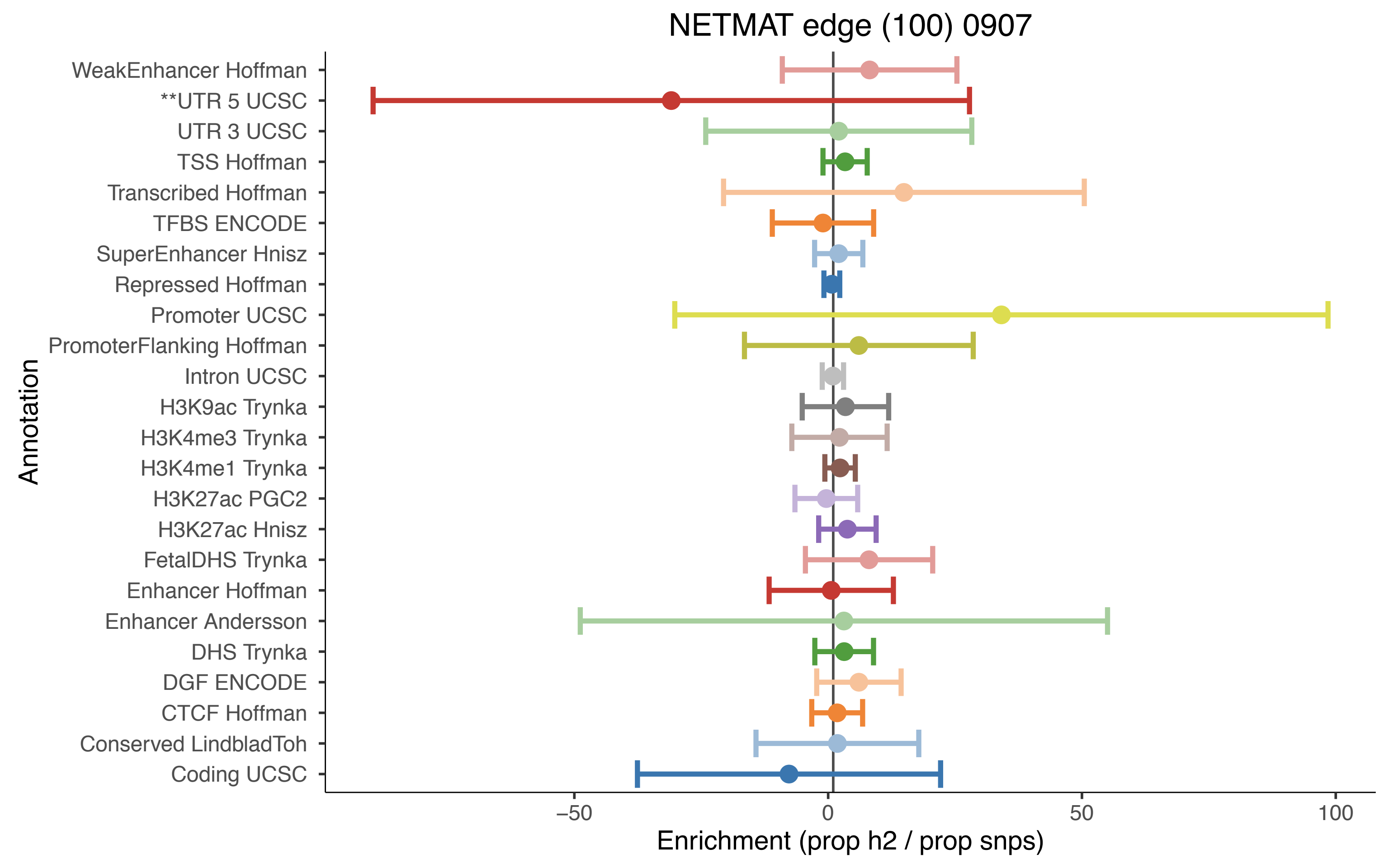
NETMAT edge (100) 0907
WeakEnhancer Hoffman
**UTR 5 UCSC
UTR 3 UCSC
TSS Hoffman
Transcribed Hoffman
TFBS ENCODE
SuperEnhancer Hnisz
Repressed Hoffman
Promoter UCSC
PromoterFlanking Hoffman
Intron UCSC
H3K9ac Trynka
H3K4me3 Trynka
H3K4me1 Trynka
H3K27ac PGC2
H3K27ac Hnisz
FetalDHS Trynka
Enhancer Hoffman
Enhancer Andersson
DHS Trynka
DGF ENCODE
CTCF Hoffman
Conserved LindbladToh
Coding UCSC
Annotation
−50
0
50
100
Enrichment (prop h2 / prop snps)

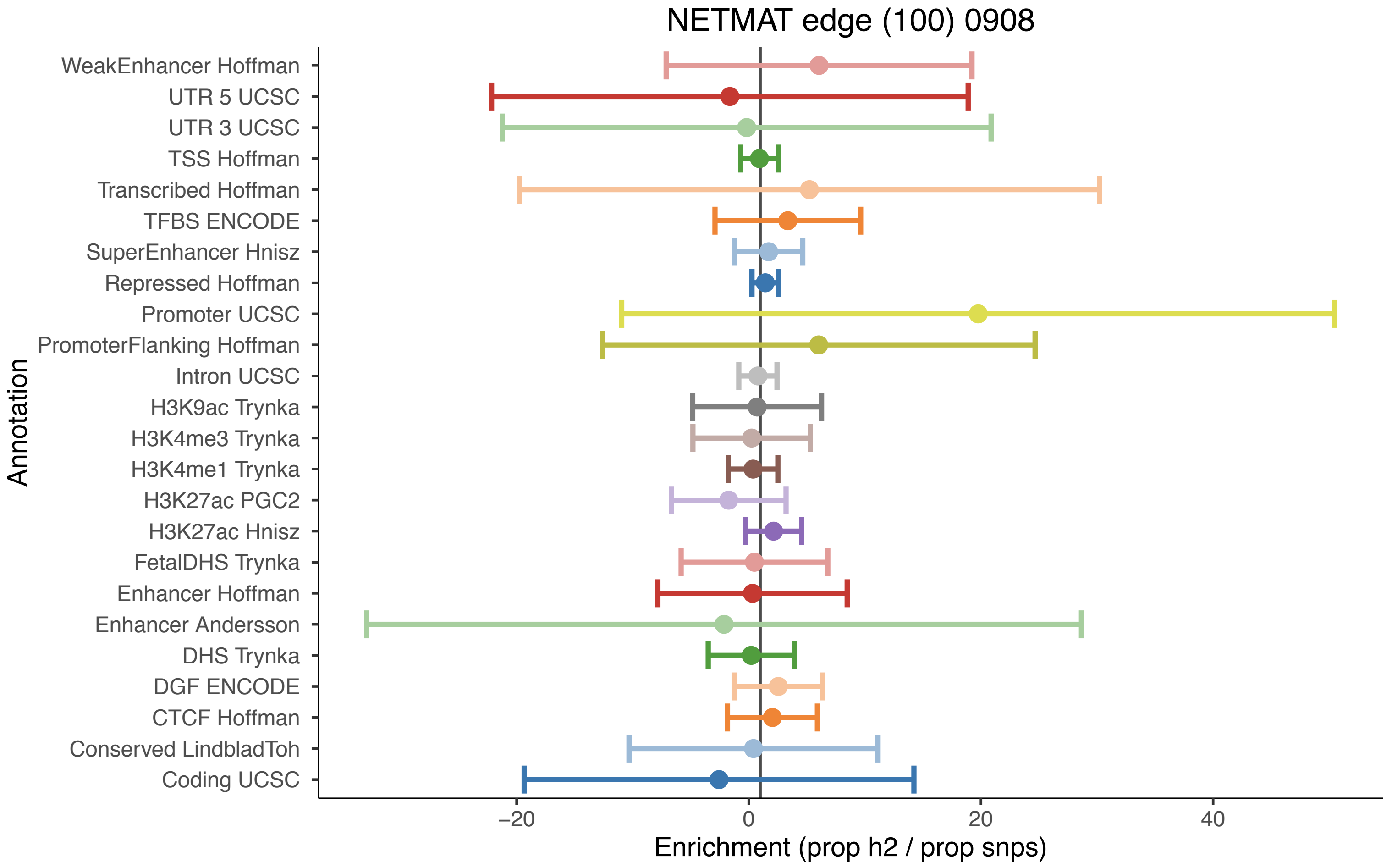

NETMAT edge (100) 0908
WeakEnhancer Hoffman
UTR 5 UCSC
UTR 3 UCSC
TSS Hoffman
Transcribed Hoffman
TFBS ENCODE
SuperEnhancer Hnisz
Repressed Hoffman
Promoter UCSC
PromoterFlanking Hoffman
Intron UCSC
H3K9ac Trynka
H3K4me3 Trynka
H3K4me1 Trynka
H3K27ac PGC2
H3K27ac Hnisz
FetalDHS Trynka
Enhancer Hoffman
Enhancer Andersson
DHS Trynka
DGF ENCODE
CTCF Hoffman
Conserved LindbladToh
Coding UCSC
Annotation
−20
0
20
40
Enrichment (prop h2 / prop snps)

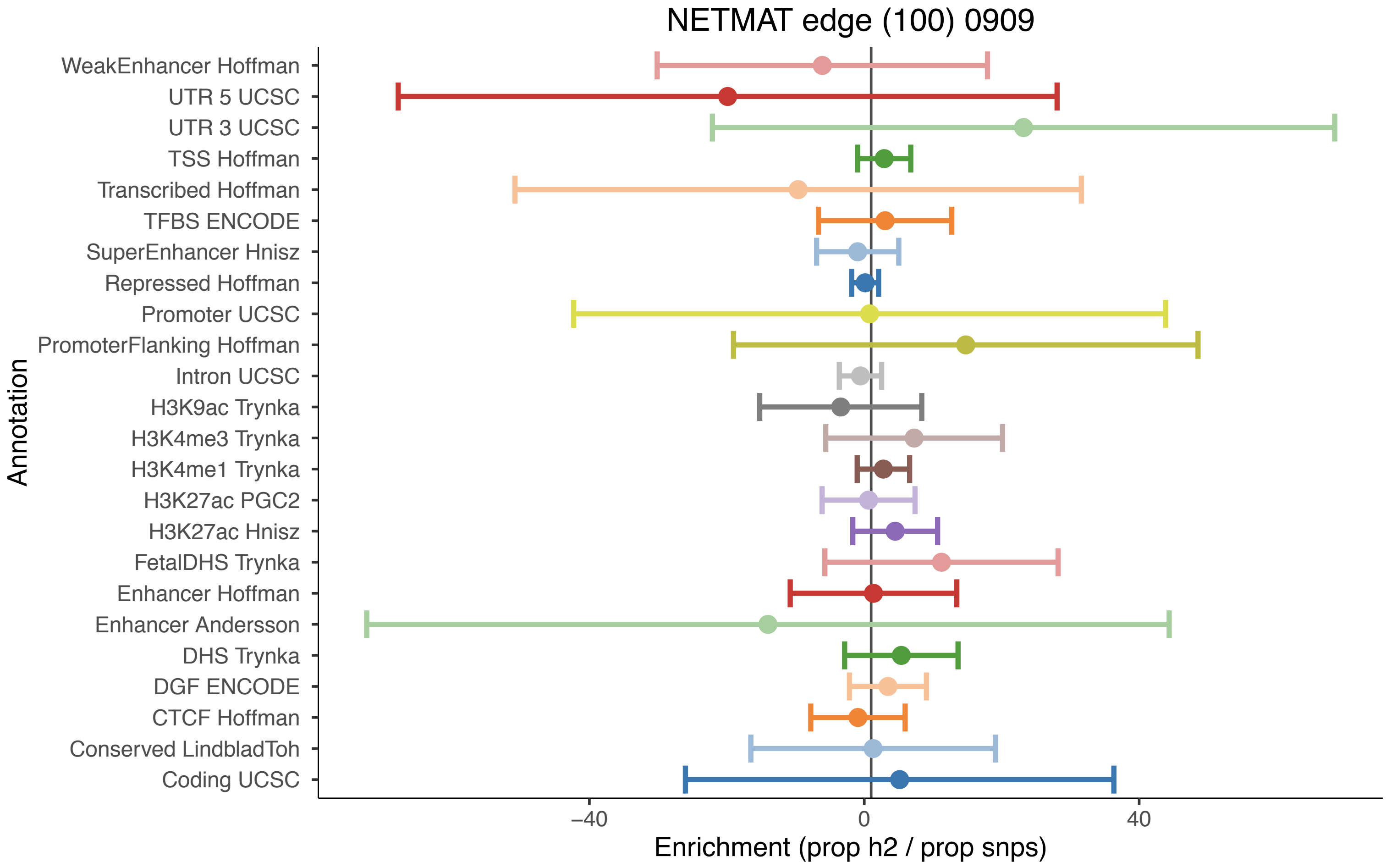
NETMAT edge (100) 0909
WeakEnhancer Hoffman
UTR 5 UCSC
UTR 3 UCSC
TSS Hoffman
Transcribed Hoffman
TFBS ENCODE
SuperEnhancer Hnisz
Repressed Hoffman
Promoter UCSC
PromoterFlanking Hoffman
Intron UCSC
H3K9ac Trynka
H3K4me3 Trynka
H3K4me1 Trynka
H3K27ac PGC2
H3K27ac Hnisz
FetalDHS Trynka
Enhancer Hoffman
Enhancer Andersson
DHS Trynka
DGF ENCODE
CTCF Hoffman
Conserved LindbladToh
Coding UCSC
Annotation
−40
0
40
Enrichment (prop h2 / prop snps)

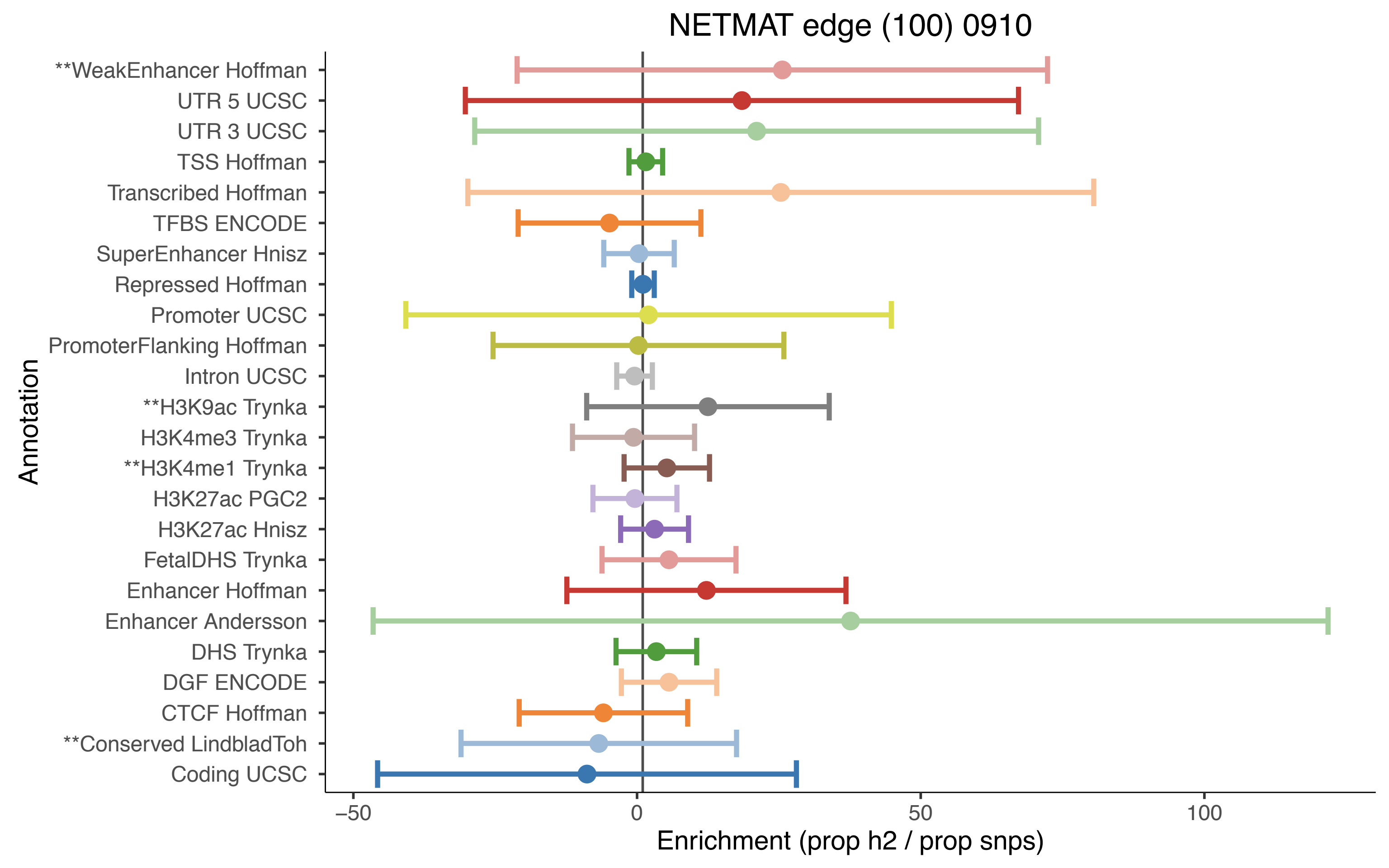
NETMAT edge (100) 0910
**WeakEnhancer Hoffman
UTR 5 UCSC
UTR 3 UCSC
TSS Hoffman
Transcribed Hoffman
TFBS ENCODE
SuperEnhancer Hnisz
Repressed Hoffman
Promoter UCSC
PromoterFlanking Hoffman
Intron UCSC
**H3K9ac Trynka
H3K4me3 Trynka
**H3K4me1 Trynka
H3K27ac PGC2
H3K27ac Hnisz
FetalDHS Trynka
Enhancer Hoffman
Enhancer Andersson
DHS Trynka
DGF ENCODE
CTCF Hoffman
**Conserved LindbladToh
Coding UCSC
Annotation
−50
0
50
100
Enrichment (prop h2 / prop snps)

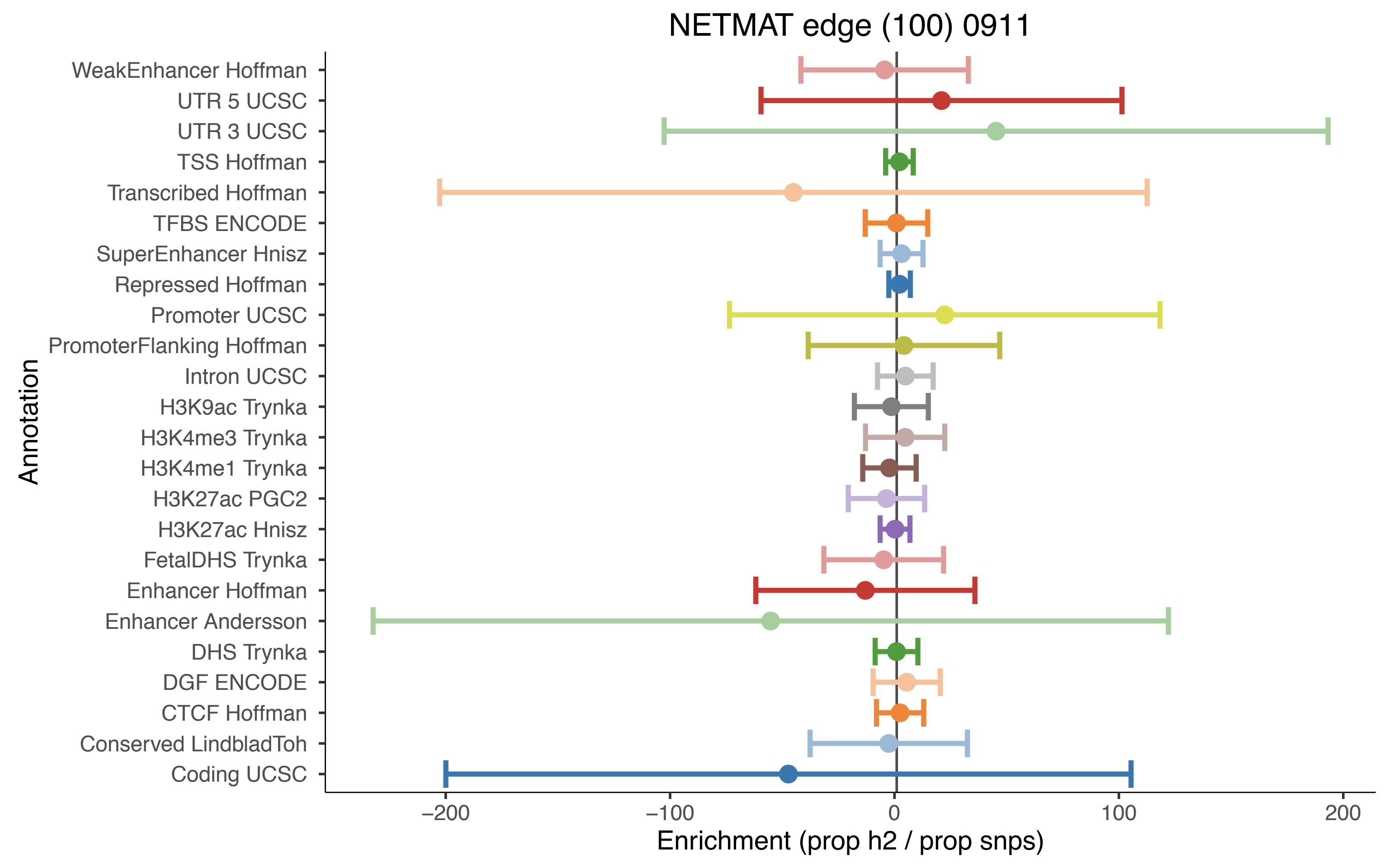
NETMAT edge (100) 0911
WeakEnhancer Hoffman
UTR 5 UCSC
UTR 3 UCSC
TSS Hoffman
Transcribed Hoffman
TFBS ENCODE
SuperEnhancer Hnisz
Repressed Hoffman
Promoter UCSC
PromoterFlanking Hoffman
Intron UCSC
H3K9ac Trynka
H3K4me3 Trynka
H3K4me1 Trynka
H3K27ac PGC2
H3K27ac Hnisz
FetalDHS Trynka
Enhancer Hoffman
Enhancer Andersson
DHS Trynka
DGF ENCODE
CTCF Hoffman
Conserved LindbladToh
Coding UCSC
Annotation
−200
−100
0
100
200
Enrichment (prop h2 / prop snps)

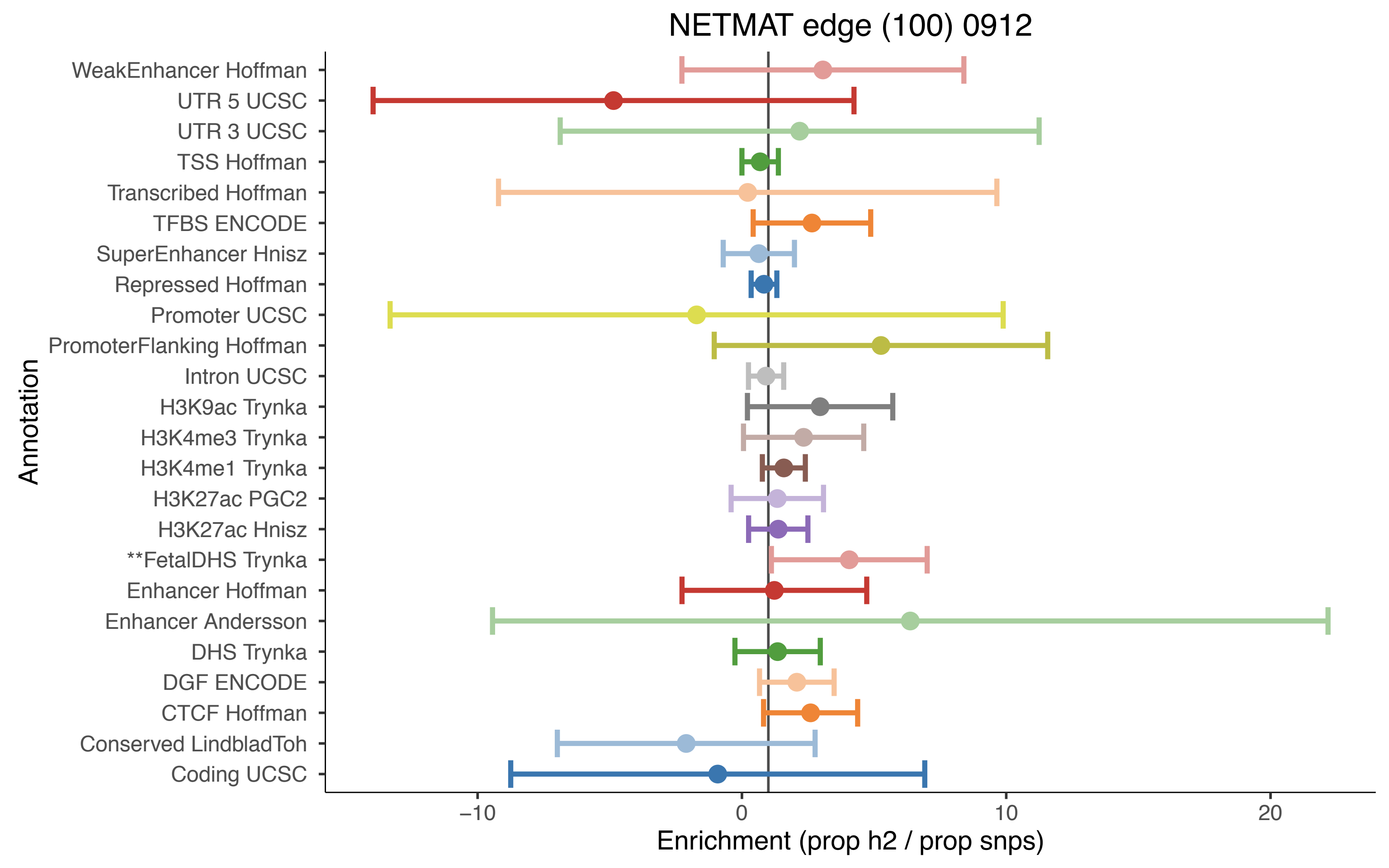
NETMAT edge (100) 0912
WeakEnhancer Hoffman
UTR 5 UCSC
UTR 3 UCSC
TSS Hoffman
Transcribed Hoffman
TFBS ENCODE
SuperEnhancer Hnisz
Repressed Hoffman
Promoter UCSC
PromoterFlanking Hoffman
Intron UCSC
H3K9ac Trynka
H3K4me3 Trynka
H3K4me1 Trynka
H3K27ac PGC2
H3K27ac Hnisz
**FetalDHS Trynka
Enhancer Hoffman
Enhancer Andersson
DHS Trynka
DGF ENCODE
CTCF Hoffman
Conserved LindbladToh
Coding UCSC
Annotation
−10
0
10
20
Enrichment (prop h2 / prop snps)

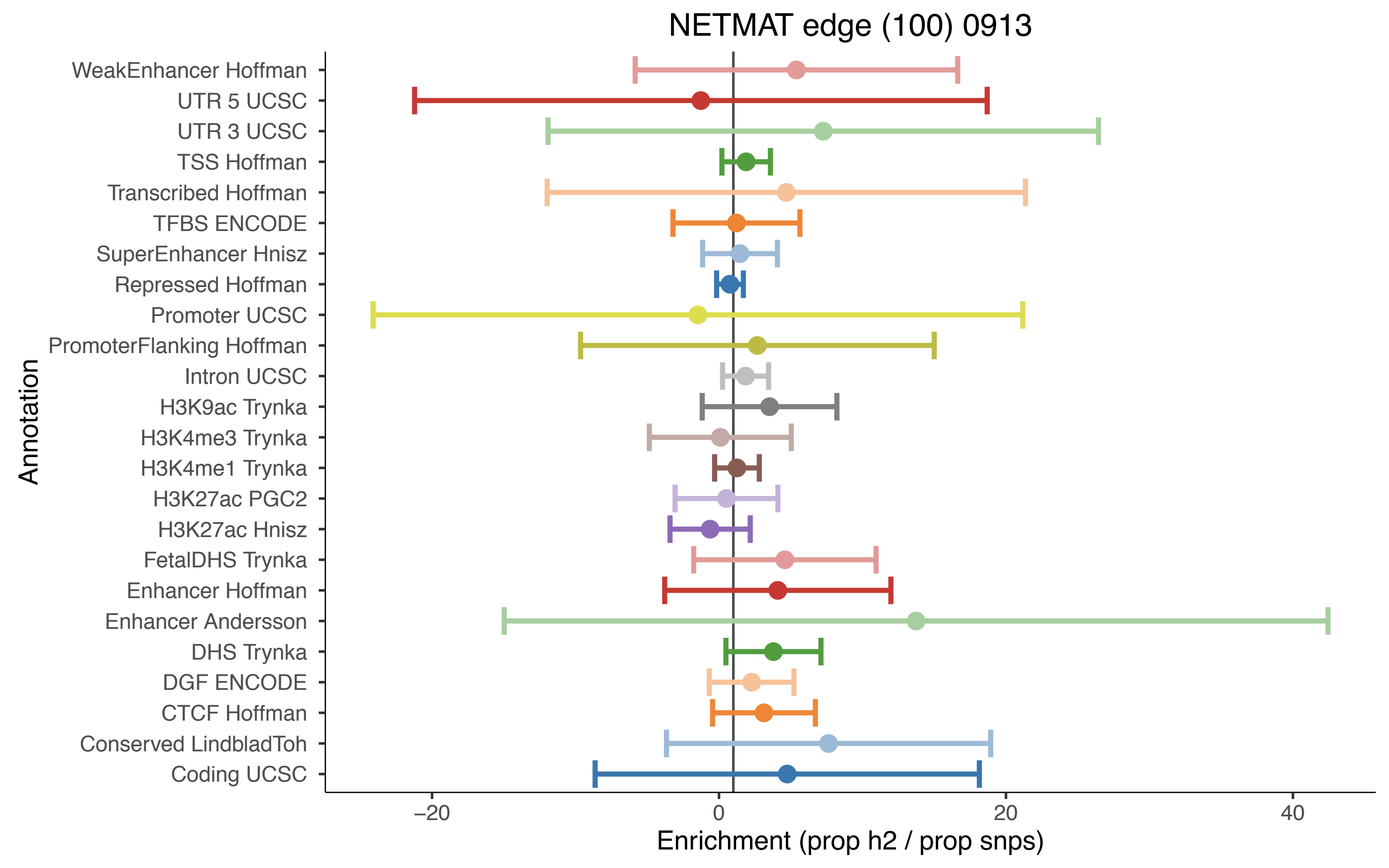
NETMAT edge (100) 0913
WeakEnhancer Hoffman
UTR 5 UCSC
UTR 3 UCSC
TSS Hoffman
Transcribed Hoffman
TFBS ENCODE
SuperEnhancer Hnisz
Repressed Hoffman
Promoter UCSC
PromoterFlanking Hoffman
Intron UCSC
H3K9ac Trynka
H3K4me3 Trynka
H3K4me1 Trynka
H3K27ac PGC2
H3K27ac Hnisz
FetalDHS Trynka
Enhancer Hoffman
Enhancer Andersson
DHS Trynka
DGF ENCODE
CTCF Hoffman
Conserved LindbladToh
Coding UCSC
Annotation
−20
0
20
40
Enrichment (prop h2 / prop snps)

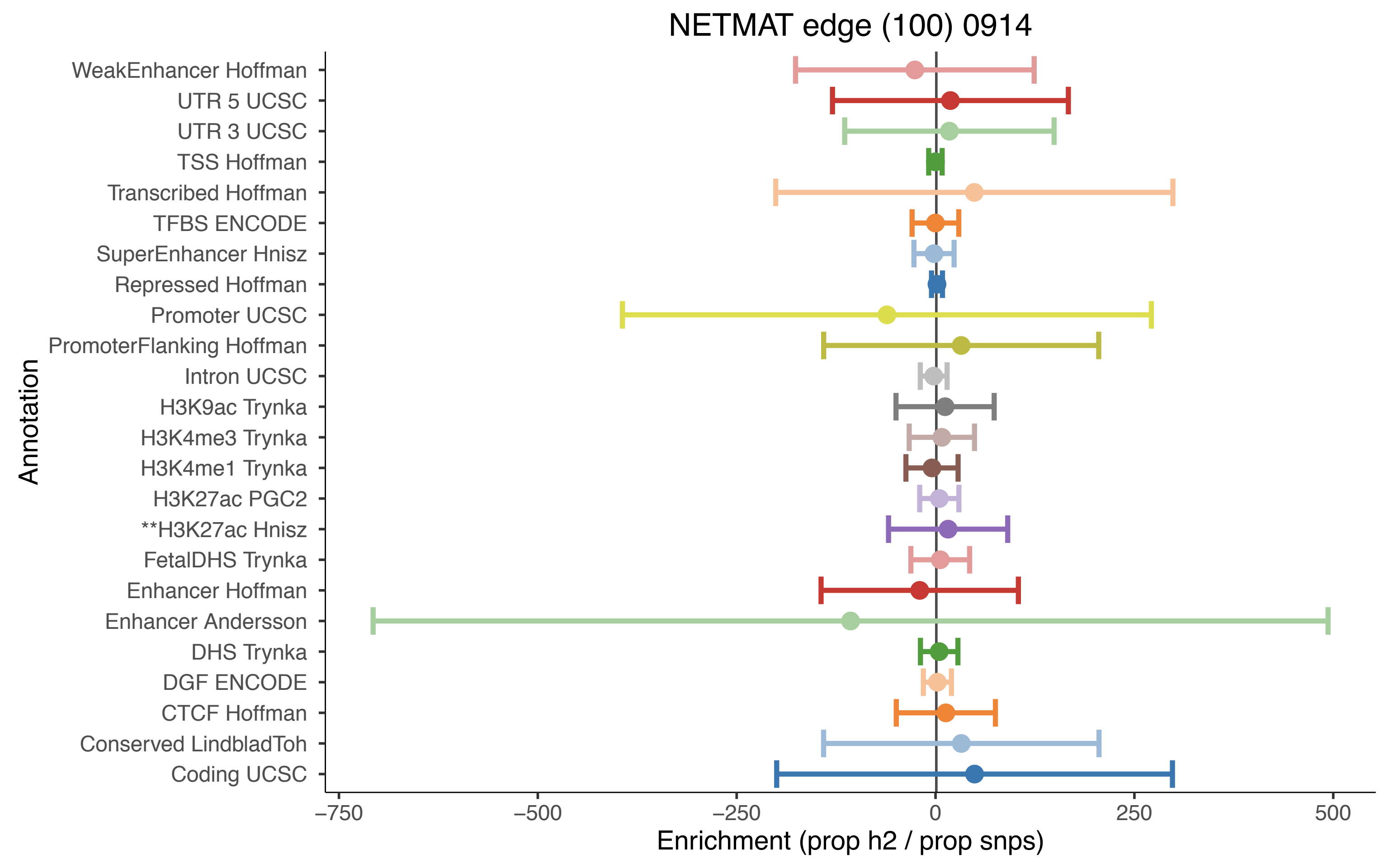
NETMAT edge (100) 0914
Annotation
WeakEnhancer Hoffman
UTR 5 UCSC
UTR 3 UCSC
TSS Hoffman
Transcribed Hoffman
TFBS ENCODE
SuperEnhancer Hnisz
Repressed Hoffman
Promoter UCSC
PromoterFlanking Hoffman
Intron UCSC
H3K9ac Trynka
H3K4me3 Trynka
H3K4me1 Trynka
H3K27ac PGC2
**H3K27ac Hnisz
FetalDHS Trynka
Enhancer Hoffman
Enhancer Andersson
DHS Trynka
DGF ENCODE
CTCF Hoffman
Conserved LindbladToh
Coding UCSC
−750
−500
−250
0
250
500
Enrichment (prop h2 / prop snps)

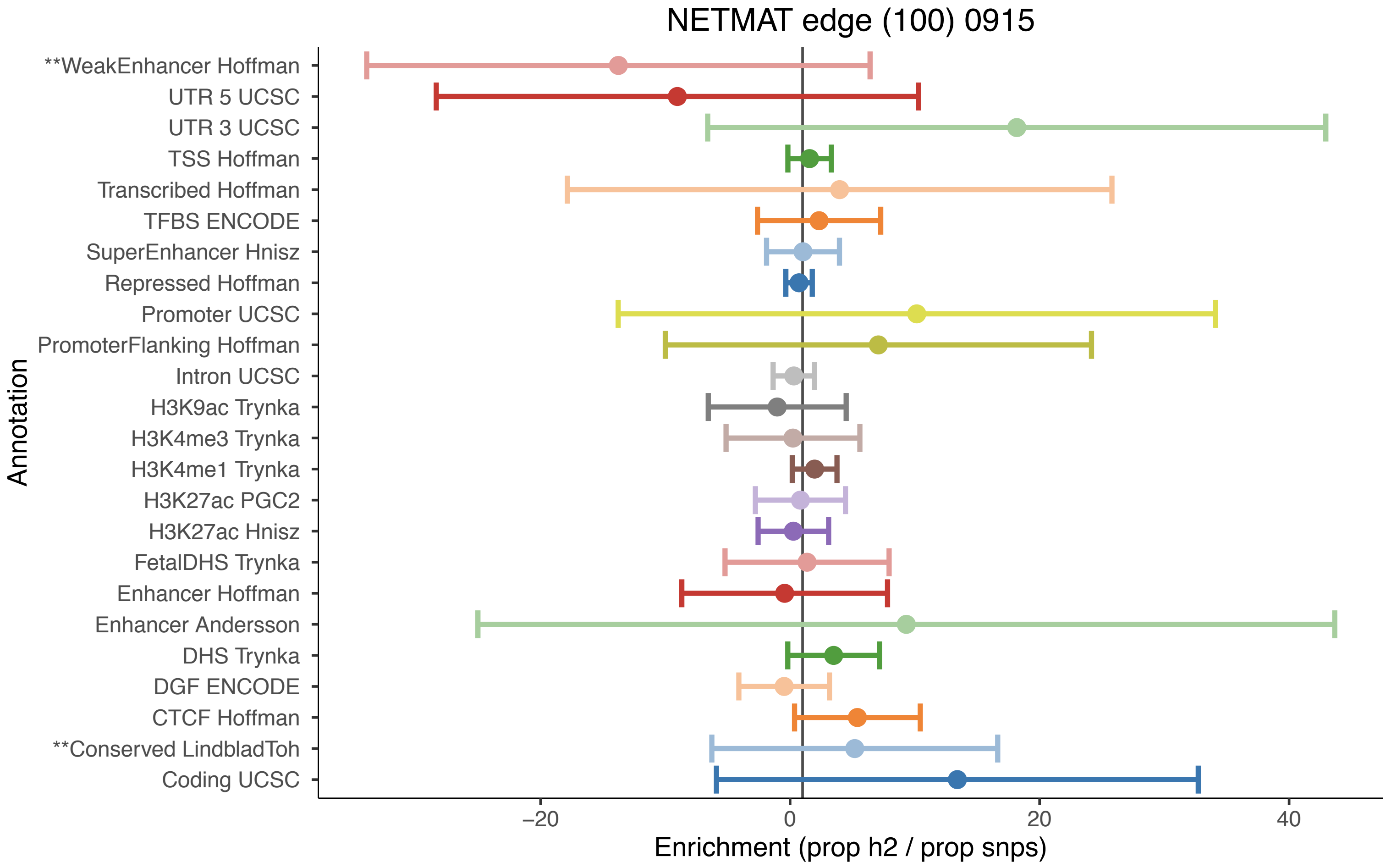

NETMAT edge (100) 0915
**WeakEnhancer Hoffman
UTR 5 UCSC
UTR 3 UCSC
TSS Hoffman
Transcribed Hoffman
TFBS ENCODE
SuperEnhancer Hnisz
Repressed Hoffman
Promoter UCSC
PromoterFlanking Hoffman
Intron UCSC
H3K9ac Trynka
H3K4me3 Trynka
H3K4me1 Trynka
H3K27ac PGC2
H3K27ac Hnisz
FetalDHS Trynka
Enhancer Hoffman
Enhancer Andersson
DHS Trynka
DGF ENCODE
CTCF Hoffman
**Conserved LindbladToh
Coding UCSC
Annotation
−20
0
20
40
Enrichment (prop h2 / prop snps)

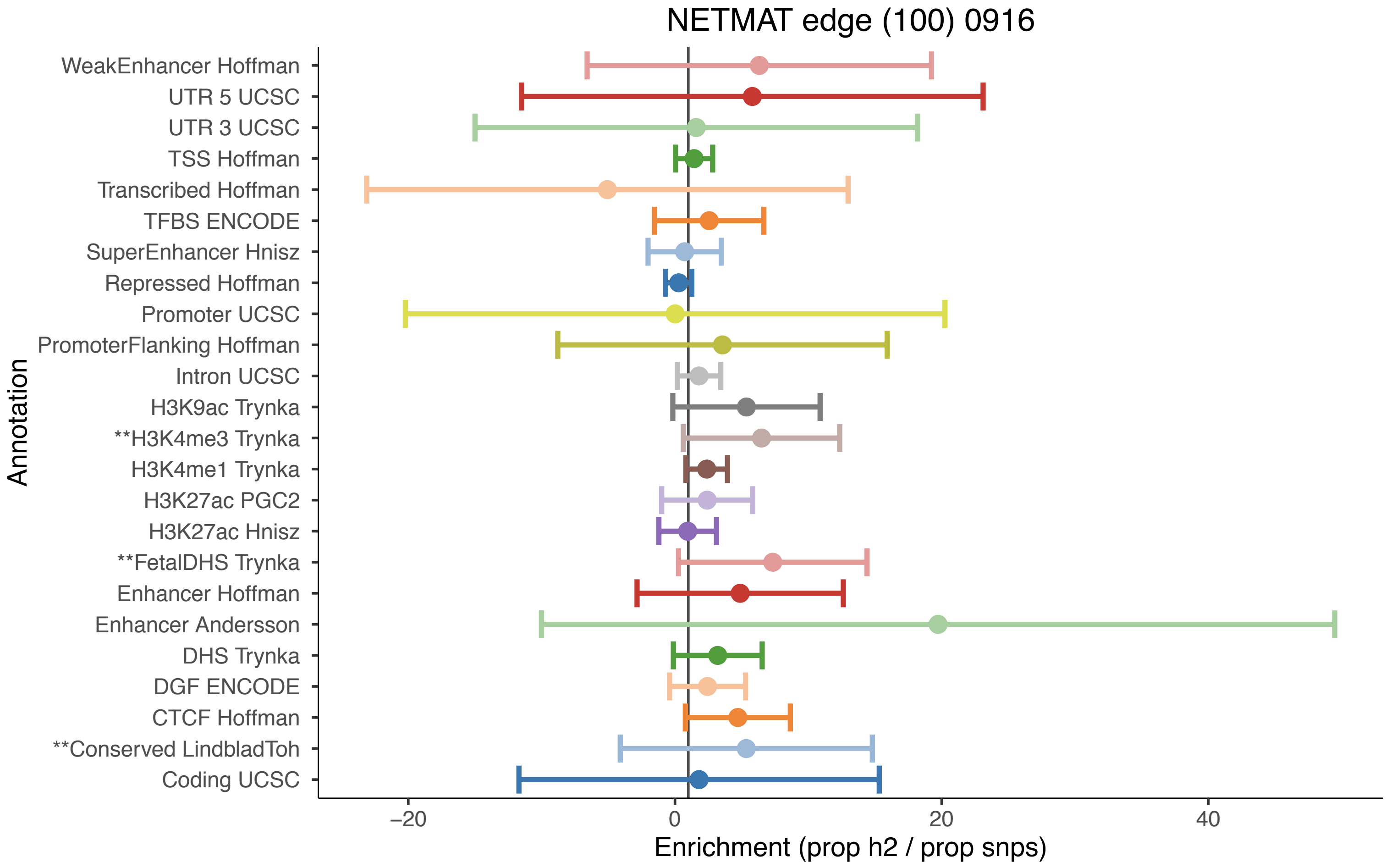
NETMAT edge (100) 0916
WeakEnhancer Hoffman
UTR 5 UCSC
UTR 3 UCSC
TSS Hoffman
Transcribed Hoffman
TFBS ENCODE
SuperEnhancer Hnisz
Repressed Hoffman
Promoter UCSC
PromoterFlanking Hoffman
Intron UCSC
H3K9ac Trynka
**H3K4me3 Trynka
H3K4me1 Trynka
H3K27ac PGC2
H3K27ac Hnisz
**FetalDHS Trynka
Enhancer Hoffman
Enhancer Andersson
DHS Trynka
DGF ENCODE
CTCF Hoffman
**Conserved LindbladToh
Coding UCSC
Annotation
−20
0
20
40
Enrichment (prop h2 / prop snps)

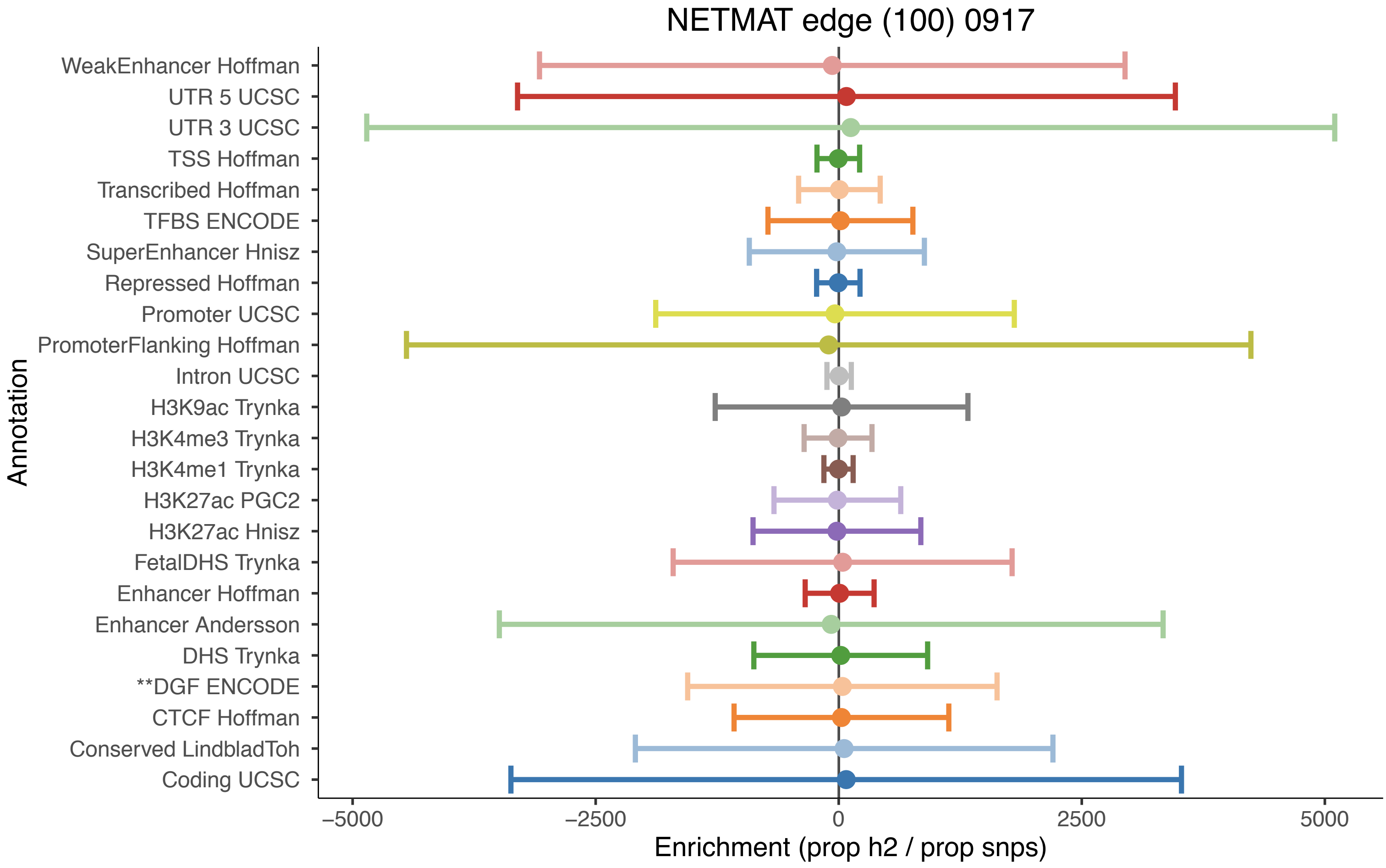
NETMAT edge (100) 0917
WeakEnhancer Hoffman
UTR 5 UCSC
UTR 3 UCSC
TSS Hoffman
Transcribed Hoffman
TFBS ENCODE
SuperEnhancer Hnisz
Repressed Hoffman
Promoter UCSC
PromoterFlanking Hoffman
Intron UCSC
H3K9ac Trynka
H3K4me3 Trynka
H3K4me1 Trynka
H3K27ac PGC2
H3K27ac Hnisz
FetalDHS Trynka
Enhancer Hoffman
Enhancer Andersson
DHS Trynka
**DGF ENCODE
CTCF Hoffman
Conserved LindbladToh
Coding UCSC
Annotation
−5000
−2500
0
2500
5000
Enrichment (prop h2 / prop snps)

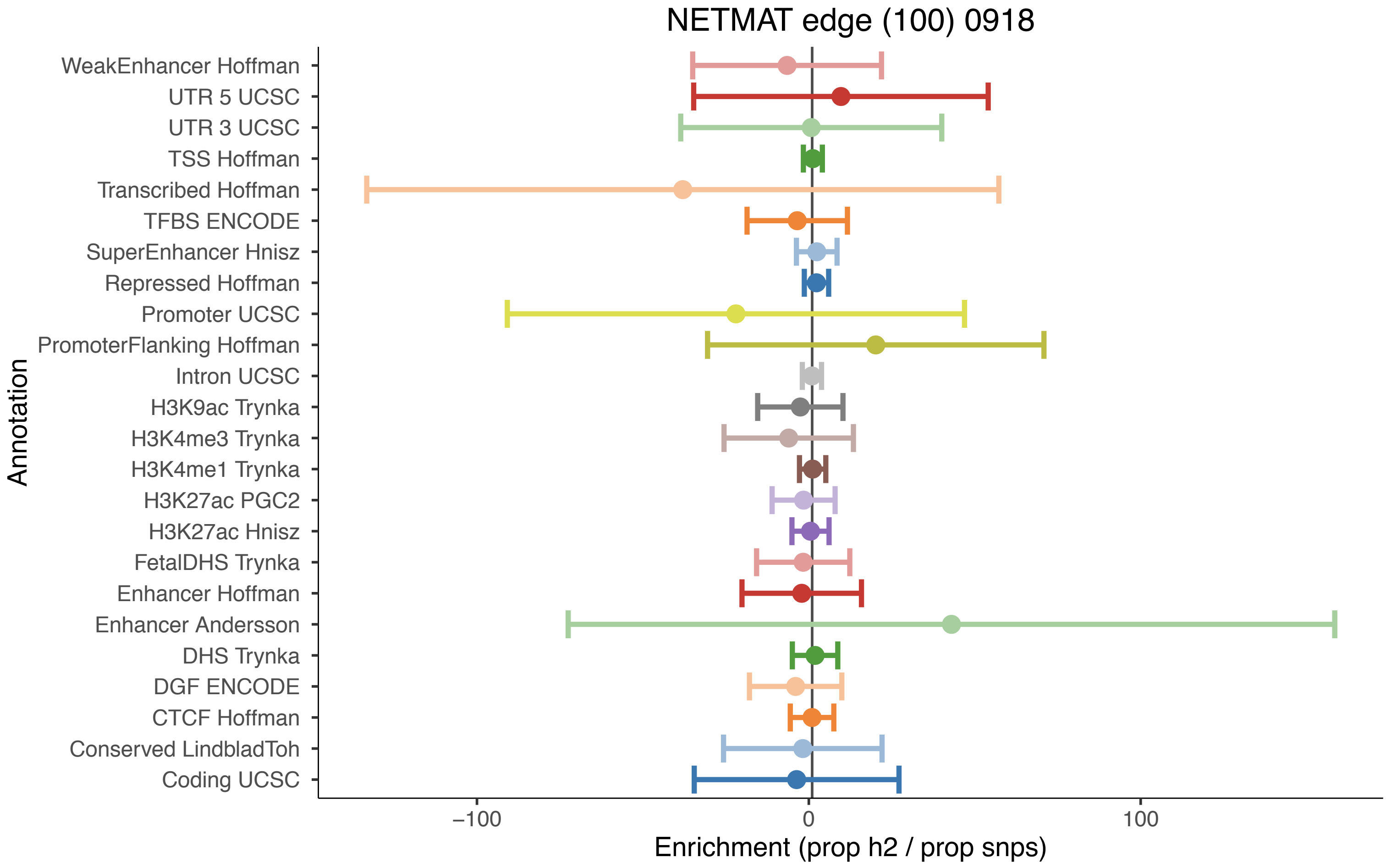
NETMAT edge (100) 0918
WeakEnhancer Hoffman
UTR 5 UCSC
UTR 3 UCSC
TSS Hoffman
Transcribed Hoffman
TFBS ENCODE
SuperEnhancer Hnisz
Repressed Hoffman
Promoter UCSC
PromoterFlanking Hoffman
Intron UCSC
H3K9ac Trynka
H3K4me3 Trynka
H3K4me1 Trynka
H3K27ac PGC2
H3K27ac Hnisz
FetalDHS Trynka
Enhancer Hoffman
Enhancer Andersson
DHS Trynka
DGF ENCODE
CTCF Hoffman
Conserved LindbladToh
Coding UCSC
Annotation
−100
0
100
Enrichment (prop h2 / prop snps)

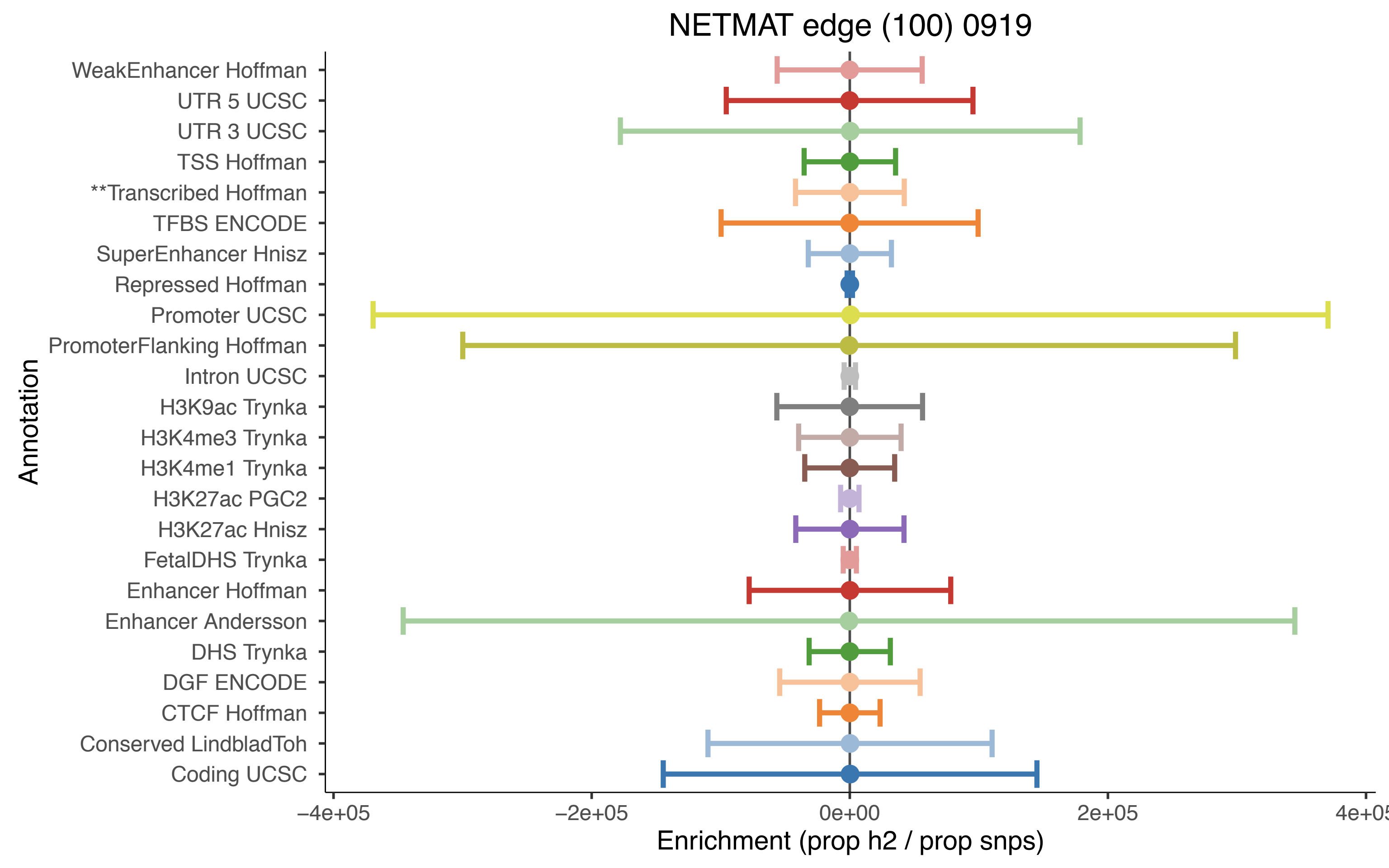
NETMAT edge (100) 0919
Annotation
WeakEnhancer Hoffman
UTR 5 UCSC
UTR 3 UCSC
TSS Hoffman
**Transcribed Hoffman
TFBS ENCODE
SuperEnhancer Hnisz
Repressed Hoffman
Promoter UCSC
PromoterFlanking Hoffman
Intron UCSC
H3K9ac Trynka
H3K4me3 Trynka
H3K4me1 Trynka
H3K27ac PGC2
H3K27ac Hnisz
FetalDHS Trynka
Enhancer Hoffman
Enhancer Andersson
DHS Trynka
DGF ENCODE
CTCF Hoffman
Conserved LindbladToh
Coding UCSC
−4e+05
−2e+05
0e+00
2e+05
Enrichment (prop h2 / prop snps)

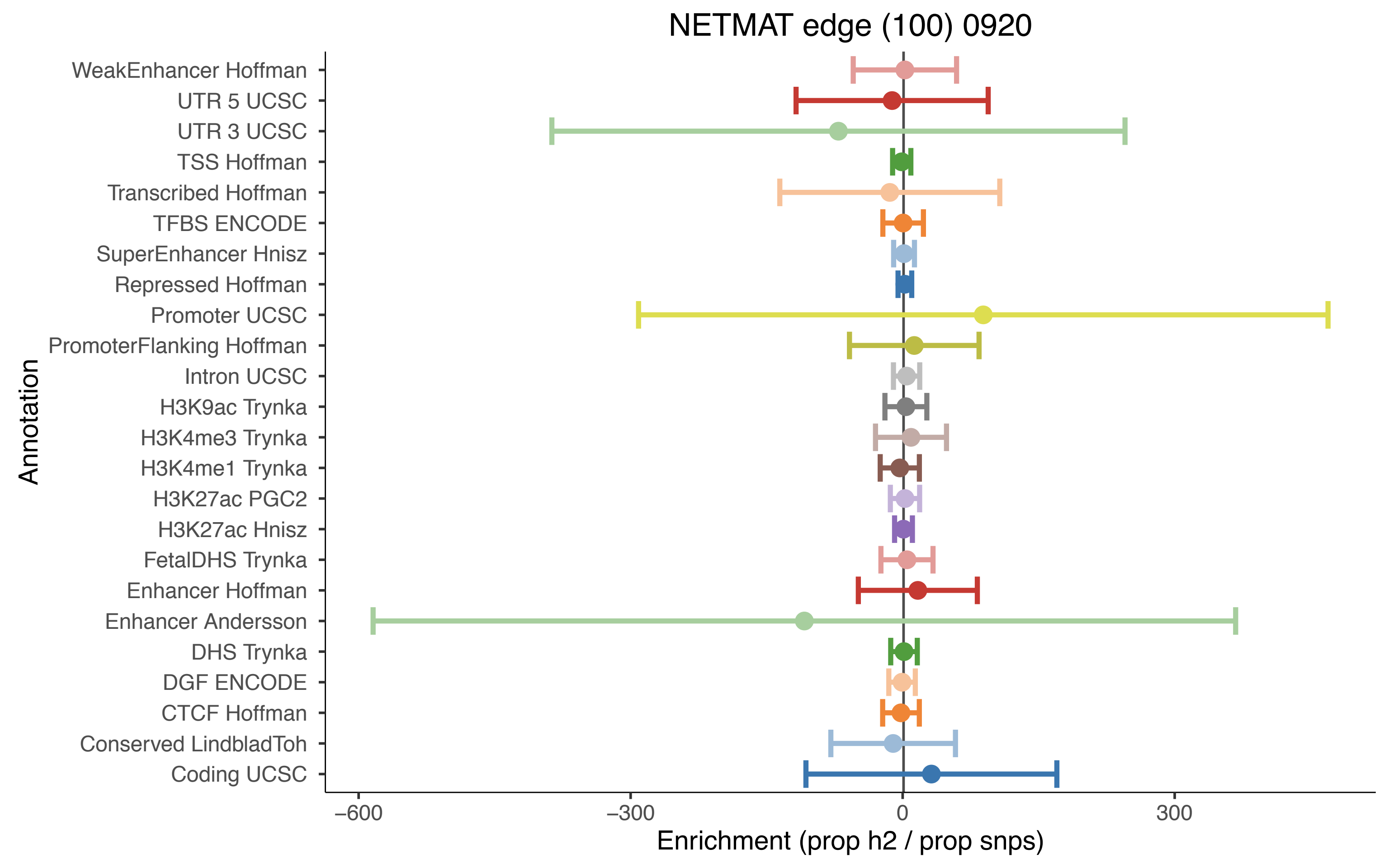
NETMAT edge (100) 0920
WeakEnhancer Hoffman
UTR 5 UCSC
UTR 3 UCSC
TSS Hoffman
Transcribed Hoffman
TFBS ENCODE
SuperEnhancer Hnisz
Repressed Hoffman
Promoter UCSC
PromoterFlanking Hoffman
Intron UCSC
H3K9ac Trynka
H3K4me3 Trynka
H3K4me1 Trynka
H3K27ac PGC2
H3K27ac Hnisz
FetalDHS Trynka
Enhancer Hoffman
Enhancer Andersson
DHS Trynka
DGF ENCODE
CTCF Hoffman
Conserved LindbladToh
Coding UCSC
Annotation
−600
−300
0
300
Enrichment (prop h2 / prop snps)

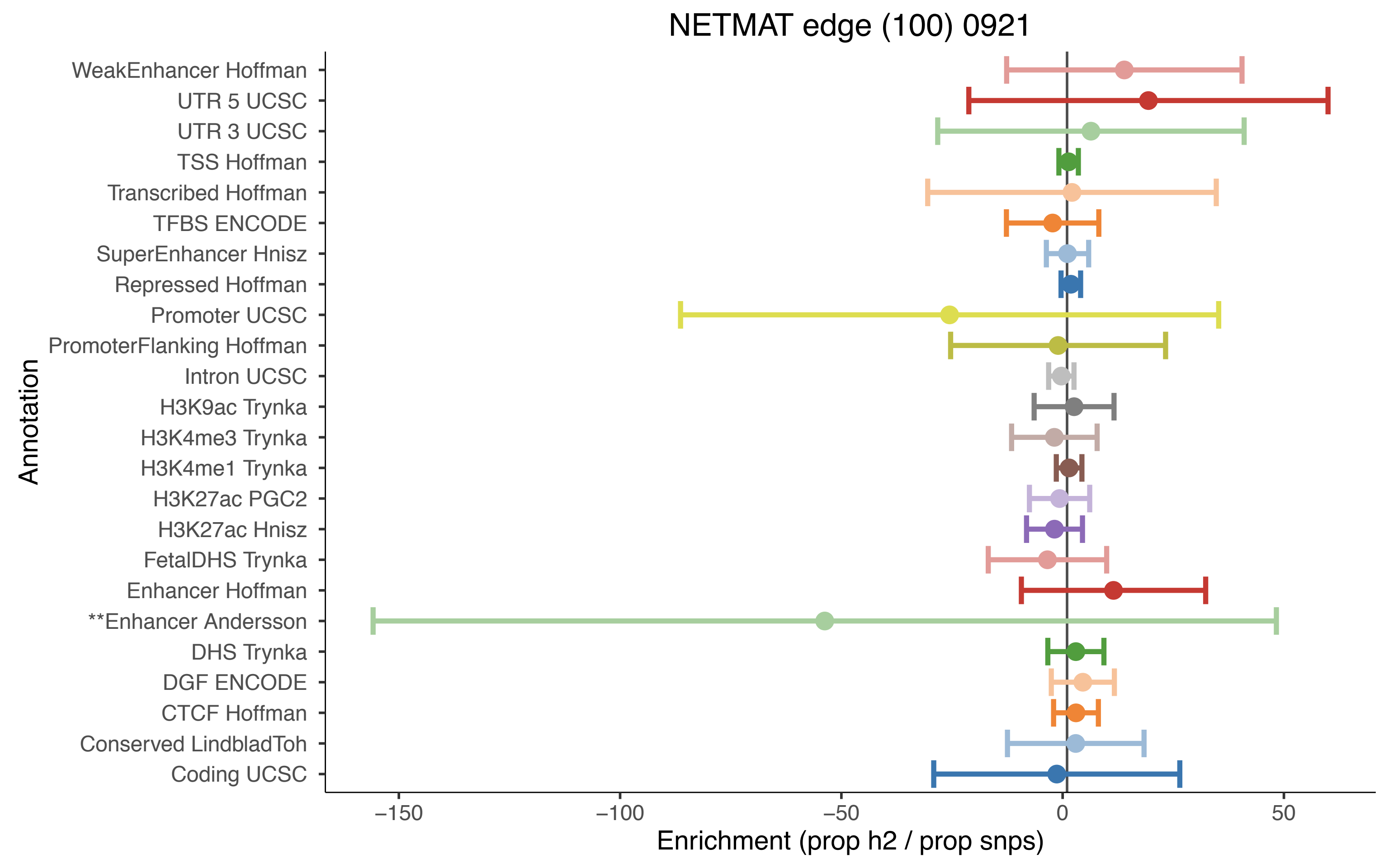

NETMAT edge (100) 0921
WeakEnhancer Hoffman
UTR 5 UCSC
UTR 3 UCSC
TSS Hoffman
Transcribed Hoffman
TFBS ENCODE
SuperEnhancer Hnisz
Repressed Hoffman
Promoter UCSC
PromoterFlanking Hoffman
Intron UCSC
H3K9ac Trynka
H3K4me3 Trynka
H3K4me1 Trynka
H3K27ac PGC2
H3K27ac Hnisz
FetalDHS Trynka
Enhancer Hoffman
**Enhancer Andersson
DHS Trynka
DGF ENCODE
CTCF Hoffman
Conserved LindbladToh
Coding UCSC
Annotation
−150
−100
−50
0
50
Enrichment (prop h2 / prop snps)

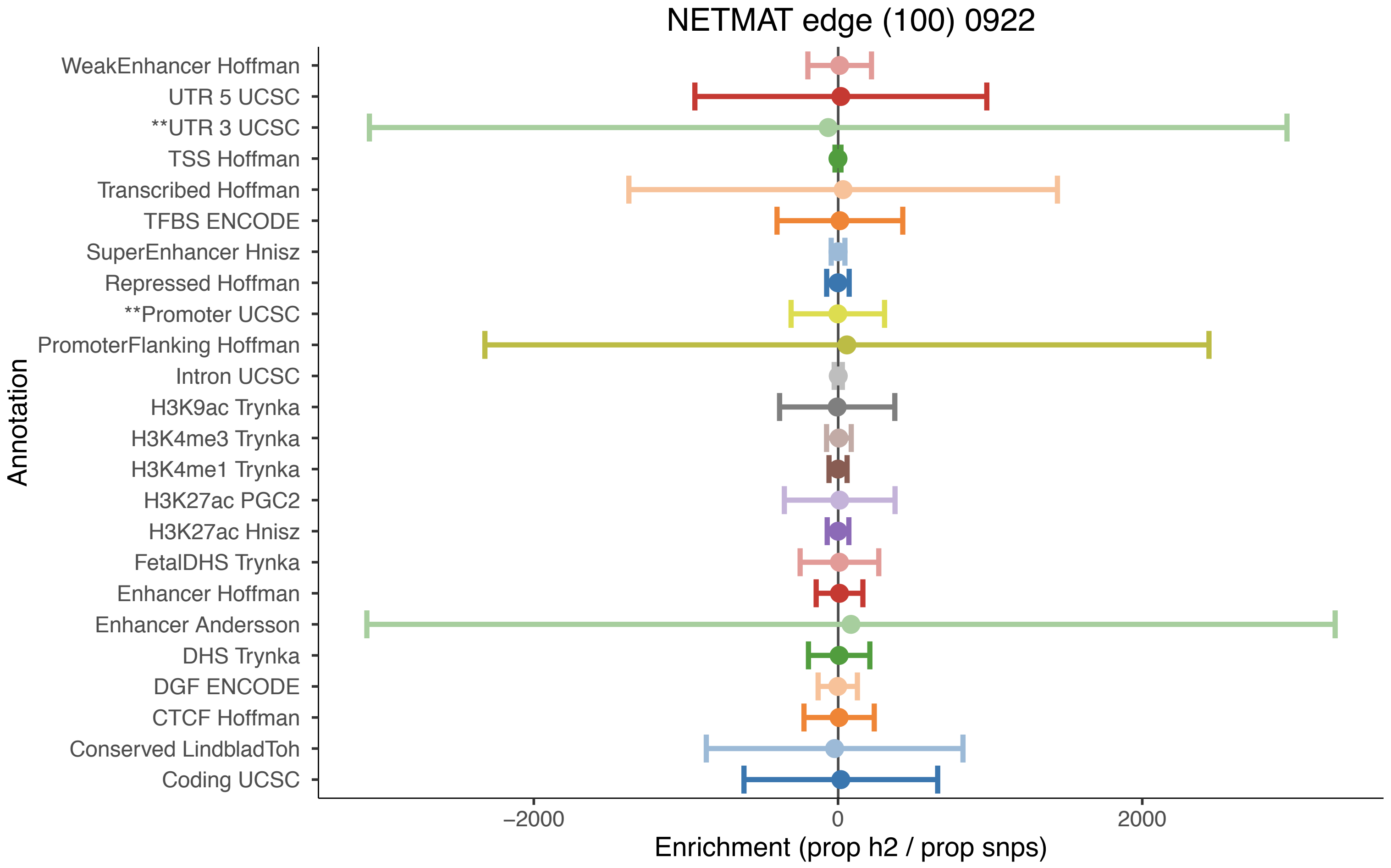

NETMAT edge (100) 0922
WeakEnhancer Hoffman
UTR 5 UCSC
**UTR 3 UCSC
TSS Hoffman
Transcribed Hoffman
TFBS ENCODE
SuperEnhancer Hnisz
Repressed Hoffman
**Promoter UCSC
PromoterFlanking Hoffman
Intron UCSC
H3K9ac Trynka
H3K4me3 Trynka
H3K4me1 Trynka
H3K27ac PGC2
H3K27ac Hnisz
FetalDHS Trynka
Enhancer Hoffman
Enhancer Andersson
DHS Trynka
DGF ENCODE
CTCF Hoffman
Conserved LindbladToh
Coding UCSC
Annotation
−2000
0
2000
Enrichment (prop h2 / prop snps)

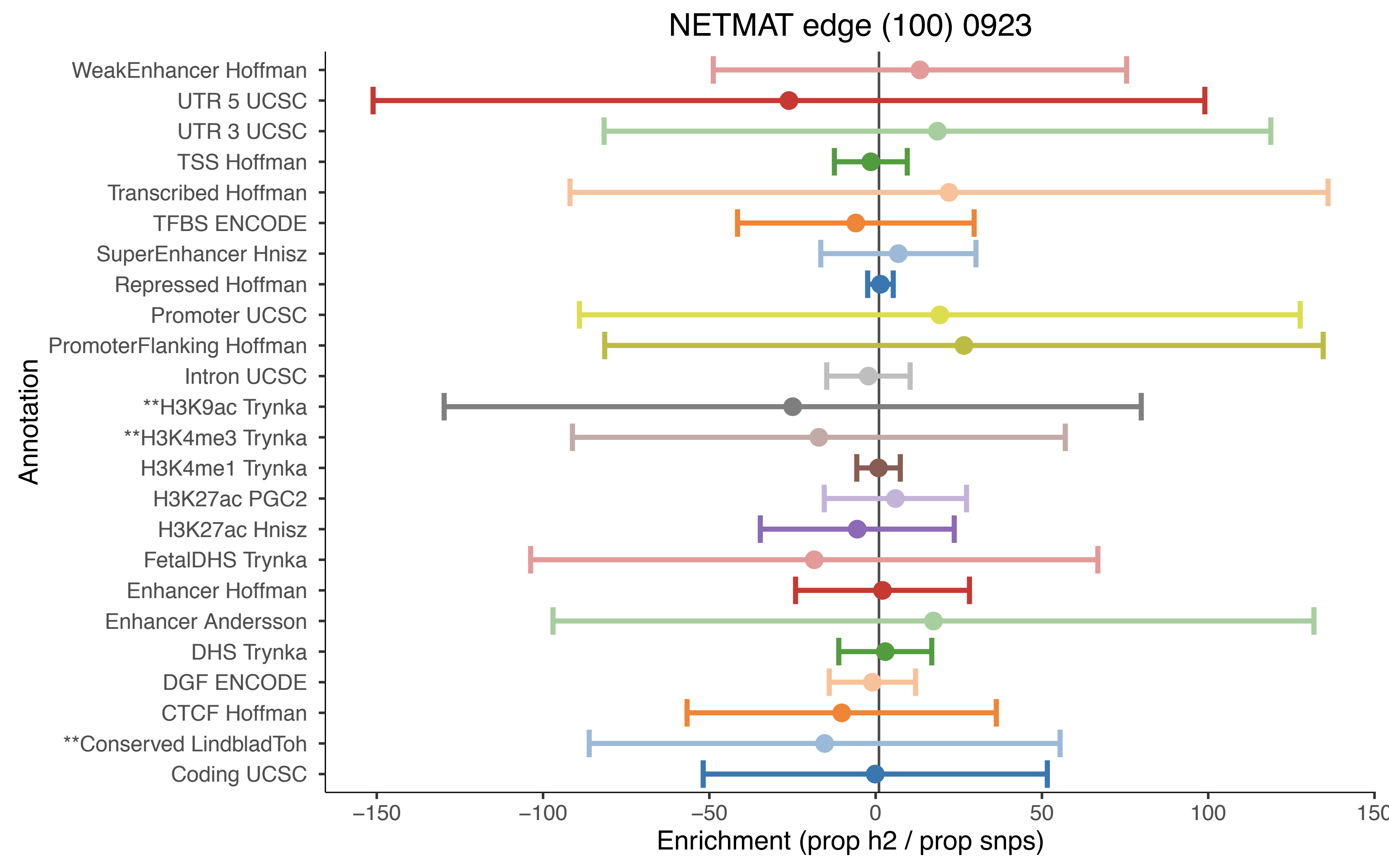
NETMAT edge (100) 0923
WeakEnhancer Hoffman
UTR 5 UCSC
UTR 3 UCSC
TSS Hoffman
Transcribed Hoffman
TFBS ENCODE
SuperEnhancer Hnisz
Repressed Hoffman
Promoter UCSC
PromoterFlanking Hoffman
Intron UCSC
**H3K9ac Trynka
**H3K4me3 Trynka
H3K4me1 Trynka
H3K27ac PGC2
H3K27ac Hnisz
FetalDHS Trynka
Enhancer Hoffman
Enhancer Andersson
DHS Trynka
DGF ENCODE
CTCF Hoffman
**Conserved LindbladToh
Coding UCSC
Annotation
−150
−100
−50
0
50
100
150
Enrichment (prop h2 / prop snps)

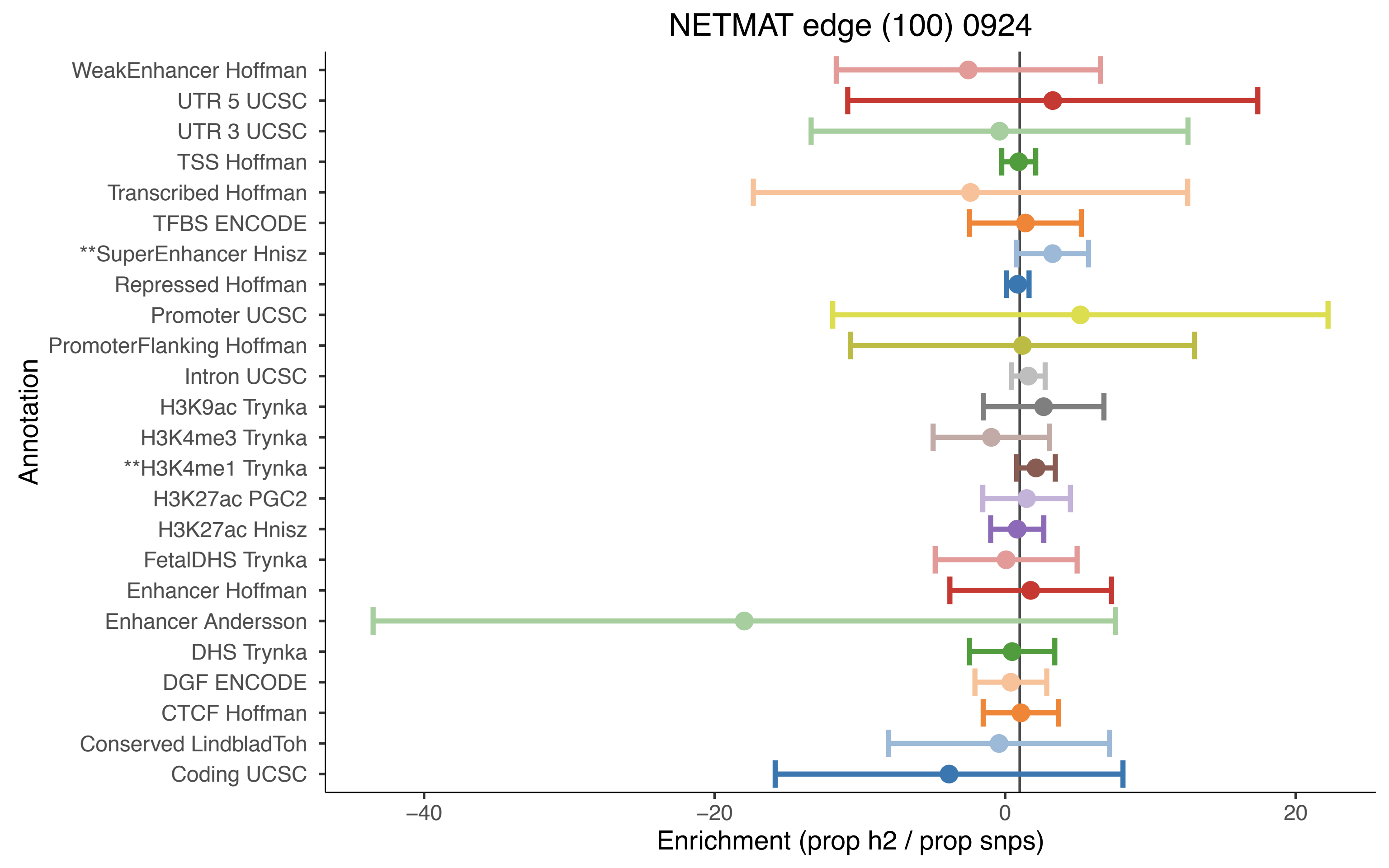
NETMAT edge (100) 0924
WeakEnhancer Hoffman
UTR 5 UCSC
UTR 3 UCSC
TSS Hoffman
Transcribed Hoffman
TFBS ENCODE
**SuperEnhancer Hnisz
Repressed Hoffman
Promoter UCSC
PromoterFlanking Hoffman
Intron UCSC
H3K9ac Trynka
H3K4me3 Trynka
**H3K4me1 Trynka
H3K27ac PGC2
H3K27ac Hnisz
FetalDHS Trynka
Enhancer Hoffman
Enhancer Andersson
DHS Trynka
DGF ENCODE
CTCF Hoffman
Conserved LindbladToh
Coding UCSC
Annotation
−40
−20
0
20
Enrichment (prop h2 / prop snps)

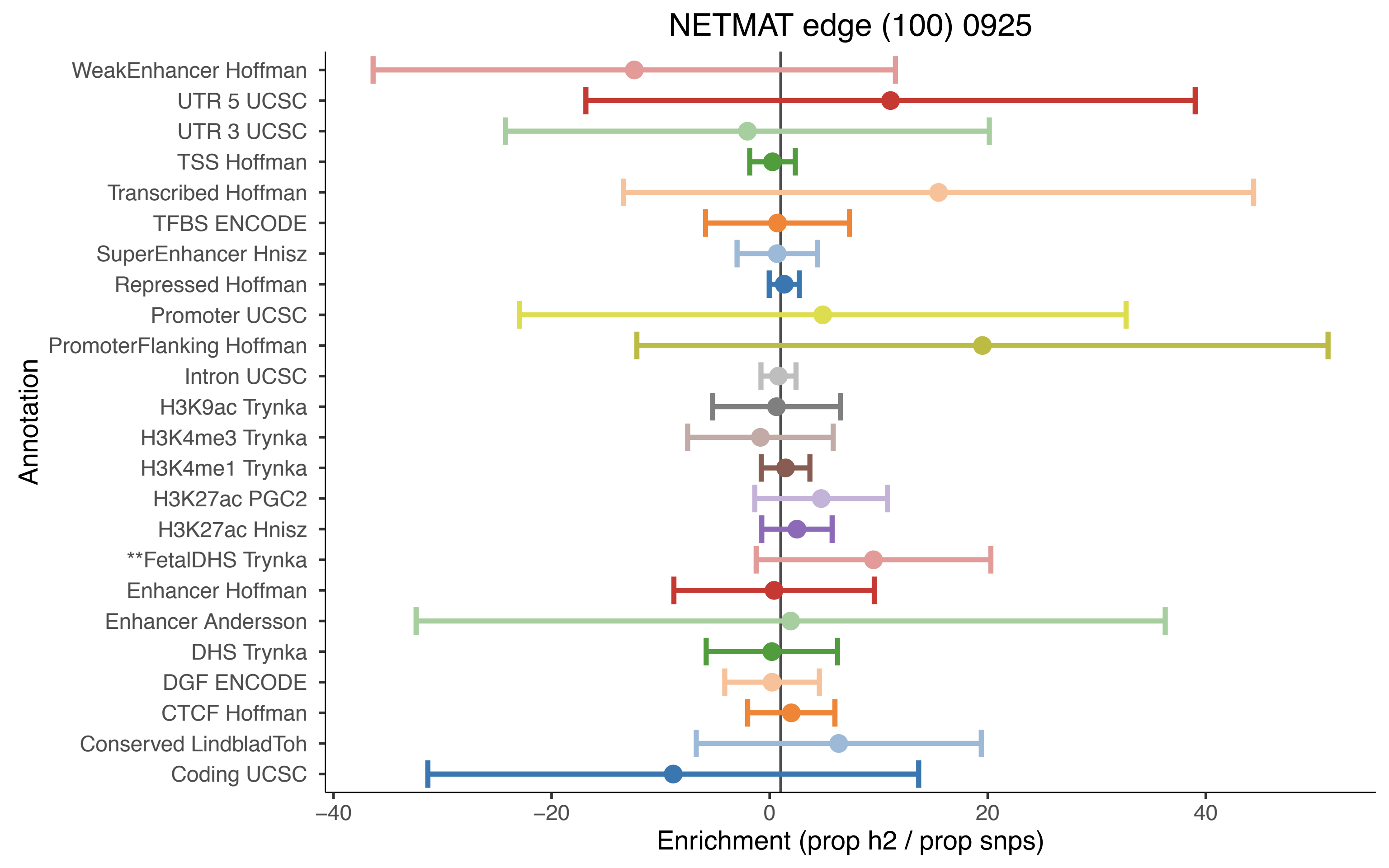
NETMAT edge (100) 0925
WeakEnhancer Hoffman
UTR 5 UCSC
UTR 3 UCSC
TSS Hoffman
Transcribed Hoffman
TFBS ENCODE
SuperEnhancer Hnisz
Repressed Hoffman
Promoter UCSC
PromoterFlanking Hoffman
Intron UCSC
H3K9ac Trynka
H3K4me3 Trynka
H3K4me1 Trynka
H3K27ac PGC2
H3K27ac Hnisz
**FetalDHS Trynka
Enhancer Hoffman
Enhancer Andersson
DHS Trynka
DGF ENCODE
CTCF Hoffman
Conserved LindbladToh
Coding UCSC
Annotation
−40
−20
0
20
40
Enrichment (prop h2 / prop snps)

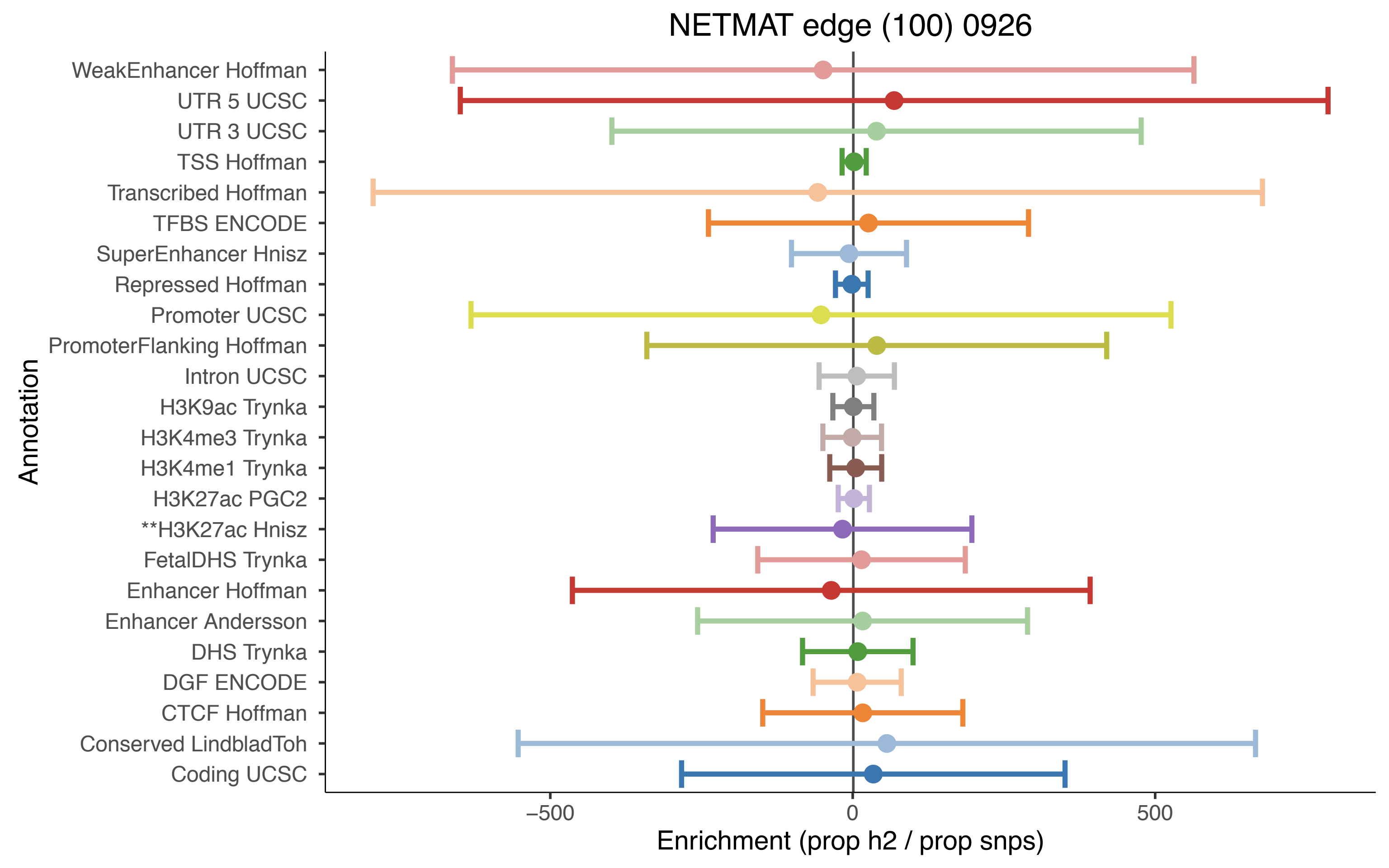

NETMAT edge (100) 0926
Annotation
WeakEnhancer Hoffman
UTR 5 UCSC
UTR 3 UCSC
TSS Hoffman
Transcribed Hoffman
TFBS ENCODE
SuperEnhancer Hnisz
Repressed Hoffman
Promoter UCSC
PromoterFlanking Hoffman
Intron UCSC
H3K9ac Trynka
H3K4me3 Trynka
H3K4me1 Trynka
H3K27ac PGC2
**H3K27ac Hnisz
FetalDHS Trynka
Enhancer Hoffman
Enhancer Andersson
DHS Trynka
DGF ENCODE
CTCF Hoffman
Conserved LindbladToh
Coding UCSC
−500
0
500
Enrichment (prop h2 / prop snps)

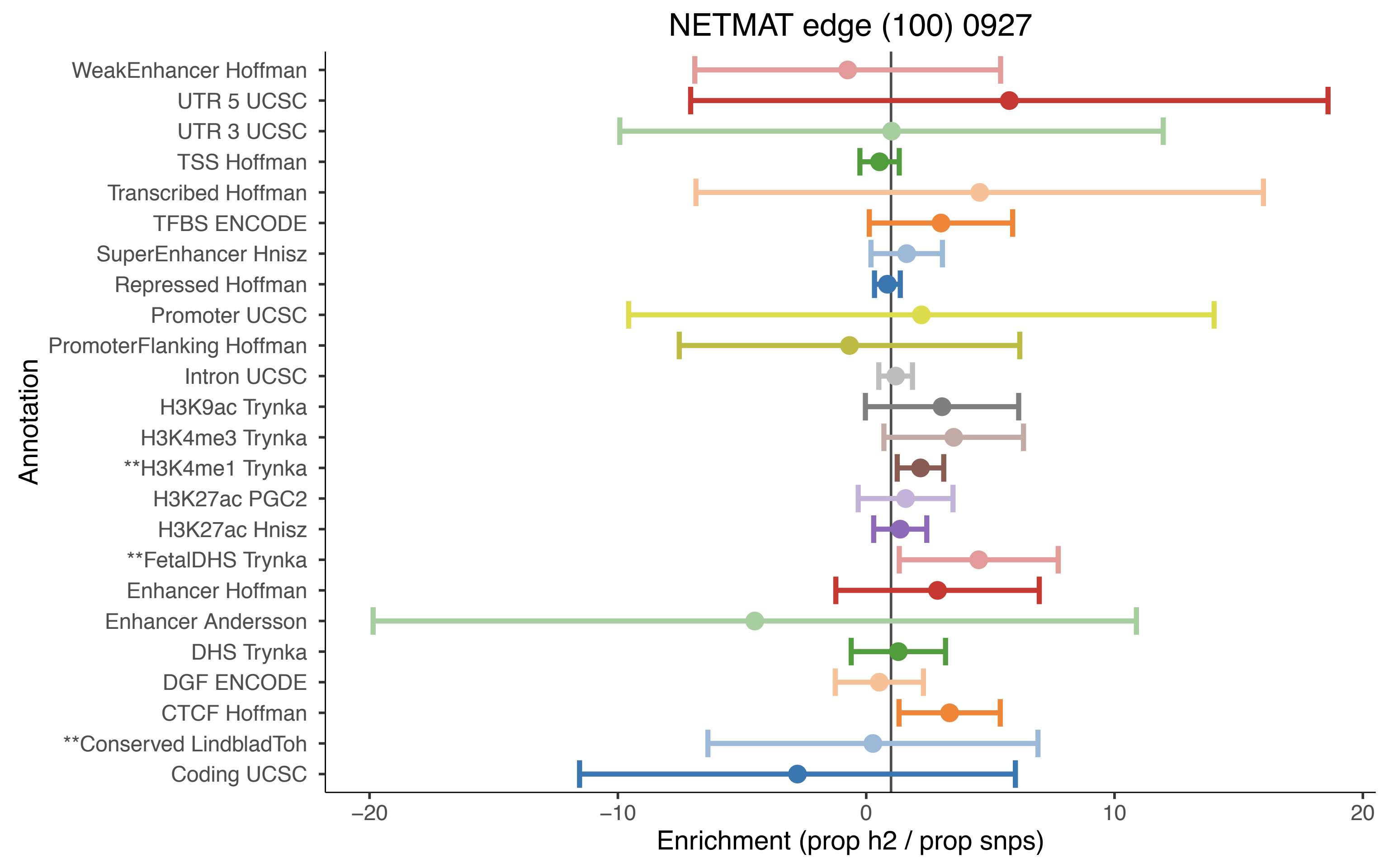
NETMAT edge (100) 0927
Annotation
WeakEnhancer Hoffman
UTR 5 UCSC
UTR 3 UCSC
TSS Hoffman
Transcribed Hoffman
TFBS ENCODE
SuperEnhancer Hnisz
Repressed Hoffman
Promoter UCSC
PromoterFlanking Hoffman
Intron UCSC
H3K9ac Trynka
H3K4me3 Trynka
**H3K4me1 Trynka
H3K27ac PGC2
H3K27ac Hnisz
**FetalDHS Trynka
Enhancer Hoffman
Enhancer Andersson
DHS Trynka
DGF ENCODE
CTCF Hoffman
**Conserved LindbladToh
Coding UCSC
−20
−10
0
10
20
Enrichment (prop h2 / prop snps)

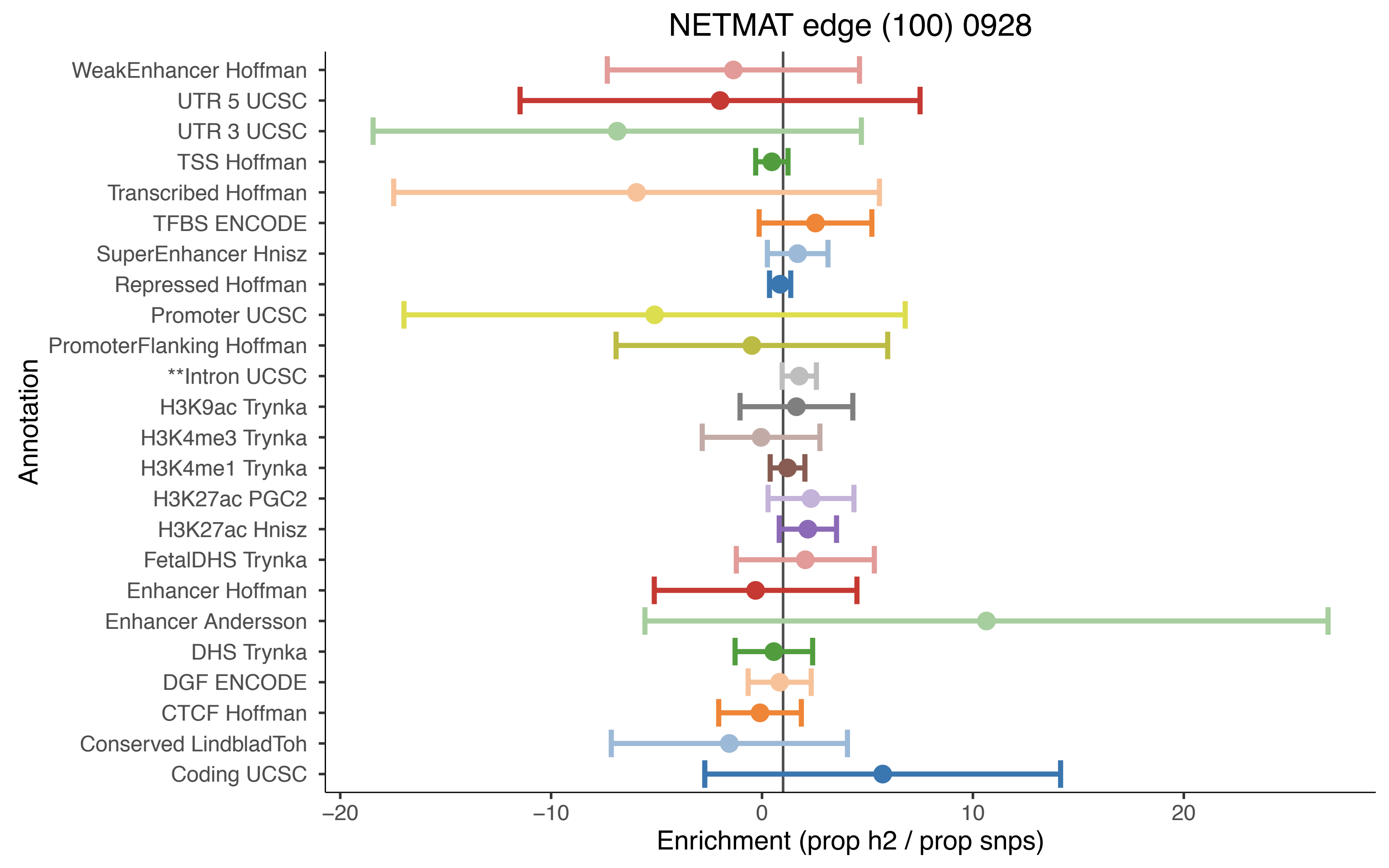
NETMAT edge (100) 0928
WeakEnhancer Hoffman
UTR 5 UCSC
UTR 3 UCSC
TSS Hoffman
Transcribed Hoffman
TFBS ENCODE
SuperEnhancer Hnisz
Repressed Hoffman
Promoter UCSC
PromoterFlanking Hoffman
**Intron UCSC
H3K9ac Trynka
H3K4me3 Trynka
H3K4me1 Trynka
H3K27ac PGC2
H3K27ac Hnisz
FetalDHS Trynka
Enhancer Hoffman
Enhancer Andersson
DHS Trynka
DGF ENCODE
CTCF Hoffman
Conserved LindbladToh
Coding UCSC
Annotation
−20
−10
0
10
20
Enrichment (prop h2 / prop snps)

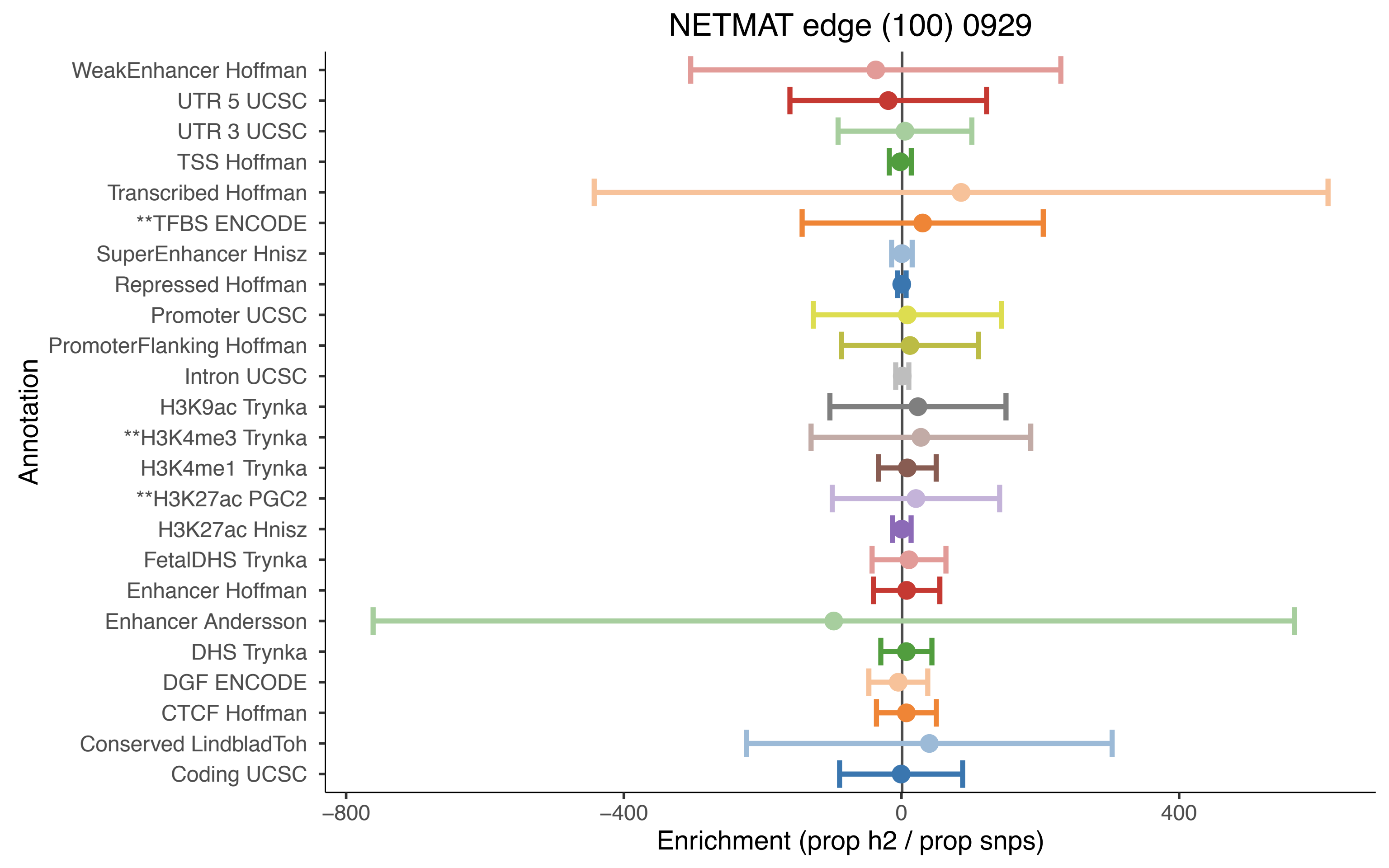

NETMAT edge (100) 0929
WeakEnhancer Hoffman
UTR 5 UCSC
UTR 3 UCSC
TSS Hoffman
Transcribed Hoffman
**TFBS ENCODE
SuperEnhancer Hnisz
Repressed Hoffman
Promoter UCSC
PromoterFlanking Hoffman
Intron UCSC
H3K9ac Trynka
**H3K4me3 Trynka
H3K4me1 Trynka
**H3K27ac PGC2
H3K27ac Hnisz
FetalDHS Trynka
Enhancer Hoffman
Enhancer Andersson
DHS Trynka
DGF ENCODE
CTCF Hoffman
Conserved LindbladToh
Coding UCSC
Annotation
−800
−400
0
400
Enrichment (prop h2 / prop snps)

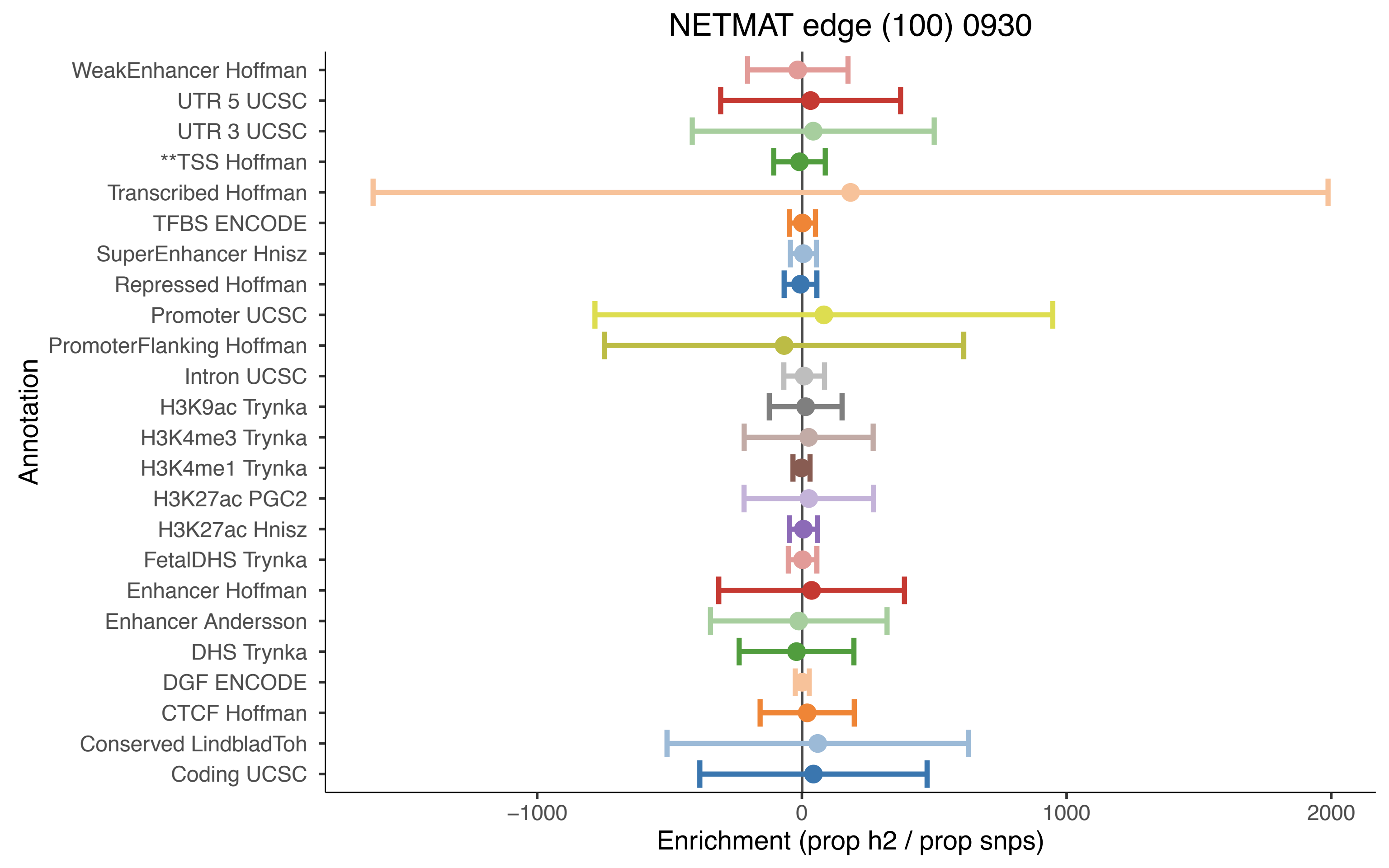
NETMAT edge (100) 0930
WeakEnhancer Hoffman
UTR 5 UCSC
UTR 3 UCSC
**TSS Hoffman
Transcribed Hoffman
TFBS ENCODE
SuperEnhancer Hnisz
Repressed Hoffman
Promoter UCSC
PromoterFlanking Hoffman
Intron UCSC
H3K9ac Trynka
H3K4me3 Trynka
H3K4me1 Trynka
H3K27ac PGC2
H3K27ac Hnisz
FetalDHS Trynka
Enhancer Hoffman
Enhancer Andersson
DHS Trynka
DGF ENCODE
CTCF Hoffman
Conserved LindbladToh
Coding UCSC
Annotation
−1000
0
1000
2000
Enrichment (prop h2 / prop snps)

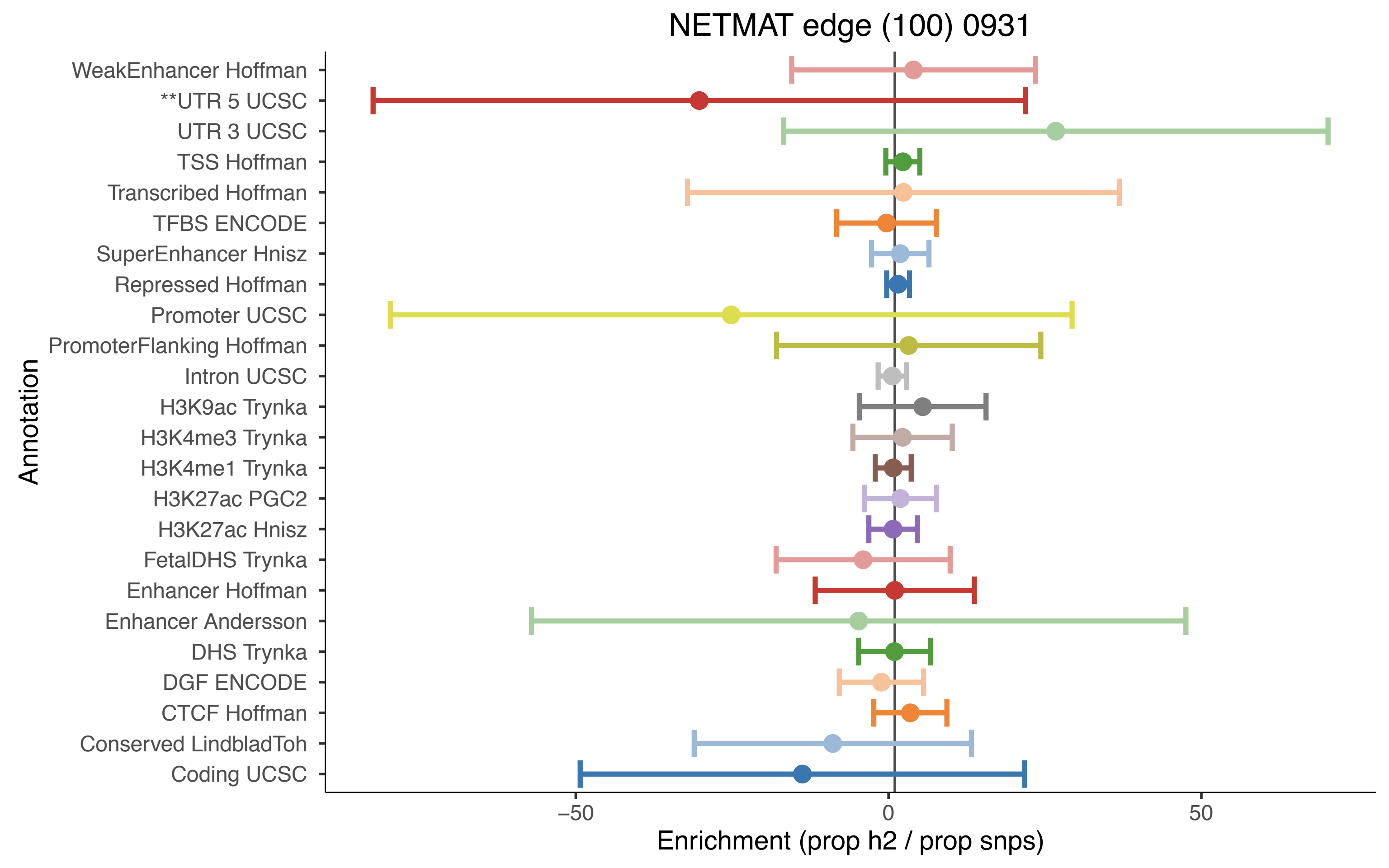

NETMAT edge (100) 0931
WeakEnhancer Hoffman
**UTR 5 UCSC
UTR 3 UCSC
TSS Hoffman
Transcribed Hoffman
TFBS ENCODE
SuperEnhancer Hnisz
Repressed Hoffman
Promoter UCSC
PromoterFlanking Hoffman
Intron UCSC
H3K9ac Trynka
H3K4me3 Trynka
H3K4me1 Trynka
H3K27ac PGC2
H3K27ac Hnisz
FetalDHS Trynka
Enhancer Hoffman
Enhancer Andersson
DHS Trynka
DGF ENCODE
CTCF Hoffman
Conserved LindbladToh
Coding UCSC
Annotation
−50
0
50
Enrichment (prop h2 / prop snps)

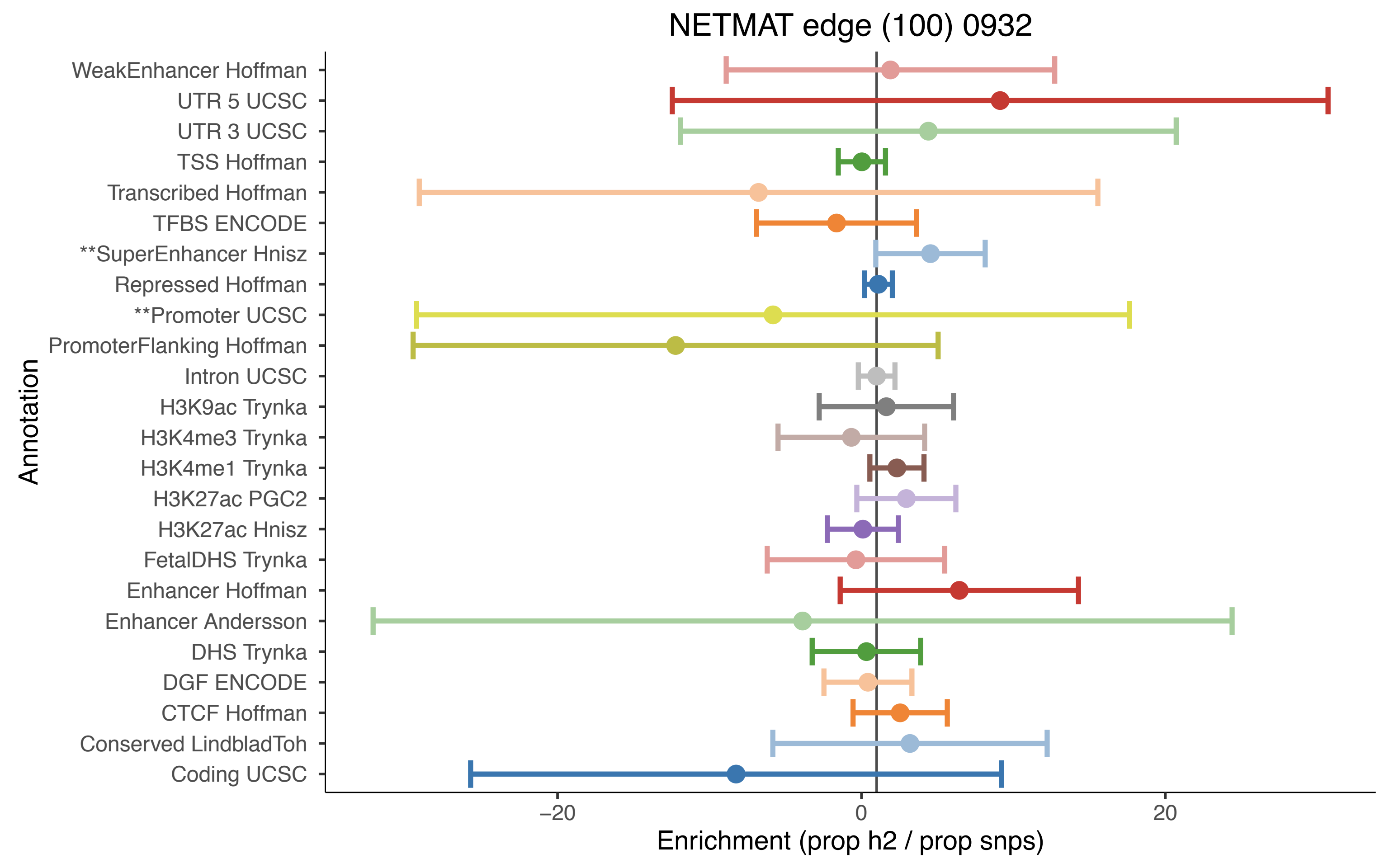
NETMAT edge (100) 0932
WeakEnhancer Hoffman
UTR 5 UCSC
UTR 3 UCSC
TSS Hoffman
Transcribed Hoffman
TFBS ENCODE
**SuperEnhancer Hnisz
Repressed Hoffman
**Promoter UCSC
PromoterFlanking Hoffman
Intron UCSC
H3K9ac Trynka
H3K4me3 Trynka
H3K4me1 Trynka
H3K27ac PGC2
H3K27ac Hnisz
FetalDHS Trynka
Enhancer Hoffman
Enhancer Andersson
DHS Trynka
DGF ENCODE
CTCF Hoffman
Conserved LindbladToh
Coding UCSC
Annotation
−20
0
20
Enrichment (prop h2 / prop snps)

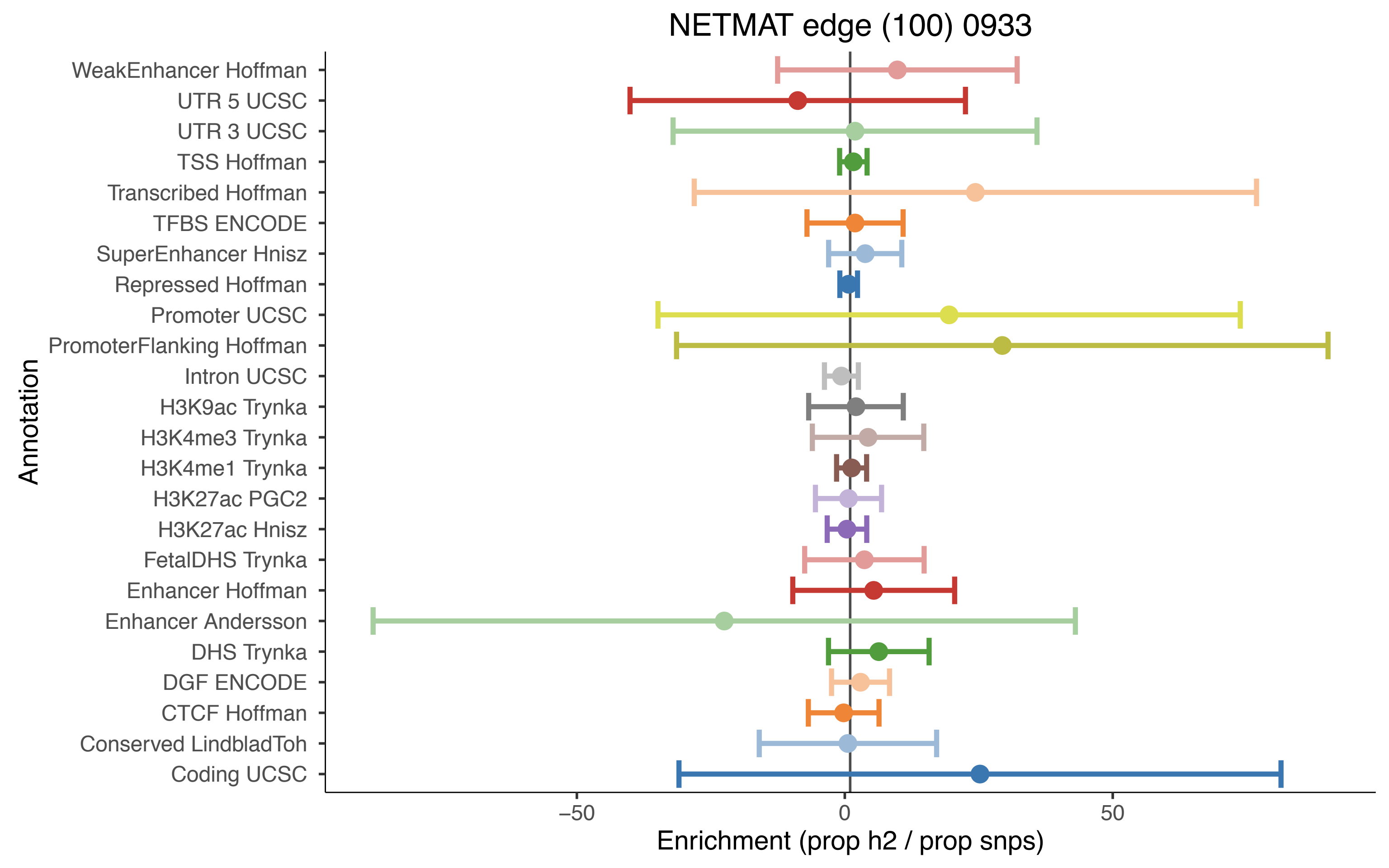
NETMAT edge (100) 0933
WeakEnhancer Hoffman
UTR 5 UCSC
UTR 3 UCSC
TSS Hoffman
Transcribed Hoffman
TFBS ENCODE
SuperEnhancer Hnisz
Repressed Hoffman
Promoter UCSC
PromoterFlanking Hoffman
Intron UCSC
H3K9ac Trynka
H3K4me3 Trynka
H3K4me1 Trynka
H3K27ac PGC2
H3K27ac Hnisz
FetalDHS Trynka
Enhancer Hoffman
Enhancer Andersson
DHS Trynka
DGF ENCODE
CTCF Hoffman
Conserved LindbladToh
Coding UCSC
Annotation
−50
0
50
Enrichment (prop h2 / prop snps)

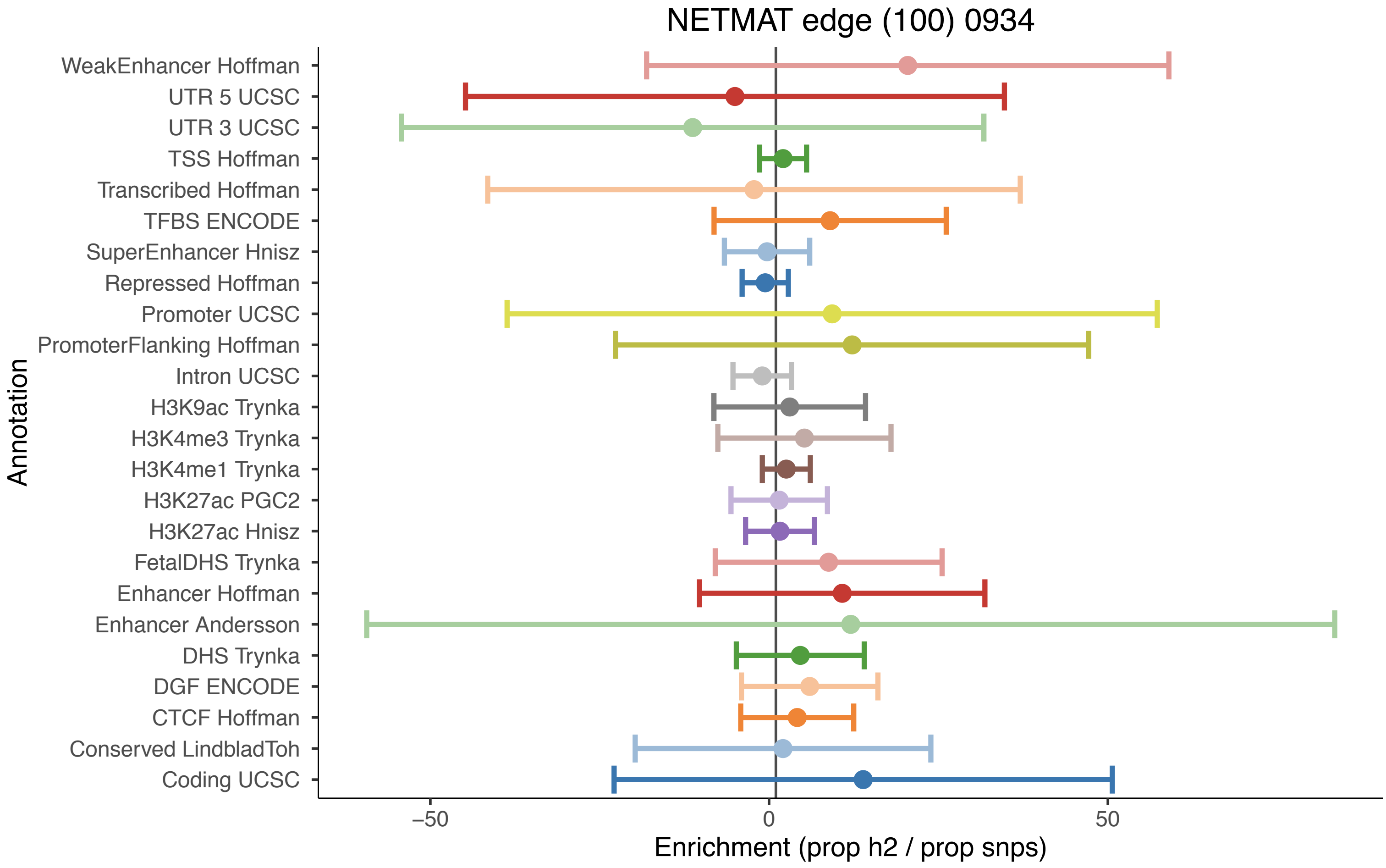
NETMAT edge (100) 0934
WeakEnhancer Hoffman
UTR 5 UCSC
UTR 3 UCSC
TSS Hoffman
Transcribed Hoffman
TFBS ENCODE
SuperEnhancer Hnisz
Repressed Hoffman
Promoter UCSC
PromoterFlanking Hoffman
Intron UCSC
H3K9ac Trynka
H3K4me3 Trynka
H3K4me1 Trynka
H3K27ac PGC2
H3K27ac Hnisz
FetalDHS Trynka
Enhancer Hoffman
Enhancer Andersson
DHS Trynka
DGF ENCODE
CTCF Hoffman
Conserved LindbladToh
Coding UCSC
Annotation
−50
0
50
Enrichment (prop h2 / prop snps)

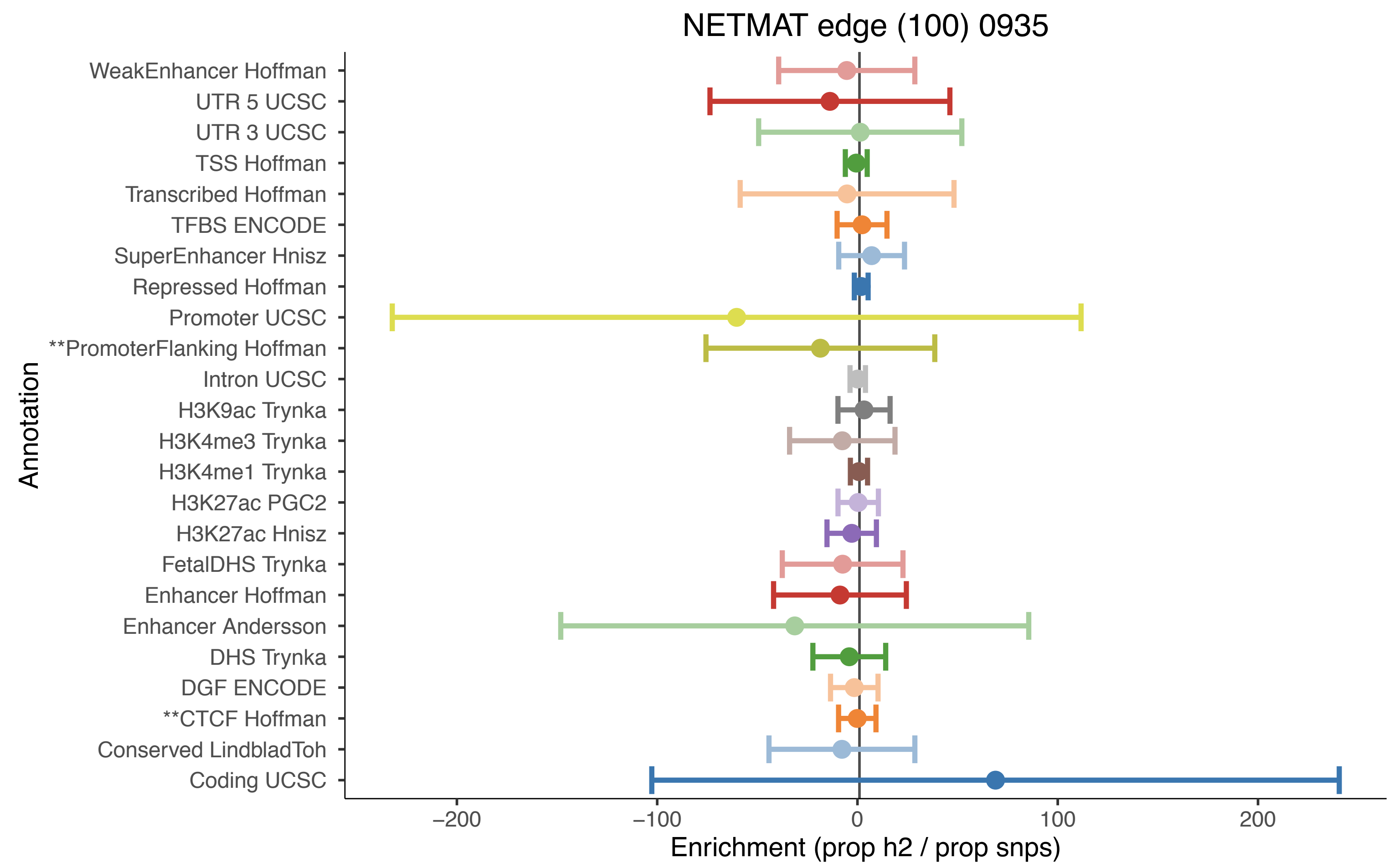

NETMAT edge (100) 0935
WeakEnhancer Hoffman
UTR 5 UCSC
UTR 3 UCSC
TSS Hoffman
Transcribed Hoffman
TFBS ENCODE
SuperEnhancer Hnisz
Repressed Hoffman
Promoter UCSC
**PromoterFlanking Hoffman
Intron UCSC
H3K9ac Trynka
H3K4me3 Trynka
H3K4me1 Trynka
H3K27ac PGC2
H3K27ac Hnisz
FetalDHS Trynka
Enhancer Hoffman
Enhancer Andersson
DHS Trynka
DGF ENCODE
**CTCF Hoffman
Conserved LindbladToh
Coding UCSC
Annotation
−200
−100
0
100
200
Enrichment (prop h2 / prop snps)

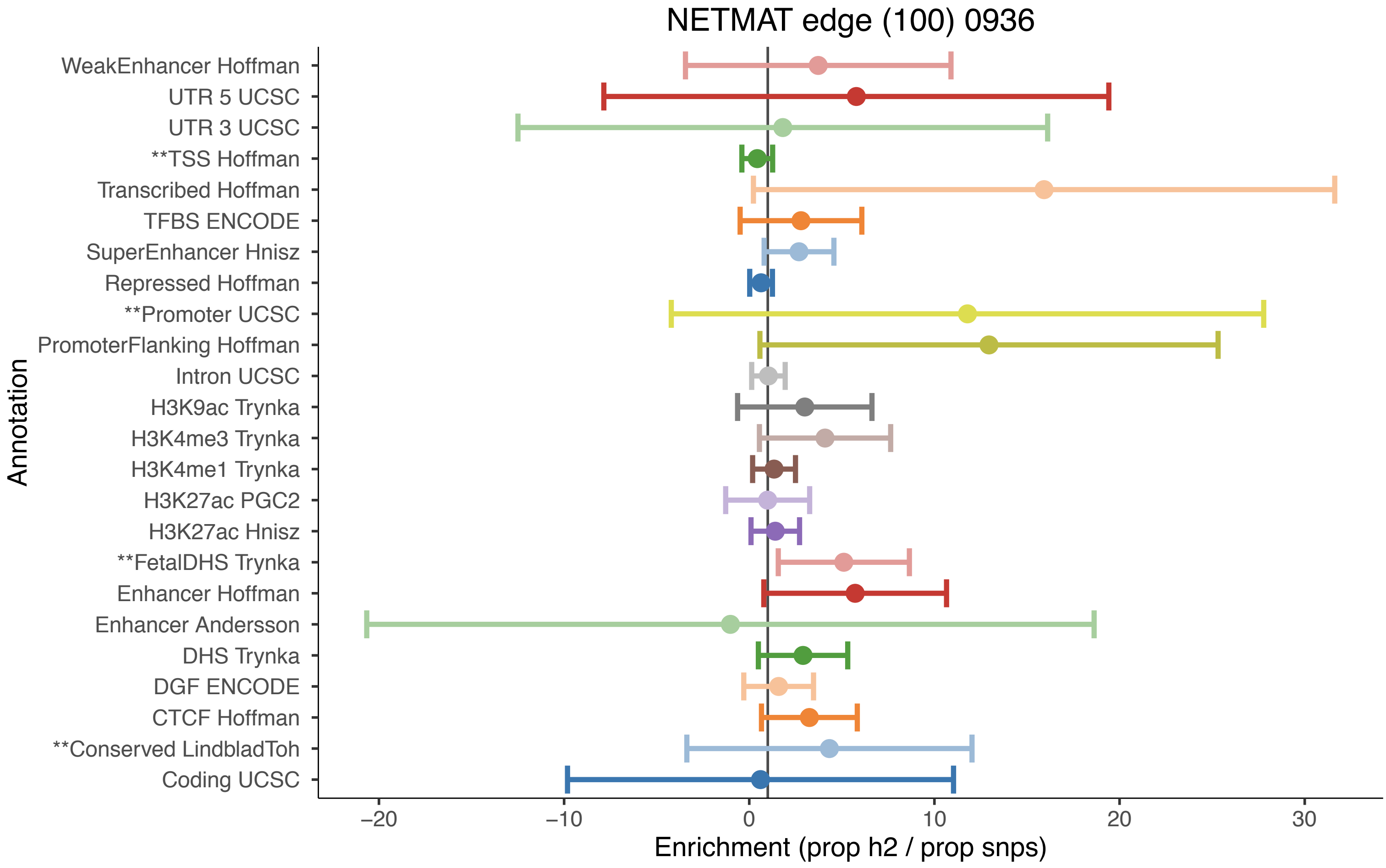

NETMAT edge (100) 0936
WeakEnhancer Hoffman
UTR 5 UCSC
UTR 3 UCSC
**TSS Hoffman
Transcribed Hoffman
TFBS ENCODE
SuperEnhancer Hnisz
Repressed Hoffman
**Promoter UCSC
PromoterFlanking Hoffman
Intron UCSC
H3K9ac Trynka
H3K4me3 Trynka
H3K4me1 Trynka
H3K27ac PGC2
H3K27ac Hnisz
**FetalDHS Trynka
Enhancer Hoffman
Enhancer Andersson
DHS Trynka
DGF ENCODE
CTCF Hoffman
**Conserved LindbladToh
Coding UCSC
Annotation
−20
−10
0
10
20
30
Enrichment (prop h2 / prop snps)

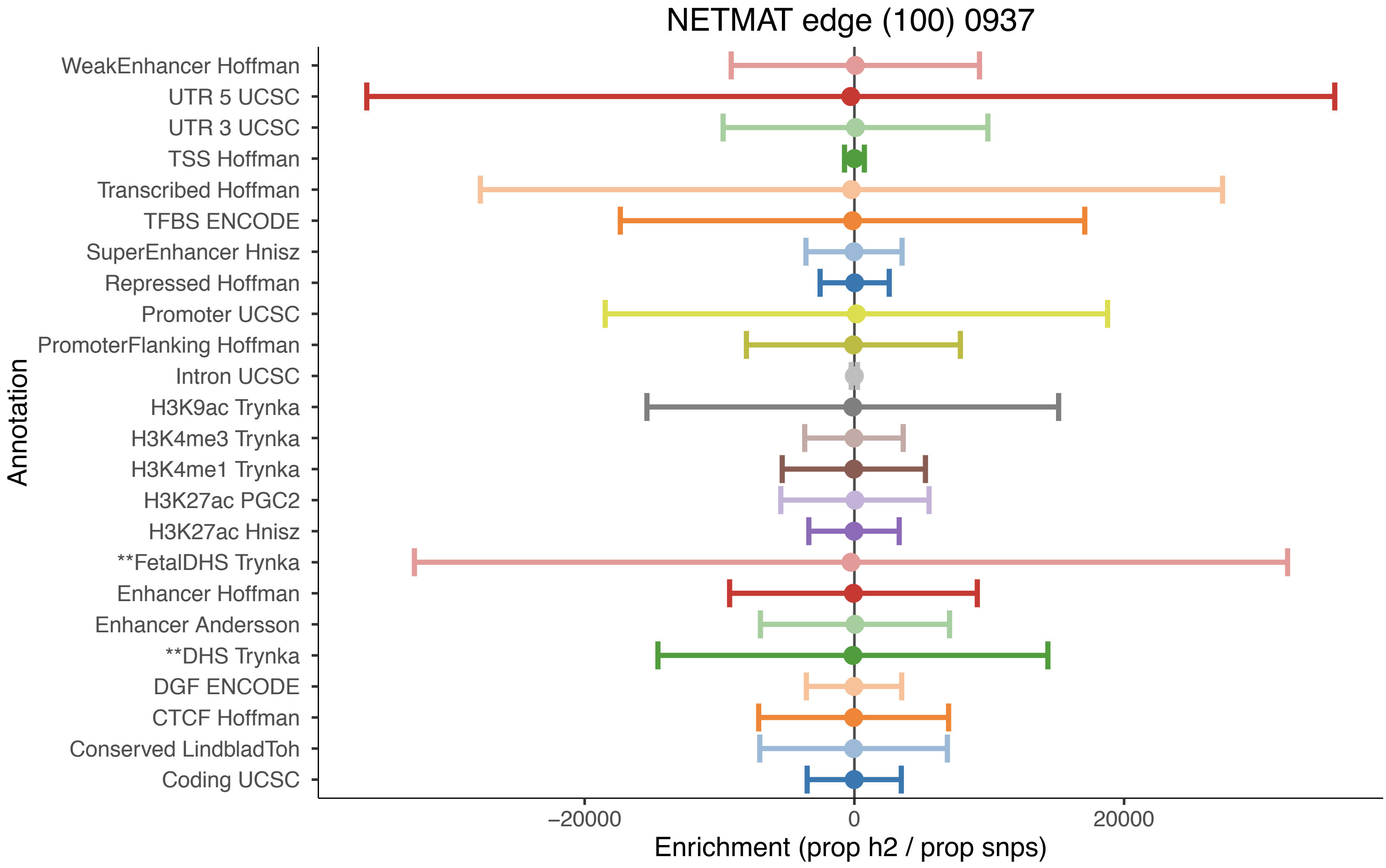
NETMAT edge (100) 0937
WeakEnhancer Hoffman
UTR 5 UCSC
UTR 3 UCSC
TSS Hoffman
Transcribed Hoffman
TFBS ENCODE
SuperEnhancer Hnisz
Repressed Hoffman
Promoter UCSC
PromoterFlanking Hoffman
Intron UCSC
H3K9ac Trynka
H3K4me3 Trynka
H3K4me1 Trynka
H3K27ac PGC2
H3K27ac Hnisz
**FetalDHS Trynka
Enhancer Hoffman
Enhancer Andersson
**DHS Trynka
DGF ENCODE
CTCF Hoffman
Conserved LindbladToh
Coding UCSC
Annotation
−20000
0
20000
Enrichment (prop h2 / prop snps)

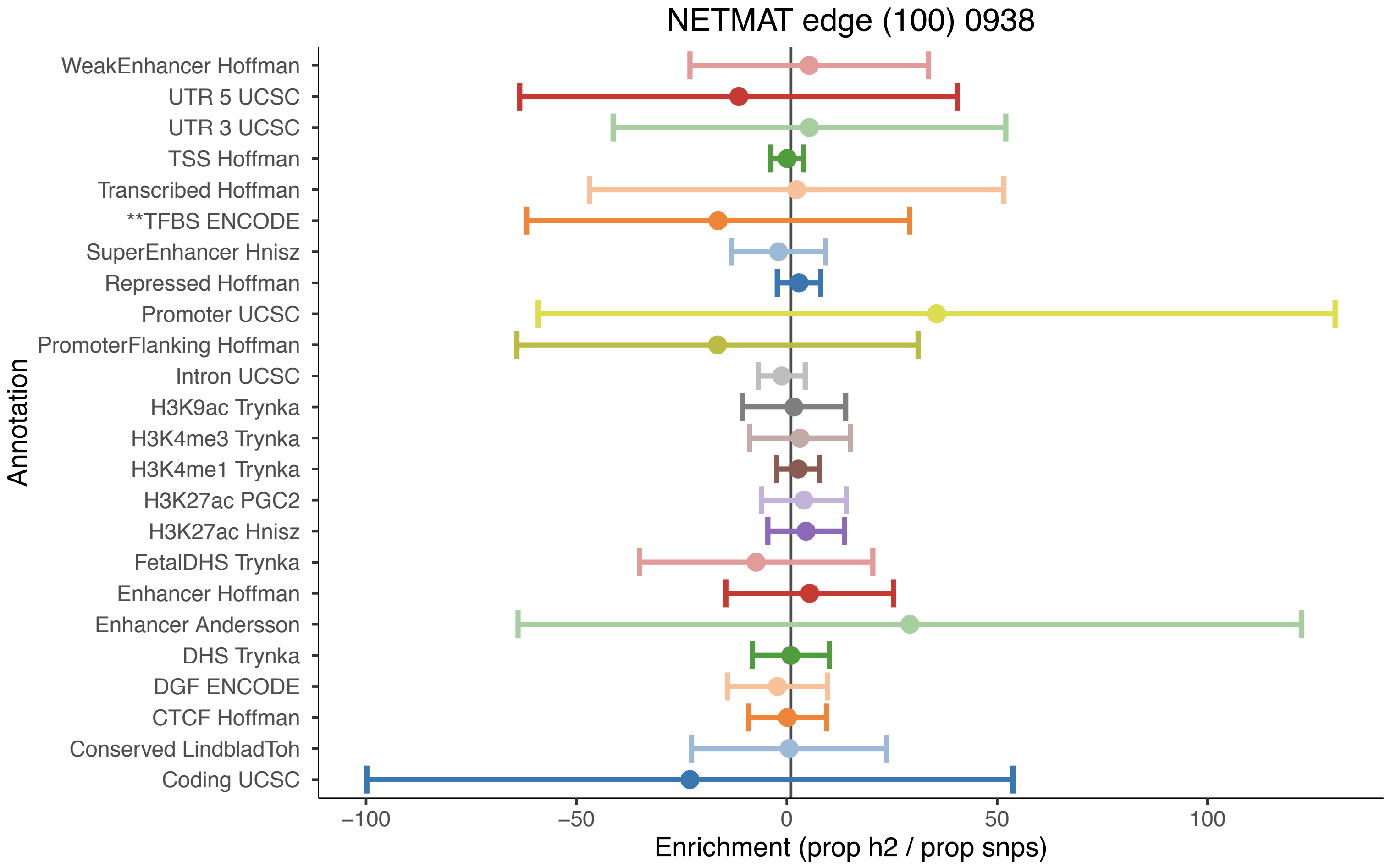

NETMAT edge (100) 0938
Annotation
WeakEnhancer Hoffman
UTR 5 UCSC
UTR 3 UCSC
TSS Hoffman
Transcribed Hoffman
**TFBS ENCODE
SuperEnhancer Hnisz
Repressed Hoffman
Promoter UCSC
PromoterFlanking Hoffman
Intron UCSC
H3K9ac Trynka
H3K4me3 Trynka
H3K4me1 Trynka
H3K27ac PGC2
H3K27ac Hnisz
FetalDHS Trynka
Enhancer Hoffman
Enhancer Andersson
DHS Trynka
DGF ENCODE
CTCF Hoffman
Conserved LindbladToh
Coding UCSC
−100
−50
0
50
100
Enrichment (prop h2 / prop snps)

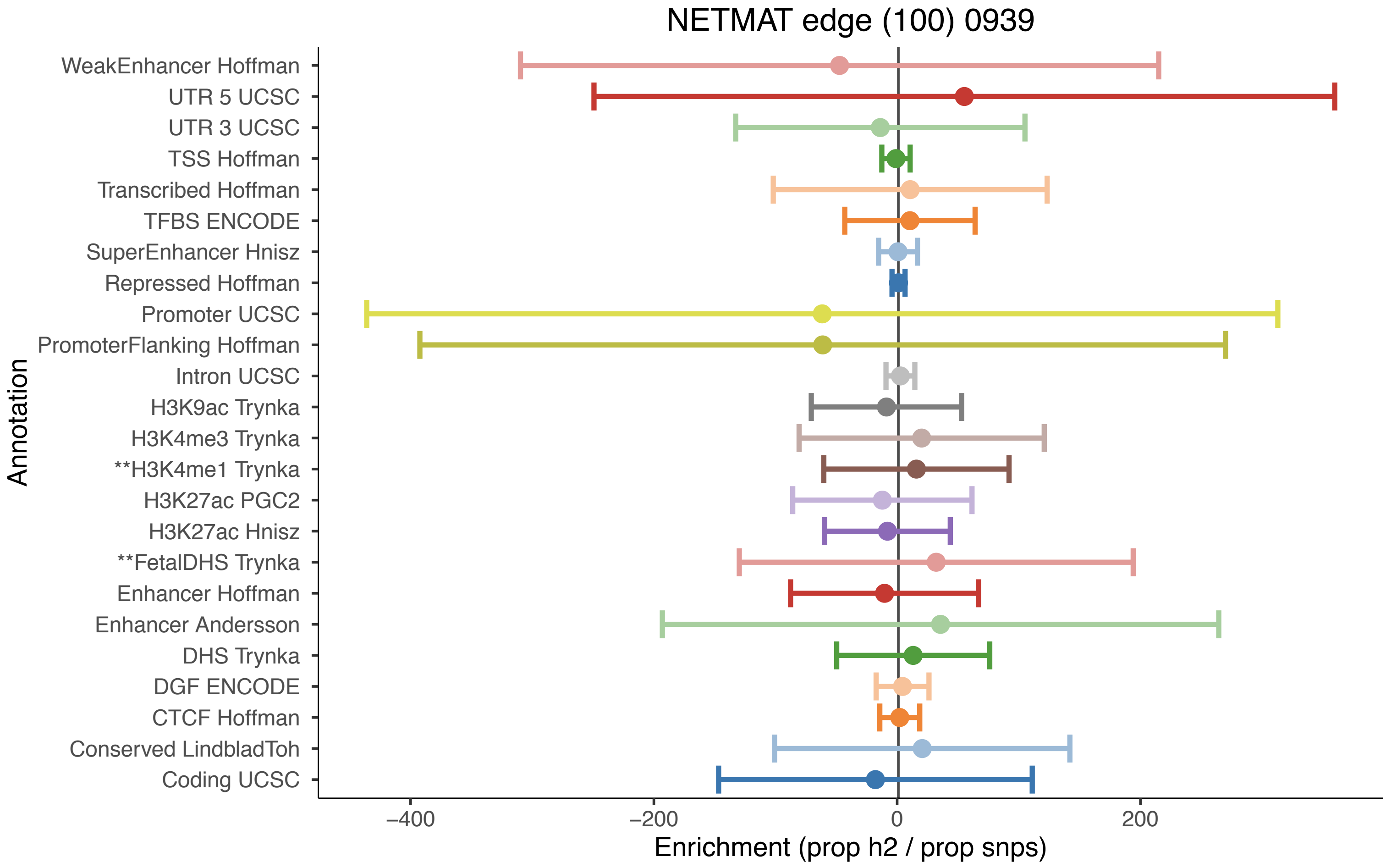
NETMAT edge (100) 0939
WeakEnhancer Hoffman
UTR 5 UCSC
UTR 3 UCSC
TSS Hoffman
Transcribed Hoffman
TFBS ENCODE
SuperEnhancer Hnisz
Repressed Hoffman
Promoter UCSC
PromoterFlanking Hoffman
Intron UCSC
H3K9ac Trynka
H3K4me3 Trynka
**H3K4me1 Trynka
H3K27ac PGC2
H3K27ac Hnisz
**FetalDHS Trynka
Enhancer Hoffman
Enhancer Andersson
DHS Trynka
DGF ENCODE
CTCF Hoffman
Conserved LindbladToh
Coding UCSC
Annotation
−400
−200
0
200
Enrichment (prop h2 / prop snps)

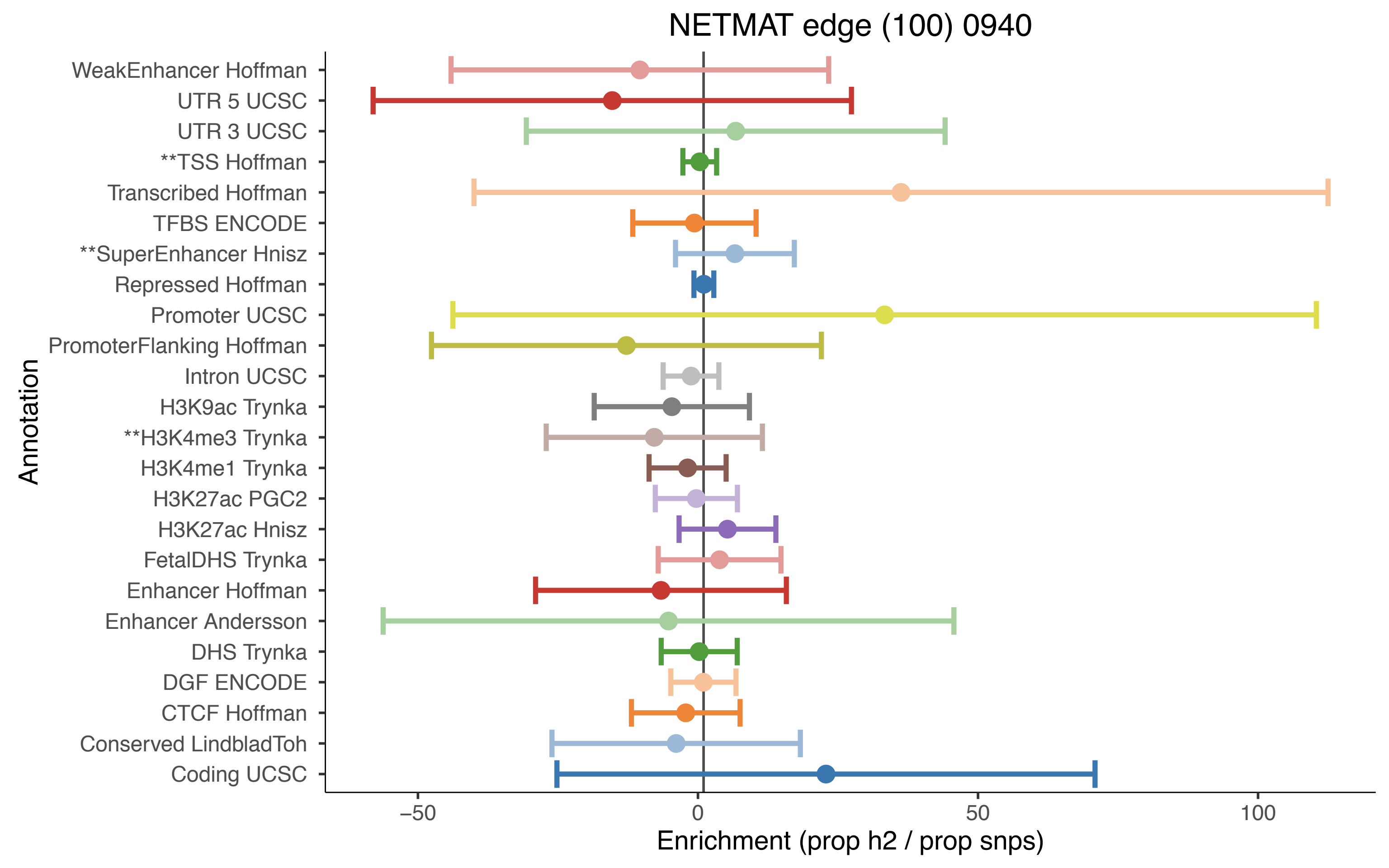

NETMAT edge (100) 0940
WeakEnhancer Hoffman
UTR 5 UCSC
UTR 3 UCSC
**TSS Hoffman
Transcribed Hoffman
TFBS ENCODE
**SuperEnhancer Hnisz
Repressed Hoffman
Promoter UCSC
PromoterFlanking Hoffman
Intron UCSC
H3K9ac Trynka
**H3K4me3 Trynka
H3K4me1 Trynka
H3K27ac PGC2
H3K27ac Hnisz
FetalDHS Trynka
Enhancer Hoffman
Enhancer Andersson
DHS Trynka
DGF ENCODE
CTCF Hoffman
Conserved LindbladToh
Coding UCSC
Annotation
−50
0
50
100
Enrichment (prop h2 / prop snps)

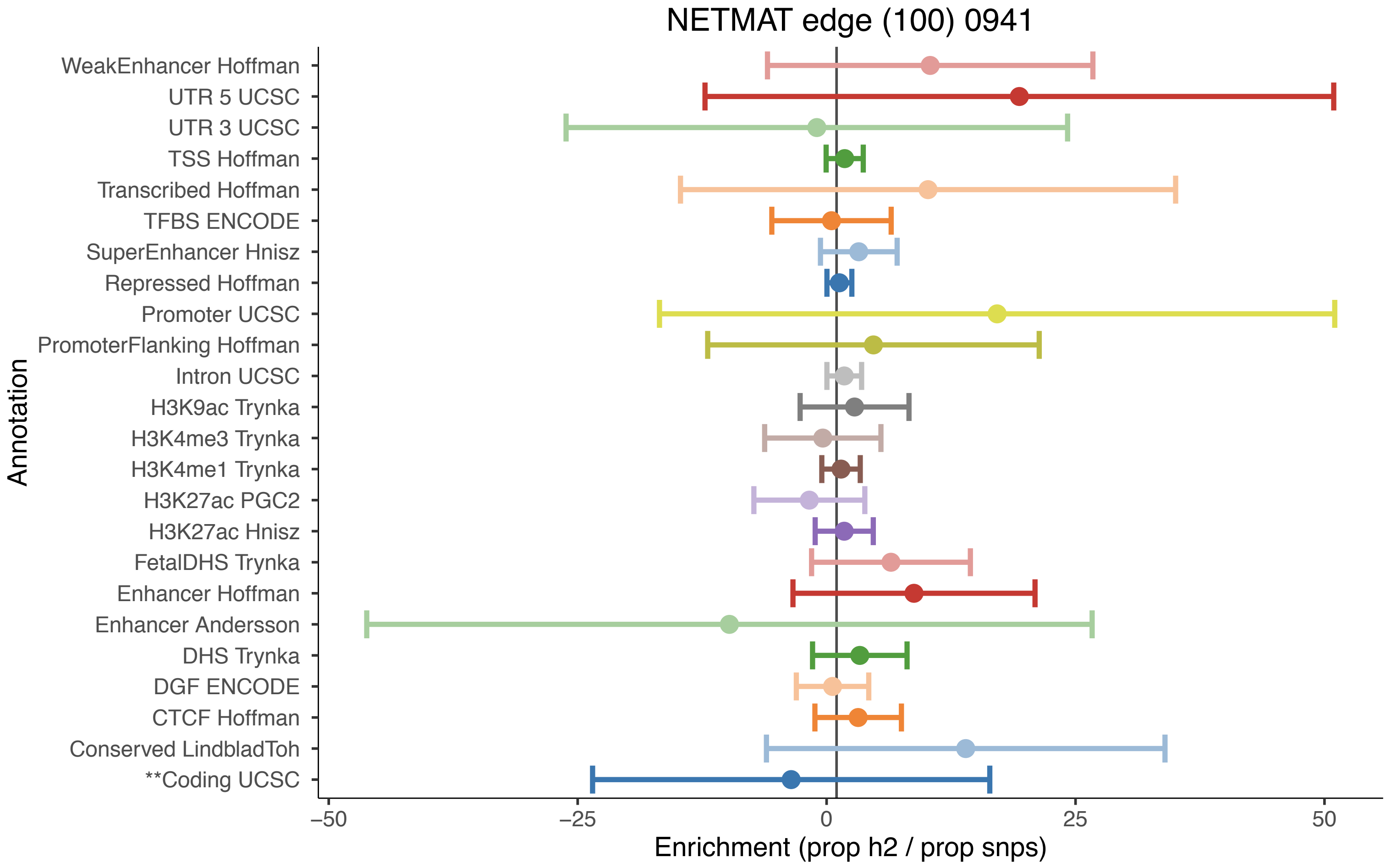

NETMAT edge (100) 0941
WeakEnhancer Hoffman
UTR 5 UCSC
UTR 3 UCSC
TSS Hoffman
Transcribed Hoffman
TFBS ENCODE
SuperEnhancer Hnisz
Repressed Hoffman
Promoter UCSC
PromoterFlanking Hoffman
Intron UCSC
H3K9ac Trynka
H3K4me3 Trynka
H3K4me1 Trynka
H3K27ac PGC2
H3K27ac Hnisz
FetalDHS Trynka
Enhancer Hoffman
Enhancer Andersson
DHS Trynka
DGF ENCODE
CTCF Hoffman
Conserved LindbladToh
**Coding UCSC
Annotation
−50
−25
0
25
50
Enrichment (prop h2 / prop snps)

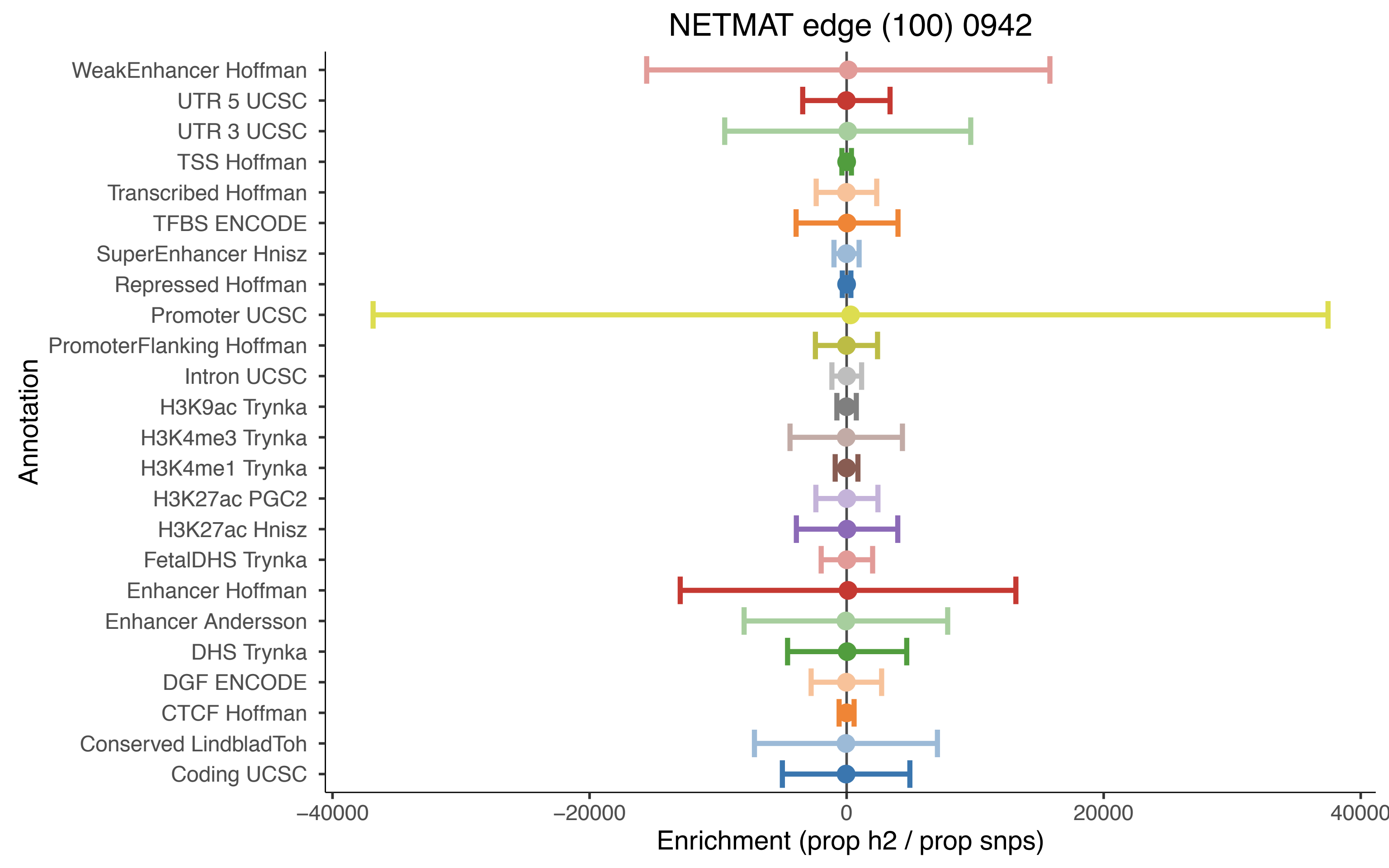

NETMAT edge (100) 0942
WeakEnhancer Hoffman
UTR 5 UCSC
UTR 3 UCSC
TSS Hoffman
Transcribed Hoffman
TFBS ENCODE
SuperEnhancer Hnisz
Repressed Hoffman
Promoter UCSC
PromoterFlanking Hoffman
Intron UCSC
H3K9ac Trynka
H3K4me3 Trynka
H3K4me1 Trynka
H3K27ac PGC2
H3K27ac Hnisz
FetalDHS Trynka
Enhancer Hoffman
Enhancer Andersson
DHS Trynka
DGF ENCODE
CTCF Hoffman
Conserved LindbladToh
Coding UCSC
Annotation
−40000
−20000
0
20000
40000
Enrichment (prop h2 / prop snps)

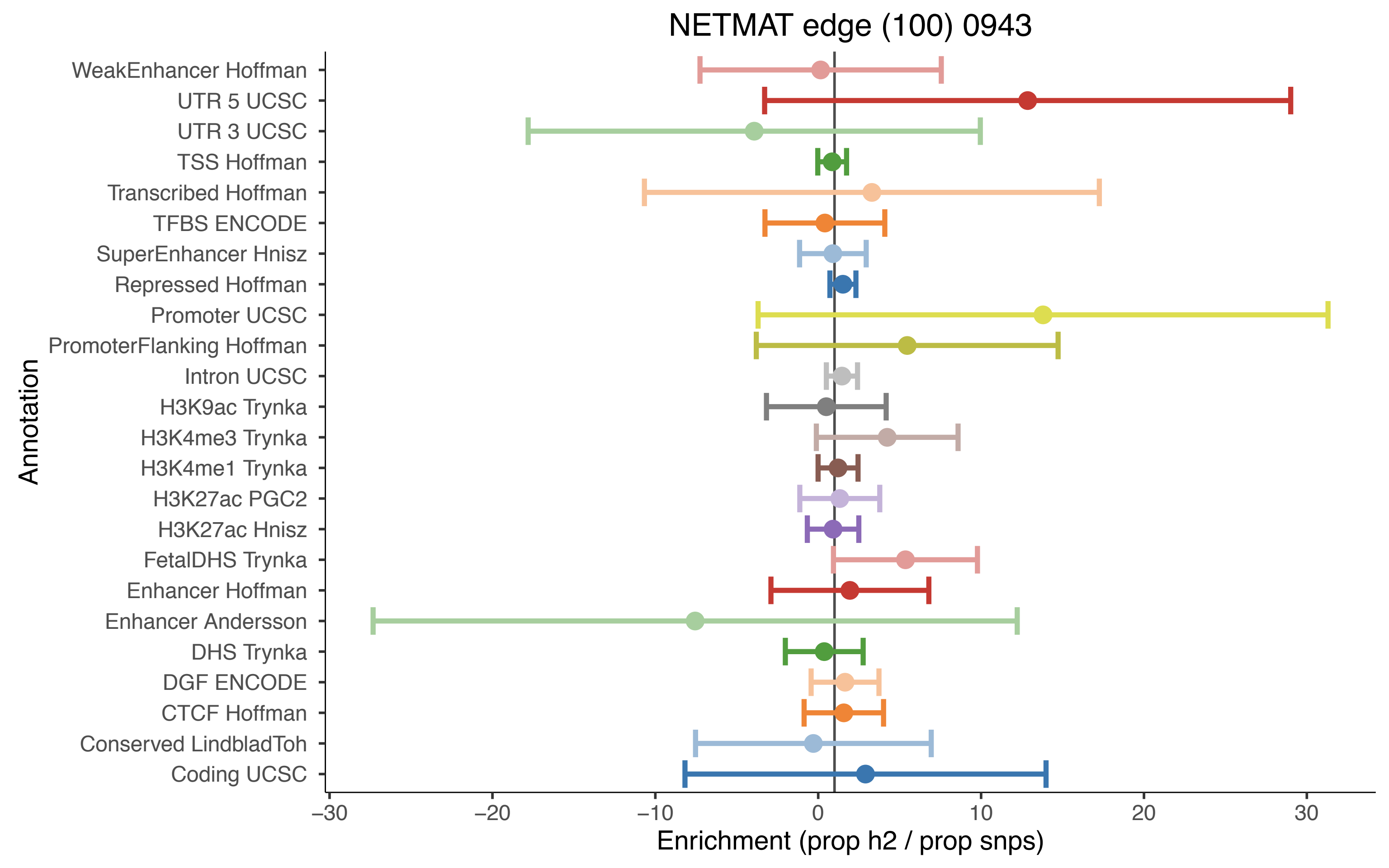

NETMAT edge (100) 0943
Annotation
WeakEnhancer Hoffman
UTR 5 UCSC
UTR 3 UCSC
TSS Hoffman
Transcribed Hoffman
TFBS ENCODE
SuperEnhancer Hnisz
Repressed Hoffman
Promoter UCSC
PromoterFlanking Hoffman
Intron UCSC
H3K9ac Trynka
H3K4me3 Trynka
H3K4me1 Trynka
H3K27ac PGC2
H3K27ac Hnisz
FetalDHS Trynka
Enhancer Hoffman
Enhancer Andersson
DHS Trynka
DGF ENCODE
CTCF Hoffman
Conserved LindbladToh
Coding UCSC
−30
−20
−10
0
10
20
30
Enrichment (prop h2 / prop snps)

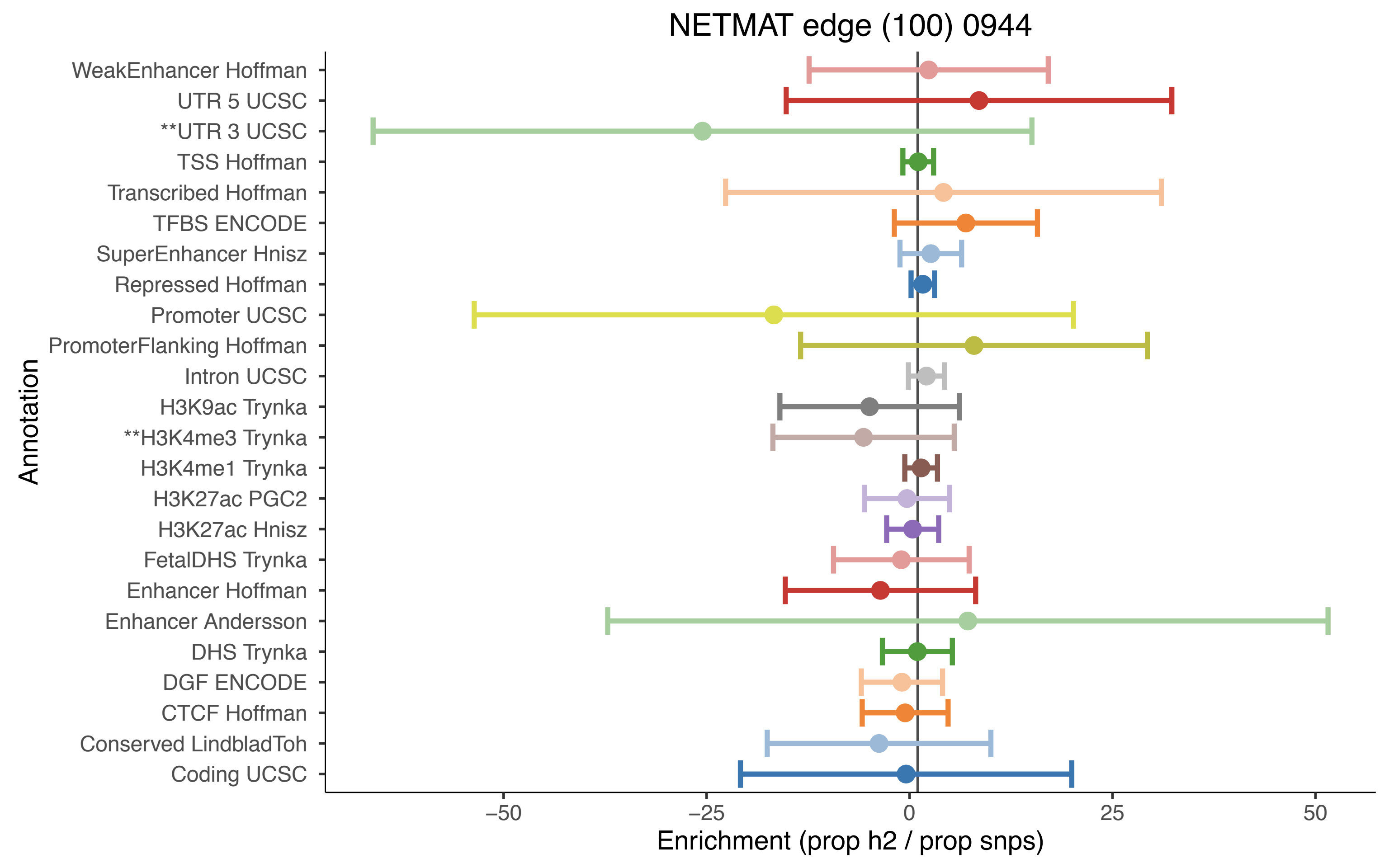

NETMAT edge (100) 0944
WeakEnhancer Hoffman
UTR 5 UCSC
**UTR 3 UCSC
TSS Hoffman
Transcribed Hoffman
TFBS ENCODE
SuperEnhancer Hnisz
Repressed Hoffman
Promoter UCSC
PromoterFlanking Hoffman
Intron UCSC
H3K9ac Trynka
**H3K4me3 Trynka
H3K4me1 Trynka
H3K27ac PGC2
H3K27ac Hnisz
FetalDHS Trynka
Enhancer Hoffman
Enhancer Andersson
DHS Trynka
DGF ENCODE
CTCF Hoffman
Conserved LindbladToh
Coding UCSC
Annotation
−50
−25
0
25
50
Enrichment (prop h2 / prop snps)

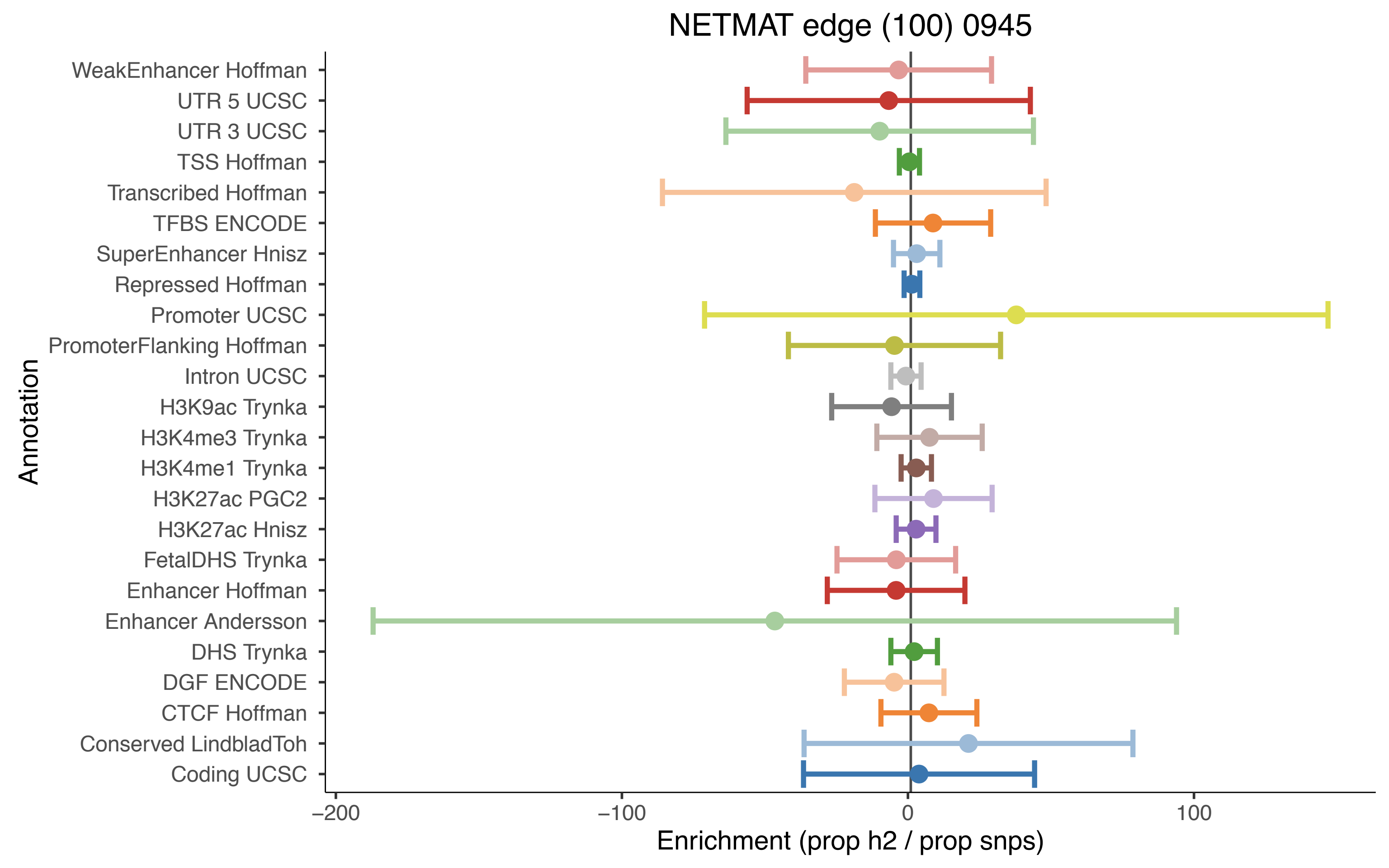

NETMAT edge (100) 0945
Annotation
WeakEnhancer Hoffman
UTR 5 UCSC
UTR 3 UCSC
TSS Hoffman
Transcribed Hoffman
TFBS ENCODE
SuperEnhancer Hnisz
Repressed Hoffman
Promoter UCSC
PromoterFlanking Hoffman
Intron UCSC
H3K9ac Trynka
H3K4me3 Trynka
H3K4me1 Trynka
H3K27ac PGC2
H3K27ac Hnisz
FetalDHS Trynka
Enhancer Hoffman
Enhancer Andersson
DHS Trynka
DGF ENCODE
CTCF Hoffman
Conserved LindbladToh
Coding UCSC
−200
−100
0
100
Enrichment (prop h2 / prop snps)

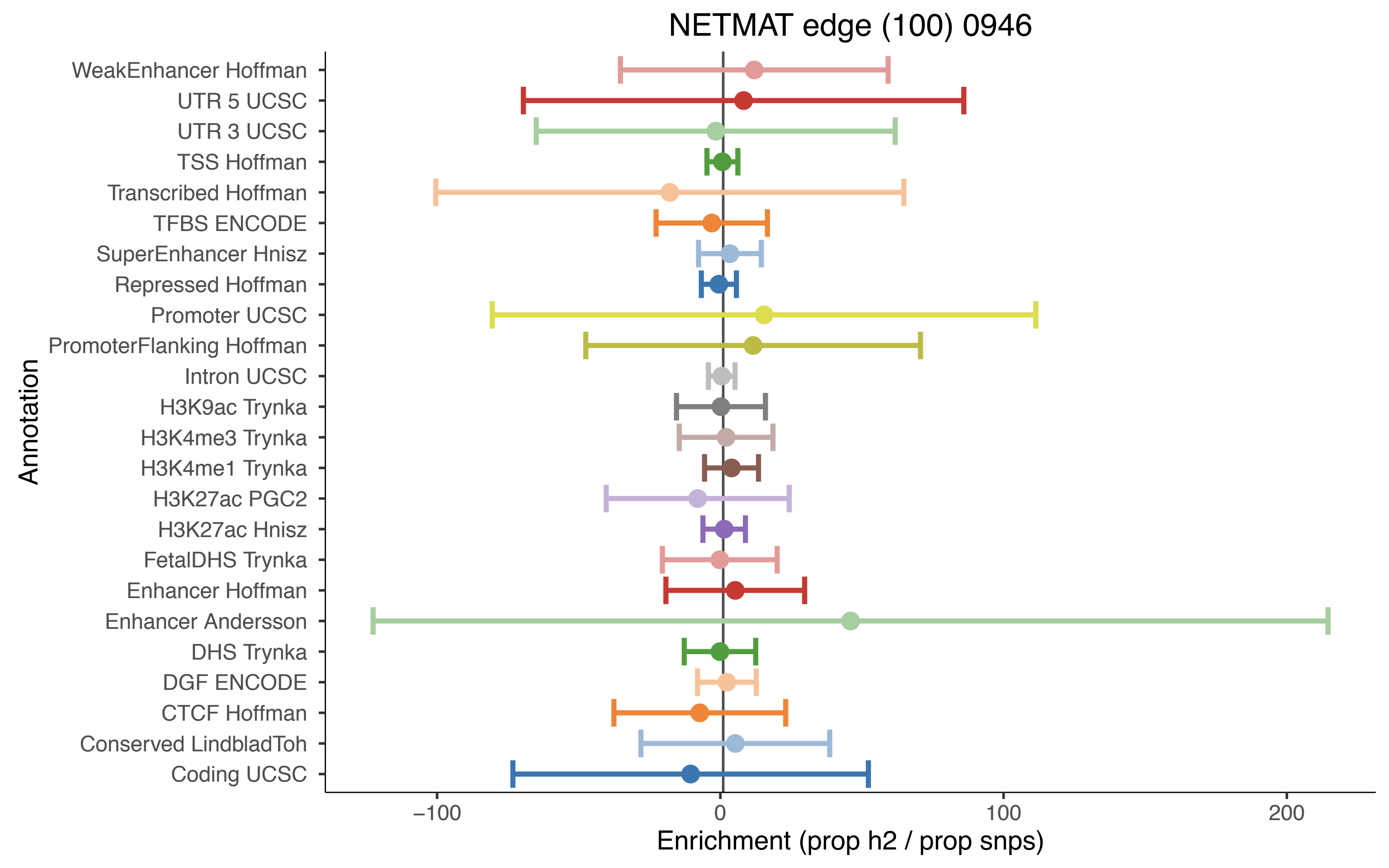

NETMAT edge (100) 0946
WeakEnhancer Hoffman
UTR 5 UCSC
UTR 3 UCSC
TSS Hoffman
Transcribed Hoffman
TFBS ENCODE
SuperEnhancer Hnisz
Repressed Hoffman
Promoter UCSC
PromoterFlanking Hoffman
Intron UCSC
H3K9ac Trynka
H3K4me3 Trynka
H3K4me1 Trynka
H3K27ac PGC2
H3K27ac Hnisz
FetalDHS Trynka
Enhancer Hoffman
Enhancer Andersson
DHS Trynka
DGF ENCODE
CTCF Hoffman
Conserved LindbladToh
Coding UCSC
Annotation
−100
0
100
200
Enrichment (prop h2 / prop snps)

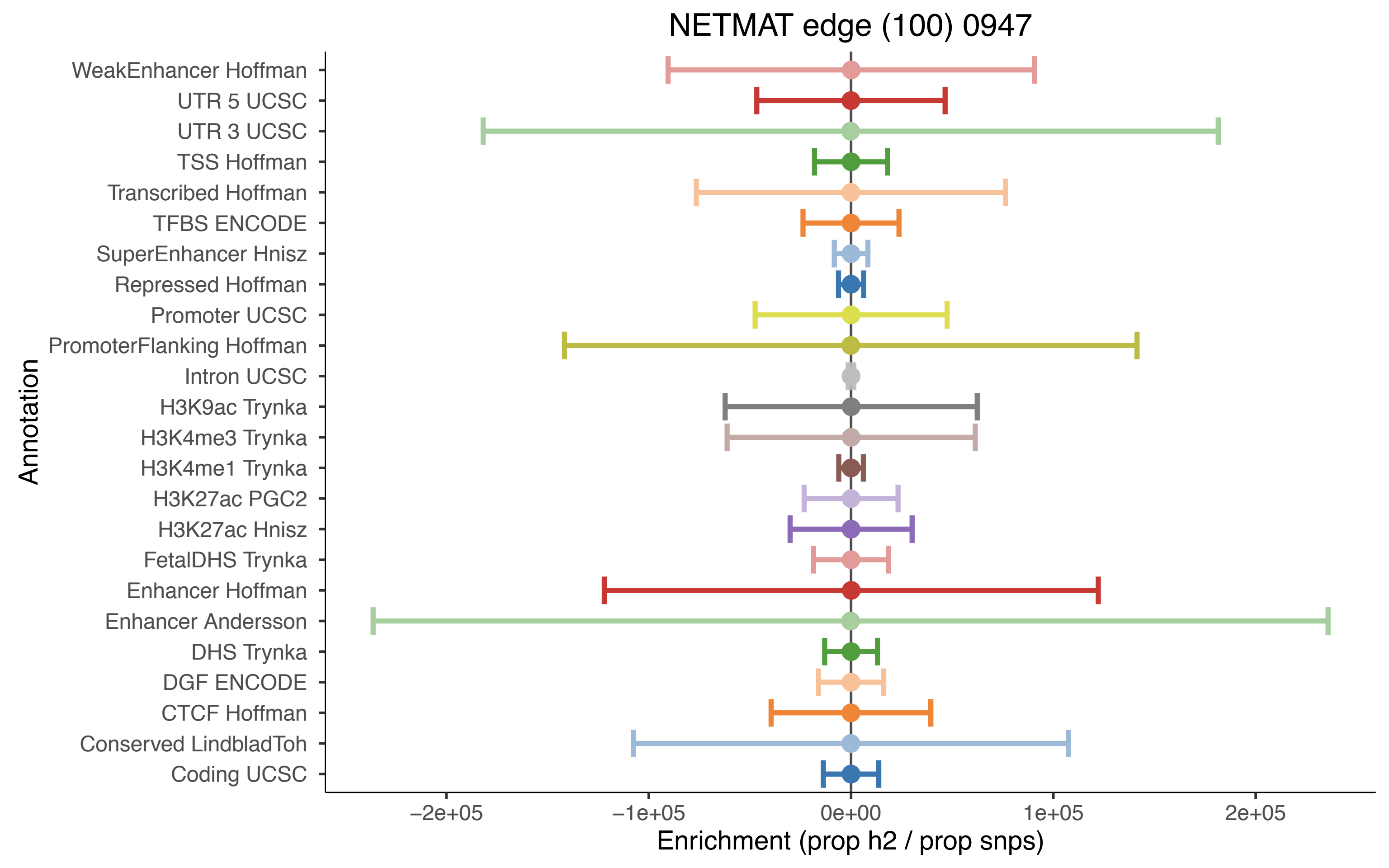
NETMAT edge (100) 0947
WeakEnhancer Hoffman
UTR 5 UCSC
UTR 3 UCSC
TSS Hoffman
Transcribed Hoffman
TFBS ENCODE
SuperEnhancer Hnisz
Repressed Hoffman
Promoter UCSC
PromoterFlanking Hoffman
Intron UCSC
H3K9ac Trynka
H3K4me3 Trynka
H3K4me1 Trynka
H3K27ac PGC2
H3K27ac Hnisz
FetalDHS Trynka
Enhancer Hoffman
Enhancer Andersson
DHS Trynka
DGF ENCODE
CTCF Hoffman
Conserved LindbladToh
Coding UCSC
Annotation
−2e+05
−1e+05
0e+00
1e+05
2e+05
Enrichment (prop h2 / prop snps)

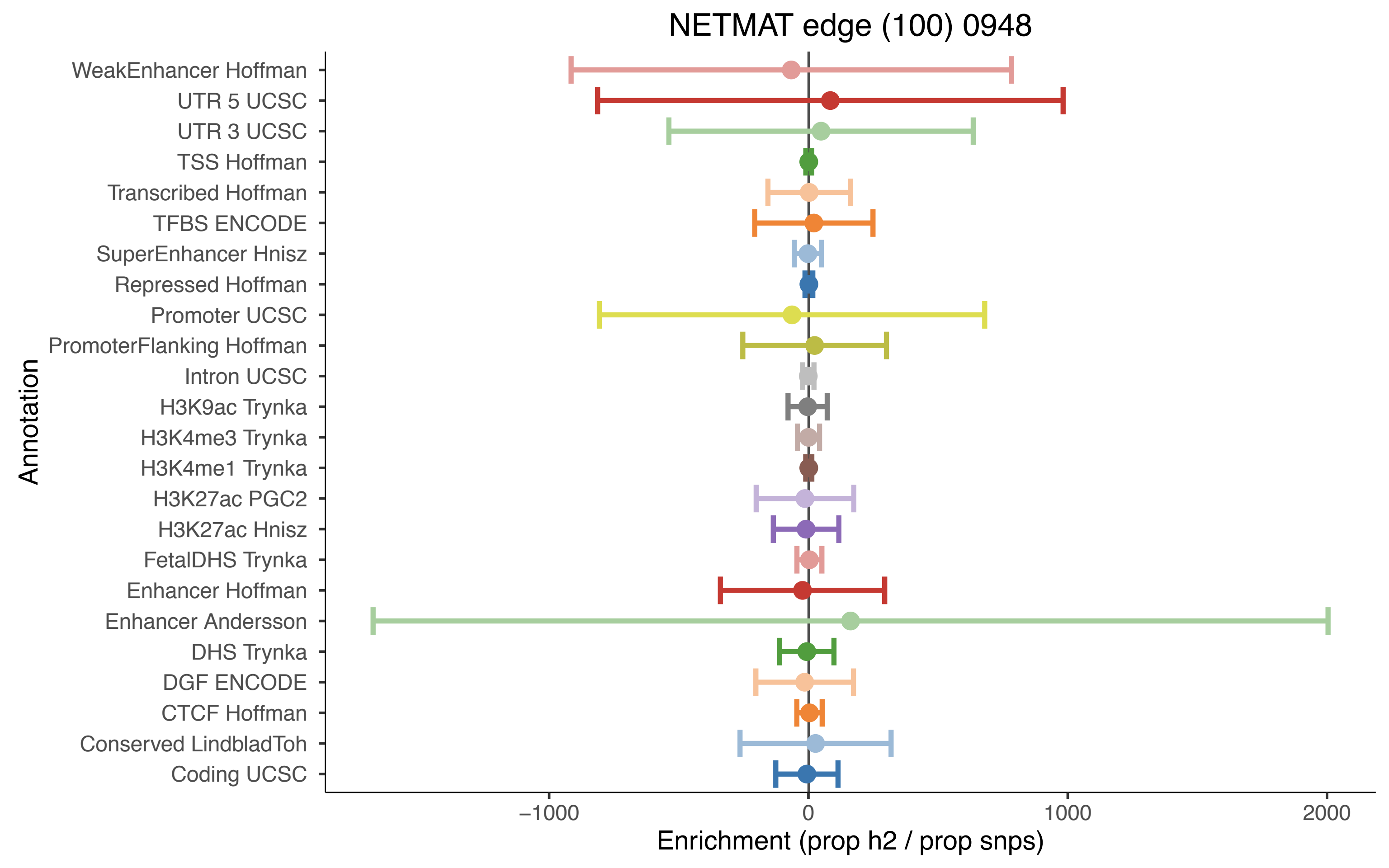

NETMAT edge (100) 0948
WeakEnhancer Hoffman
UTR 5 UCSC
UTR 3 UCSC
TSS Hoffman
Transcribed Hoffman
TFBS ENCODE
SuperEnhancer Hnisz
Repressed Hoffman
Promoter UCSC
PromoterFlanking Hoffman
Intron UCSC
H3K9ac Trynka
H3K4me3 Trynka
H3K4me1 Trynka
H3K27ac PGC2
H3K27ac Hnisz
FetalDHS Trynka
Enhancer Hoffman
Enhancer Andersson
DHS Trynka
DGF ENCODE
CTCF Hoffman
Conserved LindbladToh
Coding UCSC
Annotation
−1000
0
1000
2000
Enrichment (prop h2 / prop snps)

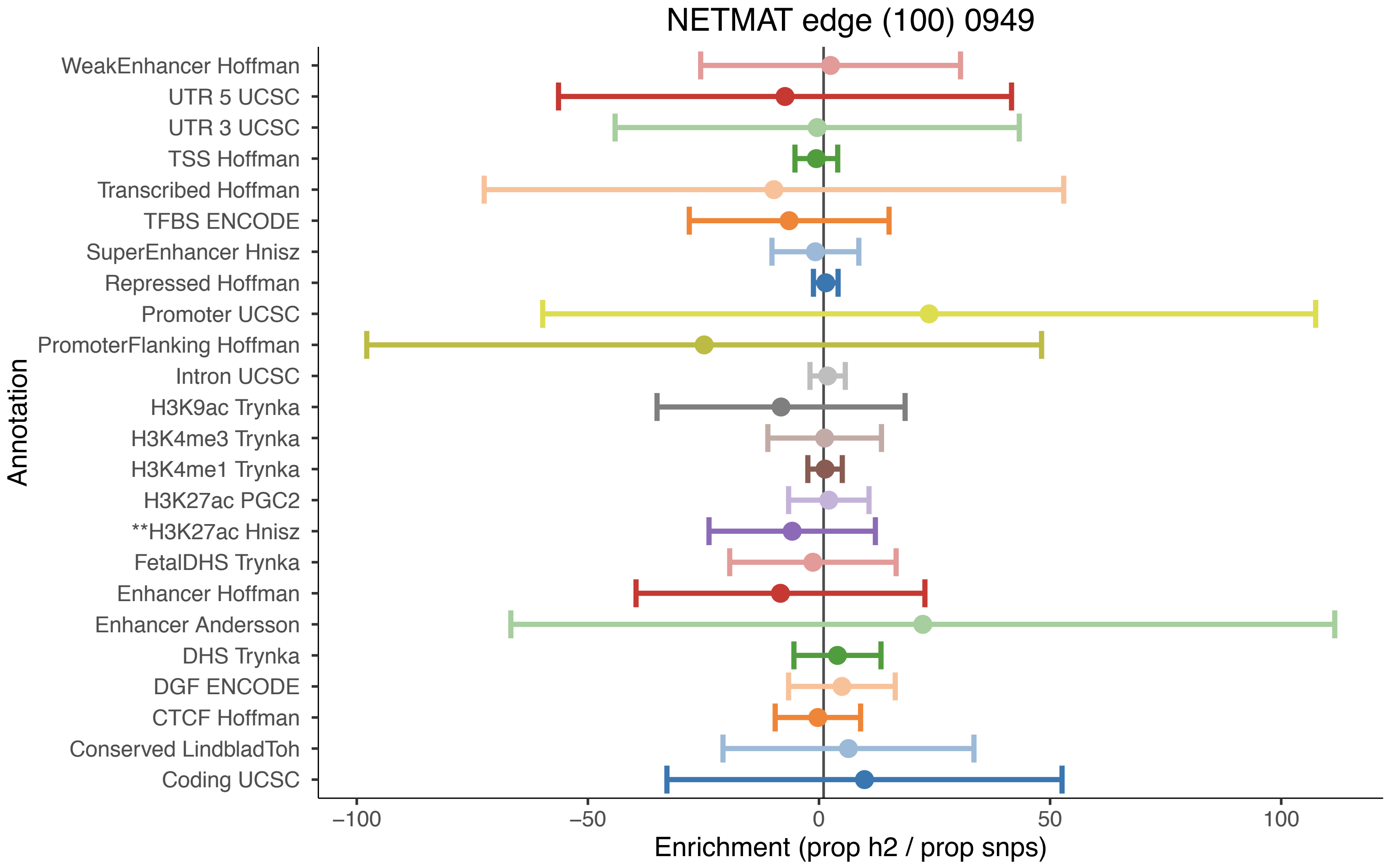
NETMAT edge (100) 0949
WeakEnhancer Hoffman
UTR 5 UCSC
UTR 3 UCSC
TSS Hoffman
Transcribed Hoffman
TFBS ENCODE
SuperEnhancer Hnisz
Repressed Hoffman
Promoter UCSC
PromoterFlanking Hoffman
Intron UCSC
H3K9ac Trynka
H3K4me3 Trynka
H3K4me1 Trynka
H3K27ac PGC2
**H3K27ac Hnisz
FetalDHS Trynka
Enhancer Hoffman
Enhancer Andersson
DHS Trynka
DGF ENCODE
CTCF Hoffman
Conserved LindbladToh
Coding UCSC
Annotation
−100
−50
0
50
100
Enrichment (prop h2 / prop snps)

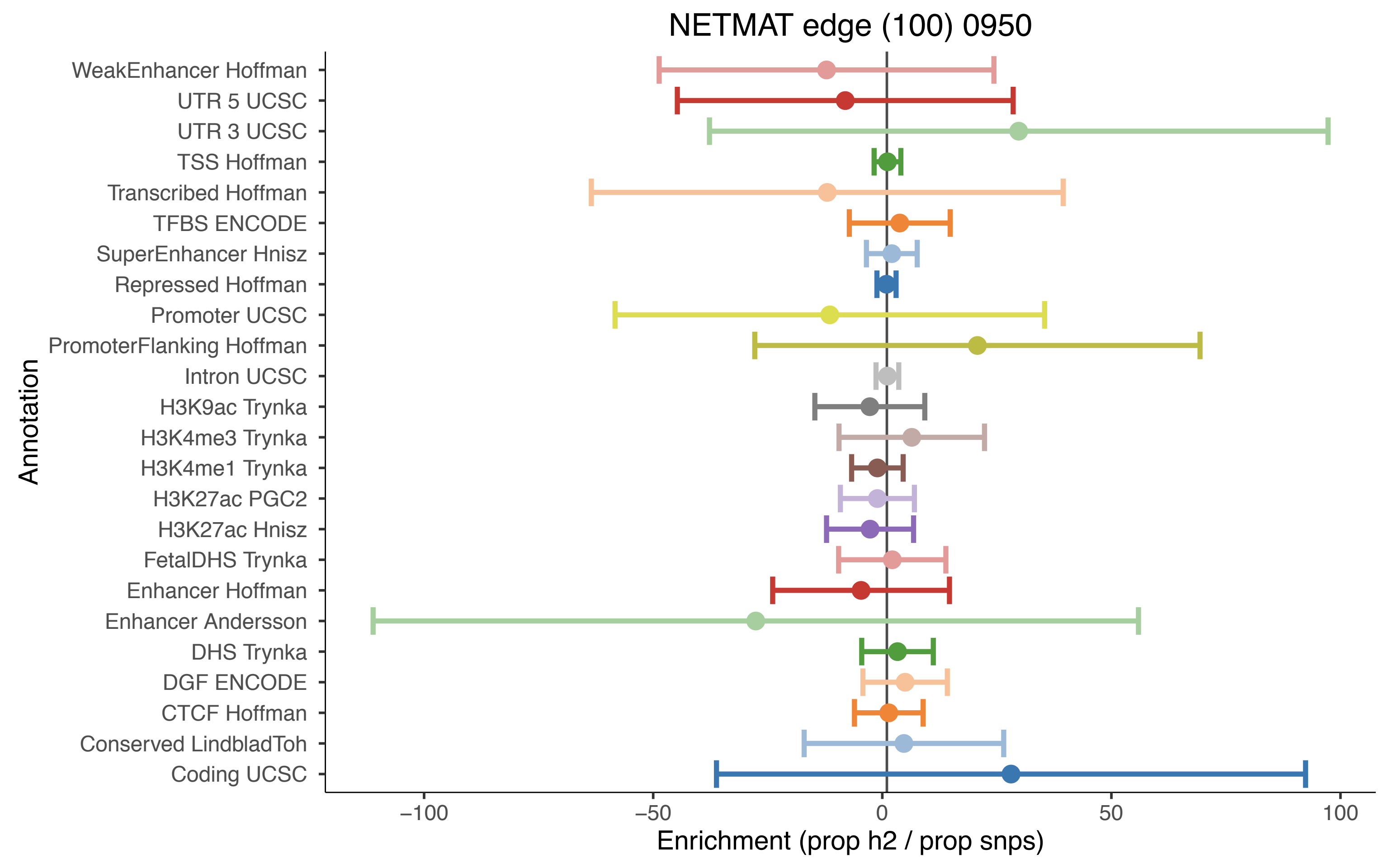

NETMAT edge (100) 0950
Annotation
WeakEnhancer Hoffman
UTR 5 UCSC
UTR 3 UCSC
TSS Hoffman
Transcribed Hoffman
TFBS ENCODE
SuperEnhancer Hnisz
Repressed Hoffman
Promoter UCSC
PromoterFlanking Hoffman
Intron UCSC
H3K9ac Trynka
H3K4me3 Trynka
H3K4me1 Trynka
H3K27ac PGC2
H3K27ac Hnisz
FetalDHS Trynka
Enhancer Hoffman
Enhancer Andersson
DHS Trynka
DGF ENCODE
CTCF Hoffman
Conserved LindbladToh
Coding UCSC
−100
−50
0
50
100
Enrichment (prop h2 / prop snps)

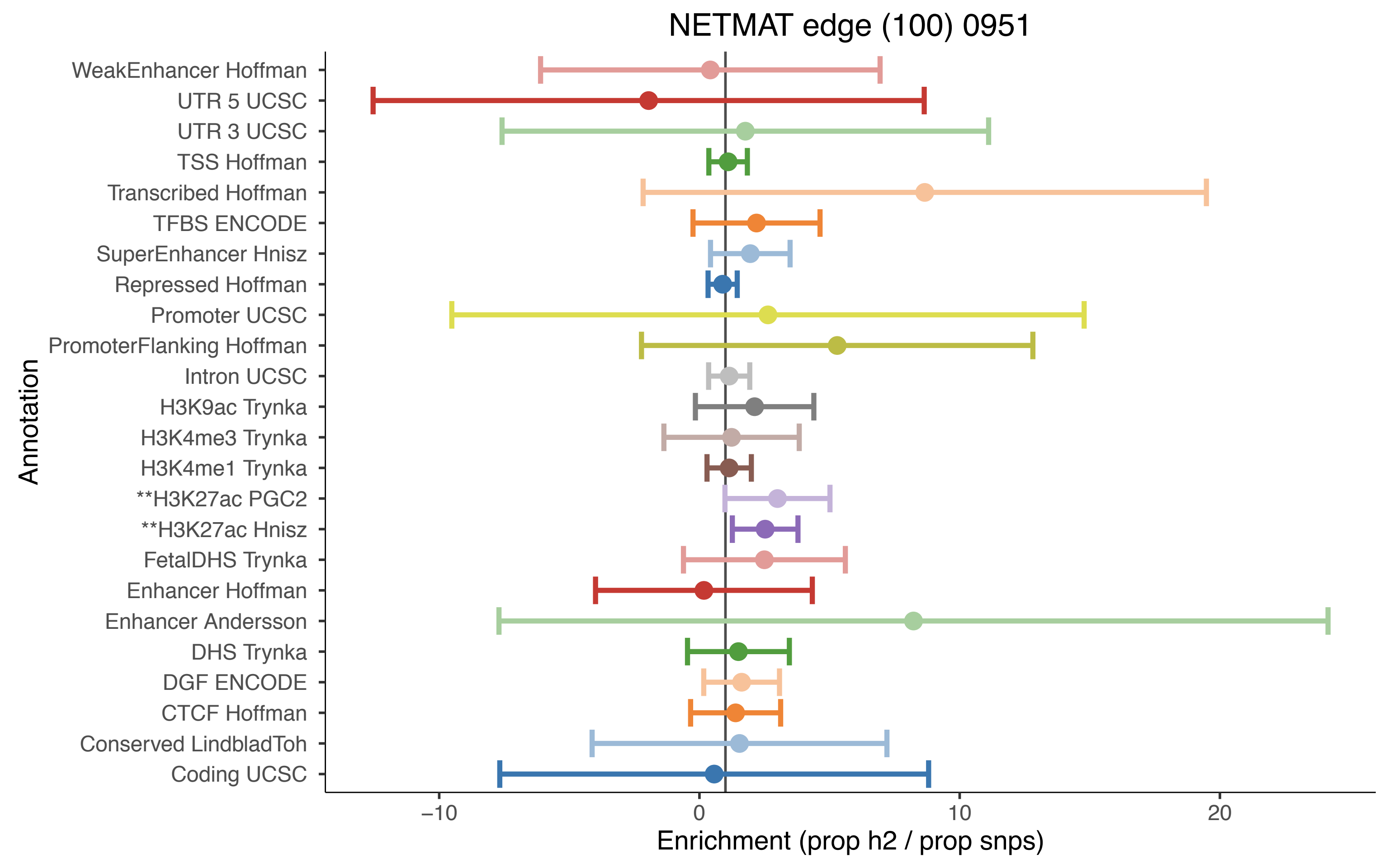

NETMAT edge (100) 0951
WeakEnhancer Hoffman
UTR 5 UCSC
UTR 3 UCSC
TSS Hoffman
Transcribed Hoffman
TFBS ENCODE
SuperEnhancer Hnisz
Repressed Hoffman
Promoter UCSC
PromoterFlanking Hoffman
Intron UCSC
H3K9ac Trynka
H3K4me3 Trynka
H3K4me1 Trynka
**H3K27ac PGC2
**H3K27ac Hnisz
FetalDHS Trynka
Enhancer Hoffman
Enhancer Andersson
DHS Trynka
DGF ENCODE
CTCF Hoffman
Conserved LindbladToh
Coding UCSC
Annotation
−10
0
10
20
Enrichment (prop h2 / prop snps)

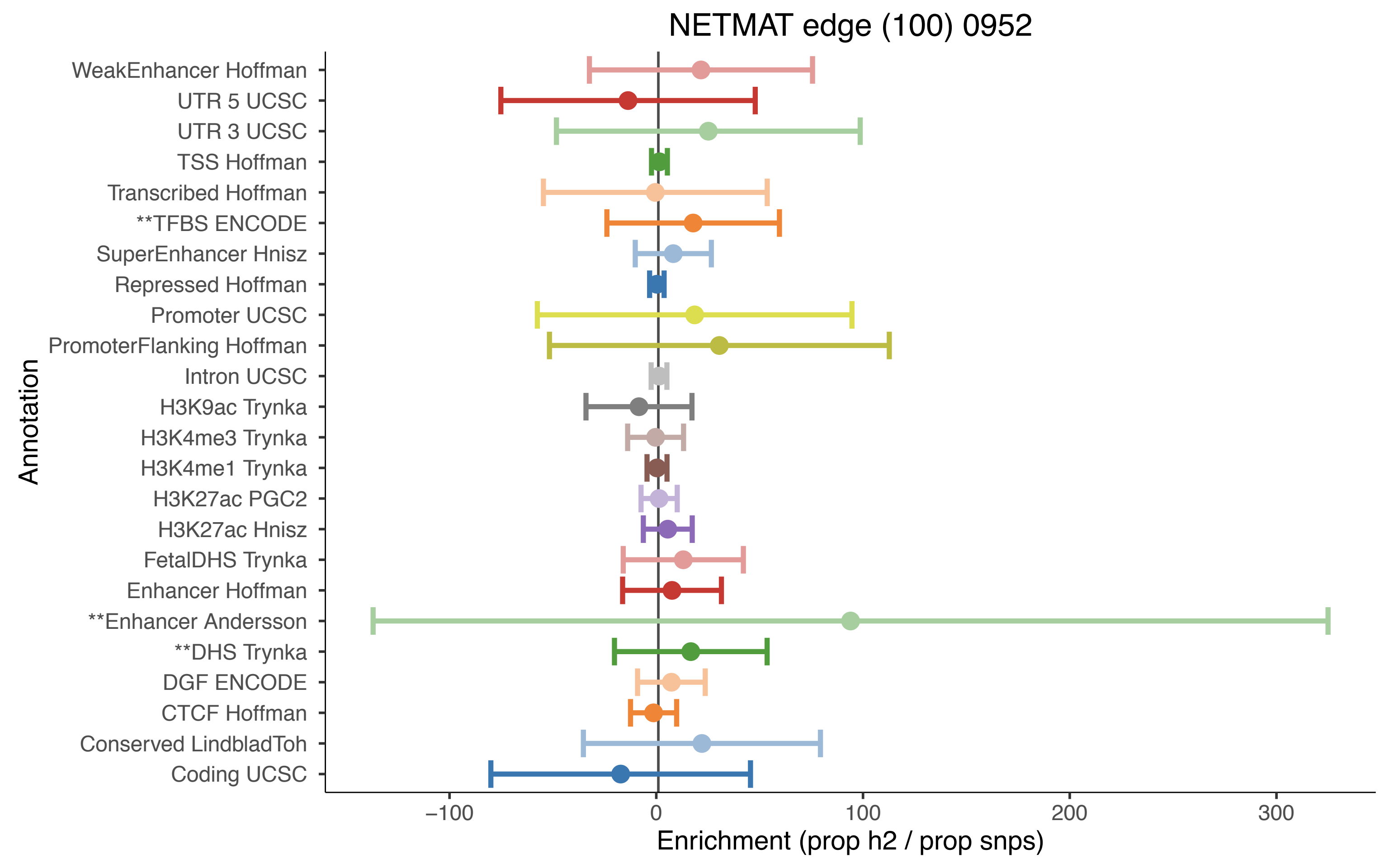

NETMAT edge (100) 0952
Annotation
WeakEnhancer Hoffman
UTR 5 UCSC
UTR 3 UCSC
TSS Hoffman
Transcribed Hoffman
**TFBS ENCODE
SuperEnhancer Hnisz
Repressed Hoffman
Promoter UCSC
PromoterFlanking Hoffman
Intron UCSC
H3K9ac Trynka
H3K4me3 Trynka
H3K4me1 Trynka
H3K27ac PGC2
H3K27ac Hnisz
FetalDHS Trynka
Enhancer Hoffman
**Enhancer Andersson
**DHS Trynka
DGF ENCODE
CTCF Hoffman
Conserved LindbladToh
Coding UCSC
−100
0
100
200
300
Enrichment (prop h2 / prop snps)

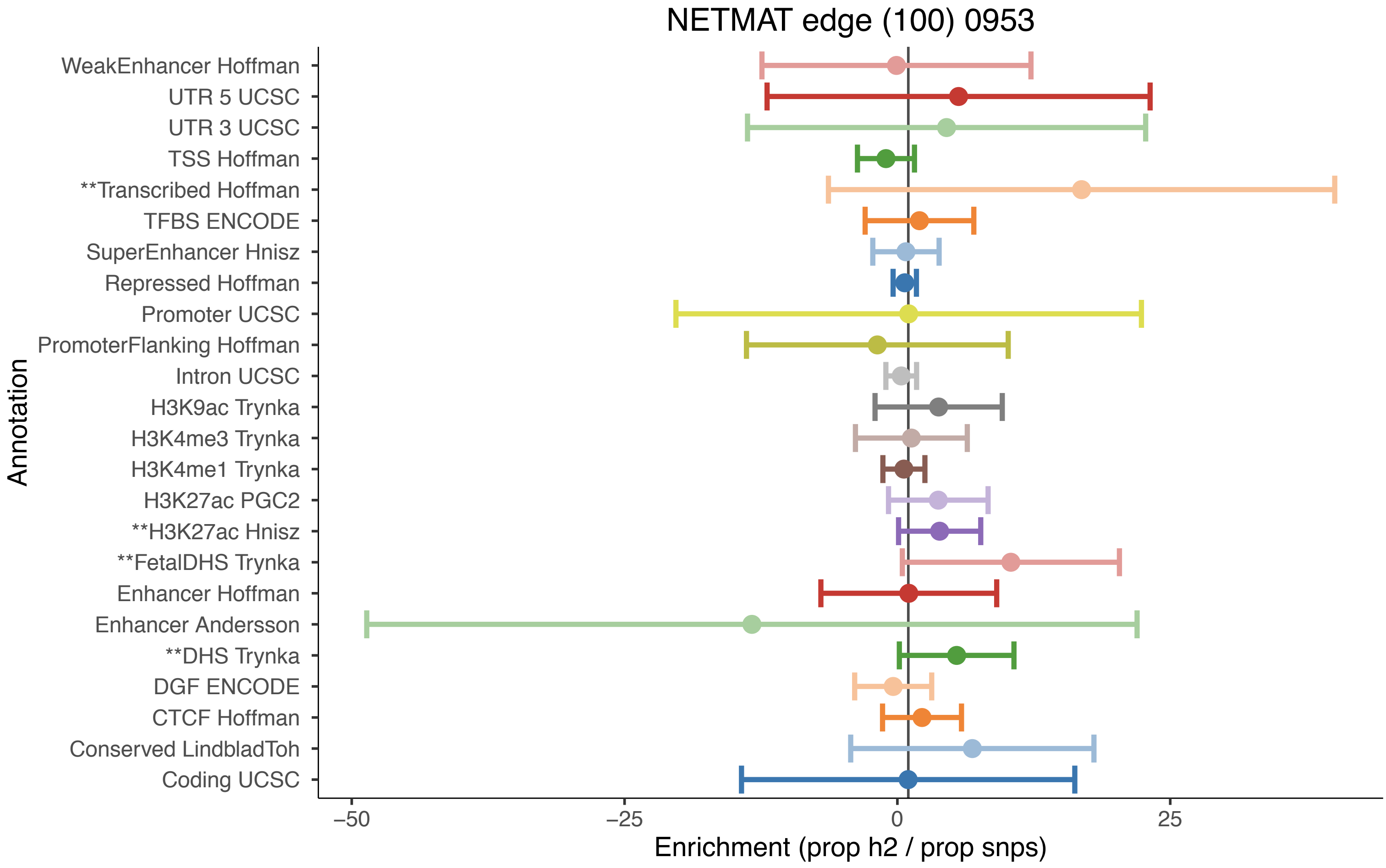
NETMAT edge (100) 0953
Annotation
WeakEnhancer Hoffman
UTR 5 UCSC
UTR 3 UCSC
TSS Hoffman
**Transcribed Hoffman
TFBS ENCODE
SuperEnhancer Hnisz
Repressed Hoffman
Promoter UCSC
PromoterFlanking Hoffman
Intron UCSC
H3K9ac Trynka
H3K4me3 Trynka
H3K4me1 Trynka
H3K27ac PGC2
**H3K27ac Hnisz
**FetalDHS Trynka
Enhancer Hoffman
Enhancer Andersson
**DHS Trynka
DGF ENCODE
CTCF Hoffman
Conserved LindbladToh
Coding UCSC
−50
−25
0
25
Enrichment (prop h2 / prop snps)

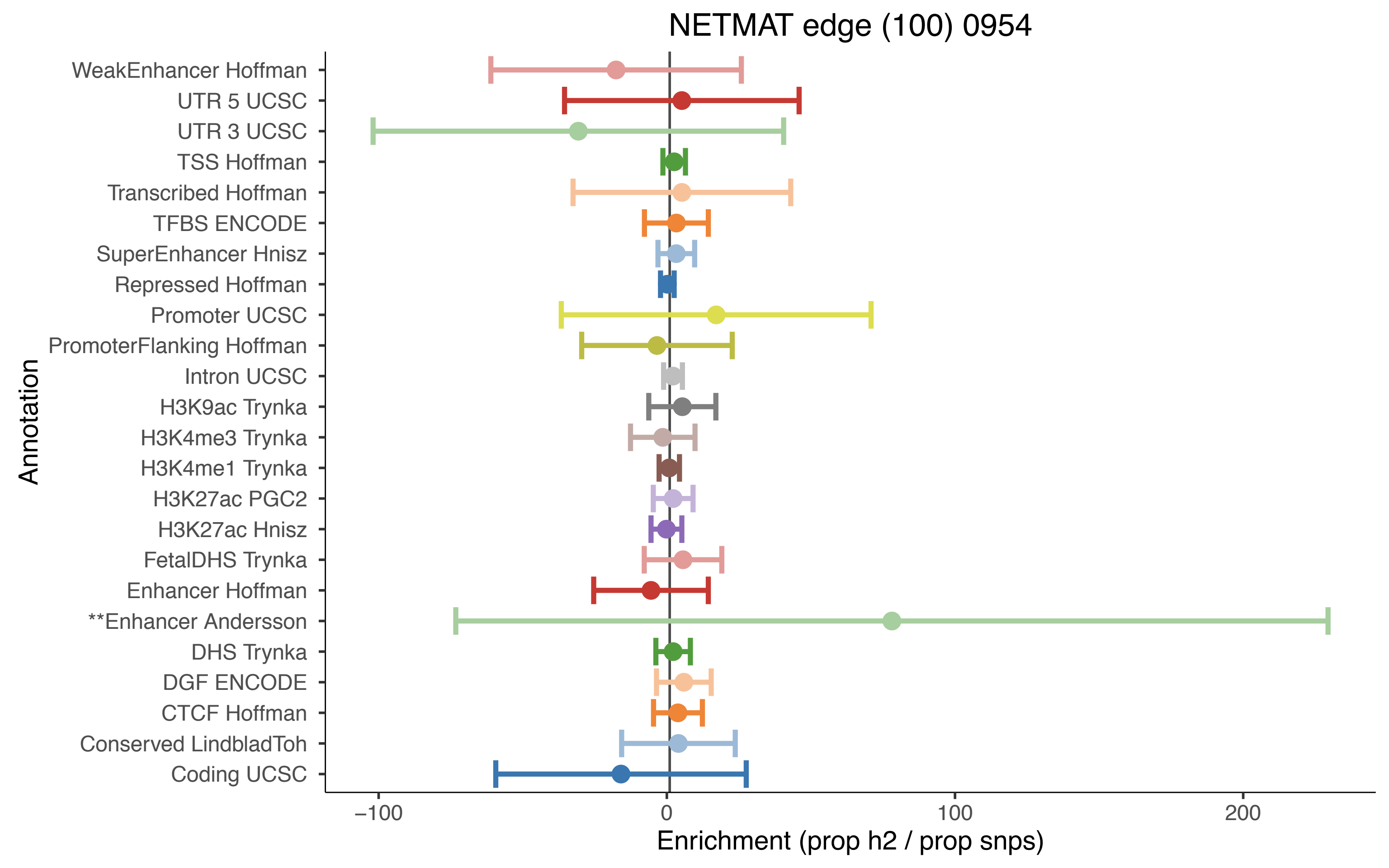
NETMAT edge (100) 0954
Annotation
WeakEnhancer Hoffman
UTR 5 UCSC
UTR 3 UCSC
TSS Hoffman
Transcribed Hoffman
TFBS ENCODE
SuperEnhancer Hnisz
Repressed Hoffman
Promoter UCSC
PromoterFlanking Hoffman
Intron UCSC
H3K9ac Trynka
H3K4me3 Trynka
H3K4me1 Trynka
H3K27ac PGC2
H3K27ac Hnisz
FetalDHS Trynka
Enhancer Hoffman
**Enhancer Andersson
DHS Trynka
DGF ENCODE
CTCF Hoffman
Conserved LindbladToh
Coding UCSC
−100
0
100
200
Enrichment (prop h2 / prop snps)

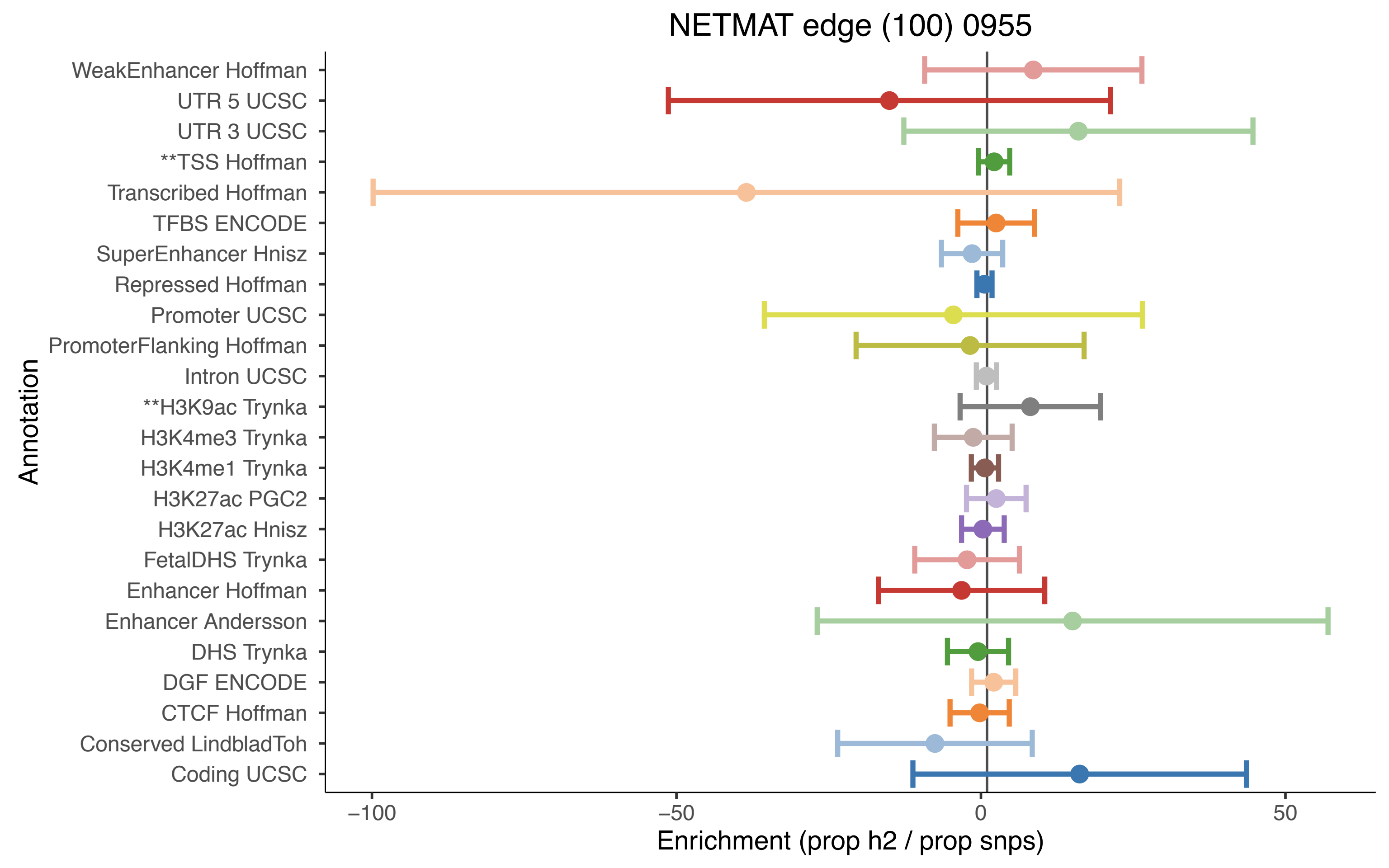

NETMAT edge (100) 0955
WeakEnhancer Hoffman
UTR 5 UCSC
UTR 3 UCSC
**TSS Hoffman
Transcribed Hoffman
TFBS ENCODE
SuperEnhancer Hnisz
Repressed Hoffman
Promoter UCSC
PromoterFlanking Hoffman
Intron UCSC
**H3K9ac Trynka
H3K4me3 Trynka
H3K4me1 Trynka
H3K27ac PGC2
H3K27ac Hnisz
FetalDHS Trynka
Enhancer Hoffman
Enhancer Andersson
DHS Trynka
DGF ENCODE
CTCF Hoffman
Conserved LindbladToh
Coding UCSC
Annotation
−100
−50
0
50
Enrichment (prop h2 / prop snps)

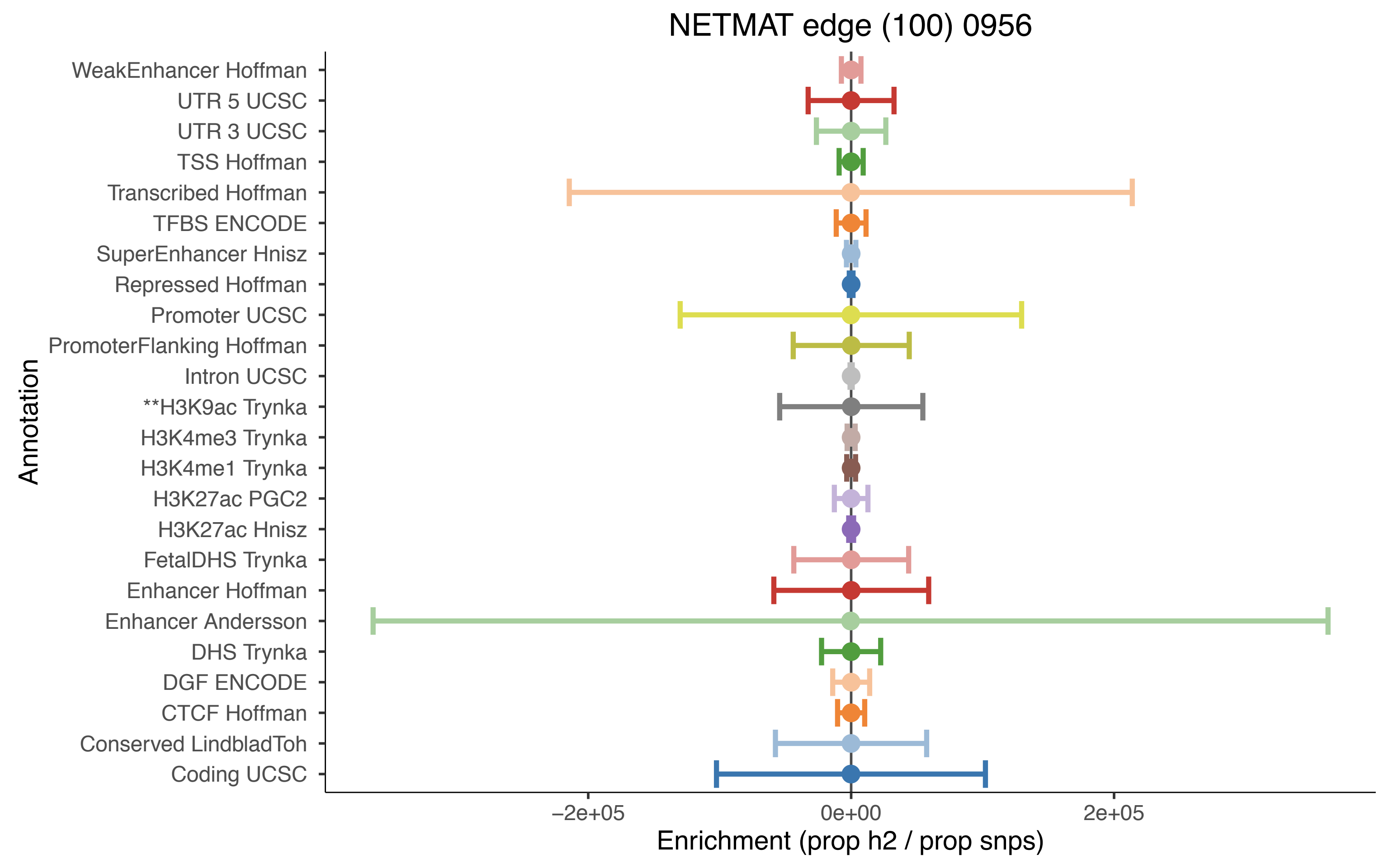

NETMAT edge (100) 0956
Annotation
WeakEnhancer Hoffman
UTR 5 UCSC
UTR 3 UCSC
TSS Hoffman
Transcribed Hoffman
TFBS ENCODE
SuperEnhancer Hnisz
Repressed Hoffman
Promoter UCSC
PromoterFlanking Hoffman
Intron UCSC
**H3K9ac Trynka
H3K4me3 Trynka
H3K4me1 Trynka
H3K27ac PGC2
H3K27ac Hnisz
FetalDHS Trynka
Enhancer Hoffman
Enhancer Andersson
DHS Trynka
DGF ENCODE
CTCF Hoffman
Conserved LindbladToh
Coding UCSC
−2e+05
0e+00
2e+05
Enrichment (prop h2 / prop snps)

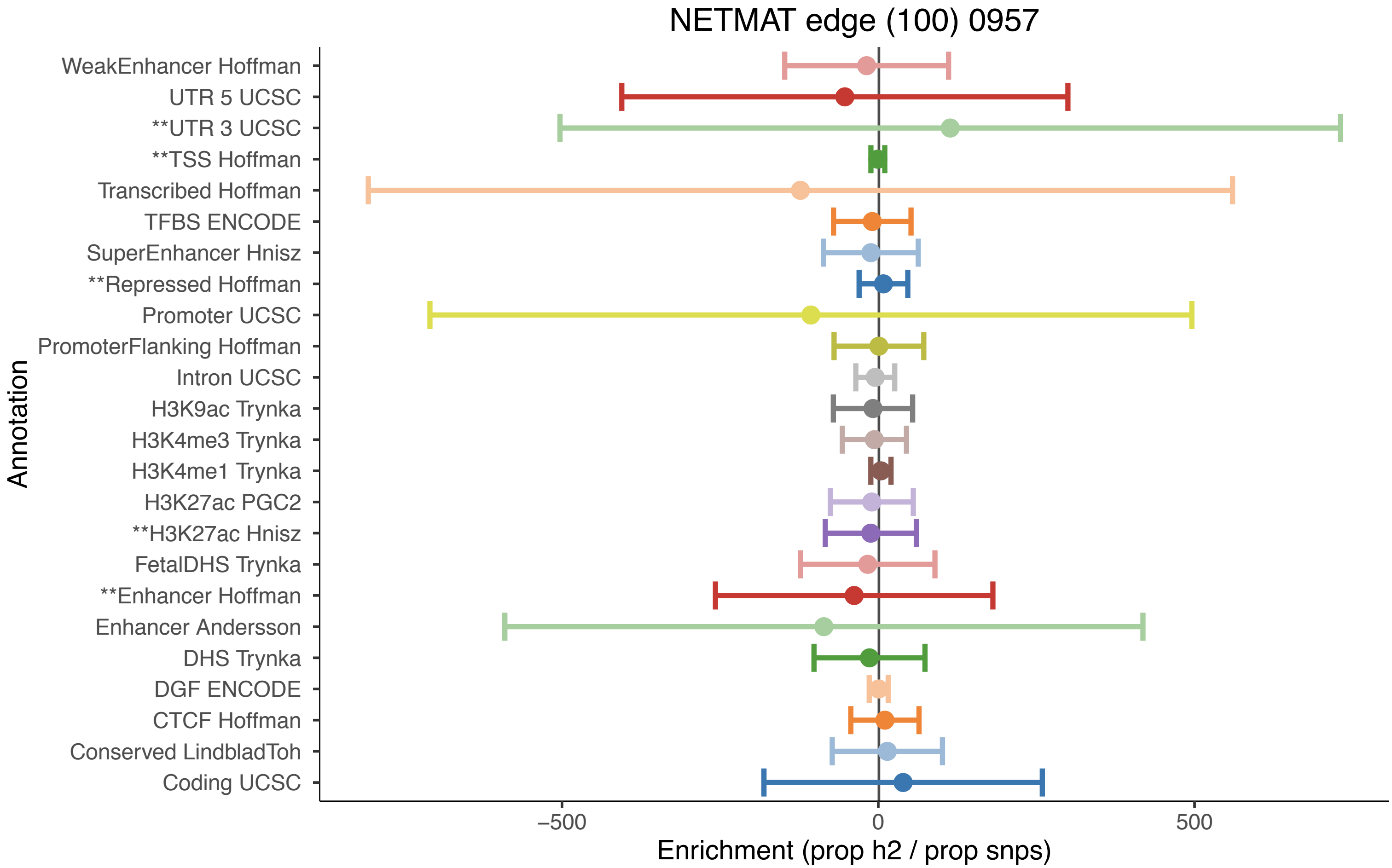
NETMAT edge (100) 0957
WeakEnhancer Hoffman
UTR 5 UCSC
**UTR 3 UCSC
**TSS Hoffman
Transcribed Hoffman
TFBS ENCODE
SuperEnhancer Hnisz
**Repressed Hoffman
Promoter UCSC
PromoterFlanking Hoffman
Intron UCSC
H3K9ac Trynka
H3K4me3 Trynka
H3K4me1 Trynka
H3K27ac PGC2
**H3K27ac Hnisz
FetalDHS Trynka
**Enhancer Hoffman
Enhancer Andersson
DHS Trynka
DGF ENCODE
CTCF Hoffman
Conserved LindbladToh
Coding UCSC
Annotation
−500
0
500
Enrichment (prop h2 / prop snps)

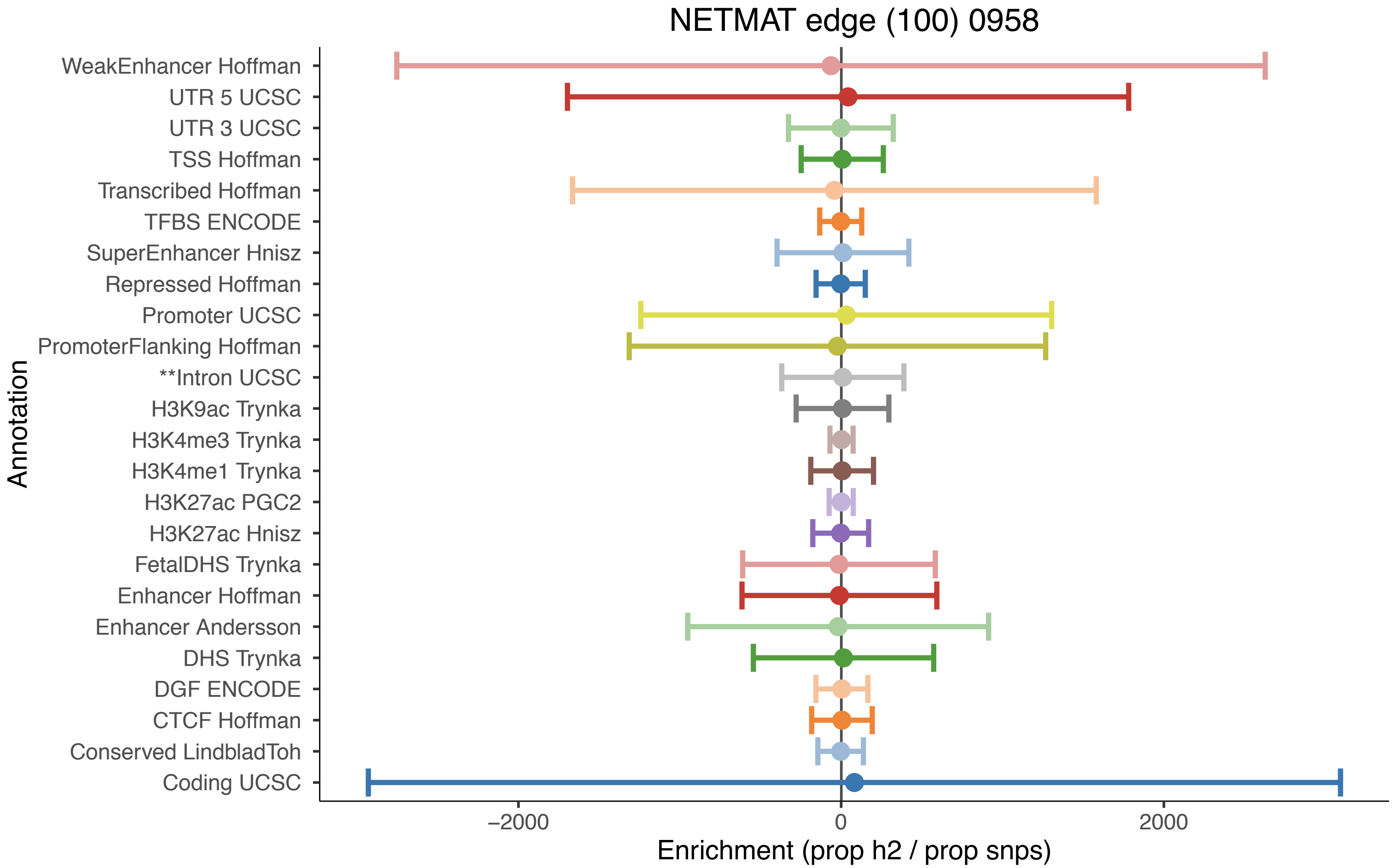
NETMAT edge (100) 0958
Annotation
WeakEnhancer Hoffman
UTR 5 UCSC
UTR 3 UCSC
TSS Hoffman
Transcribed Hoffman
TFBS ENCODE
SuperEnhancer Hnisz
Repressed Hoffman
Promoter UCSC
PromoterFlanking Hoffman
**Intron UCSC
H3K9ac Trynka
H3K4me3 Trynka
H3K4me1 Trynka
H3K27ac PGC2
H3K27ac Hnisz
FetalDHS Trynka
Enhancer Hoffman
Enhancer Andersson
DHS Trynka
DGF ENCODE
CTCF Hoffman
Conserved LindbladToh
Coding UCSC
−2000
0
2000
Enrichment (prop h2 / prop snps)

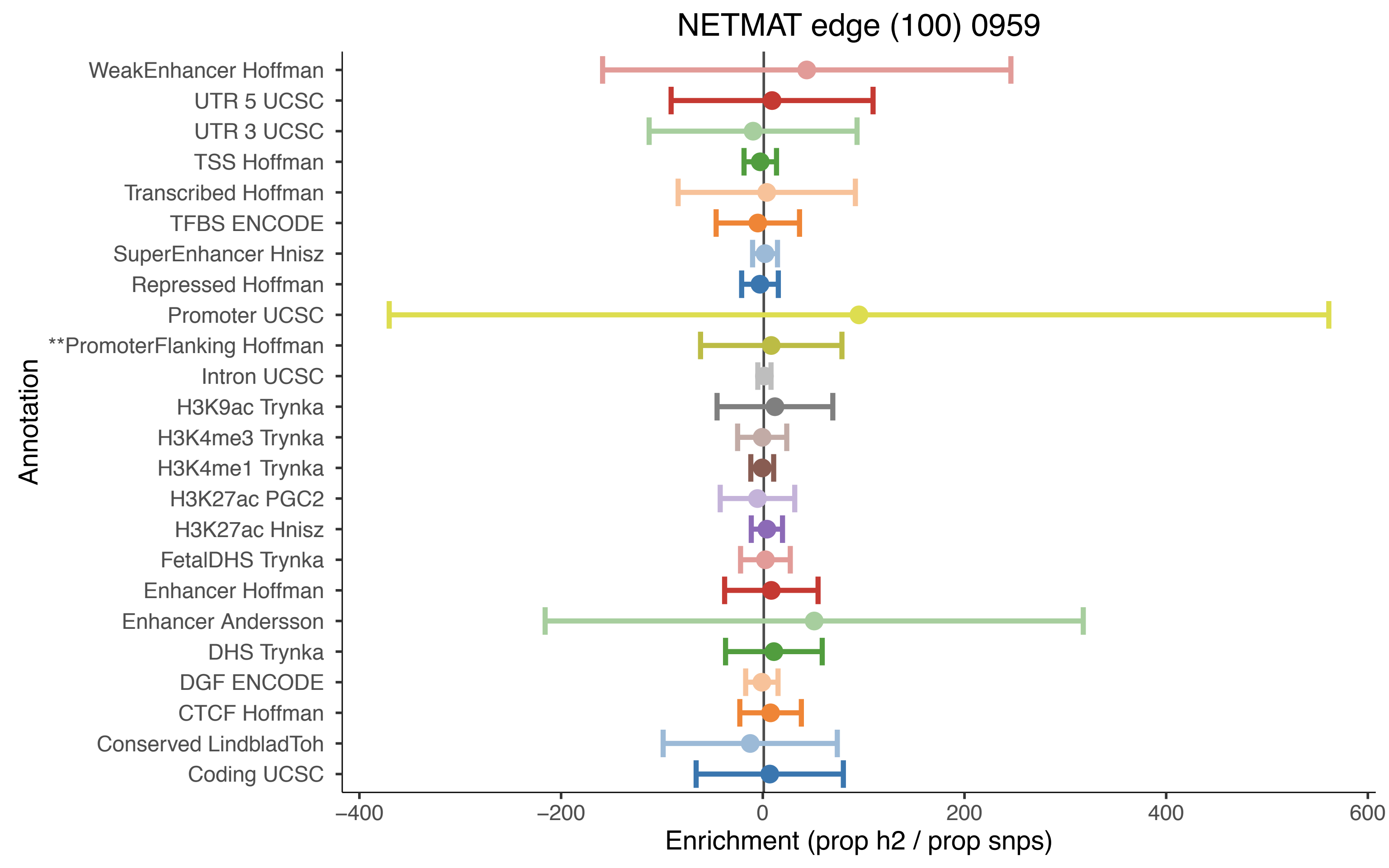

NETMAT edge (100) 0959
WeakEnhancer Hoffman
UTR 5 UCSC
UTR 3 UCSC
TSS Hoffman
Transcribed Hoffman
TFBS ENCODE
SuperEnhancer Hnisz
Repressed Hoffman
Promoter UCSC
**PromoterFlanking Hoffman
Intron UCSC
H3K9ac Trynka
H3K4me3 Trynka
H3K4me1 Trynka
H3K27ac PGC2
H3K27ac Hnisz
FetalDHS Trynka
Enhancer Hoffman
Enhancer Andersson
DHS Trynka
DGF ENCODE
CTCF Hoffman
Conserved LindbladToh
Coding UCSC
Annotation
−400
−200
0
200
400
600
Enrichment (prop h2 / prop snps)

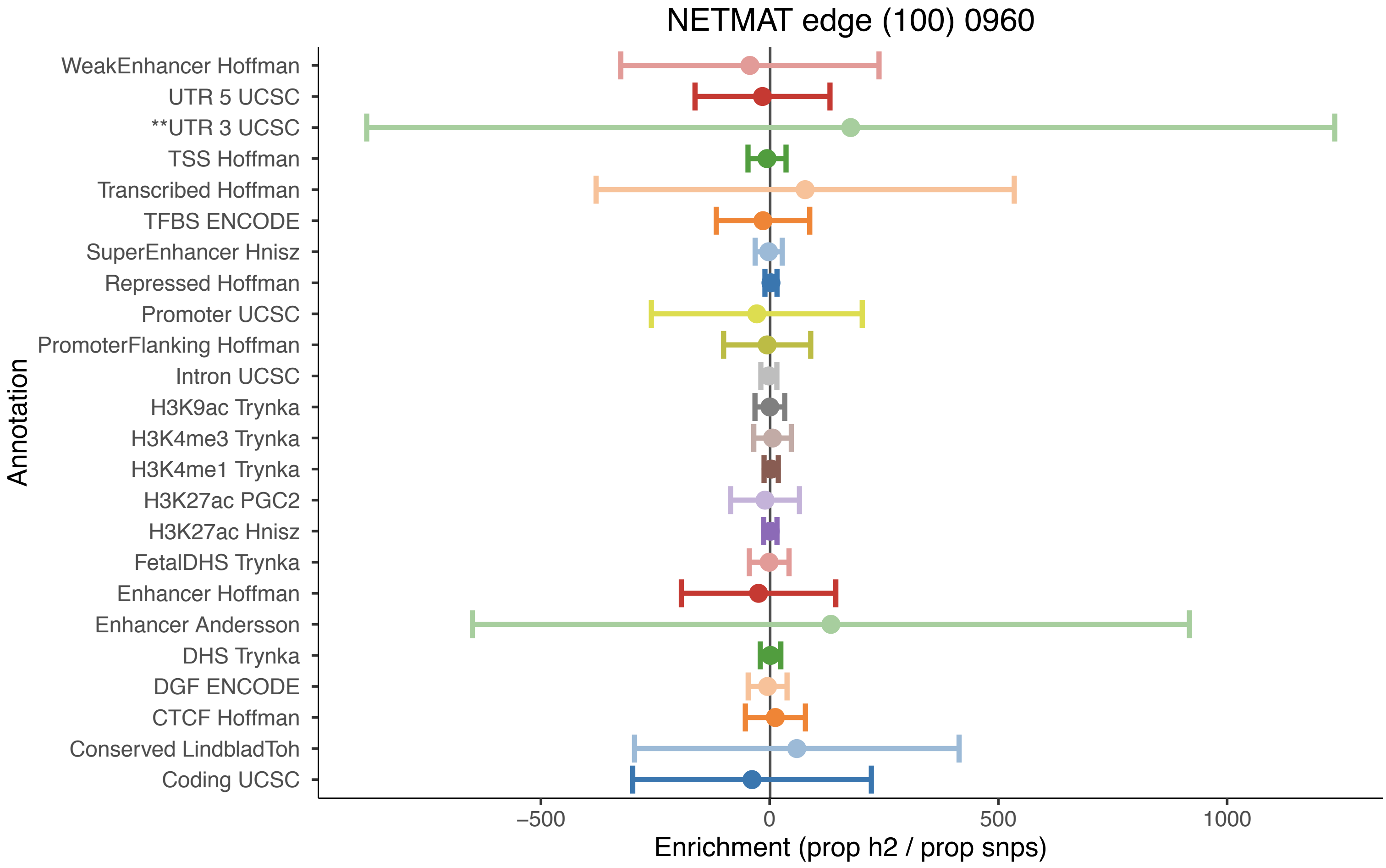

NETMAT edge (100) 0960
WeakEnhancer Hoffman
UTR 5 UCSC
**UTR 3 UCSC
TSS Hoffman
Transcribed Hoffman
TFBS ENCODE
SuperEnhancer Hnisz
Repressed Hoffman
Promoter UCSC
PromoterFlanking Hoffman
Intron UCSC
H3K9ac Trynka
H3K4me3 Trynka
H3K4me1 Trynka
H3K27ac PGC2
H3K27ac Hnisz
FetalDHS Trynka
Enhancer Hoffman
Enhancer Andersson
DHS Trynka
DGF ENCODE
CTCF Hoffman
Conserved LindbladToh
Coding UCSC
Annotation
−500
0
500
1000
Enrichment (prop h2 / prop snps)

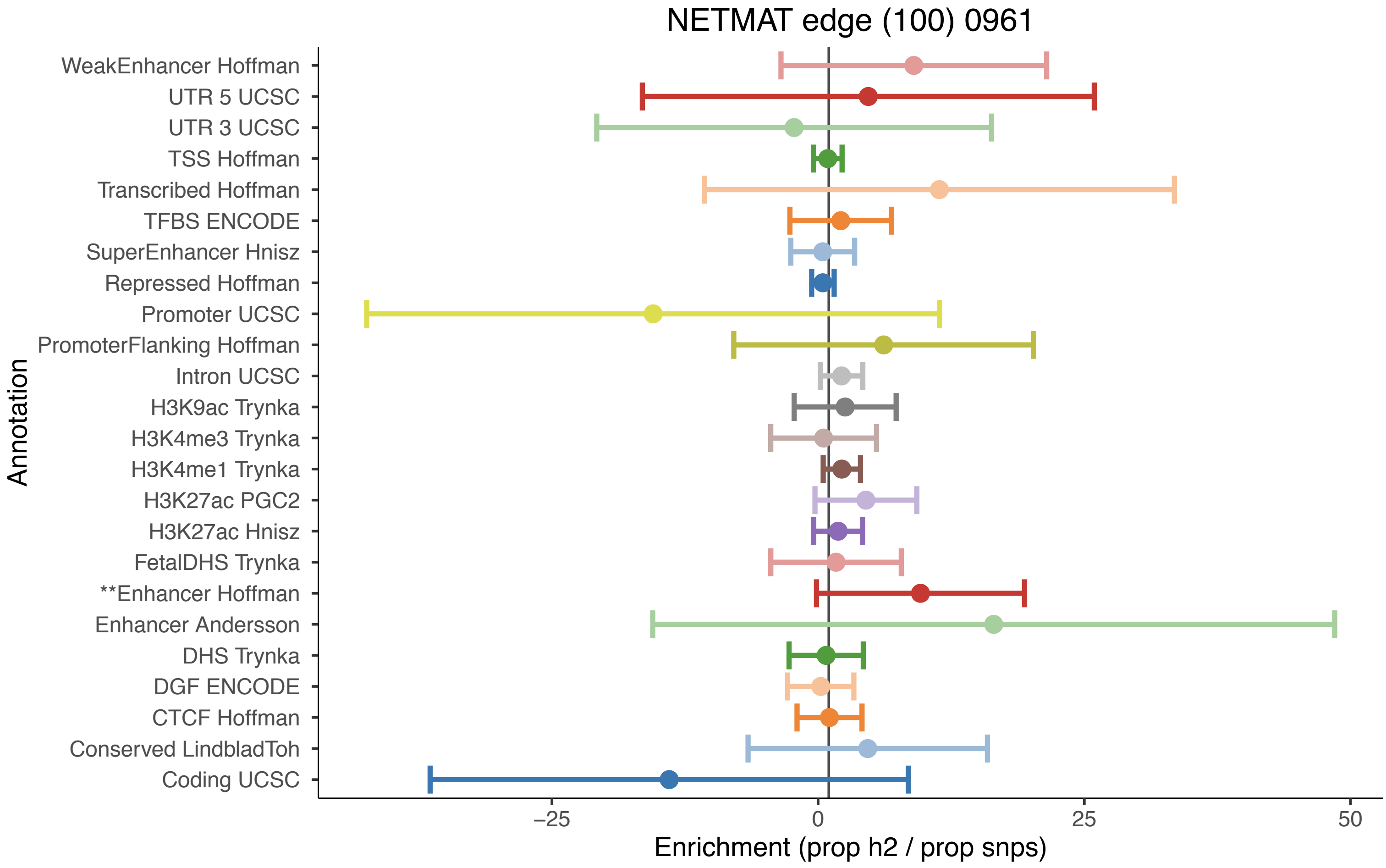
NETMAT edge (100) 0961
WeakEnhancer Hoffman
UTR 5 UCSC
UTR 3 UCSC
TSS Hoffman
Transcribed Hoffman
TFBS ENCODE
SuperEnhancer Hnisz
Repressed Hoffman
Promoter UCSC
PromoterFlanking Hoffman
Intron UCSC
H3K9ac Trynka
H3K4me3 Trynka
H3K4me1 Trynka
H3K27ac PGC2
H3K27ac Hnisz
FetalDHS Trynka
**Enhancer Hoffman
Enhancer Andersson
DHS Trynka
DGF ENCODE
CTCF Hoffman
Conserved LindbladToh
Coding UCSC
Annotation
−25
0
25
50
Enrichment (prop h2 / prop snps)

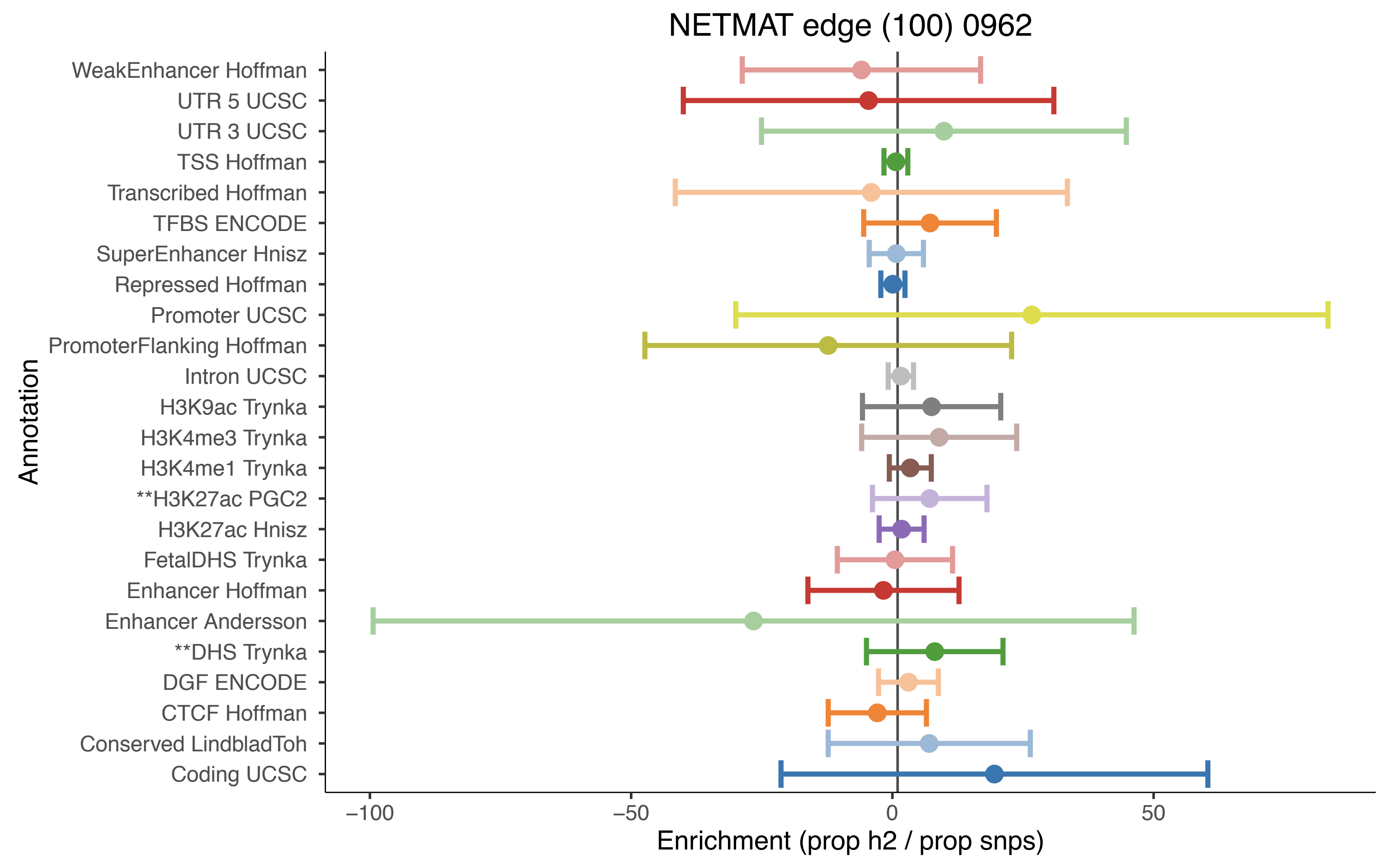
NETMAT edge (100) 0962
WeakEnhancer Hoffman
UTR 5 UCSC
UTR 3 UCSC
TSS Hoffman
Transcribed Hoffman
TFBS ENCODE
SuperEnhancer Hnisz
Repressed Hoffman
Promoter UCSC
PromoterFlanking Hoffman
Intron UCSC
H3K9ac Trynka
H3K4me3 Trynka
H3K4me1 Trynka
**H3K27ac PGC2
H3K27ac Hnisz
FetalDHS Trynka
Enhancer Hoffman
Enhancer Andersson
**DHS Trynka
DGF ENCODE
CTCF Hoffman
Conserved LindbladToh
Coding UCSC
Annotation
−100
−50
0
50
Enrichment (prop h2 / prop snps)

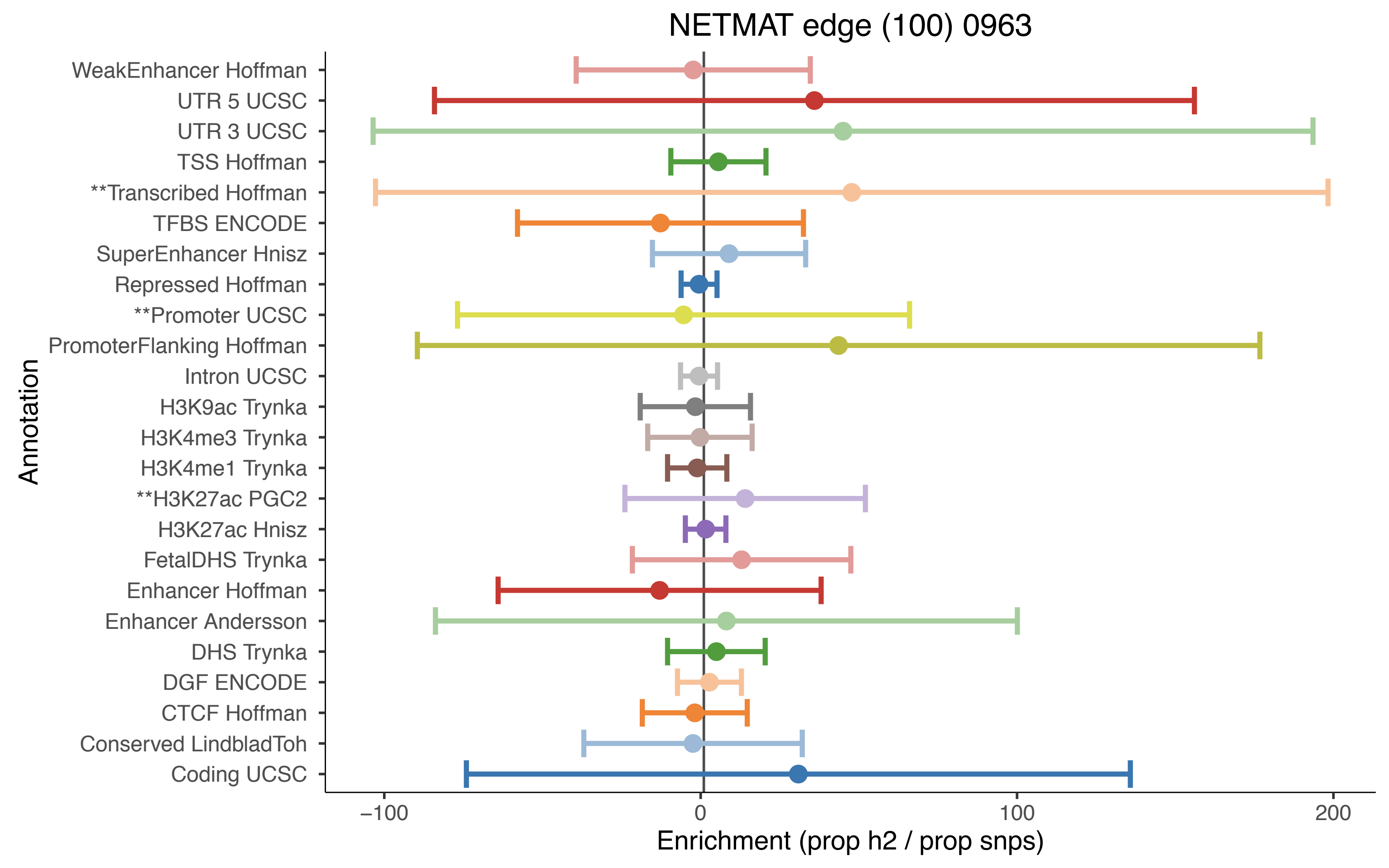

NETMAT edge (100) 0963
WeakEnhancer Hoffman
UTR 5 UCSC
UTR 3 UCSC
TSS Hoffman
**Transcribed Hoffman
TFBS ENCODE
SuperEnhancer Hnisz
Repressed Hoffman
**Promoter UCSC
PromoterFlanking Hoffman
Intron UCSC
H3K9ac Trynka
H3K4me3 Trynka
H3K4me1 Trynka
**H3K27ac PGC2
H3K27ac Hnisz
FetalDHS Trynka
Enhancer Hoffman
Enhancer Andersson
DHS Trynka
DGF ENCODE
CTCF Hoffman
Conserved LindbladToh
Coding UCSC
Annotation
−100
0
100
200
Enrichment (prop h2 / prop snps)

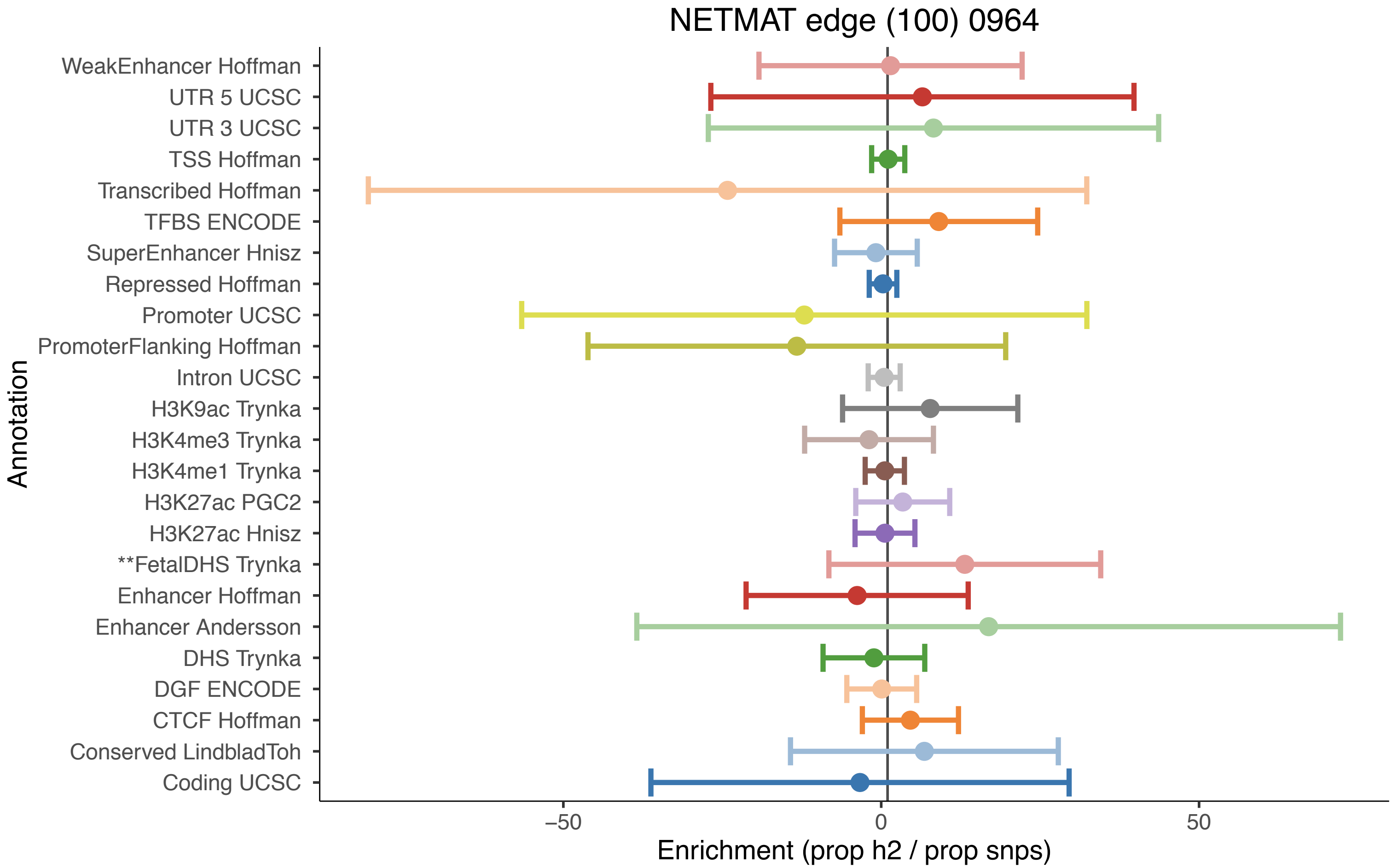

NETMAT edge (100) 0964
Annotation
WeakEnhancer Hoffman
UTR 5 UCSC
UTR 3 UCSC
TSS Hoffman
Transcribed Hoffman
TFBS ENCODE
SuperEnhancer Hnisz
Repressed Hoffman
Promoter UCSC
PromoterFlanking Hoffman
Intron UCSC
H3K9ac Trynka
H3K4me3 Trynka
H3K4me1 Trynka
H3K27ac PGC2
H3K27ac Hnisz
**FetalDHS Trynka
Enhancer Hoffman
Enhancer Andersson
DHS Trynka
DGF ENCODE
CTCF Hoffman
Conserved LindbladToh
Coding UCSC
−50
0
50
Enrichment (prop h2 / prop snps)

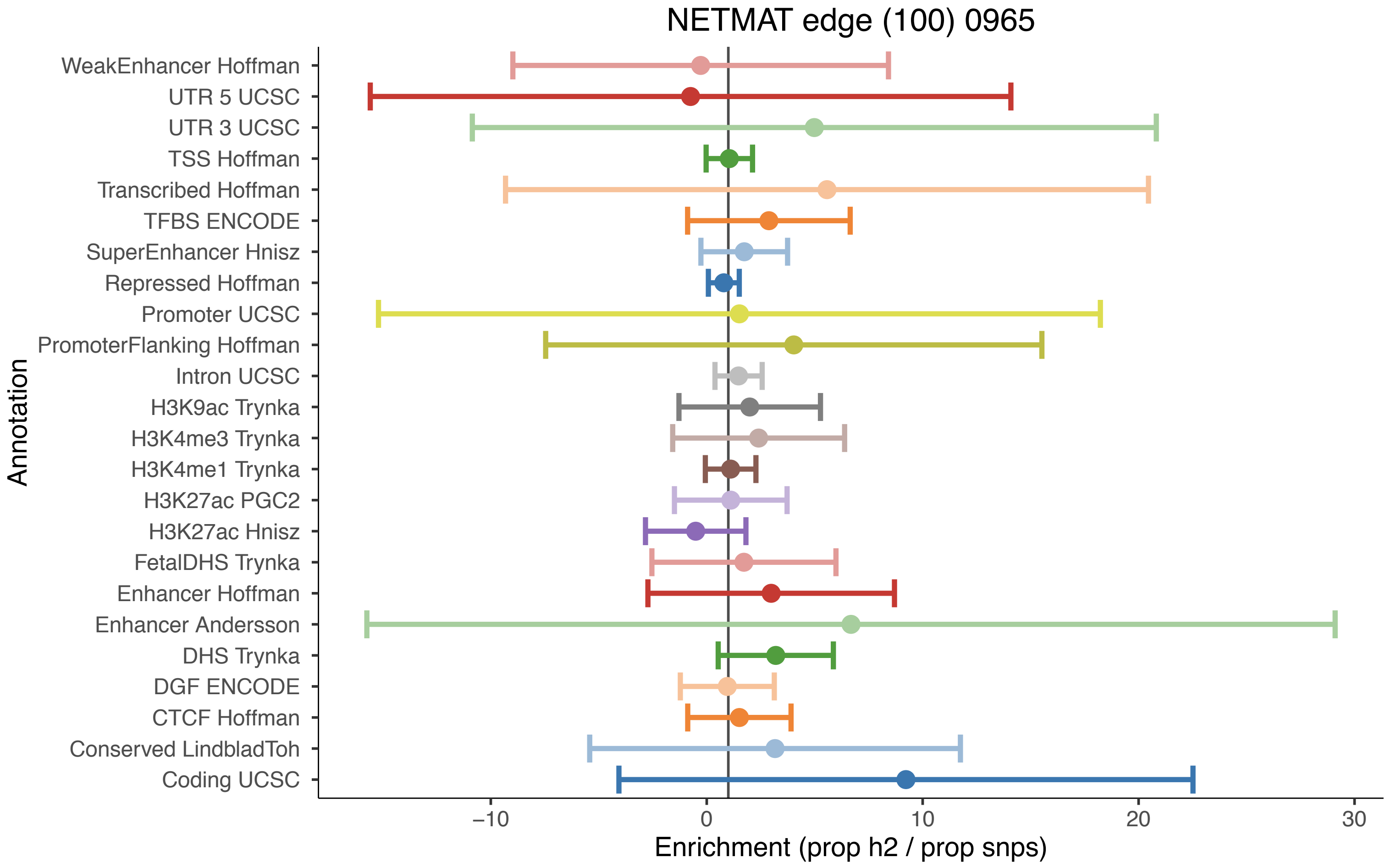

NETMAT edge (100) 0965
Annotation
WeakEnhancer Hoffman
UTR 5 UCSC
UTR 3 UCSC
TSS Hoffman
Transcribed Hoffman
TFBS ENCODE
SuperEnhancer Hnisz
Repressed Hoffman
Promoter UCSC
PromoterFlanking Hoffman
Intron UCSC
H3K9ac Trynka
H3K4me3 Trynka
H3K4me1 Trynka
H3K27ac PGC2
H3K27ac Hnisz
FetalDHS Trynka
Enhancer Hoffman
Enhancer Andersson
DHS Trynka
DGF ENCODE
CTCF Hoffman
Conserved LindbladToh
Coding UCSC
−10
0
10
20
30
Enrichment (prop h2 / prop snps)

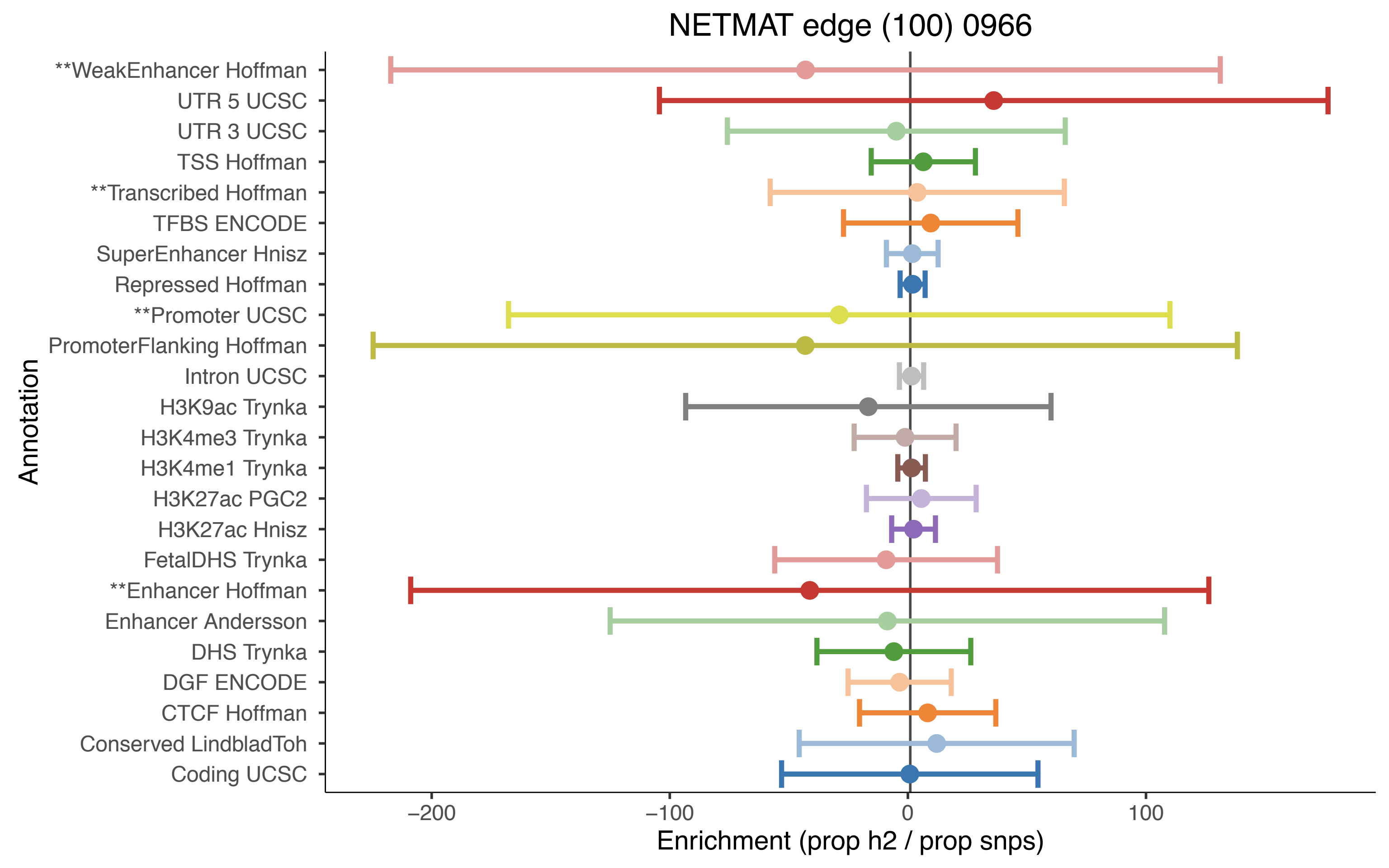
NETMAT edge (100) 0966
**WeakEnhancer Hoffman
UTR 5 UCSC
UTR 3 UCSC
TSS Hoffman
**Transcribed Hoffman
TFBS ENCODE
SuperEnhancer Hnisz
Repressed Hoffman
**Promoter UCSC
PromoterFlanking Hoffman
Intron UCSC
H3K9ac Trynka
H3K4me3 Trynka
H3K4me1 Trynka
H3K27ac PGC2
H3K27ac Hnisz
FetalDHS Trynka
**Enhancer Hoffman
Enhancer Andersson
DHS Trynka
DGF ENCODE
CTCF Hoffman
Conserved LindbladToh
Coding UCSC
Annotation
−200
−100
0
100
Enrichment (prop h2 / prop snps)

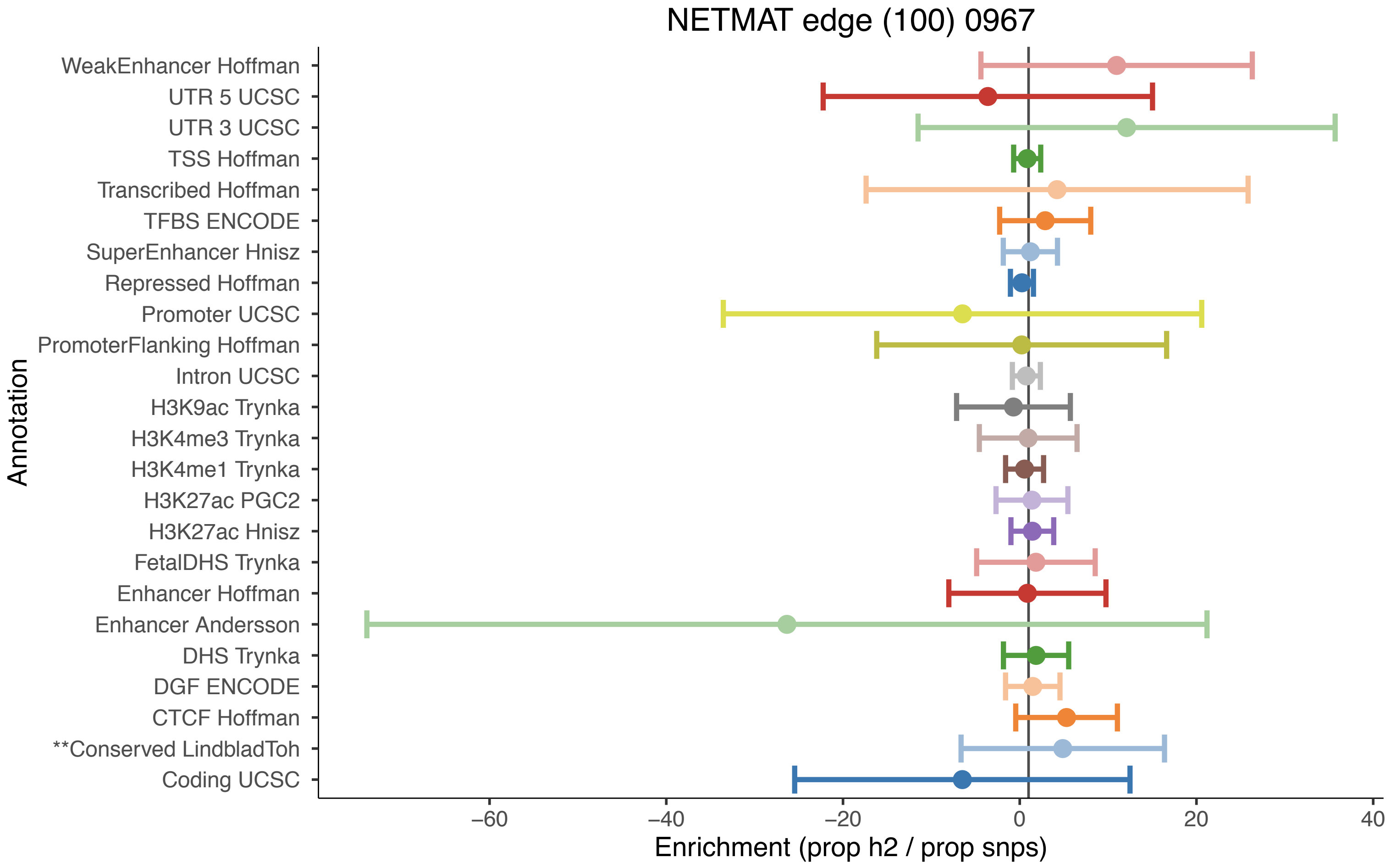

NETMAT edge (100) 0967
WeakEnhancer Hoffman
UTR 5 UCSC
UTR 3 UCSC
TSS Hoffman
Transcribed Hoffman
TFBS ENCODE
SuperEnhancer Hnisz
Repressed Hoffman
Promoter UCSC
PromoterFlanking Hoffman
Intron UCSC
H3K9ac Trynka
H3K4me3 Trynka
H3K4me1 Trynka
H3K27ac PGC2
H3K27ac Hnisz
FetalDHS Trynka
Enhancer Hoffman
Enhancer Andersson
DHS Trynka
DGF ENCODE
CTCF Hoffman
**Conserved LindbladToh
Coding UCSC
Annotation
−60
−40
−20
0
20
40
Enrichment (prop h2 / prop snps)

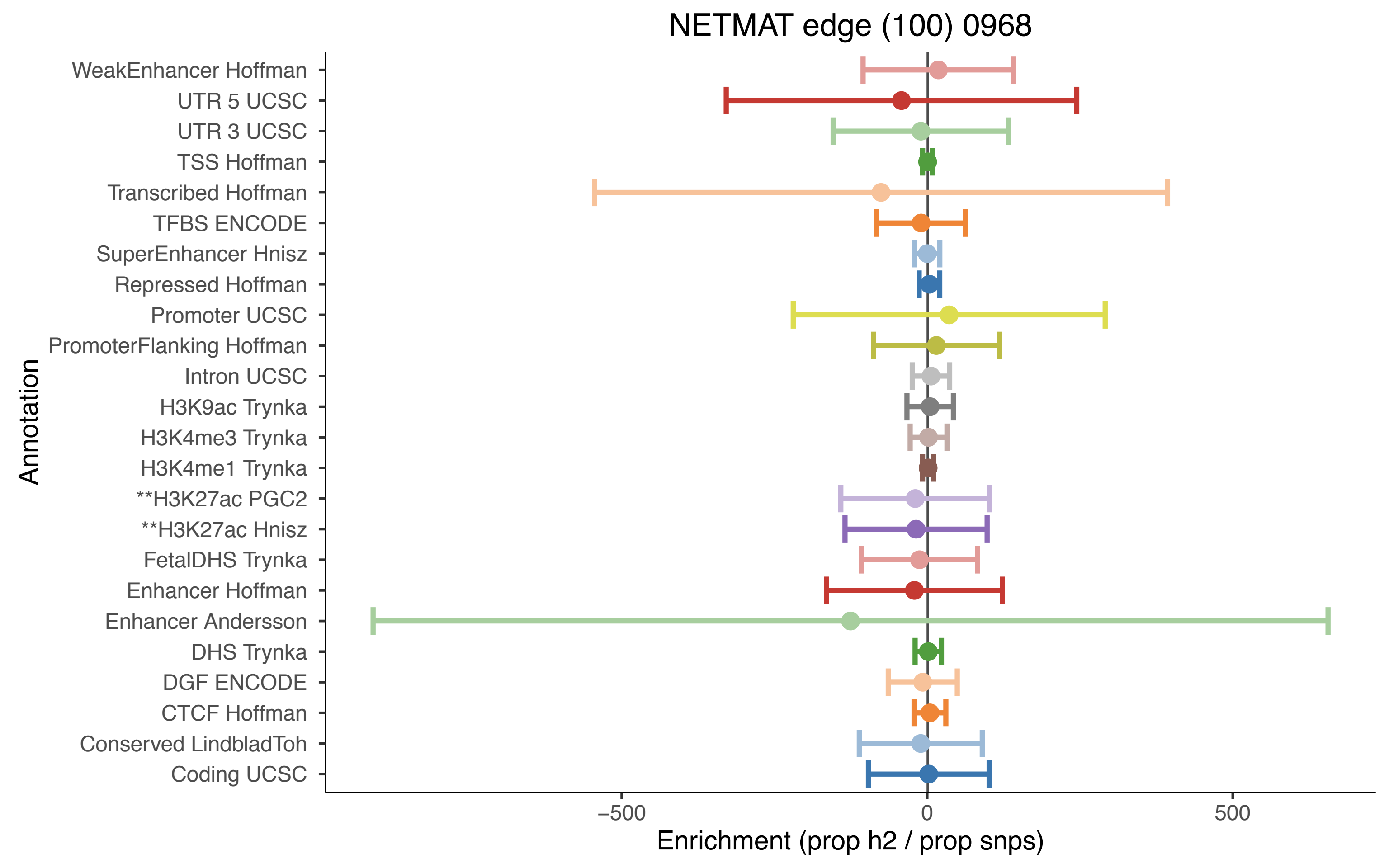

NETMAT edge (100) 0968
WeakEnhancer Hoffman
UTR 5 UCSC
UTR 3 UCSC
TSS Hoffman
Transcribed Hoffman
TFBS ENCODE
SuperEnhancer Hnisz
Repressed Hoffman
Promoter UCSC
PromoterFlanking Hoffman
Intron UCSC
H3K9ac Trynka
H3K4me3 Trynka
H3K4me1 Trynka
**H3K27ac PGC2
**H3K27ac Hnisz
FetalDHS Trynka
Enhancer Hoffman
Enhancer Andersson
DHS Trynka
DGF ENCODE
CTCF Hoffman
Conserved LindbladToh
Coding UCSC
Annotation
−500
0
500
Enrichment (prop h2 / prop snps)

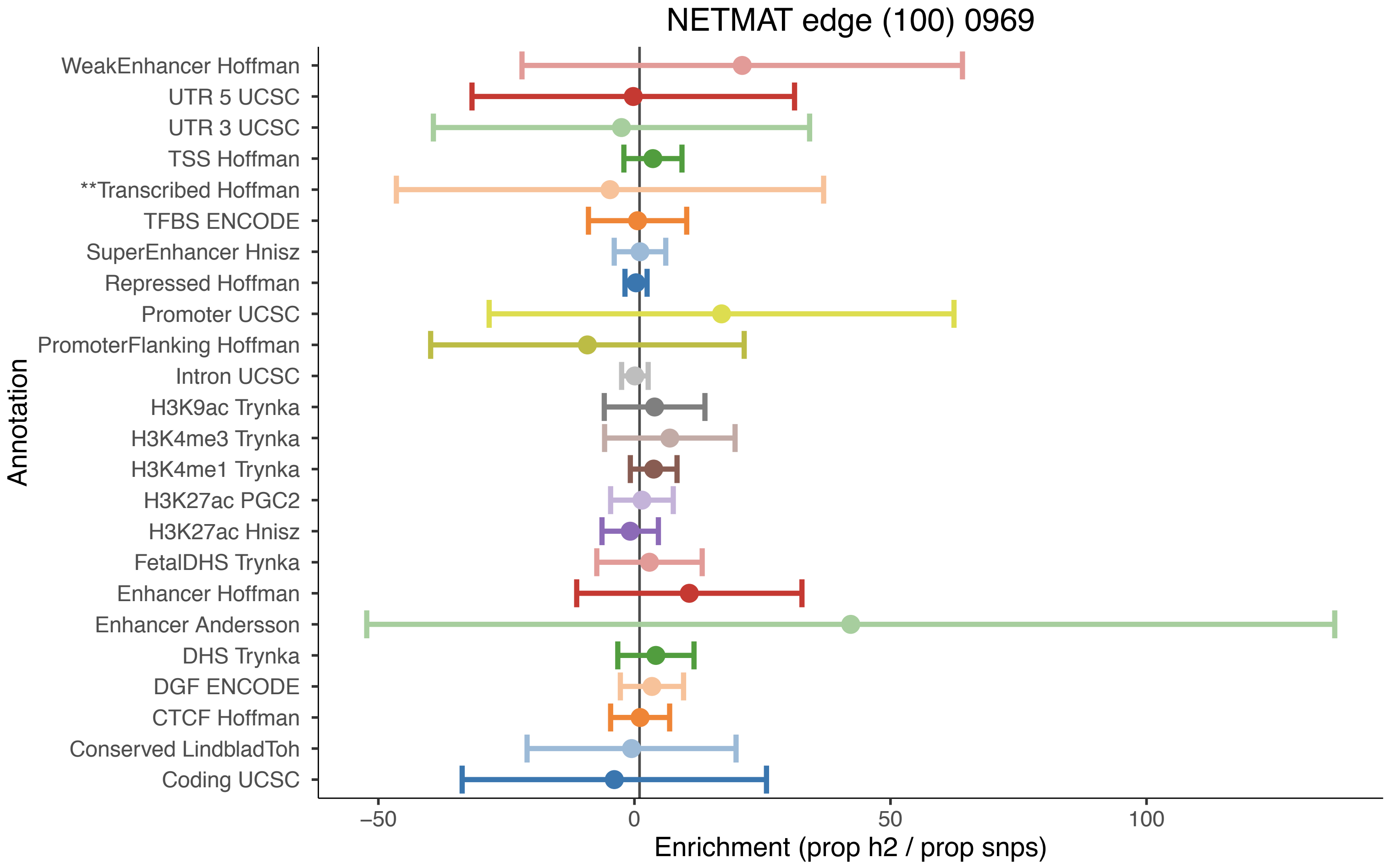

NETMAT edge (100) 0969
WeakEnhancer Hoffman
UTR 5 UCSC
UTR 3 UCSC
TSS Hoffman
**Transcribed Hoffman
TFBS ENCODE
SuperEnhancer Hnisz
Repressed Hoffman
Promoter UCSC
PromoterFlanking Hoffman
Intron UCSC
H3K9ac Trynka
H3K4me3 Trynka
H3K4me1 Trynka
H3K27ac PGC2
H3K27ac Hnisz
FetalDHS Trynka
Enhancer Hoffman
Enhancer Andersson
DHS Trynka
DGF ENCODE
CTCF Hoffman
Conserved LindbladToh
Coding UCSC
Annotation
−50
0
50
100
Enrichment (prop h2 / prop snps)

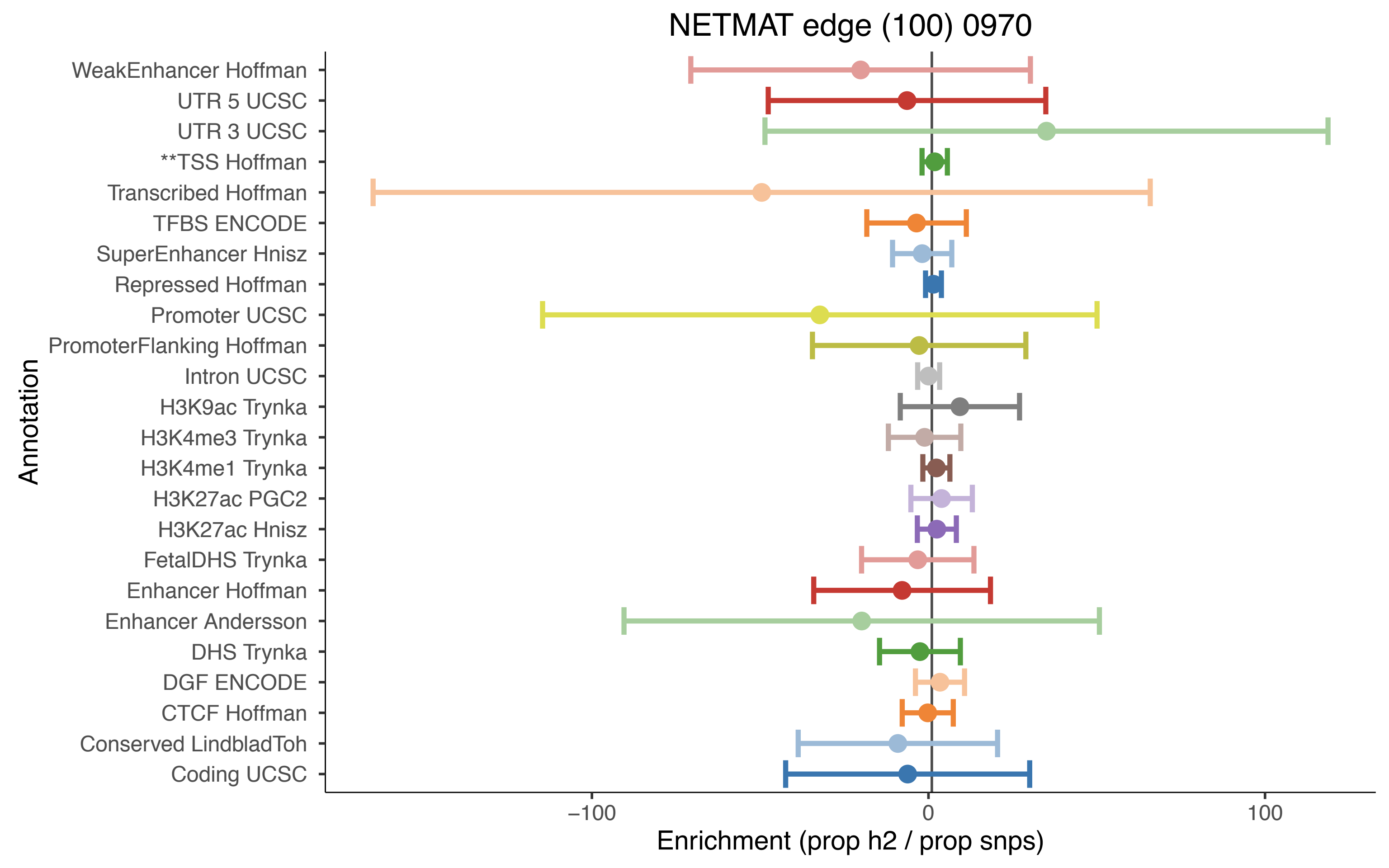
NETMAT edge (100) 0970
WeakEnhancer Hoffman
UTR 5 UCSC
UTR 3 UCSC
**TSS Hoffman
Transcribed Hoffman
TFBS ENCODE
SuperEnhancer Hnisz
Repressed Hoffman
Promoter UCSC
PromoterFlanking Hoffman
Intron UCSC
H3K9ac Trynka
H3K4me3 Trynka
H3K4me1 Trynka
H3K27ac PGC2
H3K27ac Hnisz
FetalDHS Trynka
Enhancer Hoffman
Enhancer Andersson
DHS Trynka
DGF ENCODE
CTCF Hoffman
Conserved LindbladToh
Coding UCSC
Annotation
−100
0
100
Enrichment (prop h2 / prop snps)

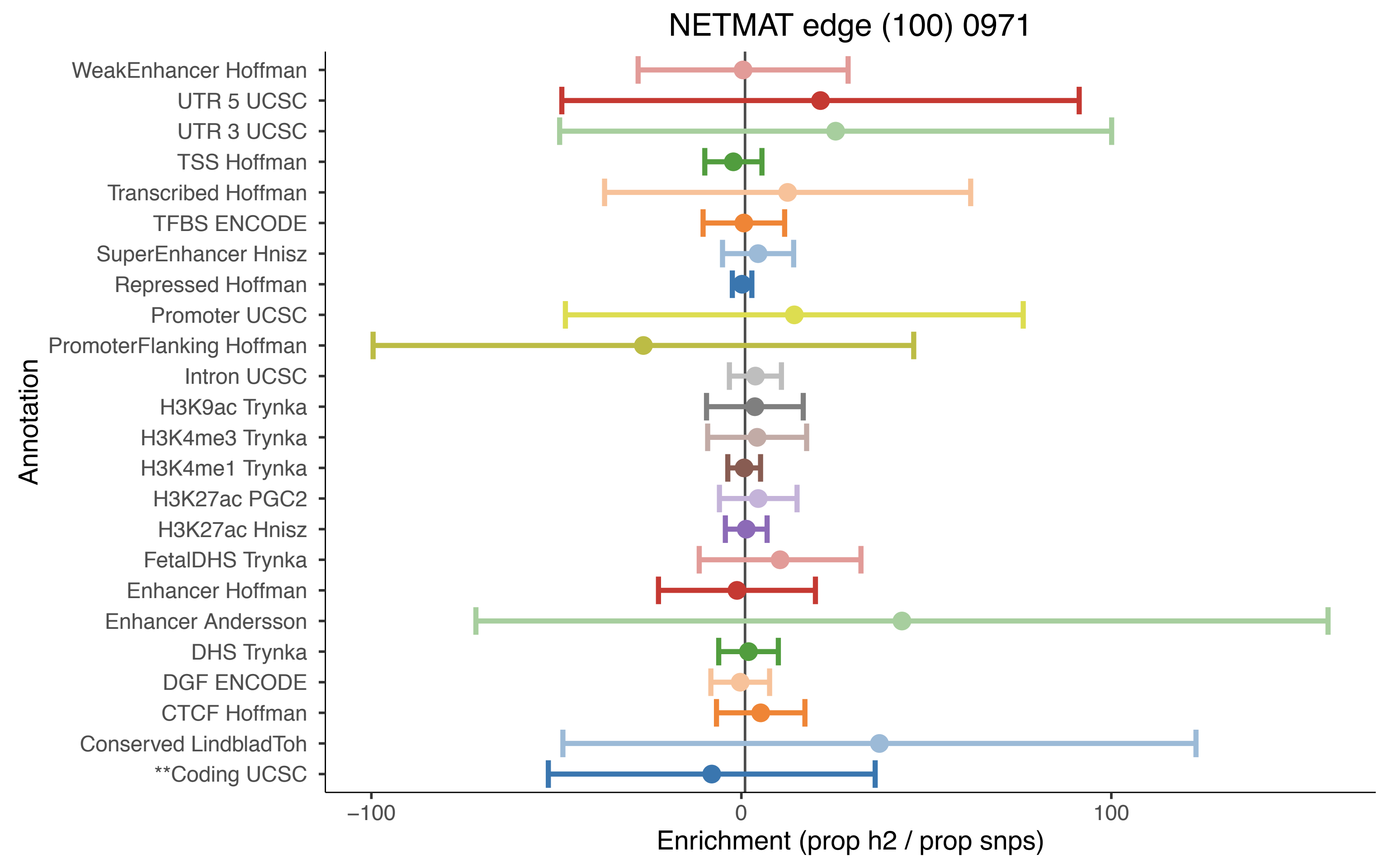
NETMAT edge (100) 0971
WeakEnhancer Hoffman
UTR 5 UCSC
UTR 3 UCSC
TSS Hoffman
Transcribed Hoffman
TFBS ENCODE
SuperEnhancer Hnisz
Repressed Hoffman
Promoter UCSC
PromoterFlanking Hoffman
Intron UCSC
H3K9ac Trynka
H3K4me3 Trynka
H3K4me1 Trynka
H3K27ac PGC2
H3K27ac Hnisz
FetalDHS Trynka
Enhancer Hoffman
Enhancer Andersson
DHS Trynka
DGF ENCODE
CTCF Hoffman
Conserved LindbladToh
**Coding UCSC
Annotation
−100
0
100
Enrichment (prop h2 / prop snps)

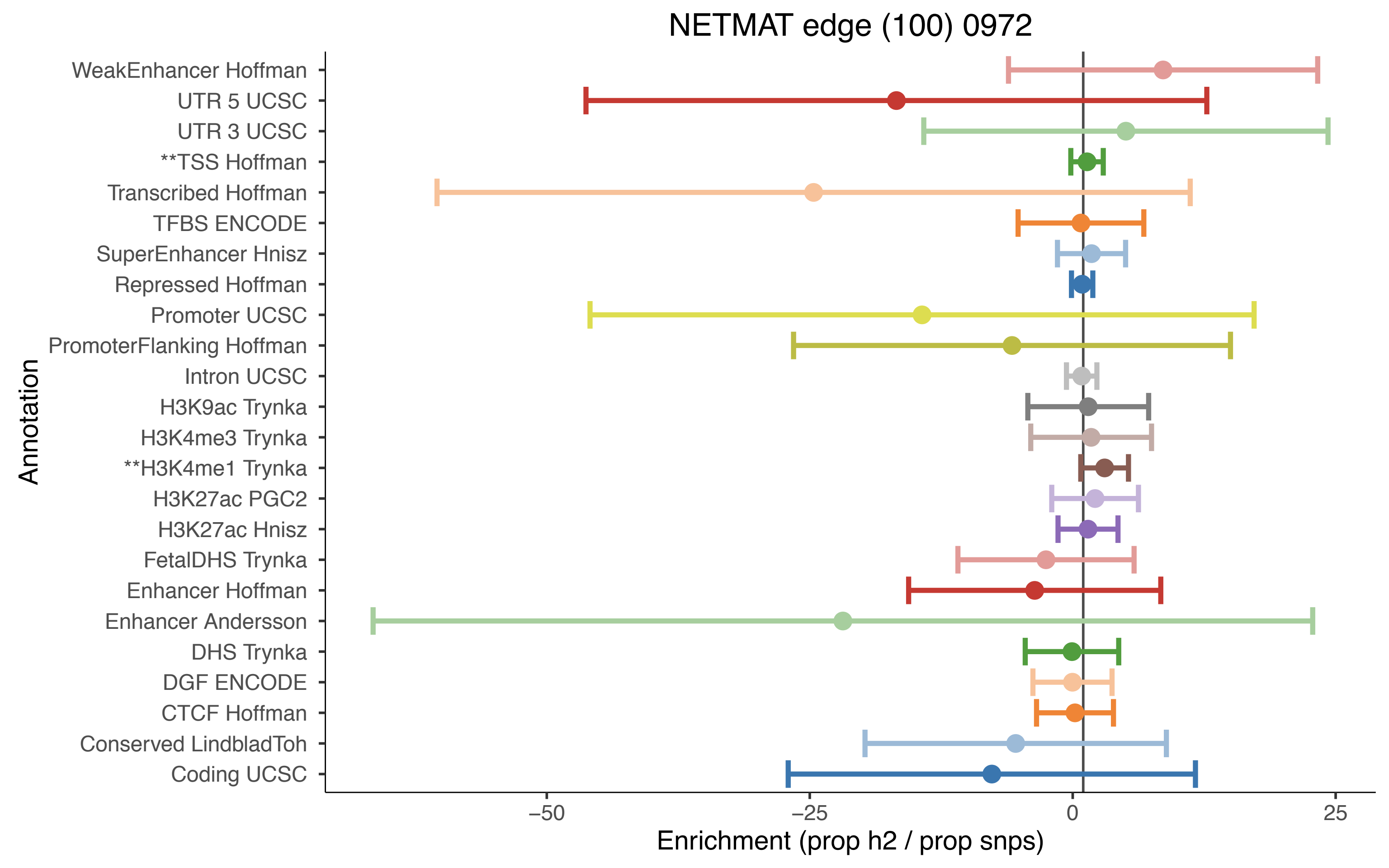
NETMAT edge (100) 0972
WeakEnhancer Hoffman
UTR 5 UCSC
UTR 3 UCSC
**TSS Hoffman
Transcribed Hoffman
TFBS ENCODE
SuperEnhancer Hnisz
Repressed Hoffman
Promoter UCSC
PromoterFlanking Hoffman
Intron UCSC
H3K9ac Trynka
H3K4me3 Trynka
**H3K4me1 Trynka
H3K27ac PGC2
H3K27ac Hnisz
FetalDHS Trynka
Enhancer Hoffman
Enhancer Andersson
DHS Trynka
DGF ENCODE
CTCF Hoffman
Conserved LindbladToh
Coding UCSC
Annotation
−50
−25
0
25
Enrichment (prop h2 / prop snps)

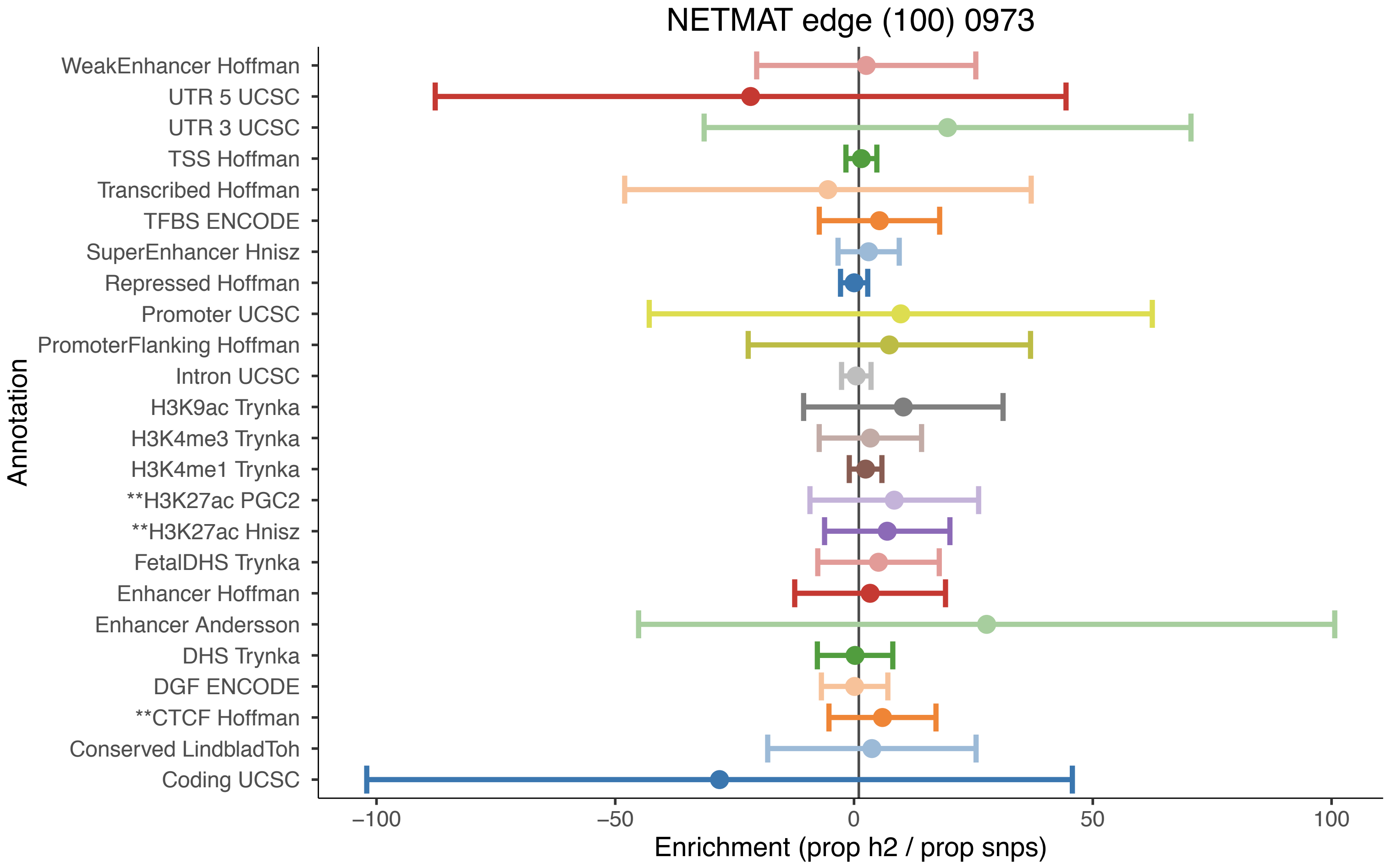

NETMAT edge (100) 0973
Annotation
WeakEnhancer Hoffman
UTR 5 UCSC
UTR 3 UCSC
TSS Hoffman
Transcribed Hoffman
TFBS ENCODE
SuperEnhancer Hnisz
Repressed Hoffman
Promoter UCSC
PromoterFlanking Hoffman
Intron UCSC
H3K9ac Trynka
H3K4me3 Trynka
H3K4me1 Trynka
**H3K27ac PGC2
**H3K27ac Hnisz
FetalDHS Trynka
Enhancer Hoffman
Enhancer Andersson
DHS Trynka
DGF ENCODE
**CTCF Hoffman
Conserved LindbladToh
Coding UCSC
−100
−50
0
50
100
Enrichment (prop h2 / prop snps)

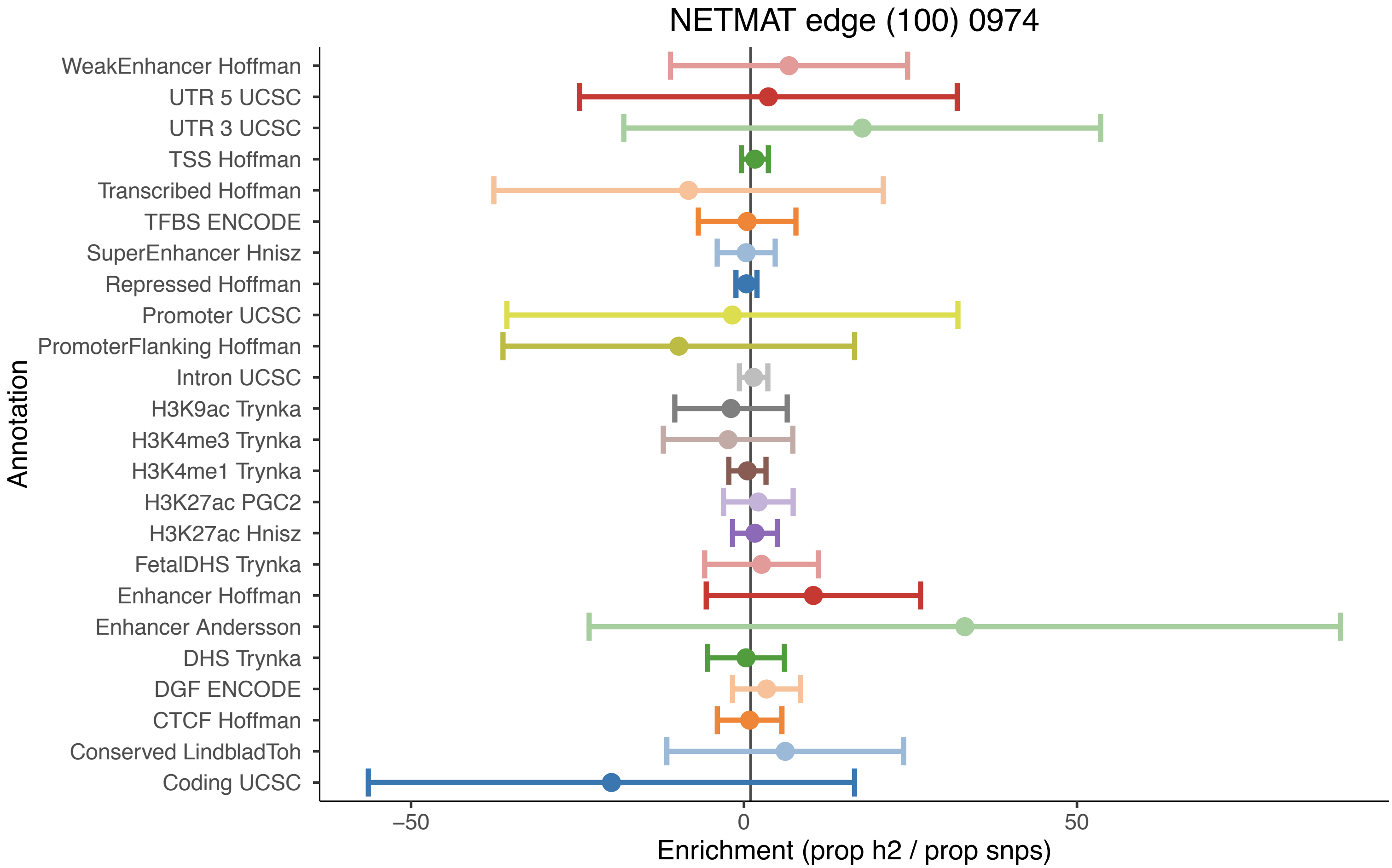

NETMAT edge (100) 0974
WeakEnhancer Hoffman
UTR 5 UCSC
UTR 3 UCSC
TSS Hoffman
Transcribed Hoffman
TFBS ENCODE
SuperEnhancer Hnisz
Repressed Hoffman
Promoter UCSC
PromoterFlanking Hoffman
Intron UCSC
H3K9ac Trynka
H3K4me3 Trynka
H3K4me1 Trynka
H3K27ac PGC2
H3K27ac Hnisz
FetalDHS Trynka
Enhancer Hoffman
Enhancer Andersson
DHS Trynka
DGF ENCODE
CTCF Hoffman
Conserved LindbladToh
Coding UCSC
Annotation
−50
0
50
Enrichment (prop h2 / prop snps)

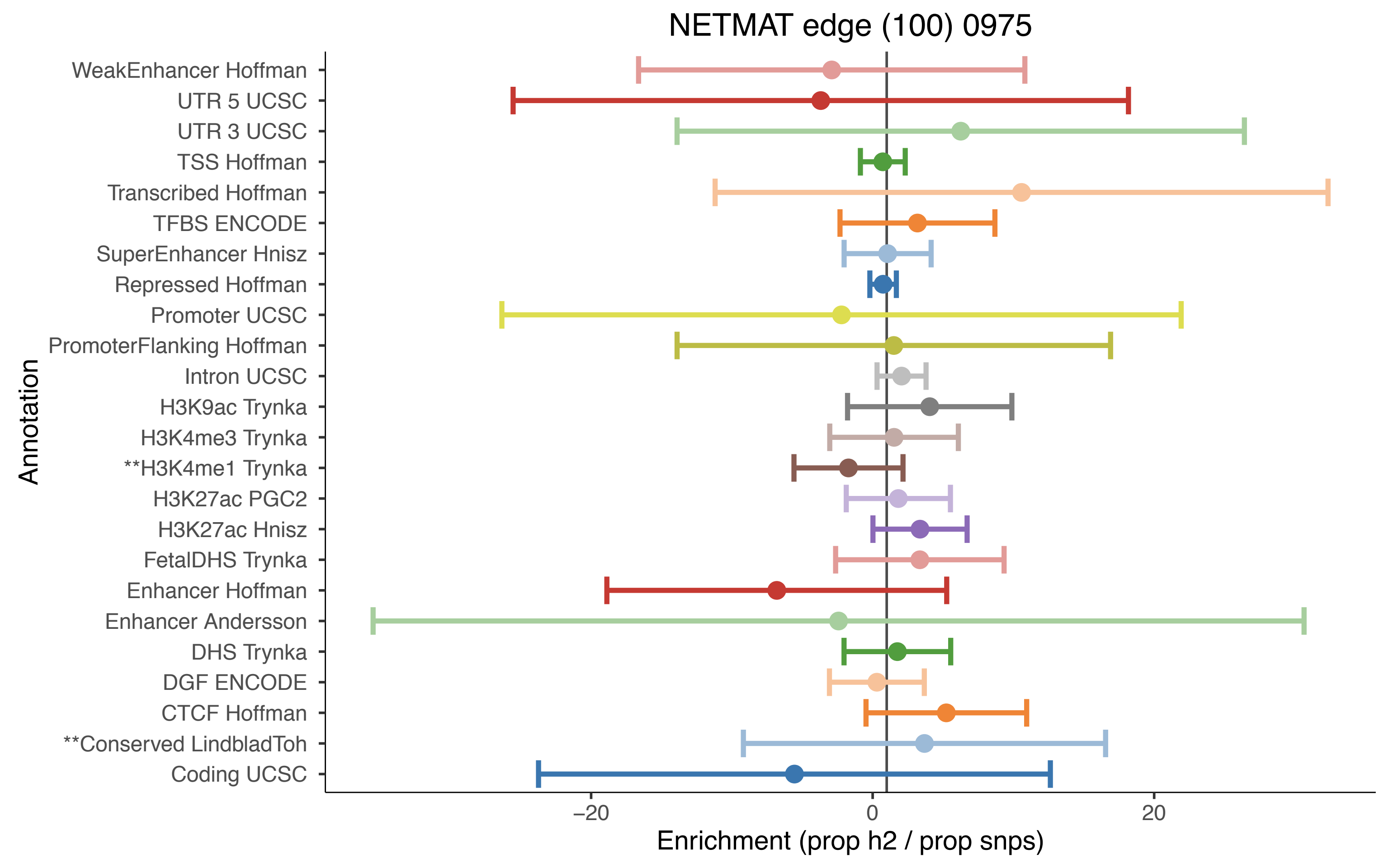
NETMAT edge (100) 0975
WeakEnhancer Hoffman
UTR 5 UCSC
UTR 3 UCSC
TSS Hoffman
Transcribed Hoffman
TFBS ENCODE
SuperEnhancer Hnisz
Repressed Hoffman
Promoter UCSC
PromoterFlanking Hoffman
Intron UCSC
H3K9ac Trynka
H3K4me3 Trynka
**H3K4me1 Trynka
H3K27ac PGC2
H3K27ac Hnisz
FetalDHS Trynka
Enhancer Hoffman
Enhancer Andersson
DHS Trynka
DGF ENCODE
CTCF Hoffman
**Conserved LindbladToh
Coding UCSC
Annotation
−20
0
20
Enrichment (prop h2 / prop snps)

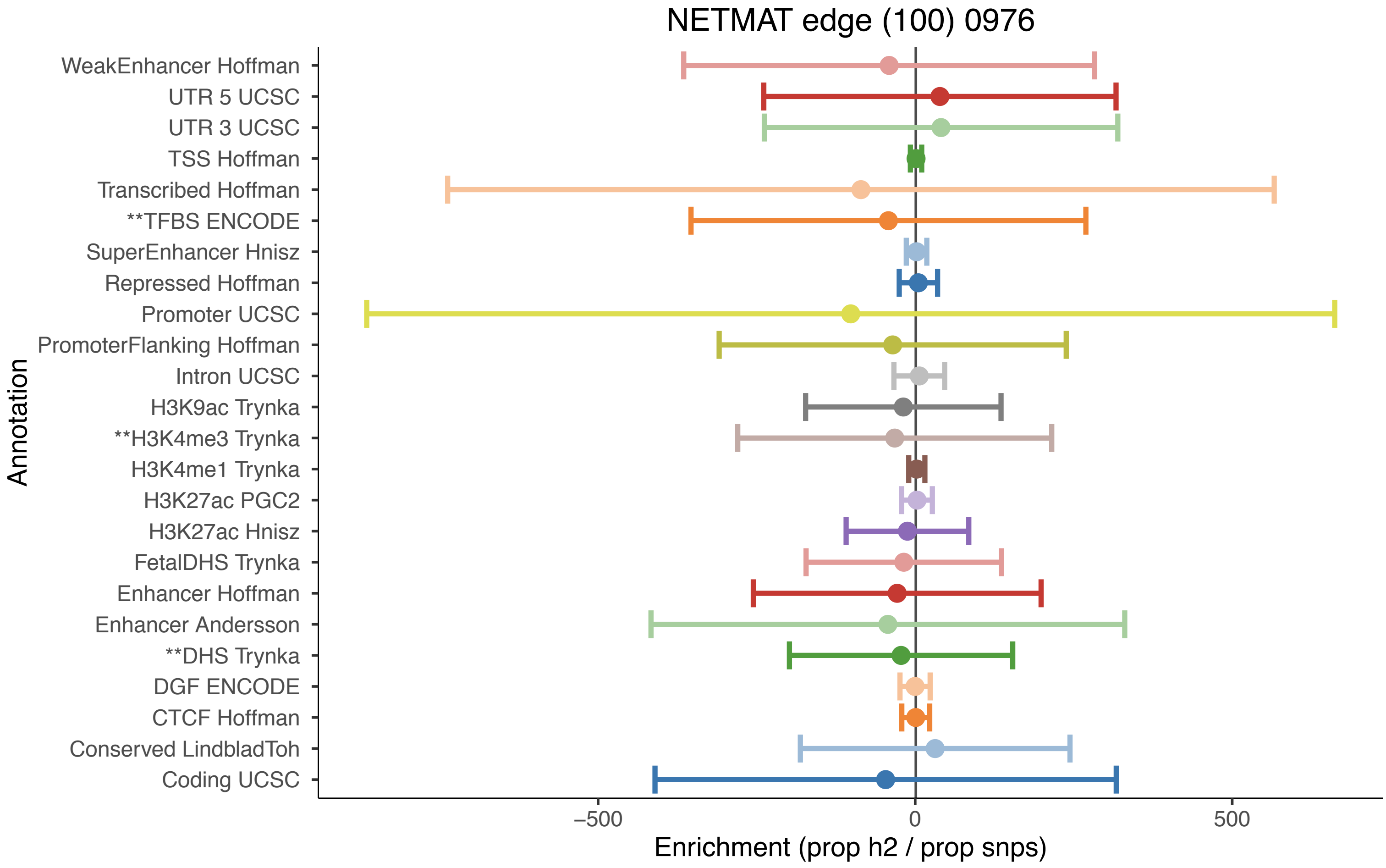

NETMAT edge (100) 0976
WeakEnhancer Hoffman
UTR 5 UCSC
UTR 3 UCSC
TSS Hoffman
Transcribed Hoffman
**TFBS ENCODE
SuperEnhancer Hnisz
Repressed Hoffman
Promoter UCSC
PromoterFlanking Hoffman
Intron UCSC
H3K9ac Trynka
**H3K4me3 Trynka
H3K4me1 Trynka
H3K27ac PGC2
H3K27ac Hnisz
FetalDHS Trynka
Enhancer Hoffman
Enhancer Andersson
**DHS Trynka
DGF ENCODE
CTCF Hoffman
Conserved LindbladToh
Coding UCSC
Annotation
−500
0
500
Enrichment (prop h2 / prop snps)

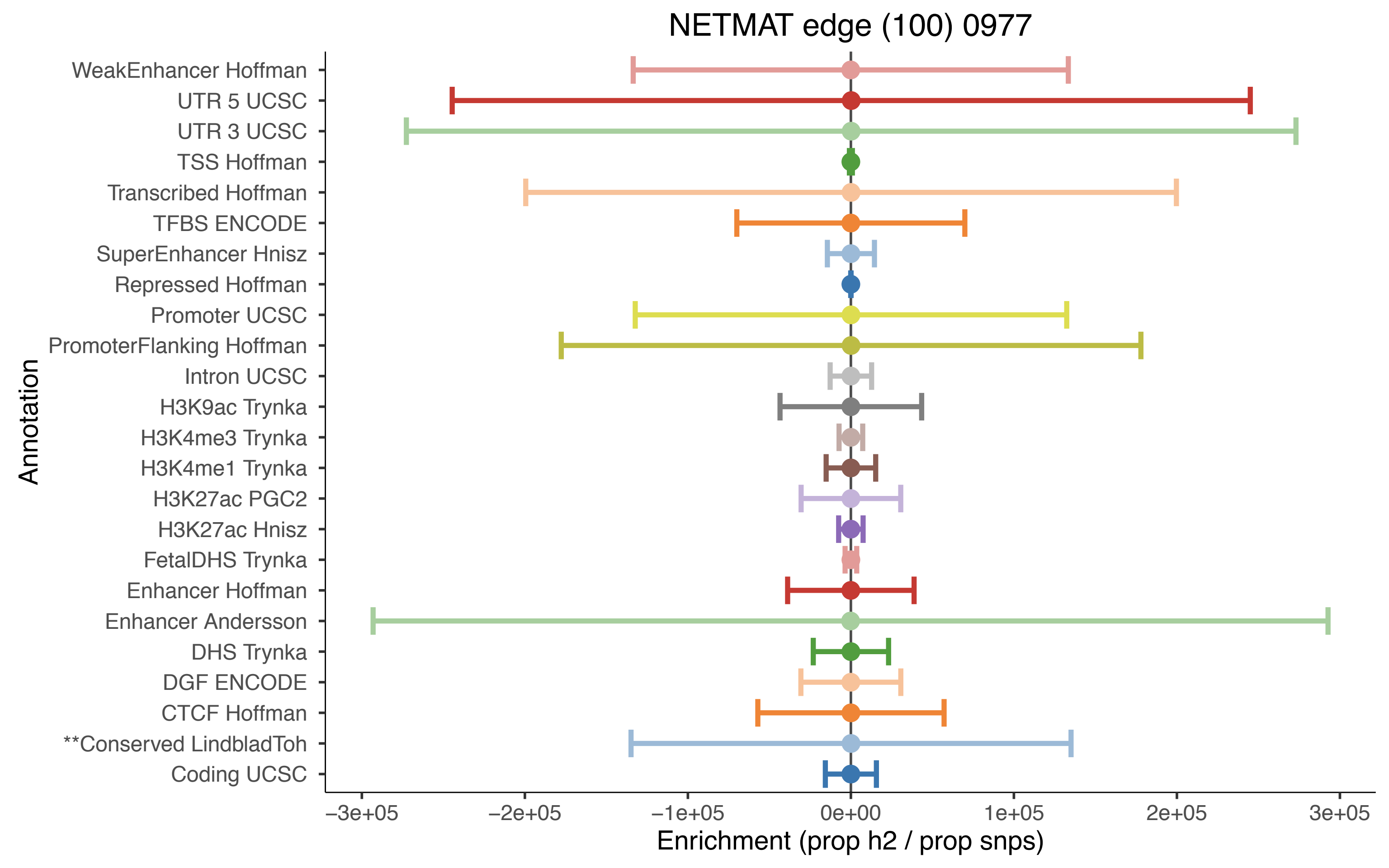

NETMAT edge (100) 0977
Annotation
WeakEnhancer Hoffman
UTR 5 UCSC
UTR 3 UCSC
TSS Hoffman
Transcribed Hoffman
TFBS ENCODE
SuperEnhancer Hnisz
Repressed Hoffman
Promoter UCSC
PromoterFlanking Hoffman
Intron UCSC
H3K9ac Trynka
H3K4me3 Trynka
H3K4me1 Trynka
H3K27ac PGC2
H3K27ac Hnisz
FetalDHS Trynka
Enhancer Hoffman
Enhancer Andersson
DHS Trynka
DGF ENCODE
CTCF Hoffman
**Conserved LindbladToh
Coding UCSC
−3e+05
−2e+05
−1e+05
0e+00
1e+05
2e+05
3e+05
Enrichment (prop h2 / prop snps)

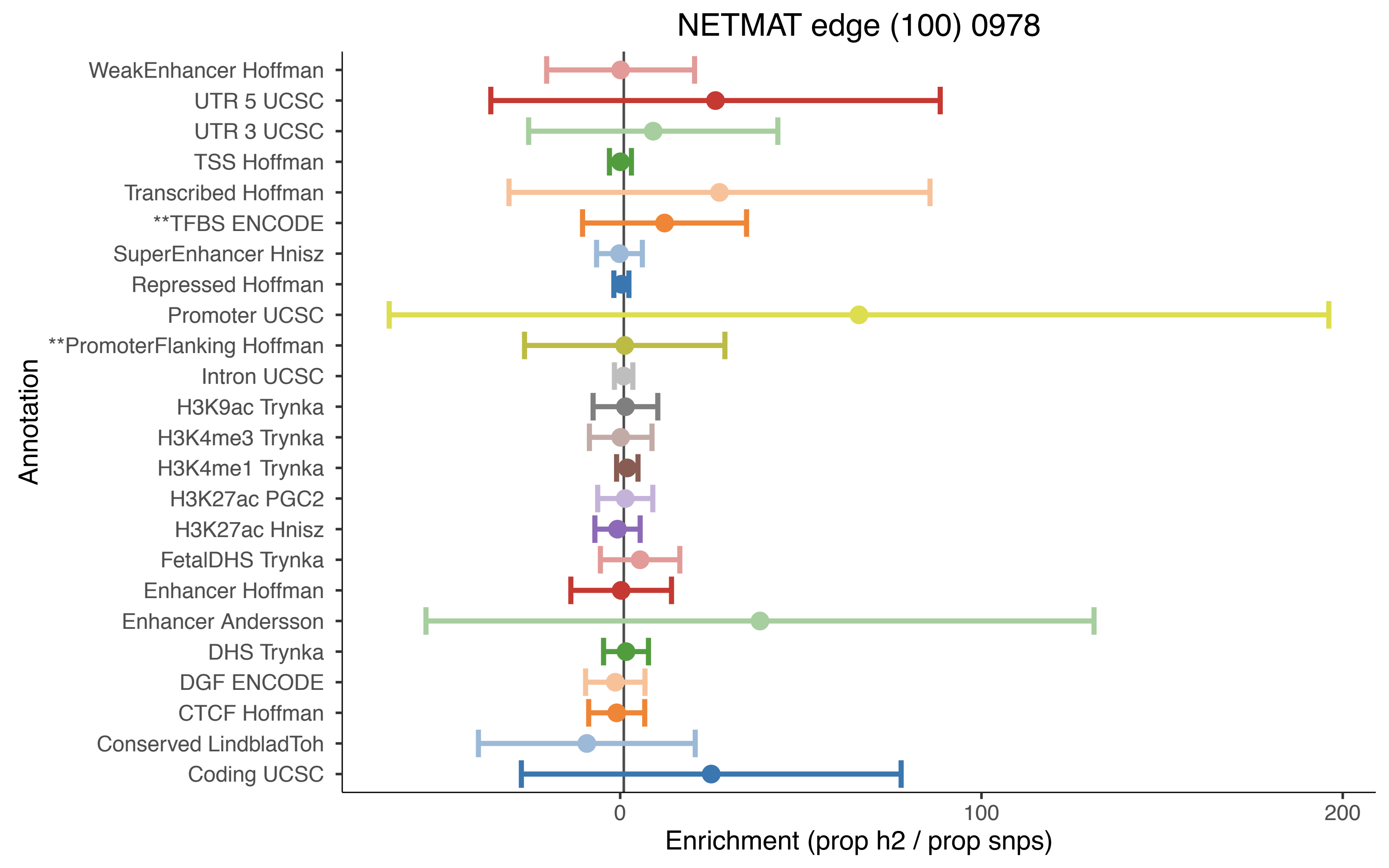

NETMAT edge (100) 0978
Annotation
WeakEnhancer Hoffman
UTR 5 UCSC
UTR 3 UCSC
TSS Hoffman
Transcribed Hoffman
**TFBS ENCODE
SuperEnhancer Hnisz
Repressed Hoffman
Promoter UCSC
**PromoterFlanking Hoffman
Intron UCSC
H3K9ac Trynka
H3K4me3 Trynka
H3K4me1 Trynka
H3K27ac PGC2
H3K27ac Hnisz
FetalDHS Trynka
Enhancer Hoffman
Enhancer Andersson
DHS Trynka
DGF ENCODE
CTCF Hoffman
Conserved LindbladToh
Coding UCSC
0
100
200
Enrichment (prop h2 / prop snps)

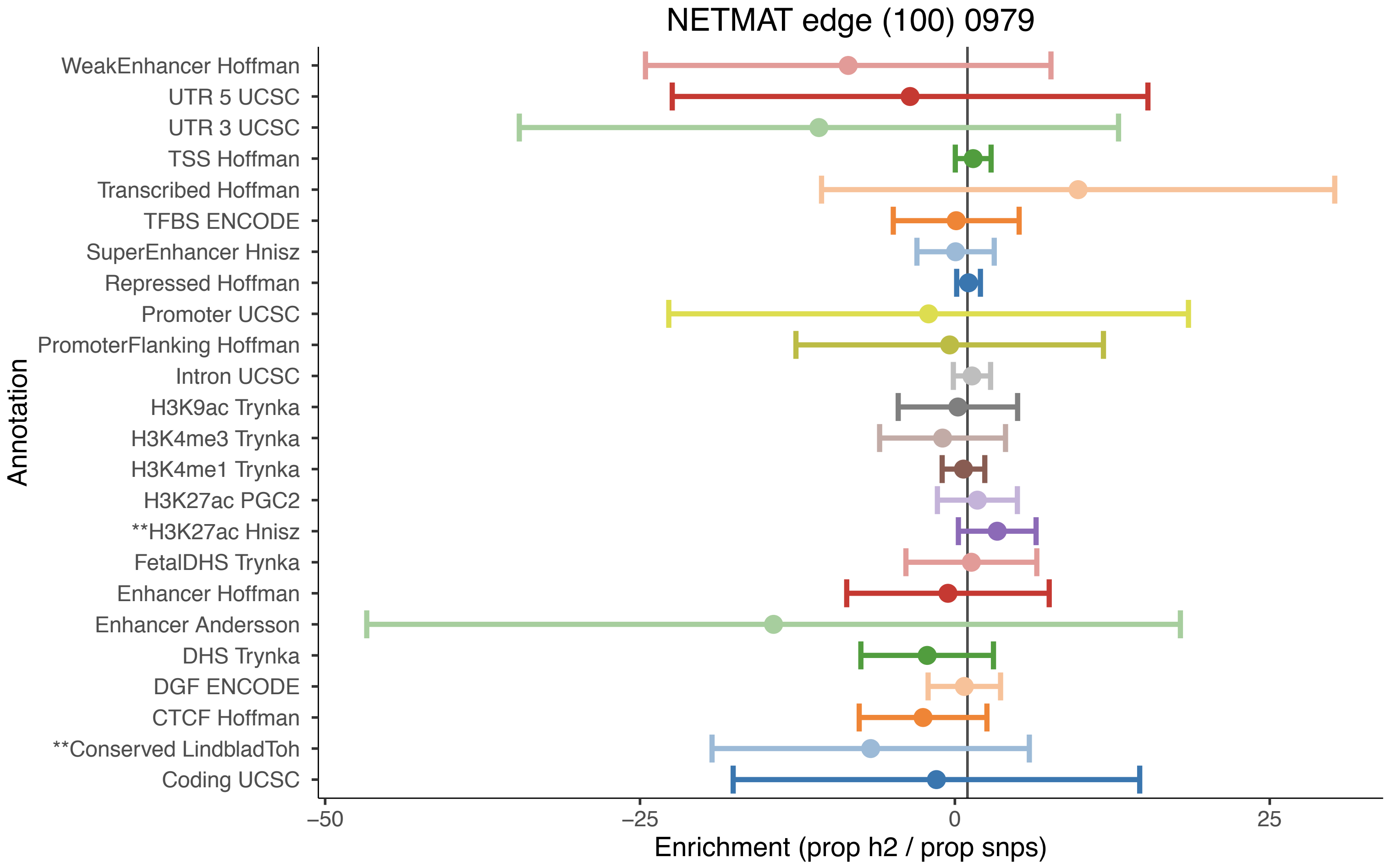

NETMAT edge (100) 0979
WeakEnhancer Hoffman
UTR 5 UCSC
UTR 3 UCSC
TSS Hoffman
Transcribed Hoffman
TFBS ENCODE
SuperEnhancer Hnisz
Repressed Hoffman
Promoter UCSC
PromoterFlanking Hoffman
Intron UCSC
H3K9ac Trynka
H3K4me3 Trynka
H3K4me1 Trynka
H3K27ac PGC2
**H3K27ac Hnisz
FetalDHS Trynka
Enhancer Hoffman
Enhancer Andersson
DHS Trynka
DGF ENCODE
CTCF Hoffman
**Conserved LindbladToh
Coding UCSC
Annotation
−50
−25
0
25
Enrichment (prop h2 / prop snps)

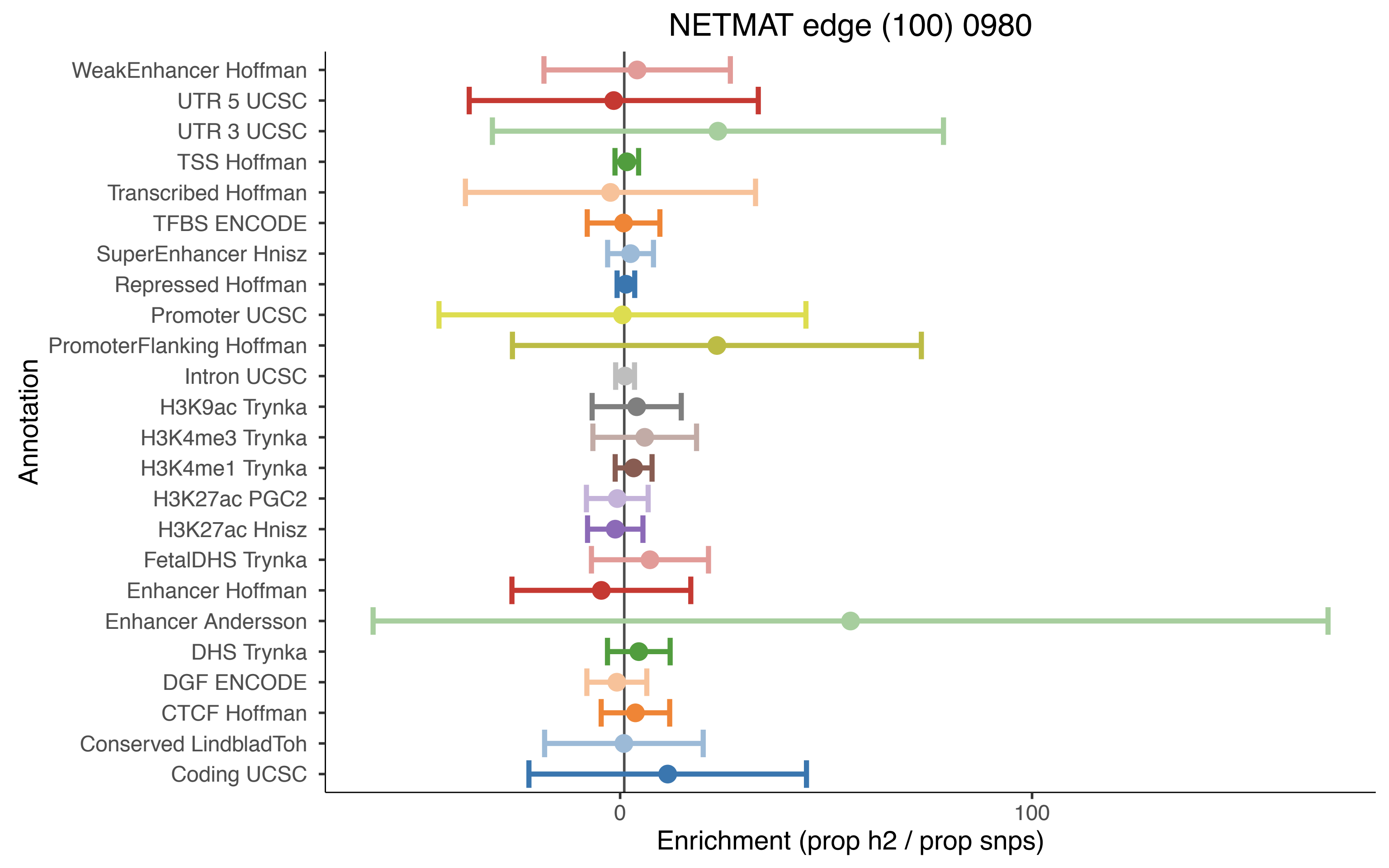
NETMAT edge (100) 0980
Annotation
WeakEnhancer Hoffman
UTR 5 UCSC
UTR 3 UCSC
TSS Hoffman
Transcribed Hoffman
TFBS ENCODE
SuperEnhancer Hnisz
Repressed Hoffman
Promoter UCSC
PromoterFlanking Hoffman
Intron UCSC
H3K9ac Trynka
H3K4me3 Trynka
H3K4me1 Trynka
H3K27ac PGC2
H3K27ac Hnisz
FetalDHS Trynka
Enhancer Hoffman
Enhancer Andersson
DHS Trynka
DGF ENCODE
CTCF Hoffman
Conserved LindbladToh
Coding UCSC
0
100
Enrichment (prop h2 / prop snps)

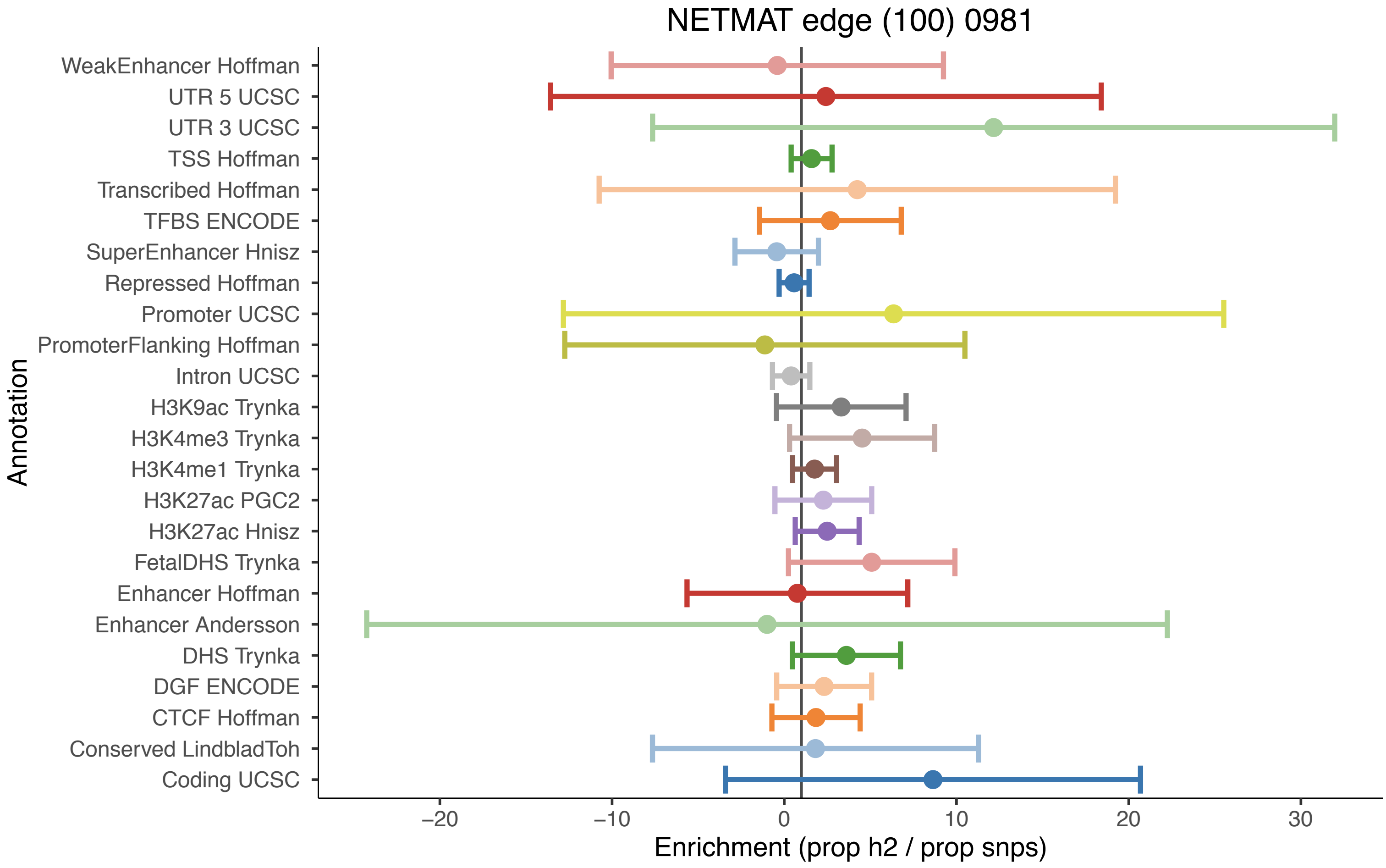

NETMAT edge (100) 0981
WeakEnhancer Hoffman
UTR 5 UCSC
UTR 3 UCSC
TSS Hoffman
Transcribed Hoffman
TFBS ENCODE
SuperEnhancer Hnisz
Repressed Hoffman
Promoter UCSC
PromoterFlanking Hoffman
Intron UCSC
H3K9ac Trynka
H3K4me3 Trynka
H3K4me1 Trynka
H3K27ac PGC2
H3K27ac Hnisz
FetalDHS Trynka
Enhancer Hoffman
Enhancer Andersson
DHS Trynka
DGF ENCODE
CTCF Hoffman
Conserved LindbladToh
Coding UCSC
Annotation
−20
−10
0
10
20
30
Enrichment (prop h2 / prop snps)

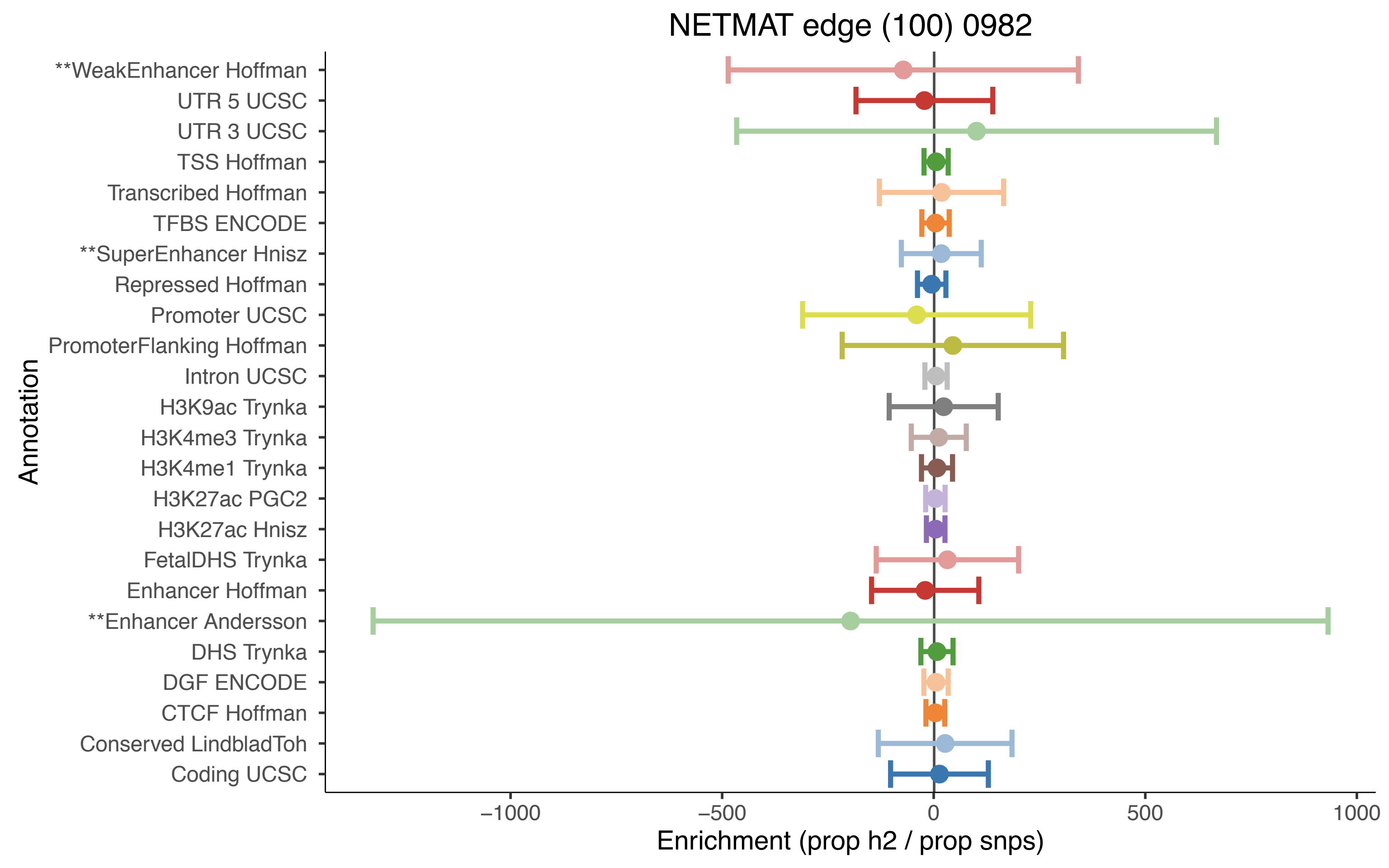
NETMAT edge (100) 0982
**WeakEnhancer Hoffman
UTR 5 UCSC
UTR 3 UCSC
TSS Hoffman
Transcribed Hoffman
TFBS ENCODE
**SuperEnhancer Hnisz
Repressed Hoffman
Promoter UCSC
PromoterFlanking Hoffman
Intron UCSC
H3K9ac Trynka
H3K4me3 Trynka
H3K4me1 Trynka
H3K27ac PGC2
H3K27ac Hnisz
FetalDHS Trynka
Enhancer Hoffman
**Enhancer Andersson
DHS Trynka
DGF ENCODE
CTCF Hoffman
Conserved LindbladToh
Coding UCSC
Annotation
−1000
−500
0
500
1000
Enrichment (prop h2 / prop snps)

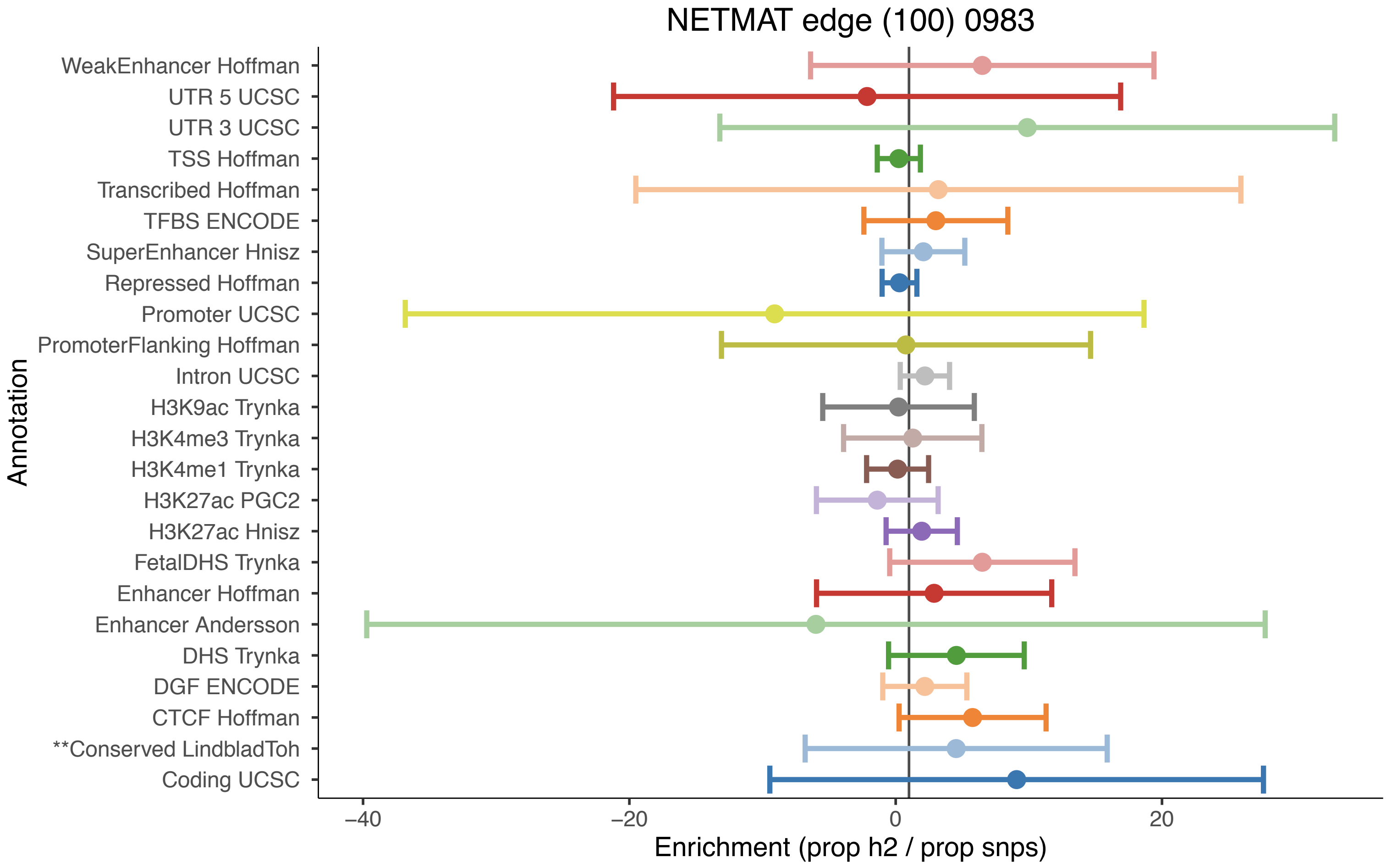
NETMAT edge (100) 0983
WeakEnhancer Hoffman
UTR 5 UCSC
UTR 3 UCSC
TSS Hoffman
Transcribed Hoffman
TFBS ENCODE
SuperEnhancer Hnisz
Repressed Hoffman
Promoter UCSC
PromoterFlanking Hoffman
Intron UCSC
H3K9ac Trynka
H3K4me3 Trynka
H3K4me1 Trynka
H3K27ac PGC2
H3K27ac Hnisz
FetalDHS Trynka
Enhancer Hoffman
Enhancer Andersson
DHS Trynka
DGF ENCODE
CTCF Hoffman
**Conserved LindbladToh
Coding UCSC
Annotation
−40
−20
0
20
Enrichment (prop h2 / prop snps)

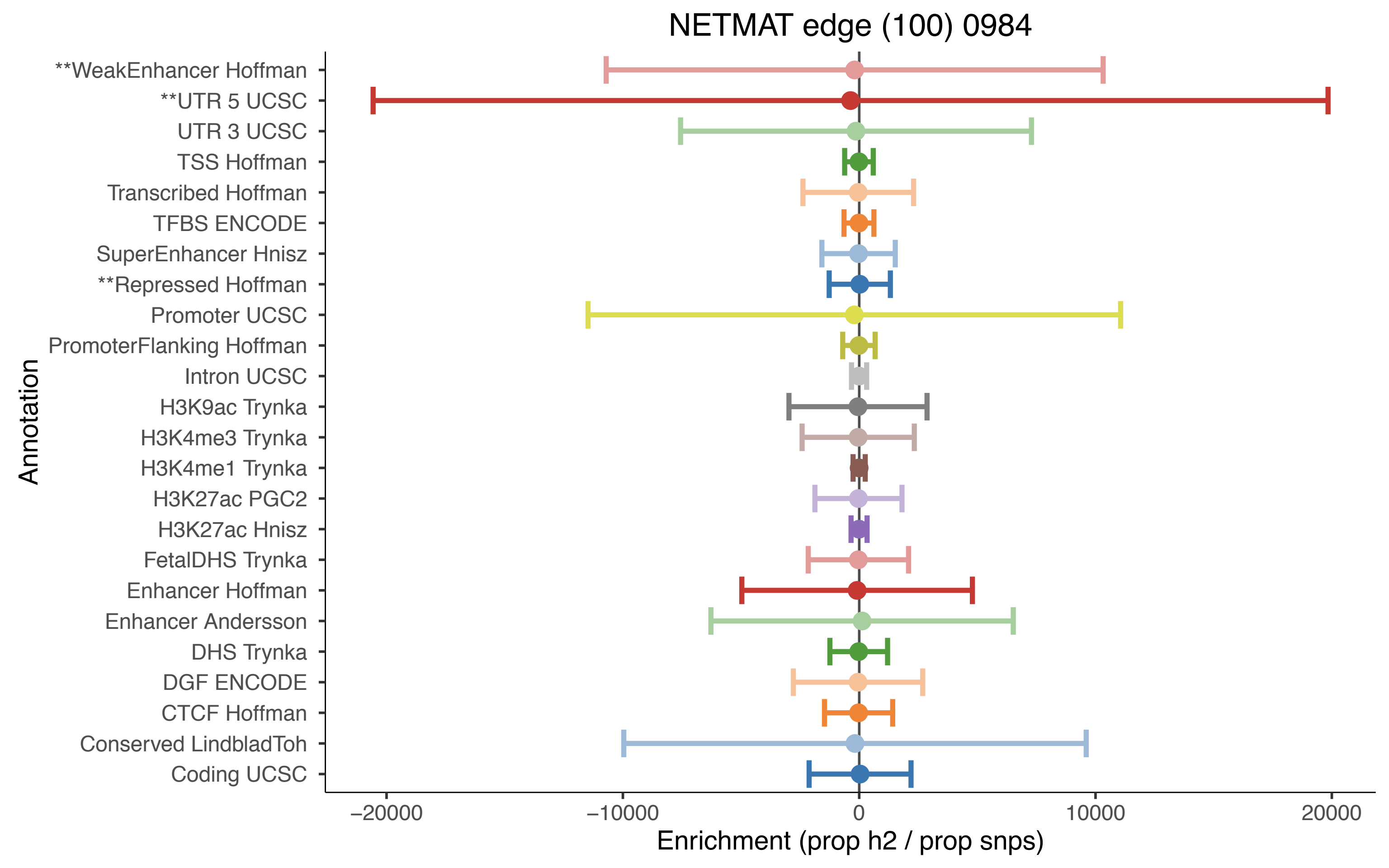

NETMAT edge (100) 0984
**WeakEnhancer Hoffman
**UTR 5 UCSC
UTR 3 UCSC
TSS Hoffman
Transcribed Hoffman
TFBS ENCODE
SuperEnhancer Hnisz
**Repressed Hoffman
Promoter UCSC
PromoterFlanking Hoffman
Intron UCSC
H3K9ac Trynka
H3K4me3 Trynka
H3K4me1 Trynka
H3K27ac PGC2
H3K27ac Hnisz
FetalDHS Trynka
Enhancer Hoffman
Enhancer Andersson
DHS Trynka
DGF ENCODE
CTCF Hoffman
Conserved LindbladToh
Coding UCSC
Annotation
−20000
−10000
0
10000
20000
Enrichment (prop h2 / prop snps)

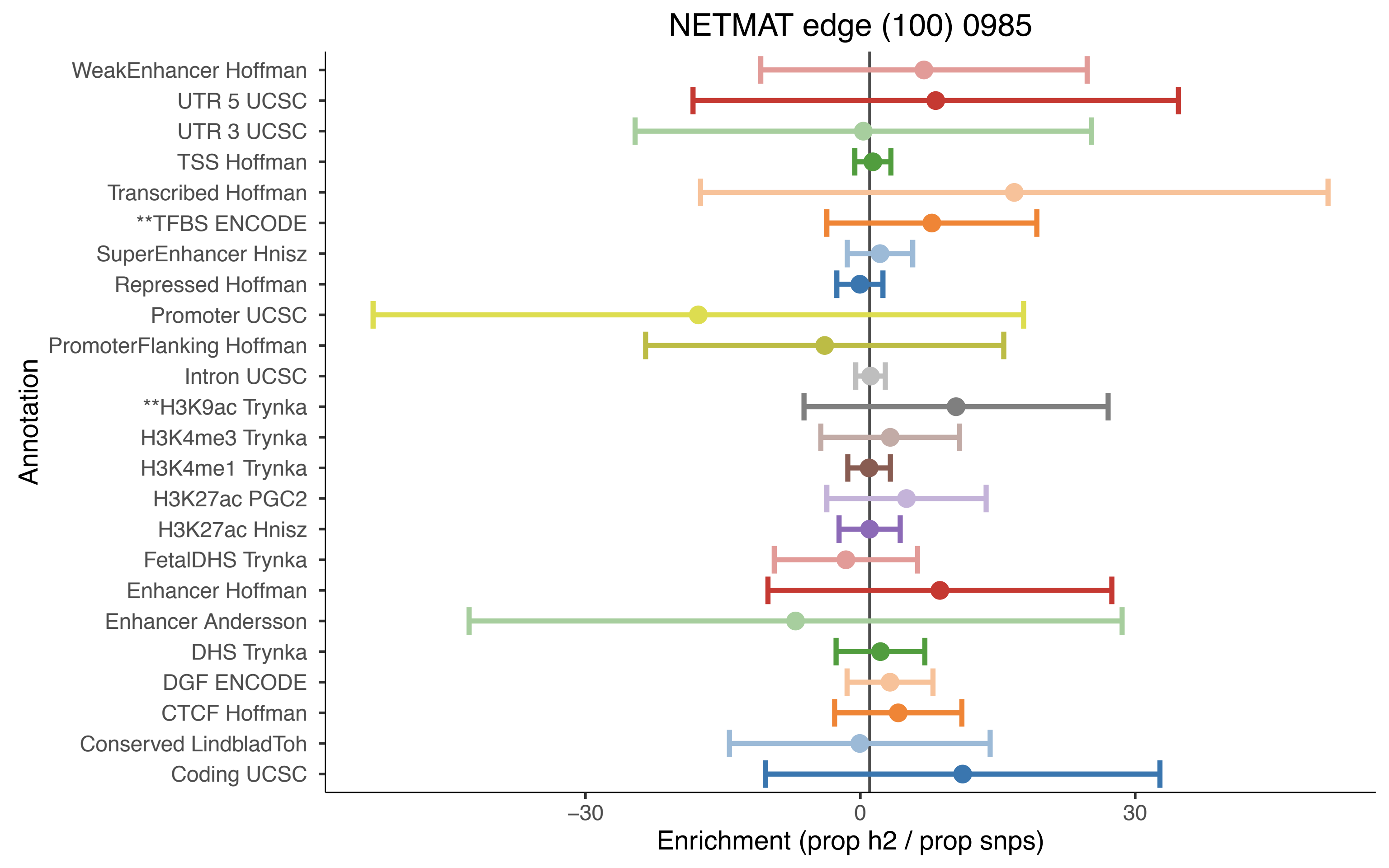

NETMAT edge (100) 0985
WeakEnhancer Hoffman
UTR 5 UCSC
UTR 3 UCSC
TSS Hoffman
Transcribed Hoffman
**TFBS ENCODE
SuperEnhancer Hnisz
Repressed Hoffman
Promoter UCSC
PromoterFlanking Hoffman
Intron UCSC
**H3K9ac Trynka
H3K4me3 Trynka
H3K4me1 Trynka
H3K27ac PGC2
H3K27ac Hnisz
FetalDHS Trynka
Enhancer Hoffman
Enhancer Andersson
DHS Trynka
DGF ENCODE
CTCF Hoffman
Conserved LindbladToh
Coding UCSC
Annotation
−30
0
30
Enrichment (prop h2 / prop snps)

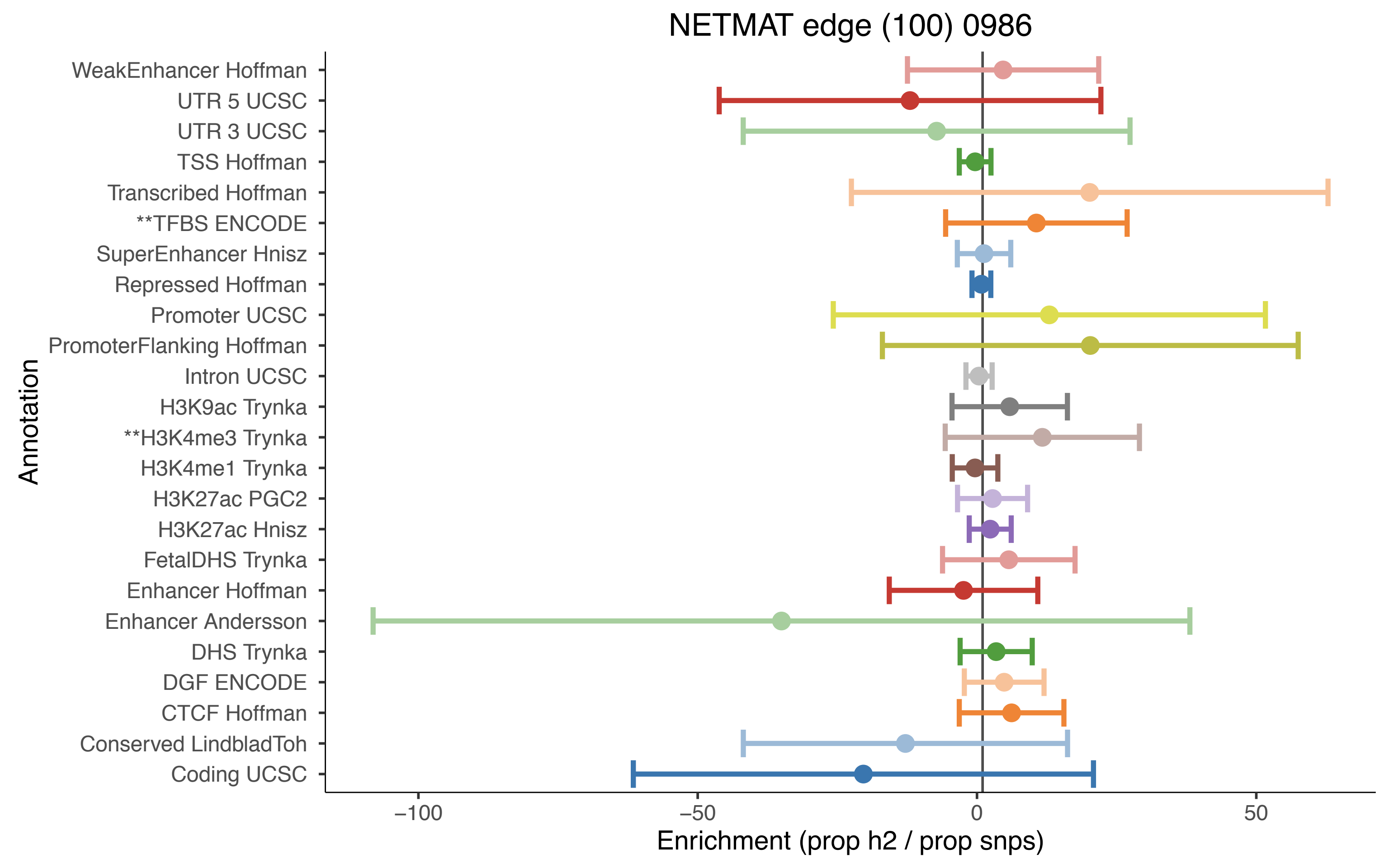
NETMAT edge (100) 0986
WeakEnhancer Hoffman
UTR 5 UCSC
UTR 3 UCSC
TSS Hoffman
Transcribed Hoffman
**TFBS ENCODE
SuperEnhancer Hnisz
Repressed Hoffman
Promoter UCSC
PromoterFlanking Hoffman
Intron UCSC
H3K9ac Trynka
**H3K4me3 Trynka
H3K4me1 Trynka
H3K27ac PGC2
H3K27ac Hnisz
FetalDHS Trynka
Enhancer Hoffman
Enhancer Andersson
DHS Trynka
DGF ENCODE
CTCF Hoffman
Conserved LindbladToh
Coding UCSC
Annotation
−100
−50
0
50
Enrichment (prop h2 / prop snps)

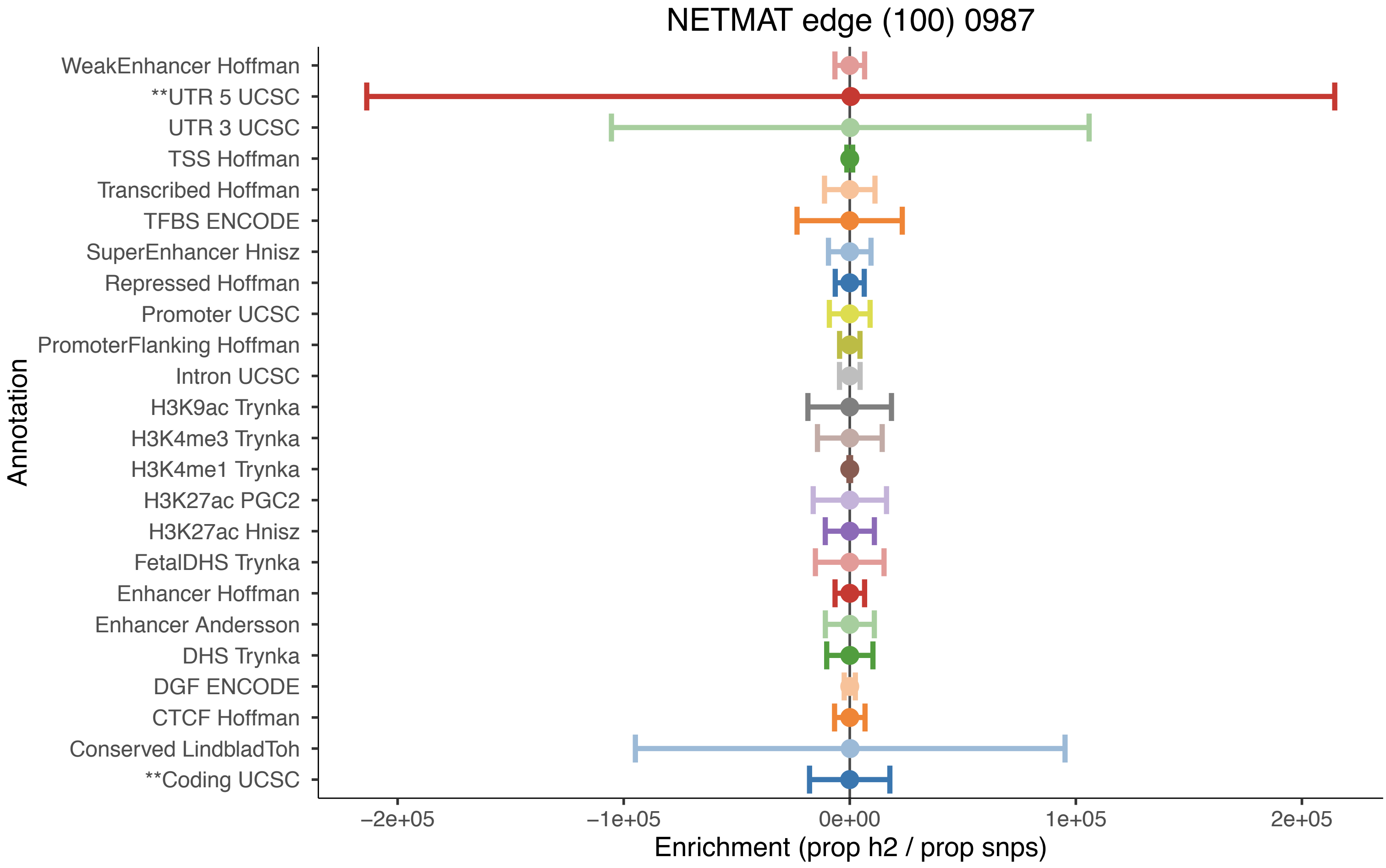
NETMAT edge (100) 0987
Annotation
WeakEnhancer Hoffman
**UTR 5 UCSC
UTR 3 UCSC
TSS Hoffman
Transcribed Hoffman
TFBS ENCODE
SuperEnhancer Hnisz
Repressed Hoffman
Promoter UCSC
PromoterFlanking Hoffman
Intron UCSC
H3K9ac Trynka
H3K4me3 Trynka
H3K4me1 Trynka
H3K27ac PGC2
H3K27ac Hnisz
FetalDHS Trynka
Enhancer Hoffman
Enhancer Andersson
DHS Trynka
DGF ENCODE
CTCF Hoffman
Conserved LindbladToh
**Coding UCSC
−2e+05
−1e+05
0e+00
1e+05
2e+05
Enrichment (prop h2 / prop snps)

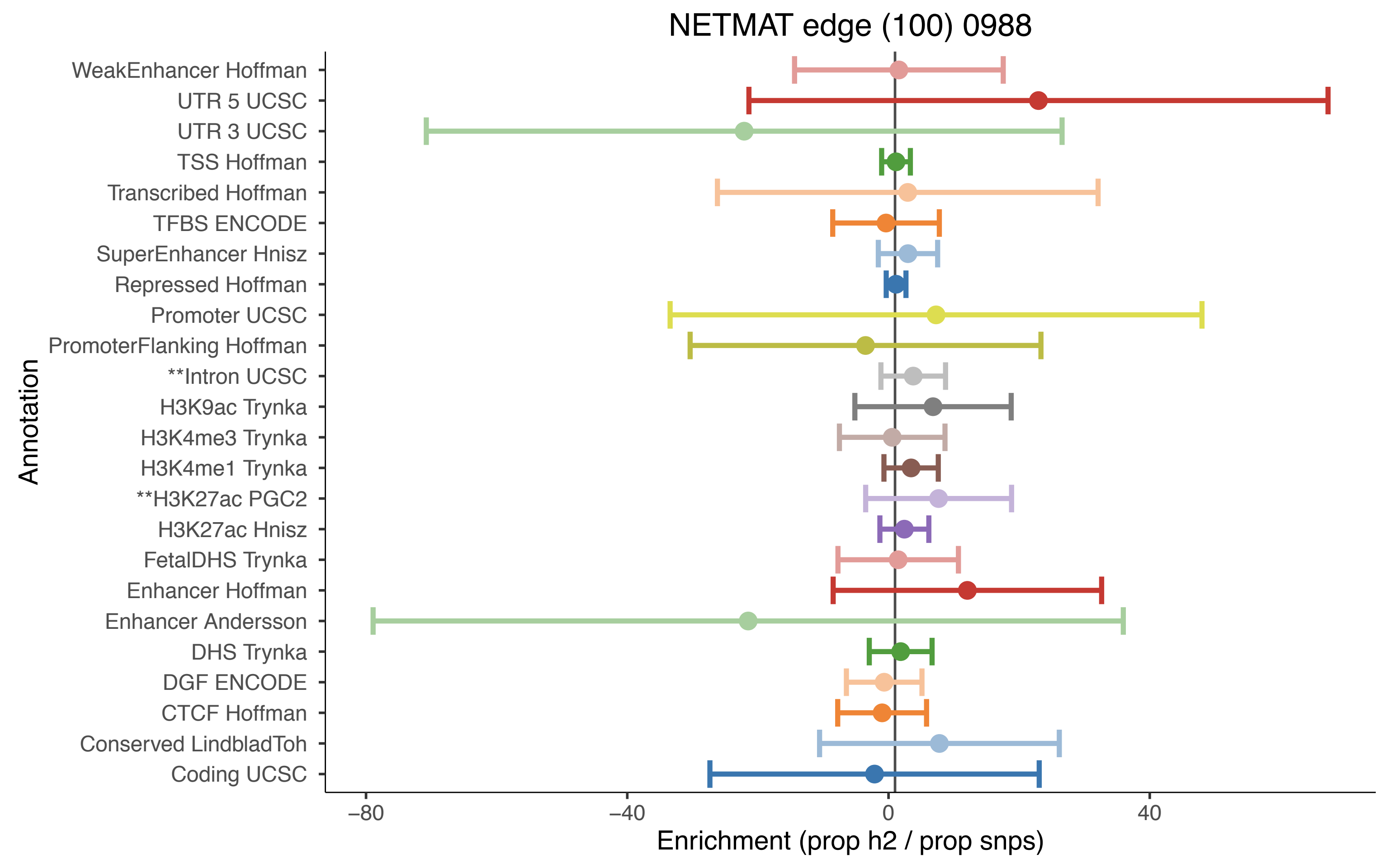
NETMAT edge (100) 0988
WeakEnhancer Hoffman
UTR 5 UCSC
UTR 3 UCSC
TSS Hoffman
Transcribed Hoffman
TFBS ENCODE
SuperEnhancer Hnisz
Repressed Hoffman
Promoter UCSC
PromoterFlanking Hoffman
**Intron UCSC
H3K9ac Trynka
H3K4me3 Trynka
H3K4me1 Trynka
**H3K27ac PGC2
H3K27ac Hnisz
FetalDHS Trynka
Enhancer Hoffman
Enhancer Andersson
DHS Trynka
DGF ENCODE
CTCF Hoffman
Conserved LindbladToh
Coding UCSC
Annotation
−80
−40
0
40
Enrichment (prop h2 / prop snps)

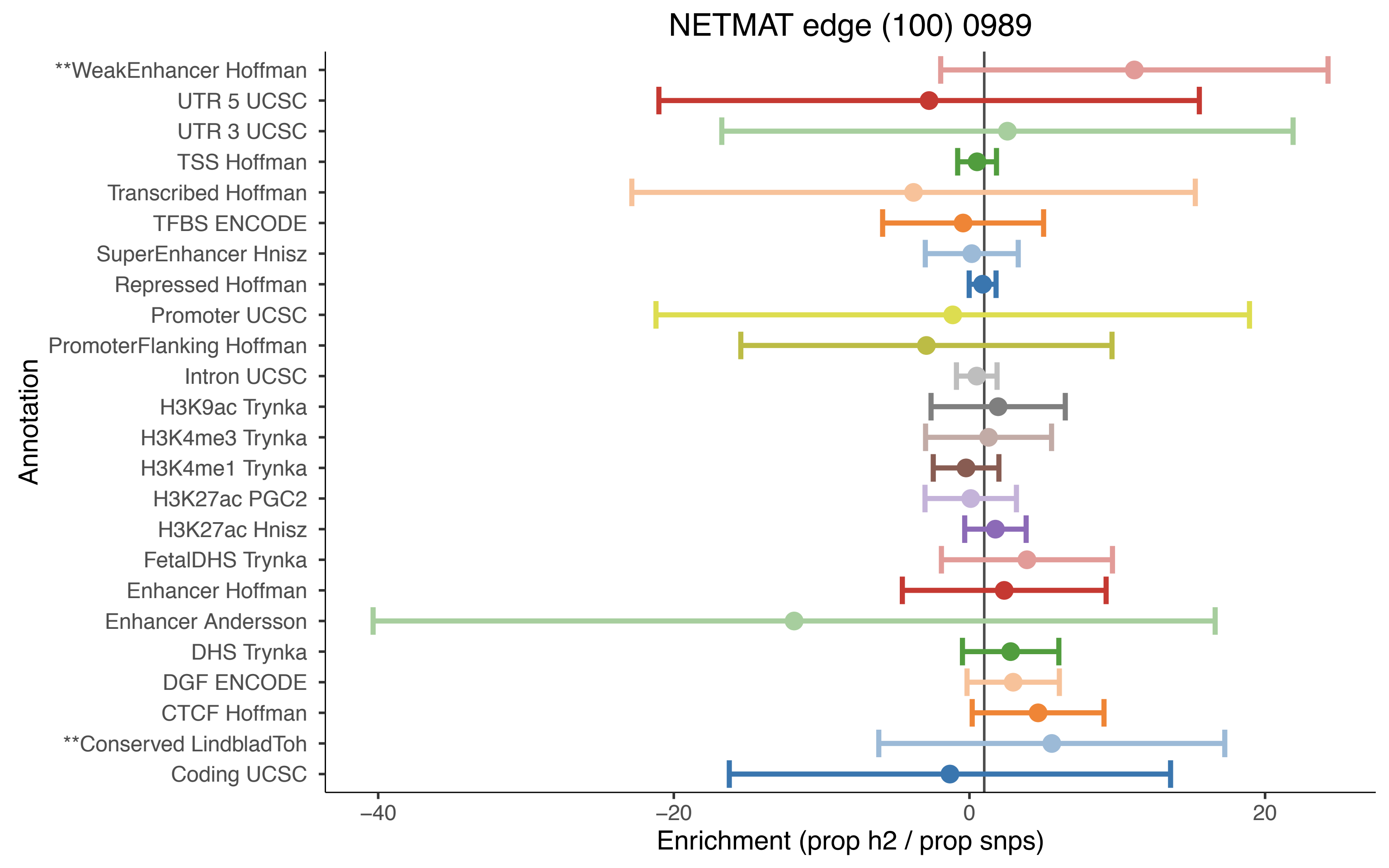
NETMAT edge (100) 0989
**WeakEnhancer Hoffman
UTR 5 UCSC
UTR 3 UCSC
TSS Hoffman
Transcribed Hoffman
TFBS ENCODE
SuperEnhancer Hnisz
Repressed Hoffman
Promoter UCSC
PromoterFlanking Hoffman
Intron UCSC
H3K9ac Trynka
H3K4me3 Trynka
H3K4me1 Trynka
H3K27ac PGC2
H3K27ac Hnisz
FetalDHS Trynka
Enhancer Hoffman
Enhancer Andersson
DHS Trynka
DGF ENCODE
CTCF Hoffman
**Conserved LindbladToh
Coding UCSC
Annotation
−40
−20
0
20
Enrichment (prop h2 / prop snps)

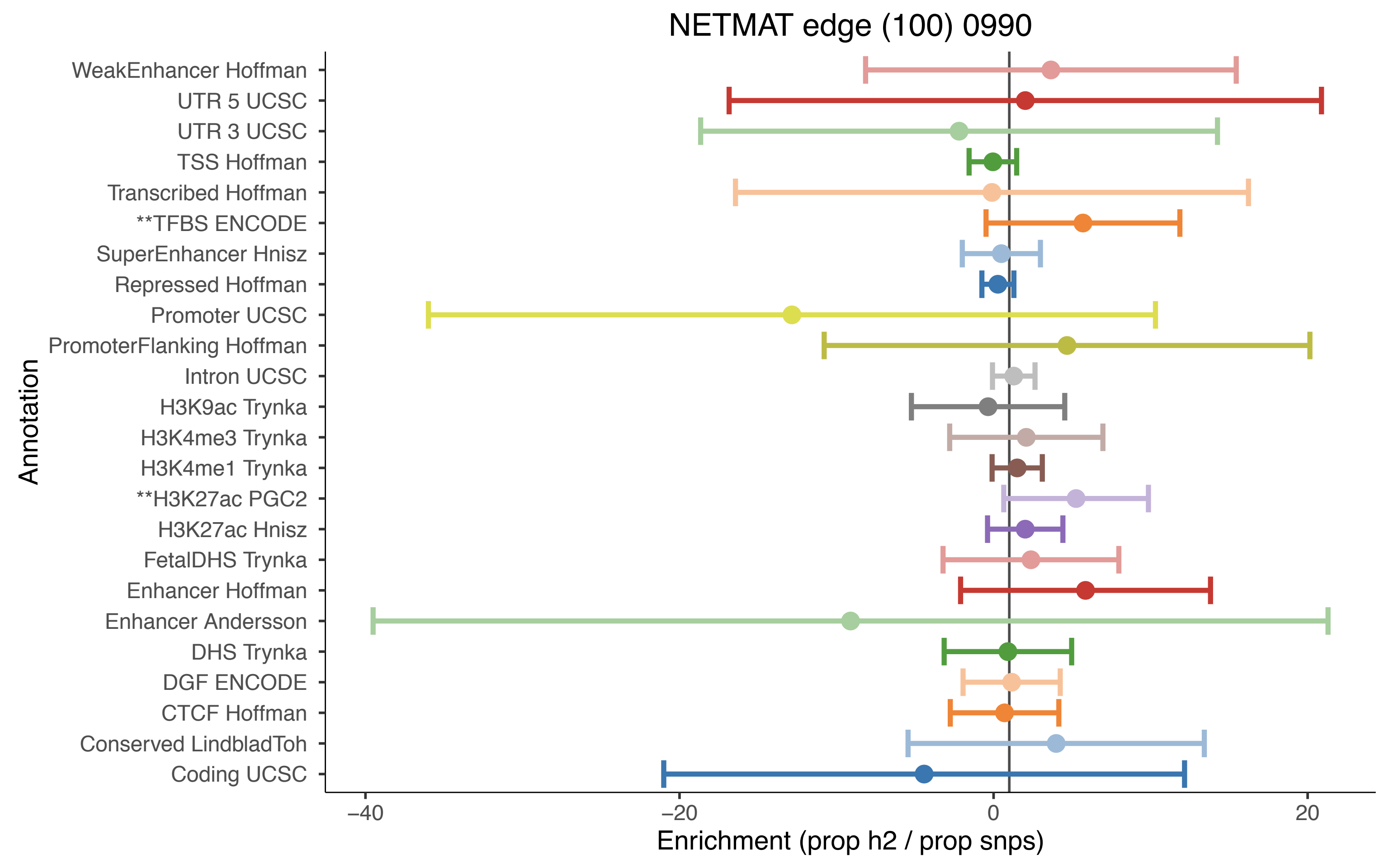
NETMAT edge (100) 0990
WeakEnhancer Hoffman
UTR 5 UCSC
UTR 3 UCSC
TSS Hoffman
Transcribed Hoffman
**TFBS ENCODE
SuperEnhancer Hnisz
Repressed Hoffman
Promoter UCSC
PromoterFlanking Hoffman
Intron UCSC
H3K9ac Trynka
H3K4me3 Trynka
H3K4me1 Trynka
**H3K27ac PGC2
H3K27ac Hnisz
FetalDHS Trynka
Enhancer Hoffman
Enhancer Andersson
DHS Trynka
DGF ENCODE
CTCF Hoffman
Conserved LindbladToh
Coding UCSC
Annotation
−40
−20
0
20
Enrichment (prop h2 / prop snps)

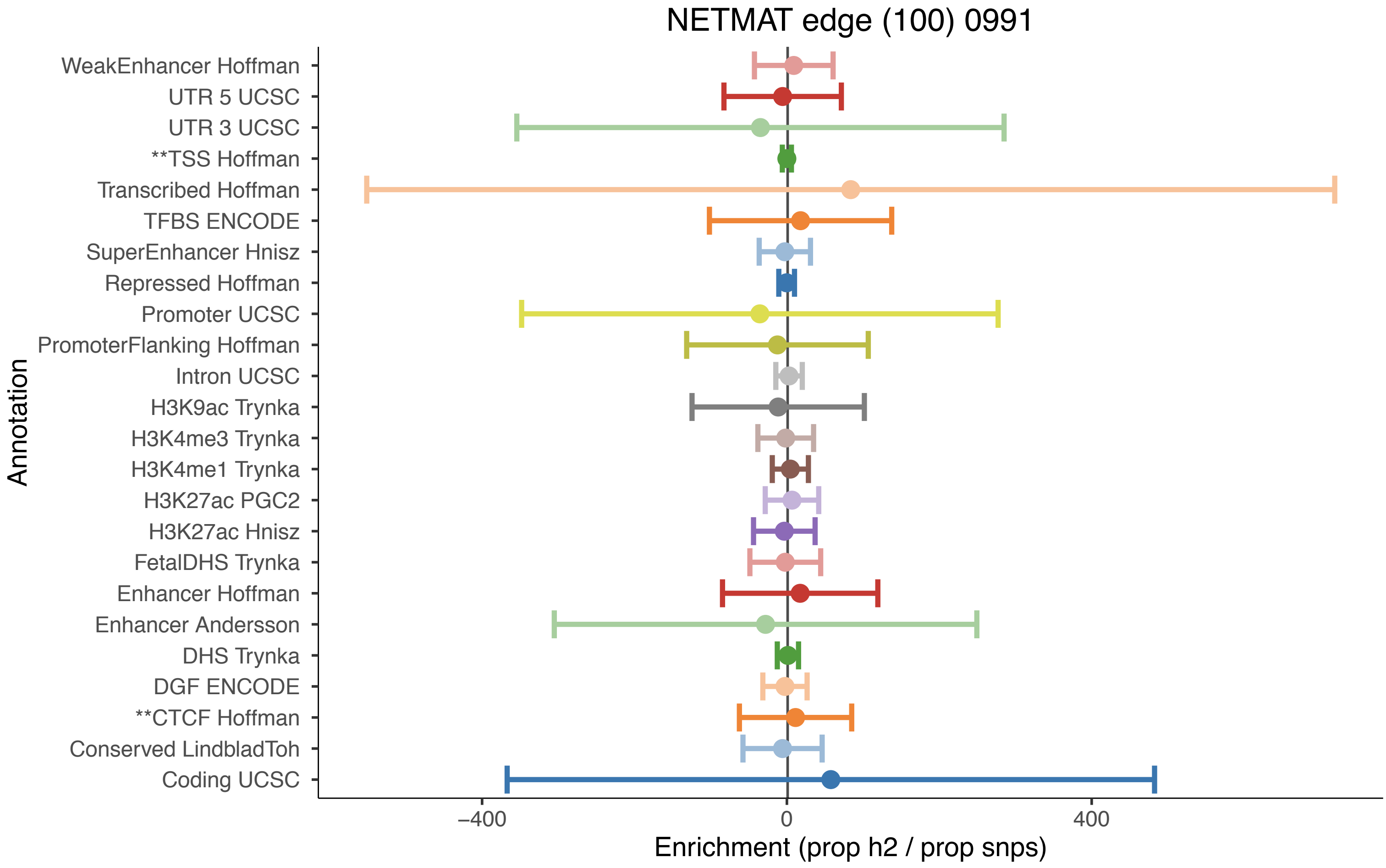
NETMAT edge (100) 0991
WeakEnhancer Hoffman
UTR 5 UCSC
UTR 3 UCSC
**TSS Hoffman
Transcribed Hoffman
TFBS ENCODE
SuperEnhancer Hnisz
Repressed Hoffman
Promoter UCSC
PromoterFlanking Hoffman
Intron UCSC
H3K9ac Trynka
H3K4me3 Trynka
H3K4me1 Trynka
H3K27ac PGC2
H3K27ac Hnisz
FetalDHS Trynka
Enhancer Hoffman
Enhancer Andersson
DHS Trynka
DGF ENCODE
**CTCF Hoffman
Conserved LindbladToh
Coding UCSC
Annotation
−400
0
400
Enrichment (prop h2 / prop snps)

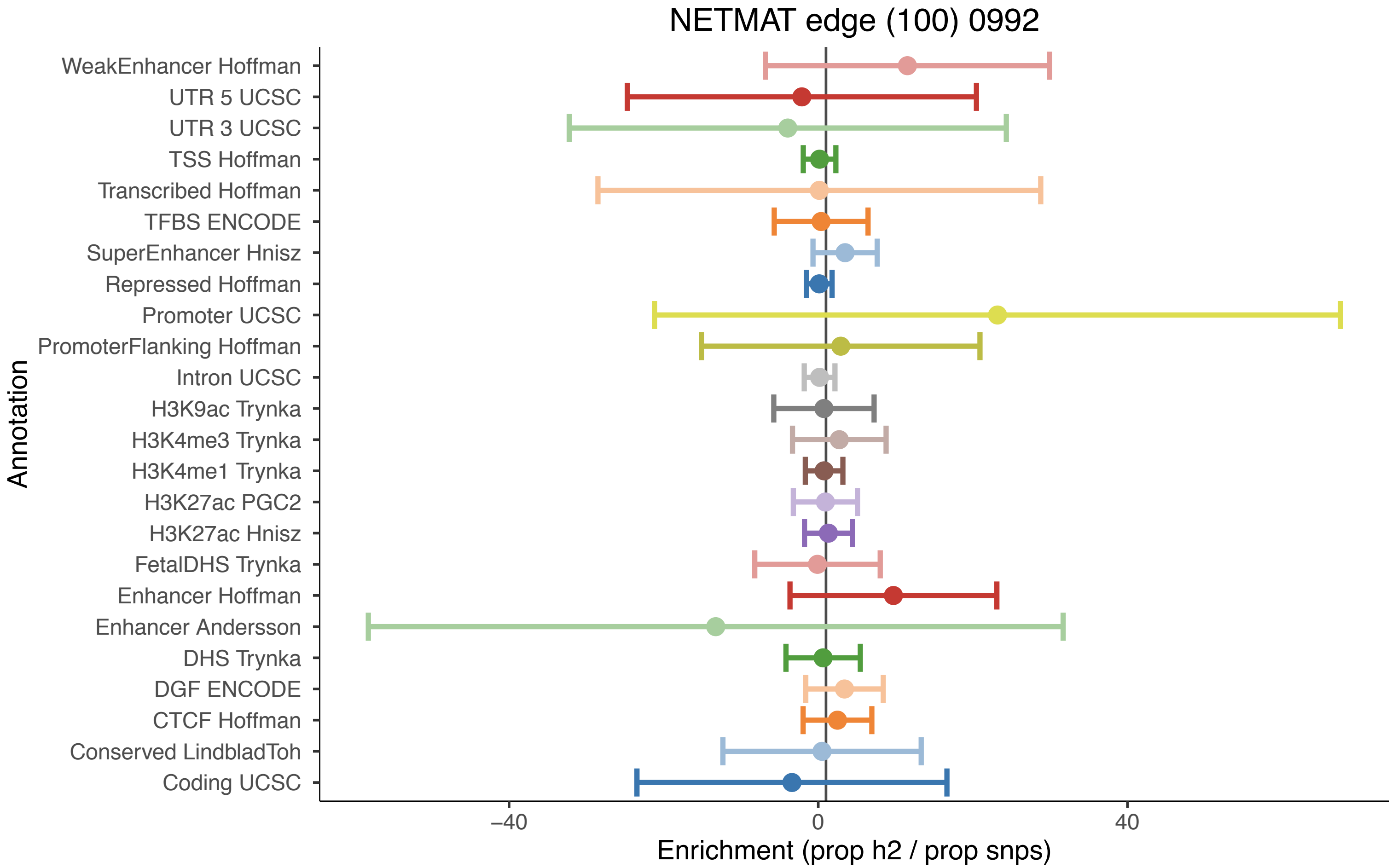

NETMAT edge (100) 0992
Annotation
WeakEnhancer Hoffman
UTR 5 UCSC
UTR 3 UCSC
TSS Hoffman
Transcribed Hoffman
TFBS ENCODE
SuperEnhancer Hnisz
Repressed Hoffman
Promoter UCSC
PromoterFlanking Hoffman
Intron UCSC
H3K9ac Trynka
H3K4me3 Trynka
H3K4me1 Trynka
H3K27ac PGC2
H3K27ac Hnisz
FetalDHS Trynka
Enhancer Hoffman
Enhancer Andersson
DHS Trynka
DGF ENCODE
CTCF Hoffman
Conserved LindbladToh
Coding UCSC
−40
0
40
Enrichment (prop h2 / prop snps)

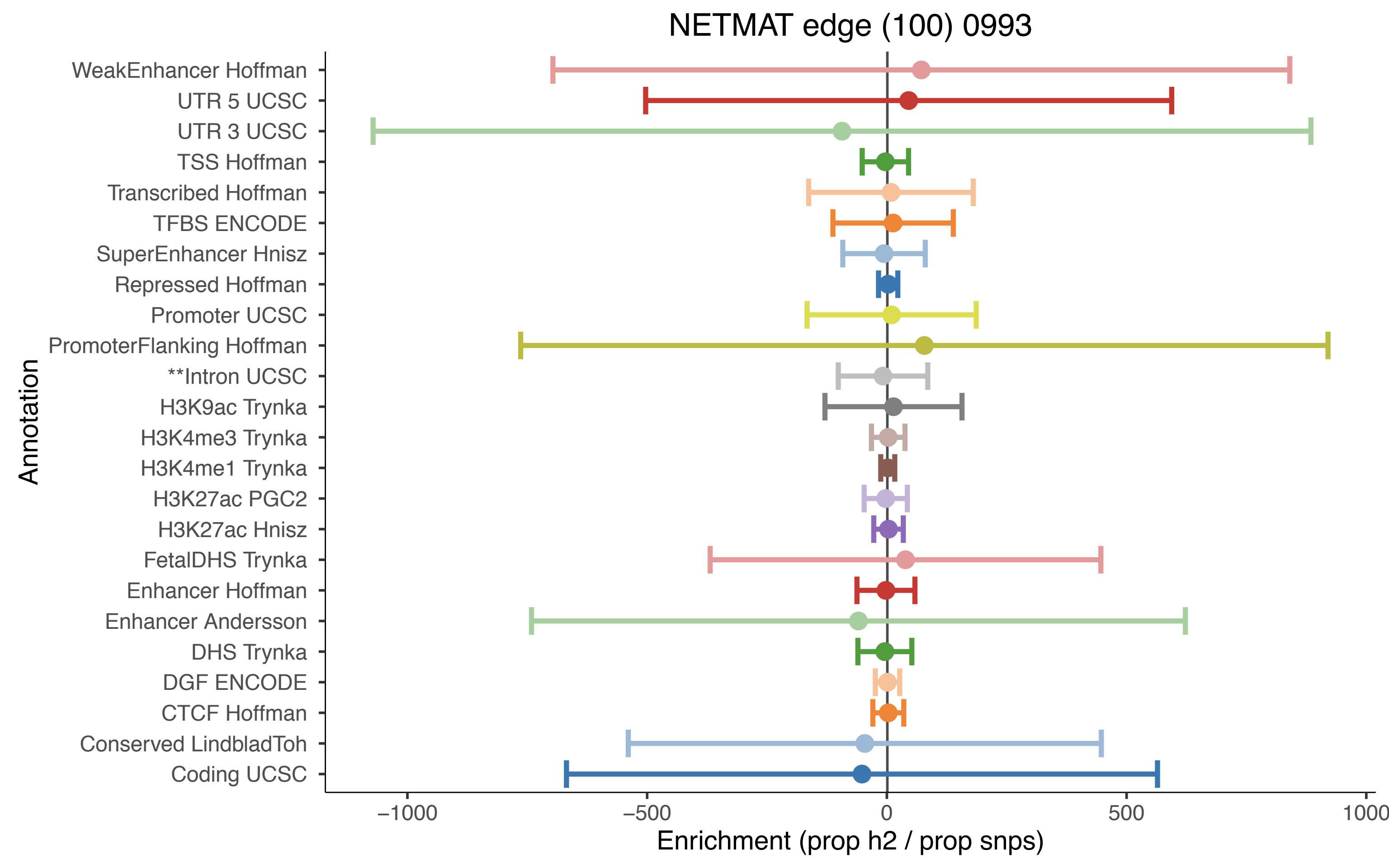
NETMAT edge (100) 0993
WeakEnhancer Hoffman
UTR 5 UCSC
UTR 3 UCSC
TSS Hoffman
Transcribed Hoffman
TFBS ENCODE
SuperEnhancer Hnisz
Repressed Hoffman
Promoter UCSC
PromoterFlanking Hoffman
**Intron UCSC
H3K9ac Trynka
H3K4me3 Trynka
H3K4me1 Trynka
H3K27ac PGC2
H3K27ac Hnisz
FetalDHS Trynka
Enhancer Hoffman
Enhancer Andersson
DHS Trynka
DGF ENCODE
CTCF Hoffman
Conserved LindbladToh
Coding UCSC
Annotation
−1000
−500
0
500
1000
Enrichment (prop h2 / prop snps)

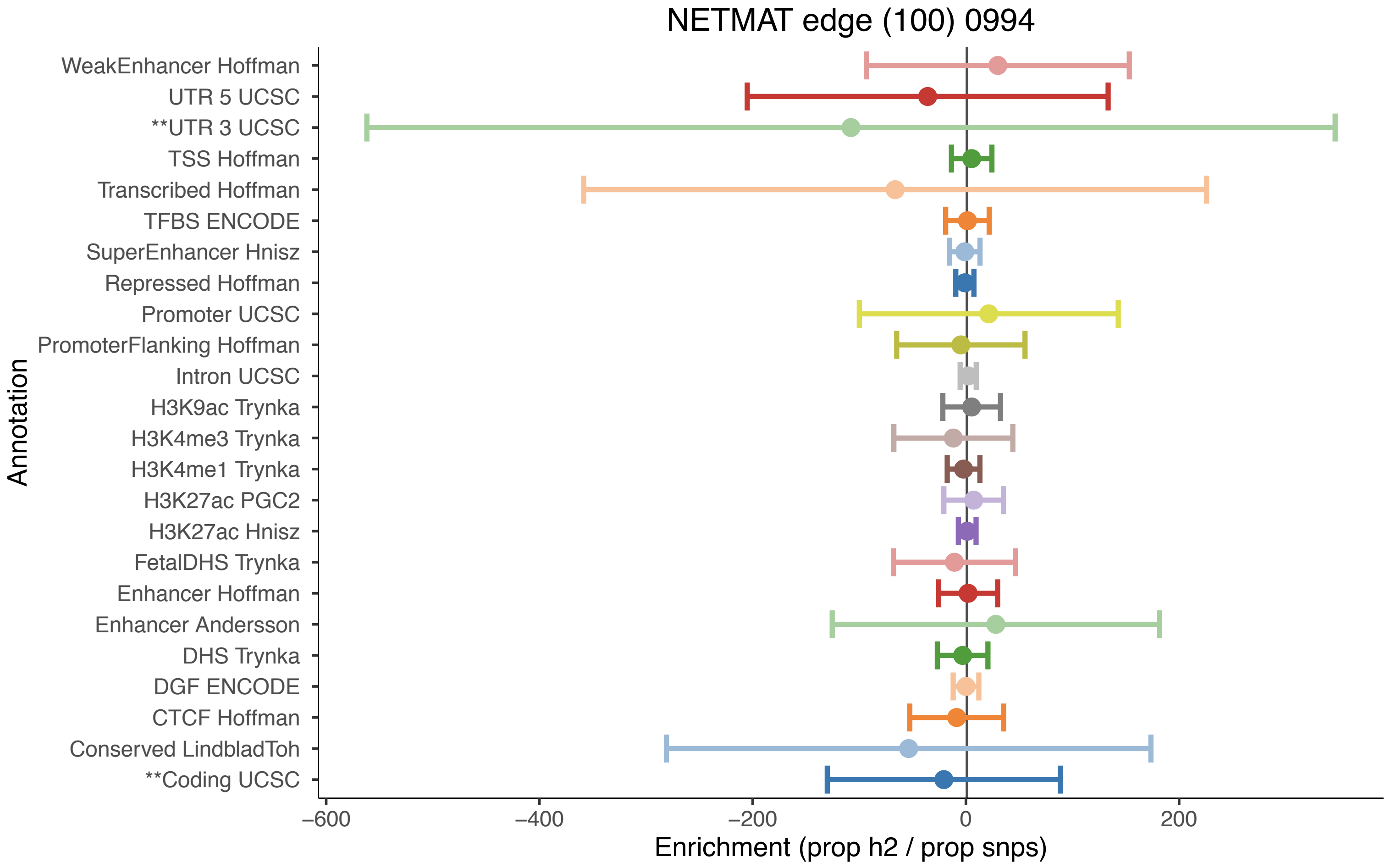
NETMAT edge (100) 0994
WeakEnhancer Hoffman
UTR 5 UCSC
**UTR 3 UCSC
TSS Hoffman
Transcribed Hoffman
TFBS ENCODE
SuperEnhancer Hnisz
Repressed Hoffman
Promoter UCSC
PromoterFlanking Hoffman
Intron UCSC
H3K9ac Trynka
H3K4me3 Trynka
H3K4me1 Trynka
H3K27ac PGC2
H3K27ac Hnisz
FetalDHS Trynka
Enhancer Hoffman
Enhancer Andersson
DHS Trynka
DGF ENCODE
CTCF Hoffman
Conserved LindbladToh
**Coding UCSC
Annotation
−600
−400
−200
0
200
Enrichment (prop h2 / prop snps)

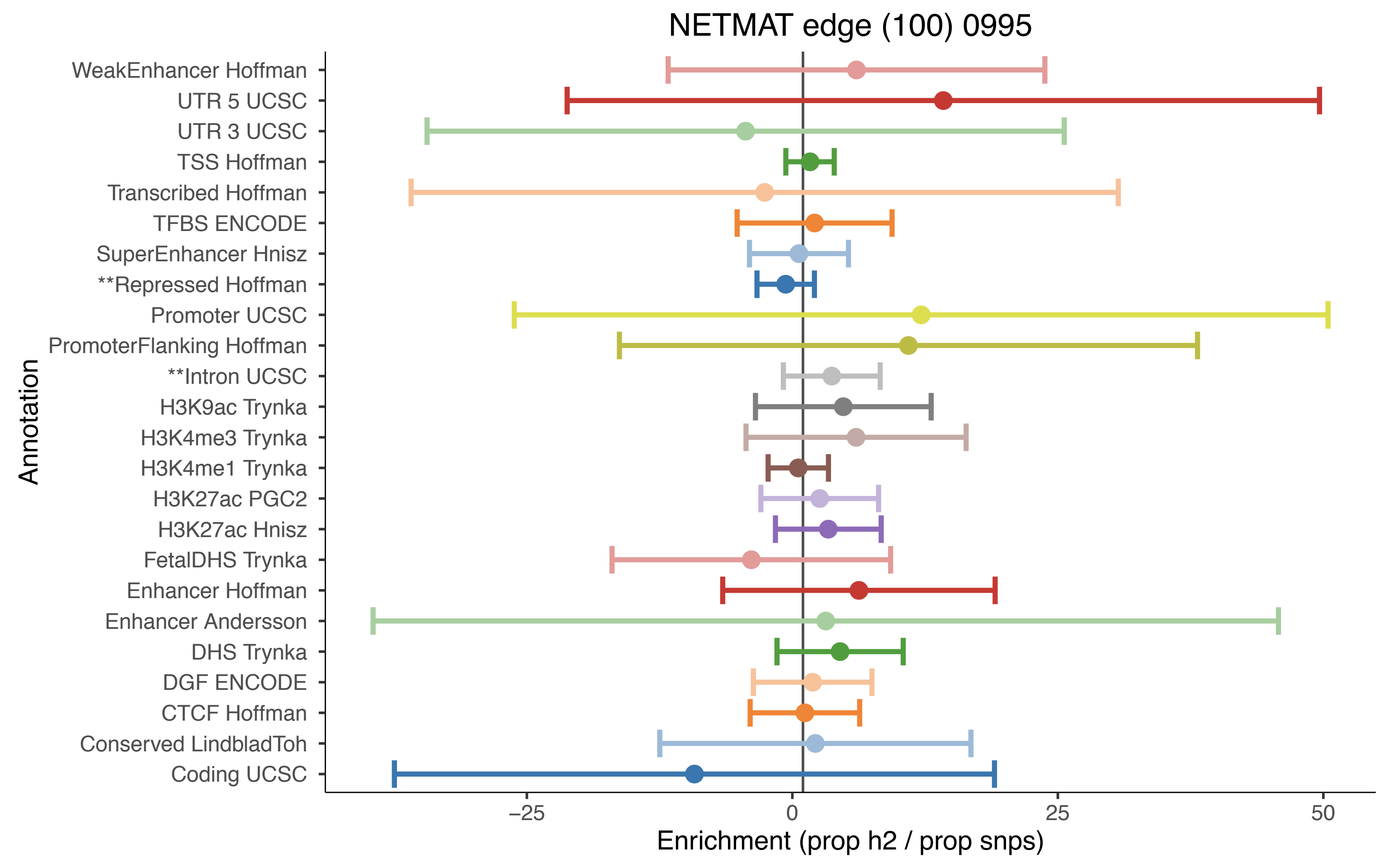
NETMAT edge (100) 0995
WeakEnhancer Hoffman
UTR 5 UCSC
UTR 3 UCSC
TSS Hoffman
Transcribed Hoffman
TFBS ENCODE
SuperEnhancer Hnisz
**Repressed Hoffman
Promoter UCSC
PromoterFlanking Hoffman
**Intron UCSC
H3K9ac Trynka
H3K4me3 Trynka
H3K4me1 Trynka
H3K27ac PGC2
H3K27ac Hnisz
FetalDHS Trynka
Enhancer Hoffman
Enhancer Andersson
DHS Trynka
DGF ENCODE
CTCF Hoffman
Conserved LindbladToh
Coding UCSC
Annotation
−25
0
25
50
Enrichment (prop h2 / prop snps)

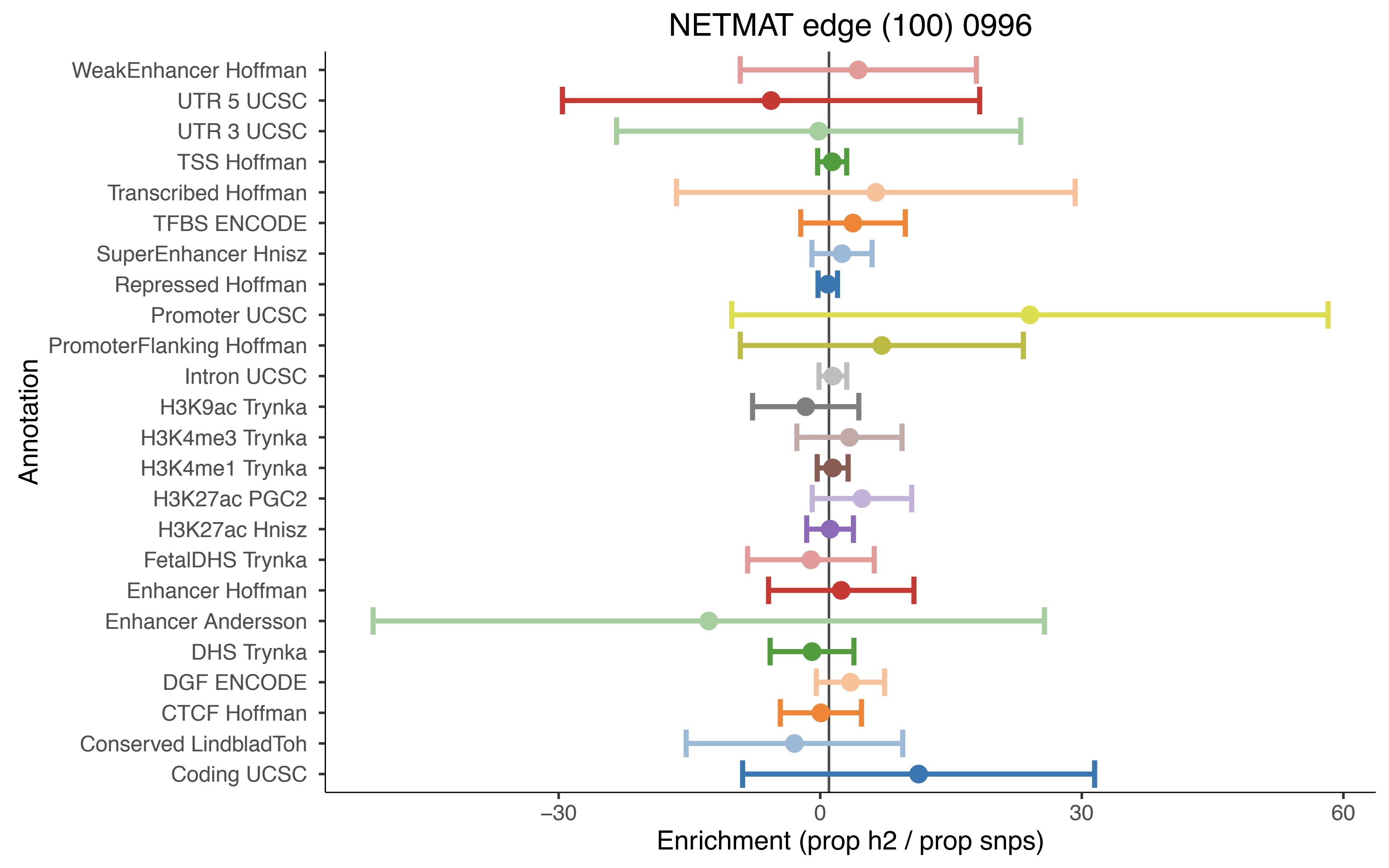
NETMAT edge (100) 0996
WeakEnhancer Hoffman
UTR 5 UCSC
UTR 3 UCSC
TSS Hoffman
Transcribed Hoffman
TFBS ENCODE
SuperEnhancer Hnisz
Repressed Hoffman
Promoter UCSC
PromoterFlanking Hoffman
Intron UCSC
H3K9ac Trynka
H3K4me3 Trynka
H3K4me1 Trynka
H3K27ac PGC2
H3K27ac Hnisz
FetalDHS Trynka
Enhancer Hoffman
Enhancer Andersson
DHS Trynka
DGF ENCODE
CTCF Hoffman
Conserved LindbladToh
Coding UCSC
Annotation
−30
0
30
60
Enrichment (prop h2 / prop snps)

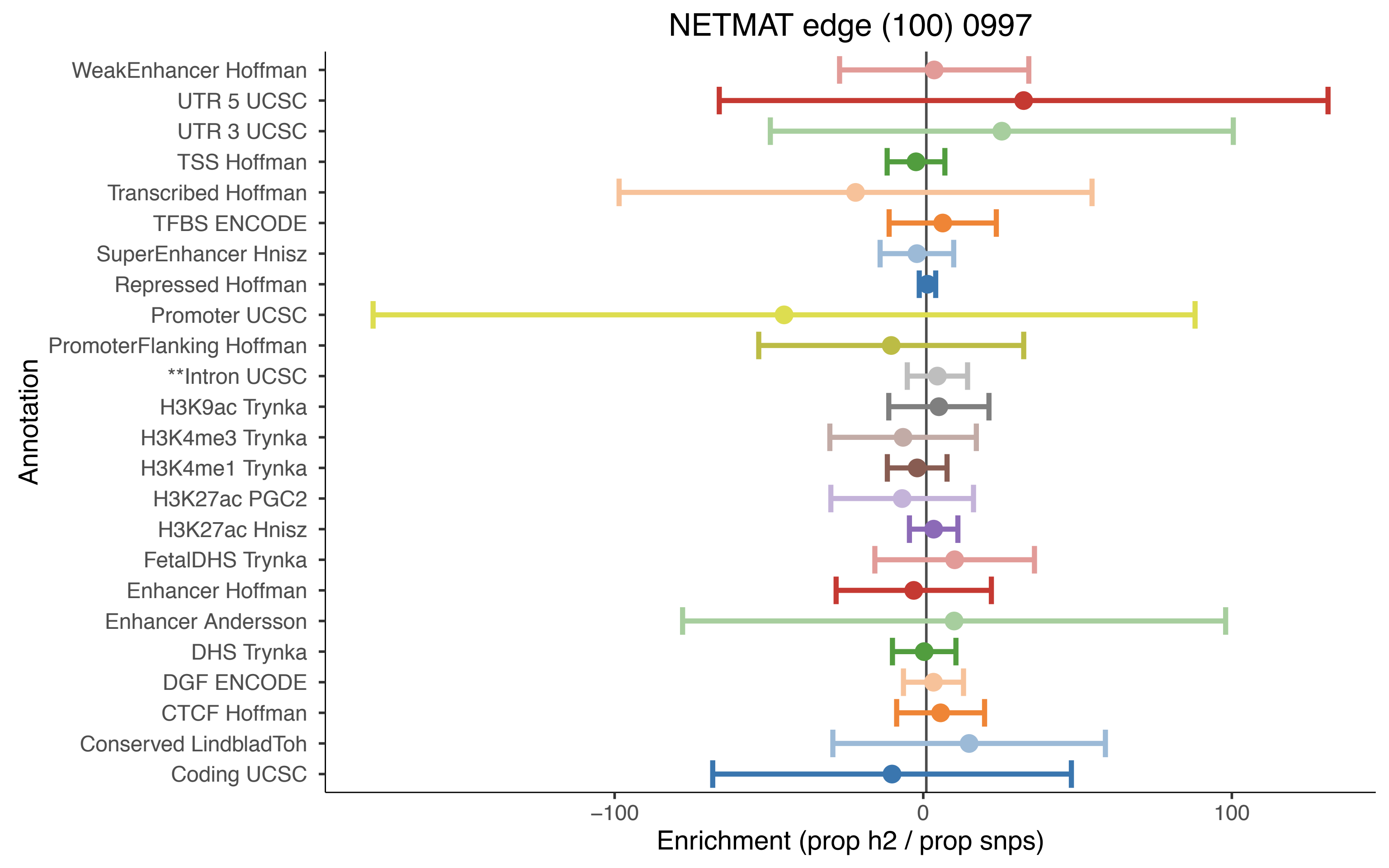

NETMAT edge (100) 0997
WeakEnhancer Hoffman
UTR 5 UCSC
UTR 3 UCSC
TSS Hoffman
Transcribed Hoffman
TFBS ENCODE
SuperEnhancer Hnisz
Repressed Hoffman
Promoter UCSC
PromoterFlanking Hoffman
**Intron UCSC
H3K9ac Trynka
H3K4me3 Trynka
H3K4me1 Trynka
H3K27ac PGC2
H3K27ac Hnisz
FetalDHS Trynka
Enhancer Hoffman
Enhancer Andersson
DHS Trynka
DGF ENCODE
CTCF Hoffman
Conserved LindbladToh
Coding UCSC
Annotation
−100
0
100
Enrichment (prop h2 / prop snps)

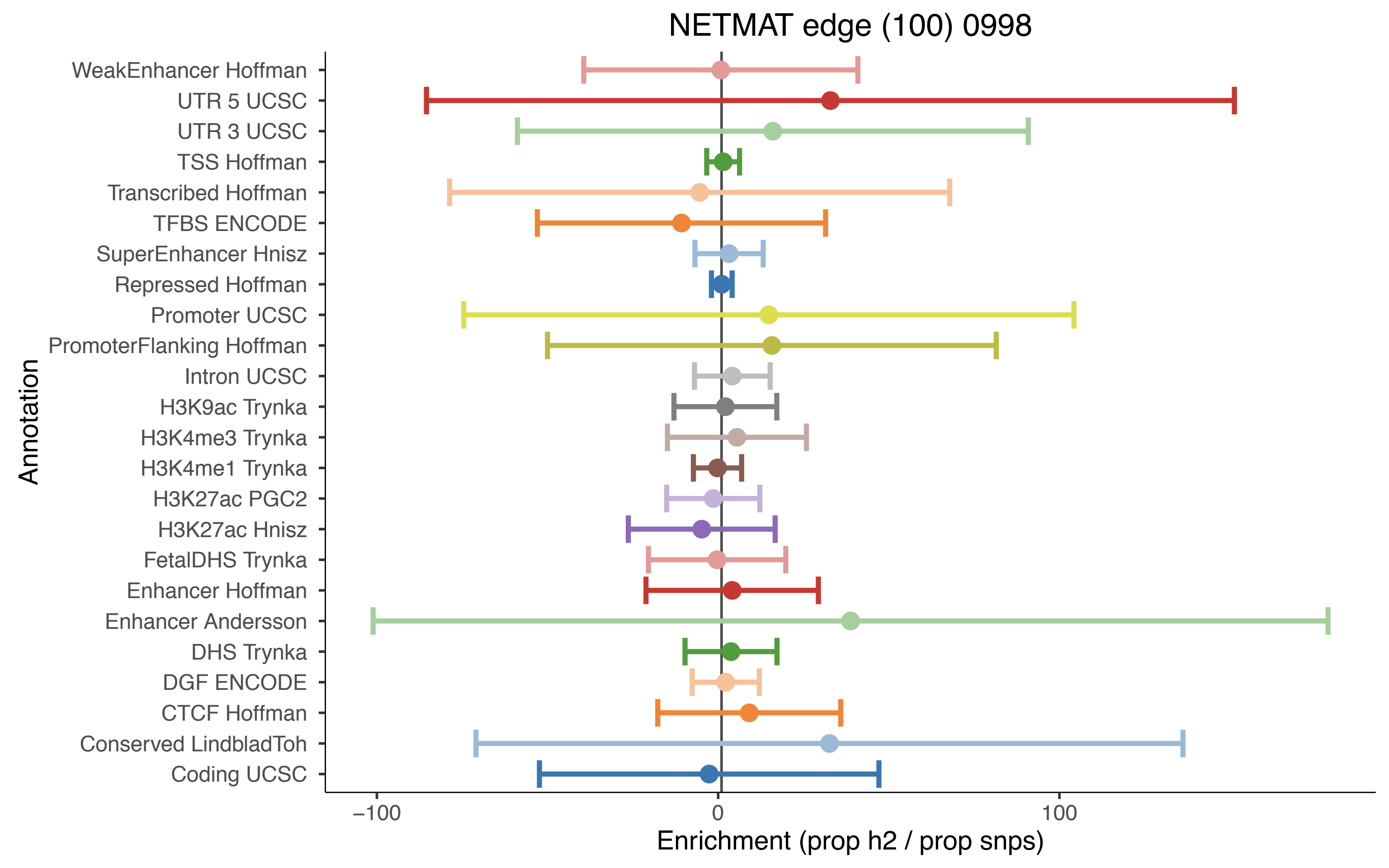

NETMAT edge (100) 0998
WeakEnhancer Hoffman
UTR 5 UCSC
UTR 3 UCSC
TSS Hoffman
Transcribed Hoffman
TFBS ENCODE
SuperEnhancer Hnisz
Repressed Hoffman
Promoter UCSC
PromoterFlanking Hoffman
Intron UCSC
H3K9ac Trynka
H3K4me3 Trynka
H3K4me1 Trynka
H3K27ac PGC2
H3K27ac Hnisz
FetalDHS Trynka
Enhancer Hoffman
Enhancer Andersson
DHS Trynka
DGF ENCODE
CTCF Hoffman
Conserved LindbladToh
Coding UCSC
Annotation
−100
0
100
Enrichment (prop h2 / prop snps)

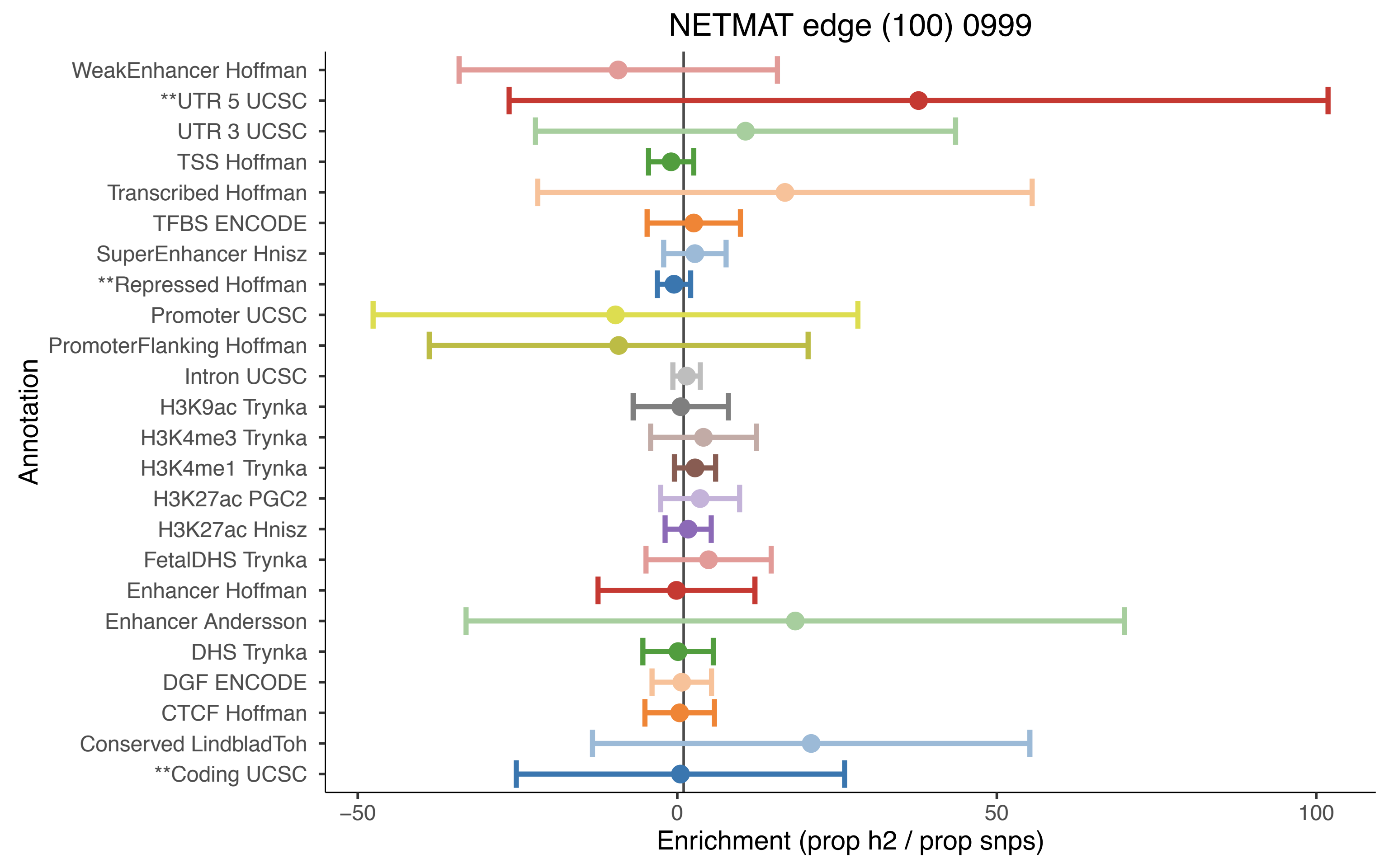
NETMAT edge (100) 0999
WeakEnhancer Hoffman
**UTR 5 UCSC
UTR 3 UCSC
TSS Hoffman
Transcribed Hoffman
TFBS ENCODE
SuperEnhancer Hnisz
**Repressed Hoffman
Promoter UCSC
PromoterFlanking Hoffman
Intron UCSC
H3K9ac Trynka
H3K4me3 Trynka
H3K4me1 Trynka
H3K27ac PGC2
H3K27ac Hnisz
FetalDHS Trynka
Enhancer Hoffman
Enhancer Andersson
DHS Trynka
DGF ENCODE
CTCF Hoffman
Conserved LindbladToh
**Coding UCSC
Annotation
−50
0
50
100
Enrichment (prop h2 / prop snps)

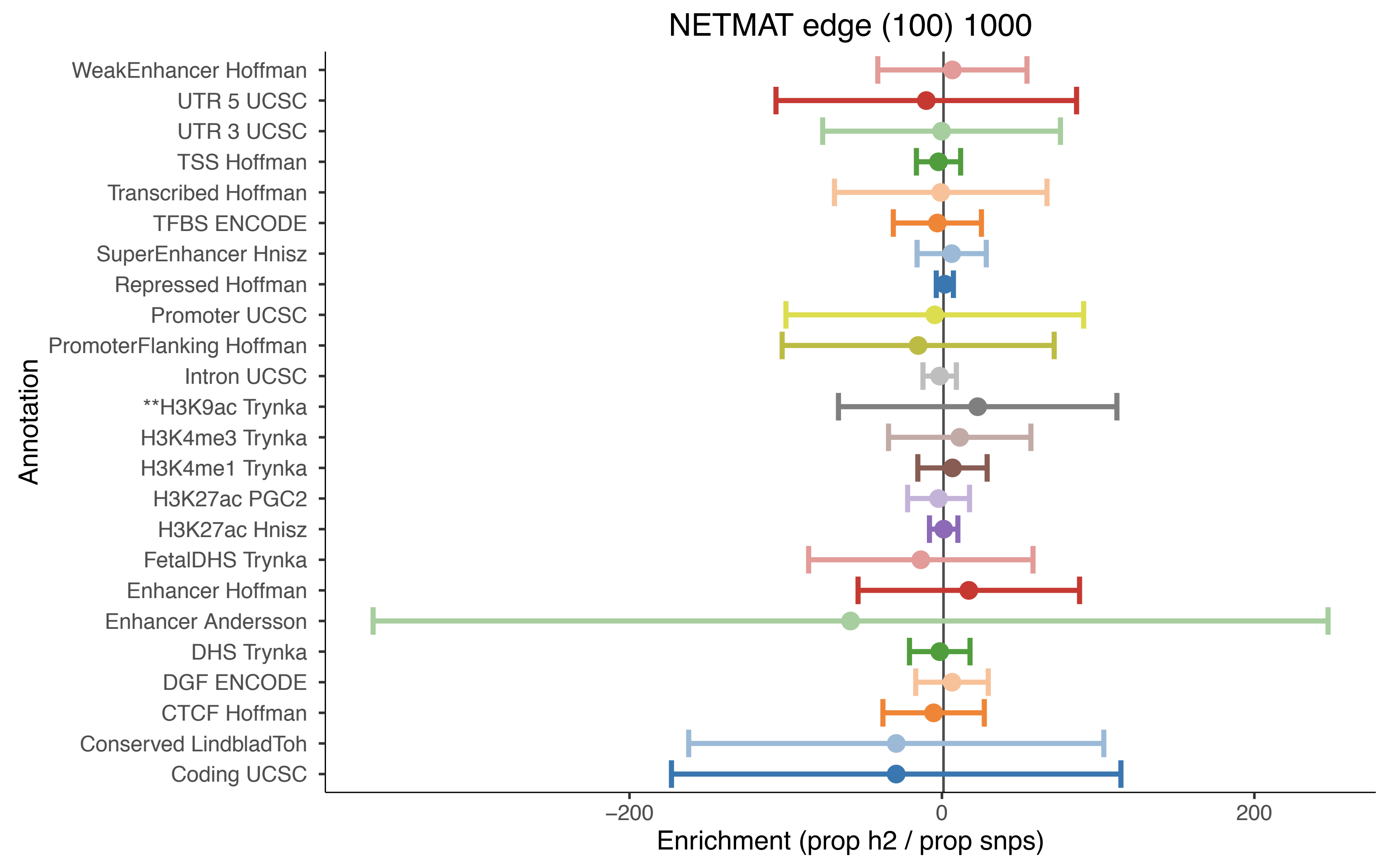

NETMAT edge (100) 1000
WeakEnhancer Hoffman
UTR 5 UCSC
UTR 3 UCSC
TSS Hoffman
Transcribed Hoffman
TFBS ENCODE
SuperEnhancer Hnisz
Repressed Hoffman
Promoter UCSC
PromoterFlanking Hoffman
Intron UCSC
**H3K9ac Trynka
H3K4me3 Trynka
H3K4me1 Trynka
H3K27ac PGC2
H3K27ac Hnisz
FetalDHS Trynka
Enhancer Hoffman
Enhancer Andersson
DHS Trynka
DGF ENCODE
CTCF Hoffman
Conserved LindbladToh
Coding UCSC
Annotation
−200
0
200
Enrichment (prop h2 / prop snps)

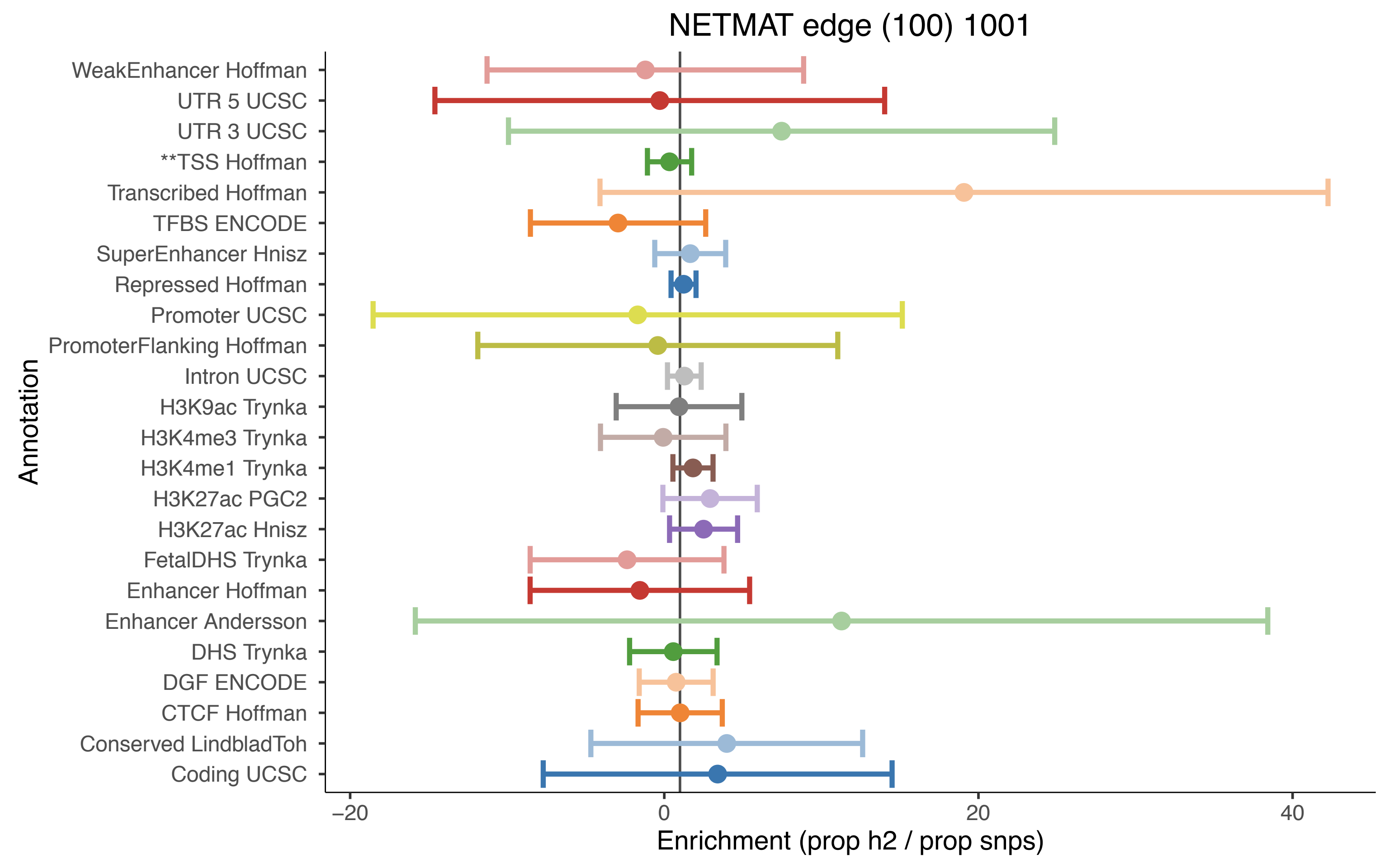
NETMAT edge (100) 1001
WeakEnhancer Hoffman
UTR 5 UCSC
UTR 3 UCSC
**TSS Hoffman
Transcribed Hoffman
TFBS ENCODE
SuperEnhancer Hnisz
Repressed Hoffman
Promoter UCSC
PromoterFlanking Hoffman
Intron UCSC
H3K9ac Trynka
H3K4me3 Trynka
H3K4me1 Trynka
H3K27ac PGC2
H3K27ac Hnisz
FetalDHS Trynka
Enhancer Hoffman
Enhancer Andersson
DHS Trynka
DGF ENCODE
CTCF Hoffman
Conserved LindbladToh
Coding UCSC
Annotation
−20
0
20
40
Enrichment (prop h2 / prop snps)

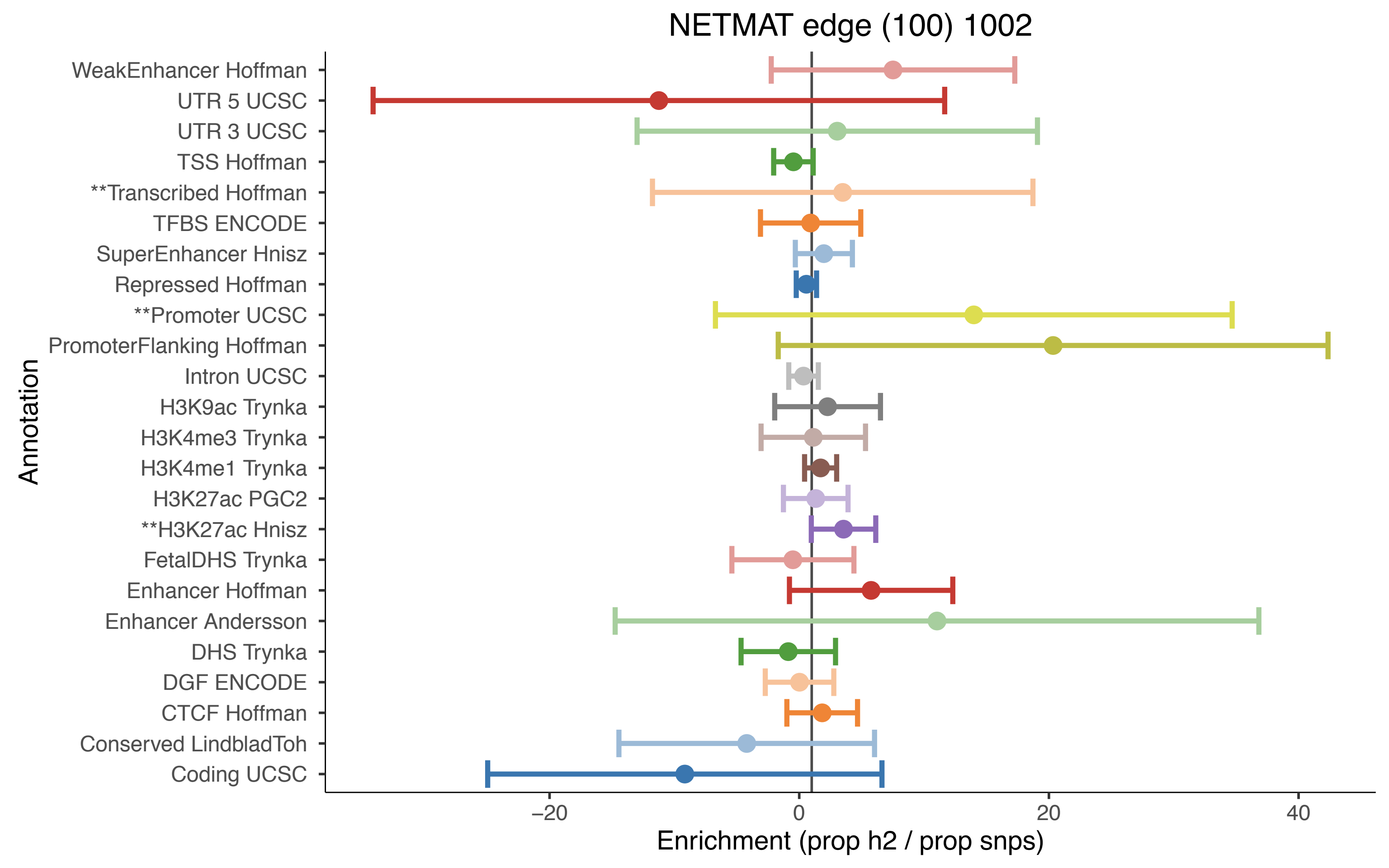
NETMAT edge (100) 1002
WeakEnhancer Hoffman
UTR 5 UCSC
UTR 3 UCSC
TSS Hoffman
**Transcribed Hoffman
TFBS ENCODE
SuperEnhancer Hnisz
Repressed Hoffman
**Promoter UCSC
PromoterFlanking Hoffman
Intron UCSC
H3K9ac Trynka
H3K4me3 Trynka
H3K4me1 Trynka
H3K27ac PGC2
**H3K27ac Hnisz
FetalDHS Trynka
Enhancer Hoffman
Enhancer Andersson
DHS Trynka
DGF ENCODE
CTCF Hoffman
Conserved LindbladToh
Coding UCSC
Annotation
−20
0
20
40
Enrichment (prop h2 / prop snps)

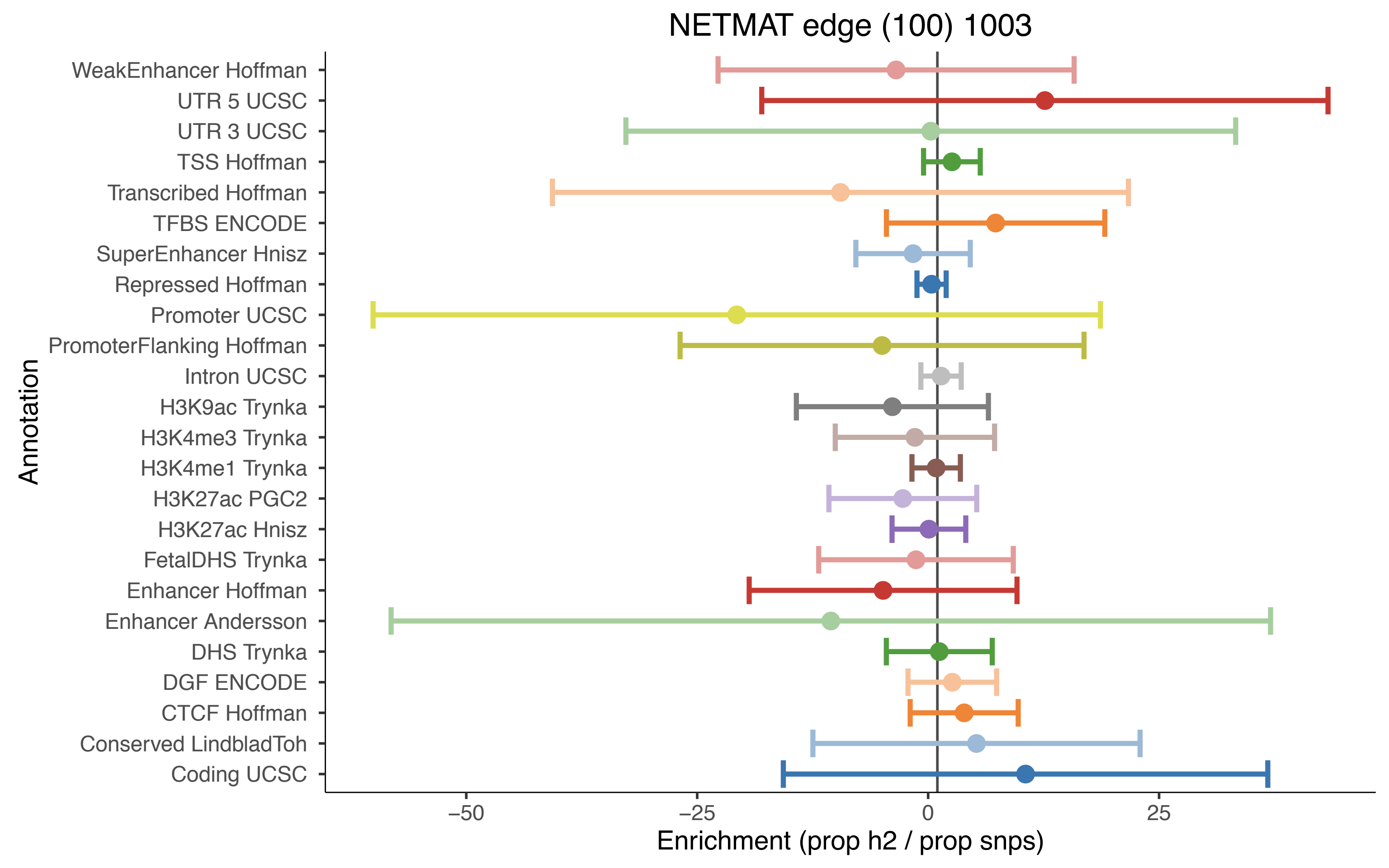
NETMAT edge (100) 1003
WeakEnhancer Hoffman
UTR 5 UCSC
UTR 3 UCSC
TSS Hoffman
Transcribed Hoffman
TFBS ENCODE
SuperEnhancer Hnisz
Repressed Hoffman
Promoter UCSC
PromoterFlanking Hoffman
Intron UCSC
H3K9ac Trynka
H3K4me3 Trynka
H3K4me1 Trynka
H3K27ac PGC2
H3K27ac Hnisz
FetalDHS Trynka
Enhancer Hoffman
Enhancer Andersson
DHS Trynka
DGF ENCODE
CTCF Hoffman
Conserved LindbladToh
Coding UCSC
Annotation
−50
−25
0
25
Enrichment (prop h2 / prop snps)

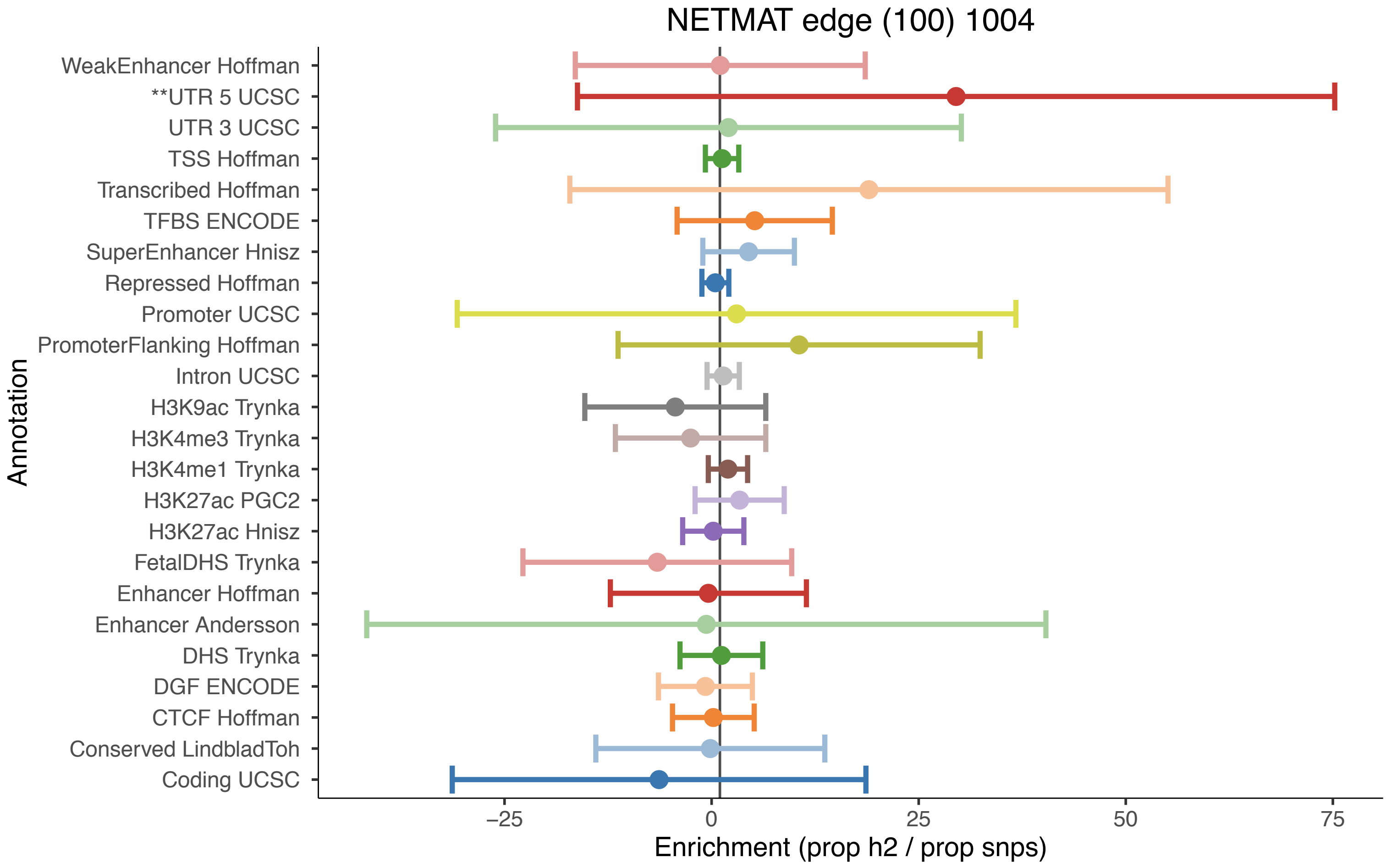
NETMAT edge (100) 1004
WeakEnhancer Hoffman
**UTR 5 UCSC
UTR 3 UCSC
TSS Hoffman
Transcribed Hoffman
TFBS ENCODE
SuperEnhancer Hnisz
Repressed Hoffman
Promoter UCSC
PromoterFlanking Hoffman
Intron UCSC
H3K9ac Trynka
H3K4me3 Trynka
H3K4me1 Trynka
H3K27ac PGC2
H3K27ac Hnisz
FetalDHS Trynka
Enhancer Hoffman
Enhancer Andersson
DHS Trynka
DGF ENCODE
CTCF Hoffman
Conserved LindbladToh
Coding UCSC
Annotation
−25
0
25
50
75
Enrichment (prop h2 / prop snps)

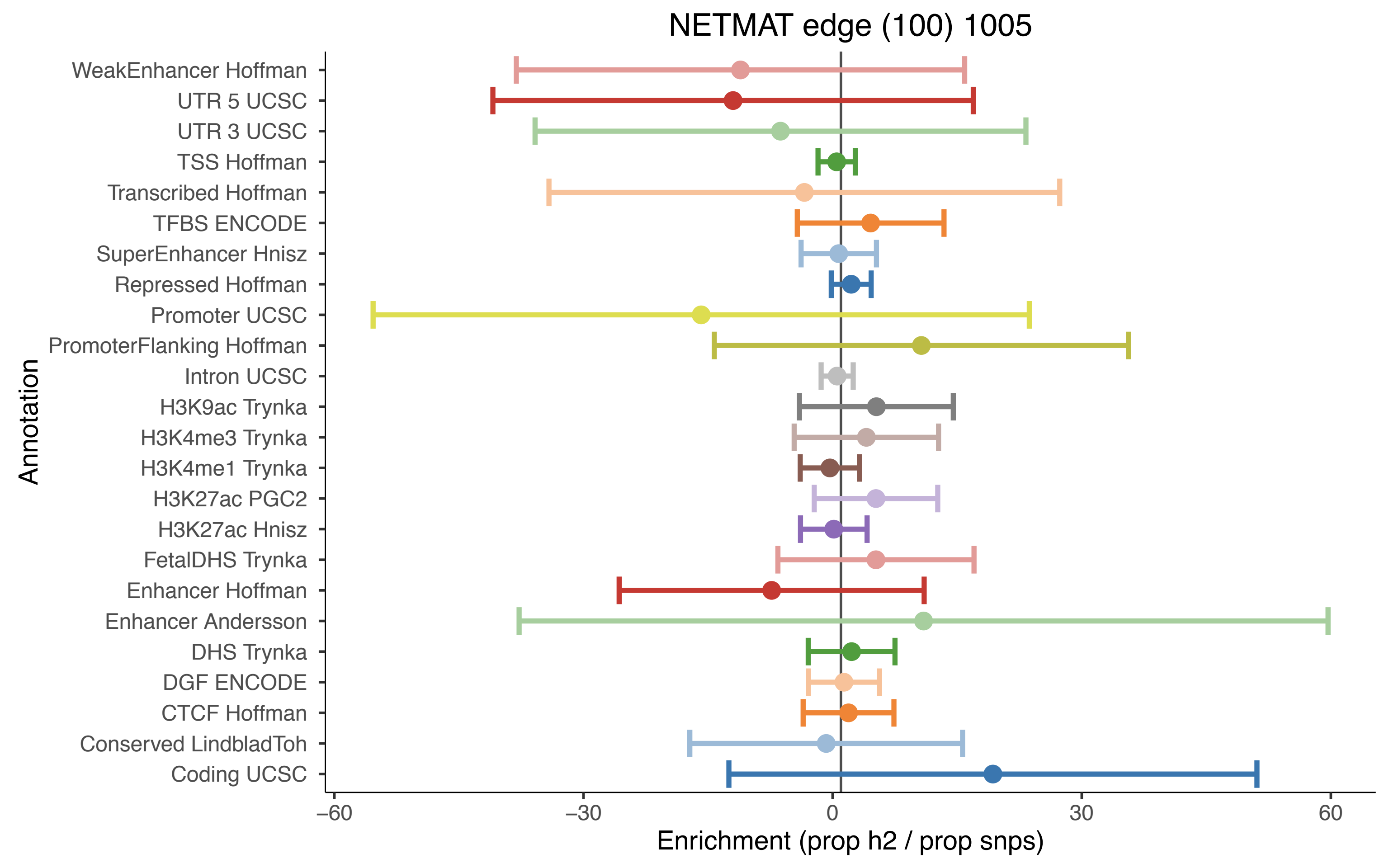
NETMAT edge (100) 1005
WeakEnhancer Hoffman
UTR 5 UCSC
UTR 3 UCSC
TSS Hoffman
Transcribed Hoffman
TFBS ENCODE
SuperEnhancer Hnisz
Repressed Hoffman
Promoter UCSC
PromoterFlanking Hoffman
Intron UCSC
H3K9ac Trynka
H3K4me3 Trynka
H3K4me1 Trynka
H3K27ac PGC2
H3K27ac Hnisz
FetalDHS Trynka
Enhancer Hoffman
Enhancer Andersson
DHS Trynka
DGF ENCODE
CTCF Hoffman
Conserved LindbladToh
Coding UCSC
Annotation
−60
−30
0
30
60
Enrichment (prop h2 / prop snps)

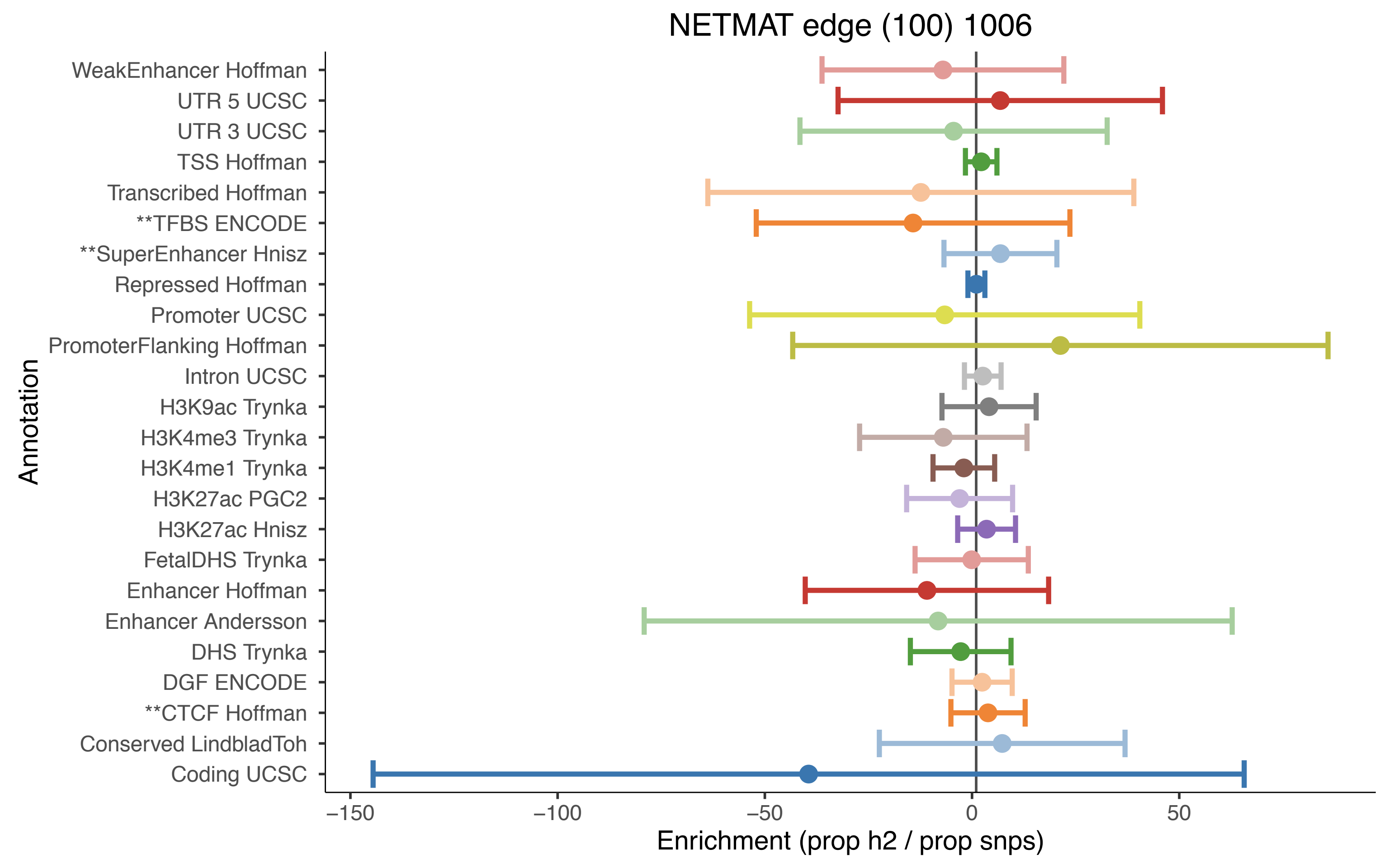

NETMAT edge (100) 1006
WeakEnhancer Hoffman
UTR 5 UCSC
UTR 3 UCSC
TSS Hoffman
Transcribed Hoffman
**TFBS ENCODE
**SuperEnhancer Hnisz
Repressed Hoffman
Promoter UCSC
PromoterFlanking Hoffman
Intron UCSC
H3K9ac Trynka
H3K4me3 Trynka
H3K4me1 Trynka
H3K27ac PGC2
H3K27ac Hnisz
FetalDHS Trynka
Enhancer Hoffman
Enhancer Andersson
DHS Trynka
DGF ENCODE
**CTCF Hoffman
Conserved LindbladToh
Coding UCSC
Annotation
−150
−100
−50
0
50
Enrichment (prop h2 / prop snps)

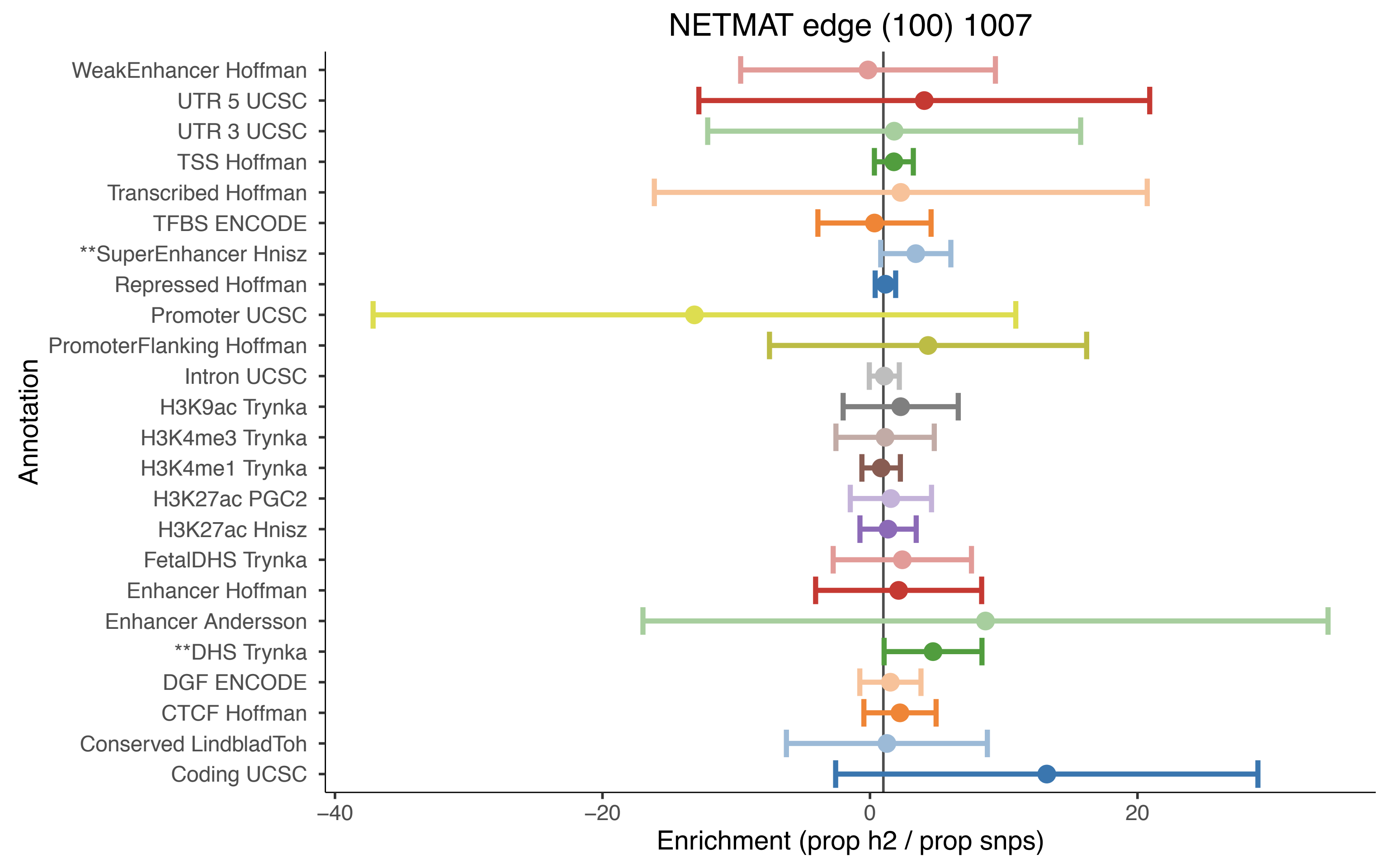
NETMAT edge (100) 1007
WeakEnhancer Hoffman
UTR 5 UCSC
UTR 3 UCSC
TSS Hoffman
Transcribed Hoffman
TFBS ENCODE
**SuperEnhancer Hnisz
Repressed Hoffman
Promoter UCSC
PromoterFlanking Hoffman
Intron UCSC
H3K9ac Trynka
H3K4me3 Trynka
H3K4me1 Trynka
H3K27ac PGC2
H3K27ac Hnisz
FetalDHS Trynka
Enhancer Hoffman
Enhancer Andersson
**DHS Trynka
DGF ENCODE
CTCF Hoffman
Conserved LindbladToh
Coding UCSC
Annotation
−40
−20
0
20
Enrichment (prop h2 / prop snps)

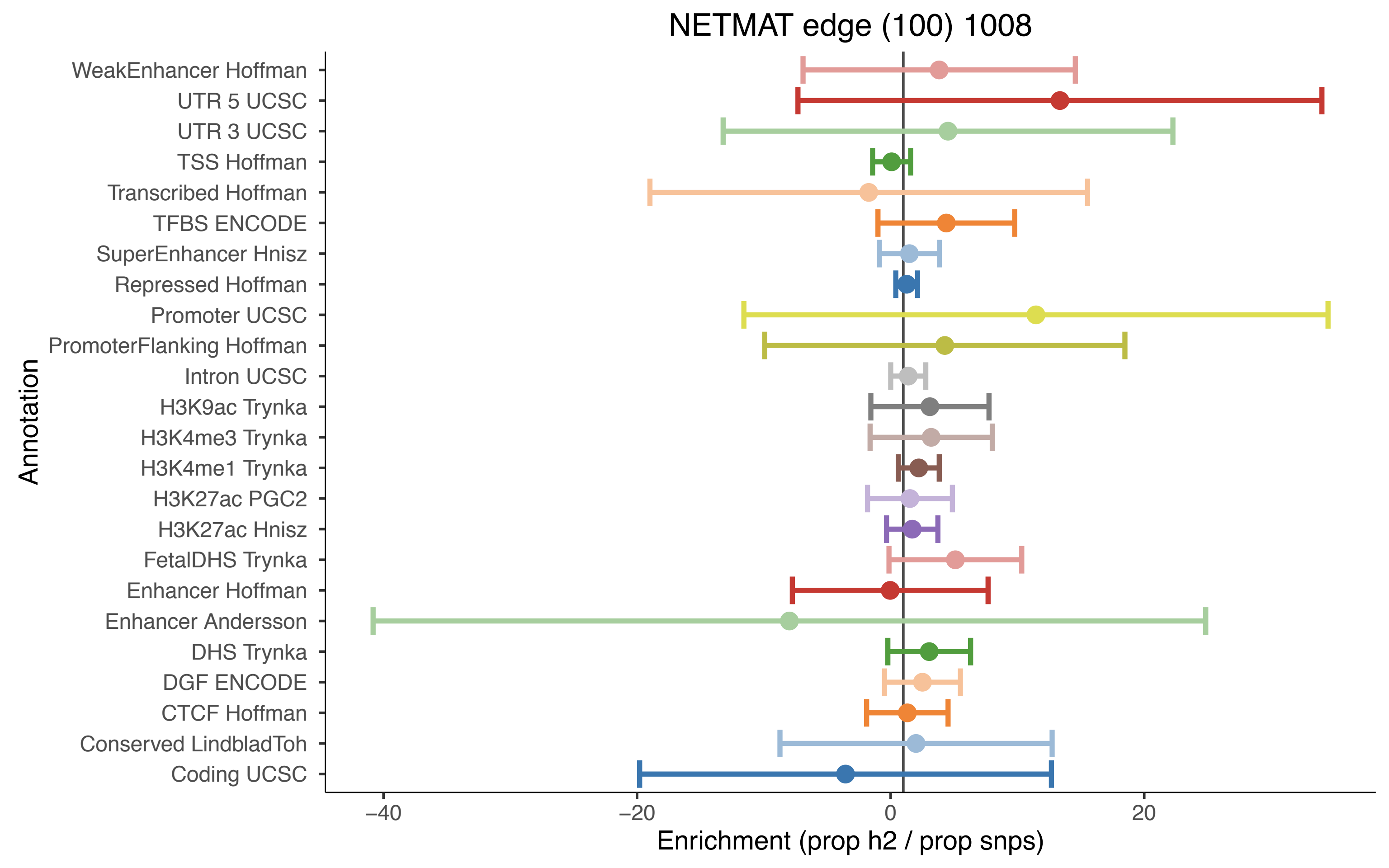

NETMAT edge (100) 1008
WeakEnhancer Hoffman
UTR 5 UCSC
UTR 3 UCSC
TSS Hoffman
Transcribed Hoffman
TFBS ENCODE
SuperEnhancer Hnisz
Repressed Hoffman
Promoter UCSC
PromoterFlanking Hoffman
Intron UCSC
H3K9ac Trynka
H3K4me3 Trynka
H3K4me1 Trynka
H3K27ac PGC2
H3K27ac Hnisz
FetalDHS Trynka
Enhancer Hoffman
Enhancer Andersson
DHS Trynka
DGF ENCODE
CTCF Hoffman
Conserved LindbladToh
Coding UCSC
Annotation
−40
−20
0
20
Enrichment (prop h2 / prop snps)

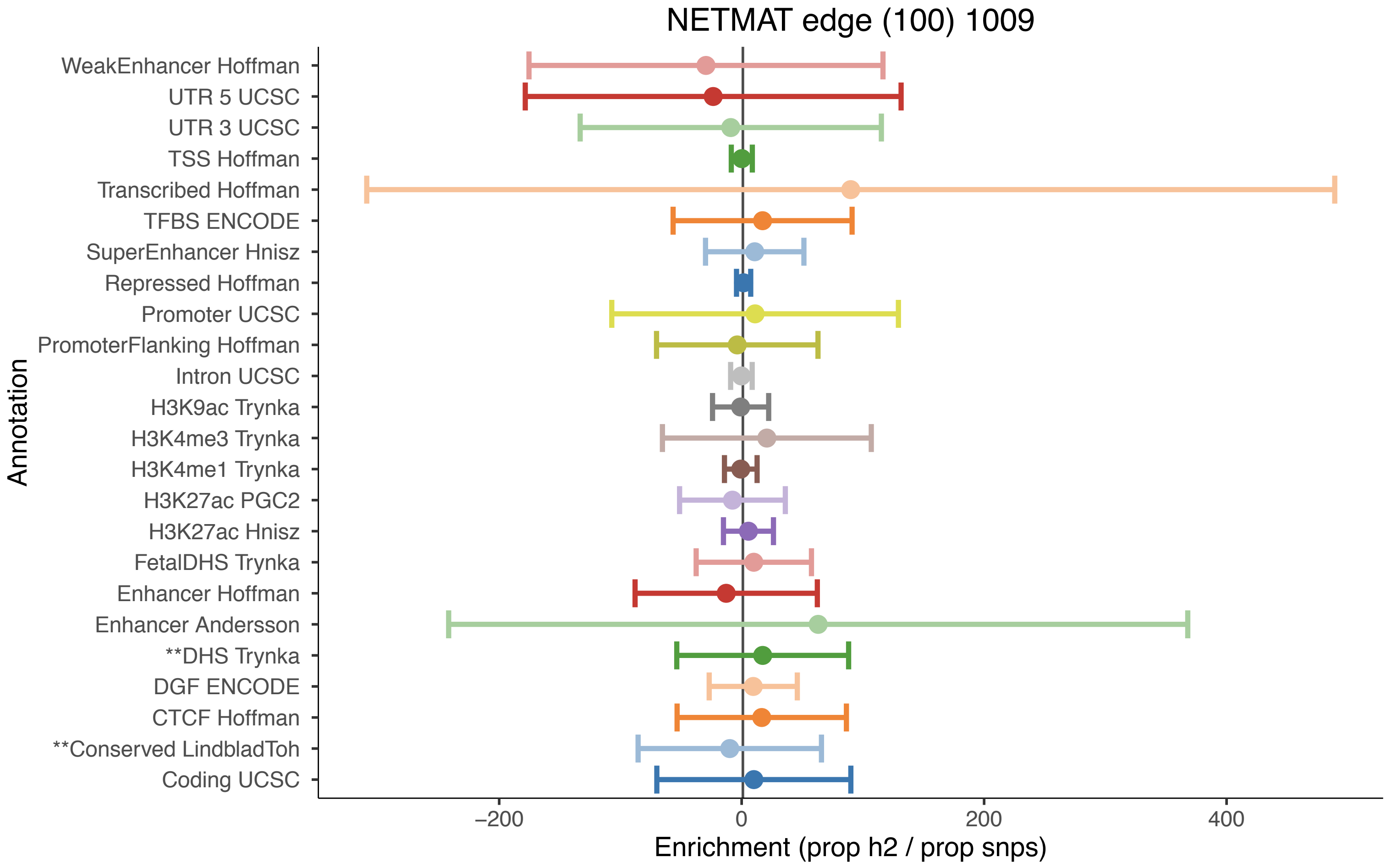
NETMAT edge (100) 1009
WeakEnhancer Hoffman
UTR 5 UCSC
UTR 3 UCSC
TSS Hoffman
Transcribed Hoffman
TFBS ENCODE
SuperEnhancer Hnisz
Repressed Hoffman
Promoter UCSC
PromoterFlanking Hoffman
Intron UCSC
H3K9ac Trynka
H3K4me3 Trynka
H3K4me1 Trynka
H3K27ac PGC2
H3K27ac Hnisz
FetalDHS Trynka
Enhancer Hoffman
Enhancer Andersson
**DHS Trynka
DGF ENCODE
CTCF Hoffman
**Conserved LindbladToh
Coding UCSC
Annotation
−200
0
200
400
Enrichment (prop h2 / prop snps)

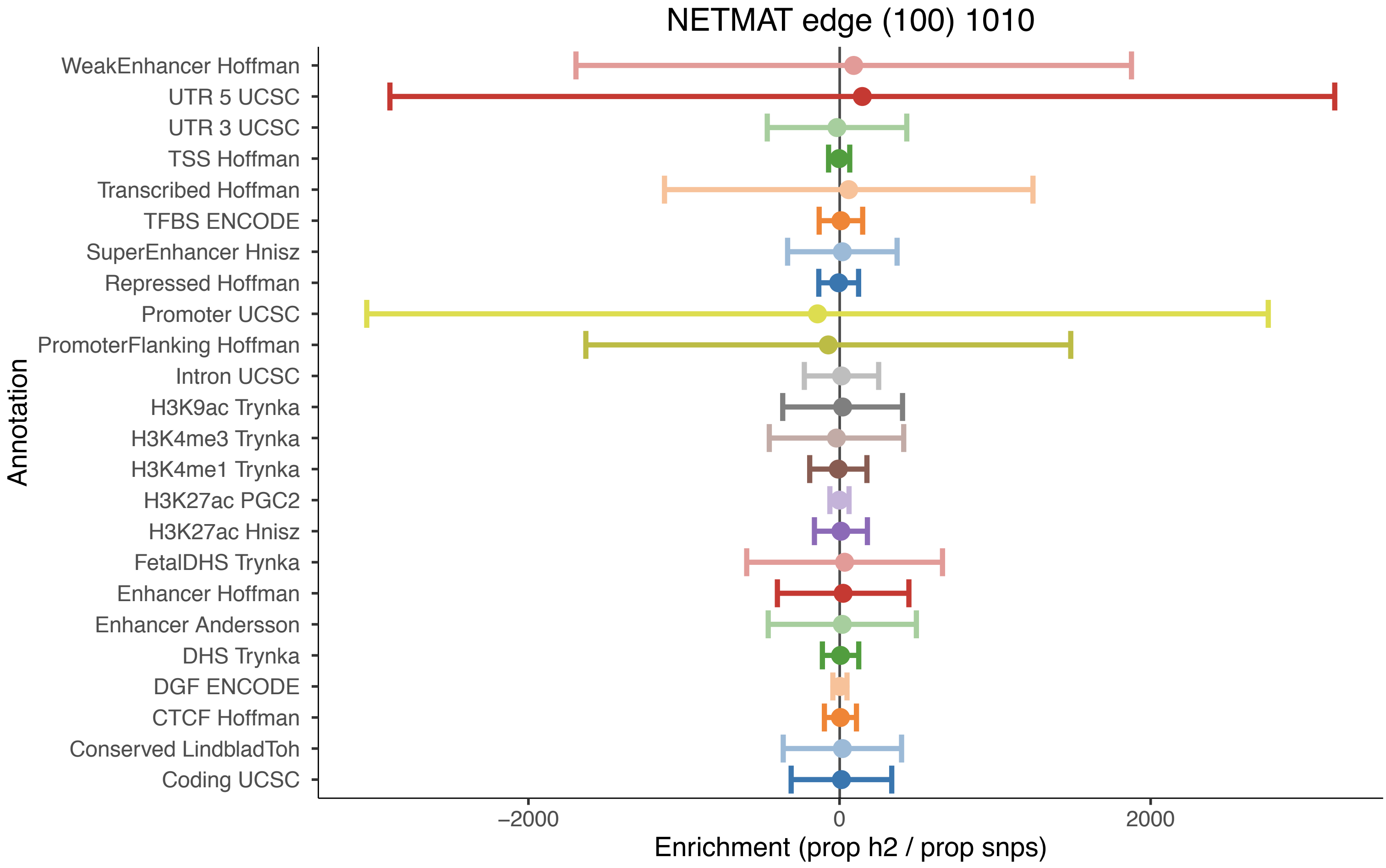
NETMAT edge (100) 1010
WeakEnhancer Hoffman
UTR 5 UCSC
UTR 3 UCSC
TSS Hoffman
Transcribed Hoffman
TFBS ENCODE
SuperEnhancer Hnisz
Repressed Hoffman
Promoter UCSC
PromoterFlanking Hoffman
Intron UCSC
H3K9ac Trynka
H3K4me3 Trynka
H3K4me1 Trynka
H3K27ac PGC2
H3K27ac Hnisz
FetalDHS Trynka
Enhancer Hoffman
Enhancer Andersson
DHS Trynka
DGF ENCODE
CTCF Hoffman
Conserved LindbladToh
Coding UCSC
Annotation
−2000
0
2000
Enrichment (prop h2 / prop snps)

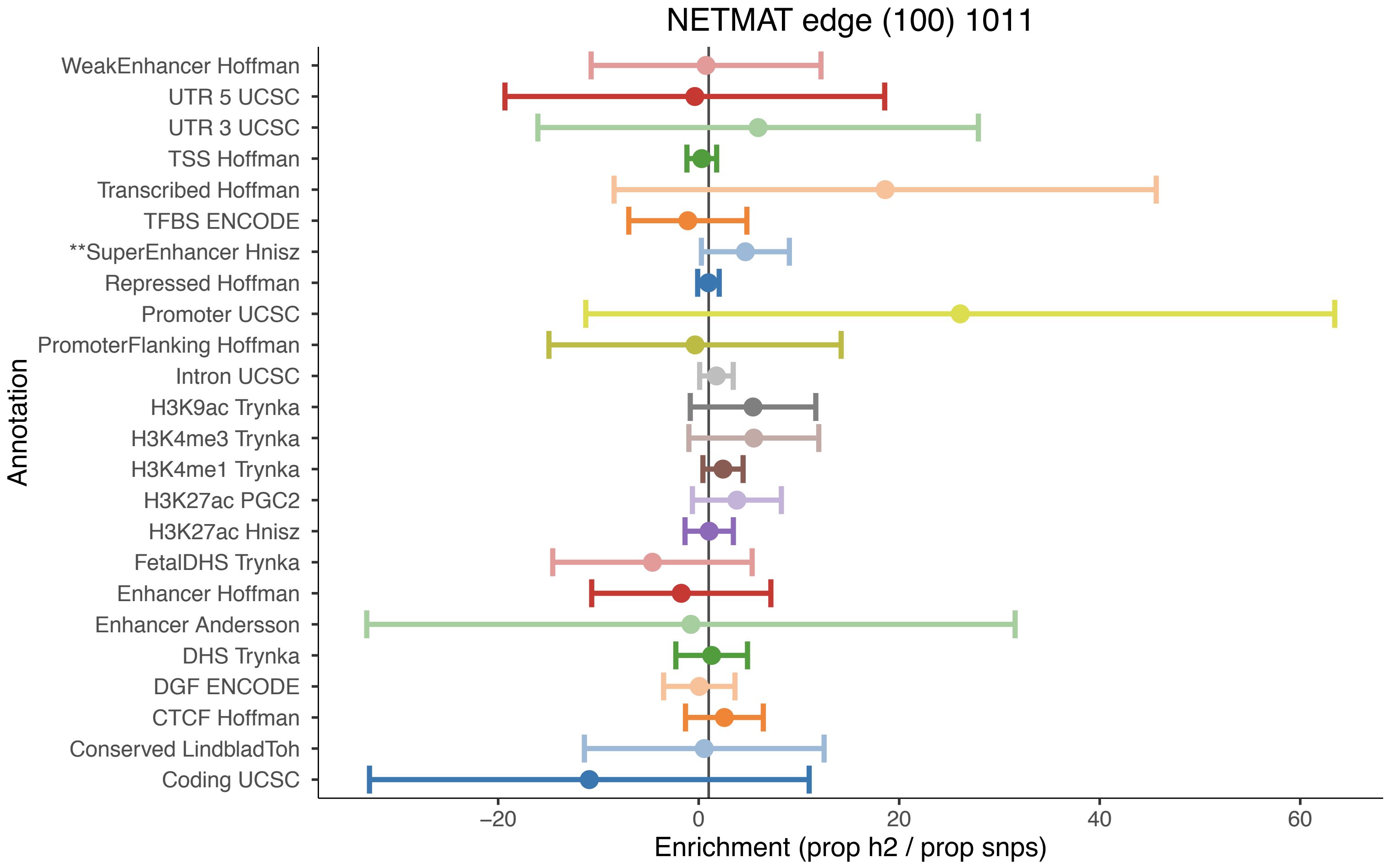
NETMAT edge (100) 1011
WeakEnhancer Hoffman
UTR 5 UCSC
UTR 3 UCSC
TSS Hoffman
Transcribed Hoffman
TFBS ENCODE
**SuperEnhancer Hnisz
Repressed Hoffman
Promoter UCSC
PromoterFlanking Hoffman
Intron UCSC
H3K9ac Trynka
H3K4me3 Trynka
H3K4me1 Trynka
H3K27ac PGC2
H3K27ac Hnisz
FetalDHS Trynka
Enhancer Hoffman
Enhancer Andersson
DHS Trynka
DGF ENCODE
CTCF Hoffman
Conserved LindbladToh
Coding UCSC
Annotation
−20
0
20
40
60
Enrichment (prop h2 / prop snps)

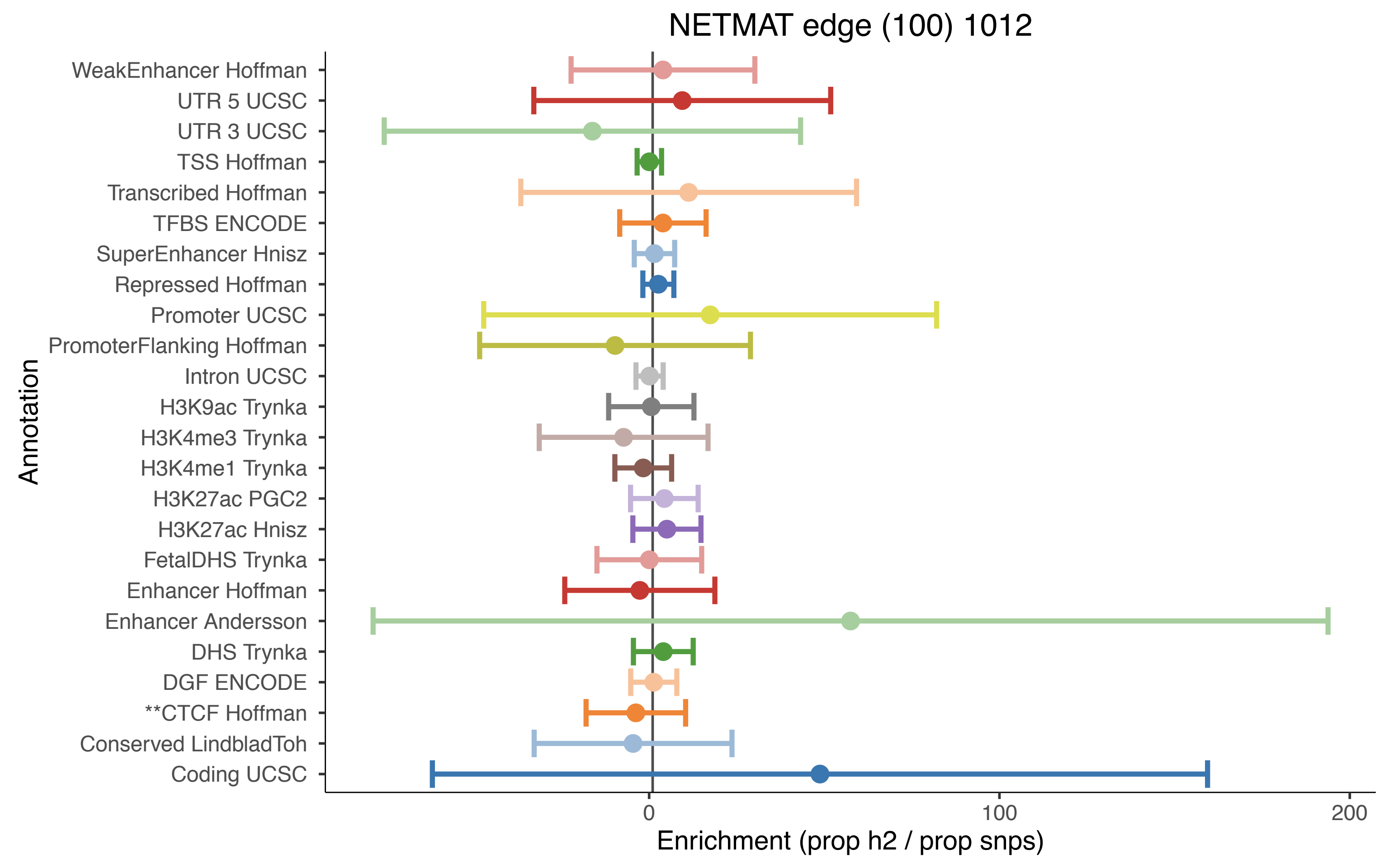

NETMAT edge (100) 1012
Annotation
WeakEnhancer Hoffman
UTR 5 UCSC
UTR 3 UCSC
TSS Hoffman
Transcribed Hoffman
TFBS ENCODE
SuperEnhancer Hnisz
Repressed Hoffman
Promoter UCSC
PromoterFlanking Hoffman
Intron UCSC
H3K9ac Trynka
H3K4me3 Trynka
H3K4me1 Trynka
H3K27ac PGC2
H3K27ac Hnisz
FetalDHS Trynka
Enhancer Hoffman
Enhancer Andersson
DHS Trynka
DGF ENCODE
**CTCF Hoffman
Conserved LindbladToh
Coding UCSC
0
100
200
Enrichment (prop h2 / prop snps)

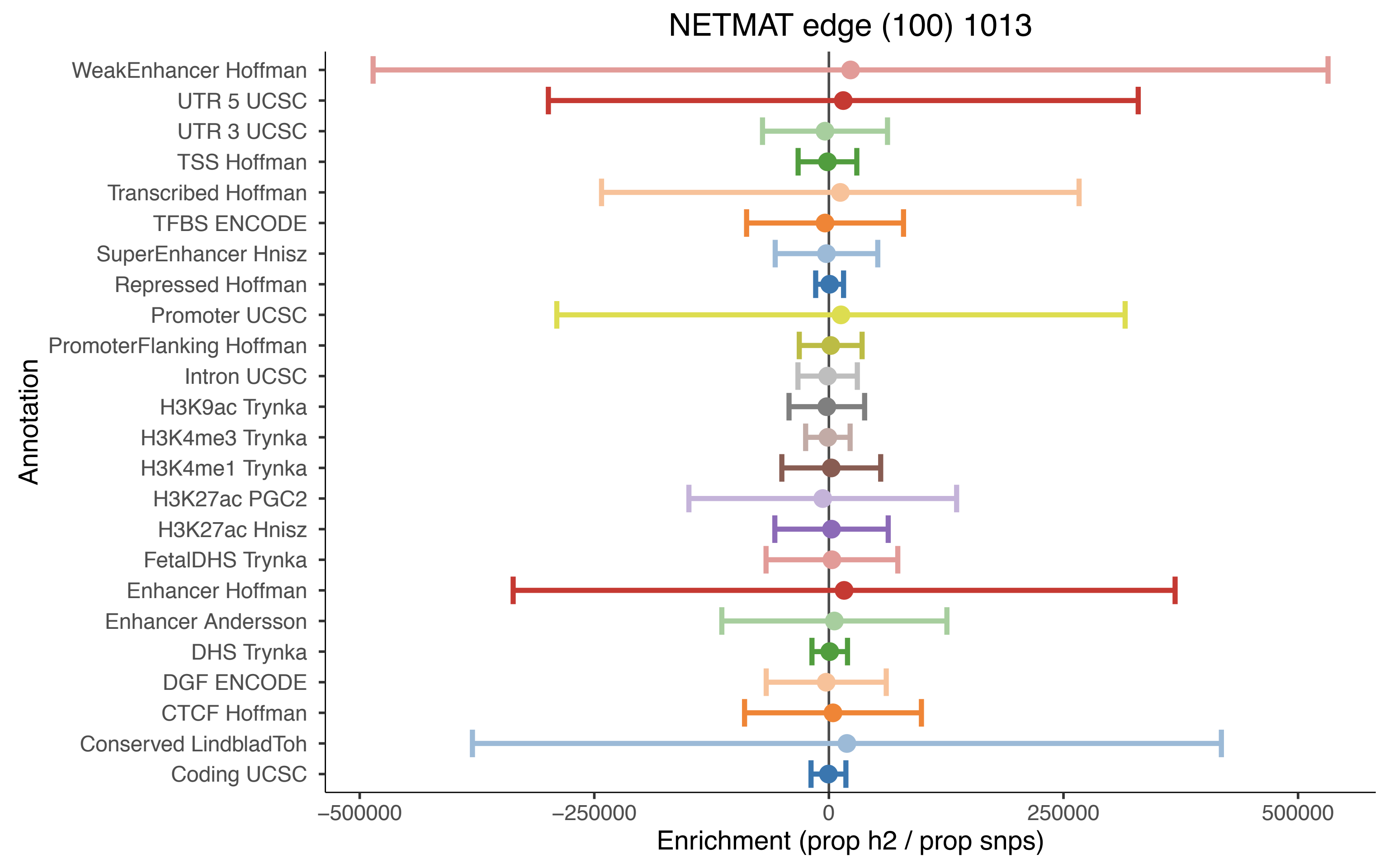
NETMAT edge (100) 1013
WeakEnhancer Hoffman
UTR 5 UCSC
UTR 3 UCSC
TSS Hoffman
Transcribed Hoffman
TFBS ENCODE
SuperEnhancer Hnisz
Repressed Hoffman
Promoter UCSC
PromoterFlanking Hoffman
Intron UCSC
H3K9ac Trynka
H3K4me3 Trynka
H3K4me1 Trynka
H3K27ac PGC2
H3K27ac Hnisz
FetalDHS Trynka
Enhancer Hoffman
Enhancer Andersson
DHS Trynka
DGF ENCODE
CTCF Hoffman
Conserved LindbladToh
Coding UCSC
Annotation
−500000
−250000
0
250000
500000
Enrichment (prop h2 / prop snps)

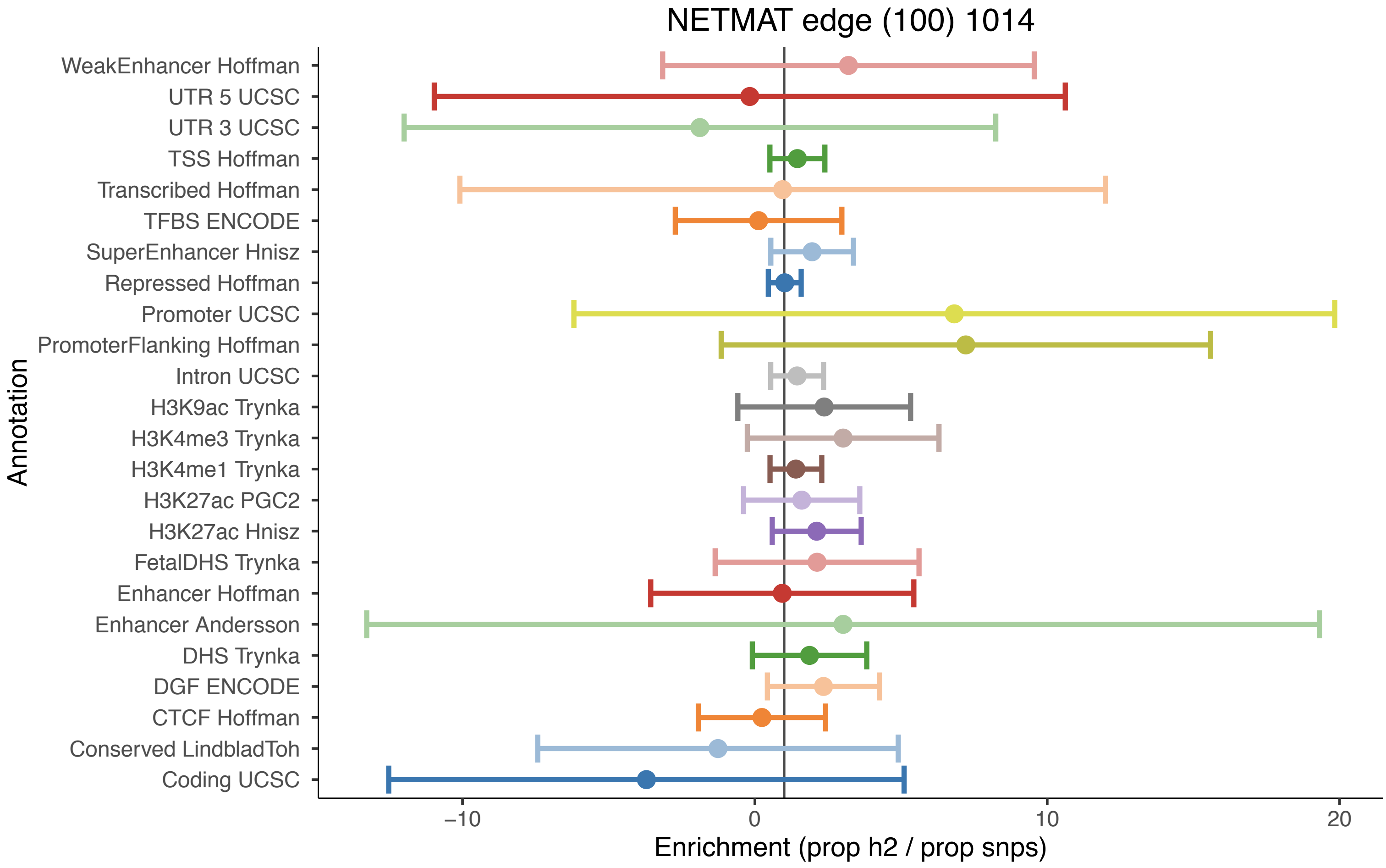

NETMAT edge (100) 1014
WeakEnhancer Hoffman
UTR 5 UCSC
UTR 3 UCSC
TSS Hoffman
Transcribed Hoffman
TFBS ENCODE
SuperEnhancer Hnisz
Repressed Hoffman
Promoter UCSC
PromoterFlanking Hoffman
Intron UCSC
H3K9ac Trynka
H3K4me3 Trynka
H3K4me1 Trynka
H3K27ac PGC2
H3K27ac Hnisz
FetalDHS Trynka
Enhancer Hoffman
Enhancer Andersson
DHS Trynka
DGF ENCODE
CTCF Hoffman
Conserved LindbladToh
Coding UCSC
Annotation
−10
0
10
20
Enrichment (prop h2 / prop snps)

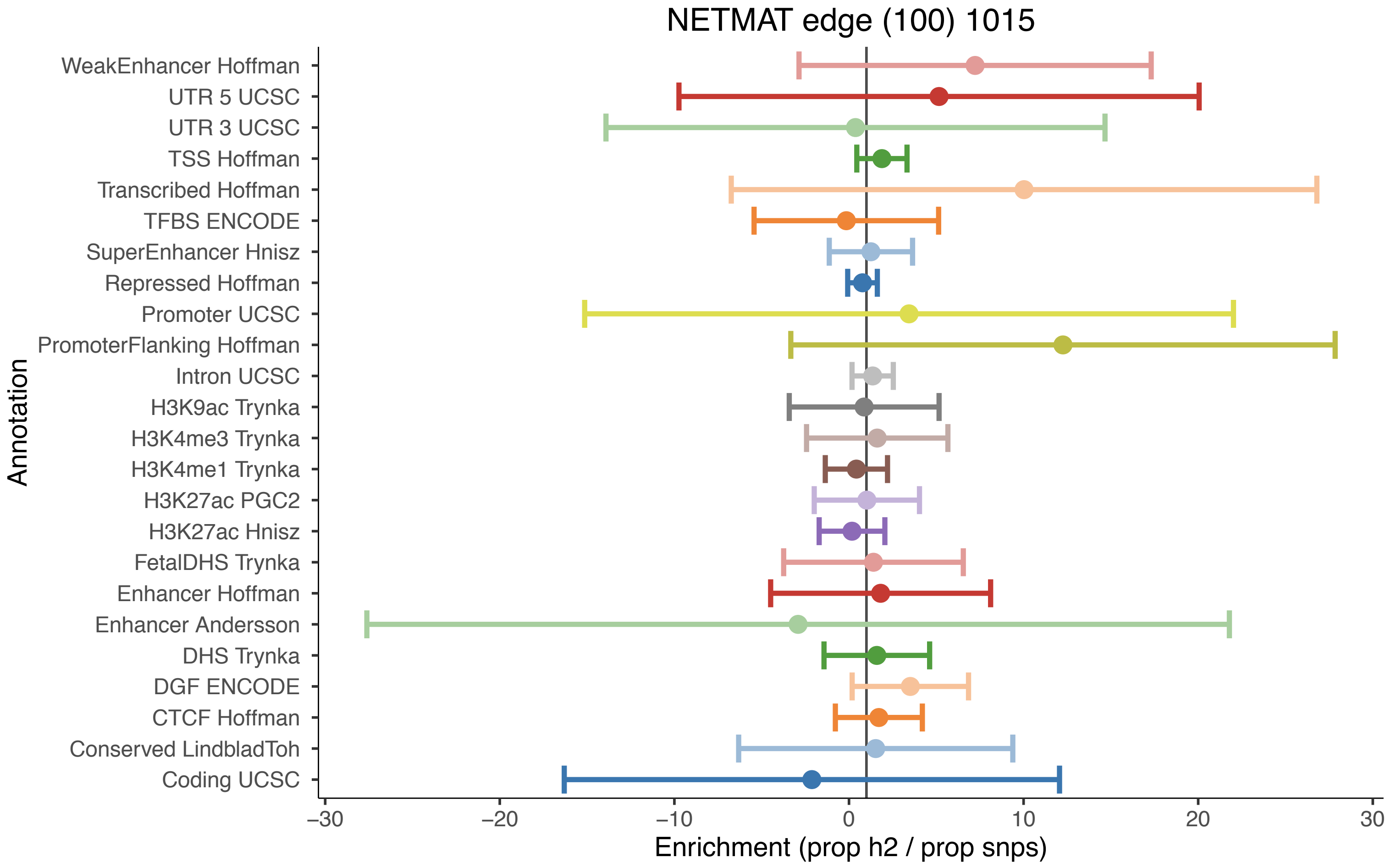
NETMAT edge (100) 1015
WeakEnhancer Hoffman
UTR 5 UCSC
UTR 3 UCSC
TSS Hoffman
Transcribed Hoffman
TFBS ENCODE
SuperEnhancer Hnisz
Repressed Hoffman
Promoter UCSC
PromoterFlanking Hoffman
Intron UCSC
H3K9ac Trynka
H3K4me3 Trynka
H3K4me1 Trynka
H3K27ac PGC2
H3K27ac Hnisz
FetalDHS Trynka
Enhancer Hoffman
Enhancer Andersson
DHS Trynka
DGF ENCODE
CTCF Hoffman
Conserved LindbladToh
Coding UCSC
Annotation
−30
−20
−10
0
10
20
30
Enrichment (prop h2 / prop snps)

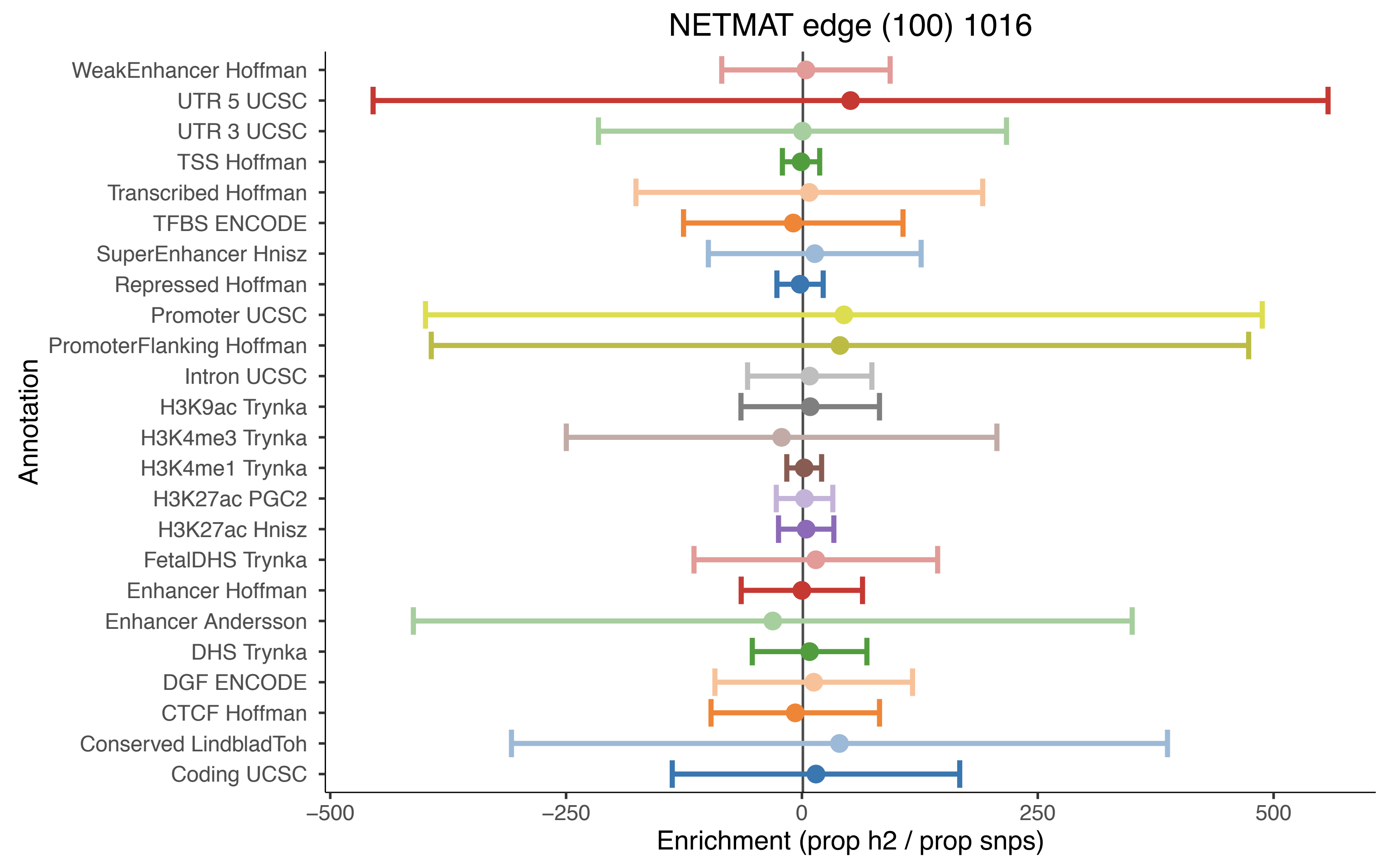
NETMAT edge (100) 1016
WeakEnhancer Hoffman
UTR 5 UCSC
UTR 3 UCSC
TSS Hoffman
Transcribed Hoffman
TFBS ENCODE
SuperEnhancer Hnisz
Repressed Hoffman
Promoter UCSC
PromoterFlanking Hoffman
Intron UCSC
H3K9ac Trynka
H3K4me3 Trynka
H3K4me1 Trynka
H3K27ac PGC2
H3K27ac Hnisz
FetalDHS Trynka
Enhancer Hoffman
Enhancer Andersson
DHS Trynka
DGF ENCODE
CTCF Hoffman
Conserved LindbladToh
Coding UCSC
Annotation
−500
−250
0
250
500
Enrichment (prop h2 / prop snps)

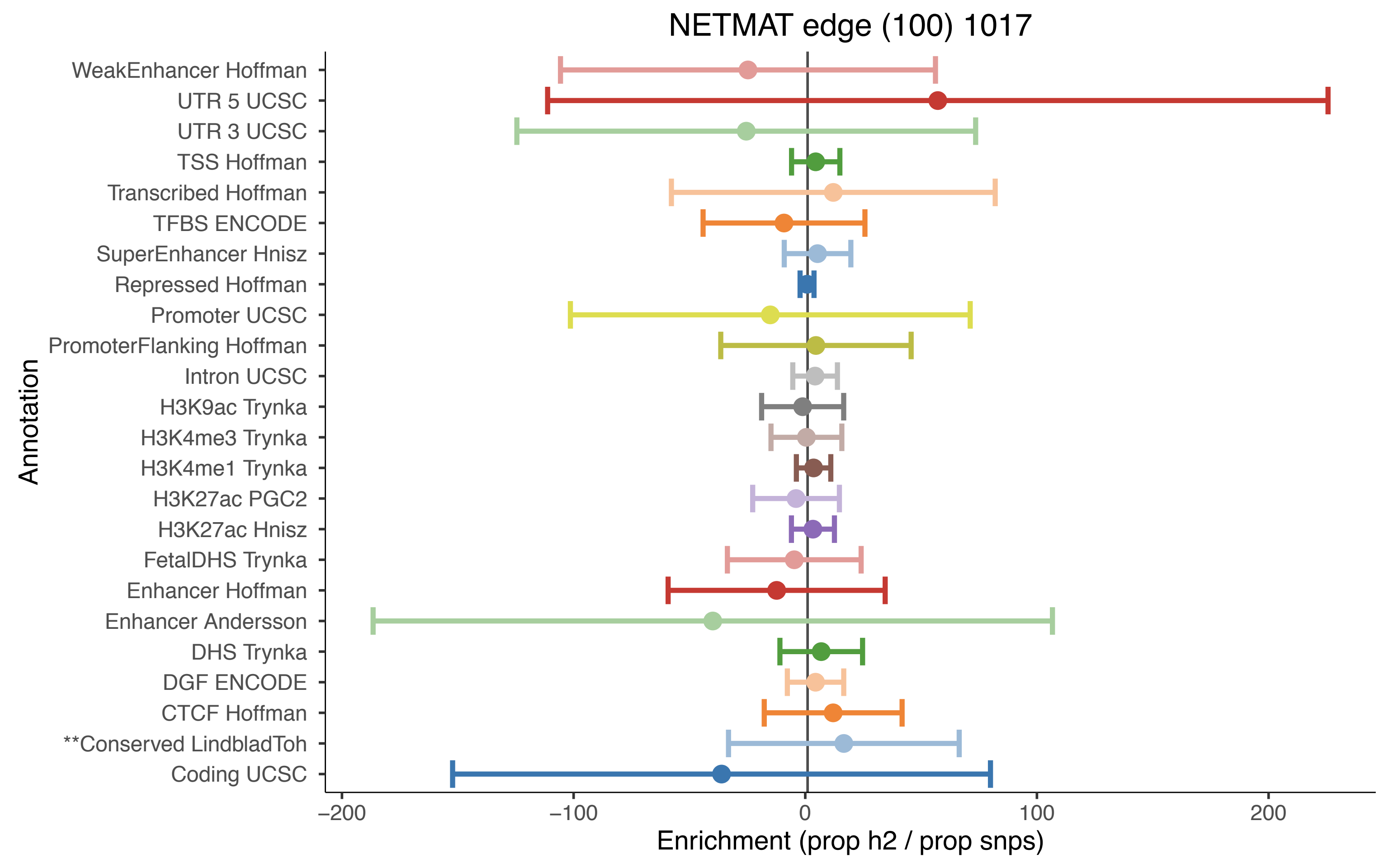

NETMAT edge (100) 1017
WeakEnhancer Hoffman
UTR 5 UCSC
UTR 3 UCSC
TSS Hoffman
Transcribed Hoffman
TFBS ENCODE
SuperEnhancer Hnisz
Repressed Hoffman
Promoter UCSC
PromoterFlanking Hoffman
Intron UCSC
H3K9ac Trynka
H3K4me3 Trynka
H3K4me1 Trynka
H3K27ac PGC2
H3K27ac Hnisz
FetalDHS Trynka
Enhancer Hoffman
Enhancer Andersson
DHS Trynka
DGF ENCODE
CTCF Hoffman
**Conserved LindbladToh
Coding UCSC
Annotation
−200
−100
0
100
200
Enrichment (prop h2 / prop snps)

NETMAT edge (100) 1018

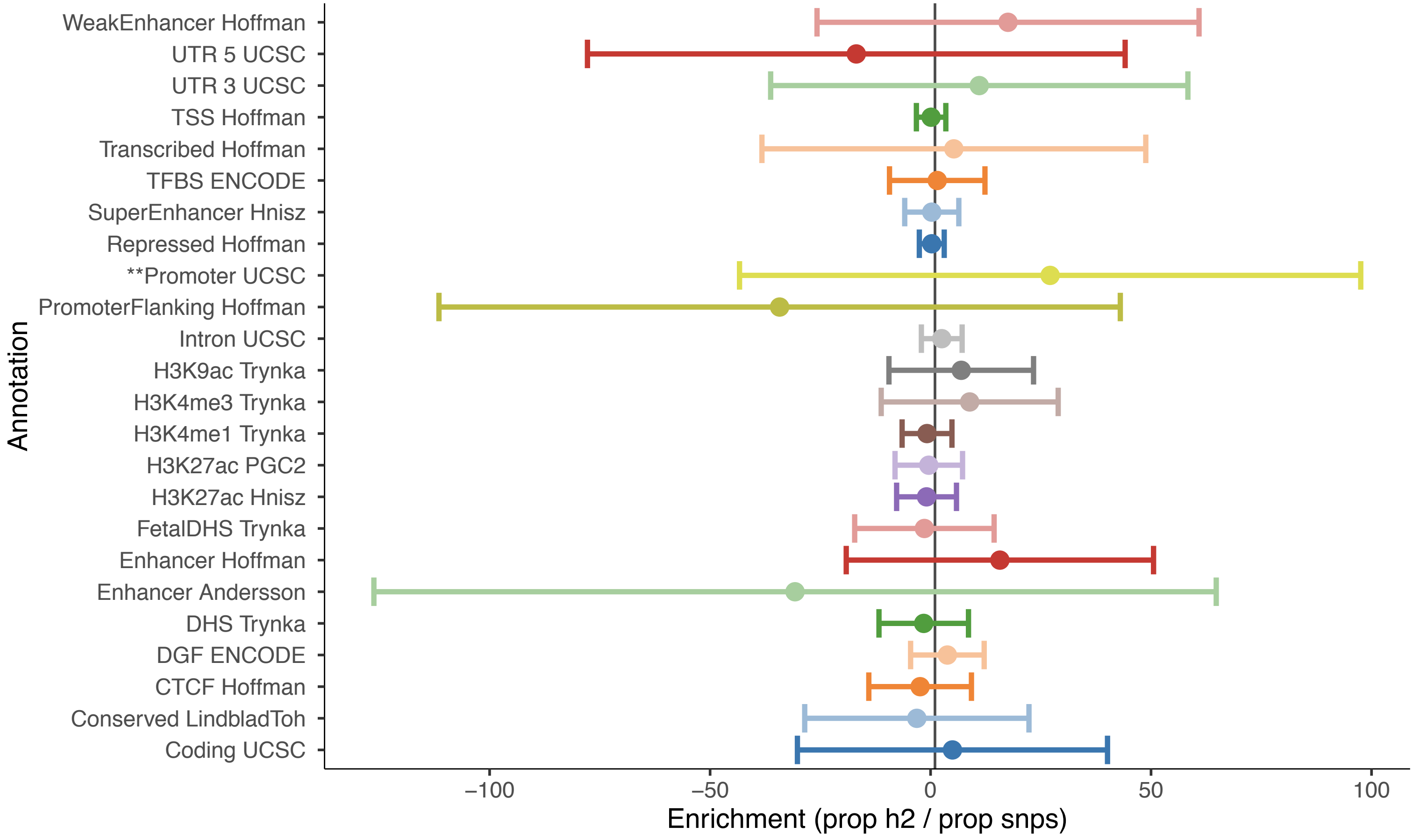

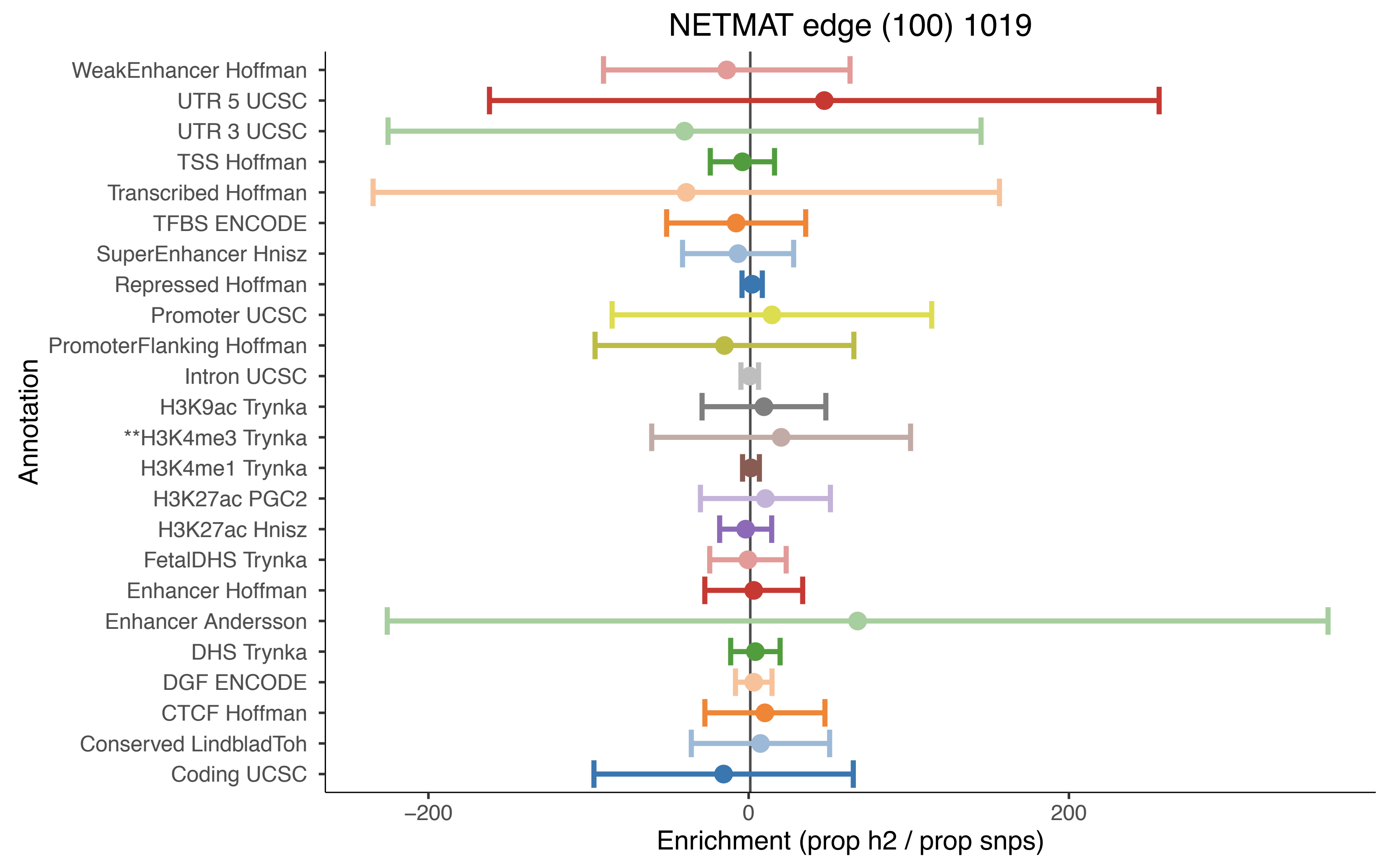
NETMAT edge (100) 1019
WeakEnhancer Hoffman
UTR 5 UCSC
UTR 3 UCSC
TSS Hoffman
Transcribed Hoffman
TFBS ENCODE
SuperEnhancer Hnisz
Repressed Hoffman
Promoter UCSC
PromoterFlanking Hoffman
Intron UCSC
H3K9ac Trynka
**H3K4me3 Trynka
H3K4me1 Trynka
H3K27ac PGC2
H3K27ac Hnisz
FetalDHS Trynka
Enhancer Hoffman
Enhancer Andersson
DHS Trynka
DGF ENCODE
CTCF Hoffman
Conserved LindbladToh
Coding UCSC
Annotation
−200
0
200
Enrichment (prop h2 / prop snps)

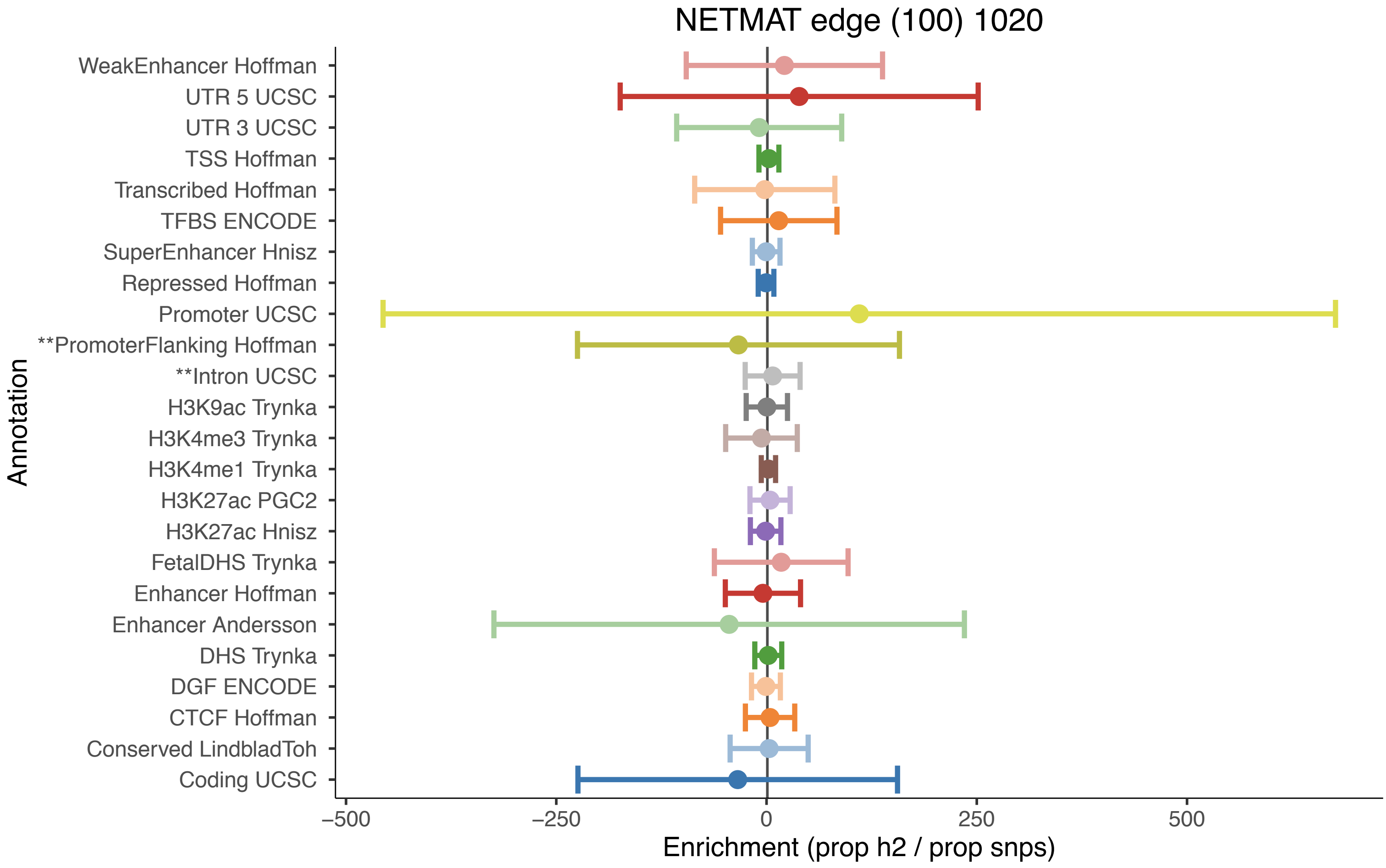
NETMAT edge (100) 1020
WeakEnhancer Hoffman
UTR 5 UCSC
UTR 3 UCSC
TSS Hoffman
Transcribed Hoffman
TFBS ENCODE
SuperEnhancer Hnisz
Repressed Hoffman
Promoter UCSC
**PromoterFlanking Hoffman
**Intron UCSC
H3K9ac Trynka
H3K4me3 Trynka
H3K4me1 Trynka
H3K27ac PGC2
H3K27ac Hnisz
FetalDHS Trynka
Enhancer Hoffman
Enhancer Andersson
DHS Trynka
DGF ENCODE
CTCF Hoffman
Conserved LindbladToh
Coding UCSC
Annotation
−500
−250
0
250
500
Enrichment (prop h2 / prop snps)

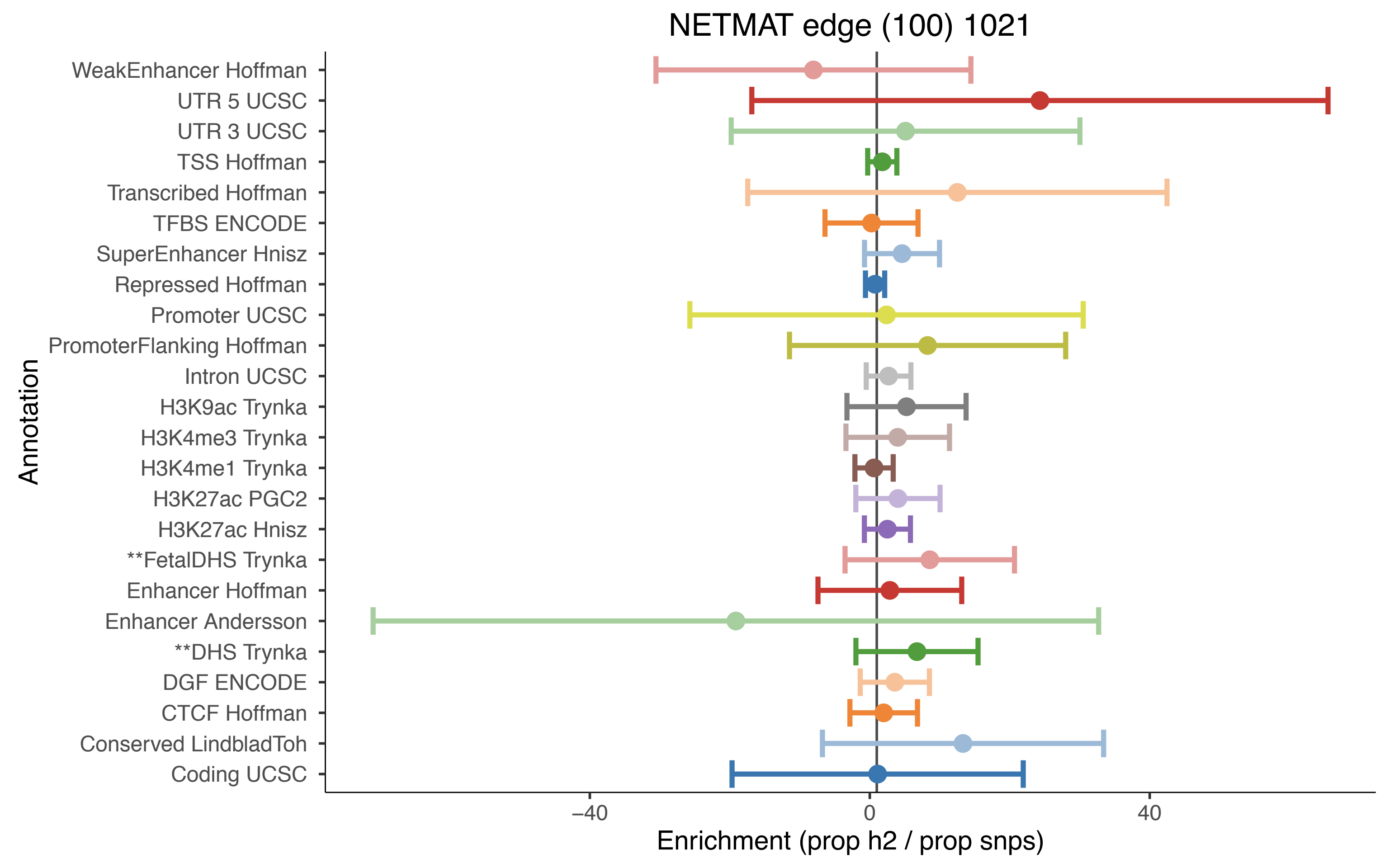
NETMAT edge (100) 1021
WeakEnhancer Hoffman
UTR 5 UCSC
UTR 3 UCSC
TSS Hoffman
Transcribed Hoffman
TFBS ENCODE
SuperEnhancer Hnisz
Repressed Hoffman
Promoter UCSC
PromoterFlanking Hoffman
Intron UCSC
H3K9ac Trynka
H3K4me3 Trynka
H3K4me1 Trynka
H3K27ac PGC2
H3K27ac Hnisz
**FetalDHS Trynka
Enhancer Hoffman
Enhancer Andersson
**DHS Trynka
DGF ENCODE
CTCF Hoffman
Conserved LindbladToh
Coding UCSC
Annotation
−40
0
40
Enrichment (prop h2 / prop snps)

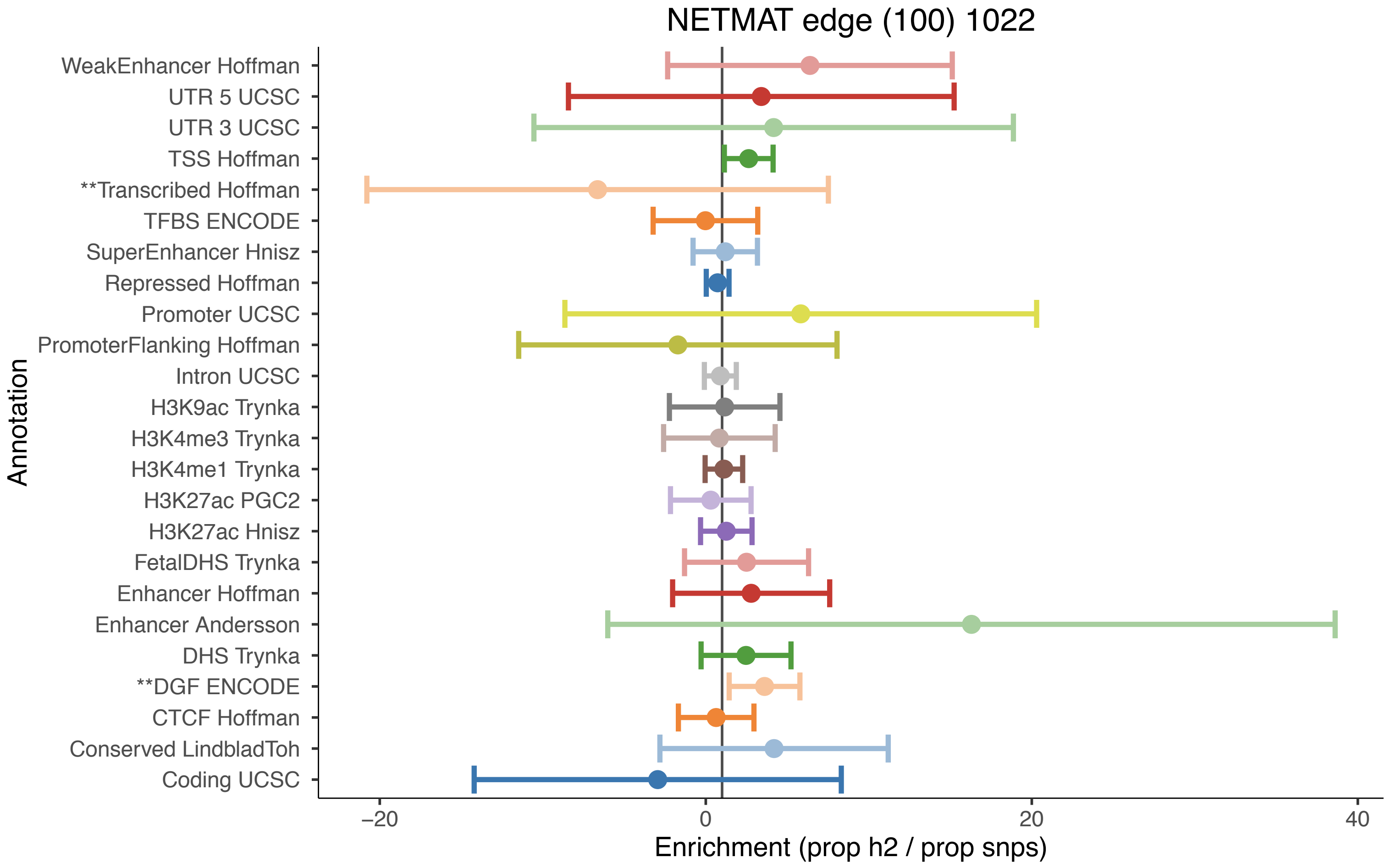
NETMAT edge (100) 1022
WeakEnhancer Hoffman
UTR 5 UCSC
UTR 3 UCSC
TSS Hoffman
**Transcribed Hoffman
TFBS ENCODE
SuperEnhancer Hnisz
Repressed Hoffman
Promoter UCSC
PromoterFlanking Hoffman
Intron UCSC
H3K9ac Trynka
H3K4me3 Trynka
H3K4me1 Trynka
H3K27ac PGC2
H3K27ac Hnisz
FetalDHS Trynka
Enhancer Hoffman
Enhancer Andersson
DHS Trynka
**DGF ENCODE
CTCF Hoffman
Conserved LindbladToh
Coding UCSC
Annotation
−20
0
20
40
Enrichment (prop h2 / prop snps)

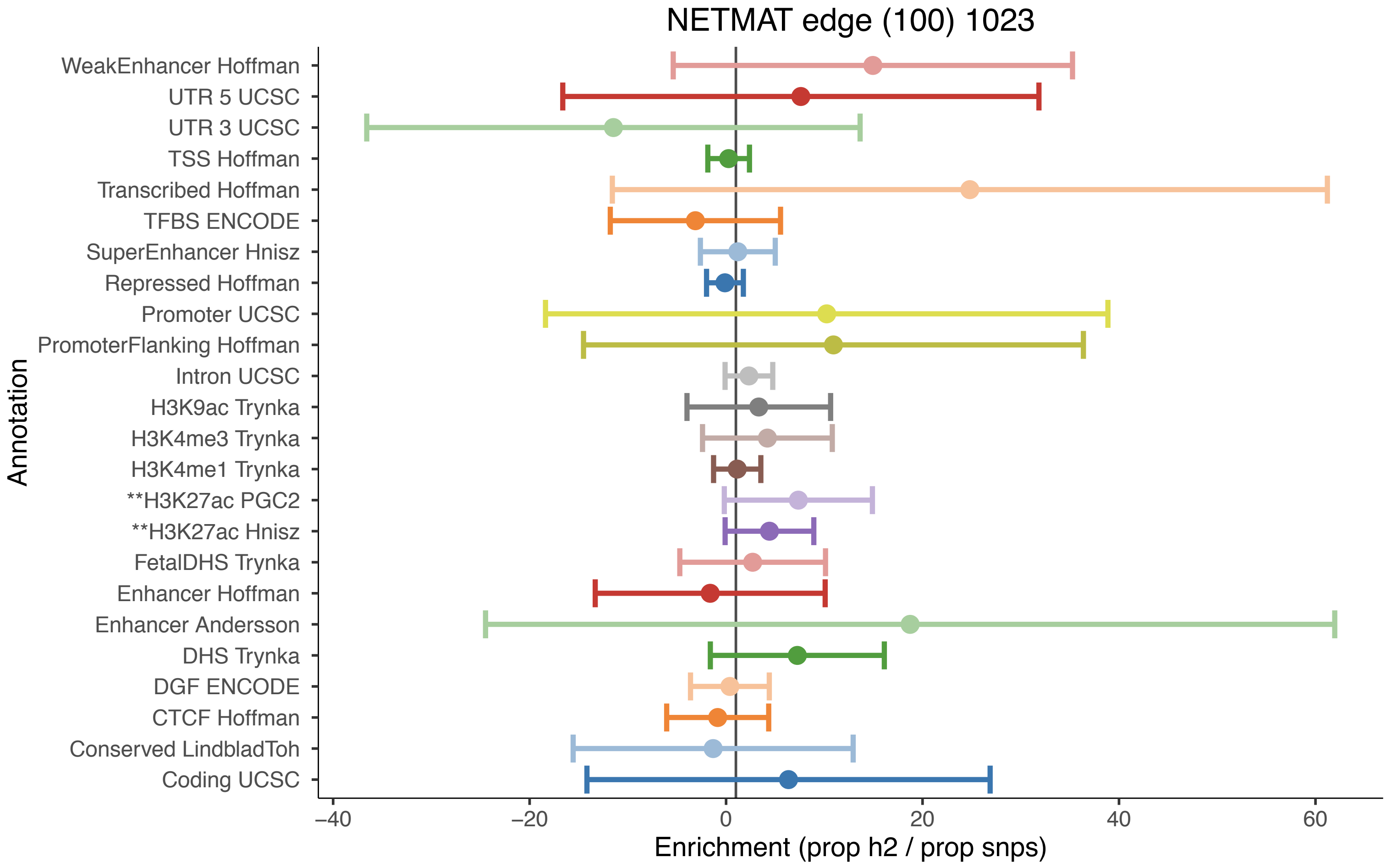
NETMAT edge (100) 1023
WeakEnhancer Hoffman
UTR 5 UCSC
UTR 3 UCSC
TSS Hoffman
Transcribed Hoffman
TFBS ENCODE
SuperEnhancer Hnisz
Repressed Hoffman
Promoter UCSC
PromoterFlanking Hoffman
Intron UCSC
H3K9ac Trynka
H3K4me3 Trynka
H3K4me1 Trynka
**H3K27ac PGC2
**H3K27ac Hnisz
FetalDHS Trynka
Enhancer Hoffman
Enhancer Andersson
DHS Trynka
DGF ENCODE
CTCF Hoffman
Conserved LindbladToh
Coding UCSC
Annotation
−40
−20
0
20
40
60
Enrichment (prop h2 / prop snps)

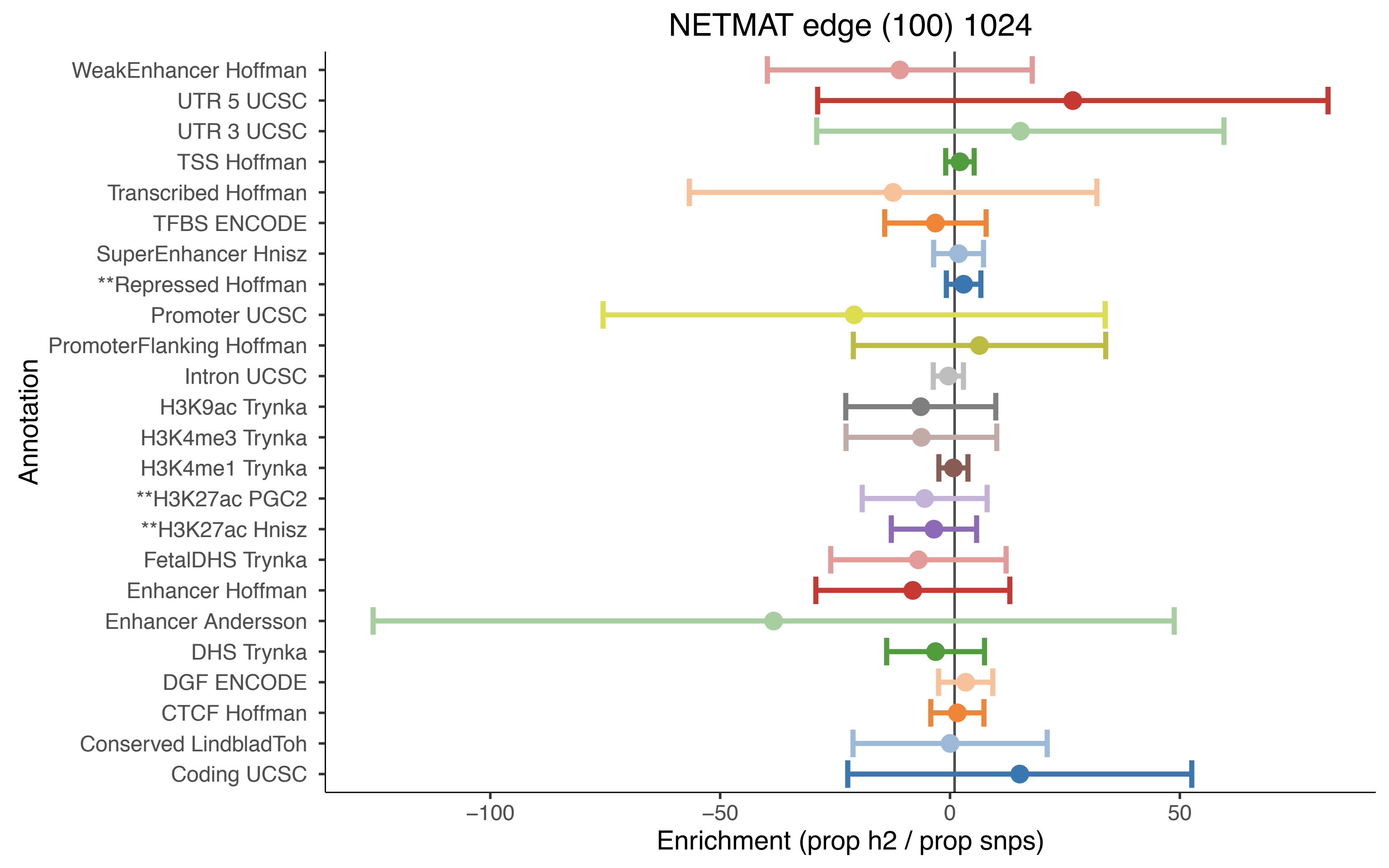

NETMAT edge (100) 1024
WeakEnhancer Hoffman
UTR 5 UCSC
UTR 3 UCSC
TSS Hoffman
Transcribed Hoffman
TFBS ENCODE
SuperEnhancer Hnisz
**Repressed Hoffman
Promoter UCSC
PromoterFlanking Hoffman
Intron UCSC
H3K9ac Trynka
H3K4me3 Trynka
H3K4me1 Trynka
**H3K27ac PGC2
**H3K27ac Hnisz
FetalDHS Trynka
Enhancer Hoffman
Enhancer Andersson
DHS Trynka
DGF ENCODE
CTCF Hoffman
Conserved LindbladToh
Coding UCSC
Annotation
−100
−50
0
50
Enrichment (prop h2 / prop snps)

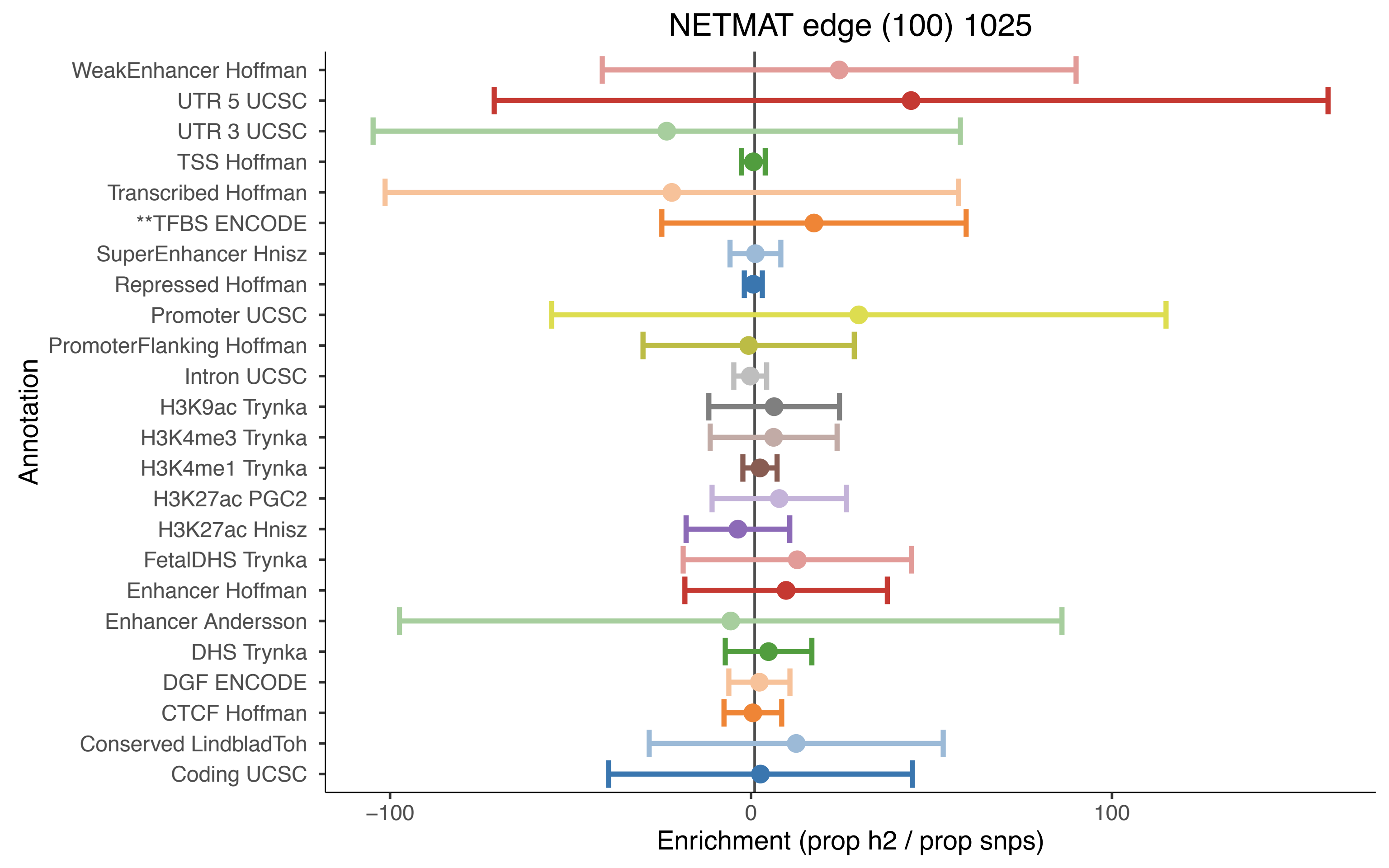
NETMAT edge (100) 1025
WeakEnhancer Hoffman
UTR 5 UCSC
UTR 3 UCSC
TSS Hoffman
Transcribed Hoffman
**TFBS ENCODE
SuperEnhancer Hnisz
Repressed Hoffman
Promoter UCSC
PromoterFlanking Hoffman
Intron UCSC
H3K9ac Trynka
H3K4me3 Trynka
H3K4me1 Trynka
H3K27ac PGC2
H3K27ac Hnisz
FetalDHS Trynka
Enhancer Hoffman
Enhancer Andersson
DHS Trynka
DGF ENCODE
CTCF Hoffman
Conserved LindbladToh
Coding UCSC
Annotation
−100
0
100
Enrichment (prop h2 / prop snps)

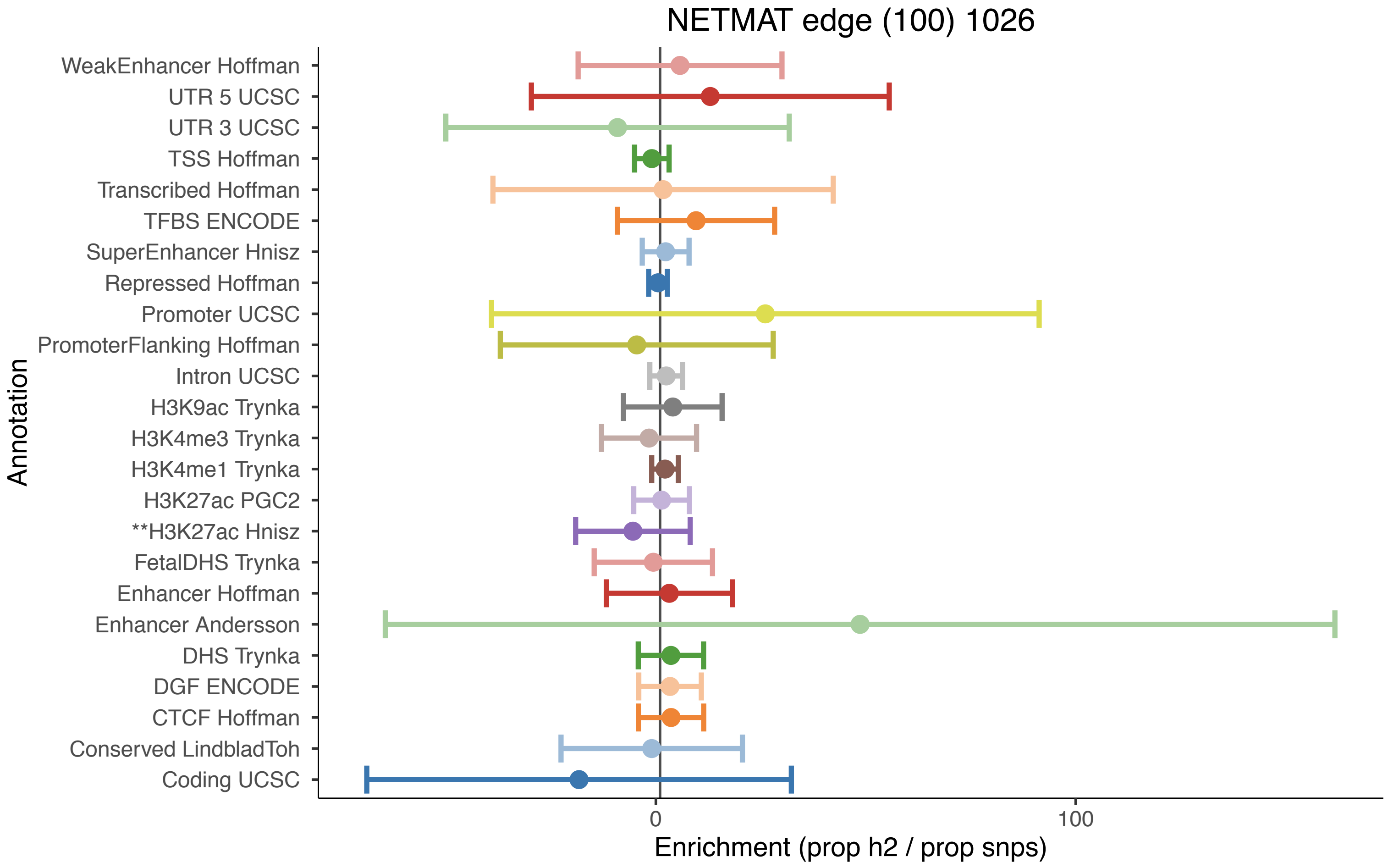
NETMAT edge (100) 1026
WeakEnhancer Hoffman
UTR 5 UCSC
UTR 3 UCSC
TSS Hoffman
Transcribed Hoffman
TFBS ENCODE
SuperEnhancer Hnisz
Repressed Hoffman
Promoter UCSC
PromoterFlanking Hoffman
Intron UCSC
H3K9ac Trynka
H3K4me3 Trynka
H3K4me1 Trynka
H3K27ac PGC2
**H3K27ac Hnisz
FetalDHS Trynka
Enhancer Hoffman
Enhancer Andersson
DHS Trynka
DGF ENCODE
CTCF Hoffman
Conserved LindbladToh
Coding UCSC
Annotation
0
100
Enrichment (prop h2 / prop snps)

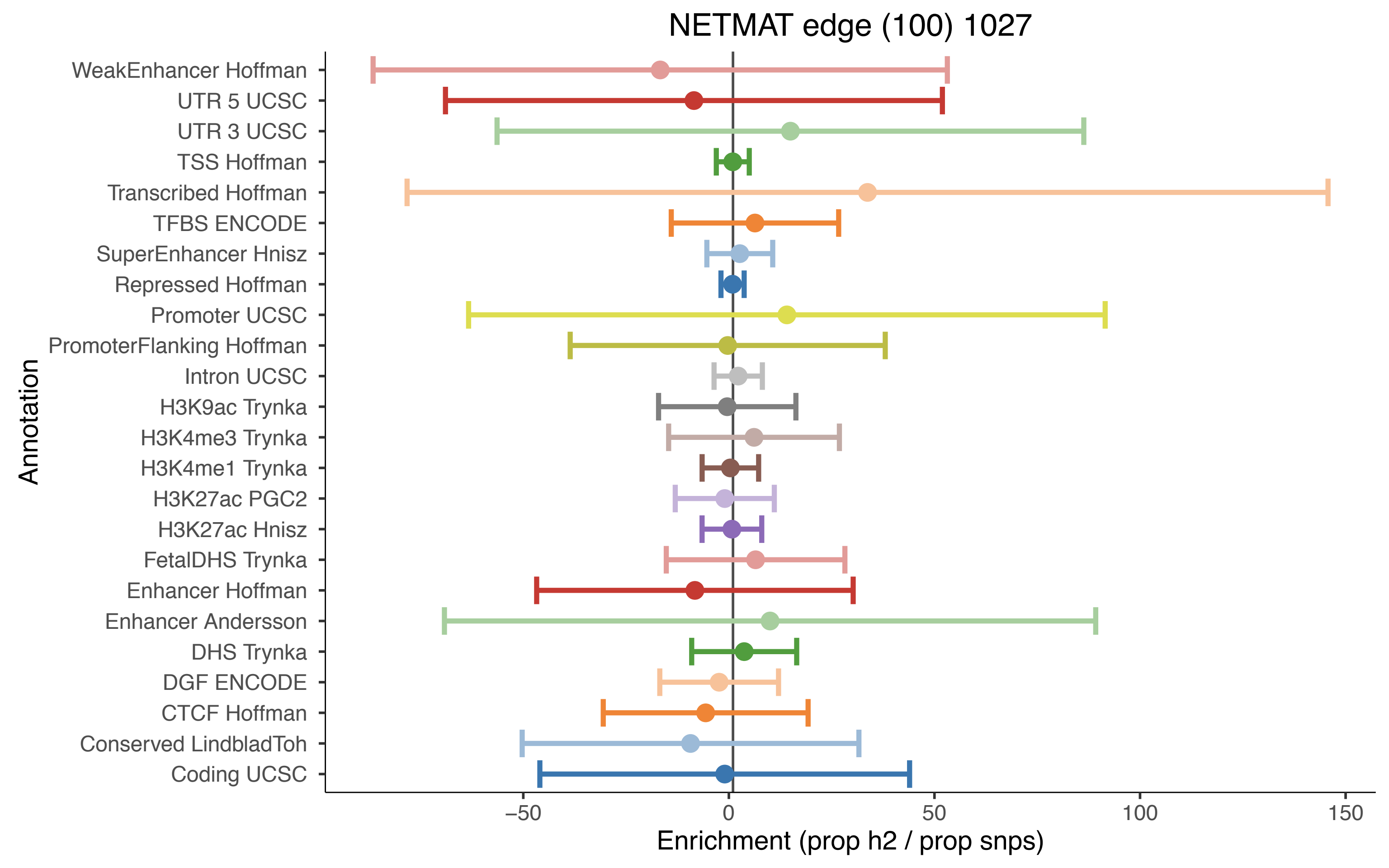

NETMAT edge (100) 1027
WeakEnhancer Hoffman
UTR 5 UCSC
UTR 3 UCSC
TSS Hoffman
Transcribed Hoffman
TFBS ENCODE
SuperEnhancer Hnisz
Repressed Hoffman
Promoter UCSC
PromoterFlanking Hoffman
Intron UCSC
H3K9ac Trynka
H3K4me3 Trynka
H3K4me1 Trynka
H3K27ac PGC2
H3K27ac Hnisz
FetalDHS Trynka
Enhancer Hoffman
Enhancer Andersson
DHS Trynka
DGF ENCODE
CTCF Hoffman
Conserved LindbladToh
Coding UCSC
Annotation
−50
0
50
100
150
Enrichment (prop h2 / prop snps)

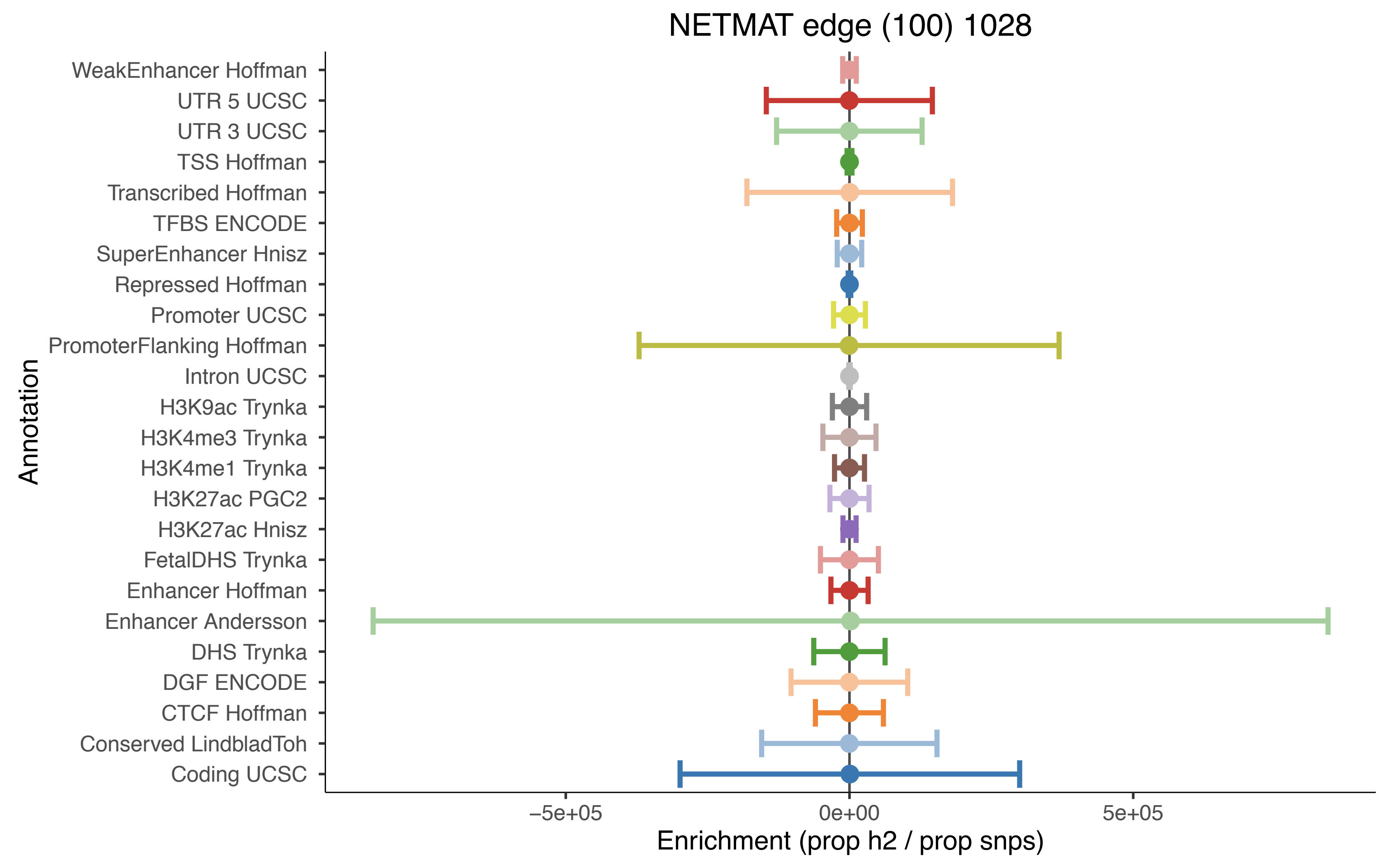
NETMAT edge (100) 1028
WeakEnhancer Hoffman
UTR 5 UCSC
UTR 3 UCSC
TSS Hoffman
Transcribed Hoffman
TFBS ENCODE
SuperEnhancer Hnisz
Repressed Hoffman
Promoter UCSC
PromoterFlanking Hoffman
Intron UCSC
H3K9ac Trynka
H3K4me3 Trynka
H3K4me1 Trynka
H3K27ac PGC2
H3K27ac Hnisz
FetalDHS Trynka
Enhancer Hoffman
Enhancer Andersson
DHS Trynka
DGF ENCODE
CTCF Hoffman
Conserved LindbladToh
Coding UCSC
Annotation
−5e+05
0e+00
5e+05
Enrichment (prop h2 / prop snps)

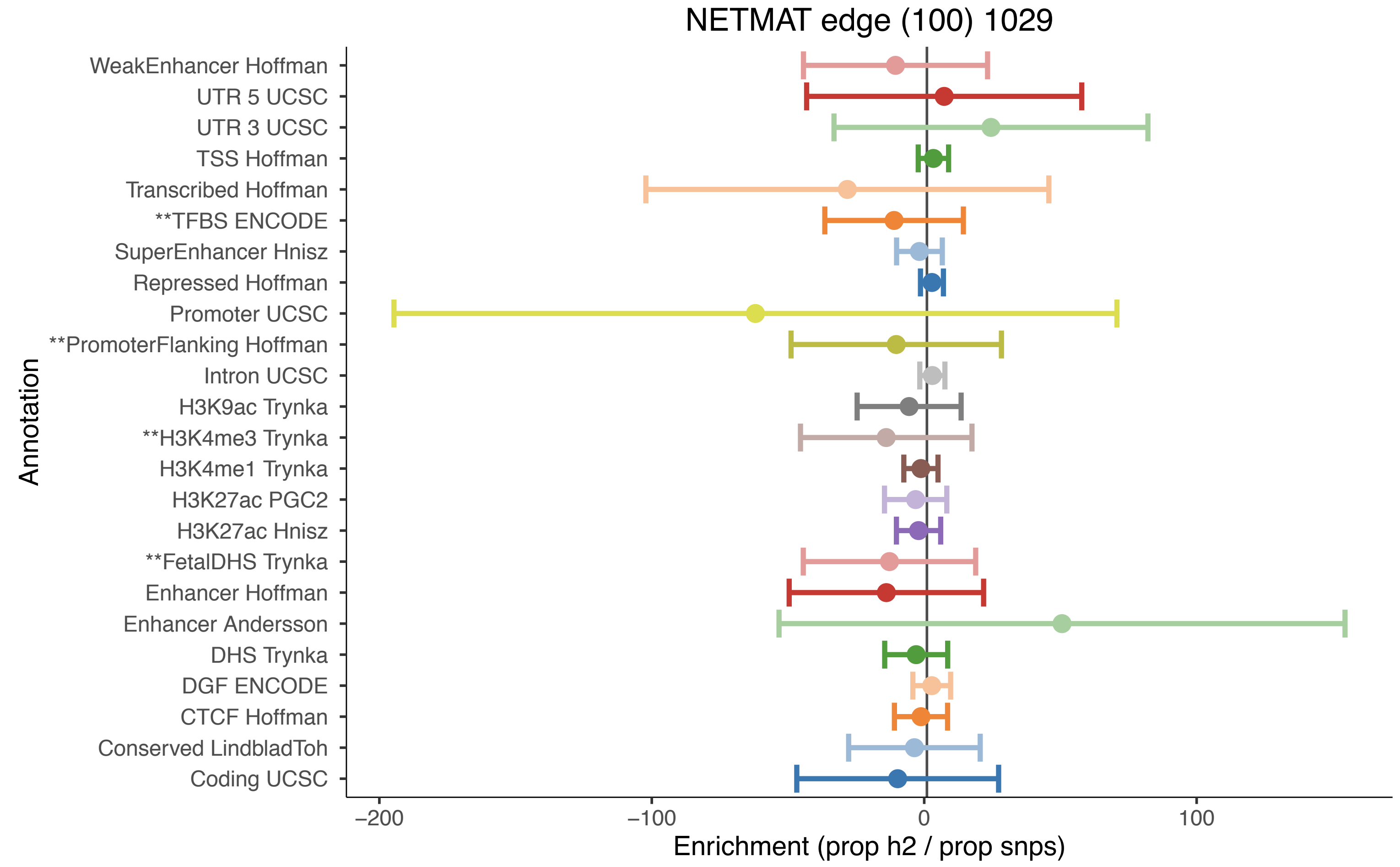

NETMAT edge (100) 1029
WeakEnhancer Hoffman
UTR 5 UCSC
UTR 3 UCSC
TSS Hoffman
Transcribed Hoffman
**TFBS ENCODE
SuperEnhancer Hnisz
Repressed Hoffman
Promoter UCSC
**PromoterFlanking Hoffman
Intron UCSC
H3K9ac Trynka
**H3K4me3 Trynka
H3K4me1 Trynka
H3K27ac PGC2
H3K27ac Hnisz
**FetalDHS Trynka
Enhancer Hoffman
Enhancer Andersson
DHS Trynka
DGF ENCODE
CTCF Hoffman
Conserved LindbladToh
Coding UCSC
Annotation
−200
−100
0
100
Enrichment (prop h2 / prop snps)

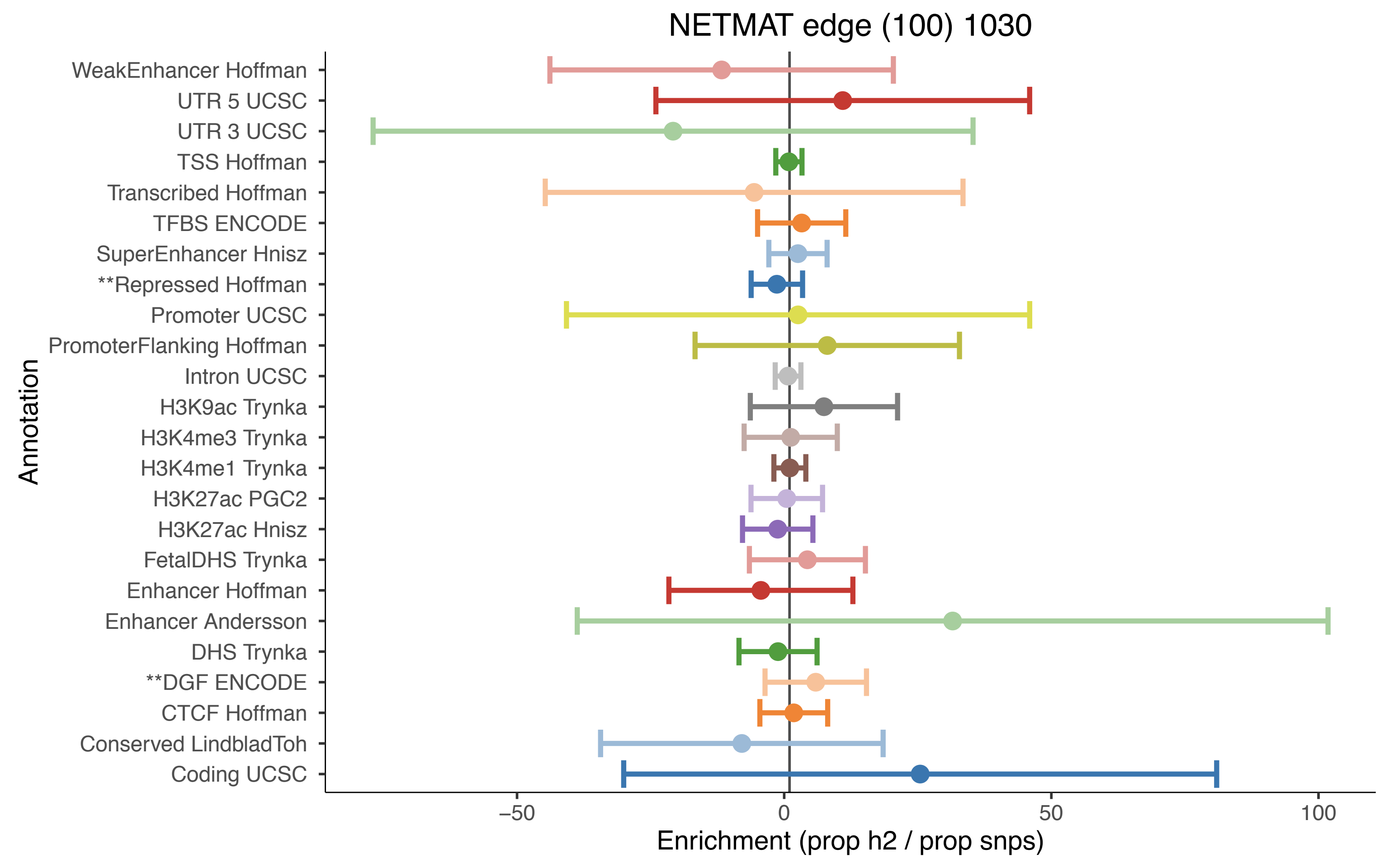

NETMAT edge (100) 1030
WeakEnhancer Hoffman
UTR 5 UCSC
UTR 3 UCSC
TSS Hoffman
Transcribed Hoffman
TFBS ENCODE
SuperEnhancer Hnisz
**Repressed Hoffman
Promoter UCSC
PromoterFlanking Hoffman
Intron UCSC
H3K9ac Trynka
H3K4me3 Trynka
H3K4me1 Trynka
H3K27ac PGC2
H3K27ac Hnisz
FetalDHS Trynka
Enhancer Hoffman
Enhancer Andersson
DHS Trynka
**DGF ENCODE
CTCF Hoffman
Conserved LindbladToh
Coding UCSC
Annotation
−50
0
50
100
Enrichment (prop h2 / prop snps)

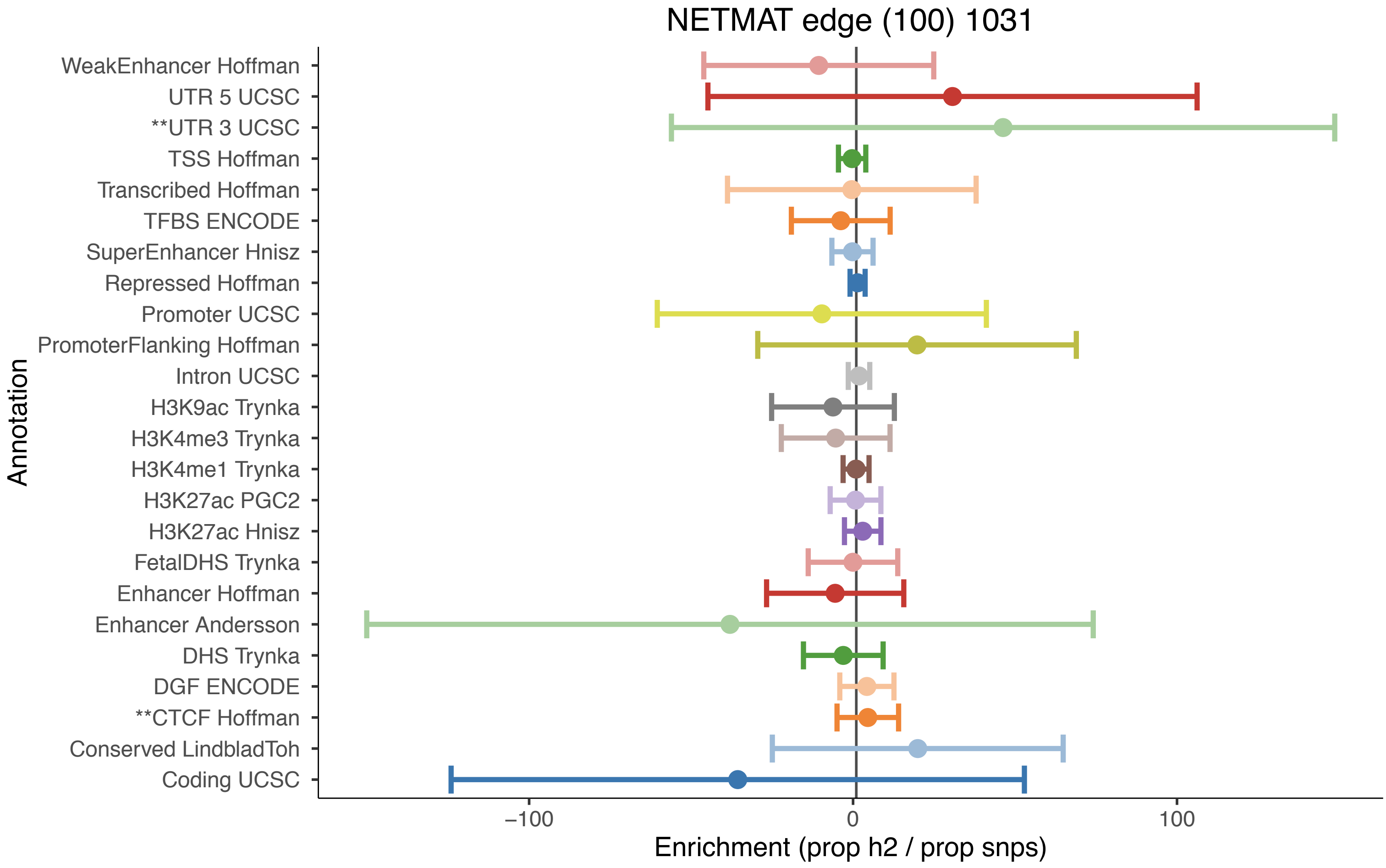
NETMAT edge (100) 1031
WeakEnhancer Hoffman
UTR 5 UCSC
**UTR 3 UCSC
TSS Hoffman
Transcribed Hoffman
TFBS ENCODE
SuperEnhancer Hnisz
Repressed Hoffman
Promoter UCSC
PromoterFlanking Hoffman
Intron UCSC
H3K9ac Trynka
H3K4me3 Trynka
H3K4me1 Trynka
H3K27ac PGC2
H3K27ac Hnisz
FetalDHS Trynka
Enhancer Hoffman
Enhancer Andersson
DHS Trynka
DGF ENCODE
**CTCF Hoffman
Conserved LindbladToh
Coding UCSC
Annotation
−100
0
100
Enrichment (prop h2 / prop snps)

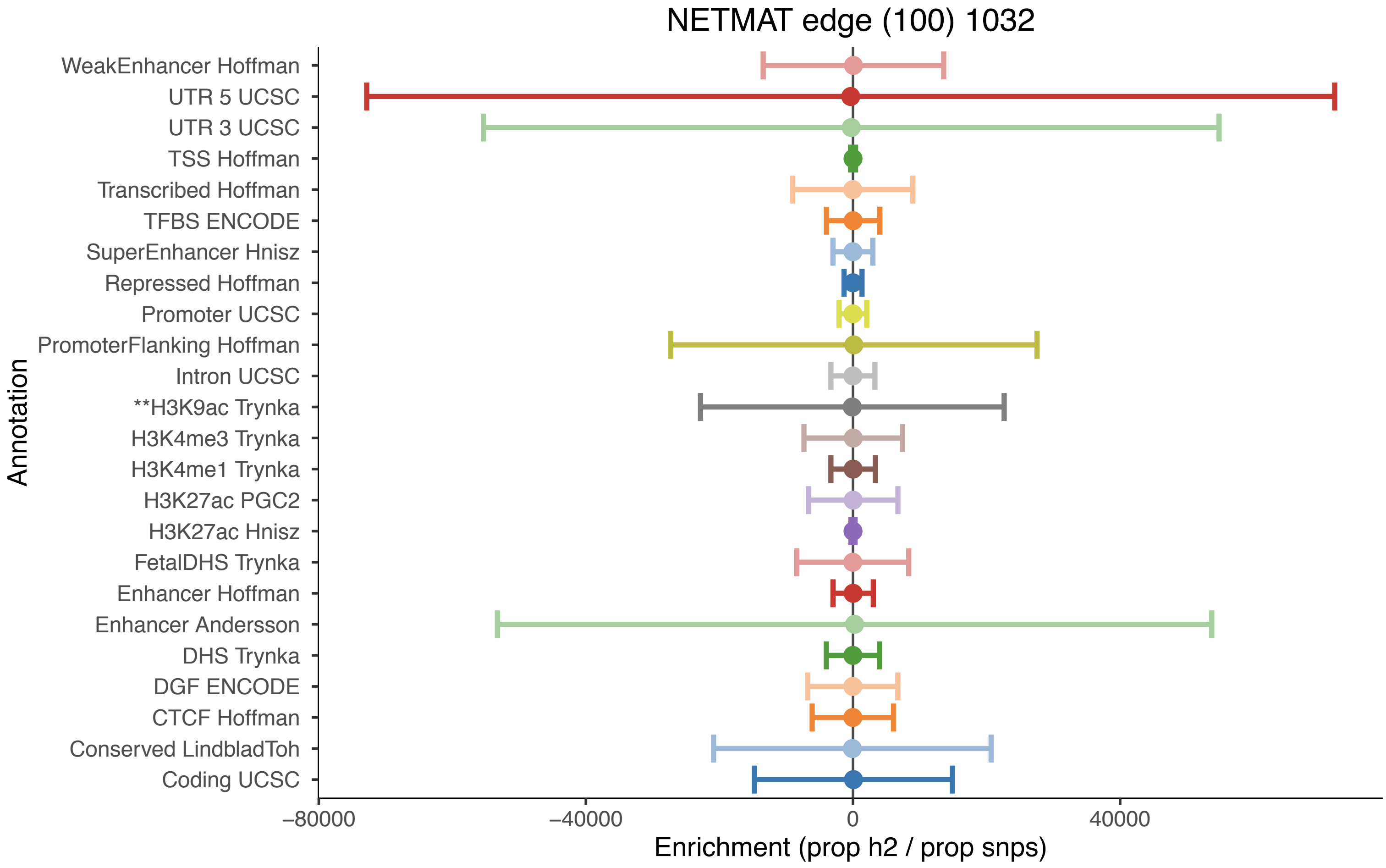

NETMAT edge (100) 1032
WeakEnhancer Hoffman
UTR 5 UCSC
UTR 3 UCSC
TSS Hoffman
Transcribed Hoffman
TFBS ENCODE
SuperEnhancer Hnisz
Repressed Hoffman
Promoter UCSC
PromoterFlanking Hoffman
Intron UCSC
**H3K9ac Trynka
H3K4me3 Trynka
H3K4me1 Trynka
H3K27ac PGC2
H3K27ac Hnisz
FetalDHS Trynka
Enhancer Hoffman
Enhancer Andersson
DHS Trynka
DGF ENCODE
CTCF Hoffman
Conserved LindbladToh
Coding UCSC
Annotation
−80000
−40000
0
40000
Enrichment (prop h2 / prop snps)

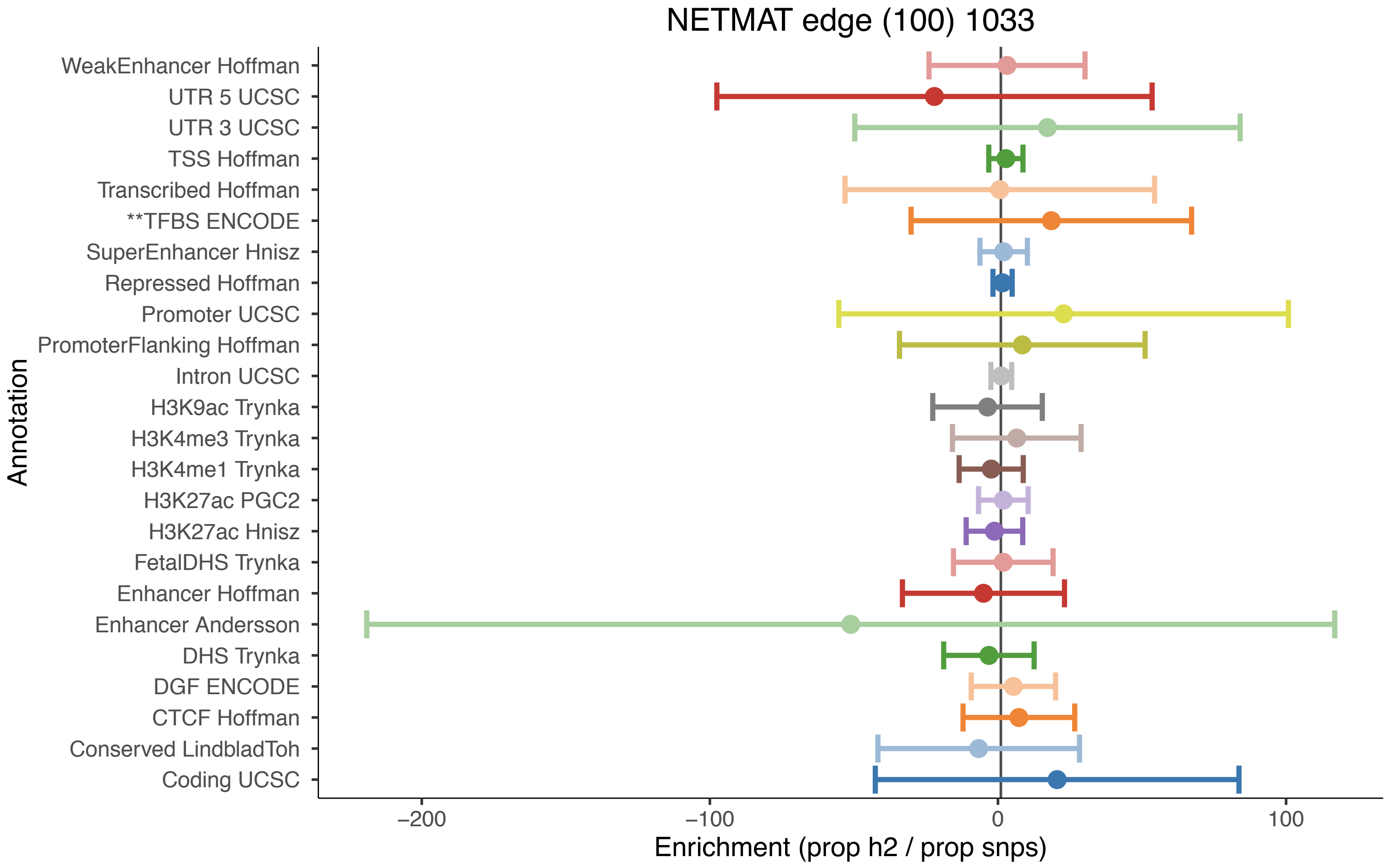

NETMAT edge (100) 1033
WeakEnhancer Hoffman
UTR 5 UCSC
UTR 3 UCSC
TSS Hoffman
Transcribed Hoffman
**TFBS ENCODE
SuperEnhancer Hnisz
Repressed Hoffman
Promoter UCSC
PromoterFlanking Hoffman
Intron UCSC
H3K9ac Trynka
H3K4me3 Trynka
H3K4me1 Trynka
H3K27ac PGC2
H3K27ac Hnisz
FetalDHS Trynka
Enhancer Hoffman
Enhancer Andersson
DHS Trynka
DGF ENCODE
CTCF Hoffman
Conserved LindbladToh
Coding UCSC
Annotation
−200
−100
0
100
Enrichment (prop h2 / prop snps)

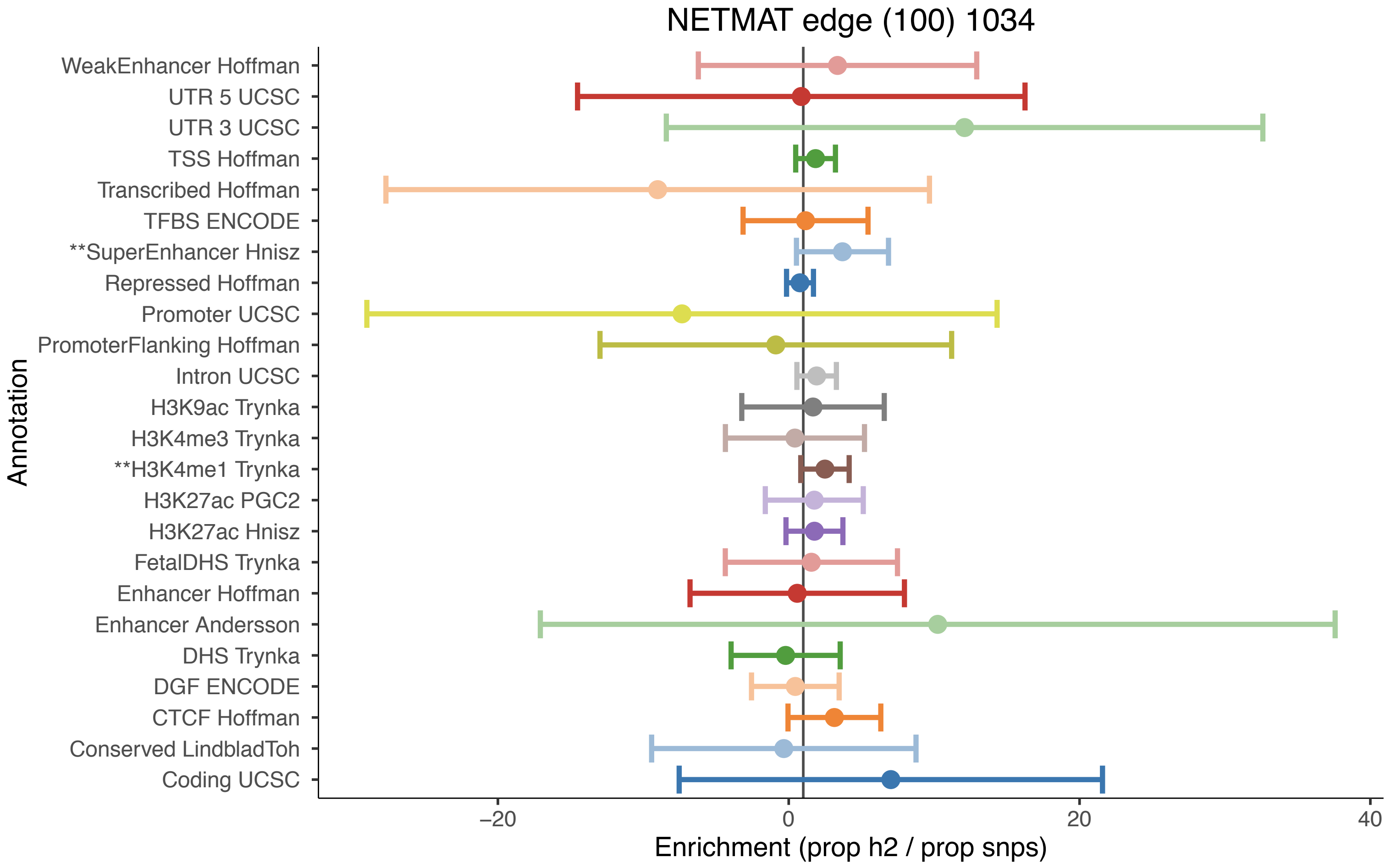

NETMAT edge (100) 1034
WeakEnhancer Hoffman
UTR 5 UCSC
UTR 3 UCSC
TSS Hoffman
Transcribed Hoffman
TFBS ENCODE
**SuperEnhancer Hnisz
Repressed Hoffman
Promoter UCSC
PromoterFlanking Hoffman
Intron UCSC
H3K9ac Trynka
H3K4me3 Trynka
**H3K4me1 Trynka
H3K27ac PGC2
H3K27ac Hnisz
FetalDHS Trynka
Enhancer Hoffman
Enhancer Andersson
DHS Trynka
DGF ENCODE
CTCF Hoffman
Conserved LindbladToh
Coding UCSC
Annotation
−20
0
20
40
Enrichment (prop h2 / prop snps)

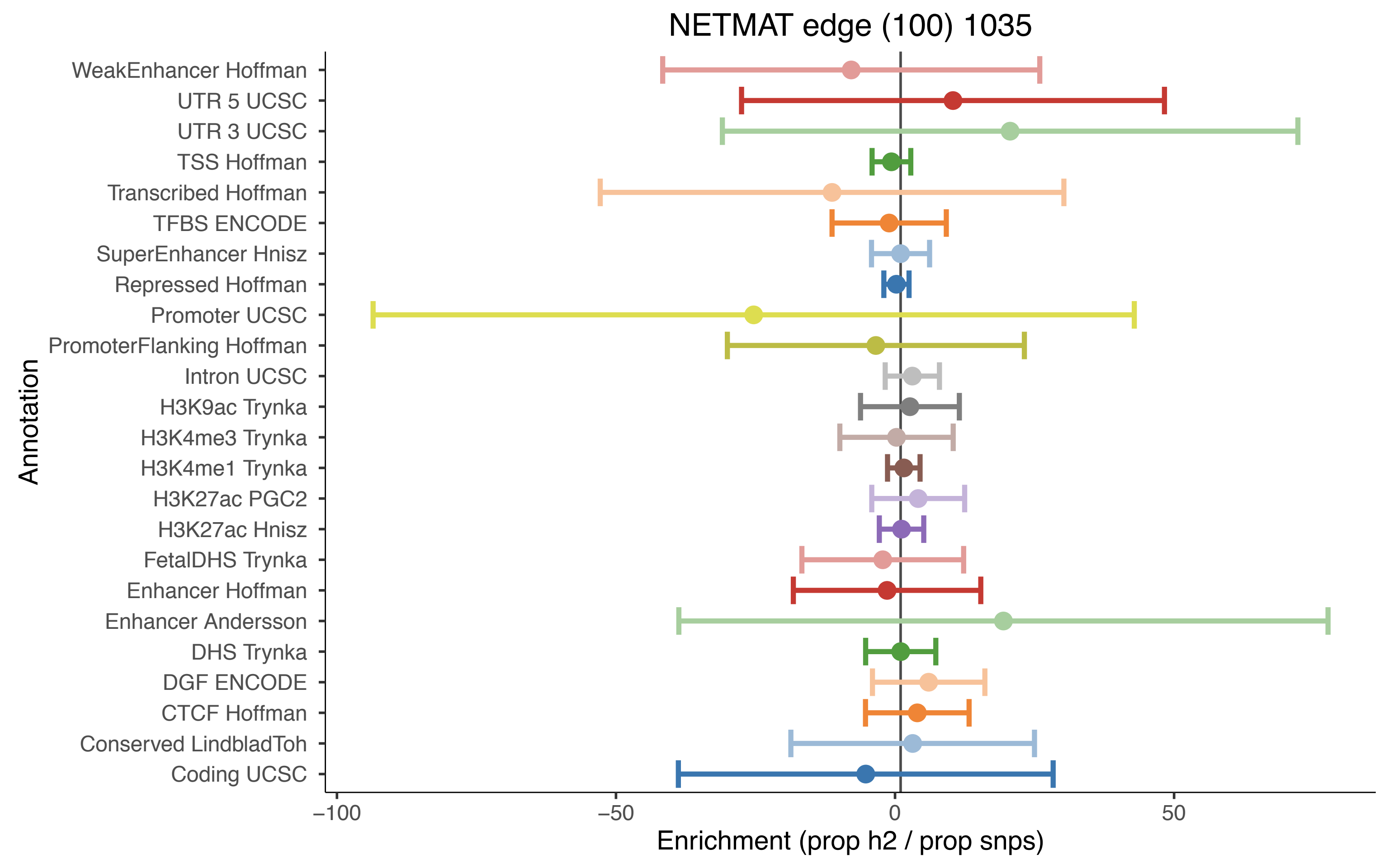
NETMAT edge (100) 1035
WeakEnhancer Hoffman
UTR 5 UCSC
UTR 3 UCSC
TSS Hoffman
Transcribed Hoffman
TFBS ENCODE
SuperEnhancer Hnisz
Repressed Hoffman
Promoter UCSC
PromoterFlanking Hoffman
Intron UCSC
H3K9ac Trynka
H3K4me3 Trynka
H3K4me1 Trynka
H3K27ac PGC2
H3K27ac Hnisz
FetalDHS Trynka
Enhancer Hoffman
Enhancer Andersson
DHS Trynka
DGF ENCODE
CTCF Hoffman
Conserved LindbladToh
Coding UCSC
Annotation
−100
−50
0
50
Enrichment (prop h2 / prop snps)

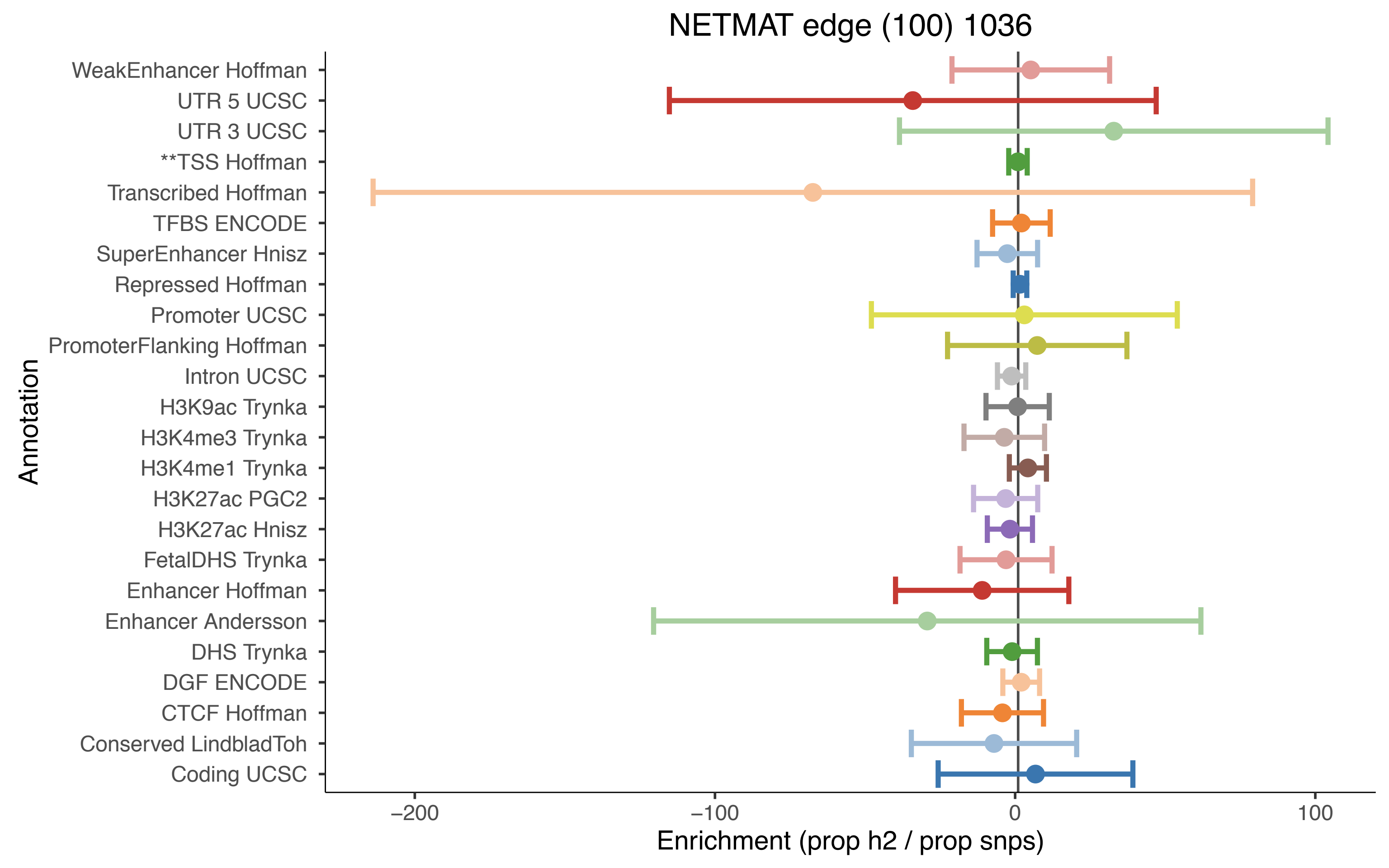

NETMAT edge (100) 1036
WeakEnhancer Hoffman
UTR 5 UCSC
UTR 3 UCSC
**TSS Hoffman
Transcribed Hoffman
TFBS ENCODE
SuperEnhancer Hnisz
Repressed Hoffman
Promoter UCSC
PromoterFlanking Hoffman
Intron UCSC
H3K9ac Trynka
H3K4me3 Trynka
H3K4me1 Trynka
H3K27ac PGC2
H3K27ac Hnisz
FetalDHS Trynka
Enhancer Hoffman
Enhancer Andersson
DHS Trynka
DGF ENCODE
CTCF Hoffman
Conserved LindbladToh
Coding UCSC
Annotation
−200
−100
0
100
Enrichment (prop h2 / prop snps)

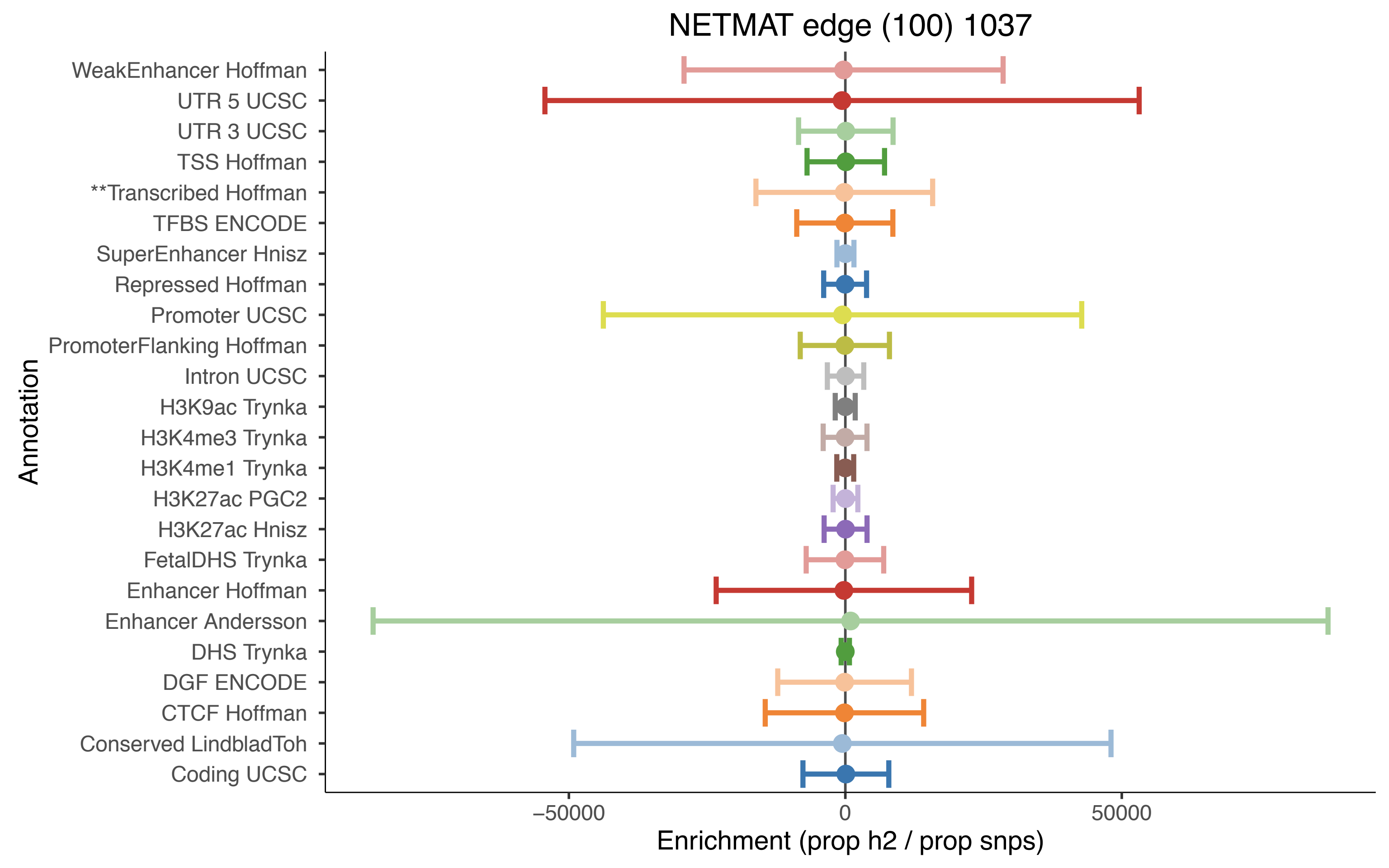

NETMAT edge (100) 1037
WeakEnhancer Hoffman
UTR 5 UCSC
UTR 3 UCSC
TSS Hoffman
**Transcribed Hoffman
TFBS ENCODE
SuperEnhancer Hnisz
Repressed Hoffman
Promoter UCSC
PromoterFlanking Hoffman
Intron UCSC
H3K9ac Trynka
H3K4me3 Trynka
H3K4me1 Trynka
H3K27ac PGC2
H3K27ac Hnisz
FetalDHS Trynka
Enhancer Hoffman
Enhancer Andersson
DHS Trynka
DGF ENCODE
CTCF Hoffman
Conserved LindbladToh
Coding UCSC
Annotation
−50000
0
50000
Enrichment (prop h2 / prop snps)

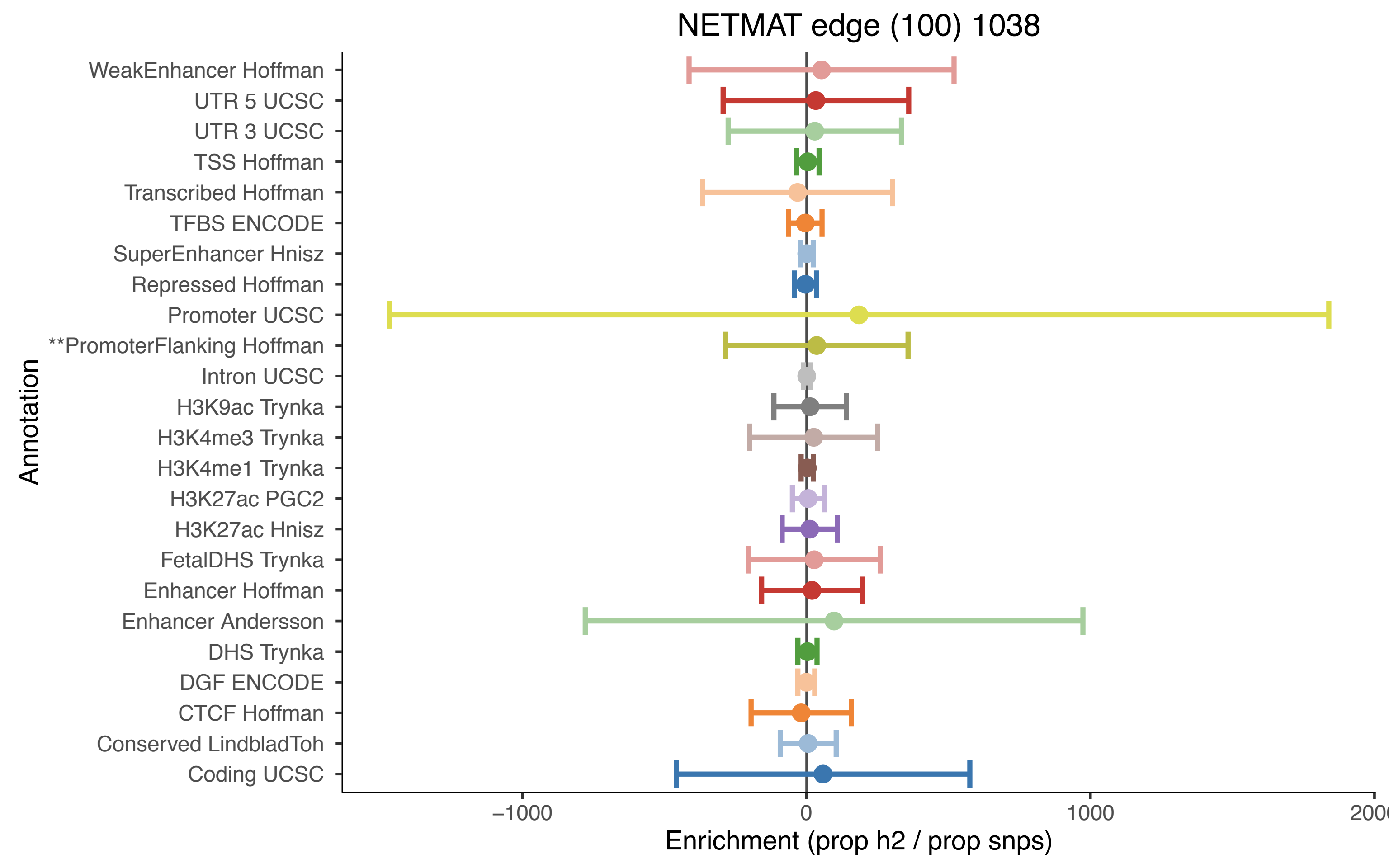

NETMAT edge (100) 1038
WeakEnhancer Hoffman
UTR 5 UCSC
UTR 3 UCSC
TSS Hoffman
Transcribed Hoffman
TFBS ENCODE
SuperEnhancer Hnisz
Repressed Hoffman
Promoter UCSC
**PromoterFlanking Hoffman
Intron UCSC
H3K9ac Trynka
H3K4me3 Trynka
H3K4me1 Trynka
H3K27ac PGC2
H3K27ac Hnisz
FetalDHS Trynka
Enhancer Hoffman
Enhancer Andersson
DHS Trynka
DGF ENCODE
CTCF Hoffman
Conserved LindbladToh
Coding UCSC
Annotation
−1000
0
1000
Enrichment (prop h2 / prop snps)

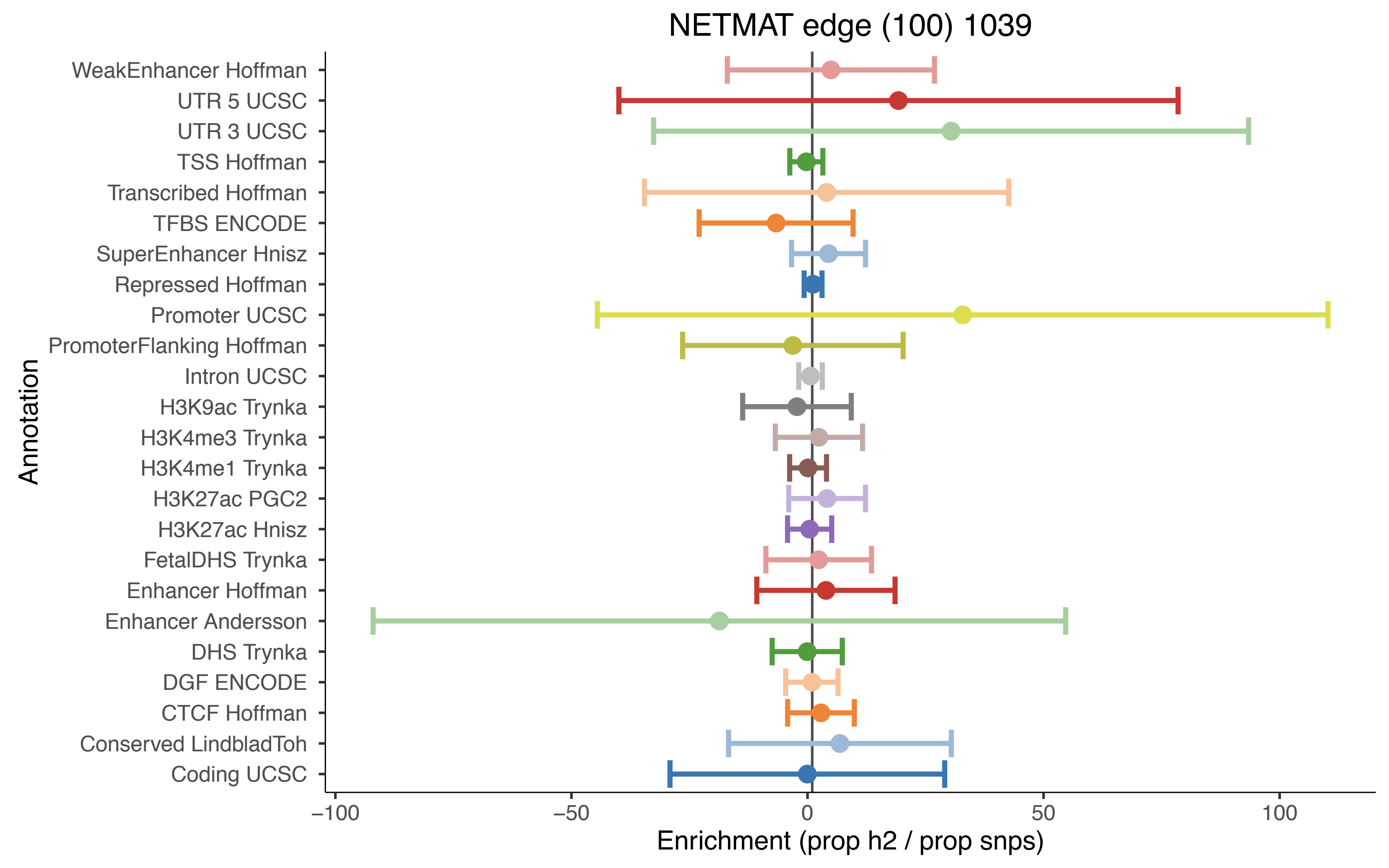
NETMAT edge (100) 1039
WeakEnhancer Hoffman
UTR 5 UCSC
UTR 3 UCSC
TSS Hoffman
Transcribed Hoffman
TFBS ENCODE
SuperEnhancer Hnisz
Repressed Hoffman
Promoter UCSC
PromoterFlanking Hoffman
Intron UCSC
H3K9ac Trynka
H3K4me3 Trynka
H3K4me1 Trynka
H3K27ac PGC2
H3K27ac Hnisz
FetalDHS Trynka
Enhancer Hoffman
Enhancer Andersson
DHS Trynka
DGF ENCODE
CTCF Hoffman
Conserved LindbladToh
Coding UCSC
Annotation
−100
−50
0
50
100
Enrichment (prop h2 / prop snps)

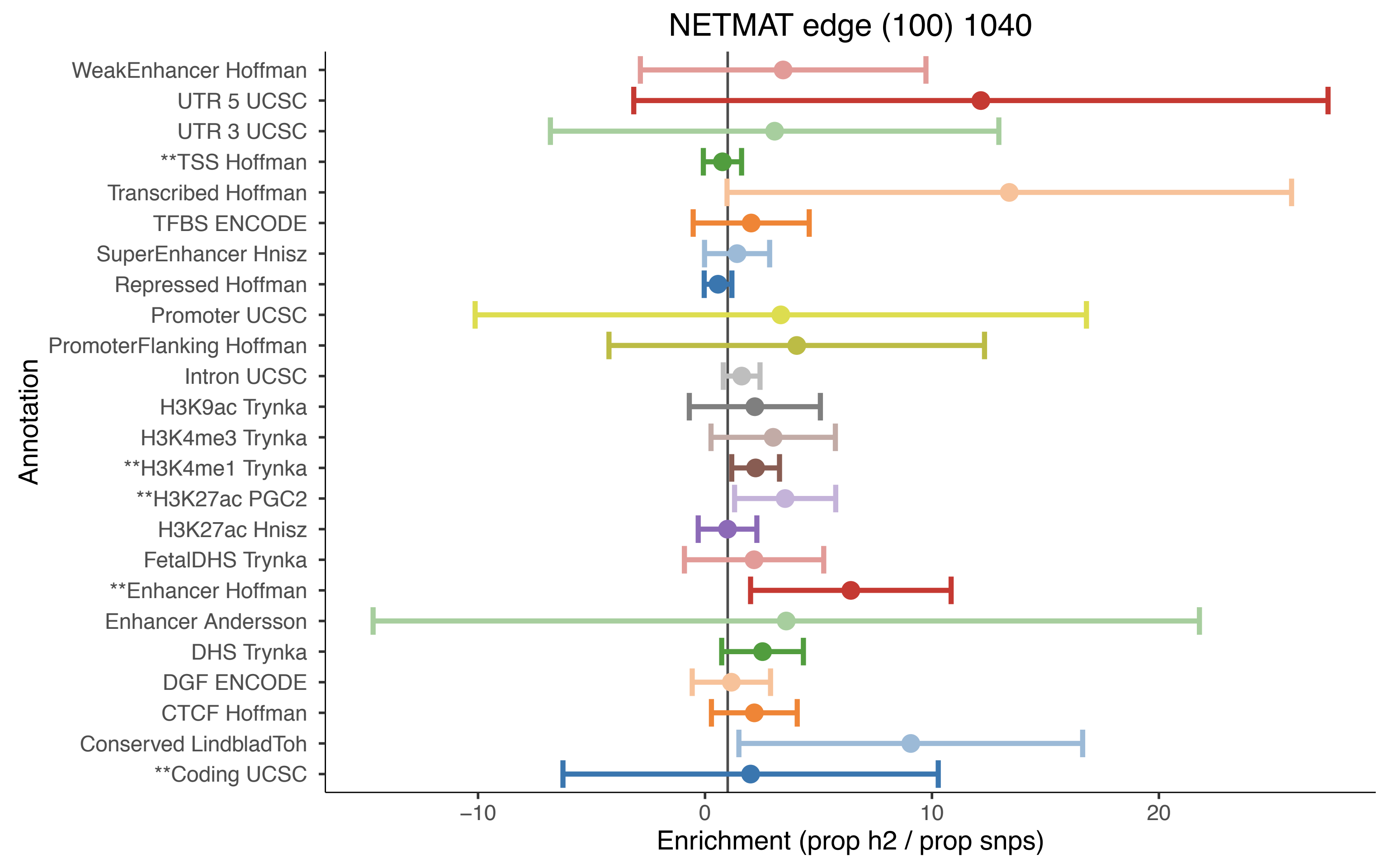

NETMAT edge (100) 1040
WeakEnhancer Hoffman
UTR 5 UCSC
UTR 3 UCSC
**TSS Hoffman
Transcribed Hoffman
TFBS ENCODE
SuperEnhancer Hnisz
Repressed Hoffman
Promoter UCSC
PromoterFlanking Hoffman
Intron UCSC
H3K9ac Trynka
H3K4me3 Trynka
**H3K4me1 Trynka
**H3K27ac PGC2
H3K27ac Hnisz
FetalDHS Trynka
**Enhancer Hoffman
Enhancer Andersson
DHS Trynka
DGF ENCODE
CTCF Hoffman
Conserved LindbladToh
**Coding UCSC
Annotation
−10
0
10
20
Enrichment (prop h2 / prop snps)

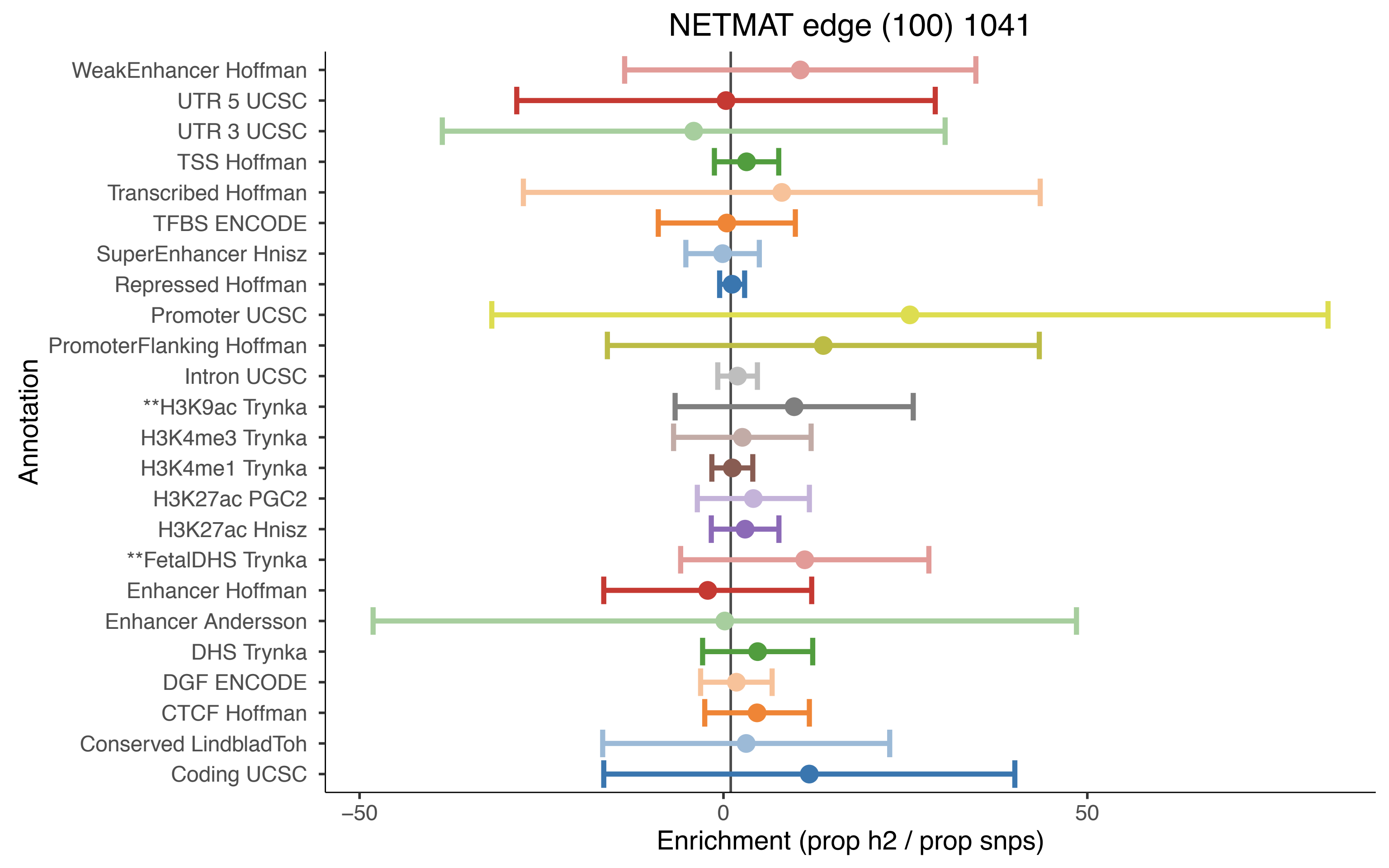
NETMAT edge (100) 1041
WeakEnhancer Hoffman
UTR 5 UCSC
UTR 3 UCSC
TSS Hoffman
Transcribed Hoffman
TFBS ENCODE
SuperEnhancer Hnisz
Repressed Hoffman
Promoter UCSC
PromoterFlanking Hoffman
Intron UCSC
**H3K9ac Trynka
H3K4me3 Trynka
H3K4me1 Trynka
H3K27ac PGC2
H3K27ac Hnisz
**FetalDHS Trynka
Enhancer Hoffman
Enhancer Andersson
DHS Trynka
DGF ENCODE
CTCF Hoffman
Conserved LindbladToh
Coding UCSC
Annotation
−50
0
50
Enrichment (prop h2 / prop snps)

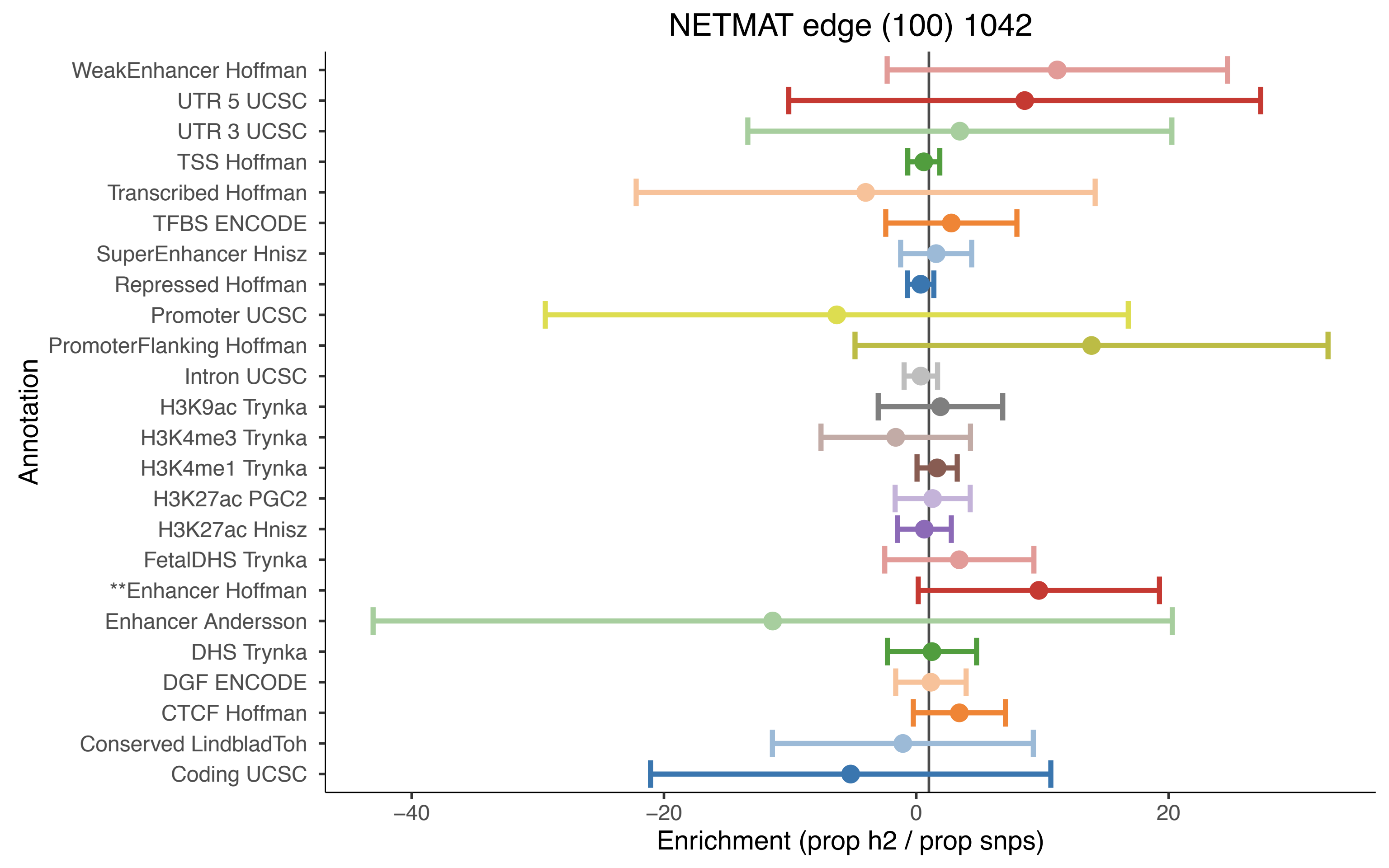

NETMAT edge (100) 1042
WeakEnhancer Hoffman
UTR 5 UCSC
UTR 3 UCSC
TSS Hoffman
Transcribed Hoffman
TFBS ENCODE
SuperEnhancer Hnisz
Repressed Hoffman
Promoter UCSC
PromoterFlanking Hoffman
Intron UCSC
H3K9ac Trynka
H3K4me3 Trynka
H3K4me1 Trynka
H3K27ac PGC2
H3K27ac Hnisz
FetalDHS Trynka
**Enhancer Hoffman
Enhancer Andersson
DHS Trynka
DGF ENCODE
CTCF Hoffman
Conserved LindbladToh
Coding UCSC
Annotation
−40
−20
0
20
Enrichment (prop h2 / prop snps)

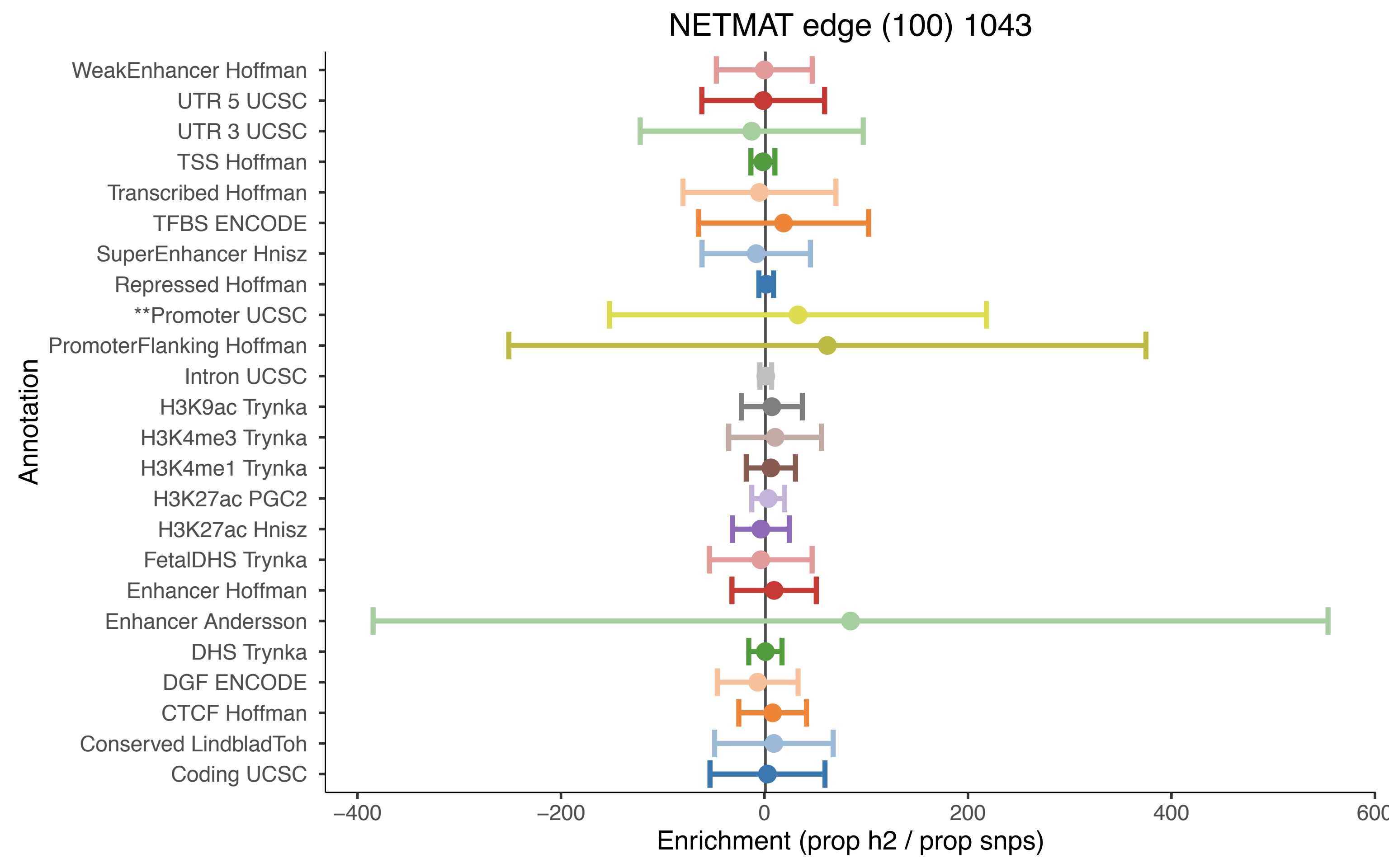
NETMAT edge (100) 1043
WeakEnhancer Hoffman
UTR 5 UCSC
UTR 3 UCSC
TSS Hoffman
Transcribed Hoffman
TFBS ENCODE
SuperEnhancer Hnisz
Repressed Hoffman
**Promoter UCSC
PromoterFlanking Hoffman
Intron UCSC
H3K9ac Trynka
H3K4me3 Trynka
H3K4me1 Trynka
H3K27ac PGC2
H3K27ac Hnisz
FetalDHS Trynka
Enhancer Hoffman
Enhancer Andersson
DHS Trynka
DGF ENCODE
CTCF Hoffman
Conserved LindbladToh
Coding UCSC
Annotation
−400
−200
0
200
400
600
Enrichment (prop h2 / prop snps)

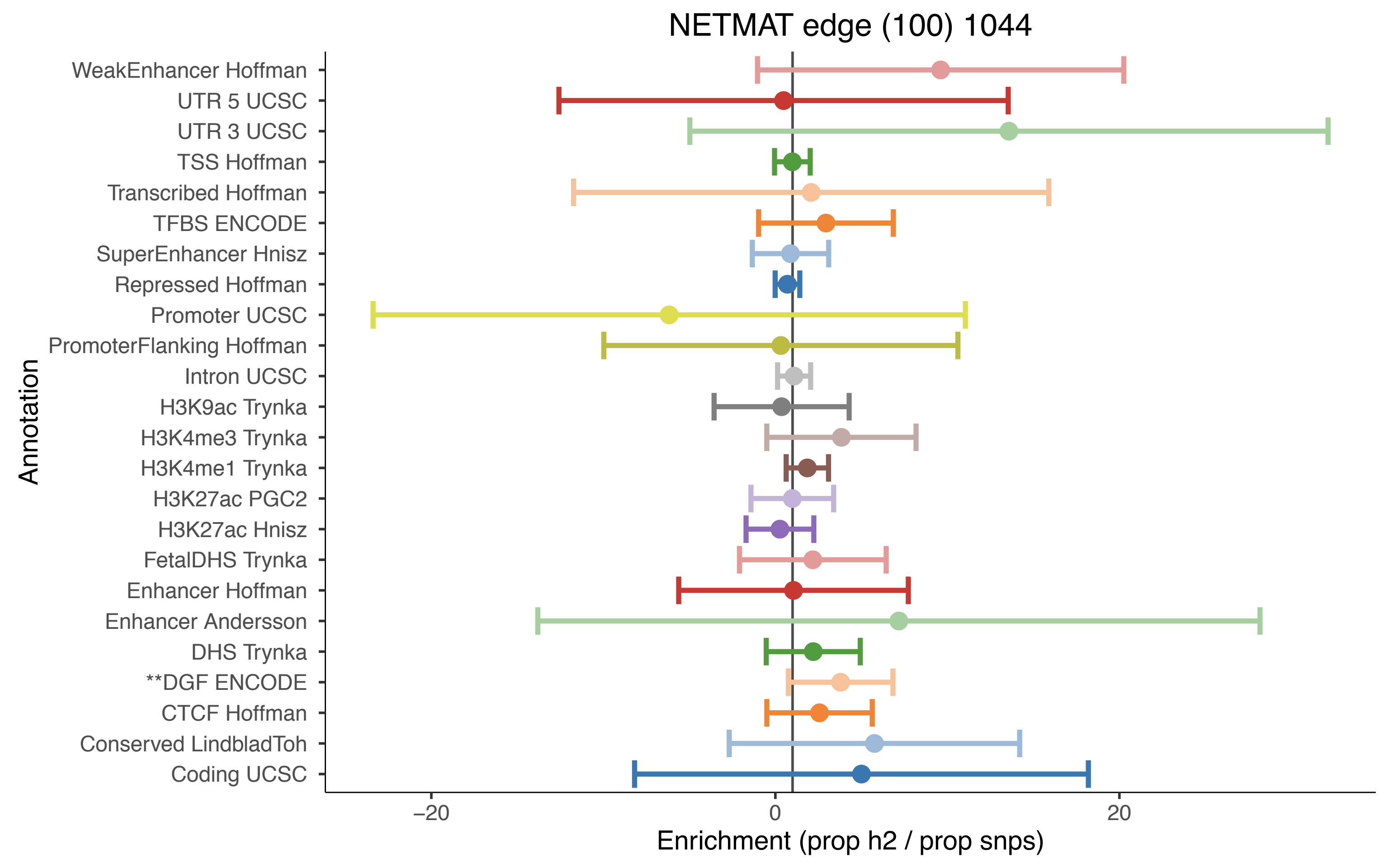

NETMAT edge (100) 1044
WeakEnhancer Hoffman
UTR 5 UCSC
UTR 3 UCSC
TSS Hoffman
Transcribed Hoffman
TFBS ENCODE
SuperEnhancer Hnisz
Repressed Hoffman
Promoter UCSC
PromoterFlanking Hoffman
Intron UCSC
H3K9ac Trynka
H3K4me3 Trynka
H3K4me1 Trynka
H3K27ac PGC2
H3K27ac Hnisz
FetalDHS Trynka
Enhancer Hoffman
Enhancer Andersson
DHS Trynka
**DGF ENCODE
CTCF Hoffman
Conserved LindbladToh
Coding UCSC
Annotation
−20
0
20
Enrichment (prop h2 / prop snps)

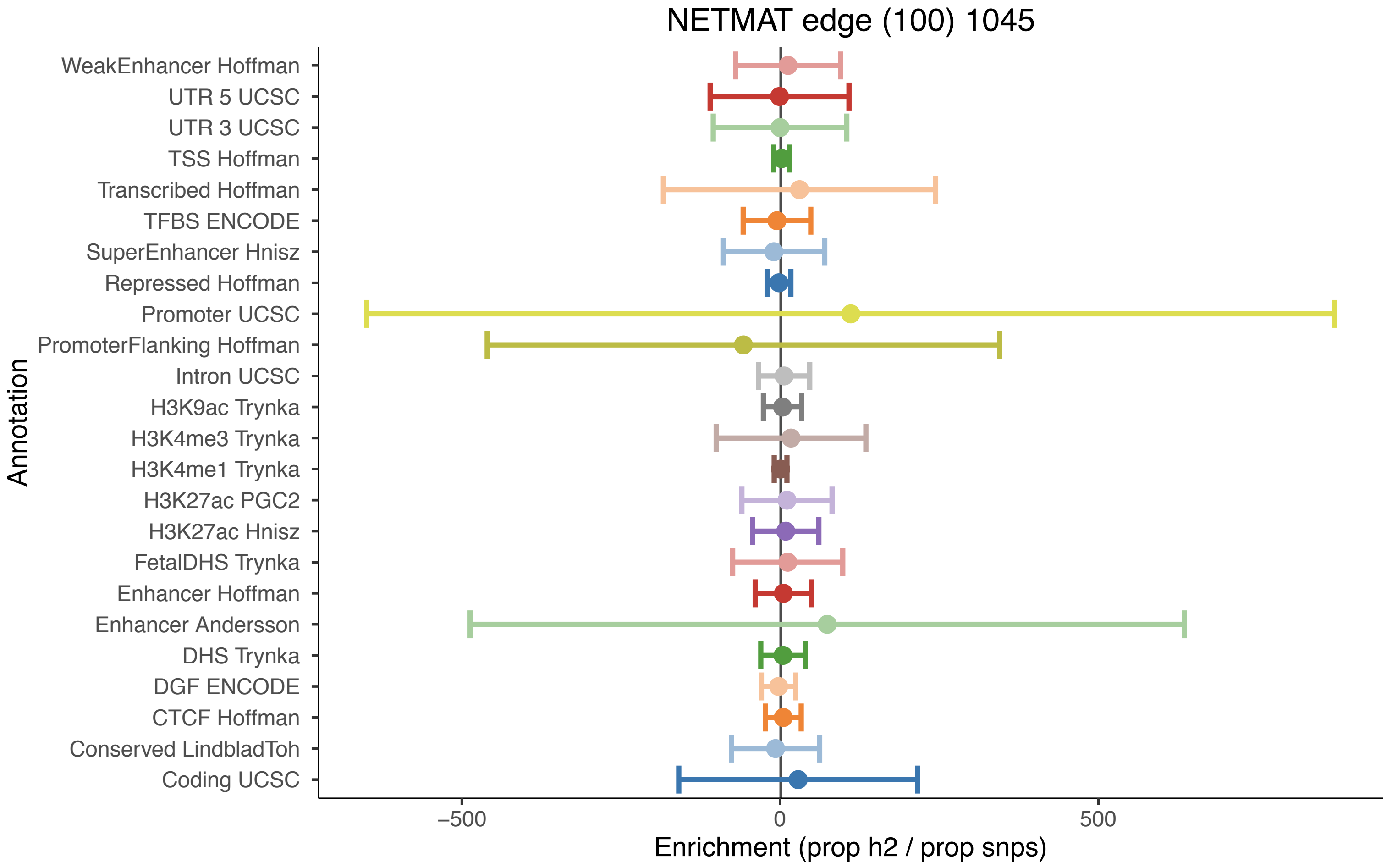

NETMAT edge (100) 1045
WeakEnhancer Hoffman
UTR 5 UCSC
UTR 3 UCSC
TSS Hoffman
Transcribed Hoffman
TFBS ENCODE
SuperEnhancer Hnisz
Repressed Hoffman
Promoter UCSC
PromoterFlanking Hoffman
Intron UCSC
H3K9ac Trynka
H3K4me3 Trynka
H3K4me1 Trynka
H3K27ac PGC2
H3K27ac Hnisz
FetalDHS Trynka
Enhancer Hoffman
Enhancer Andersson
DHS Trynka
DGF ENCODE
CTCF Hoffman
Conserved LindbladToh
Coding UCSC
Annotation
−500
0
500
Enrichment (prop h2 / prop snps)

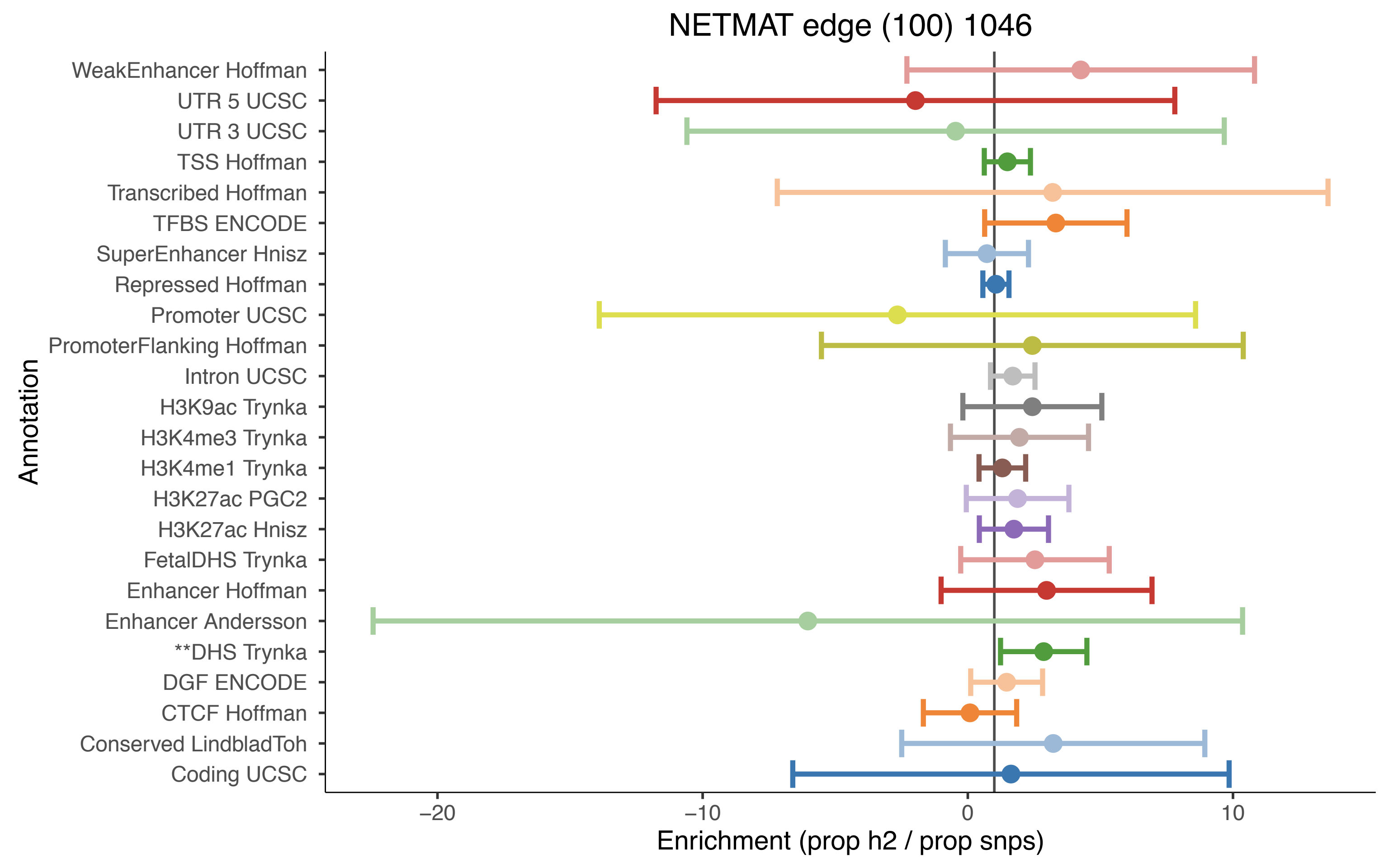

NETMAT edge (100) 1046
WeakEnhancer Hoffman
UTR 5 UCSC
UTR 3 UCSC
TSS Hoffman
Transcribed Hoffman
TFBS ENCODE
SuperEnhancer Hnisz
Repressed Hoffman
Promoter UCSC
PromoterFlanking Hoffman
Intron UCSC
H3K9ac Trynka
H3K4me3 Trynka
H3K4me1 Trynka
H3K27ac PGC2
H3K27ac Hnisz
FetalDHS Trynka
Enhancer Hoffman
Enhancer Andersson
**DHS Trynka
DGF ENCODE
CTCF Hoffman
Conserved LindbladToh
Coding UCSC
Annotation
−20
−10
0
10
Enrichment (prop h2 / prop snps)

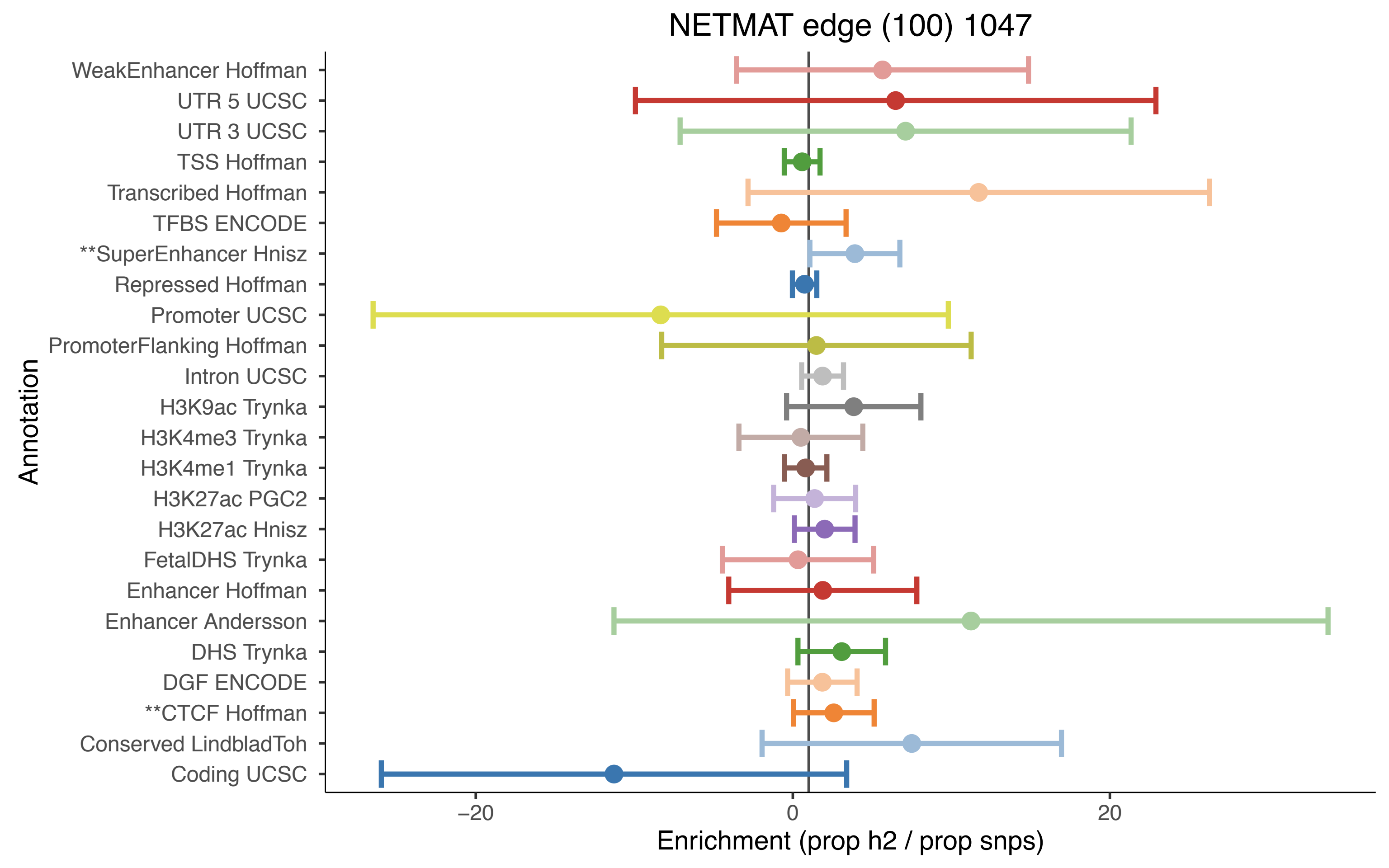

NETMAT edge (100) 1047
WeakEnhancer Hoffman
UTR 5 UCSC
UTR 3 UCSC
TSS Hoffman
Transcribed Hoffman
TFBS ENCODE
**SuperEnhancer Hnisz
Repressed Hoffman
Promoter UCSC
PromoterFlanking Hoffman
Intron UCSC
H3K9ac Trynka
H3K4me3 Trynka
H3K4me1 Trynka
H3K27ac PGC2
H3K27ac Hnisz
FetalDHS Trynka
Enhancer Hoffman
Enhancer Andersson
DHS Trynka
DGF ENCODE
**CTCF Hoffman
Conserved LindbladToh
Coding UCSC
Annotation
−20
0
20
Enrichment (prop h2 / prop snps)

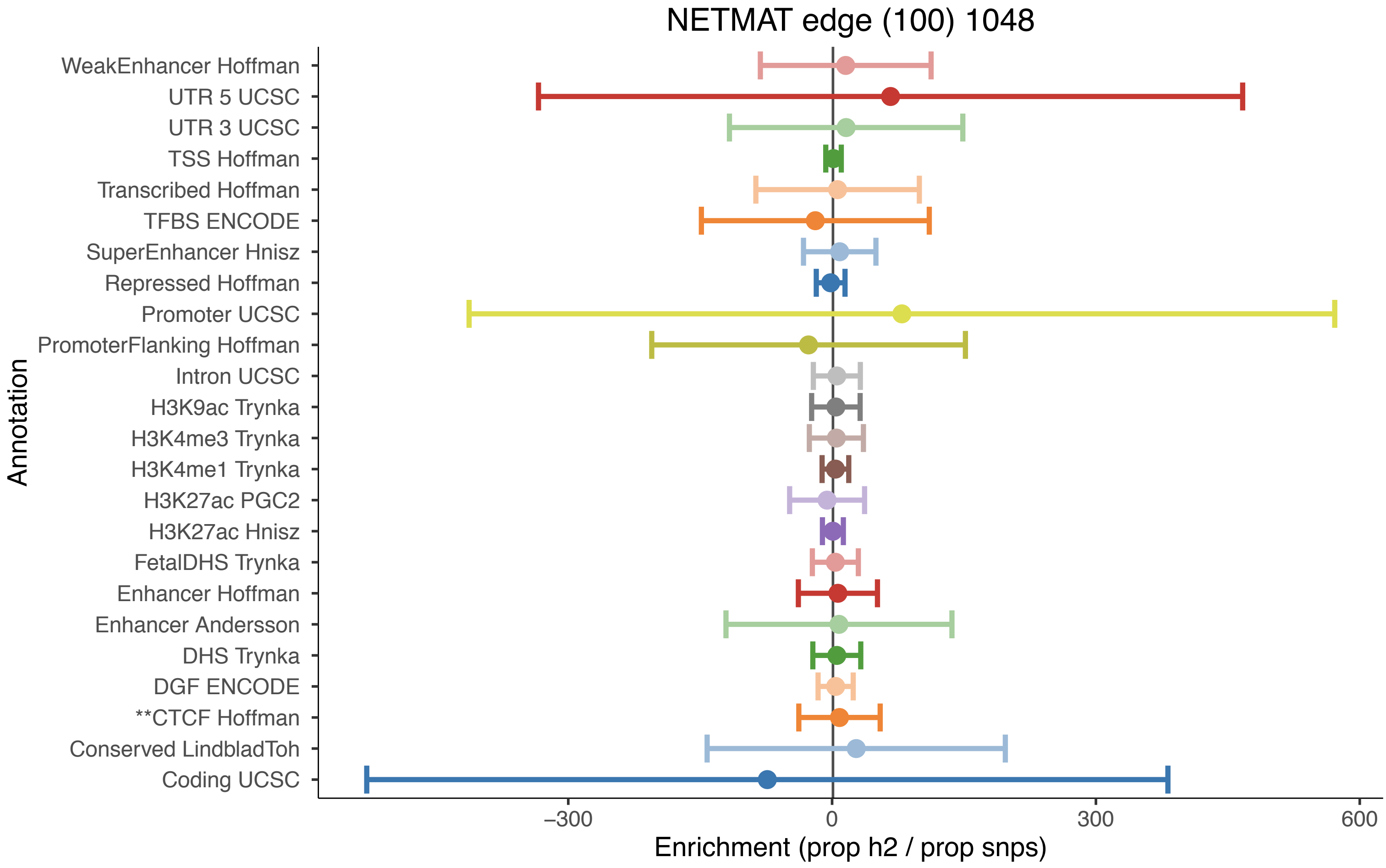

NETMAT edge (100) 1048
Annotation
WeakEnhancer Hoffman
UTR 5 UCSC
UTR 3 UCSC
TSS Hoffman
Transcribed Hoffman
TFBS ENCODE
SuperEnhancer Hnisz
Repressed Hoffman
Promoter UCSC
PromoterFlanking Hoffman
Intron UCSC
H3K9ac Trynka
H3K4me3 Trynka
H3K4me1 Trynka
H3K27ac PGC2
H3K27ac Hnisz
FetalDHS Trynka
Enhancer Hoffman
Enhancer Andersson
DHS Trynka
DGF ENCODE
**CTCF Hoffman
Conserved LindbladToh
Coding UCSC
–300
0
300
600
Enrichment (prop h2 / prop snps)

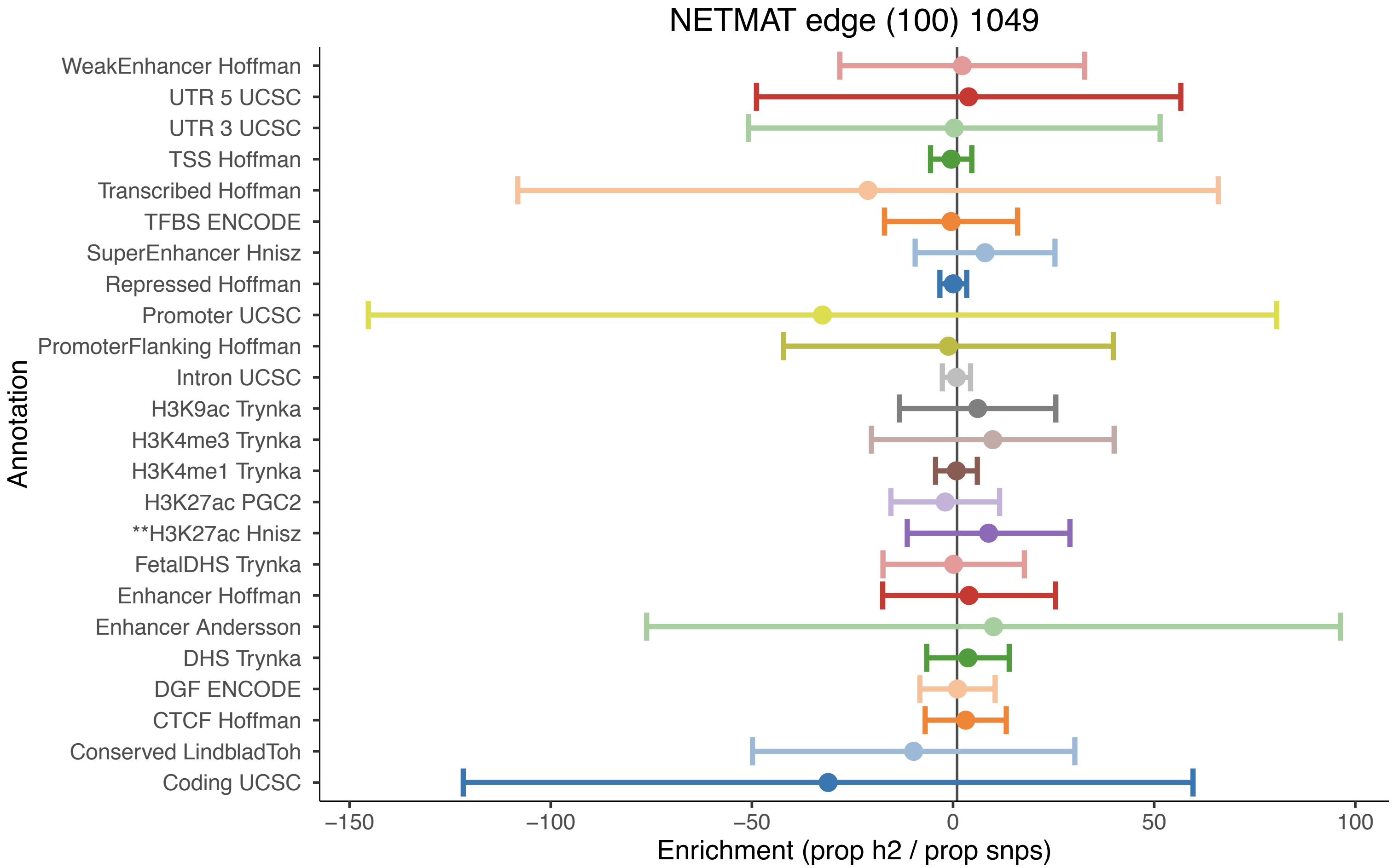
NETMAT edge (100) 1049
WeakEnhancer Hoffman
UTR 5 UCSC
UTR 3 UCSC
TSS Hoffman
Transcribed Hoffman
TFBS ENCODE
SuperEnhancer Hnisz
Repressed Hoffman
Promoter UCSC
PromoterFlanking Hoffman
Intron UCSC
H3K9ac Trynka
H3K4me3 Trynka
H3K4me1 Trynka
H3K27ac PGC2
**H3K27ac Hnisz
FetalDHS Trynka
Enhancer Hoffman
Enhancer Andersson
DHS Trynka
DGF ENCODE
CTCF Hoffman
Conserved LindbladToh
Coding UCSC
Annotation
−150
−100
−50
0
50
100
Enrichment (prop h2 / prop snps)

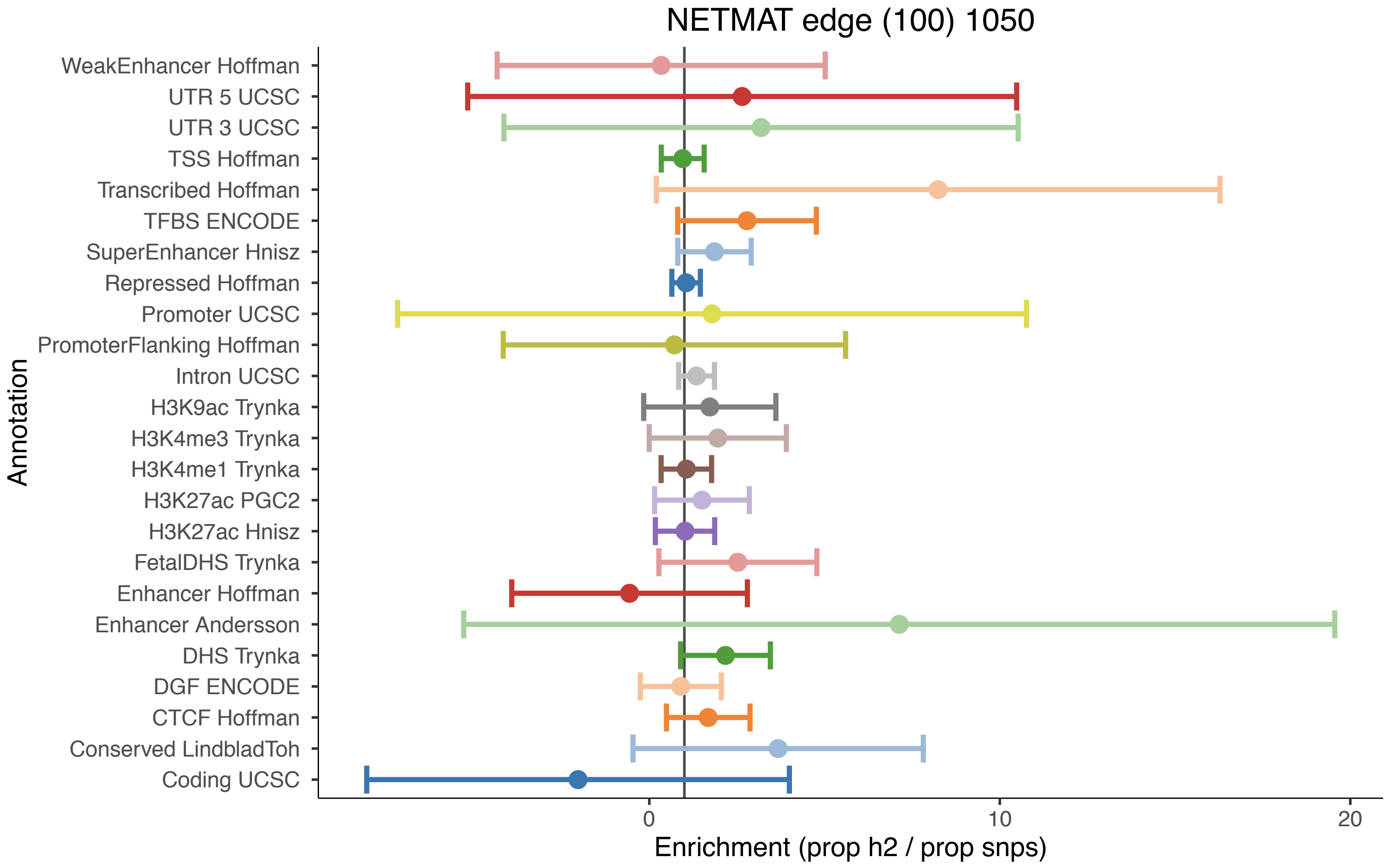
NETMAT edge (100) 1050
WeakEnhancer Hoffman
UTR 5 UCSC
UTR 3 UCSC
TSS Hoffman
Transcribed Hoffman
TFBS ENCODE
SuperEnhancer Hnisz
Repressed Hoffman
Promoter UCSC
PromoterFlanking Hoffman
Intron UCSC
H3K9ac Trynka
H3K4me3 Trynka
H3K4me1 Trynka
H3K27ac PGC2
H3K27ac Hnisz
FetalDHS Trynka
Enhancer Hoffman
Enhancer Andersson
DHS Trynka
DGF ENCODE
CTCF Hoffman
Conserved LindbladToh
Coding UCSC
Annotation
0
10
20
Enrichment (prop h2 / prop snps)

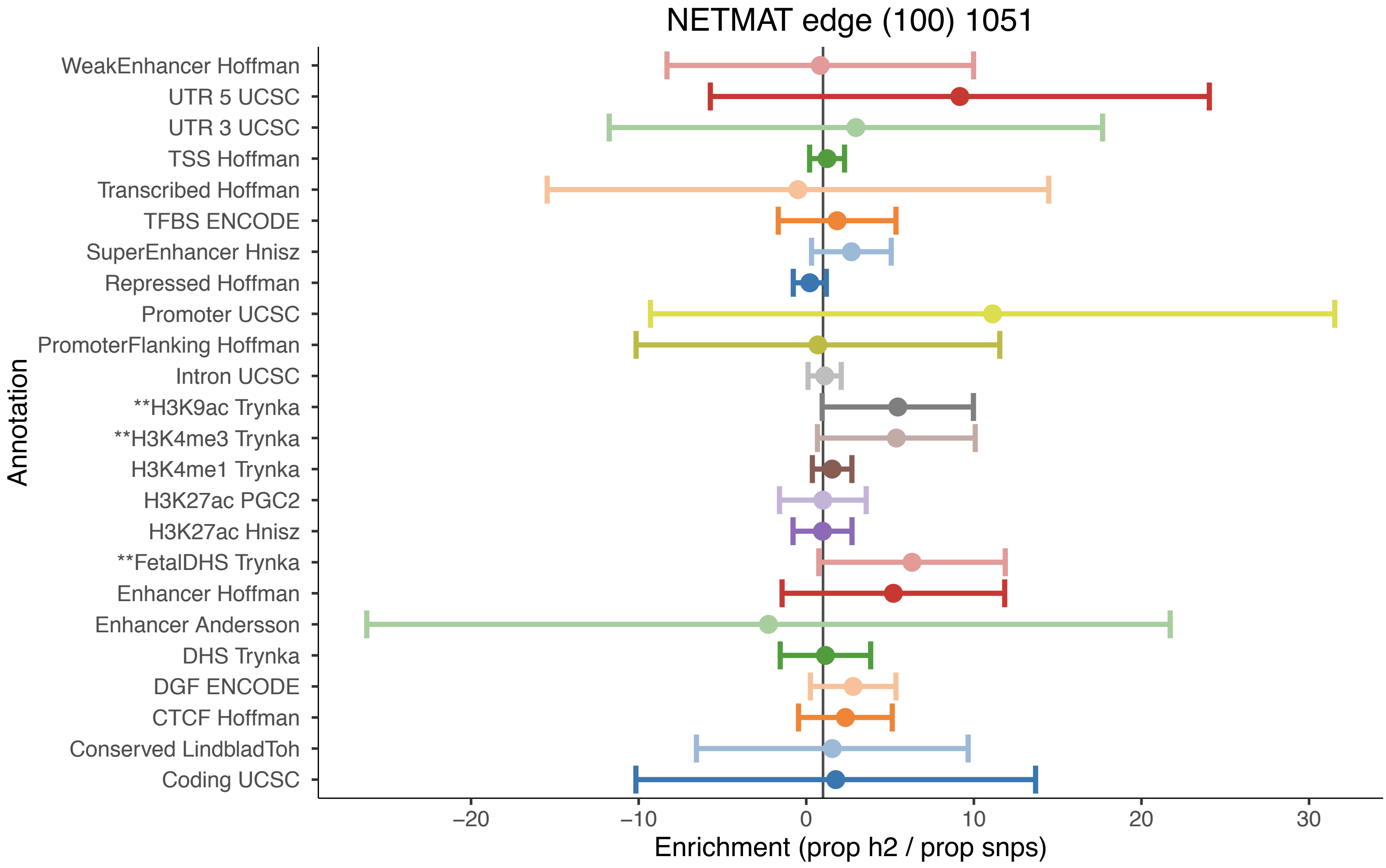
NETMAT edge (100) 1051
WeakEnhancer Hoffman
UTR 5 UCSC
UTR 3 UCSC
TSS Hoffman
Transcribed Hoffman
TFBS ENCODE
SuperEnhancer Hnisz
Repressed Hoffman
Promoter UCSC
PromoterFlanking Hoffman
Intron UCSC
**H3K9ac Trynka
**H3K4me3 Trynka
H3K4me1 Trynka
H3K27ac PGC2
H3K27ac Hnisz
**FetalDHS Trynka
Enhancer Hoffman
Enhancer Andersson
DHS Trynka
DGF ENCODE
CTCF Hoffman
Conserved LindbladToh
Coding UCSC
Annotation
−20
−10
0
10
20
30
Enrichment (prop h2 / prop snps)

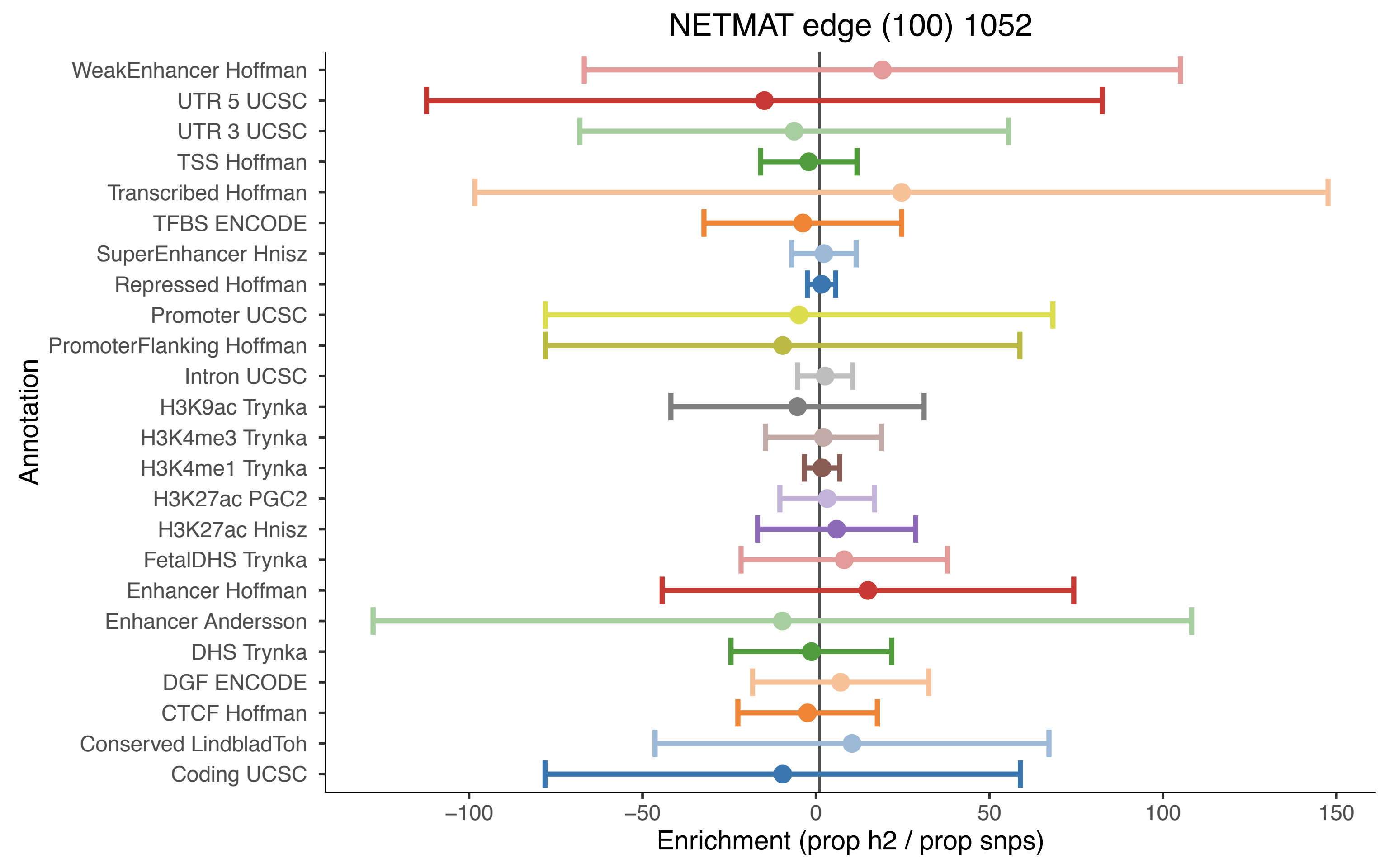

NETMAT edge (100) 1052
WeakEnhancer Hoffman
UTR 5 UCSC
UTR 3 UCSC
TSS Hoffman
Transcribed Hoffman
TFBS ENCODE
SuperEnhancer Hnisz
Repressed Hoffman
Promoter UCSC
PromoterFlanking Hoffman
Intron UCSC
H3K9ac Trynka
H3K4me3 Trynka
H3K4me1 Trynka
H3K27ac PGC2
H3K27ac Hnisz
FetalDHS Trynka
Enhancer Hoffman
Enhancer Andersson
DHS Trynka
DGF ENCODE
CTCF Hoffman
Conserved LindbladToh
Coding UCSC
Annotation
−100
−50
0
50
100
150
Enrichment (prop h2 / prop snps)

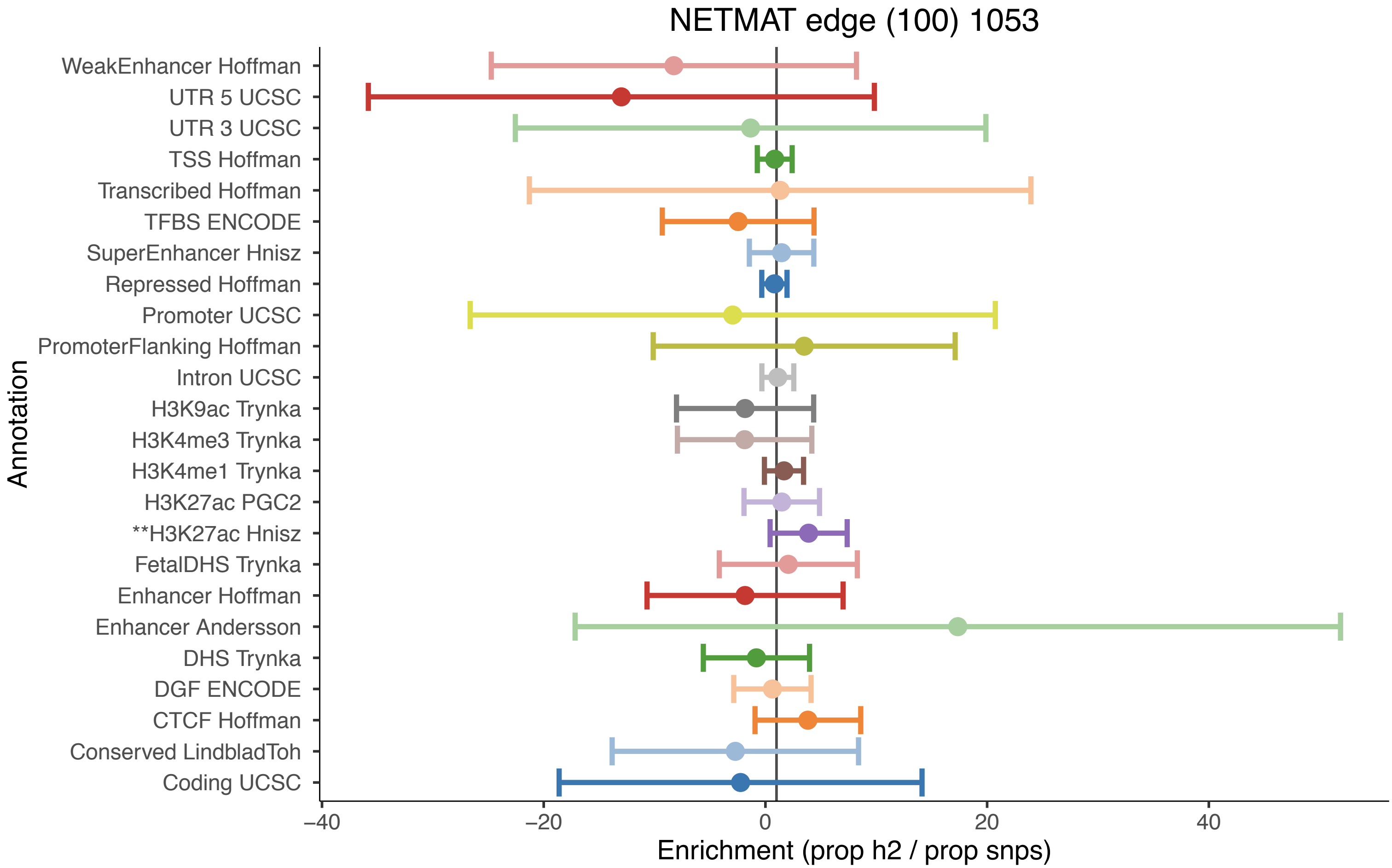

NETMAT edge (100) 1053
Annotation
WeakEnhancer Hoffman
UTR 5 UCSC
UTR 3 UCSC
TSS Hoffman
Transcribed Hoffman
TFBS ENCODE
SuperEnhancer Hnisz
Repressed Hoffman
Promoter UCSC
PromoterFlanking Hoffman
Intron UCSC
H3K9ac Trynka
H3K4me3 Trynka
H3K4me1 Trynka
H3K27ac PGC2
**H3K27ac Hnisz
FetalDHS Trynka
Enhancer Hoffman
Enhancer Andersson
DHS Trynka
DGF ENCODE
CTCF Hoffman
Conserved LindbladToh
Coding UCSC
−40
−20
0
20
40
Enrichment (prop h2 / prop snps)

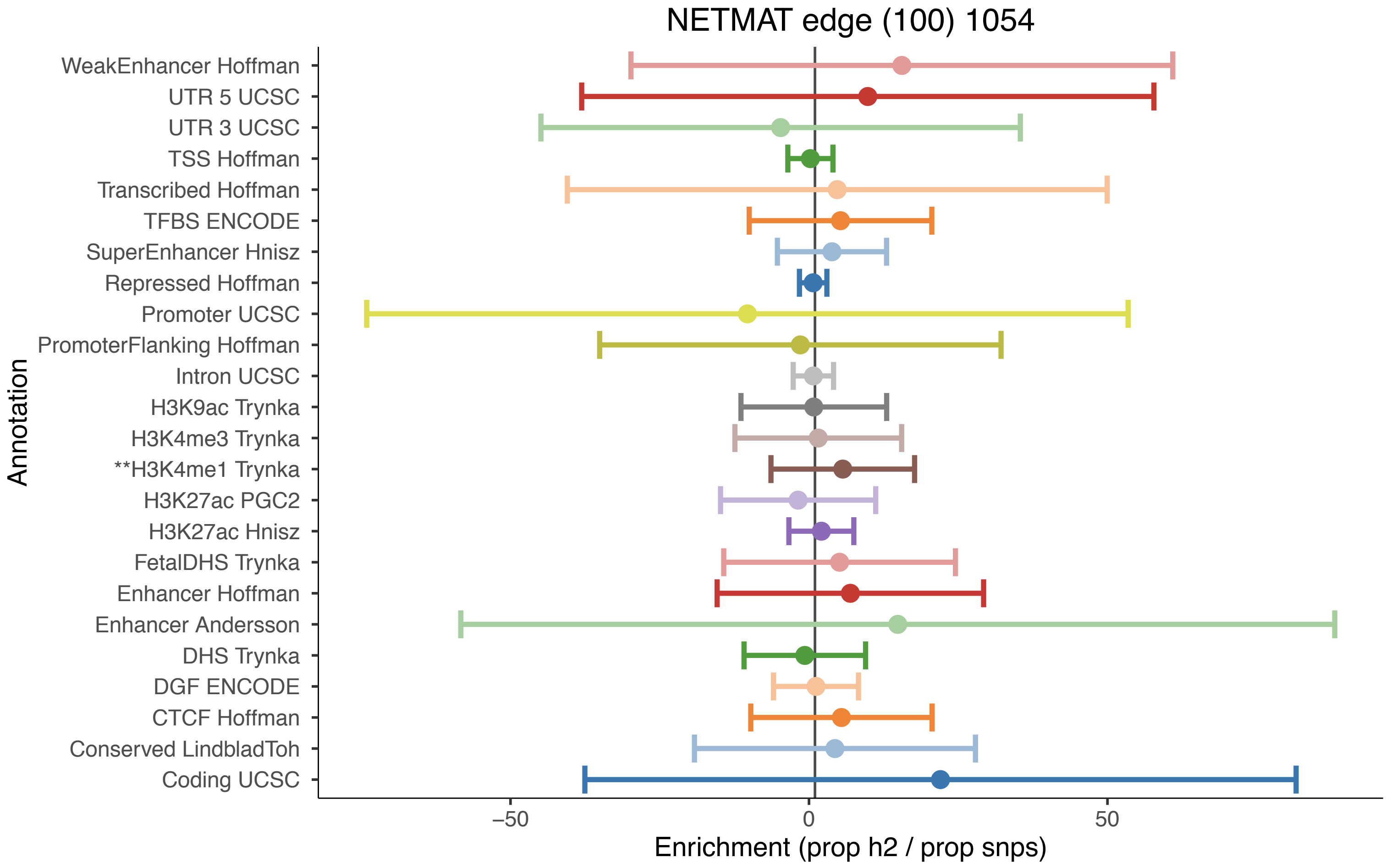

NETMAT edge (100) 1054
WeakEnhancer Hoffman
UTR 5 UCSC
UTR 3 UCSC
TSS Hoffman
Transcribed Hoffman
TFBS ENCODE
SuperEnhancer Hnisz
Repressed Hoffman
Promoter UCSC
PromoterFlanking Hoffman
Intron UCSC
H3K9ac Trynka
H3K4me3 Trynka
**H3K4me1 Trynka
H3K27ac PGC2
H3K27ac Hnisz
FetalDHS Trynka
Enhancer Hoffman
Enhancer Andersson
DHS Trynka
DGF ENCODE
CTCF Hoffman
Conserved LindbladToh
Coding UCSC
Annotation
−50
0
50
Enrichment (prop h2 / prop snps)

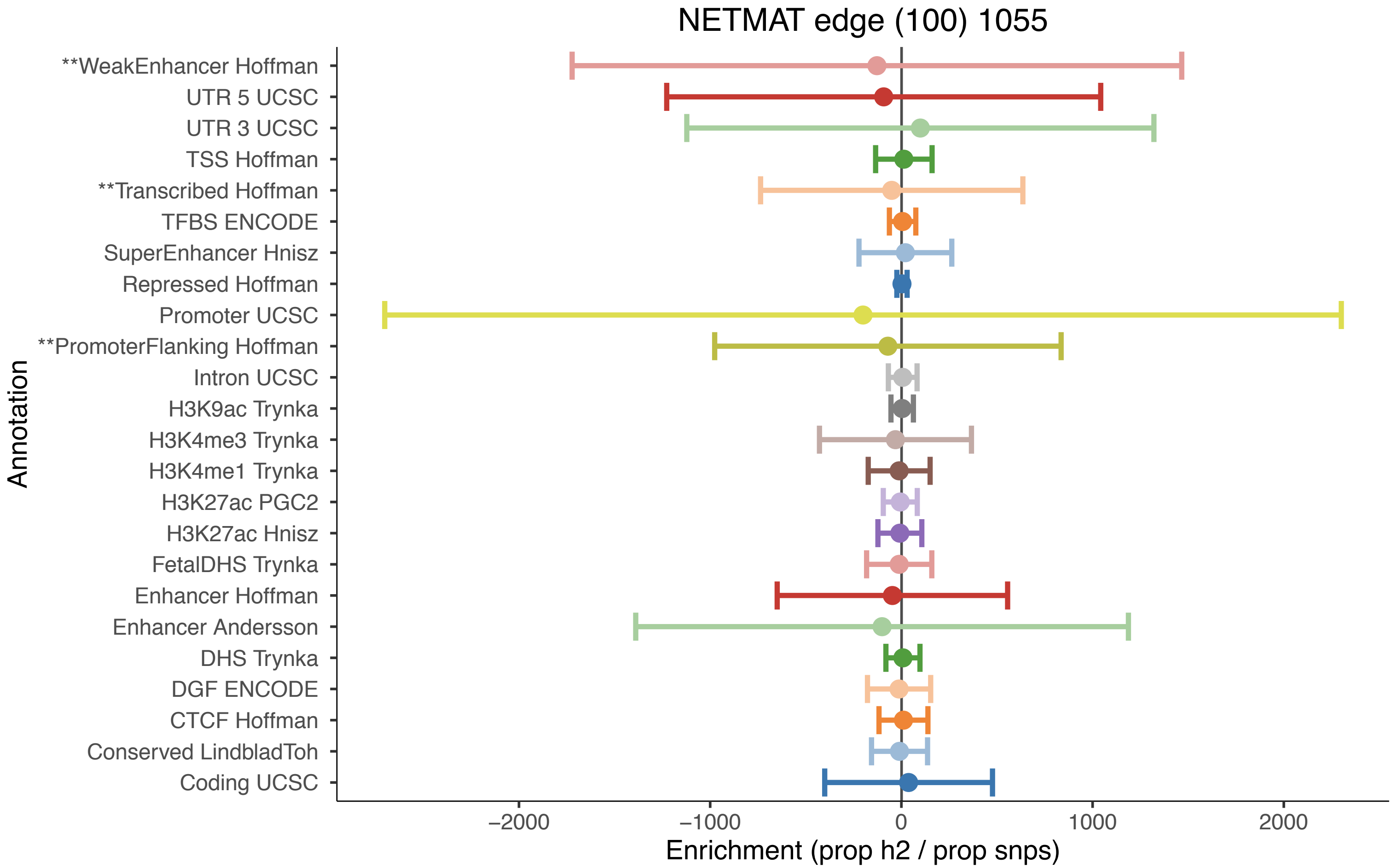
NETMAT edge (100) 1055
**WeakEnhancer Hoffman
UTR 5 UCSC
UTR 3 UCSC
TSS Hoffman
**Transcribed Hoffman
TFBS ENCODE
SuperEnhancer Hnisz
Repressed Hoffman
Promoter UCSC
**PromoterFlanking Hoffman
Intron UCSC
H3K9ac Trynka
H3K4me3 Trynka
H3K4me1 Trynka
H3K27ac PGC2
H3K27ac Hnisz
FetalDHS Trynka
Enhancer Hoffman
Enhancer Andersson
DHS Trynka
DGF ENCODE
CTCF Hoffman
Conserved LindbladToh
Coding UCSC
Annotation
−2000
−1000
0
1000
2000
Enrichment (prop h2 / prop snps)

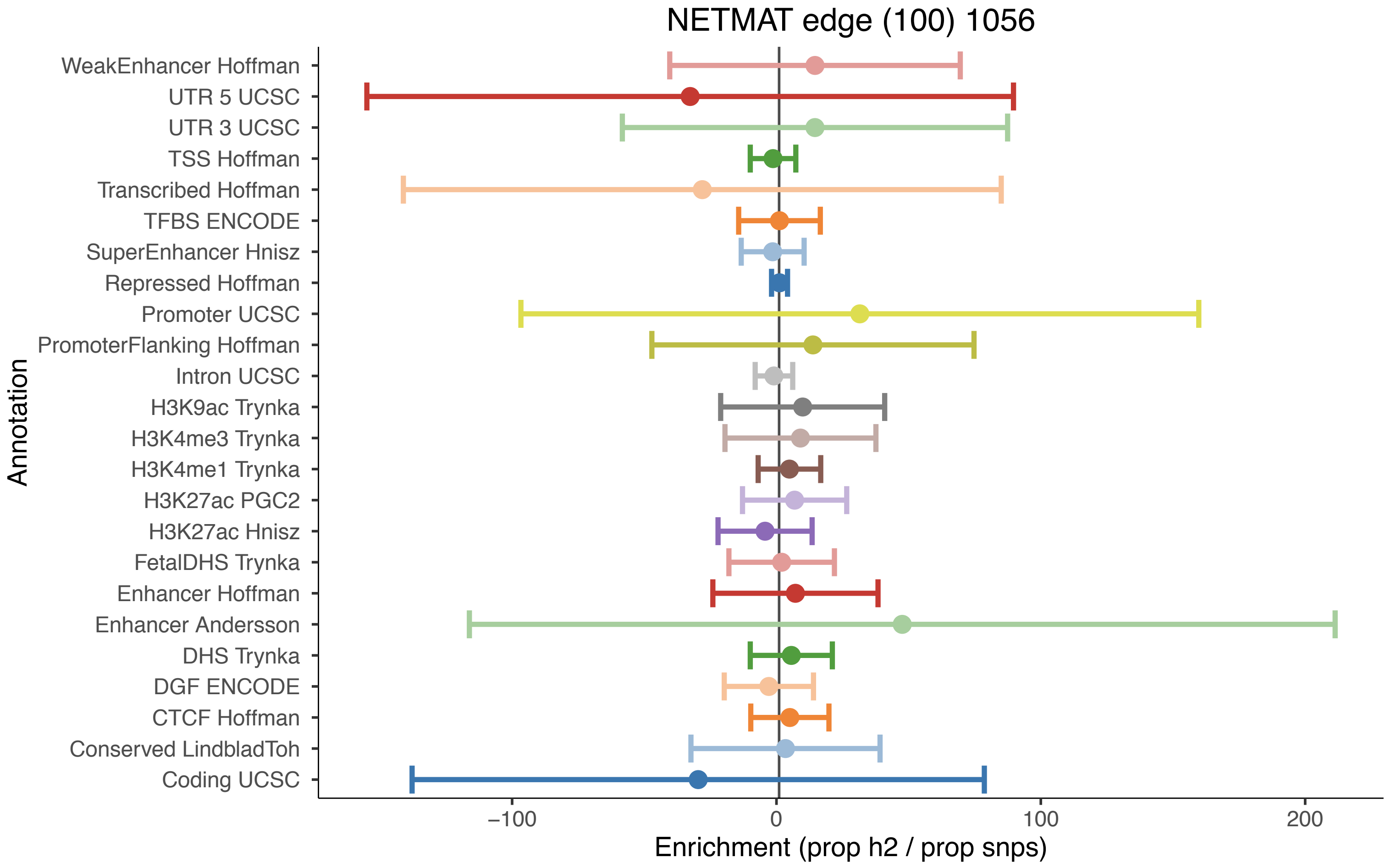
NETMAT edge (100) 1056
WeakEnhancer Hoffman
UTR 5 UCSC
UTR 3 UCSC
TSS Hoffman
Transcribed Hoffman
TFBS ENCODE
SuperEnhancer Hnisz
Repressed Hoffman
Promoter UCSC
PromoterFlanking Hoffman
Intron UCSC
H3K9ac Trynka
H3K4me3 Trynka
H3K4me1 Trynka
H3K27ac PGC2
H3K27ac Hnisz
FetalDHS Trynka
Enhancer Hoffman
Enhancer Andersson
DHS Trynka
DGF ENCODE
CTCF Hoffman
Conserved LindbladToh
Coding UCSC
Annotation
−100
0
100
200
Enrichment (prop h2 / prop snps)

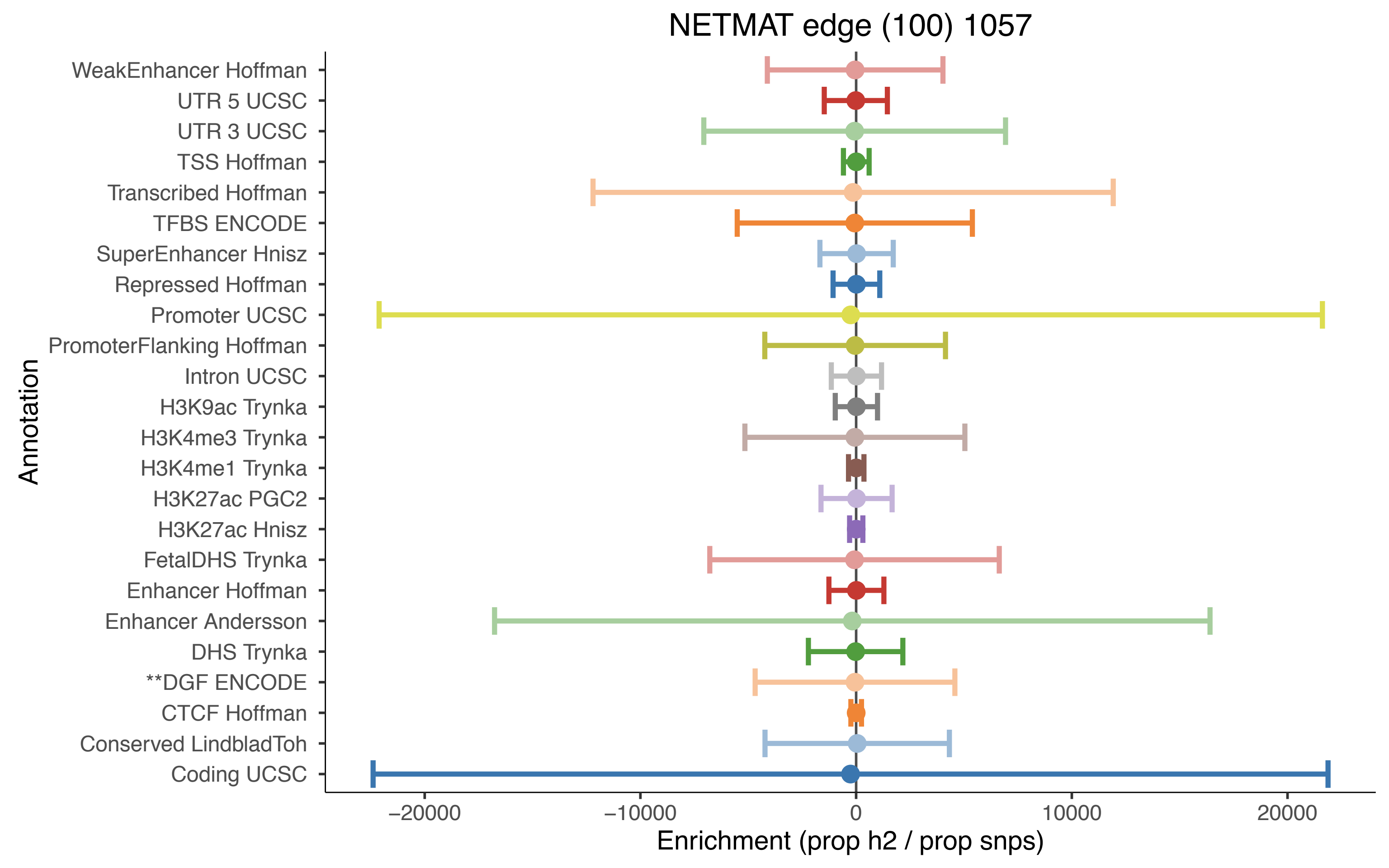
NETMAT edge (100) 1057
WeakEnhancer Hoffman
UTR 5 UCSC
UTR 3 UCSC
TSS Hoffman
Transcribed Hoffman
TFBS ENCODE
SuperEnhancer Hnisz
Repressed Hoffman
Promoter UCSC
PromoterFlanking Hoffman
Intron UCSC
H3K9ac Trynka
H3K4me3 Trynka
H3K4me1 Trynka
H3K27ac PGC2
H3K27ac Hnisz
FetalDHS Trynka
Enhancer Hoffman
Enhancer Andersson
DHS Trynka
**DGF ENCODE
CTCF Hoffman
Conserved LindbladToh
Coding UCSC
Annotation
−20000
−10000
0
10000
20000
Enrichment (prop h2 / prop snps)

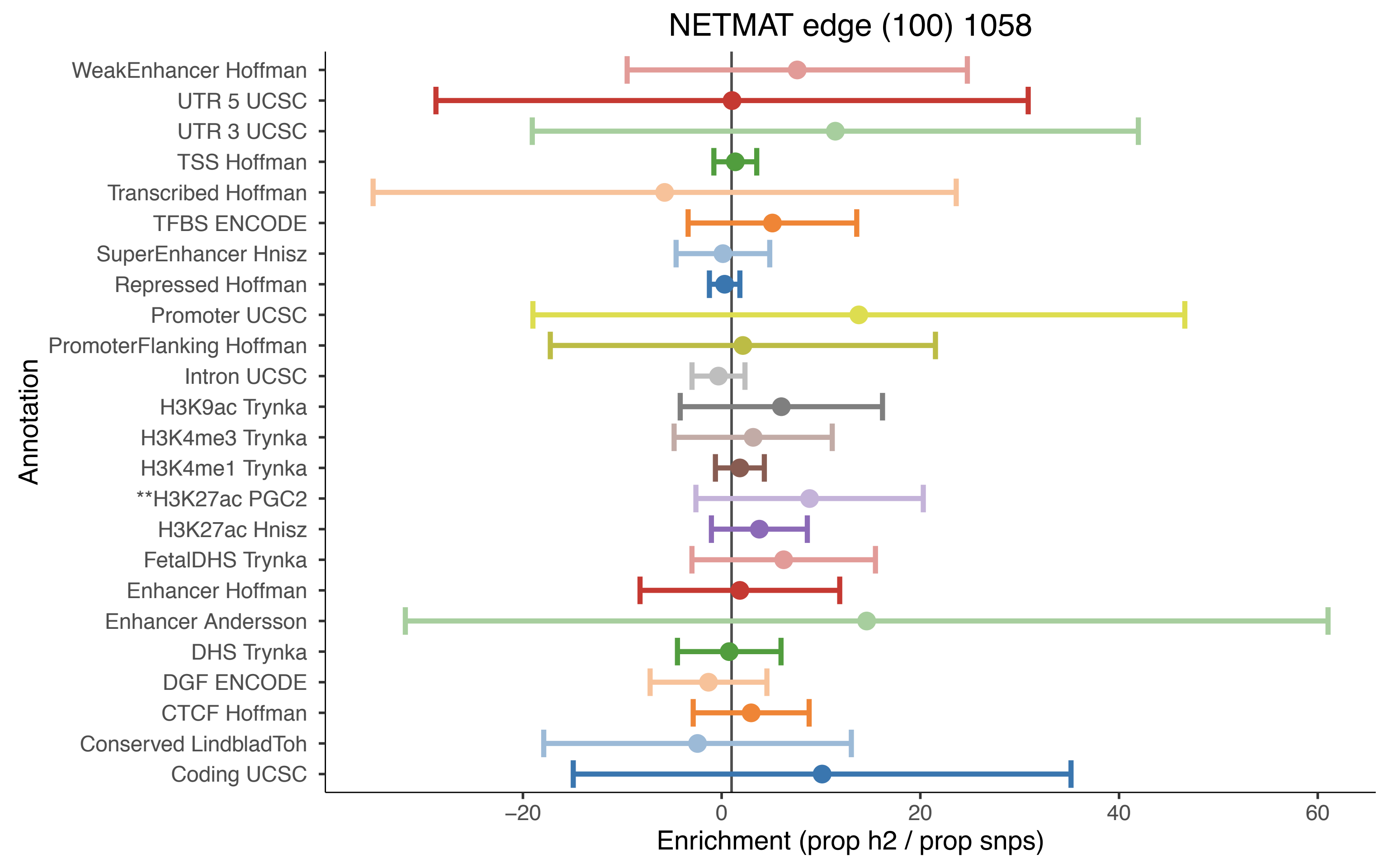

NETMAT edge (100) 1058
Annotation
WeakEnhancer Hoffman
UTR 5 UCSC
UTR 3 UCSC
TSS Hoffman
Transcribed Hoffman
TFBS ENCODE
SuperEnhancer Hnisz
Repressed Hoffman
Promoter UCSC
PromoterFlanking Hoffman
Intron UCSC
H3K9ac Trynka
H3K4me3 Trynka
H3K4me1 Trynka
**H3K27ac PGC2
H3K27ac Hnisz
FetalDHS Trynka
Enhancer Hoffman
Enhancer Andersson
DHS Trynka
DGF ENCODE
CTCF Hoffman
Conserved LindbladToh
Coding UCSC
−20
0
20
40
60
Enrichment (prop h2 / prop snps)

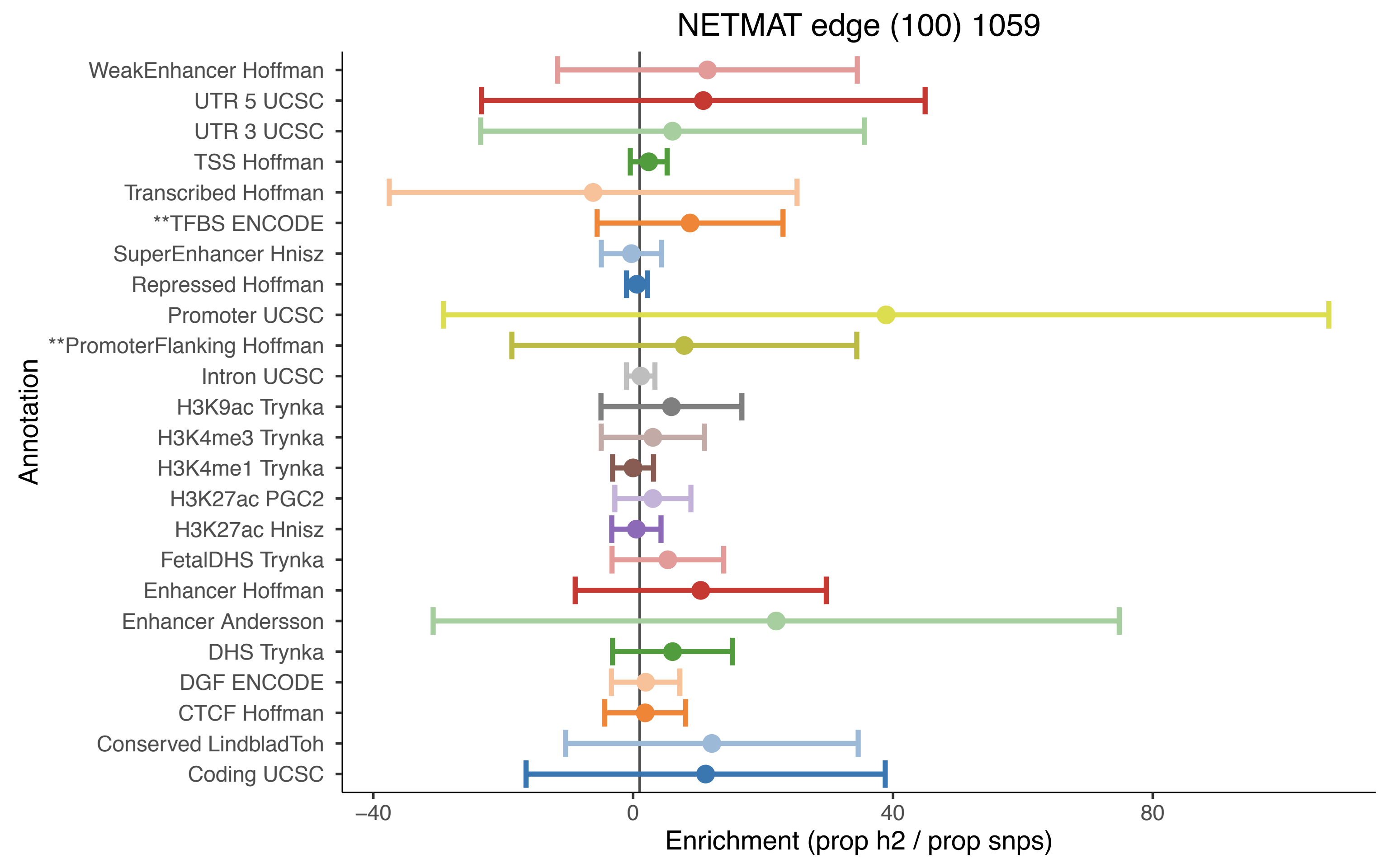

NETMAT edge (100) 1059
WeakEnhancer Hoffman
UTR 5 UCSC
UTR 3 UCSC
TSS Hoffman
Transcribed Hoffman
**TFBS ENCODE
SuperEnhancer Hnisz
Repressed Hoffman
Promoter UCSC
**PromoterFlanking Hoffman
Intron UCSC
H3K9ac Trynka
H3K4me3 Trynka
H3K4me1 Trynka
H3K27ac PGC2
H3K27ac Hnisz
FetalDHS Trynka
Enhancer Hoffman
Enhancer Andersson
DHS Trynka
DGF ENCODE
CTCF Hoffman
Conserved LindbladToh
Coding UCSC
Annotation
−40
0
40
80
Enrichment (prop h2 / prop snps)

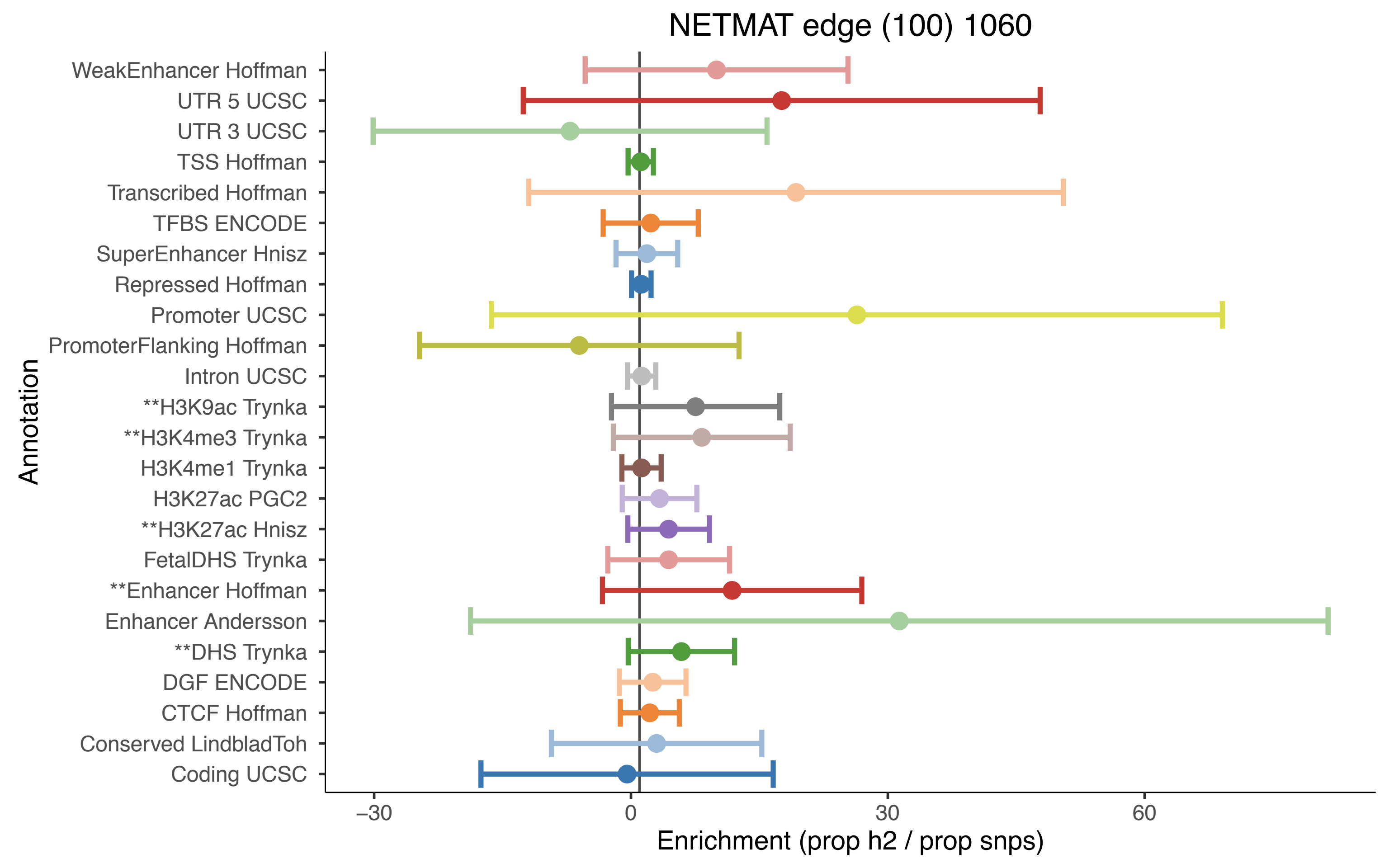
NETMAT edge (100) 1060
WeakEnhancer Hoffman
UTR 5 UCSC
UTR 3 UCSC
TSS Hoffman
Transcribed Hoffman
TFBS ENCODE
SuperEnhancer Hnisz
Repressed Hoffman
Promoter UCSC
PromoterFlanking Hoffman
Intron UCSC
**H3K9ac Trynka
**H3K4me3 Trynka
H3K4me1 Trynka
H3K27ac PGC2
**H3K27ac Hnisz
FetalDHS Trynka
**Enhancer Hoffman
Enhancer Andersson
**DHS Trynka
DGF ENCODE
CTCF Hoffman
Conserved LindbladToh
Coding UCSC
Annotation
−30
0
30
60
Enrichment (prop h2 / prop snps)

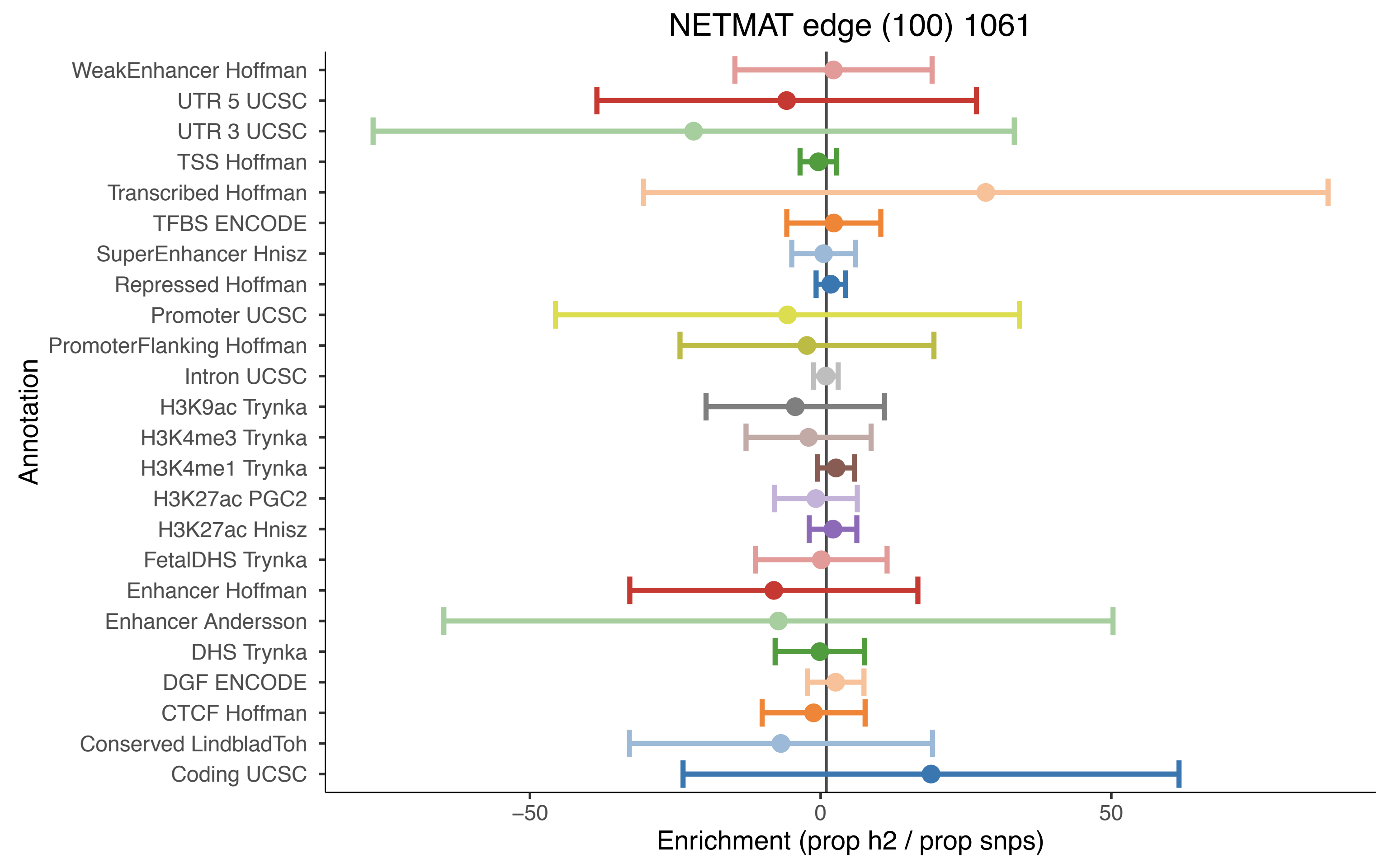
NETMAT edge (100) 1061
Annotation
WeakEnhancer Hoffman
UTR 5 UCSC
UTR 3 UCSC
TSS Hoffman
Transcribed Hoffman
TFBS ENCODE
SuperEnhancer Hnisz
Repressed Hoffman
Promoter UCSC
PromoterFlanking Hoffman
Intron UCSC
H3K9ac Trynka
H3K4me3 Trynka
H3K4me1 Trynka
H3K27ac PGC2
H3K27ac Hnisz
FetalDHS Trynka
Enhancer Hoffman
Enhancer Andersson
DHS Trynka
DGF ENCODE
CTCF Hoffman
Conserved LindbladToh
Coding UCSC
−50
0
50
Enrichment (prop h2 / prop snps)

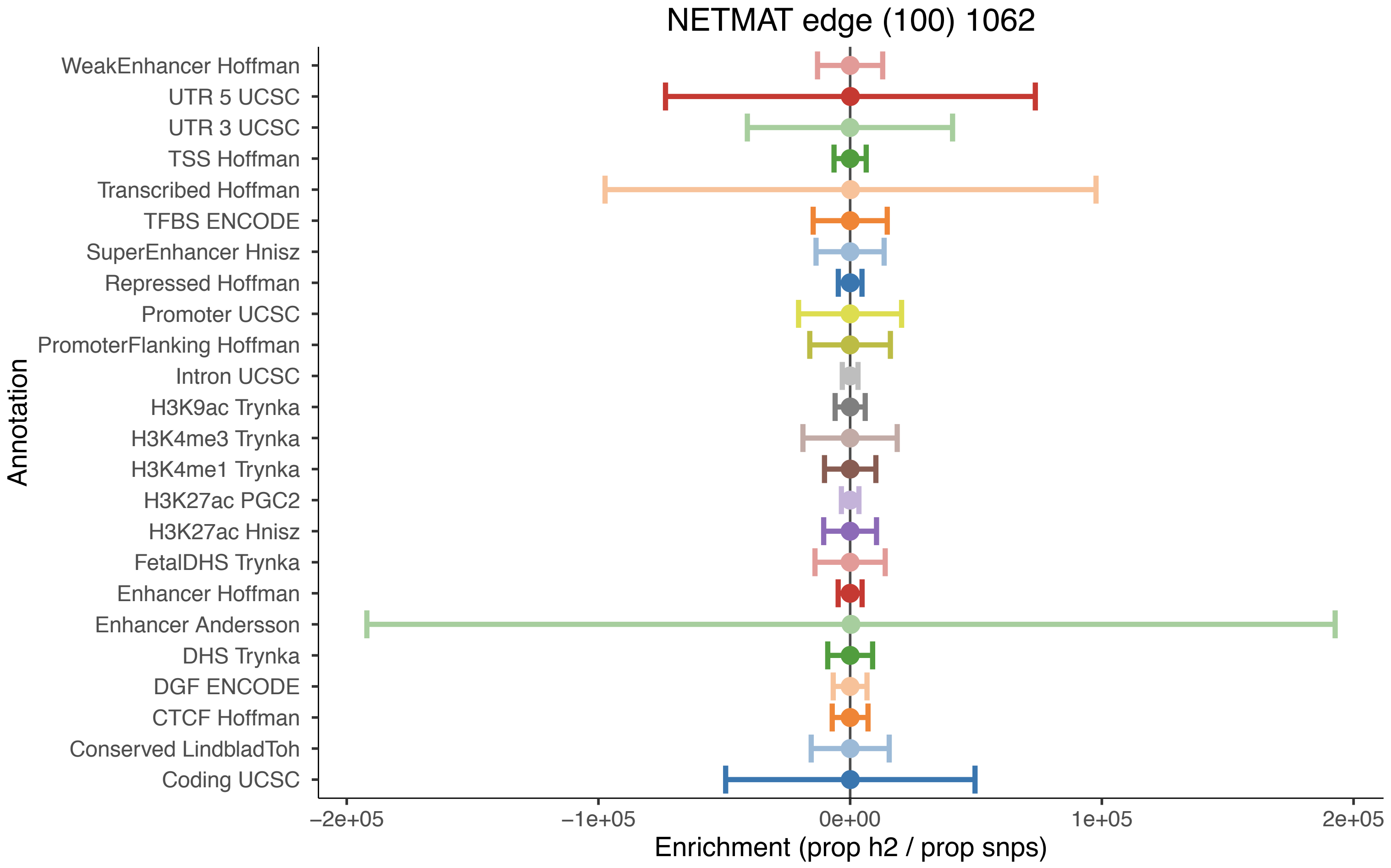
NETMAT edge (100) 1062
Annotation
WeakEnhancer Hoffman
UTR 5 UCSC
UTR 3 UCSC
TSS Hoffman
Transcribed Hoffman
TFBS ENCODE
SuperEnhancer Hnisz
Repressed Hoffman
Promoter UCSC
PromoterFlanking Hoffman
Intron UCSC
H3K9ac Trynka
H3K4me3 Trynka
H3K4me1 Trynka
H3K27ac PGC2
H3K27ac Hnisz
FetalDHS Trynka
Enhancer Hoffman
Enhancer Andersson
DHS Trynka
DGF ENCODE
CTCF Hoffman
Conserved LindbladToh
Coding UCSC
−2e+05
−1e+05
0e+00
1e+05
2e+05
Enrichment (prop h2 / prop snps)

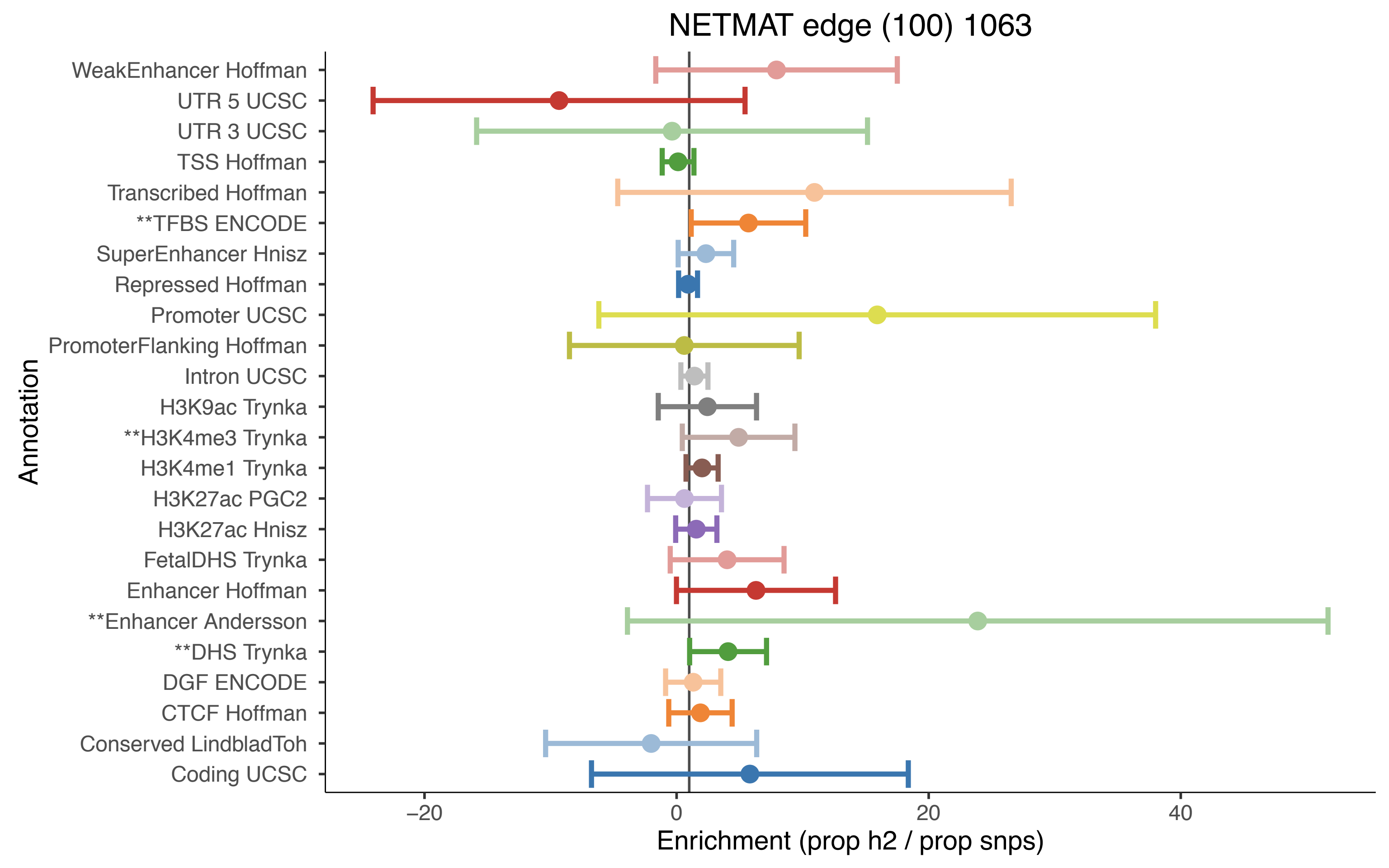

NETMAT edge (100) 1063
WeakEnhancer Hoffman
UTR 5 UCSC
UTR 3 UCSC
TSS Hoffman
Transcribed Hoffman
**TFBS ENCODE
SuperEnhancer Hnisz
Repressed Hoffman
Promoter UCSC
PromoterFlanking Hoffman
Intron UCSC
H3K9ac Trynka
**H3K4me3 Trynka
H3K4me1 Trynka
H3K27ac PGC2
H3K27ac Hnisz
FetalDHS Trynka
Enhancer Hoffman
**Enhancer Andersson
**DHS Trynka
DGF ENCODE
CTCF Hoffman
Conserved LindbladToh
Coding UCSC
Annotation
−20
0
20
40
Enrichment (prop h2 / prop snps)

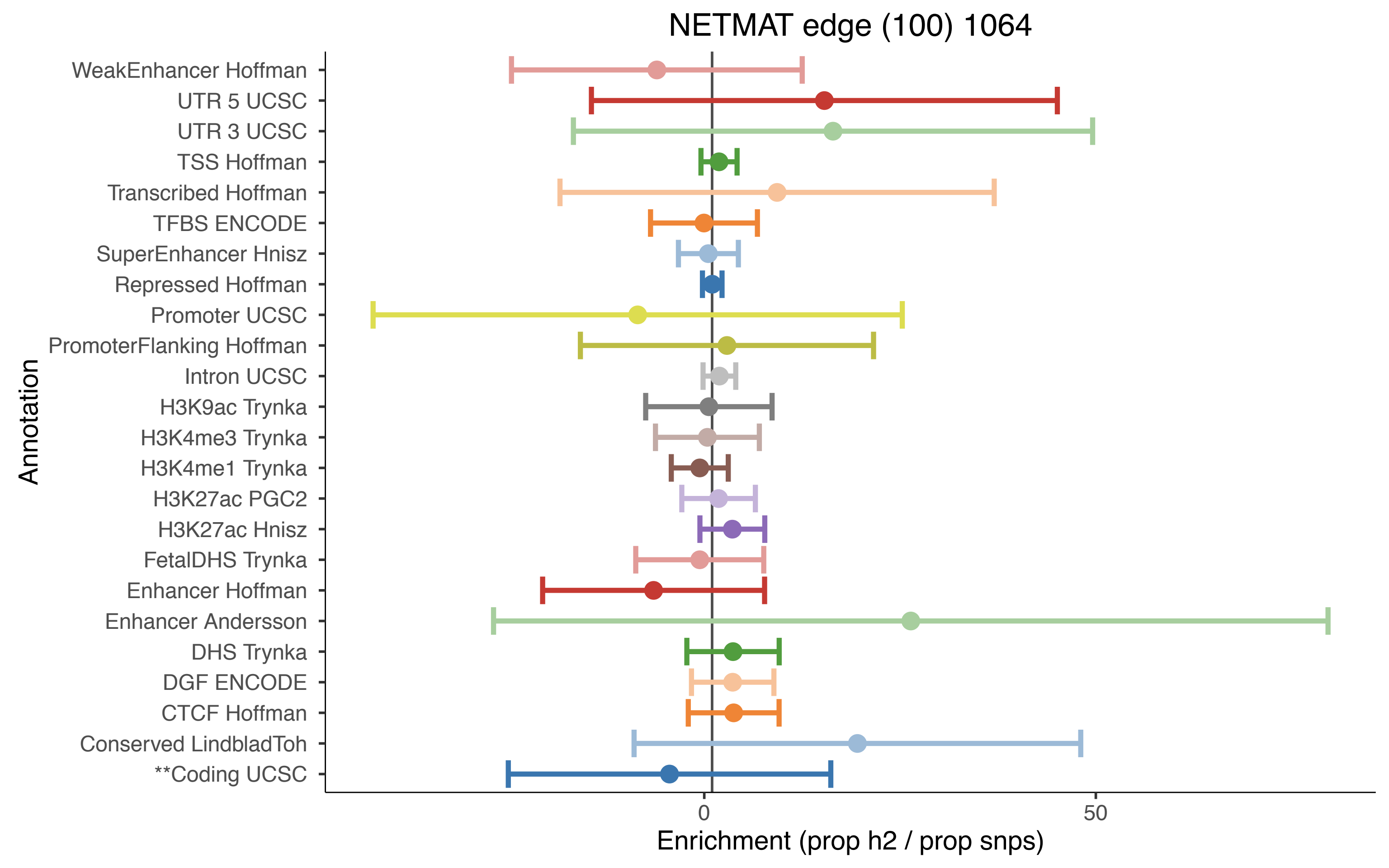
NETMAT edge (100) 1064
WeakEnhancer Hoffman
UTR 5 UCSC
UTR 3 UCSC
TSS Hoffman
Transcribed Hoffman
TFBS ENCODE
SuperEnhancer Hnisz
Repressed Hoffman
Promoter UCSC
PromoterFlanking Hoffman
Intron UCSC
H3K9ac Trynka
H3K4me3 Trynka
H3K4me1 Trynka
H3K27ac PGC2
H3K27ac Hnisz
FetalDHS Trynka
Enhancer Hoffman
Enhancer Andersson
DHS Trynka
DGF ENCODE
CTCF Hoffman
Conserved LindbladToh
**Coding UCSC
Annotation
0
50
Enrichment (prop h2 / prop snps)

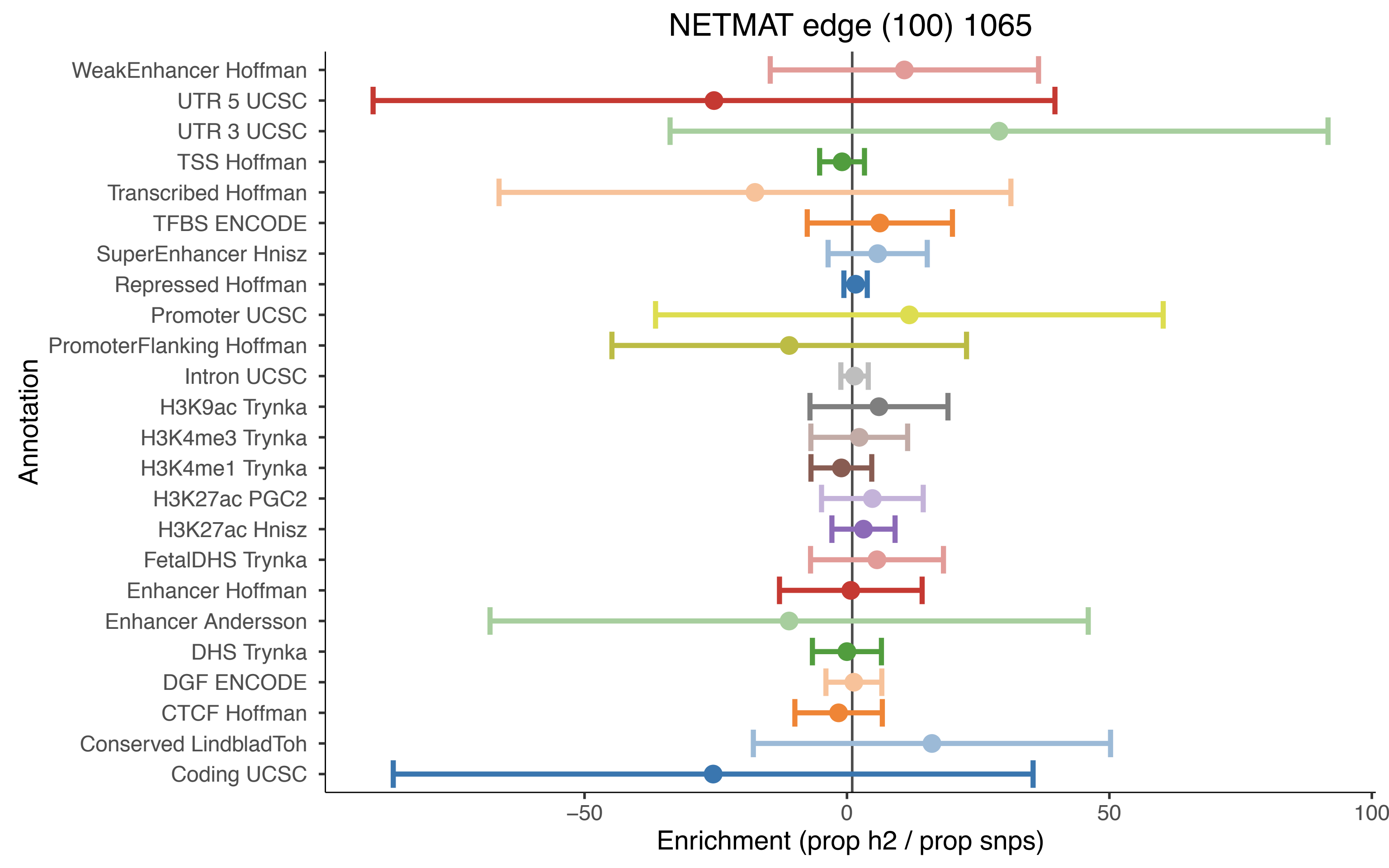

NETMAT edge (100) 1065
WeakEnhancer Hoffman
UTR 5 UCSC
UTR 3 UCSC
TSS Hoffman
Transcribed Hoffman
TFBS ENCODE
SuperEnhancer Hnisz
Repressed Hoffman
Promoter UCSC
PromoterFlanking Hoffman
Intron UCSC
H3K9ac Trynka
H3K4me3 Trynka
H3K4me1 Trynka
H3K27ac PGC2
H3K27ac Hnisz
FetalDHS Trynka
Enhancer Hoffman
Enhancer Andersson
DHS Trynka
DGF ENCODE
CTCF Hoffman
Conserved LindbladToh
Coding UCSC
Annotation
−50
0
50
100
Enrichment (prop h2 / prop snps)

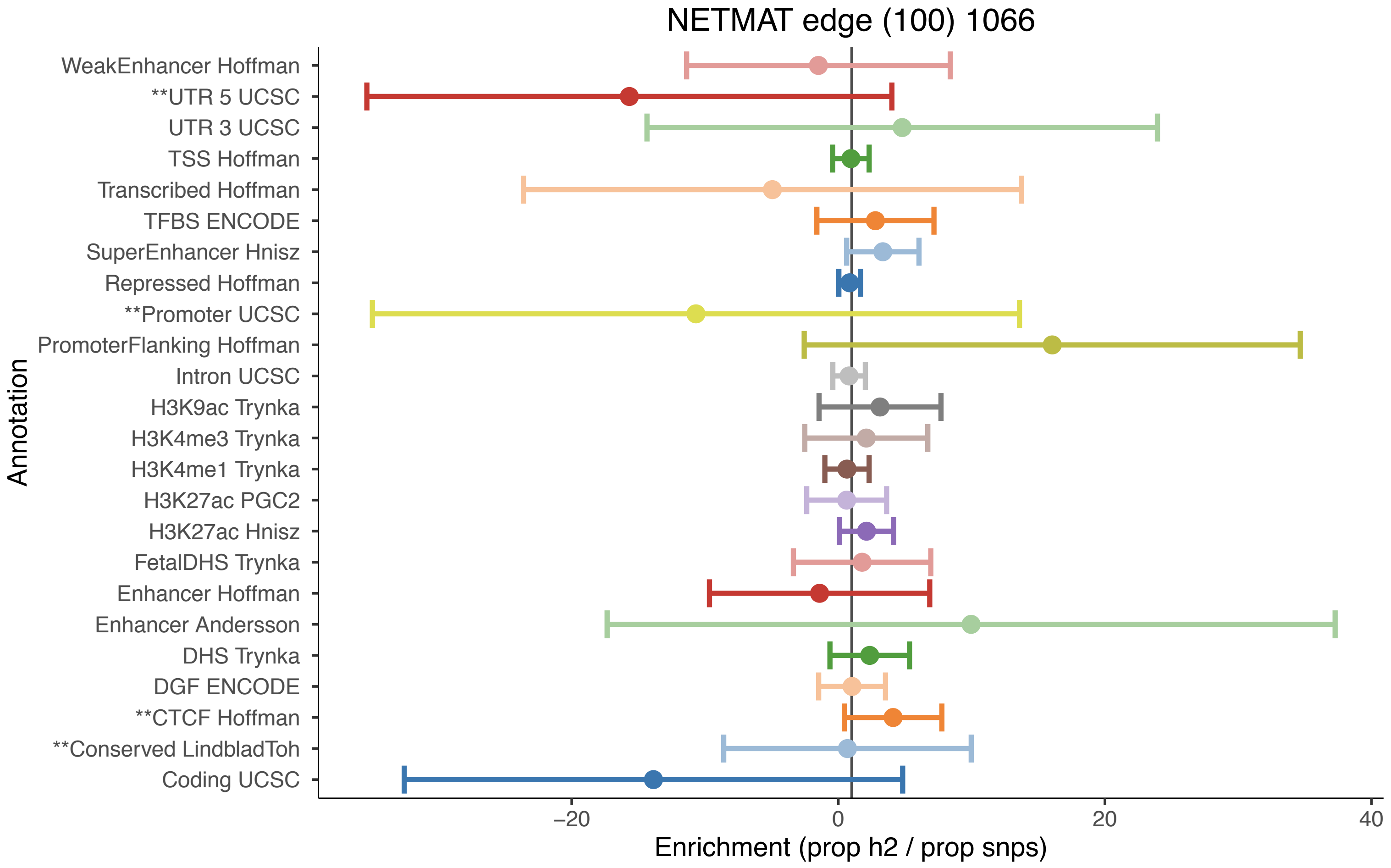
NETMAT edge (100) 1066
WeakEnhancer Hoffman
**UTR 5 UCSC
UTR 3 UCSC
TSS Hoffman
Transcribed Hoffman
TFBS ENCODE
SuperEnhancer Hnisz
Repressed Hoffman
**Promoter UCSC
PromoterFlanking Hoffman
Intron UCSC
H3K9ac Trynka
H3K4me3 Trynka
H3K4me1 Trynka
H3K27ac PGC2
H3K27ac Hnisz
FetalDHS Trynka
Enhancer Hoffman
Enhancer Andersson
DHS Trynka
DGF ENCODE
**CTCF Hoffman
**Conserved LindbladToh
Coding UCSC
Annotation
−20
0
20
40
Enrichment (prop h2 / prop snps)

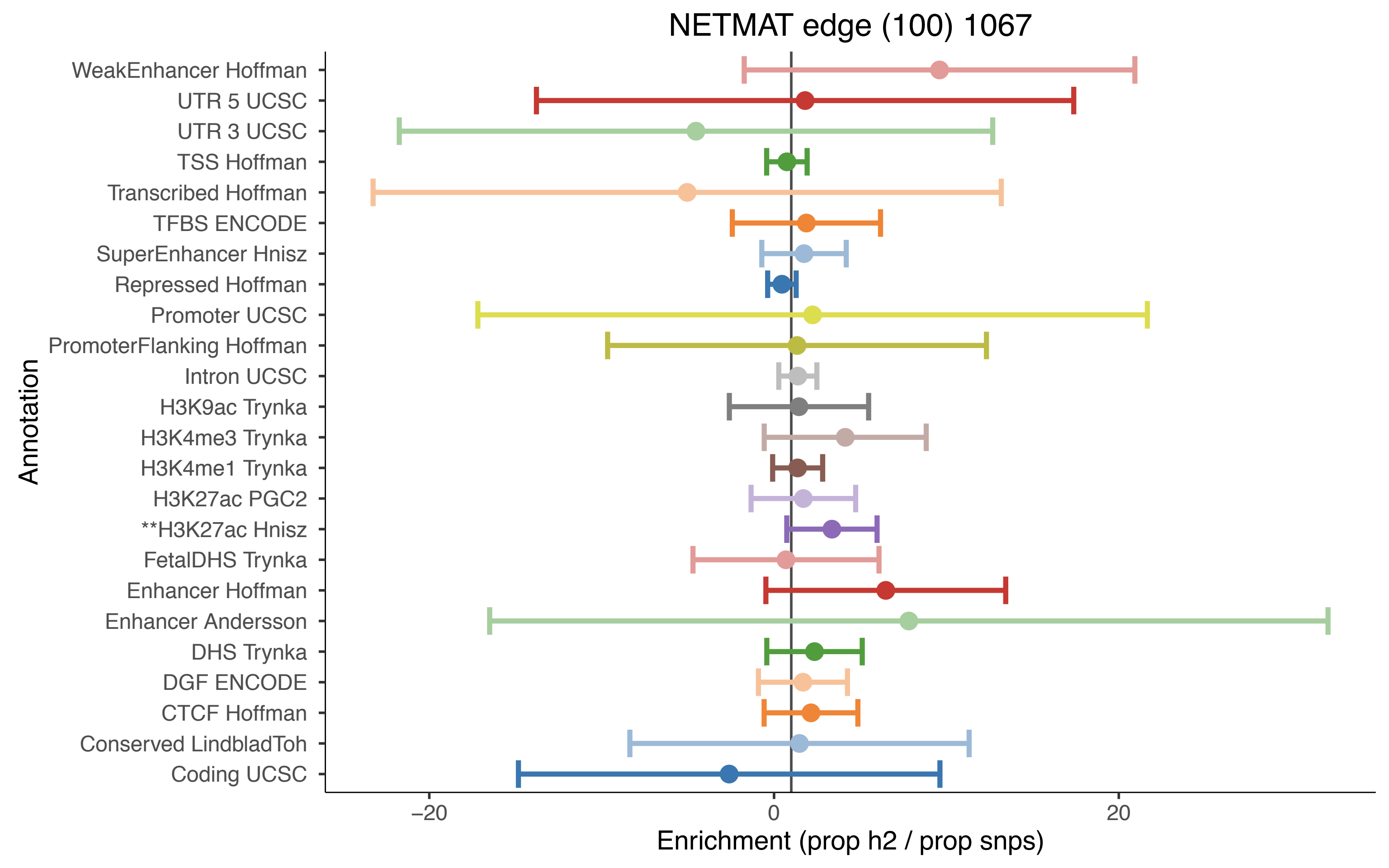
NETMAT edge (100) 1067
WeakEnhancer Hoffman
UTR 5 UCSC
UTR 3 UCSC
TSS Hoffman
Transcribed Hoffman
TFBS ENCODE
SuperEnhancer Hnisz
Repressed Hoffman
Promoter UCSC
PromoterFlanking Hoffman
Intron UCSC
H3K9ac Trynka
H3K4me3 Trynka
H3K4me1 Trynka
H3K27ac PGC2
**H3K27ac Hnisz
FetalDHS Trynka
Enhancer Hoffman
Enhancer Andersson
DHS Trynka
DGF ENCODE
CTCF Hoffman
Conserved LindbladToh
Coding UCSC
Annotation
−20
0
20
Enrichment (prop h2 / prop snps)

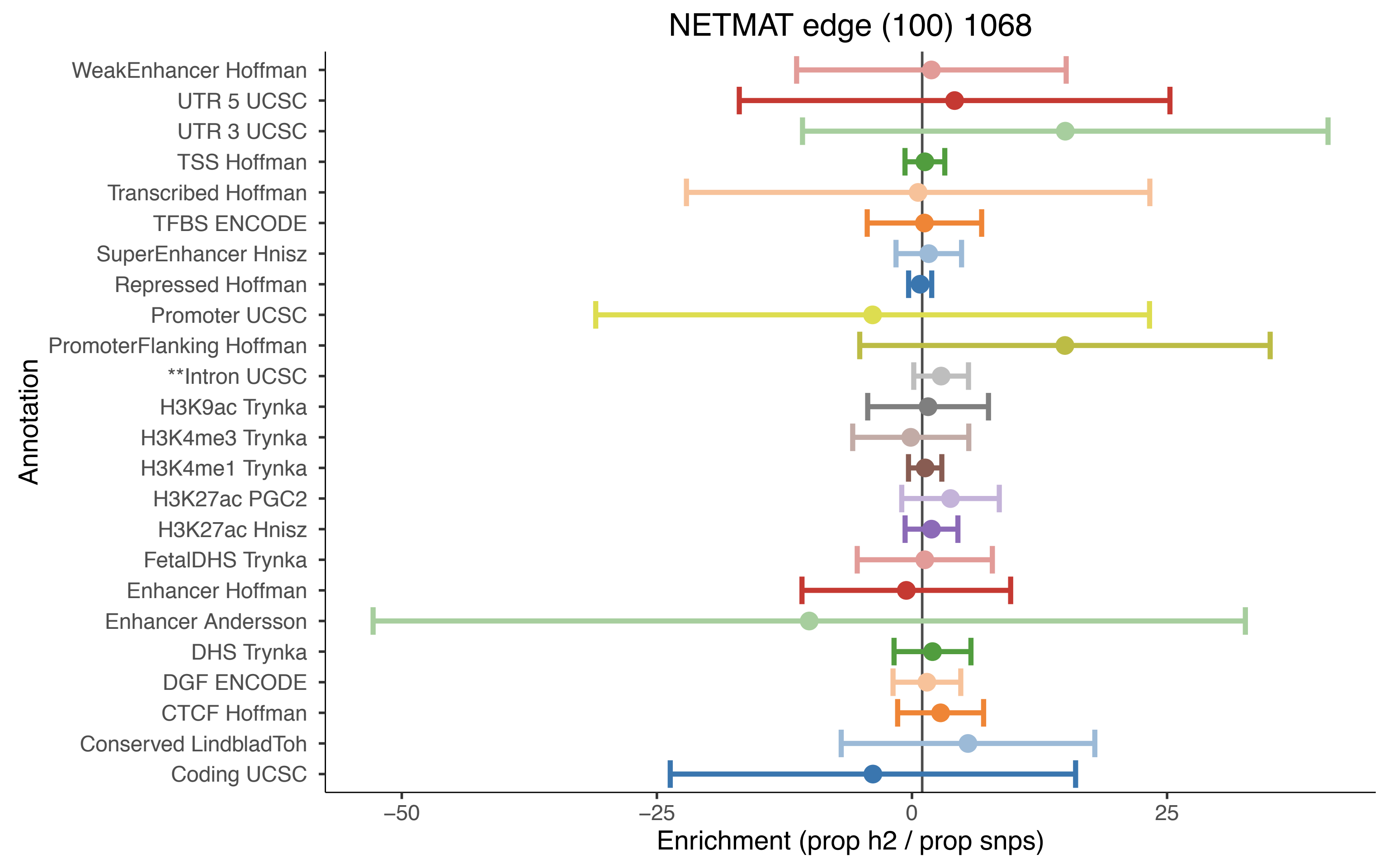

NETMAT edge (100) 1068
WeakEnhancer Hoffman
UTR 5 UCSC
UTR 3 UCSC
TSS Hoffman
Transcribed Hoffman
TFBS ENCODE
SuperEnhancer Hnisz
Repressed Hoffman
Promoter UCSC
PromoterFlanking Hoffman
**Intron UCSC
H3K9ac Trynka
H3K4me3 Trynka
H3K4me1 Trynka
H3K27ac PGC2
H3K27ac Hnisz
FetalDHS Trynka
Enhancer Hoffman
Enhancer Andersson
DHS Trynka
DGF ENCODE
CTCF Hoffman
Conserved LindbladToh
Coding UCSC
Annotation
−50
−25
0
25
Enrichment (prop h2 / prop snps)

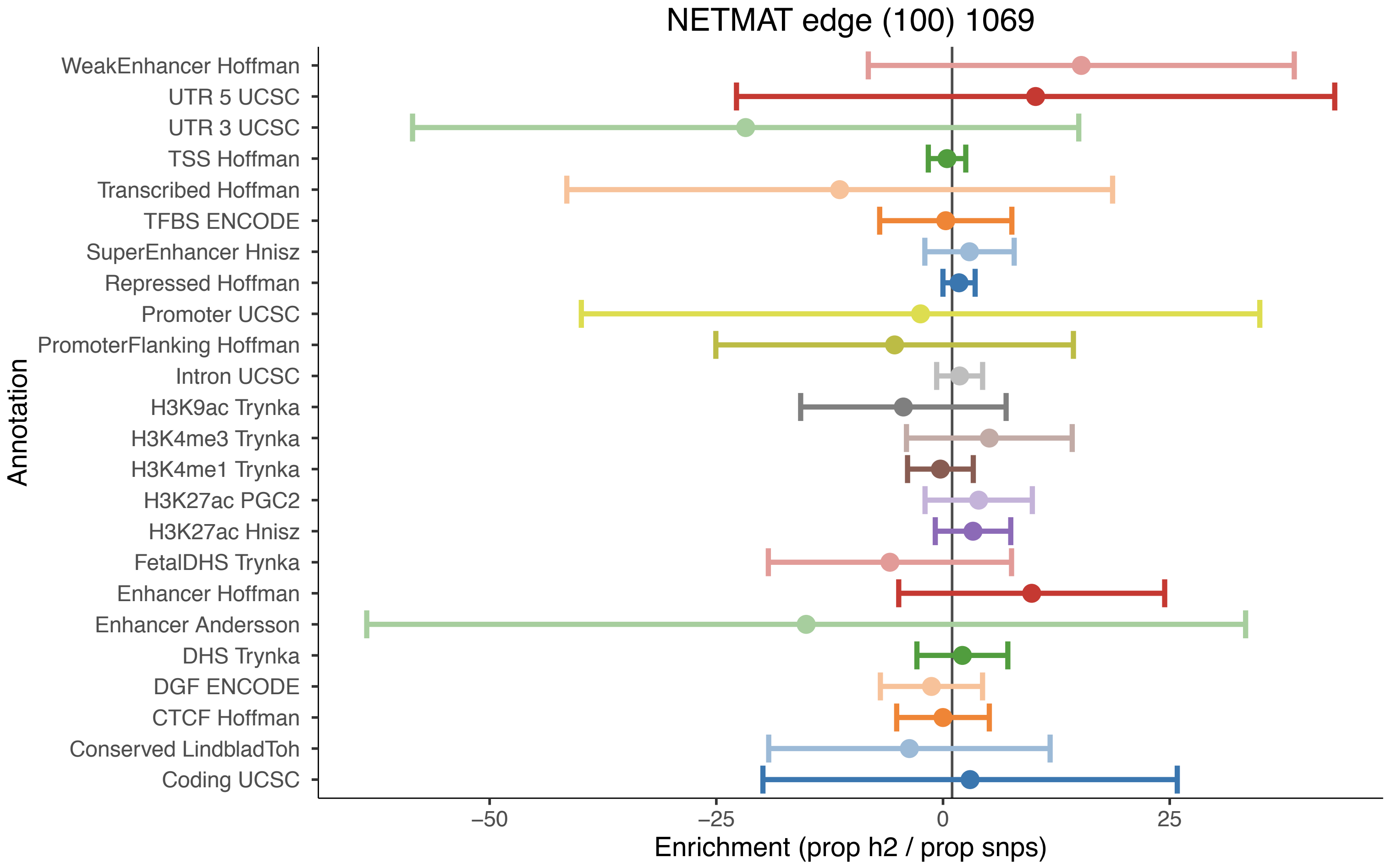
NETMAT edge (100) 1069
WeakEnhancer Hoffman
UTR 5 UCSC
UTR 3 UCSC
TSS Hoffman
Transcribed Hoffman
TFBS ENCODE
SuperEnhancer Hnisz
Repressed Hoffman
Promoter UCSC
PromoterFlanking Hoffman
Intron UCSC
H3K9ac Trynka
H3K4me3 Trynka
H3K4me1 Trynka
H3K27ac PGC2
H3K27ac Hnisz
FetalDHS Trynka
Enhancer Hoffman
Enhancer Andersson
DHS Trynka
DGF ENCODE
CTCF Hoffman
Conserved LindbladToh
Coding UCSC
Annotation
−50
−25
0
25
Enrichment (prop h2 / prop snps)

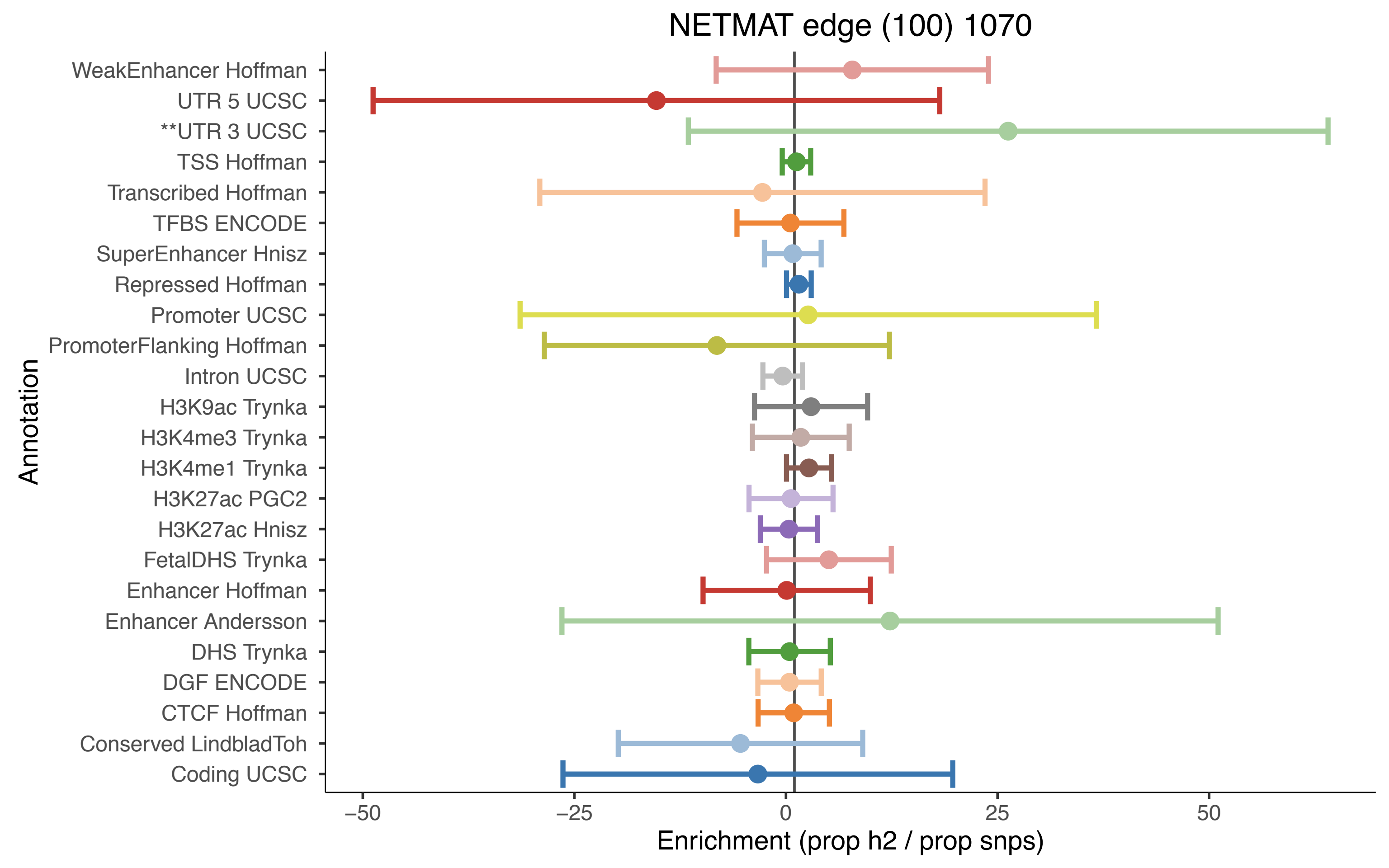

NETMAT edge (100) 1070
WeakEnhancer Hoffman
UTR 5 UCSC
**UTR 3 UCSC
TSS Hoffman
Transcribed Hoffman
TFBS ENCODE
SuperEnhancer Hnisz
Repressed Hoffman
Promoter UCSC
PromoterFlanking Hoffman
Intron UCSC
H3K9ac Trynka
H3K4me3 Trynka
H3K4me1 Trynka
H3K27ac PGC2
H3K27ac Hnisz
FetalDHS Trynka
Enhancer Hoffman
Enhancer Andersson
DHS Trynka
DGF ENCODE
CTCF Hoffman
Conserved LindbladToh
Coding UCSC
Annotation
−50
−25
0
25
50
Enrichment (prop h2 / prop snps)

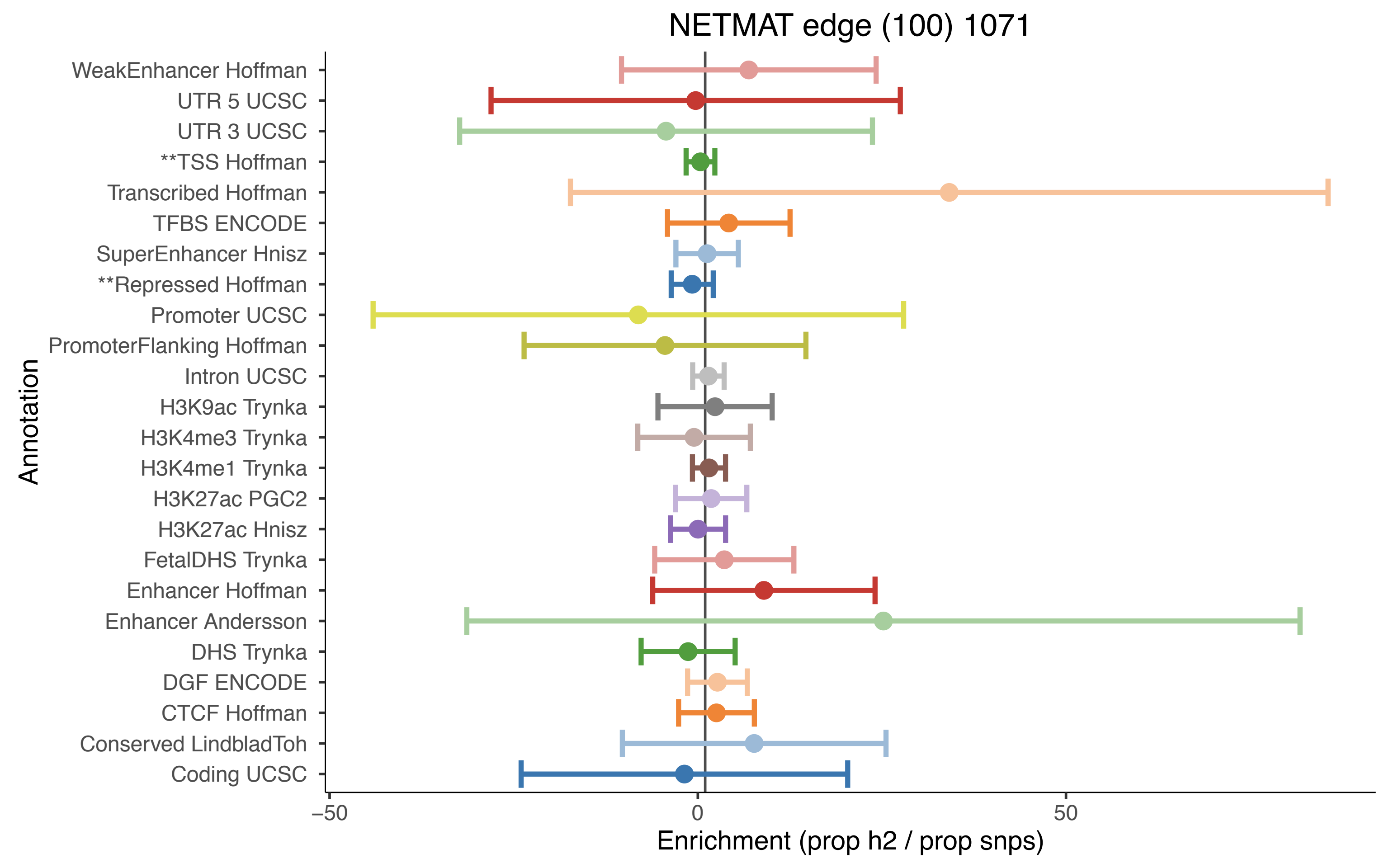
NETMAT edge (100) 1071
WeakEnhancer Hoffman
UTR 5 UCSC
UTR 3 UCSC
**TSS Hoffman
Transcribed Hoffman
TFBS ENCODE
SuperEnhancer Hnisz
**Repressed Hoffman
Promoter UCSC
PromoterFlanking Hoffman
Intron UCSC
H3K9ac Trynka
H3K4me3 Trynka
H3K4me1 Trynka
H3K27ac PGC2
H3K27ac Hnisz
FetalDHS Trynka
Enhancer Hoffman
Enhancer Andersson
DHS Trynka
DGF ENCODE
CTCF Hoffman
Conserved LindbladToh
Coding UCSC
Annotation
−50
0
50
Enrichment (prop h2 / prop snps)

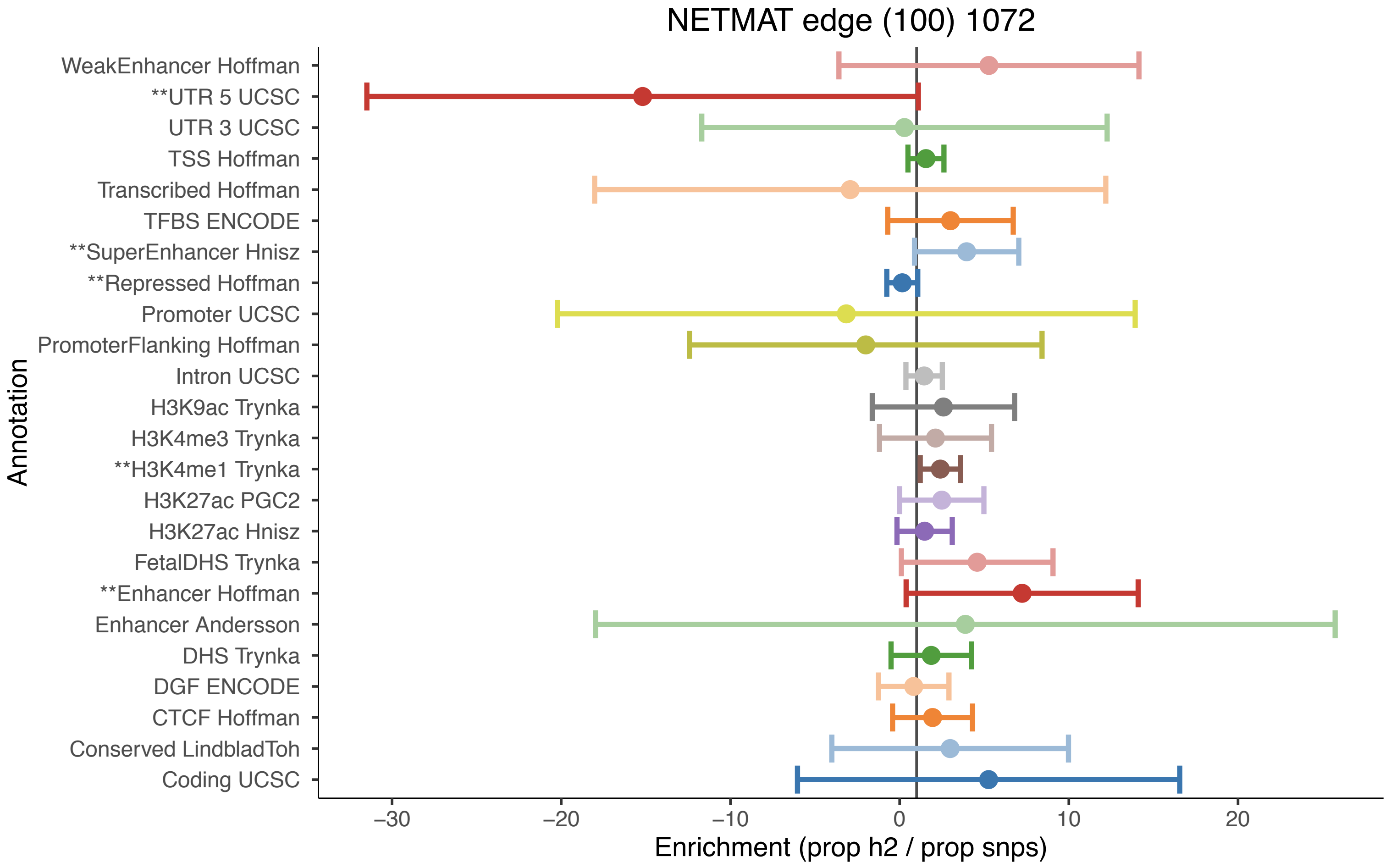
NETMAT edge (100) 1072
WeakEnhancer Hoffman
**UTR 5 UCSC
UTR 3 UCSC
TSS Hoffman
Transcribed Hoffman
TFBS ENCODE
**SuperEnhancer Hnisz
**Repressed Hoffman
Promoter UCSC
PromoterFlanking Hoffman
Intron UCSC
H3K9ac Trynka
H3K4me3 Trynka
**H3K4me1 Trynka
H3K27ac PGC2
H3K27ac Hnisz
FetalDHS Trynka
**Enhancer Hoffman
Enhancer Andersson
DHS Trynka
DGF ENCODE
CTCF Hoffman
Conserved LindbladToh
Coding UCSC
Annotation
−30
−20
−10
0
10
20
Enrichment (prop h2 / prop snps)

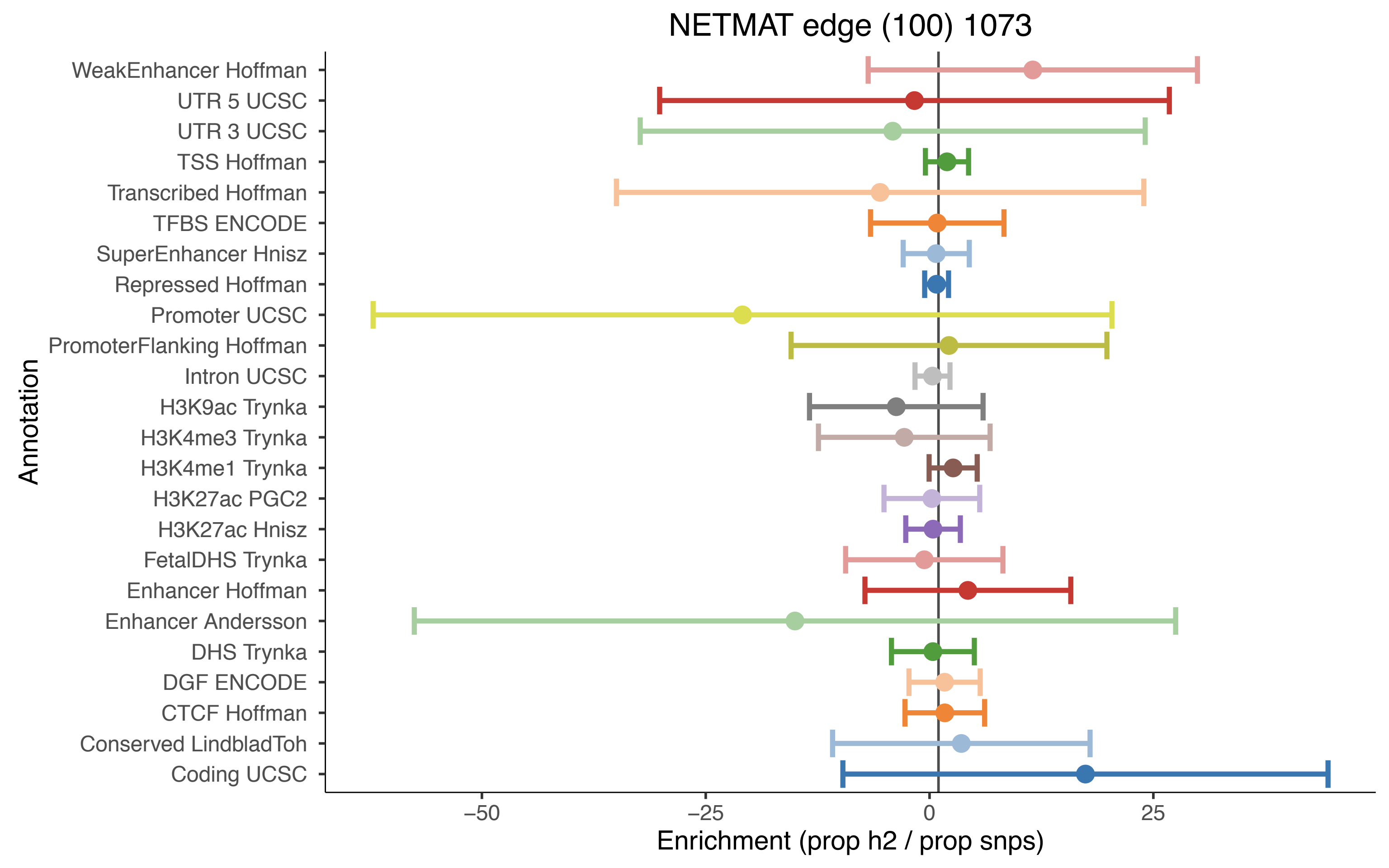

NETMAT edge (100) 1073
WeakEnhancer Hoffman
UTR 5 UCSC
UTR 3 UCSC
TSS Hoffman
Transcribed Hoffman
TFBS ENCODE
SuperEnhancer Hnisz
Repressed Hoffman
Promoter UCSC
PromoterFlanking Hoffman
Intron UCSC
H3K9ac Trynka
H3K4me3 Trynka
H3K4me1 Trynka
H3K27ac PGC2
H3K27ac Hnisz
FetalDHS Trynka
Enhancer Hoffman
Enhancer Andersson
DHS Trynka
DGF ENCODE
CTCF Hoffman
Conserved LindbladToh
Coding UCSC
Annotation
−50
−25
0
25
Enrichment (prop h2 / prop snps)

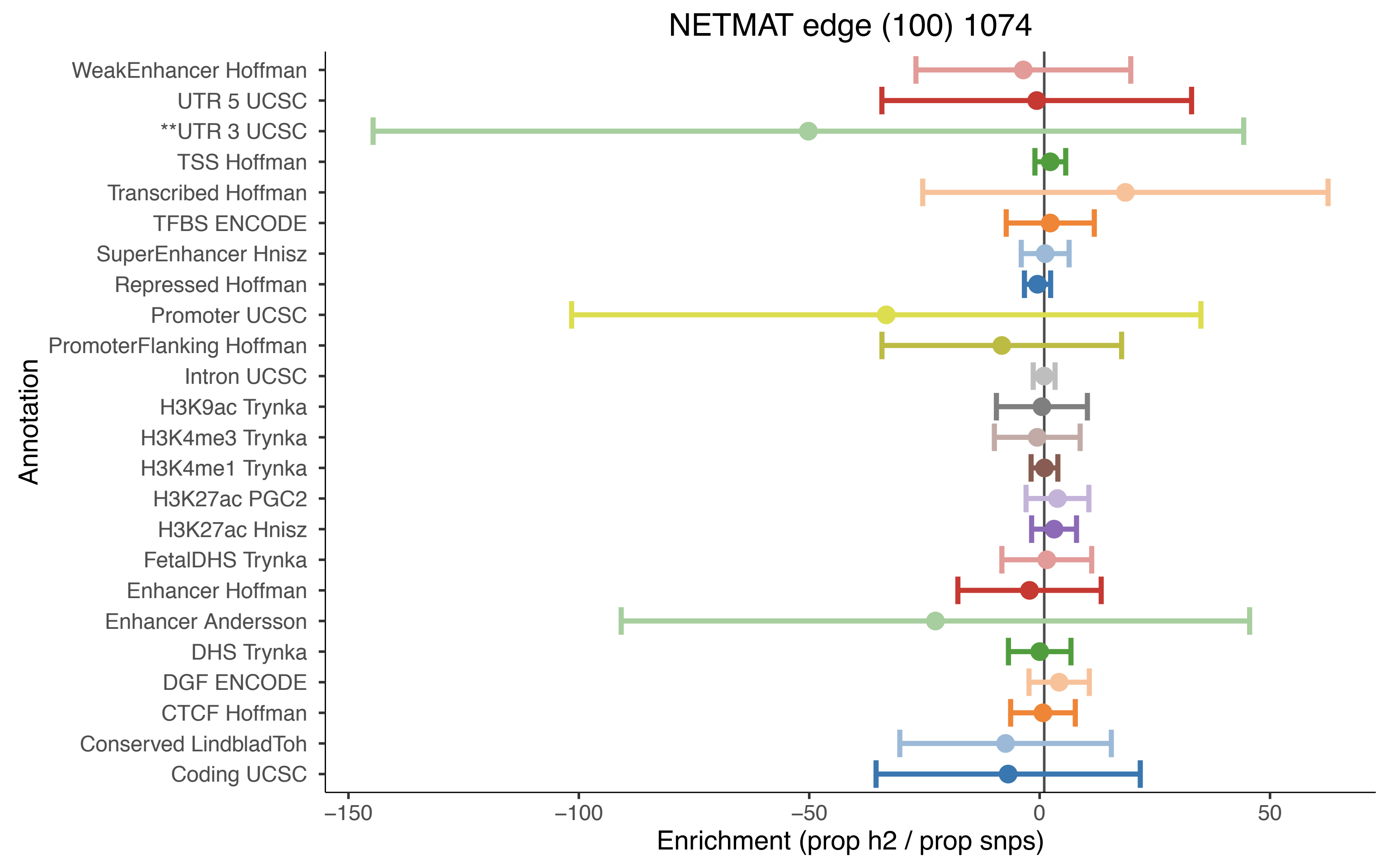
NETMAT edge (100) 1074
WeakEnhancer Hoffman
UTR 5 UCSC
**UTR 3 UCSC
TSS Hoffman
Transcribed Hoffman
TFBS ENCODE
SuperEnhancer Hnisz
Repressed Hoffman
Promoter UCSC
PromoterFlanking Hoffman
Intron UCSC
H3K9ac Trynka
H3K4me3 Trynka
H3K4me1 Trynka
H3K27ac PGC2
H3K27ac Hnisz
FetalDHS Trynka
Enhancer Hoffman
Enhancer Andersson
DHS Trynka
DGF ENCODE
CTCF Hoffman
Conserved LindbladToh
Coding UCSC
Annotation
−150
−100
−50
0
50
Enrichment (prop h2 / prop snps)

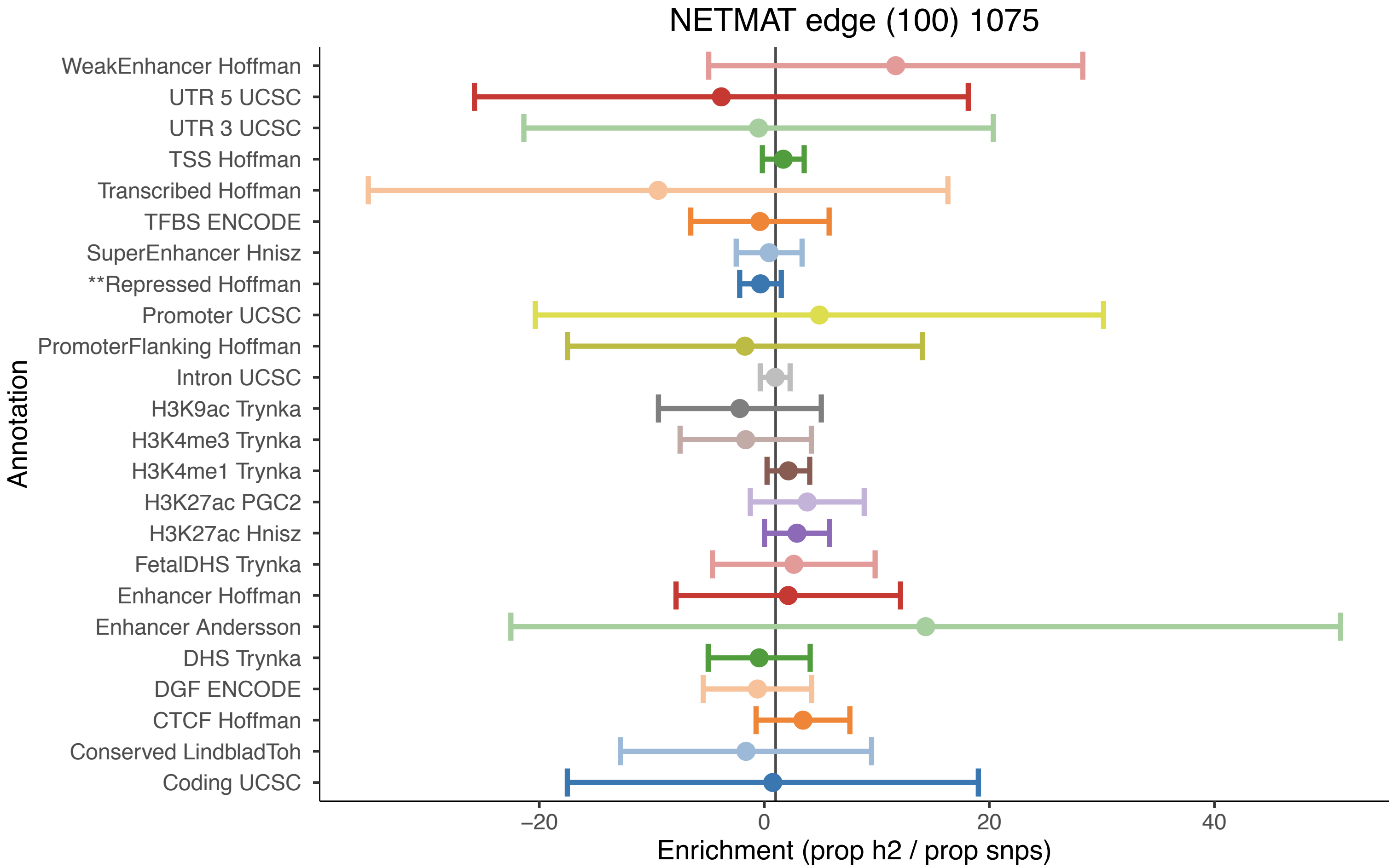
NETMAT edge (100) 1075
WeakEnhancer Hoffman
UTR 5 UCSC
UTR 3 UCSC
TSS Hoffman
Transcribed Hoffman
TFBS ENCODE
SuperEnhancer Hnisz
**Repressed Hoffman
Promoter UCSC
PromoterFlanking Hoffman
Intron UCSC
H3K9ac Trynka
H3K4me3 Trynka
H3K4me1 Trynka
H3K27ac PGC2
H3K27ac Hnisz
FetalDHS Trynka
Enhancer Hoffman
Enhancer Andersson
DHS Trynka
DGF ENCODE
CTCF Hoffman
Conserved LindbladToh
Coding UCSC
Annotation
−20
0
20
40
Enrichment (prop h2 / prop snps)

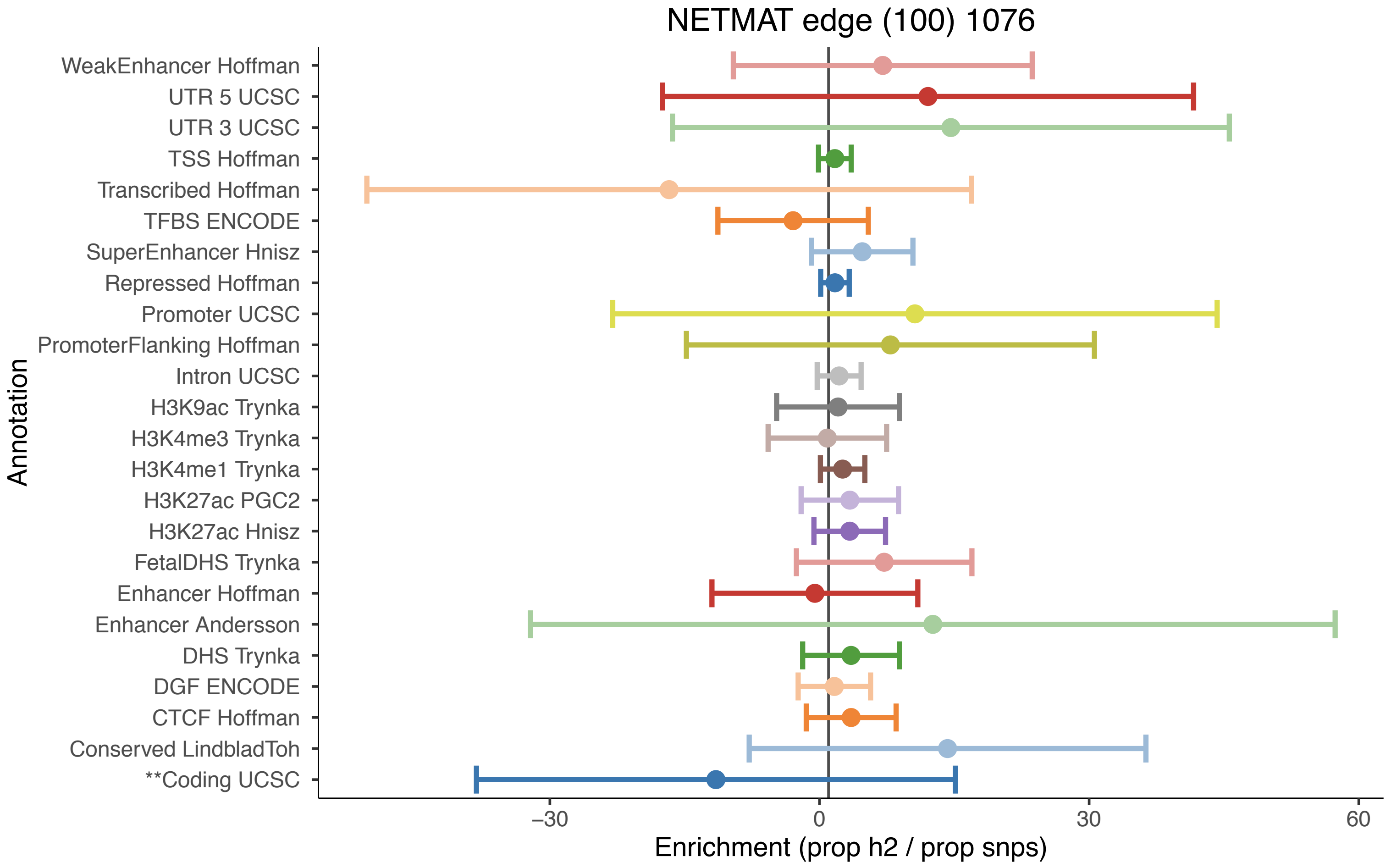
NETMAT edge (100) 1076
Annotation
WeakEnhancer Hoffman
UTR 5 UCSC
UTR 3 UCSC
TSS Hoffman
Transcribed Hoffman
TFBS ENCODE
SuperEnhancer Hnisz
Repressed Hoffman
Promoter UCSC
PromoterFlanking Hoffman
Intron UCSC
H3K9ac Trynka
H3K4me3 Trynka
H3K4me1 Trynka
H3K27ac PGC2
H3K27ac Hnisz
FetalDHS Trynka
Enhancer Hoffman
Enhancer Andersson
DHS Trynka
DGF ENCODE
CTCF Hoffman
Conserved LindbladToh
**Coding UCSC
−30
0
30
60
Enrichment (prop h2 / prop snps)

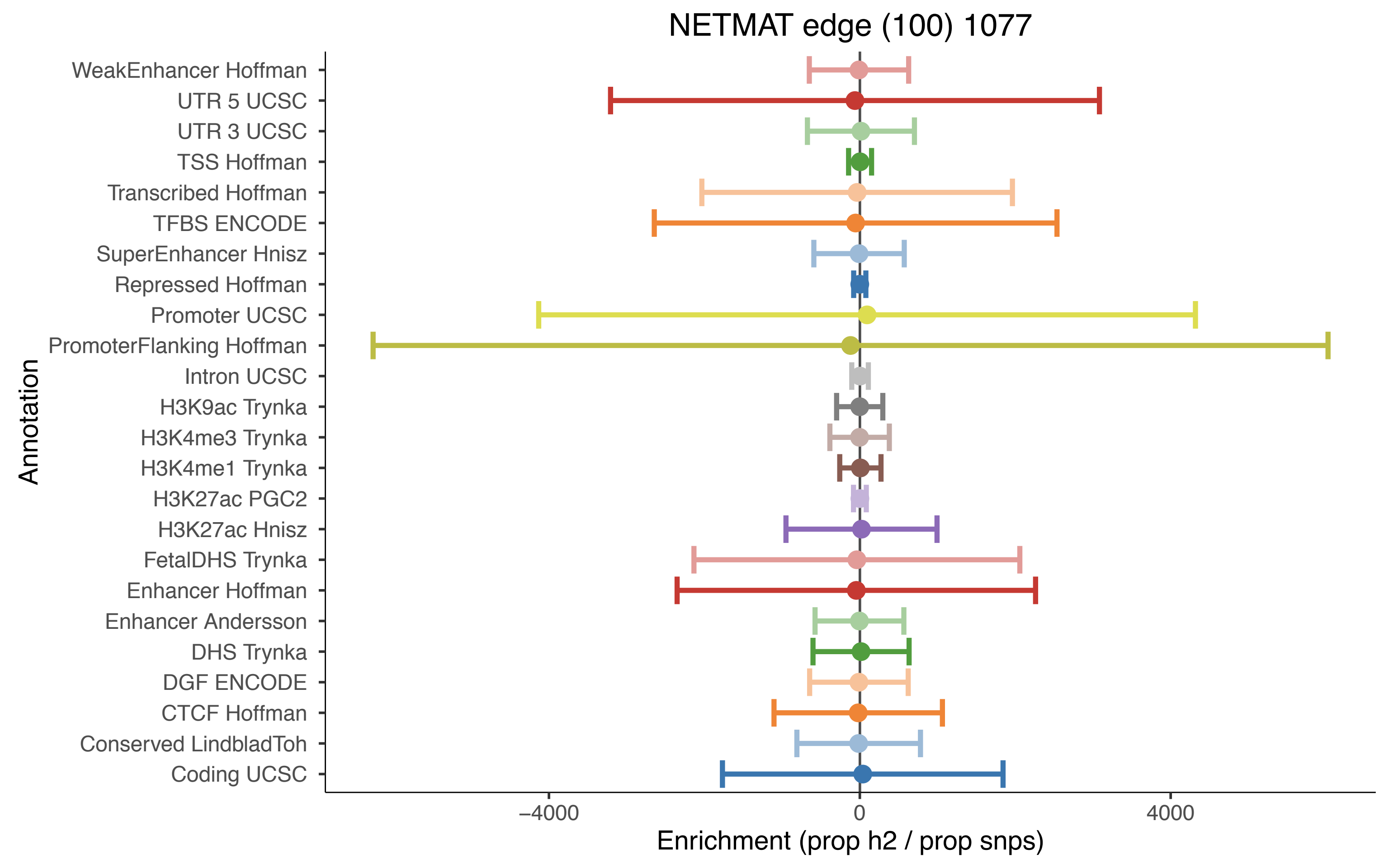
NETMAT edge (100) 1077
WeakEnhancer Hoffman
UTR 5 UCSC
UTR 3 UCSC
TSS Hoffman
Transcribed Hoffman
TFBS ENCODE
SuperEnhancer Hnisz
Repressed Hoffman
Promoter UCSC
PromoterFlanking Hoffman
Intron UCSC
H3K9ac Trynka
H3K4me3 Trynka
H3K4me1 Trynka
H3K27ac PGC2
H3K27ac Hnisz
FetalDHS Trynka
Enhancer Hoffman
Enhancer Andersson
DHS Trynka
DGF ENCODE
CTCF Hoffman
Conserved LindbladToh
Coding UCSC
Annotation
−4000
0
4000
Enrichment (prop h2 / prop snps)

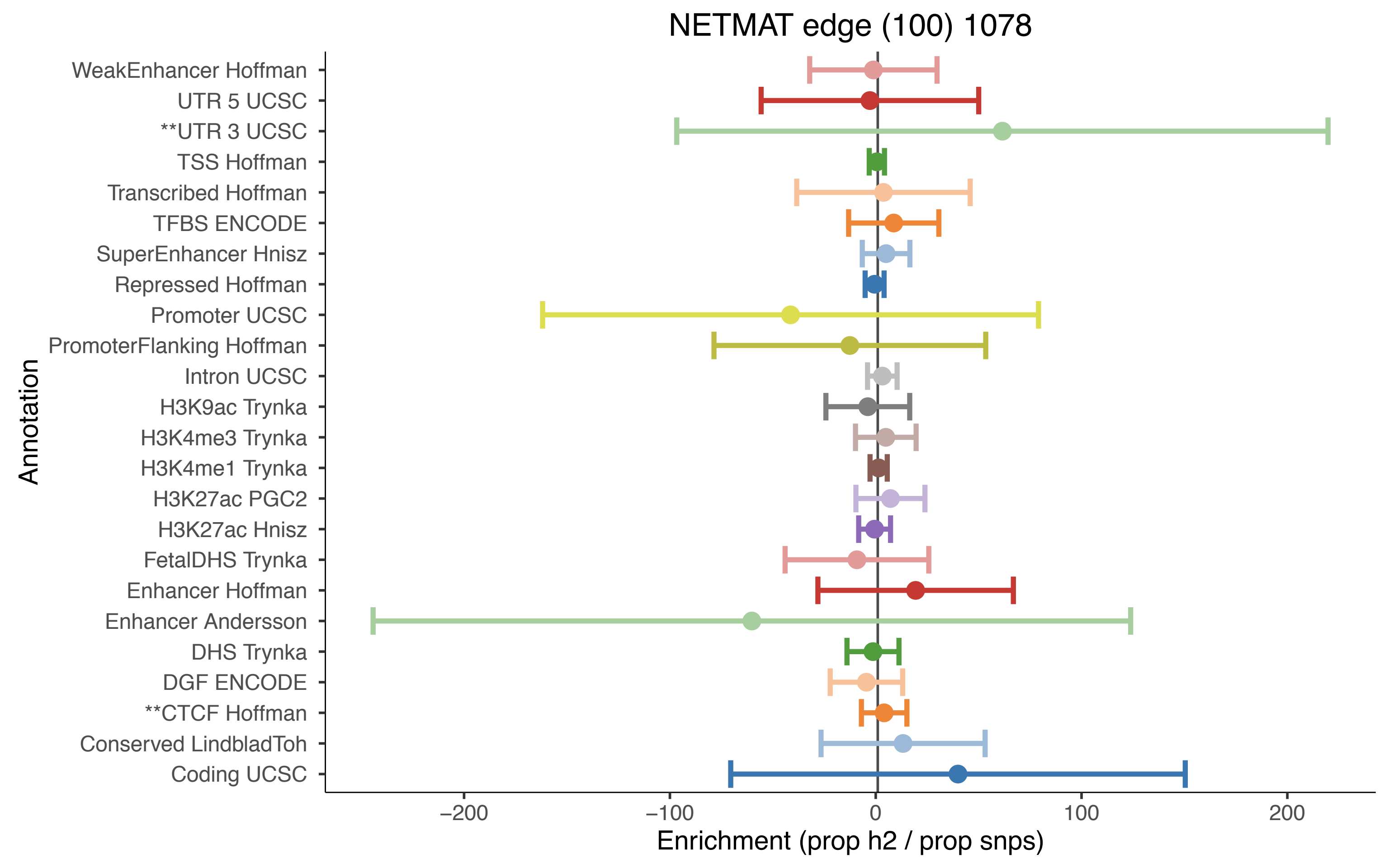

NETMAT edge (100) 1078
WeakEnhancer Hoffman
UTR 5 UCSC
**UTR 3 UCSC
TSS Hoffman
Transcribed Hoffman
TFBS ENCODE
SuperEnhancer Hnisz
Repressed Hoffman
Promoter UCSC
PromoterFlanking Hoffman
Intron UCSC
H3K9ac Trynka
H3K4me3 Trynka
H3K4me1 Trynka
H3K27ac PGC2
H3K27ac Hnisz
FetalDHS Trynka
Enhancer Hoffman
Enhancer Andersson
DHS Trynka
DGF ENCODE
**CTCF Hoffman
Conserved LindbladToh
Coding UCSC
Annotation
−200
−100
0
100
200
Enrichment (prop h2 / prop snps)

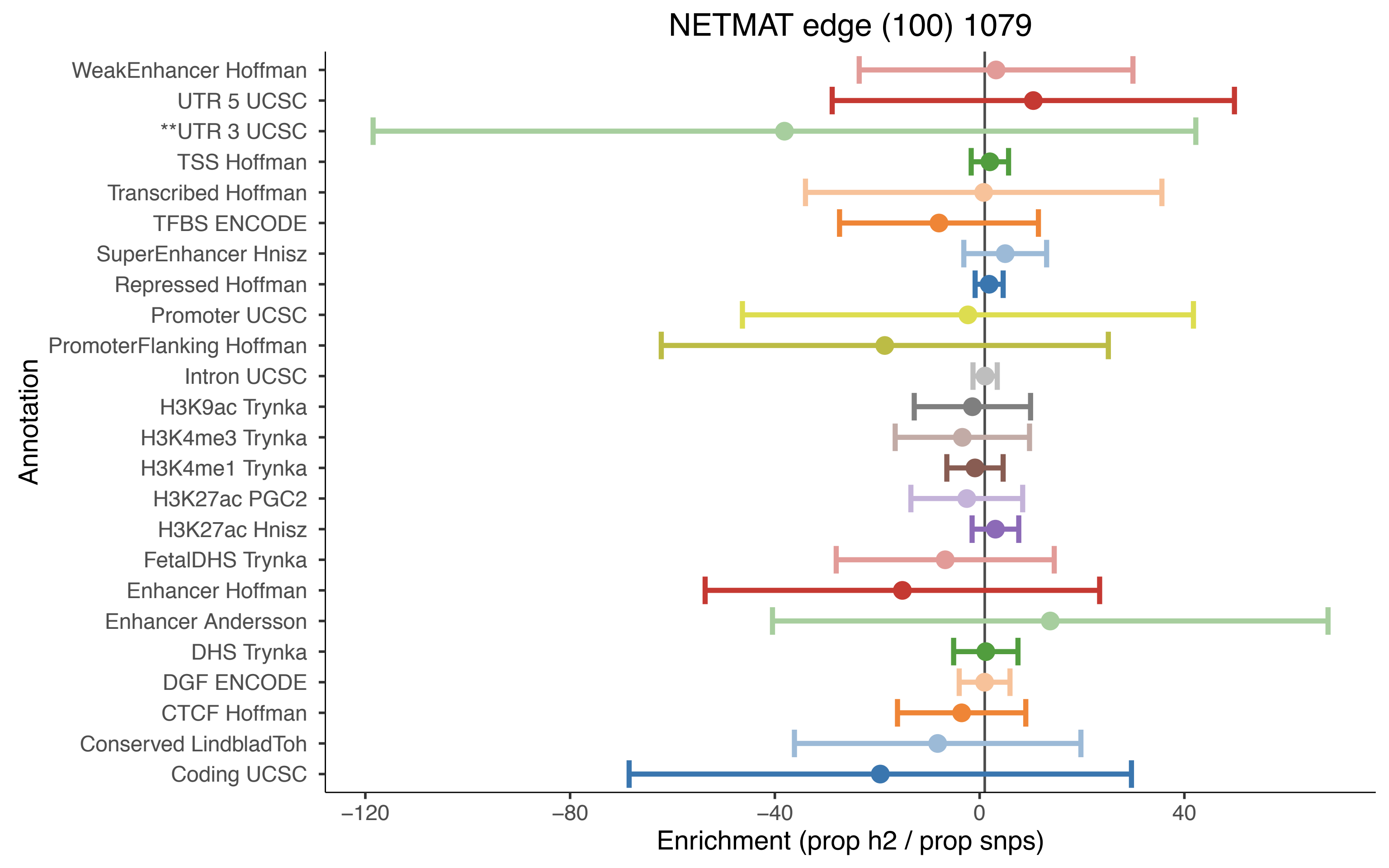
NETMAT edge (100) 1079
WeakEnhancer Hoffman
UTR 5 UCSC
**UTR 3 UCSC
TSS Hoffman
Transcribed Hoffman
TFBS ENCODE
SuperEnhancer Hnisz
Repressed Hoffman
Promoter UCSC
PromoterFlanking Hoffman
Intron UCSC
H3K9ac Trynka
H3K4me3 Trynka
H3K4me1 Trynka
H3K27ac PGC2
H3K27ac Hnisz
FetalDHS Trynka
Enhancer Hoffman
Enhancer Andersson
DHS Trynka
DGF ENCODE
CTCF Hoffman
Conserved LindbladToh
Coding UCSC
Annotation
−120
−80
−40
0
40
Enrichment (prop h2 / prop snps)

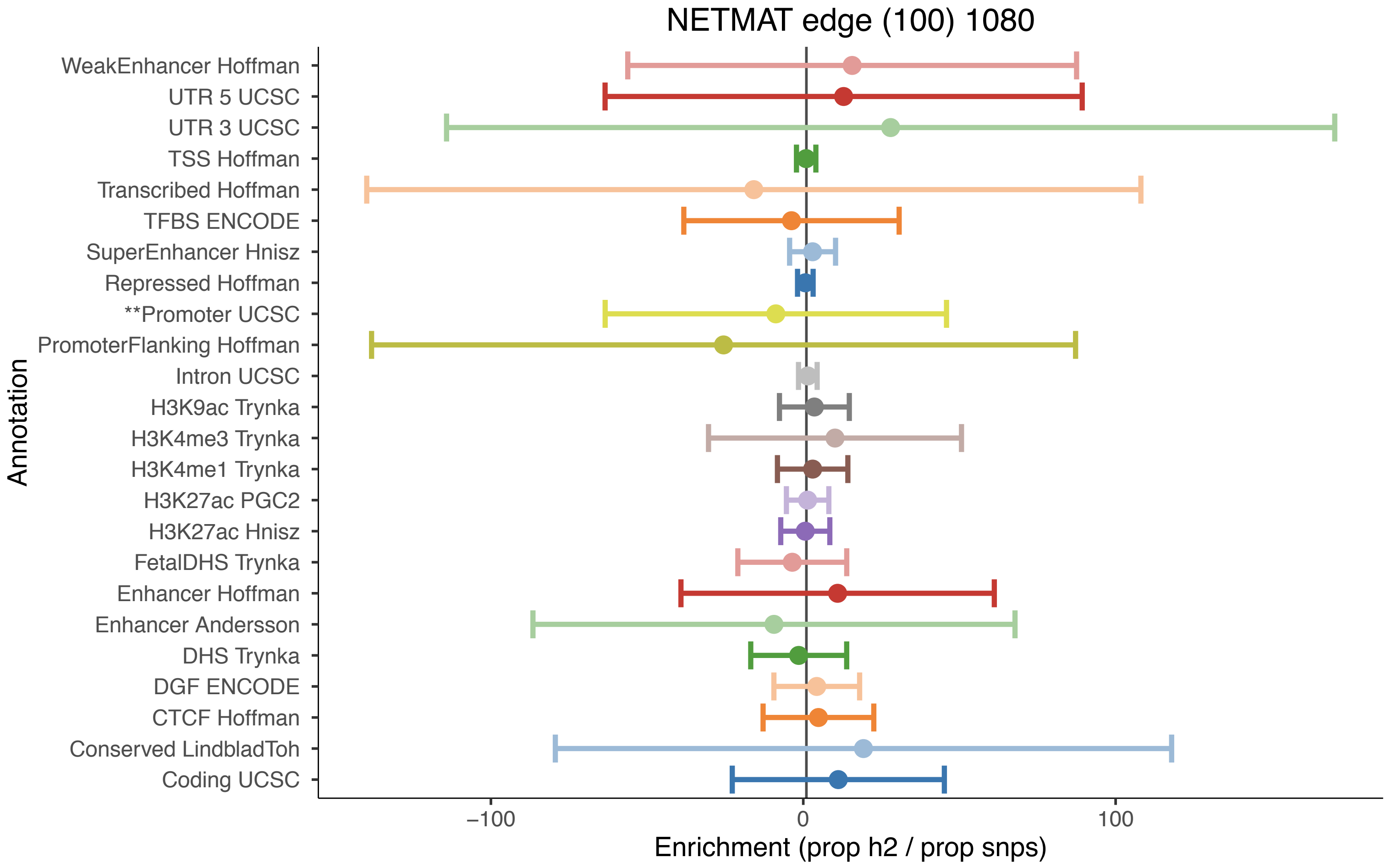
NETMAT edge (100) 1080
WeakEnhancer Hoffman
UTR 5 UCSC
UTR 3 UCSC
TSS Hoffman
Transcribed Hoffman
TFBS ENCODE
SuperEnhancer Hnisz
Repressed Hoffman
**Promoter UCSC
PromoterFlanking Hoffman
Intron UCSC
H3K9ac Trynka
H3K4me3 Trynka
H3K4me1 Trynka
H3K27ac PGC2
H3K27ac Hnisz
FetalDHS Trynka
Enhancer Hoffman
Enhancer Andersson
DHS Trynka
DGF ENCODE
CTCF Hoffman
Conserved LindbladToh
Coding UCSC
Annotation
−100
0
100
Enrichment (prop h2 / prop snps)

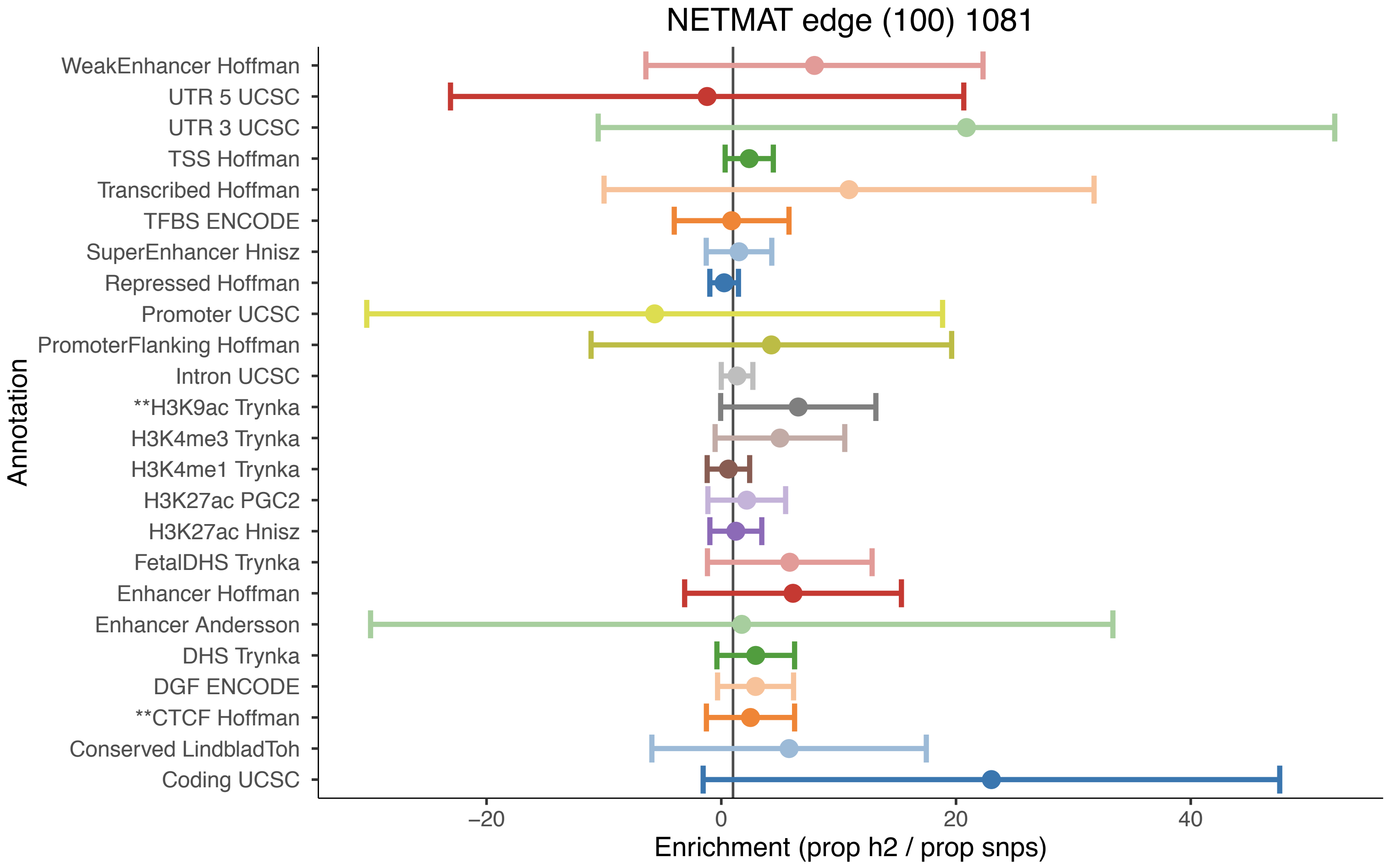

NETMAT edge (100) 1081
WeakEnhancer Hoffman
UTR 5 UCSC
UTR 3 UCSC
TSS Hoffman
Transcribed Hoffman
TFBS ENCODE
SuperEnhancer Hnisz
Repressed Hoffman
Promoter UCSC
PromoterFlanking Hoffman
Intron UCSC
**H3K9ac Trynka
H3K4me3 Trynka
H3K4me1 Trynka
H3K27ac PGC2
H3K27ac Hnisz
FetalDHS Trynka
Enhancer Hoffman
Enhancer Andersson
DHS Trynka
DGF ENCODE
**CTCF Hoffman
Conserved LindbladToh
Coding UCSC
Annotation
−20
0
20
40
Enrichment (prop h2 / prop snps)

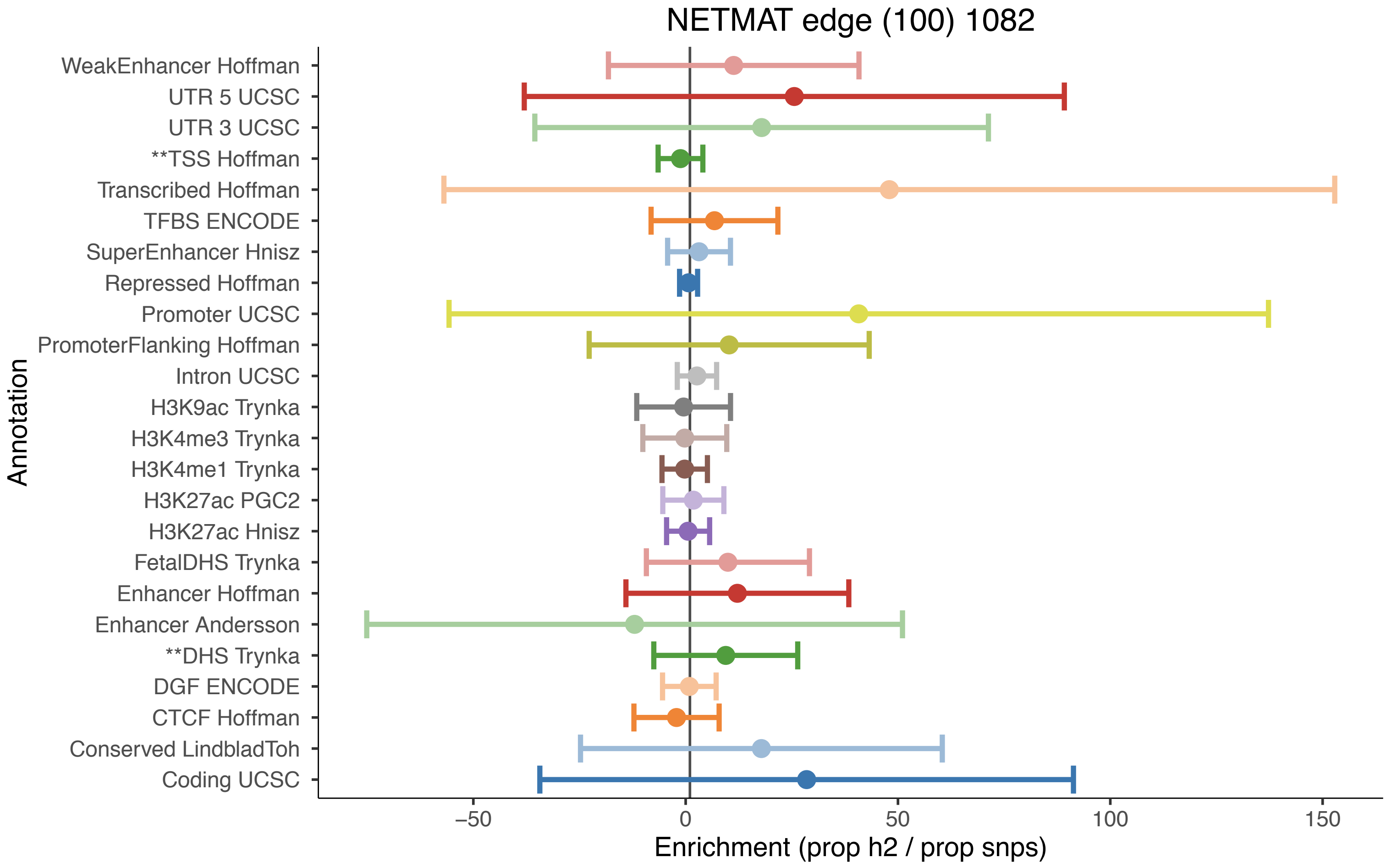

NETMAT edge (100) 1082
WeakEnhancer Hoffman
UTR 5 UCSC
UTR 3 UCSC
**TSS Hoffman
Transcribed Hoffman
TFBS ENCODE
SuperEnhancer Hnisz
Repressed Hoffman
Promoter UCSC
PromoterFlanking Hoffman
Intron UCSC
H3K9ac Trynka
H3K4me3 Trynka
H3K4me1 Trynka
H3K27ac PGC2
H3K27ac Hnisz
FetalDHS Trynka
Enhancer Hoffman
Enhancer Andersson
**DHS Trynka
DGF ENCODE
CTCF Hoffman
Conserved LindbladToh
Coding UCSC
Annotation
−50
0
50
100
150
Enrichment (prop h2 / prop snps)

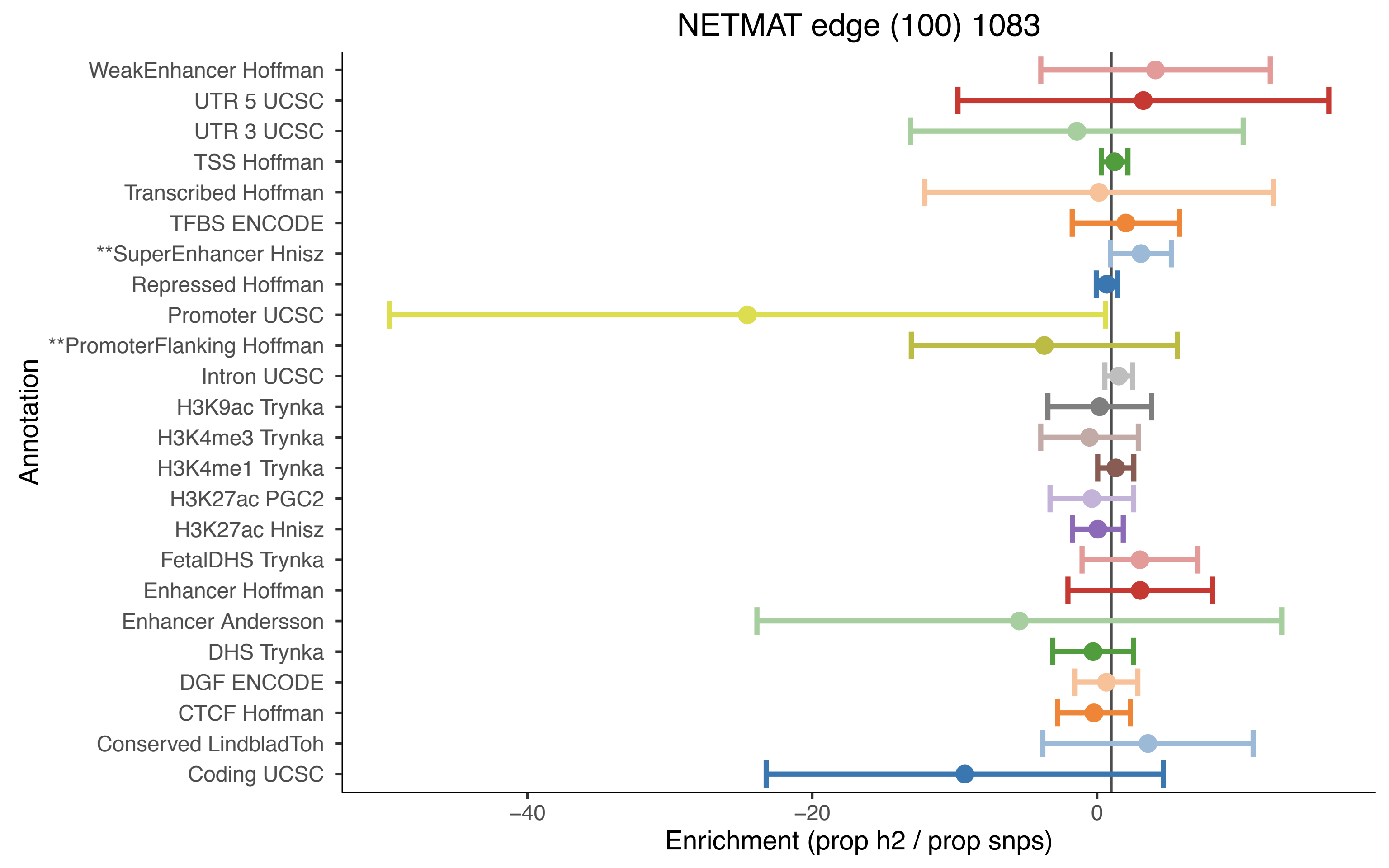
NETMAT edge (100) 1083
WeakEnhancer Hoffman
UTR 5 UCSC
UTR 3 UCSC
TSS Hoffman
Transcribed Hoffman
TFBS ENCODE
**SuperEnhancer Hnisz
Repressed Hoffman
Promoter UCSC
**PromoterFlanking Hoffman
Intron UCSC
H3K9ac Trynka
H3K4me3 Trynka
H3K4me1 Trynka
H3K27ac PGC2
H3K27ac Hnisz
FetalDHS Trynka
Enhancer Hoffman
Enhancer Andersson
DHS Trynka
DGF ENCODE
CTCF Hoffman
Conserved LindbladToh
Coding UCSC
Annotation
−40
−20
0
Enrichment (prop h2 / prop snps)

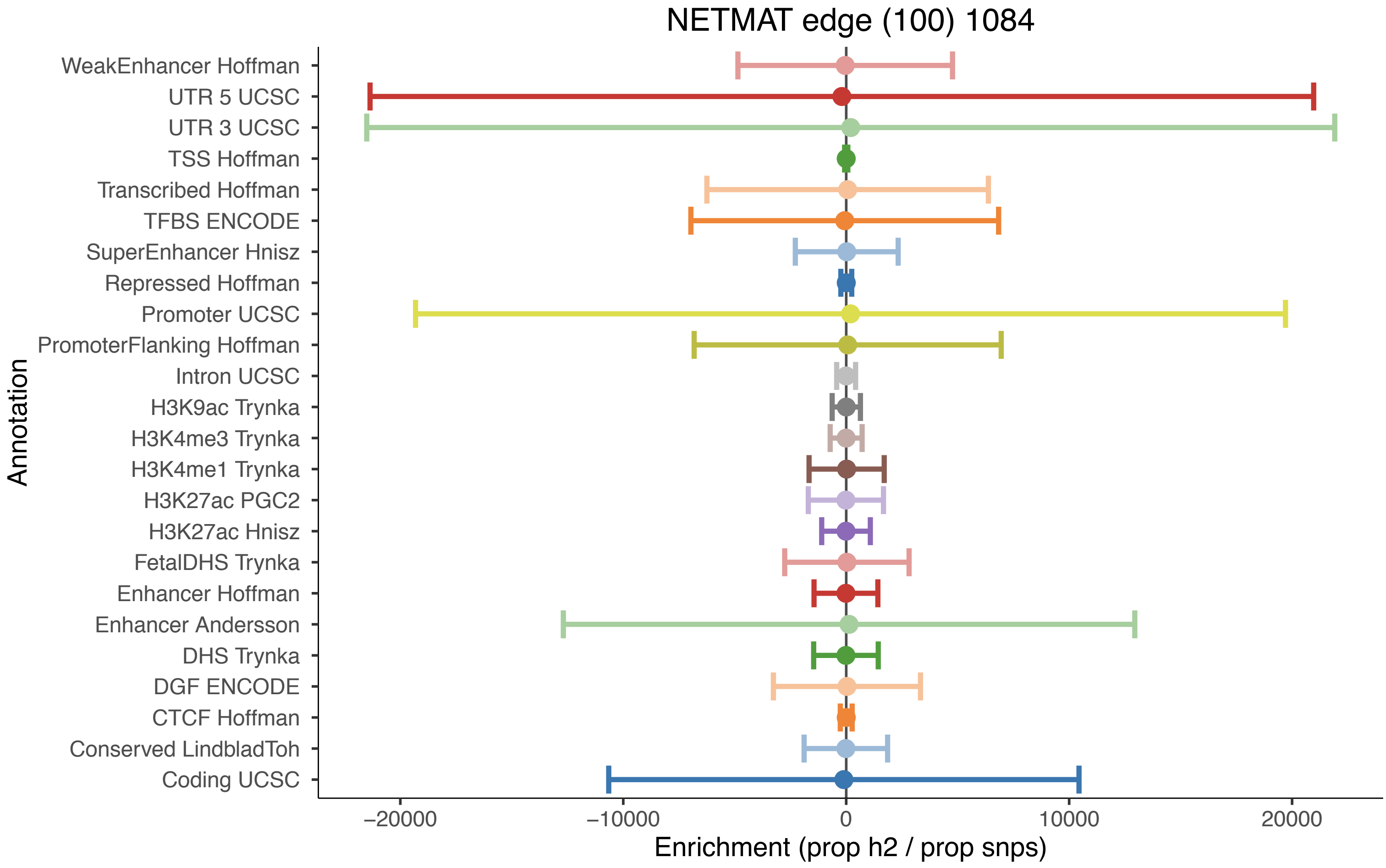

NETMAT edge (100) 1084
WeakEnhancer Hoffman
UTR 5 UCSC
UTR 3 UCSC
TSS Hoffman
Transcribed Hoffman
TFBS ENCODE
SuperEnhancer Hnisz
Repressed Hoffman
Promoter UCSC
PromoterFlanking Hoffman
Intron UCSC
H3K9ac Trynka
H3K4me3 Trynka
H3K4me1 Trynka
H3K27ac PGC2
H3K27ac Hnisz
FetalDHS Trynka
Enhancer Hoffman
Enhancer Andersson
DHS Trynka
DGF ENCODE
CTCF Hoffman
Conserved LindbladToh
Coding UCSC
Annotation
−20000
−10000
0
10000
20000
Enrichment (prop h2 / prop snps)

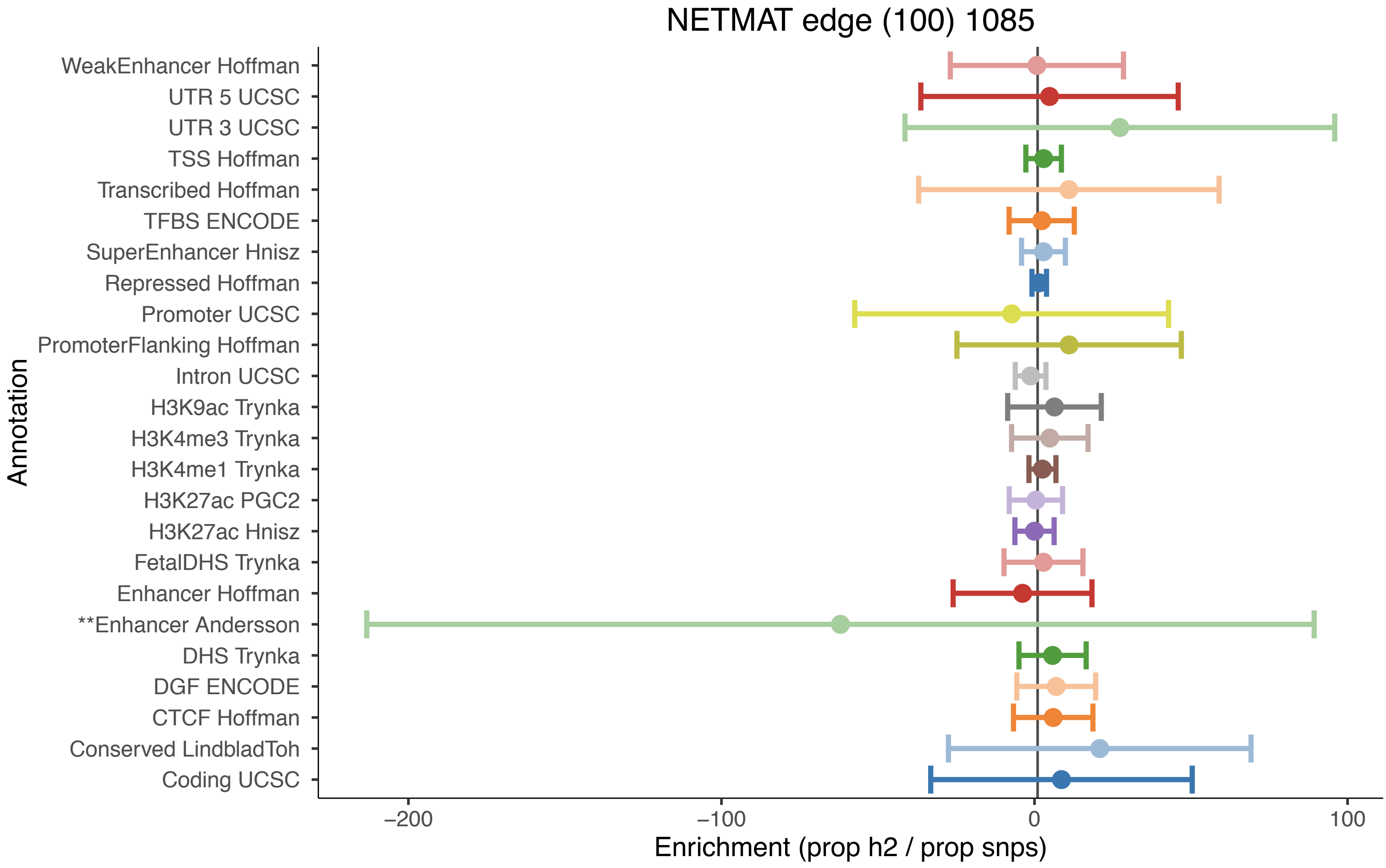

NETMAT edge (100) 1085
WeakEnhancer Hoffman
UTR 5 UCSC
UTR 3 UCSC
TSS Hoffman
Transcribed Hoffman
TFBS ENCODE
SuperEnhancer Hnisz
Repressed Hoffman
Promoter UCSC
PromoterFlanking Hoffman
Intron UCSC
H3K9ac Trynka
H3K4me3 Trynka
H3K4me1 Trynka
H3K27ac PGC2
H3K27ac Hnisz
FetalDHS Trynka
Enhancer Hoffman
**Enhancer Andersson
DHS Trynka
DGF ENCODE
CTCF Hoffman
Conserved LindbladToh
Coding UCSC
Annotation
−200
−100
0
100
Enrichment (prop h2 / prop snps)

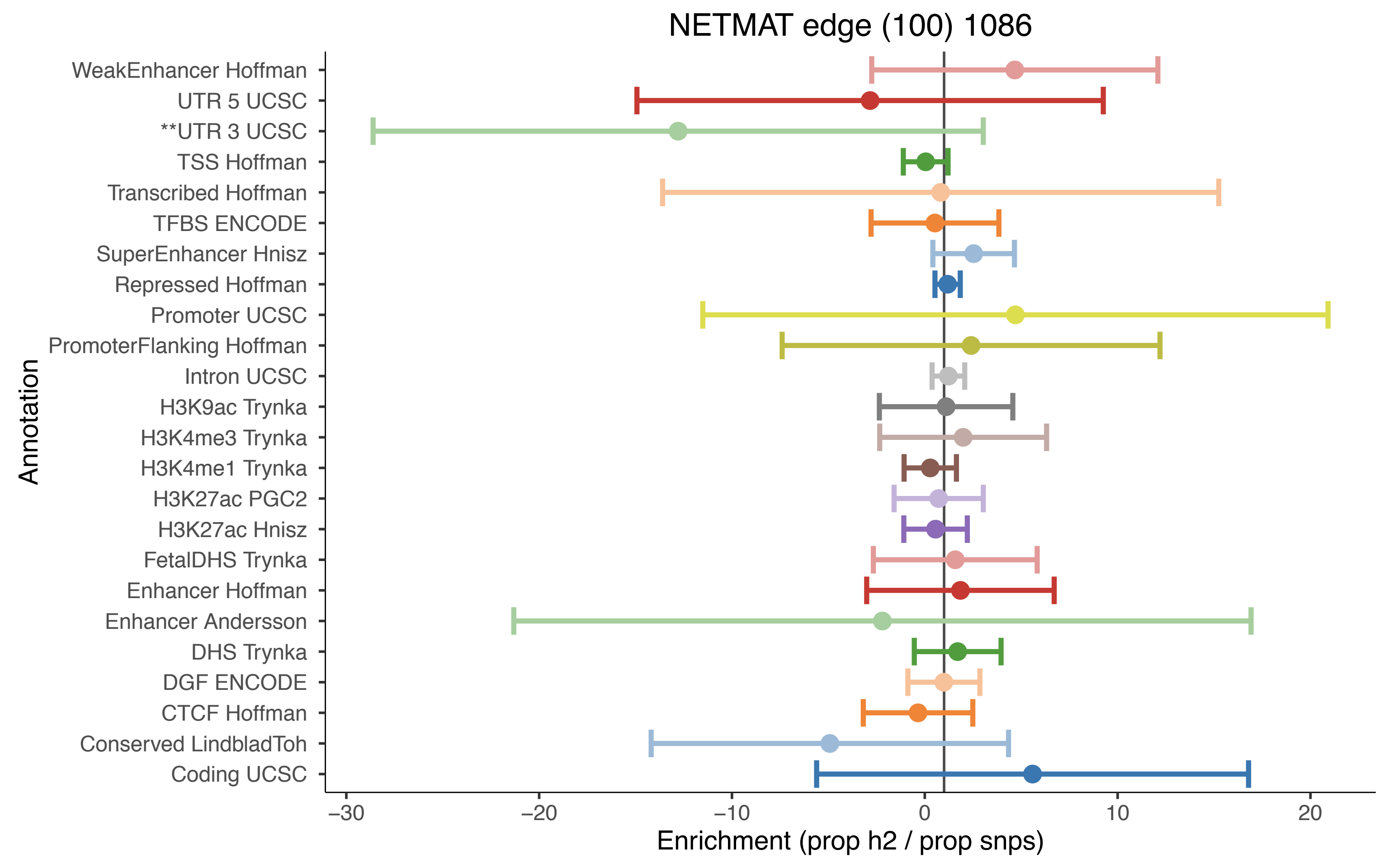

NETMAT edge (100) 1086
Annotation
WeakEnhancer Hoffman
UTR 5 UCSC
**UTR 3 UCSC
TSS Hoffman
Transcribed Hoffman
TFBS ENCODE
SuperEnhancer Hnisz
Repressed Hoffman
Promoter UCSC
PromoterFlanking Hoffman
Intron UCSC
H3K9ac Trynka
H3K4me3 Trynka
H3K4me1 Trynka
H3K27ac PGC2
H3K27ac Hnisz
FetalDHS Trynka
Enhancer Hoffman
Enhancer Andersson
DHS Trynka
DGF ENCODE
CTCF Hoffman
Conserved LindbladToh
Coding UCSC
−30
−20
−10
0
10
20
Enrichment (prop h2 / prop snps)

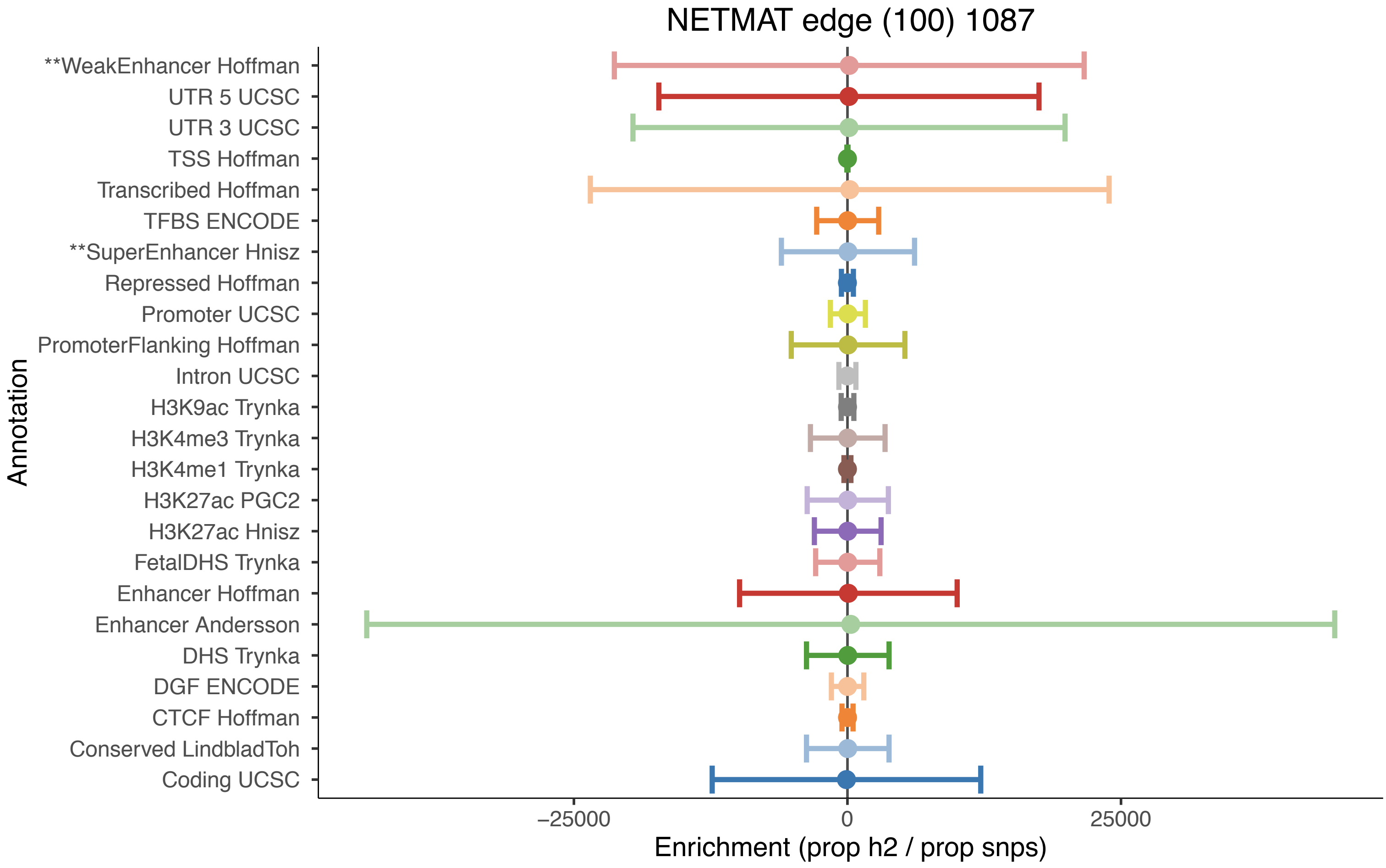
NETMAT edge (100) 1087
Annotation
**WeakEnhancer Hoffman
UTR 5 UCSC
UTR 3 UCSC
TSS Hoffman
Transcribed Hoffman
TFBS ENCODE
**SuperEnhancer Hnisz
Repressed Hoffman
Promoter UCSC
PromoterFlanking Hoffman
Intron UCSC
H3K9ac Trynka
H3K4me3 Trynka
H3K4me1 Trynka
H3K27ac PGC2
H3K27ac Hnisz
FetalDHS Trynka
Enhancer Hoffman
Enhancer Andersson
DHS Trynka
DGF ENCODE
CTCF Hoffman
Conserved LindbladToh
Coding UCSC
−25000
0
25000
Enrichment (prop h2 / prop snps)

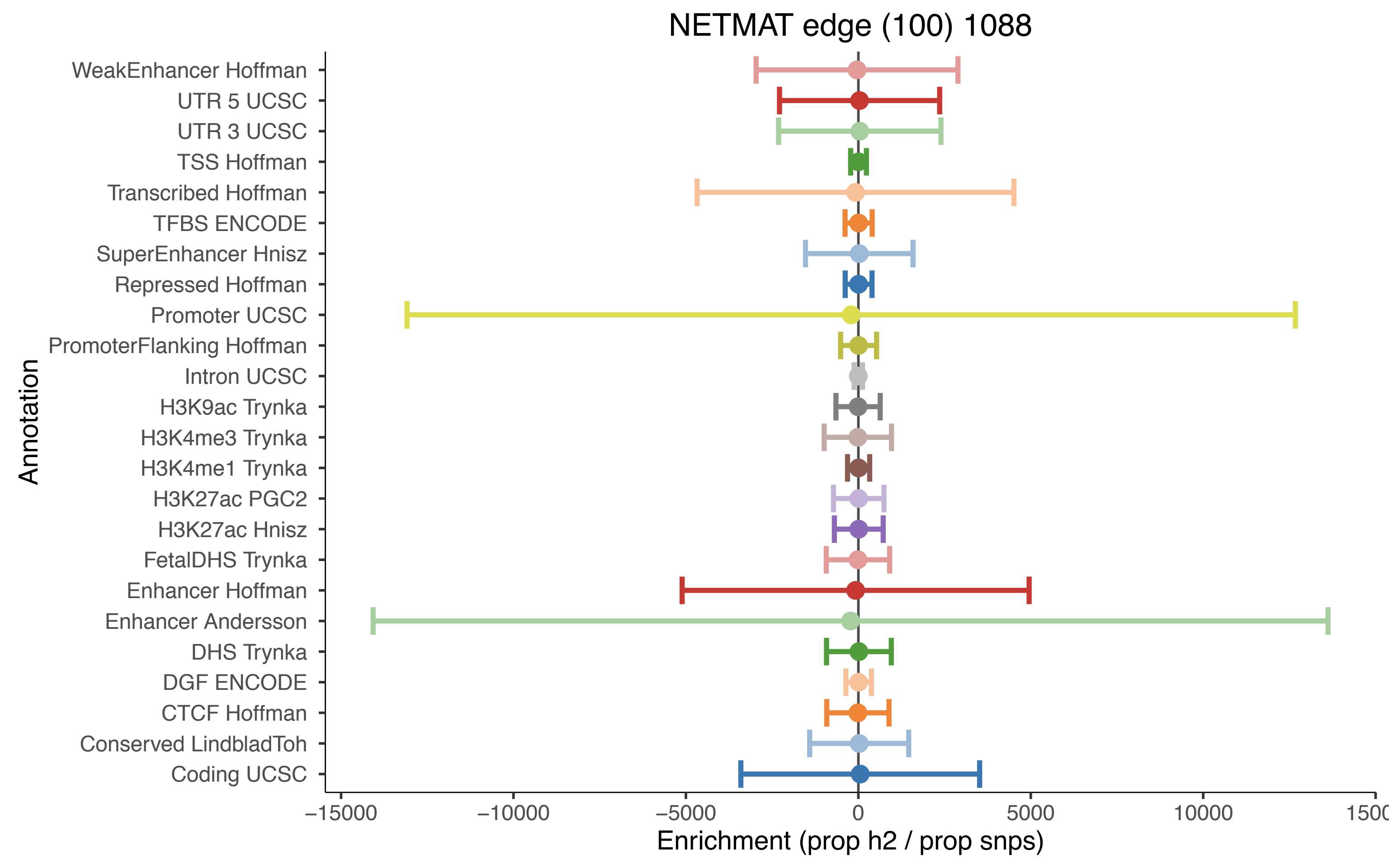
NETMAT edge (100) 1088
WeakEnhancer Hoffman
UTR 5 UCSC
UTR 3 UCSC
TSS Hoffman
Transcribed Hoffman
TFBS ENCODE
SuperEnhancer Hnisz
Repressed Hoffman
Promoter UCSC
PromoterFlanking Hoffman
Intron UCSC
H3K9ac Trynka
H3K4me3 Trynka
H3K4me1 Trynka
H3K27ac PGC2
H3K27ac Hnisz
FetalDHS Trynka
Enhancer Hoffman
Enhancer Andersson
DHS Trynka
DGF ENCODE
CTCF Hoffman
Conserved LindbladToh
Coding UCSC
Annotation
−15000
−10000
−5000
0
5000
10000
Enrichment (prop h2 / prop snps)

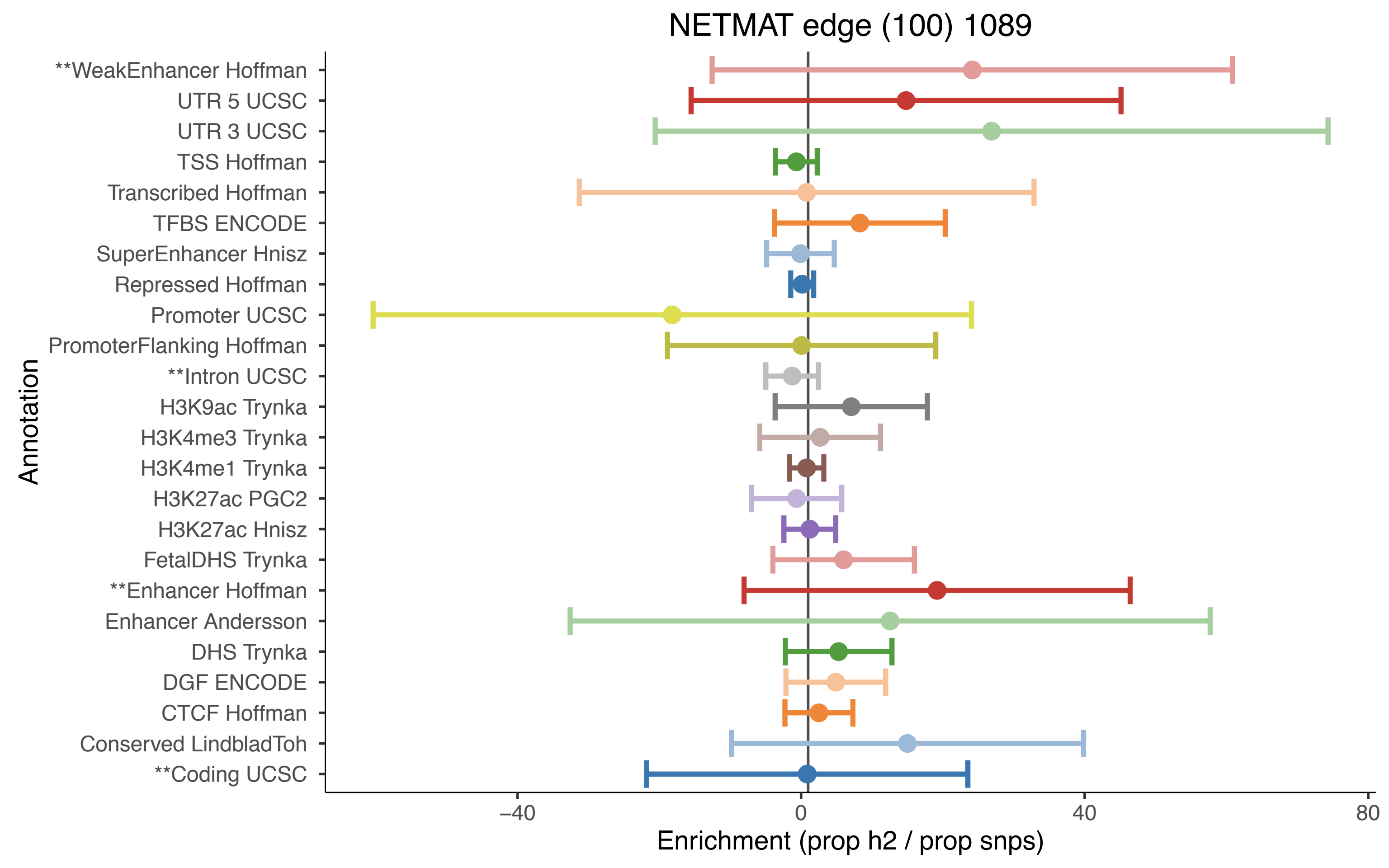
NETMAT edge (100) 1089
**WeakEnhancer Hoffman
UTR 5 UCSC
UTR 3 UCSC
TSS Hoffman
Transcribed Hoffman
TFBS ENCODE
SuperEnhancer Hnisz
Repressed Hoffman
Promoter UCSC
PromoterFlanking Hoffman
**Intron UCSC
H3K9ac Trynka
H3K4me3 Trynka
H3K4me1 Trynka
H3K27ac PGC2
H3K27ac Hnisz
FetalDHS Trynka
**Enhancer Hoffman
Enhancer Andersson
DHS Trynka
DGF ENCODE
CTCF Hoffman
Conserved LindbladToh
**Coding UCSC
Annotation
−40
0
40
80
Enrichment (prop h2 / prop snps)

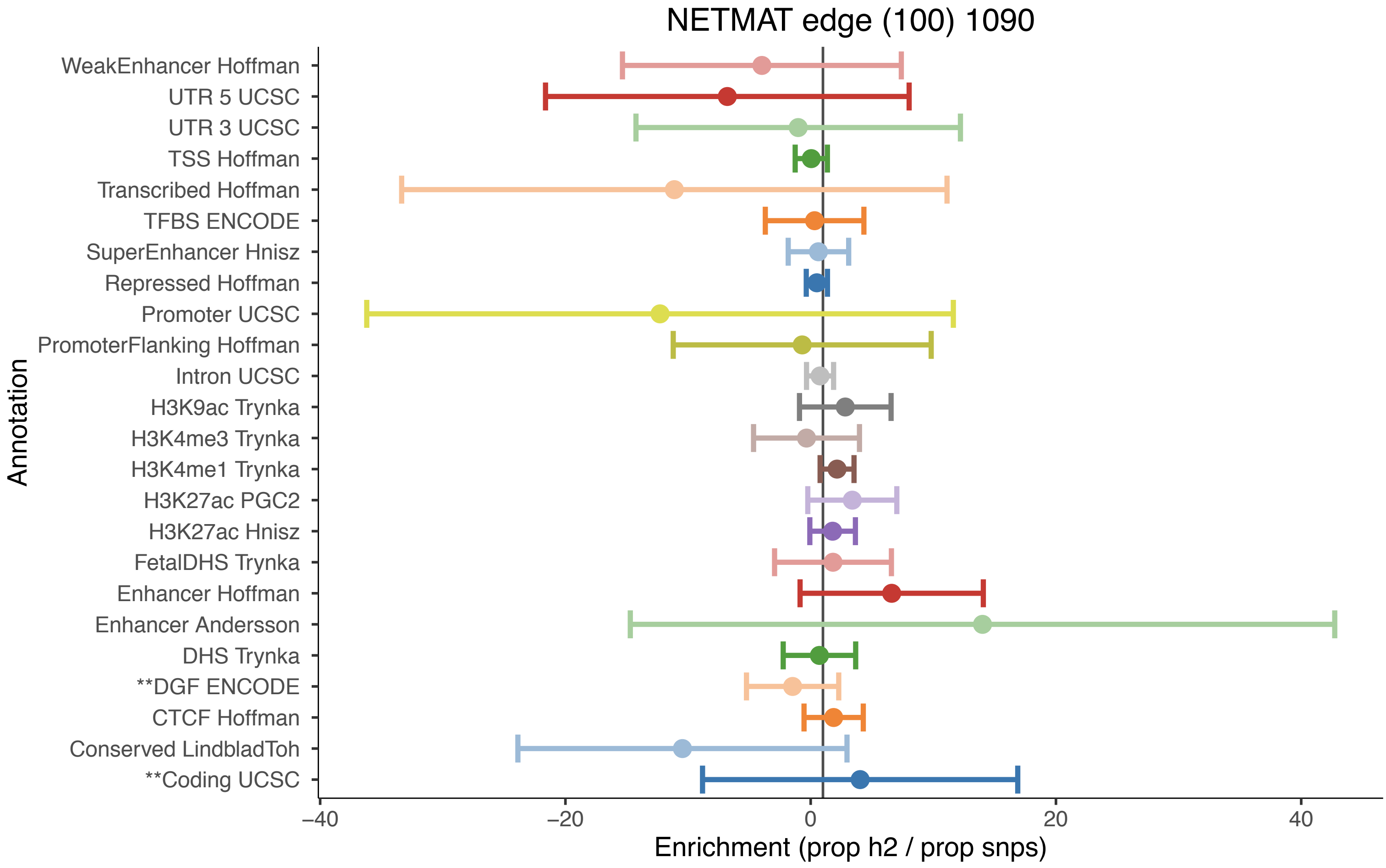
NETMAT edge (100) 1090
WeakEnhancer Hoffman
UTR 5 UCSC
UTR 3 UCSC
TSS Hoffman
Transcribed Hoffman
TFBS ENCODE
SuperEnhancer Hnisz
Repressed Hoffman
Promoter UCSC
PromoterFlanking Hoffman
Intron UCSC
H3K9ac Trynka
H3K4me3 Trynka
H3K4me1 Trynka
H3K27ac PGC2
H3K27ac Hnisz
FetalDHS Trynka
Enhancer Hoffman
Enhancer Andersson
DHS Trynka
**DGF ENCODE
CTCF Hoffman
Conserved LindbladToh
**Coding UCSC
Annotation
−40
−20
0
20
40
Enrichment (prop h2 / prop snps)

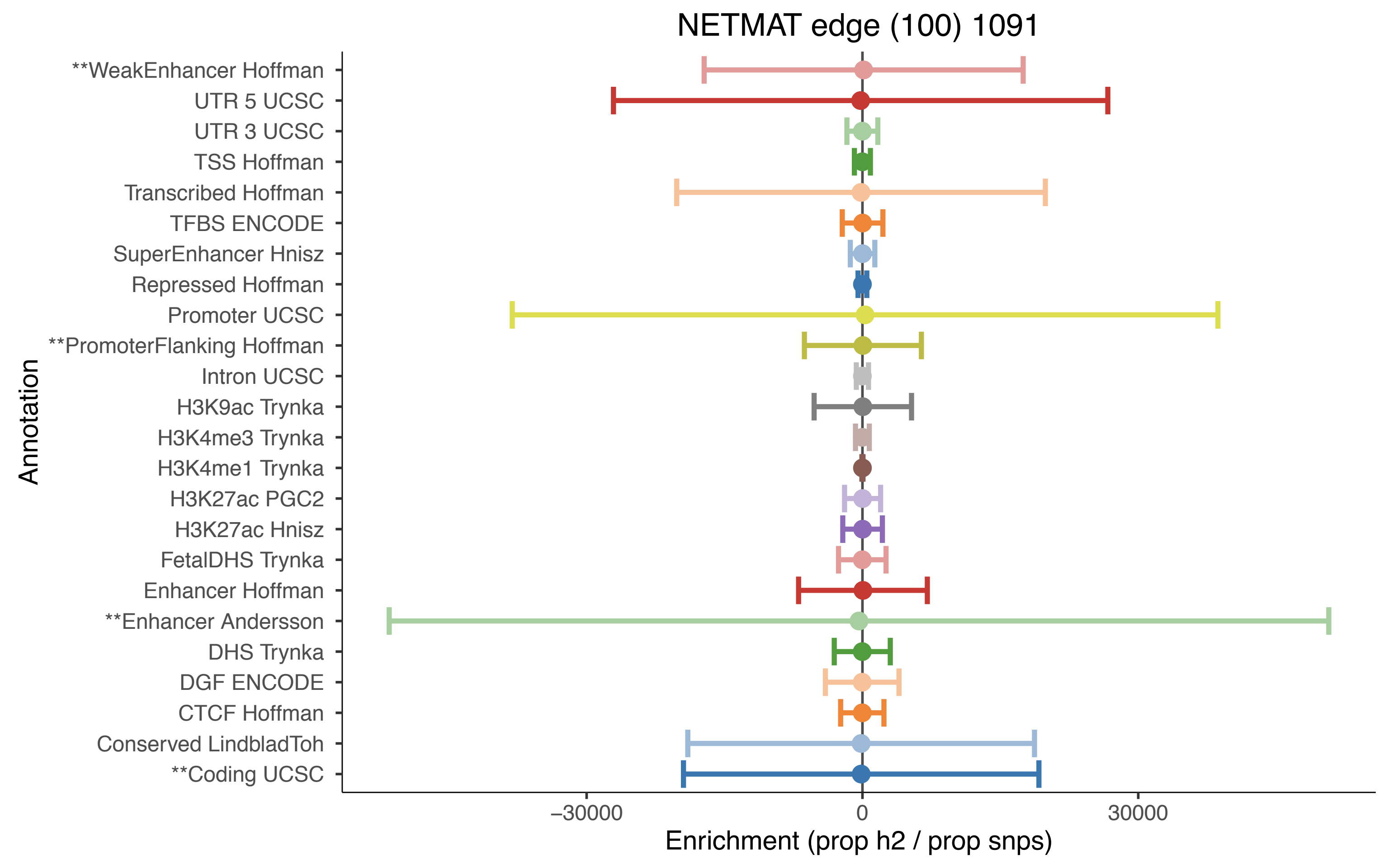
NETMAT edge (100) 1091
Annotation
**WeakEnhancer Hoffman
UTR 5 UCSC
UTR 3 UCSC
TSS Hoffman
Transcribed Hoffman
TFBS ENCODE
SuperEnhancer Hnisz
Repressed Hoffman
Promoter UCSC
**PromoterFlanking Hoffman
Intron UCSC
H3K9ac Trynka
H3K4me3 Trynka
H3K4me1 Trynka
H3K27ac PGC2
H3K27ac Hnisz
FetalDHS Trynka
Enhancer Hoffman
**Enhancer Andersson
DHS Trynka
DGF ENCODE
CTCF Hoffman
Conserved LindbladToh
**Coding UCSC
−30000
0
30000
Enrichment (prop h2 / prop snps)

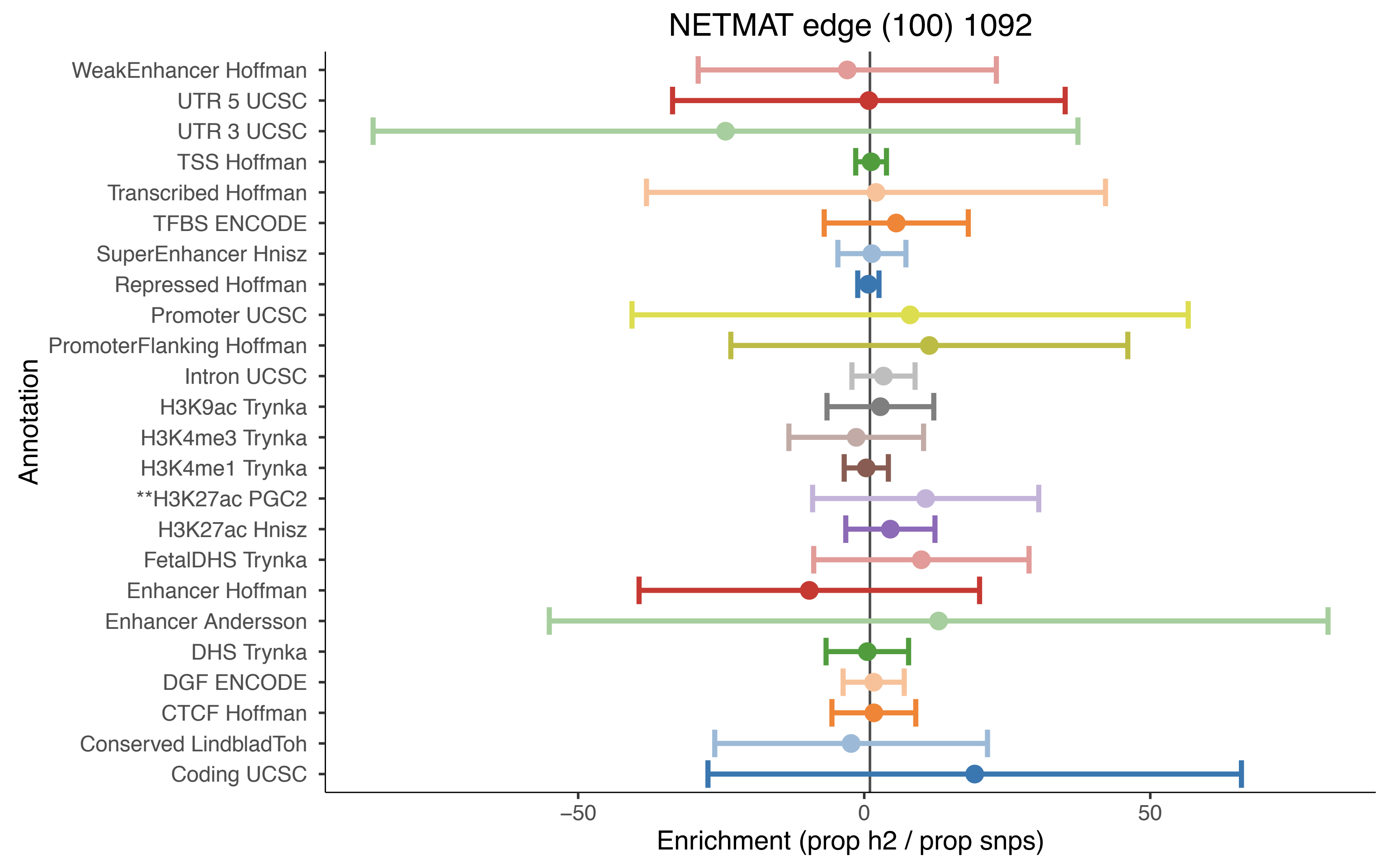

NETMAT edge (100) 1092
WeakEnhancer Hoffman
UTR 5 UCSC
UTR 3 UCSC
TSS Hoffman
Transcribed Hoffman
TFBS ENCODE
SuperEnhancer Hnisz
Repressed Hoffman
Promoter UCSC
PromoterFlanking Hoffman
Intron UCSC
H3K9ac Trynka
H3K4me3 Trynka
H3K4me1 Trynka
**H3K27ac PGC2
H3K27ac Hnisz
FetalDHS Trynka
Enhancer Hoffman
Enhancer Andersson
DHS Trynka
DGF ENCODE
CTCF Hoffman
Conserved LindbladToh
Coding UCSC
Annotation
−50
0
50
Enrichment (prop h2 / prop snps)

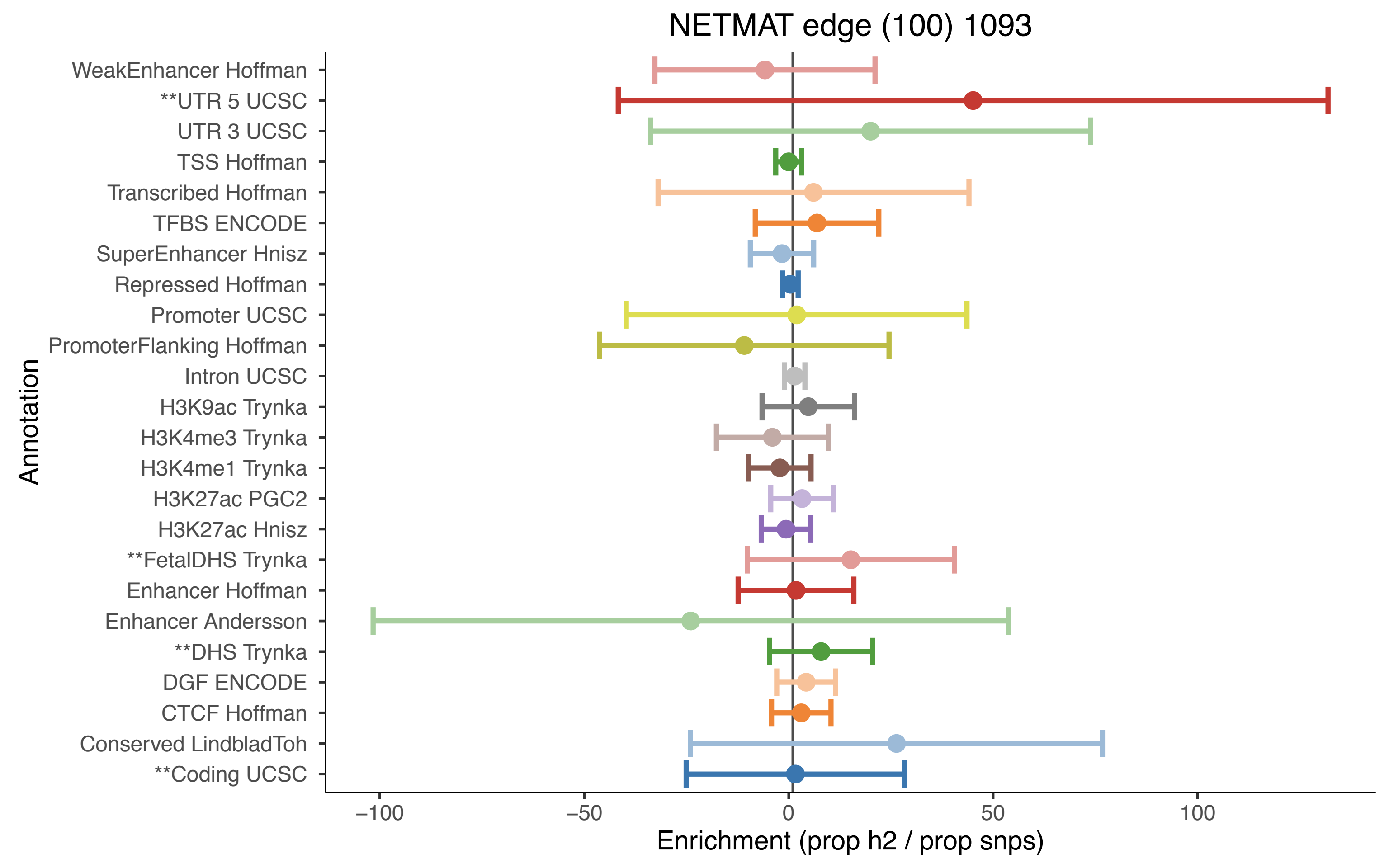
NETMAT edge (100) 1093
WeakEnhancer Hoffman
**UTR 5 UCSC
UTR 3 UCSC
TSS Hoffman
Transcribed Hoffman
TFBS ENCODE
SuperEnhancer Hnisz
Repressed Hoffman
Promoter UCSC
PromoterFlanking Hoffman
Intron UCSC
H3K9ac Trynka
H3K4me3 Trynka
H3K4me1 Trynka
H3K27ac PGC2
H3K27ac Hnisz
**FetalDHS Trynka
Enhancer Hoffman
Enhancer Andersson
**DHS Trynka
DGF ENCODE
CTCF Hoffman
Conserved LindbladToh
**Coding UCSC
Annotation
−100
−50
0
50
100
Enrichment (prop h2 / prop snps)

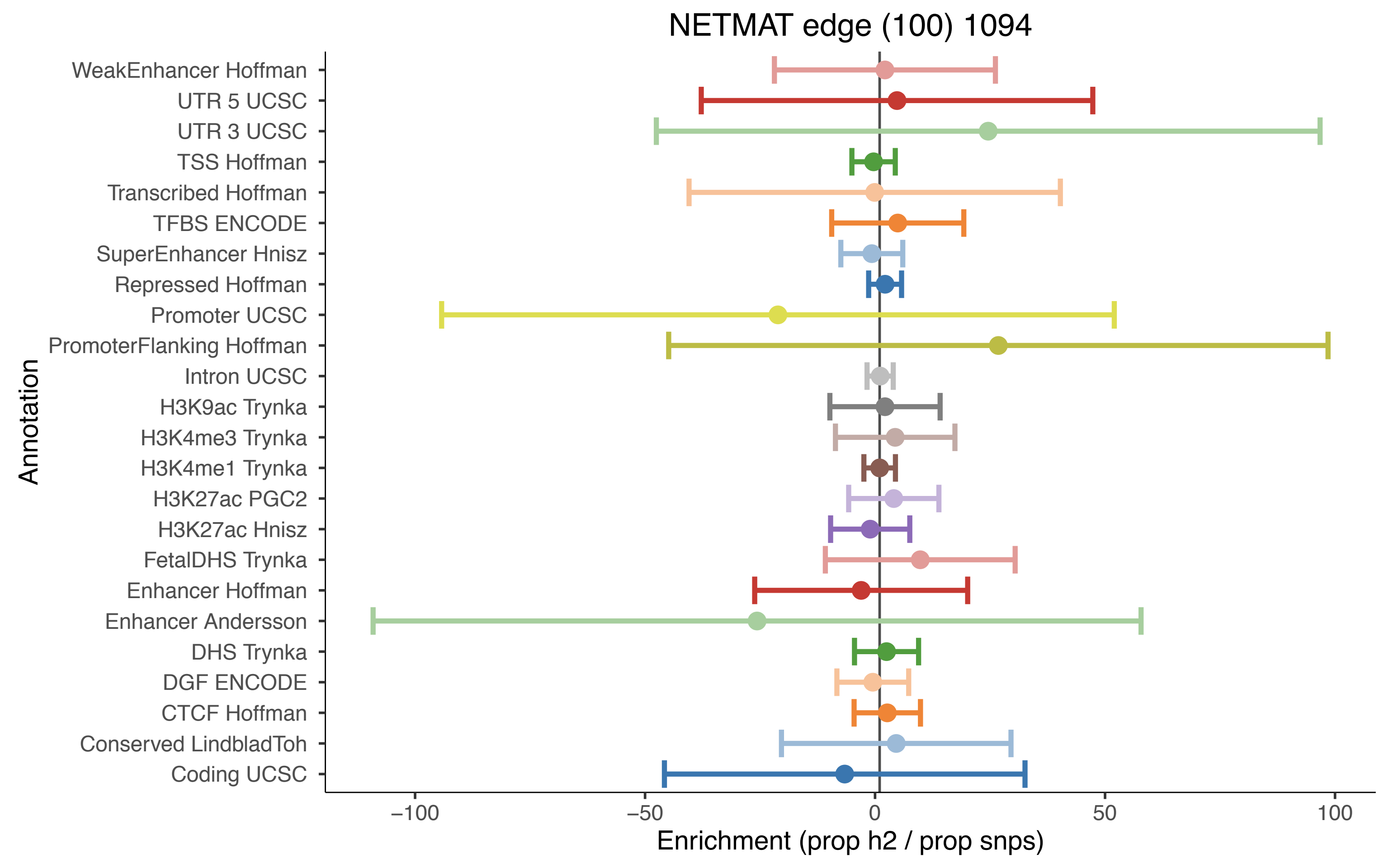

NETMAT edge (100) 1094
WeakEnhancer Hoffman
UTR 5 UCSC
UTR 3 UCSC
TSS Hoffman
Transcribed Hoffman
TFBS ENCODE
SuperEnhancer Hnisz
Repressed Hoffman
Promoter UCSC
PromoterFlanking Hoffman
Intron UCSC
H3K9ac Trynka
H3K4me3 Trynka
H3K4me1 Trynka
H3K27ac PGC2
H3K27ac Hnisz
FetalDHS Trynka
Enhancer Hoffman
Enhancer Andersson
DHS Trynka
DGF ENCODE
CTCF Hoffman
Conserved LindbladToh
Coding UCSC
Annotation
−100
−50
0
50
100
Enrichment (prop h2 / prop snps)

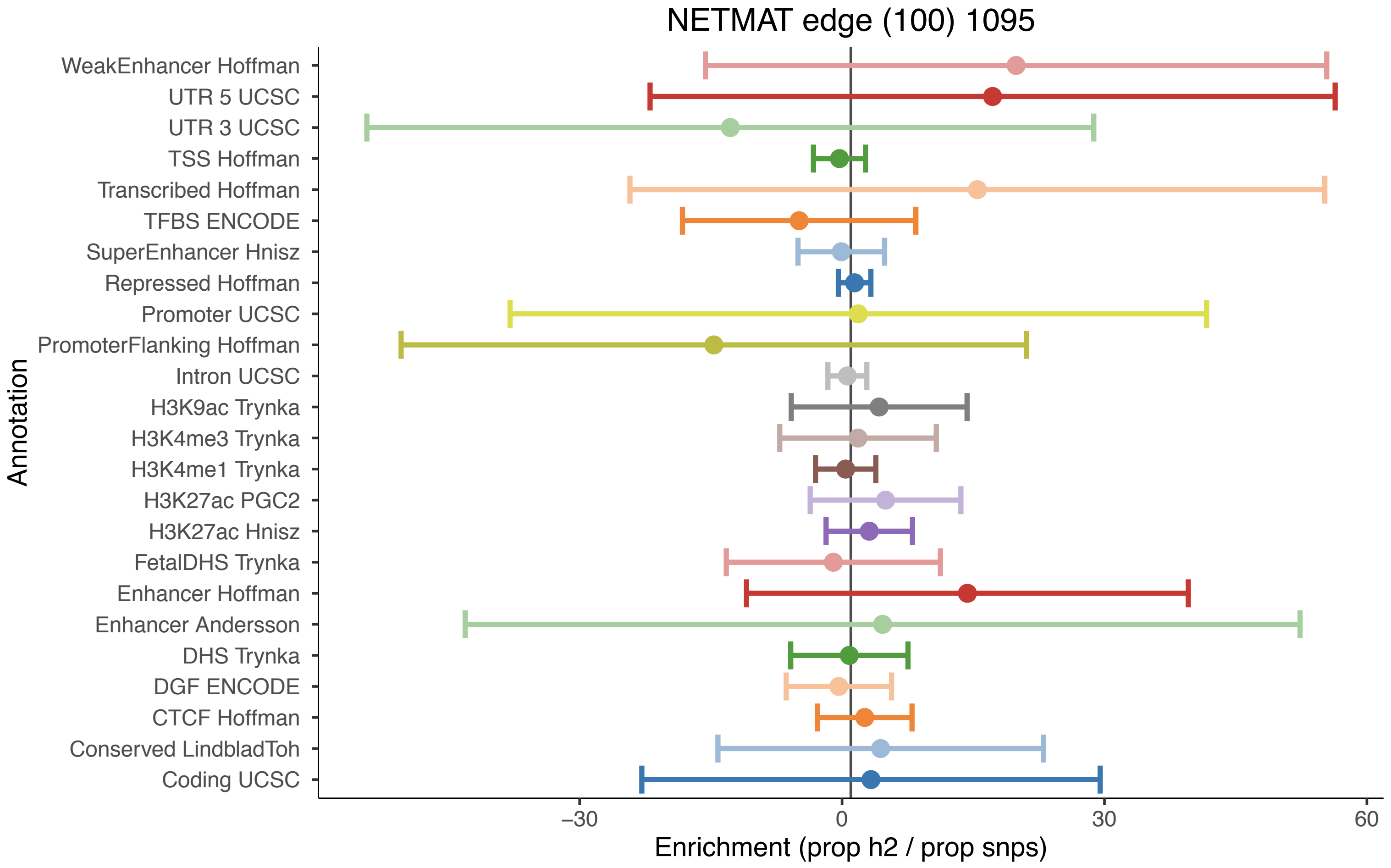
NETMAT edge (100) 1095
WeakEnhancer Hoffman
UTR 5 UCSC
UTR 3 UCSC
TSS Hoffman
Transcribed Hoffman
TFBS ENCODE
SuperEnhancer Hnisz
Repressed Hoffman
Promoter UCSC
PromoterFlanking Hoffman
Intron UCSC
H3K9ac Trynka
H3K4me3 Trynka
H3K4me1 Trynka
H3K27ac PGC2
H3K27ac Hnisz
FetalDHS Trynka
Enhancer Hoffman
Enhancer Andersson
DHS Trynka
DGF ENCODE
CTCF Hoffman
Conserved LindbladToh
Coding UCSC
Annotation
−30
0
30
60
Enrichment (prop h2 / prop snps)

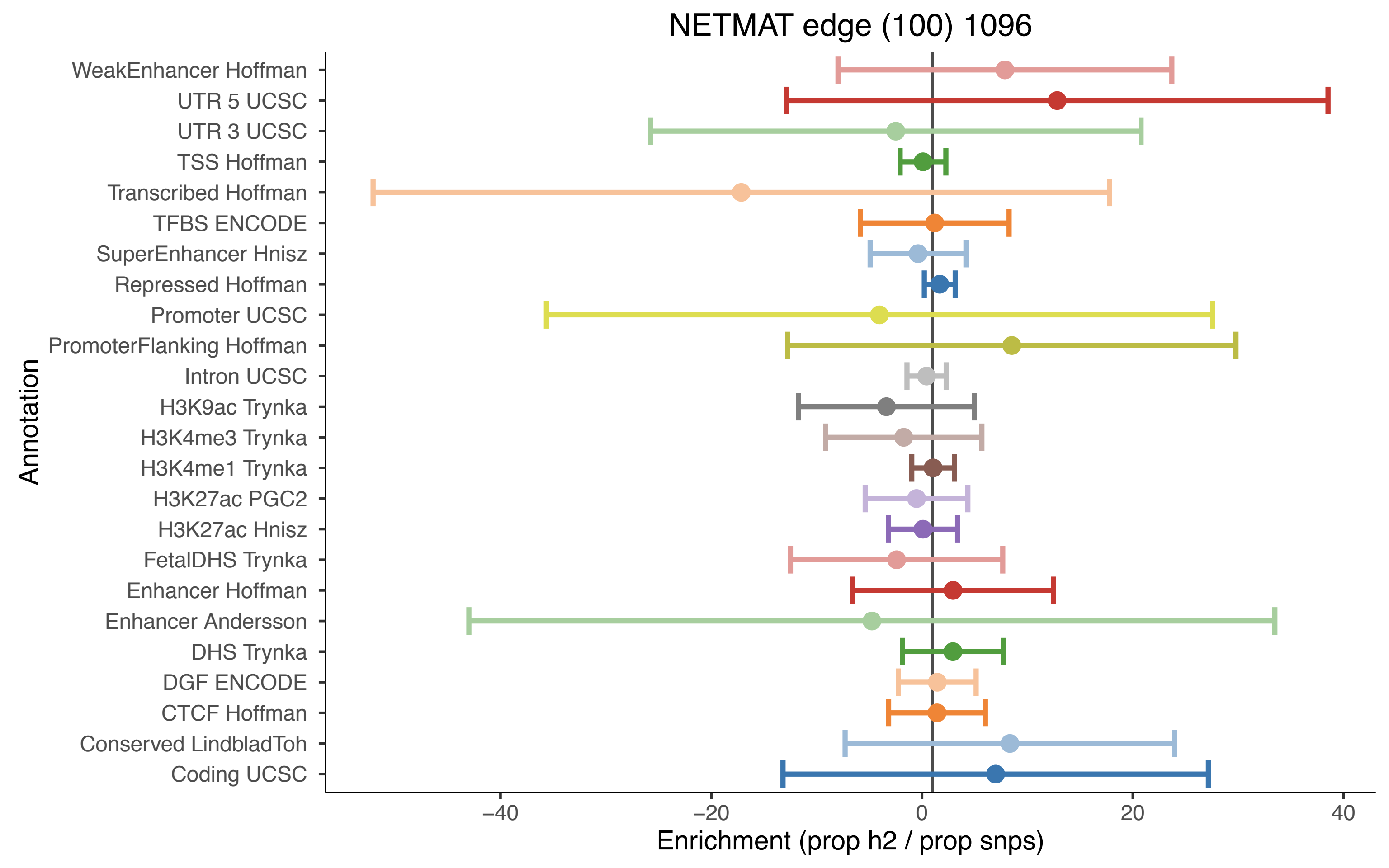
NETMAT edge (100) 1096
WeakEnhancer Hoffman
UTR 5 UCSC
UTR 3 UCSC
TSS Hoffman
Transcribed Hoffman
TFBS ENCODE
SuperEnhancer Hnisz
Repressed Hoffman
Promoter UCSC
PromoterFlanking Hoffman
Intron UCSC
H3K9ac Trynka
H3K4me3 Trynka
H3K4me1 Trynka
H3K27ac PGC2
H3K27ac Hnisz
FetalDHS Trynka
Enhancer Hoffman
Enhancer Andersson
DHS Trynka
DGF ENCODE
CTCF Hoffman
Conserved LindbladToh
Coding UCSC
Annotation
−40
−20
0
20
40
Enrichment (prop h2 / prop snps)

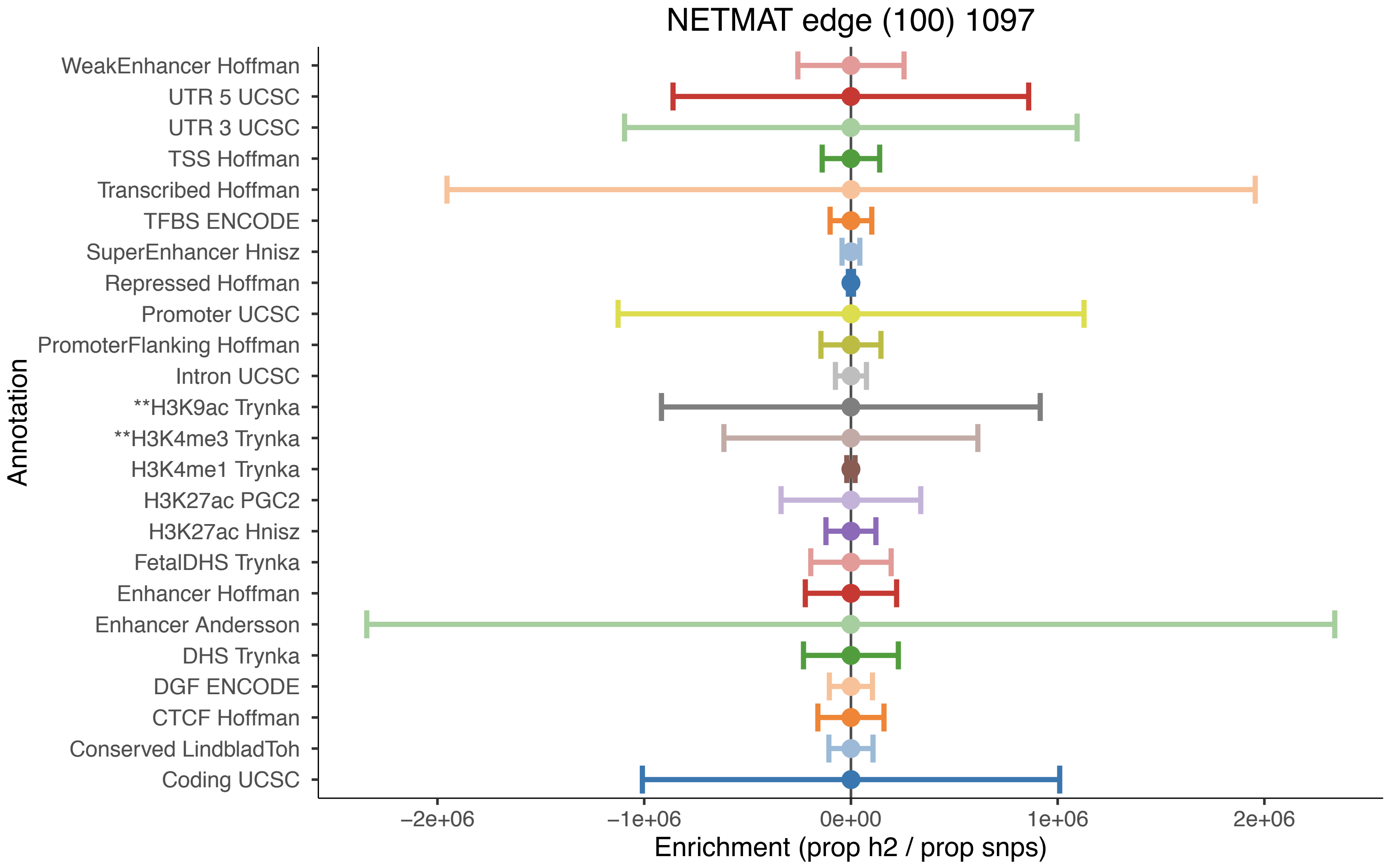
NETMAT edge (100) 1097
WeakEnhancer Hoffman
UTR 5 UCSC
UTR 3 UCSC
TSS Hoffman
Transcribed Hoffman
TFBS ENCODE
SuperEnhancer Hnisz
Repressed Hoffman
Promoter UCSC
PromoterFlanking Hoffman
Intron UCSC
**H3K9ac Trynka
**H3K4me3 Trynka
H3K4me1 Trynka
H3K27ac PGC2
H3K27ac Hnisz
FetalDHS Trynka
Enhancer Hoffman
Enhancer Andersson
DHS Trynka
DGF ENCODE
CTCF Hoffman
Conserved LindbladToh
Coding UCSC
Annotation
−2e+06
−1e+06
0e+00
1e+06
2e+06
Enrichment (prop h2 / prop snps)

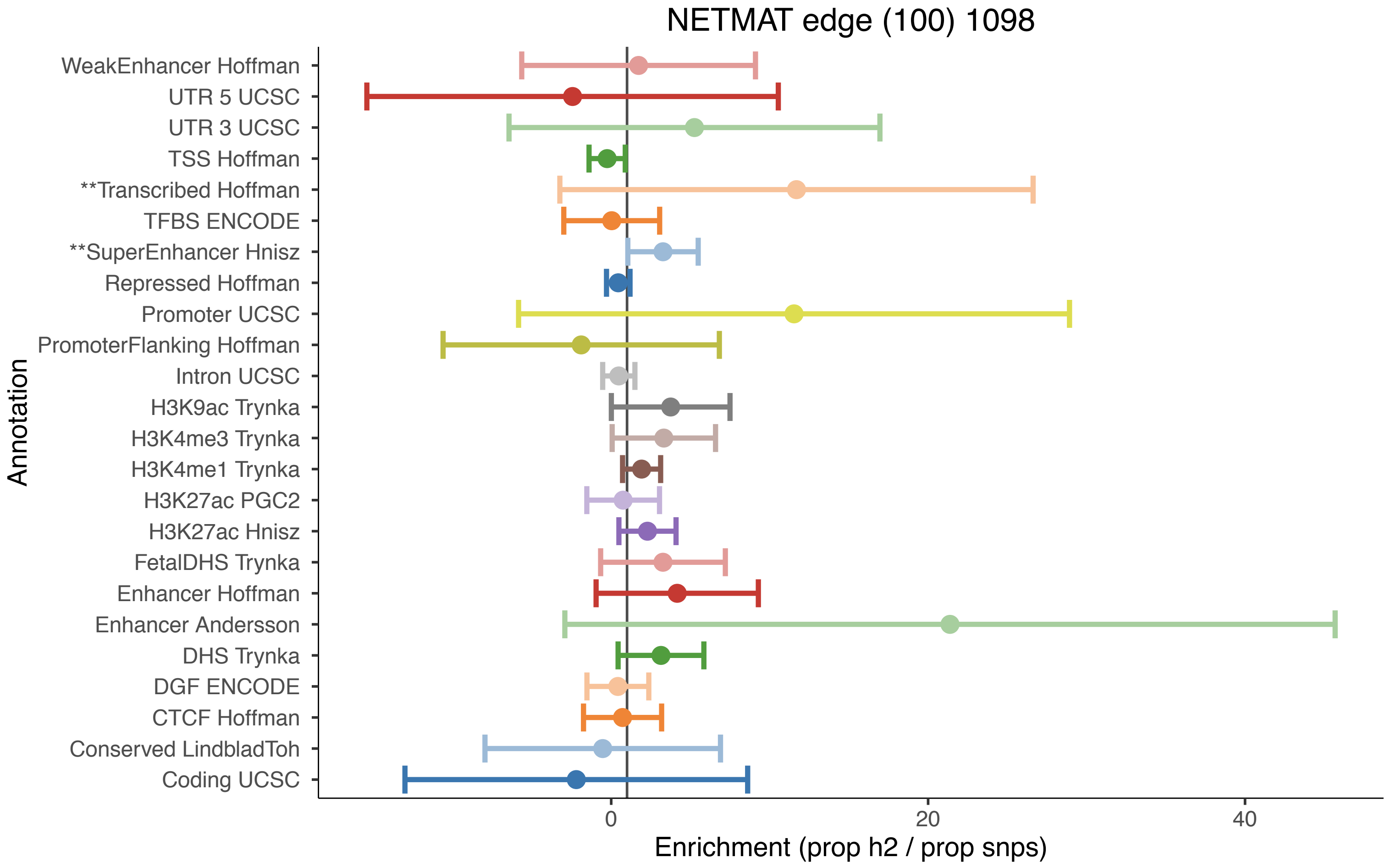
NETMAT edge (100) 1098
WeakEnhancer Hoffman
UTR 5 UCSC
UTR 3 UCSC
TSS Hoffman
**Transcribed Hoffman
TFBS ENCODE
**SuperEnhancer Hnisz
Repressed Hoffman
Promoter UCSC
PromoterFlanking Hoffman
Intron UCSC
H3K9ac Trynka
H3K4me3 Trynka
H3K4me1 Trynka
H3K27ac PGC2
H3K27ac Hnisz
FetalDHS Trynka
Enhancer Hoffman
Enhancer Andersson
DHS Trynka
DGF ENCODE
CTCF Hoffman
Conserved LindbladToh
Coding UCSC
Annotation
0
20
40
Enrichment (prop h2 / prop snps)

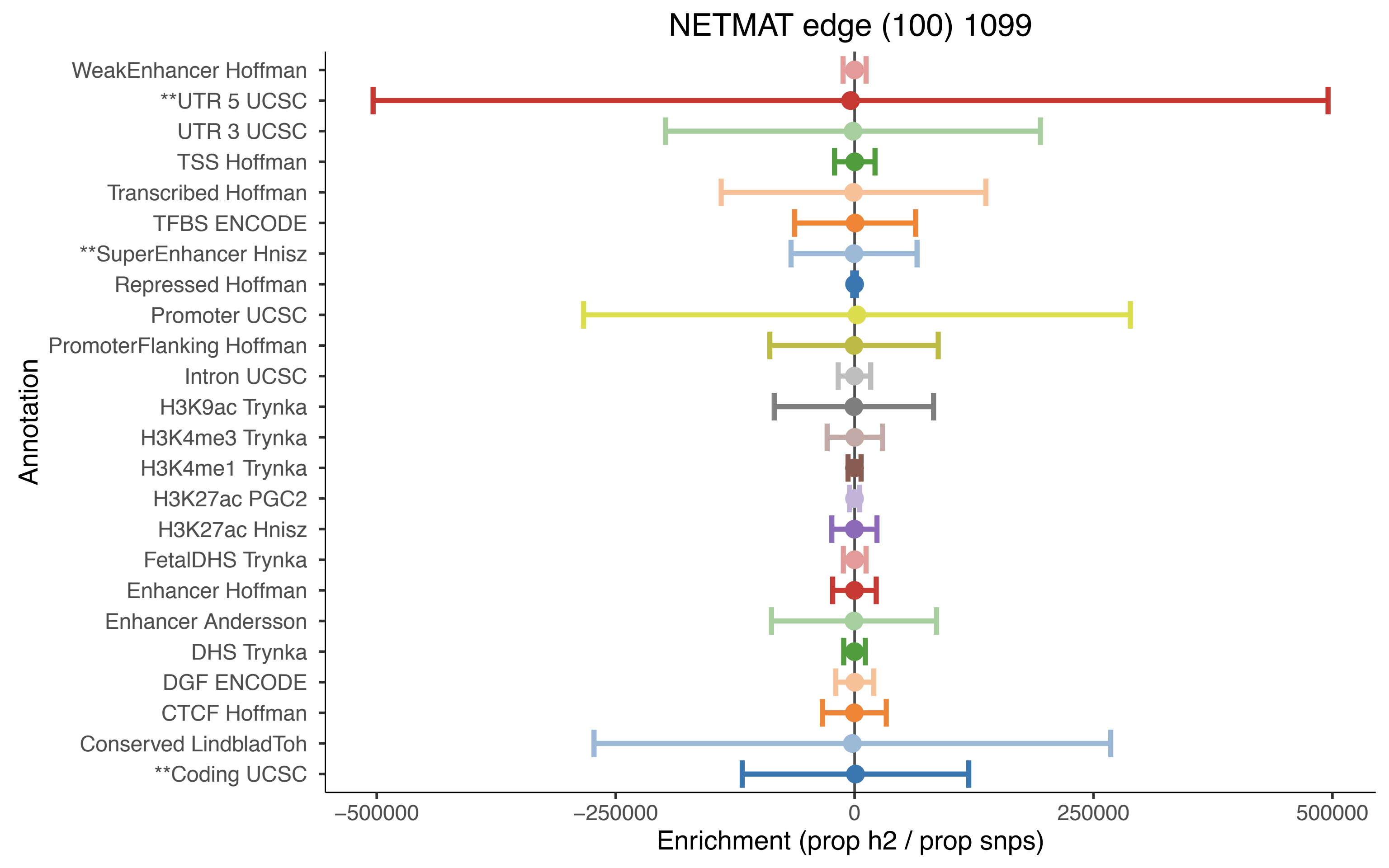
NETMAT edge (100) 1099
WeakEnhancer Hoffman
**UTR 5 UCSC
UTR 3 UCSC
TSS Hoffman
Transcribed Hoffman
TFBS ENCODE
**SuperEnhancer Hnisz
Repressed Hoffman
Promoter UCSC
PromoterFlanking Hoffman
Intron UCSC
H3K9ac Trynka
H3K4me3 Trynka
H3K4me1 Trynka
H3K27ac PGC2
H3K27ac Hnisz
FetalDHS Trynka
Enhancer Hoffman
Enhancer Andersson
DHS Trynka
DGF ENCODE
CTCF Hoffman
Conserved LindbladToh
**Coding UCSC
Annotation
−500000
−250000
0
250000
500000
Enrichment (prop h2 / prop snps)

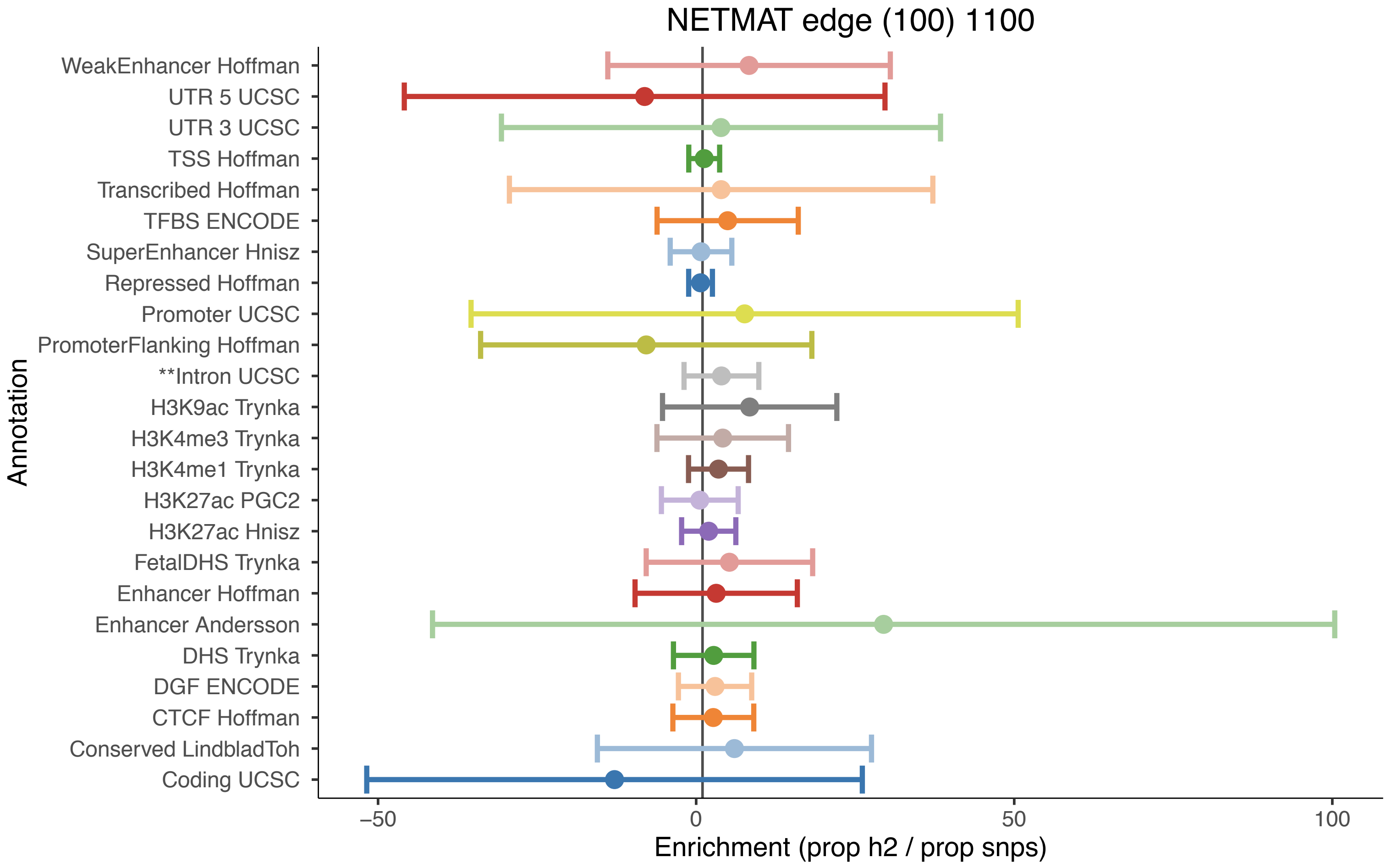

NETMAT edge (100) 1100
WeakEnhancer Hoffman
UTR 5 UCSC
UTR 3 UCSC
TSS Hoffman
Transcribed Hoffman
TFBS ENCODE
SuperEnhancer Hnisz
Repressed Hoffman
Promoter UCSC
PromoterFlanking Hoffman
**Intron UCSC
H3K9ac Trynka
H3K4me3 Trynka
H3K4me1 Trynka
H3K27ac PGC2
H3K27ac Hnisz
FetalDHS Trynka
Enhancer Hoffman
Enhancer Andersson
DHS Trynka
DGF ENCODE
CTCF Hoffman
Conserved LindbladToh
Coding UCSC
Annotation
−50
0
50
100
Enrichment (prop h2 / prop snps)

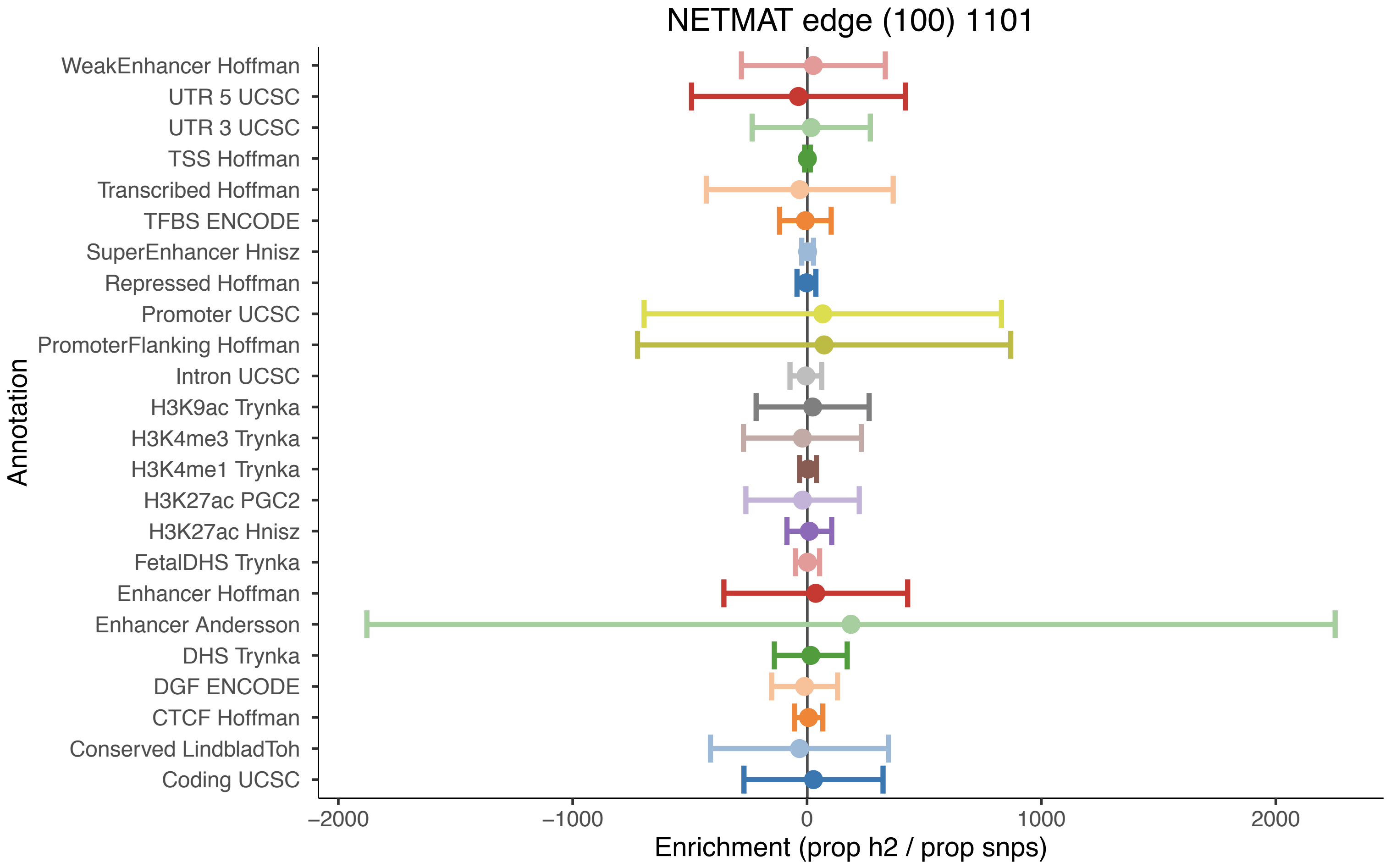
NETMAT edge (100) 1101
WeakEnhancer Hoffman
UTR 5 UCSC
UTR 3 UCSC
TSS Hoffman
Transcribed Hoffman
TFBS ENCODE
SuperEnhancer Hnisz
Repressed Hoffman
Promoter UCSC
PromoterFlanking Hoffman
Intron UCSC
H3K9ac Trynka
H3K4me3 Trynka
H3K4me1 Trynka
H3K27ac PGC2
H3K27ac Hnisz
FetalDHS Trynka
Enhancer Hoffman
Enhancer Andersson
DHS Trynka
DGF ENCODE
CTCF Hoffman
Conserved LindbladToh
Coding UCSC
Annotation
−2000
−1000
0
1000
2000
Enrichment (prop h2 / prop snps)

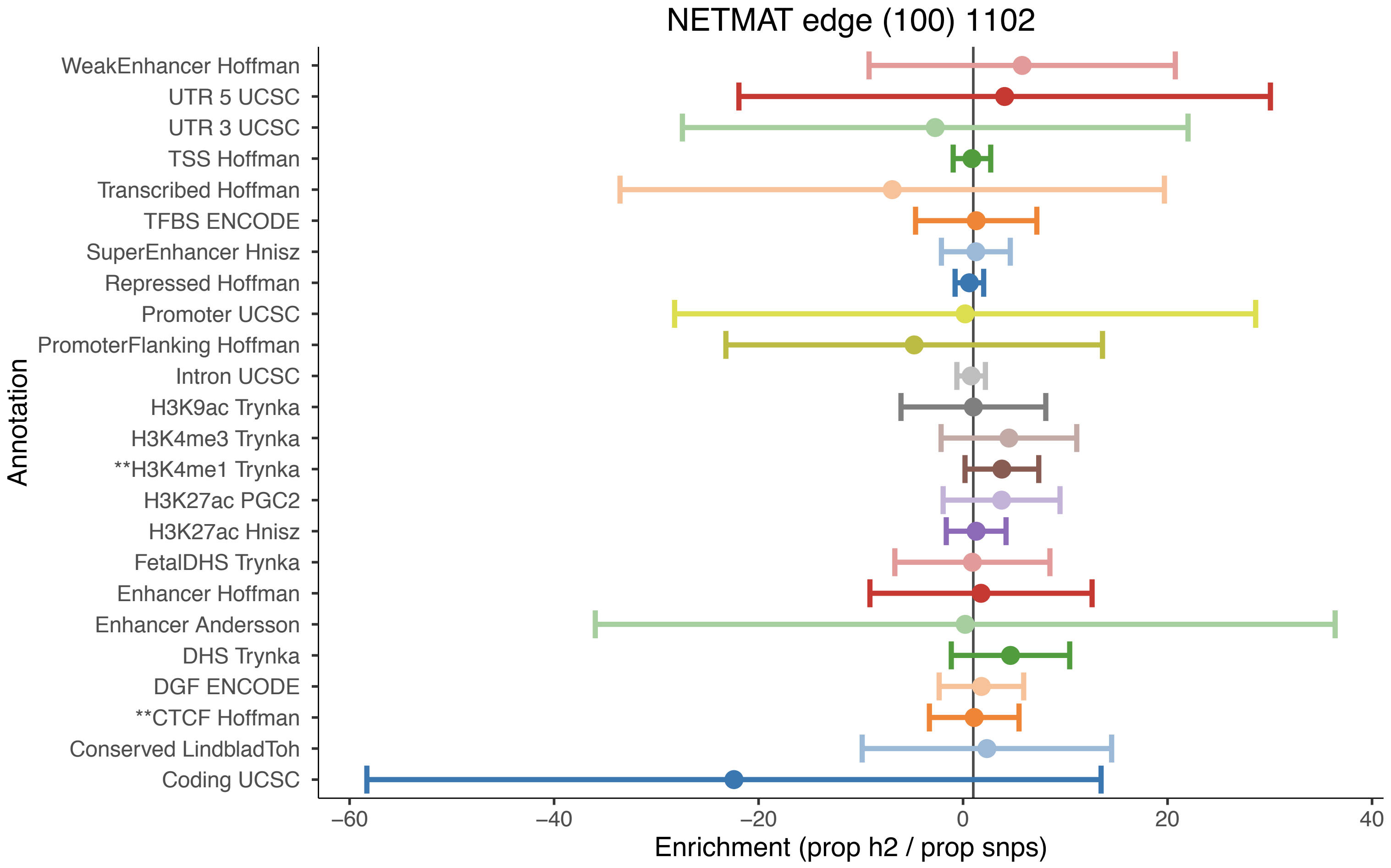

NETMAT edge (100) 1102
WeakEnhancer Hoffman
UTR 5 UCSC
UTR 3 UCSC
TSS Hoffman
Transcribed Hoffman
TFBS ENCODE
SuperEnhancer Hnisz
Repressed Hoffman
Promoter UCSC
PromoterFlanking Hoffman
Intron UCSC
H3K9ac Trynka
H3K4me3 Trynka
**H3K4me1 Trynka
H3K27ac PGC2
H3K27ac Hnisz
FetalDHS Trynka
Enhancer Hoffman
Enhancer Andersson
DHS Trynka
DGF ENCODE
**CTCF Hoffman
Conserved LindbladToh
Coding UCSC
Annotation
−60
−40
−20
0
20
40
Enrichment (prop h2 / prop snps)

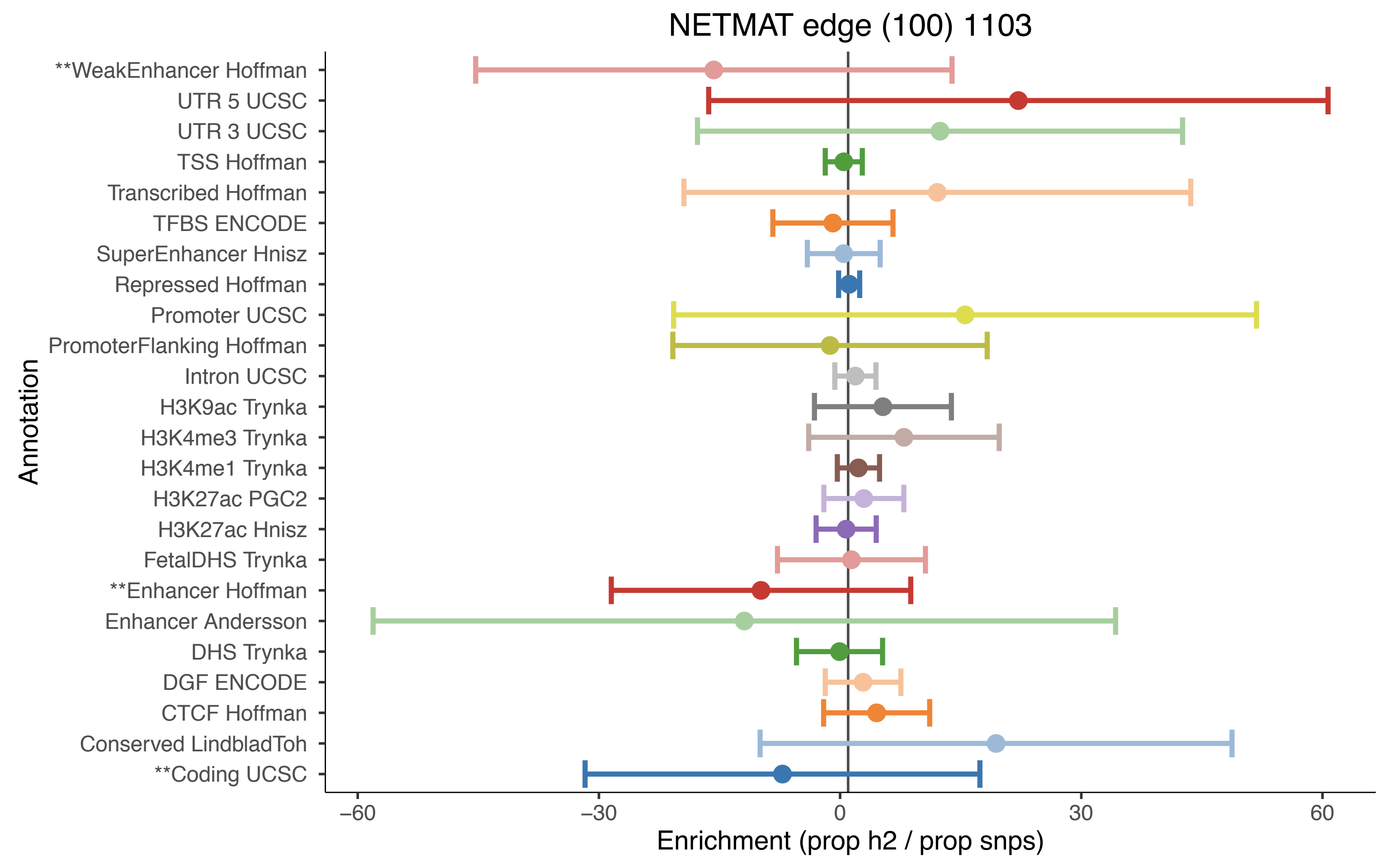
NETMAT edge (100) 1103
**WeakEnhancer Hoffman
UTR 5 UCSC
UTR 3 UCSC
TSS Hoffman
Transcribed Hoffman
TFBS ENCODE
SuperEnhancer Hnisz
Repressed Hoffman
Promoter UCSC
PromoterFlanking Hoffman
Intron UCSC
H3K9ac Trynka
H3K4me3 Trynka
H3K4me1 Trynka
H3K27ac PGC2
H3K27ac Hnisz
FetalDHS Trynka
**Enhancer Hoffman
Enhancer Andersson
DHS Trynka
DGF ENCODE
CTCF Hoffman
Conserved LindbladToh
**Coding UCSC
Annotation
−60
−30
0
30
60
Enrichment (prop h2 / prop snps)

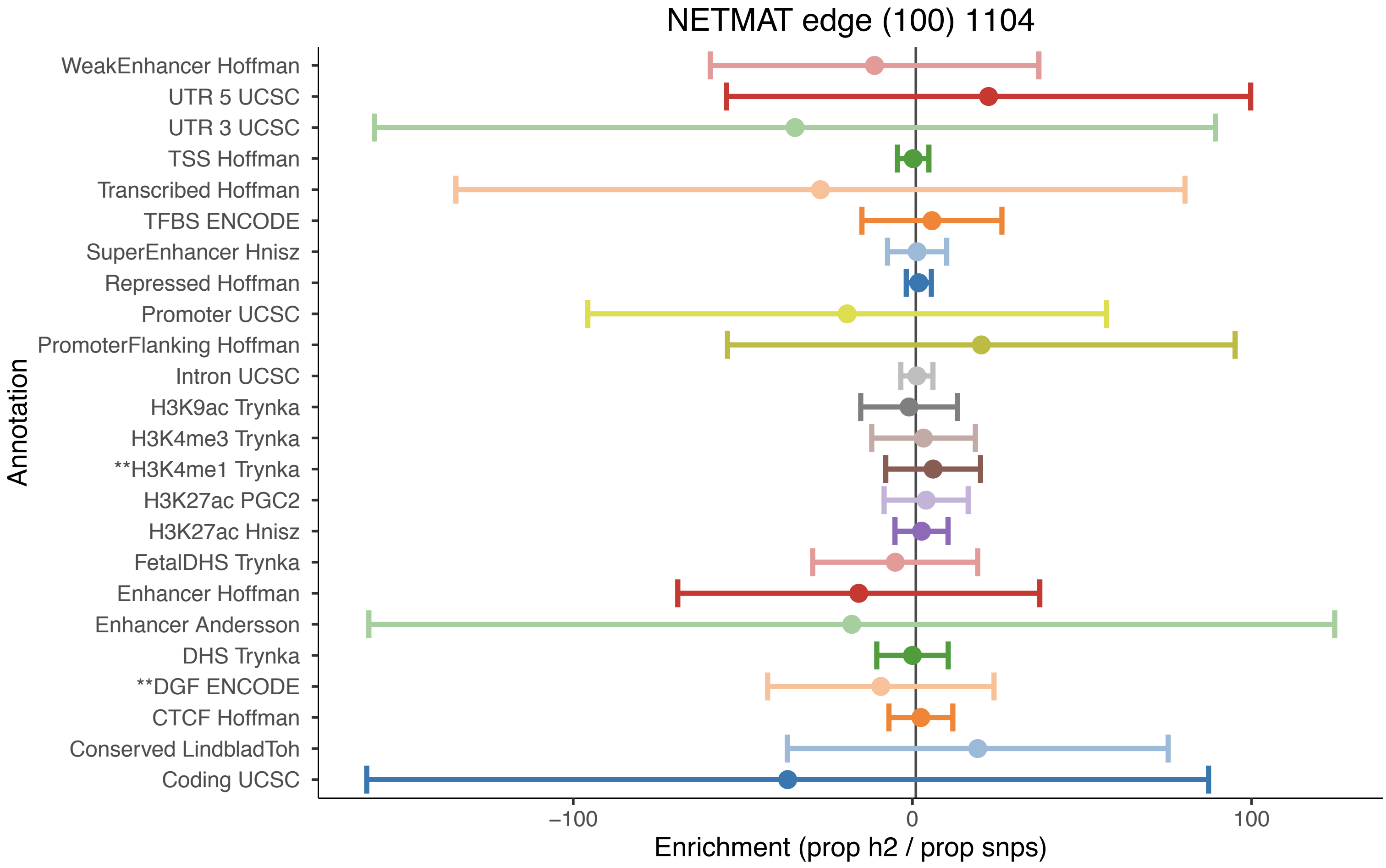

NETMAT edge (100) 1104
WeakEnhancer Hoffman
UTR 5 UCSC
UTR 3 UCSC
TSS Hoffman
Transcribed Hoffman
TFBS ENCODE
SuperEnhancer Hnisz
Repressed Hoffman
Promoter UCSC
PromoterFlanking Hoffman
Intron UCSC
H3K9ac Trynka
H3K4me3 Trynka
**H3K4me1 Trynka
H3K27ac PGC2
H3K27ac Hnisz
FetalDHS Trynka
Enhancer Hoffman
Enhancer Andersson
DHS Trynka
**DGF ENCODE
CTCF Hoffman
Conserved LindbladToh
Coding UCSC
Annotation
−100
0
100
Enrichment (prop h2 / prop snps)

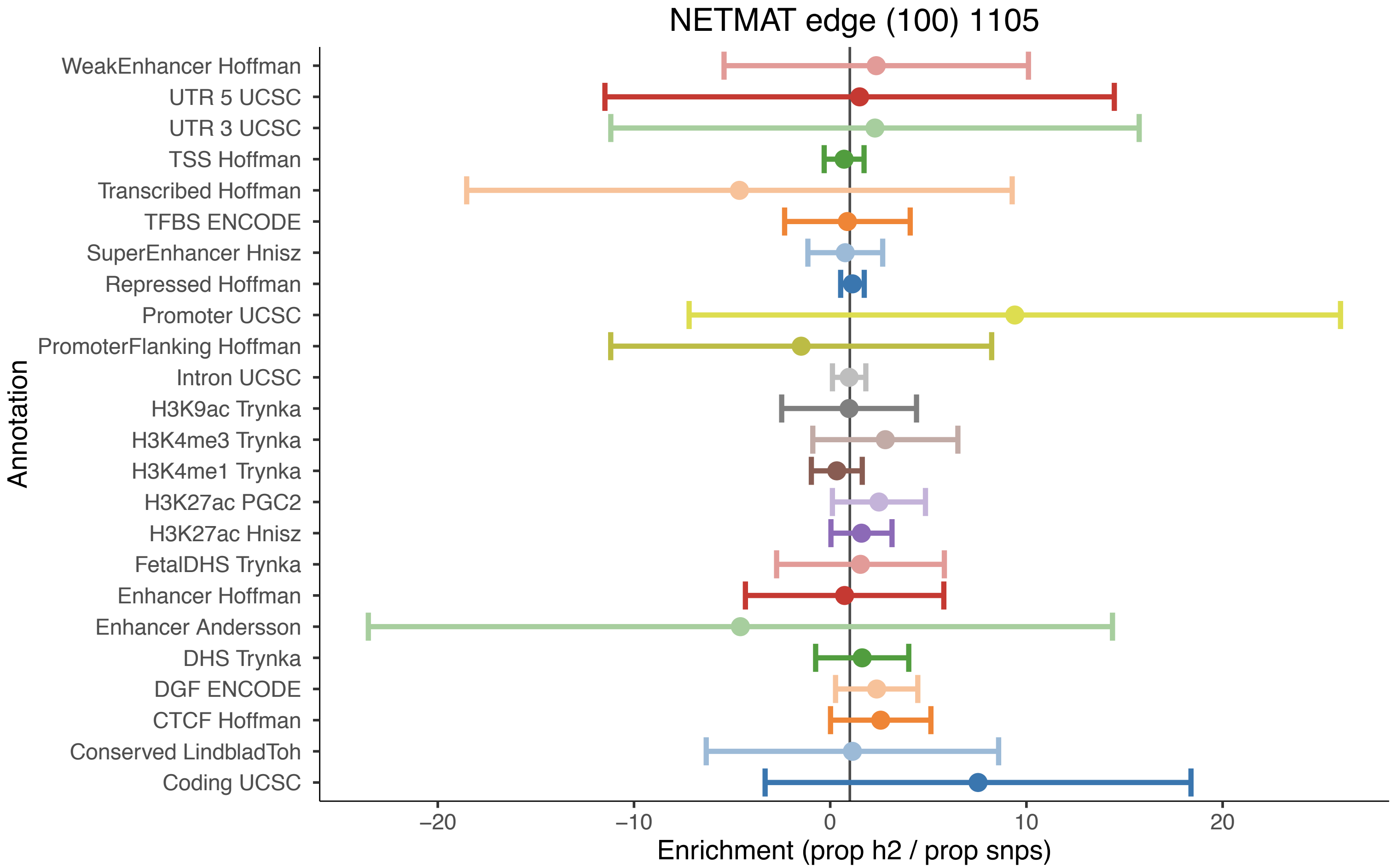
NETMAT edge (100) 1105
Annotation
WeakEnhancer Hoffman
UTR 5 UCSC
UTR 3 UCSC
TSS Hoffman
Transcribed Hoffman
TFBS ENCODE
SuperEnhancer Hnisz
Repressed Hoffman
Promoter UCSC
PromoterFlanking Hoffman
Intron UCSC
H3K9ac Trynka
H3K4me3 Trynka
H3K4me1 Trynka
H3K27ac PGC2
H3K27ac Hnisz
FetalDHS Trynka
Enhancer Hoffman
Enhancer Andersson
DHS Trynka
DGF ENCODE
CTCF Hoffman
Conserved LindbladToh
Coding UCSC
−20
−10
0
10
20
Enrichment (prop h2 / prop snps)

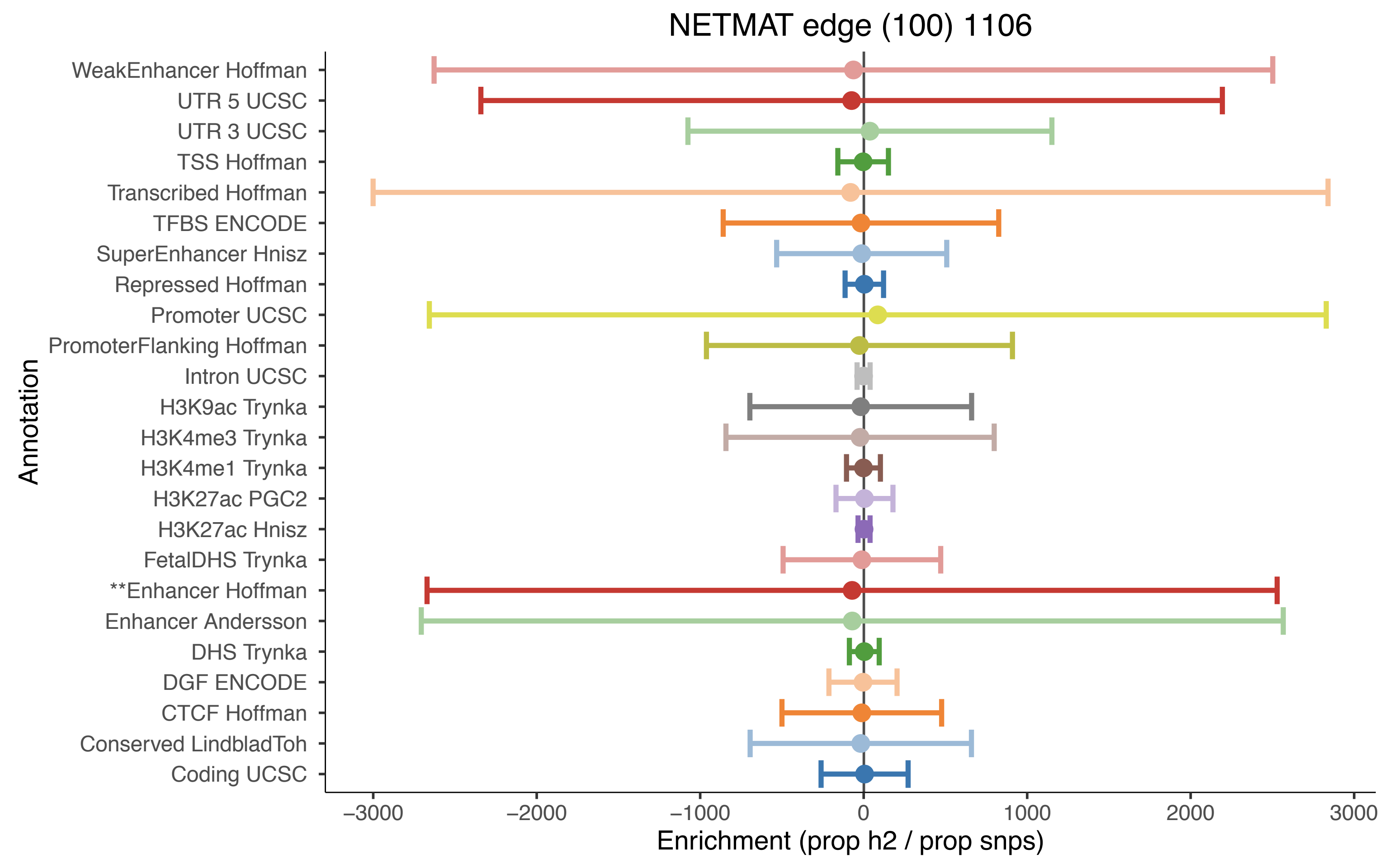

NETMAT edge (100) 1106
Annotation
WeakEnhancer Hoffman
UTR 5 UCSC
UTR 3 UCSC
TSS Hoffman
Transcribed Hoffman
TFBS ENCODE
SuperEnhancer Hnisz
Repressed Hoffman
Promoter UCSC
PromoterFlanking Hoffman
Intron UCSC
H3K9ac Trynka
H3K4me3 Trynka
H3K4me1 Trynka
H3K27ac PGC2
H3K27ac Hnisz
FetalDHS Trynka
**Enhancer Hoffman
Enhancer Andersson
DHS Trynka
DGF ENCODE
CTCF Hoffman
Conserved LindbladToh
Coding UCSC
−3000
−2000
−1000
0
1000
2000
3000
Enrichment (prop h2 / prop snps)

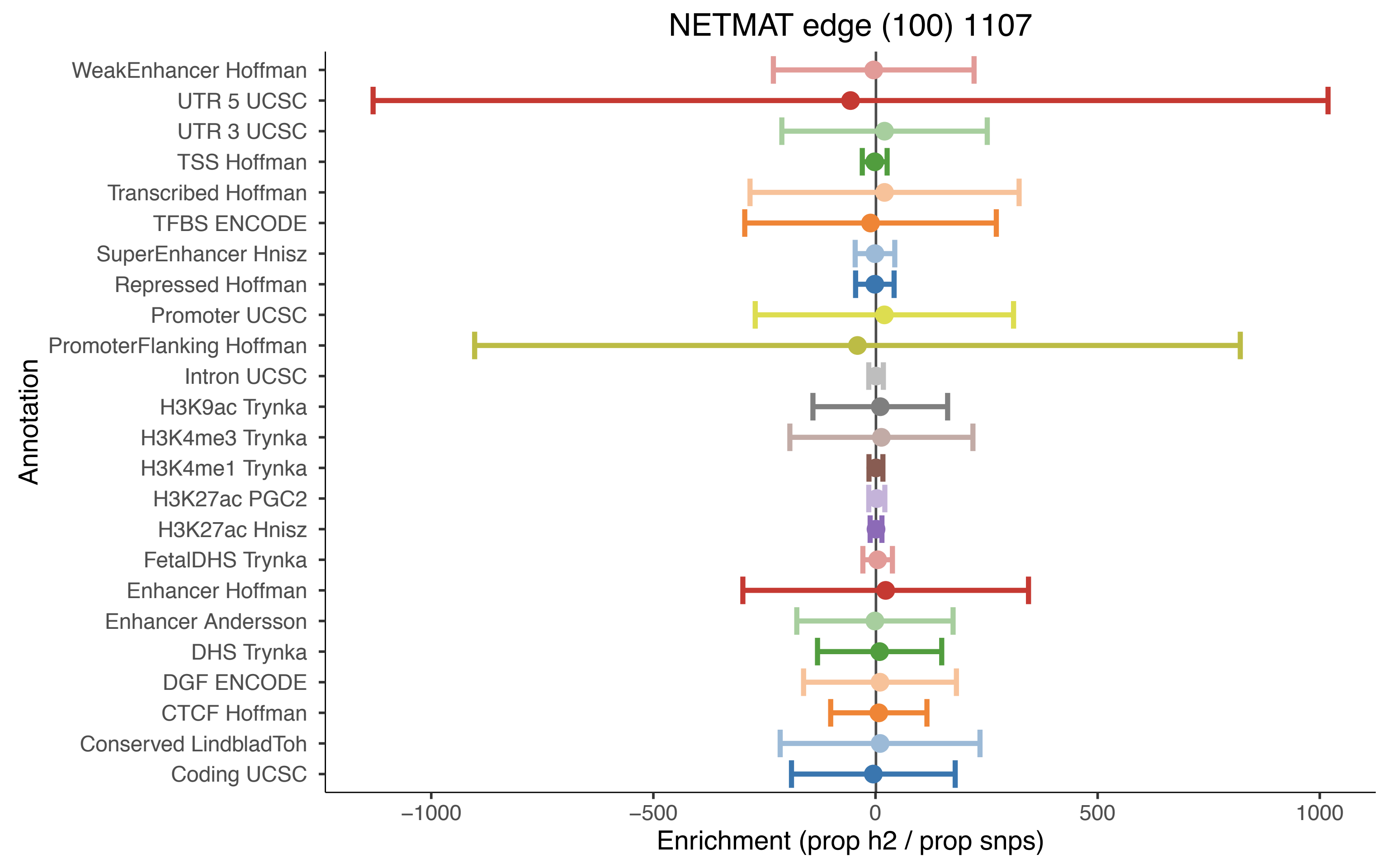

NETMAT edge (100) 1107
WeakEnhancer Hoffman
UTR 5 UCSC
UTR 3 UCSC
TSS Hoffman
Transcribed Hoffman
TFBS ENCODE
SuperEnhancer Hnisz
Repressed Hoffman
Promoter UCSC
PromoterFlanking Hoffman
Intron UCSC
H3K9ac Trynka
H3K4me3 Trynka
H3K4me1 Trynka
H3K27ac PGC2
H3K27ac Hnisz
FetalDHS Trynka
Enhancer Hoffman
Enhancer Andersson
DHS Trynka
DGF ENCODE
CTCF Hoffman
Conserved LindbladToh
Coding UCSC
Annotation
−1000
−500
0
500
1000
Enrichment (prop h2 / prop snps)

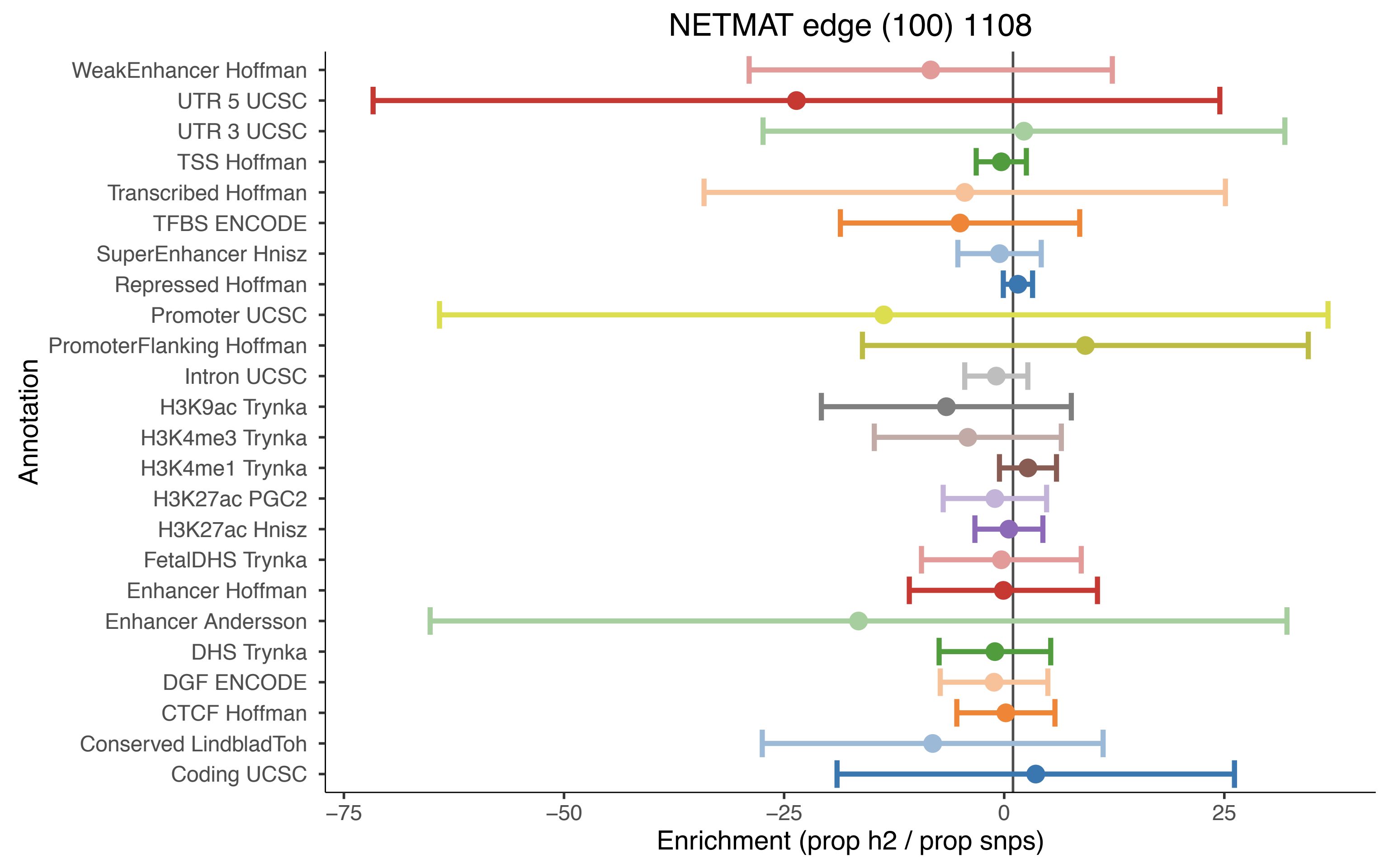
NETMAT edge (100) 1108
WeakEnhancer Hoffman
UTR 5 UCSC
UTR 3 UCSC
TSS Hoffman
Transcribed Hoffman
TFBS ENCODE
SuperEnhancer Hnisz
Repressed Hoffman
Promoter UCSC
PromoterFlanking Hoffman
Intron UCSC
H3K9ac Trynka
H3K4me3 Trynka
H3K4me1 Trynka
H3K27ac PGC2
H3K27ac Hnisz
FetalDHS Trynka
Enhancer Hoffman
Enhancer Andersson
DHS Trynka
DGF ENCODE
CTCF Hoffman
Conserved LindbladToh
Coding UCSC
Annotation
−75
−50
−25
0
25
Enrichment (prop h2 / prop snps)

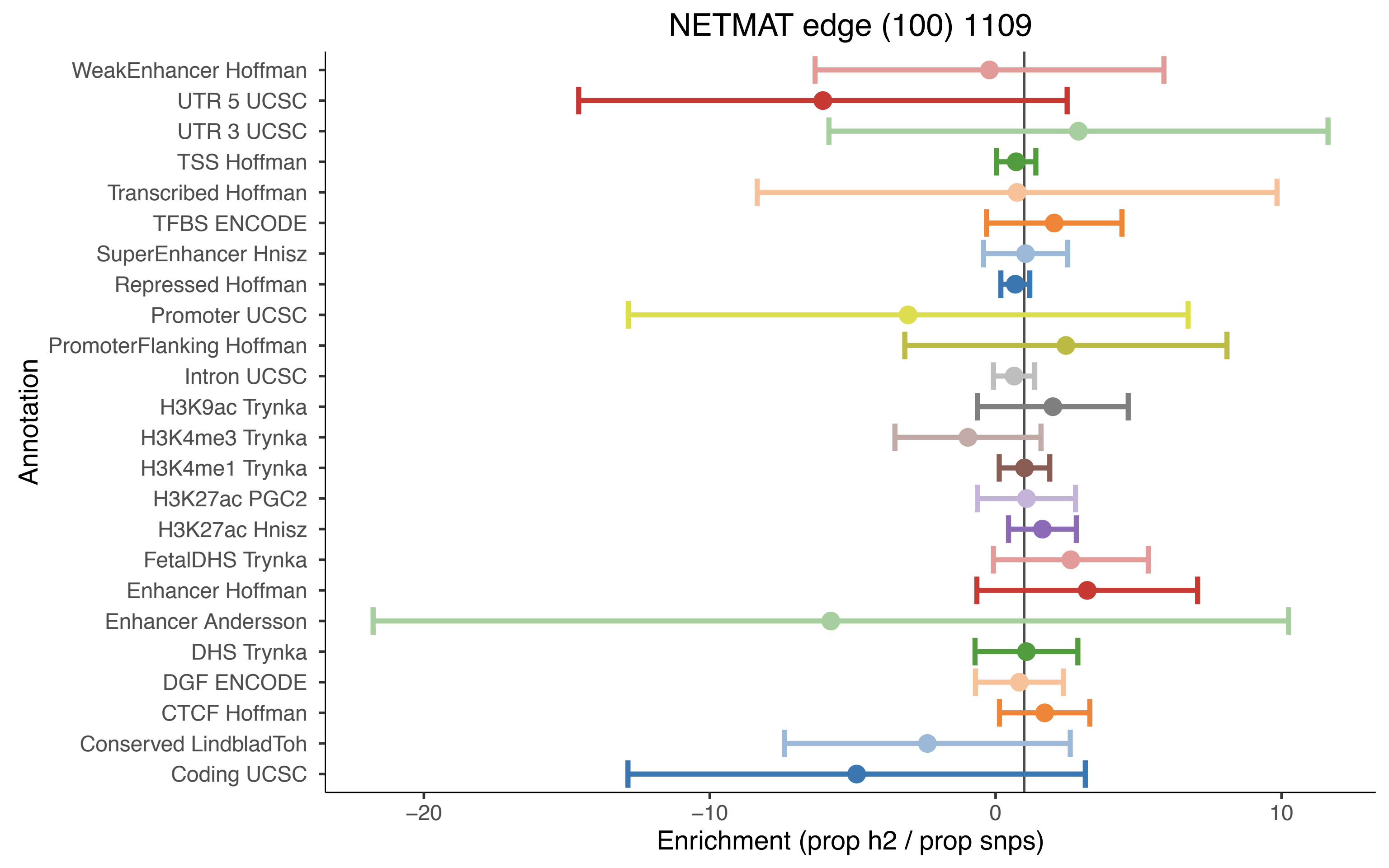

NETMAT edge (100) 1109
Annotation
WeakEnhancer Hoffman
UTR 5 UCSC
UTR 3 UCSC
TSS Hoffman
Transcribed Hoffman
TFBS ENCODE
SuperEnhancer Hnisz
Repressed Hoffman
Promoter UCSC
PromoterFlanking Hoffman
Intron UCSC
H3K9ac Trynka
H3K4me3 Trynka
H3K4me1 Trynka
H3K27ac PGC2
H3K27ac Hnisz
FetalDHS Trynka
Enhancer Hoffman
Enhancer Andersson
DHS Trynka
DGF ENCODE
CTCF Hoffman
Conserved LindbladToh
Coding UCSC
−20
−10
0
10
Enrichment (prop h2 / prop snps)

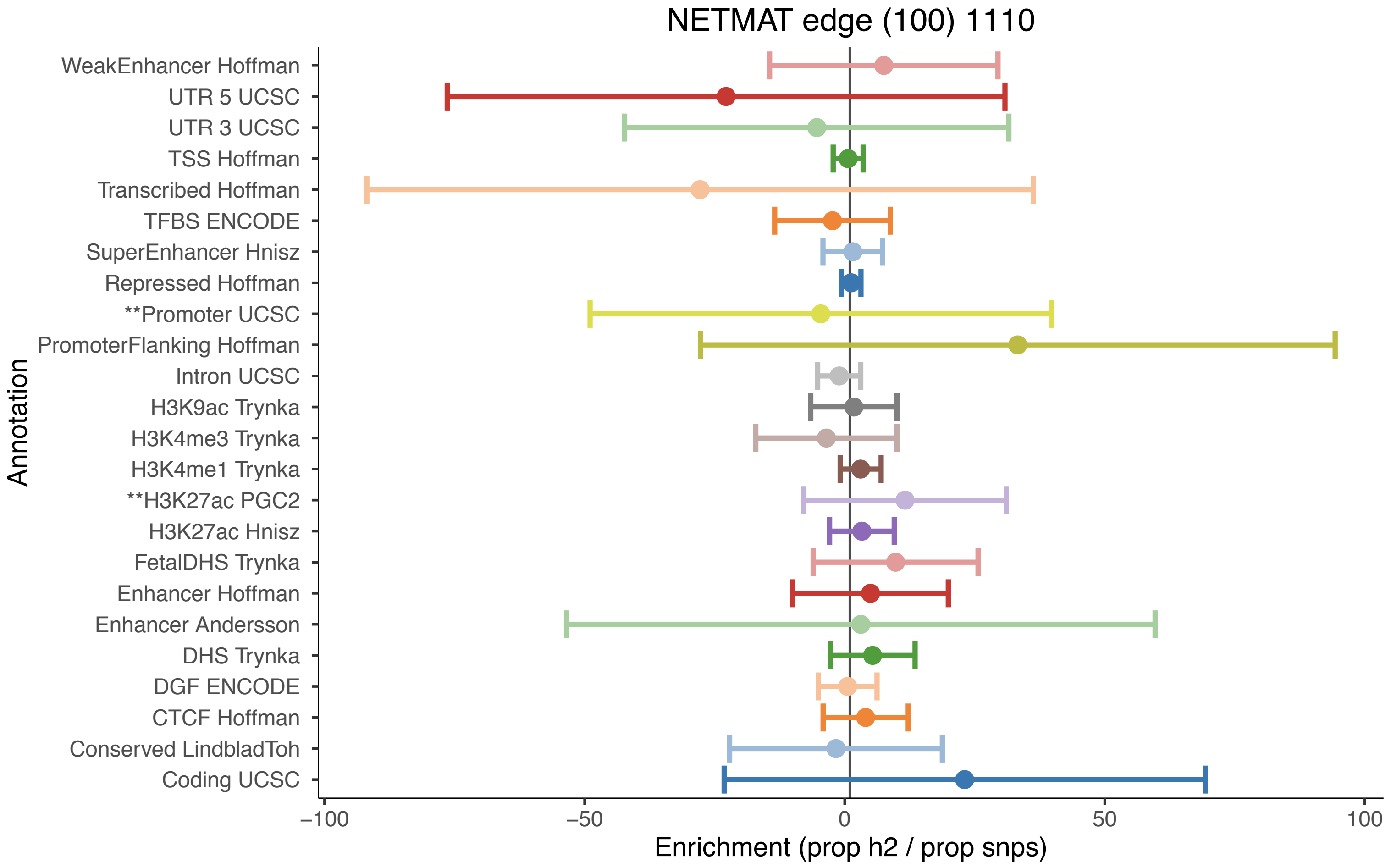

NETMAT edge (100) 1110
WeakEnhancer Hoffman
UTR 5 UCSC
UTR 3 UCSC
TSS Hoffman
Transcribed Hoffman
TFBS ENCODE
SuperEnhancer Hnisz
Repressed Hoffman
**Promoter UCSC
PromoterFlanking Hoffman
Intron UCSC
H3K9ac Trynka
H3K4me3 Trynka
H3K4me1 Trynka
**H3K27ac PGC2
H3K27ac Hnisz
FetalDHS Trynka
Enhancer Hoffman
Enhancer Andersson
DHS Trynka
DGF ENCODE
CTCF Hoffman
Conserved LindbladToh
Coding UCSC
Annotation
−100
−50
0
50
100
Enrichment (prop h2 / prop snps)

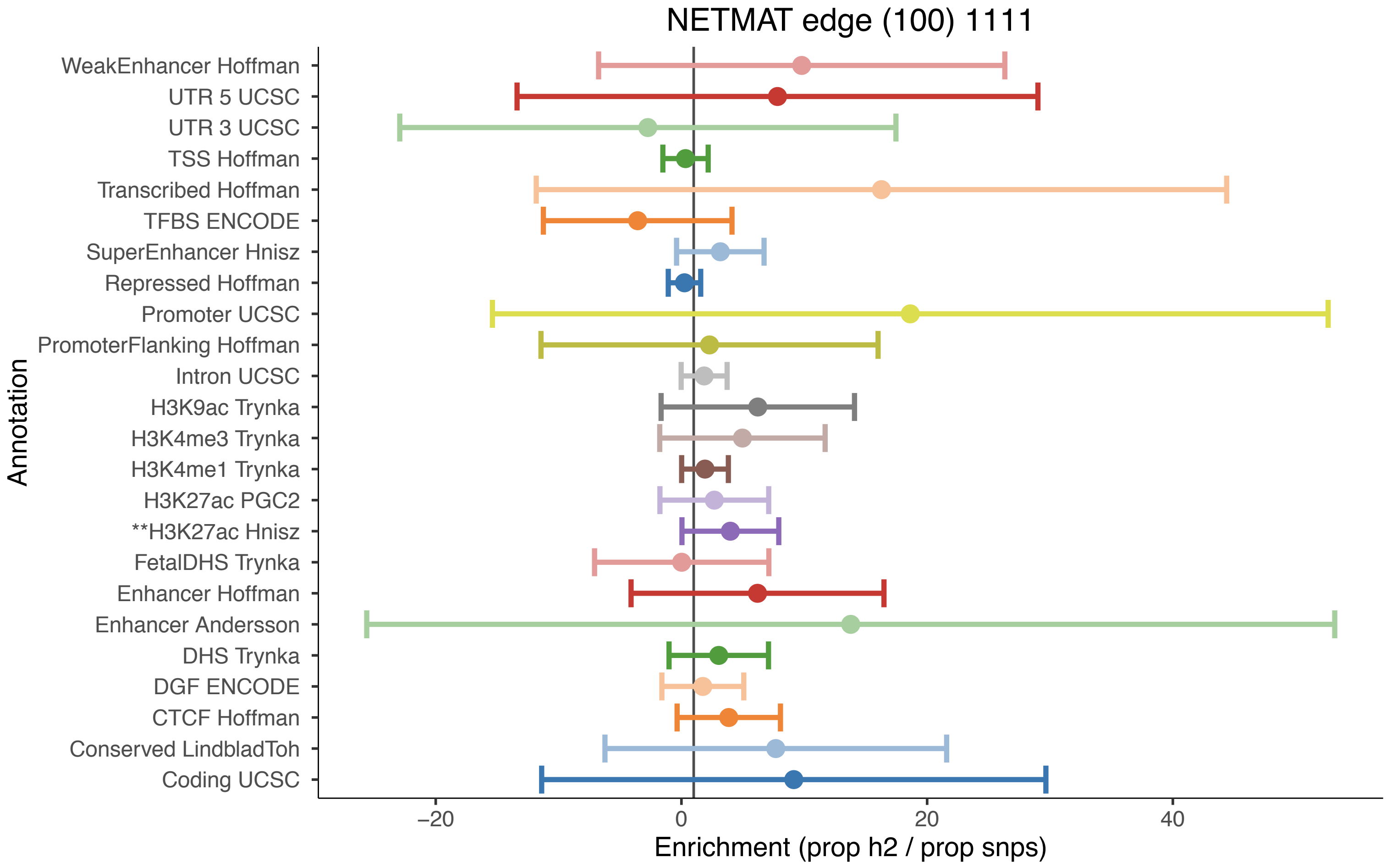
NETMAT edge (100) 1111
WeakEnhancer Hoffman
UTR 5 UCSC
UTR 3 UCSC
TSS Hoffman
Transcribed Hoffman
TFBS ENCODE
SuperEnhancer Hnisz
Repressed Hoffman
Promoter UCSC
PromoterFlanking Hoffman
Intron UCSC
H3K9ac Trynka
H3K4me3 Trynka
H3K4me1 Trynka
H3K27ac PGC2
**H3K27ac Hnisz
FetalDHS Trynka
Enhancer Hoffman
Enhancer Andersson
DHS Trynka
DGF ENCODE
CTCF Hoffman
Conserved LindbladToh
Coding UCSC
Annotation
−20
0
20
40
Enrichment (prop h2 / prop snps)

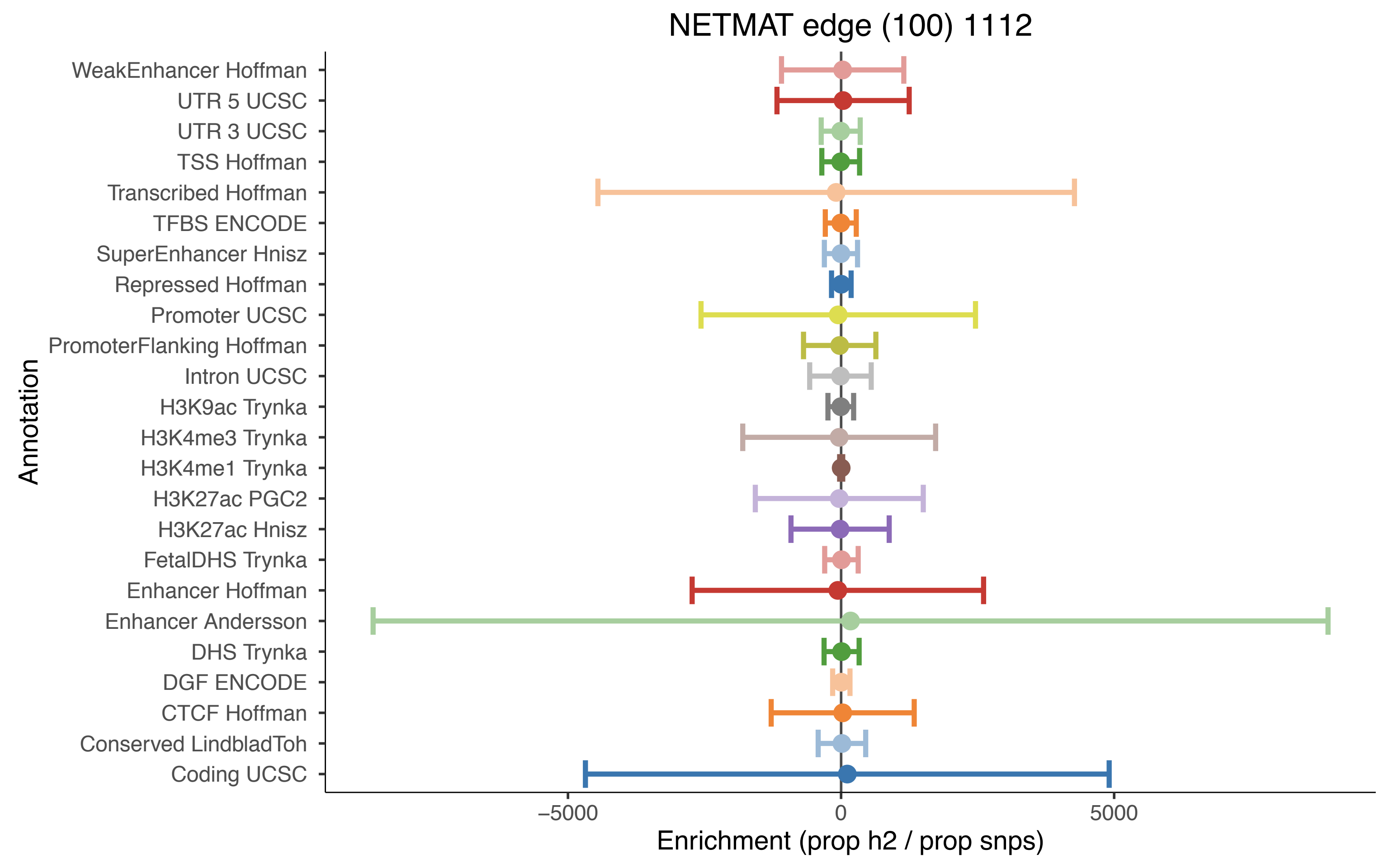
NETMAT edge (100) 1112
WeakEnhancer Hoffman
UTR 5 UCSC
UTR 3 UCSC
TSS Hoffman
Transcribed Hoffman
TFBS ENCODE
SuperEnhancer Hnisz
Repressed Hoffman
Promoter UCSC
PromoterFlanking Hoffman
Intron UCSC
H3K9ac Trynka
H3K4me3 Trynka
H3K4me1 Trynka
H3K27ac PGC2
H3K27ac Hnisz
FetalDHS Trynka
Enhancer Hoffman
Enhancer Andersson
DHS Trynka
DGF ENCODE
CTCF Hoffman
Conserved LindbladToh
Coding UCSC
Annotation
−5000
0
5000
Enrichment (prop h2 / prop snps)

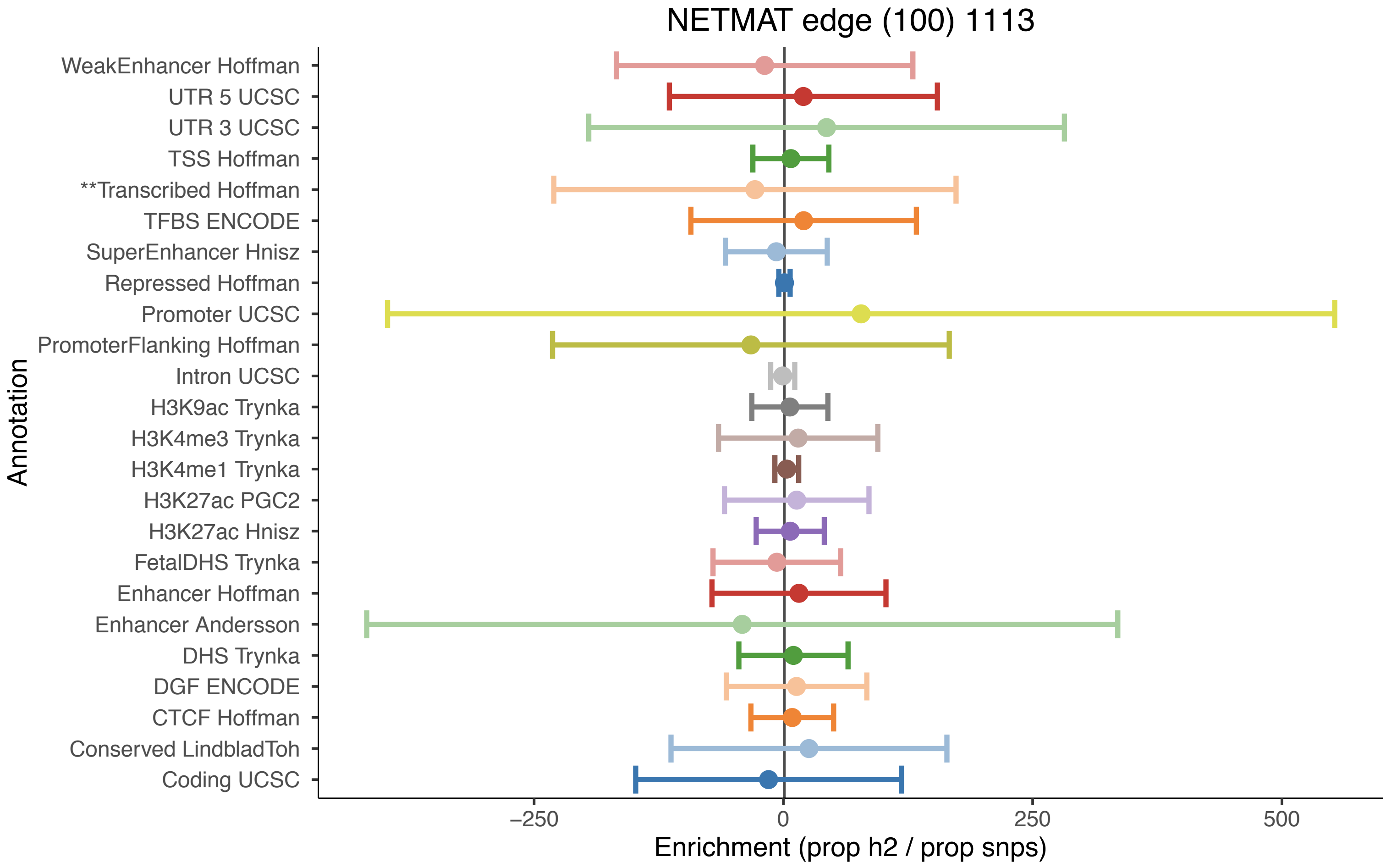

NETMAT edge (100) 1113
WeakEnhancer Hoffman
UTR 5 UCSC
UTR 3 UCSC
TSS Hoffman
**Transcribed Hoffman
TFBS ENCODE
SuperEnhancer Hnisz
Repressed Hoffman
Promoter UCSC
PromoterFlanking Hoffman
Intron UCSC
H3K9ac Trynka
H3K4me3 Trynka
H3K4me1 Trynka
H3K27ac PGC2
H3K27ac Hnisz
FetalDHS Trynka
Enhancer Hoffman
Enhancer Andersson
DHS Trynka
DGF ENCODE
CTCF Hoffman
Conserved LindbladToh
Coding UCSC
Annotation
−250
0
250
500
Enrichment (prop h2 / prop snps)

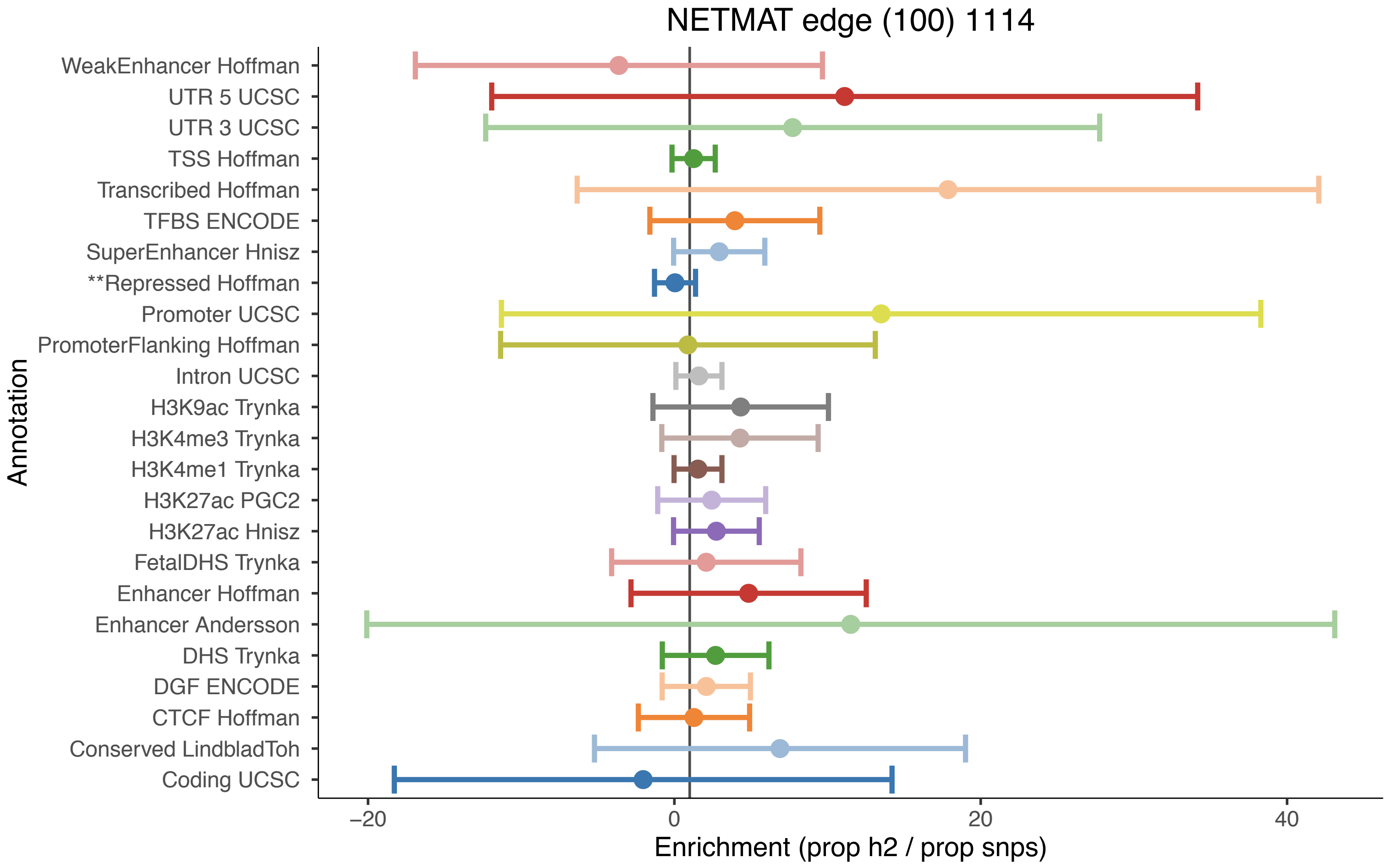
NETMAT edge (100) 1114
WeakEnhancer Hoffman
UTR 5 UCSC
UTR 3 UCSC
TSS Hoffman
Transcribed Hoffman
TFBS ENCODE
SuperEnhancer Hnisz
**Repressed Hoffman
Promoter UCSC
PromoterFlanking Hoffman
Intron UCSC
H3K9ac Trynka
H3K4me3 Trynka
H3K4me1 Trynka
H3K27ac PGC2
H3K27ac Hnisz
FetalDHS Trynka
Enhancer Hoffman
Enhancer Andersson
DHS Trynka
DGF ENCODE
CTCF Hoffman
Conserved LindbladToh
Coding UCSC
Annotation
−20
0
20
40
Enrichment (prop h2 / prop snps)

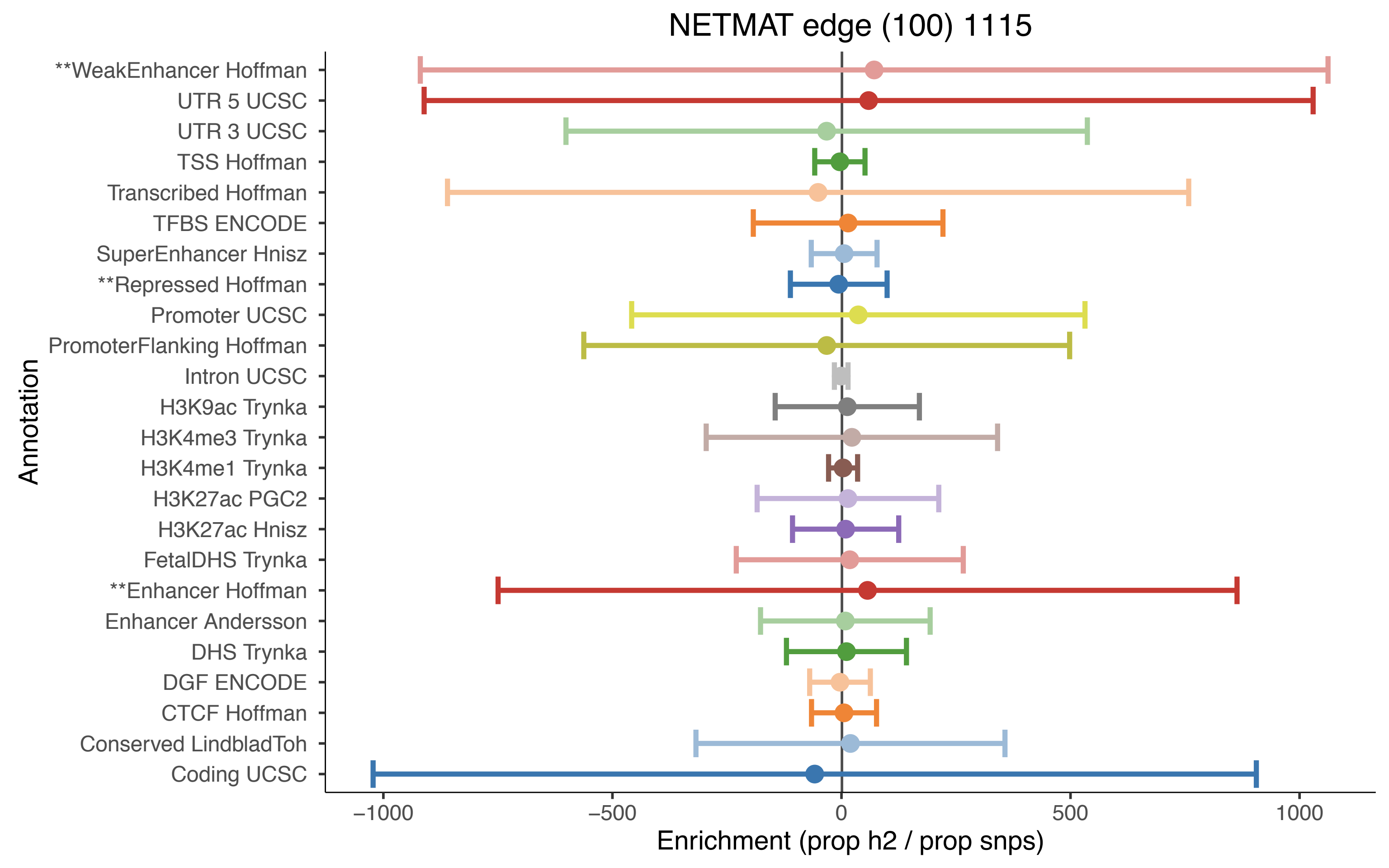
NETMAT edge (100) 1115
**WeakEnhancer Hoffman
UTR 5 UCSC
UTR 3 UCSC
TSS Hoffman
Transcribed Hoffman
TFBS ENCODE
SuperEnhancer Hnisz
**Repressed Hoffman
Promoter UCSC
PromoterFlanking Hoffman
Intron UCSC
H3K9ac Trynka
H3K4me3 Trynka
H3K4me1 Trynka
H3K27ac PGC2
H3K27ac Hnisz
FetalDHS Trynka
**Enhancer Hoffman
Enhancer Andersson
DHS Trynka
DGF ENCODE
CTCF Hoffman
Conserved LindbladToh
Coding UCSC
Annotation
−1000
−500
0
500
1000
Enrichment (prop h2 / prop snps)

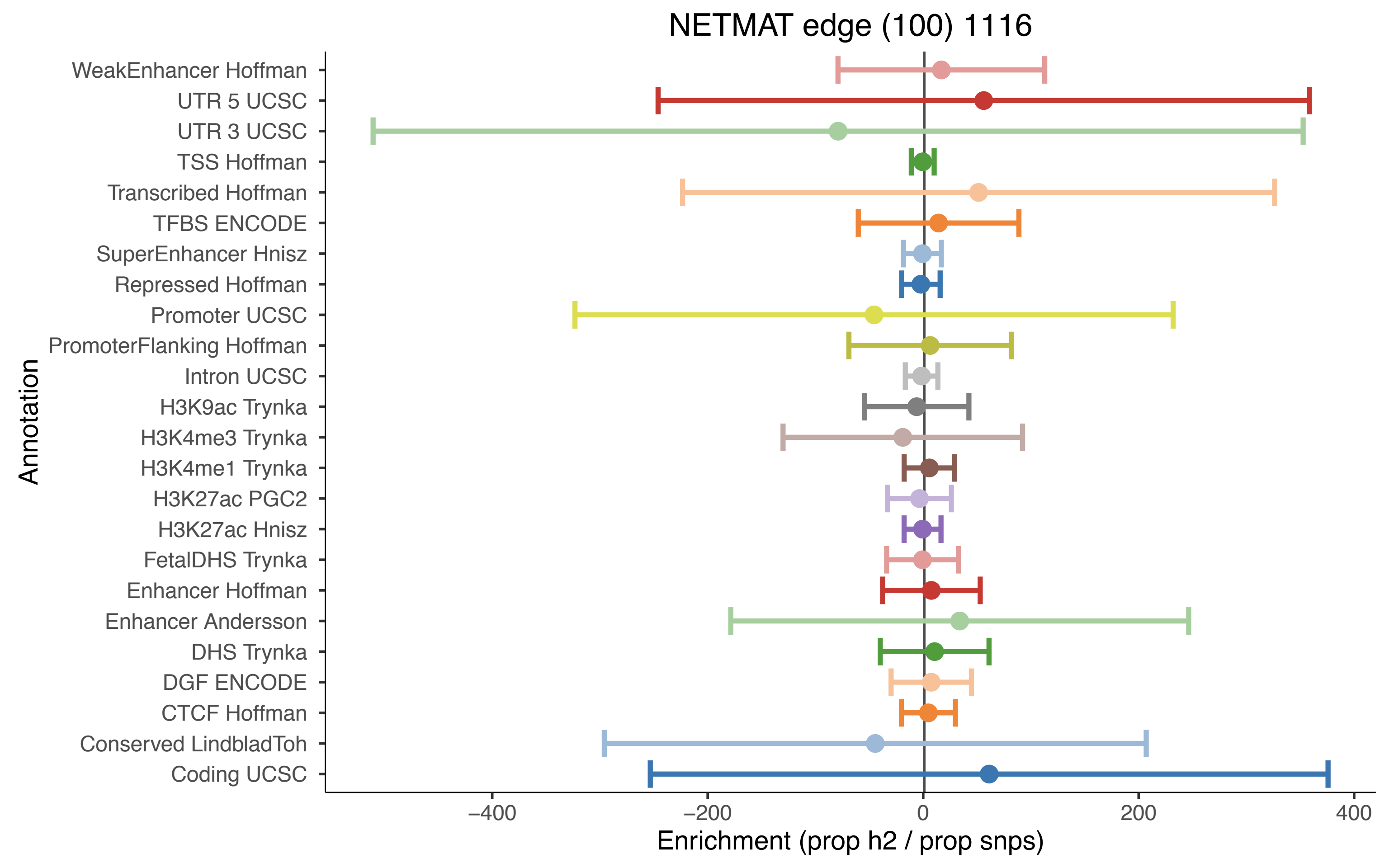

NETMAT edge (100) 1116
WeakEnhancer Hoffman
UTR 5 UCSC
UTR 3 UCSC
TSS Hoffman
Transcribed Hoffman
TFBS ENCODE
SuperEnhancer Hnisz
Repressed Hoffman
Promoter UCSC
PromoterFlanking Hoffman
Intron UCSC
H3K9ac Trynka
H3K4me3 Trynka
H3K4me1 Trynka
H3K27ac PGC2
H3K27ac Hnisz
FetalDHS Trynka
Enhancer Hoffman
Enhancer Andersson
DHS Trynka
DGF ENCODE
CTCF Hoffman
Conserved LindbladToh
Coding UCSC
Annotation
−400
−200
0
200
400
Enrichment (prop h2 / prop snps)

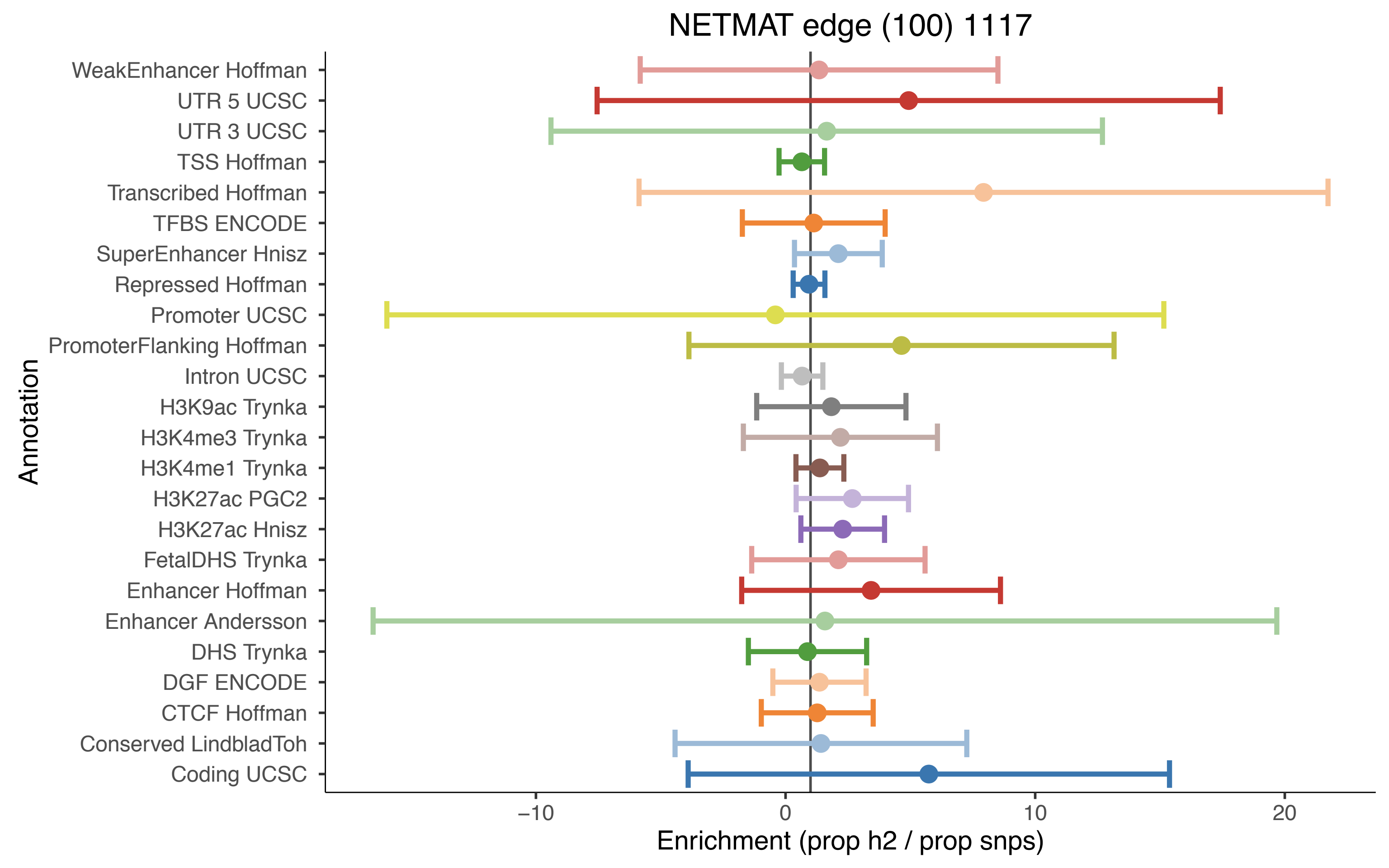
NETMAT edge (100) 1117
Annotation
WeakEnhancer Hoffman
UTR 5 UCSC
UTR 3 UCSC
TSS Hoffman
Transcribed Hoffman
TFBS ENCODE
SuperEnhancer Hnisz
Repressed Hoffman
Promoter UCSC
PromoterFlanking Hoffman
Intron UCSC
H3K9ac Trynka
H3K4me3 Trynka
H3K4me1 Trynka
H3K27ac PGC2
H3K27ac Hnisz
FetalDHS Trynka
Enhancer Hoffman
Enhancer Andersson
DHS Trynka
DGF ENCODE
CTCF Hoffman
Conserved LindbladToh
Coding UCSC
−10
0
10
20
Enrichment (prop h2 / prop snps)

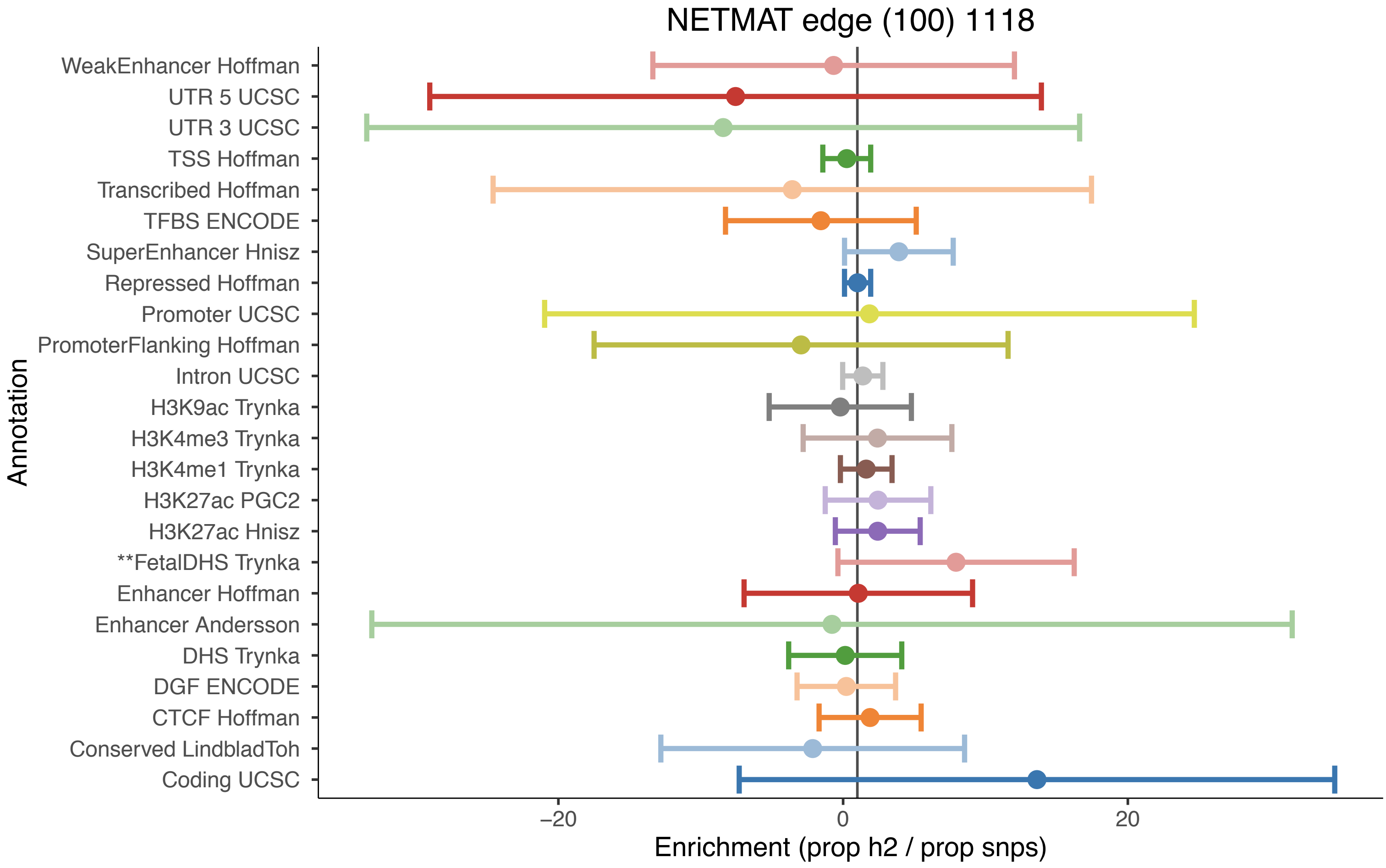
NETMAT edge (100) 1118
WeakEnhancer Hoffman
UTR 5 UCSC
UTR 3 UCSC
TSS Hoffman
Transcribed Hoffman
TFBS ENCODE
SuperEnhancer Hnisz
Repressed Hoffman
Promoter UCSC
PromoterFlanking Hoffman
Intron UCSC
H3K9ac Trynka
H3K4me3 Trynka
H3K4me1 Trynka
H3K27ac PGC2
H3K27ac Hnisz
**FetalDHS Trynka
Enhancer Hoffman
Enhancer Andersson
DHS Trynka
DGF ENCODE
CTCF Hoffman
Conserved LindbladToh
Coding UCSC
Annotation
−20
0
20
Enrichment (prop h2 / prop snps)

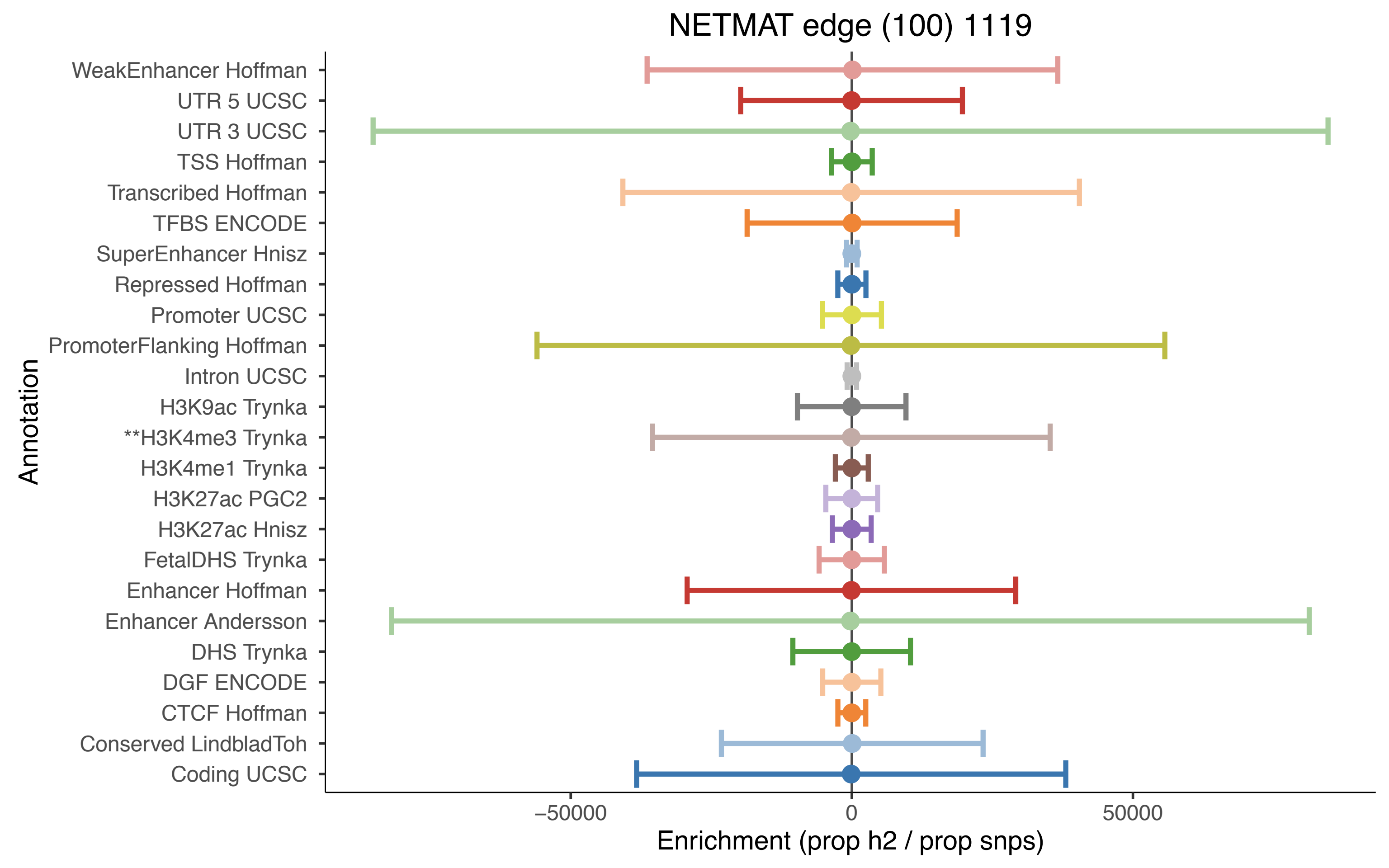
NETMAT edge (100) 1119
Annotation
WeakEnhancer Hoffman
UTR 5 UCSC
UTR 3 UCSC
TSS Hoffman
Transcribed Hoffman
TFBS ENCODE
SuperEnhancer Hnisz
Repressed Hoffman
Promoter UCSC
PromoterFlanking Hoffman
Intron UCSC
H3K9ac Trynka
**H3K4me3 Trynka
H3K4me1 Trynka
H3K27ac PGC2
H3K27ac Hnisz
FetalDHS Trynka
Enhancer Hoffman
Enhancer Andersson
DHS Trynka
DGF ENCODE
CTCF Hoffman
Conserved LindbladToh
Coding UCSC
−50000
0
50000
Enrichment (prop h2 / prop snps)

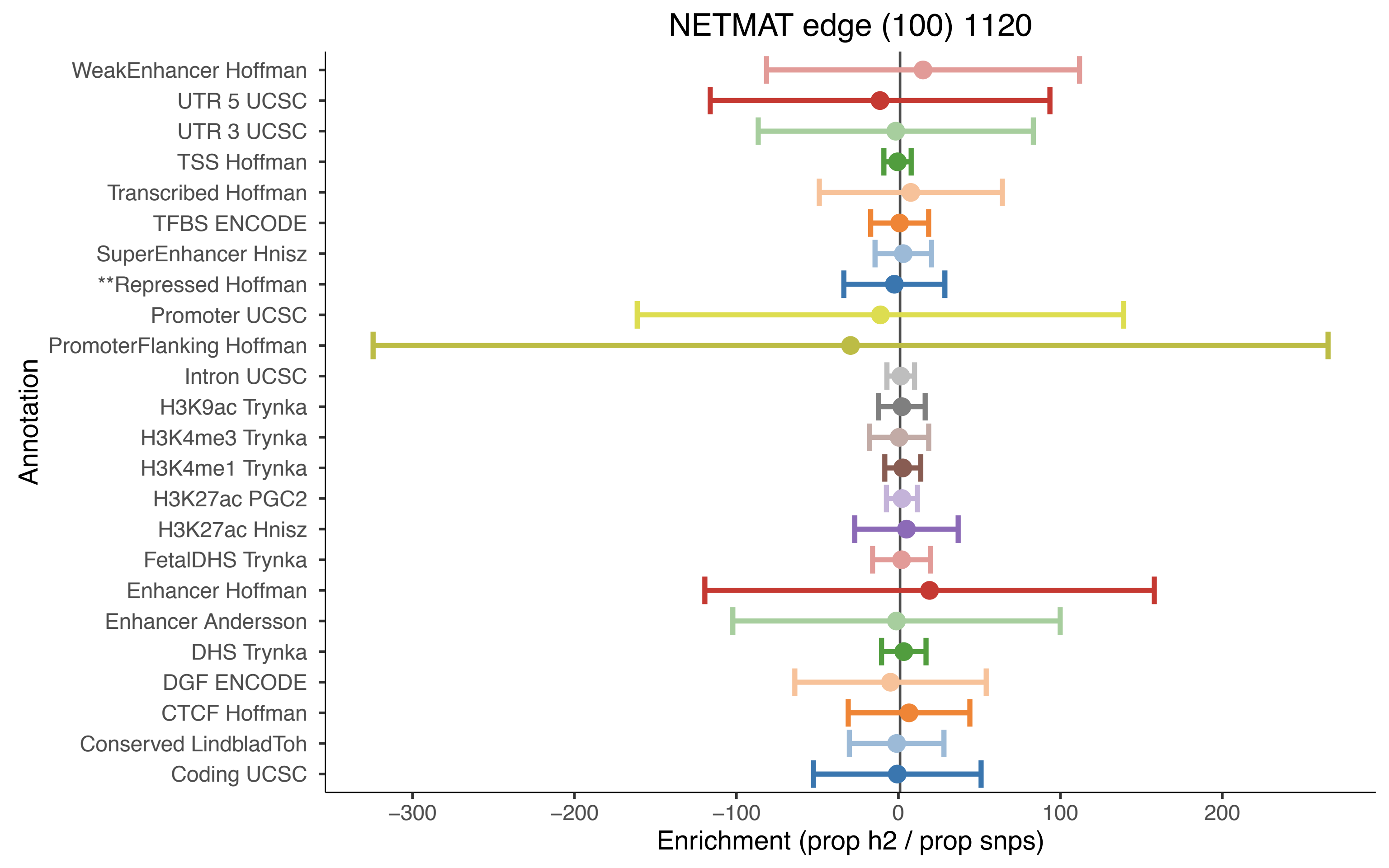
NETMAT edge (100) 1120
WeakEnhancer Hoffman
UTR 5 UCSC
UTR 3 UCSC
TSS Hoffman
Transcribed Hoffman
TFBS ENCODE
SuperEnhancer Hnisz
**Repressed Hoffman
Promoter UCSC
PromoterFlanking Hoffman
Intron UCSC
H3K9ac Trynka
H3K4me3 Trynka
H3K4me1 Trynka
H3K27ac PGC2
H3K27ac Hnisz
FetalDHS Trynka
Enhancer Hoffman
Enhancer Andersson
DHS Trynka
DGF ENCODE
CTCF Hoffman
Conserved LindbladToh
Coding UCSC
Annotation
−300
−200
−100
0
100
200
Enrichment (prop h2 / prop snps)

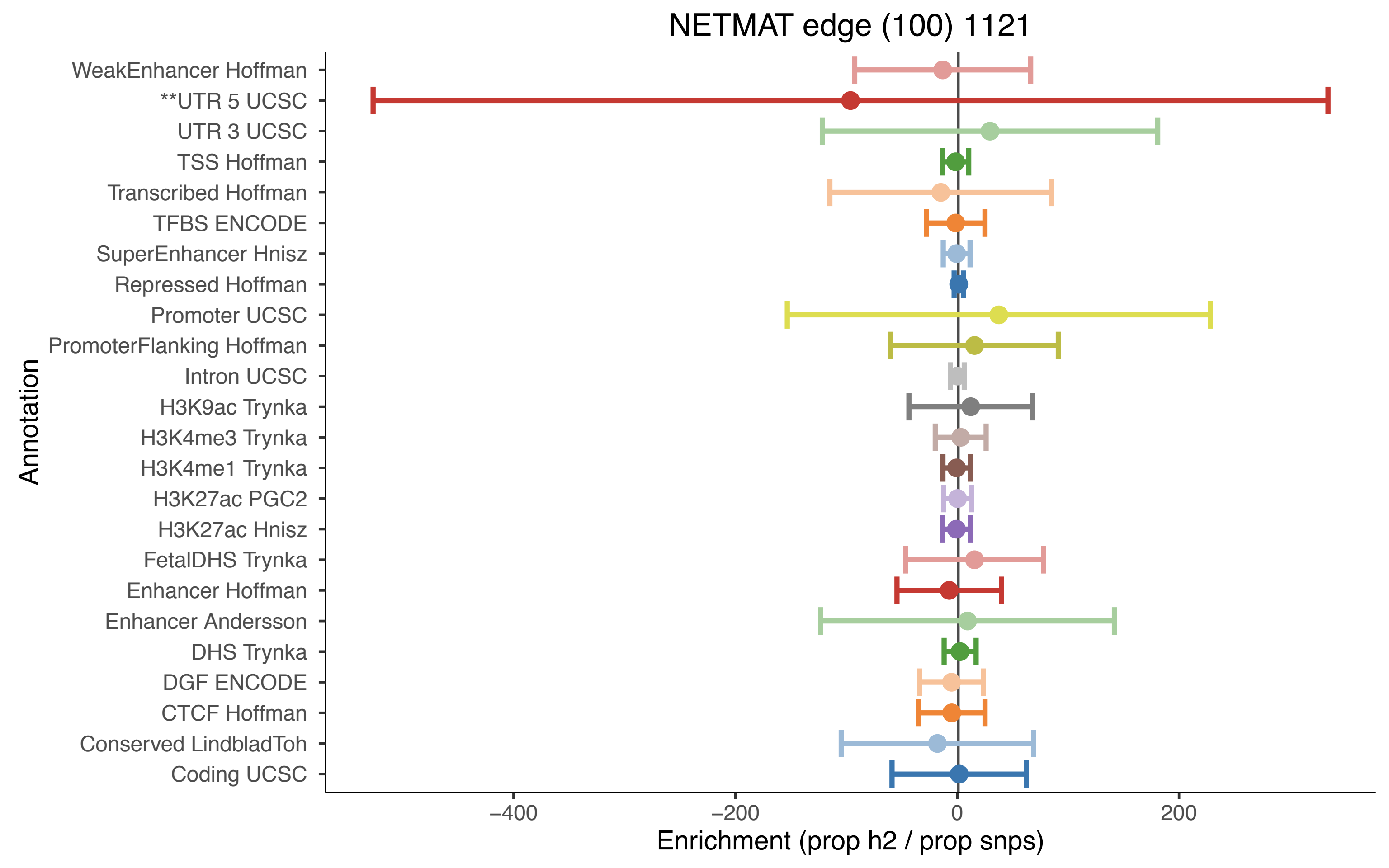

NETMAT edge (100) 1121
WeakEnhancer Hoffman
**UTR 5 UCSC
UTR 3 UCSC
TSS Hoffman
Transcribed Hoffman
TFBS ENCODE
SuperEnhancer Hnisz
Repressed Hoffman
Promoter UCSC
PromoterFlanking Hoffman
Intron UCSC
H3K9ac Trynka
H3K4me3 Trynka
H3K4me1 Trynka
H3K27ac PGC2
H3K27ac Hnisz
FetalDHS Trynka
Enhancer Hoffman
Enhancer Andersson
DHS Trynka
DGF ENCODE
CTCF Hoffman
Conserved LindbladToh
Coding UCSC
Annotation
−400
−200
0
200
Enrichment (prop h2 / prop snps)

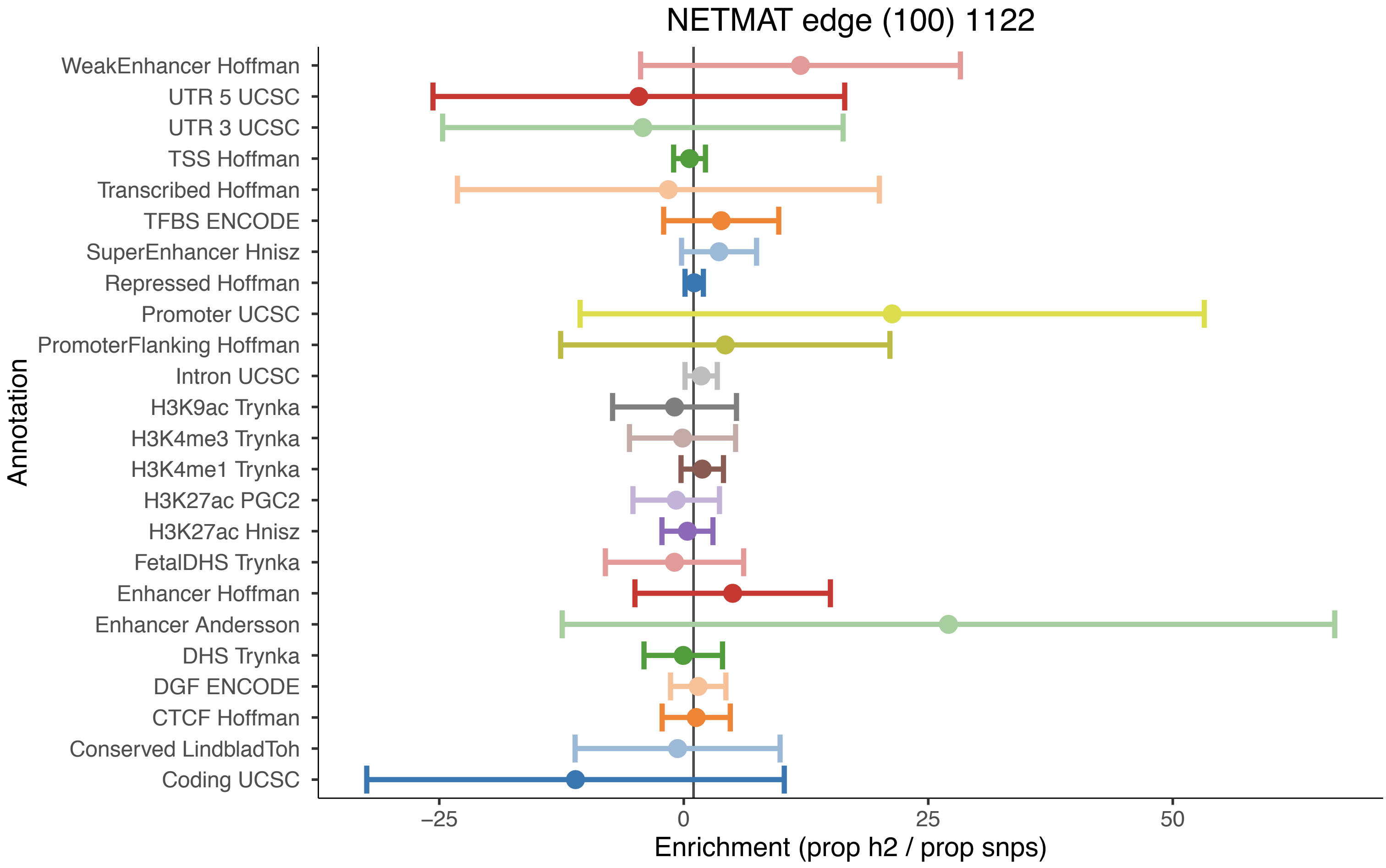
NETMAT edge (100) 1122
WeakEnhancer Hoffman
UTR 5 UCSC
UTR 3 UCSC
TSS Hoffman
Transcribed Hoffman
TFBS ENCODE
SuperEnhancer Hnisz
Repressed Hoffman
Promoter UCSC
PromoterFlanking Hoffman
Intron UCSC
H3K9ac Trynka
H3K4me3 Trynka
H3K4me1 Trynka
H3K27ac PGC2
H3K27ac Hnisz
FetalDHS Trynka
Enhancer Hoffman
Enhancer Andersson
DHS Trynka
DGF ENCODE
CTCF Hoffman
Conserved LindbladToh
Coding UCSC
Annotation
−25
0
25
50
Enrichment (prop h2 / prop snps)

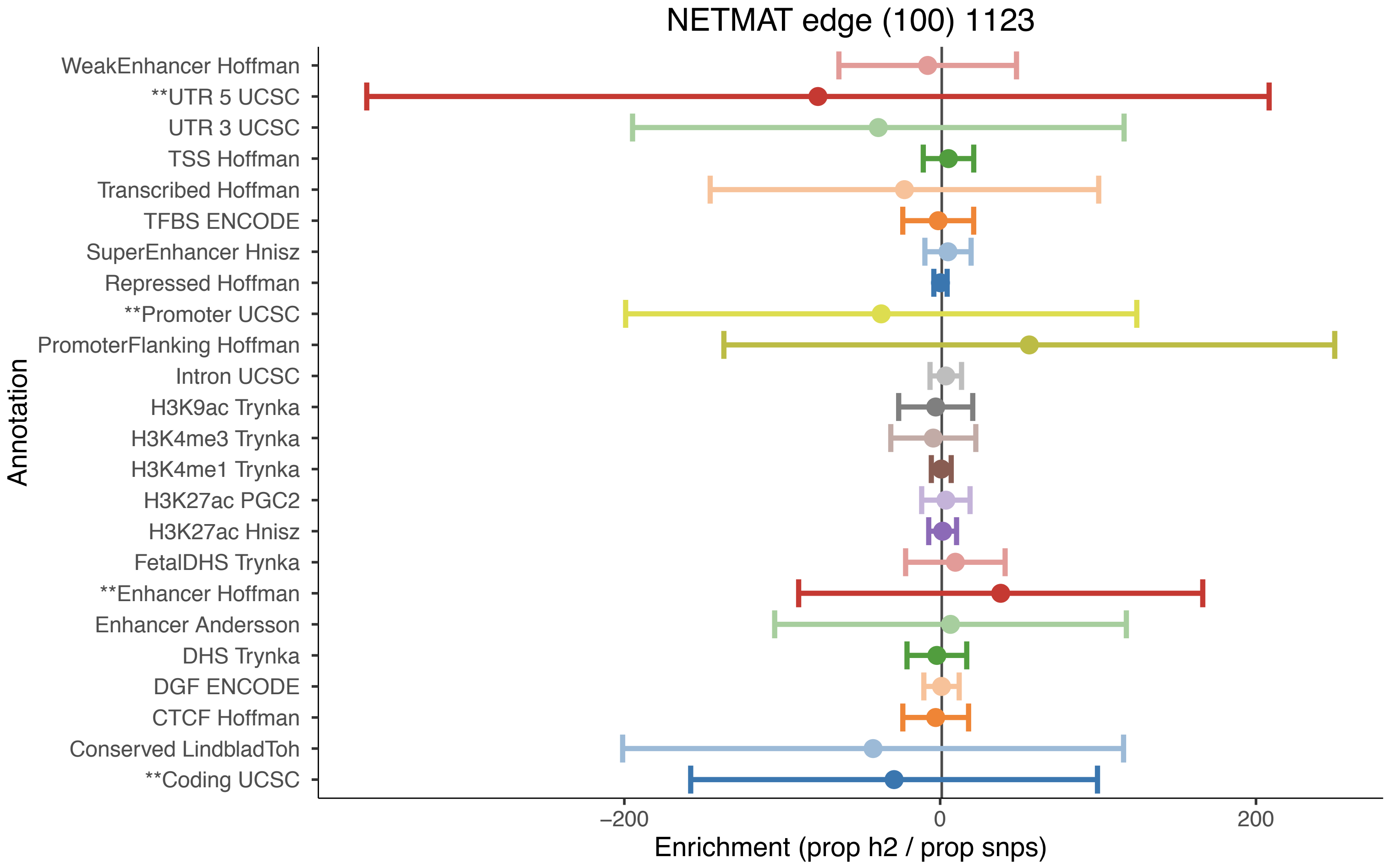

NETMAT edge (100) 1123
WeakEnhancer Hoffman
**UTR 5 UCSC
UTR 3 UCSC
TSS Hoffman
Transcribed Hoffman
TFBS ENCODE
SuperEnhancer Hnisz
Repressed Hoffman
**Promoter UCSC
PromoterFlanking Hoffman
Intron UCSC
H3K9ac Trynka
H3K4me3 Trynka
H3K4me1 Trynka
H3K27ac PGC2
H3K27ac Hnisz
FetalDHS Trynka
**Enhancer Hoffman
Enhancer Andersson
DHS Trynka
DGF ENCODE
CTCF Hoffman
Conserved LindbladToh
**Coding UCSC
Annotation
−200
0
200
Enrichment (prop h2 / prop snps)

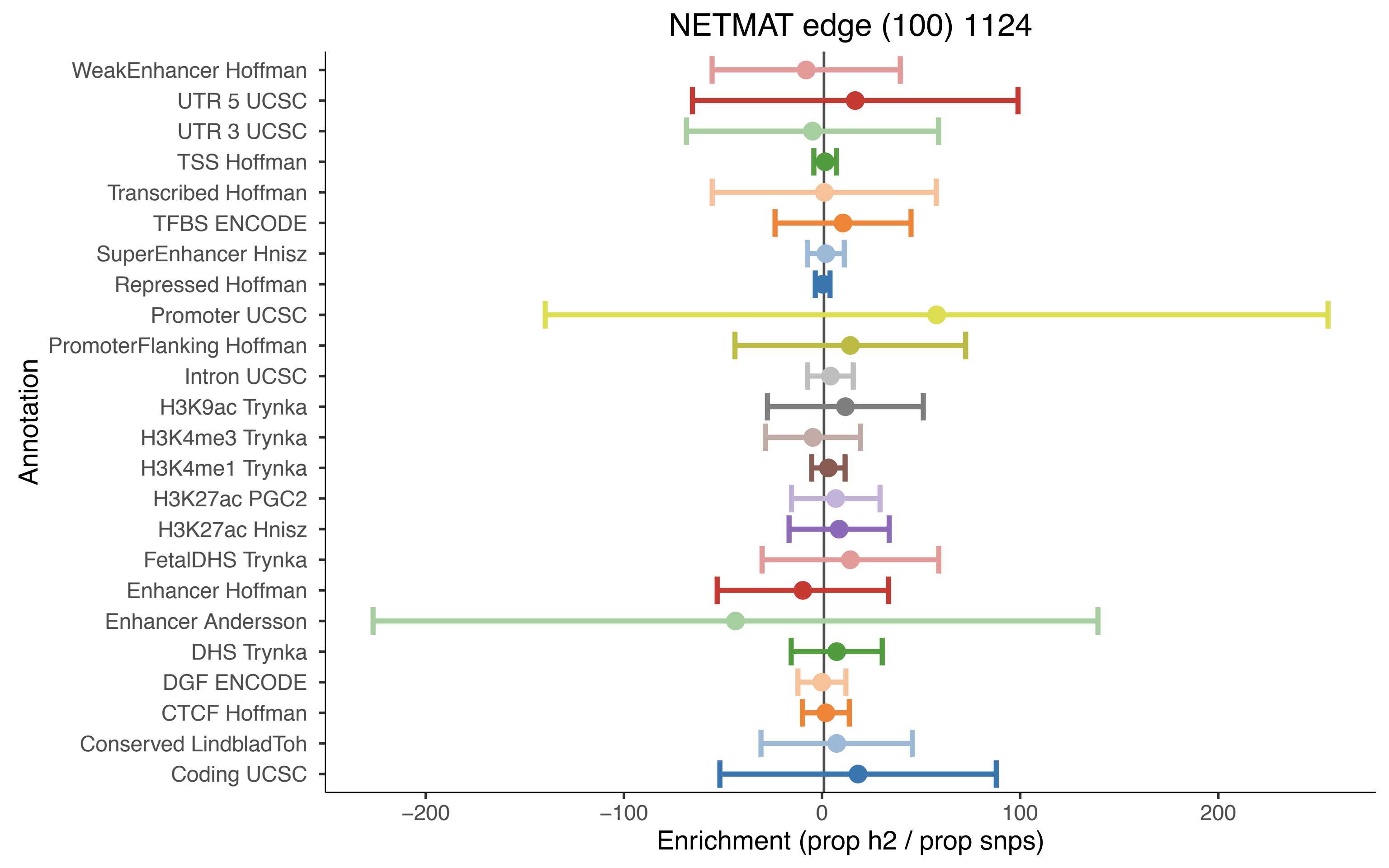
NETMAT edge (100) 1124
WeakEnhancer Hoffman
UTR 5 UCSC
UTR 3 UCSC
TSS Hoffman
Transcribed Hoffman
TFBS ENCODE
SuperEnhancer Hnisz
Repressed Hoffman
Promoter UCSC
PromoterFlanking Hoffman
Intron UCSC
H3K9ac Trynka
H3K4me3 Trynka
H3K4me1 Trynka
H3K27ac PGC2
H3K27ac Hnisz
FetalDHS Trynka
Enhancer Hoffman
Enhancer Andersson
DHS Trynka
DGF ENCODE
CTCF Hoffman
Conserved LindbladToh
Coding UCSC
Annotation
−200
−100
0
100
200
Enrichment (prop h2 / prop snps)

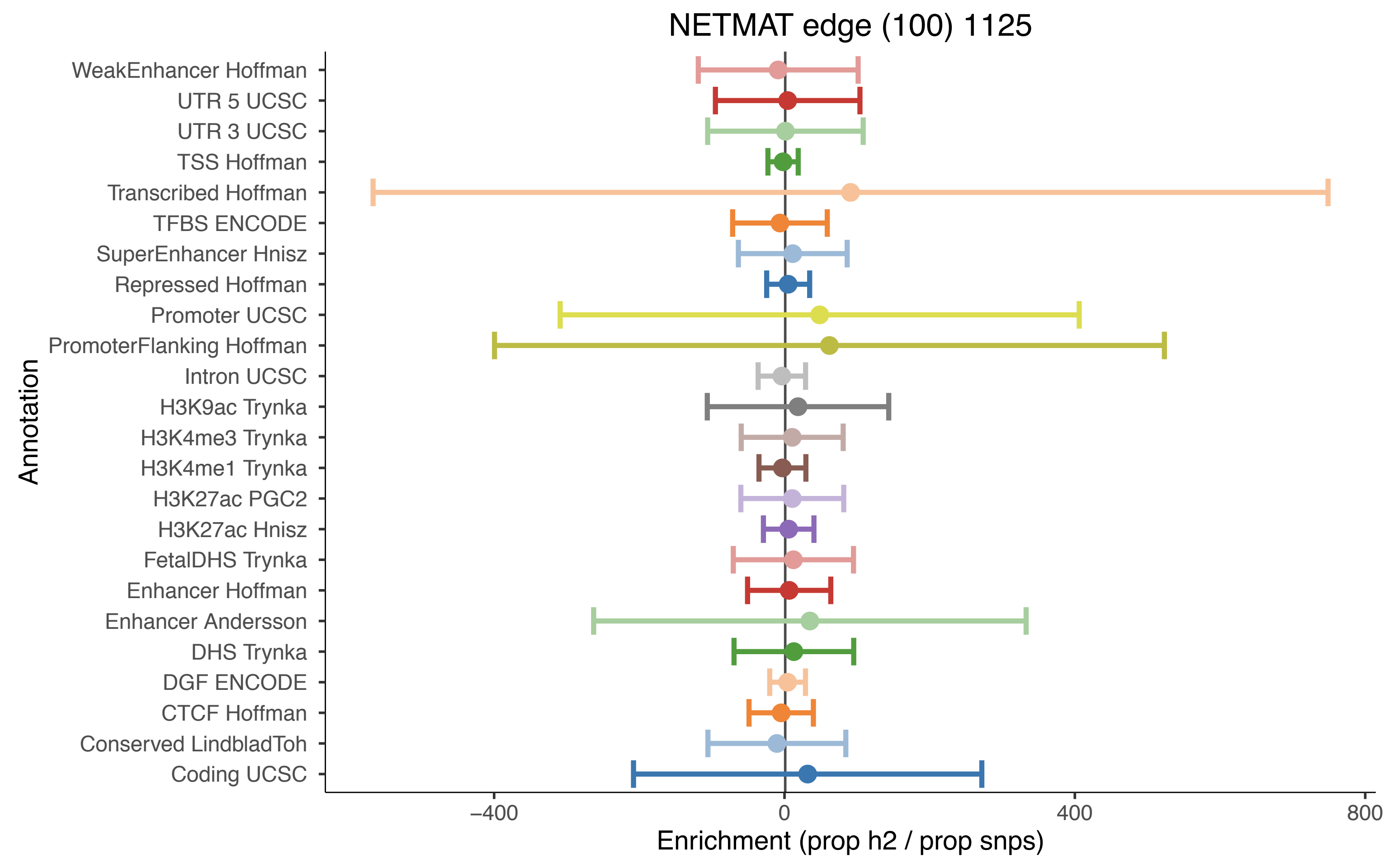

NETMAT edge (100) 1125
WeakEnhancer Hoffman
UTR 5 UCSC
UTR 3 UCSC
TSS Hoffman
Transcribed Hoffman
TFBS ENCODE
SuperEnhancer Hnisz
Repressed Hoffman
Promoter UCSC
PromoterFlanking Hoffman
Intron UCSC
H3K9ac Trynka
H3K4me3 Trynka
H3K4me1 Trynka
H3K27ac PGC2
H3K27ac Hnisz
FetalDHS Trynka
Enhancer Hoffman
Enhancer Andersson
DHS Trynka
DGF ENCODE
CTCF Hoffman
Conserved LindbladToh
Coding UCSC
Annotation
−400
0
400
800
Enrichment (prop h2 / prop snps)

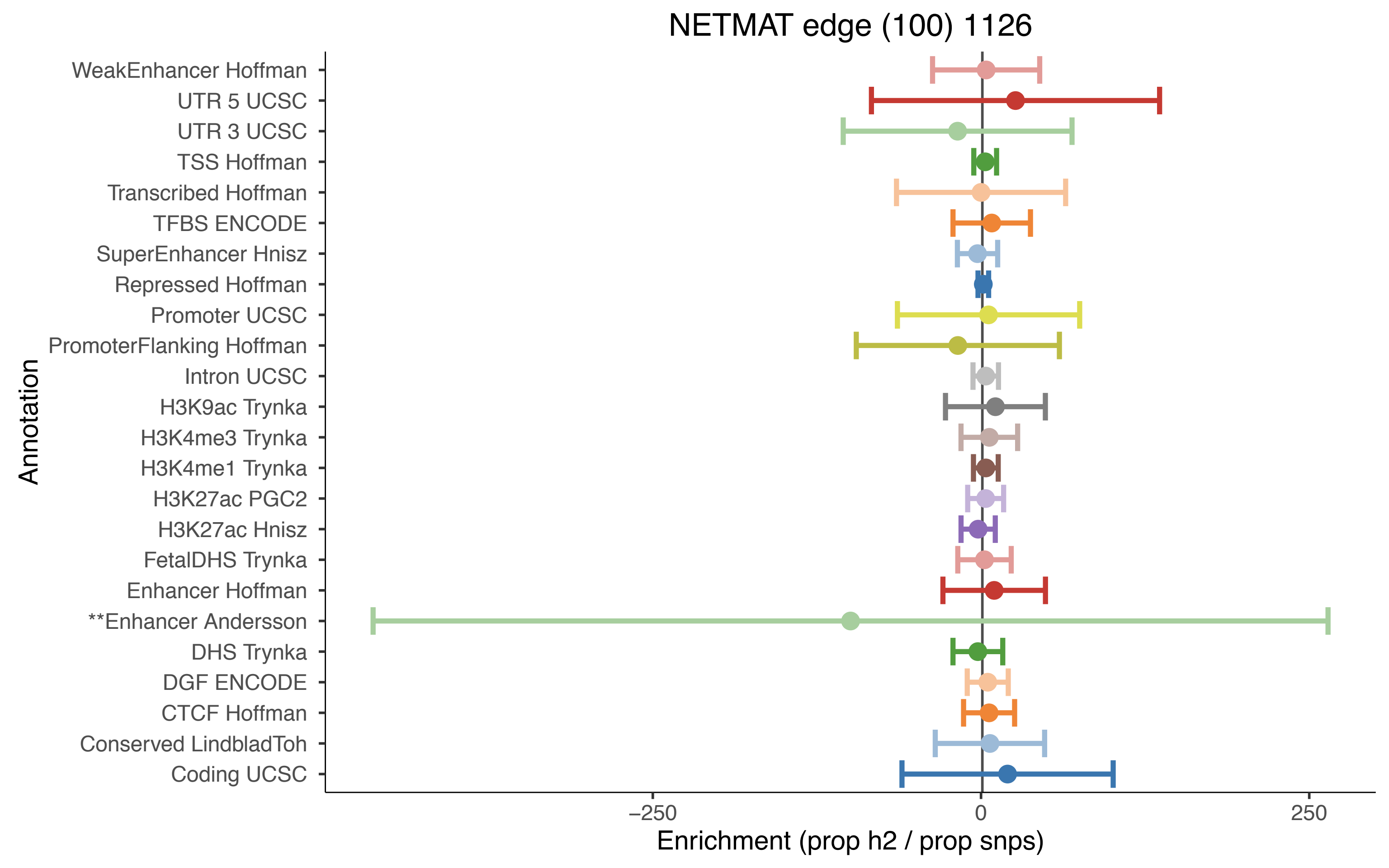
NETMAT edge (100) 1126
WeakEnhancer Hoffman
UTR 5 UCSC
UTR 3 UCSC
TSS Hoffman
Transcribed Hoffman
TFBS ENCODE
SuperEnhancer Hnisz
Repressed Hoffman
Promoter UCSC
PromoterFlanking Hoffman
Intron UCSC
H3K9ac Trynka
H3K4me3 Trynka
H3K4me1 Trynka
H3K27ac PGC2
H3K27ac Hnisz
FetalDHS Trynka
Enhancer Hoffman
**Enhancer Andersson
DHS Trynka
DGF ENCODE
CTCF Hoffman
Conserved LindbladToh
Coding UCSC
Annotation
−250
0
250
Enrichment (prop h2 / prop snps)

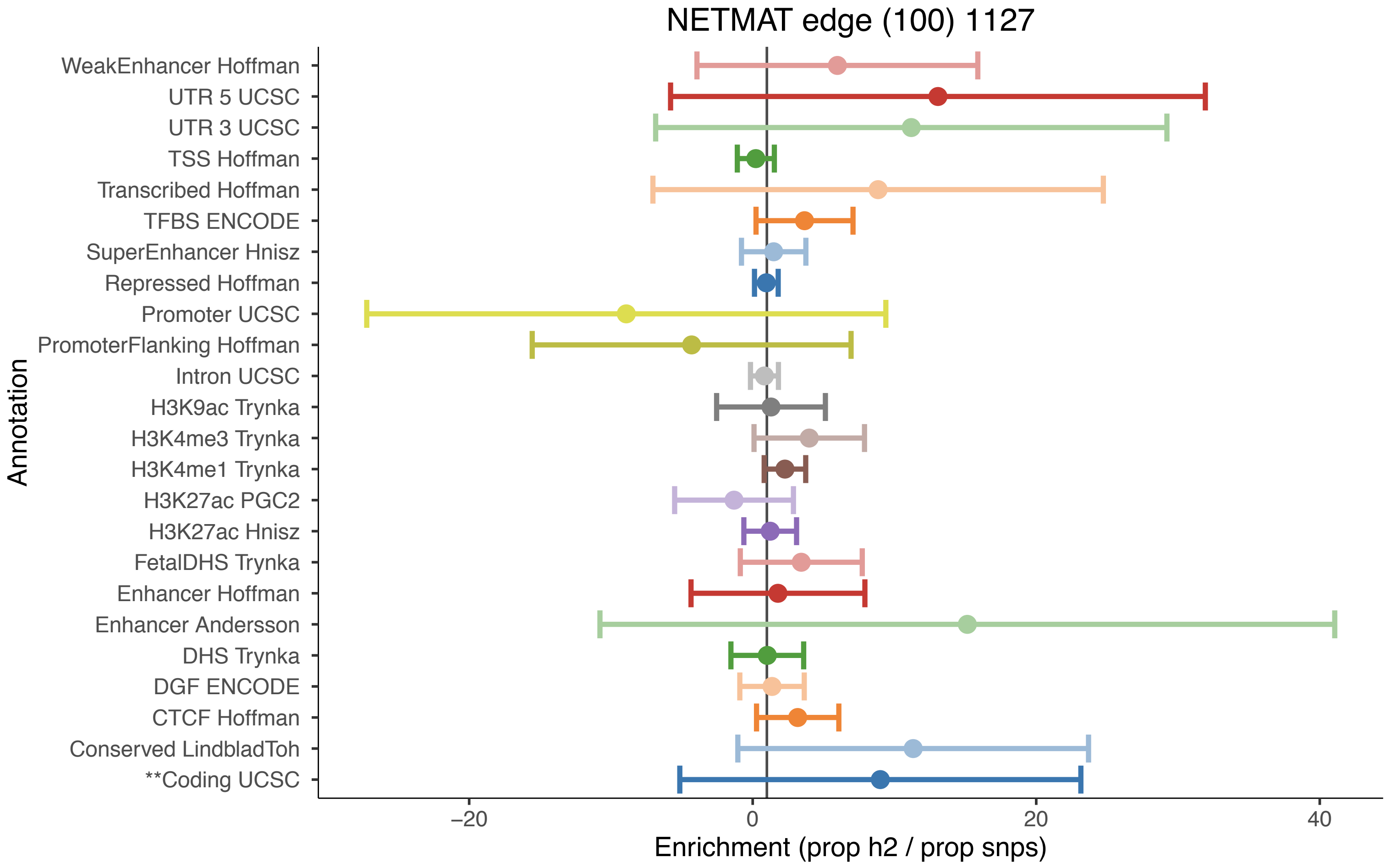
NETMAT edge (100) 1127
WeakEnhancer Hoffman
UTR 5 UCSC
UTR 3 UCSC
TSS Hoffman
Transcribed Hoffman
TFBS ENCODE
SuperEnhancer Hnisz
Repressed Hoffman
Promoter UCSC
PromoterFlanking Hoffman
Intron UCSC
H3K9ac Trynka
H3K4me3 Trynka
H3K4me1 Trynka
H3K27ac PGC2
H3K27ac Hnisz
FetalDHS Trynka
Enhancer Hoffman
Enhancer Andersson
DHS Trynka
DGF ENCODE
CTCF Hoffman
Conserved LindbladToh
**Coding UCSC
Annotation
−20
0
20
40
Enrichment (prop h2 / prop snps)

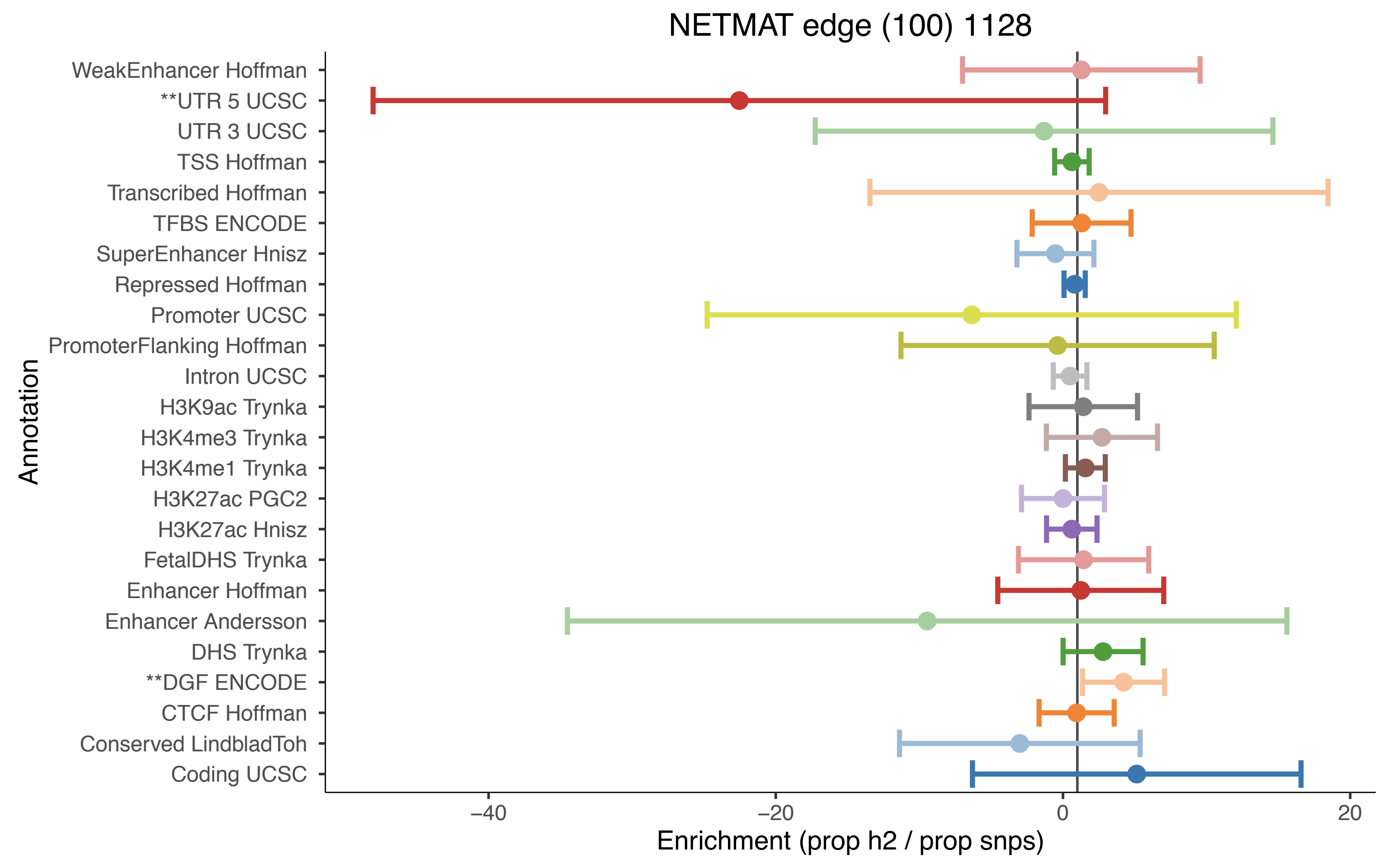
NETMAT edge (100) 1128
WeakEnhancer Hoffman
**UTR 5 UCSC
UTR 3 UCSC
TSS Hoffman
Transcribed Hoffman
TFBS ENCODE
SuperEnhancer Hnisz
Repressed Hoffman
Promoter UCSC
PromoterFlanking Hoffman
Intron UCSC
H3K9ac Trynka
H3K4me3 Trynka
H3K4me1 Trynka
H3K27ac PGC2
H3K27ac Hnisz
FetalDHS Trynka
Enhancer Hoffman
Enhancer Andersson
DHS Trynka
**DGF ENCODE
CTCF Hoffman
Conserved LindbladToh
Coding UCSC
Annotation
−40
−20
0
20
Enrichment (prop h2 / prop snps)

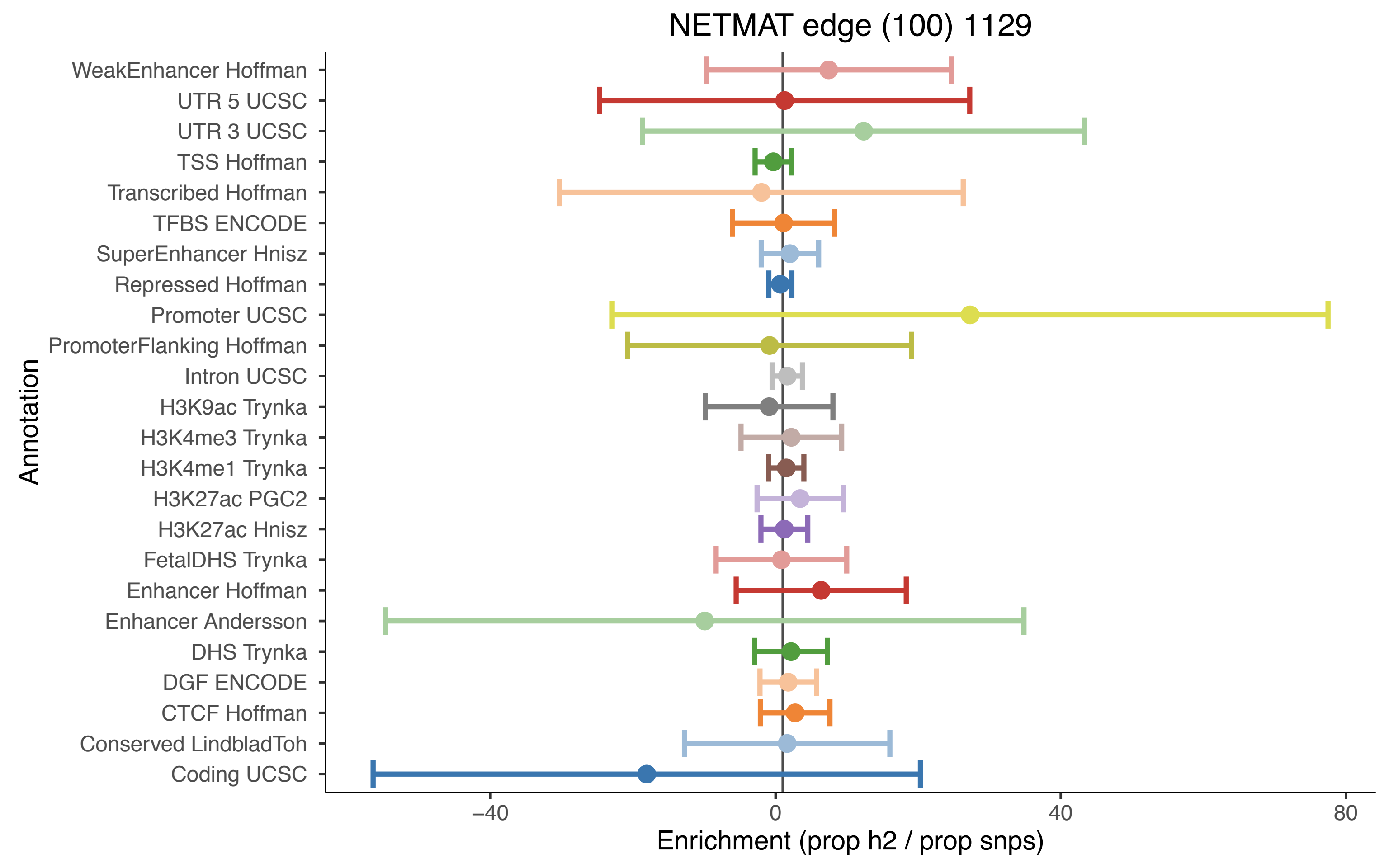

NETMAT edge (100) 1129
WeakEnhancer Hoffman
UTR 5 UCSC
UTR 3 UCSC
TSS Hoffman
Transcribed Hoffman
TFBS ENCODE
SuperEnhancer Hnisz
Repressed Hoffman
Promoter UCSC
PromoterFlanking Hoffman
Intron UCSC
H3K9ac Trynka
H3K4me3 Trynka
H3K4me1 Trynka
H3K27ac PGC2
H3K27ac Hnisz
FetalDHS Trynka
Enhancer Hoffman
Enhancer Andersson
DHS Trynka
DGF ENCODE
CTCF Hoffman
Conserved LindbladToh
Coding UCSC
Annotation
−40
0
40
80
Enrichment (prop h2 / prop snps)

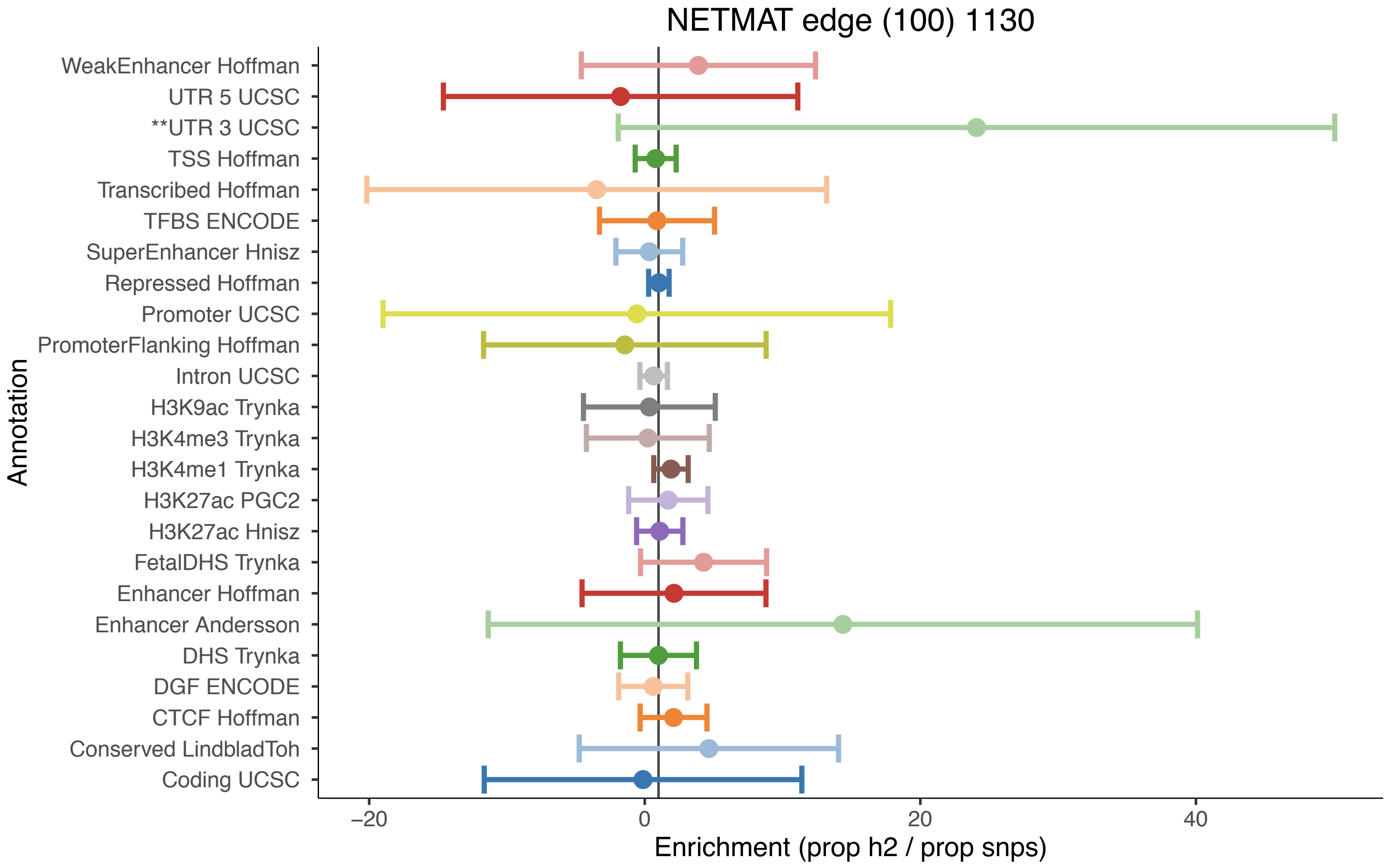
NETMAT edge (100) 1130
WeakEnhancer Hoffman
UTR 5 UCSC
**UTR 3 UCSC
TSS Hoffman
Transcribed Hoffman
TFBS ENCODE
SuperEnhancer Hnisz
Repressed Hoffman
Promoter UCSC
PromoterFlanking Hoffman
Intron UCSC
H3K9ac Trynka
H3K4me3 Trynka
H3K4me1 Trynka
H3K27ac PGC2
H3K27ac Hnisz
FetalDHS Trynka
Enhancer Hoffman
Enhancer Andersson
DHS Trynka
DGF ENCODE
CTCF Hoffman
Conserved LindbladToh
Coding UCSC
Annotation
−20
0
20
40
Enrichment (prop h2 / prop snps)

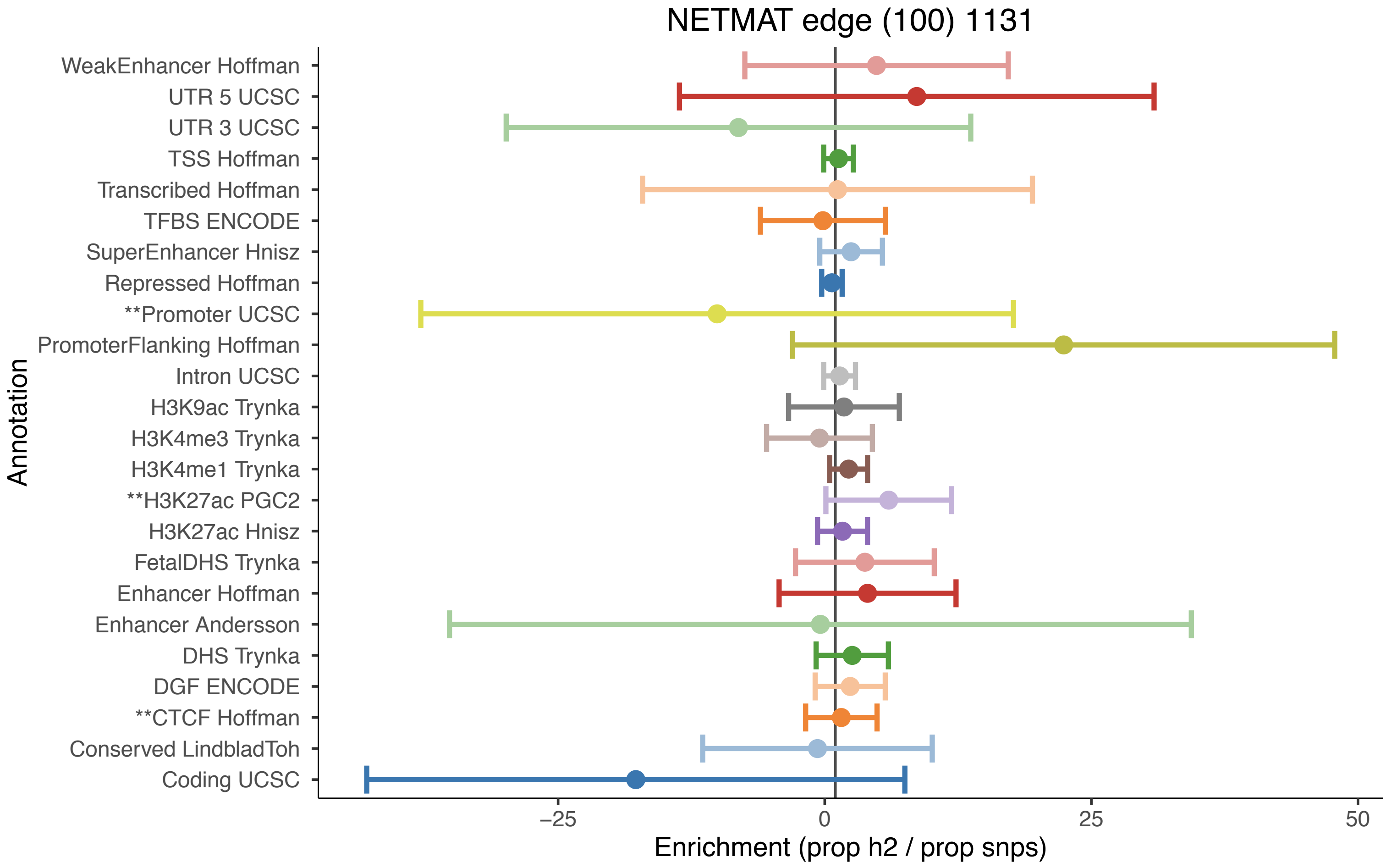

NETMAT edge (100) 1131
WeakEnhancer Hoffman
UTR 5 UCSC
UTR 3 UCSC
TSS Hoffman
Transcribed Hoffman
TFBS ENCODE
SuperEnhancer Hnisz
Repressed Hoffman
**Promoter UCSC
PromoterFlanking Hoffman
Intron UCSC
H3K9ac Trynka
H3K4me3 Trynka
H3K4me1 Trynka
**H3K27ac PGC2
H3K27ac Hnisz
FetalDHS Trynka
Enhancer Hoffman
Enhancer Andersson
DHS Trynka
DGF ENCODE
**CTCF Hoffman
Conserved LindbladToh
Coding UCSC
Annotation
−25
0
25
50
Enrichment (prop h2 / prop snps)

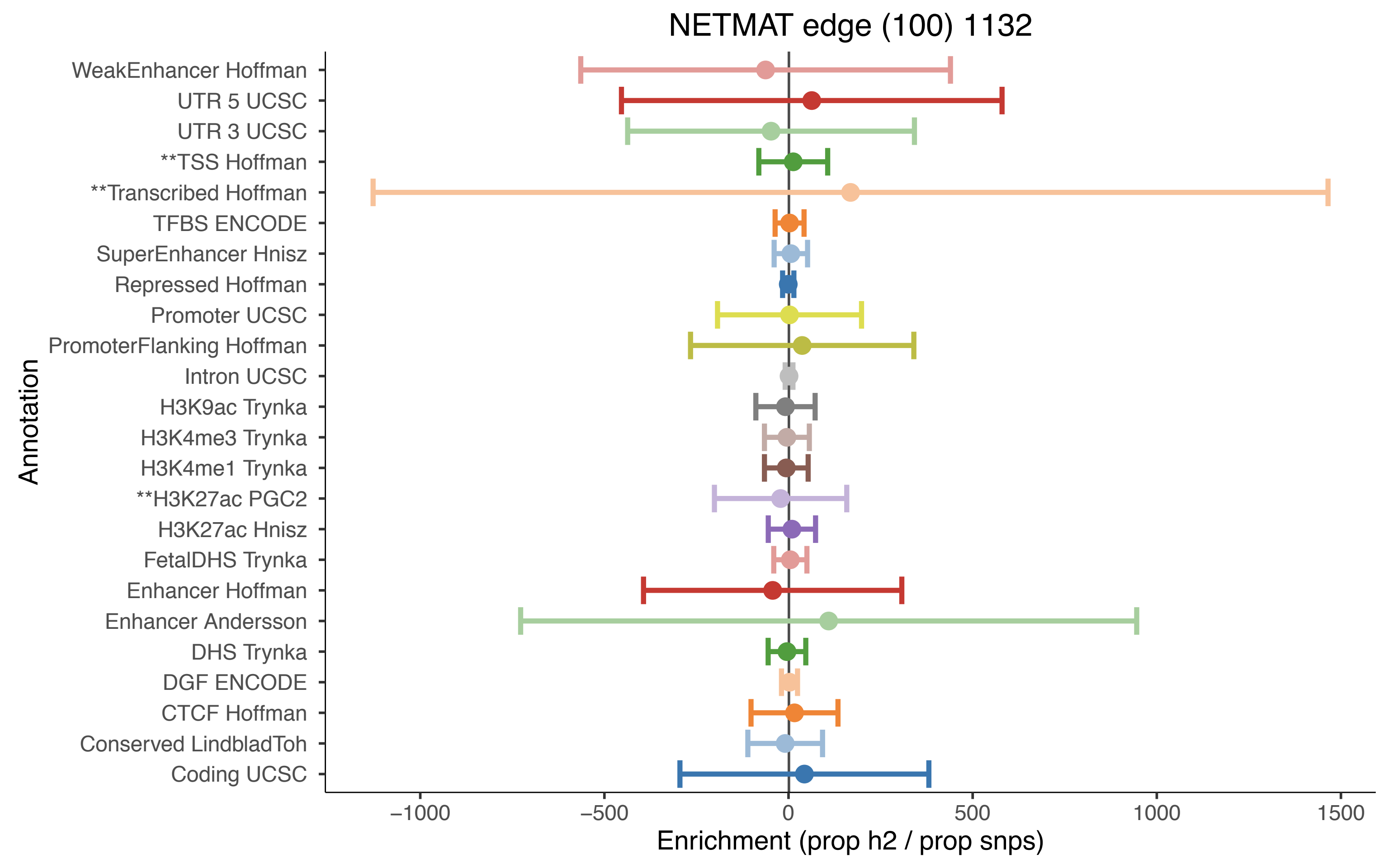
NETMAT edge (100) 1132
Annotation
WeakEnhancer Hoffman
UTR 5 UCSC
UTR 3 UCSC
**TSS Hoffman
**Transcribed Hoffman
TFBS ENCODE
SuperEnhancer Hnisz
Repressed Hoffman
Promoter UCSC
PromoterFlanking Hoffman
Intron UCSC
H3K9ac Trynka
H3K4me3 Trynka
H3K4me1 Trynka
**H3K27ac PGC2
H3K27ac Hnisz
FetalDHS Trynka
Enhancer Hoffman
Enhancer Andersson
DHS Trynka
DGF ENCODE
CTCF Hoffman
Conserved LindbladToh
Coding UCSC
−1000
−500
0
500
1000
1500
Enrichment (prop h2 / prop snps)

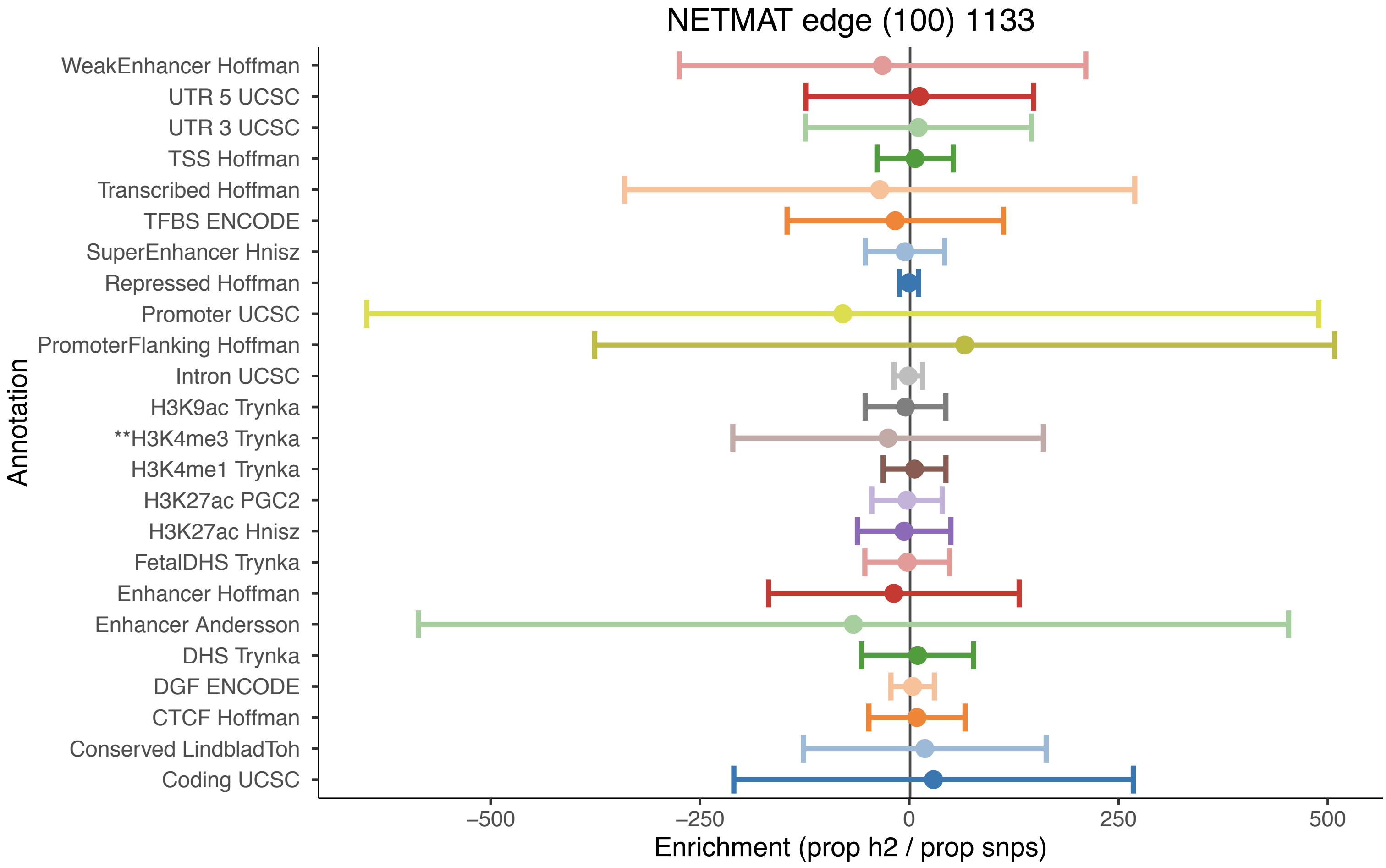
NETMAT edge (100) 1133
WeakEnhancer Hoffman
UTR 5 UCSC
UTR 3 UCSC
TSS Hoffman
Transcribed Hoffman
TFBS ENCODE
SuperEnhancer Hnisz
Repressed Hoffman
Promoter UCSC
PromoterFlanking Hoffman
Intron UCSC
H3K9ac Trynka
**H3K4me3 Trynka
H3K4me1 Trynka
H3K27ac PGC2
H3K27ac Hnisz
FetalDHS Trynka
Enhancer Hoffman
Enhancer Andersson
DHS Trynka
DGF ENCODE
CTCF Hoffman
Conserved LindbladToh
Coding UCSC
Annotation
−500
−250
0
250
500
Enrichment (prop h2 / prop snps)

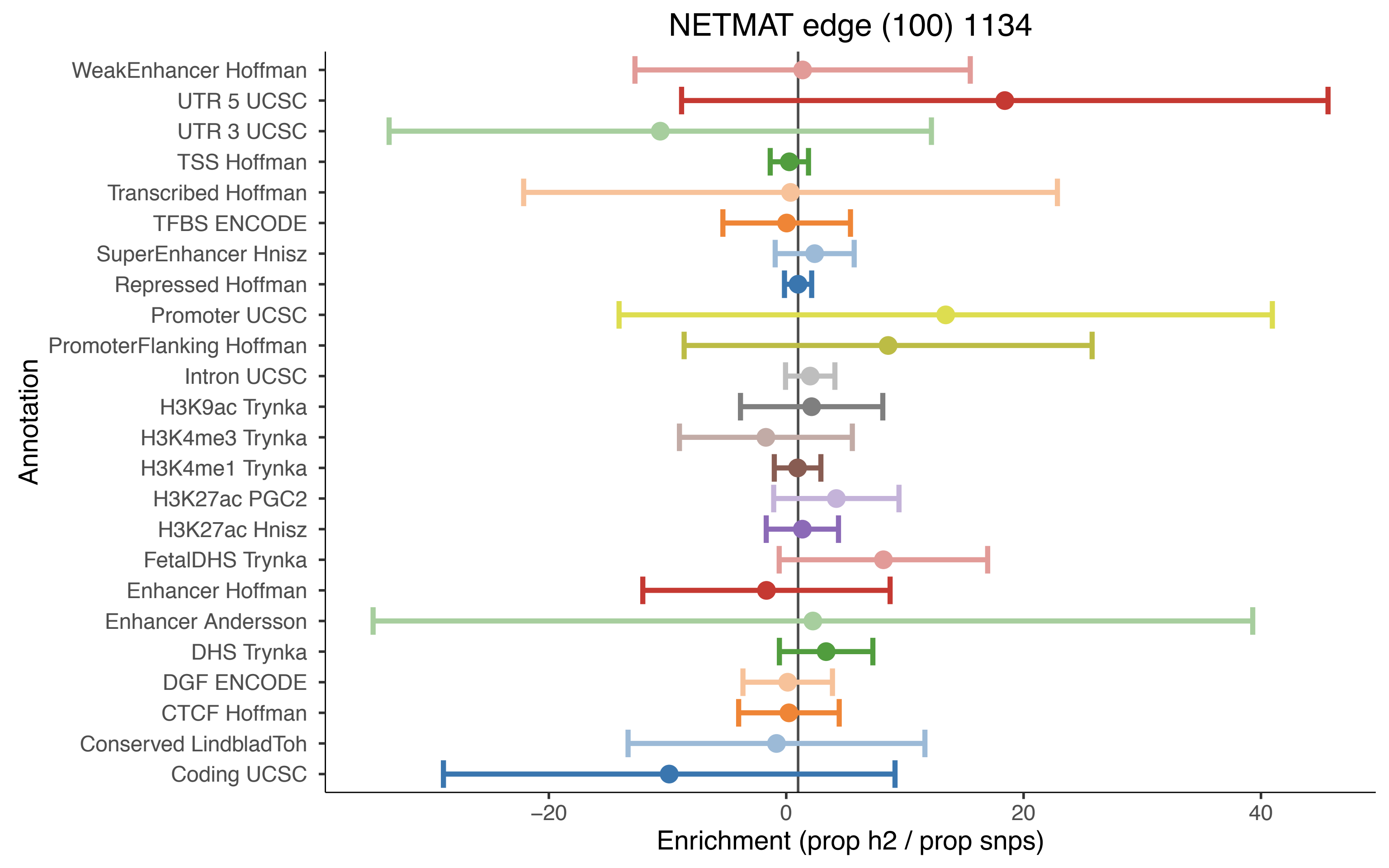

NETMAT edge (100) 1134
WeakEnhancer Hoffman
UTR 5 UCSC
UTR 3 UCSC
TSS Hoffman
Transcribed Hoffman
TFBS ENCODE
SuperEnhancer Hnisz
Repressed Hoffman
Promoter UCSC
PromoterFlanking Hoffman
Intron UCSC
H3K9ac Trynka
H3K4me3 Trynka
H3K4me1 Trynka
H3K27ac PGC2
H3K27ac Hnisz
FetalDHS Trynka
Enhancer Hoffman
Enhancer Andersson
DHS Trynka
DGF ENCODE
CTCF Hoffman
Conserved LindbladToh
Coding UCSC
Annotation
−20
0
20
40
Enrichment (prop h2 / prop snps)

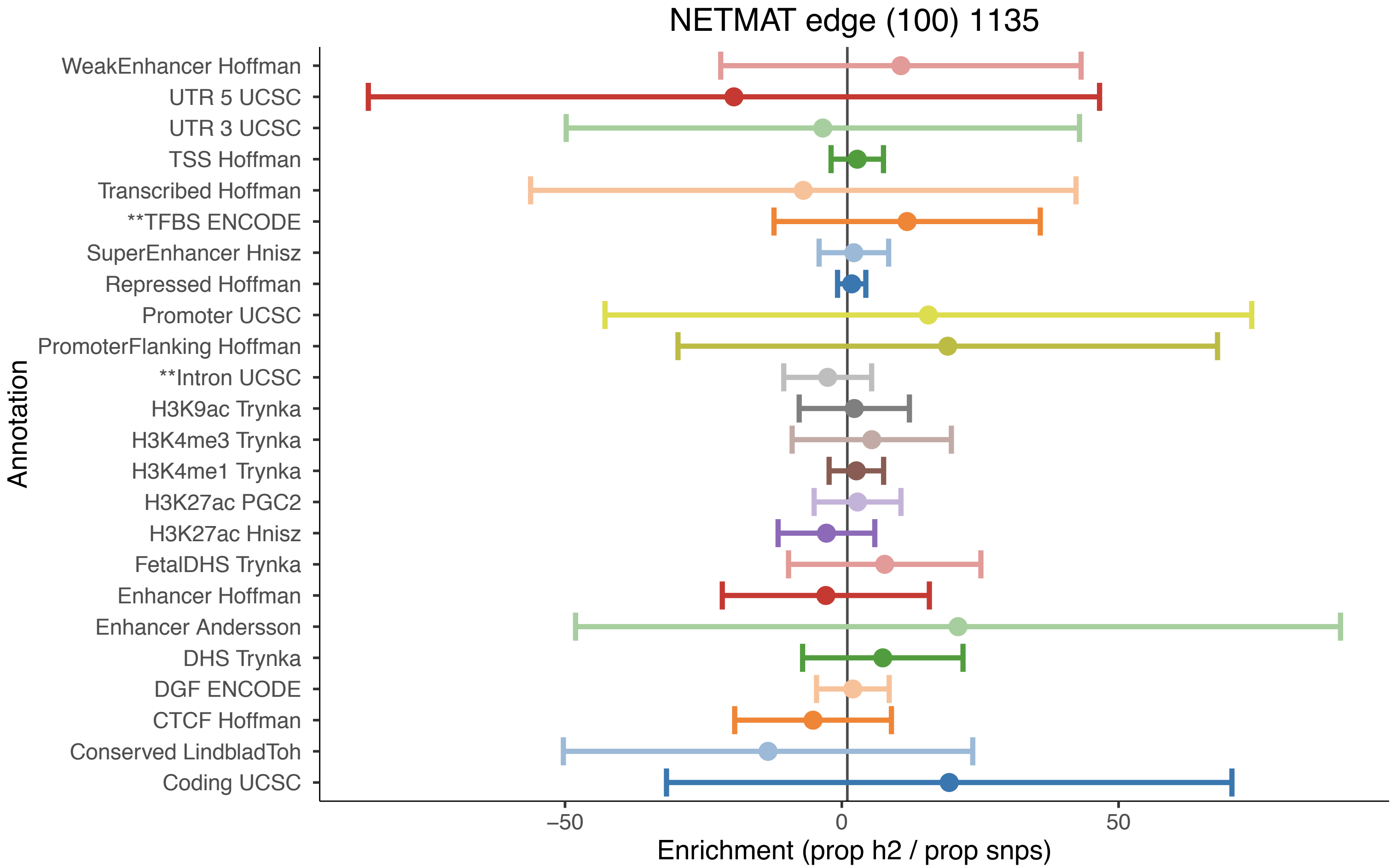

NETMAT edge (100) 1135
WeakEnhancer Hoffman
UTR 5 UCSC
UTR 3 UCSC
TSS Hoffman
Transcribed Hoffman
**TFBS ENCODE
SuperEnhancer Hnisz
Repressed Hoffman
Promoter UCSC
PromoterFlanking Hoffman
**Intron UCSC
H3K9ac Trynka
H3K4me3 Trynka
H3K4me1 Trynka
H3K27ac PGC2
H3K27ac Hnisz
FetalDHS Trynka
Enhancer Hoffman
Enhancer Andersson
DHS Trynka
DGF ENCODE
CTCF Hoffman
Conserved LindbladToh
Coding UCSC
Annotation
−50
0
50
Enrichment (prop h2 / prop snps)

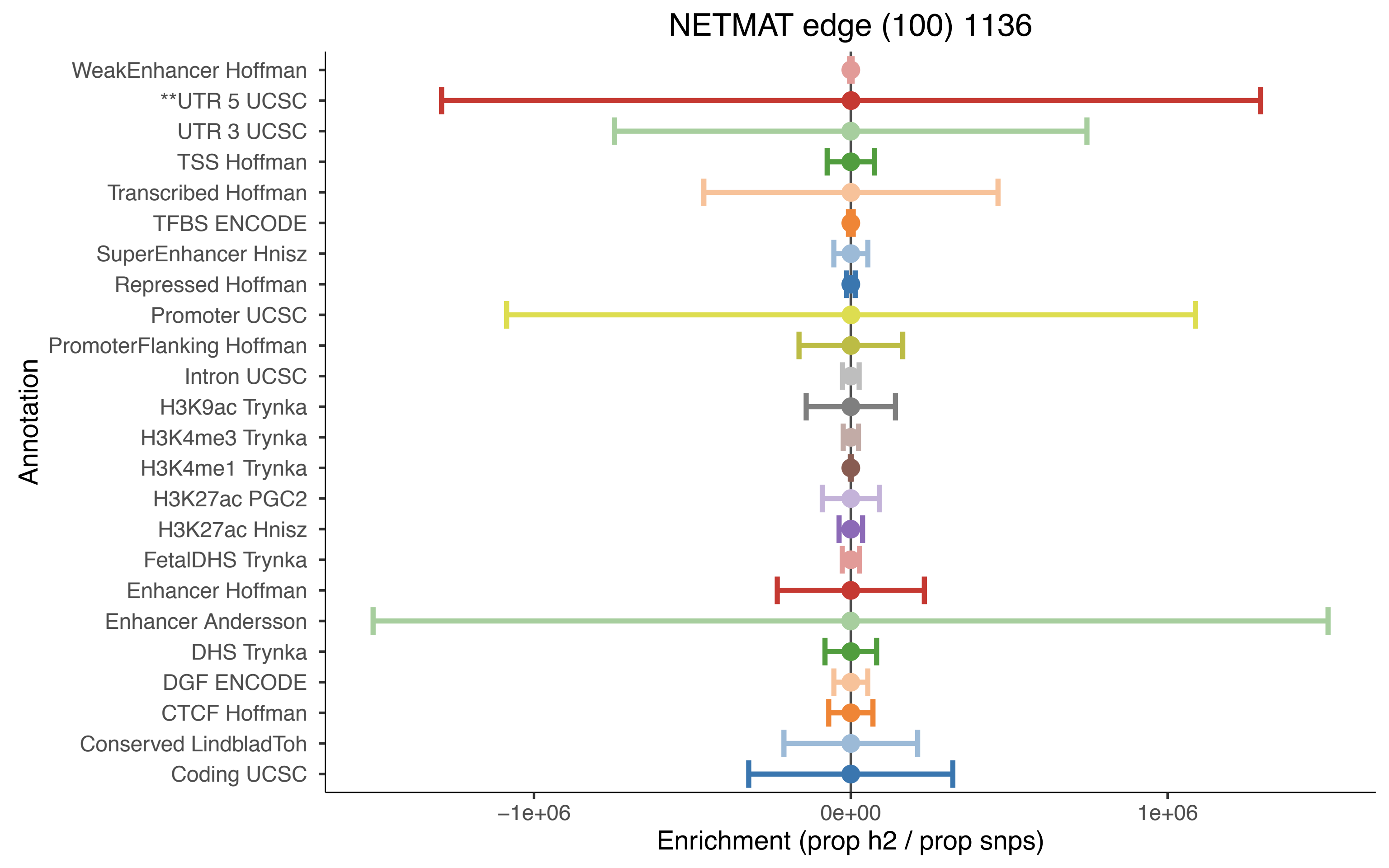
NETMAT edge (100) 1136
WeakEnhancer Hoffman
**UTR 5 UCSC
UTR 3 UCSC
TSS Hoffman
Transcribed Hoffman
TFBS ENCODE
SuperEnhancer Hnisz
Repressed Hoffman
Promoter UCSC
PromoterFlanking Hoffman
Intron UCSC
H3K9ac Trynka
H3K4me3 Trynka
H3K4me1 Trynka
H3K27ac PGC2
H3K27ac Hnisz
FetalDHS Trynka
Enhancer Hoffman
Enhancer Andersson
DHS Trynka
DGF ENCODE
CTCF Hoffman
Conserved LindbladToh
Coding UCSC
Annotation
−1e+06
0e+00
1e+06
Enrichment (prop h2 / prop snps)

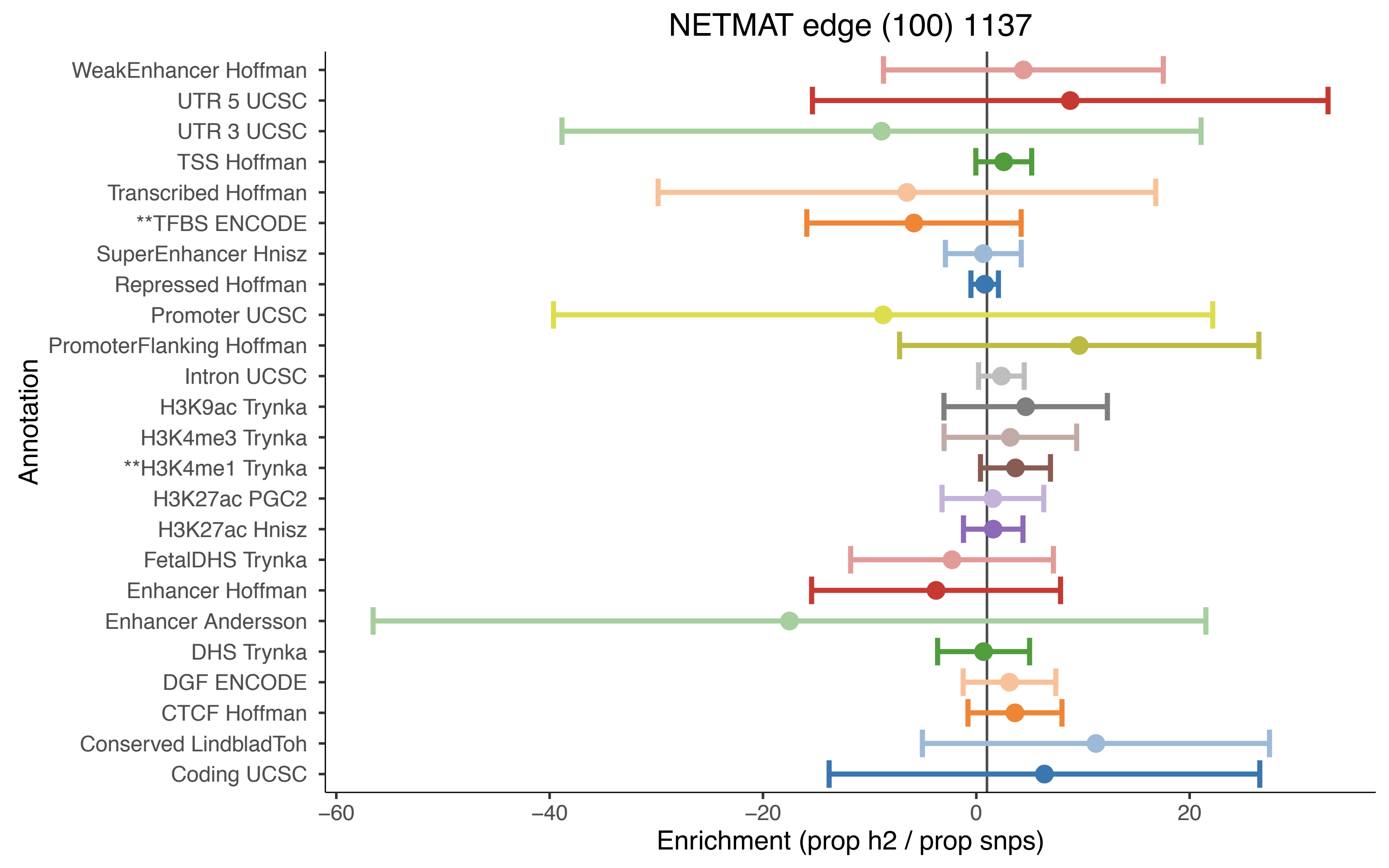
NETMAT edge (100) 1137
WeakEnhancer Hoffman
UTR 5 UCSC
UTR 3 UCSC
TSS Hoffman
Transcribed Hoffman
**TFBS ENCODE
SuperEnhancer Hnisz
Repressed Hoffman
Promoter UCSC
PromoterFlanking Hoffman
Intron UCSC
H3K9ac Trynka
H3K4me3 Trynka
**H3K4me1 Trynka
H3K27ac PGC2
H3K27ac Hnisz
FetalDHS Trynka
Enhancer Hoffman
Enhancer Andersson
DHS Trynka
DGF ENCODE
CTCF Hoffman
Conserved LindbladToh
Coding UCSC
Annotation
−60
−40
−20
0
20
Enrichment (prop h2 / prop snps)

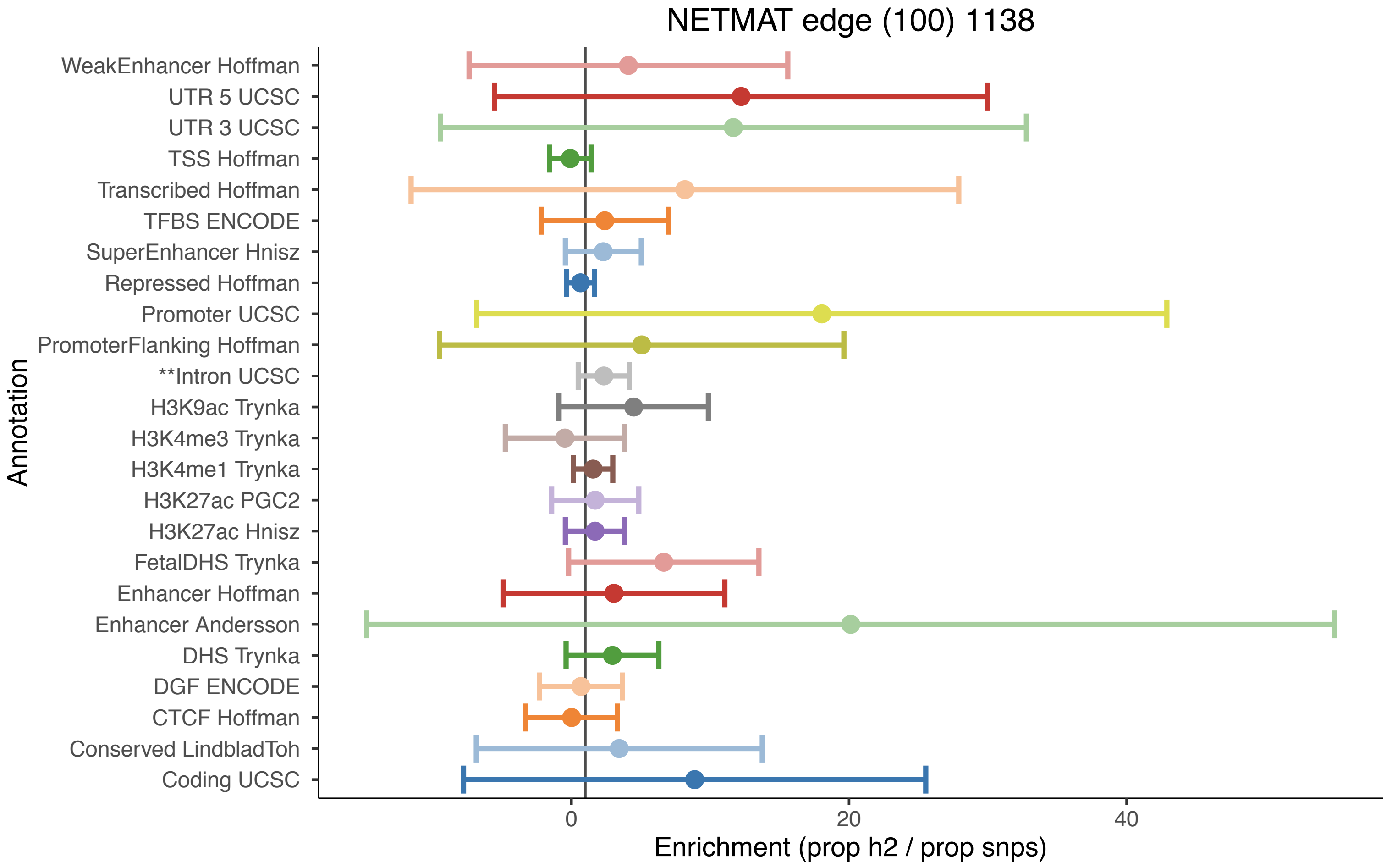
NETMAT edge (100) 1138
WeakEnhancer Hoffman
UTR 5 UCSC
UTR 3 UCSC
TSS Hoffman
Transcribed Hoffman
TFBS ENCODE
SuperEnhancer Hnisz
Repressed Hoffman
Promoter UCSC
PromoterFlanking Hoffman
**Intron UCSC
H3K9ac Trynka
H3K4me3 Trynka
H3K4me1 Trynka
H3K27ac PGC2
H3K27ac Hnisz
FetalDHS Trynka
Enhancer Hoffman
Enhancer Andersson
DHS Trynka
DGF ENCODE
CTCF Hoffman
Conserved LindbladToh
Coding UCSC
Annotation
0
20
40
Enrichment (prop h2 / prop snps)

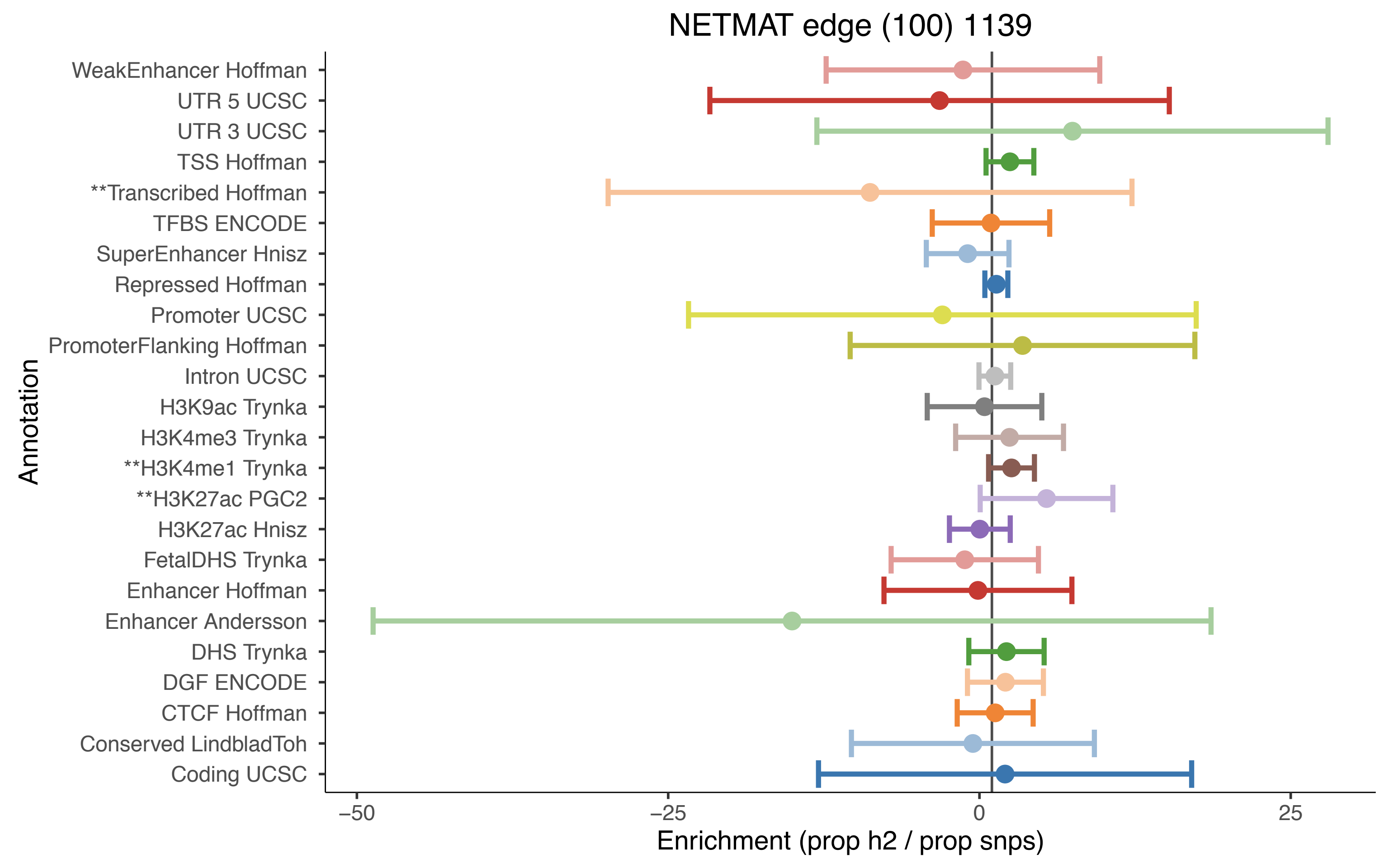
NETMAT edge (100) 1139
Annotation
WeakEnhancer Hoffman
UTR 5 UCSC
UTR 3 UCSC
TSS Hoffman
**Transcribed Hoffman
TFBS ENCODE
SuperEnhancer Hnisz
Repressed Hoffman
Promoter UCSC
PromoterFlanking Hoffman
Intron UCSC
H3K9ac Trynka
H3K4me3 Trynka
**H3K4me1 Trynka
**H3K27ac PGC2
H3K27ac Hnisz
FetalDHS Trynka
Enhancer Hoffman
Enhancer Andersson
DHS Trynka
DGF ENCODE
CTCF Hoffman
Conserved LindbladToh
Coding UCSC
−50
−25
0
25
Enrichment (prop h2 / prop snps)

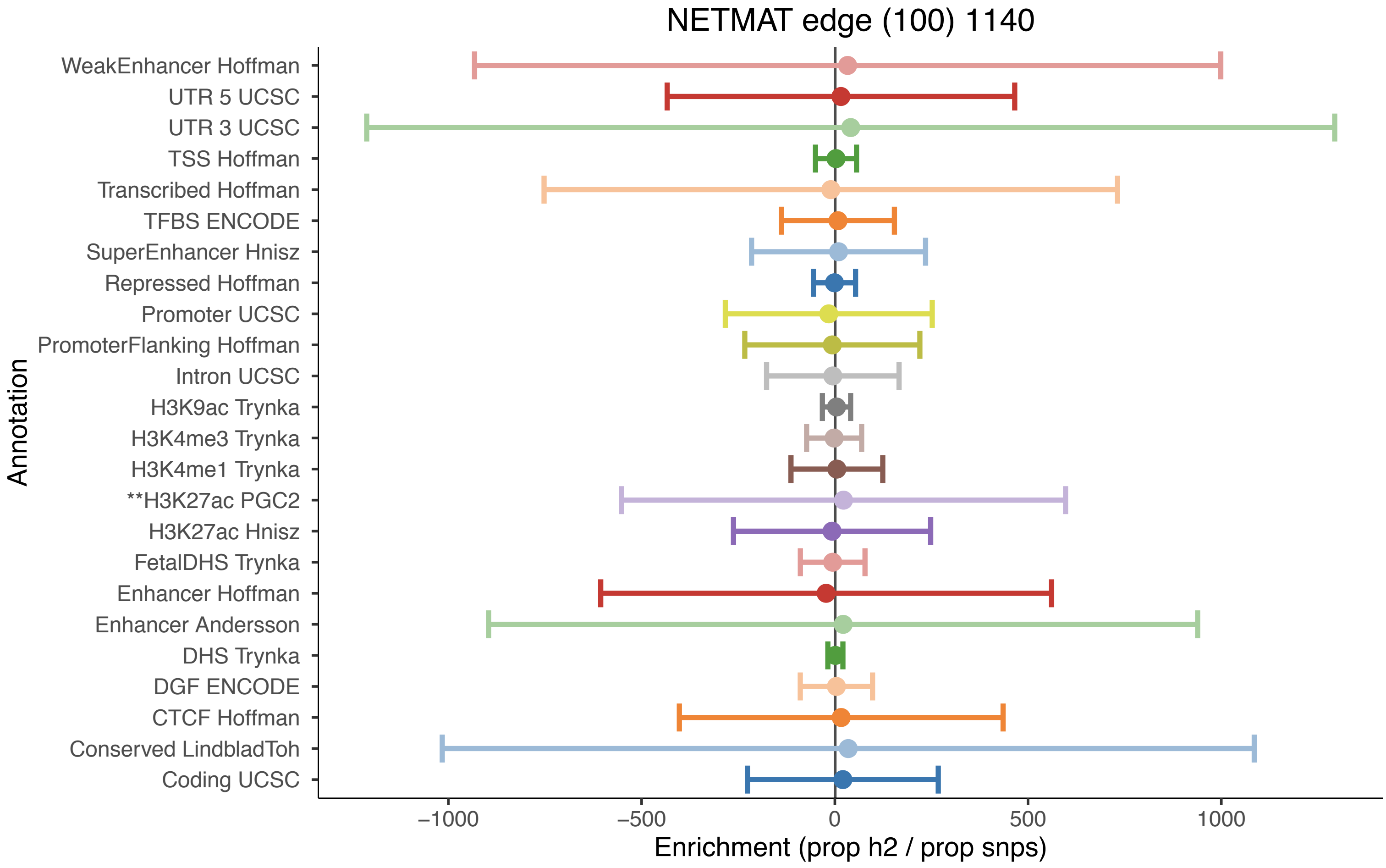
NETMAT edge (100) 1140
WeakEnhancer Hoffman
UTR 5 UCSC
UTR 3 UCSC
TSS Hoffman
Transcribed Hoffman
TFBS ENCODE
SuperEnhancer Hnisz
Repressed Hoffman
Promoter UCSC
PromoterFlanking Hoffman
Intron UCSC
H3K9ac Trynka
H3K4me3 Trynka
H3K4me1 Trynka
**H3K27ac PGC2
H3K27ac Hnisz
FetalDHS Trynka
Enhancer Hoffman
Enhancer Andersson
DHS Trynka
DGF ENCODE
CTCF Hoffman
Conserved LindbladToh
Coding UCSC
Annotation
−1000
−500
0
500
1000
Enrichment (prop h2 / prop snps)

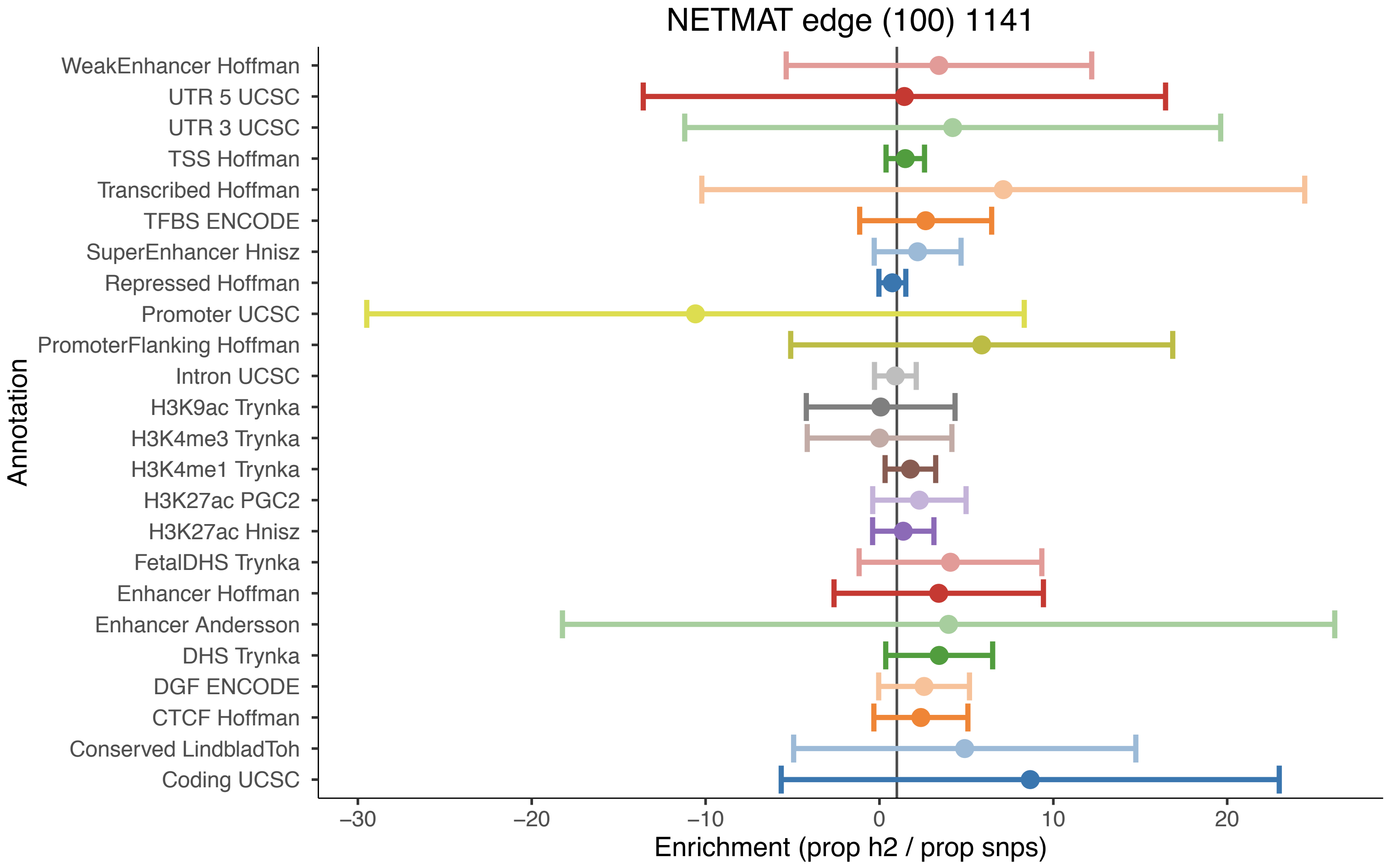
NETMAT edge (100) 1141
WeakEnhancer Hoffman
UTR 5 UCSC
UTR 3 UCSC
TSS Hoffman
Transcribed Hoffman
TFBS ENCODE
SuperEnhancer Hnisz
Repressed Hoffman
Promoter UCSC
PromoterFlanking Hoffman
Intron UCSC
H3K9ac Trynka
H3K4me3 Trynka
H3K4me1 Trynka
H3K27ac PGC2
H3K27ac Hnisz
FetalDHS Trynka
Enhancer Hoffman
Enhancer Andersson
DHS Trynka
DGF ENCODE
CTCF Hoffman
Conserved LindbladToh
Coding UCSC
Annotation
−30
−20
−10
0
10
20
Enrichment (prop h2 / prop snps)

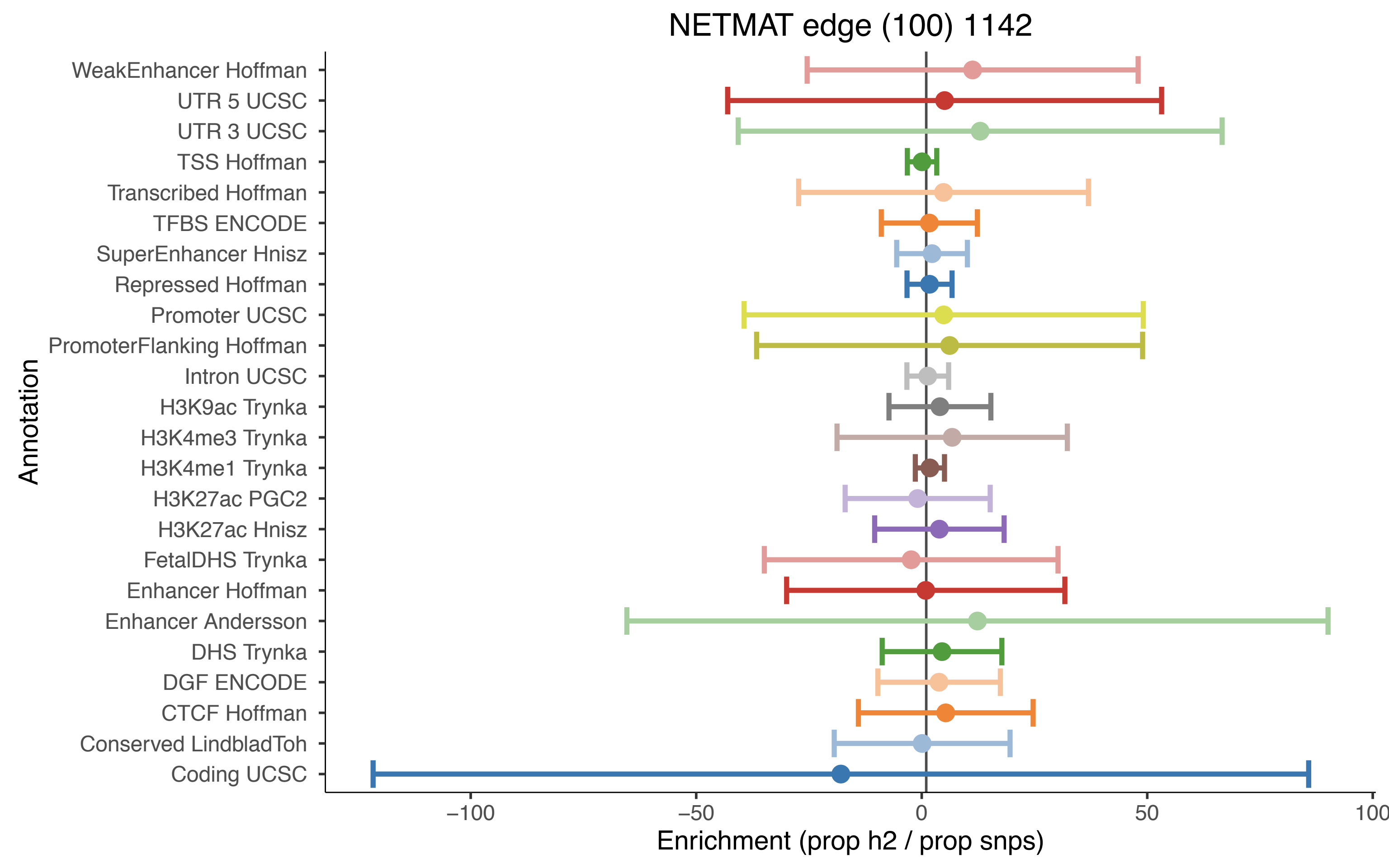
NETMAT edge (100) 1142
WeakEnhancer Hoffman
UTR 5 UCSC
UTR 3 UCSC
TSS Hoffman
Transcribed Hoffman
TFBS ENCODE
SuperEnhancer Hnisz
Repressed Hoffman
Promoter UCSC
PromoterFlanking Hoffman
Intron UCSC
H3K9ac Trynka
H3K4me3 Trynka
H3K4me1 Trynka
H3K27ac PGC2
H3K27ac Hnisz
FetalDHS Trynka
Enhancer Hoffman
Enhancer Andersson
DHS Trynka
DGF ENCODE
CTCF Hoffman
Conserved LindbladToh
Coding UCSC
Annotation
−100
−50
0
50
100
Enrichment (prop h2 / prop snps)

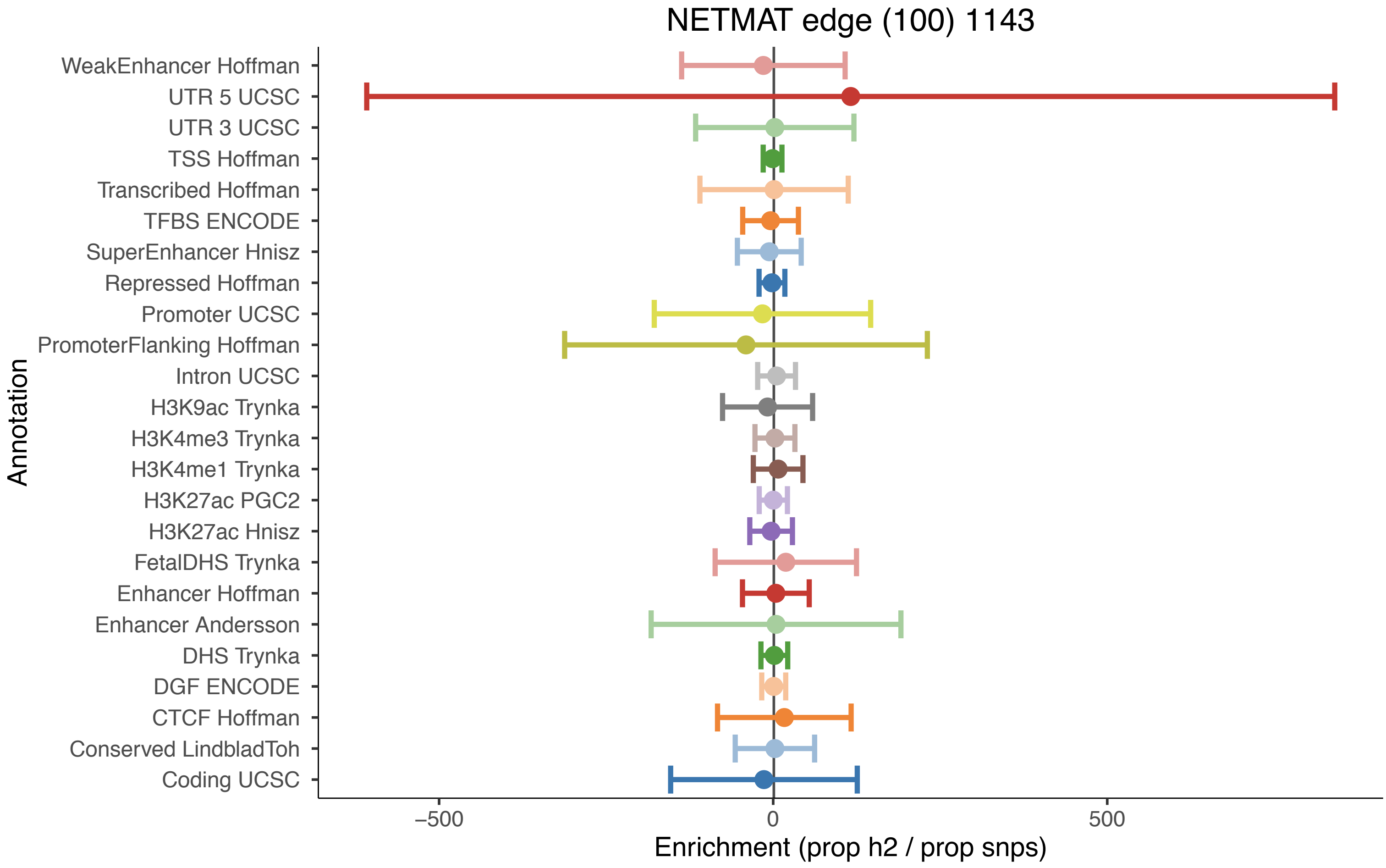

NETMAT edge (100) 1143
WeakEnhancer Hoffman
UTR 5 UCSC
UTR 3 UCSC
TSS Hoffman
Transcribed Hoffman
TFBS ENCODE
SuperEnhancer Hnisz
Repressed Hoffman
Promoter UCSC
PromoterFlanking Hoffman
Intron UCSC
H3K9ac Trynka
H3K4me3 Trynka
H3K4me1 Trynka
H3K27ac PGC2
H3K27ac Hnisz
FetalDHS Trynka
Enhancer Hoffman
Enhancer Andersson
DHS Trynka
DGF ENCODE
CTCF Hoffman
Conserved LindbladToh
Coding UCSC
Annotation
−500
0
500
Enrichment (prop h2 / prop snps)

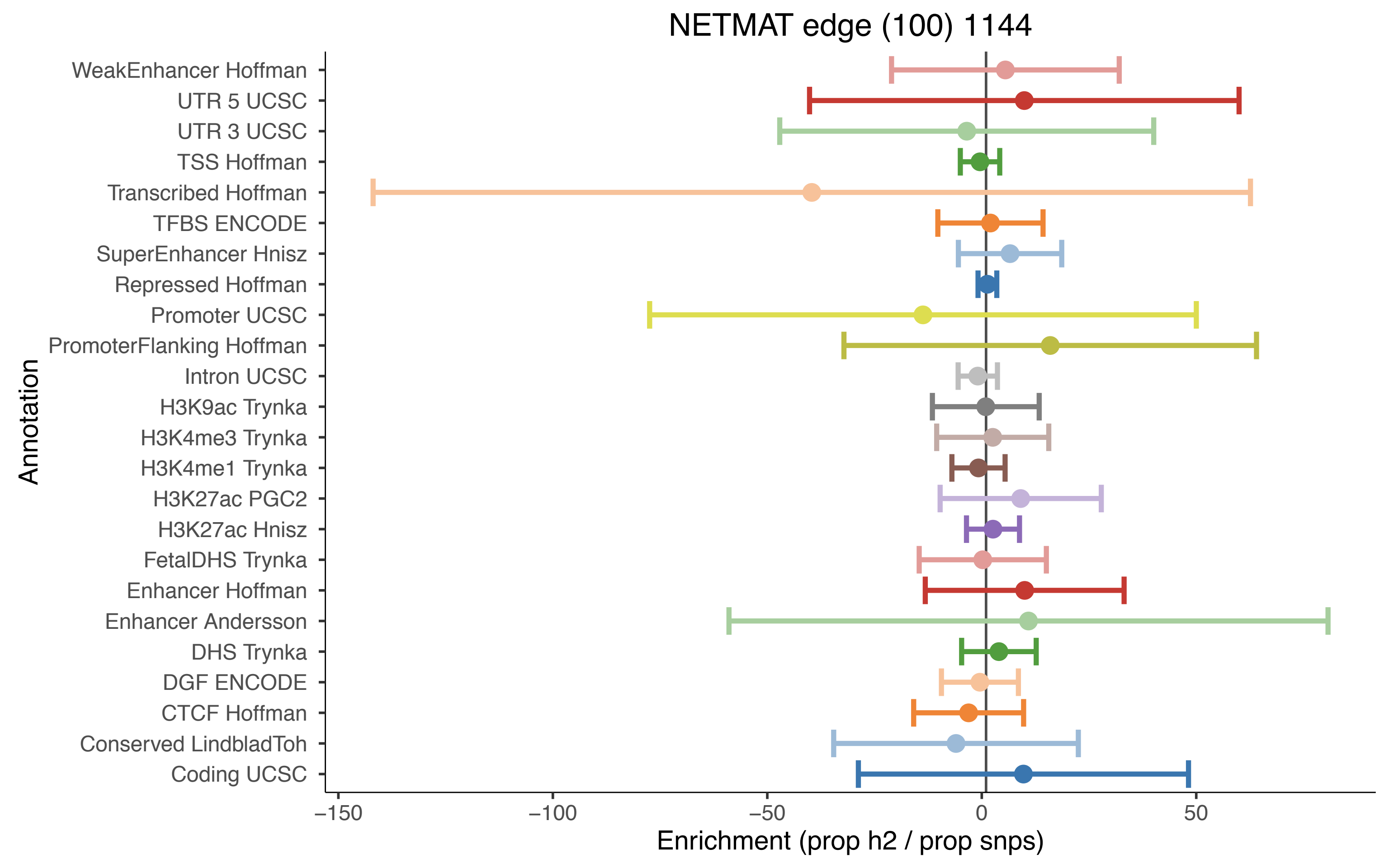

NETMAT edge (100) 1144
WeakEnhancer Hoffman
UTR 5 UCSC
UTR 3 UCSC
TSS Hoffman
Transcribed Hoffman
TFBS ENCODE
SuperEnhancer Hnisz
Repressed Hoffman
Promoter UCSC
PromoterFlanking Hoffman
Intron UCSC
H3K9ac Trynka
H3K4me3 Trynka
H3K4me1 Trynka
H3K27ac PGC2
H3K27ac Hnisz
FetalDHS Trynka
Enhancer Hoffman
Enhancer Andersson
DHS Trynka
DGF ENCODE
CTCF Hoffman
Conserved LindbladToh
Coding UCSC
Annotation
−150
−100
−50
0
50
Enrichment (prop h2 / prop snps)

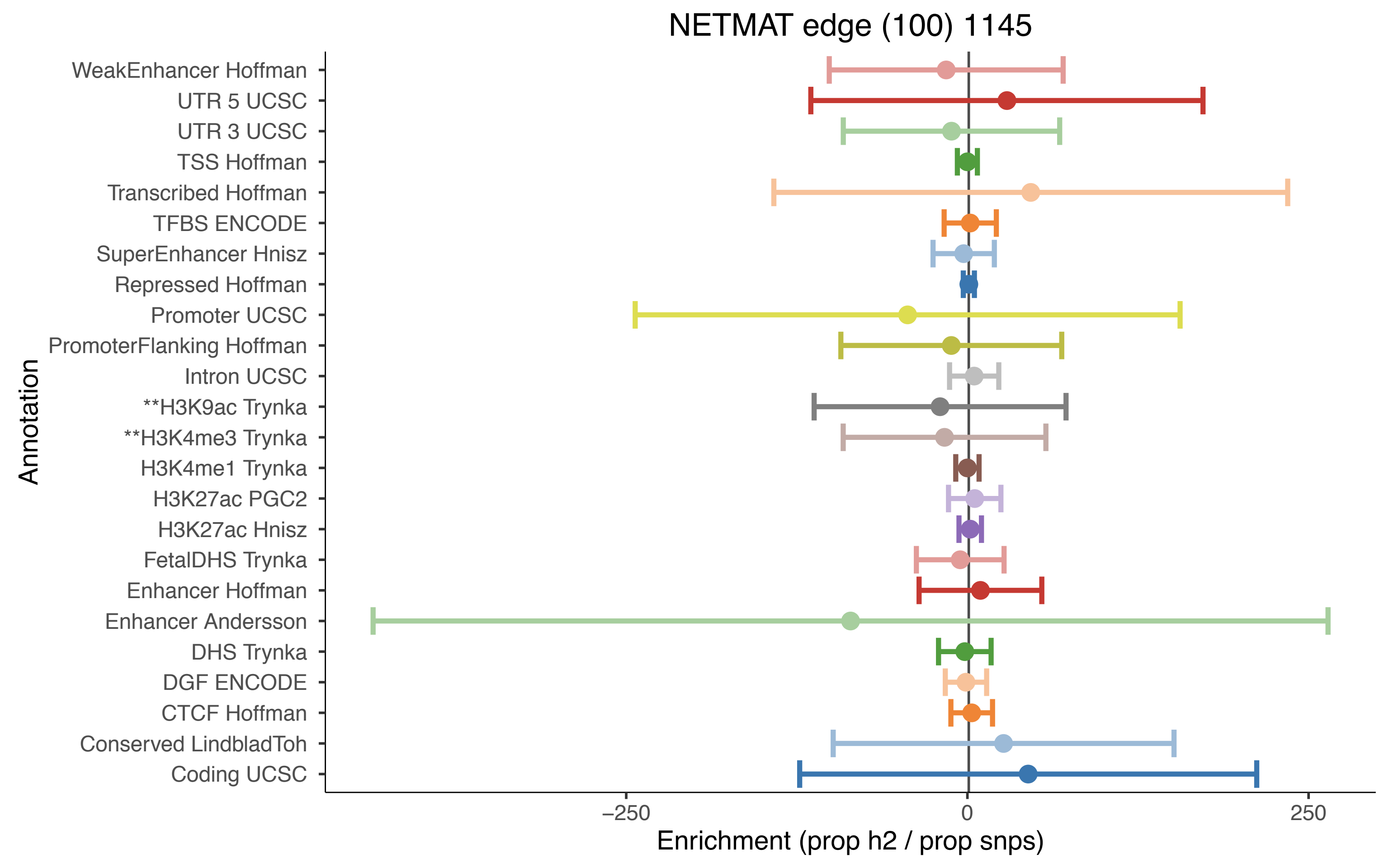
NETMAT edge (100) 1145
WeakEnhancer Hoffman
UTR 5 UCSC
UTR 3 UCSC
TSS Hoffman
Transcribed Hoffman
TFBS ENCODE
SuperEnhancer Hnisz
Repressed Hoffman
Promoter UCSC
PromoterFlanking Hoffman
Intron UCSC
**H3K9ac Trynka
**H3K4me3 Trynka
H3K4me1 Trynka
H3K27ac PGC2
H3K27ac Hnisz
FetalDHS Trynka
Enhancer Hoffman
Enhancer Andersson
DHS Trynka
DGF ENCODE
CTCF Hoffman
Conserved LindbladToh
Coding UCSC
Annotation
−250
0
250
Enrichment (prop h2 / prop snps)

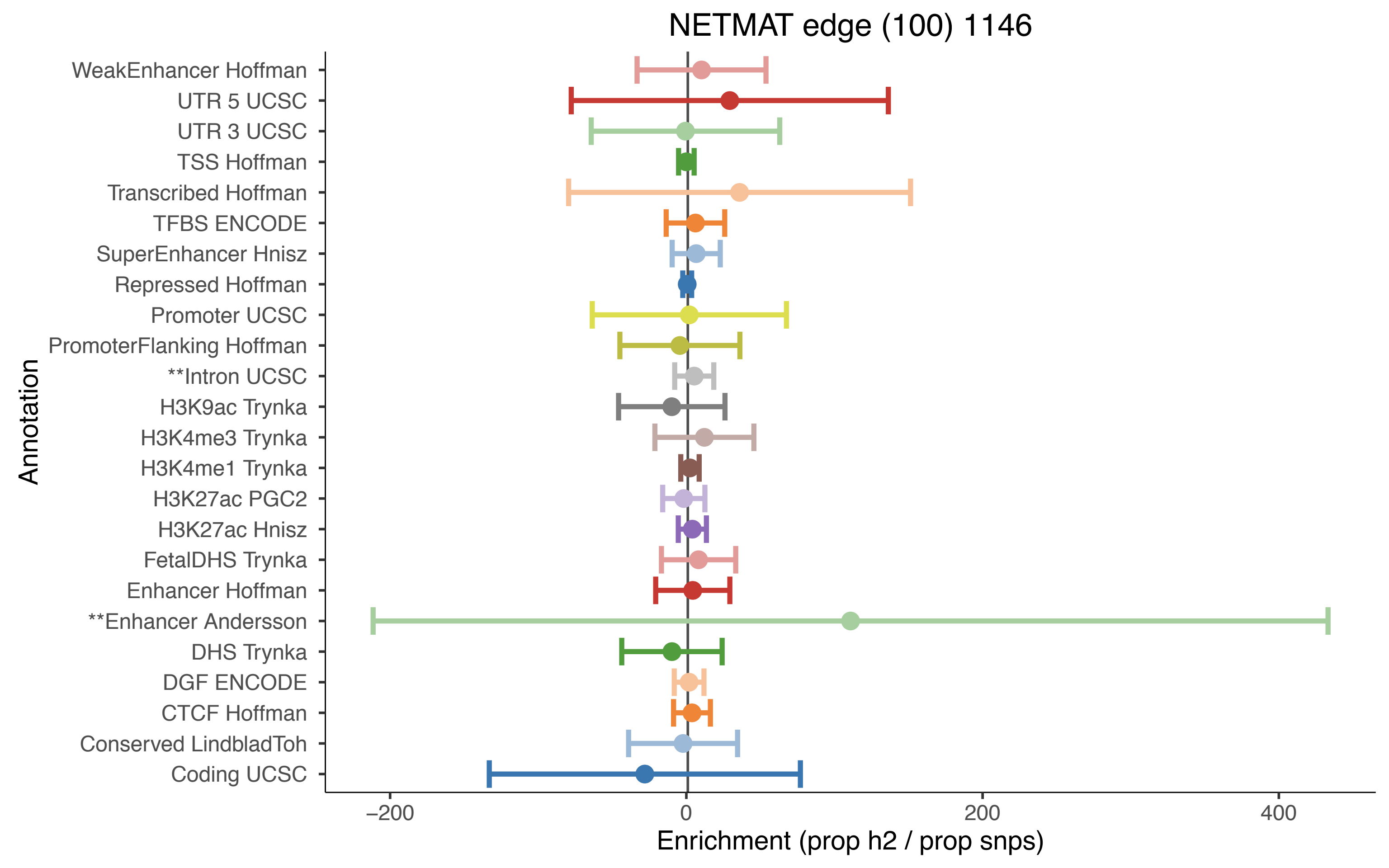
NETMAT edge (100) 1146
WeakEnhancer Hoffman
UTR 5 UCSC
UTR 3 UCSC
TSS Hoffman
Transcribed Hoffman
TFBS ENCODE
SuperEnhancer Hnisz
Repressed Hoffman
Promoter UCSC
PromoterFlanking Hoffman
**Intron UCSC
H3K9ac Trynka
H3K4me3 Trynka
H3K4me1 Trynka
H3K27ac PGC2
H3K27ac Hnisz
FetalDHS Trynka
Enhancer Hoffman
**Enhancer Andersson
DHS Trynka
DGF ENCODE
CTCF Hoffman
Conserved LindbladToh
Coding UCSC
Annotation
−200
0
200
400
Enrichment (prop h2 / prop snps)

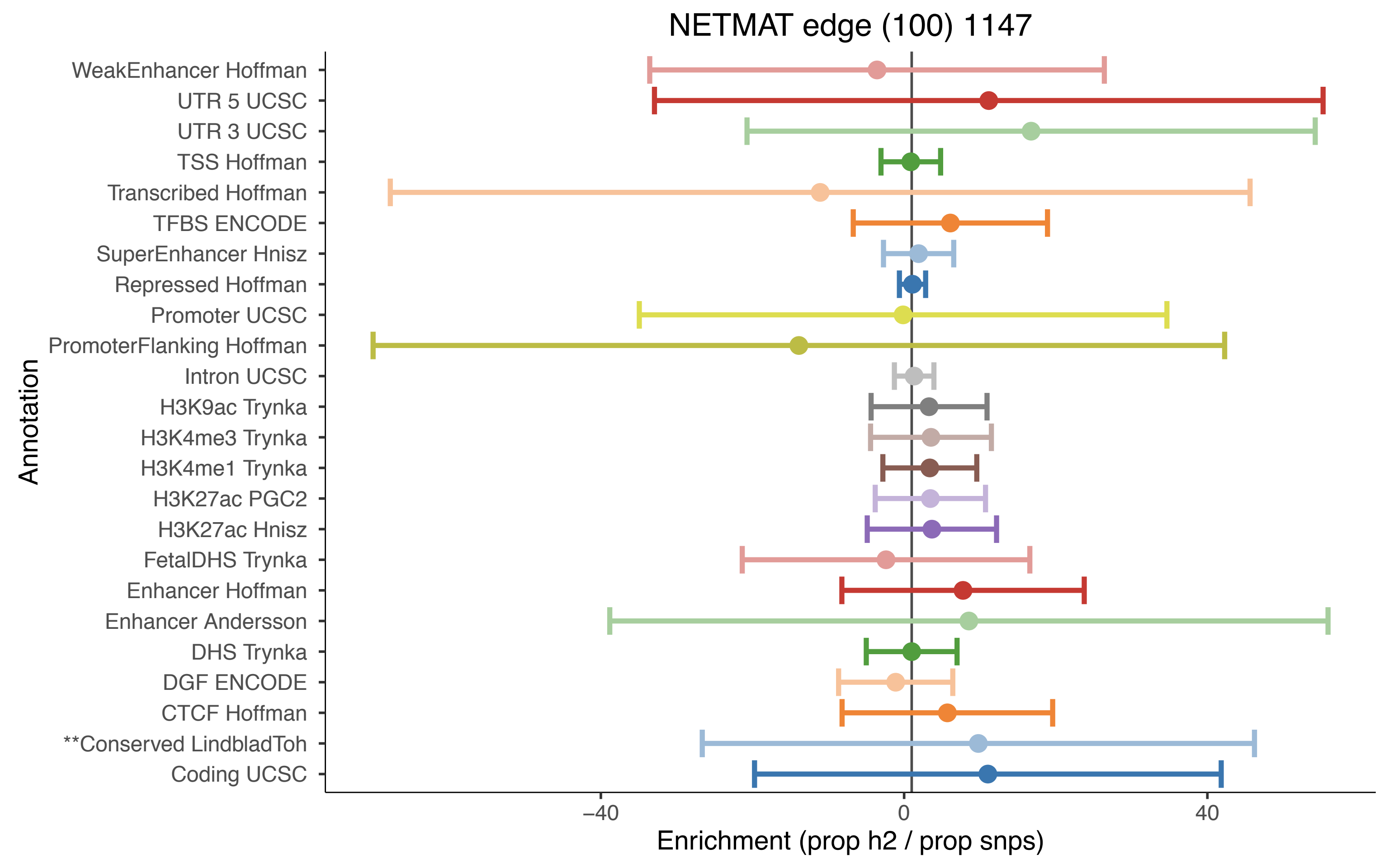
NETMAT edge (100) 1147
WeakEnhancer Hoffman
UTR 5 UCSC
UTR 3 UCSC
TSS Hoffman
Transcribed Hoffman
TFBS ENCODE
SuperEnhancer Hnisz
Repressed Hoffman
Promoter UCSC
PromoterFlanking Hoffman
Intron UCSC
H3K9ac Trynka
H3K4me3 Trynka
H3K4me1 Trynka
H3K27ac PGC2
H3K27ac Hnisz
FetalDHS Trynka
Enhancer Hoffman
Enhancer Andersson
DHS Trynka
DGF ENCODE
CTCF Hoffman
**Conserved LindbladToh
Coding UCSC
Annotation
−40
0
40
Enrichment (prop h2 / prop snps)

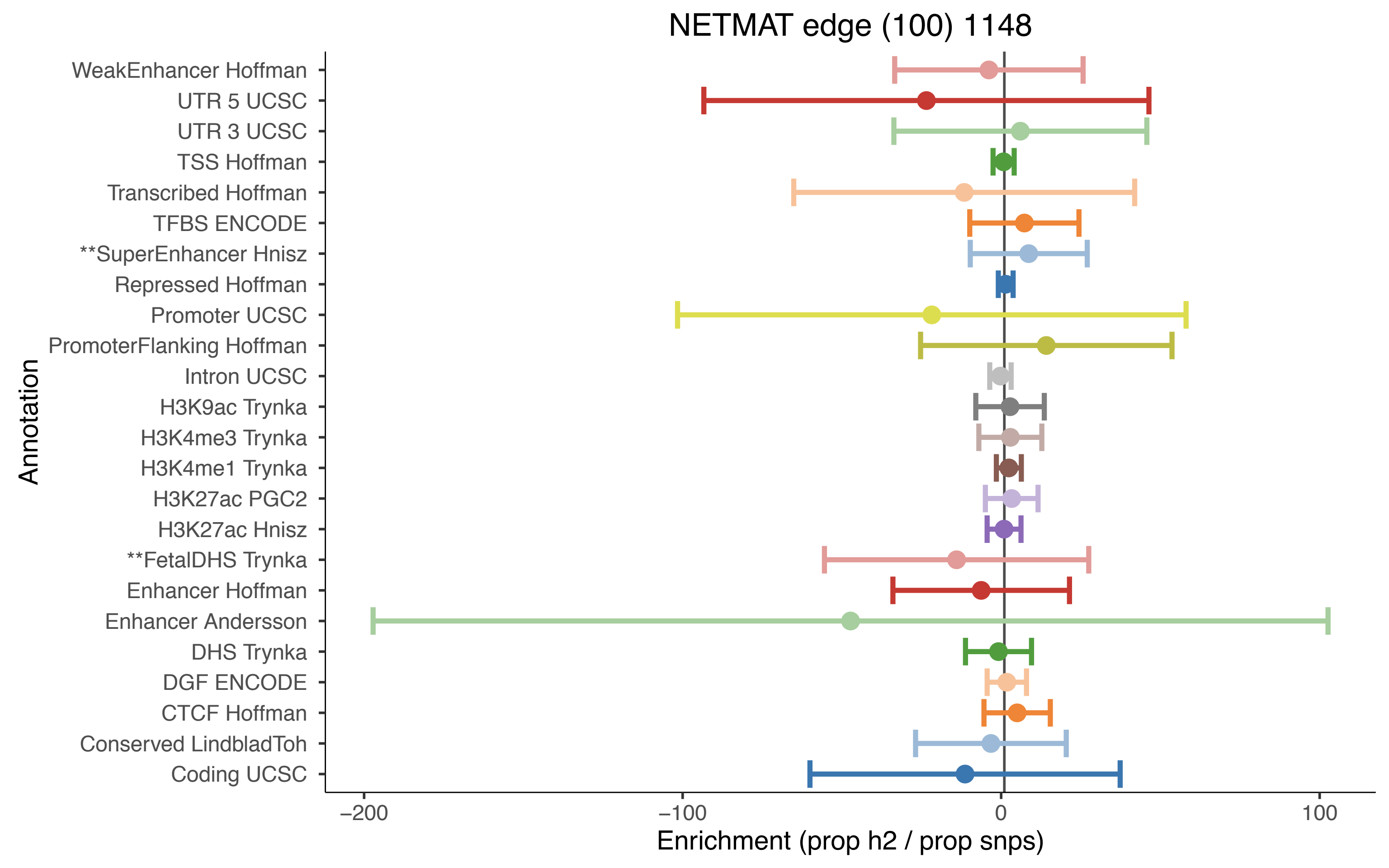
NETMAT edge (100) 1148
WeakEnhancer Hoffman
UTR 5 UCSC
UTR 3 UCSC
TSS Hoffman
Transcribed Hoffman
TFBS ENCODE
**SuperEnhancer Hnisz
Repressed Hoffman
Promoter UCSC
PromoterFlanking Hoffman
Intron UCSC
H3K9ac Trynka
H3K4me3 Trynka
H3K4me1 Trynka
H3K27ac PGC2
H3K27ac Hnisz
**FetalDHS Trynka
Enhancer Hoffman
Enhancer Andersson
DHS Trynka
DGF ENCODE
CTCF Hoffman
Conserved LindbladToh
Coding UCSC
Annotation
−200
−100
0
100
Enrichment (prop h2 / prop snps)

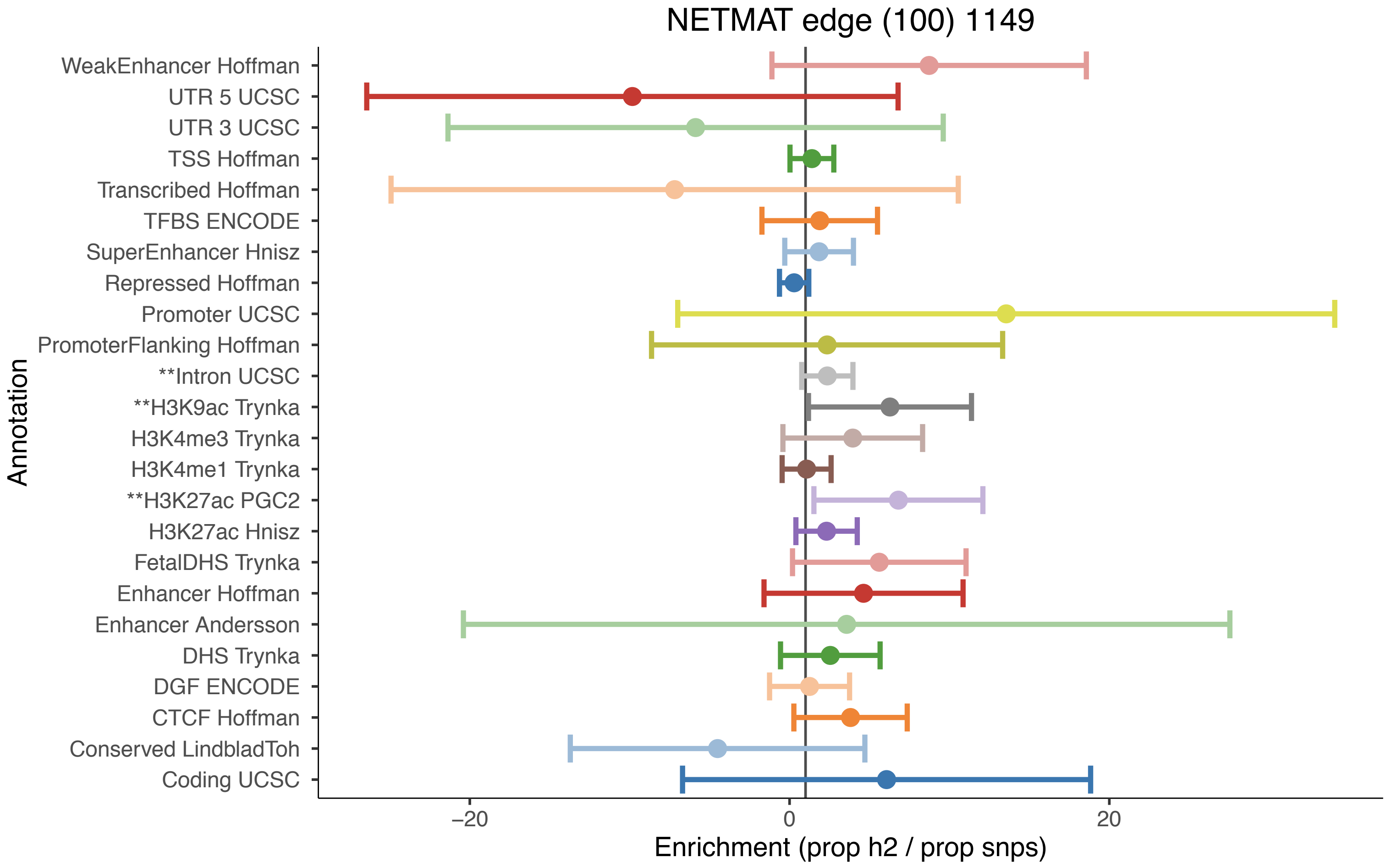

NETMAT edge (100) 1149
WeakEnhancer Hoffman
UTR 5 UCSC
UTR 3 UCSC
TSS Hoffman
Transcribed Hoffman
TFBS ENCODE
SuperEnhancer Hnisz
Repressed Hoffman
Promoter UCSC
PromoterFlanking Hoffman
**Intron UCSC
**H3K9ac Trynka
H3K4me3 Trynka
H3K4me1 Trynka
**H3K27ac PGC2
H3K27ac Hnisz
FetalDHS Trynka
Enhancer Hoffman
Enhancer Andersson
DHS Trynka
DGF ENCODE
CTCF Hoffman
Conserved LindbladToh
Coding UCSC
Annotation
−20
0
20
Enrichment (prop h2 / prop snps)

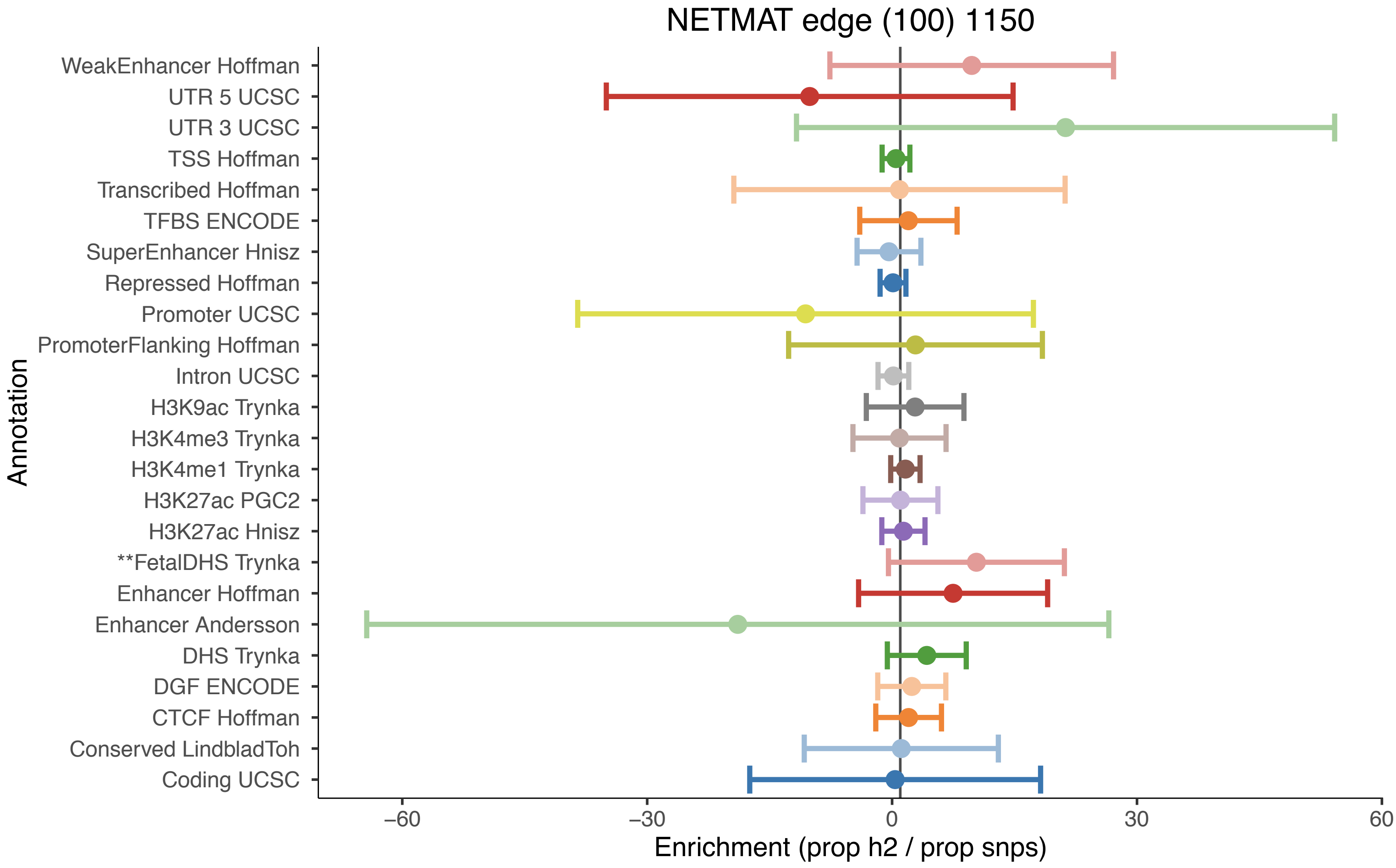
NETMAT edge (100) 1150
WeakEnhancer Hoffman
UTR 5 UCSC
UTR 3 UCSC
TSS Hoffman
Transcribed Hoffman
TFBS ENCODE
SuperEnhancer Hnisz
Repressed Hoffman
Promoter UCSC
PromoterFlanking Hoffman
Intron UCSC
H3K9ac Trynka
H3K4me3 Trynka
H3K4me1 Trynka
H3K27ac PGC2
H3K27ac Hnisz
**FetalDHS Trynka
Enhancer Hoffman
Enhancer Andersson
DHS Trynka
DGF ENCODE
CTCF Hoffman
Conserved LindbladToh
Coding UCSC
Annotation
−60
−30
0
30
60
Enrichment (prop h2 / prop snps)

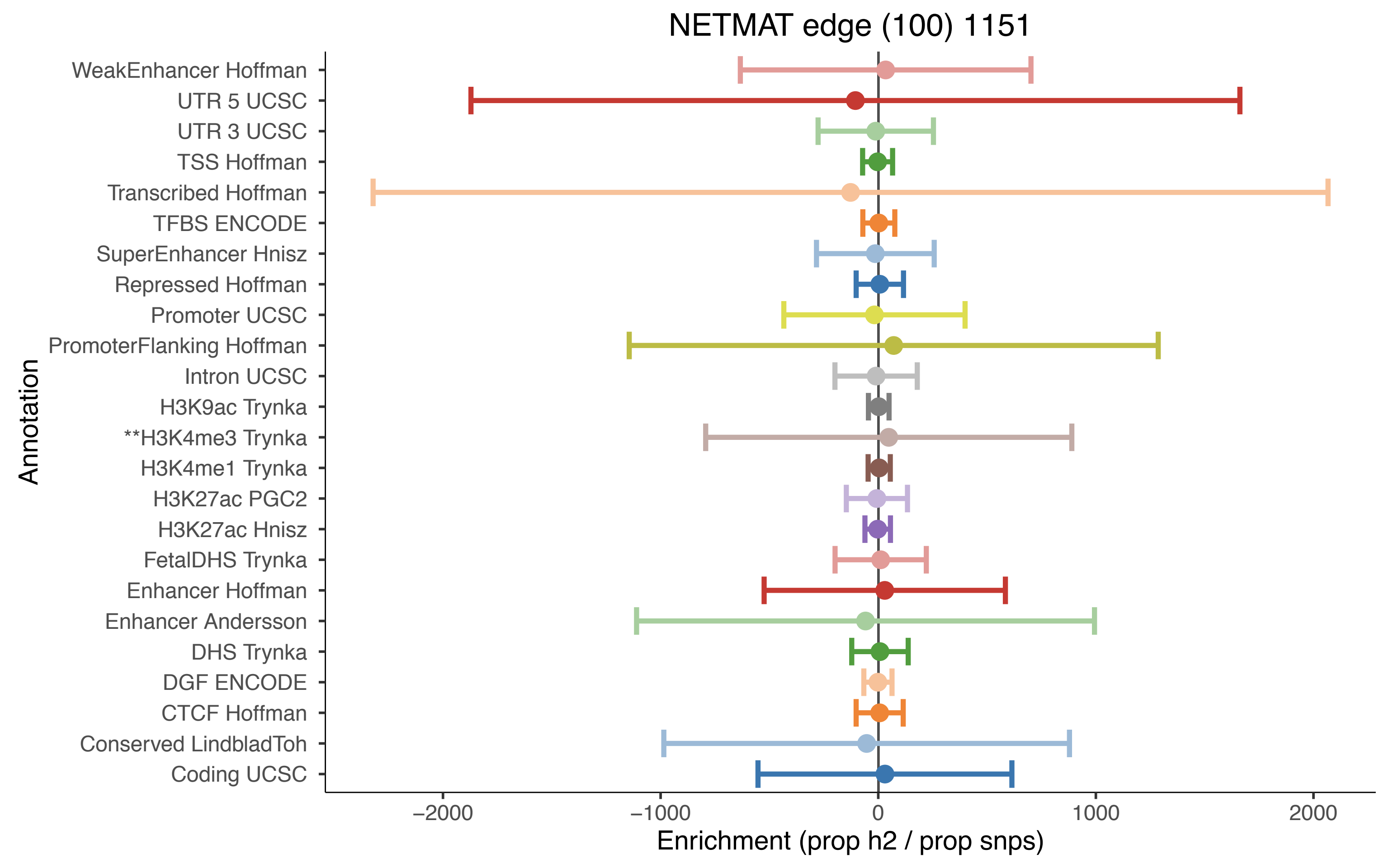

NETMAT edge (100) 1151
WeakEnhancer Hoffman
UTR 5 UCSC
UTR 3 UCSC
TSS Hoffman
Transcribed Hoffman
TFBS ENCODE
SuperEnhancer Hnisz
Repressed Hoffman
Promoter UCSC
PromoterFlanking Hoffman
Intron UCSC
H3K9ac Trynka
**H3K4me3 Trynka
H3K4me1 Trynka
H3K27ac PGC2
H3K27ac Hnisz
FetalDHS Trynka
Enhancer Hoffman
Enhancer Andersson
DHS Trynka
DGF ENCODE
CTCF Hoffman
Conserved LindbladToh
Coding UCSC
Annotation
−2000
−1000
0
1000
2000
Enrichment (prop h2 / prop snps)

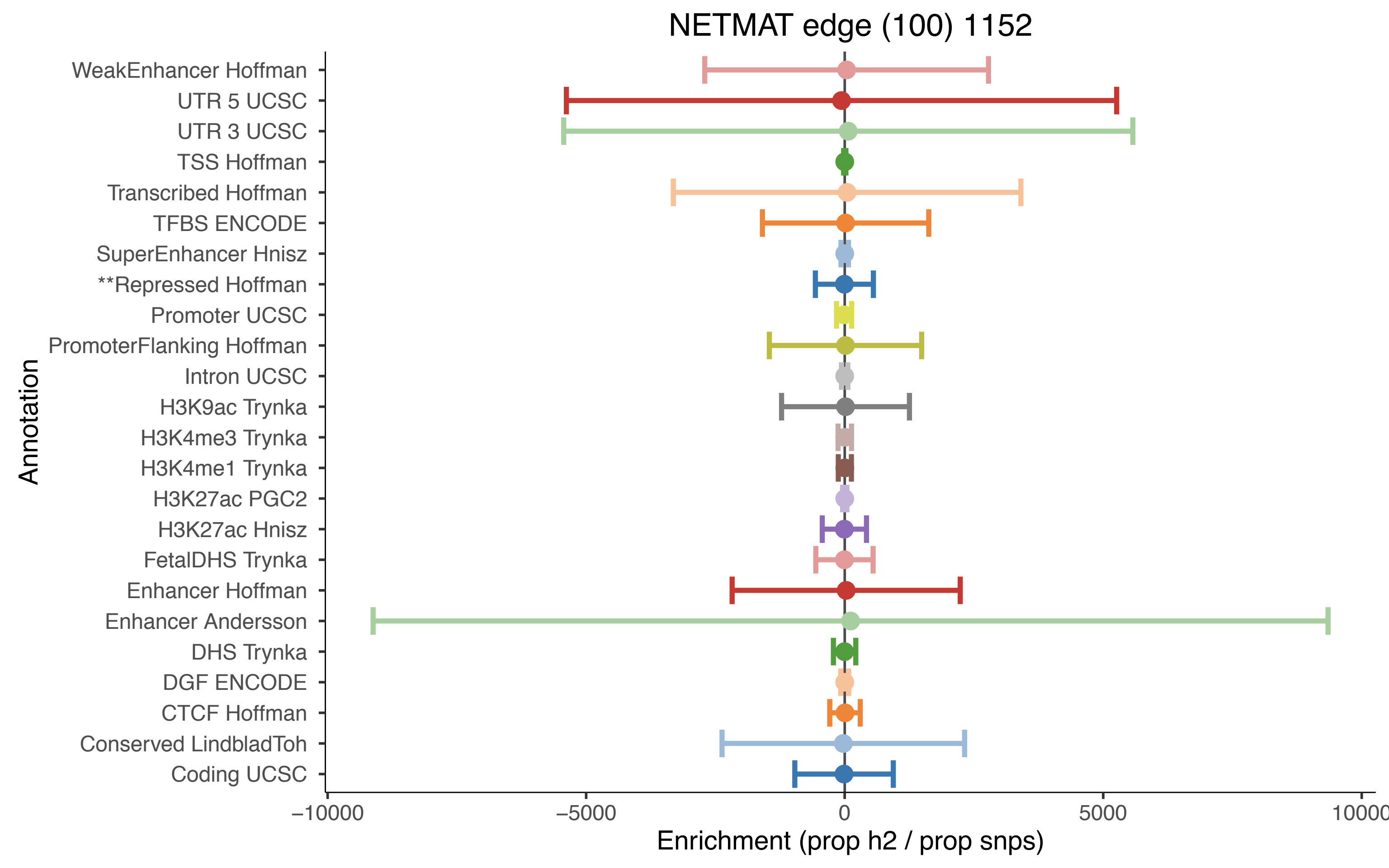

NETMAT edge (100) 1152
WeakEnhancer Hoffman
UTR 5 UCSC
UTR 3 UCSC
TSS Hoffman
Transcribed Hoffman
TFBS ENCODE
SuperEnhancer Hnisz
**Repressed Hoffman
Promoter UCSC
PromoterFlanking Hoffman
Intron UCSC
H3K9ac Trynka
H3K4me3 Trynka
H3K4me1 Trynka
H3K27ac PGC2
H3K27ac Hnisz
FetalDHS Trynka
Enhancer Hoffman
Enhancer Andersson
DHS Trynka
DGF ENCODE
CTCF Hoffman
Conserved LindbladToh
Coding UCSC
Annotation
−10000
−5000
0
5000
10000
Enrichment (prop h2 / prop snps)

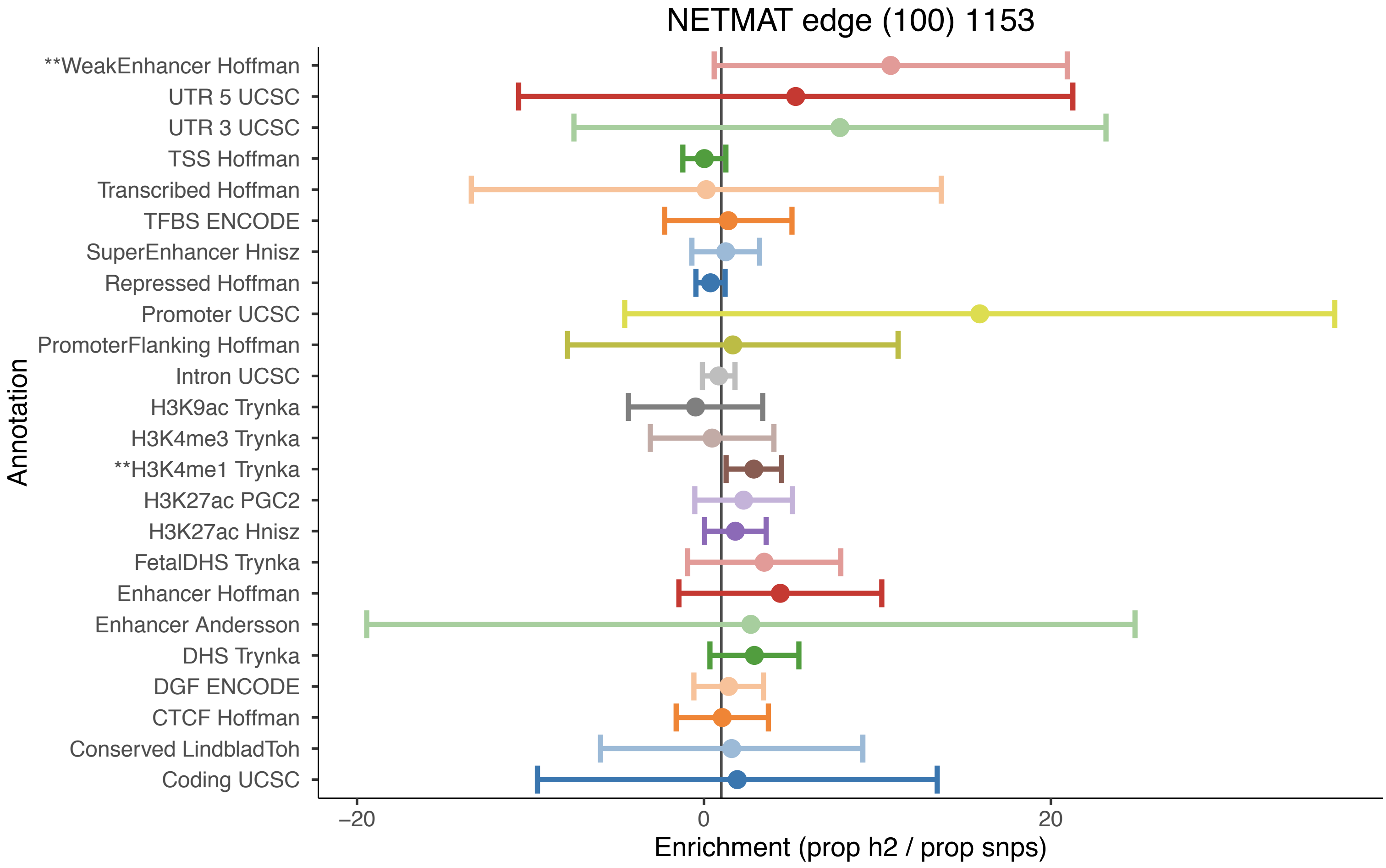
NETMAT edge (100) 1153
Annotation
**WeakEnhancer Hoffman
UTR 5 UCSC
UTR 3 UCSC
TSS Hoffman
Transcribed Hoffman
TFBS ENCODE
SuperEnhancer Hnisz
Repressed Hoffman
Promoter UCSC
PromoterFlanking Hoffman
Intron UCSC
H3K9ac Trynka
H3K4me3 Trynka
**H3K4me1 Trynka
H3K27ac PGC2
H3K27ac Hnisz
FetalDHS Trynka
Enhancer Hoffman
Enhancer Andersson
DHS Trynka
DGF ENCODE
CTCF Hoffman
Conserved LindbladToh
Coding UCSC
−20
0
20
Enrichment (prop h2 / prop snps)

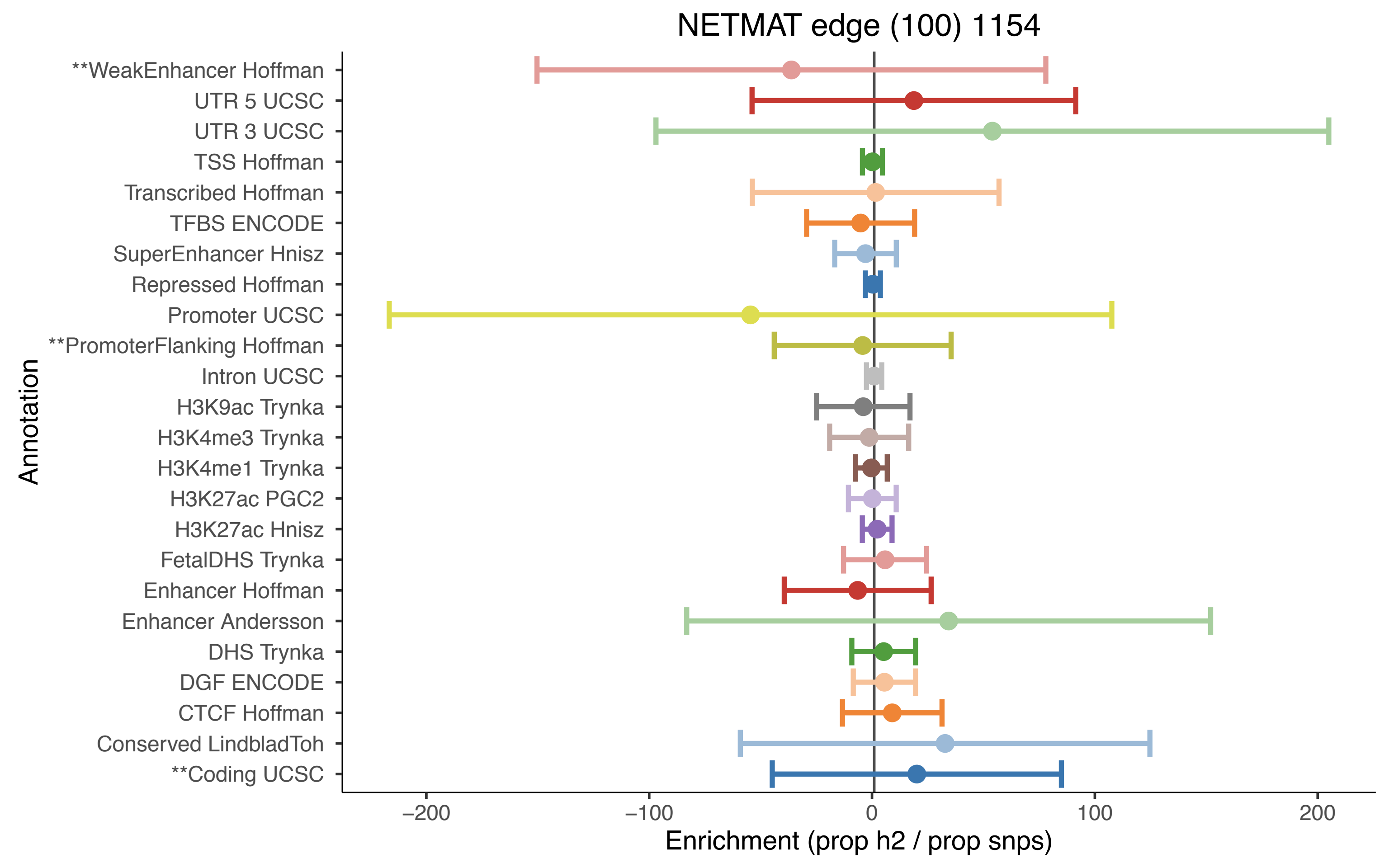
NETMAT edge (100) 1154
**WeakEnhancer Hoffman
UTR 5 UCSC
UTR 3 UCSC
TSS Hoffman
Transcribed Hoffman
TFBS ENCODE
SuperEnhancer Hnisz
Repressed Hoffman
Promoter UCSC
**PromoterFlanking Hoffman
Intron UCSC
H3K9ac Trynka
H3K4me3 Trynka
H3K4me1 Trynka
H3K27ac PGC2
H3K27ac Hnisz
FetalDHS Trynka
Enhancer Hoffman
Enhancer Andersson
DHS Trynka
DGF ENCODE
CTCF Hoffman
Conserved LindbladToh
**Coding UCSC
Annotation
−200
−100
0
100
200
Enrichment (prop h2 / prop snps)

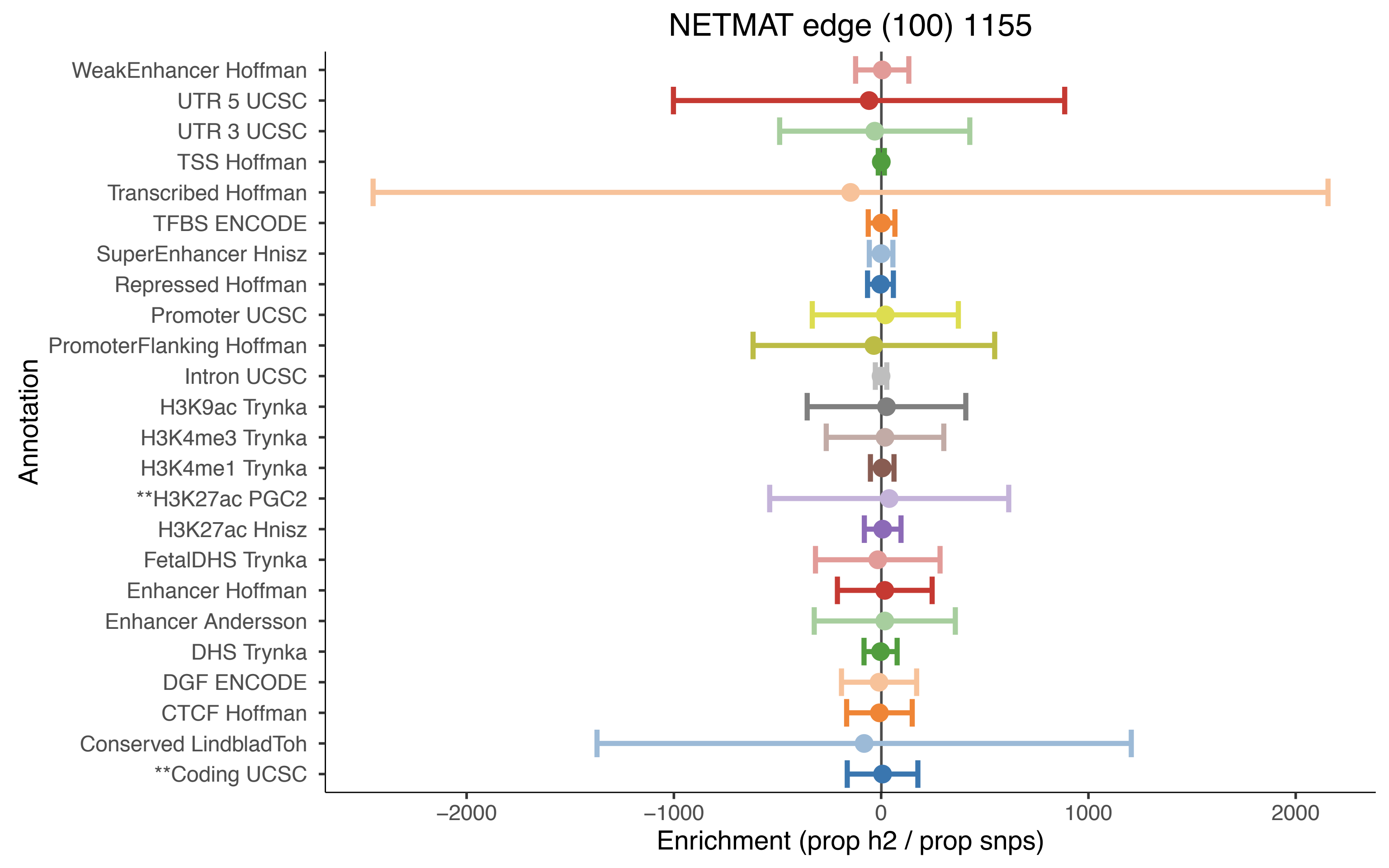

NETMAT edge (100) 1155
WeakEnhancer Hoffman
UTR 5 UCSC
UTR 3 UCSC
TSS Hoffman
Transcribed Hoffman
TFBS ENCODE
SuperEnhancer Hnisz
Repressed Hoffman
Promoter UCSC
PromoterFlanking Hoffman
Intron UCSC
H3K9ac Trynka
H3K4me3 Trynka
H3K4me1 Trynka
**H3K27ac PGC2
H3K27ac Hnisz
FetalDHS Trynka
Enhancer Hoffman
Enhancer Andersson
DHS Trynka
DGF ENCODE
CTCF Hoffman
Conserved LindbladToh
**Coding UCSC
Annotation
−2000
−1000
0
1000
2000
Enrichment (prop h2 / prop snps)

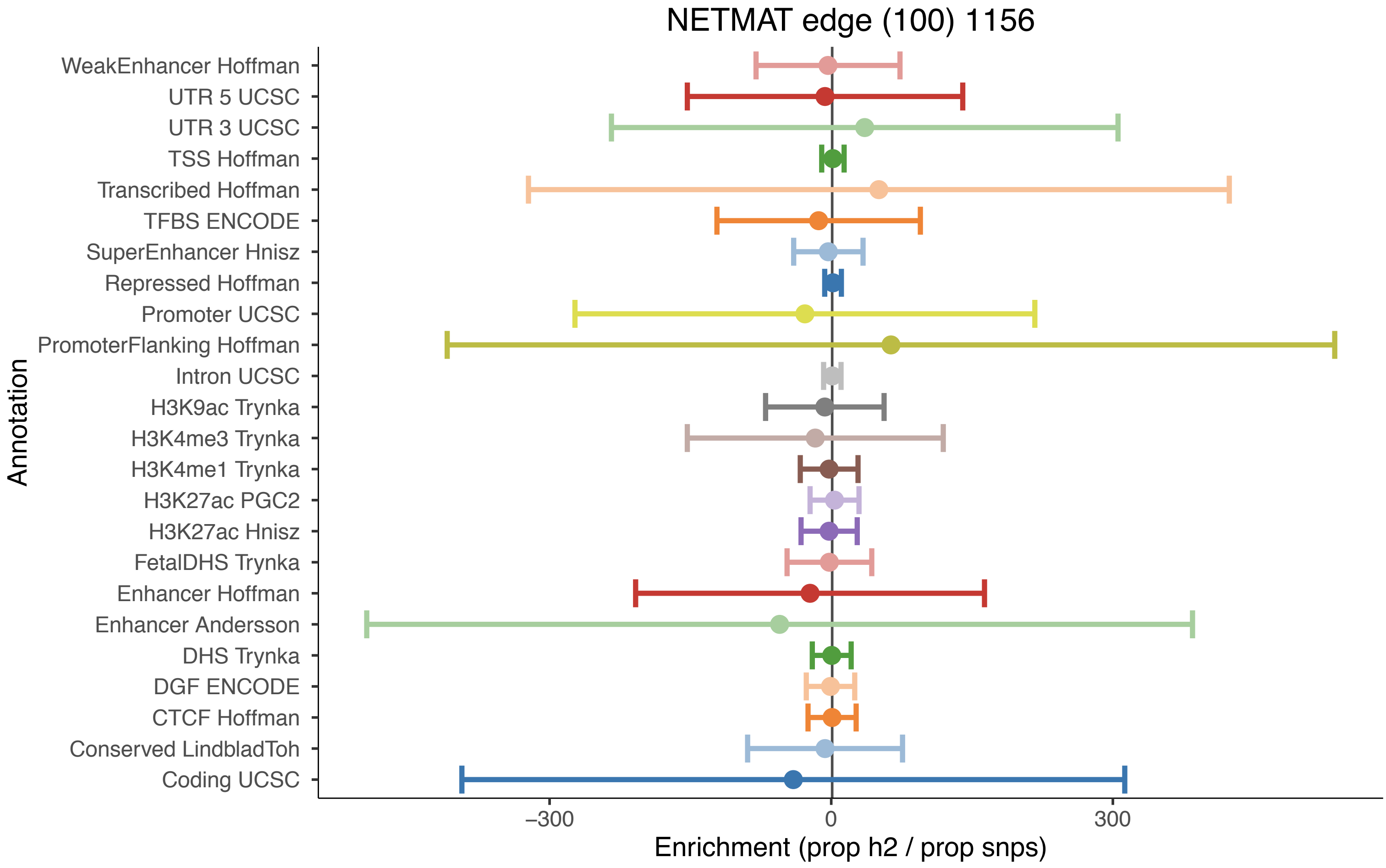
NETMAT edge (100) 1156
WeakEnhancer Hoffman
UTR 5 UCSC
UTR 3 UCSC
TSS Hoffman
Transcribed Hoffman
TFBS ENCODE
SuperEnhancer Hnisz
Repressed Hoffman
Promoter UCSC
PromoterFlanking Hoffman
Intron UCSC
H3K9ac Trynka
H3K4me3 Trynka
H3K4me1 Trynka
H3K27ac PGC2
H3K27ac Hnisz
FetalDHS Trynka
Enhancer Hoffman
Enhancer Andersson
DHS Trynka
DGF ENCODE
CTCF Hoffman
Conserved LindbladToh
Coding UCSC
Annotation
−300
0
300
Enrichment (prop h2 / prop snps)

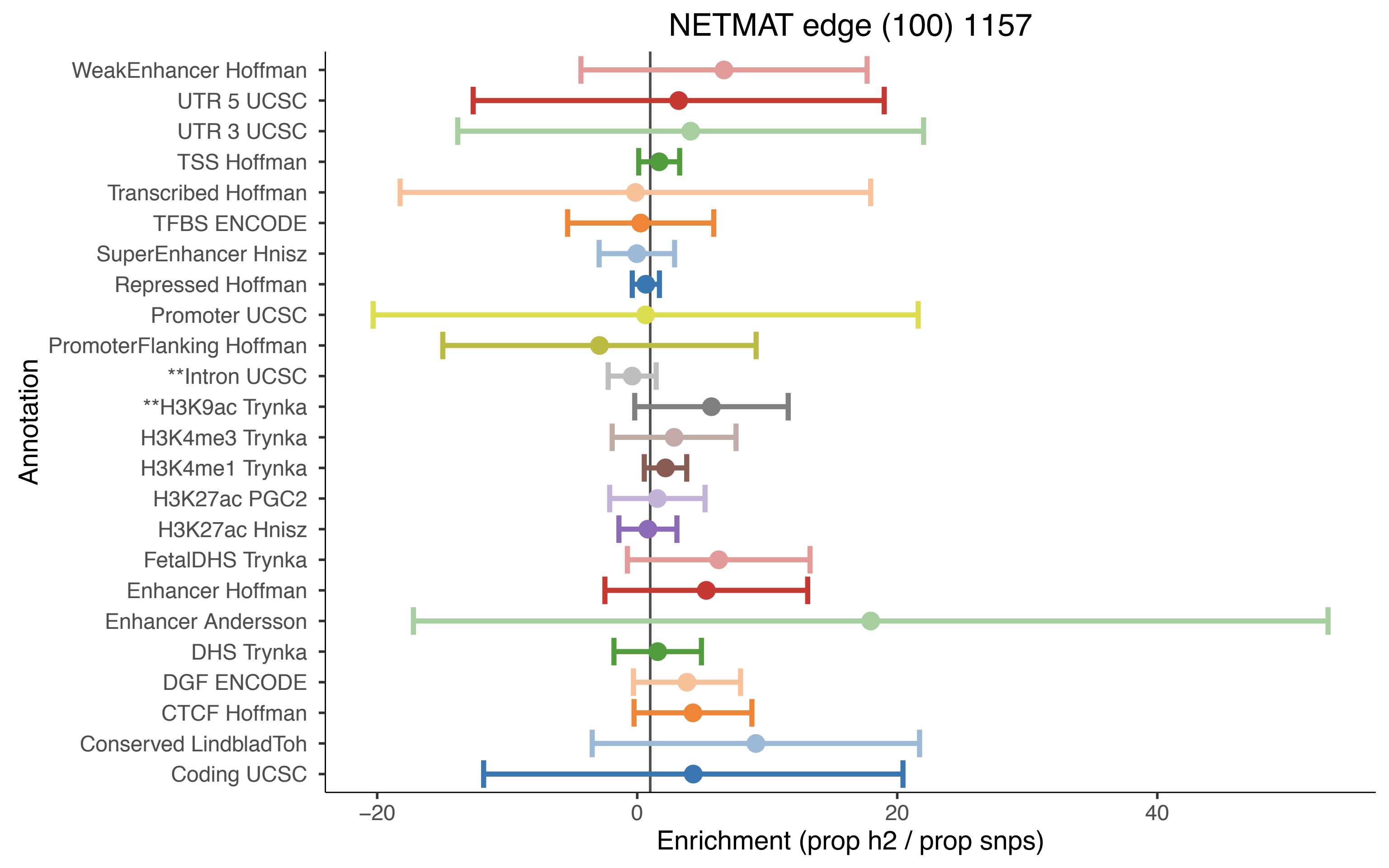
NETMAT edge (100) 1157
WeakEnhancer Hoffman
UTR 5 UCSC
UTR 3 UCSC
TSS Hoffman
Transcribed Hoffman
TFBS ENCODE
SuperEnhancer Hnisz
Repressed Hoffman
Promoter UCSC
PromoterFlanking Hoffman
**Intron UCSC
**H3K9ac Trynka
H3K4me3 Trynka
H3K4me1 Trynka
H3K27ac PGC2
H3K27ac Hnisz
FetalDHS Trynka
Enhancer Hoffman
Enhancer Andersson
DHS Trynka
DGF ENCODE
CTCF Hoffman
Conserved LindbladToh
Coding UCSC
Annotation
−20
0
20
40
Enrichment (prop h2 / prop snps)

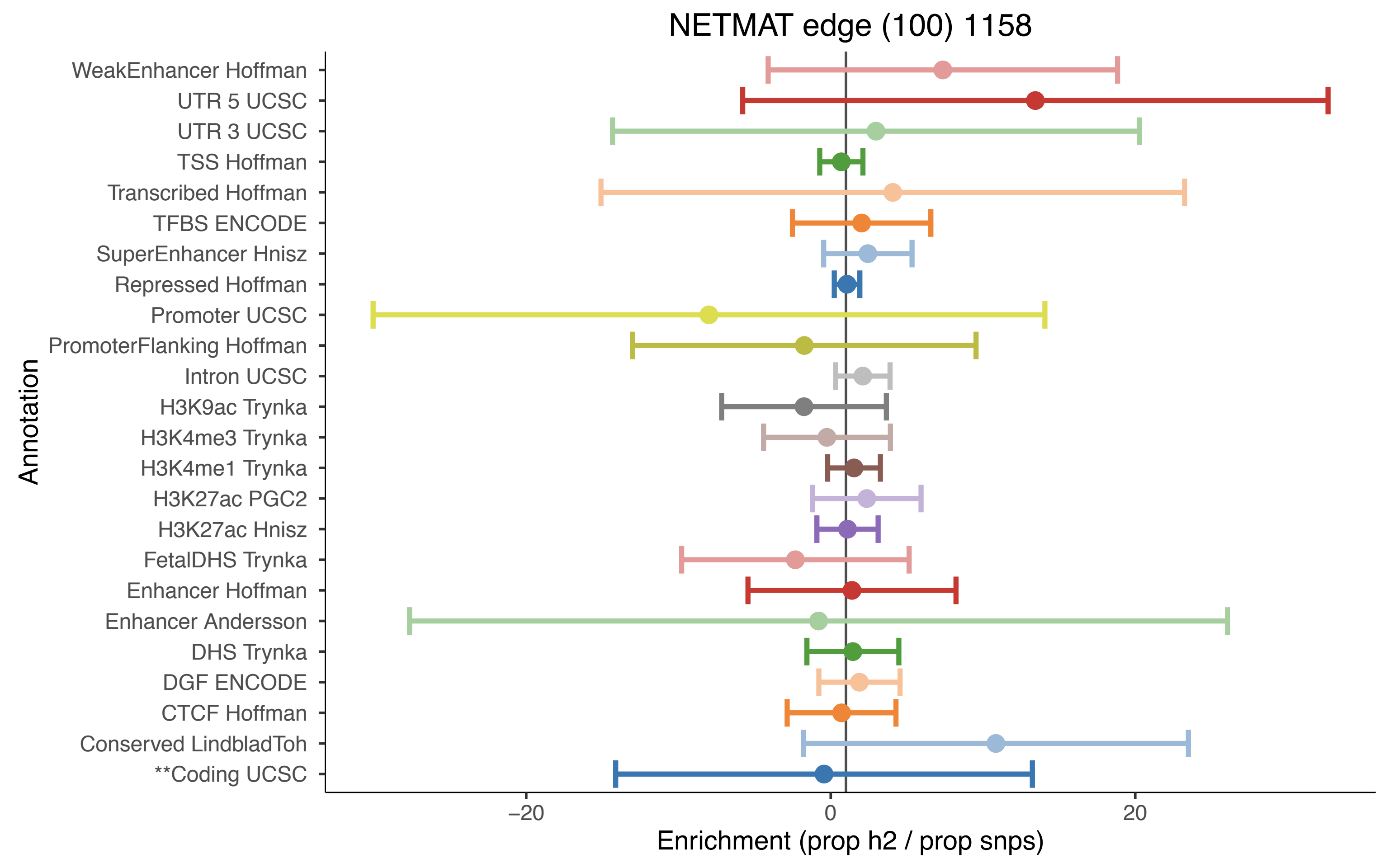
NETMAT edge (100) 1158
WeakEnhancer Hoffman
UTR 5 UCSC
UTR 3 UCSC
TSS Hoffman
Transcribed Hoffman
TFBS ENCODE
SuperEnhancer Hnisz
Repressed Hoffman
Promoter UCSC
PromoterFlanking Hoffman
Intron UCSC
H3K9ac Trynka
H3K4me3 Trynka
H3K4me1 Trynka
H3K27ac PGC2
H3K27ac Hnisz
FetalDHS Trynka
Enhancer Hoffman
Enhancer Andersson
DHS Trynka
DGF ENCODE
CTCF Hoffman
Conserved LindbladToh
**Coding UCSC
Annotation
−20
0
20
Enrichment (prop h2 / prop snps)

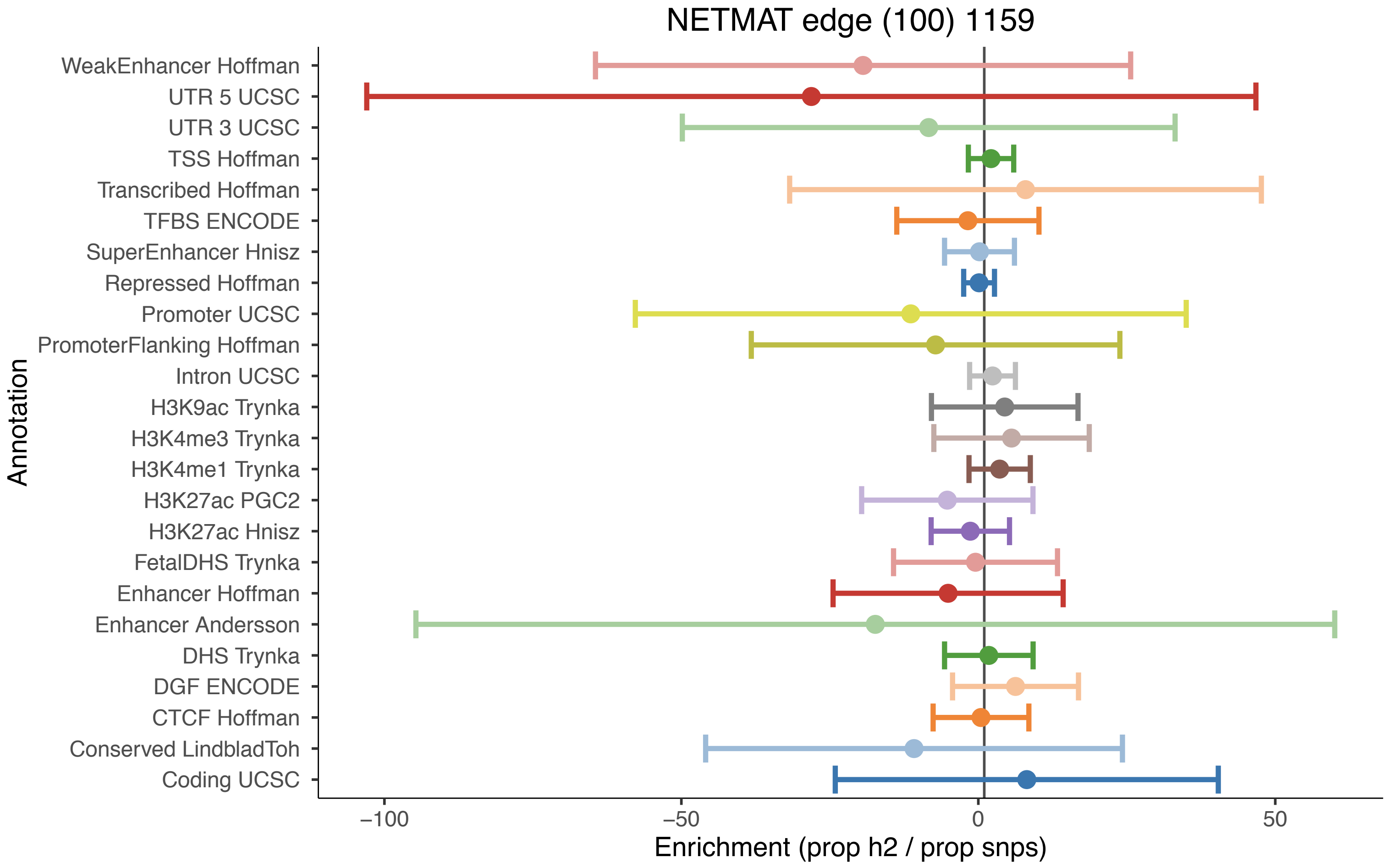

NETMAT edge (100) 1159
WeakEnhancer Hoffman
UTR 5 UCSC
UTR 3 UCSC
TSS Hoffman
Transcribed Hoffman
TFBS ENCODE
SuperEnhancer Hnisz
Repressed Hoffman
Promoter UCSC
PromoterFlanking Hoffman
Intron UCSC
H3K9ac Trynka
H3K4me3 Trynka
H3K4me1 Trynka
H3K27ac PGC2
H3K27ac Hnisz
FetalDHS Trynka
Enhancer Hoffman
Enhancer Andersson
DHS Trynka
DGF ENCODE
CTCF Hoffman
Conserved LindbladToh
Coding UCSC
Annotation
−100
−50
0
50
Enrichment (prop h2 / prop snps)

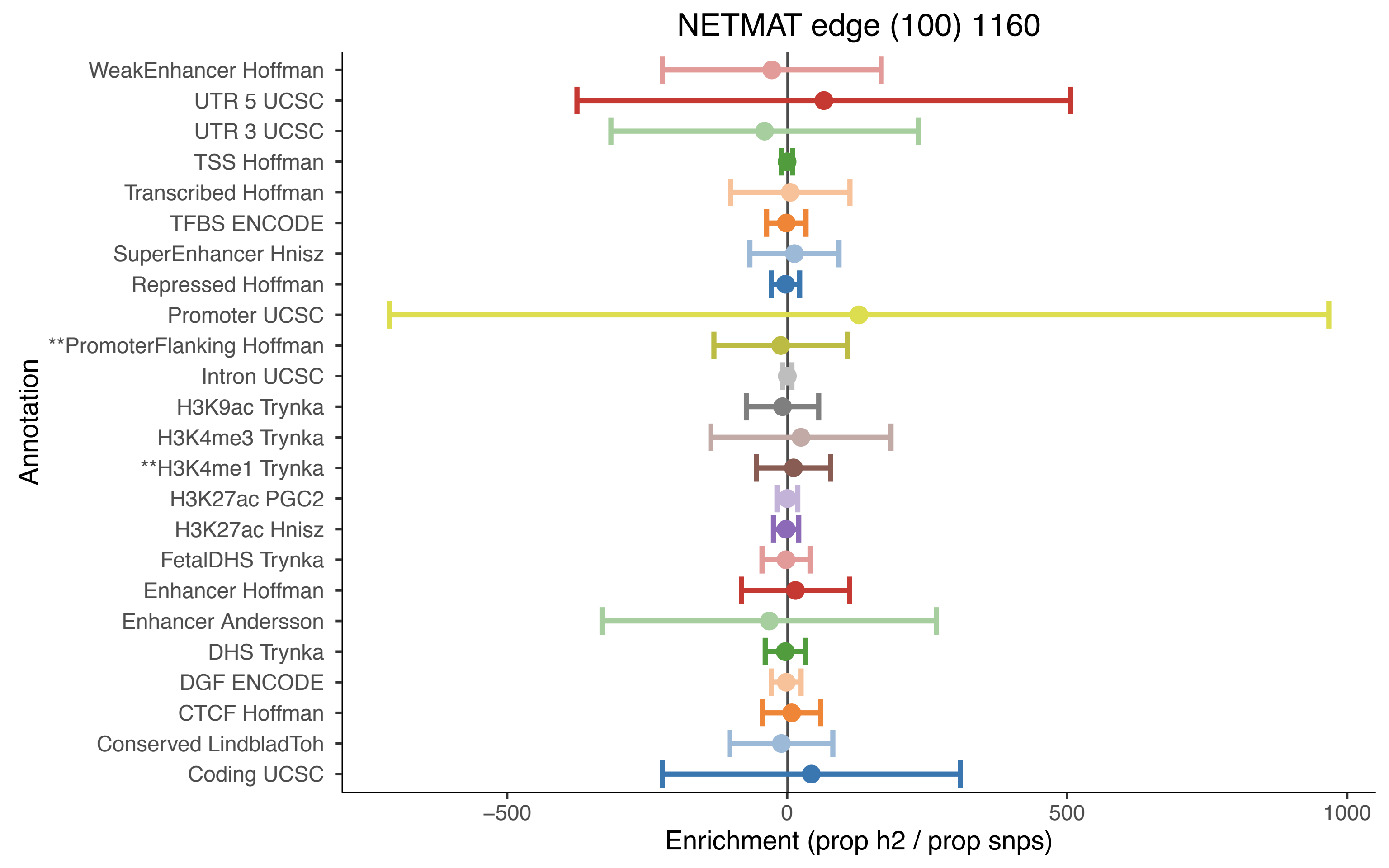

NETMAT edge (100) 1160
WeakEnhancer Hoffman
UTR 5 UCSC
UTR 3 UCSC
TSS Hoffman
Transcribed Hoffman
TFBS ENCODE
SuperEnhancer Hnisz
Repressed Hoffman
Promoter UCSC
**PromoterFlanking Hoffman
Intron UCSC
H3K9ac Trynka
H3K4me3 Trynka
**H3K4me1 Trynka
H3K27ac PGC2
H3K27ac Hnisz
FetalDHS Trynka
Enhancer Hoffman
Enhancer Andersson
DHS Trynka
DGF ENCODE
CTCF Hoffman
Conserved LindbladToh
Coding UCSC
Annotation
−500
0
500
1000
Enrichment (prop h2 / prop snps)

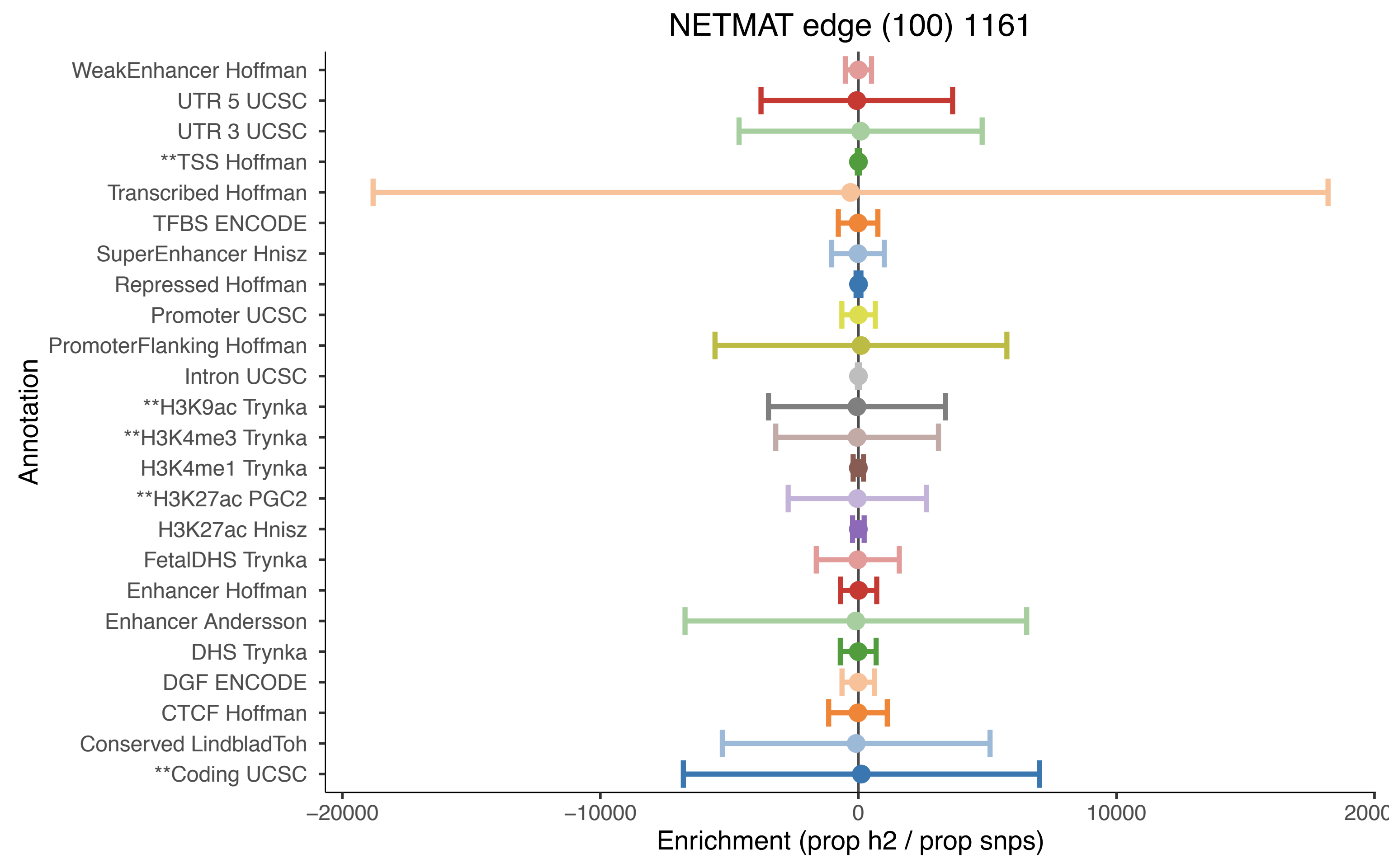

NETMAT edge (100) 1161
WeakEnhancer Hoffman
UTR 5 UCSC
UTR 3 UCSC
**TSS Hoffman
Transcribed Hoffman
TFBS ENCODE
SuperEnhancer Hnisz
Repressed Hoffman
Promoter UCSC
PromoterFlanking Hoffman
Intron UCSC
**H3K9ac Trynka
**H3K4me3 Trynka
H3K4me1 Trynka
**H3K27ac PGC2
H3K27ac Hnisz
FetalDHS Trynka
Enhancer Hoffman
Enhancer Andersson
DHS Trynka
DGF ENCODE
CTCF Hoffman
Conserved LindbladToh
**Coding UCSC
Annotation
−20000
−10000
0
10000
2000
Enrichment (prop h2 / prop snps)

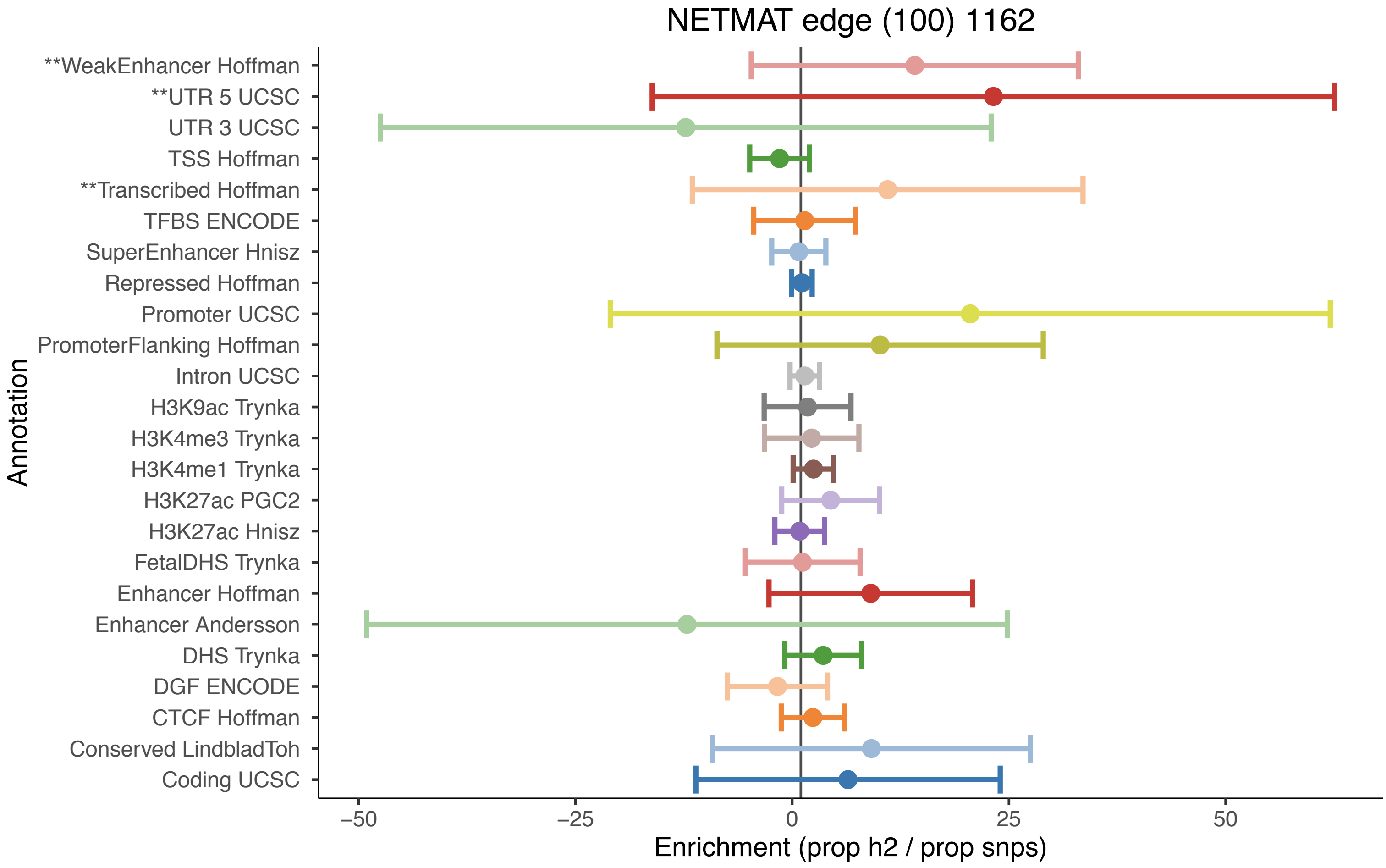
NETMAT edge (100) 1162
**WeakEnhancer Hoffman
**UTR 5 UCSC
UTR 3 UCSC
TSS Hoffman
**Transcribed Hoffman
TFBS ENCODE
SuperEnhancer Hnisz
Repressed Hoffman
Promoter UCSC
PromoterFlanking Hoffman
Intron UCSC
H3K9ac Trynka
H3K4me3 Trynka
H3K4me1 Trynka
H3K27ac PGC2
H3K27ac Hnisz
FetalDHS Trynka
Enhancer Hoffman
Enhancer Andersson
DHS Trynka
DGF ENCODE
CTCF Hoffman
Conserved LindbladToh
Coding UCSC
Annotation
−50
−25
0
25
50
Enrichment (prop h2 / prop snps)

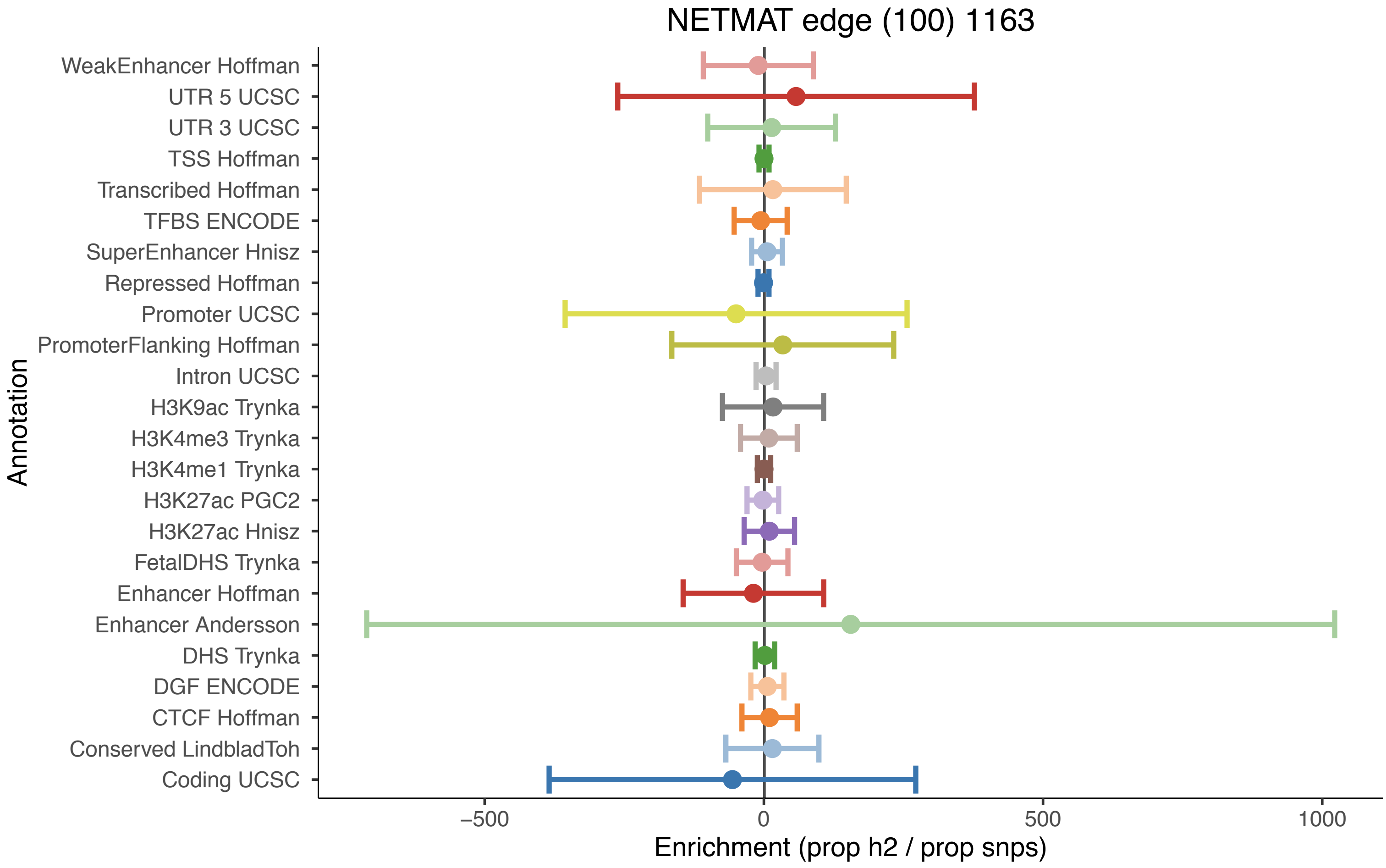

NETMAT edge (100) 1163
WeakEnhancer Hoffman
UTR 5 UCSC
UTR 3 UCSC
TSS Hoffman
Transcribed Hoffman
TFBS ENCODE
SuperEnhancer Hnisz
Repressed Hoffman
Promoter UCSC
PromoterFlanking Hoffman
Intron UCSC
H3K9ac Trynka
H3K4me3 Trynka
H3K4me1 Trynka
H3K27ac PGC2
H3K27ac Hnisz
FetalDHS Trynka
Enhancer Hoffman
Enhancer Andersson
DHS Trynka
DGF ENCODE
CTCF Hoffman
Conserved LindbladToh
Coding UCSC
Annotation
−500
0
500
1000
Enrichment (prop h2 / prop snps)

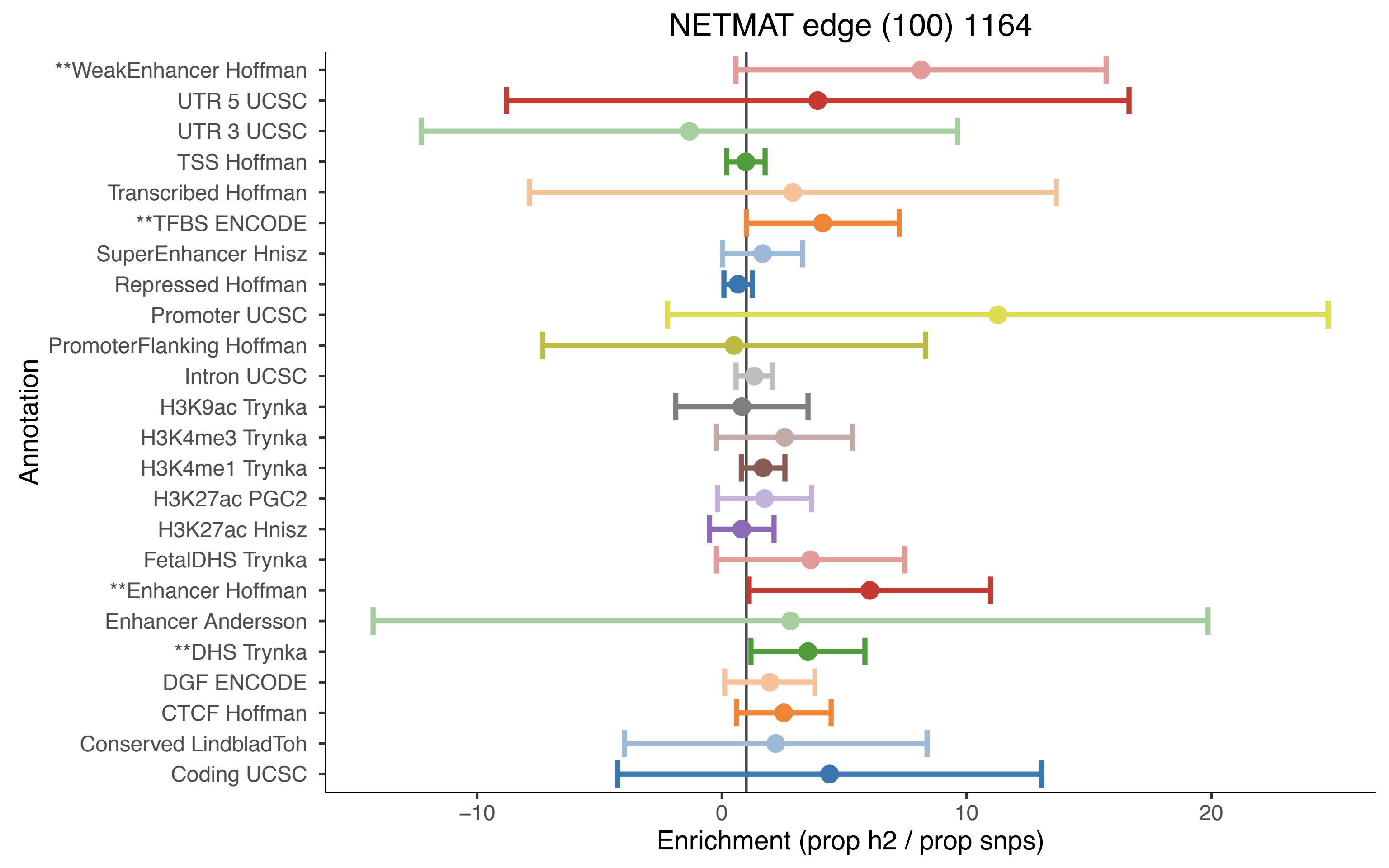

NETMAT edge (100) 1164
Annotation
**WeakEnhancer Hoffman
UTR 5 UCSC
UTR 3 UCSC
TSS Hoffman
Transcribed Hoffman
**TFBS ENCODE
SuperEnhancer Hnisz
Repressed Hoffman
Promoter UCSC
PromoterFlanking Hoffman
Intron UCSC
H3K9ac Trynka
H3K4me3 Trynka
H3K4me1 Trynka
H3K27ac PGC2
H3K27ac Hnisz
FetalDHS Trynka
**Enhancer Hoffman
Enhancer Andersson
**DHS Trynka
DGF ENCODE
CTCF Hoffman
Conserved LindbladToh
Coding UCSC
−10
0
10
20
Enrichment (prop h2 / prop snps)

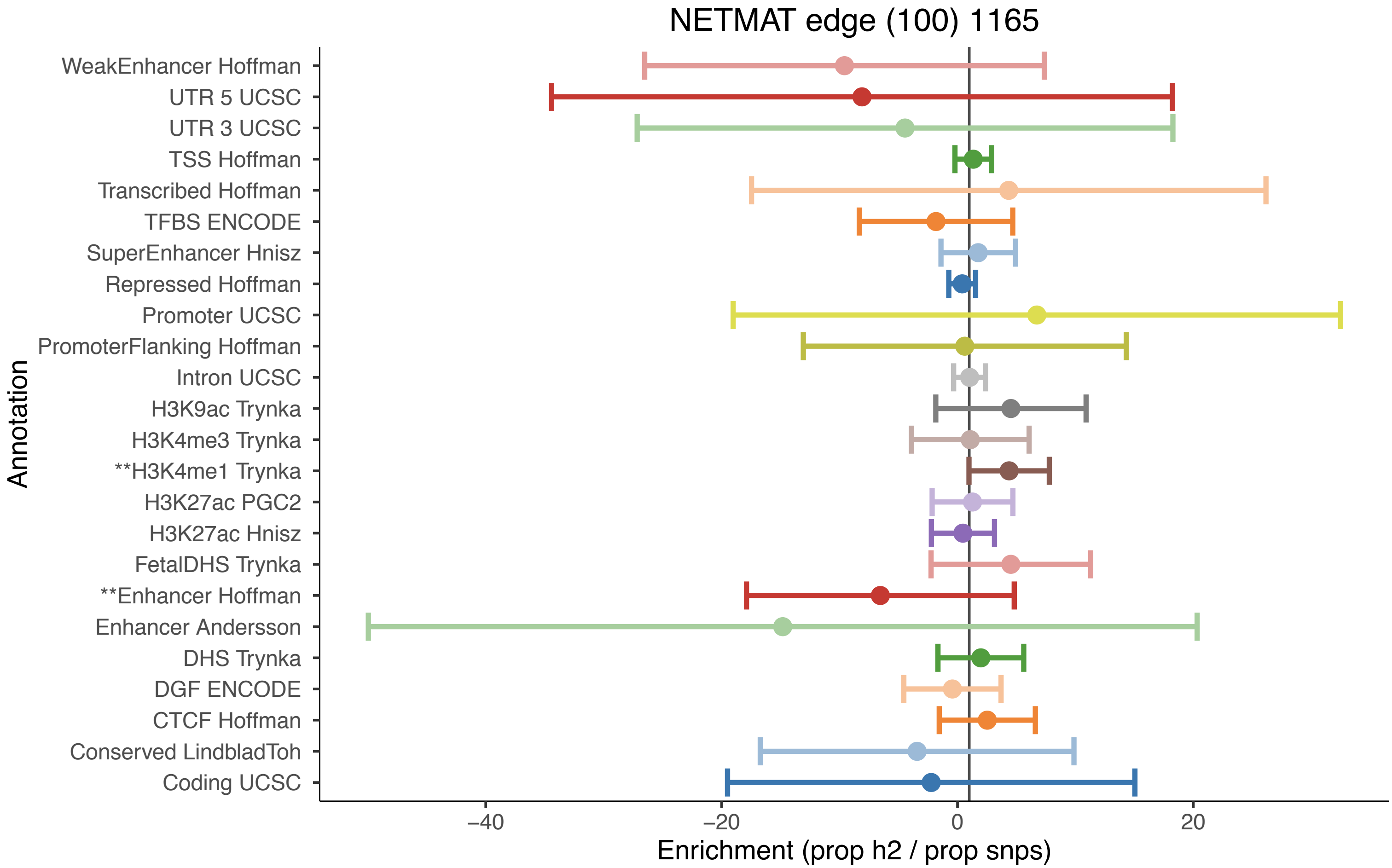

NETMAT edge (100) 1165
WeakEnhancer Hoffman
UTR 5 UCSC
UTR 3 UCSC
TSS Hoffman
Transcribed Hoffman
TFBS ENCODE
SuperEnhancer Hnisz
Repressed Hoffman
Promoter UCSC
PromoterFlanking Hoffman
Intron UCSC
H3K9ac Trynka
H3K4me3 Trynka
**H3K4me1 Trynka
H3K27ac PGC2
H3K27ac Hnisz
FetalDHS Trynka
**Enhancer Hoffman
Enhancer Andersson
DHS Trynka
DGF ENCODE
CTCF Hoffman
Conserved LindbladToh
Coding UCSC
Annotation
−40
−20
0
20
Enrichment (prop h2 / prop snps)

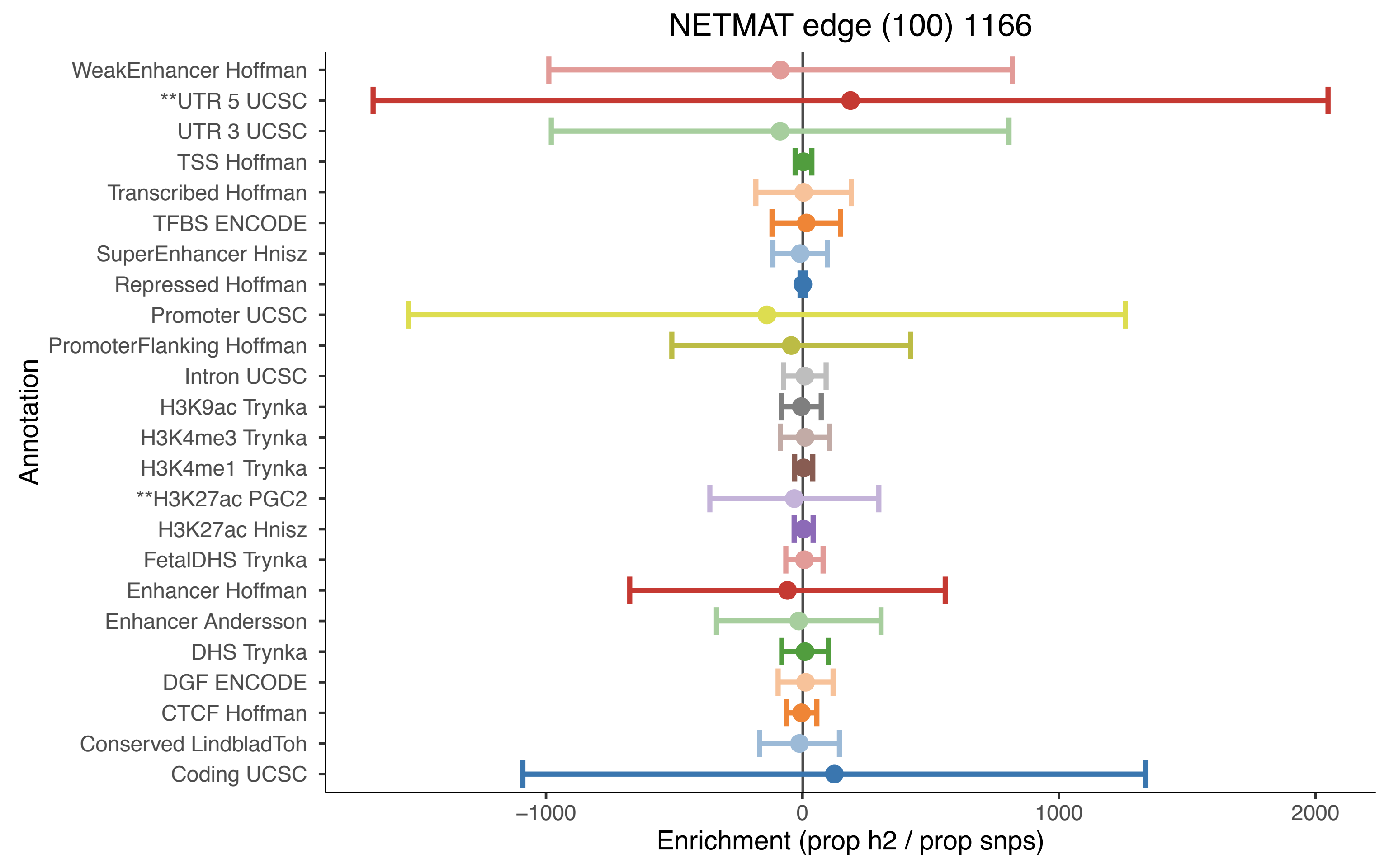

NETMAT edge (100) 1166
WeakEnhancer Hoffman
**UTR 5 UCSC
UTR 3 UCSC
TSS Hoffman
Transcribed Hoffman
TFBS ENCODE
SuperEnhancer Hnisz
Repressed Hoffman
Promoter UCSC
PromoterFlanking Hoffman
Intron UCSC
H3K9ac Trynka
H3K4me3 Trynka
H3K4me1 Trynka
**H3K27ac PGC2
H3K27ac Hnisz
FetalDHS Trynka
Enhancer Hoffman
Enhancer Andersson
DHS Trynka
DGF ENCODE
CTCF Hoffman
Conserved LindbladToh
Coding UCSC
Annotation
−1000
0
1000
2000
Enrichment (prop h2 / prop snps)

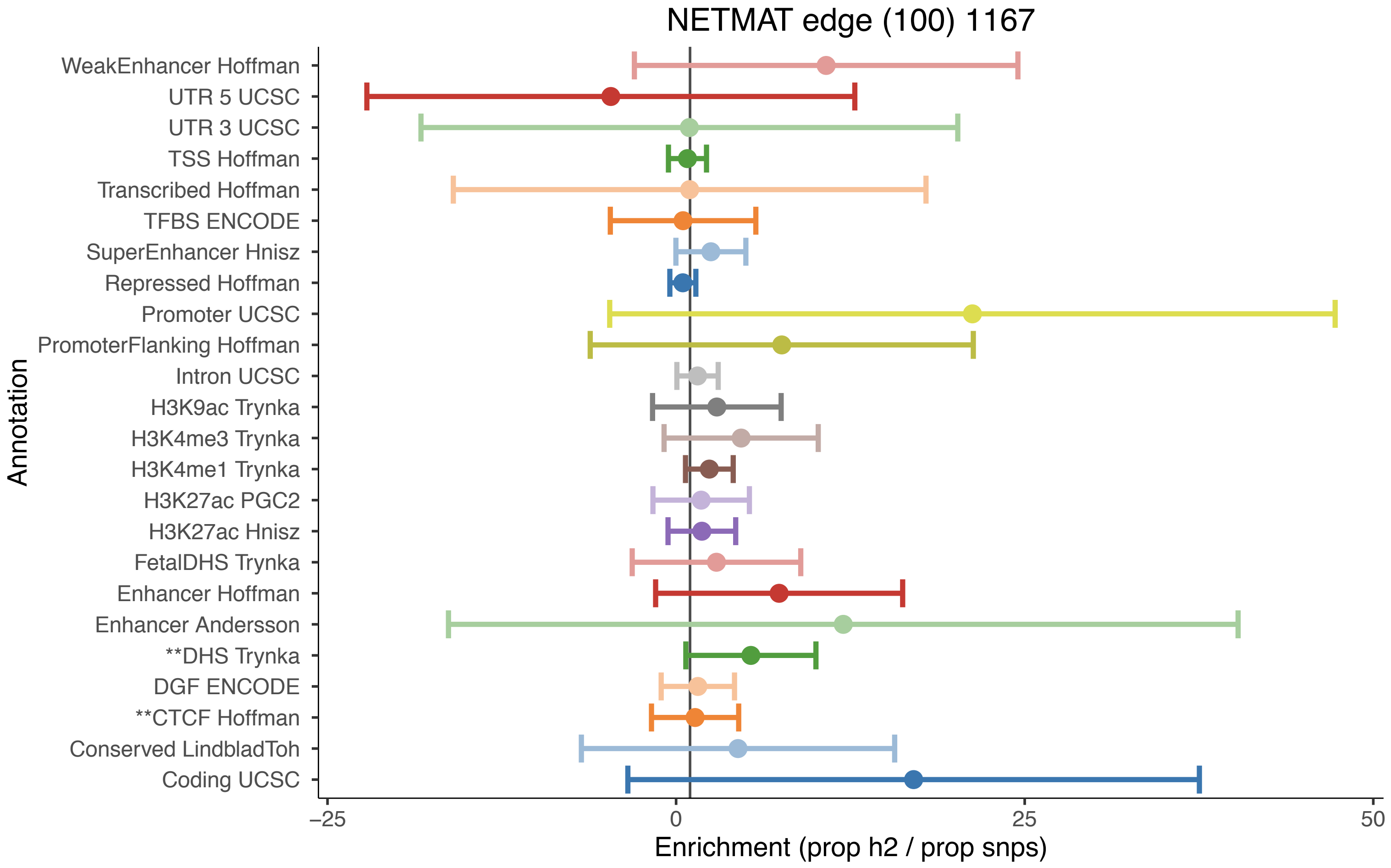

NETMAT edge (100) 1167
WeakEnhancer Hoffman
UTR 5 UCSC
UTR 3 UCSC
TSS Hoffman
Transcribed Hoffman
TFBS ENCODE
SuperEnhancer Hnisz
Repressed Hoffman
Promoter UCSC
PromoterFlanking Hoffman
Intron UCSC
H3K9ac Trynka
H3K4me3 Trynka
H3K4me1 Trynka
H3K27ac PGC2
H3K27ac Hnisz
FetalDHS Trynka
Enhancer Hoffman
Enhancer Andersson
**DHS Trynka
DGF ENCODE
**CTCF Hoffman
Conserved LindbladToh
Coding UCSC
Annotation
−25
0
25
50
Enrichment (prop h2 / prop snps)

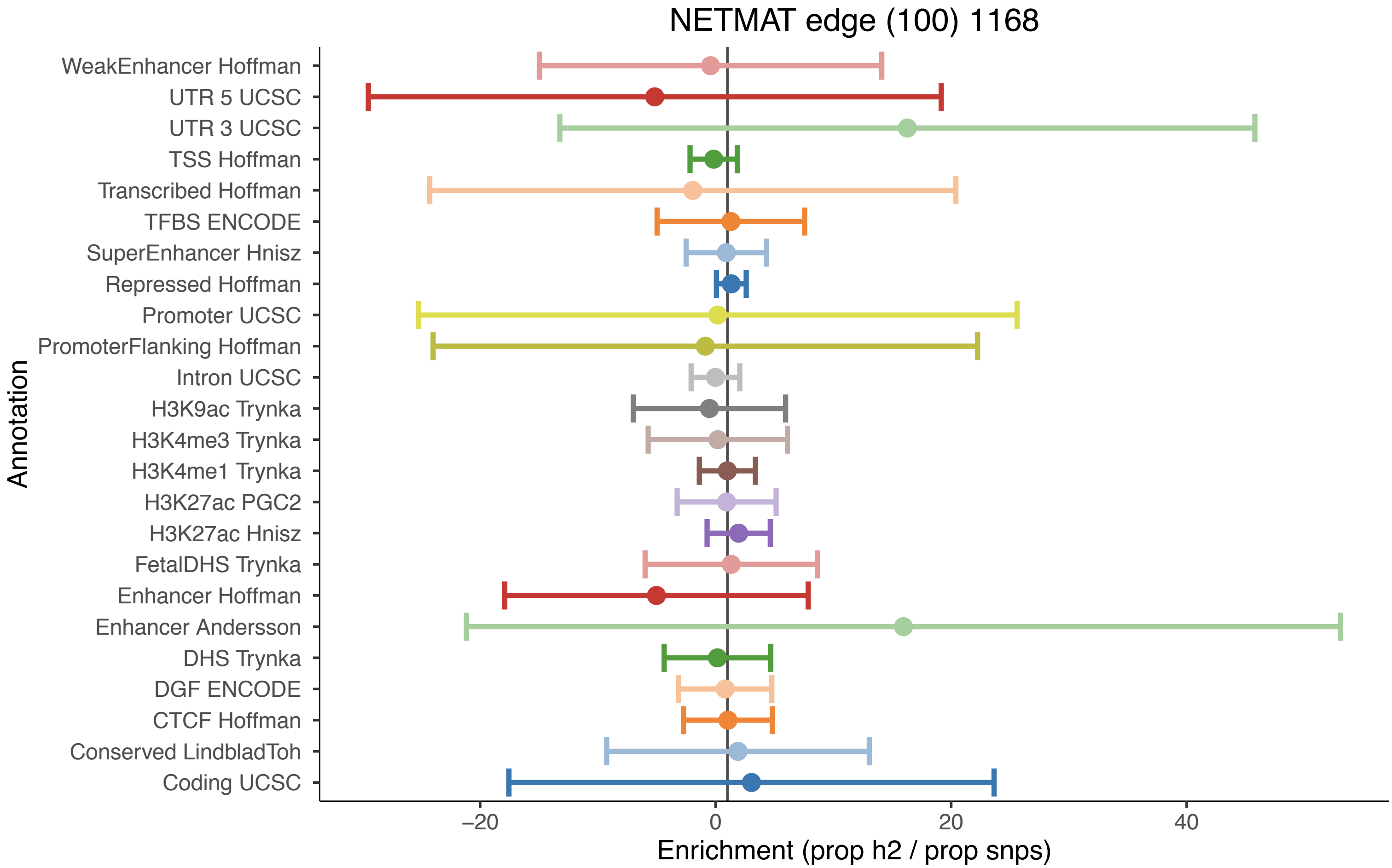

NETMAT edge (100) 1168
WeakEnhancer Hoffman
UTR 5 UCSC
UTR 3 UCSC
TSS Hoffman
Transcribed Hoffman
TFBS ENCODE
SuperEnhancer Hnisz
Repressed Hoffman
Promoter UCSC
PromoterFlanking Hoffman
Intron UCSC
H3K9ac Trynka
H3K4me3 Trynka
H3K4me1 Trynka
H3K27ac PGC2
H3K27ac Hnisz
FetalDHS Trynka
Enhancer Hoffman
Enhancer Andersson
DHS Trynka
DGF ENCODE
CTCF Hoffman
Conserved LindbladToh
Coding UCSC
Annotation
−20
0
20
40
Enrichment (prop h2 / prop snps)

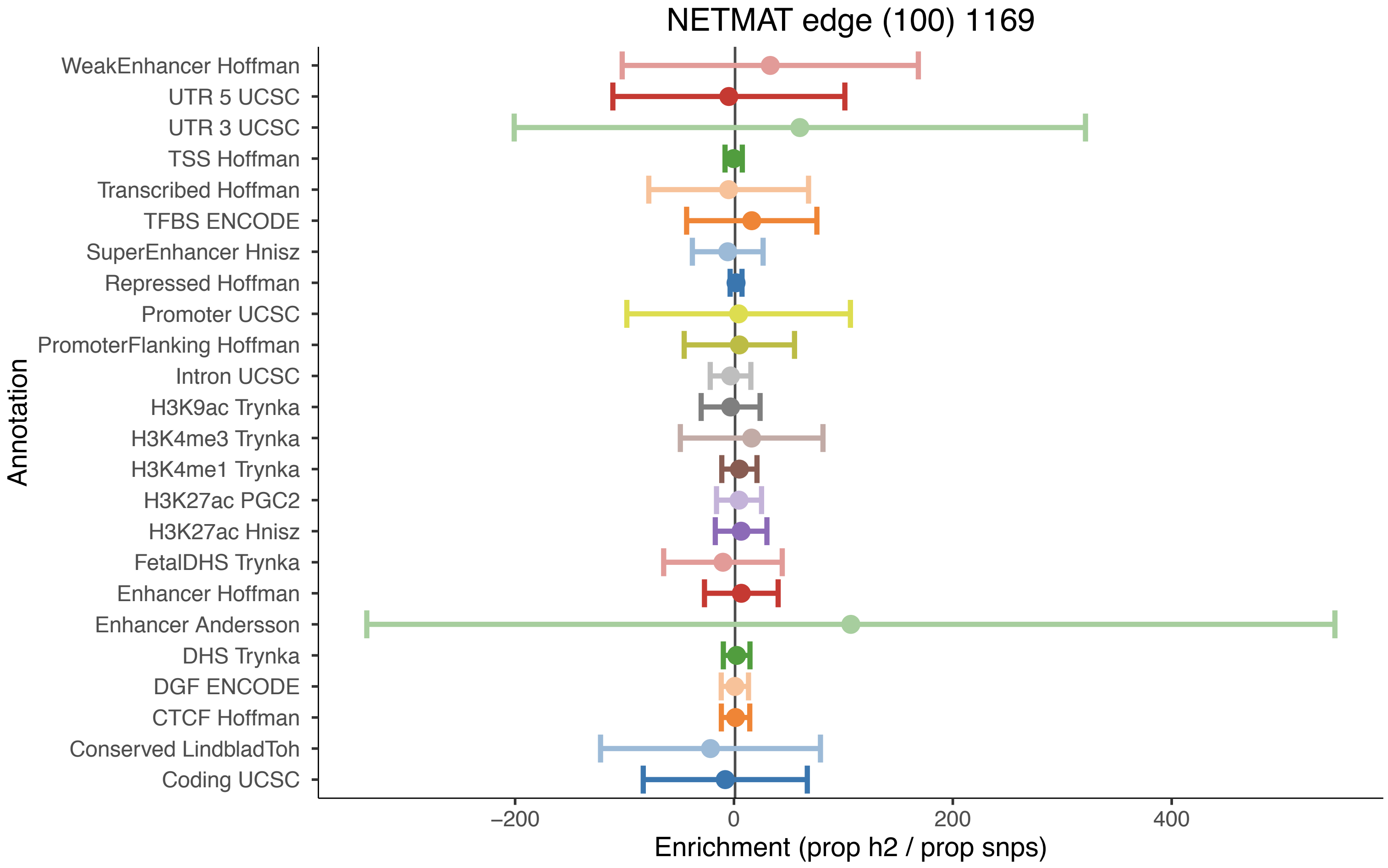
NETMAT edge (100) 1169
Annotation
WeakEnhancer Hoffman
UTR 5 UCSC
UTR 3 UCSC
TSS Hoffman
Transcribed Hoffman
TFBS ENCODE
SuperEnhancer Hnisz
Repressed Hoffman
Promoter UCSC
PromoterFlanking Hoffman
Intron UCSC
H3K9ac Trynka
H3K4me3 Trynka
H3K4me1 Trynka
H3K27ac PGC2
H3K27ac Hnisz
FetalDHS Trynka
Enhancer Hoffman
Enhancer Andersson
DHS Trynka
DGF ENCODE
CTCF Hoffman
Conserved LindbladToh
Coding UCSC
−200
0
200
400
Enrichment (prop h2 / prop snps)

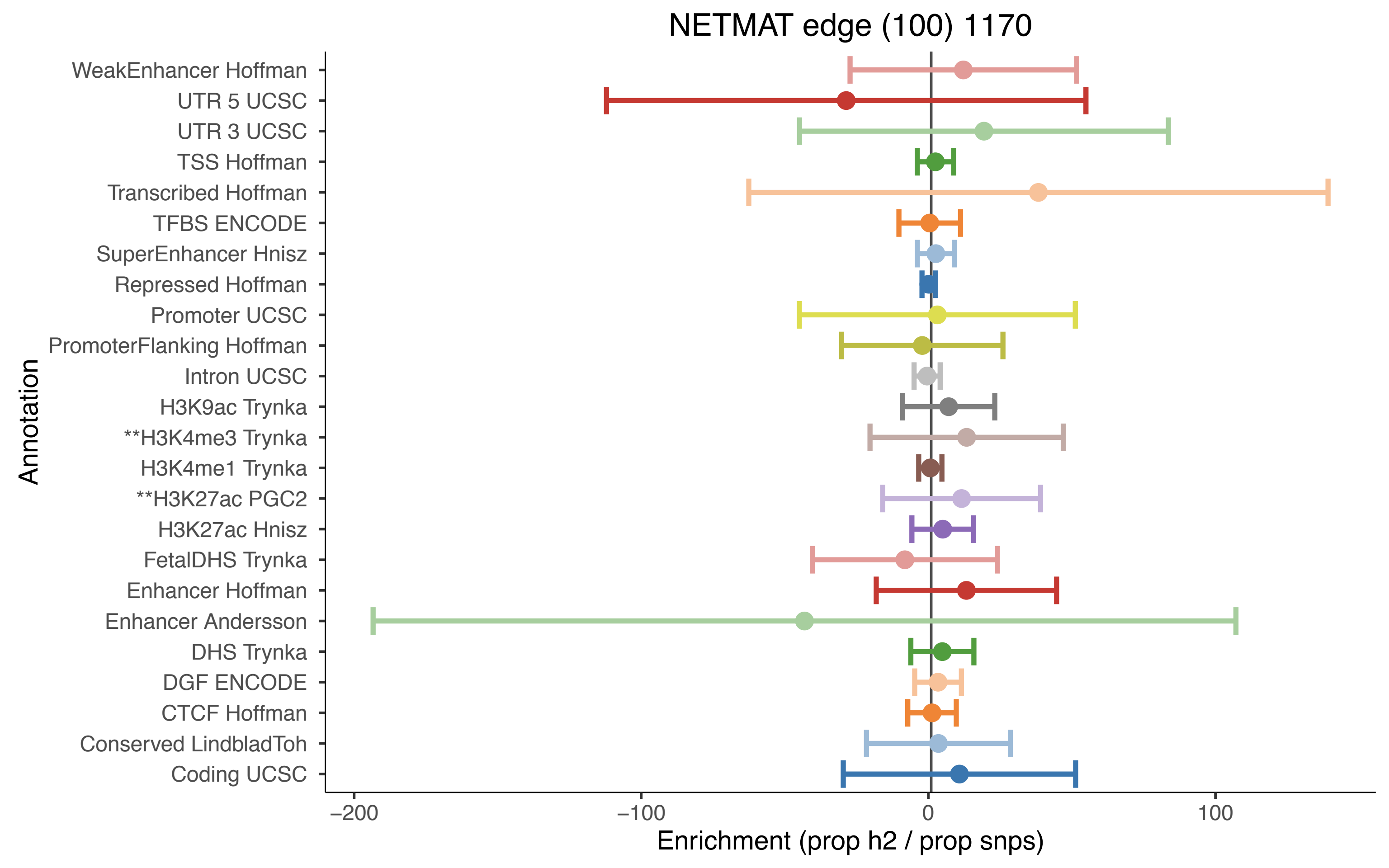
NETMAT edge (100) 1170
Annotation
WeakEnhancer Hoffman
UTR 5 UCSC
UTR 3 UCSC
TSS Hoffman
Transcribed Hoffman
TFBS ENCODE
SuperEnhancer Hnisz
Repressed Hoffman
Promoter UCSC
PromoterFlanking Hoffman
Intron UCSC
H3K9ac Trynka
**H3K4me3 Trynka
H3K4me1 Trynka
**H3K27ac PGC2
H3K27ac Hnisz
FetalDHS Trynka
Enhancer Hoffman
Enhancer Andersson
DHS Trynka
DGF ENCODE
CTCF Hoffman
Conserved LindbladToh
Coding UCSC
−200
−100
0
100
Enrichment (prop h2 / prop snps)

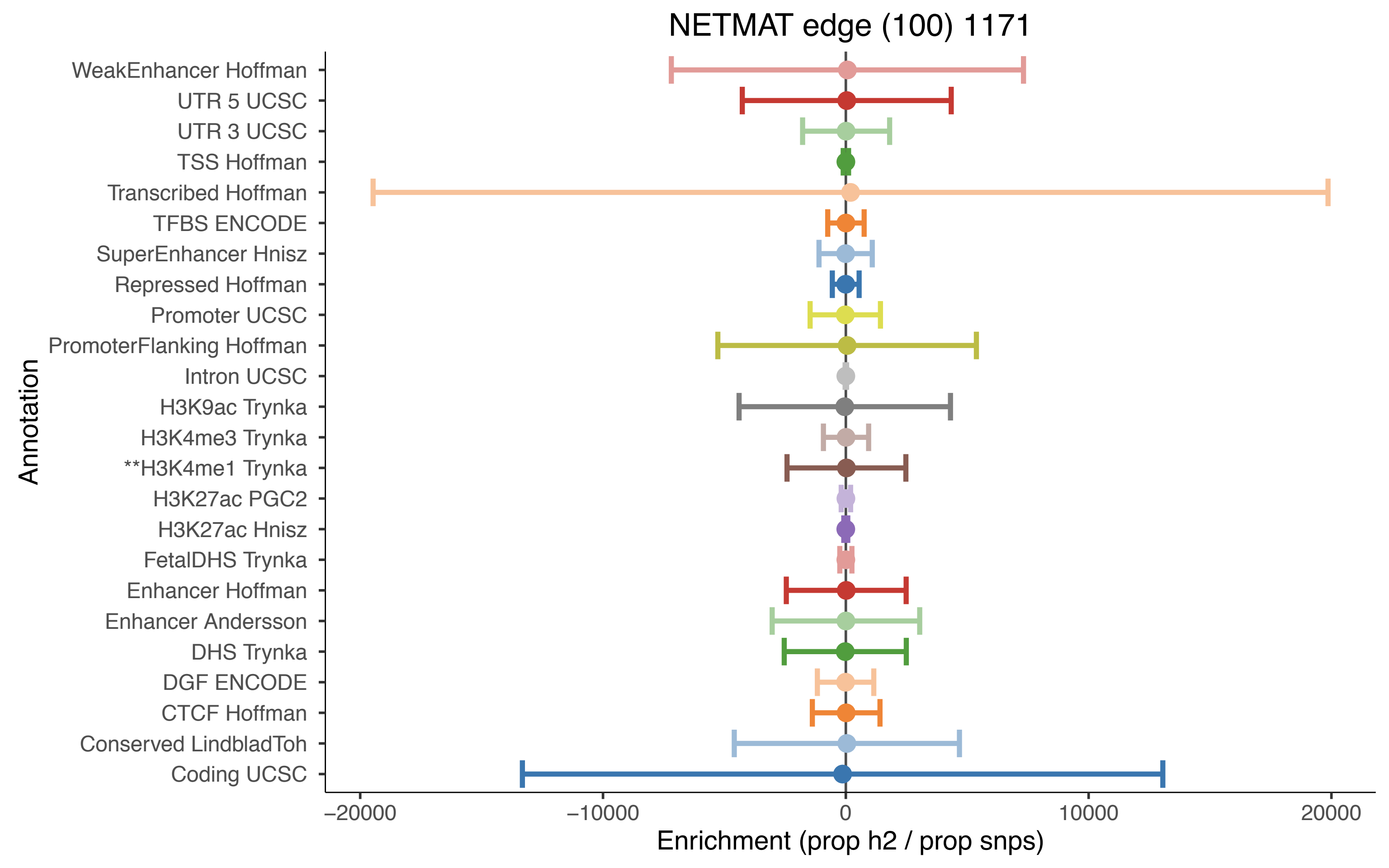

NETMAT edge (100) 1171
Annotation
WeakEnhancer Hoffman
UTR 5 UCSC
UTR 3 UCSC
TSS Hoffman
Transcribed Hoffman
TFBS ENCODE
SuperEnhancer Hnisz
Repressed Hoffman
Promoter UCSC
PromoterFlanking Hoffman
Intron UCSC
H3K9ac Trynka
H3K4me3 Trynka
**H3K4me1 Trynka
H3K27ac PGC2
H3K27ac Hnisz
FetalDHS Trynka
Enhancer Hoffman
Enhancer Andersson
DHS Trynka
DGF ENCODE
CTCF Hoffman
Conserved LindbladToh
Coding UCSC
−20000
−10000
0
10000
20000
Enrichment (prop h2 / prop snps)

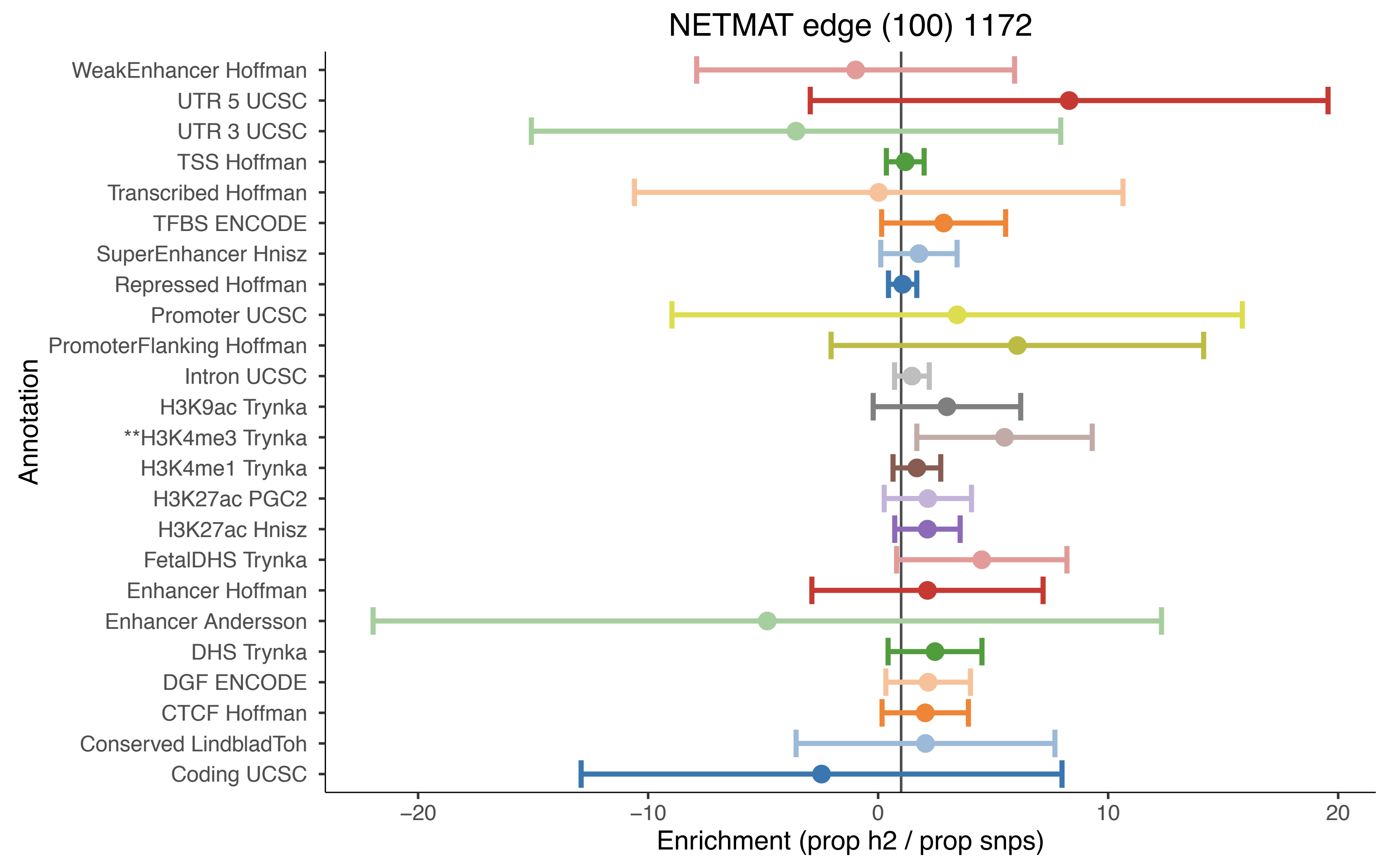
NETMAT edge (100) 1172
WeakEnhancer Hoffman
UTR 5 UCSC
UTR 3 UCSC
TSS Hoffman
Transcribed Hoffman
TFBS ENCODE
SuperEnhancer Hnisz
Repressed Hoffman
Promoter UCSC
PromoterFlanking Hoffman
Intron UCSC
H3K9ac Trynka
**H3K4me3 Trynka
H3K4me1 Trynka
H3K27ac PGC2
H3K27ac Hnisz
FetalDHS Trynka
Enhancer Hoffman
Enhancer Andersson
DHS Trynka
DGF ENCODE
CTCF Hoffman
Conserved LindbladToh
Coding UCSC
Annotation
−20
−10
0
10
20
Enrichment (prop h2 / prop snps)

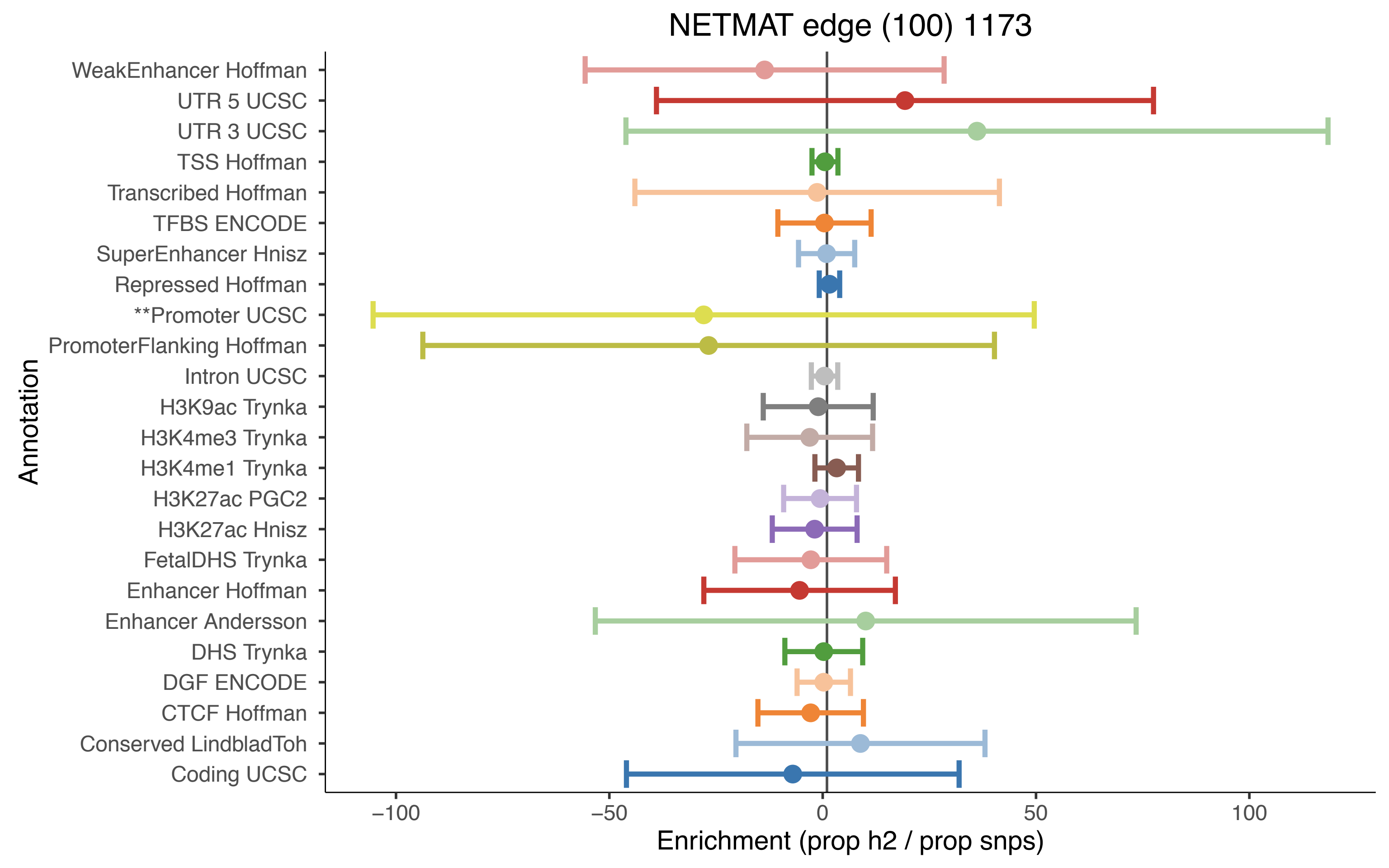

NETMAT edge (100) 1173
WeakEnhancer Hoffman
UTR 5 UCSC
UTR 3 UCSC
TSS Hoffman
Transcribed Hoffman
TFBS ENCODE
SuperEnhancer Hnisz
Repressed Hoffman
**Promoter UCSC
PromoterFlanking Hoffman
Intron UCSC
H3K9ac Trynka
H3K4me3 Trynka
H3K4me1 Trynka
H3K27ac PGC2
H3K27ac Hnisz
FetalDHS Trynka
Enhancer Hoffman
Enhancer Andersson
DHS Trynka
DGF ENCODE
CTCF Hoffman
Conserved LindbladToh
Coding UCSC
Annotation
−100
−50
0
50
100
Enrichment (prop h2 / prop snps)

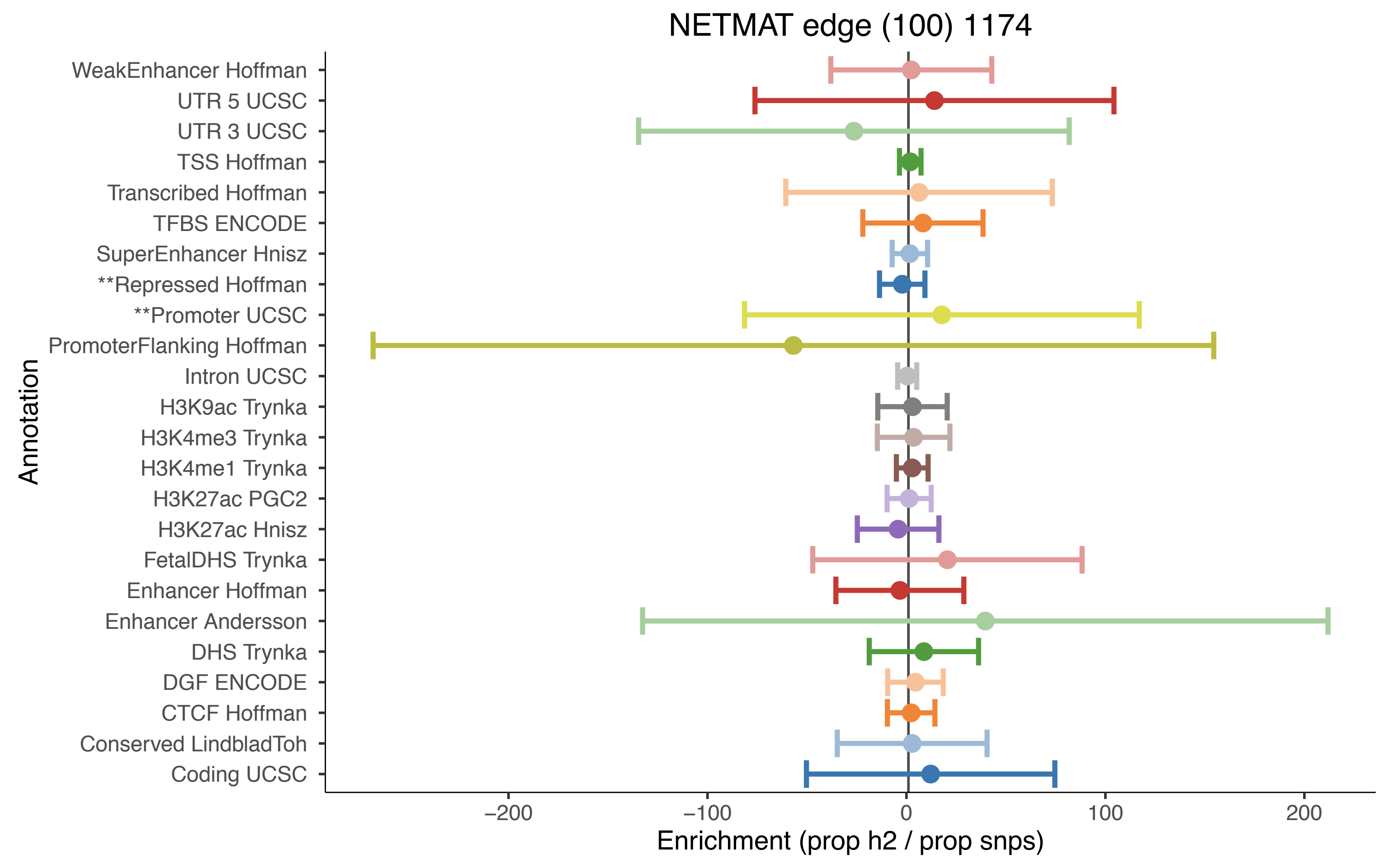

NETMAT edge (100) 1174
WeakEnhancer Hoffman
UTR 5 UCSC
UTR 3 UCSC
TSS Hoffman
Transcribed Hoffman
TFBS ENCODE
SuperEnhancer Hnisz
**Repressed Hoffman
**Promoter UCSC
PromoterFlanking Hoffman
Intron UCSC
H3K9ac Trynka
H3K4me3 Trynka
H3K4me1 Trynka
H3K27ac PGC2
H3K27ac Hnisz
FetalDHS Trynka
Enhancer Hoffman
Enhancer Andersson
DHS Trynka
DGF ENCODE
CTCF Hoffman
Conserved LindbladToh
Coding UCSC
Annotation
−200
−100
0
100
200
Enrichment (prop h2 / prop snps)

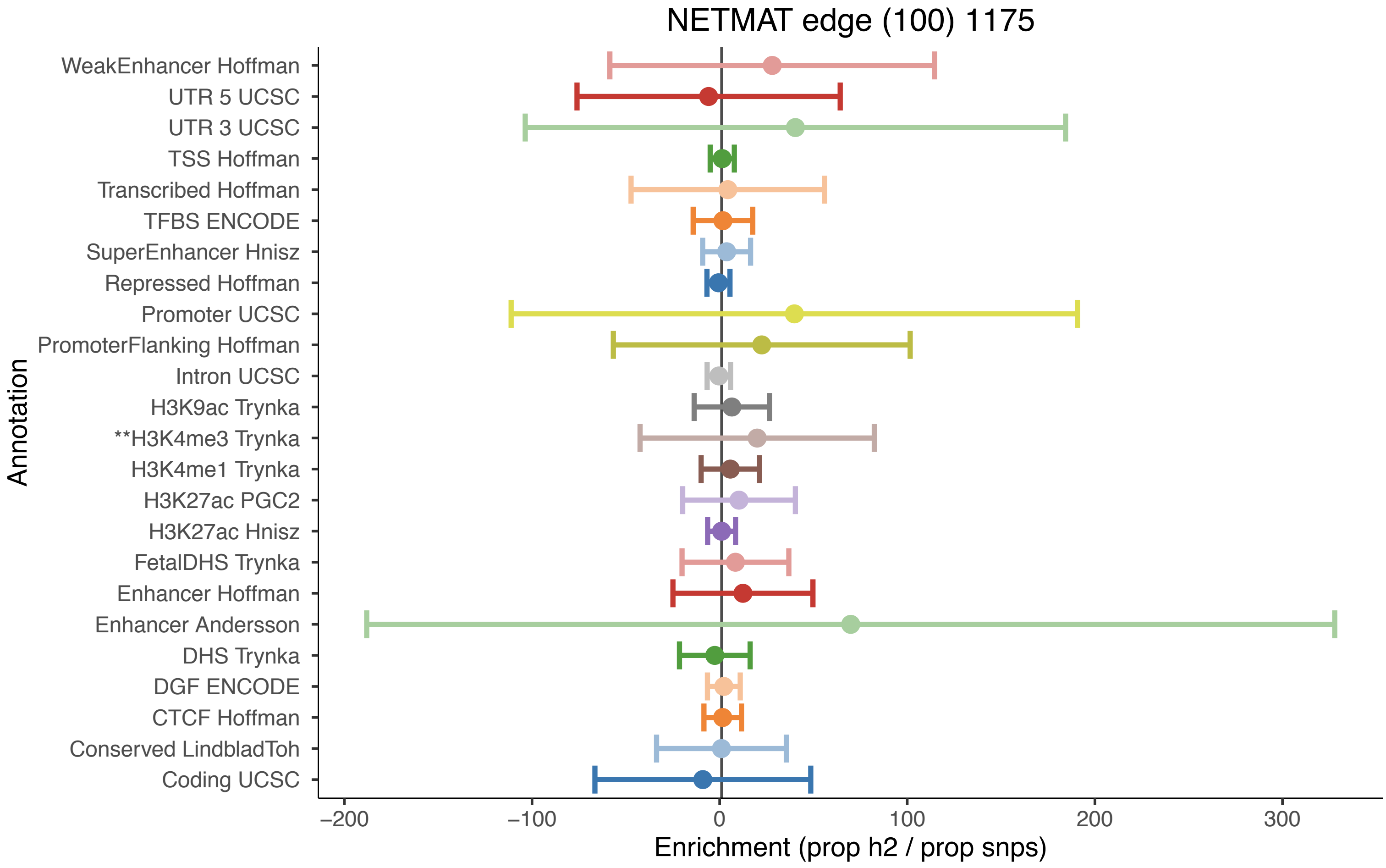
NETMAT edge (100) 1175
Annotation
WeakEnhancer Hoffman
UTR 5 UCSC
UTR 3 UCSC
TSS Hoffman
Transcribed Hoffman
TFBS ENCODE
SuperEnhancer Hnisz
Repressed Hoffman
Promoter UCSC
PromoterFlanking Hoffman
Intron UCSC
H3K9ac Trynka
**H3K4me3 Trynka
H3K4me1 Trynka
H3K27ac PGC2
H3K27ac Hnisz
FetalDHS Trynka
Enhancer Hoffman
Enhancer Andersson
DHS Trynka
DGF ENCODE
CTCF Hoffman
Conserved LindbladToh
Coding UCSC
−200
−100
0
100
200
300
Enrichment (prop h2 / prop snps)

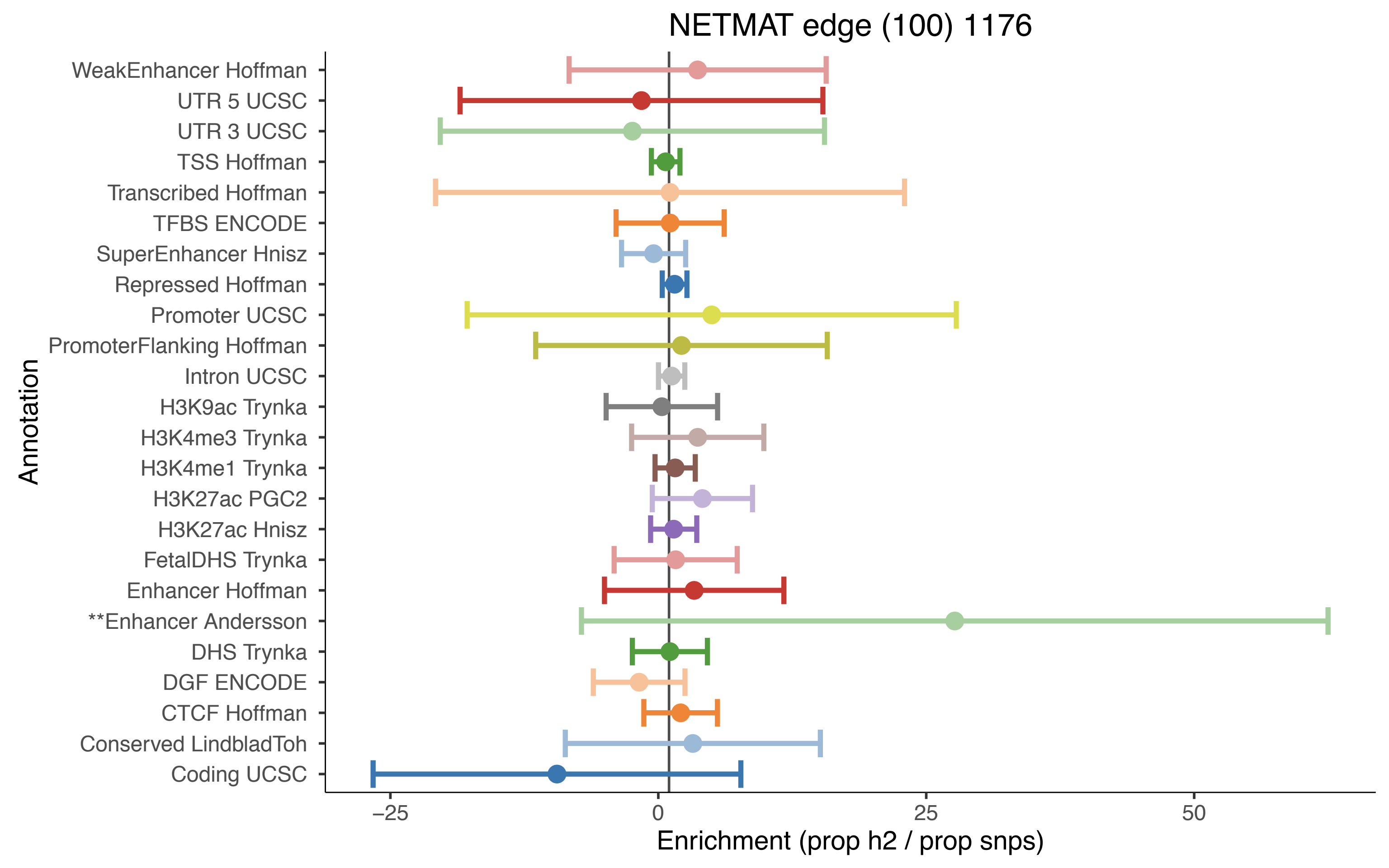
NETMAT edge (100) 1176
WeakEnhancer Hoffman
UTR 5 UCSC
UTR 3 UCSC
TSS Hoffman
Transcribed Hoffman
TFBS ENCODE
SuperEnhancer Hnisz
Repressed Hoffman
Promoter UCSC
PromoterFlanking Hoffman
Intron UCSC
H3K9ac Trynka
H3K4me3 Trynka
H3K4me1 Trynka
H3K27ac PGC2
H3K27ac Hnisz
FetalDHS Trynka
Enhancer Hoffman
**Enhancer Andersson
DHS Trynka
DGF ENCODE
CTCF Hoffman
Conserved LindbladToh
Coding UCSC
Annotation
−25
0
25
50
Enrichment (prop h2 / prop snps)

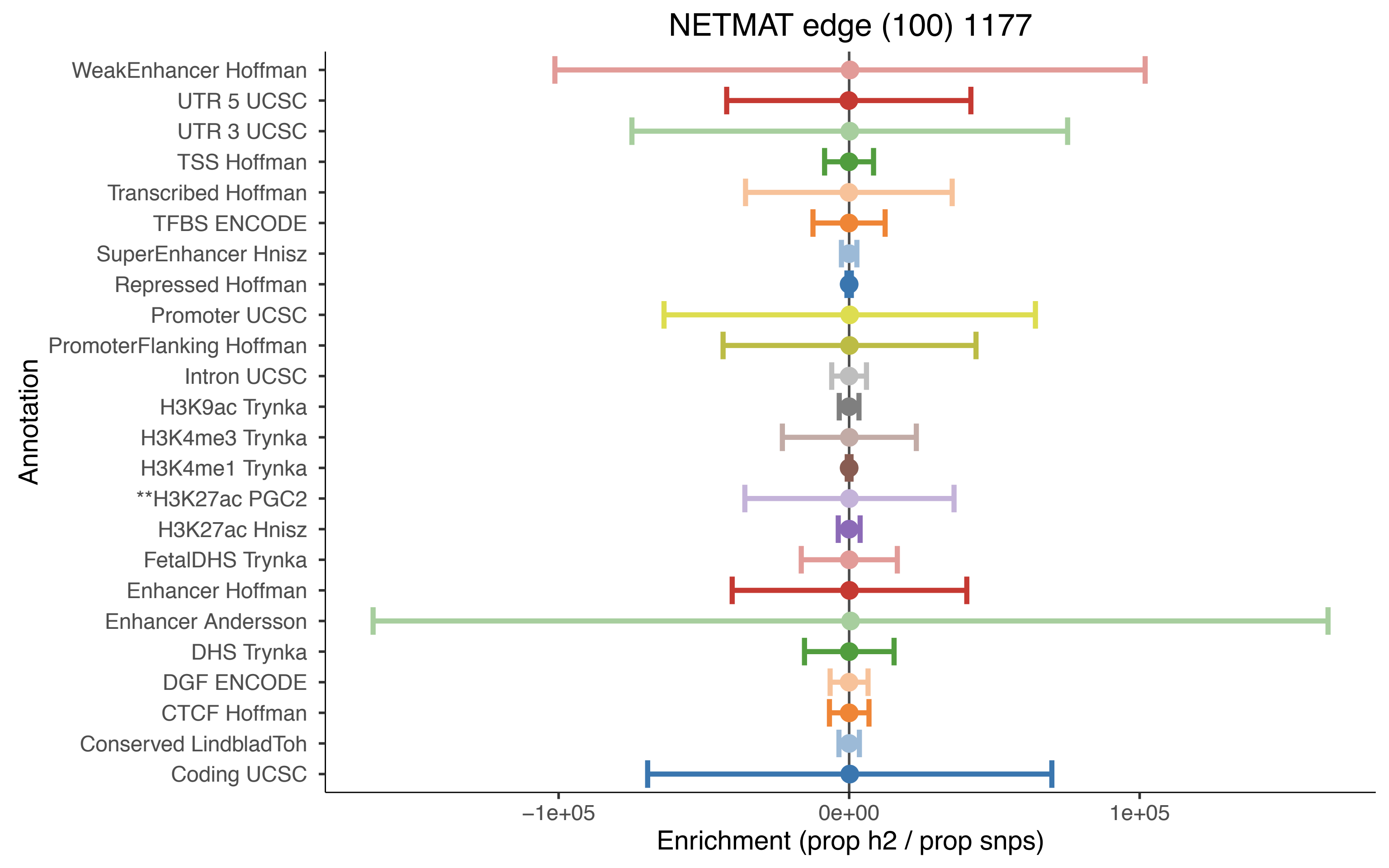

NETMAT edge (100) 1177
WeakEnhancer Hoffman
UTR 5 UCSC
UTR 3 UCSC
TSS Hoffman
Transcribed Hoffman
TFBS ENCODE
SuperEnhancer Hnisz
Repressed Hoffman
Promoter UCSC
PromoterFlanking Hoffman
Intron UCSC
H3K9ac Trynka
H3K4me3 Trynka
H3K4me1 Trynka
**H3K27ac PGC2
H3K27ac Hnisz
FetalDHS Trynka
Enhancer Hoffman
Enhancer Andersson
DHS Trynka
DGF ENCODE
CTCF Hoffman
Conserved LindbladToh
Coding UCSC
Annotation
−1e+05
0e+00
1e+05
Enrichment (prop h2 / prop snps)

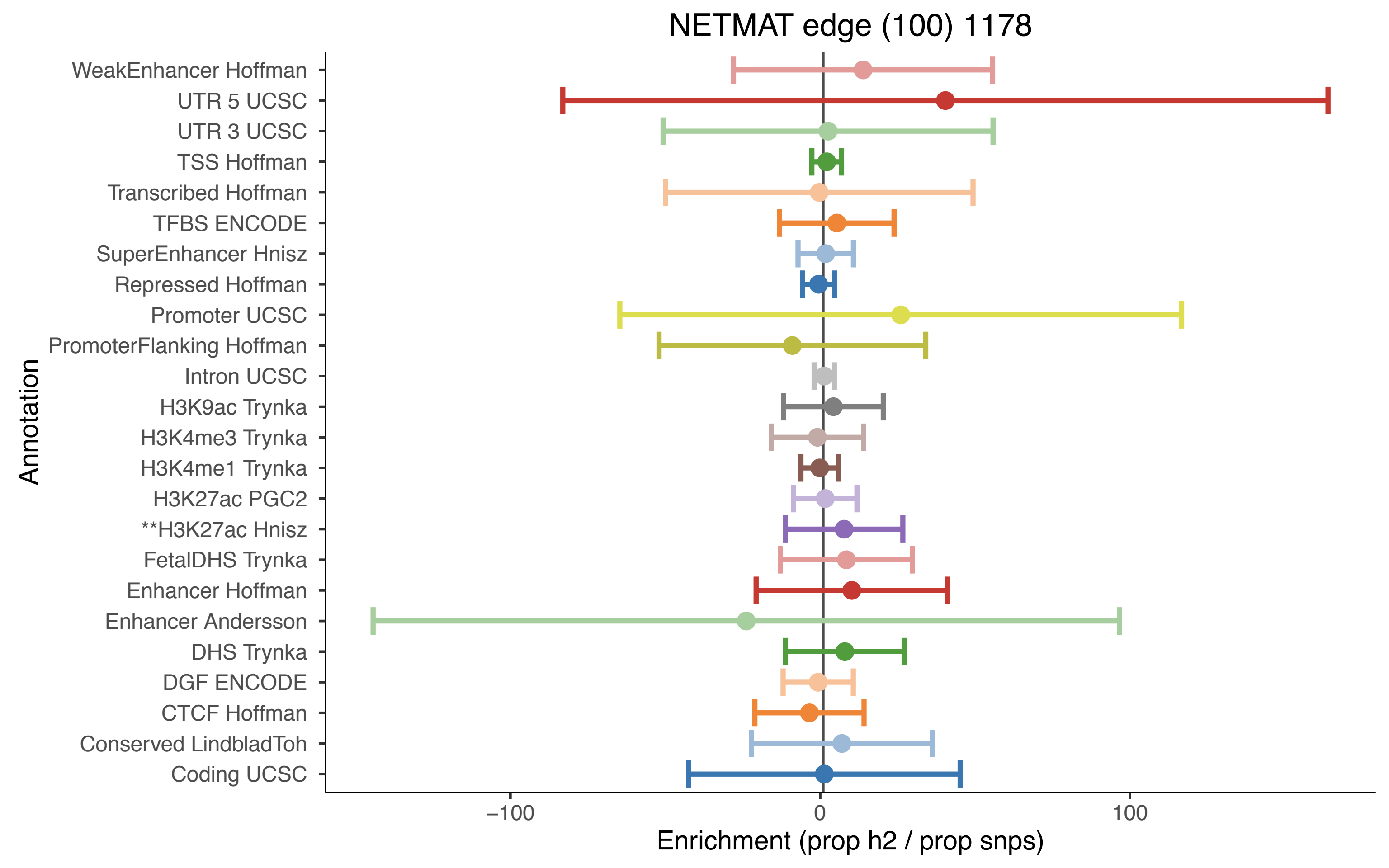

NETMAT edge (100) 1178
WeakEnhancer Hoffman
UTR 5 UCSC
UTR 3 UCSC
TSS Hoffman
Transcribed Hoffman
TFBS ENCODE
SuperEnhancer Hnisz
Repressed Hoffman
Promoter UCSC
PromoterFlanking Hoffman
Intron UCSC
H3K9ac Trynka
H3K4me3 Trynka
H3K4me1 Trynka
H3K27ac PGC2
**H3K27ac Hnisz
FetalDHS Trynka
Enhancer Hoffman
Enhancer Andersson
DHS Trynka
DGF ENCODE
CTCF Hoffman
Conserved LindbladToh
Coding UCSC
Annotation
−100
0
100
Enrichment (prop h2 / prop snps)

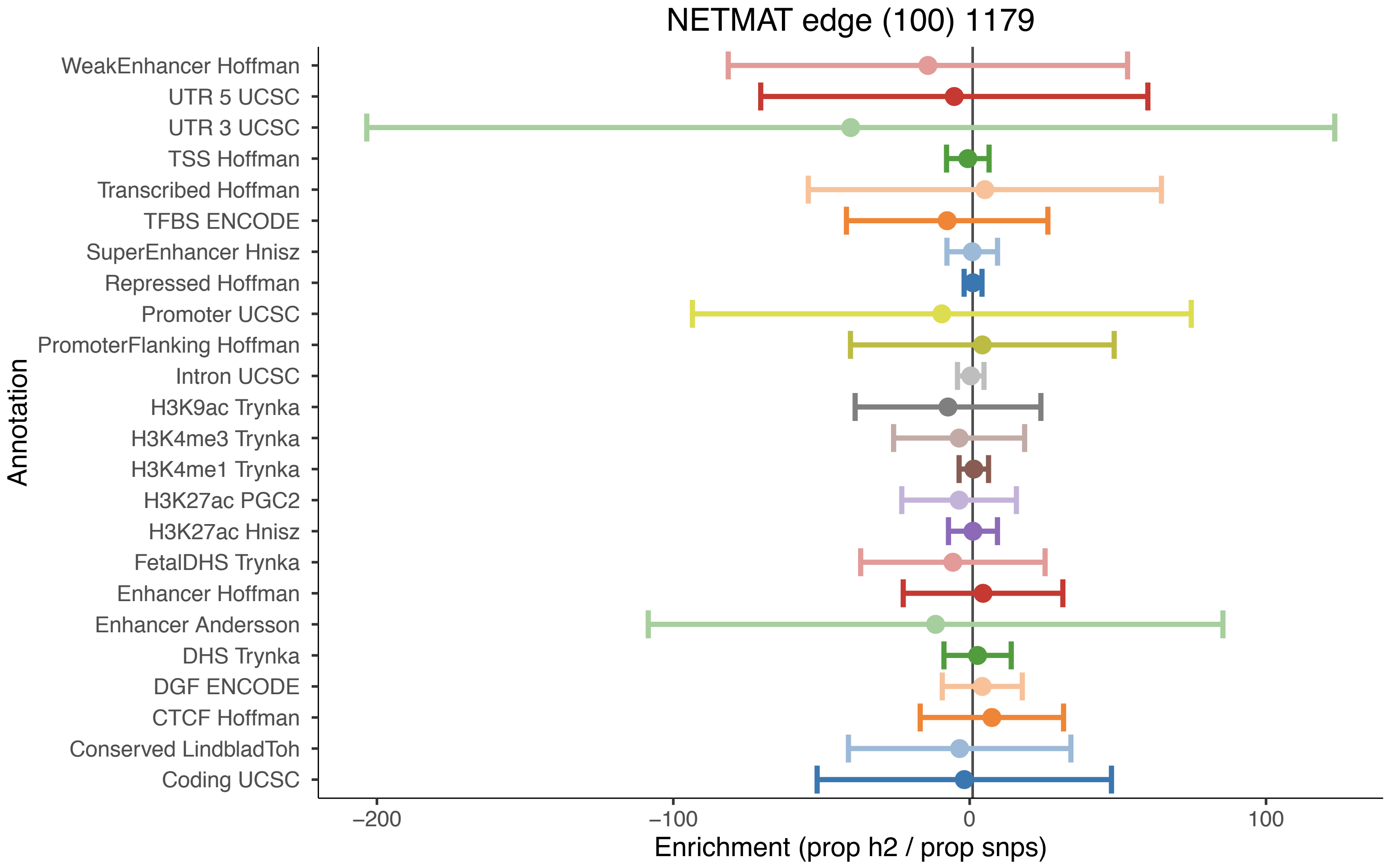
NETMAT edge (100) 1179
Annotation
WeakEnhancer Hoffman
UTR 5 UCSC
UTR 3 UCSC
TSS Hoffman
Transcribed Hoffman
TFBS ENCODE
SuperEnhancer Hnisz
Repressed Hoffman
Promoter UCSC
PromoterFlanking Hoffman
Intron UCSC
H3K9ac Trynka
H3K4me3 Trynka
H3K4me1 Trynka
H3K27ac PGC2
H3K27ac Hnisz
FetalDHS Trynka
Enhancer Hoffman
Enhancer Andersson
DHS Trynka
DGF ENCODE
CTCF Hoffman
Conserved LindbladToh
Coding UCSC
−200
−100
0
100
Enrichment (prop h2 / prop snps)

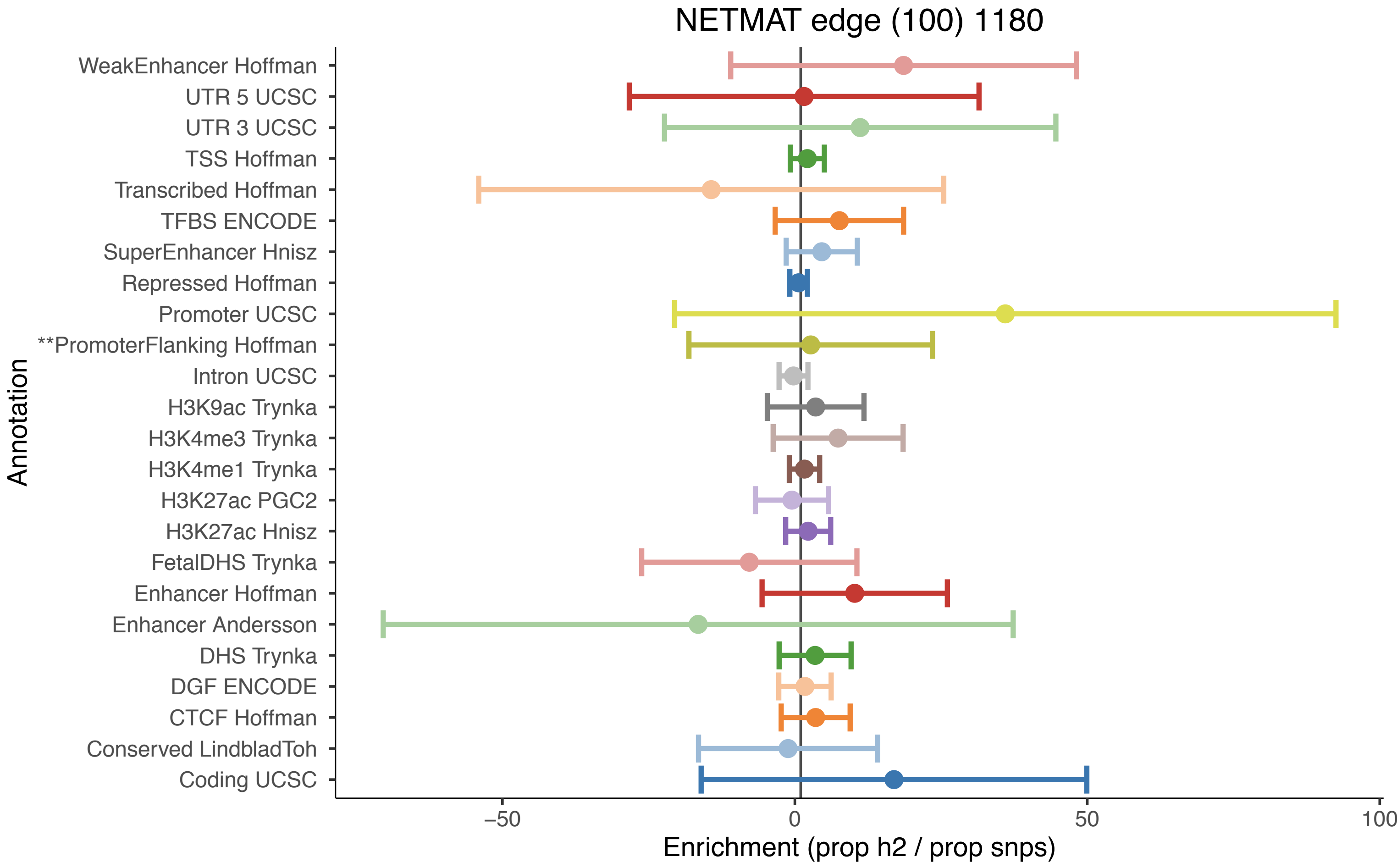
NETMAT edge (100) 1180
WeakEnhancer Hoffman
UTR 5 UCSC
UTR 3 UCSC
TSS Hoffman
Transcribed Hoffman
TFBS ENCODE
SuperEnhancer Hnisz
Repressed Hoffman
Promoter UCSC
**PromoterFlanking Hoffman
Intron UCSC
H3K9ac Trynka
H3K4me3 Trynka
H3K4me1 Trynka
H3K27ac PGC2
H3K27ac Hnisz
FetalDHS Trynka
Enhancer Hoffman
Enhancer Andersson
DHS Trynka
DGF ENCODE
CTCF Hoffman
Conserved LindbladToh
Coding UCSC
Annotation
−50
0
50
100
Enrichment (prop h2 / prop snps)

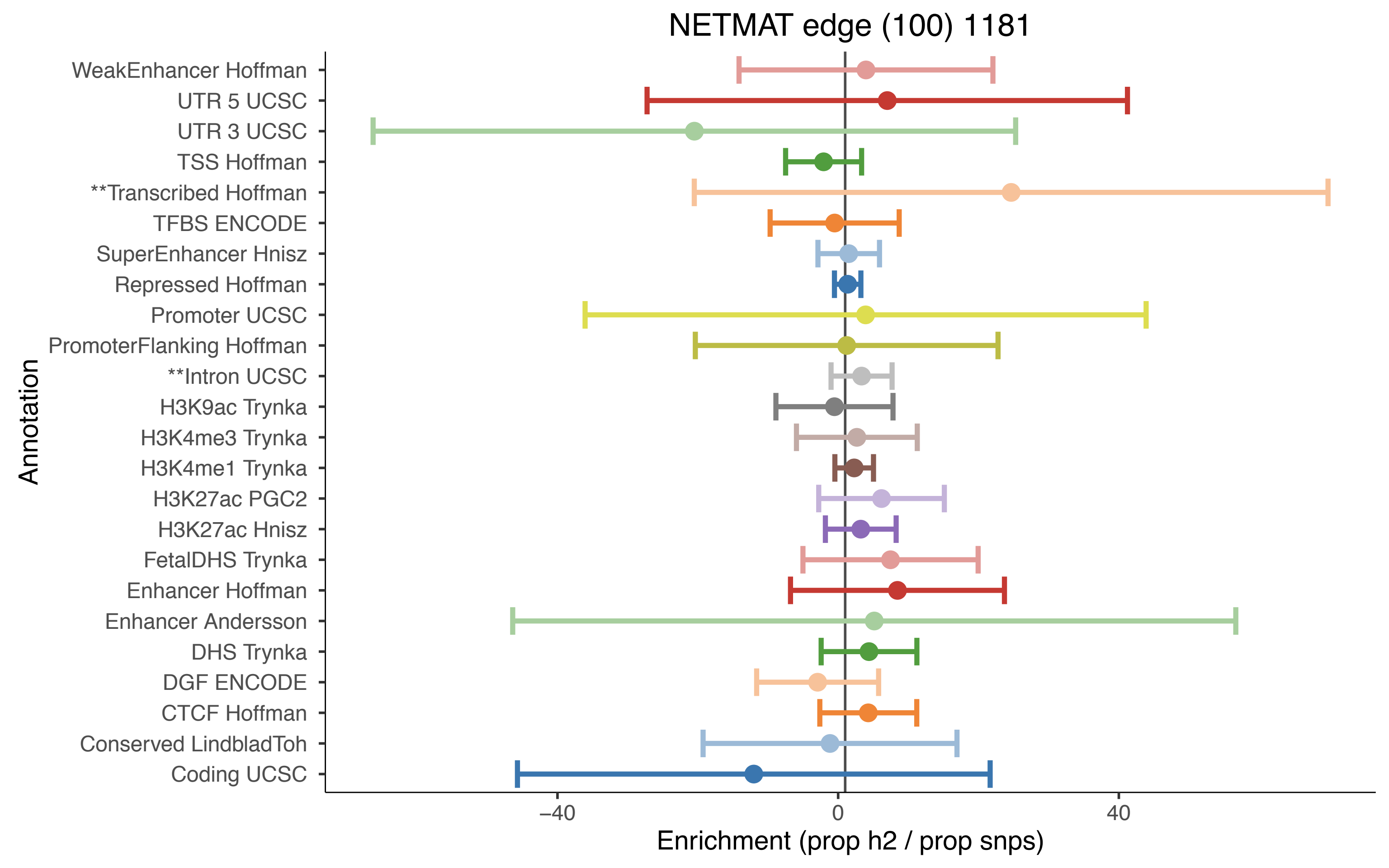
NETMAT edge (100) 1181
WeakEnhancer Hoffman
UTR 5 UCSC
UTR 3 UCSC
TSS Hoffman
**Transcribed Hoffman
TFBS ENCODE
SuperEnhancer Hnisz
Repressed Hoffman
Promoter UCSC
PromoterFlanking Hoffman
**Intron UCSC
H3K9ac Trynka
H3K4me3 Trynka
H3K4me1 Trynka
H3K27ac PGC2
H3K27ac Hnisz
FetalDHS Trynka
Enhancer Hoffman
Enhancer Andersson
DHS Trynka
DGF ENCODE
CTCF Hoffman
Conserved LindbladToh
Coding UCSC
Annotation
−40
0
40
Enrichment (prop h2 / prop snps)

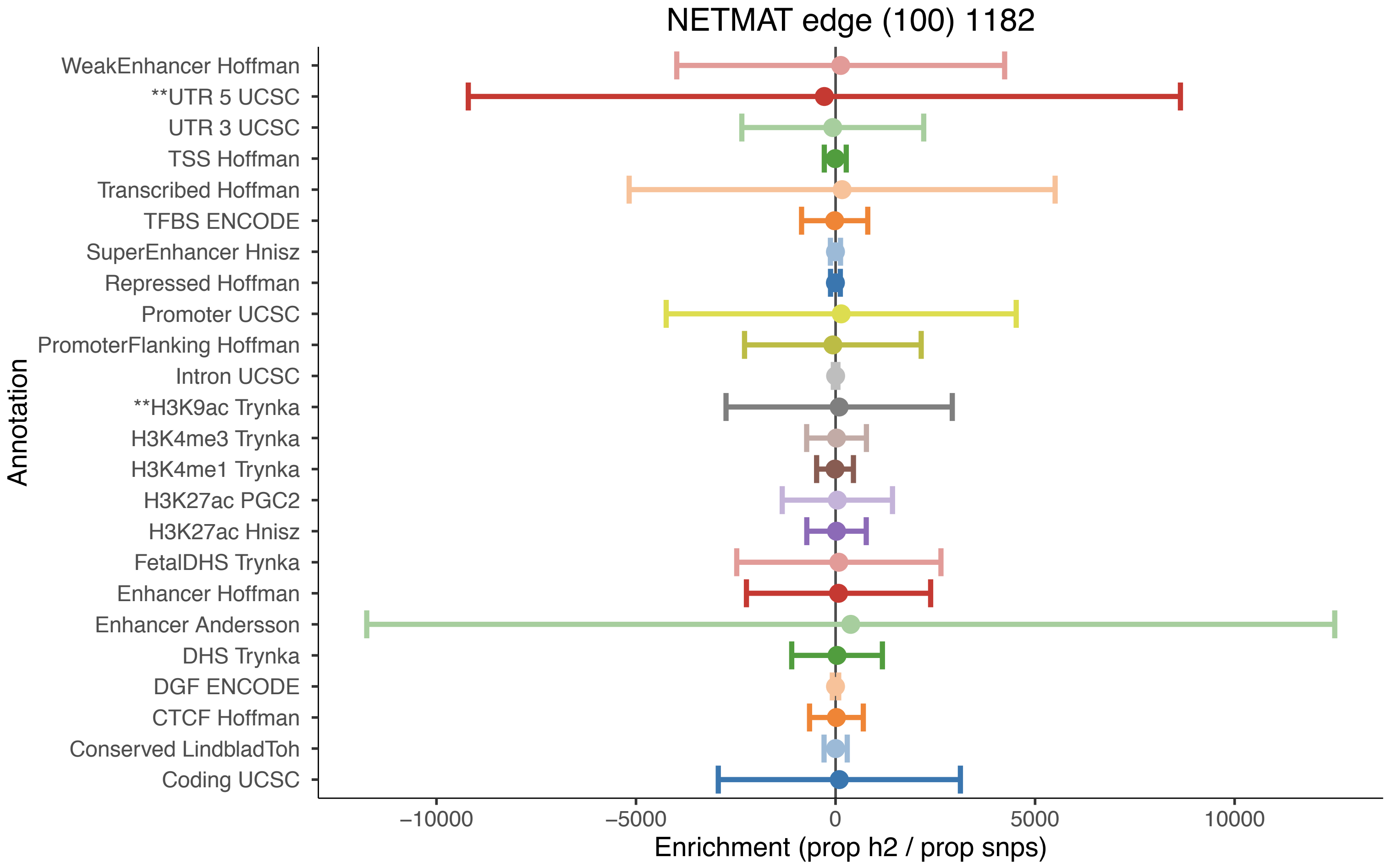

NETMAT edge (100) 1182
Annotation
WeakEnhancer Hoffman
**UTR 5 UCSC
UTR 3 UCSC
TSS Hoffman
Transcribed Hoffman
TFBS ENCODE
SuperEnhancer Hnisz
Repressed Hoffman
Promoter UCSC
PromoterFlanking Hoffman
Intron UCSC
**H3K9ac Trynka
H3K4me3 Trynka
H3K4me1 Trynka
H3K27ac PGC2
H3K27ac Hnisz
FetalDHS Trynka
Enhancer Hoffman
Enhancer Andersson
DHS Trynka
DGF ENCODE
CTCF Hoffman
Conserved LindbladToh
Coding UCSC
−10000
−5000
0
5000
10000
Enrichment (prop h2 / prop snps)

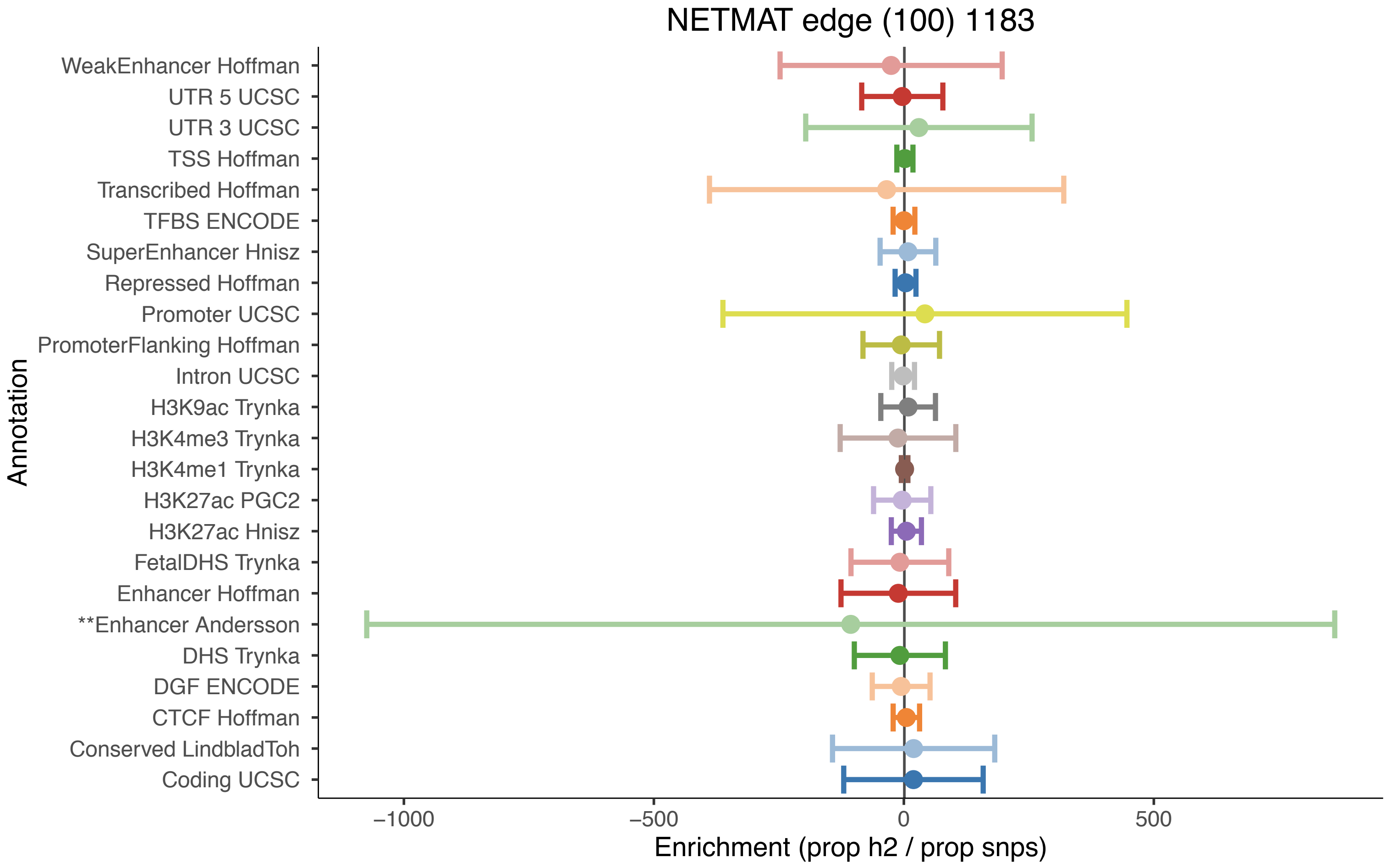

NETMAT edge (100) 1183
WeakEnhancer Hoffman
UTR 5 UCSC
UTR 3 UCSC
TSS Hoffman
Transcribed Hoffman
TFBS ENCODE
SuperEnhancer Hnisz
Repressed Hoffman
Promoter UCSC
PromoterFlanking Hoffman
Intron UCSC
H3K9ac Trynka
H3K4me3 Trynka
H3K4me1 Trynka
H3K27ac PGC2
H3K27ac Hnisz
FetalDHS Trynka
Enhancer Hoffman
**Enhancer Andersson
DHS Trynka
DGF ENCODE
CTCF Hoffman
Conserved LindbladToh
Coding UCSC
Annotation
−1000
−500
0
500
Enrichment (prop h2 / prop snps)

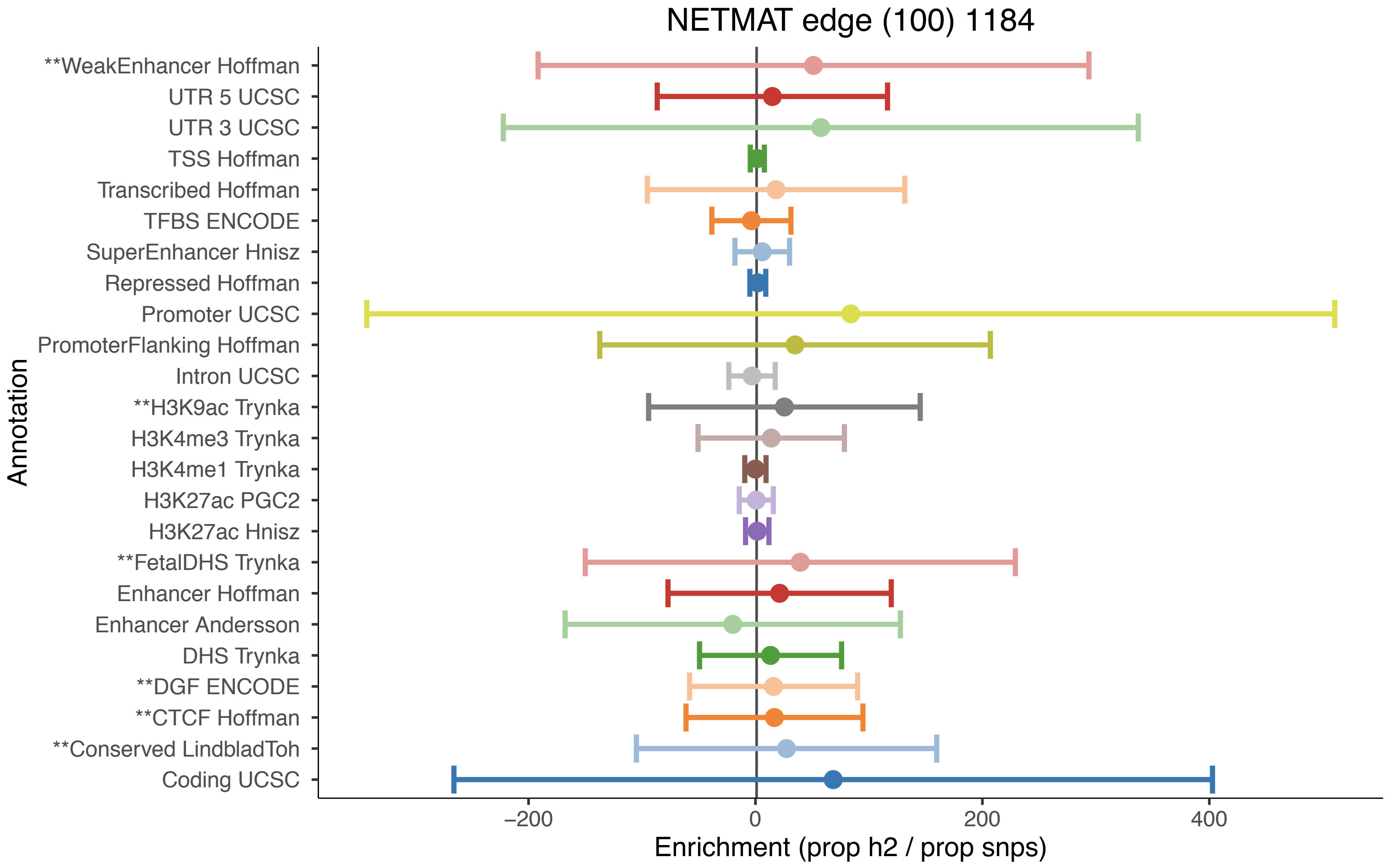
NETMAT edge (100) 1184
**WeakEnhancer Hoffman
UTR 5 UCSC
UTR 3 UCSC
TSS Hoffman
Transcribed Hoffman
TFBS ENCODE
SuperEnhancer Hnisz
Repressed Hoffman
Promoter UCSC
PromoterFlanking Hoffman
Intron UCSC
**H3K9ac Trynka
H3K4me3 Trynka
H3K4me1 Trynka
H3K27ac PGC2
H3K27ac Hnisz
**FetalDHS Trynka
Enhancer Hoffman
Enhancer Andersson
DHS Trynka
**DGF ENCODE
**CTCF Hoffman
**Conserved LindbladToh
Coding UCSC
Annotation
−200
0
200
400
Enrichment (prop h2 / prop snps)

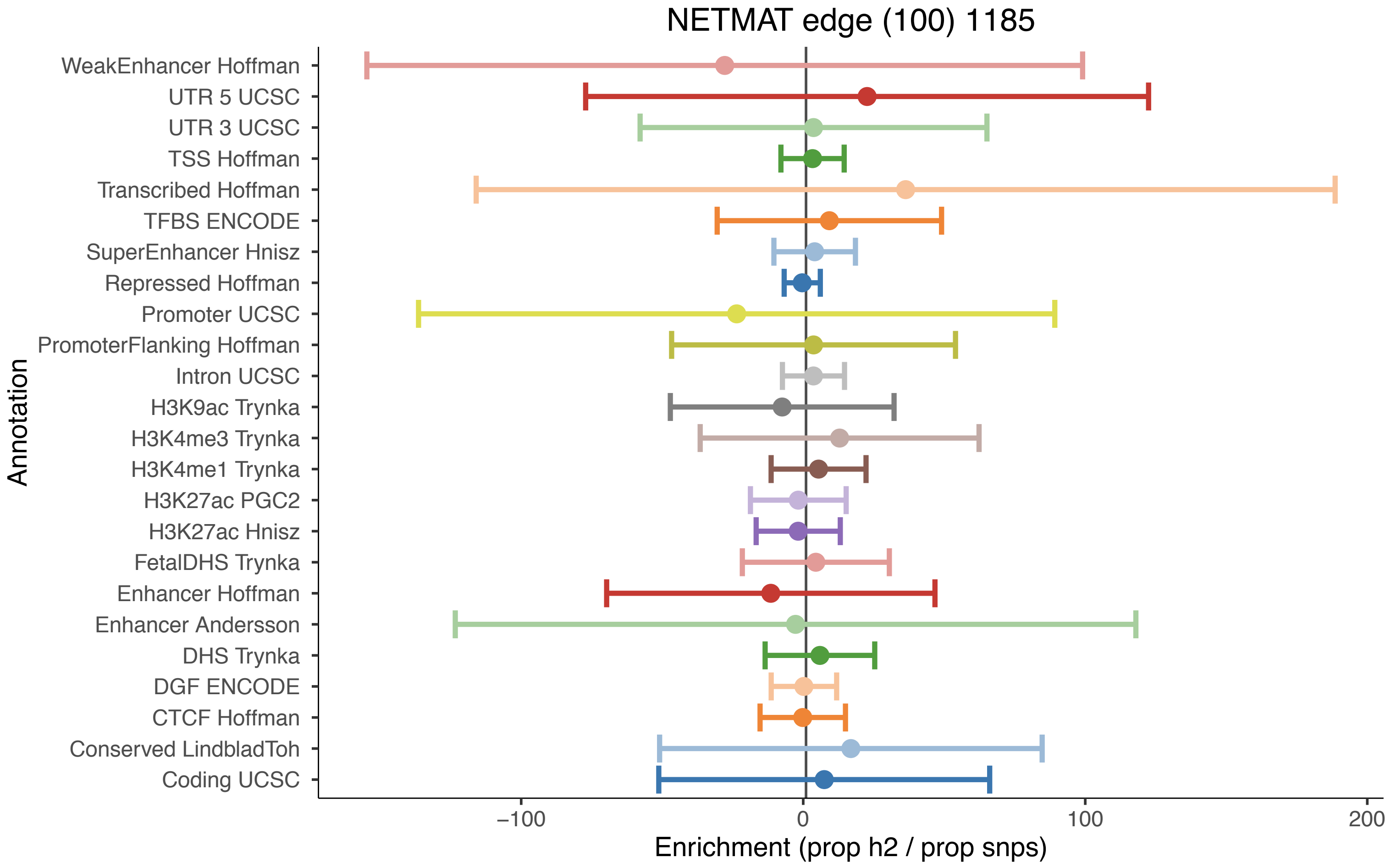
NETMAT edge (100) 1185
WeakEnhancer Hoffman
UTR 5 UCSC
UTR 3 UCSC
TSS Hoffman
Transcribed Hoffman
TFBS ENCODE
SuperEnhancer Hnisz
Repressed Hoffman
Promoter UCSC
PromoterFlanking Hoffman
Intron UCSC
H3K9ac Trynka
H3K4me3 Trynka
H3K4me1 Trynka
H3K27ac PGC2
H3K27ac Hnisz
FetalDHS Trynka
Enhancer Hoffman
Enhancer Andersson
DHS Trynka
DGF ENCODE
CTCF Hoffman
Conserved LindbladToh
Coding UCSC
Annotation
−100
0
100
200
Enrichment (prop h2 / prop snps)

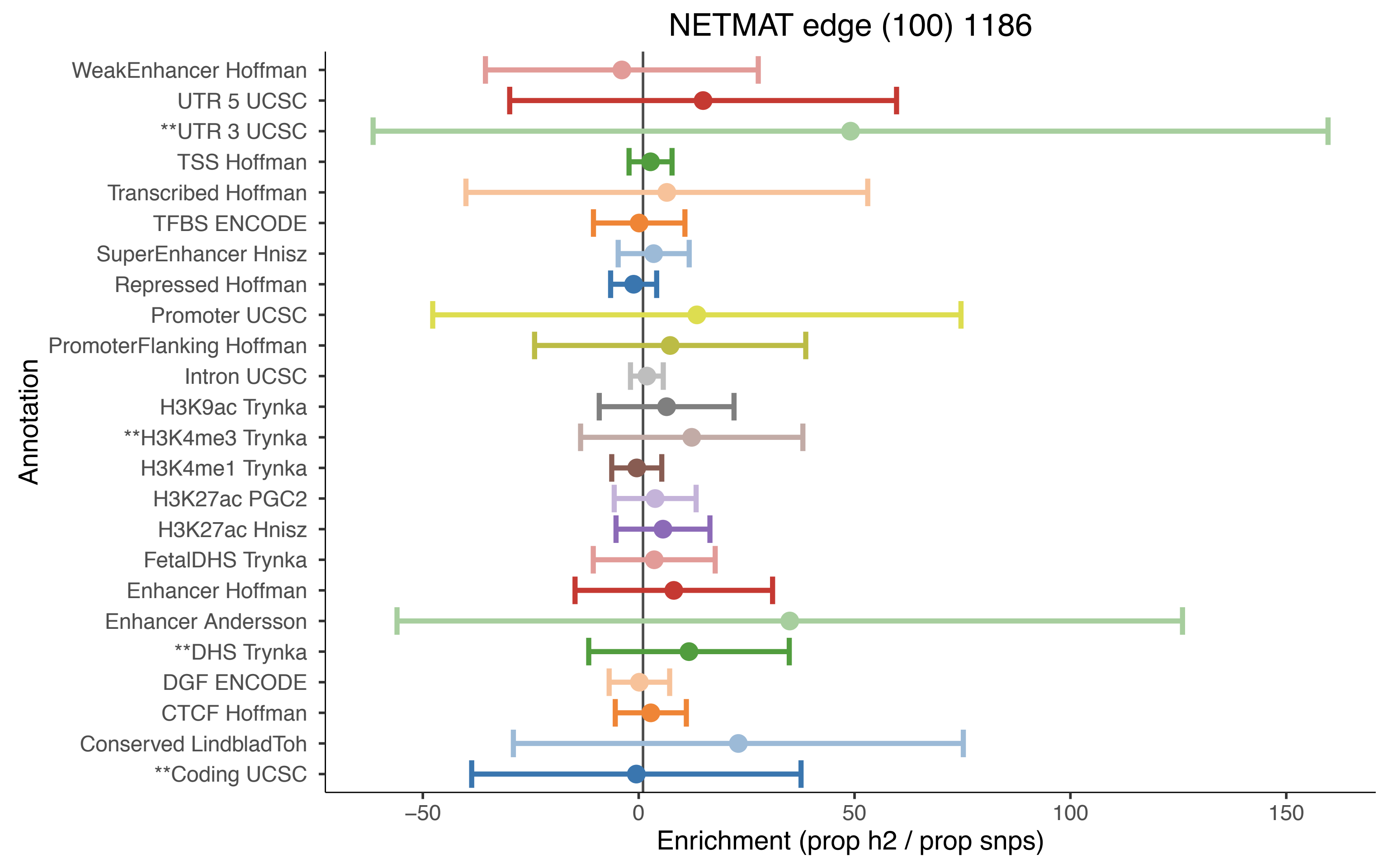

NETMAT edge (100) 1186
WeakEnhancer Hoffman
UTR 5 UCSC
**UTR 3 UCSC
TSS Hoffman
Transcribed Hoffman
TFBS ENCODE
SuperEnhancer Hnisz
Repressed Hoffman
Promoter UCSC
PromoterFlanking Hoffman
Intron UCSC
H3K9ac Trynka
**H3K4me3 Trynka
H3K4me1 Trynka
H3K27ac PGC2
H3K27ac Hnisz
FetalDHS Trynka
Enhancer Hoffman
Enhancer Andersson
**DHS Trynka
DGF ENCODE
CTCF Hoffman
Conserved LindbladToh
**Coding UCSC
Annotation
−50
0
50
100
150
Enrichment (prop h2 / prop snps)

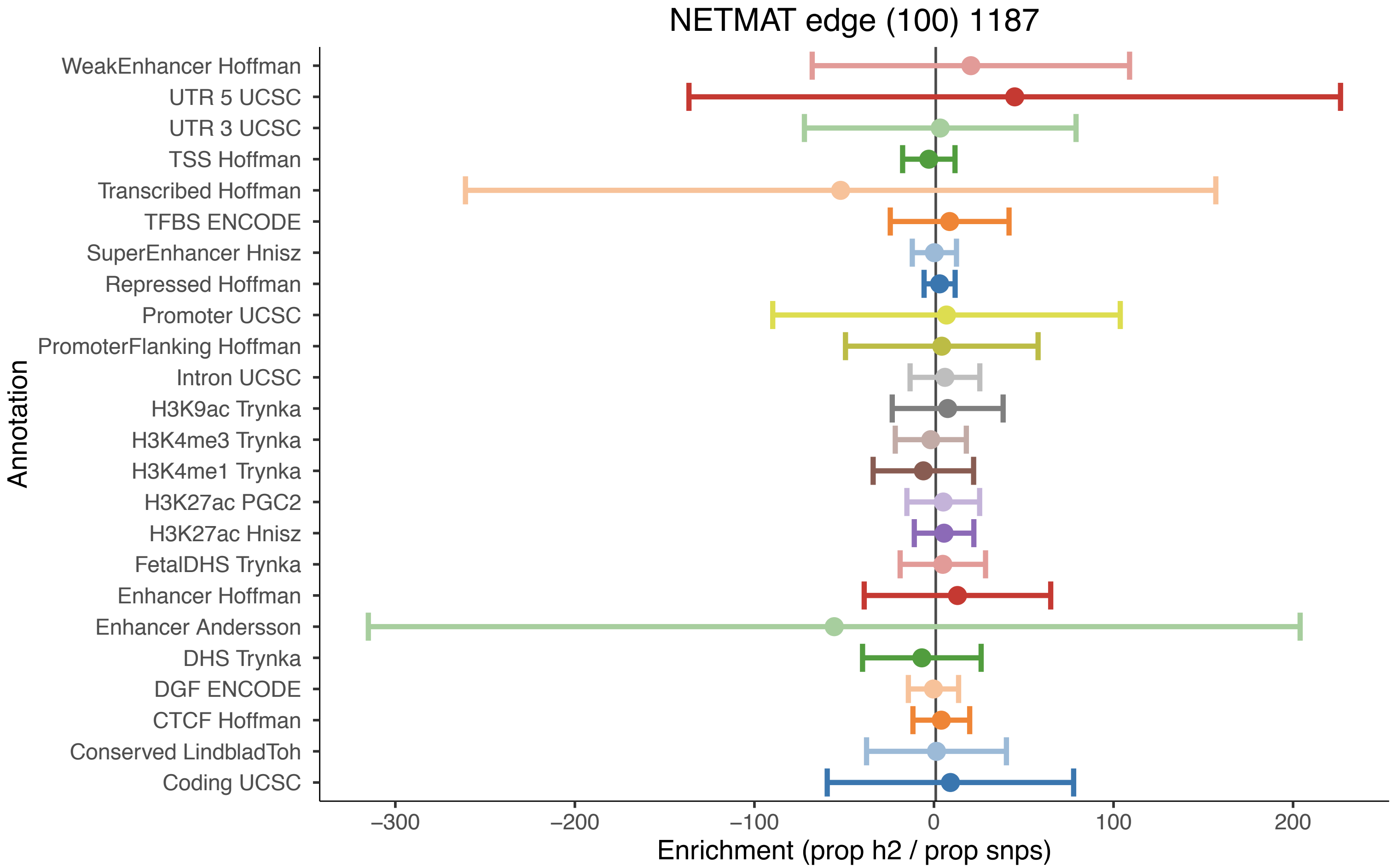
NETMAT edge (100) 1187
WeakEnhancer Hoffman
UTR 5 UCSC
UTR 3 UCSC
TSS Hoffman
Transcribed Hoffman
TFBS ENCODE
SuperEnhancer Hnisz
Repressed Hoffman
Promoter UCSC
PromoterFlanking Hoffman
Intron UCSC
H3K9ac Trynka
H3K4me3 Trynka
H3K4me1 Trynka
H3K27ac PGC2
H3K27ac Hnisz
FetalDHS Trynka
Enhancer Hoffman
Enhancer Andersson
DHS Trynka
DGF ENCODE
CTCF Hoffman
Conserved LindbladToh
Coding UCSC
Annotation
−300
−200
−100
0
100
200
Enrichment (prop h2 / prop snps)

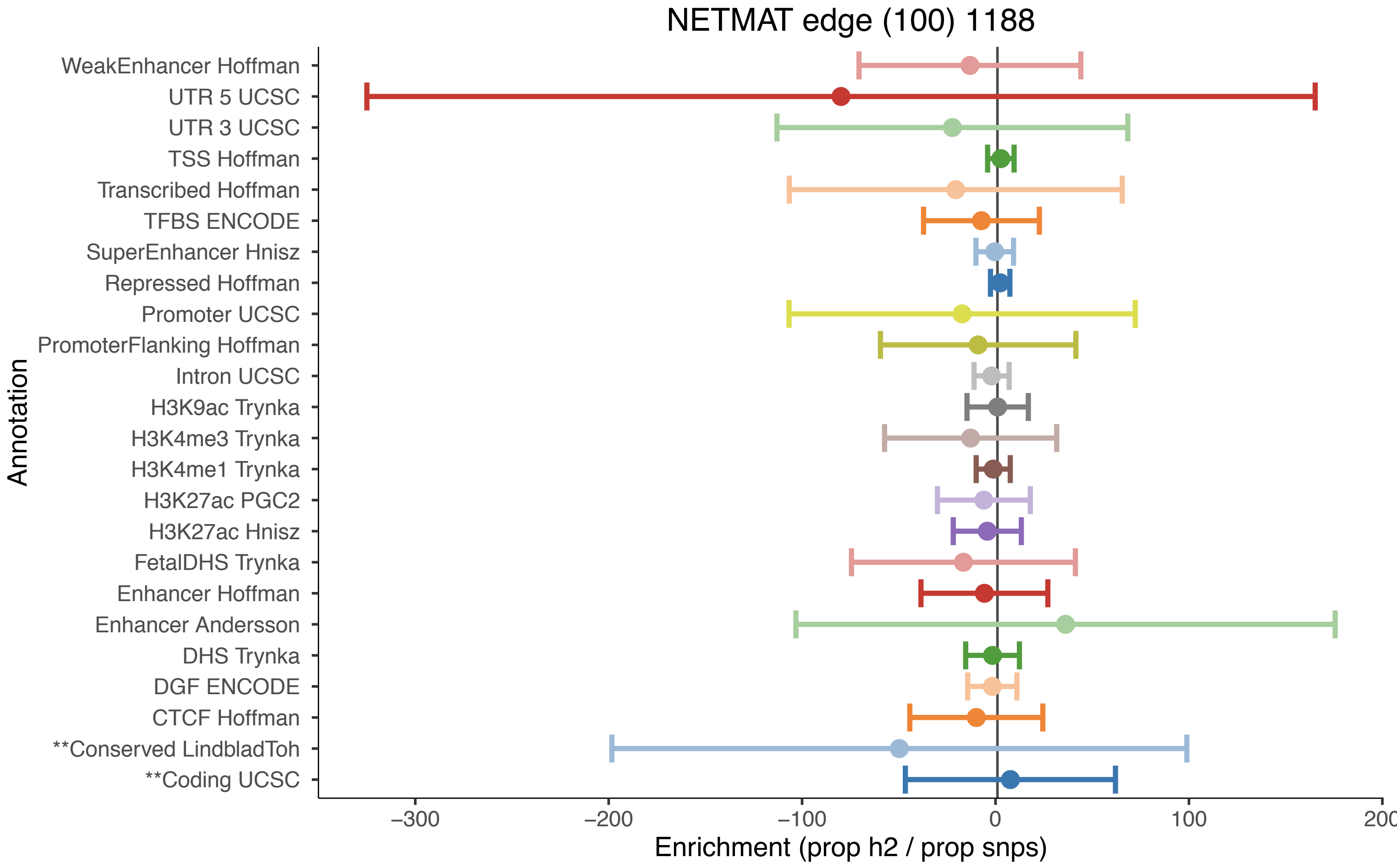

NETMAT edge (100) 1188
WeakEnhancer Hoffman
UTR 5 UCSC
UTR 3 UCSC
TSS Hoffman
Transcribed Hoffman
TFBS ENCODE
SuperEnhancer Hnisz
Repressed Hoffman
Promoter UCSC
PromoterFlanking Hoffman
Intron UCSC
H3K9ac Trynka
H3K4me3 Trynka
H3K4me1 Trynka
H3K27ac PGC2
H3K27ac Hnisz
FetalDHS Trynka
Enhancer Hoffman
Enhancer Andersson
DHS Trynka
DGF ENCODE
CTCF Hoffman
**Conserved LindbladToh
**Coding UCSC
Annotation
−300
−200
−100
0
100
200
Enrichment (prop h2 / prop snps)

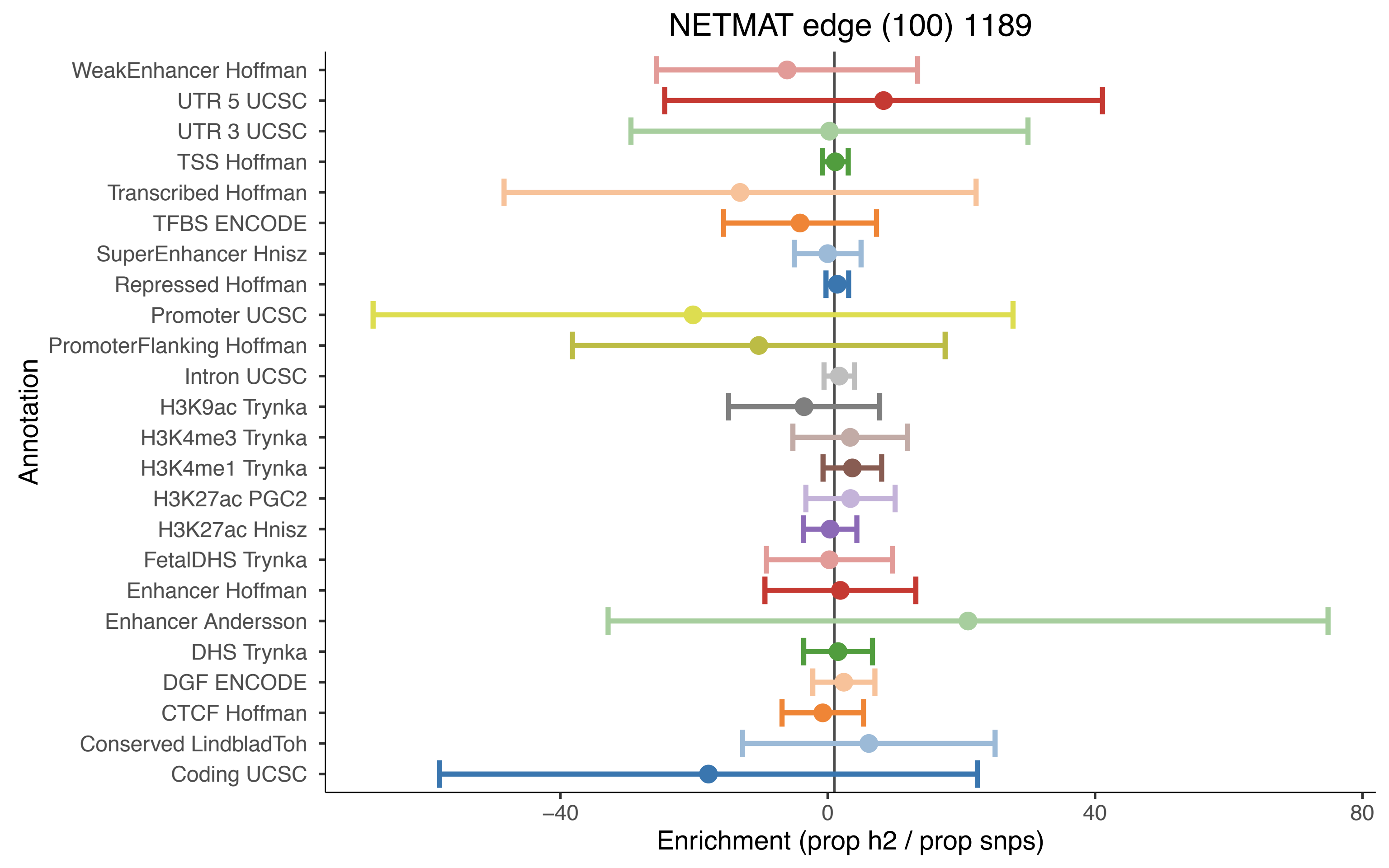
NETMAT edge (100) 1189
WeakEnhancer Hoffman
UTR 5 UCSC
UTR 3 UCSC
TSS Hoffman
Transcribed Hoffman
TFBS ENCODE
SuperEnhancer Hnisz
Repressed Hoffman
Promoter UCSC
PromoterFlanking Hoffman
Intron UCSC
H3K9ac Trynka
H3K4me3 Trynka
H3K4me1 Trynka
H3K27ac PGC2
H3K27ac Hnisz
FetalDHS Trynka
Enhancer Hoffman
Enhancer Andersson
DHS Trynka
DGF ENCODE
CTCF Hoffman
Conserved LindbladToh
Coding UCSC
Annotation
−40
0
40
80
Enrichment (prop h2 / prop snps)

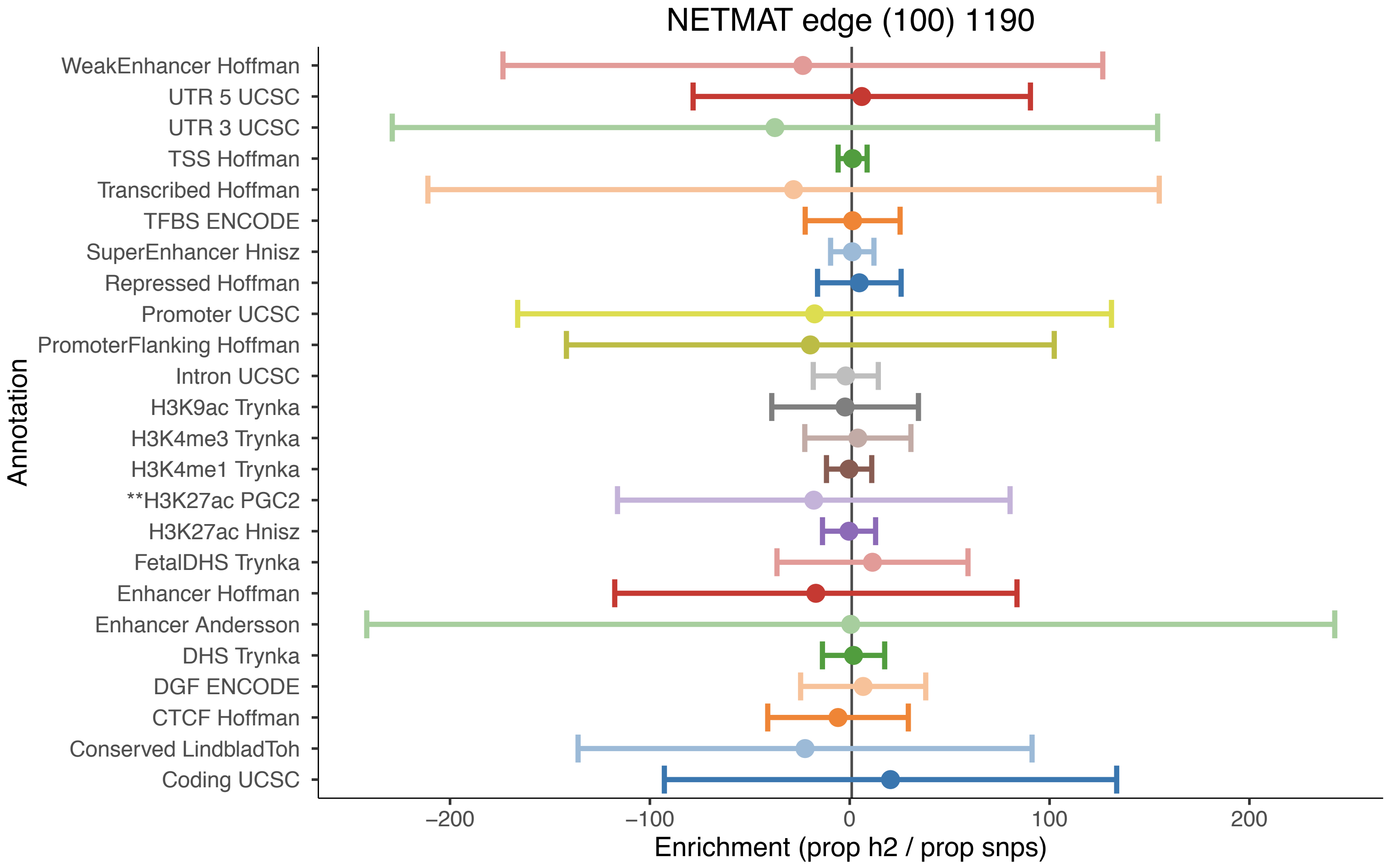

NETMAT edge (100) 1190
WeakEnhancer Hoffman
UTR 5 UCSC
UTR 3 UCSC
TSS Hoffman
Transcribed Hoffman
TFBS ENCODE
SuperEnhancer Hnisz
Repressed Hoffman
Promoter UCSC
PromoterFlanking Hoffman
Intron UCSC
H3K9ac Trynka
H3K4me3 Trynka
H3K4me1 Trynka
**H3K27ac PGC2
H3K27ac Hnisz
FetalDHS Trynka
Enhancer Hoffman
Enhancer Andersson
DHS Trynka
DGF ENCODE
CTCF Hoffman
Conserved LindbladToh
Coding UCSC
Annotation
−200
−100
0
100
200
Enrichment (prop h2 / prop snps)

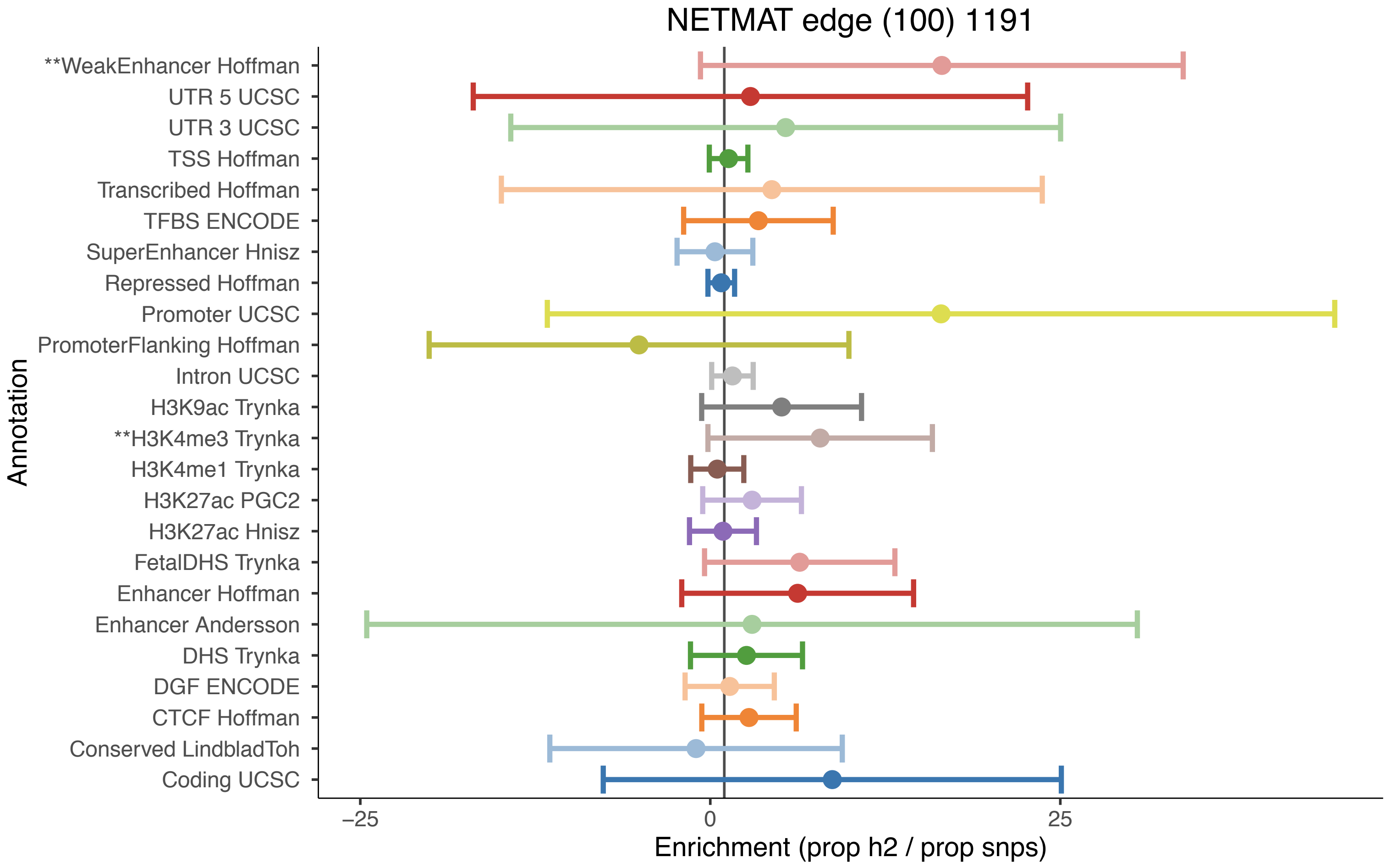
NETMAT edge (100) 1191
**WeakEnhancer Hoffman
UTR 5 UCSC
UTR 3 UCSC
TSS Hoffman
Transcribed Hoffman
TFBS ENCODE
SuperEnhancer Hnisz
Repressed Hoffman
Promoter UCSC
PromoterFlanking Hoffman
Intron UCSC
H3K9ac Trynka
**H3K4me3 Trynka
H3K4me1 Trynka
H3K27ac PGC2
H3K27ac Hnisz
FetalDHS Trynka
Enhancer Hoffman
Enhancer Andersson
DHS Trynka
DGF ENCODE
CTCF Hoffman
Conserved LindbladToh
Coding UCSC
Annotation
−25
0
25
Enrichment (prop h2 / prop snps)

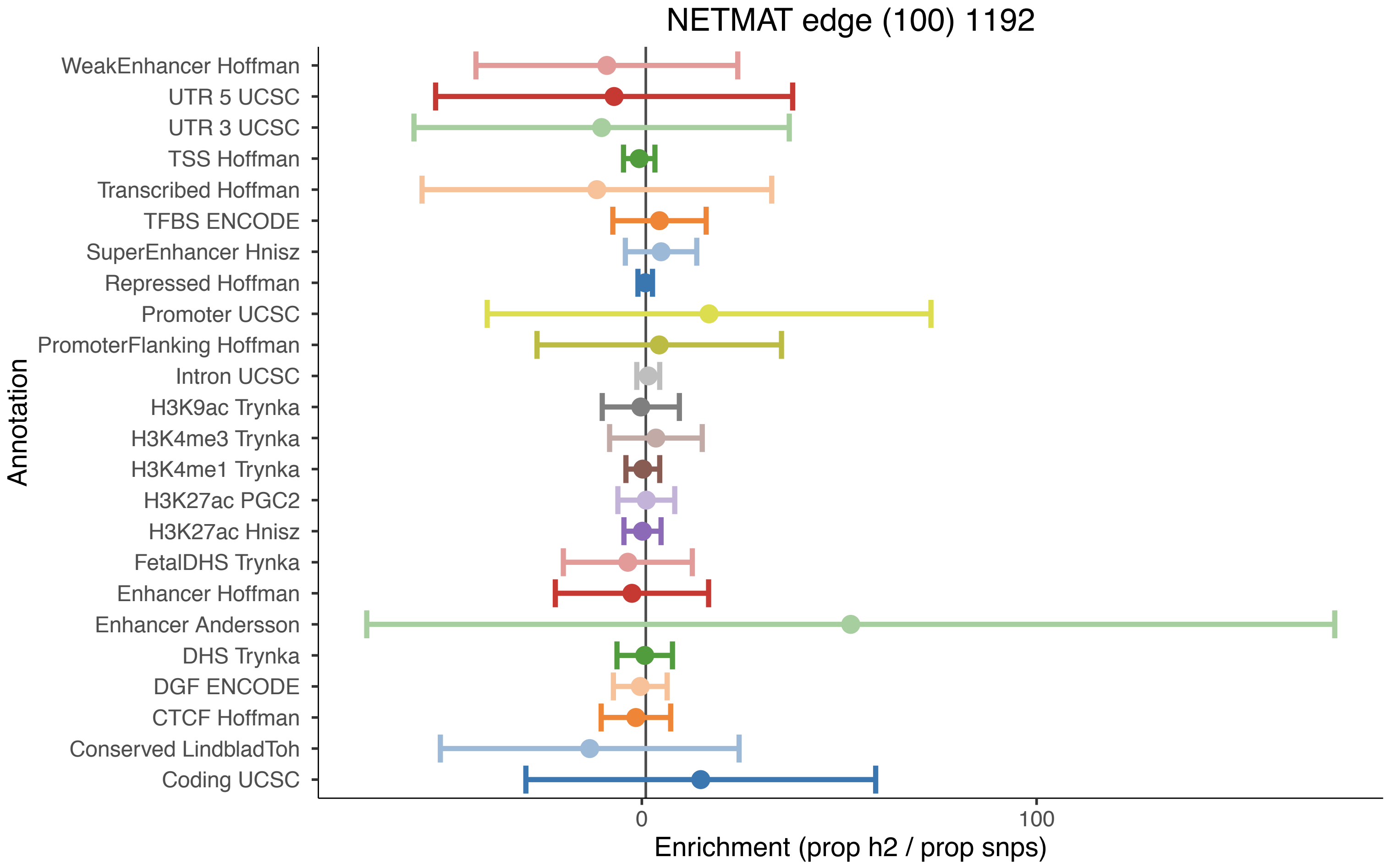
NETMAT edge (100) 1192
Annotation
WeakEnhancer Hoffman
UTR 5 UCSC
UTR 3 UCSC
TSS Hoffman
Transcribed Hoffman
TFBS ENCODE
SuperEnhancer Hnisz
Repressed Hoffman
Promoter UCSC
PromoterFlanking Hoffman
Intron UCSC
H3K9ac Trynka
H3K4me3 Trynka
H3K4me1 Trynka
H3K27ac PGC2
H3K27ac Hnisz
FetalDHS Trynka
Enhancer Hoffman
Enhancer Andersson
DHS Trynka
DGF ENCODE
CTCF Hoffman
Conserved LindbladToh
Coding UCSC
0
100
Enrichment (prop h2 / prop snps)

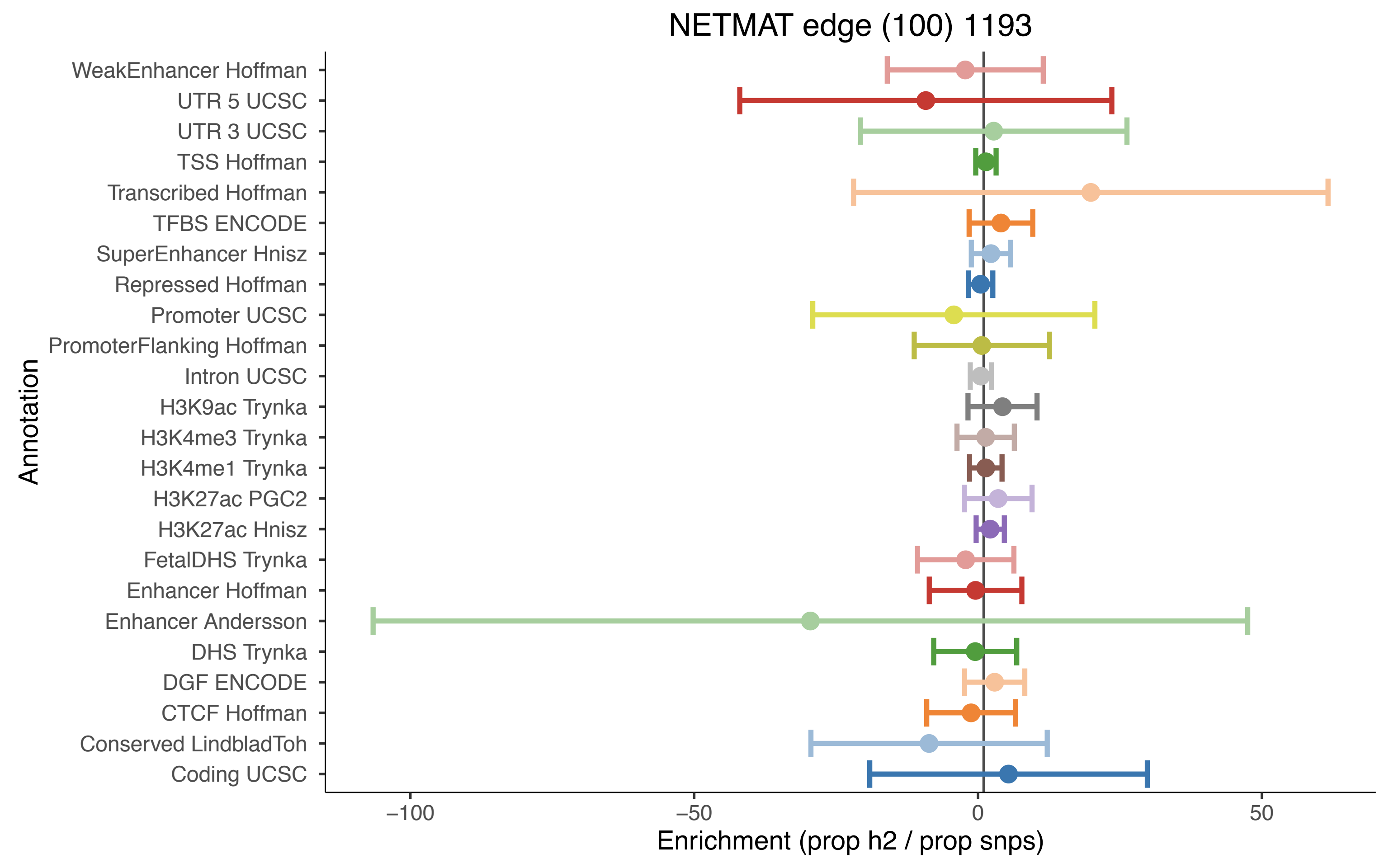

NETMAT edge (100) 1193
Annotation
WeakEnhancer Hoffman
UTR 5 UCSC
UTR 3 UCSC
TSS Hoffman
Transcribed Hoffman
TFBS ENCODE
SuperEnhancer Hnisz
Repressed Hoffman
Promoter UCSC
PromoterFlanking Hoffman
Intron UCSC
H3K9ac Trynka
H3K4me3 Trynka
H3K4me1 Trynka
H3K27ac PGC2
H3K27ac Hnisz
FetalDHS Trynka
Enhancer Hoffman
Enhancer Andersson
DHS Trynka
DGF ENCODE
CTCF Hoffman
Conserved LindbladToh
Coding UCSC
−100
−50
0
50
Enrichment (prop h2 / prop snps)

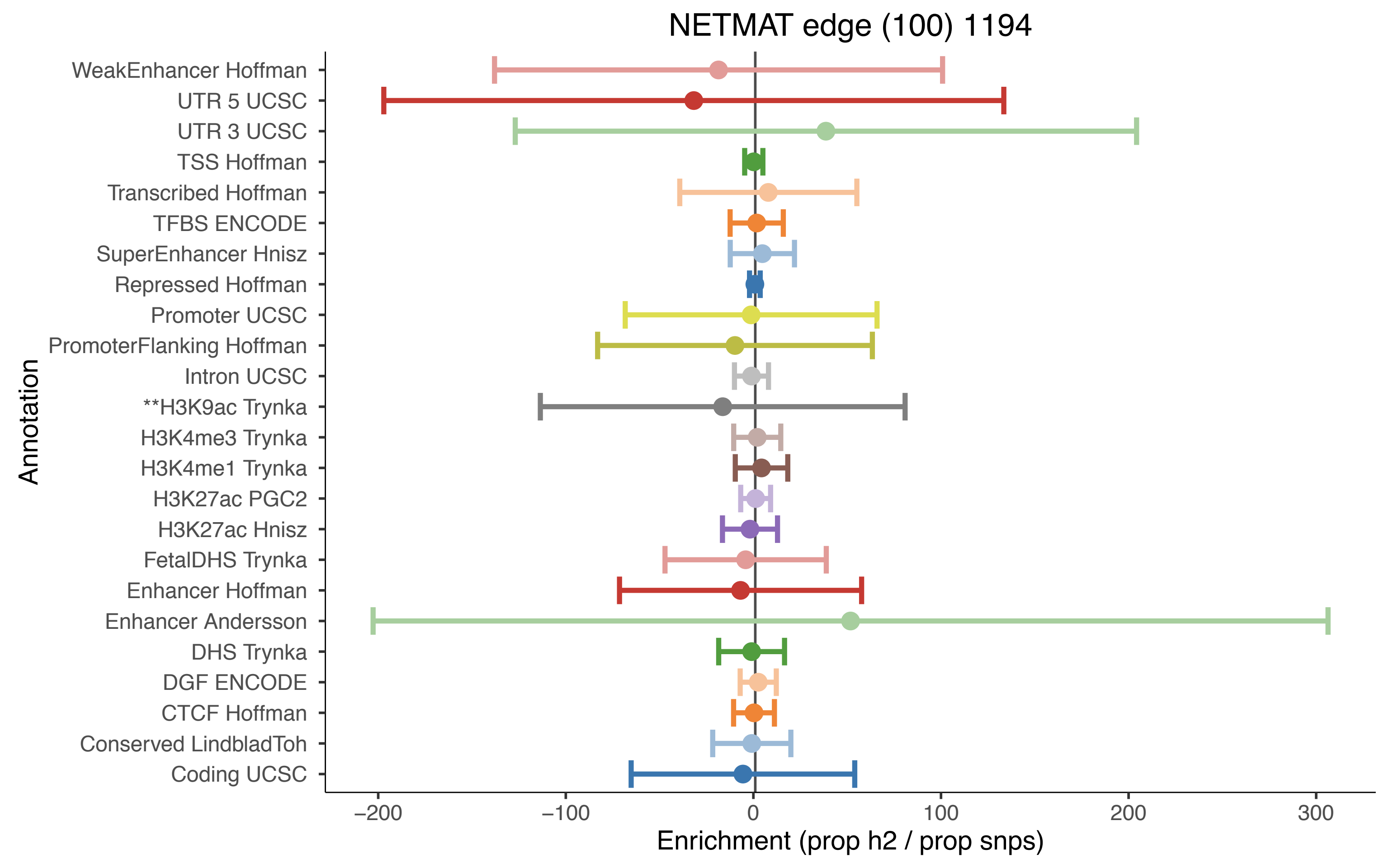
NETMAT edge (100) 1194
WeakEnhancer Hoffman
UTR 5 UCSC
UTR 3 UCSC
TSS Hoffman
Transcribed Hoffman
TFBS ENCODE
SuperEnhancer Hnisz
Repressed Hoffman
Promoter UCSC
PromoterFlanking Hoffman
Intron UCSC
**H3K9ac Trynka
H3K4me3 Trynka
H3K4me1 Trynka
H3K27ac PGC2
H3K27ac Hnisz
FetalDHS Trynka
Enhancer Hoffman
Enhancer Andersson
DHS Trynka
DGF ENCODE
CTCF Hoffman
Conserved LindbladToh
Coding UCSC
Annotation
−200
−100
0
100
200
300
Enrichment (prop h2 / prop snps)

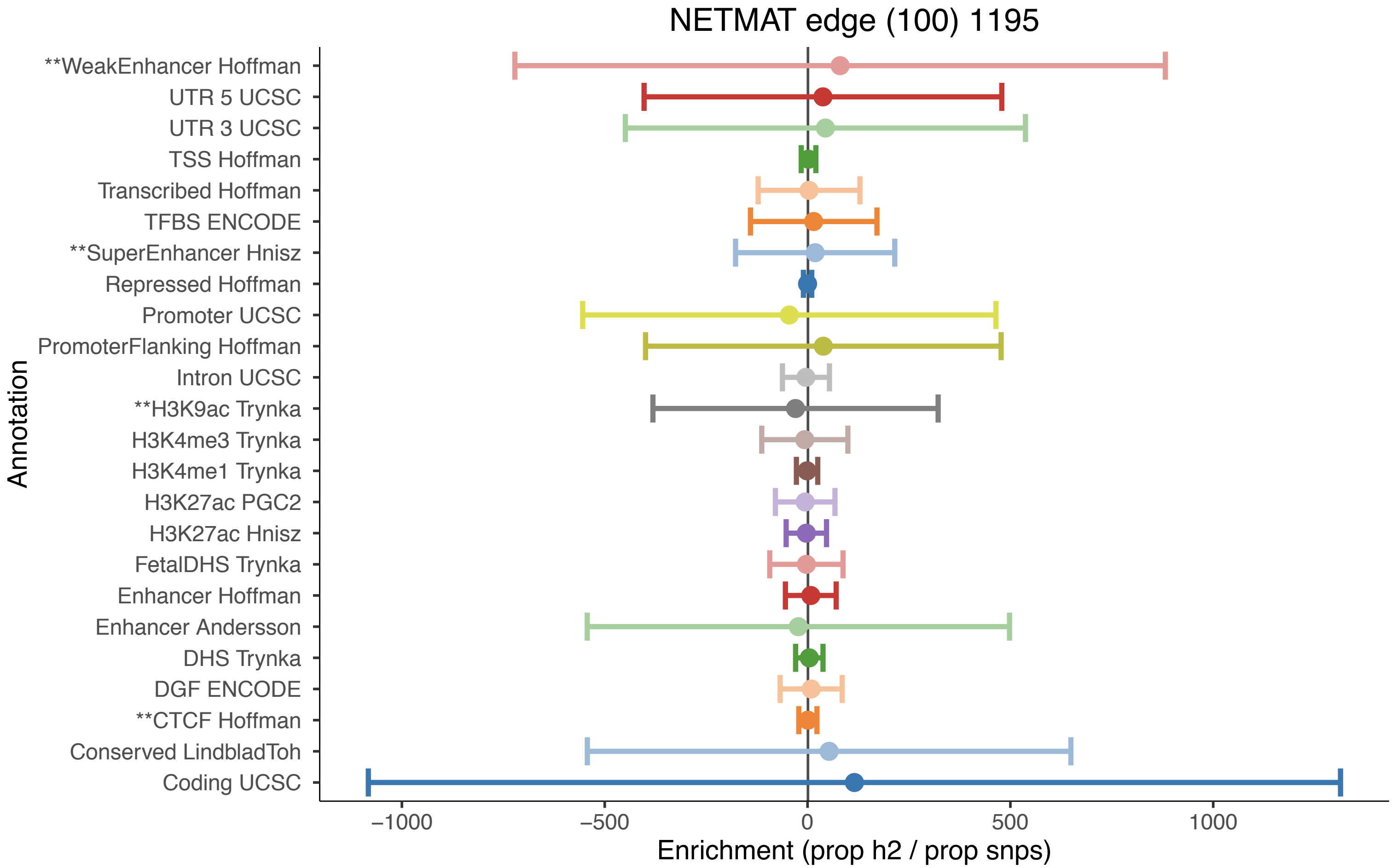
NETMAT edge (100) 1195
**WeakEnhancer Hoffman
UTR 5 UCSC
UTR 3 UCSC
TSS Hoffman
Transcribed Hoffman
TFBS ENCODE
**SuperEnhancer Hnisz
Repressed Hoffman
Promoter UCSC
PromoterFlanking Hoffman
Intron UCSC
**H3K9ac Trynka
H3K4me3 Trynka
H3K4me1 Trynka
H3K27ac PGC2
H3K27ac Hnisz
FetalDHS Trynka
Enhancer Hoffman
Enhancer Andersson
DHS Trynka
DGF ENCODE
**CTCF Hoffman
Conserved LindbladToh
Coding UCSC
Annotation
−1000
−500
0
500
1000
Enrichment (prop h2 / prop snps)

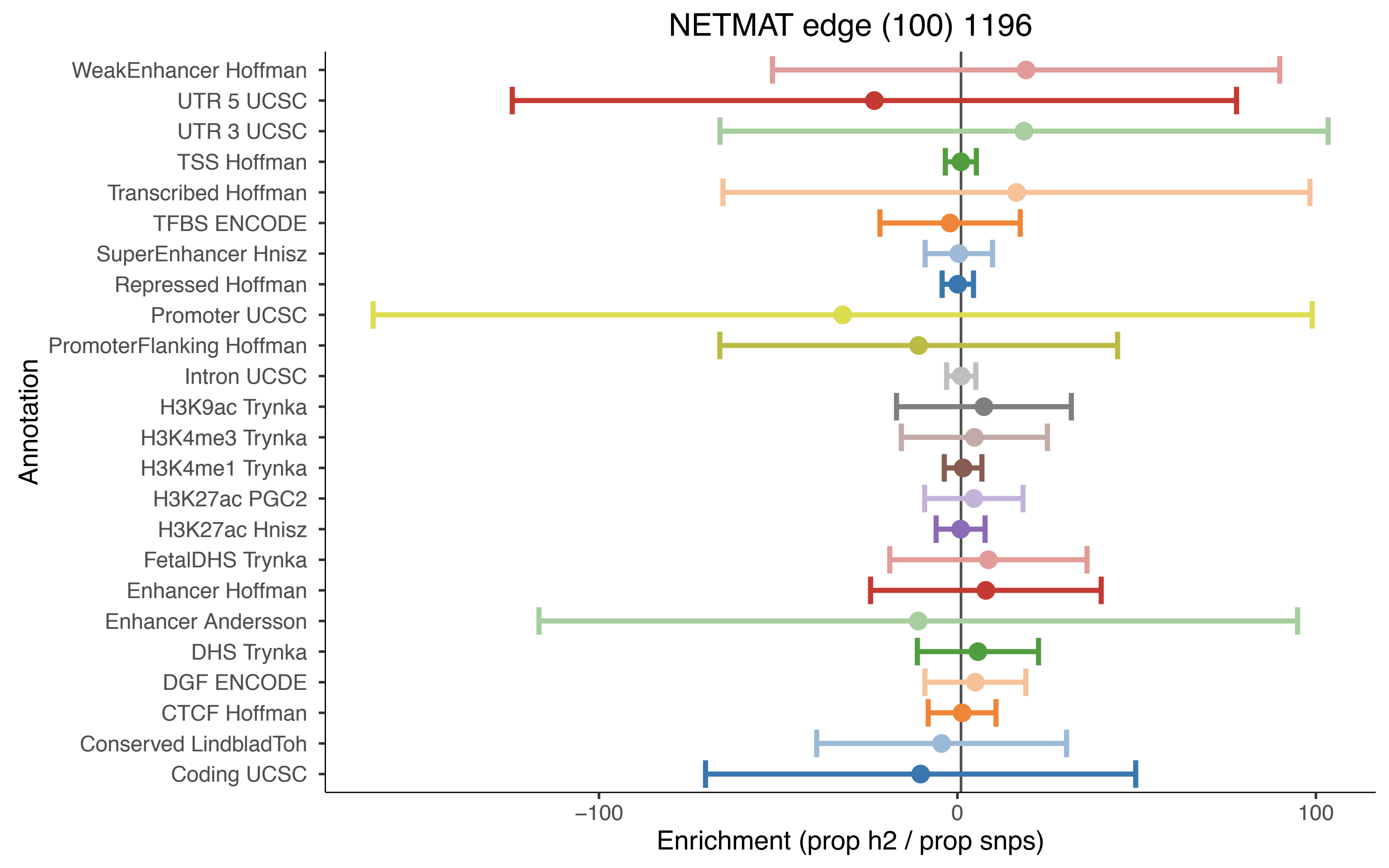
NETMAT edge (100) 1196
WeakEnhancer Hoffman
UTR 5 UCSC
UTR 3 UCSC
TSS Hoffman
Transcribed Hoffman
TFBS ENCODE
SuperEnhancer Hnisz
Repressed Hoffman
Promoter UCSC
PromoterFlanking Hoffman
Intron UCSC
H3K9ac Trynka
H3K4me3 Trynka
H3K4me1 Trynka
H3K27ac PGC2
H3K27ac Hnisz
FetalDHS Trynka
Enhancer Hoffman
Enhancer Andersson
DHS Trynka
DGF ENCODE
CTCF Hoffman
Conserved LindbladToh
Coding UCSC
Annotation
−100
0
100
Enrichment (prop h2 / prop snps)

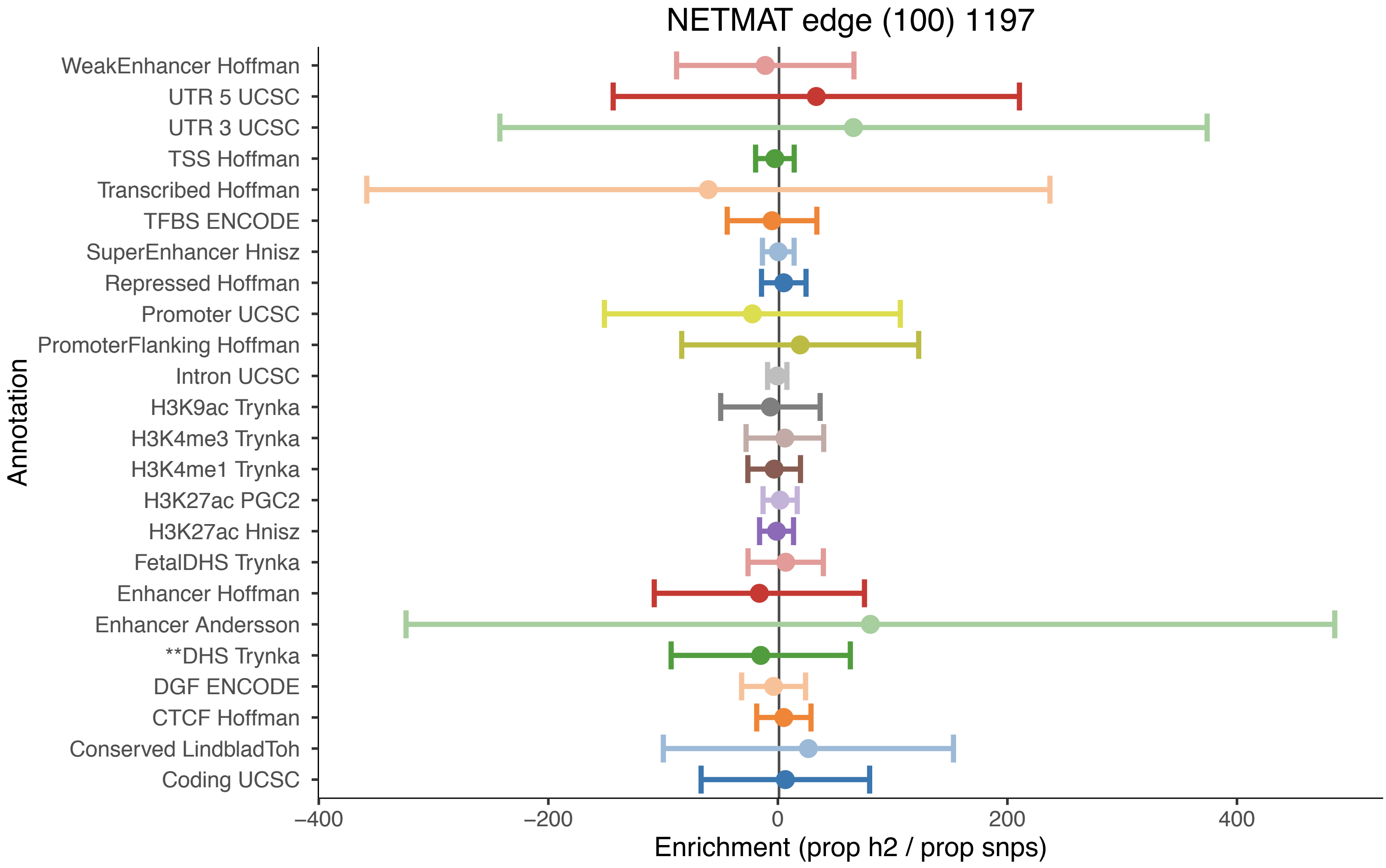

NETMAT edge (100) 1197
WeakEnhancer Hoffman
UTR 5 UCSC
UTR 3 UCSC
TSS Hoffman
Transcribed Hoffman
TFBS ENCODE
SuperEnhancer Hnisz
Repressed Hoffman
Promoter UCSC
PromoterFlanking Hoffman
Intron UCSC
H3K9ac Trynka
H3K4me3 Trynka
H3K4me1 Trynka
H3K27ac PGC2
H3K27ac Hnisz
FetalDHS Trynka
Enhancer Hoffman
Enhancer Andersson
**DHS Trynka
DGF ENCODE
CTCF Hoffman
Conserved LindbladToh
Coding UCSC
Annotation
−400
−200
0
200
400
Enrichment (prop h2 / prop snps)

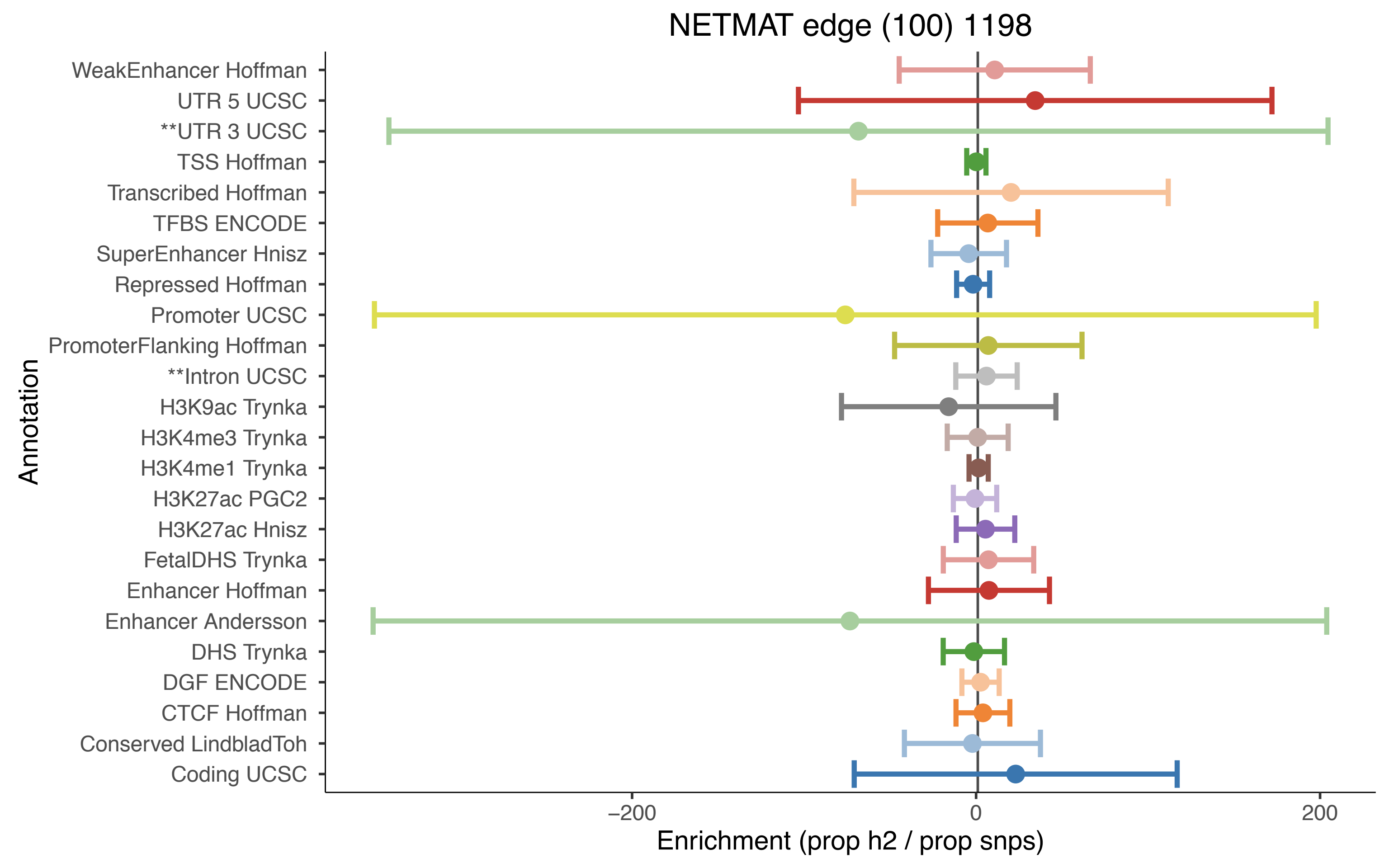
NETMAT edge (100) 1198
WeakEnhancer Hoffman
UTR 5 UCSC
**UTR 3 UCSC
TSS Hoffman
Transcribed Hoffman
TFBS ENCODE
SuperEnhancer Hnisz
Repressed Hoffman
Promoter UCSC
PromoterFlanking Hoffman
**Intron UCSC
H3K9ac Trynka
H3K4me3 Trynka
H3K4me1 Trynka
H3K27ac PGC2
H3K27ac Hnisz
FetalDHS Trynka
Enhancer Hoffman
Enhancer Andersson
DHS Trynka
DGF ENCODE
CTCF Hoffman
Conserved LindbladToh
Coding UCSC
Annotation
–200
0
200
Enrichment (prop h2 / prop snps)

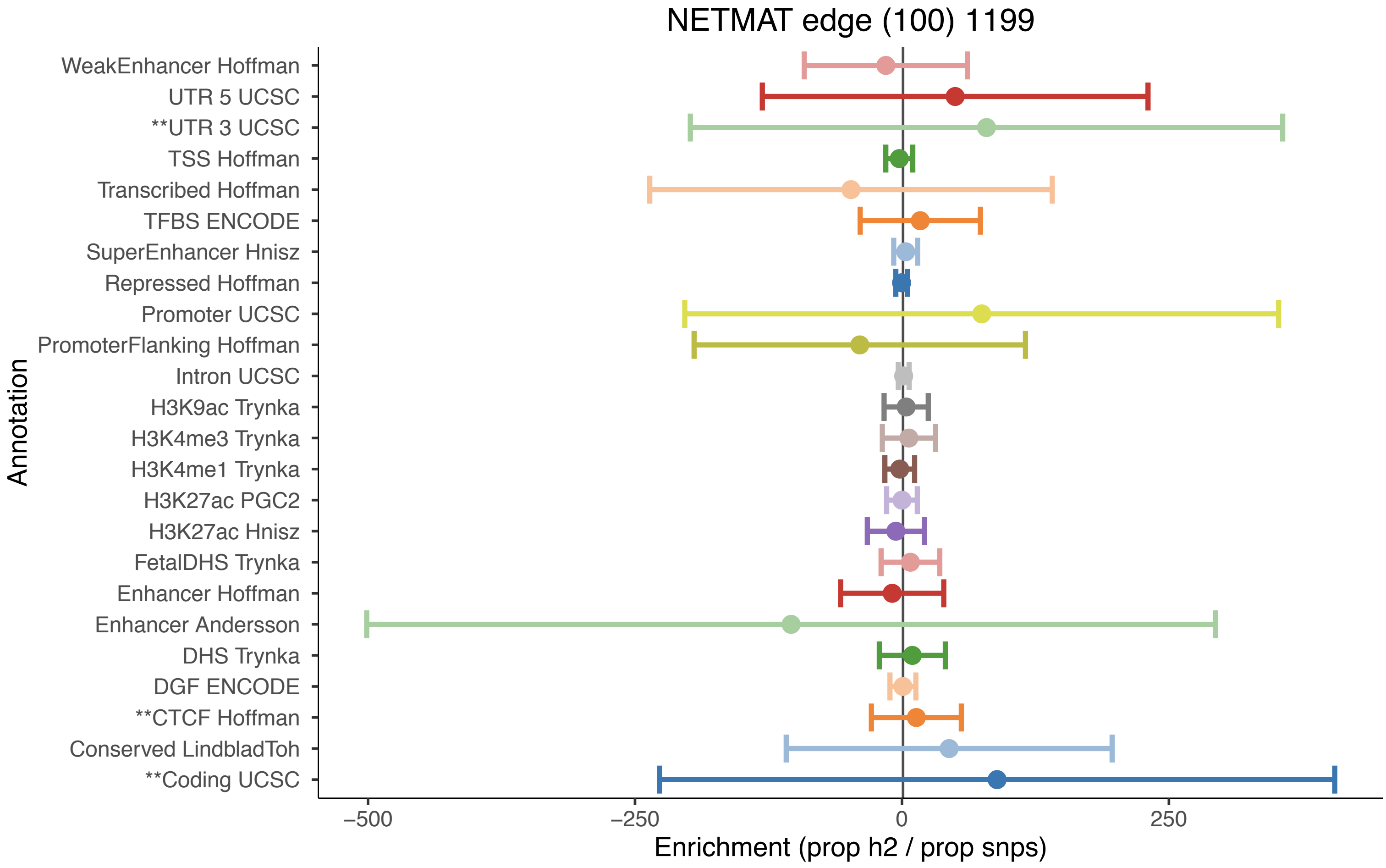
NETMAT edge (100) 1199
WeakEnhancer Hoffman
UTR 5 UCSC
**UTR 3 UCSC
TSS Hoffman
Transcribed Hoffman
TFBS ENCODE
SuperEnhancer Hnisz
Repressed Hoffman
Promoter UCSC
PromoterFlanking Hoffman
Intron UCSC
H3K9ac Trynka
H3K4me3 Trynka
H3K4me1 Trynka
H3K27ac PGC2
H3K27ac Hnisz
FetalDHS Trynka
Enhancer Hoffman
Enhancer Andersson
DHS Trynka
DGF ENCODE
**CTCF Hoffman
Conserved LindbladToh
**Coding UCSC
Annotation
−500
−250
0
250
Enrichment (prop h2 / prop snps)

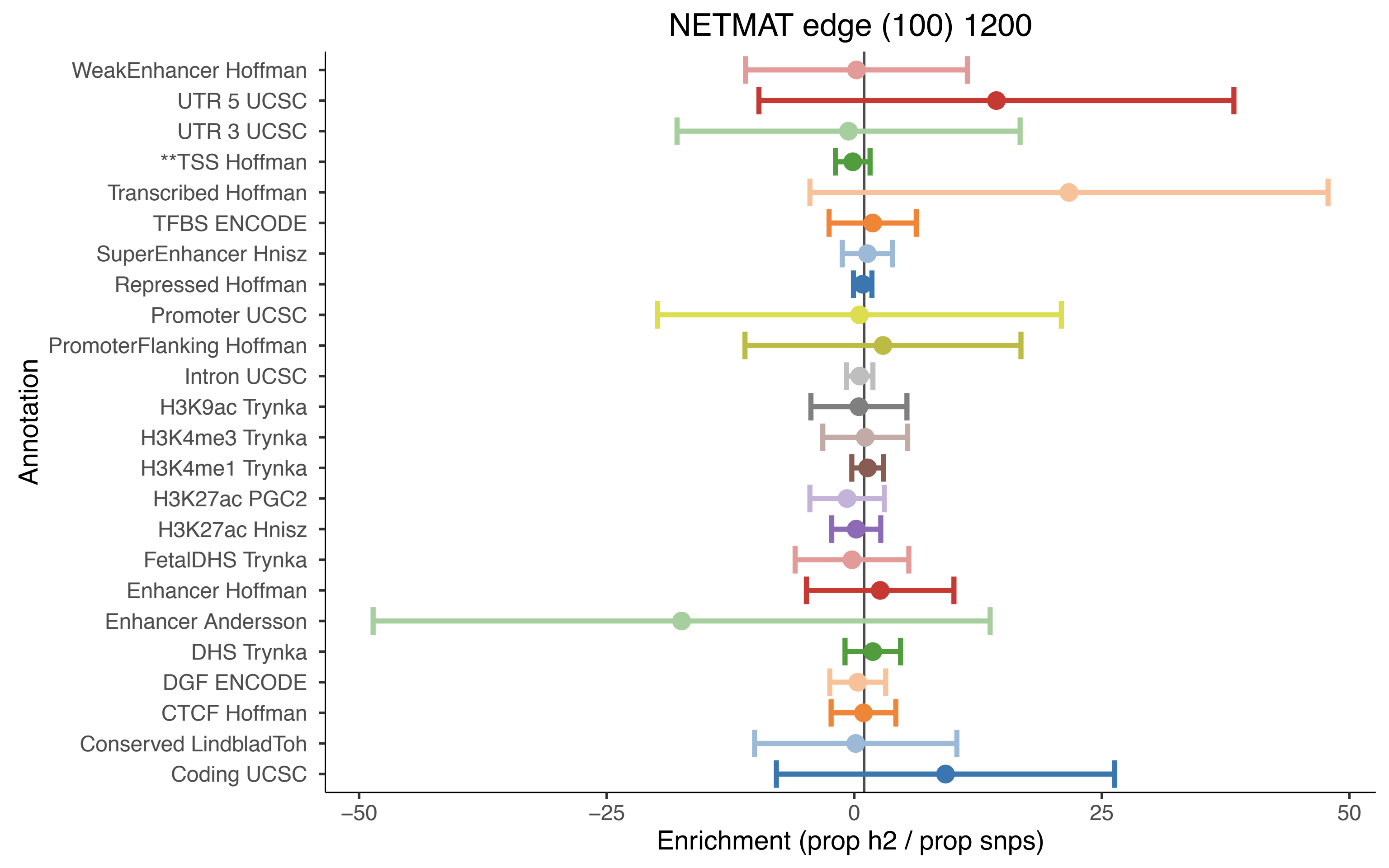
NETMAT edge (100) 1200
WeakEnhancer Hoffman
UTR 5 UCSC
UTR 3 UCSC
**TSS Hoffman
Transcribed Hoffman
TFBS ENCODE
SuperEnhancer Hnisz
Repressed Hoffman
Promoter UCSC
PromoterFlanking Hoffman
Intron UCSC
H3K9ac Trynka
H3K4me3 Trynka
H3K4me1 Trynka
H3K27ac PGC2
H3K27ac Hnisz
FetalDHS Trynka
Enhancer Hoffman
Enhancer Andersson
DHS Trynka
DGF ENCODE
CTCF Hoffman
Conserved LindbladToh
Coding UCSC
Annotation
−50
−25
0
25
50
Enrichment (prop h2 / prop snps)

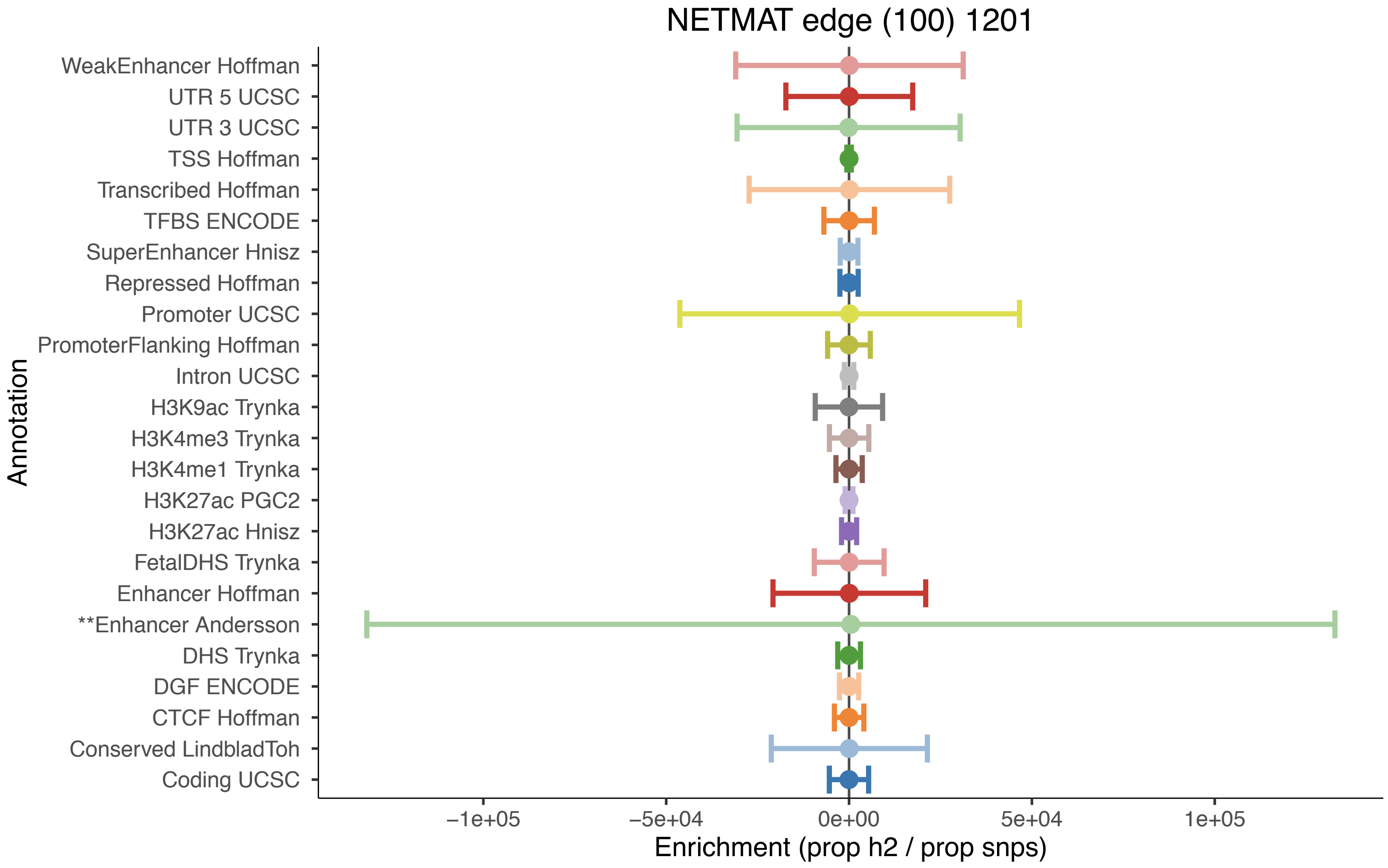

NETMAT edge (100) 1201
Annotation
WeakEnhancer Hoffman
UTR 5 UCSC
UTR 3 UCSC
TSS Hoffman
Transcribed Hoffman
TFBS ENCODE
SuperEnhancer Hnisz
Repressed Hoffman
Promoter UCSC
PromoterFlanking Hoffman
Intron UCSC
H3K9ac Trynka
H3K4me3 Trynka
H3K4me1 Trynka
H3K27ac PGC2
H3K27ac Hnisz
FetalDHS Trynka
Enhancer Hoffman
**Enhancer Andersson
DHS Trynka
DGF ENCODE
CTCF Hoffman
Conserved LindbladToh
Coding UCSC
−1e+05
−5e+04
0e+00
5e+04
1e+05
Enrichment (prop h2 / prop snps)

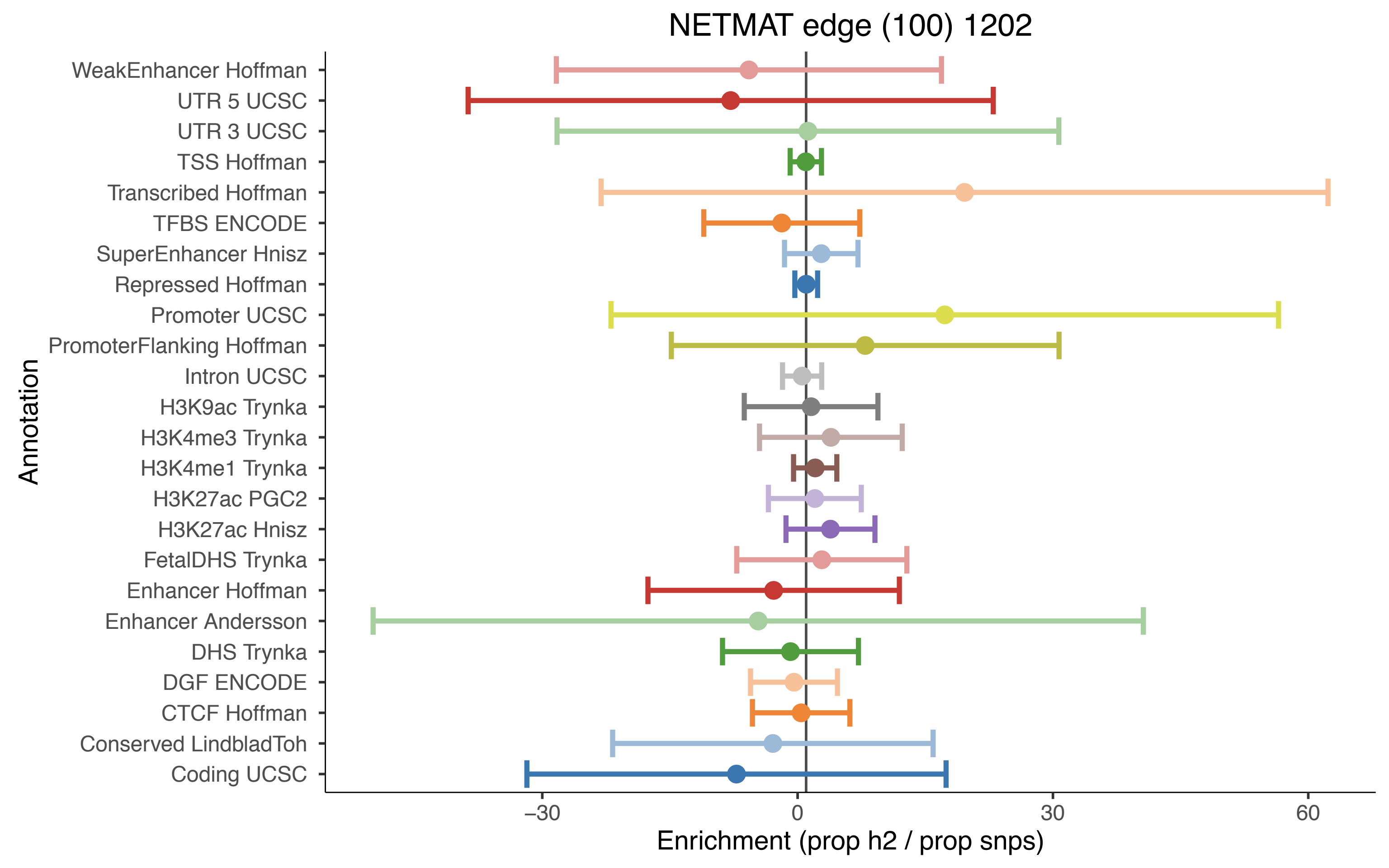
NETMAT edge (100) 1202
WeakEnhancer Hoffman
UTR 5 UCSC
UTR 3 UCSC
TSS Hoffman
Transcribed Hoffman
TFBS ENCODE
SuperEnhancer Hnisz
Repressed Hoffman
Promoter UCSC
PromoterFlanking Hoffman
Intron UCSC
H3K9ac Trynka
H3K4me3 Trynka
H3K4me1 Trynka
H3K27ac PGC2
H3K27ac Hnisz
FetalDHS Trynka
Enhancer Hoffman
Enhancer Andersson
DHS Trynka
DGF ENCODE
CTCF Hoffman
Conserved LindbladToh
Coding UCSC
Annotation
−30
0
30
60
Enrichment (prop h2 / prop snps)

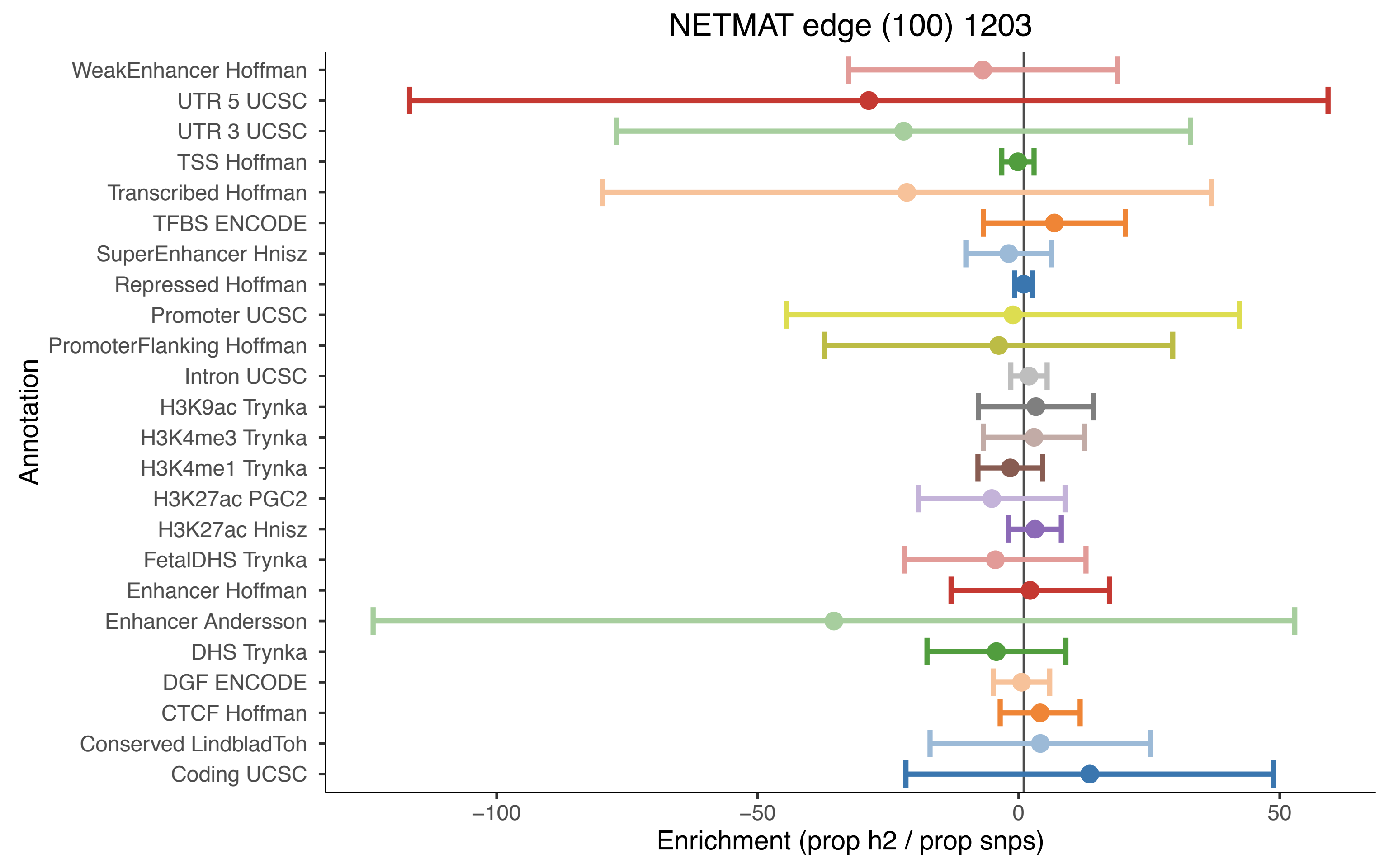

NETMAT edge (100) 1203
WeakEnhancer Hoffman
UTR 5 UCSC
UTR 3 UCSC
TSS Hoffman
Transcribed Hoffman
TFBS ENCODE
SuperEnhancer Hnisz
Repressed Hoffman
Promoter UCSC
PromoterFlanking Hoffman
Intron UCSC
H3K9ac Trynka
H3K4me3 Trynka
H3K4me1 Trynka
H3K27ac PGC2
H3K27ac Hnisz
FetalDHS Trynka
Enhancer Hoffman
Enhancer Andersson
DHS Trynka
DGF ENCODE
CTCF Hoffman
Conserved LindbladToh
Coding UCSC
Annotation
−100
−50
0
50
Enrichment (prop h2 / prop snps)

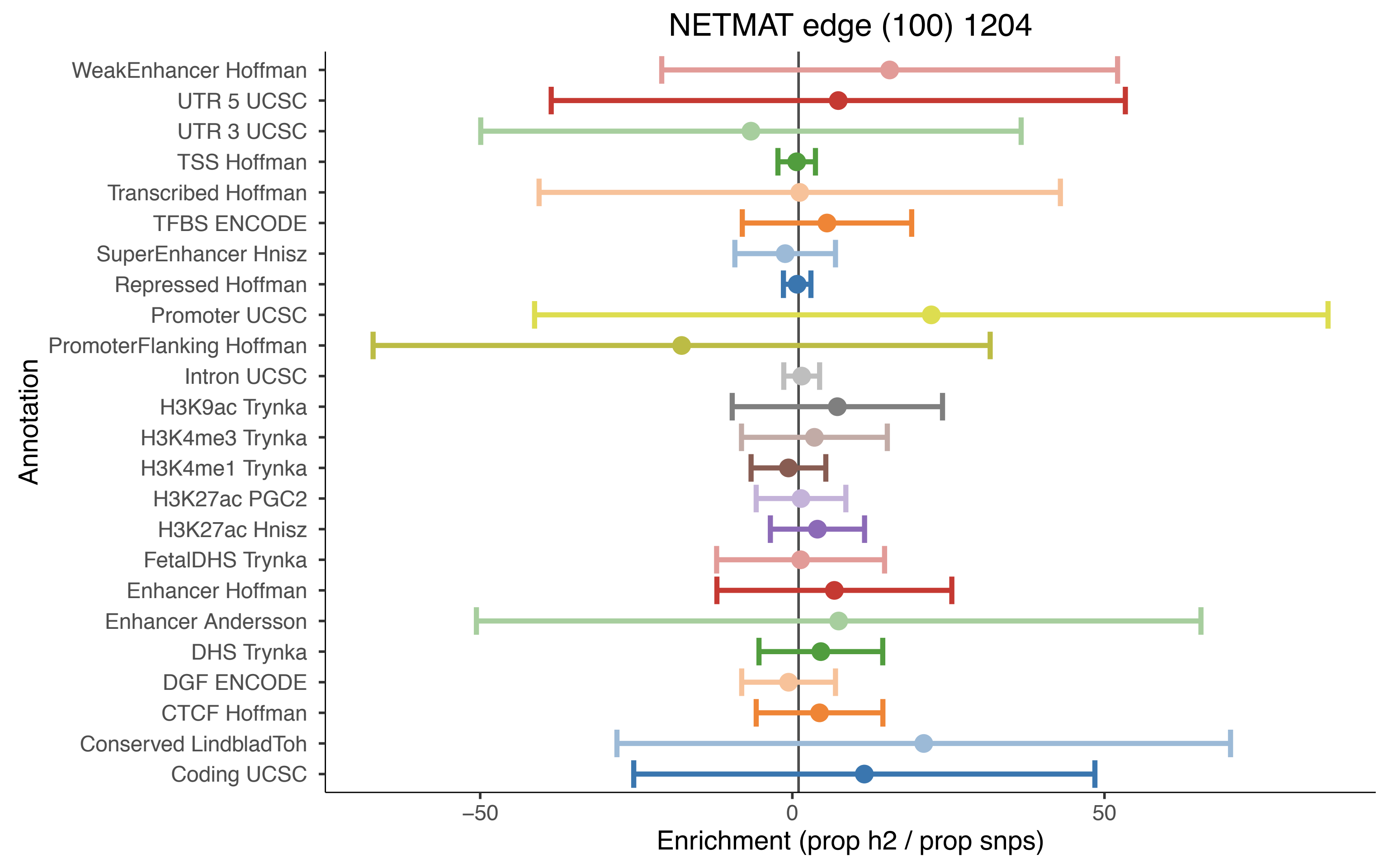

NETMAT edge (100) 1204
WeakEnhancer Hoffman
UTR 5 UCSC
UTR 3 UCSC
TSS Hoffman
Transcribed Hoffman
TFBS ENCODE
SuperEnhancer Hnisz
Repressed Hoffman
Promoter UCSC
PromoterFlanking Hoffman
Intron UCSC
H3K9ac Trynka
H3K4me3 Trynka
H3K4me1 Trynka
H3K27ac PGC2
H3K27ac Hnisz
FetalDHS Trynka
Enhancer Hoffman
Enhancer Andersson
DHS Trynka
DGF ENCODE
CTCF Hoffman
Conserved LindbladToh
Coding UCSC
Annotation
−50
0
50
Enrichment (prop h2 / prop snps)

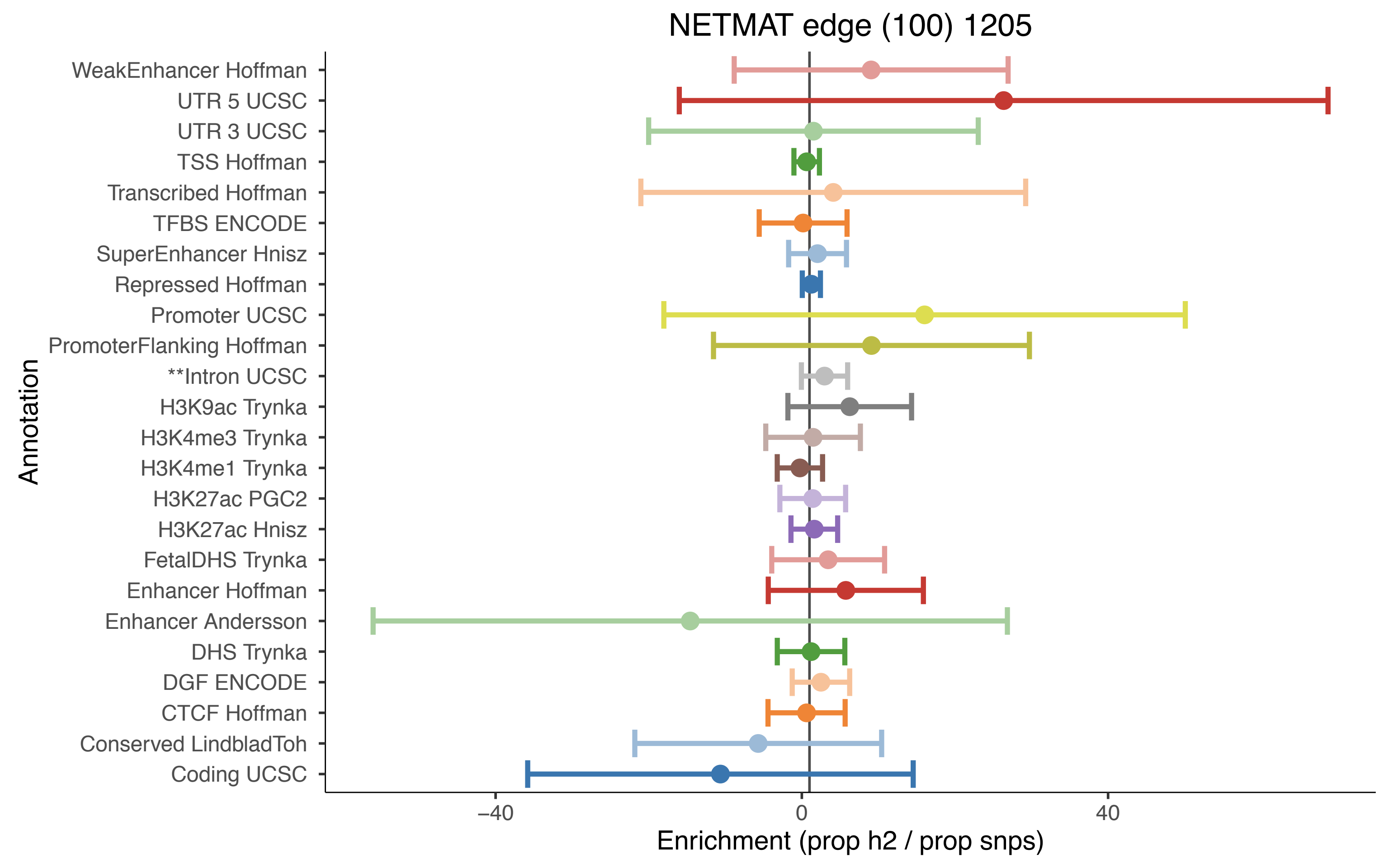
NETMAT edge (100) 1205
WeakEnhancer Hoffman
UTR 5 UCSC
UTR 3 UCSC
TSS Hoffman
Transcribed Hoffman
TFBS ENCODE
SuperEnhancer Hnisz
Repressed Hoffman
Promoter UCSC
PromoterFlanking Hoffman
**Intron UCSC
H3K9ac Trynka
H3K4me3 Trynka
H3K4me1 Trynka
H3K27ac PGC2
H3K27ac Hnisz
FetalDHS Trynka
Enhancer Hoffman
Enhancer Andersson
DHS Trynka
DGF ENCODE
CTCF Hoffman
Conserved LindbladToh
Coding UCSC
Annotation
−40
0
40
Enrichment (prop h2 / prop snps)

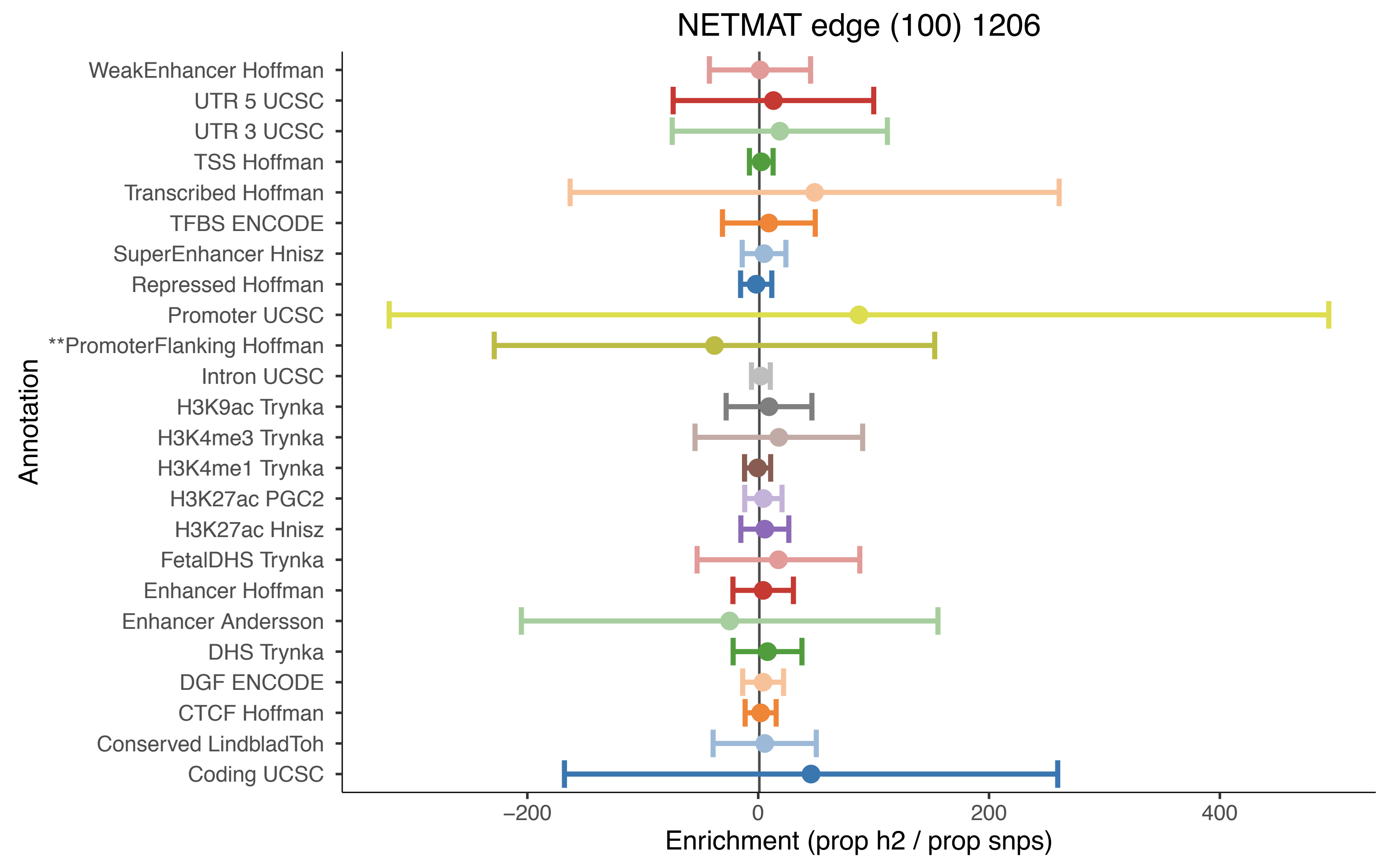
NETMAT edge (100) 1206
WeakEnhancer Hoffman
UTR 5 UCSC
UTR 3 UCSC
TSS Hoffman
Transcribed Hoffman
TFBS ENCODE
SuperEnhancer Hnisz
Repressed Hoffman
Promoter UCSC
**PromoterFlanking Hoffman
Intron UCSC
H3K9ac Trynka
H3K4me3 Trynka
H3K4me1 Trynka
H3K27ac PGC2
H3K27ac Hnisz
FetalDHS Trynka
Enhancer Hoffman
Enhancer Andersson
DHS Trynka
DGF ENCODE
CTCF Hoffman
Conserved LindbladToh
Coding UCSC
Annotation
−200
0
200
400
Enrichment (prop h2 / prop snps)

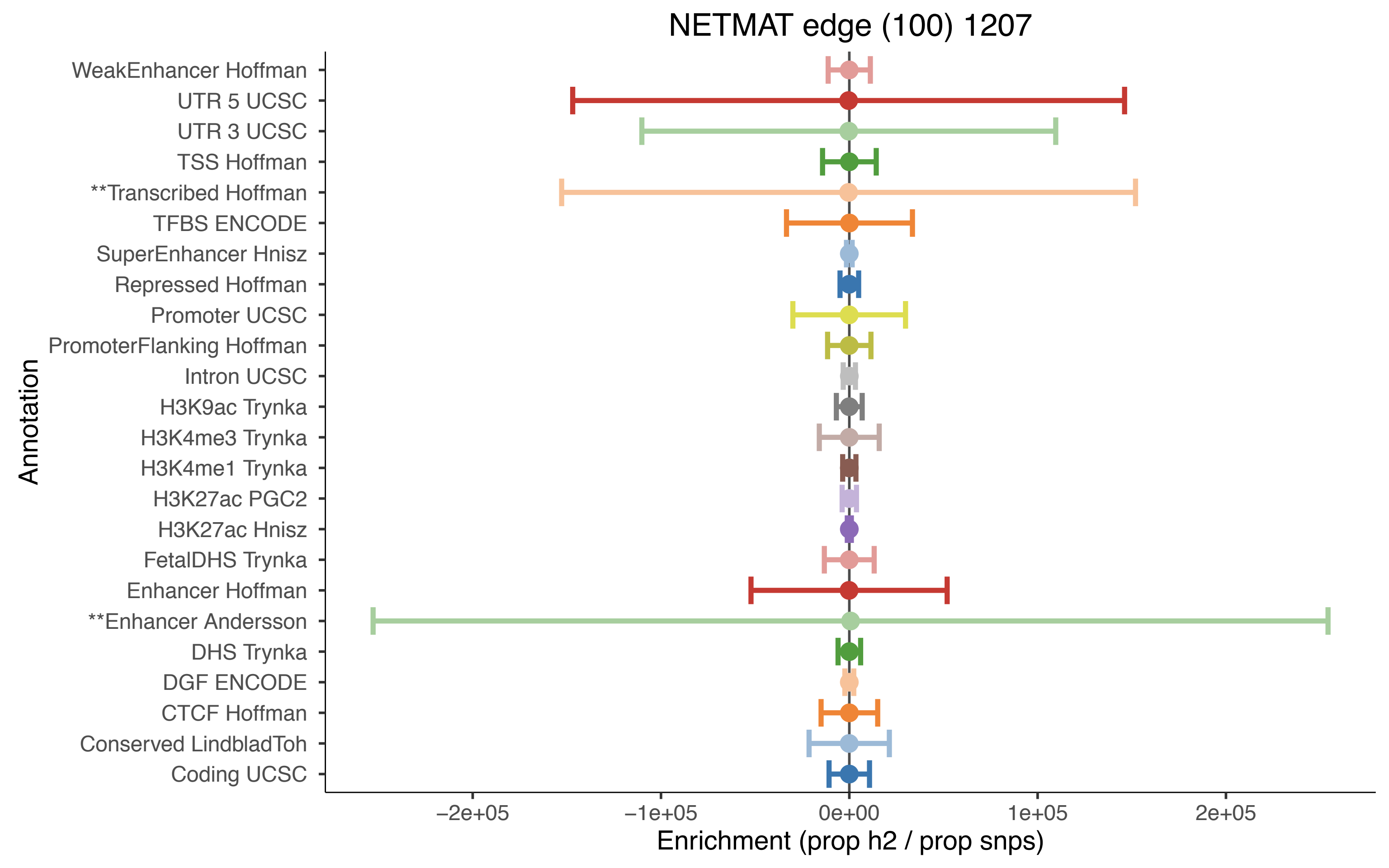

NETMAT edge (100) 1207
WeakEnhancer Hoffman
UTR 5 UCSC
UTR 3 UCSC
TSS Hoffman
**Transcribed Hoffman
TFBS ENCODE
SuperEnhancer Hnisz
Repressed Hoffman
Promoter UCSC
PromoterFlanking Hoffman
Intron UCSC
H3K9ac Trynka
H3K4me3 Trynka
H3K4me1 Trynka
H3K27ac PGC2
H3K27ac Hnisz
FetalDHS Trynka
Enhancer Hoffman
**Enhancer Andersson
DHS Trynka
DGF ENCODE
CTCF Hoffman
Conserved LindbladToh
Coding UCSC
Annotation
−2e+05
−1e+05
0e+00
1e+05
2e+05
Enrichment (prop h2 / prop snps)

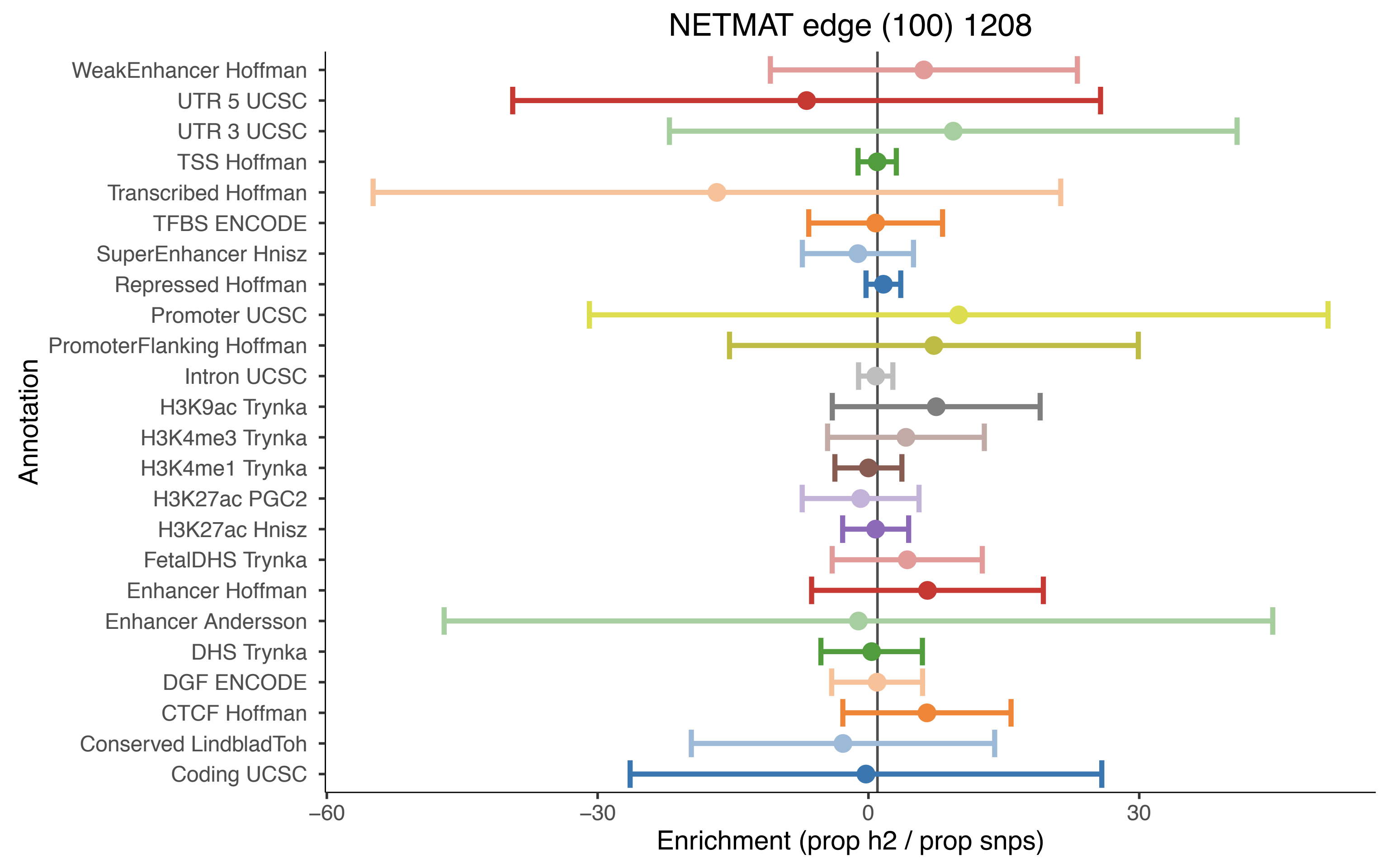
NETMAT edge (100) 1208
WeakEnhancer Hoffman
UTR 5 UCSC
UTR 3 UCSC
TSS Hoffman
Transcribed Hoffman
TFBS ENCODE
SuperEnhancer Hnisz
Repressed Hoffman
Promoter UCSC
PromoterFlanking Hoffman
Intron UCSC
H3K9ac Trynka
H3K4me3 Trynka
H3K4me1 Trynka
H3K27ac PGC2
H3K27ac Hnisz
FetalDHS Trynka
Enhancer Hoffman
Enhancer Andersson
DHS Trynka
DGF ENCODE
CTCF Hoffman
Conserved LindbladToh
Coding UCSC
Annotation
−60
−30
0
30
Enrichment (prop h2 / prop snps)

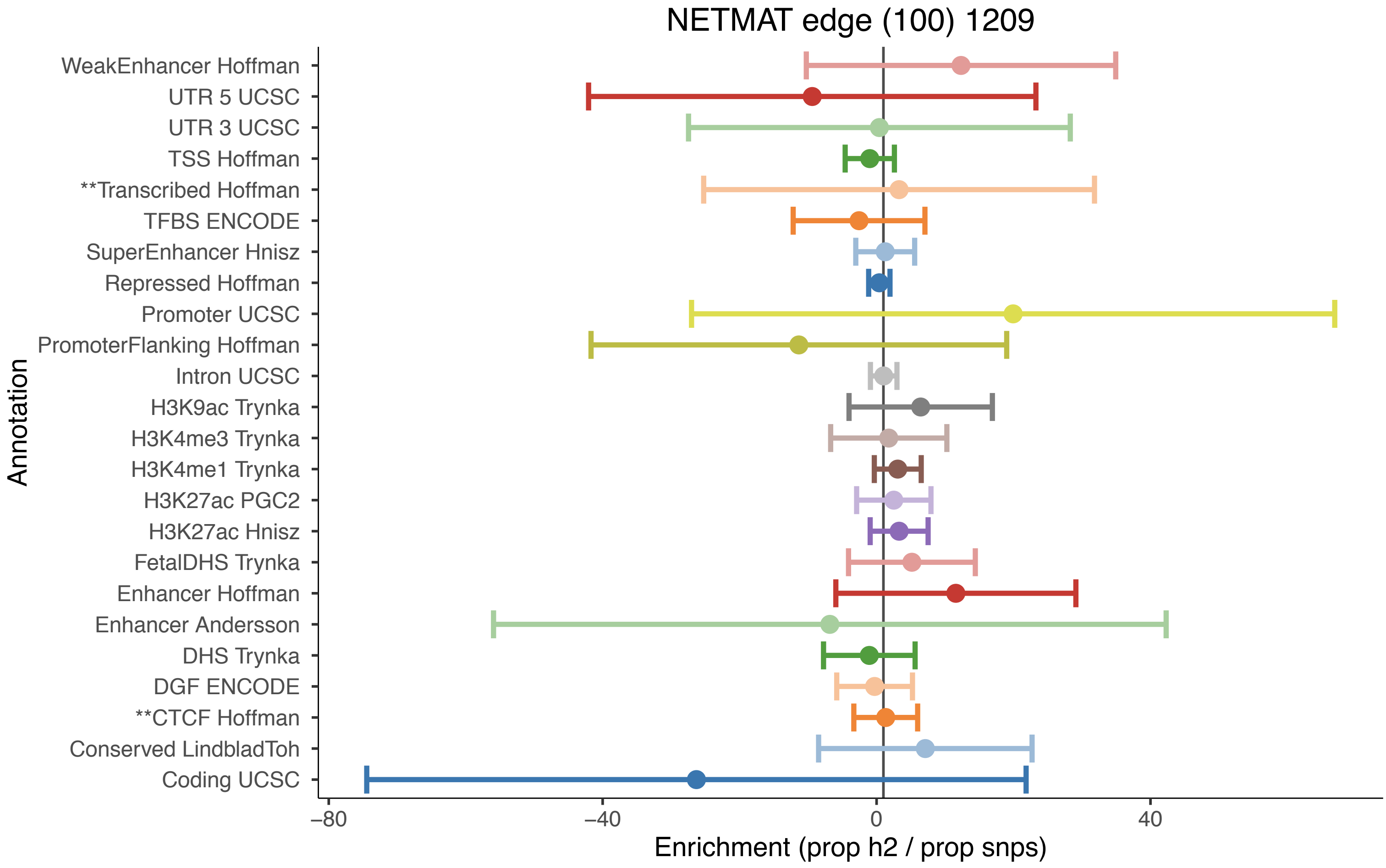
NETMAT edge (100) 1209
WeakEnhancer Hoffman
UTR 5 UCSC
UTR 3 UCSC
TSS Hoffman
**Transcribed Hoffman
TFBS ENCODE
SuperEnhancer Hnisz
Repressed Hoffman
Promoter UCSC
PromoterFlanking Hoffman
Intron UCSC
H3K9ac Trynka
H3K4me3 Trynka
H3K4me1 Trynka
H3K27ac PGC2
H3K27ac Hnisz
FetalDHS Trynka
Enhancer Hoffman
Enhancer Andersson
DHS Trynka
DGF ENCODE
**CTCF Hoffman
Conserved LindbladToh
Coding UCSC
Annotation
−80
−40
0
40
Enrichment (prop h2 / prop snps)

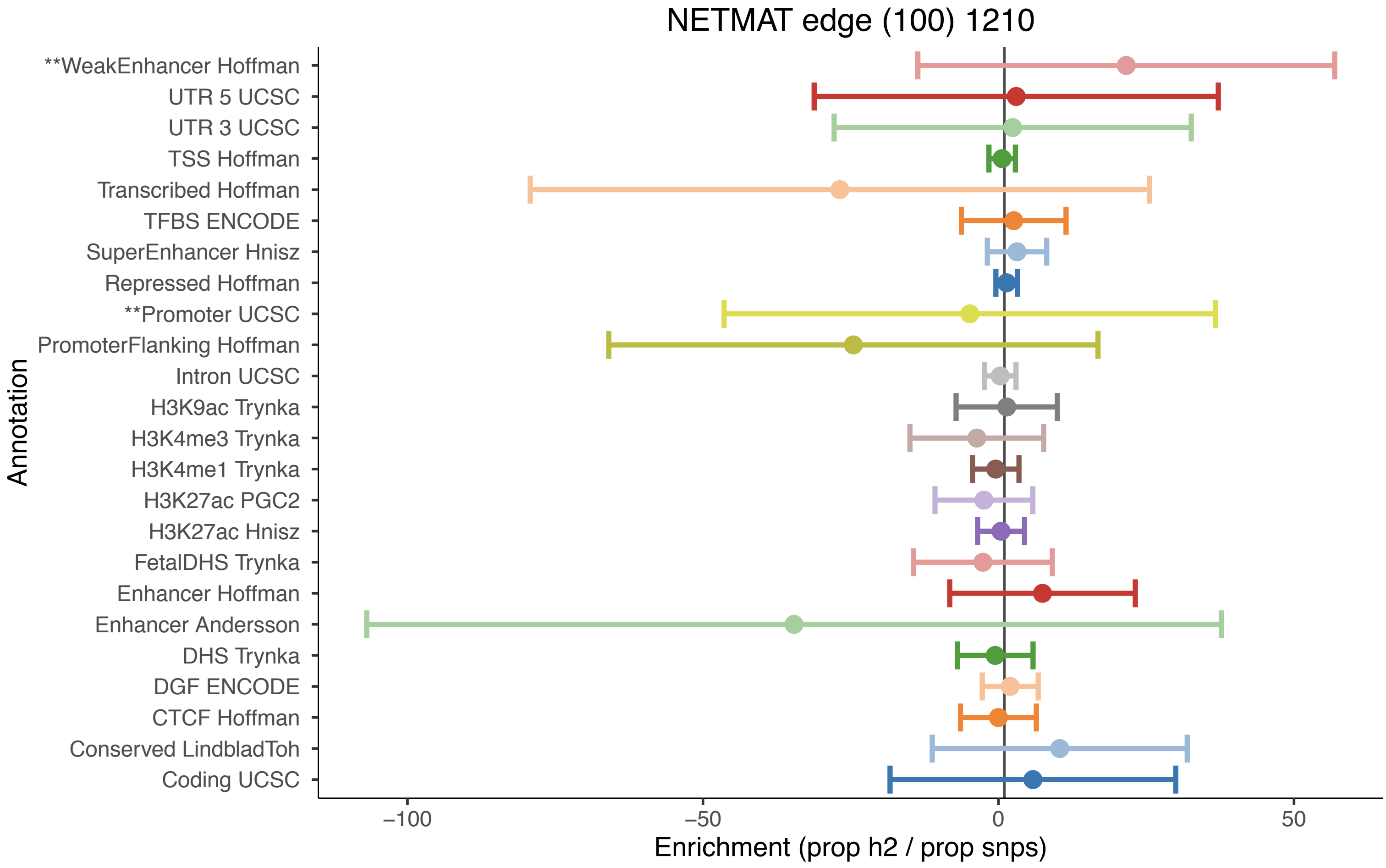

NETMAT edge (100) 1210
**WeakEnhancer Hoffman
UTR 5 UCSC
UTR 3 UCSC
TSS Hoffman
Transcribed Hoffman
TFBS ENCODE
SuperEnhancer Hnisz
Repressed Hoffman
**Promoter UCSC
PromoterFlanking Hoffman
Intron UCSC
H3K9ac Trynka
H3K4me3 Trynka
H3K4me1 Trynka
H3K27ac PGC2
H3K27ac Hnisz
FetalDHS Trynka
Enhancer Hoffman
Enhancer Andersson
DHS Trynka
DGF ENCODE
CTCF Hoffman
Conserved LindbladToh
Coding UCSC
Annotation
−100
−50
0
50
Enrichment (prop h2 / prop snps)

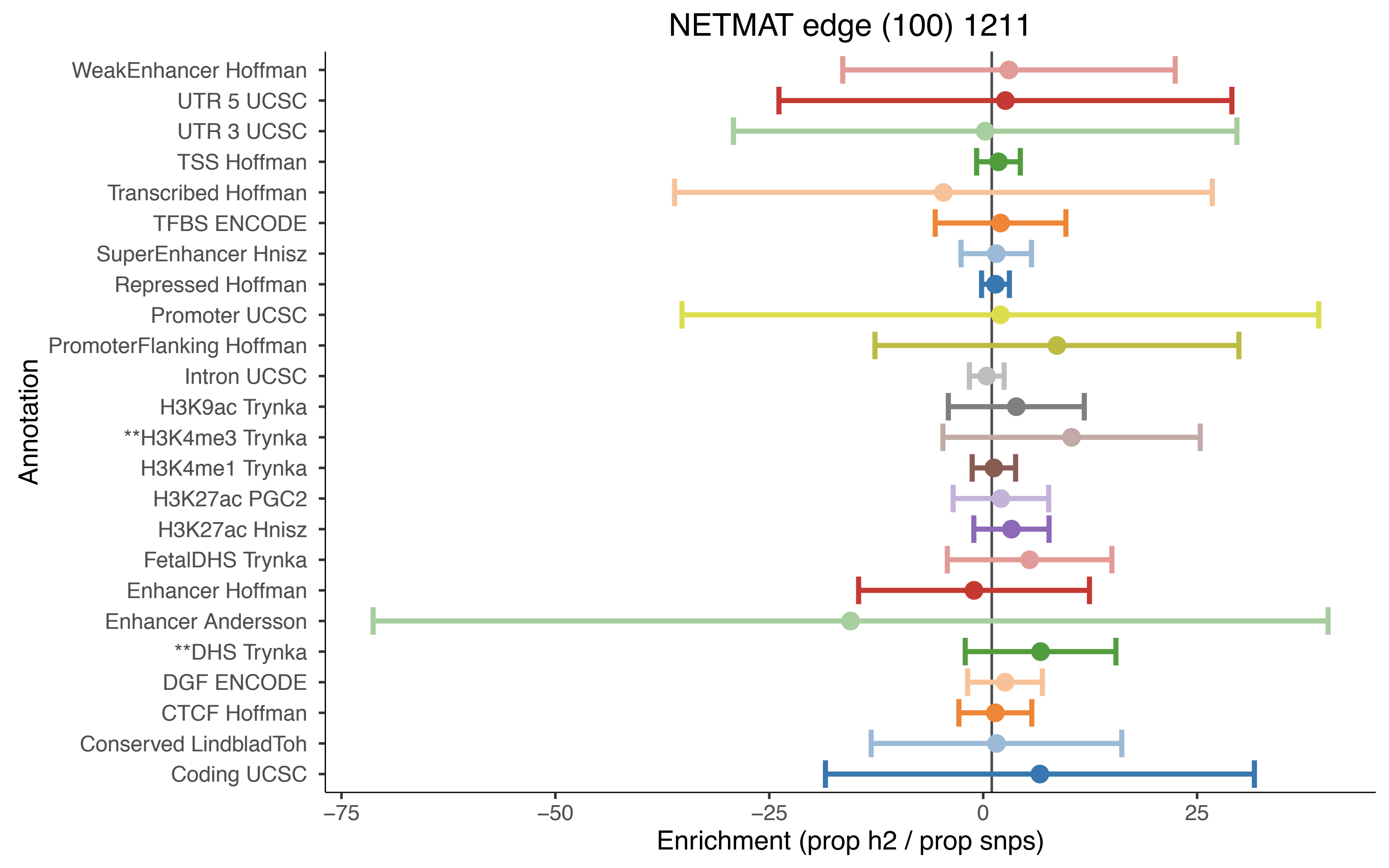

NETMAT edge (100) 1211
WeakEnhancer Hoffman
UTR 5 UCSC
UTR 3 UCSC
TSS Hoffman
Transcribed Hoffman
TFBS ENCODE
SuperEnhancer Hnisz
Repressed Hoffman
Promoter UCSC
PromoterFlanking Hoffman
Intron UCSC
H3K9ac Trynka
**H3K4me3 Trynka
H3K4me1 Trynka
H3K27ac PGC2
H3K27ac Hnisz
FetalDHS Trynka
Enhancer Hoffman
Enhancer Andersson
**DHS Trynka
DGF ENCODE
CTCF Hoffman
Conserved LindbladToh
Coding UCSC
Annotation
−75
−50
−25
0
25
Enrichment (prop h2 / prop snps)

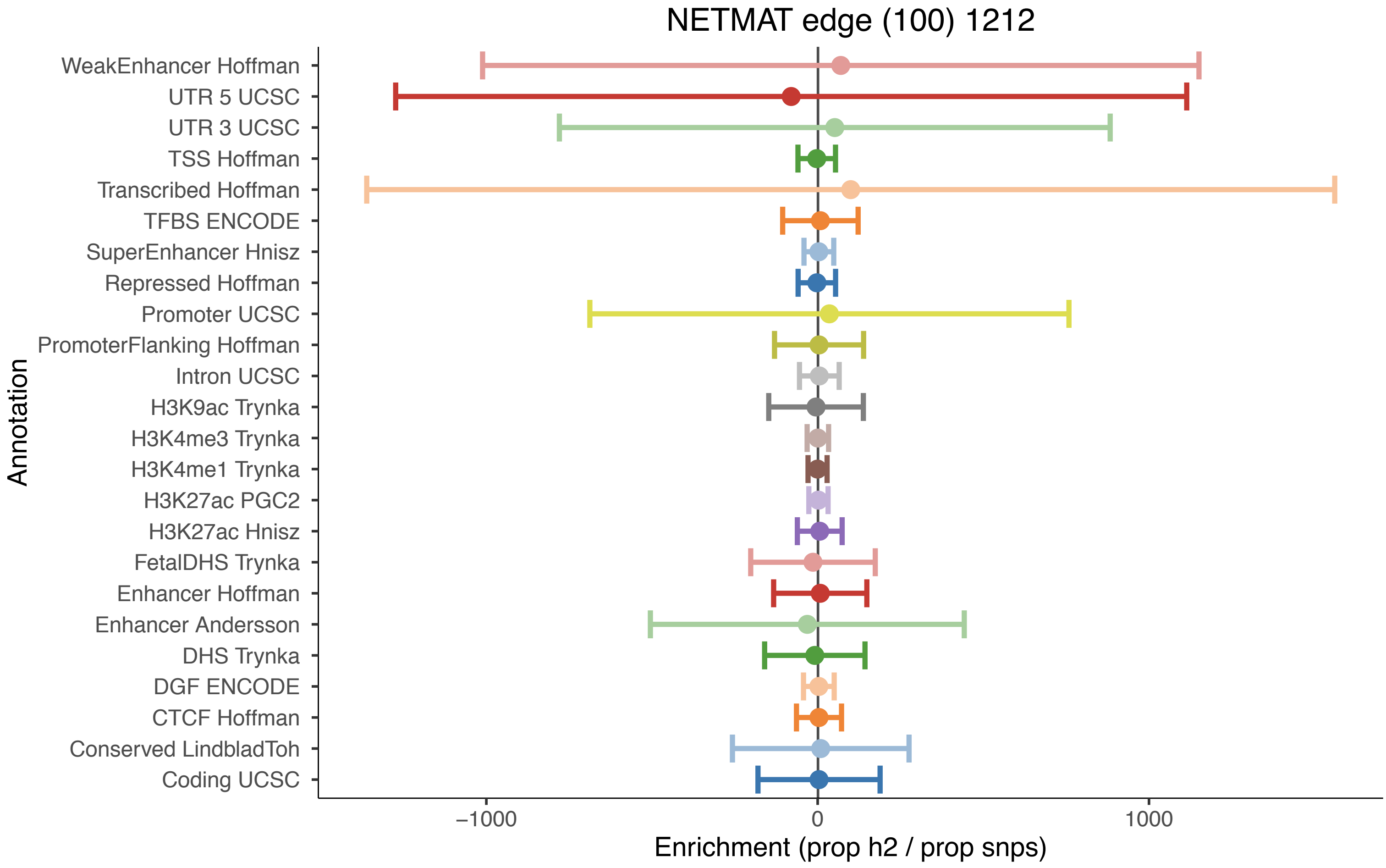

NETMAT edge (100) 1212
Annotation
WeakEnhancer Hoffman
UTR 5 UCSC
UTR 3 UCSC
TSS Hoffman
Transcribed Hoffman
TFBS ENCODE
SuperEnhancer Hnisz
Repressed Hoffman
Promoter UCSC
PromoterFlanking Hoffman
Intron UCSC
H3K9ac Trynka
H3K4me3 Trynka
H3K4me1 Trynka
H3K27ac PGC2
H3K27ac Hnisz
FetalDHS Trynka
Enhancer Hoffman
Enhancer Andersson
DHS Trynka
DGF ENCODE
CTCF Hoffman
Conserved LindbladToh
Coding UCSC
−1000
0
1000
Enrichment (prop h2 / prop snps)

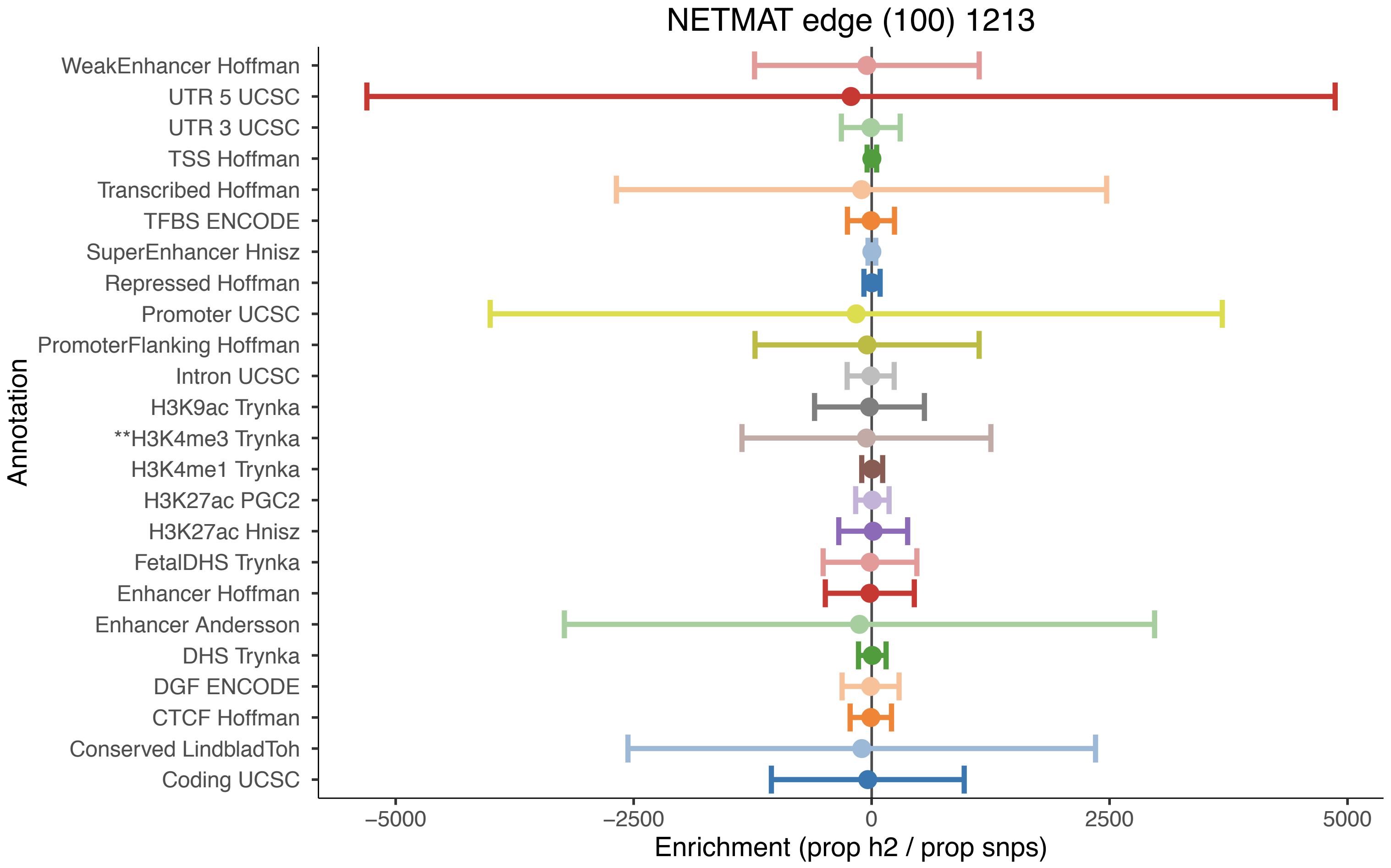

NETMAT edge (100) 1213
WeakEnhancer Hoffman
UTR 5 UCSC
UTR 3 UCSC
TSS Hoffman
Transcribed Hoffman
TFBS ENCODE
SuperEnhancer Hnisz
Repressed Hoffman
Promoter UCSC
PromoterFlanking Hoffman
Intron UCSC
H3K9ac Trynka
**H3K4me3 Trynka
H3K4me1 Trynka
H3K27ac PGC2
H3K27ac Hnisz
FetalDHS Trynka
Enhancer Hoffman
Enhancer Andersson
DHS Trynka
DGF ENCODE
CTCF Hoffman
Conserved LindbladToh
Coding UCSC
Annotation
−5000
−2500
0
2500
5000
Enrichment (prop h2 / prop snps)

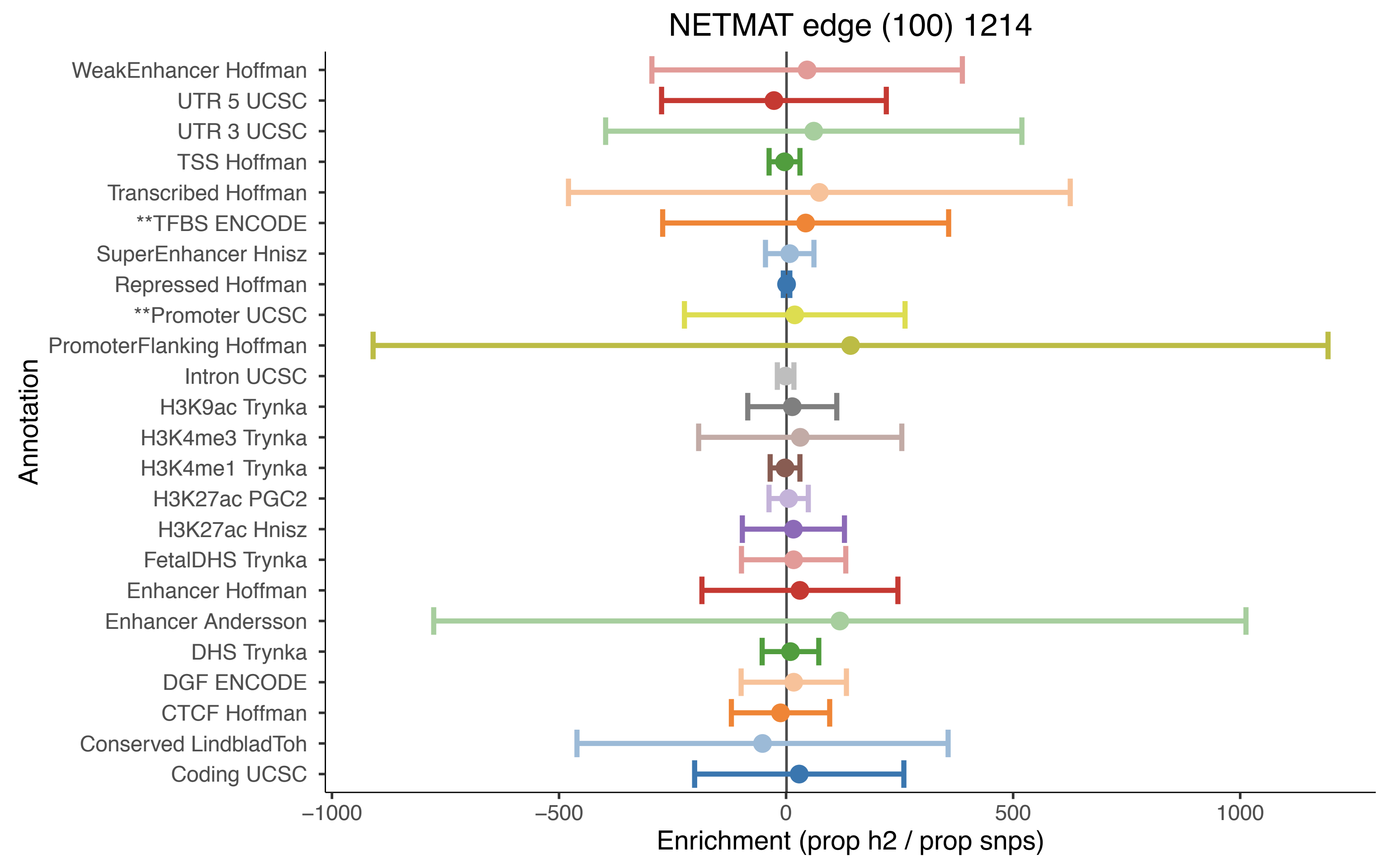

NETMAT edge (100) 1214
WeakEnhancer Hoffman
UTR 5 UCSC
UTR 3 UCSC
TSS Hoffman
Transcribed Hoffman
**TFBS ENCODE
SuperEnhancer Hnisz
Repressed Hoffman
**Promoter UCSC
PromoterFlanking Hoffman
Intron UCSC
H3K9ac Trynka
H3K4me3 Trynka
H3K4me1 Trynka
H3K27ac PGC2
H3K27ac Hnisz
FetalDHS Trynka
Enhancer Hoffman
Enhancer Andersson
DHS Trynka
DGF ENCODE
CTCF Hoffman
Conserved LindbladToh
Coding UCSC
Annotation
−1000
−500
0
500
1000
Enrichment (prop h2 / prop snps)

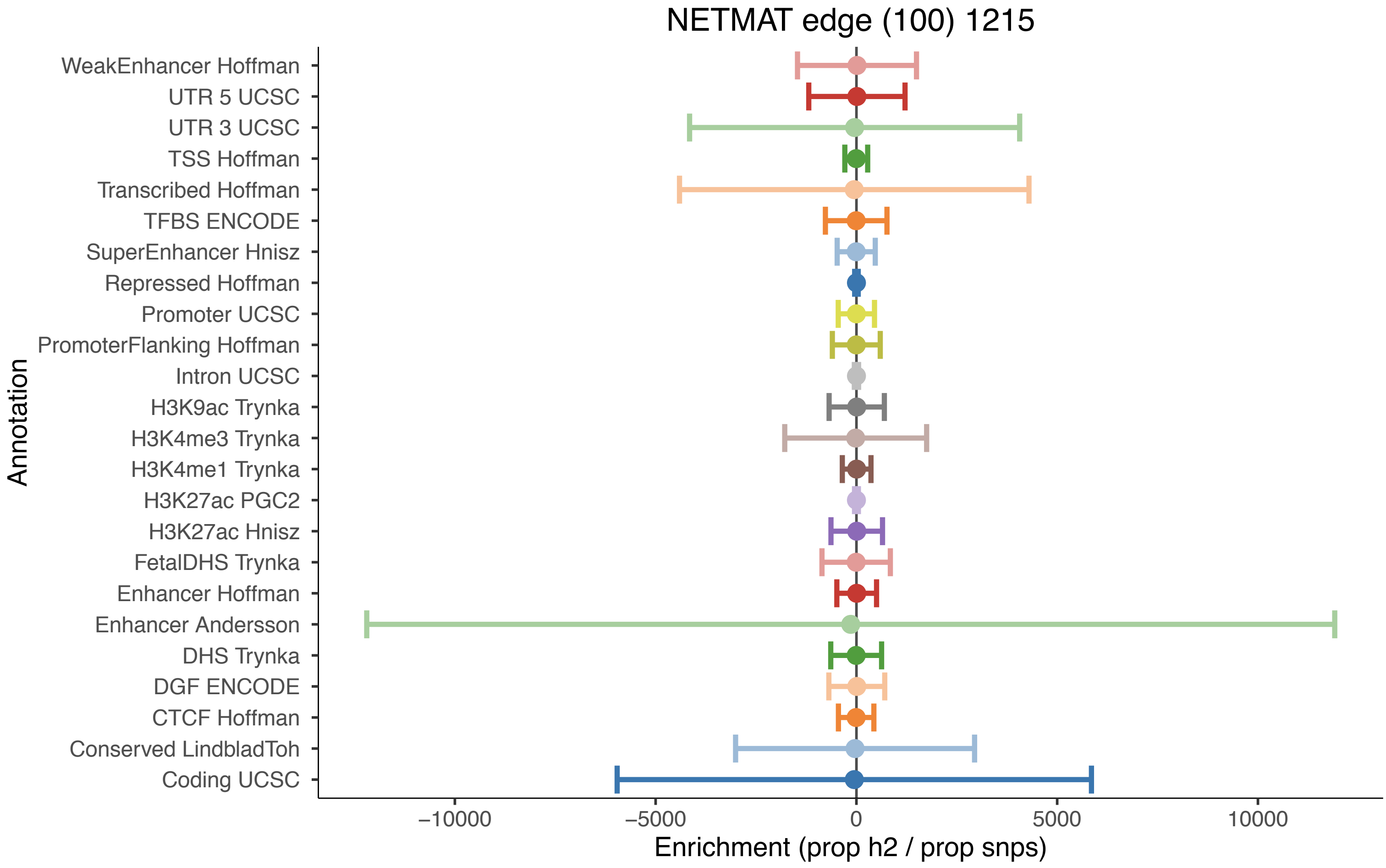

NETMAT edge (100) 1215
WeakEnhancer Hoffman
UTR 5 UCSC
UTR 3 UCSC
TSS Hoffman
Transcribed Hoffman
TFBS ENCODE
SuperEnhancer Hnisz
Repressed Hoffman
Promoter UCSC
PromoterFlanking Hoffman
Intron UCSC
H3K9ac Trynka
H3K4me3 Trynka
H3K4me1 Trynka
H3K27ac PGC2
H3K27ac Hnisz
FetalDHS Trynka
Enhancer Hoffman
Enhancer Andersson
DHS Trynka
DGF ENCODE
CTCF Hoffman
Conserved LindbladToh
Coding UCSC
Annotation
−10000
−5000
0
5000
10000
Enrichment (prop h2 / prop snps)

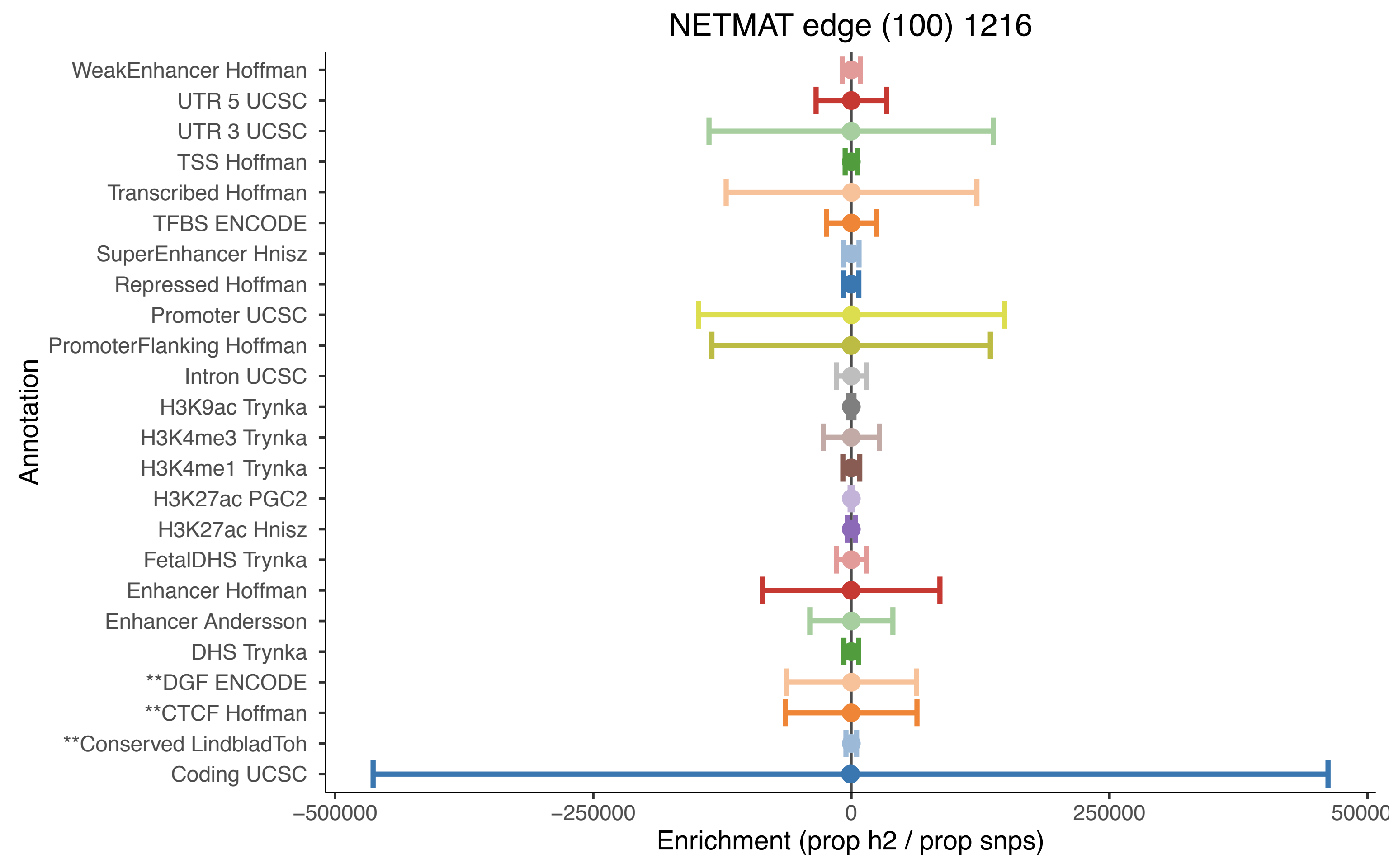

NETMAT edge (100) 1216
WeakEnhancer Hoffman
UTR 5 UCSC
UTR 3 UCSC
TSS Hoffman
Transcribed Hoffman
TFBS ENCODE
SuperEnhancer Hnisz
Repressed Hoffman
Promoter UCSC
PromoterFlanking Hoffman
Intron UCSC
H3K9ac Trynka
H3K4me3 Trynka
H3K4me1 Trynka
H3K27ac PGC2
H3K27ac Hnisz
FetalDHS Trynka
Enhancer Hoffman
Enhancer Andersson
DHS Trynka
**DGF ENCODE
**CTCF Hoffman
**Conserved LindbladToh
Coding UCSC
Annotation
−500000
−250000
0
250000
Enrichment (prop h2 / prop snps)

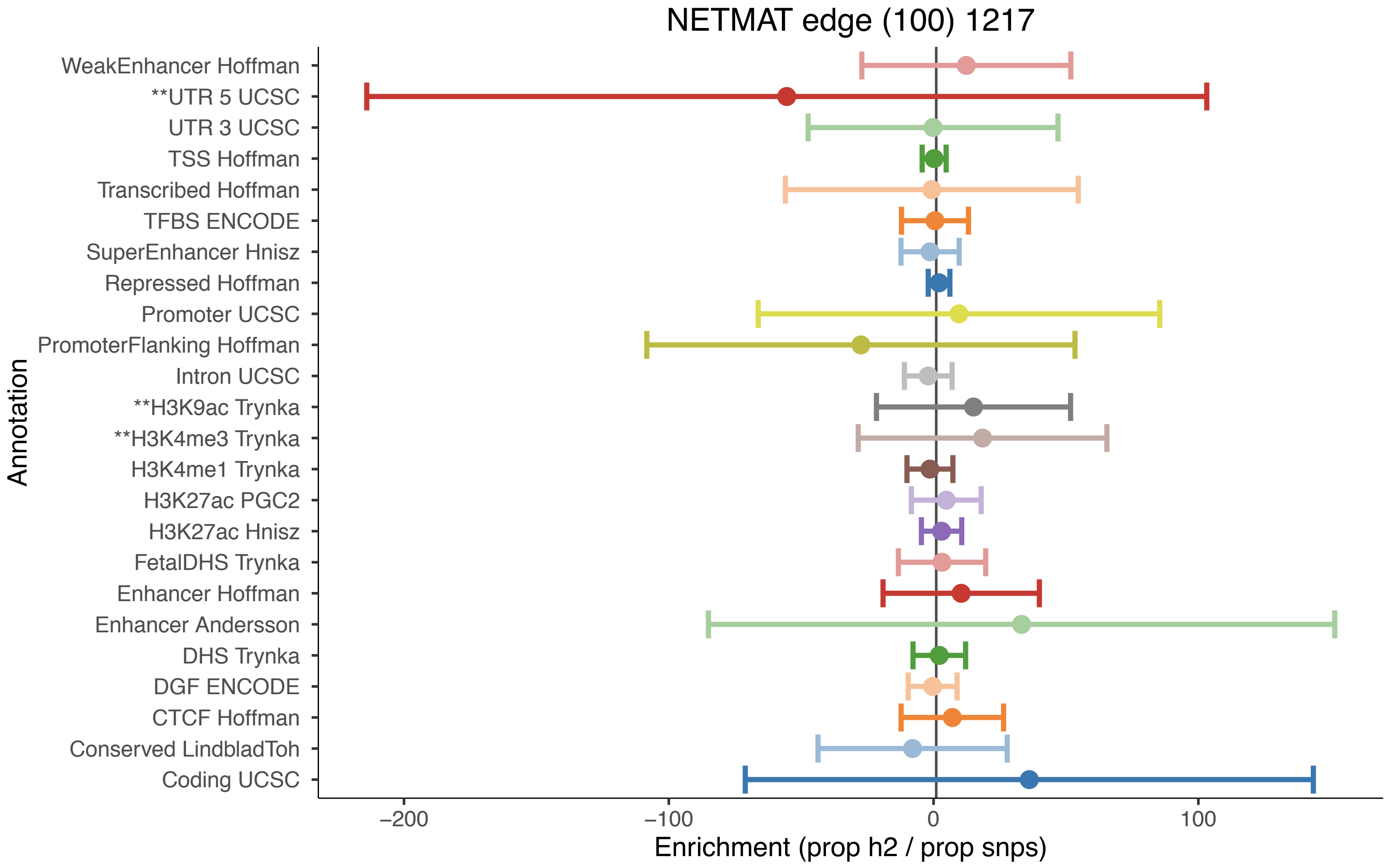

NETMAT edge (100) 1217
WeakEnhancer Hoffman
**UTR 5 UCSC
UTR 3 UCSC
TSS Hoffman
Transcribed Hoffman
TFBS ENCODE
SuperEnhancer Hnisz
Repressed Hoffman
Promoter UCSC
PromoterFlanking Hoffman
Intron UCSC
**H3K9ac Trynka
**H3K4me3 Trynka
H3K4me1 Trynka
H3K27ac PGC2
H3K27ac Hnisz
FetalDHS Trynka
Enhancer Hoffman
Enhancer Andersson
DHS Trynka
DGF ENCODE
CTCF Hoffman
Conserved LindbladToh
Coding UCSC
Annotation
−200
−100
0
100
Enrichment (prop h2 / prop snps)

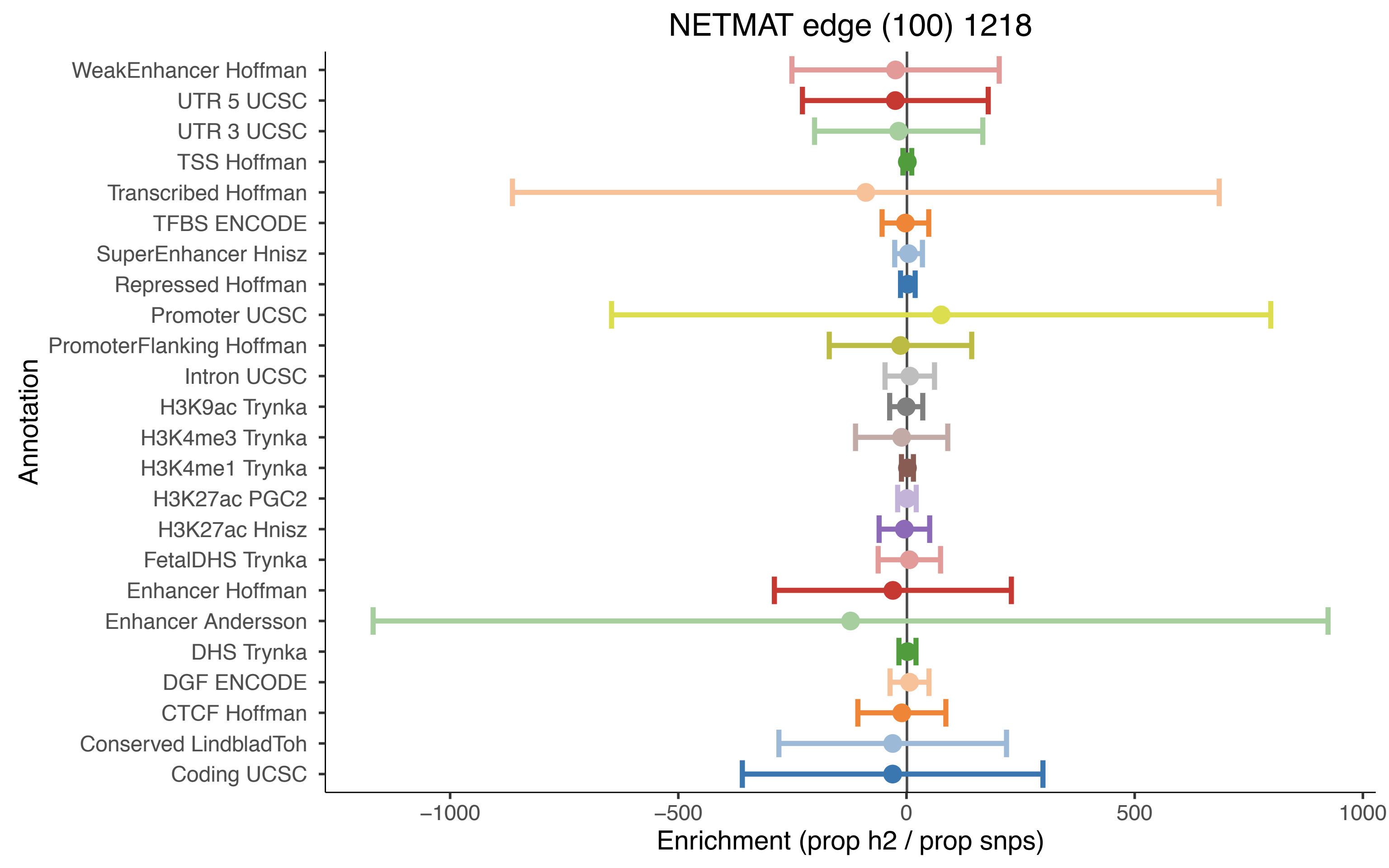
NETMAT edge (100) 1218
Annotation
WeakEnhancer Hoffman
UTR 5 UCSC
UTR 3 UCSC
TSS Hoffman
Transcribed Hoffman
TFBS ENCODE
SuperEnhancer Hnisz
Repressed Hoffman
Promoter UCSC
PromoterFlanking Hoffman
Intron UCSC
H3K9ac Trynka
H3K4me3 Trynka
H3K4me1 Trynka
H3K27ac PGC2
H3K27ac Hnisz
FetalDHS Trynka
Enhancer Hoffman
Enhancer Andersson
DHS Trynka
DGF ENCODE
CTCF Hoffman
Conserved LindbladToh
Coding UCSC
−1000
−500
0
500
1000
Enrichment (prop h2 / prop snps)

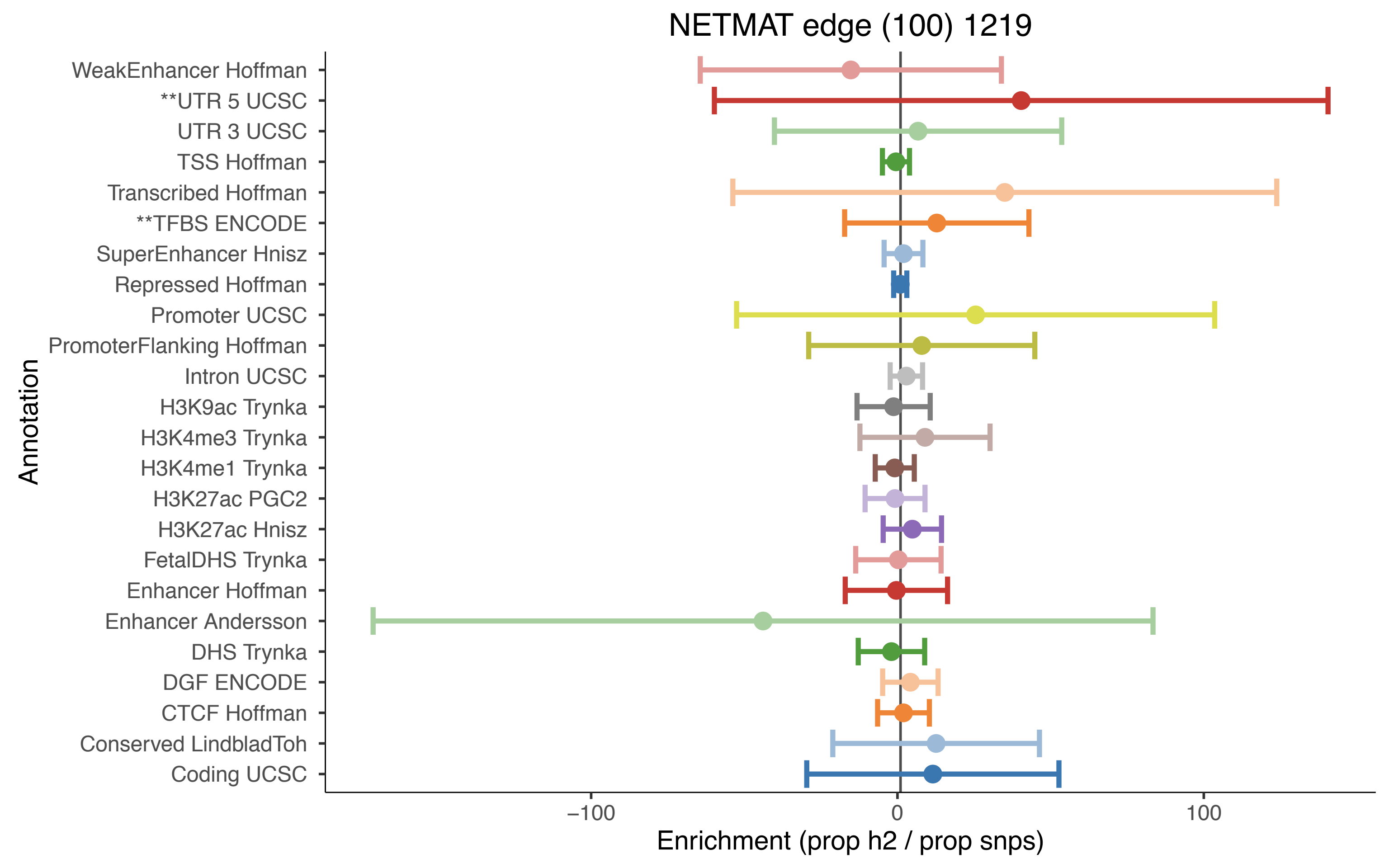

NETMAT edge (100) 1219
WeakEnhancer Hoffman
**UTR 5 UCSC
UTR 3 UCSC
TSS Hoffman
Transcribed Hoffman
**TFBS ENCODE
SuperEnhancer Hnisz
Repressed Hoffman
Promoter UCSC
PromoterFlanking Hoffman
Intron UCSC
H3K9ac Trynka
H3K4me3 Trynka
H3K4me1 Trynka
H3K27ac PGC2
H3K27ac Hnisz
FetalDHS Trynka
Enhancer Hoffman
Enhancer Andersson
DHS Trynka
DGF ENCODE
CTCF Hoffman
Conserved LindbladToh
Coding UCSC
Annotation
−100
0
100
Enrichment (prop h2 / prop snps)

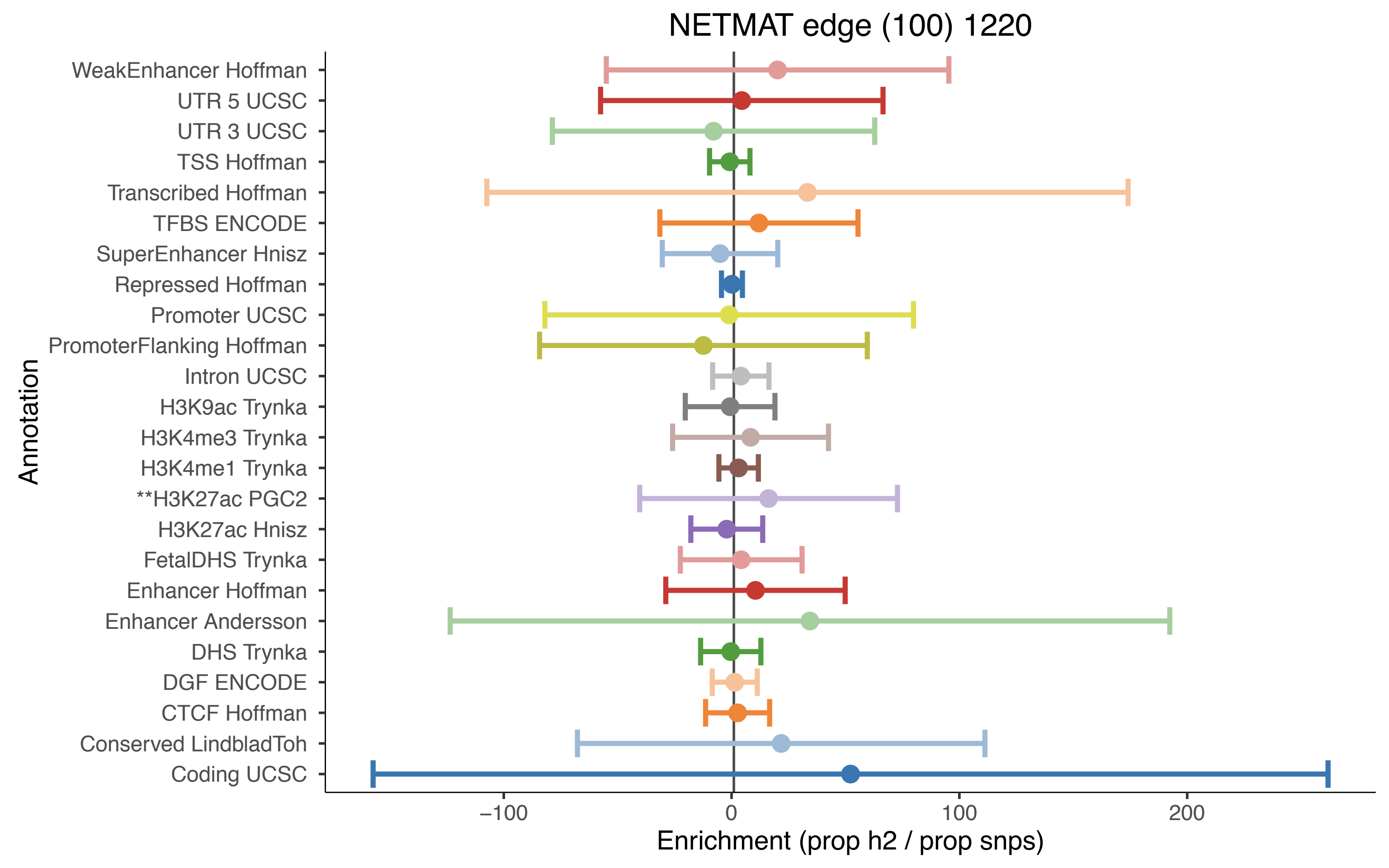
NETMAT edge (100) 1220
WeakEnhancer Hoffman
UTR 5 UCSC
UTR 3 UCSC
TSS Hoffman
Transcribed Hoffman
TFBS ENCODE
SuperEnhancer Hnisz
Repressed Hoffman
Promoter UCSC
PromoterFlanking Hoffman
Intron UCSC
H3K9ac Trynka
H3K4me3 Trynka
H3K4me1 Trynka
**H3K27ac PGC2
H3K27ac Hnisz
FetalDHS Trynka
Enhancer Hoffman
Enhancer Andersson
DHS Trynka
DGF ENCODE
CTCF Hoffman
Conserved LindbladToh
Coding UCSC
Annotation
−100
0
100
200
Enrichment (prop h2 / prop snps)

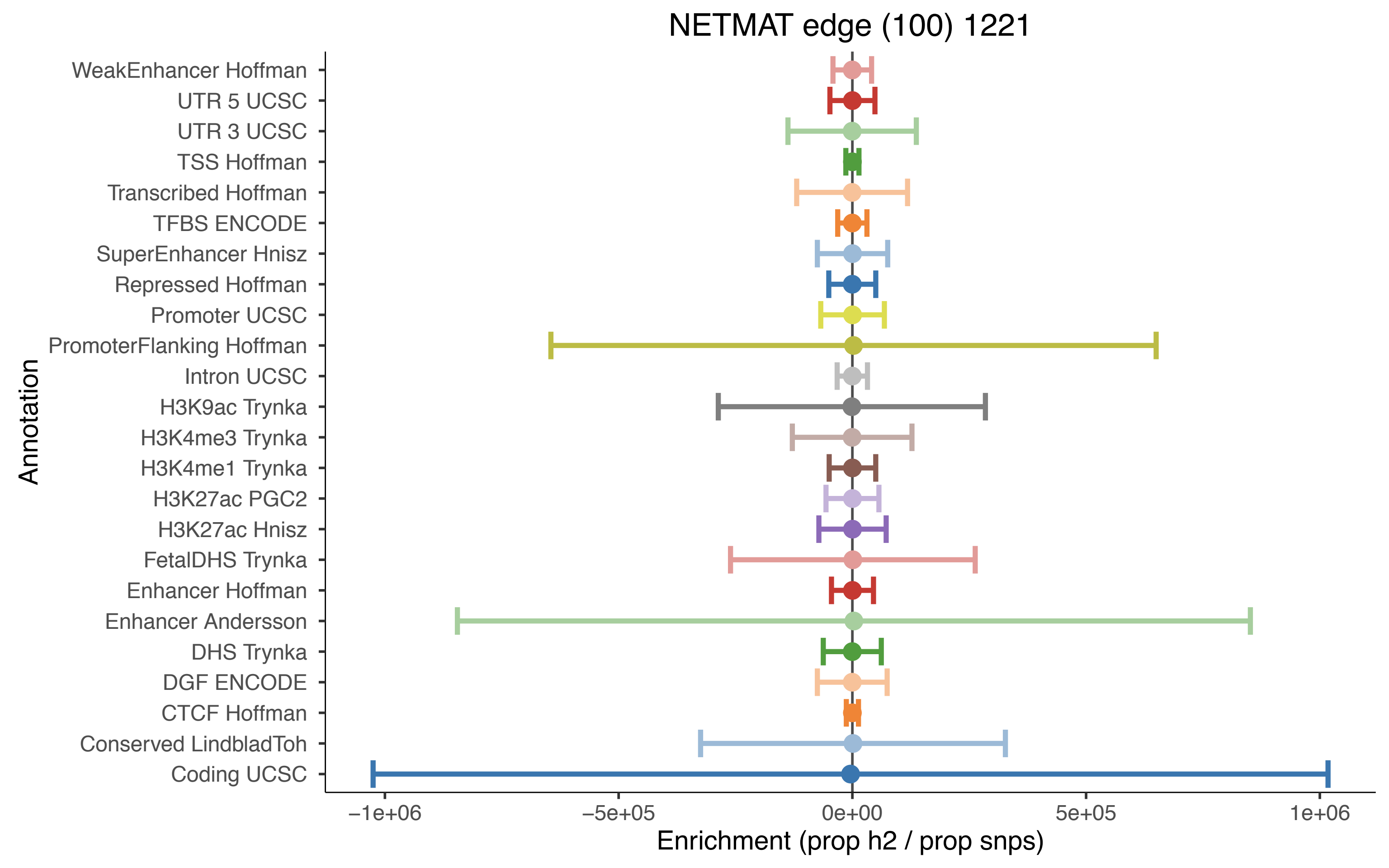
NETMAT edge (100) 1221
Annotation
WeakEnhancer Hoffman
UTR 5 UCSC
UTR 3 UCSC
TSS Hoffman
Transcribed Hoffman
TFBS ENCODE
SuperEnhancer Hnisz
Repressed Hoffman
Promoter UCSC
PromoterFlanking Hoffman
Intron UCSC
H3K9ac Trynka
H3K4me3 Trynka
H3K4me1 Trynka
H3K27ac PGC2
H3K27ac Hnisz
FetalDHS Trynka
Enhancer Hoffman
Enhancer Andersson
DHS Trynka
DGF ENCODE
CTCF Hoffman
Conserved LindbladToh
Coding UCSC
−1e+06
−5e+05
0e+00
5e+05
1e+06
Enrichment (prop h2 / prop snps)

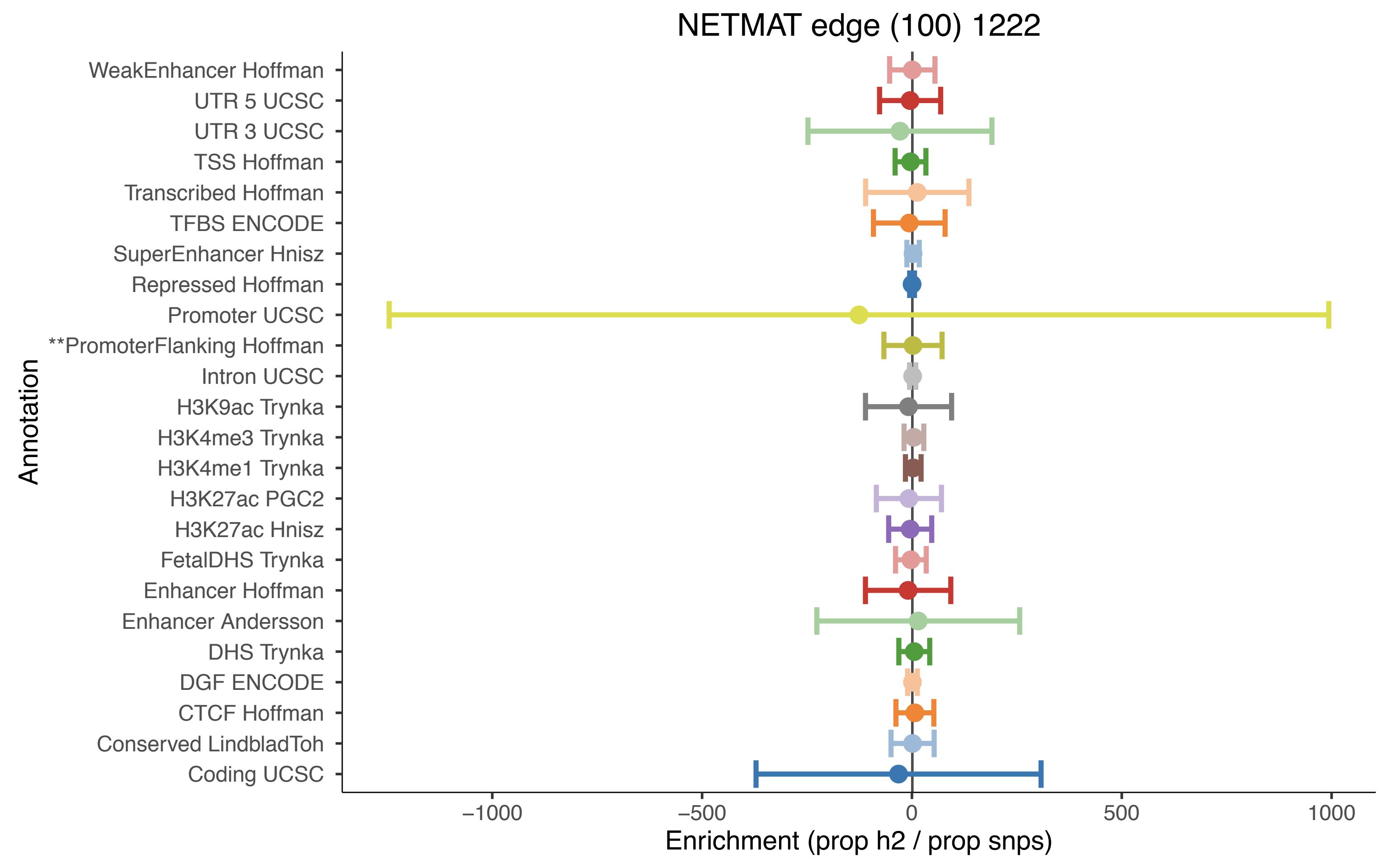
NETMAT edge (100) 1222
WeakEnhancer Hoffman
UTR 5 UCSC
UTR 3 UCSC
TSS Hoffman
Transcribed Hoffman
TFBS ENCODE
SuperEnhancer Hnisz
Repressed Hoffman
Promoter UCSC
**PromoterFlanking Hoffman
Intron UCSC
H3K9ac Trynka
H3K4me3 Trynka
H3K4me1 Trynka
H3K27ac PGC2
H3K27ac Hnisz
FetalDHS Trynka
Enhancer Hoffman
Enhancer Andersson
DHS Trynka
DGF ENCODE
CTCF Hoffman
Conserved LindbladToh
Coding UCSC
Annotation
−1000
−500
0
500
1000
Enrichment (prop h2 / prop snps)

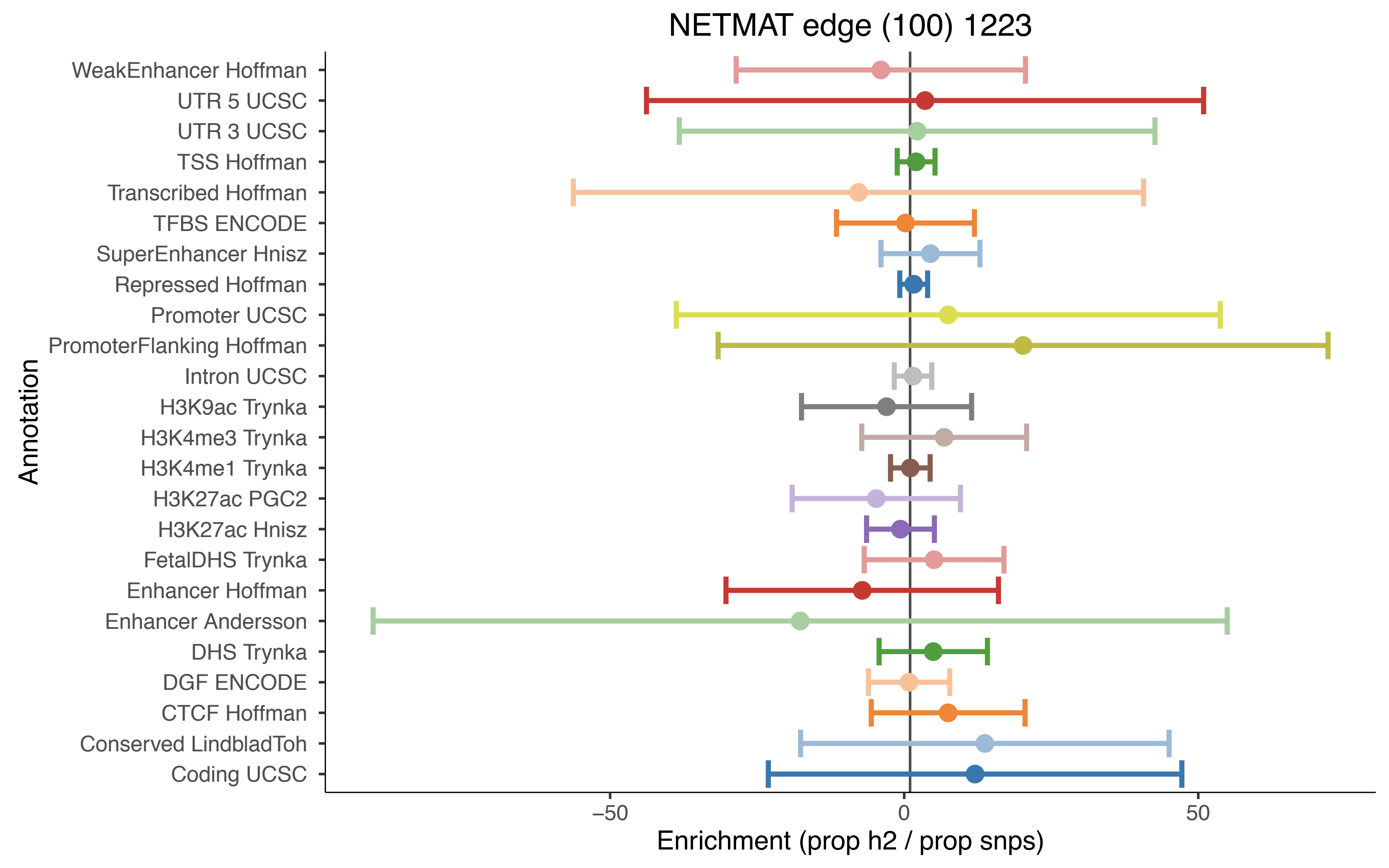
NETMAT edge (100) 1223
WeakEnhancer Hoffman
UTR 5 UCSC
UTR 3 UCSC
TSS Hoffman
Transcribed Hoffman
TFBS ENCODE
SuperEnhancer Hnisz
Repressed Hoffman
Promoter UCSC
PromoterFlanking Hoffman
Intron UCSC
H3K9ac Trynka
H3K4me3 Trynka
H3K4me1 Trynka
H3K27ac PGC2
H3K27ac Hnisz
FetalDHS Trynka
Enhancer Hoffman
Enhancer Andersson
DHS Trynka
DGF ENCODE
CTCF Hoffman
Conserved LindbladToh
Coding UCSC
Annotation
−50
0
50
Enrichment (prop h2 / prop snps)

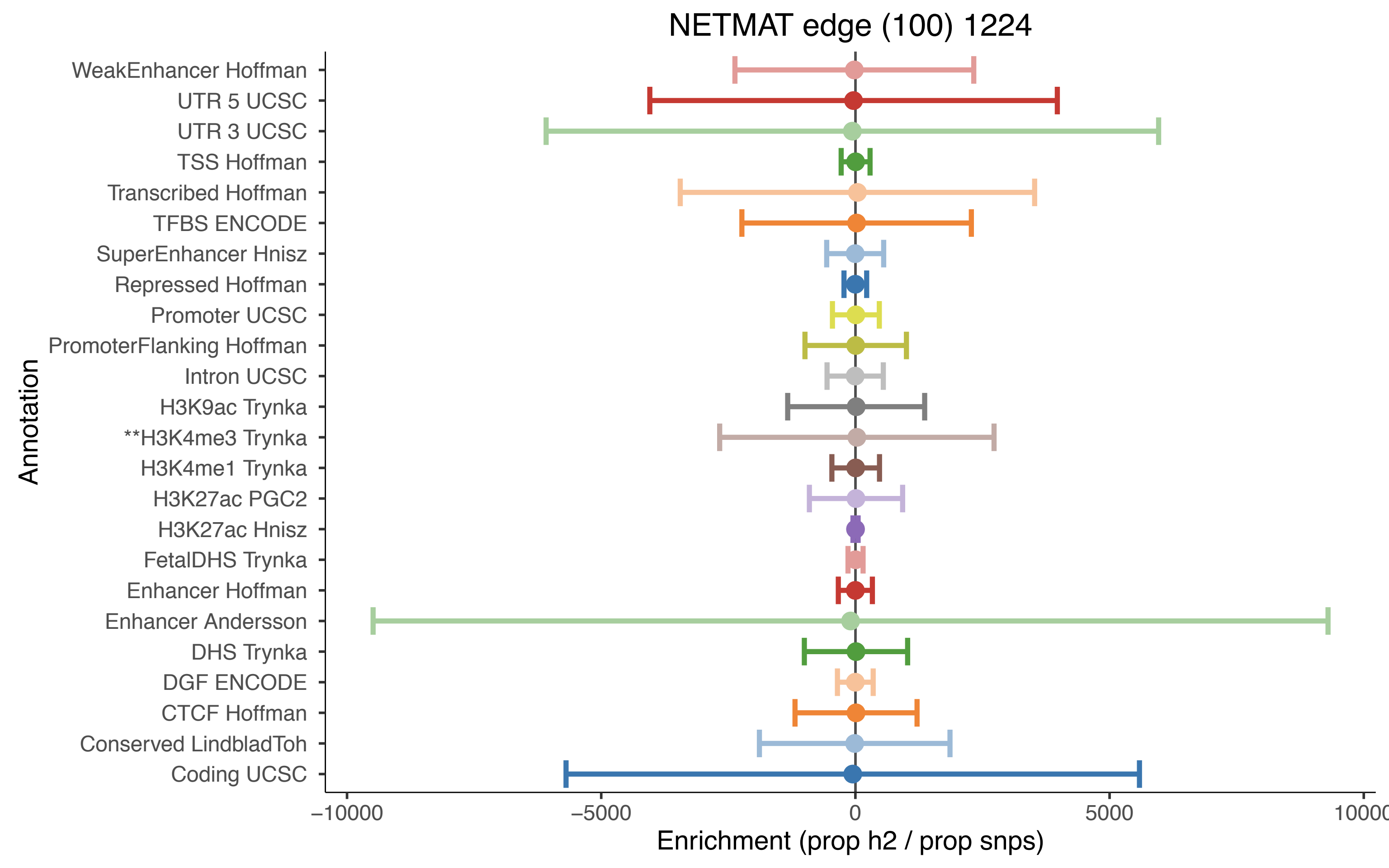

NETMAT edge (100) 1224
Annotation
WeakEnhancer Hoffman
UTR 5 UCSC
UTR 3 UCSC
TSS Hoffman
Transcribed Hoffman
TFBS ENCODE
SuperEnhancer Hnisz
Repressed Hoffman
Promoter UCSC
PromoterFlanking Hoffman
Intron UCSC
H3K9ac Trynka
**H3K4me3 Trynka
H3K4me1 Trynka
H3K27ac PGC2
H3K27ac Hnisz
FetalDHS Trynka
Enhancer Hoffman
Enhancer Andersson
DHS Trynka
DGF ENCODE
CTCF Hoffman
Conserved LindbladToh
Coding UCSC
−10000
−5000
0
5000
10000
Enrichment (prop h2 / prop snps)

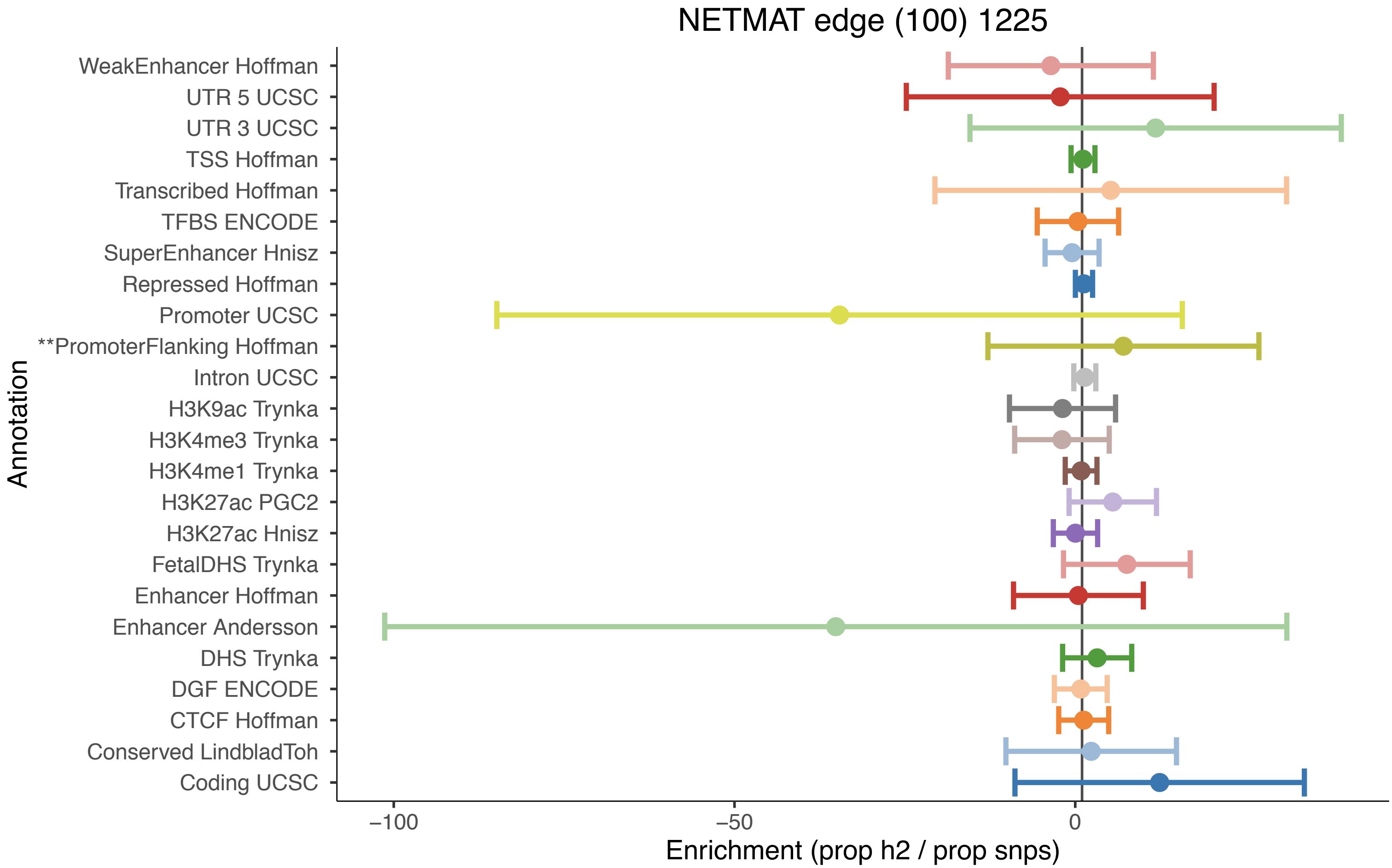
NETMAT edge (100) 1225
Annotation
WeakEnhancer Hoffman
UTR 5 UCSC
UTR 3 UCSC
TSS Hoffman
Transcribed Hoffman
TFBS ENCODE
SuperEnhancer Hnisz
Repressed Hoffman
Promoter UCSC
**PromoterFlanking Hoffman
Intron UCSC
H3K9ac Trynka
H3K4me3 Trynka
H3K4me1 Trynka
H3K27ac PGC2
H3K27ac Hnisz
FetalDHS Trynka
Enhancer Hoffman
Enhancer Andersson
DHS Trynka
DGF ENCODE
CTCF Hoffman
Conserved LindbladToh
Coding UCSC
−100
−50
0
Enrichment (prop h2 / prop snps)

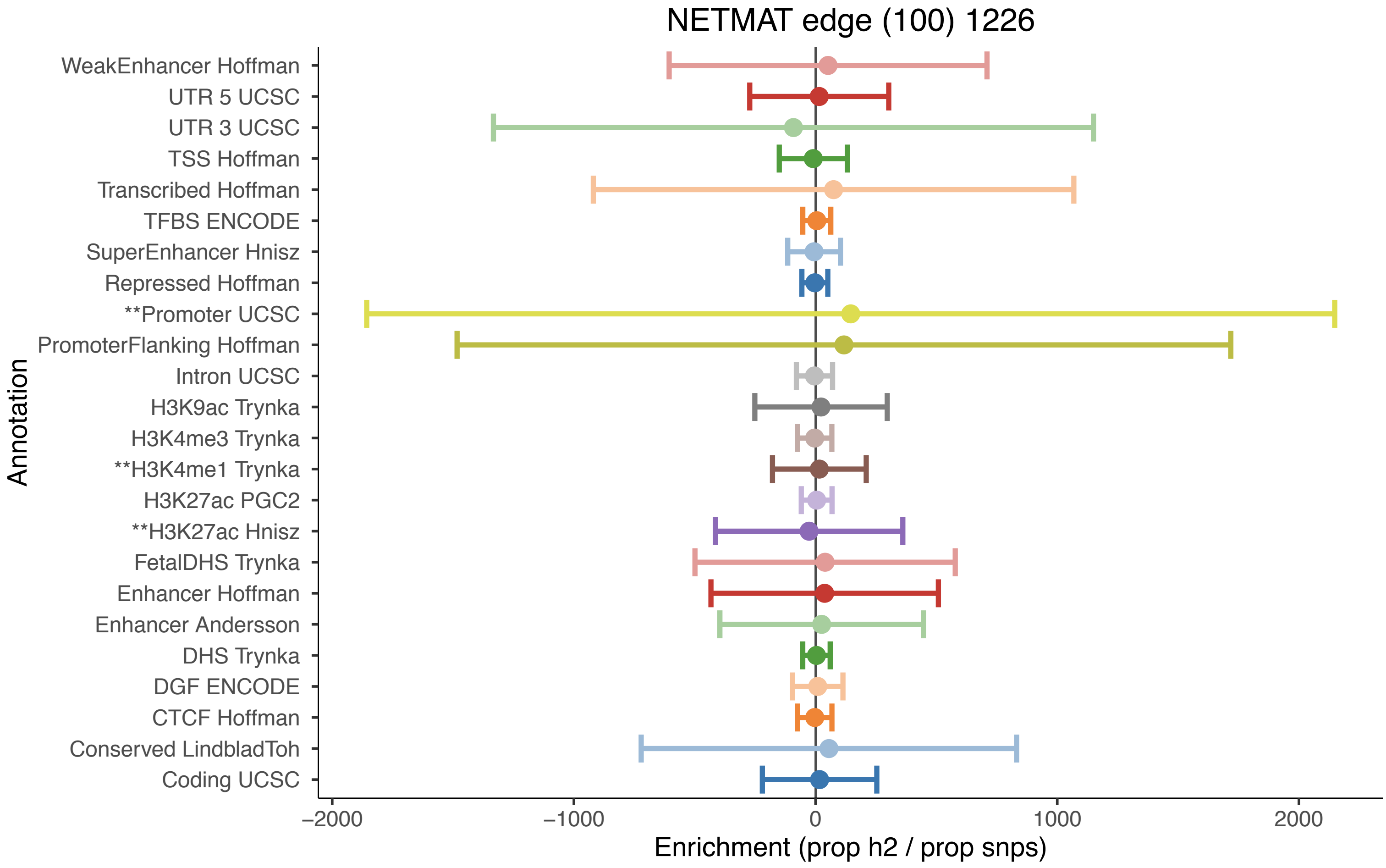
NETMAT edge (100) 1226
WeakEnhancer Hoffman
UTR 5 UCSC
UTR 3 UCSC
TSS Hoffman
Transcribed Hoffman
TFBS ENCODE
SuperEnhancer Hnisz
Repressed Hoffman
**Promoter UCSC
PromoterFlanking Hoffman
Intron UCSC
H3K9ac Trynka
H3K4me3 Trynka
**H3K4me1 Trynka
H3K27ac PGC2
**H3K27ac Hnisz
FetalDHS Trynka
Enhancer Hoffman
Enhancer Andersson
DHS Trynka
DGF ENCODE
CTCF Hoffman
Conserved LindbladToh
Coding UCSC
Annotation
−2000
−1000
0
1000
2000
Enrichment (prop h2 / prop snps)

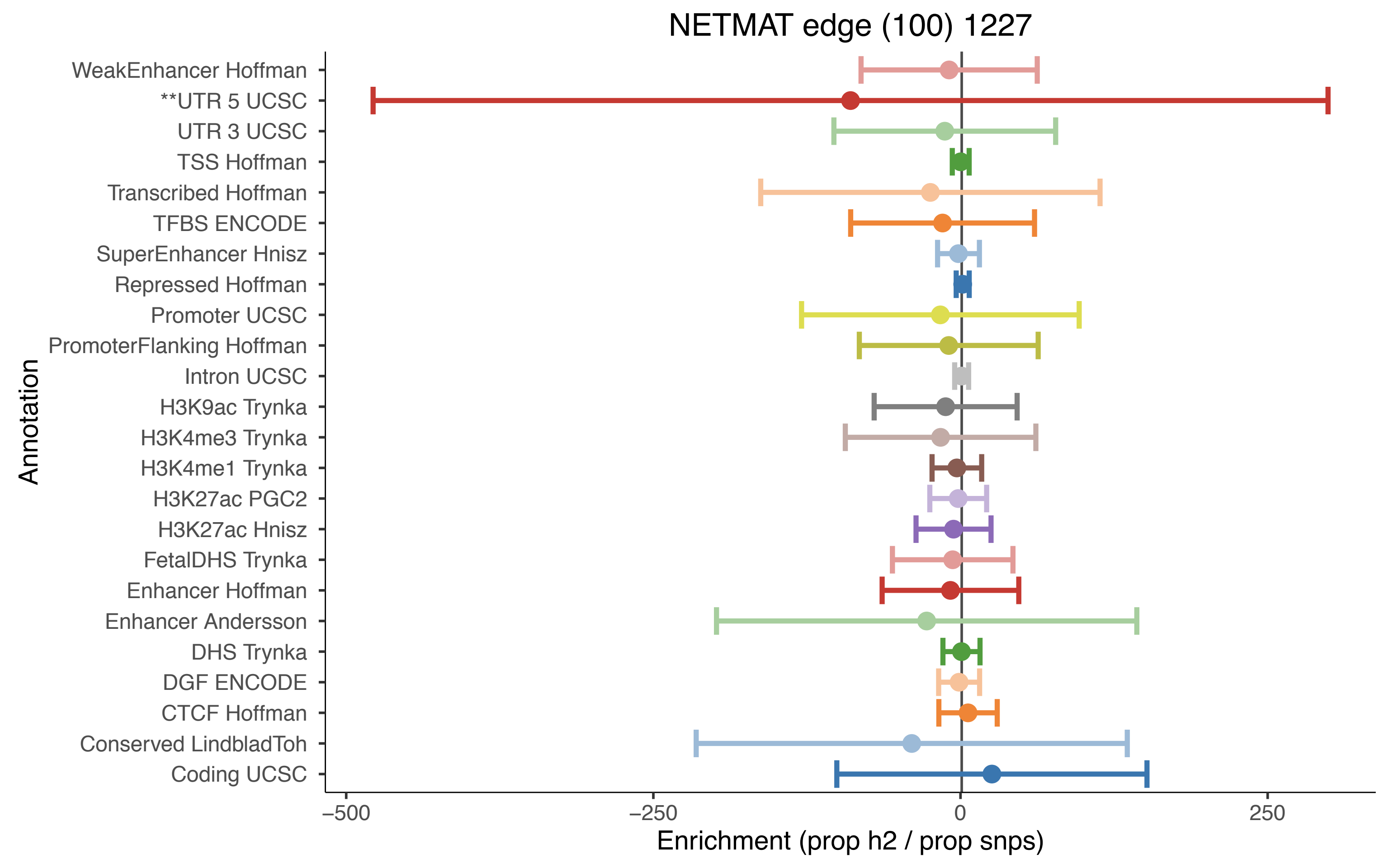
NETMAT edge (100) 1227
WeakEnhancer Hoffman
**UTR 5 UCSC
UTR 3 UCSC
TSS Hoffman
Transcribed Hoffman
TFBS ENCODE
SuperEnhancer Hnisz
Repressed Hoffman
Promoter UCSC
PromoterFlanking Hoffman
Intron UCSC
H3K9ac Trynka
H3K4me3 Trynka
H3K4me1 Trynka
H3K27ac PGC2
H3K27ac Hnisz
FetalDHS Trynka
Enhancer Hoffman
Enhancer Andersson
DHS Trynka
DGF ENCODE
CTCF Hoffman
Conserved LindbladToh
Coding UCSC
Annotation
−500
−250
0
250
Enrichment (prop h2 / prop snps)

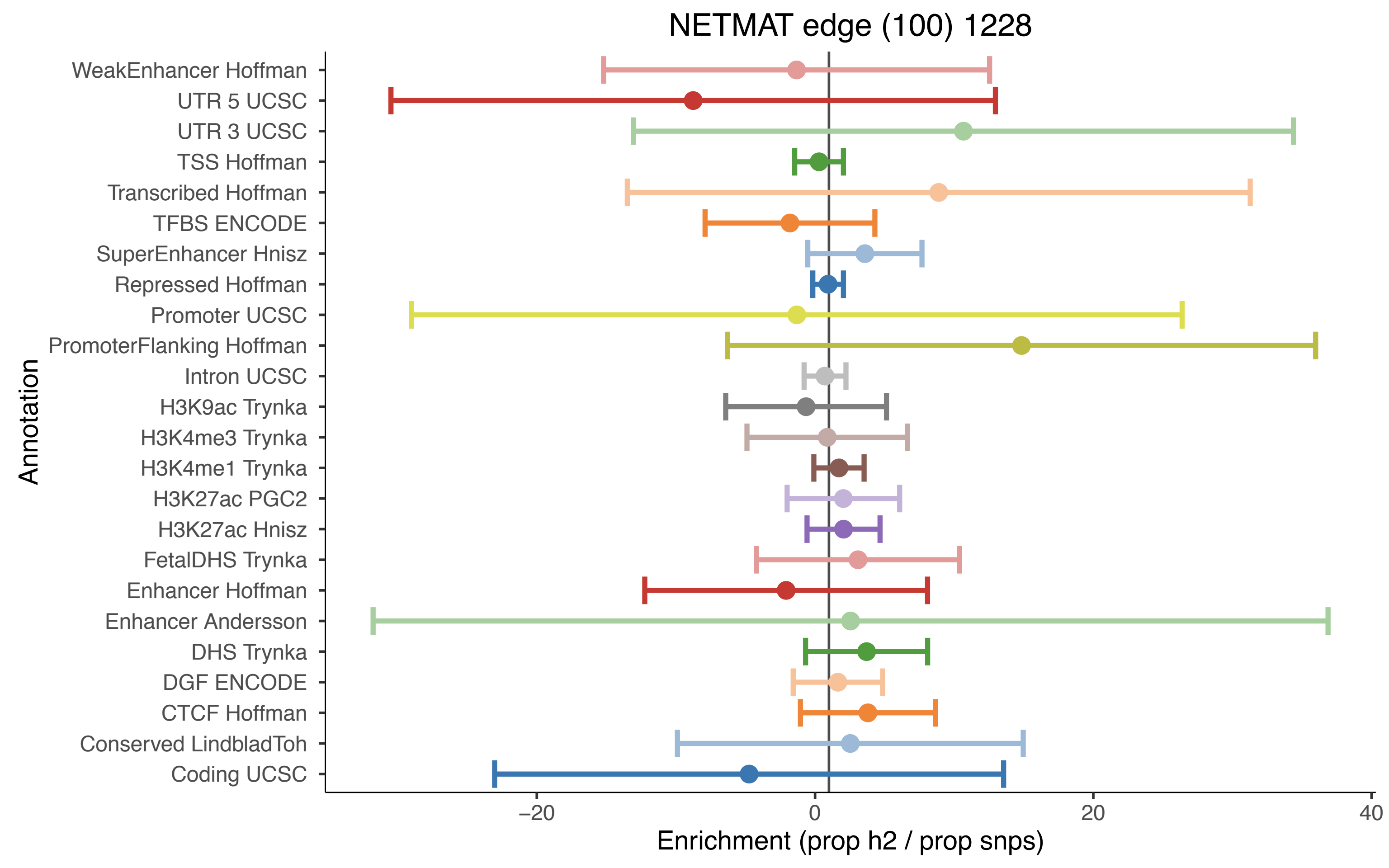
NETMAT edge (100) 1228
Annotation
WeakEnhancer Hoffman
UTR 5 UCSC
UTR 3 UCSC
TSS Hoffman
Transcribed Hoffman
TFBS ENCODE
SuperEnhancer Hnisz
Repressed Hoffman
Promoter UCSC
PromoterFlanking Hoffman
Intron UCSC
H3K9ac Trynka
H3K4me3 Trynka
H3K4me1 Trynka
H3K27ac PGC2
H3K27ac Hnisz
FetalDHS Trynka
Enhancer Hoffman
Enhancer Andersson
DHS Trynka
DGF ENCODE
CTCF Hoffman
Conserved LindbladToh
Coding UCSC
−20
0
20
40
Enrichment (prop h2 / prop snps)

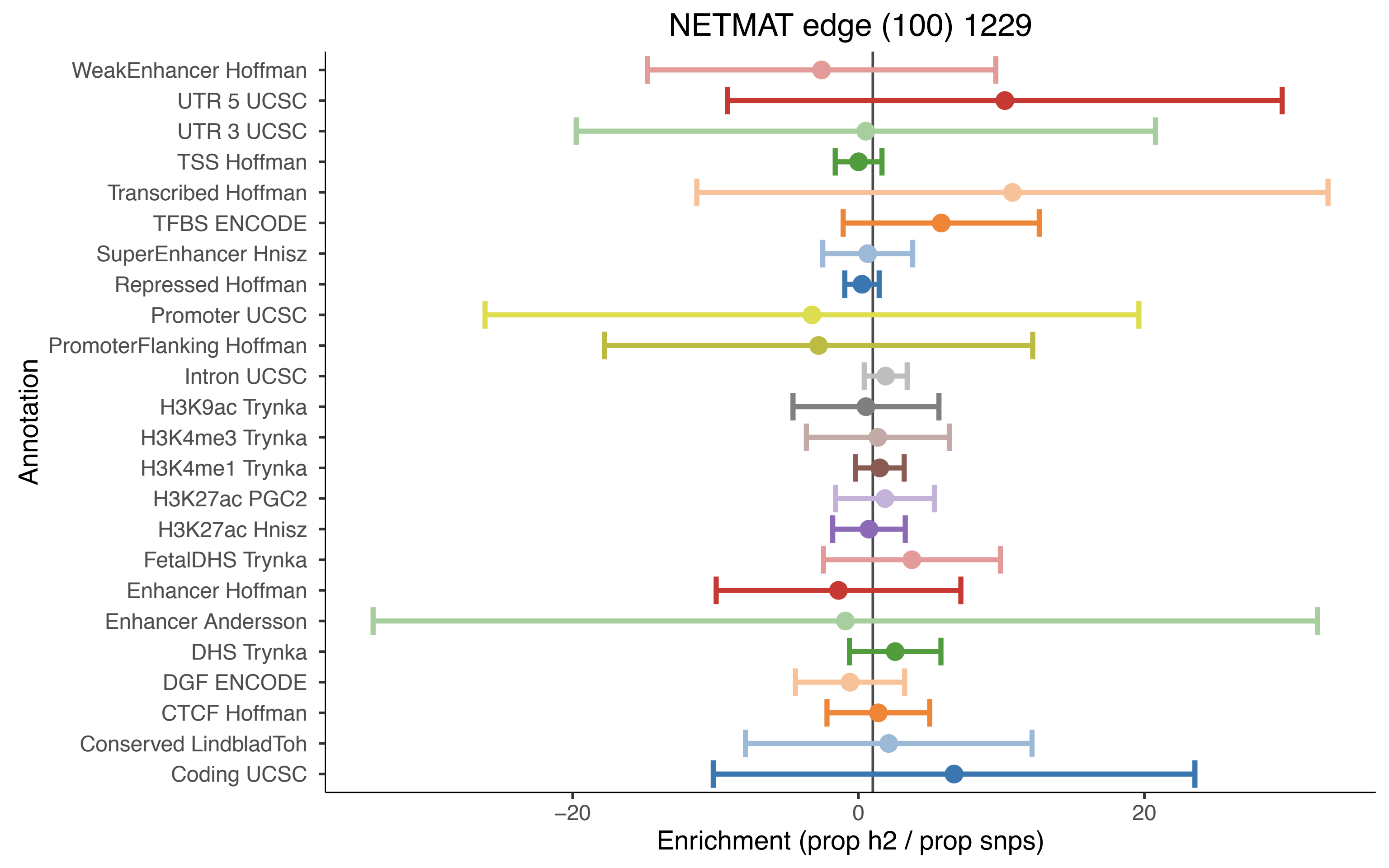
NETMAT edge (100) 1229
WeakEnhancer Hoffman
UTR 5 UCSC
UTR 3 UCSC
TSS Hoffman
Transcribed Hoffman
TFBS ENCODE
SuperEnhancer Hnisz
Repressed Hoffman
Promoter UCSC
PromoterFlanking Hoffman
Intron UCSC
H3K9ac Trynka
H3K4me3 Trynka
H3K4me1 Trynka
H3K27ac PGC2
H3K27ac Hnisz
FetalDHS Trynka
Enhancer Hoffman
Enhancer Andersson
DHS Trynka
DGF ENCODE
CTCF Hoffman
Conserved LindbladToh
Coding UCSC
Annotation
−20
0
20
Enrichment (prop h2 / prop snps)

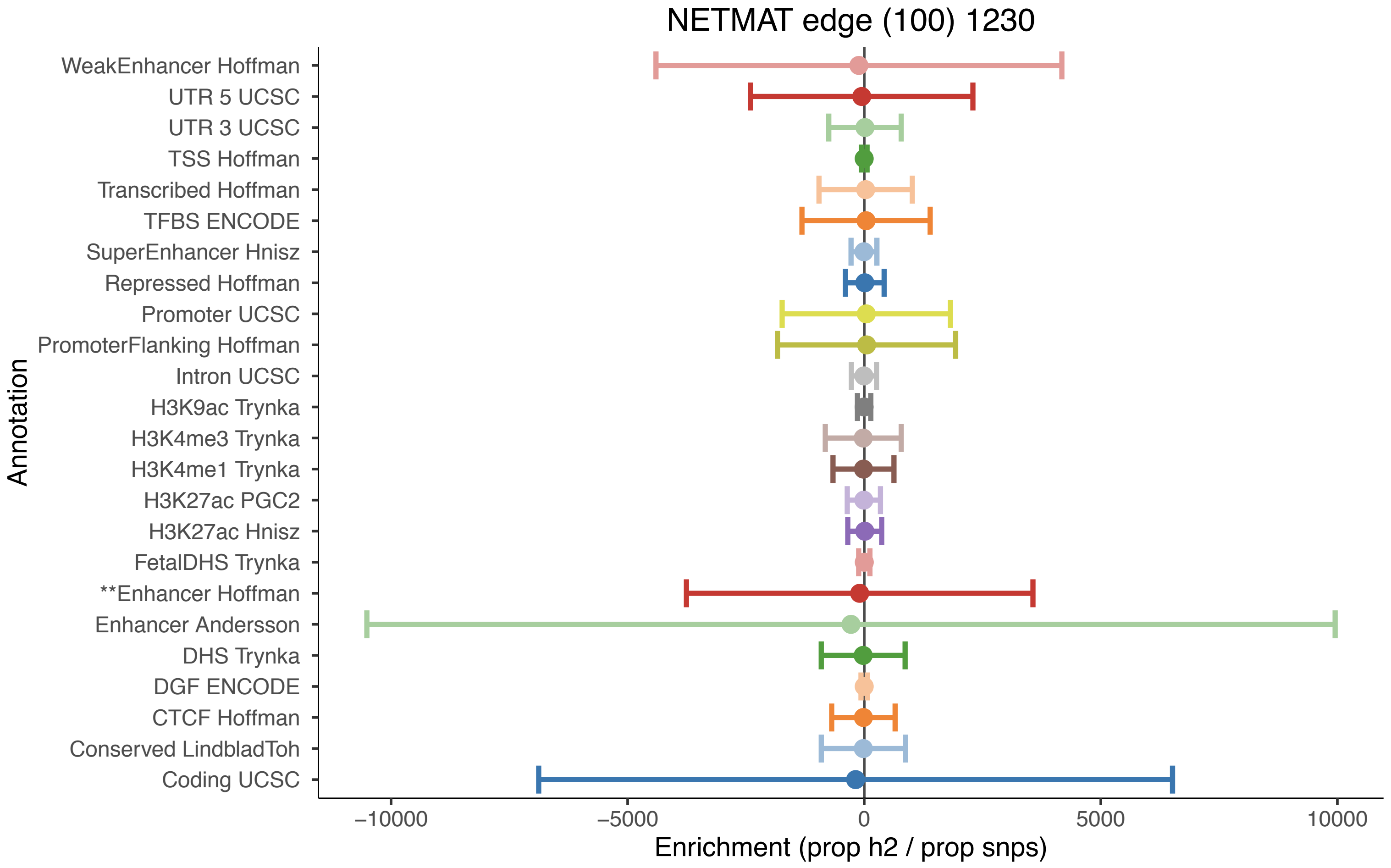
NETMAT edge (100) 1230
WeakEnhancer Hoffman
UTR 5 UCSC
UTR 3 UCSC
TSS Hoffman
Transcribed Hoffman
TFBS ENCODE
SuperEnhancer Hnisz
Repressed Hoffman
Promoter UCSC
PromoterFlanking Hoffman
Intron UCSC
H3K9ac Trynka
H3K4me3 Trynka
H3K4me1 Trynka
H3K27ac PGC2
H3K27ac Hnisz
FetalDHS Trynka
**Enhancer Hoffman
Enhancer Andersson
DHS Trynka
DGF ENCODE
CTCF Hoffman
Conserved LindbladToh
Coding UCSC
Annotation
−10000
−5000
0
5000
10000
Enrichment (prop h2 / prop snps)

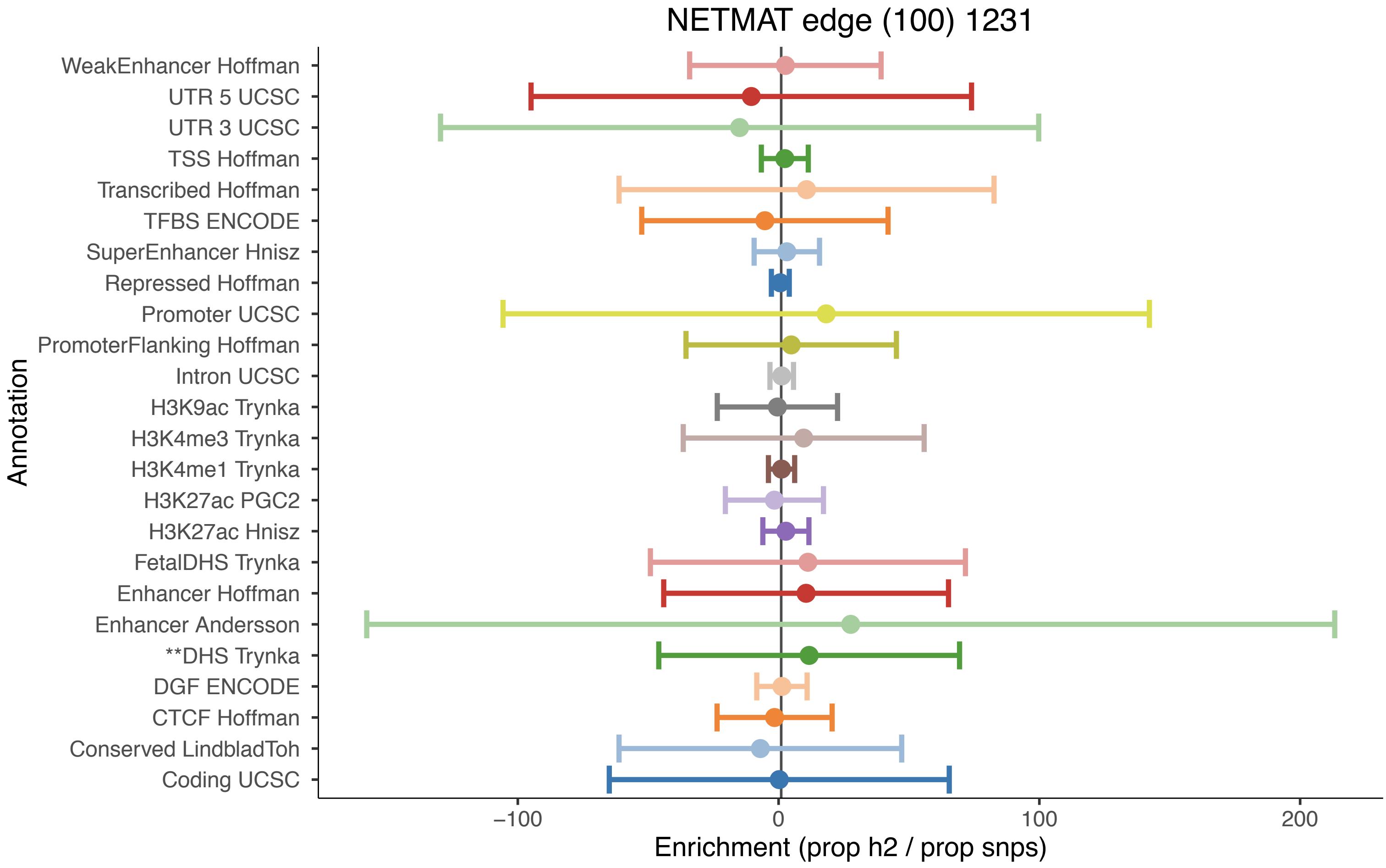
NETMAT edge (100) 1231
Annotation
WeakEnhancer Hoffman
UTR 5 UCSC
UTR 3 UCSC
TSS Hoffman
Transcribed Hoffman
TFBS ENCODE
SuperEnhancer Hnisz
Repressed Hoffman
Promoter UCSC
PromoterFlanking Hoffman
Intron UCSC
H3K9ac Trynka
H3K4me3 Trynka
H3K4me1 Trynka
H3K27ac PGC2
H3K27ac Hnisz
FetalDHS Trynka
Enhancer Hoffman
Enhancer Andersson
**DHS Trynka
DGF ENCODE
CTCF Hoffman
Conserved LindbladToh
Coding UCSC
−100
0
100
200
Enrichment (prop h2 / prop snps)

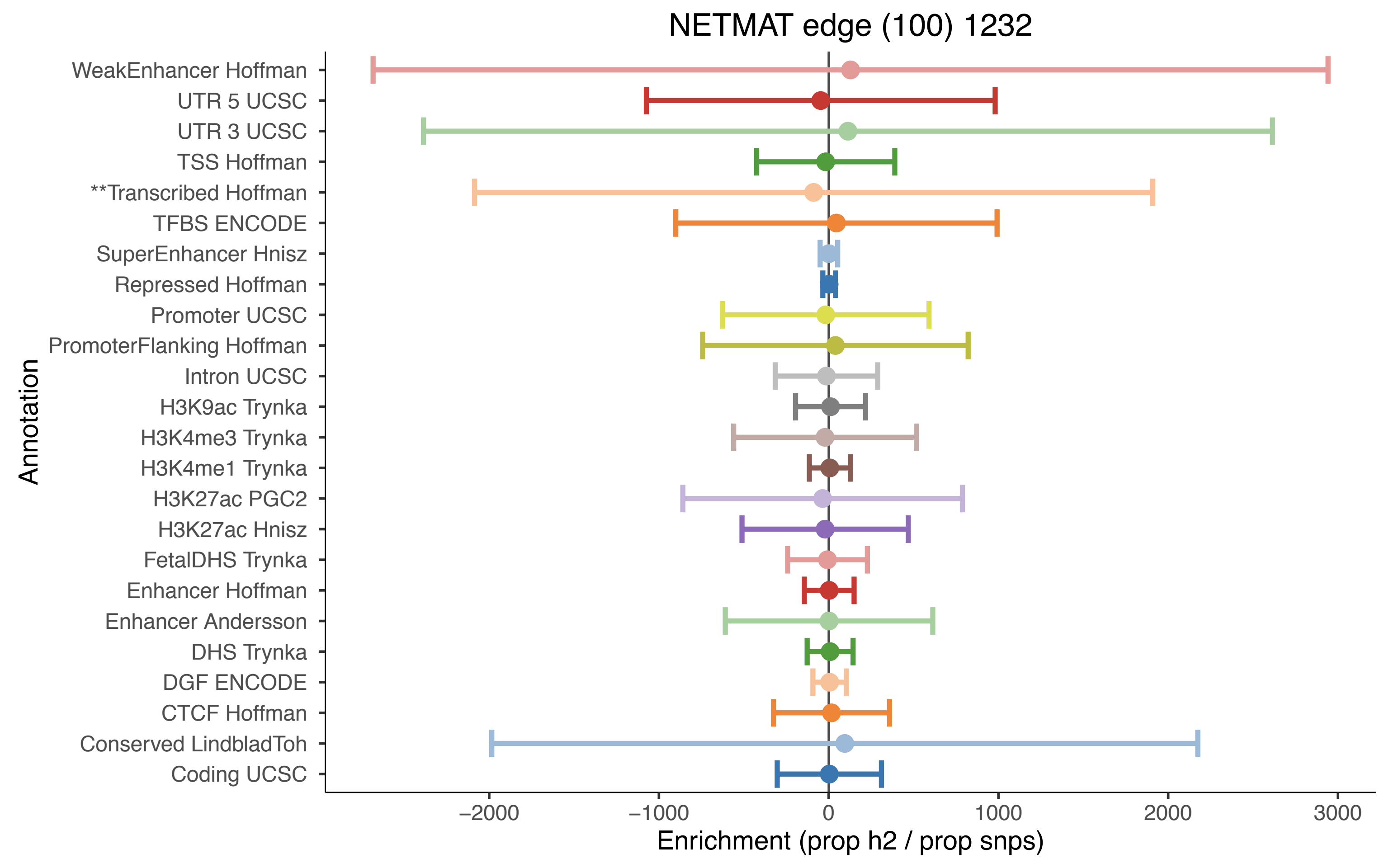
NETMAT edge (100) 1232
WeakEnhancer Hoffman
UTR 5 UCSC
UTR 3 UCSC
TSS Hoffman
**Transcribed Hoffman
TFBS ENCODE
SuperEnhancer Hnisz
Repressed Hoffman
Promoter UCSC
PromoterFlanking Hoffman
Intron UCSC
H3K9ac Trynka
H3K4me3 Trynka
H3K4me1 Trynka
H3K27ac PGC2
H3K27ac Hnisz
FetalDHS Trynka
Enhancer Hoffman
Enhancer Andersson
DHS Trynka
DGF ENCODE
CTCF Hoffman
Conserved LindbladToh
Coding UCSC
Annotation
−2000
−1000
0
1000
2000
3000
Enrichment (prop h2 / prop snps)

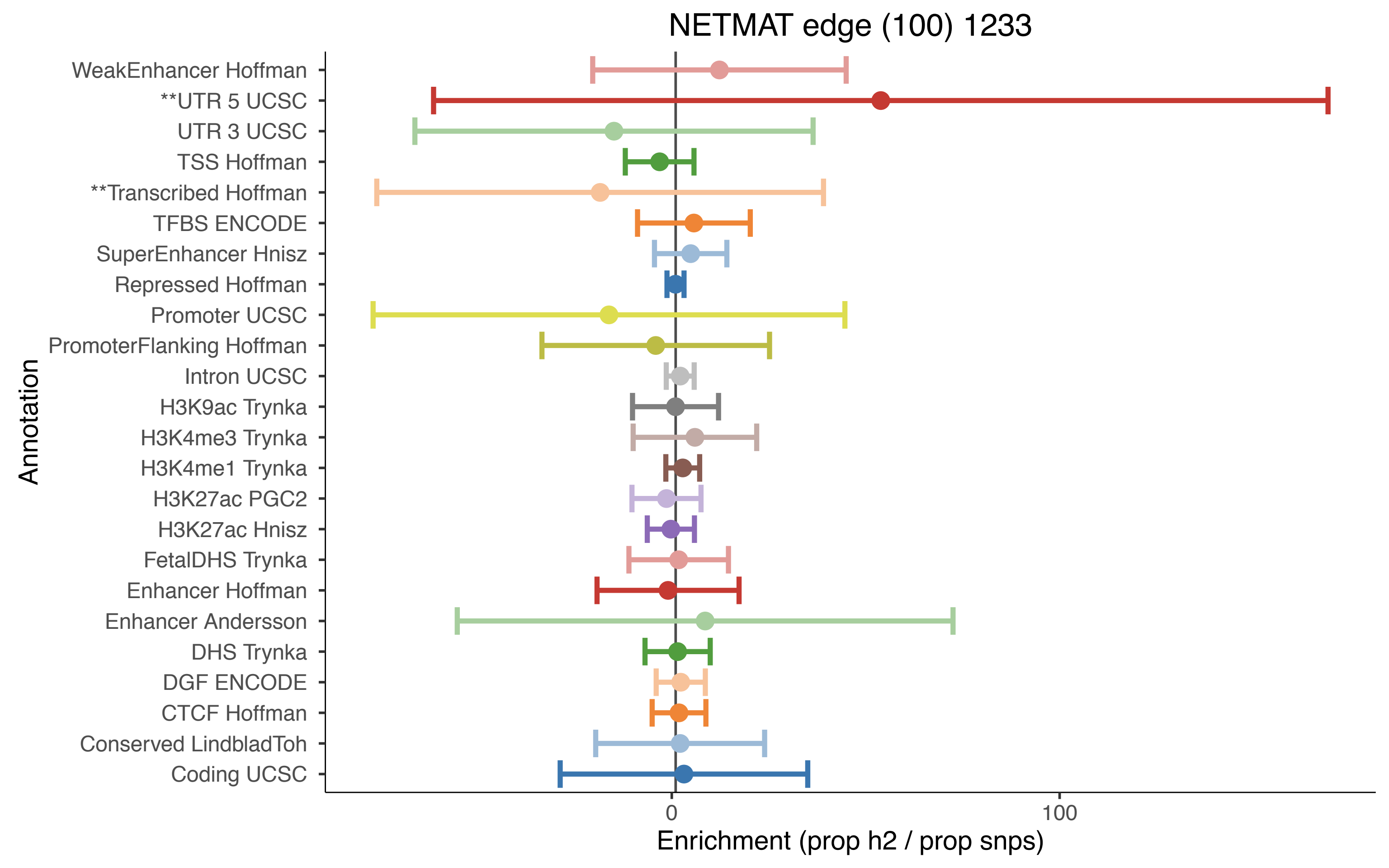
NETMAT edge (100) 1233
WeakEnhancer Hoffman
**UTR 5 UCSC
UTR 3 UCSC
TSS Hoffman
**Transcribed Hoffman
TFBS ENCODE
SuperEnhancer Hnisz
Repressed Hoffman
Promoter UCSC
PromoterFlanking Hoffman
Intron UCSC
H3K9ac Trynka
H3K4me3 Trynka
H3K4me1 Trynka
H3K27ac PGC2
H3K27ac Hnisz
FetalDHS Trynka
Enhancer Hoffman
Enhancer Andersson
DHS Trynka
DGF ENCODE
CTCF Hoffman
Conserved LindbladToh
Coding UCSC
Annotation
0
100
Enrichment (prop h2 / prop snps)

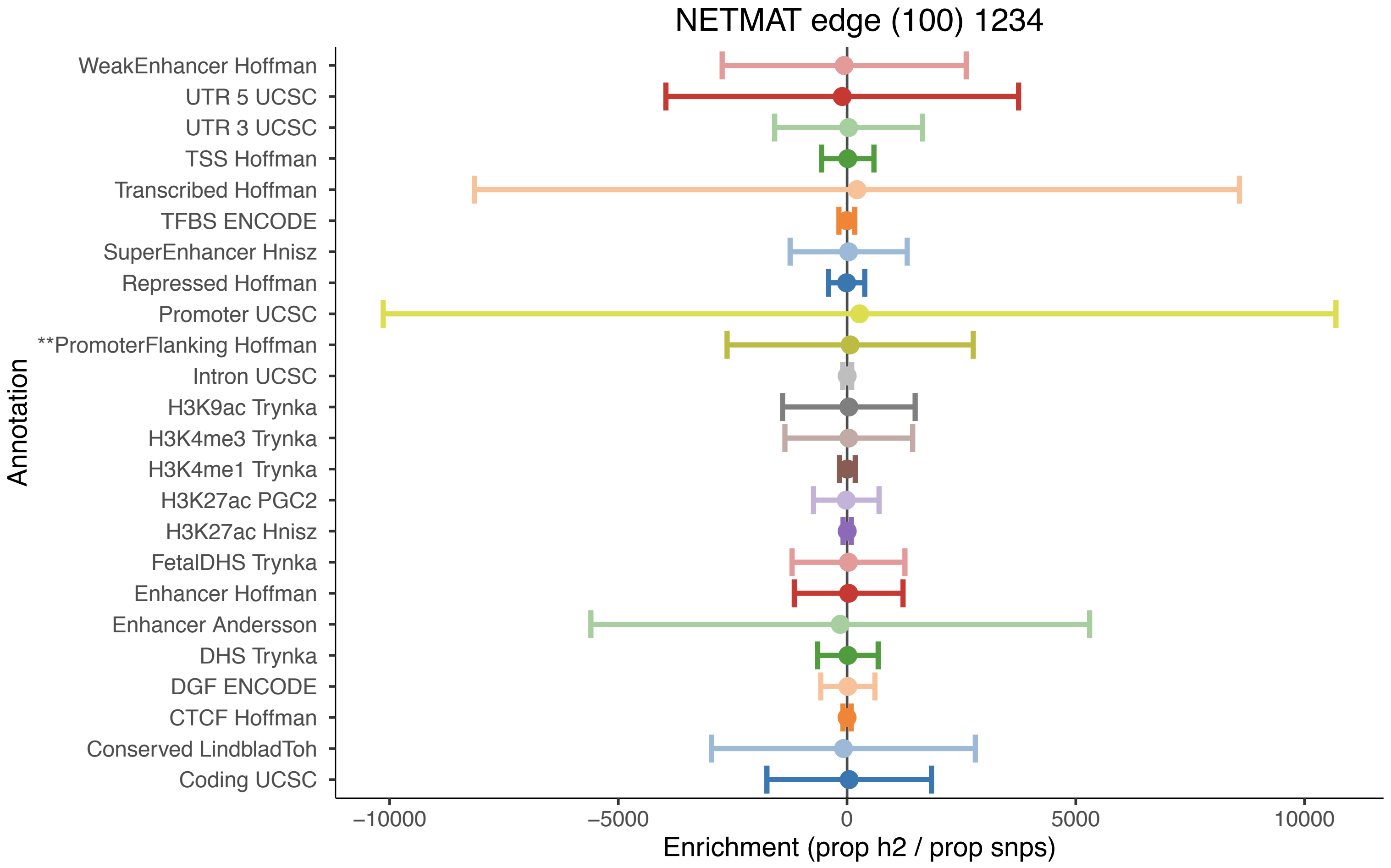

NETMAT edge (100) 1234
WeakEnhancer Hoffman
UTR 5 UCSC
UTR 3 UCSC
TSS Hoffman
Transcribed Hoffman
TFBS ENCODE
SuperEnhancer Hnisz
Repressed Hoffman
Promoter UCSC
**PromoterFlanking Hoffman
Intron UCSC
H3K9ac Trynka
H3K4me3 Trynka
H3K4me1 Trynka
H3K27ac PGC2
H3K27ac Hnisz
FetalDHS Trynka
Enhancer Hoffman
Enhancer Andersson
DHS Trynka
DGF ENCODE
CTCF Hoffman
Conserved LindbladToh
Coding UCSC
Annotation
−10000
−5000
0
5000
10000
Enrichment (prop h2 / prop snps)

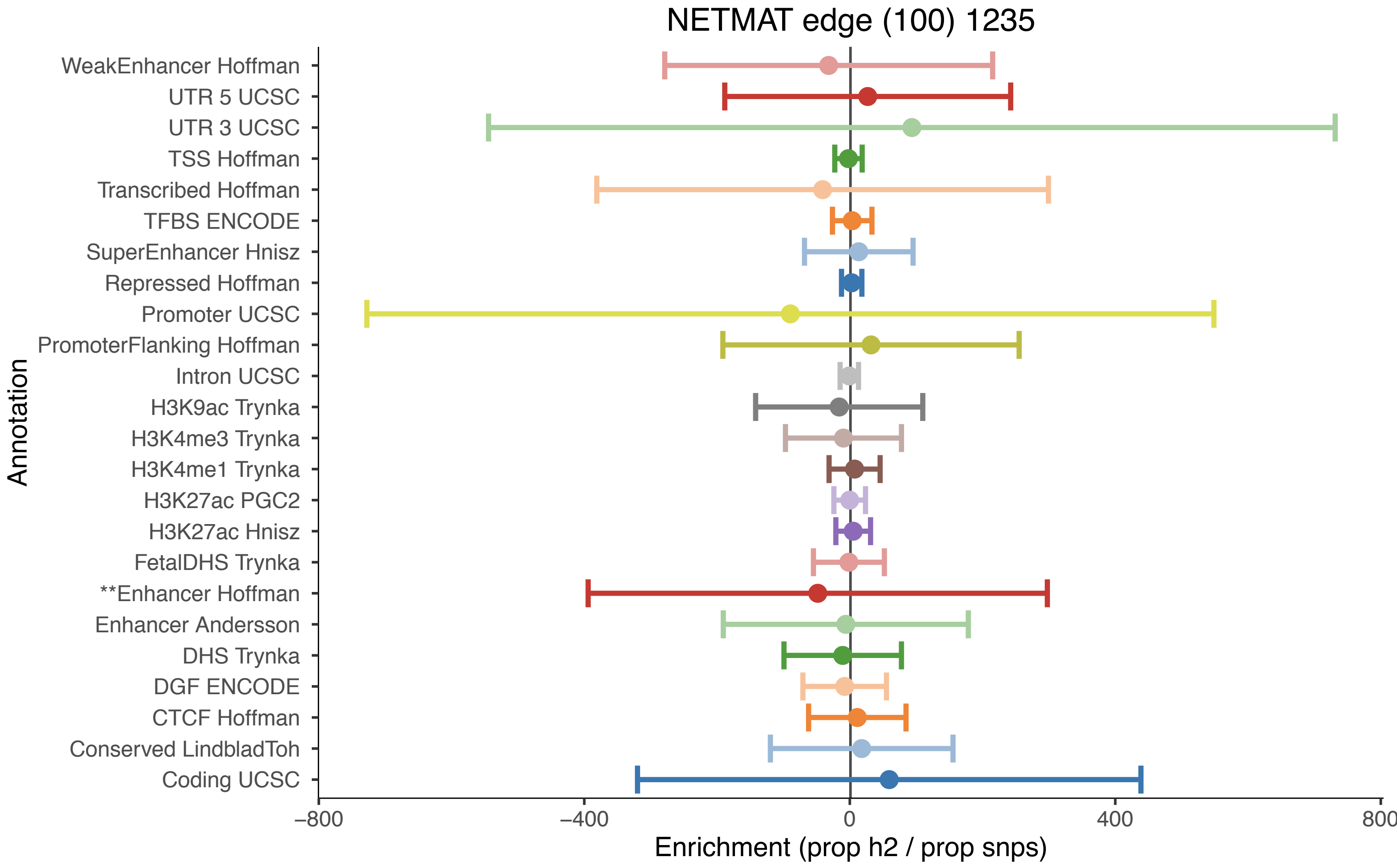

NETMAT edge (100) 1235
Annotation
WeakEnhancer Hoffman
UTR 5 UCSC
UTR 3 UCSC
TSS Hoffman
Transcribed Hoffman
TFBS ENCODE
SuperEnhancer Hnisz
Repressed Hoffman
Promoter UCSC
PromoterFlanking Hoffman
Intron UCSC
H3K9ac Trynka
H3K4me3 Trynka
H3K4me1 Trynka
H3K27ac PGC2
H3K27ac Hnisz
FetalDHS Trynka
**Enhancer Hoffman
Enhancer Andersson
DHS Trynka
DGF ENCODE
CTCF Hoffman
Conserved LindbladToh
Coding UCSC
−800
−400
0
400
800
Enrichment (prop h2 / prop snps)

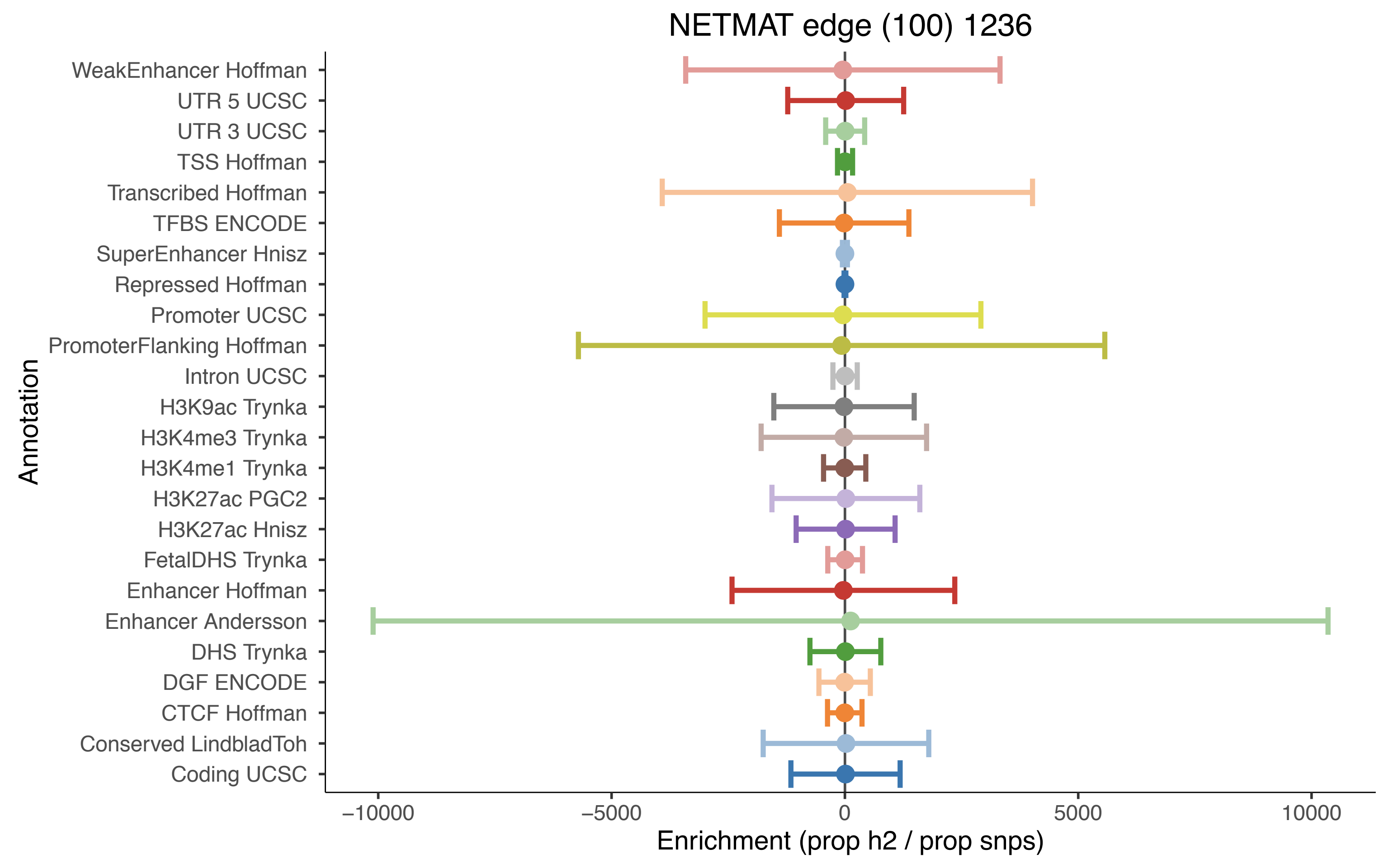
NETMAT edge (100) 1236
WeakEnhancer Hoffman
UTR 5 UCSC
UTR 3 UCSC
TSS Hoffman
Transcribed Hoffman
TFBS ENCODE
SuperEnhancer Hnisz
Repressed Hoffman
Promoter UCSC
PromoterFlanking Hoffman
Intron UCSC
H3K9ac Trynka
H3K4me3 Trynka
H3K4me1 Trynka
H3K27ac PGC2
H3K27ac Hnisz
FetalDHS Trynka
Enhancer Hoffman
Enhancer Andersson
DHS Trynka
DGF ENCODE
CTCF Hoffman
Conserved LindbladToh
Coding UCSC
Annotation
−10000
−5000
0
5000
10000
Enrichment (prop h2 / prop snps)

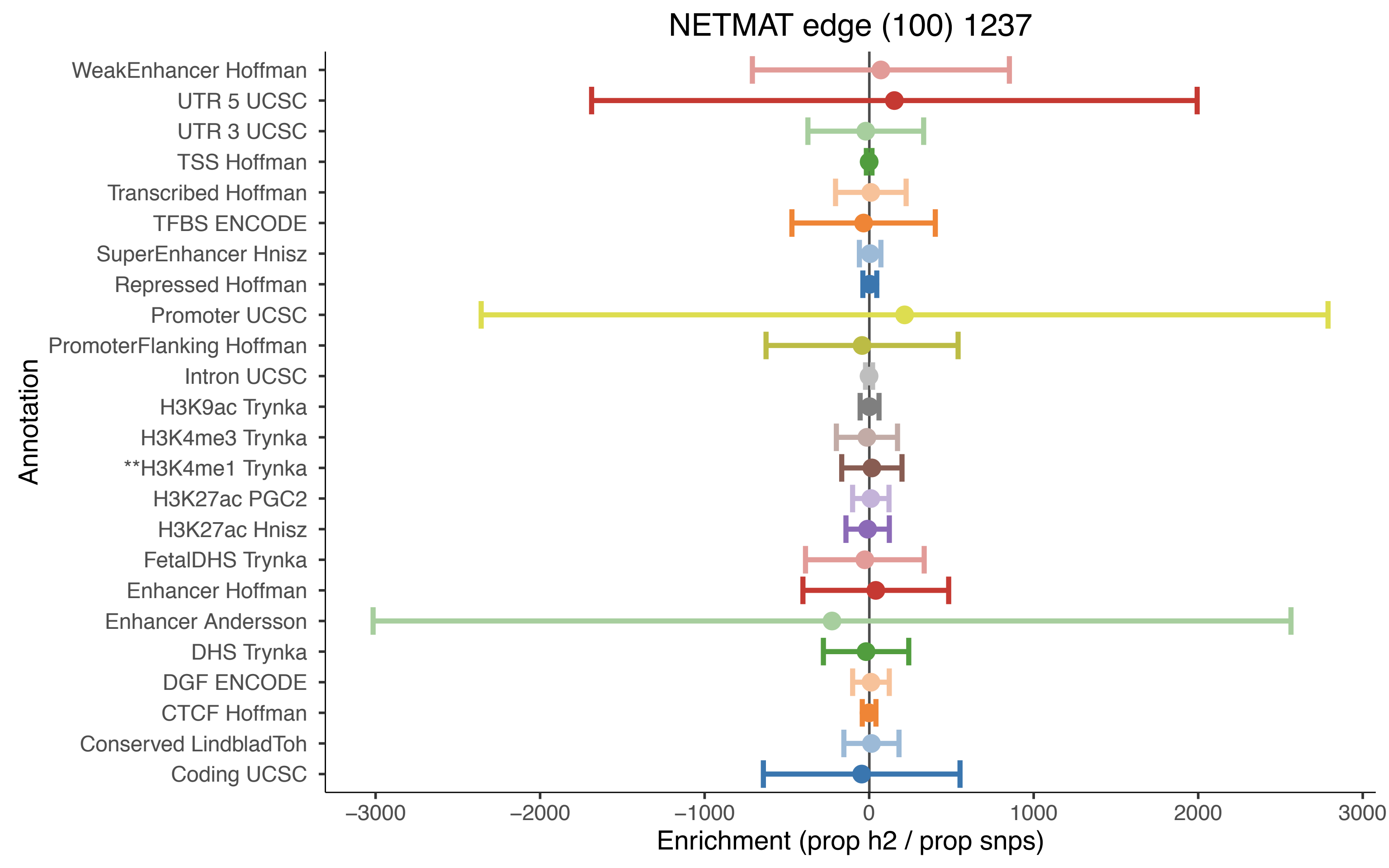

NETMAT edge (100) 1237
WeakEnhancer Hoffman
UTR 5 UCSC
UTR 3 UCSC
TSS Hoffman
Transcribed Hoffman
TFBS ENCODE
SuperEnhancer Hnisz
Repressed Hoffman
Promoter UCSC
PromoterFlanking Hoffman
Intron UCSC
H3K9ac Trynka
H3K4me3 Trynka
**H3K4me1 Trynka
H3K27ac PGC2
H3K27ac Hnisz
FetalDHS Trynka
Enhancer Hoffman
Enhancer Andersson
DHS Trynka
DGF ENCODE
CTCF Hoffman
Conserved LindbladToh
Coding UCSC
Annotation
−3000
−2000
−1000
0
1000
2000
3000
Enrichment (prop h2 / prop snps)

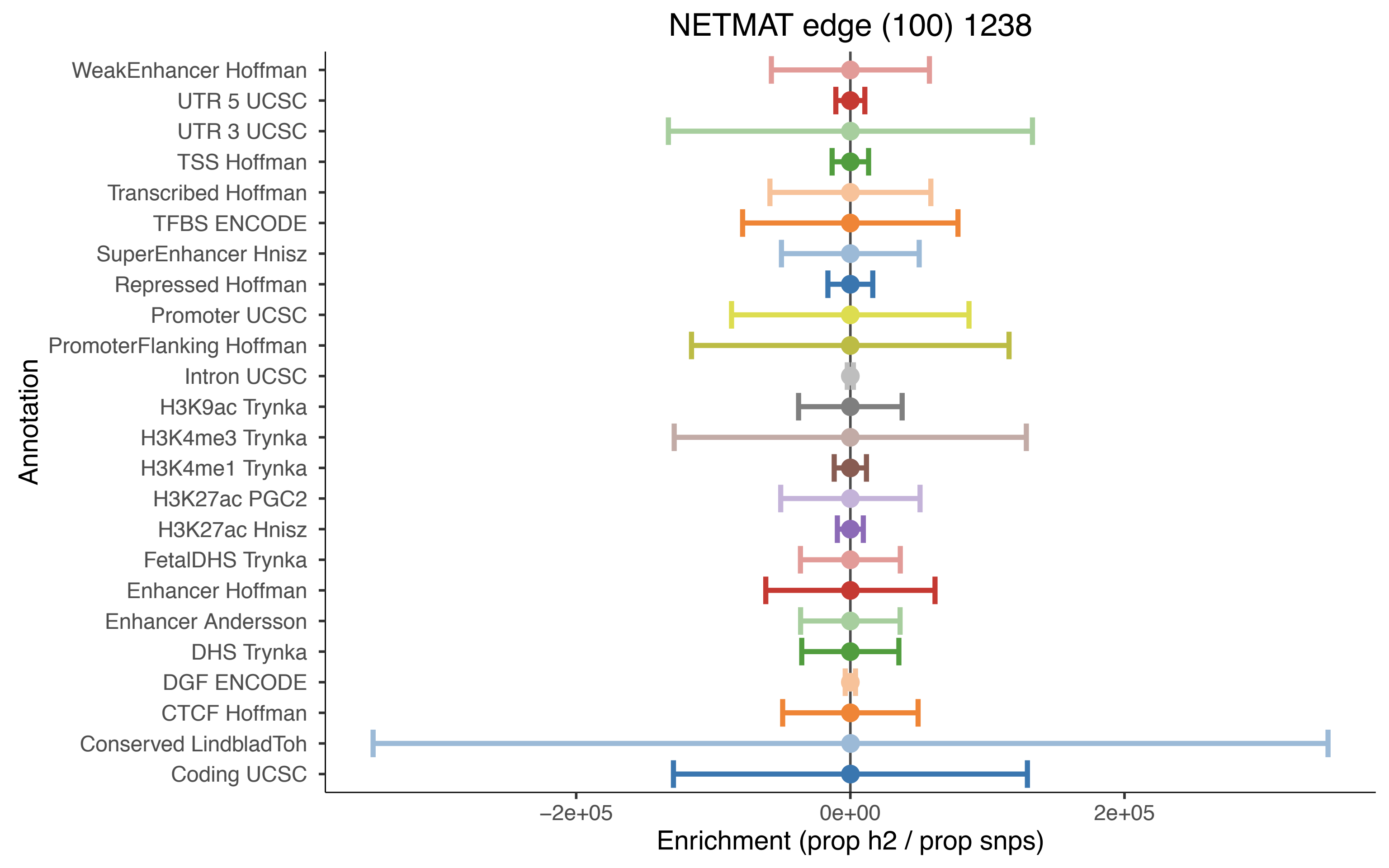
NETMAT edge (100) 1238
WeakEnhancer Hoffman
UTR 5 UCSC
UTR 3 UCSC
TSS Hoffman
Transcribed Hoffman
TFBS ENCODE
SuperEnhancer Hnisz
Repressed Hoffman
Promoter UCSC
PromoterFlanking Hoffman
Intron UCSC
H3K9ac Trynka
H3K4me3 Trynka
H3K4me1 Trynka
H3K27ac PGC2
H3K27ac Hnisz
FetalDHS Trynka
Enhancer Hoffman
Enhancer Andersson
DHS Trynka
DGF ENCODE
CTCF Hoffman
Conserved LindbladToh
Coding UCSC
Annotation
−2e+05
0e+00
2e+05
Enrichment (prop h2 / prop snps)

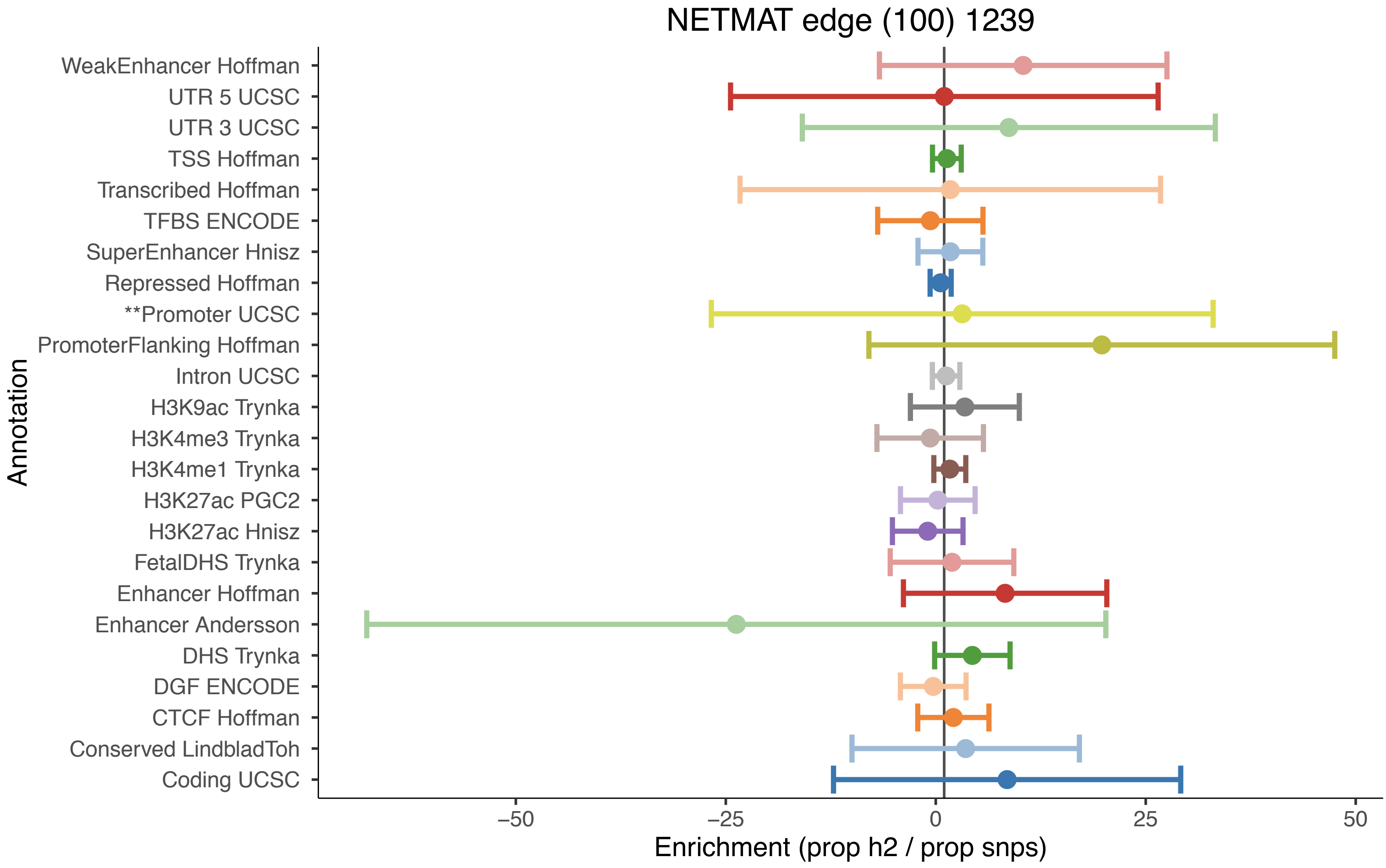
NETMAT edge (100) 1239
WeakEnhancer Hoffman
UTR 5 UCSC
UTR 3 UCSC
TSS Hoffman
Transcribed Hoffman
TFBS ENCODE
SuperEnhancer Hnisz
Repressed Hoffman
**Promoter UCSC
PromoterFlanking Hoffman
Intron UCSC
H3K9ac Trynka
H3K4me3 Trynka
H3K4me1 Trynka
H3K27ac PGC2
H3K27ac Hnisz
FetalDHS Trynka
Enhancer Hoffman
Enhancer Andersson
DHS Trynka
DGF ENCODE
CTCF Hoffman
Conserved LindbladToh
Coding UCSC
Annotation
−50
−25
0
25
50
Enrichment (prop h2 / prop snps)

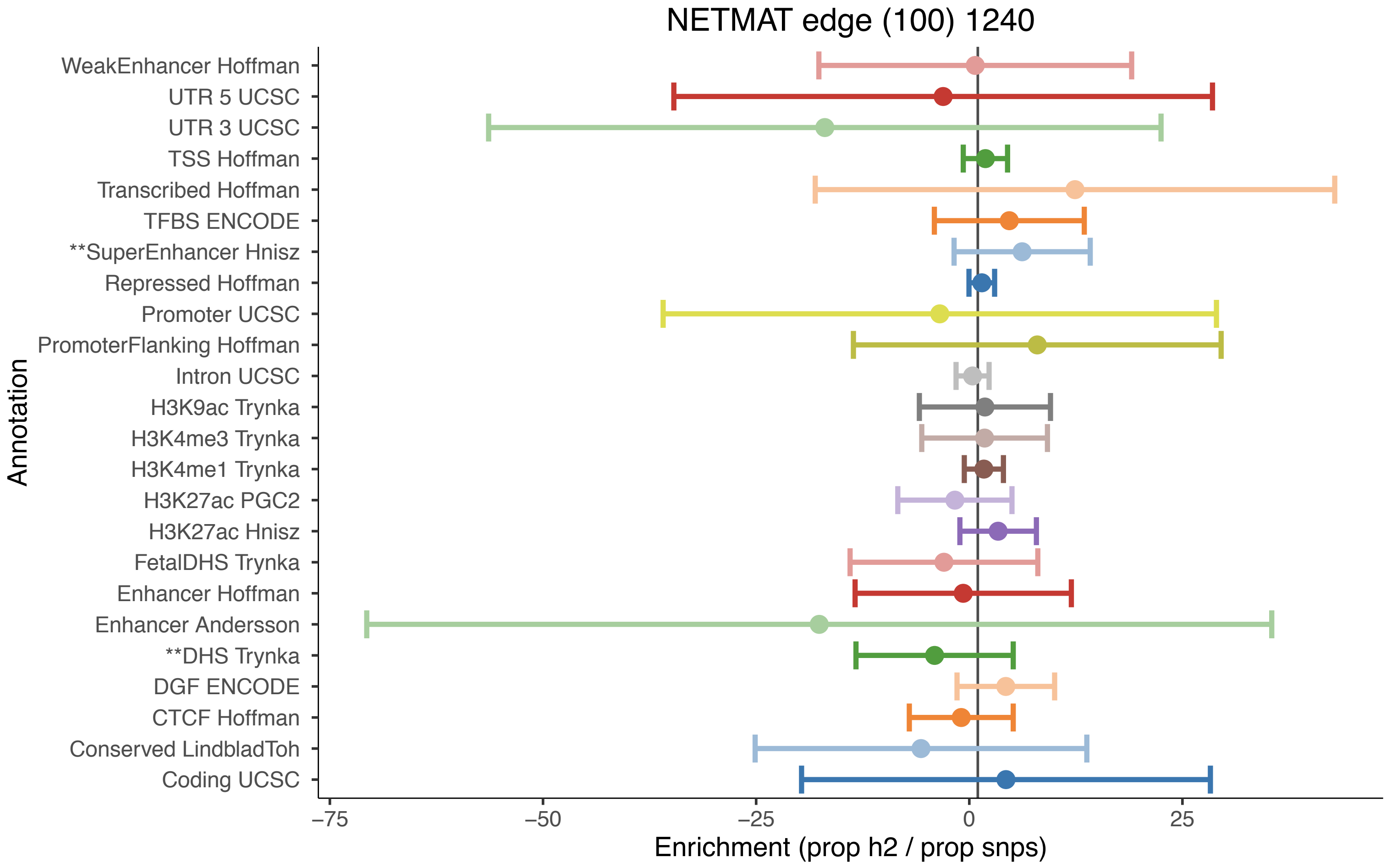

NETMAT edge (100) 1240
WeakEnhancer Hoffman
UTR 5 UCSC
UTR 3 UCSC
TSS Hoffman
Transcribed Hoffman
TFBS ENCODE
**SuperEnhancer Hnisz
Repressed Hoffman
Promoter UCSC
PromoterFlanking Hoffman
Intron UCSC
H3K9ac Trynka
H3K4me3 Trynka
H3K4me1 Trynka
H3K27ac PGC2
H3K27ac Hnisz
FetalDHS Trynka
Enhancer Hoffman
Enhancer Andersson
**DHS Trynka
DGF ENCODE
CTCF Hoffman
Conserved LindbladToh
Coding UCSC
Annotation
−75
−50
−25
0
25
Enrichment (prop h2 / prop snps)

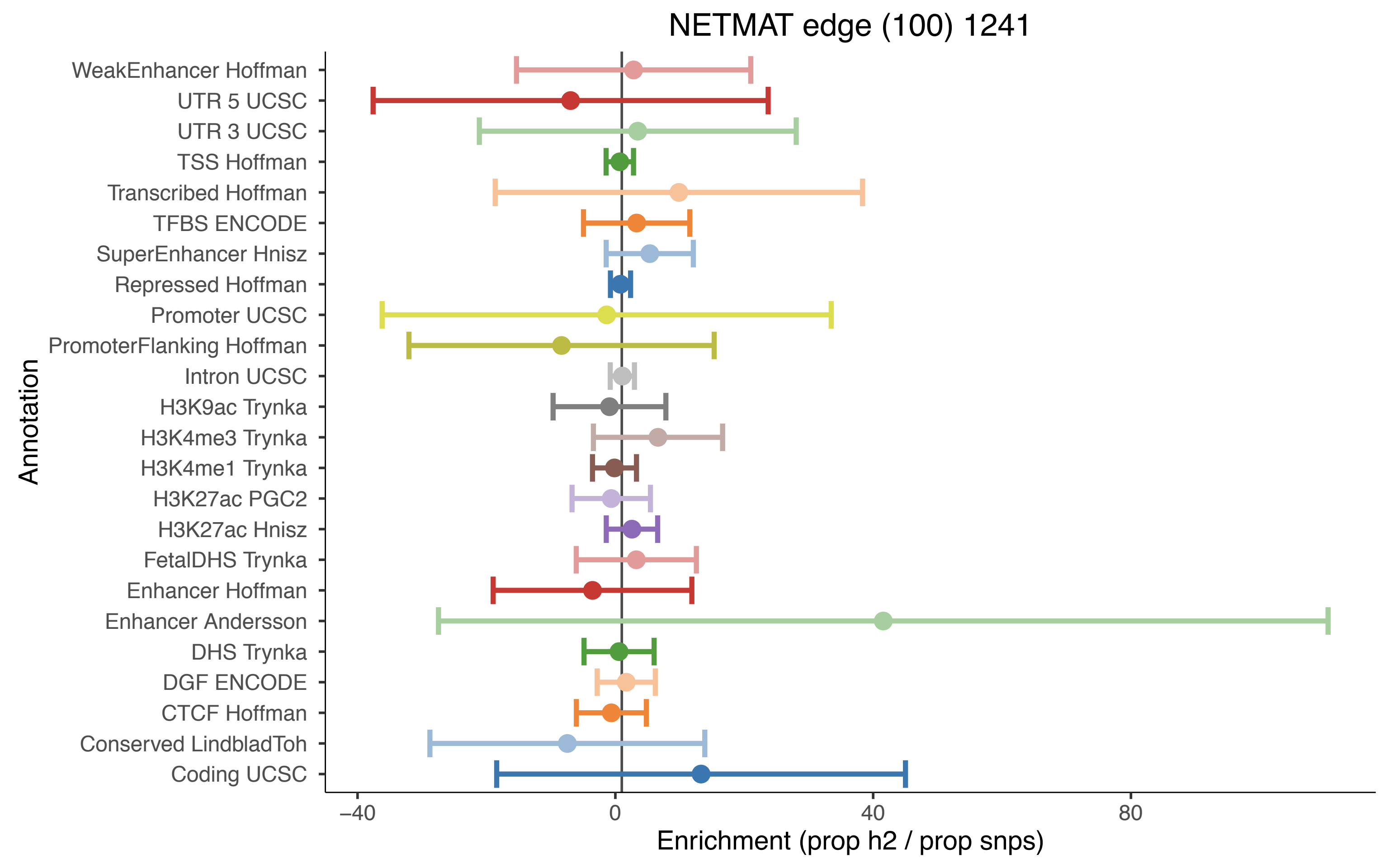

NETMAT edge (100) 1241
WeakEnhancer Hoffman
UTR 5 UCSC
UTR 3 UCSC
TSS Hoffman
Transcribed Hoffman
TFBS ENCODE
SuperEnhancer Hnisz
Repressed Hoffman
Promoter UCSC
PromoterFlanking Hoffman
Intron UCSC
H3K9ac Trynka
H3K4me3 Trynka
H3K4me1 Trynka
H3K27ac PGC2
H3K27ac Hnisz
FetalDHS Trynka
Enhancer Hoffman
Enhancer Andersson
DHS Trynka
DGF ENCODE
CTCF Hoffman
Conserved LindbladToh
Coding UCSC
Annotation
−40
0
40
80
Enrichment (prop h2 / prop snps)

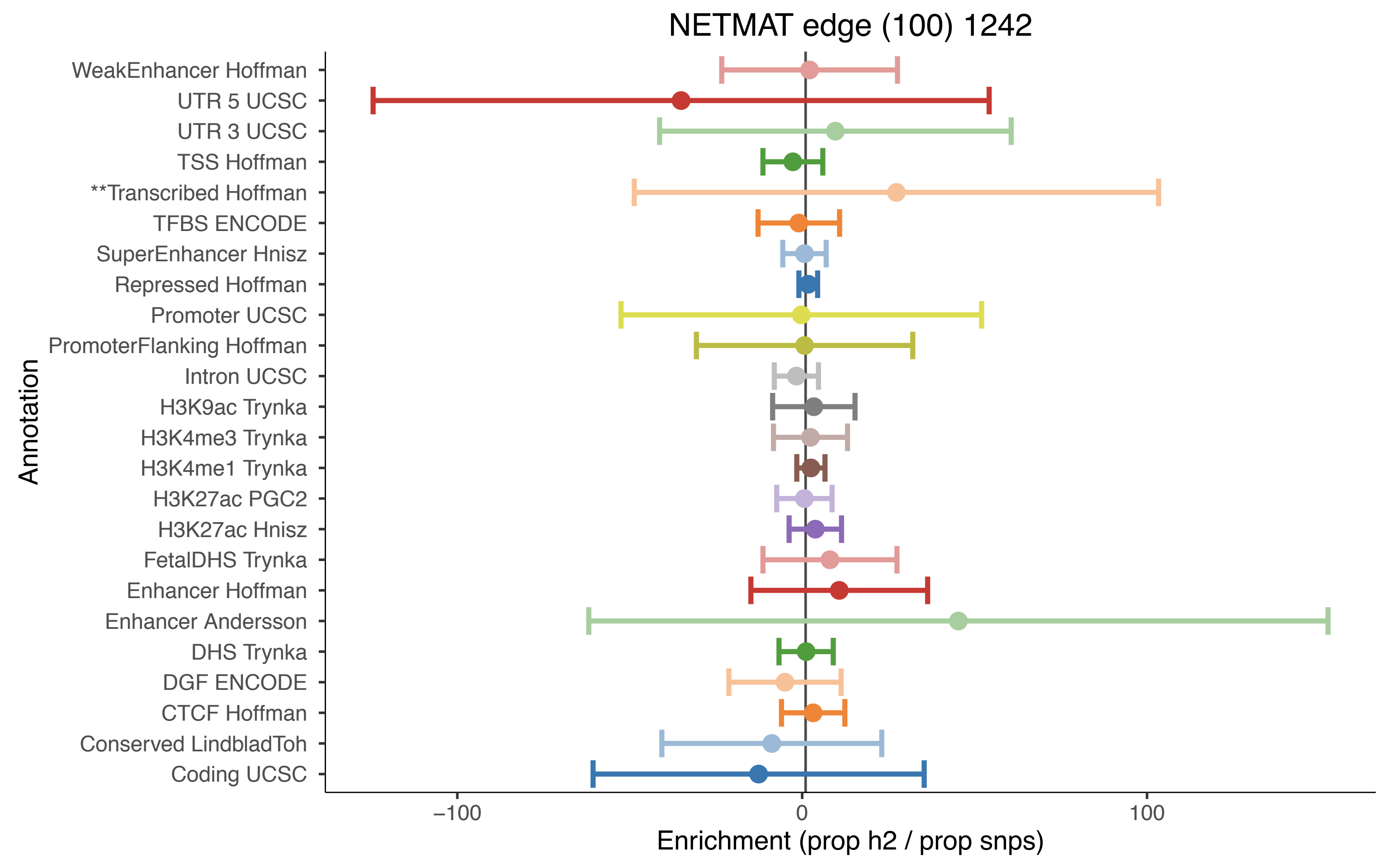
NETMAT edge (100) 1242
WeakEnhancer Hoffman
UTR 5 UCSC
UTR 3 UCSC
TSS Hoffman
**Transcribed Hoffman
TFBS ENCODE
SuperEnhancer Hnisz
Repressed Hoffman
Promoter UCSC
PromoterFlanking Hoffman
Intron UCSC
H3K9ac Trynka
H3K4me3 Trynka
H3K4me1 Trynka
H3K27ac PGC2
H3K27ac Hnisz
FetalDHS Trynka
Enhancer Hoffman
Enhancer Andersson
DHS Trynka
DGF ENCODE
CTCF Hoffman
Conserved LindbladToh
Coding UCSC
Annotation
−100
0
100
Enrichment (prop h2 / prop snps)

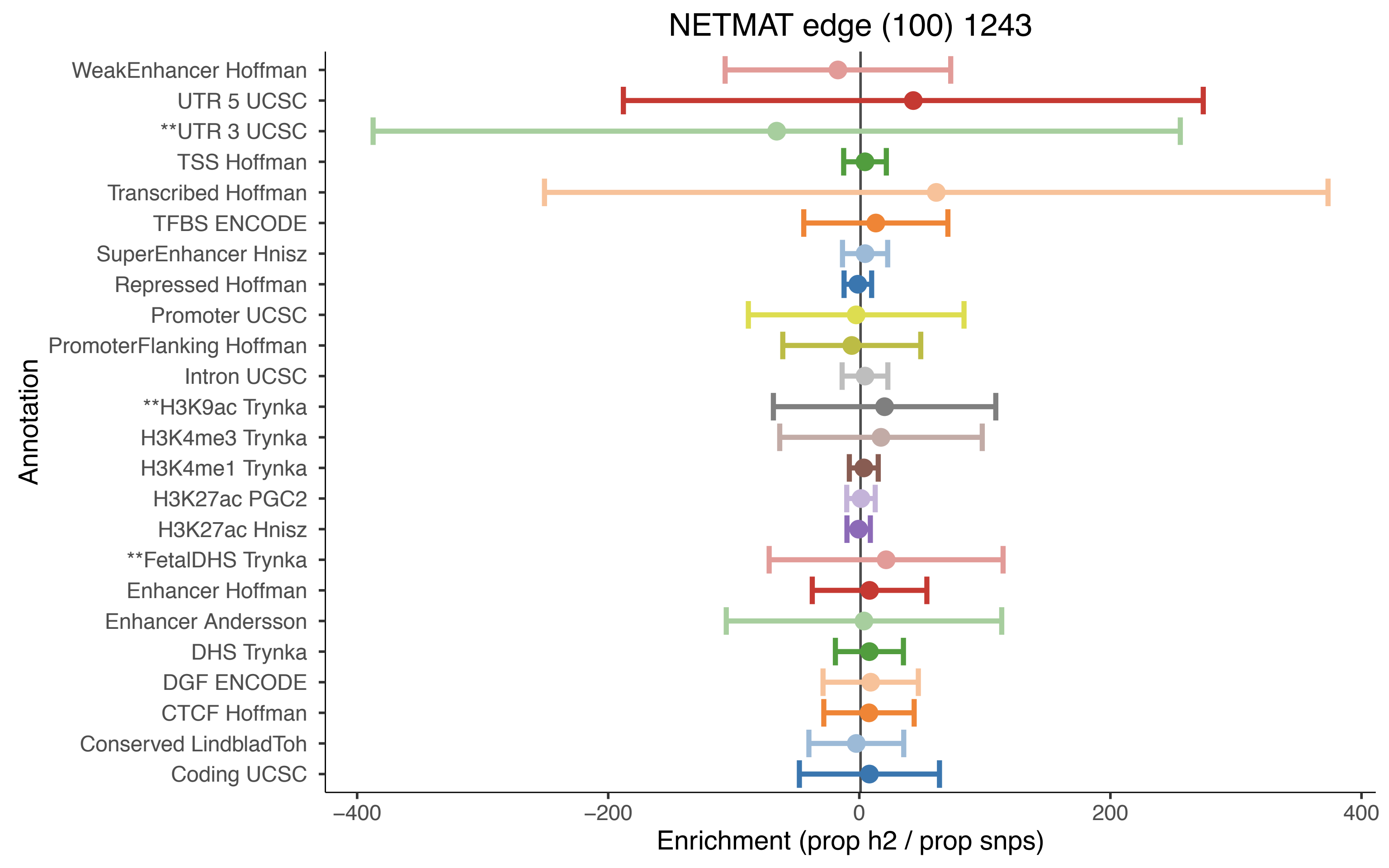
NETMAT edge (100) 1243
WeakEnhancer Hoffman
UTR 5 UCSC
**UTR 3 UCSC
TSS Hoffman
Transcribed Hoffman
TFBS ENCODE
SuperEnhancer Hnisz
Repressed Hoffman
Promoter UCSC
PromoterFlanking Hoffman
Intron UCSC
**H3K9ac Trynka
H3K4me3 Trynka
H3K4me1 Trynka
H3K27ac PGC2
H3K27ac Hnisz
**FetalDHS Trynka
Enhancer Hoffman
Enhancer Andersson
DHS Trynka
DGF ENCODE
CTCF Hoffman
Conserved LindbladToh
Coding UCSC
Annotation
−400
−200
0
200
400
Enrichment (prop h2 / prop snps)

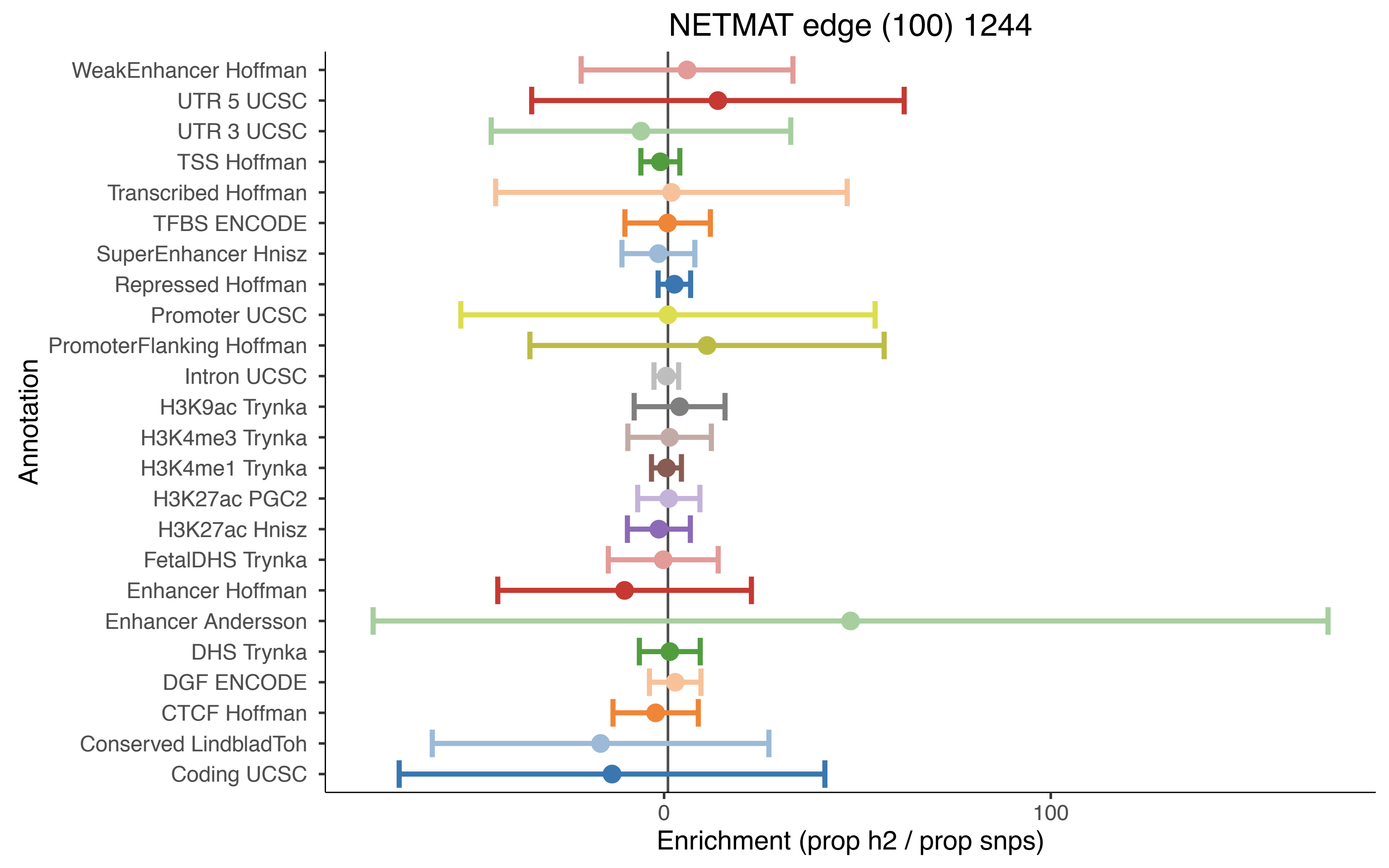
NETMAT edge (100) 1244
WeakEnhancer Hoffman
UTR 5 UCSC
UTR 3 UCSC
TSS Hoffman
Transcribed Hoffman
TFBS ENCODE
SuperEnhancer Hnisz
Repressed Hoffman
Promoter UCSC
PromoterFlanking Hoffman
Intron UCSC
H3K9ac Trynka
H3K4me3 Trynka
H3K4me1 Trynka
H3K27ac PGC2
H3K27ac Hnisz
FetalDHS Trynka
Enhancer Hoffman
Enhancer Andersson
DHS Trynka
DGF ENCODE
CTCF Hoffman
Conserved LindbladToh
Coding UCSC
Annotation
0
100
Enrichment (prop h2 / prop snps)

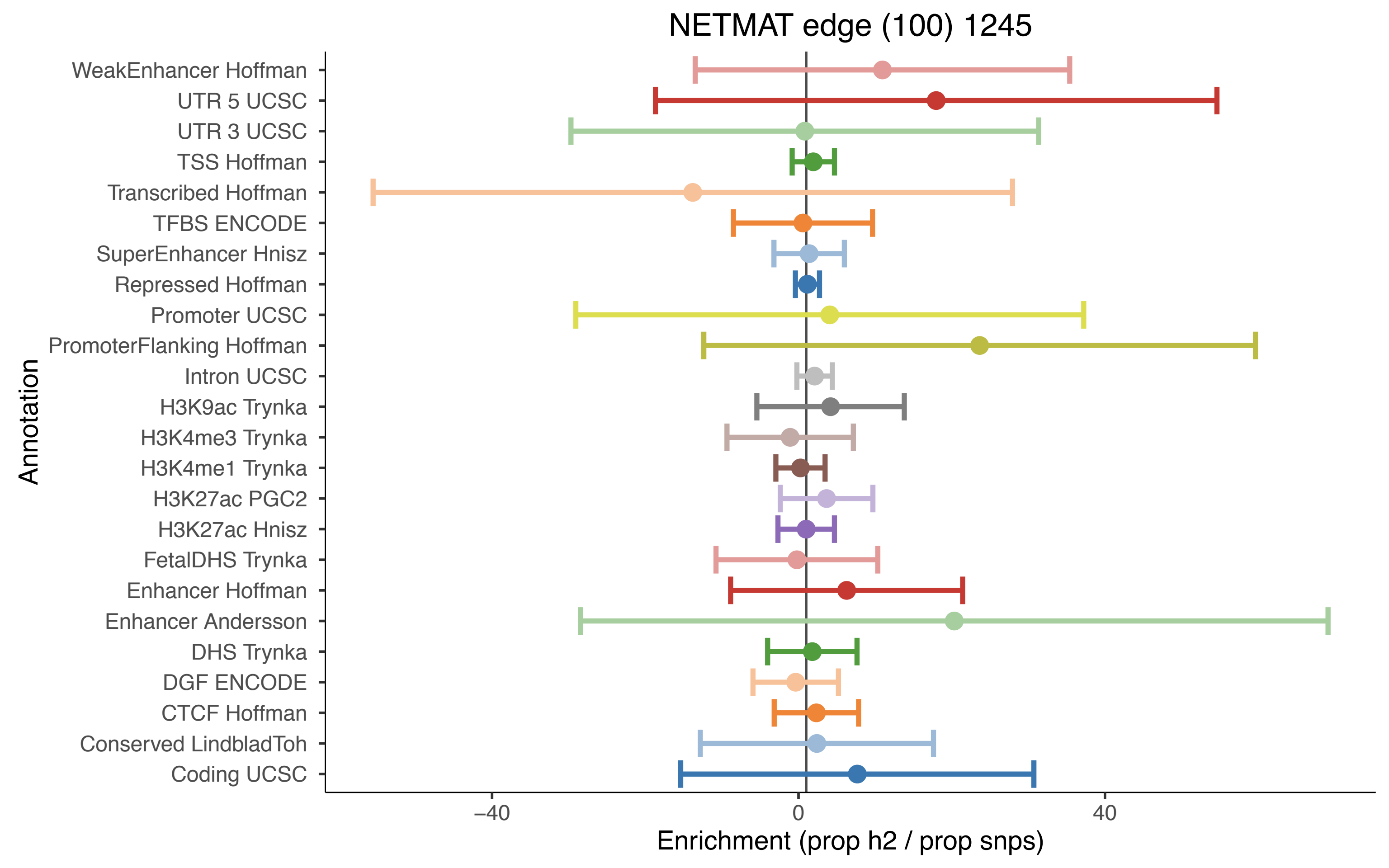
NETMAT edge (100) 1245
WeakEnhancer Hoffman
UTR 5 UCSC
UTR 3 UCSC
TSS Hoffman
Transcribed Hoffman
TFBS ENCODE
SuperEnhancer Hnisz
Repressed Hoffman
Promoter UCSC
PromoterFlanking Hoffman
Intron UCSC
H3K9ac Trynka
H3K4me3 Trynka
H3K4me1 Trynka
H3K27ac PGC2
H3K27ac Hnisz
FetalDHS Trynka
Enhancer Hoffman
Enhancer Andersson
DHS Trynka
DGF ENCODE
CTCF Hoffman
Conserved LindbladToh
Coding UCSC
Annotation
−40
0
40
Enrichment (prop h2 / prop snps)

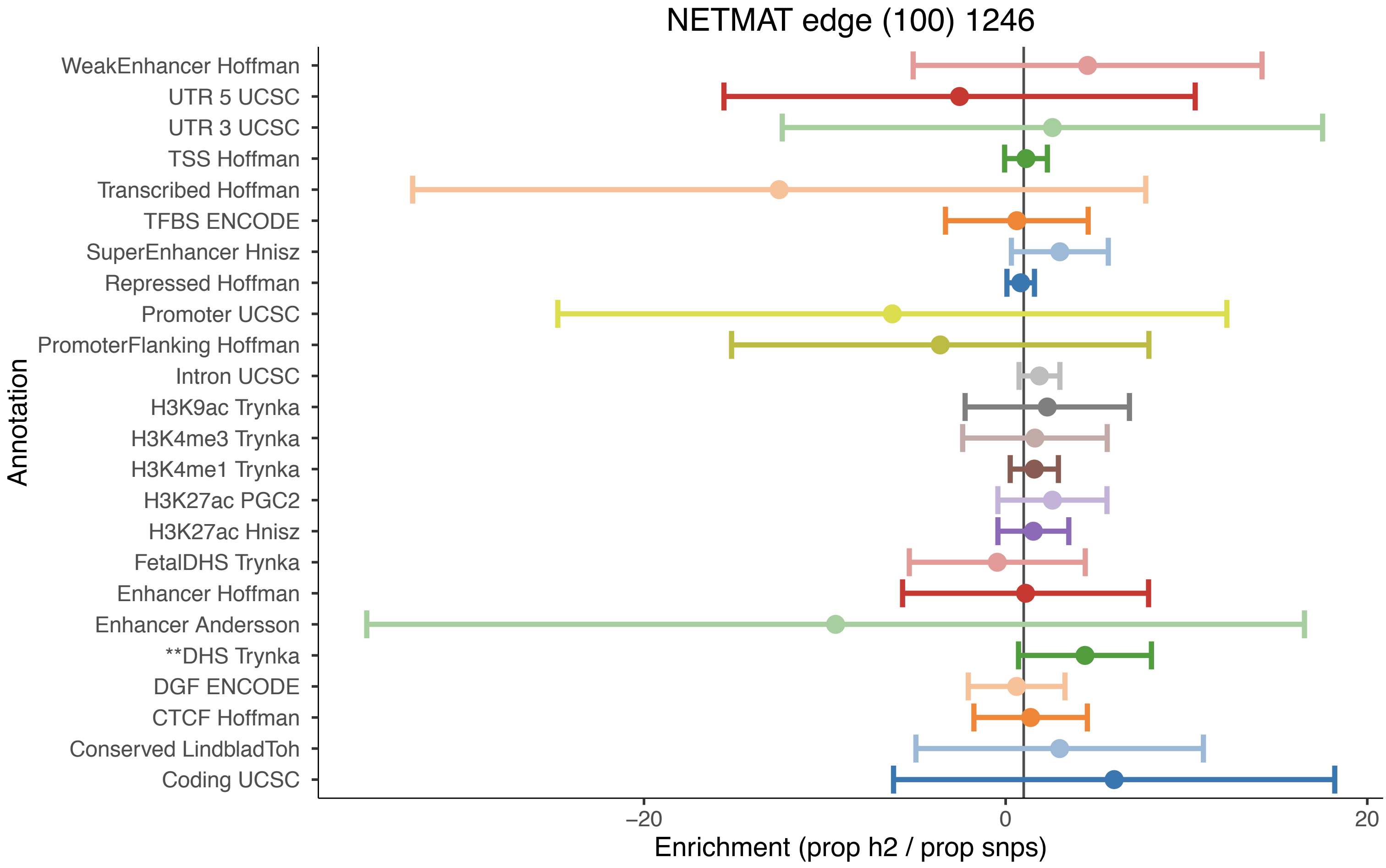
NETMAT edge (100) 1246
WeakEnhancer Hoffman
UTR 5 UCSC
UTR 3 UCSC
TSS Hoffman
Transcribed Hoffman
TFBS ENCODE
SuperEnhancer Hnisz
Repressed Hoffman
Promoter UCSC
PromoterFlanking Hoffman
Intron UCSC
H3K9ac Trynka
H3K4me3 Trynka
H3K4me1 Trynka
H3K27ac PGC2
H3K27ac Hnisz
FetalDHS Trynka
Enhancer Hoffman
Enhancer Andersson
**DHS Trynka
DGF ENCODE
CTCF Hoffman
Conserved LindbladToh
Coding UCSC
Annotation
−20
0
20
Enrichment (prop h2 / prop snps)

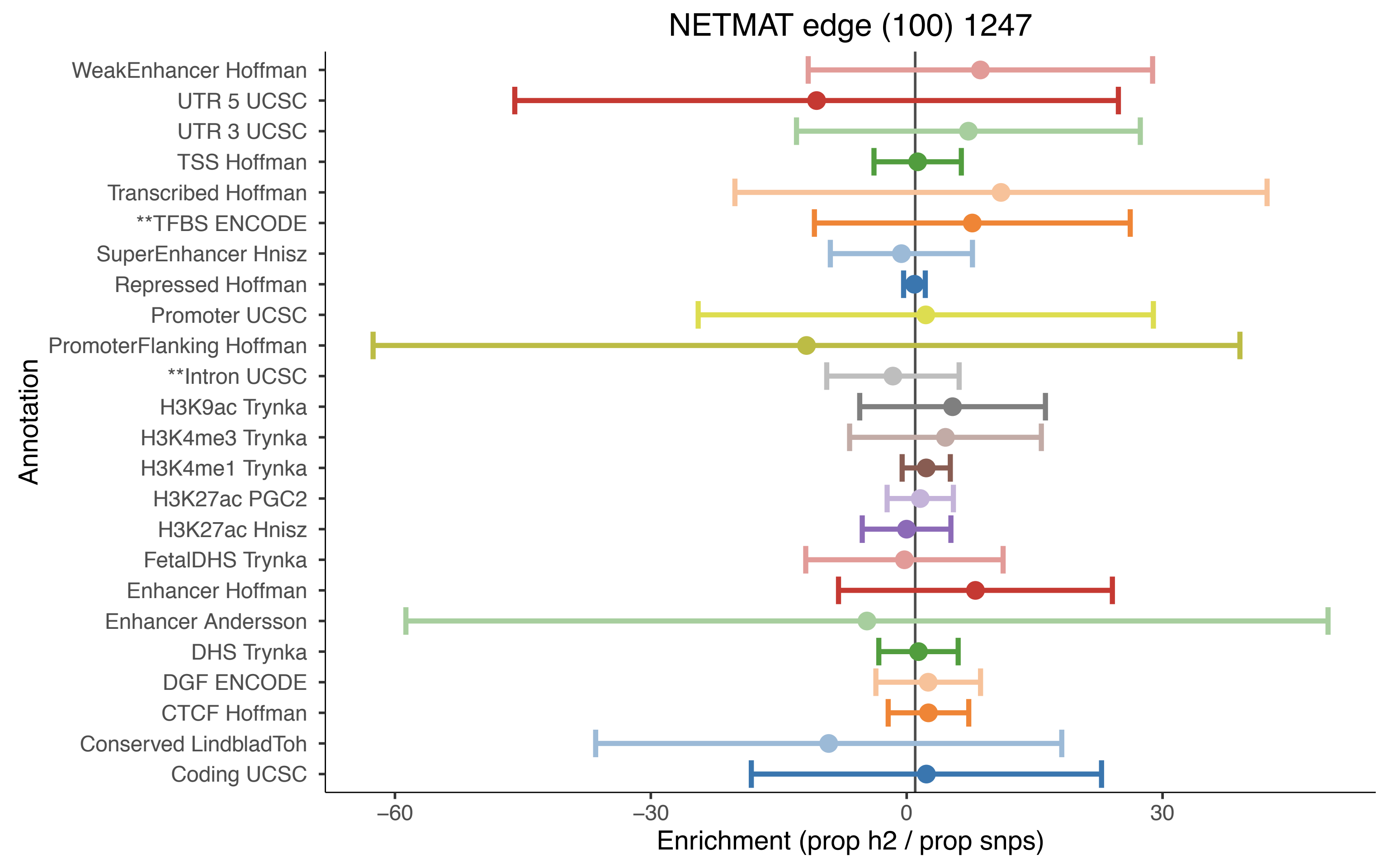

NETMAT edge (100) 1247
WeakEnhancer Hoffman
UTR 5 UCSC
UTR 3 UCSC
TSS Hoffman
Transcribed Hoffman
**TFBS ENCODE
SuperEnhancer Hnisz
Repressed Hoffman
Promoter UCSC
PromoterFlanking Hoffman
**Intron UCSC
H3K9ac Trynka
H3K4me3 Trynka
H3K4me1 Trynka
H3K27ac PGC2
H3K27ac Hnisz
FetalDHS Trynka
Enhancer Hoffman
Enhancer Andersson
DHS Trynka
DGF ENCODE
CTCF Hoffman
Conserved LindbladToh
Coding UCSC
Annotation
−60
−30
0
30
Enrichment (prop h2 / prop snps)

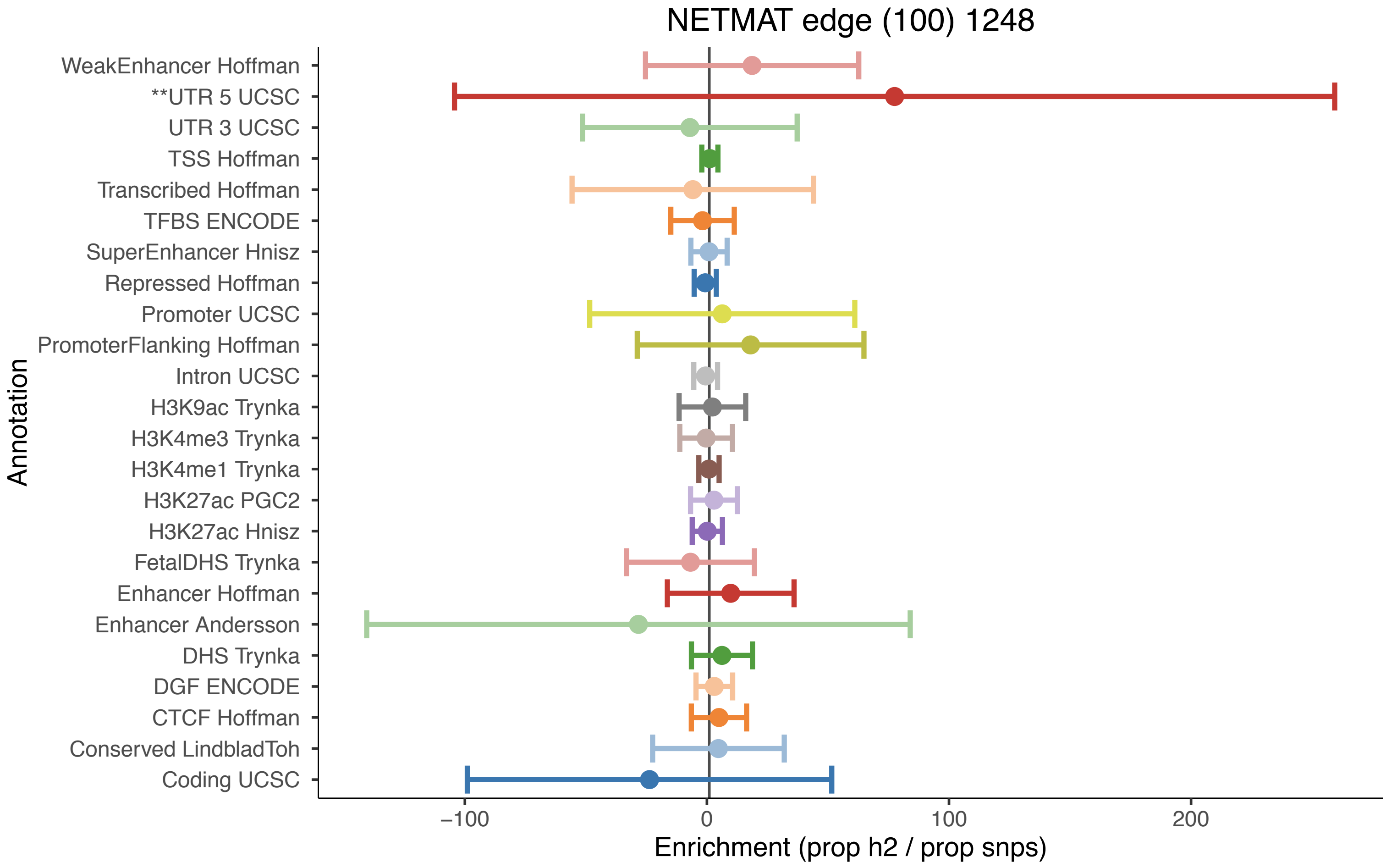

NETMAT edge (100) 1248
WeakEnhancer Hoffman
**UTR 5 UCSC
UTR 3 UCSC
TSS Hoffman
Transcribed Hoffman
TFBS ENCODE
SuperEnhancer Hnisz
Repressed Hoffman
Promoter UCSC
PromoterFlanking Hoffman
Intron UCSC
H3K9ac Trynka
H3K4me3 Trynka
H3K4me1 Trynka
H3K27ac PGC2
H3K27ac Hnisz
FetalDHS Trynka
Enhancer Hoffman
Enhancer Andersson
DHS Trynka
DGF ENCODE
CTCF Hoffman
Conserved LindbladToh
Coding UCSC
Annotation
−100
0
100
200
Enrichment (prop h2 / prop snps)

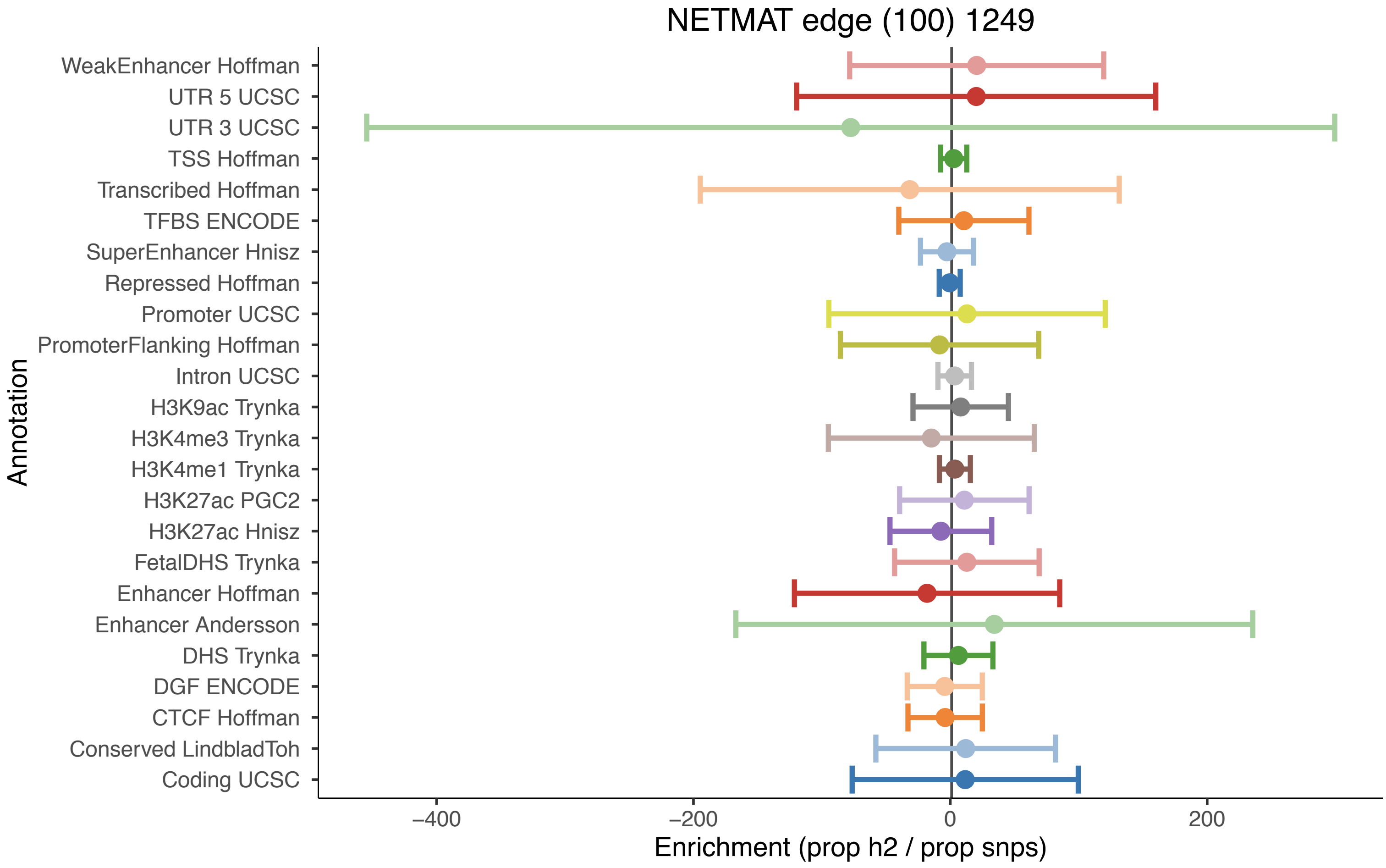
NETMAT edge (100) 1249
Annotation
WeakEnhancer Hoffman
UTR 5 UCSC
UTR 3 UCSC
TSS Hoffman
Transcribed Hoffman
TFBS ENCODE
SuperEnhancer Hnisz
Repressed Hoffman
Promoter UCSC
PromoterFlanking Hoffman
Intron UCSC
H3K9ac Trynka
H3K4me3 Trynka
H3K4me1 Trynka
H3K27ac PGC2
H3K27ac Hnisz
FetalDHS Trynka
Enhancer Hoffman
Enhancer Andersson
DHS Trynka
DGF ENCODE
CTCF Hoffman
Conserved LindbladToh
Coding UCSC
−400
−200
0
200
Enrichment (prop h2 / prop snps)

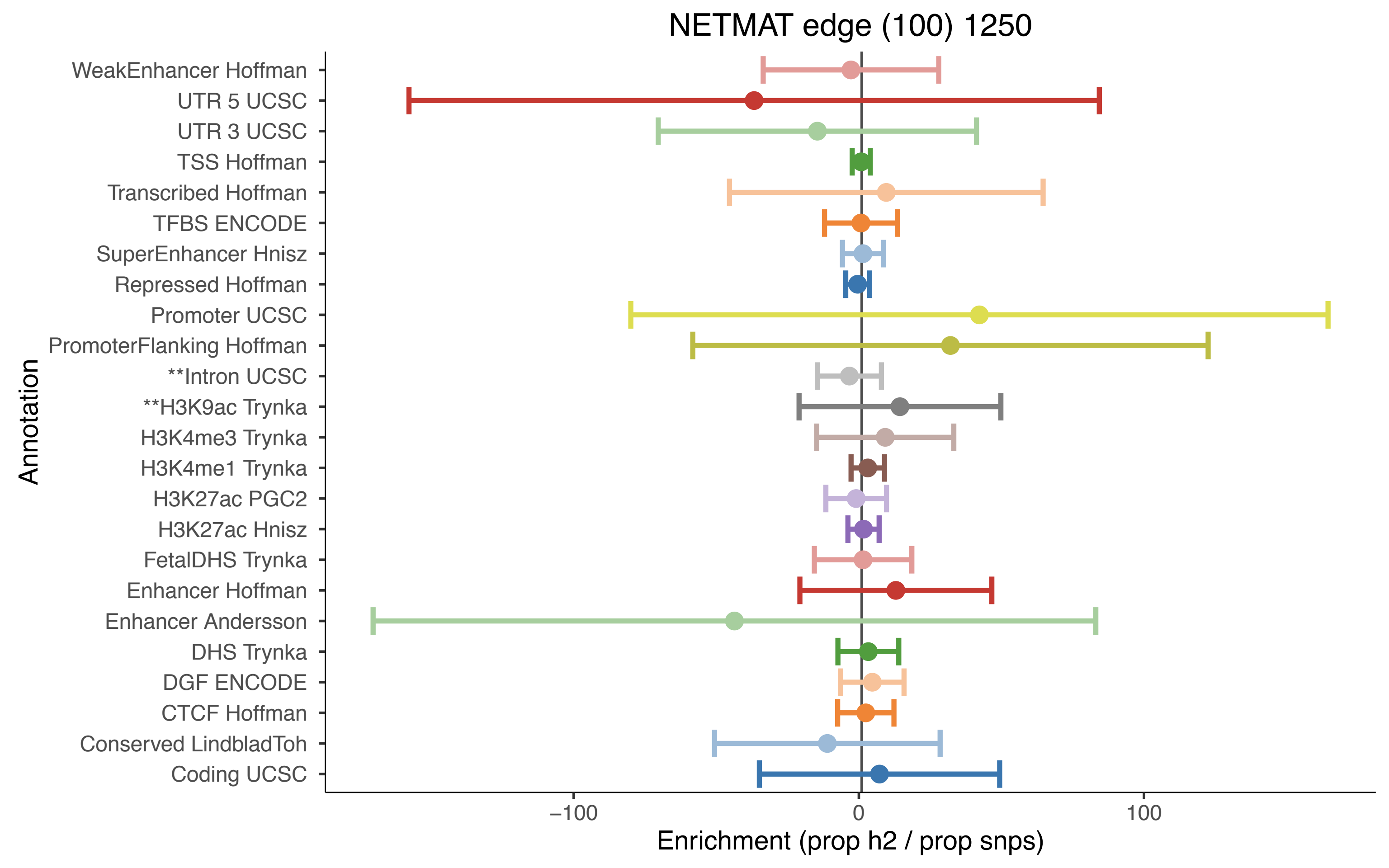
NETMAT edge (100) 1250
WeakEnhancer Hoffman
UTR 5 UCSC
UTR 3 UCSC
TSS Hoffman
Transcribed Hoffman
TFBS ENCODE
SuperEnhancer Hnisz
Repressed Hoffman
Promoter UCSC
PromoterFlanking Hoffman
**Intron UCSC
**H3K9ac Trynka
H3K4me3 Trynka
H3K4me1 Trynka
H3K27ac PGC2
H3K27ac Hnisz
FetalDHS Trynka
Enhancer Hoffman
Enhancer Andersson
DHS Trynka
DGF ENCODE
CTCF Hoffman
Conserved LindbladToh
Coding UCSC
Annotation
−100
0
100
Enrichment (prop h2 / prop snps)

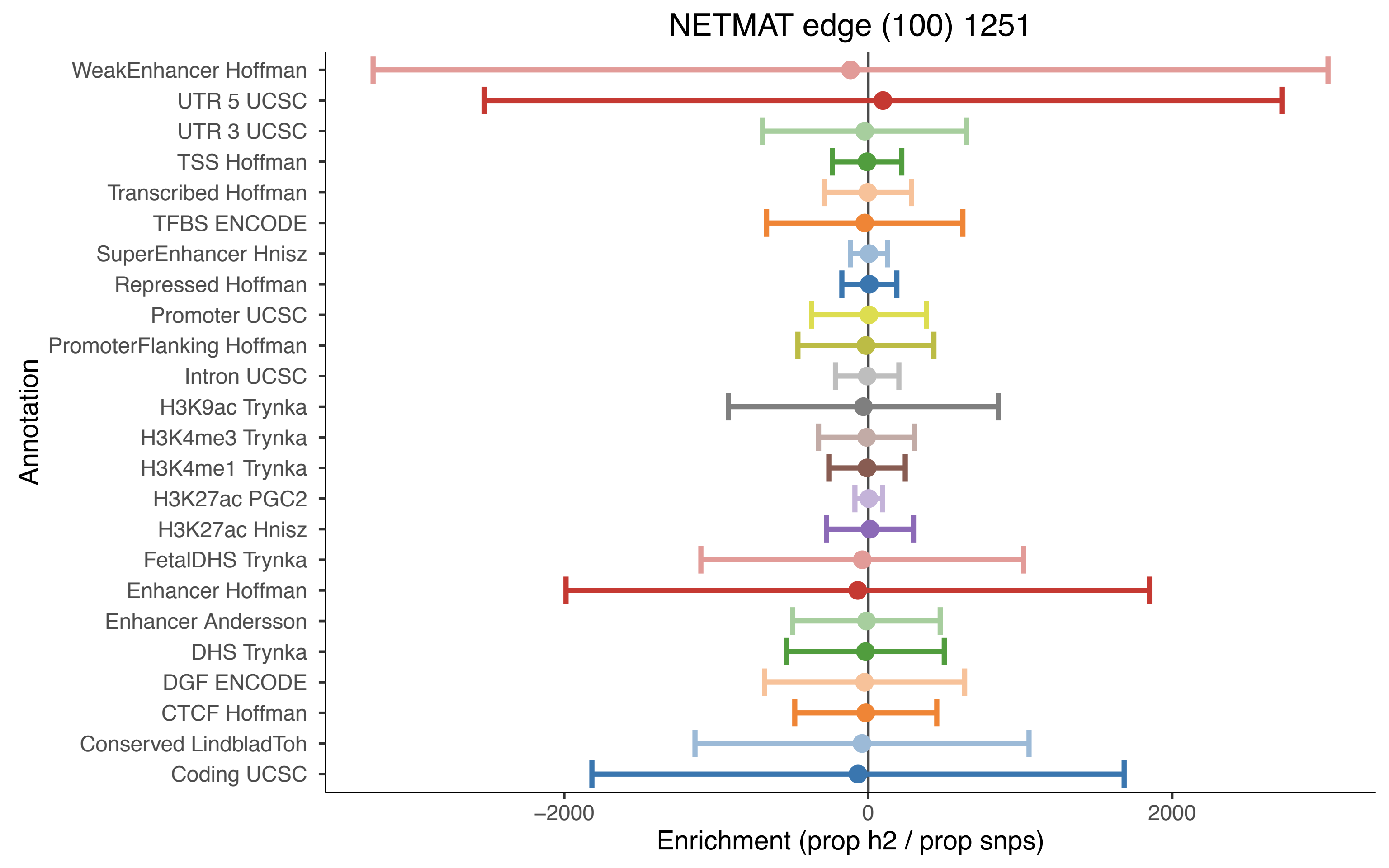
NETMAT edge (100) 1251
WeakEnhancer Hoffman
UTR 5 UCSC
UTR 3 UCSC
TSS Hoffman
Transcribed Hoffman
TFBS ENCODE
SuperEnhancer Hnisz
Repressed Hoffman
Promoter UCSC
PromoterFlanking Hoffman
Intron UCSC
H3K9ac Trynka
H3K4me3 Trynka
H3K4me1 Trynka
H3K27ac PGC2
H3K27ac Hnisz
FetalDHS Trynka
Enhancer Hoffman
Enhancer Andersson
DHS Trynka
DGF ENCODE
CTCF Hoffman
Conserved LindbladToh
Coding UCSC
Annotation
−2000
0
2000
Enrichment (prop h2 / prop snps)

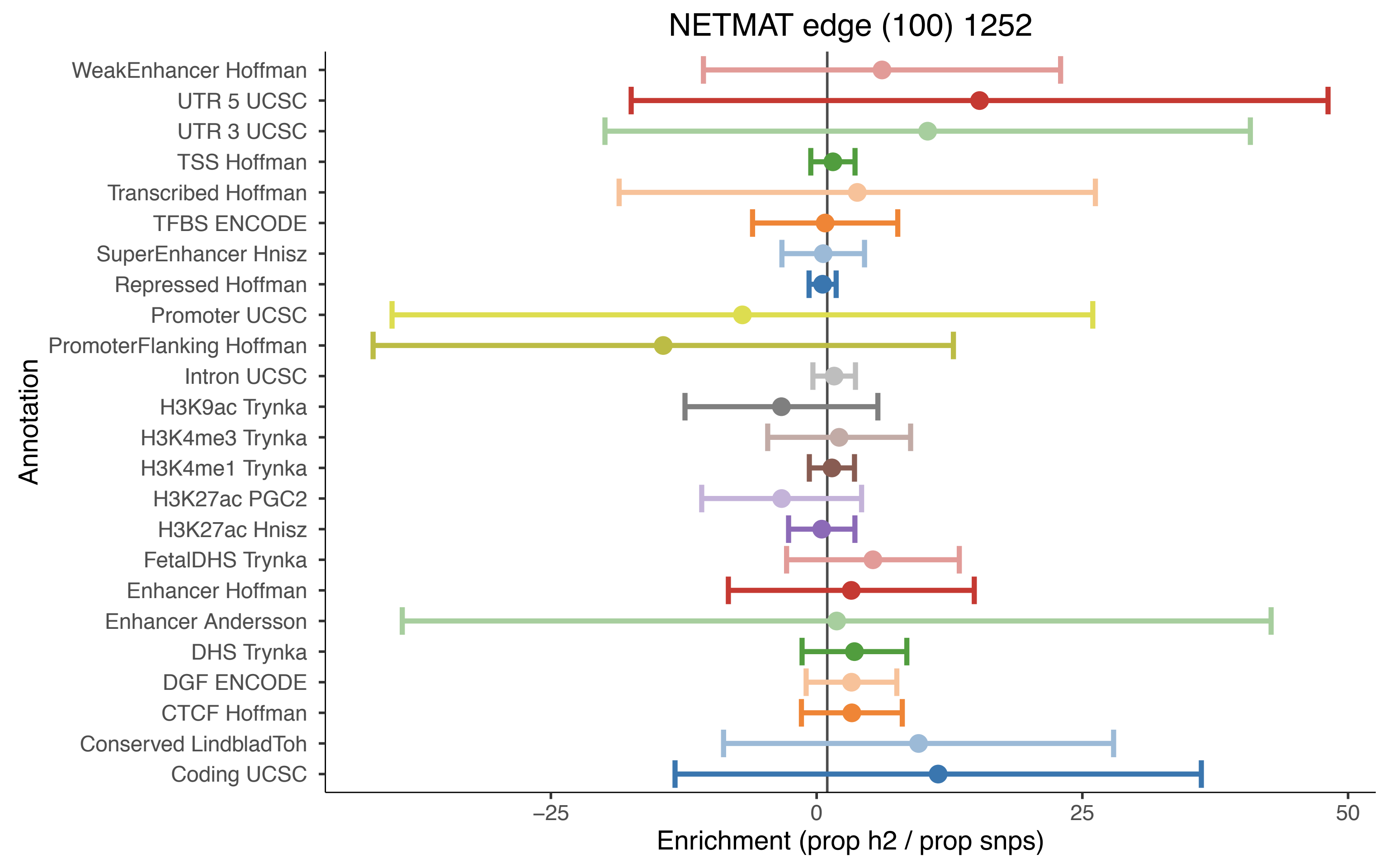

NETMAT edge (100) 1252
Annotation
WeakEnhancer Hoffman
UTR 5 UCSC
UTR 3 UCSC
TSS Hoffman
Transcribed Hoffman
TFBS ENCODE
SuperEnhancer Hnisz
Repressed Hoffman
Promoter UCSC
PromoterFlanking Hoffman
Intron UCSC
H3K9ac Trynka
H3K4me3 Trynka
H3K4me1 Trynka
H3K27ac PGC2
H3K27ac Hnisz
FetalDHS Trynka
Enhancer Hoffman
Enhancer Andersson
DHS Trynka
DGF ENCODE
CTCF Hoffman
Conserved LindbladToh
Coding UCSC
−25
0
25
50
Enrichment (prop h2 / prop snps)

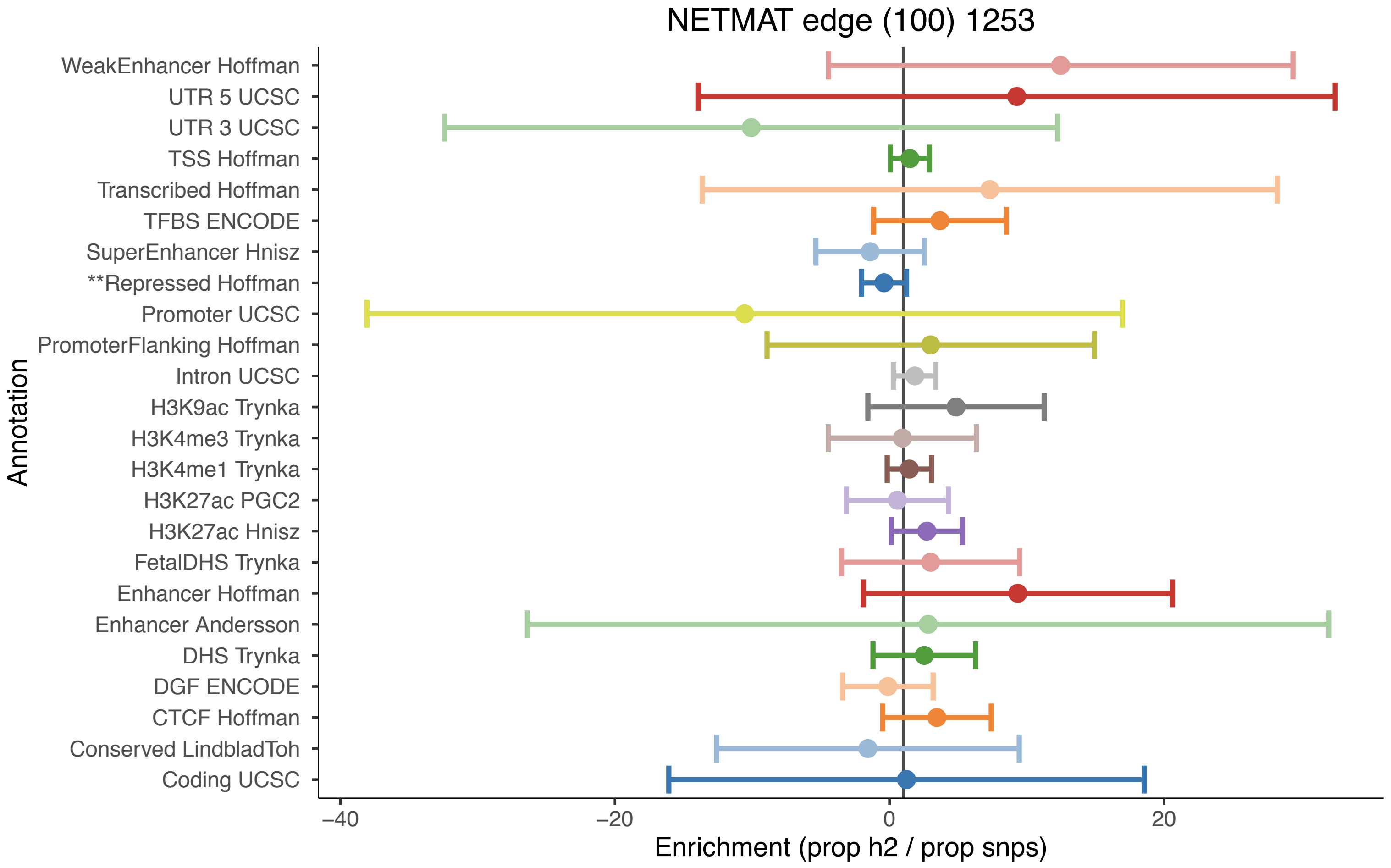

NETMAT edge (100) 1253
WeakEnhancer Hoffman
UTR 5 UCSC
UTR 3 UCSC
TSS Hoffman
Transcribed Hoffman
TFBS ENCODE
SuperEnhancer Hnisz
**Repressed Hoffman
Promoter UCSC
PromoterFlanking Hoffman
Intron UCSC
H3K9ac Trynka
H3K4me3 Trynka
H3K4me1 Trynka
H3K27ac PGC2
H3K27ac Hnisz
FetalDHS Trynka
Enhancer Hoffman
Enhancer Andersson
DHS Trynka
DGF ENCODE
CTCF Hoffman
Conserved LindbladToh
Coding UCSC
Annotation
−40
−20
0
20
Enrichment (prop h2 / prop snps)

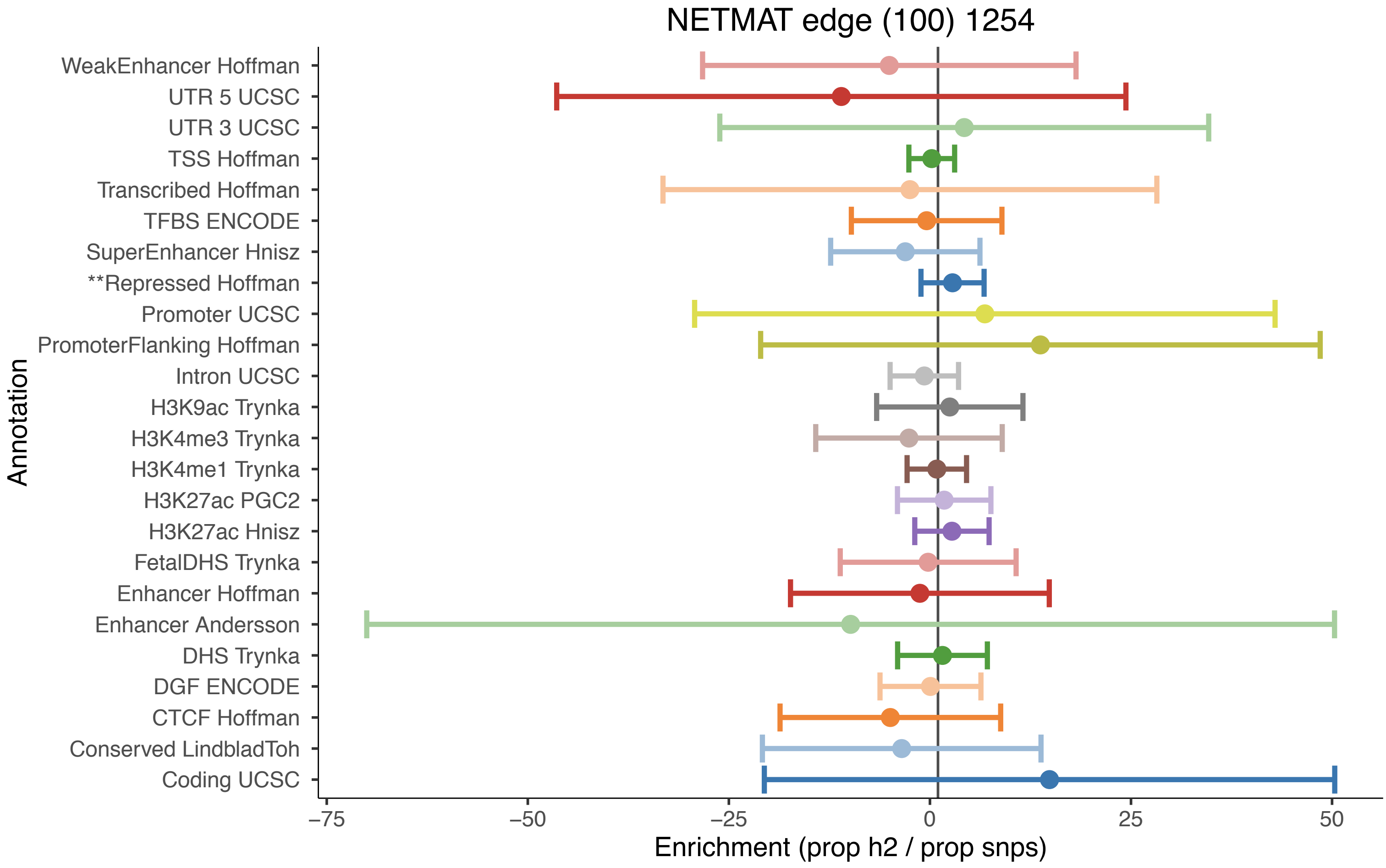
NETMAT edge (100) 1254
WeakEnhancer Hoffman
UTR 5 UCSC
UTR 3 UCSC
TSS Hoffman
Transcribed Hoffman
TFBS ENCODE
SuperEnhancer Hnisz
**Repressed Hoffman
Promoter UCSC
PromoterFlanking Hoffman
Intron UCSC
H3K9ac Trynka
H3K4me3 Trynka
H3K4me1 Trynka
H3K27ac PGC2
H3K27ac Hnisz
FetalDHS Trynka
Enhancer Hoffman
Enhancer Andersson
DHS Trynka
DGF ENCODE
CTCF Hoffman
Conserved LindbladToh
Coding UCSC
Annotation
−75
−50
−25
0
25
50
Enrichment (prop h2 / prop snps)

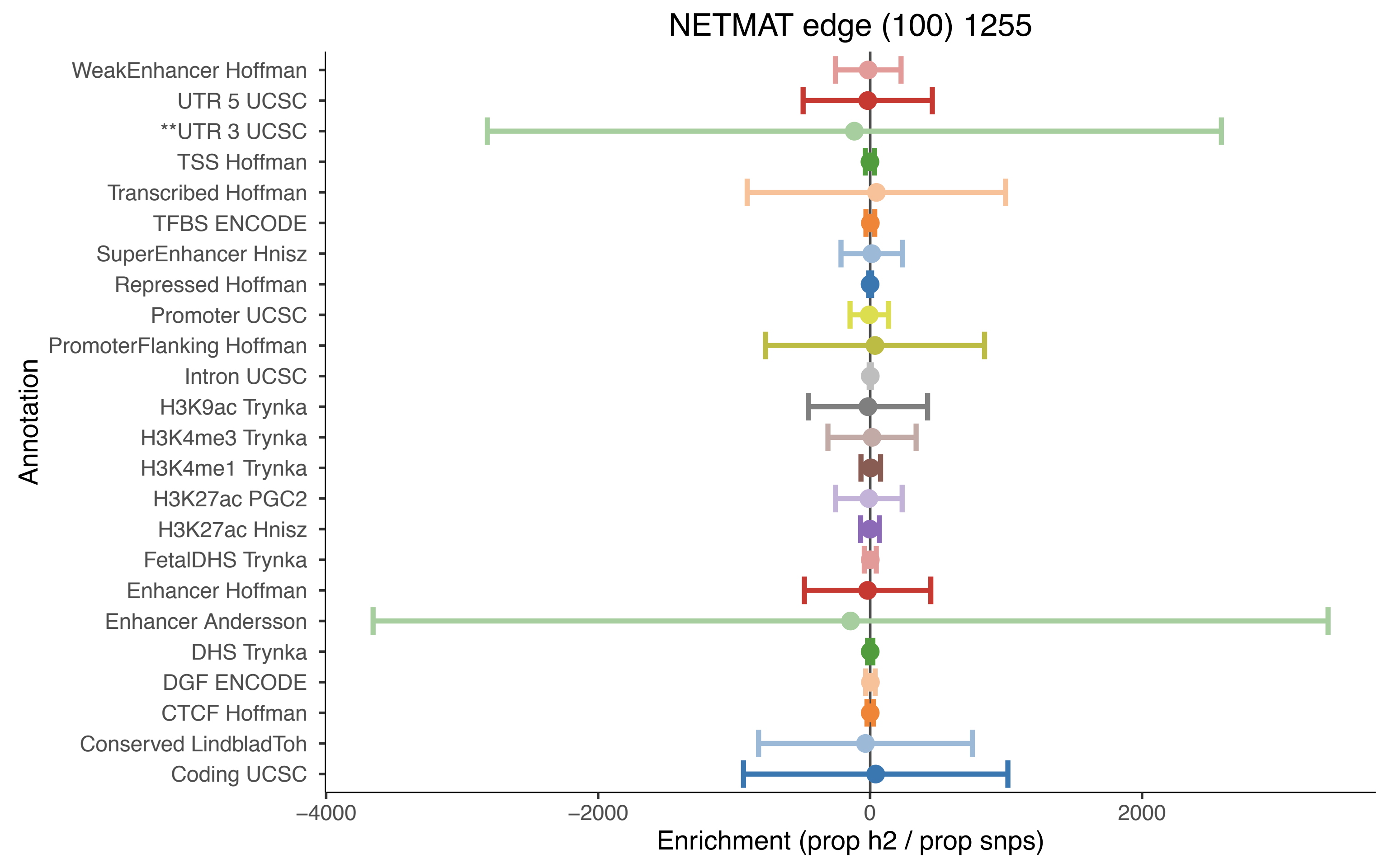
NETMAT edge (100) 1255
WeakEnhancer Hoffman
UTR 5 UCSC
**UTR 3 UCSC
TSS Hoffman
Transcribed Hoffman
TFBS ENCODE
SuperEnhancer Hnisz
Repressed Hoffman
Promoter UCSC
PromoterFlanking Hoffman
Intron UCSC
H3K9ac Trynka
H3K4me3 Trynka
H3K4me1 Trynka
H3K27ac PGC2
H3K27ac Hnisz
FetalDHS Trynka
Enhancer Hoffman
Enhancer Andersson
DHS Trynka
DGF ENCODE
CTCF Hoffman
Conserved LindbladToh
Coding UCSC
Annotation
−4000
−2000
0
2000
Enrichment (prop h2 / prop snps)

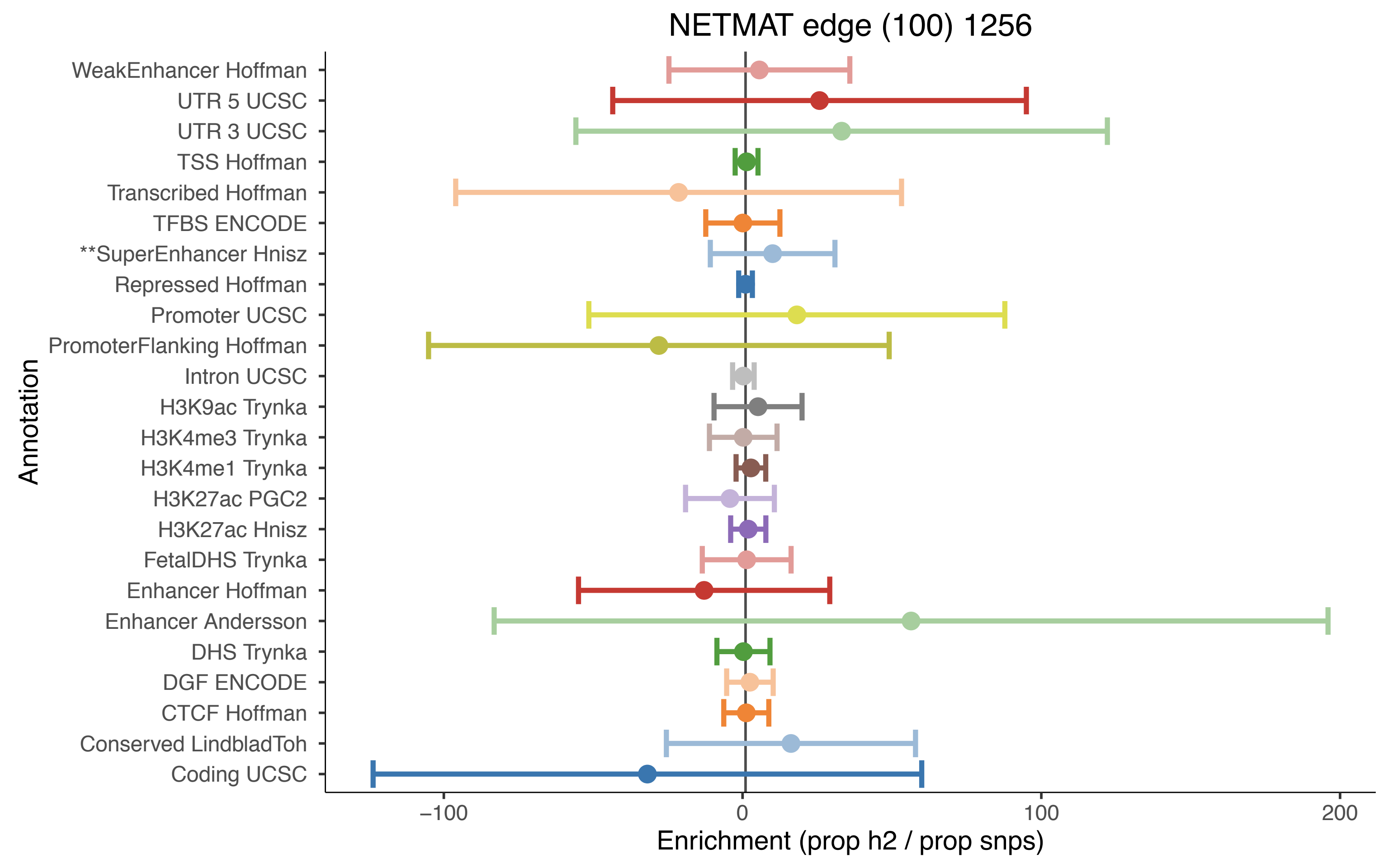
NETMAT edge (100) 1256
WeakEnhancer Hoffman
UTR 5 UCSC
UTR 3 UCSC
TSS Hoffman
Transcribed Hoffman
TFBS ENCODE
**SuperEnhancer Hnisz
Repressed Hoffman
Promoter UCSC
PromoterFlanking Hoffman
Intron UCSC
H3K9ac Trynka
H3K4me3 Trynka
H3K4me1 Trynka
H3K27ac PGC2
H3K27ac Hnisz
FetalDHS Trynka
Enhancer Hoffman
Enhancer Andersson
DHS Trynka
DGF ENCODE
CTCF Hoffman
Conserved LindbladToh
Coding UCSC
Annotation
−100
0
100
200
Enrichment (prop h2 / prop snps)

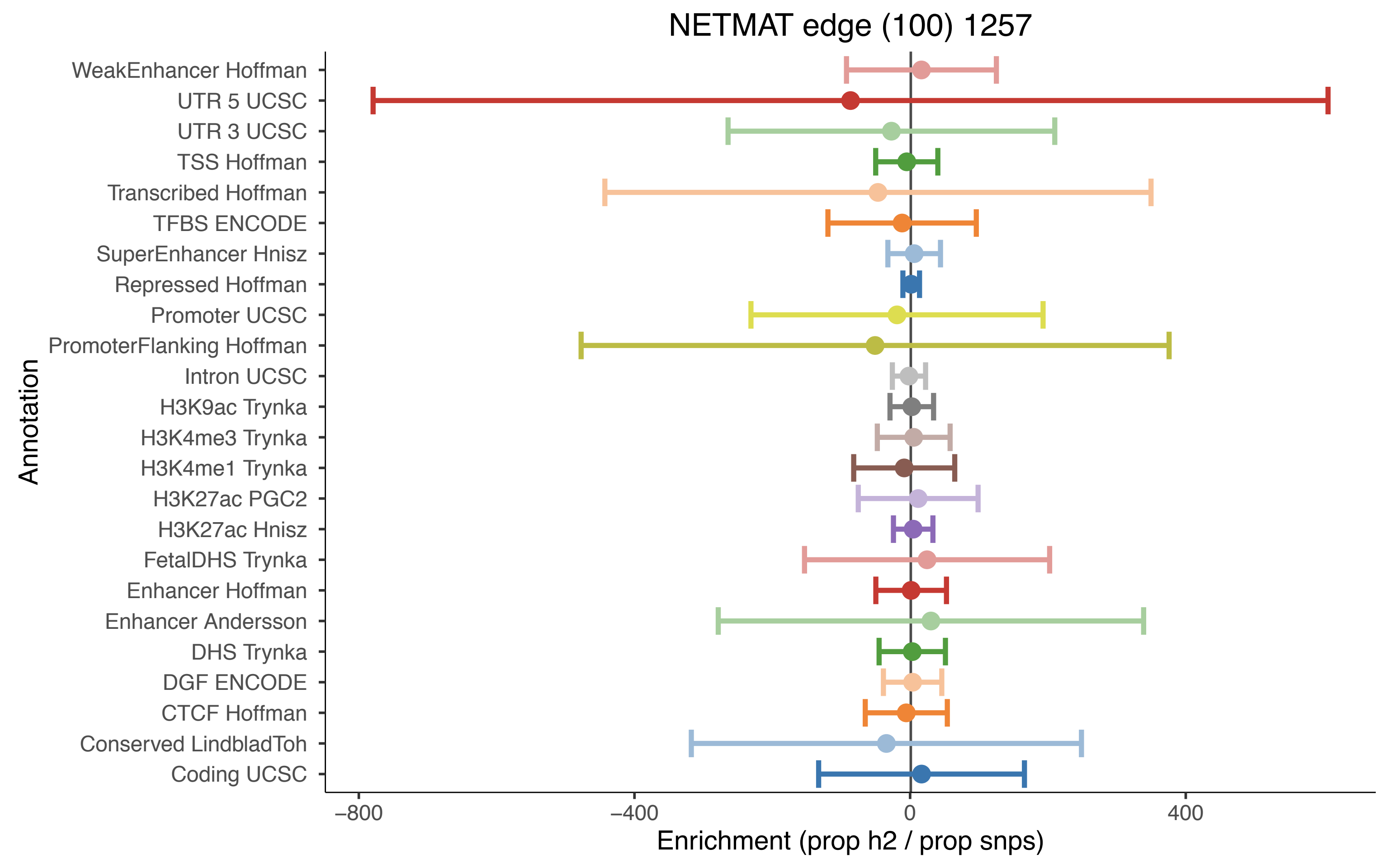
NETMAT edge (100) 1257
WeakEnhancer Hoffman
UTR 5 UCSC
UTR 3 UCSC
TSS Hoffman
Transcribed Hoffman
TFBS ENCODE
SuperEnhancer Hnisz
Repressed Hoffman
Promoter UCSC
PromoterFlanking Hoffman
Intron UCSC
H3K9ac Trynka
H3K4me3 Trynka
H3K4me1 Trynka
H3K27ac PGC2
H3K27ac Hnisz
FetalDHS Trynka
Enhancer Hoffman
Enhancer Andersson
DHS Trynka
DGF ENCODE
CTCF Hoffman
Conserved LindbladToh
Coding UCSC
Annotation
−800
−400
0
400
Enrichment (prop h2 / prop snps)

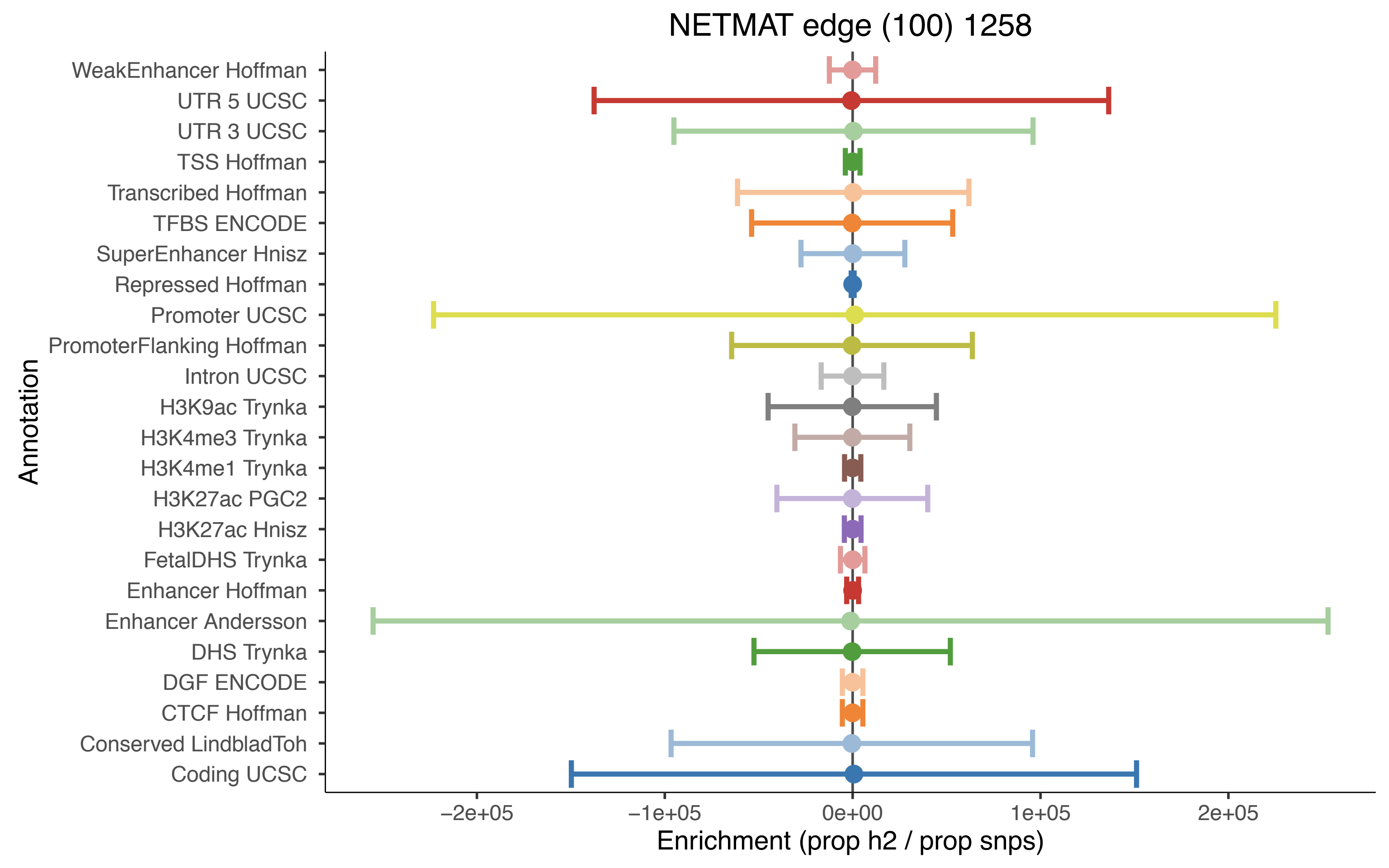
NETMAT edge (100) 1258
WeakEnhancer Hoffman
UTR 5 UCSC
UTR 3 UCSC
TSS Hoffman
Transcribed Hoffman
TFBS ENCODE
SuperEnhancer Hnisz
Repressed Hoffman
Promoter UCSC
PromoterFlanking Hoffman
Intron UCSC
H3K9ac Trynka
H3K4me3 Trynka
H3K4me1 Trynka
H3K27ac PGC2
H3K27ac Hnisz
FetalDHS Trynka
Enhancer Hoffman
Enhancer Andersson
DHS Trynka
DGF ENCODE
CTCF Hoffman
Conserved LindbladToh
Coding UCSC
Annotation
−2e+05
−1e+05
0e+00
1e+05
2e+05
Enrichment (prop h2 / prop snps)

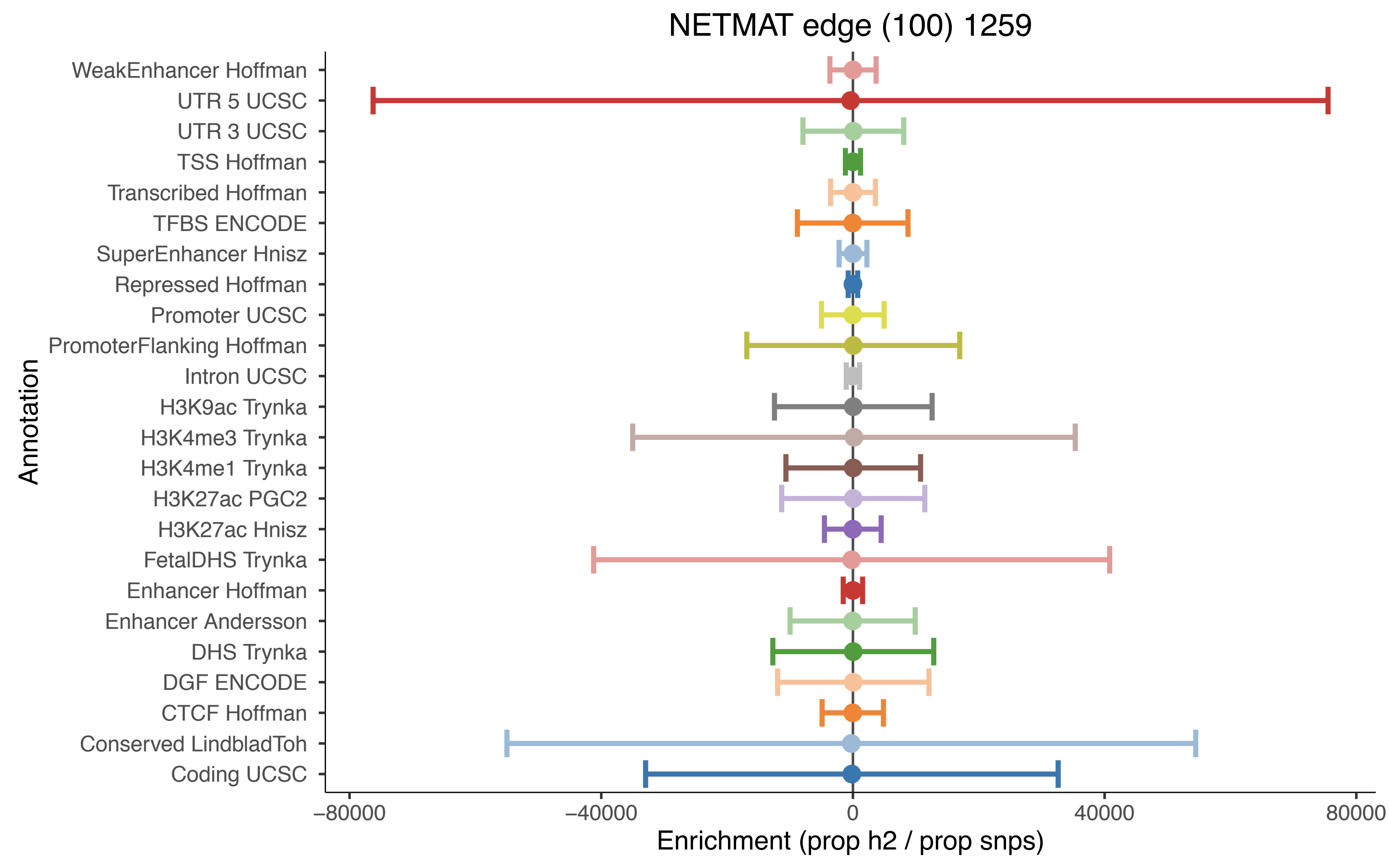

NETMAT edge (100) 1259
WeakEnhancer Hoffman
UTR 5 UCSC
UTR 3 UCSC
TSS Hoffman
Transcribed Hoffman
TFBS ENCODE
SuperEnhancer Hnisz
Repressed Hoffman
Promoter UCSC
PromoterFlanking Hoffman
Intron UCSC
H3K9ac Trynka
H3K4me3 Trynka
H3K4me1 Trynka
H3K27ac PGC2
H3K27ac Hnisz
FetalDHS Trynka
Enhancer Hoffman
Enhancer Andersson
DHS Trynka
DGF ENCODE
CTCF Hoffman
Conserved LindbladToh
Coding UCSC
Annotation
−80000
−40000
0
40000
80000
Enrichment (prop h2 / prop snps)

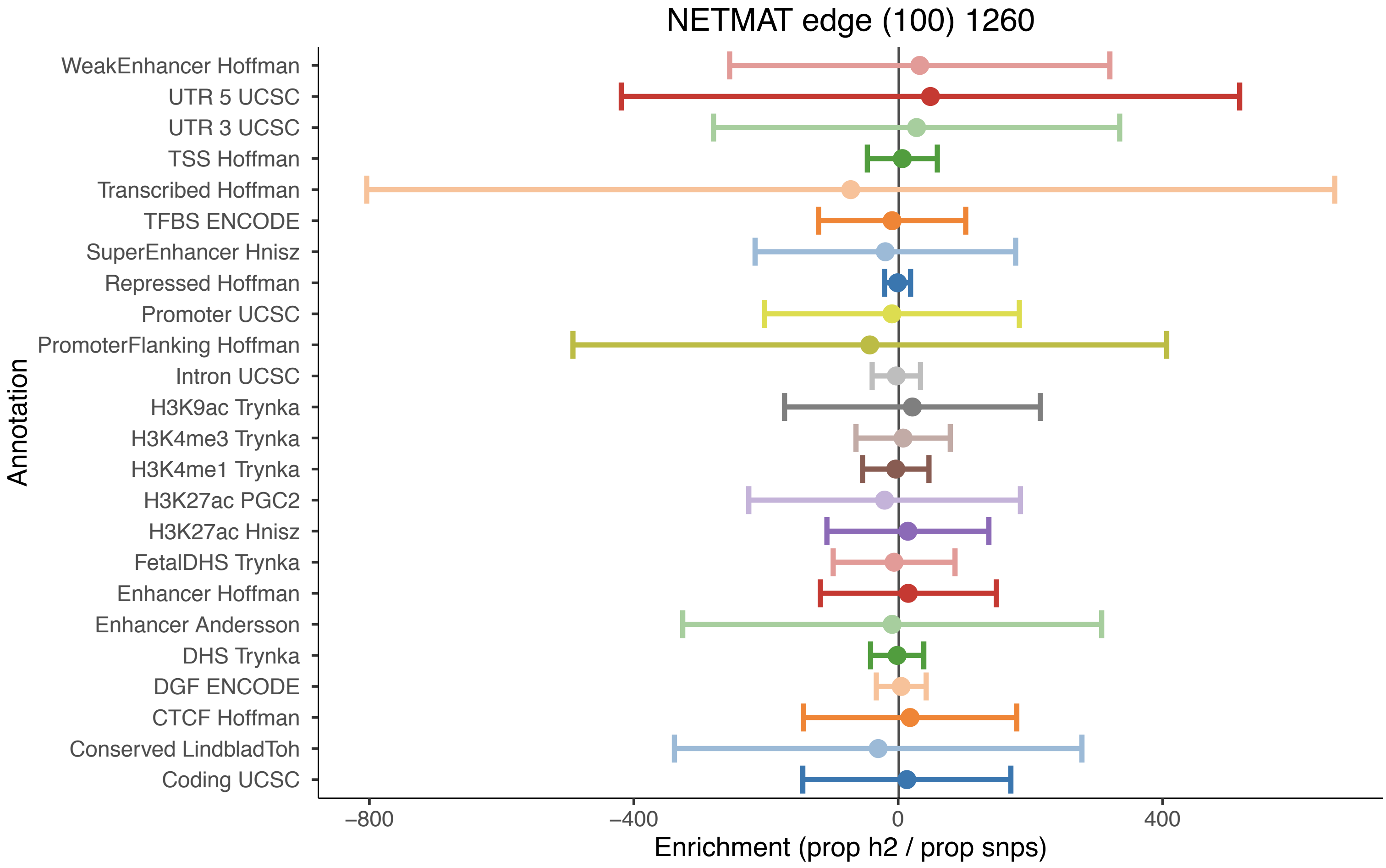
NETMAT edge (100) 1260
WeakEnhancer Hoffman
UTR 5 UCSC
UTR 3 UCSC
TSS Hoffman
Transcribed Hoffman
TFBS ENCODE
SuperEnhancer Hnisz
Repressed Hoffman
Promoter UCSC
PromoterFlanking Hoffman
Intron UCSC
H3K9ac Trynka
H3K4me3 Trynka
H3K4me1 Trynka
H3K27ac PGC2
H3K27ac Hnisz
FetalDHS Trynka
Enhancer Hoffman
Enhancer Andersson
DHS Trynka
DGF ENCODE
CTCF Hoffman
Conserved LindbladToh
Coding UCSC
Annotation
−800
−400
0
400
Enrichment (prop h2 / prop snps)

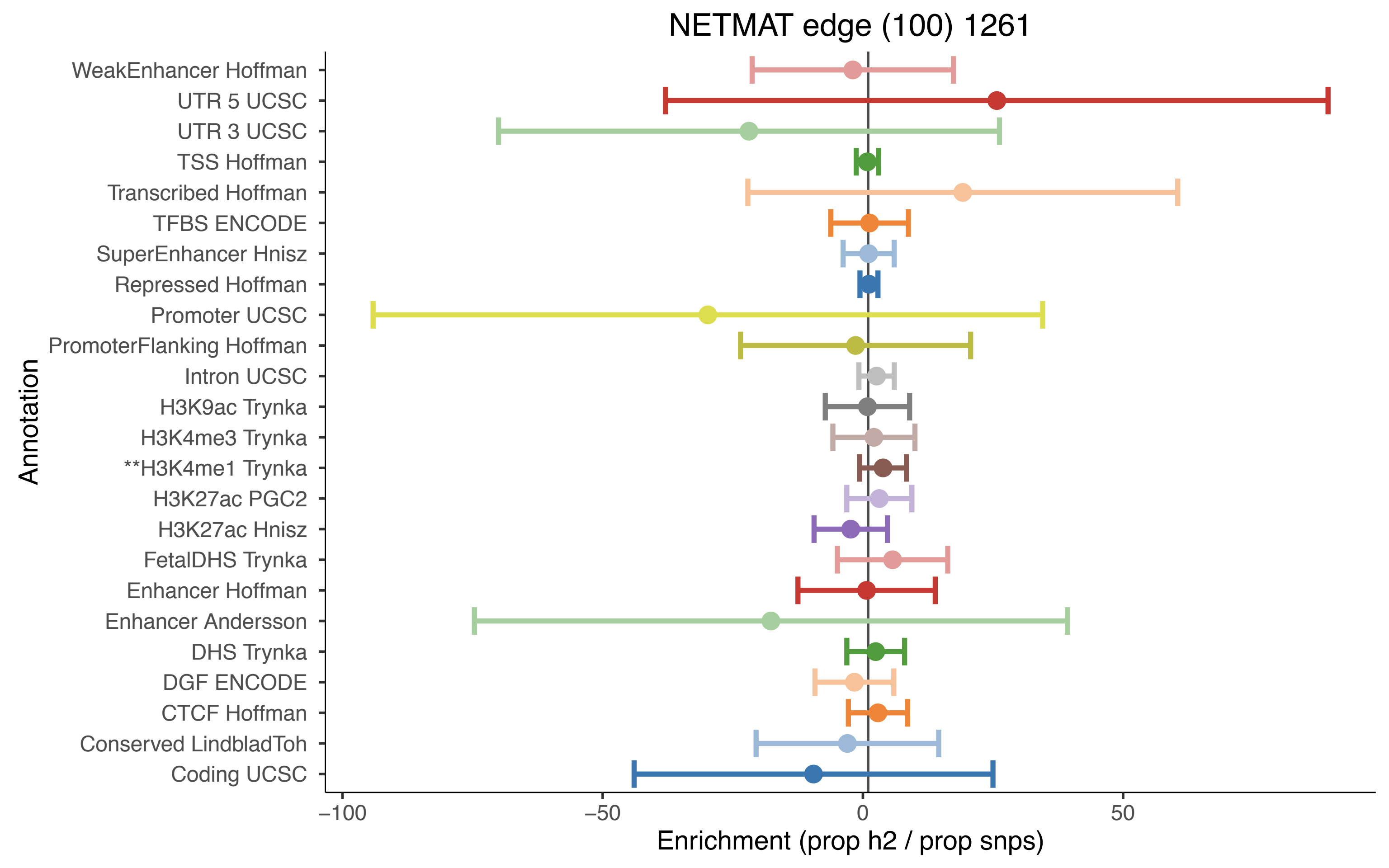
NETMAT edge (100) 1261
WeakEnhancer Hoffman
UTR 5 UCSC
UTR 3 UCSC
TSS Hoffman
Transcribed Hoffman
TFBS ENCODE
SuperEnhancer Hnisz
Repressed Hoffman
Promoter UCSC
PromoterFlanking Hoffman
Intron UCSC
H3K9ac Trynka
H3K4me3 Trynka
**H3K4me1 Trynka
H3K27ac PGC2
H3K27ac Hnisz
FetalDHS Trynka
Enhancer Hoffman
Enhancer Andersson
DHS Trynka
DGF ENCODE
CTCF Hoffman
Conserved LindbladToh
Coding UCSC
Annotation
−100
−50
0
50
Enrichment (prop h2 / prop snps)

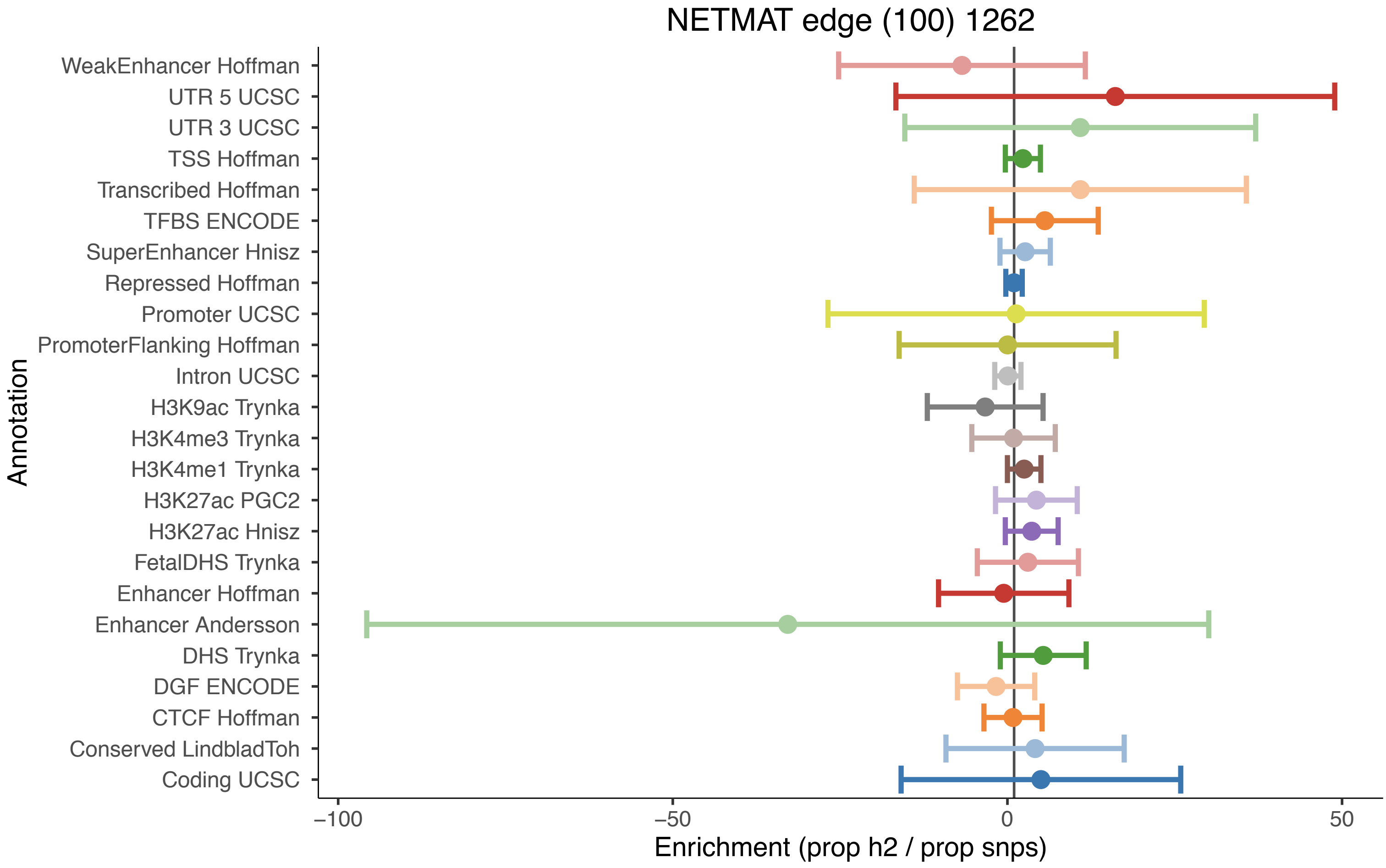
NETMAT edge (100) 1262
WeakEnhancer Hoffman
UTR 5 UCSC
UTR 3 UCSC
TSS Hoffman
Transcribed Hoffman
TFBS ENCODE
SuperEnhancer Hnisz
Repressed Hoffman
Promoter UCSC
PromoterFlanking Hoffman
Intron UCSC
H3K9ac Trynka
H3K4me3 Trynka
H3K4me1 Trynka
H3K27ac PGC2
H3K27ac Hnisz
FetalDHS Trynka
Enhancer Hoffman
Enhancer Andersson
DHS Trynka
DGF ENCODE
CTCF Hoffman
Conserved LindbladToh
Coding UCSC
Annotation
−100
−50
0
50
Enrichment (prop h2 / prop snps)

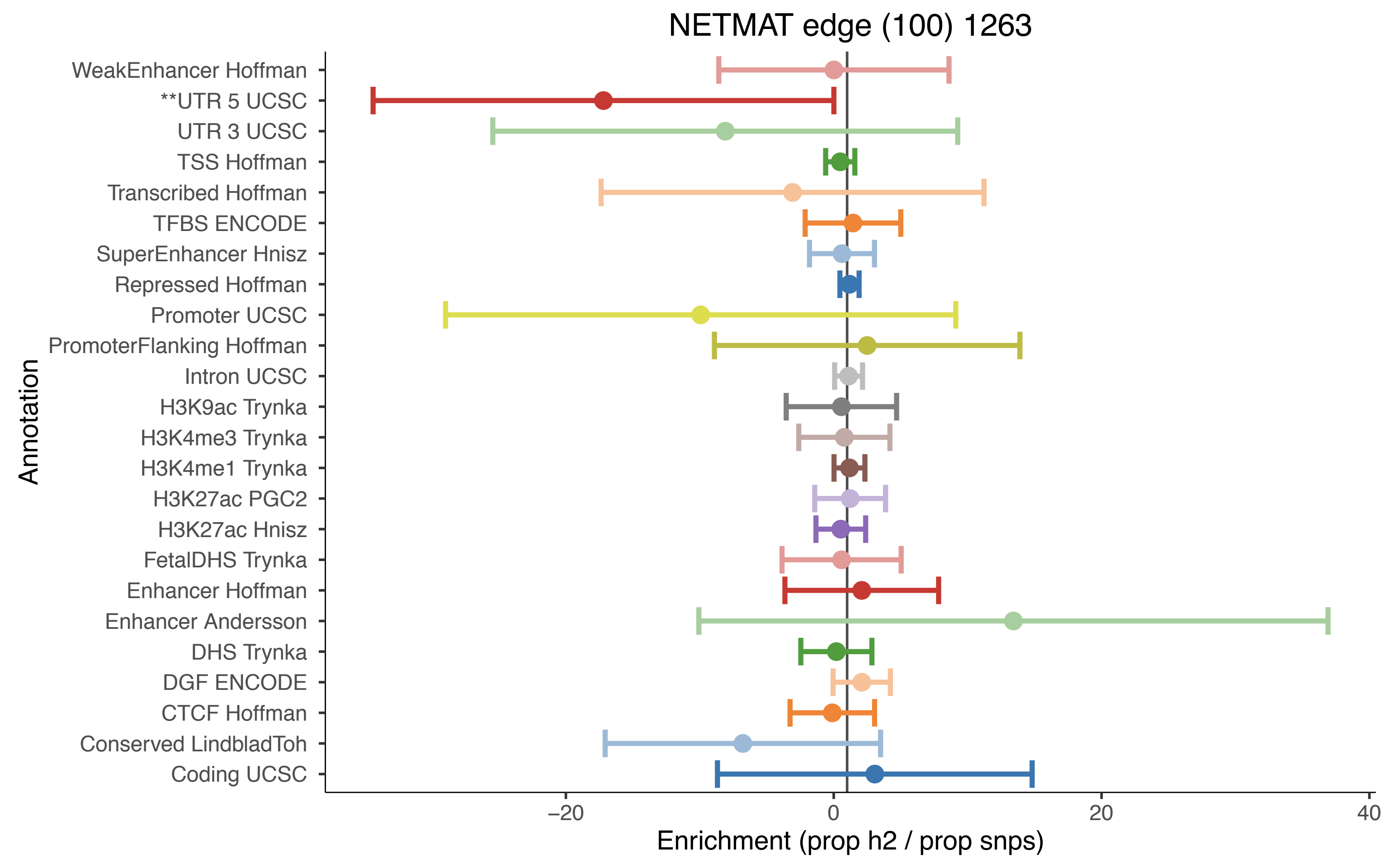
NETMAT edge (100) 1263
WeakEnhancer Hoffman
**UTR 5 UCSC
UTR 3 UCSC
TSS Hoffman
Transcribed Hoffman
TFBS ENCODE
SuperEnhancer Hnisz
Repressed Hoffman
Promoter UCSC
PromoterFlanking Hoffman
Intron UCSC
H3K9ac Trynka
H3K4me3 Trynka
H3K4me1 Trynka
H3K27ac PGC2
H3K27ac Hnisz
FetalDHS Trynka
Enhancer Hoffman
Enhancer Andersson
DHS Trynka
DGF ENCODE
CTCF Hoffman
Conserved LindbladToh
Coding UCSC
Annotation
−20
0
20
40
Enrichment (prop h2 / prop snps)

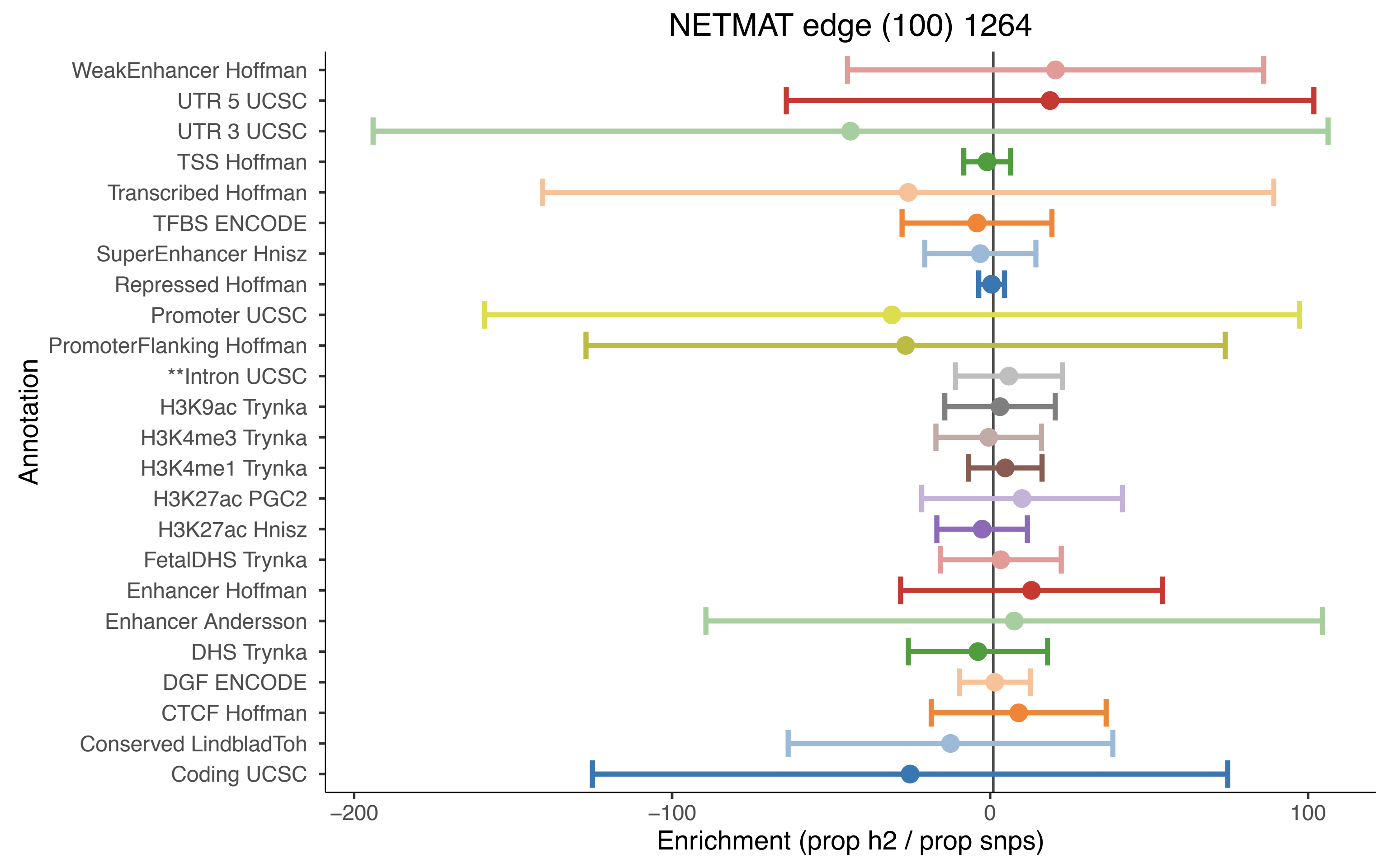
NETMAT edge (100) 1264
Annotation
WeakEnhancer Hoffman
UTR 5 UCSC
UTR 3 UCSC
TSS Hoffman
Transcribed Hoffman
TFBS ENCODE
SuperEnhancer Hnisz
Repressed Hoffman
Promoter UCSC
PromoterFlanking Hoffman
**Intron UCSC
H3K9ac Trynka
H3K4me3 Trynka
H3K4me1 Trynka
H3K27ac PGC2
H3K27ac Hnisz
FetalDHS Trynka
Enhancer Hoffman
Enhancer Andersson
DHS Trynka
DGF ENCODE
CTCF Hoffman
Conserved LindbladToh
Coding UCSC
−200
−100
0
100
Enrichment (prop h2 / prop snps)

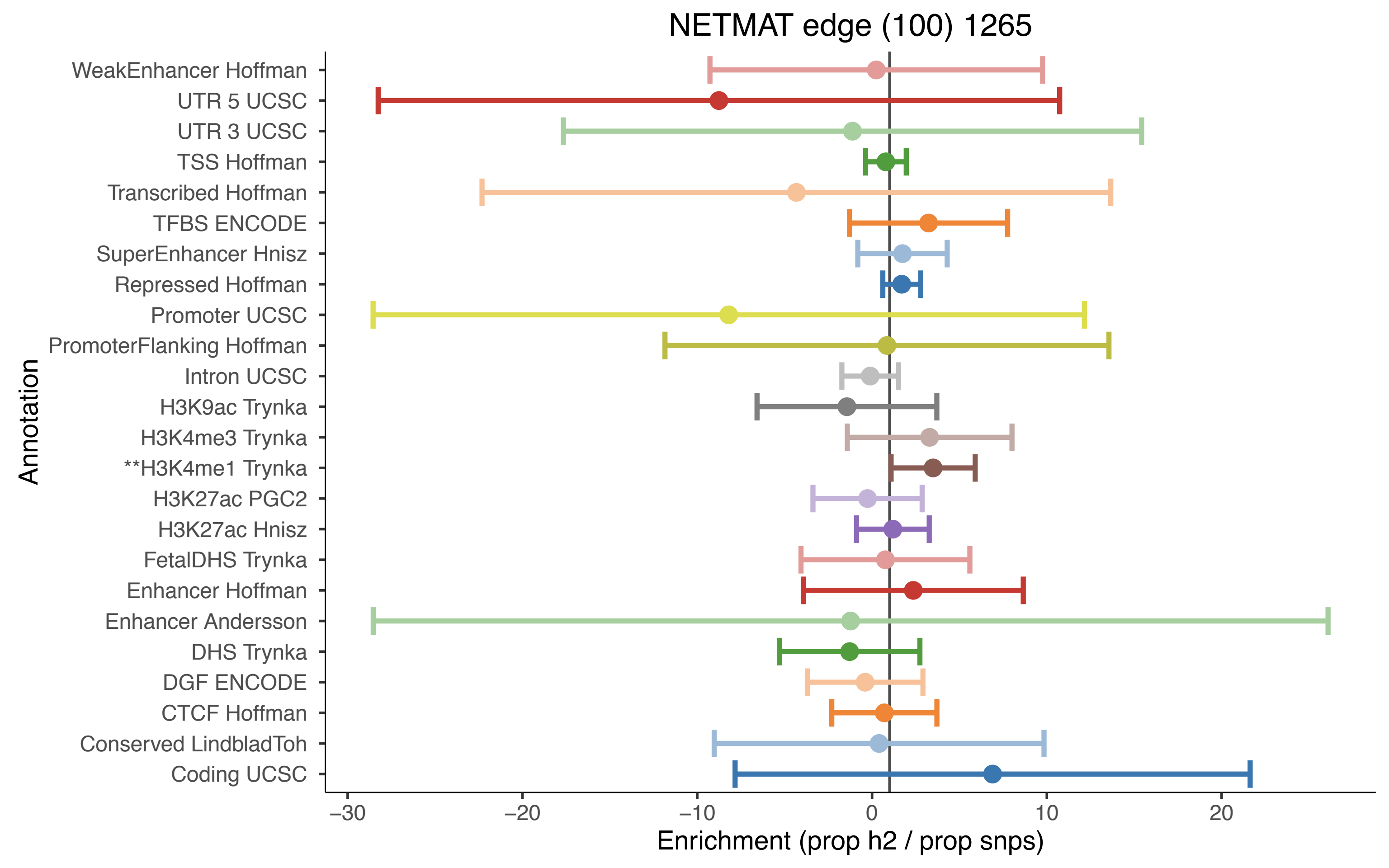

NETMAT edge (100) 1265
Annotation
WeakEnhancer Hoffman
UTR 5 UCSC
UTR 3 UCSC
TSS Hoffman
Transcribed Hoffman
TFBS ENCODE
SuperEnhancer Hnisz
Repressed Hoffman
Promoter UCSC
PromoterFlanking Hoffman
Intron UCSC
H3K9ac Trynka
H3K4me3 Trynka
**H3K4me1 Trynka
H3K27ac PGC2
H3K27ac Hnisz
FetalDHS Trynka
Enhancer Hoffman
Enhancer Andersson
DHS Trynka
DGF ENCODE
CTCF Hoffman
Conserved LindbladToh
Coding UCSC
−30
−20
−10
0
10
20
Enrichment (prop h2 / prop snps)

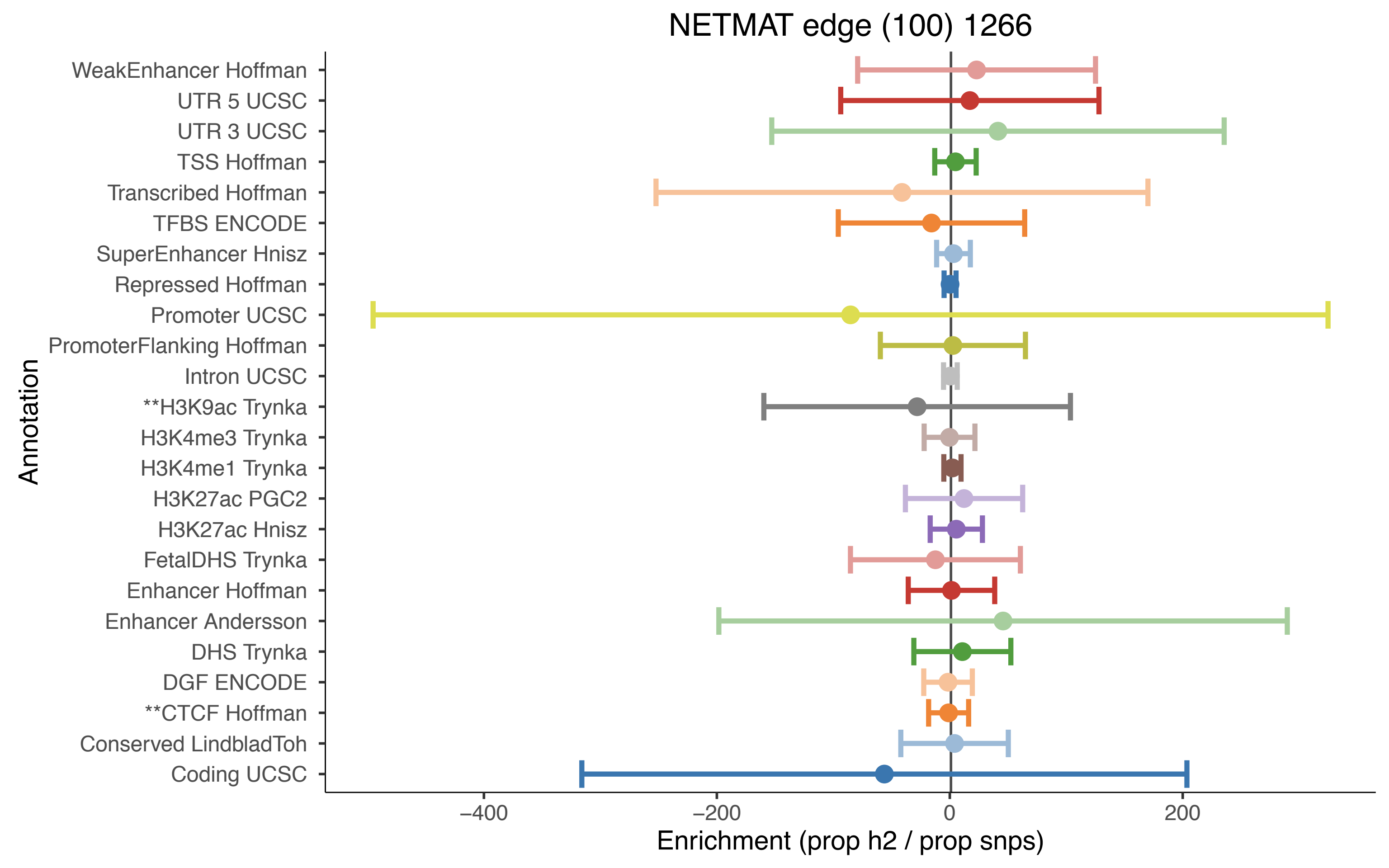
NETMAT edge (100) 1266
WeakEnhancer Hoffman
UTR 5 UCSC
UTR 3 UCSC
TSS Hoffman
Transcribed Hoffman
TFBS ENCODE
SuperEnhancer Hnisz
Repressed Hoffman
Promoter UCSC
PromoterFlanking Hoffman
Intron UCSC
**H3K9ac Trynka
H3K4me3 Trynka
H3K4me1 Trynka
H3K27ac PGC2
H3K27ac Hnisz
FetalDHS Trynka
Enhancer Hoffman
Enhancer Andersson
DHS Trynka
DGF ENCODE
**CTCF Hoffman
Conserved LindbladToh
Coding UCSC
Annotation
−400
−200
0
200
Enrichment (prop h2 / prop snps)

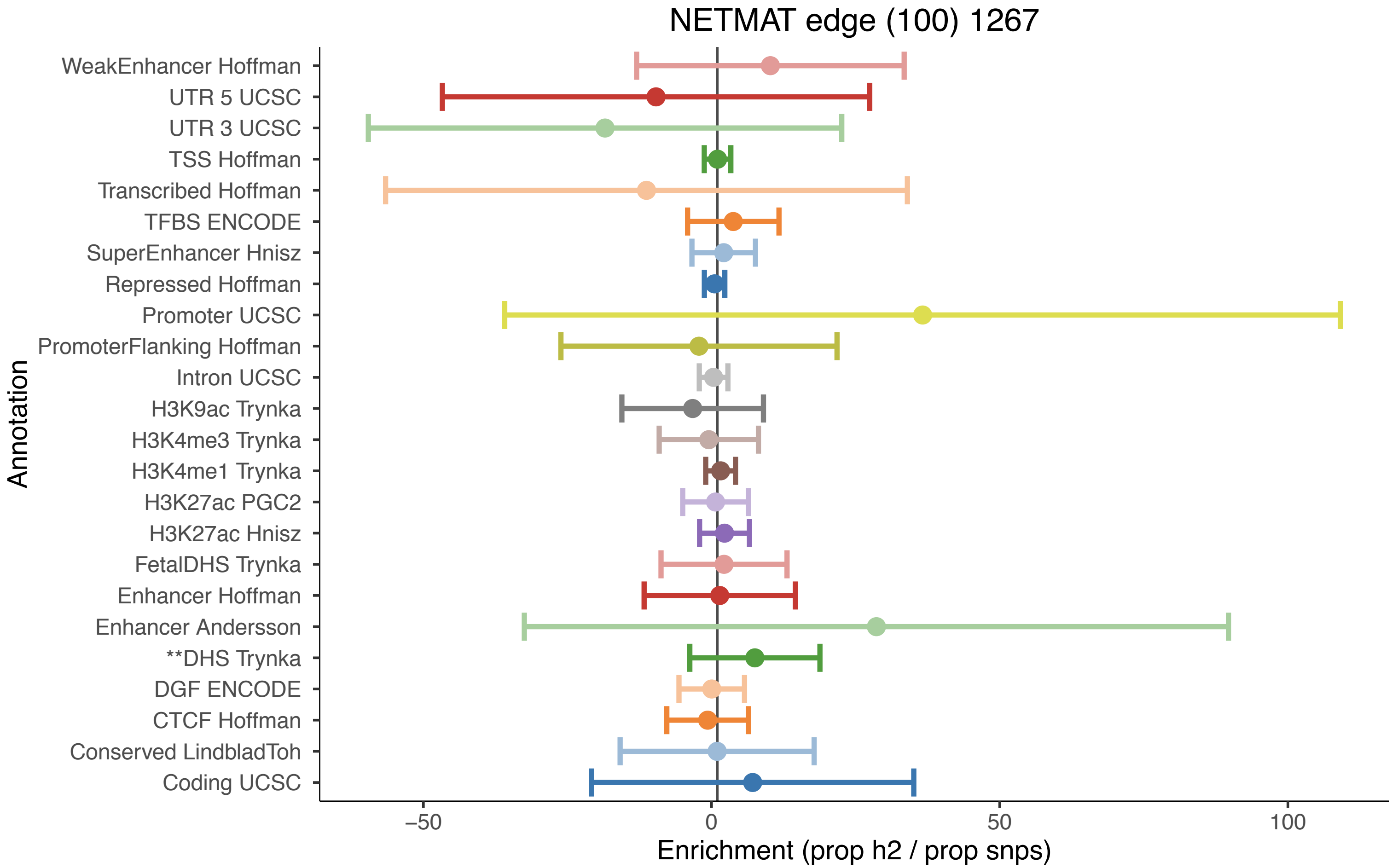
NETMAT edge (100) 1267
WeakEnhancer Hoffman
UTR 5 UCSC
UTR 3 UCSC
TSS Hoffman
Transcribed Hoffman
TFBS ENCODE
SuperEnhancer Hnisz
Repressed Hoffman
Promoter UCSC
PromoterFlanking Hoffman
Intron UCSC
H3K9ac Trynka
H3K4me3 Trynka
H3K4me1 Trynka
H3K27ac PGC2
H3K27ac Hnisz
FetalDHS Trynka
Enhancer Hoffman
Enhancer Andersson
**DHS Trynka
DGF ENCODE
CTCF Hoffman
Conserved LindbladToh
Coding UCSC
Annotation
−50
0
50
100
Enrichment (prop h2 / prop snps)

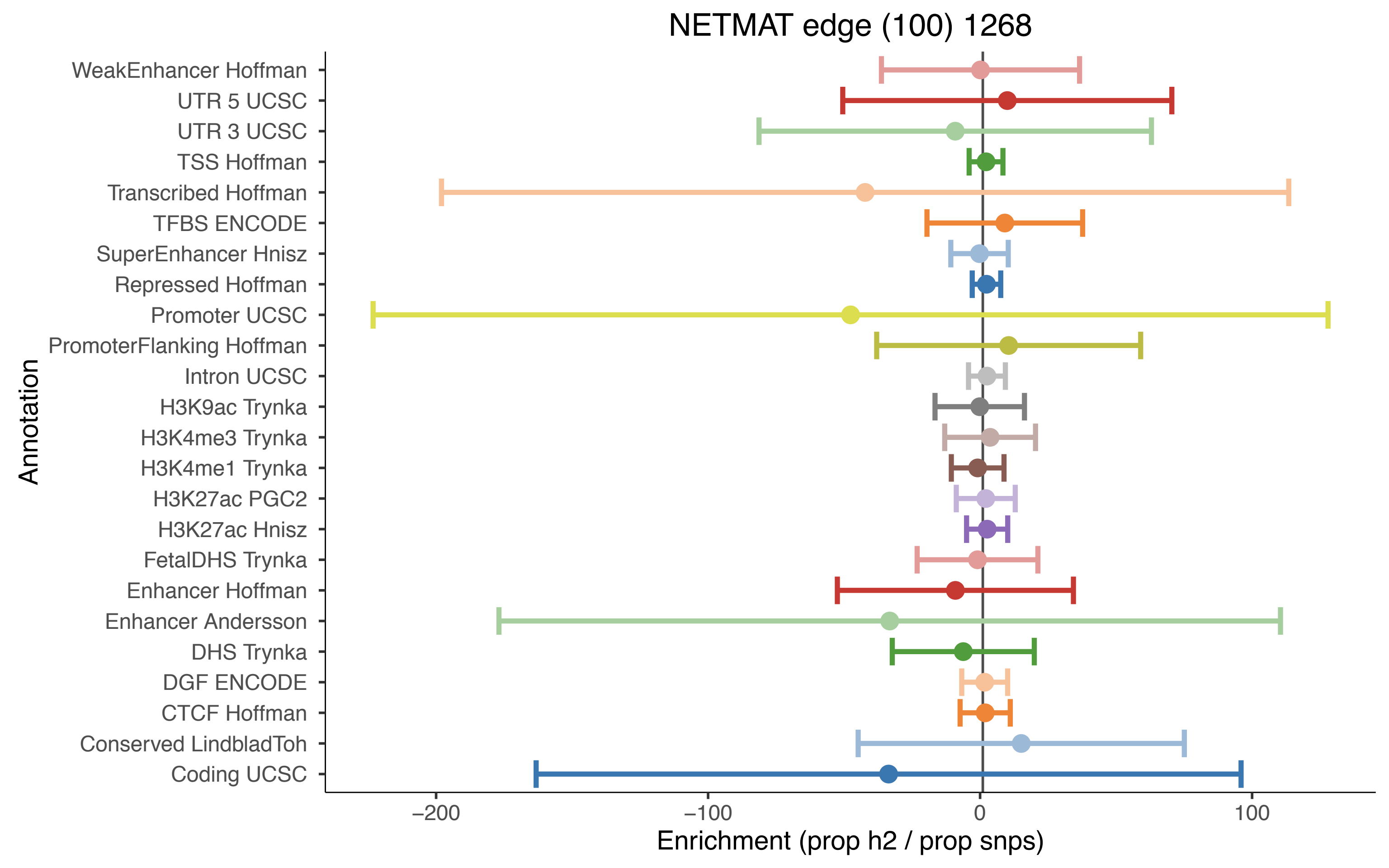
NETMAT edge (100) 1268
WeakEnhancer Hoffman
UTR 5 UCSC
UTR 3 UCSC
TSS Hoffman
Transcribed Hoffman
TFBS ENCODE
SuperEnhancer Hnisz
Repressed Hoffman
Promoter UCSC
PromoterFlanking Hoffman
Intron UCSC
H3K9ac Trynka
H3K4me3 Trynka
H3K4me1 Trynka
H3K27ac PGC2
H3K27ac Hnisz
FetalDHS Trynka
Enhancer Hoffman
Enhancer Andersson
DHS Trynka
DGF ENCODE
CTCF Hoffman
Conserved LindbladToh
Coding UCSC
Annotation
−200
−100
0
100
Enrichment (prop h2 / prop snps)

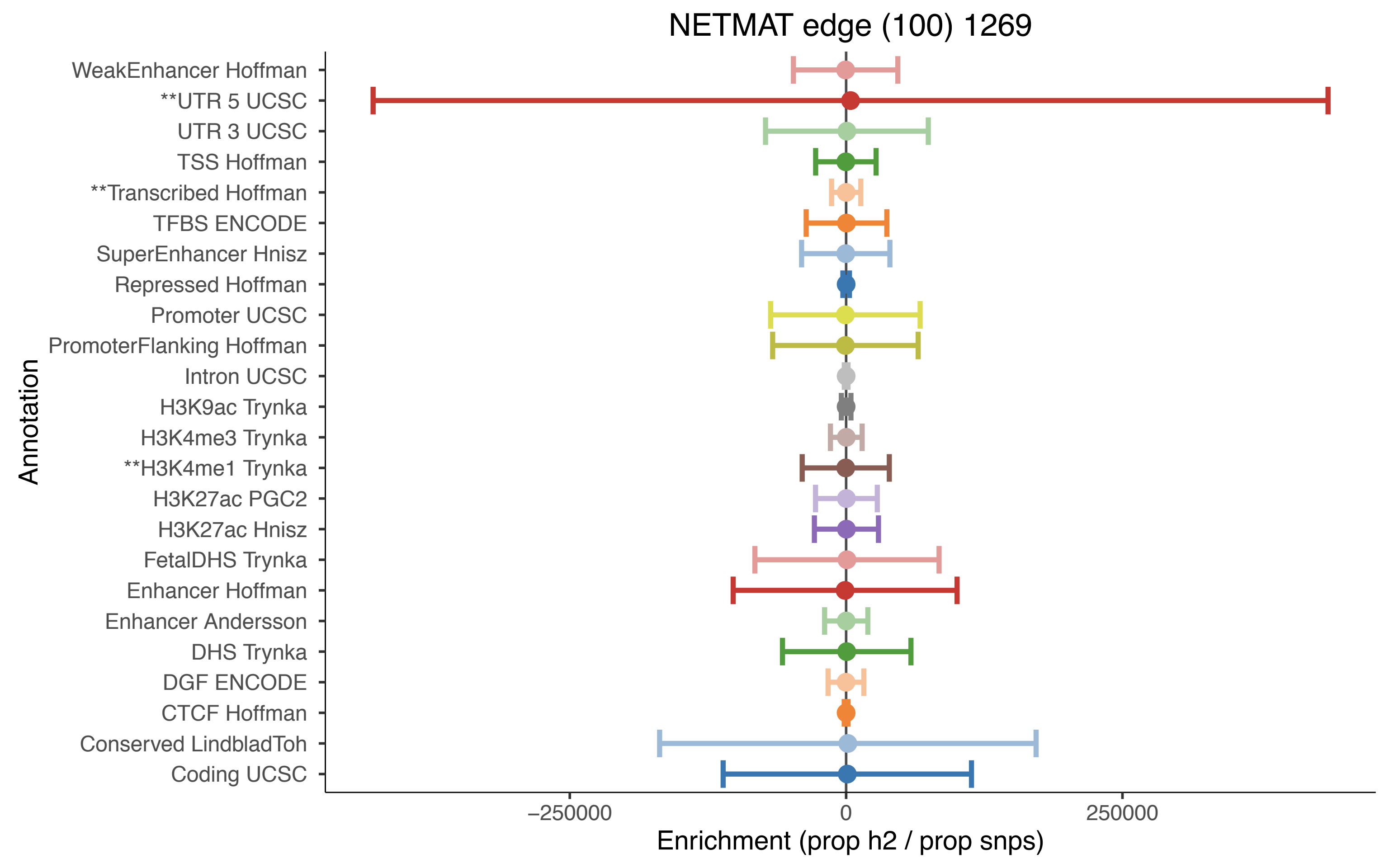

NETMAT edge (100) 1269
Annotation
WeakEnhancer Hoffman
**UTR 5 UCSC
UTR 3 UCSC
TSS Hoffman
**Transcribed Hoffman
TFBS ENCODE
SuperEnhancer Hnisz
Repressed Hoffman
Promoter UCSC
PromoterFlanking Hoffman
Intron UCSC
H3K9ac Trynka
H3K4me3 Trynka
**H3K4me1 Trynka
H3K27ac PGC2
H3K27ac Hnisz
FetalDHS Trynka
Enhancer Hoffman
Enhancer Andersson
DHS Trynka
DGF ENCODE
CTCF Hoffman
Conserved LindbladToh
Coding UCSC
–250000
0
250000
Enrichment (prop h2 / prop snps)

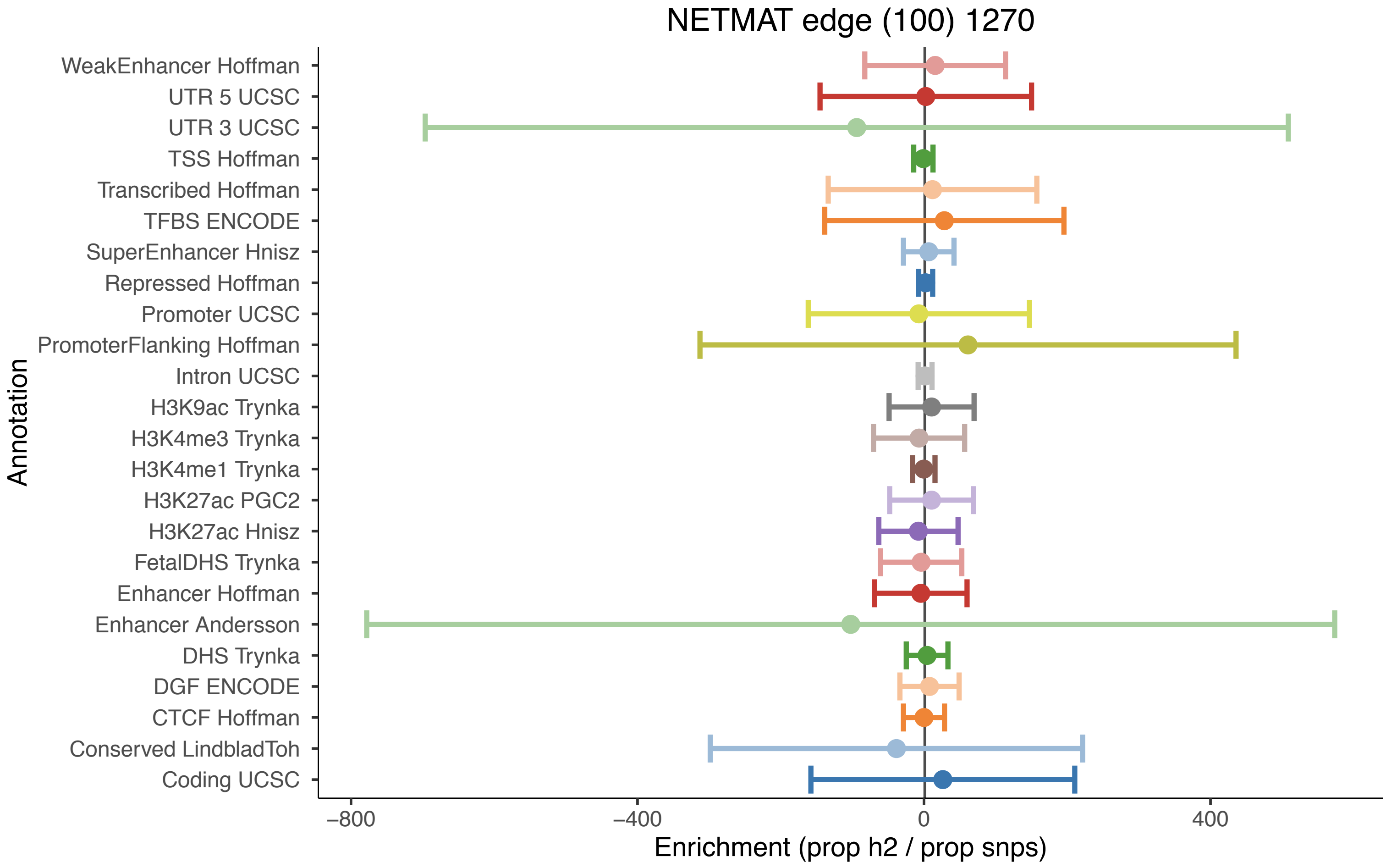

NETMAT edge (100) 1270
WeakEnhancer Hoffman
UTR 5 UCSC
UTR 3 UCSC
TSS Hoffman
Transcribed Hoffman
TFBS ENCODE
SuperEnhancer Hnisz
Repressed Hoffman
Promoter UCSC
PromoterFlanking Hoffman
Intron UCSC
H3K9ac Trynka
H3K4me3 Trynka
H3K4me1 Trynka
H3K27ac PGC2
H3K27ac Hnisz
FetalDHS Trynka
Enhancer Hoffman
Enhancer Andersson
DHS Trynka
DGF ENCODE
CTCF Hoffman
Conserved LindbladToh
Coding UCSC
Annotation
−800
−400
0
400
Enrichment (prop h2 / prop snps)

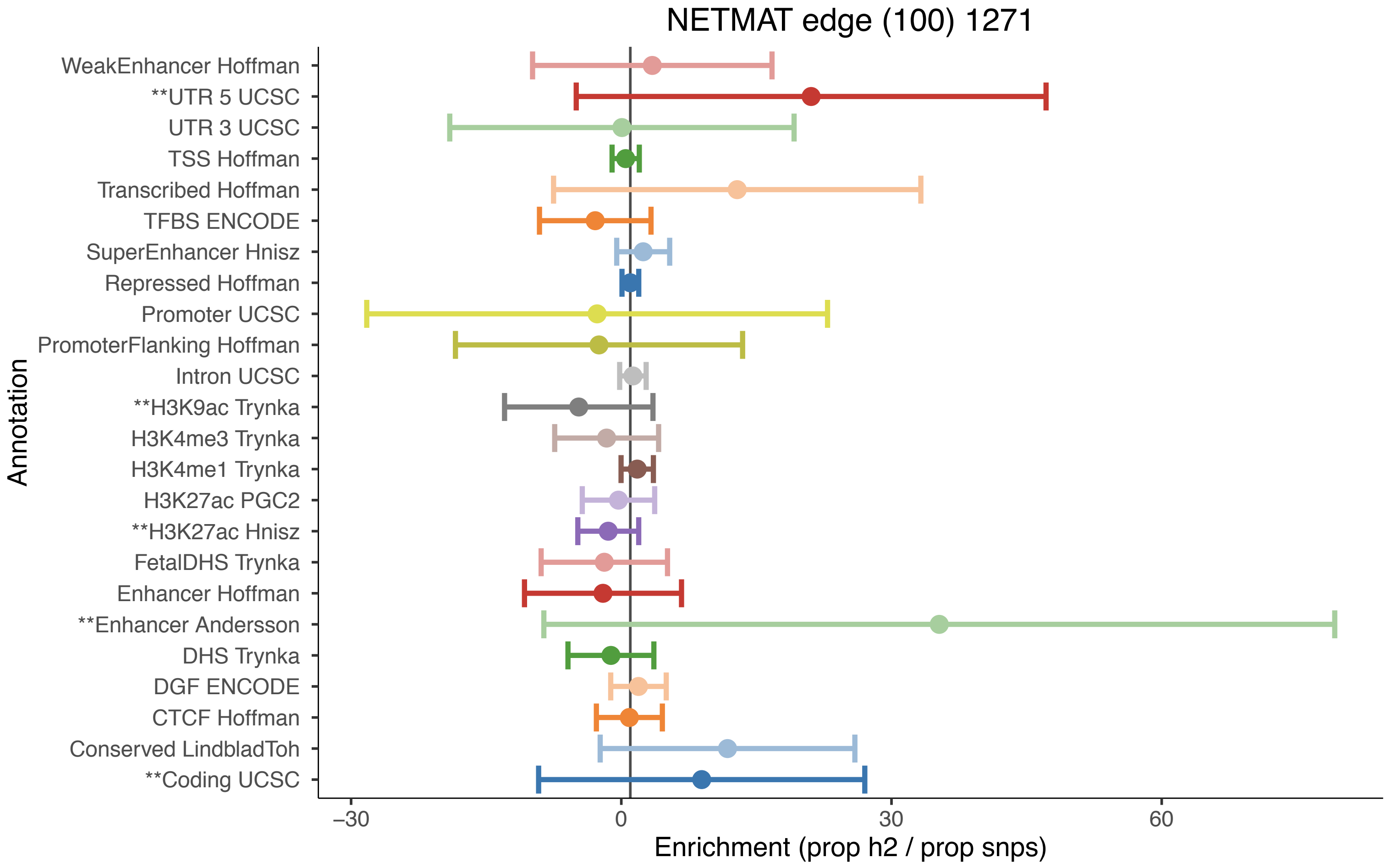
NETMAT edge (100) 1271
WeakEnhancer Hoffman
**UTR 5 UCSC
UTR 3 UCSC
TSS Hoffman
Transcribed Hoffman
TFBS ENCODE
SuperEnhancer Hnisz
Repressed Hoffman
Promoter UCSC
PromoterFlanking Hoffman
Intron UCSC
**H3K9ac Trynka
H3K4me3 Trynka
H3K4me1 Trynka
H3K27ac PGC2
**H3K27ac Hnisz
FetalDHS Trynka
Enhancer Hoffman
**Enhancer Andersson
DHS Trynka
DGF ENCODE
CTCF Hoffman
Conserved LindbladToh
**Coding UCSC
Annotation
–30
0
30
60
Enrichment (prop h2 / prop snps)

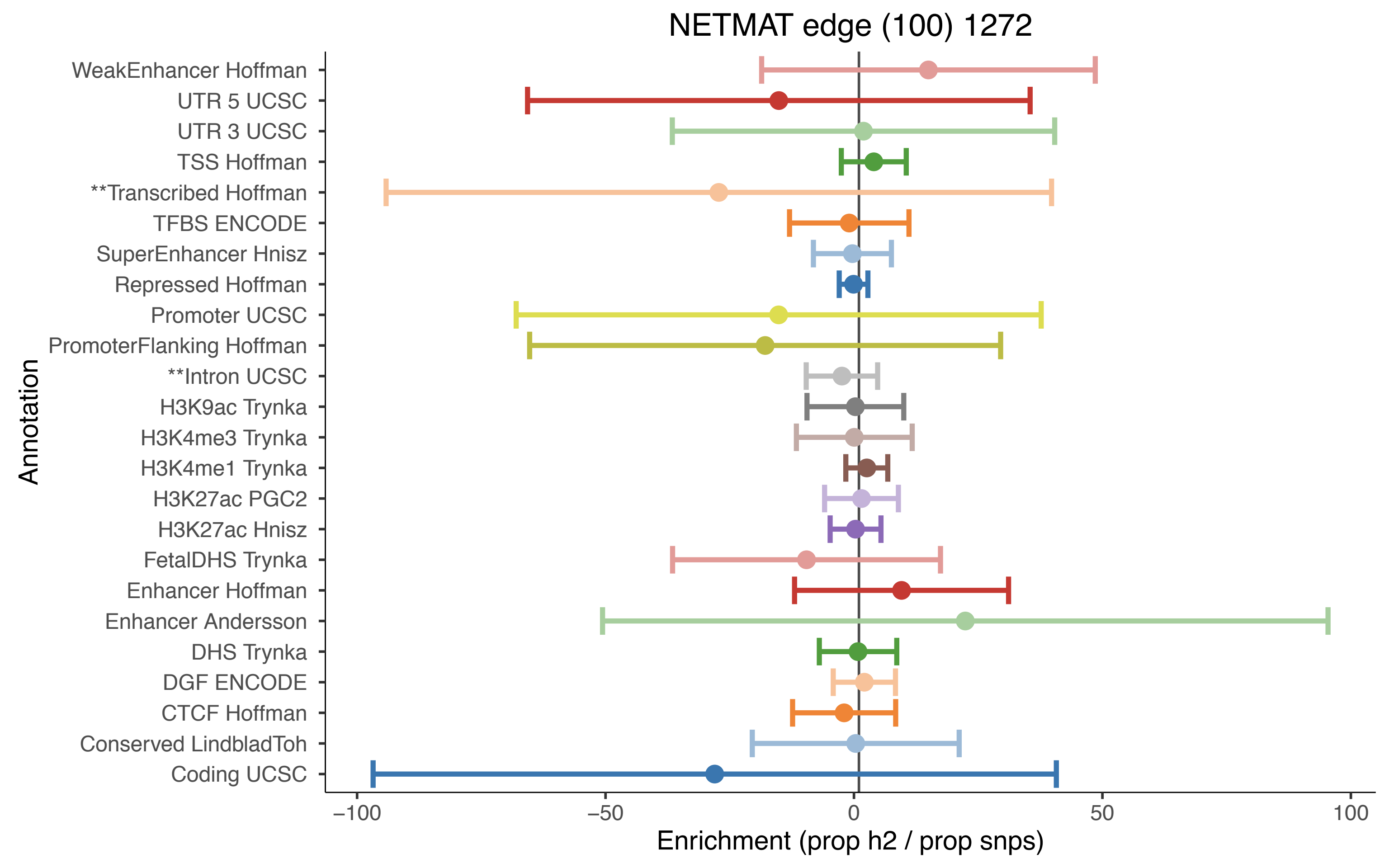
NETMAT edge (100) 1272
WeakEnhancer Hoffman
UTR 5 UCSC
UTR 3 UCSC
TSS Hoffman
**Transcribed Hoffman
TFBS ENCODE
SuperEnhancer Hnisz
Repressed Hoffman
Promoter UCSC
PromoterFlanking Hoffman
**Intron UCSC
H3K9ac Trynka
H3K4me3 Trynka
H3K4me1 Trynka
H3K27ac PGC2
H3K27ac Hnisz
FetalDHS Trynka
Enhancer Hoffman
Enhancer Andersson
DHS Trynka
DGF ENCODE
CTCF Hoffman
Conserved LindbladToh
Coding UCSC
Annotation
−100
−50
0
50
100
Enrichment (prop h2 / prop snps)

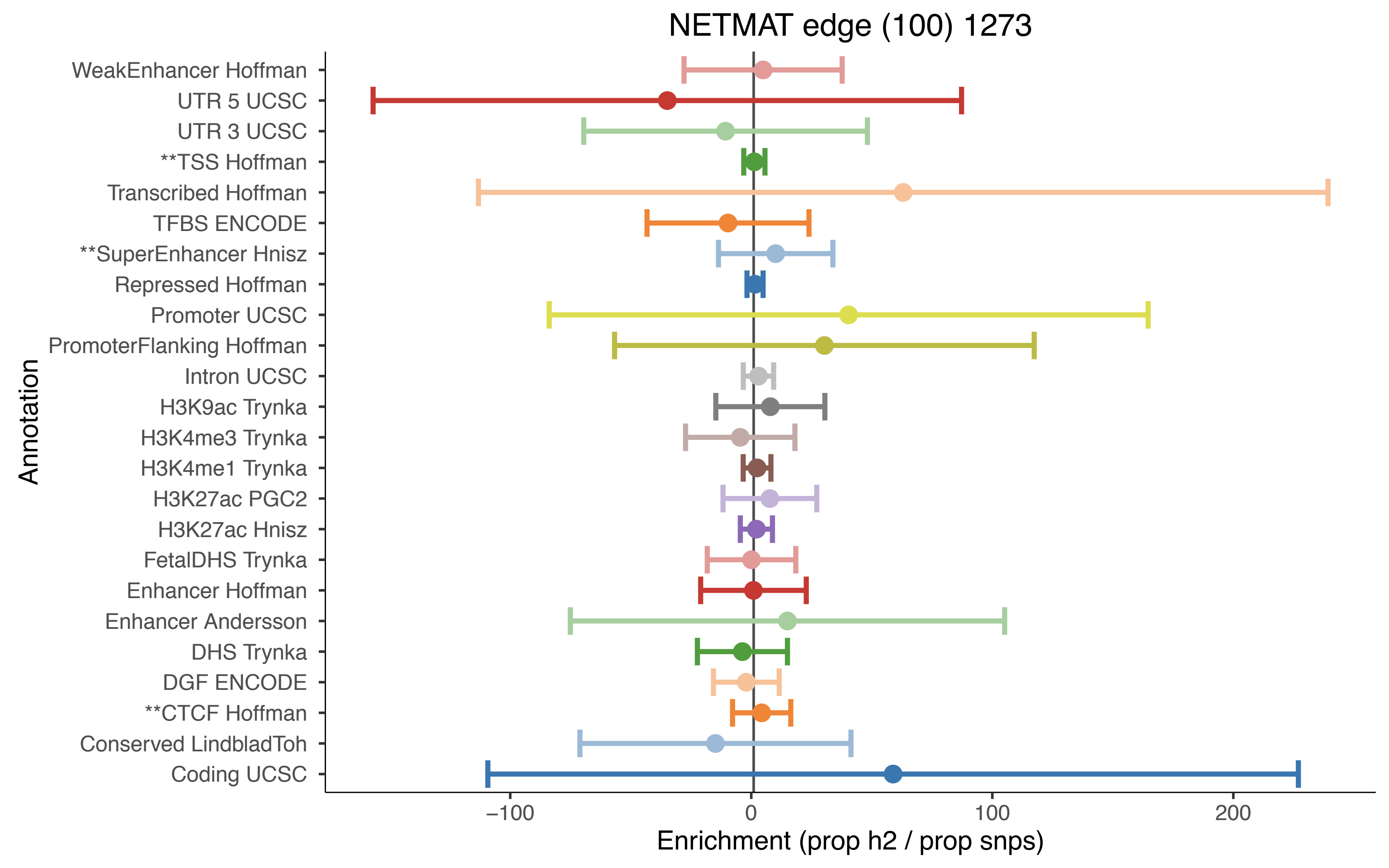

NETMAT edge (100) 1273
WeakEnhancer Hoffman
UTR 5 UCSC
UTR 3 UCSC
**TSS Hoffman
Transcribed Hoffman
TFBS ENCODE
**SuperEnhancer Hnisz
Repressed Hoffman
Promoter UCSC
PromoterFlanking Hoffman
Intron UCSC
H3K9ac Trynka
H3K4me3 Trynka
H3K4me1 Trynka
H3K27ac PGC2
H3K27ac Hnisz
FetalDHS Trynka
Enhancer Hoffman
Enhancer Andersson
DHS Trynka
DGF ENCODE
**CTCF Hoffman
Conserved LindbladToh
Coding UCSC
Annotation
−100
0
100
200
Enrichment (prop h2 / prop snps)

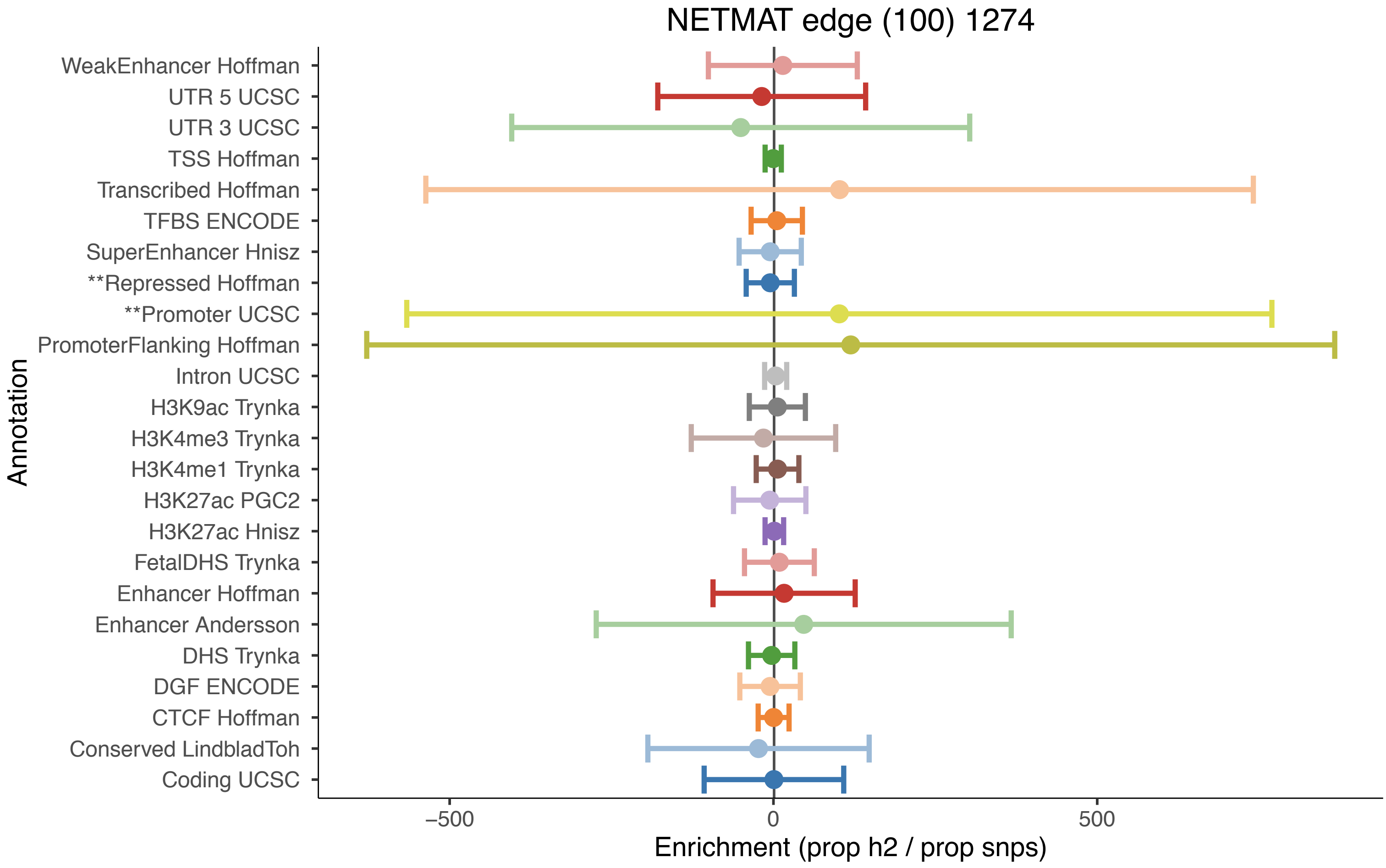
NETMAT edge (100) 1274
WeakEnhancer Hoffman
UTR 5 UCSC
UTR 3 UCSC
TSS Hoffman
Transcribed Hoffman
TFBS ENCODE
SuperEnhancer Hnisz
**Repressed Hoffman
**Promoter UCSC
PromoterFlanking Hoffman
Intron UCSC
H3K9ac Trynka
H3K4me3 Trynka
H3K4me1 Trynka
H3K27ac PGC2
H3K27ac Hnisz
FetalDHS Trynka
Enhancer Hoffman
Enhancer Andersson
DHS Trynka
DGF ENCODE
CTCF Hoffman
Conserved LindbladToh
Coding UCSC
Annotation
−500
0
500
Enrichment (prop h2 / prop snps)

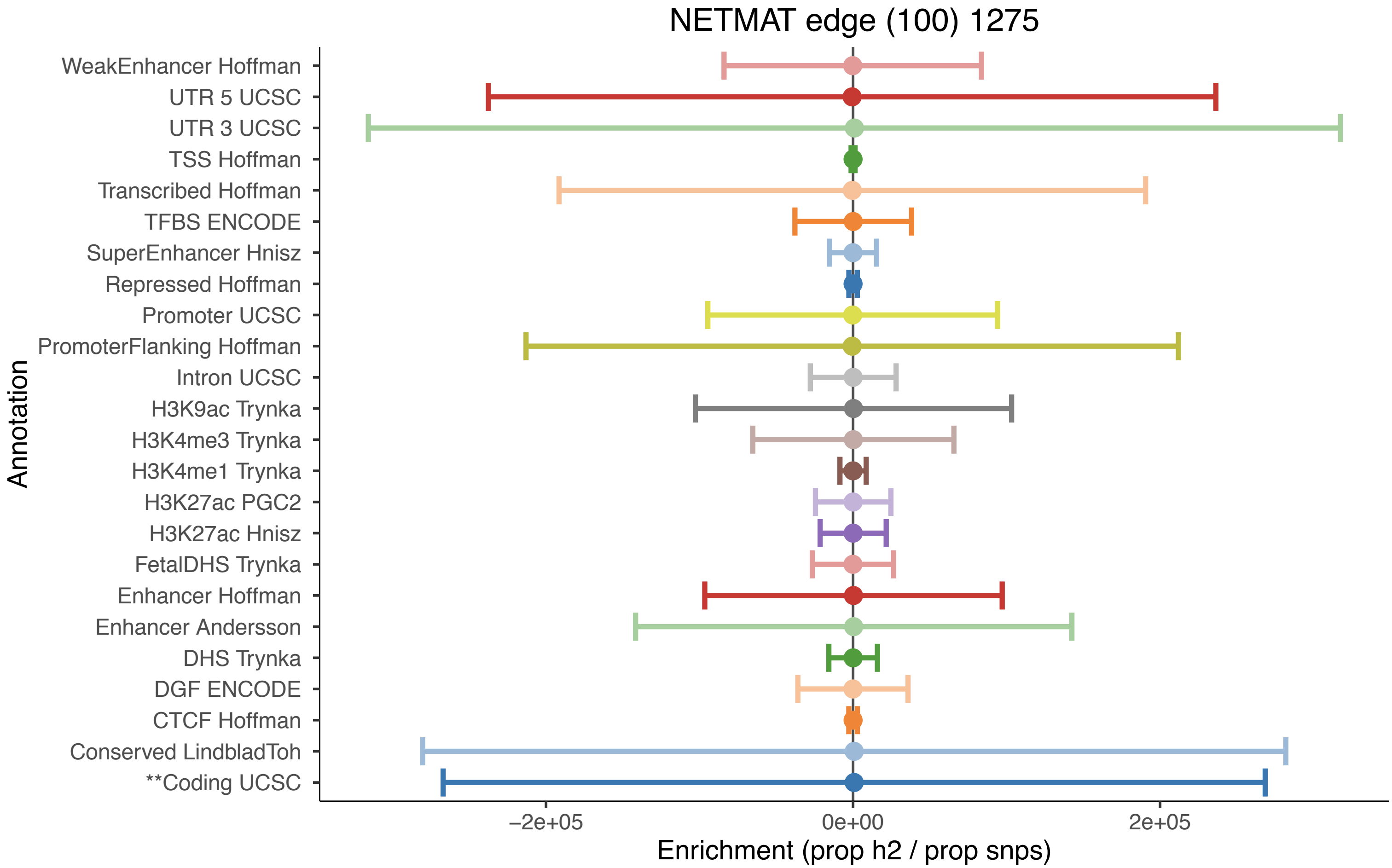
NETMAT edge (100) 1275
WeakEnhancer Hoffman
UTR 5 UCSC
UTR 3 UCSC
TSS Hoffman
Transcribed Hoffman
TFBS ENCODE
SuperEnhancer Hnisz
Repressed Hoffman
Promoter UCSC
PromoterFlanking Hoffman
Intron UCSC
H3K9ac Trynka
H3K4me3 Trynka
H3K4me1 Trynka
H3K27ac PGC2
H3K27ac Hnisz
FetalDHS Trynka
Enhancer Hoffman
Enhancer Andersson
DHS Trynka
DGF ENCODE
CTCF Hoffman
Conserved LindbladToh
**Coding UCSC
Annotation
−2e+05
0e+00
2e+05
Enrichment (prop h2 / prop snps)

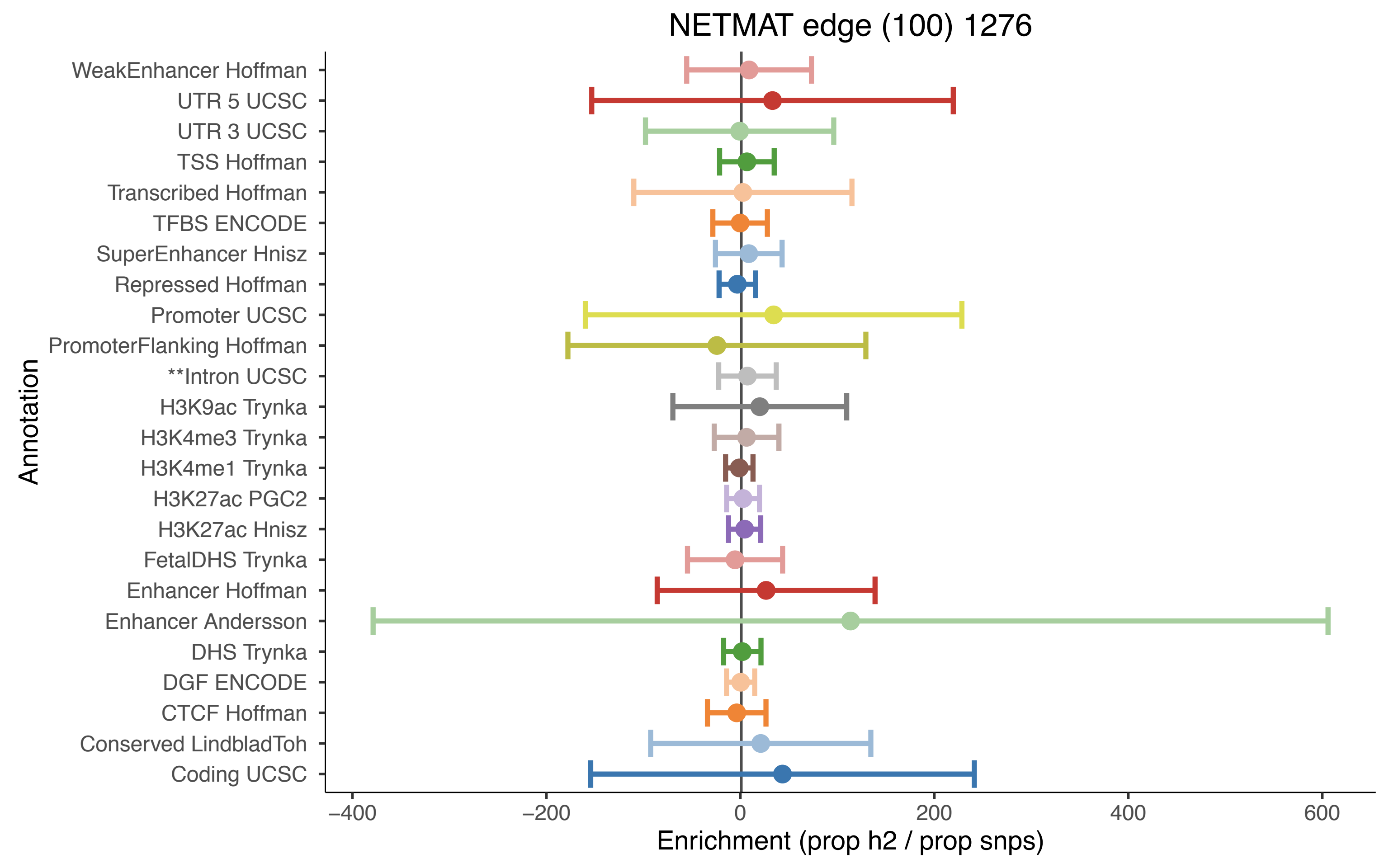
NETMAT edge (100) 1276
WeakEnhancer Hoffman
UTR 5 UCSC
UTR 3 UCSC
TSS Hoffman
Transcribed Hoffman
TFBS ENCODE
SuperEnhancer Hnisz
Repressed Hoffman
Promoter UCSC
PromoterFlanking Hoffman
**Intron UCSC
H3K9ac Trynka
H3K4me3 Trynka
H3K4me1 Trynka
H3K27ac PGC2
H3K27ac Hnisz
FetalDHS Trynka
Enhancer Hoffman
Enhancer Andersson
DHS Trynka
DGF ENCODE
CTCF Hoffman
Conserved LindbladToh
Coding UCSC
Annotation
−400
−200
0
200
400
600
Enrichment (prop h2 / prop snps)

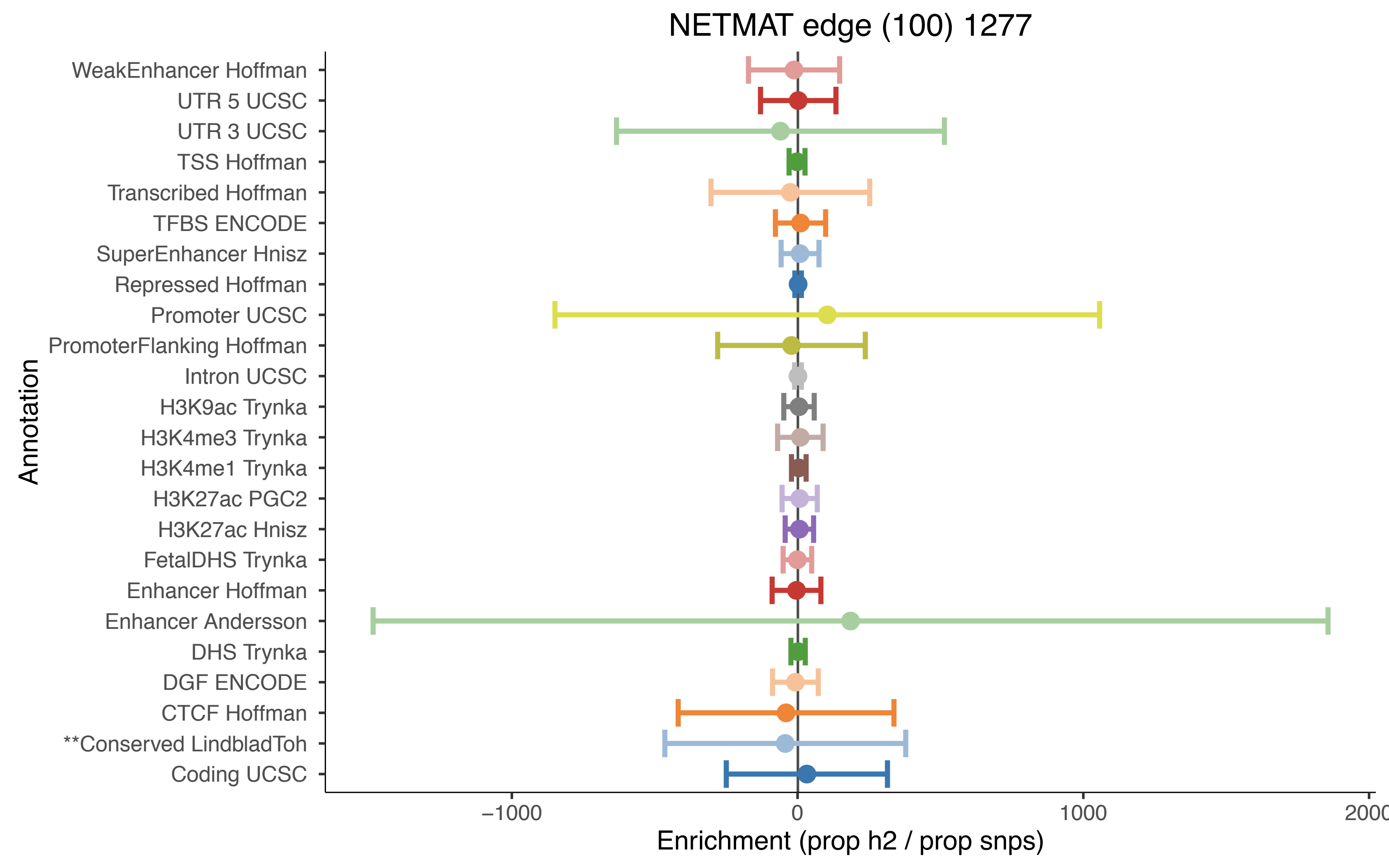
NETMAT edge (100) 1277
WeakEnhancer Hoffman
UTR 5 UCSC
UTR 3 UCSC
TSS Hoffman
Transcribed Hoffman
TFBS ENCODE
SuperEnhancer Hnisz
Repressed Hoffman
Promoter UCSC
PromoterFlanking Hoffman
Intron UCSC
H3K9ac Trynka
H3K4me3 Trynka
H3K4me1 Trynka
H3K27ac PGC2
H3K27ac Hnisz
FetalDHS Trynka
Enhancer Hoffman
Enhancer Andersson
DHS Trynka
DGF ENCODE
CTCF Hoffman
**Conserved LindbladToh
Coding UCSC
Annotation
−1000
0
1000
Enrichment (prop h2 / prop snps)

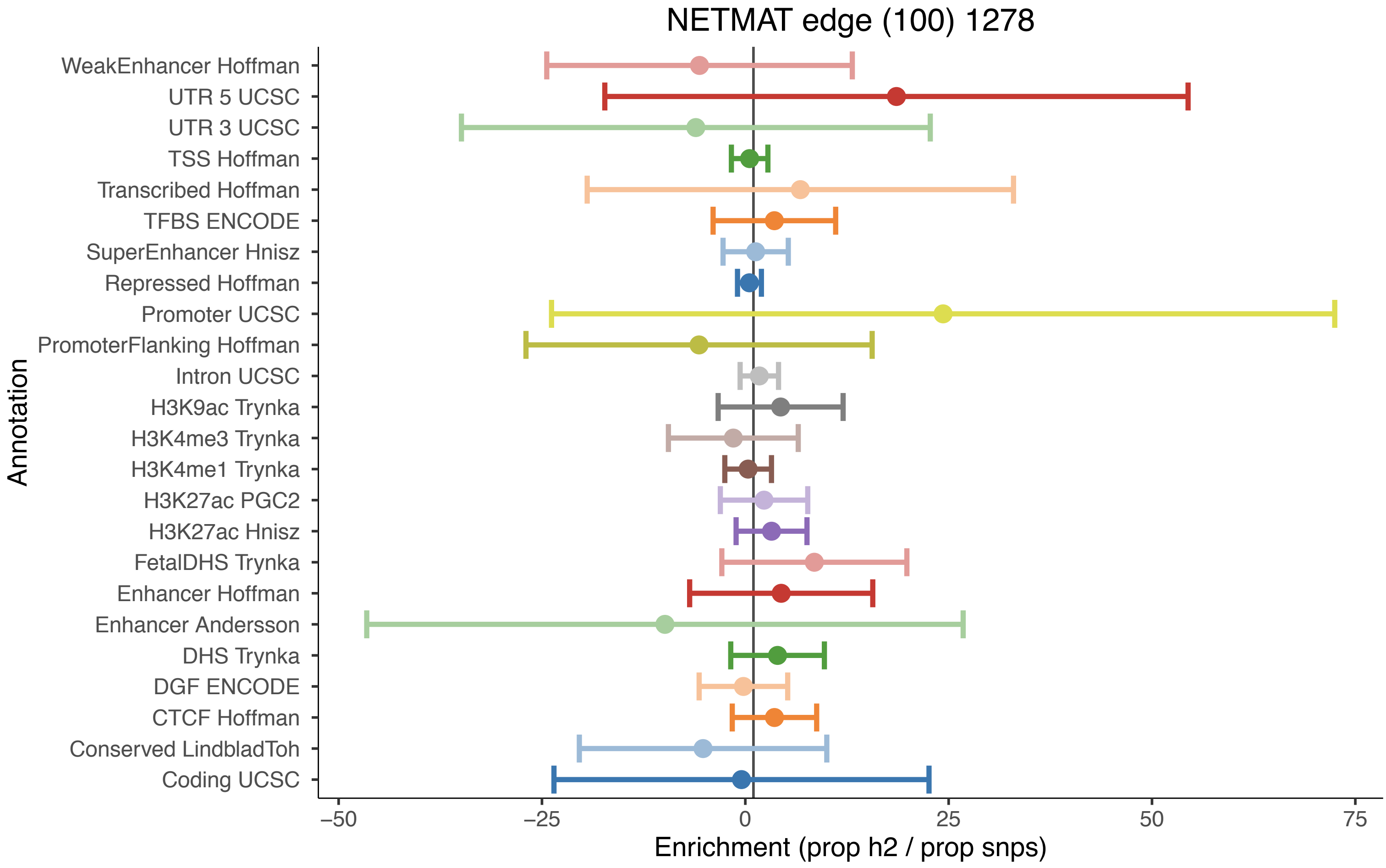
NETMAT edge (100) 1278
WeakEnhancer Hoffman
UTR 5 UCSC
UTR 3 UCSC
TSS Hoffman
Transcribed Hoffman
TFBS ENCODE
SuperEnhancer Hnisz
Repressed Hoffman
Promoter UCSC
PromoterFlanking Hoffman
Intron UCSC
H3K9ac Trynka
H3K4me3 Trynka
H3K4me1 Trynka
H3K27ac PGC2
H3K27ac Hnisz
FetalDHS Trynka
Enhancer Hoffman
Enhancer Andersson
DHS Trynka
DGF ENCODE
CTCF Hoffman
Conserved LindbladToh
Coding UCSC
Annotation
−50
−25
0
25
50
75
Enrichment (prop h2 / prop snps)

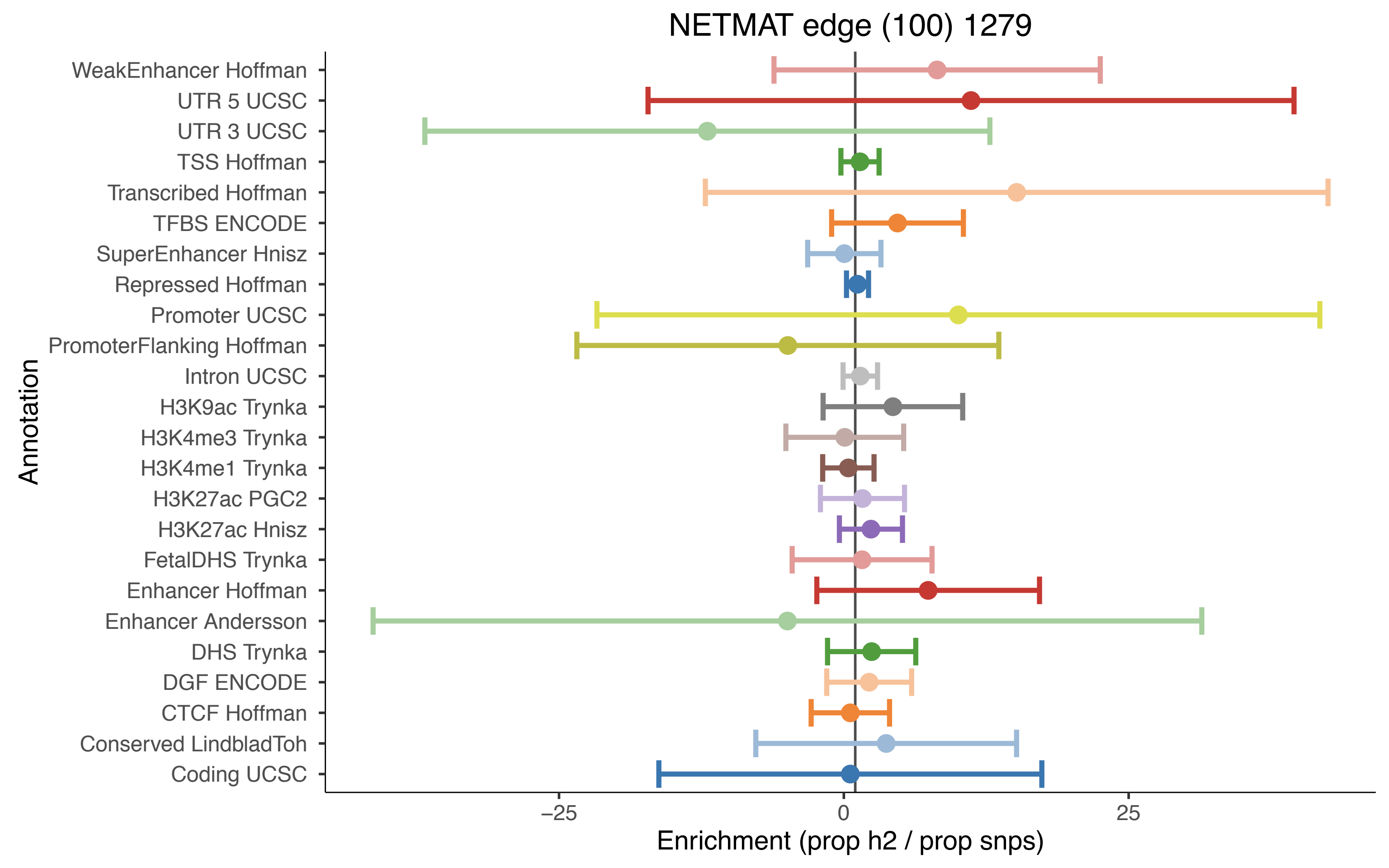
NETMAT edge (100) 1279
WeakEnhancer Hoffman
UTR 5 UCSC
UTR 3 UCSC
TSS Hoffman
Transcribed Hoffman
TFBS ENCODE
SuperEnhancer Hnisz
Repressed Hoffman
Promoter UCSC
PromoterFlanking Hoffman
Intron UCSC
H3K9ac Trynka
H3K4me3 Trynka
H3K4me1 Trynka
H3K27ac PGC2
H3K27ac Hnisz
FetalDHS Trynka
Enhancer Hoffman
Enhancer Andersson
DHS Trynka
DGF ENCODE
CTCF Hoffman
Conserved LindbladToh
Coding UCSC
Annotation
−25
0
25
Enrichment (prop h2 / prop snps)

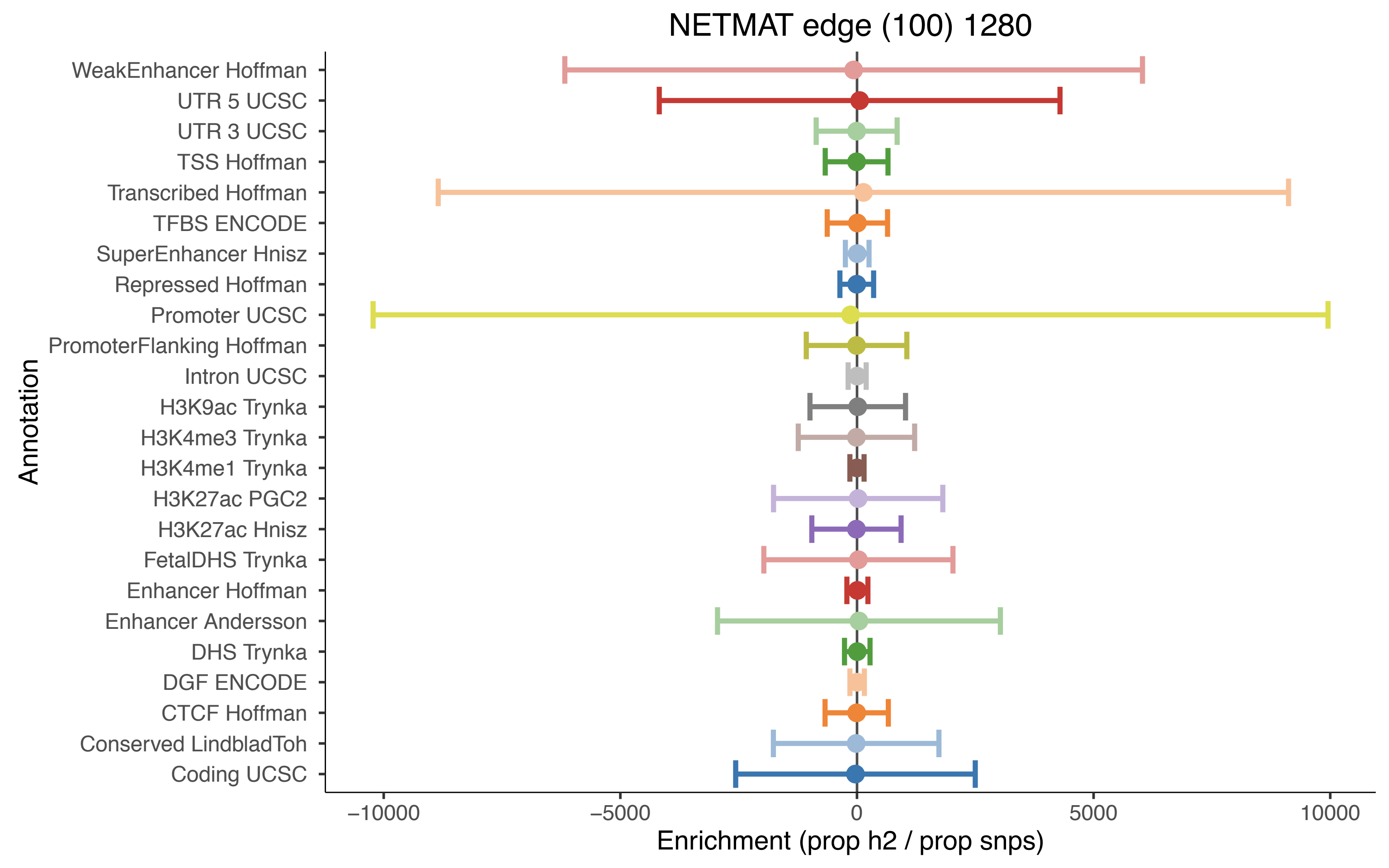
NETMAT edge (100) 1280
WeakEnhancer Hoffman
UTR 5 UCSC
UTR 3 UCSC
TSS Hoffman
Transcribed Hoffman
TFBS ENCODE
SuperEnhancer Hnisz
Repressed Hoffman
Promoter UCSC
PromoterFlanking Hoffman
Intron UCSC
H3K9ac Trynka
H3K4me3 Trynka
H3K4me1 Trynka
H3K27ac PGC2
H3K27ac Hnisz
FetalDHS Trynka
Enhancer Hoffman
Enhancer Andersson
DHS Trynka
DGF ENCODE
CTCF Hoffman
Conserved LindbladToh
Coding UCSC
Annotation
−10000
−5000
0
5000
10000
Enrichment (prop h2 / prop snps)

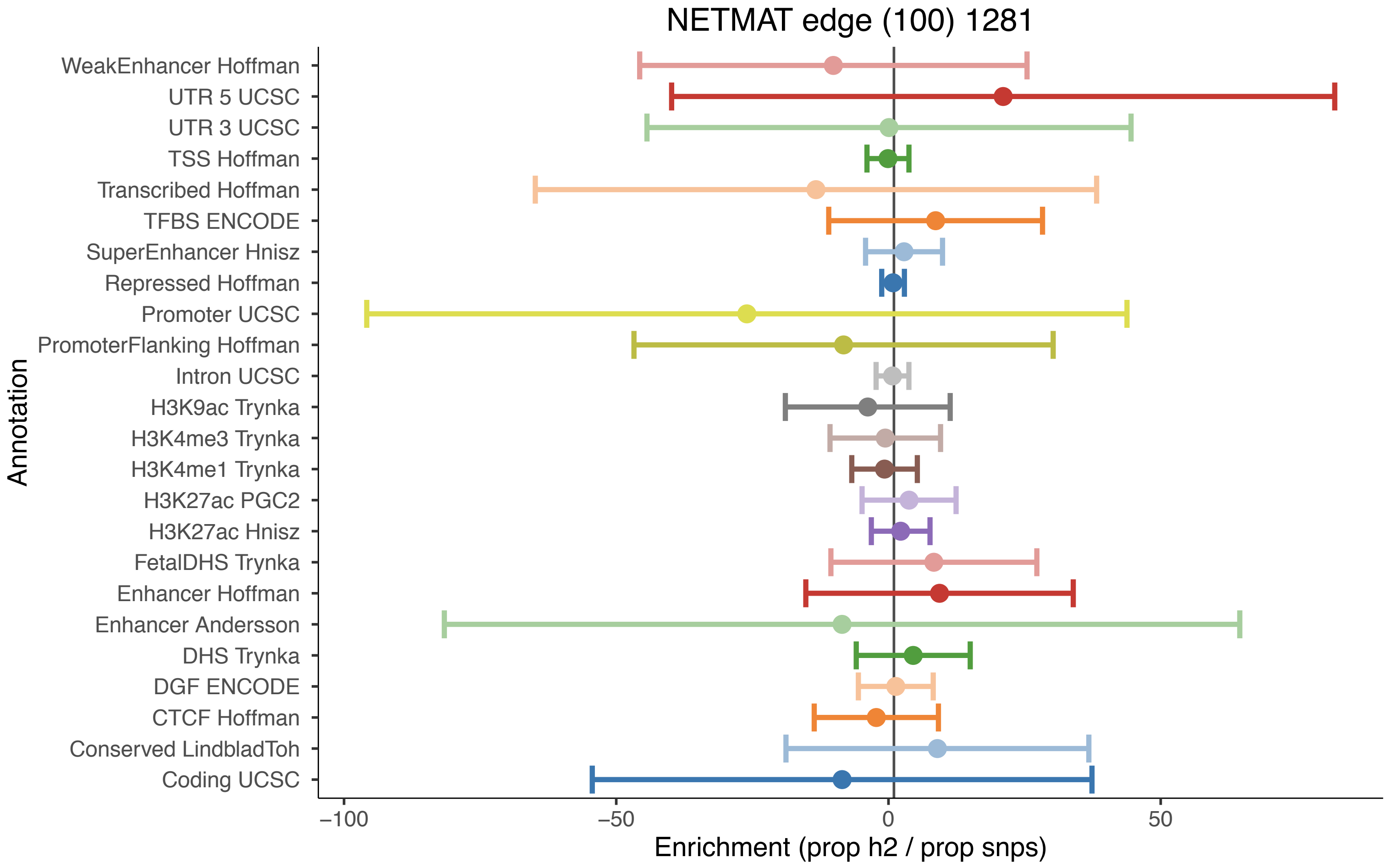
NETMAT edge (100) 1281
WeakEnhancer Hoffman
UTR 5 UCSC
UTR 3 UCSC
TSS Hoffman
Transcribed Hoffman
TFBS ENCODE
SuperEnhancer Hnisz
Repressed Hoffman
Promoter UCSC
PromoterFlanking Hoffman
Intron UCSC
H3K9ac Trynka
H3K4me3 Trynka
H3K4me1 Trynka
H3K27ac PGC2
H3K27ac Hnisz
FetalDHS Trynka
Enhancer Hoffman
Enhancer Andersson
DHS Trynka
DGF ENCODE
CTCF Hoffman
Conserved LindbladToh
Coding UCSC
Annotation
−100
−50
0
50
Enrichment (prop h2 / prop snps)

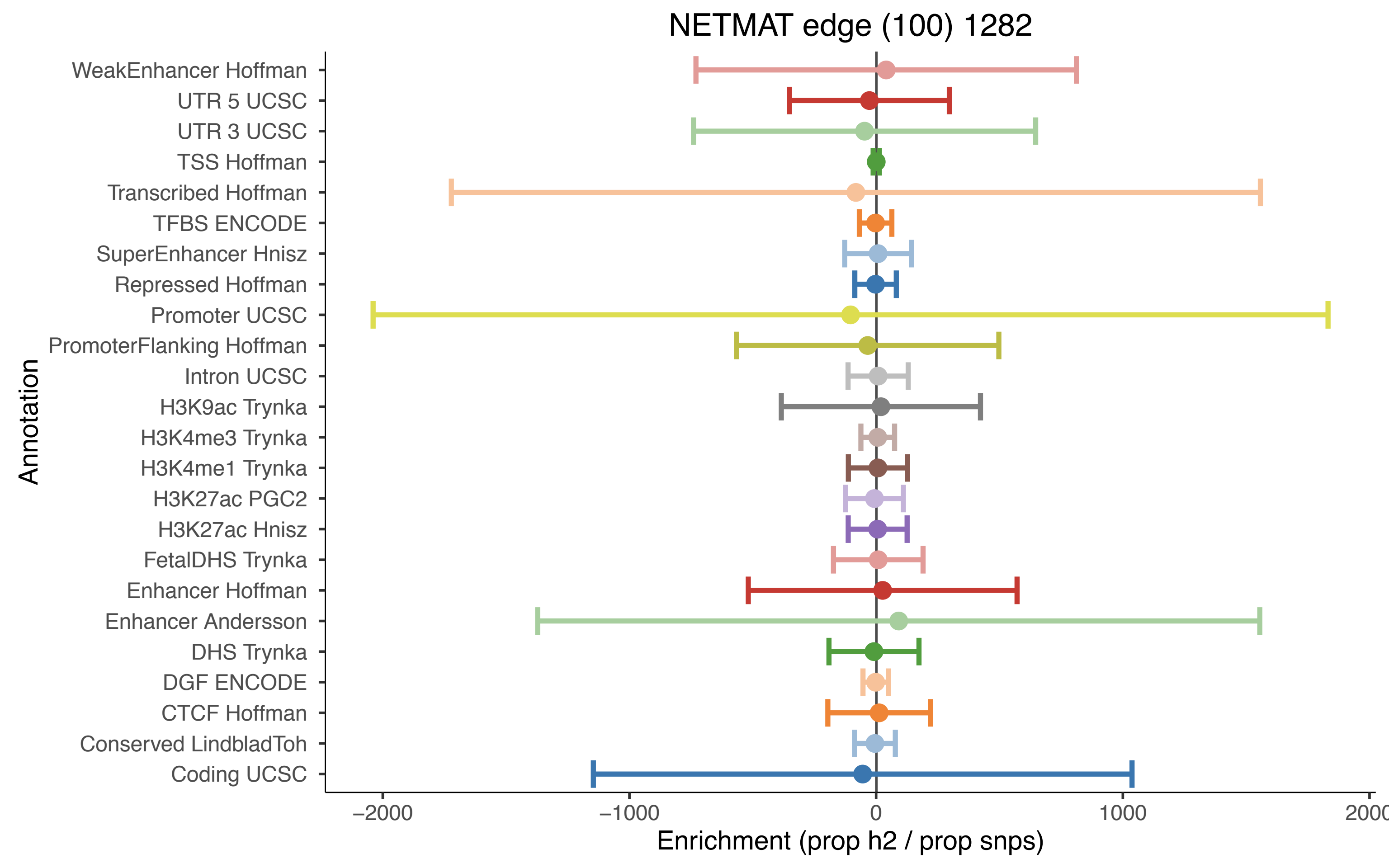
NETMAT edge (100) 1282
WeakEnhancer Hoffman
UTR 5 UCSC
UTR 3 UCSC
TSS Hoffman
Transcribed Hoffman
TFBS ENCODE
SuperEnhancer Hnisz
Repressed Hoffman
Promoter UCSC
PromoterFlanking Hoffman
Intron UCSC
H3K9ac Trynka
H3K4me3 Trynka
H3K4me1 Trynka
H3K27ac PGC2
H3K27ac Hnisz
FetalDHS Trynka
Enhancer Hoffman
Enhancer Andersson
DHS Trynka
DGF ENCODE
CTCF Hoffman
Conserved LindbladToh
Coding UCSC
Annotation
−2000
−1000
0
1000
Enrichment (prop h2 / prop snps)

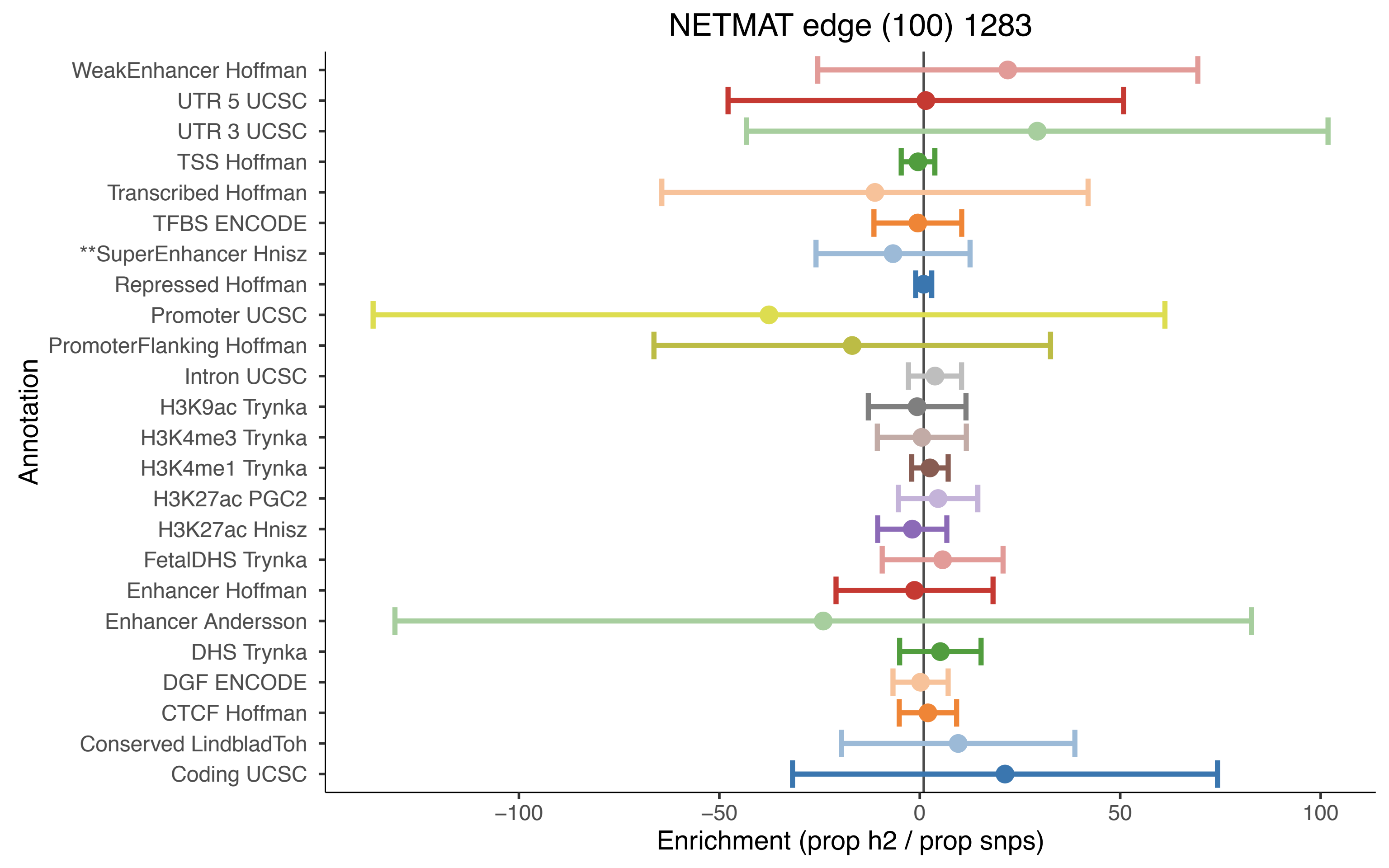

NETMAT edge (100) 1283
WeakEnhancer Hoffman
UTR 5 UCSC
UTR 3 UCSC
TSS Hoffman
Transcribed Hoffman
TFBS ENCODE
**SuperEnhancer Hnisz
Repressed Hoffman
Promoter UCSC
PromoterFlanking Hoffman
Intron UCSC
H3K9ac Trynka
H3K4me3 Trynka
H3K4me1 Trynka
H3K27ac PGC2
H3K27ac Hnisz
FetalDHS Trynka
Enhancer Hoffman
Enhancer Andersson
DHS Trynka
DGF ENCODE
CTCF Hoffman
Conserved LindbladToh
Coding UCSC
Annotation
−100
−50
0
50
100
Enrichment (prop h2 / prop snps)

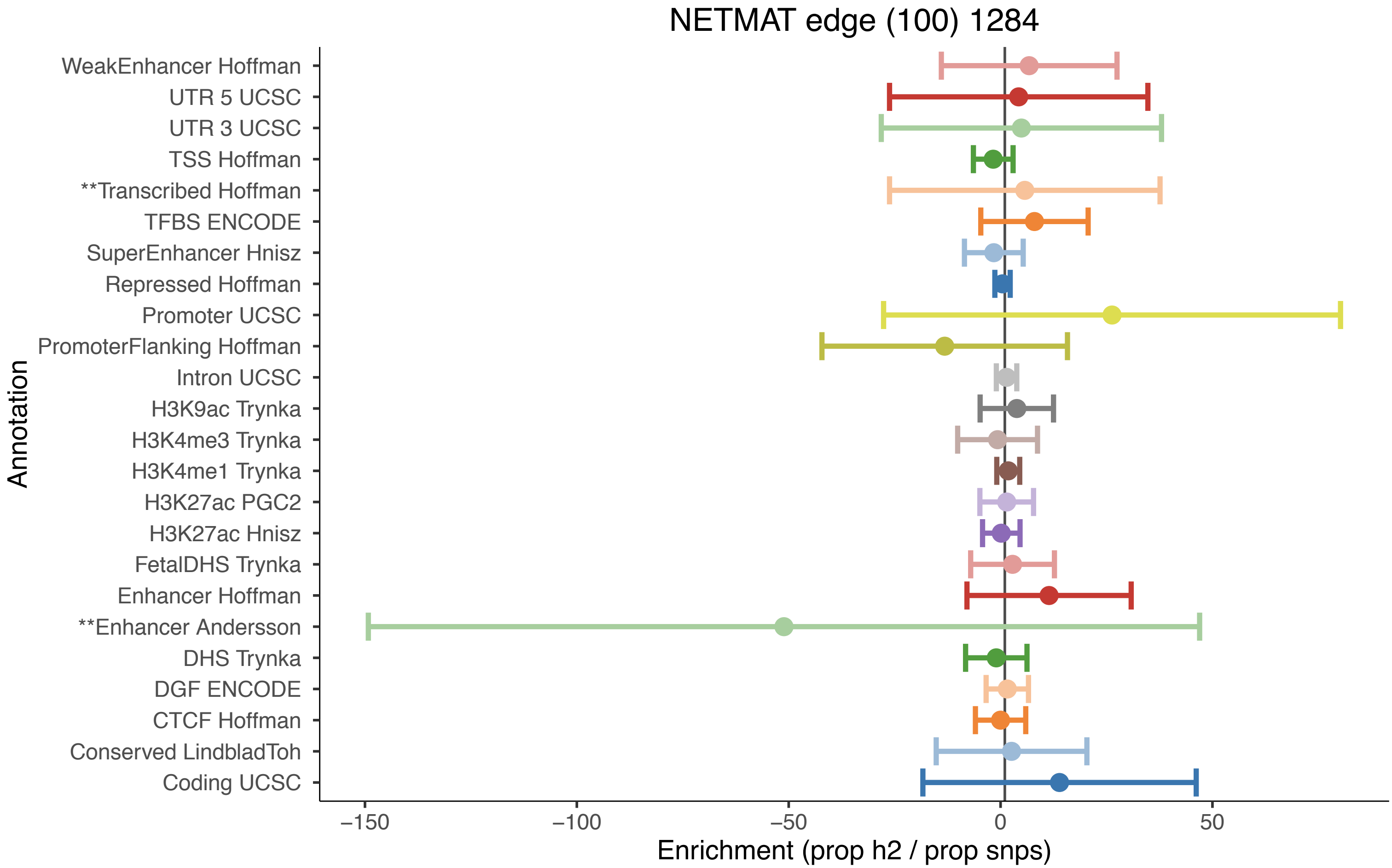
NETMAT edge (100) 1284
WeakEnhancer Hoffman
UTR 5 UCSC
UTR 3 UCSC
TSS Hoffman
**Transcribed Hoffman
TFBS ENCODE
SuperEnhancer Hnisz
Repressed Hoffman
Promoter UCSC
PromoterFlanking Hoffman
Intron UCSC
H3K9ac Trynka
H3K4me3 Trynka
H3K4me1 Trynka
H3K27ac PGC2
H3K27ac Hnisz
FetalDHS Trynka
Enhancer Hoffman
**Enhancer Andersson
DHS Trynka
DGF ENCODE
CTCF Hoffman
Conserved LindbladToh
Coding UCSC
Annotation
−150
−100
−50
0
50
Enrichment (prop h2 / prop snps)

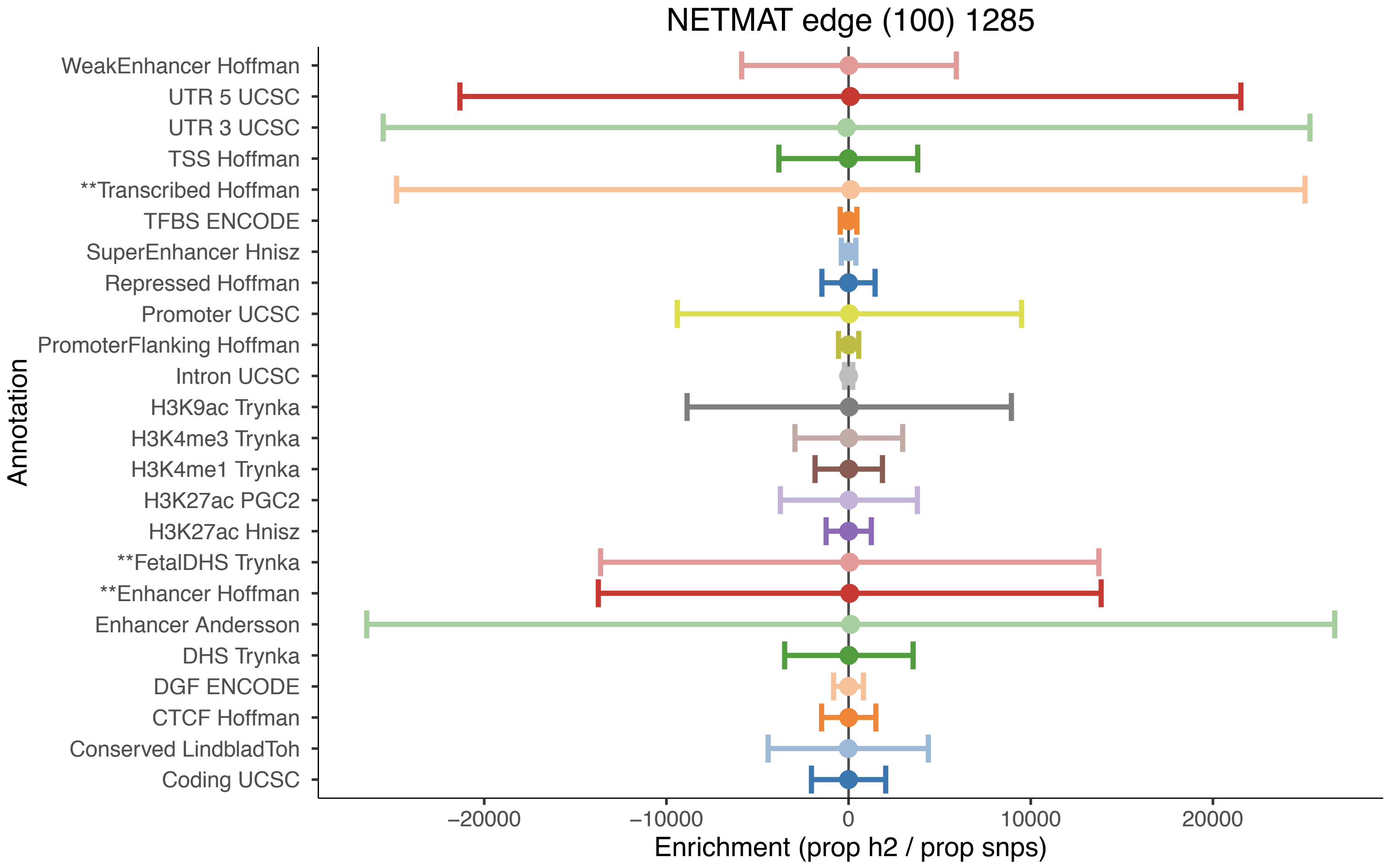
NETMAT edge (100) 1285
WeakEnhancer Hoffman
UTR 5 UCSC
UTR 3 UCSC
TSS Hoffman
**Transcribed Hoffman
TFBS ENCODE
SuperEnhancer Hnisz
Repressed Hoffman
Promoter UCSC
PromoterFlanking Hoffman
Intron UCSC
H3K9ac Trynka
H3K4me3 Trynka
H3K4me1 Trynka
H3K27ac PGC2
H3K27ac Hnisz
**FetalDHS Trynka
**Enhancer Hoffman
Enhancer Andersson
DHS Trynka
DGF ENCODE
CTCF Hoffman
Conserved LindbladToh
Coding UCSC
Annotation
−20000
−10000
0
10000
20000
Enrichment (prop h2 / prop snps)

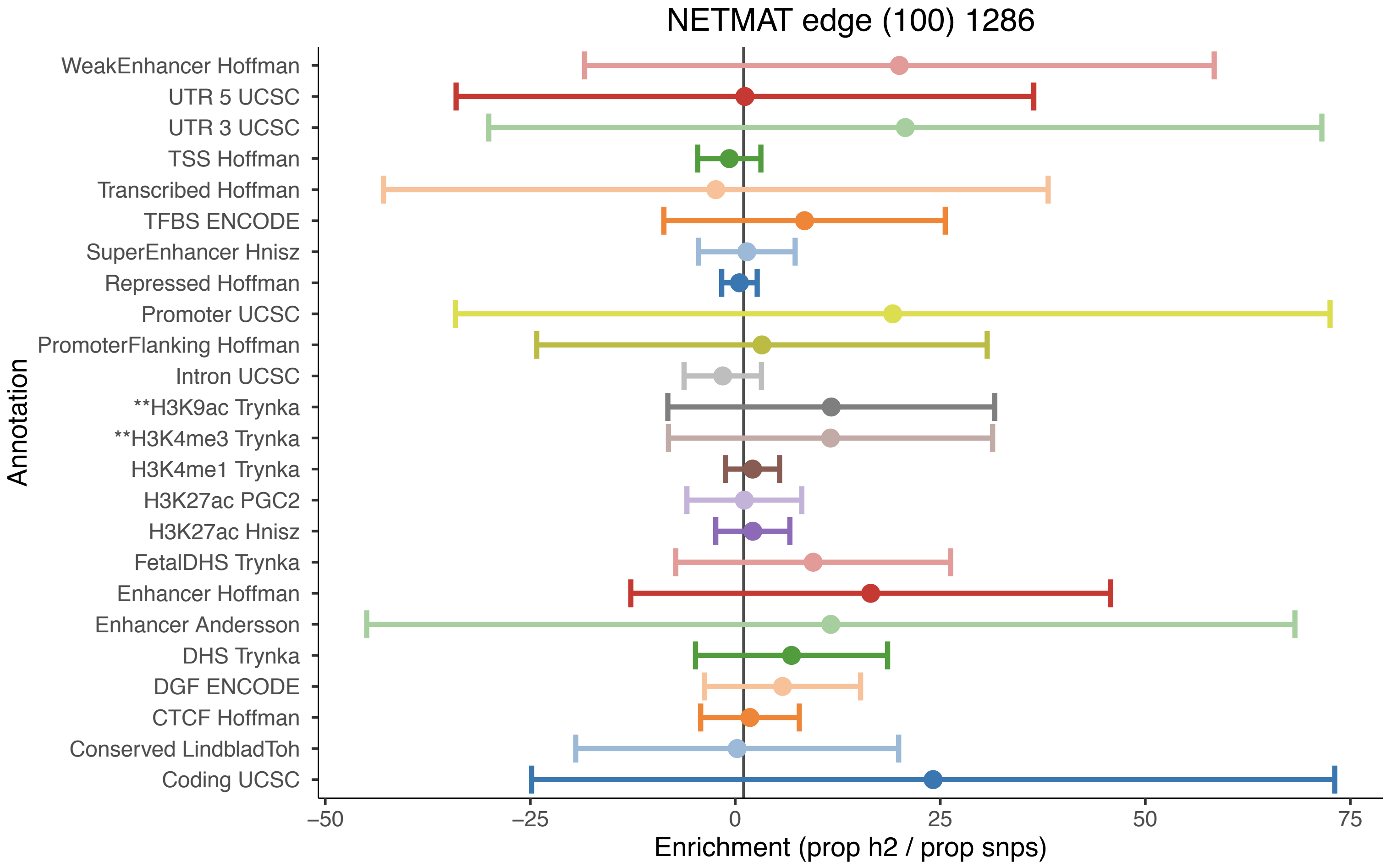

NETMAT edge (100) 1286
WeakEnhancer Hoffman
UTR 5 UCSC
UTR 3 UCSC
TSS Hoffman
Transcribed Hoffman
TFBS ENCODE
SuperEnhancer Hnisz
Repressed Hoffman
Promoter UCSC
PromoterFlanking Hoffman
Intron UCSC
**H3K9ac Trynka
**H3K4me3 Trynka
H3K4me1 Trynka
H3K27ac PGC2
H3K27ac Hnisz
FetalDHS Trynka
Enhancer Hoffman
Enhancer Andersson
DHS Trynka
DGF ENCODE
CTCF Hoffman
Conserved LindbladToh
Coding UCSC
Annotation
−50
−25
0
25
50
75
Enrichment (prop h2 / prop snps)

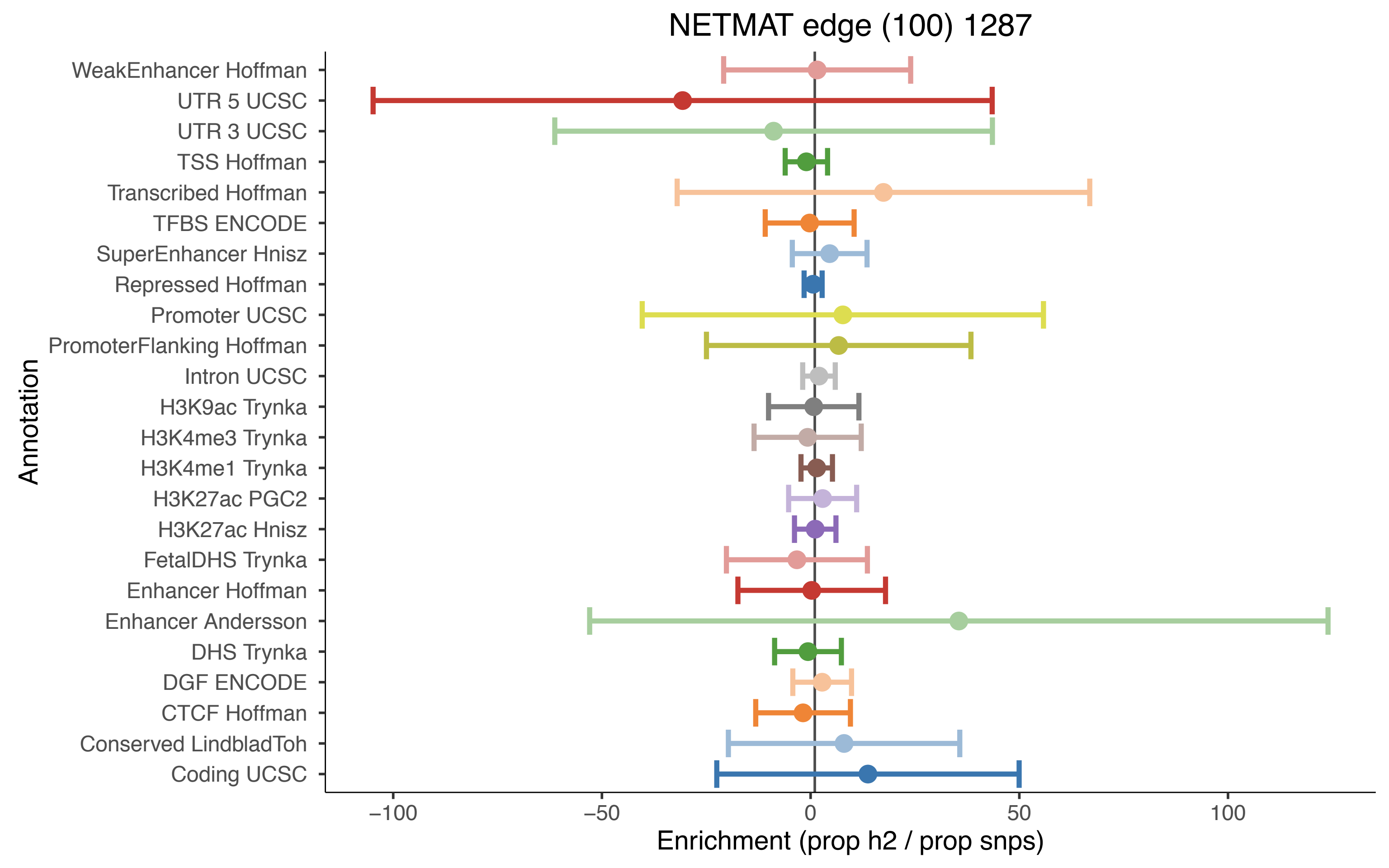

NETMAT edge (100) 1287
WeakEnhancer Hoffman
UTR 5 UCSC
UTR 3 UCSC
TSS Hoffman
Transcribed Hoffman
TFBS ENCODE
SuperEnhancer Hnisz
Repressed Hoffman
Promoter UCSC
PromoterFlanking Hoffman
Intron UCSC
H3K9ac Trynka
H3K4me3 Trynka
H3K4me1 Trynka
H3K27ac PGC2
H3K27ac Hnisz
FetalDHS Trynka
Enhancer Hoffman
Enhancer Andersson
DHS Trynka
DGF ENCODE
CTCF Hoffman
Conserved LindbladToh
Coding UCSC
Annotation
−100
−50
0
50
100
Enrichment (prop h2 / prop snps)

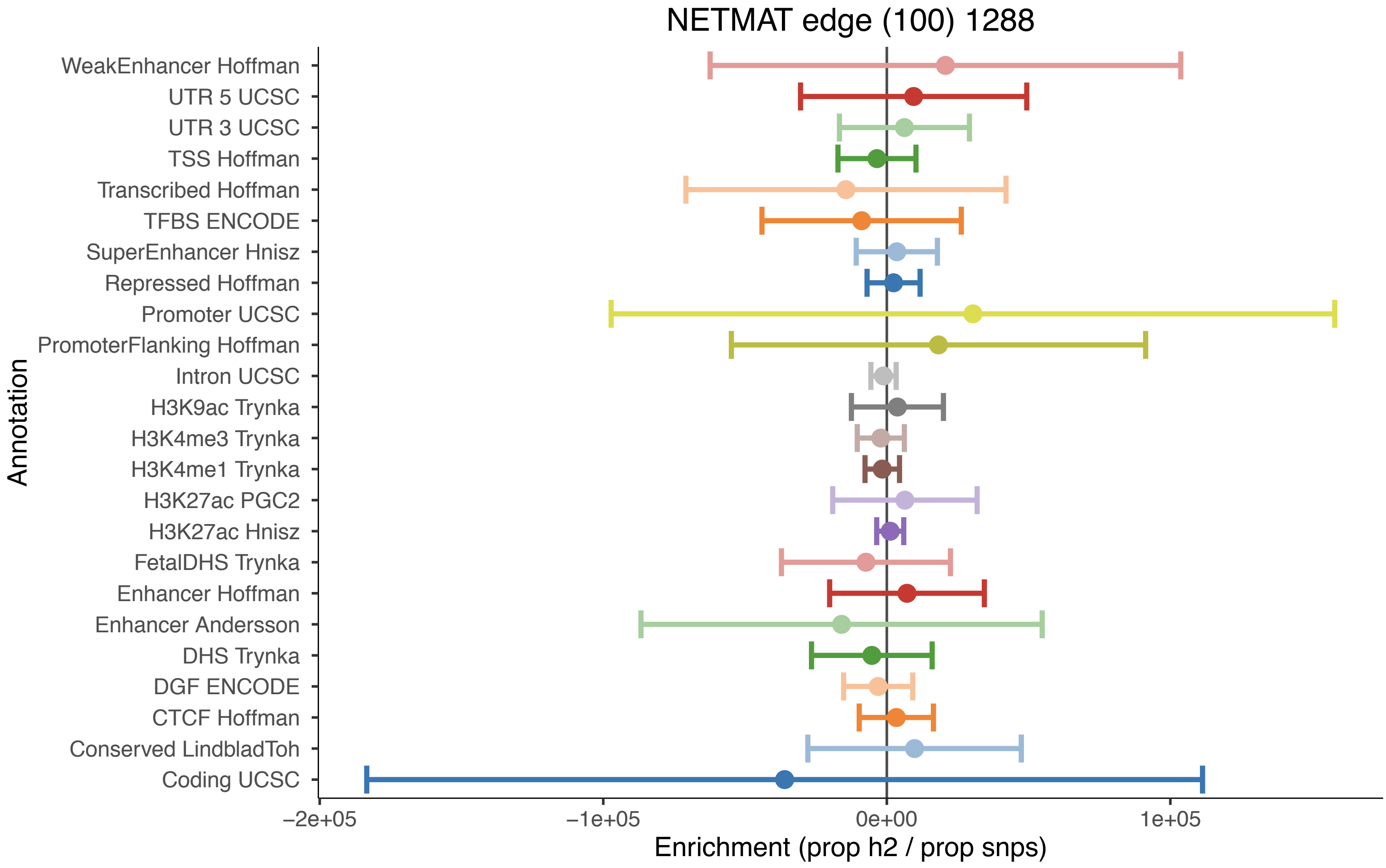

NETMAT edge (100) 1288
WeakEnhancer Hoffman
UTR 5 UCSC
UTR 3 UCSC
TSS Hoffman
Transcribed Hoffman
TFBS ENCODE
SuperEnhancer Hnisz
Repressed Hoffman
Promoter UCSC
PromoterFlanking Hoffman
Intron UCSC
H3K9ac Trynka
H3K4me3 Trynka
H3K4me1 Trynka
H3K27ac PGC2
H3K27ac Hnisz
FetalDHS Trynka
Enhancer Hoffman
Enhancer Andersson
DHS Trynka
DGF ENCODE
CTCF Hoffman
Conserved LindbladToh
Coding UCSC
Annotation
−2e+05
−1e+05
0e+00
1e+05
Enrichment (prop h2 / prop snps)

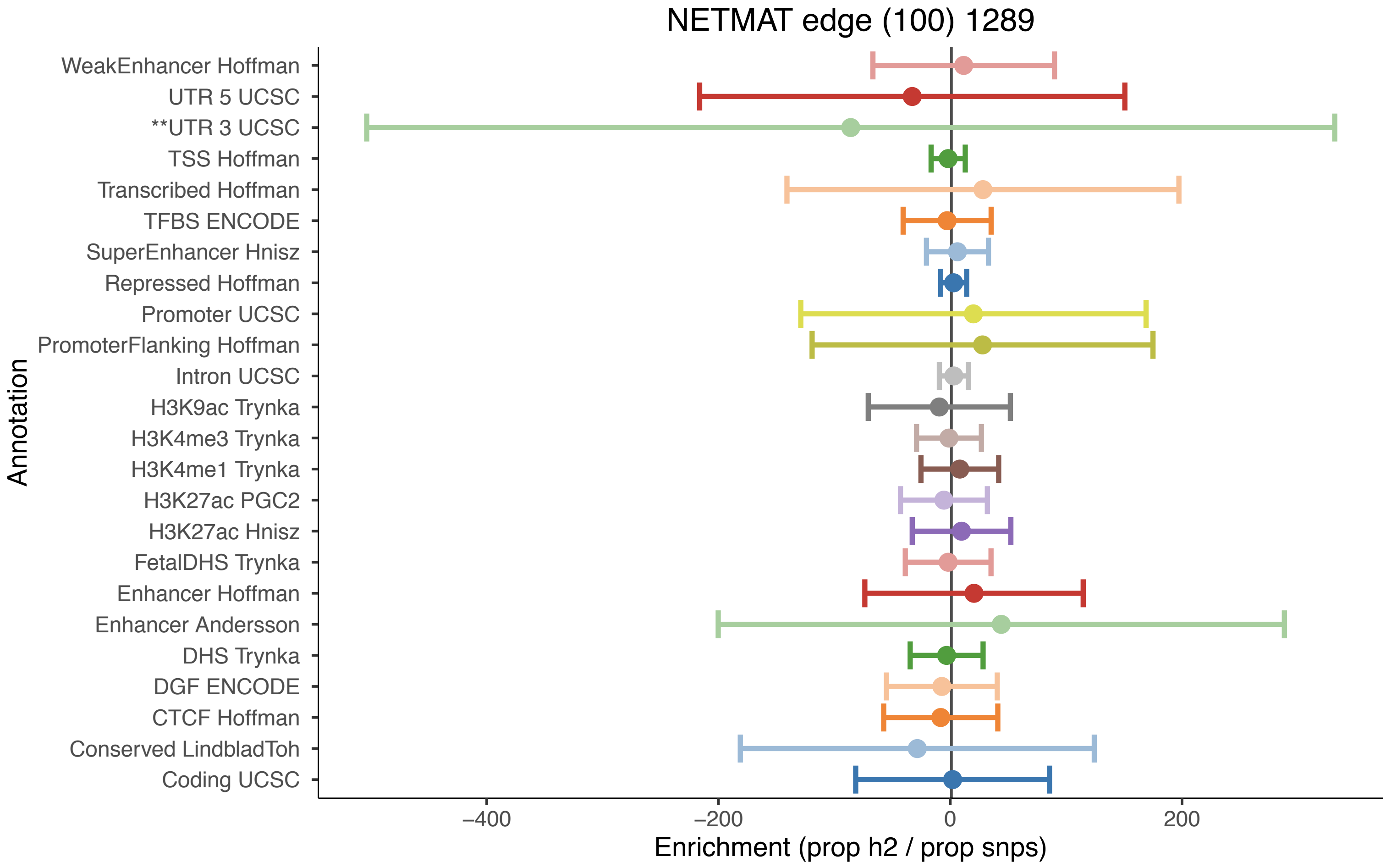
NETMAT edge (100) 1289
WeakEnhancer Hoffman
UTR 5 UCSC
**UTR 3 UCSC
TSS Hoffman
Transcribed Hoffman
TFBS ENCODE
SuperEnhancer Hnisz
Repressed Hoffman
Promoter UCSC
PromoterFlanking Hoffman
Intron UCSC
H3K9ac Trynka
H3K4me3 Trynka
H3K4me1 Trynka
H3K27ac PGC2
H3K27ac Hnisz
FetalDHS Trynka
Enhancer Hoffman
Enhancer Andersson
DHS Trynka
DGF ENCODE
CTCF Hoffman
Conserved LindbladToh
Coding UCSC
Annotation
−400
−200
0
200
Enrichment (prop h2 / prop snps)

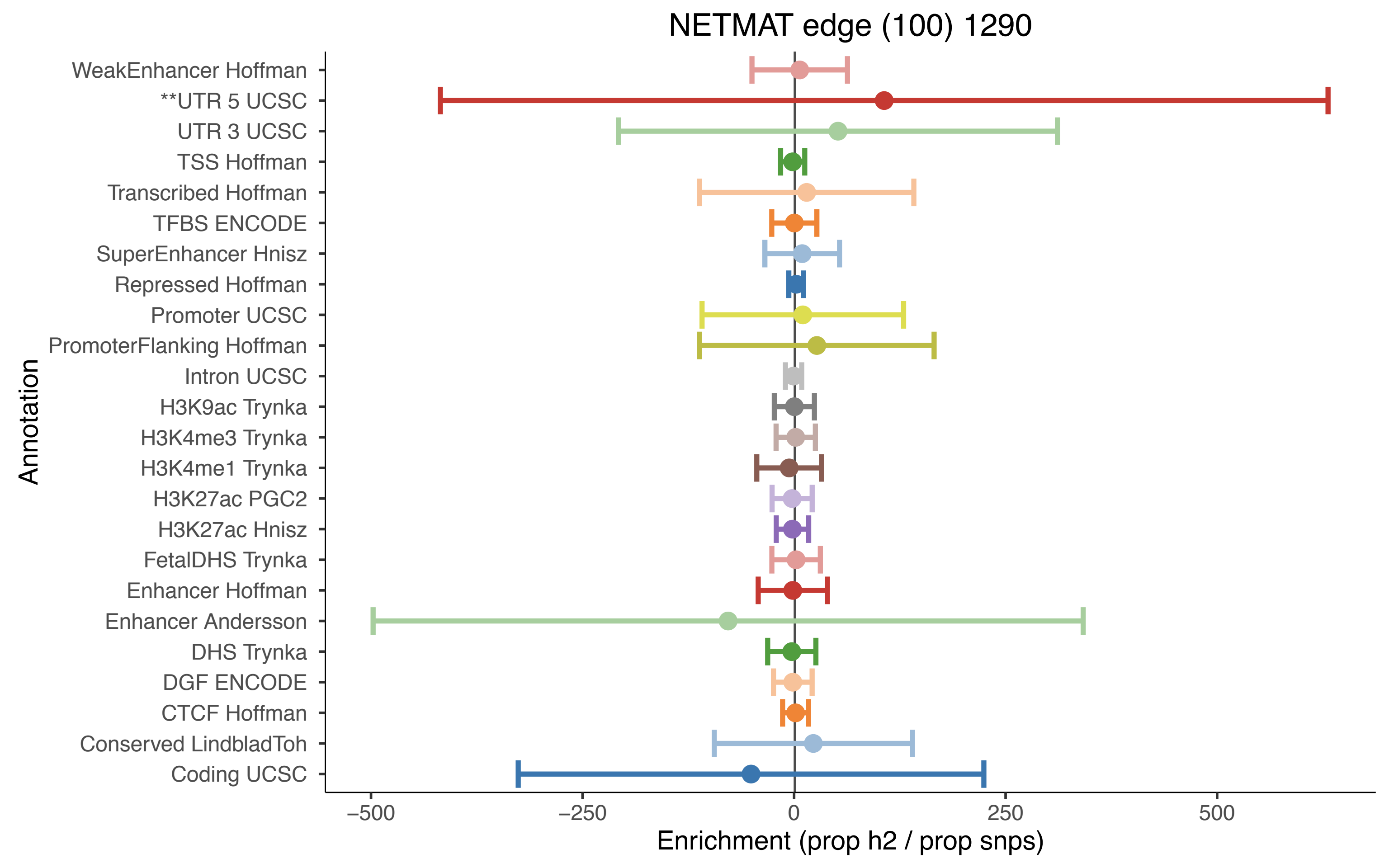
NETMAT edge (100) 1290
WeakEnhancer Hoffman
**UTR 5 UCSC
UTR 3 UCSC
TSS Hoffman
Transcribed Hoffman
TFBS ENCODE
SuperEnhancer Hnisz
Repressed Hoffman
Promoter UCSC
PromoterFlanking Hoffman
Intron UCSC
H3K9ac Trynka
H3K4me3 Trynka
H3K4me1 Trynka
H3K27ac PGC2
H3K27ac Hnisz
FetalDHS Trynka
Enhancer Hoffman
Enhancer Andersson
DHS Trynka
DGF ENCODE
CTCF Hoffman
Conserved LindbladToh
Coding UCSC
Annotation
−500
−250
0
250
500
Enrichment (prop h2 / prop snps)

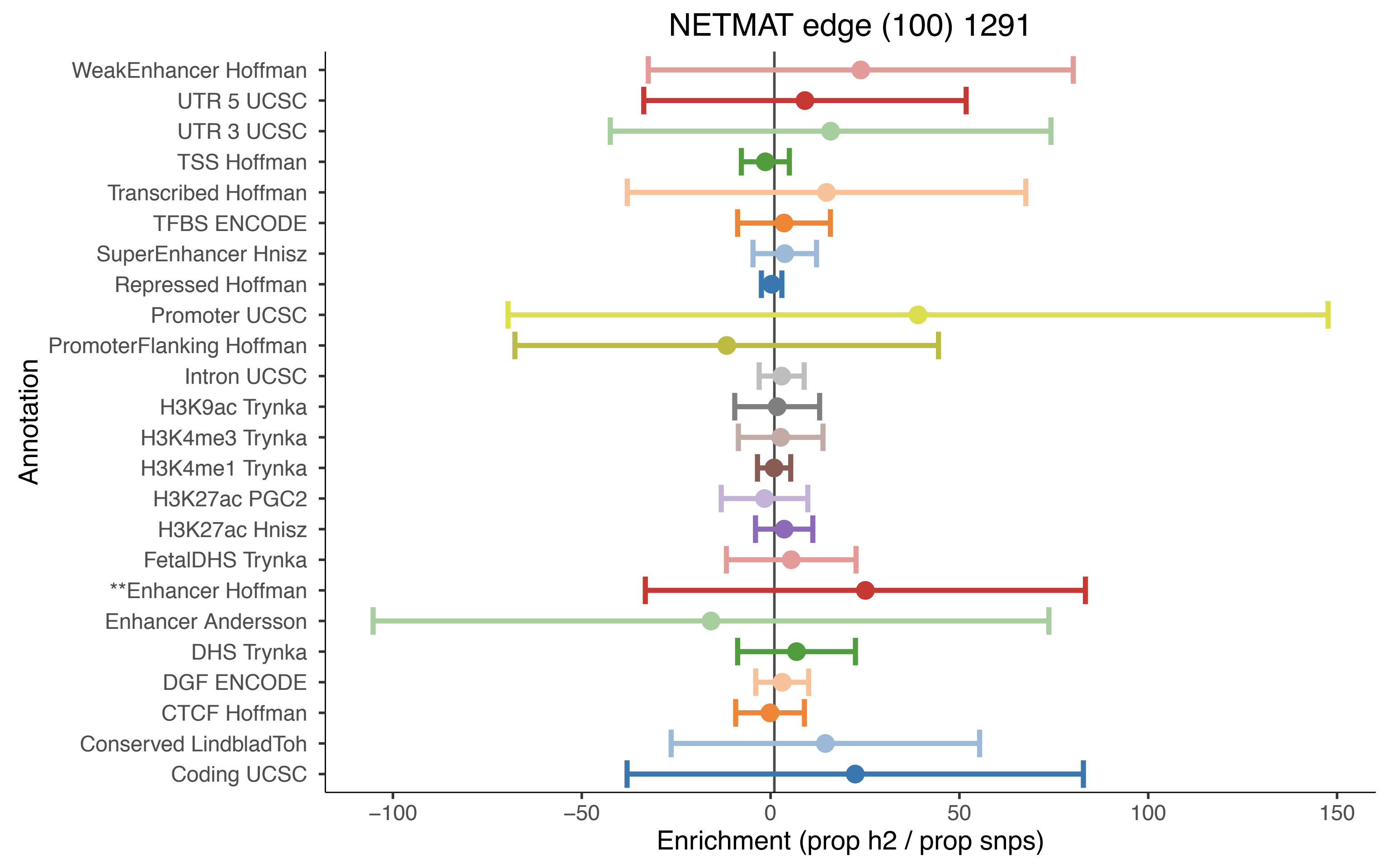

NETMAT edge (100) 1291
WeakEnhancer Hoffman
UTR 5 UCSC
UTR 3 UCSC
TSS Hoffman
Transcribed Hoffman
TFBS ENCODE
SuperEnhancer Hnisz
Repressed Hoffman
Promoter UCSC
PromoterFlanking Hoffman
Intron UCSC
H3K9ac Trynka
H3K4me3 Trynka
H3K4me1 Trynka
H3K27ac PGC2
H3K27ac Hnisz
FetalDHS Trynka
**Enhancer Hoffman
Enhancer Andersson
DHS Trynka
DGF ENCODE
CTCF Hoffman
Conserved LindbladToh
Coding UCSC
Annotation
−100
−50
0
50
100
150
Enrichment (prop h2 / prop snps)

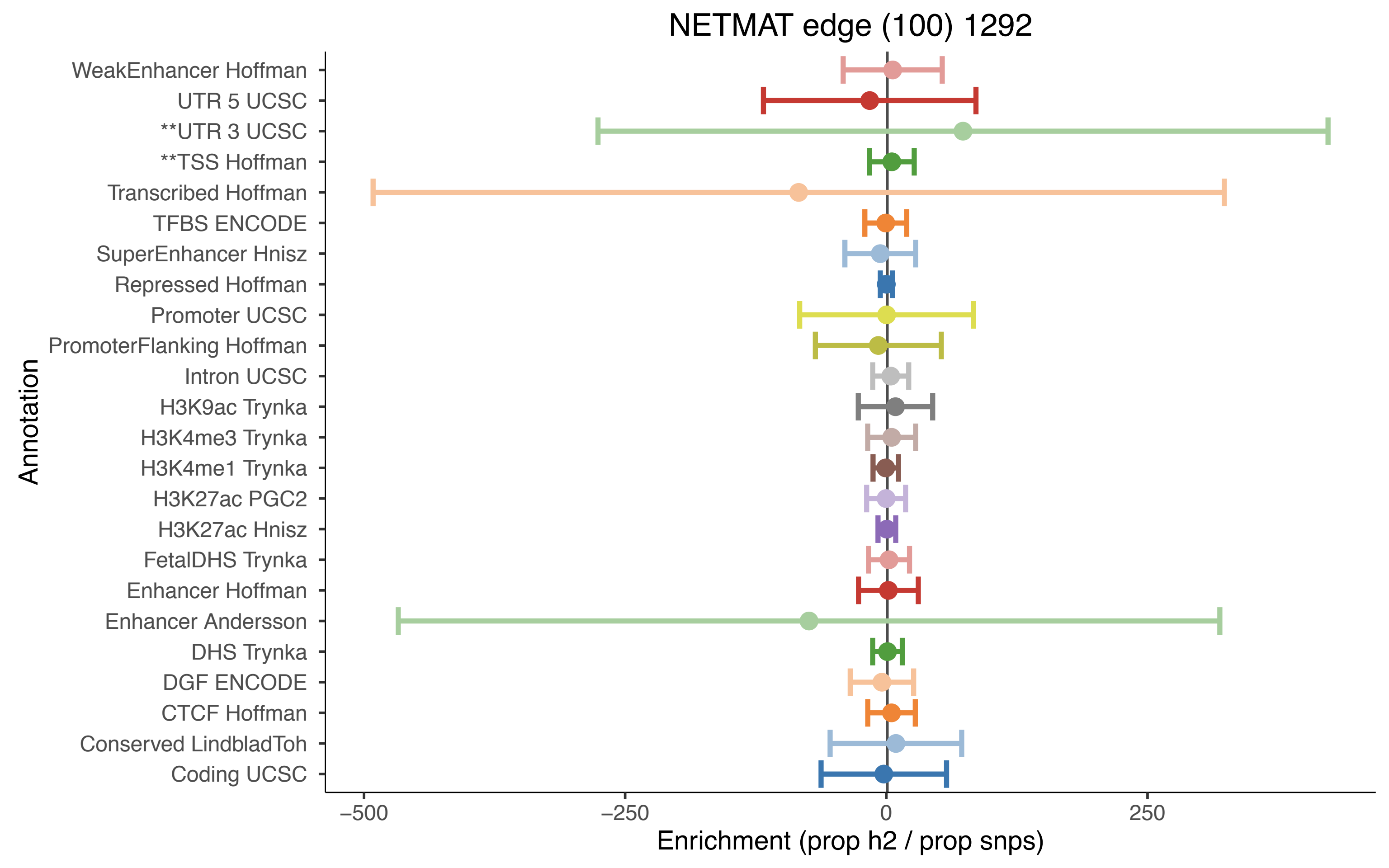
NETMAT edge (100) 1292
WeakEnhancer Hoffman
UTR 5 UCSC
**UTR 3 UCSC
**TSS Hoffman
Transcribed Hoffman
TFBS ENCODE
SuperEnhancer Hnisz
Repressed Hoffman
Promoter UCSC
PromoterFlanking Hoffman
Intron UCSC
H3K9ac Trynka
H3K4me3 Trynka
H3K4me1 Trynka
H3K27ac PGC2
H3K27ac Hnisz
FetalDHS Trynka
Enhancer Hoffman
Enhancer Andersson
DHS Trynka
DGF ENCODE
CTCF Hoffman
Conserved LindbladToh
Coding UCSC
Annotation
−500
−250
0
250
Enrichment (prop h2 / prop snps)

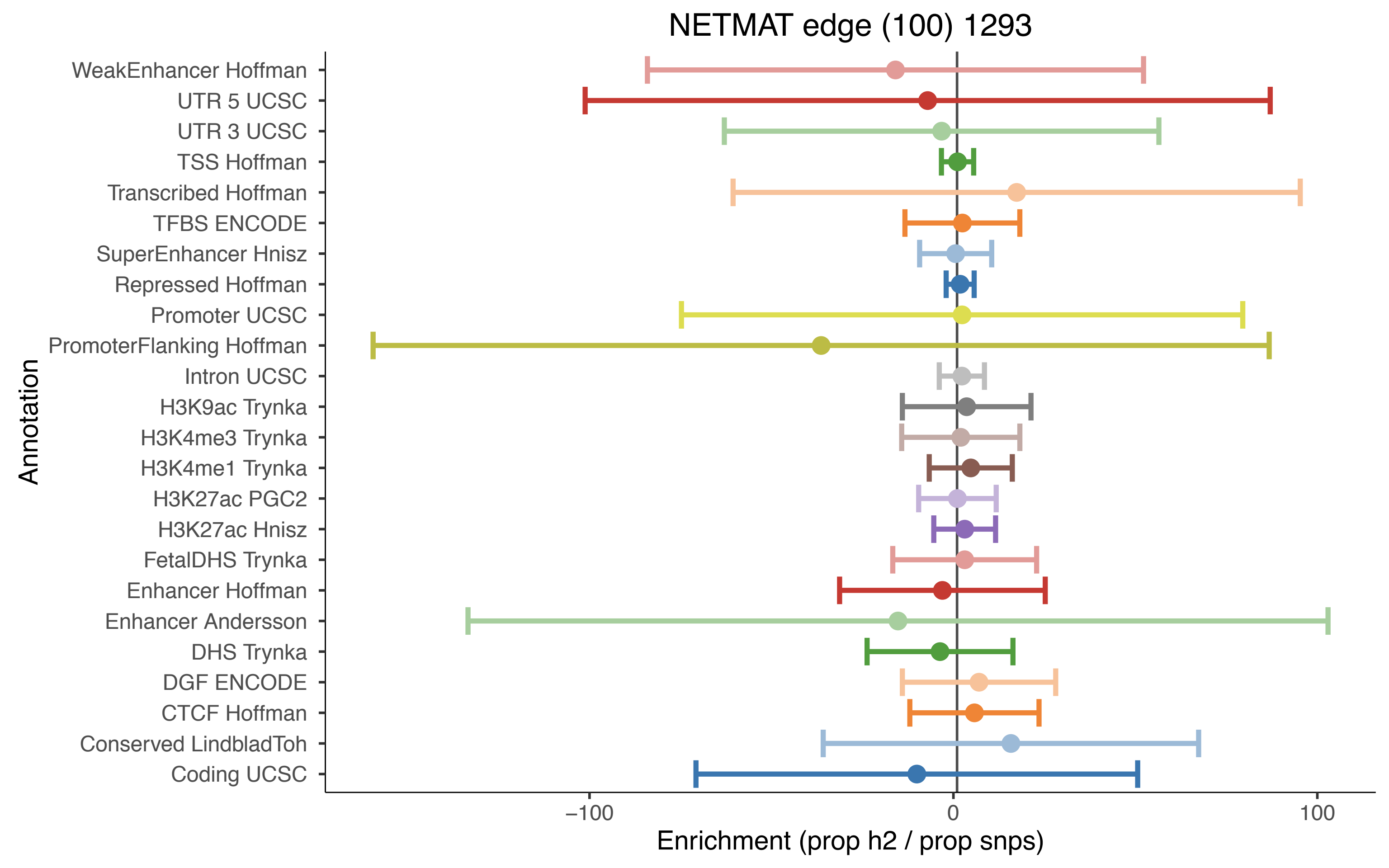
NETMAT edge (100) 1293
WeakEnhancer Hoffman
UTR 5 UCSC
UTR 3 UCSC
TSS Hoffman
Transcribed Hoffman
TFBS ENCODE
SuperEnhancer Hnisz
Repressed Hoffman
Promoter UCSC
PromoterFlanking Hoffman
Intron UCSC
H3K9ac Trynka
H3K4me3 Trynka
H3K4me1 Trynka
H3K27ac PGC2
H3K27ac Hnisz
FetalDHS Trynka
Enhancer Hoffman
Enhancer Andersson
DHS Trynka
DGF ENCODE
CTCF Hoffman
Conserved LindbladToh
Coding UCSC
Annotation
−100
0
100
Enrichment (prop h2 / prop snps)

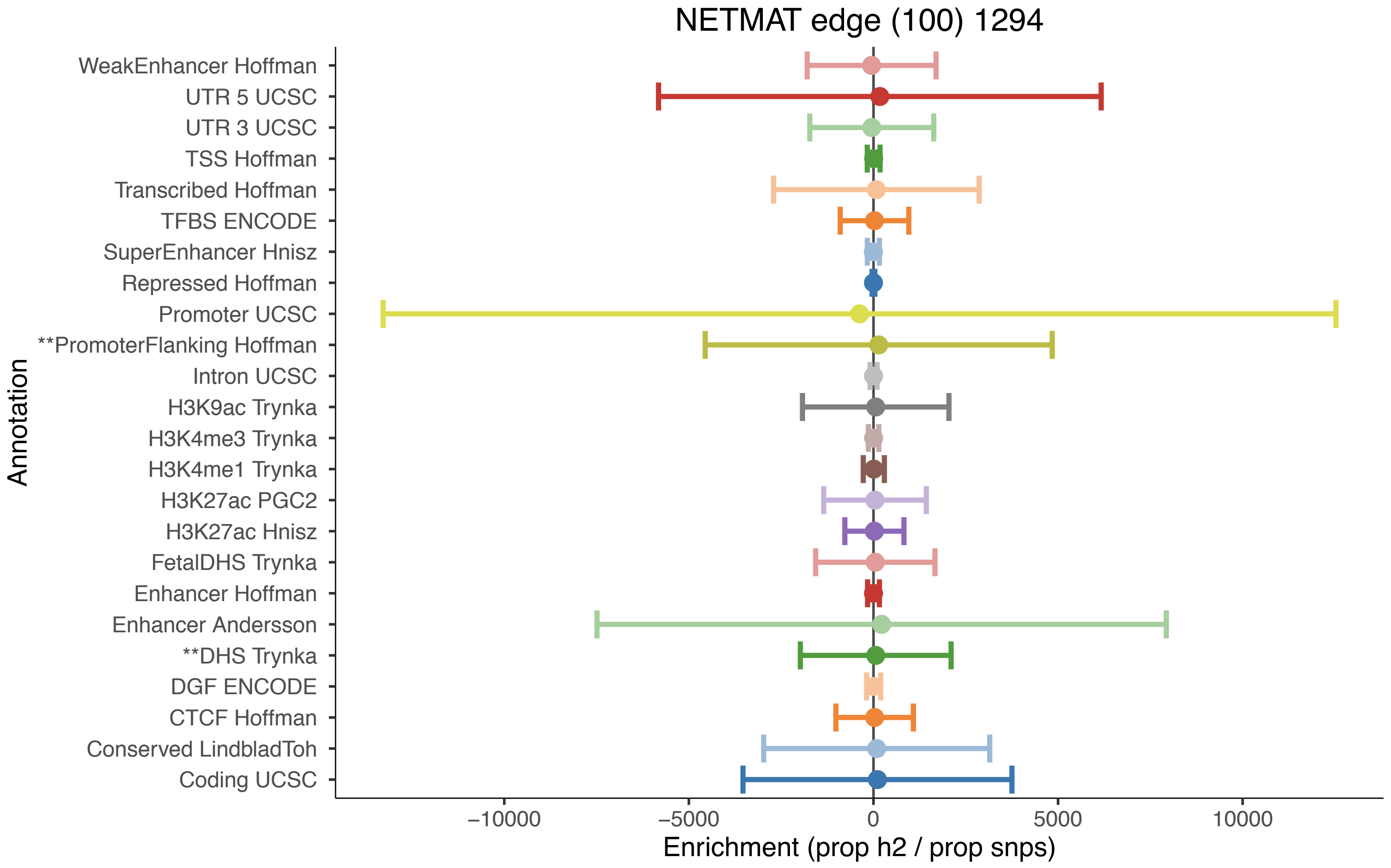

NETMAT edge (100) 1294
WeakEnhancer Hoffman
UTR 5 UCSC
UTR 3 UCSC
TSS Hoffman
Transcribed Hoffman
TFBS ENCODE
SuperEnhancer Hnisz
Repressed Hoffman
Promoter UCSC
**PromoterFlanking Hoffman
Intron UCSC
H3K9ac Trynka
H3K4me3 Trynka
H3K4me1 Trynka
H3K27ac PGC2
H3K27ac Hnisz
FetalDHS Trynka
Enhancer Hoffman
Enhancer Andersson
**DHS Trynka
DGF ENCODE
CTCF Hoffman
Conserved LindbladToh
Coding UCSC
Annotation
−10000
−5000
0
5000
10000
Enrichment (prop h2 / prop snps)

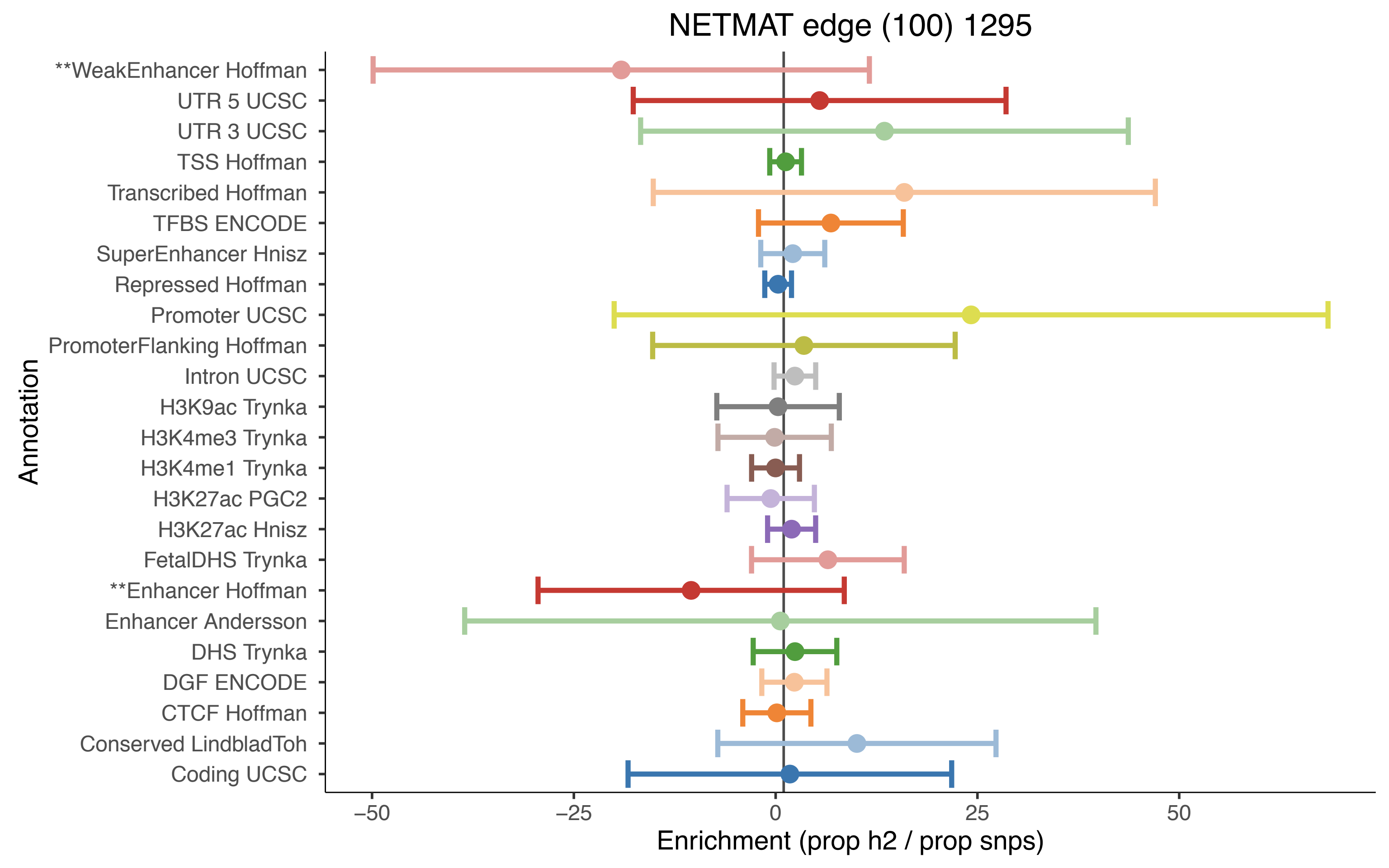
NETMAT edge (100) 1295
**WeakEnhancer Hoffman
UTR 5 UCSC
UTR 3 UCSC
TSS Hoffman
Transcribed Hoffman
TFBS ENCODE
SuperEnhancer Hnisz
Repressed Hoffman
Promoter UCSC
PromoterFlanking Hoffman
Intron UCSC
H3K9ac Trynka
H3K4me3 Trynka
H3K4me1 Trynka
H3K27ac PGC2
H3K27ac Hnisz
FetalDHS Trynka
**Enhancer Hoffman
Enhancer Andersson
DHS Trynka
DGF ENCODE
CTCF Hoffman
Conserved LindbladToh
Coding UCSC
Annotation
−50
−25
0
25
50
Enrichment (prop h2 / prop snps)

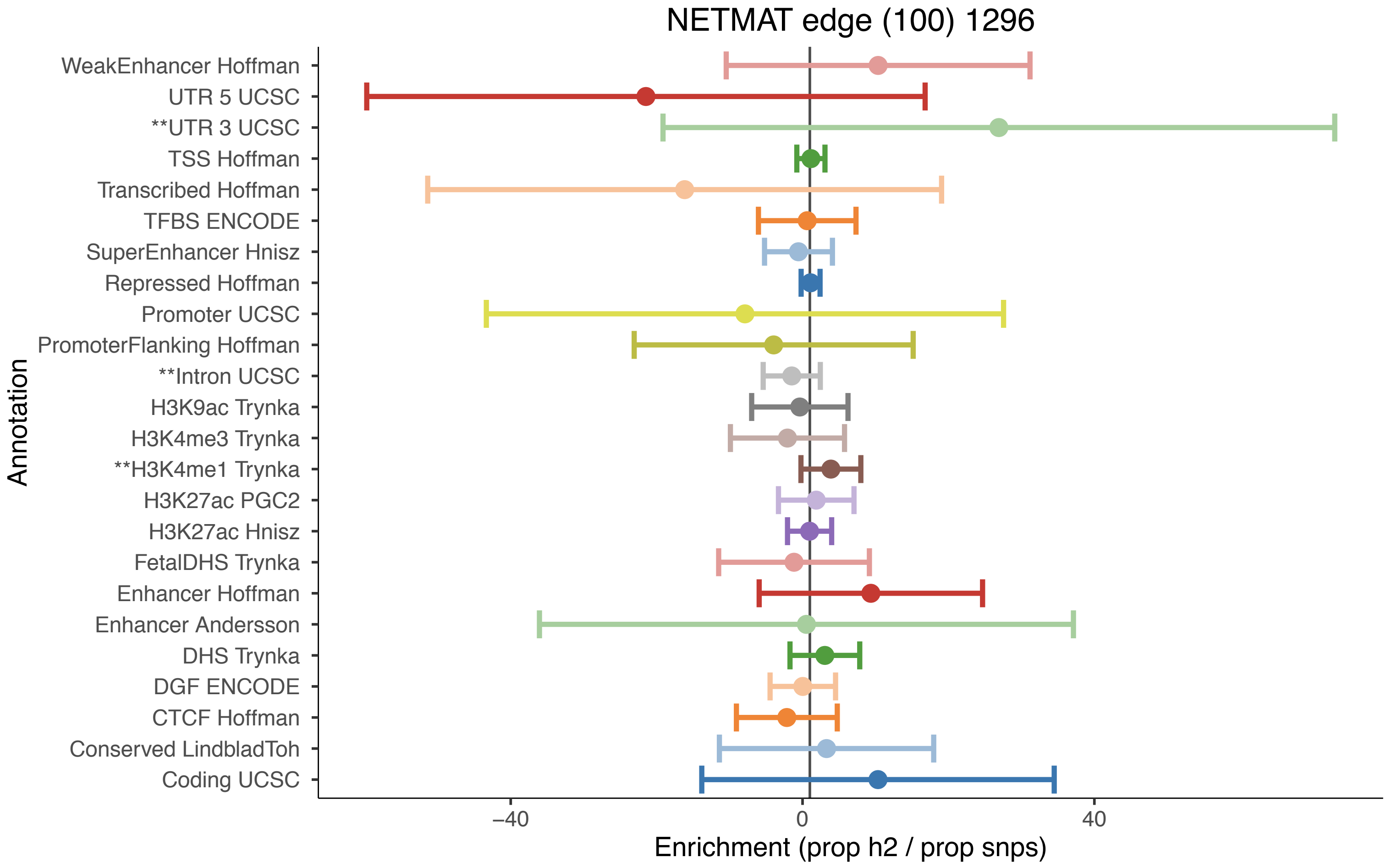
NETMAT edge (100) 1296
WeakEnhancer Hoffman
UTR 5 UCSC
**UTR 3 UCSC
TSS Hoffman
Transcribed Hoffman
TFBS ENCODE
SuperEnhancer Hnisz
Repressed Hoffman
Promoter UCSC
PromoterFlanking Hoffman
**Intron UCSC
H3K9ac Trynka
H3K4me3 Trynka
**H3K4me1 Trynka
H3K27ac PGC2
H3K27ac Hnisz
FetalDHS Trynka
Enhancer Hoffman
Enhancer Andersson
DHS Trynka
DGF ENCODE
CTCF Hoffman
Conserved LindbladToh
Coding UCSC
Annotation
−40
0
40
Enrichment (prop h2 / prop snps)

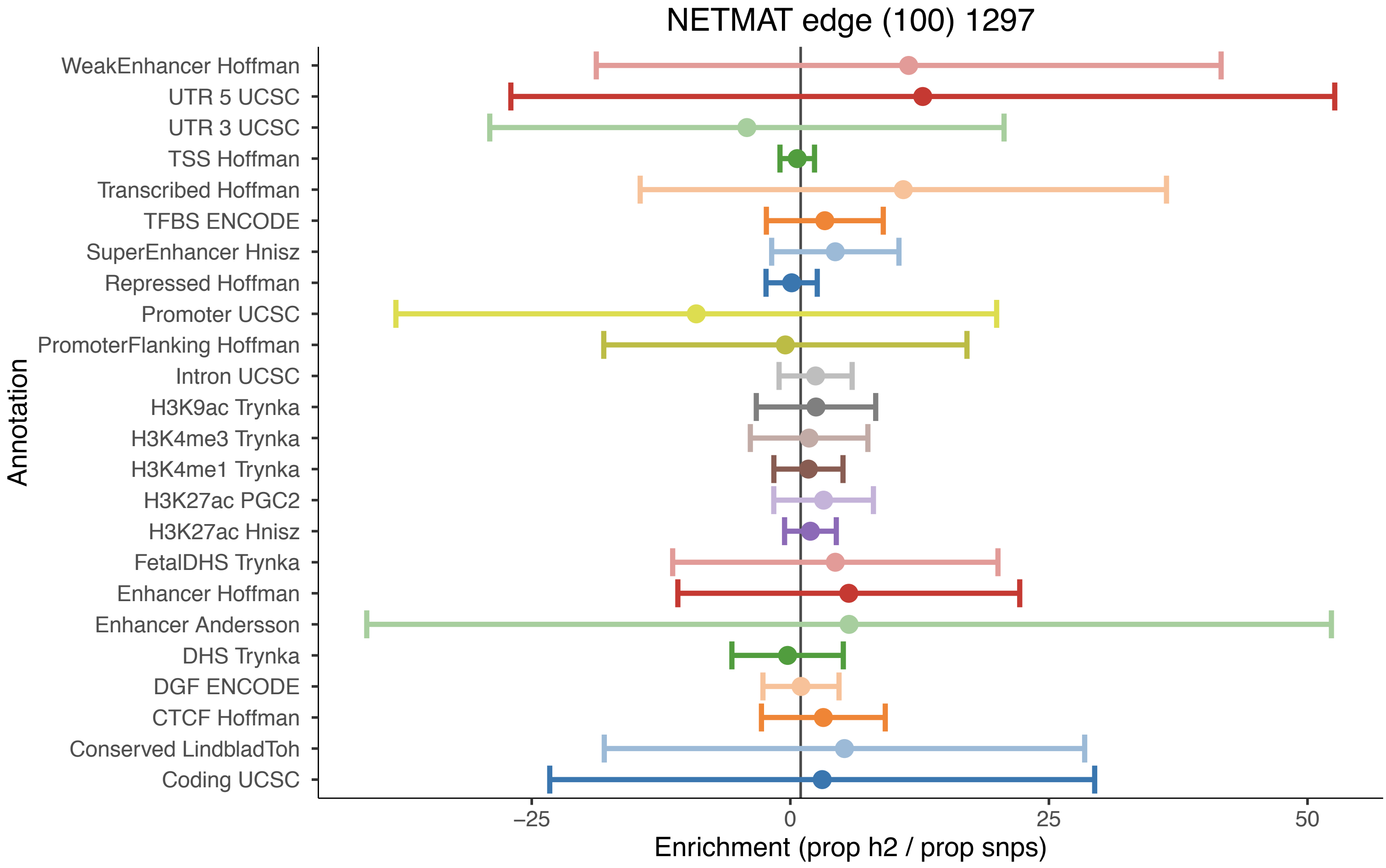
NETMAT edge (100) 1297
WeakEnhancer Hoffman
UTR 5 UCSC
UTR 3 UCSC
TSS Hoffman
Transcribed Hoffman
TFBS ENCODE
SuperEnhancer Hnisz
Repressed Hoffman
Promoter UCSC
PromoterFlanking Hoffman
Intron UCSC
H3K9ac Trynka
H3K4me3 Trynka
H3K4me1 Trynka
H3K27ac PGC2
H3K27ac Hnisz
FetalDHS Trynka
Enhancer Hoffman
Enhancer Andersson
DHS Trynka
DGF ENCODE
CTCF Hoffman
Conserved LindbladToh
Coding UCSC
Annotation
−25
0
25
50
Enrichment (prop h2 / prop snps)

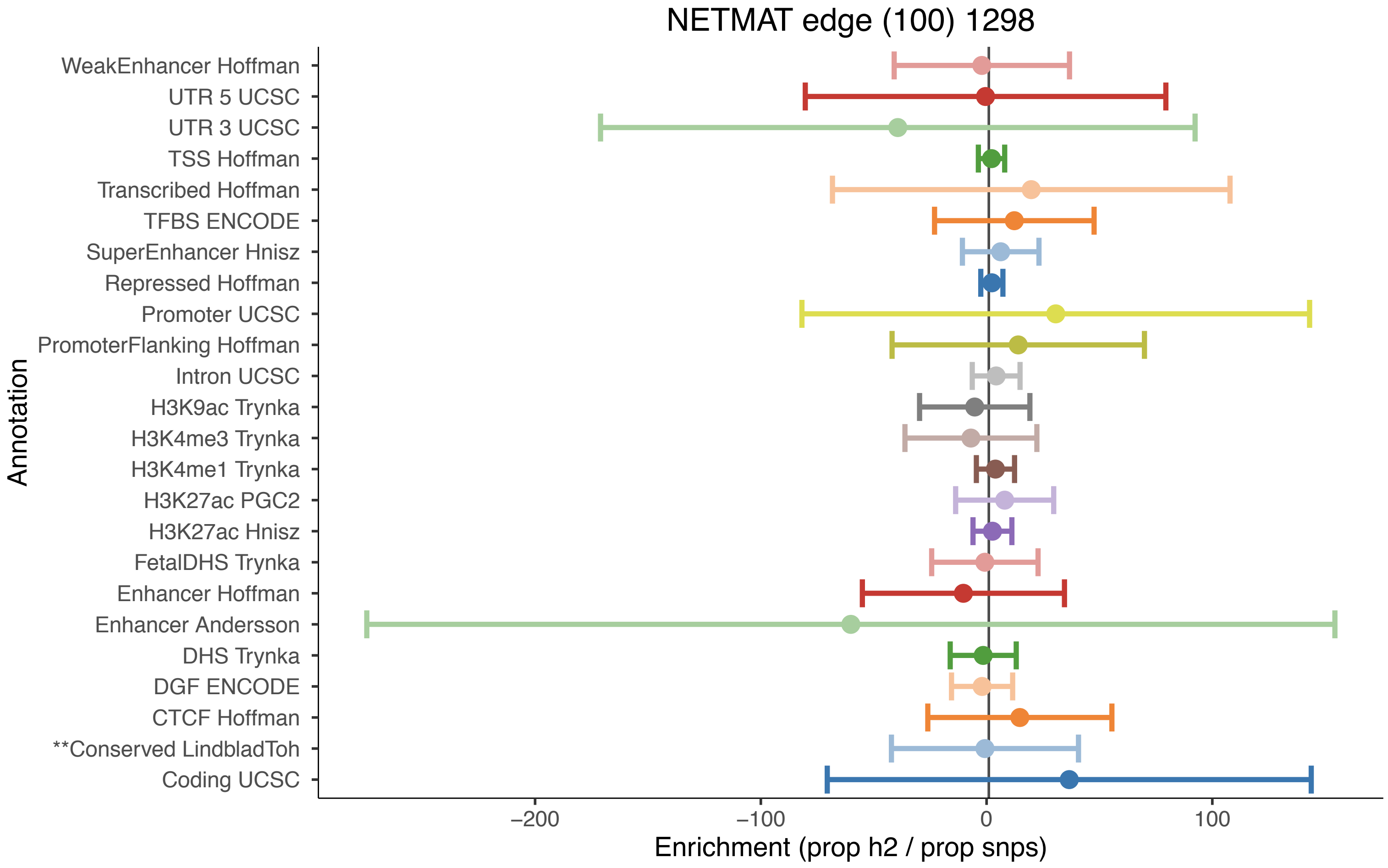
NETMAT edge (100) 1298
WeakEnhancer Hoffman
UTR 5 UCSC
UTR 3 UCSC
TSS Hoffman
Transcribed Hoffman
TFBS ENCODE
SuperEnhancer Hnisz
Repressed Hoffman
Promoter UCSC
PromoterFlanking Hoffman
Intron UCSC
H3K9ac Trynka
H3K4me3 Trynka
H3K4me1 Trynka
H3K27ac PGC2
H3K27ac Hnisz
FetalDHS Trynka
Enhancer Hoffman
Enhancer Andersson
DHS Trynka
DGF ENCODE
CTCF Hoffman
**Conserved LindbladToh
Coding UCSC
Annotation
−200
−100
0
100
Enrichment (prop h2 / prop snps)

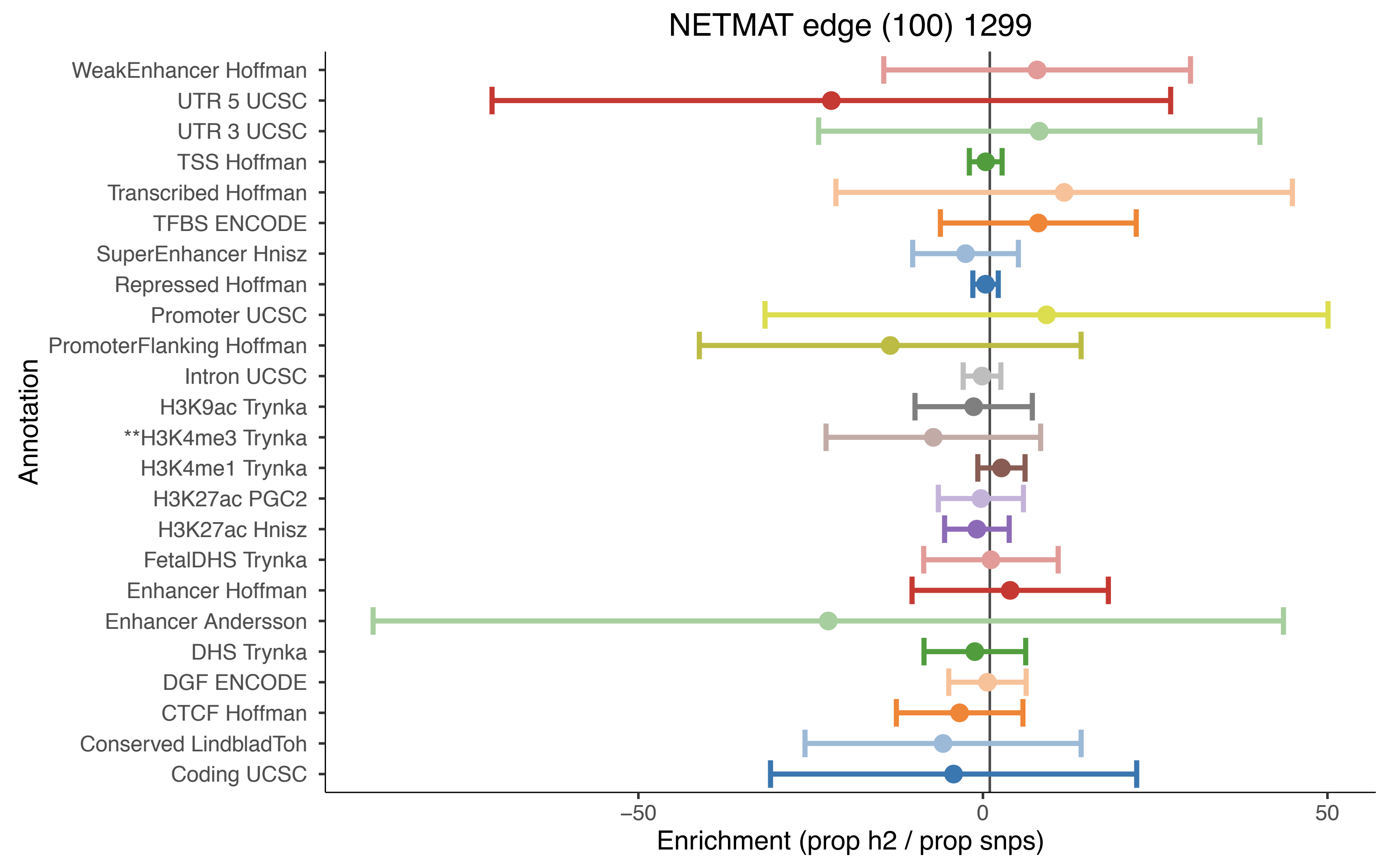

NETMAT edge (100) 1299
WeakEnhancer Hoffman
UTR 5 UCSC
UTR 3 UCSC
TSS Hoffman
Transcribed Hoffman
TFBS ENCODE
SuperEnhancer Hnisz
Repressed Hoffman
Promoter UCSC
PromoterFlanking Hoffman
Intron UCSC
H3K9ac Trynka
**H3K4me3 Trynka
H3K4me1 Trynka
H3K27ac PGC2
H3K27ac Hnisz
FetalDHS Trynka
Enhancer Hoffman
Enhancer Andersson
DHS Trynka
DGF ENCODE
CTCF Hoffman
Conserved LindbladToh
Coding UCSC
Annotation
−50
0
50
Enrichment (prop h2 / prop snps)

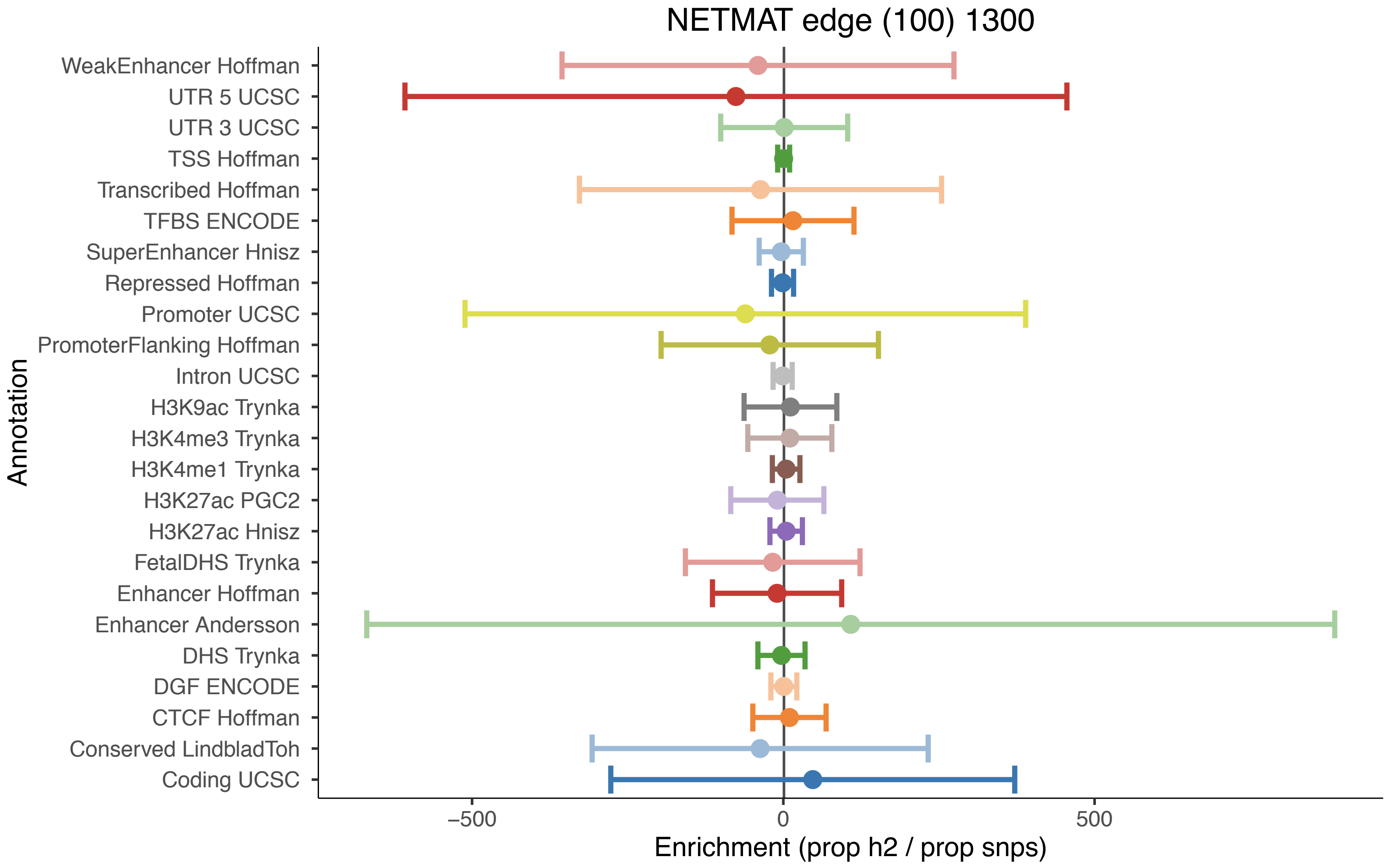
NETMAT edge (100) 1300
WeakEnhancer Hoffman
UTR 5 UCSC
UTR 3 UCSC
TSS Hoffman
Transcribed Hoffman
TFBS ENCODE
SuperEnhancer Hnisz
Repressed Hoffman
Promoter UCSC
PromoterFlanking Hoffman
Intron UCSC
H3K9ac Trynka
H3K4me3 Trynka
H3K4me1 Trynka
H3K27ac PGC2
H3K27ac Hnisz
FetalDHS Trynka
Enhancer Hoffman
Enhancer Andersson
DHS Trynka
DGF ENCODE
CTCF Hoffman
Conserved LindbladToh
Coding UCSC
Annotation
−500
0
500
Enrichment (prop h2 / prop snps)

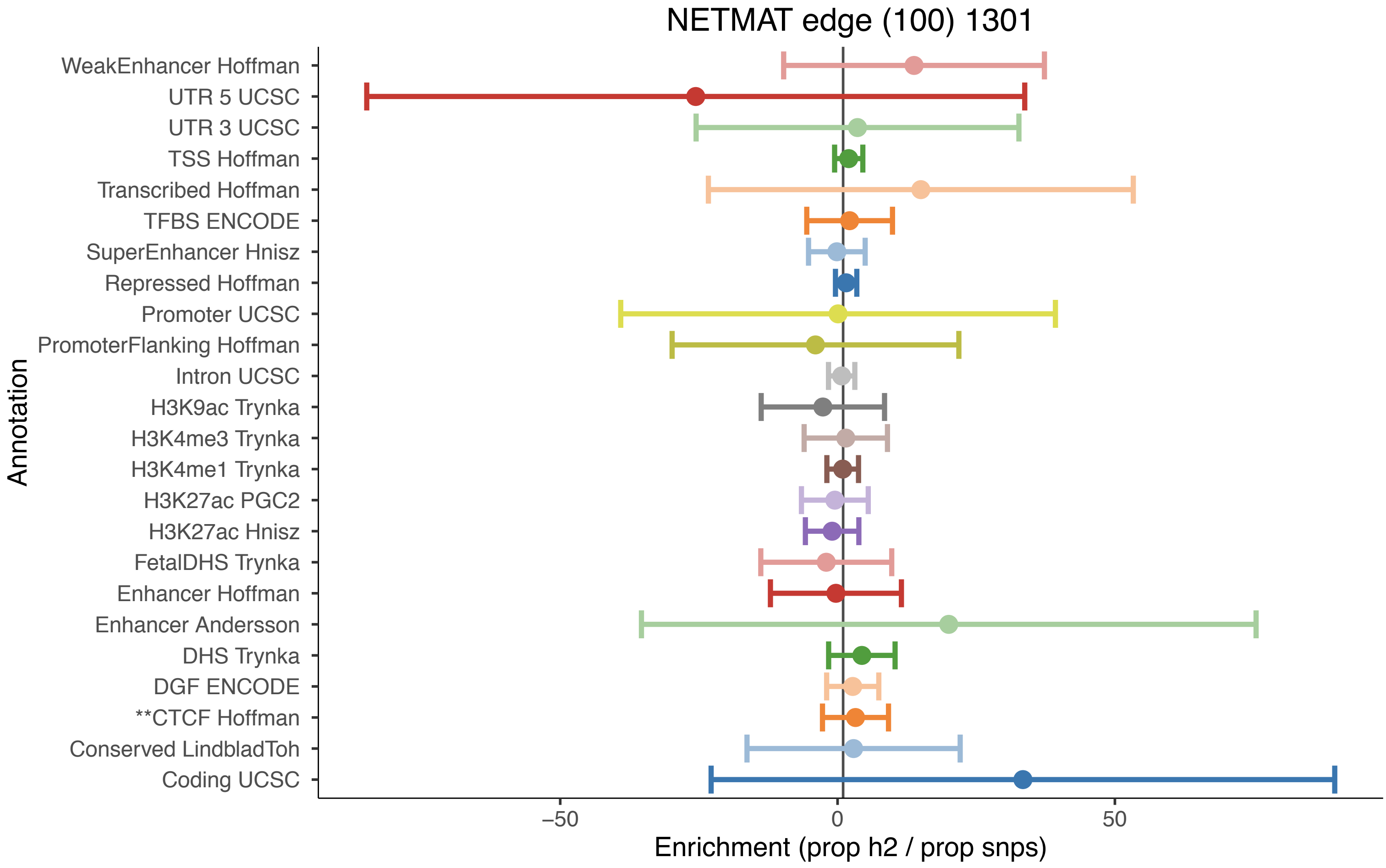

NETMAT edge (100) 1301
WeakEnhancer Hoffman
UTR 5 UCSC
UTR 3 UCSC
TSS Hoffman
Transcribed Hoffman
TFBS ENCODE
SuperEnhancer Hnisz
Repressed Hoffman
Promoter UCSC
PromoterFlanking Hoffman
Intron UCSC
H3K9ac Trynka
H3K4me3 Trynka
H3K4me1 Trynka
H3K27ac PGC2
H3K27ac Hnisz
FetalDHS Trynka
Enhancer Hoffman
Enhancer Andersson
DHS Trynka
DGF ENCODE
**CTCF Hoffman
Conserved LindbladToh
Coding UCSC
Annotation
−50
0
50
Enrichment (prop h2 / prop snps)

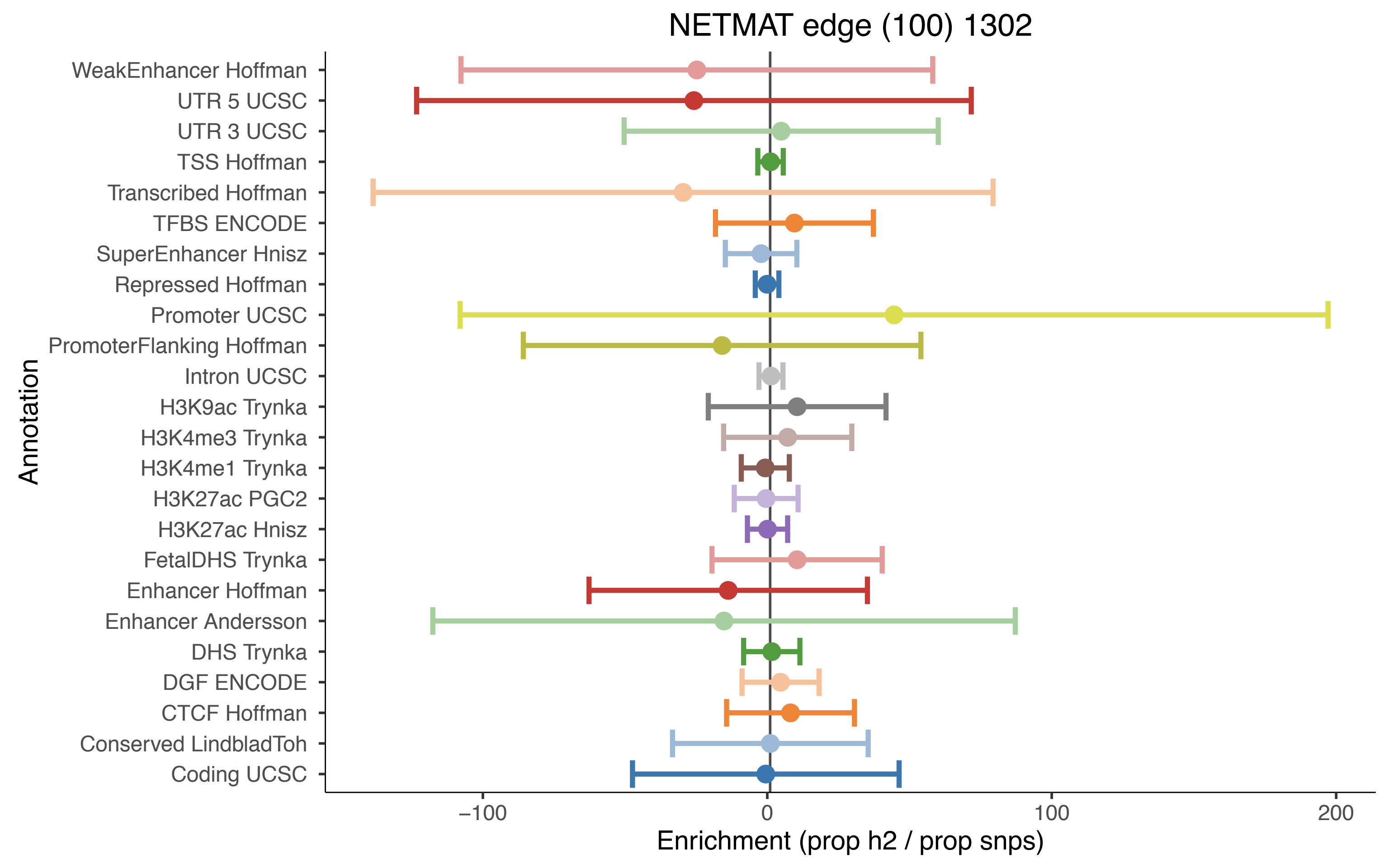
NETMAT edge (100) 1302
WeakEnhancer Hoffman
UTR 5 UCSC
UTR 3 UCSC
TSS Hoffman
Transcribed Hoffman
TFBS ENCODE
SuperEnhancer Hnisz
Repressed Hoffman
Promoter UCSC
PromoterFlanking Hoffman
Intron UCSC
H3K9ac Trynka
H3K4me3 Trynka
H3K4me1 Trynka
H3K27ac PGC2
H3K27ac Hnisz
FetalDHS Trynka
Enhancer Hoffman
Enhancer Andersson
DHS Trynka
DGF ENCODE
CTCF Hoffman
Conserved LindbladToh
Coding UCSC
Annotation
−100
0
100
200
Enrichment (prop h2 / prop snps)

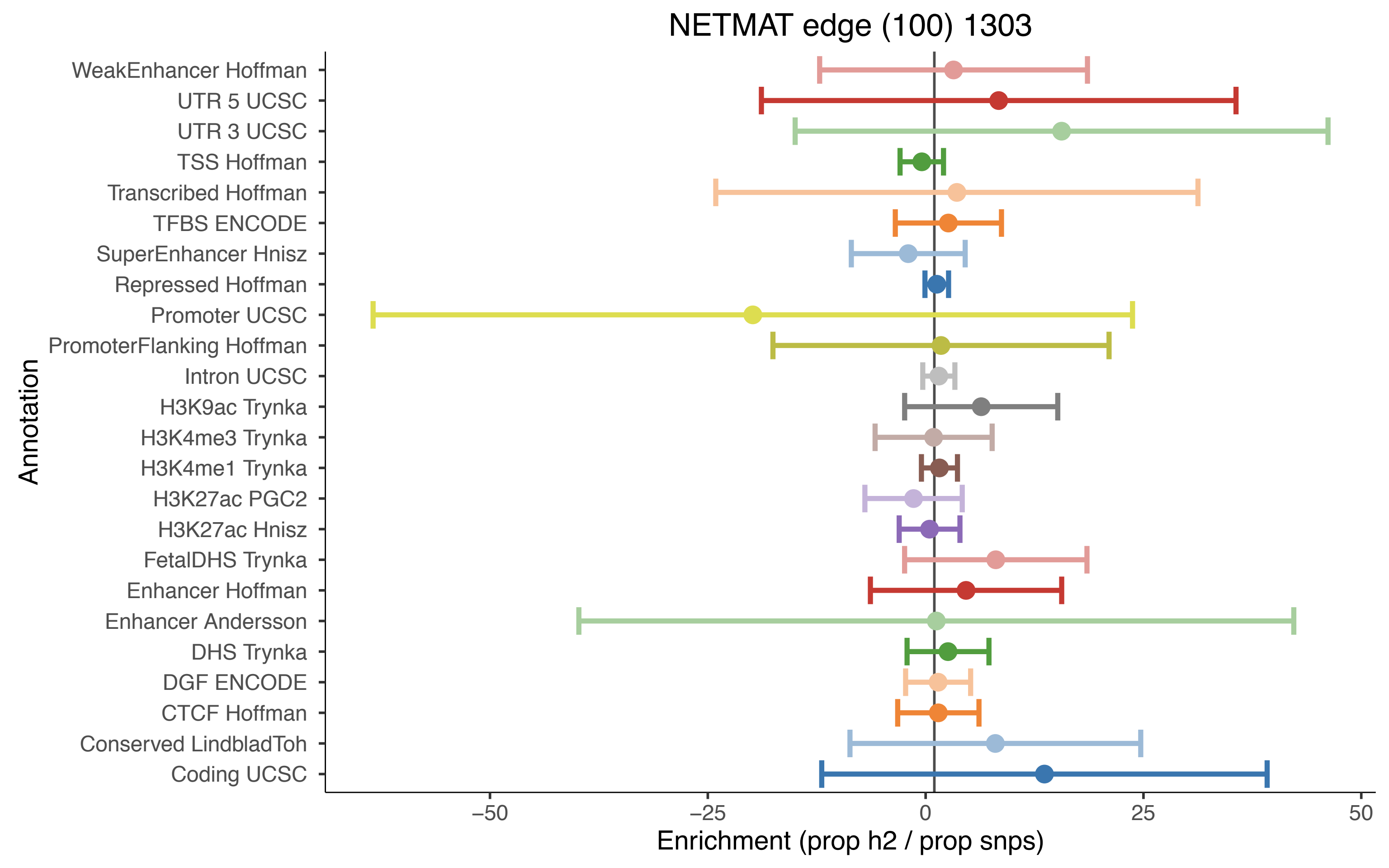

NETMAT edge (100) 1303
WeakEnhancer Hoffman
UTR 5 UCSC
UTR 3 UCSC
TSS Hoffman
Transcribed Hoffman
TFBS ENCODE
SuperEnhancer Hnisz
Repressed Hoffman
Promoter UCSC
PromoterFlanking Hoffman
Intron UCSC
H3K9ac Trynka
H3K4me3 Trynka
H3K4me1 Trynka
H3K27ac PGC2
H3K27ac Hnisz
FetalDHS Trynka
Enhancer Hoffman
Enhancer Andersson
DHS Trynka
DGF ENCODE
CTCF Hoffman
Conserved LindbladToh
Coding UCSC
Annotation
−50
−25
0
25
50
Enrichment (prop h2 / prop snps)

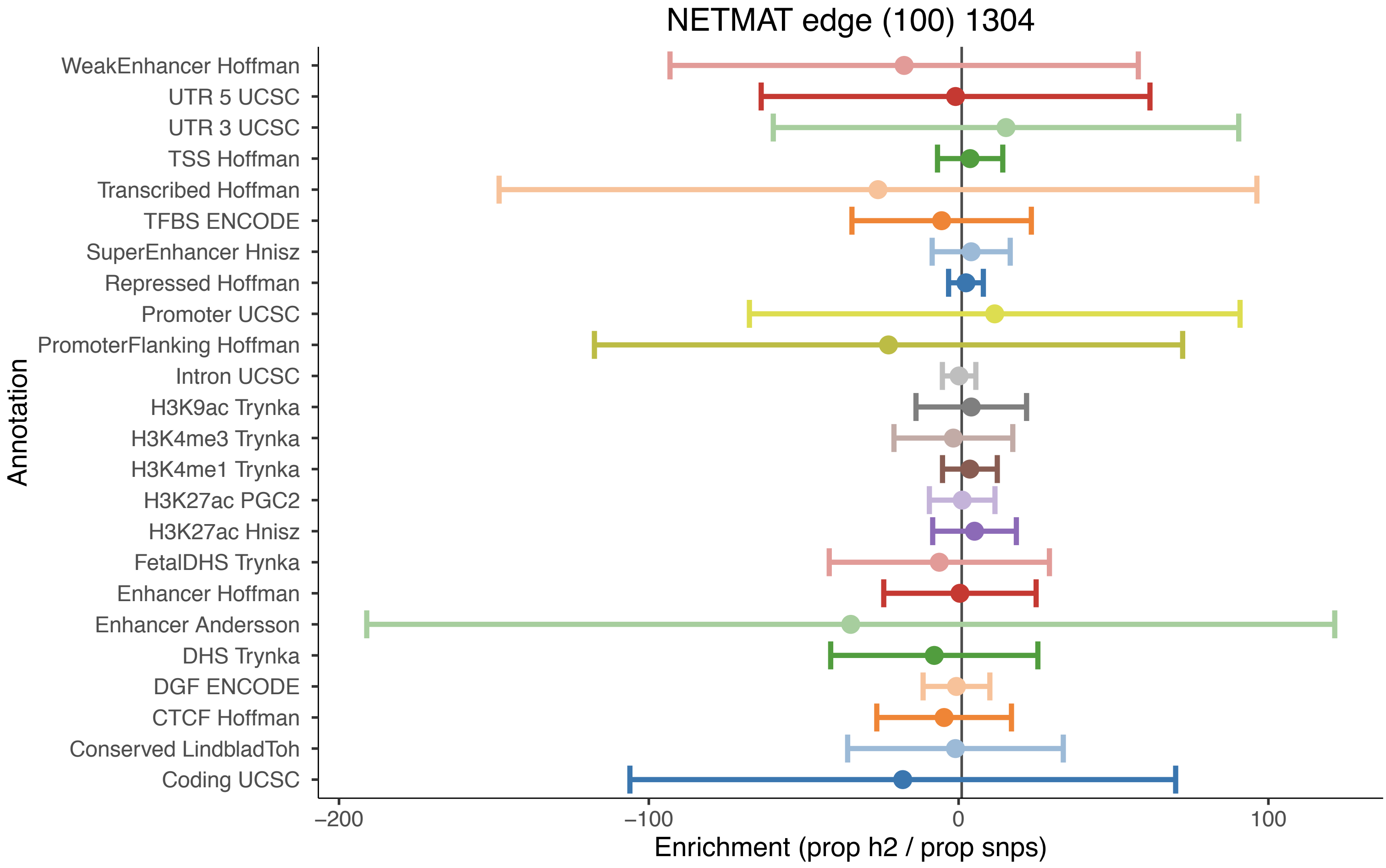
NETMAT edge (100) 1304
WeakEnhancer Hoffman
UTR 5 UCSC
UTR 3 UCSC
TSS Hoffman
Transcribed Hoffman
TFBS ENCODE
SuperEnhancer Hnisz
Repressed Hoffman
Promoter UCSC
PromoterFlanking Hoffman
Intron UCSC
H3K9ac Trynka
H3K4me3 Trynka
H3K4me1 Trynka
H3K27ac PGC2
H3K27ac Hnisz
FetalDHS Trynka
Enhancer Hoffman
Enhancer Andersson
DHS Trynka
DGF ENCODE
CTCF Hoffman
Conserved LindbladToh
Coding UCSC
Annotation
−200
−100
0
100
Enrichment (prop h2 / prop snps)

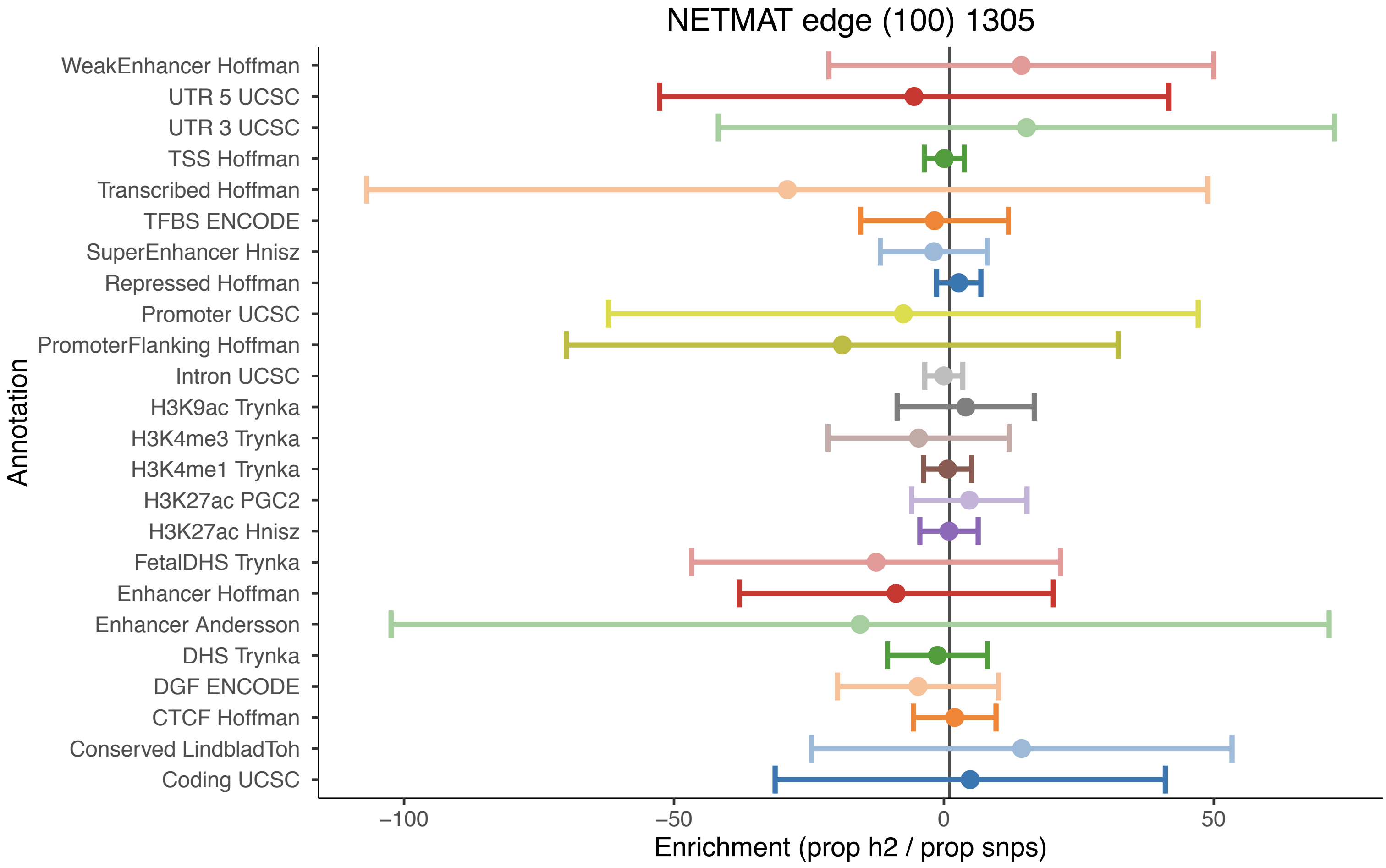
NETMAT edge (100) 1305
WeakEnhancer Hoffman
UTR 5 UCSC
UTR 3 UCSC
TSS Hoffman
Transcribed Hoffman
TFBS ENCODE
SuperEnhancer Hnisz
Repressed Hoffman
Promoter UCSC
PromoterFlanking Hoffman
Intron UCSC
H3K9ac Trynka
H3K4me3 Trynka
H3K4me1 Trynka
H3K27ac PGC2
H3K27ac Hnisz
FetalDHS Trynka
Enhancer Hoffman
Enhancer Andersson
DHS Trynka
DGF ENCODE
CTCF Hoffman
Conserved LindbladToh
Coding UCSC
Annotation
−100
−50
0
50
Enrichment (prop h2 / prop snps)

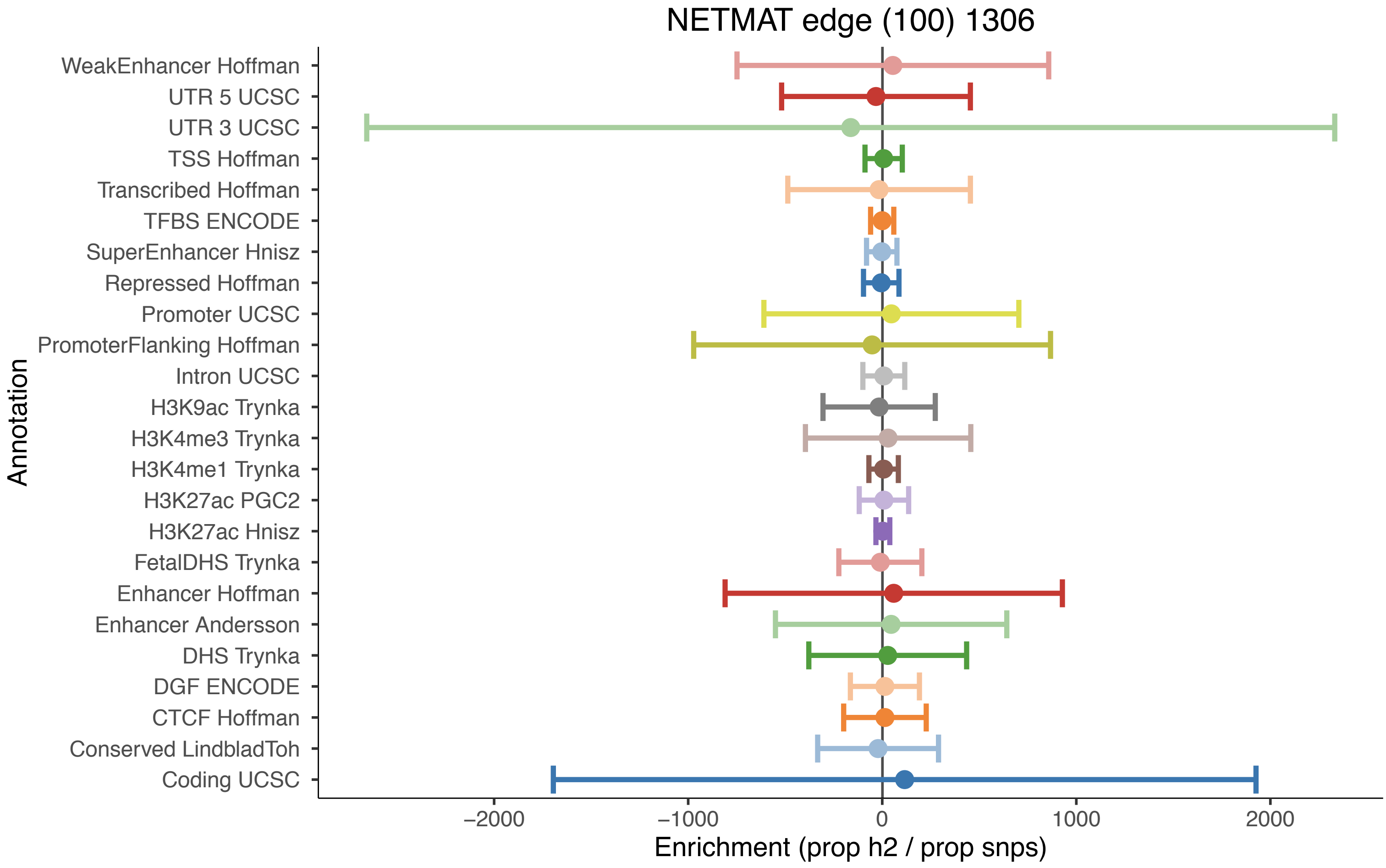
NETMAT edge (100) 1306
WeakEnhancer Hoffman
UTR 5 UCSC
UTR 3 UCSC
TSS Hoffman
Transcribed Hoffman
TFBS ENCODE
SuperEnhancer Hnisz
Repressed Hoffman
Promoter UCSC
PromoterFlanking Hoffman
Intron UCSC
H3K9ac Trynka
H3K4me3 Trynka
H3K4me1 Trynka
H3K27ac PGC2
H3K27ac Hnisz
FetalDHS Trynka
Enhancer Hoffman
Enhancer Andersson
DHS Trynka
DGF ENCODE
CTCF Hoffman
Conserved LindbladToh
Coding UCSC
Annotation
−2000
−1000
0
1000
2000
Enrichment (prop h2 / prop snps)

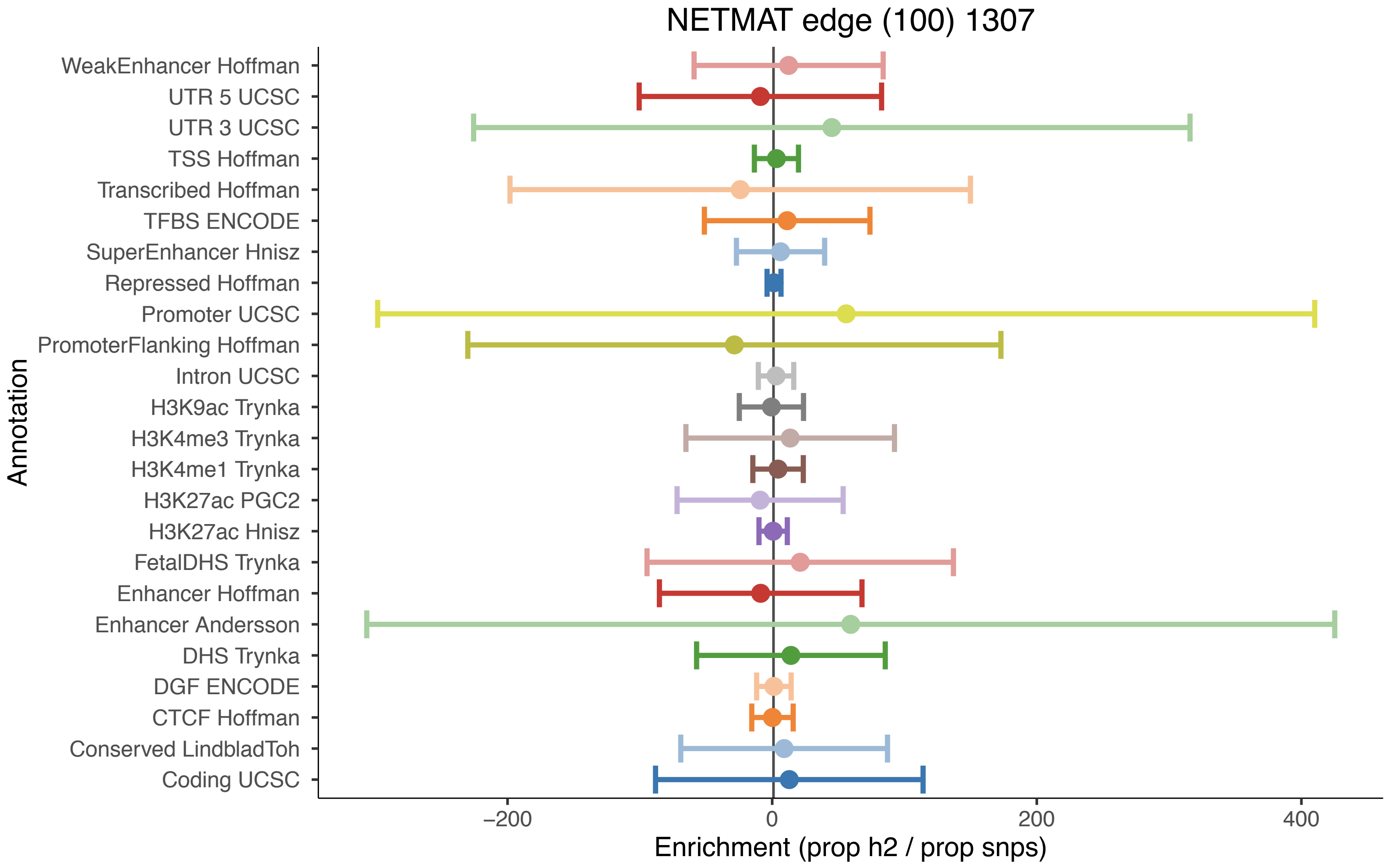

NETMAT edge (100) 1307
WeakEnhancer Hoffman
UTR 5 UCSC
UTR 3 UCSC
TSS Hoffman
Transcribed Hoffman
TFBS ENCODE
SuperEnhancer Hnisz
Repressed Hoffman
Promoter UCSC
PromoterFlanking Hoffman
Intron UCSC
H3K9ac Trynka
H3K4me3 Trynka
H3K4me1 Trynka
H3K27ac PGC2
H3K27ac Hnisz
FetalDHS Trynka
Enhancer Hoffman
Enhancer Andersson
DHS Trynka
DGF ENCODE
CTCF Hoffman
Conserved LindbladToh
Coding UCSC
Annotation
−200
0
200
400
Enrichment (prop h2 / prop snps)

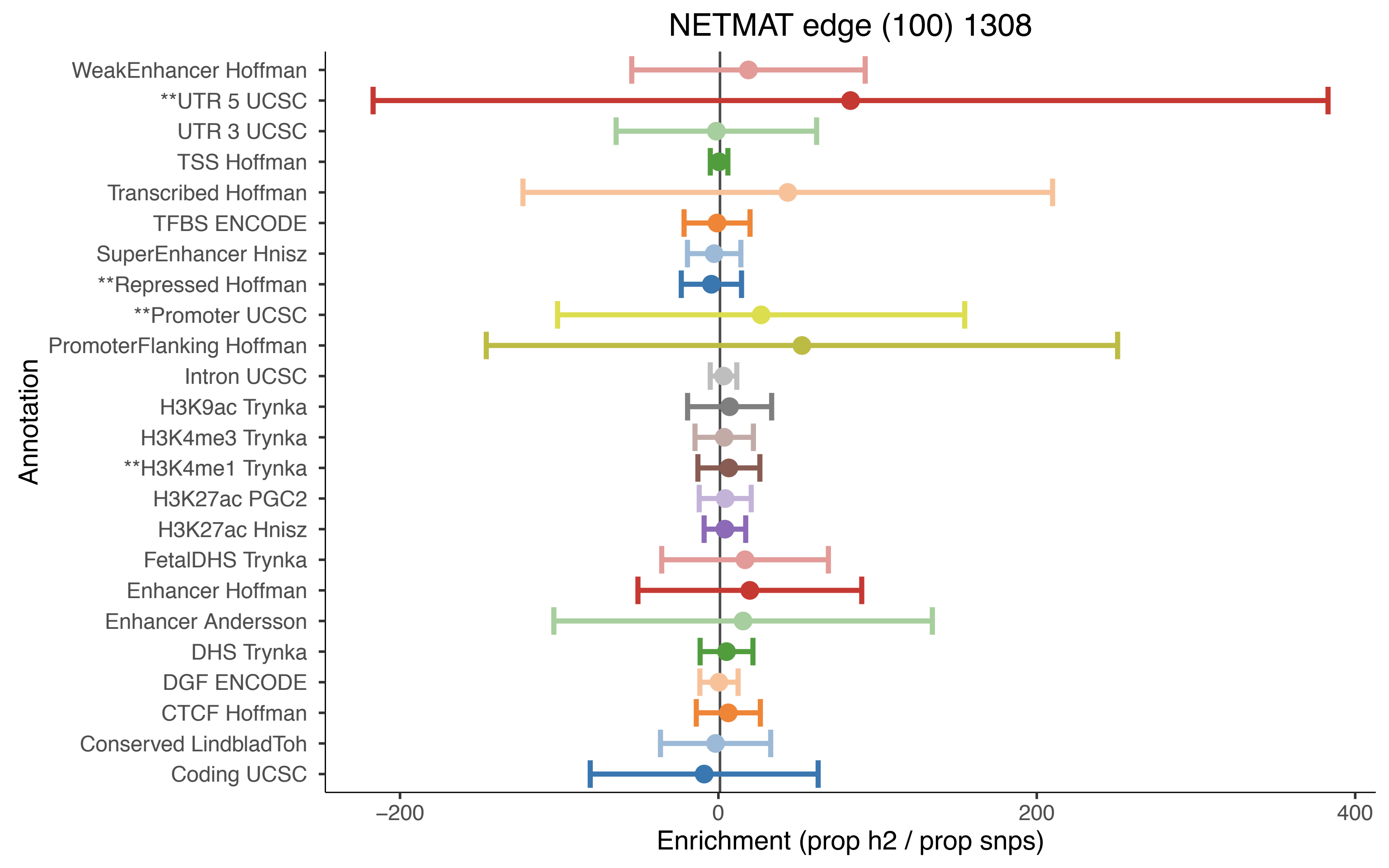
NETMAT edge (100) 1308
WeakEnhancer Hoffman
**UTR 5 UCSC
UTR 3 UCSC
TSS Hoffman
Transcribed Hoffman
TFBS ENCODE
SuperEnhancer Hnisz
**Repressed Hoffman
**Promoter UCSC
PromoterFlanking Hoffman
Intron UCSC
H3K9ac Trynka
H3K4me3 Trynka
**H3K4me1 Trynka
H3K27ac PGC2
H3K27ac Hnisz
FetalDHS Trynka
Enhancer Hoffman
Enhancer Andersson
DHS Trynka
DGF ENCODE
CTCF Hoffman
Conserved LindbladToh
Coding UCSC
Annotation
−200
0
200
400
Enrichment (prop h2 / prop snps)

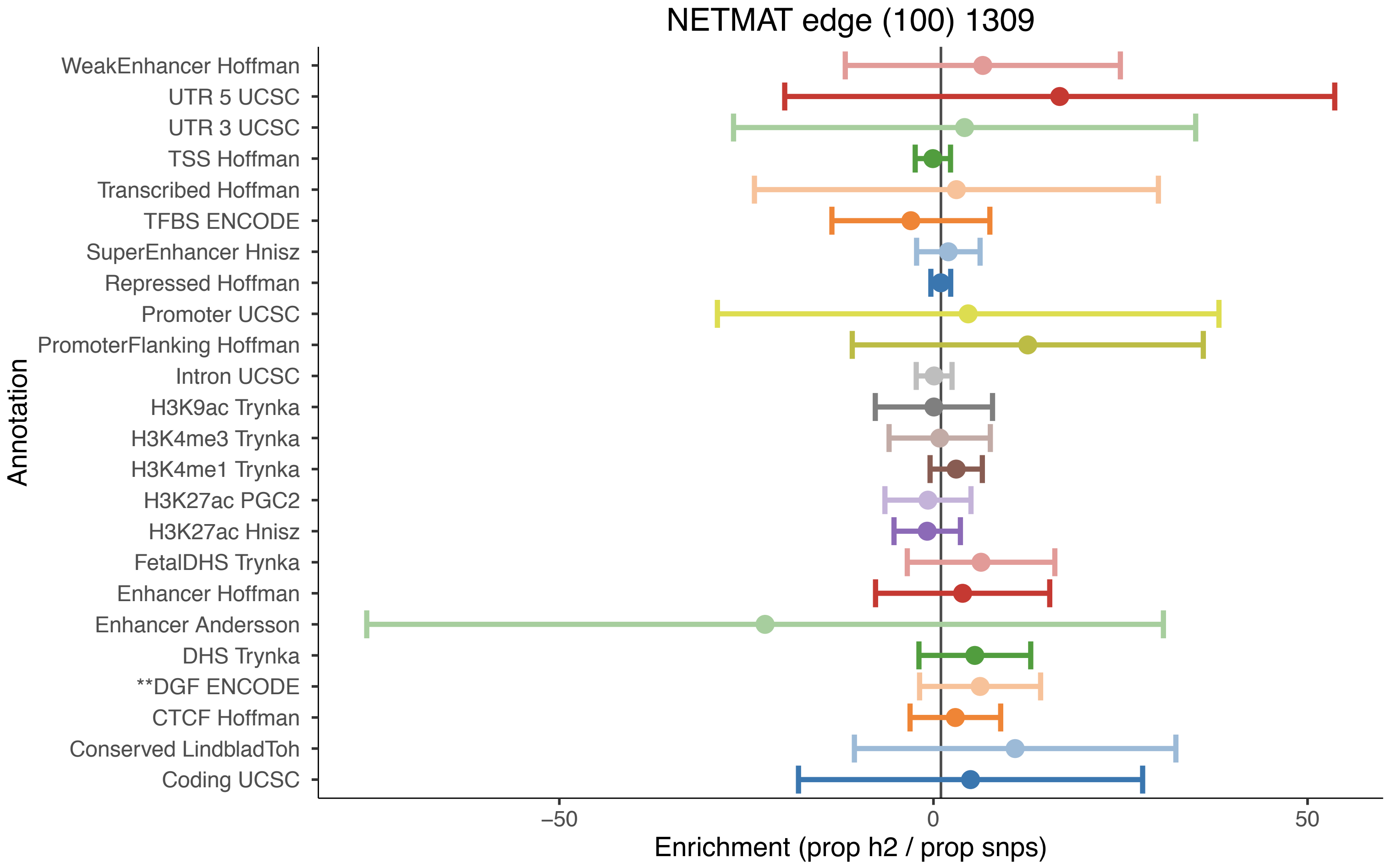

NETMAT edge (100) 1309
WeakEnhancer Hoffman
UTR 5 UCSC
UTR 3 UCSC
TSS Hoffman
Transcribed Hoffman
TFBS ENCODE
SuperEnhancer Hnisz
Repressed Hoffman
Promoter UCSC
PromoterFlanking Hoffman
Intron UCSC
H3K9ac Trynka
H3K4me3 Trynka
H3K4me1 Trynka
H3K27ac PGC2
H3K27ac Hnisz
FetalDHS Trynka
Enhancer Hoffman
Enhancer Andersson
DHS Trynka
**DGF ENCODE
CTCF Hoffman
Conserved LindbladToh
Coding UCSC
Annotation
−50
0
50
Enrichment (prop h2 / prop snps)

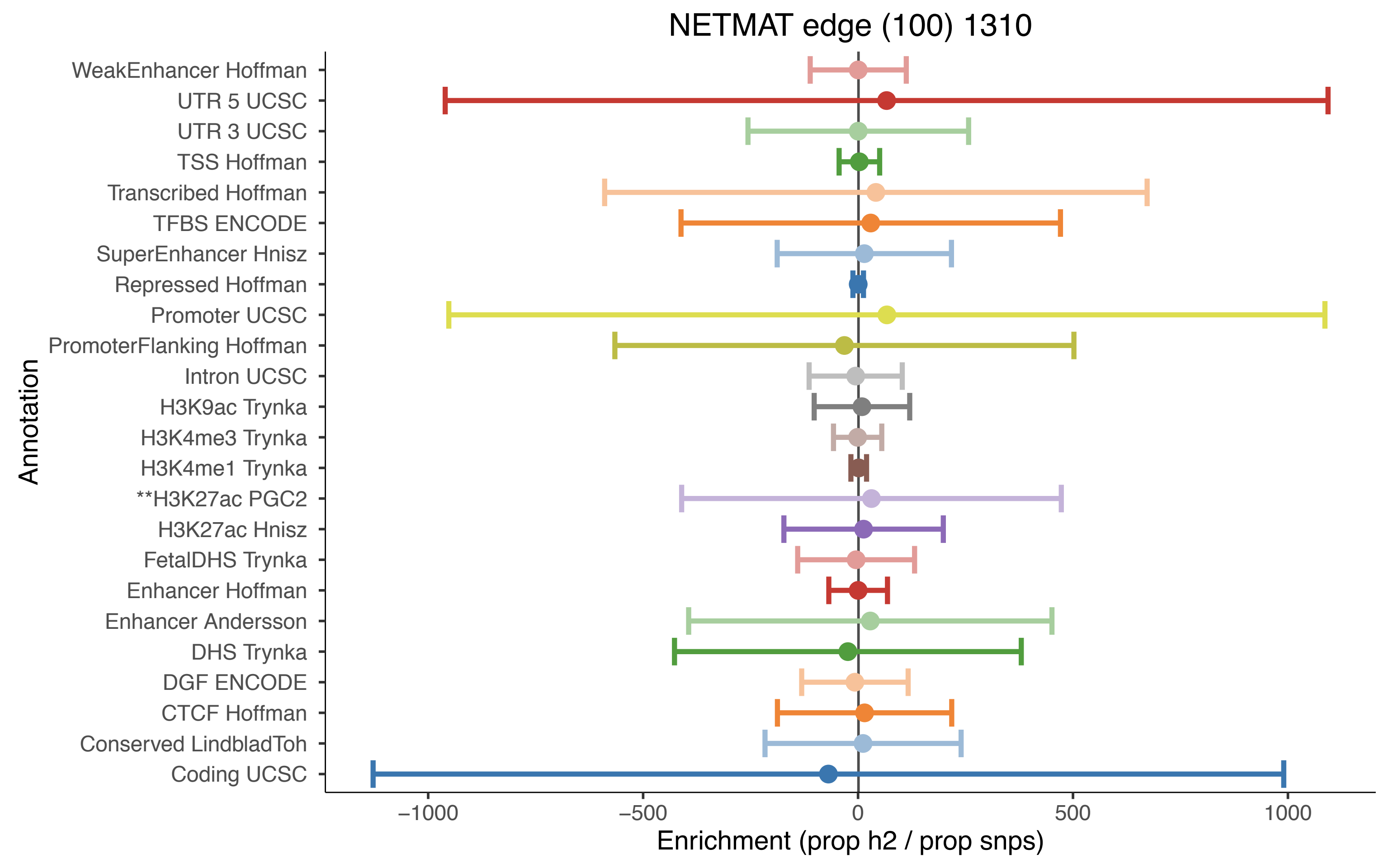

NETMAT edge (100) 1310
Annotation
WeakEnhancer Hoffman
UTR 5 UCSC
UTR 3 UCSC
TSS Hoffman
Transcribed Hoffman
TFBS ENCODE
SuperEnhancer Hnisz
Repressed Hoffman
Promoter UCSC
PromoterFlanking Hoffman
Intron UCSC
H3K9ac Trynka
H3K4me3 Trynka
H3K4me1 Trynka
**H3K27ac PGC2
H3K27ac Hnisz
FetalDHS Trynka
Enhancer Hoffman
Enhancer Andersson
DHS Trynka
DGF ENCODE
CTCF Hoffman
Conserved LindbladToh
Coding UCSC
−1000
−500
0
500
1000
Enrichment (prop h2 / prop snps)

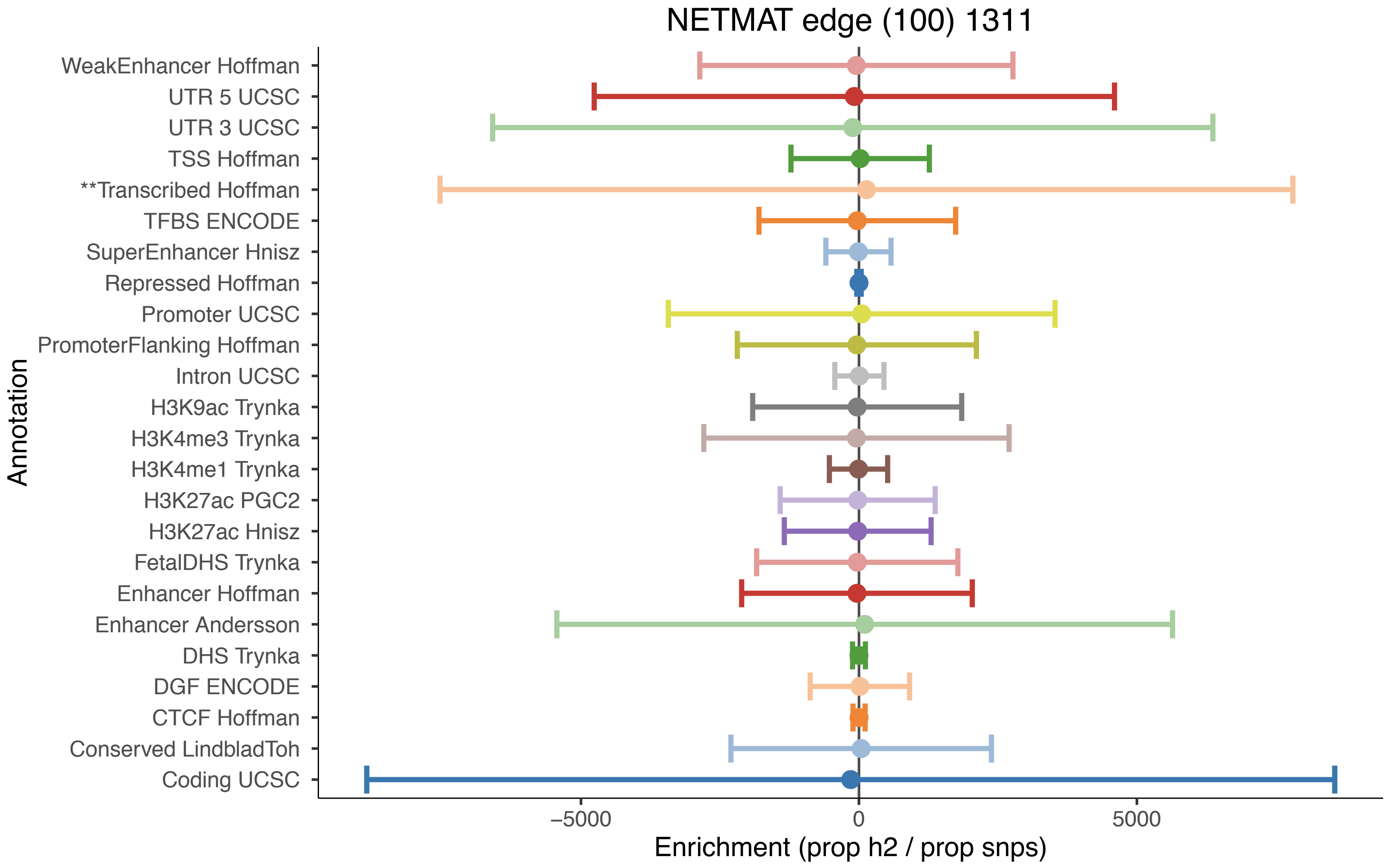
NETMAT edge (100) 1311
WeakEnhancer Hoffman
UTR 5 UCSC
UTR 3 UCSC
TSS Hoffman
**Transcribed Hoffman
TFBS ENCODE
SuperEnhancer Hnisz
Repressed Hoffman
Promoter UCSC
PromoterFlanking Hoffman
Intron UCSC
H3K9ac Trynka
H3K4me3 Trynka
H3K4me1 Trynka
H3K27ac PGC2
H3K27ac Hnisz
FetalDHS Trynka
Enhancer Hoffman
Enhancer Andersson
DHS Trynka
DGF ENCODE
CTCF Hoffman
Conserved LindbladToh
Coding UCSC
Annotation
−5000
0
5000
Enrichment (prop h2 / prop snps)

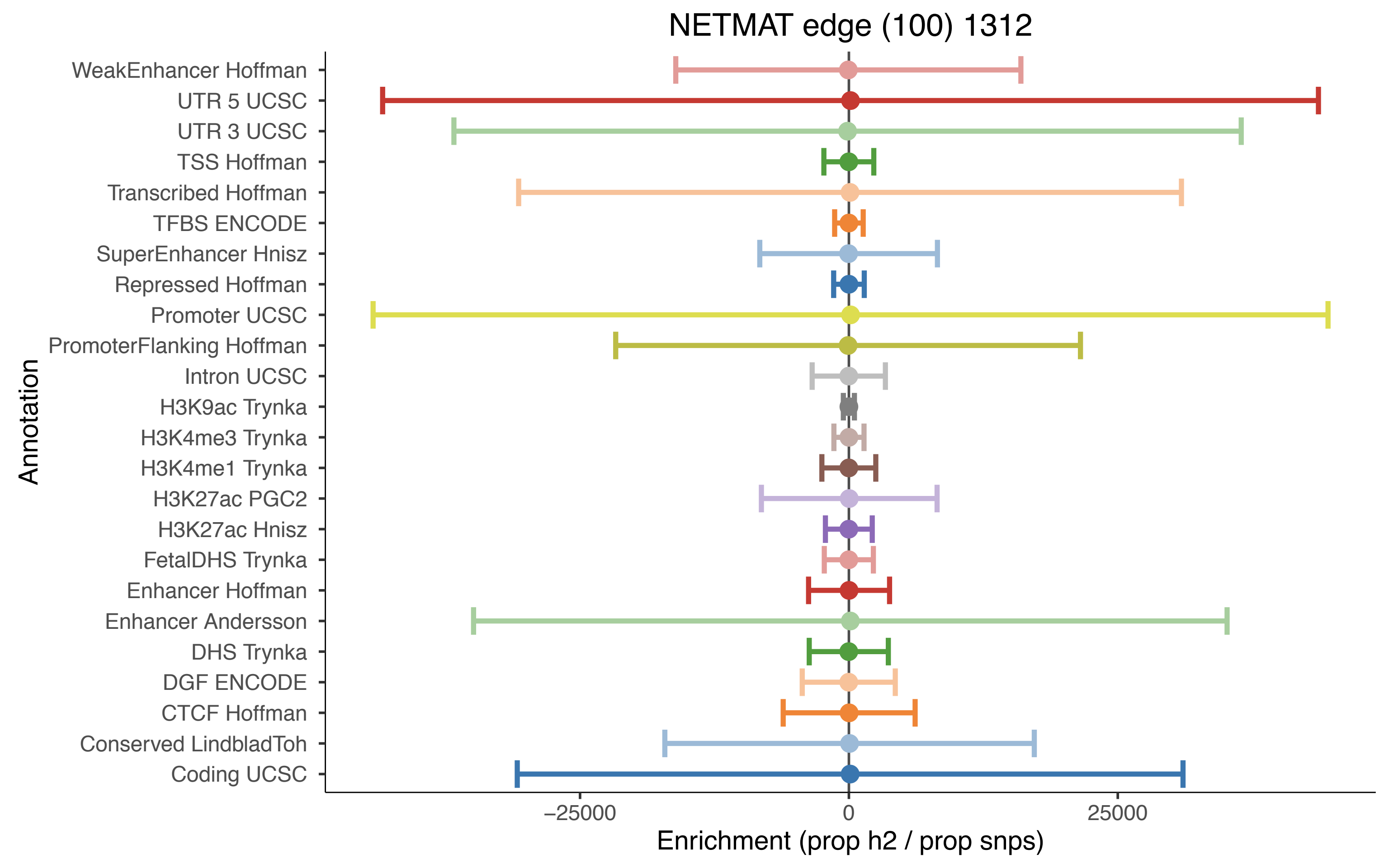
NETMAT edge (100) 1312
WeakEnhancer Hoffman
UTR 5 UCSC
UTR 3 UCSC
TSS Hoffman
Transcribed Hoffman
TFBS ENCODE
SuperEnhancer Hnisz
Repressed Hoffman
Promoter UCSC
PromoterFlanking Hoffman
Intron UCSC
H3K9ac Trynka
H3K4me3 Trynka
H3K4me1 Trynka
H3K27ac PGC2
H3K27ac Hnisz
FetalDHS Trynka
Enhancer Hoffman
Enhancer Andersson
DHS Trynka
DGF ENCODE
CTCF Hoffman
Conserved LindbladToh
Coding UCSC
Annotation
−25000
0
25000
Enrichment (prop h2 / prop snps)

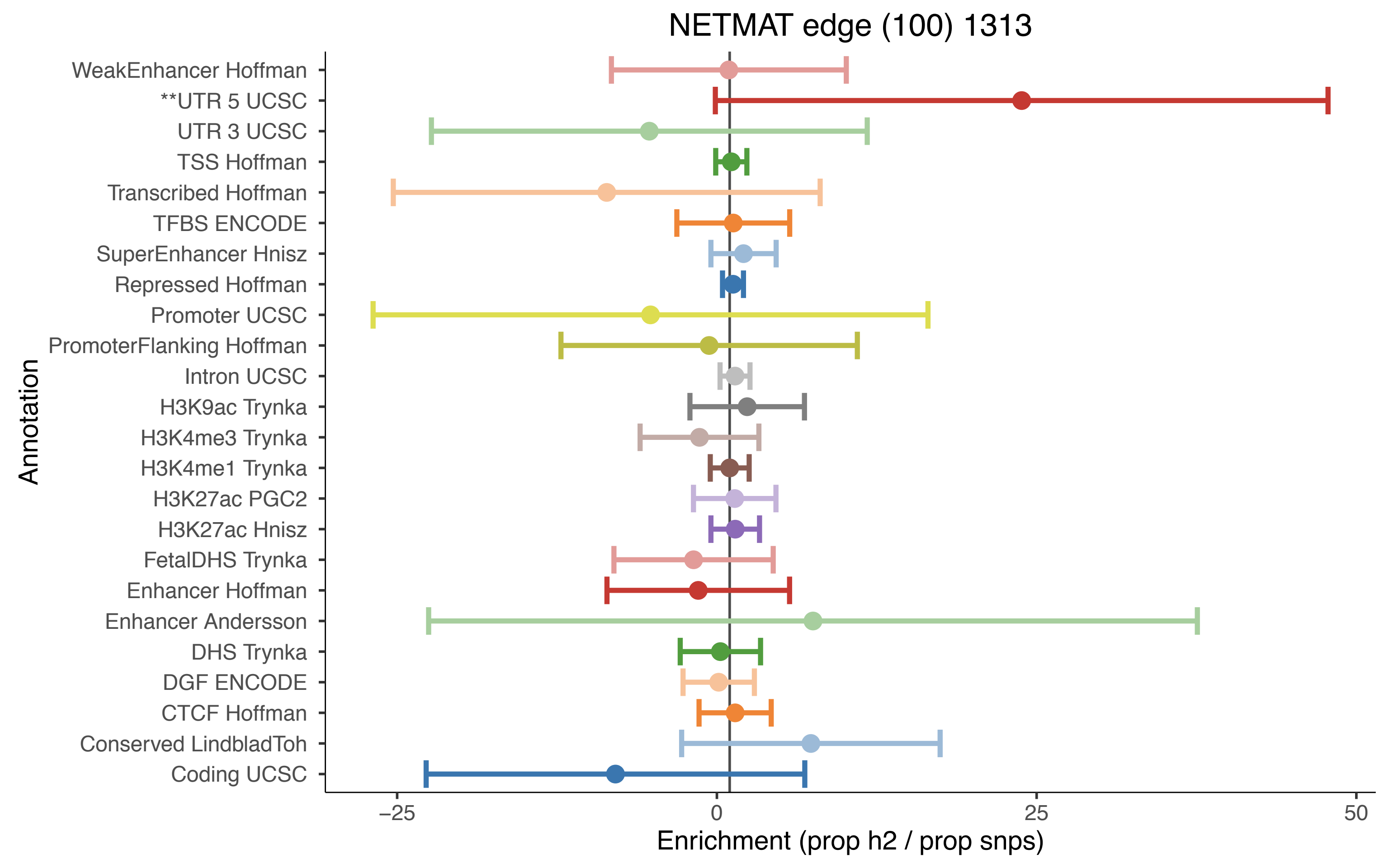

NETMAT edge (100) 1313
WeakEnhancer Hoffman
**UTR 5 UCSC
UTR 3 UCSC
TSS Hoffman
Transcribed Hoffman
TFBS ENCODE
SuperEnhancer Hnisz
Repressed Hoffman
Promoter UCSC
PromoterFlanking Hoffman
Intron UCSC
H3K9ac Trynka
H3K4me3 Trynka
H3K4me1 Trynka
H3K27ac PGC2
H3K27ac Hnisz
FetalDHS Trynka
Enhancer Hoffman
Enhancer Andersson
DHS Trynka
DGF ENCODE
CTCF Hoffman
Conserved LindbladToh
Coding UCSC
Annotation
−25
0
25
50
Enrichment (prop h2 / prop snps)

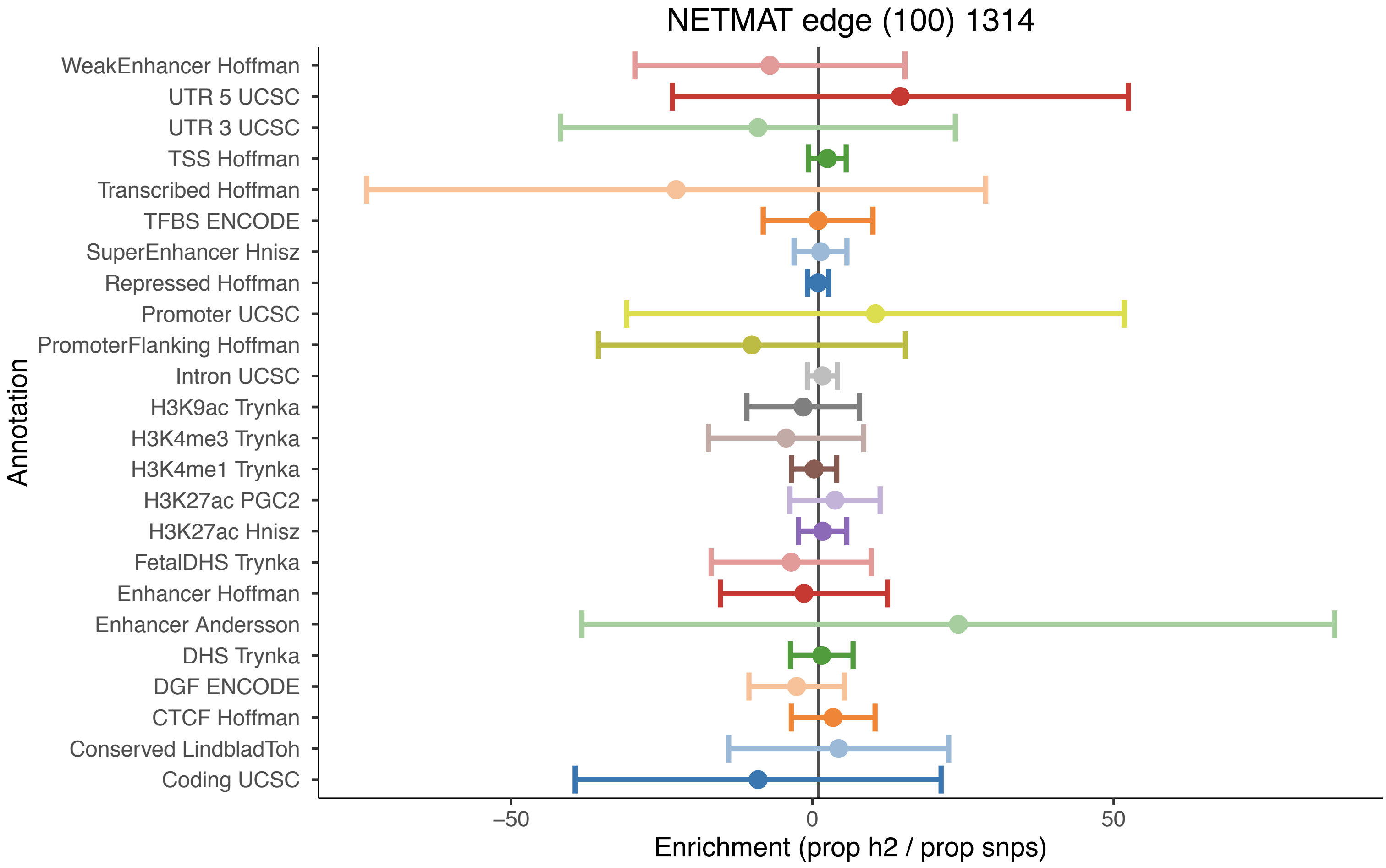
NETMAT edge (100) 1314
Annotation
WeakEnhancer Hoffman
UTR 5 UCSC
UTR 3 UCSC
TSS Hoffman
Transcribed Hoffman
TFBS ENCODE
SuperEnhancer Hnisz
Repressed Hoffman
Promoter UCSC
PromoterFlanking Hoffman
Intron UCSC
H3K9ac Trynka
H3K4me3 Trynka
H3K4me1 Trynka
H3K27ac PGC2
H3K27ac Hnisz
FetalDHS Trynka
Enhancer Hoffman
Enhancer Andersson
DHS Trynka
DGF ENCODE
CTCF Hoffman
Conserved LindbladToh
Coding UCSC
−50
0
50
Enrichment (prop h2 / prop snps)

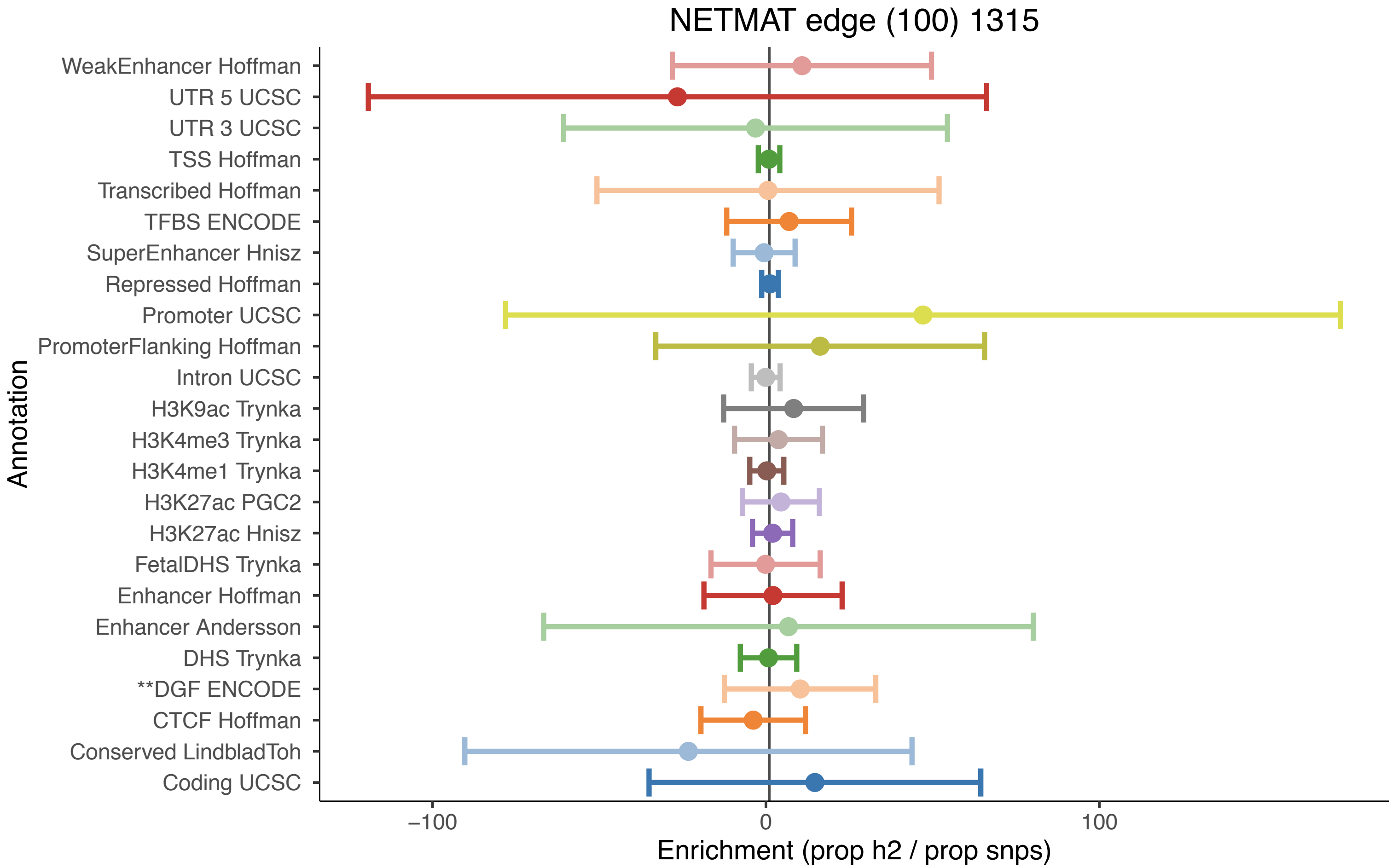

NETMAT edge (100) 1315
WeakEnhancer Hoffman
UTR 5 UCSC
UTR 3 UCSC
TSS Hoffman
Transcribed Hoffman
TFBS ENCODE
SuperEnhancer Hnisz
Repressed Hoffman
Promoter UCSC
PromoterFlanking Hoffman
Intron UCSC
H3K9ac Trynka
H3K4me3 Trynka
H3K4me1 Trynka
H3K27ac PGC2
H3K27ac Hnisz
FetalDHS Trynka
Enhancer Hoffman
Enhancer Andersson
DHS Trynka
**DGF ENCODE
CTCF Hoffman
Conserved LindbladToh
Coding UCSC
Annotation
−100
0
100
Enrichment (prop h2 / prop snps)

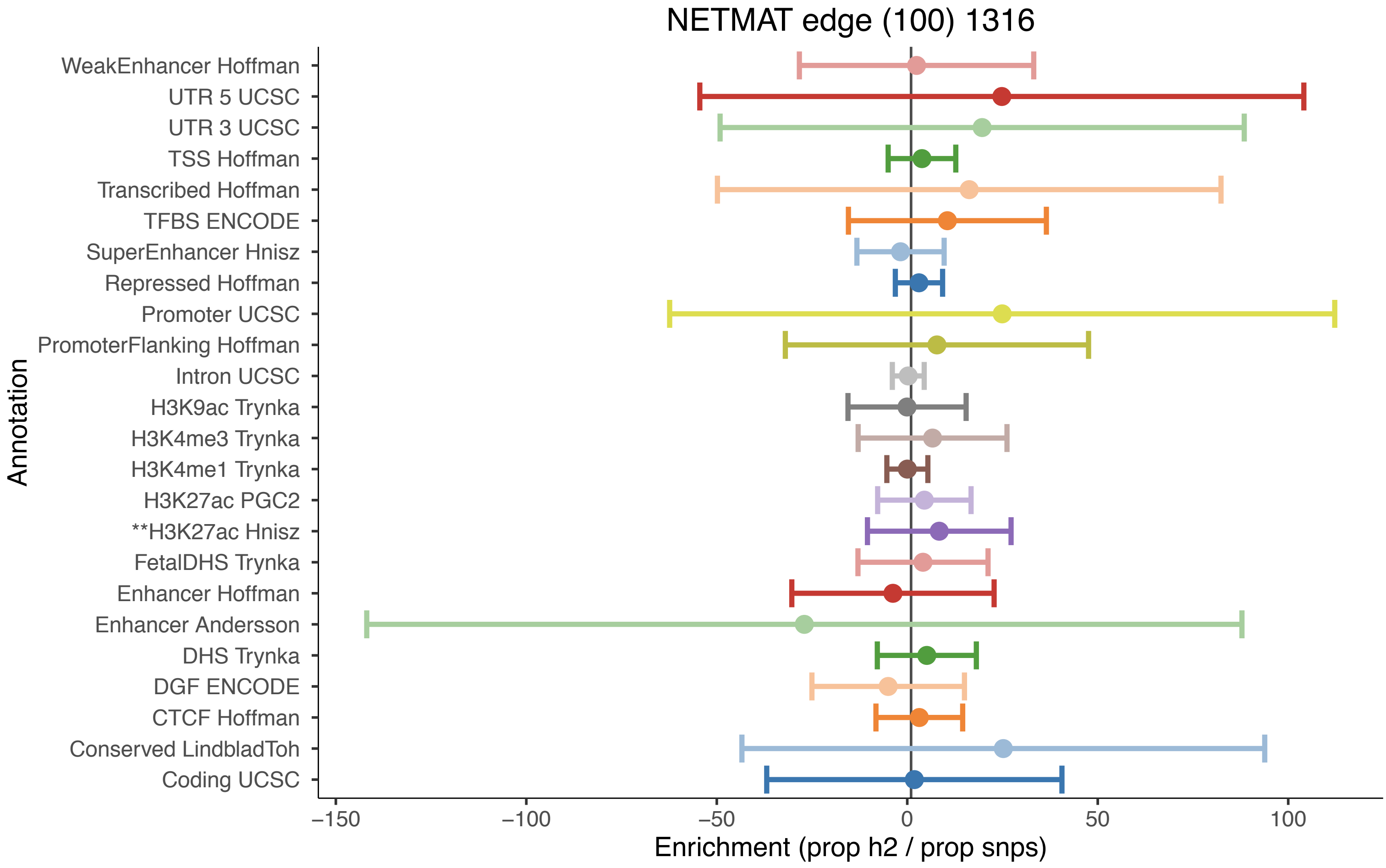

NETMAT edge (100) 1316
WeakEnhancer Hoffman
UTR 5 UCSC
UTR 3 UCSC
TSS Hoffman
Transcribed Hoffman
TFBS ENCODE
SuperEnhancer Hnisz
Repressed Hoffman
Promoter UCSC
PromoterFlanking Hoffman
Intron UCSC
H3K9ac Trynka
H3K4me3 Trynka
H3K4me1 Trynka
H3K27ac PGC2
**H3K27ac Hnisz
FetalDHS Trynka
Enhancer Hoffman
Enhancer Andersson
DHS Trynka
DGF ENCODE
CTCF Hoffman
Conserved LindbladToh
Coding UCSC
Annotation
−150
−100
−50
0
50
100
Enrichment (prop h2 / prop snps)

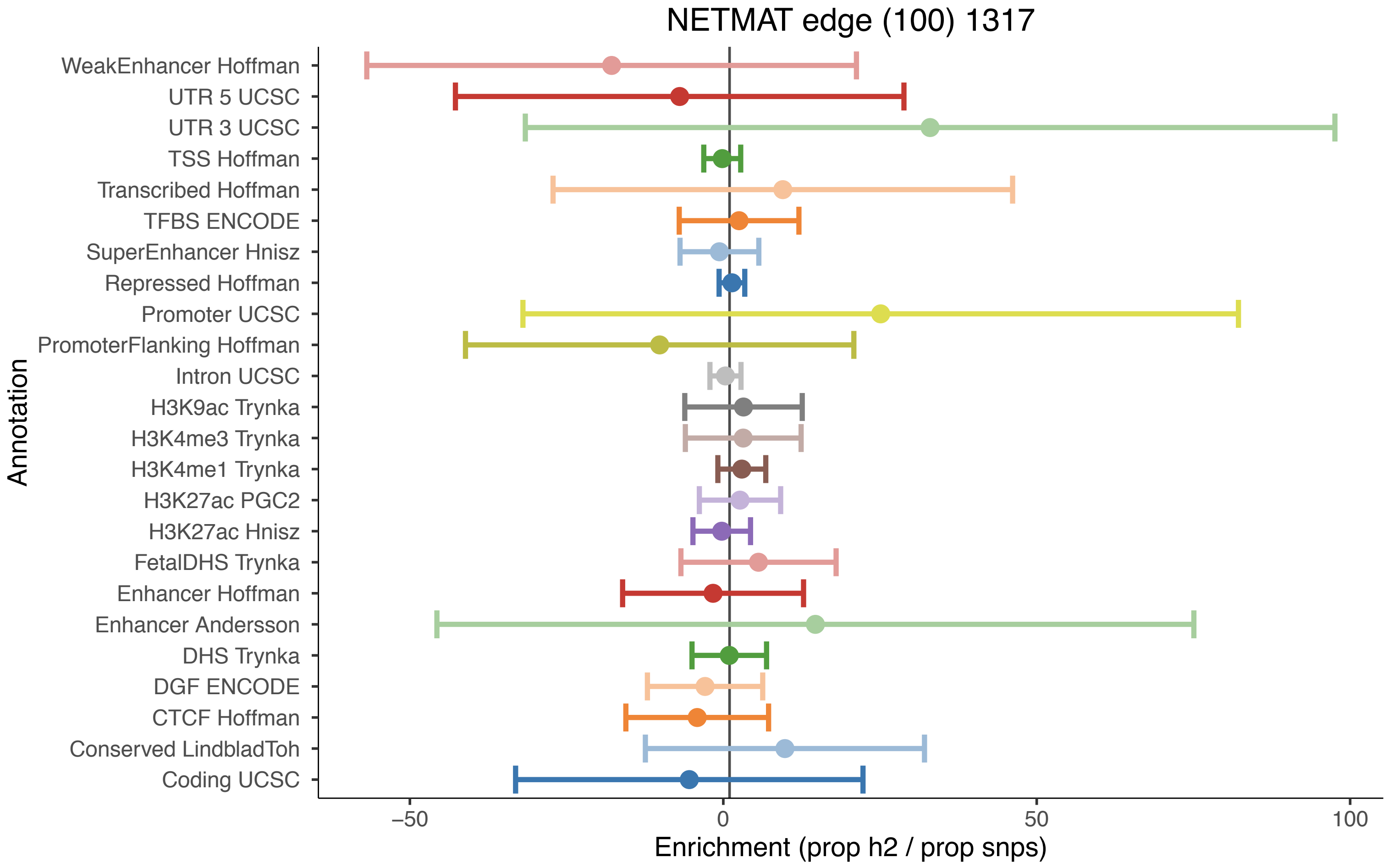
NETMAT edge (100) 1317
WeakEnhancer Hoffman
UTR 5 UCSC
UTR 3 UCSC
TSS Hoffman
Transcribed Hoffman
TFBS ENCODE
SuperEnhancer Hnisz
Repressed Hoffman
Promoter UCSC
PromoterFlanking Hoffman
Intron UCSC
H3K9ac Trynka
H3K4me3 Trynka
H3K4me1 Trynka
H3K27ac PGC2
H3K27ac Hnisz
FetalDHS Trynka
Enhancer Hoffman
Enhancer Andersson
DHS Trynka
DGF ENCODE
CTCF Hoffman
Conserved LindbladToh
Coding UCSC
Annotation
−50
0
50
100
Enrichment (prop h2 / prop snps)

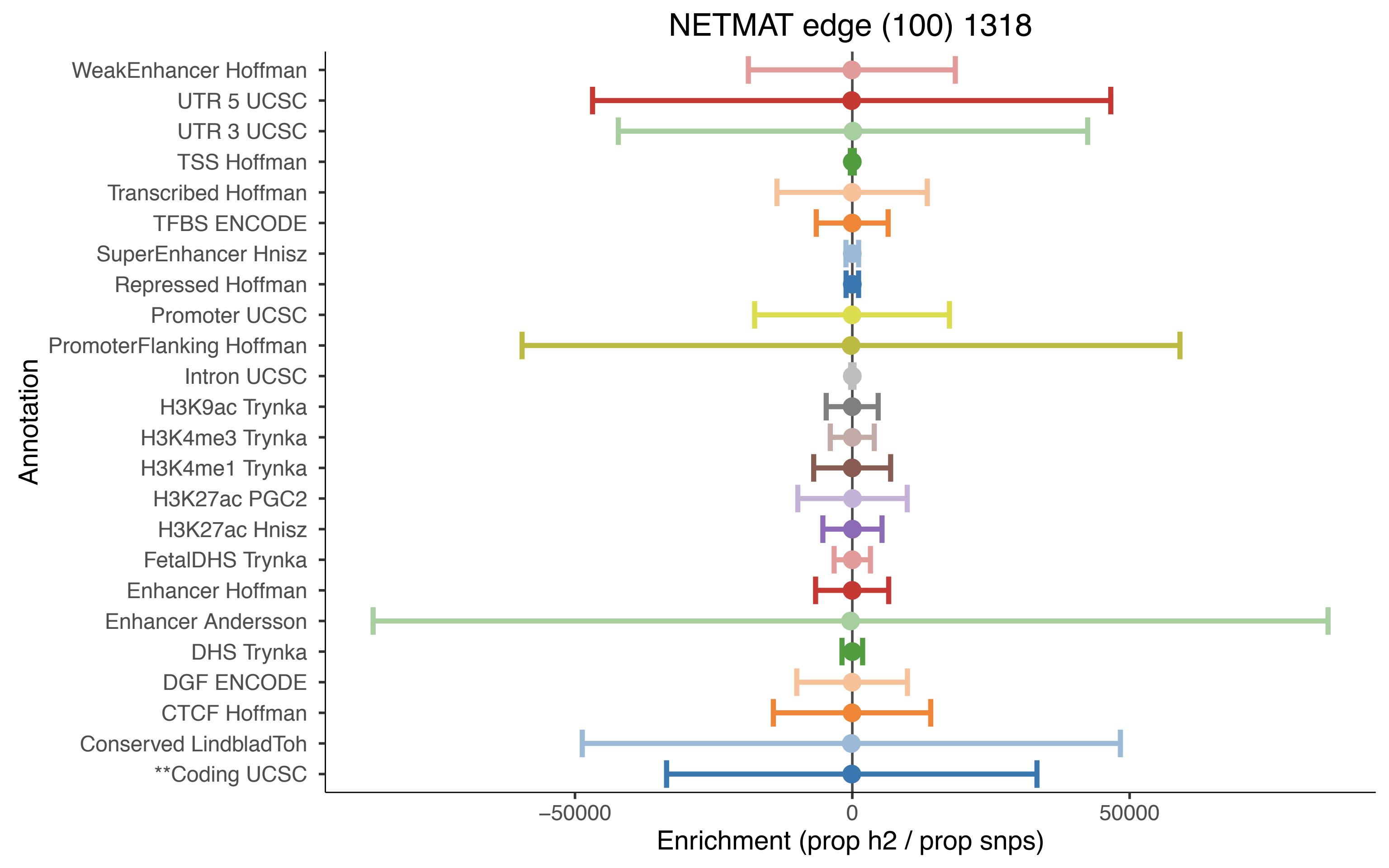
NETMAT edge (100) 1318
WeakEnhancer Hoffman
UTR 5 UCSC
UTR 3 UCSC
TSS Hoffman
Transcribed Hoffman
TFBS ENCODE
SuperEnhancer Hnisz
Repressed Hoffman
Promoter UCSC
PromoterFlanking Hoffman
Intron UCSC
H3K9ac Trynka
H3K4me3 Trynka
H3K4me1 Trynka
H3K27ac PGC2
H3K27ac Hnisz
FetalDHS Trynka
Enhancer Hoffman
Enhancer Andersson
DHS Trynka
DGF ENCODE
CTCF Hoffman
Conserved LindbladToh
**Coding UCSC
Annotation
−50000
0
50000
Enrichment (prop h2 / prop snps)

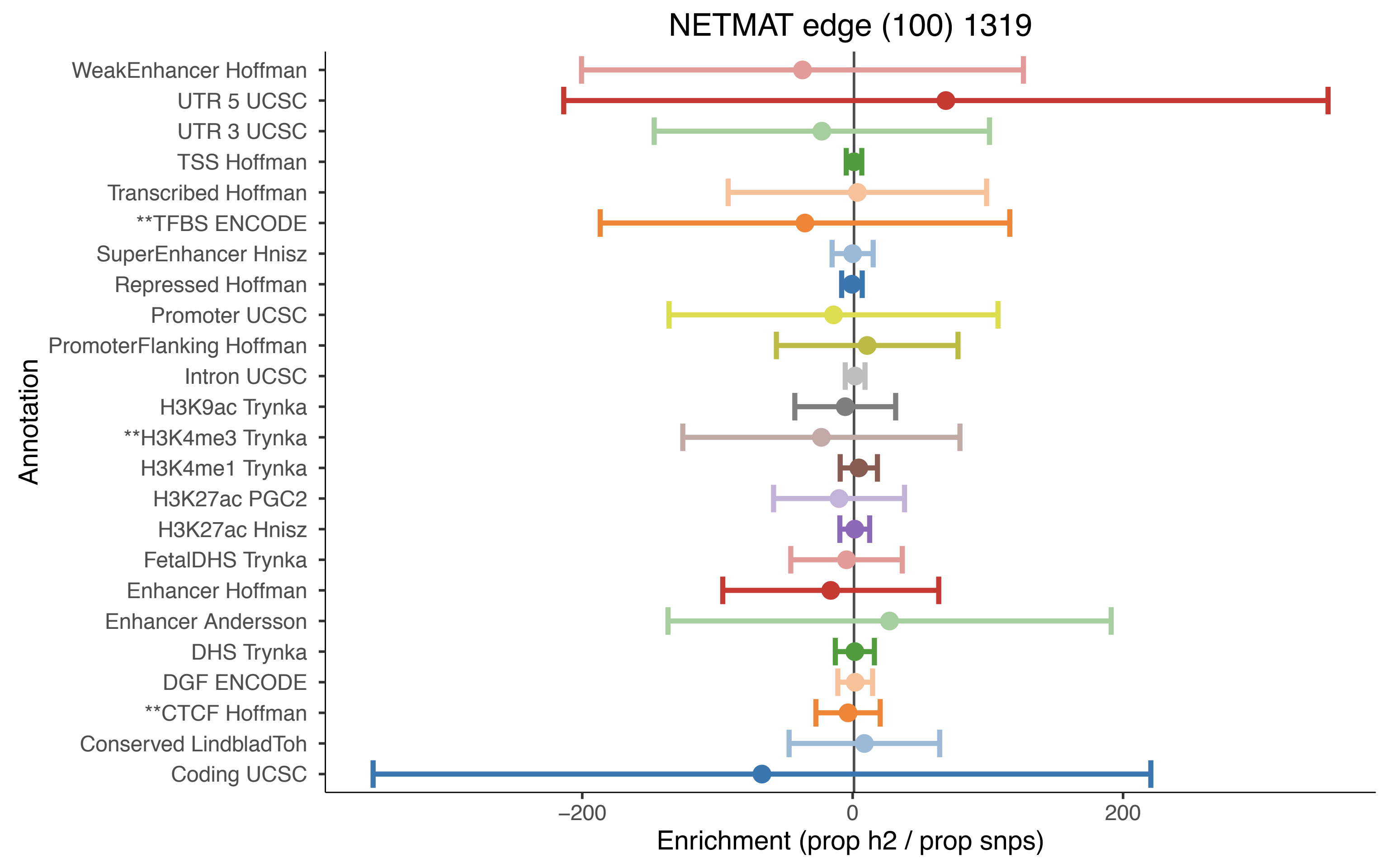

NETMAT edge (100) 1319
WeakEnhancer Hoffman
UTR 5 UCSC
UTR 3 UCSC
TSS Hoffman
Transcribed Hoffman
**TFBS ENCODE
SuperEnhancer Hnisz
Repressed Hoffman
Promoter UCSC
PromoterFlanking Hoffman
Intron UCSC
H3K9ac Trynka
**H3K4me3 Trynka
H3K4me1 Trynka
H3K27ac PGC2
H3K27ac Hnisz
FetalDHS Trynka
Enhancer Hoffman
Enhancer Andersson
DHS Trynka
DGF ENCODE
**CTCF Hoffman
Conserved LindbladToh
Coding UCSC
Annotation
−200
0
200
Enrichment (prop h2 / prop snps)

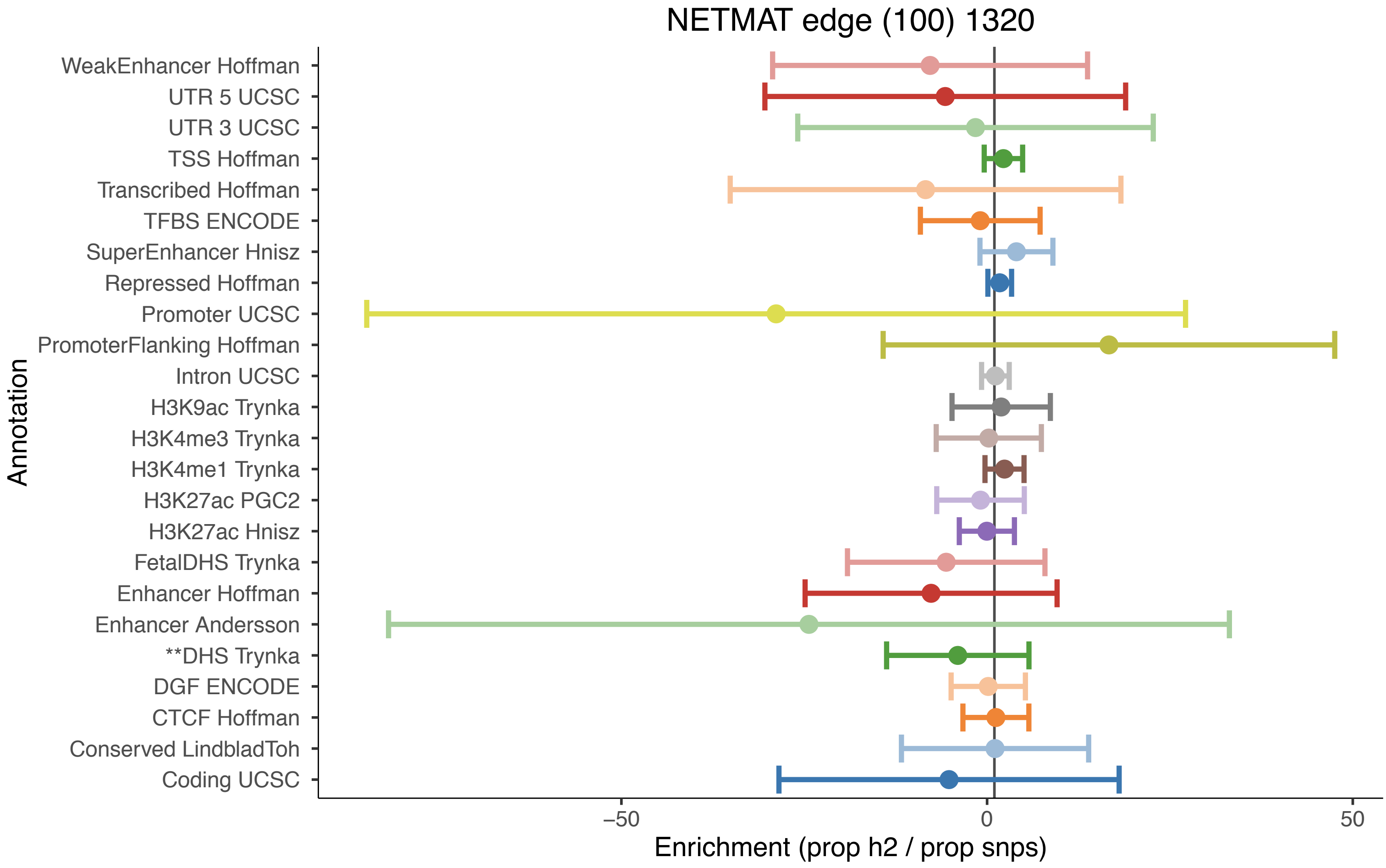

NETMAT edge (100) 1320
WeakEnhancer Hoffman
UTR 5 UCSC
UTR 3 UCSC
TSS Hoffman
Transcribed Hoffman
TFBS ENCODE
SuperEnhancer Hnisz
Repressed Hoffman
Promoter UCSC
PromoterFlanking Hoffman
Intron UCSC
H3K9ac Trynka
H3K4me3 Trynka
H3K4me1 Trynka
H3K27ac PGC2
H3K27ac Hnisz
FetalDHS Trynka
Enhancer Hoffman
Enhancer Andersson
**DHS Trynka
DGF ENCODE
CTCF Hoffman
Conserved LindbladToh
Coding UCSC
Annotation
−50
0
50
Enrichment (prop h2 / prop snps)

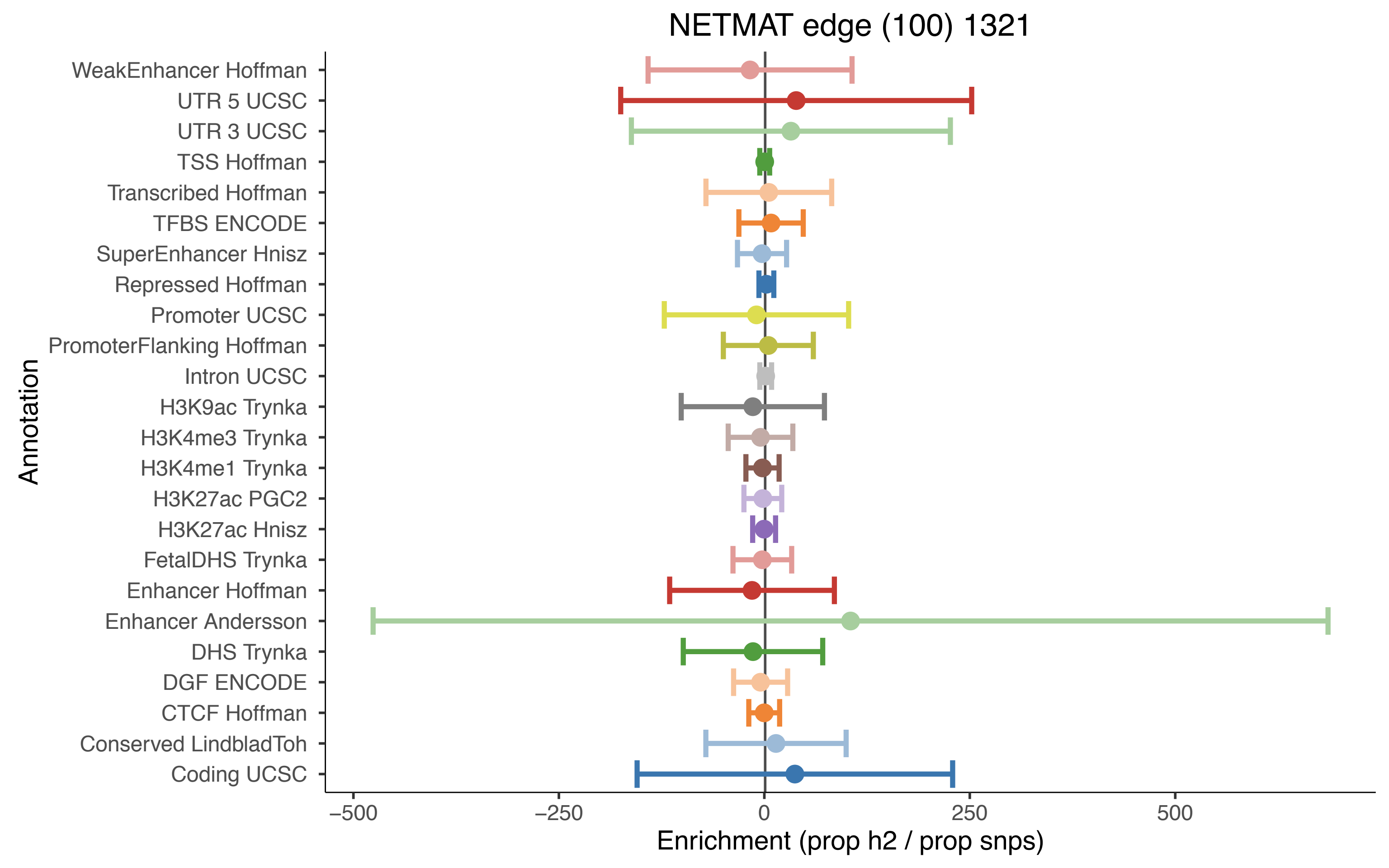
NETMAT edge (100) 1321
Annotation
WeakEnhancer Hoffman
UTR 5 UCSC
UTR 3 UCSC
TSS Hoffman
Transcribed Hoffman
TFBS ENCODE
SuperEnhancer Hnisz
Repressed Hoffman
Promoter UCSC
PromoterFlanking Hoffman
Intron UCSC
H3K9ac Trynka
H3K4me3 Trynka
H3K4me1 Trynka
H3K27ac PGC2
H3K27ac Hnisz
FetalDHS Trynka
Enhancer Hoffman
Enhancer Andersson
DHS Trynka
DGF ENCODE
CTCF Hoffman
Conserved LindbladToh
Coding UCSC
−500
−250
0
250
500
Enrichment (prop h2 / prop snps)

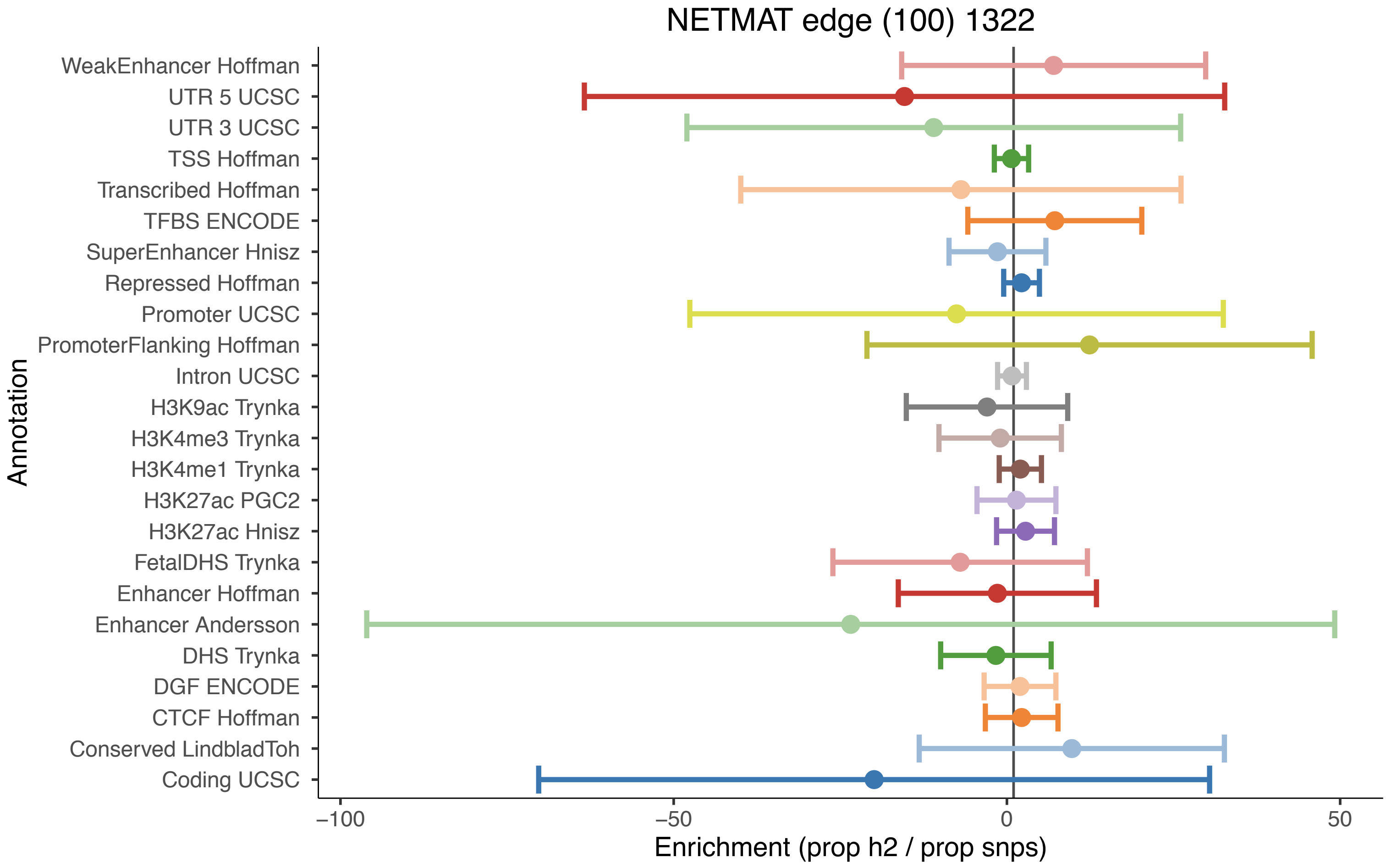

NETMAT edge (100) 1322
WeakEnhancer Hoffman
UTR 5 UCSC
UTR 3 UCSC
TSS Hoffman
Transcribed Hoffman
TFBS ENCODE
SuperEnhancer Hnisz
Repressed Hoffman
Promoter UCSC
PromoterFlanking Hoffman
Intron UCSC
H3K9ac Trynka
H3K4me3 Trynka
H3K4me1 Trynka
H3K27ac PGC2
H3K27ac Hnisz
FetalDHS Trynka
Enhancer Hoffman
Enhancer Andersson
DHS Trynka
DGF ENCODE
CTCF Hoffman
Conserved LindbladToh
Coding UCSC
Annotation
−100
−50
0
50
Enrichment (prop h2 / prop snps)

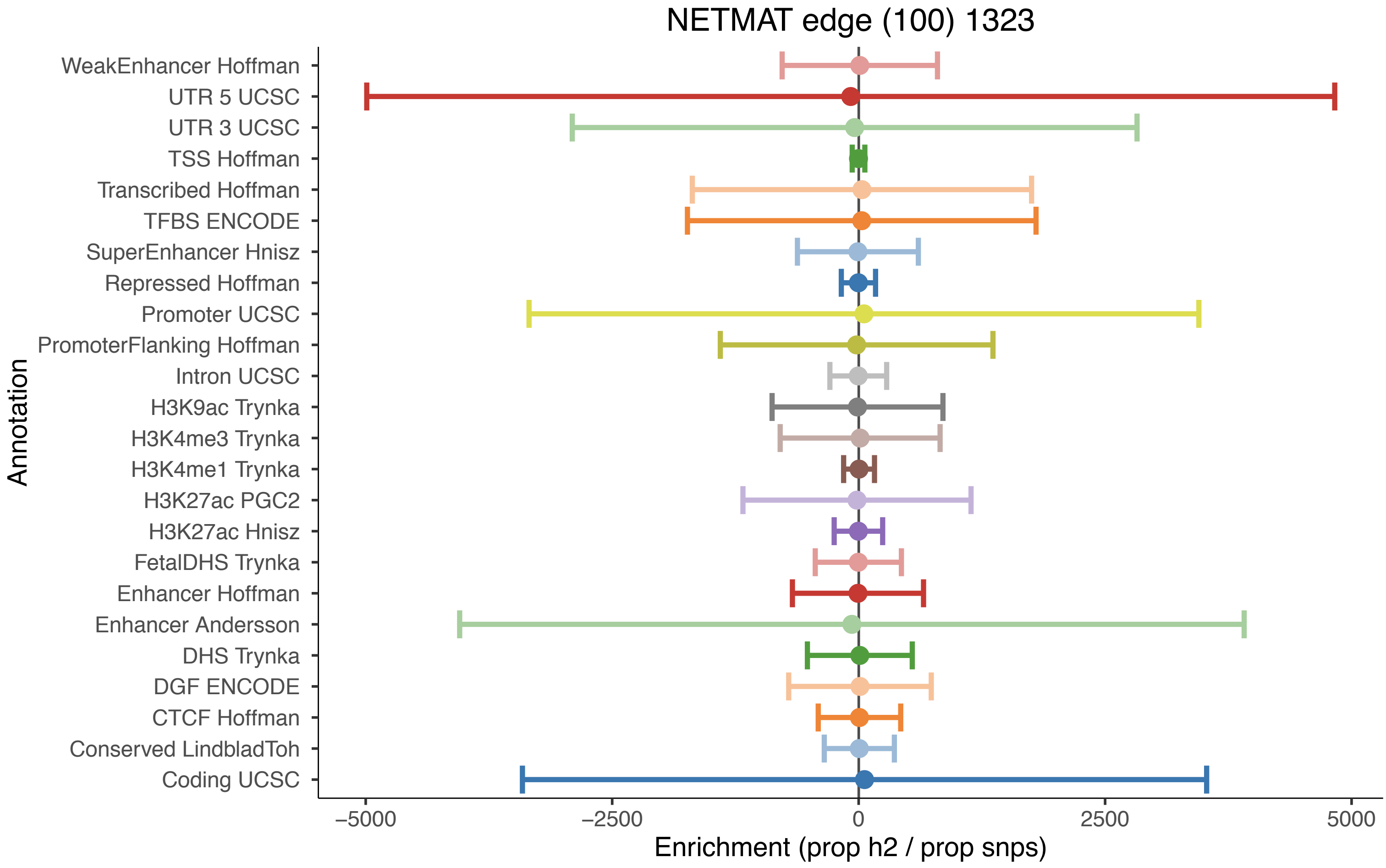

NETMAT edge (100) 1323
WeakEnhancer Hoffman
UTR 5 UCSC
UTR 3 UCSC
TSS Hoffman
Transcribed Hoffman
TFBS ENCODE
SuperEnhancer Hnisz
Repressed Hoffman
Promoter UCSC
PromoterFlanking Hoffman
Intron UCSC
H3K9ac Trynka
H3K4me3 Trynka
H3K4me1 Trynka
H3K27ac PGC2
H3K27ac Hnisz
FetalDHS Trynka
Enhancer Hoffman
Enhancer Andersson
DHS Trynka
DGF ENCODE
CTCF Hoffman
Conserved LindbladToh
Coding UCSC
Annotation
−5000
−2500
0
2500
5000
Enrichment (prop h2 / prop snps)

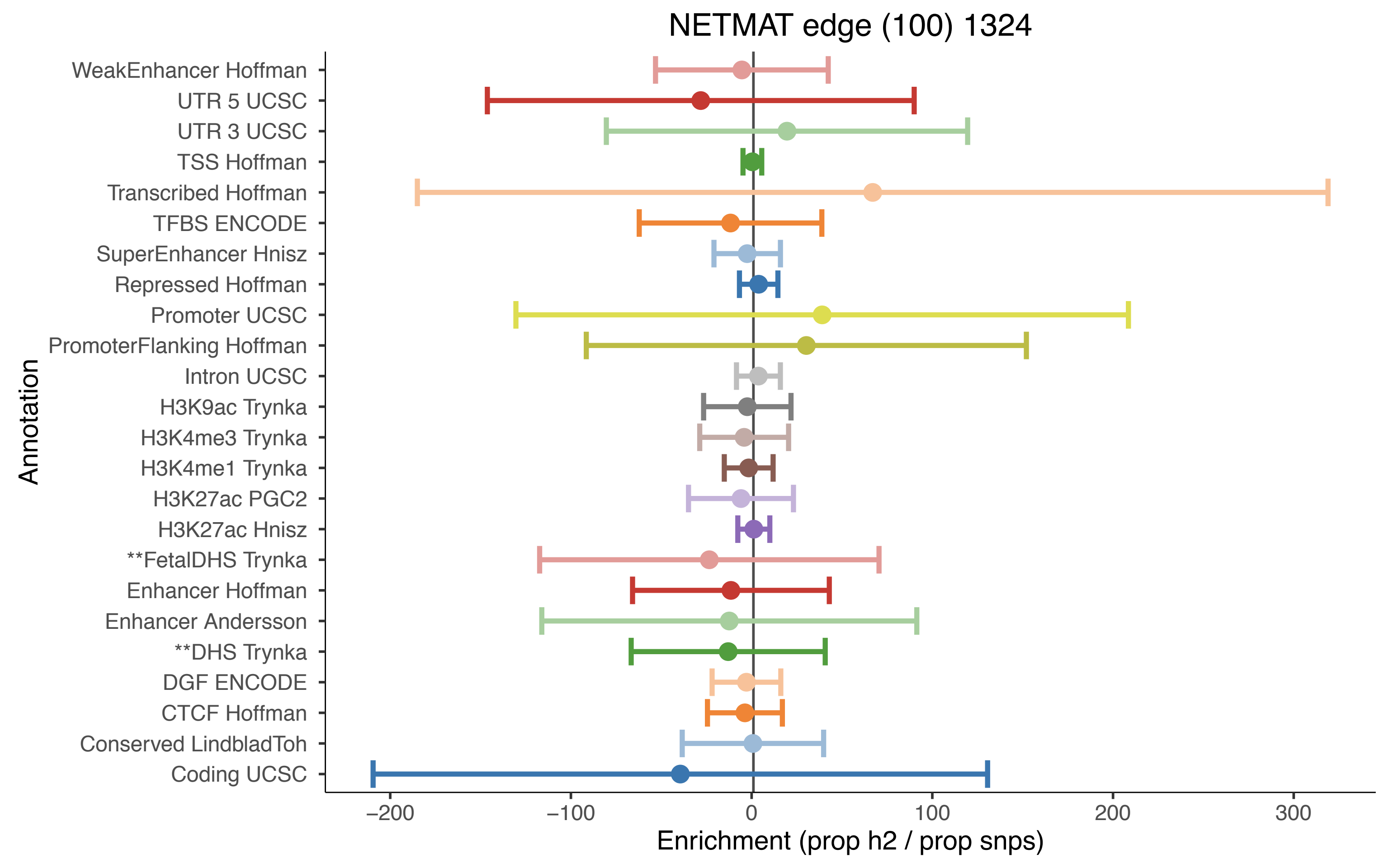
NETMAT edge (100) 1324
WeakEnhancer Hoffman
UTR 5 UCSC
UTR 3 UCSC
TSS Hoffman
Transcribed Hoffman
TFBS ENCODE
SuperEnhancer Hnisz
Repressed Hoffman
Promoter UCSC
PromoterFlanking Hoffman
Intron UCSC
H3K9ac Trynka
H3K4me3 Trynka
H3K4me1 Trynka
H3K27ac PGC2
H3K27ac Hnisz
**FetalDHS Trynka
Enhancer Hoffman
Enhancer Andersson
**DHS Trynka
DGF ENCODE
CTCF Hoffman
Conserved LindbladToh
Coding UCSC
Annotation
−200
−100
0
100
200
300
Enrichment (prop h2 / prop snps)

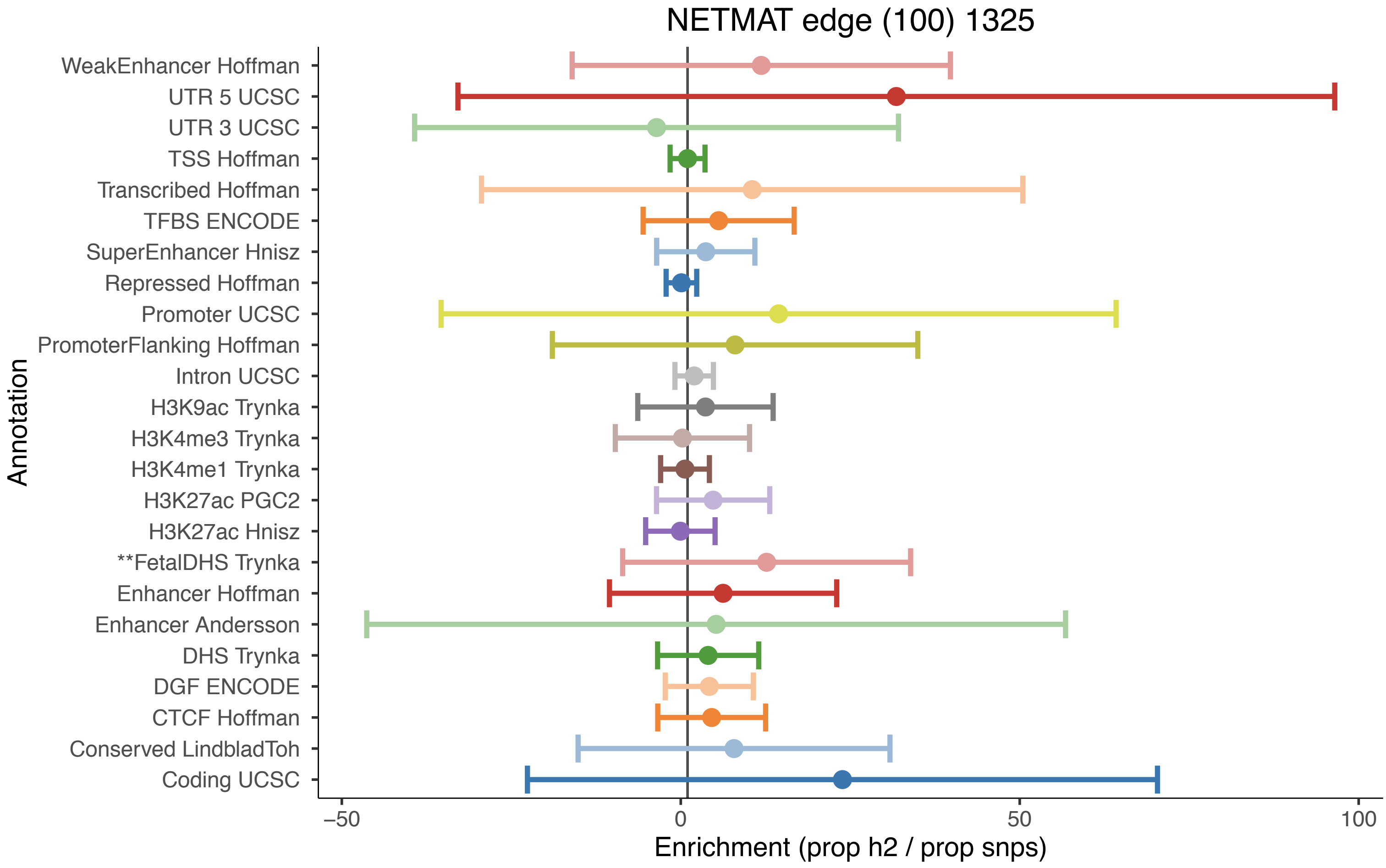
NETMAT edge (100) 1325
Annotation
WeakEnhancer Hoffman
UTR 5 UCSC
UTR 3 UCSC
TSS Hoffman
Transcribed Hoffman
TFBS ENCODE
SuperEnhancer Hnisz
Repressed Hoffman
Promoter UCSC
PromoterFlanking Hoffman
Intron UCSC
H3K9ac Trynka
H3K4me3 Trynka
H3K4me1 Trynka
H3K27ac PGC2
H3K27ac Hnisz
**FetalDHS Trynka
Enhancer Hoffman
Enhancer Andersson
DHS Trynka
DGF ENCODE
CTCF Hoffman
Conserved LindbladToh
Coding UCSC
−50
0
50
100
Enrichment (prop h2 / prop snps)

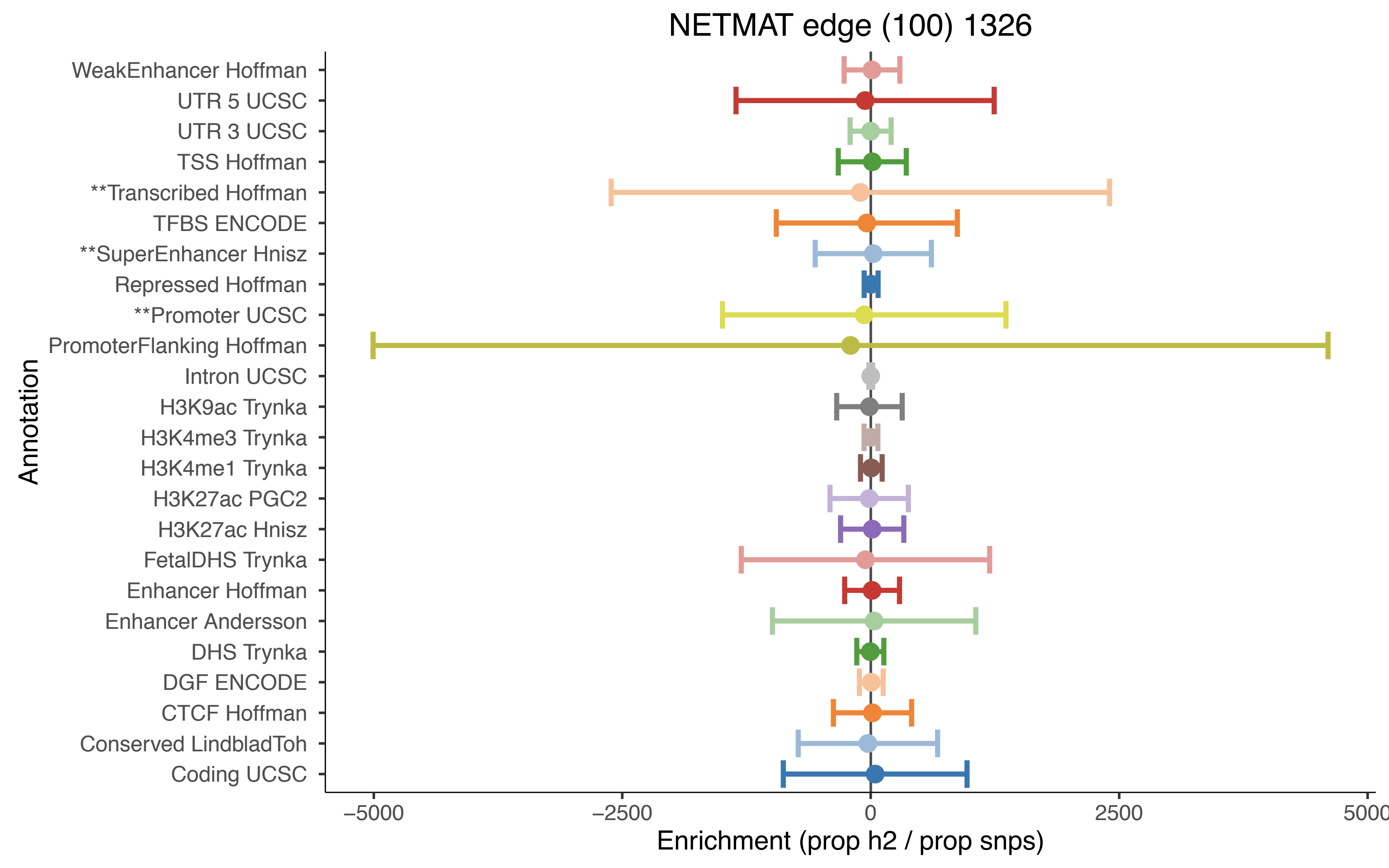
NETMAT edge (100) 1326
WeakEnhancer Hoffman
UTR 5 UCSC
UTR 3 UCSC
TSS Hoffman
**Transcribed Hoffman
TFBS ENCODE
**SuperEnhancer Hnisz
Repressed Hoffman
**Promoter UCSC
PromoterFlanking Hoffman
Intron UCSC
H3K9ac Trynka
H3K4me3 Trynka
H3K4me1 Trynka
H3K27ac PGC2
H3K27ac Hnisz
FetalDHS Trynka
Enhancer Hoffman
Enhancer Andersson
DHS Trynka
DGF ENCODE
CTCF Hoffman
Conserved LindbladToh
Coding UCSC
Annotation
−5000
−2500
0
2500
5000
Enrichment (prop h2 / prop snps)

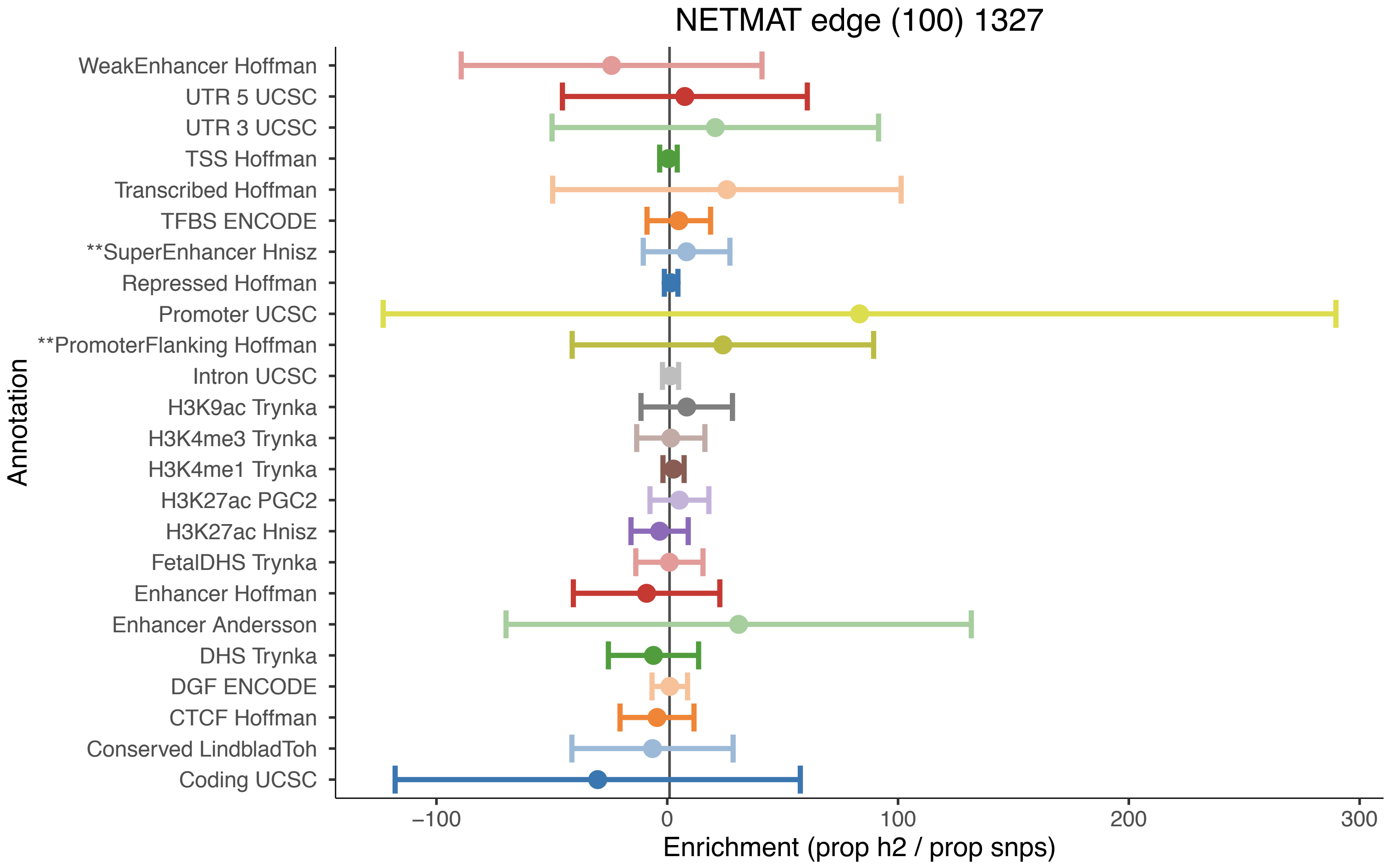
NETMAT edge (100) 1327
WeakEnhancer Hoffman
UTR 5 UCSC
UTR 3 UCSC
TSS Hoffman
Transcribed Hoffman
TFBS ENCODE
**SuperEnhancer Hnisz
Repressed Hoffman
Promoter UCSC
**PromoterFlanking Hoffman
Intron UCSC
H3K9ac Trynka
H3K4me3 Trynka
H3K4me1 Trynka
H3K27ac PGC2
H3K27ac Hnisz
FetalDHS Trynka
Enhancer Hoffman
Enhancer Andersson
DHS Trynka
DGF ENCODE
CTCF Hoffman
Conserved LindbladToh
Coding UCSC
Annotation
−100
0
100
200
300
Enrichment (prop h2 / prop snps)

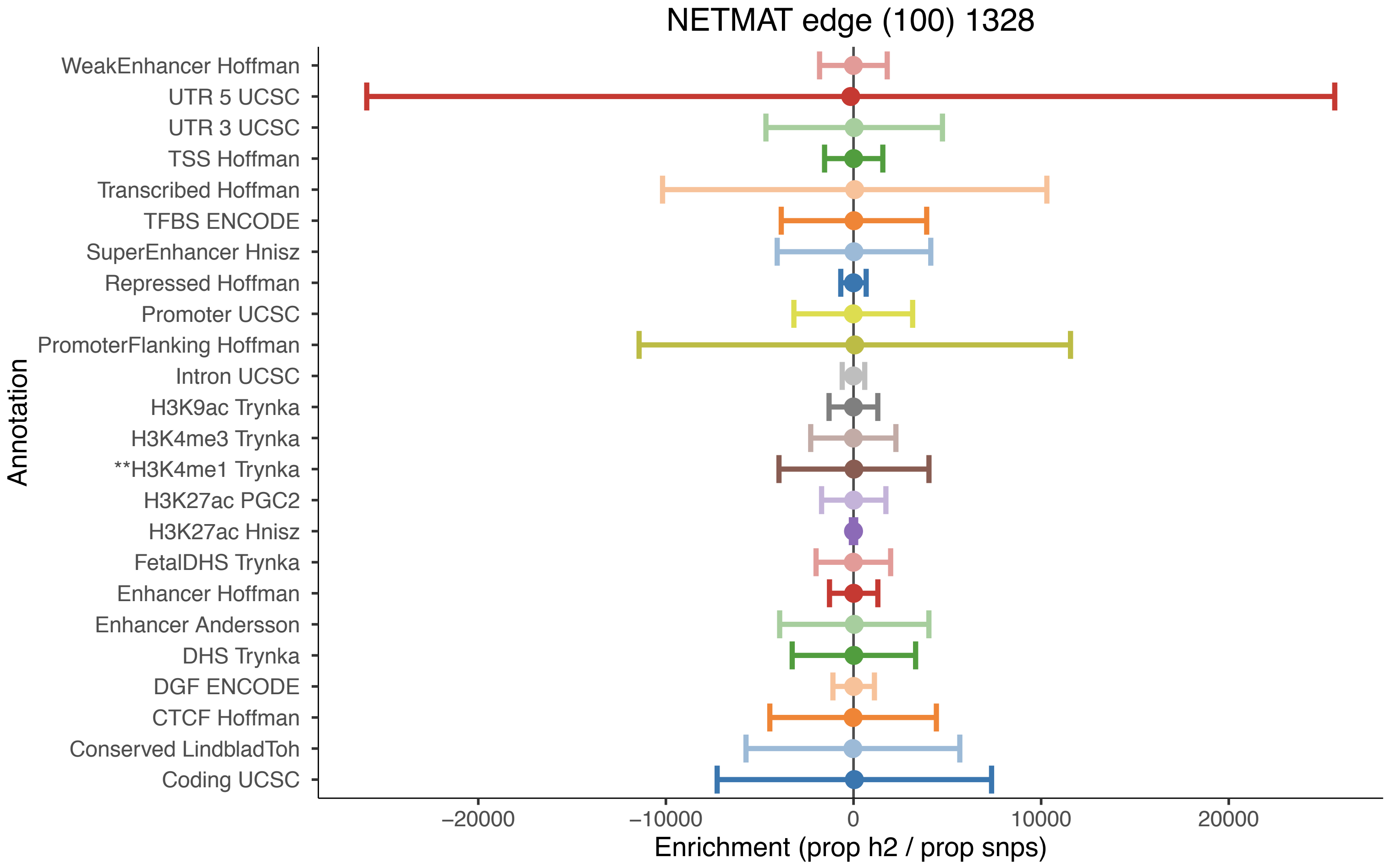
NETMAT edge (100) 1328
WeakEnhancer Hoffman
UTR 5 UCSC
UTR 3 UCSC
TSS Hoffman
Transcribed Hoffman
TFBS ENCODE
SuperEnhancer Hnisz
Repressed Hoffman
Promoter UCSC
PromoterFlanking Hoffman
Intron UCSC
H3K9ac Trynka
H3K4me3 Trynka
**H3K4me1 Trynka
H3K27ac PGC2
H3K27ac Hnisz
FetalDHS Trynka
Enhancer Hoffman
Enhancer Andersson
DHS Trynka
DGF ENCODE
CTCF Hoffman
Conserved LindbladToh
Coding UCSC
Annotation
−20000
−10000
0
10000
20000
Enrichment (prop h2 / prop snps)

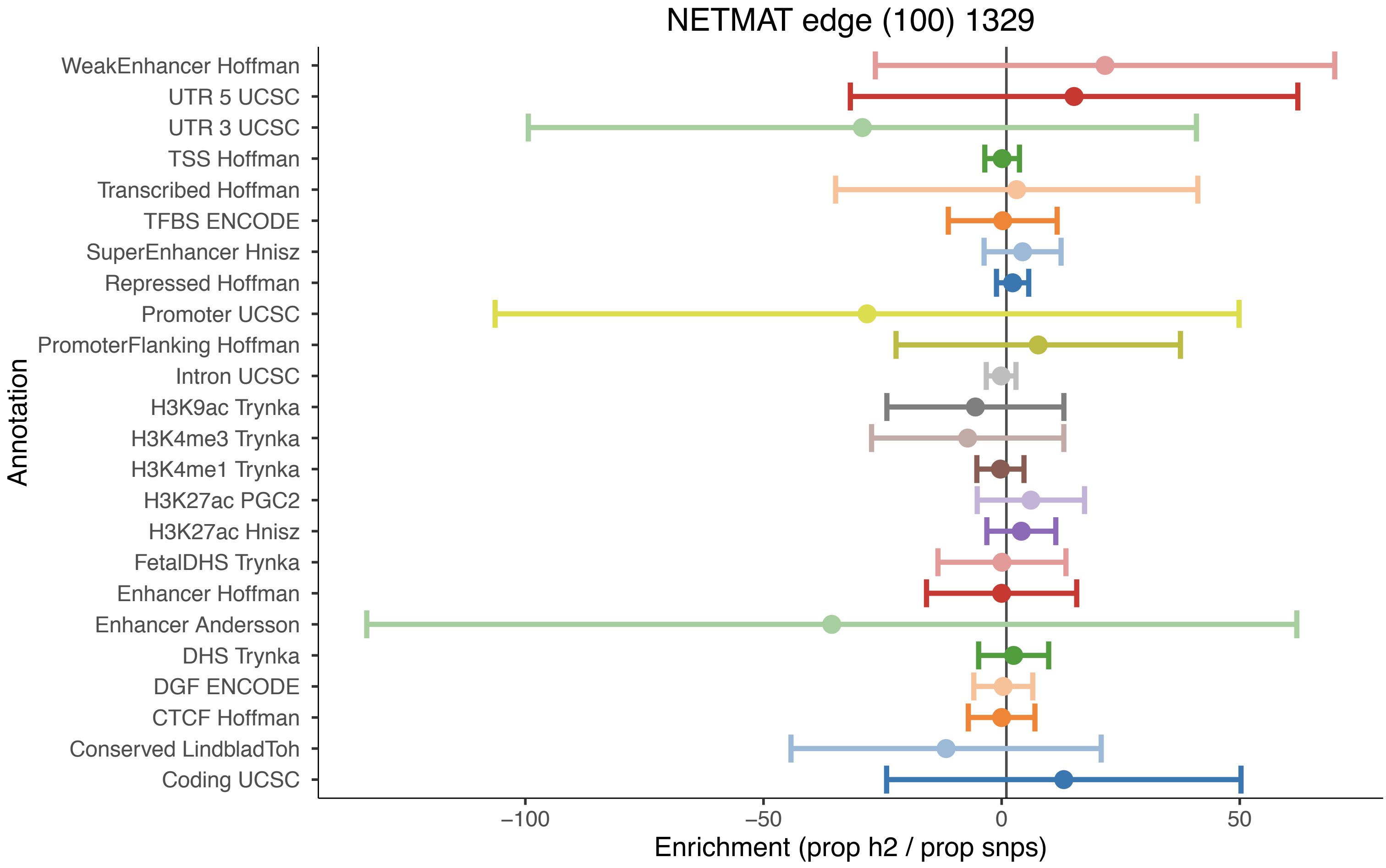
NETMAT edge (100) 1329
WeakEnhancer Hoffman
UTR 5 UCSC
UTR 3 UCSC
TSS Hoffman
Transcribed Hoffman
TFBS ENCODE
SuperEnhancer Hnisz
Repressed Hoffman
Promoter UCSC
PromoterFlanking Hoffman
Intron UCSC
H3K9ac Trynka
H3K4me3 Trynka
H3K4me1 Trynka
H3K27ac PGC2
H3K27ac Hnisz
FetalDHS Trynka
Enhancer Hoffman
Enhancer Andersson
DHS Trynka
DGF ENCODE
CTCF Hoffman
Conserved LindbladToh
Coding UCSC
Annotation
−100
−50
0
50
Enrichment (prop h2 / prop snps)

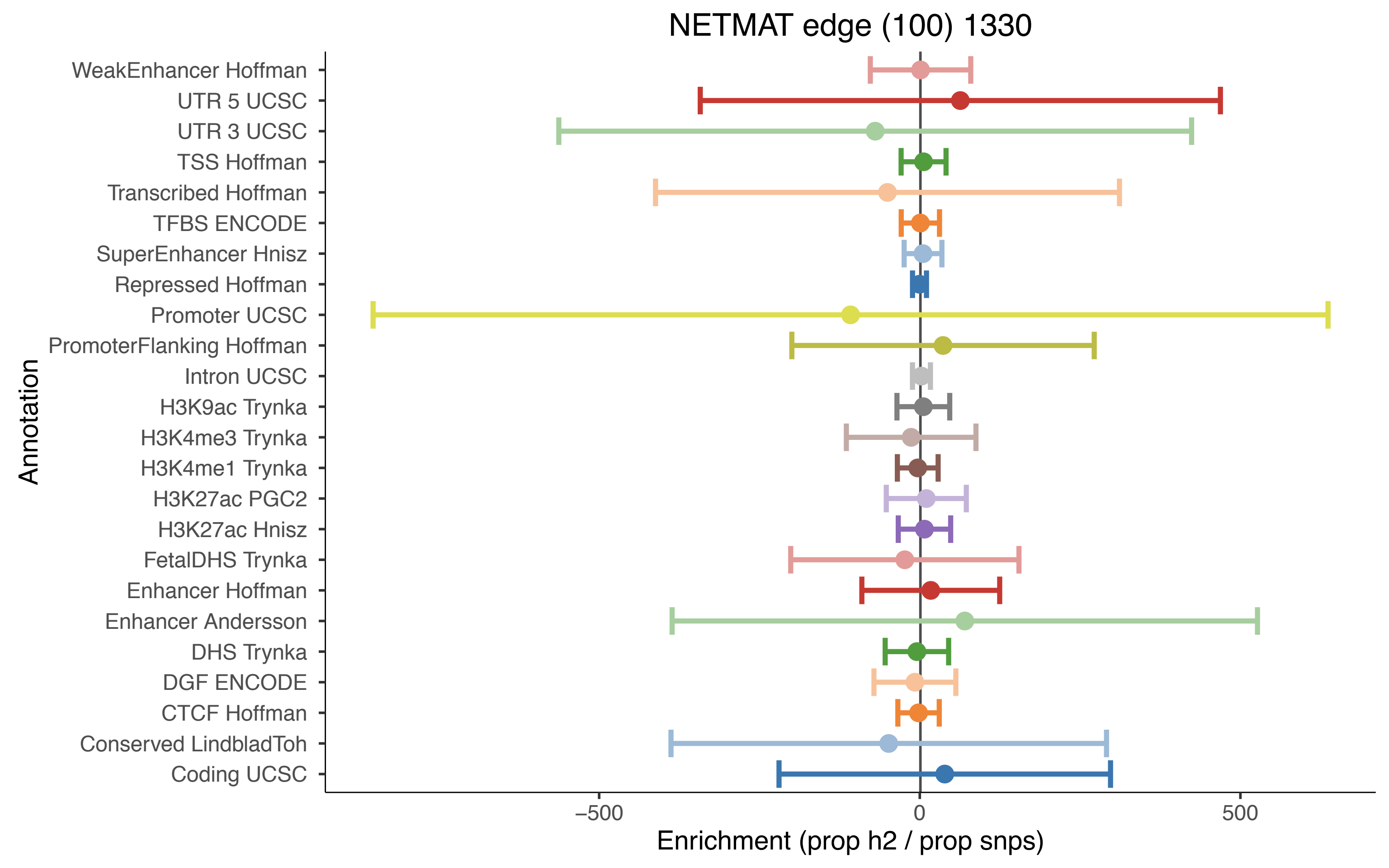
NETMAT edge (100) 1330
WeakEnhancer Hoffman
UTR 5 UCSC
UTR 3 UCSC
TSS Hoffman
Transcribed Hoffman
TFBS ENCODE
SuperEnhancer Hnisz
Repressed Hoffman
Promoter UCSC
PromoterFlanking Hoffman
Intron UCSC
H3K9ac Trynka
H3K4me3 Trynka
H3K4me1 Trynka
H3K27ac PGC2
H3K27ac Hnisz
FetalDHS Trynka
Enhancer Hoffman
Enhancer Andersson
DHS Trynka
DGF ENCODE
CTCF Hoffman
Conserved LindbladToh
Coding UCSC
Annotation
−500
0
500
Enrichment (prop h2 / prop snps)

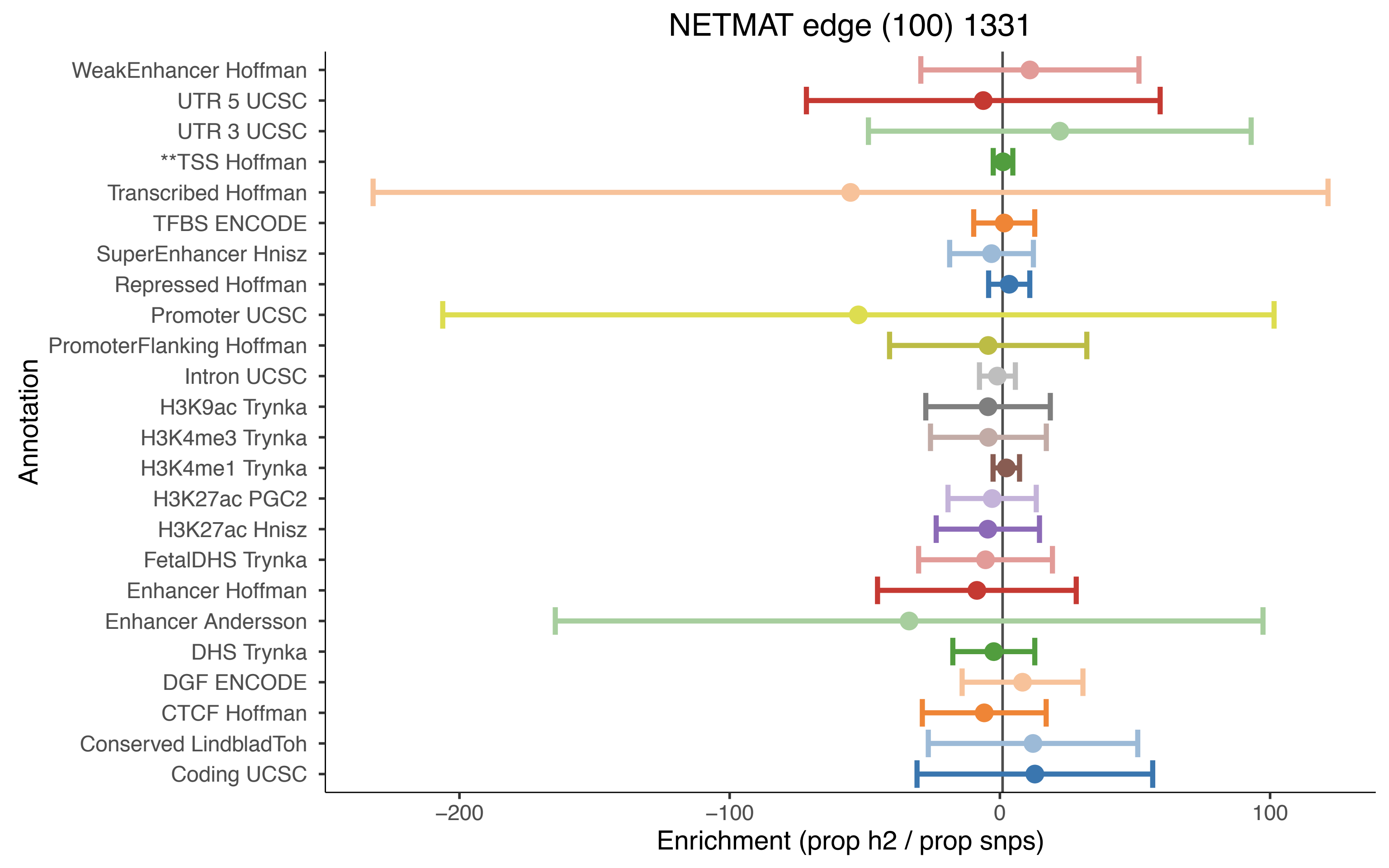
NETMAT edge (100) 1331
WeakEnhancer Hoffman
UTR 5 UCSC
UTR 3 UCSC
**TSS Hoffman
Transcribed Hoffman
TFBS ENCODE
SuperEnhancer Hnisz
Repressed Hoffman
Promoter UCSC
PromoterFlanking Hoffman
Intron UCSC
H3K9ac Trynka
H3K4me3 Trynka
H3K4me1 Trynka
H3K27ac PGC2
H3K27ac Hnisz
FetalDHS Trynka
Enhancer Hoffman
Enhancer Andersson
DHS Trynka
DGF ENCODE
CTCF Hoffman
Conserved LindbladToh
Coding UCSC
Annotation
−200
−100
0
100
Enrichment (prop h2 / prop snps)

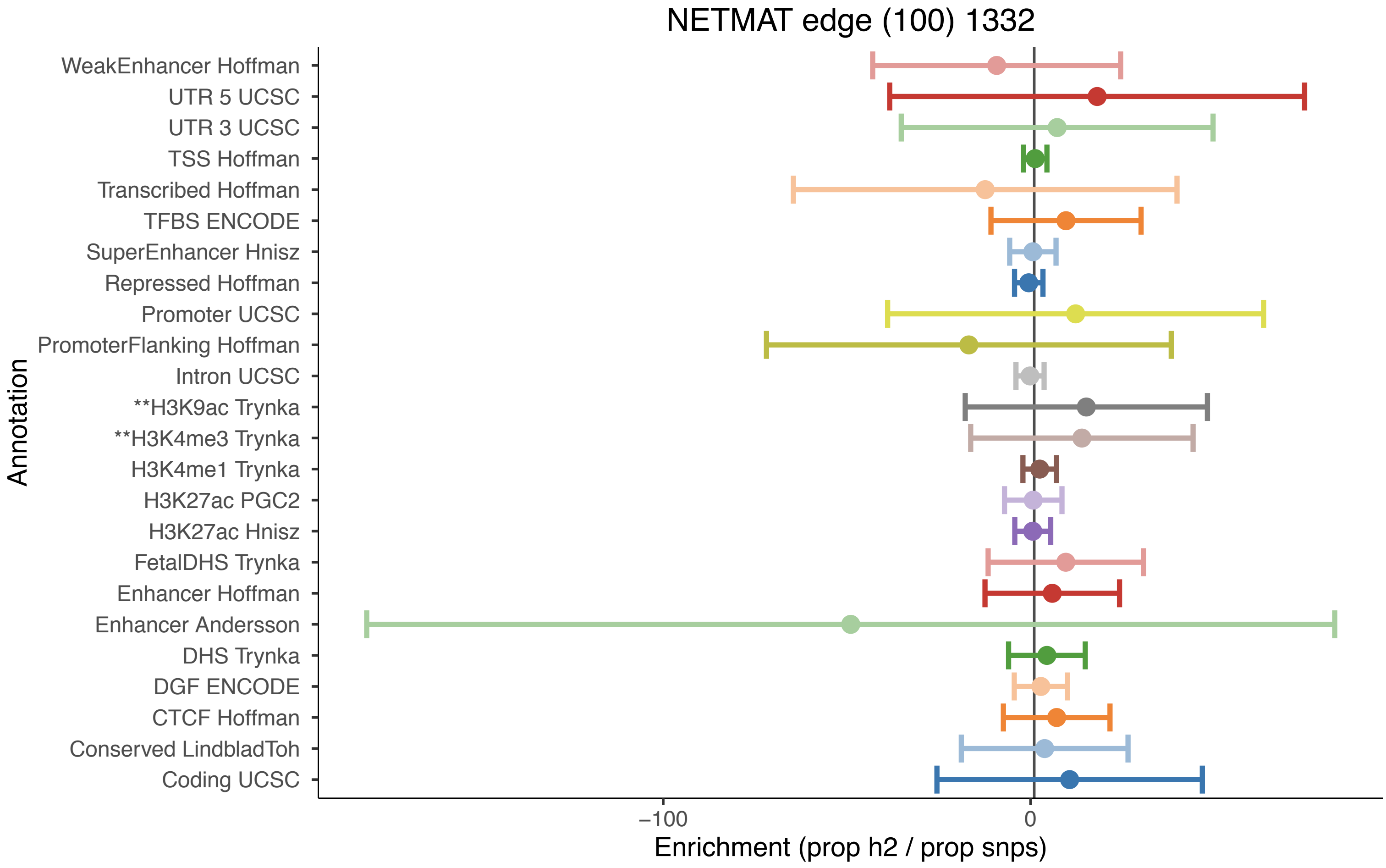

NETMAT edge (100) 1332
WeakEnhancer Hoffman
UTR 5 UCSC
UTR 3 UCSC
TSS Hoffman
Transcribed Hoffman
TFBS ENCODE
SuperEnhancer Hnisz
Repressed Hoffman
Promoter UCSC
PromoterFlanking Hoffman
Intron UCSC
**H3K9ac Trynka
**H3K4me3 Trynka
H3K4me1 Trynka
H3K27ac PGC2
H3K27ac Hnisz
FetalDHS Trynka
Enhancer Hoffman
Enhancer Andersson
DHS Trynka
DGF ENCODE
CTCF Hoffman
Conserved LindbladToh
Coding UCSC
Annotation
−100
0
Enrichment (prop h2 / prop snps)

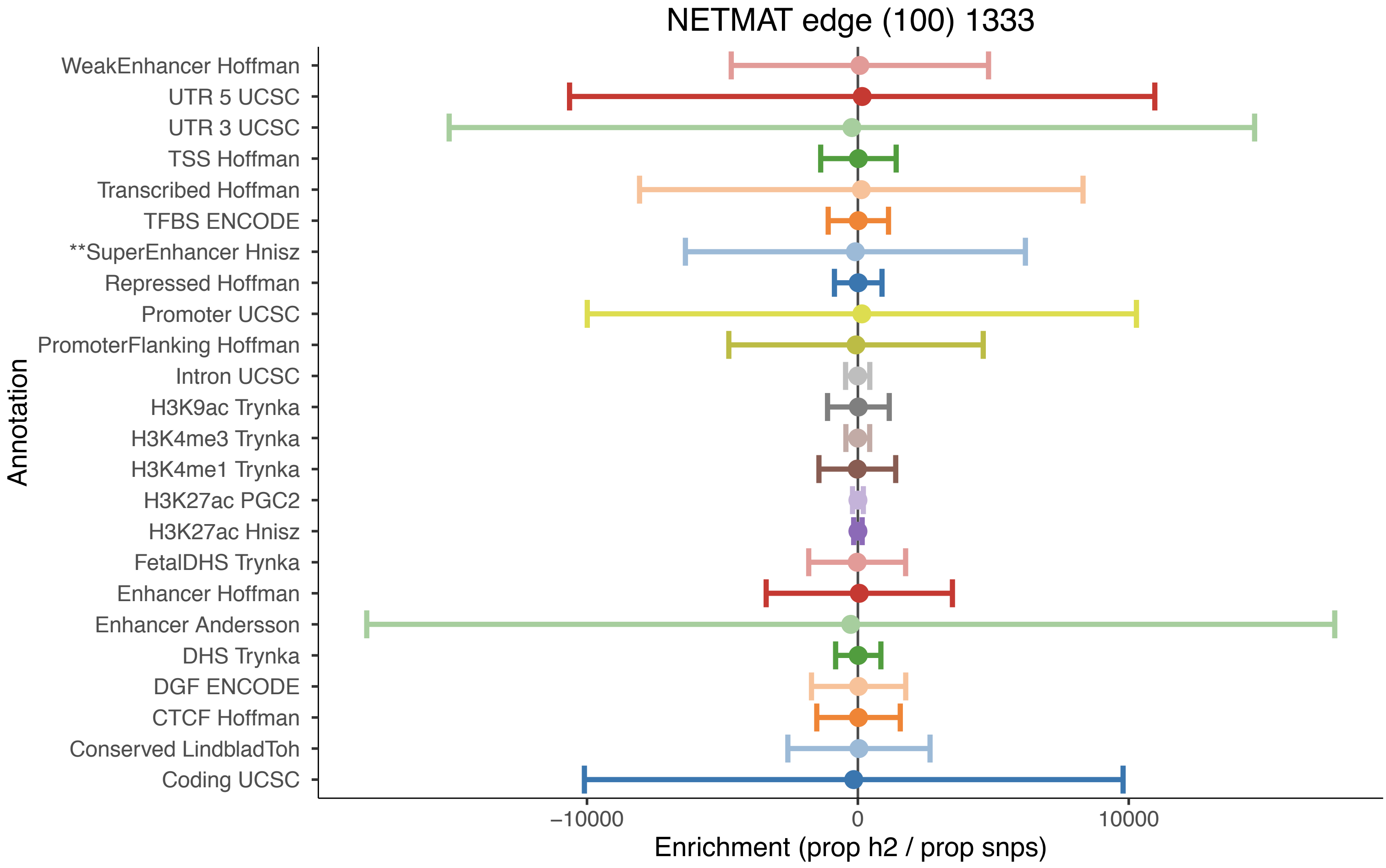
NETMAT edge (100) 1333
WeakEnhancer Hoffman
UTR 5 UCSC
UTR 3 UCSC
TSS Hoffman
Transcribed Hoffman
TFBS ENCODE
**SuperEnhancer Hnisz
Repressed Hoffman
Promoter UCSC
PromoterFlanking Hoffman
Intron UCSC
H3K9ac Trynka
H3K4me3 Trynka
H3K4me1 Trynka
H3K27ac PGC2
H3K27ac Hnisz
FetalDHS Trynka
Enhancer Hoffman
Enhancer Andersson
DHS Trynka
DGF ENCODE
CTCF Hoffman
Conserved LindbladToh
Coding UCSC
Annotation
−10000
0
10000
Enrichment (prop h2 / prop snps)

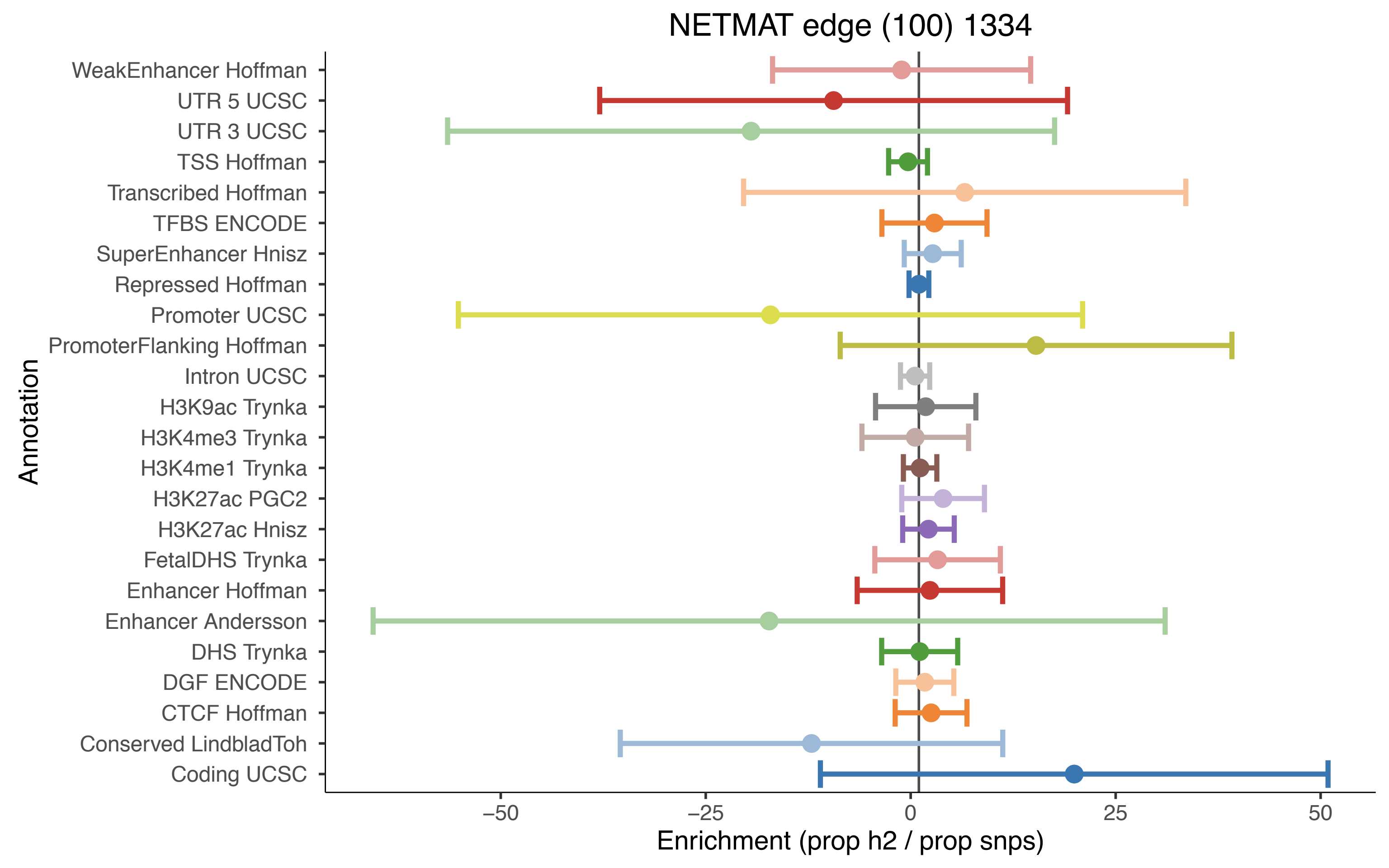
NETMAT edge (100) 1334
Annotation
WeakEnhancer Hoffman
UTR 5 UCSC
UTR 3 UCSC
TSS Hoffman
Transcribed Hoffman
TFBS ENCODE
SuperEnhancer Hnisz
Repressed Hoffman
Promoter UCSC
PromoterFlanking Hoffman
Intron UCSC
H3K9ac Trynka
H3K4me3 Trynka
H3K4me1 Trynka
H3K27ac PGC2
H3K27ac Hnisz
FetalDHS Trynka
Enhancer Hoffman
Enhancer Andersson
DHS Trynka
DGF ENCODE
CTCF Hoffman
Conserved LindbladToh
Coding UCSC
−50
−25
0
25
50
Enrichment (prop h2 / prop snps)

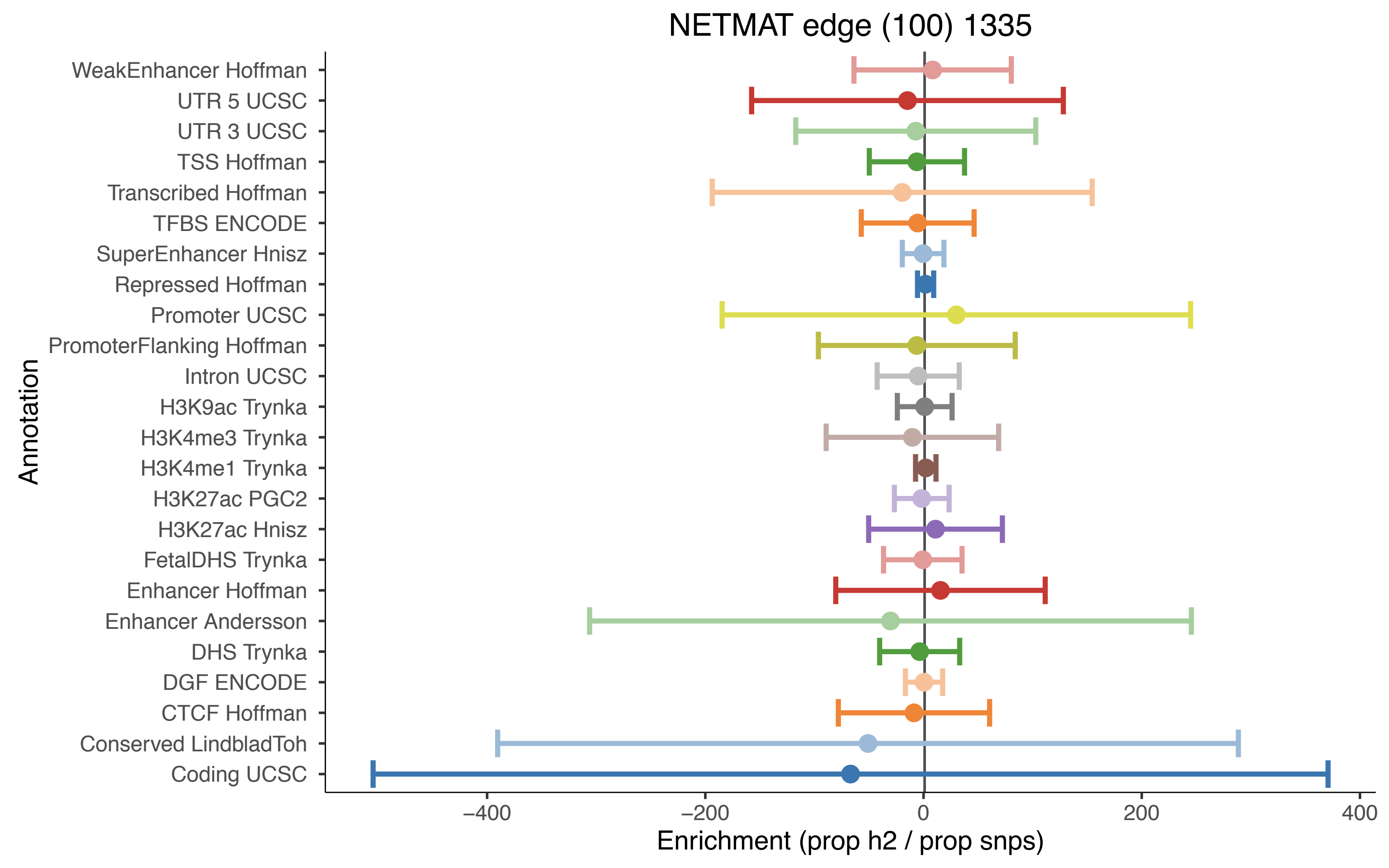

NETMAT edge (100) 1335
WeakEnhancer Hoffman
UTR 5 UCSC
UTR 3 UCSC
TSS Hoffman
Transcribed Hoffman
TFBS ENCODE
SuperEnhancer Hnisz
Repressed Hoffman
Promoter UCSC
PromoterFlanking Hoffman
Intron UCSC
H3K9ac Trynka
H3K4me3 Trynka
H3K4me1 Trynka
H3K27ac PGC2
H3K27ac Hnisz
FetalDHS Trynka
Enhancer Hoffman
Enhancer Andersson
DHS Trynka
DGF ENCODE
CTCF Hoffman
Conserved LindbladToh
Coding UCSC
Annotation
−400
−200
0
200
400
Enrichment (prop h2 / prop snps)

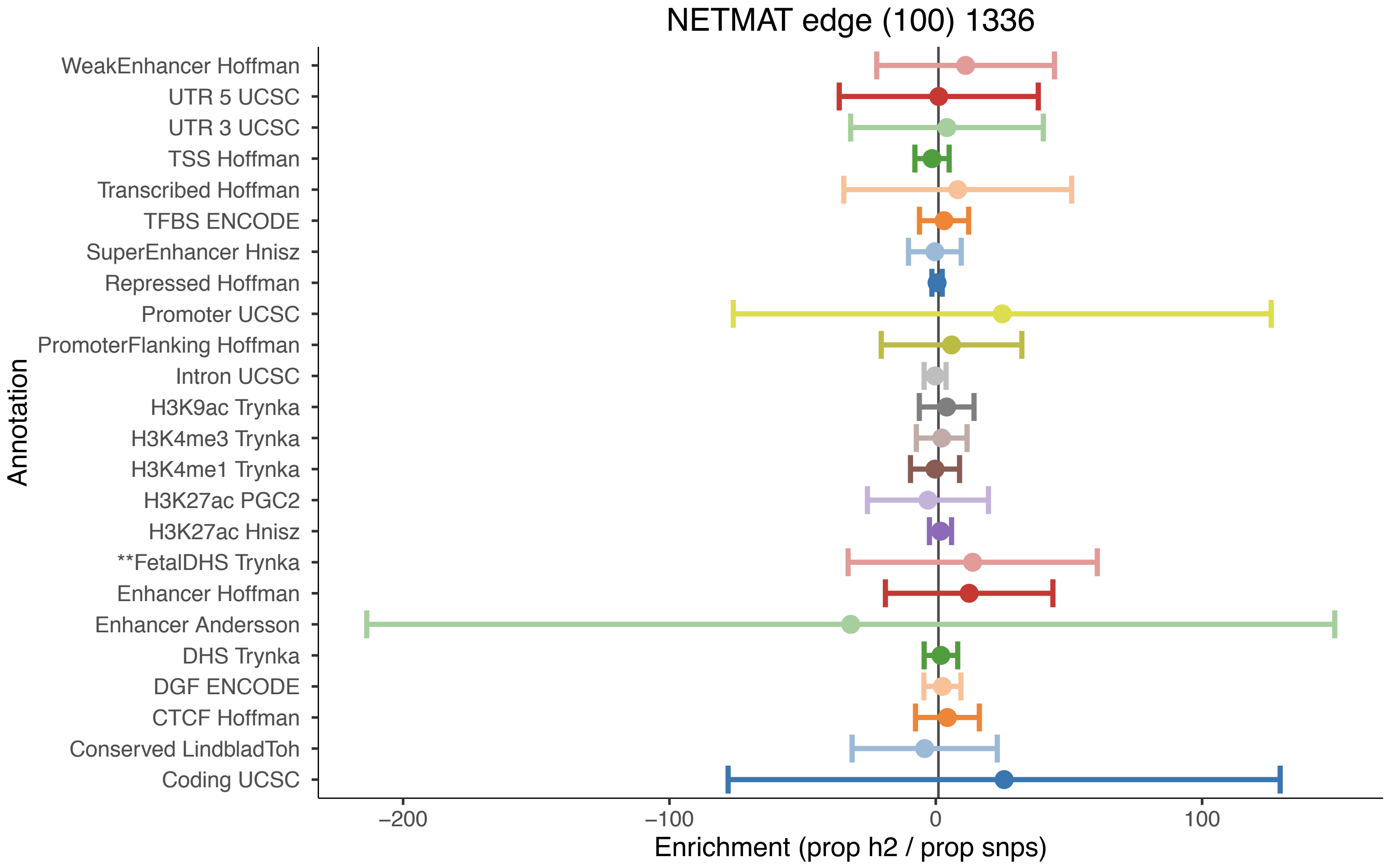
NETMAT edge (100) 1336
WeakEnhancer Hoffman
UTR 5 UCSC
UTR 3 UCSC
TSS Hoffman
Transcribed Hoffman
TFBS ENCODE
SuperEnhancer Hnisz
Repressed Hoffman
Promoter UCSC
PromoterFlanking Hoffman
Intron UCSC
H3K9ac Trynka
H3K4me3 Trynka
H3K4me1 Trynka
H3K27ac PGC2
H3K27ac Hnisz
**FetalDHS Trynka
Enhancer Hoffman
Enhancer Andersson
DHS Trynka
DGF ENCODE
CTCF Hoffman
Conserved LindbladToh
Coding UCSC
Annotation
−200
−100
0
100
Enrichment (prop h2 / prop snps)

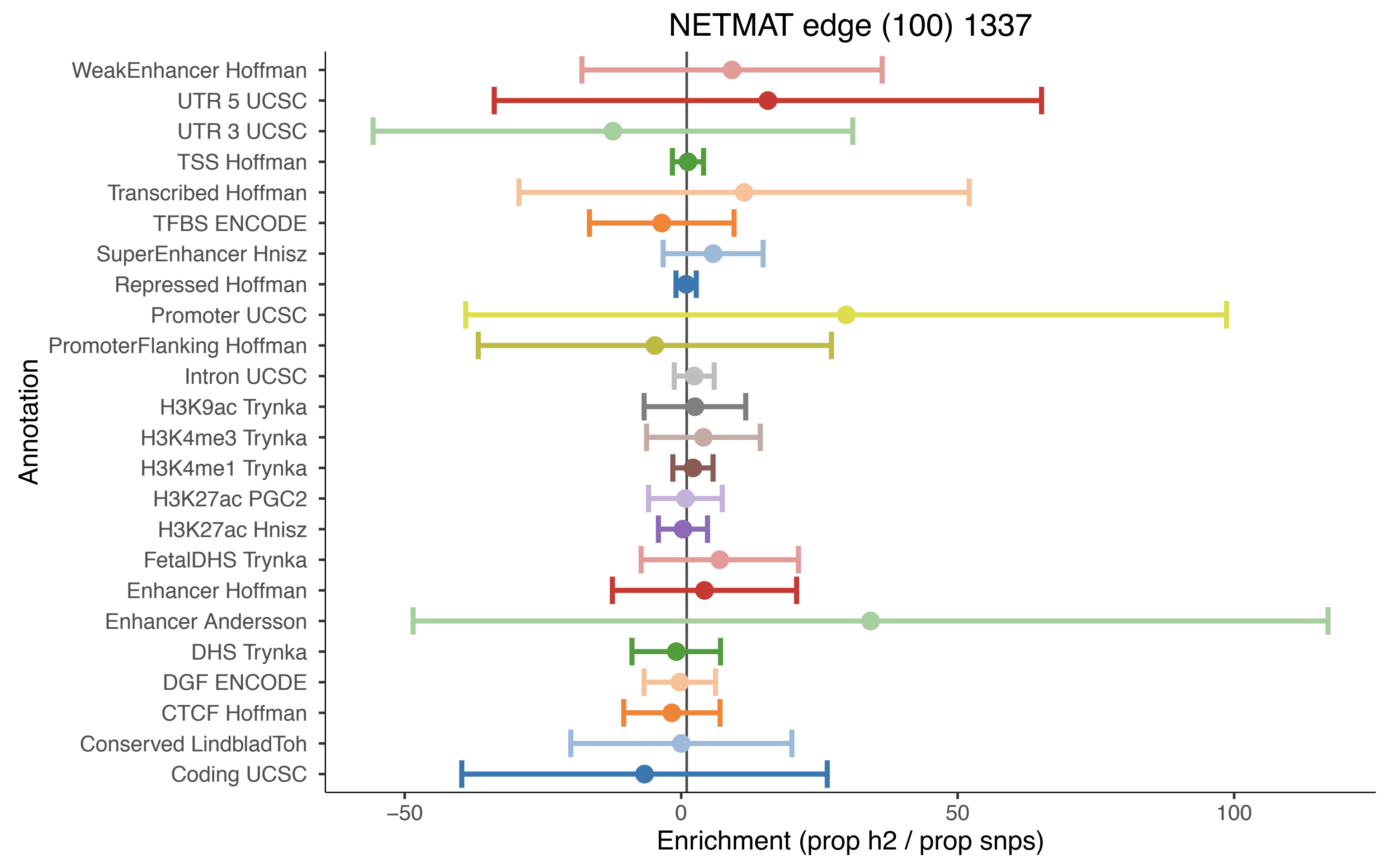
NETMAT edge (100) 1337
WeakEnhancer Hoffman
UTR 5 UCSC
UTR 3 UCSC
TSS Hoffman
Transcribed Hoffman
TFBS ENCODE
SuperEnhancer Hnisz
Repressed Hoffman
Promoter UCSC
PromoterFlanking Hoffman
Intron UCSC
H3K9ac Trynka
H3K4me3 Trynka
H3K4me1 Trynka
H3K27ac PGC2
H3K27ac Hnisz
FetalDHS Trynka
Enhancer Hoffman
Enhancer Andersson
DHS Trynka
DGF ENCODE
CTCF Hoffman
Conserved LindbladToh
Coding UCSC
Annotation
−50
0
50
100
Enrichment (prop h2 / prop snps)

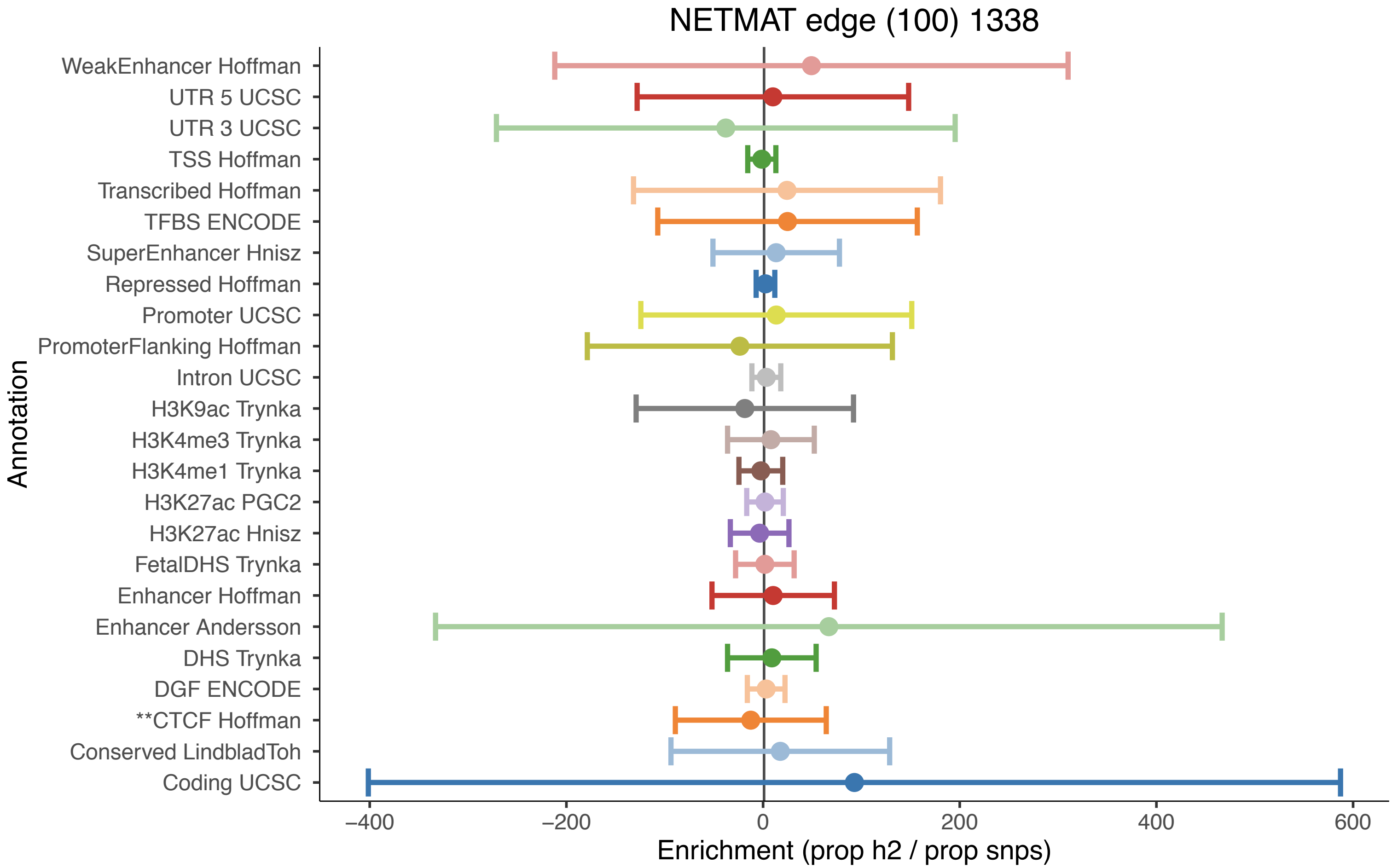
NETMAT edge (100) 1338
WeakEnhancer Hoffman
UTR 5 UCSC
UTR 3 UCSC
TSS Hoffman
Transcribed Hoffman
TFBS ENCODE
SuperEnhancer Hnisz
Repressed Hoffman
Promoter UCSC
PromoterFlanking Hoffman
Intron UCSC
H3K9ac Trynka
H3K4me3 Trynka
H3K4me1 Trynka
H3K27ac PGC2
H3K27ac Hnisz
FetalDHS Trynka
Enhancer Hoffman
Enhancer Andersson
DHS Trynka
DGF ENCODE
**CTCF Hoffman
Conserved LindbladToh
Coding UCSC
Annotation
−400
−200
0
200
400
600
Enrichment (prop h2 / prop snps)

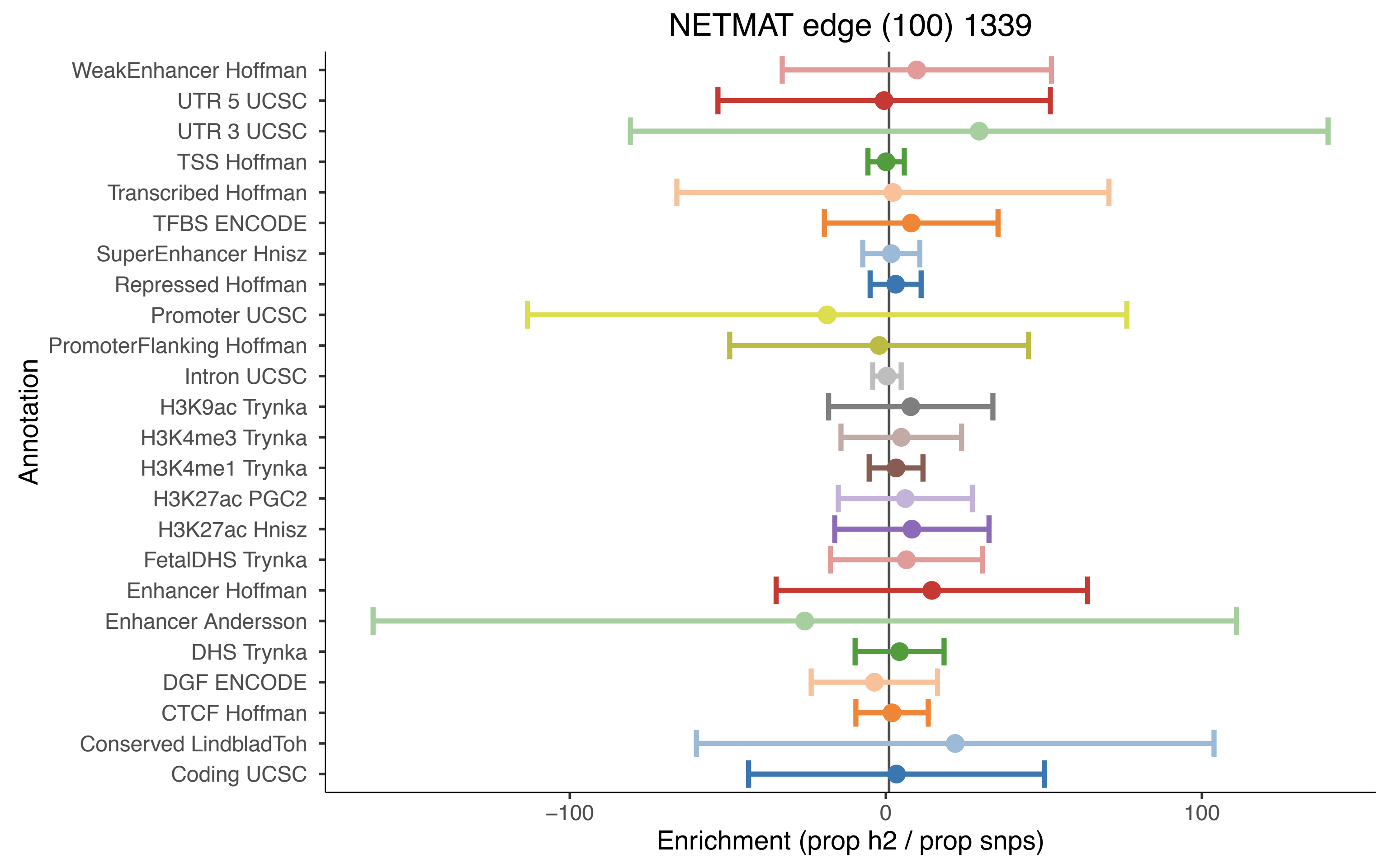

NETMAT edge (100) 1339
WeakEnhancer Hoffman
UTR 5 UCSC
UTR 3 UCSC
TSS Hoffman
Transcribed Hoffman
TFBS ENCODE
SuperEnhancer Hnisz
Repressed Hoffman
Promoter UCSC
PromoterFlanking Hoffman
Intron UCSC
H3K9ac Trynka
H3K4me3 Trynka
H3K4me1 Trynka
H3K27ac PGC2
H3K27ac Hnisz
FetalDHS Trynka
Enhancer Hoffman
Enhancer Andersson
DHS Trynka
DGF ENCODE
CTCF Hoffman
Conserved LindbladToh
Coding UCSC
Annotation
−100
0
100
Enrichment (prop h2 / prop snps)

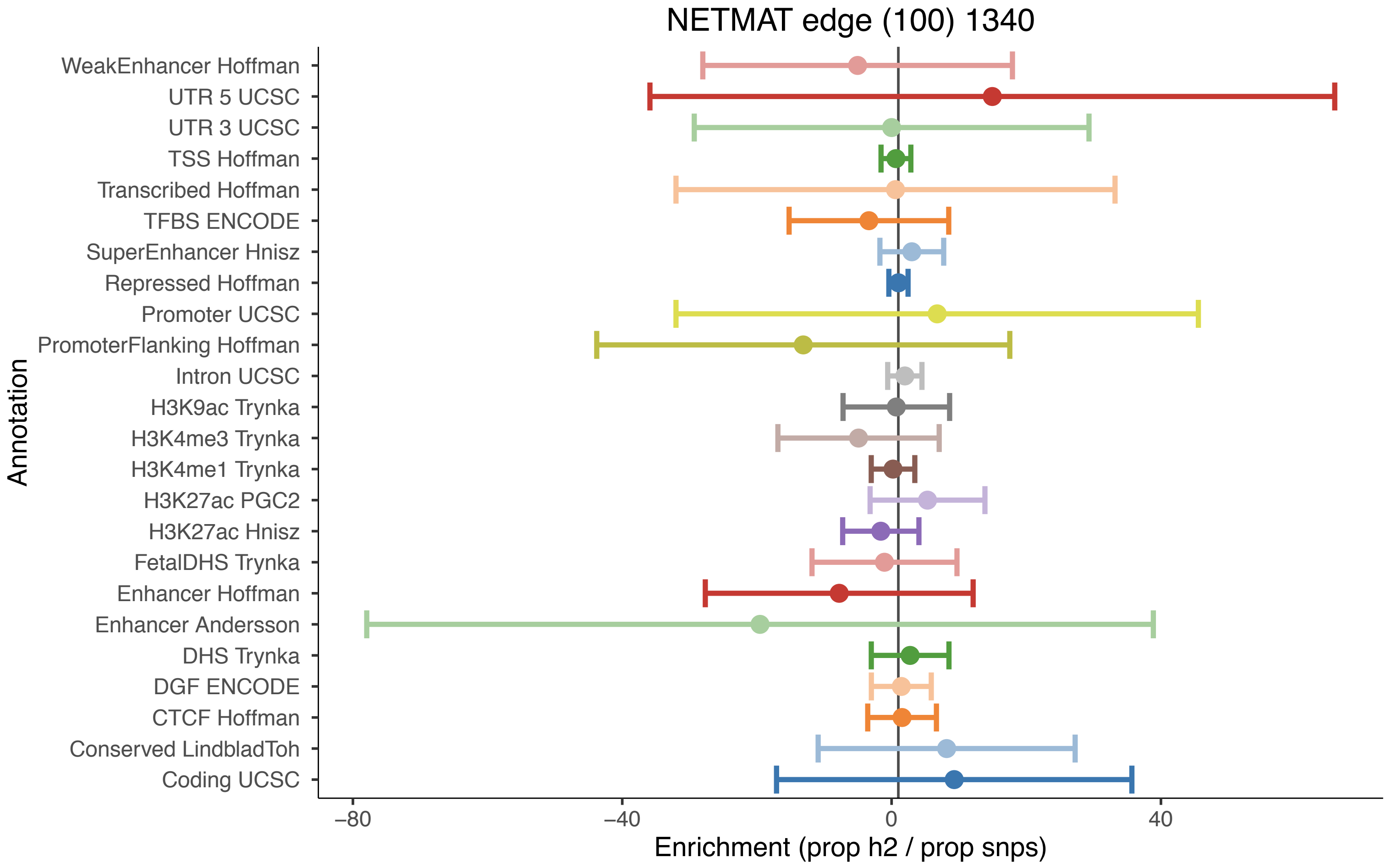

NETMAT edge (100) 1340
WeakEnhancer Hoffman
UTR 5 UCSC
UTR 3 UCSC
TSS Hoffman
Transcribed Hoffman
TFBS ENCODE
SuperEnhancer Hnisz
Repressed Hoffman
Promoter UCSC
PromoterFlanking Hoffman
Intron UCSC
H3K9ac Trynka
H3K4me3 Trynka
H3K4me1 Trynka
H3K27ac PGC2
H3K27ac Hnisz
FetalDHS Trynka
Enhancer Hoffman
Enhancer Andersson
DHS Trynka
DGF ENCODE
CTCF Hoffman
Conserved LindbladToh
Coding UCSC
Annotation
−80
−40
0
40
Enrichment (prop h2 / prop snps)

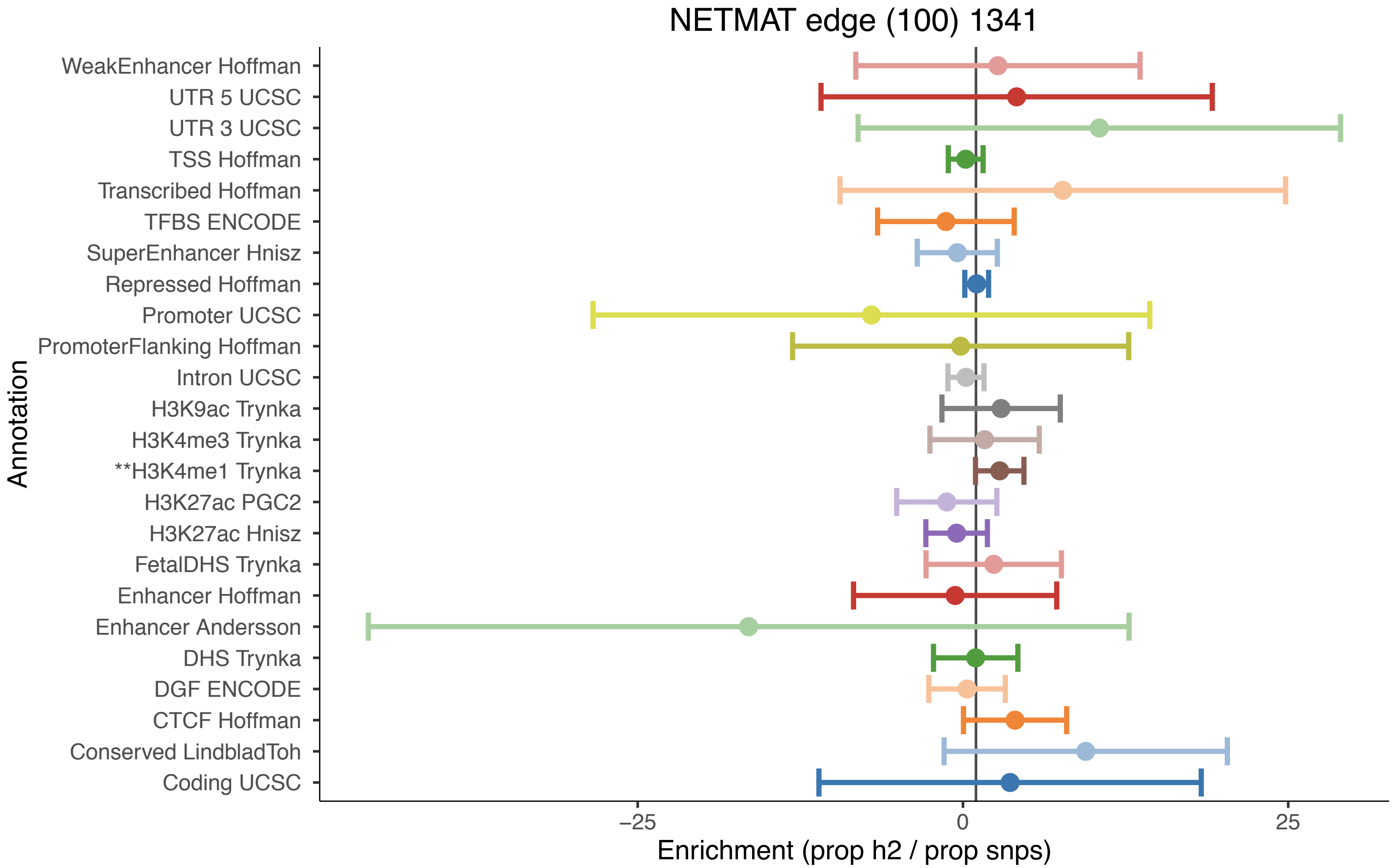
NETMAT edge (100) 1341
WeakEnhancer Hoffman
UTR 5 UCSC
UTR 3 UCSC
TSS Hoffman
Transcribed Hoffman
TFBS ENCODE
SuperEnhancer Hnisz
Repressed Hoffman
Promoter UCSC
PromoterFlanking Hoffman
Intron UCSC
H3K9ac Trynka
H3K4me3 Trynka
**H3K4me1 Trynka
H3K27ac PGC2
H3K27ac Hnisz
FetalDHS Trynka
Enhancer Hoffman
Enhancer Andersson
DHS Trynka
DGF ENCODE
CTCF Hoffman
Conserved LindbladToh
Coding UCSC
Annotation
−25
0
25
Enrichment (prop h2 / prop snps)

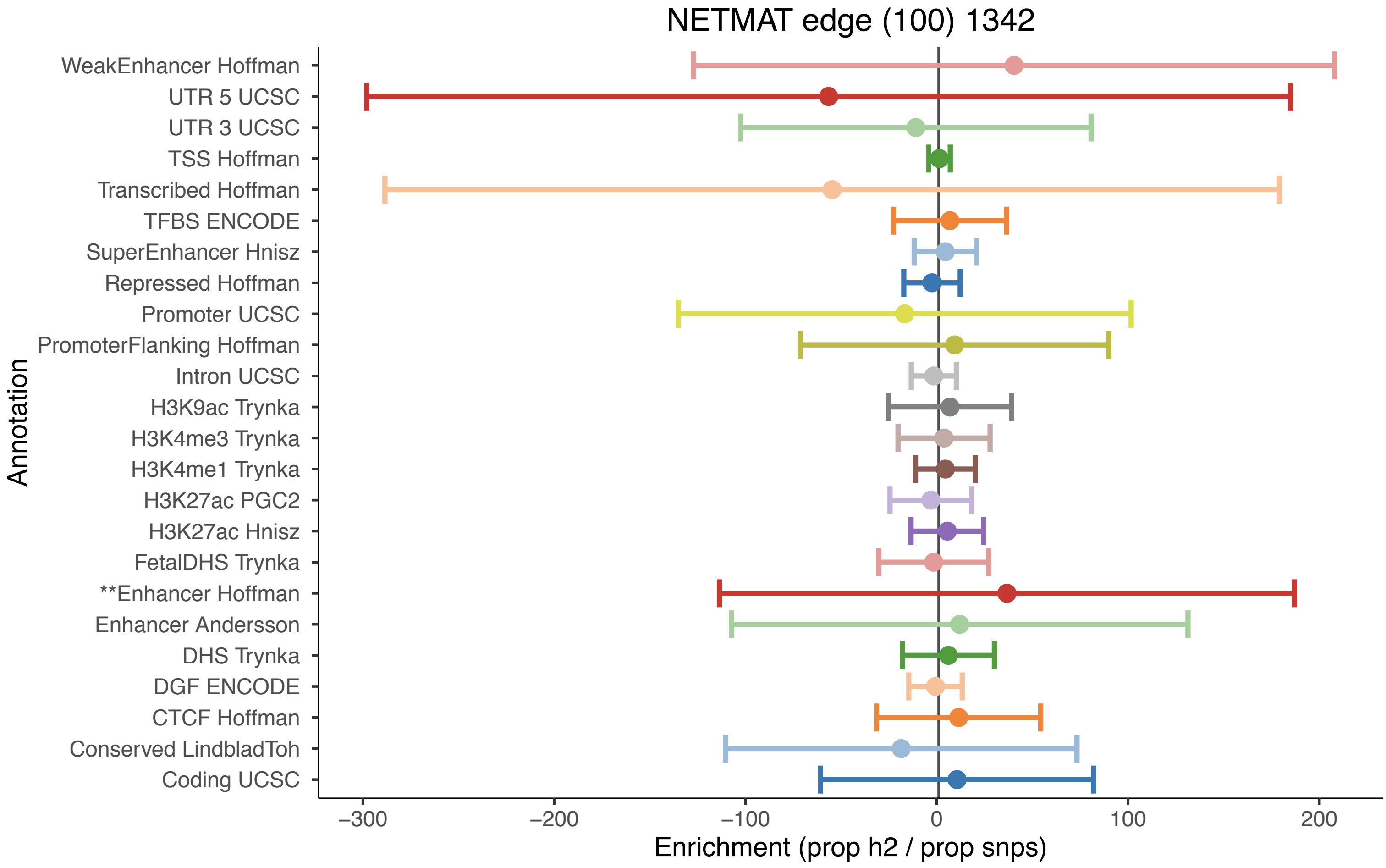

NETMAT edge (100) 1342
WeakEnhancer Hoffman
UTR 5 UCSC
UTR 3 UCSC
TSS Hoffman
Transcribed Hoffman
TFBS ENCODE
SuperEnhancer Hnisz
Repressed Hoffman
Promoter UCSC
PromoterFlanking Hoffman
Intron UCSC
H3K9ac Trynka
H3K4me3 Trynka
H3K4me1 Trynka
H3K27ac PGC2
H3K27ac Hnisz
FetalDHS Trynka
**Enhancer Hoffman
Enhancer Andersson
DHS Trynka
DGF ENCODE
CTCF Hoffman
Conserved LindbladToh
Coding UCSC
Annotation
−300
−200
−100
0
100
200
Enrichment (prop h2 / prop snps)

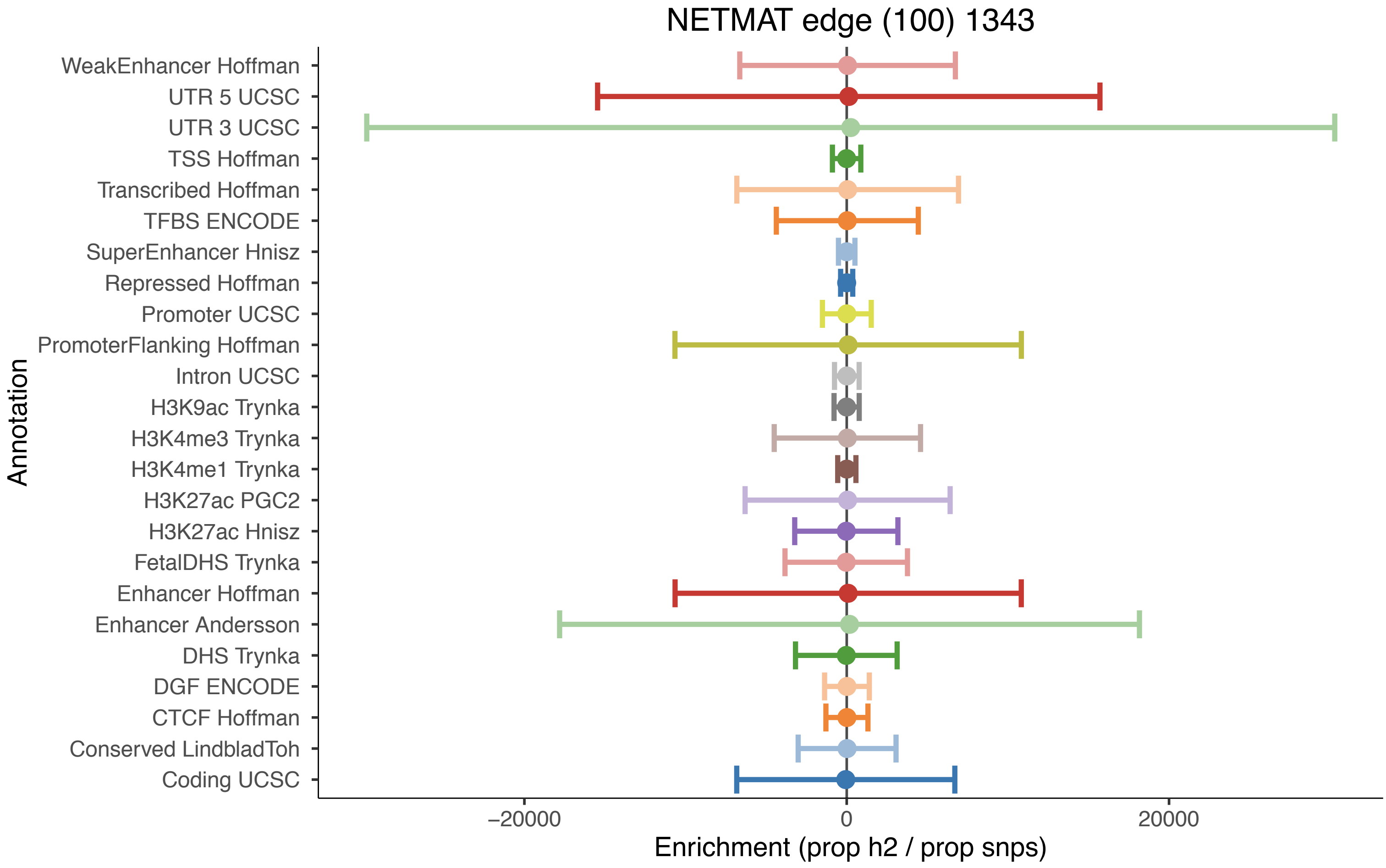

NETMAT edge (100) 1343
WeakEnhancer Hoffman
UTR 5 UCSC
UTR 3 UCSC
TSS Hoffman
Transcribed Hoffman
TFBS ENCODE
SuperEnhancer Hnisz
Repressed Hoffman
Promoter UCSC
PromoterFlanking Hoffman
Intron UCSC
H3K9ac Trynka
H3K4me3 Trynka
H3K4me1 Trynka
H3K27ac PGC2
H3K27ac Hnisz
FetalDHS Trynka
Enhancer Hoffman
Enhancer Andersson
DHS Trynka
DGF ENCODE
CTCF Hoffman
Conserved LindbladToh
Coding UCSC
Annotation
−20000
0
20000
Enrichment (prop h2 / prop snps)

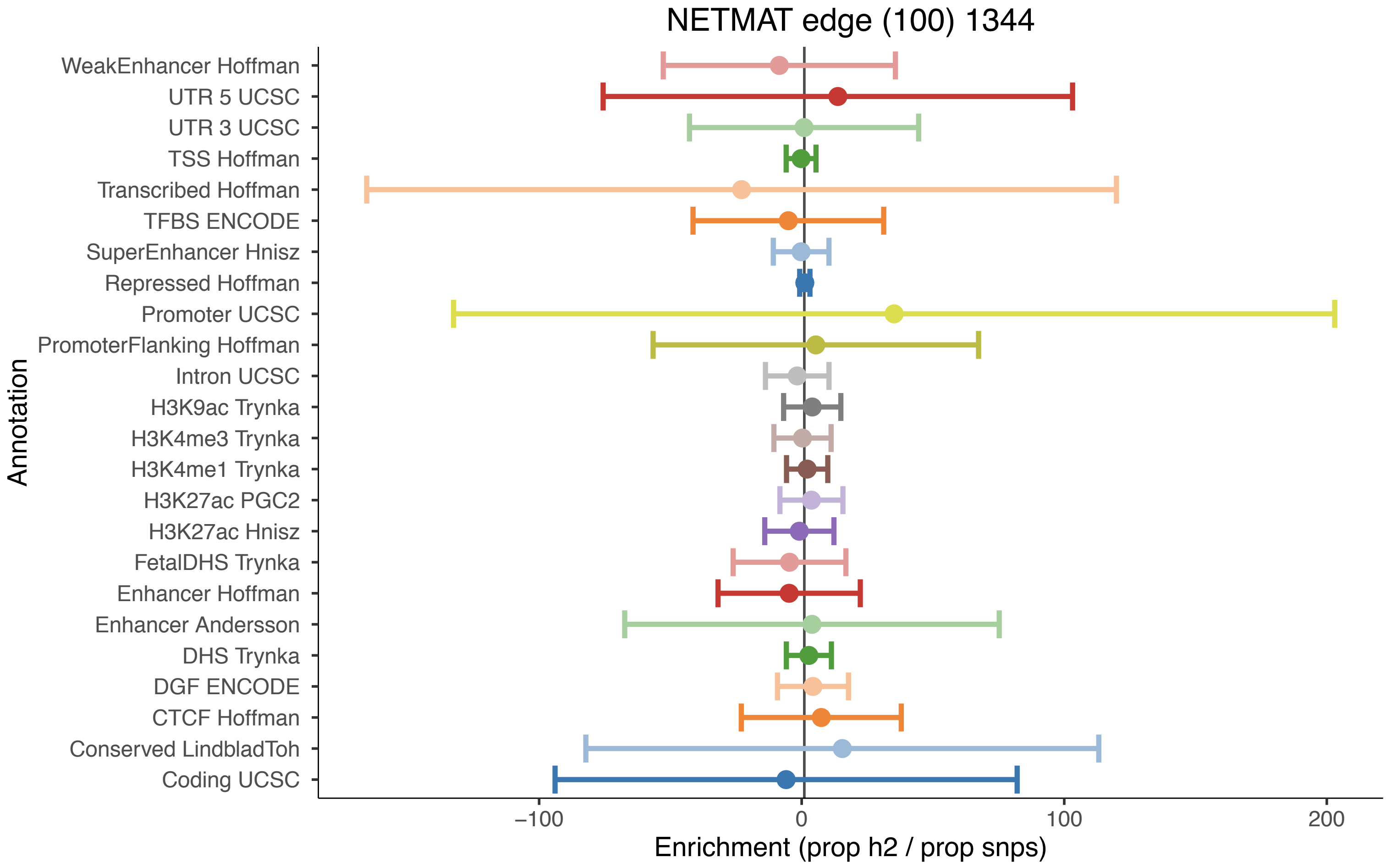
NETMAT edge (100) 1344
Annotation
WeakEnhancer Hoffman
UTR 5 UCSC
UTR 3 UCSC
TSS Hoffman
Transcribed Hoffman
TFBS ENCODE
SuperEnhancer Hnisz
Repressed Hoffman
Promoter UCSC
PromoterFlanking Hoffman
Intron UCSC
H3K9ac Trynka
H3K4me3 Trynka
H3K4me1 Trynka
H3K27ac PGC2
H3K27ac Hnisz
FetalDHS Trynka
Enhancer Hoffman
Enhancer Andersson
DHS Trynka
DGF ENCODE
CTCF Hoffman
Conserved LindbladToh
Coding UCSC
−100
0
100
200
Enrichment (prop h2 / prop snps)

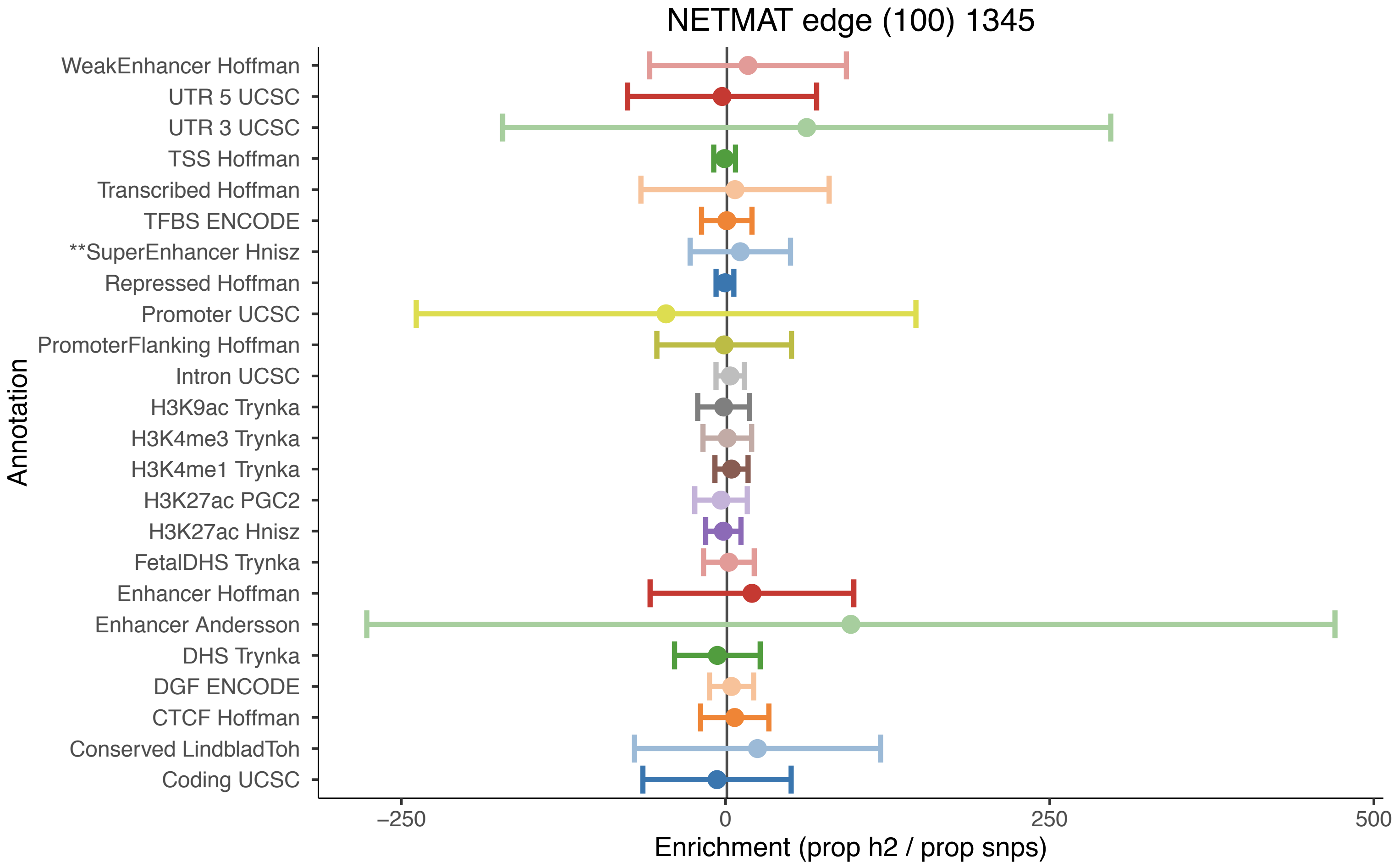
NETMAT edge (100) 1345
WeakEnhancer Hoffman
UTR 5 UCSC
UTR 3 UCSC
TSS Hoffman
Transcribed Hoffman
TFBS ENCODE
**SuperEnhancer Hnisz
Repressed Hoffman
Promoter UCSC
PromoterFlanking Hoffman
Intron UCSC
H3K9ac Trynka
H3K4me3 Trynka
H3K4me1 Trynka
H3K27ac PGC2
H3K27ac Hnisz
FetalDHS Trynka
Enhancer Hoffman
Enhancer Andersson
DHS Trynka
DGF ENCODE
CTCF Hoffman
Conserved LindbladToh
Coding UCSC
Annotation
−250
0
250
500
Enrichment (prop h2 / prop snps)

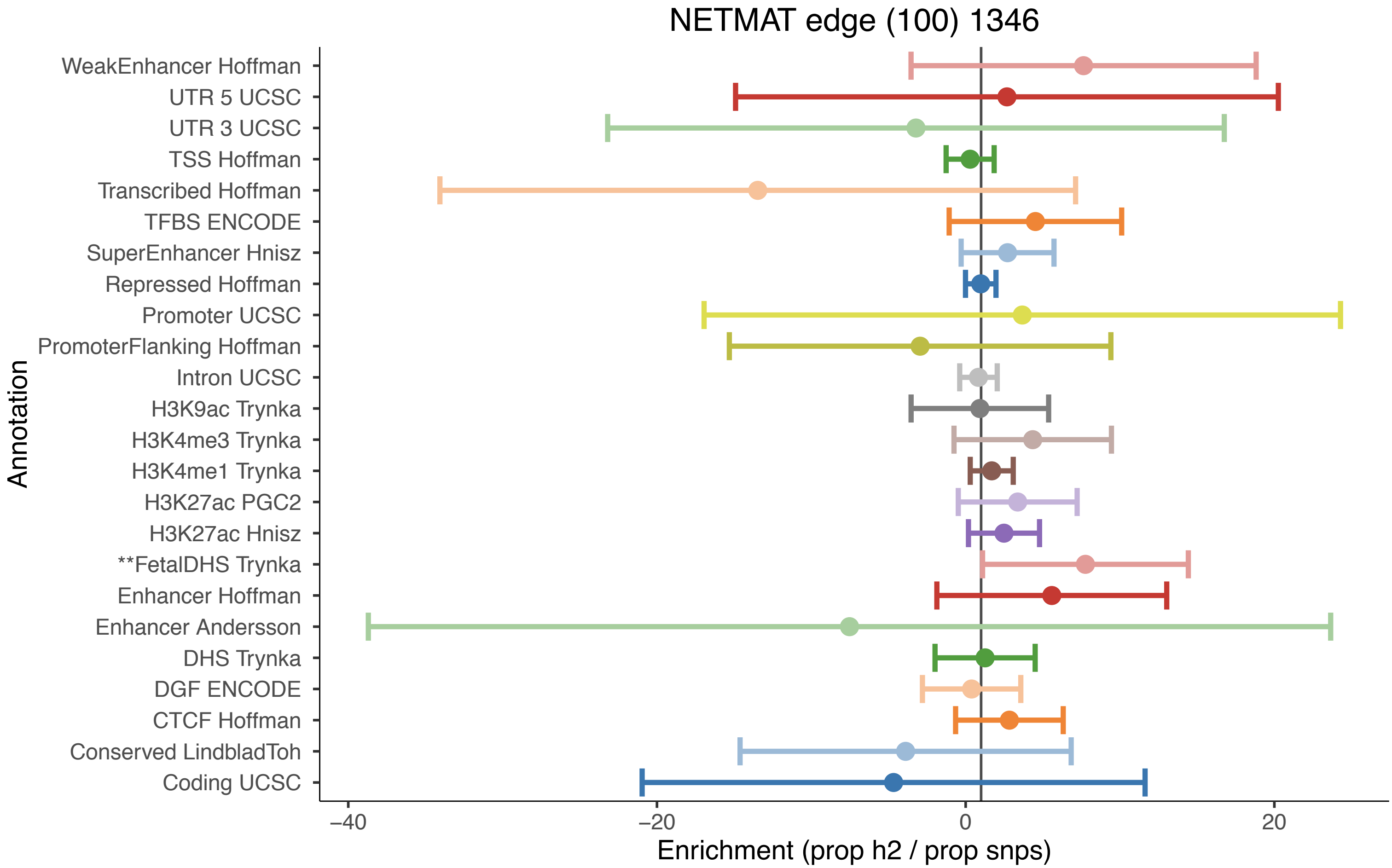

NETMAT edge (100) 1346
WeakEnhancer Hoffman
UTR 5 UCSC
UTR 3 UCSC
TSS Hoffman
Transcribed Hoffman
TFBS ENCODE
SuperEnhancer Hnisz
Repressed Hoffman
Promoter UCSC
PromoterFlanking Hoffman
Intron UCSC
H3K9ac Trynka
H3K4me3 Trynka
H3K4me1 Trynka
H3K27ac PGC2
H3K27ac Hnisz
**FetalDHS Trynka
Enhancer Hoffman
Enhancer Andersson
DHS Trynka
DGF ENCODE
CTCF Hoffman
Conserved LindbladToh
Coding UCSC
Annotation
−40
−20
0
20
Enrichment (prop h2 / prop snps)

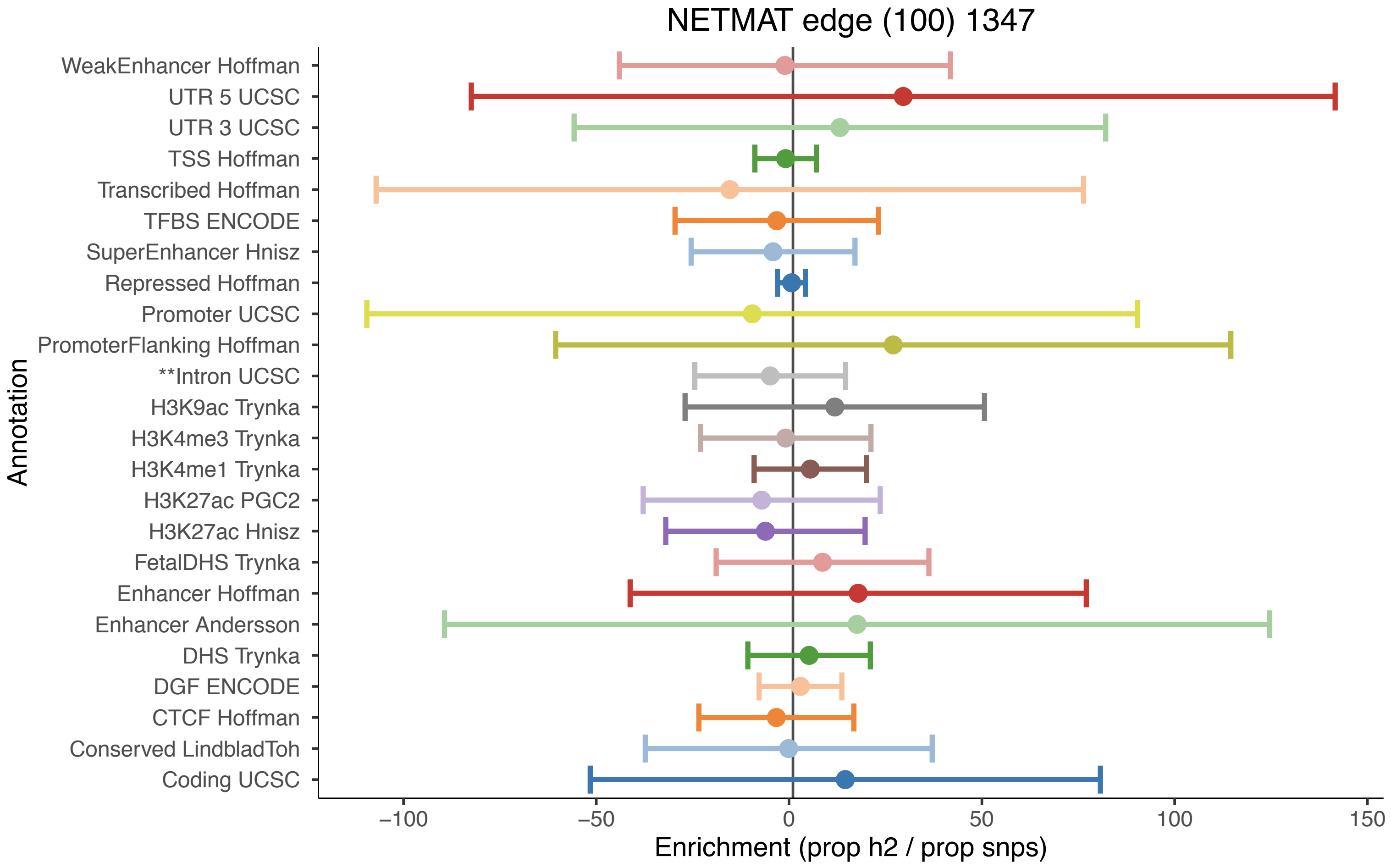
NETMAT edge (100) 1347
WeakEnhancer Hoffman
UTR 5 UCSC
UTR 3 UCSC
TSS Hoffman
Transcribed Hoffman
TFBS ENCODE
SuperEnhancer Hnisz
Repressed Hoffman
Promoter UCSC
PromoterFlanking Hoffman
**Intron UCSC
H3K9ac Trynka
H3K4me3 Trynka
H3K4me1 Trynka
H3K27ac PGC2
H3K27ac Hnisz
FetalDHS Trynka
Enhancer Hoffman
Enhancer Andersson
DHS Trynka
DGF ENCODE
CTCF Hoffman
Conserved LindbladToh
Coding UCSC
Annotation
−100
−50
0
50
100
150
Enrichment (prop h2 / prop snps)

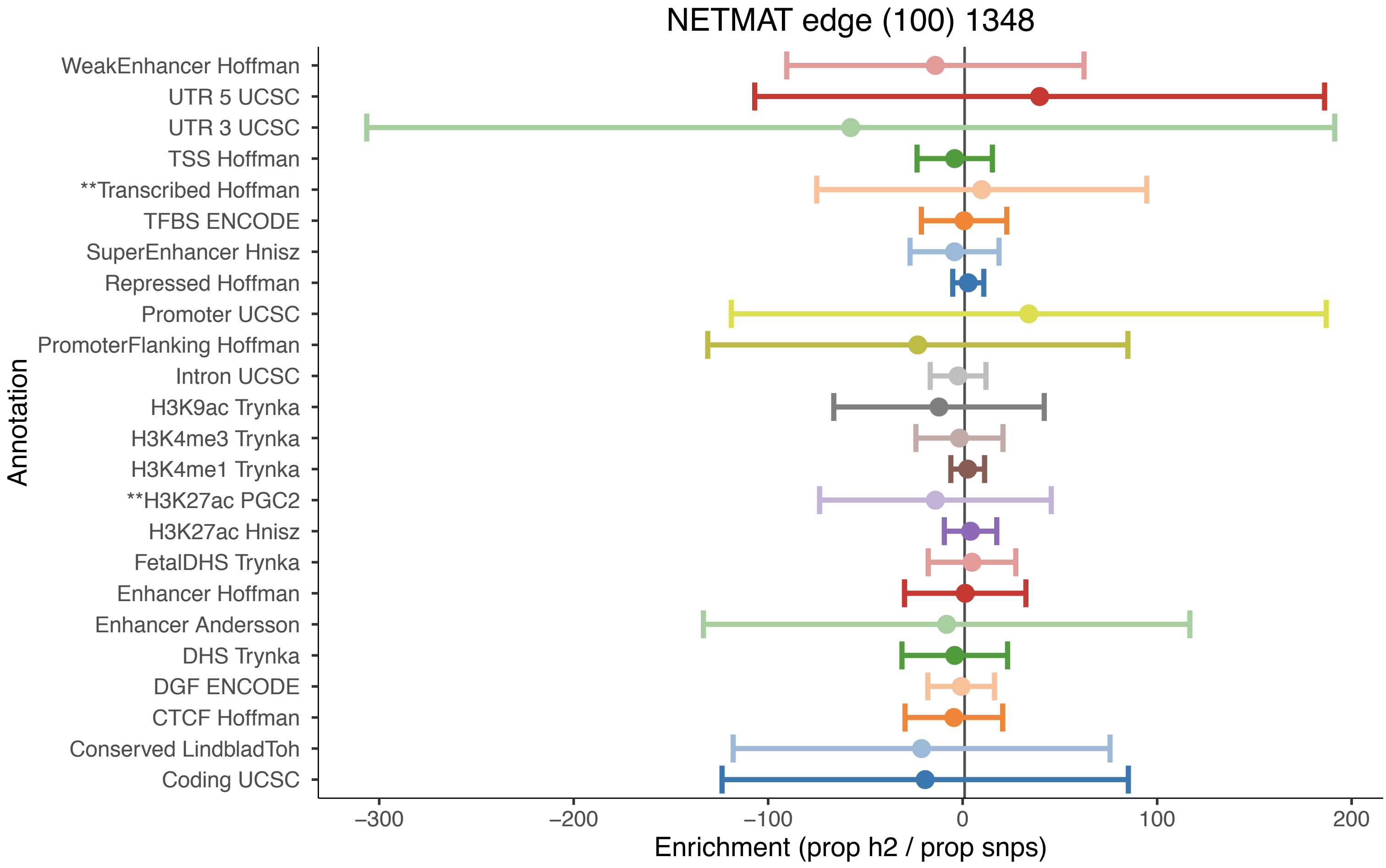
NETMAT edge (100) 1348
WeakEnhancer Hoffman
UTR 5 UCSC
UTR 3 UCSC
TSS Hoffman
**Transcribed Hoffman
TFBS ENCODE
SuperEnhancer Hnisz
Repressed Hoffman
Promoter UCSC
PromoterFlanking Hoffman
Intron UCSC
H3K9ac Trynka
H3K4me3 Trynka
H3K4me1 Trynka
**H3K27ac PGC2
H3K27ac Hnisz
FetalDHS Trynka
Enhancer Hoffman
Enhancer Andersson
DHS Trynka
DGF ENCODE
CTCF Hoffman
Conserved LindbladToh
Coding UCSC
Annotation
−300
−200
−100
0
100
200
Enrichment (prop h2 / prop snps)

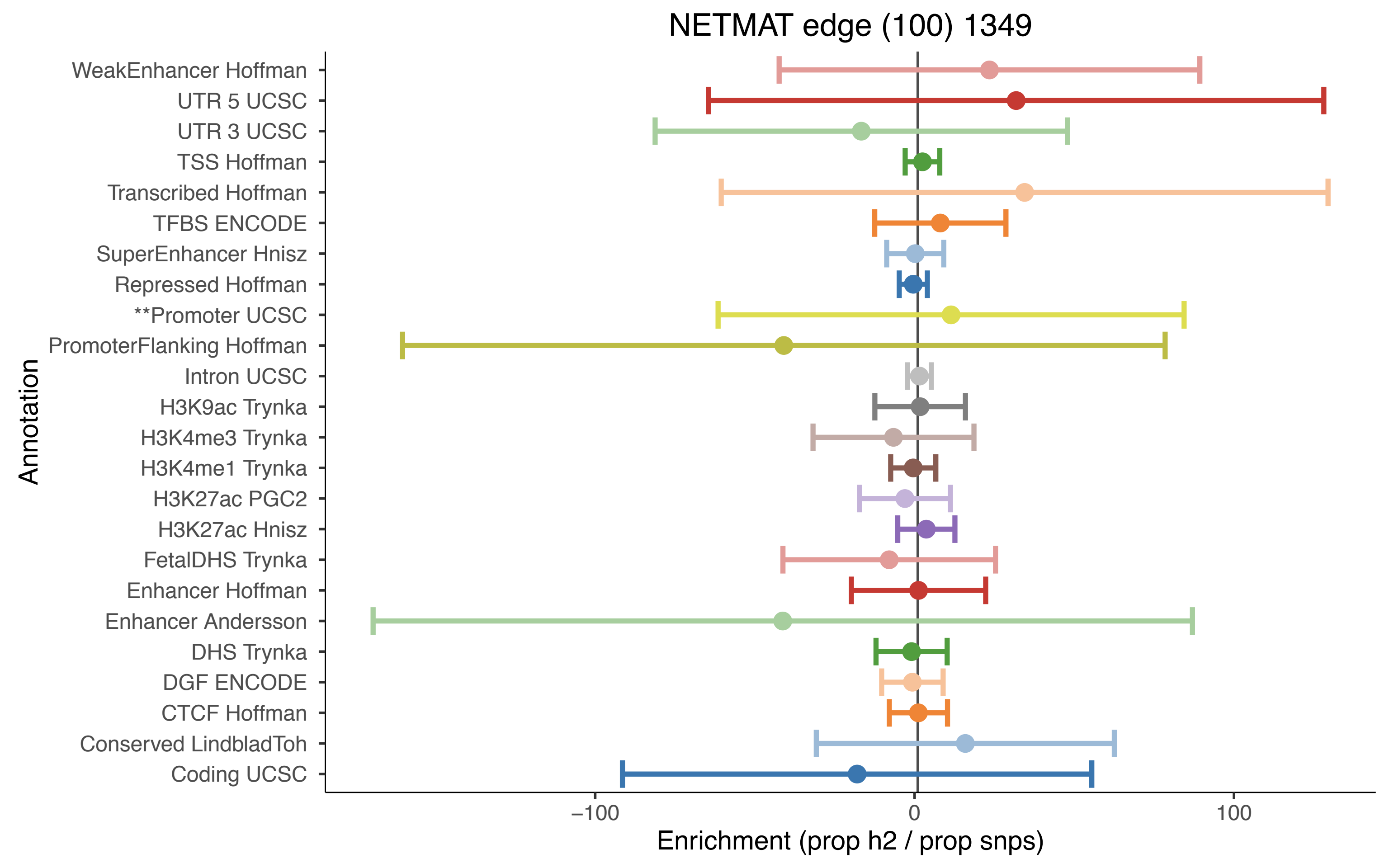
NETMAT edge (100) 1349
WeakEnhancer Hoffman
UTR 5 UCSC
UTR 3 UCSC
TSS Hoffman
Transcribed Hoffman
TFBS ENCODE
SuperEnhancer Hnisz
Repressed Hoffman
**Promoter UCSC
PromoterFlanking Hoffman
Intron UCSC
H3K9ac Trynka
H3K4me3 Trynka
H3K4me1 Trynka
H3K27ac PGC2
H3K27ac Hnisz
FetalDHS Trynka
Enhancer Hoffman
Enhancer Andersson
DHS Trynka
DGF ENCODE
CTCF Hoffman
Conserved LindbladToh
Coding UCSC
Annotation
−100
0
100
Enrichment (prop h2 / prop snps)

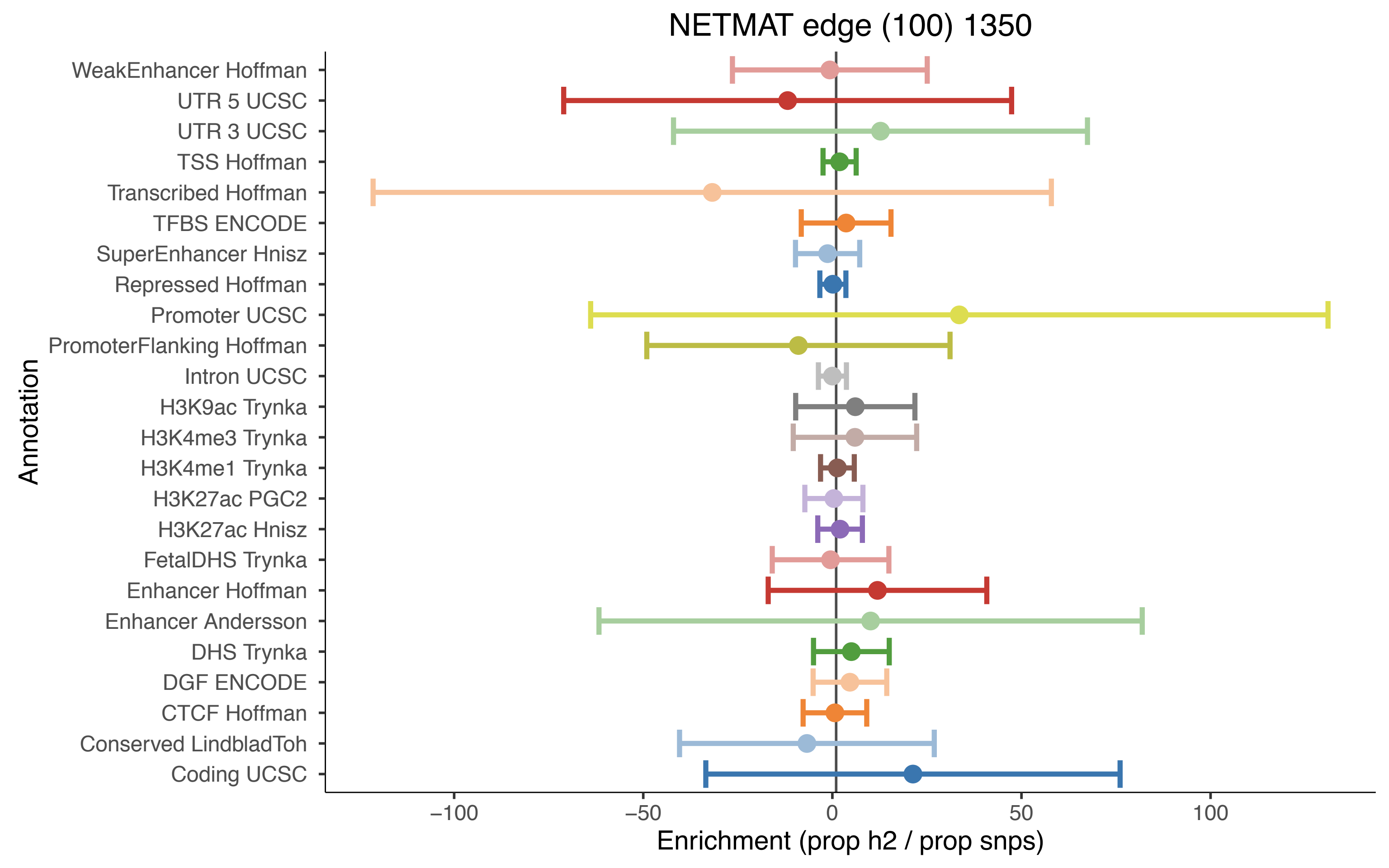
NETMAT edge (100) 1350
WeakEnhancer Hoffman
UTR 5 UCSC
UTR 3 UCSC
TSS Hoffman
Transcribed Hoffman
TFBS ENCODE
SuperEnhancer Hnisz
Repressed Hoffman
Promoter UCSC
PromoterFlanking Hoffman
Intron UCSC
H3K9ac Trynka
H3K4me3 Trynka
H3K4me1 Trynka
H3K27ac PGC2
H3K27ac Hnisz
FetalDHS Trynka
Enhancer Hoffman
Enhancer Andersson
DHS Trynka
DGF ENCODE
CTCF Hoffman
Conserved LindbladToh
Coding UCSC
Annotation
−100
−50
0
50
100
Enrichment (prop h2 / prop snps)

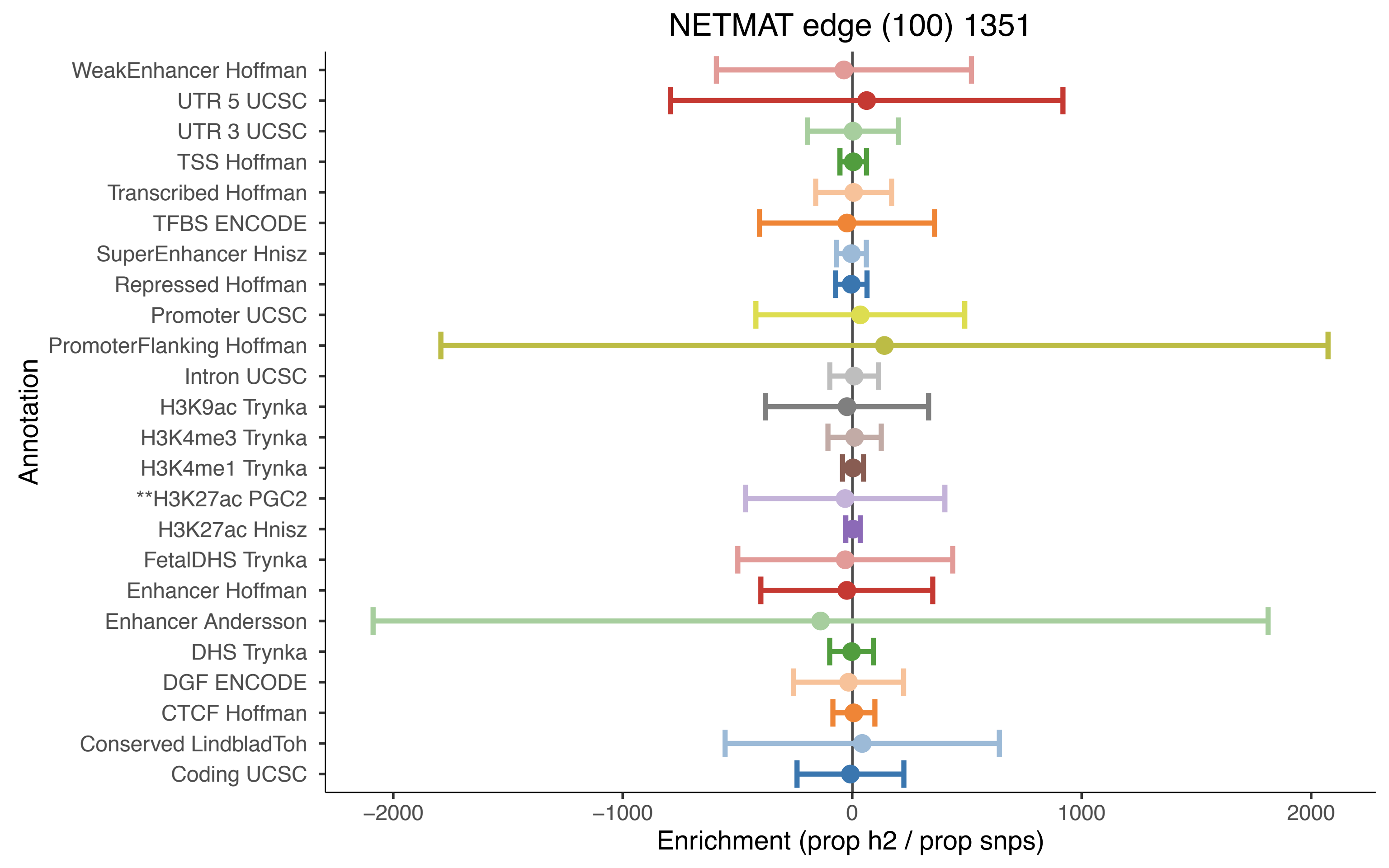

NETMAT edge (100) 1351
WeakEnhancer Hoffman
UTR 5 UCSC
UTR 3 UCSC
TSS Hoffman
Transcribed Hoffman
TFBS ENCODE
SuperEnhancer Hnisz
Repressed Hoffman
Promoter UCSC
PromoterFlanking Hoffman
Intron UCSC
H3K9ac Trynka
H3K4me3 Trynka
H3K4me1 Trynka
**H3K27ac PGC2
H3K27ac Hnisz
FetalDHS Trynka
Enhancer Hoffman
Enhancer Andersson
DHS Trynka
DGF ENCODE
CTCF Hoffman
Conserved LindbladToh
Coding UCSC
Annotation
−2000
−1000
0
1000
2000
Enrichment (prop h2 / prop snps)

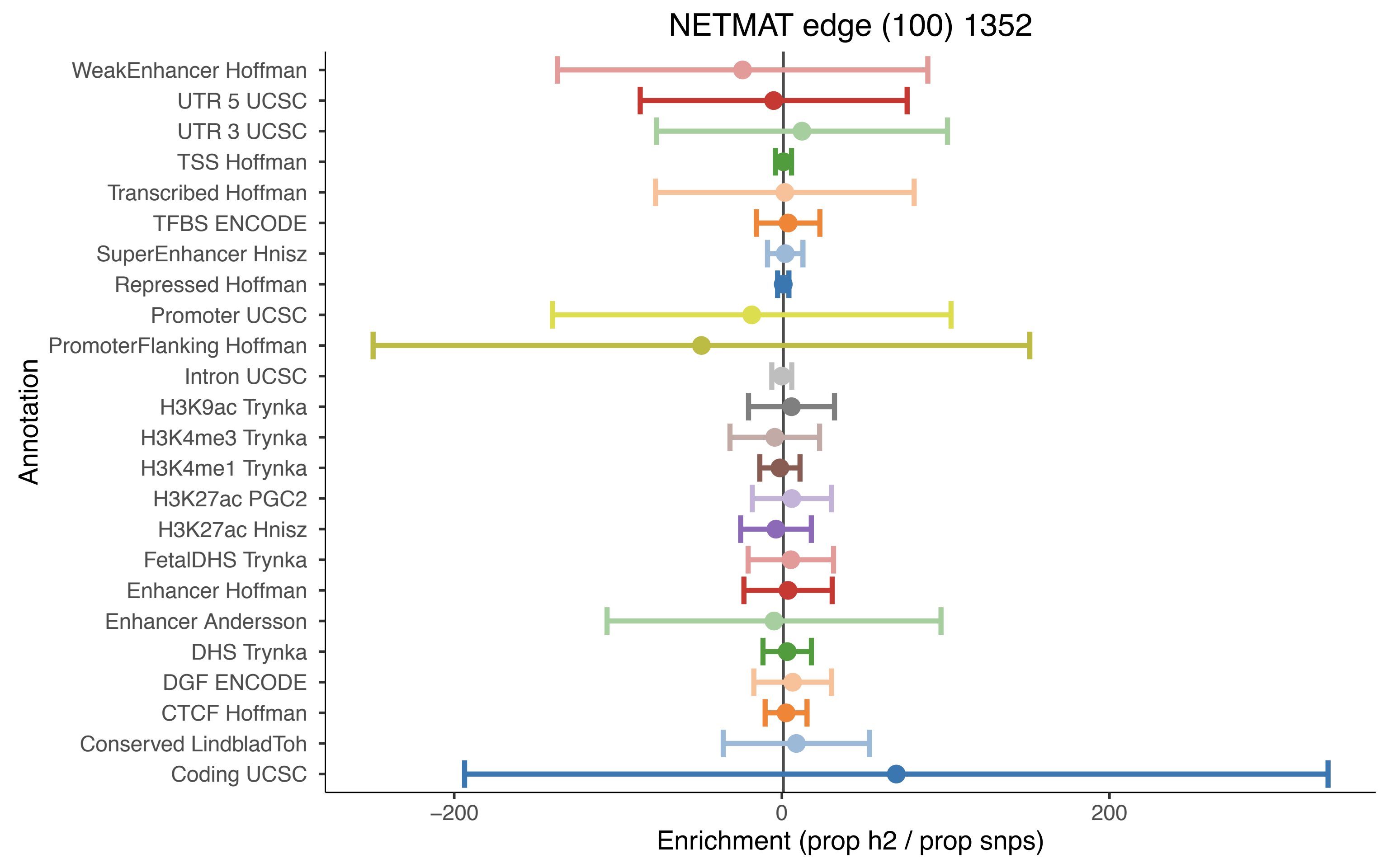
NETMAT edge (100) 1352
WeakEnhancer Hoffman
UTR 5 UCSC
UTR 3 UCSC
TSS Hoffman
Transcribed Hoffman
TFBS ENCODE
SuperEnhancer Hnisz
Repressed Hoffman
Promoter UCSC
PromoterFlanking Hoffman
Intron UCSC
H3K9ac Trynka
H3K4me3 Trynka
H3K4me1 Trynka
H3K27ac PGC2
H3K27ac Hnisz
FetalDHS Trynka
Enhancer Hoffman
Enhancer Andersson
DHS Trynka
DGF ENCODE
CTCF Hoffman
Conserved LindbladToh
Coding UCSC
Annotation
−200
0
200
Enrichment (prop h2 / prop snps)

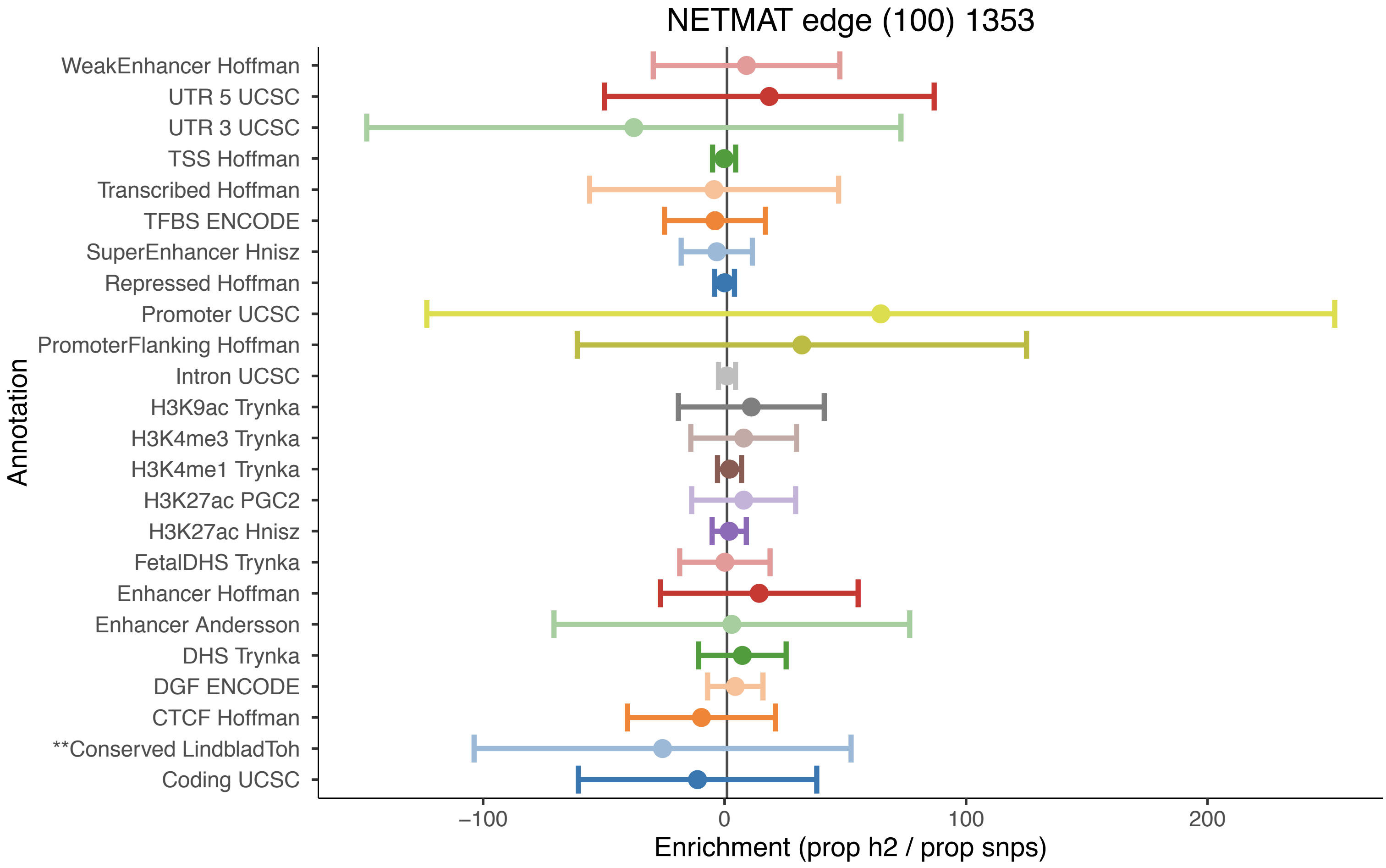
NETMAT edge (100) 1353
WeakEnhancer Hoffman
UTR 5 UCSC
UTR 3 UCSC
TSS Hoffman
Transcribed Hoffman
TFBS ENCODE
SuperEnhancer Hnisz
Repressed Hoffman
Promoter UCSC
PromoterFlanking Hoffman
Intron UCSC
H3K9ac Trynka
H3K4me3 Trynka
H3K4me1 Trynka
H3K27ac PGC2
H3K27ac Hnisz
FetalDHS Trynka
Enhancer Hoffman
Enhancer Andersson
DHS Trynka
DGF ENCODE
CTCF Hoffman
**Conserved LindbladToh
Coding UCSC
Annotation
−100
0
100
200
Enrichment (prop h2 / prop snps)

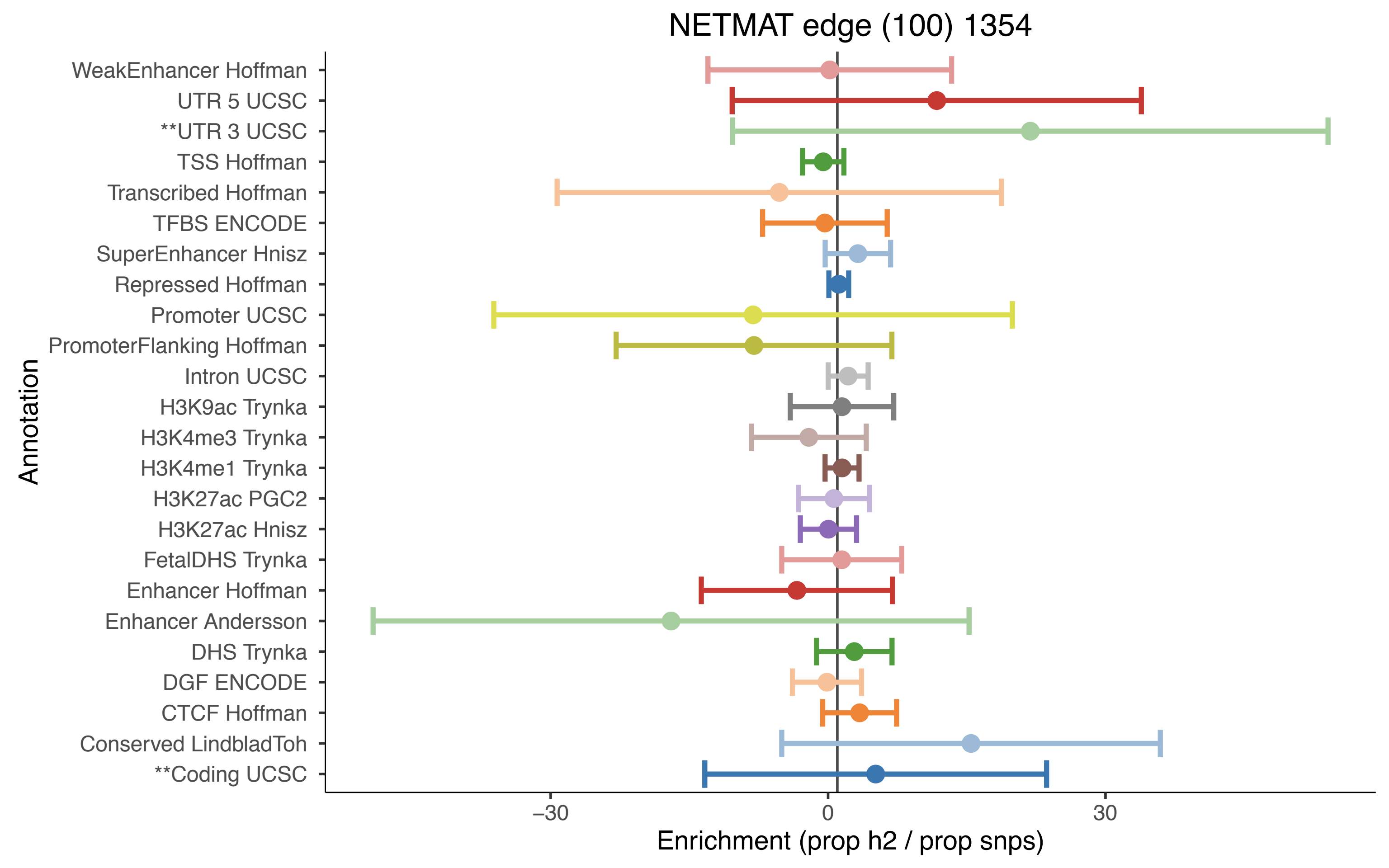

NETMAT edge (100) 1354
WeakEnhancer Hoffman
UTR 5 UCSC
**UTR 3 UCSC
TSS Hoffman
Transcribed Hoffman
TFBS ENCODE
SuperEnhancer Hnisz
Repressed Hoffman
Promoter UCSC
PromoterFlanking Hoffman
Intron UCSC
H3K9ac Trynka
H3K4me3 Trynka
H3K4me1 Trynka
H3K27ac PGC2
H3K27ac Hnisz
FetalDHS Trynka
Enhancer Hoffman
Enhancer Andersson
DHS Trynka
DGF ENCODE
CTCF Hoffman
Conserved LindbladToh
**Coding UCSC
Annotation
–30
0
30
Enrichment (prop h2 / prop snps)

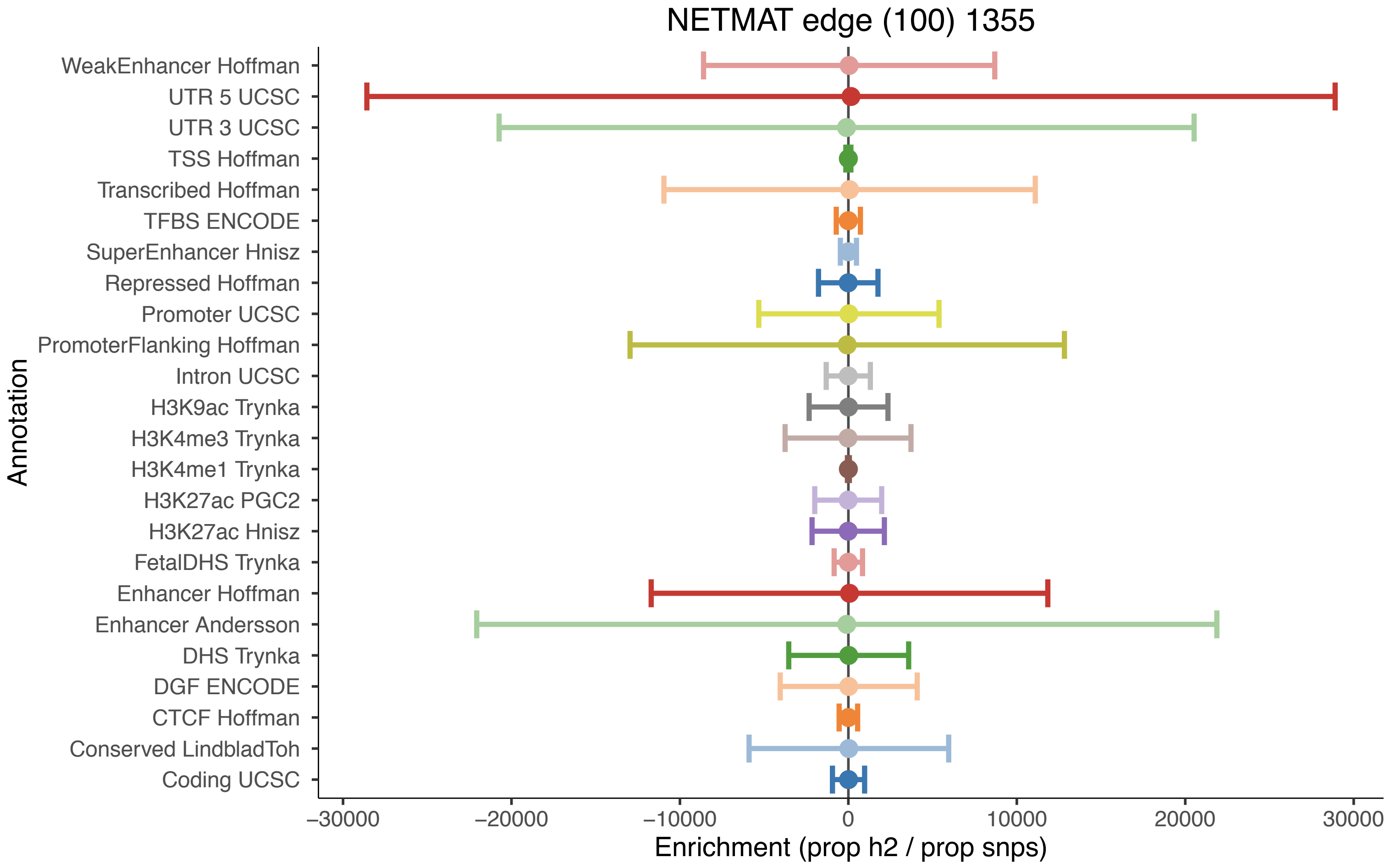
NETMAT edge (100) 1355
Annotation
WeakEnhancer Hoffman
UTR 5 UCSC
UTR 3 UCSC
TSS Hoffman
Transcribed Hoffman
TFBS ENCODE
SuperEnhancer Hnisz
Repressed Hoffman
Promoter UCSC
PromoterFlanking Hoffman
Intron UCSC
H3K9ac Trynka
H3K4me3 Trynka
H3K4me1 Trynka
H3K27ac PGC2
H3K27ac Hnisz
FetalDHS Trynka
Enhancer Hoffman
Enhancer Andersson
DHS Trynka
DGF ENCODE
CTCF Hoffman
Conserved LindbladToh
Coding UCSC
−30000
−20000
−10000
0
10000
20000
30000
Enrichment (prop h2 / prop snps)

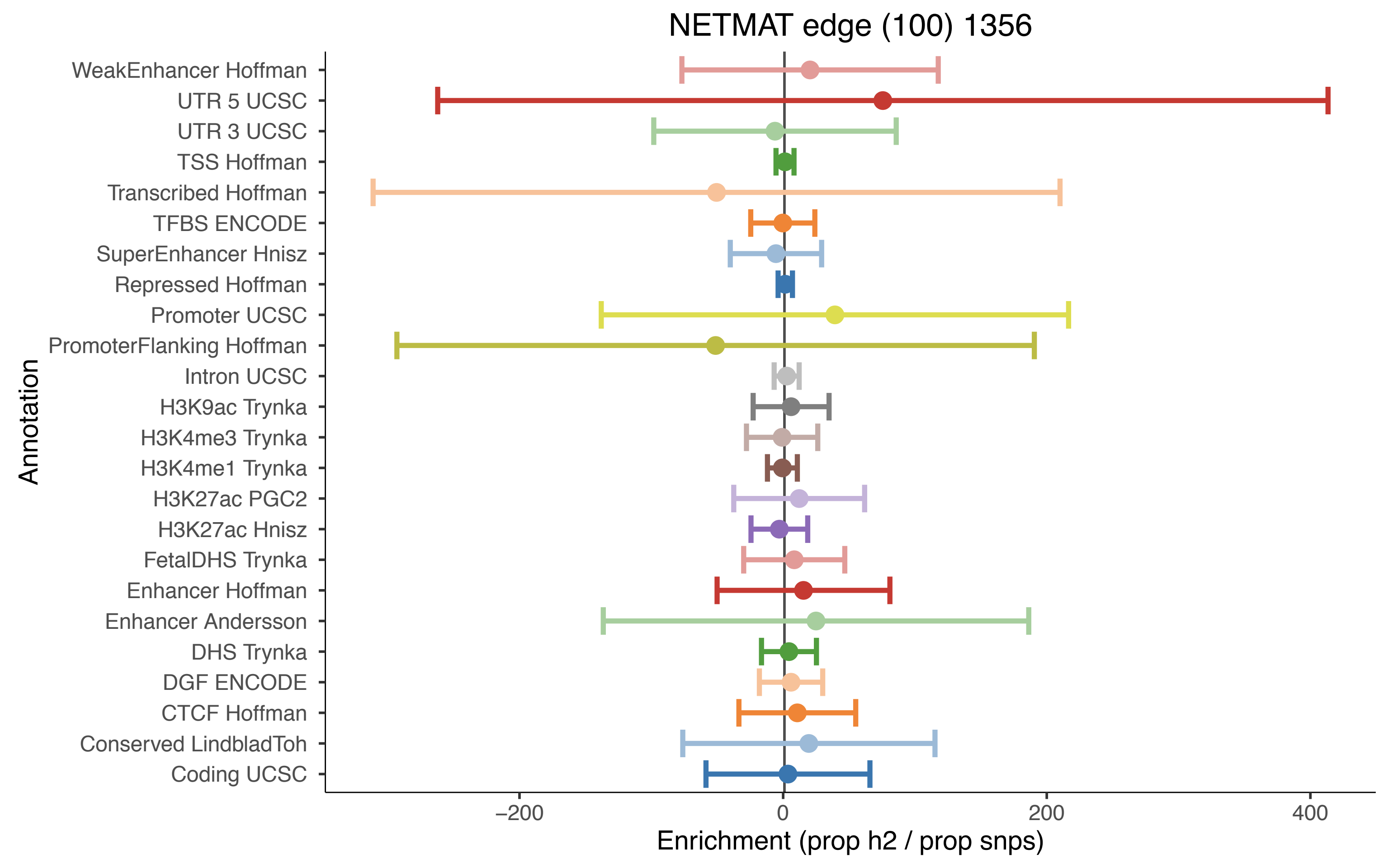
NETMAT edge (100) 1356
WeakEnhancer Hoffman
UTR 5 UCSC
UTR 3 UCSC
TSS Hoffman
Transcribed Hoffman
TFBS ENCODE
SuperEnhancer Hnisz
Repressed Hoffman
Promoter UCSC
PromoterFlanking Hoffman
Intron UCSC
H3K9ac Trynka
H3K4me3 Trynka
H3K4me1 Trynka
H3K27ac PGC2
H3K27ac Hnisz
FetalDHS Trynka
Enhancer Hoffman
Enhancer Andersson
DHS Trynka
DGF ENCODE
CTCF Hoffman
Conserved LindbladToh
Coding UCSC
Annotation
−200
0
200
400
Enrichment (prop h2 / prop snps)

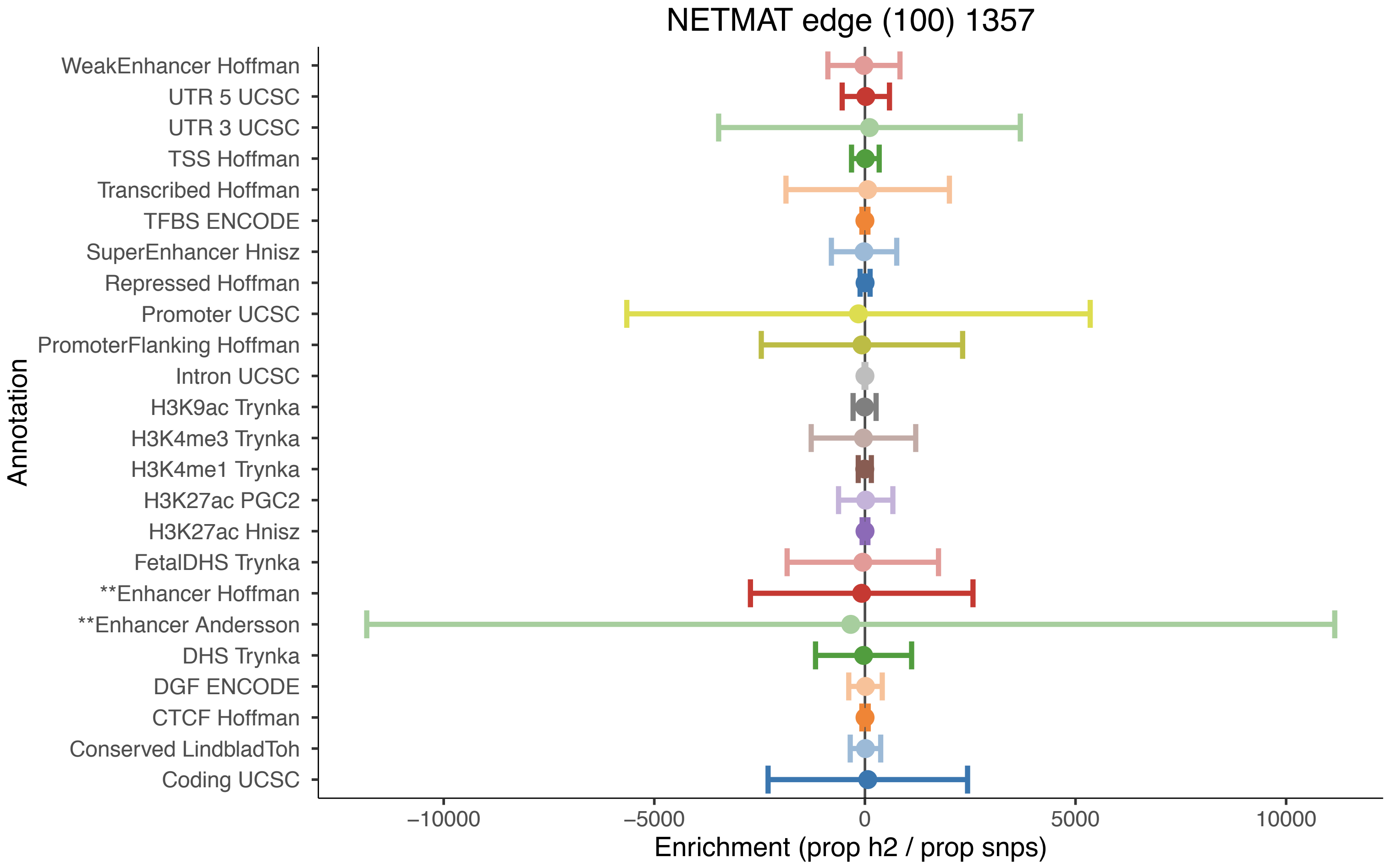
NETMAT edge (100) 1357
WeakEnhancer Hoffman
UTR 5 UCSC
UTR 3 UCSC
TSS Hoffman
Transcribed Hoffman
TFBS ENCODE
SuperEnhancer Hnisz
Repressed Hoffman
Promoter UCSC
PromoterFlanking Hoffman
Intron UCSC
H3K9ac Trynka
H3K4me3 Trynka
H3K4me1 Trynka
H3K27ac PGC2
H3K27ac Hnisz
FetalDHS Trynka
**Enhancer Hoffman
**Enhancer Andersson
DHS Trynka
DGF ENCODE
CTCF Hoffman
Conserved LindbladToh
Coding UCSC
Annotation
−10000
−5000
0
5000
10000
Enrichment (prop h2 / prop snps)

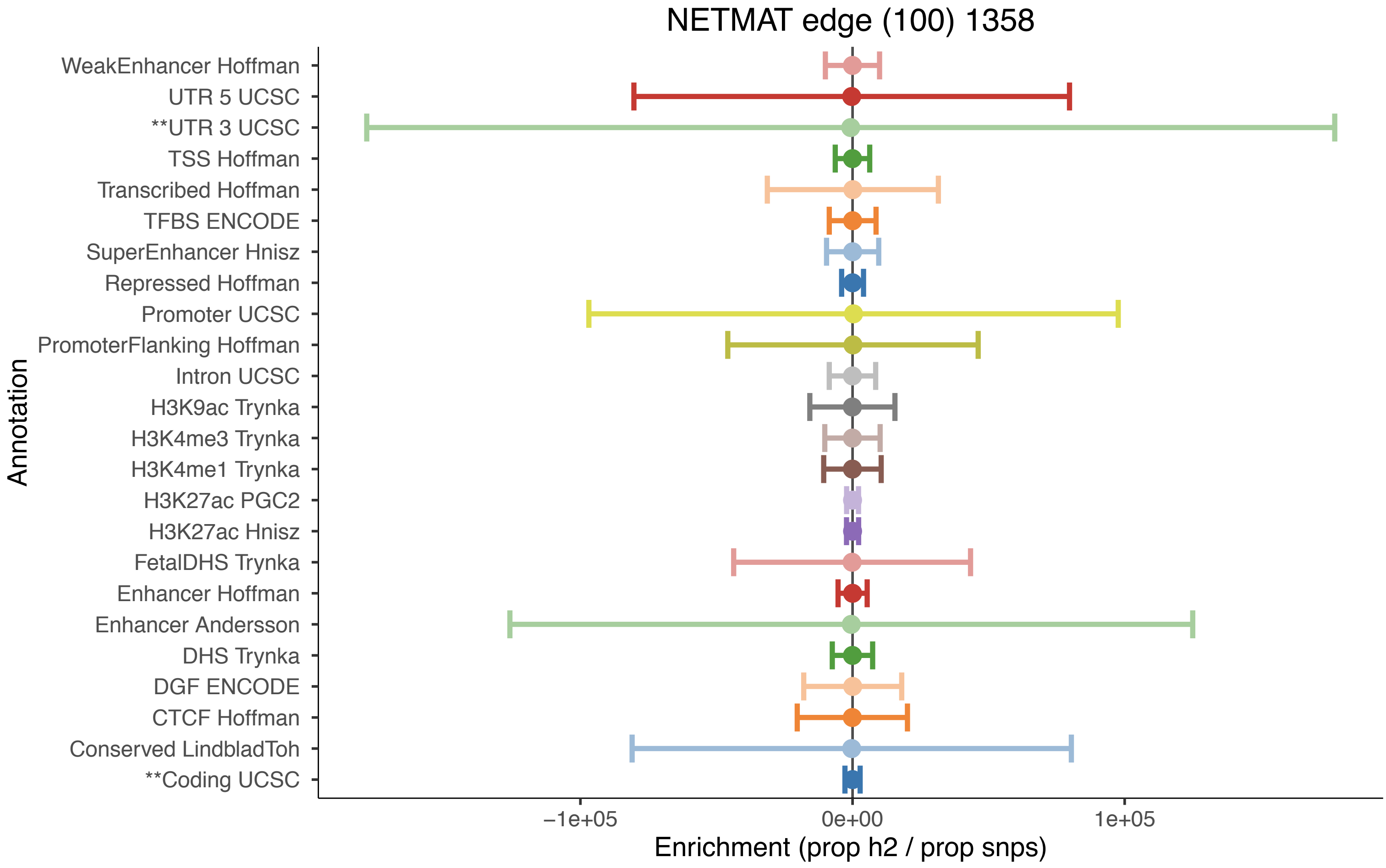

NETMAT edge (100) 1358
WeakEnhancer Hoffman
UTR 5 UCSC
**UTR 3 UCSC
TSS Hoffman
Transcribed Hoffman
TFBS ENCODE
SuperEnhancer Hnisz
Repressed Hoffman
Promoter UCSC
PromoterFlanking Hoffman
Intron UCSC
H3K9ac Trynka
H3K4me3 Trynka
H3K4me1 Trynka
H3K27ac PGC2
H3K27ac Hnisz
FetalDHS Trynka
Enhancer Hoffman
Enhancer Andersson
DHS Trynka
DGF ENCODE
CTCF Hoffman
Conserved LindbladToh
**Coding UCSC
Annotation
−1e+05
0e+00
1e+05
Enrichment (prop h2 / prop snps)

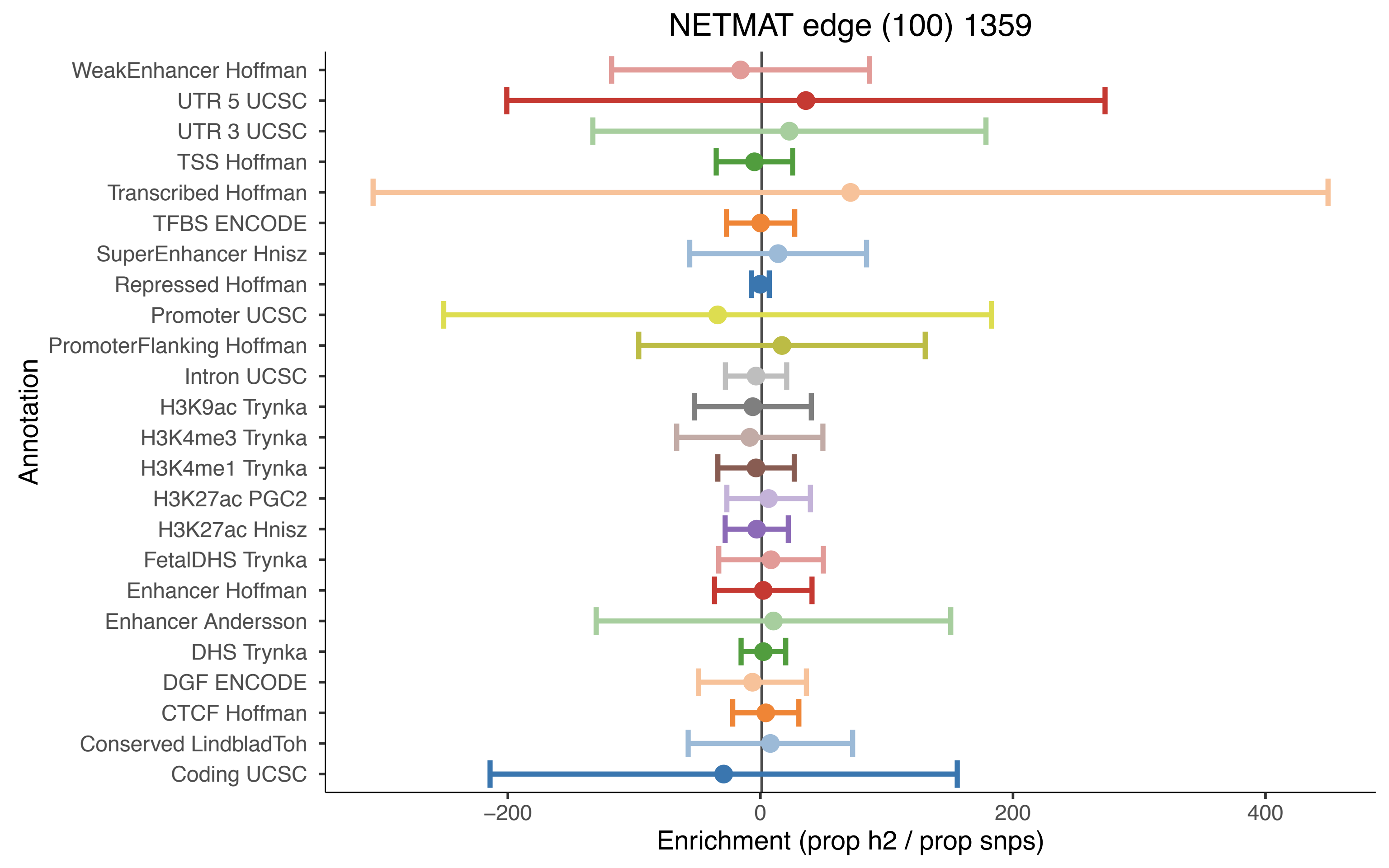
NETMAT edge (100) 1359
WeakEnhancer Hoffman
UTR 5 UCSC
UTR 3 UCSC
TSS Hoffman
Transcribed Hoffman
TFBS ENCODE
SuperEnhancer Hnisz
Repressed Hoffman
Promoter UCSC
PromoterFlanking Hoffman
Intron UCSC
H3K9ac Trynka
H3K4me3 Trynka
H3K4me1 Trynka
H3K27ac PGC2
H3K27ac Hnisz
FetalDHS Trynka
Enhancer Hoffman
Enhancer Andersson
DHS Trynka
DGF ENCODE
CTCF Hoffman
Conserved LindbladToh
Coding UCSC
Annotation
−200
0
200
400
Enrichment (prop h2 / prop snps)

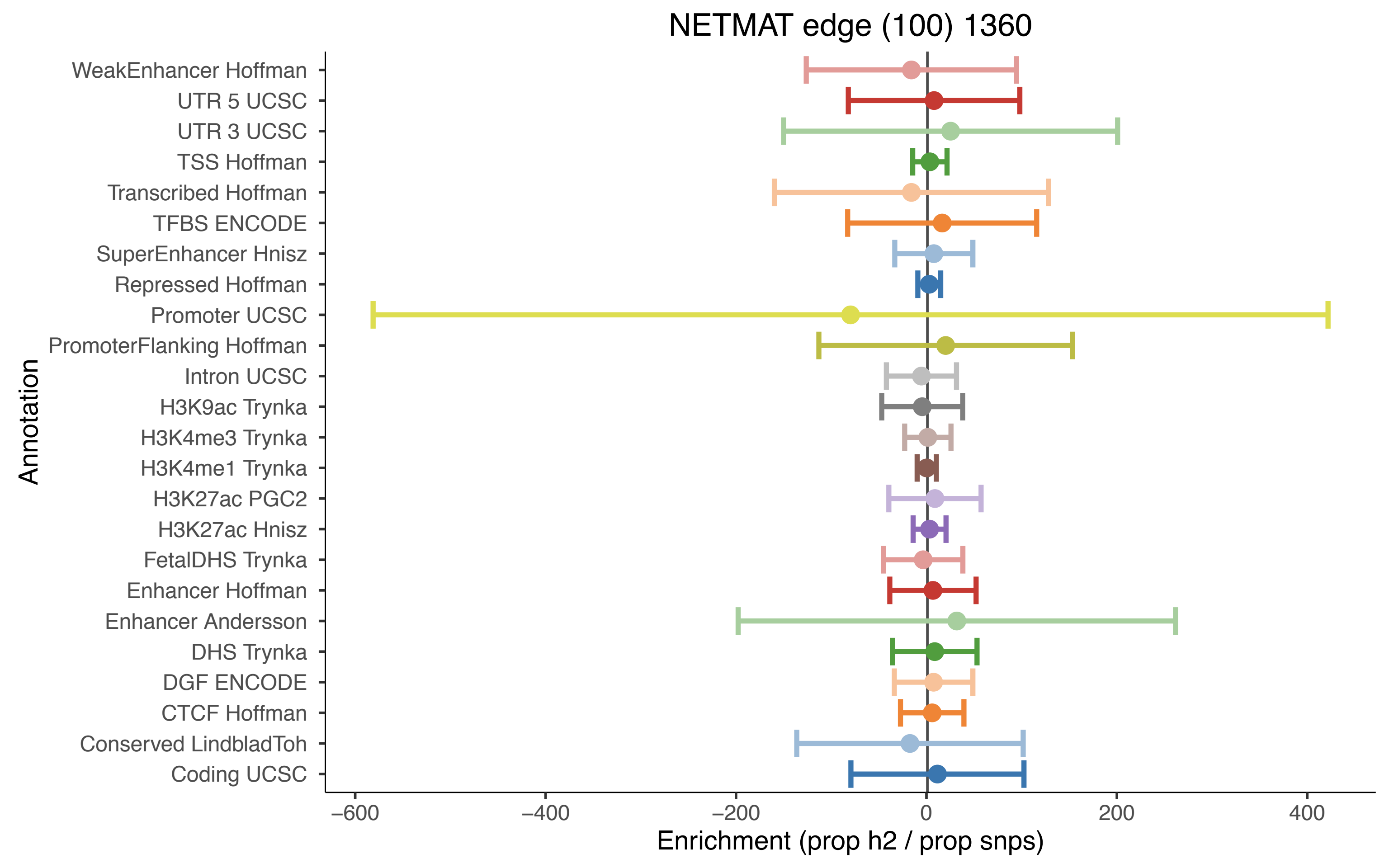

NETMAT edge (100) 1360
WeakEnhancer Hoffman
UTR 5 UCSC
UTR 3 UCSC
TSS Hoffman
Transcribed Hoffman
TFBS ENCODE
SuperEnhancer Hnisz
Repressed Hoffman
Promoter UCSC
PromoterFlanking Hoffman
Intron UCSC
H3K9ac Trynka
H3K4me3 Trynka
H3K4me1 Trynka
H3K27ac PGC2
H3K27ac Hnisz
FetalDHS Trynka
Enhancer Hoffman
Enhancer Andersson
DHS Trynka
DGF ENCODE
CTCF Hoffman
Conserved LindbladToh
Coding UCSC
Annotation
−600
−400
−200
0
200
400
Enrichment (prop h2 / prop snps)

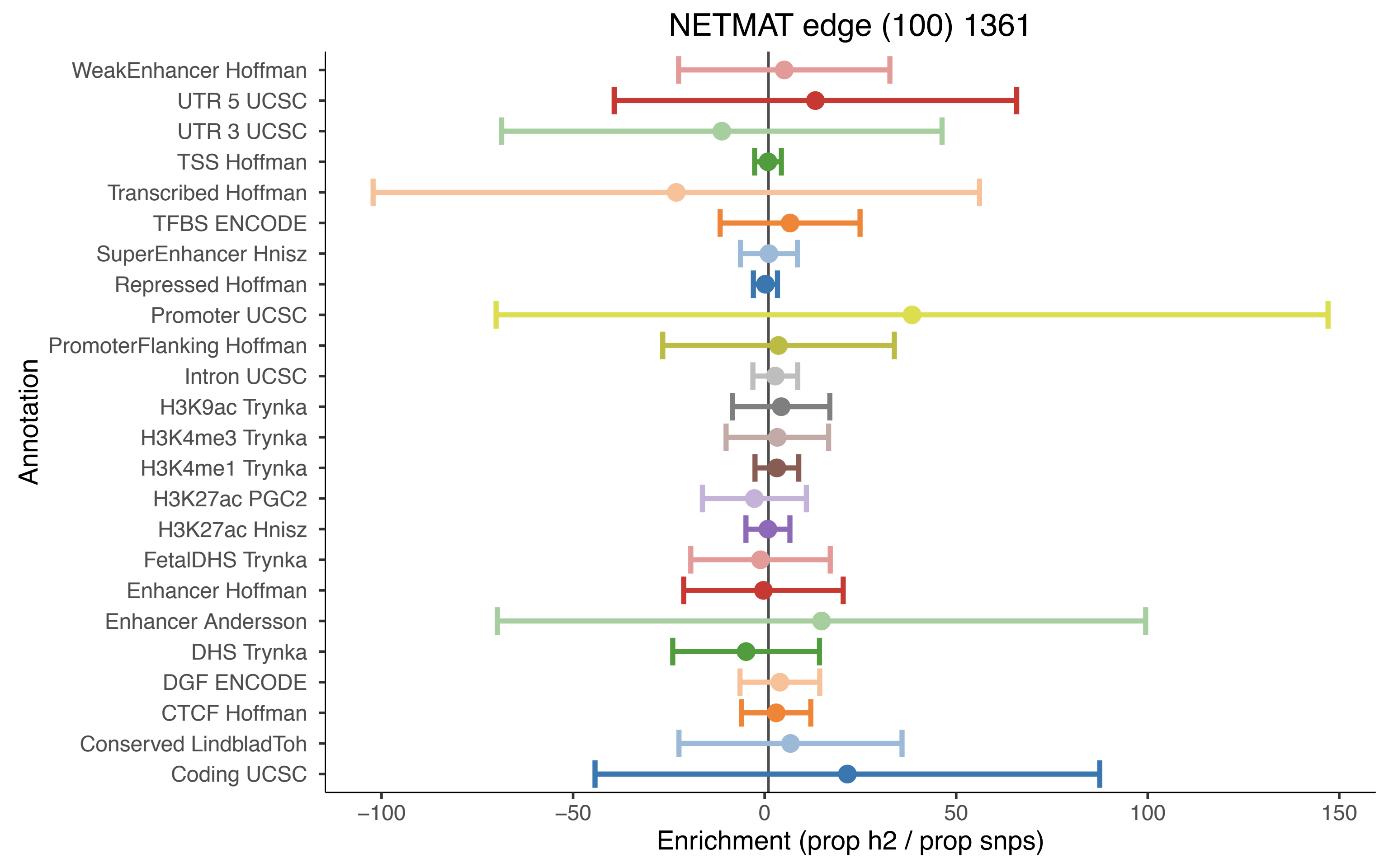
NETMAT edge (100) 1361
WeakEnhancer Hoffman
UTR 5 UCSC
UTR 3 UCSC
TSS Hoffman
Transcribed Hoffman
TFBS ENCODE
SuperEnhancer Hnisz
Repressed Hoffman
Promoter UCSC
PromoterFlanking Hoffman
Intron UCSC
H3K9ac Trynka
H3K4me3 Trynka
H3K4me1 Trynka
H3K27ac PGC2
H3K27ac Hnisz
FetalDHS Trynka
Enhancer Hoffman
Enhancer Andersson
DHS Trynka
DGF ENCODE
CTCF Hoffman
Conserved LindbladToh
Coding UCSC
Annotation
−100
−50
0
50
100
150
Enrichment (prop h2 / prop snps)

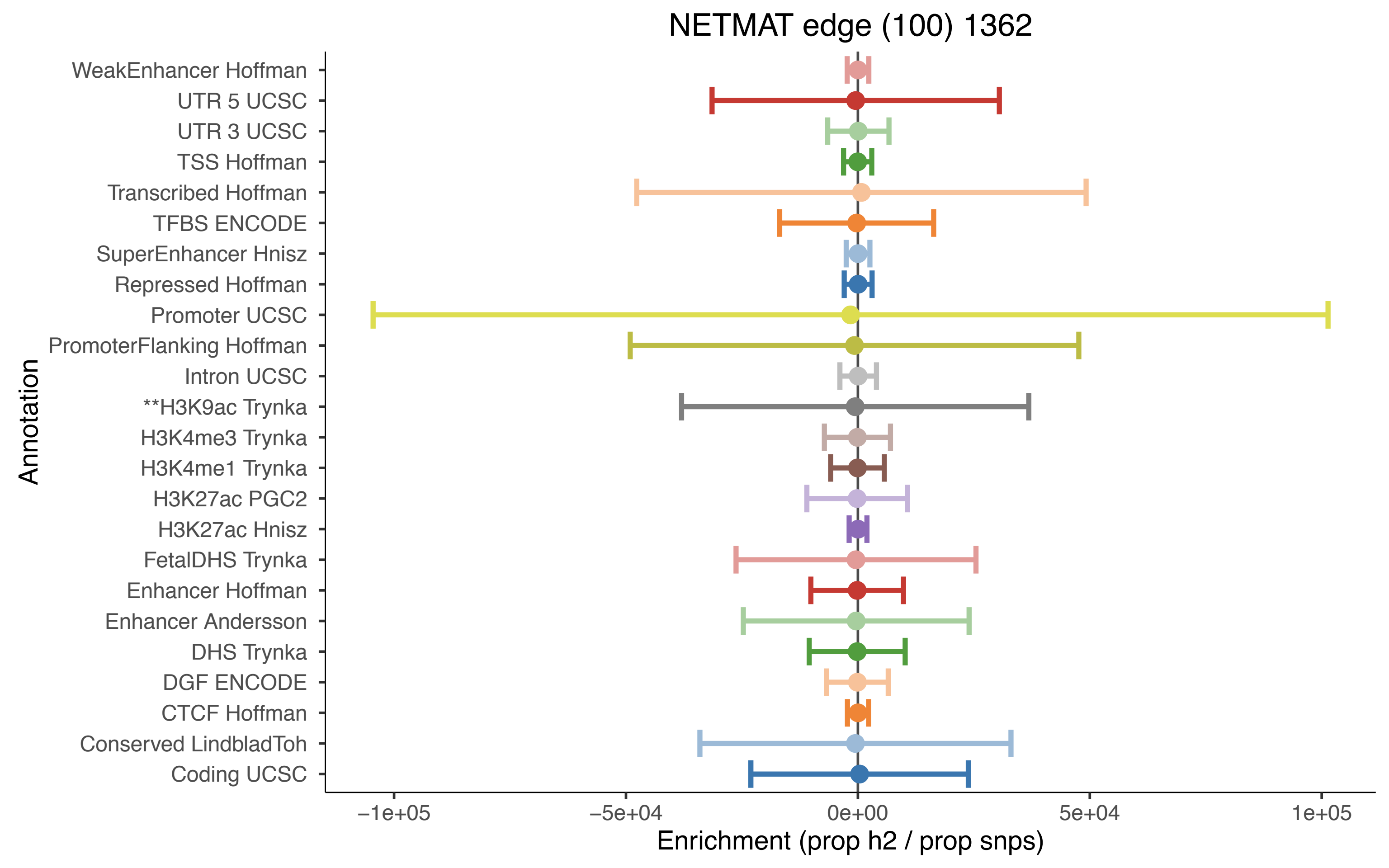
NETMAT edge (100) 1362
WeakEnhancer Hoffman
UTR 5 UCSC
UTR 3 UCSC
TSS Hoffman
Transcribed Hoffman
TFBS ENCODE
SuperEnhancer Hnisz
Repressed Hoffman
Promoter UCSC
PromoterFlanking Hoffman
Intron UCSC
**H3K9ac Trynka
H3K4me3 Trynka
H3K4me1 Trynka
H3K27ac PGC2
H3K27ac Hnisz
FetalDHS Trynka
Enhancer Hoffman
Enhancer Andersson
DHS Trynka
DGF ENCODE
CTCF Hoffman
Conserved LindbladToh
Coding UCSC
Annotation
−1e+05
−5e+04
0e+00
5e+04
1e+05
Enrichment (prop h2 / prop snps)

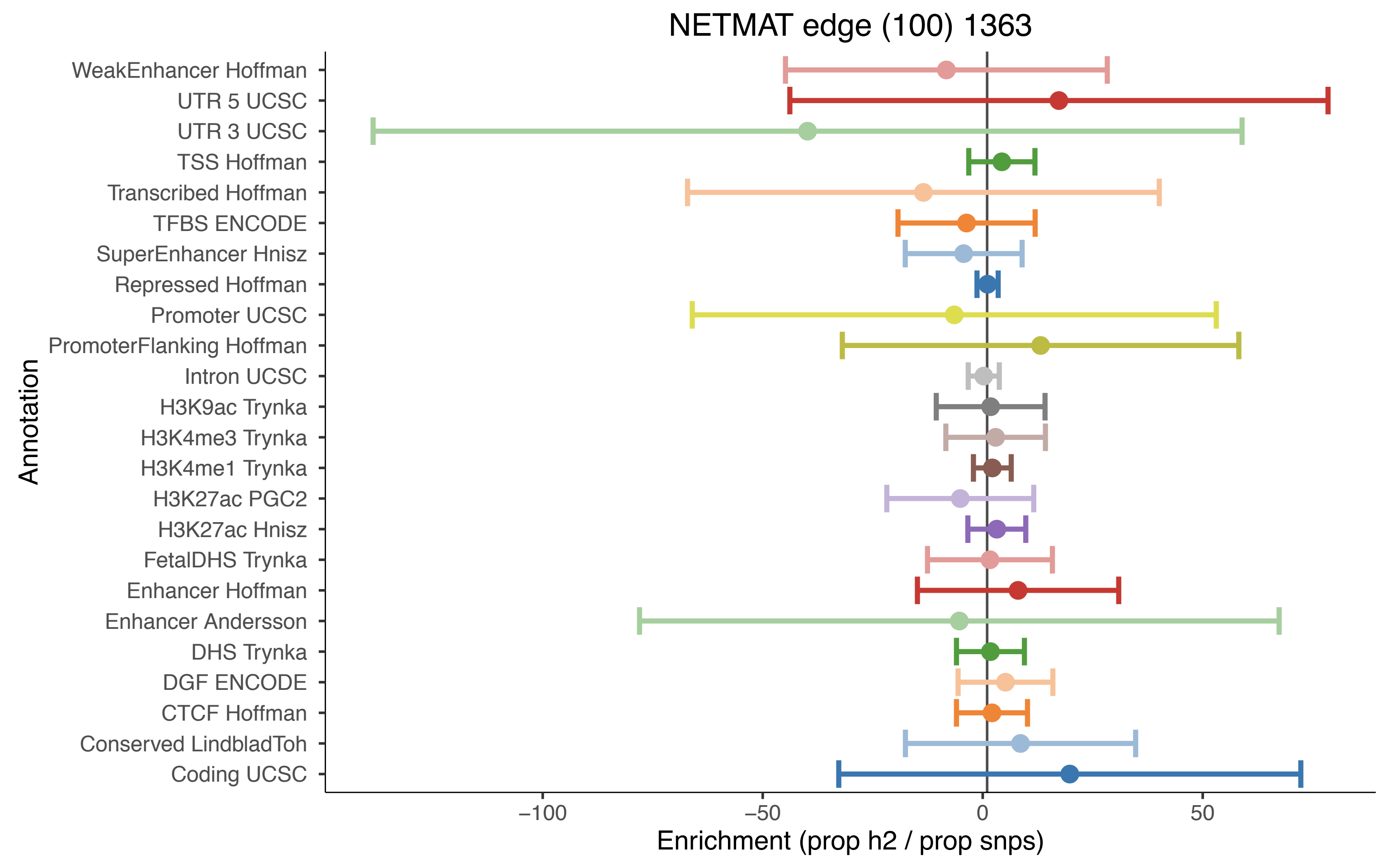
NETMAT edge (100) 1363
WeakEnhancer Hoffman
UTR 5 UCSC
UTR 3 UCSC
TSS Hoffman
Transcribed Hoffman
TFBS ENCODE
SuperEnhancer Hnisz
Repressed Hoffman
Promoter UCSC
PromoterFlanking Hoffman
Intron UCSC
H3K9ac Trynka
H3K4me3 Trynka
H3K4me1 Trynka
H3K27ac PGC2
H3K27ac Hnisz
FetalDHS Trynka
Enhancer Hoffman
Enhancer Andersson
DHS Trynka
DGF ENCODE
CTCF Hoffman
Conserved LindbladToh
Coding UCSC
Annotation
−100
−50
0
50
Enrichment (prop h2 / prop snps)

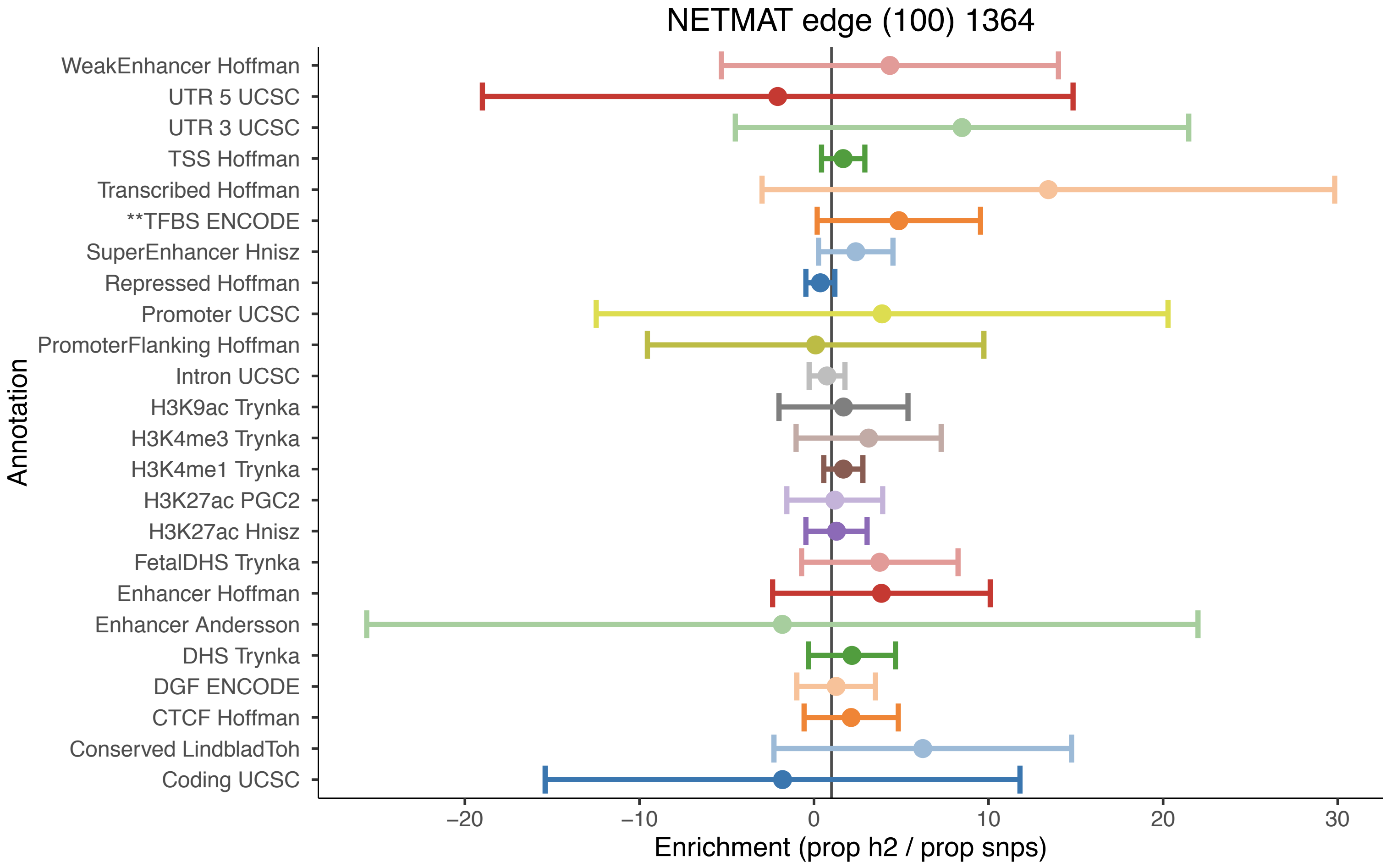
NETMAT edge (100) 1364
Annotation
WeakEnhancer Hoffman
UTR 5 UCSC
UTR 3 UCSC
TSS Hoffman
Transcribed Hoffman
**TFBS ENCODE
SuperEnhancer Hnisz
Repressed Hoffman
Promoter UCSC
PromoterFlanking Hoffman
Intron UCSC
H3K9ac Trynka
H3K4me3 Trynka
H3K4me1 Trynka
H3K27ac PGC2
H3K27ac Hnisz
FetalDHS Trynka
Enhancer Hoffman
Enhancer Andersson
DHS Trynka
DGF ENCODE
CTCF Hoffman
Conserved LindbladToh
Coding UCSC
−20
−10
0
10
20
30
Enrichment (prop h2 / prop snps)

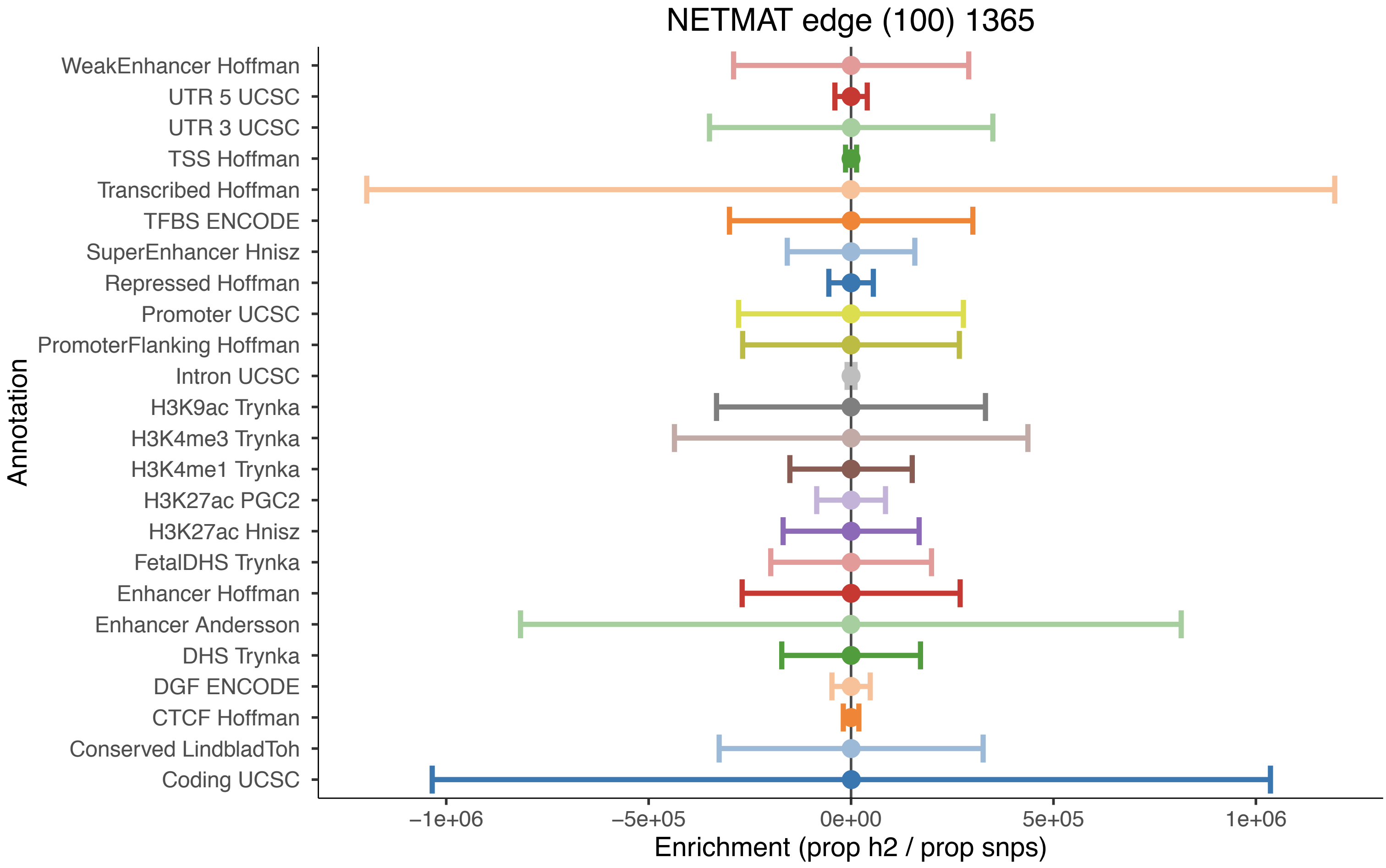

NETMAT edge (100) 1365
Annotation
WeakEnhancer Hoffman
UTR 5 UCSC
UTR 3 UCSC
TSS Hoffman
Transcribed Hoffman
TFBS ENCODE
SuperEnhancer Hnisz
Repressed Hoffman
Promoter UCSC
PromoterFlanking Hoffman
Intron UCSC
H3K9ac Trynka
H3K4me3 Trynka
H3K4me1 Trynka
H3K27ac PGC2
H3K27ac Hnisz
FetalDHS Trynka
Enhancer Hoffman
Enhancer Andersson
DHS Trynka
DGF ENCODE
CTCF Hoffman
Conserved LindbladToh
Coding UCSC
−1e+06
−5e+05
0e+00
5e+05
1e+06
Enrichment (prop h2 / prop snps)

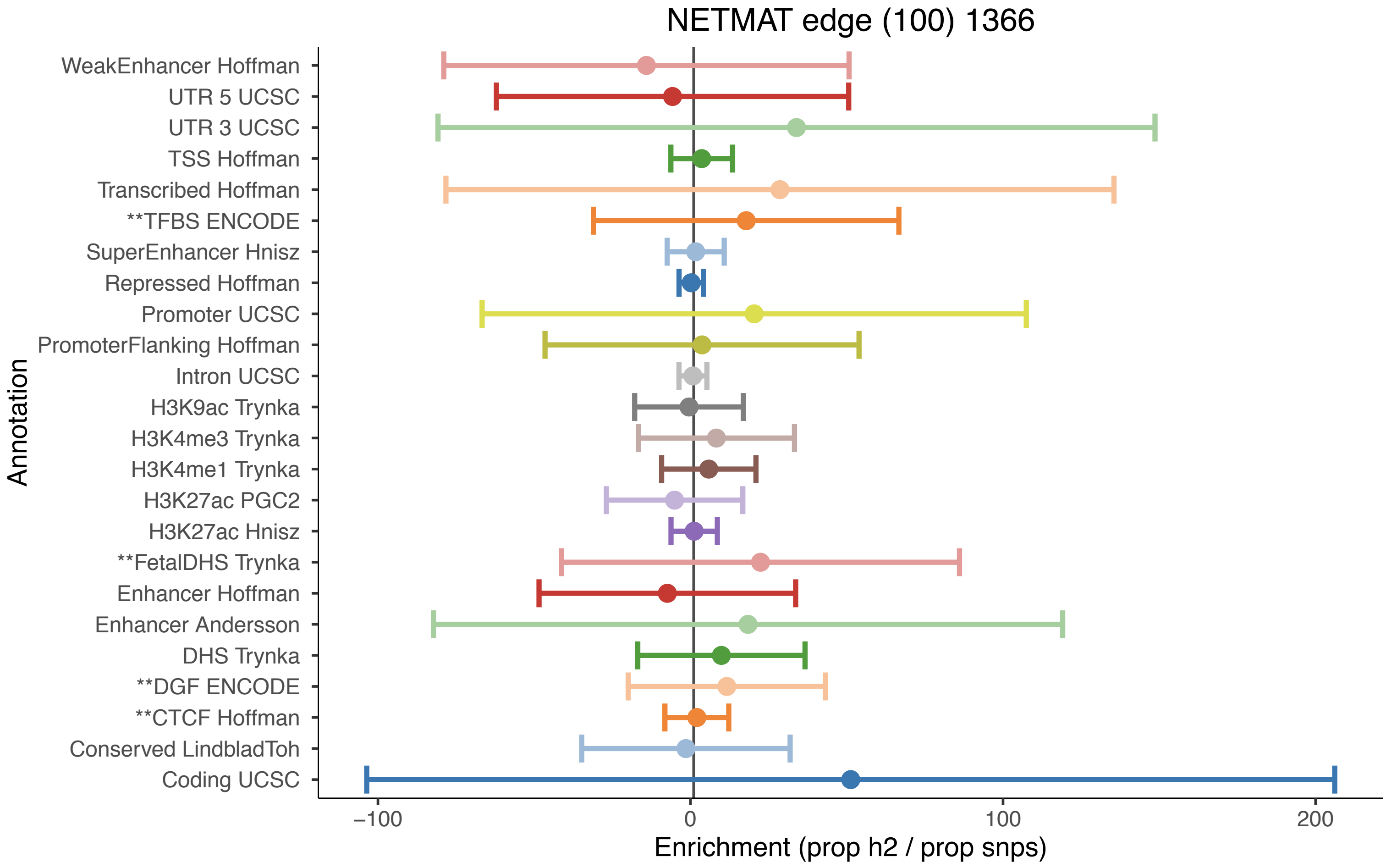
NETMAT edge (100) 1366
WeakEnhancer Hoffman
UTR 5 UCSC
UTR 3 UCSC
TSS Hoffman
Transcribed Hoffman
**TFBS ENCODE
SuperEnhancer Hnisz
Repressed Hoffman
Promoter UCSC
PromoterFlanking Hoffman
Intron UCSC
H3K9ac Trynka
H3K4me3 Trynka
H3K4me1 Trynka
H3K27ac PGC2
H3K27ac Hnisz
**FetalDHS Trynka
Enhancer Hoffman
Enhancer Andersson
DHS Trynka
**DGF ENCODE
**CTCF Hoffman
Conserved LindbladToh
Coding UCSC
Annotation
−100
0
100
200
Enrichment (prop h2 / prop snps)

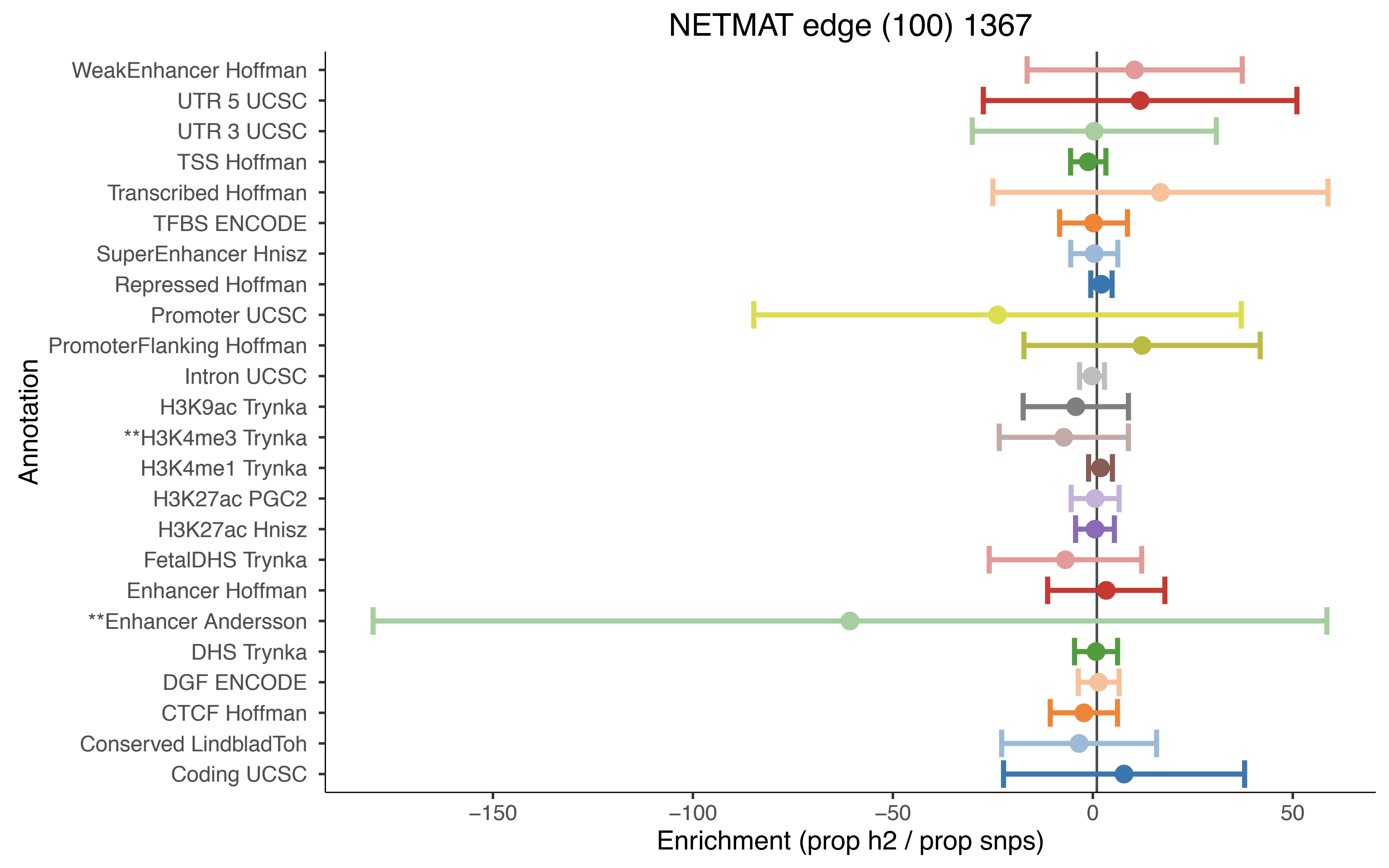
NETMAT edge (100) 1367
WeakEnhancer Hoffman
UTR 5 UCSC
UTR 3 UCSC
TSS Hoffman
Transcribed Hoffman
TFBS ENCODE
SuperEnhancer Hnisz
Repressed Hoffman
Promoter UCSC
PromoterFlanking Hoffman
Intron UCSC
H3K9ac Trynka
**H3K4me3 Trynka
H3K4me1 Trynka
H3K27ac PGC2
H3K27ac Hnisz
FetalDHS Trynka
Enhancer Hoffman
**Enhancer Andersson
DHS Trynka
DGF ENCODE
CTCF Hoffman
Conserved LindbladToh
Coding UCSC
Annotation
−150
−100
−50
0
50
Enrichment (prop h2 / prop snps)

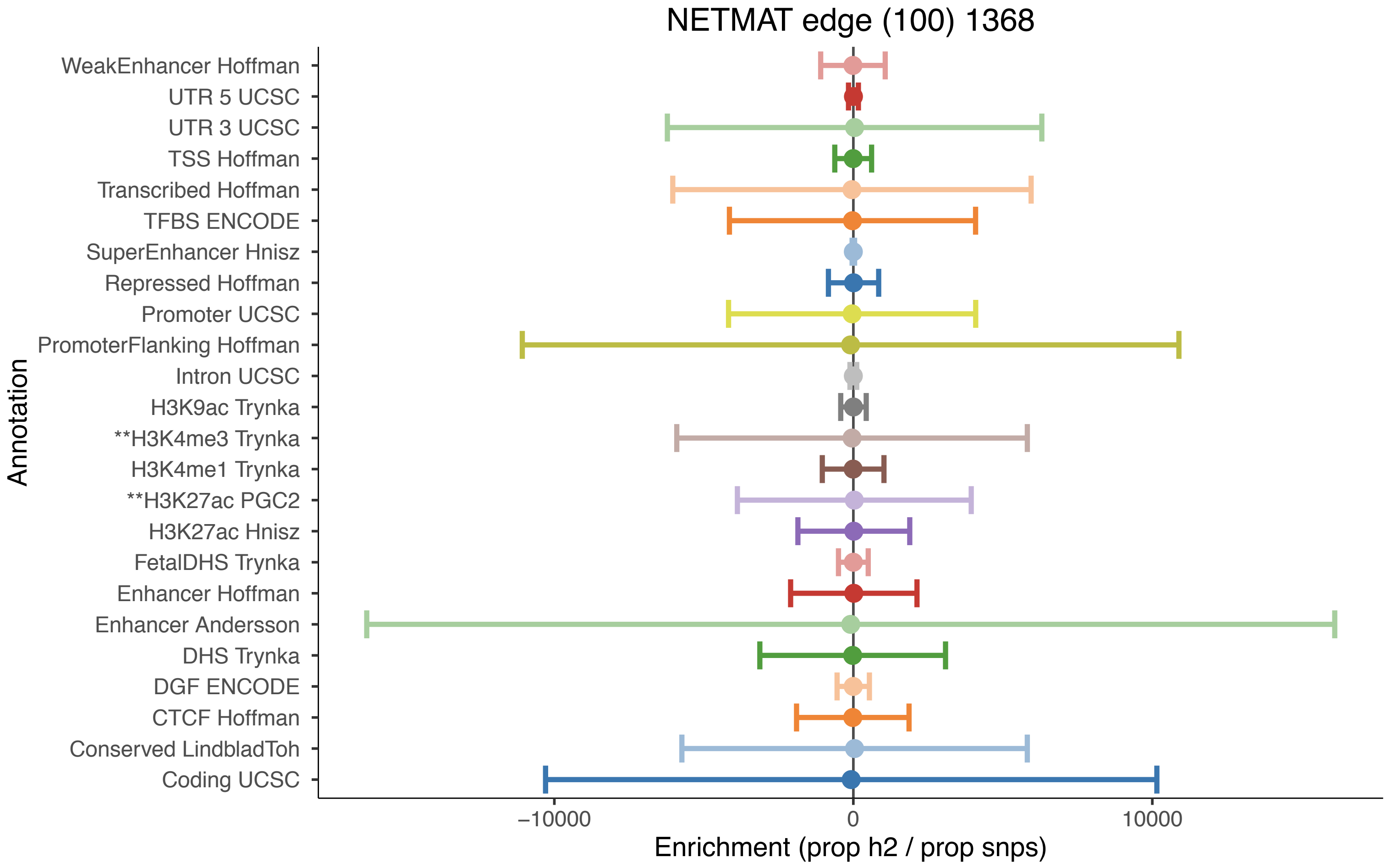
NETMAT edge (100) 1368
WeakEnhancer Hoffman
UTR 5 UCSC
UTR 3 UCSC
TSS Hoffman
Transcribed Hoffman
TFBS ENCODE
SuperEnhancer Hnisz
Repressed Hoffman
Promoter UCSC
PromoterFlanking Hoffman
Intron UCSC
H3K9ac Trynka
**H3K4me3 Trynka
H3K4me1 Trynka
**H3K27ac PGC2
H3K27ac Hnisz
FetalDHS Trynka
Enhancer Hoffman
Enhancer Andersson
DHS Trynka
DGF ENCODE
CTCF Hoffman
Conserved LindbladToh
Coding UCSC
Annotation
−10000
0
10000
Enrichment (prop h2 / prop snps)

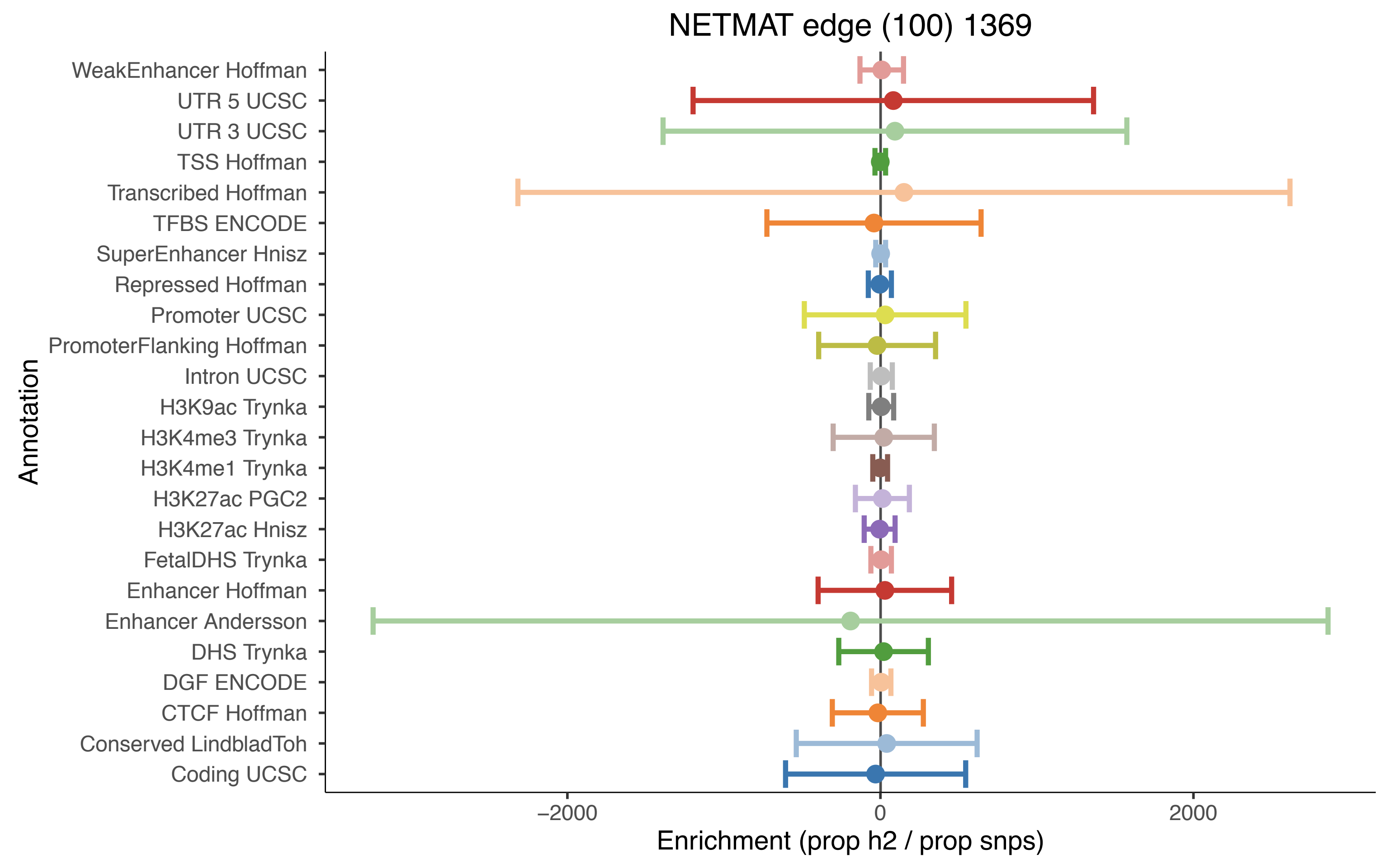

NETMAT edge (100) 1369
WeakEnhancer Hoffman
UTR 5 UCSC
UTR 3 UCSC
TSS Hoffman
Transcribed Hoffman
TFBS ENCODE
SuperEnhancer Hnisz
Repressed Hoffman
Promoter UCSC
PromoterFlanking Hoffman
Intron UCSC
H3K9ac Trynka
H3K4me3 Trynka
H3K4me1 Trynka
H3K27ac PGC2
H3K27ac Hnisz
FetalDHS Trynka
Enhancer Hoffman
Enhancer Andersson
DHS Trynka
DGF ENCODE
CTCF Hoffman
Conserved LindbladToh
Coding UCSC
Annotation
−2000
0
2000
Enrichment (prop h2 / prop snps)

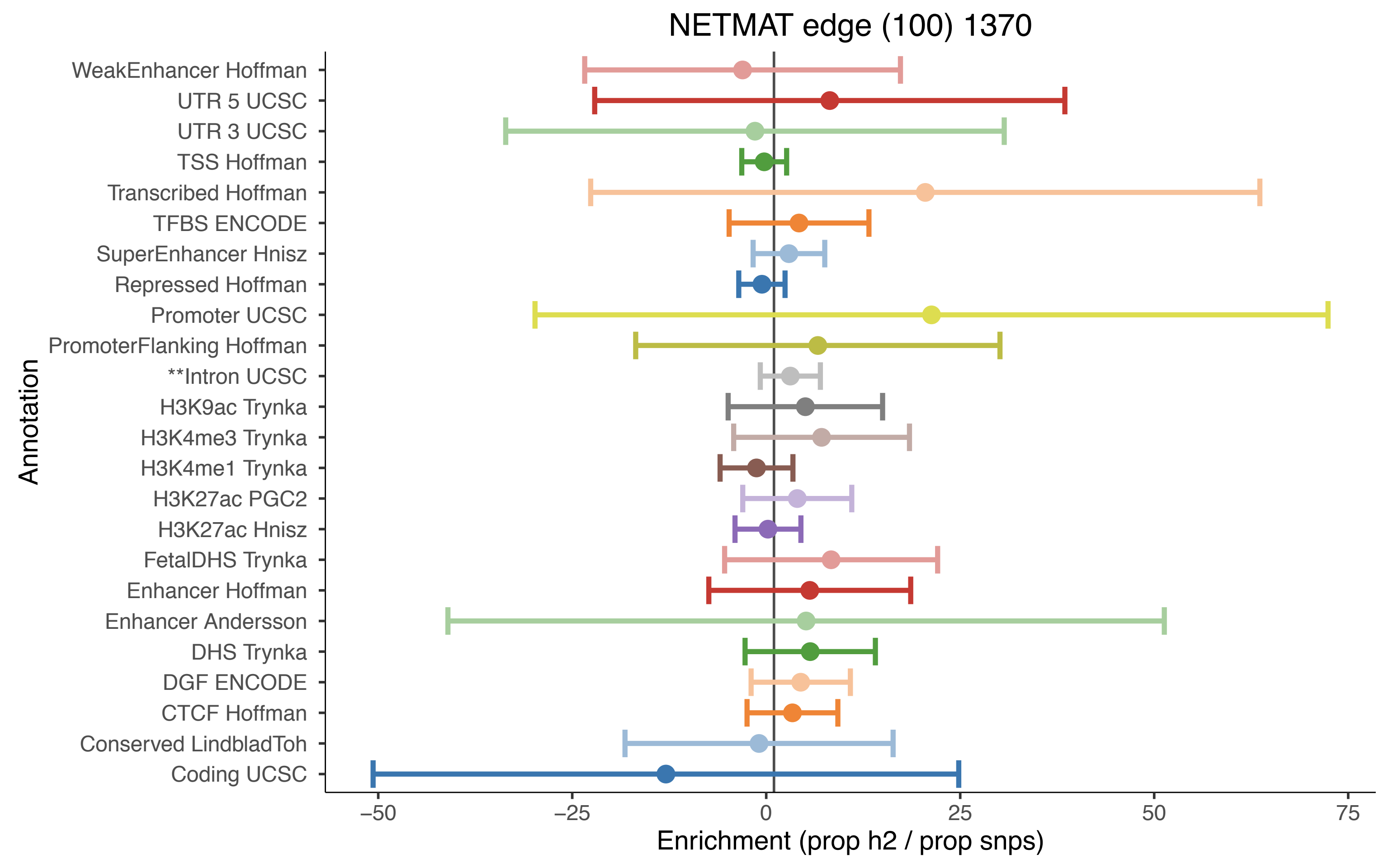
NETMAT edge (100) 1370
Annotation
WeakEnhancer Hoffman
UTR 5 UCSC
UTR 3 UCSC
TSS Hoffman
Transcribed Hoffman
TFBS ENCODE
SuperEnhancer Hnisz
Repressed Hoffman
Promoter UCSC
PromoterFlanking Hoffman
**Intron UCSC
H3K9ac Trynka
H3K4me3 Trynka
H3K4me1 Trynka
H3K27ac PGC2
H3K27ac Hnisz
FetalDHS Trynka
Enhancer Hoffman
Enhancer Andersson
DHS Trynka
DGF ENCODE
CTCF Hoffman
Conserved LindbladToh
Coding UCSC
−50
−25
0
25
50
75
Enrichment (prop h2 / prop snps)

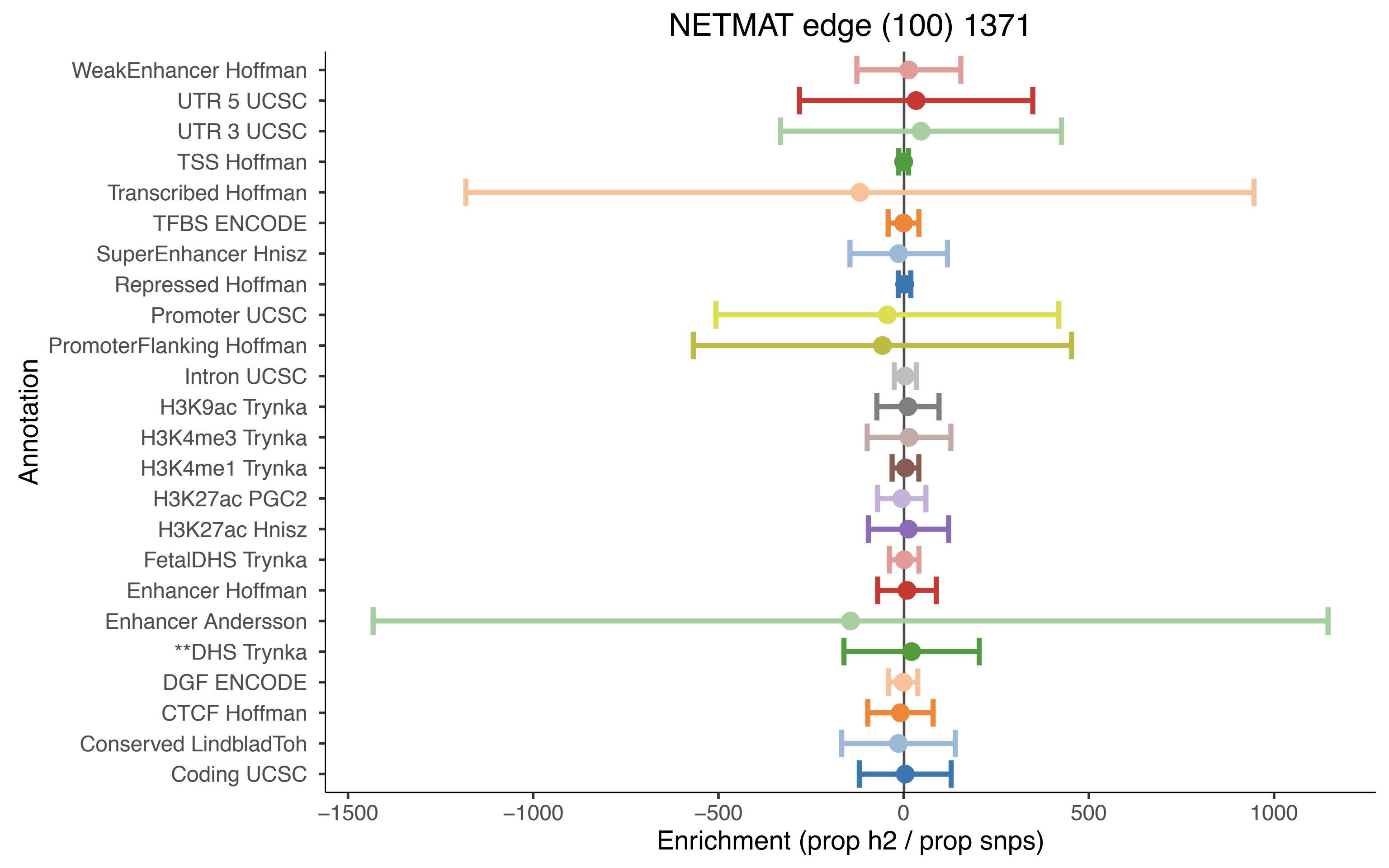
NETMAT edge (100) 1371
WeakEnhancer Hoffman
UTR 5 UCSC
UTR 3 UCSC
TSS Hoffman
Transcribed Hoffman
TFBS ENCODE
SuperEnhancer Hnisz
Repressed Hoffman
Promoter UCSC
PromoterFlanking Hoffman
Intron UCSC
H3K9ac Trynka
H3K4me3 Trynka
H3K4me1 Trynka
H3K27ac PGC2
H3K27ac Hnisz
FetalDHS Trynka
Enhancer Hoffman
Enhancer Andersson
**DHS Trynka
DGF ENCODE
CTCF Hoffman
Conserved LindbladToh
Coding UCSC
Annotation
−1500
−1000
−500
0
500
1000
Enrichment (prop h2 / prop snps)

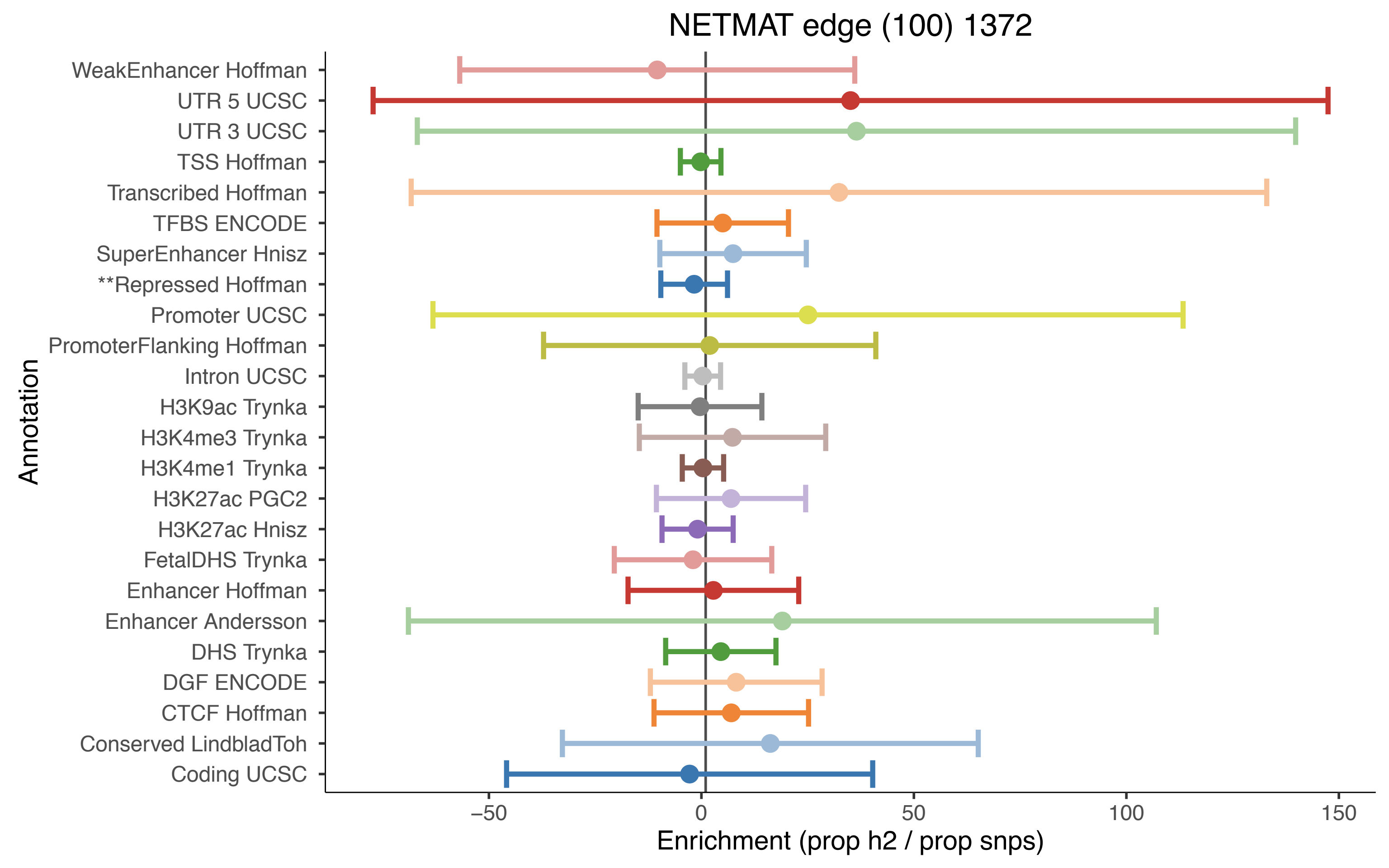

NETMAT edge (100) 1372
WeakEnhancer Hoffman
UTR 5 UCSC
UTR 3 UCSC
TSS Hoffman
Transcribed Hoffman
TFBS ENCODE
SuperEnhancer Hnisz
**Repressed Hoffman
Promoter UCSC
PromoterFlanking Hoffman
Intron UCSC
H3K9ac Trynka
H3K4me3 Trynka
H3K4me1 Trynka
H3K27ac PGC2
H3K27ac Hnisz
FetalDHS Trynka
Enhancer Hoffman
Enhancer Andersson
DHS Trynka
DGF ENCODE
CTCF Hoffman
Conserved LindbladToh
Coding UCSC
Annotation
−50
0
50
100
150
Enrichment (prop h2 / prop snps)

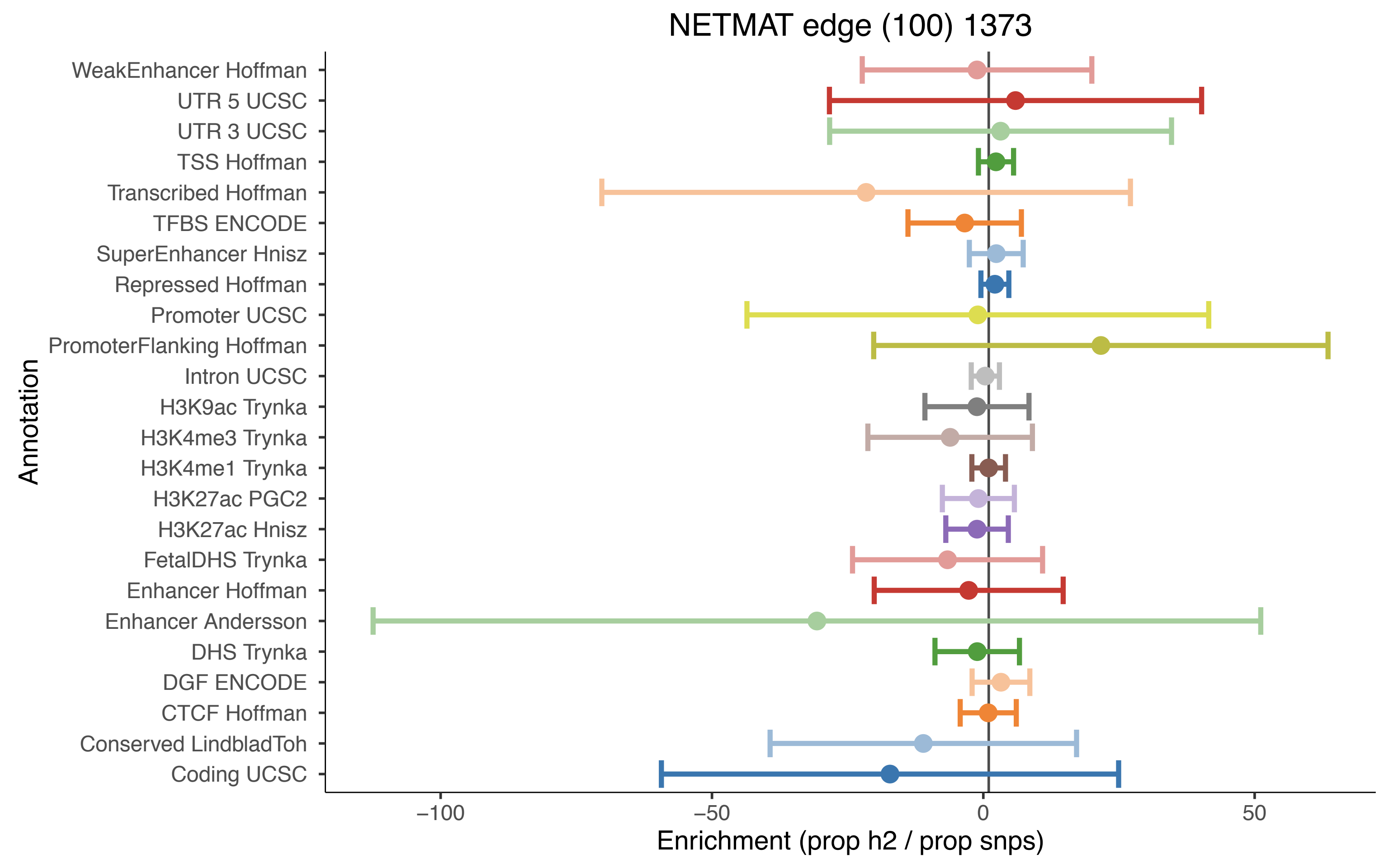
NETMAT edge (100) 1373
Annotation
WeakEnhancer Hoffman
UTR 5 UCSC
UTR 3 UCSC
TSS Hoffman
Transcribed Hoffman
TFBS ENCODE
SuperEnhancer Hnisz
Repressed Hoffman
Promoter UCSC
PromoterFlanking Hoffman
Intron UCSC
H3K9ac Trynka
H3K4me3 Trynka
H3K4me1 Trynka
H3K27ac PGC2
H3K27ac Hnisz
FetalDHS Trynka
Enhancer Hoffman
Enhancer Andersson
DHS Trynka
DGF ENCODE
CTCF Hoffman
Conserved LindbladToh
Coding UCSC
−100
−50
0
50
Enrichment (prop h2 / prop snps)

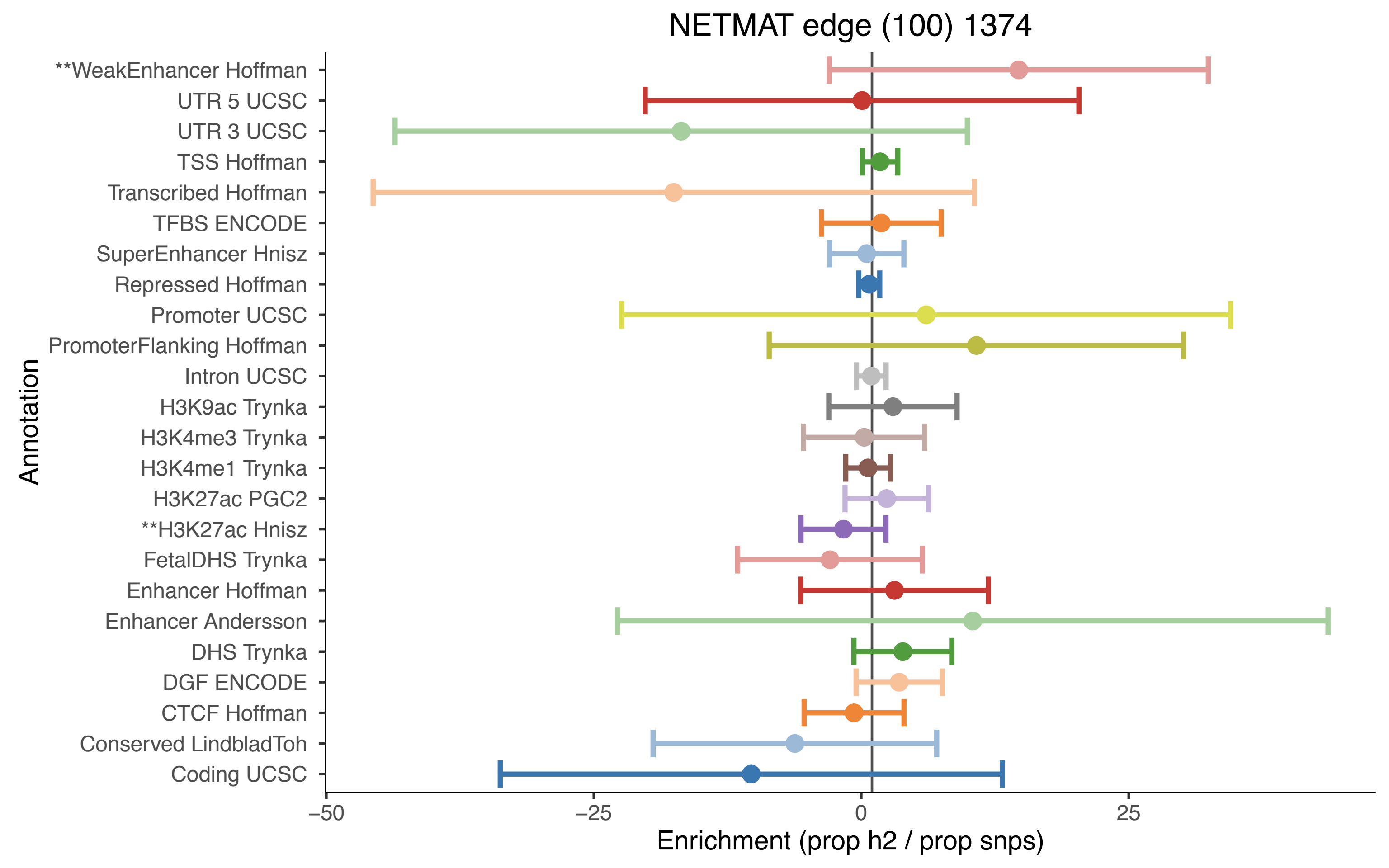
NETMAT edge (100) 1374
**WeakEnhancer Hoffman
UTR 5 UCSC
UTR 3 UCSC
TSS Hoffman
Transcribed Hoffman
TFBS ENCODE
SuperEnhancer Hnisz
Repressed Hoffman
Promoter UCSC
PromoterFlanking Hoffman
Intron UCSC
H3K9ac Trynka
H3K4me3 Trynka
H3K4me1 Trynka
H3K27ac PGC2
**H3K27ac Hnisz
FetalDHS Trynka
Enhancer Hoffman
Enhancer Andersson
DHS Trynka
DGF ENCODE
CTCF Hoffman
Conserved LindbladToh
Coding UCSC
Annotation
−50
−25
0
25
Enrichment (prop h2 / prop snps)

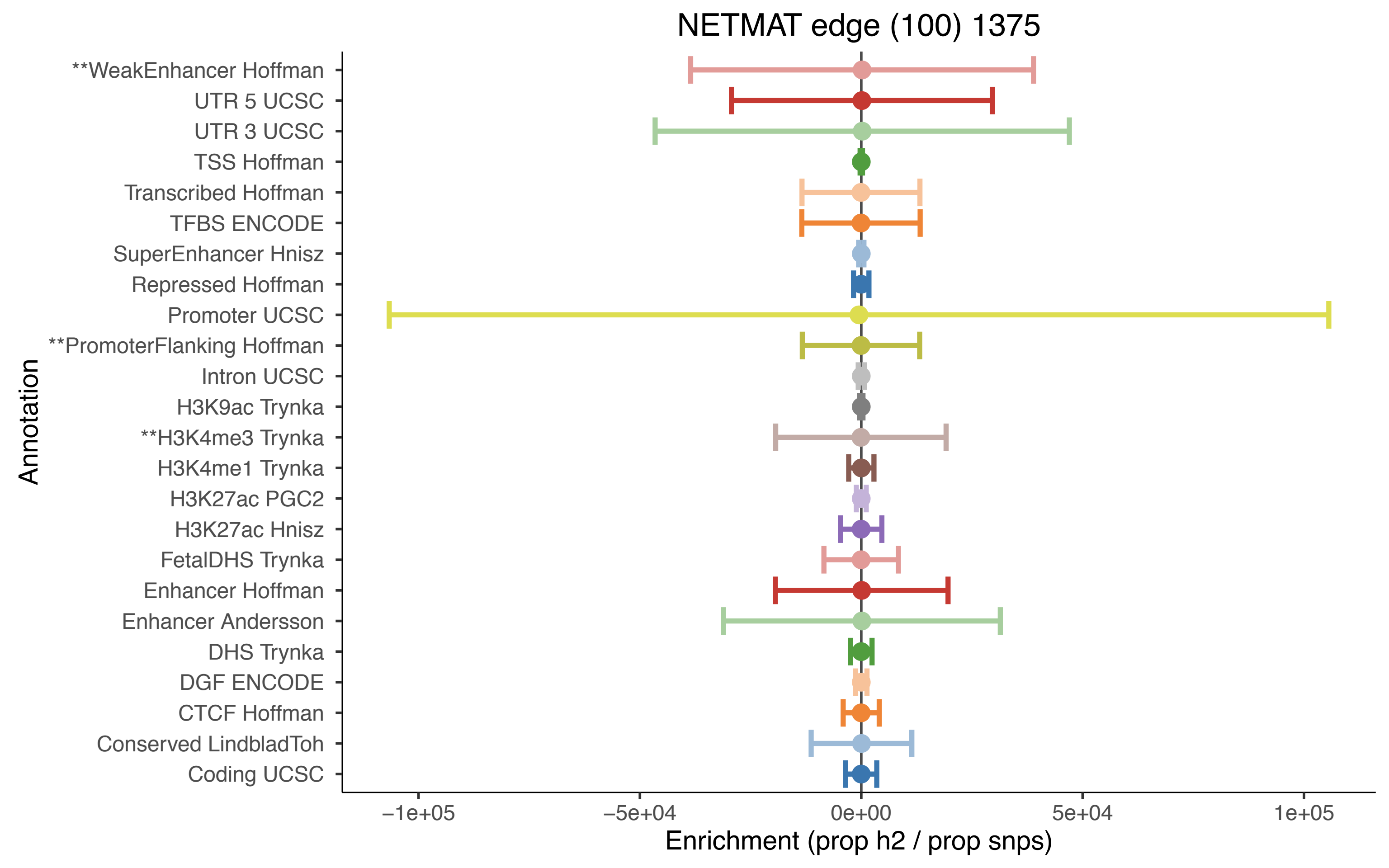
NETMAT edge (100) 1375
**WeakEnhancer Hoffman
UTR 5 UCSC
UTR 3 UCSC
TSS Hoffman
Transcribed Hoffman
TFBS ENCODE
SuperEnhancer Hnisz
Repressed Hoffman
Promoter UCSC
**PromoterFlanking Hoffman
Intron UCSC
H3K9ac Trynka
**H3K4me3 Trynka
H3K4me1 Trynka
H3K27ac PGC2
H3K27ac Hnisz
FetalDHS Trynka
Enhancer Hoffman
Enhancer Andersson
DHS Trynka
DGF ENCODE
CTCF Hoffman
Conserved LindbladToh
Coding UCSC
Annotation
−1e+05
−5e+04
0e+00
5e+04
1e+05
Enrichment (prop h2 / prop snps)

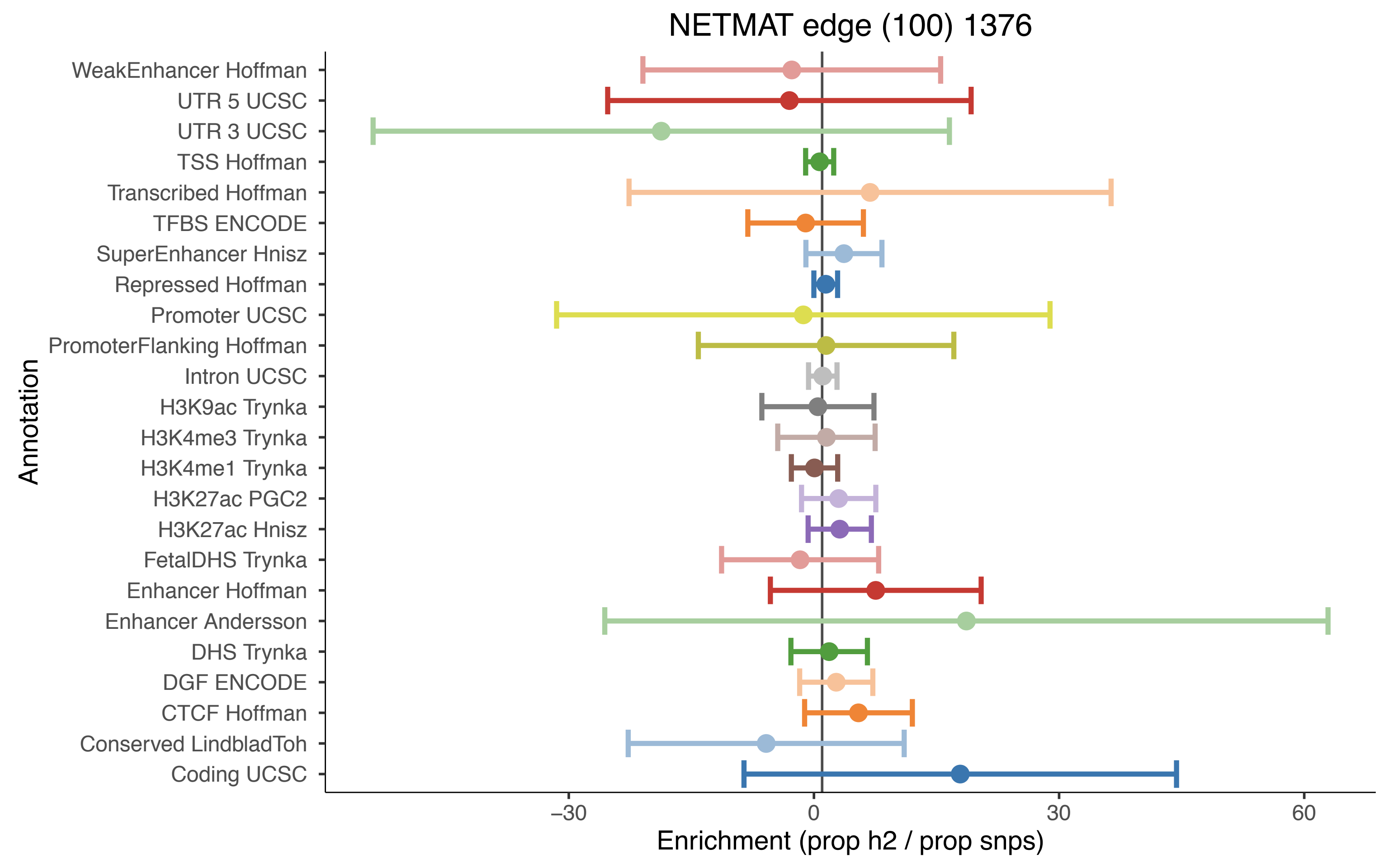
NETMAT edge (100) 1376
WeakEnhancer Hoffman
UTR 5 UCSC
UTR 3 UCSC
TSS Hoffman
Transcribed Hoffman
TFBS ENCODE
SuperEnhancer Hnisz
Repressed Hoffman
Promoter UCSC
PromoterFlanking Hoffman
Intron UCSC
H3K9ac Trynka
H3K4me3 Trynka
H3K4me1 Trynka
H3K27ac PGC2
H3K27ac Hnisz
FetalDHS Trynka
Enhancer Hoffman
Enhancer Andersson
DHS Trynka
DGF ENCODE
CTCF Hoffman
Conserved LindbladToh
Coding UCSC
Annotation
−30
0
30
60
Enrichment (prop h2 / prop snps)

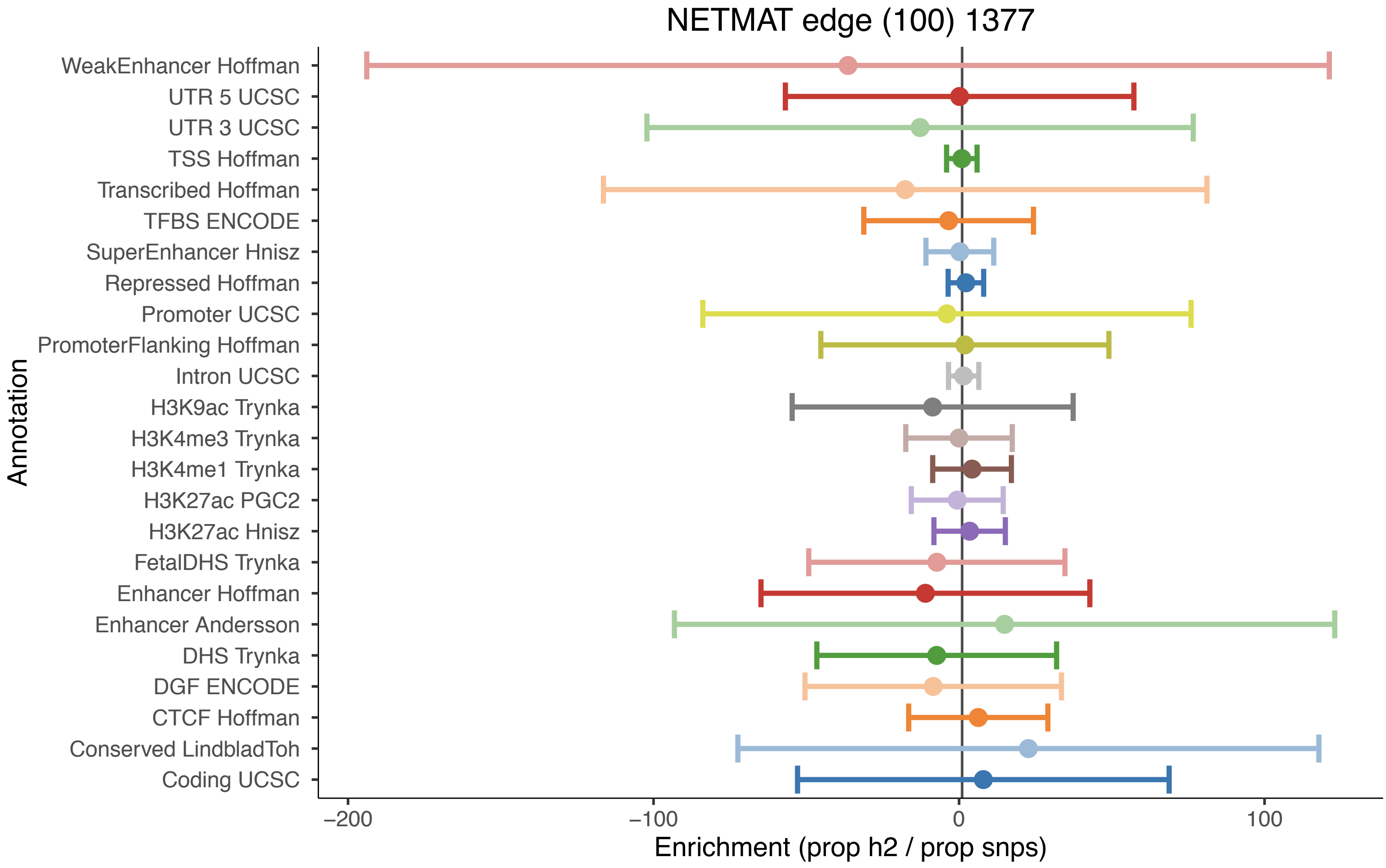

NETMAT edge (100) 1377
WeakEnhancer Hoffman
UTR 5 UCSC
UTR 3 UCSC
TSS Hoffman
Transcribed Hoffman
TFBS ENCODE
SuperEnhancer Hnisz
Repressed Hoffman
Promoter UCSC
PromoterFlanking Hoffman
Intron UCSC
H3K9ac Trynka
H3K4me3 Trynka
H3K4me1 Trynka
H3K27ac PGC2
H3K27ac Hnisz
FetalDHS Trynka
Enhancer Hoffman
Enhancer Andersson
DHS Trynka
DGF ENCODE
CTCF Hoffman
Conserved LindbladToh
Coding UCSC
Annotation
−200
−100
0
100
Enrichment (prop h2 / prop snps)

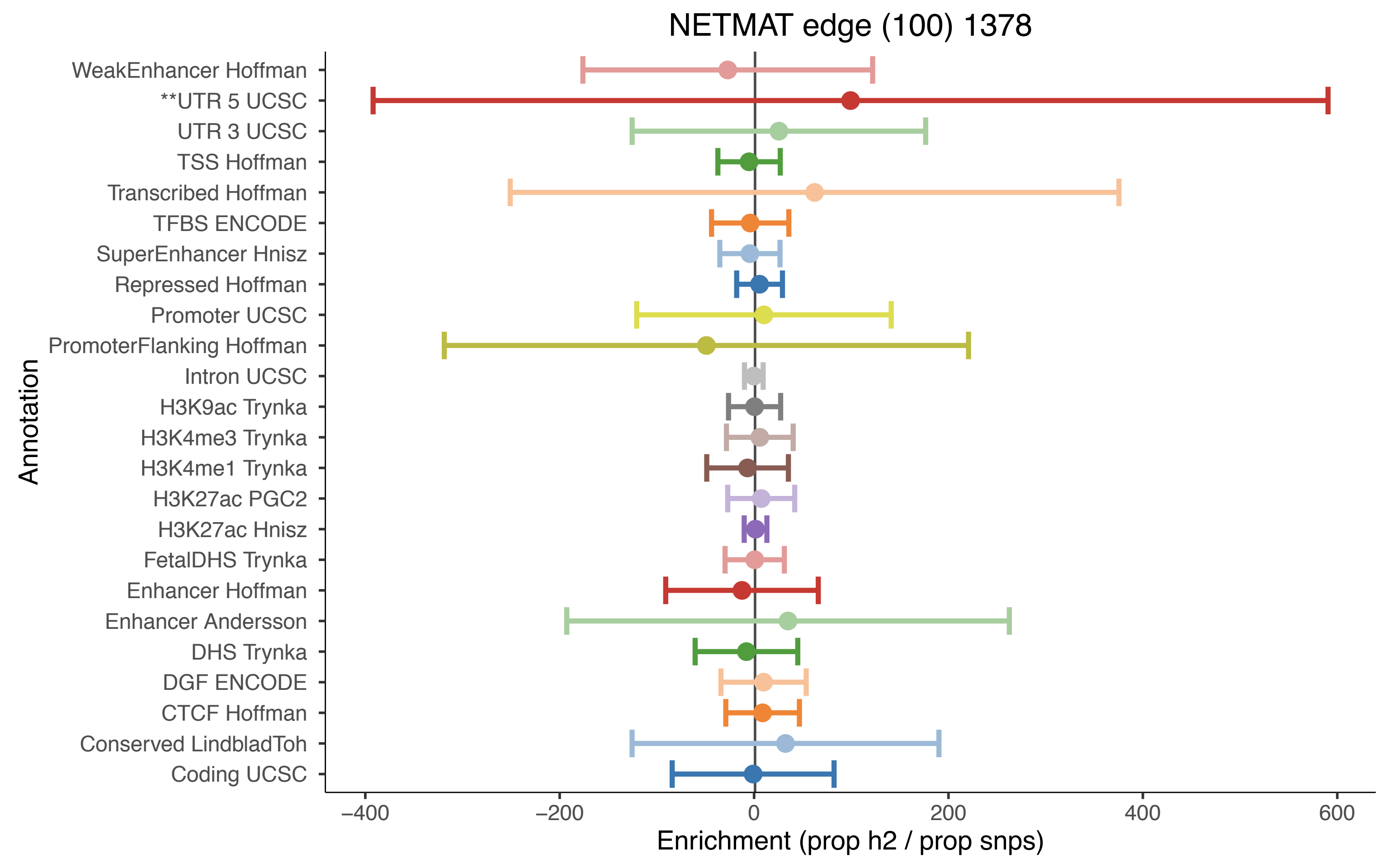

NETMAT edge (100) 1378
WeakEnhancer Hoffman
**UTR 5 UCSC
UTR 3 UCSC
TSS Hoffman
Transcribed Hoffman
TFBS ENCODE
SuperEnhancer Hnisz
Repressed Hoffman
Promoter UCSC
PromoterFlanking Hoffman
Intron UCSC
H3K9ac Trynka
H3K4me3 Trynka
H3K4me1 Trynka
H3K27ac PGC2
H3K27ac Hnisz
FetalDHS Trynka
Enhancer Hoffman
Enhancer Andersson
DHS Trynka
DGF ENCODE
CTCF Hoffman
Conserved LindbladToh
Coding UCSC
Annotation
−400
−200
0
200
400
600
Enrichment (prop h2 / prop snps)

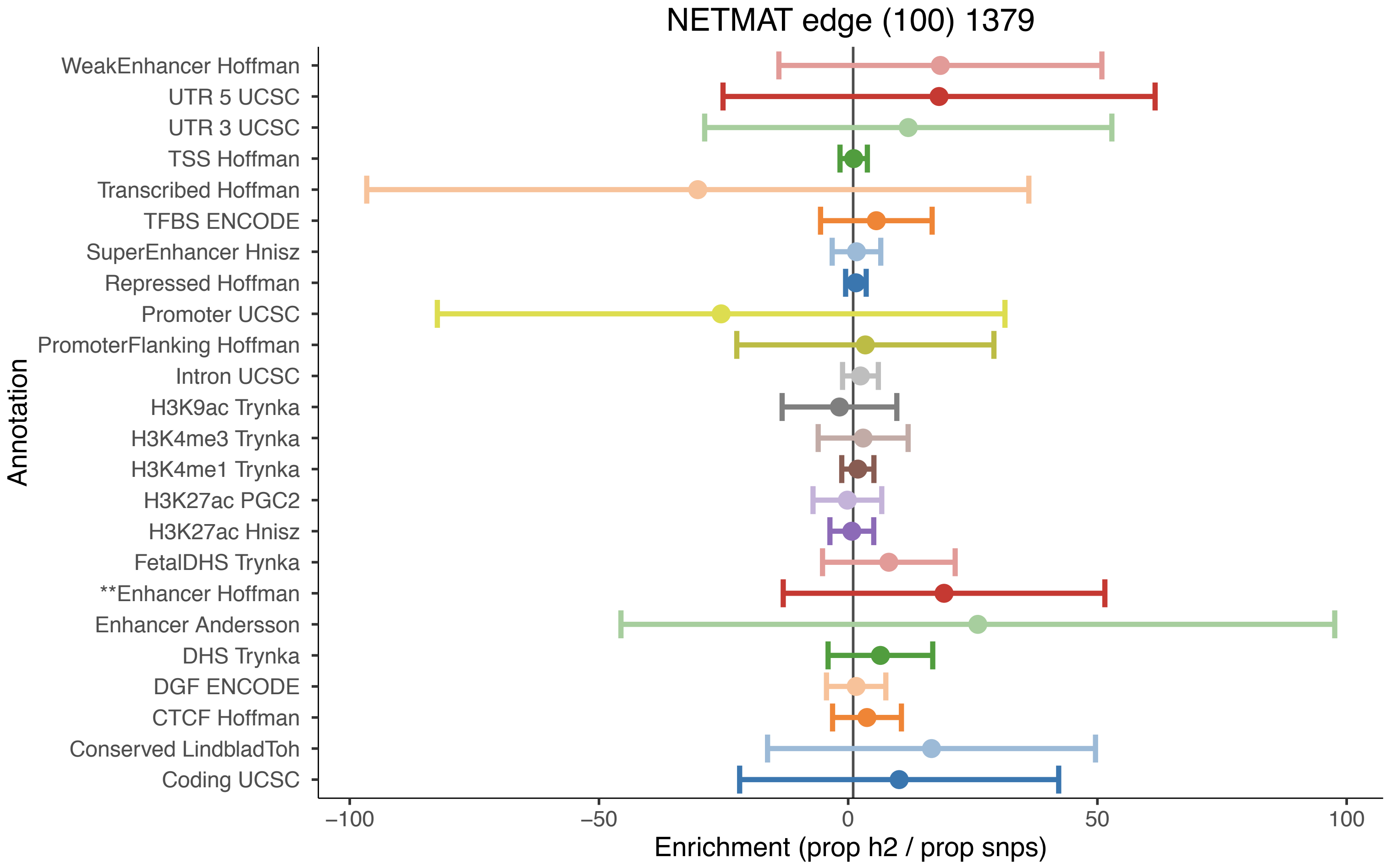
NETMAT edge (100) 1379
WeakEnhancer Hoffman
UTR 5 UCSC
UTR 3 UCSC
TSS Hoffman
Transcribed Hoffman
TFBS ENCODE
SuperEnhancer Hnisz
Repressed Hoffman
Promoter UCSC
PromoterFlanking Hoffman
Intron UCSC
H3K9ac Trynka
H3K4me3 Trynka
H3K4me1 Trynka
H3K27ac PGC2
H3K27ac Hnisz
FetalDHS Trynka
**Enhancer Hoffman
Enhancer Andersson
DHS Trynka
DGF ENCODE
CTCF Hoffman
Conserved LindbladToh
Coding UCSC
Annotation
−100
−50
0
50
100
Enrichment (prop h2 / prop snps)

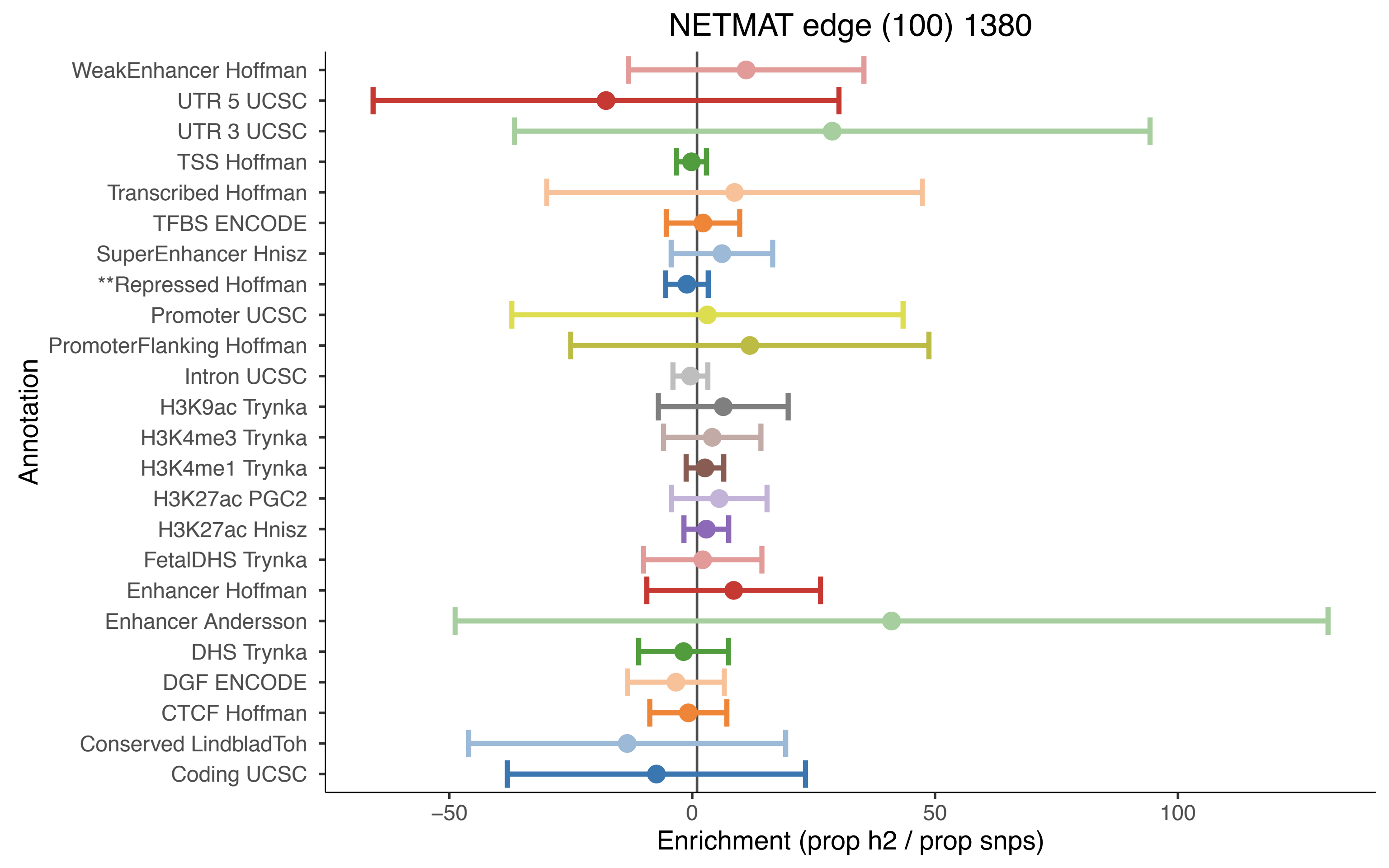
NETMAT edge (100) 1380
WeakEnhancer Hoffman
UTR 5 UCSC
UTR 3 UCSC
TSS Hoffman
Transcribed Hoffman
TFBS ENCODE
SuperEnhancer Hnisz
**Repressed Hoffman
Promoter UCSC
PromoterFlanking Hoffman
Intron UCSC
H3K9ac Trynka
H3K4me3 Trynka
H3K4me1 Trynka
H3K27ac PGC2
H3K27ac Hnisz
FetalDHS Trynka
Enhancer Hoffman
Enhancer Andersson
DHS Trynka
DGF ENCODE
CTCF Hoffman
Conserved LindbladToh
Coding UCSC
Annotation
−50
0
50
100
Enrichment (prop h2 / prop snps)

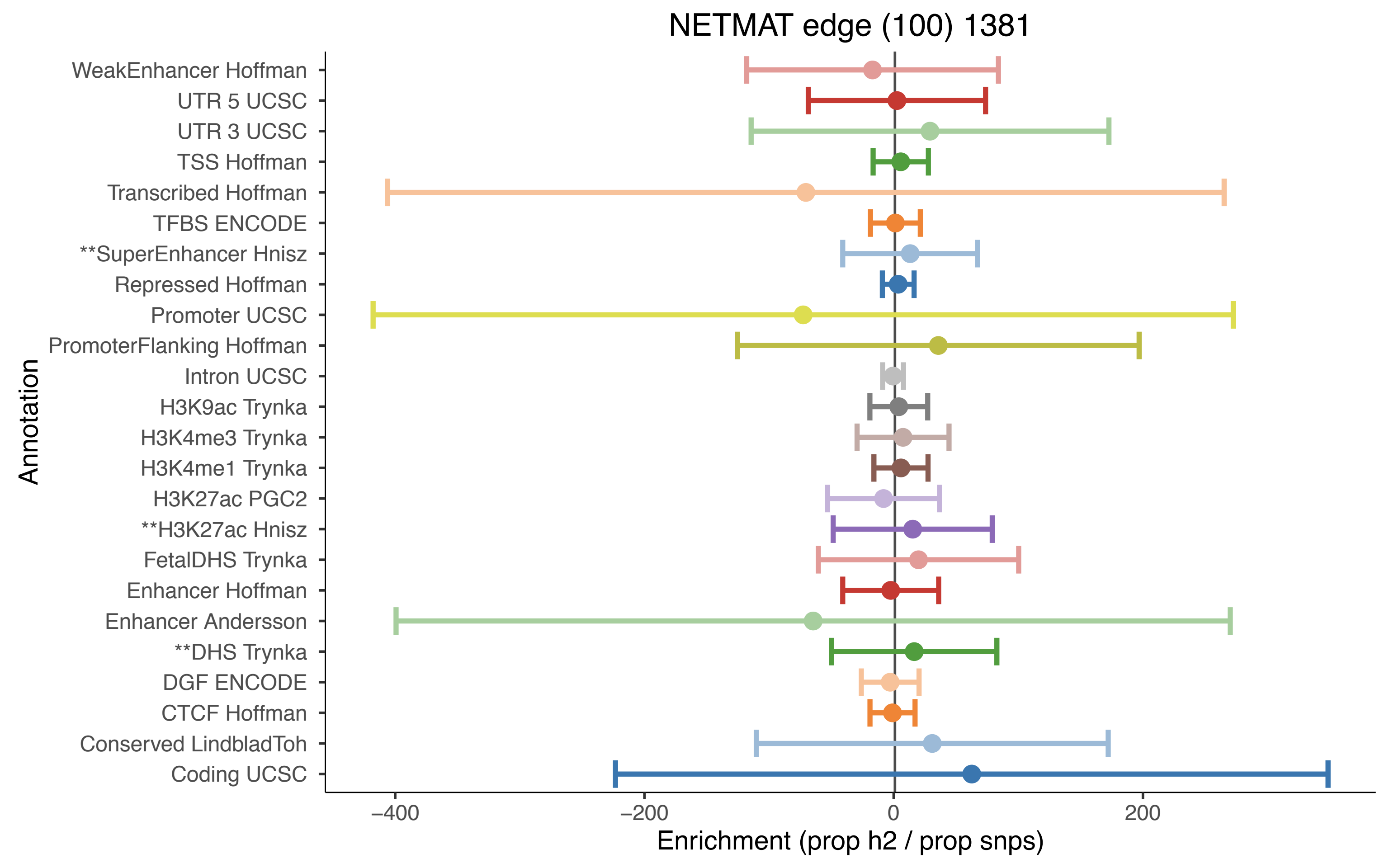
NETMAT edge (100) 1381
WeakEnhancer Hoffman
UTR 5 UCSC
UTR 3 UCSC
TSS Hoffman
Transcribed Hoffman
TFBS ENCODE
**SuperEnhancer Hnisz
Repressed Hoffman
Promoter UCSC
PromoterFlanking Hoffman
Intron UCSC
H3K9ac Trynka
H3K4me3 Trynka
H3K4me1 Trynka
H3K27ac PGC2
**H3K27ac Hnisz
FetalDHS Trynka
Enhancer Hoffman
Enhancer Andersson
**DHS Trynka
DGF ENCODE
CTCF Hoffman
Conserved LindbladToh
Coding UCSC
Annotation
−400
−200
0
200
Enrichment (prop h2 / prop snps)

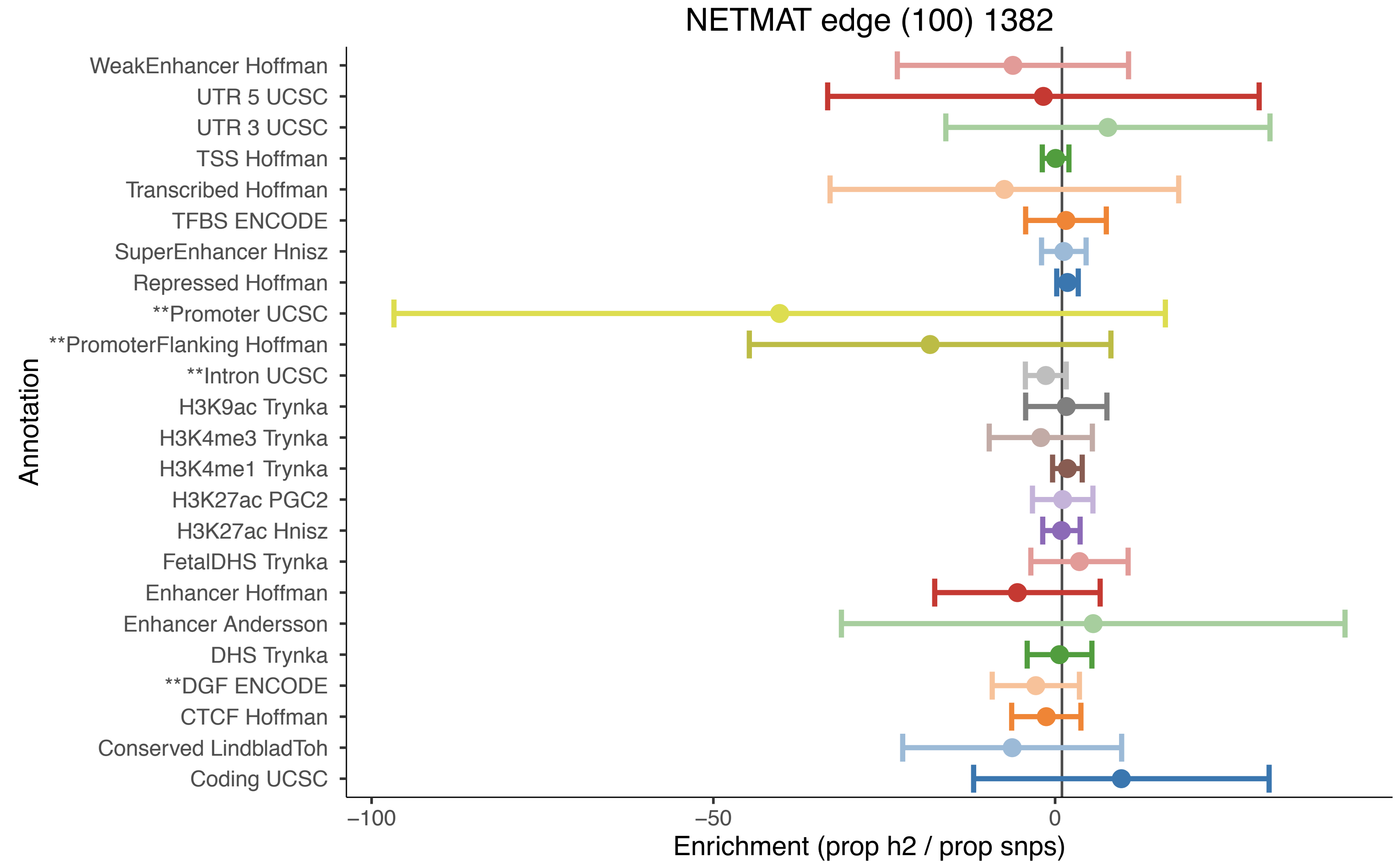
NETMAT edge (100) 1382
WeakEnhancer Hoffman
UTR 5 UCSC
UTR 3 UCSC
TSS Hoffman
Transcribed Hoffman
TFBS ENCODE
SuperEnhancer Hnisz
Repressed Hoffman
**Promoter UCSC
**PromoterFlanking Hoffman
**Intron UCSC
H3K9ac Trynka
H3K4me3 Trynka
H3K4me1 Trynka
H3K27ac PGC2
H3K27ac Hnisz
FetalDHS Trynka
Enhancer Hoffman
Enhancer Andersson
DHS Trynka
**DGF ENCODE
CTCF Hoffman
Conserved LindbladToh
Coding UCSC
Annotation
−100
−50
0
Enrichment (prop h2 / prop snps)

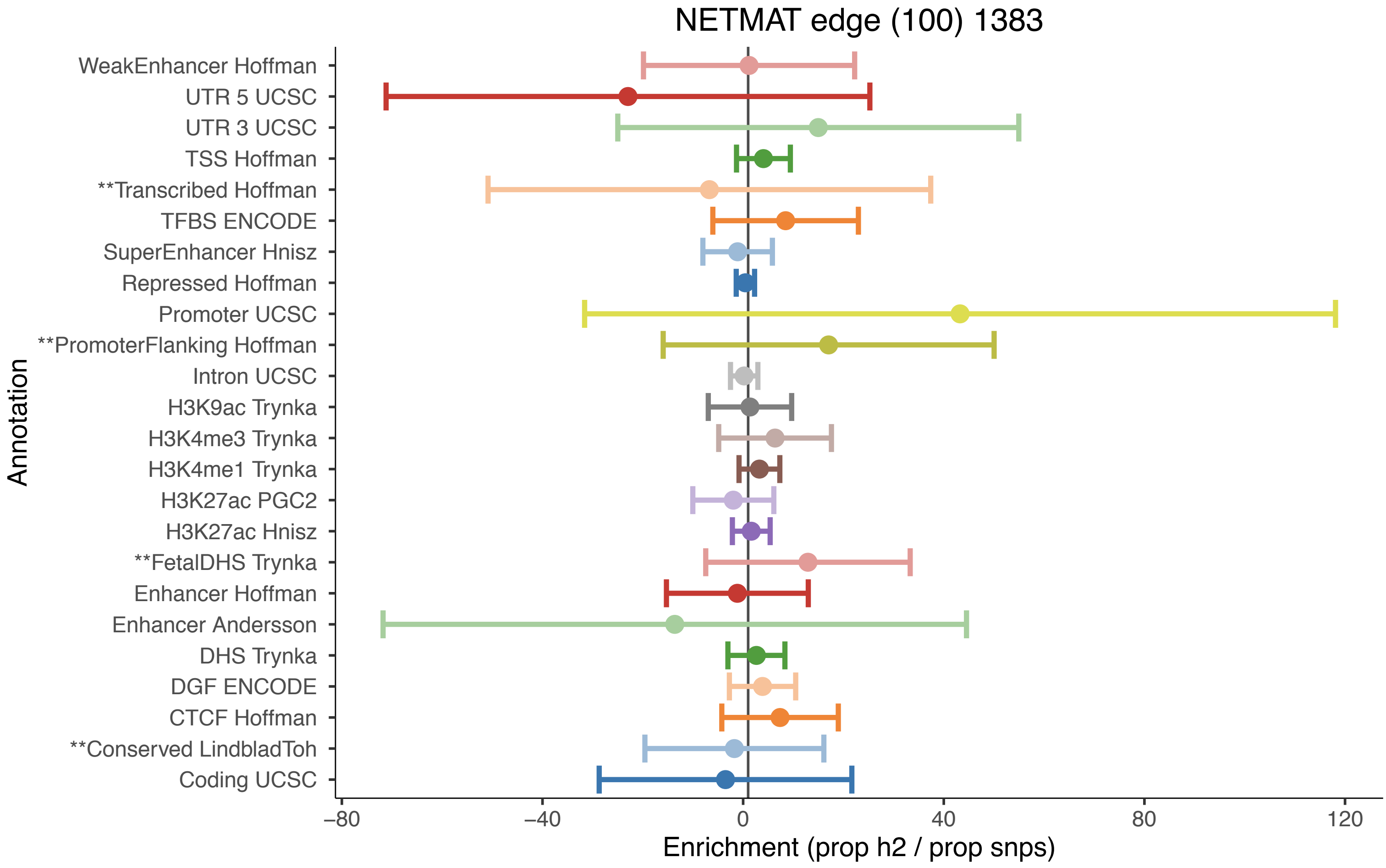
NETMAT edge (100) 1383
WeakEnhancer Hoffman
UTR 5 UCSC
UTR 3 UCSC
TSS Hoffman
**Transcribed Hoffman
TFBS ENCODE
SuperEnhancer Hnisz
Repressed Hoffman
Promoter UCSC
**PromoterFlanking Hoffman
Intron UCSC
H3K9ac Trynka
H3K4me3 Trynka
H3K4me1 Trynka
H3K27ac PGC2
H3K27ac Hnisz
**FetalDHS Trynka
Enhancer Hoffman
Enhancer Andersson
DHS Trynka
DGF ENCODE
CTCF Hoffman
**Conserved LindbladToh
Coding UCSC
Annotation
−80
−40
0
40
80
120
Enrichment (prop h2 / prop snps)

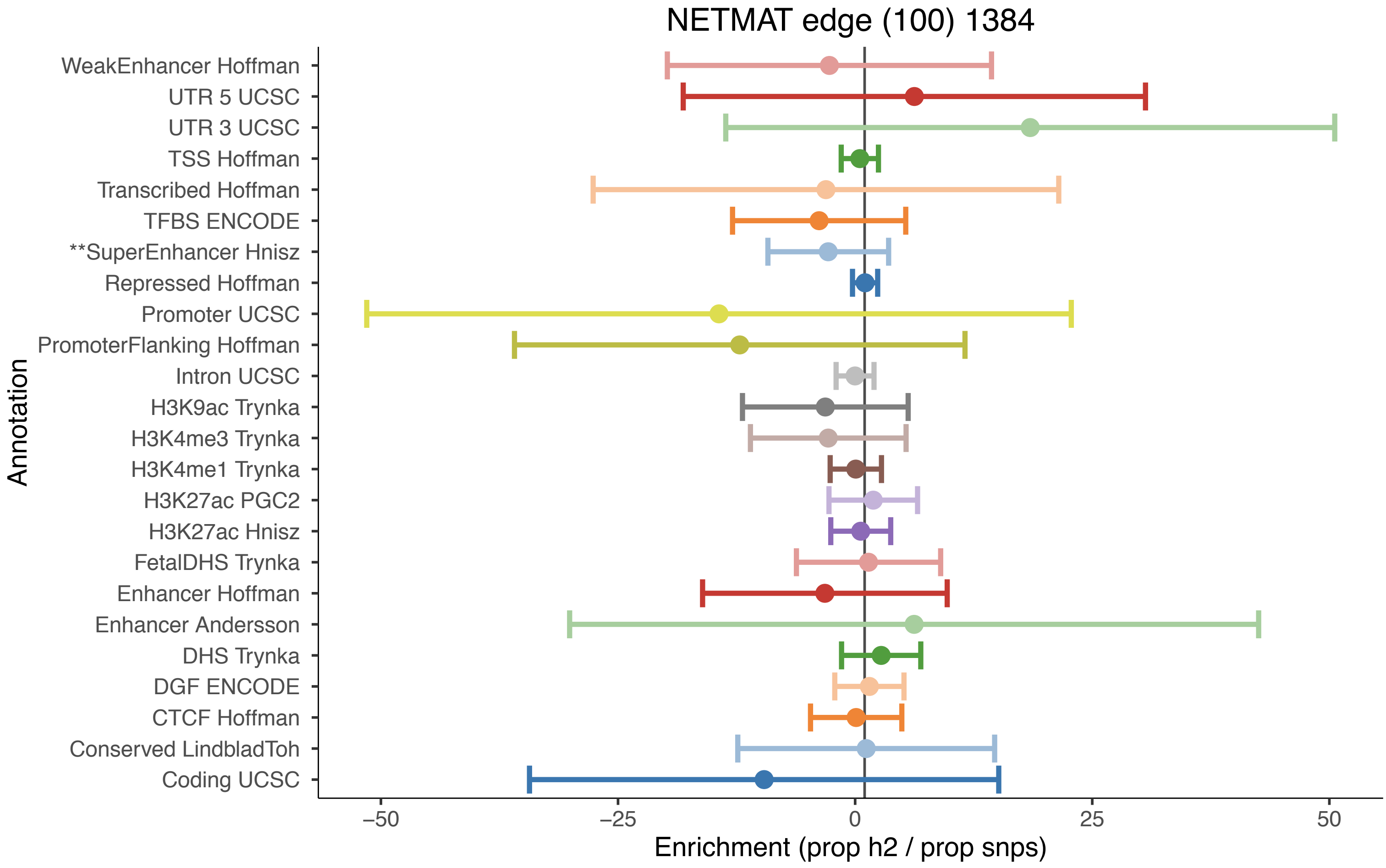

NETMAT edge (100) 1384
WeakEnhancer Hoffman
UTR 5 UCSC
UTR 3 UCSC
TSS Hoffman
Transcribed Hoffman
TFBS ENCODE
**SuperEnhancer Hnisz
Repressed Hoffman
Promoter UCSC
PromoterFlanking Hoffman
Intron UCSC
H3K9ac Trynka
H3K4me3 Trynka
H3K4me1 Trynka
H3K27ac PGC2
H3K27ac Hnisz
FetalDHS Trynka
Enhancer Hoffman
Enhancer Andersson
DHS Trynka
DGF ENCODE
CTCF Hoffman
Conserved LindbladToh
Coding UCSC
Annotation
−50
−25
0
25
50
Enrichment (prop h2 / prop snps)

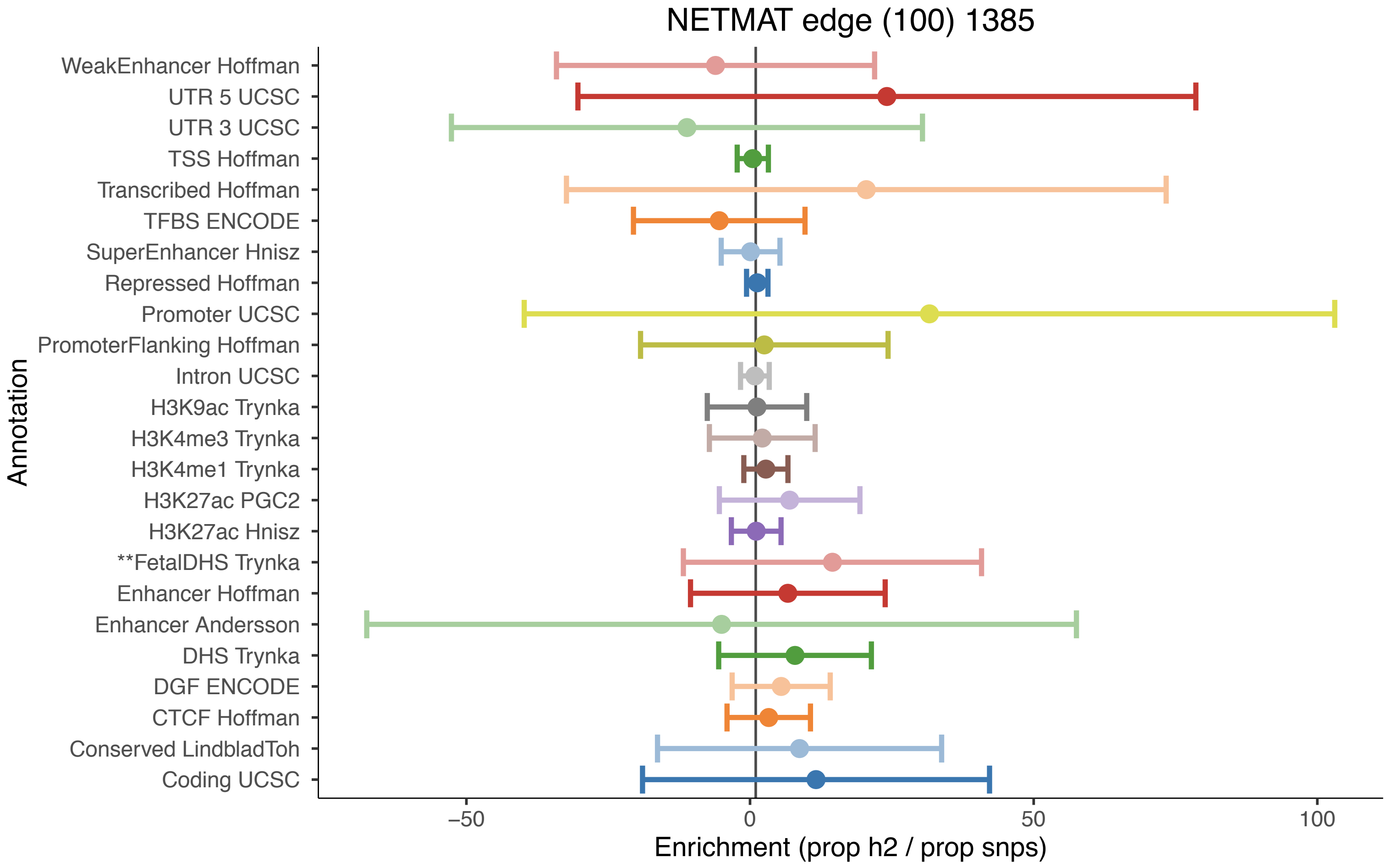

NETMAT edge (100) 1385
WeakEnhancer Hoffman
UTR 5 UCSC
UTR 3 UCSC
TSS Hoffman
Transcribed Hoffman
TFBS ENCODE
SuperEnhancer Hnisz
Repressed Hoffman
Promoter UCSC
PromoterFlanking Hoffman
Intron UCSC
H3K9ac Trynka
H3K4me3 Trynka
H3K4me1 Trynka
H3K27ac PGC2
H3K27ac Hnisz
**FetalDHS Trynka
Enhancer Hoffman
Enhancer Andersson
DHS Trynka
DGF ENCODE
CTCF Hoffman
Conserved LindbladToh
Coding UCSC
Annotation
−50
0
50
100
Enrichment (prop h2 / prop snps)

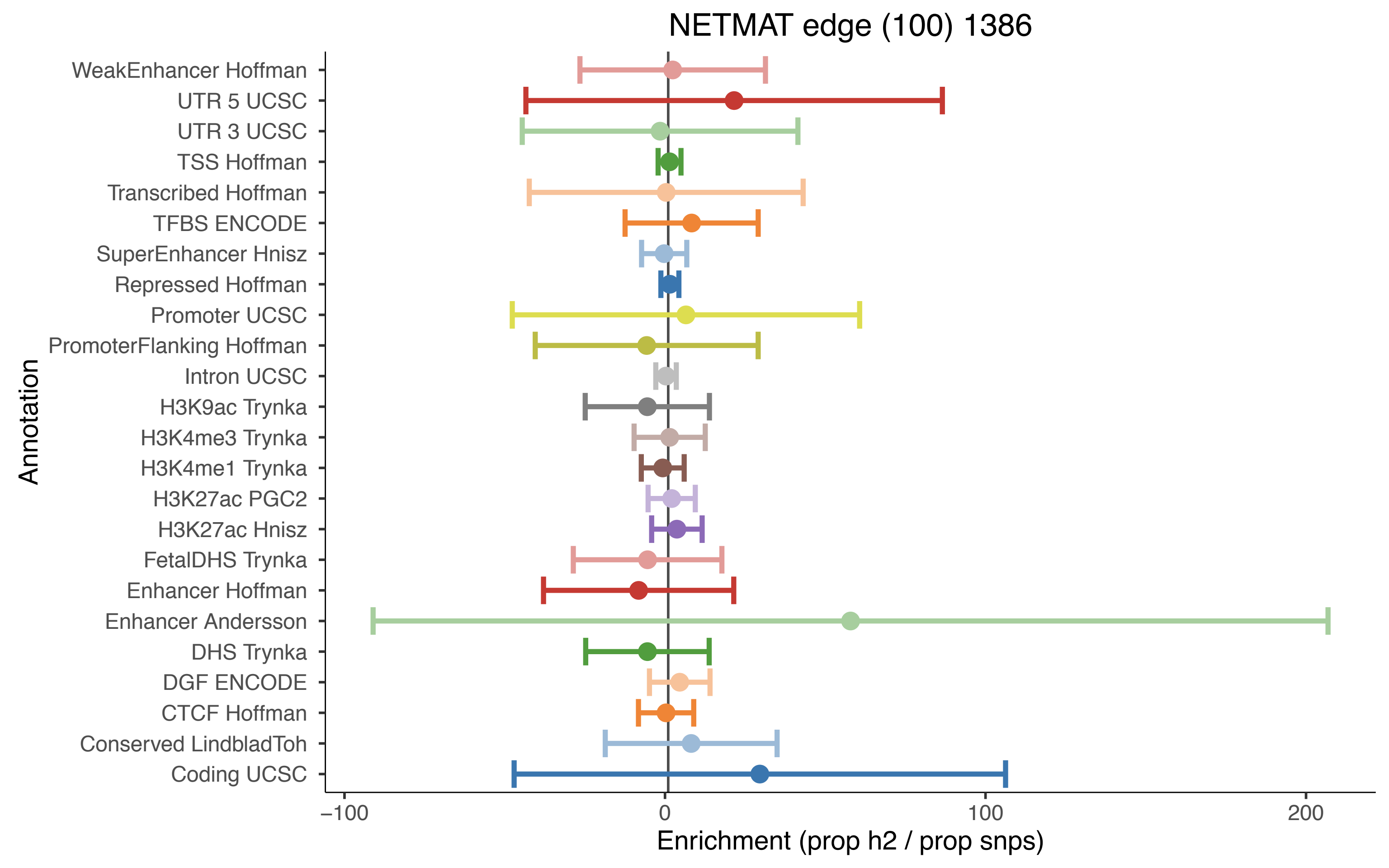

NETMAT edge (100) 1386
WeakEnhancer Hoffman
UTR 5 UCSC
UTR 3 UCSC
TSS Hoffman
Transcribed Hoffman
TFBS ENCODE
SuperEnhancer Hnisz
Repressed Hoffman
Promoter UCSC
PromoterFlanking Hoffman
Intron UCSC
H3K9ac Trynka
H3K4me3 Trynka
H3K4me1 Trynka
H3K27ac PGC2
H3K27ac Hnisz
FetalDHS Trynka
Enhancer Hoffman
Enhancer Andersson
DHS Trynka
DGF ENCODE
CTCF Hoffman
Conserved LindbladToh
Coding UCSC
Annotation
−100
0
100
200
Enrichment (prop h2 / prop snps)

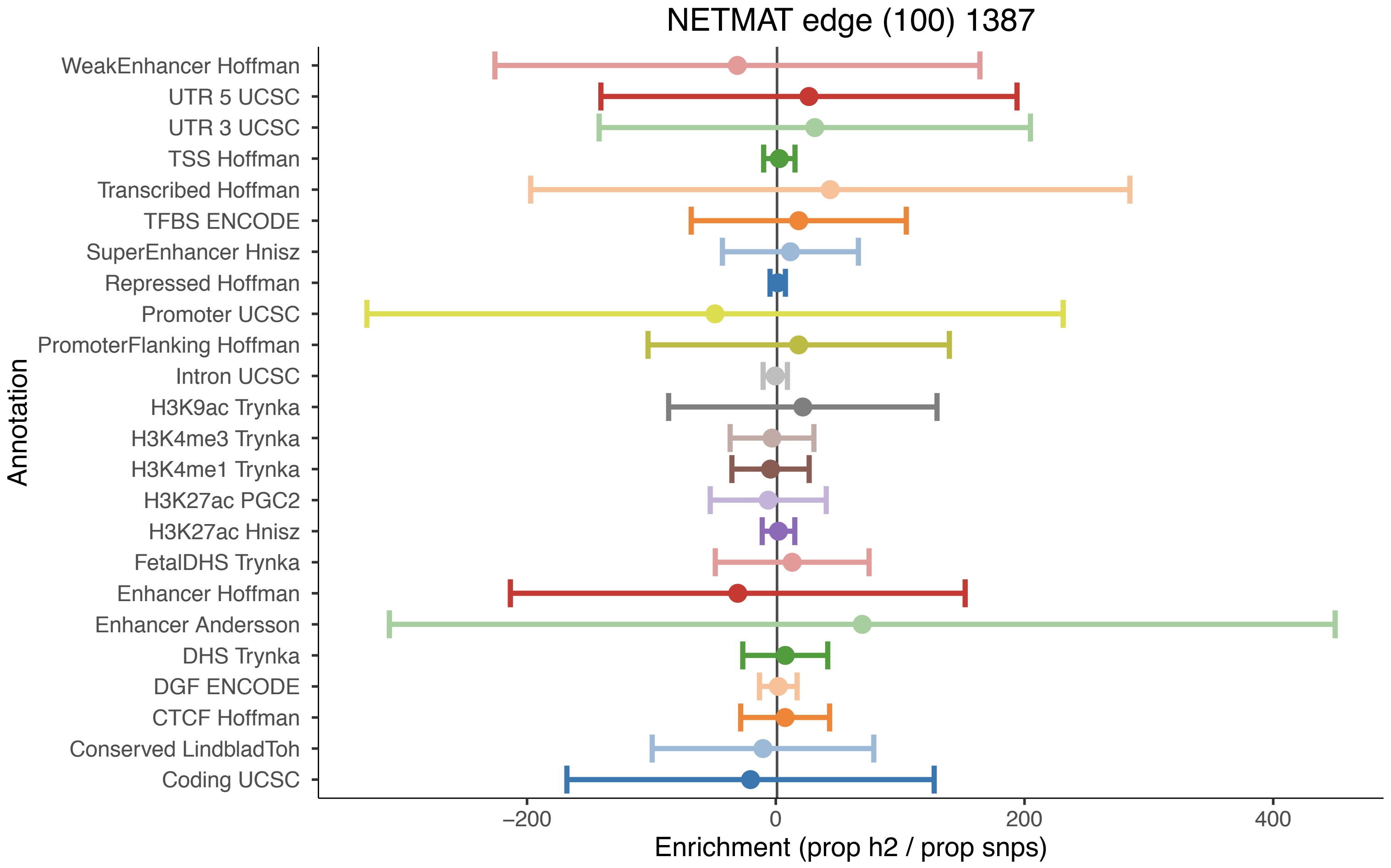
NETMAT edge (100) 1387
WeakEnhancer Hoffman
UTR 5 UCSC
UTR 3 UCSC
TSS Hoffman
Transcribed Hoffman
TFBS ENCODE
SuperEnhancer Hnisz
Repressed Hoffman
Promoter UCSC
PromoterFlanking Hoffman
Intron UCSC
H3K9ac Trynka
H3K4me3 Trynka
H3K4me1 Trynka
H3K27ac PGC2
H3K27ac Hnisz
FetalDHS Trynka
Enhancer Hoffman
Enhancer Andersson
DHS Trynka
DGF ENCODE
CTCF Hoffman
Conserved LindbladToh
Coding UCSC
Annotation
−200
0
200
400
Enrichment (prop h2 / prop snps)

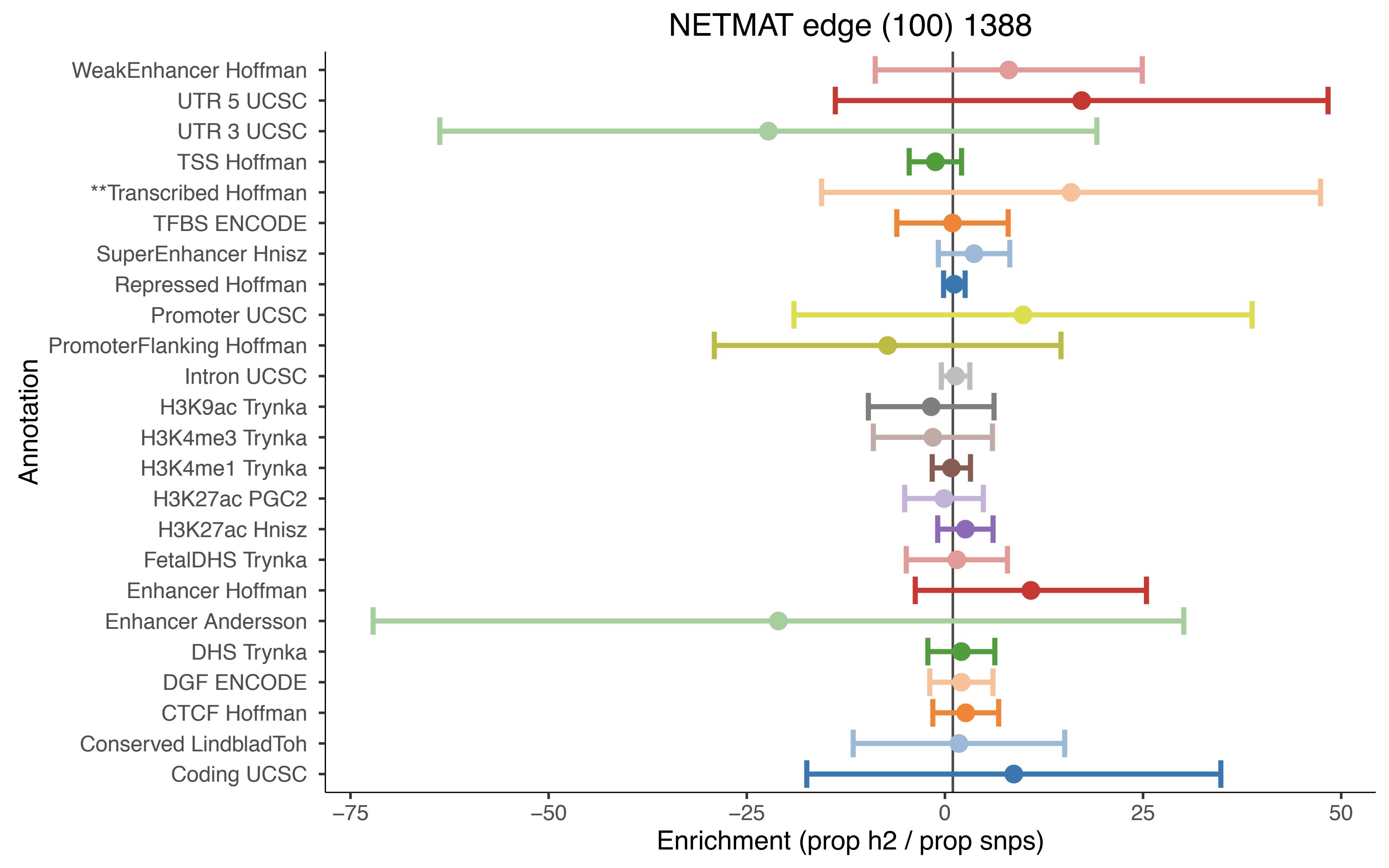
NETMAT edge (100) 1388
WeakEnhancer Hoffman
UTR 5 UCSC
UTR 3 UCSC
TSS Hoffman
**Transcribed Hoffman
TFBS ENCODE
SuperEnhancer Hnisz
Repressed Hoffman
Promoter UCSC
PromoterFlanking Hoffman
Intron UCSC
H3K9ac Trynka
H3K4me3 Trynka
H3K4me1 Trynka
H3K27ac PGC2
H3K27ac Hnisz
FetalDHS Trynka
Enhancer Hoffman
Enhancer Andersson
DHS Trynka
DGF ENCODE
CTCF Hoffman
Conserved LindbladToh
Coding UCSC
Annotation
−75
−50
−25
0
25
50
Enrichment (prop h2 / prop snps)

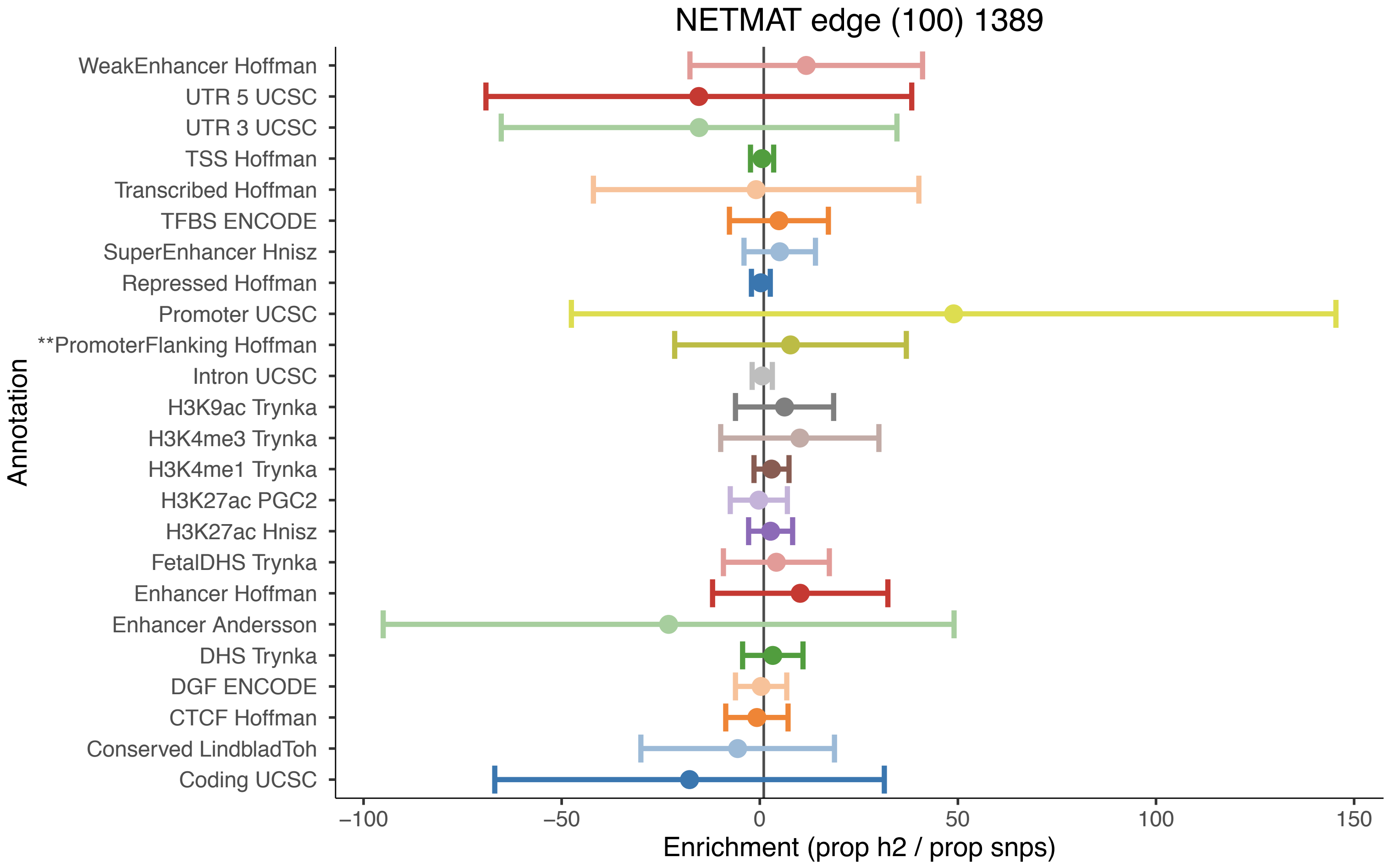
NETMAT edge (100) 1389
WeakEnhancer Hoffman
UTR 5 UCSC
UTR 3 UCSC
TSS Hoffman
Transcribed Hoffman
TFBS ENCODE
SuperEnhancer Hnisz
Repressed Hoffman
Promoter UCSC
**PromoterFlanking Hoffman
Intron UCSC
H3K9ac Trynka
H3K4me3 Trynka
H3K4me1 Trynka
H3K27ac PGC2
H3K27ac Hnisz
FetalDHS Trynka
Enhancer Hoffman
Enhancer Andersson
DHS Trynka
DGF ENCODE
CTCF Hoffman
Conserved LindbladToh
Coding UCSC
Annotation
−100
−50
0
50
100
150
Enrichment (prop h2 / prop snps)

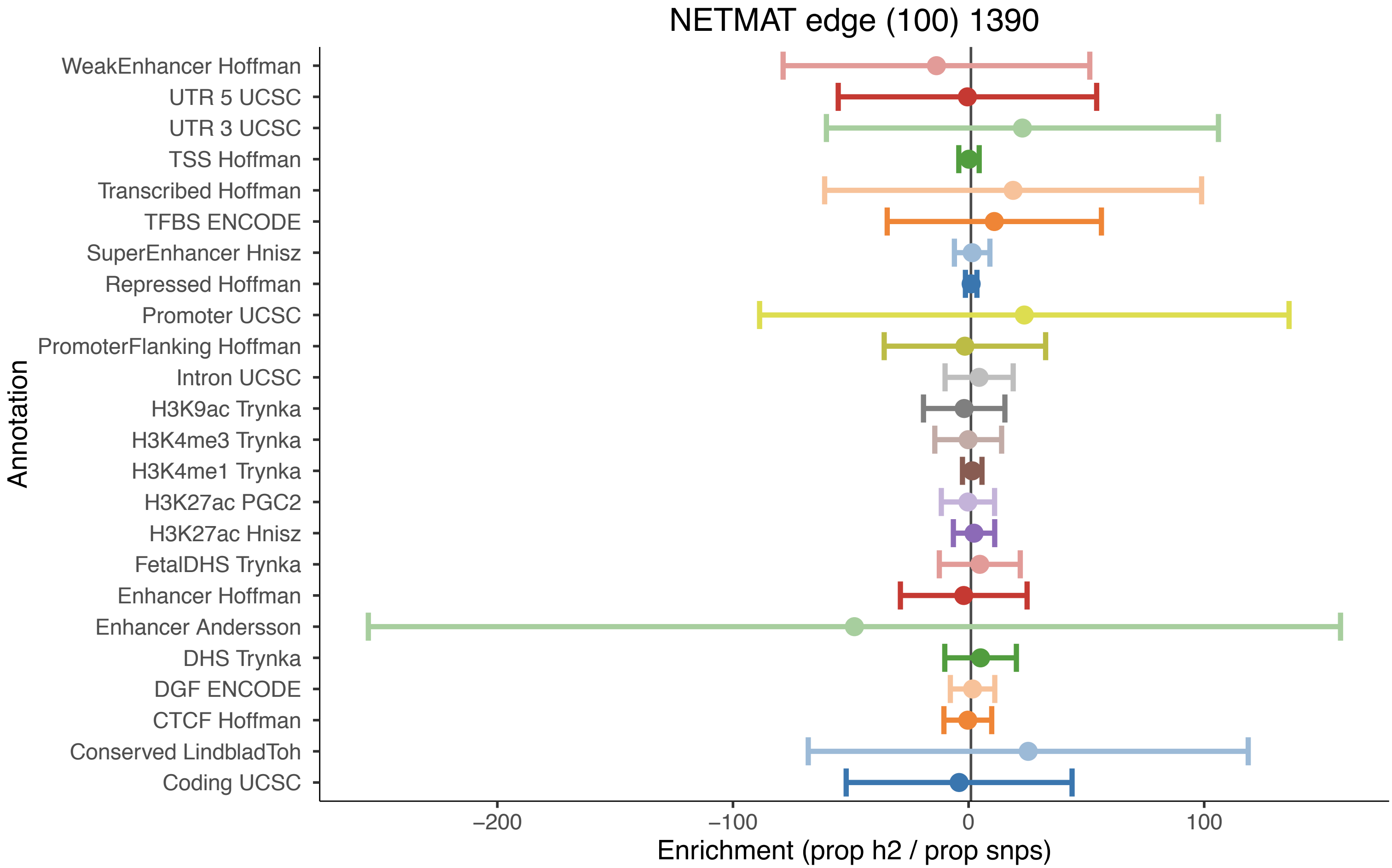

NETMAT edge (100) 1390
WeakEnhancer Hoffman
UTR 5 UCSC
UTR 3 UCSC
TSS Hoffman
Transcribed Hoffman
TFBS ENCODE
SuperEnhancer Hnisz
Repressed Hoffman
Promoter UCSC
PromoterFlanking Hoffman
Intron UCSC
H3K9ac Trynka
H3K4me3 Trynka
H3K4me1 Trynka
H3K27ac PGC2
H3K27ac Hnisz
FetalDHS Trynka
Enhancer Hoffman
Enhancer Andersson
DHS Trynka
DGF ENCODE
CTCF Hoffman
Conserved LindbladToh
Coding UCSC
Annotation
−200
−100
0
100
Enrichment (prop h2 / prop snps)

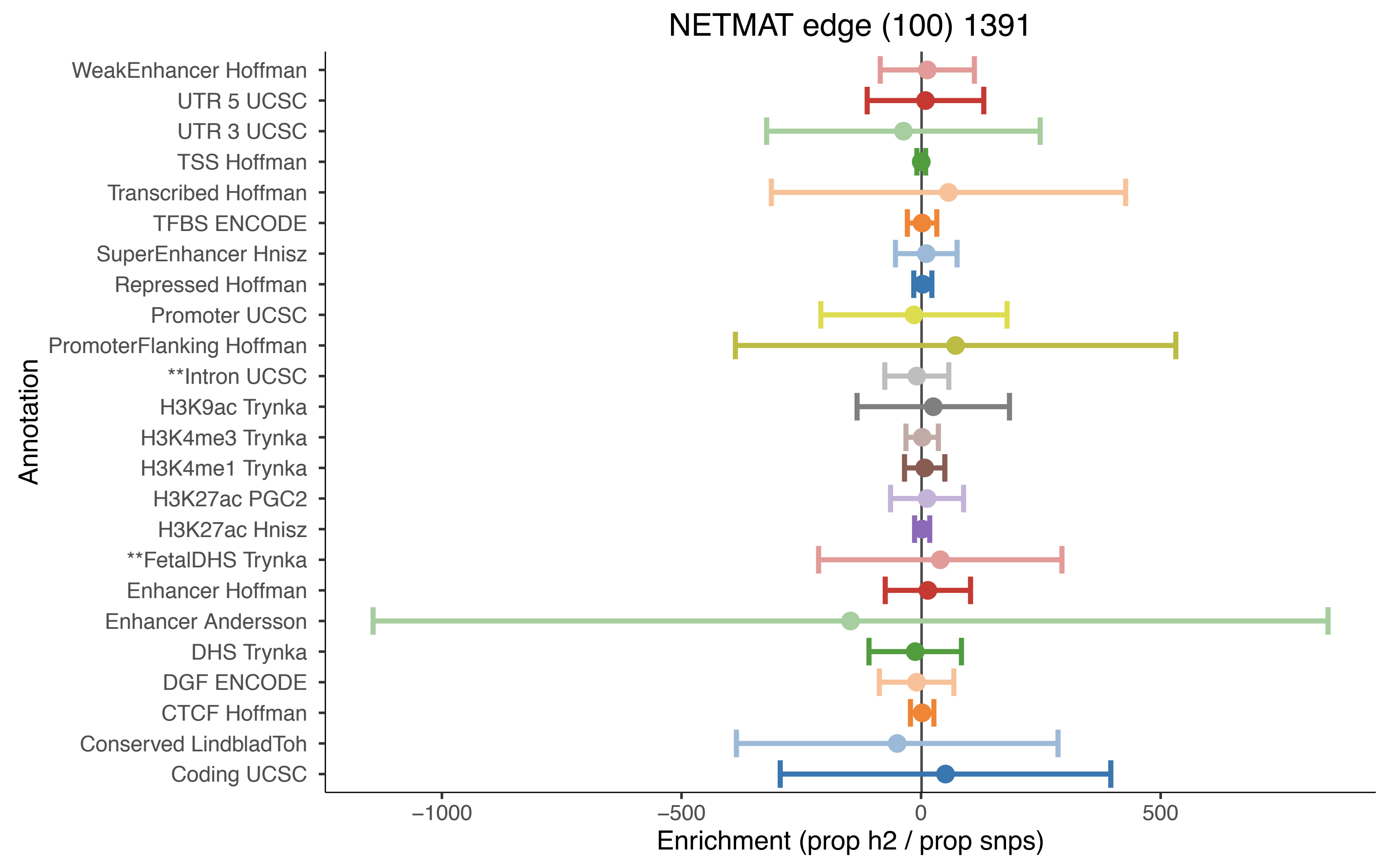
NETMAT edge (100) 1391
WeakEnhancer Hoffman
UTR 5 UCSC
UTR 3 UCSC
TSS Hoffman
Transcribed Hoffman
TFBS ENCODE
SuperEnhancer Hnisz
Repressed Hoffman
Promoter UCSC
PromoterFlanking Hoffman
**Intron UCSC
H3K9ac Trynka
H3K4me3 Trynka
H3K4me1 Trynka
H3K27ac PGC2
H3K27ac Hnisz
**FetalDHS Trynka
Enhancer Hoffman
Enhancer Andersson
DHS Trynka
DGF ENCODE
CTCF Hoffman
Conserved LindbladToh
Coding UCSC
Annotation
−1000
−500
0
500
Enrichment (prop h2 / prop snps)

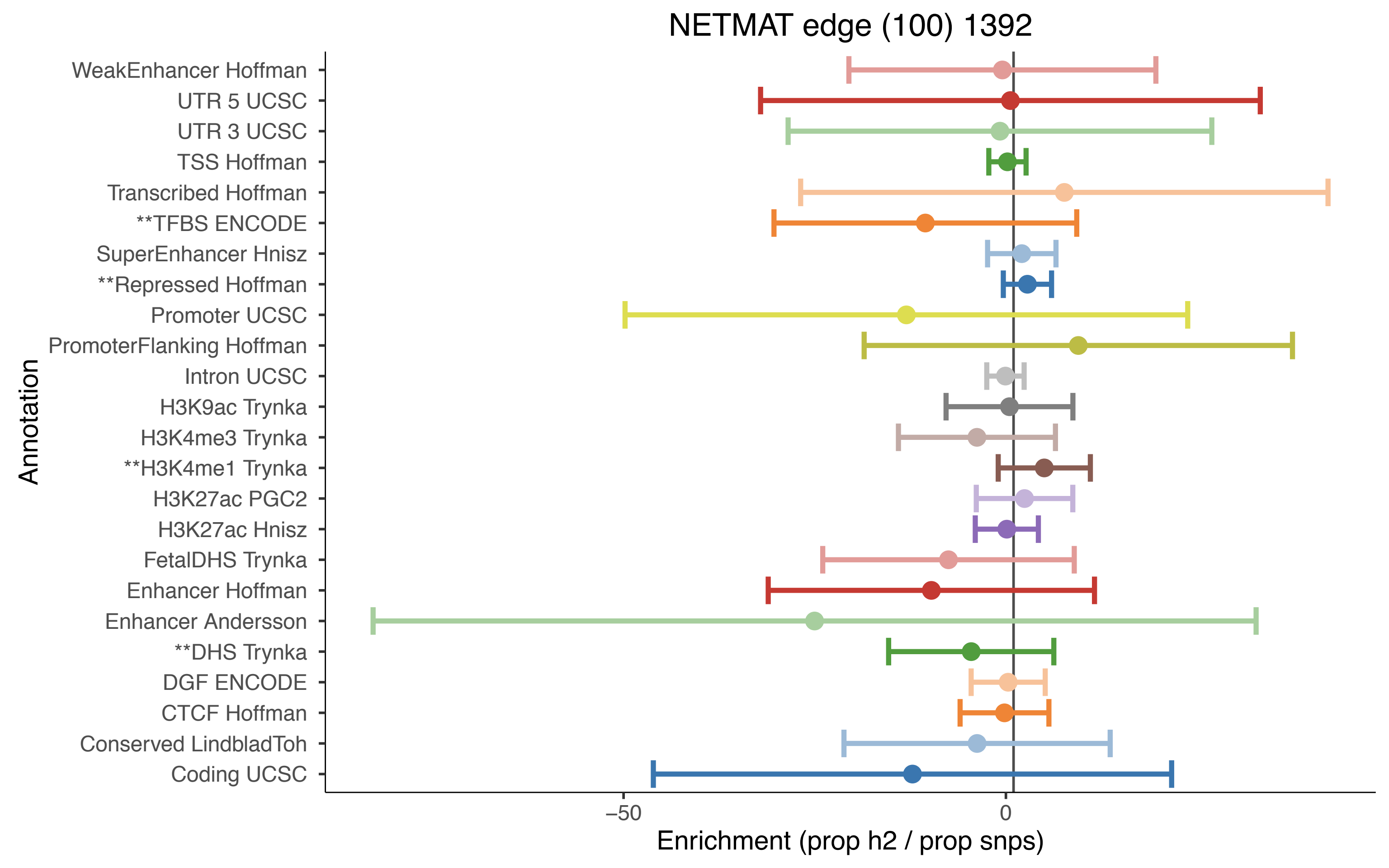
NETMAT edge (100) 1392
Annotation
WeakEnhancer Hoffman
UTR 5 UCSC
UTR 3 UCSC
TSS Hoffman
Transcribed Hoffman
**TFBS ENCODE
SuperEnhancer Hnisz
**Repressed Hoffman
Promoter UCSC
PromoterFlanking Hoffman
Intron UCSC
H3K9ac Trynka
H3K4me3 Trynka
**H3K4me1 Trynka
H3K27ac PGC2
H3K27ac Hnisz
FetalDHS Trynka
Enhancer Hoffman
Enhancer Andersson
**DHS Trynka
DGF ENCODE
CTCF Hoffman
Conserved LindbladToh
Coding UCSC
−50
0
Enrichment (prop h2 / prop snps)

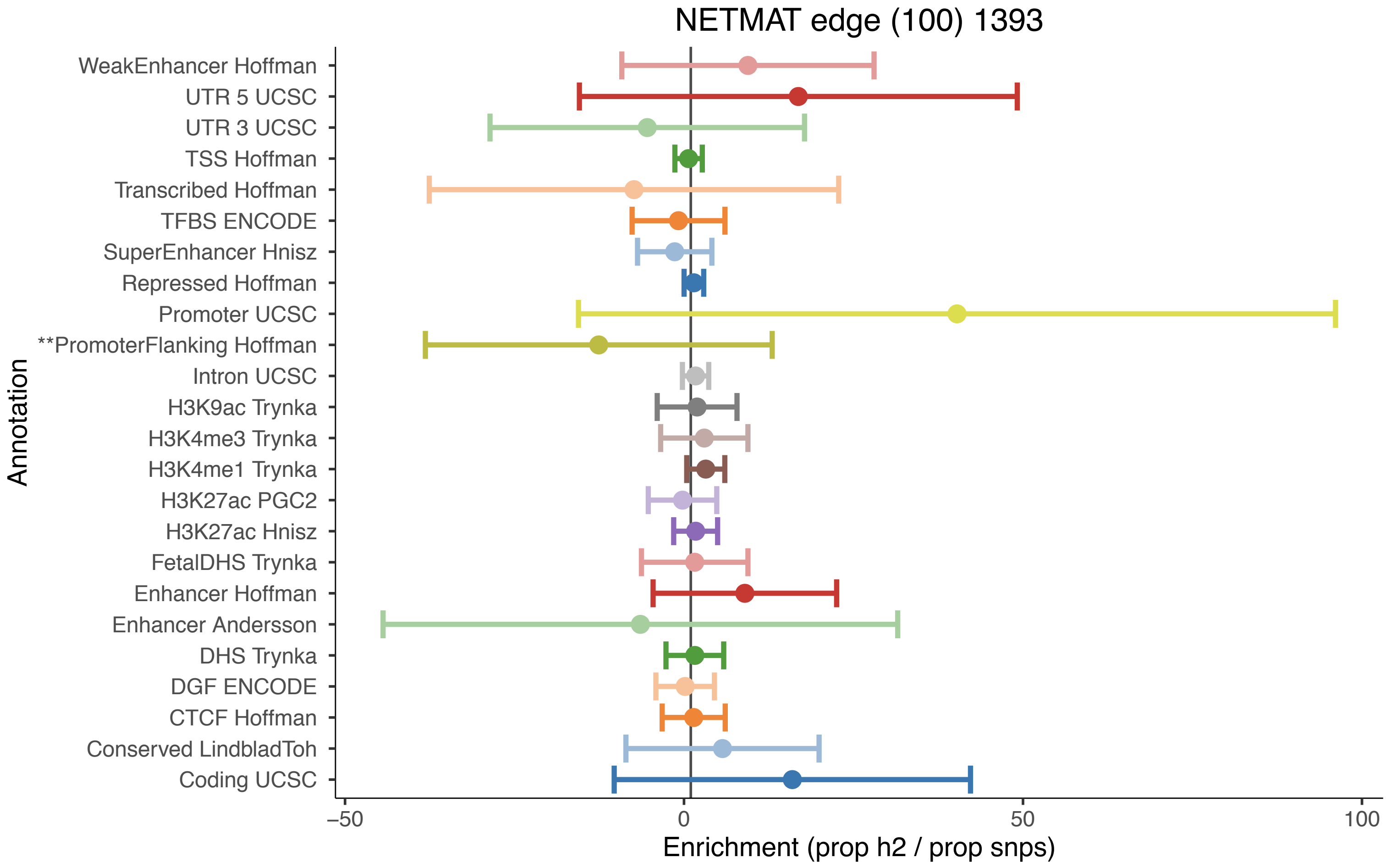

NETMAT edge (100) 1393
WeakEnhancer Hoffman
UTR 5 UCSC
UTR 3 UCSC
TSS Hoffman
Transcribed Hoffman
TFBS ENCODE
SuperEnhancer Hnisz
Repressed Hoffman
Promoter UCSC
**PromoterFlanking Hoffman
Intron UCSC
H3K9ac Trynka
H3K4me3 Trynka
H3K4me1 Trynka
H3K27ac PGC2
H3K27ac Hnisz
FetalDHS Trynka
Enhancer Hoffman
Enhancer Andersson
DHS Trynka
DGF ENCODE
CTCF Hoffman
Conserved LindbladToh
Coding UCSC
Annotation
−50
0
50
100
Enrichment (prop h2 / prop snps)

NETMAT edge (100) 1394

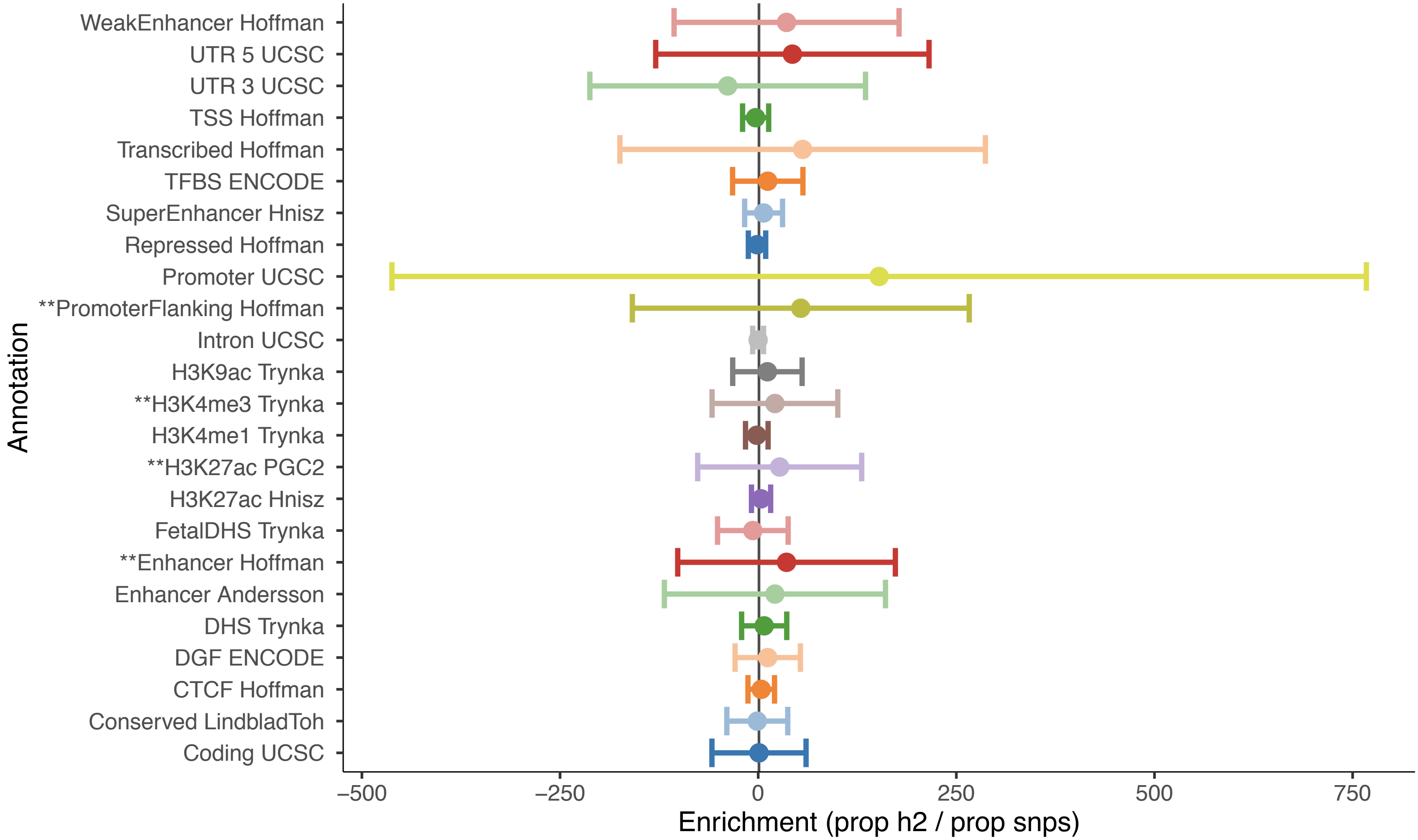

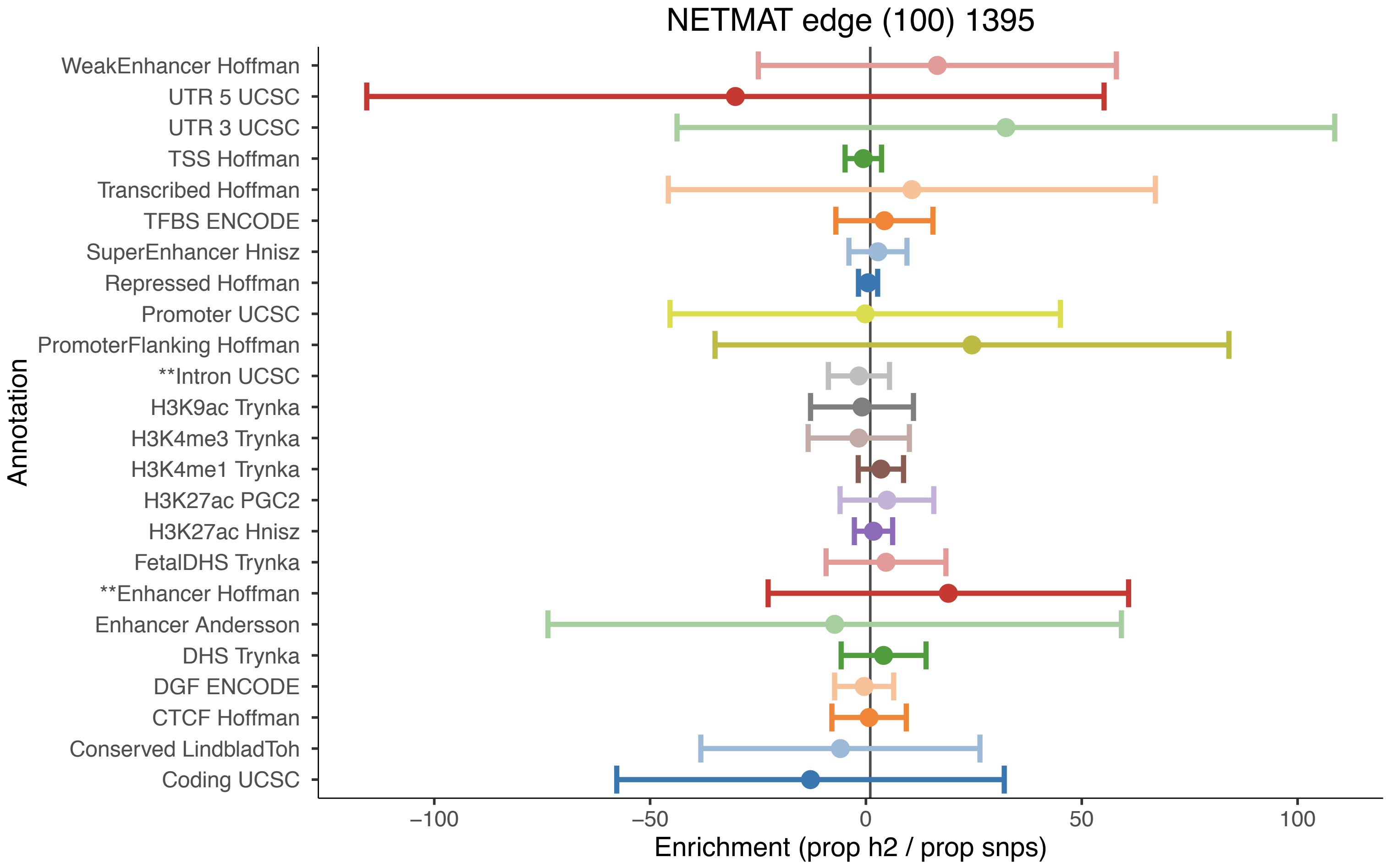

NETMAT edge (100) 1395
WeakEnhancer Hoffman
UTR 5 UCSC
UTR 3 UCSC
TSS Hoffman
Transcribed Hoffman
TFBS ENCODE
SuperEnhancer Hnisz
Repressed Hoffman
Promoter UCSC
PromoterFlanking Hoffman
**Intron UCSC
H3K9ac Trynka
H3K4me3 Trynka
H3K4me1 Trynka
H3K27ac PGC2
H3K27ac Hnisz
FetalDHS Trynka
**Enhancer Hoffman
Enhancer Andersson
DHS Trynka
DGF ENCODE
CTCF Hoffman
Conserved LindbladToh
Coding UCSC
Annotation
−100
−50
0
50
100
Enrichment (prop h2 / prop snps)

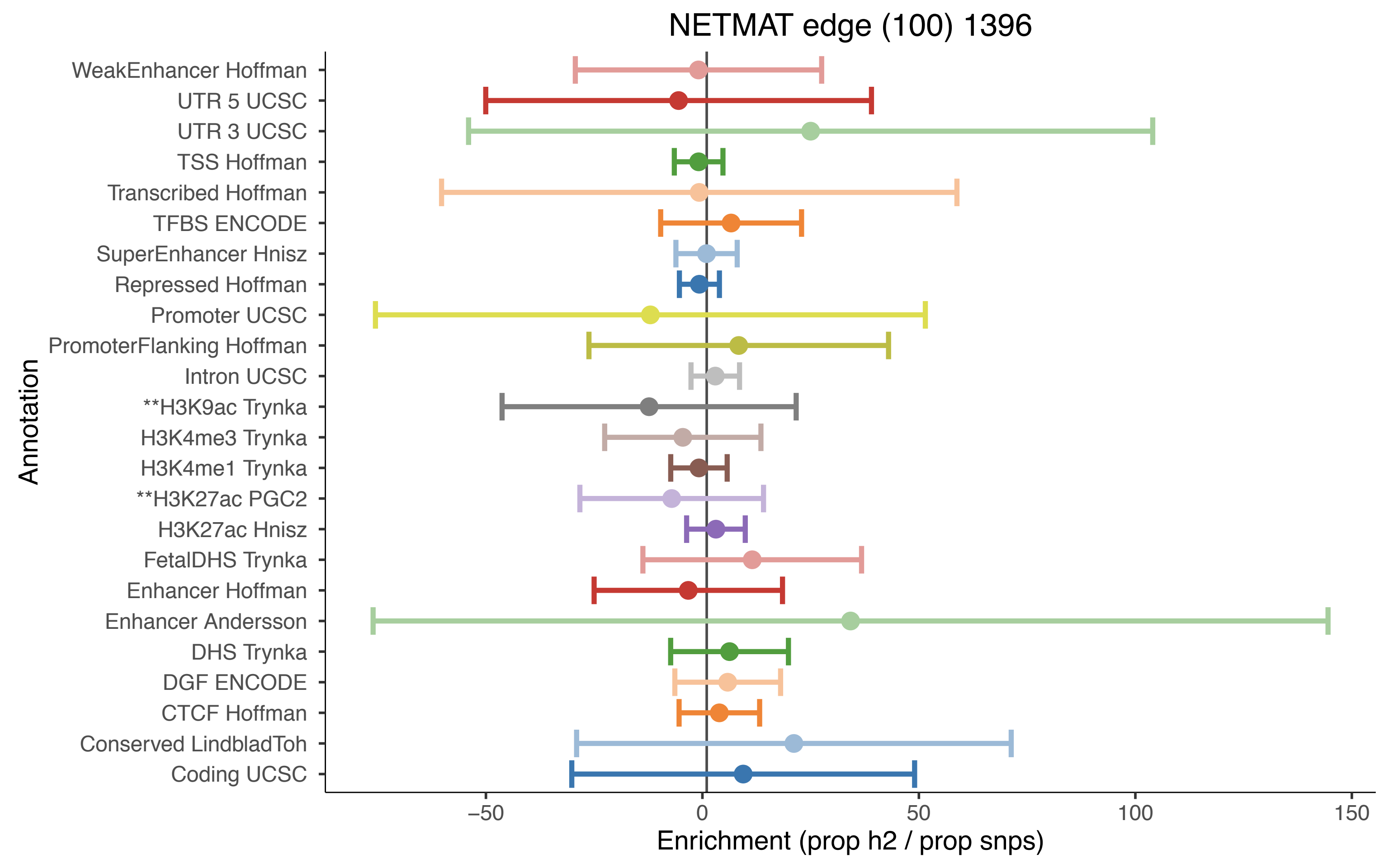

NETMAT edge (100) 1396
WeakEnhancer Hoffman
UTR 5 UCSC
UTR 3 UCSC
TSS Hoffman
Transcribed Hoffman
TFBS ENCODE
SuperEnhancer Hnisz
Repressed Hoffman
Promoter UCSC
PromoterFlanking Hoffman
Intron UCSC
**H3K9ac Trynka
H3K4me3 Trynka
H3K4me1 Trynka
**H3K27ac PGC2
H3K27ac Hnisz
FetalDHS Trynka
Enhancer Hoffman
Enhancer Andersson
DHS Trynka
DGF ENCODE
CTCF Hoffman
Conserved LindbladToh
Coding UCSC
Annotation
−50
0
50
100
150
Enrichment (prop h2 / prop snps)

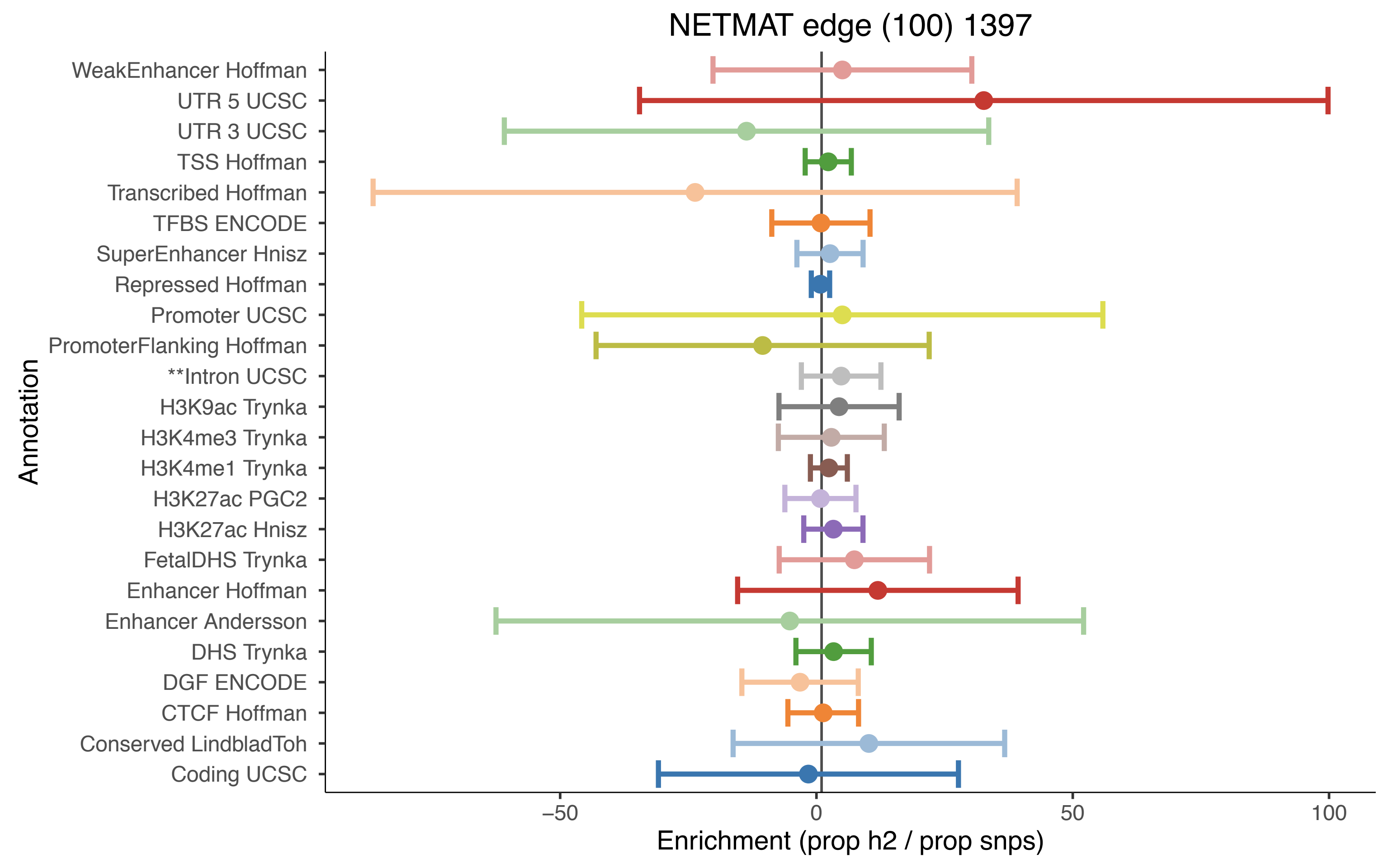

NETMAT edge (100) 1397
WeakEnhancer Hoffman
UTR 5 UCSC
UTR 3 UCSC
TSS Hoffman
Transcribed Hoffman
TFBS ENCODE
SuperEnhancer Hnisz
Repressed Hoffman
Promoter UCSC
PromoterFlanking Hoffman
**Intron UCSC
H3K9ac Trynka
H3K4me3 Trynka
H3K4me1 Trynka
H3K27ac PGC2
H3K27ac Hnisz
FetalDHS Trynka
Enhancer Hoffman
Enhancer Andersson
DHS Trynka
DGF ENCODE
CTCF Hoffman
Conserved LindbladToh
Coding UCSC
Annotation
−50
0
50
100
Enrichment (prop h2 / prop snps)

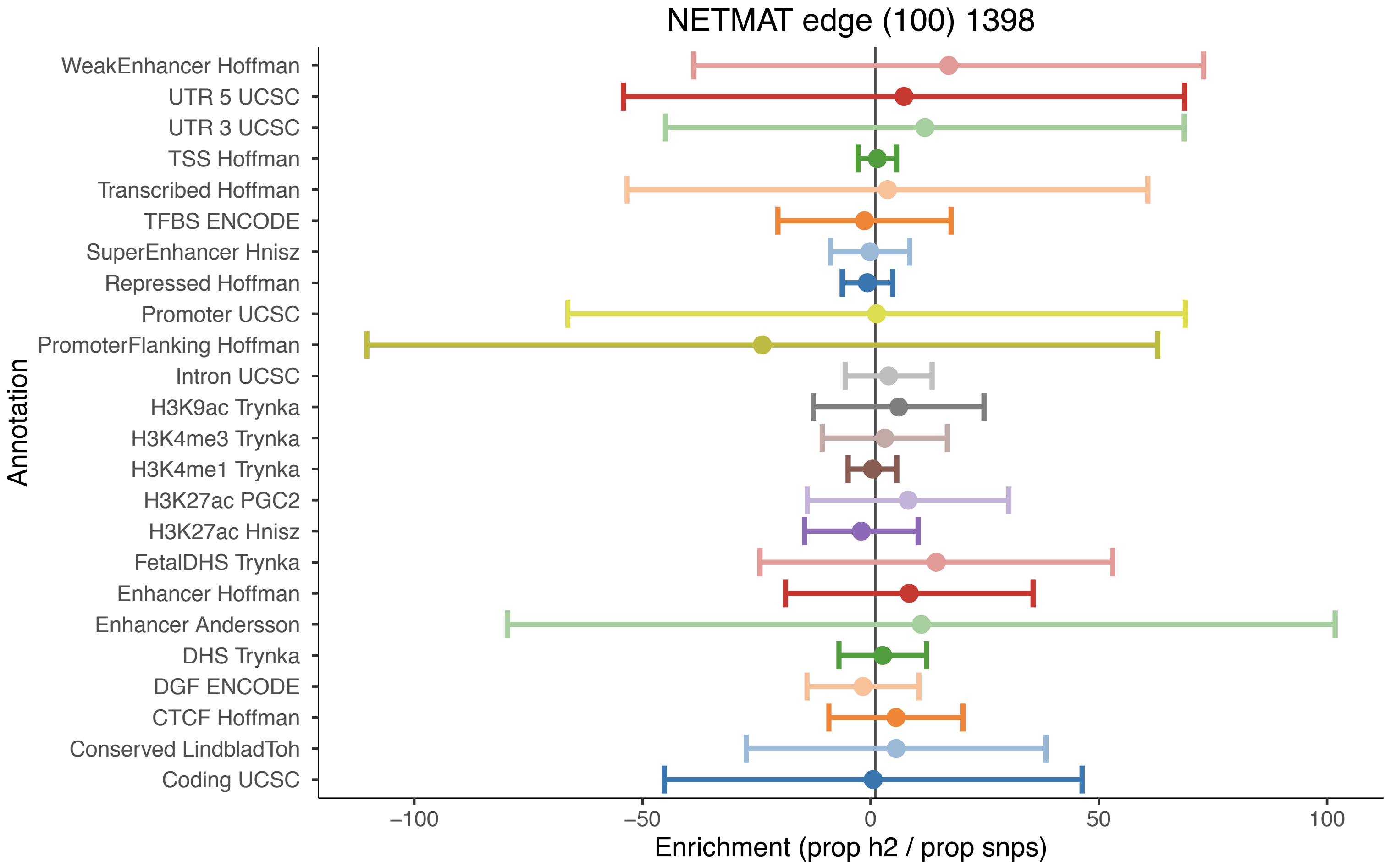

NETMAT edge (100) 1398
Annotation
WeakEnhancer Hoffman
UTR 5 UCSC
UTR 3 UCSC
TSS Hoffman
Transcribed Hoffman
TFBS ENCODE
SuperEnhancer Hnisz
Repressed Hoffman
Promoter UCSC
PromoterFlanking Hoffman
Intron UCSC
H3K9ac Trynka
H3K4me3 Trynka
H3K4me1 Trynka
H3K27ac PGC2
H3K27ac Hnisz
FetalDHS Trynka
Enhancer Hoffman
Enhancer Andersson
DHS Trynka
DGF ENCODE
CTCF Hoffman
Conserved LindbladToh
Coding UCSC
−100
−50
0
50
100
Enrichment (prop h2 / prop snps)

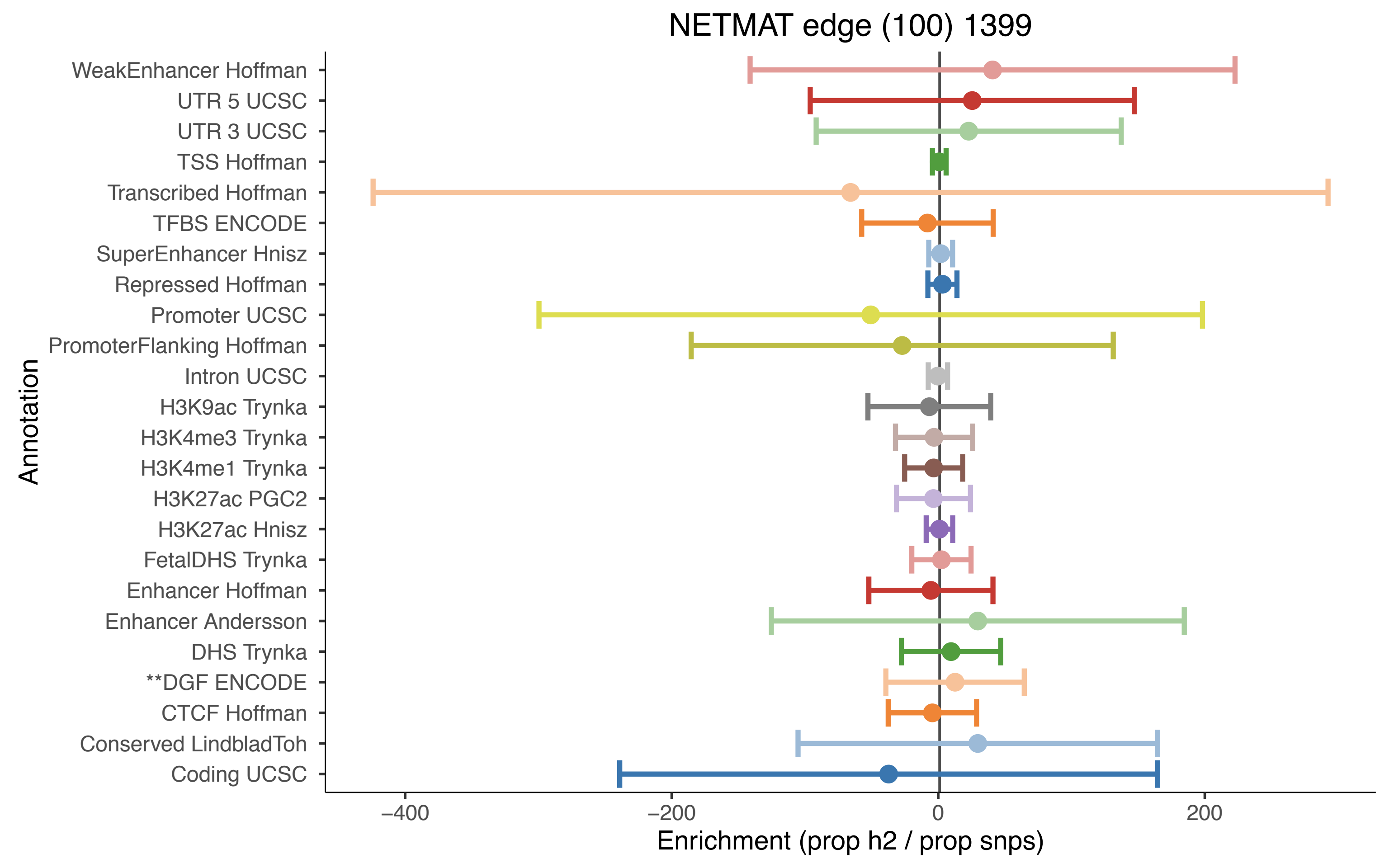

NETMAT edge (100) 1399
WeakEnhancer Hoffman
UTR 5 UCSC
UTR 3 UCSC
TSS Hoffman
Transcribed Hoffman
TFBS ENCODE
SuperEnhancer Hnisz
Repressed Hoffman
Promoter UCSC
PromoterFlanking Hoffman
Intron UCSC
H3K9ac Trynka
H3K4me3 Trynka
H3K4me1 Trynka
H3K27ac PGC2
H3K27ac Hnisz
FetalDHS Trynka
Enhancer Hoffman
Enhancer Andersson
DHS Trynka
**DGF ENCODE
CTCF Hoffman
Conserved LindbladToh
Coding UCSC
Annotation
−400
−200
0
200
Enrichment (prop h2 / prop snps)

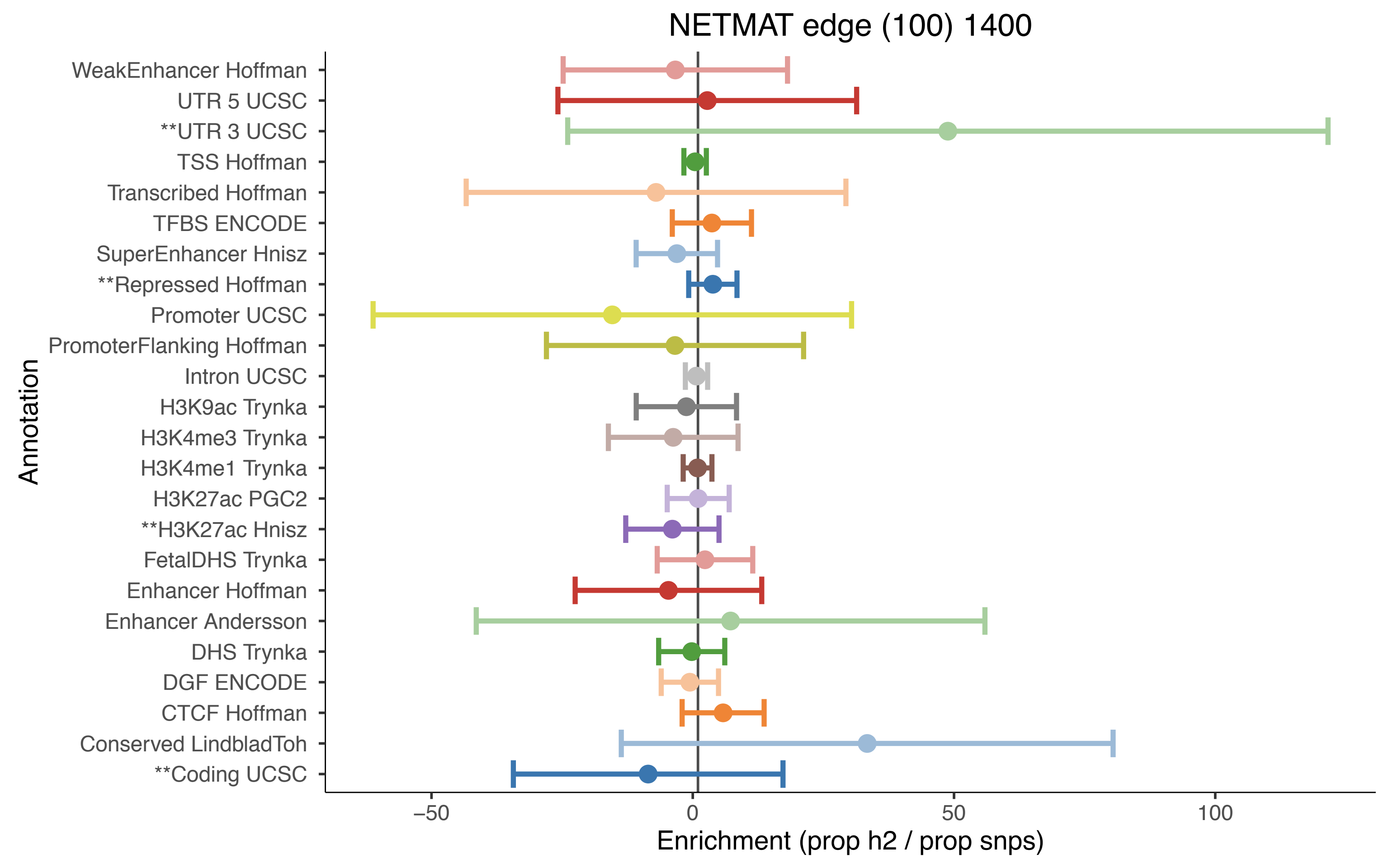
NETMAT edge (100) 1400
Annotation
WeakEnhancer Hoffman
UTR 5 UCSC
**UTR 3 UCSC
TSS Hoffman
Transcribed Hoffman
TFBS ENCODE
SuperEnhancer Hnisz
**Repressed Hoffman
Promoter UCSC
PromoterFlanking Hoffman
Intron UCSC
H3K9ac Trynka
H3K4me3 Trynka
H3K4me1 Trynka
H3K27ac PGC2
**H3K27ac Hnisz
FetalDHS Trynka
Enhancer Hoffman
Enhancer Andersson
DHS Trynka
DGF ENCODE
CTCF Hoffman
Conserved LindbladToh
**Coding UCSC
−50
0
50
100
Enrichment (prop h2 / prop snps)

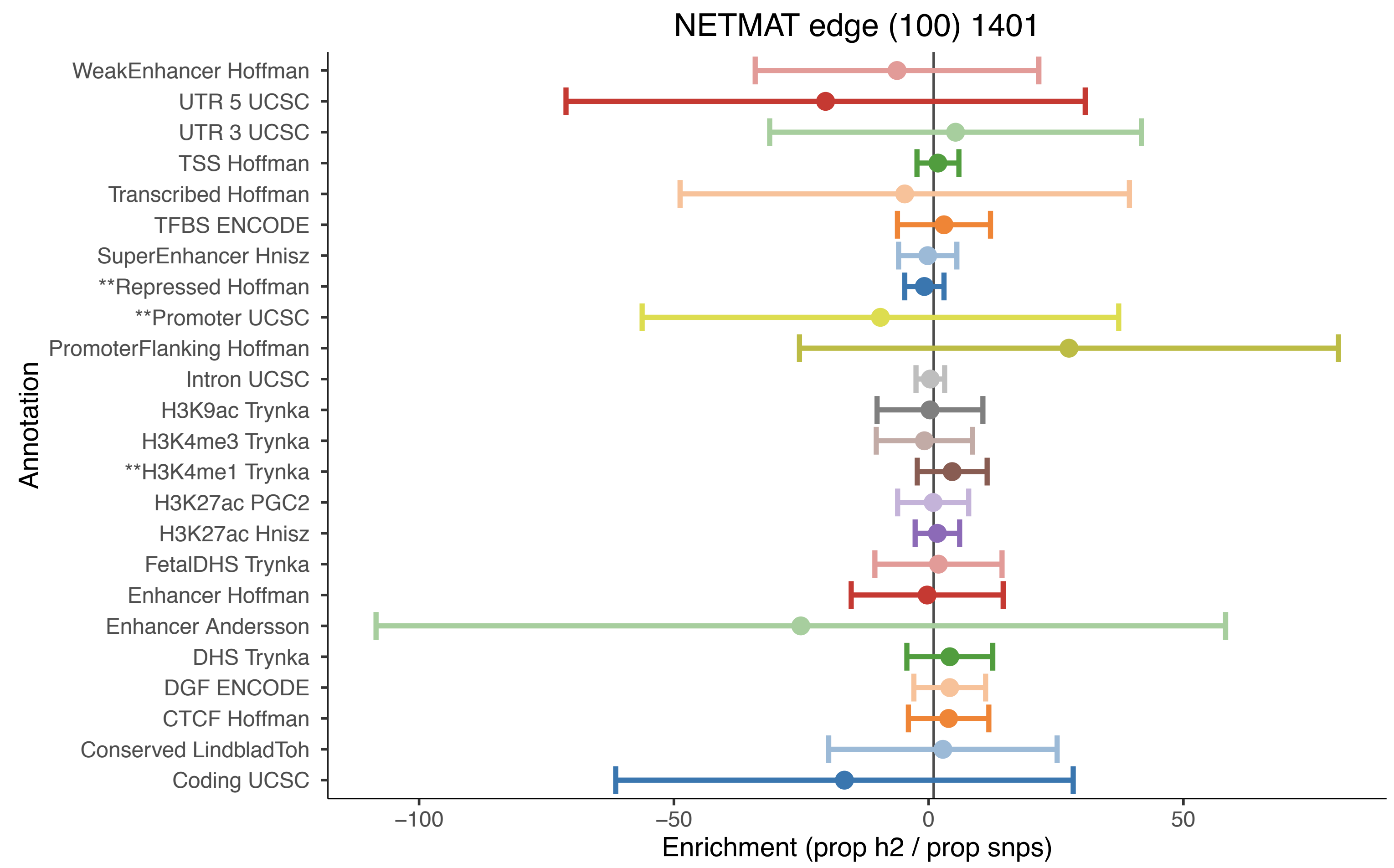

NETMAT edge (100) 1401
WeakEnhancer Hoffman
UTR 5 UCSC
UTR 3 UCSC
TSS Hoffman
Transcribed Hoffman
TFBS ENCODE
SuperEnhancer Hnisz
**Repressed Hoffman
**Promoter UCSC
PromoterFlanking Hoffman
Intron UCSC
H3K9ac Trynka
H3K4me3 Trynka
**H3K4me1 Trynka
H3K27ac PGC2
H3K27ac Hnisz
FetalDHS Trynka
Enhancer Hoffman
Enhancer Andersson
DHS Trynka
DGF ENCODE
CTCF Hoffman
Conserved LindbladToh
Coding UCSC
Annotation
−100
−50
0
50
Enrichment (prop h2 / prop snps)

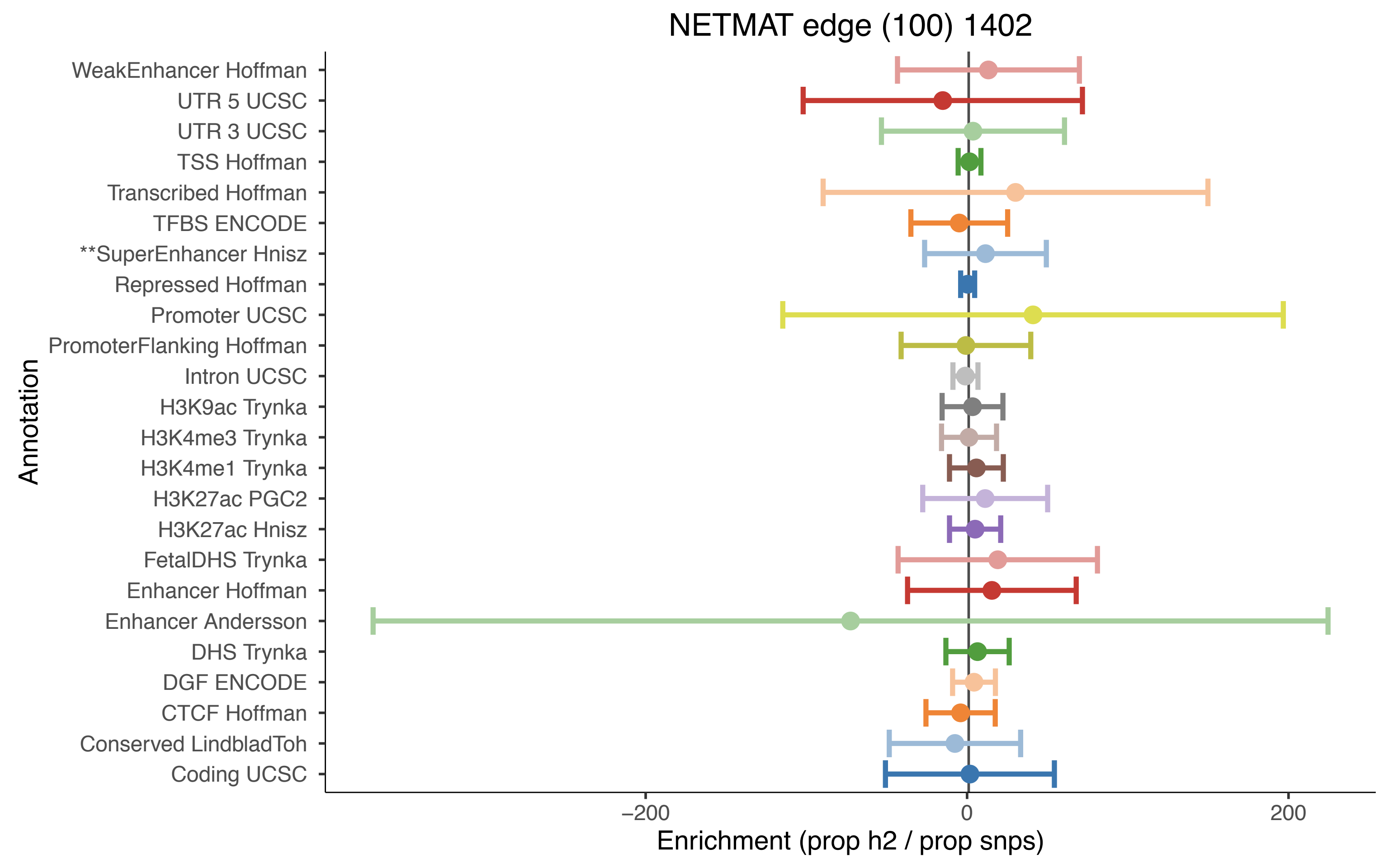
NETMAT edge (100) 1402
WeakEnhancer Hoffman
UTR 5 UCSC
UTR 3 UCSC
TSS Hoffman
Transcribed Hoffman
TFBS ENCODE
**SuperEnhancer Hnisz
Repressed Hoffman
Promoter UCSC
PromoterFlanking Hoffman
Intron UCSC
H3K9ac Trynka
H3K4me3 Trynka
H3K4me1 Trynka
H3K27ac PGC2
H3K27ac Hnisz
FetalDHS Trynka
Enhancer Hoffman
Enhancer Andersson
DHS Trynka
DGF ENCODE
CTCF Hoffman
Conserved LindbladToh
Coding UCSC
Annotation
−200
0
200
Enrichment (prop h2 / prop snps)

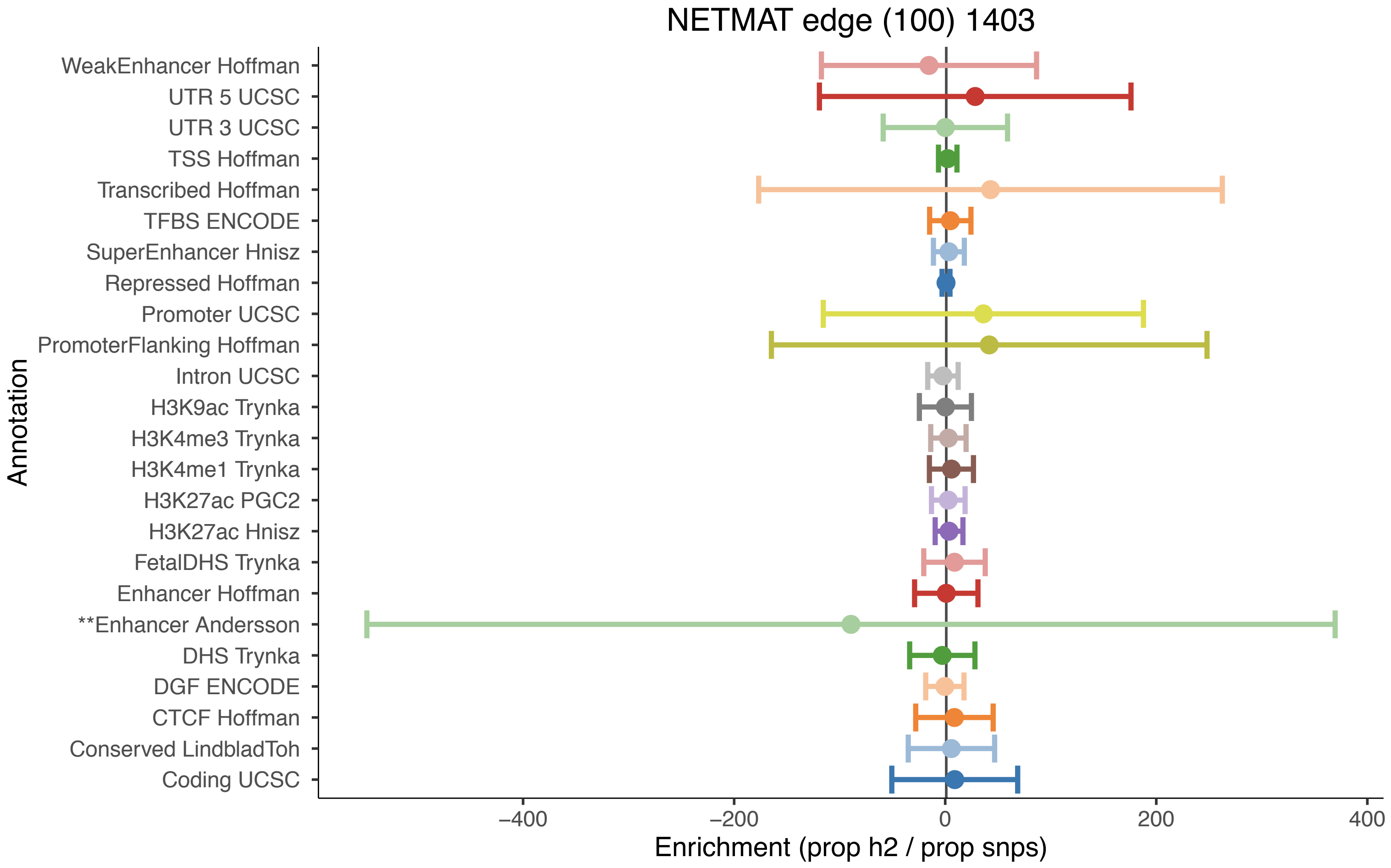

NETMAT edge (100) 1403
WeakEnhancer Hoffman
UTR 5 UCSC
UTR 3 UCSC
TSS Hoffman
Transcribed Hoffman
TFBS ENCODE
SuperEnhancer Hnisz
Repressed Hoffman
Promoter UCSC
PromoterFlanking Hoffman
Intron UCSC
H3K9ac Trynka
H3K4me3 Trynka
H3K4me1 Trynka
H3K27ac PGC2
H3K27ac Hnisz
FetalDHS Trynka
Enhancer Hoffman
**Enhancer Andersson
DHS Trynka
DGF ENCODE
CTCF Hoffman
Conserved LindbladToh
Coding UCSC
Annotation
−400
−200
0
200
400
Enrichment (prop h2 / prop snps)

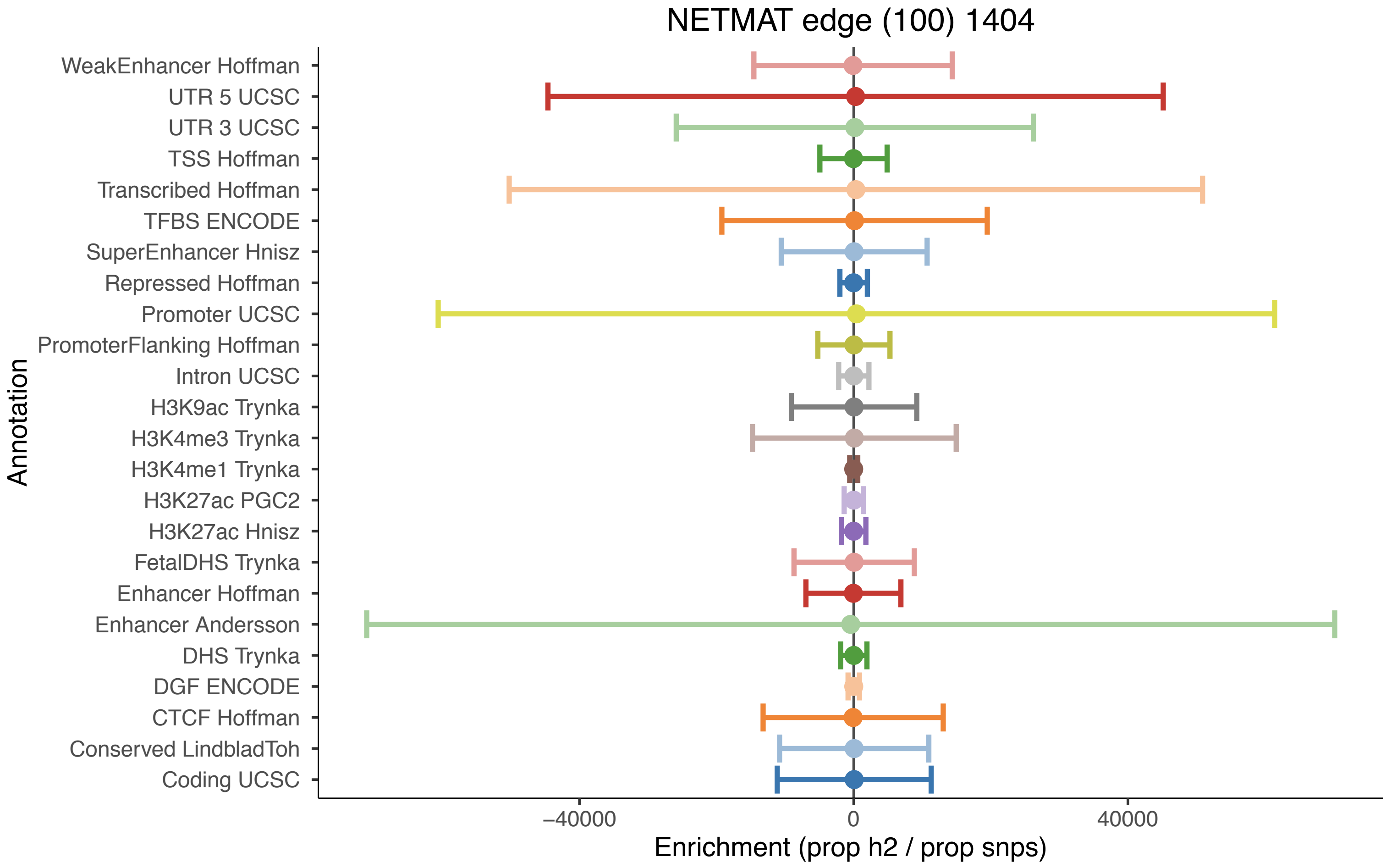

NETMAT edge (100) 1404
WeakEnhancer Hoffman
UTR 5 UCSC
UTR 3 UCSC
TSS Hoffman
Transcribed Hoffman
TFBS ENCODE
SuperEnhancer Hnisz
Repressed Hoffman
Promoter UCSC
PromoterFlanking Hoffman
Intron UCSC
H3K9ac Trynka
H3K4me3 Trynka
H3K4me1 Trynka
H3K27ac PGC2
H3K27ac Hnisz
FetalDHS Trynka
Enhancer Hoffman
Enhancer Andersson
DHS Trynka
DGF ENCODE
CTCF Hoffman
Conserved LindbladToh
Coding UCSC
Annotation
−40000
0
40000
Enrichment (prop h2 / prop snps)

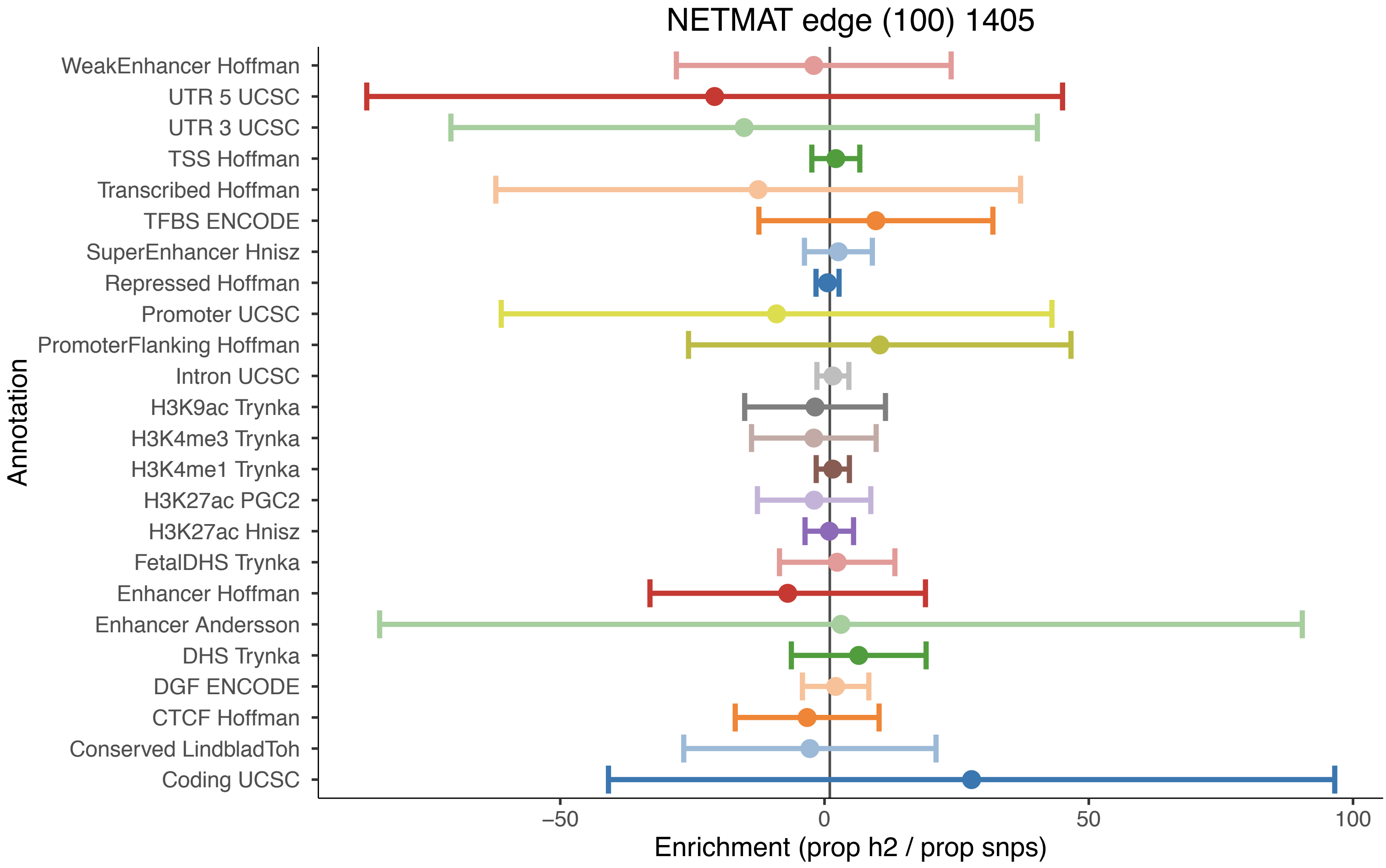

NETMAT edge (100) 1405
WeakEnhancer Hoffman
UTR 5 UCSC
UTR 3 UCSC
TSS Hoffman
Transcribed Hoffman
TFBS ENCODE
SuperEnhancer Hnisz
Repressed Hoffman
Promoter UCSC
PromoterFlanking Hoffman
Intron UCSC
H3K9ac Trynka
H3K4me3 Trynka
H3K4me1 Trynka
H3K27ac PGC2
H3K27ac Hnisz
FetalDHS Trynka
Enhancer Hoffman
Enhancer Andersson
DHS Trynka
DGF ENCODE
CTCF Hoffman
Conserved LindbladToh
Coding UCSC
Annotation
−50
0
50
100
Enrichment (prop h2 / prop snps)

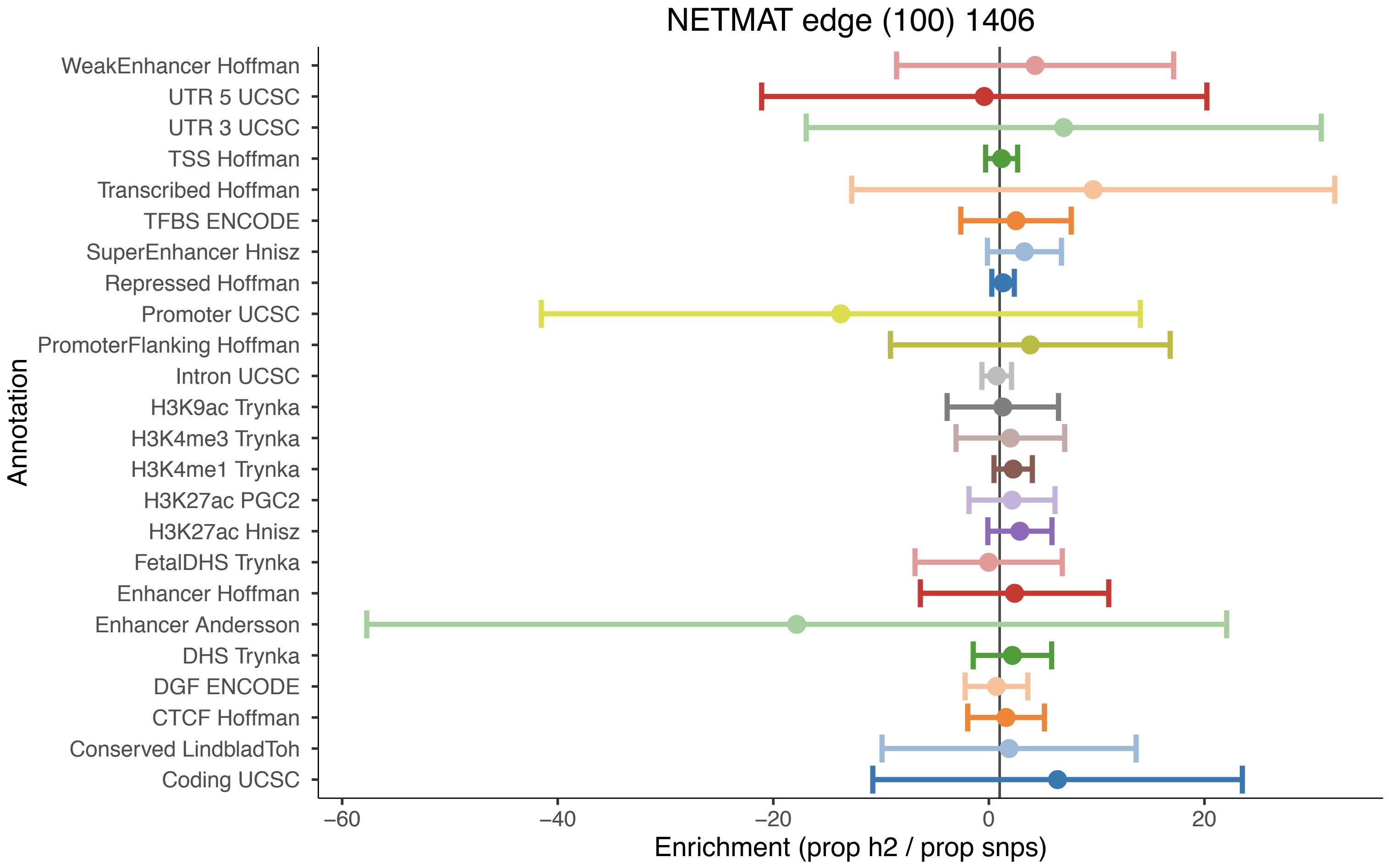

NETMAT edge (100) 1406
WeakEnhancer Hoffman
UTR 5 UCSC
UTR 3 UCSC
TSS Hoffman
Transcribed Hoffman
TFBS ENCODE
SuperEnhancer Hnisz
Repressed Hoffman
Promoter UCSC
PromoterFlanking Hoffman
Intron UCSC
H3K9ac Trynka
H3K4me3 Trynka
H3K4me1 Trynka
H3K27ac PGC2
H3K27ac Hnisz
FetalDHS Trynka
Enhancer Hoffman
Enhancer Andersson
DHS Trynka
DGF ENCODE
CTCF Hoffman
Conserved LindbladToh
Coding UCSC
Annotation
−60
−40
−20
0
20
Enrichment (prop h2 / prop snps)

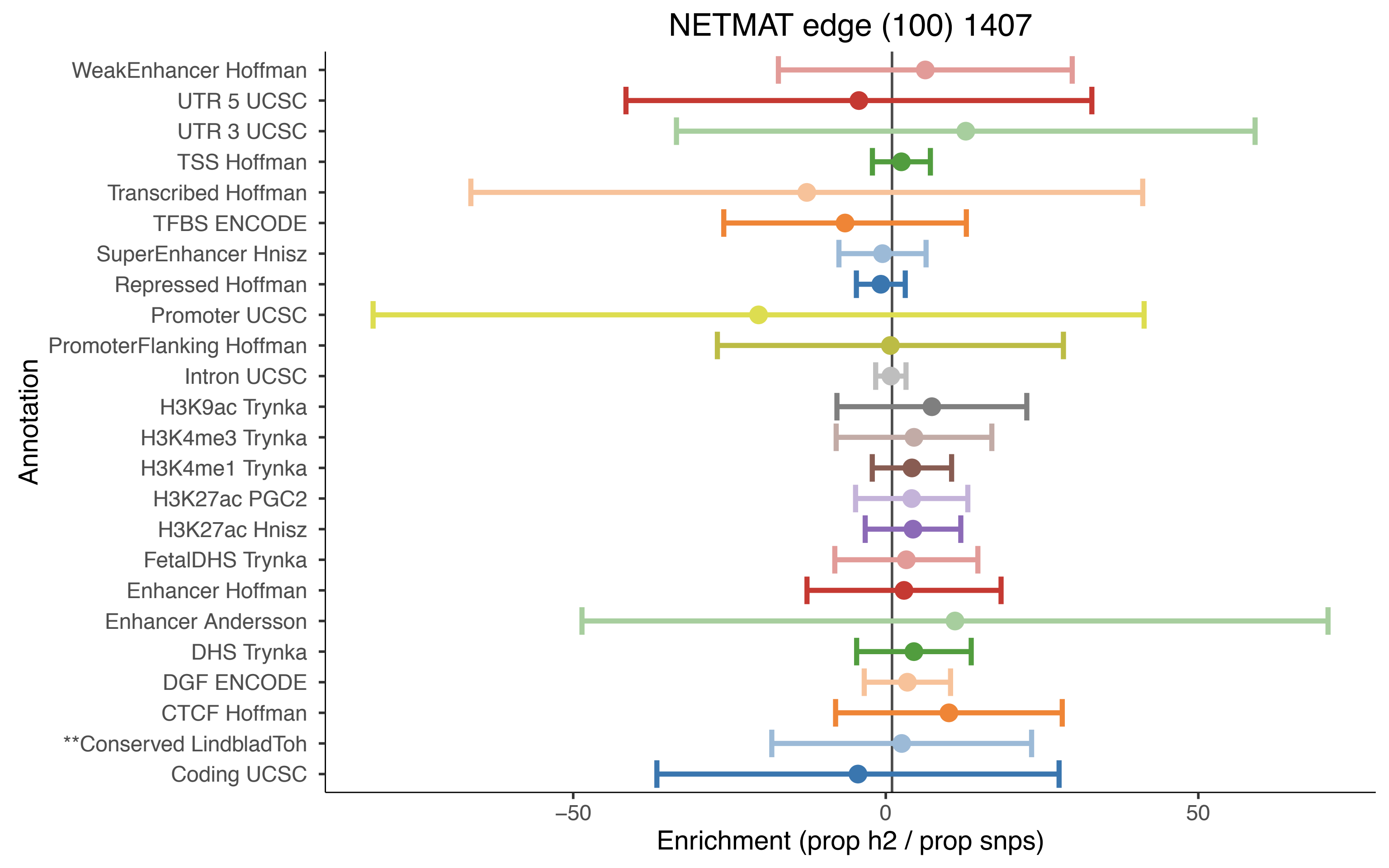

NETMAT edge (100) 1407
Annotation
WeakEnhancer Hoffman
UTR 5 UCSC
UTR 3 UCSC
TSS Hoffman
Transcribed Hoffman
TFBS ENCODE
SuperEnhancer Hnisz
Repressed Hoffman
Promoter UCSC
PromoterFlanking Hoffman
Intron UCSC
H3K9ac Trynka
H3K4me3 Trynka
H3K4me1 Trynka
H3K27ac PGC2
H3K27ac Hnisz
FetalDHS Trynka
Enhancer Hoffman
Enhancer Andersson
DHS Trynka
DGF ENCODE
CTCF Hoffman
**Conserved LindbladToh
Coding UCSC
−50
0
50
Enrichment (prop h2 / prop snps)

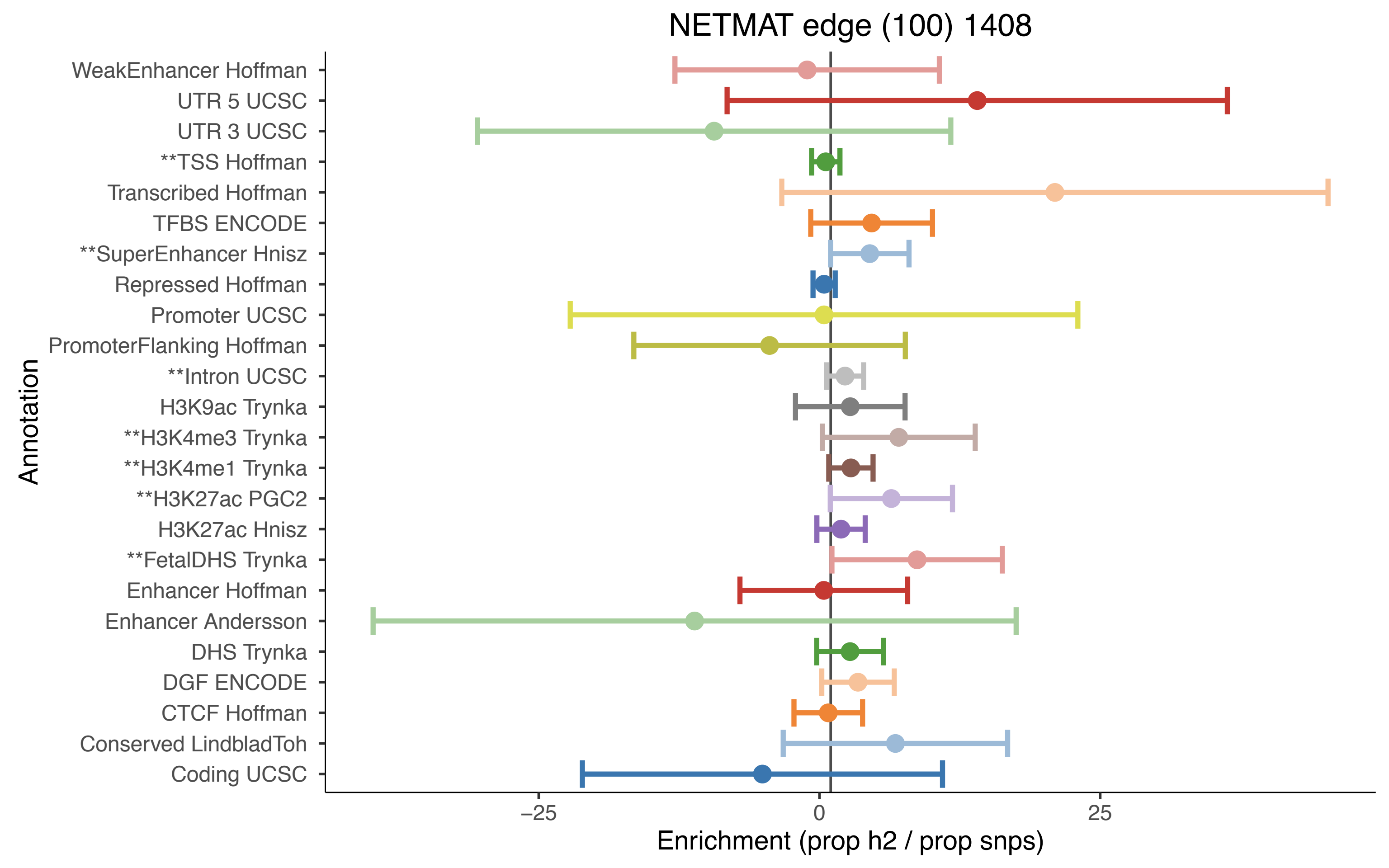
NETMAT edge (100) 1408
WeakEnhancer Hoffman
UTR 5 UCSC
UTR 3 UCSC
**TSS Hoffman
Transcribed Hoffman
TFBS ENCODE
**SuperEnhancer Hnisz
Repressed Hoffman
Promoter UCSC
PromoterFlanking Hoffman
**Intron UCSC
H3K9ac Trynka
**H3K4me3 Trynka
**H3K4me1 Trynka
**H3K27ac PGC2
H3K27ac Hnisz
**FetalDHS Trynka
Enhancer Hoffman
Enhancer Andersson
DHS Trynka
DGF ENCODE
CTCF Hoffman
Conserved LindbladToh
Coding UCSC
Annotation
−25
0
25
Enrichment (prop h2 / prop snps)

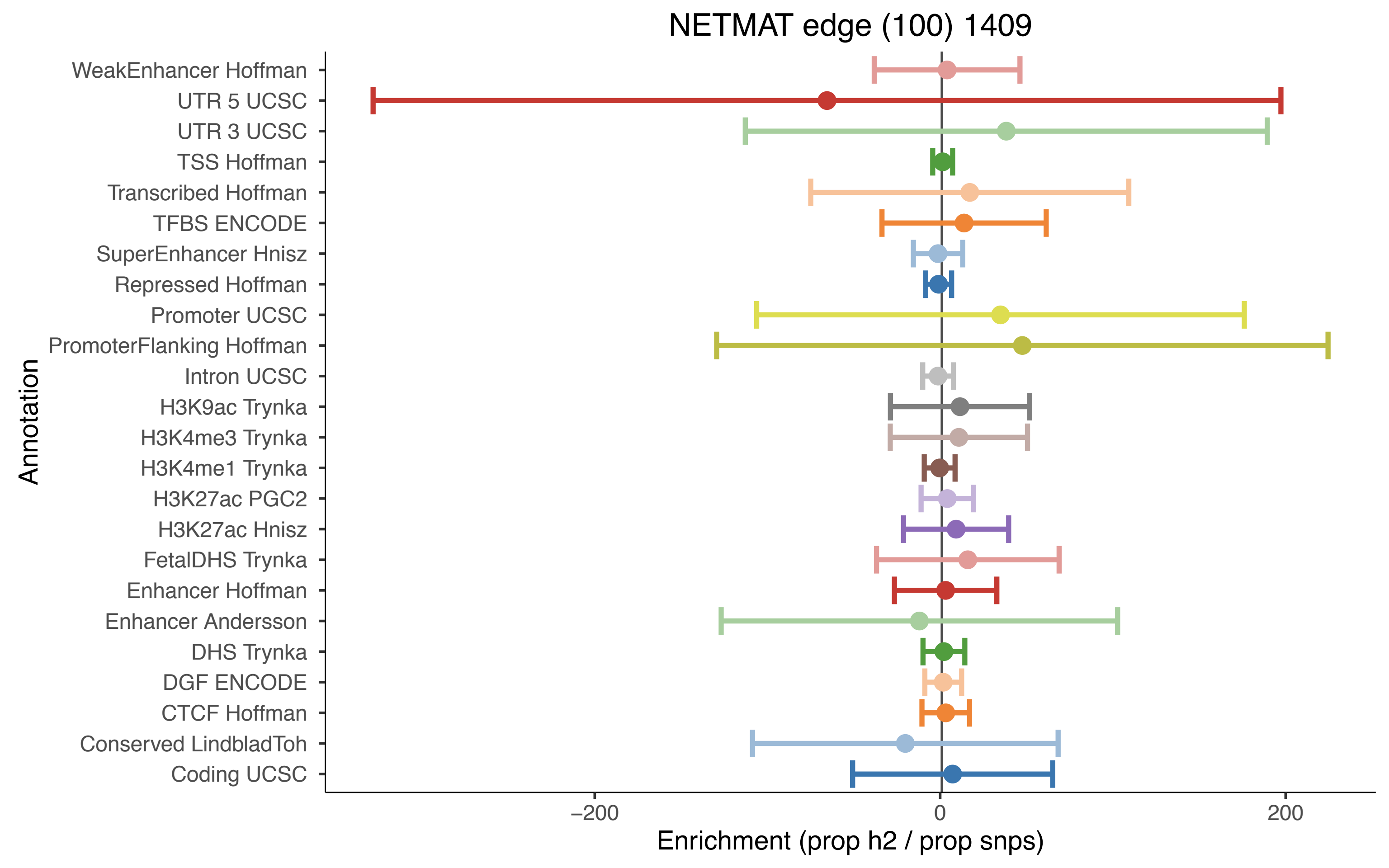
NETMAT edge (100) 1409
WeakEnhancer Hoffman
UTR 5 UCSC
UTR 3 UCSC
TSS Hoffman
Transcribed Hoffman
TFBS ENCODE
SuperEnhancer Hnisz
Repressed Hoffman
Promoter UCSC
PromoterFlanking Hoffman
Intron UCSC
H3K9ac Trynka
H3K4me3 Trynka
H3K4me1 Trynka
H3K27ac PGC2
H3K27ac Hnisz
FetalDHS Trynka
Enhancer Hoffman
Enhancer Andersson
DHS Trynka
DGF ENCODE
CTCF Hoffman
Conserved LindbladToh
Coding UCSC
Annotation
−200
0
200
Enrichment (prop h2 / prop snps)

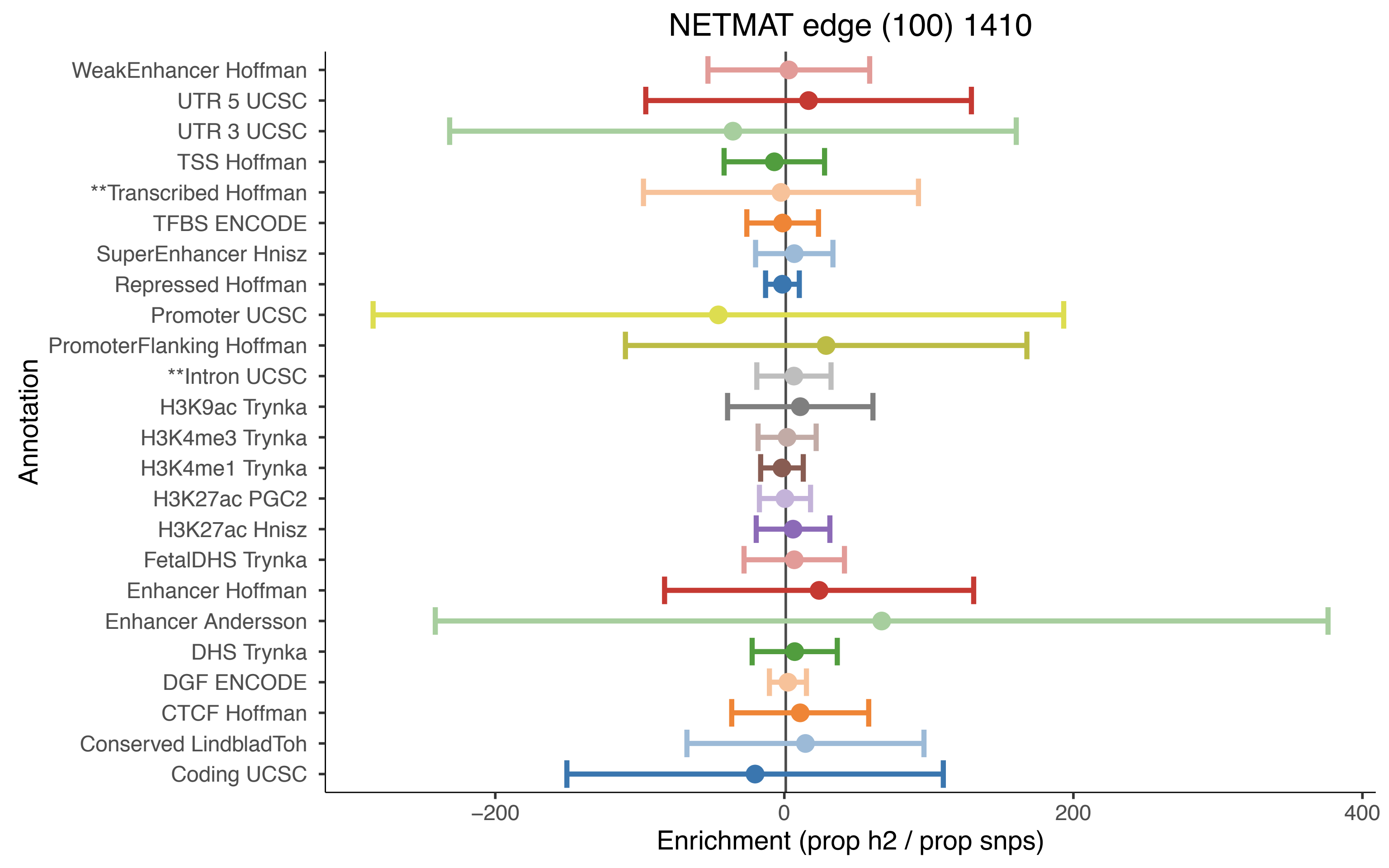

NETMAT edge (100) 1410
WeakEnhancer Hoffman
UTR 5 UCSC
UTR 3 UCSC
TSS Hoffman
**Transcribed Hoffman
TFBS ENCODE
SuperEnhancer Hnisz
Repressed Hoffman
Promoter UCSC
PromoterFlanking Hoffman
**Intron UCSC
H3K9ac Trynka
H3K4me3 Trynka
H3K4me1 Trynka
H3K27ac PGC2
H3K27ac Hnisz
FetalDHS Trynka
Enhancer Hoffman
Enhancer Andersson
DHS Trynka
DGF ENCODE
CTCF Hoffman
Conserved LindbladToh
Coding UCSC
Annotation
−200
0
200
400
Enrichment (prop h2 / prop snps)

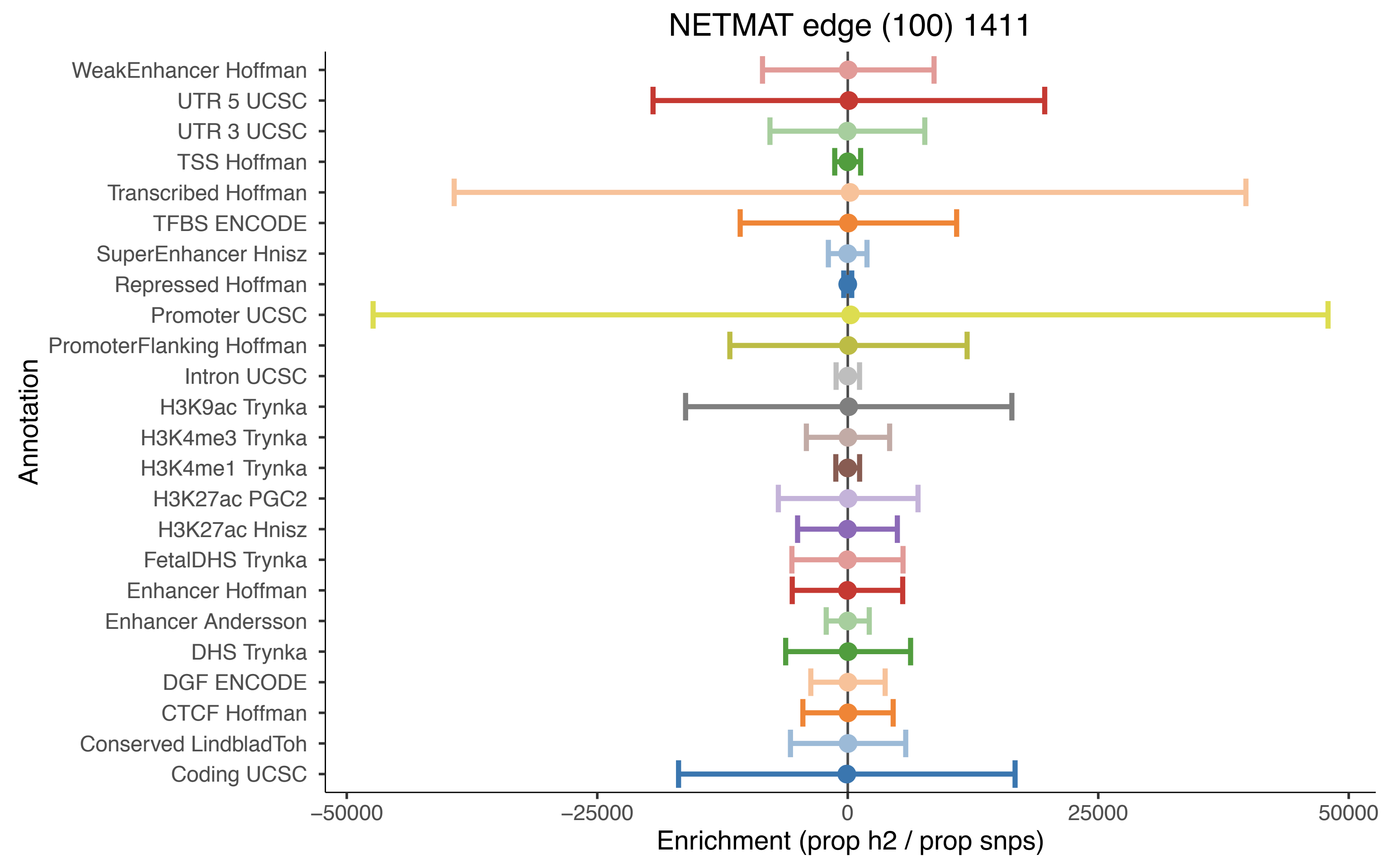

NETMAT edge (100) 1411
WeakEnhancer Hoffman
UTR 5 UCSC
UTR 3 UCSC
TSS Hoffman
Transcribed Hoffman
TFBS ENCODE
SuperEnhancer Hnisz
Repressed Hoffman
Promoter UCSC
PromoterFlanking Hoffman
Intron UCSC
H3K9ac Trynka
H3K4me3 Trynka
H3K4me1 Trynka
H3K27ac PGC2
H3K27ac Hnisz
FetalDHS Trynka
Enhancer Hoffman
Enhancer Andersson
DHS Trynka
DGF ENCODE
CTCF Hoffman
Conserved LindbladToh
Coding UCSC
Annotation
−50000
−25000
0
25000
50000
Enrichment (prop h2 / prop snps)

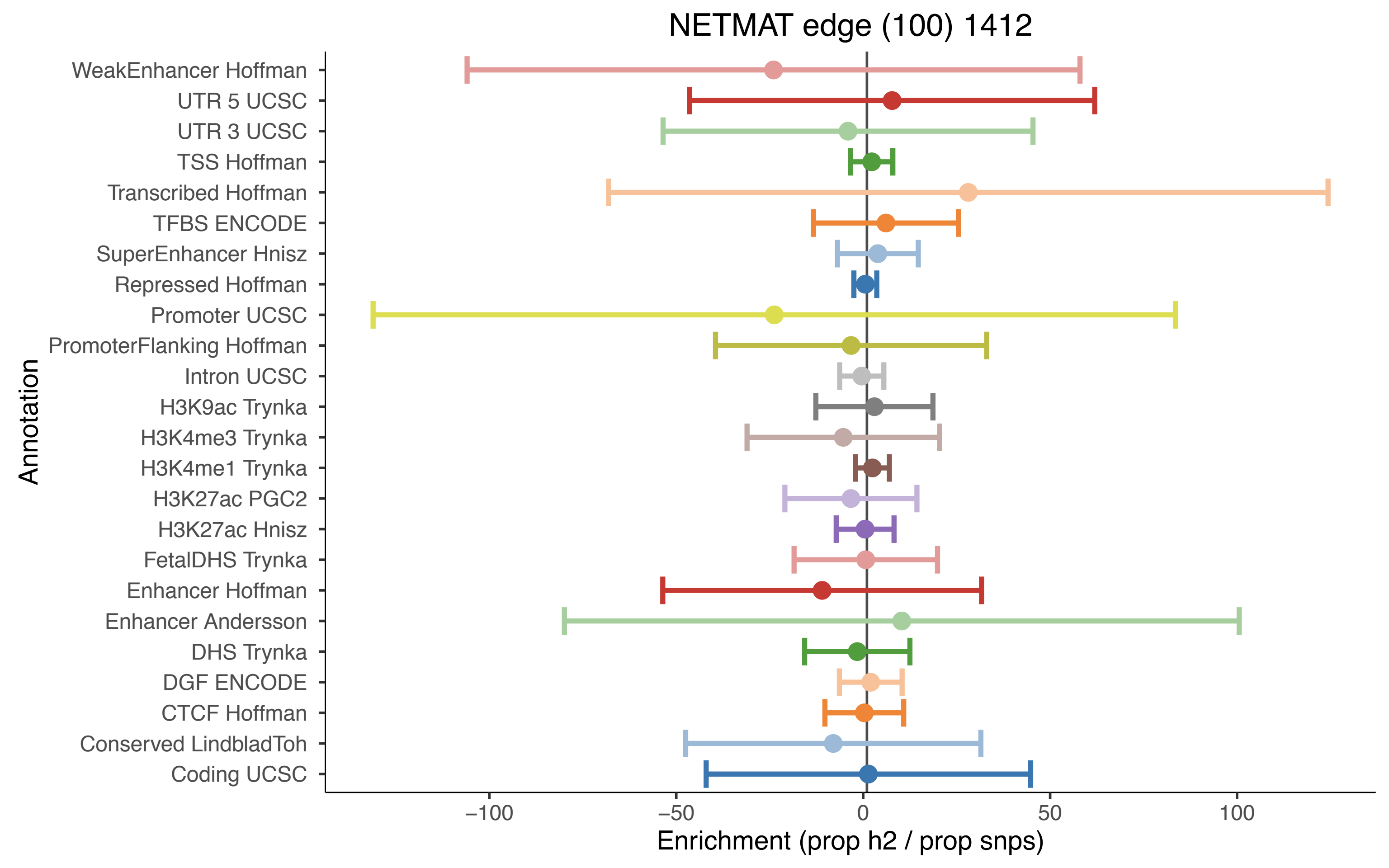
NETMAT edge (100) 1412
WeakEnhancer Hoffman
UTR 5 UCSC
UTR 3 UCSC
TSS Hoffman
Transcribed Hoffman
TFBS ENCODE
SuperEnhancer Hnisz
Repressed Hoffman
Promoter UCSC
PromoterFlanking Hoffman
Intron UCSC
H3K9ac Trynka
H3K4me3 Trynka
H3K4me1 Trynka
H3K27ac PGC2
H3K27ac Hnisz
FetalDHS Trynka
Enhancer Hoffman
Enhancer Andersson
DHS Trynka
DGF ENCODE
CTCF Hoffman
Conserved LindbladToh
Coding UCSC
Annotation
−100
−50
0
50
100
Enrichment (prop h2 / prop snps)

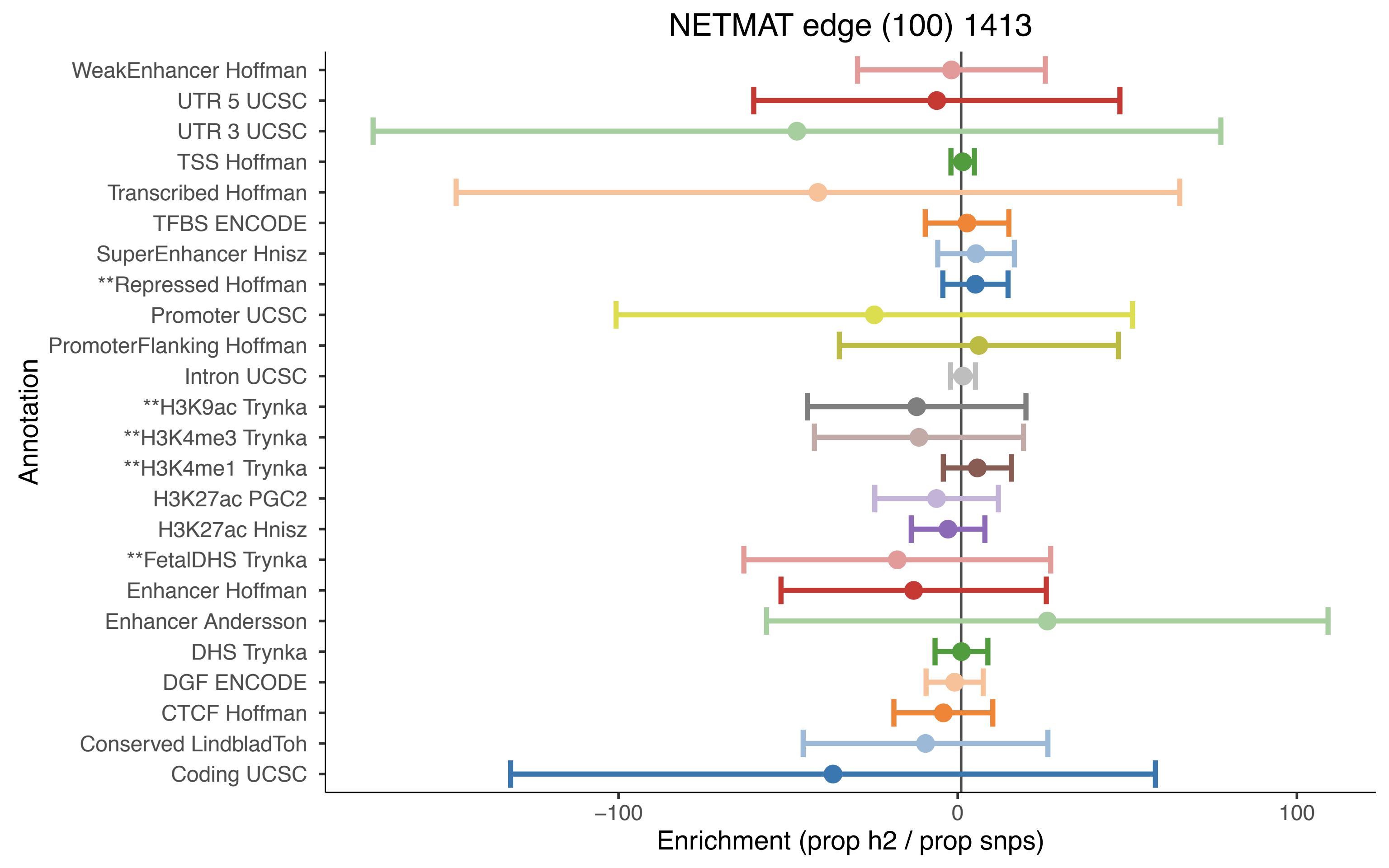
NETMAT edge (100) 1413
WeakEnhancer Hoffman
UTR 5 UCSC
UTR 3 UCSC
TSS Hoffman
Transcribed Hoffman
TFBS ENCODE
SuperEnhancer Hnisz
**Repressed Hoffman
Promoter UCSC
PromoterFlanking Hoffman
Intron UCSC
**H3K9ac Trynka
**H3K4me3 Trynka
**H3K4me1 Trynka
H3K27ac PGC2
H3K27ac Hnisz
**FetalDHS Trynka
Enhancer Hoffman
Enhancer Andersson
DHS Trynka
DGF ENCODE
CTCF Hoffman
Conserved LindbladToh
Coding UCSC
Annotation
−100
0
100
Enrichment (prop h2 / prop snps)

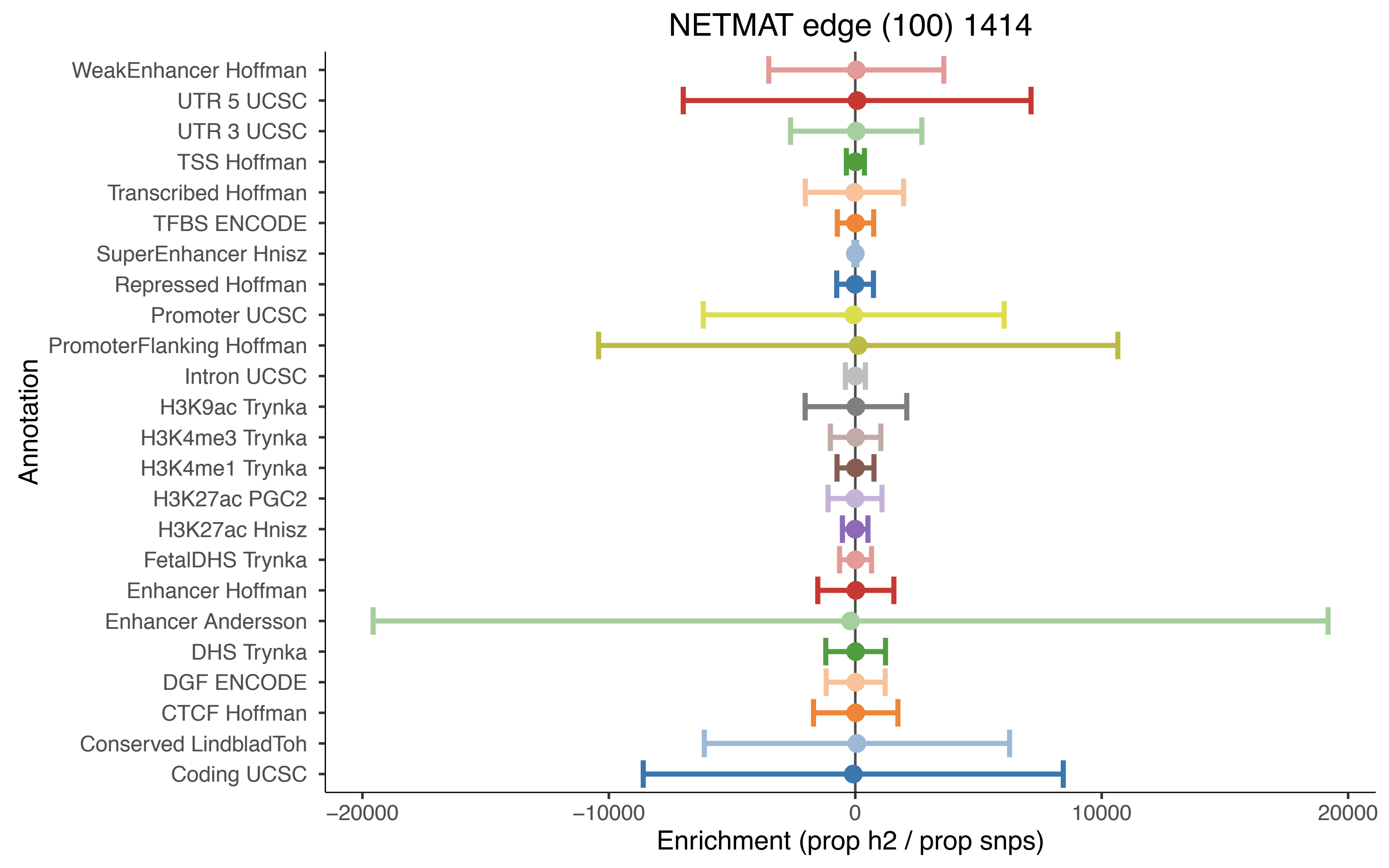
NETMAT edge (100) 1414
WeakEnhancer Hoffman
UTR 5 UCSC
UTR 3 UCSC
TSS Hoffman
Transcribed Hoffman
TFBS ENCODE
SuperEnhancer Hnisz
Repressed Hoffman
Promoter UCSC
PromoterFlanking Hoffman
Intron UCSC
H3K9ac Trynka
H3K4me3 Trynka
H3K4me1 Trynka
H3K27ac PGC2
H3K27ac Hnisz
FetalDHS Trynka
Enhancer Hoffman
Enhancer Andersson
DHS Trynka
DGF ENCODE
CTCF Hoffman
Conserved LindbladToh
Coding UCSC
Annotation
−20000
−10000
0
10000
20000
Enrichment (prop h2 / prop snps)

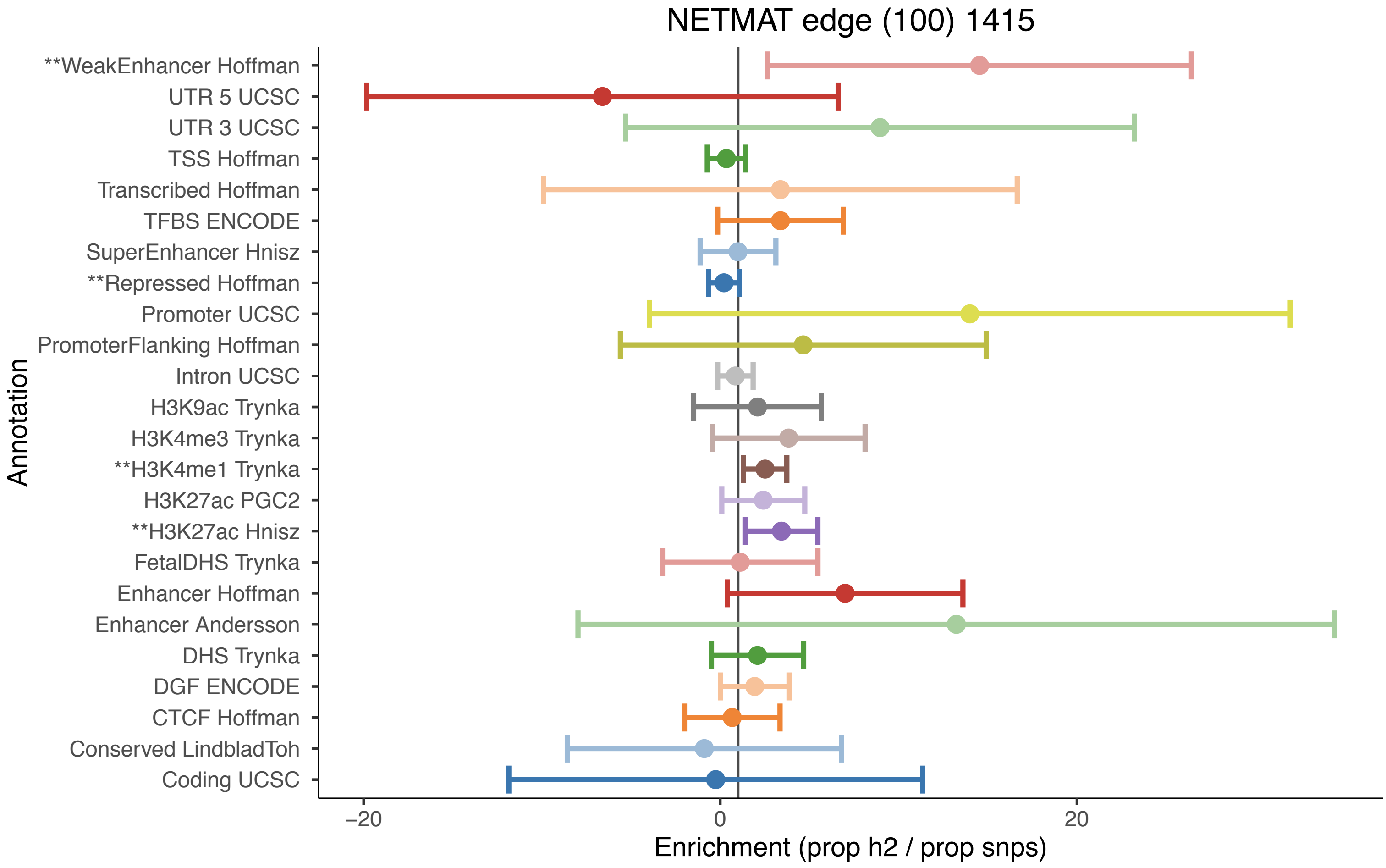

NETMAT edge (100) 1415
**WeakEnhancer Hoffman
UTR 5 UCSC
UTR 3 UCSC
TSS Hoffman
Transcribed Hoffman
TFBS ENCODE
SuperEnhancer Hnisz
**Repressed Hoffman
Promoter UCSC
PromoterFlanking Hoffman
Intron UCSC
H3K9ac Trynka
H3K4me3 Trynka
**H3K4me1 Trynka
H3K27ac PGC2
**H3K27ac Hnisz
FetalDHS Trynka
Enhancer Hoffman
Enhancer Andersson
DHS Trynka
DGF ENCODE
CTCF Hoffman
Conserved LindbladToh
Coding UCSC
Annotation
−20
0
20
Enrichment (prop h2 / prop snps)

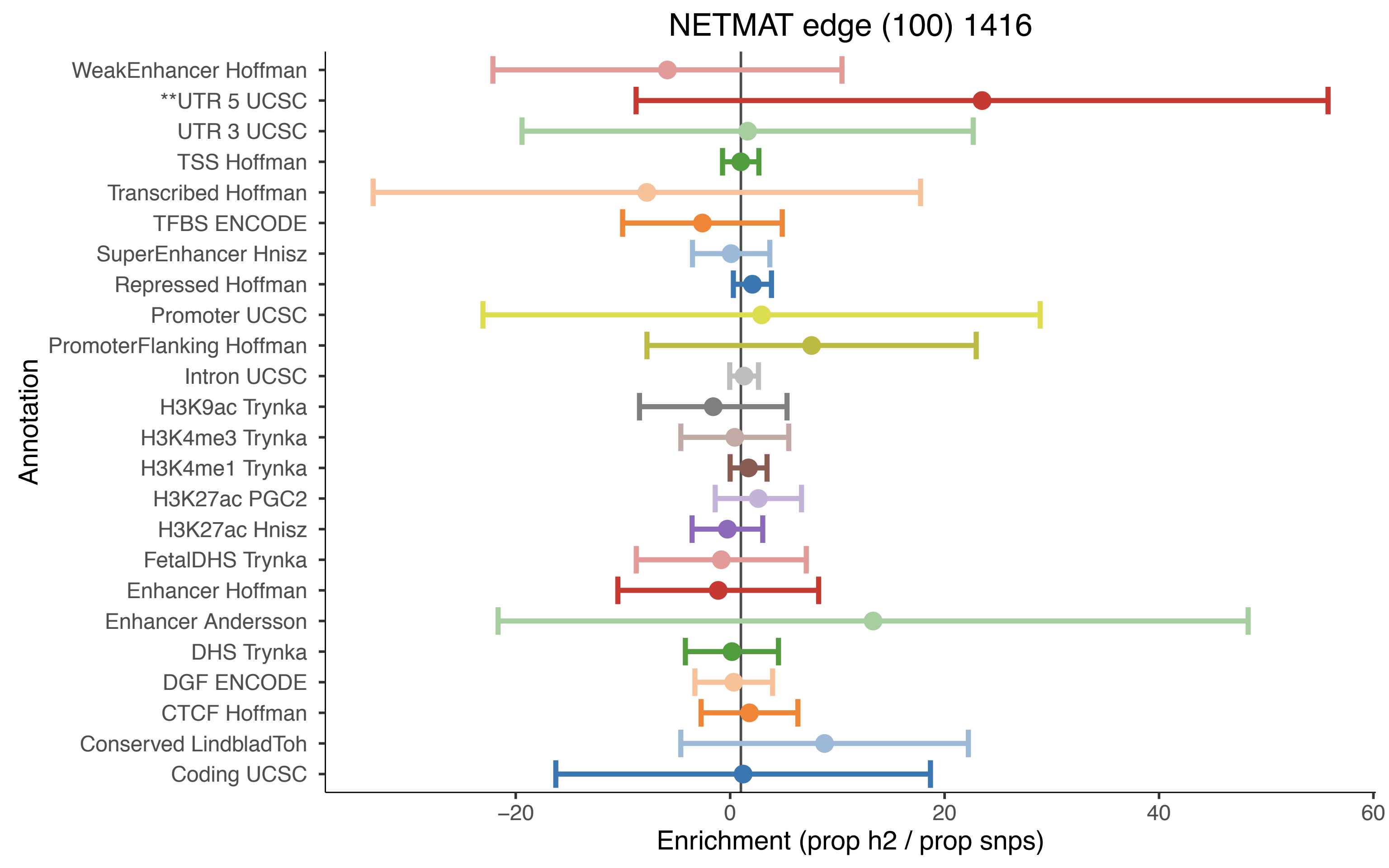
NETMAT edge (100) 1416
WeakEnhancer Hoffman
**UTR 5 UCSC
UTR 3 UCSC
TSS Hoffman
Transcribed Hoffman
TFBS ENCODE
SuperEnhancer Hnisz
Repressed Hoffman
Promoter UCSC
PromoterFlanking Hoffman
Intron UCSC
H3K9ac Trynka
H3K4me3 Trynka
H3K4me1 Trynka
H3K27ac PGC2
H3K27ac Hnisz
FetalDHS Trynka
Enhancer Hoffman
Enhancer Andersson
DHS Trynka
DGF ENCODE
CTCF Hoffman
Conserved LindbladToh
Coding UCSC
Annotation
−20
0
20
40
60
Enrichment (prop h2 / prop snps)

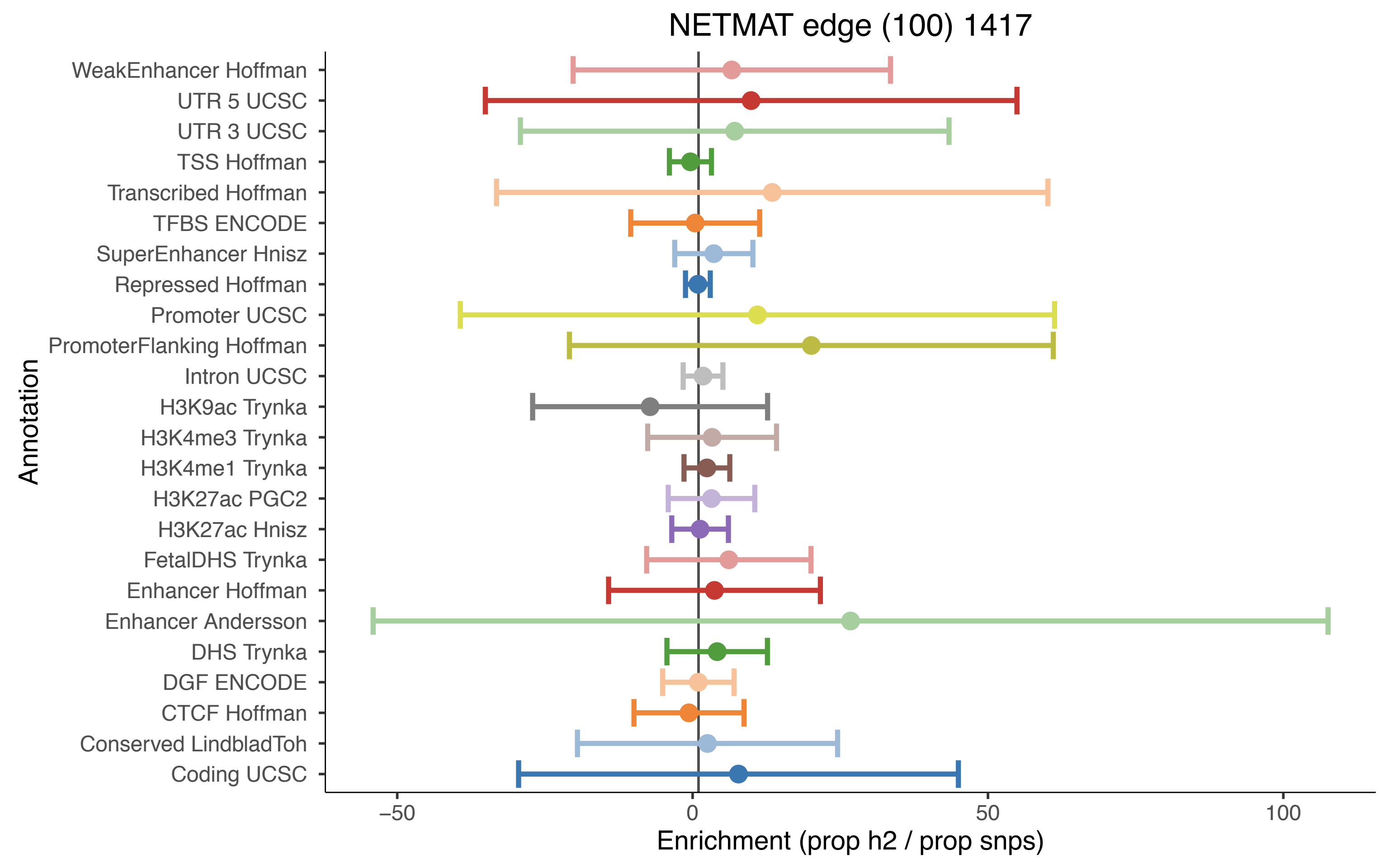
NETMAT edge (100) 1417
WeakEnhancer Hoffman
UTR 5 UCSC
UTR 3 UCSC
TSS Hoffman
Transcribed Hoffman
TFBS ENCODE
SuperEnhancer Hnisz
Repressed Hoffman
Promoter UCSC
PromoterFlanking Hoffman
Intron UCSC
H3K9ac Trynka
H3K4me3 Trynka
H3K4me1 Trynka
H3K27ac PGC2
H3K27ac Hnisz
FetalDHS Trynka
Enhancer Hoffman
Enhancer Andersson
DHS Trynka
DGF ENCODE
CTCF Hoffman
Conserved LindbladToh
Coding UCSC
Annotation
−50
0
50
100
Enrichment (prop h2 / prop snps)

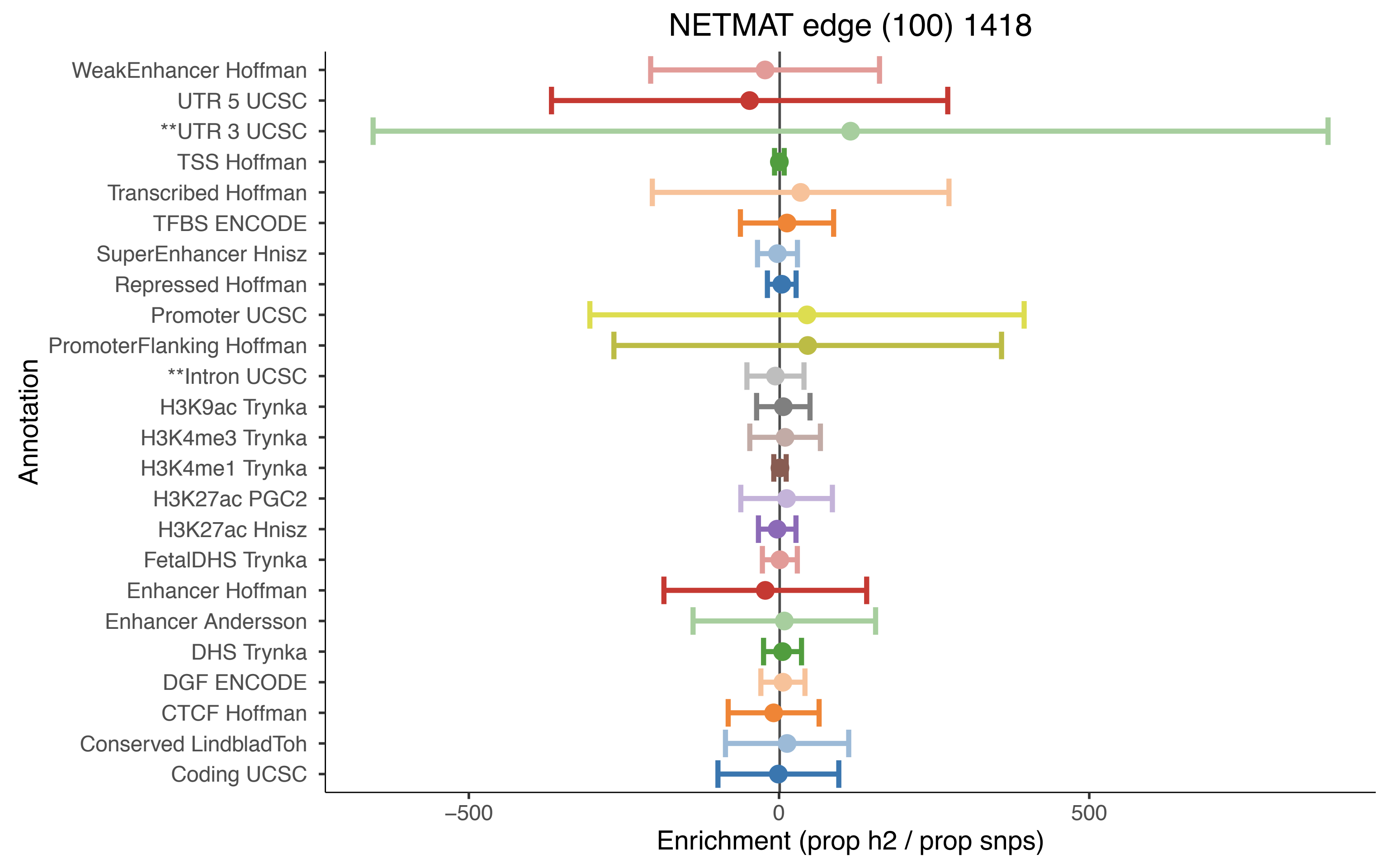

NETMAT edge (100) 1418
WeakEnhancer Hoffman
UTR 5 UCSC
**UTR 3 UCSC
TSS Hoffman
Transcribed Hoffman
TFBS ENCODE
SuperEnhancer Hnisz
Repressed Hoffman
Promoter UCSC
PromoterFlanking Hoffman
**Intron UCSC
H3K9ac Trynka
H3K4me3 Trynka
H3K4me1 Trynka
H3K27ac PGC2
H3K27ac Hnisz
FetalDHS Trynka
Enhancer Hoffman
Enhancer Andersson
DHS Trynka
DGF ENCODE
CTCF Hoffman
Conserved LindbladToh
Coding UCSC
Annotation
−500
0
500
Enrichment (prop h2 / prop snps)

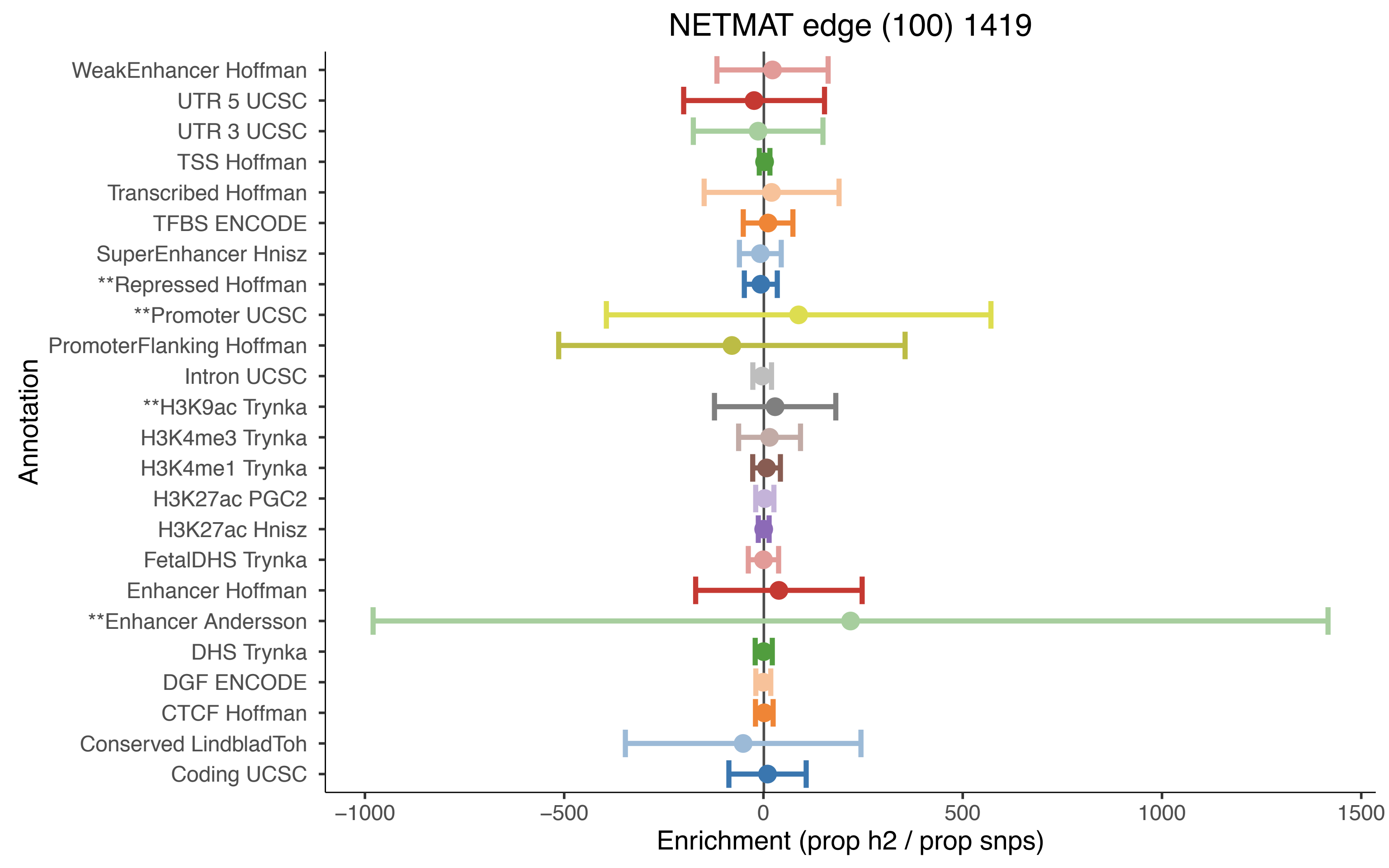
NETMAT edge (100) 1419
Annotation
WeakEnhancer Hoffman
UTR 5 UCSC
UTR 3 UCSC
TSS Hoffman
Transcribed Hoffman
TFBS ENCODE
SuperEnhancer Hnisz
**Repressed Hoffman
**Promoter UCSC
PromoterFlanking Hoffman
Intron UCSC
**H3K9ac Trynka
H3K4me3 Trynka
H3K4me1 Trynka
H3K27ac PGC2
H3K27ac Hnisz
FetalDHS Trynka
Enhancer Hoffman
**Enhancer Andersson
DHS Trynka
DGF ENCODE
CTCF Hoffman
Conserved LindbladToh
Coding UCSC
−1000
−500
0
500
1000
1500
Enrichment (prop h2 / prop snps)

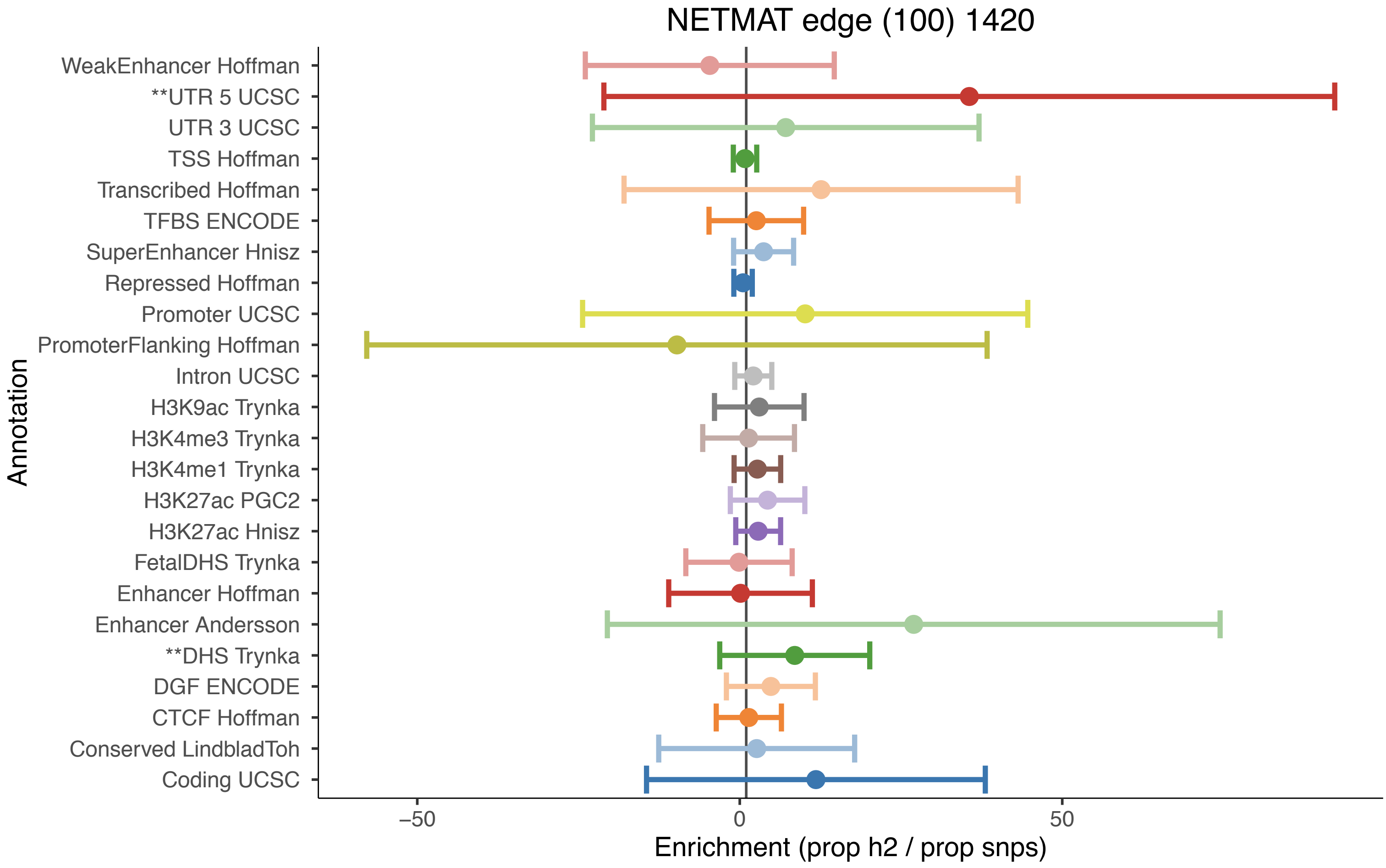

NETMAT edge (100) 1420
WeakEnhancer Hoffman
**UTR 5 UCSC
UTR 3 UCSC
TSS Hoffman
Transcribed Hoffman
TFBS ENCODE
SuperEnhancer Hnisz
Repressed Hoffman
Promoter UCSC
PromoterFlanking Hoffman
Intron UCSC
H3K9ac Trynka
H3K4me3 Trynka
H3K4me1 Trynka
H3K27ac PGC2
H3K27ac Hnisz
FetalDHS Trynka
Enhancer Hoffman
Enhancer Andersson
**DHS Trynka
DGF ENCODE
CTCF Hoffman
Conserved LindbladToh
Coding UCSC
Annotation
−50
0
50
Enrichment (prop h2 / prop snps)

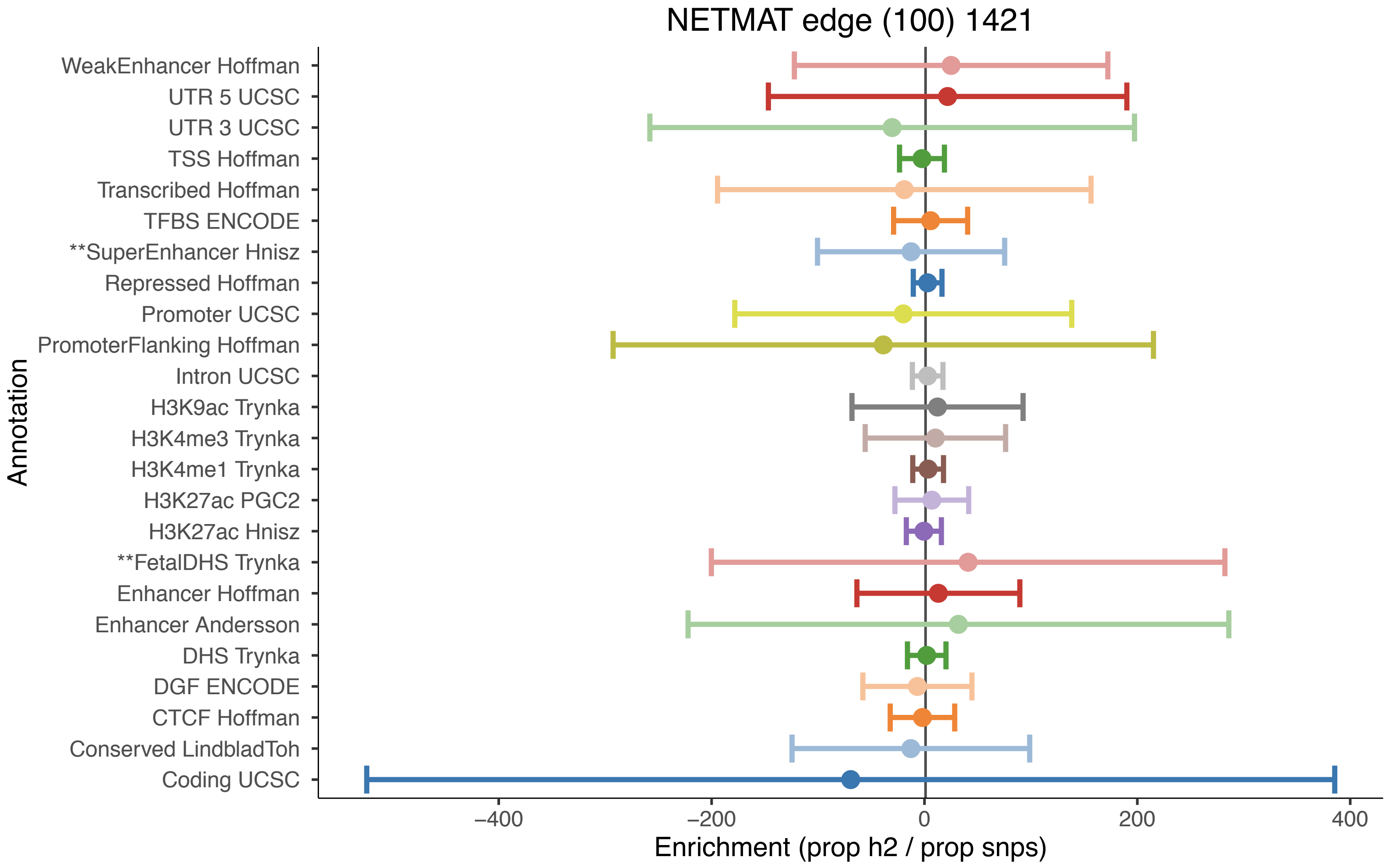
NETMAT edge (100) 1421
Annotation
WeakEnhancer Hoffman
UTR 5 UCSC
UTR 3 UCSC
TSS Hoffman
Transcribed Hoffman
TFBS ENCODE
**SuperEnhancer Hnisz
Repressed Hoffman
Promoter UCSC
PromoterFlanking Hoffman
Intron UCSC
H3K9ac Trynka
H3K4me3 Trynka
H3K4me1 Trynka
H3K27ac PGC2
H3K27ac Hnisz
**FetalDHS Trynka
Enhancer Hoffman
Enhancer Andersson
DHS Trynka
DGF ENCODE
CTCF Hoffman
Conserved LindbladToh
Coding UCSC
−400
−200
0
200
400
Enrichment (prop h2 / prop snps)

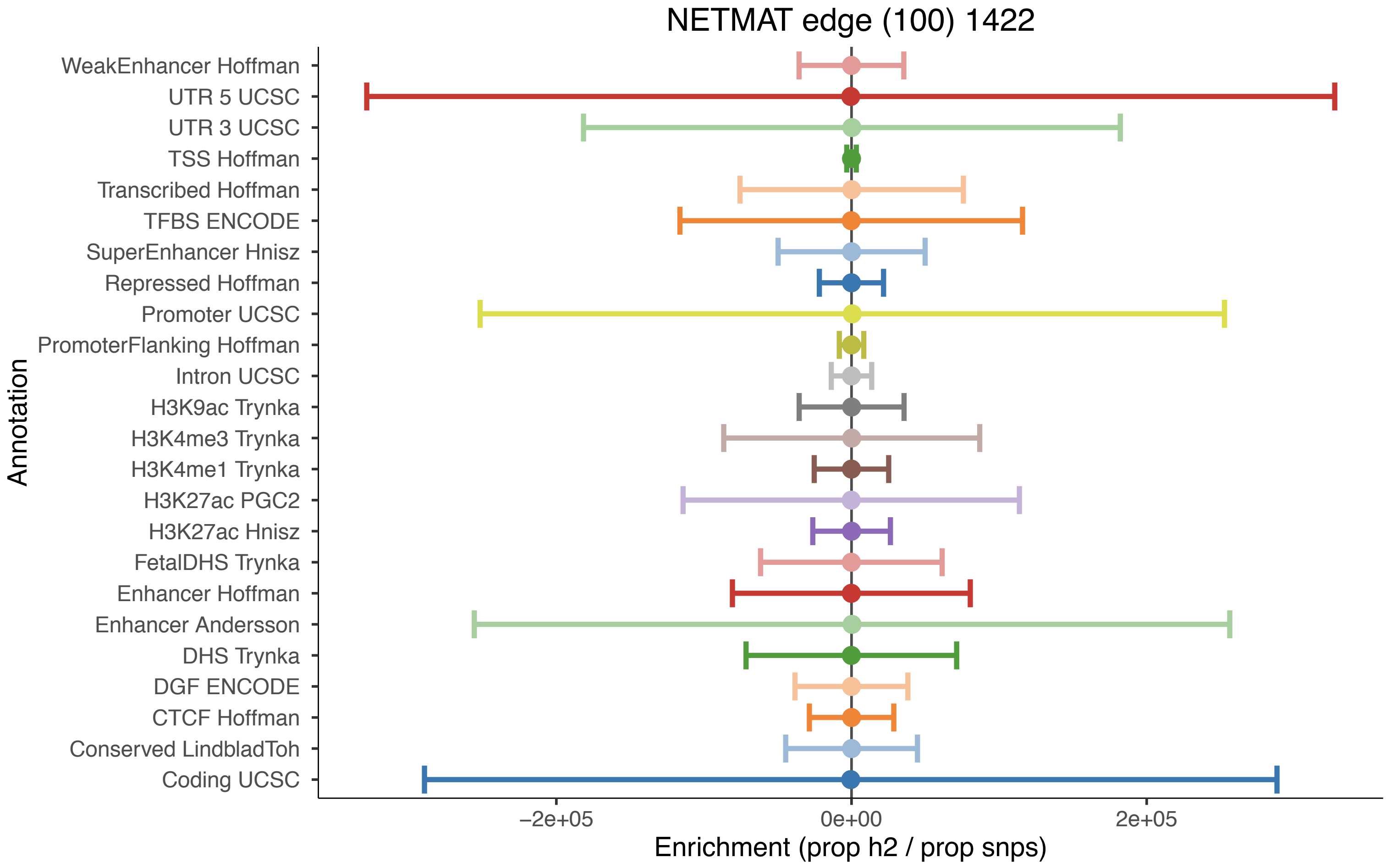
NETMAT edge (100) 1422
WeakEnhancer Hoffman
UTR 5 UCSC
UTR 3 UCSC
TSS Hoffman
Transcribed Hoffman
TFBS ENCODE
SuperEnhancer Hnisz
Repressed Hoffman
Promoter UCSC
PromoterFlanking Hoffman
Intron UCSC
H3K9ac Trynka
H3K4me3 Trynka
H3K4me1 Trynka
H3K27ac PGC2
H3K27ac Hnisz
FetalDHS Trynka
Enhancer Hoffman
Enhancer Andersson
DHS Trynka
DGF ENCODE
CTCF Hoffman
Conserved LindbladToh
Coding UCSC
Annotation
−2e+05
0e+00
2e+05
Enrichment (prop h2 / prop snps)

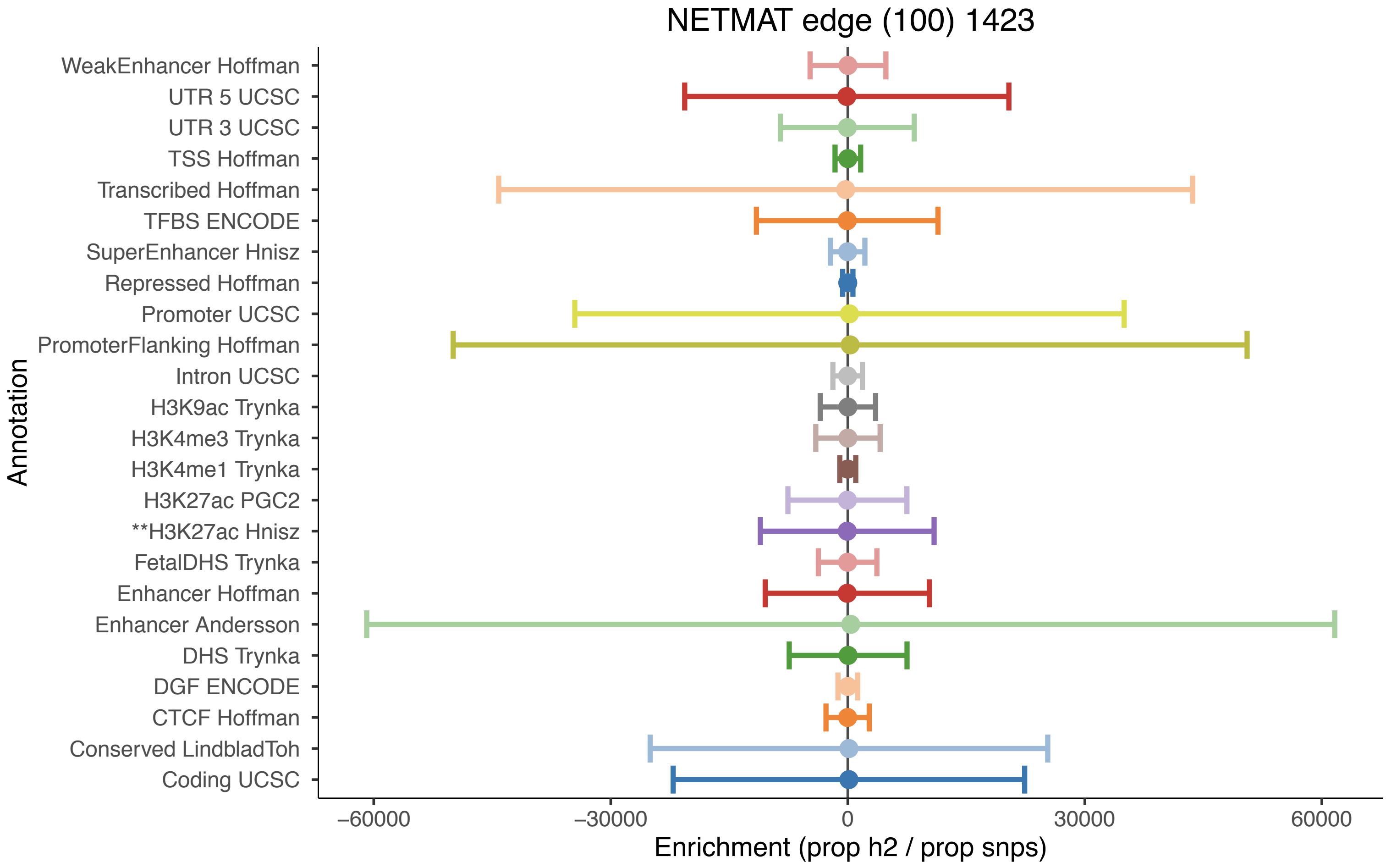
NETMAT edge (100) 1423
WeakEnhancer Hoffman
UTR 5 UCSC
UTR 3 UCSC
TSS Hoffman
Transcribed Hoffman
TFBS ENCODE
SuperEnhancer Hnisz
Repressed Hoffman
Promoter UCSC
PromoterFlanking Hoffman
Intron UCSC
H3K9ac Trynka
H3K4me3 Trynka
H3K4me1 Trynka
H3K27ac PGC2
**H3K27ac Hnisz
FetalDHS Trynka
Enhancer Hoffman
Enhancer Andersson
DHS Trynka
DGF ENCODE
CTCF Hoffman
Conserved LindbladToh
Coding UCSC
Annotation
−60000
−30000
0
30000
60000
Enrichment (prop h2 / prop snps)

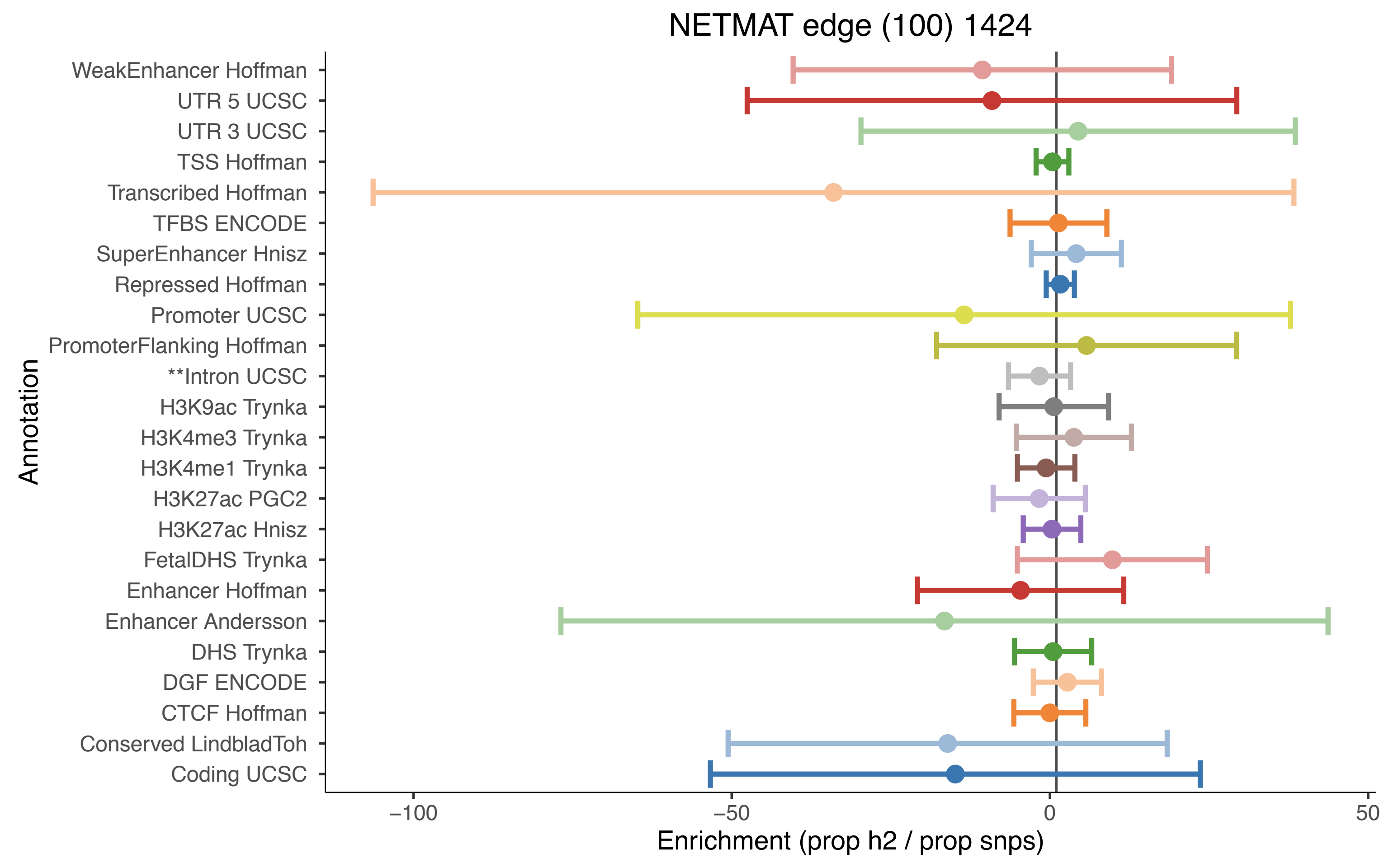
NETMAT edge (100) 1424
WeakEnhancer Hoffman
UTR 5 UCSC
UTR 3 UCSC
TSS Hoffman
Transcribed Hoffman
TFBS ENCODE
SuperEnhancer Hnisz
Repressed Hoffman
Promoter UCSC
PromoterFlanking Hoffman
**Intron UCSC
H3K9ac Trynka
H3K4me3 Trynka
H3K4me1 Trynka
H3K27ac PGC2
H3K27ac Hnisz
FetalDHS Trynka
Enhancer Hoffman
Enhancer Andersson
DHS Trynka
DGF ENCODE
CTCF Hoffman
Conserved LindbladToh
Coding UCSC
Annotation
−100
−50
0
50
Enrichment (prop h2 / prop snps)

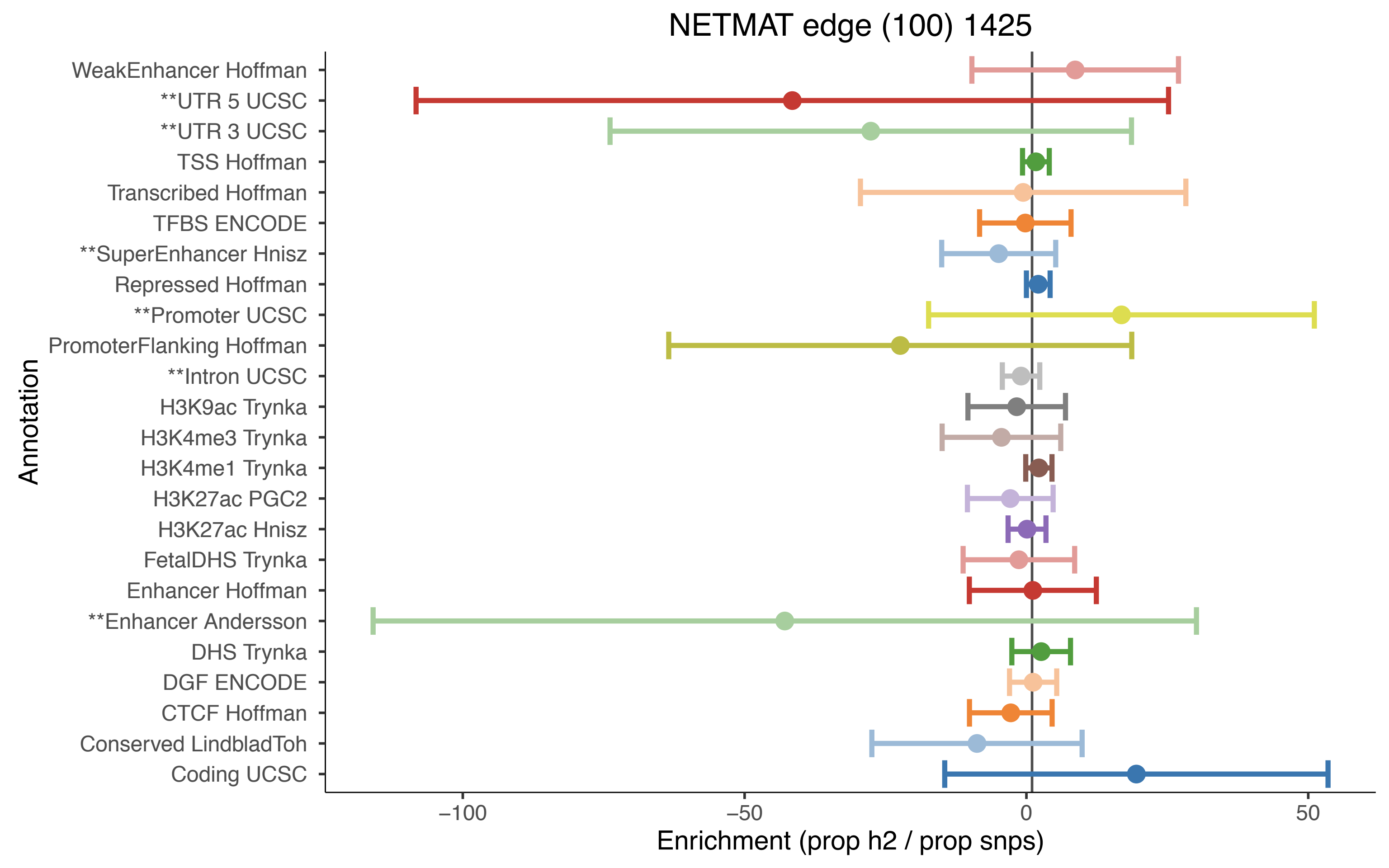
NETMAT edge (100) 1425
WeakEnhancer Hoffman
**UTR 5 UCSC
**UTR 3 UCSC
TSS Hoffman
Transcribed Hoffman
TFBS ENCODE
**SuperEnhancer Hnisz
Repressed Hoffman
**Promoter UCSC
PromoterFlanking Hoffman
**Intron UCSC
H3K9ac Trynka
H3K4me3 Trynka
H3K4me1 Trynka
H3K27ac PGC2
H3K27ac Hnisz
FetalDHS Trynka
Enhancer Hoffman
**Enhancer Andersson
DHS Trynka
DGF ENCODE
CTCF Hoffman
Conserved LindbladToh
Coding UCSC
Annotation
−100
−50
0
50
Enrichment (prop h2 / prop snps)

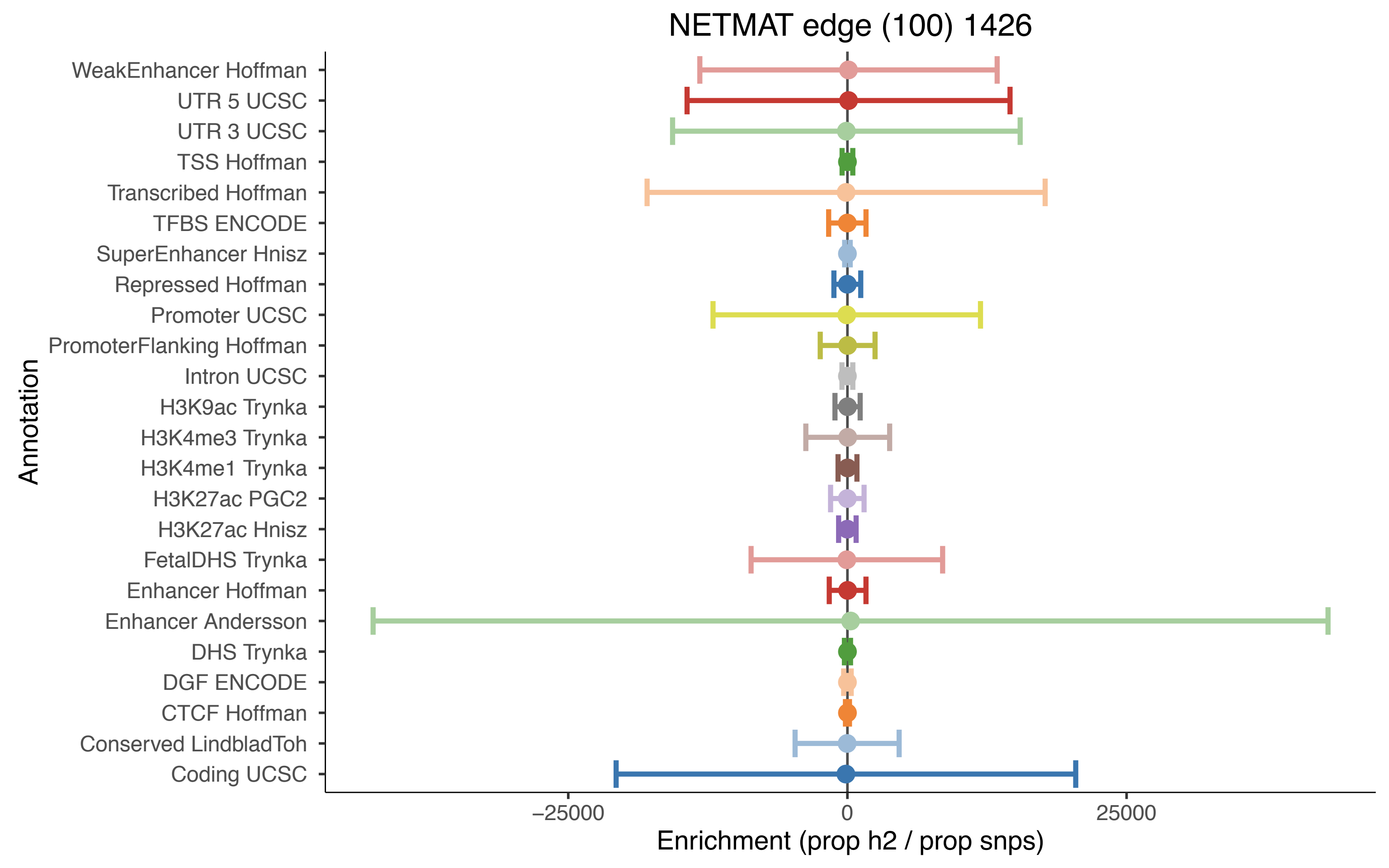
NETMAT edge (100) 1426
Annotation
WeakEnhancer Hoffman
UTR 5 UCSC
UTR 3 UCSC
TSS Hoffman
Transcribed Hoffman
TFBS ENCODE
SuperEnhancer Hnisz
Repressed Hoffman
Promoter UCSC
PromoterFlanking Hoffman
Intron UCSC
H3K9ac Trynka
H3K4me3 Trynka
H3K4me1 Trynka
H3K27ac PGC2
H3K27ac Hnisz
FetalDHS Trynka
Enhancer Hoffman
Enhancer Andersson
DHS Trynka
DGF ENCODE
CTCF Hoffman
Conserved LindbladToh
Coding UCSC
−25000
0
25000
Enrichment (prop h2 / prop snps)

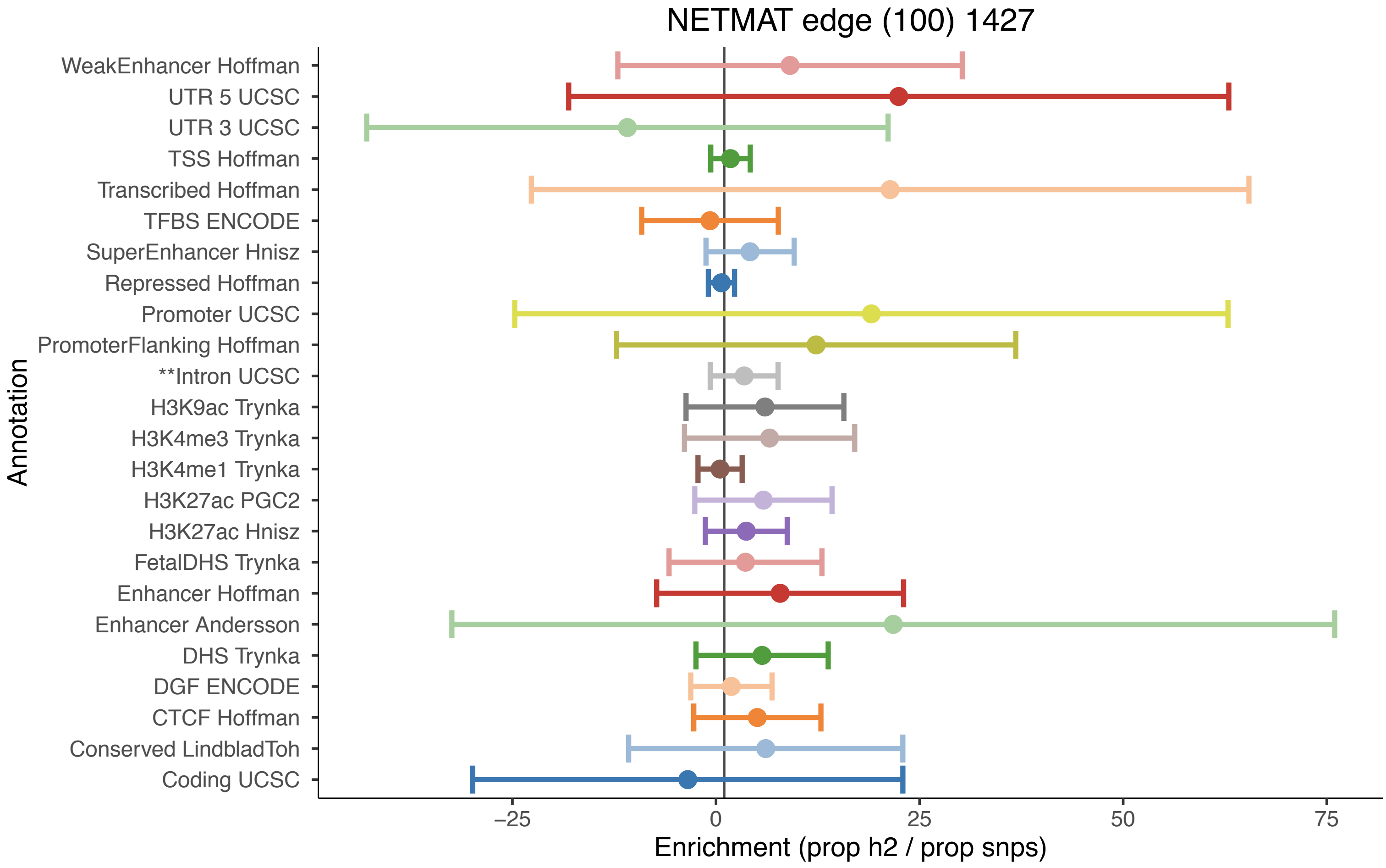

NETMAT edge (100) 1427
WeakEnhancer Hoffman
UTR 5 UCSC
UTR 3 UCSC
TSS Hoffman
Transcribed Hoffman
TFBS ENCODE
SuperEnhancer Hnisz
Repressed Hoffman
Promoter UCSC
PromoterFlanking Hoffman
**Intron UCSC
H3K9ac Trynka
H3K4me3 Trynka
H3K4me1 Trynka
H3K27ac PGC2
H3K27ac Hnisz
FetalDHS Trynka
Enhancer Hoffman
Enhancer Andersson
DHS Trynka
DGF ENCODE
CTCF Hoffman
Conserved LindbladToh
Coding UCSC
Annotation
−25
0
25
50
75
Enrichment (prop h2 / prop snps)

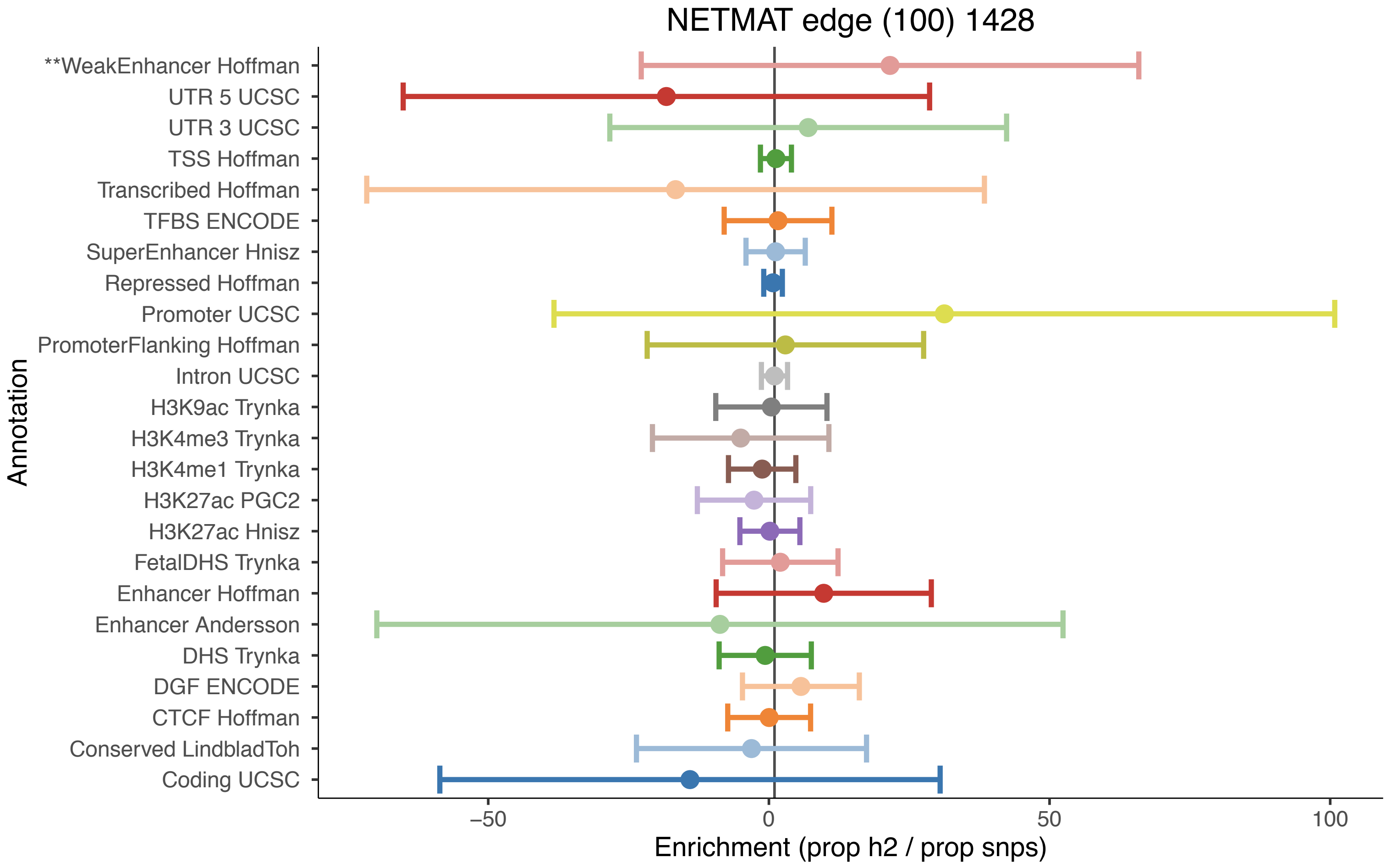

NETMAT edge (100) 1428
**WeakEnhancer Hoffman
UTR 5 UCSC
UTR 3 UCSC
TSS Hoffman
Transcribed Hoffman
TFBS ENCODE
SuperEnhancer Hnisz
Repressed Hoffman
Promoter UCSC
PromoterFlanking Hoffman
Intron UCSC
H3K9ac Trynka
H3K4me3 Trynka
H3K4me1 Trynka
H3K27ac PGC2
H3K27ac Hnisz
FetalDHS Trynka
Enhancer Hoffman
Enhancer Andersson
DHS Trynka
DGF ENCODE
CTCF Hoffman
Conserved LindbladToh
Coding UCSC
Annotation
−50
0
50
100
Enrichment (prop h2 / prop snps)

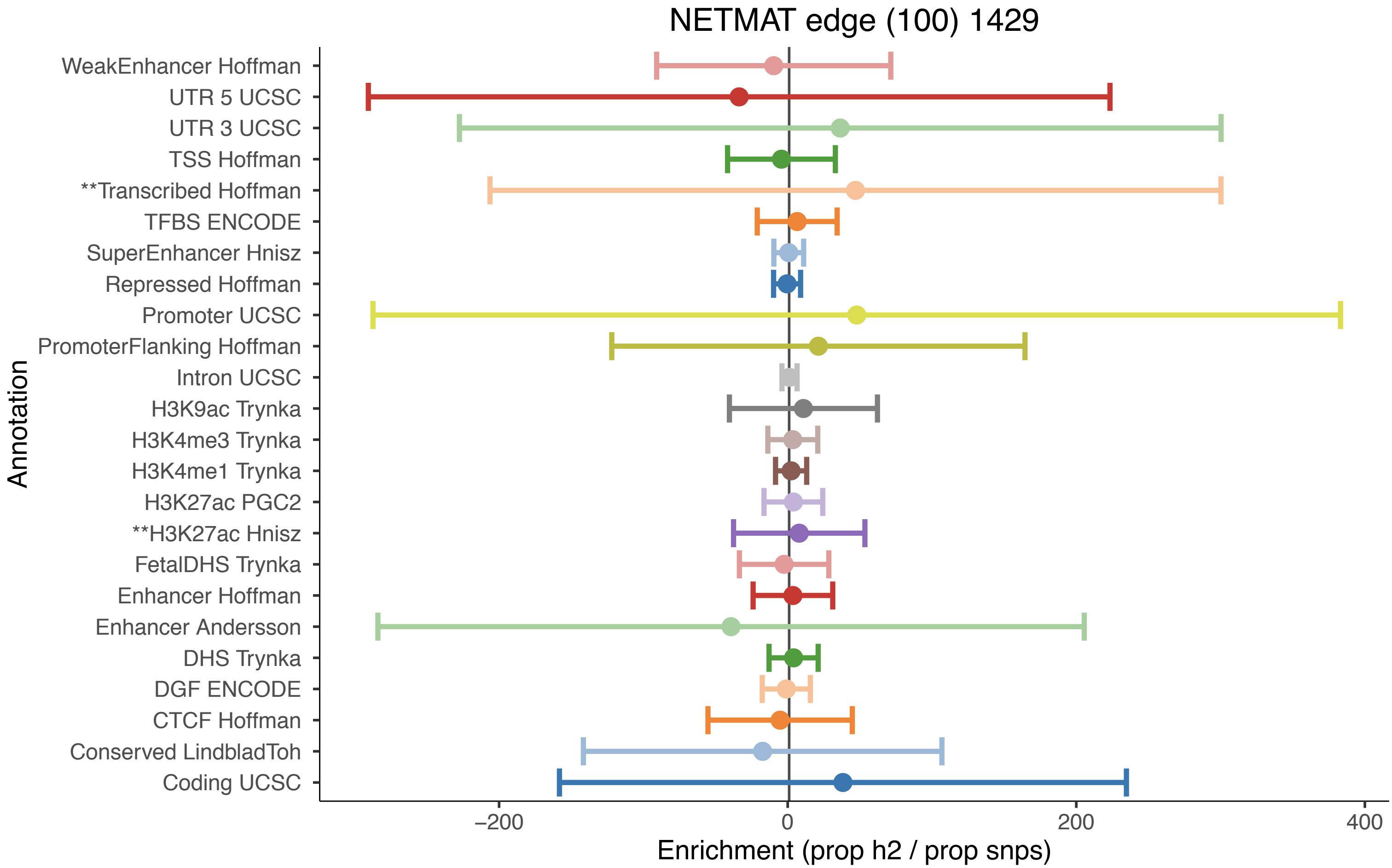
NETMAT edge (100) 1429
WeakEnhancer Hoffman
UTR 5 UCSC
UTR 3 UCSC
TSS Hoffman
**Transcribed Hoffman
TFBS ENCODE
SuperEnhancer Hnisz
Repressed Hoffman
Promoter UCSC
PromoterFlanking Hoffman
Intron UCSC
H3K9ac Trynka
H3K4me3 Trynka
H3K4me1 Trynka
H3K27ac PGC2
**H3K27ac Hnisz
FetalDHS Trynka
Enhancer Hoffman
Enhancer Andersson
DHS Trynka
DGF ENCODE
CTCF Hoffman
Conserved LindbladToh
Coding UCSC
Annotation
−200
0
200
400
Enrichment (prop h2 / prop snps)

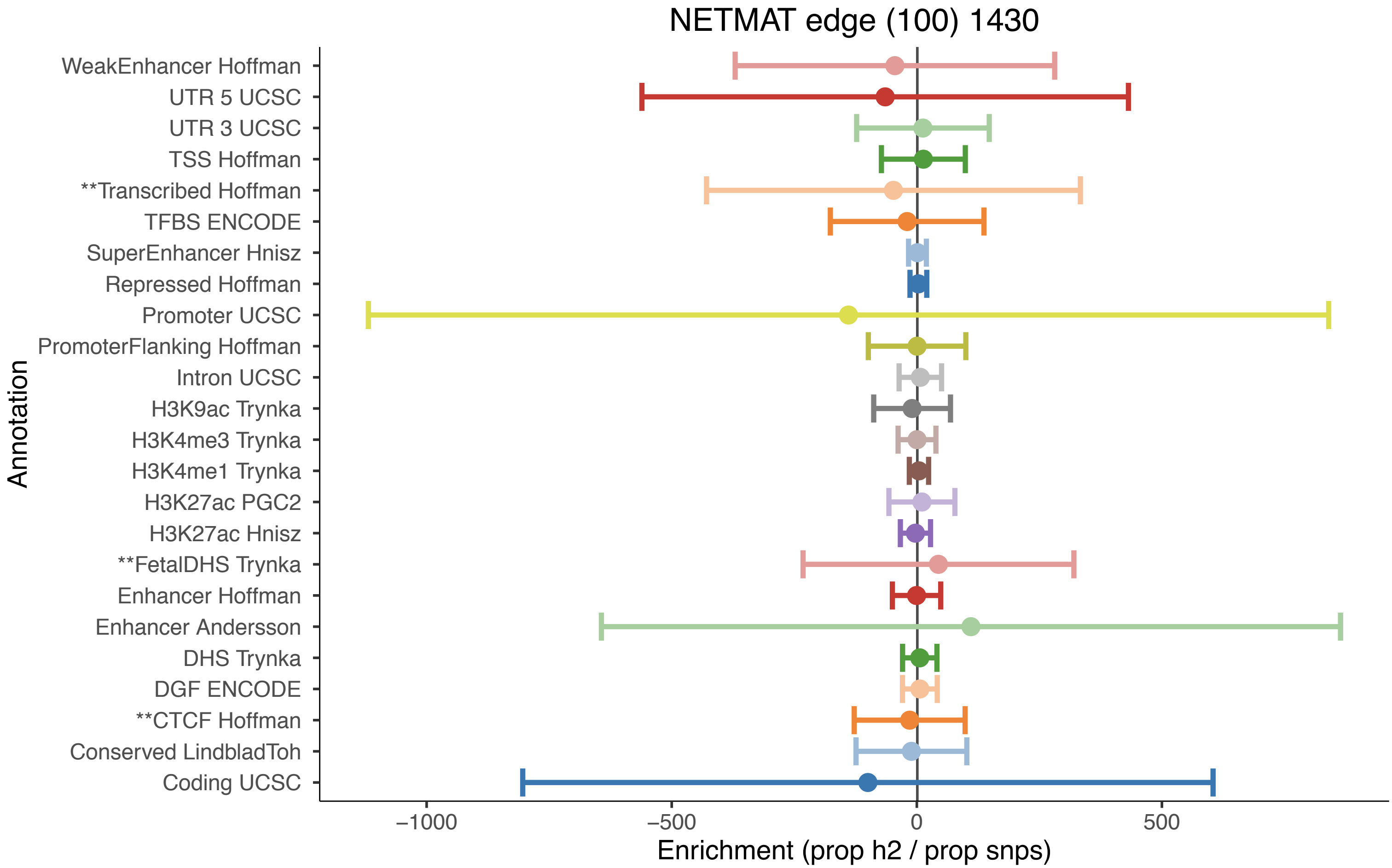
NETMAT edge (100) 1430
WeakEnhancer Hoffman
UTR 5 UCSC
UTR 3 UCSC
TSS Hoffman
**Transcribed Hoffman
TFBS ENCODE
SuperEnhancer Hnisz
Repressed Hoffman
Promoter UCSC
PromoterFlanking Hoffman
Intron UCSC
H3K9ac Trynka
H3K4me3 Trynka
H3K4me1 Trynka
H3K27ac PGC2
H3K27ac Hnisz
**FetalDHS Trynka
Enhancer Hoffman
Enhancer Andersson
DHS Trynka
DGF ENCODE
**CTCF Hoffman
Conserved LindbladToh
Coding UCSC
Annotation
−1000
−500
0
500
Enrichment (prop h2 / prop snps)

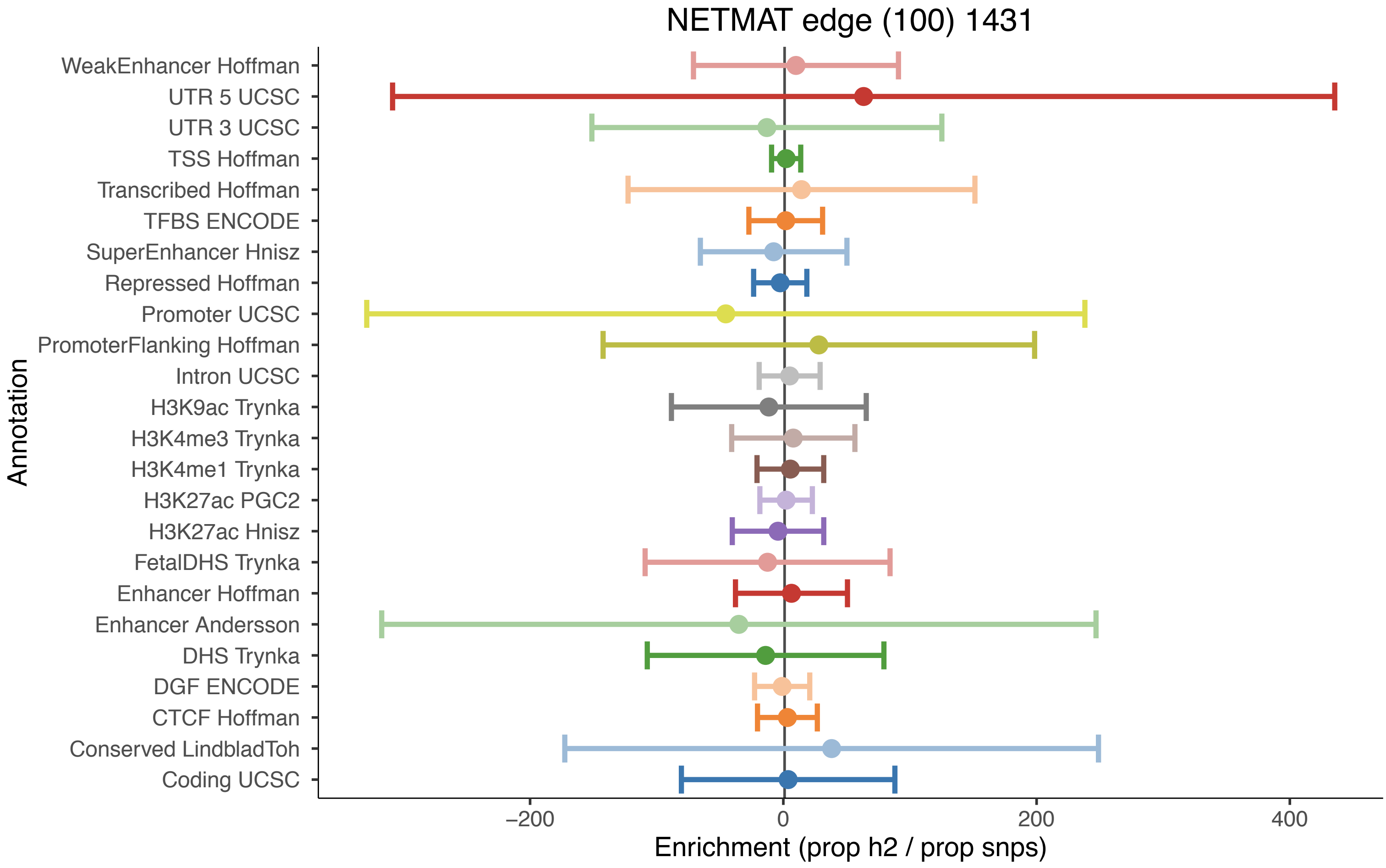

NETMAT edge (100) 1431
Annotation
WeakEnhancer Hoffman
UTR 5 UCSC
UTR 3 UCSC
TSS Hoffman
Transcribed Hoffman
TFBS ENCODE
SuperEnhancer Hnisz
Repressed Hoffman
Promoter UCSC
PromoterFlanking Hoffman
Intron UCSC
H3K9ac Trynka
H3K4me3 Trynka
H3K4me1 Trynka
H3K27ac PGC2
H3K27ac Hnisz
FetalDHS Trynka
Enhancer Hoffman
Enhancer Andersson
DHS Trynka
DGF ENCODE
CTCF Hoffman
Conserved LindbladToh
Coding UCSC
−200
0
200
400
Enrichment (prop h2 / prop snps)

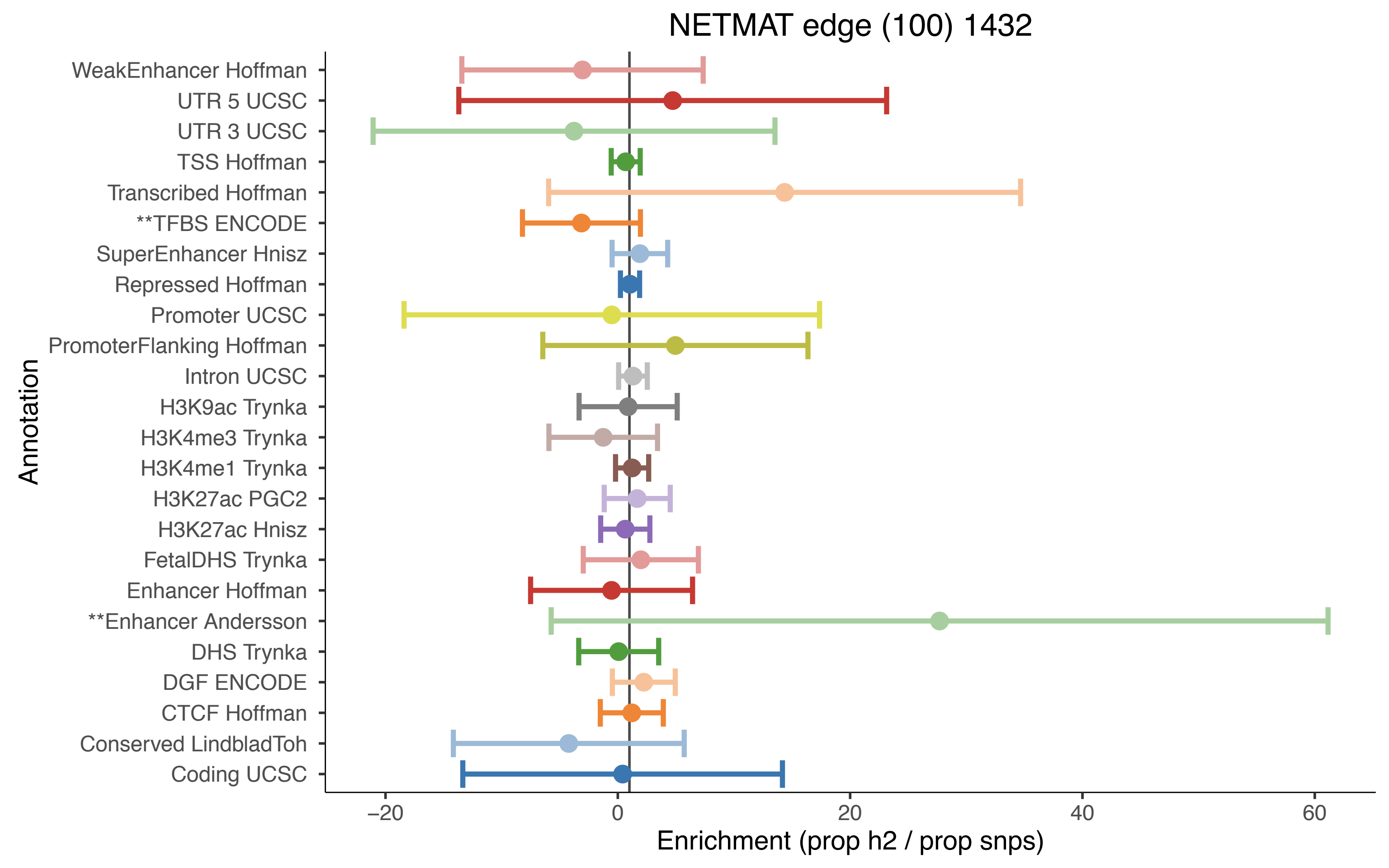
NETMAT edge (100) 1432
WeakEnhancer Hoffman
UTR 5 UCSC
UTR 3 UCSC
TSS Hoffman
Transcribed Hoffman
**TFBS ENCODE
SuperEnhancer Hnisz
Repressed Hoffman
Promoter UCSC
PromoterFlanking Hoffman
Intron UCSC
H3K9ac Trynka
H3K4me3 Trynka
H3K4me1 Trynka
H3K27ac PGC2
H3K27ac Hnisz
FetalDHS Trynka
Enhancer Hoffman
**Enhancer Andersson
DHS Trynka
DGF ENCODE
CTCF Hoffman
Conserved LindbladToh
Coding UCSC
Annotation
−20
0
20
40
60
Enrichment (prop h2 / prop snps)

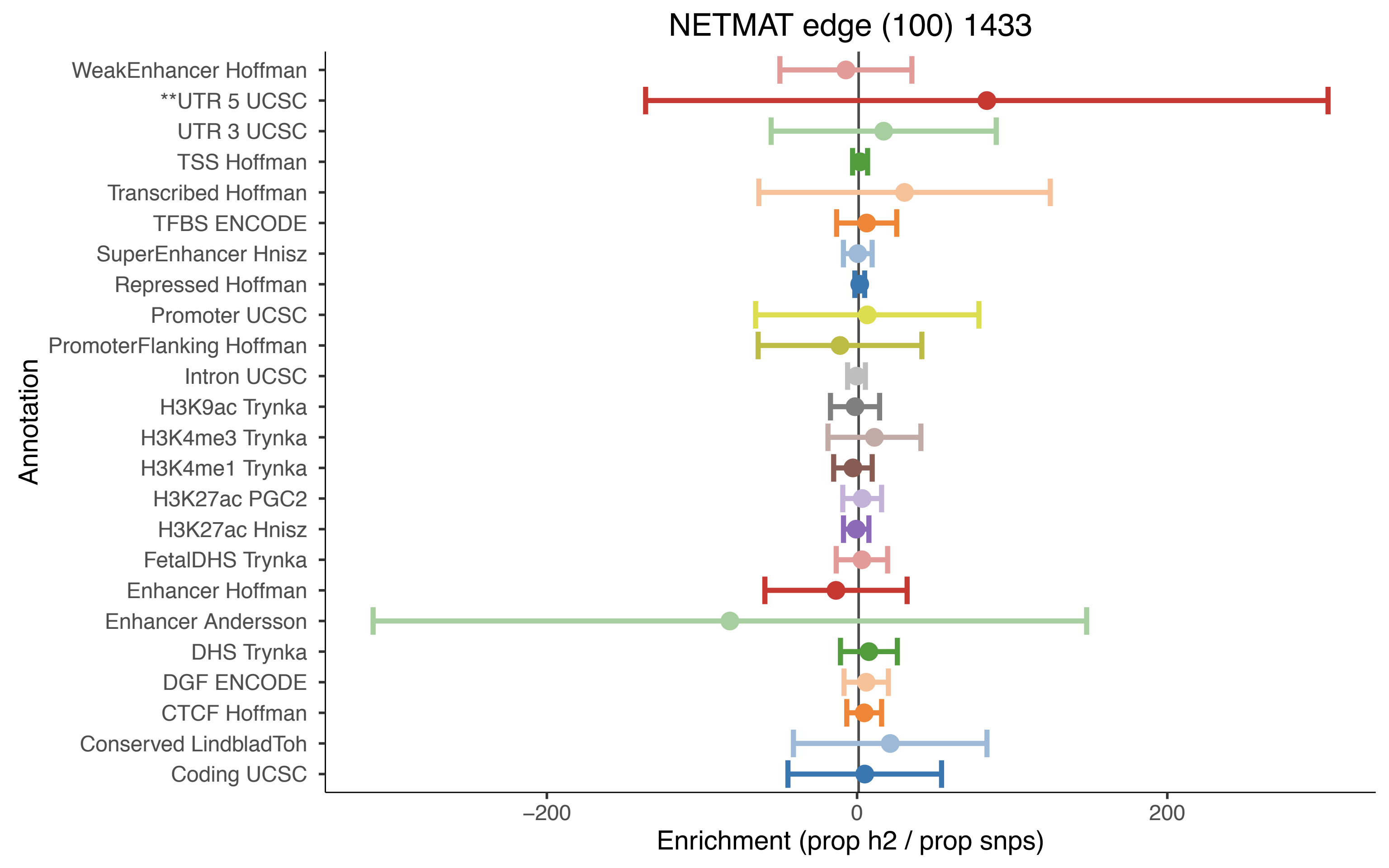
NETMAT edge (100) 1433
WeakEnhancer Hoffman
**UTR 5 UCSC
UTR 3 UCSC
TSS Hoffman
Transcribed Hoffman
TFBS ENCODE
SuperEnhancer Hnisz
Repressed Hoffman
Promoter UCSC
PromoterFlanking Hoffman
Intron UCSC
H3K9ac Trynka
H3K4me3 Trynka
H3K4me1 Trynka
H3K27ac PGC2
H3K27ac Hnisz
FetalDHS Trynka
Enhancer Hoffman
Enhancer Andersson
DHS Trynka
DGF ENCODE
CTCF Hoffman
Conserved LindbladToh
Coding UCSC
Annotation
−200
0
200
Enrichment (prop h2 / prop snps)

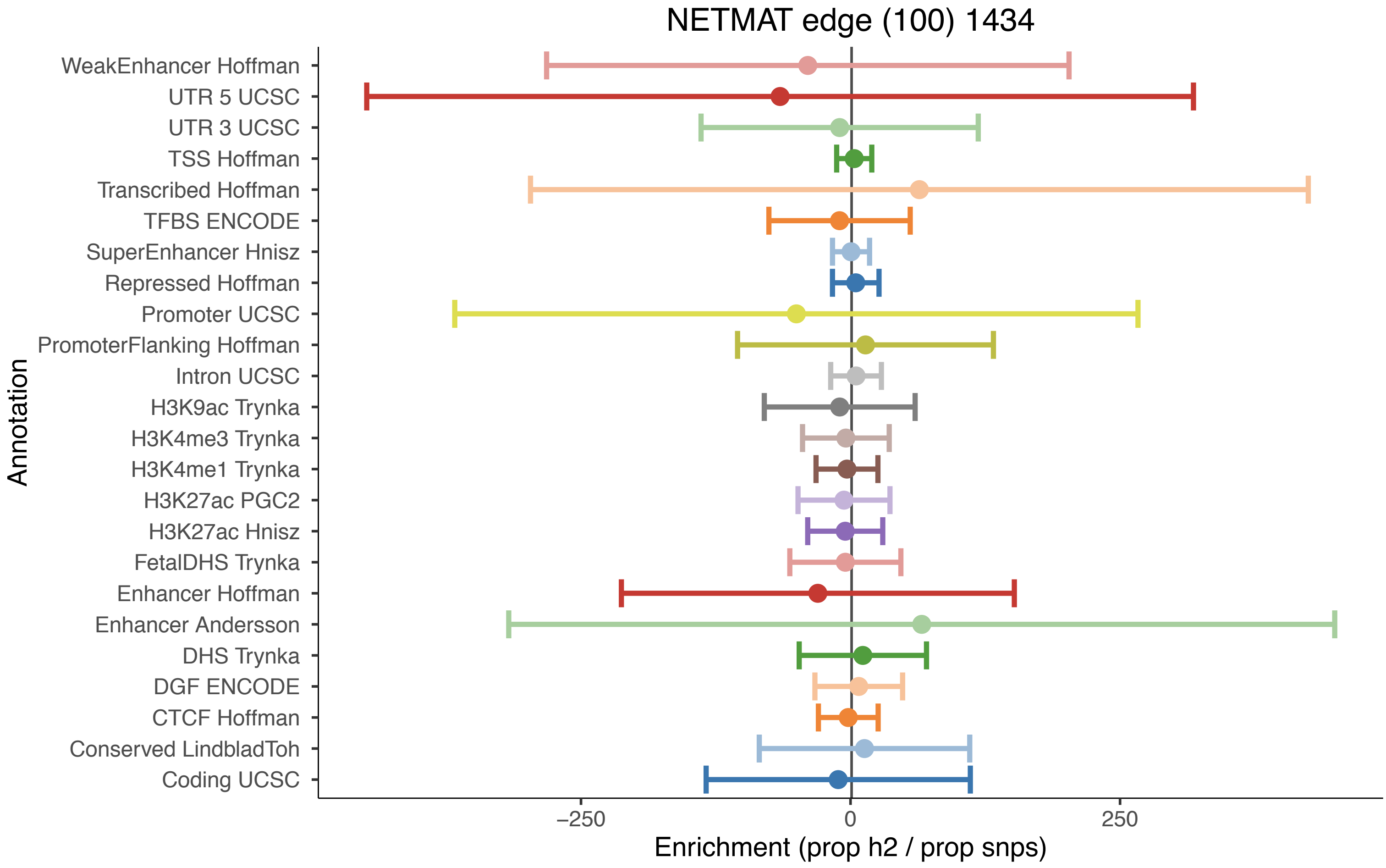

NETMAT edge (100) 1434
WeakEnhancer Hoffman
UTR 5 UCSC
UTR 3 UCSC
TSS Hoffman
Transcribed Hoffman
TFBS ENCODE
SuperEnhancer Hnisz
Repressed Hoffman
Promoter UCSC
PromoterFlanking Hoffman
Intron UCSC
H3K9ac Trynka
H3K4me3 Trynka
H3K4me1 Trynka
H3K27ac PGC2
H3K27ac Hnisz
FetalDHS Trynka
Enhancer Hoffman
Enhancer Andersson
DHS Trynka
DGF ENCODE
CTCF Hoffman
Conserved LindbladToh
Coding UCSC
Annotation
−250
0
250
Enrichment (prop h2 / prop snps)

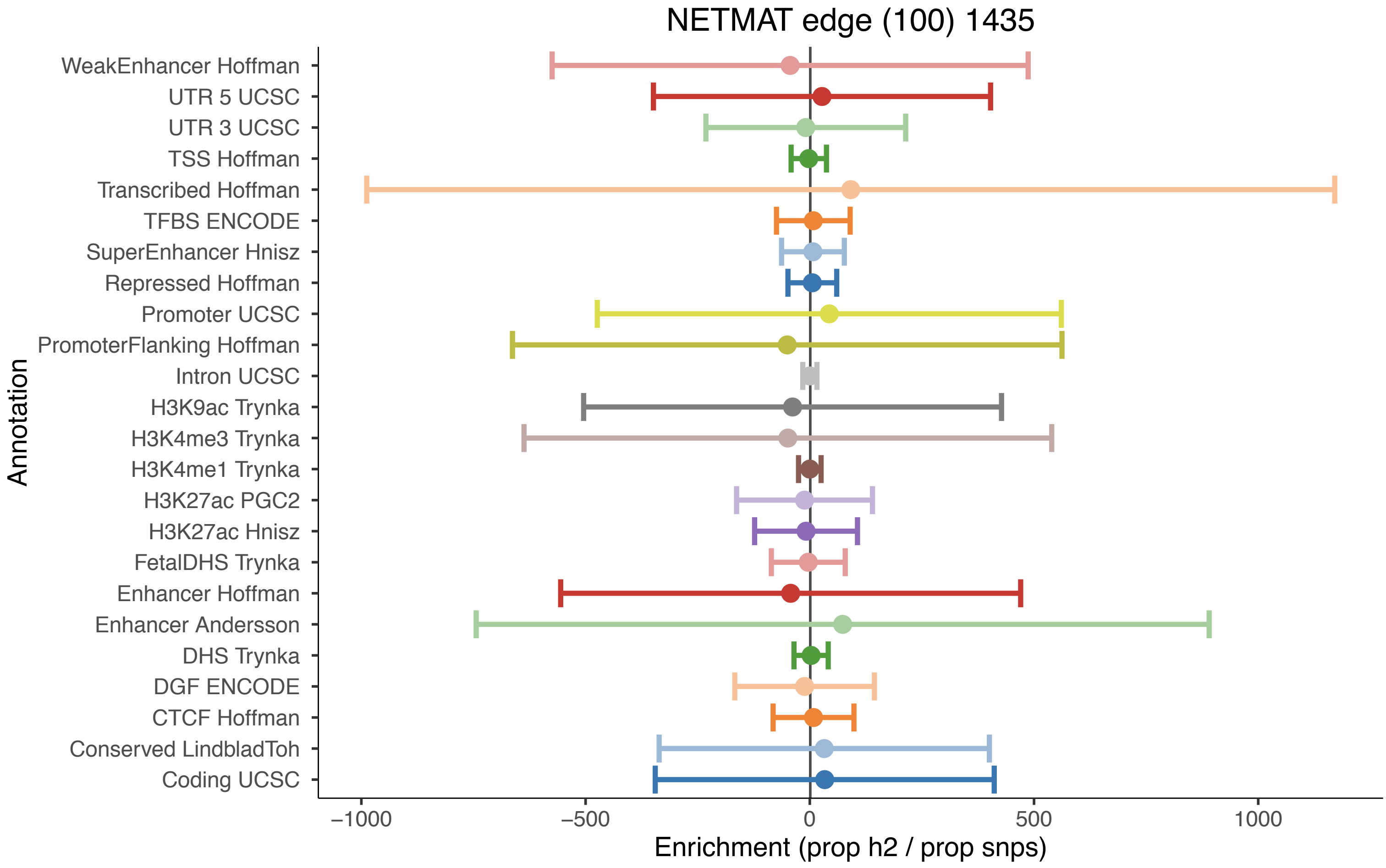
NETMAT edge (100) 1435
Annotation
WeakEnhancer Hoffman
UTR 5 UCSC
UTR 3 UCSC
TSS Hoffman
Transcribed Hoffman
TFBS ENCODE
SuperEnhancer Hnisz
Repressed Hoffman
Promoter UCSC
PromoterFlanking Hoffman
Intron UCSC
H3K9ac Trynka
H3K4me3 Trynka
H3K4me1 Trynka
H3K27ac PGC2
H3K27ac Hnisz
FetalDHS Trynka
Enhancer Hoffman
Enhancer Andersson
DHS Trynka
DGF ENCODE
CTCF Hoffman
Conserved LindbladToh
Coding UCSC
−1000
−500
0
500
1000
Enrichment (prop h2 / prop snps)

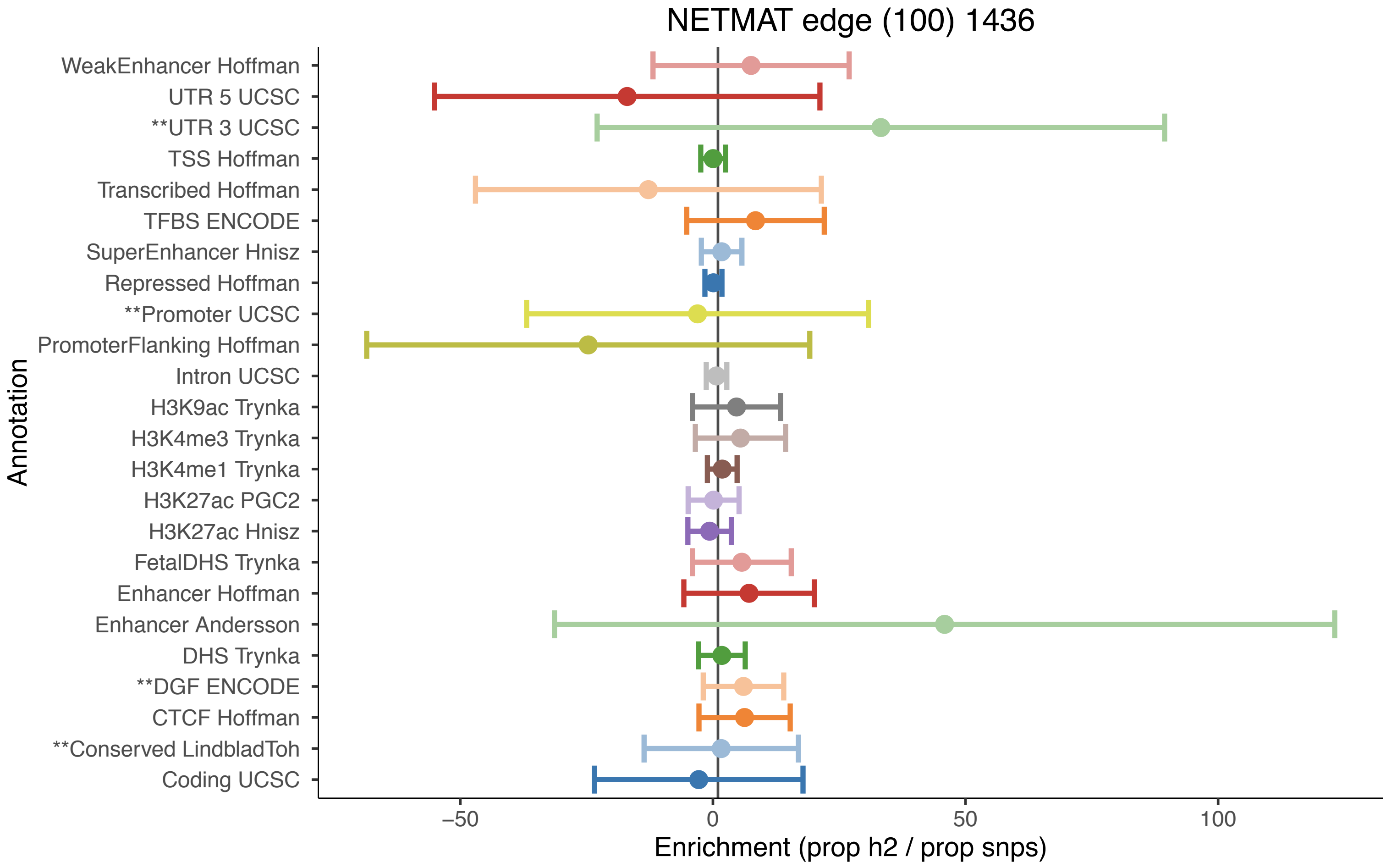

NETMAT edge (100) 1436
WeakEnhancer Hoffman
UTR 5 UCSC
**UTR 3 UCSC
TSS Hoffman
Transcribed Hoffman
TFBS ENCODE
SuperEnhancer Hnisz
Repressed Hoffman
**Promoter UCSC
PromoterFlanking Hoffman
Intron UCSC
H3K9ac Trynka
H3K4me3 Trynka
H3K4me1 Trynka
H3K27ac PGC2
H3K27ac Hnisz
FetalDHS Trynka
Enhancer Hoffman
Enhancer Andersson
DHS Trynka
**DGF ENCODE
CTCF Hoffman
**Conserved LindbladToh
Coding UCSC
Annotation
−50
0
50
100
Enrichment (prop h2 / prop snps)

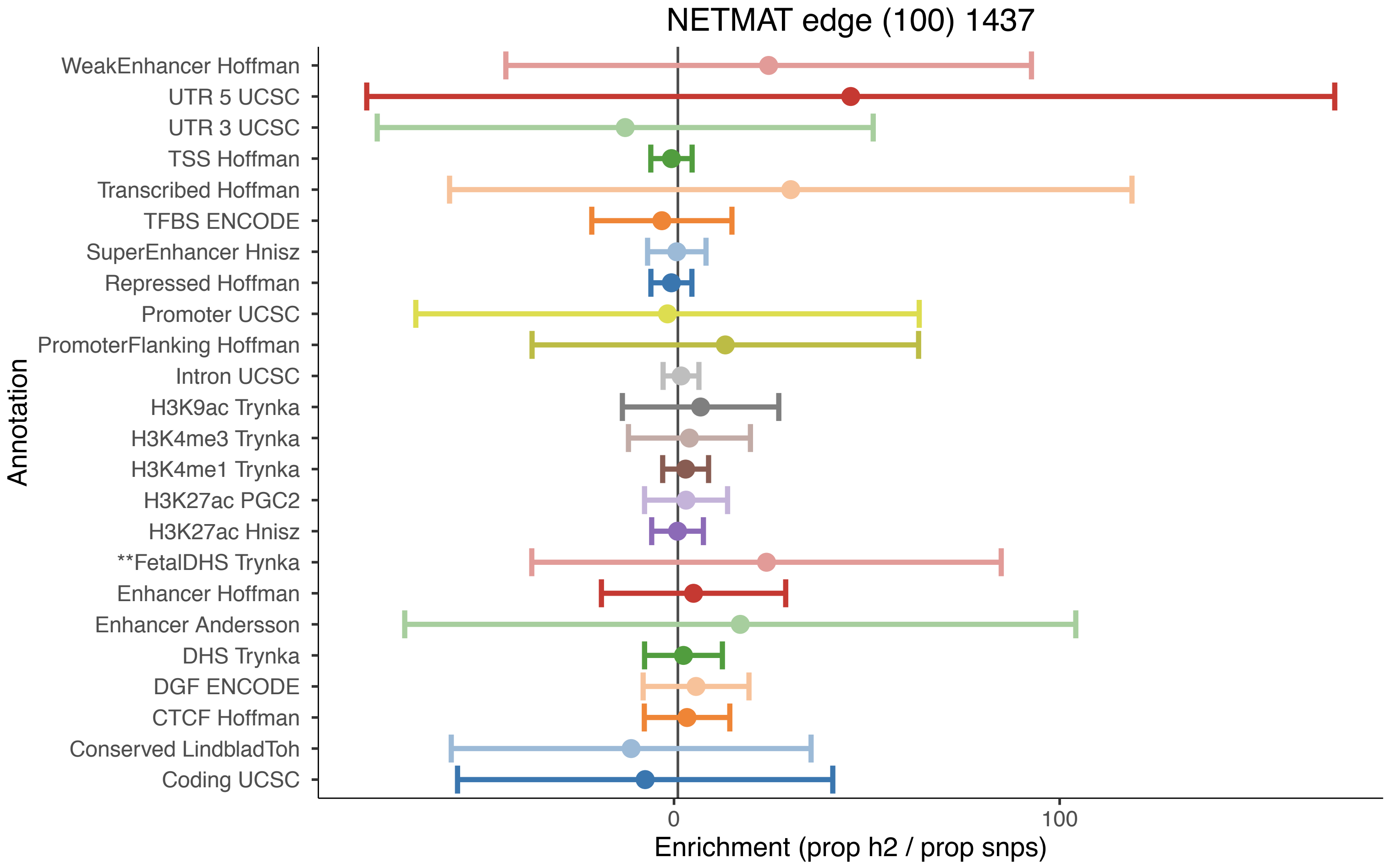
NETMAT edge (100) 1437
WeakEnhancer Hoffman
UTR 5 UCSC
UTR 3 UCSC
TSS Hoffman
Transcribed Hoffman
TFBS ENCODE
SuperEnhancer Hnisz
Repressed Hoffman
Promoter UCSC
PromoterFlanking Hoffman
Intron UCSC
H3K9ac Trynka
H3K4me3 Trynka
H3K4me1 Trynka
H3K27ac PGC2
H3K27ac Hnisz
**FetalDHS Trynka
Enhancer Hoffman
Enhancer Andersson
DHS Trynka
DGF ENCODE
CTCF Hoffman
Conserved LindbladToh
Coding UCSC
Annotation
0
100
Enrichment (prop h2 / prop snps)

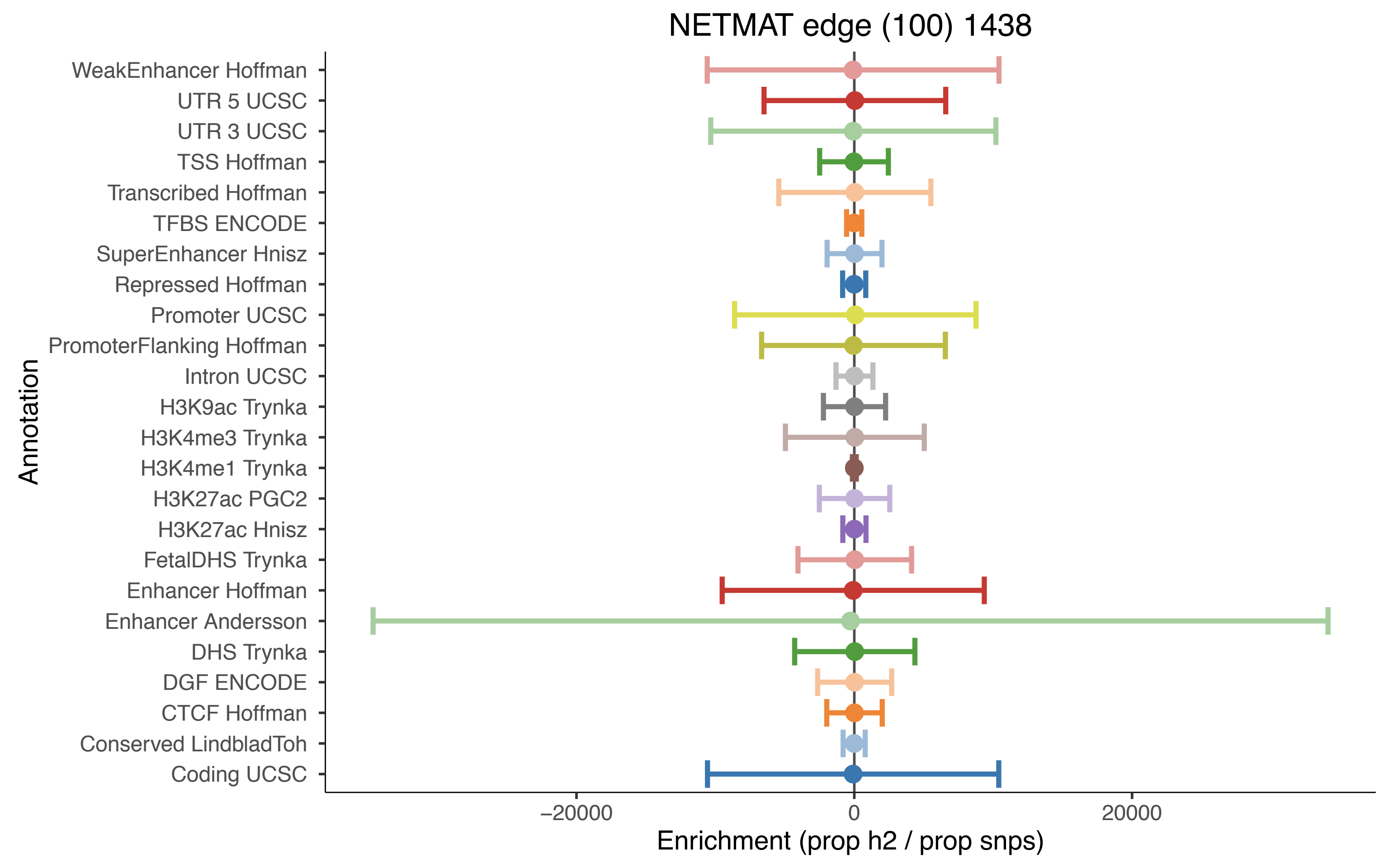

NETMAT edge (100) 1438
WeakEnhancer Hoffman
UTR 5 UCSC
UTR 3 UCSC
TSS Hoffman
Transcribed Hoffman
TFBS ENCODE
SuperEnhancer Hnisz
Repressed Hoffman
Promoter UCSC
PromoterFlanking Hoffman
Intron UCSC
H3K9ac Trynka
H3K4me3 Trynka
H3K4me1 Trynka
H3K27ac PGC2
H3K27ac Hnisz
FetalDHS Trynka
Enhancer Hoffman
Enhancer Andersson
DHS Trynka
DGF ENCODE
CTCF Hoffman
Conserved LindbladToh
Coding UCSC
Annotation
−20000
0
20000
Enrichment (prop h2 / prop snps)

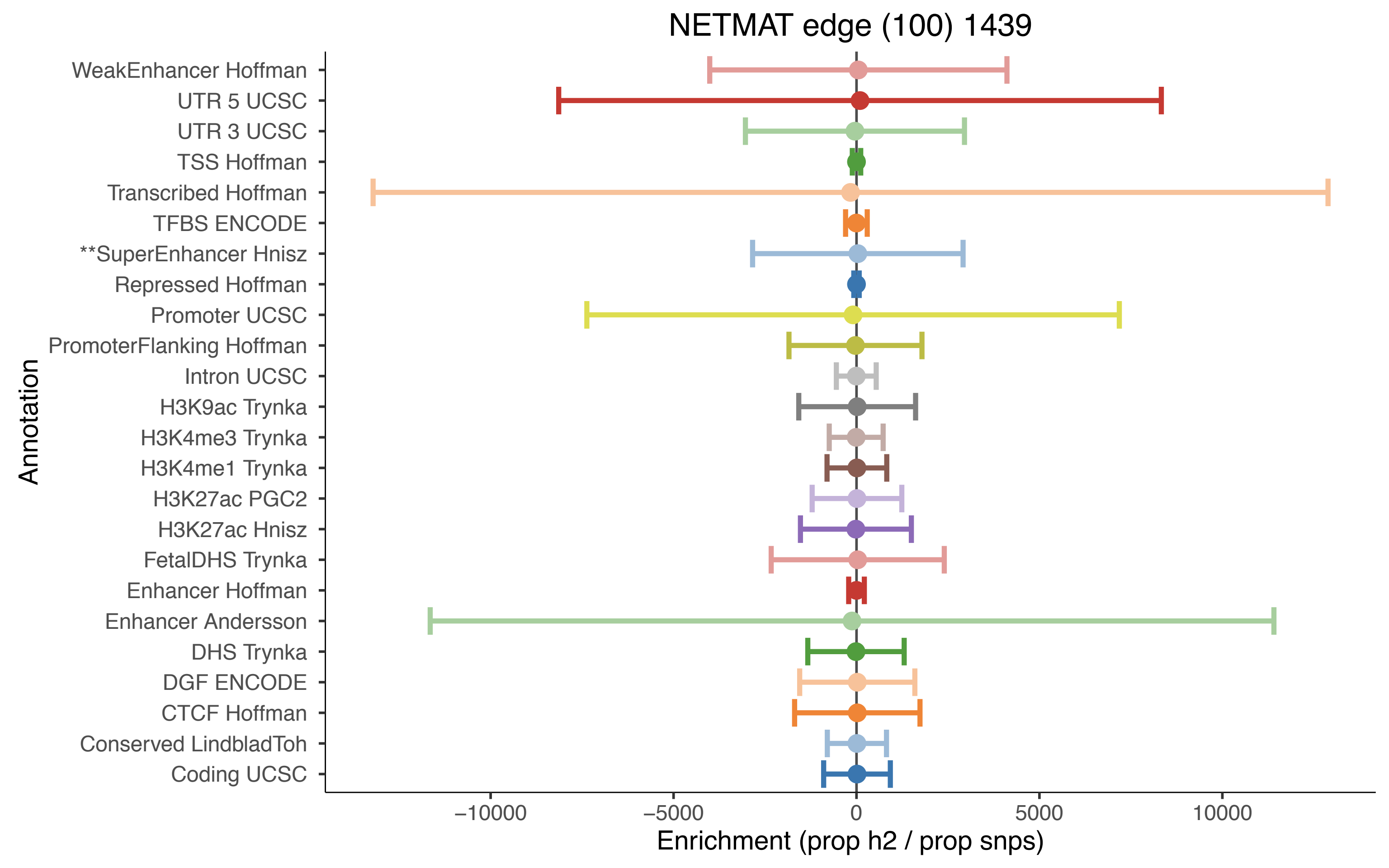
NETMAT edge (100) 1439
WeakEnhancer Hoffman
UTR 5 UCSC
UTR 3 UCSC
TSS Hoffman
Transcribed Hoffman
TFBS ENCODE
**SuperEnhancer Hnisz
Repressed Hoffman
Promoter UCSC
PromoterFlanking Hoffman
Intron UCSC
H3K9ac Trynka
H3K4me3 Trynka
H3K4me1 Trynka
H3K27ac PGC2
H3K27ac Hnisz
FetalDHS Trynka
Enhancer Hoffman
Enhancer Andersson
DHS Trynka
DGF ENCODE
CTCF Hoffman
Conserved LindbladToh
Coding UCSC
Annotation
−10000
−5000
0
5000
10000
Enrichment (prop h2 / prop snps)

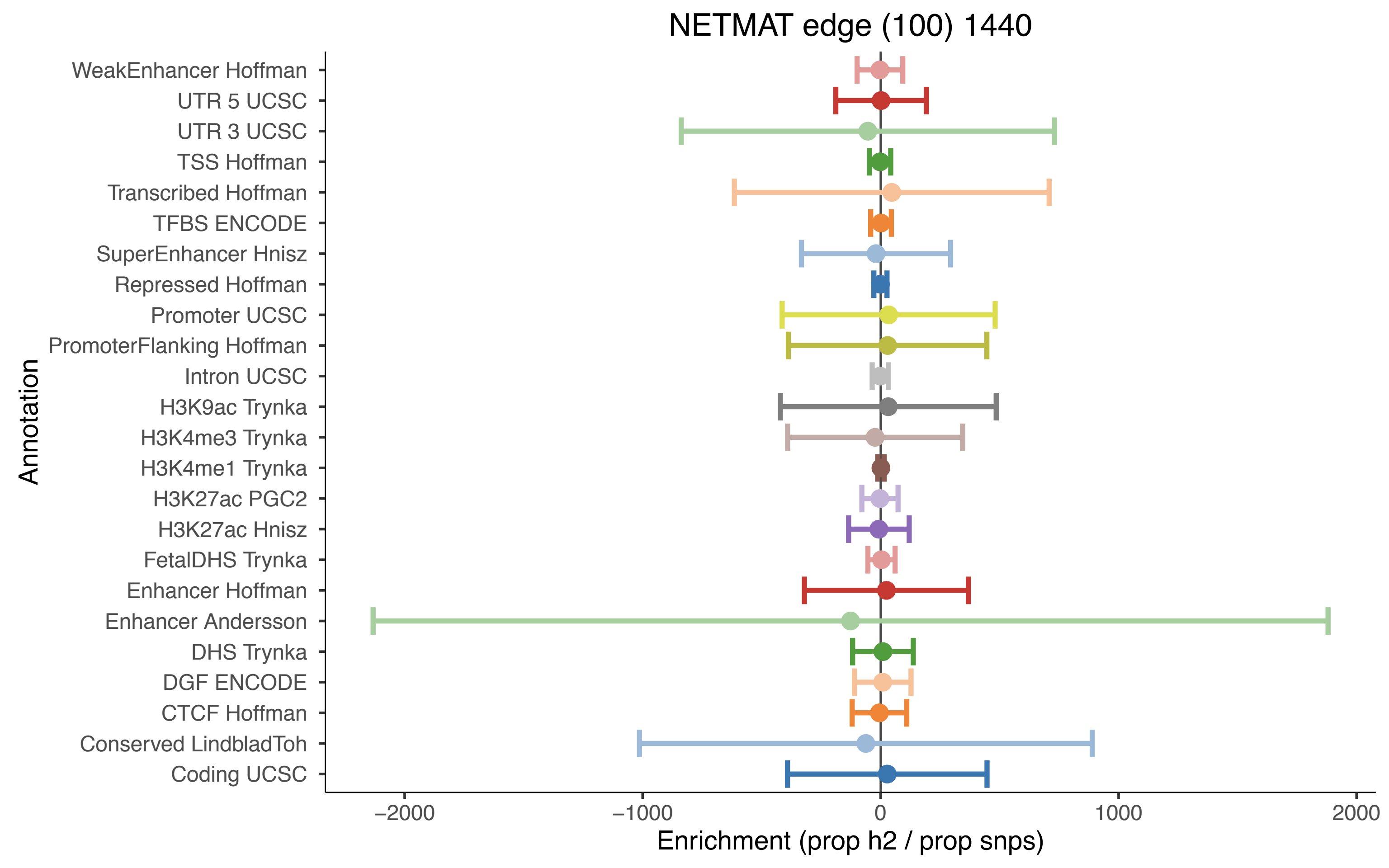
NETMAT edge (100) 1440
Annotation
WeakEnhancer Hoffman
UTR 5 UCSC
UTR 3 UCSC
TSS Hoffman
Transcribed Hoffman
TFBS ENCODE
SuperEnhancer Hnisz
Repressed Hoffman
Promoter UCSC
PromoterFlanking Hoffman
Intron UCSC
H3K9ac Trynka
H3K4me3 Trynka
H3K4me1 Trynka
H3K27ac PGC2
H3K27ac Hnisz
FetalDHS Trynka
Enhancer Hoffman
Enhancer Andersson
DHS Trynka
DGF ENCODE
CTCF Hoffman
Conserved LindbladToh
Coding UCSC
−2000
−1000
0
1000
2000
Enrichment (prop h2 / prop snps)

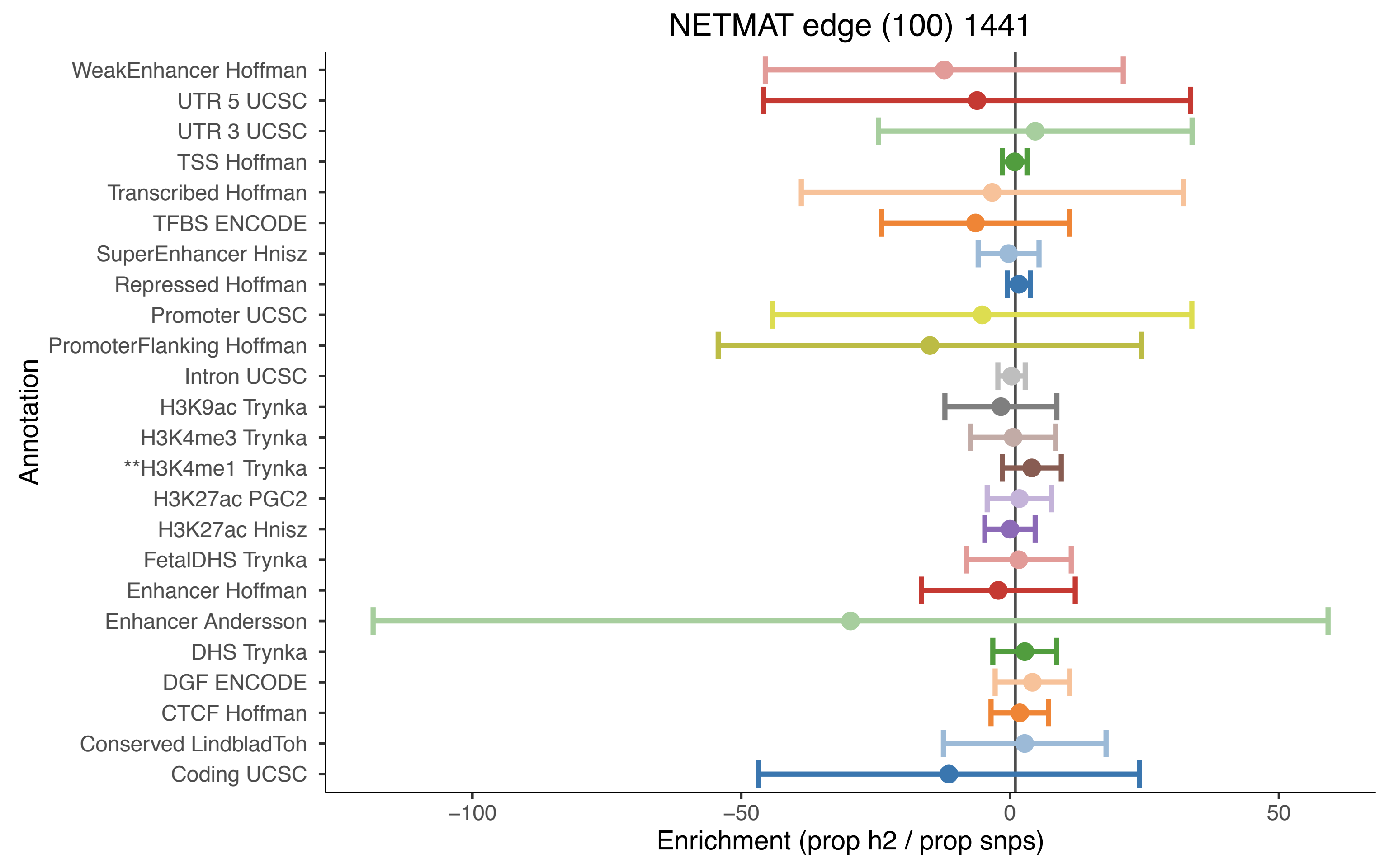
NETMAT edge (100) 1441
WeakEnhancer Hoffman
UTR 5 UCSC
UTR 3 UCSC
TSS Hoffman
Transcribed Hoffman
TFBS ENCODE
SuperEnhancer Hnisz
Repressed Hoffman
Promoter UCSC
PromoterFlanking Hoffman
Intron UCSC
H3K9ac Trynka
H3K4me3 Trynka
**H3K4me1 Trynka
H3K27ac PGC2
H3K27ac Hnisz
FetalDHS Trynka
Enhancer Hoffman
Enhancer Andersson
DHS Trynka
DGF ENCODE
CTCF Hoffman
Conserved LindbladToh
Coding UCSC
Annotation
−100
−50
0
50
Enrichment (prop h2 / prop snps)

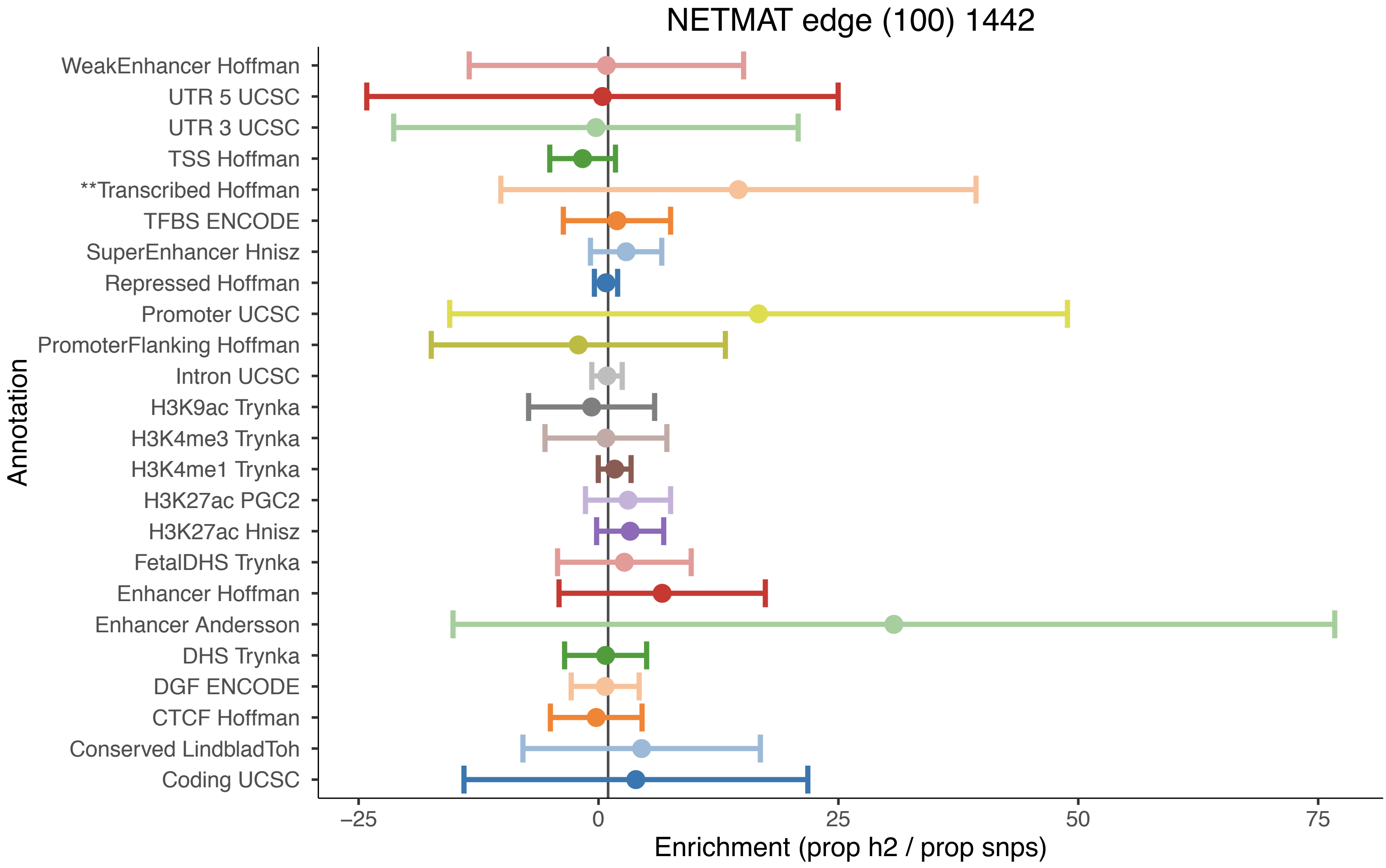
NETMAT edge (100) 1442
Annotation
WeakEnhancer Hoffman
UTR 5 UCSC
UTR 3 UCSC
TSS Hoffman
**Transcribed Hoffman
TFBS ENCODE
SuperEnhancer Hnisz
Repressed Hoffman
Promoter UCSC
PromoterFlanking Hoffman
Intron UCSC
H3K9ac Trynka
H3K4me3 Trynka
H3K4me1 Trynka
H3K27ac PGC2
H3K27ac Hnisz
FetalDHS Trynka
Enhancer Hoffman
Enhancer Andersson
DHS Trynka
DGF ENCODE
CTCF Hoffman
Conserved LindbladToh
Coding UCSC
−25
0
25
50
75
Enrichment (prop h2 / prop snps)

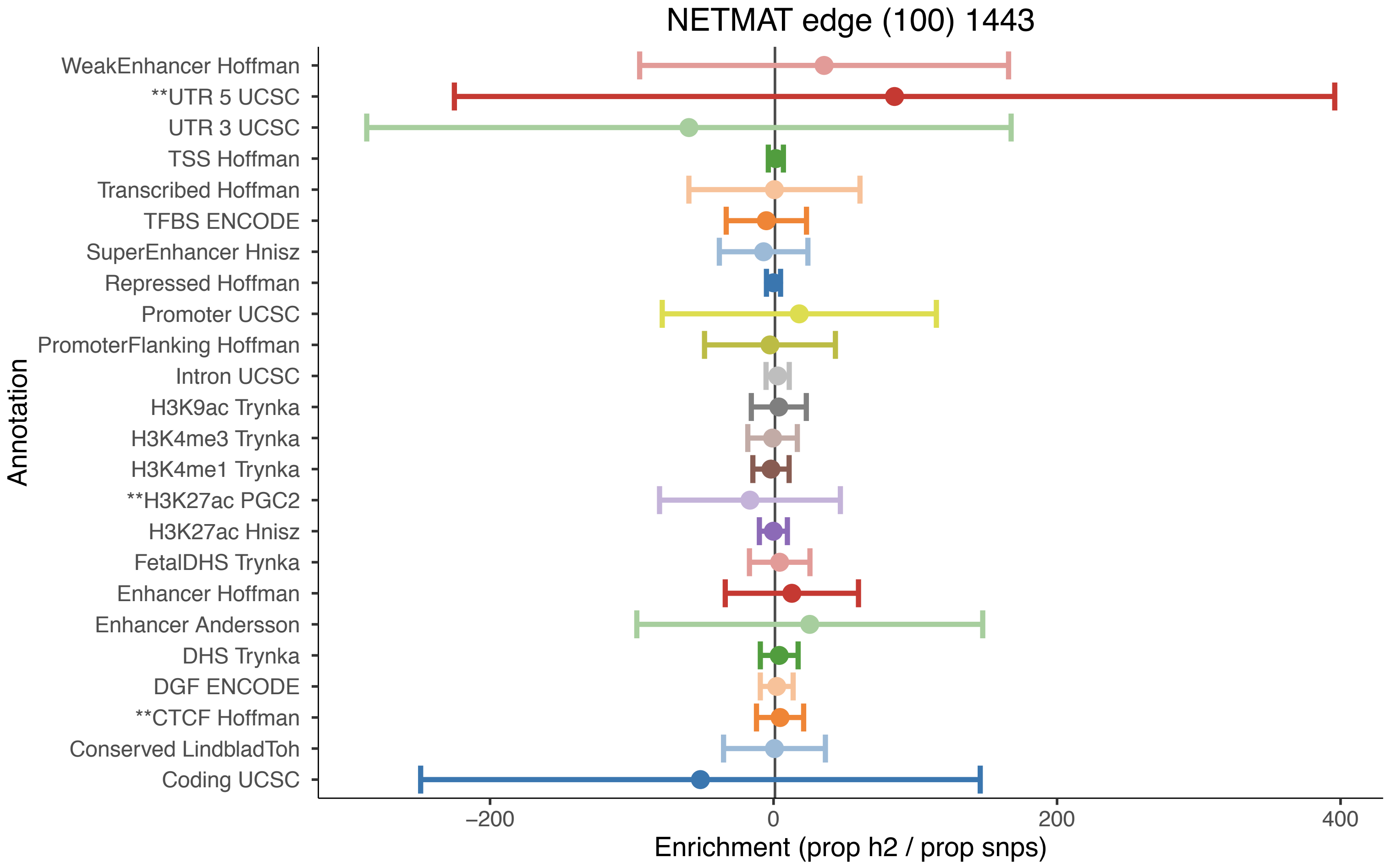

NETMAT edge (100) 1443
WeakEnhancer Hoffman
**UTR 5 UCSC
UTR 3 UCSC
TSS Hoffman
Transcribed Hoffman
TFBS ENCODE
SuperEnhancer Hnisz
Repressed Hoffman
Promoter UCSC
PromoterFlanking Hoffman
Intron UCSC
H3K9ac Trynka
H3K4me3 Trynka
H3K4me1 Trynka
**H3K27ac PGC2
H3K27ac Hnisz
FetalDHS Trynka
Enhancer Hoffman
Enhancer Andersson
DHS Trynka
DGF ENCODE
**CTCF Hoffman
Conserved LindbladToh
Coding UCSC
Annotation
−200
0
200
400
Enrichment (prop h2 / prop snps)

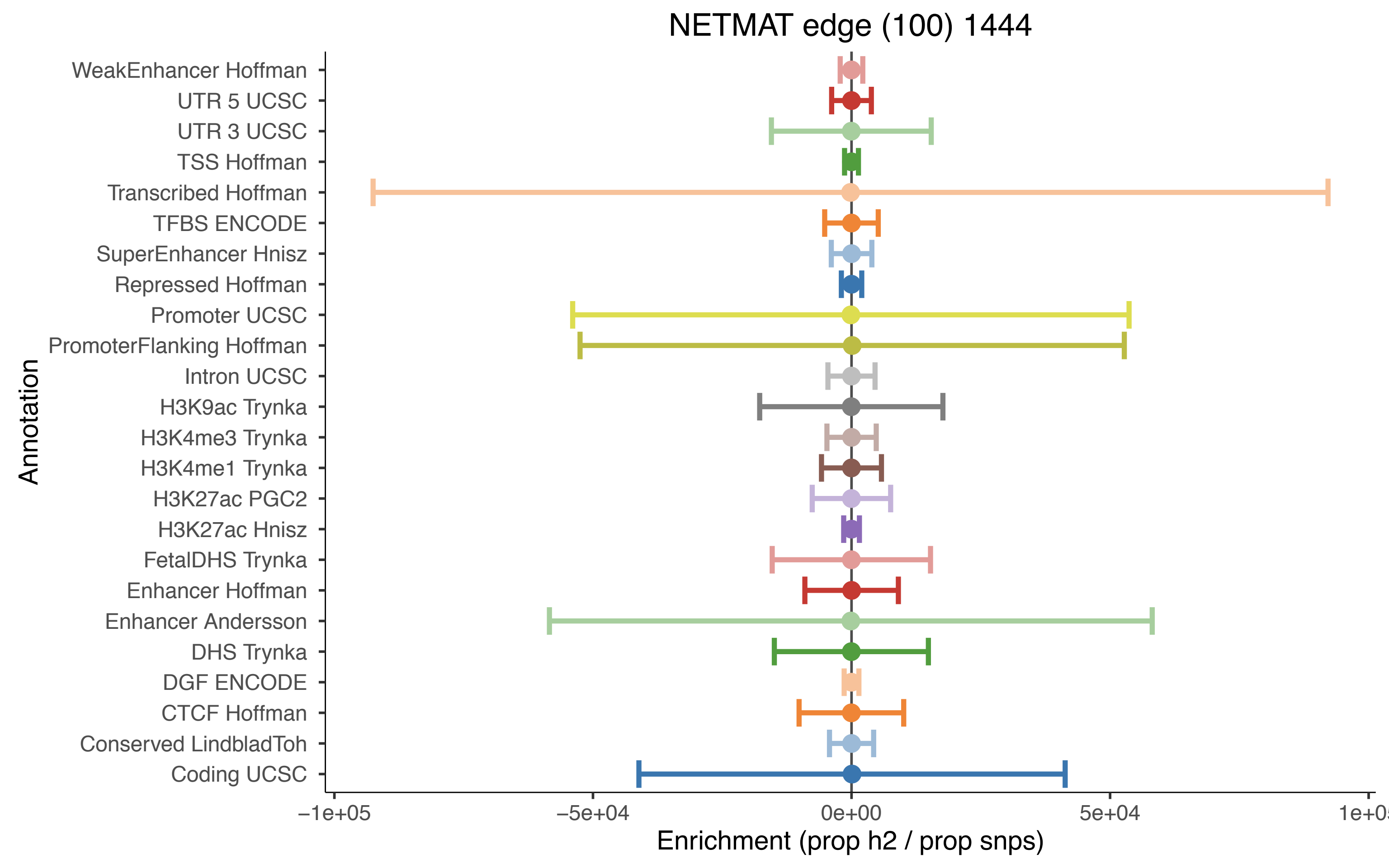

NETMAT edge (100) 1444
WeakEnhancer Hoffman
UTR 5 UCSC
UTR 3 UCSC
TSS Hoffman
Transcribed Hoffman
TFBS ENCODE
SuperEnhancer Hnisz
Repressed Hoffman
Promoter UCSC
PromoterFlanking Hoffman
Intron UCSC
H3K9ac Trynka
H3K4me3 Trynka
H3K4me1 Trynka
H3K27ac PGC2
H3K27ac Hnisz
FetalDHS Trynka
Enhancer Hoffman
Enhancer Andersson
DHS Trynka
DGF ENCODE
CTCF Hoffman
Conserved LindbladToh
Coding UCSC
Annotation
−1e+05
−5e+04
0e+00
5e+04
Enrichment (prop h2 / prop snps)

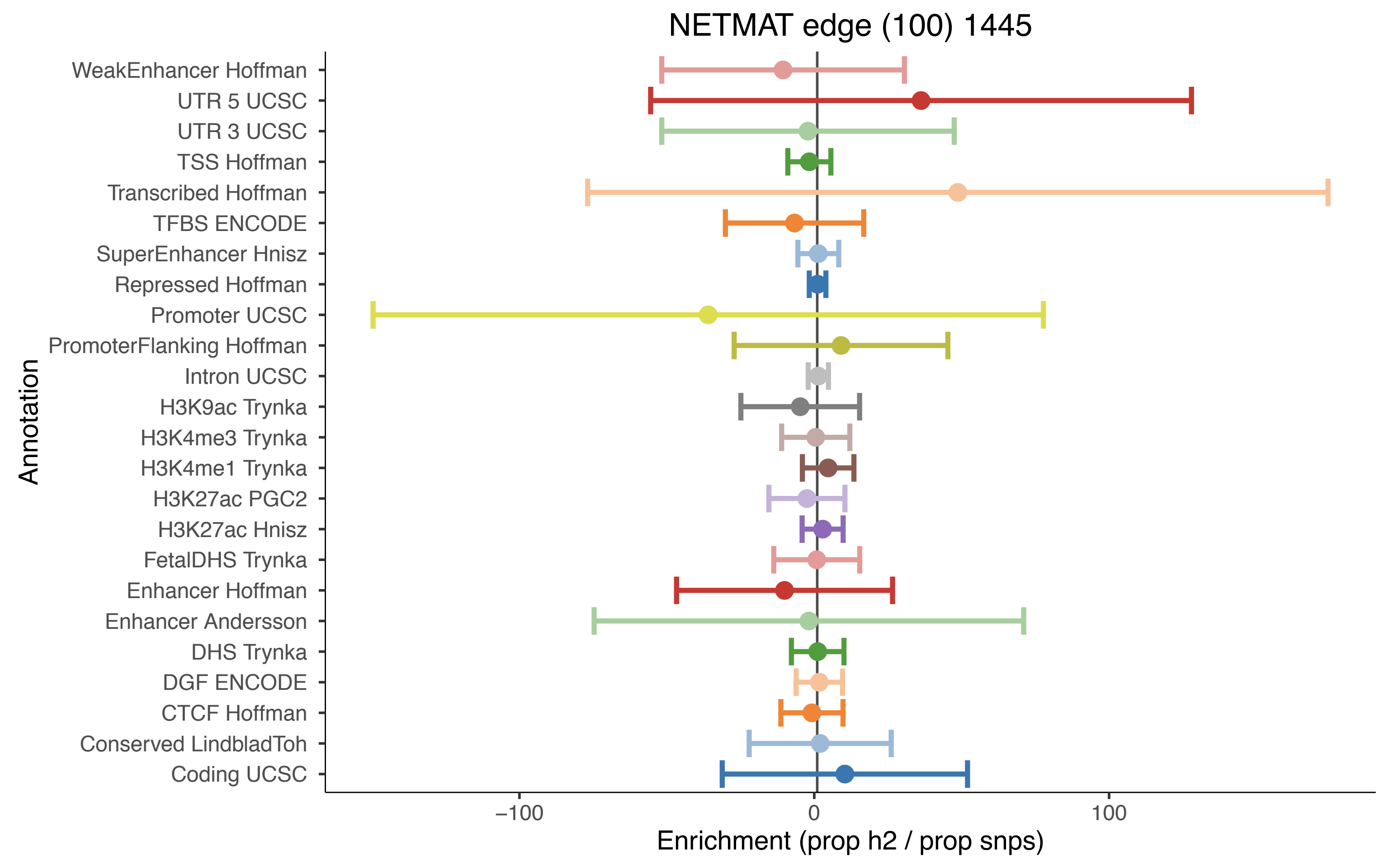
NETMAT edge (100) 1445
Annotation
WeakEnhancer Hoffman
UTR 5 UCSC
UTR 3 UCSC
TSS Hoffman
Transcribed Hoffman
TFBS ENCODE
SuperEnhancer Hnisz
Repressed Hoffman
Promoter UCSC
PromoterFlanking Hoffman
Intron UCSC
H3K9ac Trynka
H3K4me3 Trynka
H3K4me1 Trynka
H3K27ac PGC2
H3K27ac Hnisz
FetalDHS Trynka
Enhancer Hoffman
Enhancer Andersson
DHS Trynka
DGF ENCODE
CTCF Hoffman
Conserved LindbladToh
Coding UCSC
−100
0
100
Enrichment (prop h2 / prop snps)

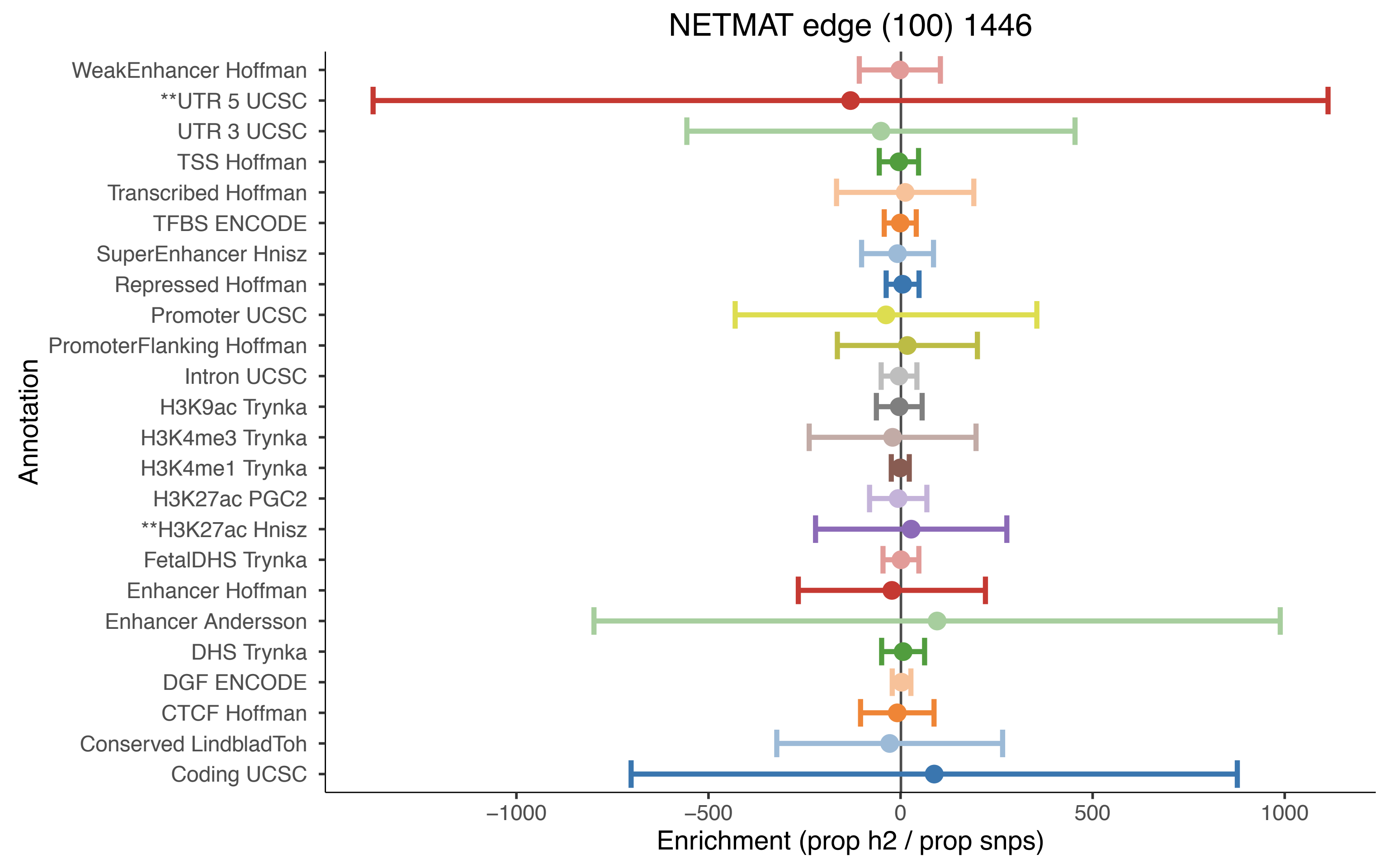

NETMAT edge (100) 1446
WeakEnhancer Hoffman
**UTR 5 UCSC
UTR 3 UCSC
TSS Hoffman
Transcribed Hoffman
TFBS ENCODE
SuperEnhancer Hnisz
Repressed Hoffman
Promoter UCSC
PromoterFlanking Hoffman
Intron UCSC
H3K9ac Trynka
H3K4me3 Trynka
H3K4me1 Trynka
H3K27ac PGC2
**H3K27ac Hnisz
FetalDHS Trynka
Enhancer Hoffman
Enhancer Andersson
DHS Trynka
DGF ENCODE
CTCF Hoffman
Conserved LindbladToh
Coding UCSC
Annotation
−1000
−500
0
500
1000
Enrichment (prop h2 / prop snps)

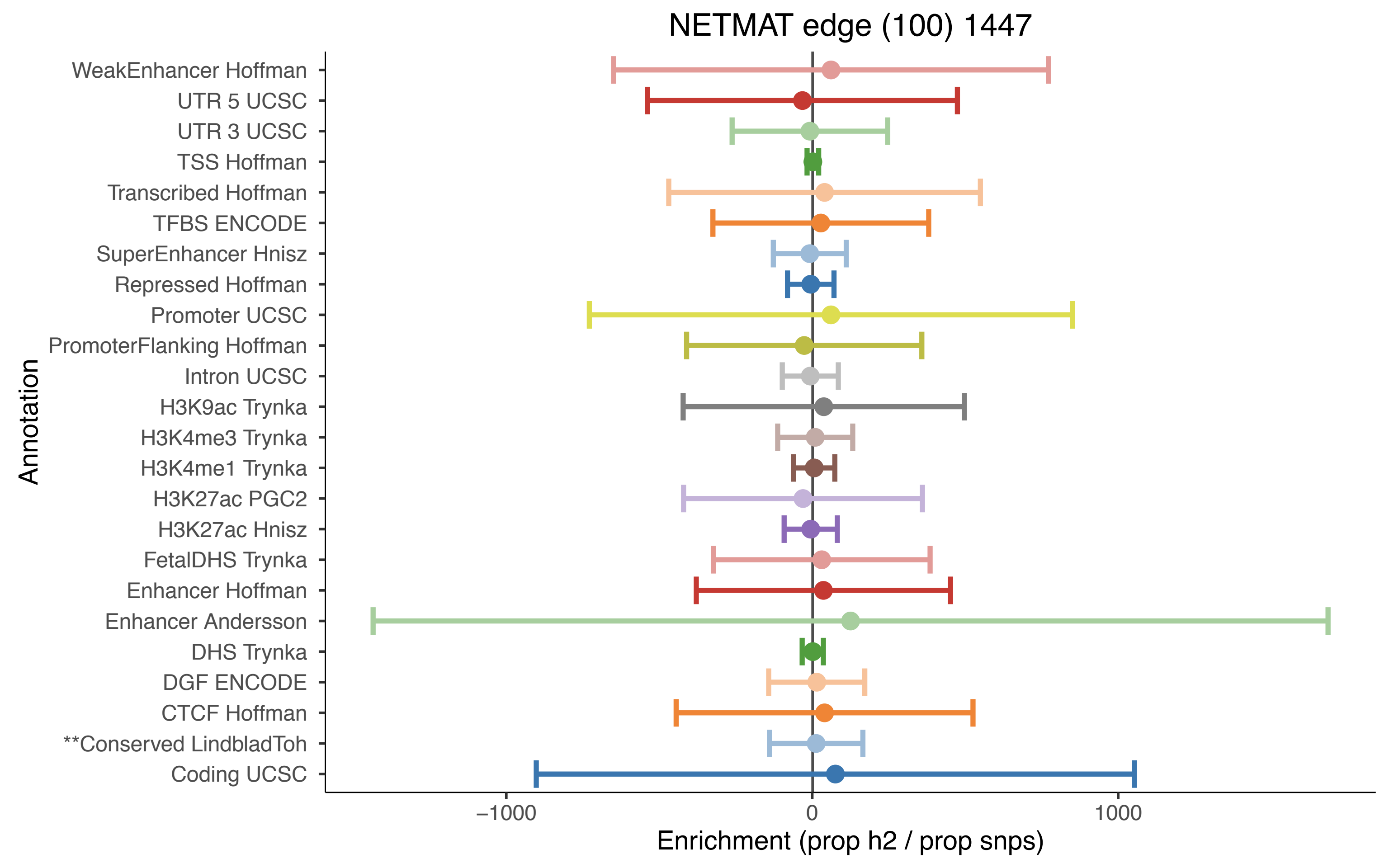

NETMAT edge (100) 1447
WeakEnhancer Hoffman
UTR 5 UCSC
UTR 3 UCSC
TSS Hoffman
Transcribed Hoffman
TFBS ENCODE
SuperEnhancer Hnisz
Repressed Hoffman
Promoter UCSC
PromoterFlanking Hoffman
Intron UCSC
H3K9ac Trynka
H3K4me3 Trynka
H3K4me1 Trynka
H3K27ac PGC2
H3K27ac Hnisz
FetalDHS Trynka
Enhancer Hoffman
Enhancer Andersson
DHS Trynka
DGF ENCODE
CTCF Hoffman
**Conserved LindbladToh
Coding UCSC
Annotation
−1000
0
1000
Enrichment (prop h2 / prop snps)

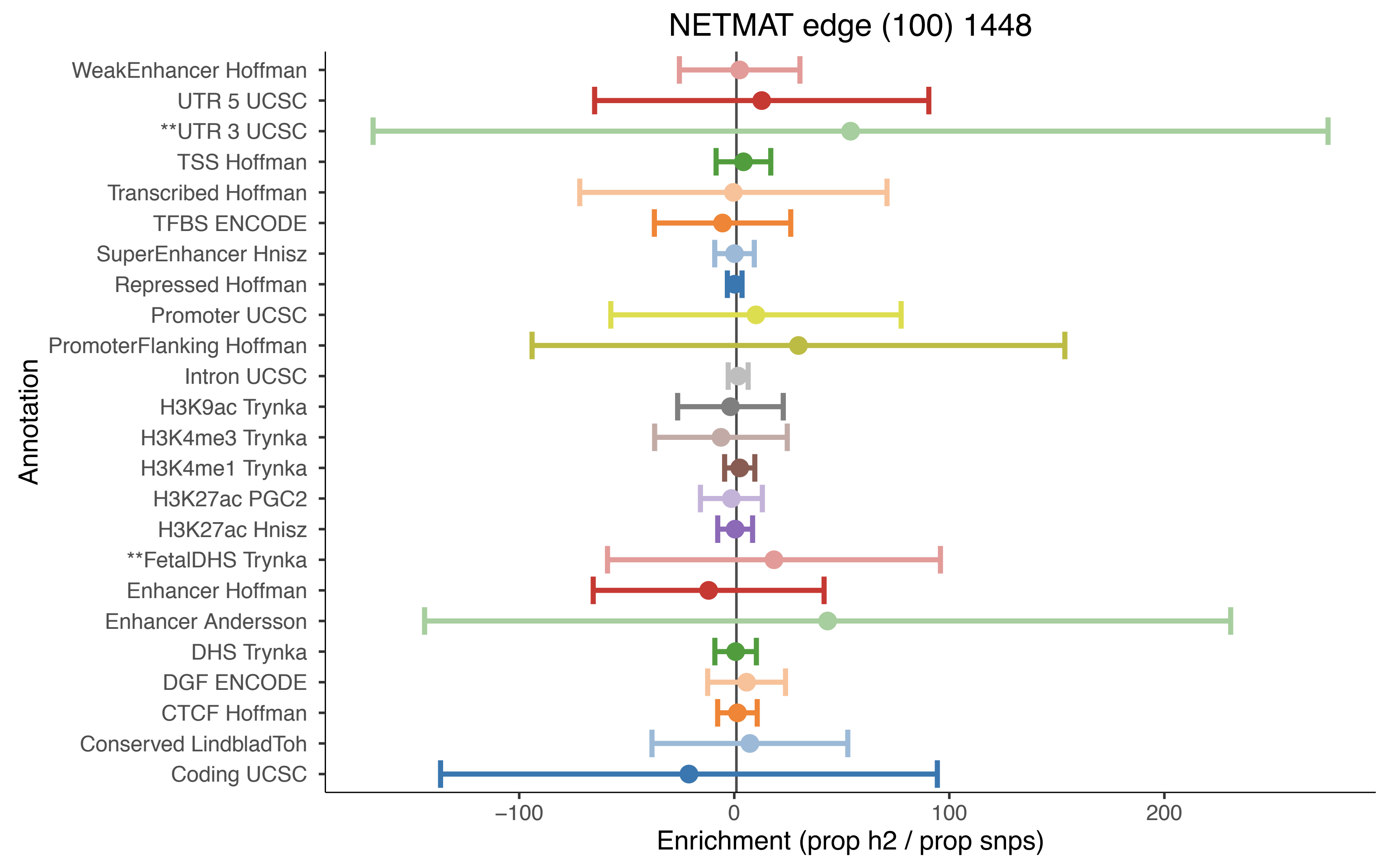

NETMAT edge (100) 1448
Annotation
WeakEnhancer Hoffman
UTR 5 UCSC
**UTR 3 UCSC
TSS Hoffman
Transcribed Hoffman
TFBS ENCODE
SuperEnhancer Hnisz
Repressed Hoffman
Promoter UCSC
PromoterFlanking Hoffman
Intron UCSC
H3K9ac Trynka
H3K4me3 Trynka
H3K4me1 Trynka
H3K27ac PGC2
H3K27ac Hnisz
**FetalDHS Trynka
Enhancer Hoffman
Enhancer Andersson
DHS Trynka
DGF ENCODE
CTCF Hoffman
Conserved LindbladToh
Coding UCSC
−100
0
100
200
Enrichment (prop h2 / prop snps)

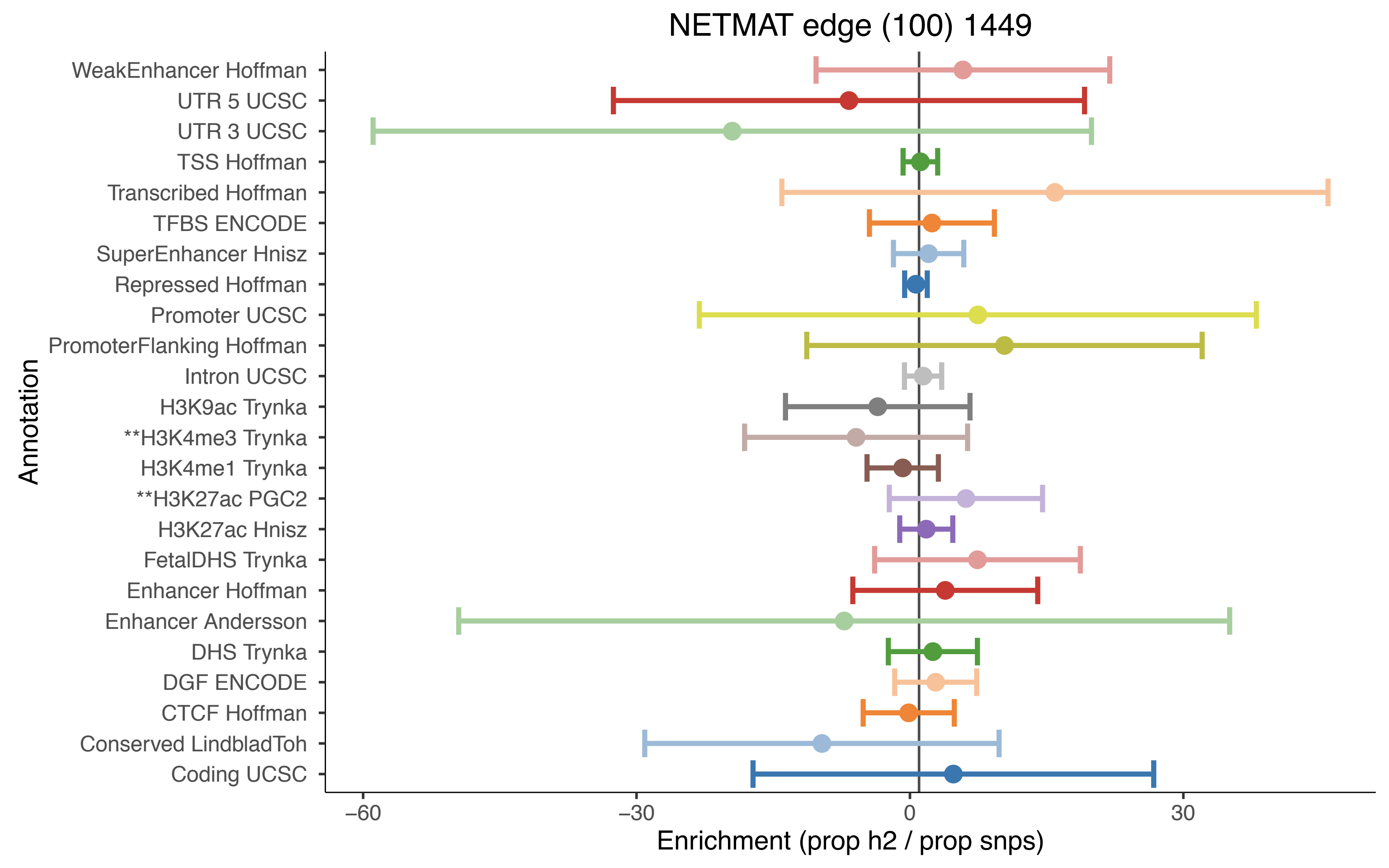

NETMAT edge (100) 1449
WeakEnhancer Hoffman
UTR 5 UCSC
UTR 3 UCSC
TSS Hoffman
Transcribed Hoffman
TFBS ENCODE
SuperEnhancer Hnisz
Repressed Hoffman
Promoter UCSC
PromoterFlanking Hoffman
Intron UCSC
H3K9ac Trynka
**H3K4me3 Trynka
H3K4me1 Trynka
**H3K27ac PGC2
H3K27ac Hnisz
FetalDHS Trynka
Enhancer Hoffman
Enhancer Andersson
DHS Trynka
DGF ENCODE
CTCF Hoffman
Conserved LindbladToh
Coding UCSC
Annotation
−60
−30
0
30
Enrichment (prop h2 / prop snps)

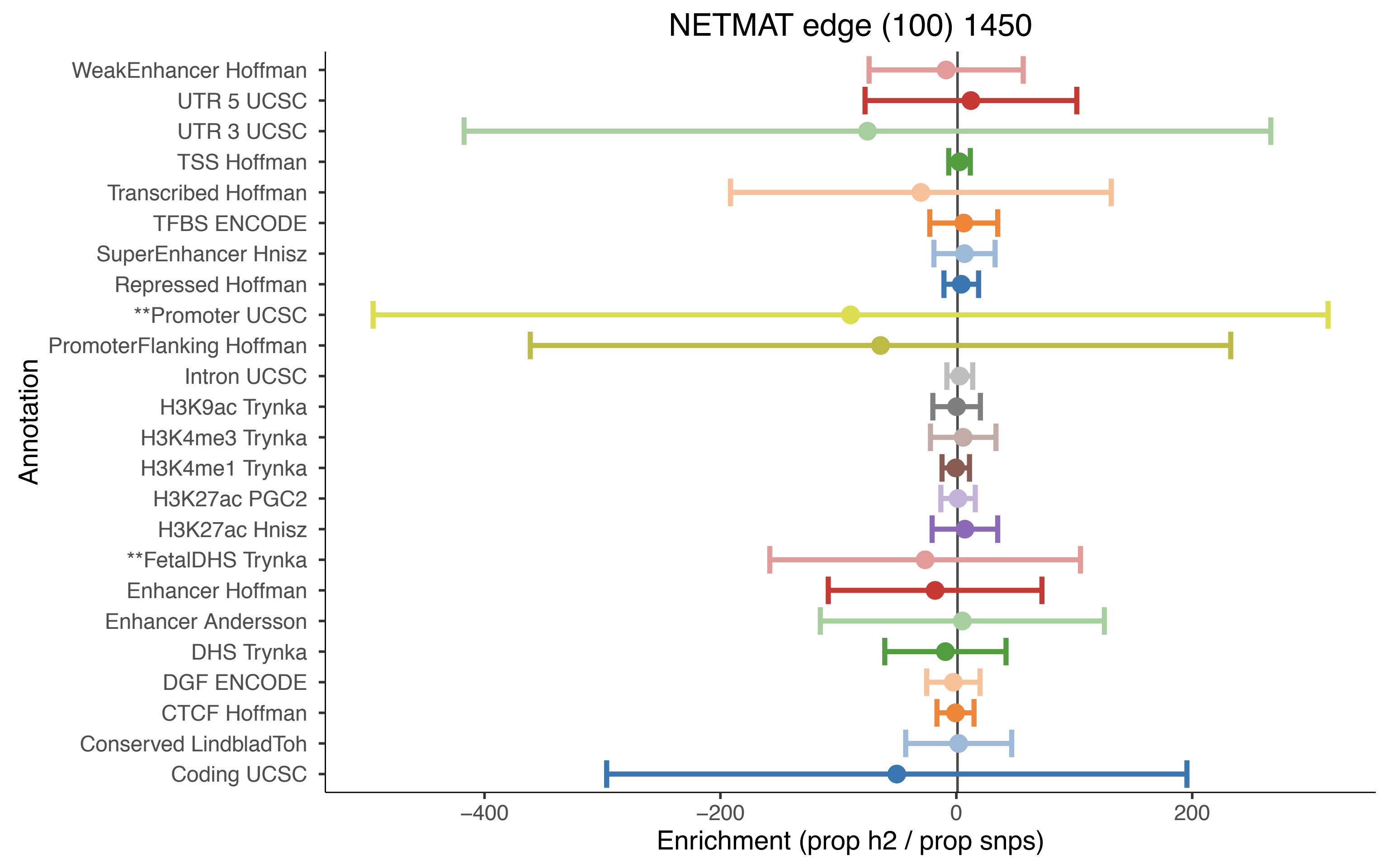
NETMAT edge (100) 1450
WeakEnhancer Hoffman
UTR 5 UCSC
UTR 3 UCSC
TSS Hoffman
Transcribed Hoffman
TFBS ENCODE
SuperEnhancer Hnisz
Repressed Hoffman
**Promoter UCSC
PromoterFlanking Hoffman
Intron UCSC
H3K9ac Trynka
H3K4me3 Trynka
H3K4me1 Trynka
H3K27ac PGC2
H3K27ac Hnisz
**FetalDHS Trynka
Enhancer Hoffman
Enhancer Andersson
DHS Trynka
DGF ENCODE
CTCF Hoffman
Conserved LindbladToh
Coding UCSC
Annotation
−400
−200
0
200
Enrichment (prop h2 / prop snps)

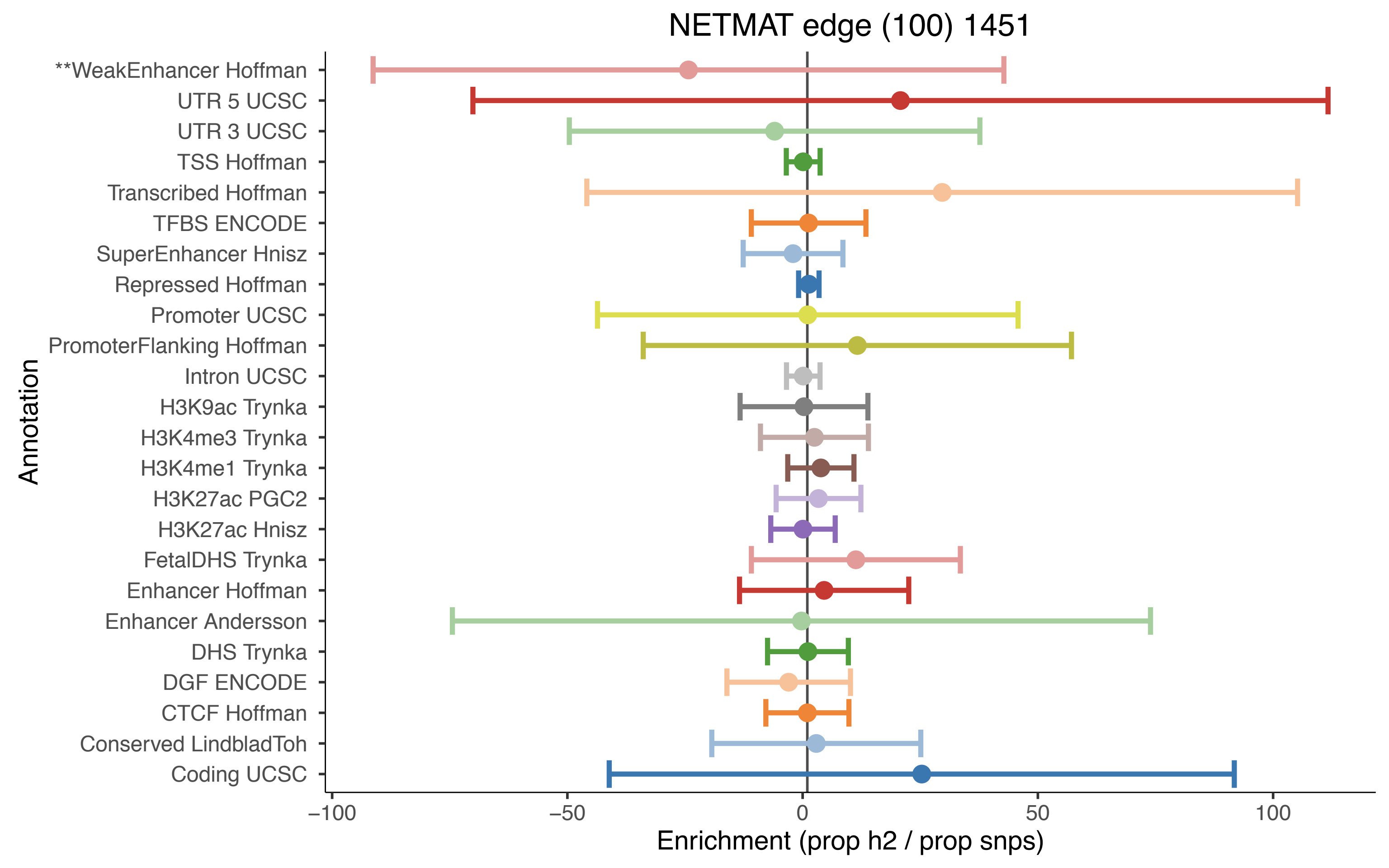
NETMAT edge (100) 1451
**WeakEnhancer Hoffman
UTR 5 UCSC
UTR 3 UCSC
TSS Hoffman
Transcribed Hoffman
TFBS ENCODE
SuperEnhancer Hnisz
Repressed Hoffman
Promoter UCSC
PromoterFlanking Hoffman
Intron UCSC
H3K9ac Trynka
H3K4me3 Trynka
H3K4me1 Trynka
H3K27ac PGC2
H3K27ac Hnisz
FetalDHS Trynka
Enhancer Hoffman
Enhancer Andersson
DHS Trynka
DGF ENCODE
CTCF Hoffman
Conserved LindbladToh
Coding UCSC
Annotation
−100
−50
0
50
100
Enrichment (prop h2 / prop snps)

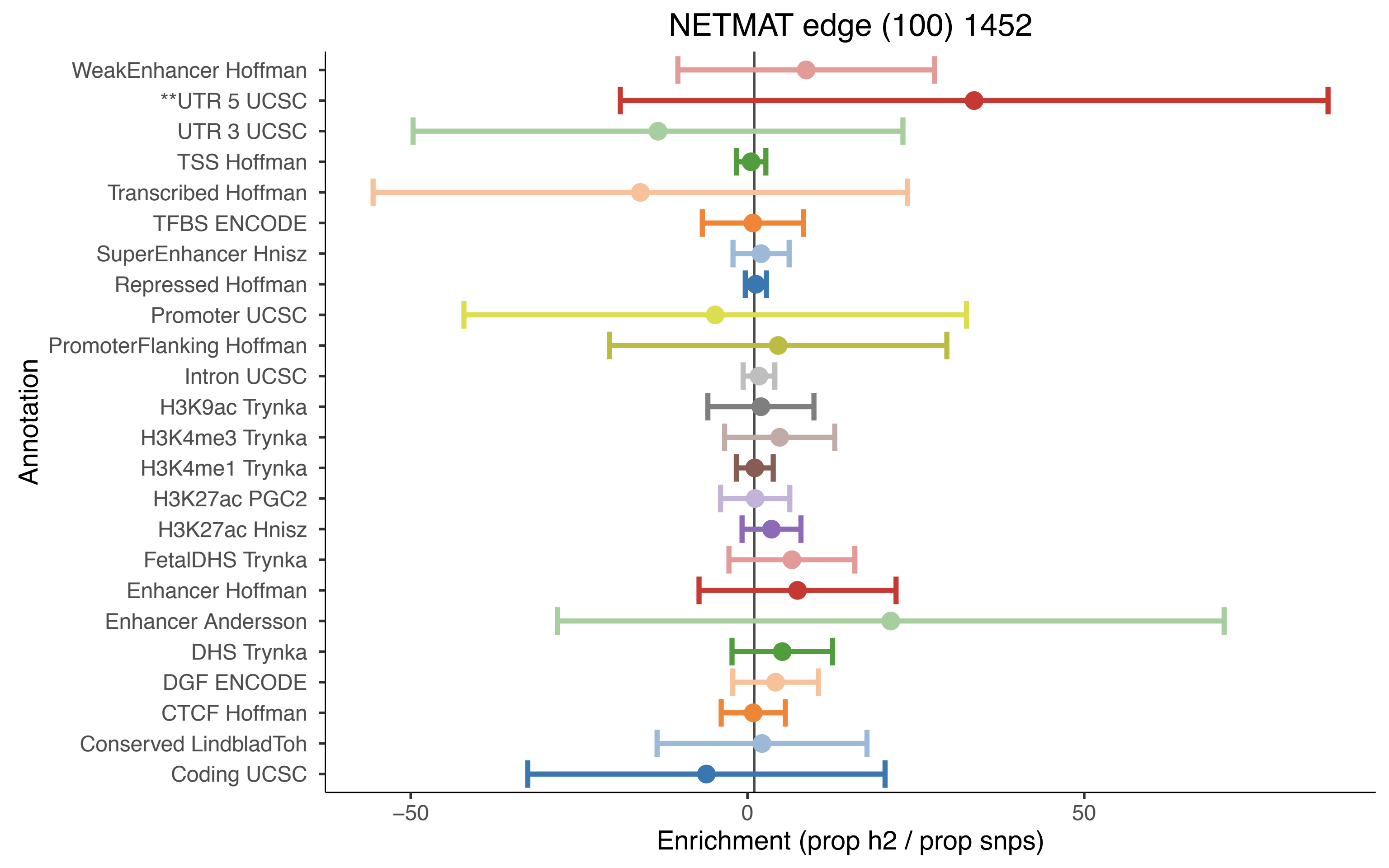

NETMAT edge (100) 1452
WeakEnhancer Hoffman
**UTR 5 UCSC
UTR 3 UCSC
TSS Hoffman
Transcribed Hoffman
TFBS ENCODE
SuperEnhancer Hnisz
Repressed Hoffman
Promoter UCSC
PromoterFlanking Hoffman
Intron UCSC
H3K9ac Trynka
H3K4me3 Trynka
H3K4me1 Trynka
H3K27ac PGC2
H3K27ac Hnisz
FetalDHS Trynka
Enhancer Hoffman
Enhancer Andersson
DHS Trynka
DGF ENCODE
CTCF Hoffman
Conserved LindbladToh
Coding UCSC
Annotation
−50
0
50
Enrichment (prop h2 / prop snps)

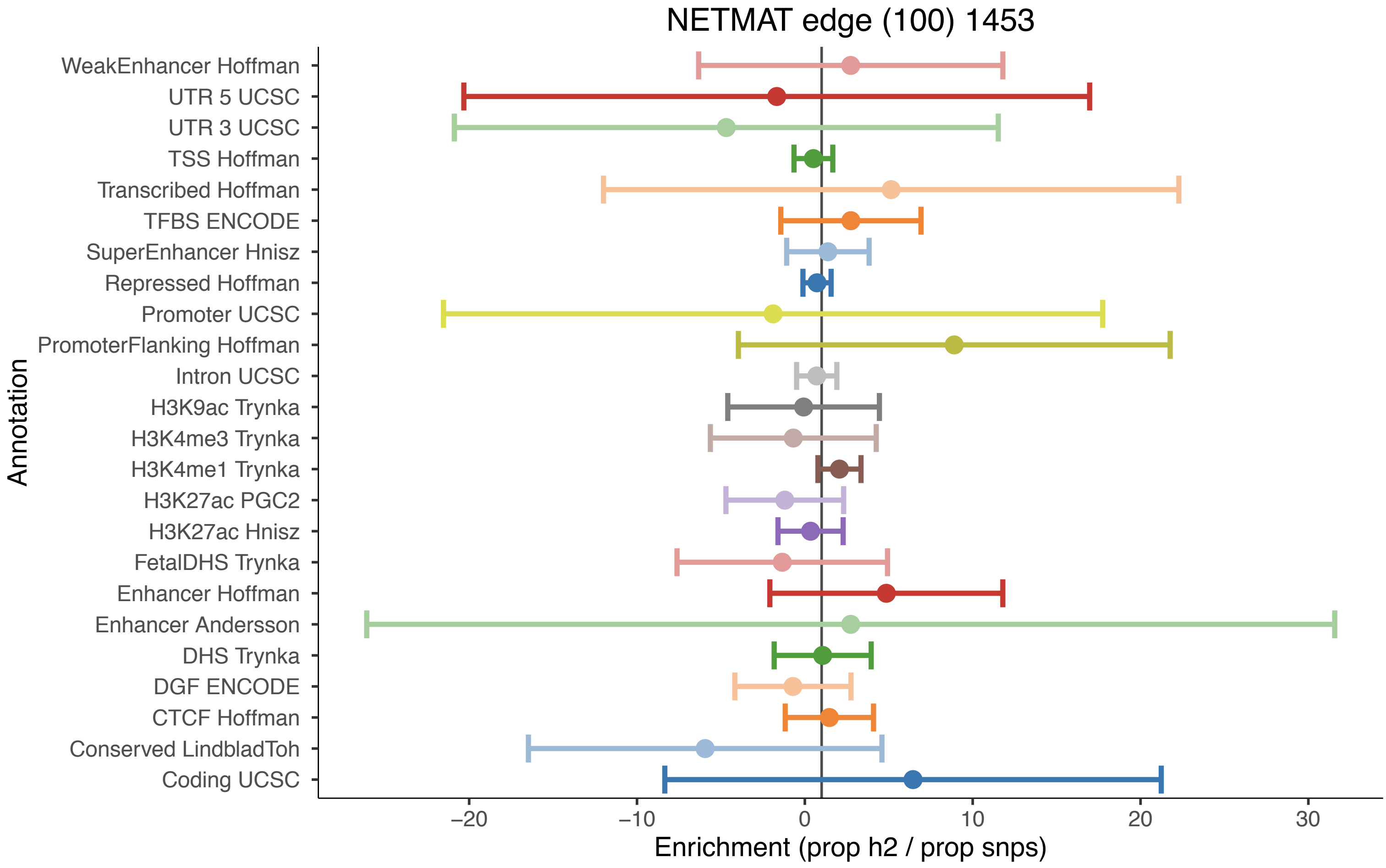
NETMAT edge (100) 1453
WeakEnhancer Hoffman
UTR 5 UCSC
UTR 3 UCSC
TSS Hoffman
Transcribed Hoffman
TFBS ENCODE
SuperEnhancer Hnisz
Repressed Hoffman
Promoter UCSC
PromoterFlanking Hoffman
Intron UCSC
H3K9ac Trynka
H3K4me3 Trynka
H3K4me1 Trynka
H3K27ac PGC2
H3K27ac Hnisz
FetalDHS Trynka
Enhancer Hoffman
Enhancer Andersson
DHS Trynka
DGF ENCODE
CTCF Hoffman
Conserved LindbladToh
Coding UCSC
Annotation
−20
−10
0
10
20
30
Enrichment (prop h2 / prop snps)

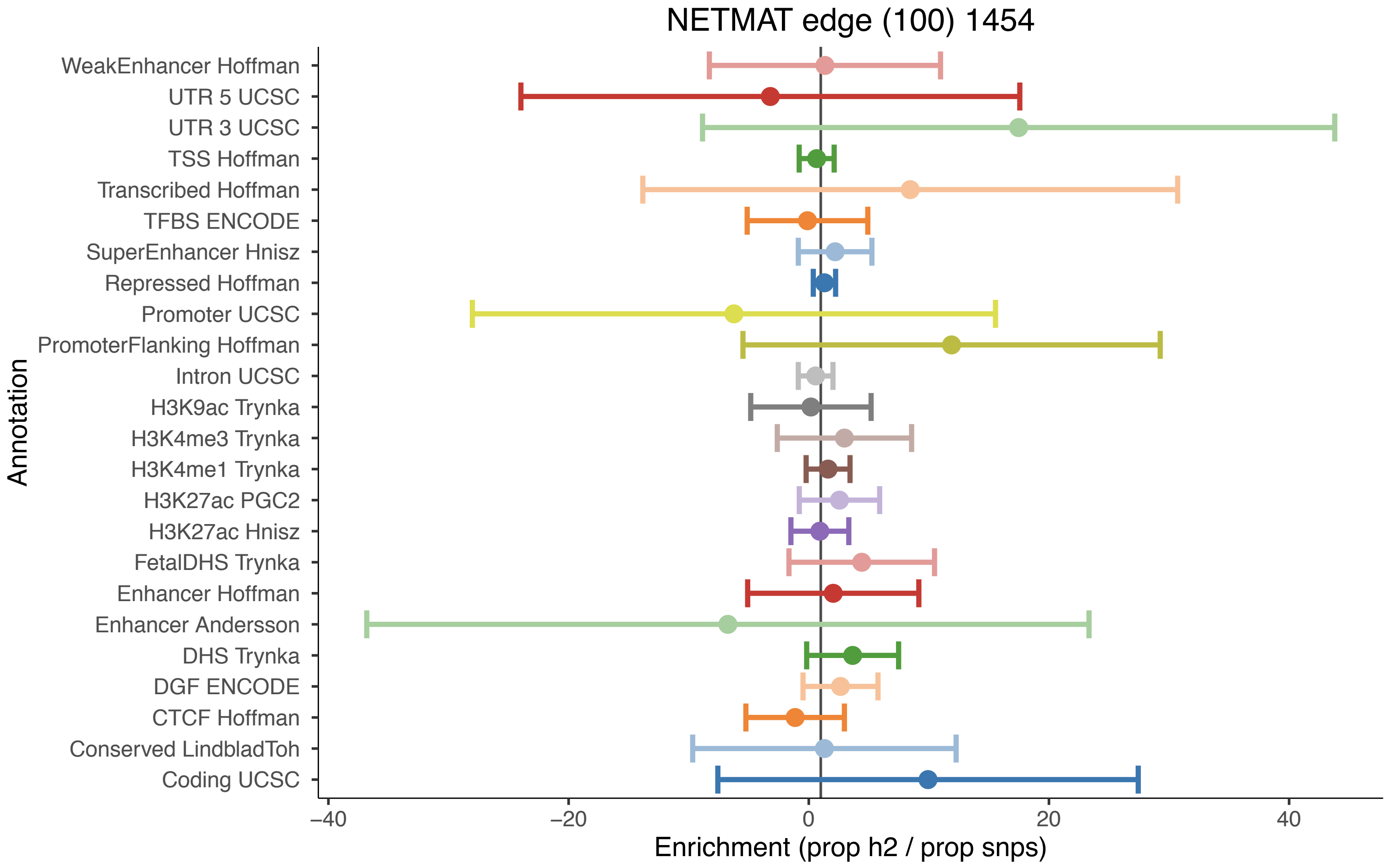
NETMAT edge (100) 1454
WeakEnhancer Hoffman
UTR 5 UCSC
UTR 3 UCSC
TSS Hoffman
Transcribed Hoffman
TFBS ENCODE
SuperEnhancer Hnisz
Repressed Hoffman
Promoter UCSC
PromoterFlanking Hoffman
Intron UCSC
H3K9ac Trynka
H3K4me3 Trynka
H3K4me1 Trynka
H3K27ac PGC2
H3K27ac Hnisz
FetalDHS Trynka
Enhancer Hoffman
Enhancer Andersson
DHS Trynka
DGF ENCODE
CTCF Hoffman
Conserved LindbladToh
Coding UCSC
Annotation
−40
−20
0
20
40
Enrichment (prop h2 / prop snps)

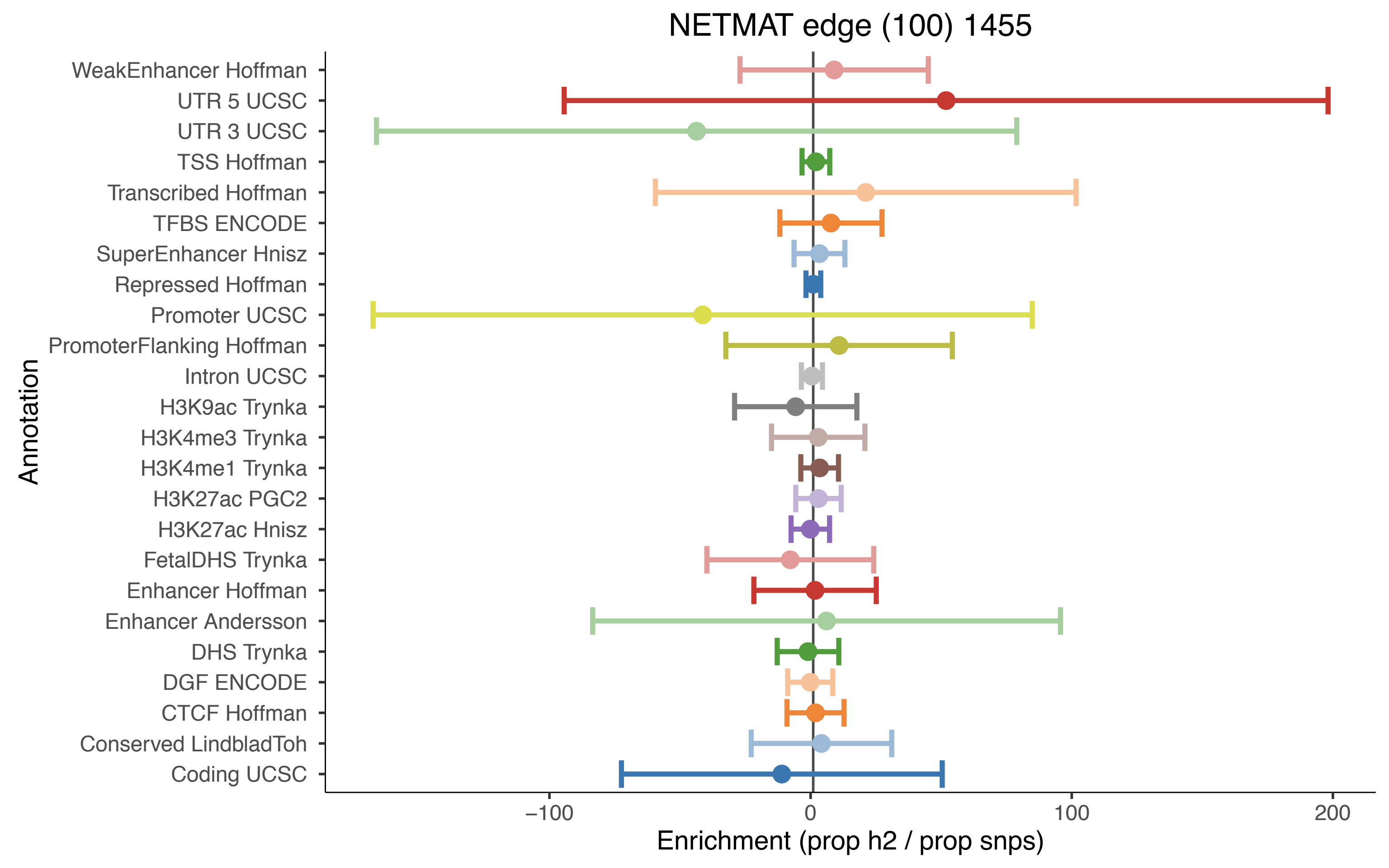

NETMAT edge (100) 1455
WeakEnhancer Hoffman
UTR 5 UCSC
UTR 3 UCSC
TSS Hoffman
Transcribed Hoffman
TFBS ENCODE
SuperEnhancer Hnisz
Repressed Hoffman
Promoter UCSC
PromoterFlanking Hoffman
Intron UCSC
H3K9ac Trynka
H3K4me3 Trynka
H3K4me1 Trynka
H3K27ac PGC2
H3K27ac Hnisz
FetalDHS Trynka
Enhancer Hoffman
Enhancer Andersson
DHS Trynka
DGF ENCODE
CTCF Hoffman
Conserved LindbladToh
Coding UCSC
Annotation
−100
0
100
200
Enrichment (prop h2 / prop snps)

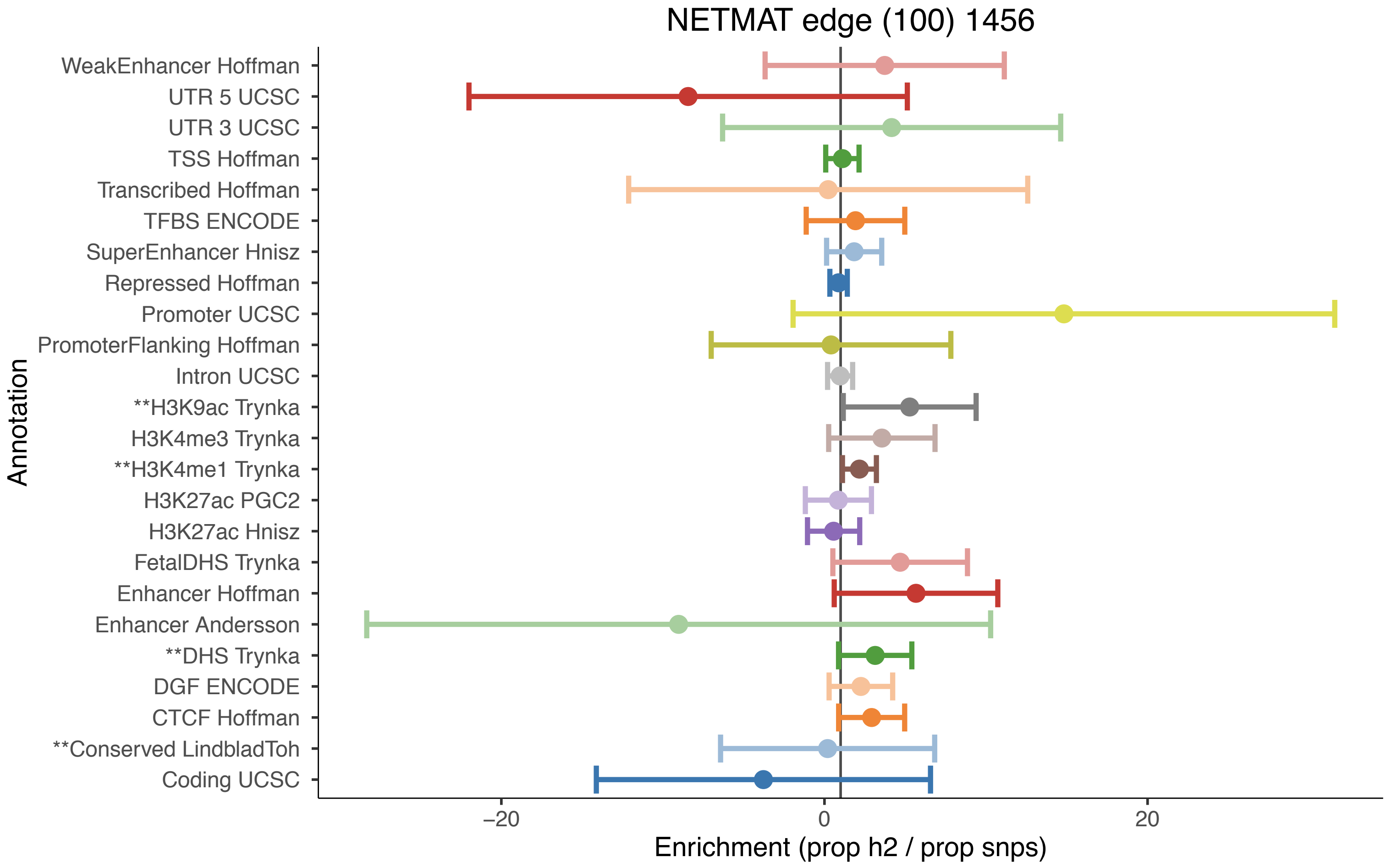

NETMAT edge (100) 1456
WeakEnhancer Hoffman
UTR 5 UCSC
UTR 3 UCSC
TSS Hoffman
Transcribed Hoffman
TFBS ENCODE
SuperEnhancer Hnisz
Repressed Hoffman
Promoter UCSC
PromoterFlanking Hoffman
Intron UCSC
**H3K9ac Trynka
H3K4me3 Trynka
**H3K4me1 Trynka
H3K27ac PGC2
H3K27ac Hnisz
FetalDHS Trynka
Enhancer Hoffman
Enhancer Andersson
**DHS Trynka
DGF ENCODE
CTCF Hoffman
**Conserved LindbladToh
Coding UCSC
Annotation
−20
0
20
Enrichment (prop h2 / prop snps)

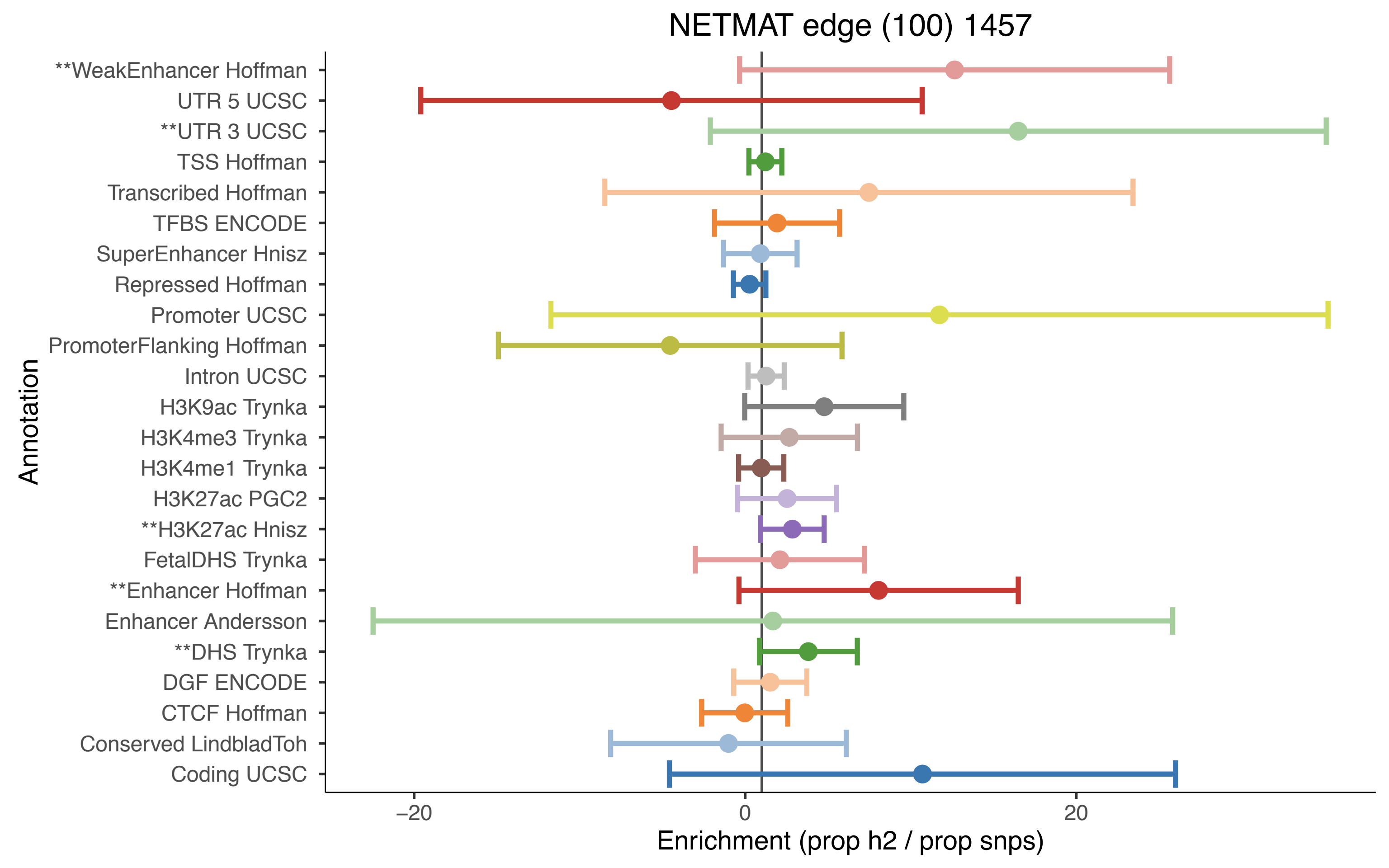
NETMAT edge (100) 1457
**WeakEnhancer Hoffman
UTR 5 UCSC
**UTR 3 UCSC
TSS Hoffman
Transcribed Hoffman
TFBS ENCODE
SuperEnhancer Hnisz
Repressed Hoffman
Promoter UCSC
PromoterFlanking Hoffman
Intron UCSC
H3K9ac Trynka
H3K4me3 Trynka
H3K4me1 Trynka
H3K27ac PGC2
**H3K27ac Hnisz
FetalDHS Trynka
**Enhancer Hoffman
Enhancer Andersson
**DHS Trynka
DGF ENCODE
CTCF Hoffman
Conserved LindbladToh
Coding UCSC
Annotation
−20
0
20
Enrichment (prop h2 / prop snps)

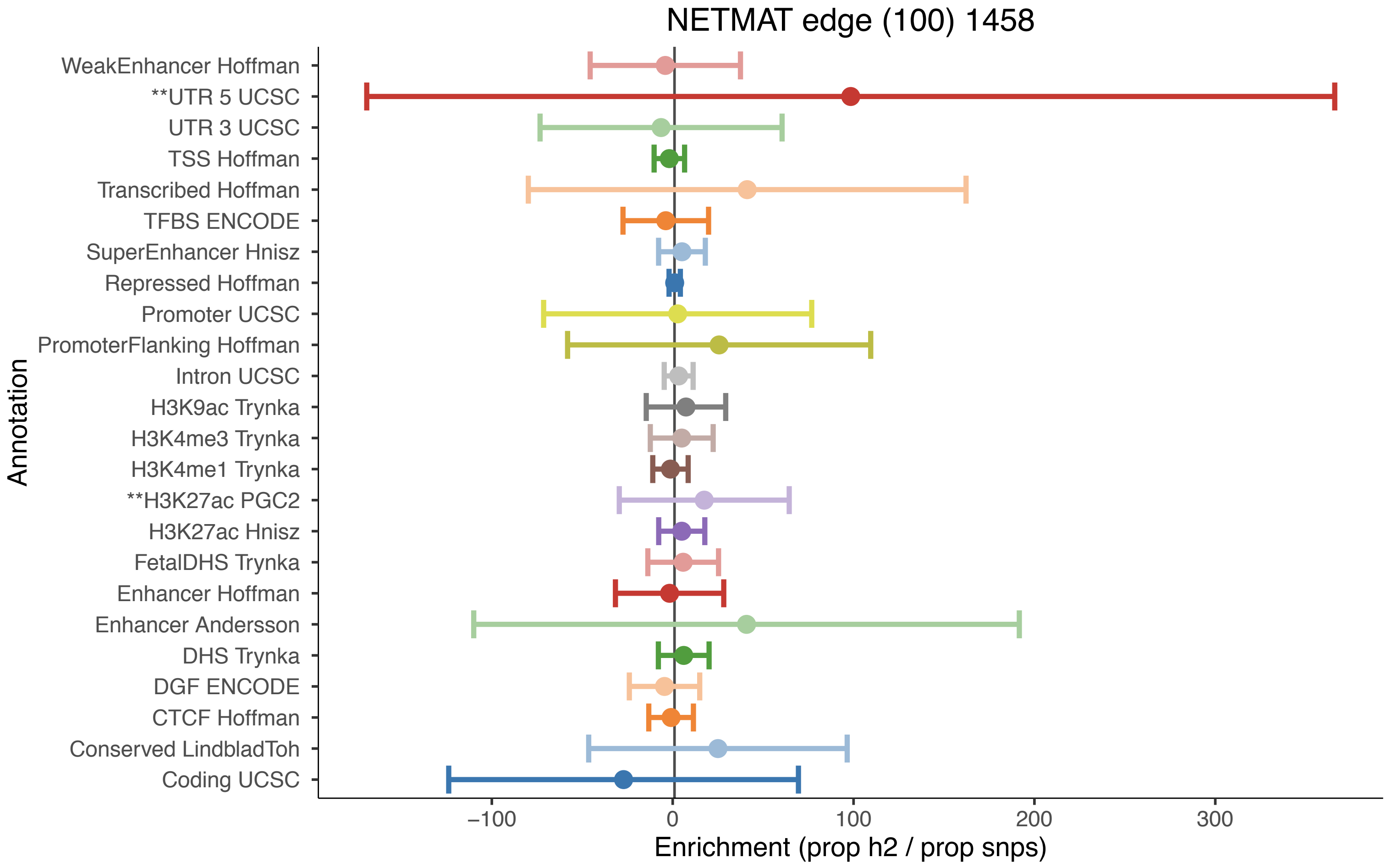

NETMAT edge (100) 1458
WeakEnhancer Hoffman
**UTR 5 UCSC
UTR 3 UCSC
TSS Hoffman
Transcribed Hoffman
TFBS ENCODE
SuperEnhancer Hnisz
Repressed Hoffman
Promoter UCSC
PromoterFlanking Hoffman
Intron UCSC
H3K9ac Trynka
H3K4me3 Trynka
H3K4me1 Trynka
**H3K27ac PGC2
H3K27ac Hnisz
FetalDHS Trynka
Enhancer Hoffman
Enhancer Andersson
DHS Trynka
DGF ENCODE
CTCF Hoffman
Conserved LindbladToh
Coding UCSC
Annotation
−100
0
100
200
300
Enrichment (prop h2 / prop snps)

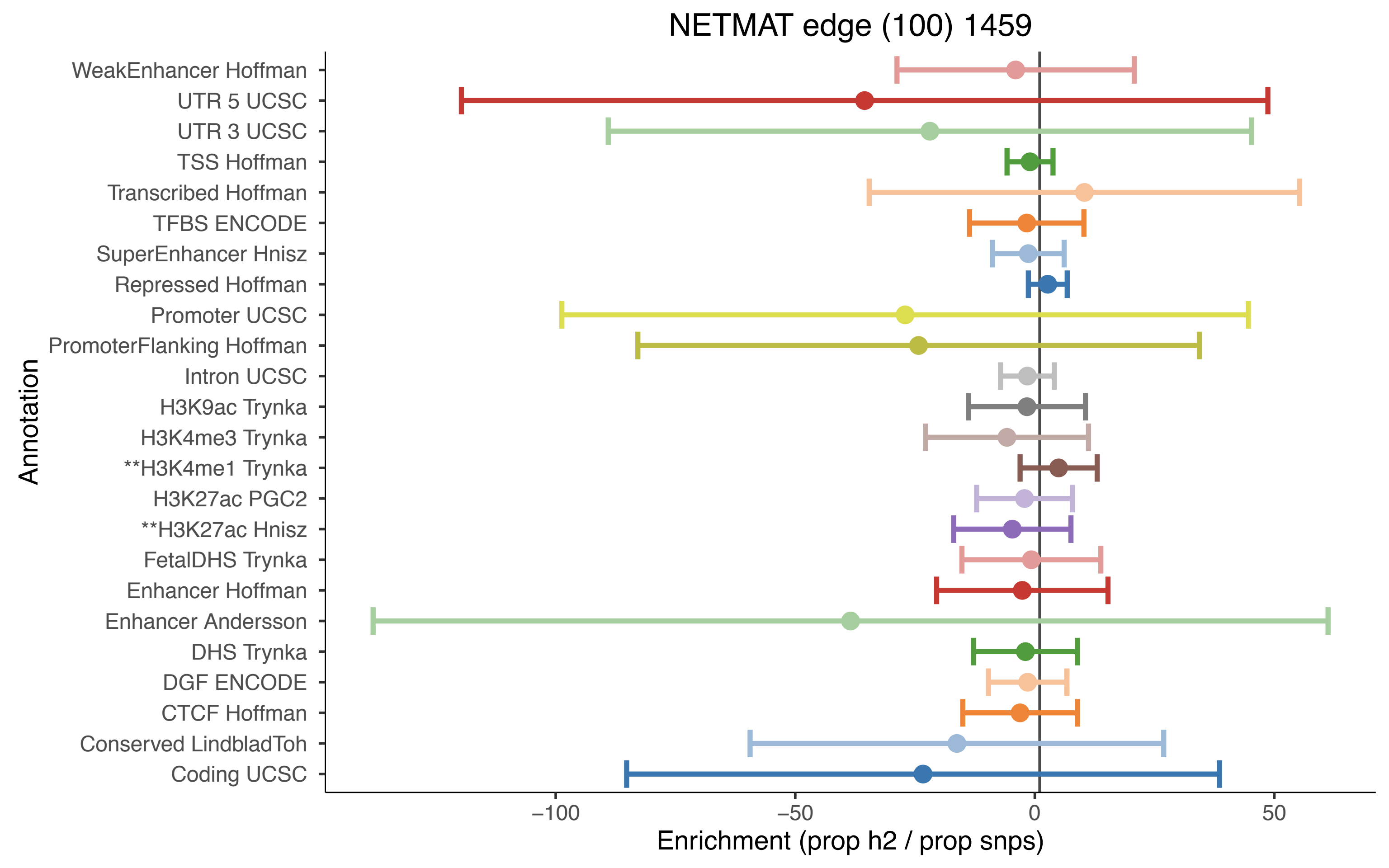
NETMAT edge (100) 1459
WeakEnhancer Hoffman
UTR 5 UCSC
UTR 3 UCSC
TSS Hoffman
Transcribed Hoffman
TFBS ENCODE
SuperEnhancer Hnisz
Repressed Hoffman
Promoter UCSC
PromoterFlanking Hoffman
Intron UCSC
H3K9ac Trynka
H3K4me3 Trynka
**H3K4me1 Trynka
H3K27ac PGC2
**H3K27ac Hnisz
FetalDHS Trynka
Enhancer Hoffman
Enhancer Andersson
DHS Trynka
DGF ENCODE
CTCF Hoffman
Conserved LindbladToh
Coding UCSC
Annotation
−100
−50
0
50
Enrichment (prop h2 / prop snps)

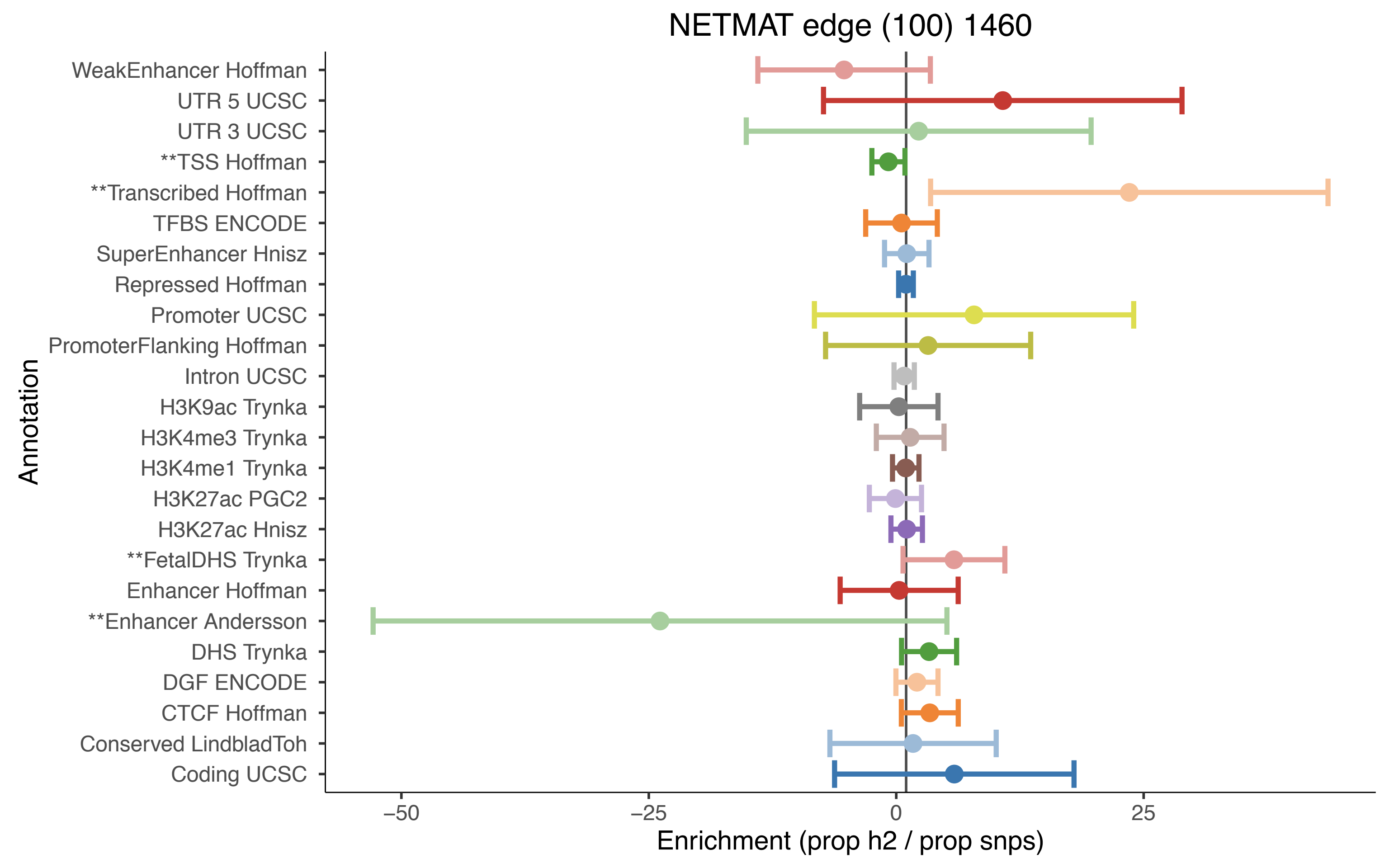

NETMAT edge (100) 1460
WeakEnhancer Hoffman
UTR 5 UCSC
UTR 3 UCSC
**TSS Hoffman
**Transcribed Hoffman
TFBS ENCODE
SuperEnhancer Hnisz
Repressed Hoffman
Promoter UCSC
PromoterFlanking Hoffman
Intron UCSC
H3K9ac Trynka
H3K4me3 Trynka
H3K4me1 Trynka
H3K27ac PGC2
H3K27ac Hnisz
**FetalDHS Trynka
Enhancer Hoffman
**Enhancer Andersson
DHS Trynka
DGF ENCODE
CTCF Hoffman
Conserved LindbladToh
Coding UCSC
Annotation
−50
−25
0
25
Enrichment (prop h2 / prop snps)

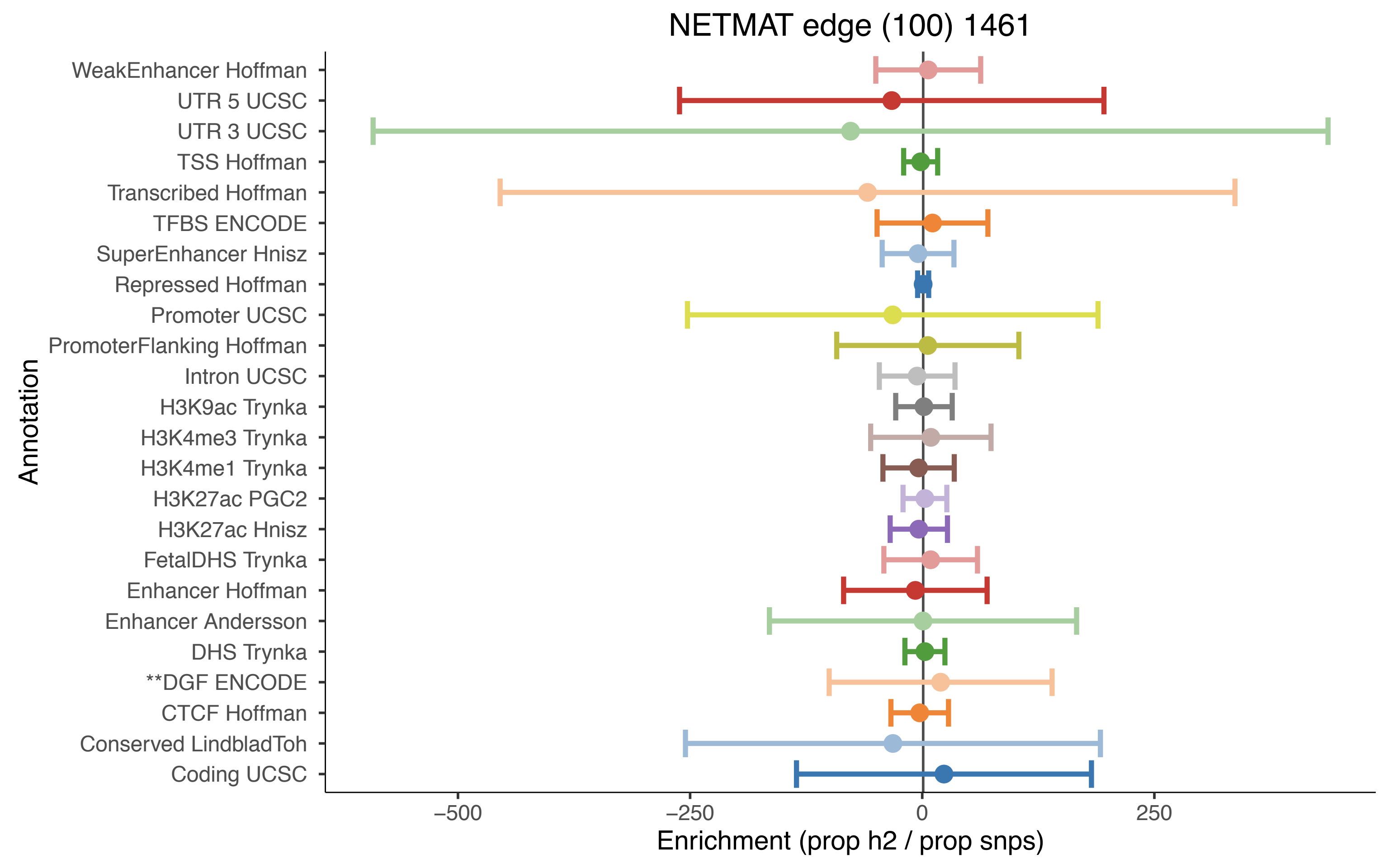

NETMAT edge (100) 1461
WeakEnhancer Hoffman
UTR 5 UCSC
UTR 3 UCSC
TSS Hoffman
Transcribed Hoffman
TFBS ENCODE
SuperEnhancer Hnisz
Repressed Hoffman
Promoter UCSC
PromoterFlanking Hoffman
Intron UCSC
H3K9ac Trynka
H3K4me3 Trynka
H3K4me1 Trynka
H3K27ac PGC2
H3K27ac Hnisz
FetalDHS Trynka
Enhancer Hoffman
Enhancer Andersson
DHS Trynka
**DGF ENCODE
CTCF Hoffman
Conserved LindbladToh
Coding UCSC
Annotation
−500
−250
0
250
Enrichment (prop h2 / prop snps)

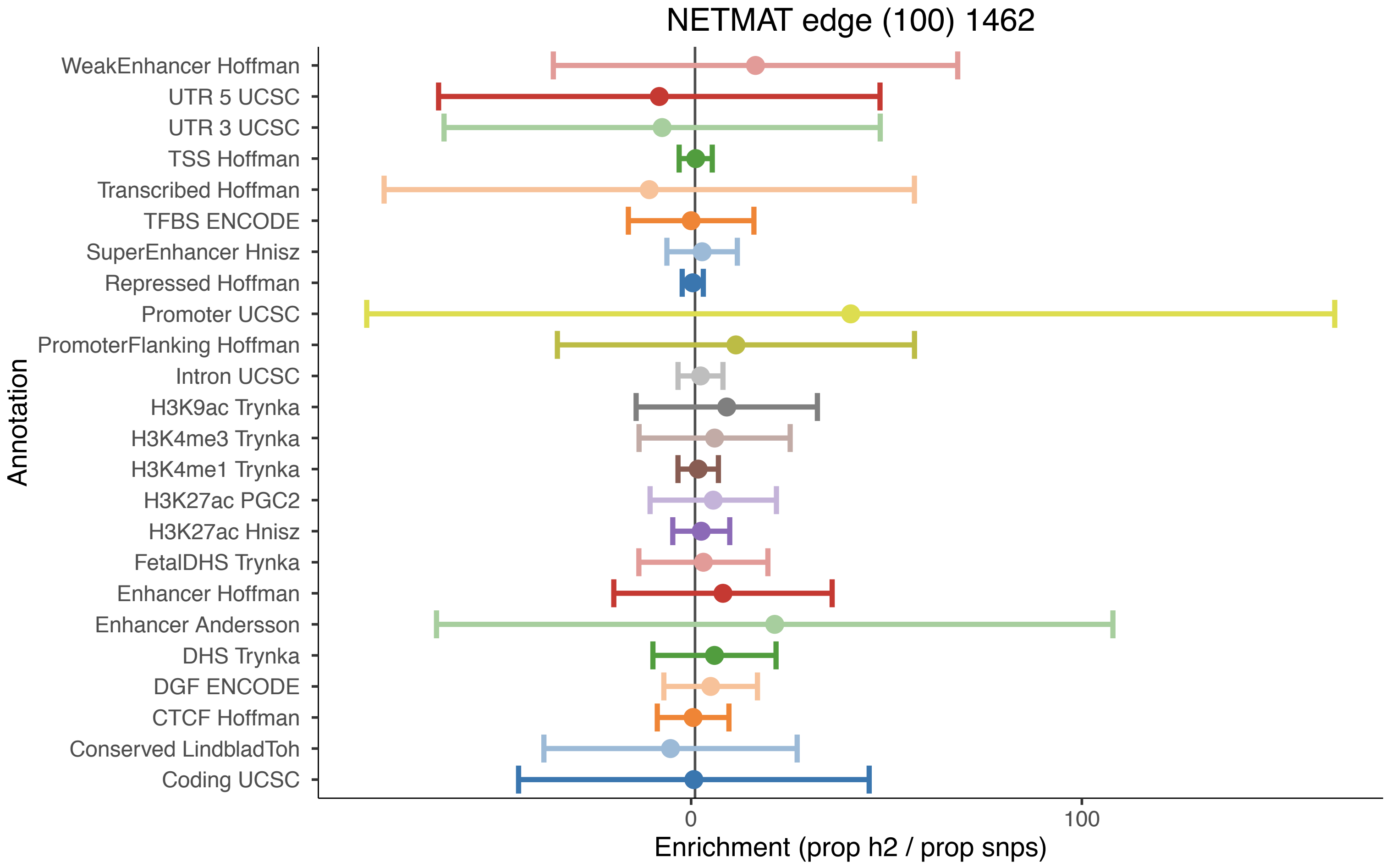

NETMAT edge (100) 1462
WeakEnhancer Hoffman
UTR 5 UCSC
UTR 3 UCSC
TSS Hoffman
Transcribed Hoffman
TFBS ENCODE
SuperEnhancer Hnisz
Repressed Hoffman
Promoter UCSC
PromoterFlanking Hoffman
Intron UCSC
H3K9ac Trynka
H3K4me3 Trynka
H3K4me1 Trynka
H3K27ac PGC2
H3K27ac Hnisz
FetalDHS Trynka
Enhancer Hoffman
Enhancer Andersson
DHS Trynka
DGF ENCODE
CTCF Hoffman
Conserved LindbladToh
Coding UCSC
Annotation
0
100
Enrichment (prop h2 / prop snps)

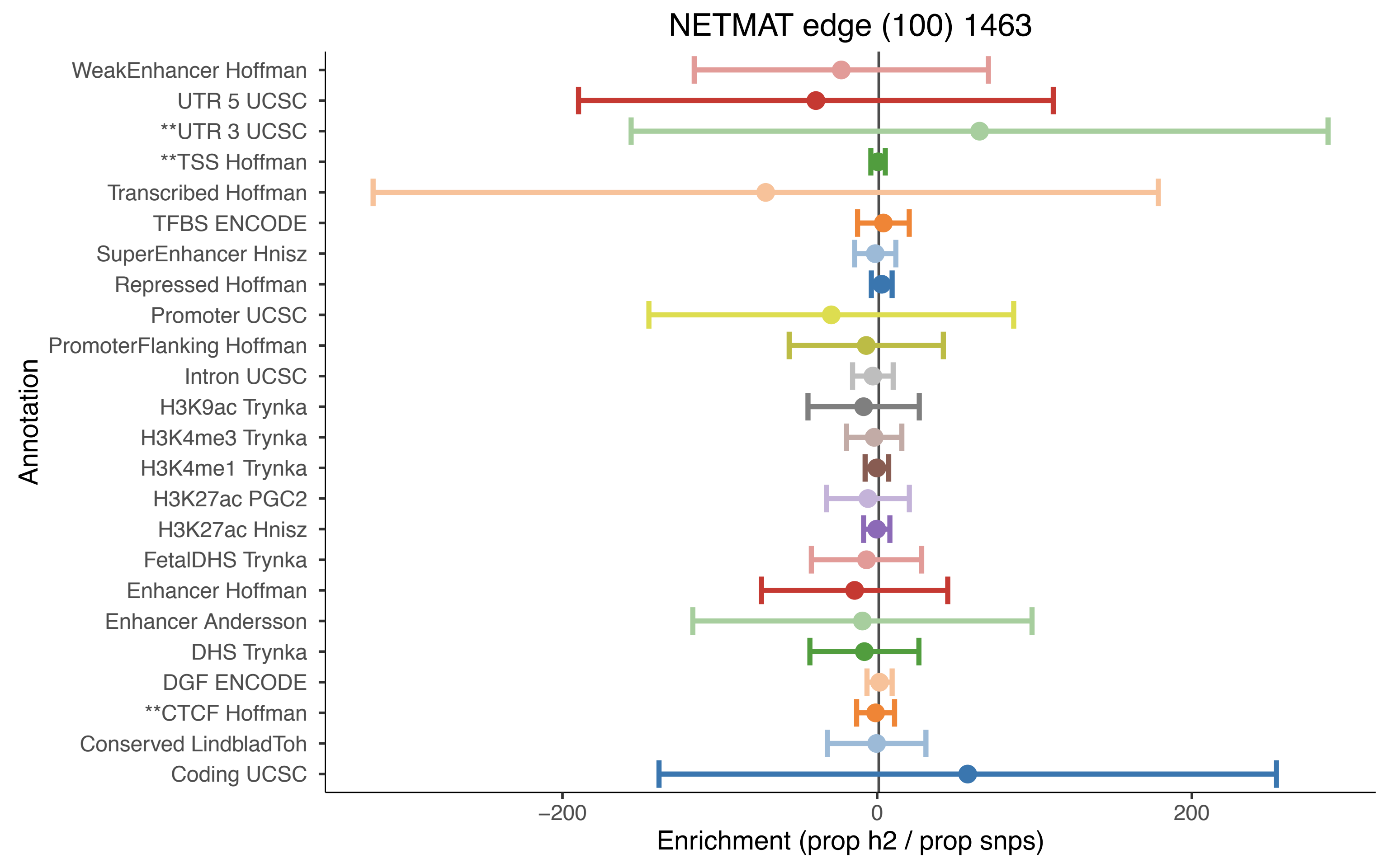
NETMAT edge (100) 1463
WeakEnhancer Hoffman
UTR 5 UCSC
**UTR 3 UCSC
**TSS Hoffman
Transcribed Hoffman
TFBS ENCODE
SuperEnhancer Hnisz
Repressed Hoffman
Promoter UCSC
PromoterFlanking Hoffman
Intron UCSC
H3K9ac Trynka
H3K4me3 Trynka
H3K4me1 Trynka
H3K27ac PGC2
H3K27ac Hnisz
FetalDHS Trynka
Enhancer Hoffman
Enhancer Andersson
DHS Trynka
DGF ENCODE
**CTCF Hoffman
Conserved LindbladToh
Coding UCSC
Annotation
−200
0
200
Enrichment (prop h2 / prop snps)

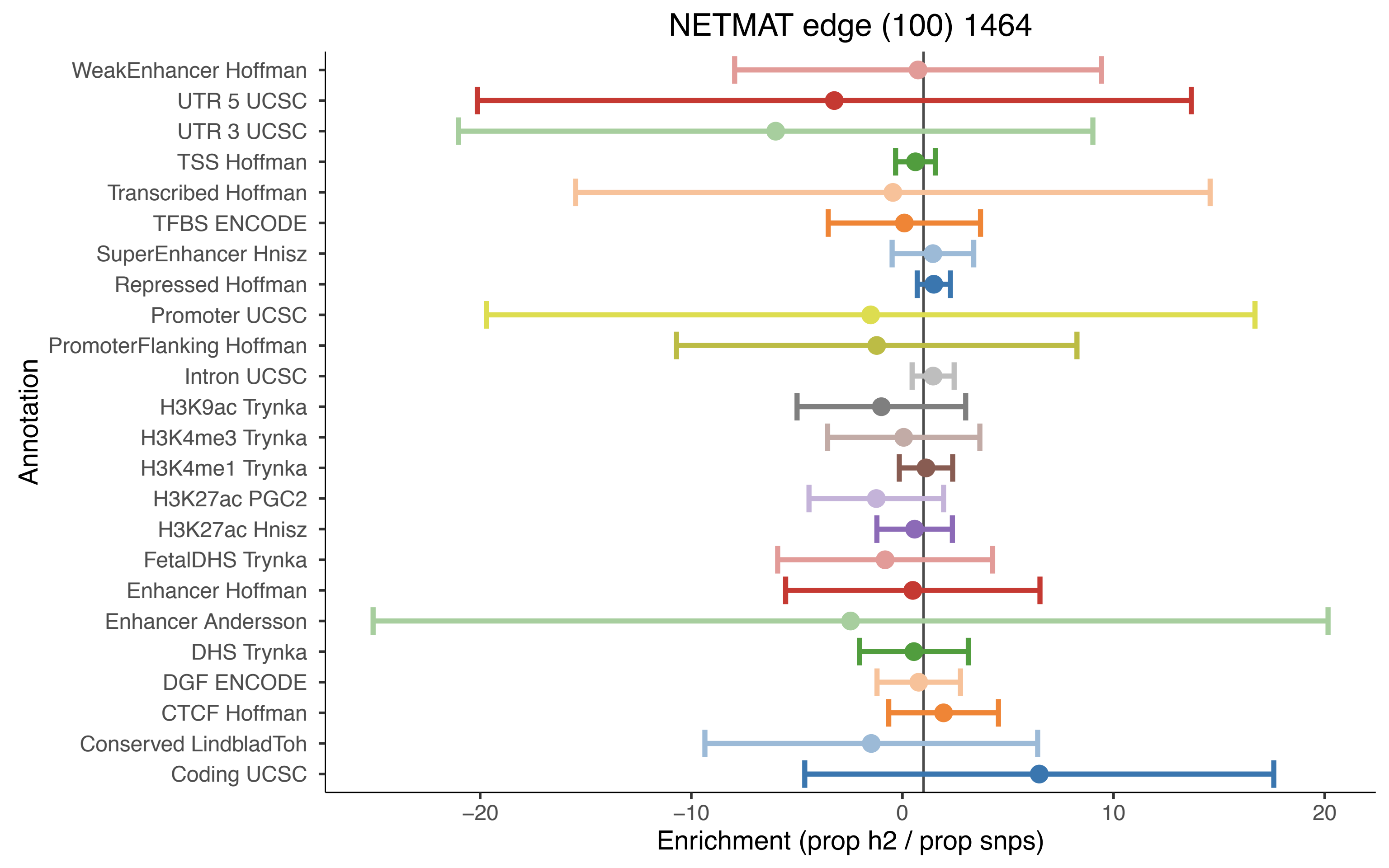
NETMAT edge (100) 1464
WeakEnhancer Hoffman
UTR 5 UCSC
UTR 3 UCSC
TSS Hoffman
Transcribed Hoffman
TFBS ENCODE
SuperEnhancer Hnisz
Repressed Hoffman
Promoter UCSC
PromoterFlanking Hoffman
Intron UCSC
H3K9ac Trynka
H3K4me3 Trynka
H3K4me1 Trynka
H3K27ac PGC2
H3K27ac Hnisz
FetalDHS Trynka
Enhancer Hoffman
Enhancer Andersson
DHS Trynka
DGF ENCODE
CTCF Hoffman
Conserved LindbladToh
Coding UCSC
Annotation
−20
−10
0
10
20
Enrichment (prop h2 / prop snps)

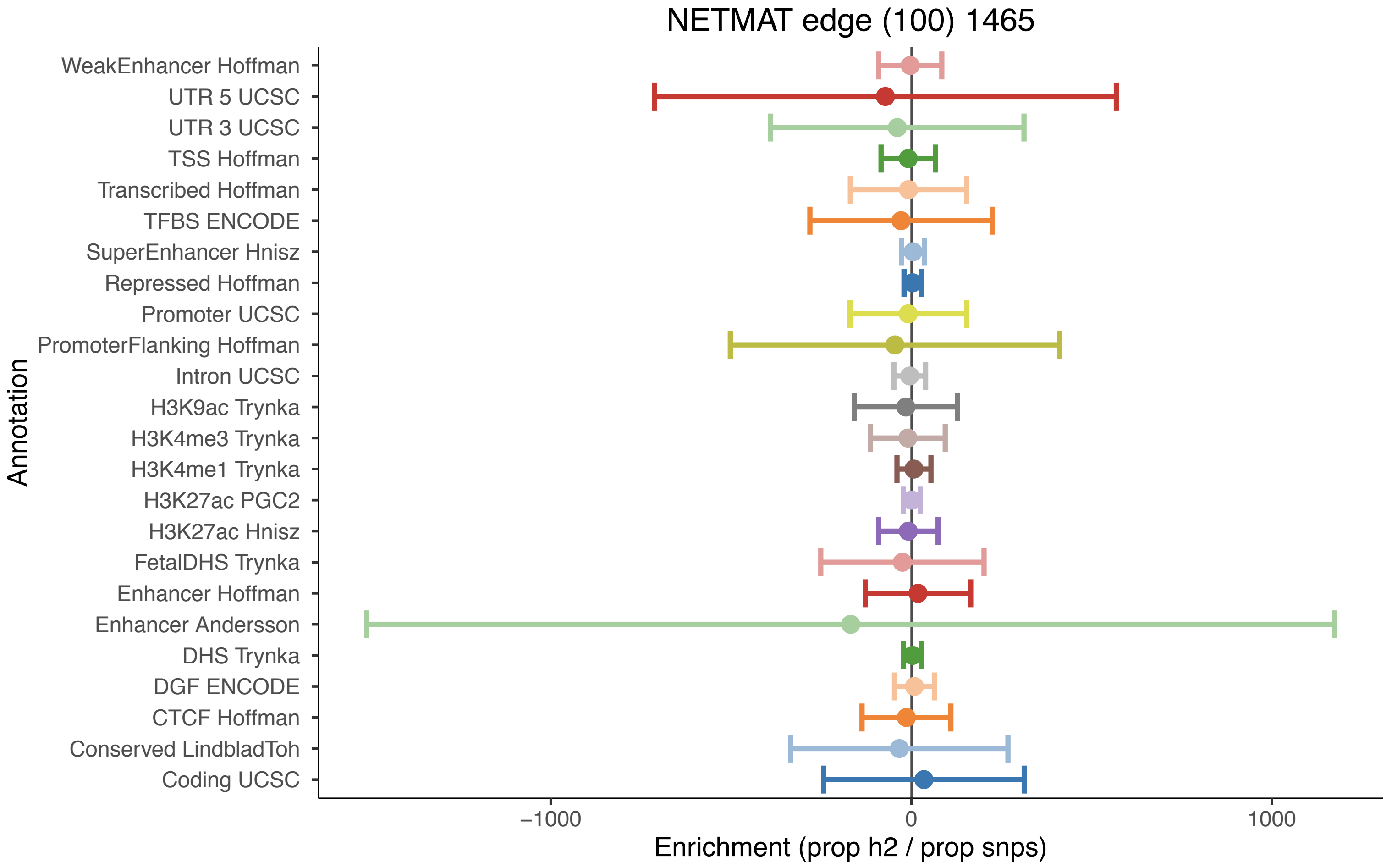
NETMAT edge (100) 1465
WeakEnhancer Hoffman
UTR 5 UCSC
UTR 3 UCSC
TSS Hoffman
Transcribed Hoffman
TFBS ENCODE
SuperEnhancer Hnisz
Repressed Hoffman
Promoter UCSC
PromoterFlanking Hoffman
Intron UCSC
H3K9ac Trynka
H3K4me3 Trynka
H3K4me1 Trynka
H3K27ac PGC2
H3K27ac Hnisz
FetalDHS Trynka
Enhancer Hoffman
Enhancer Andersson
DHS Trynka
DGF ENCODE
CTCF Hoffman
Conserved LindbladToh
Coding UCSC
Annotation
−1000
0
1000
Enrichment (prop h2 / prop snps)

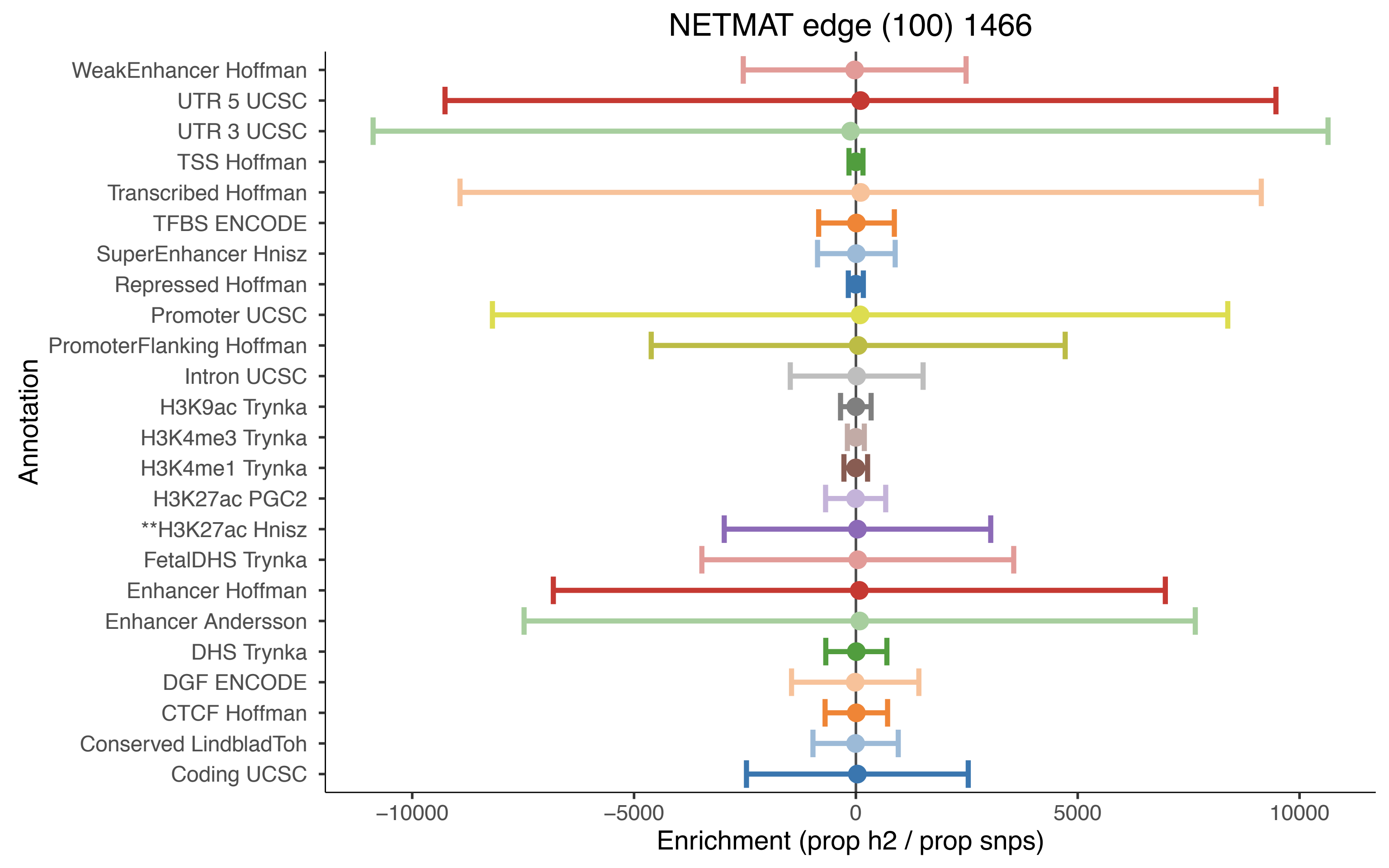
NETMAT edge (100) 1466
WeakEnhancer Hoffman
UTR 5 UCSC
UTR 3 UCSC
TSS Hoffman
Transcribed Hoffman
TFBS ENCODE
SuperEnhancer Hnisz
Repressed Hoffman
Promoter UCSC
PromoterFlanking Hoffman
Intron UCSC
H3K9ac Trynka
H3K4me3 Trynka
H3K4me1 Trynka
H3K27ac PGC2
**H3K27ac Hnisz
FetalDHS Trynka
Enhancer Hoffman
Enhancer Andersson
DHS Trynka
DGF ENCODE
CTCF Hoffman
Conserved LindbladToh
Coding UCSC
Annotation
−10000
−5000
0
5000
10000
Enrichment (prop h2 / prop snps)

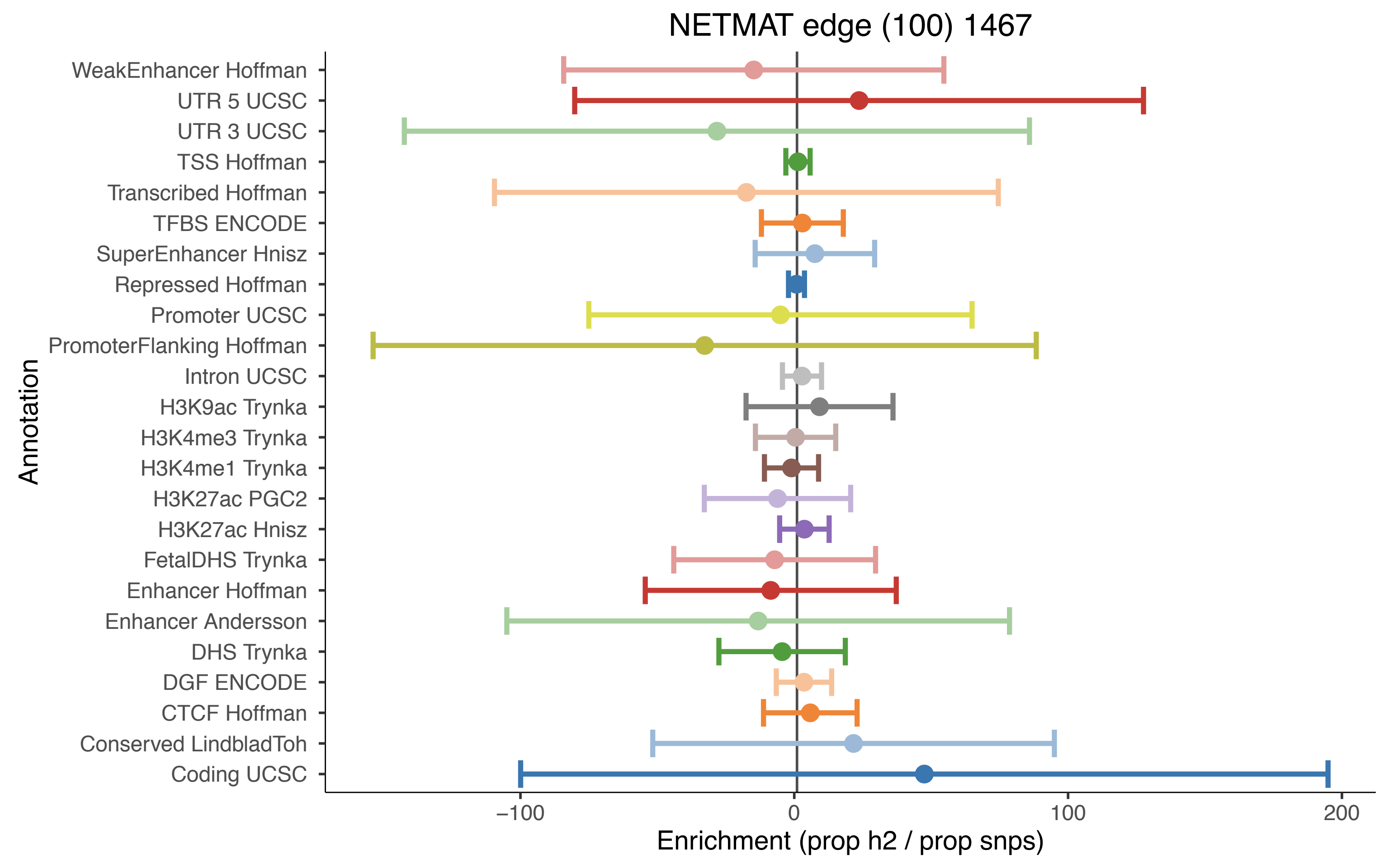
NETMAT edge (100) 1467
WeakEnhancer Hoffman
UTR 5 UCSC
UTR 3 UCSC
TSS Hoffman
Transcribed Hoffman
TFBS ENCODE
SuperEnhancer Hnisz
Repressed Hoffman
Promoter UCSC
PromoterFlanking Hoffman
Intron UCSC
H3K9ac Trynka
H3K4me3 Trynka
H3K4me1 Trynka
H3K27ac PGC2
H3K27ac Hnisz
FetalDHS Trynka
Enhancer Hoffman
Enhancer Andersson
DHS Trynka
DGF ENCODE
CTCF Hoffman
Conserved LindbladToh
Coding UCSC
Annotation
−100
0
100
200
Enrichment (prop h2 / prop snps)

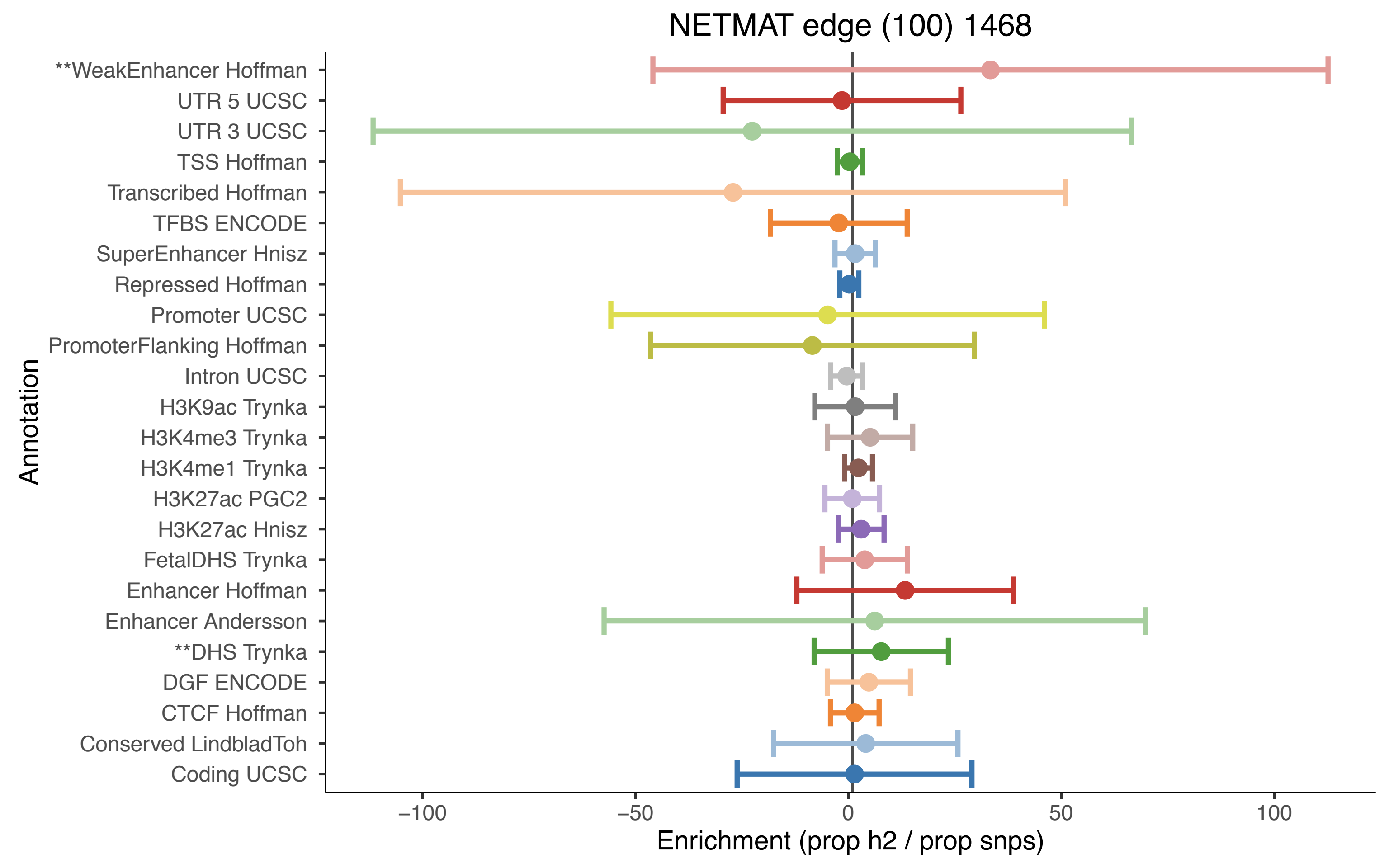
NETMAT edge (100) 1468
**WeakEnhancer Hoffman
UTR 5 UCSC
UTR 3 UCSC
TSS Hoffman
Transcribed Hoffman
TFBS ENCODE
SuperEnhancer Hnisz
Repressed Hoffman
Promoter UCSC
PromoterFlanking Hoffman
Intron UCSC
H3K9ac Trynka
H3K4me3 Trynka
H3K4me1 Trynka
H3K27ac PGC2
H3K27ac Hnisz
FetalDHS Trynka
Enhancer Hoffman
Enhancer Andersson
**DHS Trynka
DGF ENCODE
CTCF Hoffman
Conserved LindbladToh
Coding UCSC
Annotation
−100
−50
0
50
100
Enrichment (prop h2 / prop snps)

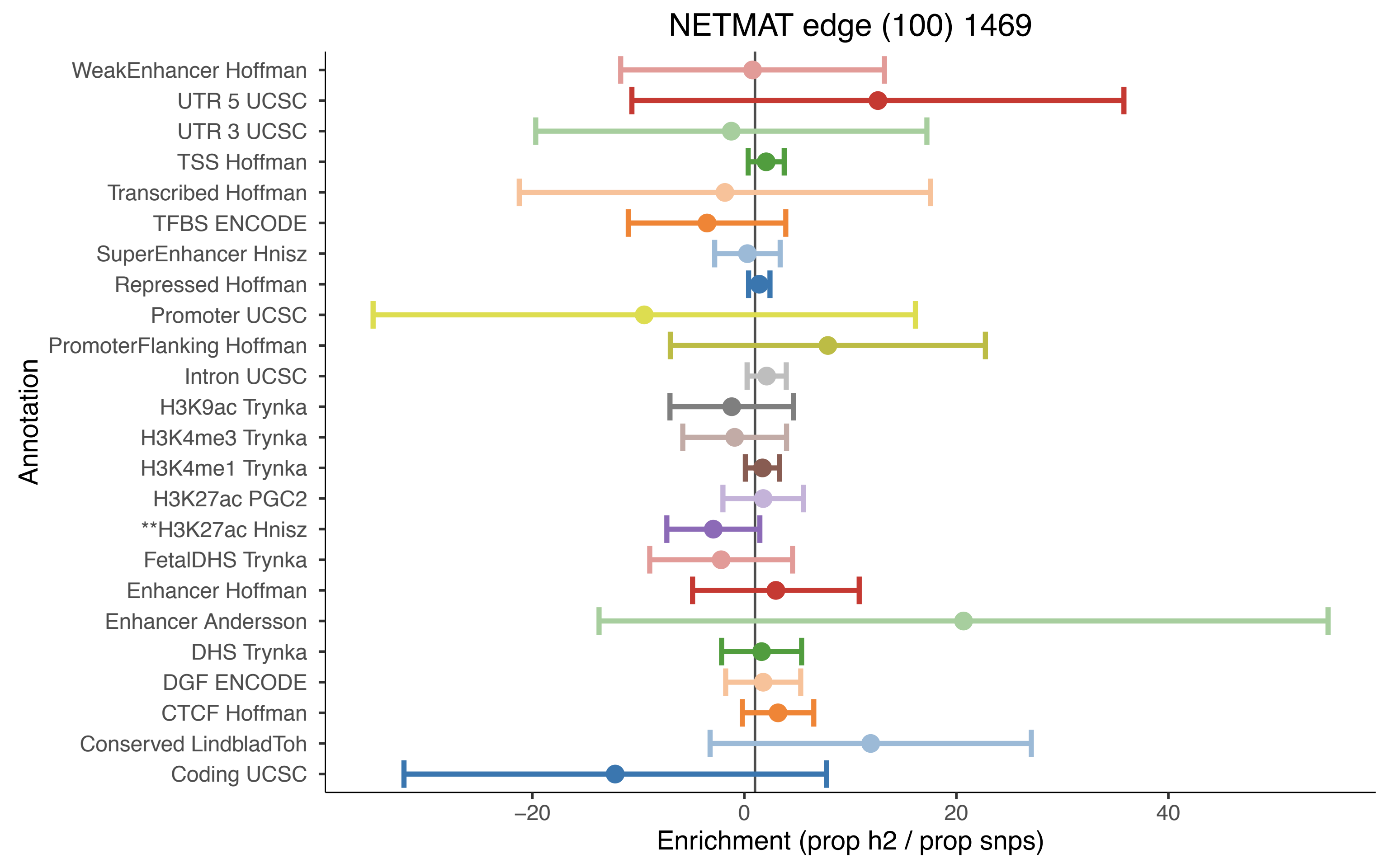
NETMAT edge (100) 1469
Annotation
WeakEnhancer Hoffman
UTR 5 UCSC
UTR 3 UCSC
TSS Hoffman
Transcribed Hoffman
TFBS ENCODE
SuperEnhancer Hnisz
Repressed Hoffman
Promoter UCSC
PromoterFlanking Hoffman
Intron UCSC
H3K9ac Trynka
H3K4me3 Trynka
H3K4me1 Trynka
H3K27ac PGC2
**H3K27ac Hnisz
FetalDHS Trynka
Enhancer Hoffman
Enhancer Andersson
DHS Trynka
DGF ENCODE
CTCF Hoffman
Conserved LindbladToh
Coding UCSC
−20
0
20
40
Enrichment (prop h2 / prop snps)

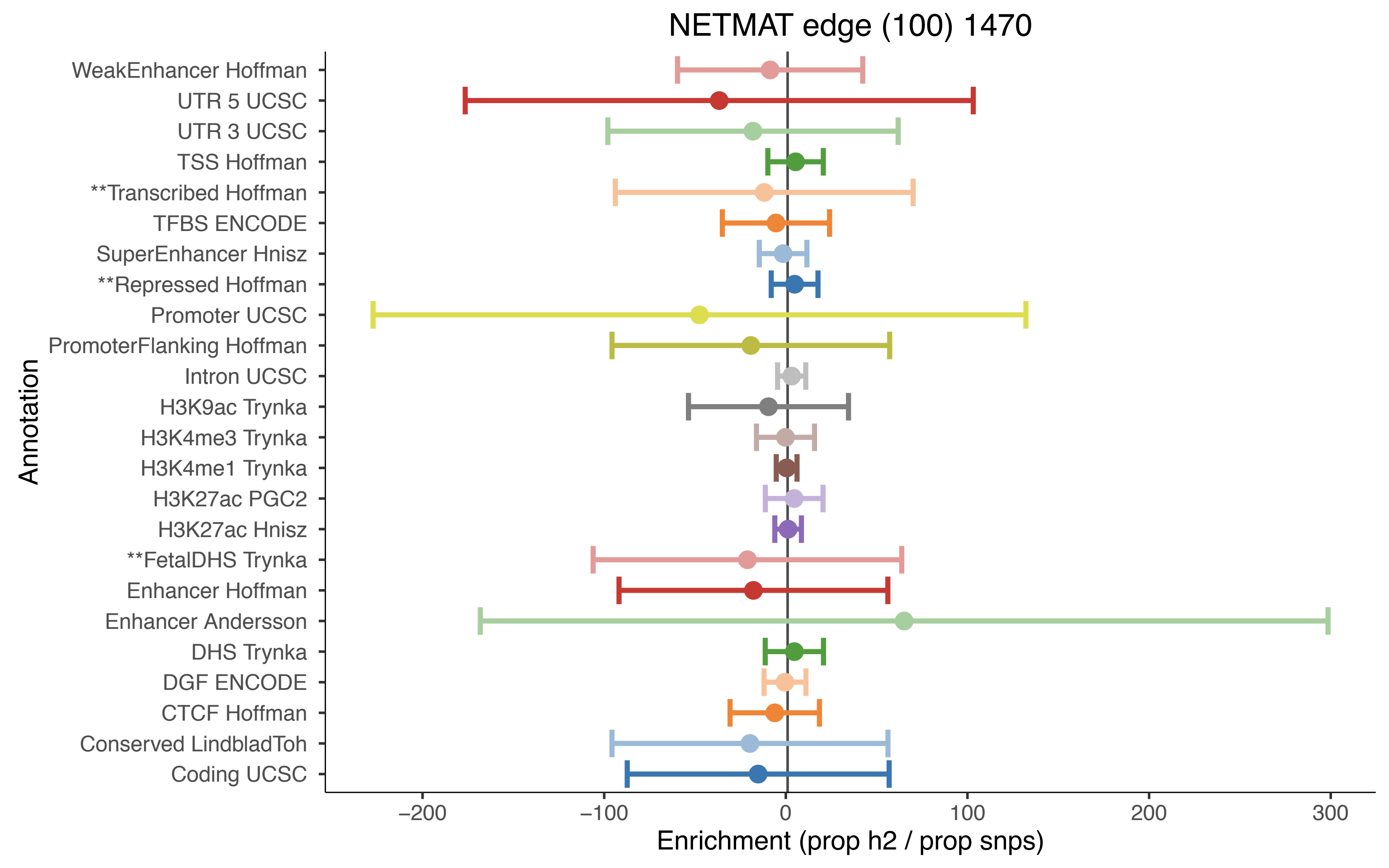

NETMAT edge (100) 1470
Annotation
WeakEnhancer Hoffman
UTR 5 UCSC
UTR 3 UCSC
TSS Hoffman
**Transcribed Hoffman
TFBS ENCODE
SuperEnhancer Hnisz
**Repressed Hoffman
Promoter UCSC
PromoterFlanking Hoffman
Intron UCSC
H3K9ac Trynka
H3K4me3 Trynka
H3K4me1 Trynka
H3K27ac PGC2
H3K27ac Hnisz
**FetalDHS Trynka
Enhancer Hoffman
Enhancer Andersson
DHS Trynka
DGF ENCODE
CTCF Hoffman
Conserved LindbladToh
Coding UCSC
−200
−100
0
100
200
300
Enrichment (prop h2 / prop snps)

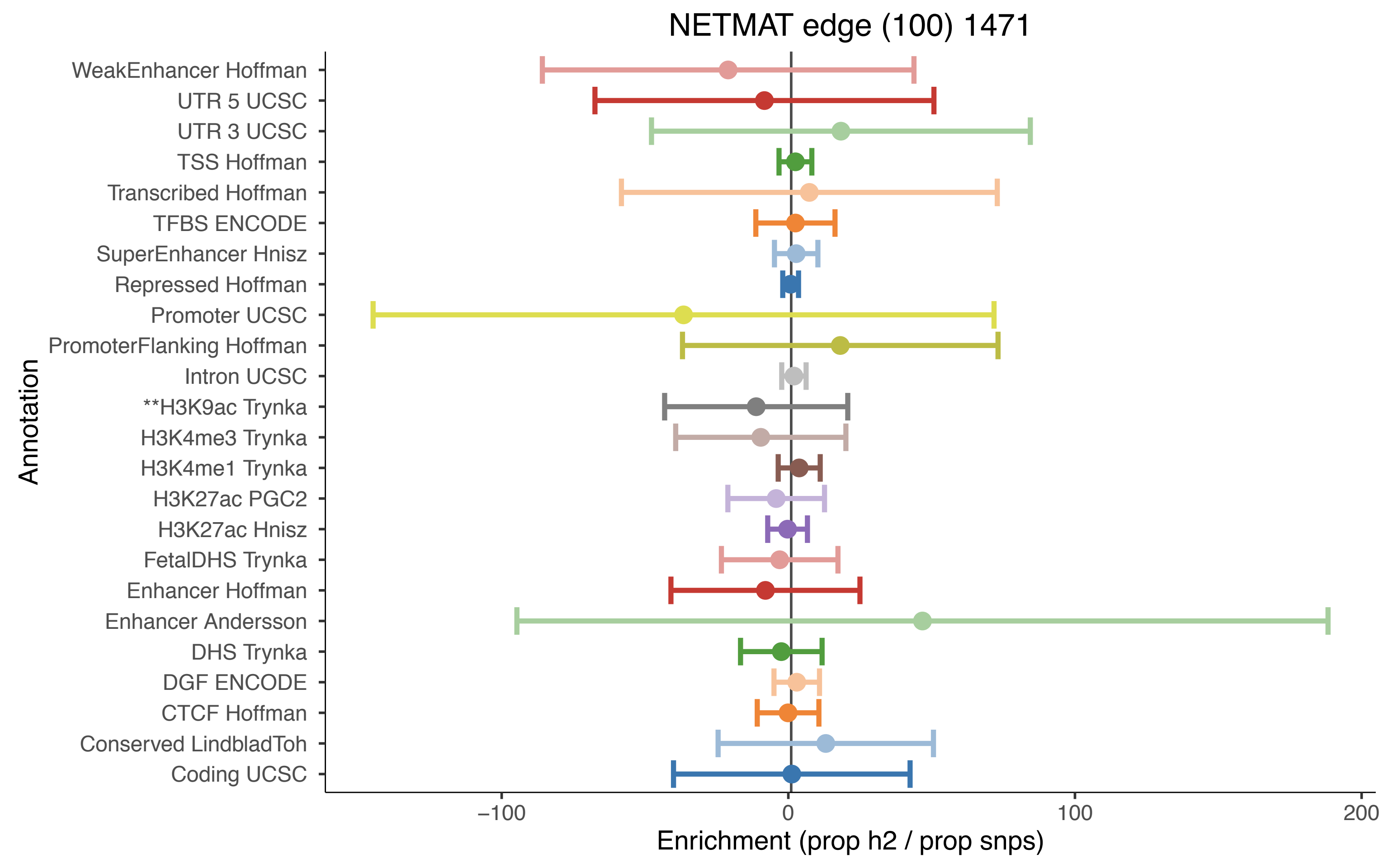
NETMAT edge (100) 1471
WeakEnhancer Hoffman
UTR 5 UCSC
UTR 3 UCSC
TSS Hoffman
Transcribed Hoffman
TFBS ENCODE
SuperEnhancer Hnisz
Repressed Hoffman
Promoter UCSC
PromoterFlanking Hoffman
Intron UCSC
**H3K9ac Trynka
H3K4me3 Trynka
H3K4me1 Trynka
H3K27ac PGC2
H3K27ac Hnisz
FetalDHS Trynka
Enhancer Hoffman
Enhancer Andersson
DHS Trynka
DGF ENCODE
CTCF Hoffman
Conserved LindbladToh
Coding UCSC
Annotation
−100
0
100
200
Enrichment (prop h2 / prop snps)

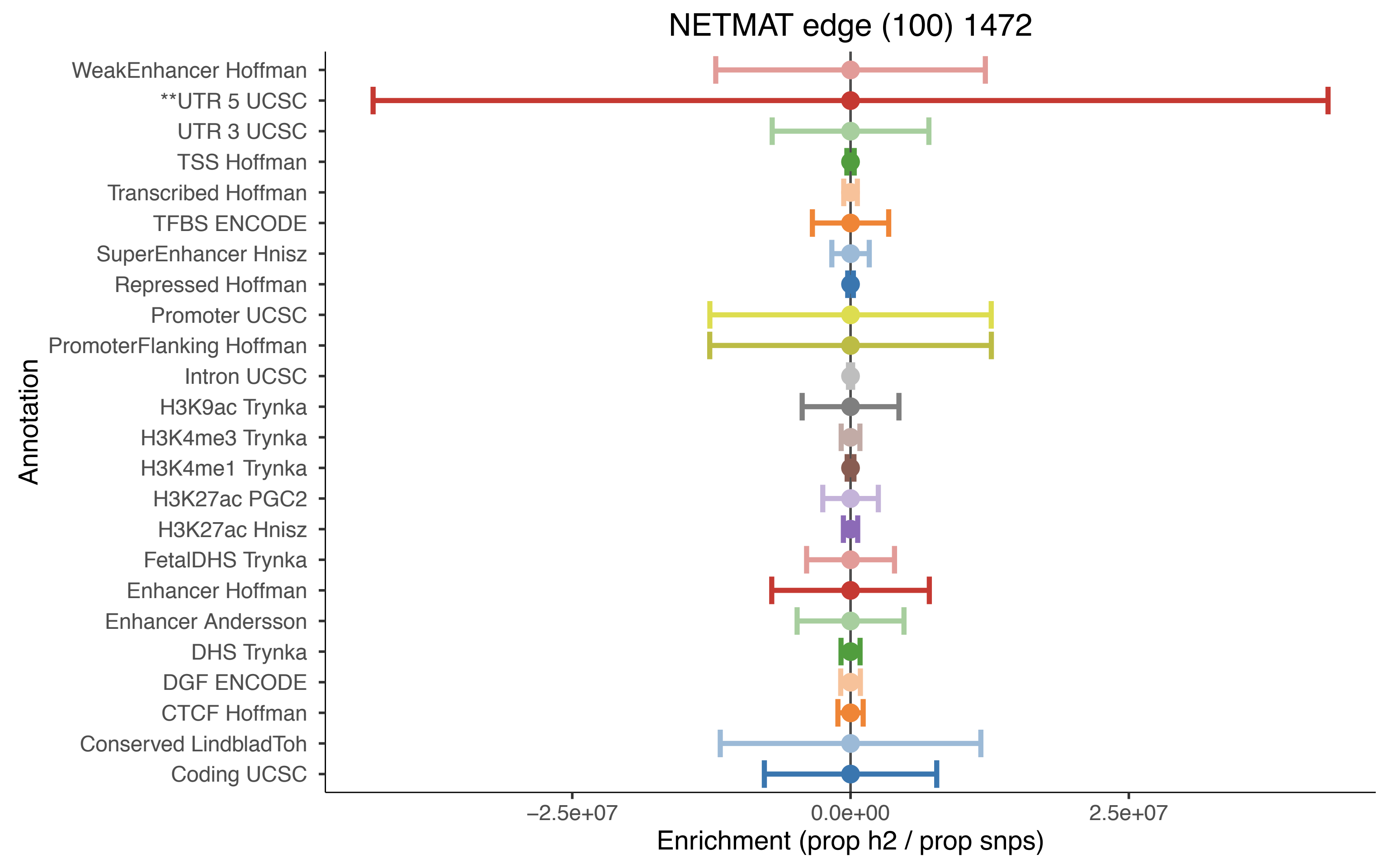

NETMAT edge (100) 1472
WeakEnhancer Hoffman
**UTR 5 UCSC
UTR 3 UCSC
TSS Hoffman
Transcribed Hoffman
TFBS ENCODE
SuperEnhancer Hnisz
Repressed Hoffman
Promoter UCSC
PromoterFlanking Hoffman
Intron UCSC
H3K9ac Trynka
H3K4me3 Trynka
H3K4me1 Trynka
H3K27ac PGC2
H3K27ac Hnisz
FetalDHS Trynka
Enhancer Hoffman
Enhancer Andersson
DHS Trynka
DGF ENCODE
CTCF Hoffman
Conserved LindbladToh
Coding UCSC
Annotation
−2.5e+07
0.0e+00
2.5e+07
Enrichment (prop h2 / prop snps)

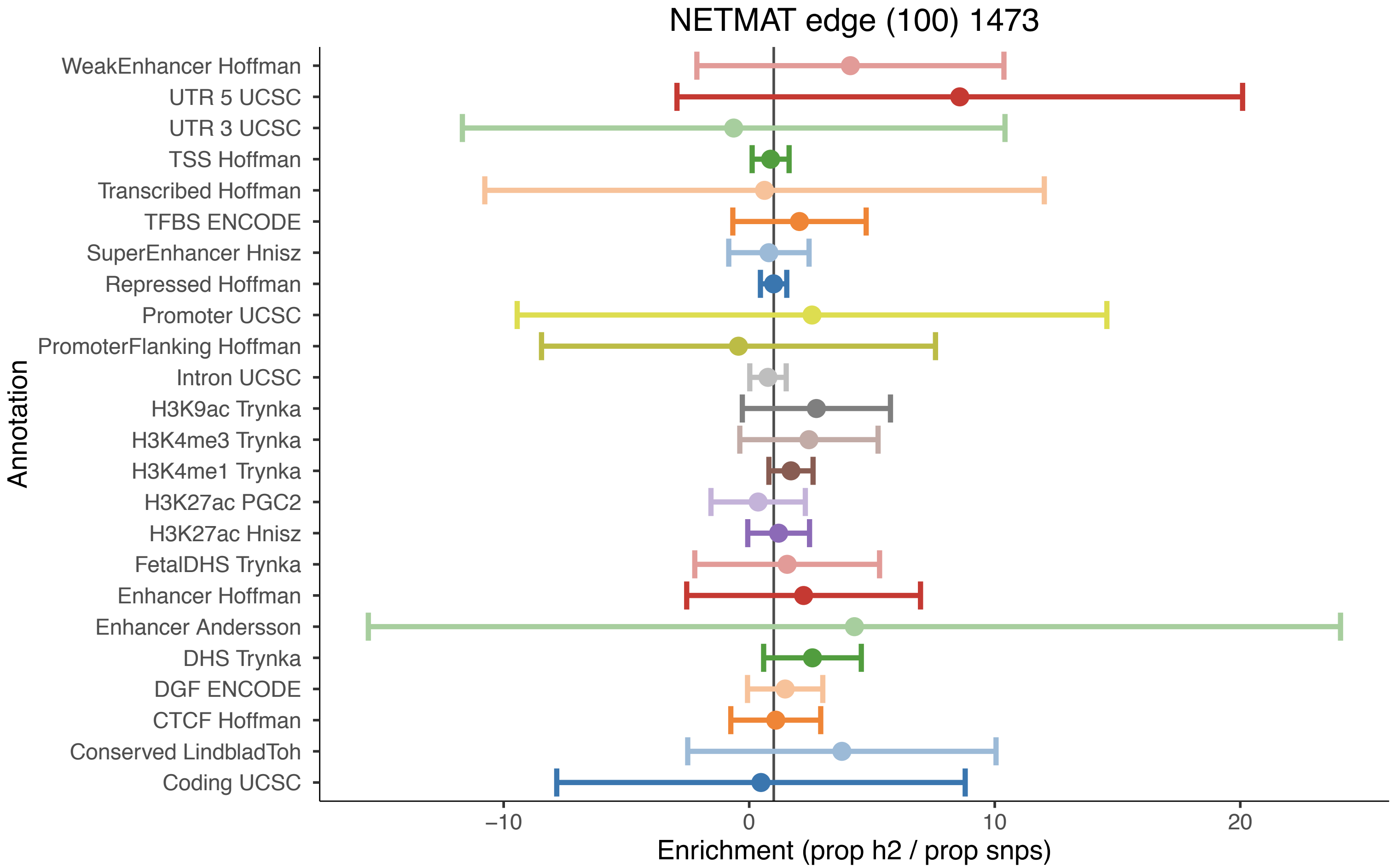
NETMAT edge (100) 1473
WeakEnhancer Hoffman
UTR 5 UCSC
UTR 3 UCSC
TSS Hoffman
Transcribed Hoffman
TFBS ENCODE
SuperEnhancer Hnisz
Repressed Hoffman
Promoter UCSC
PromoterFlanking Hoffman
Intron UCSC
H3K9ac Trynka
H3K4me3 Trynka
H3K4me1 Trynka
H3K27ac PGC2
H3K27ac Hnisz
FetalDHS Trynka
Enhancer Hoffman
Enhancer Andersson
DHS Trynka
DGF ENCODE
CTCF Hoffman
Conserved LindbladToh
Coding UCSC
Annotation
−10
0
10
20
Enrichment (prop h2 / prop snps)

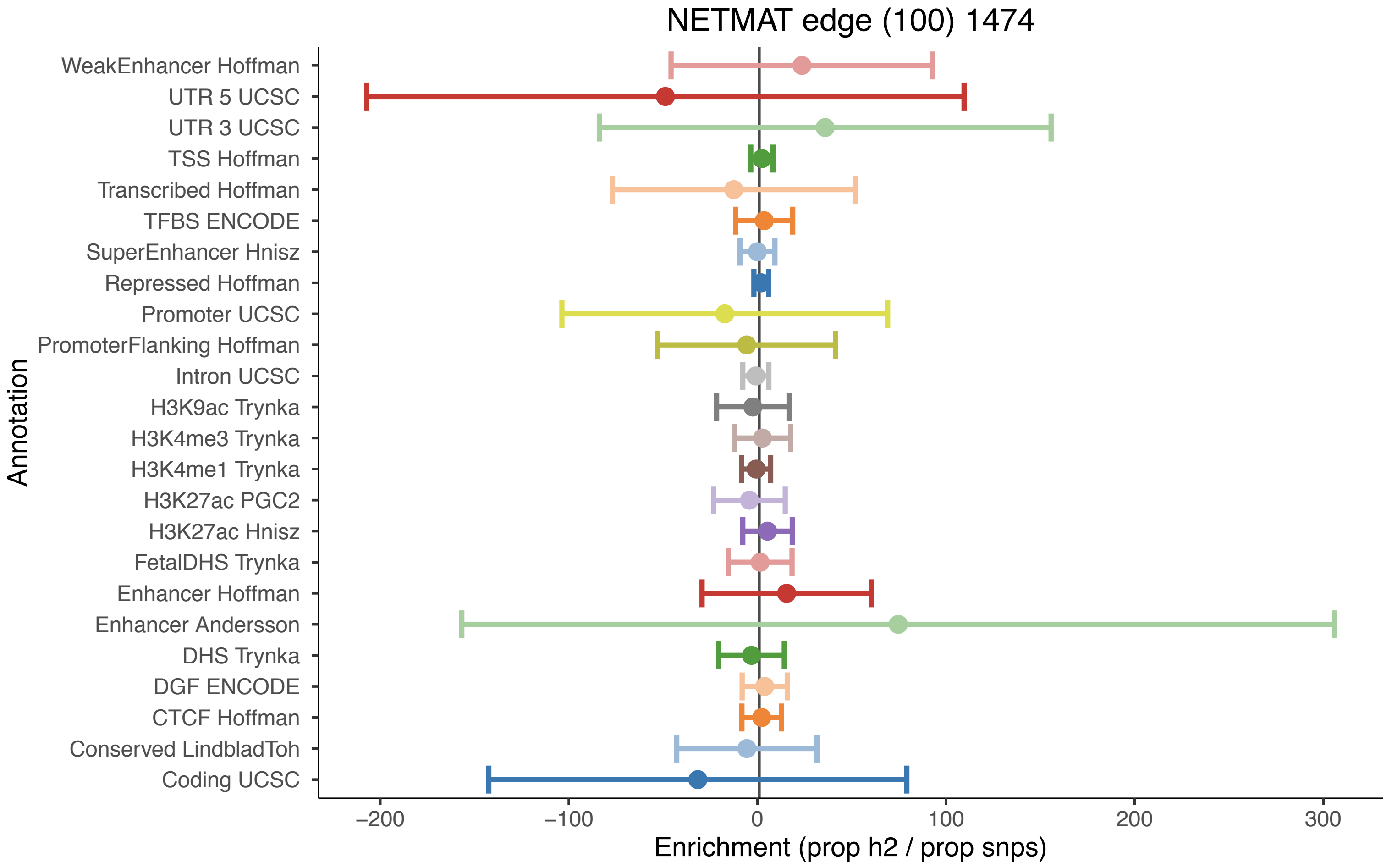
NETMAT edge (100) 1474
WeakEnhancer Hoffman
UTR 5 UCSC
UTR 3 UCSC
TSS Hoffman
Transcribed Hoffman
TFBS ENCODE
SuperEnhancer Hnisz
Repressed Hoffman
Promoter UCSC
PromoterFlanking Hoffman
Intron UCSC
H3K9ac Trynka
H3K4me3 Trynka
H3K4me1 Trynka
H3K27ac PGC2
H3K27ac Hnisz
FetalDHS Trynka
Enhancer Hoffman
Enhancer Andersson
DHS Trynka
DGF ENCODE
CTCF Hoffman
Conserved LindbladToh
Coding UCSC
Annotation
−200
−100
0
100
200
300
Enrichment (prop h2 / prop snps)

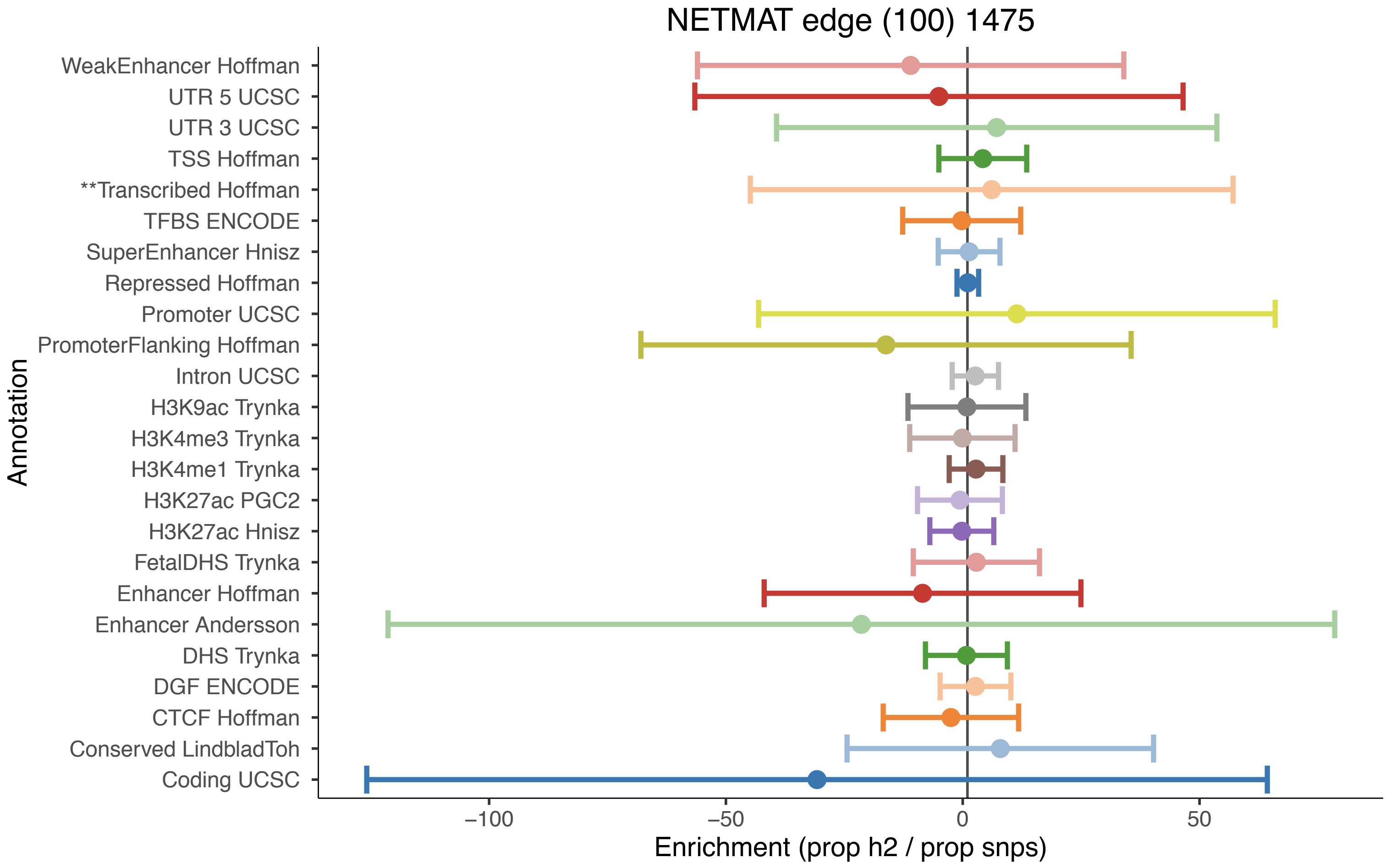

NETMAT edge (100) 1475
WeakEnhancer Hoffman
UTR 5 UCSC
UTR 3 UCSC
TSS Hoffman
**Transcribed Hoffman
TFBS ENCODE
SuperEnhancer Hnisz
Repressed Hoffman
Promoter UCSC
PromoterFlanking Hoffman
Intron UCSC
H3K9ac Trynka
H3K4me3 Trynka
H3K4me1 Trynka
H3K27ac PGC2
H3K27ac Hnisz
FetalDHS Trynka
Enhancer Hoffman
Enhancer Andersson
DHS Trynka
DGF ENCODE
CTCF Hoffman
Conserved LindbladToh
Coding UCSC
Annotation
−100
−50
0
50
Enrichment (prop h2 / prop snps)

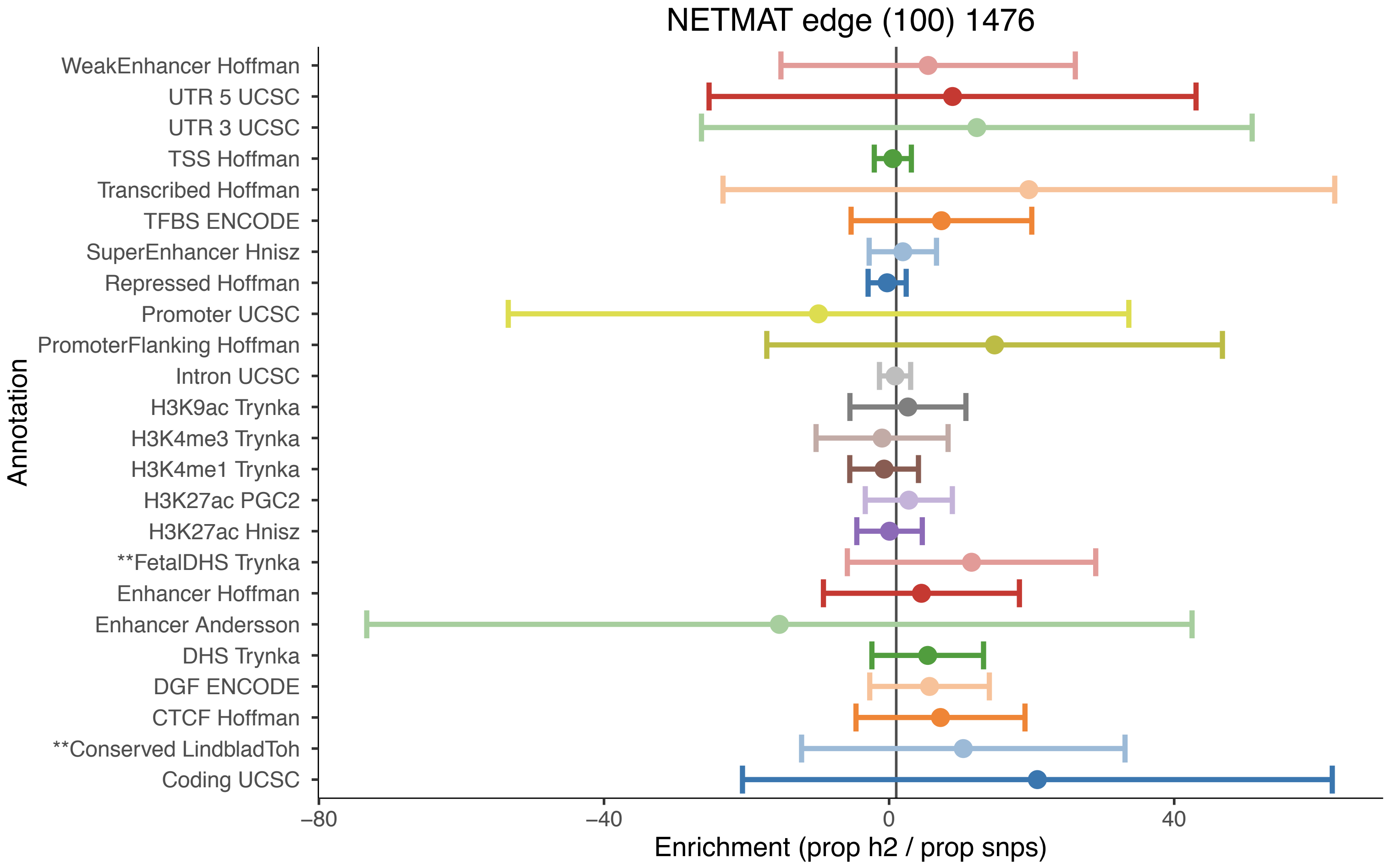
NETMAT edge (100) 1476
WeakEnhancer Hoffman
UTR 5 UCSC
UTR 3 UCSC
TSS Hoffman
Transcribed Hoffman
TFBS ENCODE
SuperEnhancer Hnisz
Repressed Hoffman
Promoter UCSC
PromoterFlanking Hoffman
Intron UCSC
H3K9ac Trynka
H3K4me3 Trynka
H3K4me1 Trynka
H3K27ac PGC2
H3K27ac Hnisz
**FetalDHS Trynka
Enhancer Hoffman
Enhancer Andersson
DHS Trynka
DGF ENCODE
CTCF Hoffman
**Conserved LindbladToh
Coding UCSC
Annotation
−80
−40
0
40
Enrichment (prop h2 / prop snps)

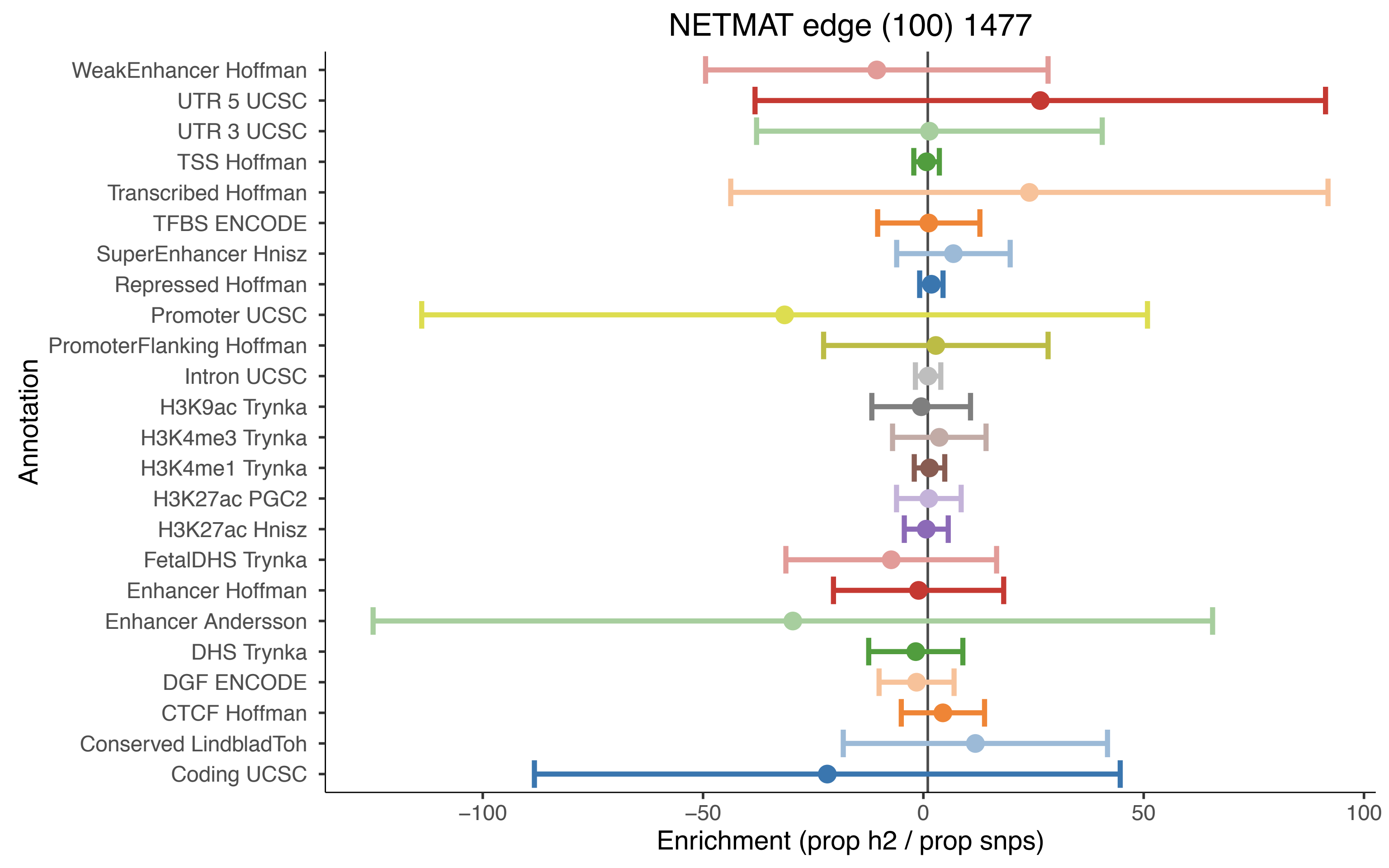

NETMAT edge (100) 1477
WeakEnhancer Hoffman
UTR 5 UCSC
UTR 3 UCSC
TSS Hoffman
Transcribed Hoffman
TFBS ENCODE
SuperEnhancer Hnisz
Repressed Hoffman
Promoter UCSC
PromoterFlanking Hoffman
Intron UCSC
H3K9ac Trynka
H3K4me3 Trynka
H3K4me1 Trynka
H3K27ac PGC2
H3K27ac Hnisz
FetalDHS Trynka
Enhancer Hoffman
Enhancer Andersson
DHS Trynka
DGF ENCODE
CTCF Hoffman
Conserved LindbladToh
Coding UCSC
Annotation
−100
−50
0
50
100
Enrichment (prop h2 / prop snps)

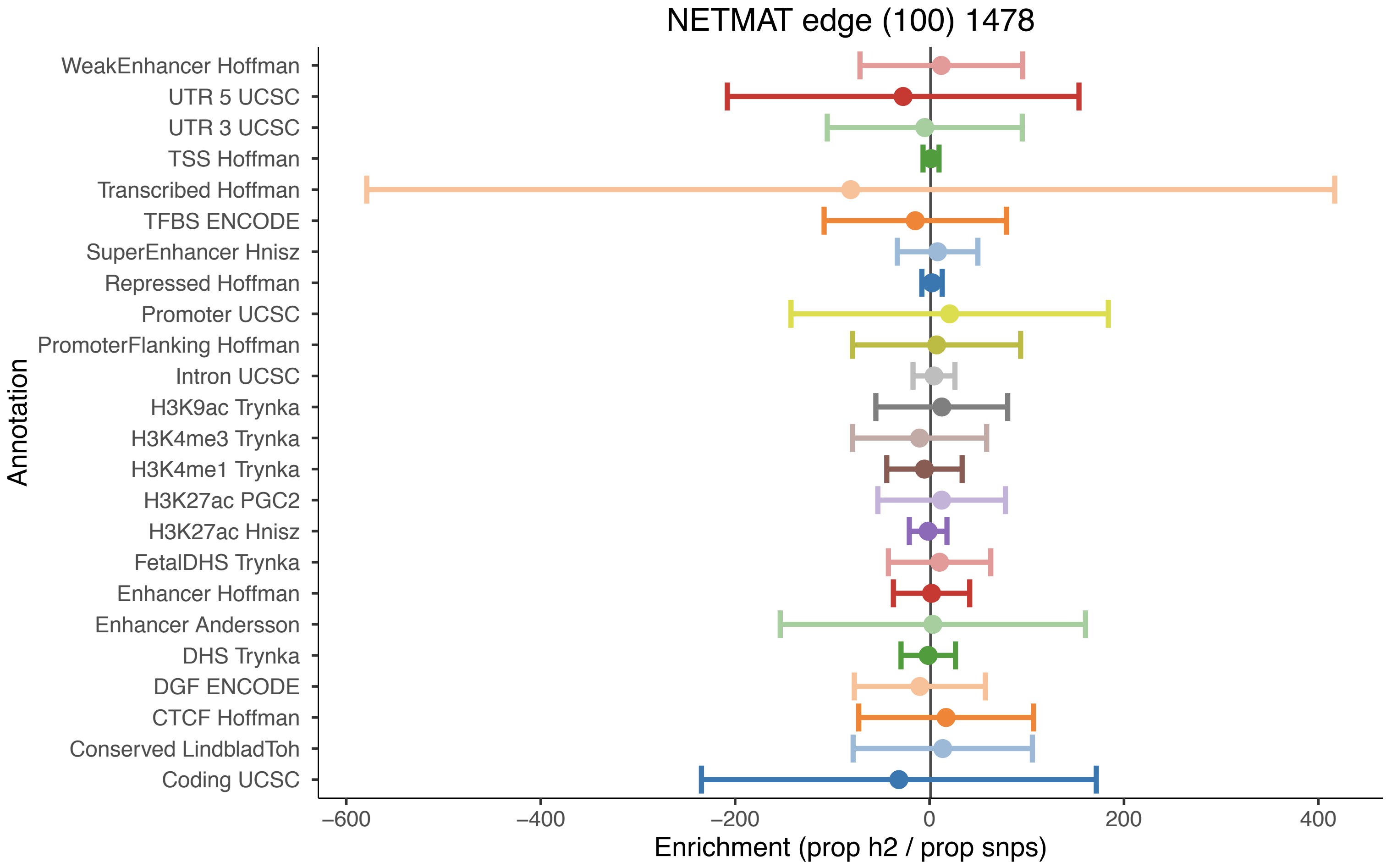
NETMAT edge (100) 1478
WeakEnhancer Hoffman
UTR 5 UCSC
UTR 3 UCSC
TSS Hoffman
Transcribed Hoffman
TFBS ENCODE
SuperEnhancer Hnisz
Repressed Hoffman
Promoter UCSC
PromoterFlanking Hoffman
Intron UCSC
H3K9ac Trynka
H3K4me3 Trynka
H3K4me1 Trynka
H3K27ac PGC2
H3K27ac Hnisz
FetalDHS Trynka
Enhancer Hoffman
Enhancer Andersson
DHS Trynka
DGF ENCODE
CTCF Hoffman
Conserved LindbladToh
Coding UCSC
Annotation
−600
−400
−200
0
200
400
Enrichment (prop h2 / prop snps)

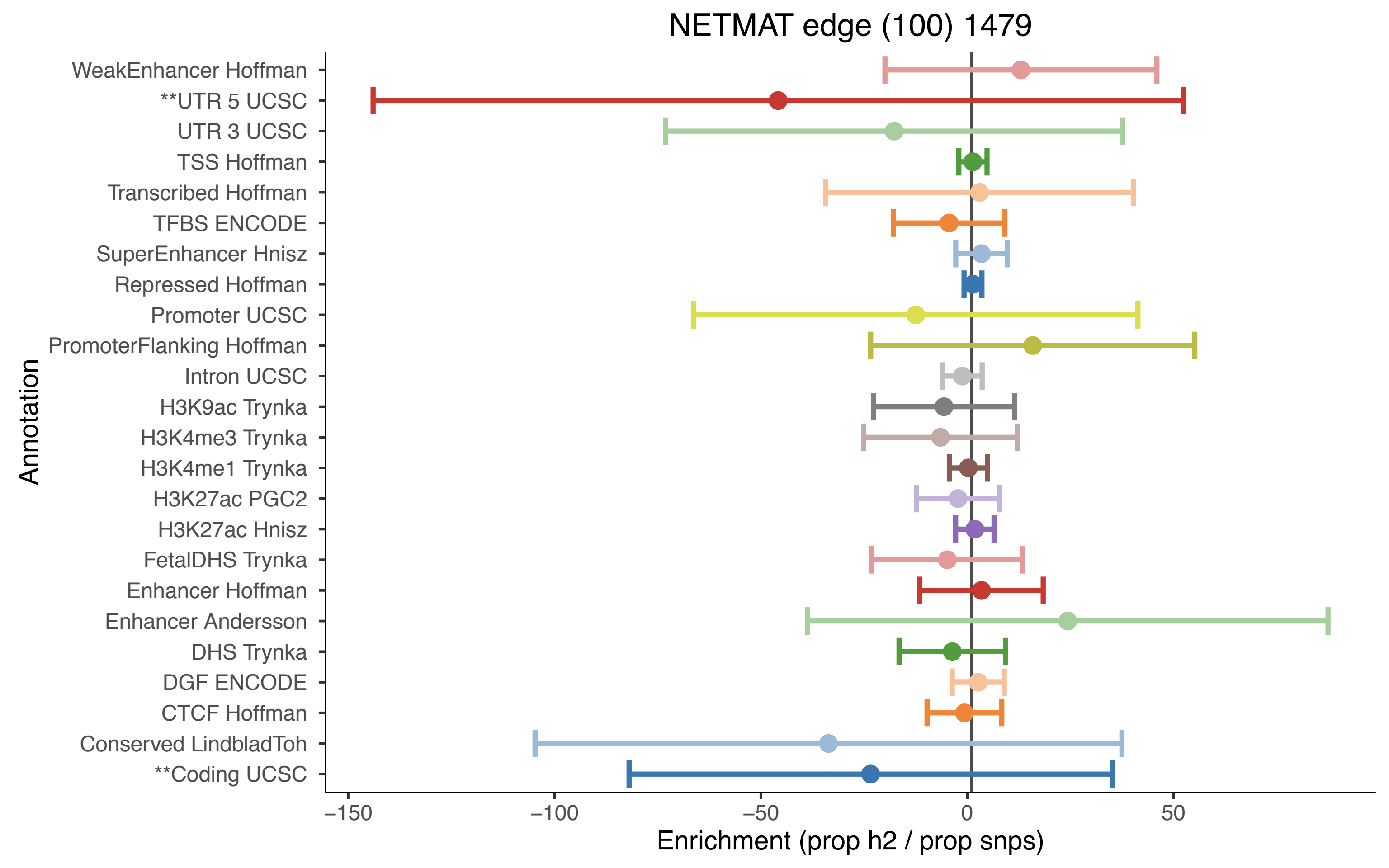
NETMAT edge (100) 1479
WeakEnhancer Hoffman
**UTR 5 UCSC
UTR 3 UCSC
TSS Hoffman
Transcribed Hoffman
TFBS ENCODE
SuperEnhancer Hnisz
Repressed Hoffman
Promoter UCSC
PromoterFlanking Hoffman
Intron UCSC
H3K9ac Trynka
H3K4me3 Trynka
H3K4me1 Trynka
H3K27ac PGC2
H3K27ac Hnisz
FetalDHS Trynka
Enhancer Hoffman
Enhancer Andersson
DHS Trynka
DGF ENCODE
CTCF Hoffman
Conserved LindbladToh
**Coding UCSC
Annotation
−150
−100
−50
0
50
Enrichment (prop h2 / prop snps)

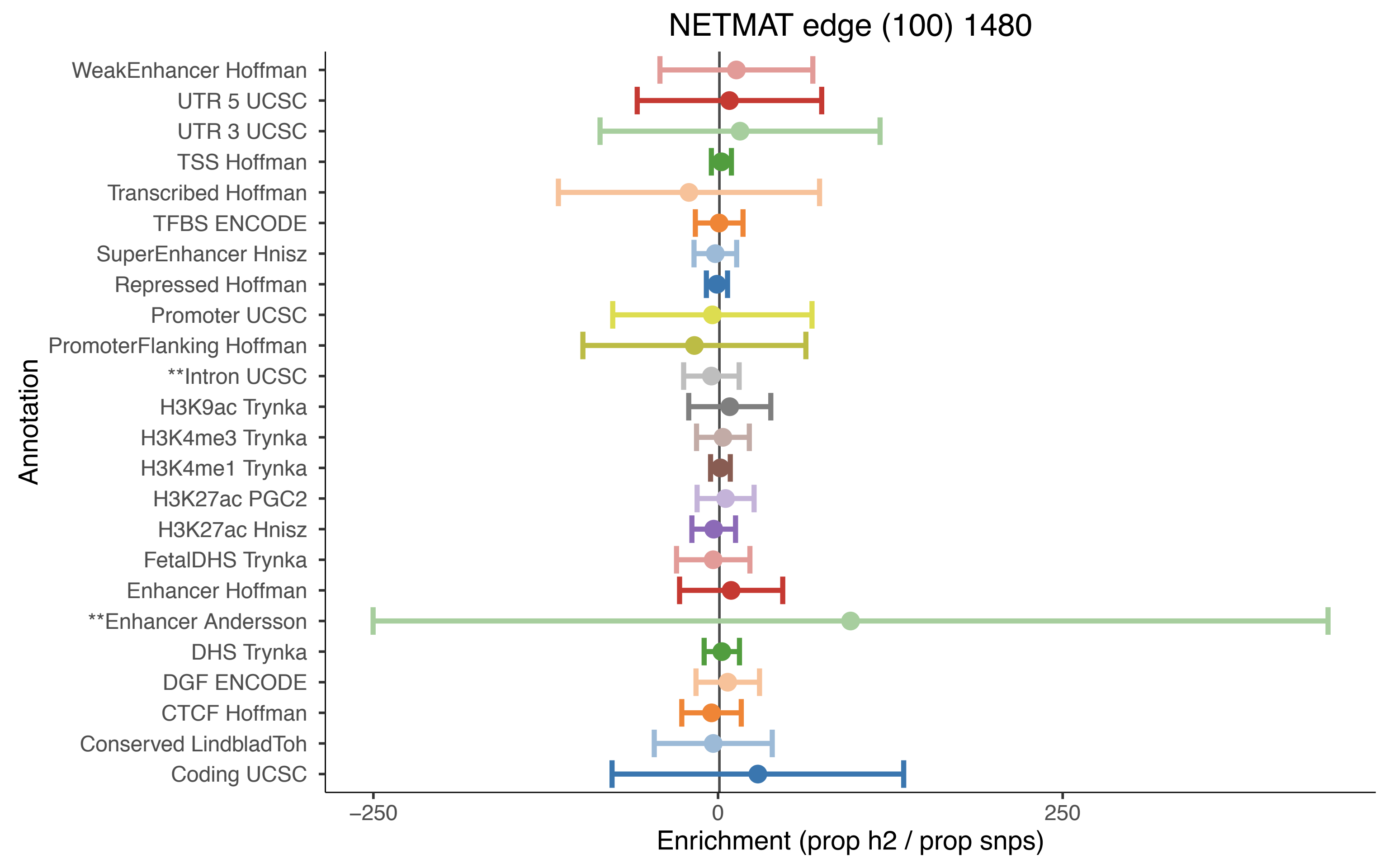
NETMAT edge (100) 1480
WeakEnhancer Hoffman
UTR 5 UCSC
UTR 3 UCSC
TSS Hoffman
Transcribed Hoffman
TFBS ENCODE
SuperEnhancer Hnisz
Repressed Hoffman
Promoter UCSC
PromoterFlanking Hoffman
**Intron UCSC
H3K9ac Trynka
H3K4me3 Trynka
H3K4me1 Trynka
H3K27ac PGC2
H3K27ac Hnisz
FetalDHS Trynka
Enhancer Hoffman
**Enhancer Andersson
DHS Trynka
DGF ENCODE
CTCF Hoffman
Conserved LindbladToh
Coding UCSC
Annotation
−250
0
250
Enrichment (prop h2 / prop snps)

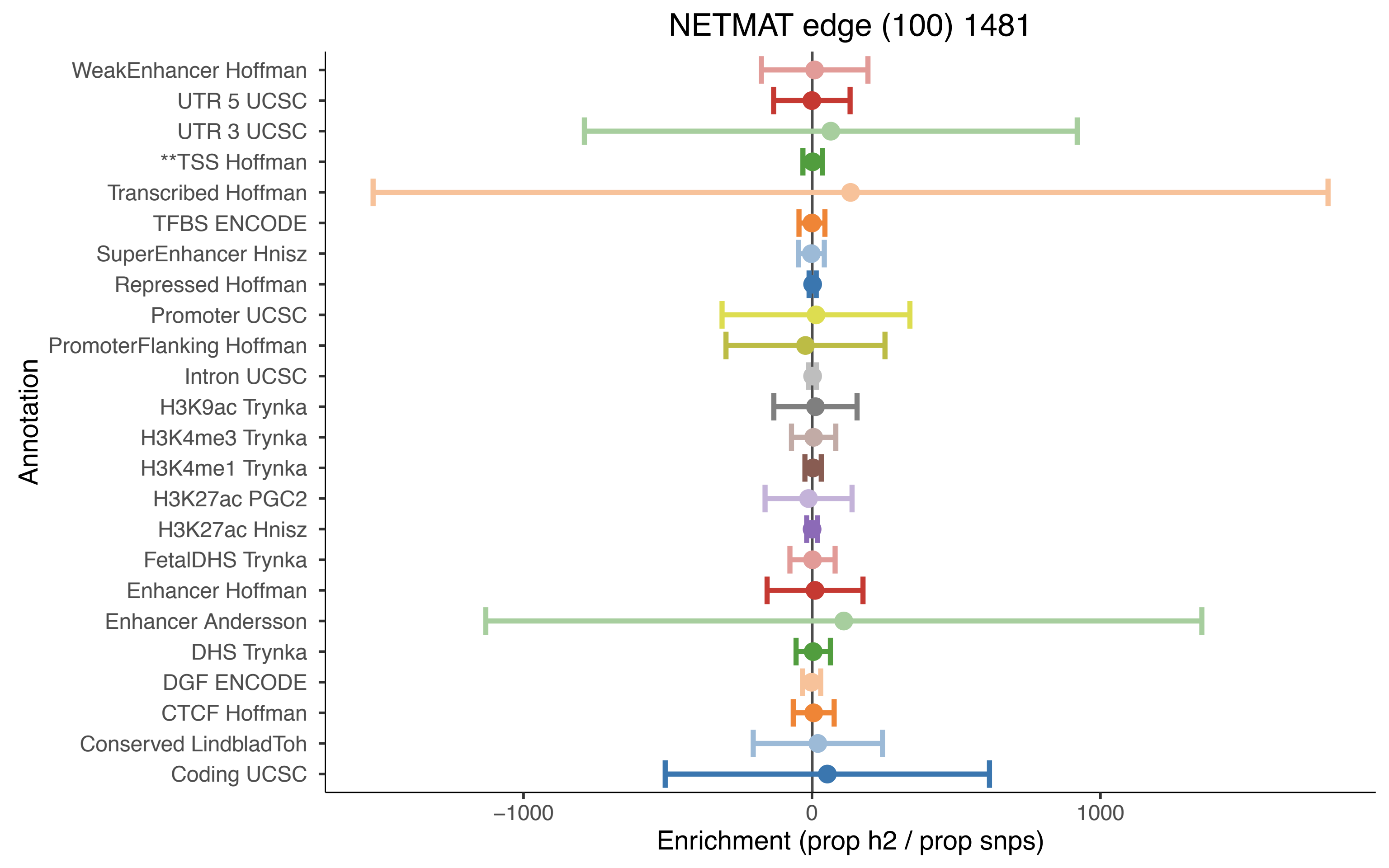
NETMAT edge (100) 1481
WeakEnhancer Hoffman
UTR 5 UCSC
UTR 3 UCSC
**TSS Hoffman
Transcribed Hoffman
TFBS ENCODE
SuperEnhancer Hnisz
Repressed Hoffman
Promoter UCSC
PromoterFlanking Hoffman
Intron UCSC
H3K9ac Trynka
H3K4me3 Trynka
H3K4me1 Trynka
H3K27ac PGC2
H3K27ac Hnisz
FetalDHS Trynka
Enhancer Hoffman
Enhancer Andersson
DHS Trynka
DGF ENCODE
CTCF Hoffman
Conserved LindbladToh
Coding UCSC
Annotation
−1000
0
1000
Enrichment (prop h2 / prop snps)

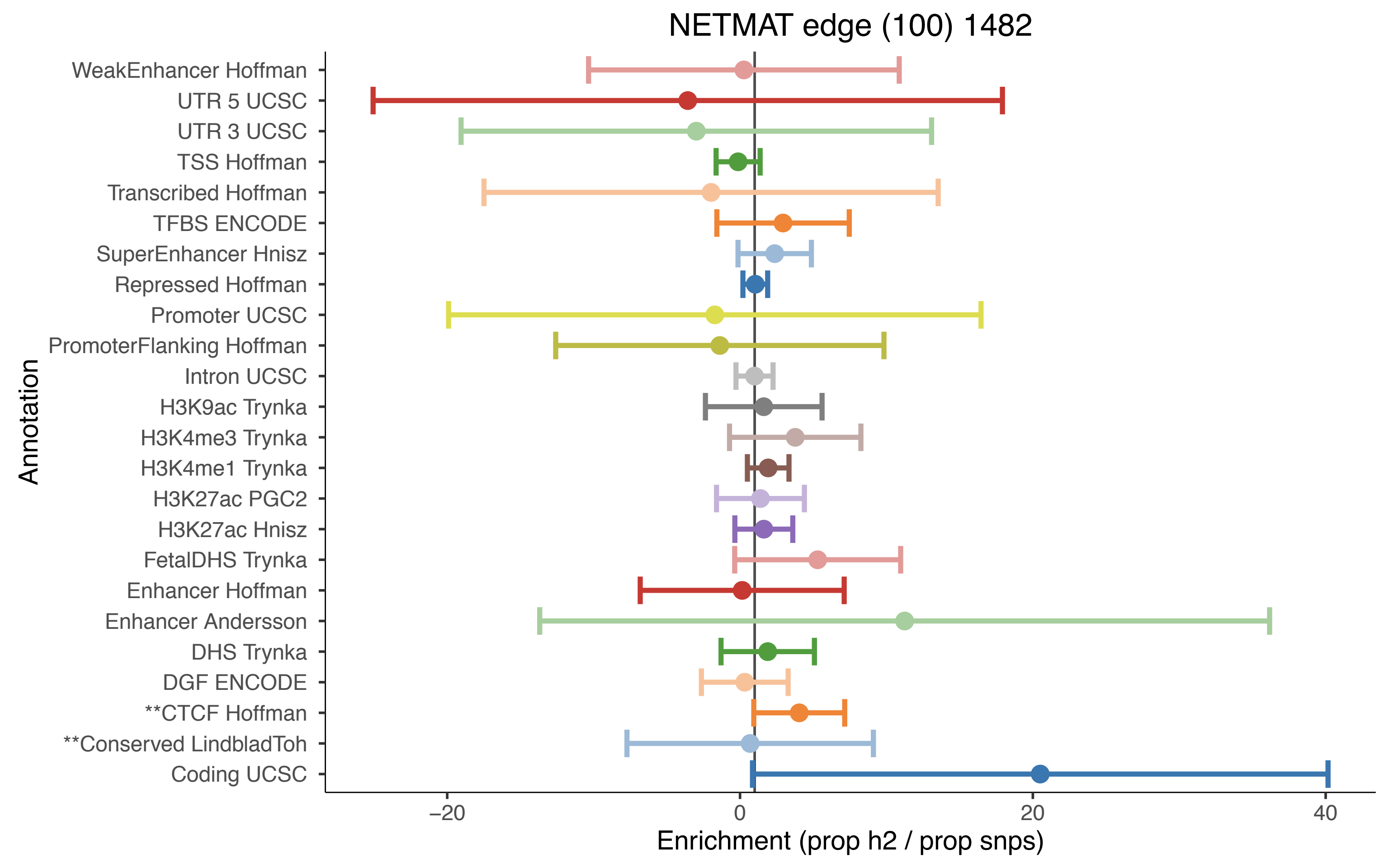
NETMAT edge (100) 1482
WeakEnhancer Hoffman
UTR 5 UCSC
UTR 3 UCSC
TSS Hoffman
Transcribed Hoffman
TFBS ENCODE
SuperEnhancer Hnisz
Repressed Hoffman
Promoter UCSC
PromoterFlanking Hoffman
Intron UCSC
H3K9ac Trynka
H3K4me3 Trynka
H3K4me1 Trynka
H3K27ac PGC2
H3K27ac Hnisz
FetalDHS Trynka
Enhancer Hoffman
Enhancer Andersson
DHS Trynka
DGF ENCODE
**CTCF Hoffman
**Conserved LindbladToh
Coding UCSC
Annotation
−20
0
20
40
Enrichment (prop h2 / prop snps)

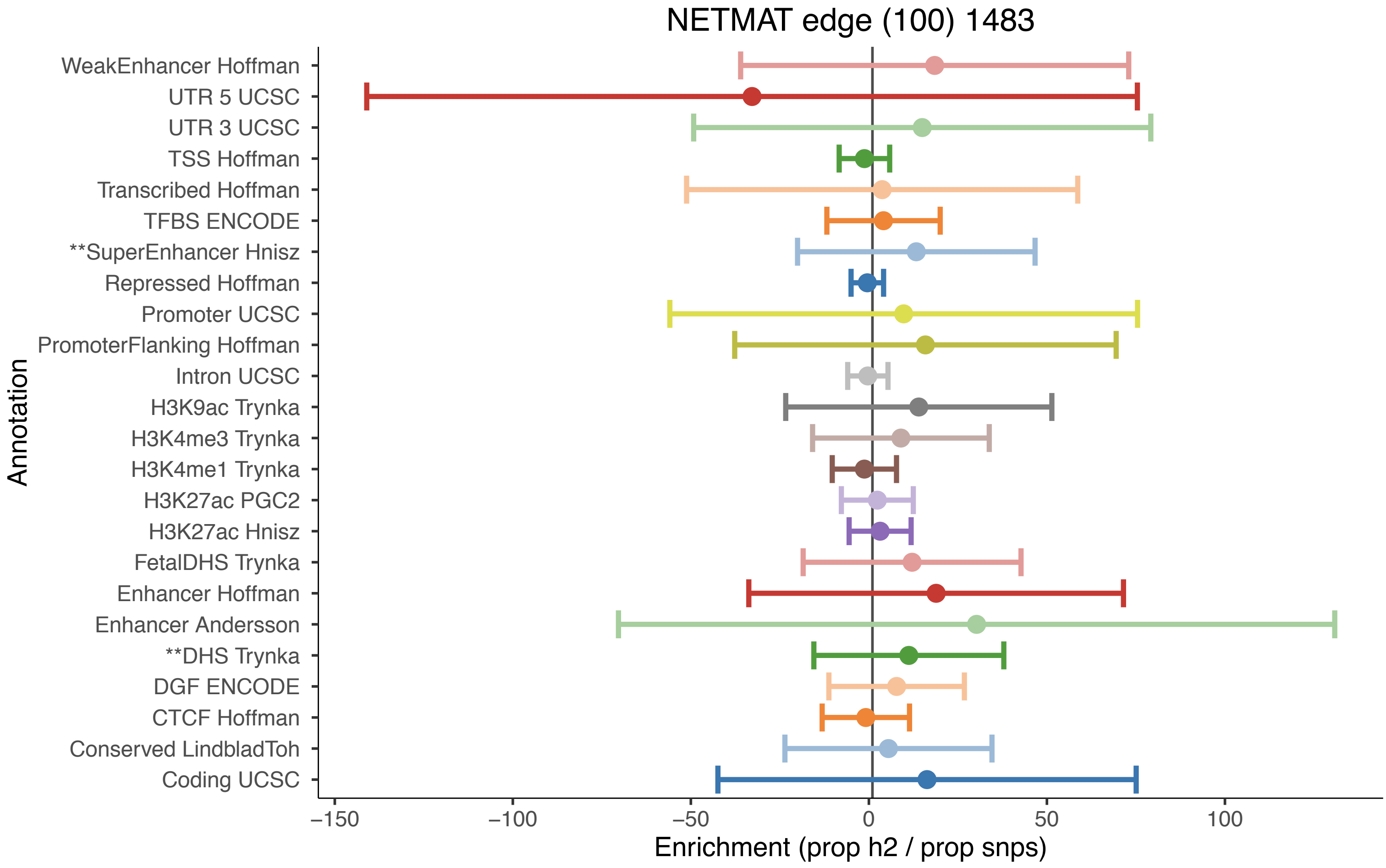
NETMAT edge (100) 1483
WeakEnhancer Hoffman
UTR 5 UCSC
UTR 3 UCSC
TSS Hoffman
Transcribed Hoffman
TFBS ENCODE
**SuperEnhancer Hnisz
Repressed Hoffman
Promoter UCSC
PromoterFlanking Hoffman
Intron UCSC
H3K9ac Trynka
H3K4me3 Trynka
H3K4me1 Trynka
H3K27ac PGC2
H3K27ac Hnisz
FetalDHS Trynka
Enhancer Hoffman
Enhancer Andersson
**DHS Trynka
DGF ENCODE
CTCF Hoffman
Conserved LindbladToh
Coding UCSC
Annotation
−150
−100
−50
0
50
100
Enrichment (prop h2 / prop snps)

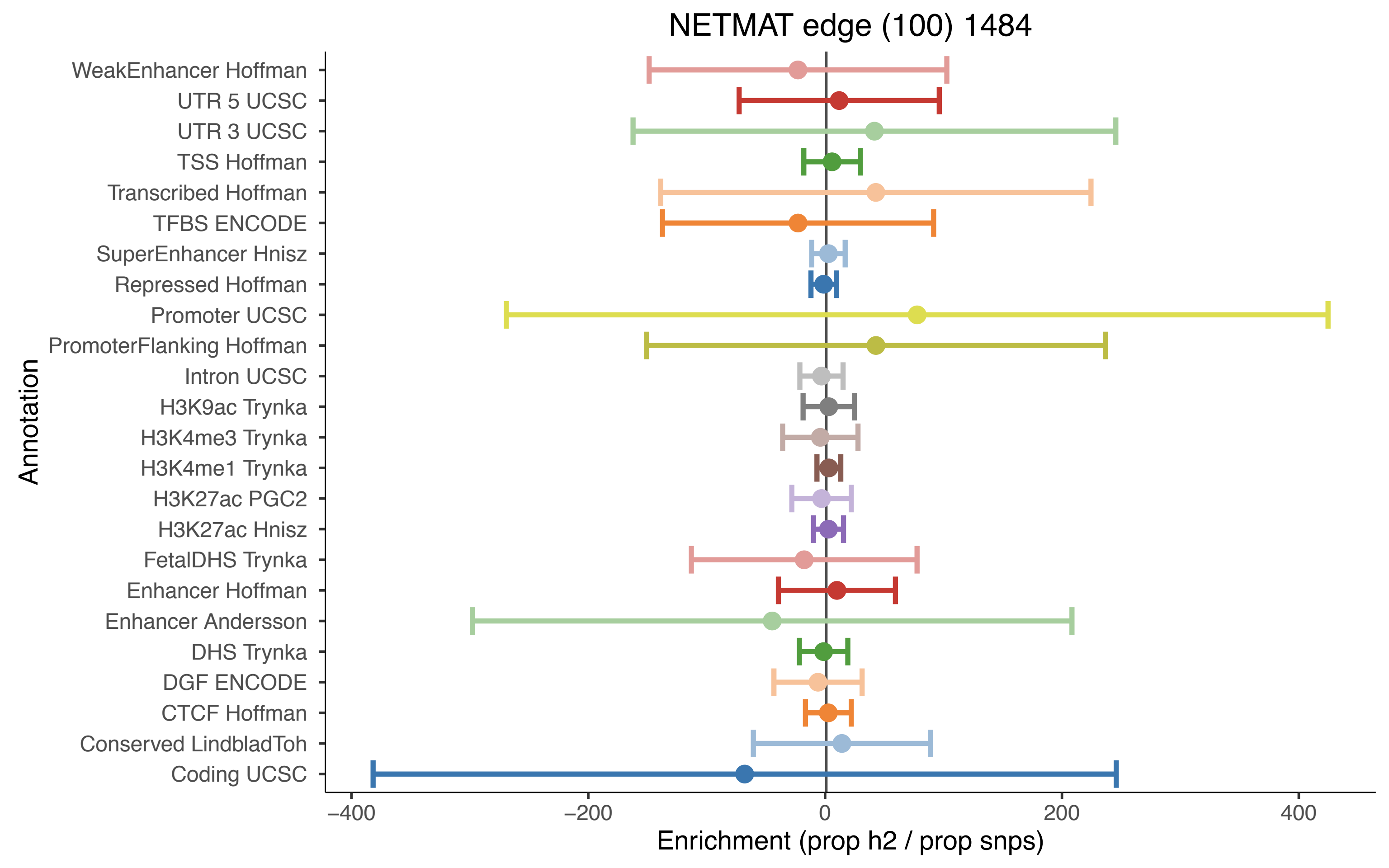
NETMAT edge (100) 1484
WeakEnhancer Hoffman
UTR 5 UCSC
UTR 3 UCSC
TSS Hoffman
Transcribed Hoffman
TFBS ENCODE
SuperEnhancer Hnisz
Repressed Hoffman
Promoter UCSC
PromoterFlanking Hoffman
Intron UCSC
H3K9ac Trynka
H3K4me3 Trynka
H3K4me1 Trynka
H3K27ac PGC2
H3K27ac Hnisz
FetalDHS Trynka
Enhancer Hoffman
Enhancer Andersson
DHS Trynka
DGF ENCODE
CTCF Hoffman
Conserved LindbladToh
Coding UCSC
Annotation
−400
−200
0
200
400
Enrichment (prop h2 / prop snps)

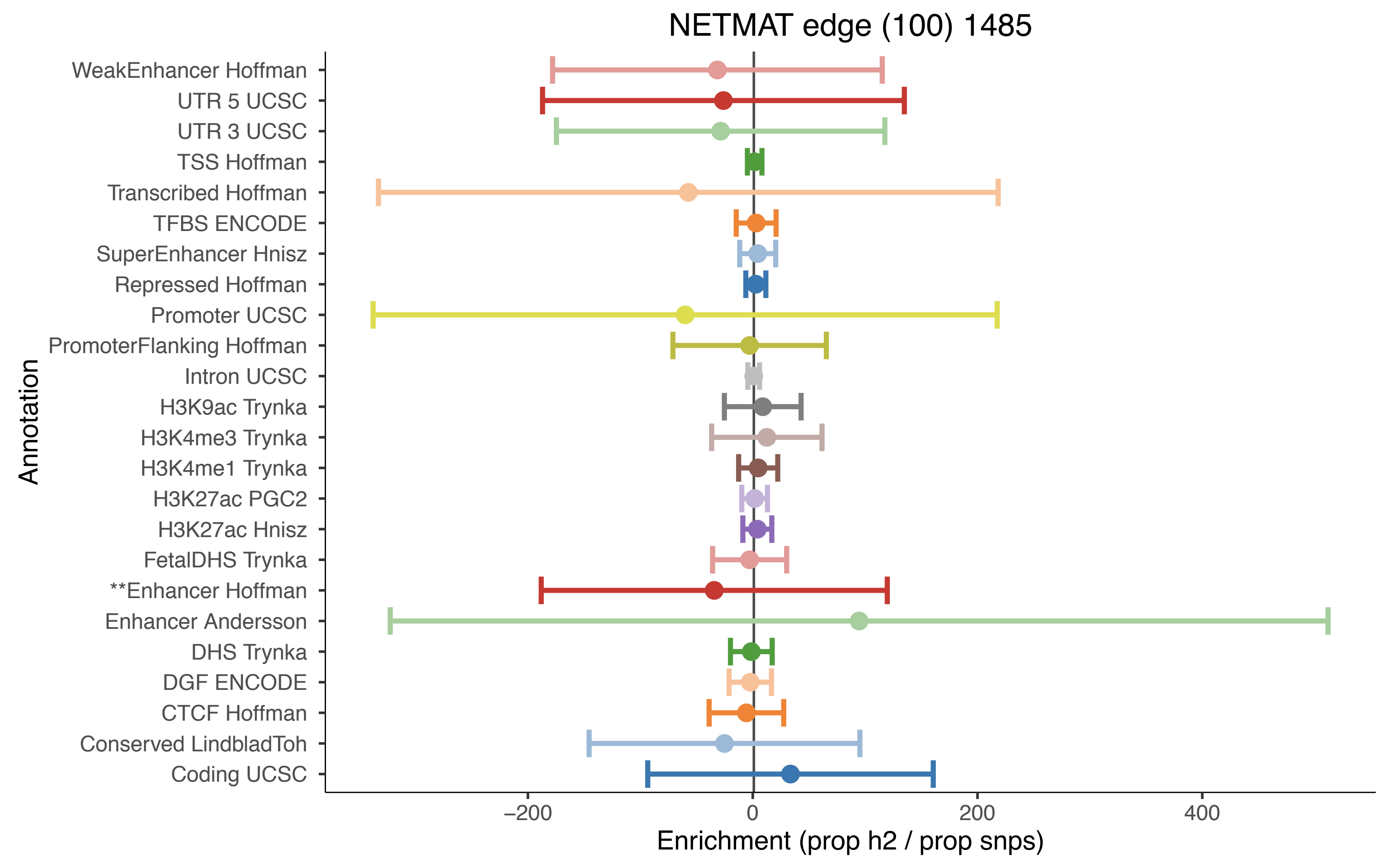
NETMAT edge (100) 1485
WeakEnhancer Hoffman
UTR 5 UCSC
UTR 3 UCSC
TSS Hoffman
Transcribed Hoffman
TFBS ENCODE
SuperEnhancer Hnisz
Repressed Hoffman
Promoter UCSC
PromoterFlanking Hoffman
Intron UCSC
H3K9ac Trynka
H3K4me3 Trynka
H3K4me1 Trynka
H3K27ac PGC2
H3K27ac Hnisz
FetalDHS Trynka
**Enhancer Hoffman
Enhancer Andersson
DHS Trynka
DGF ENCODE
CTCF Hoffman
Conserved LindbladToh
Coding UCSC
Annotation
−200
0
200
400
Enrichment (prop h2 / prop snps)

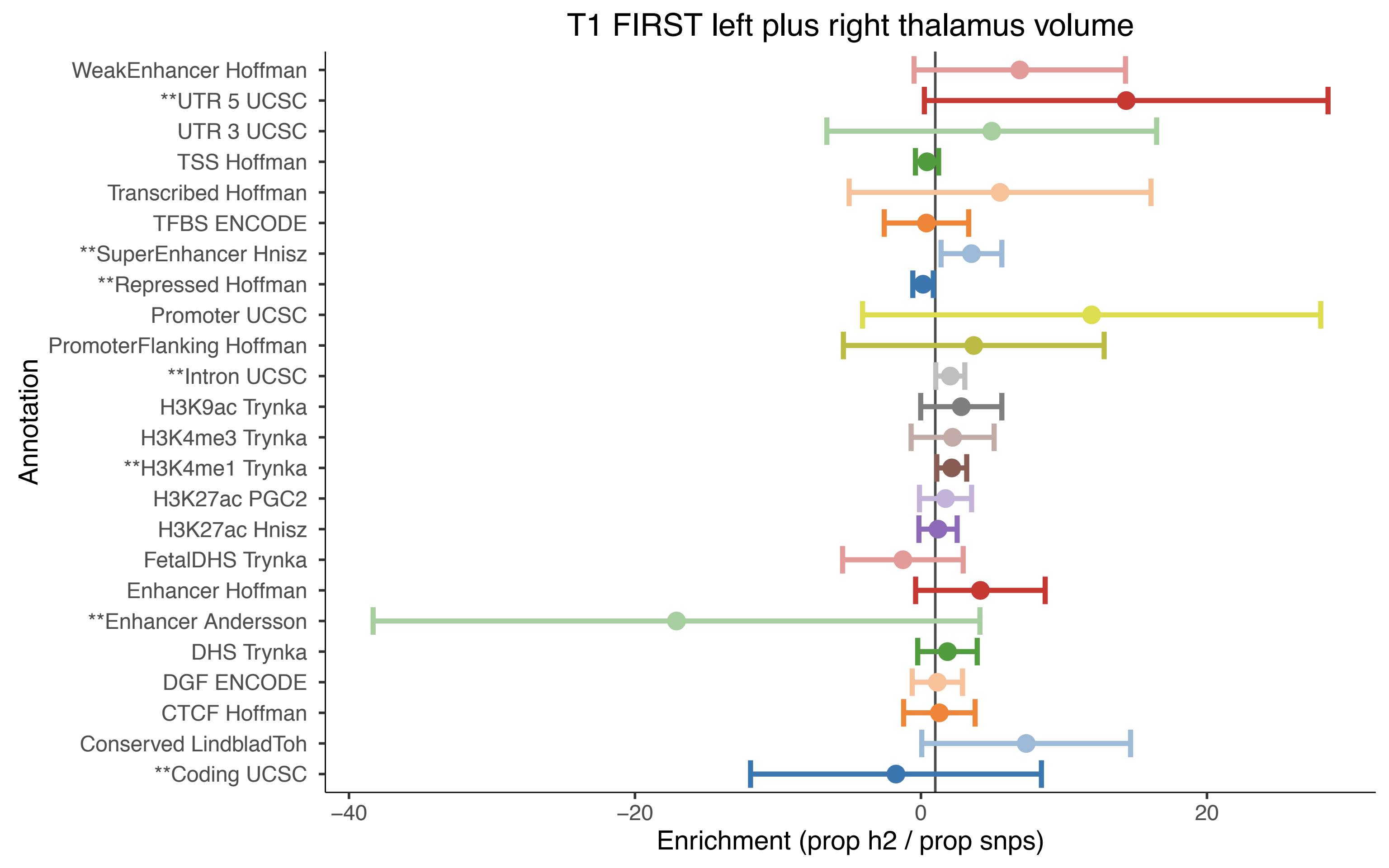
T1 FIRST left plus right thalamus volume
WeakEnhancer Hoffman
**UTR 5 UCSC
UTR 3 UCSC
TSS Hoffman
Transcribed Hoffman
TFBS ENCODE
**SuperEnhancer Hnisz
**Repressed Hoffman
Promoter UCSC
PromoterFlanking Hoffman
**Intron UCSC
H3K9ac Trynka
H3K4me3 Trynka
**H3K4me1 Trynka
H3K27ac PGC2
H3K27ac Hnisz
FetalDHS Trynka
Enhancer Hoffman
**Enhancer Andersson
DHS Trynka
DGF ENCODE
CTCF Hoffman
Conserved LindbladToh
**Coding UCSC
Annotation
−40
−20
0
20
Enrichment (prop h2 / prop snps)

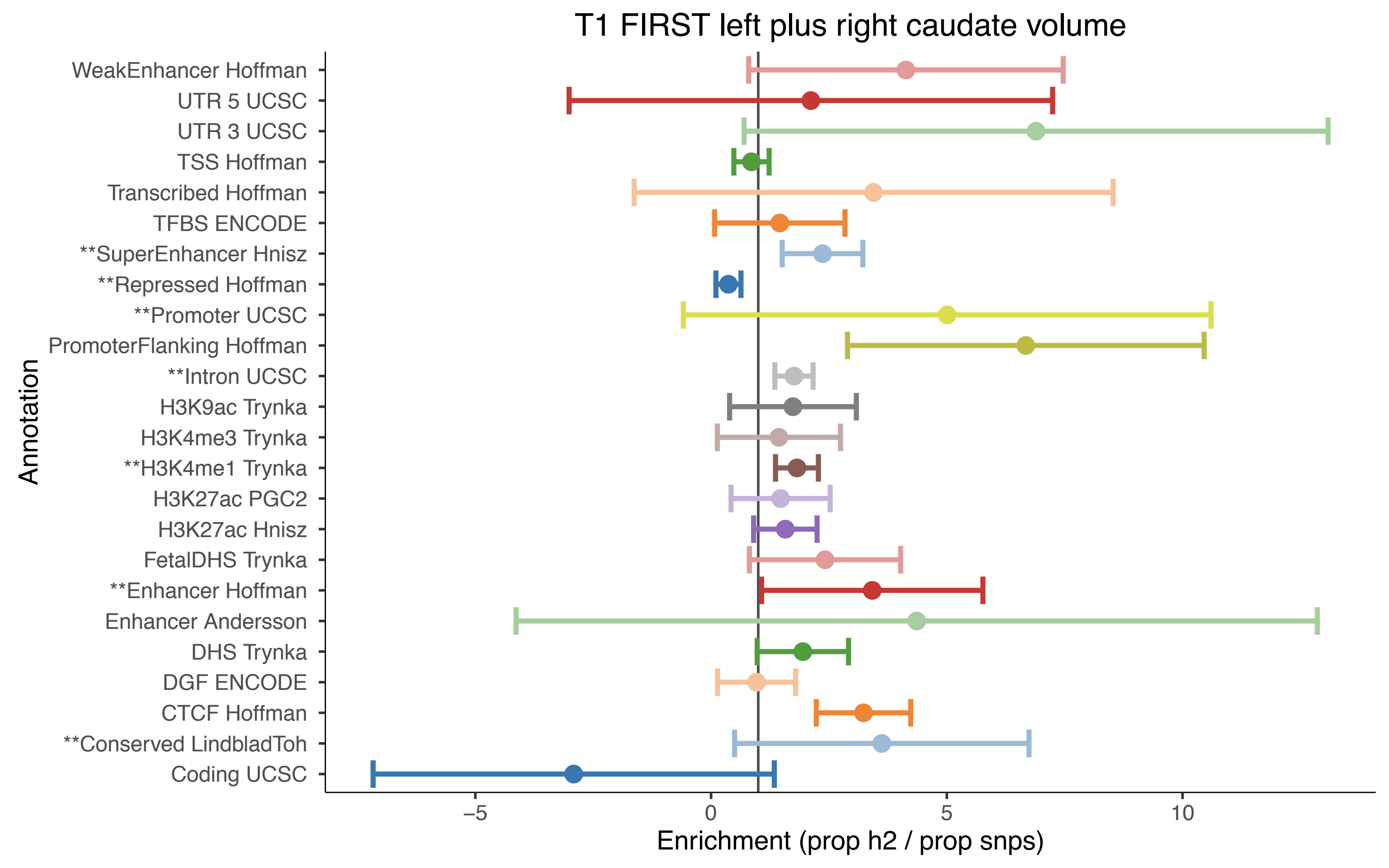

T1 FIRST left plus right caudate volume
WeakEnhancer Hoffman
UTR 5 UCSC
UTR 3 UCSC
TSS Hoffman
Transcribed Hoffman
TFBS ENCODE
**SuperEnhancer Hnisz
**Repressed Hoffman
**Promoter UCSC
PromoterFlanking Hoffman
**Intron UCSC
H3K9ac Trynka
H3K4me3 Trynka
**H3K4me1 Trynka
H3K27ac PGC2
H3K27ac Hnisz
FetalDHS Trynka
**Enhancer Hoffman
Enhancer Andersson
DHS Trynka
DGF ENCODE
CTCF Hoffman
**Conserved LindbladToh
Coding UCSC
Annotation
−5
0
5
10
Enrichment (prop h2 / prop snps)

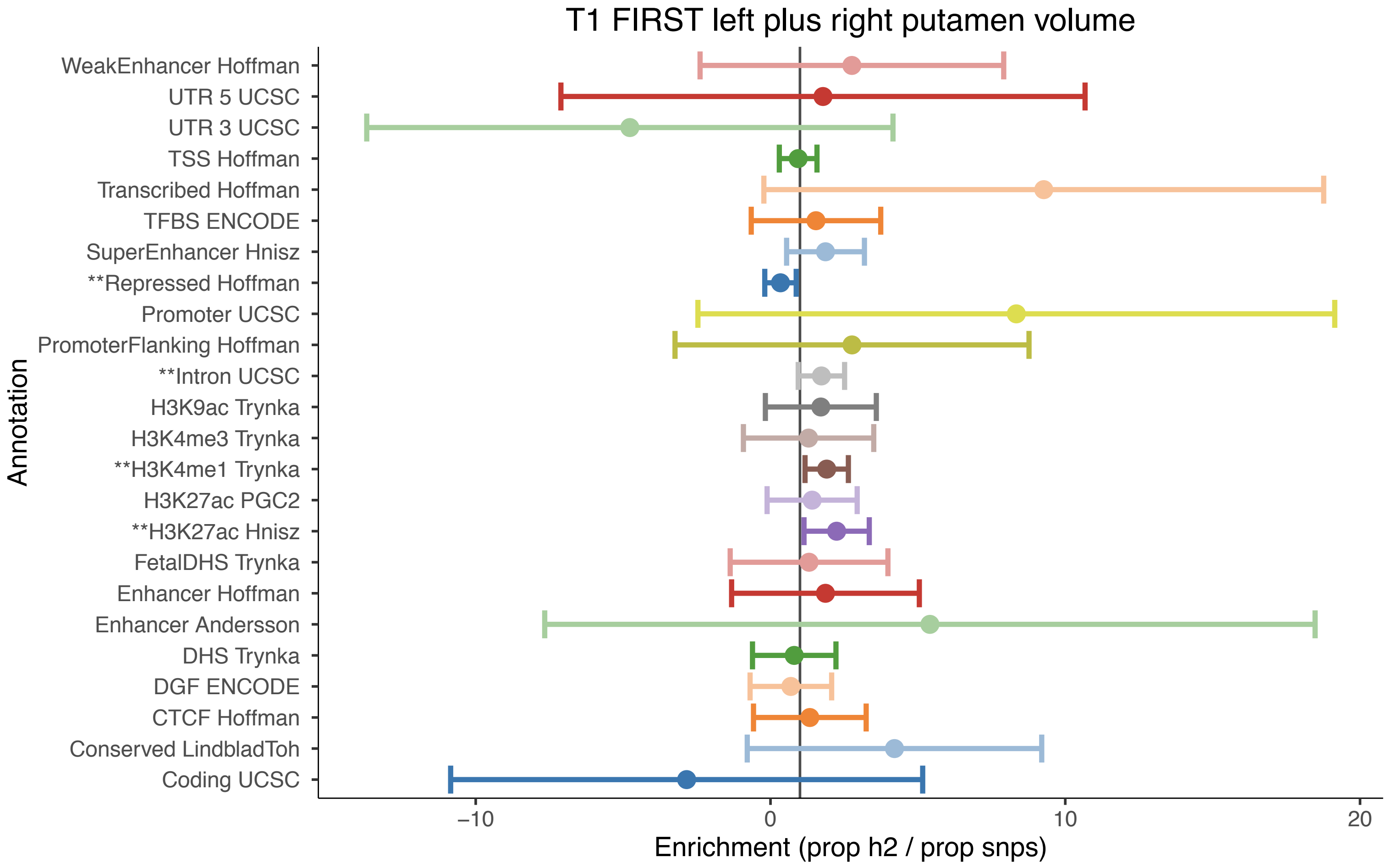

T1 FIRST left plus right putamen volume
WeakEnhancer Hoffman
UTR 5 UCSC
UTR 3 UCSC
TSS Hoffman
Transcribed Hoffman
TFBS ENCODE
SuperEnhancer Hnisz
**Repressed Hoffman
Promoter UCSC
PromoterFlanking Hoffman
**Intron UCSC
H3K9ac Trynka
H3K4me3 Trynka
**H3K4me1 Trynka
H3K27ac PGC2
**H3K27ac Hnisz
FetalDHS Trynka
Enhancer Hoffman
Enhancer Andersson
DHS Trynka
DGF ENCODE
CTCF Hoffman
Conserved LindbladToh
Coding UCSC
Annotation
−10
0
10
20
Enrichment (prop h2 / prop snps)

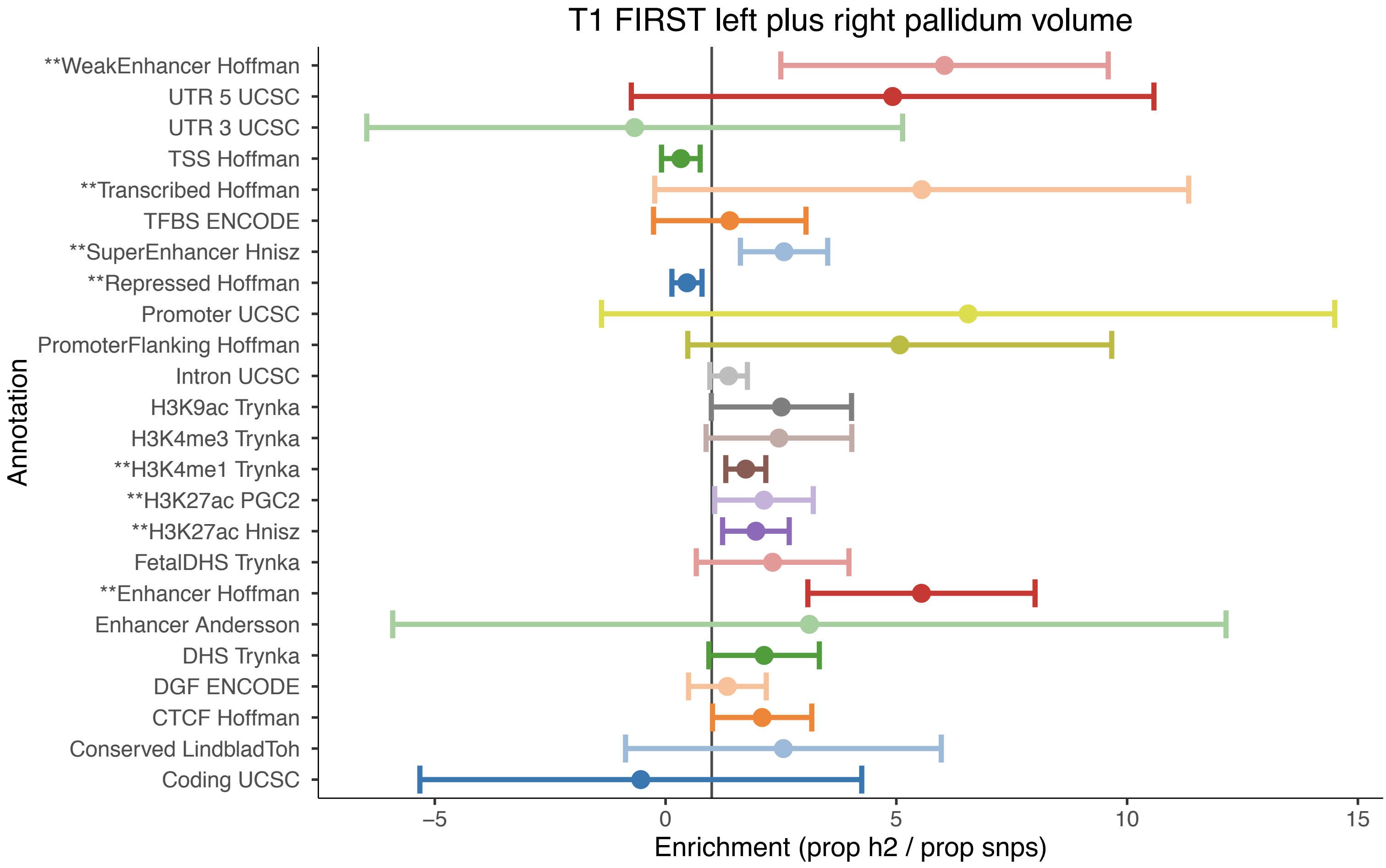
T1 FIRST left plus right pallidum volume
**WeakEnhancer Hoffman
UTR 5 UCSC
UTR 3 UCSC
TSS Hoffman
**Transcribed Hoffman
TFBS ENCODE
**SuperEnhancer Hnisz
**Repressed Hoffman
Promoter UCSC
PromoterFlanking Hoffman
Intron UCSC
H3K9ac Trynka
H3K4me3 Trynka
**H3K4me1 Trynka
**H3K27ac PGC2
**H3K27ac Hnisz
FetalDHS Trynka
**Enhancer Hoffman
Enhancer Andersson
DHS Trynka
DGF ENCODE
CTCF Hoffman
Conserved LindbladToh
Coding UCSC
Annotation
−5
0
5
10
15
Enrichment (prop h2 / prop snps)

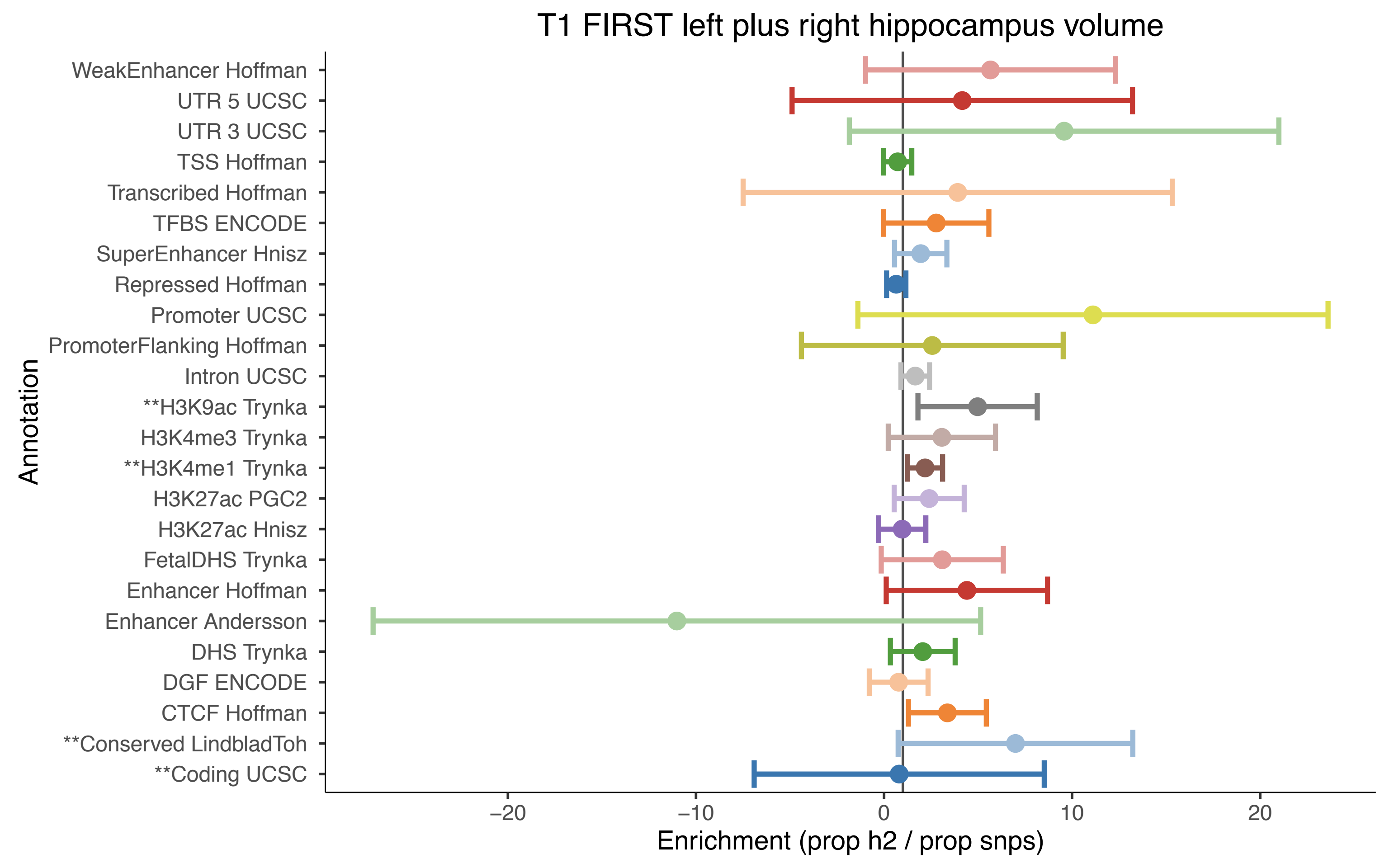
T1 FIRST left plus right hippocampus volume
WeakEnhancer Hoffman
UTR 5 UCSC
UTR 3 UCSC
TSS Hoffman
Transcribed Hoffman
TFBS ENCODE
SuperEnhancer Hnisz
Repressed Hoffman
Promoter UCSC
PromoterFlanking Hoffman
Intron UCSC
**H3K9ac Trynka
H3K4me3 Trynka
**H3K4me1 Trynka
H3K27ac PGC2
H3K27ac Hnisz
FetalDHS Trynka
Enhancer Hoffman
Enhancer Andersson
DHS Trynka
DGF ENCODE
CTCF Hoffman
**Conserved LindbladToh
**Coding UCSC
Annotation
−20
−10
0
10
20
Enrichment (prop h2 / prop snps)

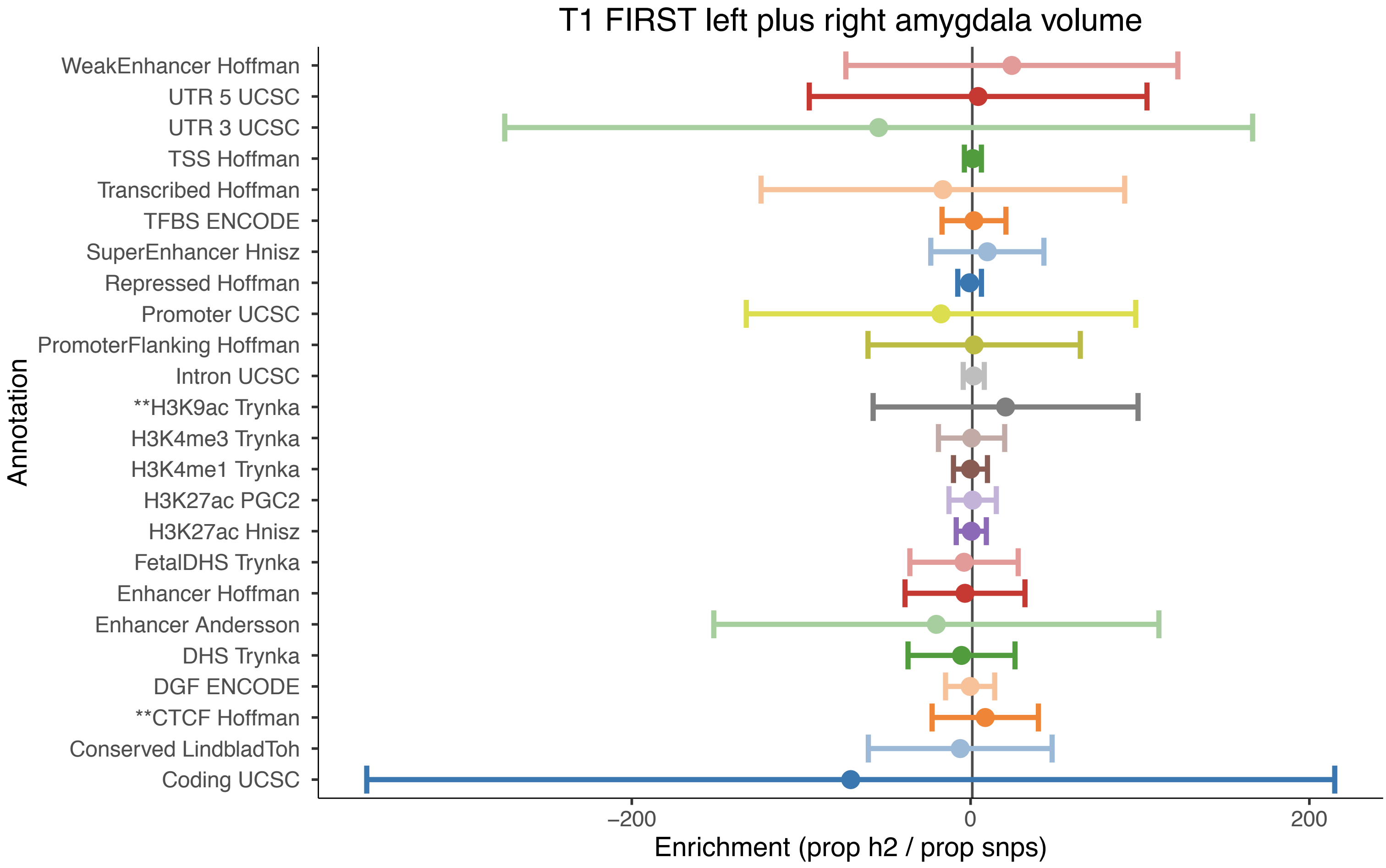

T1 FIRST left plus right amygdala volume
WeakEnhancer Hoffman
UTR 5 UCSC
UTR 3 UCSC
TSS Hoffman
Transcribed Hoffman
TFBS ENCODE
SuperEnhancer Hnisz
Repressed Hoffman
Promoter UCSC
PromoterFlanking Hoffman
Intron UCSC
**H3K9ac Trynka
H3K4me3 Trynka
H3K4me1 Trynka
H3K27ac PGC2
H3K27ac Hnisz
FetalDHS Trynka
Enhancer Hoffman
Enhancer Andersson
DHS Trynka
DGF ENCODE
**CTCF Hoffman
Conserved LindbladToh
Coding UCSC
Annotation
−200
0
200
Enrichment (prop h2 / prop snps)

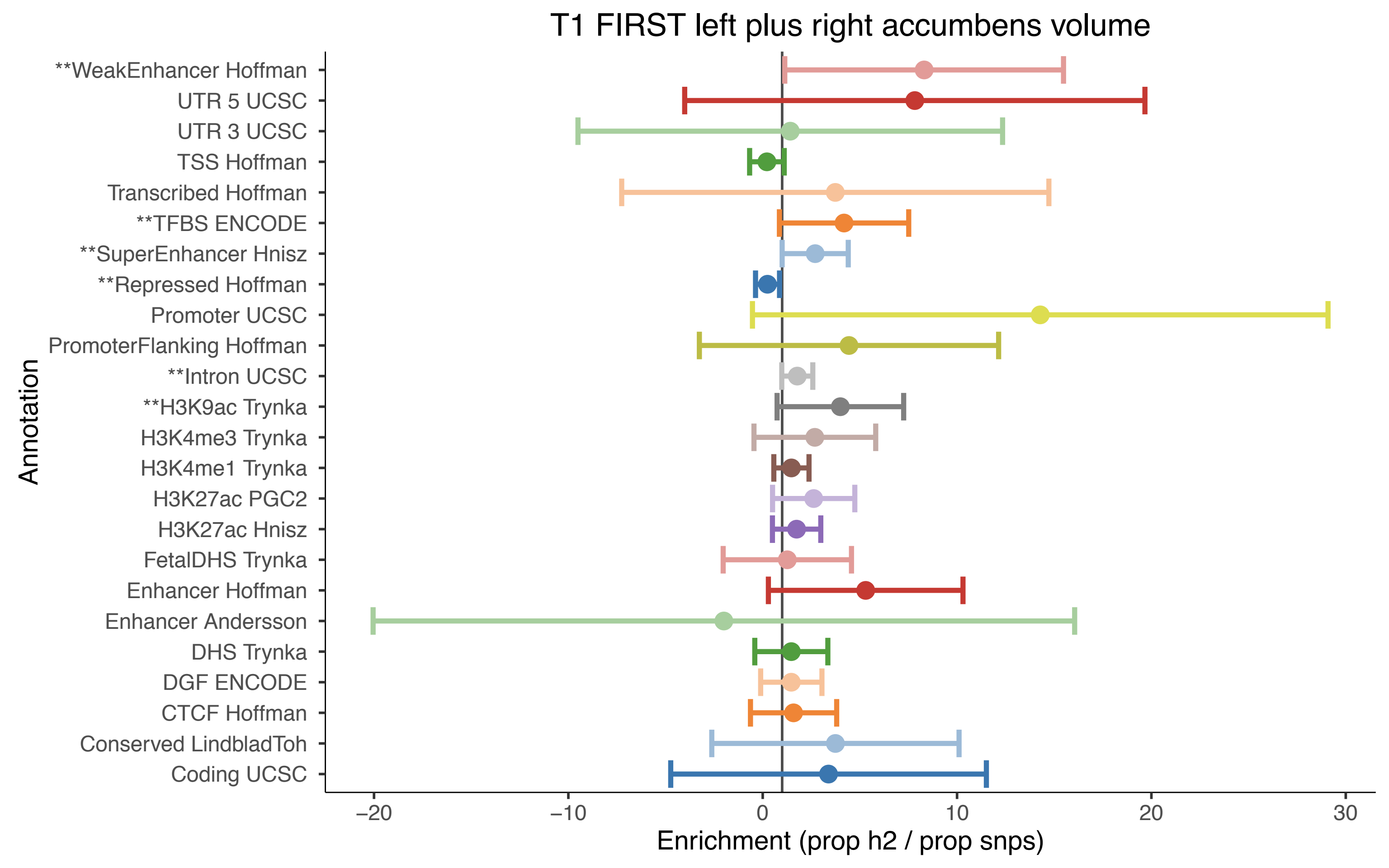

T1 FIRST left plus right accumbens volume
**WeakEnhancer Hoffman
UTR 5 UCSC
UTR 3 UCSC
TSS Hoffman
Transcribed Hoffman
**TFBS ENCODE
**SuperEnhancer Hnisz
**Repressed Hoffman
Promoter UCSC
PromoterFlanking Hoffman
**Intron UCSC
**H3K9ac Trynka
H3K4me3 Trynka
H3K4me1 Trynka
H3K27ac PGC2
H3K27ac Hnisz
FetalDHS Trynka
Enhancer Hoffman
Enhancer Andersson
DHS Trynka
DGF ENCODE
CTCF Hoffman
Conserved LindbladToh
Coding UCSC
Annotation
−20
−10
0
10
20
30
Enrichment (prop h2 / prop snps)

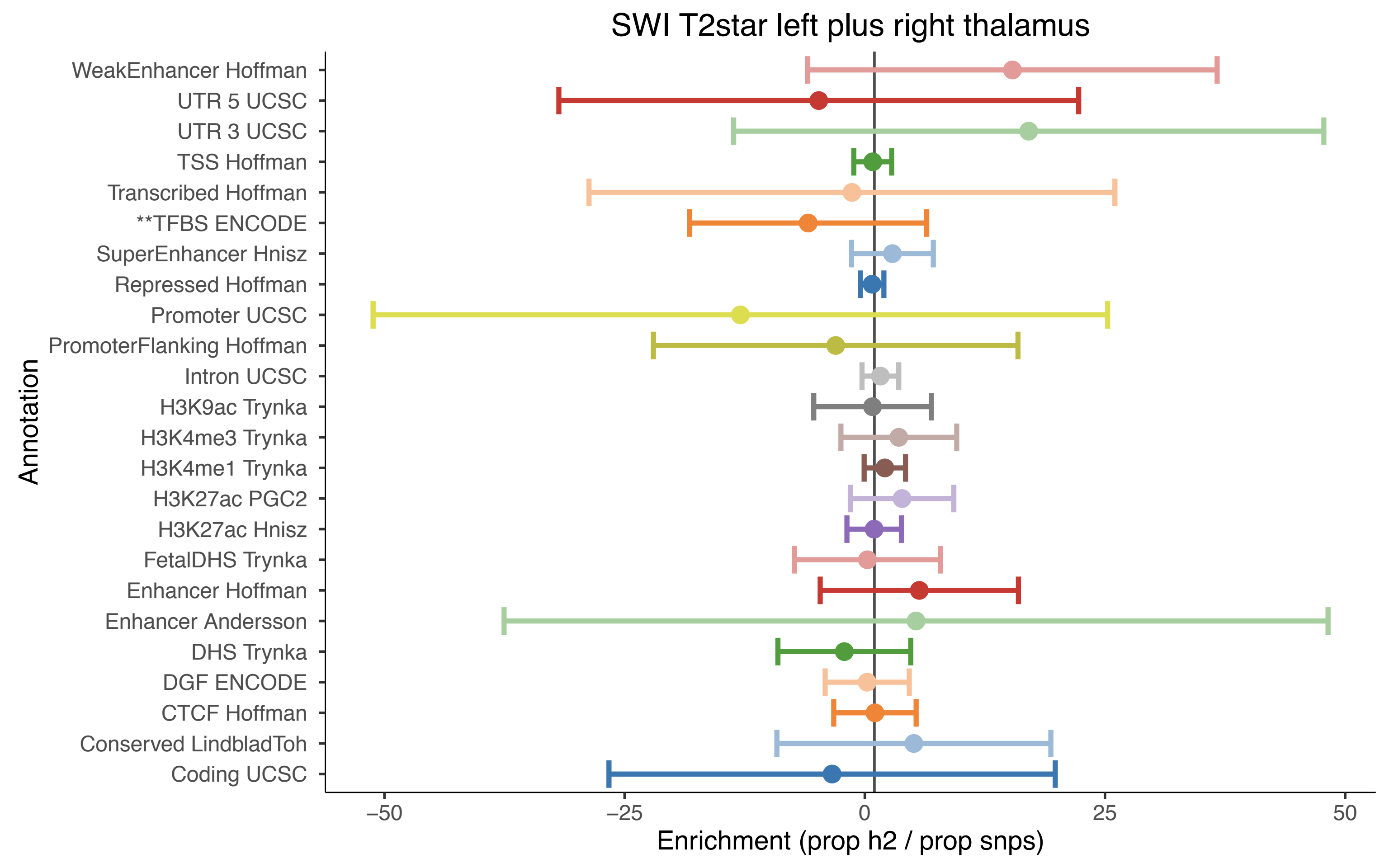

SWI T2star left plus right thalamus
WeakEnhancer Hoffman
UTR 5 UCSC
UTR 3 UCSC
TSS Hoffman
Transcribed Hoffman
**TFBS ENCODE
SuperEnhancer Hnisz
Repressed Hoffman
Promoter UCSC
PromoterFlanking Hoffman
Intron UCSC
H3K9ac Trynka
H3K4me3 Trynka
H3K4me1 Trynka
H3K27ac PGC2
H3K27ac Hnisz
FetalDHS Trynka
Enhancer Hoffman
Enhancer Andersson
DHS Trynka
DGF ENCODE
CTCF Hoffman
Conserved LindbladToh
Coding UCSC
Annotation
−50
−25
0
25
50
Enrichment (prop h2 / prop snps)

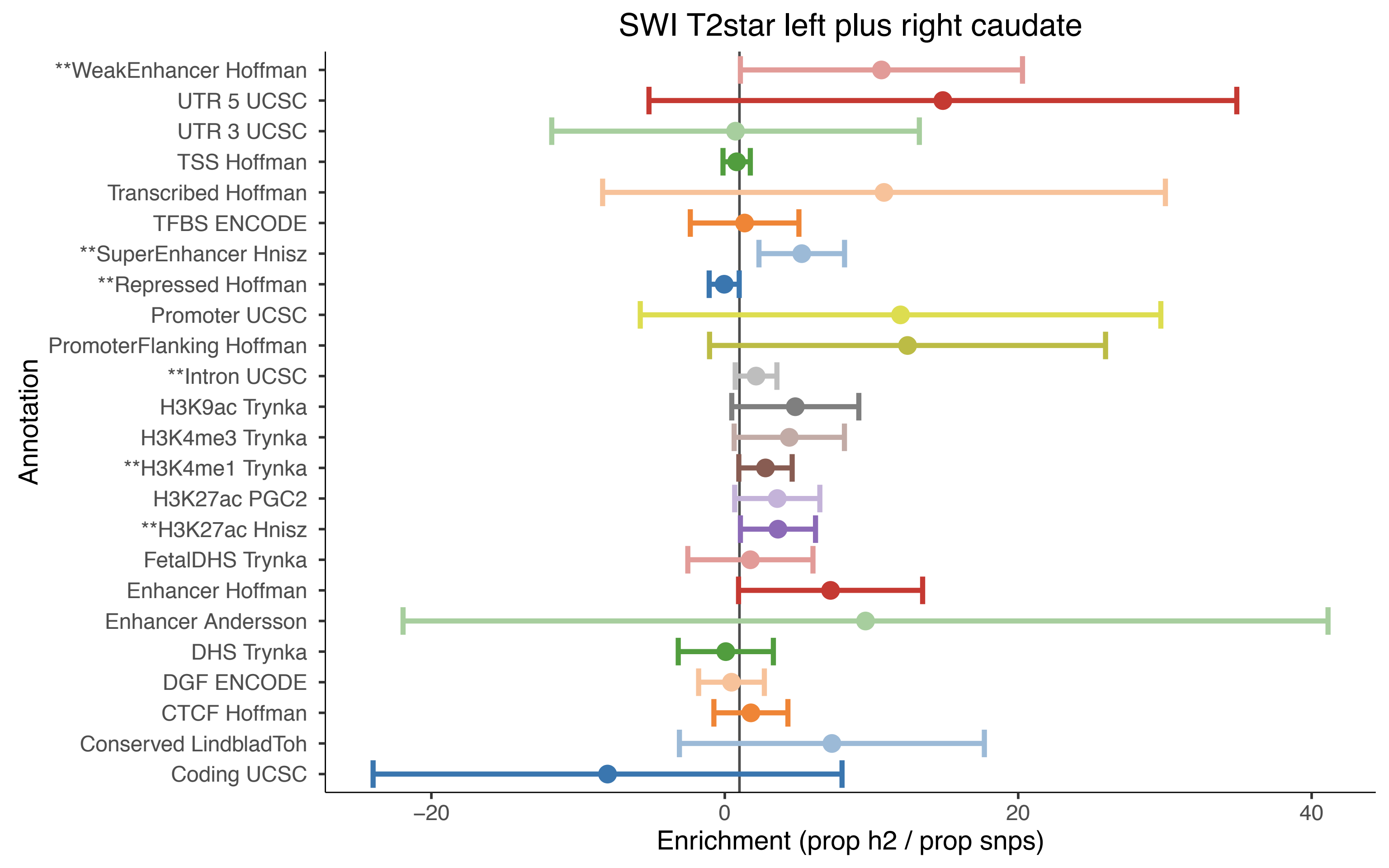

SWI T2star left plus right caudate
**WeakEnhancer Hoffman
UTR 5 UCSC
UTR 3 UCSC
TSS Hoffman
Transcribed Hoffman
TFBS ENCODE
**SuperEnhancer Hnisz
**Repressed Hoffman
Promoter UCSC
PromoterFlanking Hoffman
**Intron UCSC
H3K9ac Trynka
H3K4me3 Trynka
**H3K4me1 Trynka
H3K27ac PGC2
**H3K27ac Hnisz
FetalDHS Trynka
Enhancer Hoffman
Enhancer Andersson
DHS Trynka
DGF ENCODE
CTCF Hoffman
Conserved LindbladToh
Coding UCSC
Annotation
−20
0
20
40
Enrichment (prop h2 / prop snps)

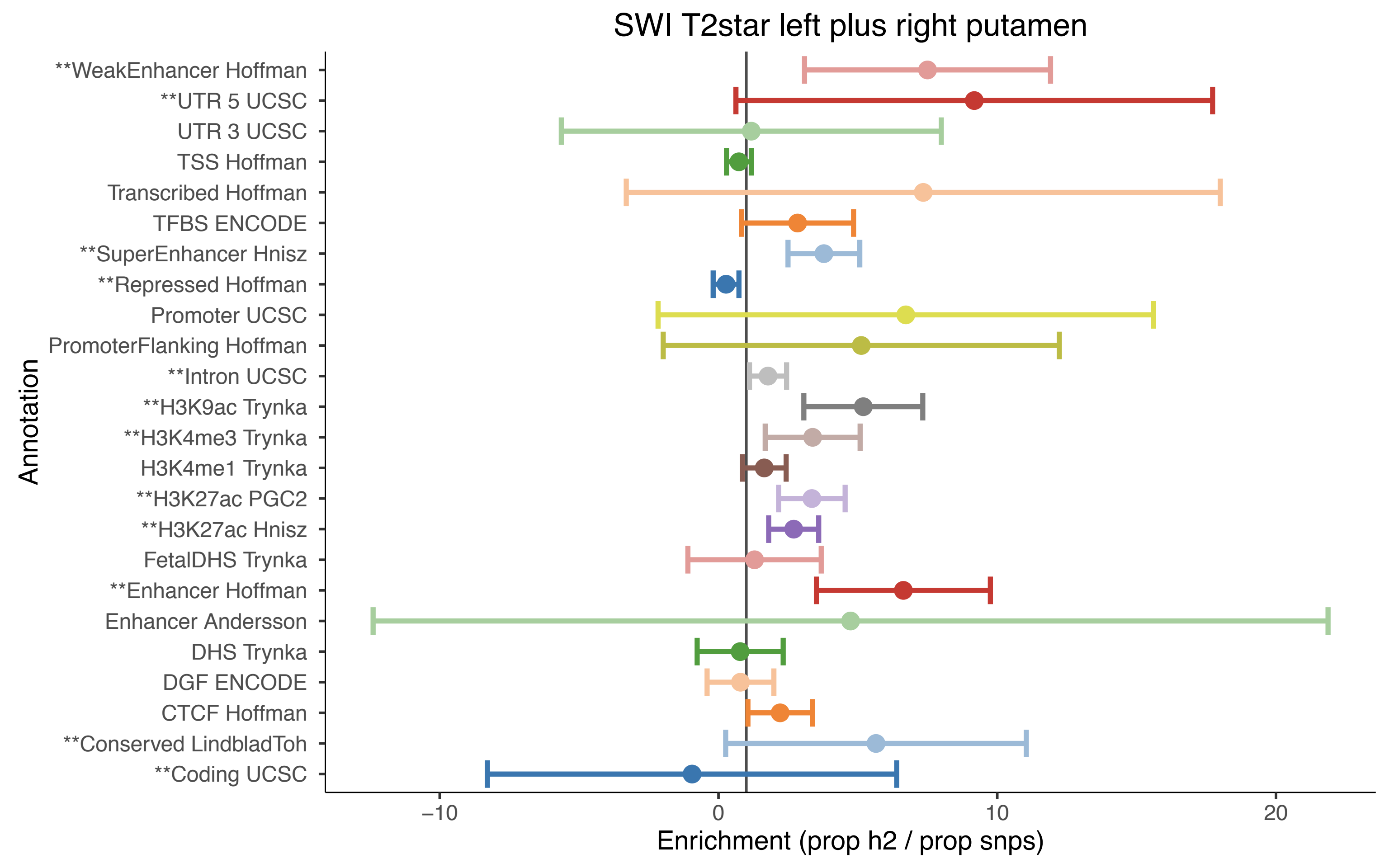
SWI T2star left plus right putamen
Annotation
**WeakEnhancer Hoffman
**UTR 5 UCSC
UTR 3 UCSC
TSS Hoffman
Transcribed Hoffman
TFBS ENCODE
**SuperEnhancer Hnisz
**Repressed Hoffman
Promoter UCSC
PromoterFlanking Hoffman
**Intron UCSC
**H3K9ac Trynka
**H3K4me3 Trynka
H3K4me1 Trynka
**H3K27ac PGC2
**H3K27ac Hnisz
FetalDHS Trynka
**Enhancer Hoffman
Enhancer Andersson
DHS Trynka
DGF ENCODE
CTCF Hoffman
**Conserved LindbladToh
**Coding UCSC
−10
0
10
20
Enrichment (prop h2 / prop snps)

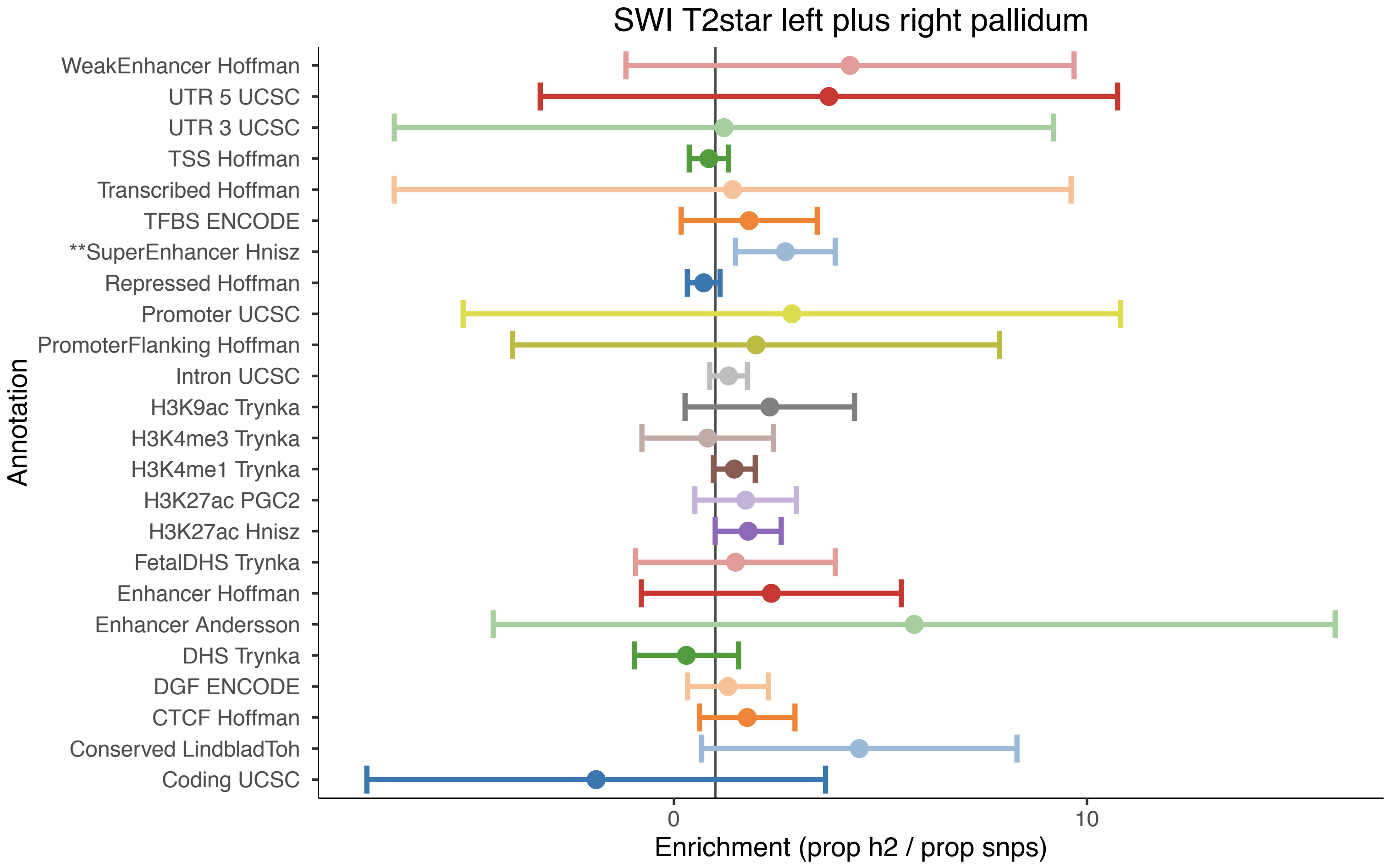

SWI T2star left plus right pallidum
WeakEnhancer Hoffman
UTR 5 UCSC
UTR 3 UCSC
TSS Hoffman
Transcribed Hoffman
TFBS ENCODE
**SuperEnhancer Hnisz
Repressed Hoffman
Promoter UCSC
PromoterFlanking Hoffman
Intron UCSC
H3K9ac Trynka
H3K4me3 Trynka
H3K4me1 Trynka
H3K27ac PGC2
H3K27ac Hnisz
FetalDHS Trynka
Enhancer Hoffman
Enhancer Andersson
DHS Trynka
DGF ENCODE
CTCF Hoffman
Conserved LindbladToh
Coding UCSC
Annotation
0
10
Enrichment (prop h2 / prop snps)

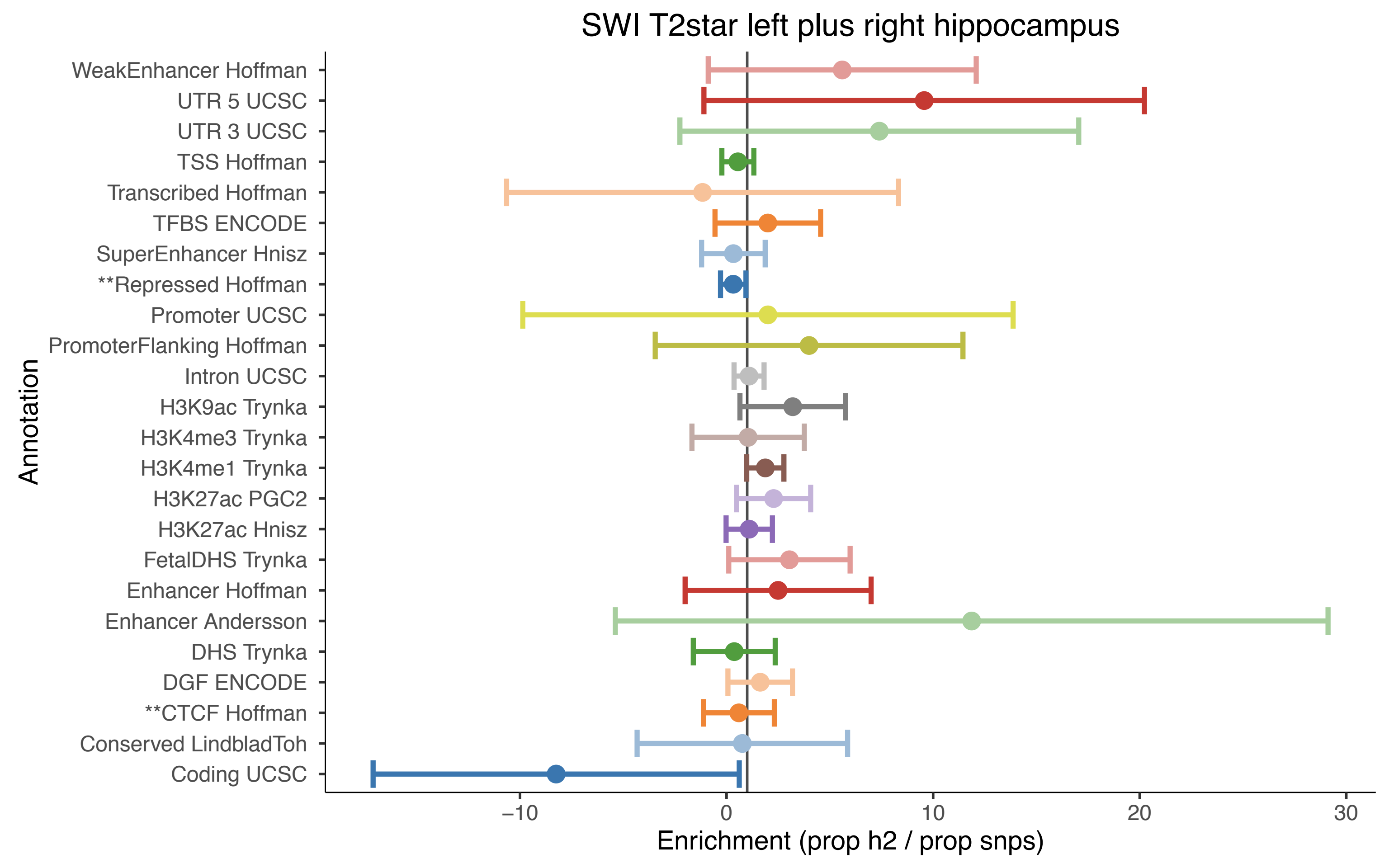

SWI T2star left plus right hippocampus
WeakEnhancer Hoffman
UTR 5 UCSC
UTR 3 UCSC
TSS Hoffman
Transcribed Hoffman
TFBS ENCODE
SuperEnhancer Hnisz
**Repressed Hoffman
Promoter UCSC
PromoterFlanking Hoffman
Intron UCSC
H3K9ac Trynka
H3K4me3 Trynka
H3K4me1 Trynka
H3K27ac PGC2
H3K27ac Hnisz
FetalDHS Trynka
Enhancer Hoffman
Enhancer Andersson
DHS Trynka
DGF ENCODE
**CTCF Hoffman
Conserved LindbladToh
Coding UCSC
Annotation
−10
0
10
20
30
Enrichment (prop h2 / prop snps)

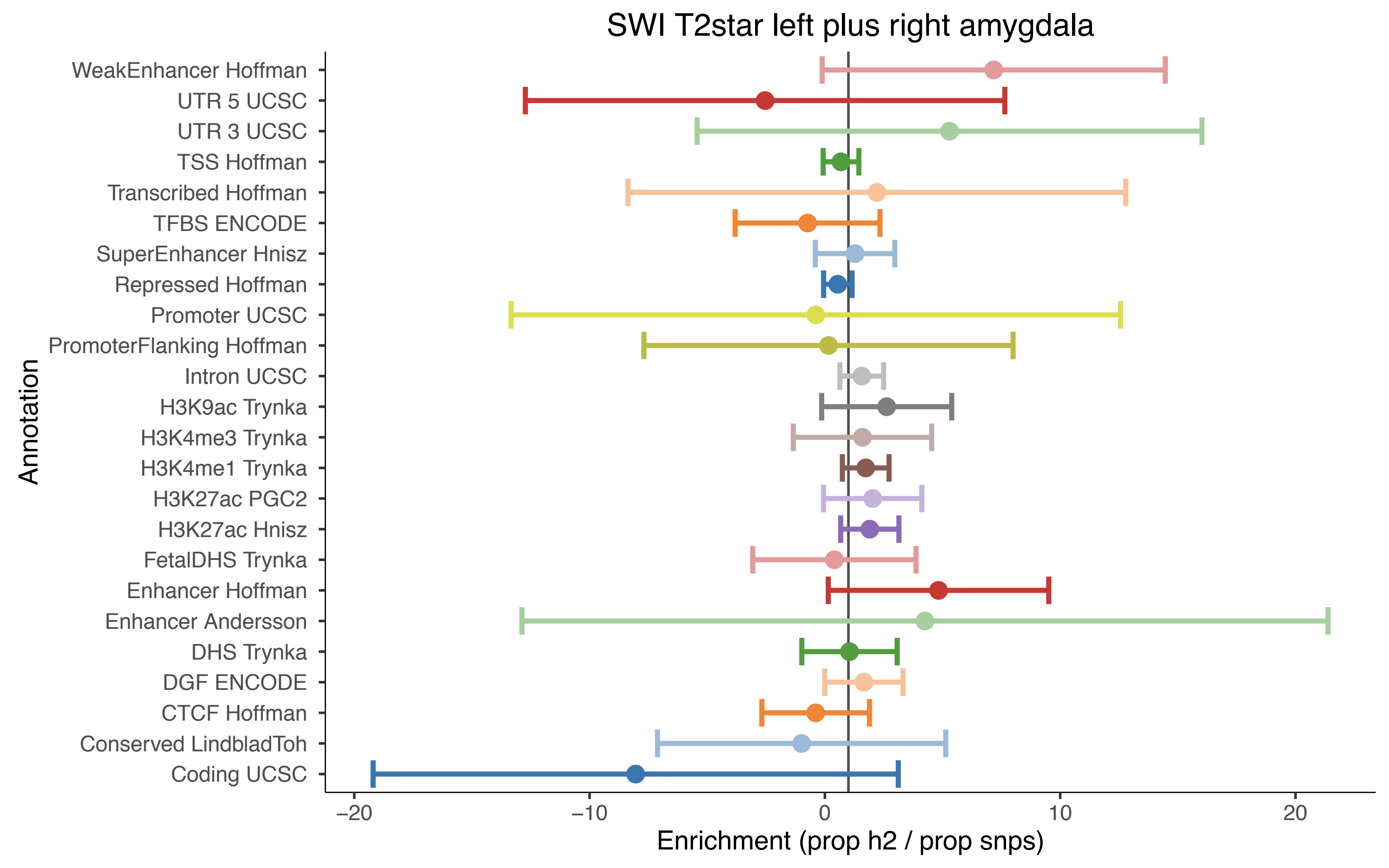

SWI T2star left plus right amygdala
Annotation
WeakEnhancer Hoffman
UTR 5 UCSC
UTR 3 UCSC
TSS Hoffman
Transcribed Hoffman
TFBS ENCODE
SuperEnhancer Hnisz
Repressed Hoffman
Promoter UCSC
PromoterFlanking Hoffman
Intron UCSC
H3K9ac Trynka
H3K4me3 Trynka
H3K4me1 Trynka
H3K27ac PGC2
H3K27ac Hnisz
FetalDHS Trynka
Enhancer Hoffman
Enhancer Andersson
DHS Trynka
DGF ENCODE
CTCF Hoffman
Conserved LindbladToh
Coding UCSC
−20
−10
0
10
20
Enrichment (prop h2 / prop snps)

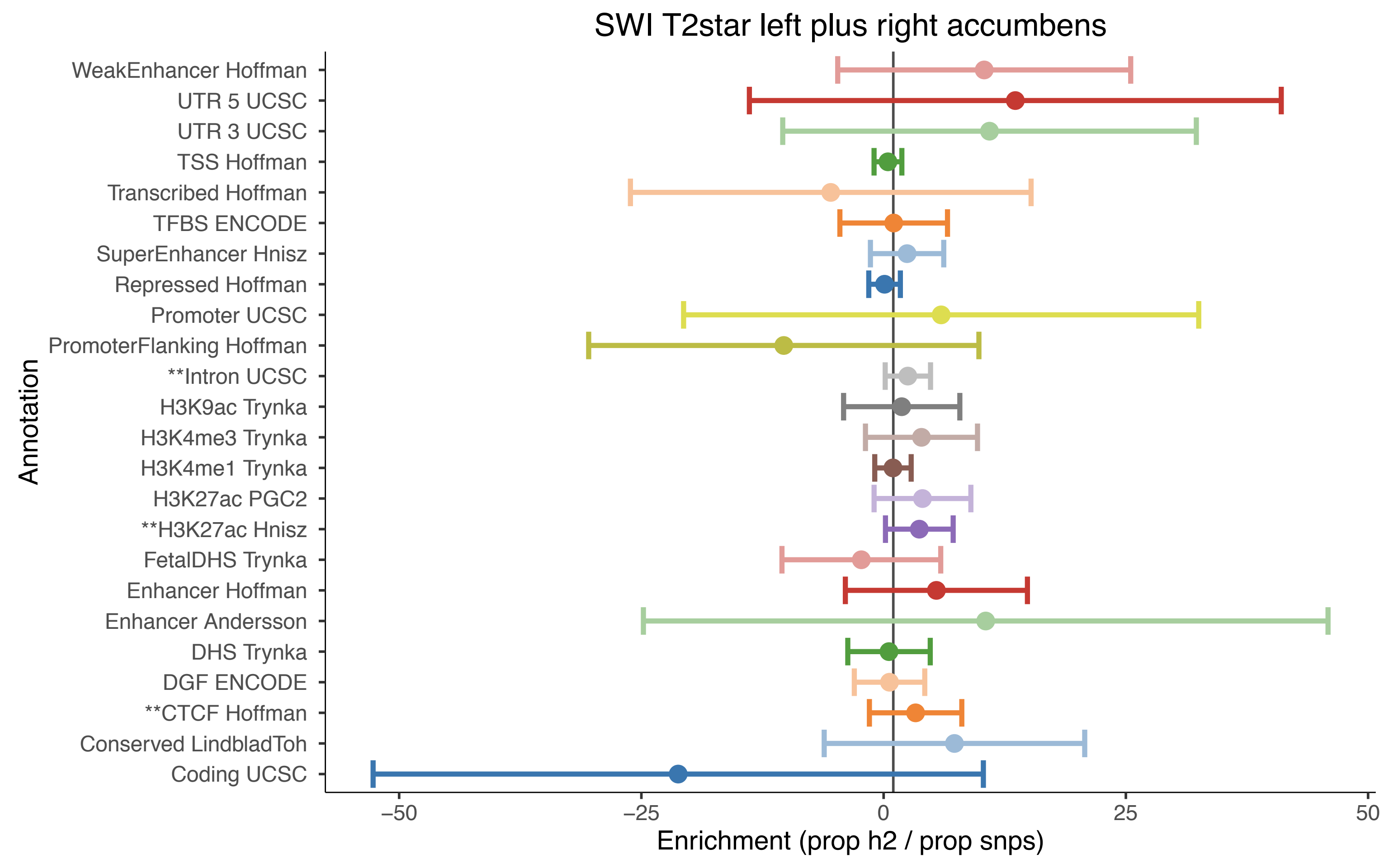
SWI T2star left plus right accumbens
WeakEnhancer Hoffman
UTR 5 UCSC
UTR 3 UCSC
TSS Hoffman
Transcribed Hoffman
TFBS ENCODE
SuperEnhancer Hnisz
Repressed Hoffman
Promoter UCSC
PromoterFlanking Hoffman
**Intron UCSC
H3K9ac Trynka
H3K4me3 Trynka
H3K4me1 Trynka
H3K27ac PGC2
**H3K27ac Hnisz
FetalDHS Trynka
Enhancer Hoffman
Enhancer Andersson
DHS Trynka
DGF ENCODE
**CTCF Hoffman
Conserved LindbladToh
Coding UCSC
Annotation
−50
−25
0
25
50
Enrichment (prop h2 / prop snps)

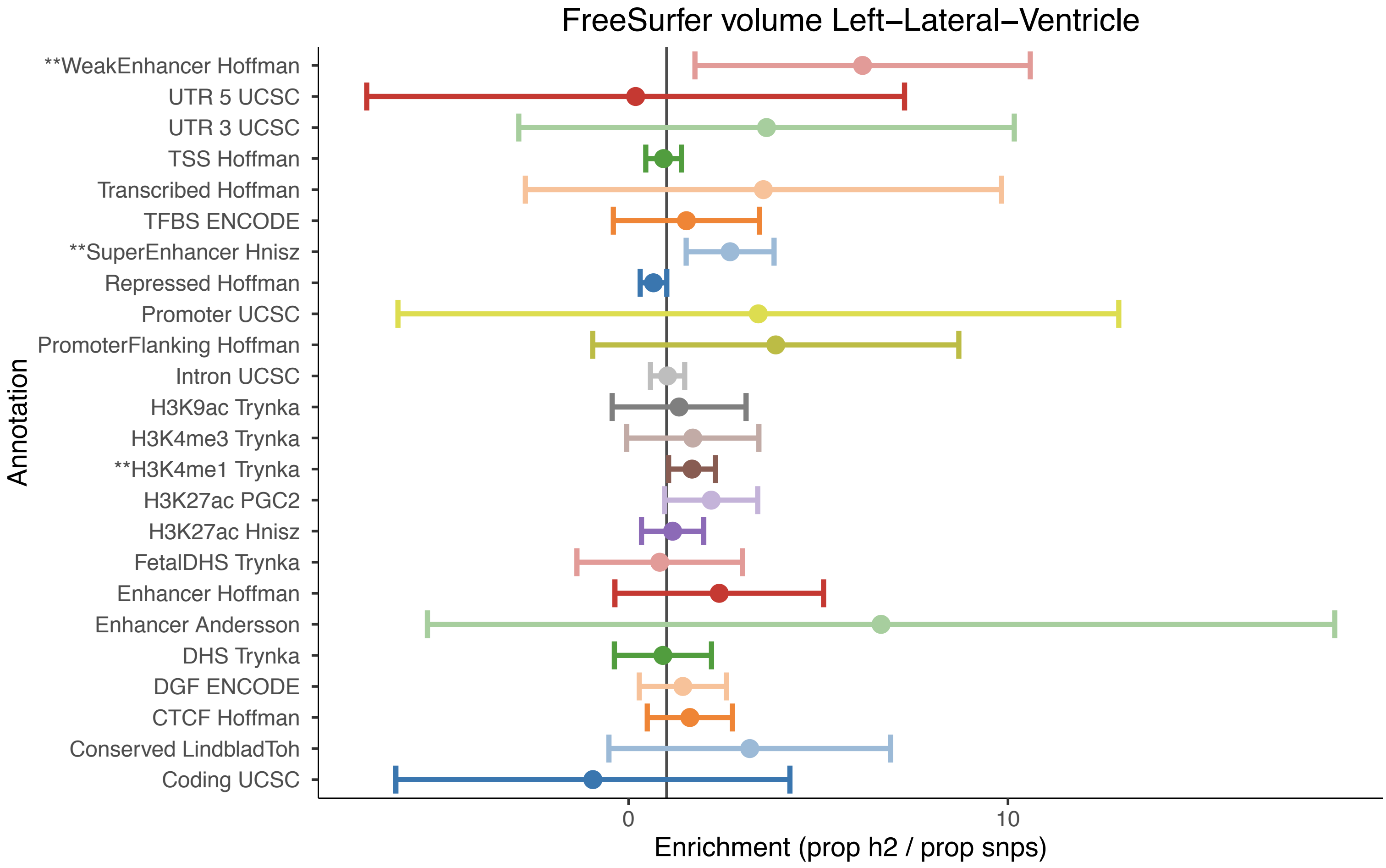

FreeSurfer volume Left−Lateral−Ventricle
**WeakEnhancer Hoffman
UTR 5 UCSC
UTR 3 UCSC
TSS Hoffman
Transcribed Hoffman
TFBS ENCODE
**SuperEnhancer Hnisz
Repressed Hoffman
Promoter UCSC
PromoterFlanking Hoffman
Intron UCSC
H3K9ac Trynka
H3K4me3 Trynka
**H3K4me1 Trynka
H3K27ac PGC2
H3K27ac Hnisz
FetalDHS Trynka
Enhancer Hoffman
Enhancer Andersson
DHS Trynka
DGF ENCODE
CTCF Hoffman
Conserved LindbladToh
Coding UCSC
Annotation
0
10
Enrichment (prop h2 / prop snps)

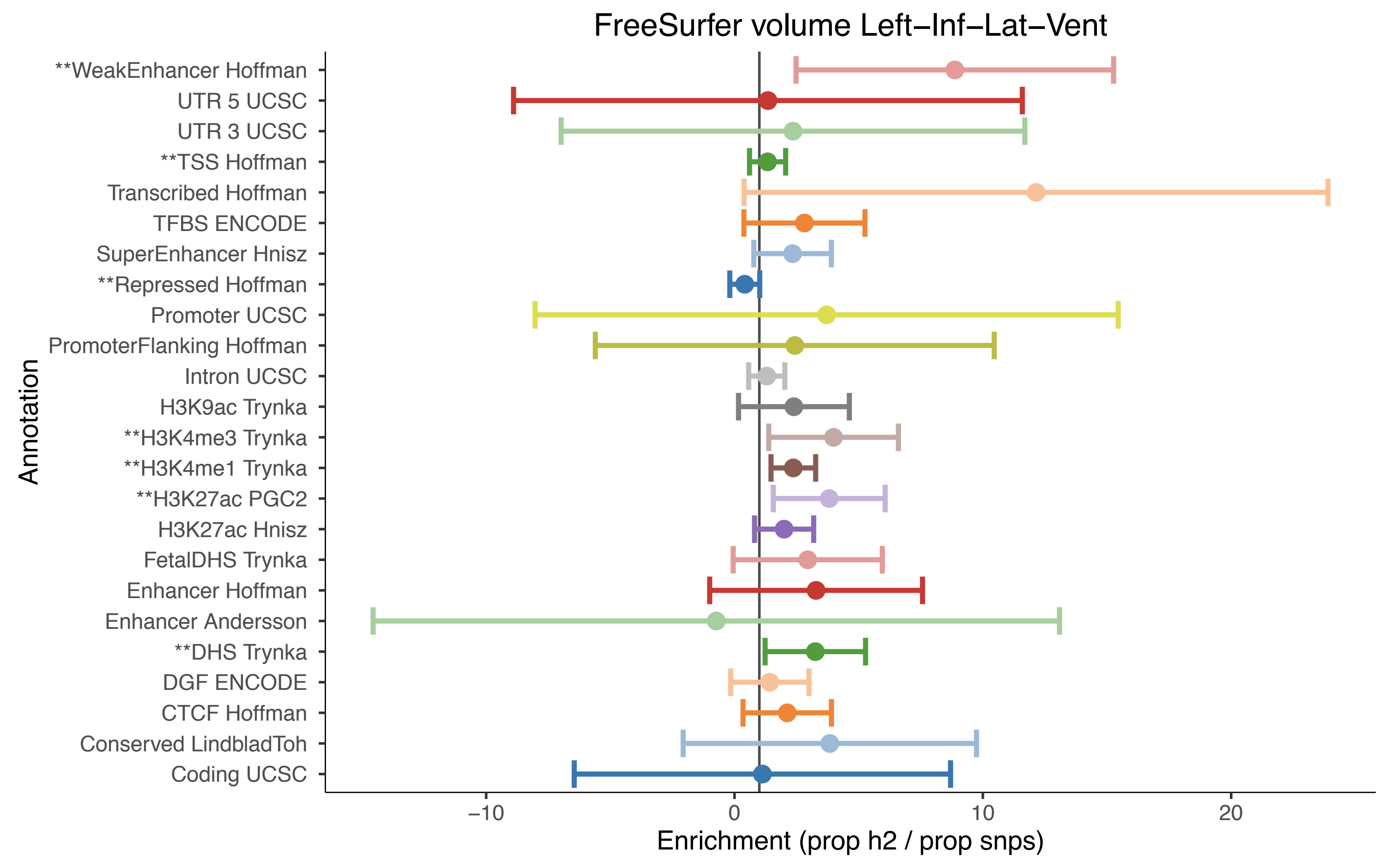
FreeSurfer volume Left−Inf−Lat−Vent
**WeakEnhancer Hoffman
UTR 5 UCSC
UTR 3 UCSC
**TSS Hoffman
Transcribed Hoffman
TFBS ENCODE
SuperEnhancer Hnisz
**Repressed Hoffman
Promoter UCSC
PromoterFlanking Hoffman
Intron UCSC
H3K9ac Trynka
**H3K4me3 Trynka
**H3K4me1 Trynka
**H3K27ac PGC2
H3K27ac Hnisz
FetalDHS Trynka
Enhancer Hoffman
Enhancer Andersson
**DHS Trynka
DGF ENCODE
CTCF Hoffman
Conserved LindbladToh
Coding UCSC
Annotation
−10
0
10
20
Enrichment (prop h2 / prop snps)

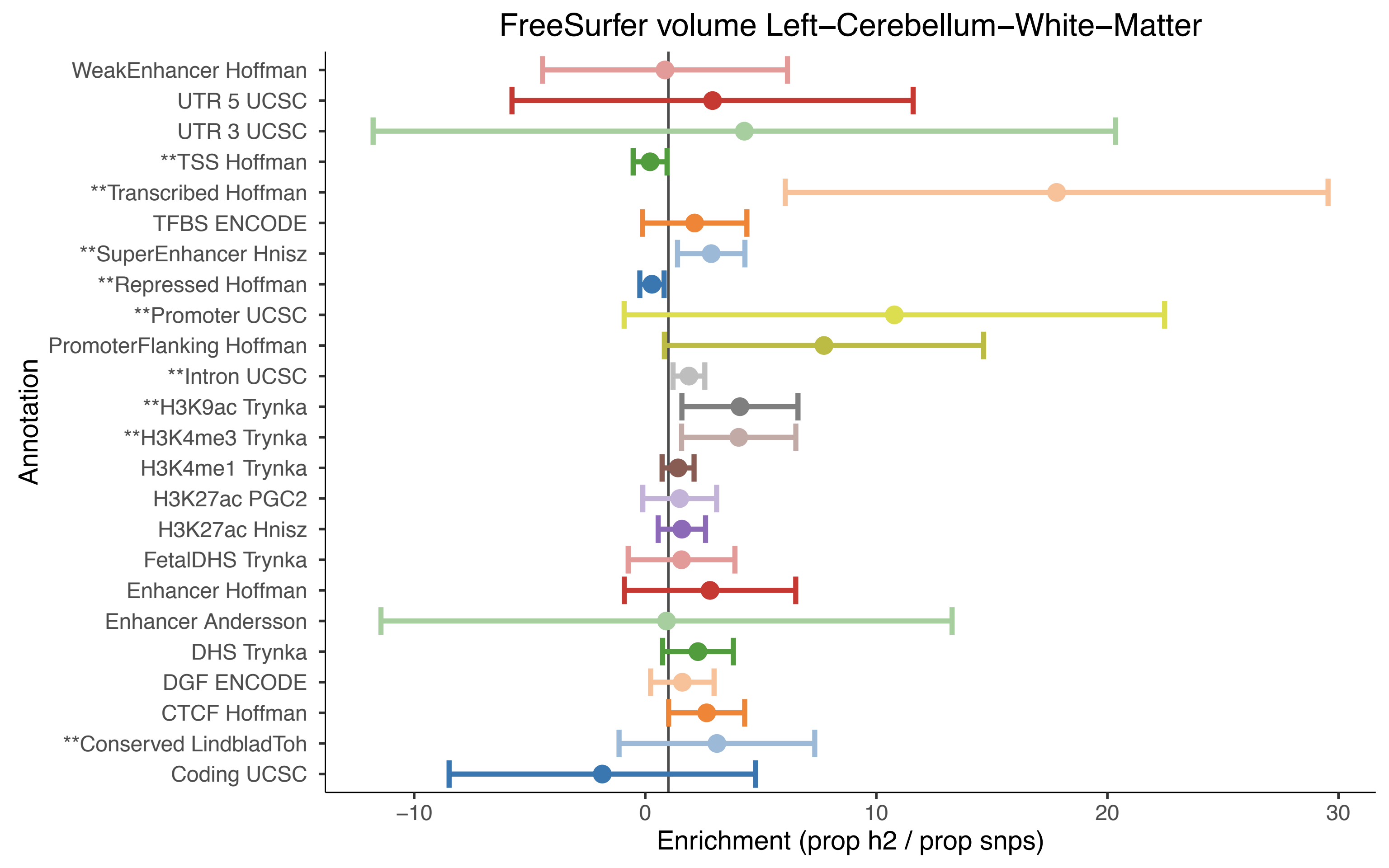
FreeSurfer volume Left−Cerebellum−White−Matter
WeakEnhancer Hoffman
UTR 5 UCSC
UTR 3 UCSC
**TSS Hoffman
**Transcribed Hoffman
TFBS ENCODE
**SuperEnhancer Hnisz
**Repressed Hoffman
**Promoter UCSC
PromoterFlanking Hoffman
**Intron UCSC
**H3K9ac Trynka
**H3K4me3 Trynka
H3K4me1 Trynka
H3K27ac PGC2
H3K27ac Hnisz
FetalDHS Trynka
Enhancer Hoffman
Enhancer Andersson
DHS Trynka
DGF ENCODE
CTCF Hoffman
**Conserved LindbladToh
Coding UCSC
Annotation
−10
0
10
20
30
Enrichment (prop h2 / prop snps)

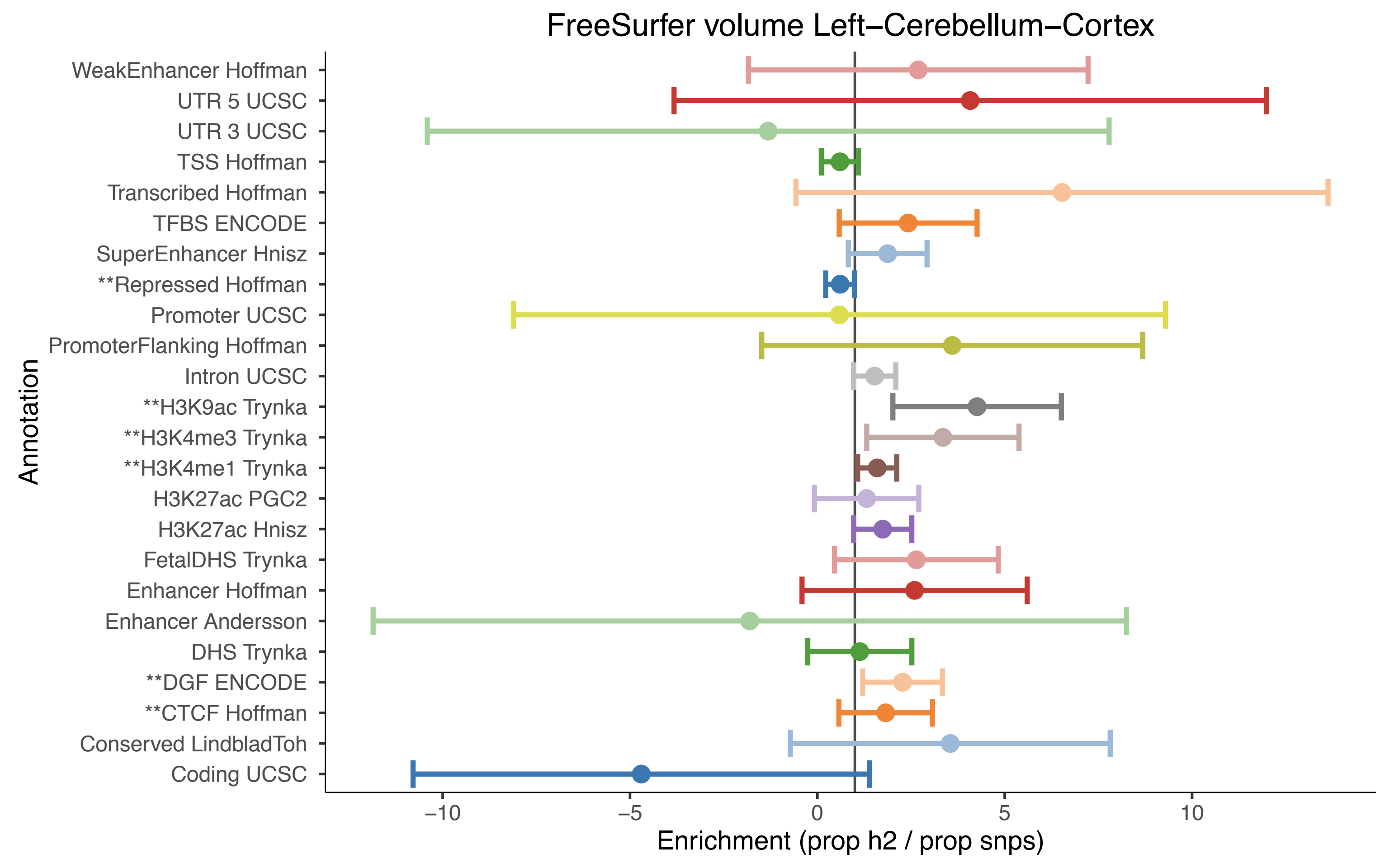

FreeSurfer volume Left−Cerebellum−Cortex
WeakEnhancer Hoffman
UTR 5 UCSC
UTR 3 UCSC
TSS Hoffman
Transcribed Hoffman
TFBS ENCODE
SuperEnhancer Hnisz
**Repressed Hoffman
Promoter UCSC
PromoterFlanking Hoffman
Intron UCSC
**H3K9ac Trynka
**H3K4me3 Trynka
**H3K4me1 Trynka
H3K27ac PGC2
H3K27ac Hnisz
FetalDHS Trynka
Enhancer Hoffman
Enhancer Andersson
DHS Trynka
**DGF ENCODE
**CTCF Hoffman
Conserved LindbladToh
Coding UCSC
Annotation
−10
−5
0
5
10
Enrichment (prop h2 / prop snps)

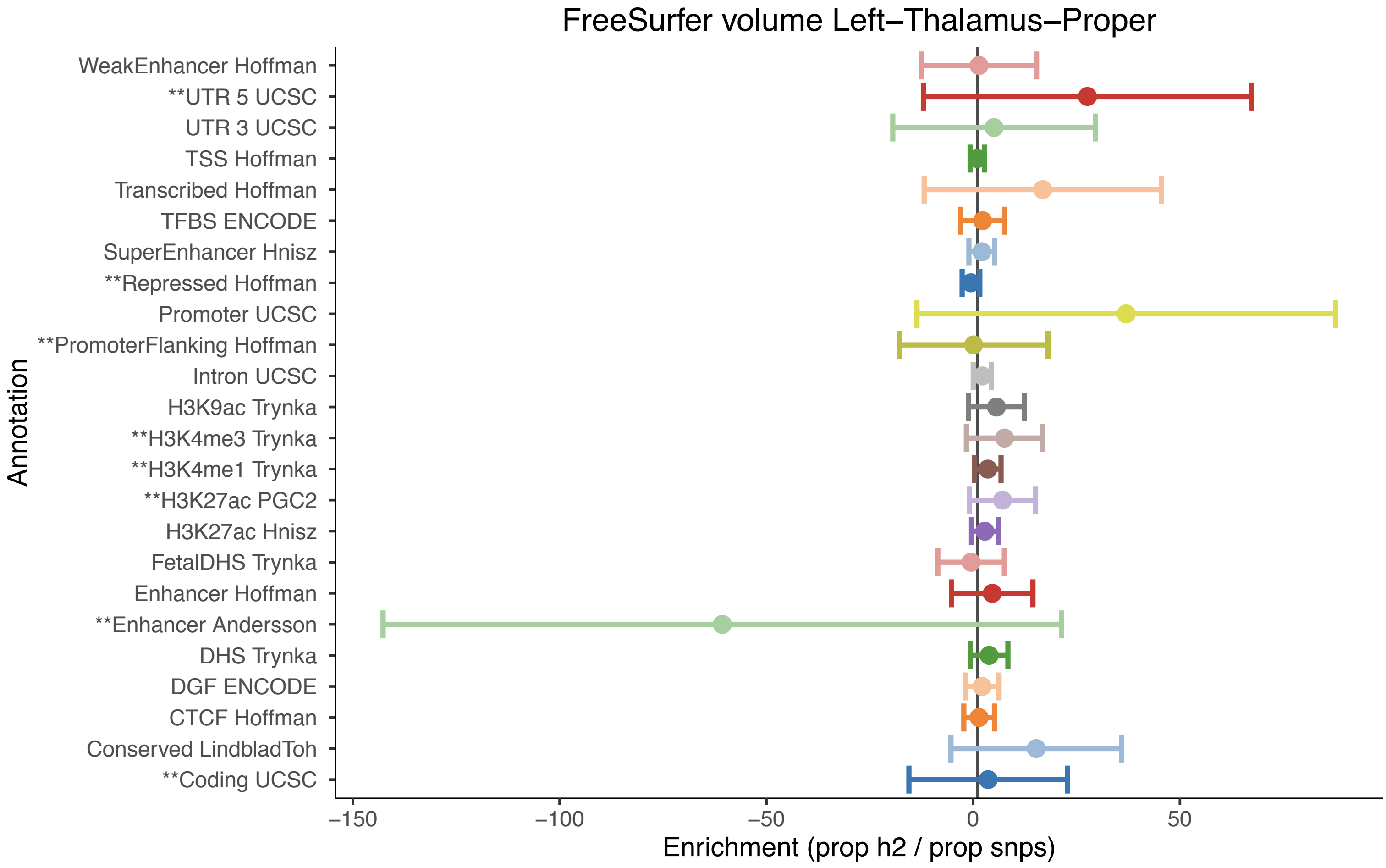
FreeSurfer volume Left−Thalamus−Proper
WeakEnhancer Hoffman
**UTR 5 UCSC
UTR 3 UCSC
TSS Hoffman
Transcribed Hoffman
TFBS ENCODE
SuperEnhancer Hnisz
**Repressed Hoffman
Promoter UCSC
**PromoterFlanking Hoffman
Intron UCSC
H3K9ac Trynka
**H3K4me3 Trynka
**H3K4me1 Trynka
**H3K27ac PGC2
H3K27ac Hnisz
FetalDHS Trynka
Enhancer Hoffman
**Enhancer Andersson
DHS Trynka
DGF ENCODE
CTCF Hoffman
Conserved LindbladToh
**Coding UCSC
Annotation
−150
−100
−50
0
50
Enrichment (prop h2 / prop snps)

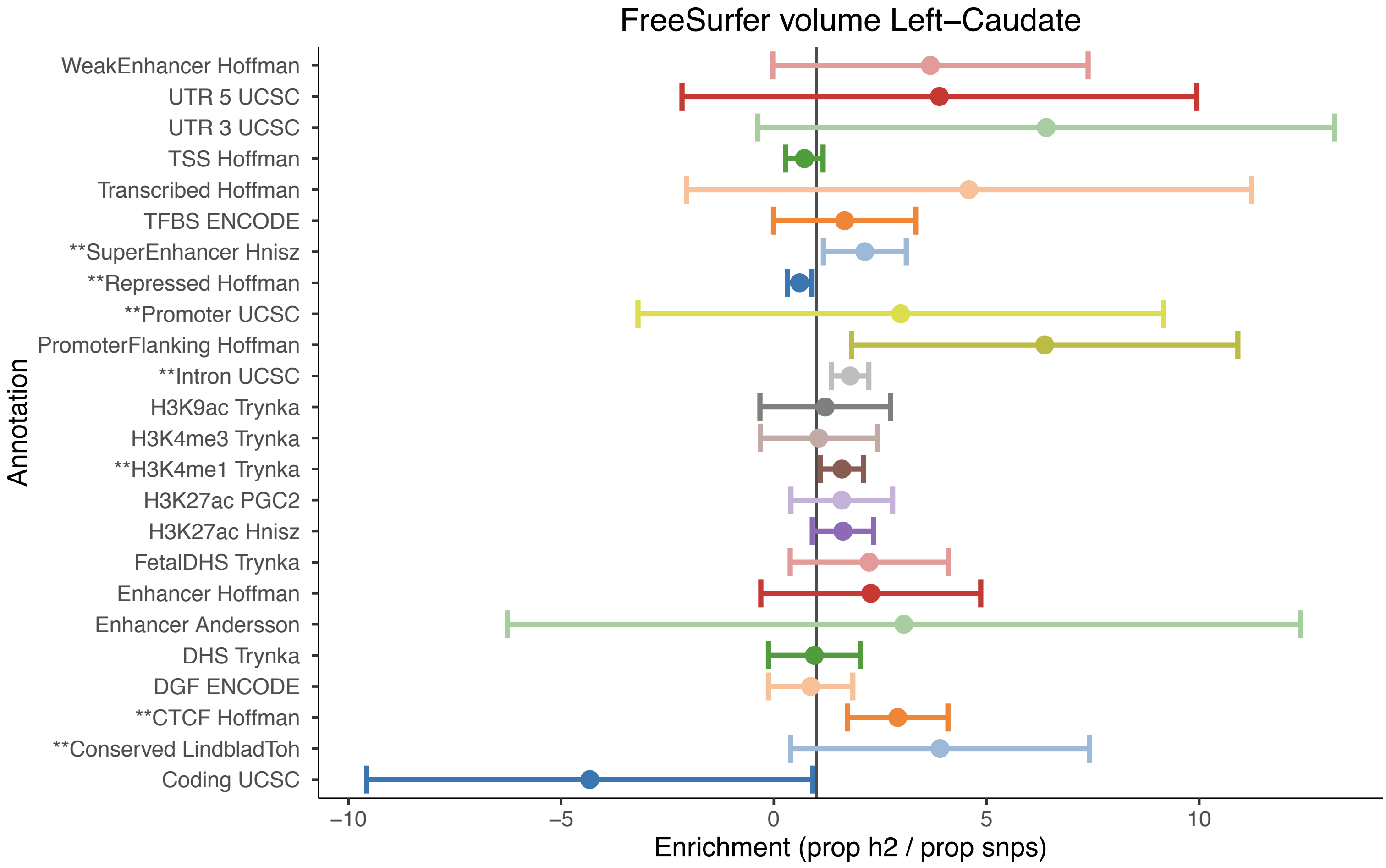
FreeSurfer volume Left–Caudate
WeakEnhancer Hoffman
UTR 5 UCSC
UTR 3 UCSC
TSS Hoffman
Transcribed Hoffman
TFBS ENCODE
**SuperEnhancer Hnisz
**Repressed Hoffman
**Promoter UCSC
PromoterFlanking Hoffman
**Intron UCSC
H3K9ac Trynka
H3K4me3 Trynka
**H3K4me1 Trynka
H3K27ac PGC2
H3K27ac Hnisz
FetalDHS Trynka
Enhancer Hoffman
Enhancer Andersson
DHS Trynka
DGF ENCODE
**CTCF Hoffman
**Conserved LindbladToh
Coding UCSC
Annotation
−10
−5
0
5
10
Enrichment (prop h2 / prop snps)

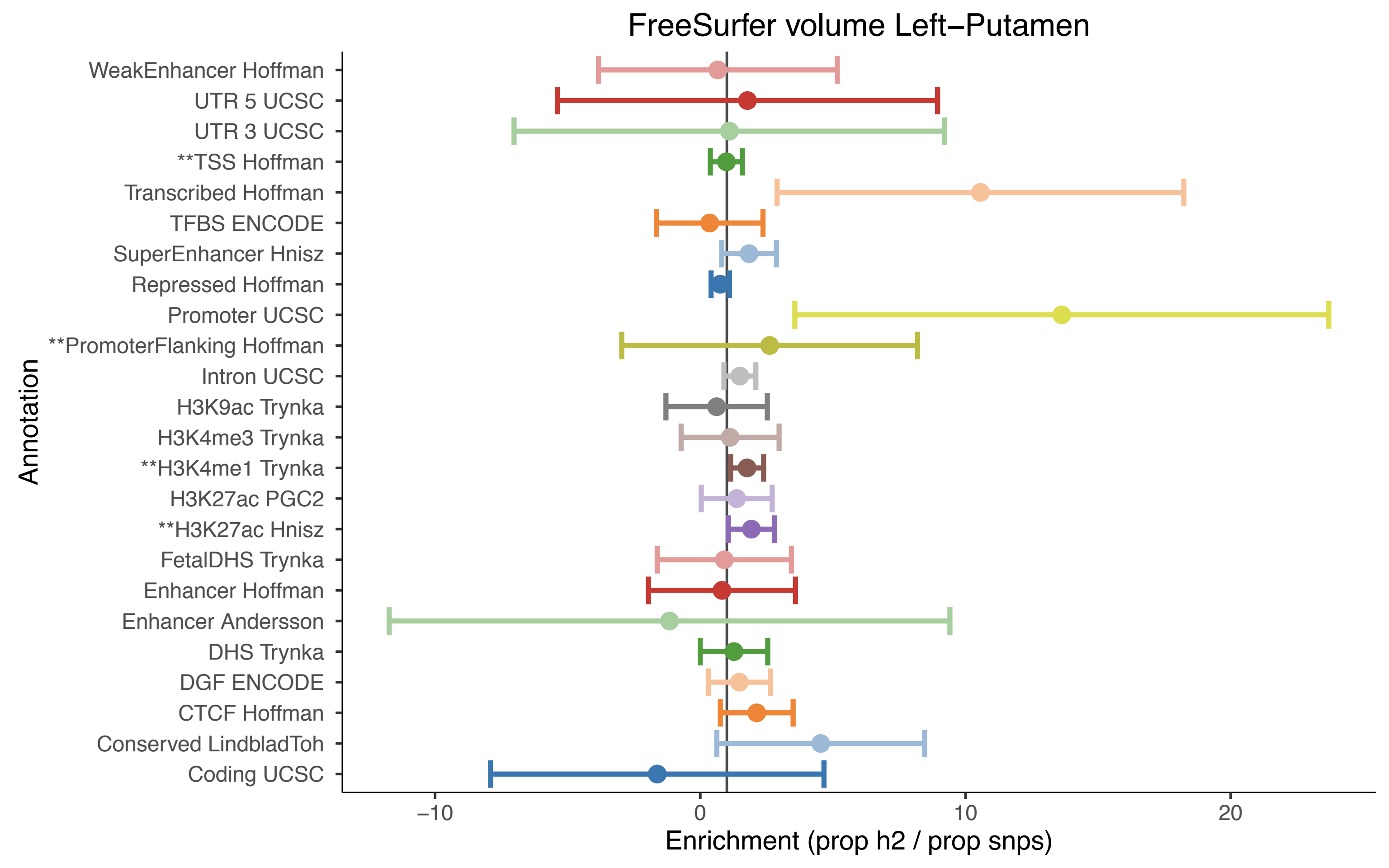

FreeSurfer volume Left−Putamen
WeakEnhancer Hoffman
UTR 5 UCSC
UTR 3 UCSC
**TSS Hoffman
Transcribed Hoffman
TFBS ENCODE
SuperEnhancer Hnisz
Repressed Hoffman
Promoter UCSC
**PromoterFlanking Hoffman
Intron UCSC
H3K9ac Trynka
H3K4me3 Trynka
**H3K4me1 Trynka
H3K27ac PGC2
**H3K27ac Hnisz
FetalDHS Trynka
Enhancer Hoffman
Enhancer Andersson
DHS Trynka
DGF ENCODE
CTCF Hoffman
Conserved LindbladToh
Coding UCSC
Annotation
−10
0
10
20
Enrichment (prop h2 / prop snps)

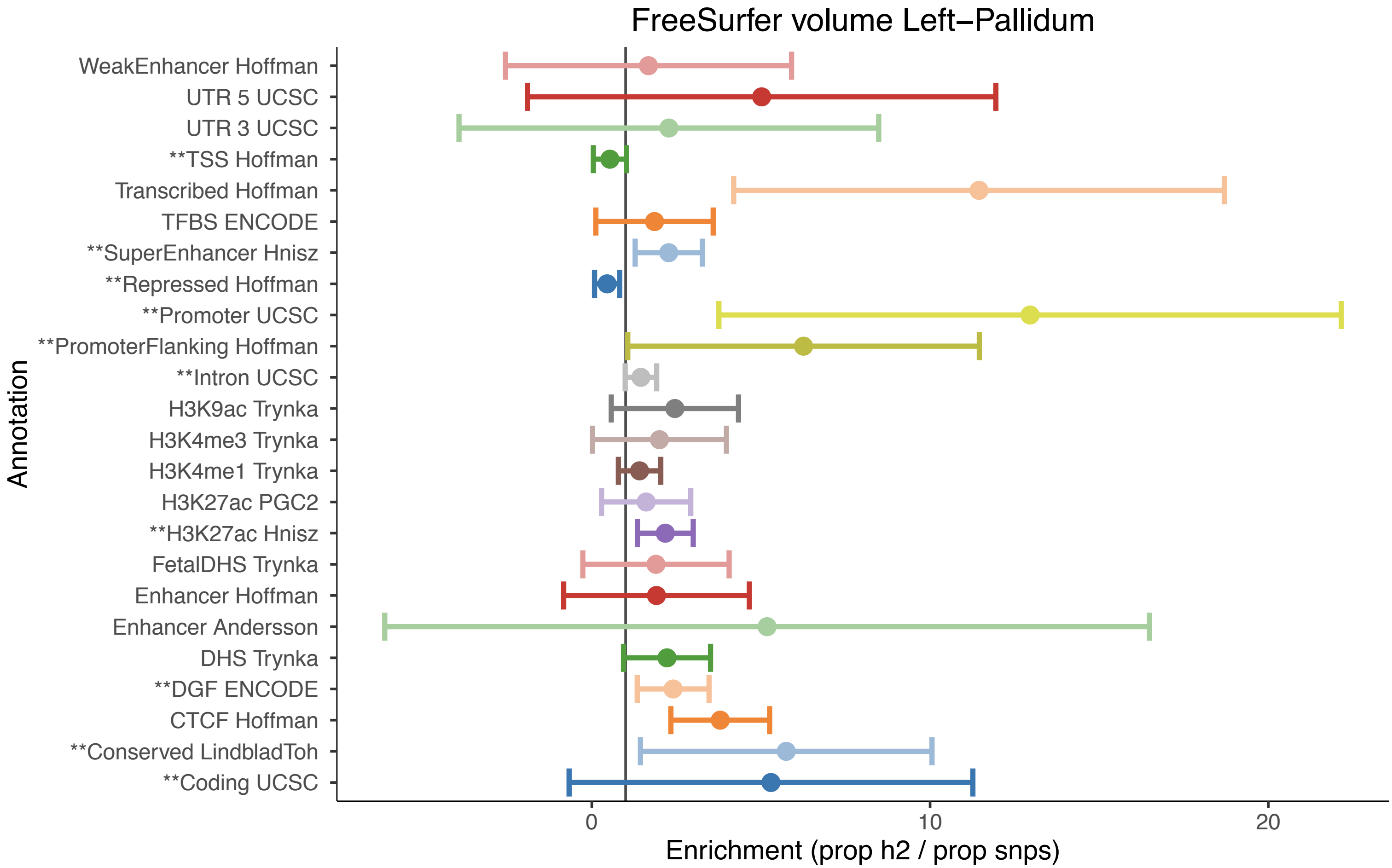
FreeSurfer volume Left−Pallidum
WeakEnhancer Hoffman
UTR 5 UCSC
UTR 3 UCSC
**TSS Hoffman
Transcribed Hoffman
TFBS ENCODE
**SuperEnhancer Hnisz
**Repressed Hoffman
**Promoter UCSC
**PromoterFlanking Hoffman
**Intron UCSC
H3K9ac Trynka
H3K4me3 Trynka
H3K4me1 Trynka
H3K27ac PGC2
**H3K27ac Hnisz
FetalDHS Trynka
Enhancer Hoffman
Enhancer Andersson
DHS Trynka
**DGF ENCODE
CTCF Hoffman
**Conserved LindbladToh
**Coding UCSC
Annotation
0
10
20
Enrichment (prop h2 / prop snps)

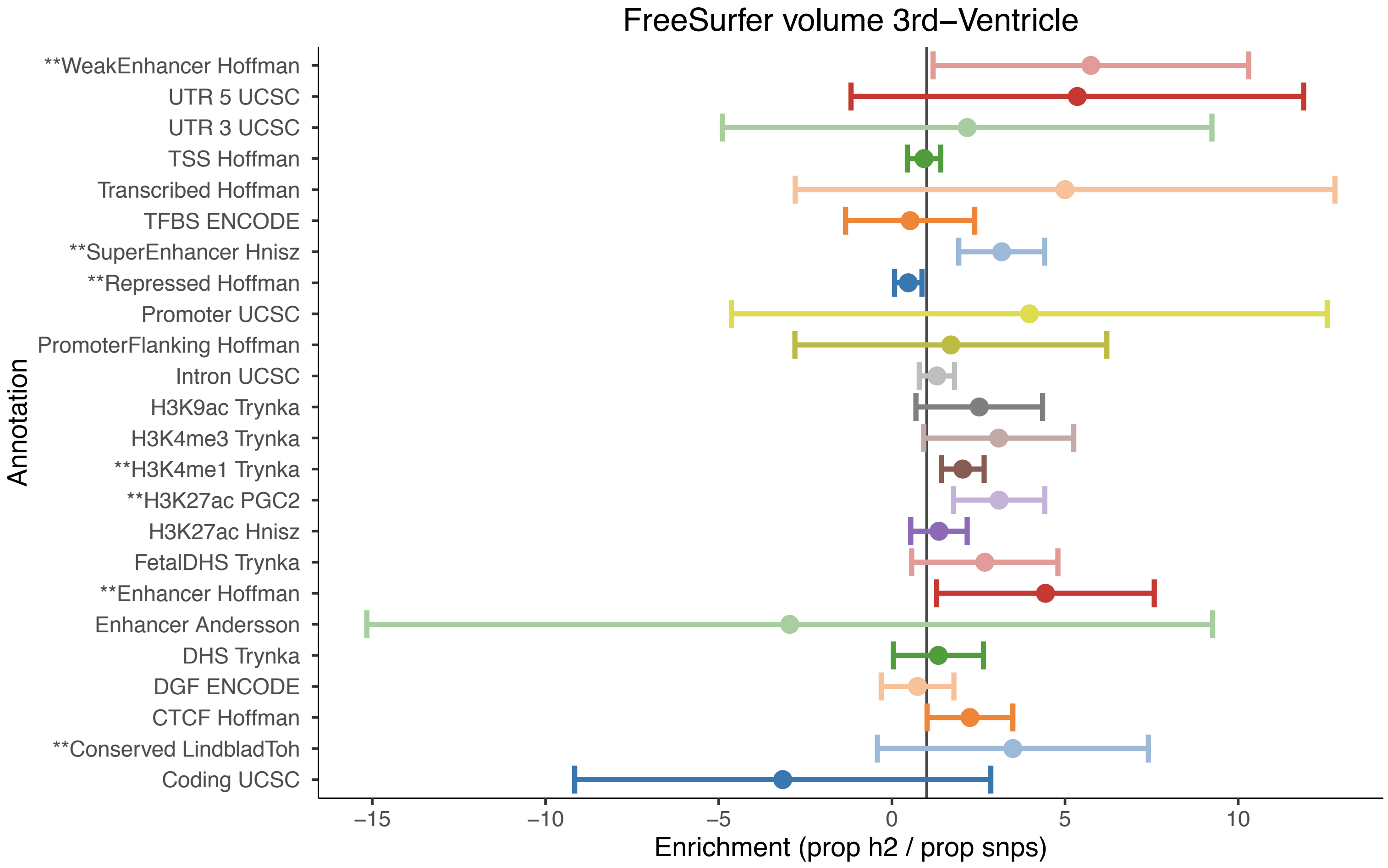
FreeSurfer volume 3rd−Ventricle
**WeakEnhancer Hoffman
UTR 5 UCSC
UTR 3 UCSC
TSS Hoffman
Transcribed Hoffman
TFBS ENCODE
**SuperEnhancer Hnisz
**Repressed Hoffman
Promoter UCSC
PromoterFlanking Hoffman
Intron UCSC
H3K9ac Trynka
H3K4me3 Trynka
**H3K4me1 Trynka
**H3K27ac PGC2
H3K27ac Hnisz
FetalDHS Trynka
**Enhancer Hoffman
Enhancer Andersson
DHS Trynka
DGF ENCODE
CTCF Hoffman
**Conserved LindbladToh
Coding UCSC
Annotation
−15
−10
−5
0
5
10
Enrichment (prop h2 / prop snps)

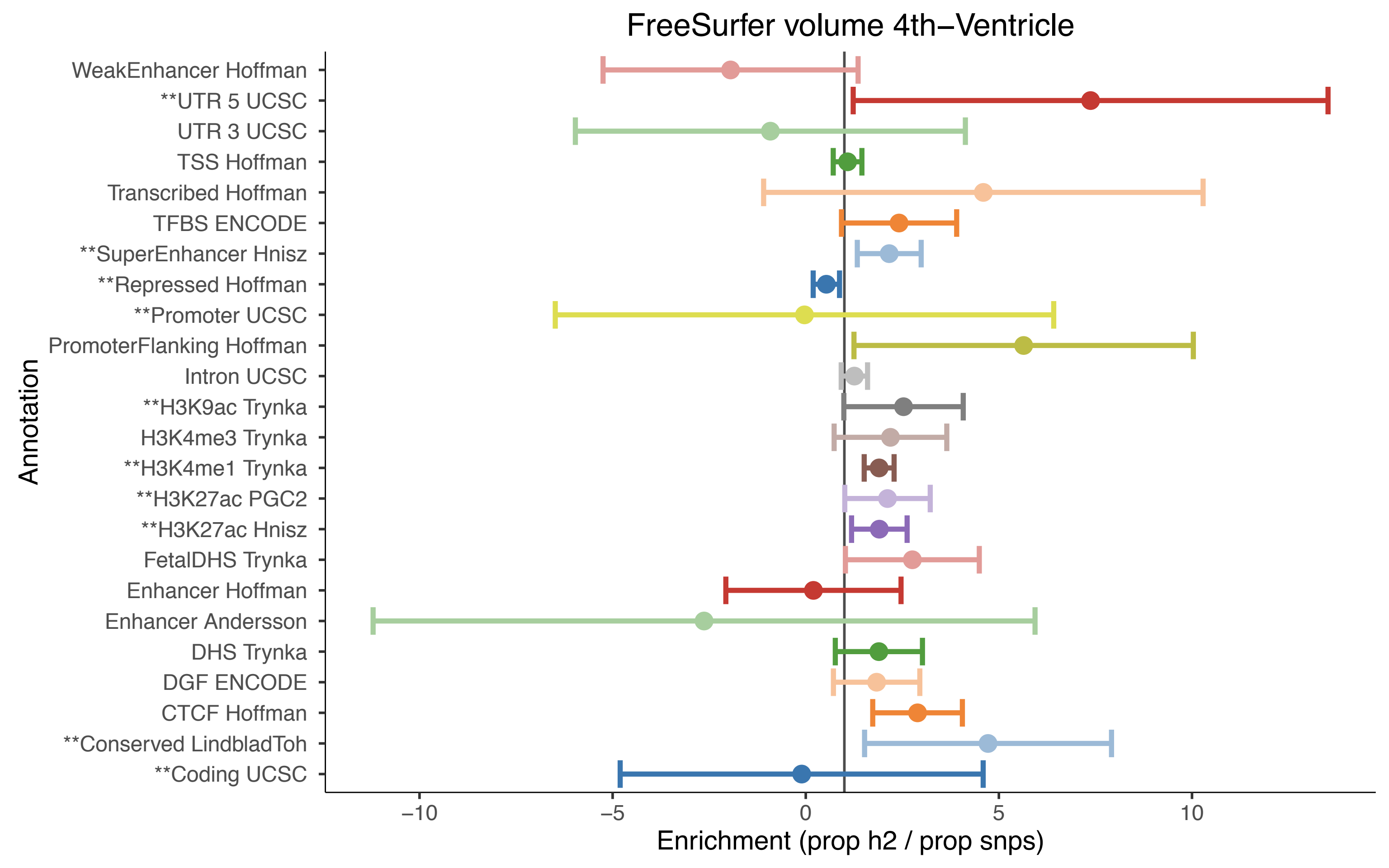
FreeSurfer volume 4th−Ventricle
WeakEnhancer Hoffman
**UTR 5 UCSC
UTR 3 UCSC
TSS Hoffman
Transcribed Hoffman
TFBS ENCODE
**SuperEnhancer Hnisz
**Repressed Hoffman
**Promoter UCSC
PromoterFlanking Hoffman
Intron UCSC
**H3K9ac Trynka
H3K4me3 Trynka
**H3K4me1 Trynka
**H3K27ac PGC2
**H3K27ac Hnisz
FetalDHS Trynka
Enhancer Hoffman
Enhancer Andersson
DHS Trynka
DGF ENCODE
CTCF Hoffman
**Conserved LindbladToh
**Coding UCSC
Annotation
−10
−5
0
5
10
Enrichment (prop h2 / prop snps)

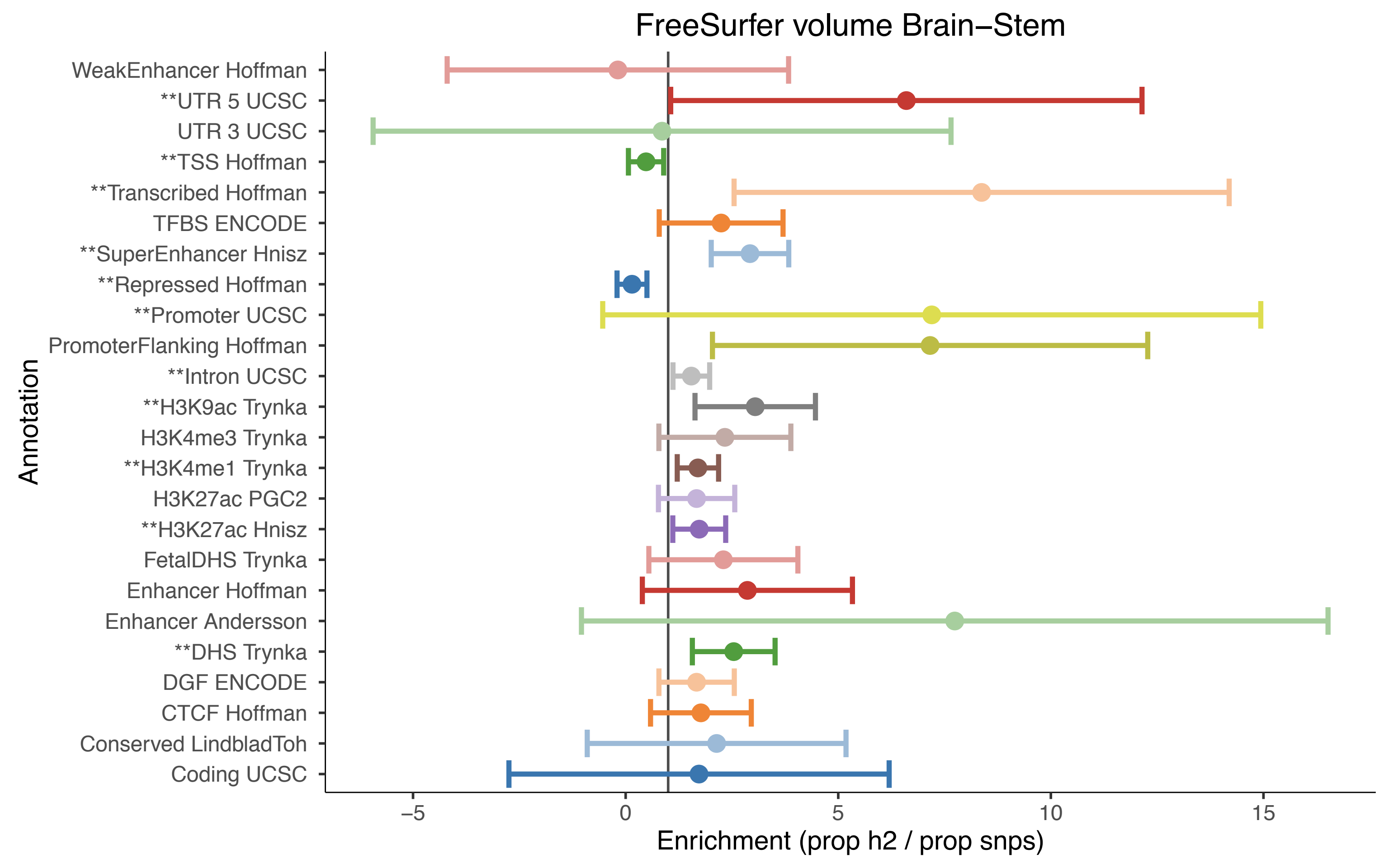

FreeSurfer volume Brain−Stem
WeakEnhancer Hoffman
**UTR 5 UCSC
UTR 3 UCSC
**TSS Hoffman
**Transcribed Hoffman
TFBS ENCODE
**SuperEnhancer Hnisz
**Repressed Hoffman
**Promoter UCSC
PromoterFlanking Hoffman
**Intron UCSC
**H3K9ac Trynka
H3K4me3 Trynka
**H3K4me1 Trynka
H3K27ac PGC2
**H3K27ac Hnisz
FetalDHS Trynka
Enhancer Hoffman
Enhancer Andersson
**DHS Trynka
DGF ENCODE
CTCF Hoffman
Conserved LindbladToh
Coding UCSC
Annotation
−5
0
5
10
15
Enrichment (prop h2 / prop snps)

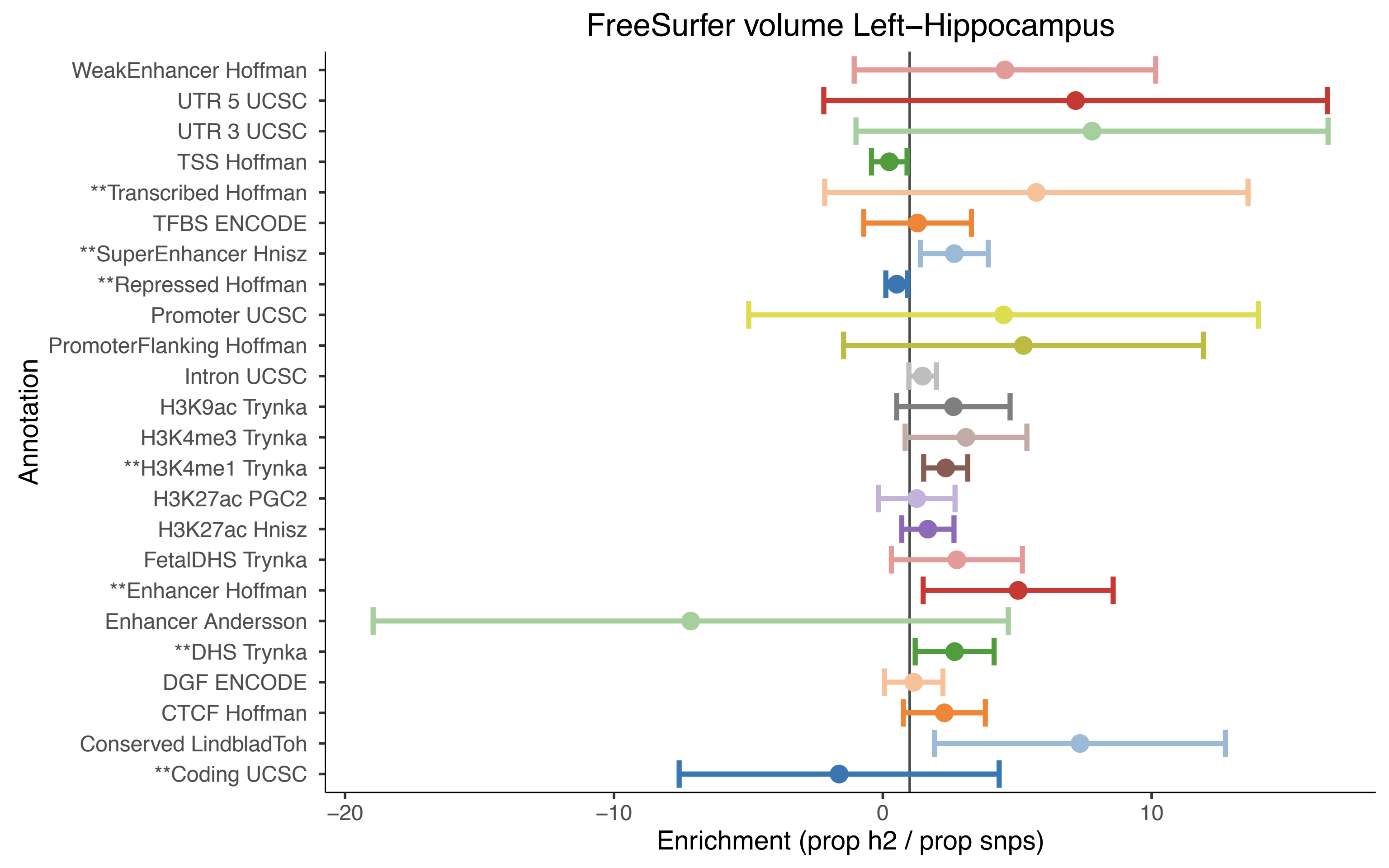
FreeSurfer volume Left−Hippocampus
WeakEnhancer Hoffman
UTR 5 UCSC
UTR 3 UCSC
TSS Hoffman
**Transcribed Hoffman
TFBS ENCODE
**SuperEnhancer Hnisz
**Repressed Hoffman
Promoter UCSC
PromoterFlanking Hoffman
Intron UCSC
H3K9ac Trynka
H3K4me3 Trynka
**H3K4me1 Trynka
H3K27ac PGC2
H3K27ac Hnisz
FetalDHS Trynka
**Enhancer Hoffman
Enhancer Andersson
**DHS Trynka
DGF ENCODE
CTCF Hoffman
Conserved LindbladToh
**Coding UCSC
Annotation
−20
−10
0
10
Enrichment (prop h2 / prop snps)

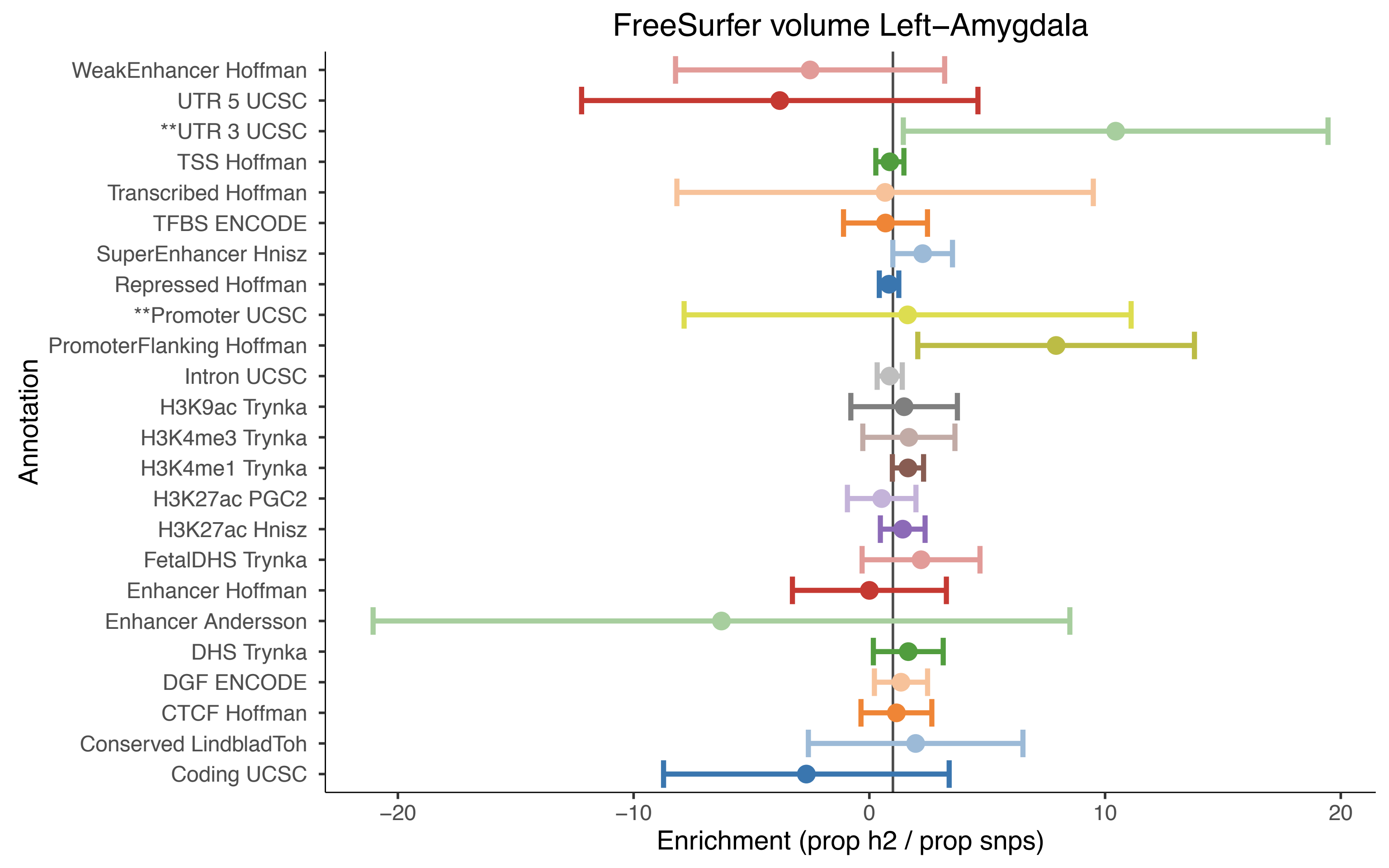

FreeSurfer volume Left−Amygdala
Annotation
WeakEnhancer Hoffman
UTR 5 UCSC
**UTR 3 UCSC
TSS Hoffman
Transcribed Hoffman
TFBS ENCODE
SuperEnhancer Hnisz
Repressed Hoffman
**Promoter UCSC
PromoterFlanking Hoffman
Intron UCSC
H3K9ac Trynka
H3K4me3 Trynka
H3K4me1 Trynka
H3K27ac PGC2
H3K27ac Hnisz
FetalDHS Trynka
Enhancer Hoffman
Enhancer Andersson
DHS Trynka
DGF ENCODE
CTCF Hoffman
Conserved LindbladToh
Coding UCSC
−20
−10
0
10
20
Enrichment (prop h2 / prop snps)

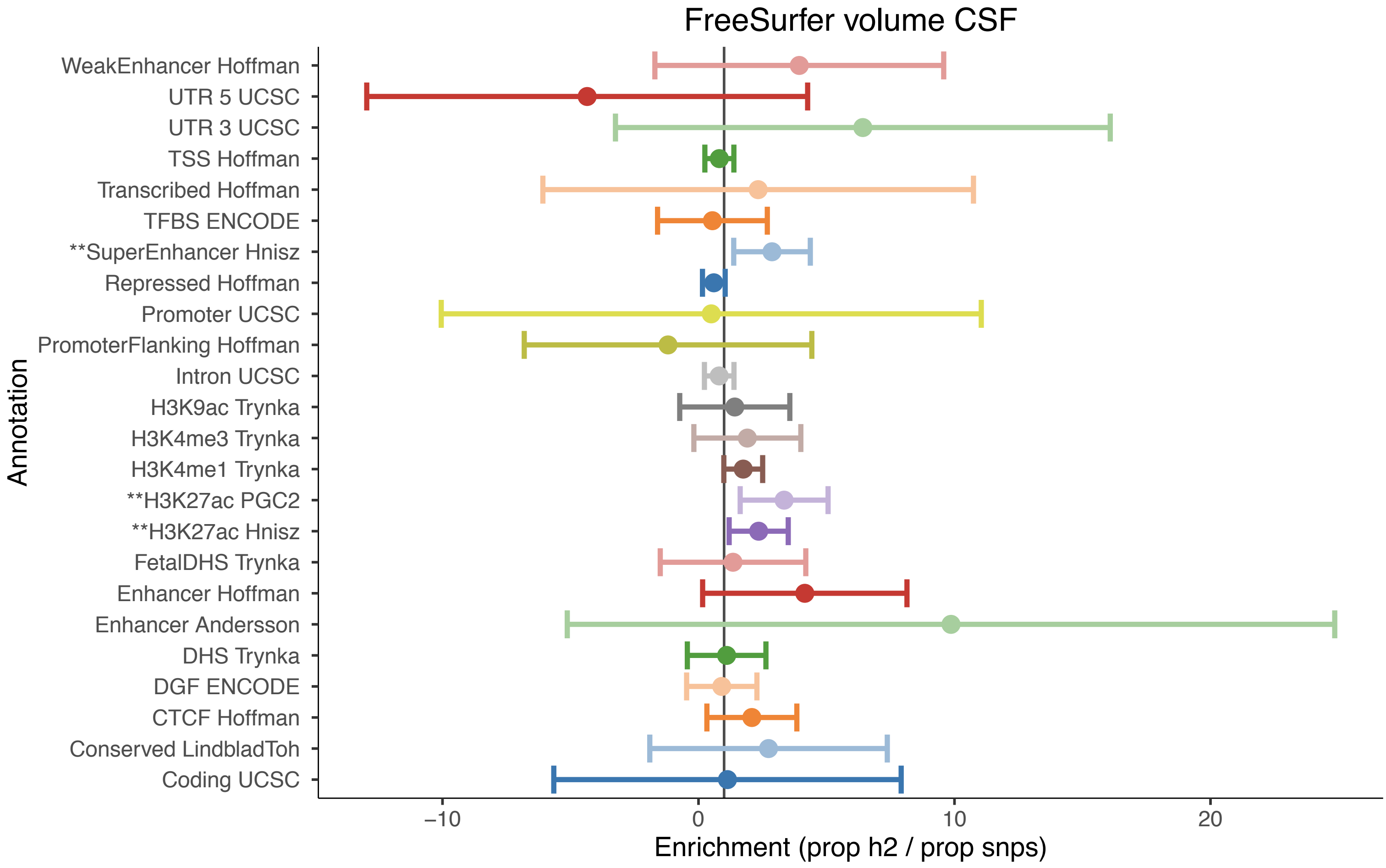
FreeSurfer volume CSF
WeakEnhancer Hoffman
UTR 5 UCSC
UTR 3 UCSC
TSS Hoffman
Transcribed Hoffman
TFBS ENCODE
**SuperEnhancer Hnisz
Repressed Hoffman
Promoter UCSC
PromoterFlanking Hoffman
Intron UCSC
H3K9ac Trynka
H3K4me3 Trynka
H3K4me1 Trynka
**H3K27ac PGC2
**H3K27ac Hnisz
FetalDHS Trynka
Enhancer Hoffman
Enhancer Andersson
DHS Trynka
DGF ENCODE
CTCF Hoffman
Conserved LindbladToh
Coding UCSC
Annotation
−10
0
10
20
Enrichment (prop h2 / prop snps)

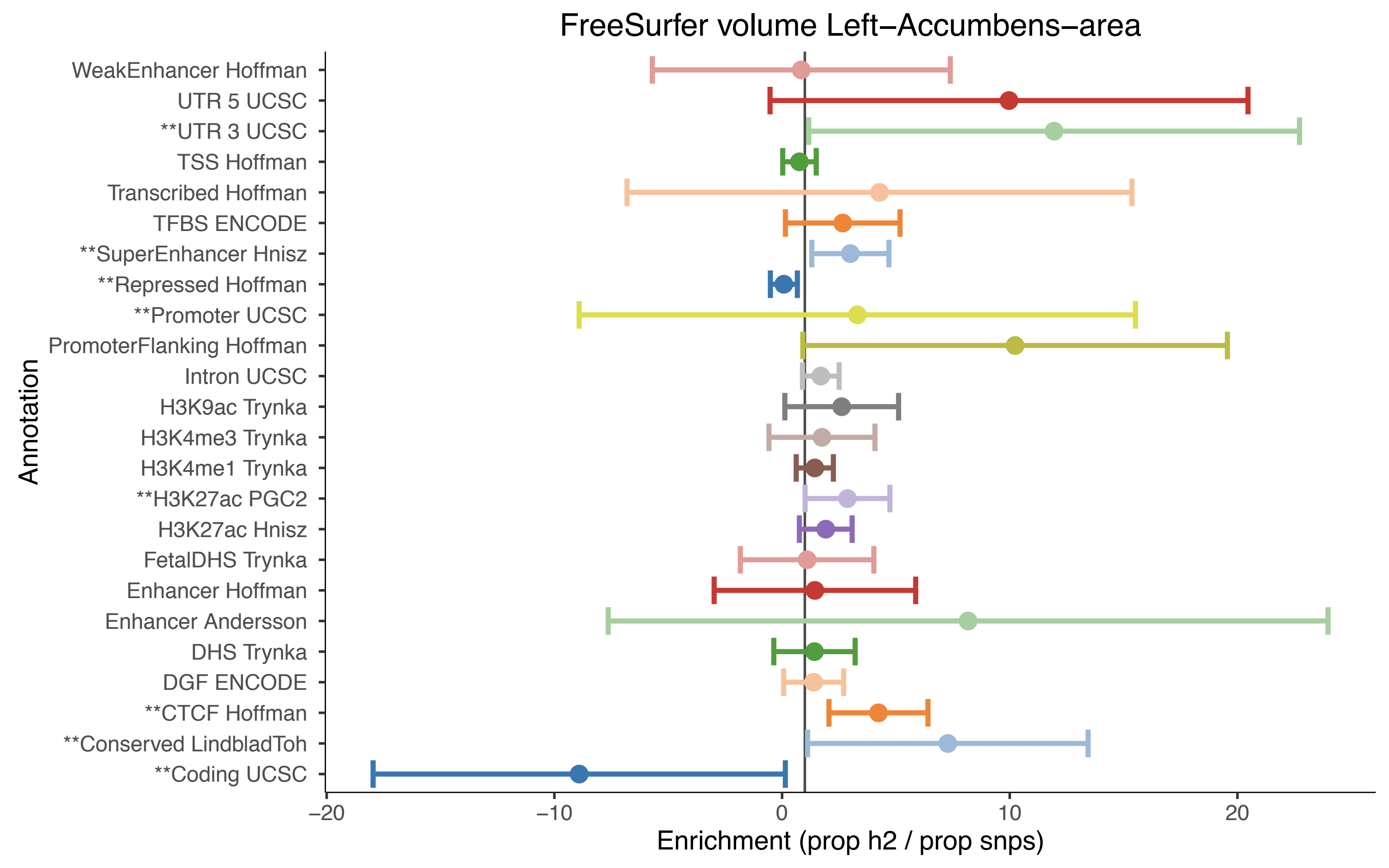

FreeSurfer volume Left−Accumbens−area
WeakEnhancer Hoffman
UTR 5 UCSC
**UTR 3 UCSC
TSS Hoffman
Transcribed Hoffman
TFBS ENCODE
**SuperEnhancer Hnisz
**Repressed Hoffman
**Promoter UCSC
PromoterFlanking Hoffman
Intron UCSC
H3K9ac Trynka
H3K4me3 Trynka
H3K4me1 Trynka
**H3K27ac PGC2
H3K27ac Hnisz
FetalDHS Trynka
Enhancer Hoffman
Enhancer Andersson
DHS Trynka
DGF ENCODE
**CTCF Hoffman
**Conserved LindbladToh
**Coding UCSC
Annotation
−20
−10
0
10
20
Enrichment (prop h2 / prop snps)

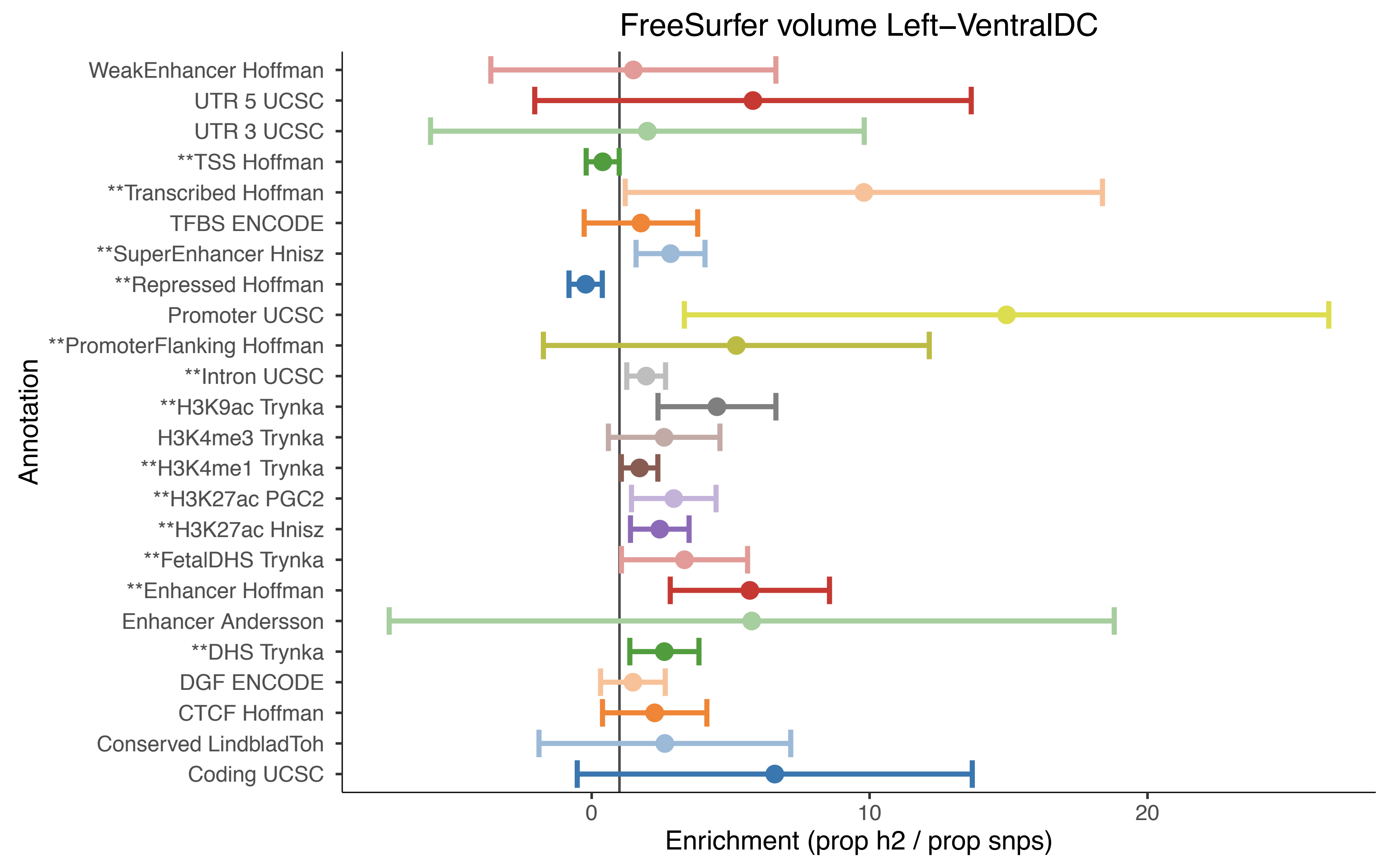
FreeSurfer volume Left−VentralDC
WeakEnhancer Hoffman
UTR 5 UCSC
UTR 3 UCSC
**TSS Hoffman
**Transcribed Hoffman
TFBS ENCODE
**SuperEnhancer Hnisz
**Repressed Hoffman
Promoter UCSC
**PromoterFlanking Hoffman
**Intron UCSC
**H3K9ac Trynka
H3K4me3 Trynka
**H3K4me1 Trynka
**H3K27ac PGC2
**H3K27ac Hnisz
**FetalDHS Trynka
**Enhancer Hoffman
Enhancer Andersson
**DHS Trynka
DGF ENCODE
CTCF Hoffman
Conserved LindbladToh
Coding UCSC
Annotation
0
10
20
Enrichment (prop h2 / prop snps)

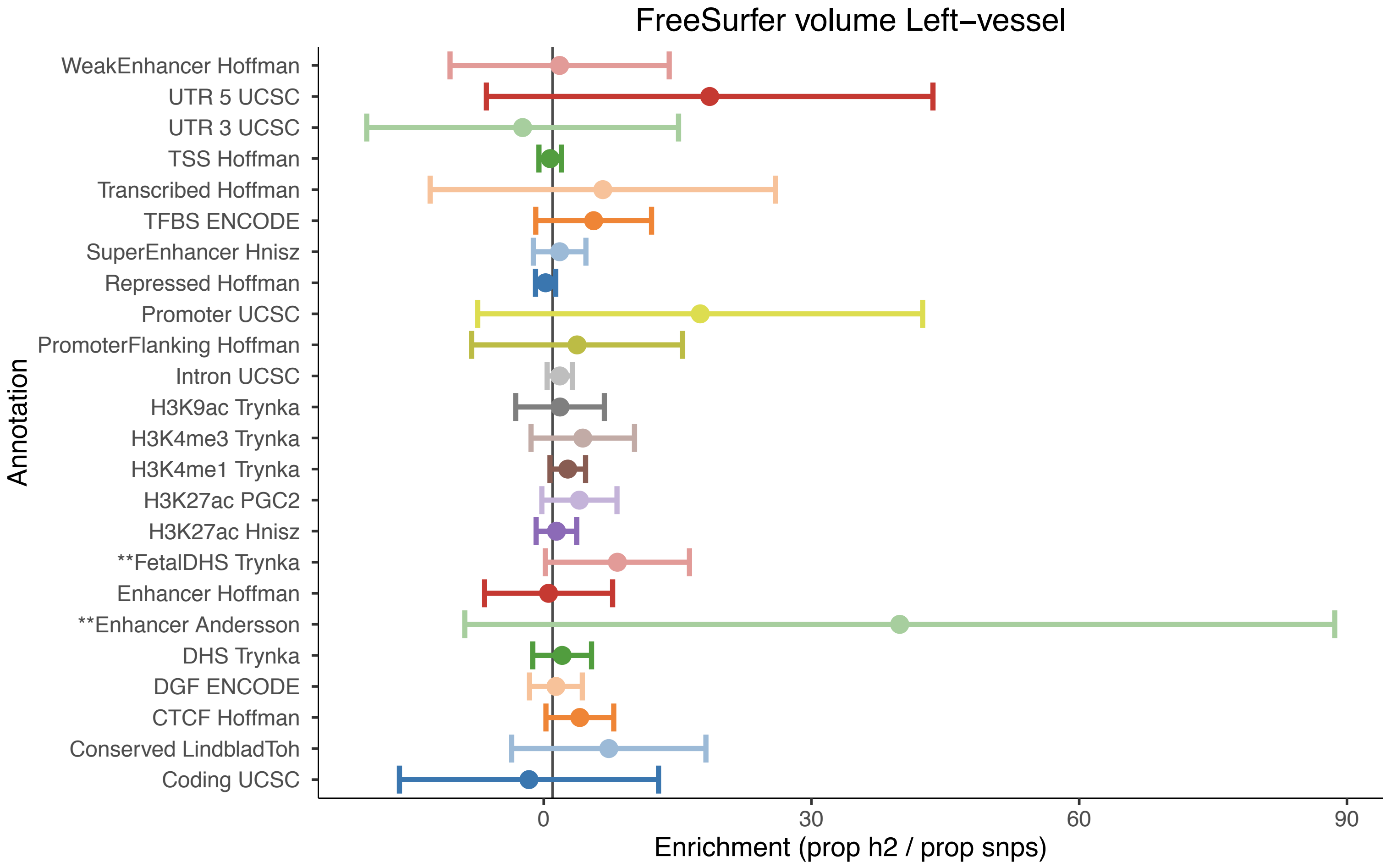

FreeSurfer volume Left−vessel
WeakEnhancer Hoffman
UTR 5 UCSC
UTR 3 UCSC
TSS Hoffman
Transcribed Hoffman
TFBS ENCODE
SuperEnhancer Hnisz
Repressed Hoffman
Promoter UCSC
PromoterFlanking Hoffman
Intron UCSC
H3K9ac Trynka
H3K4me3 Trynka
H3K4me1 Trynka
H3K27ac PGC2
H3K27ac Hnisz
**FetalDHS Trynka
Enhancer Hoffman
**Enhancer Andersson
DHS Trynka
DGF ENCODE
CTCF Hoffman
Conserved LindbladToh
Coding UCSC
Annotation
0
30
60
90
Enrichment (prop h2 / prop snps)

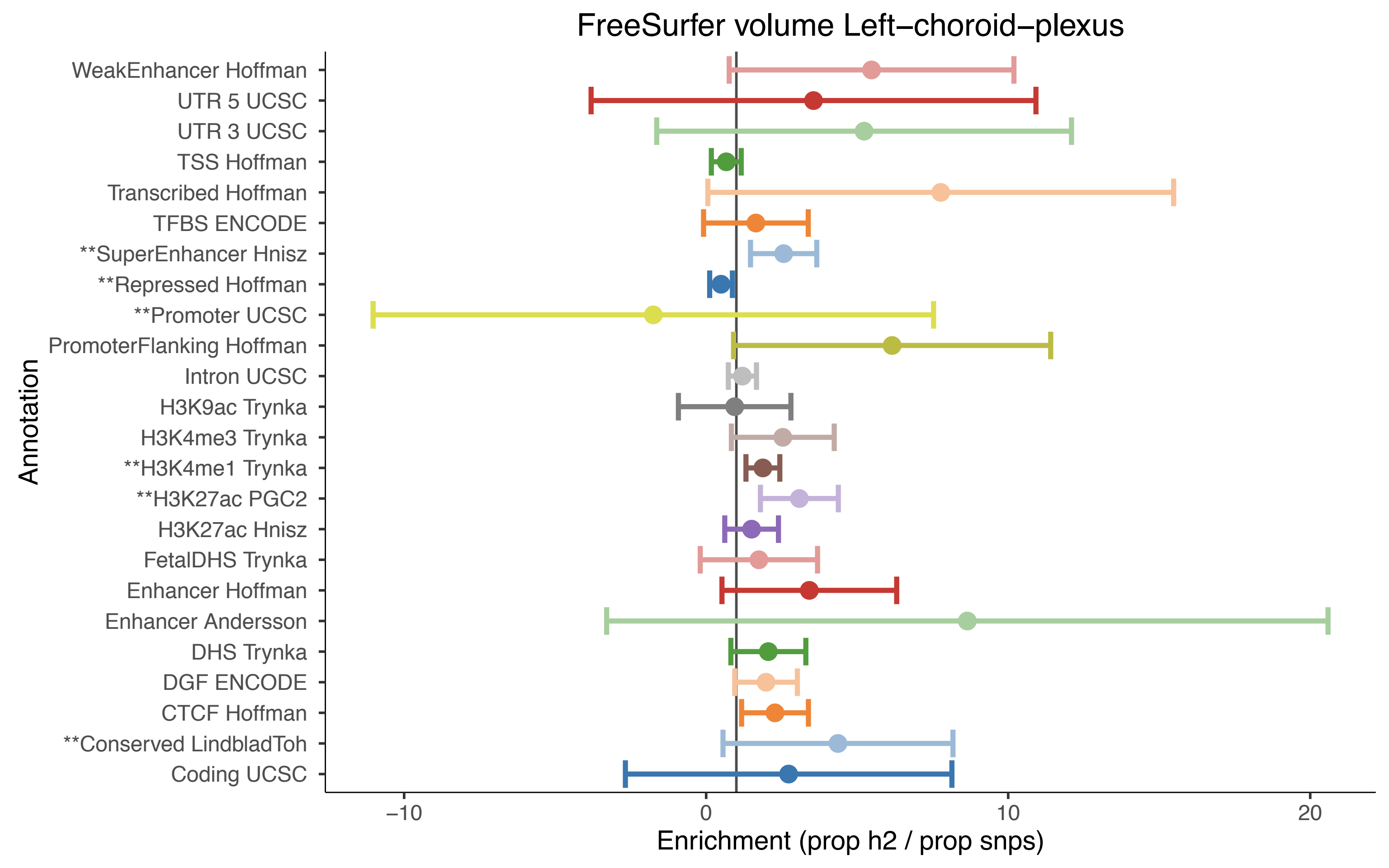

FreeSurfer volume Left−choroid−plexus
WeakEnhancer Hoffman
UTR 5 UCSC
UTR 3 UCSC
TSS Hoffman
Transcribed Hoffman
TFBS ENCODE
**SuperEnhancer Hnisz
**Repressed Hoffman
**Promoter UCSC
PromoterFlanking Hoffman
Intron UCSC
H3K9ac Trynka
H3K4me3 Trynka
**H3K4me1 Trynka
**H3K27ac PGC2
H3K27ac Hnisz
FetalDHS Trynka
Enhancer Hoffman
Enhancer Andersson
DHS Trynka
DGF ENCODE
CTCF Hoffman
**Conserved LindbladToh
Coding UCSC
Annotation
−10
0
10
20
Enrichment (prop h2 / prop snps)

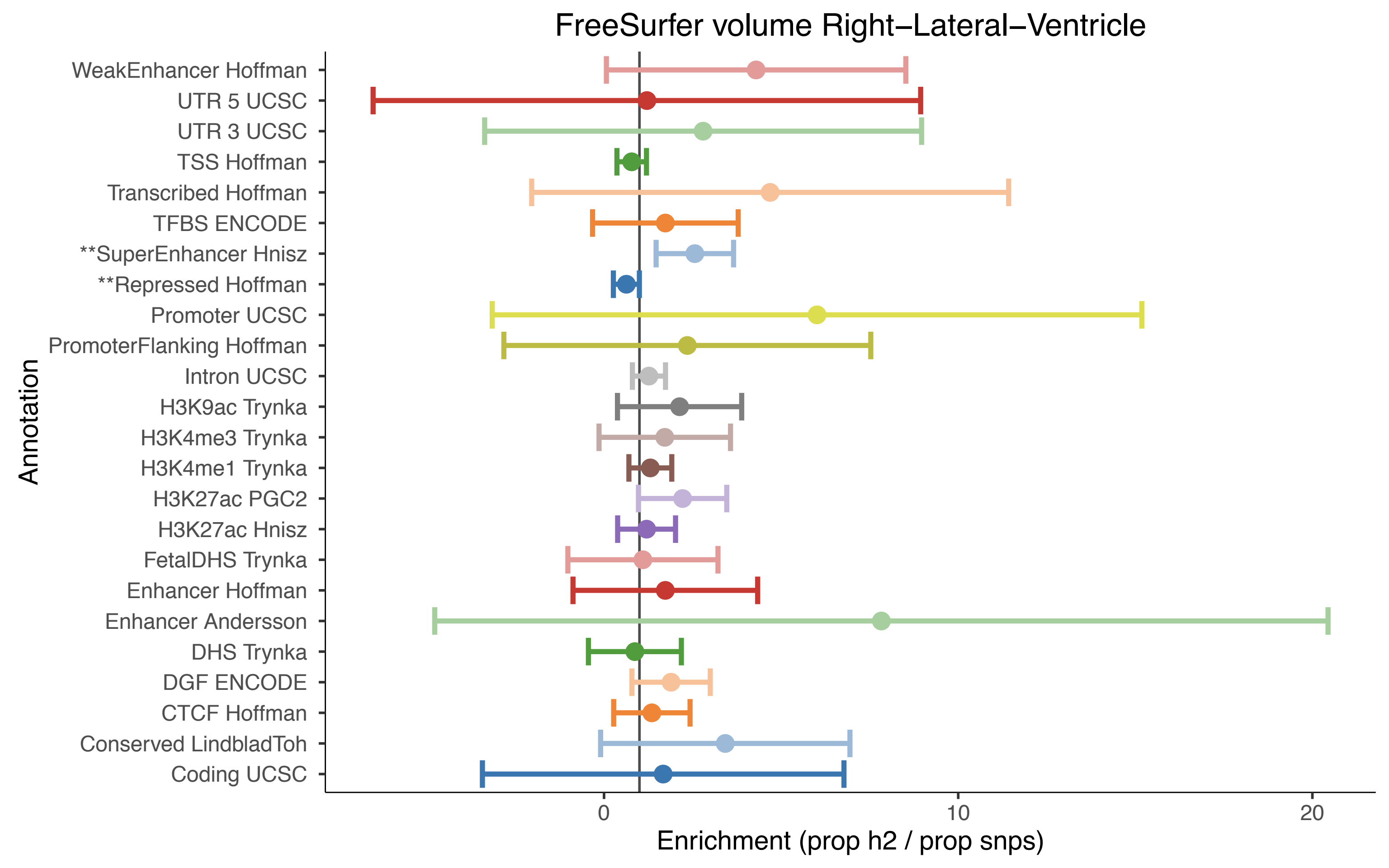

FreeSurfer volume Right−Lateral−Ventricle
WeakEnhancer Hoffman
UTR 5 UCSC
UTR 3 UCSC
TSS Hoffman
Transcribed Hoffman
TFBS ENCODE
**SuperEnhancer Hnisz
**Repressed Hoffman
Promoter UCSC
PromoterFlanking Hoffman
Intron UCSC
H3K9ac Trynka
H3K4me3 Trynka
H3K4me1 Trynka
H3K27ac PGC2
H3K27ac Hnisz
FetalDHS Trynka
Enhancer Hoffman
Enhancer Andersson
DHS Trynka
DGF ENCODE
CTCF Hoffman
Conserved LindbladToh
Coding UCSC
Annotation
0
10
20
Enrichment (prop h2 / prop snps)

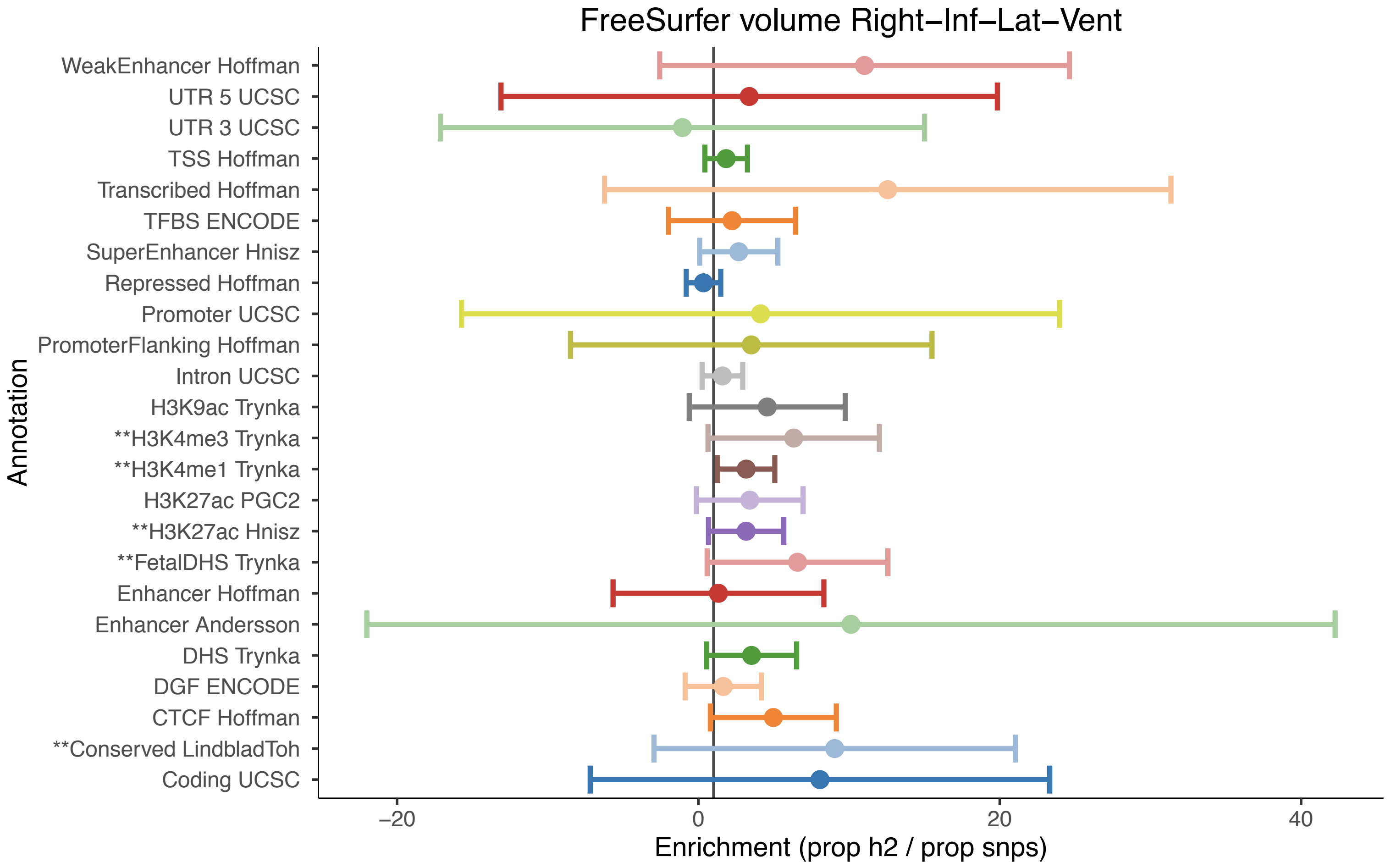
FreeSurfer volume Right−Inf−Lat−Vent
WeakEnhancer Hoffman
UTR 5 UCSC
UTR 3 UCSC
TSS Hoffman
Transcribed Hoffman
TFBS ENCODE
SuperEnhancer Hnisz
Repressed Hoffman
Promoter UCSC
PromoterFlanking Hoffman
Intron UCSC
H3K9ac Trynka
**H3K4me3 Trynka
**H3K4me1 Trynka
H3K27ac PGC2
**H3K27ac Hnisz
**FetalDHS Trynka
Enhancer Hoffman
Enhancer Andersson
DHS Trynka
DGF ENCODE
CTCF Hoffman
**Conserved LindbladToh
Coding UCSC
Annotation
−20
0
20
40
Enrichment (prop h2 / prop snps)

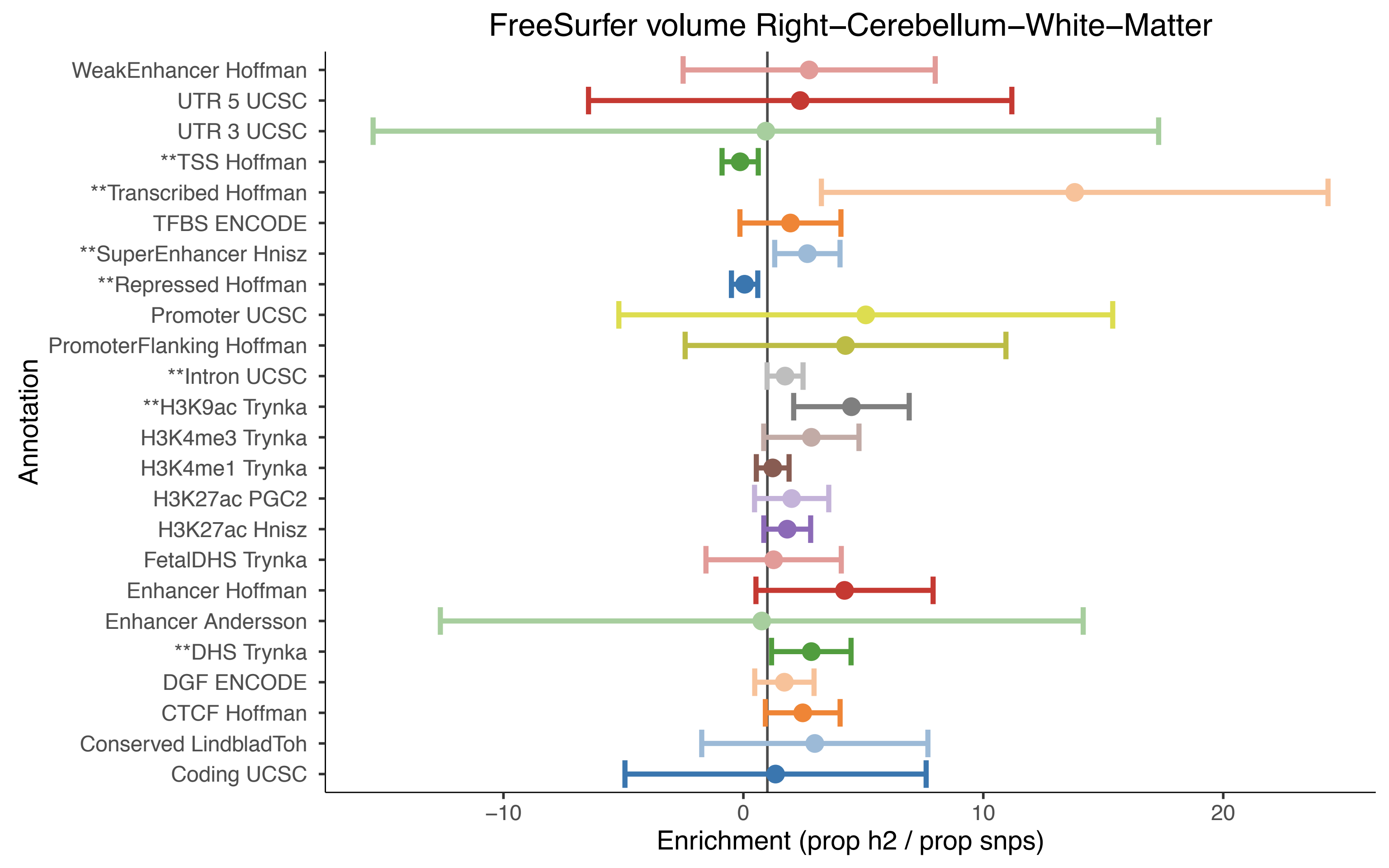

FreeSurfer volume Right−Cerebellum−White−Matter
WeakEnhancer Hoffman
UTR 5 UCSC
UTR 3 UCSC
**TSS Hoffman
**Transcribed Hoffman
TFBS ENCODE
**SuperEnhancer Hnisz
**Repressed Hoffman
Promoter UCSC
PromoterFlanking Hoffman
**Intron UCSC
**H3K9ac Trynka
H3K4me3 Trynka
H3K4me1 Trynka
H3K27ac PGC2
H3K27ac Hnisz
FetalDHS Trynka
Enhancer Hoffman
Enhancer Andersson
**DHS Trynka
DGF ENCODE
CTCF Hoffman
Conserved LindbladToh
Coding UCSC
Annotation
−10
0
10
20
Enrichment (prop h2 / prop snps)

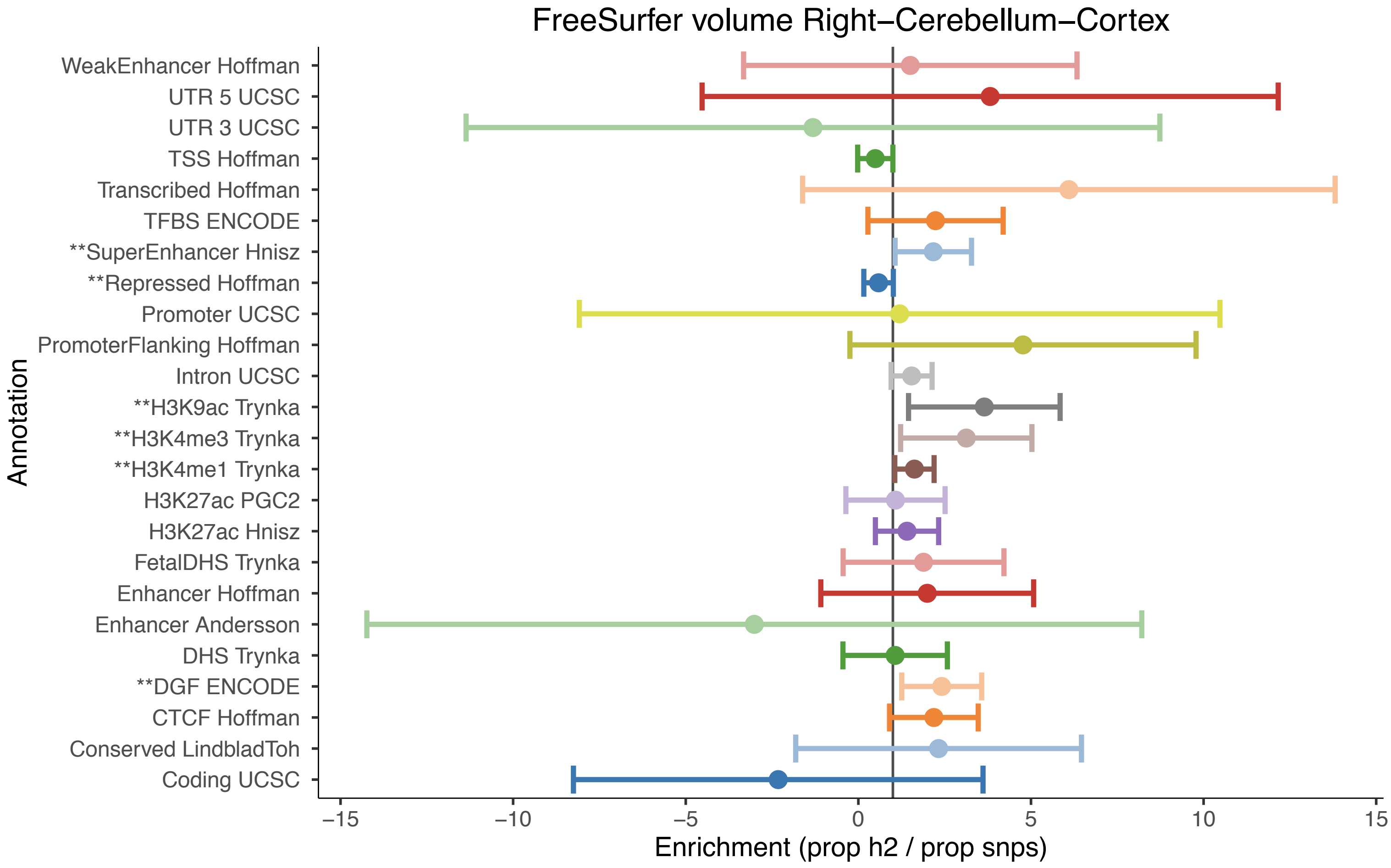
FreeSurfer volume Right−Cerebellum−Cortex
WeakEnhancer Hoffman
UTR 5 UCSC
UTR 3 UCSC
TSS Hoffman
Transcribed Hoffman
TFBS ENCODE
**SuperEnhancer Hnisz
**Repressed Hoffman
Promoter UCSC
PromoterFlanking Hoffman
Intron UCSC
**H3K9ac Trynka
**H3K4me3 Trynka
**H3K4me1 Trynka
H3K27ac PGC2
H3K27ac Hnisz
FetalDHS Trynka
Enhancer Hoffman
Enhancer Andersson
DHS Trynka
**DGF ENCODE
CTCF Hoffman
Conserved LindbladToh
Coding UCSC
Annotation
−15
−10
−5
0
5
10
15
Enrichment (prop h2 / prop snps)

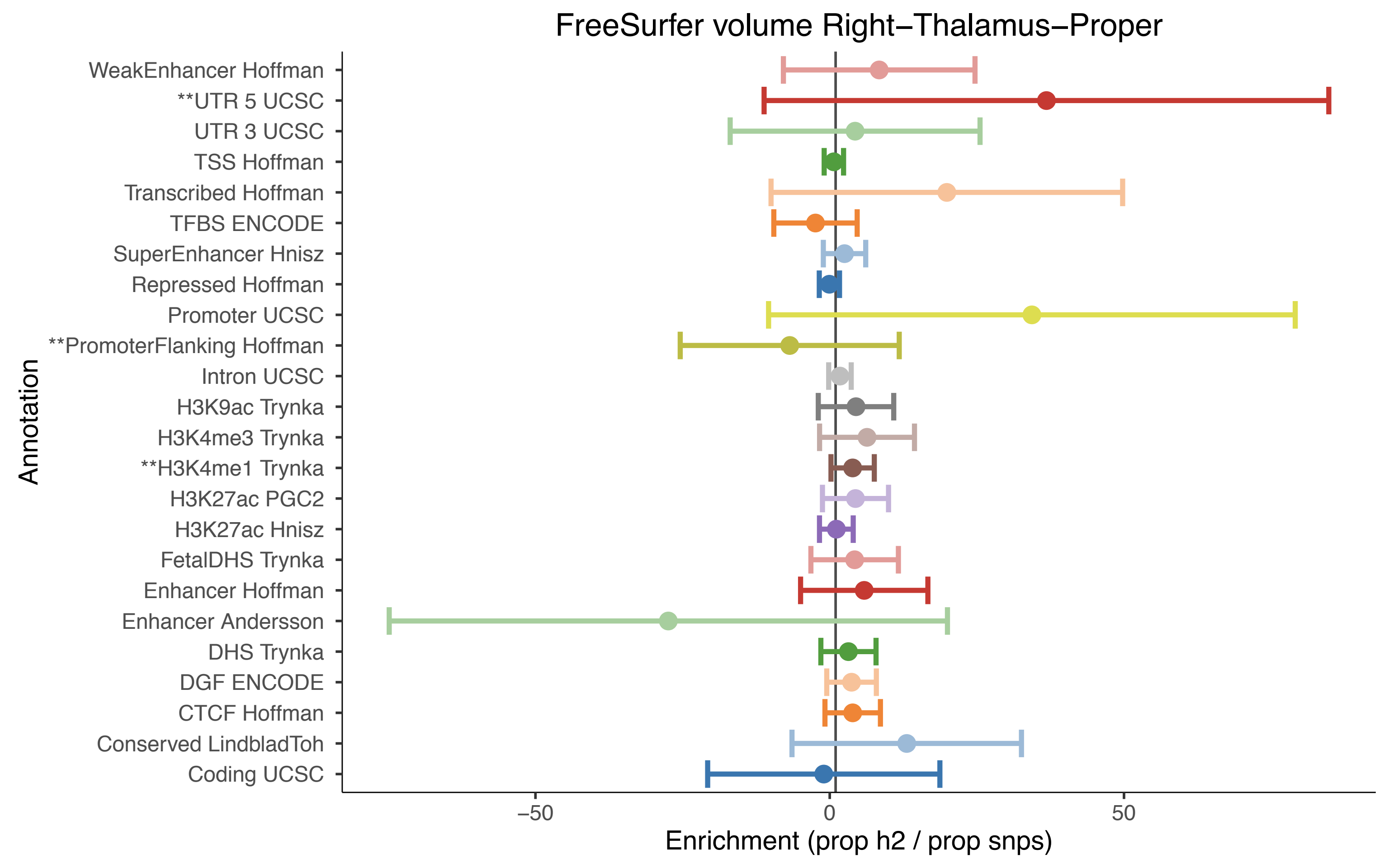
FreeSurfer volume Right−Thalamus−Proper
WeakEnhancer Hoffman
**UTR 5 UCSC
UTR 3 UCSC
TSS Hoffman
Transcribed Hoffman
TFBS ENCODE
SuperEnhancer Hnisz
Repressed Hoffman
Promoter UCSC
**PromoterFlanking Hoffman
Intron UCSC
H3K9ac Trynka
H3K4me3 Trynka
**H3K4me1 Trynka
H3K27ac PGC2
H3K27ac Hnisz
FetalDHS Trynka
Enhancer Hoffman
Enhancer Andersson
DHS Trynka
DGF ENCODE
CTCF Hoffman
Conserved LindbladToh
Coding UCSC
Annotation
−50
0
50
Enrichment (prop h2 / prop snps)

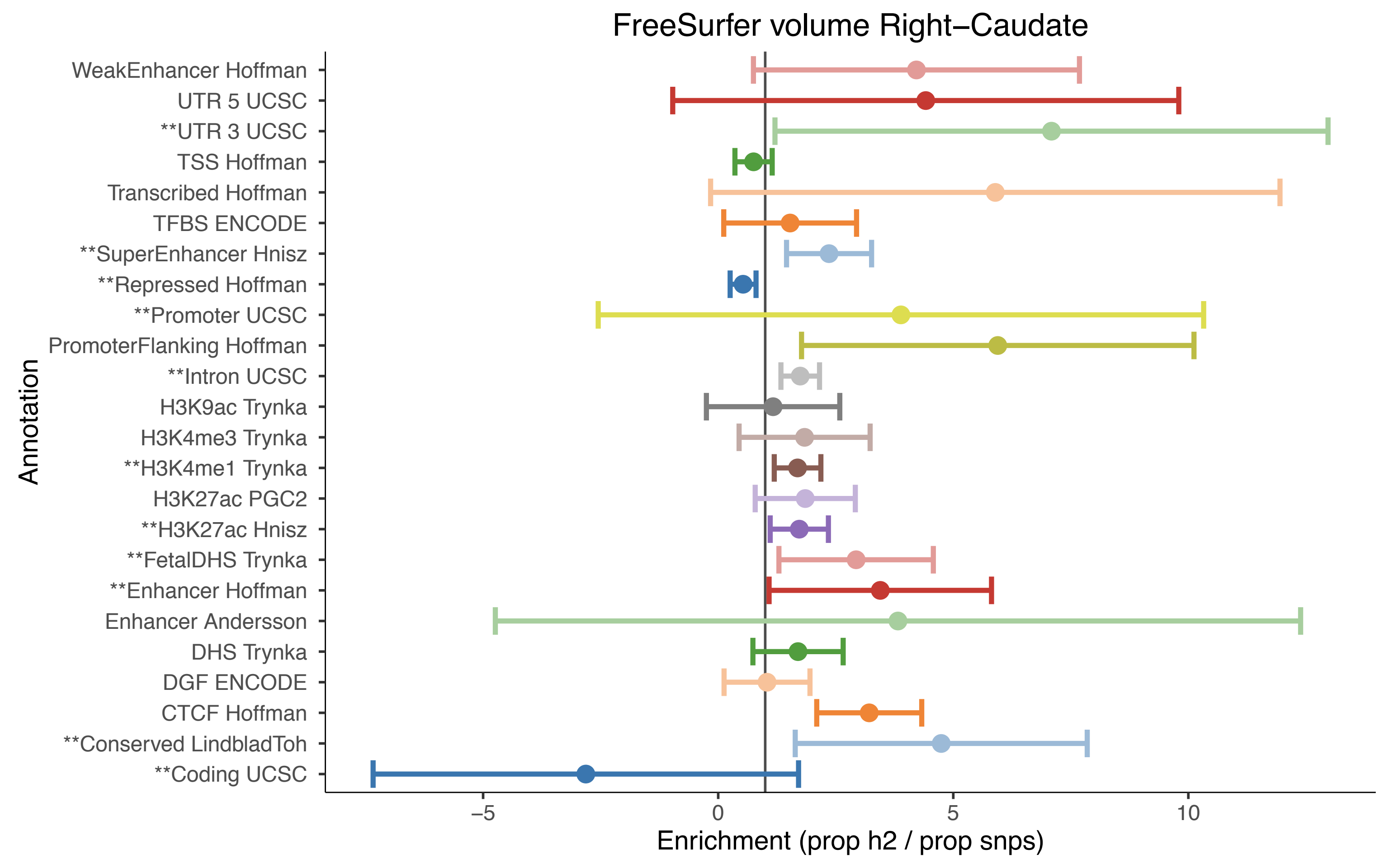

FreeSurfer volume Right−Caudate
WeakEnhancer Hoffman
UTR 5 UCSC
**UTR 3 UCSC
TSS Hoffman
Transcribed Hoffman
TFBS ENCODE
**SuperEnhancer Hnisz
**Repressed Hoffman
**Promoter UCSC
PromoterFlanking Hoffman
**Intron UCSC
H3K9ac Trynka
H3K4me3 Trynka
**H3K4me1 Trynka
H3K27ac PGC2
**H3K27ac Hnisz
**FetalDHS Trynka
**Enhancer Hoffman
Enhancer Andersson
DHS Trynka
DGF ENCODE
CTCF Hoffman
**Conserved LindbladToh
**Coding UCSC
Annotation
−5
0
5
10
Enrichment (prop h2 / prop snps)

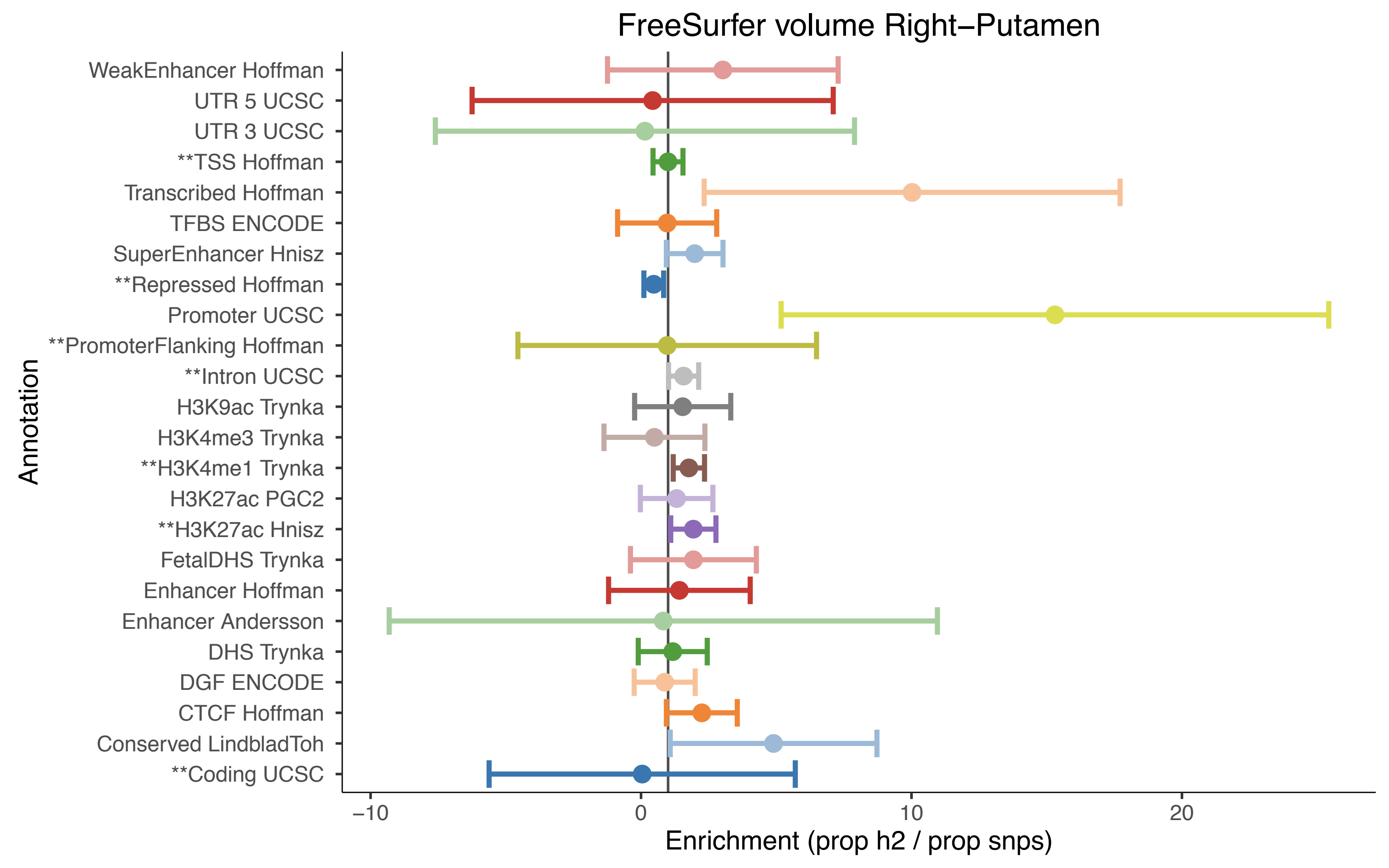

FreeSurfer volume Right−Putamen
WeakEnhancer Hoffman
UTR 5 UCSC
UTR 3 UCSC
**TSS Hoffman
Transcribed Hoffman
TFBS ENCODE
SuperEnhancer Hnisz
**Repressed Hoffman
Promoter UCSC
**PromoterFlanking Hoffman
**Intron UCSC
H3K9ac Trynka
H3K4me3 Trynka
**H3K4me1 Trynka
H3K27ac PGC2
**H3K27ac Hnisz
FetalDHS Trynka
Enhancer Hoffman
Enhancer Andersson
DHS Trynka
DGF ENCODE
CTCF Hoffman
Conserved LindbladToh
**Coding UCSC
Annotation
−10
0
10
20
Enrichment (prop h2 / prop snps)

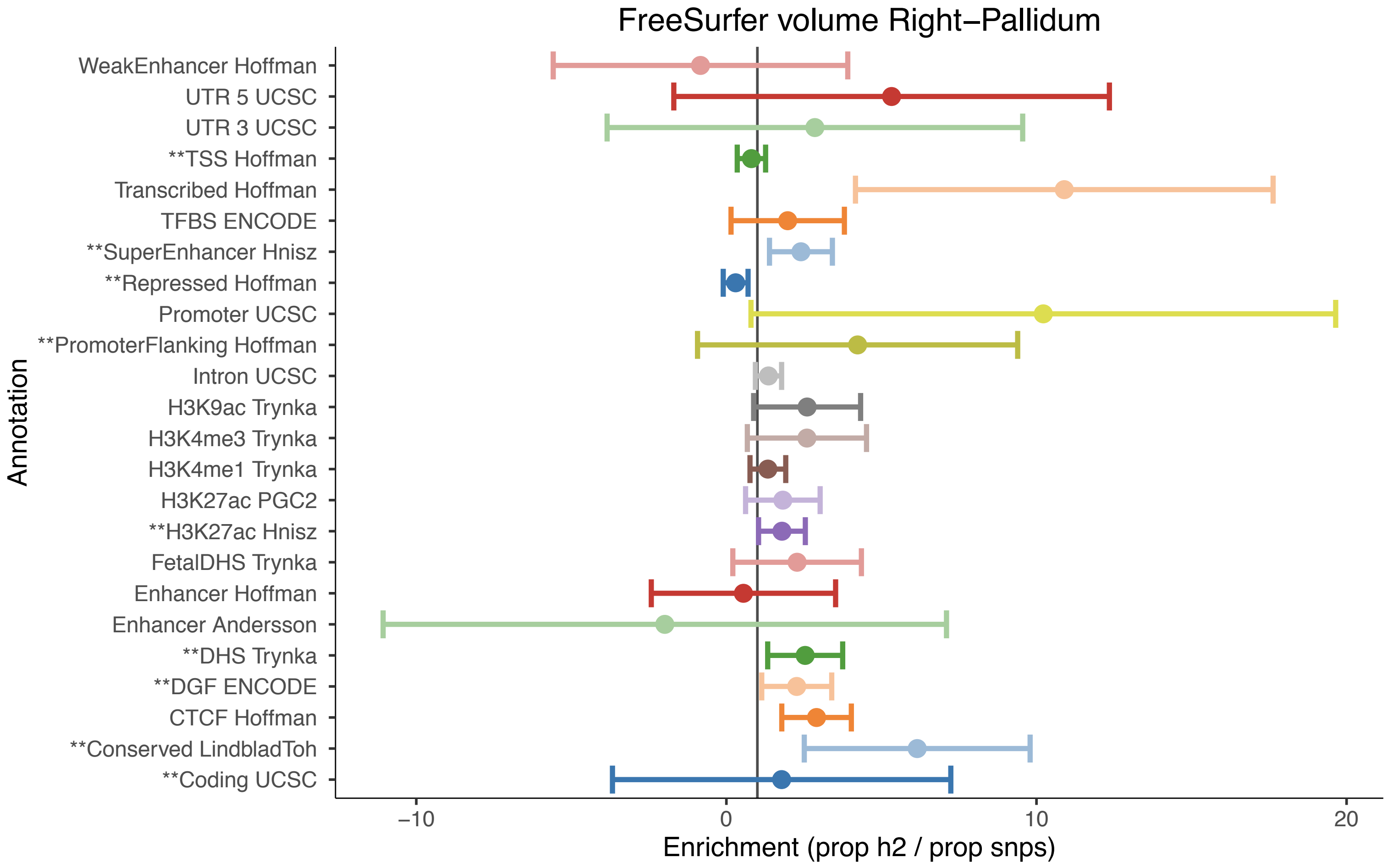
FreeSurfer volume Right–Pallidum
WeakEnhancer Hoffman
UTR 5 UCSC
UTR 3 UCSC
**TSS Hoffman
Transcribed Hoffman
TFBS ENCODE
**SuperEnhancer Hnisz
**Repressed Hoffman
Promoter UCSC
**PromoterFlanking Hoffman
Intron UCSC
H3K9ac Trynka
H3K4me3 Trynka
H3K4me1 Trynka
H3K27ac PGC2
**H3K27ac Hnisz
FetalDHS Trynka
Enhancer Hoffman
Enhancer Andersson
**DHS Trynka
**DGF ENCODE
CTCF Hoffman
**Conserved LindbladToh
**Coding UCSC
Annotation
−10
0
10
20
Enrichment (prop h2 / prop snps)

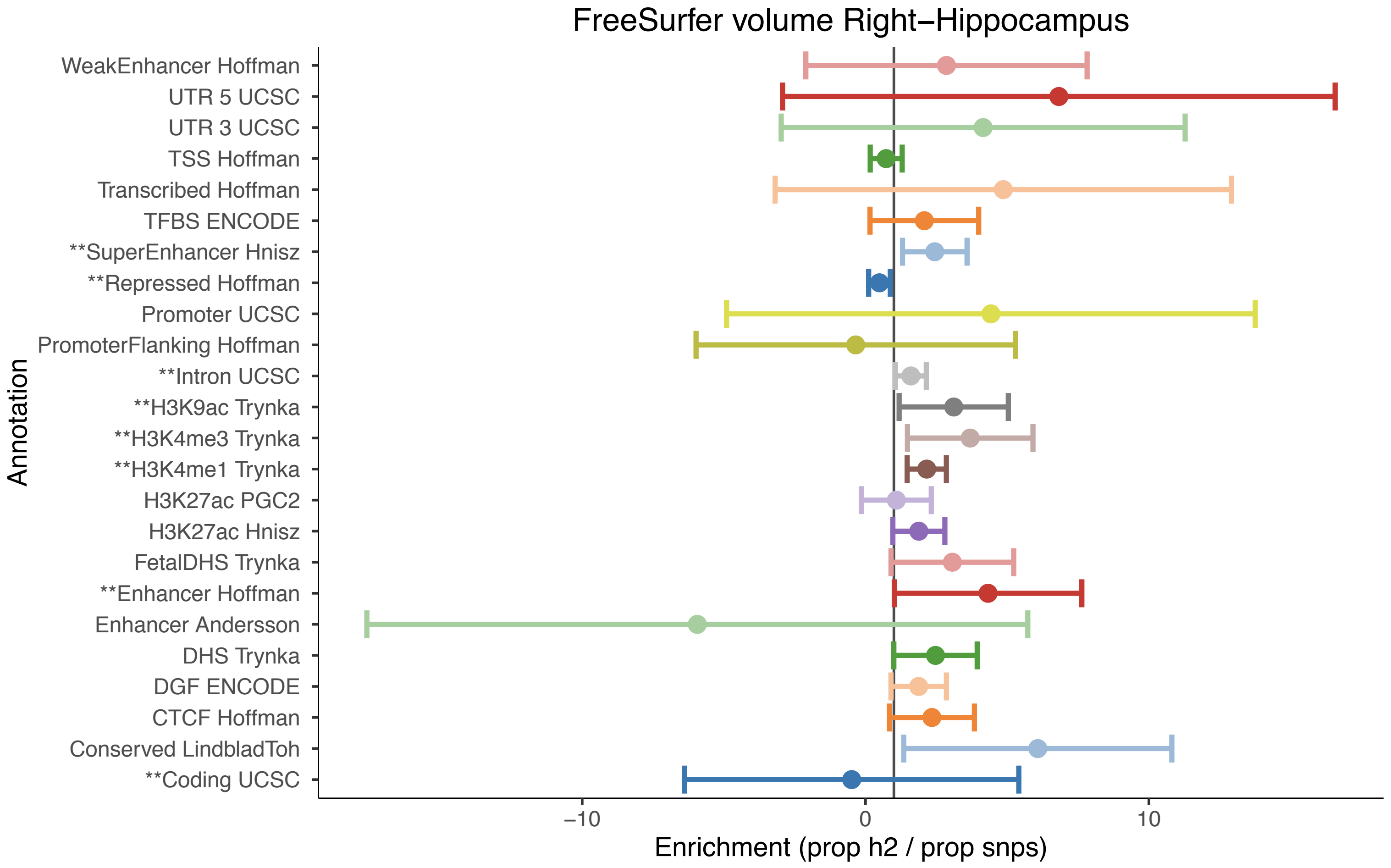
FreeSurfer volume Right−Hippocampus
WeakEnhancer Hoffman
UTR 5 UCSC
UTR 3 UCSC
TSS Hoffman
Transcribed Hoffman
TFBS ENCODE
**SuperEnhancer Hnisz
**Repressed Hoffman
Promoter UCSC
PromoterFlanking Hoffman
**Intron UCSC
**H3K9ac Trynka
**H3K4me3 Trynka
**H3K4me1 Trynka
H3K27ac PGC2
H3K27ac Hnisz
FetalDHS Trynka
**Enhancer Hoffman
Enhancer Andersson
DHS Trynka
DGF ENCODE
CTCF Hoffman
Conserved LindbladToh
**Coding UCSC
Annotation
−10
0
10
Enrichment (prop h2 / prop snps)

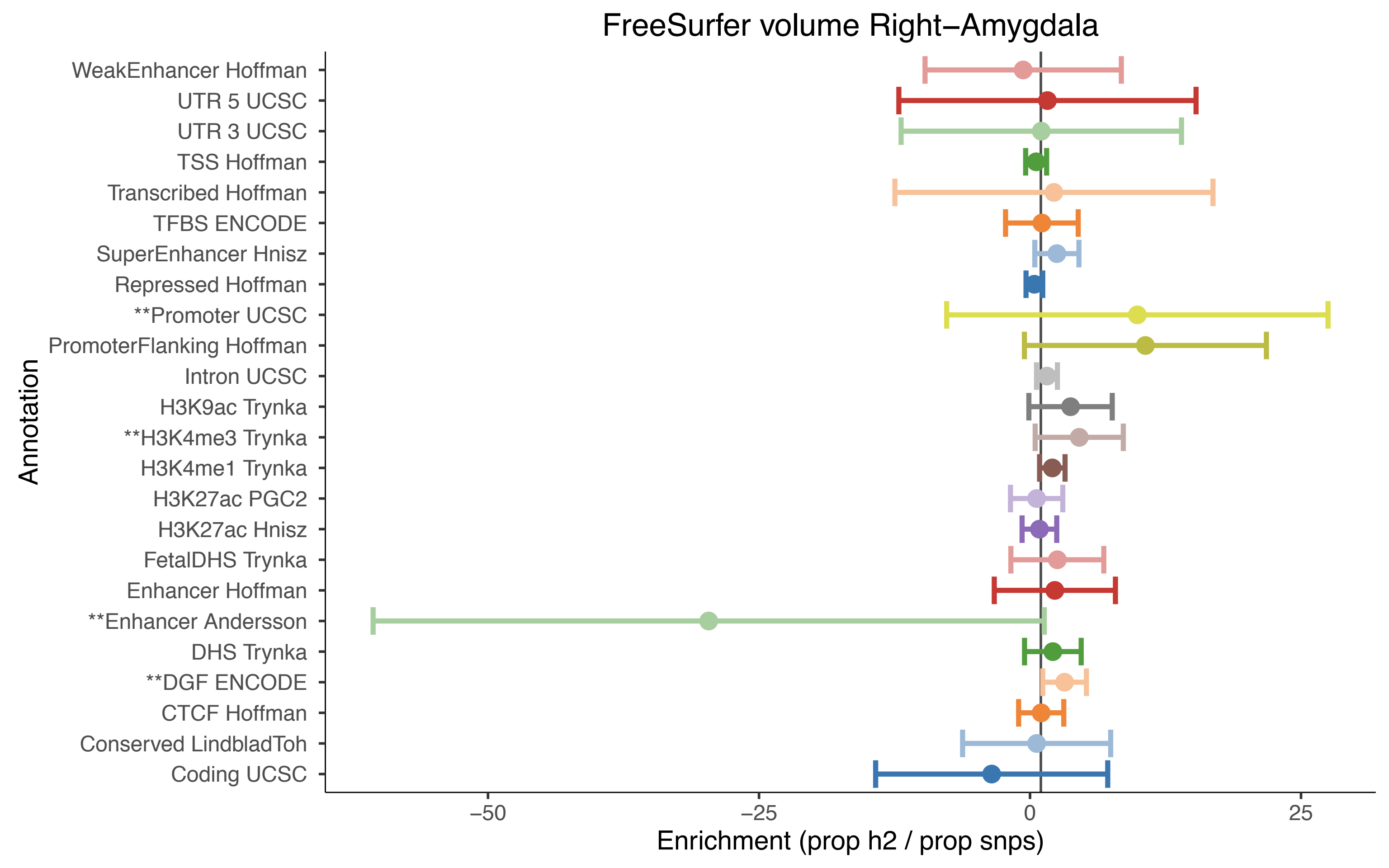
FreeSurfer volume Right−Amygdala
Annotation
WeakEnhancer Hoffman
UTR 5 UCSC
UTR 3 UCSC
TSS Hoffman
Transcribed Hoffman
TFBS ENCODE
SuperEnhancer Hnisz
Repressed Hoffman
**Promoter UCSC
PromoterFlanking Hoffman
Intron UCSC
H3K9ac Trynka
**H3K4me3 Trynka
H3K4me1 Trynka
H3K27ac PGC2
H3K27ac Hnisz
FetalDHS Trynka
Enhancer Hoffman
**Enhancer Andersson
DHS Trynka
**DGF ENCODE
CTCF Hoffman
Conserved LindbladToh
Coding UCSC
−50
−25
0
25
Enrichment (prop h2 / prop snps)

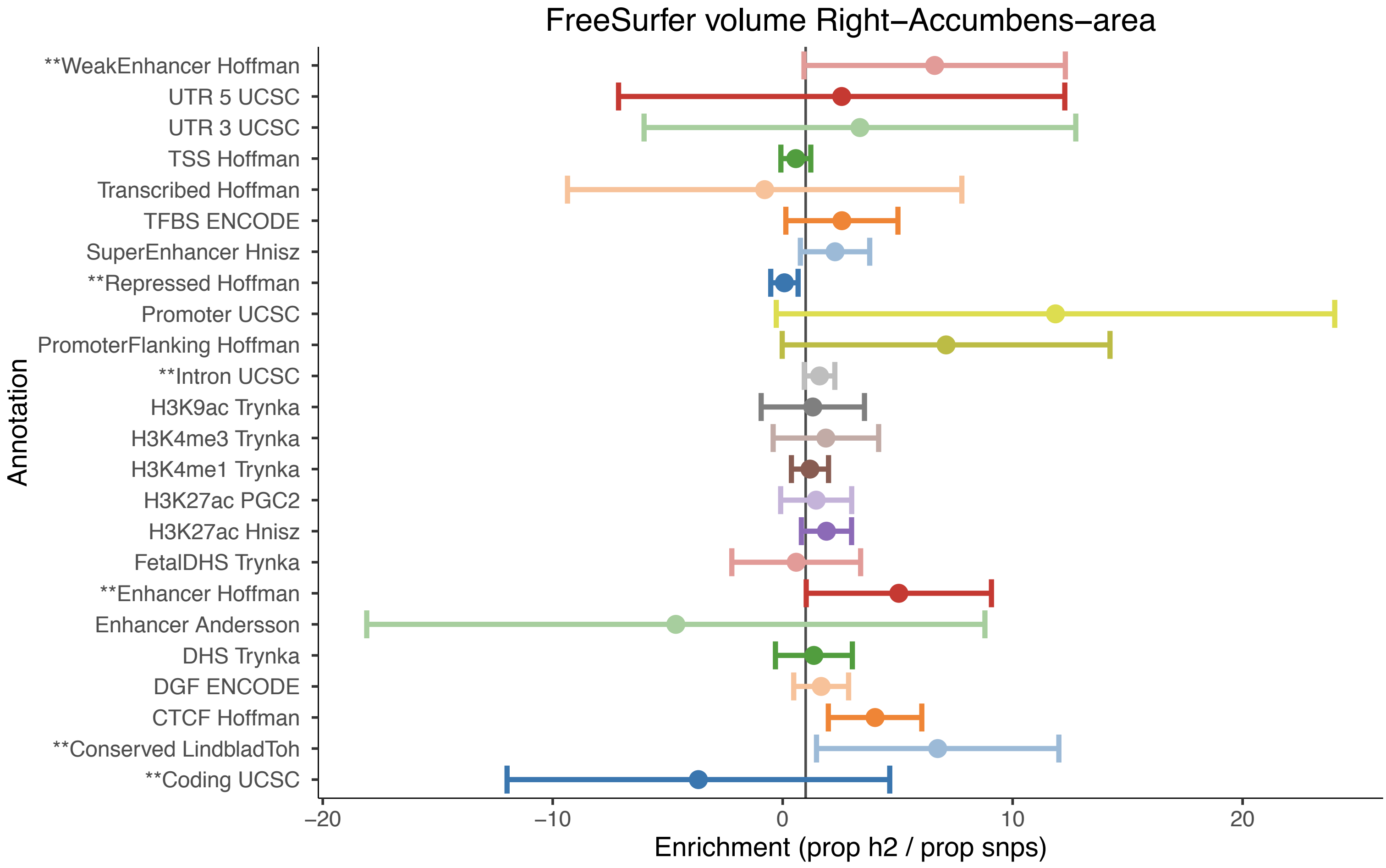
FreeSurfer volume Right−Accumbens−area
**WeakEnhancer Hoffman
UTR 5 UCSC
UTR 3 UCSC
TSS Hoffman
Transcribed Hoffman
TFBS ENCODE
SuperEnhancer Hnisz
**Repressed Hoffman
Promoter UCSC
PromoterFlanking Hoffman
**Intron UCSC
H3K9ac Trynka
H3K4me3 Trynka
H3K4me1 Trynka
H3K27ac PGC2
H3K27ac Hnisz
FetalDHS Trynka
**Enhancer Hoffman
Enhancer Andersson
DHS Trynka
DGF ENCODE
CTCF Hoffman
**Conserved LindbladToh
**Coding UCSC
Annotation
−20
−10
0
10
20
Enrichment (prop h2 / prop snps)

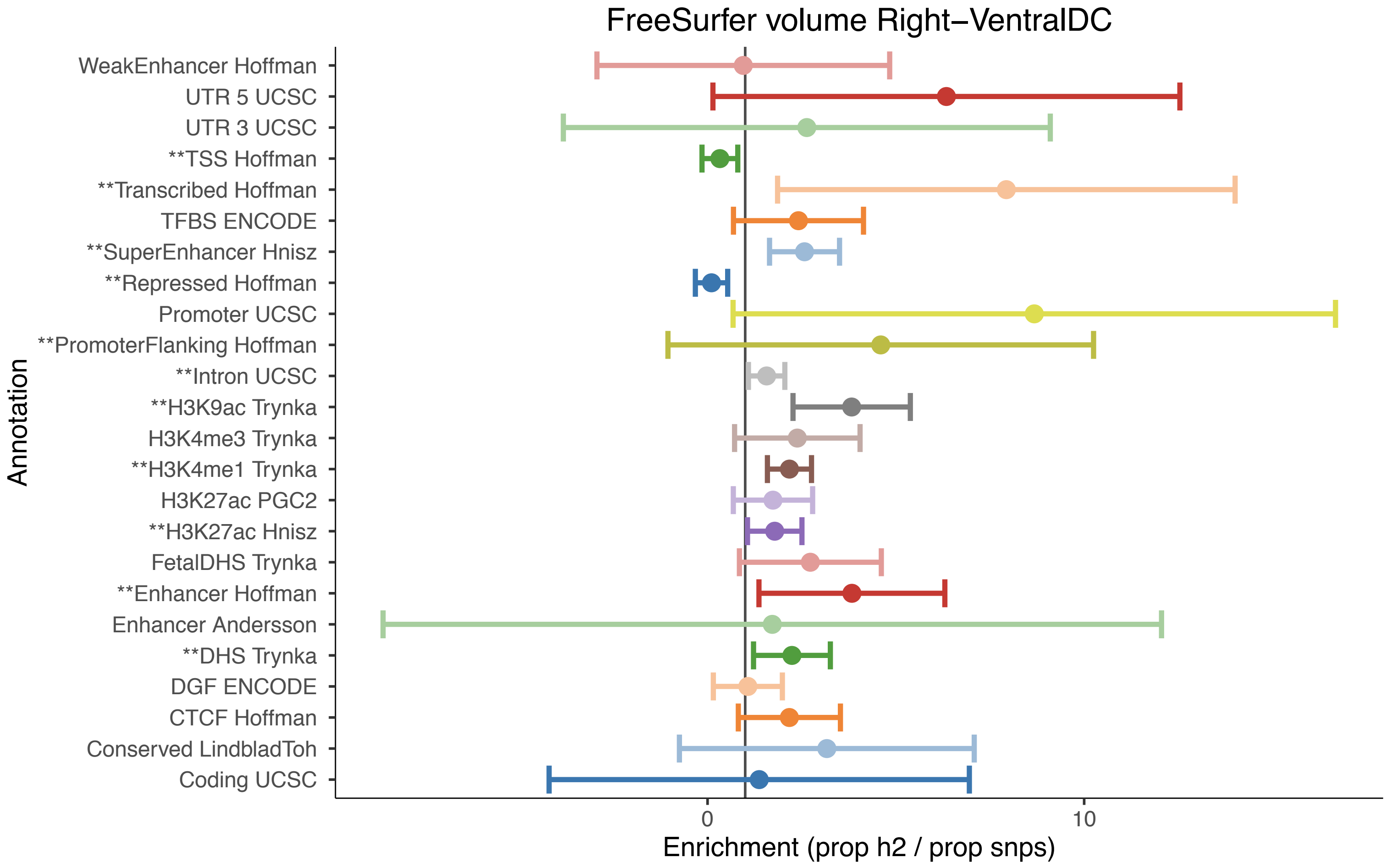
FreeSurfer volume Right−VentralDC
WeakEnhancer Hoffman
UTR 5 UCSC
UTR 3 UCSC
**TSS Hoffman
**Transcribed Hoffman
TFBS ENCODE
**SuperEnhancer Hnisz
**Repressed Hoffman
Promoter UCSC
**PromoterFlanking Hoffman
**Intron UCSC
**H3K9ac Trynka
H3K4me3 Trynka
**H3K4me1 Trynka
H3K27ac PGC2
**H3K27ac Hnisz
FetalDHS Trynka
**Enhancer Hoffman
Enhancer Andersson
**DHS Trynka
DGF ENCODE
CTCF Hoffman
Conserved LindbladToh
Coding UCSC
Annotation
0
10
Enrichment (prop h2 / prop snps)

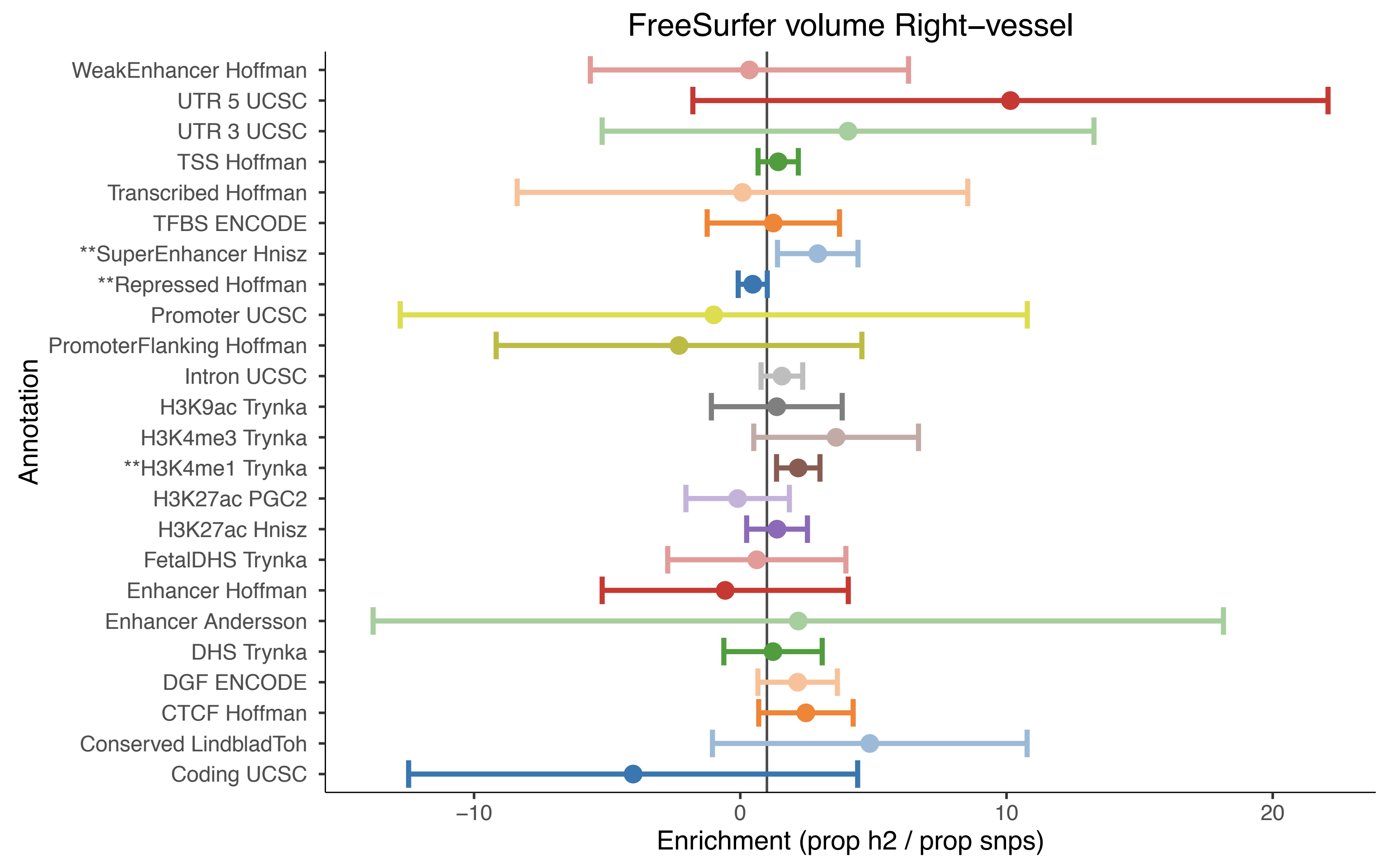

FreeSurfer volume Right–vessel
WeakEnhancer Hoffman
UTR 5 UCSC
UTR 3 UCSC
TSS Hoffman
Transcribed Hoffman
TFBS ENCODE
**SuperEnhancer Hnisz
**Repressed Hoffman
Promoter UCSC
PromoterFlanking Hoffman
Intron UCSC
H3K9ac Trynka
H3K4me3 Trynka
**H3K4me1 Trynka
H3K27ac PGC2
H3K27ac Hnisz
FetalDHS Trynka
Enhancer Hoffman
Enhancer Andersson
DHS Trynka
DGF ENCODE
CTCF Hoffman
Conserved LindbladToh
Coding UCSC
Annotation
−10
0
10
20
Enrichment (prop h2 / prop snps)

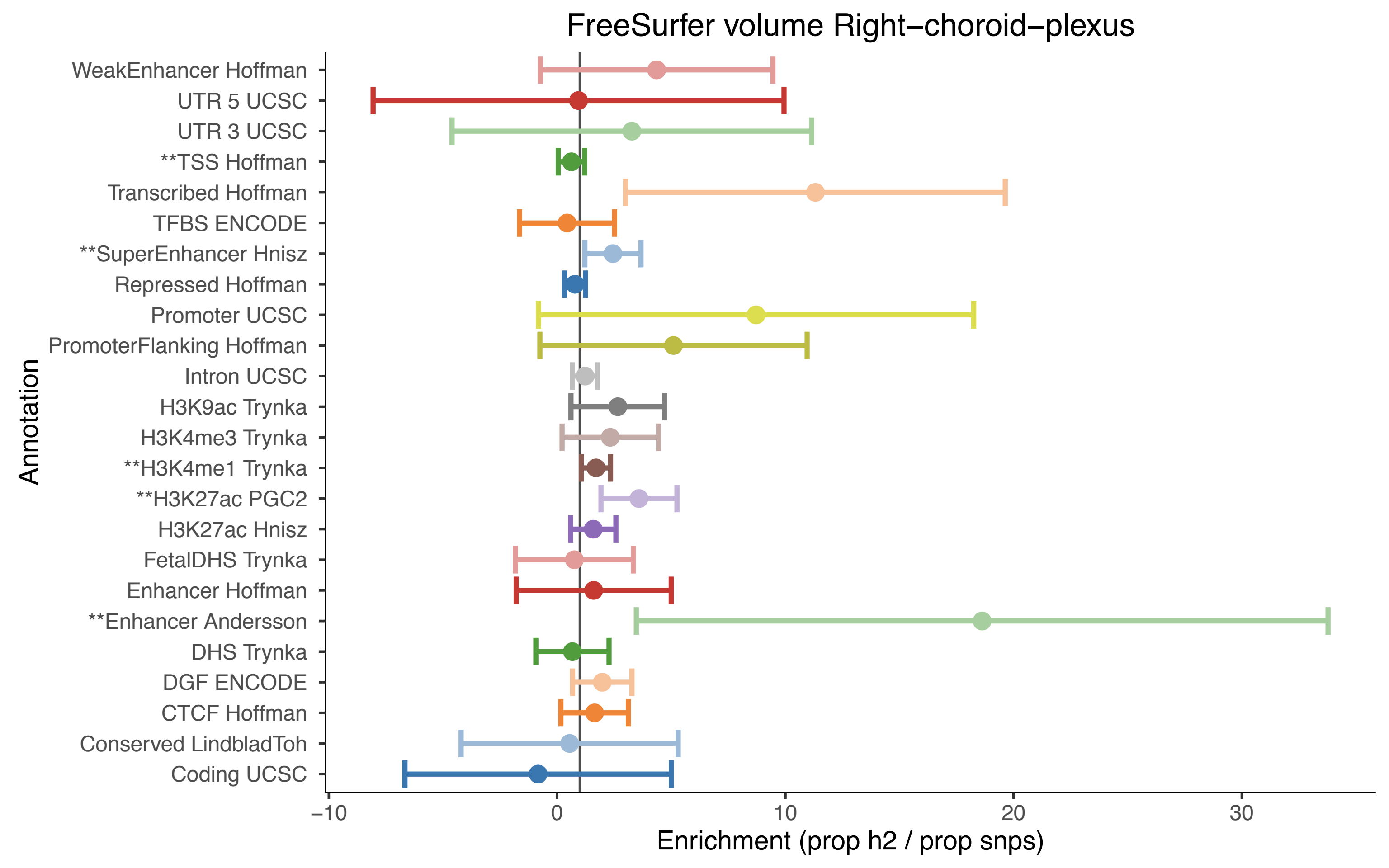

FreeSurfer volume Right−choroid−plexus
WeakEnhancer Hoffman
UTR 5 UCSC
UTR 3 UCSC
**TSS Hoffman
Transcribed Hoffman
TFBS ENCODE
**SuperEnhancer Hnisz
Repressed Hoffman
Promoter UCSC
PromoterFlanking Hoffman
Intron UCSC
H3K9ac Trynka
H3K4me3 Trynka
**H3K4me1 Trynka
**H3K27ac PGC2
H3K27ac Hnisz
FetalDHS Trynka
Enhancer Hoffman
**Enhancer Andersson
DHS Trynka
DGF ENCODE
CTCF Hoffman
Conserved LindbladToh
Coding UCSC
Annotation
−10
0
10
20
30
Enrichment (prop h2 / prop snps)

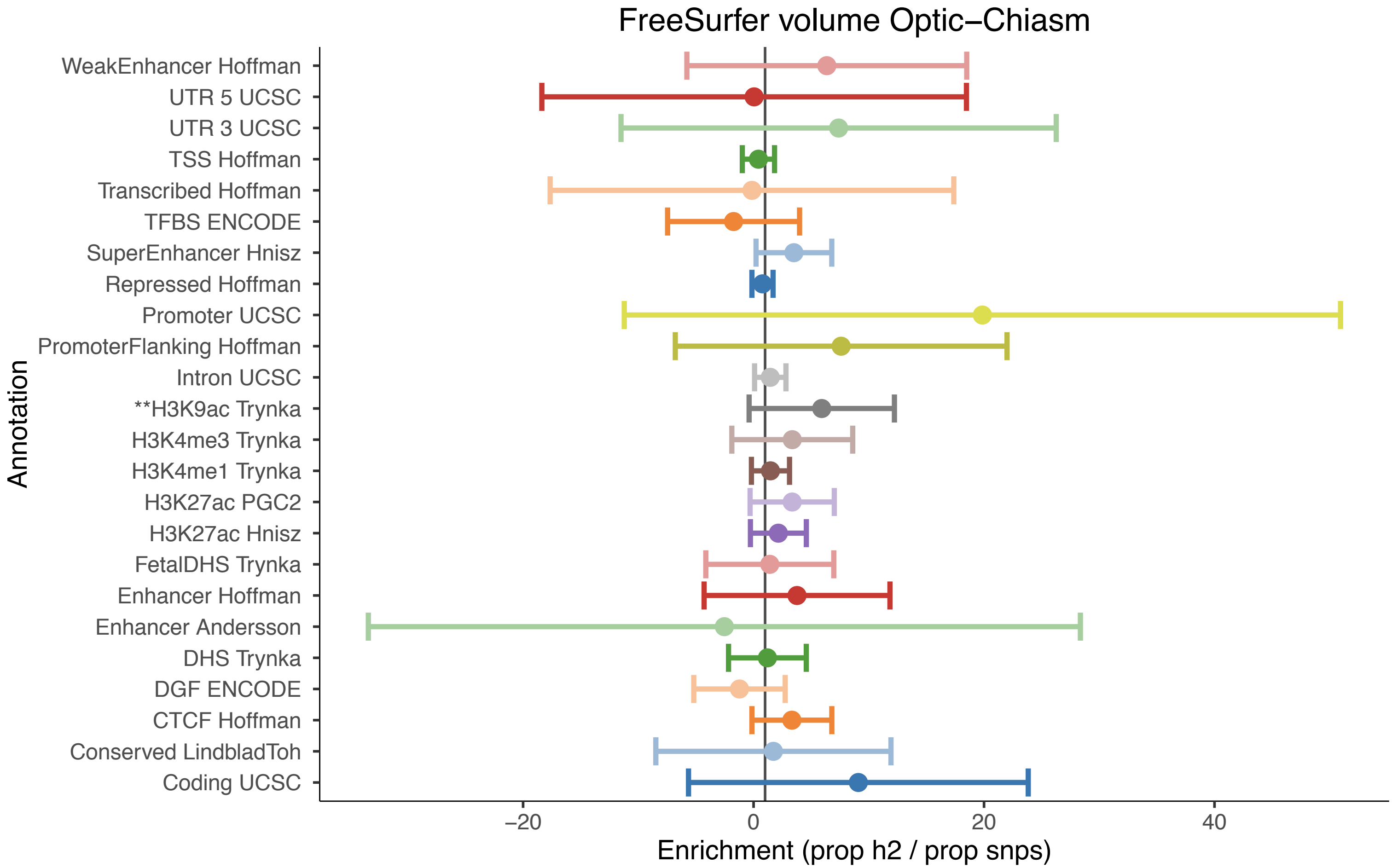

FreeSurfer volume Optic−Chiasm
Annotation
WeakEnhancer Hoffman
UTR 5 UCSC
UTR 3 UCSC
TSS Hoffman
Transcribed Hoffman
TFBS ENCODE
SuperEnhancer Hnisz
Repressed Hoffman
Promoter UCSC
PromoterFlanking Hoffman
Intron UCSC
**H3K9ac Trynka
H3K4me3 Trynka
H3K4me1 Trynka
H3K27ac PGC2
H3K27ac Hnisz
FetalDHS Trynka
Enhancer Hoffman
Enhancer Andersson
DHS Trynka
DGF ENCODE
CTCF Hoffman
Conserved LindbladToh
Coding UCSC
−20
0
20
40
Enrichment (prop h2 / prop snps)

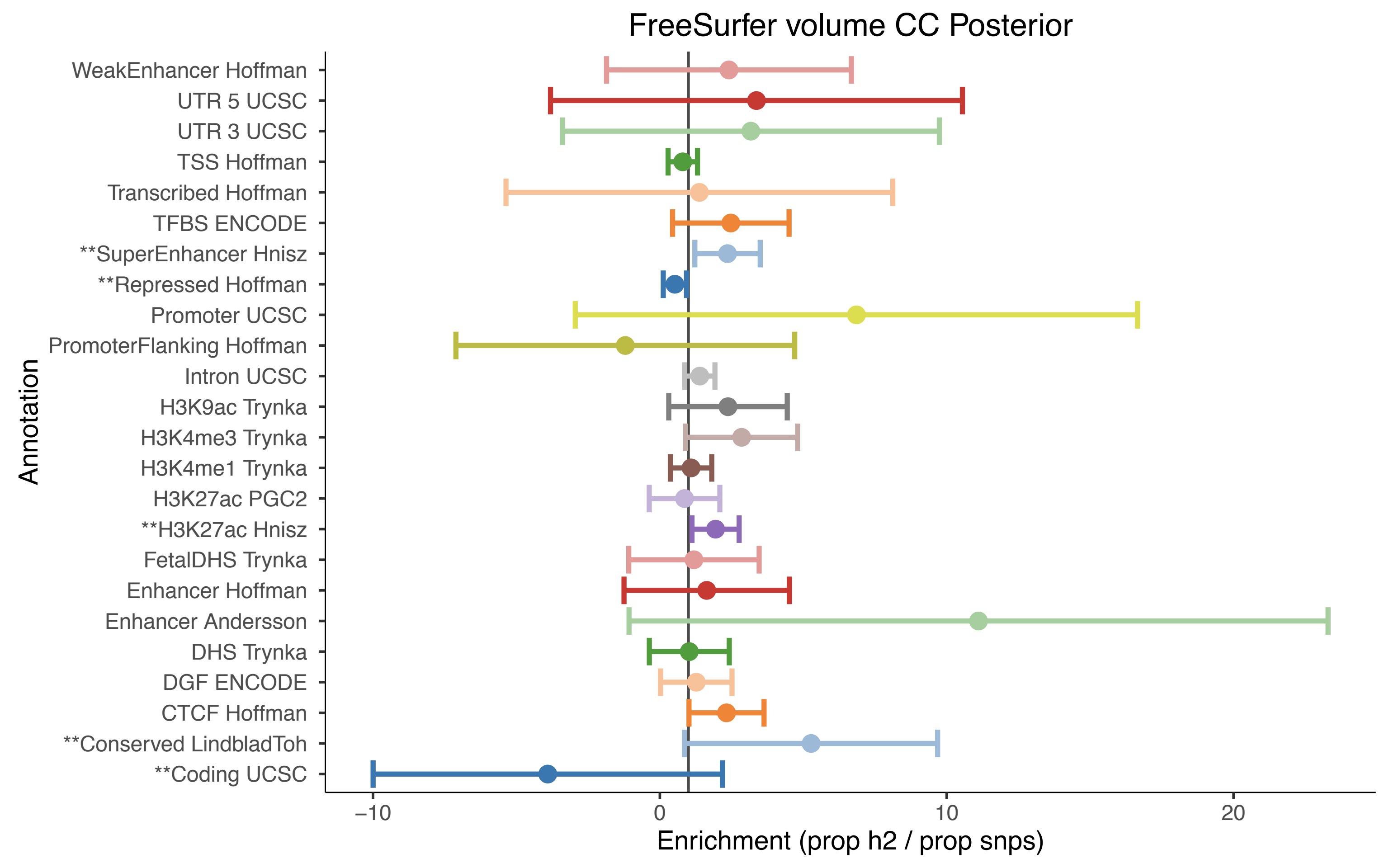

FreeSurfer volume CC Posterior
WeakEnhancer Hoffman
UTR 5 UCSC
UTR 3 UCSC
TSS Hoffman
Transcribed Hoffman
TFBS ENCODE
**SuperEnhancer Hnisz
**Repressed Hoffman
Promoter UCSC
PromoterFlanking Hoffman
Intron UCSC
H3K9ac Trynka
H3K4me3 Trynka
H3K4me1 Trynka
H3K27ac PGC2
**H3K27ac Hnisz
FetalDHS Trynka
Enhancer Hoffman
Enhancer Andersson
DHS Trynka
DGF ENCODE
CTCF Hoffman
**Conserved LindbladToh
**Coding UCSC
Annotation
−10
0
10
20
Enrichment (prop h2 / prop snps)

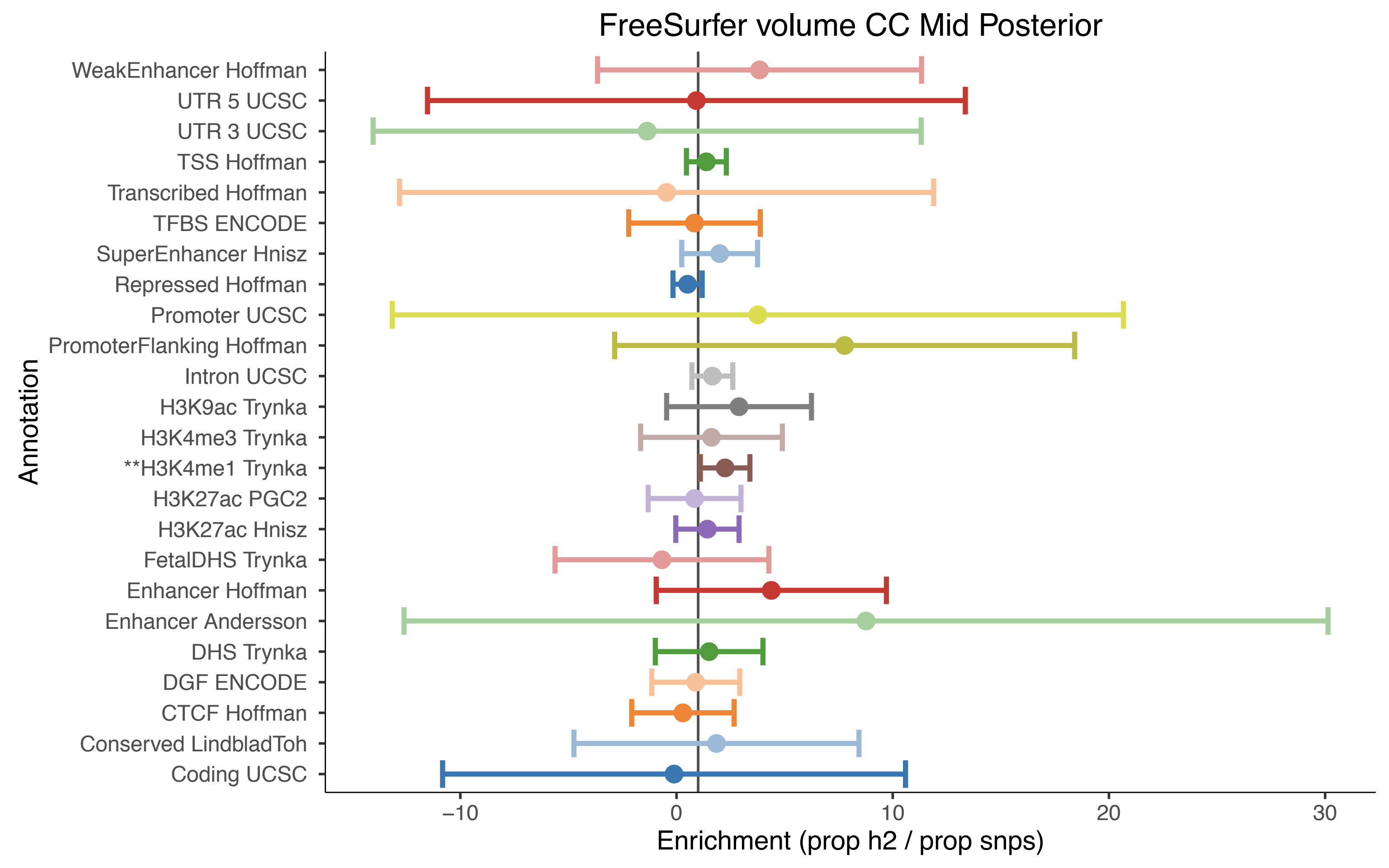
FreeSurfer volume CC Mid Posterior
WeakEnhancer Hoffman
UTR 5 UCSC
UTR 3 UCSC
TSS Hoffman
Transcribed Hoffman
TFBS ENCODE
SuperEnhancer Hnisz
Repressed Hoffman
Promoter UCSC
PromoterFlanking Hoffman
Intron UCSC
H3K9ac Trynka
H3K4me3 Trynka
**H3K4me1 Trynka
H3K27ac PGC2
H3K27ac Hnisz
FetalDHS Trynka
Enhancer Hoffman
Enhancer Andersson
DHS Trynka
DGF ENCODE
CTCF Hoffman
Conserved LindbladToh
Coding UCSC
Annotation
−10
0
10
20
30
Enrichment (prop h2 / prop snps)

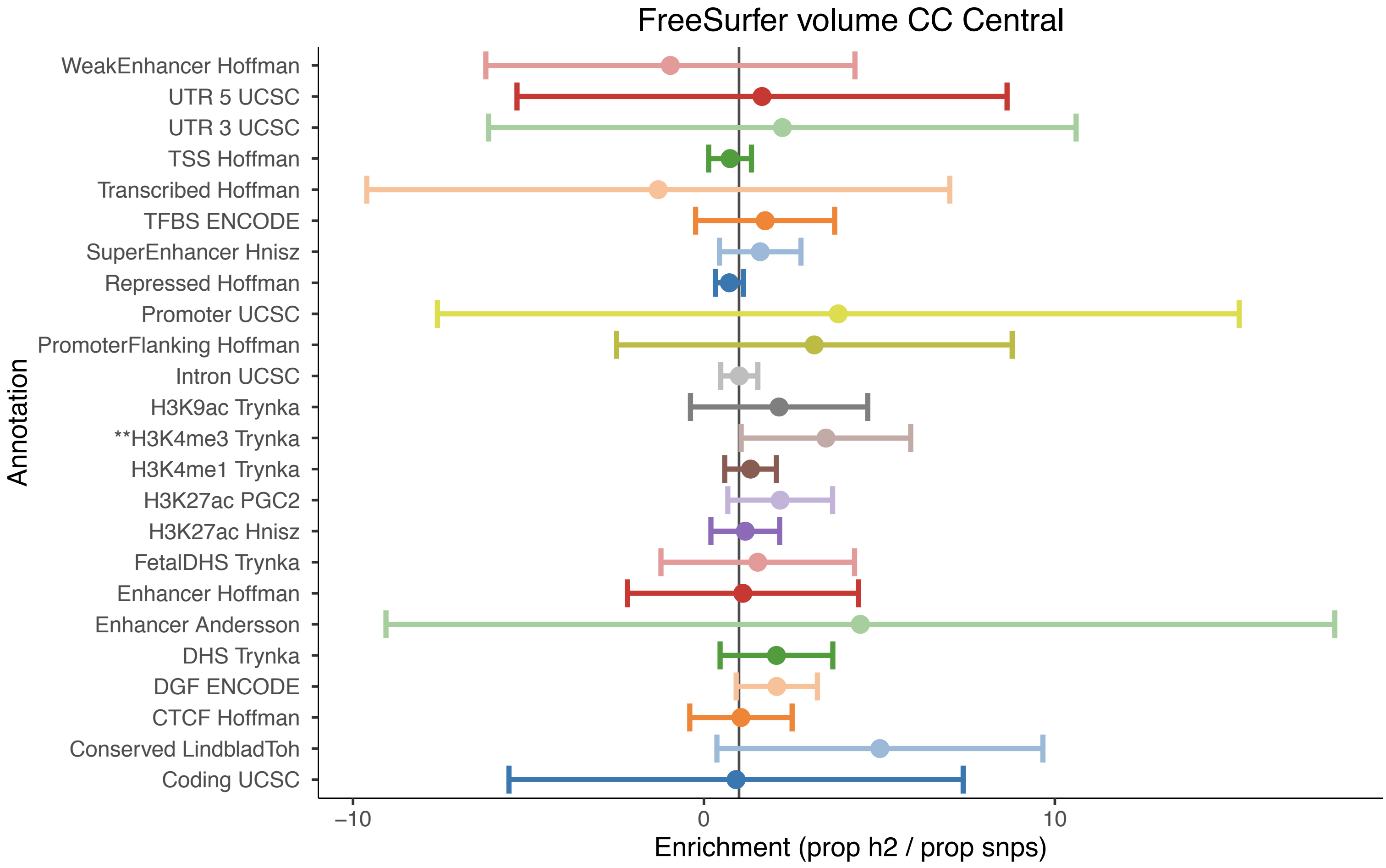
FreeSurfer volume CC Central
WeakEnhancer Hoffman
UTR 5 UCSC
UTR 3 UCSC
TSS Hoffman
Transcribed Hoffman
TFBS ENCODE
SuperEnhancer Hnisz
Repressed Hoffman
Promoter UCSC
PromoterFlanking Hoffman
Intron UCSC
H3K9ac Trynka
**H3K4me3 Trynka
H3K4me1 Trynka
H3K27ac PGC2
H3K27ac Hnisz
FetalDHS Trynka
Enhancer Hoffman
Enhancer Andersson
DHS Trynka
DGF ENCODE
CTCF Hoffman
Conserved LindbladToh
Coding UCSC
Annotation
−10
0
10
Enrichment (prop h2 / prop snps)

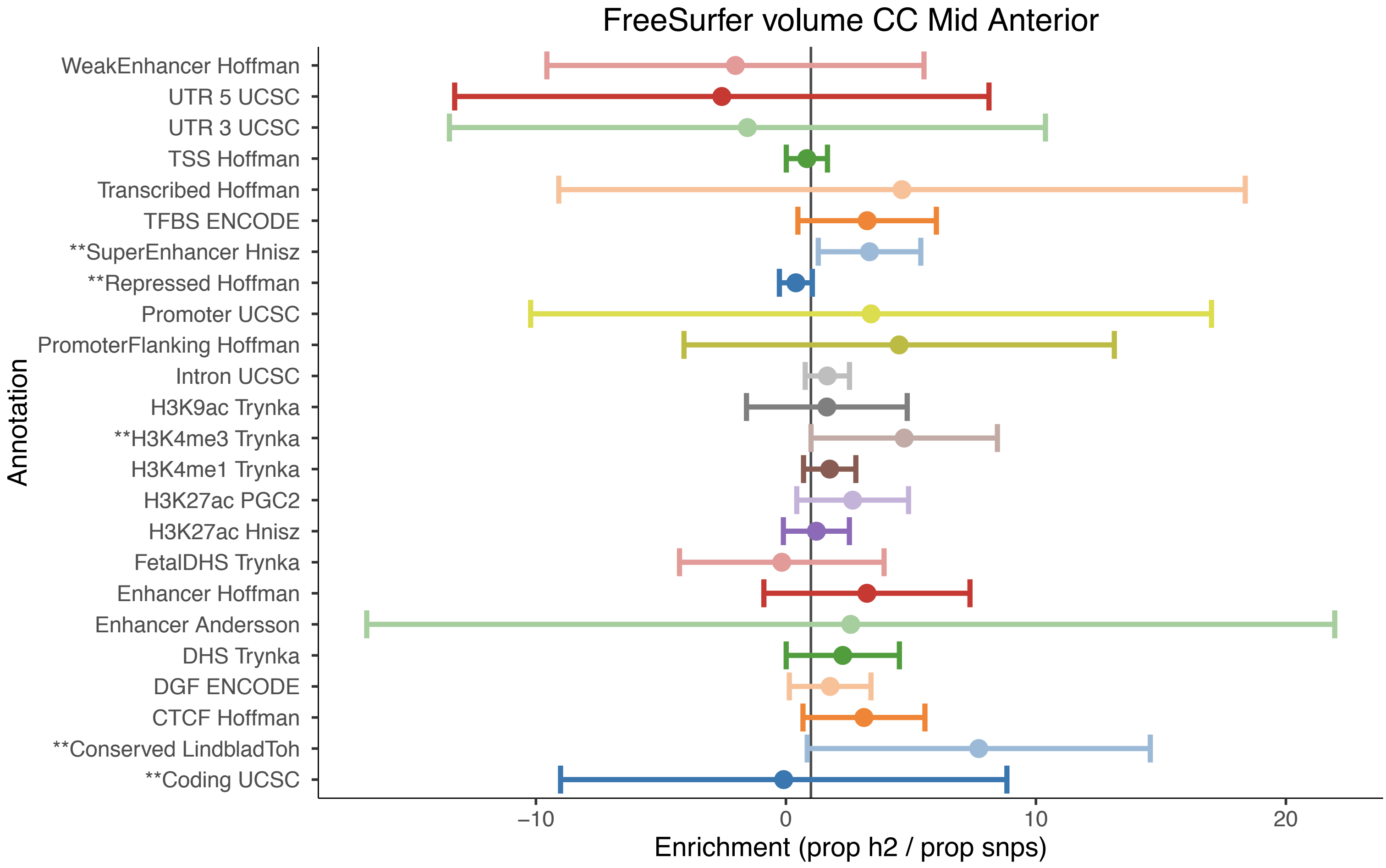
FreeSurfer volume CC Mid Anterior
WeakEnhancer Hoffman
UTR 5 UCSC
UTR 3 UCSC
TSS Hoffman
Transcribed Hoffman
TFBS ENCODE
**SuperEnhancer Hnisz
**Repressed Hoffman
Promoter UCSC
PromoterFlanking Hoffman
Intron UCSC
H3K9ac Trynka
**H3K4me3 Trynka
H3K4me1 Trynka
H3K27ac PGC2
H3K27ac Hnisz
FetalDHS Trynka
Enhancer Hoffman
Enhancer Andersson
DHS Trynka
DGF ENCODE
CTCF Hoffman
**Conserved LindbladToh
**Coding UCSC
Annotation
−10
0
10
20
Enrichment (prop h2 / prop snps)

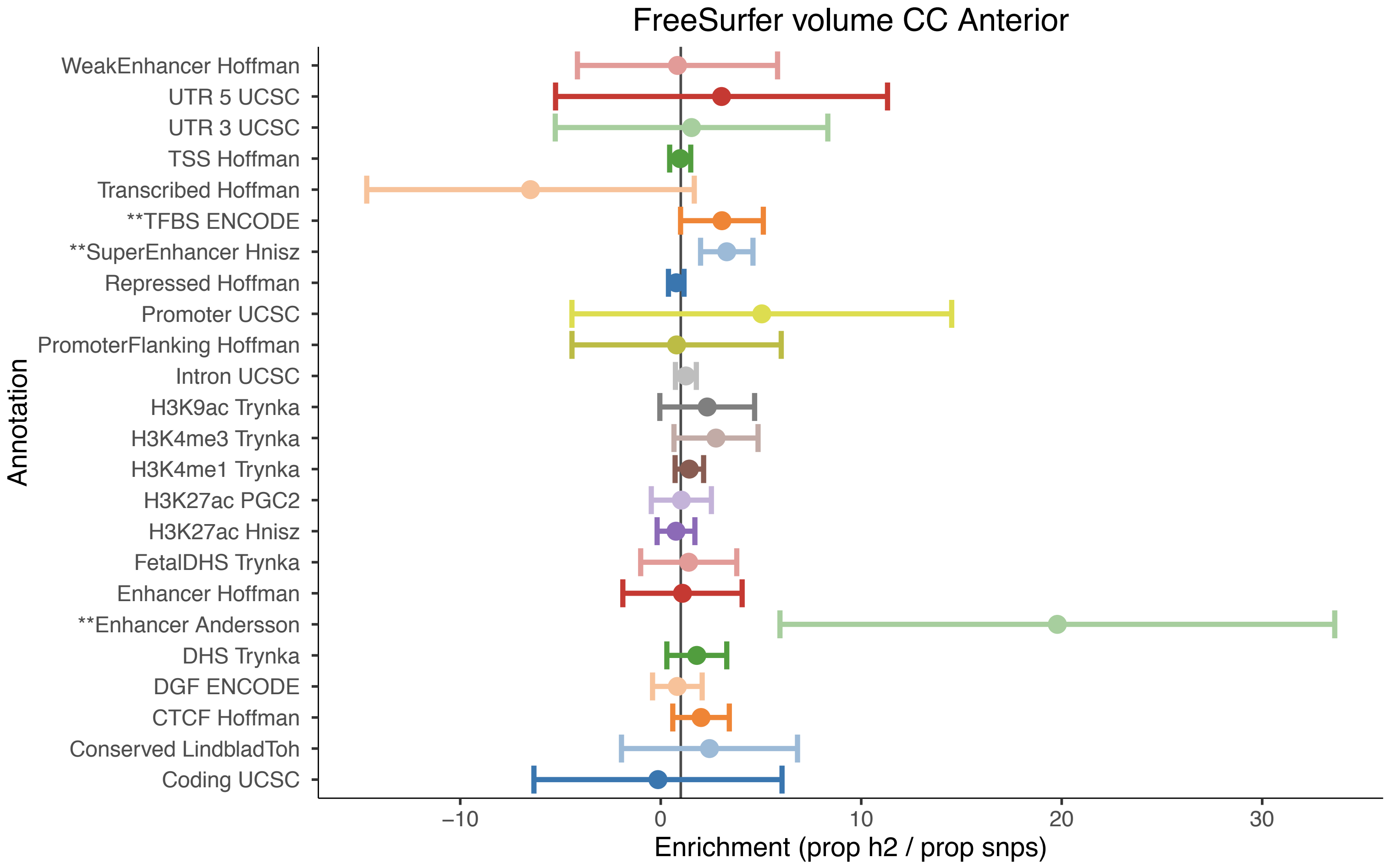

FreeSurfer volume CC Anterior
WeakEnhancer Hoffman
UTR 5 UCSC
UTR 3 UCSC
TSS Hoffman
Transcribed Hoffman
**TFBS ENCODE
**SuperEnhancer Hnisz
Repressed Hoffman
Promoter UCSC
PromoterFlanking Hoffman
Intron UCSC
H3K9ac Trynka
H3K4me3 Trynka
H3K4me1 Trynka
H3K27ac PGC2
H3K27ac Hnisz
FetalDHS Trynka
Enhancer Hoffman
**Enhancer Andersson
DHS Trynka
DGF ENCODE
CTCF Hoffman
Conserved LindbladToh
Coding UCSC
Annotation
−10
0
10
20
30
Enrichment (prop h2 / prop snps)

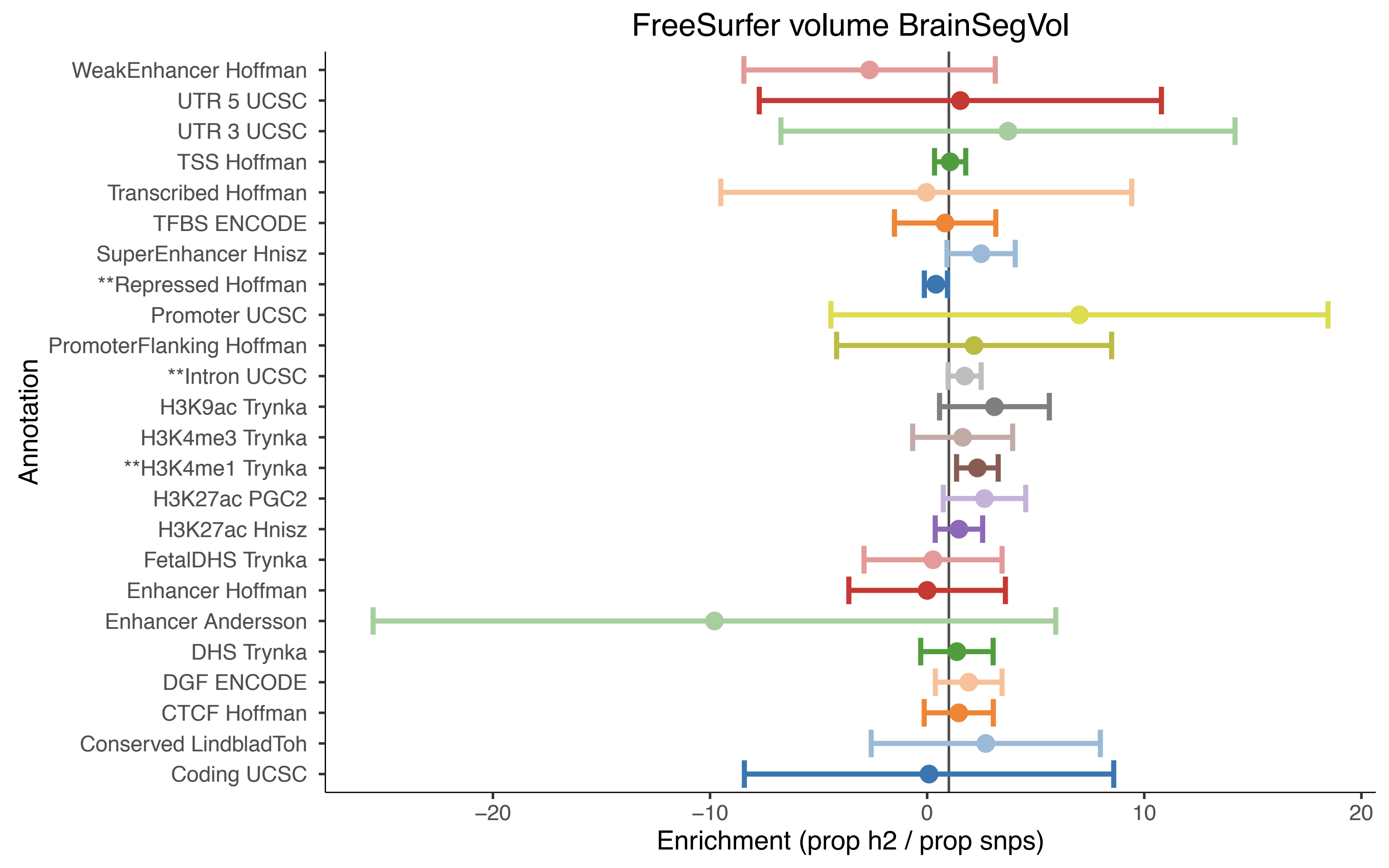
FreeSurfer volume BrainSegVol
WeakEnhancer Hoffman
UTR 5 UCSC
UTR 3 UCSC
TSS Hoffman
Transcribed Hoffman
TFBS ENCODE
SuperEnhancer Hnisz
**Repressed Hoffman
Promoter UCSC
PromoterFlanking Hoffman
**Intron UCSC
H3K9ac Trynka
H3K4me3 Trynka
**H3K4me1 Trynka
H3K27ac PGC2
H3K27ac Hnisz
FetalDHS Trynka
Enhancer Hoffman
Enhancer Andersson
DHS Trynka
DGF ENCODE
CTCF Hoffman
Conserved LindbladToh
Coding UCSC
Annotation
−20
−10
0
10
20
Enrichment (prop h2 / prop snps)

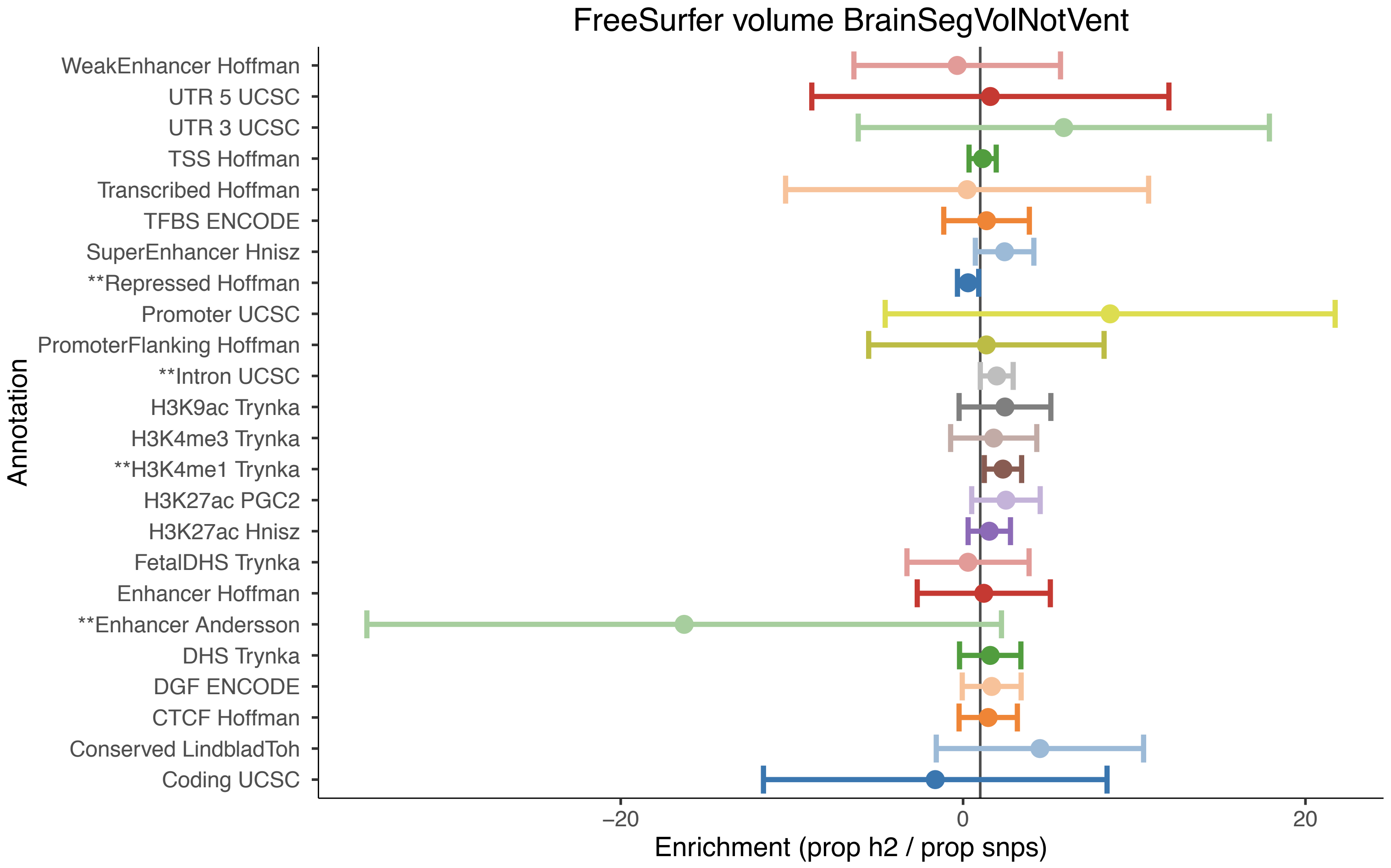

FreeSurfer volume BrainSegVolNotVent
Annotation
WeakEnhancer Hoffman
UTR 5 UCSC
UTR 3 UCSC
TSS Hoffman
Transcribed Hoffman
TFBS ENCODE
SuperEnhancer Hnisz
**Repressed Hoffman
Promoter UCSC
PromoterFlanking Hoffman
**Intron UCSC
H3K9ac Trynka
H3K4me3 Trynka
**H3K4me1 Trynka
H3K27ac PGC2
H3K27ac Hnisz
FetalDHS Trynka
Enhancer Hoffman
**Enhancer Andersson
DHS Trynka
DGF ENCODE
CTCF Hoffman
Conserved LindbladToh
Coding UCSC
−20
0
20
Enrichment (prop h2 / prop snps)

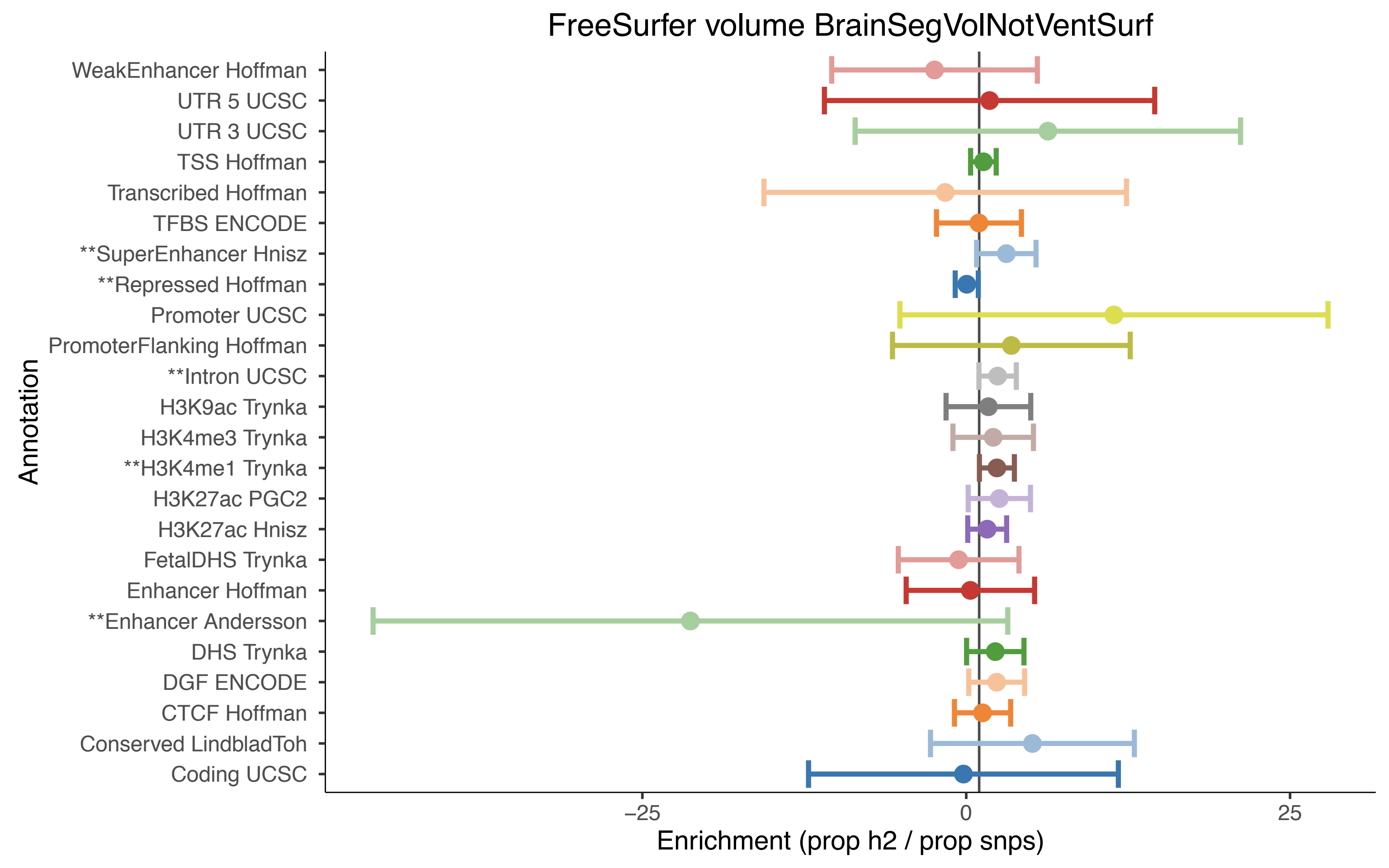
FreeSurfer volume BrainSegVolNotVentSurf
WeakEnhancer Hoffman
UTR 5 UCSC
UTR 3 UCSC
TSS Hoffman
Transcribed Hoffman
TFBS ENCODE
**SuperEnhancer Hnisz
**Repressed Hoffman
Promoter UCSC
PromoterFlanking Hoffman
**Intron UCSC
H3K9ac Trynka
H3K4me3 Trynka
**H3K4me1 Trynka
H3K27ac PGC2
H3K27ac Hnisz
FetalDHS Trynka
Enhancer Hoffman
**Enhancer Andersson
DHS Trynka
DGF ENCODE
CTCF Hoffman
Conserved LindbladToh
Coding UCSC
Annotation
−25
0
25
Enrichment (prop h2 / prop snps)

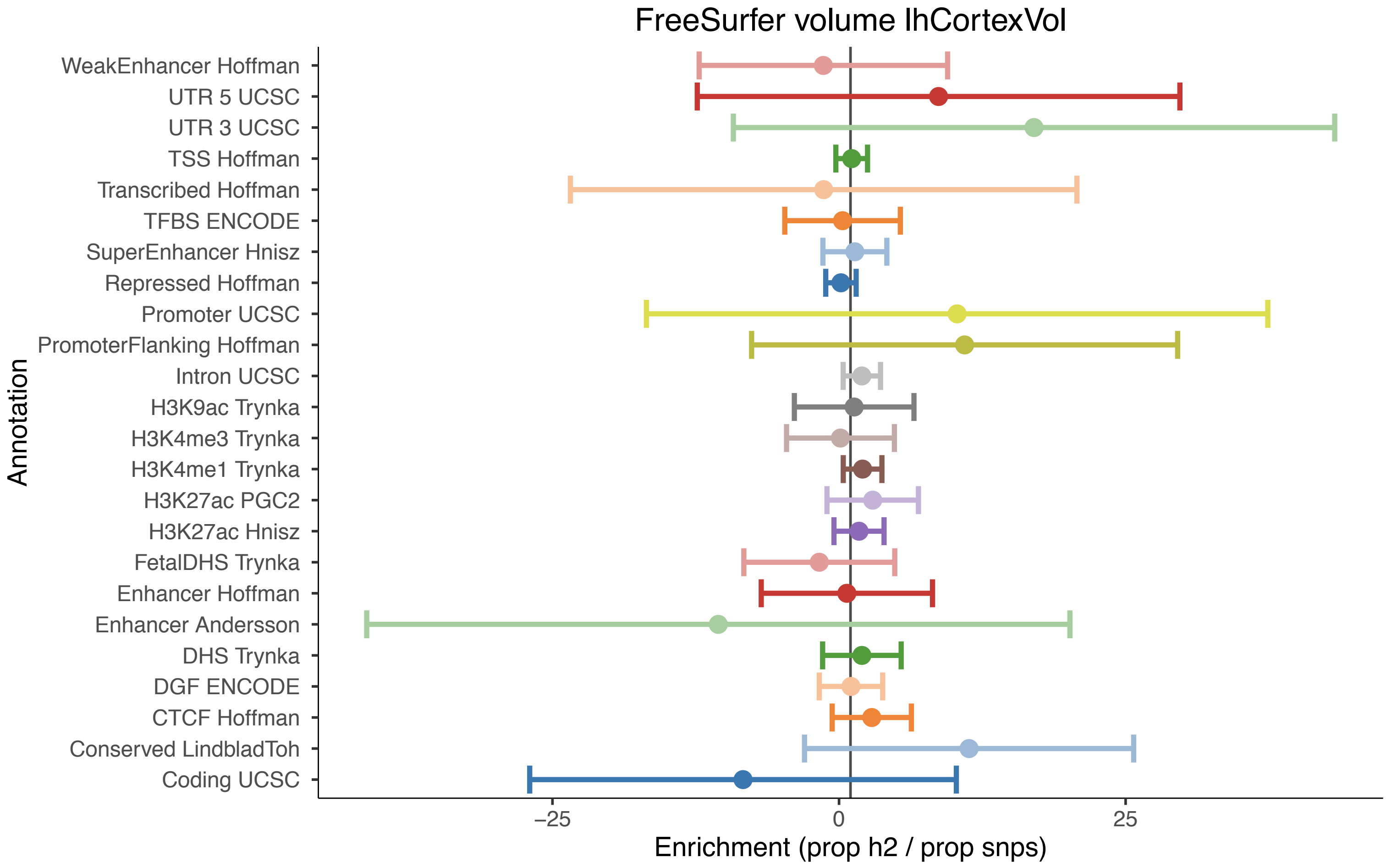

FreeSurfer volume lhCortexVol
WeakEnhancer Hoffman
UTR 5 UCSC
UTR 3 UCSC
TSS Hoffman
Transcribed Hoffman
TFBS ENCODE
SuperEnhancer Hnisz
Repressed Hoffman
Promoter UCSC
PromoterFlanking Hoffman
Intron UCSC
H3K9ac Trynka
H3K4me3 Trynka
H3K4me1 Trynka
H3K27ac PGC2
H3K27ac Hnisz
FetalDHS Trynka
Enhancer Hoffman
Enhancer Andersson
DHS Trynka
DGF ENCODE
CTCF Hoffman
Conserved LindbladToh
Coding UCSC
Annotation
−25
0
25
Enrichment (prop h2 / prop snps)

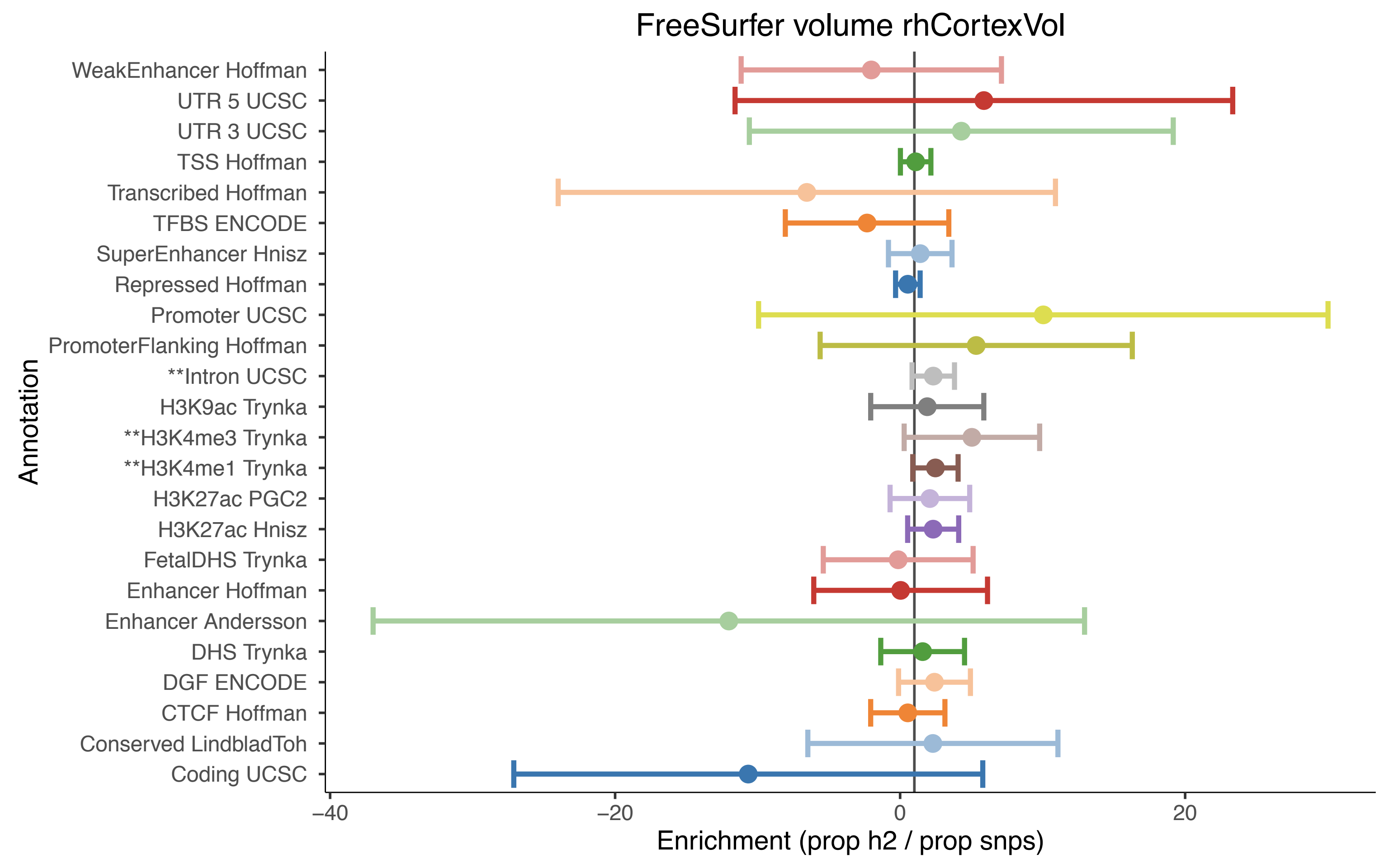
FreeSurfer volume rhCortexVol
Annotation
WeakEnhancer Hoffman
UTR 5 UCSC
UTR 3 UCSC
TSS Hoffman
Transcribed Hoffman
TFBS ENCODE
SuperEnhancer Hnisz
Repressed Hoffman
Promoter UCSC
PromoterFlanking Hoffman
**Intron UCSC
H3K9ac Trynka
**H3K4me3 Trynka
**H3K4me1 Trynka
H3K27ac PGC2
H3K27ac Hnisz
FetalDHS Trynka
Enhancer Hoffman
Enhancer Andersson
DHS Trynka
DGF ENCODE
CTCF Hoffman
Conserved LindbladToh
Coding UCSC
−40
−20
0
20
Enrichment (prop h2 / prop snps)

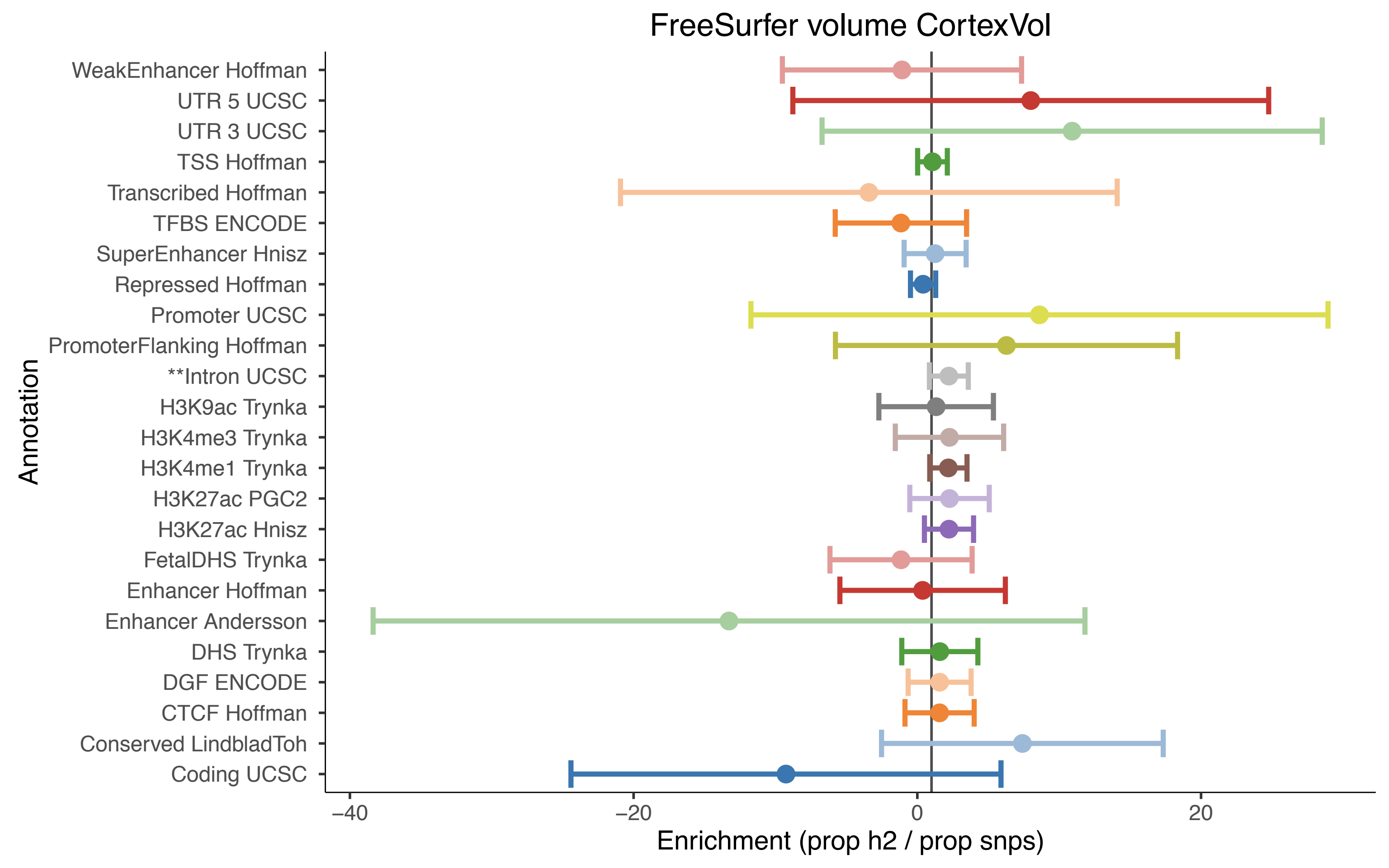
FreeSurfer volume CortexVol
Annotation
WeakEnhancer Hoffman
UTR 5 UCSC
UTR 3 UCSC
TSS Hoffman
Transcribed Hoffman
TFBS ENCODE
SuperEnhancer Hnisz
Repressed Hoffman
Promoter UCSC
PromoterFlanking Hoffman
**Intron UCSC
H3K9ac Trynka
H3K4me3 Trynka
H3K4me1 Trynka
H3K27ac PGC2
H3K27ac Hnisz
FetalDHS Trynka
Enhancer Hoffman
Enhancer Andersson
DHS Trynka
DGF ENCODE
CTCF Hoffman
Conserved LindbladToh
Coding UCSC
−40
−20
0
20
Enrichment (prop h2 / prop snps)

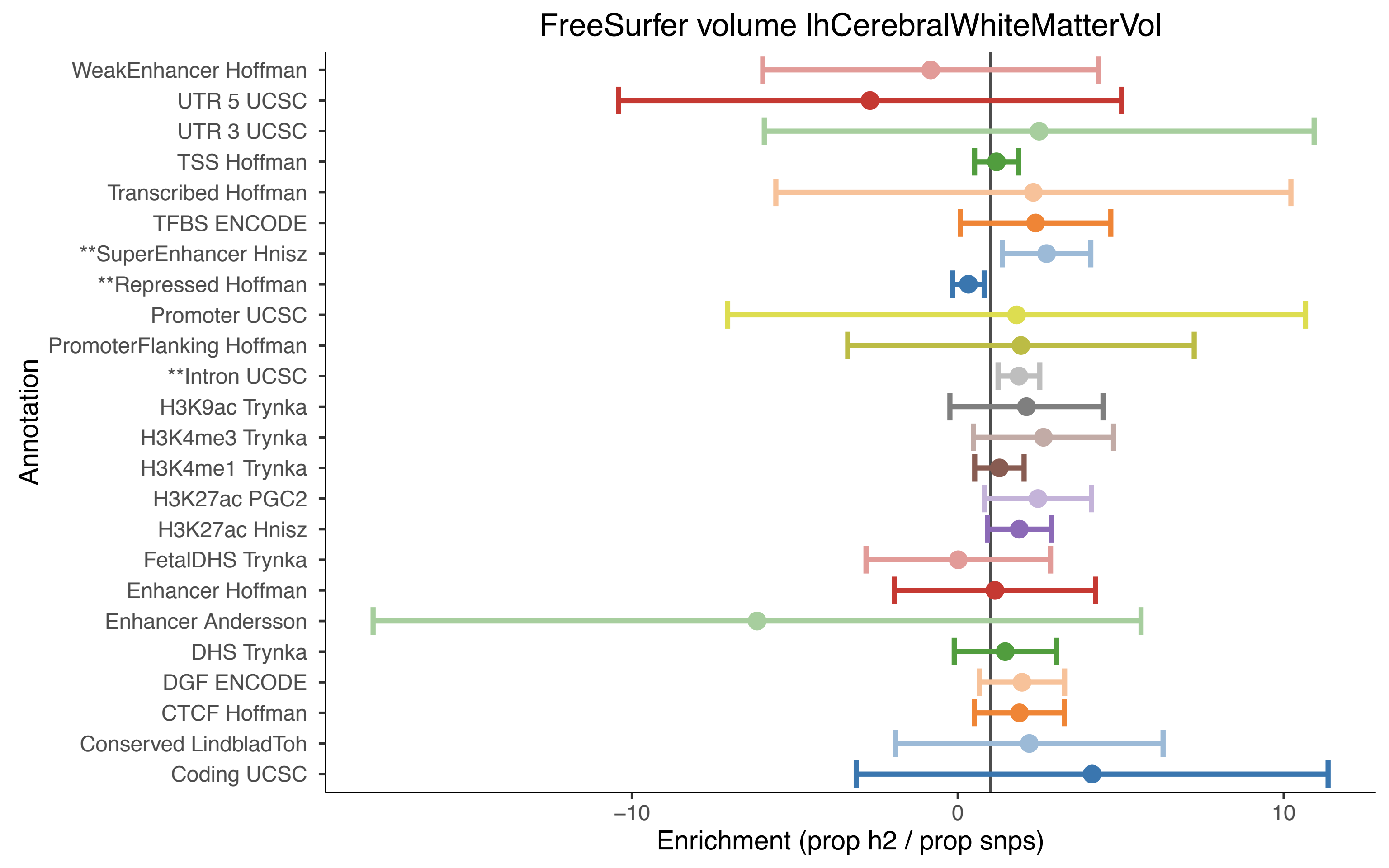
FreeSurfer volume lhCerebralWhiteMatterVol
WeakEnhancer Hoffman
UTR 5 UCSC
UTR 3 UCSC
TSS Hoffman
Transcribed Hoffman
TFBS ENCODE
**SuperEnhancer Hnisz
**Repressed Hoffman
Promoter UCSC
PromoterFlanking Hoffman
**Intron UCSC
H3K9ac Trynka
H3K4me3 Trynka
H3K4me1 Trynka
H3K27ac PGC2
H3K27ac Hnisz
FetalDHS Trynka
Enhancer Hoffman
Enhancer Andersson
DHS Trynka
DGF ENCODE
CTCF Hoffman
Conserved LindbladToh
Coding UCSC
Annotation
−10
0
10
Enrichment (prop h2 / prop snps)

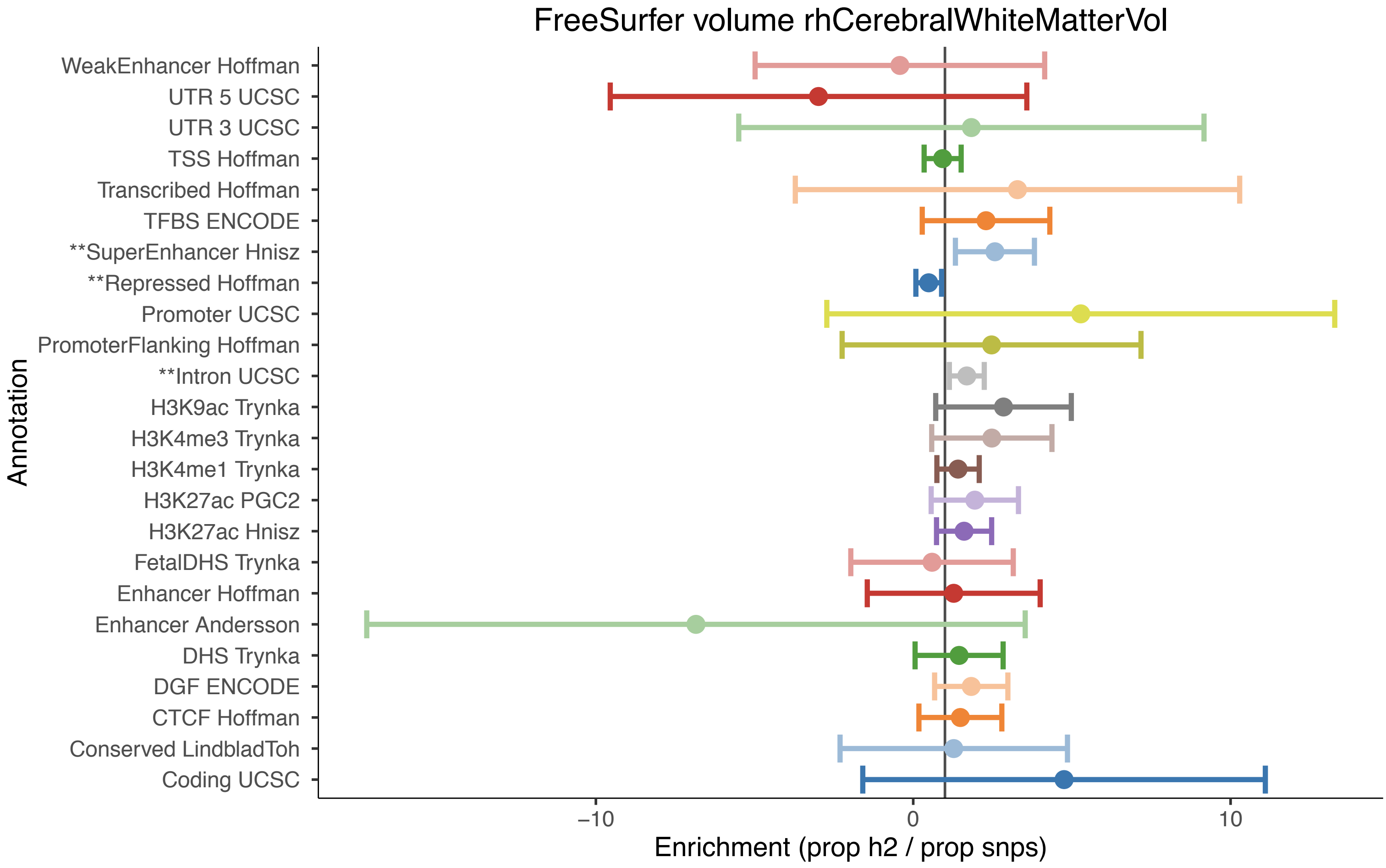

FreeSurfer volume rhCerebralWhiteMatterVol
WeakEnhancer Hoffman
UTR 5 UCSC
UTR 3 UCSC
TSS Hoffman
Transcribed Hoffman
TFBS ENCODE
**SuperEnhancer Hnisz
**Repressed Hoffman
Promoter UCSC
PromoterFlanking Hoffman
**Intron UCSC
H3K9ac Trynka
H3K4me3 Trynka
H3K4me1 Trynka
H3K27ac PGC2
H3K27ac Hnisz
FetalDHS Trynka
Enhancer Hoffman
Enhancer Andersson
DHS Trynka
DGF ENCODE
CTCF Hoffman
Conserved LindbladToh
Coding UCSC
Annotation
−10
0
10
Enrichment (prop h2 / prop snps)

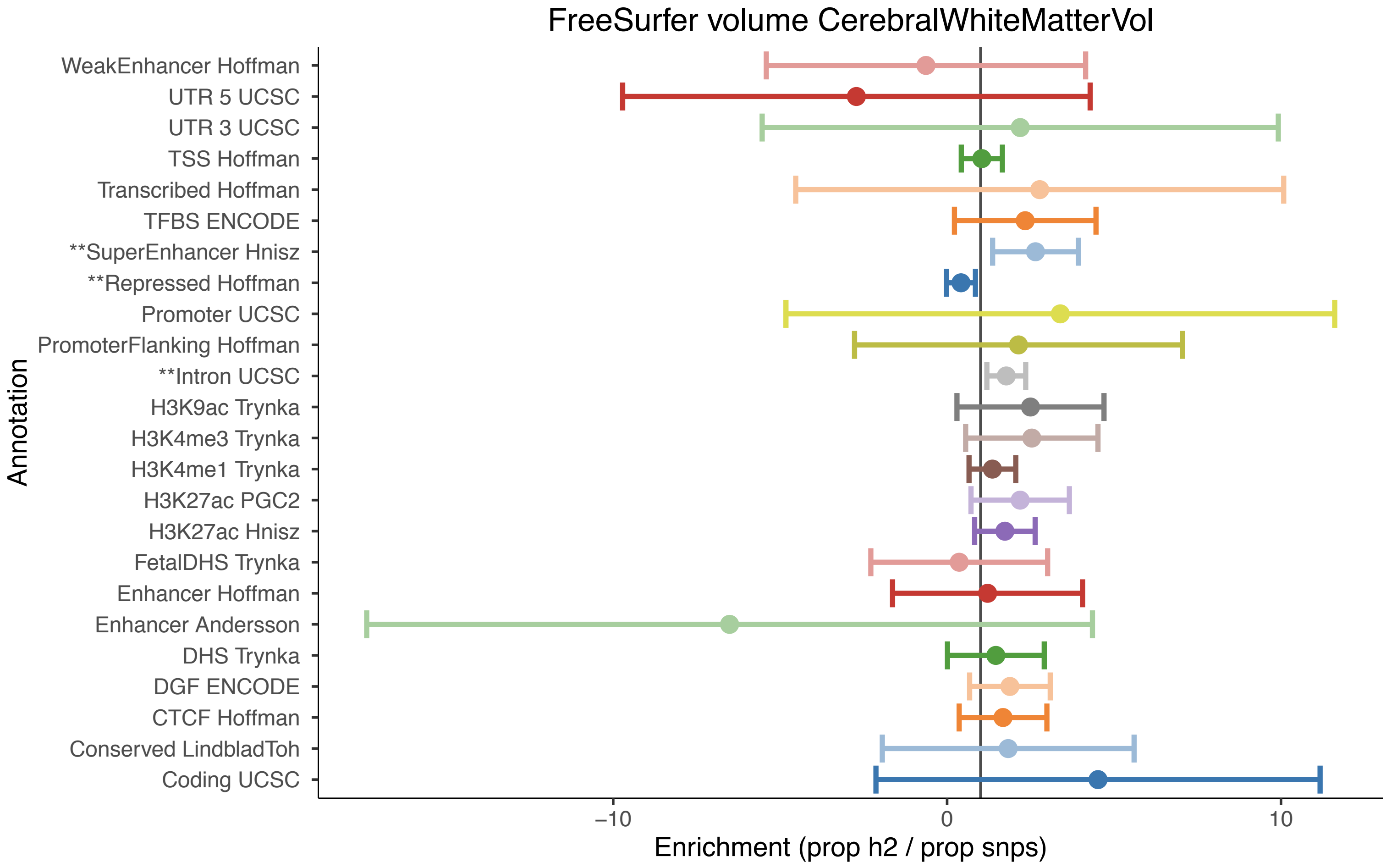
FreeSurfer volume CerebralWhiteMatterVol
WeakEnhancer Hoffman
UTR 5 UCSC
UTR 3 UCSC
TSS Hoffman
Transcribed Hoffman
TFBS ENCODE
**SuperEnhancer Hnisz
**Repressed Hoffman
Promoter UCSC
PromoterFlanking Hoffman
**Intron UCSC
H3K9ac Trynka
H3K4me3 Trynka
H3K4me1 Trynka
H3K27ac PGC2
H3K27ac Hnisz
FetalDHS Trynka
Enhancer Hoffman
Enhancer Andersson
DHS Trynka
DGF ENCODE
CTCF Hoffman
Conserved LindbladToh
Coding UCSC
Annotation
−10
0
10
Enrichment (prop h2 / prop snps)

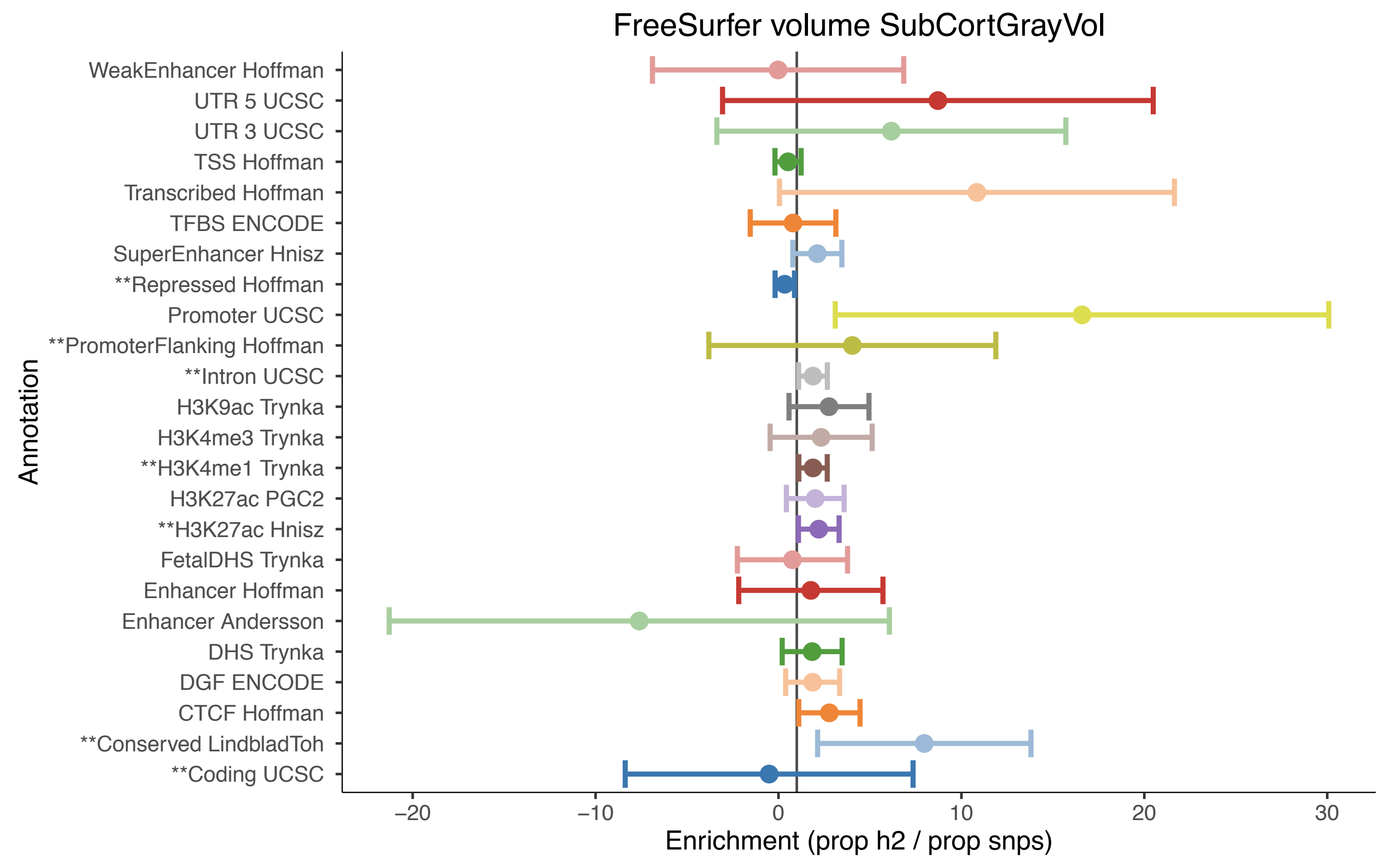

FreeSurfer volume SubCortGrayVol
Annotation
WeakEnhancer Hoffman
UTR 5 UCSC
UTR 3 UCSC
TSS Hoffman
Transcribed Hoffman
TFBS ENCODE
SuperEnhancer Hnisz
**Repressed Hoffman
Promoter UCSC
**PromoterFlanking Hoffman
**Intron UCSC
H3K9ac Trynka
H3K4me3 Trynka
**H3K4me1 Trynka
H3K27ac PGC2
**H3K27ac Hnisz
FetalDHS Trynka
Enhancer Hoffman
Enhancer Andersson
DHS Trynka
DGF ENCODE
CTCF Hoffman
**Conserved LindbladToh
**Coding UCSC
−20
−10
0
10
20
30
Enrichment (prop h2 / prop snps)

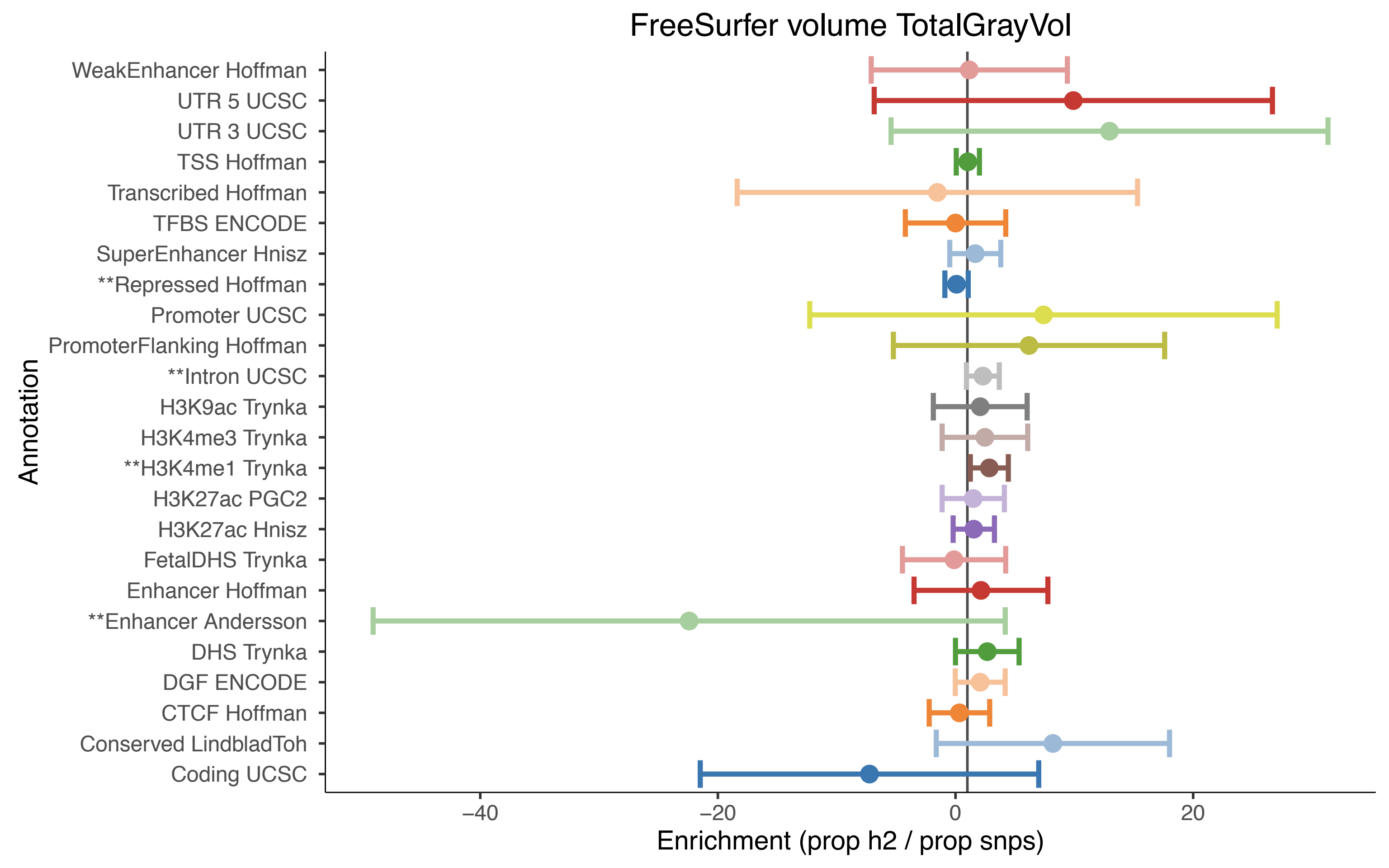
FreeSurfer volume TotalGrayVol
WeakEnhancer Hoffman
UTR 5 UCSC
UTR 3 UCSC
TSS Hoffman
Transcribed Hoffman
TFBS ENCODE
SuperEnhancer Hnisz
**Repressed Hoffman
Promoter UCSC
PromoterFlanking Hoffman
**Intron UCSC
H3K9ac Trynka
H3K4me3 Trynka
**H3K4me1 Trynka
H3K27ac PGC2
H3K27ac Hnisz
FetalDHS Trynka
Enhancer Hoffman
**Enhancer Andersson
DHS Trynka
DGF ENCODE
CTCF Hoffman
Conserved LindbladToh
Coding UCSC
Annotation
−40
−20
0
20
Enrichment (prop h2 / prop snps)

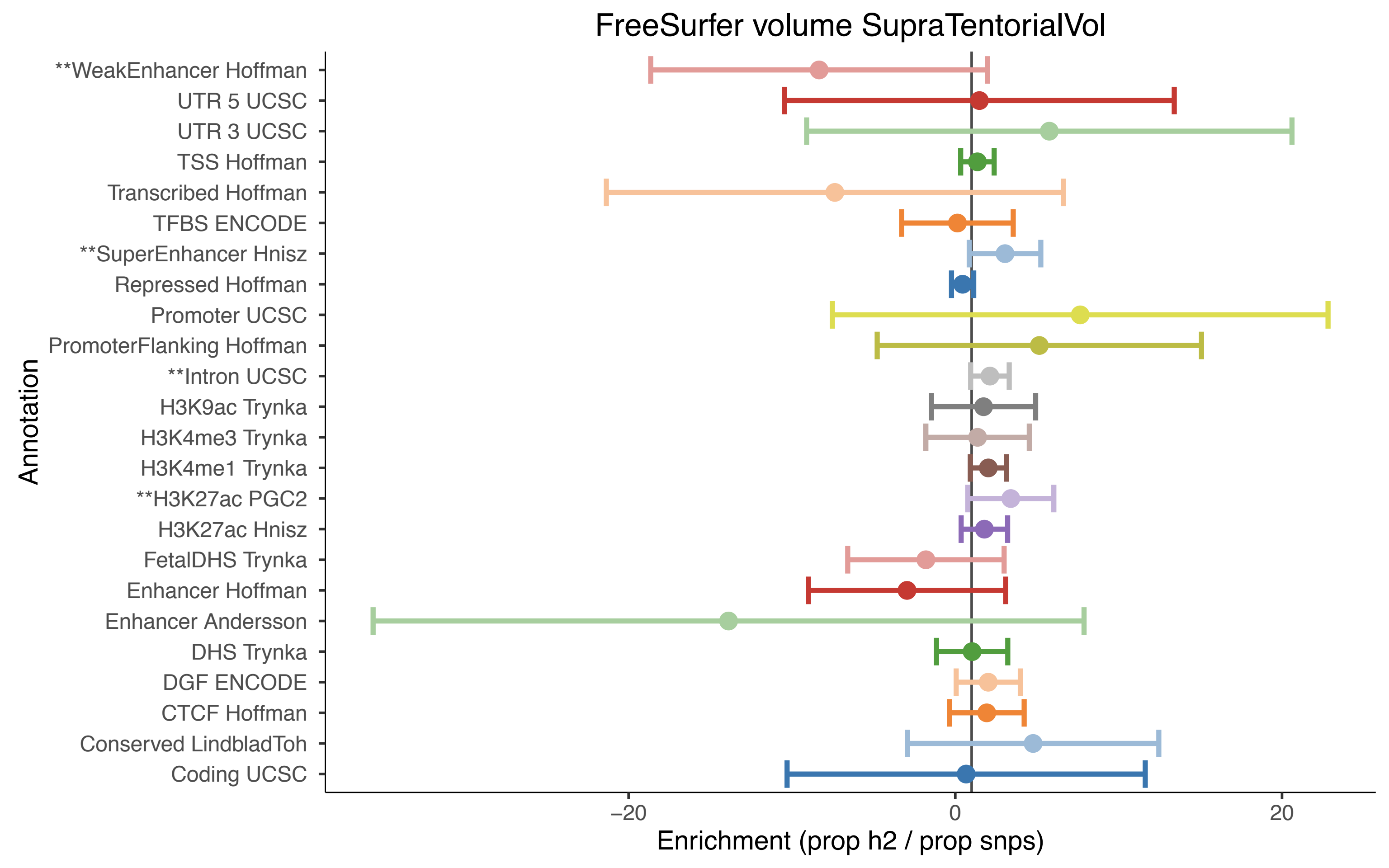
FreeSurfer volume SupraTentorialVol
**WeakEnhancer Hoffman
UTR 5 UCSC
UTR 3 UCSC
TSS Hoffman
Transcribed Hoffman
TFBS ENCODE
**SuperEnhancer Hnisz
Repressed Hoffman
Promoter UCSC
PromoterFlanking Hoffman
**Intron UCSC
H3K9ac Trynka
H3K4me3 Trynka
H3K4me1 Trynka
**H3K27ac PGC2
H3K27ac Hnisz
FetalDHS Trynka
Enhancer Hoffman
Enhancer Andersson
DHS Trynka
DGF ENCODE
CTCF Hoffman
Conserved LindbladToh
Coding UCSC
Annotation
−20
0
20
Enrichment (prop h2 / prop snps)

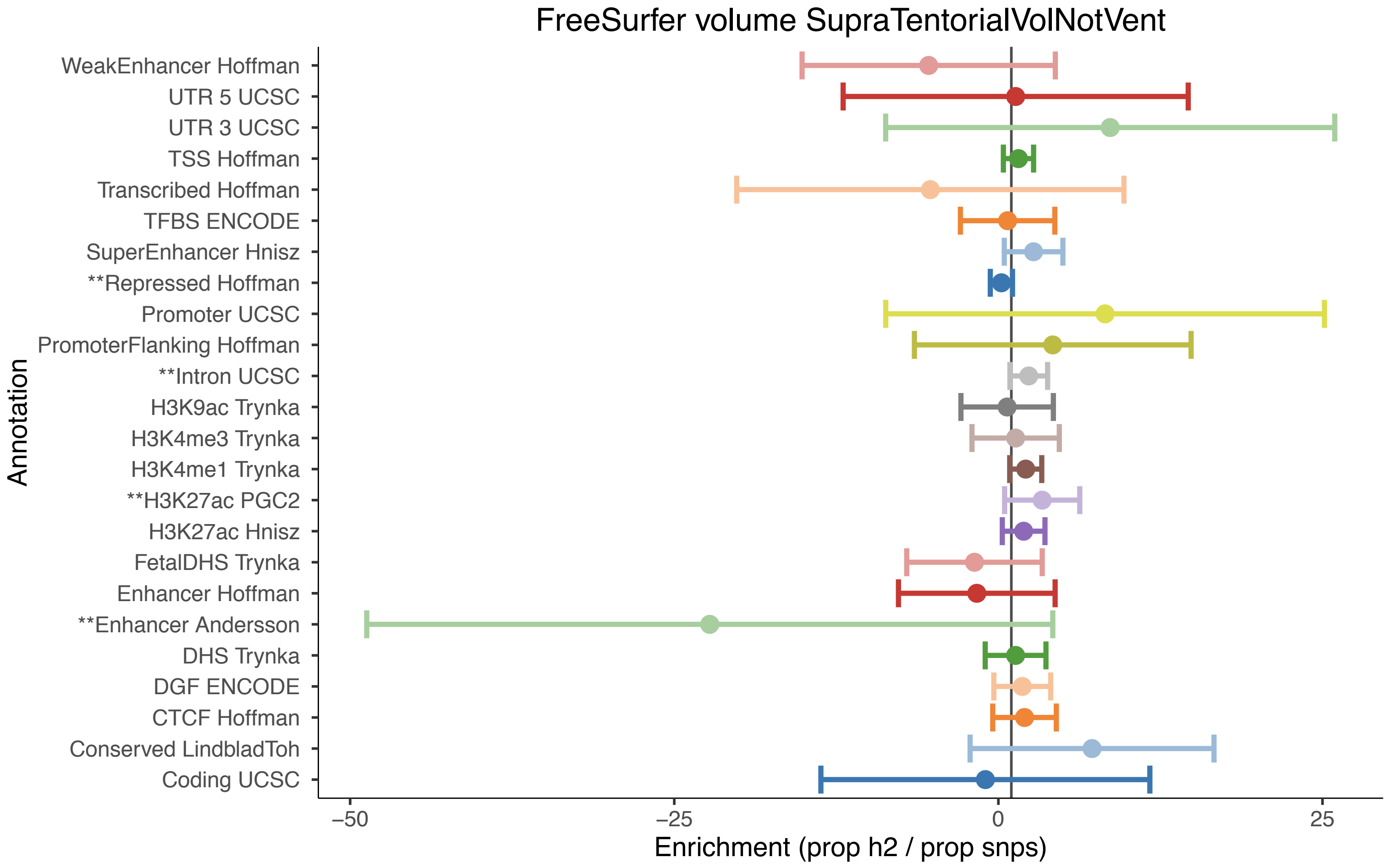

FreeSurfer volume SupraTentorialVolNotVent
WeakEnhancer Hoffman
UTR 5 UCSC
UTR 3 UCSC
TSS Hoffman
Transcribed Hoffman
TFBS ENCODE
SuperEnhancer Hnisz
**Repressed Hoffman
Promoter UCSC
PromoterFlanking Hoffman
**Intron UCSC
H3K9ac Trynka
H3K4me3 Trynka
H3K4me1 Trynka
**H3K27ac PGC2
H3K27ac Hnisz
FetalDHS Trynka
Enhancer Hoffman
**Enhancer Andersson
DHS Trynka
DGF ENCODE
CTCF Hoffman
Conserved LindbladToh
Coding UCSC
Annotation
−50
−25
0
25
Enrichment (prop h2 / prop snps)

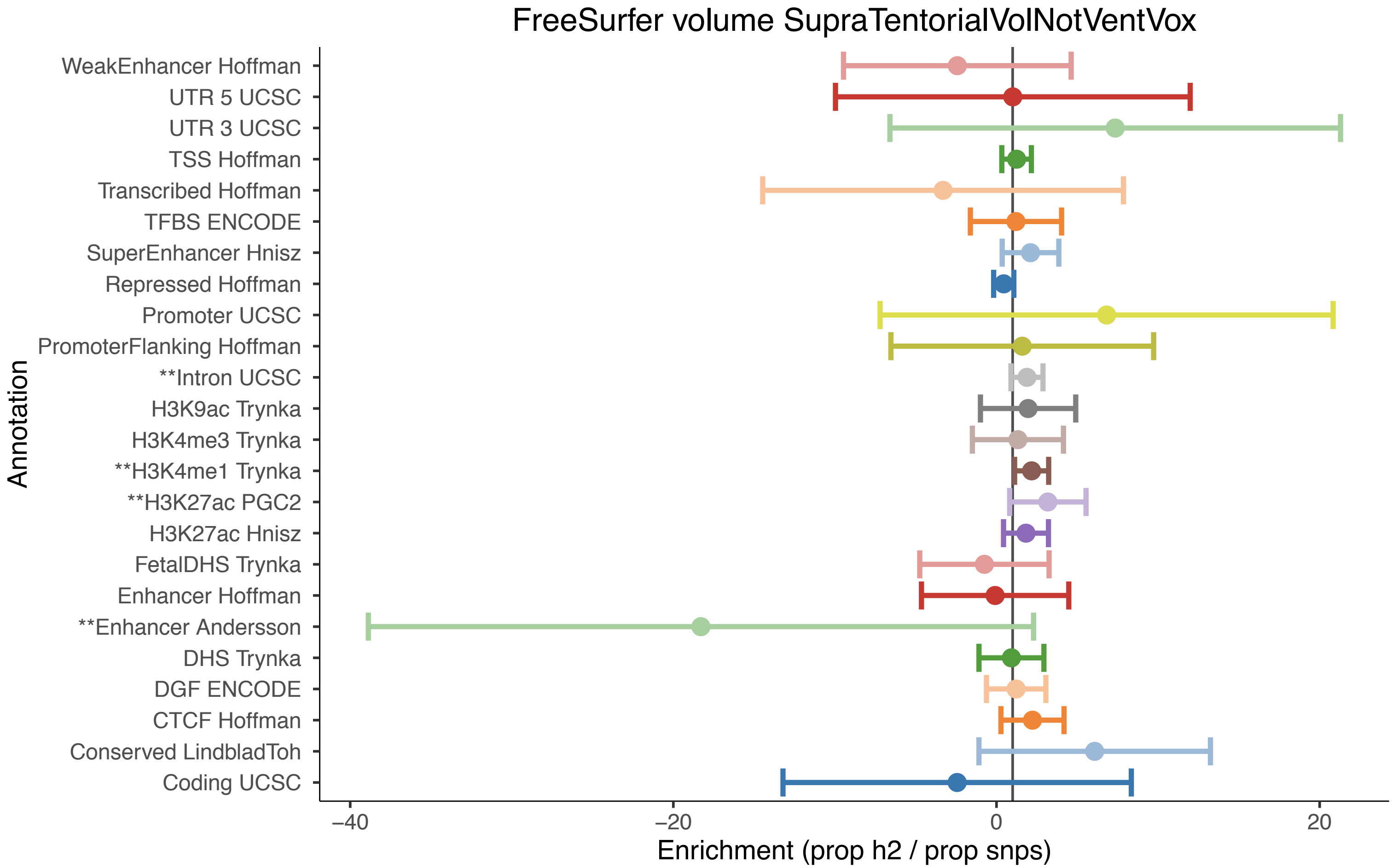

FreeSurfer volume SupraTentorialVolNotVentVox
WeakEnhancer Hoffman
UTR 5 UCSC
UTR 3 UCSC
TSS Hoffman
Transcribed Hoffman
TFBS ENCODE
SuperEnhancer Hnisz
Repressed Hoffman
Promoter UCSC
PromoterFlanking Hoffman
**Intron UCSC
H3K9ac Trynka
H3K4me3 Trynka
**H3K4me1 Trynka
**H3K27ac PGC2
H3K27ac Hnisz
FetalDHS Trynka
Enhancer Hoffman
**Enhancer Andersson
DHS Trynka
DGF ENCODE
CTCF Hoffman
Conserved LindbladToh
Coding UCSC
Annotation
−40
−20
0
20
Enrichment (prop h2 / prop snps)

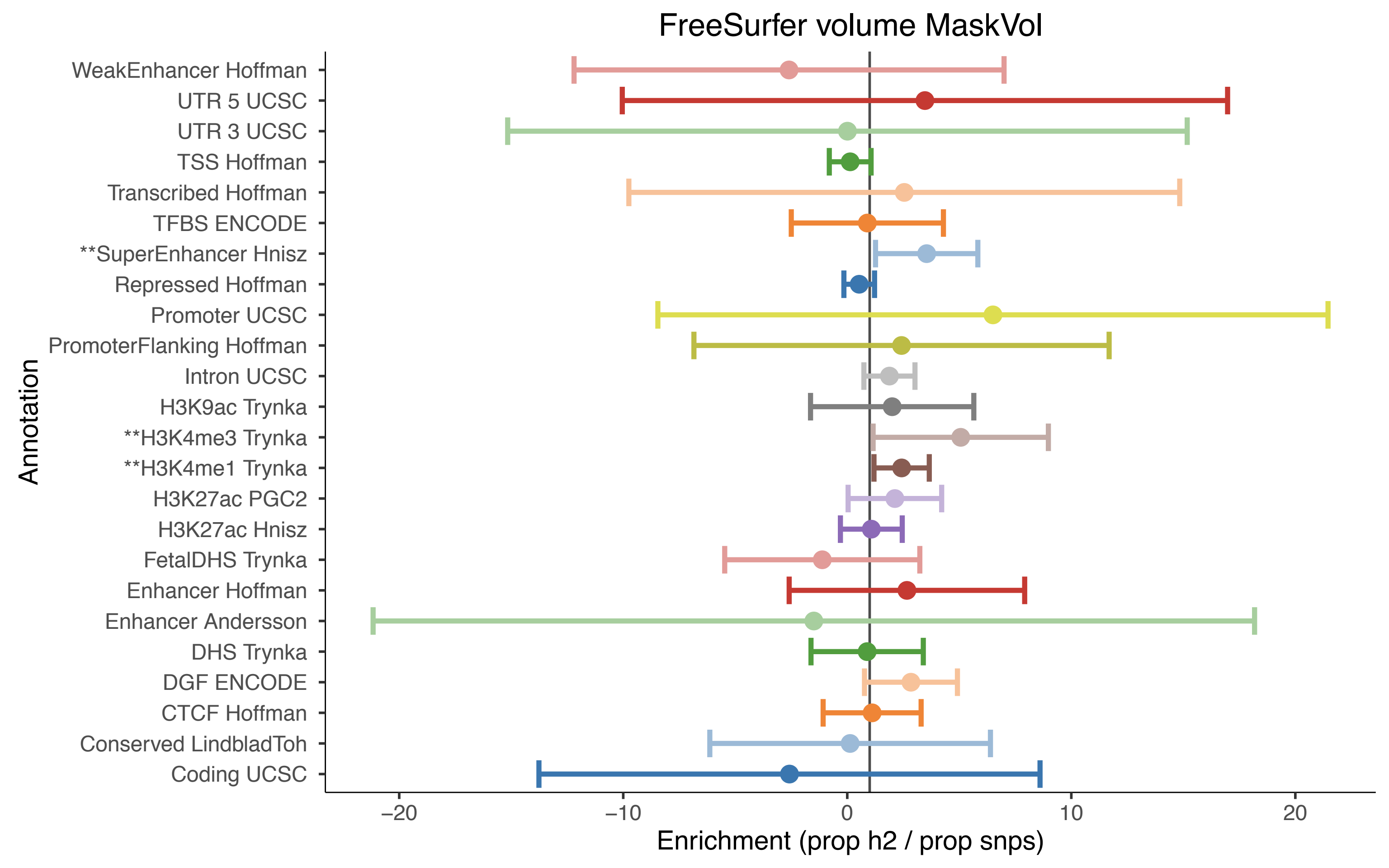

FreeSurfer volume MaskVol
WeakEnhancer Hoffman
UTR 5 UCSC
UTR 3 UCSC
TSS Hoffman
Transcribed Hoffman
TFBS ENCODE
**SuperEnhancer Hnisz
Repressed Hoffman
Promoter UCSC
PromoterFlanking Hoffman
Intron UCSC
H3K9ac Trynka
**H3K4me3 Trynka
**H3K4me1 Trynka
H3K27ac PGC2
H3K27ac Hnisz
FetalDHS Trynka
Enhancer Hoffman
Enhancer Andersson
DHS Trynka
DGF ENCODE
CTCF Hoffman
Conserved LindbladToh
Coding UCSC
Annotation
−20
−10
0
10
20
Enrichment (prop h2 / prop snps)

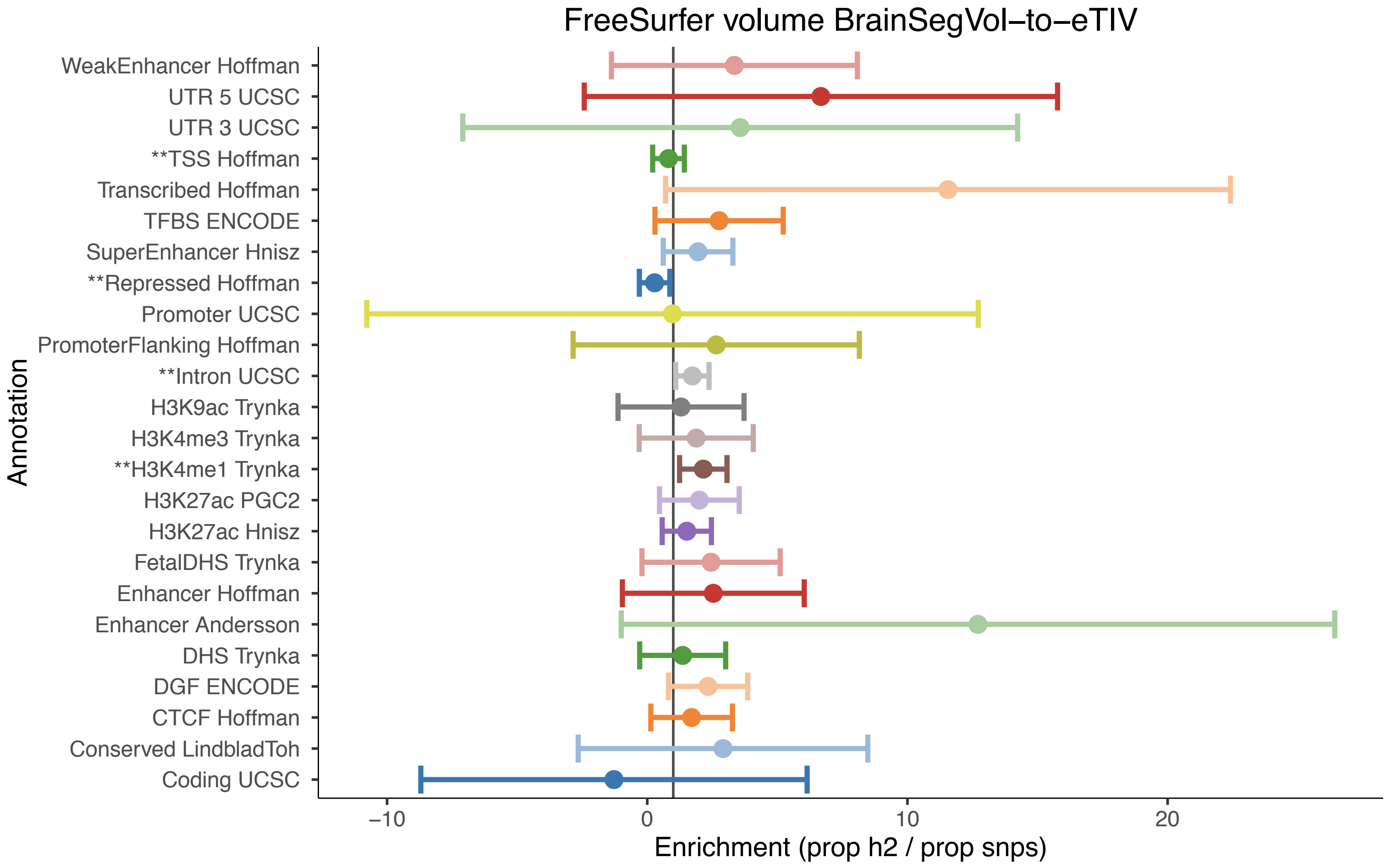
FreeSurfer volume BrainSegVol−to−eTIV
WeakEnhancer Hoffman
UTR 5 UCSC
UTR 3 UCSC
**TSS Hoffman
Transcribed Hoffman
TFBS ENCODE
SuperEnhancer Hnisz
**Repressed Hoffman
Promoter UCSC
PromoterFlanking Hoffman
**Intron UCSC
H3K9ac Trynka
H3K4me3 Trynka
**H3K4me1 Trynka
H3K27ac PGC2
H3K27ac Hnisz
FetalDHS Trynka
Enhancer Hoffman
Enhancer Andersson
DHS Trynka
DGF ENCODE
CTCF Hoffman
Conserved LindbladToh
Coding UCSC
Annotation
−10
0
10
20
Enrichment (prop h2 / prop snps)

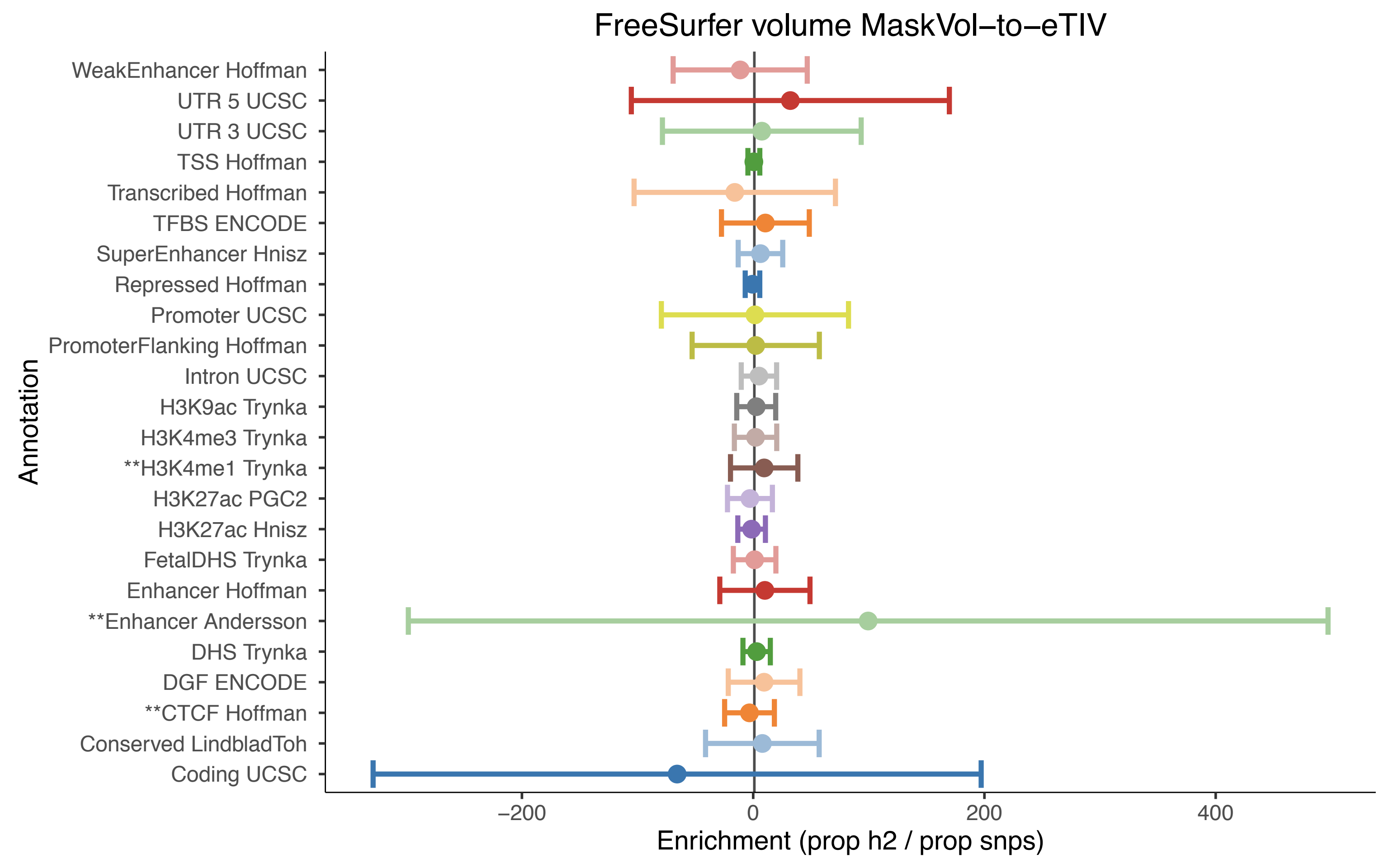
FreeSurfer volume MaskVol–to–eTIV
Annotation
WeakEnhancer Hoffman
UTR 5 UCSC
UTR 3 UCSC
TSS Hoffman
Transcribed Hoffman
TFBS ENCODE
SuperEnhancer Hnisz
Repressed Hoffman
Promoter UCSC
PromoterFlanking Hoffman
Intron UCSC
H3K9ac Trynka
H3K4me3 Trynka
**H3K4me1 Trynka
H3K27ac PGC2
H3K27ac Hnisz
FetalDHS Trynka
Enhancer Hoffman
**Enhancer Andersson
DHS Trynka
DGF ENCODE
**CTCF Hoffman
Conserved LindbladToh
Coding UCSC
−200
0
200
400
Enrichment (prop h2 / prop snps)

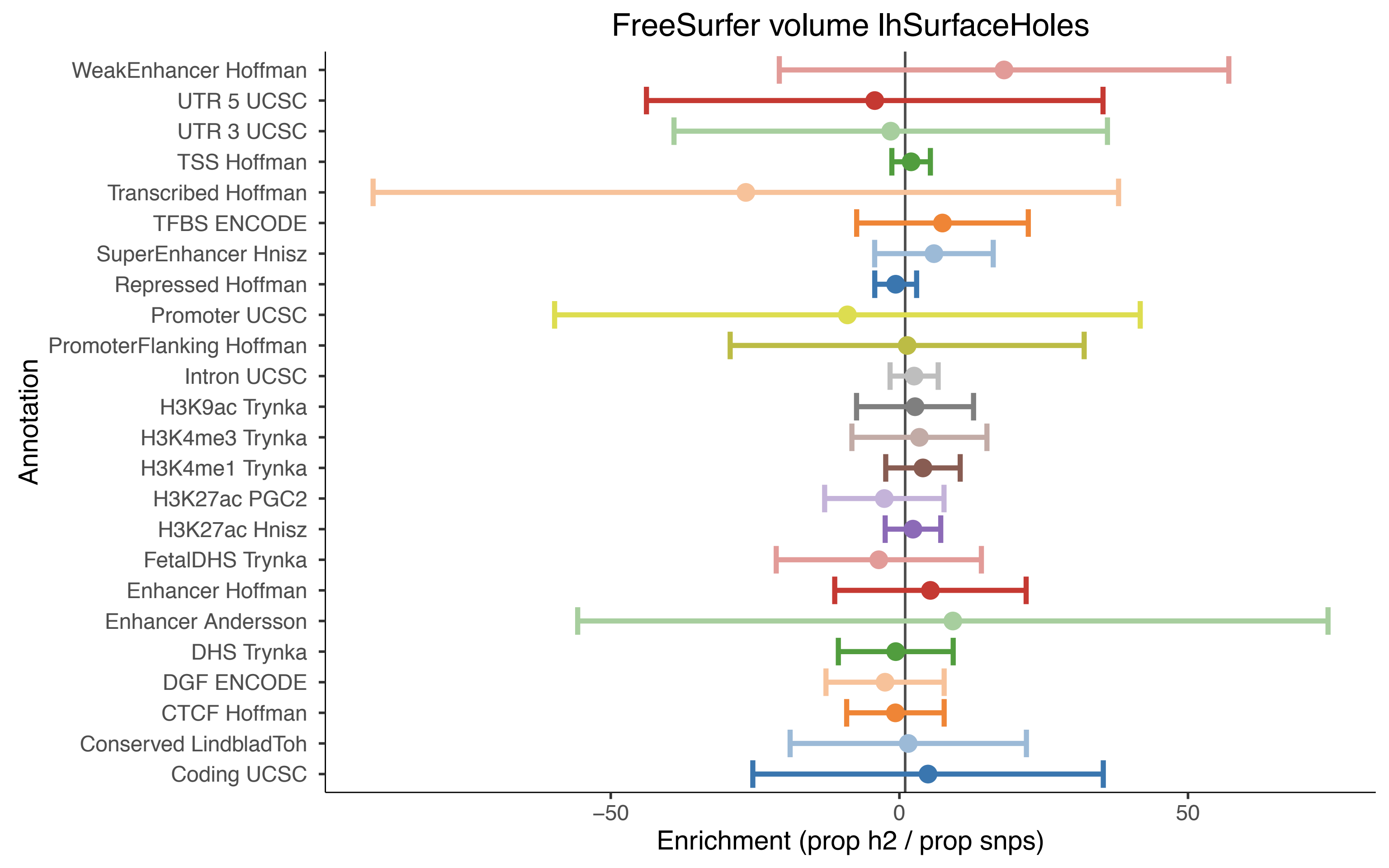
FreeSurfer volume lhSurfaceHoles
WeakEnhancer Hoffman
UTR 5 UCSC
UTR 3 UCSC
TSS Hoffman
Transcribed Hoffman
TFBS ENCODE
SuperEnhancer Hnisz
Repressed Hoffman
Promoter UCSC
PromoterFlanking Hoffman
Intron UCSC
H3K9ac Trynka
H3K4me3 Trynka
H3K4me1 Trynka
H3K27ac PGC2
H3K27ac Hnisz
FetalDHS Trynka
Enhancer Hoffman
Enhancer Andersson
DHS Trynka
DGF ENCODE
CTCF Hoffman
Conserved LindbladToh
Coding UCSC
Annotation
−50
0
50
Enrichment (prop h2 / prop snps)

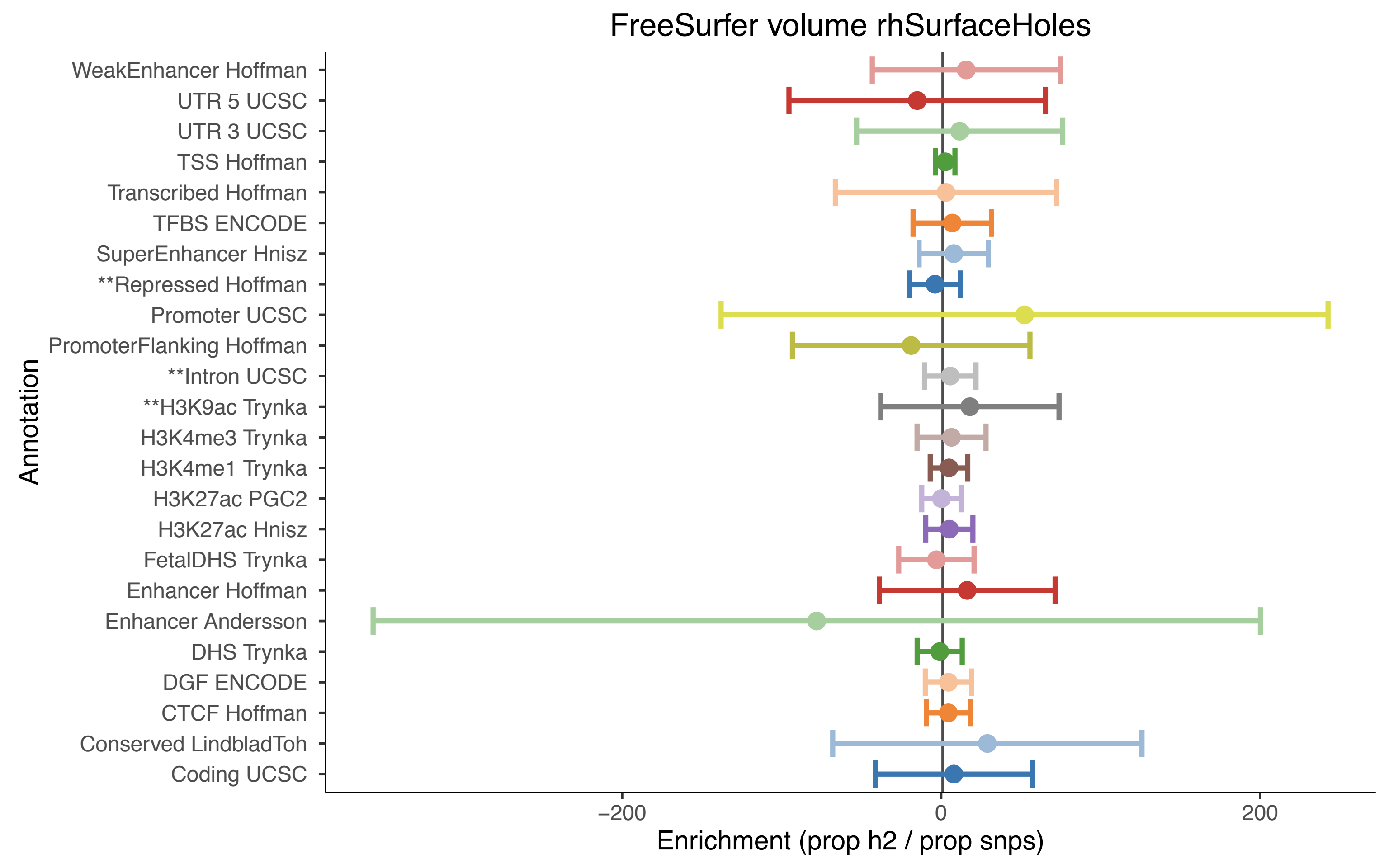
FreeSurfer volume rhSurfaceHoles
WeakEnhancer Hoffman
UTR 5 UCSC
UTR 3 UCSC
TSS Hoffman
Transcribed Hoffman
TFBS ENCODE
SuperEnhancer Hnisz
**Repressed Hoffman
Promoter UCSC
PromoterFlanking Hoffman
**Intron UCSC
**H3K9ac Trynka
H3K4me3 Trynka
H3K4me1 Trynka
H3K27ac PGC2
H3K27ac Hnisz
FetalDHS Trynka
Enhancer Hoffman
Enhancer Andersson
DHS Trynka
DGF ENCODE
CTCF Hoffman
Conserved LindbladToh
Coding UCSC
Annotation
−200
0
200
Enrichment (prop h2 / prop snps)

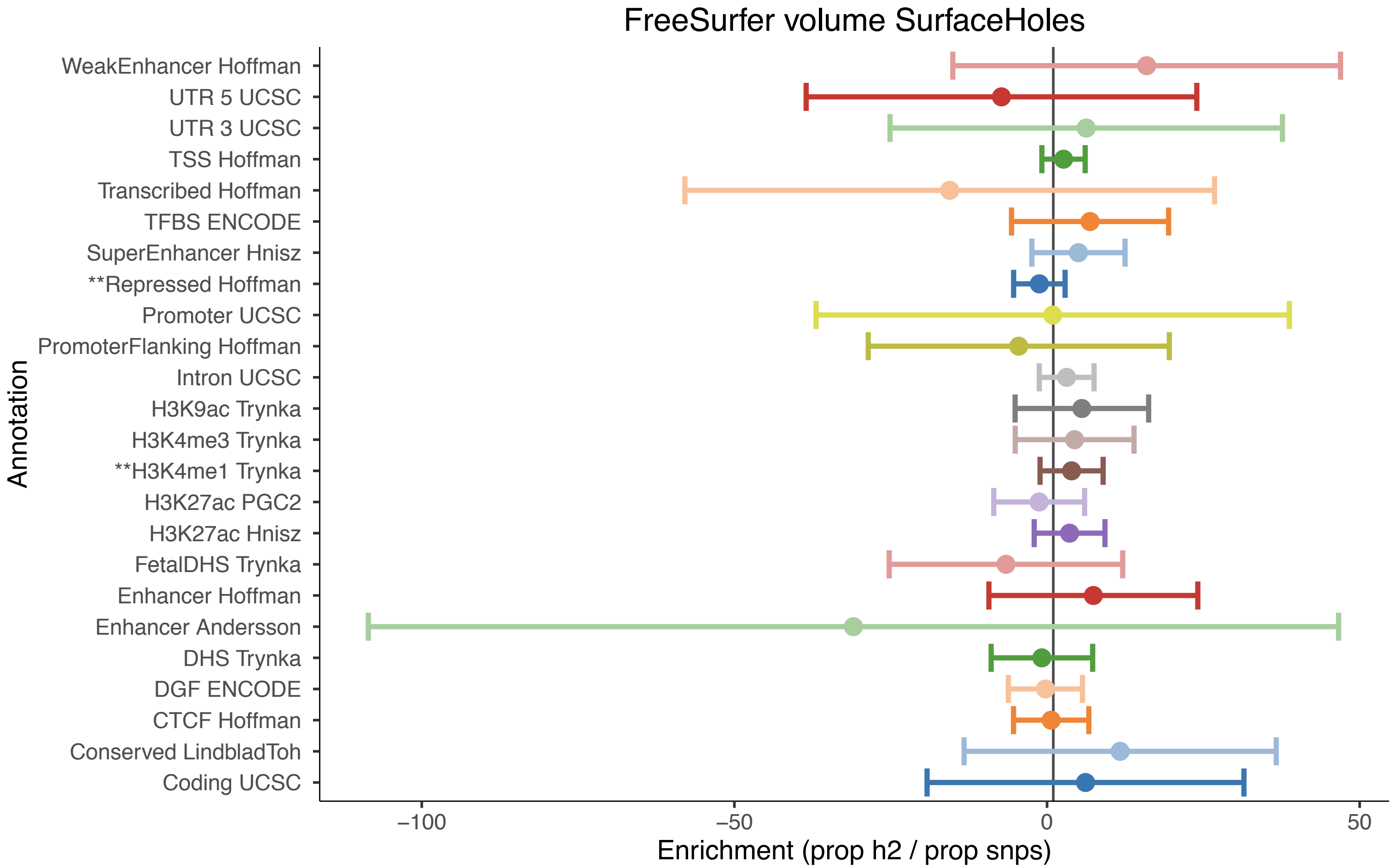
FreeSurfer volume SurfaceHoles
Annotation
WeakEnhancer Hoffman
UTR 5 UCSC
UTR 3 UCSC
TSS Hoffman
Transcribed Hoffman
TFBS ENCODE
SuperEnhancer Hnisz
**Repressed Hoffman
Promoter UCSC
PromoterFlanking Hoffman
Intron UCSC
H3K9ac Trynka
H3K4me3 Trynka
**H3K4me1 Trynka
H3K27ac PGC2
H3K27ac Hnisz
FetalDHS Trynka
Enhancer Hoffman
Enhancer Andersson
DHS Trynka
DGF ENCODE
CTCF Hoffman
Conserved LindbladToh
Coding UCSC
−100
−50
0
50
Enrichment (prop h2 / prop snps)

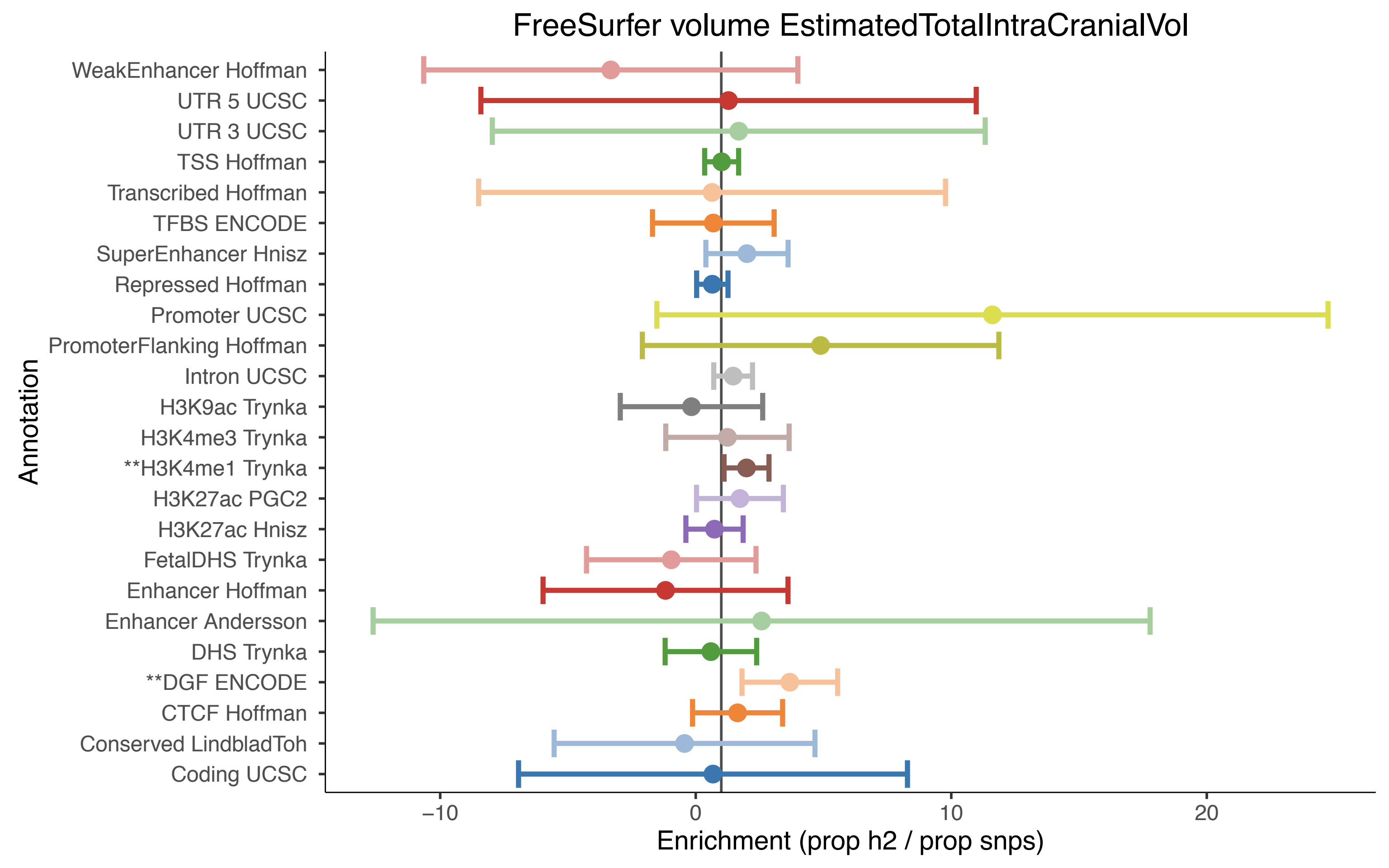
FreeSurfer volume EstimatedTotalIntraCranialVol
Annotation
WeakEnhancer Hoffman
UTR 5 UCSC
UTR 3 UCSC
TSS Hoffman
Transcribed Hoffman
TFBS ENCODE
SuperEnhancer Hnisz
Repressed Hoffman
Promoter UCSC
PromoterFlanking Hoffman
Intron UCSC
H3K9ac Trynka
H3K4me3 Trynka
**H3K4me1 Trynka
H3K27ac PGC2
H3K27ac Hnisz
FetalDHS Trynka
Enhancer Hoffman
Enhancer Andersson
DHS Trynka
**DGF ENCODE
CTCF Hoffman
Conserved LindbladToh
Coding UCSC
−10
0
10
20
Enrichment (prop h2 / prop snps)

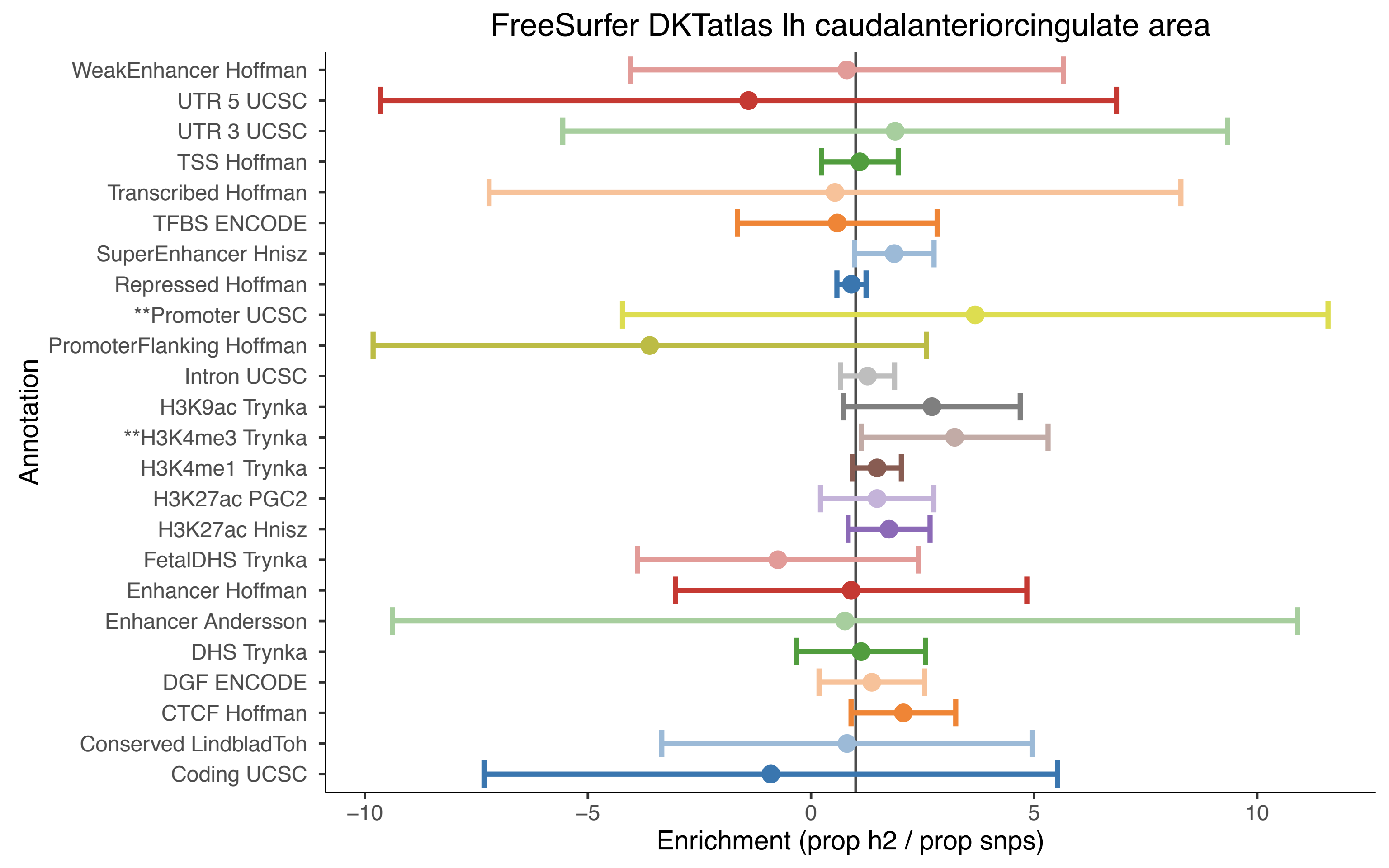
FreeSurfer DKTatlas lh caudalanteriorcingulate area
WeakEnhancer Hoffman
UTR 5 UCSC
UTR 3 UCSC
TSS Hoffman
Transcribed Hoffman
TFBS ENCODE
SuperEnhancer Hnisz
Repressed Hoffman
**Promoter UCSC
PromoterFlanking Hoffman
Intron UCSC
H3K9ac Trynka
**H3K4me3 Trynka
H3K4me1 Trynka
H3K27ac PGC2
H3K27ac Hnisz
FetalDHS Trynka
Enhancer Hoffman
Enhancer Andersson
DHS Trynka
DGF ENCODE
CTCF Hoffman
Conserved LindbladToh
Coding UCSC
Annotation
−10
−5
0
5
10
Enrichment (prop h2 / prop snps)

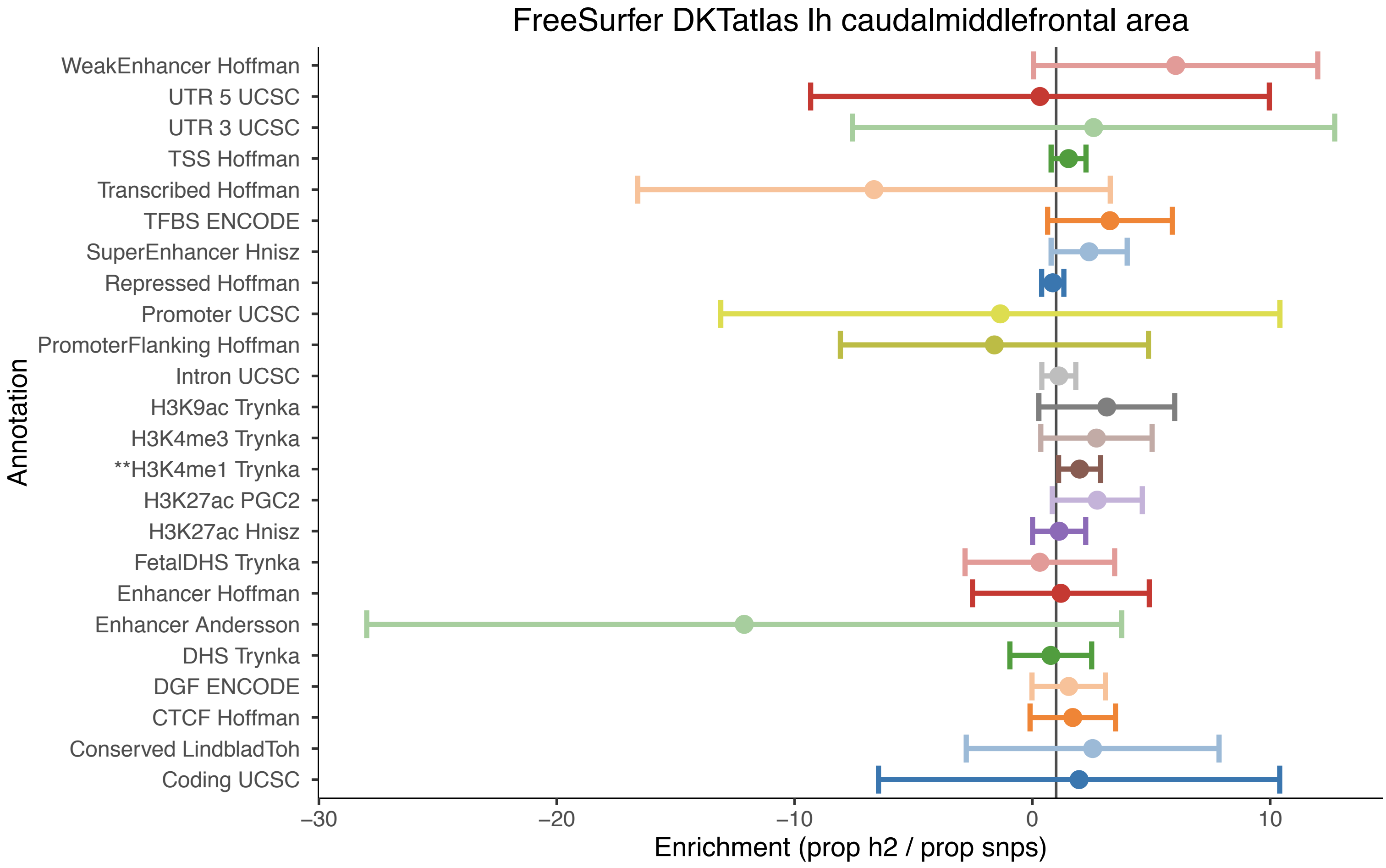

FreeSurfer DKTatlas lh caudalmiddlefrontal area
WeakEnhancer Hoffman
UTR 5 UCSC
UTR 3 UCSC
TSS Hoffman
Transcribed Hoffman
TFBS ENCODE
SuperEnhancer Hnisz
Repressed Hoffman
Promoter UCSC
PromoterFlanking Hoffman
Intron UCSC
H3K9ac Trynka
H3K4me3 Trynka
**H3K4me1 Trynka
H3K27ac PGC2
H3K27ac Hnisz
FetalDHS Trynka
Enhancer Hoffman
Enhancer Andersson
DHS Trynka
DGF ENCODE
CTCF Hoffman
Conserved LindbladToh
Coding UCSC
Annotation
−30
−20
−10
0
10
Enrichment (prop h2 / prop snps)

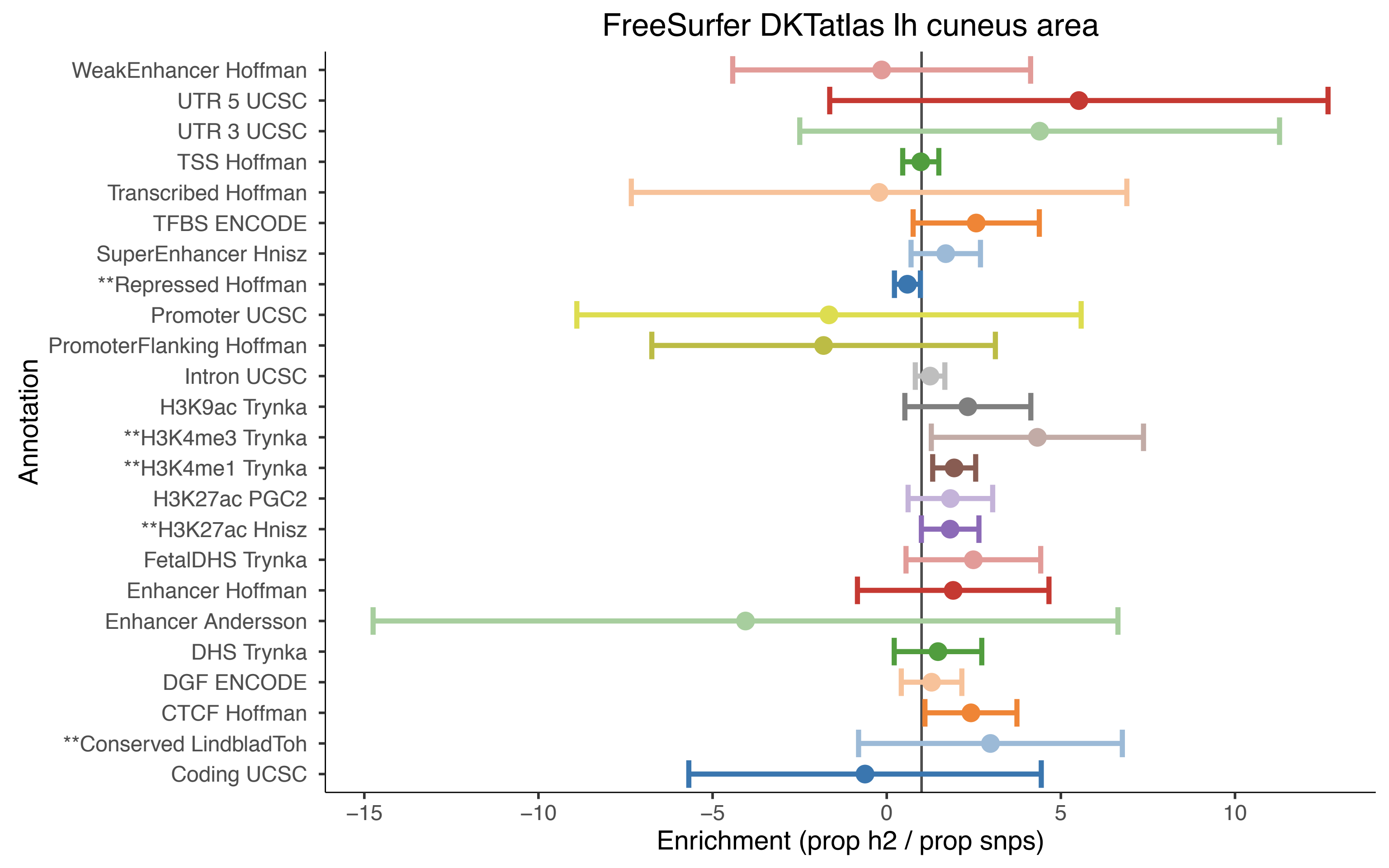
FreeSurfer DKTatlas lh cuneus area
Annotation
WeakEnhancer Hoffman
UTR 5 UCSC
UTR 3 UCSC
TSS Hoffman
Transcribed Hoffman
TFBS ENCODE
SuperEnhancer Hnisz
**Repressed Hoffman
Promoter UCSC
PromoterFlanking Hoffman
Intron UCSC
H3K9ac Trynka
**H3K4me3 Trynka
**H3K4me1 Trynka
H3K27ac PGC2
**H3K27ac Hnisz
FetalDHS Trynka
Enhancer Hoffman
Enhancer Andersson
DHS Trynka
DGF ENCODE
CTCF Hoffman
**Conserved LindbladToh
Coding UCSC
−15
−10
−5
0
5
10
Enrichment (prop h2 / prop snps)

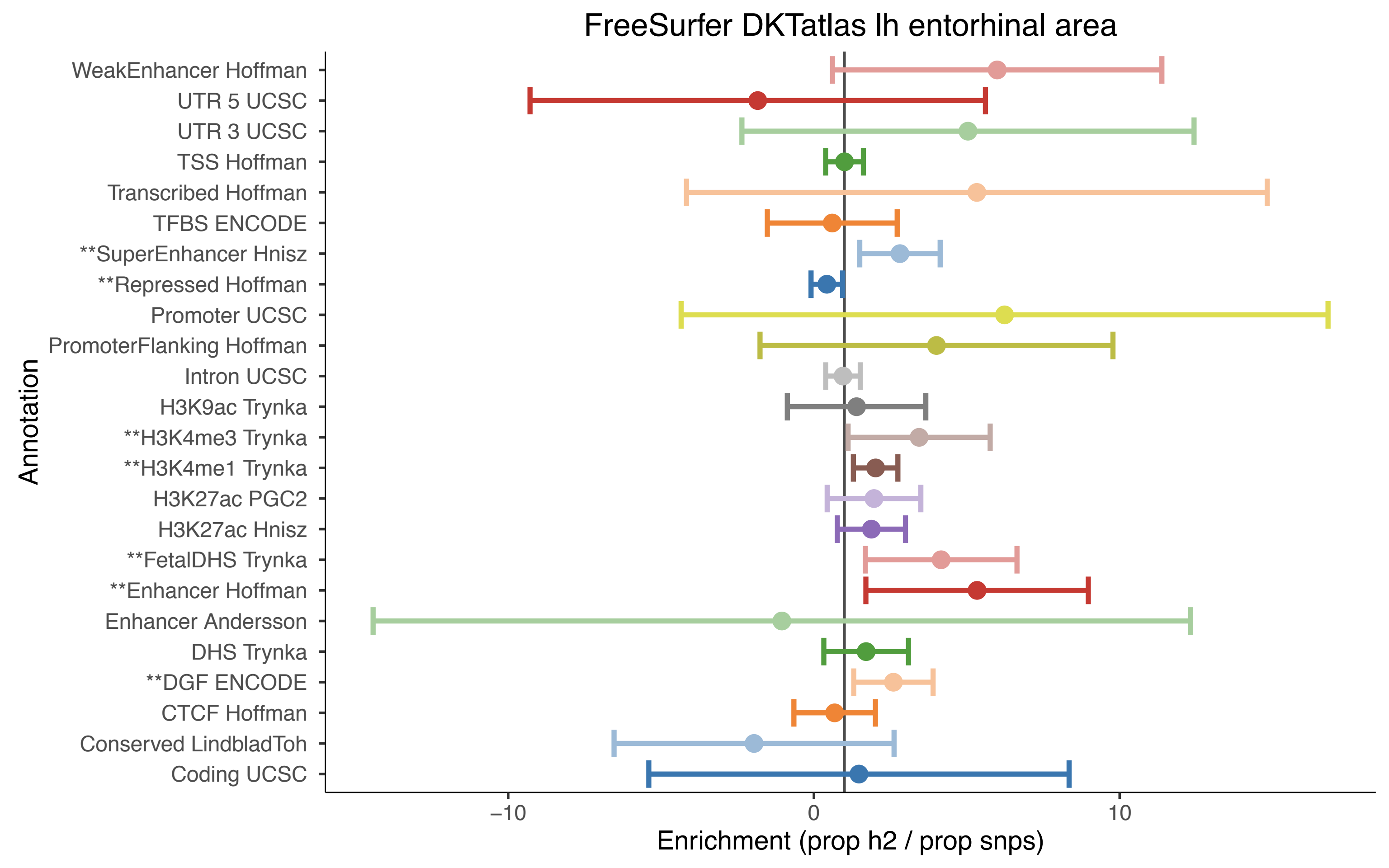
FreeSurfer DKTatlas lh entorhinal area
WeakEnhancer Hoffman
UTR 5 UCSC
UTR 3 UCSC
TSS Hoffman
Transcribed Hoffman
TFBS ENCODE
**SuperEnhancer Hnisz
**Repressed Hoffman
Promoter UCSC
PromoterFlanking Hoffman
Intron UCSC
H3K9ac Trynka
**H3K4me3 Trynka
**H3K4me1 Trynka
H3K27ac PGC2
H3K27ac Hnisz
**FetalDHS Trynka
**Enhancer Hoffman
Enhancer Andersson
DHS Trynka
**DGF ENCODE
CTCF Hoffman
Conserved LindbladToh
Coding UCSC
Annotation
−10
0
10
Enrichment (prop h2 / prop snps)

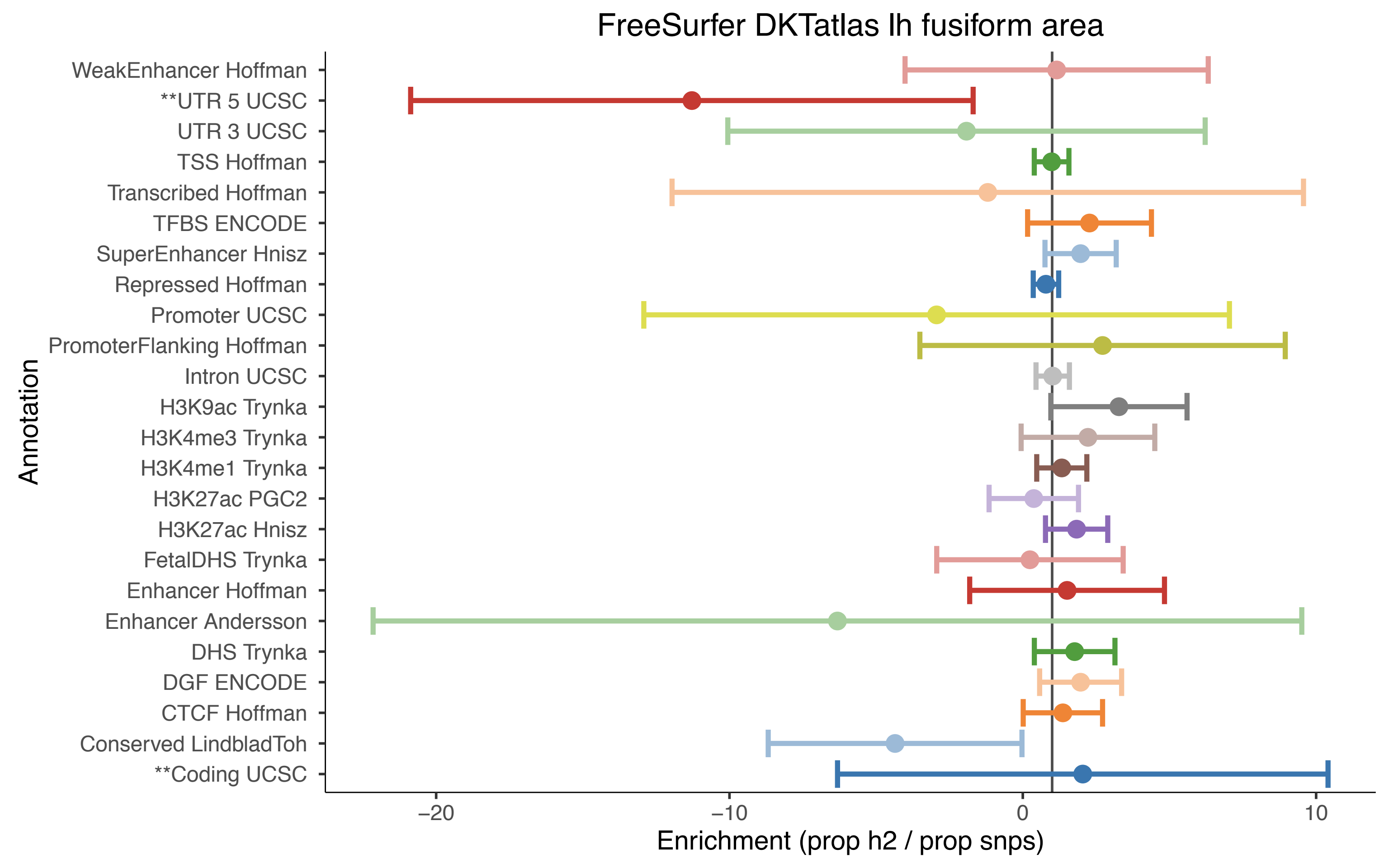
FreeSurfer DKTatlas lh fusiform area
Annotation
WeakEnhancer Hoffman
**UTR 5 UCSC
UTR 3 UCSC
TSS Hoffman
Transcribed Hoffman
TFBS ENCODE
SuperEnhancer Hnisz
Repressed Hoffman
Promoter UCSC
PromoterFlanking Hoffman
Intron UCSC
H3K9ac Trynka
H3K4me3 Trynka
H3K4me1 Trynka
H3K27ac PGC2
H3K27ac Hnisz
FetalDHS Trynka
Enhancer Hoffman
Enhancer Andersson
DHS Trynka
DGF ENCODE
CTCF Hoffman
Conserved LindbladToh
**Coding UCSC
−20
−10
0
10
Enrichment (prop h2 / prop snps)

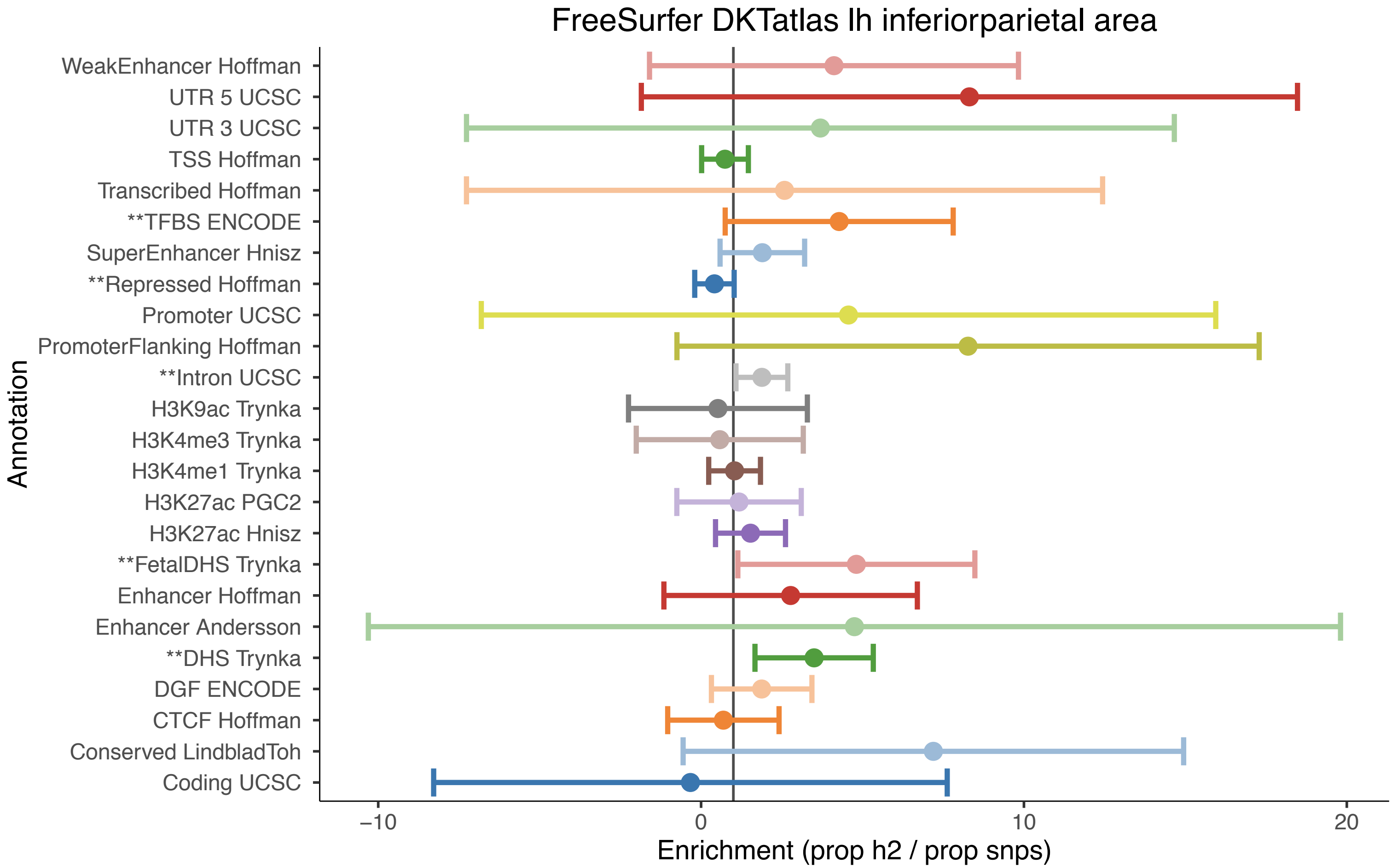

FreeSurfer DKTatlas lh inferiorparietal area
WeakEnhancer Hoffman
UTR 5 UCSC
UTR 3 UCSC
TSS Hoffman
Transcribed Hoffman
**TFBS ENCODE
SuperEnhancer Hnisz
**Repressed Hoffman
Promoter UCSC
PromoterFlanking Hoffman
**Intron UCSC
H3K9ac Trynka
H3K4me3 Trynka
H3K4me1 Trynka
H3K27ac PGC2
H3K27ac Hnisz
**FetalDHS Trynka
Enhancer Hoffman
Enhancer Andersson
**DHS Trynka
DGF ENCODE
CTCF Hoffman
Conserved LindbladToh
Coding UCSC
Annotation
−10
0
10
20
Enrichment (prop h2 / prop snps)

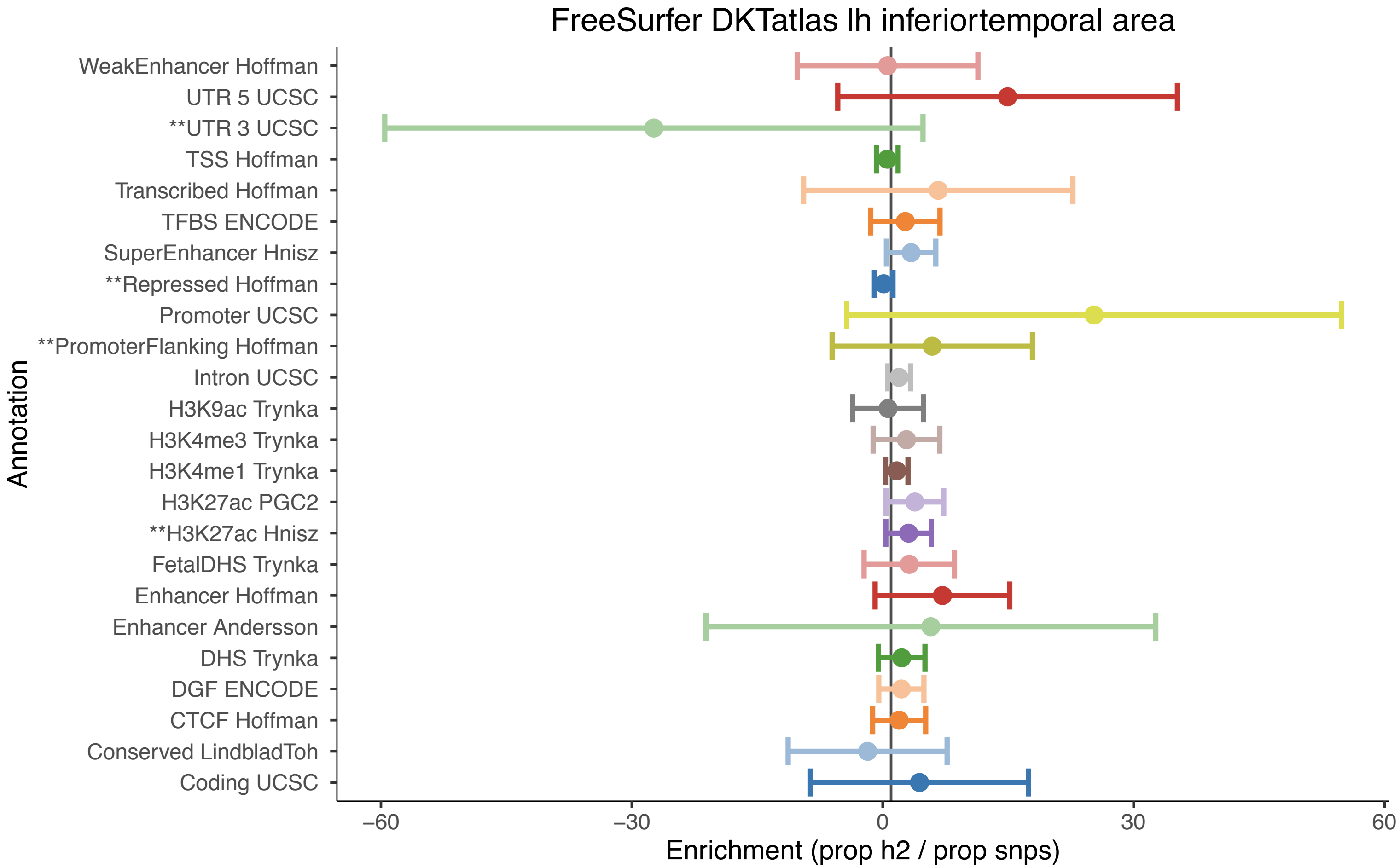
FreeSurfer DKTatlas lh inferiortemporal area
Annotation
WeakEnhancer Hoffman
UTR 5 UCSC
**UTR 3 UCSC
TSS Hoffman
Transcribed Hoffman
TFBS ENCODE
SuperEnhancer Hnisz
**Repressed Hoffman
Promoter UCSC
**PromoterFlanking Hoffman
Intron UCSC
H3K9ac Trynka
H3K4me3 Trynka
H3K4me1 Trynka
H3K27ac PGC2
**H3K27ac Hnisz
FetalDHS Trynka
Enhancer Hoffman
Enhancer Andersson
DHS Trynka
DGF ENCODE
CTCF Hoffman
Conserved LindbladToh
Coding UCSC
−60
−30
0
30
60
Enrichment (prop h2 / prop snps)

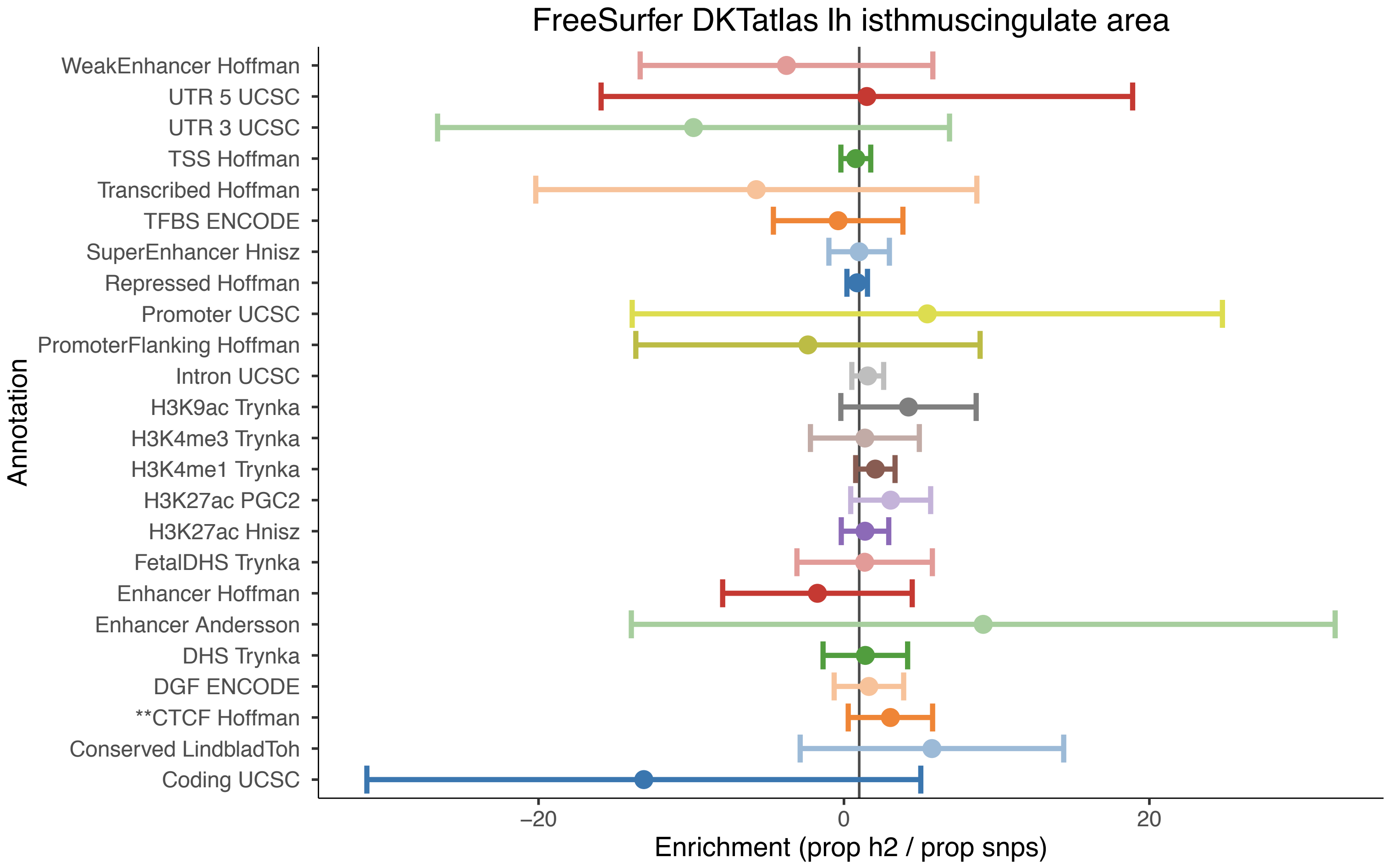
FreeSurfer DKTatlas lh isthmuscingulate area
WeakEnhancer Hoffman
UTR 5 UCSC
UTR 3 UCSC
TSS Hoffman
Transcribed Hoffman
TFBS ENCODE
SuperEnhancer Hnisz
Repressed Hoffman
Promoter UCSC
PromoterFlanking Hoffman
Intron UCSC
H3K9ac Trynka
H3K4me3 Trynka
H3K4me1 Trynka
H3K27ac PGC2
H3K27ac Hnisz
FetalDHS Trynka
Enhancer Hoffman
Enhancer Andersson
DHS Trynka
DGF ENCODE
**CTCF Hoffman
Conserved LindbladToh
Coding UCSC
Annotation
−20
0
20
Enrichment (prop h2 / prop snps)

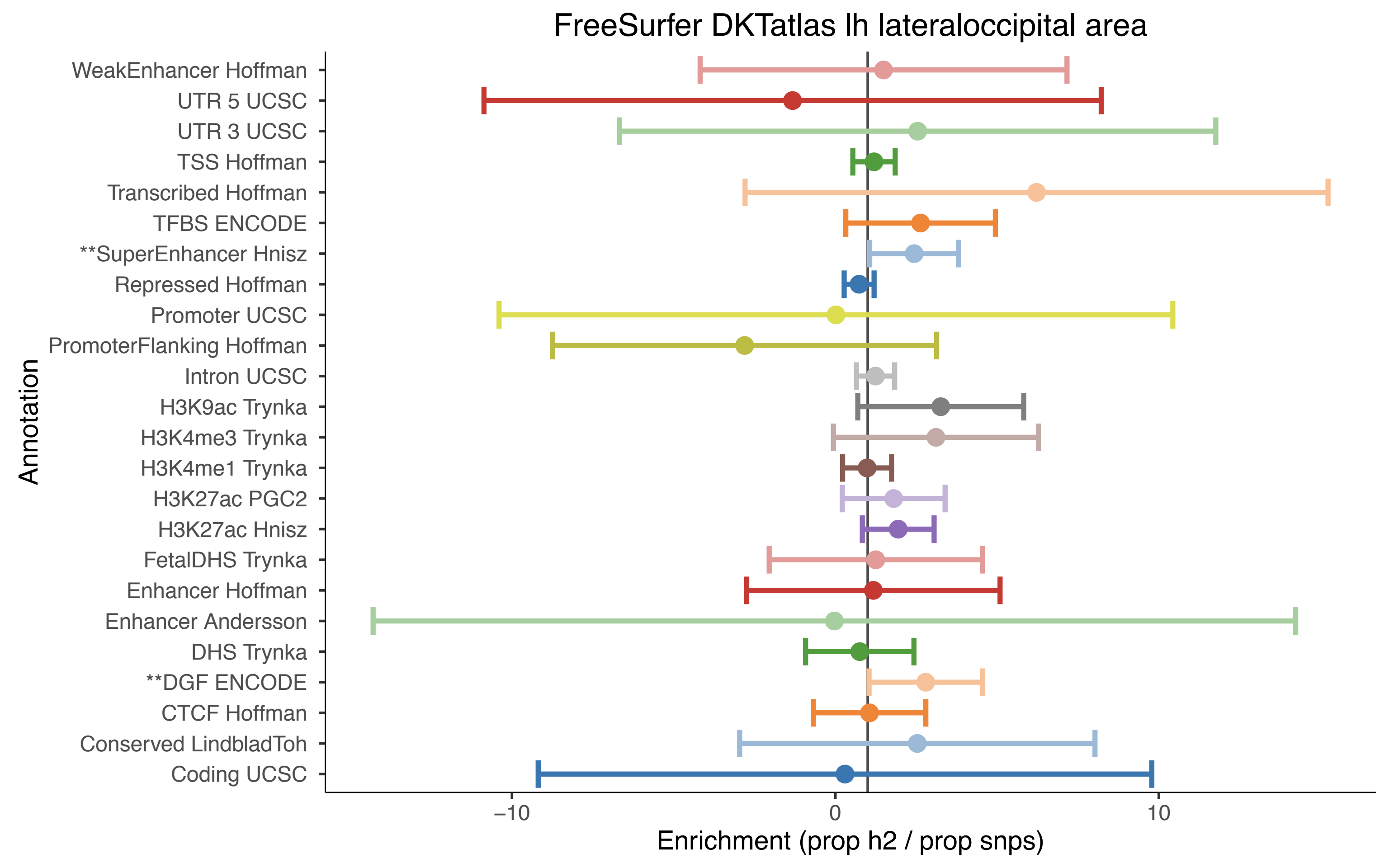
FreeSurfer DKTatlas lh lateraloccipital area
WeakEnhancer Hoffman
UTR 5 UCSC
UTR 3 UCSC
TSS Hoffman
Transcribed Hoffman
TFBS ENCODE
**SuperEnhancer Hnisz
Repressed Hoffman
Promoter UCSC
PromoterFlanking Hoffman
Intron UCSC
H3K9ac Trynka
H3K4me3 Trynka
H3K4me1 Trynka
H3K27ac PGC2
H3K27ac Hnisz
FetalDHS Trynka
Enhancer Hoffman
Enhancer Andersson
DHS Trynka
**DGF ENCODE
CTCF Hoffman
Conserved LindbladToh
Coding UCSC
Annotation
−10
0
10
Enrichment (prop h2 / prop snps)

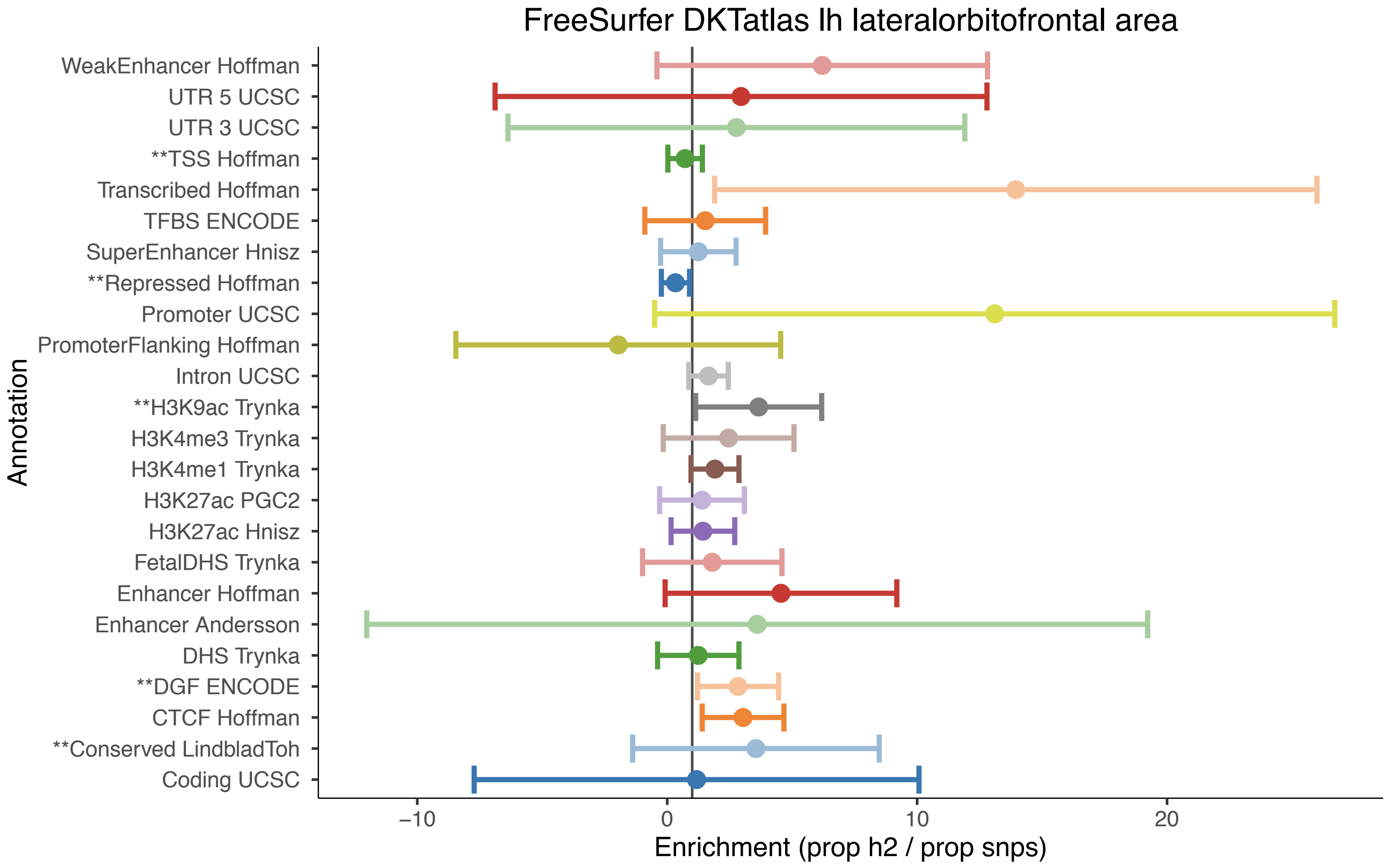
FreeSurfer DKTatlas lh lateralorbitofrontal area
WeakEnhancer Hoffman
UTR 5 UCSC
UTR 3 UCSC
**TSS Hoffman
Transcribed Hoffman
TFBS ENCODE
SuperEnhancer Hnisz
**Repressed Hoffman
Promoter UCSC
PromoterFlanking Hoffman
Intron UCSC
**H3K9ac Trynka
H3K4me3 Trynka
H3K4me1 Trynka
H3K27ac PGC2
H3K27ac Hnisz
FetalDHS Trynka
Enhancer Hoffman
Enhancer Andersson
DHS Trynka
**DGF ENCODE
CTCF Hoffman
**Conserved LindbladToh
Coding UCSC
Annotation
−10
0
10
20
Enrichment (prop h2 / prop snps)

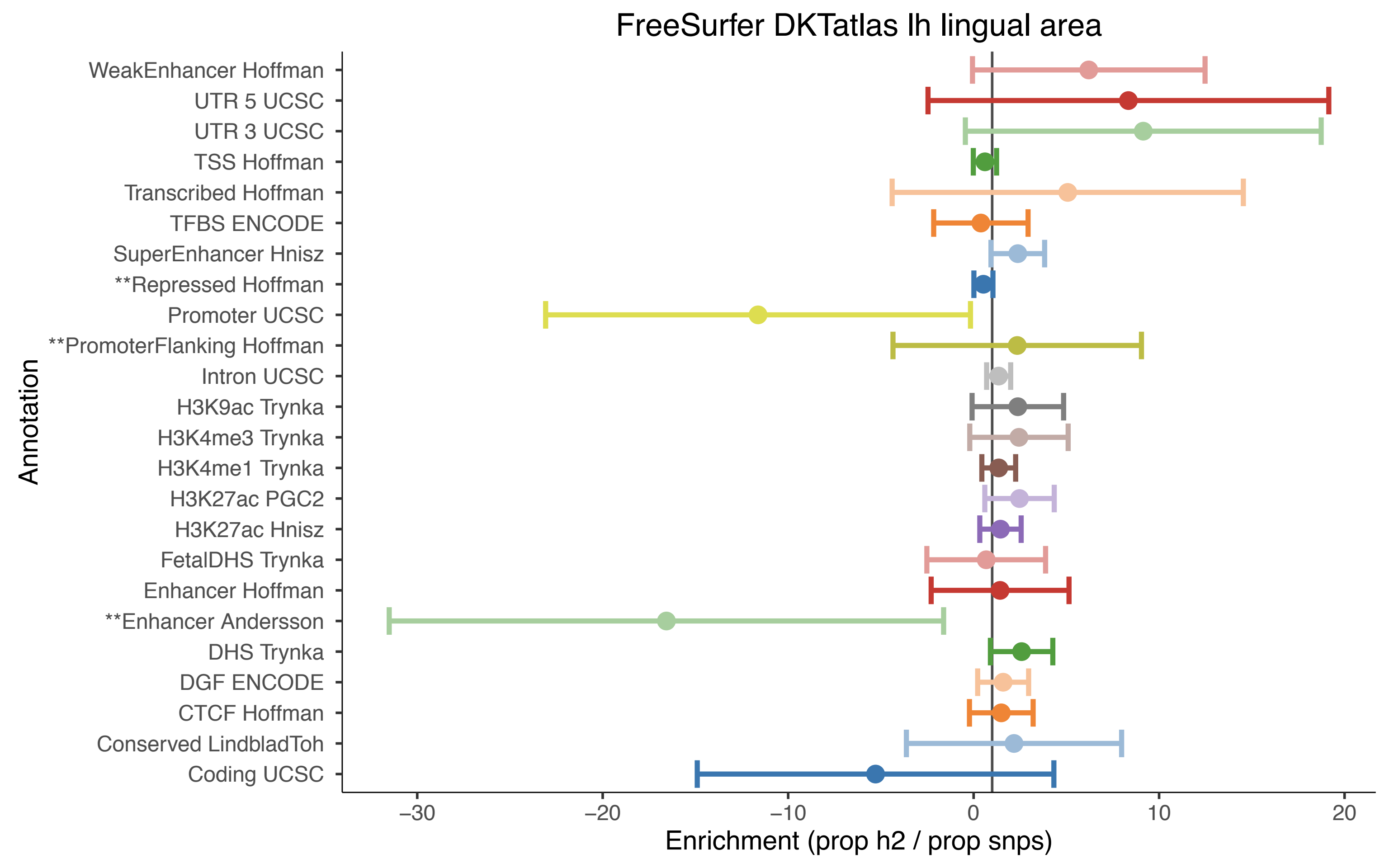

FreeSurfer DKTatlas lh lingual area
WeakEnhancer Hoffman
UTR 5 UCSC
UTR 3 UCSC
TSS Hoffman
Transcribed Hoffman
TFBS ENCODE
SuperEnhancer Hnisz
**Repressed Hoffman
Promoter UCSC
**PromoterFlanking Hoffman
Intron UCSC
H3K9ac Trynka
H3K4me3 Trynka
H3K4me1 Trynka
H3K27ac PGC2
H3K27ac Hnisz
FetalDHS Trynka
Enhancer Hoffman
**Enhancer Andersson
DHS Trynka
DGF ENCODE
CTCF Hoffman
Conserved LindbladToh
Coding UCSC
Annotation
−30
−20
−10
0
10
20
Enrichment (prop h2 / prop snps)

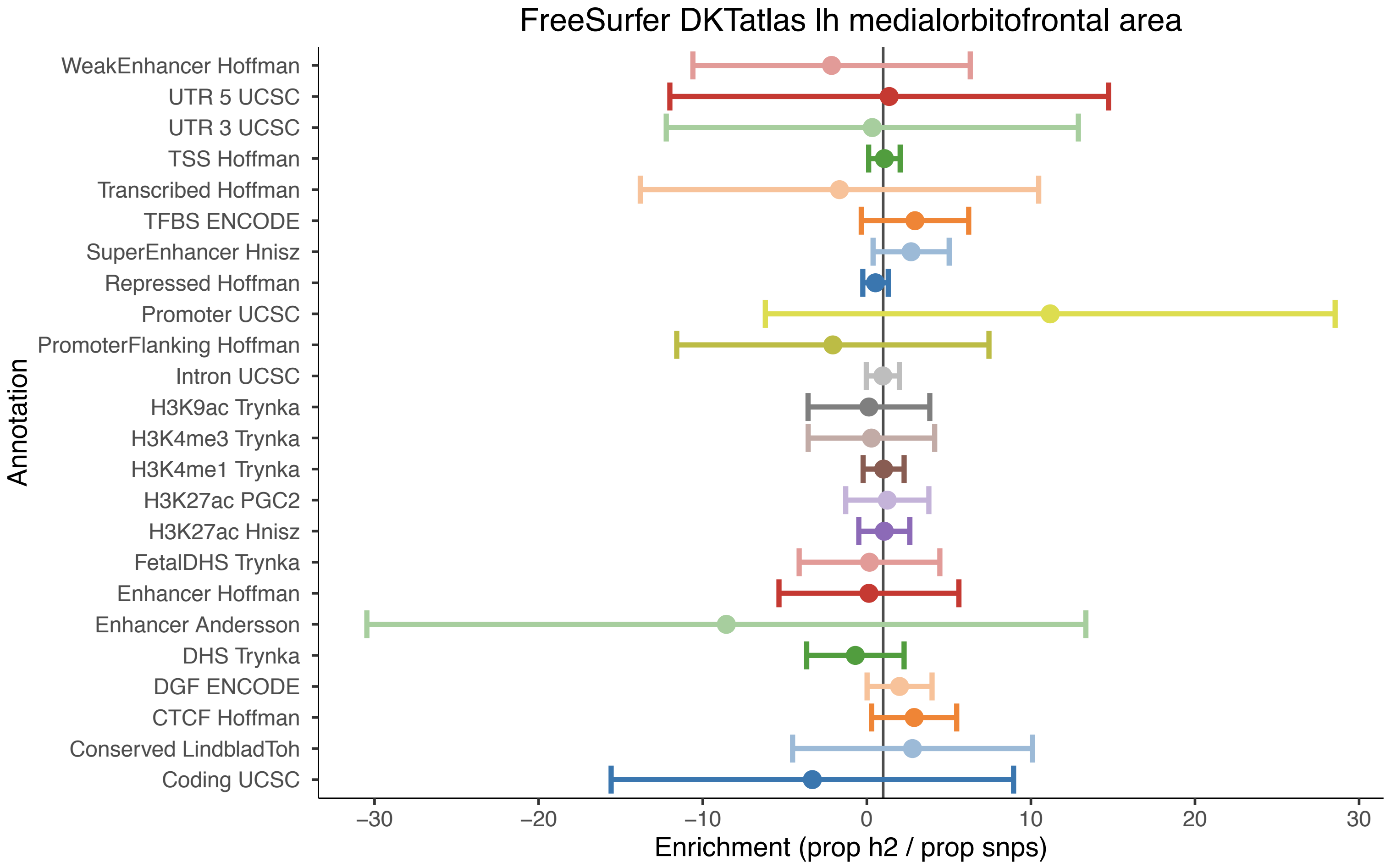
FreeSurfer DKTatlas lh medialorbitofrontal area
WeakEnhancer Hoffman
UTR 5 UCSC
UTR 3 UCSC
TSS Hoffman
Transcribed Hoffman
TFBS ENCODE
SuperEnhancer Hnisz
Repressed Hoffman
Promoter UCSC
PromoterFlanking Hoffman
Intron UCSC
H3K9ac Trynka
H3K4me3 Trynka
H3K4me1 Trynka
H3K27ac PGC2
H3K27ac Hnisz
FetalDHS Trynka
Enhancer Hoffman
Enhancer Andersson
DHS Trynka
DGF ENCODE
CTCF Hoffman
Conserved LindbladToh
Coding UCSC
Annotation
−30
−20
−10
0
10
20
30
Enrichment (prop h2 / prop snps)

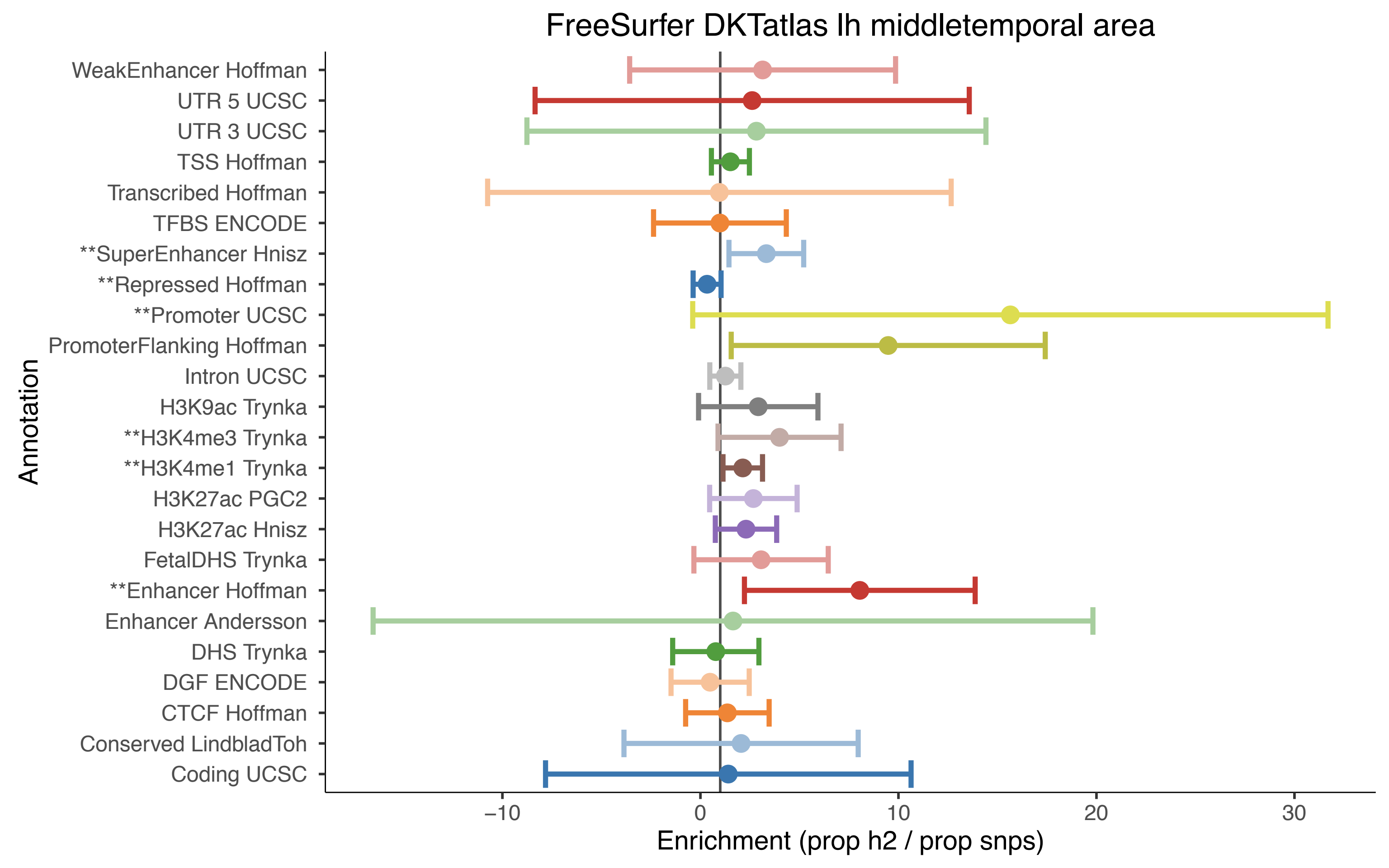
FreeSurfer DKTatlas lh middletemporal area
WeakEnhancer Hoffman
UTR 5 UCSC
UTR 3 UCSC
TSS Hoffman
Transcribed Hoffman
TFBS ENCODE
**SuperEnhancer Hnisz
**Repressed Hoffman
**Promoter UCSC
PromoterFlanking Hoffman
Intron UCSC
H3K9ac Trynka
**H3K4me3 Trynka
**H3K4me1 Trynka
H3K27ac PGC2
H3K27ac Hnisz
FetalDHS Trynka
**Enhancer Hoffman
Enhancer Andersson
DHS Trynka
DGF ENCODE
CTCF Hoffman
Conserved LindbladToh
Coding UCSC
Annotation
−10
0
10
20
30
Enrichment (prop h2 / prop snps)

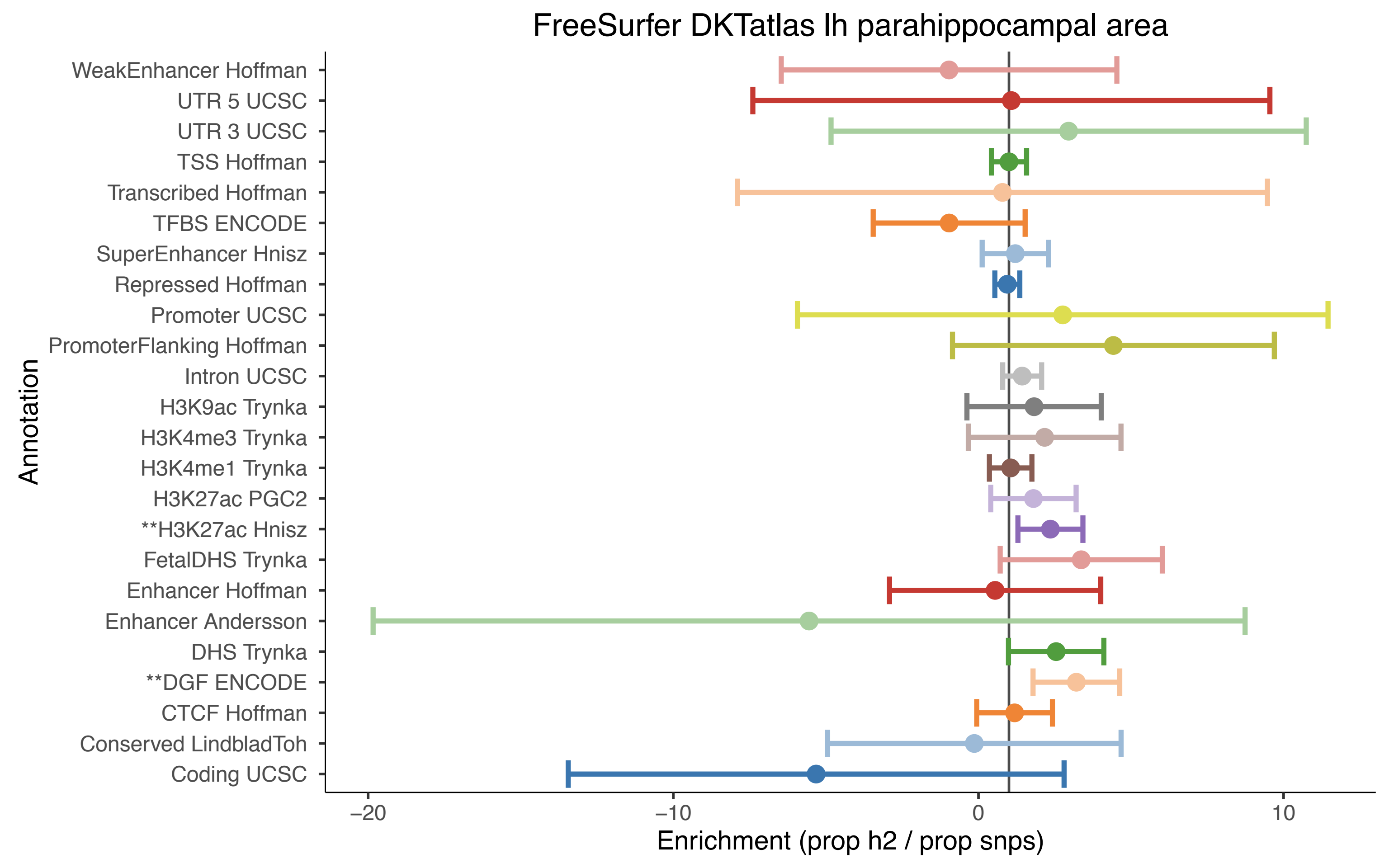
FreeSurfer DKTatlas lh parahippocampal area
WeakEnhancer Hoffman
UTR 5 UCSC
UTR 3 UCSC
TSS Hoffman
Transcribed Hoffman
TFBS ENCODE
SuperEnhancer Hnisz
Repressed Hoffman
Promoter UCSC
PromoterFlanking Hoffman
Intron UCSC
H3K9ac Trynka
H3K4me3 Trynka
H3K4me1 Trynka
H3K27ac PGC2
**H3K27ac Hnisz
FetalDHS Trynka
Enhancer Hoffman
Enhancer Andersson
DHS Trynka
**DGF ENCODE
CTCF Hoffman
Conserved LindbladToh
Coding UCSC
Annotation
−20
−10
0
10
Enrichment (prop h2 / prop snps)

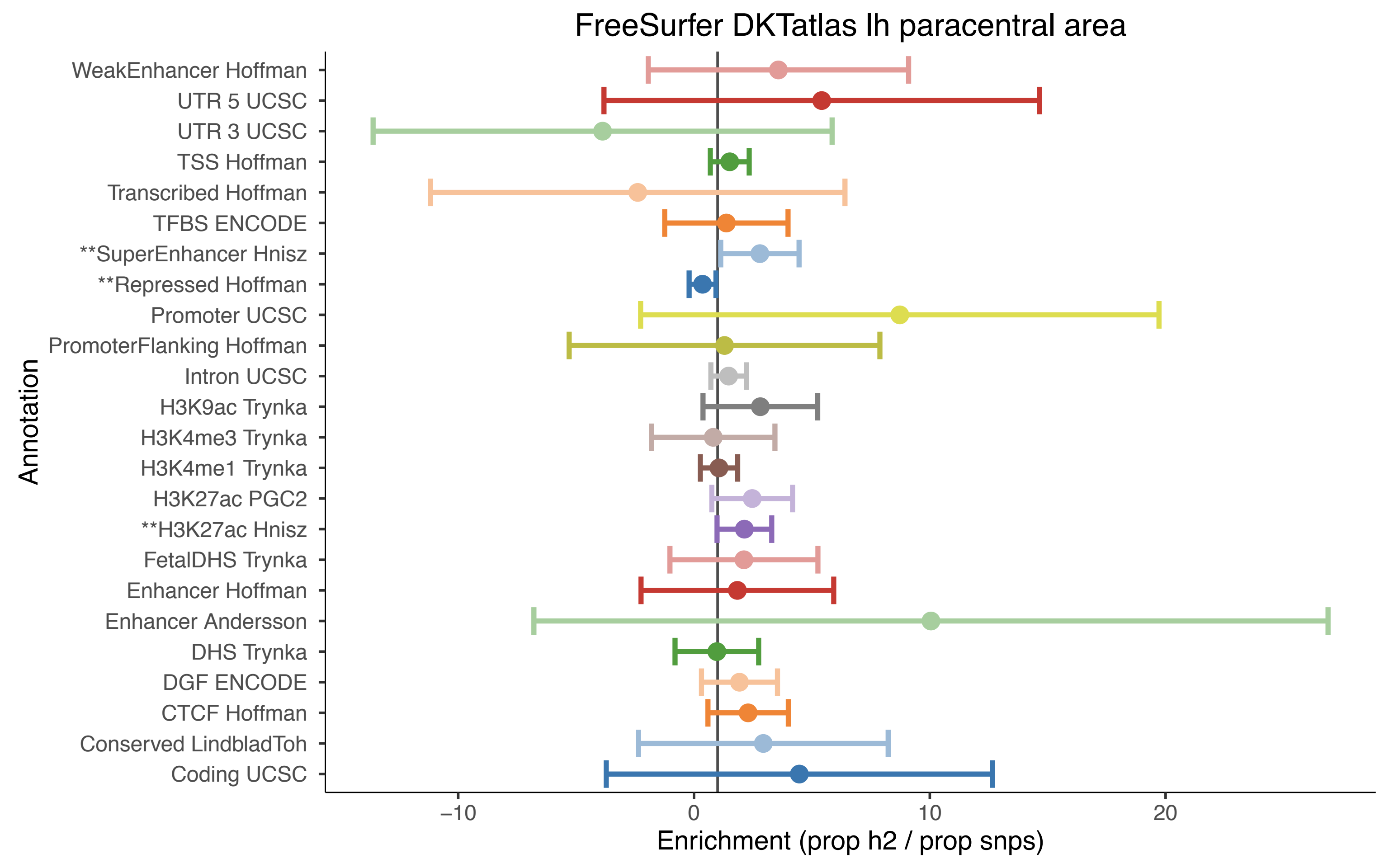
FreeSurfer DKTatlas lh paracentral area
WeakEnhancer Hoffman
UTR 5 UCSC
UTR 3 UCSC
TSS Hoffman
Transcribed Hoffman
TFBS ENCODE
**SuperEnhancer Hnisz
**Repressed Hoffman
Promoter UCSC
PromoterFlanking Hoffman
Intron UCSC
H3K9ac Trynka
H3K4me3 Trynka
H3K4me1 Trynka
H3K27ac PGC2
**H3K27ac Hnisz
FetalDHS Trynka
Enhancer Hoffman
Enhancer Andersson
DHS Trynka
DGF ENCODE
CTCF Hoffman
Conserved LindbladToh
Coding UCSC
Annotation
−10
0
10
20
Enrichment (prop h2 / prop snps)

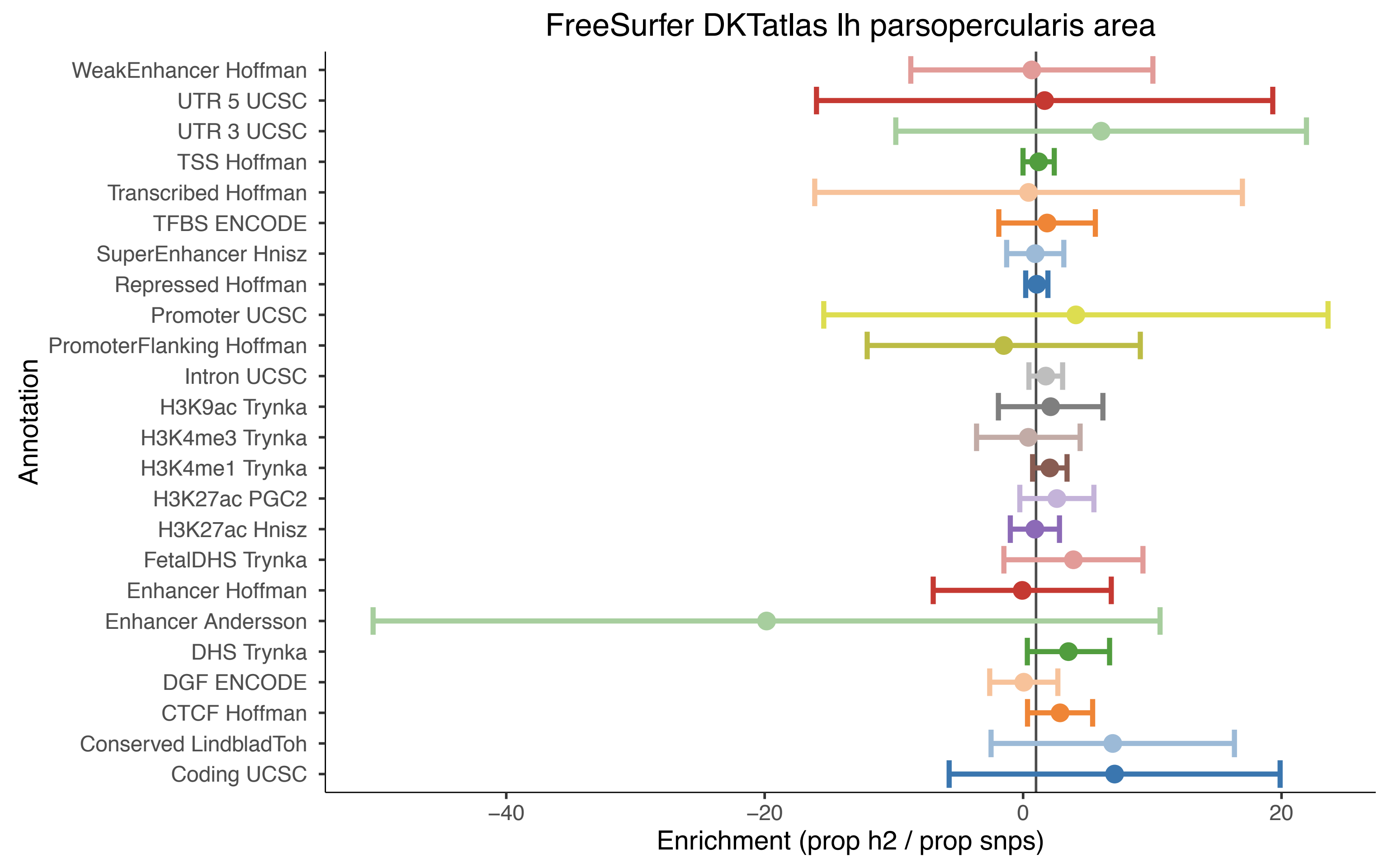

FreeSurfer DKTatlas lh parsopercularis area
WeakEnhancer Hoffman
UTR 5 UCSC
UTR 3 UCSC
TSS Hoffman
Transcribed Hoffman
TFBS ENCODE
SuperEnhancer Hnisz
Repressed Hoffman
Promoter UCSC
PromoterFlanking Hoffman
Intron UCSC
H3K9ac Trynka
H3K4me3 Trynka
H3K4me1 Trynka
H3K27ac PGC2
H3K27ac Hnisz
FetalDHS Trynka
Enhancer Hoffman
Enhancer Andersson
DHS Trynka
DGF ENCODE
CTCF Hoffman
Conserved LindbladToh
Coding UCSC
Annotation
−40
−20
0
20
Enrichment (prop h2 / prop snps)

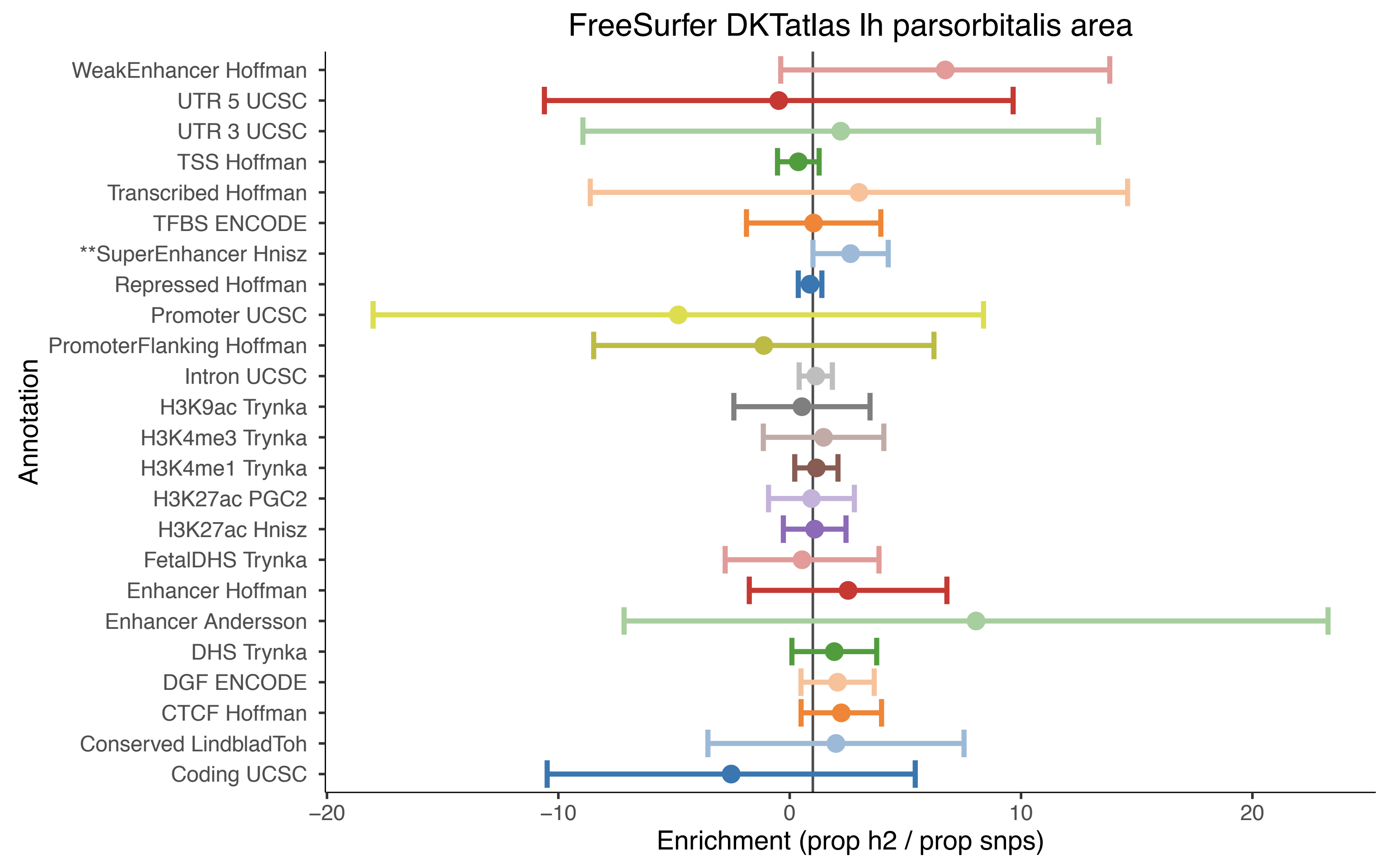
FreeSurfer DKTatlas lh parsorbitalis area
WeakEnhancer Hoffman
UTR 5 UCSC
UTR 3 UCSC
TSS Hoffman
Transcribed Hoffman
TFBS ENCODE
**SuperEnhancer Hnisz
Repressed Hoffman
Promoter UCSC
PromoterFlanking Hoffman
Intron UCSC
H3K9ac Trynka
H3K4me3 Trynka
H3K4me1 Trynka
H3K27ac PGC2
H3K27ac Hnisz
FetalDHS Trynka
Enhancer Hoffman
Enhancer Andersson
DHS Trynka
DGF ENCODE
CTCF Hoffman
Conserved LindbladToh
Coding UCSC
Annotation
−20
−10
0
10
20
Enrichment (prop h2 / prop snps)

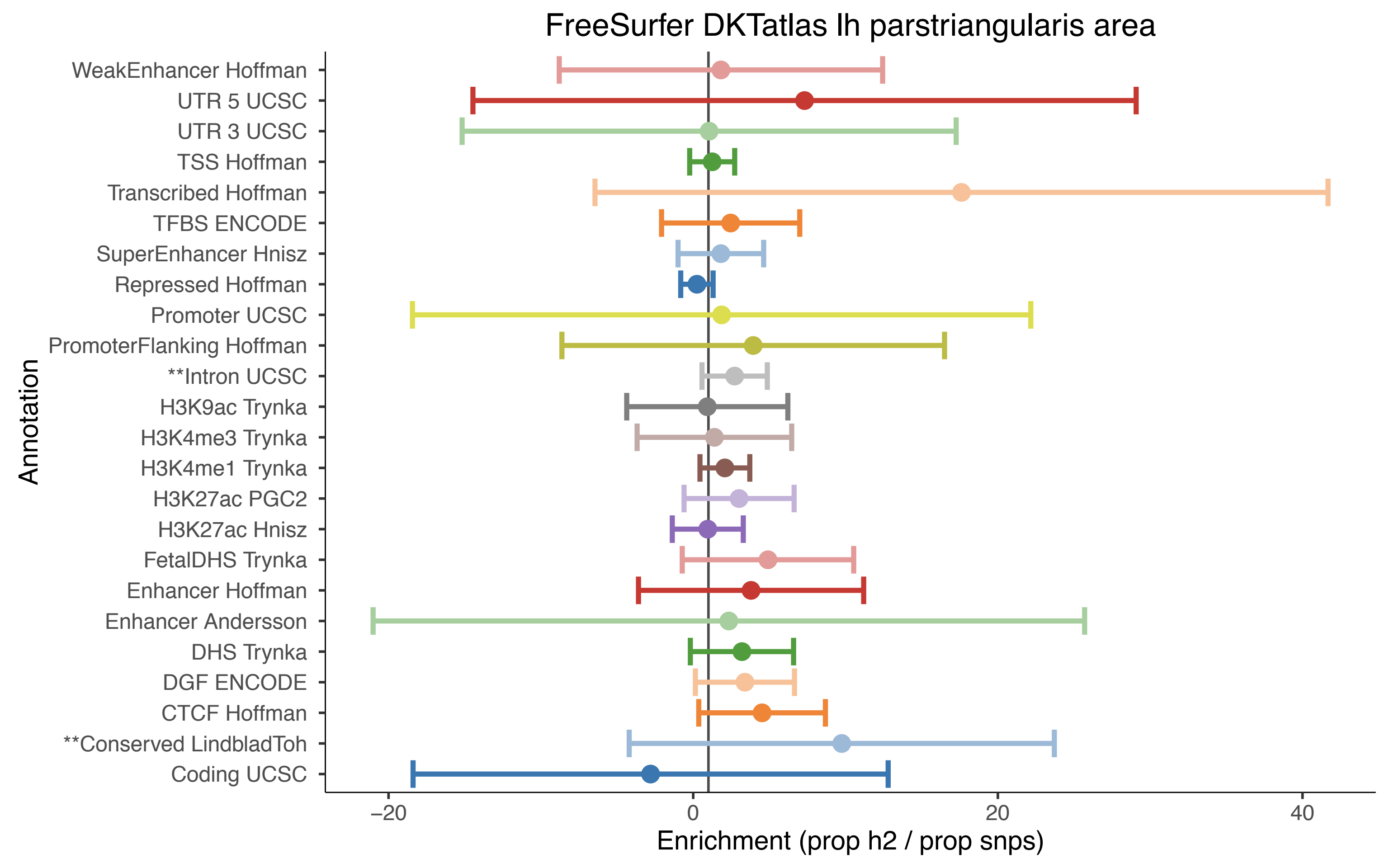

FreeSurfer DKTatlas lh parstriangularis area
WeakEnhancer Hoffman
UTR 5 UCSC
UTR 3 UCSC
TSS Hoffman
Transcribed Hoffman
TFBS ENCODE
SuperEnhancer Hnisz
Repressed Hoffman
Promoter UCSC
PromoterFlanking Hoffman
**Intron UCSC
H3K9ac Trynka
H3K4me3 Trynka
H3K4me1 Trynka
H3K27ac PGC2
H3K27ac Hnisz
FetalDHS Trynka
Enhancer Hoffman
Enhancer Andersson
DHS Trynka
DGF ENCODE
CTCF Hoffman
**Conserved LindbladToh
Coding UCSC
Annotation
−20
0
20
40
Enrichment (prop h2 / prop snps)

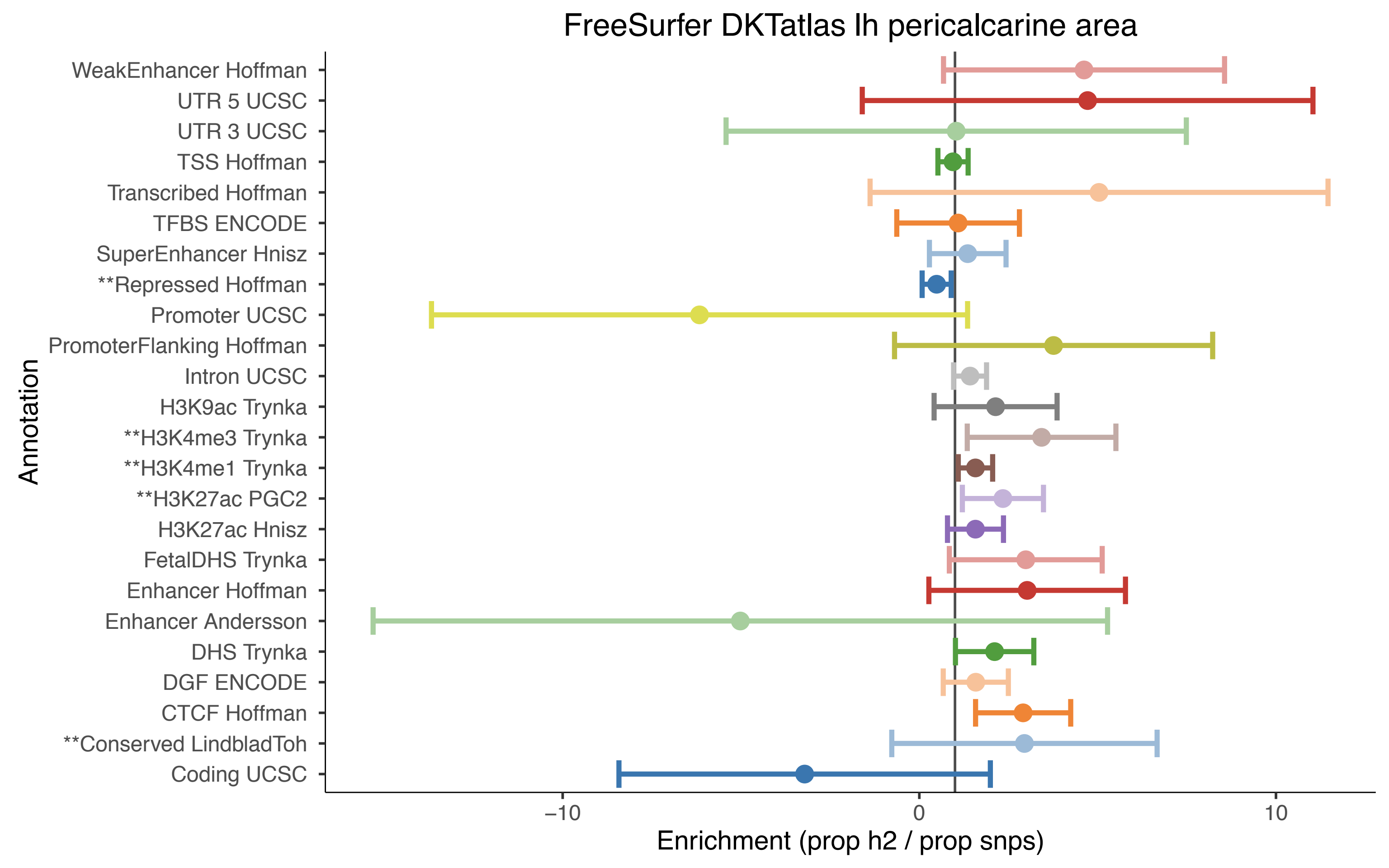
FreeSurfer DKTatlas lh pericalcarine area
WeakEnhancer Hoffman
UTR 5 UCSC
UTR 3 UCSC
TSS Hoffman
Transcribed Hoffman
TFBS ENCODE
SuperEnhancer Hnisz
**Repressed Hoffman
Promoter UCSC
PromoterFlanking Hoffman
Intron UCSC
H3K9ac Trynka
**H3K4me3 Trynka
**H3K4me1 Trynka
**H3K27ac PGC2
H3K27ac Hnisz
FetalDHS Trynka
Enhancer Hoffman
Enhancer Andersson
DHS Trynka
DGF ENCODE
CTCF Hoffman
**Conserved LindbladToh
Coding UCSC
Annotation
−10
0
10
Enrichment (prop h2 / prop snps)

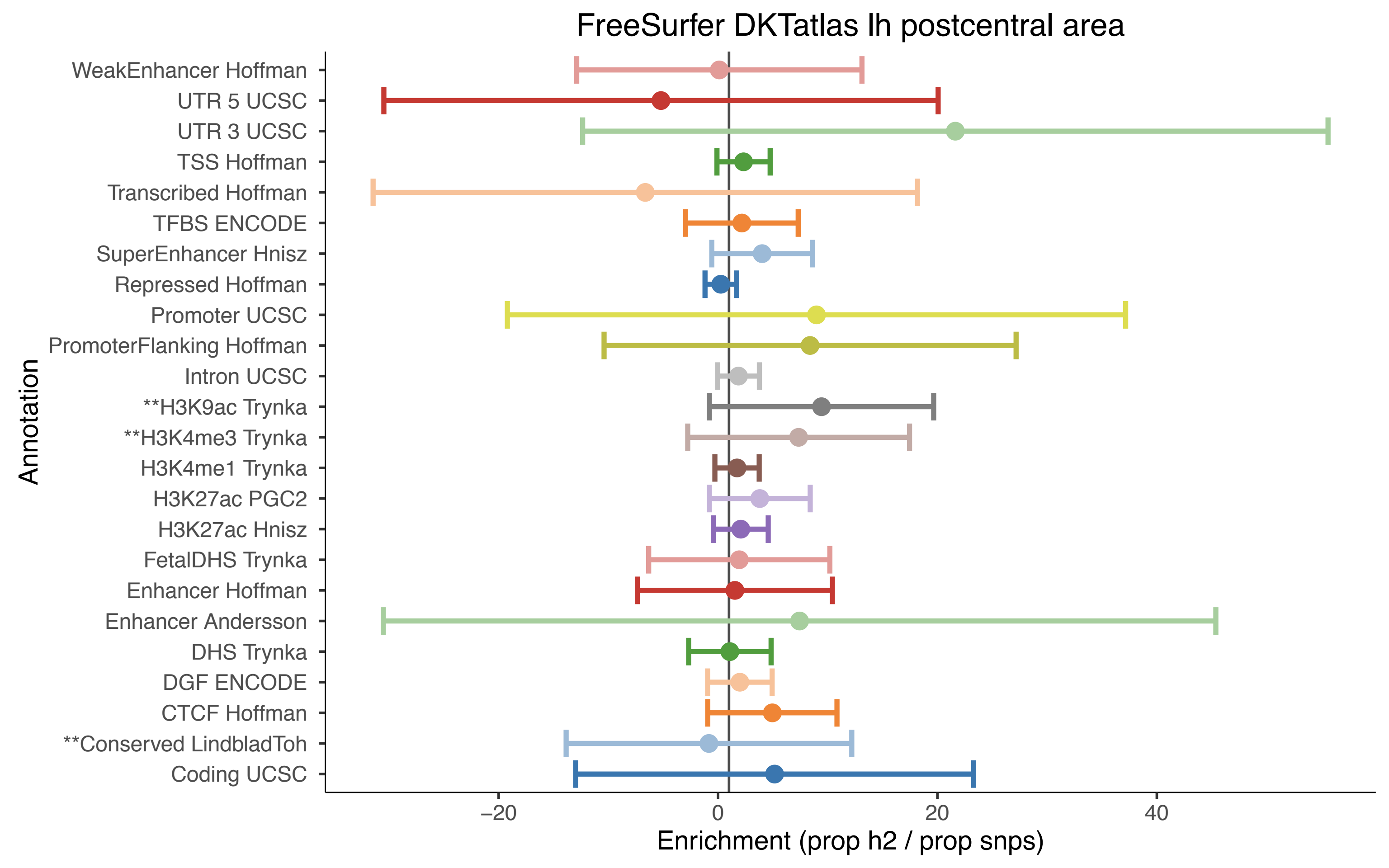
FreeSurfer DKTatlas lh postcentral area
WeakEnhancer Hoffman
UTR 5 UCSC
UTR 3 UCSC
TSS Hoffman
Transcribed Hoffman
TFBS ENCODE
SuperEnhancer Hnisz
Repressed Hoffman
Promoter UCSC
PromoterFlanking Hoffman
Intron UCSC
**H3K9ac Trynka
**H3K4me3 Trynka
H3K4me1 Trynka
H3K27ac PGC2
H3K27ac Hnisz
FetalDHS Trynka
Enhancer Hoffman
Enhancer Andersson
DHS Trynka
DGF ENCODE
CTCF Hoffman
**Conserved LindbladToh
Coding UCSC
Annotation
−20
0
20
40
Enrichment (prop h2 / prop snps)

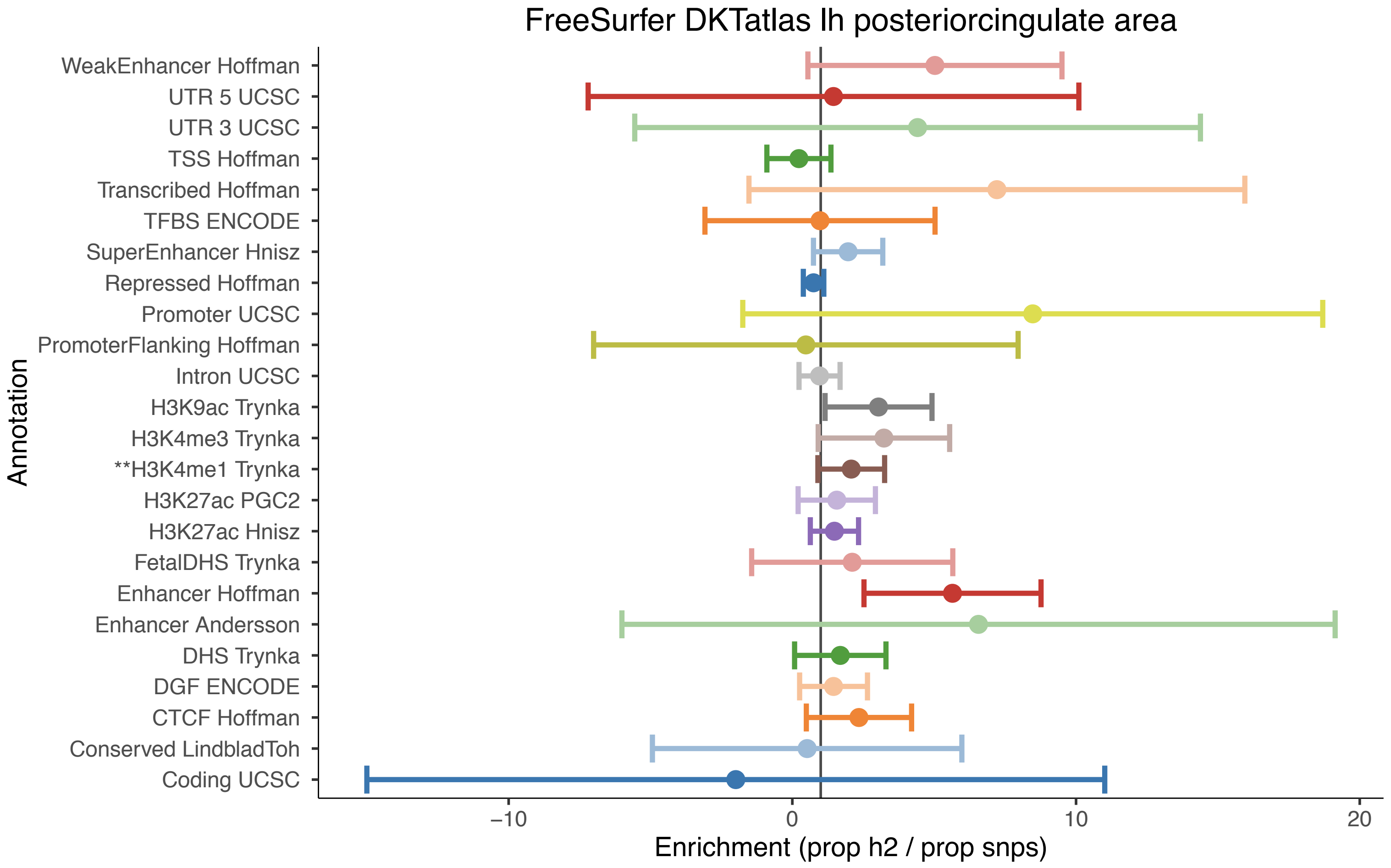

FreeSurfer DKTatlas lh posteriorcingulate area
Annotation
WeakEnhancer Hoffman
UTR 5 UCSC
UTR 3 UCSC
TSS Hoffman
Transcribed Hoffman
TFBS ENCODE
SuperEnhancer Hnisz
Repressed Hoffman
Promoter UCSC
PromoterFlanking Hoffman
Intron UCSC
H3K9ac Trynka
H3K4me3 Trynka
**H3K4me1 Trynka
H3K27ac PGC2
H3K27ac Hnisz
FetalDHS Trynka
Enhancer Hoffman
Enhancer Andersson
DHS Trynka
DGF ENCODE
CTCF Hoffman
Conserved LindbladToh
Coding UCSC
−10
0
10
20
Enrichment (prop h2 / prop snps)

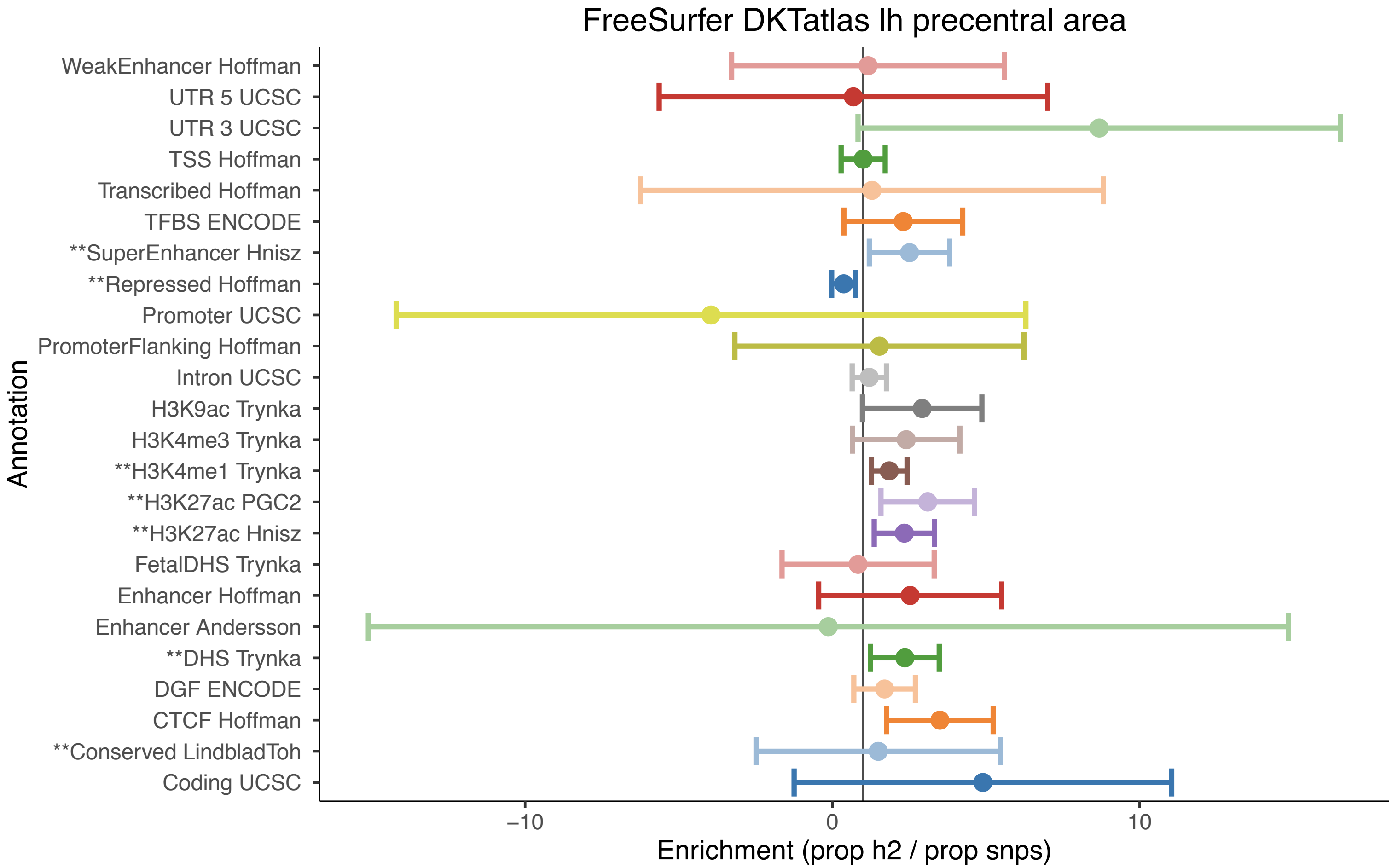
FreeSurfer DKTatlas lh precentral area
WeakEnhancer Hoffman
UTR 5 UCSC
UTR 3 UCSC
TSS Hoffman
Transcribed Hoffman
TFBS ENCODE
**SuperEnhancer Hnisz
**Repressed Hoffman
Promoter UCSC
PromoterFlanking Hoffman
Intron UCSC
H3K9ac Trynka
H3K4me3 Trynka
**H3K4me1 Trynka
**H3K27ac PGC2
**H3K27ac Hnisz
FetalDHS Trynka
Enhancer Hoffman
Enhancer Andersson
**DHS Trynka
DGF ENCODE
CTCF Hoffman
**Conserved LindbladToh
Coding UCSC
Annotation
−10
0
10
Enrichment (prop h2 / prop snps)

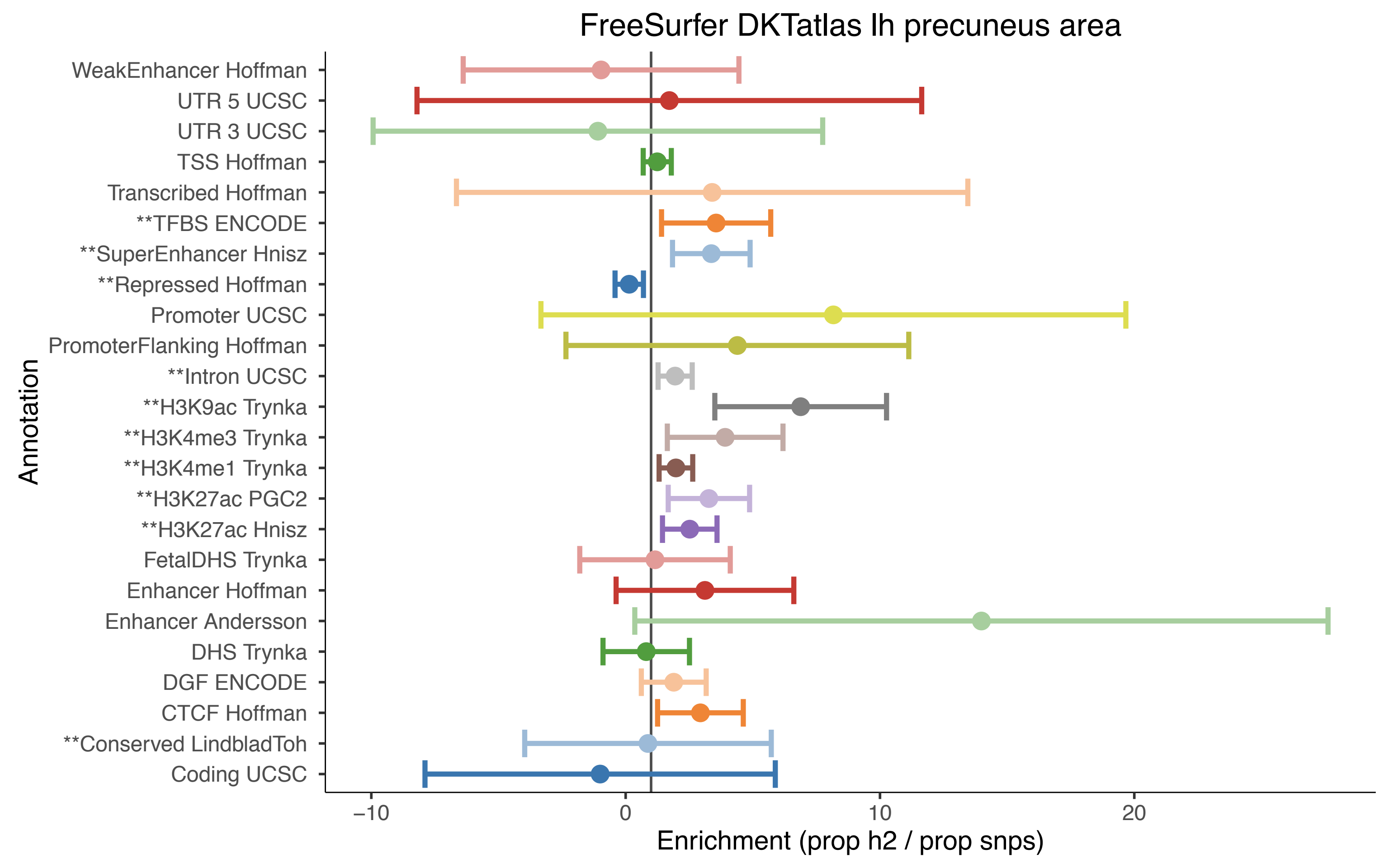

FreeSurfer DKTatlas lh precuneus area
WeakEnhancer Hoffman
UTR 5 UCSC
UTR 3 UCSC
TSS Hoffman
Transcribed Hoffman
**TFBS ENCODE
**SuperEnhancer Hnisz
**Repressed Hoffman
Promoter UCSC
PromoterFlanking Hoffman
**Intron UCSC
**H3K9ac Trynka
**H3K4me3 Trynka
**H3K4me1 Trynka
**H3K27ac PGC2
**H3K27ac Hnisz
FetalDHS Trynka
Enhancer Hoffman
Enhancer Andersson
DHS Trynka
DGF ENCODE
CTCF Hoffman
**Conserved LindbladToh
Coding UCSC
Annotation
−10
0
10
20
Enrichment (prop h2 / prop snps)

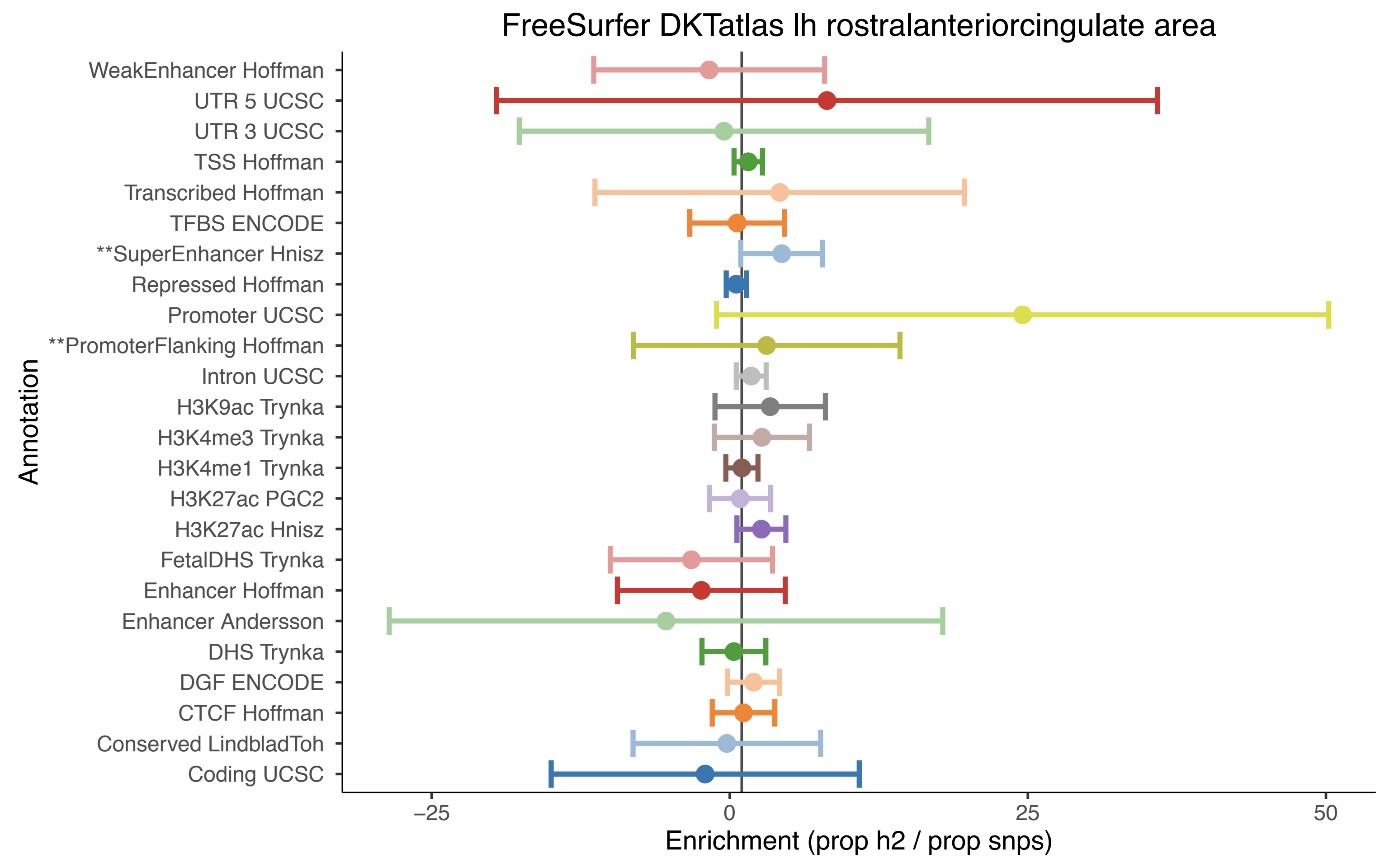
FreeSurfer DKTatlas lh rostralanteriorcingulate area
WeakEnhancer Hoffman
UTR 5 UCSC
UTR 3 UCSC
TSS Hoffman
Transcribed Hoffman
TFBS ENCODE
**SuperEnhancer Hnisz
Repressed Hoffman
Promoter UCSC
**PromoterFlanking Hoffman
Intron UCSC
H3K9ac Trynka
H3K4me3 Trynka
H3K4me1 Trynka
H3K27ac PGC2
H3K27ac Hnisz
FetalDHS Trynka
Enhancer Hoffman
Enhancer Andersson
DHS Trynka
DGF ENCODE
CTCF Hoffman
Conserved LindbladToh
Coding UCSC
Annotation
−25
0
25
50
Enrichment (prop h2 / prop snps)

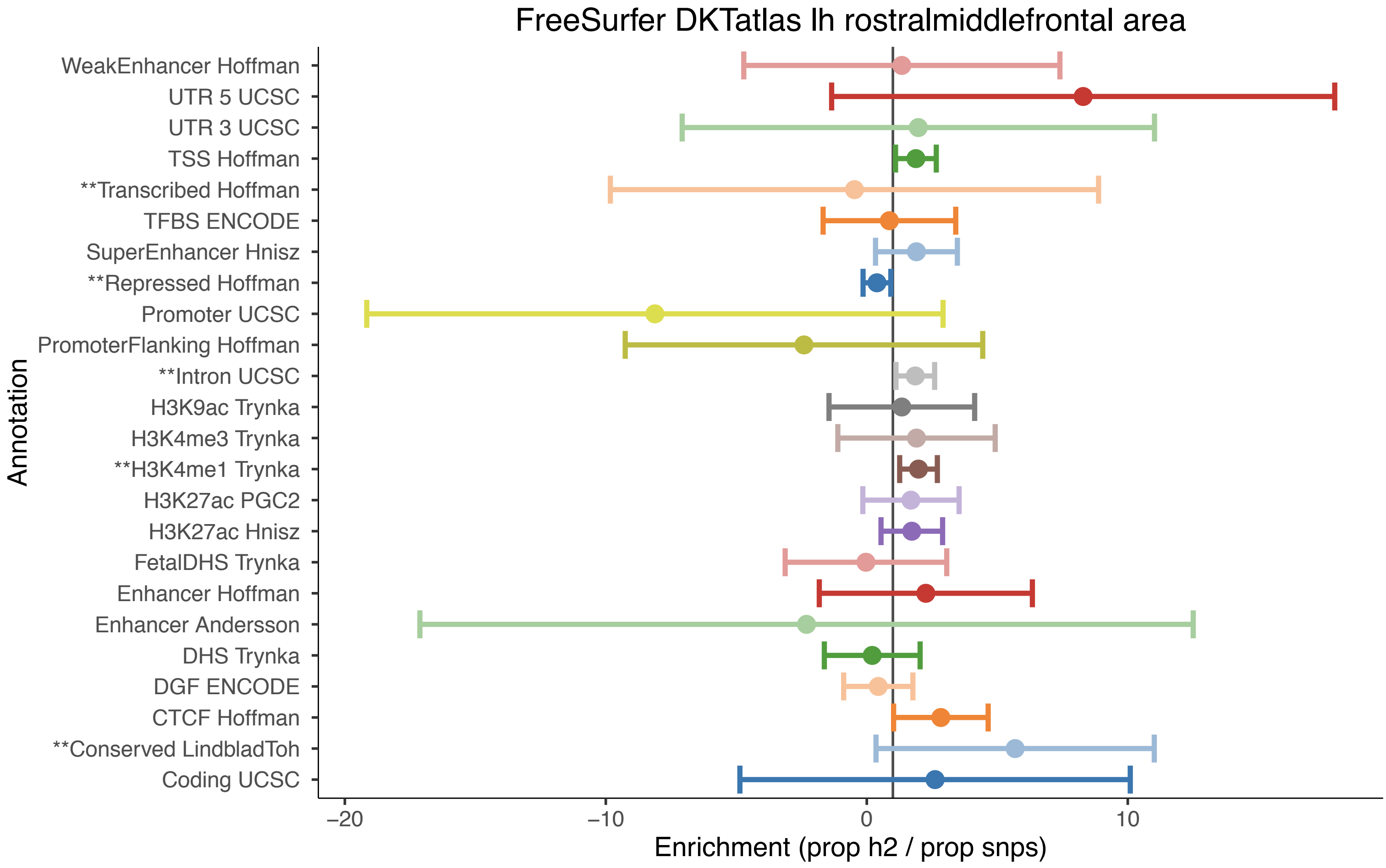

FreeSurfer DKTatlas lh rostralmiddlefrontal area
WeakEnhancer Hoffman
UTR 5 UCSC
UTR 3 UCSC
TSS Hoffman
**Transcribed Hoffman
TFBS ENCODE
SuperEnhancer Hnisz
**Repressed Hoffman
Promoter UCSC
PromoterFlanking Hoffman
**Intron UCSC
H3K9ac Trynka
H3K4me3 Trynka
**H3K4me1 Trynka
H3K27ac PGC2
H3K27ac Hnisz
FetalDHS Trynka
Enhancer Hoffman
Enhancer Andersson
DHS Trynka
DGF ENCODE
CTCF Hoffman
**Conserved LindbladToh
Coding UCSC
Annotation
−20
−10
0
10
Enrichment (prop h2 / prop snps)

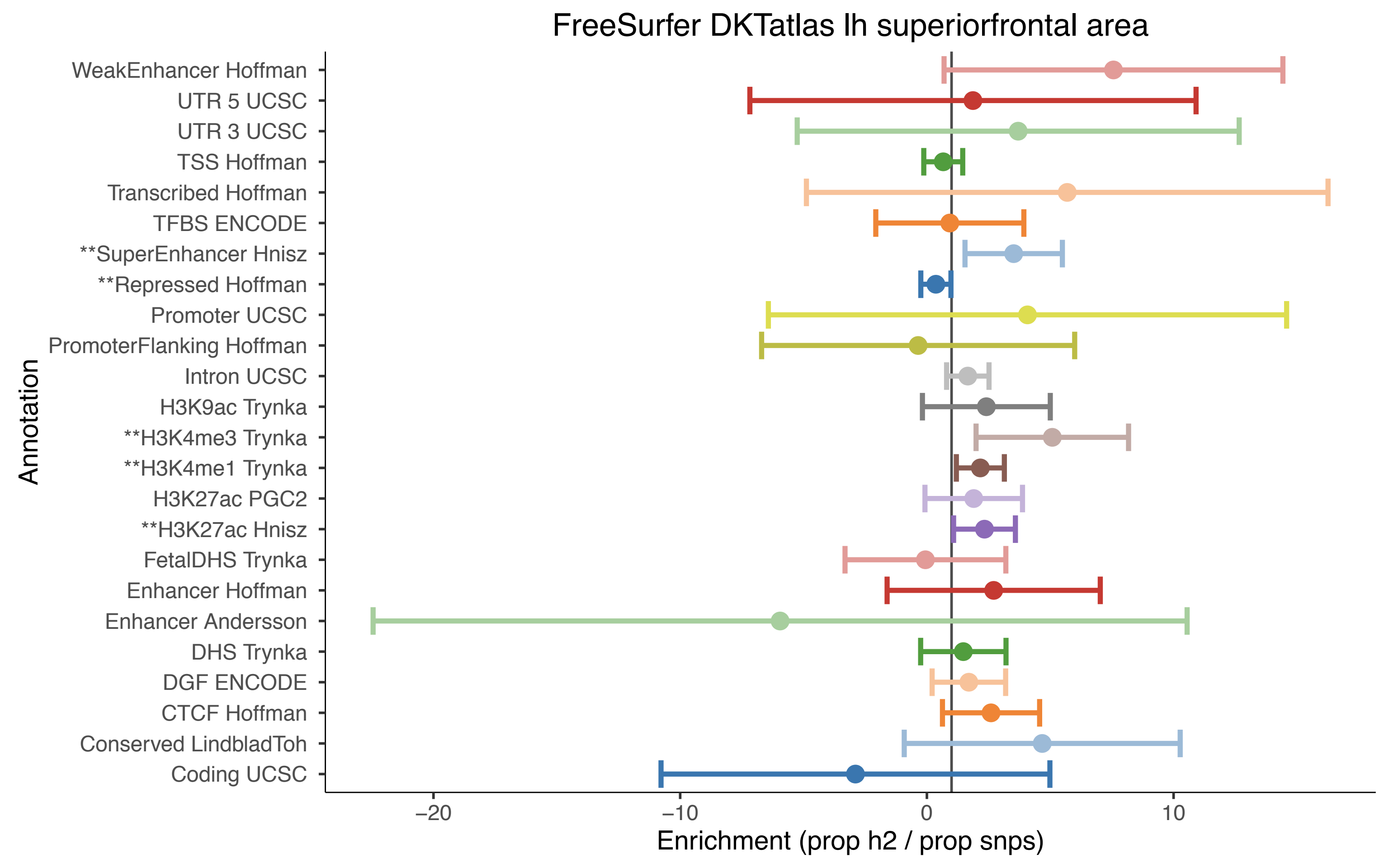

FreeSurfer DKTatlas lh superiorfrontal area
WeakEnhancer Hoffman
UTR 5 UCSC
UTR 3 UCSC
TSS Hoffman
Transcribed Hoffman
TFBS ENCODE
**SuperEnhancer Hnisz
**Repressed Hoffman
Promoter UCSC
PromoterFlanking Hoffman
Intron UCSC
H3K9ac Trynka
**H3K4me3 Trynka
**H3K4me1 Trynka
H3K27ac PGC2
**H3K27ac Hnisz
FetalDHS Trynka
Enhancer Hoffman
Enhancer Andersson
DHS Trynka
DGF ENCODE
CTCF Hoffman
Conserved LindbladToh
Coding UCSC
Annotation
−20
−10
0
10
Enrichment (prop h2 / prop snps)

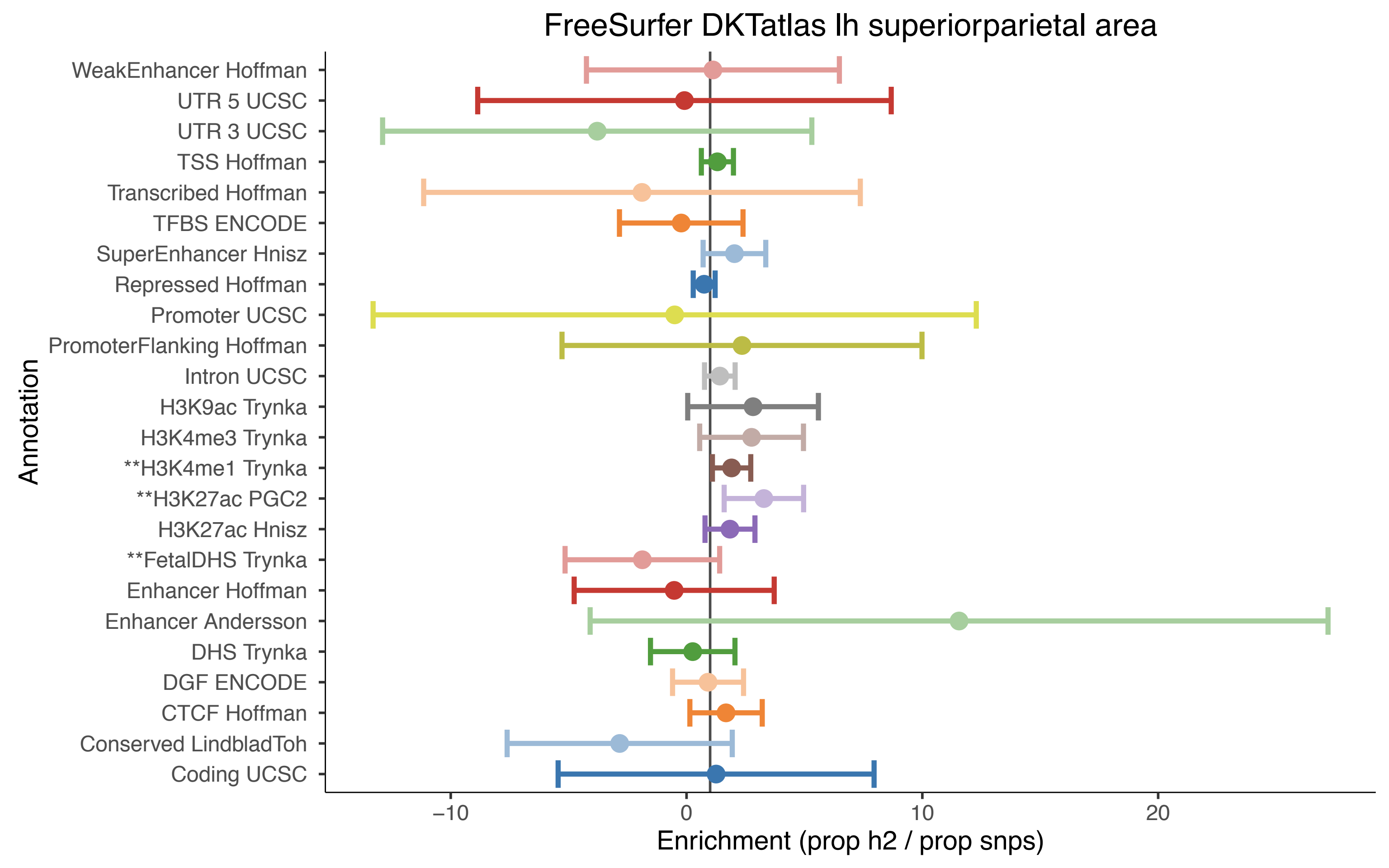
FreeSurfer DKTatlas lh superiorparietal area
WeakEnhancer Hoffman
UTR 5 UCSC
UTR 3 UCSC
TSS Hoffman
Transcribed Hoffman
TFBS ENCODE
SuperEnhancer Hnisz
Repressed Hoffman
Promoter UCSC
PromoterFlanking Hoffman
Intron UCSC
H3K9ac Trynka
H3K4me3 Trynka
**H3K4me1 Trynka
**H3K27ac PGC2
H3K27ac Hnisz
**FetalDHS Trynka
Enhancer Hoffman
Enhancer Andersson
DHS Trynka
DGF ENCODE
CTCF Hoffman
Conserved LindbladToh
Coding UCSC
Annotation
−10
0
10
20
Enrichment (prop h2 / prop snps)

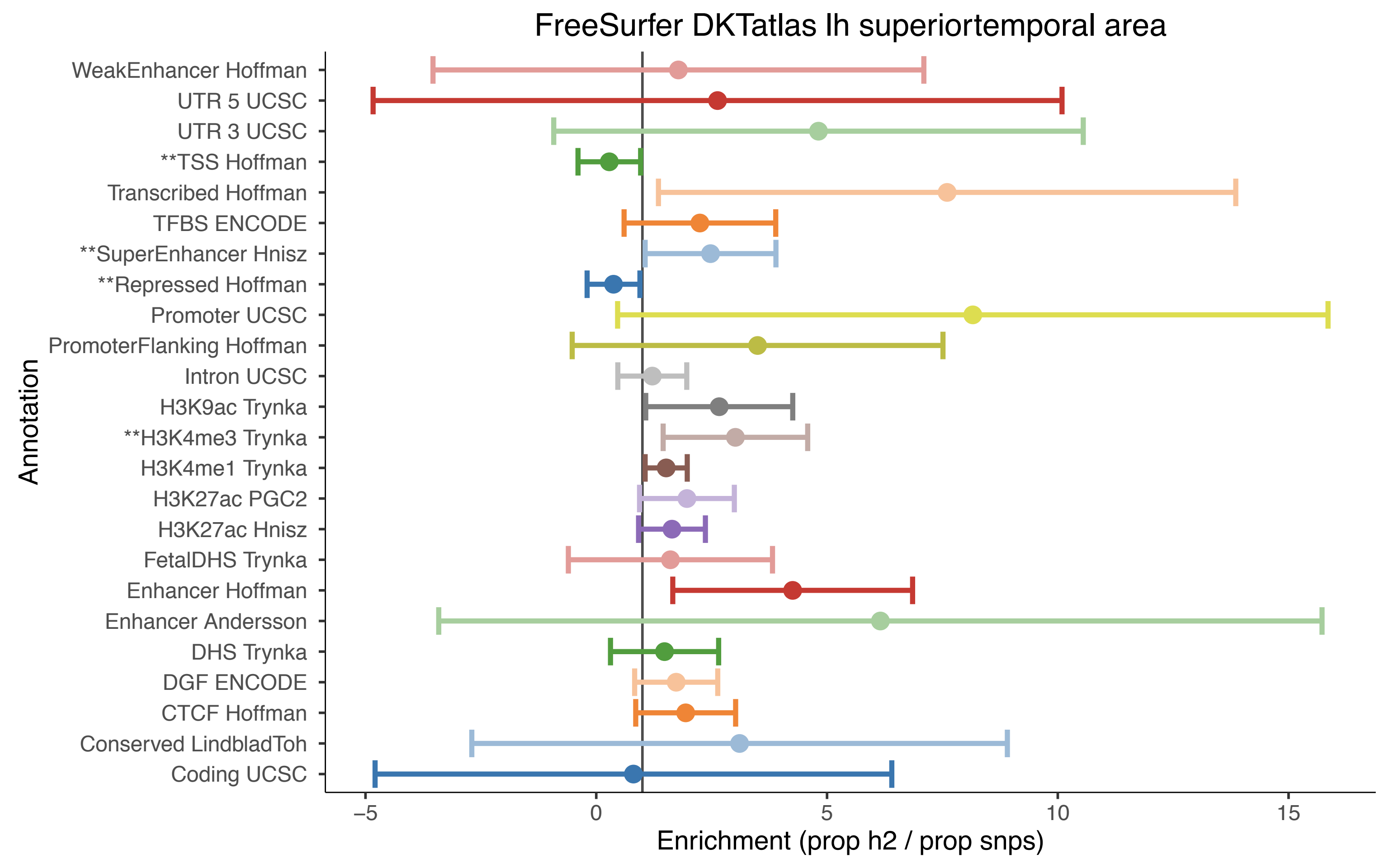
FreeSurfer DKTatlas lh superiortemporal area
WeakEnhancer Hoffman
UTR 5 UCSC
UTR 3 UCSC
**TSS Hoffman
Transcribed Hoffman
TFBS ENCODE
**SuperEnhancer Hnisz
**Repressed Hoffman
Promoter UCSC
PromoterFlanking Hoffman
Intron UCSC
H3K9ac Trynka
**H3K4me3 Trynka
H3K4me1 Trynka
H3K27ac PGC2
H3K27ac Hnisz
FetalDHS Trynka
Enhancer Hoffman
Enhancer Andersson
DHS Trynka
DGF ENCODE
CTCF Hoffman
Conserved LindbladToh
Coding UCSC
Annotation
−5
0
5
10
15
Enrichment (prop h2 / prop snps)

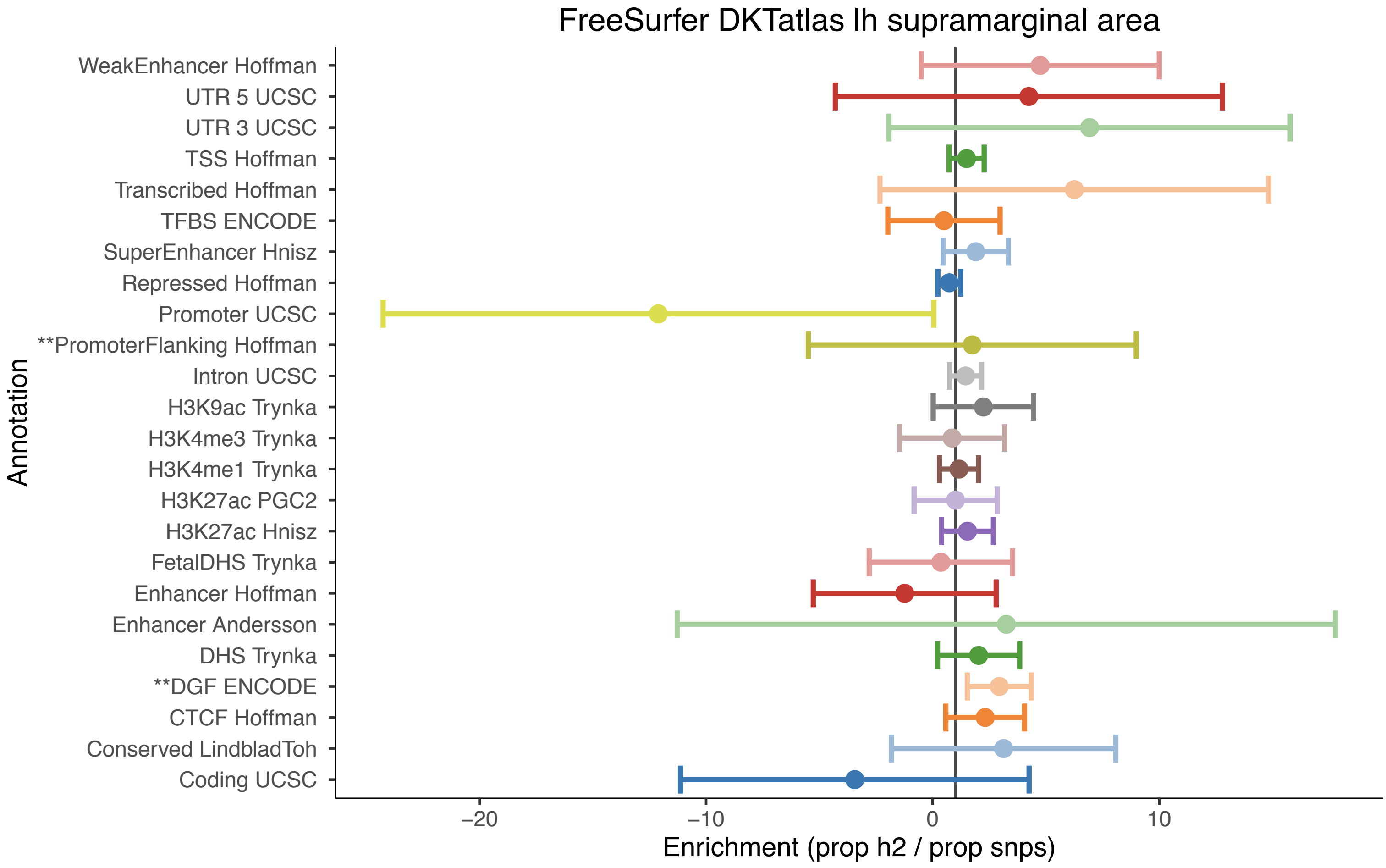
FreeSurfer DKTatlas lh supramarginal area
Annotation
WeakEnhancer Hoffman
UTR 5 UCSC
UTR 3 UCSC
TSS Hoffman
Transcribed Hoffman
TFBS ENCODE
SuperEnhancer Hnisz
Repressed Hoffman
Promoter UCSC
**PromoterFlanking Hoffman
Intron UCSC
H3K9ac Trynka
H3K4me3 Trynka
H3K4me1 Trynka
H3K27ac PGC2
H3K27ac Hnisz
FetalDHS Trynka
Enhancer Hoffman
Enhancer Andersson
DHS Trynka
**DGF ENCODE
CTCF Hoffman
Conserved LindbladToh
Coding UCSC
−20
−10
0
10
Enrichment (prop h2 / prop snps)

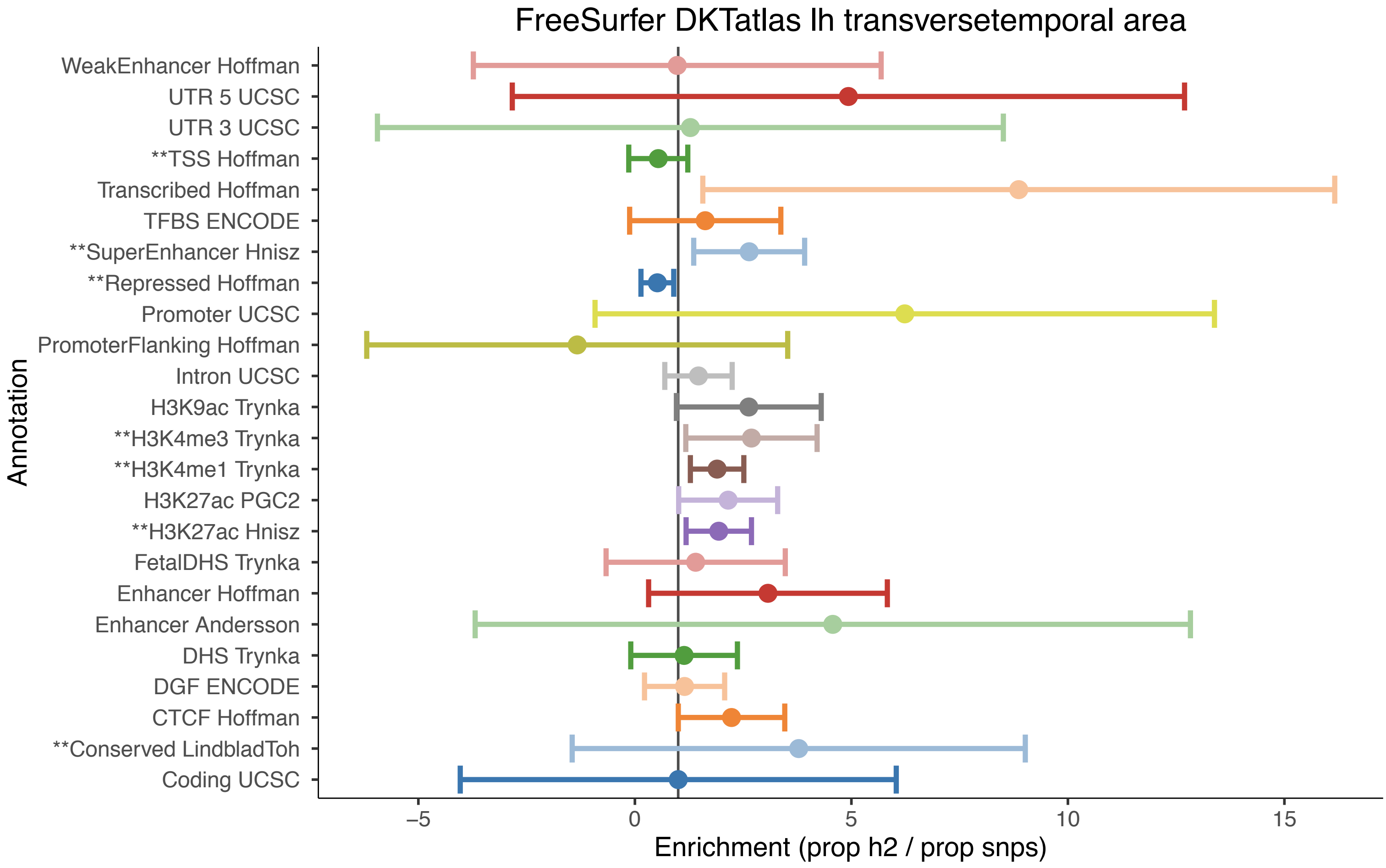

FreeSurfer DKTatlas lh transversetemporal area
WeakEnhancer Hoffman
UTR 5 UCSC
UTR 3 UCSC
**TSS Hoffman
Transcribed Hoffman
TFBS ENCODE
**SuperEnhancer Hnisz
**Repressed Hoffman
Promoter UCSC
PromoterFlanking Hoffman
Intron UCSC
H3K9ac Trynka
**H3K4me3 Trynka
**H3K4me1 Trynka
H3K27ac PGC2
**H3K27ac Hnisz
FetalDHS Trynka
Enhancer Hoffman
Enhancer Andersson
DHS Trynka
DGF ENCODE
CTCF Hoffman
**Conserved LindbladToh
Coding UCSC
Annotation
−5
0
5
10
15
Enrichment (prop h2 / prop snps)

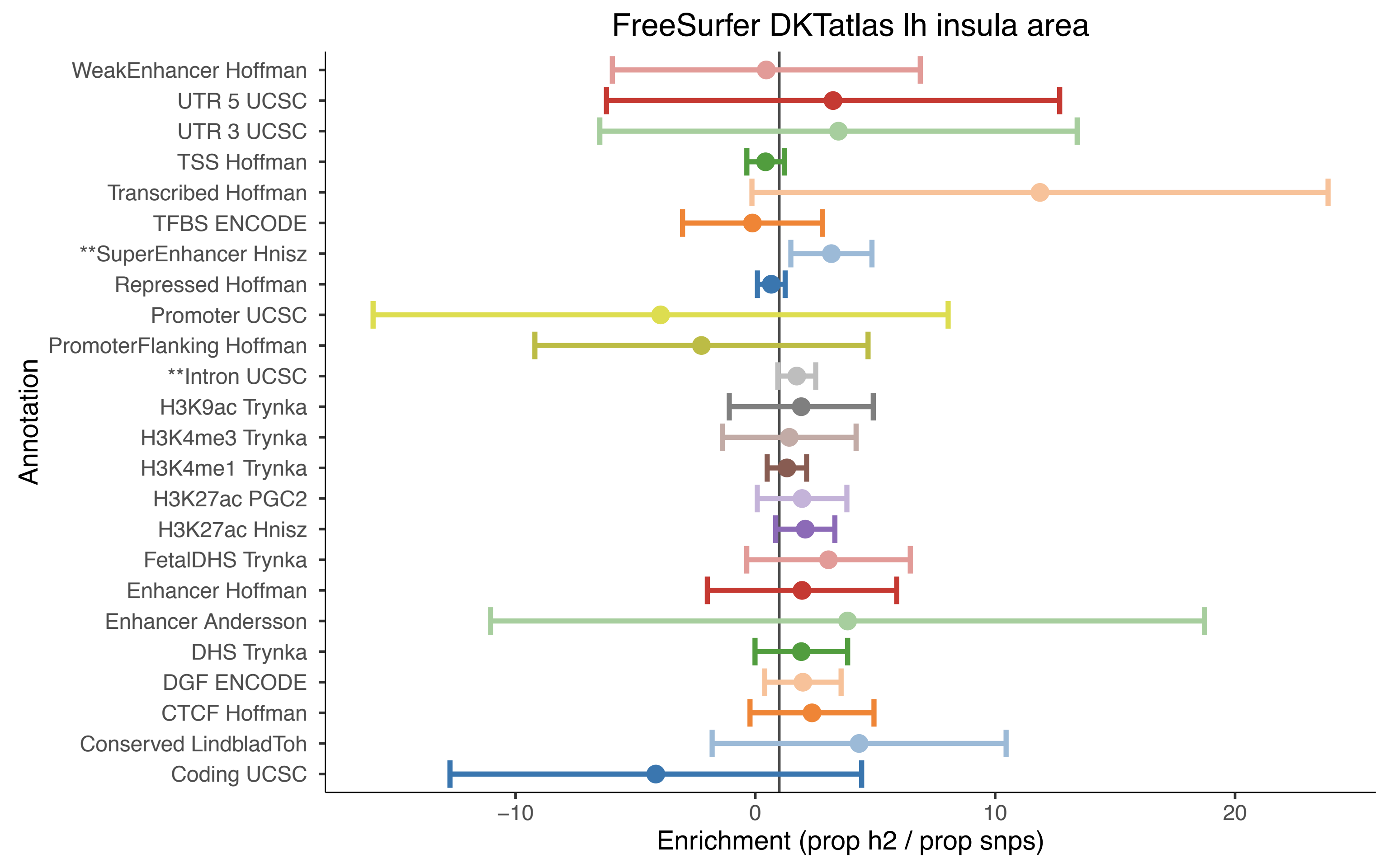
FreeSurfer DKTatlas lh insula area
WeakEnhancer Hoffman
UTR 5 UCSC
UTR 3 UCSC
TSS Hoffman
Transcribed Hoffman
TFBS ENCODE
**SuperEnhancer Hnisz
Repressed Hoffman
Promoter UCSC
PromoterFlanking Hoffman
**Intron UCSC
H3K9ac Trynka
H3K4me3 Trynka
H3K4me1 Trynka
H3K27ac PGC2
H3K27ac Hnisz
FetalDHS Trynka
Enhancer Hoffman
Enhancer Andersson
DHS Trynka
DGF ENCODE
CTCF Hoffman
Conserved LindbladToh
Coding UCSC
Annotation
−10
0
10
20
Enrichment (prop h2 / prop snps)

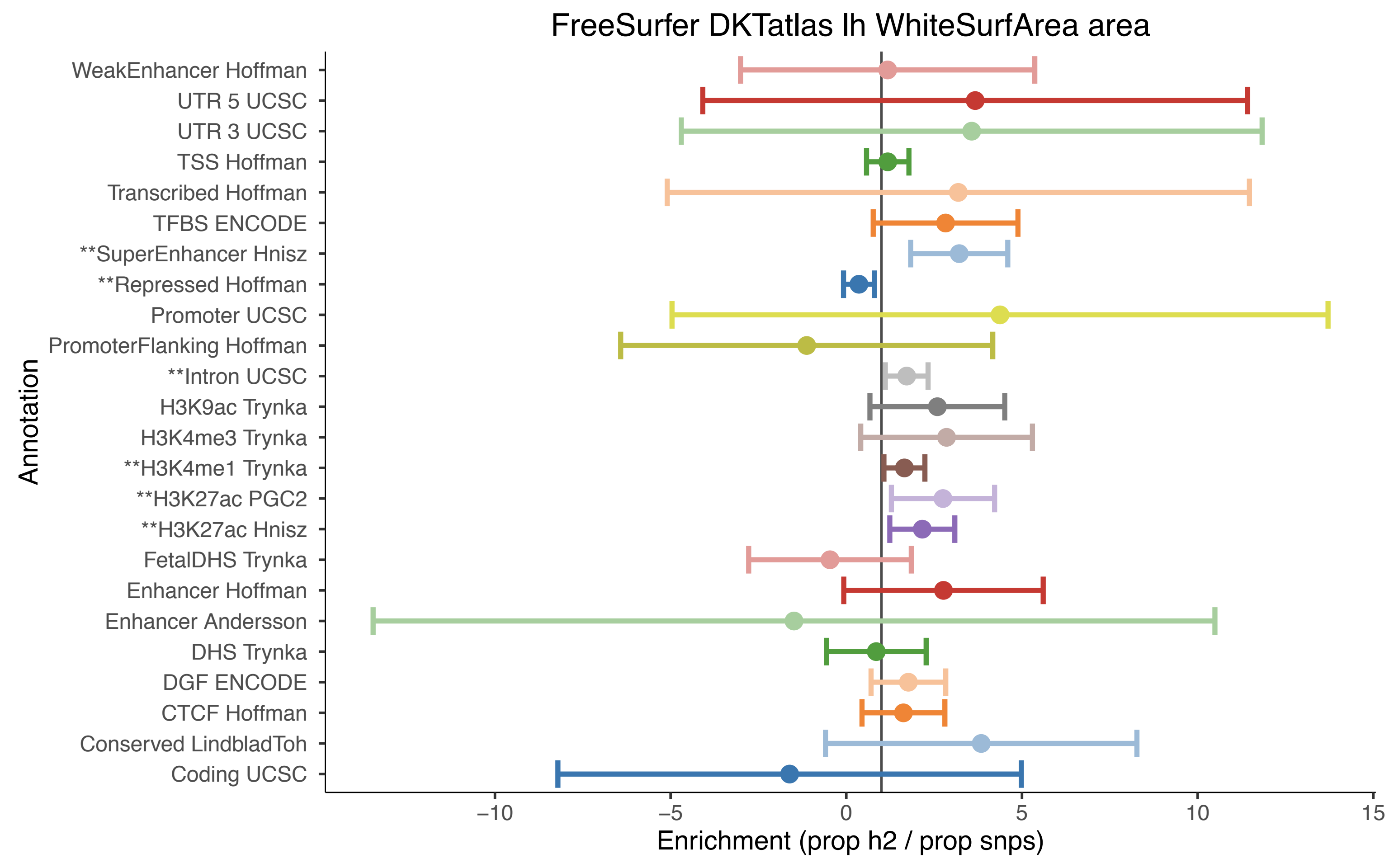
FreeSurfer DKTatlas lh WhiteSurfArea area
WeakEnhancer Hoffman
UTR 5 UCSC
UTR 3 UCSC
TSS Hoffman
Transcribed Hoffman
TFBS ENCODE
**SuperEnhancer Hnisz
**Repressed Hoffman
Promoter UCSC
PromoterFlanking Hoffman
**Intron UCSC
H3K9ac Trynka
H3K4me3 Trynka
**H3K4me1 Trynka
**H3K27ac PGC2
**H3K27ac Hnisz
FetalDHS Trynka
Enhancer Hoffman
Enhancer Andersson
DHS Trynka
DGF ENCODE
CTCF Hoffman
Conserved LindbladToh
Coding UCSC
Annotation
−10
−5
0
5
10
15
Enrichment (prop h2 / prop snps)

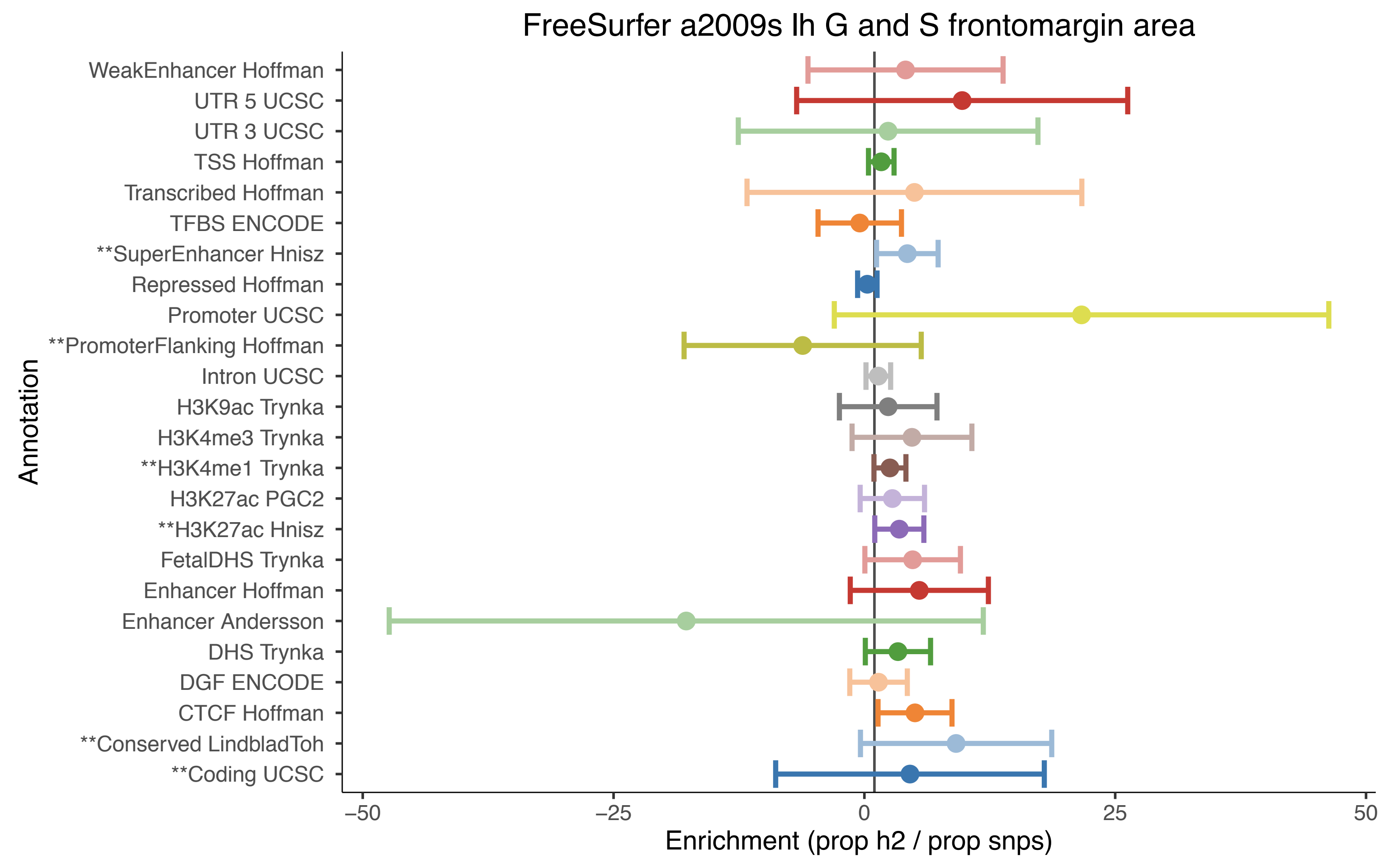
FreeSurfer a2009s lh G and S frontomargin area
WeakEnhancer Hoffman
UTR 5 UCSC
UTR 3 UCSC
TSS Hoffman
Transcribed Hoffman
TFBS ENCODE
**SuperEnhancer Hnisz
Repressed Hoffman
Promoter UCSC
**PromoterFlanking Hoffman
Intron UCSC
H3K9ac Trynka
H3K4me3 Trynka
**H3K4me1 Trynka
H3K27ac PGC2
**H3K27ac Hnisz
FetalDHS Trynka
Enhancer Hoffman
Enhancer Andersson
DHS Trynka
DGF ENCODE
CTCF Hoffman
**Conserved LindbladToh
**Coding UCSC
Annotation
−50
−25
0
25
50
Enrichment (prop h2 / prop snps)

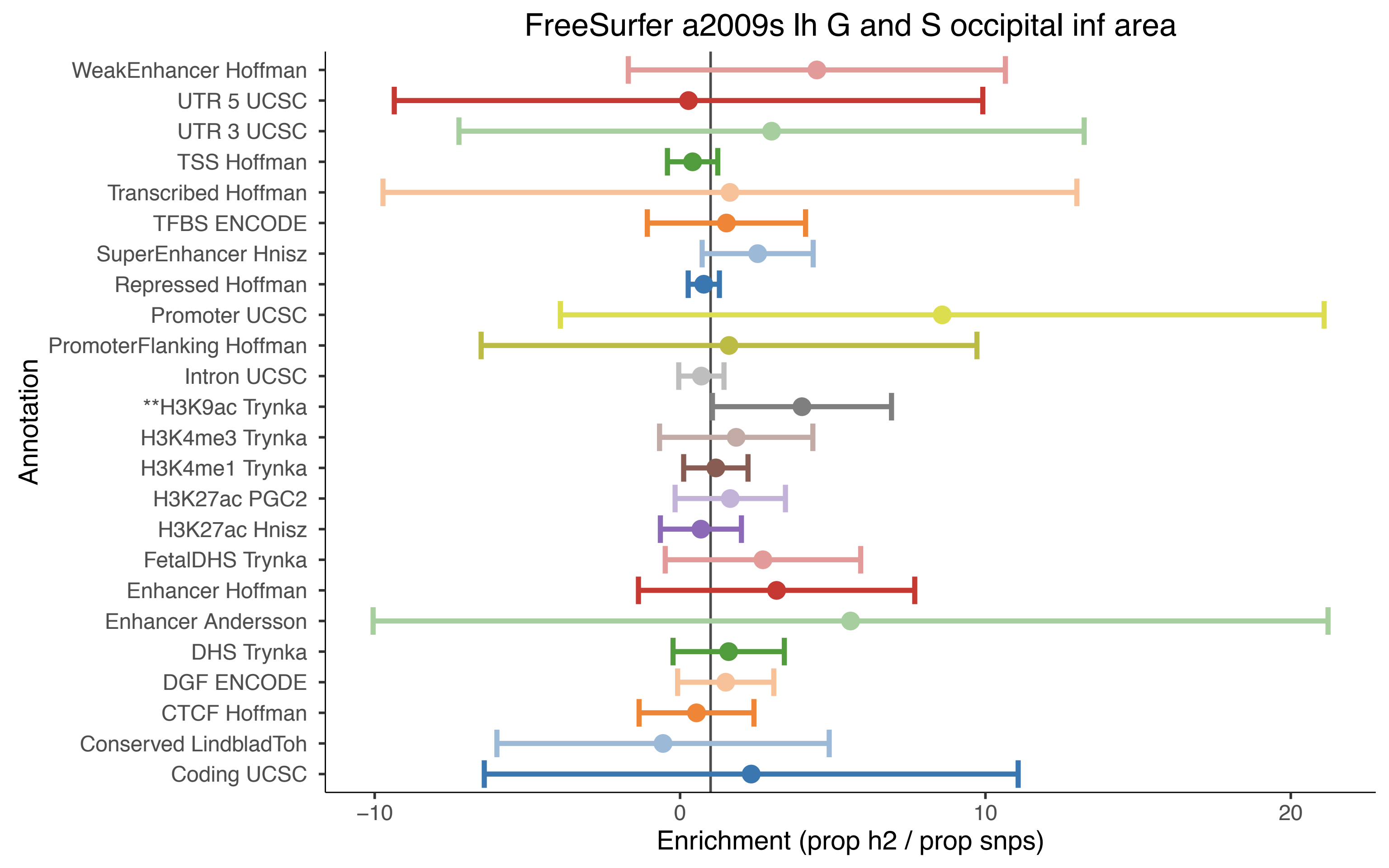

FreeSurfer a2009s lh G and S occipital inf area
WeakEnhancer Hoffman
UTR 5 UCSC
UTR 3 UCSC
TSS Hoffman
Transcribed Hoffman
TFBS ENCODE
SuperEnhancer Hnisz
Repressed Hoffman
Promoter UCSC
PromoterFlanking Hoffman
Intron UCSC
**H3K9ac Trynka
H3K4me3 Trynka
H3K4me1 Trynka
H3K27ac PGC2
H3K27ac Hnisz
FetalDHS Trynka
Enhancer Hoffman
Enhancer Andersson
DHS Trynka
DGF ENCODE
CTCF Hoffman
Conserved LindbladToh
Coding UCSC
Annotation
−10
0
10
20
Enrichment (prop h2 / prop snps)

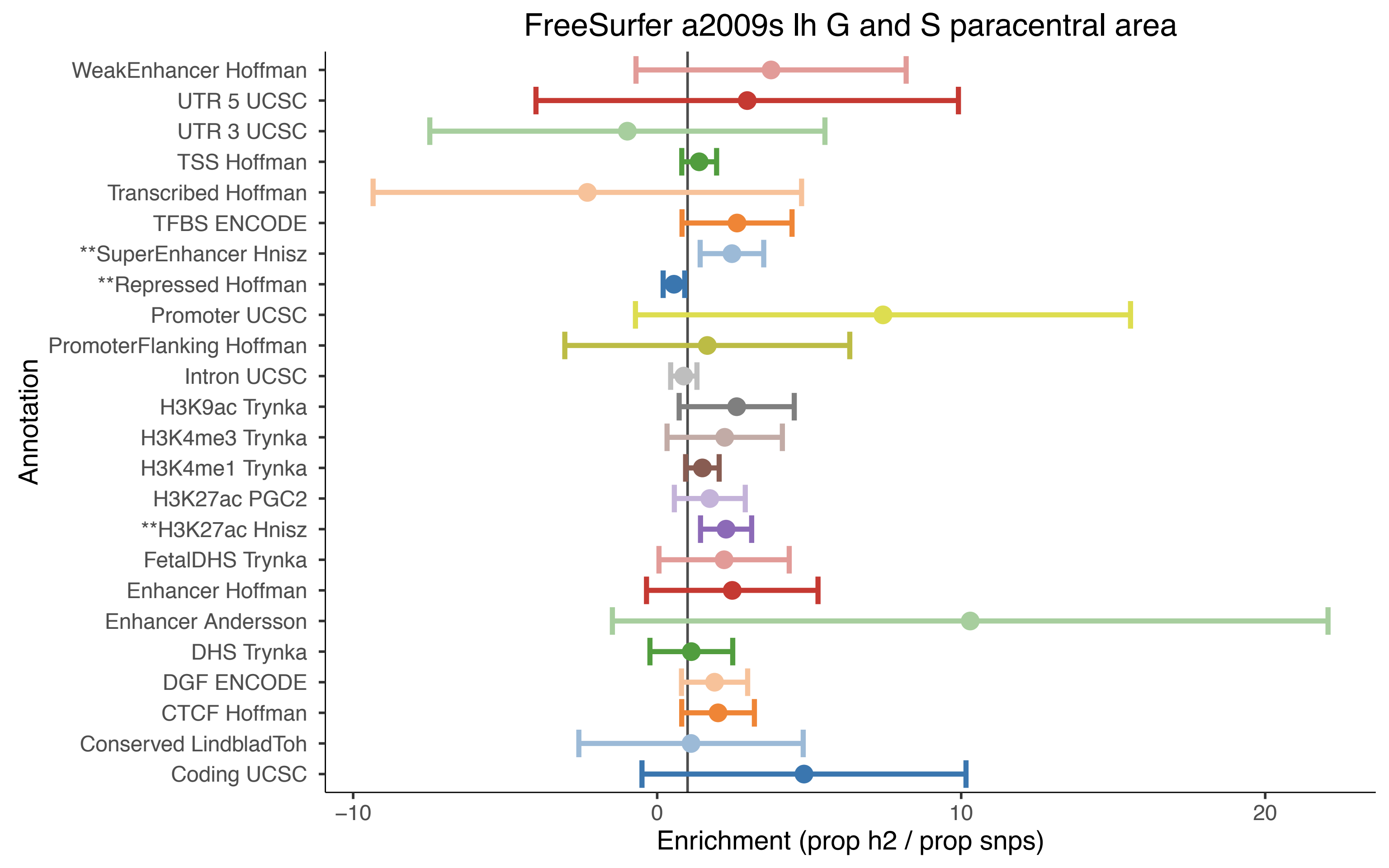
FreeSurfer a2009s lh G and S paracentral area
WeakEnhancer Hoffman
UTR 5 UCSC
UTR 3 UCSC
TSS Hoffman
Transcribed Hoffman
TFBS ENCODE
**SuperEnhancer Hnisz
**Repressed Hoffman
Promoter UCSC
PromoterFlanking Hoffman
Intron UCSC
H3K9ac Trynka
H3K4me3 Trynka
H3K4me1 Trynka
H3K27ac PGC2
**H3K27ac Hnisz
FetalDHS Trynka
Enhancer Hoffman
Enhancer Andersson
DHS Trynka
DGF ENCODE
CTCF Hoffman
Conserved LindbladToh
Coding UCSC
Annotation
−10
0
10
20
Enrichment (prop h2 / prop snps)

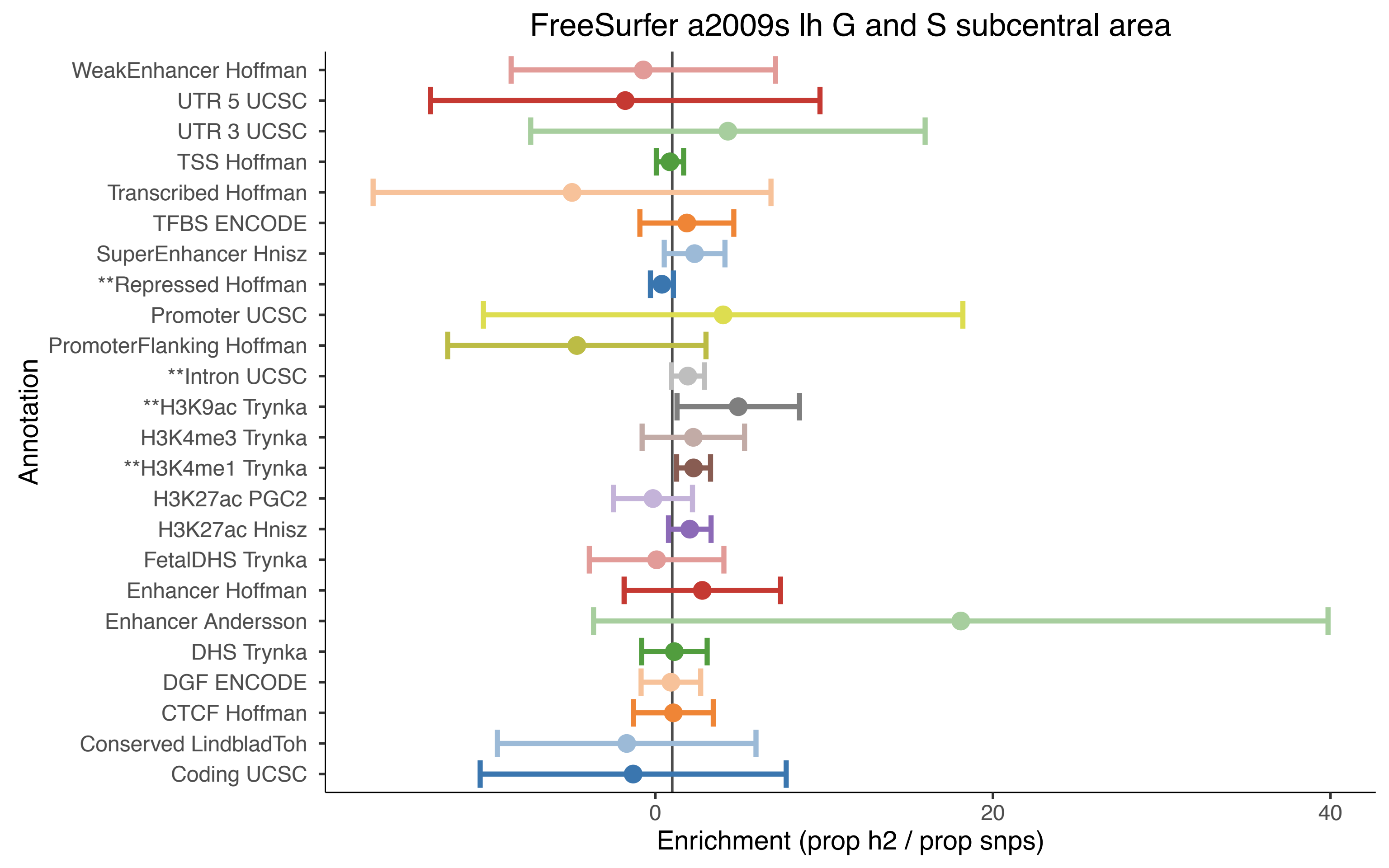
FreeSurfer a2009s lh G and S subcentral area
WeakEnhancer Hoffman
UTR 5 UCSC
UTR 3 UCSC
TSS Hoffman
Transcribed Hoffman
TFBS ENCODE
SuperEnhancer Hnisz
**Repressed Hoffman
Promoter UCSC
PromoterFlanking Hoffman
**Intron UCSC
**H3K9ac Trynka
H3K4me3 Trynka
**H3K4me1 Trynka
H3K27ac PGC2
H3K27ac Hnisz
FetalDHS Trynka
Enhancer Hoffman
Enhancer Andersson
DHS Trynka
DGF ENCODE
CTCF Hoffman
Conserved LindbladToh
Coding UCSC
Annotation
0
20
40
Enrichment (prop h2 / prop snps)

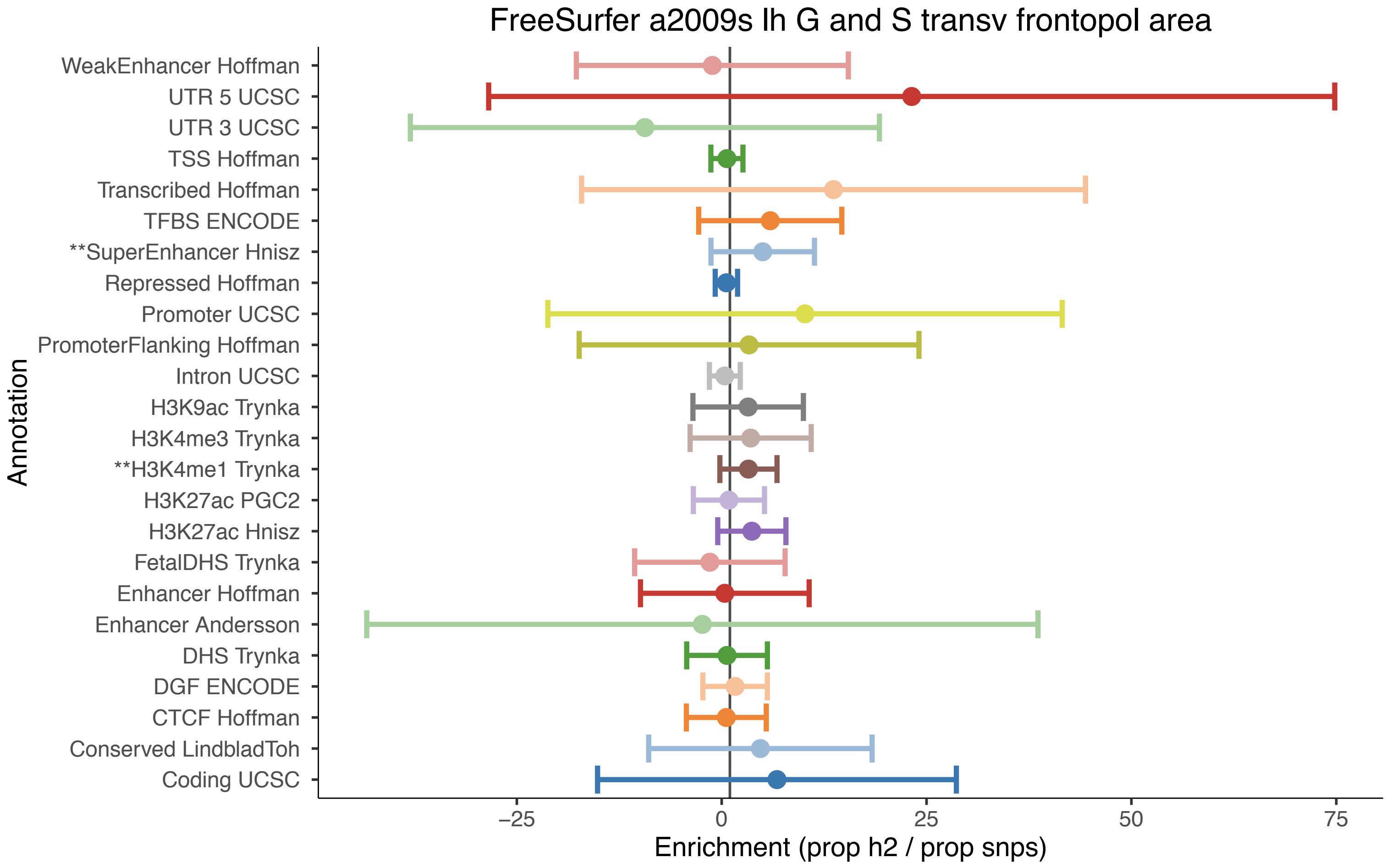

FreeSurfer a2009s lh G and S transv frontopol area
Annotation
WeakEnhancer Hoffman
UTR 5 UCSC
UTR 3 UCSC
TSS Hoffman
Transcribed Hoffman
TFBS ENCODE
**SuperEnhancer Hnisz
Repressed Hoffman
Promoter UCSC
PromoterFlanking Hoffman
Intron UCSC
H3K9ac Trynka
H3K4me3 Trynka
**H3K4me1 Trynka
H3K27ac PGC2
H3K27ac Hnisz
FetalDHS Trynka
Enhancer Hoffman
Enhancer Andersson
DHS Trynka
DGF ENCODE
CTCF Hoffman
Conserved LindbladToh
Coding UCSC
−25
0
25
50
75
Enrichment (prop h2 / prop snps)

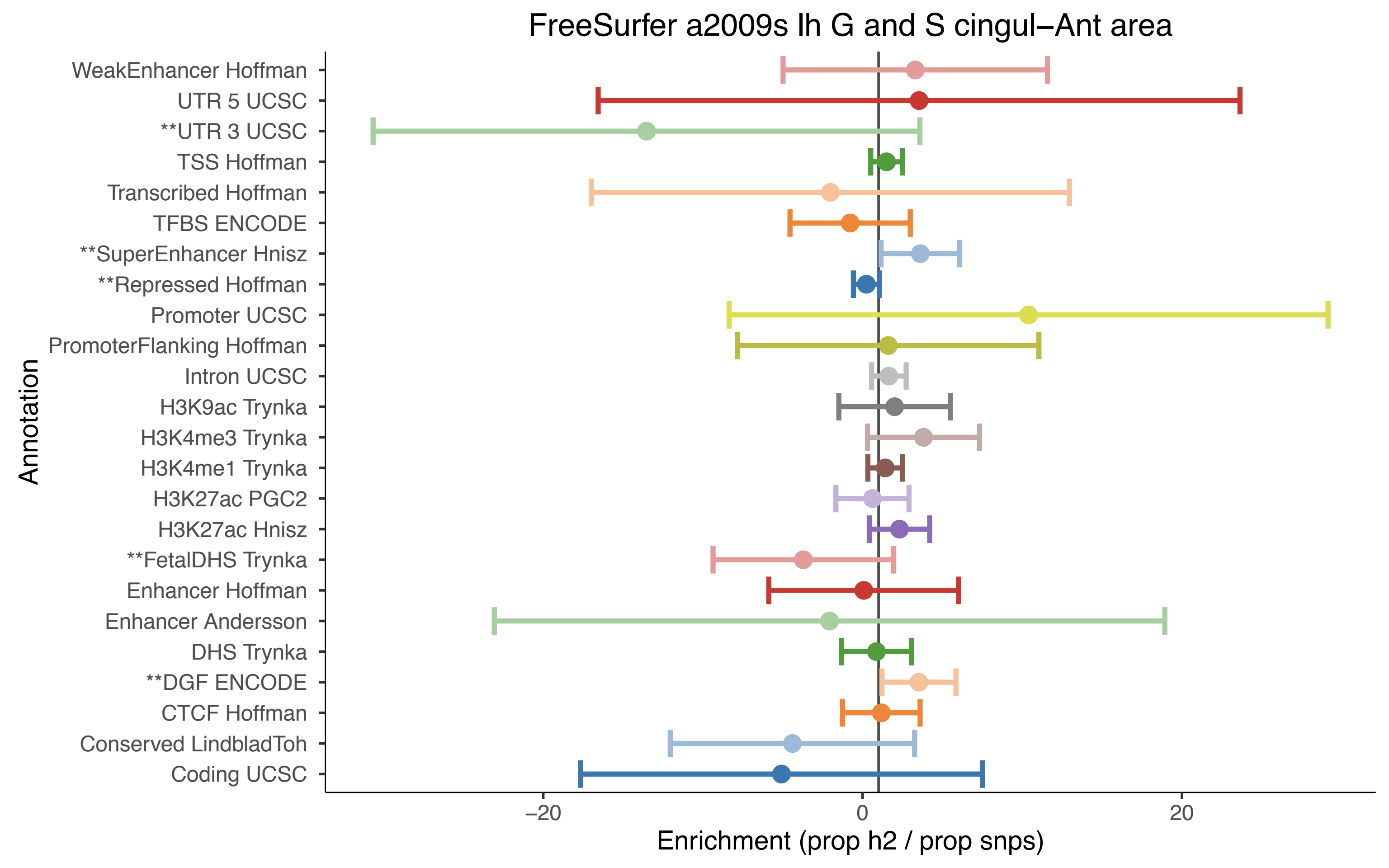
FreeSurfer a2009s lh G and S cingul−Ant area
WeakEnhancer Hoffman
UTR 5 UCSC
**UTR 3 UCSC
TSS Hoffman
Transcribed Hoffman
TFBS ENCODE
**SuperEnhancer Hnisz
**Repressed Hoffman
Promoter UCSC
PromoterFlanking Hoffman
Intron UCSC
H3K9ac Trynka
H3K4me3 Trynka
H3K4me1 Trynka
H3K27ac PGC2
H3K27ac Hnisz
**FetalDHS Trynka
Enhancer Hoffman
Enhancer Andersson
DHS Trynka
**DGF ENCODE
CTCF Hoffman
Conserved LindbladToh
Coding UCSC
Annotation
−20
0
20
Enrichment (prop h2 / prop snps)

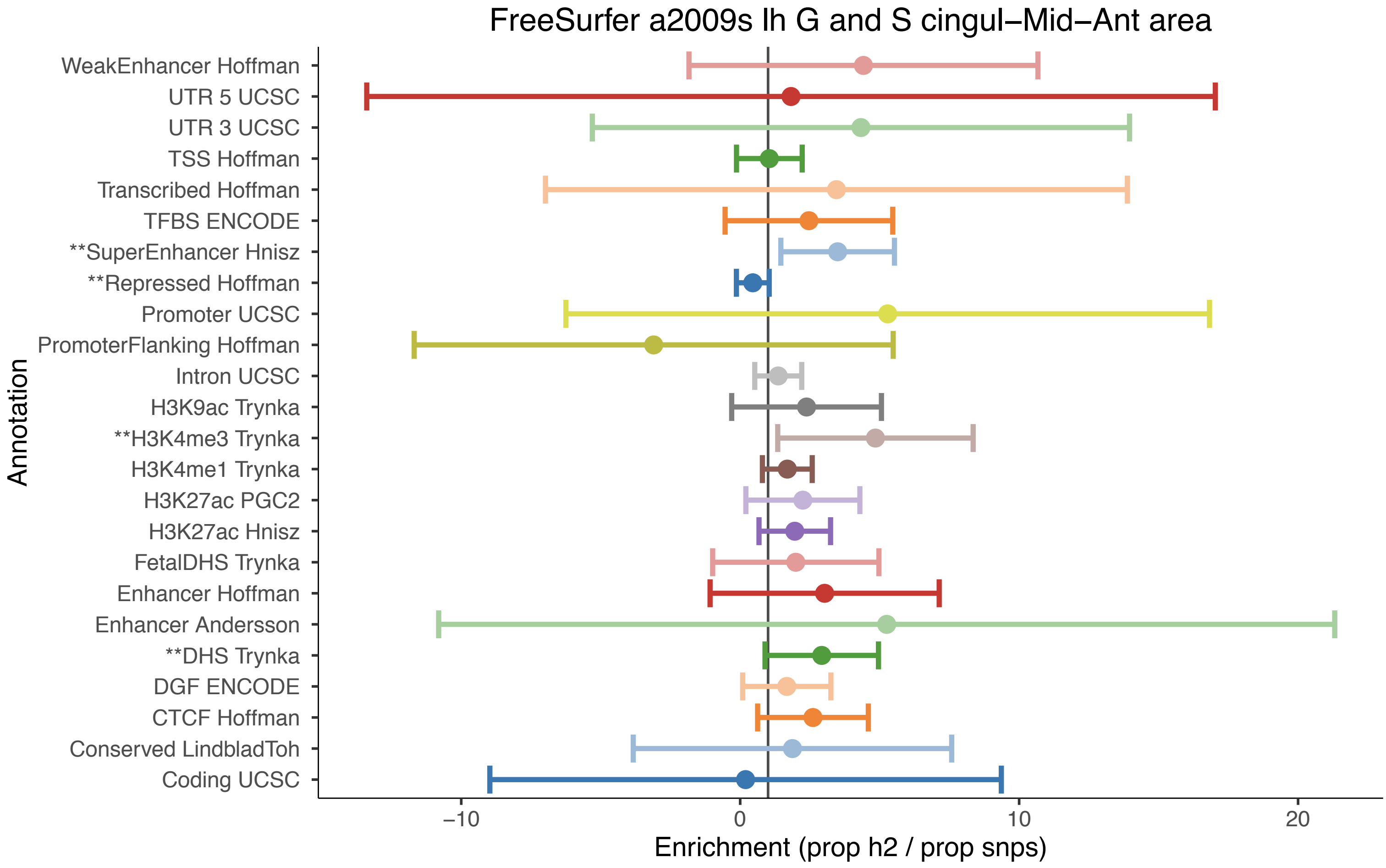

FreeSurfer a2009s lh G and S cingul−Mid−Ant area
Annotation
WeakEnhancer Hoffman
UTR 5 UCSC
UTR 3 UCSC
TSS Hoffman
Transcribed Hoffman
TFBS ENCODE
**SuperEnhancer Hnisz
**Repressed Hoffman
Promoter UCSC
PromoterFlanking Hoffman
Intron UCSC
H3K9ac Trynka
**H3K4me3 Trynka
H3K4me1 Trynka
H3K27ac PGC2
H3K27ac Hnisz
FetalDHS Trynka
Enhancer Hoffman
Enhancer Andersson
**DHS Trynka
DGF ENCODE
CTCF Hoffman
Conserved LindbladToh
Coding UCSC
−10
0
10
20
Enrichment (prop h2 / prop snps)

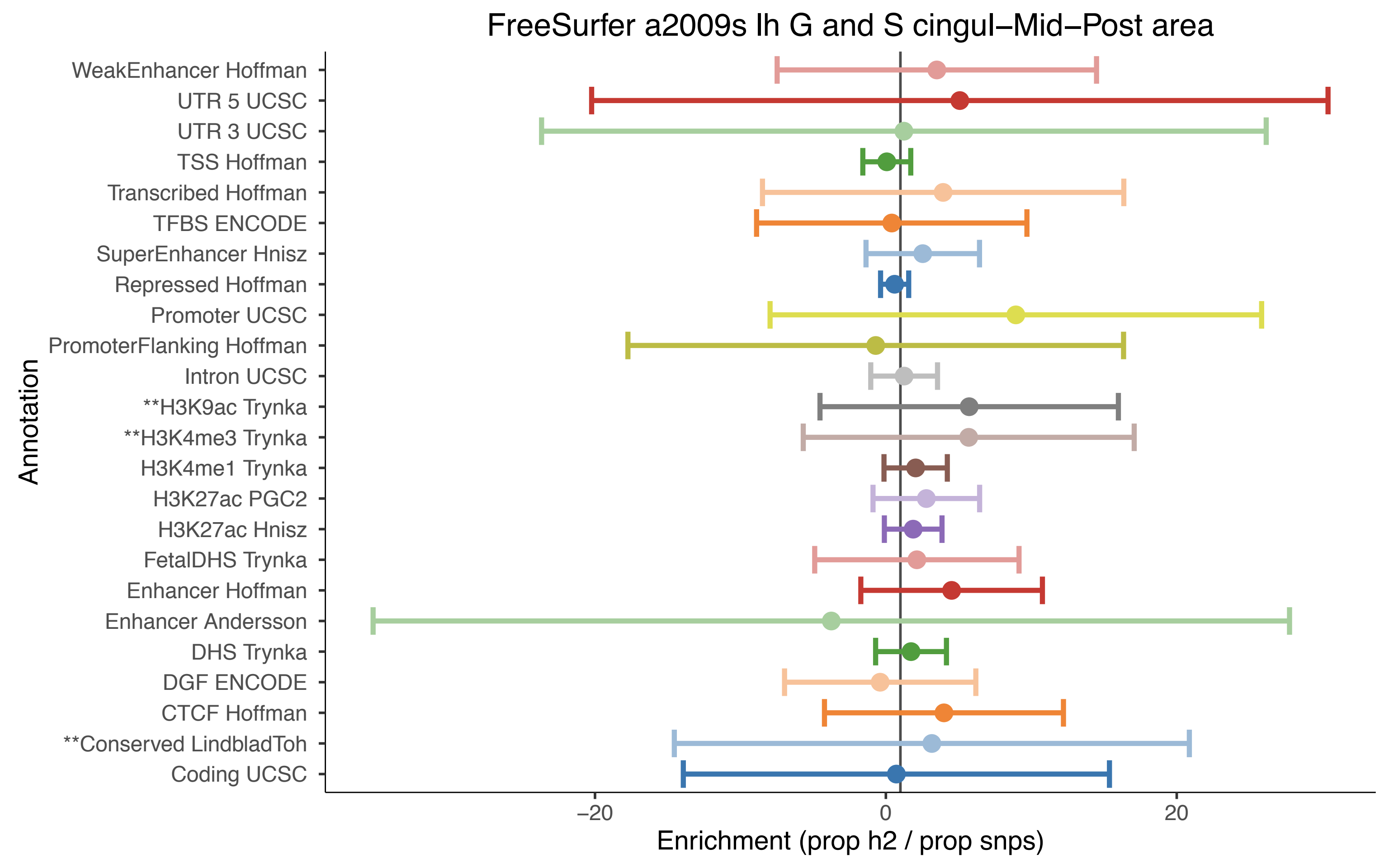

FreeSurfer a2009s lh G and S cingul−Mid−Post area
WeakEnhancer Hoffman
UTR 5 UCSC
UTR 3 UCSC
TSS Hoffman
Transcribed Hoffman
TFBS ENCODE
SuperEnhancer Hnisz
Repressed Hoffman
Promoter UCSC
PromoterFlanking Hoffman
Intron UCSC
**H3K9ac Trynka
**H3K4me3 Trynka
H3K4me1 Trynka
H3K27ac PGC2
H3K27ac Hnisz
FetalDHS Trynka
Enhancer Hoffman
Enhancer Andersson
DHS Trynka
DGF ENCODE
CTCF Hoffman
**Conserved LindbladToh
Coding UCSC
Annotation
−20
0
20
Enrichment (prop h2 / prop snps)

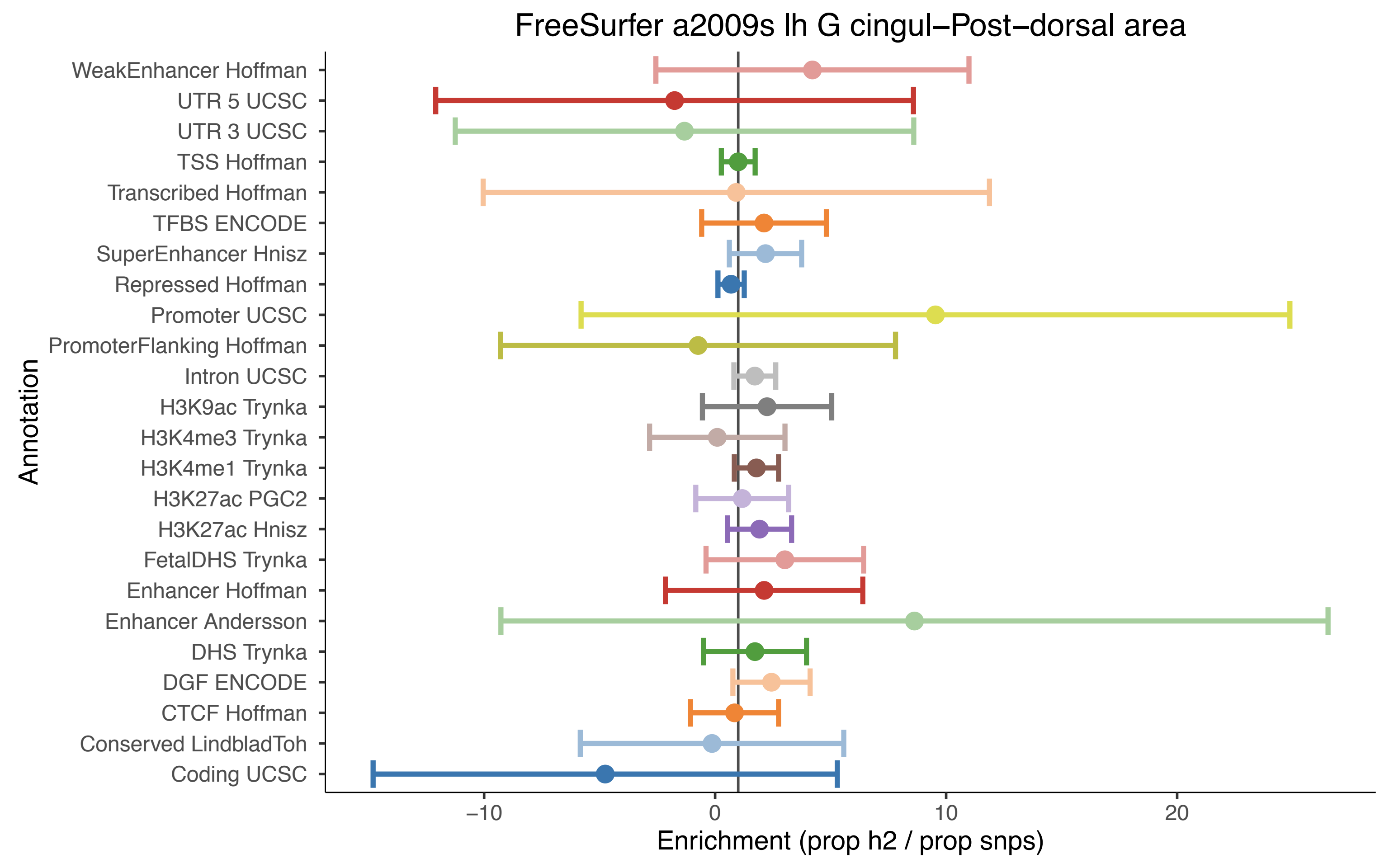
FreeSurfer a2009s lh G cingul−Post−dorsal area
WeakEnhancer Hoffman
UTR 5 UCSC
UTR 3 UCSC
TSS Hoffman
Transcribed Hoffman
TFBS ENCODE
SuperEnhancer Hnisz
Repressed Hoffman
Promoter UCSC
PromoterFlanking Hoffman
Intron UCSC
H3K9ac Trynka
H3K4me3 Trynka
H3K4me1 Trynka
H3K27ac PGC2
H3K27ac Hnisz
FetalDHS Trynka
Enhancer Hoffman
Enhancer Andersson
DHS Trynka
DGF ENCODE
CTCF Hoffman
Conserved LindbladToh
Coding UCSC
Annotation
−10
0
10
20
Enrichment (prop h2 / prop snps)

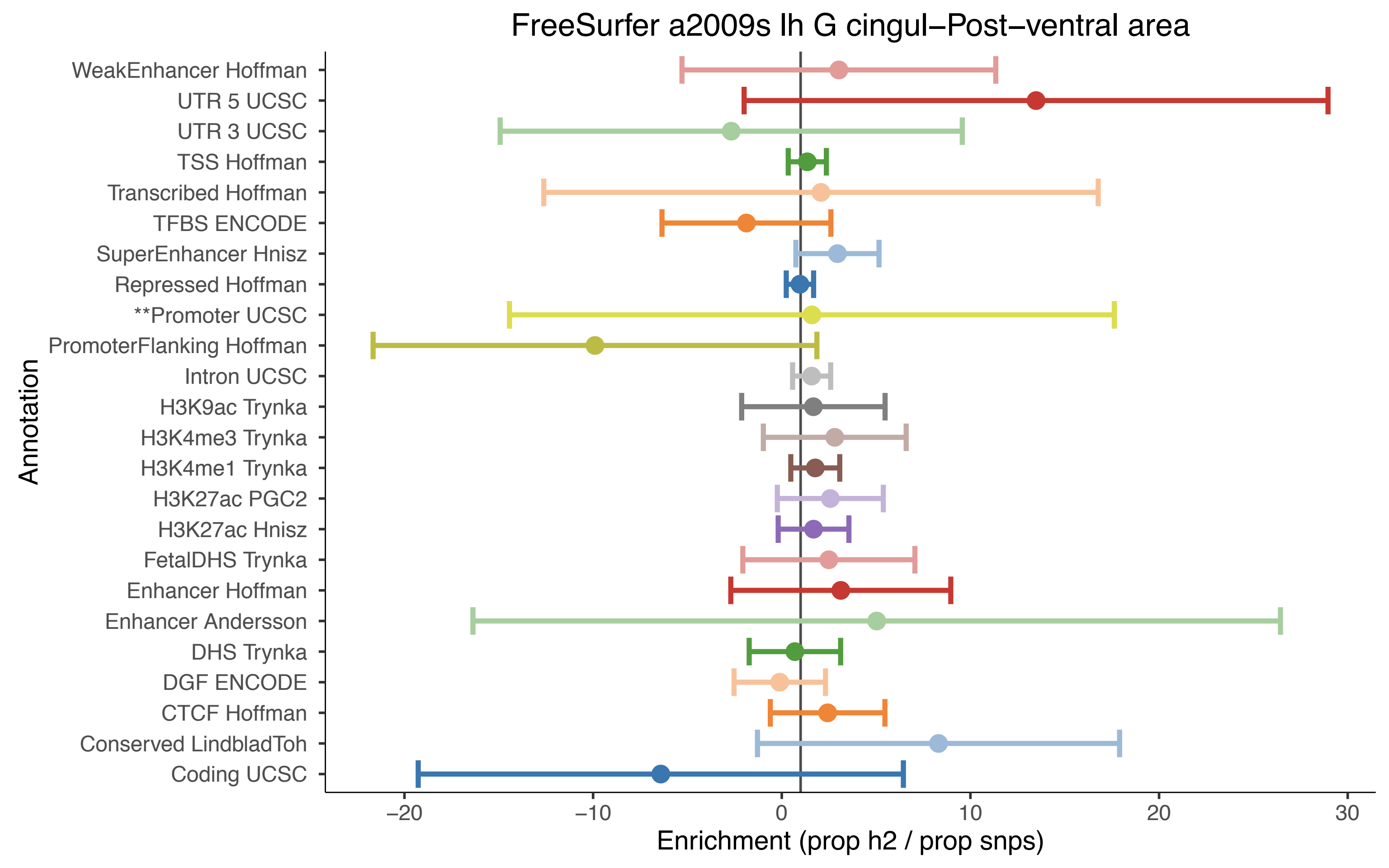
FreeSurfer a2009s lh G cingul−Post−ventral area
WeakEnhancer Hoffman
UTR 5 UCSC
UTR 3 UCSC
TSS Hoffman
Transcribed Hoffman
TFBS ENCODE
SuperEnhancer Hnisz
Repressed Hoffman
**Promoter UCSC
PromoterFlanking Hoffman
Intron UCSC
H3K9ac Trynka
H3K4me3 Trynka
H3K4me1 Trynka
H3K27ac PGC2
H3K27ac Hnisz
FetalDHS Trynka
Enhancer Hoffman
Enhancer Andersson
DHS Trynka
DGF ENCODE
CTCF Hoffman
Conserved LindbladToh
Coding UCSC
Annotation
−20
−10
0
10
20
30
Enrichment (prop h2 / prop snps)

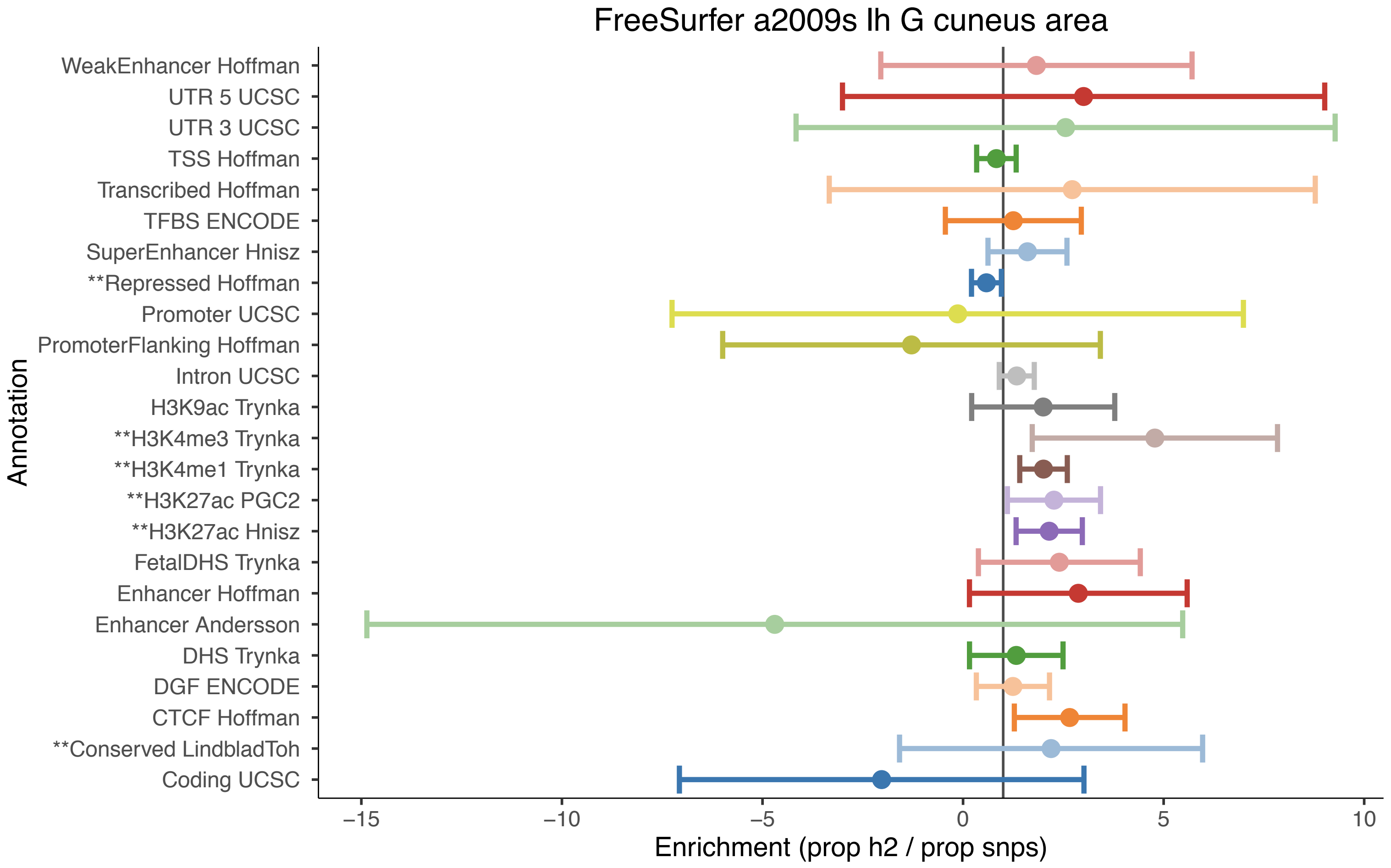
FreeSurfer a2009s lh G cuneus area
WeakEnhancer Hoffman
UTR 5 UCSC
UTR 3 UCSC
TSS Hoffman
Transcribed Hoffman
TFBS ENCODE
SuperEnhancer Hnisz
**Repressed Hoffman
Promoter UCSC
PromoterFlanking Hoffman
Intron UCSC
H3K9ac Trynka
**H3K4me3 Trynka
**H3K4me1 Trynka
**H3K27ac PGC2
**H3K27ac Hnisz
FetalDHS Trynka
Enhancer Hoffman
Enhancer Andersson
DHS Trynka
DGF ENCODE
CTCF Hoffman
**Conserved LindbladToh
Coding UCSC
Annotation
−15
−10
−5
0
5
10
Enrichment (prop h2 / prop snps)

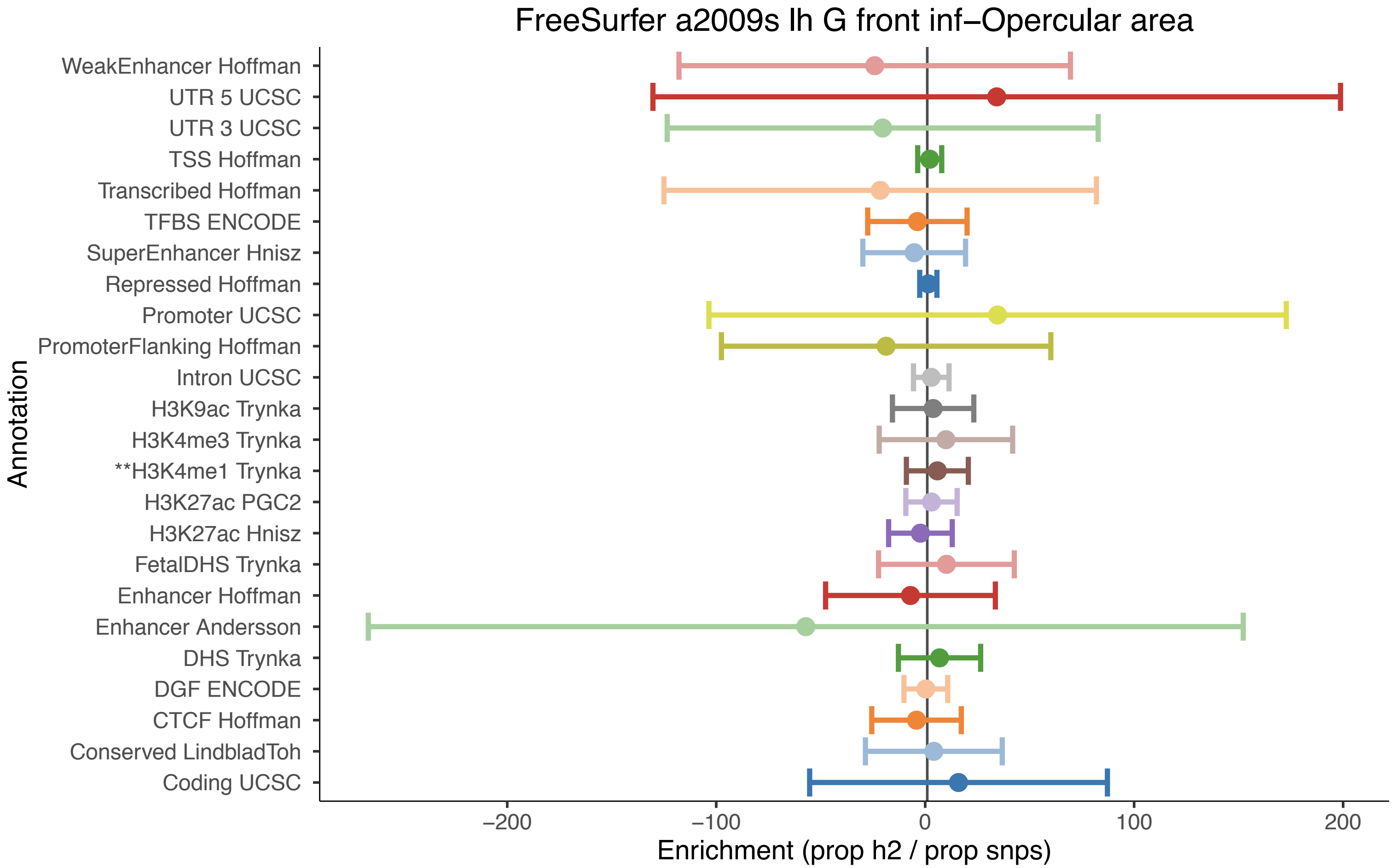

FreeSurfer a2009s lh G front inf−Opercular area
WeakEnhancer Hoffman
UTR 5 UCSC
UTR 3 UCSC
TSS Hoffman
Transcribed Hoffman
TFBS ENCODE
SuperEnhancer Hnisz
Repressed Hoffman
Promoter UCSC
PromoterFlanking Hoffman
Intron UCSC
H3K9ac Trynka
H3K4me3 Trynka
**H3K4me1 Trynka
H3K27ac PGC2
H3K27ac Hnisz
FetalDHS Trynka
Enhancer Hoffman
Enhancer Andersson
DHS Trynka
DGF ENCODE
CTCF Hoffman
Conserved LindbladToh
Coding UCSC
Annotation
−200
−100
0
100
200
Enrichment (prop h2 / prop snps)

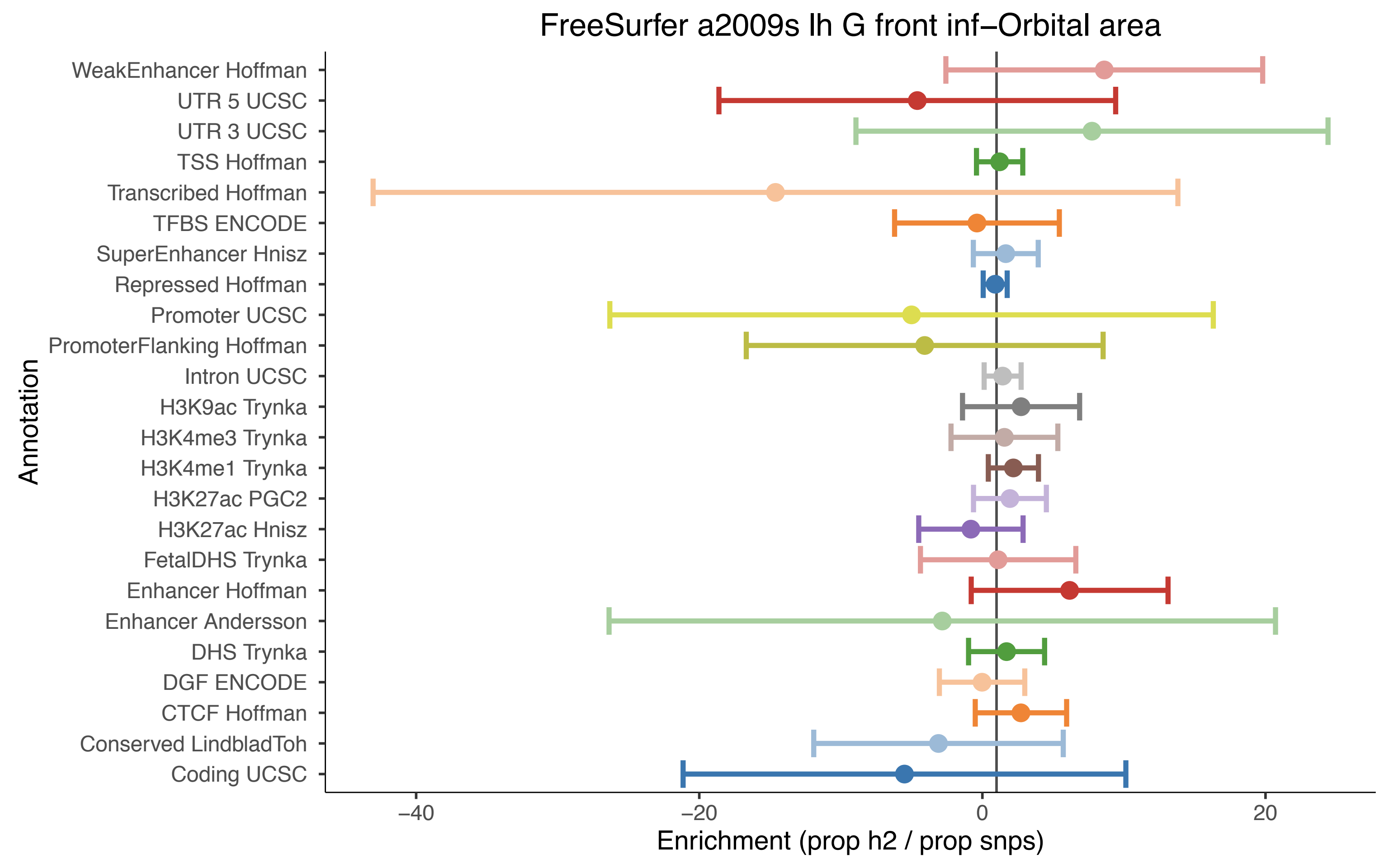
FreeSurfer a2009s lh G front inf−Orbital area
WeakEnhancer Hoffman
UTR 5 UCSC
UTR 3 UCSC
TSS Hoffman
Transcribed Hoffman
TFBS ENCODE
SuperEnhancer Hnisz
Repressed Hoffman
Promoter UCSC
PromoterFlanking Hoffman
Intron UCSC
H3K9ac Trynka
H3K4me3 Trynka
H3K4me1 Trynka
H3K27ac PGC2
H3K27ac Hnisz
FetalDHS Trynka
Enhancer Hoffman
Enhancer Andersson
DHS Trynka
DGF ENCODE
CTCF Hoffman
Conserved LindbladToh
Coding UCSC
Annotation
−40
−20
0
20
Enrichment (prop h2 / prop snps)

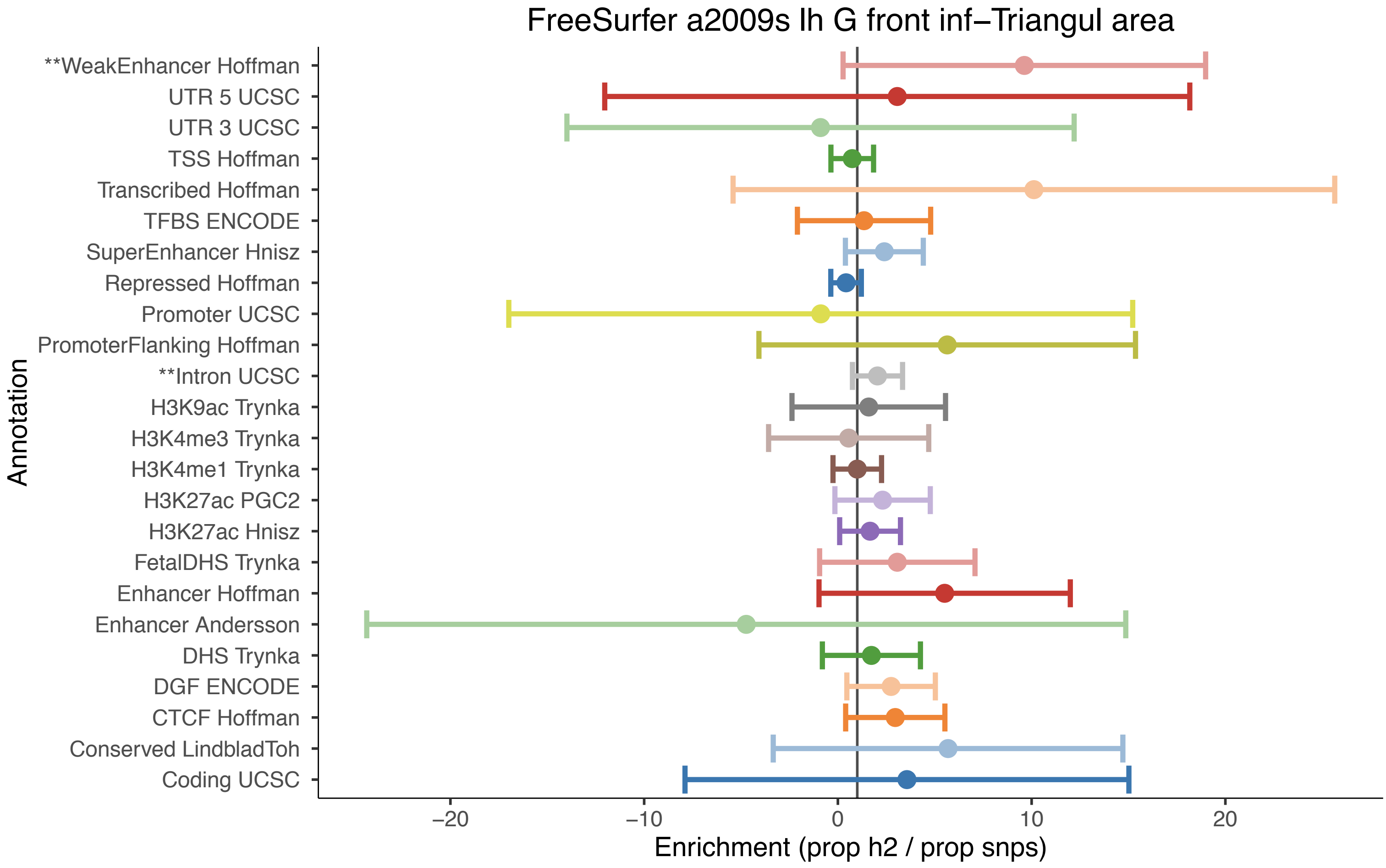

FreeSurfer a2009s lh G front inf−Triangul area
**WeakEnhancer Hoffman
UTR 5 UCSC
UTR 3 UCSC
TSS Hoffman
Transcribed Hoffman
TFBS ENCODE
SuperEnhancer Hnisz
Repressed Hoffman
Promoter UCSC
PromoterFlanking Hoffman
**Intron UCSC
H3K9ac Trynka
H3K4me3 Trynka
H3K4me1 Trynka
H3K27ac PGC2
H3K27ac Hnisz
FetalDHS Trynka
Enhancer Hoffman
Enhancer Andersson
DHS Trynka
DGF ENCODE
CTCF Hoffman
Conserved LindbladToh
Coding UCSC
Annotation
−20
−10
0
10
20
Enrichment (prop h2 / prop snps)

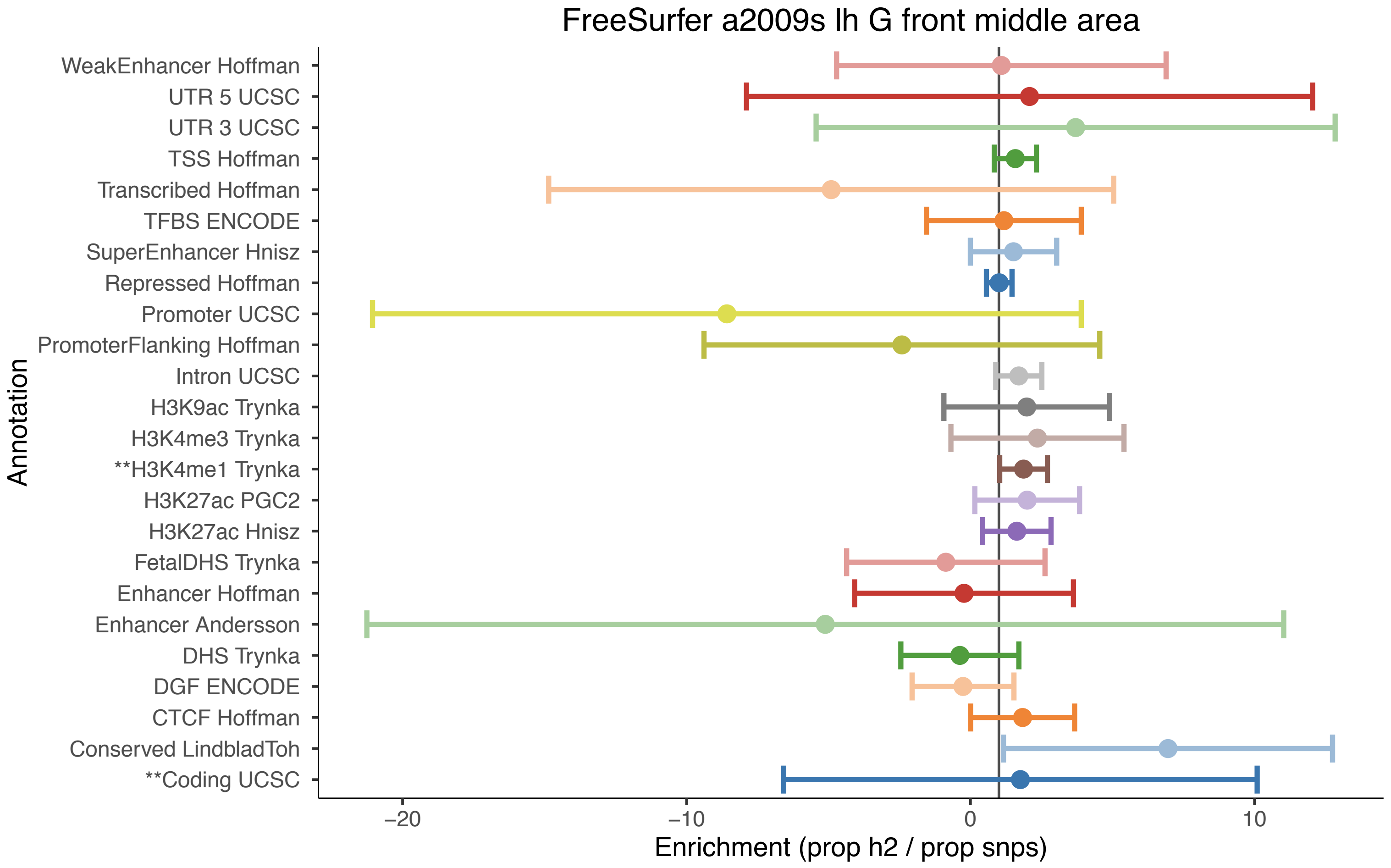
FreeSurfer a2009s lh G front middle area
WeakEnhancer Hoffman
UTR 5 UCSC
UTR 3 UCSC
TSS Hoffman
Transcribed Hoffman
TFBS ENCODE
SuperEnhancer Hnisz
Repressed Hoffman
Promoter UCSC
PromoterFlanking Hoffman
Intron UCSC
H3K9ac Trynka
H3K4me3 Trynka
**H3K4me1 Trynka
H3K27ac PGC2
H3K27ac Hnisz
FetalDHS Trynka
Enhancer Hoffman
Enhancer Andersson
DHS Trynka
DGF ENCODE
CTCF Hoffman
Conserved LindbladToh
**Coding UCSC
Annotation
−20
−10
0
10
Enrichment (prop h2 / prop snps)

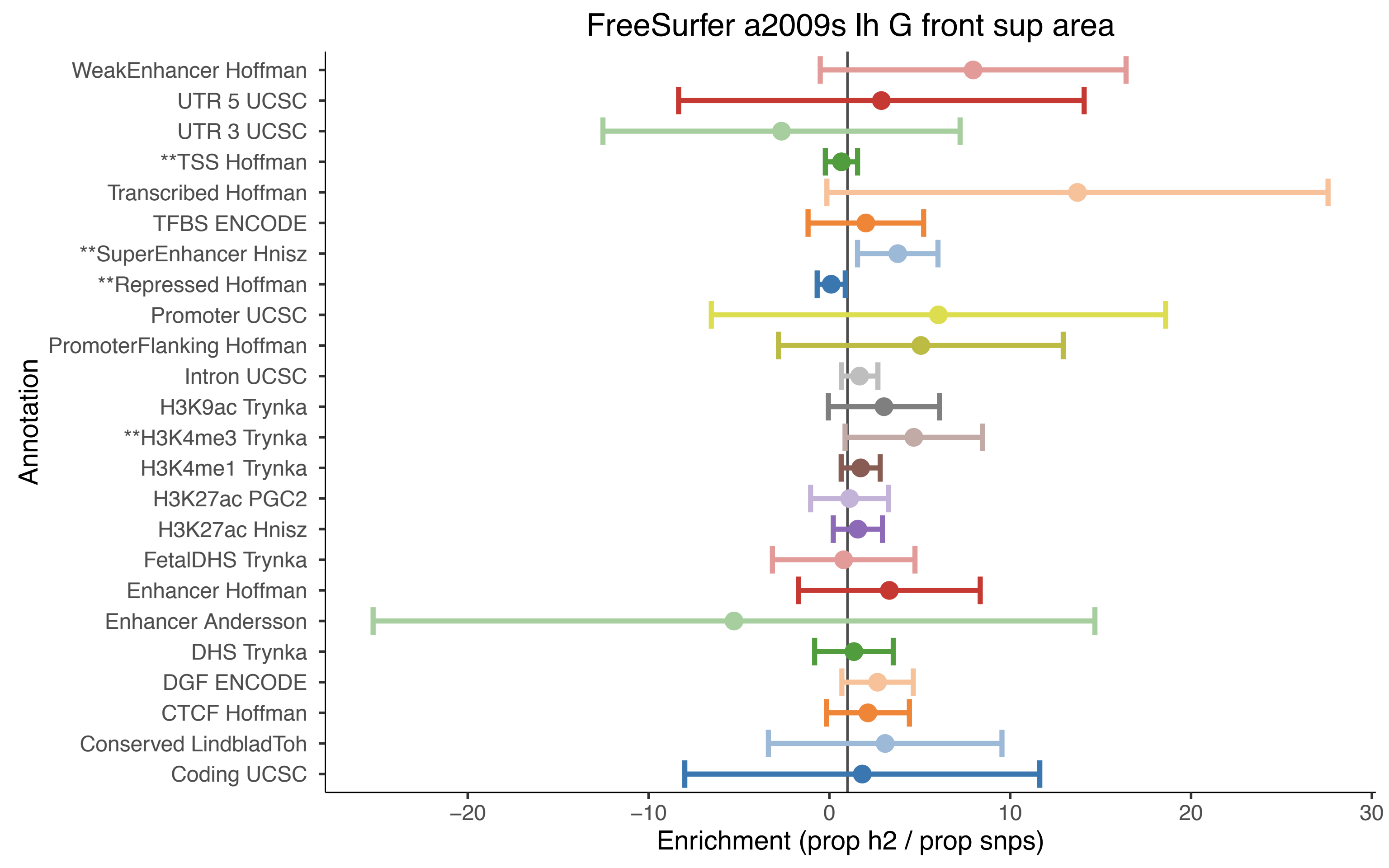

FreeSurfer a2009s lh G front sup area
WeakEnhancer Hoffman
UTR 5 UCSC
UTR 3 UCSC
**TSS Hoffman
Transcribed Hoffman
TFBS ENCODE
**SuperEnhancer Hnisz
**Repressed Hoffman
Promoter UCSC
PromoterFlanking Hoffman
Intron UCSC
H3K9ac Trynka
**H3K4me3 Trynka
H3K4me1 Trynka
H3K27ac PGC2
H3K27ac Hnisz
FetalDHS Trynka
Enhancer Hoffman
Enhancer Andersson
DHS Trynka
DGF ENCODE
CTCF Hoffman
Conserved LindbladToh
Coding UCSC
Annotation
−20
−10
0
10
20
30
Enrichment (prop h2 / prop snps)

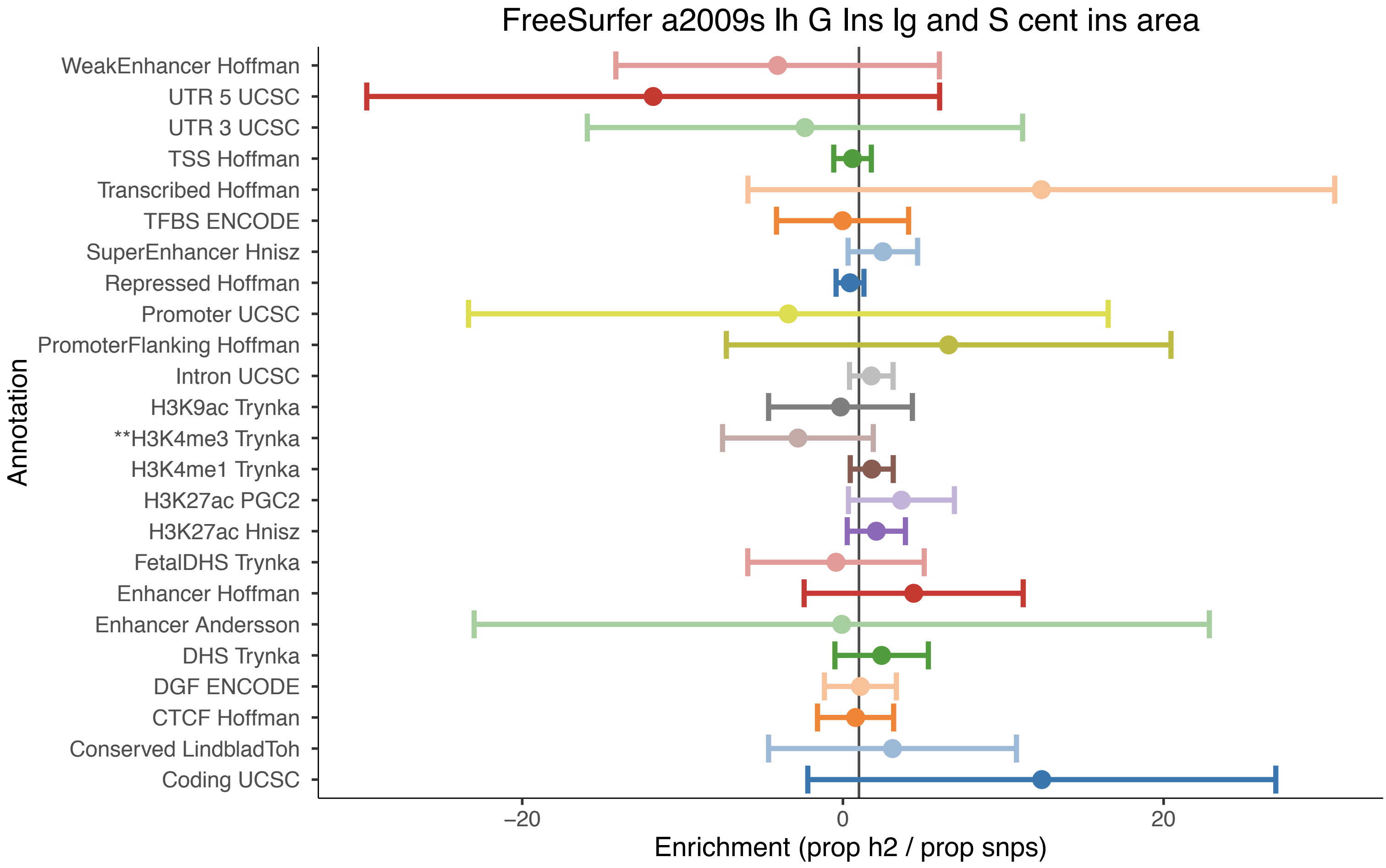

FreeSurfer a2009s lh G Ins lg and S cent ins area
WeakEnhancer Hoffman
UTR 5 UCSC
UTR 3 UCSC
TSS Hoffman
Transcribed Hoffman
TFBS ENCODE
SuperEnhancer Hnisz
Repressed Hoffman
Promoter UCSC
PromoterFlanking Hoffman
Intron UCSC
H3K9ac Trynka
**H3K4me3 Trynka
H3K4me1 Trynka
H3K27ac PGC2
H3K27ac Hnisz
FetalDHS Trynka
Enhancer Hoffman
Enhancer Andersson
DHS Trynka
DGF ENCODE
CTCF Hoffman
Conserved LindbladToh
Coding UCSC
Annotation
−20
0
20
Enrichment (prop h2 / prop snps)

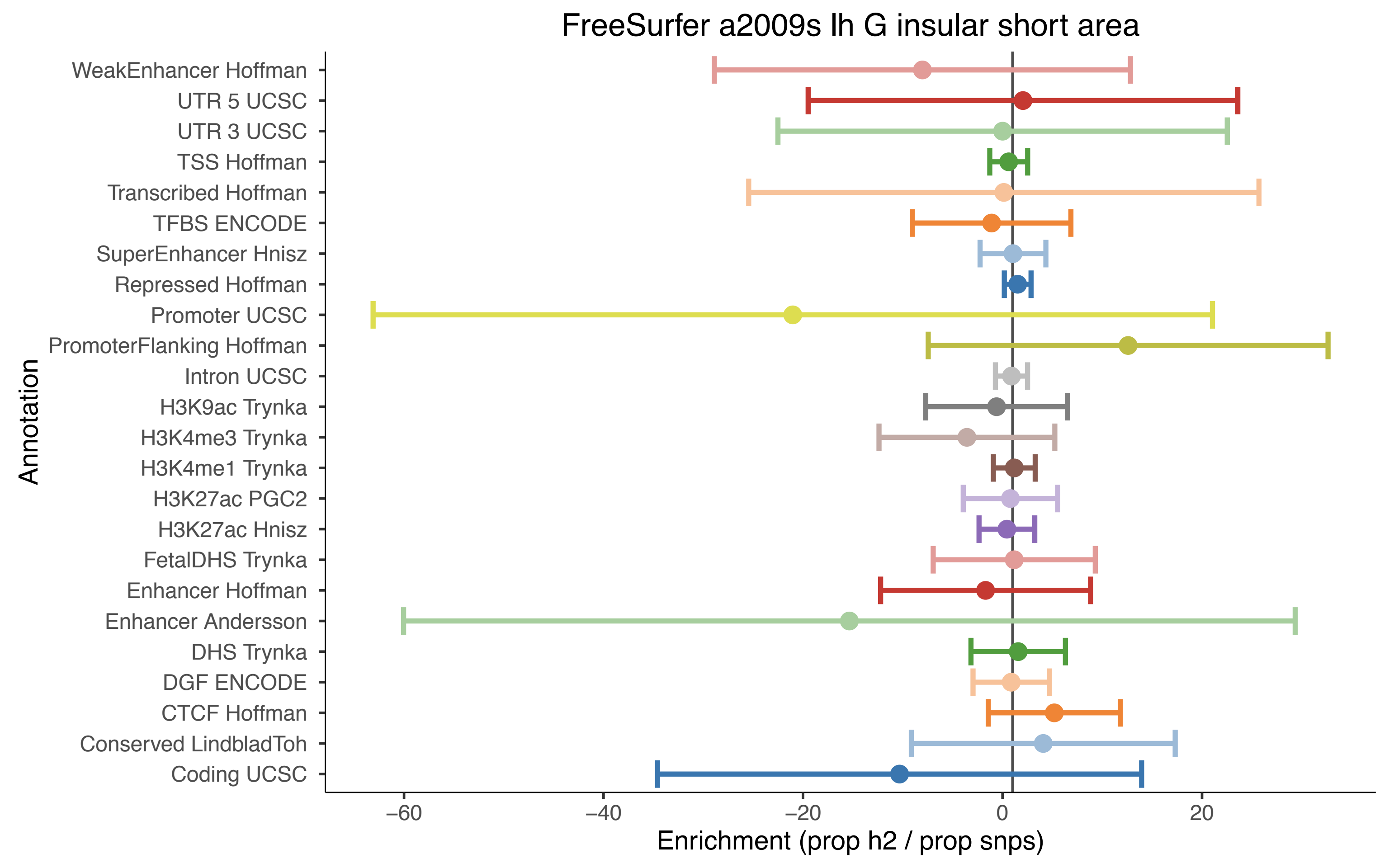

FreeSurfer a2009s lh G insular short area
WeakEnhancer Hoffman
UTR 5 UCSC
UTR 3 UCSC
TSS Hoffman
Transcribed Hoffman
TFBS ENCODE
SuperEnhancer Hnisz
Repressed Hoffman
Promoter UCSC
PromoterFlanking Hoffman
Intron UCSC
H3K9ac Trynka
H3K4me3 Trynka
H3K4me1 Trynka
H3K27ac PGC2
H3K27ac Hnisz
FetalDHS Trynka
Enhancer Hoffman
Enhancer Andersson
DHS Trynka
DGF ENCODE
CTCF Hoffman
Conserved LindbladToh
Coding UCSC
Annotation
−60
−40
−20
0
20
Enrichment (prop h2 / prop snps)

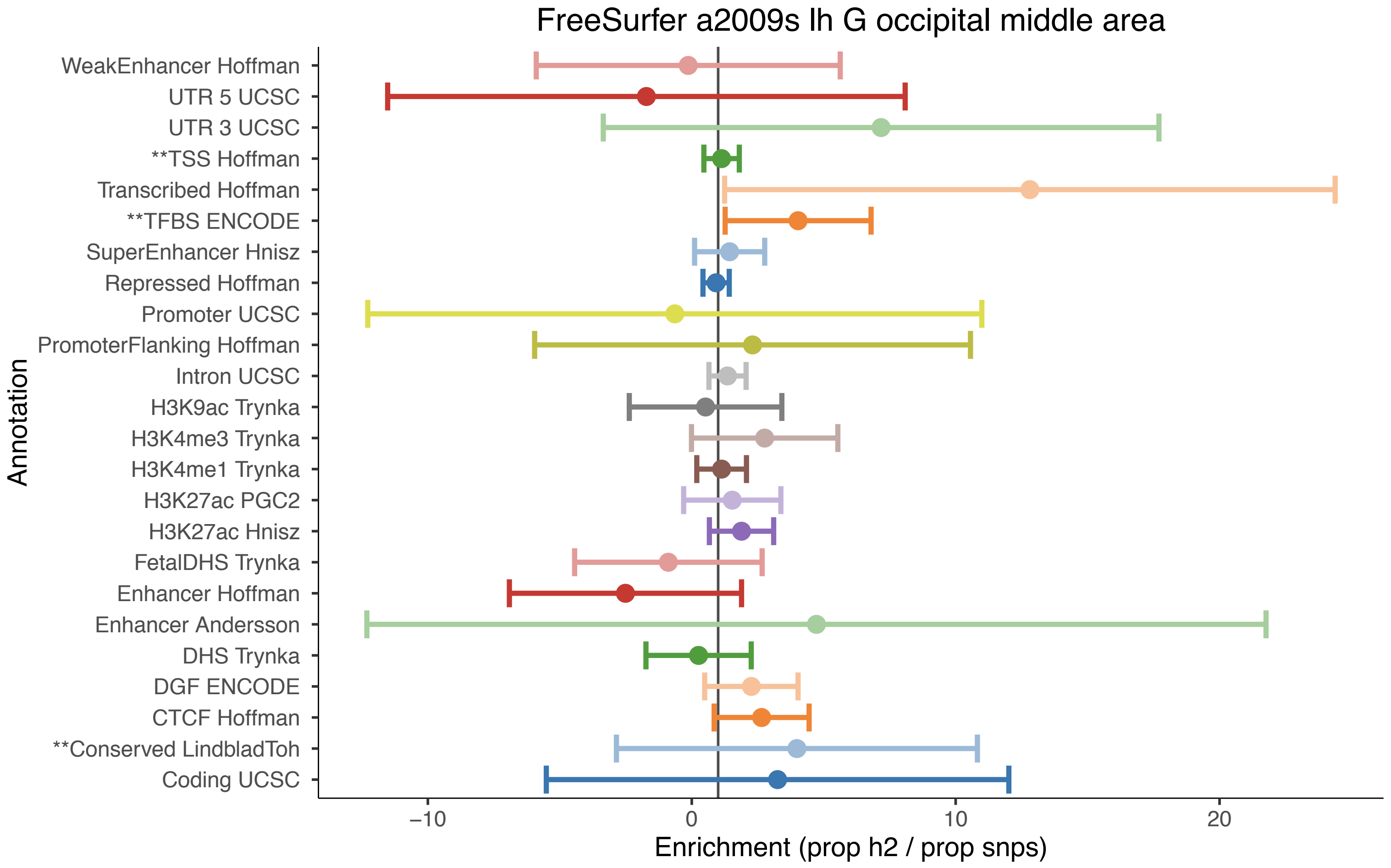
FreeSurfer a2009s lh G occipital middle area
WeakEnhancer Hoffman
UTR 5 UCSC
UTR 3 UCSC
**TSS Hoffman
Transcribed Hoffman
**TFBS ENCODE
SuperEnhancer Hnisz
Repressed Hoffman
Promoter UCSC
PromoterFlanking Hoffman
Intron UCSC
H3K9ac Trynka
H3K4me3 Trynka
H3K4me1 Trynka
H3K27ac PGC2
H3K27ac Hnisz
FetalDHS Trynka
Enhancer Hoffman
Enhancer Andersson
DHS Trynka
DGF ENCODE
CTCF Hoffman
**Conserved LindbladToh
Coding UCSC
Annotation
−10
0
10
20
Enrichment (prop h2 / prop snps)

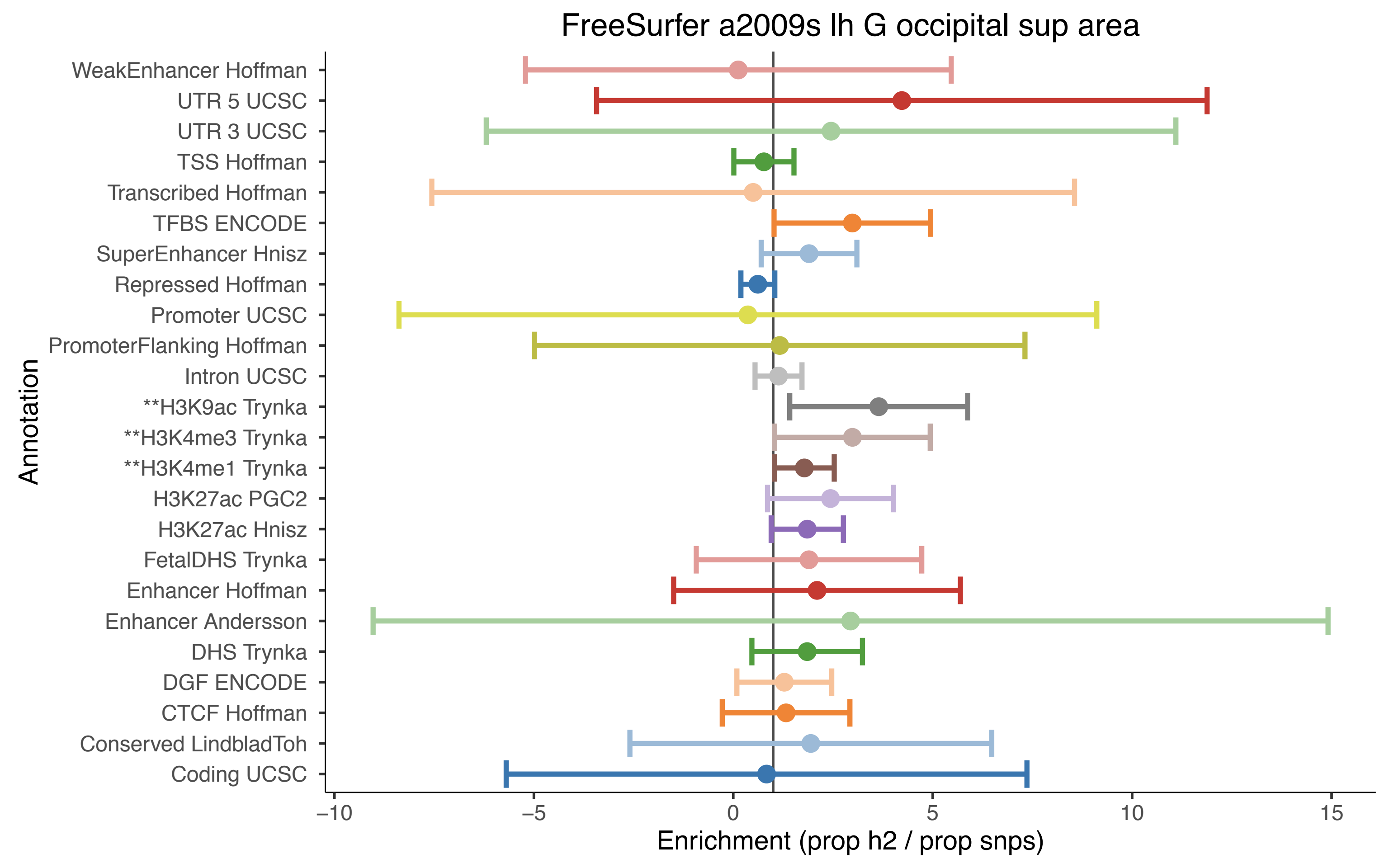
FreeSurfer a2009s lh G occipital sup area
WeakEnhancer Hoffman
UTR 5 UCSC
UTR 3 UCSC
TSS Hoffman
Transcribed Hoffman
TFBS ENCODE
SuperEnhancer Hnisz
Repressed Hoffman
Promoter UCSC
PromoterFlanking Hoffman
Intron UCSC
**H3K9ac Trynka
**H3K4me3 Trynka
**H3K4me1 Trynka
H3K27ac PGC2
H3K27ac Hnisz
FetalDHS Trynka
Enhancer Hoffman
Enhancer Andersson
DHS Trynka
DGF ENCODE
CTCF Hoffman
Conserved LindbladToh
Coding UCSC
Annotation
−10
−5
0
5
10
15
Enrichment (prop h2 / prop snps)

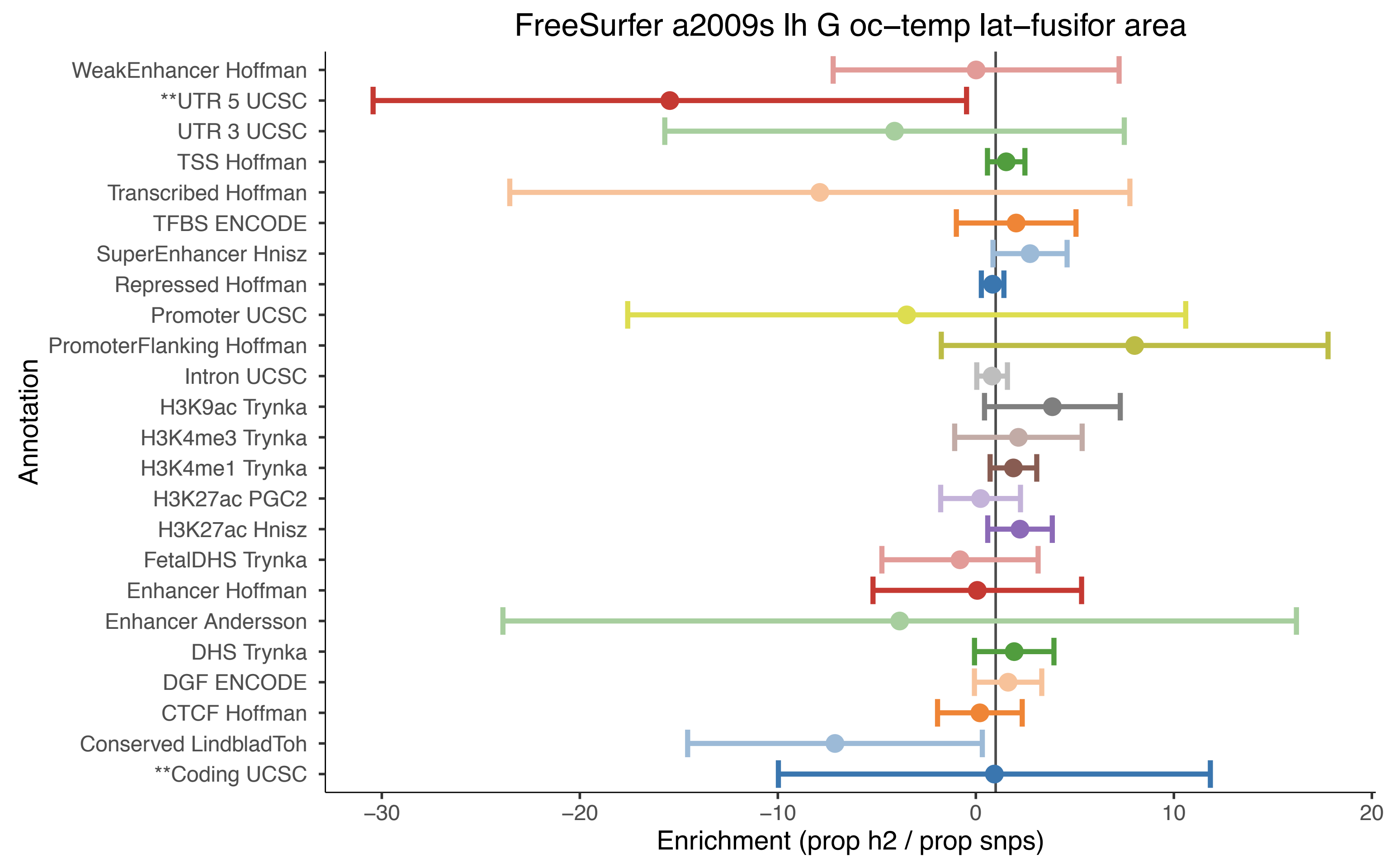
FreeSurfer a2009s lh G oc−temp lat−fusifor area
WeakEnhancer Hoffman
**UTR 5 UCSC
UTR 3 UCSC
TSS Hoffman
Transcribed Hoffman
TFBS ENCODE
SuperEnhancer Hnisz
Repressed Hoffman
Promoter UCSC
PromoterFlanking Hoffman
Intron UCSC
H3K9ac Trynka
H3K4me3 Trynka
H3K4me1 Trynka
H3K27ac PGC2
H3K27ac Hnisz
FetalDHS Trynka
Enhancer Hoffman
Enhancer Andersson
DHS Trynka
DGF ENCODE
CTCF Hoffman
Conserved LindbladToh
**Coding UCSC
Annotation
−30
−20
−10
0
10
20
Enrichment (prop h2 / prop snps)

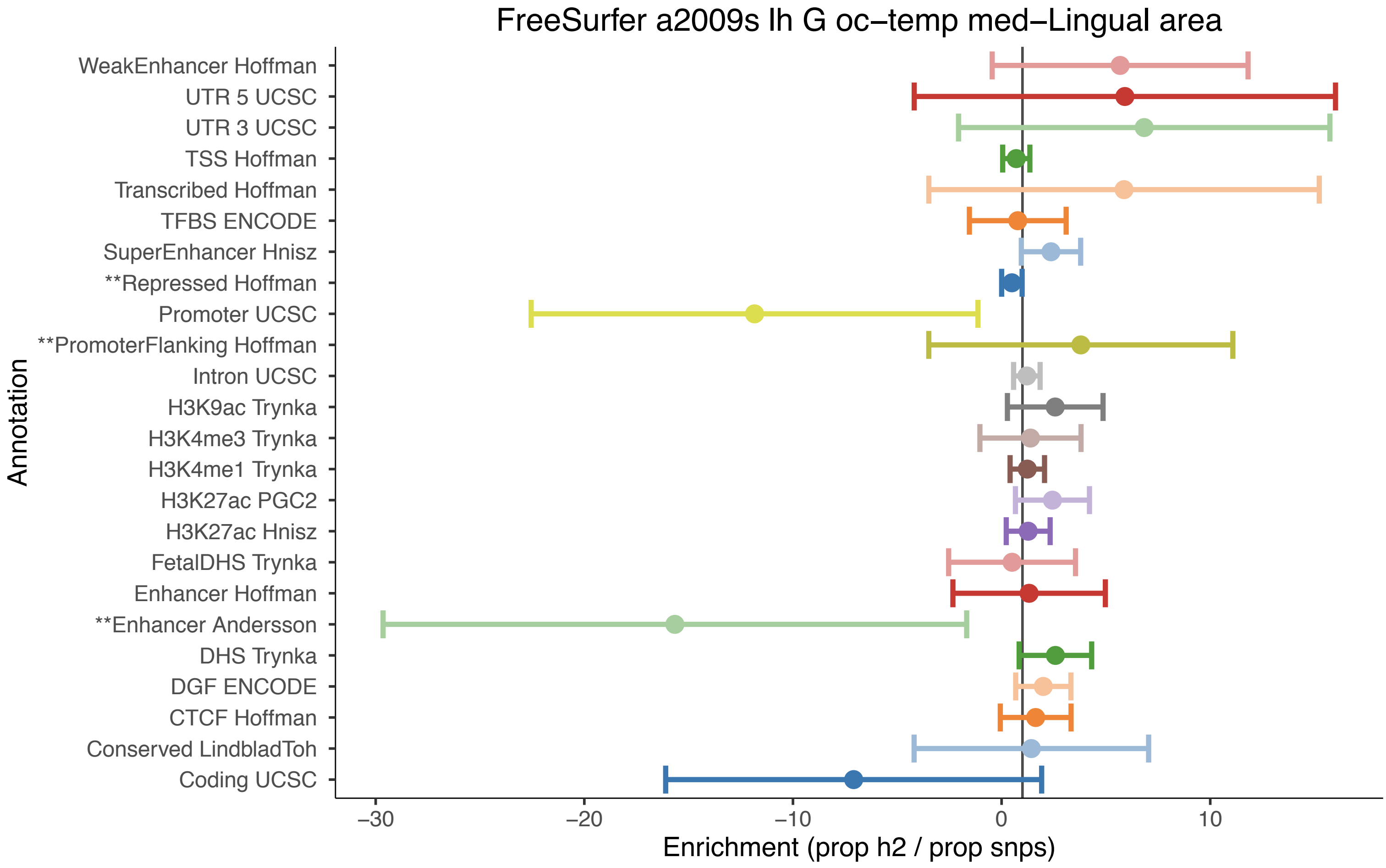

FreeSurfer a2009s lh G oc−temp med−Lingual area
Annotation
WeakEnhancer Hoffman
UTR 5 UCSC
UTR 3 UCSC
TSS Hoffman
Transcribed Hoffman
TFBS ENCODE
SuperEnhancer Hnisz
**Repressed Hoffman
Promoter UCSC
**PromoterFlanking Hoffman
Intron UCSC
H3K9ac Trynka
H3K4me3 Trynka
H3K4me1 Trynka
H3K27ac PGC2
H3K27ac Hnisz
FetalDHS Trynka
Enhancer Hoffman
**Enhancer Andersson
DHS Trynka
DGF ENCODE
CTCF Hoffman
Conserved LindbladToh
Coding UCSC
−30
−20
−10
0
10
Enrichment (prop h2 / prop snps)

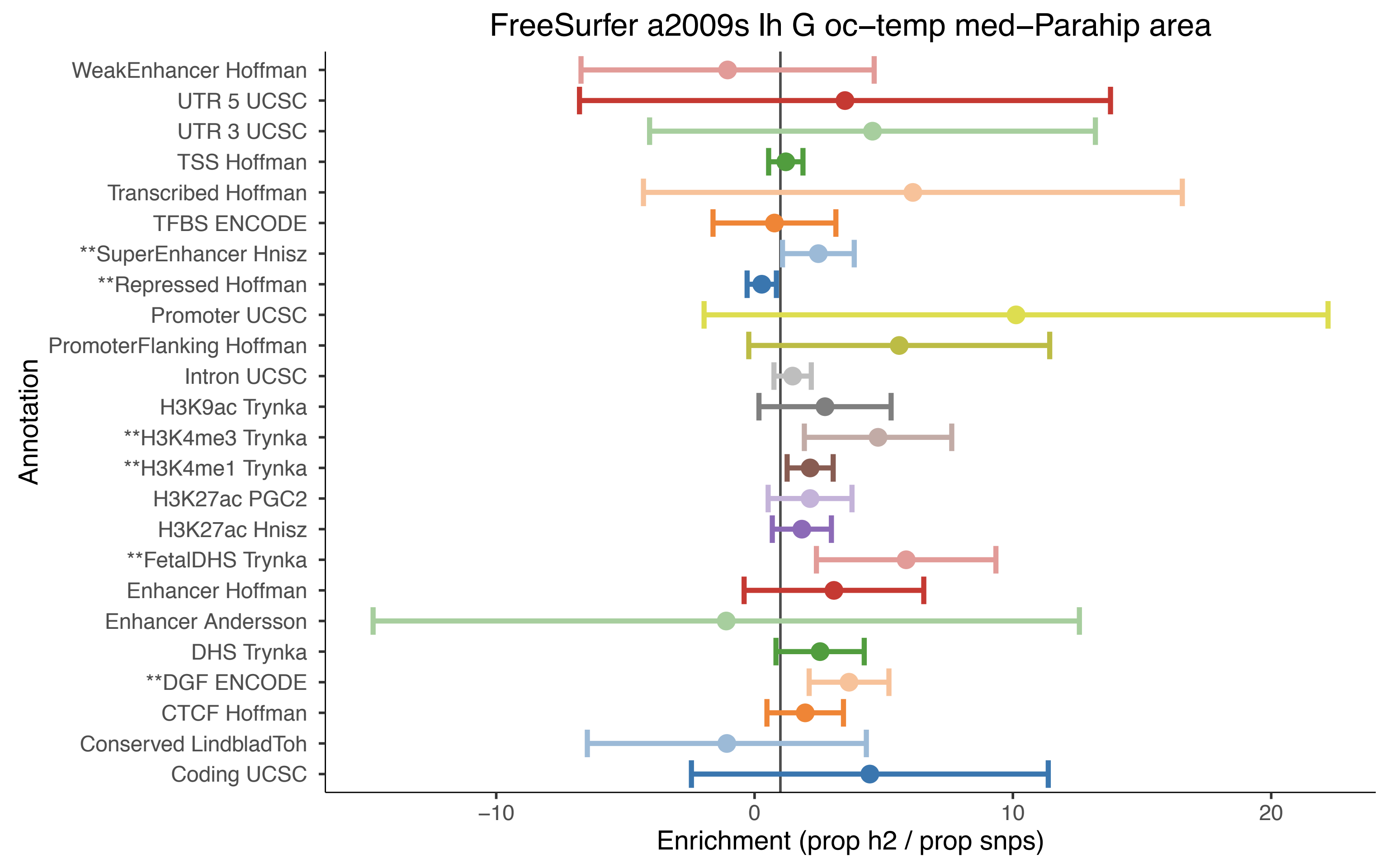
FreeSurfer a2009s lh G oc−temp med−Parahip area
WeakEnhancer Hoffman
UTR 5 UCSC
UTR 3 UCSC
TSS Hoffman
Transcribed Hoffman
TFBS ENCODE
**SuperEnhancer Hnisz
**Repressed Hoffman
Promoter UCSC
PromoterFlanking Hoffman
Intron UCSC
H3K9ac Trynka
**H3K4me3 Trynka
**H3K4me1 Trynka
H3K27ac PGC2
H3K27ac Hnisz
**FetalDHS Trynka
Enhancer Hoffman
Enhancer Andersson
DHS Trynka
**DGF ENCODE
CTCF Hoffman
Conserved LindbladToh
Coding UCSC
Annotation
−10
0
10
20
Enrichment (prop h2 / prop snps)

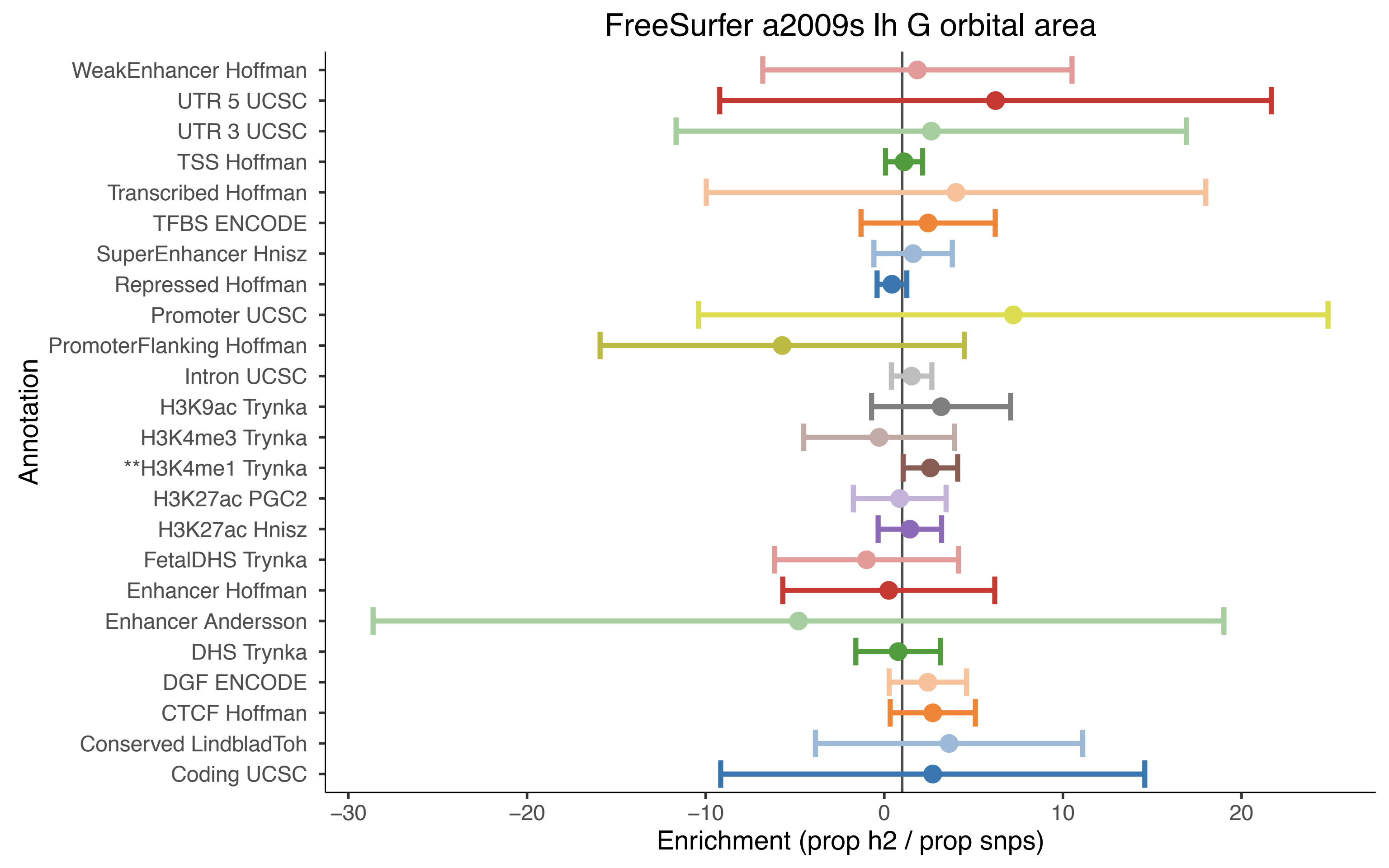
FreeSurfer a2009s lh G orbital area
WeakEnhancer Hoffman
UTR 5 UCSC
UTR 3 UCSC
TSS Hoffman
Transcribed Hoffman
TFBS ENCODE
SuperEnhancer Hnisz
Repressed Hoffman
Promoter UCSC
PromoterFlanking Hoffman
Intron UCSC
H3K9ac Trynka
H3K4me3 Trynka
**H3K4me1 Trynka
H3K27ac PGC2
H3K27ac Hnisz
FetalDHS Trynka
Enhancer Hoffman
Enhancer Andersson
DHS Trynka
DGF ENCODE
CTCF Hoffman
Conserved LindbladToh
Coding UCSC
Annotation
−30
−20
−10
0
10
20
Enrichment (prop h2 / prop snps)

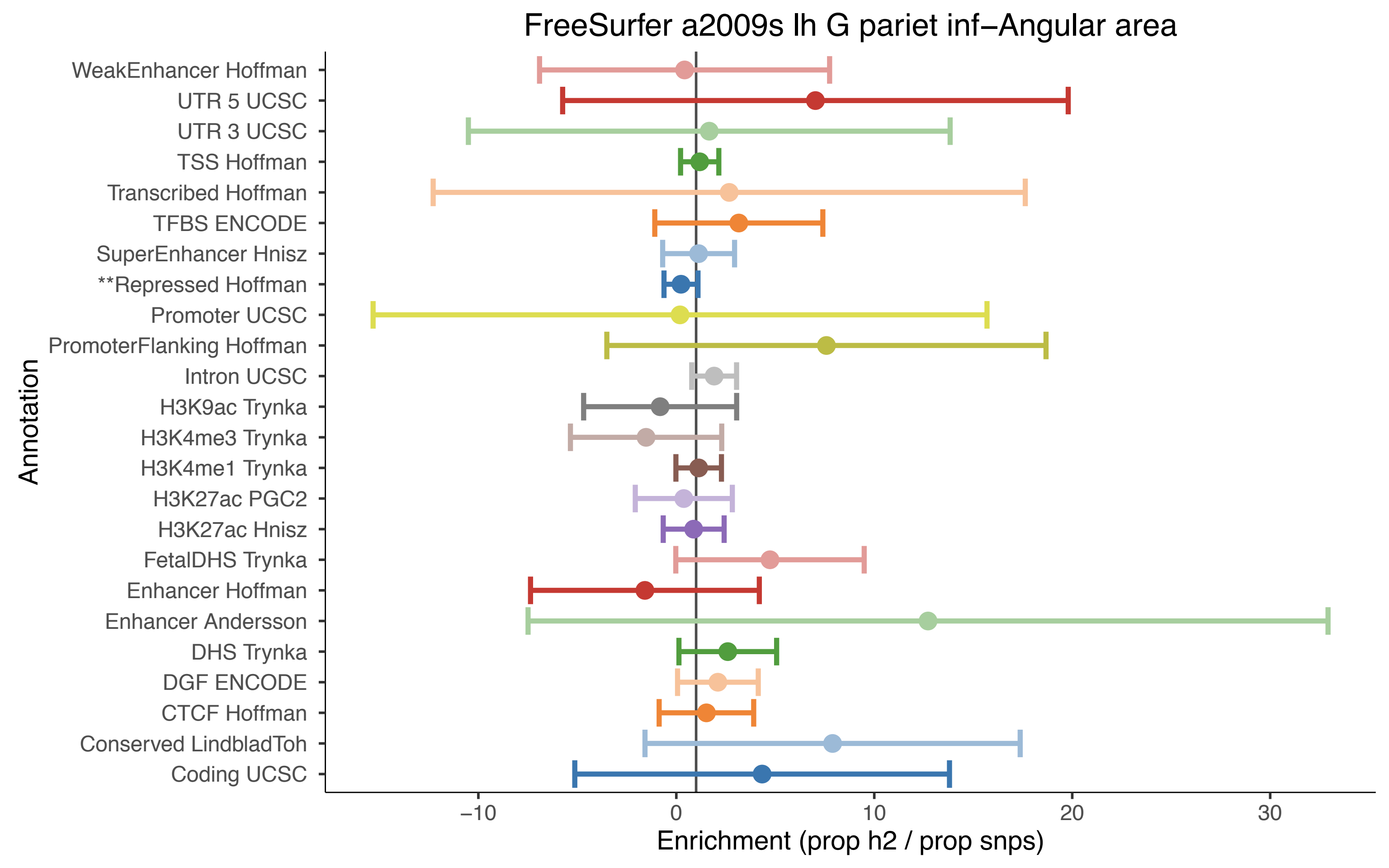
FreeSurfer a2009s lh G pariet inf−Angular area
WeakEnhancer Hoffman
UTR 5 UCSC
UTR 3 UCSC
TSS Hoffman
Transcribed Hoffman
TFBS ENCODE
SuperEnhancer Hnisz
**Repressed Hoffman
Promoter UCSC
PromoterFlanking Hoffman
Intron UCSC
H3K9ac Trynka
H3K4me3 Trynka
H3K4me1 Trynka
H3K27ac PGC2
H3K27ac Hnisz
FetalDHS Trynka
Enhancer Hoffman
Enhancer Andersson
DHS Trynka
DGF ENCODE
CTCF Hoffman
Conserved LindbladToh
Coding UCSC
Annotation
−10
0
10
20
30
Enrichment (prop h2 / prop snps)

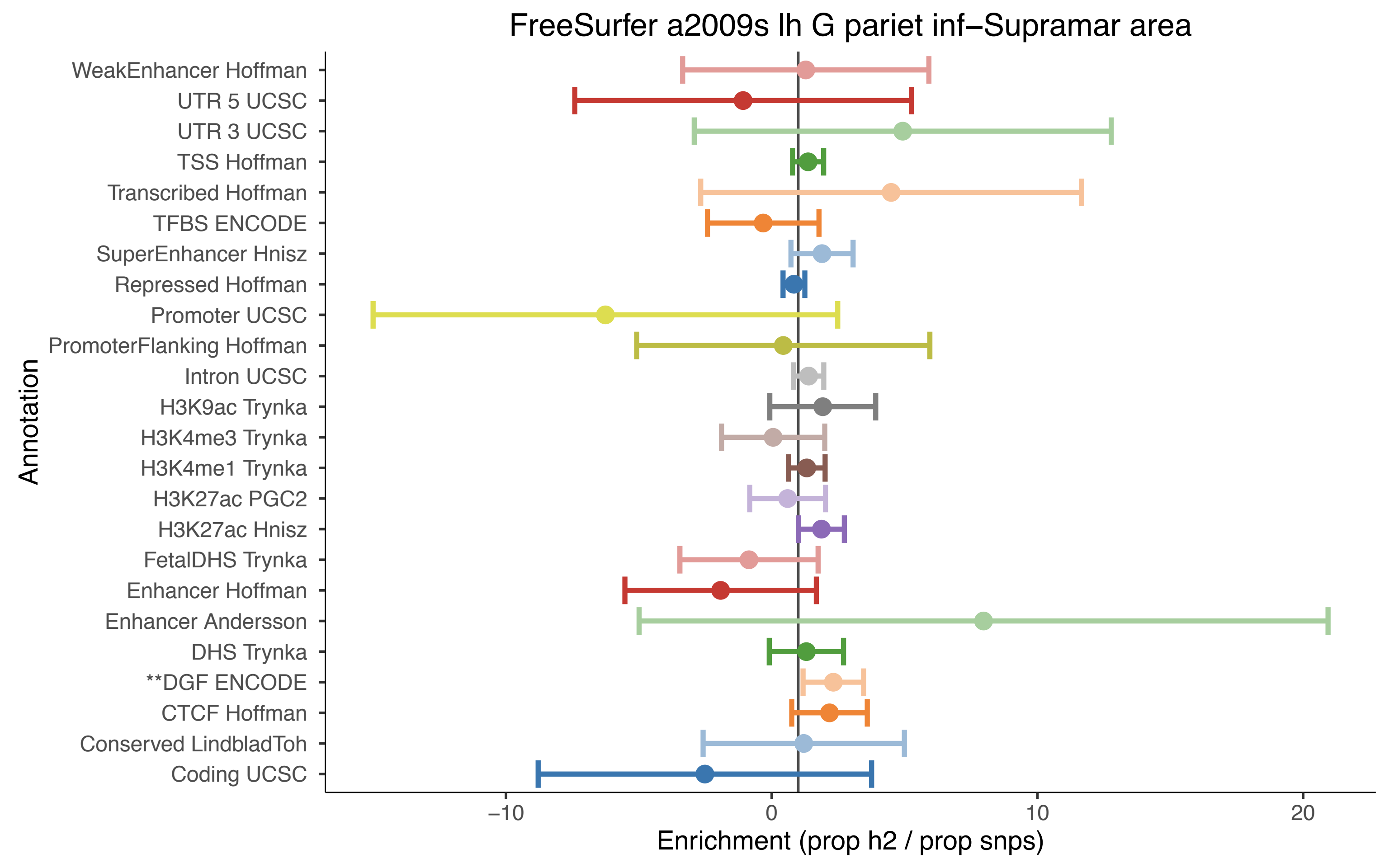
FreeSurfer a2009s lh G pariet inf−Supramar area
WeakEnhancer Hoffman
UTR 5 UCSC
UTR 3 UCSC
TSS Hoffman
Transcribed Hoffman
TFBS ENCODE
SuperEnhancer Hnisz
Repressed Hoffman
Promoter UCSC
PromoterFlanking Hoffman
Intron UCSC
H3K9ac Trynka
H3K4me3 Trynka
H3K4me1 Trynka
H3K27ac PGC2
H3K27ac Hnisz
FetalDHS Trynka
Enhancer Hoffman
Enhancer Andersson
DHS Trynka
**DGF ENCODE
CTCF Hoffman
Conserved LindbladToh
Coding UCSC
Annotation
−10
0
10
20
Enrichment (prop h2 / prop snps)

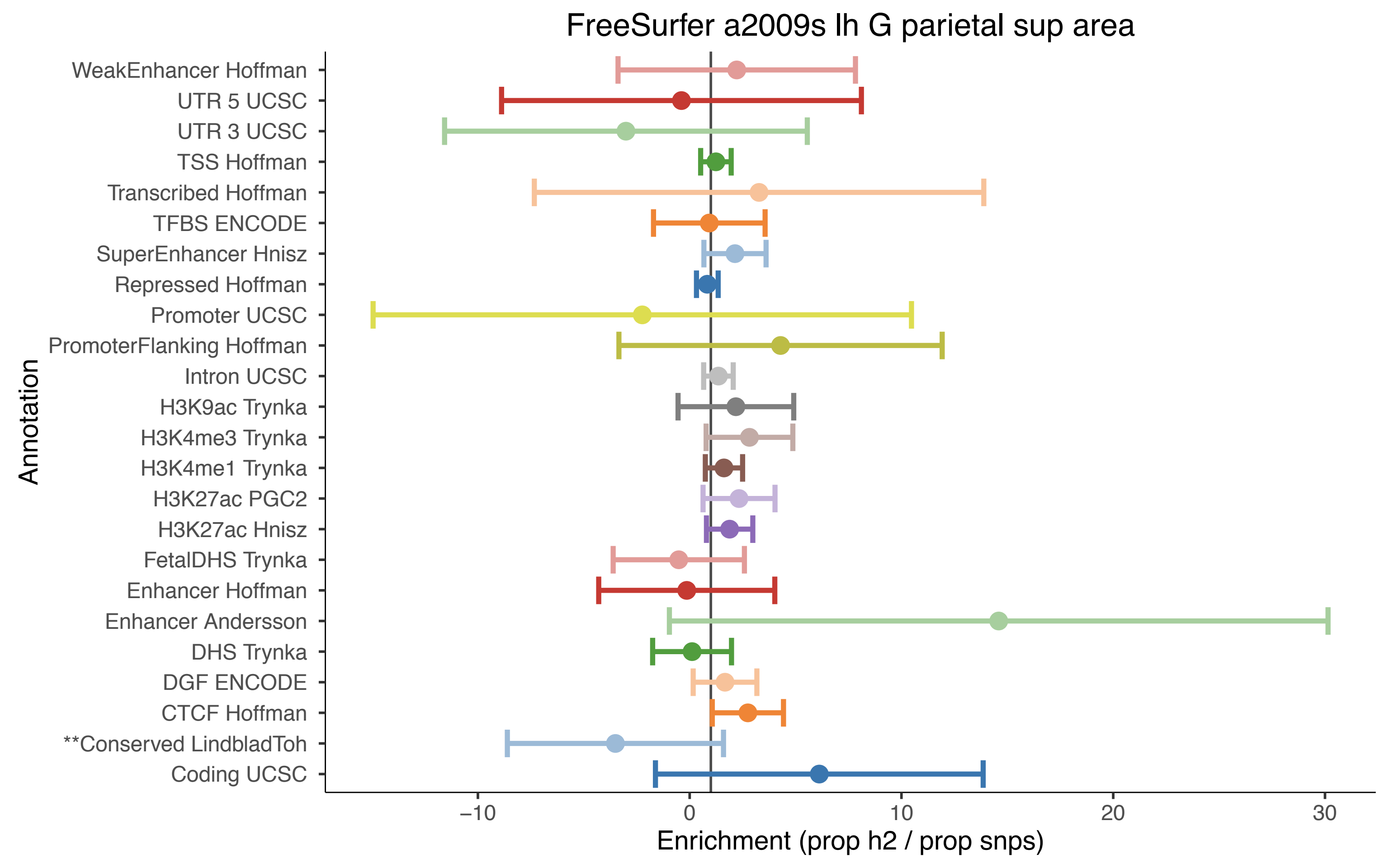

FreeSurfer a2009s lh G parietal sup area
Annotation
WeakEnhancer Hoffman
UTR 5 UCSC
UTR 3 UCSC
TSS Hoffman
Transcribed Hoffman
TFBS ENCODE
SuperEnhancer Hnisz
Repressed Hoffman
Promoter UCSC
PromoterFlanking Hoffman
Intron UCSC
H3K9ac Trynka
H3K4me3 Trynka
H3K4me1 Trynka
H3K27ac PGC2
H3K27ac Hnisz
FetalDHS Trynka
Enhancer Hoffman
Enhancer Andersson
DHS Trynka
DGF ENCODE
CTCF Hoffman
**Conserved LindbladToh
Coding UCSC
−10
0
10
20
30
Enrichment (prop h2 / prop snps)

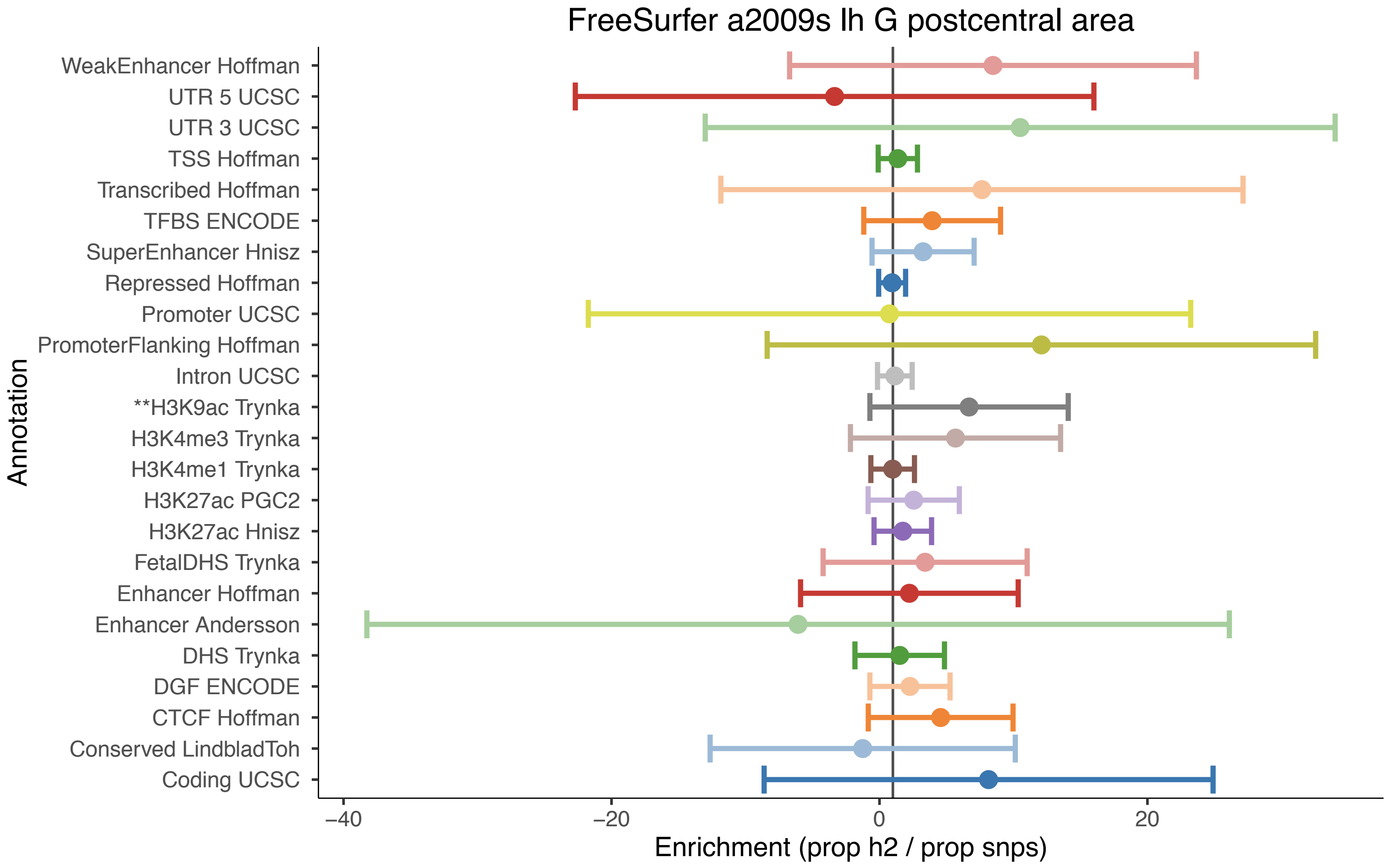

FreeSurfer a2009s lh G postcentral area
WeakEnhancer Hoffman
UTR 5 UCSC
UTR 3 UCSC
TSS Hoffman
Transcribed Hoffman
TFBS ENCODE
SuperEnhancer Hnisz
Repressed Hoffman
Promoter UCSC
PromoterFlanking Hoffman
Intron UCSC
**H3K9ac Trynka
H3K4me3 Trynka
H3K4me1 Trynka
H3K27ac PGC2
H3K27ac Hnisz
FetalDHS Trynka
Enhancer Hoffman
Enhancer Andersson
DHS Trynka
DGF ENCODE
CTCF Hoffman
Conserved LindbladToh
Coding UCSC
Annotation
−40
−20
0
20
Enrichment (prop h2 / prop snps)

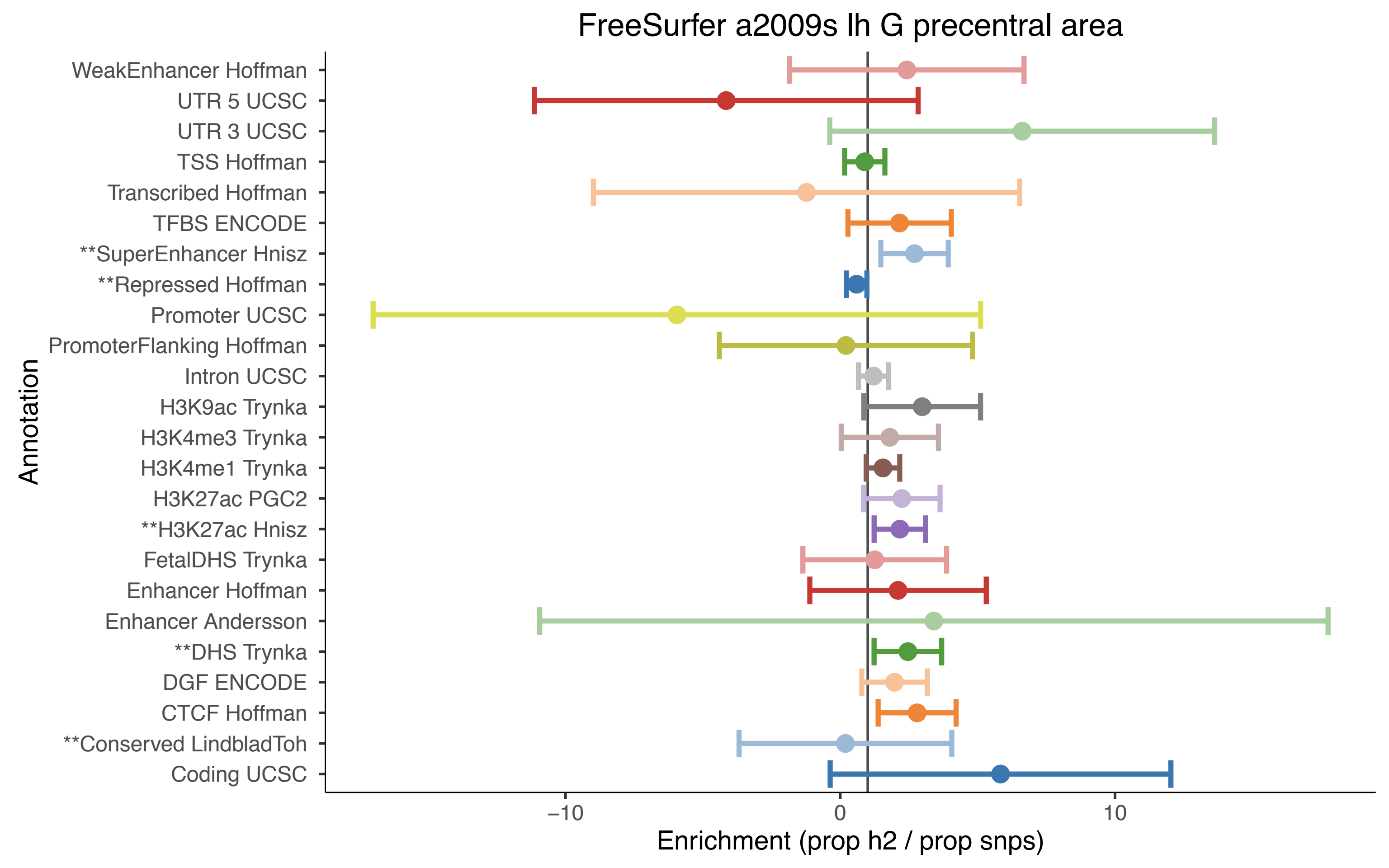
FreeSurfer a2009s lh G precentral area
WeakEnhancer Hoffman
UTR 5 UCSC
UTR 3 UCSC
TSS Hoffman
Transcribed Hoffman
TFBS ENCODE
**SuperEnhancer Hnisz
**Repressed Hoffman
Promoter UCSC
PromoterFlanking Hoffman
Intron UCSC
H3K9ac Trynka
H3K4me3 Trynka
H3K4me1 Trynka
H3K27ac PGC2
**H3K27ac Hnisz
FetalDHS Trynka
Enhancer Hoffman
Enhancer Andersson
**DHS Trynka
DGF ENCODE
CTCF Hoffman
**Conserved LindbladToh
Coding UCSC
Annotation
−10
0
10
Enrichment (prop h2 / prop snps)

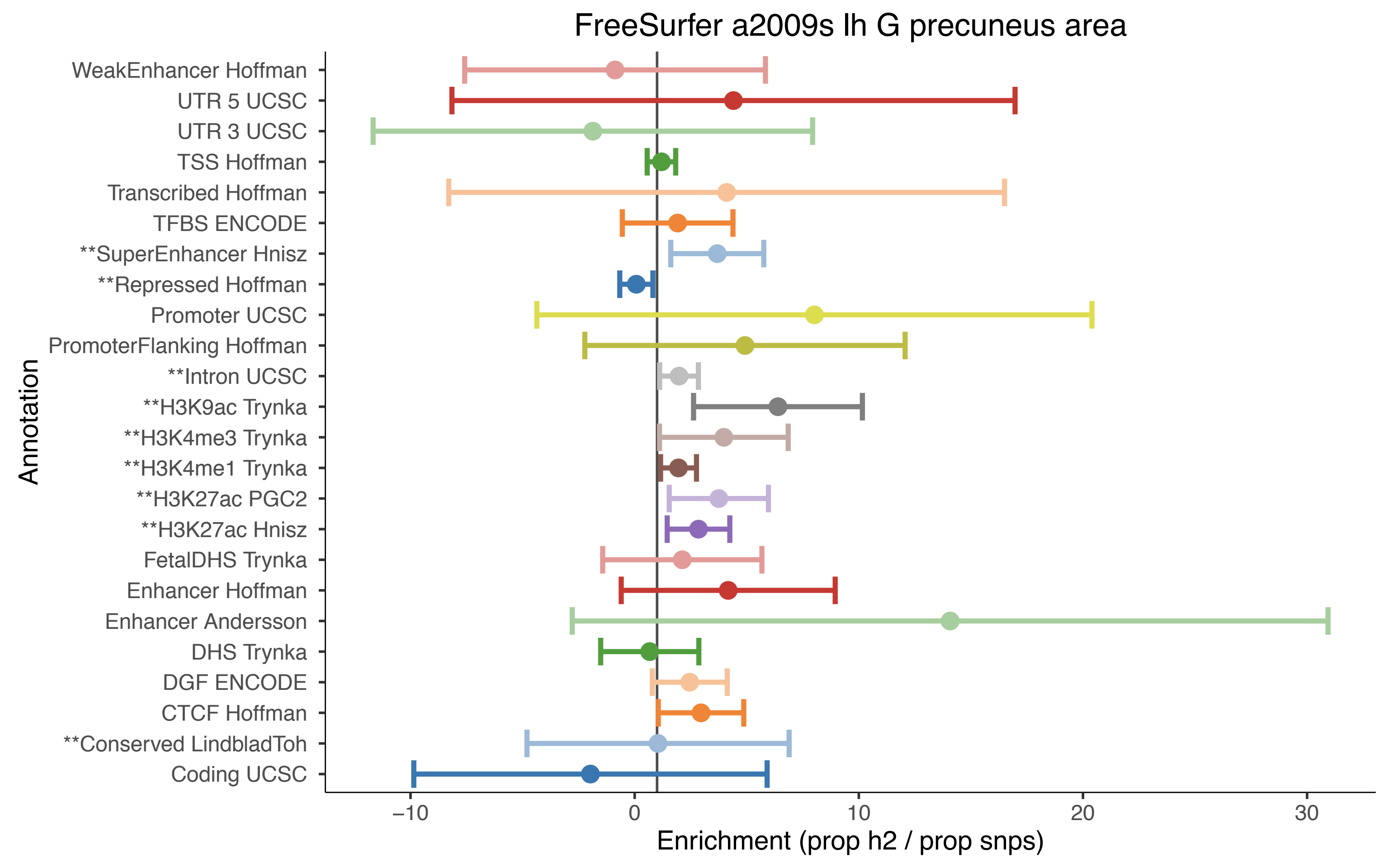
FreeSurfer a2009s lh G precuneus area
WeakEnhancer Hoffman
UTR 5 UCSC
UTR 3 UCSC
TSS Hoffman
Transcribed Hoffman
TFBS ENCODE
**SuperEnhancer Hnisz
**Repressed Hoffman
Promoter UCSC
PromoterFlanking Hoffman
**Intron UCSC
**H3K9ac Trynka
**H3K4me3 Trynka
**H3K4me1 Trynka
**H3K27ac PGC2
**H3K27ac Hnisz
FetalDHS Trynka
Enhancer Hoffman
Enhancer Andersson
DHS Trynka
DGF ENCODE
CTCF Hoffman
**Conserved LindbladToh
Coding UCSC
Annotation
−10
0
10
20
30
Enrichment (prop h2 / prop snps)

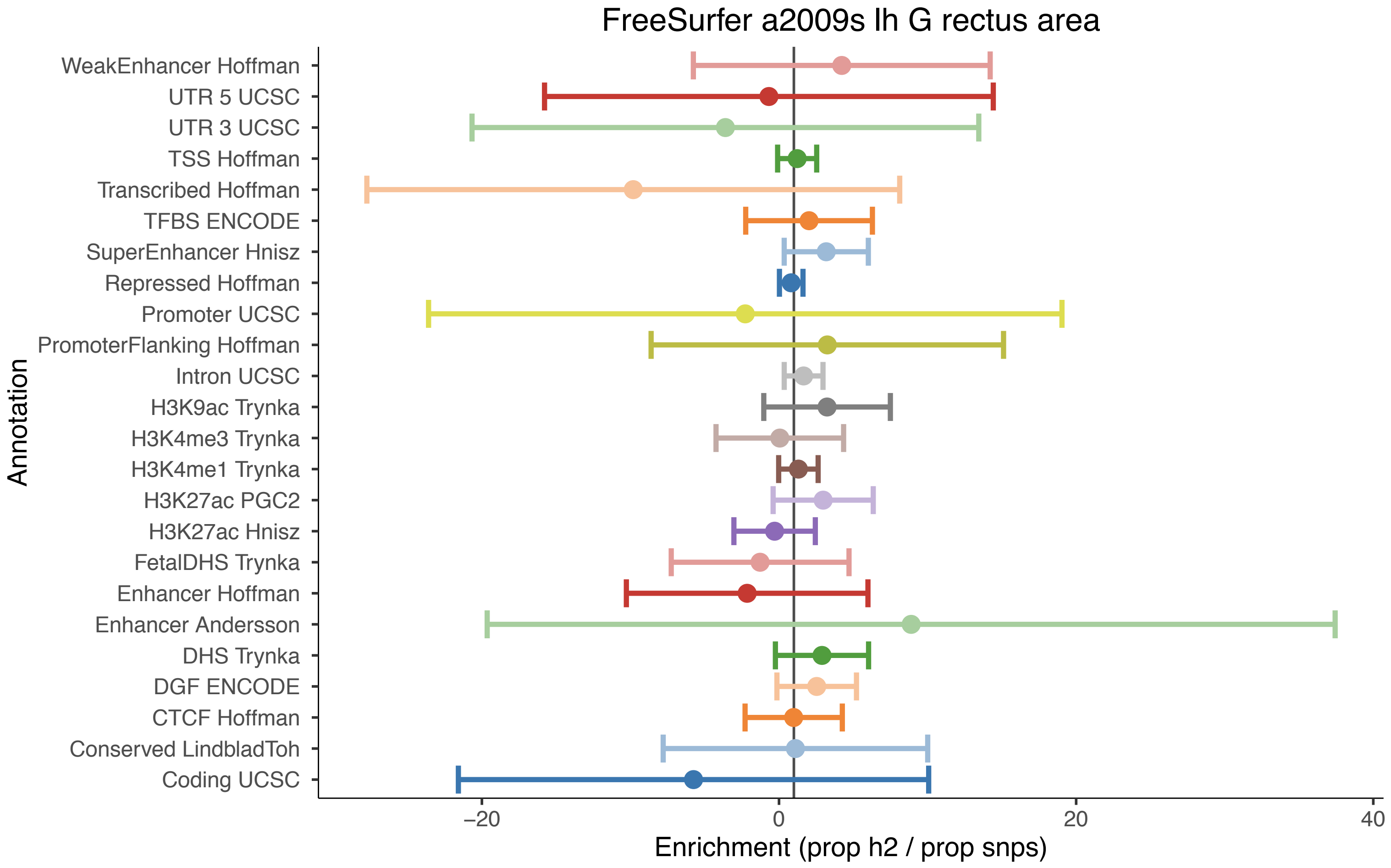
FreeSurfer a2009s lh G rectus area
WeakEnhancer Hoffman
UTR 5 UCSC
UTR 3 UCSC
TSS Hoffman
Transcribed Hoffman
TFBS ENCODE
SuperEnhancer Hnisz
Repressed Hoffman
Promoter UCSC
PromoterFlanking Hoffman
Intron UCSC
H3K9ac Trynka
H3K4me3 Trynka
H3K4me1 Trynka
H3K27ac PGC2
H3K27ac Hnisz
FetalDHS Trynka
Enhancer Hoffman
Enhancer Andersson
DHS Trynka
DGF ENCODE
CTCF Hoffman
Conserved LindbladToh
Coding UCSC
Annotation
−20
0
20
40
Enrichment (prop h2 / prop snps)

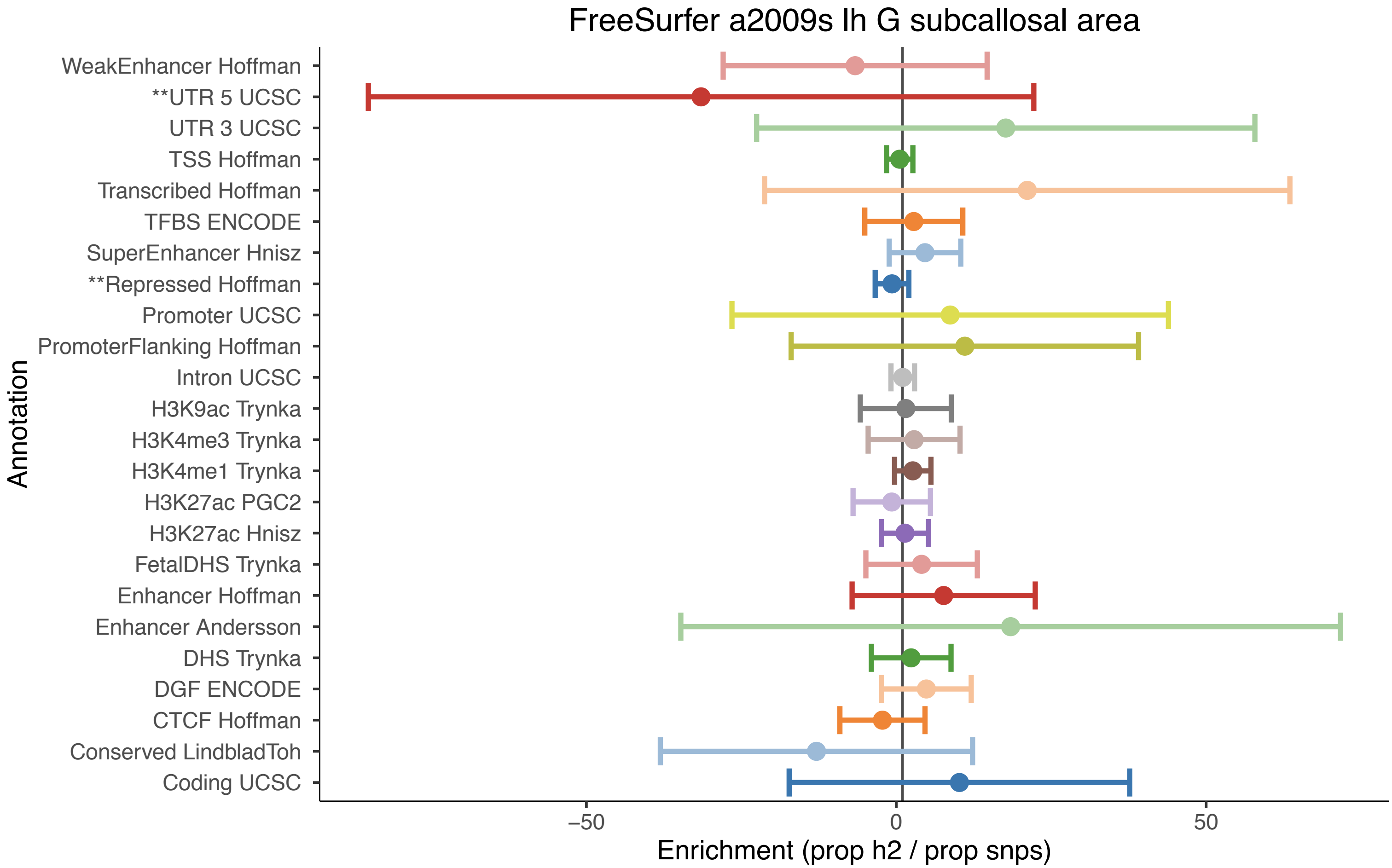

FreeSurfer a2009s lh G subcallosal area
WeakEnhancer Hoffman
**UTR 5 UCSC
UTR 3 UCSC
TSS Hoffman
Transcribed Hoffman
TFBS ENCODE
SuperEnhancer Hnisz
**Repressed Hoffman
Promoter UCSC
PromoterFlanking Hoffman
Intron UCSC
H3K9ac Trynka
H3K4me3 Trynka
H3K4me1 Trynka
H3K27ac PGC2
H3K27ac Hnisz
FetalDHS Trynka
Enhancer Hoffman
Enhancer Andersson
DHS Trynka
DGF ENCODE
CTCF Hoffman
Conserved LindbladToh
Coding UCSC
Annotation
−50
0
50
Enrichment (prop h2 / prop snps)

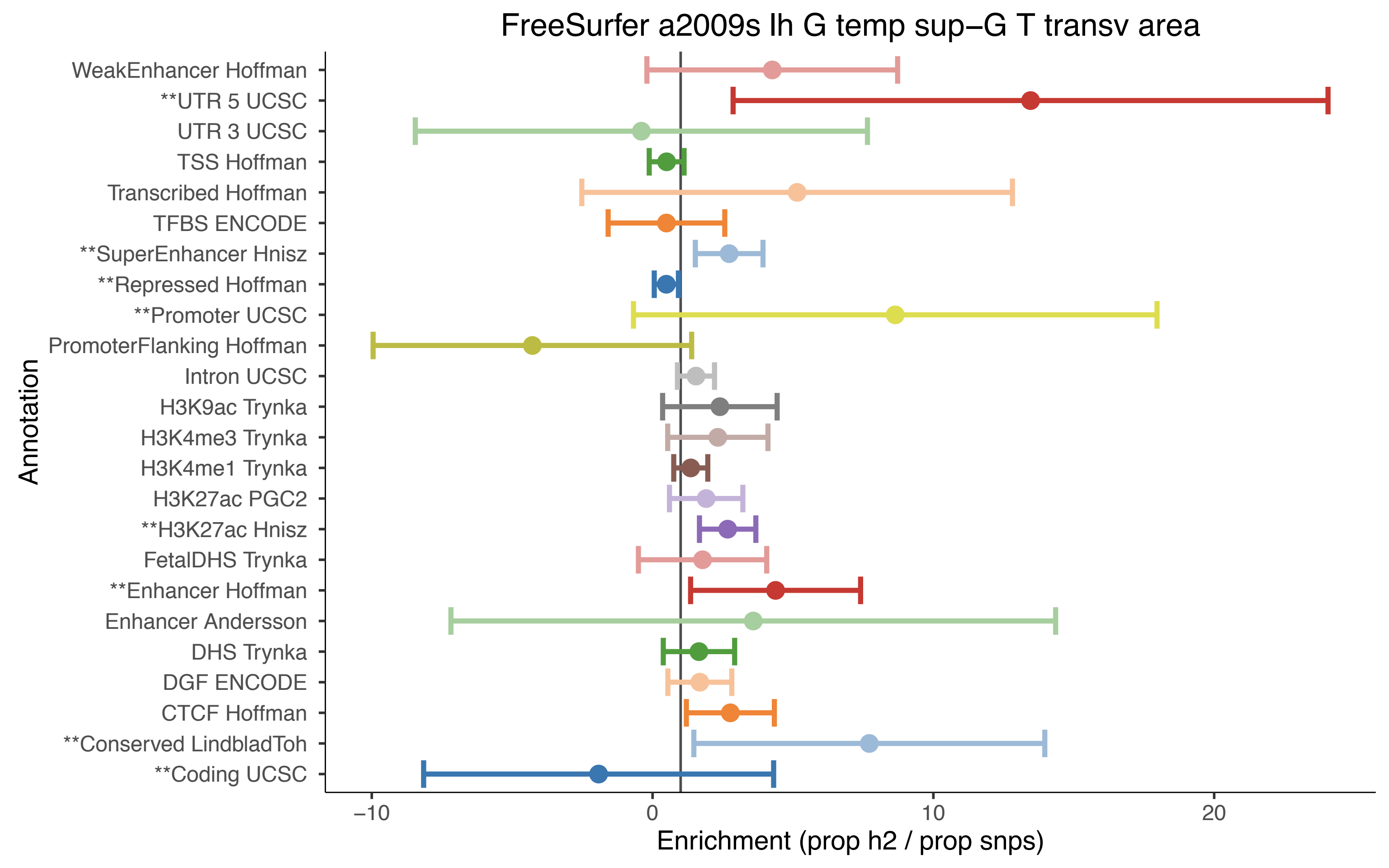
FreeSurfer a2009s lh G temp sup−G T transv area
WeakEnhancer Hoffman
**UTR 5 UCSC
UTR 3 UCSC
TSS Hoffman
Transcribed Hoffman
TFBS ENCODE
**SuperEnhancer Hnisz
**Repressed Hoffman
**Promoter UCSC
PromoterFlanking Hoffman
Intron UCSC
H3K9ac Trynka
H3K4me3 Trynka
H3K4me1 Trynka
H3K27ac PGC2
**H3K27ac Hnisz
FetalDHS Trynka
**Enhancer Hoffman
Enhancer Andersson
DHS Trynka
DGF ENCODE
CTCF Hoffman
**Conserved LindbladToh
**Coding UCSC
Annotation
−10
0
10
20
Enrichment (prop h2 / prop snps)

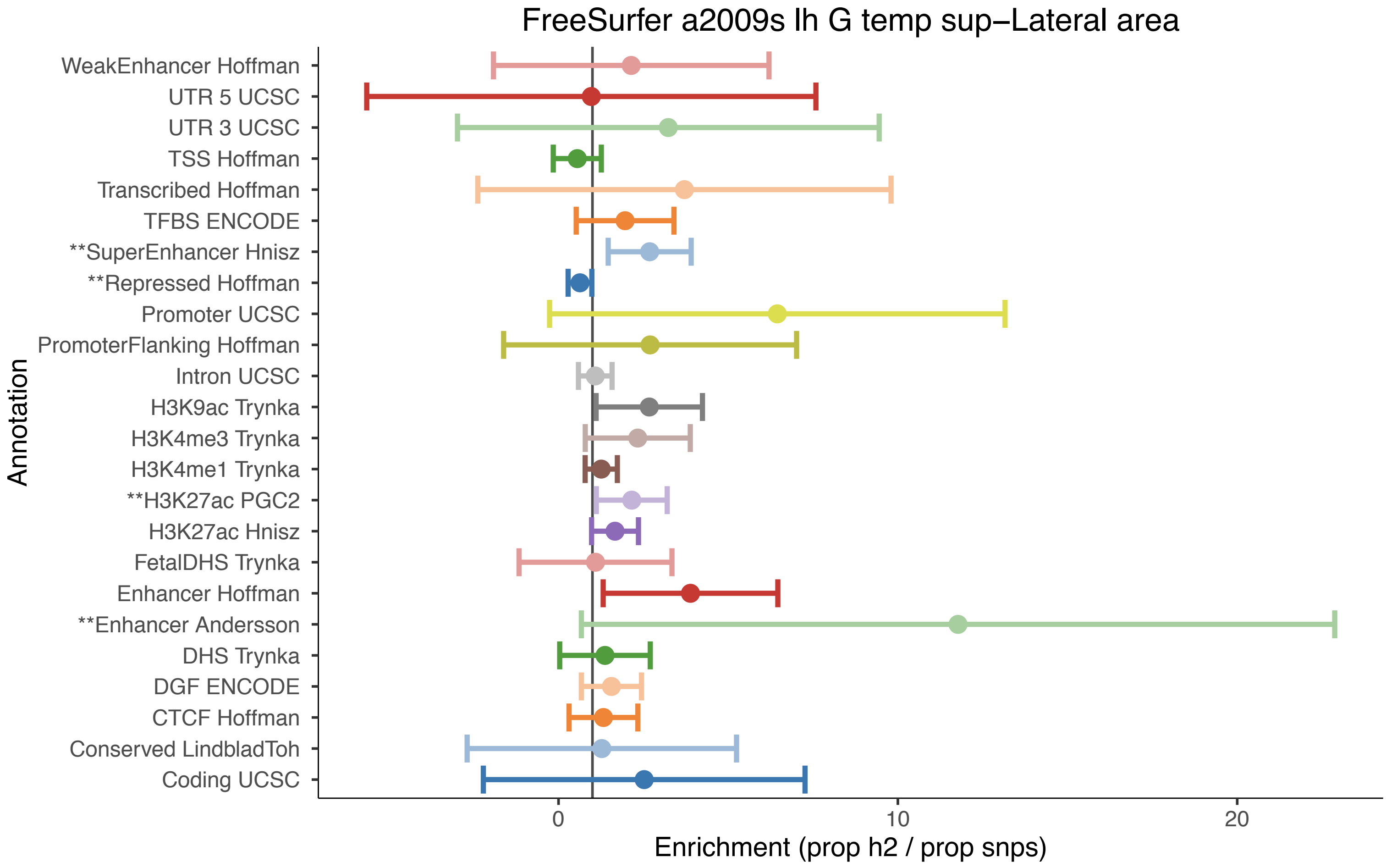
FreeSurfer a2009s lh G temp sup−Lateral area
Annotation
WeakEnhancer Hoffman
UTR 5 UCSC
UTR 3 UCSC
TSS Hoffman
Transcribed Hoffman
TFBS ENCODE
**SuperEnhancer Hnisz
**Repressed Hoffman
Promoter UCSC
PromoterFlanking Hoffman
Intron UCSC
H3K9ac Trynka
H3K4me3 Trynka
H3K4me1 Trynka
**H3K27ac PGC2
H3K27ac Hnisz
FetalDHS Trynka
Enhancer Hoffman
**Enhancer Andersson
DHS Trynka
DGF ENCODE
CTCF Hoffman
Conserved LindbladToh
Coding UCSC
0
10
20
Enrichment (prop h2 / prop snps)

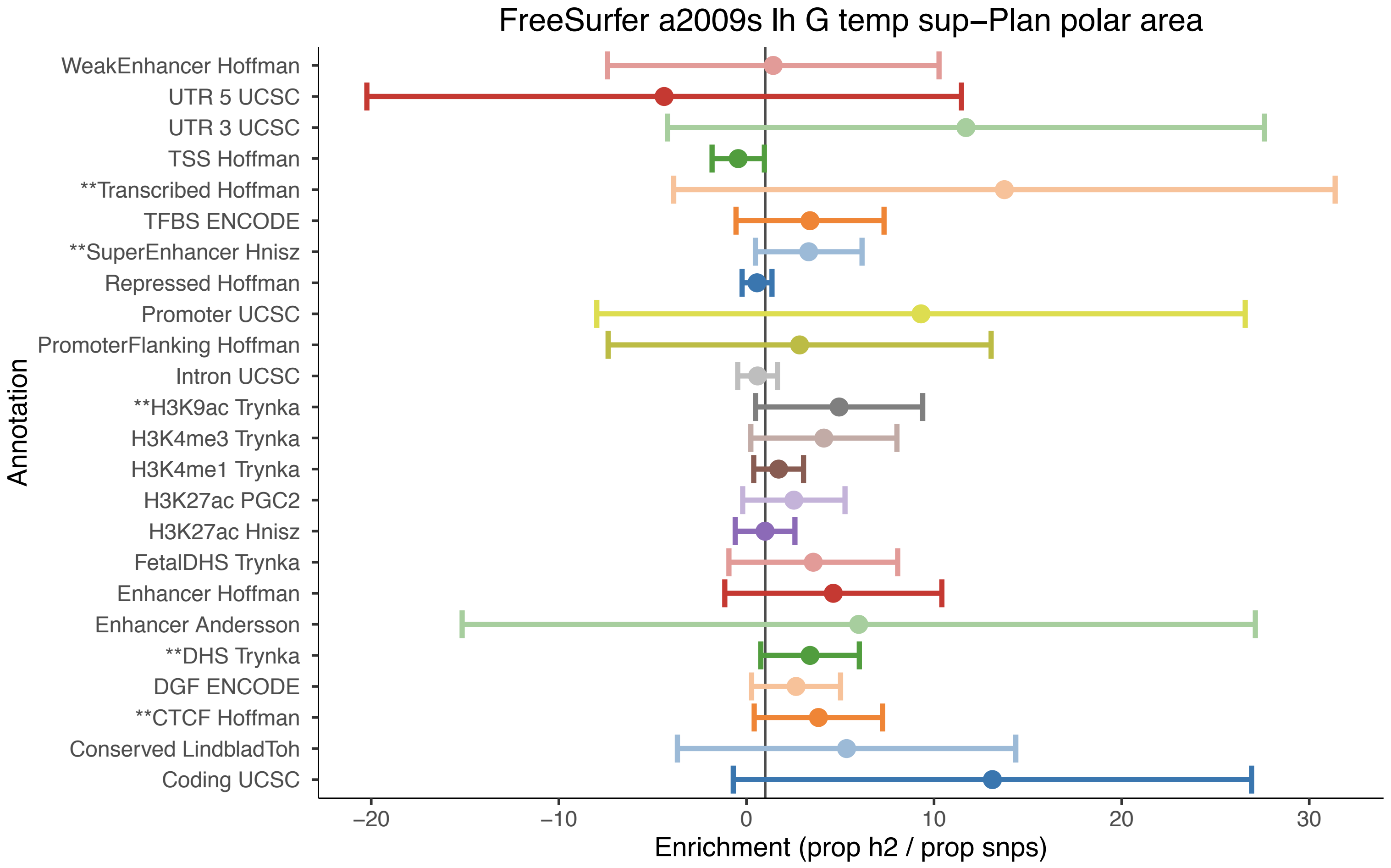
FreeSurfer a2009s lh G temp sup−Plan polar area
WeakEnhancer Hoffman
UTR 5 UCSC
UTR 3 UCSC
TSS Hoffman
**Transcribed Hoffman
TFBS ENCODE
**SuperEnhancer Hnisz
Repressed Hoffman
Promoter UCSC
PromoterFlanking Hoffman
Intron UCSC
**H3K9ac Trynka
H3K4me3 Trynka
H3K4me1 Trynka
H3K27ac PGC2
H3K27ac Hnisz
FetalDHS Trynka
Enhancer Hoffman
Enhancer Andersson
**DHS Trynka
DGF ENCODE
**CTCF Hoffman
Conserved LindbladToh
Coding UCSC
Annotation
−20
−10
0
10
20
30
Enrichment (prop h2 / prop snps)

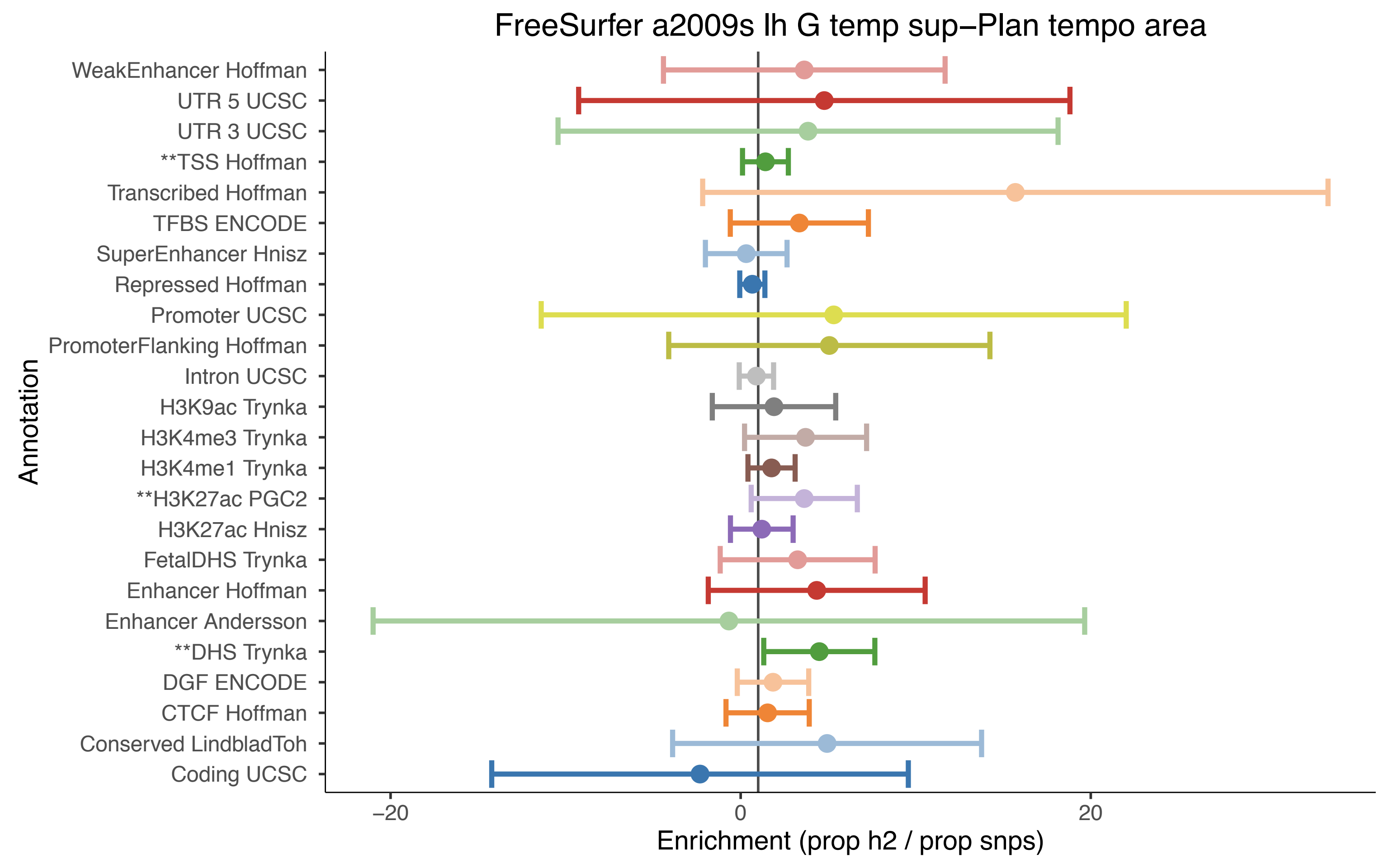
FreeSurfer a2009s lh G temp sup−Plan tempo area
WeakEnhancer Hoffman
UTR 5 UCSC
UTR 3 UCSC
**TSS Hoffman
Transcribed Hoffman
TFBS ENCODE
SuperEnhancer Hnisz
Repressed Hoffman
Promoter UCSC
PromoterFlanking Hoffman
Intron UCSC
H3K9ac Trynka
H3K4me3 Trynka
H3K4me1 Trynka
**H3K27ac PGC2
H3K27ac Hnisz
FetalDHS Trynka
Enhancer Hoffman
Enhancer Andersson
**DHS Trynka
DGF ENCODE
CTCF Hoffman
Conserved LindbladToh
Coding UCSC
Annotation
−20
0
20
Enrichment (prop h2 / prop snps)

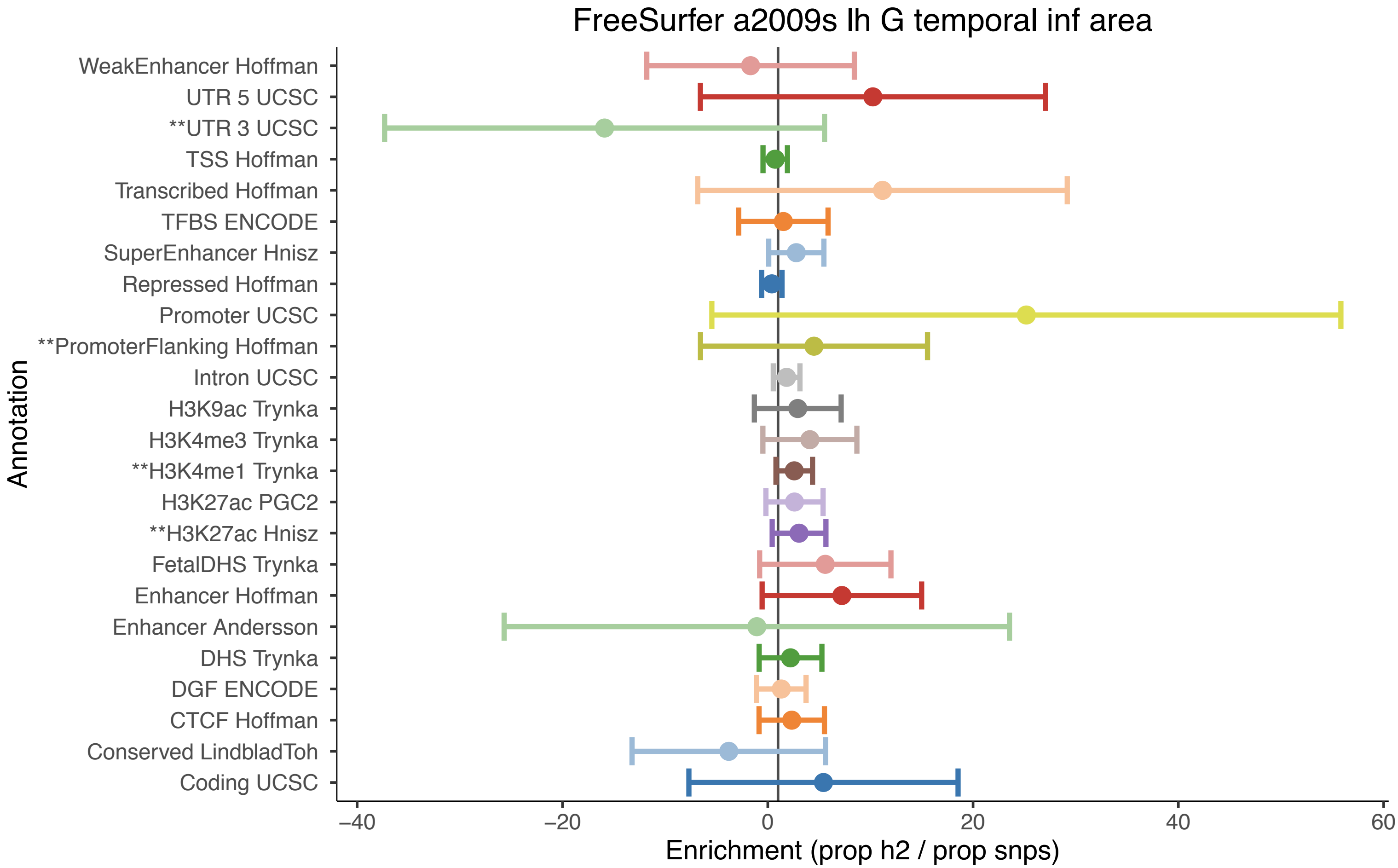
FreeSurfer a2009s lh G temporal inf area
WeakEnhancer Hoffman
UTR 5 UCSC
**UTR 3 UCSC
TSS Hoffman
Transcribed Hoffman
TFBS ENCODE
SuperEnhancer Hnisz
Repressed Hoffman
Promoter UCSC
**PromoterFlanking Hoffman
Intron UCSC
H3K9ac Trynka
H3K4me3 Trynka
**H3K4me1 Trynka
H3K27ac PGC2
**H3K27ac Hnisz
FetalDHS Trynka
Enhancer Hoffman
Enhancer Andersson
DHS Trynka
DGF ENCODE
CTCF Hoffman
Conserved LindbladToh
Coding UCSC
Annotation
−40
−20
0
20
40
60
Enrichment (prop h2 / prop snps)

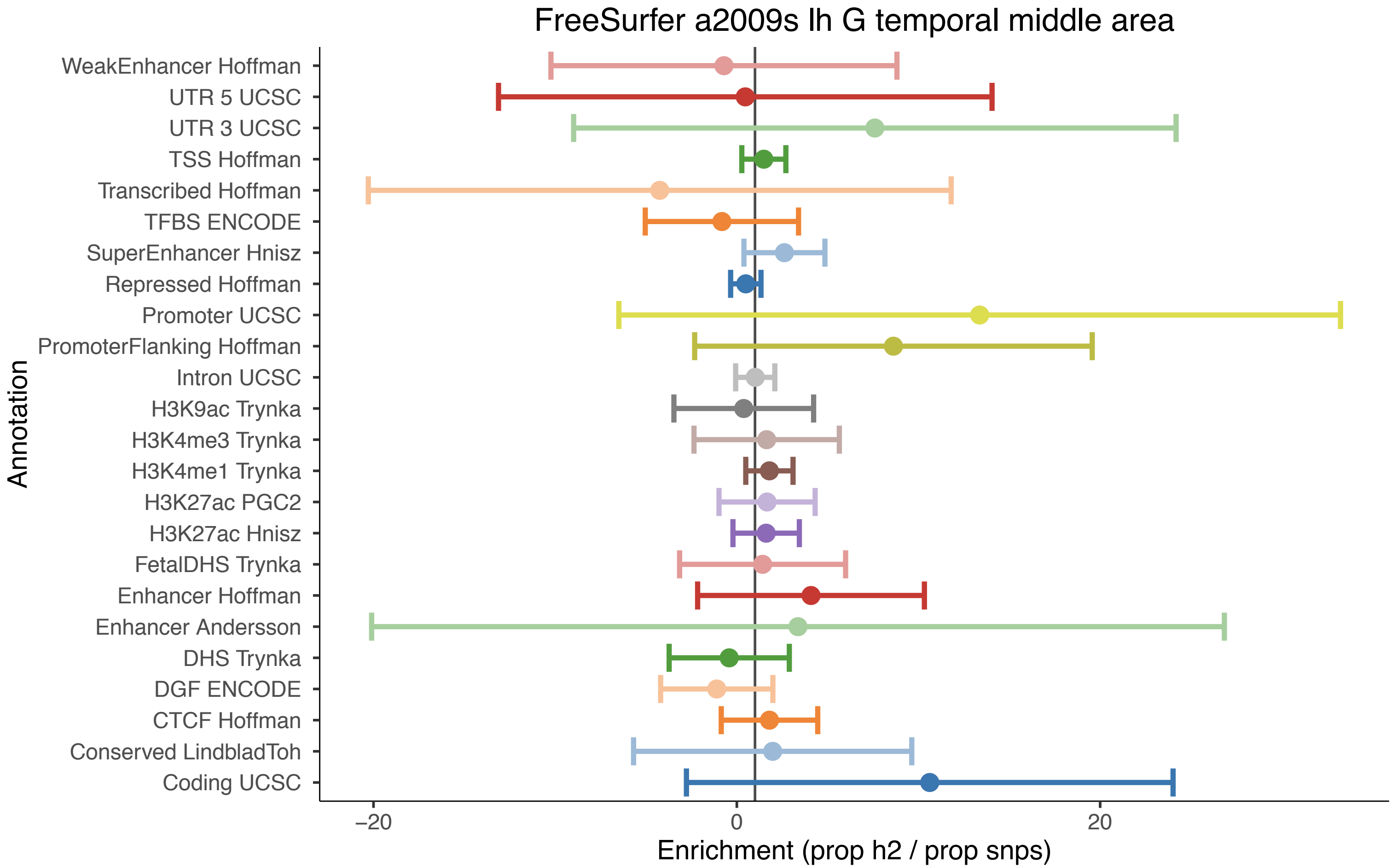

FreeSurfer a2009s lh G temporal middle area
WeakEnhancer Hoffman
UTR 5 UCSC
UTR 3 UCSC
TSS Hoffman
Transcribed Hoffman
TFBS ENCODE
SuperEnhancer Hnisz
Repressed Hoffman
Promoter UCSC
PromoterFlanking Hoffman
Intron UCSC
H3K9ac Trynka
H3K4me3 Trynka
H3K4me1 Trynka
H3K27ac PGC2
H3K27ac Hnisz
FetalDHS Trynka
Enhancer Hoffman
Enhancer Andersson
DHS Trynka
DGF ENCODE
CTCF Hoffman
Conserved LindbladToh
Coding UCSC
Annotation
−20
0
20
Enrichment (prop h2 / prop snps)

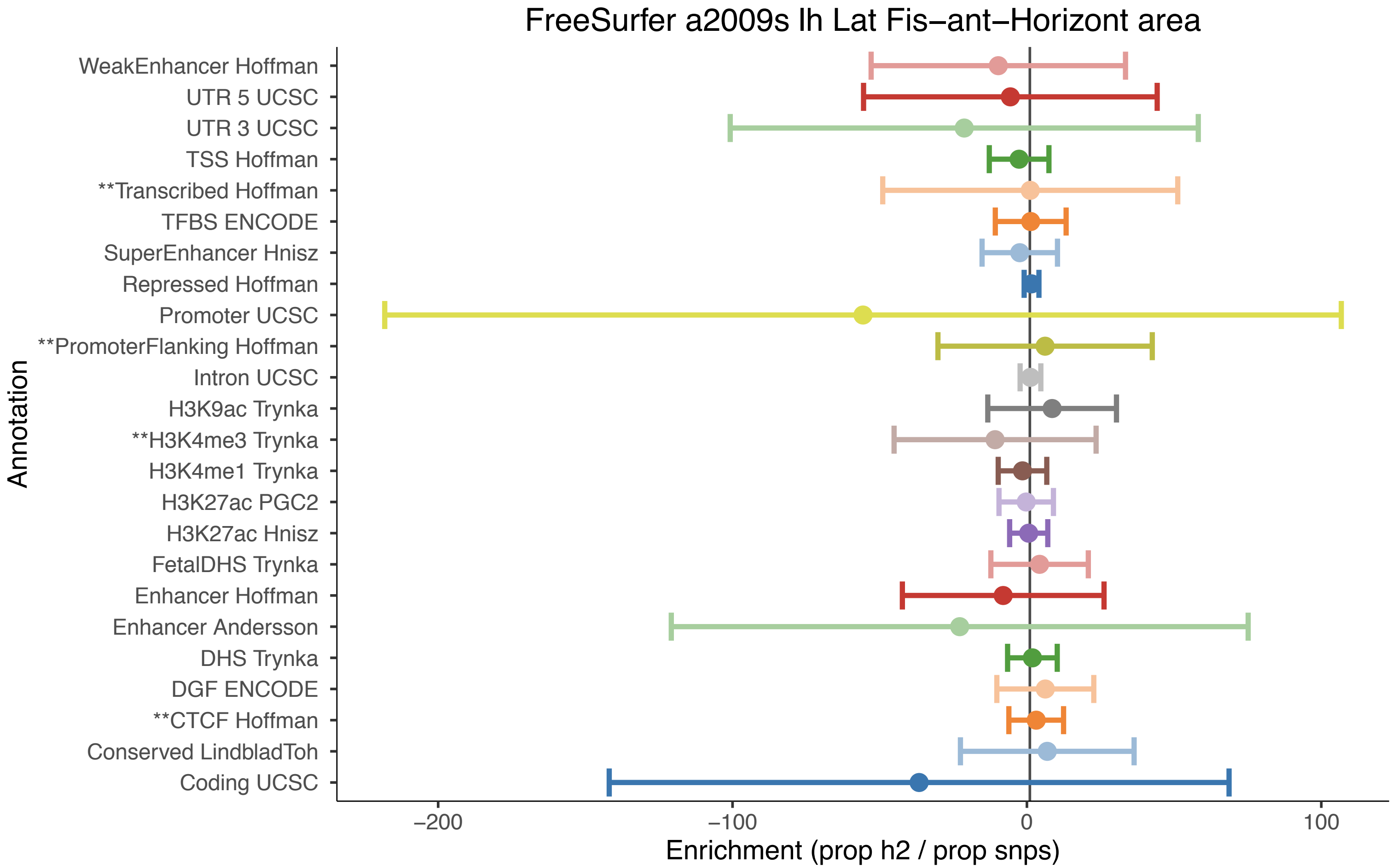

FreeSurfer a2009s lh Lat Fis−ant−Horizont area
Annotation
WeakEnhancer Hoffman
UTR 5 UCSC
UTR 3 UCSC
TSS Hoffman
**Transcribed Hoffman
TFBS ENCODE
SuperEnhancer Hnisz
Repressed Hoffman
Promoter UCSC
**PromoterFlanking Hoffman
Intron UCSC
H3K9ac Trynka
**H3K4me3 Trynka
H3K4me1 Trynka
H3K27ac PGC2
H3K27ac Hnisz
FetalDHS Trynka
Enhancer Hoffman
Enhancer Andersson
DHS Trynka
DGF ENCODE
**CTCF Hoffman
Conserved LindbladToh
Coding UCSC
−200
−100
0
100
Enrichment (prop h2 / prop snps)

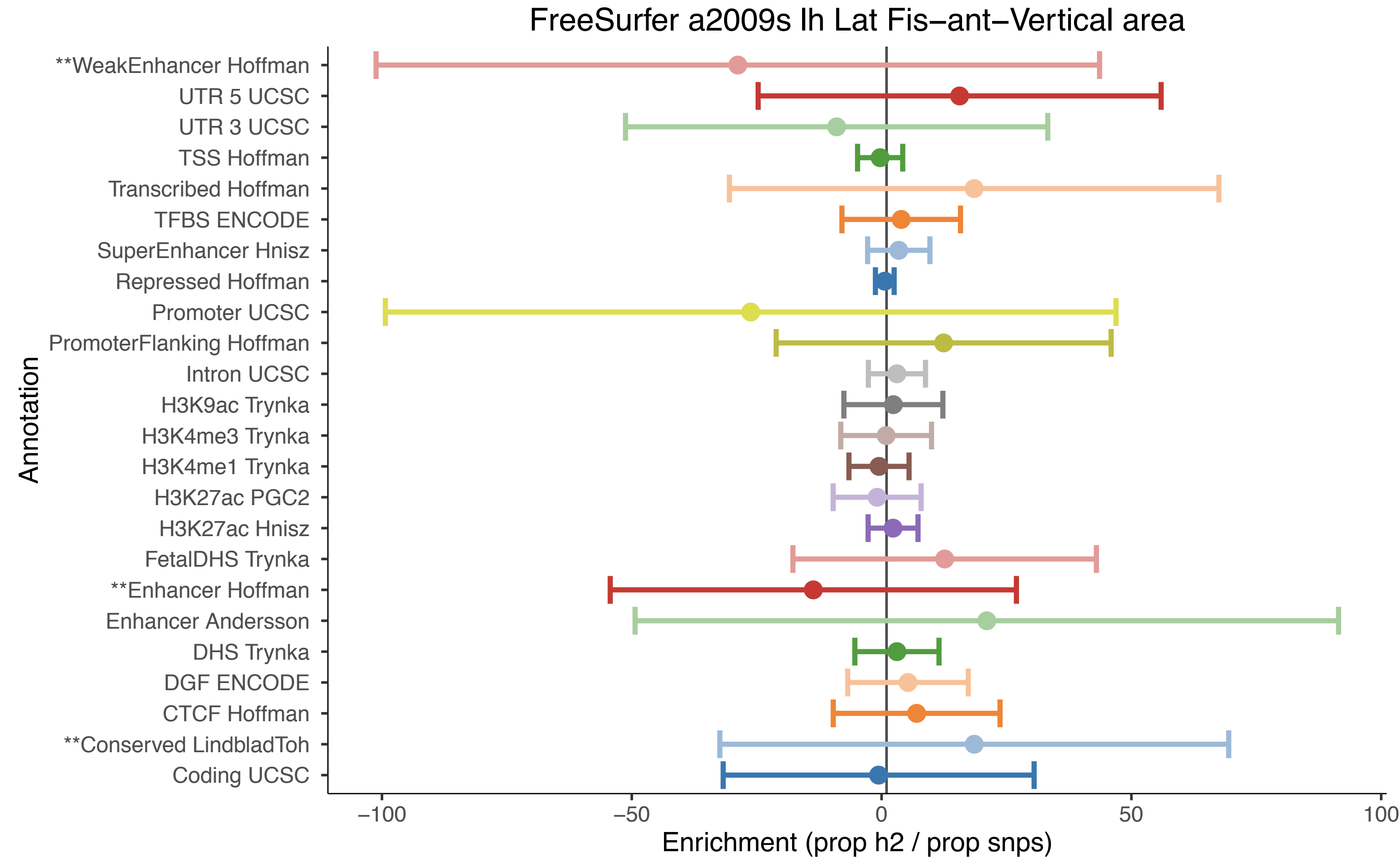

FreeSurfer a2009s lh Lat Fis−ant−Vertical area
Annotation
**WeakEnhancer Hoffman
UTR 5 UCSC
UTR 3 UCSC
TSS Hoffman
Transcribed Hoffman
TFBS ENCODE
SuperEnhancer Hnisz
Repressed Hoffman
Promoter UCSC
PromoterFlanking Hoffman
Intron UCSC
H3K9ac Trynka
H3K4me3 Trynka
H3K4me1 Trynka
H3K27ac PGC2
H3K27ac Hnisz
FetalDHS Trynka
**Enhancer Hoffman
Enhancer Andersson
DHS Trynka
DGF ENCODE
CTCF Hoffman
**Conserved LindbladToh
Coding UCSC
−100
−50
0
50
100
Enrichment (prop h2 / prop snps)

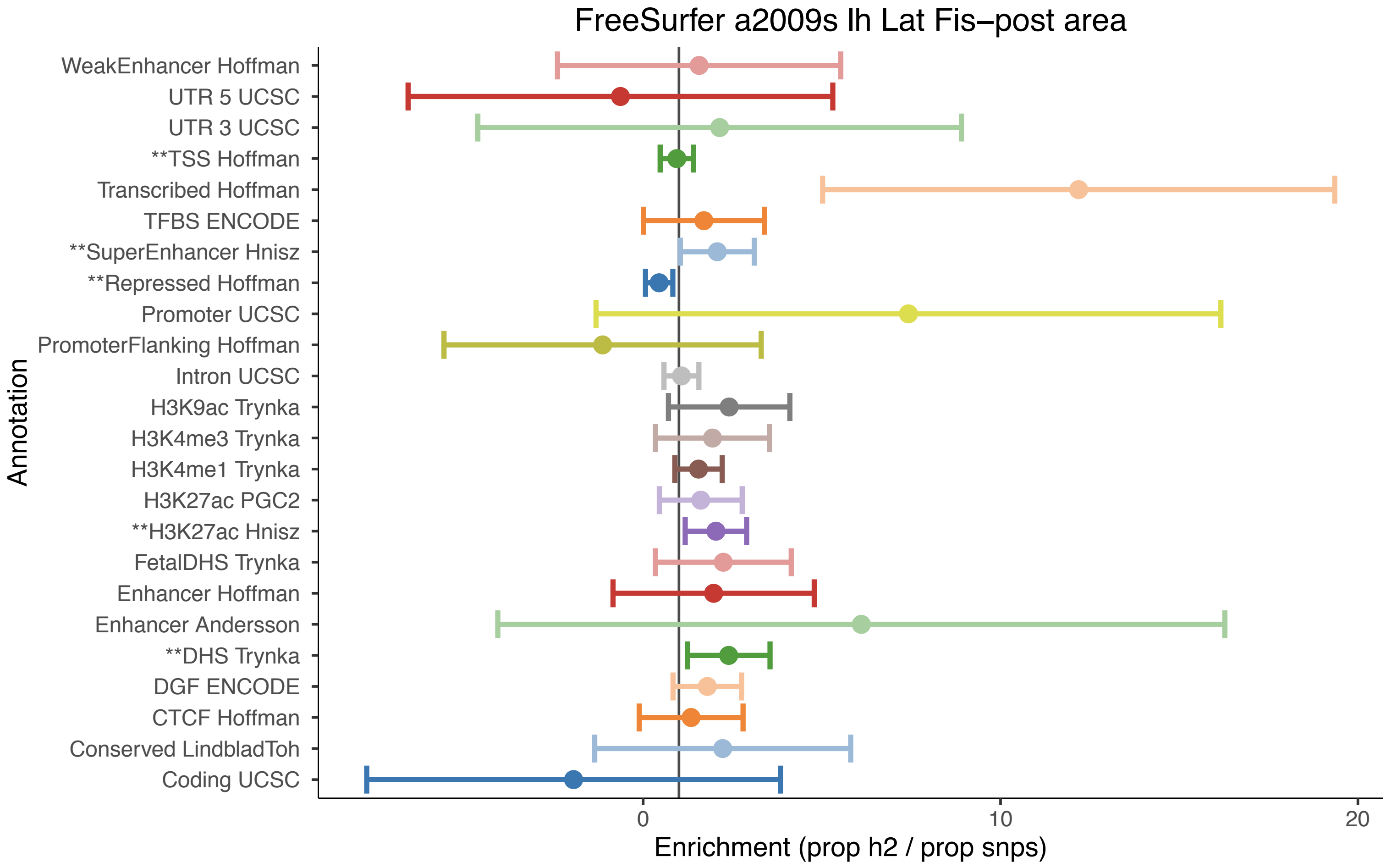

FreeSurfer a2009s lh Lat Fis−post area
WeakEnhancer Hoffman
UTR 5 UCSC
UTR 3 UCSC
**TSS Hoffman
Transcribed Hoffman
TFBS ENCODE
**SuperEnhancer Hnisz
**Repressed Hoffman
Promoter UCSC
PromoterFlanking Hoffman
Intron UCSC
H3K9ac Trynka
H3K4me3 Trynka
H3K4me1 Trynka
H3K27ac PGC2
**H3K27ac Hnisz
FetalDHS Trynka
Enhancer Hoffman
Enhancer Andersson
**DHS Trynka
DGF ENCODE
CTCF Hoffman
Conserved LindbladToh
Coding UCSC
Annotation
0
10
20
Enrichment (prop h2 / prop snps)

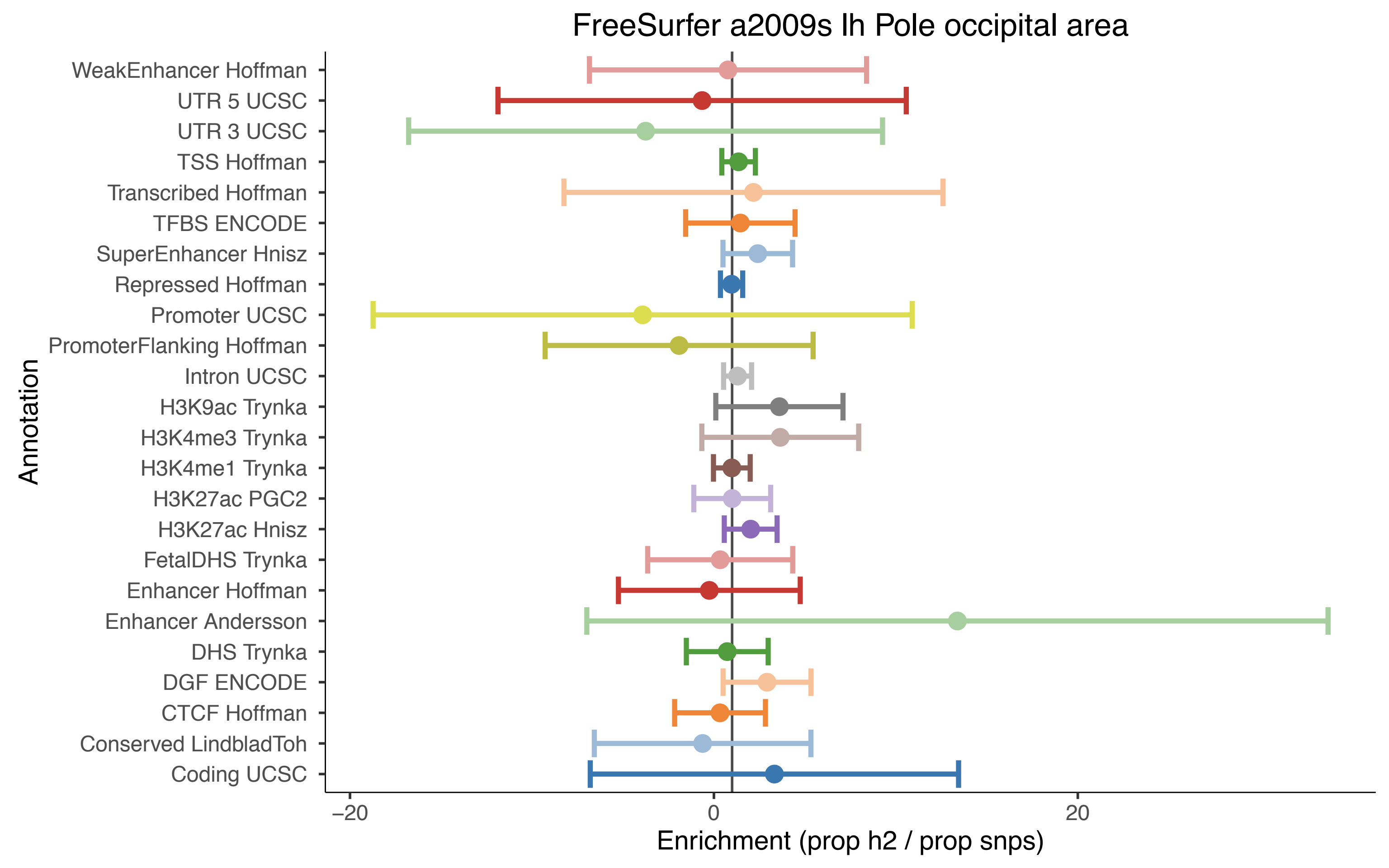

FreeSurfer a2009s lh Pole occipital area
WeakEnhancer Hoffman
UTR 5 UCSC
UTR 3 UCSC
TSS Hoffman
Transcribed Hoffman
TFBS ENCODE
SuperEnhancer Hnisz
Repressed Hoffman
Promoter UCSC
PromoterFlanking Hoffman
Intron UCSC
H3K9ac Trynka
H3K4me3 Trynka
H3K4me1 Trynka
H3K27ac PGC2
H3K27ac Hnisz
FetalDHS Trynka
Enhancer Hoffman
Enhancer Andersson
DHS Trynka
DGF ENCODE
CTCF Hoffman
Conserved LindbladToh
Coding UCSC
Annotation
−20
0
20
Enrichment (prop h2 / prop snps)

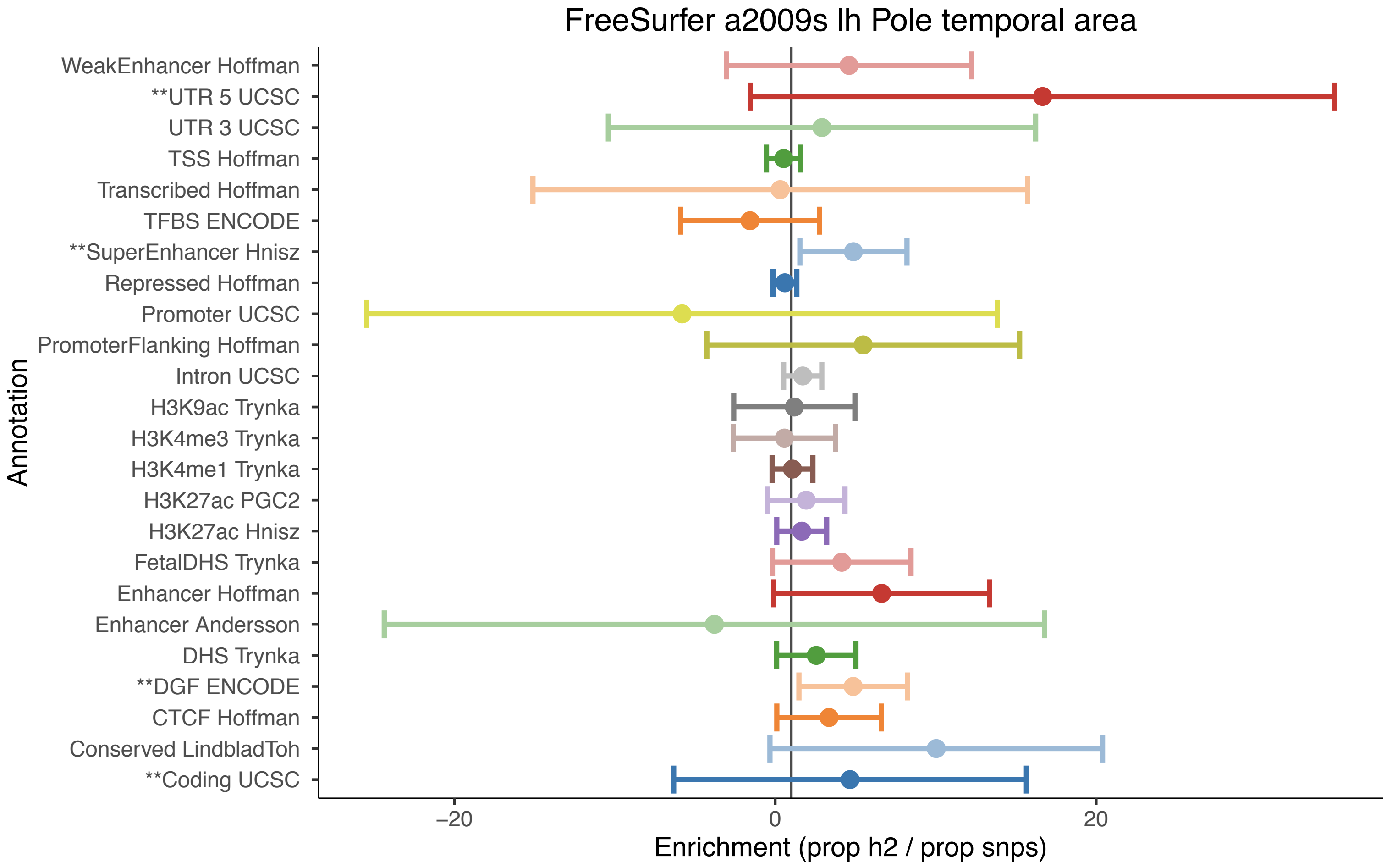

FreeSurfer a2009s lh Pole temporal area
WeakEnhancer Hoffman
**UTR 5 UCSC
UTR 3 UCSC
TSS Hoffman
Transcribed Hoffman
TFBS ENCODE
**SuperEnhancer Hnisz
Repressed Hoffman
Promoter UCSC
PromoterFlanking Hoffman
Intron UCSC
H3K9ac Trynka
H3K4me3 Trynka
H3K4me1 Trynka
H3K27ac PGC2
H3K27ac Hnisz
FetalDHS Trynka
Enhancer Hoffman
Enhancer Andersson
DHS Trynka
**DGF ENCODE
CTCF Hoffman
Conserved LindbladToh
**Coding UCSC
Annotation
−20
0
20
Enrichment (prop h2 / prop snps)

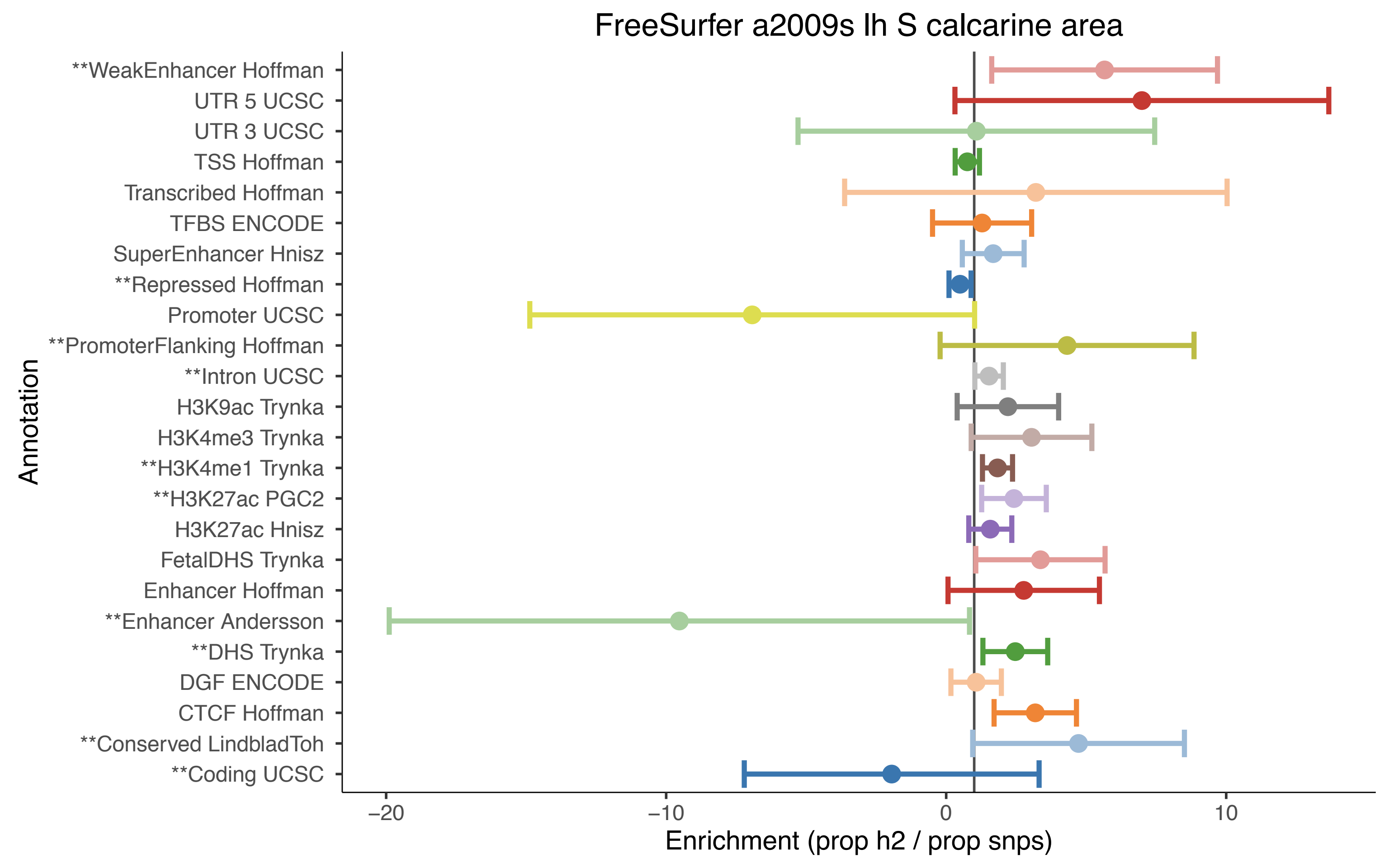
FreeSurfer a2009s lh S calcarine area
Annotation
**WeakEnhancer Hoffman
UTR 5 UCSC
UTR 3 UCSC
TSS Hoffman
Transcribed Hoffman
TFBS ENCODE
SuperEnhancer Hnisz
**Repressed Hoffman
Promoter UCSC
**PromoterFlanking Hoffman
**Intron UCSC
H3K9ac Trynka
H3K4me3 Trynka
**H3K4me1 Trynka
**H3K27ac PGC2
H3K27ac Hnisz
FetalDHS Trynka
Enhancer Hoffman
**Enhancer Andersson
**DHS Trynka
DGF ENCODE
CTCF Hoffman
**Conserved LindbladToh
**Coding UCSC
−20
−10
0
10
Enrichment (prop h2 / prop snps)

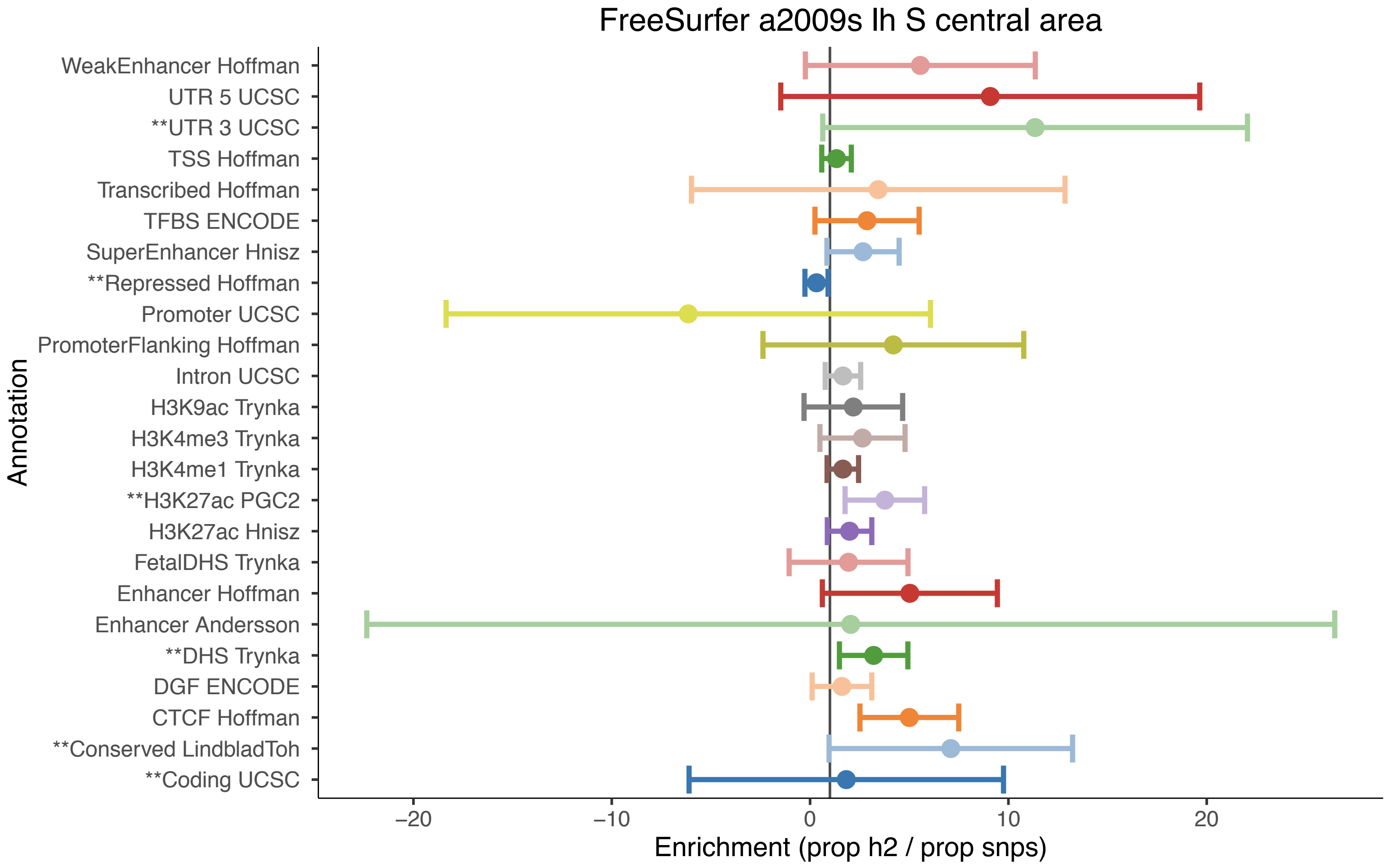

FreeSurfer a2009s lh S central area
WeakEnhancer Hoffman
UTR 5 UCSC
**UTR 3 UCSC
TSS Hoffman
Transcribed Hoffman
TFBS ENCODE
SuperEnhancer Hnisz
**Repressed Hoffman
Promoter UCSC
PromoterFlanking Hoffman
Intron UCSC
H3K9ac Trynka
H3K4me3 Trynka
H3K4me1 Trynka
**H3K27ac PGC2
H3K27ac Hnisz
FetalDHS Trynka
Enhancer Hoffman
Enhancer Andersson
**DHS Trynka
DGF ENCODE
CTCF Hoffman
**Conserved LindbladToh
**Coding UCSC
Annotation
−20
−10
0
10
20
Enrichment (prop h2 / prop snps)

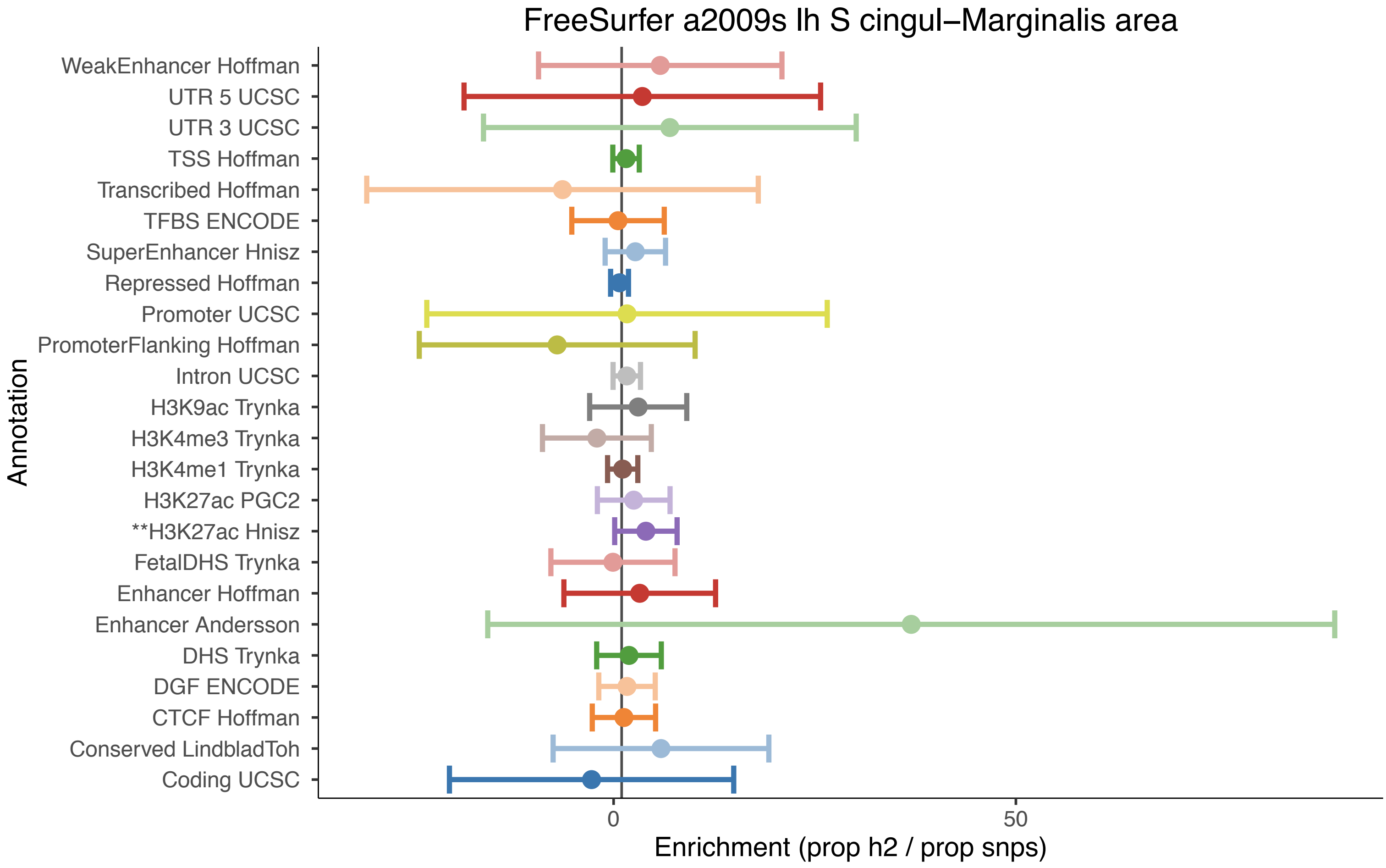

FreeSurfer a2009s lh S cingul−Marginalis area
WeakEnhancer Hoffman
UTR 5 UCSC
UTR 3 UCSC
TSS Hoffman
Transcribed Hoffman
TFBS ENCODE
SuperEnhancer Hnisz
Repressed Hoffman
Promoter UCSC
PromoterFlanking Hoffman
Intron UCSC
H3K9ac Trynka
H3K4me3 Trynka
H3K4me1 Trynka
H3K27ac PGC2
**H3K27ac Hnisz
FetalDHS Trynka
Enhancer Hoffman
Enhancer Andersson
DHS Trynka
DGF ENCODE
CTCF Hoffman
Conserved LindbladToh
Coding UCSC
Annotation
0
50
Enrichment (prop h2 / prop snps)

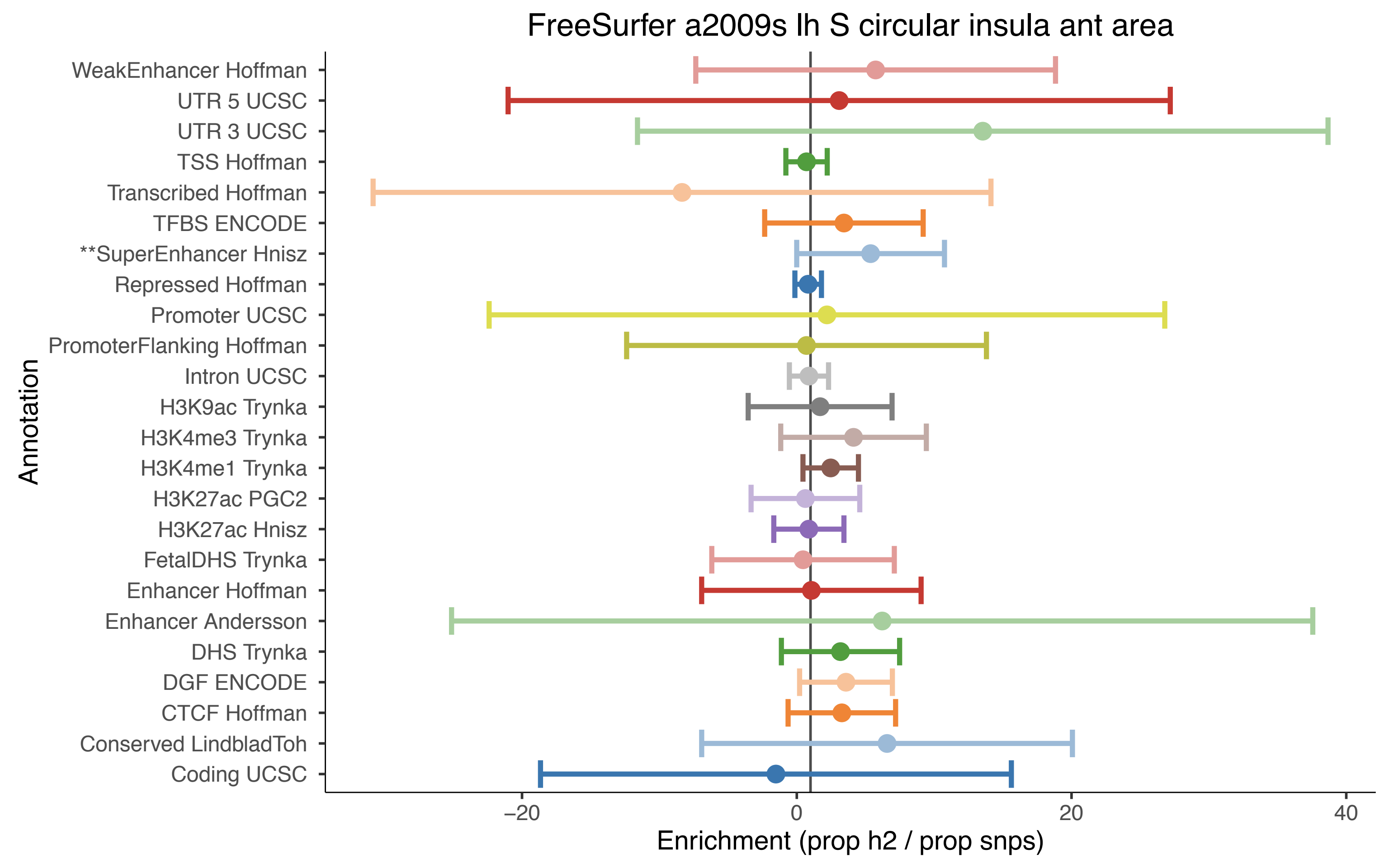
FreeSurfer a2009s lh S circular insula ant area
WeakEnhancer Hoffman
UTR 5 UCSC
UTR 3 UCSC
TSS Hoffman
Transcribed Hoffman
TFBS ENCODE
**SuperEnhancer Hnisz
Repressed Hoffman
Promoter UCSC
PromoterFlanking Hoffman
Intron UCSC
H3K9ac Trynka
H3K4me3 Trynka
H3K4me1 Trynka
H3K27ac PGC2
H3K27ac Hnisz
FetalDHS Trynka
Enhancer Hoffman
Enhancer Andersson
DHS Trynka
DGF ENCODE
CTCF Hoffman
Conserved LindbladToh
Coding UCSC
Annotation
−20
0
20
40
Enrichment (prop h2 / prop snps)

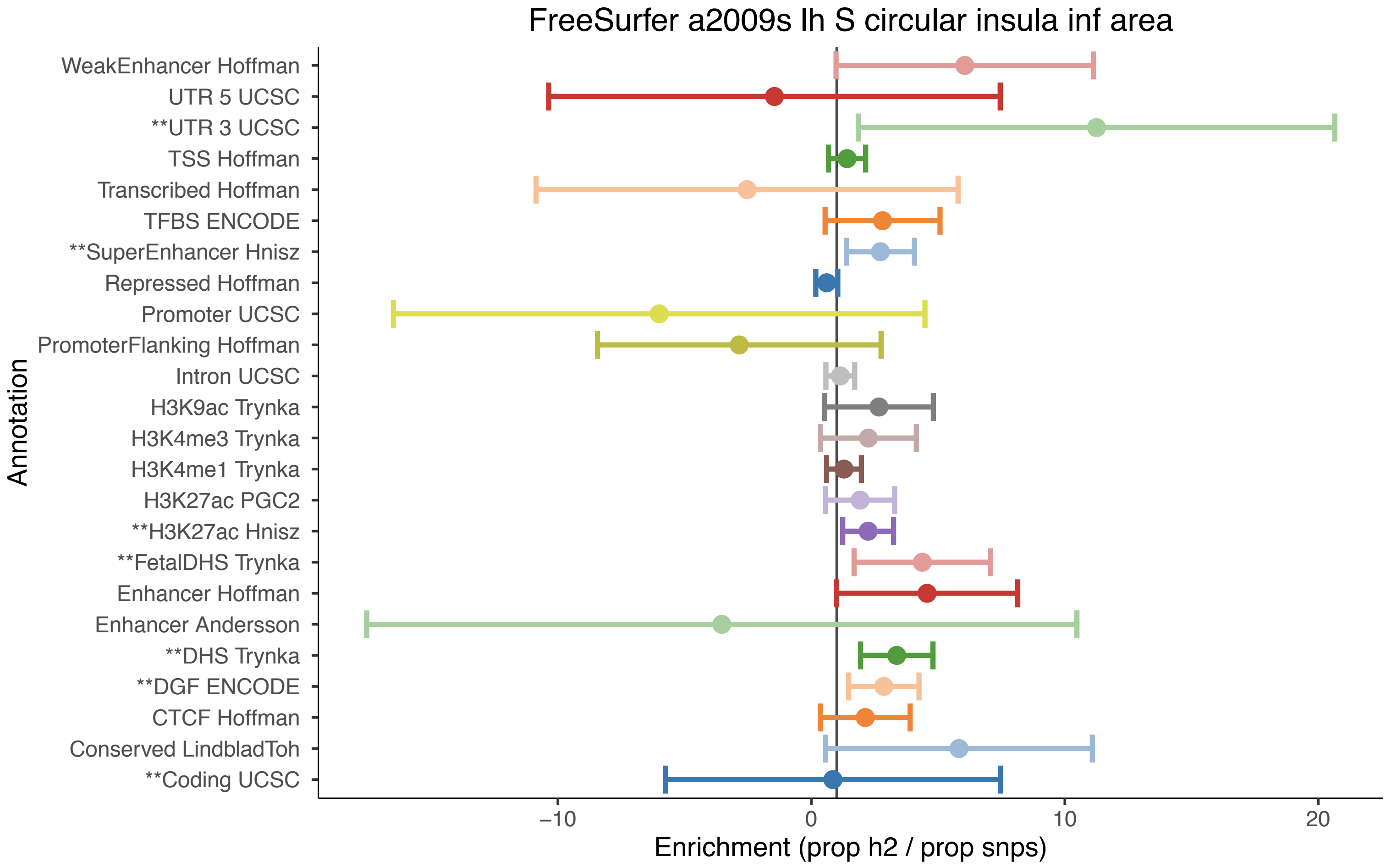
FreeSurfer a2009s lh S circular insula inf area
WeakEnhancer Hoffman
UTR 5 UCSC
**UTR 3 UCSC
TSS Hoffman
Transcribed Hoffman
TFBS ENCODE
**SuperEnhancer Hnisz
Repressed Hoffman
Promoter UCSC
PromoterFlanking Hoffman
Intron UCSC
H3K9ac Trynka
H3K4me3 Trynka
H3K4me1 Trynka
H3K27ac PGC2
**H3K27ac Hnisz
**FetalDHS Trynka
Enhancer Hoffman
Enhancer Andersson
**DHS Trynka
**DGF ENCODE
CTCF Hoffman
Conserved LindbladToh
**Coding UCSC
Annotation
−10
0
10
20
Enrichment (prop h2 / prop snps)

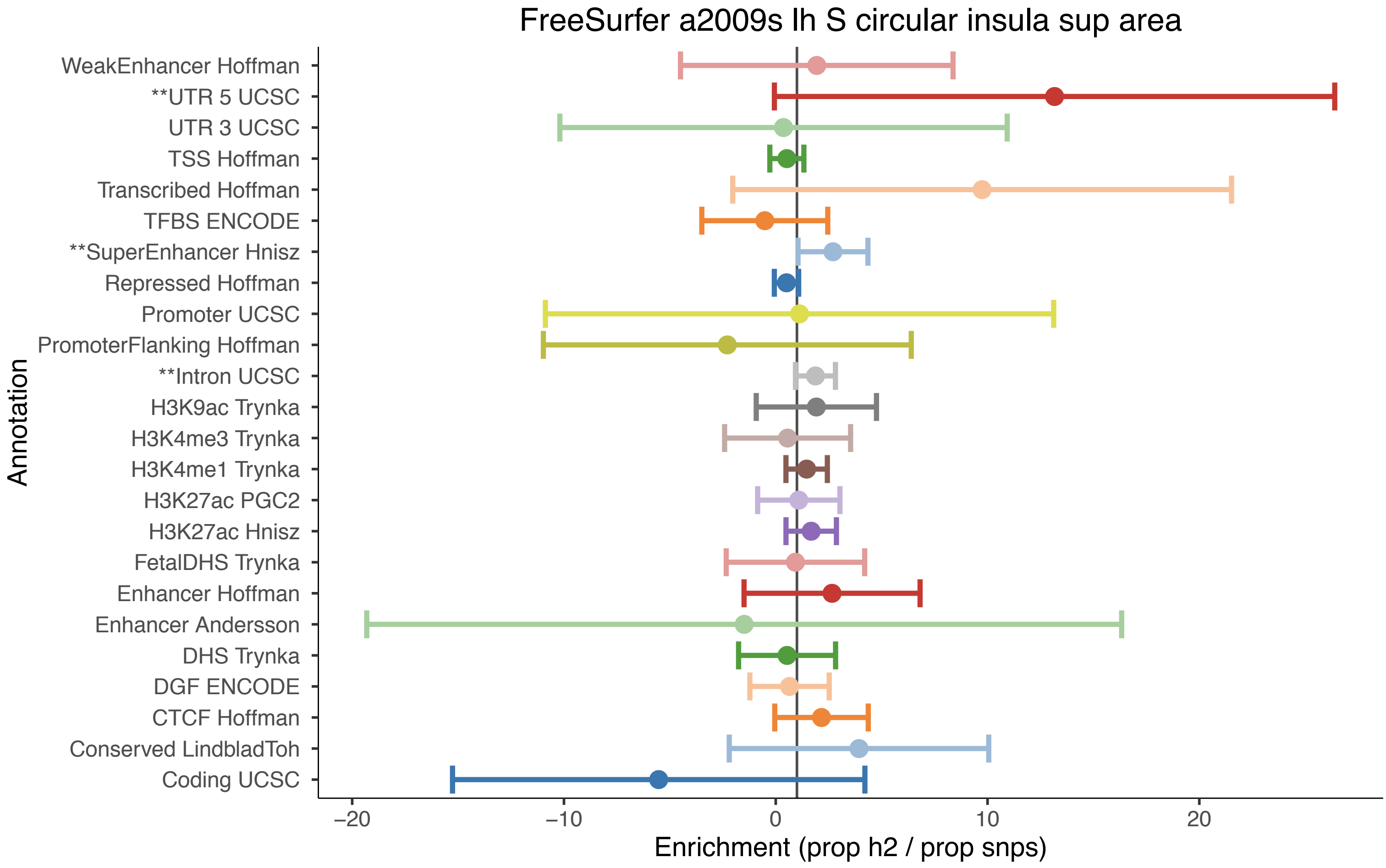
FreeSurfer a2009s lh S circular insula sup area
WeakEnhancer Hoffman
**UTR 5 UCSC
UTR 3 UCSC
TSS Hoffman
Transcribed Hoffman
TFBS ENCODE
**SuperEnhancer Hnisz
Repressed Hoffman
Promoter UCSC
PromoterFlanking Hoffman
**Intron UCSC
H3K9ac Trynka
H3K4me3 Trynka
H3K4me1 Trynka
H3K27ac PGC2
H3K27ac Hnisz
FetalDHS Trynka
Enhancer Hoffman
Enhancer Andersson
DHS Trynka
DGF ENCODE
CTCF Hoffman
Conserved LindbladToh
Coding UCSC
Annotation
−20
−10
0
10
20
Enrichment (prop h2 / prop snps)

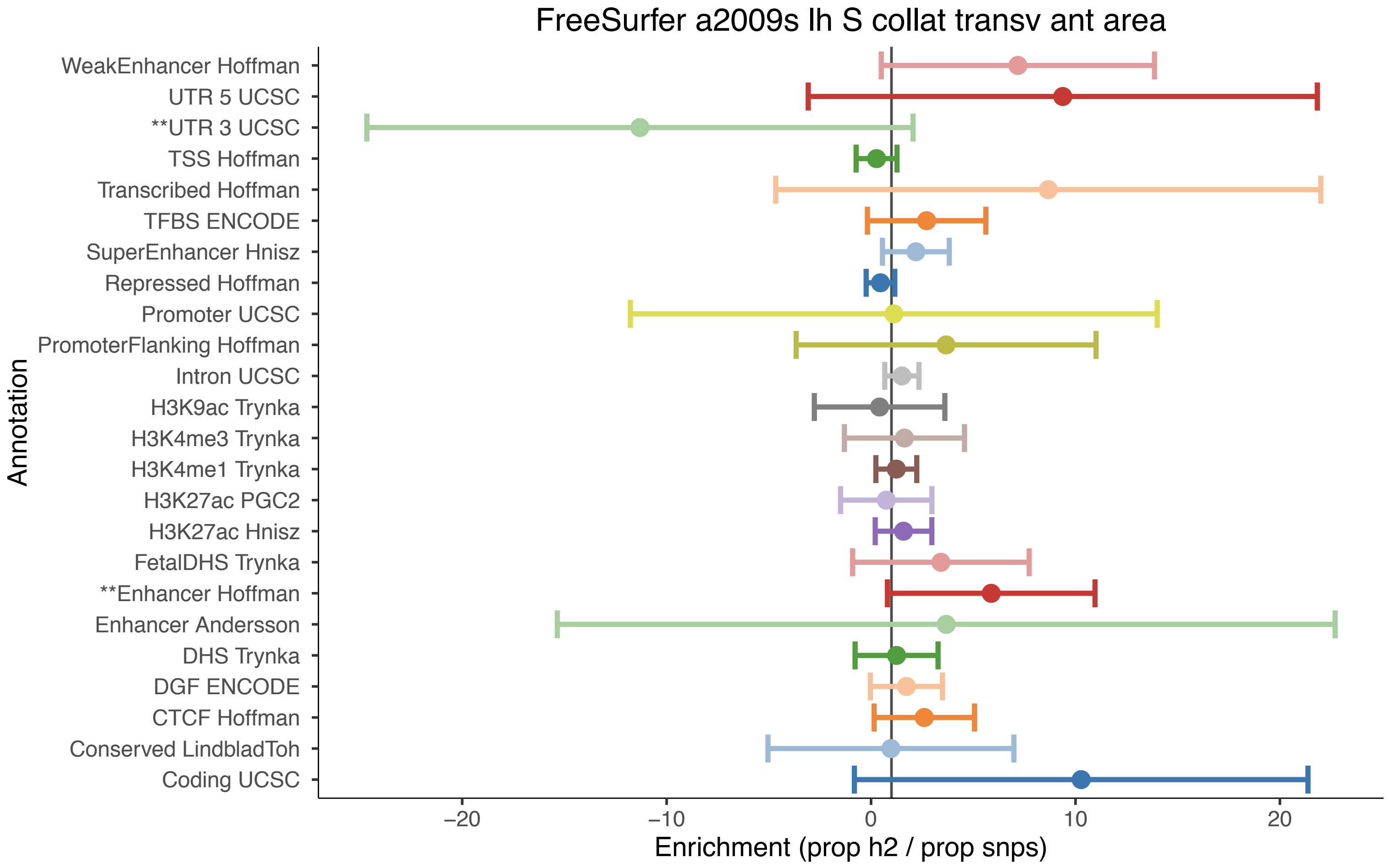
FreeSurfer a2009s lh S collat transv ant area
WeakEnhancer Hoffman
UTR 5 UCSC
**UTR 3 UCSC
TSS Hoffman
Transcribed Hoffman
TFBS ENCODE
SuperEnhancer Hnisz
Repressed Hoffman
Promoter UCSC
PromoterFlanking Hoffman
Intron UCSC
H3K9ac Trynka
H3K4me3 Trynka
H3K4me1 Trynka
H3K27ac PGC2
H3K27ac Hnisz
FetalDHS Trynka
**Enhancer Hoffman
Enhancer Andersson
DHS Trynka
DGF ENCODE
CTCF Hoffman
Conserved LindbladToh
Coding UCSC
Annotation
−20
−10
0
10
20
Enrichment (prop h2 / prop snps)

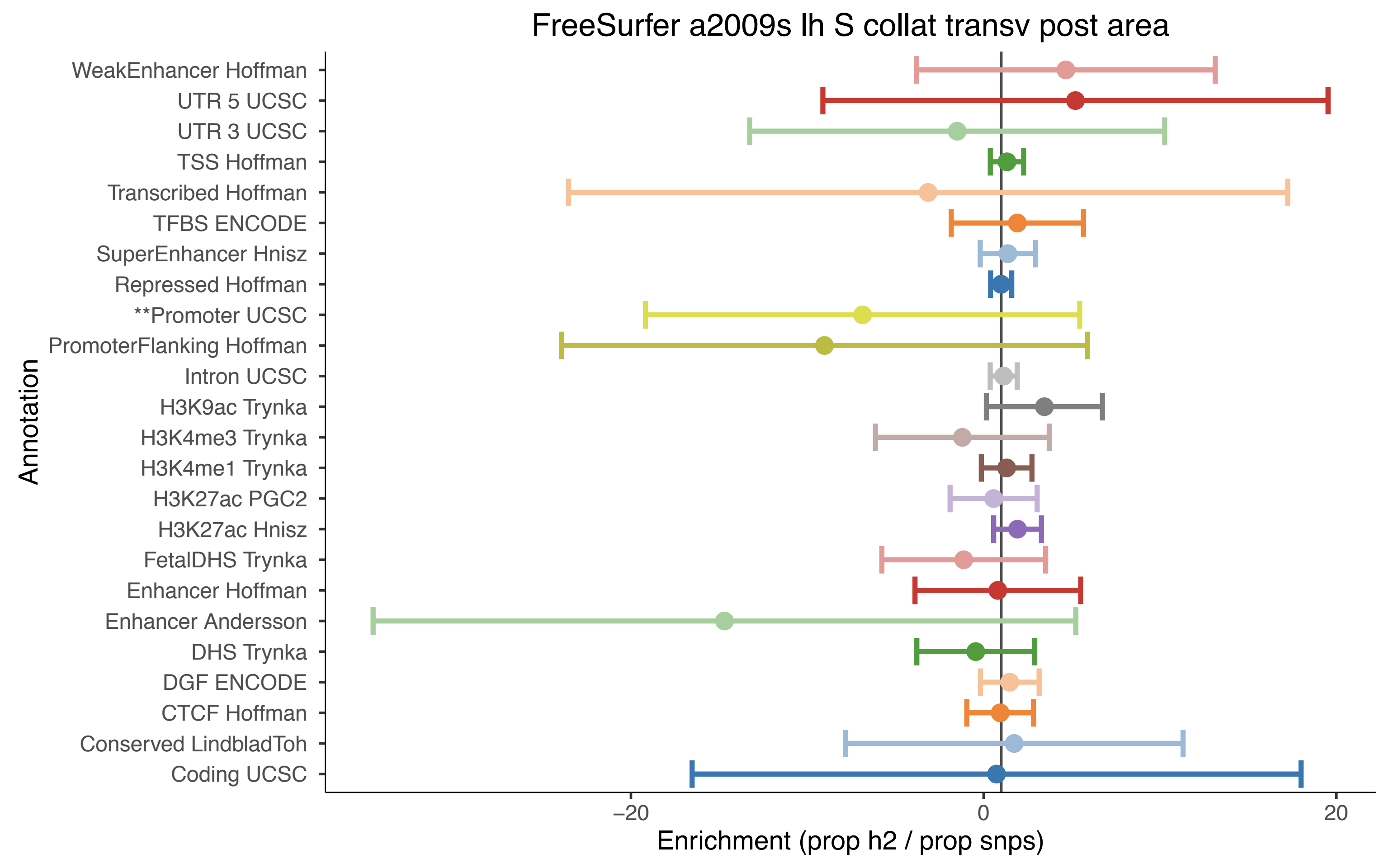
FreeSurfer a2009s lh S collat transv post area
WeakEnhancer Hoffman
UTR 5 UCSC
UTR 3 UCSC
TSS Hoffman
Transcribed Hoffman
TFBS ENCODE
SuperEnhancer Hnisz
Repressed Hoffman
**Promoter UCSC
PromoterFlanking Hoffman
Intron UCSC
H3K9ac Trynka
H3K4me3 Trynka
H3K4me1 Trynka
H3K27ac PGC2
H3K27ac Hnisz
FetalDHS Trynka
Enhancer Hoffman
Enhancer Andersson
DHS Trynka
DGF ENCODE
CTCF Hoffman
Conserved LindbladToh
Coding UCSC
Annotation
−20
0
20
Enrichment (prop h2 / prop snps)

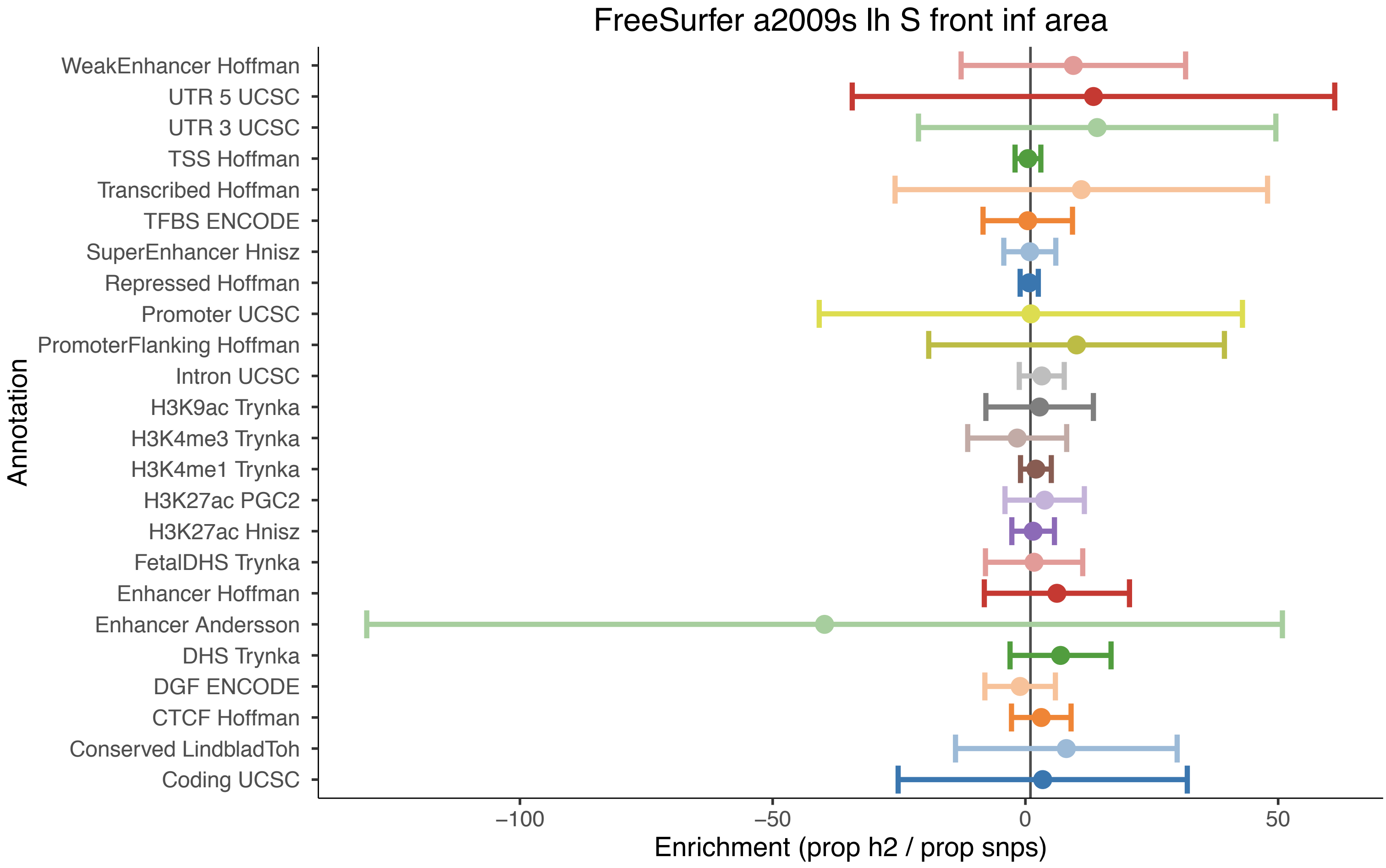
FreeSurfer a2009s lh S front inf area
WeakEnhancer Hoffman
UTR 5 UCSC
UTR 3 UCSC
TSS Hoffman
Transcribed Hoffman
TFBS ENCODE
SuperEnhancer Hnisz
Repressed Hoffman
Promoter UCSC
PromoterFlanking Hoffman
Intron UCSC
H3K9ac Trynka
H3K4me3 Trynka
H3K4me1 Trynka
H3K27ac PGC2
H3K27ac Hnisz
FetalDHS Trynka
Enhancer Hoffman
Enhancer Andersson
DHS Trynka
DGF ENCODE
CTCF Hoffman
Conserved LindbladToh
Coding UCSC
Annotation
−100
−50
0
50
Enrichment (prop h2 / prop snps)

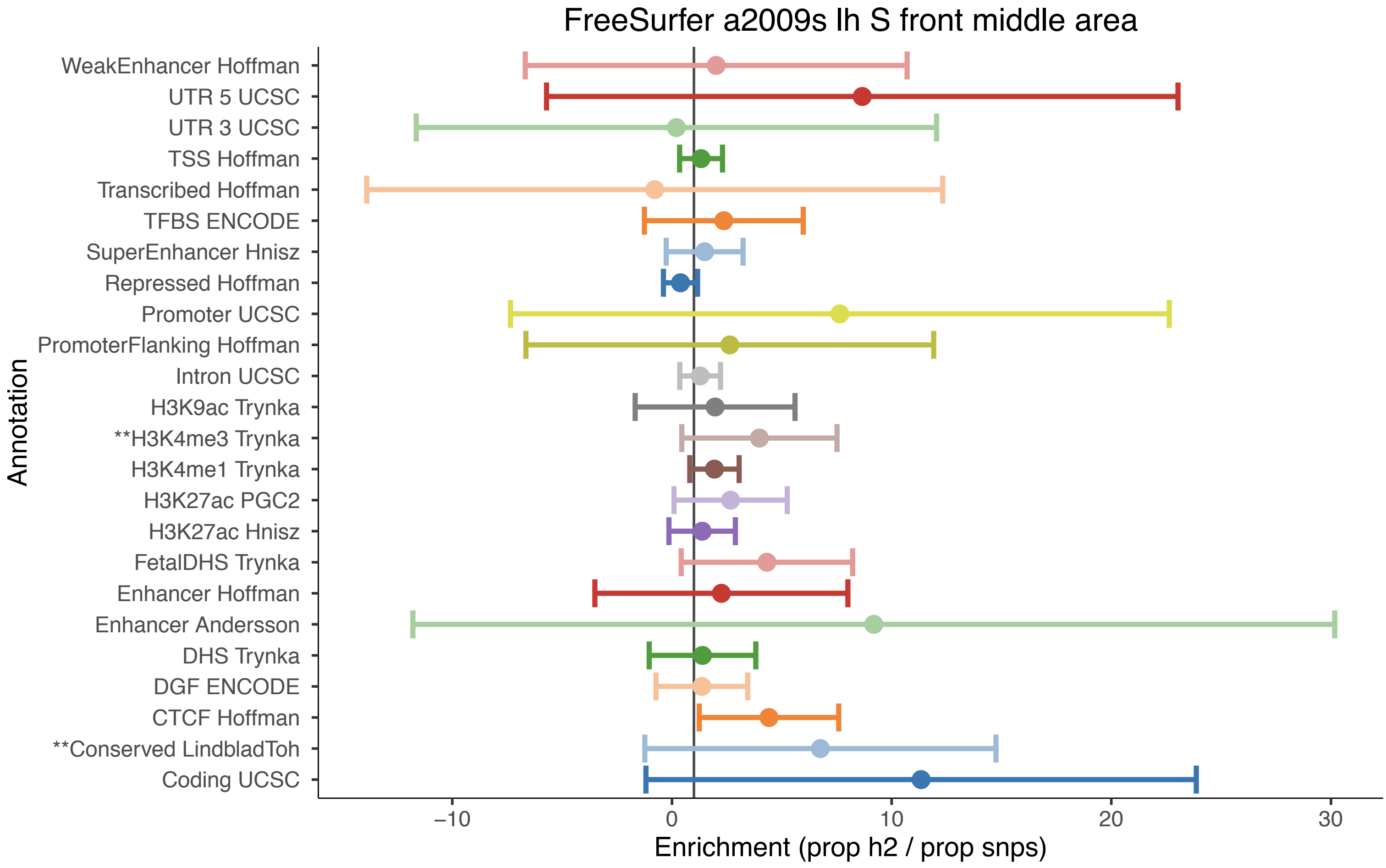

FreeSurfer a2009s lh S front middle area
WeakEnhancer Hoffman
UTR 5 UCSC
UTR 3 UCSC
TSS Hoffman
Transcribed Hoffman
TFBS ENCODE
SuperEnhancer Hnisz
Repressed Hoffman
Promoter UCSC
PromoterFlanking Hoffman
Intron UCSC
H3K9ac Trynka
**H3K4me3 Trynka
H3K4me1 Trynka
H3K27ac PGC2
H3K27ac Hnisz
FetalDHS Trynka
Enhancer Hoffman
Enhancer Andersson
DHS Trynka
DGF ENCODE
CTCF Hoffman
**Conserved LindbladToh
Coding UCSC
Annotation
−10
0
10
20
30
Enrichment (prop h2 / prop snps)

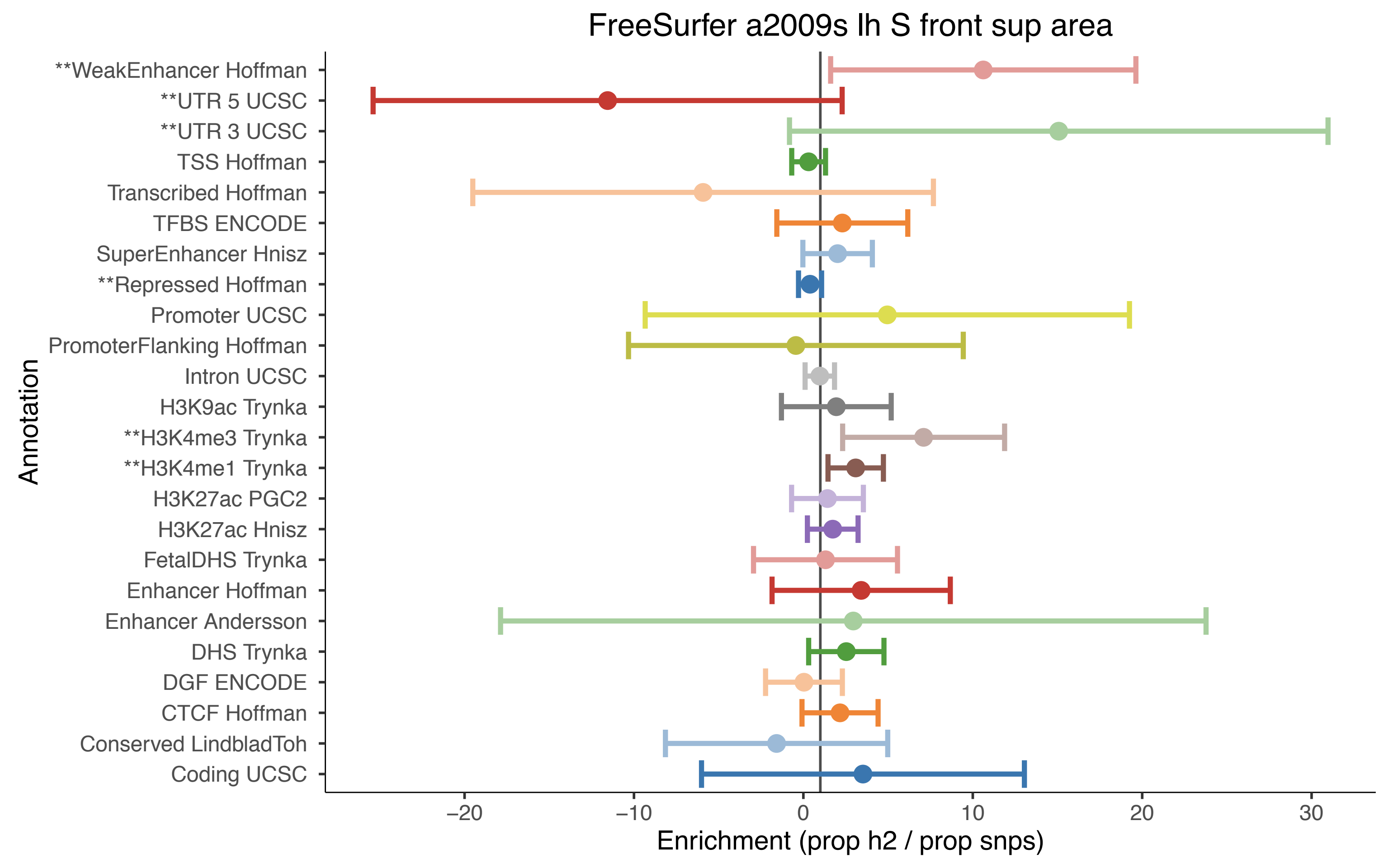

FreeSurfer a2009s lh S front sup area
**WeakEnhancer Hoffman
**UTR 5 UCSC
**UTR 3 UCSC
TSS Hoffman
Transcribed Hoffman
TFBS ENCODE
SuperEnhancer Hnisz
**Repressed Hoffman
Promoter UCSC
PromoterFlanking Hoffman
Intron UCSC
H3K9ac Trynka
**H3K4me3 Trynka
**H3K4me1 Trynka
H3K27ac PGC2
H3K27ac Hnisz
FetalDHS Trynka
Enhancer Hoffman
Enhancer Andersson
DHS Trynka
DGF ENCODE
CTCF Hoffman
Conserved LindbladToh
Coding UCSC
Annotation
−20
−10
0
10
20
30
Enrichment (prop h2 / prop snps)

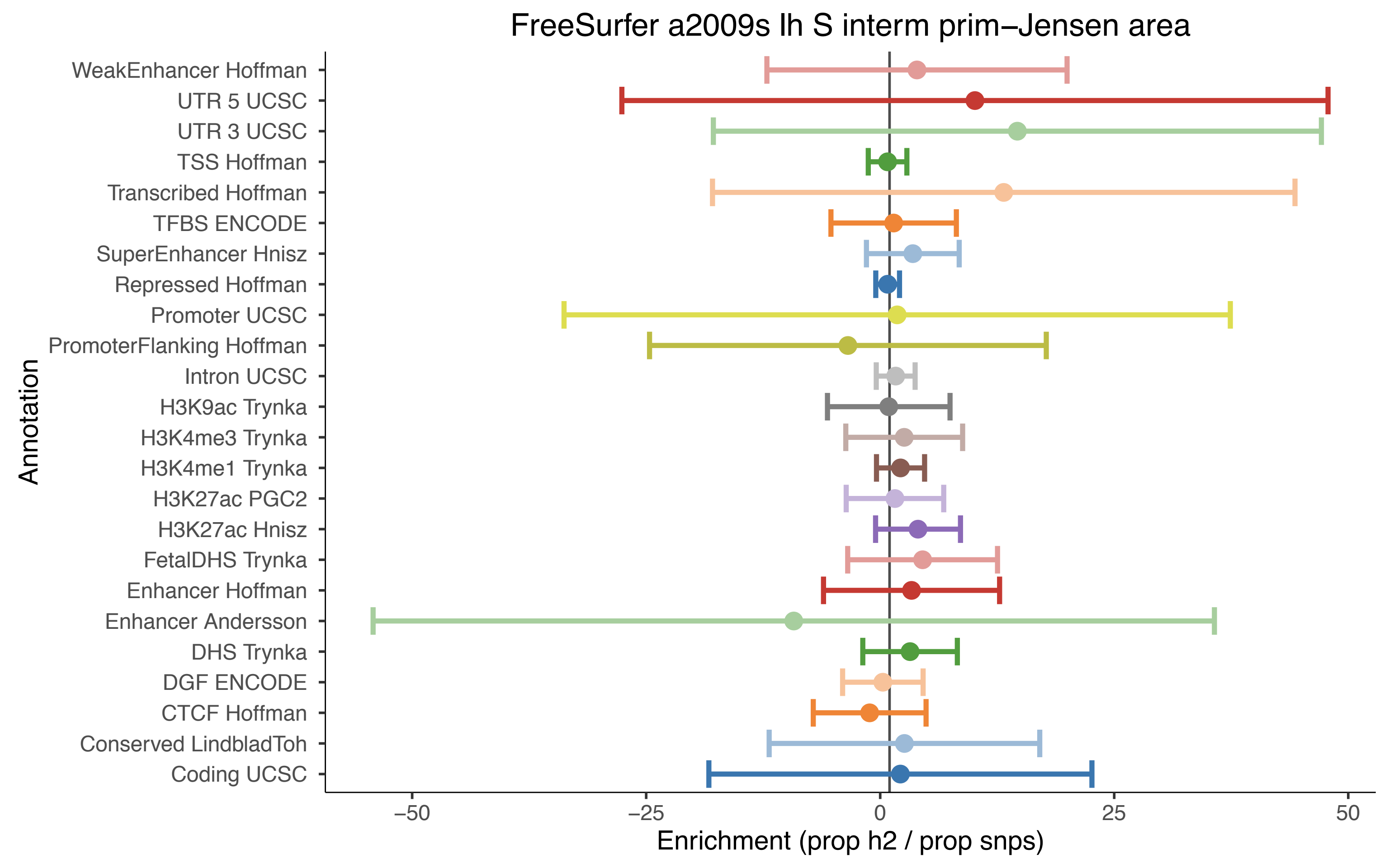

FreeSurfer a2009s lh S interm prim−Jensen area
WeakEnhancer Hoffman
UTR 5 UCSC
UTR 3 UCSC
TSS Hoffman
Transcribed Hoffman
TFBS ENCODE
SuperEnhancer Hnisz
Repressed Hoffman
Promoter UCSC
PromoterFlanking Hoffman
Intron UCSC
H3K9ac Trynka
H3K4me3 Trynka
H3K4me1 Trynka
H3K27ac PGC2
H3K27ac Hnisz
FetalDHS Trynka
Enhancer Hoffman
Enhancer Andersson
DHS Trynka
DGF ENCODE
CTCF Hoffman
Conserved LindbladToh
Coding UCSC
Annotation
−50
−25
0
25
50
Enrichment (prop h2 / prop snps)

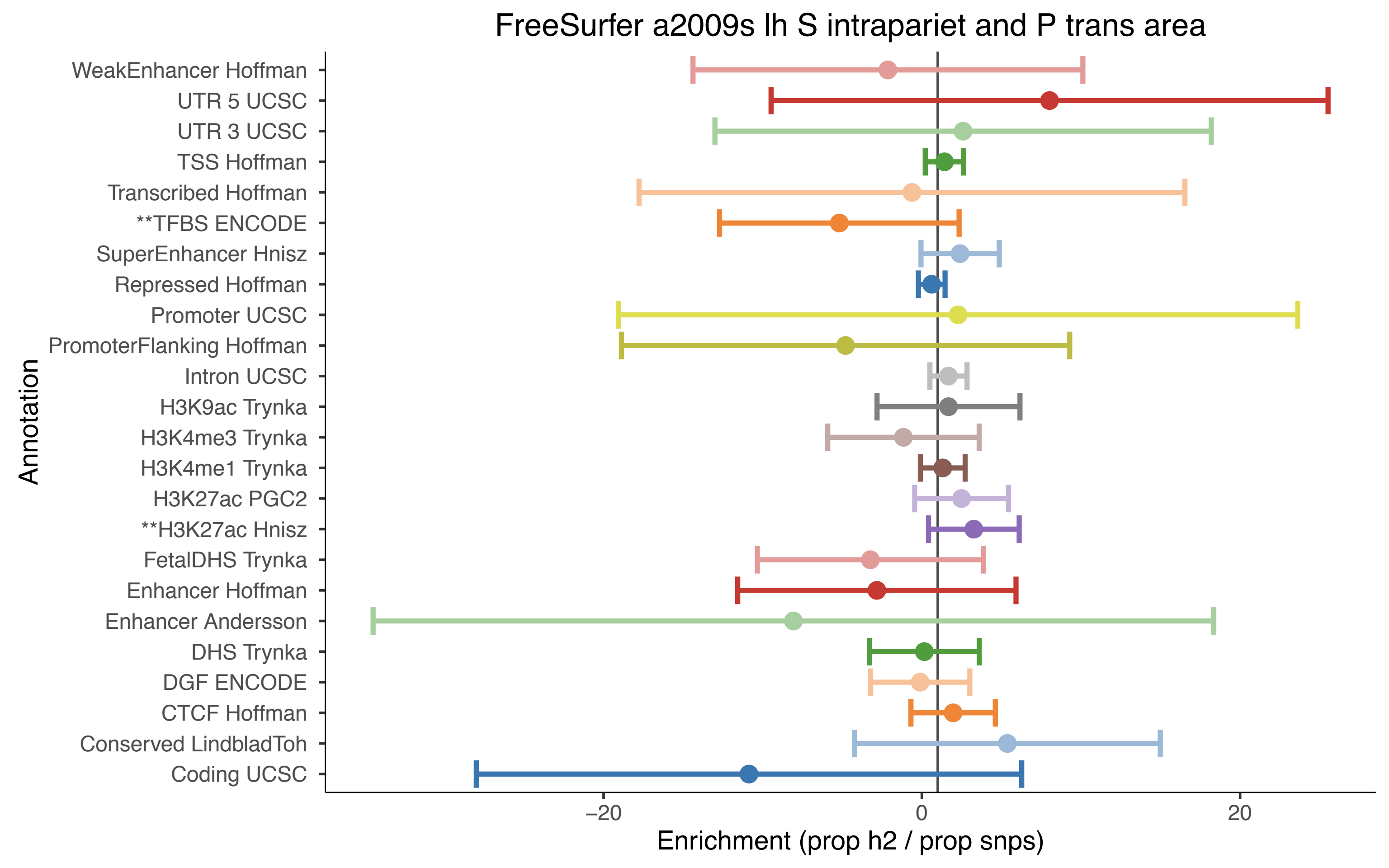

FreeSurfer a2009s lh S intrapariet and P trans area
WeakEnhancer Hoffman
UTR 5 UCSC
UTR 3 UCSC
TSS Hoffman
Transcribed Hoffman
**TFBS ENCODE
SuperEnhancer Hnisz
Repressed Hoffman
Promoter UCSC
PromoterFlanking Hoffman
Intron UCSC
H3K9ac Trynka
H3K4me3 Trynka
H3K4me1 Trynka
H3K27ac PGC2
**H3K27ac Hnisz
FetalDHS Trynka
Enhancer Hoffman
Enhancer Andersson
DHS Trynka
DGF ENCODE
CTCF Hoffman
Conserved LindbladToh
Coding UCSC
Annotation
−20
0
20
Enrichment (prop h2 / prop snps)

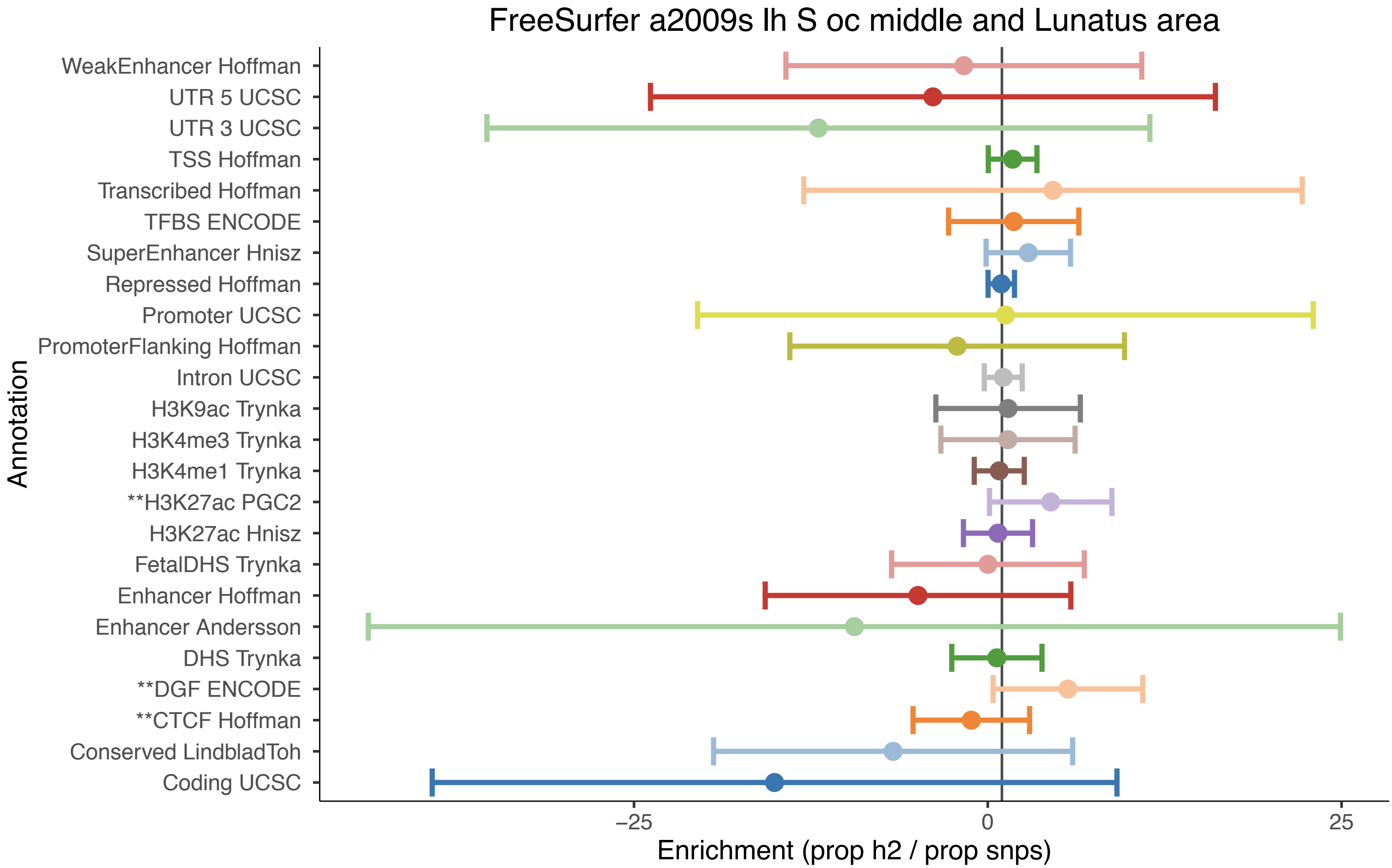

FreeSurfer a2009s lh S oc middle and Lunatus area
WeakEnhancer Hoffman
UTR 5 UCSC
UTR 3 UCSC
TSS Hoffman
Transcribed Hoffman
TFBS ENCODE
SuperEnhancer Hnisz
Repressed Hoffman
Promoter UCSC
PromoterFlanking Hoffman
Intron UCSC
H3K9ac Trynka
H3K4me3 Trynka
H3K4me1 Trynka
**H3K27ac PGC2
H3K27ac Hnisz
FetalDHS Trynka
Enhancer Hoffman
Enhancer Andersson
DHS Trynka
**DGF ENCODE
**CTCF Hoffman
Conserved LindbladToh
Coding UCSC
Annotation
−25
0
25
Enrichment (prop h2 / prop snps)

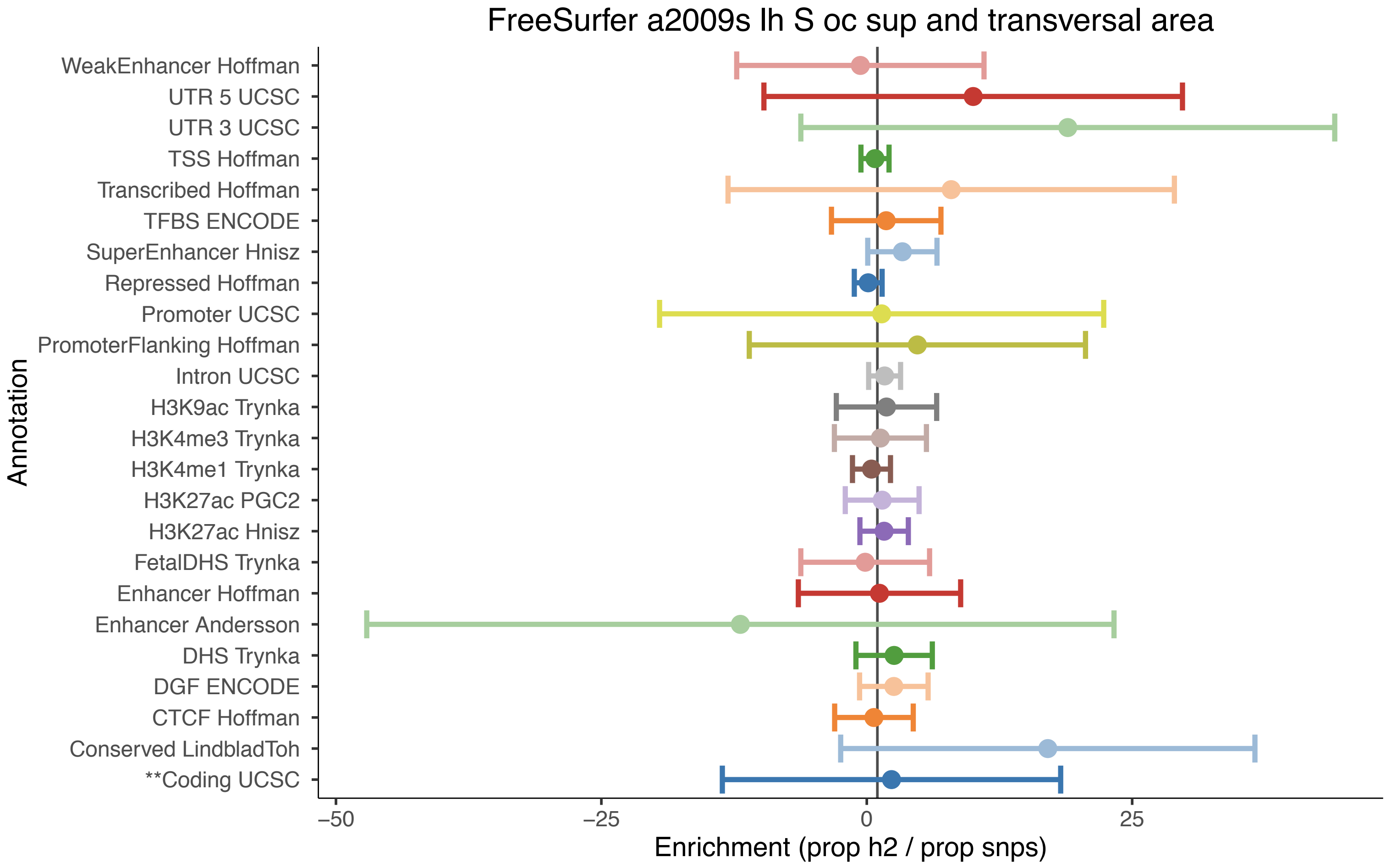

FreeSurfer a2009s lh S oc sup and transversal area
WeakEnhancer Hoffman
UTR 5 UCSC
UTR 3 UCSC
TSS Hoffman
Transcribed Hoffman
TFBS ENCODE
SuperEnhancer Hnisz
Repressed Hoffman
Promoter UCSC
PromoterFlanking Hoffman
Intron UCSC
H3K9ac Trynka
H3K4me3 Trynka
H3K4me1 Trynka
H3K27ac PGC2
H3K27ac Hnisz
FetalDHS Trynka
Enhancer Hoffman
Enhancer Andersson
DHS Trynka
DGF ENCODE
CTCF Hoffman
Conserved LindbladToh
**Coding UCSC
Annotation
−50
−25
0
25
Enrichment (prop h2 / prop snps)

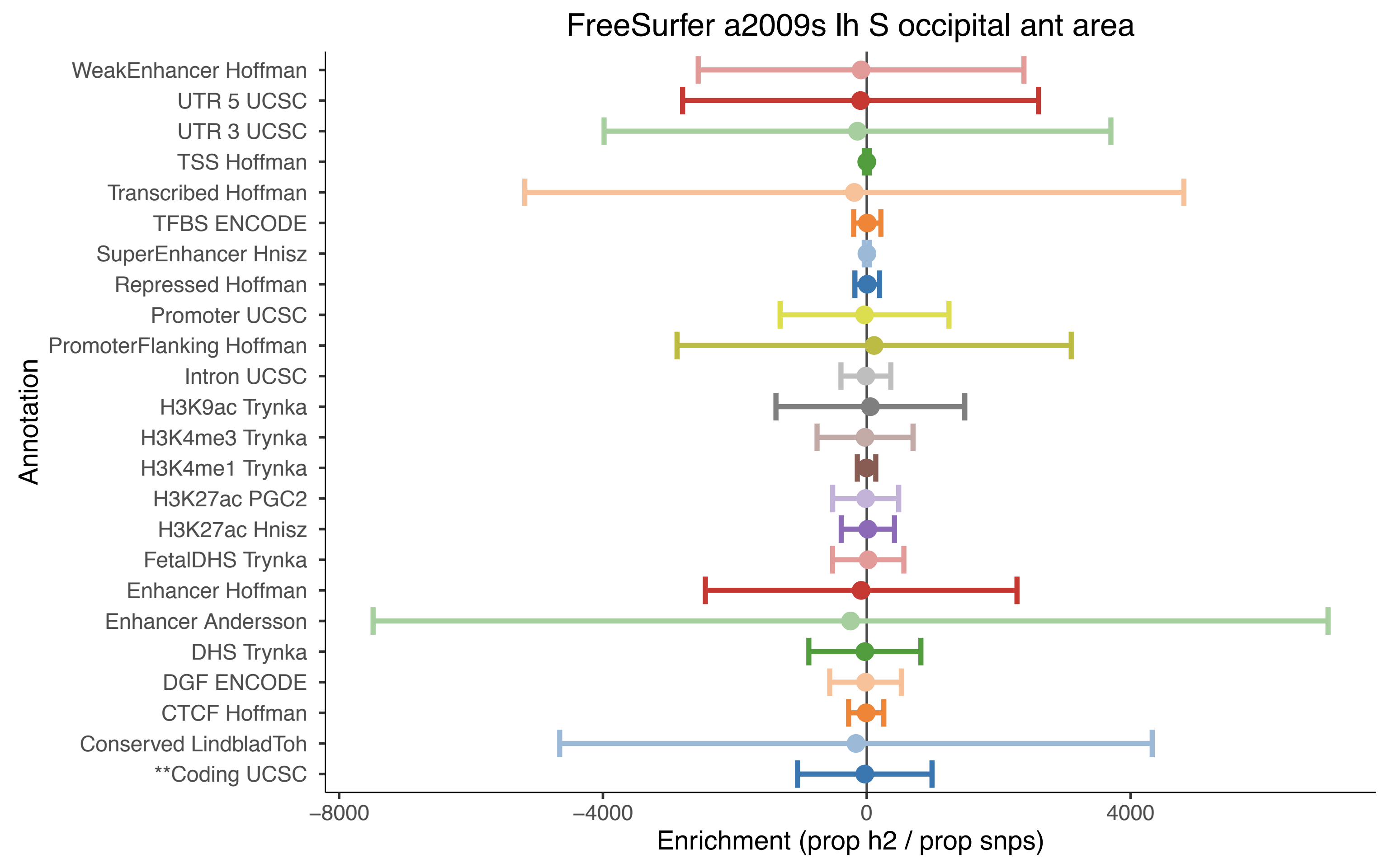
FreeSurfer a2009s lh S occipital ant area
WeakEnhancer Hoffman
UTR 5 UCSC
UTR 3 UCSC
TSS Hoffman
Transcribed Hoffman
TFBS ENCODE
SuperEnhancer Hnisz
Repressed Hoffman
Promoter UCSC
PromoterFlanking Hoffman
Intron UCSC
H3K9ac Trynka
H3K4me3 Trynka
H3K4me1 Trynka
H3K27ac PGC2
H3K27ac Hnisz
FetalDHS Trynka
Enhancer Hoffman
Enhancer Andersson
DHS Trynka
DGF ENCODE
CTCF Hoffman
Conserved LindbladToh
**Coding UCSC
Annotation
−8000
−4000
0
4000
Enrichment (prop h2 / prop snps)

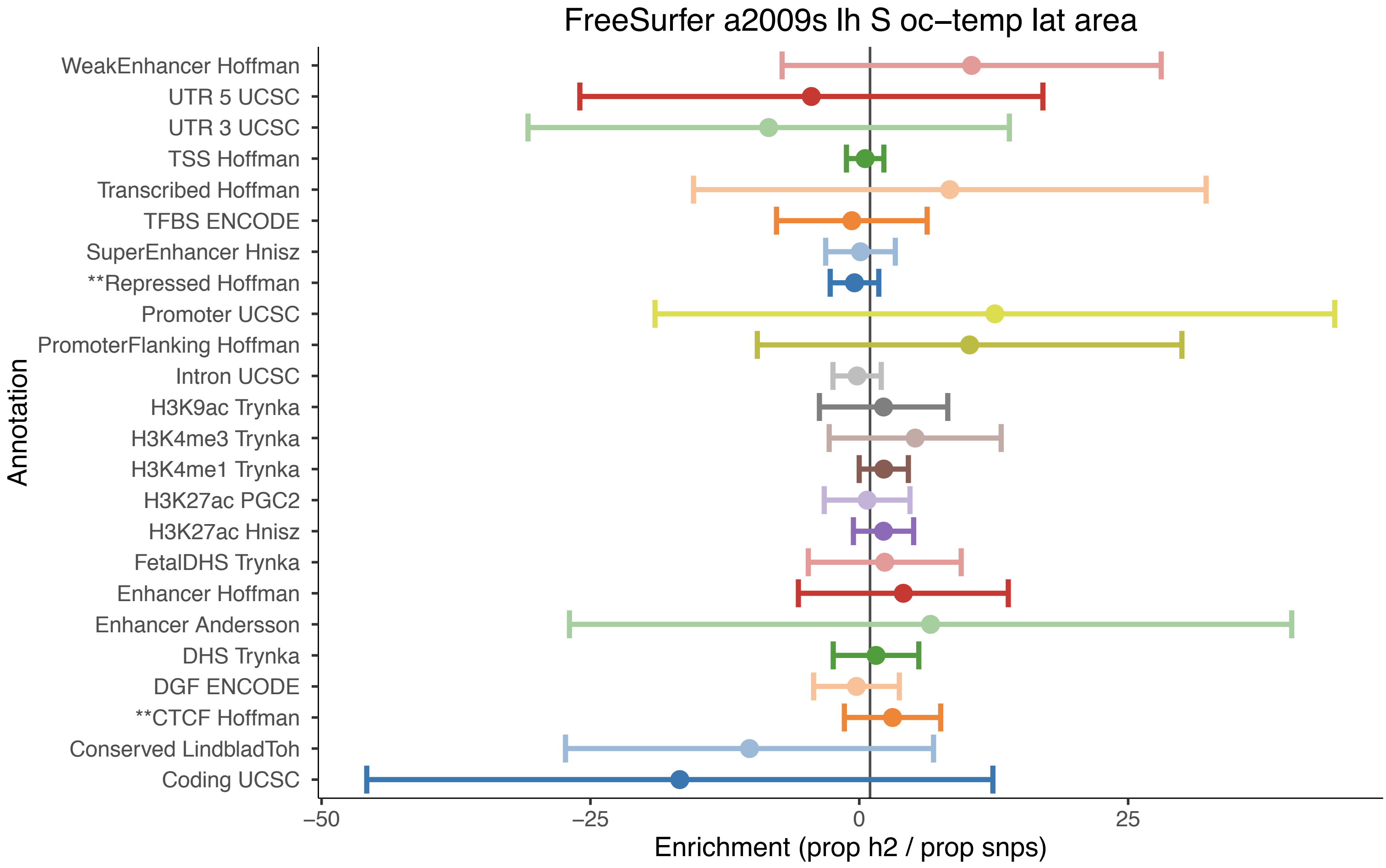

FreeSurfer a2009s lh S oc−temp lat area
WeakEnhancer Hoffman
UTR 5 UCSC
UTR 3 UCSC
TSS Hoffman
Transcribed Hoffman
TFBS ENCODE
SuperEnhancer Hnisz
**Repressed Hoffman
Promoter UCSC
PromoterFlanking Hoffman
Intron UCSC
H3K9ac Trynka
H3K4me3 Trynka
H3K4me1 Trynka
H3K27ac PGC2
H3K27ac Hnisz
FetalDHS Trynka
Enhancer Hoffman
Enhancer Andersson
DHS Trynka
DGF ENCODE
**CTCF Hoffman
Conserved LindbladToh
Coding UCSC
Annotation
−50
−25
0
25
Enrichment (prop h2 / prop snps)

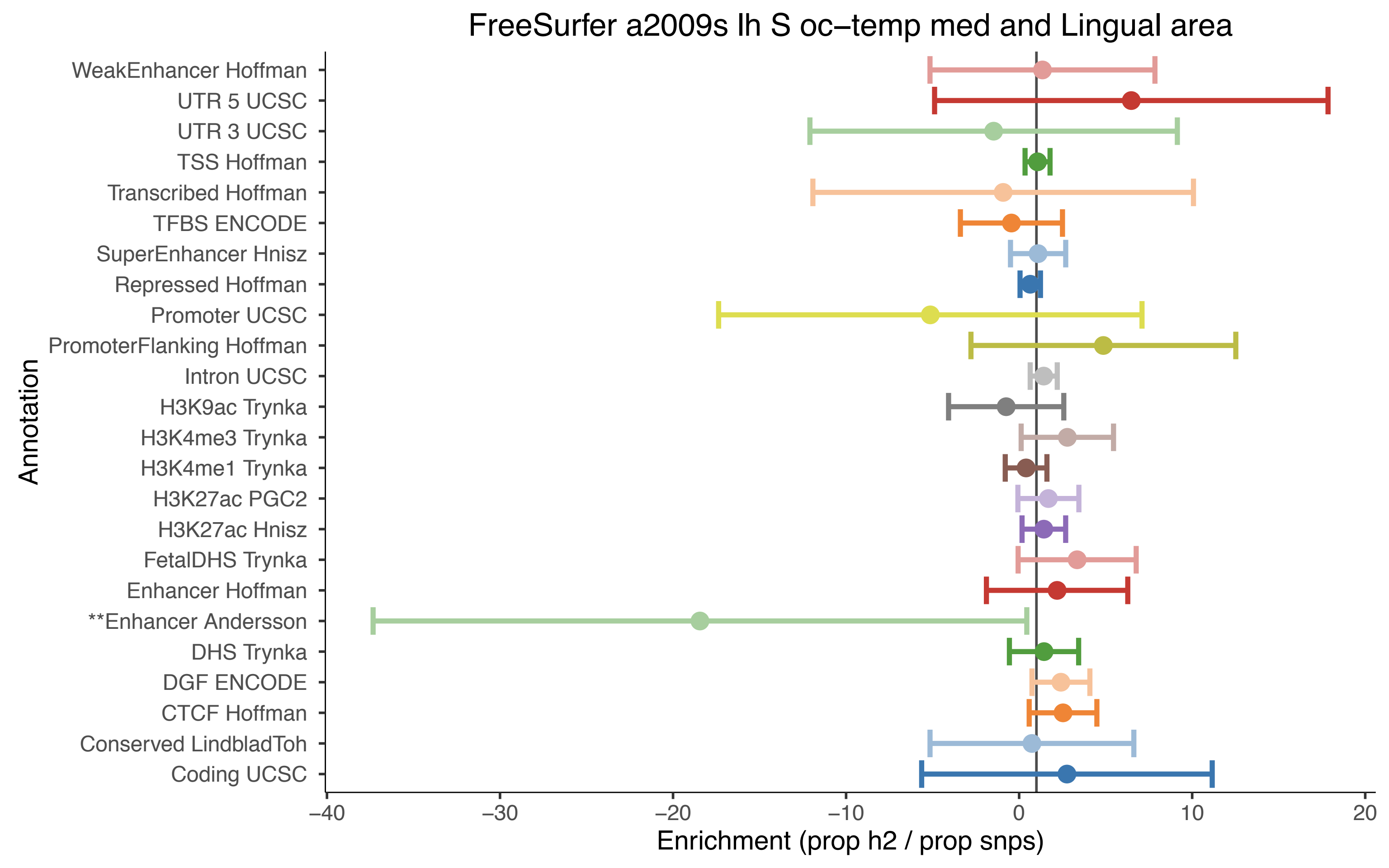
FreeSurfer a2009s lh S oc–temp med and Lingual area
WeakEnhancer Hoffman
UTR 5 UCSC
UTR 3 UCSC
TSS Hoffman
Transcribed Hoffman
TFBS ENCODE
SuperEnhancer Hnisz
Repressed Hoffman
Promoter UCSC
PromoterFlanking Hoffman
Intron UCSC
H3K9ac Trynka
H3K4me3 Trynka
H3K4me1 Trynka
H3K27ac PGC2
H3K27ac Hnisz
FetalDHS Trynka
Enhancer Hoffman
**Enhancer Andersson
DHS Trynka
DGF ENCODE
CTCF Hoffman
Conserved LindbladToh
Coding UCSC
Annotation
−40
−30
−20
−10
0
10
20
Enrichment (prop h2 / prop snps)

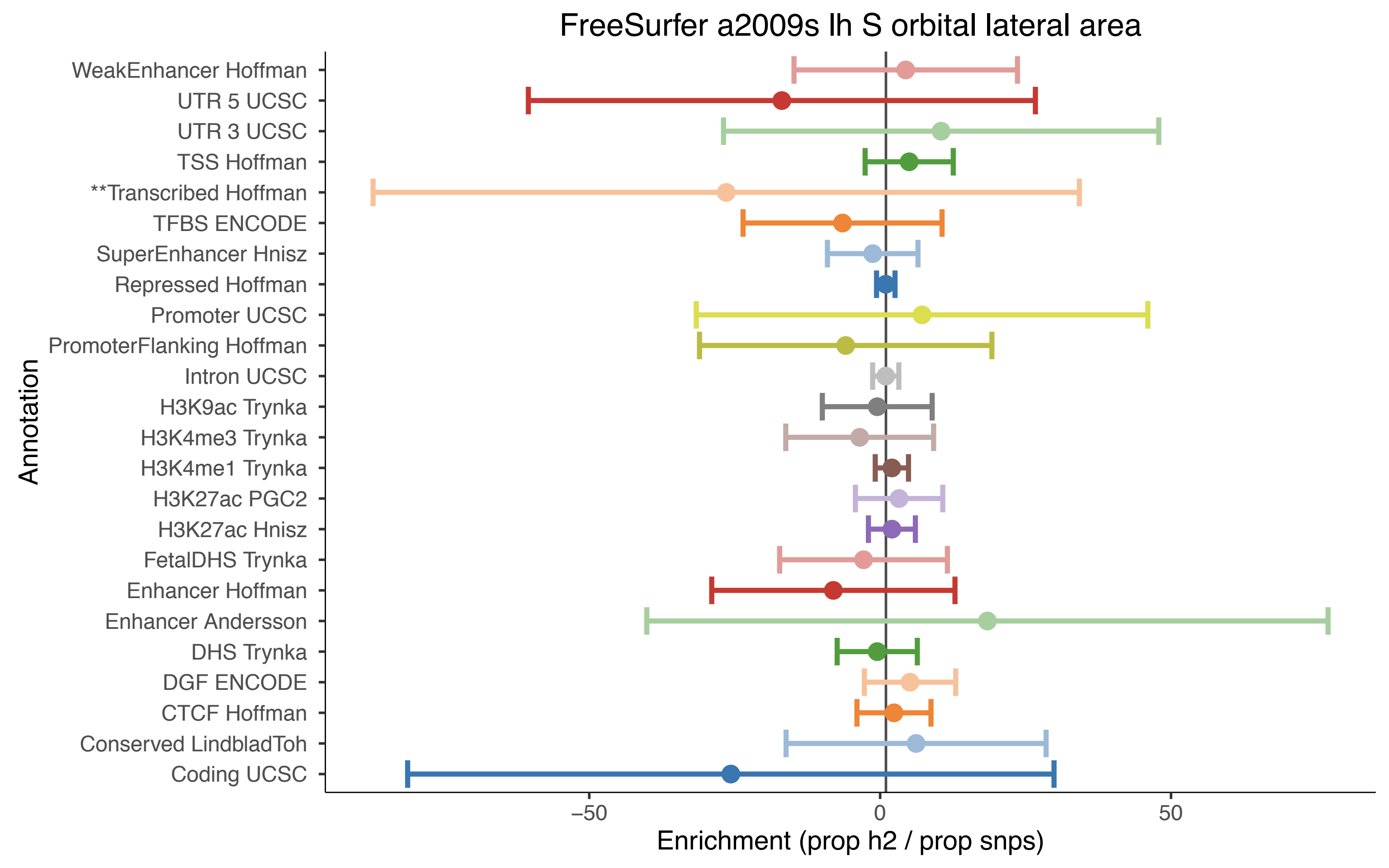
FreeSurfer a2009s lh S orbital lateral area
WeakEnhancer Hoffman
UTR 5 UCSC
UTR 3 UCSC
TSS Hoffman
**Transcribed Hoffman
TFBS ENCODE
SuperEnhancer Hnisz
Repressed Hoffman
Promoter UCSC
PromoterFlanking Hoffman
Intron UCSC
H3K9ac Trynka
H3K4me3 Trynka
H3K4me1 Trynka
H3K27ac PGC2
H3K27ac Hnisz
FetalDHS Trynka
Enhancer Hoffman
Enhancer Andersson
DHS Trynka
DGF ENCODE
CTCF Hoffman
Conserved LindbladToh
Coding UCSC
Annotation
−50
0
50
Enrichment (prop h2 / prop snps)

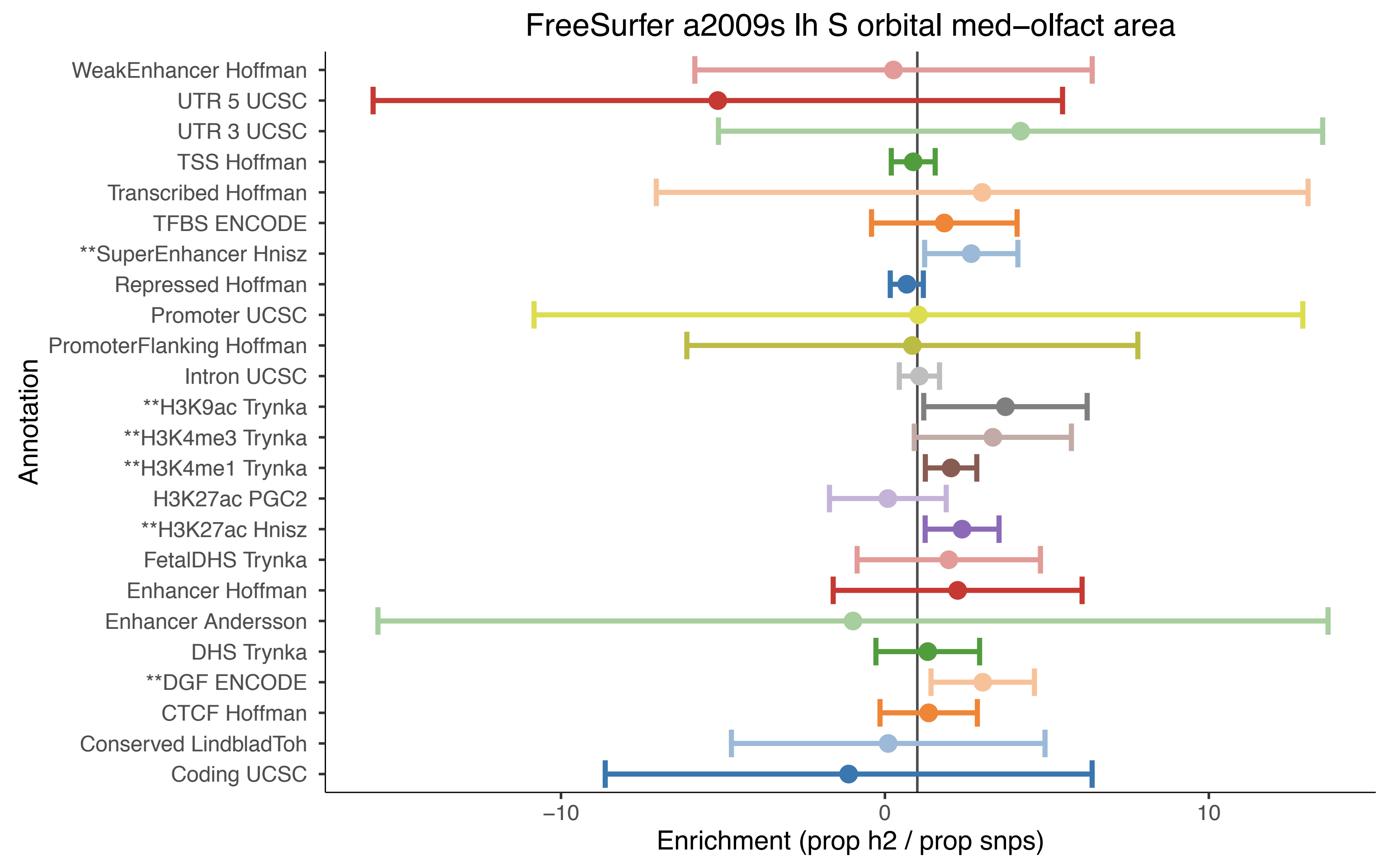

FreeSurfer a2009s lh S orbital med−olfact area
WeakEnhancer Hoffman
UTR 5 UCSC
UTR 3 UCSC
TSS Hoffman
Transcribed Hoffman
TFBS ENCODE
**SuperEnhancer Hnisz
Repressed Hoffman
Promoter UCSC
PromoterFlanking Hoffman
Intron UCSC
**H3K9ac Trynka
**H3K4me3 Trynka
**H3K4me1 Trynka
H3K27ac PGC2
**H3K27ac Hnisz
FetalDHS Trynka
Enhancer Hoffman
Enhancer Andersson
DHS Trynka
**DGF ENCODE
CTCF Hoffman
Conserved LindbladToh
Coding UCSC
Annotation
−10
0
10
Enrichment (prop h2 / prop snps)

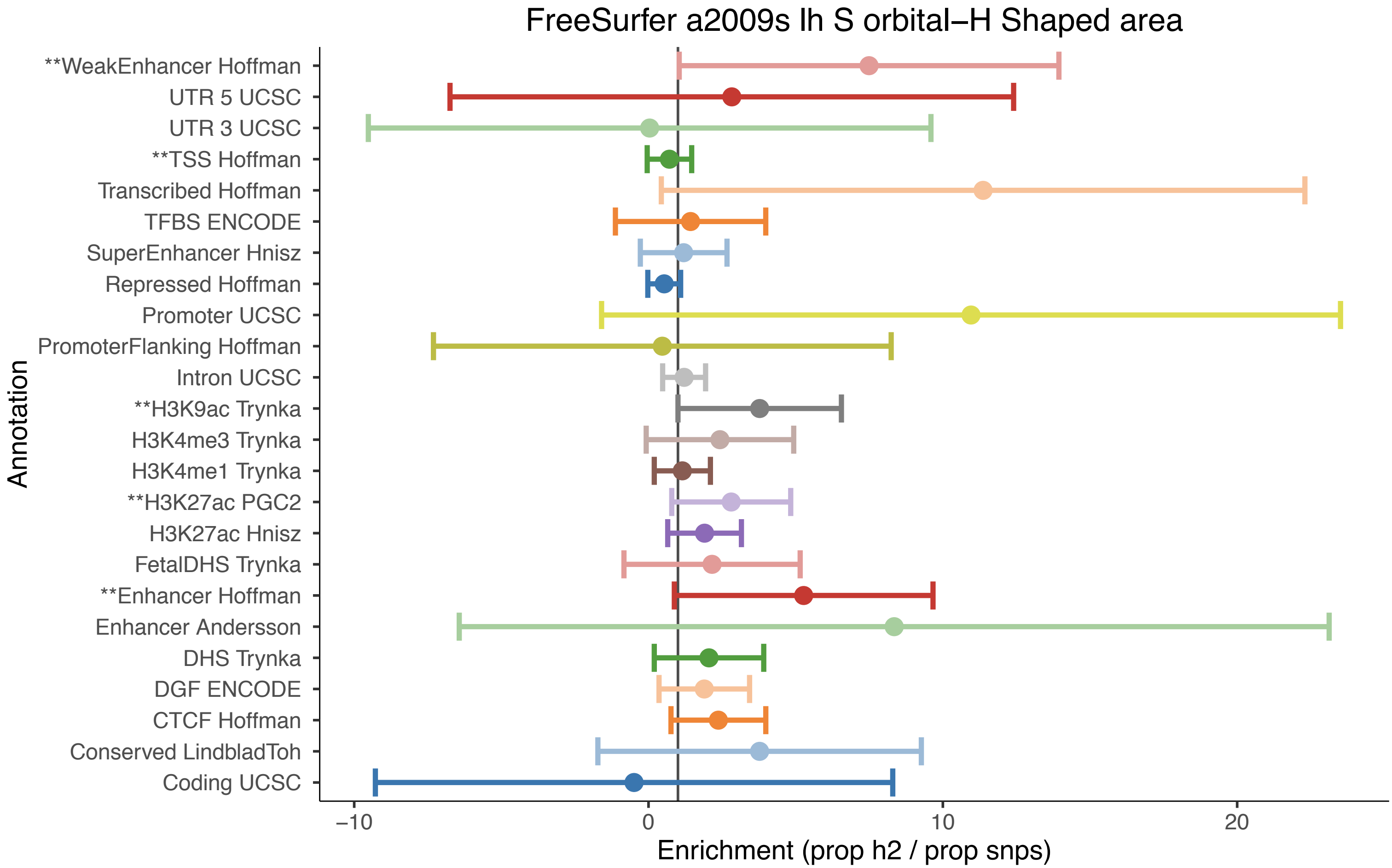
FreeSurfer a2009s lh S orbital−H Shaped area
**WeakEnhancer Hoffman
UTR 5 UCSC
UTR 3 UCSC
**TSS Hoffman
Transcribed Hoffman
TFBS ENCODE
SuperEnhancer Hnisz
Repressed Hoffman
Promoter UCSC
PromoterFlanking Hoffman
Intron UCSC
**H3K9ac Trynka
H3K4me3 Trynka
H3K4me1 Trynka
**H3K27ac PGC2
H3K27ac Hnisz
FetalDHS Trynka
**Enhancer Hoffman
Enhancer Andersson
DHS Trynka
DGF ENCODE
CTCF Hoffman
Conserved LindbladToh
Coding UCSC
Annotation
−10
0
10
20
Enrichment (prop h2 / prop snps)

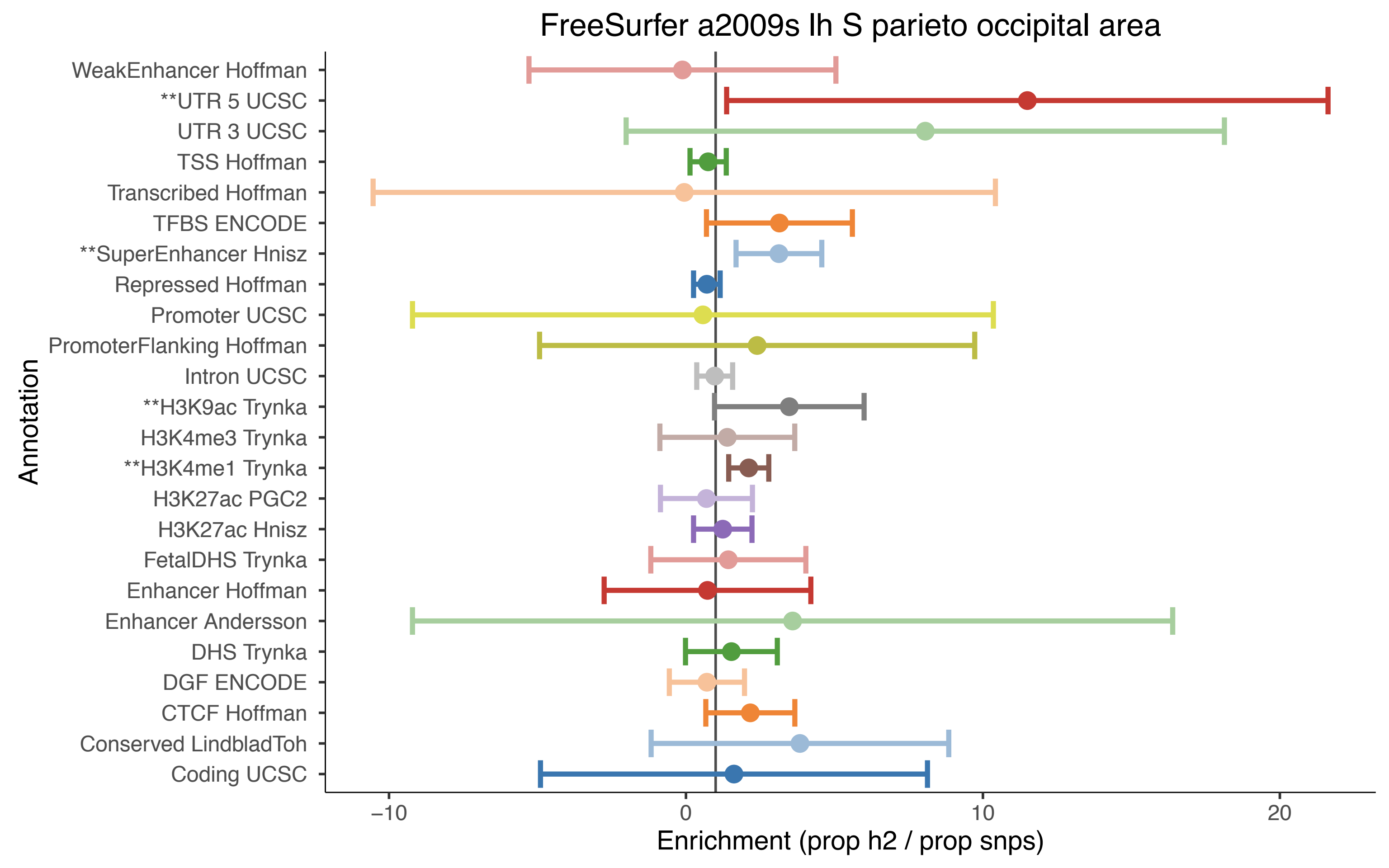
FreeSurfer a2009s lh S parieto occipital area
WeakEnhancer Hoffman
**UTR 5 UCSC
UTR 3 UCSC
TSS Hoffman
Transcribed Hoffman
TFBS ENCODE
**SuperEnhancer Hnisz
Repressed Hoffman
Promoter UCSC
PromoterFlanking Hoffman
Intron UCSC
**H3K9ac Trynka
H3K4me3 Trynka
**H3K4me1 Trynka
H3K27ac PGC2
H3K27ac Hnisz
FetalDHS Trynka
Enhancer Hoffman
Enhancer Andersson
DHS Trynka
DGF ENCODE
CTCF Hoffman
Conserved LindbladToh
Coding UCSC
Annotation
−10
0
10
20
Enrichment (prop h2 / prop snps)

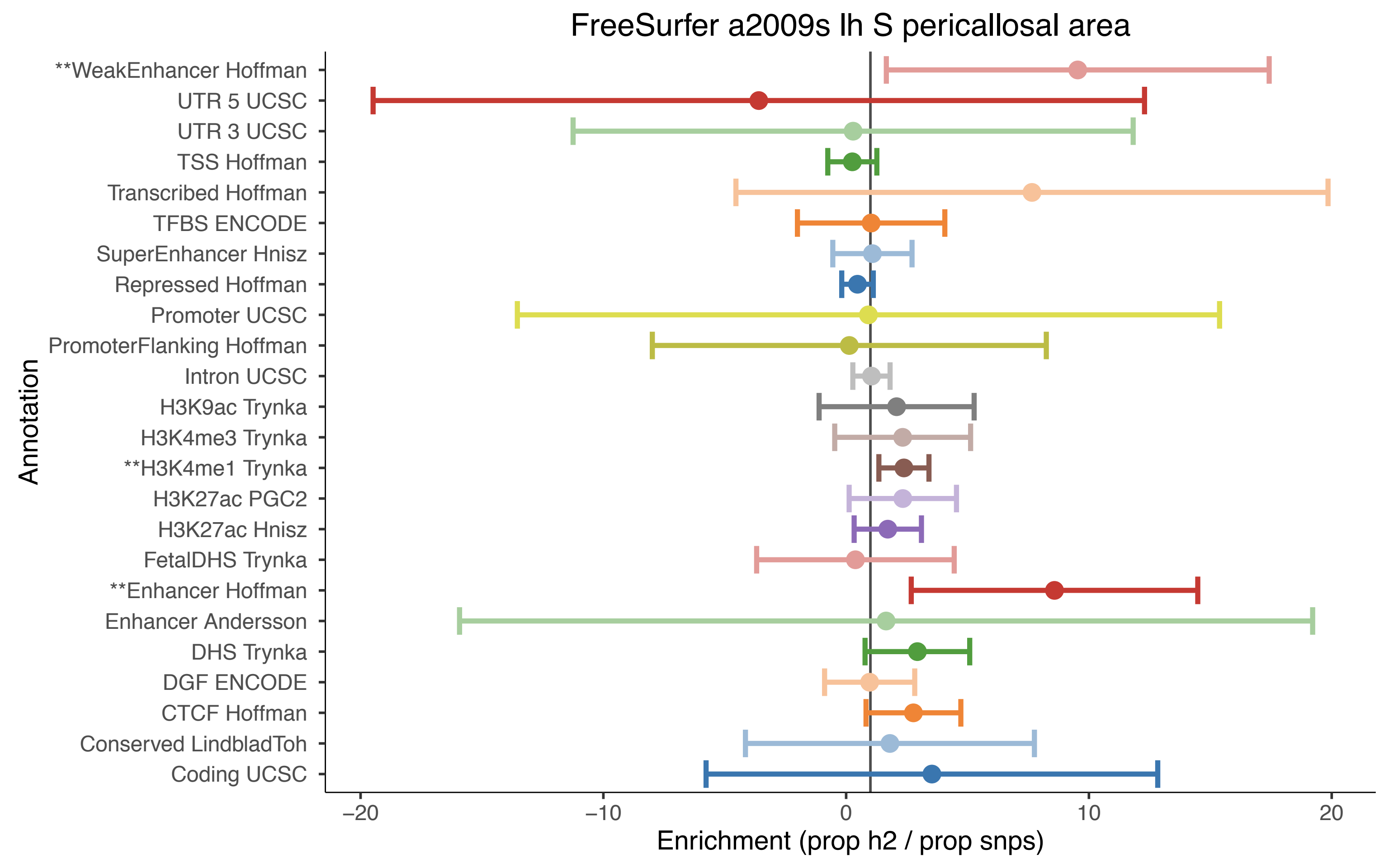
FreeSurfer a2009s lh S pericallosal area
**WeakEnhancer Hoffman
UTR 5 UCSC
UTR 3 UCSC
TSS Hoffman
Transcribed Hoffman
TFBS ENCODE
SuperEnhancer Hnisz
Repressed Hoffman
Promoter UCSC
PromoterFlanking Hoffman
Intron UCSC
H3K9ac Trynka
H3K4me3 Trynka
**H3K4me1 Trynka
H3K27ac PGC2
H3K27ac Hnisz
FetalDHS Trynka
**Enhancer Hoffman
Enhancer Andersson
DHS Trynka
DGF ENCODE
CTCF Hoffman
Conserved LindbladToh
Coding UCSC
Annotation
−20
−10
0
10
20
Enrichment (prop h2 / prop snps)

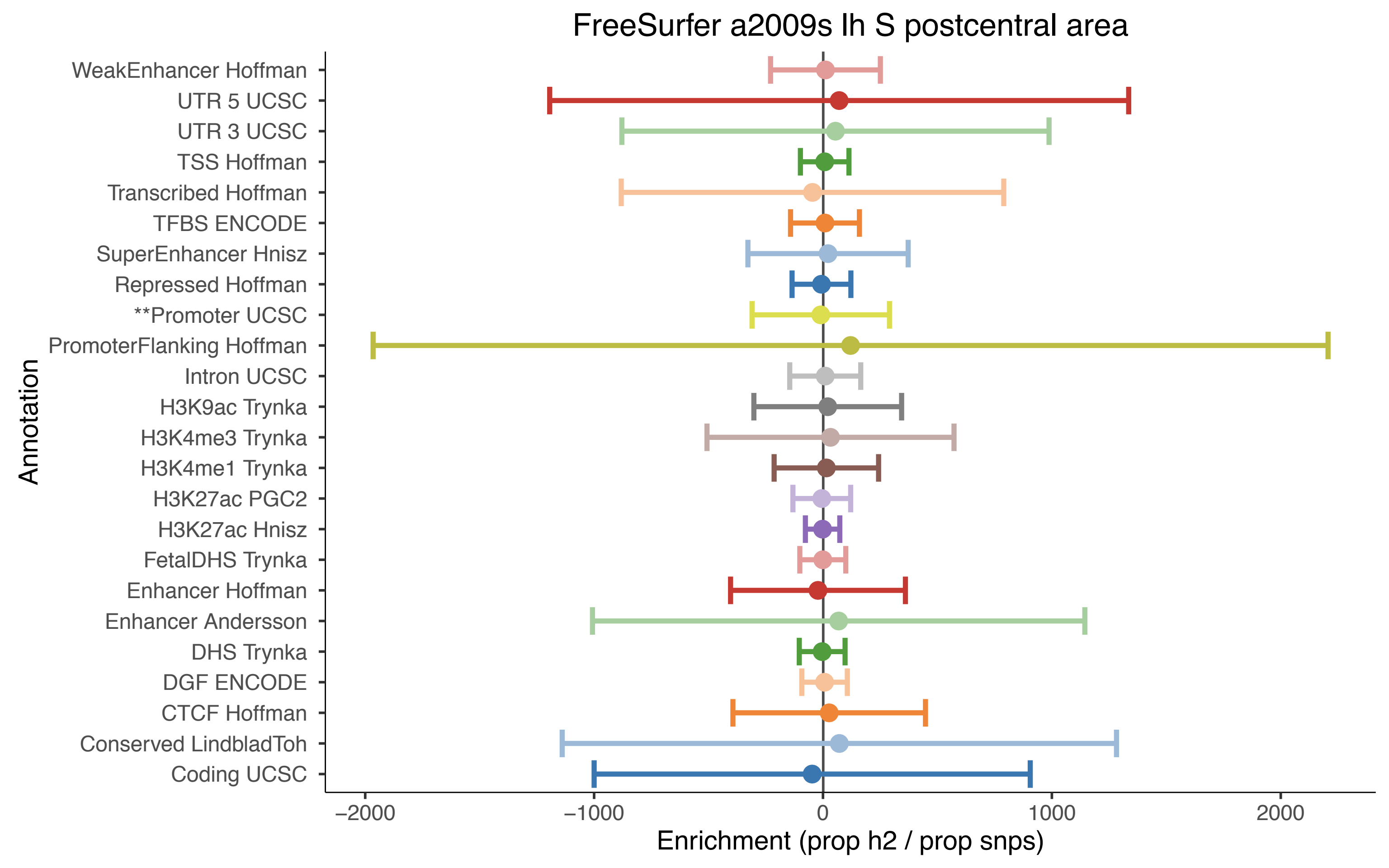

FreeSurfer a2009s lh S postcentral area
WeakEnhancer Hoffman
UTR 5 UCSC
UTR 3 UCSC
TSS Hoffman
Transcribed Hoffman
TFBS ENCODE
SuperEnhancer Hnisz
Repressed Hoffman
**Promoter UCSC
PromoterFlanking Hoffman
Intron UCSC
H3K9ac Trynka
H3K4me3 Trynka
H3K4me1 Trynka
H3K27ac PGC2
H3K27ac Hnisz
FetalDHS Trynka
Enhancer Hoffman
Enhancer Andersson
DHS Trynka
DGF ENCODE
CTCF Hoffman
Conserved LindbladToh
Coding UCSC
Annotation
−2000
−1000
0
1000
2000
Enrichment (prop h2 / prop snps)

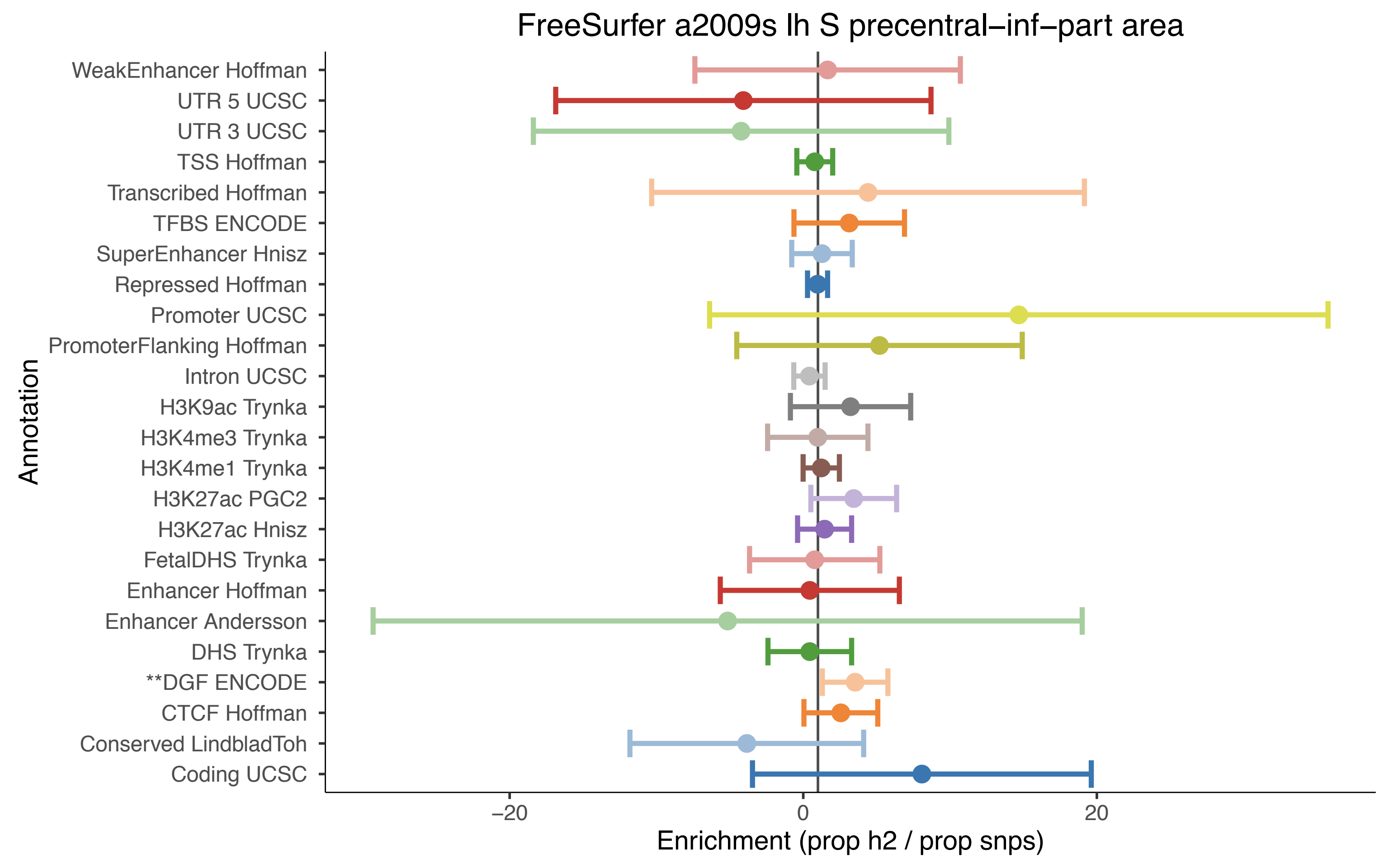

FreeSurfer a2009s lh S precentral−inf−part area
WeakEnhancer Hoffman
UTR 5 UCSC
UTR 3 UCSC
TSS Hoffman
Transcribed Hoffman
TFBS ENCODE
SuperEnhancer Hnisz
Repressed Hoffman
Promoter UCSC
PromoterFlanking Hoffman
Intron UCSC
H3K9ac Trynka
H3K4me3 Trynka
H3K4me1 Trynka
H3K27ac PGC2
H3K27ac Hnisz
FetalDHS Trynka
Enhancer Hoffman
Enhancer Andersson
DHS Trynka
**DGF ENCODE
CTCF Hoffman
Conserved LindbladToh
Coding UCSC
Annotation
−20
0
20
Enrichment (prop h2 / prop snps)

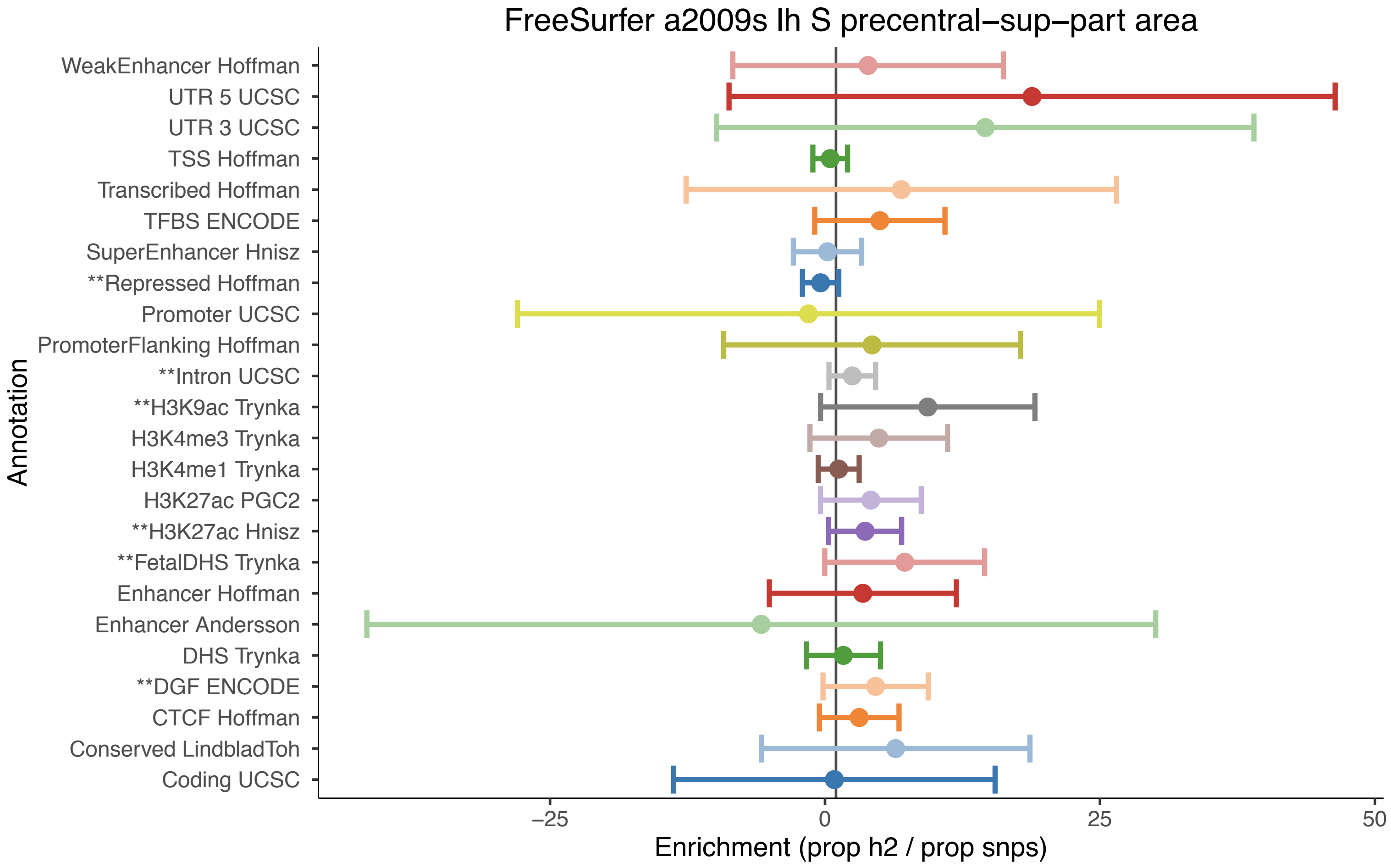

FreeSurfer a2009s lh S precentral−sup−part area
WeakEnhancer Hoffman
UTR 5 UCSC
UTR 3 UCSC
TSS Hoffman
Transcribed Hoffman
TFBS ENCODE
SuperEnhancer Hnisz
**Repressed Hoffman
Promoter UCSC
PromoterFlanking Hoffman
**Intron UCSC
**H3K9ac Trynka
H3K4me3 Trynka
H3K4me1 Trynka
H3K27ac PGC2
**H3K27ac Hnisz
**FetalDHS Trynka
Enhancer Hoffman
Enhancer Andersson
DHS Trynka
**DGF ENCODE
CTCF Hoffman
Conserved LindbladToh
Coding UCSC
Annotation
−25
0
25
50
Enrichment (prop h2 / prop snps)

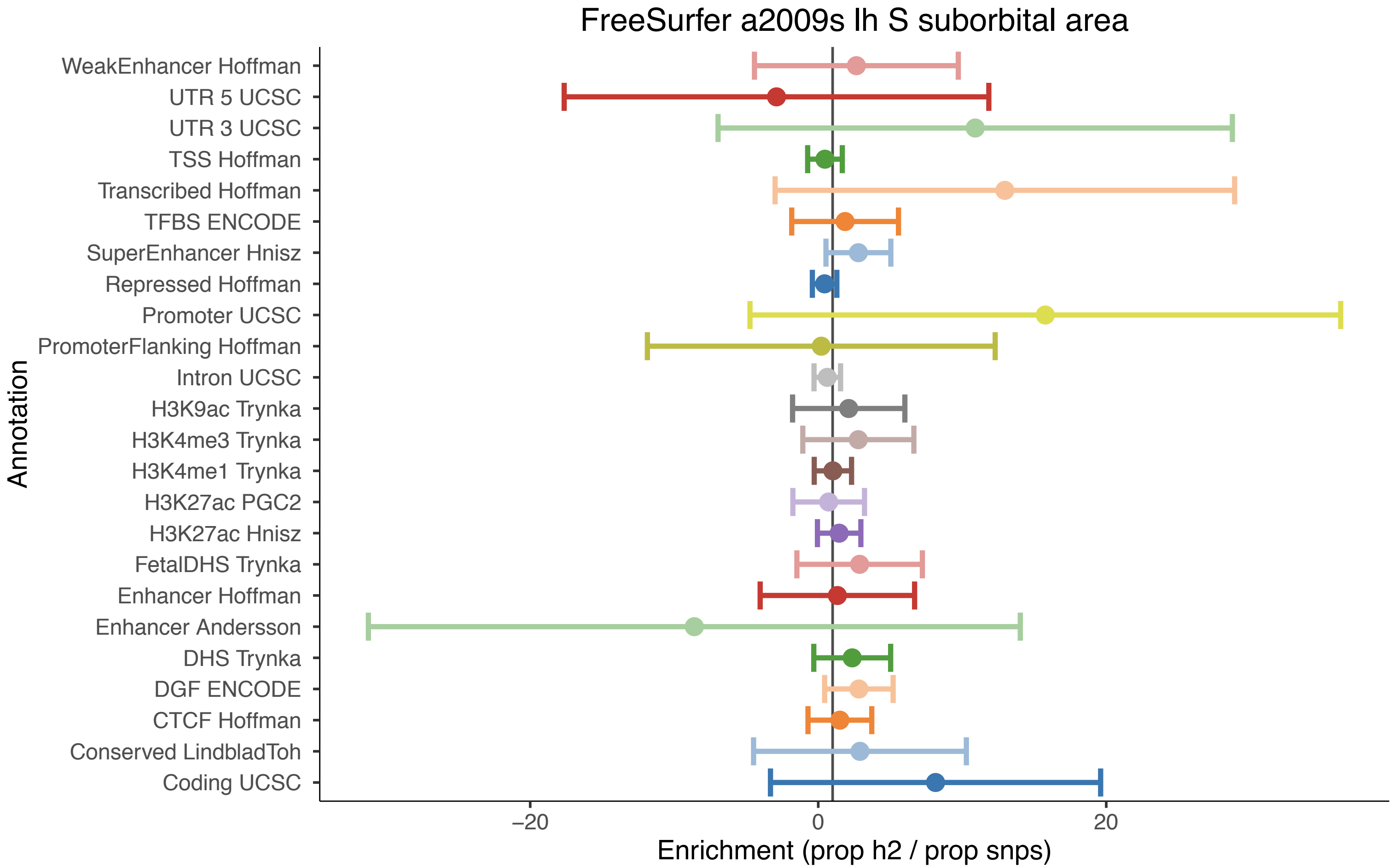
FreeSurfer a2009s lh S suborbital area
WeakEnhancer Hoffman
UTR 5 UCSC
UTR 3 UCSC
TSS Hoffman
Transcribed Hoffman
TFBS ENCODE
SuperEnhancer Hnisz
Repressed Hoffman
Promoter UCSC
PromoterFlanking Hoffman
Intron UCSC
H3K9ac Trynka
H3K4me3 Trynka
H3K4me1 Trynka
H3K27ac PGC2
H3K27ac Hnisz
FetalDHS Trynka
Enhancer Hoffman
Enhancer Andersson
DHS Trynka
DGF ENCODE
CTCF Hoffman
Conserved LindbladToh
Coding UCSC
Annotation
−20
0
20
Enrichment (prop h2 / prop snps)

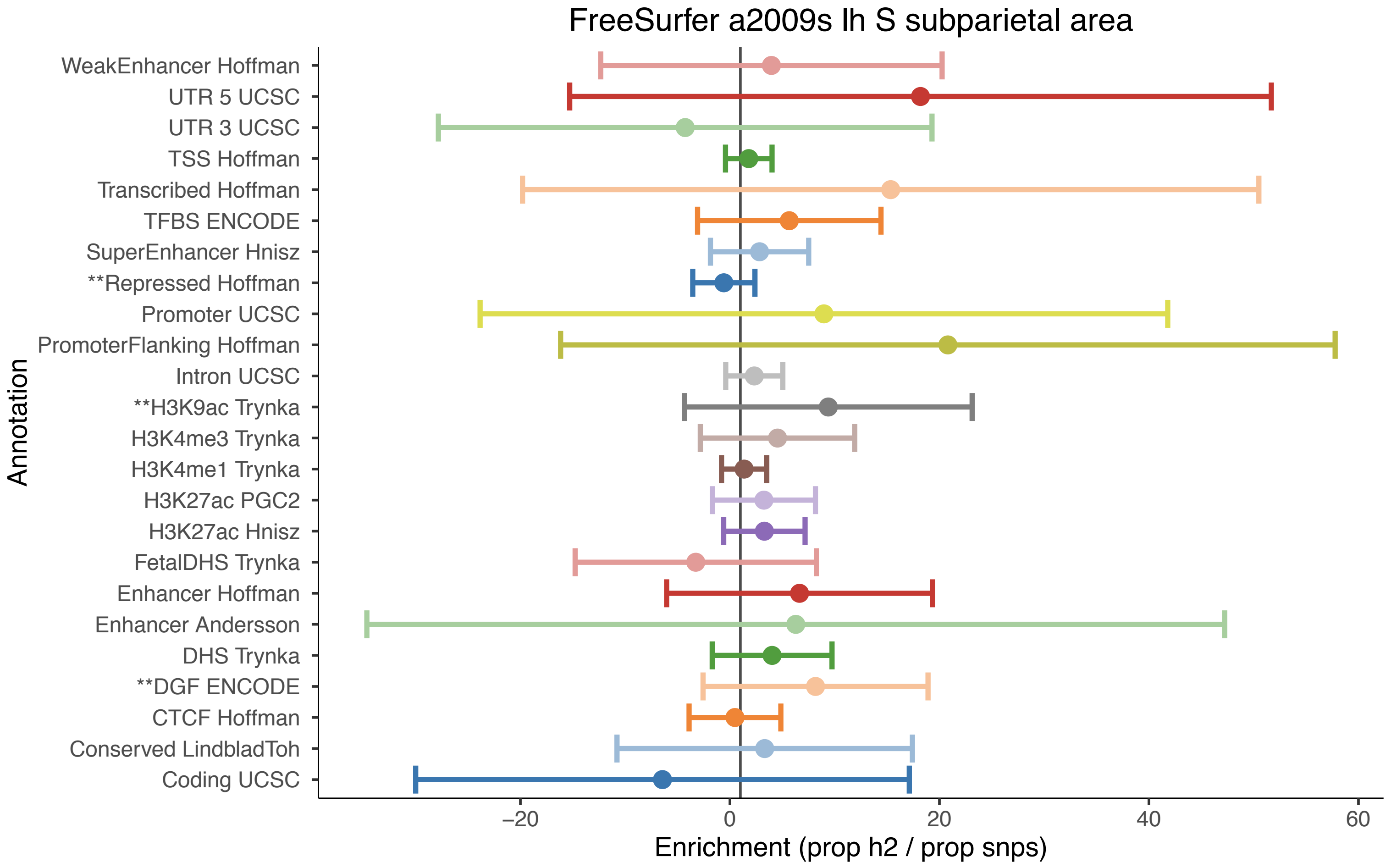
FreeSurfer a2009s lh S subparietal area
WeakEnhancer Hoffman
UTR 5 UCSC
UTR 3 UCSC
TSS Hoffman
Transcribed Hoffman
TFBS ENCODE
SuperEnhancer Hnisz
**Repressed Hoffman
Promoter UCSC
PromoterFlanking Hoffman
Intron UCSC
**H3K9ac Trynka
H3K4me3 Trynka
H3K4me1 Trynka
H3K27ac PGC2
H3K27ac Hnisz
FetalDHS Trynka
Enhancer Hoffman
Enhancer Andersson
DHS Trynka
**DGF ENCODE
CTCF Hoffman
Conserved LindbladToh
Coding UCSC
Annotation
−20
0
20
40
60
Enrichment (prop h2 / prop snps)

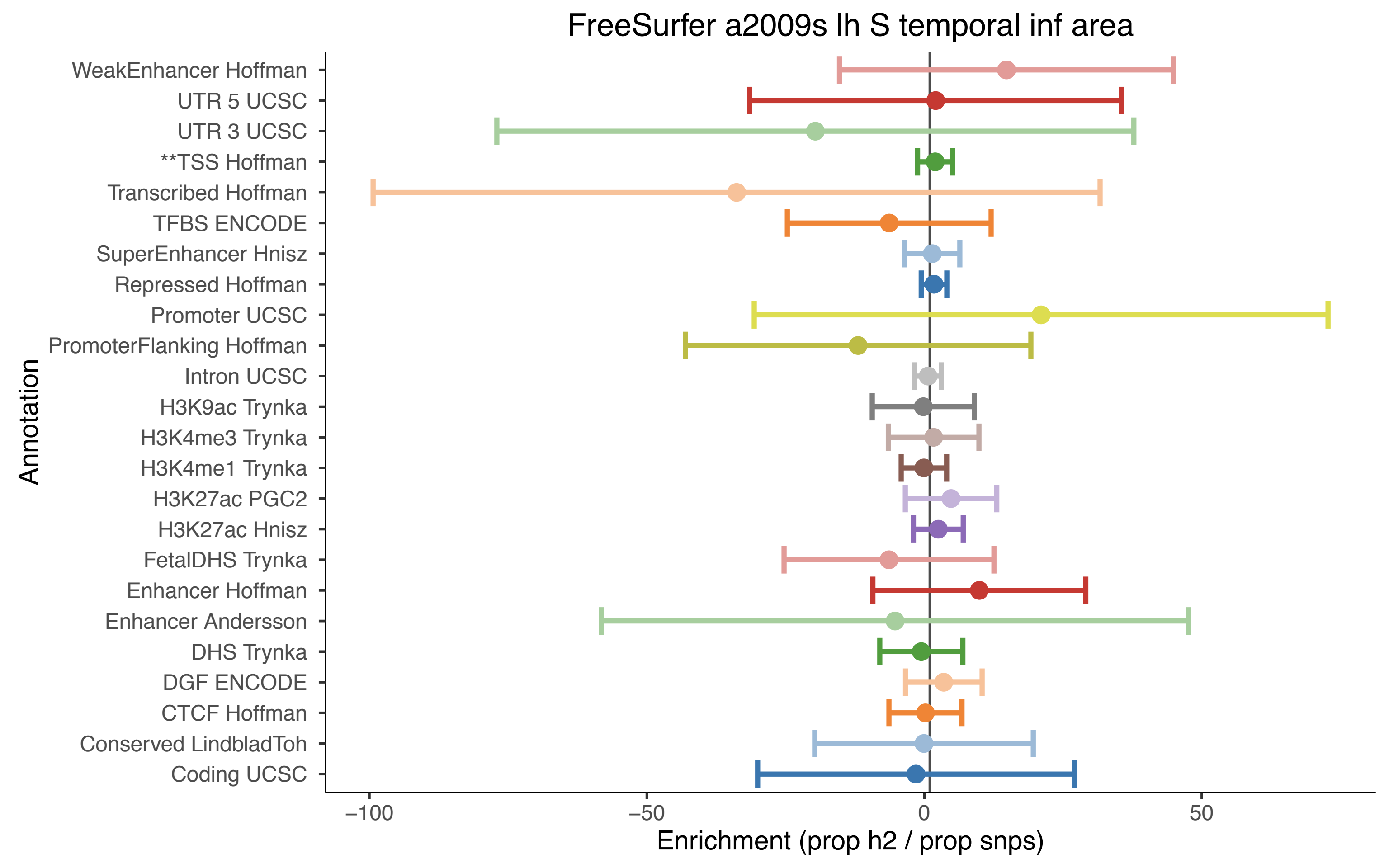

FreeSurfer a2009s lh S temporal inf area
WeakEnhancer Hoffman
UTR 5 UCSC
UTR 3 UCSC
**TSS Hoffman
Transcribed Hoffman
TFBS ENCODE
SuperEnhancer Hnisz
Repressed Hoffman
Promoter UCSC
PromoterFlanking Hoffman
Intron UCSC
H3K9ac Trynka
H3K4me3 Trynka
H3K4me1 Trynka
H3K27ac PGC2
H3K27ac Hnisz
FetalDHS Trynka
Enhancer Hoffman
Enhancer Andersson
DHS Trynka
DGF ENCODE
CTCF Hoffman
Conserved LindbladToh
Coding UCSC
Annotation
−100
−50
0
50
Enrichment (prop h2 / prop snps)

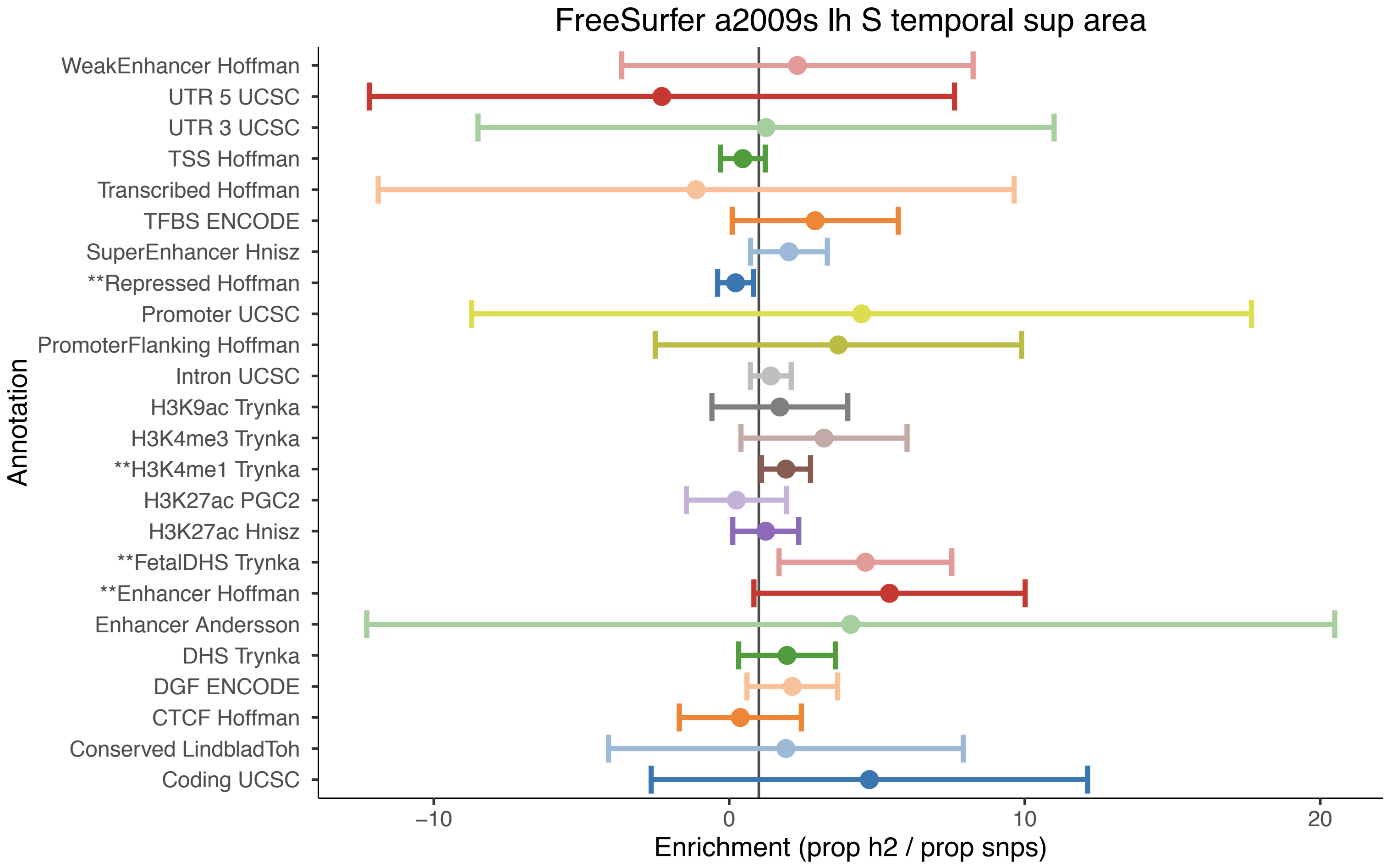
FreeSurfer a2009s lh S temporal sup area
WeakEnhancer Hoffman
UTR 5 UCSC
UTR 3 UCSC
TSS Hoffman
Transcribed Hoffman
TFBS ENCODE
SuperEnhancer Hnisz
**Repressed Hoffman
Promoter UCSC
PromoterFlanking Hoffman
Intron UCSC
H3K9ac Trynka
H3K4me3 Trynka
**H3K4me1 Trynka
H3K27ac PGC2
H3K27ac Hnisz
**FetalDHS Trynka
**Enhancer Hoffman
Enhancer Andersson
DHS Trynka
DGF ENCODE
CTCF Hoffman
Conserved LindbladToh
Coding UCSC
Annotation
−10
0
10
20
Enrichment (prop h2 / prop snps)

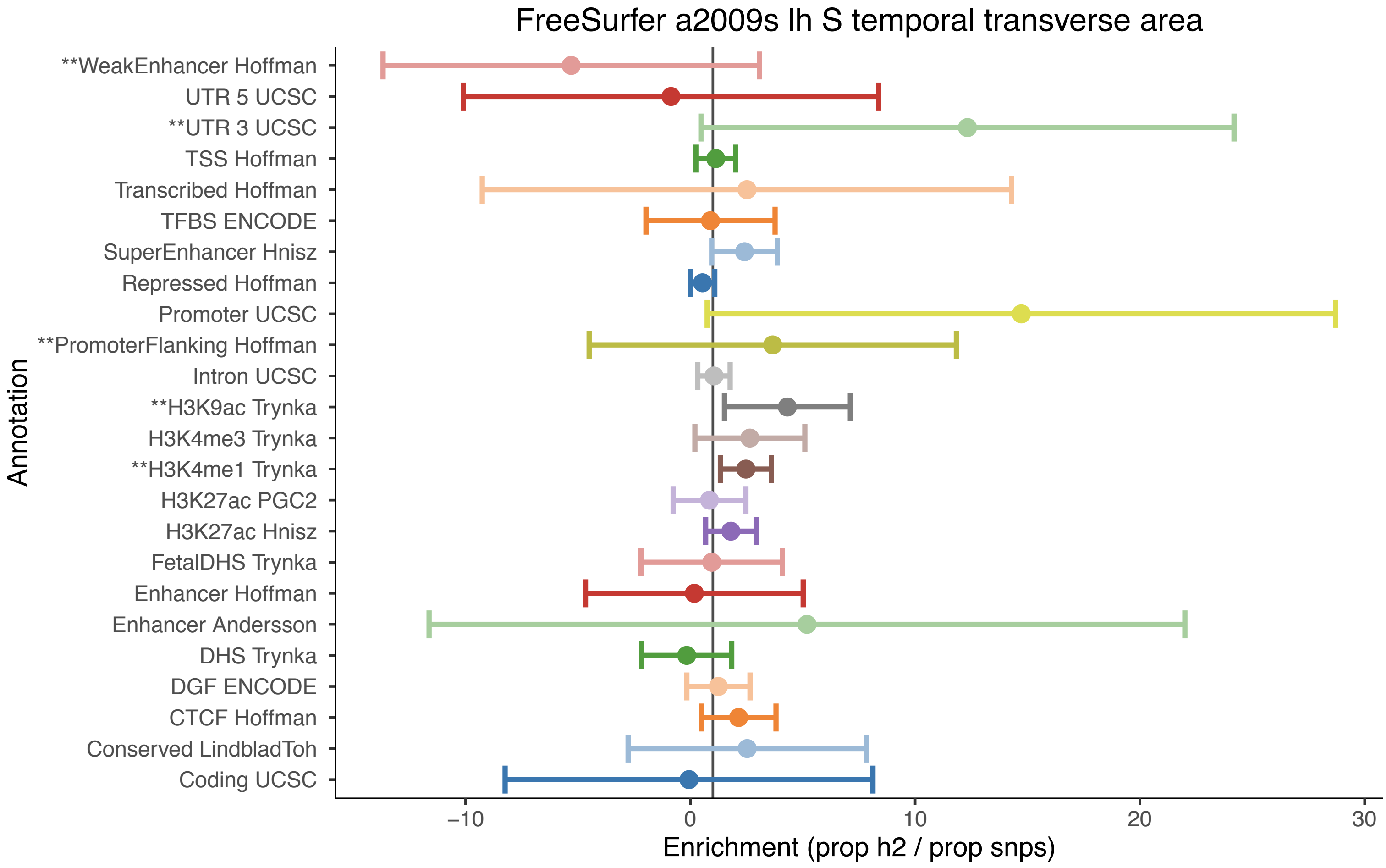

FreeSurfer a2009s lh S temporal transverse area
Annotation
**WeakEnhancer Hoffman
UTR 5 UCSC
**UTR 3 UCSC
TSS Hoffman
Transcribed Hoffman
TFBS ENCODE
SuperEnhancer Hnisz
Repressed Hoffman
Promoter UCSC
**PromoterFlanking Hoffman
Intron UCSC
**H3K9ac Trynka
H3K4me3 Trynka
**H3K4me1 Trynka
H3K27ac PGC2
H3K27ac Hnisz
FetalDHS Trynka
Enhancer Hoffman
Enhancer Andersson
DHS Trynka
DGF ENCODE
CTCF Hoffman
Conserved LindbladToh
Coding UCSC
−10
0
10
20
30
Enrichment (prop h2 / prop snps)

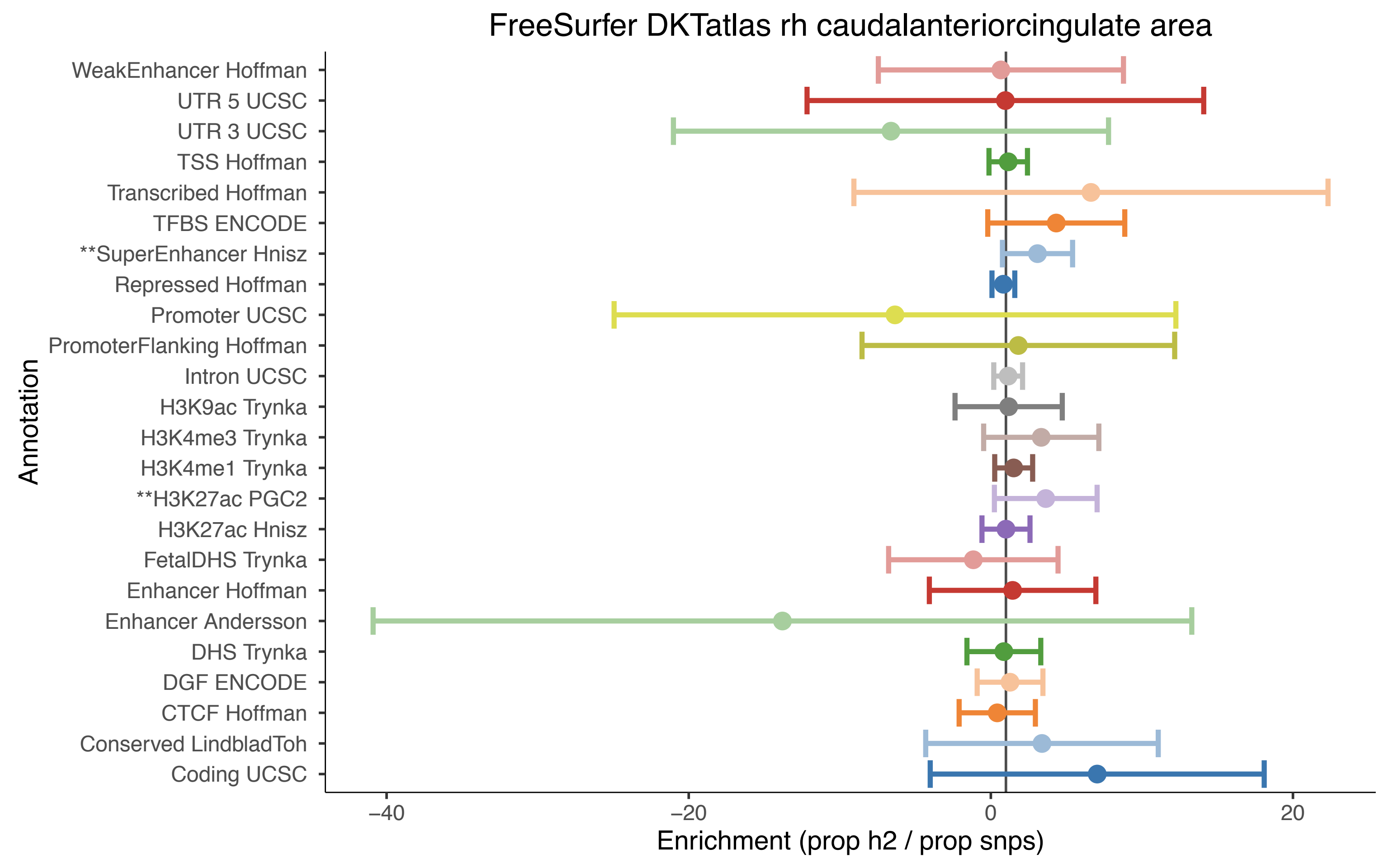

FreeSurfer DKTatlas rh caudalanteriorcingulate area
WeakEnhancer Hoffman
UTR 5 UCSC
UTR 3 UCSC
TSS Hoffman
Transcribed Hoffman
TFBS ENCODE
**SuperEnhancer Hnisz
Repressed Hoffman
Promoter UCSC
PromoterFlanking Hoffman
Intron UCSC
H3K9ac Trynka
H3K4me3 Trynka
H3K4me1 Trynka
**H3K27ac PGC2
H3K27ac Hnisz
FetalDHS Trynka
Enhancer Hoffman
Enhancer Andersson
DHS Trynka
DGF ENCODE
CTCF Hoffman
Conserved LindbladToh
Coding UCSC
Annotation
−40
−20
0
20
Enrichment (prop h2 / prop snps)

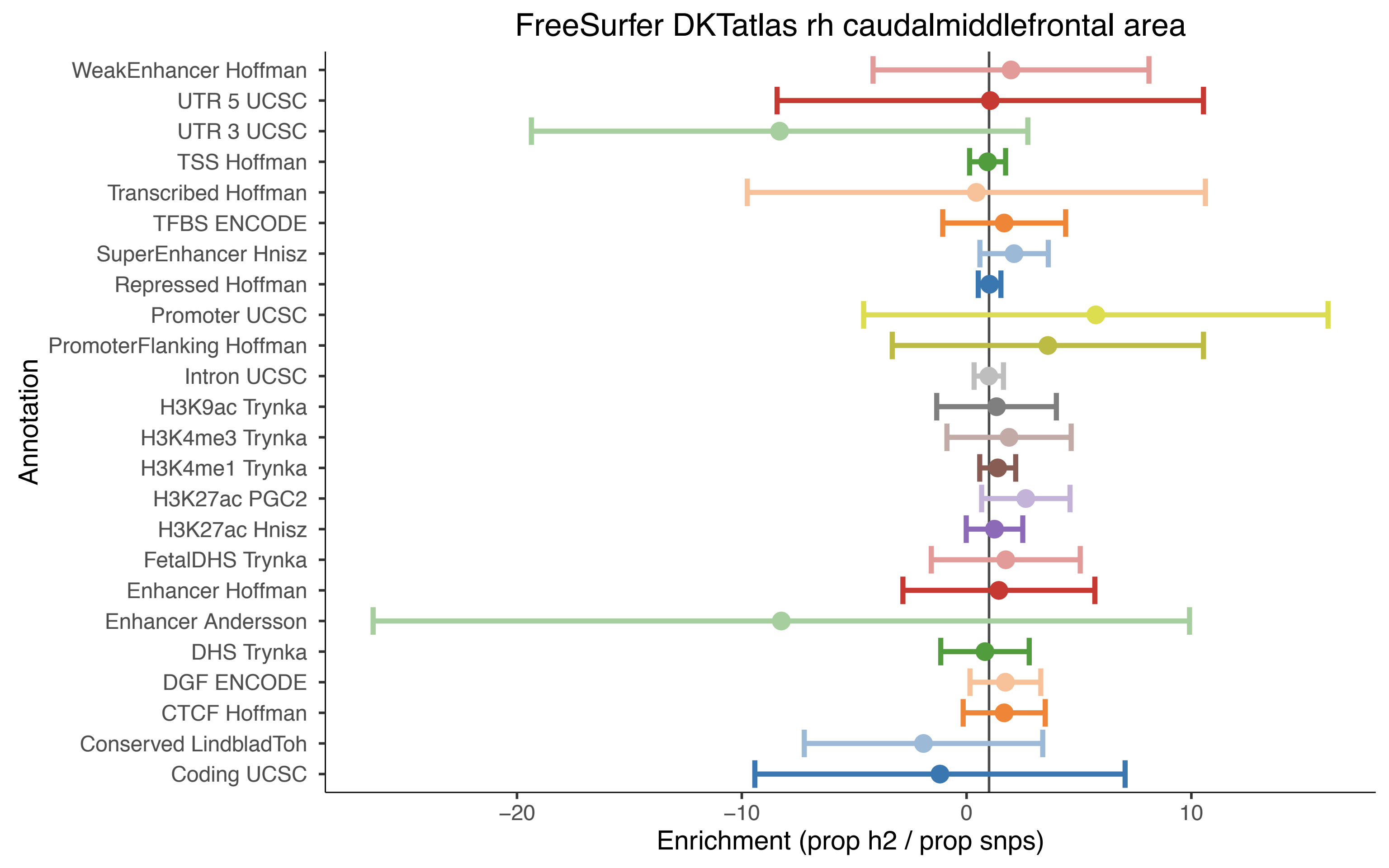
FreeSurfer DKTatlas rh caudalmiddlefrontal area
WeakEnhancer Hoffman
UTR 5 UCSC
UTR 3 UCSC
TSS Hoffman
Transcribed Hoffman
TFBS ENCODE
SuperEnhancer Hnisz
Repressed Hoffman
Promoter UCSC
PromoterFlanking Hoffman
Intron UCSC
H3K9ac Trynka
H3K4me3 Trynka
H3K4me1 Trynka
H3K27ac PGC2
H3K27ac Hnisz
FetalDHS Trynka
Enhancer Hoffman
Enhancer Andersson
DHS Trynka
DGF ENCODE
CTCF Hoffman
Conserved LindbladToh
Coding UCSC
Annotation
−20
−10
0
10
Enrichment (prop h2 / prop snps)

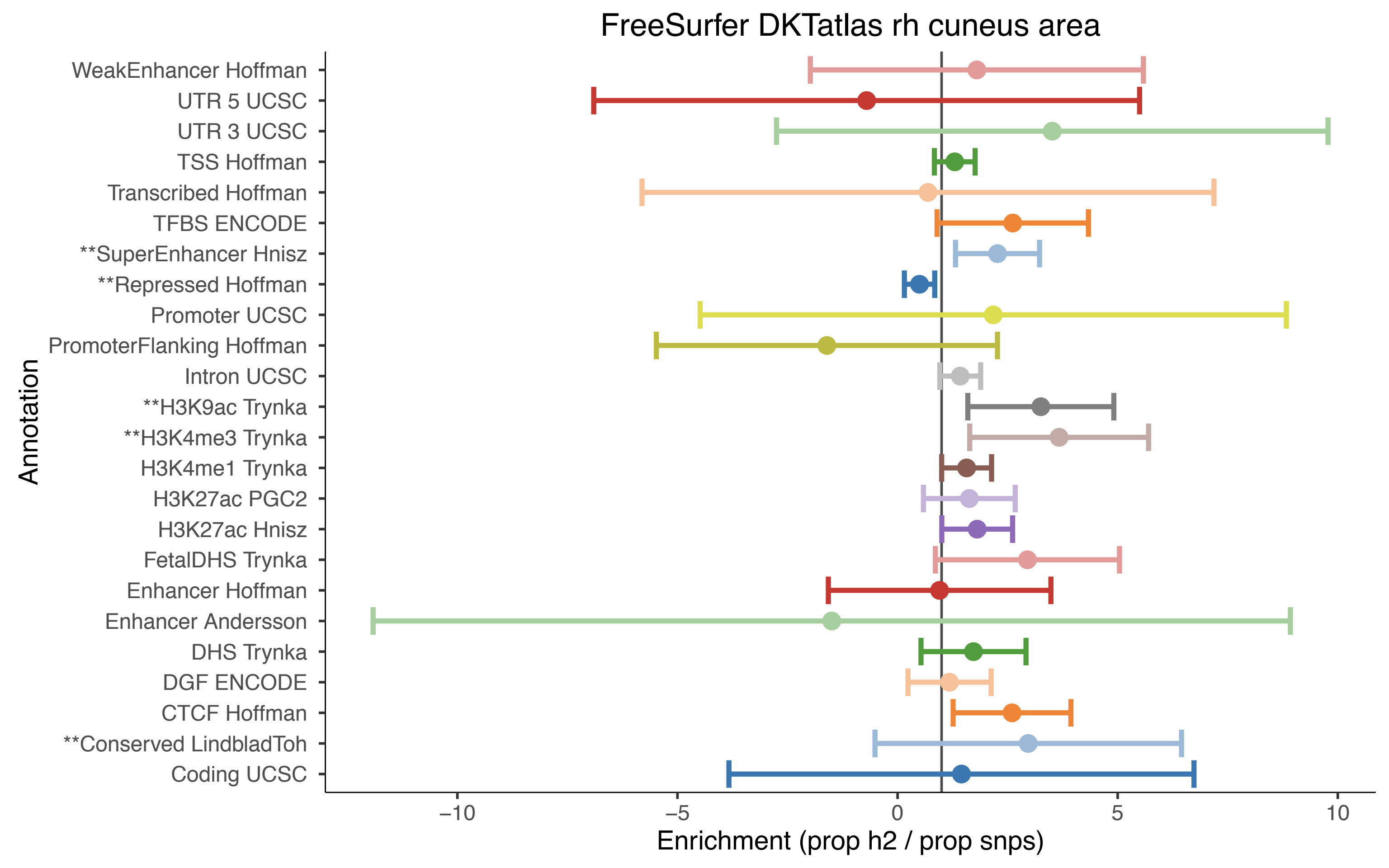

FreeSurfer DKTatlas rh cuneus area
WeakEnhancer Hoffman
UTR 5 UCSC
UTR 3 UCSC
TSS Hoffman
Transcribed Hoffman
TFBS ENCODE
**SuperEnhancer Hnisz
**Repressed Hoffman
Promoter UCSC
PromoterFlanking Hoffman
Intron UCSC
**H3K9ac Trynka
**H3K4me3 Trynka
H3K4me1 Trynka
H3K27ac PGC2
H3K27ac Hnisz
FetalDHS Trynka
Enhancer Hoffman
Enhancer Andersson
DHS Trynka
DGF ENCODE
CTCF Hoffman
**Conserved LindbladToh
Coding UCSC
Annotation
−10
−5
0
5
10
Enrichment (prop h2 / prop snps)

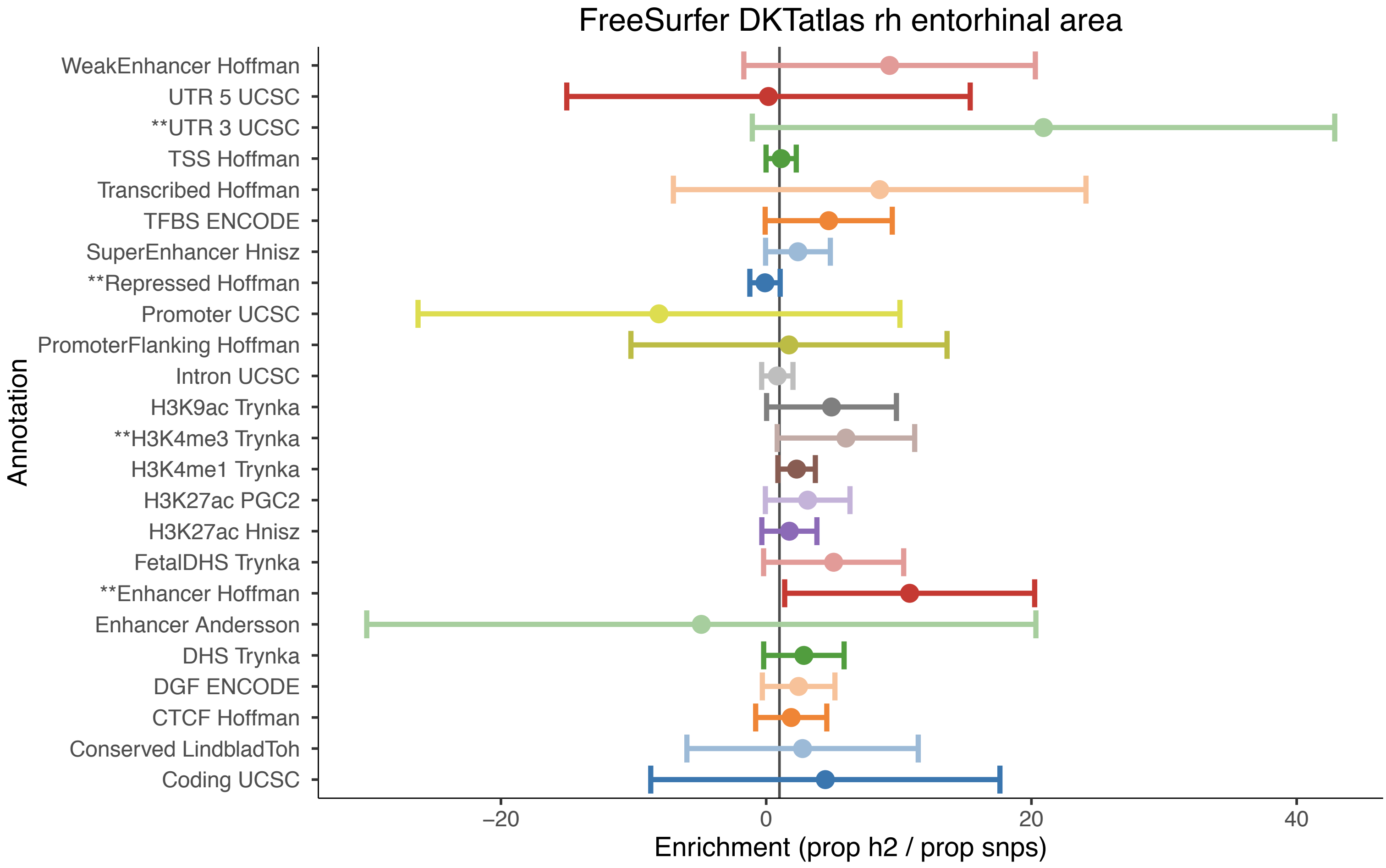

FreeSurfer DKTatlas rh entorhinal area
WeakEnhancer Hoffman
UTR 5 UCSC
**UTR 3 UCSC
TSS Hoffman
Transcribed Hoffman
TFBS ENCODE
SuperEnhancer Hnisz
**Repressed Hoffman
Promoter UCSC
PromoterFlanking Hoffman
Intron UCSC
H3K9ac Trynka
**H3K4me3 Trynka
H3K4me1 Trynka
H3K27ac PGC2
H3K27ac Hnisz
FetalDHS Trynka
**Enhancer Hoffman
Enhancer Andersson
DHS Trynka
DGF ENCODE
CTCF Hoffman
Conserved LindbladToh
Coding UCSC
Annotation
−20
0
20
40
Enrichment (prop h2 / prop snps)

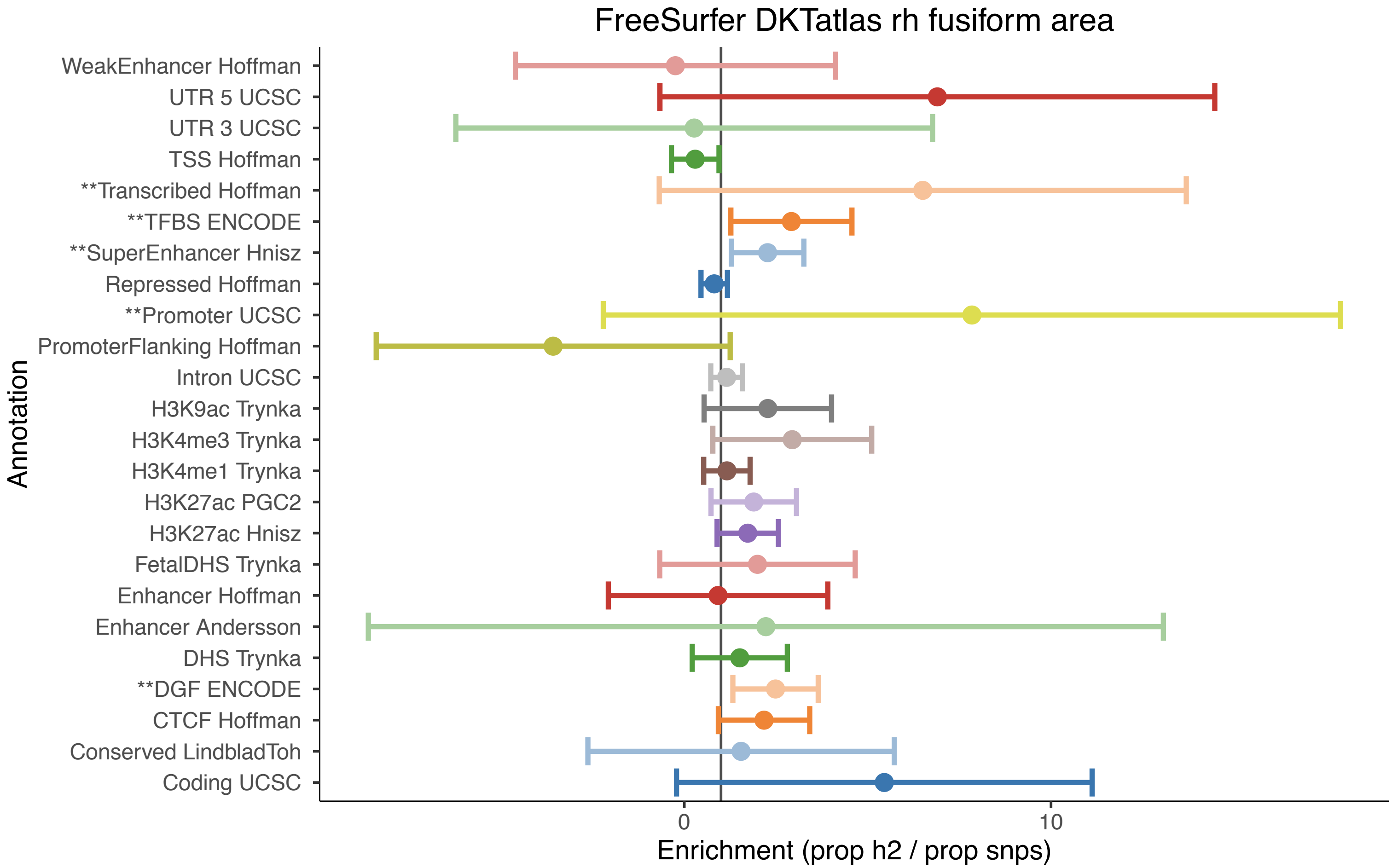

FreeSurfer DKTatlas rh fusiform area
WeakEnhancer Hoffman
UTR 5 UCSC
UTR 3 UCSC
TSS Hoffman
**Transcribed Hoffman
**TFBS ENCODE
**SuperEnhancer Hnisz
Repressed Hoffman
**Promoter UCSC
PromoterFlanking Hoffman
Intron UCSC
H3K9ac Trynka
H3K4me3 Trynka
H3K4me1 Trynka
H3K27ac PGC2
H3K27ac Hnisz
FetalDHS Trynka
Enhancer Hoffman
Enhancer Andersson
DHS Trynka
**DGF ENCODE
CTCF Hoffman
Conserved LindbladToh
Coding UCSC
Annotation
0
10
Enrichment (prop h2 / prop snps)

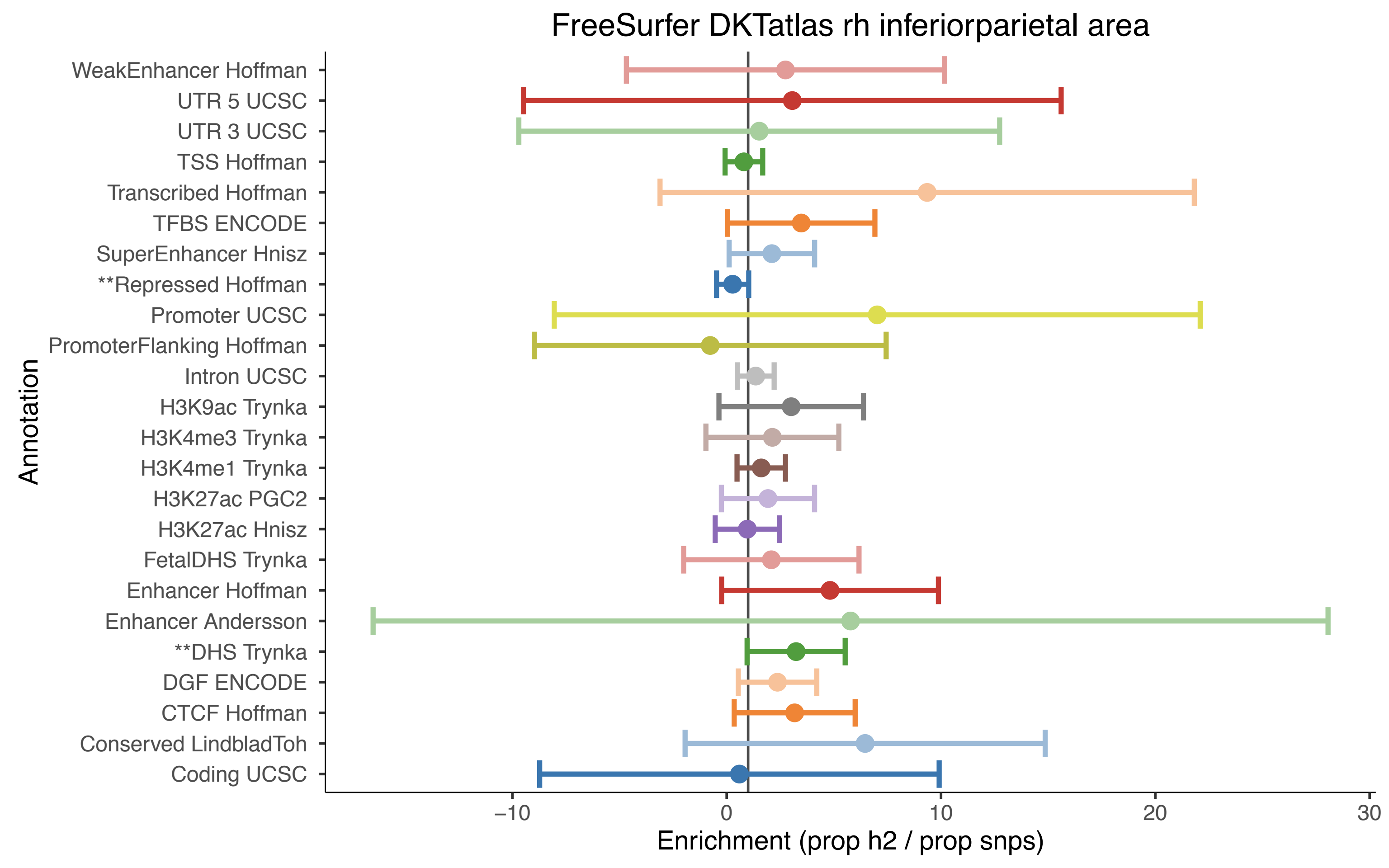

FreeSurfer DKTatlas rh inferiorparietal area
WeakEnhancer Hoffman
UTR 5 UCSC
UTR 3 UCSC
TSS Hoffman
Transcribed Hoffman
TFBS ENCODE
SuperEnhancer Hnisz
**Repressed Hoffman
Promoter UCSC
PromoterFlanking Hoffman
Intron UCSC
H3K9ac Trynka
H3K4me3 Trynka
H3K4me1 Trynka
H3K27ac PGC2
H3K27ac Hnisz
FetalDHS Trynka
Enhancer Hoffman
Enhancer Andersson
**DHS Trynka
DGF ENCODE
CTCF Hoffman
Conserved LindbladToh
Coding UCSC
Annotation
−10
0
10
20
30
Enrichment (prop h2 / prop snps)

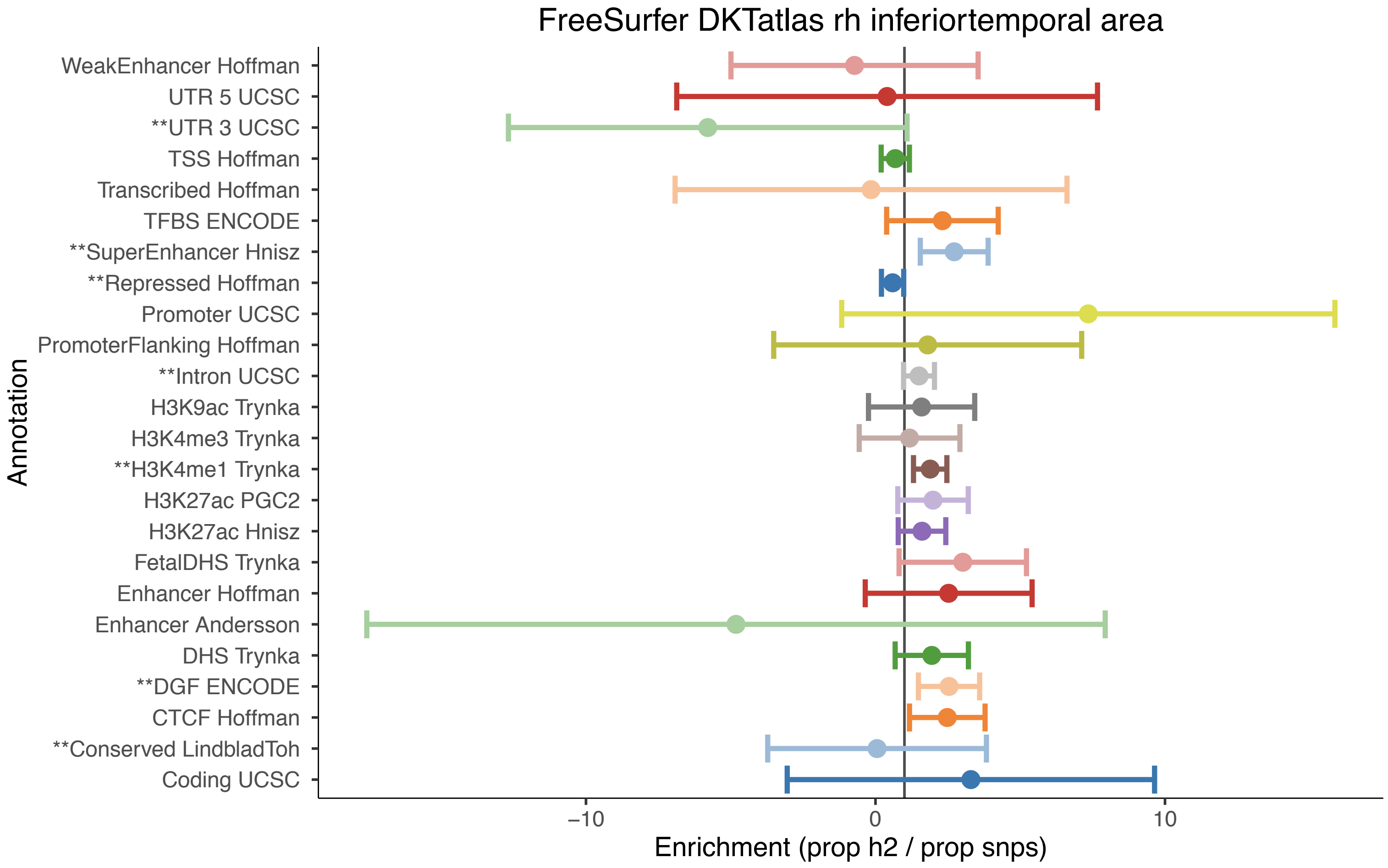

FreeSurfer DKTatlas rh inferiortemporal area
WeakEnhancer Hoffman
UTR 5 UCSC
**UTR 3 UCSC
TSS Hoffman
Transcribed Hoffman
TFBS ENCODE
**SuperEnhancer Hnisz
**Repressed Hoffman
Promoter UCSC
PromoterFlanking Hoffman
**Intron UCSC
H3K9ac Trynka
H3K4me3 Trynka
**H3K4me1 Trynka
H3K27ac PGC2
H3K27ac Hnisz
FetalDHS Trynka
Enhancer Hoffman
Enhancer Andersson
DHS Trynka
**DGF ENCODE
CTCF Hoffman
**Conserved LindbladToh
Coding UCSC
Annotation
−10
0
10
Enrichment (prop h2 / prop snps)

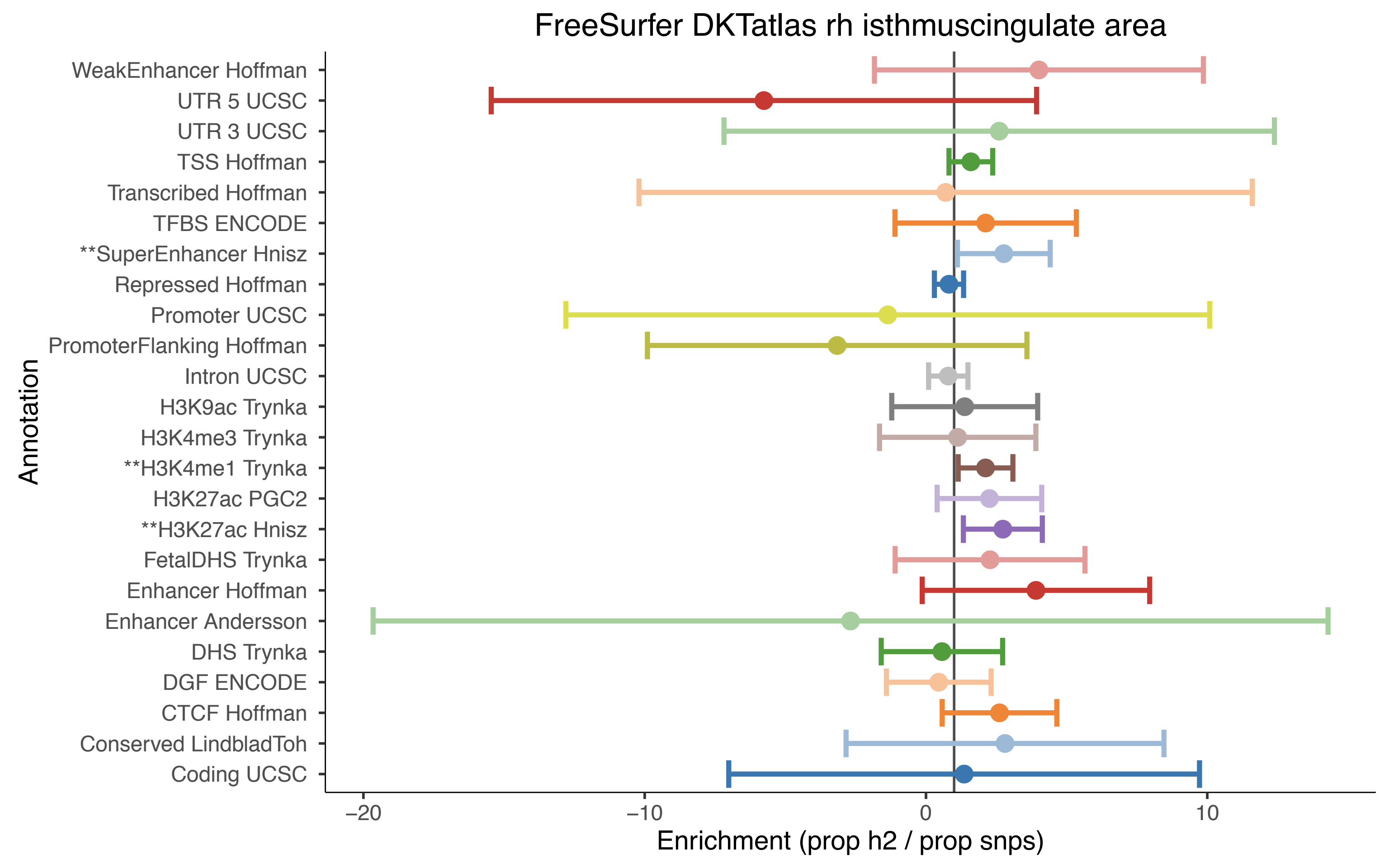

FreeSurfer DKTatlas rh isthmuscingulate area
WeakEnhancer Hoffman
UTR 5 UCSC
UTR 3 UCSC
TSS Hoffman
Transcribed Hoffman
TFBS ENCODE
**SuperEnhancer Hnisz
Repressed Hoffman
Promoter UCSC
PromoterFlanking Hoffman
Intron UCSC
H3K9ac Trynka
H3K4me3 Trynka
**H3K4me1 Trynka
H3K27ac PGC2
**H3K27ac Hnisz
FetalDHS Trynka
Enhancer Hoffman
Enhancer Andersson
DHS Trynka
DGF ENCODE
CTCF Hoffman
Conserved LindbladToh
Coding UCSC
Annotation
−20
−10
0
10
Enrichment (prop h2 / prop snps)

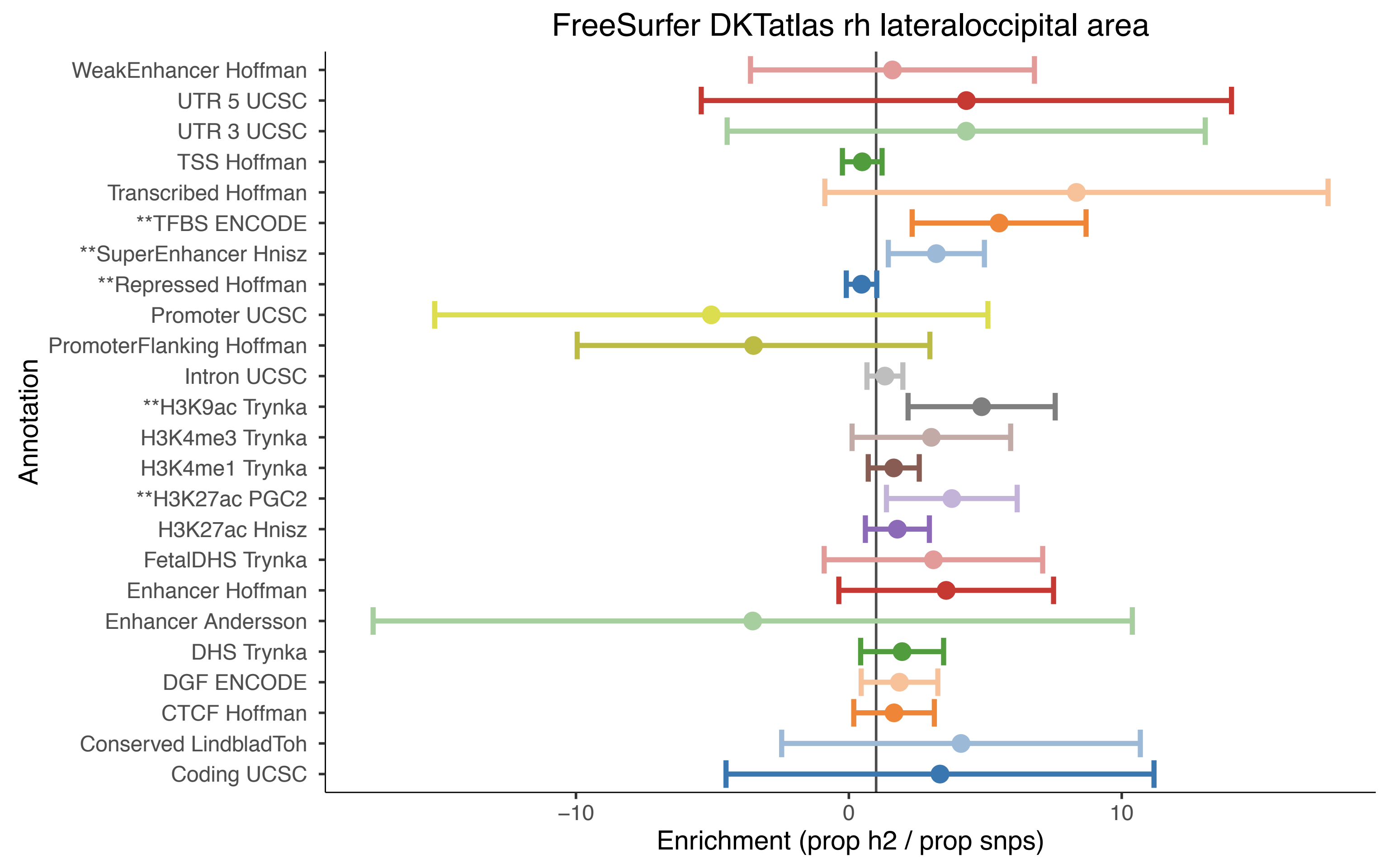
FreeSurfer DKTatlas rh lateraloccipital area
WeakEnhancer Hoffman
UTR 5 UCSC
UTR 3 UCSC
TSS Hoffman
Transcribed Hoffman
**TFBS ENCODE
**SuperEnhancer Hnisz
**Repressed Hoffman
Promoter UCSC
PromoterFlanking Hoffman
Intron UCSC
**H3K9ac Trynka
H3K4me3 Trynka
H3K4me1 Trynka
**H3K27ac PGC2
H3K27ac Hnisz
FetalDHS Trynka
Enhancer Hoffman
Enhancer Andersson
DHS Trynka
DGF ENCODE
CTCF Hoffman
Conserved LindbladToh
Coding UCSC
Annotation
−10
0
10
Enrichment (prop h2 / prop snps)

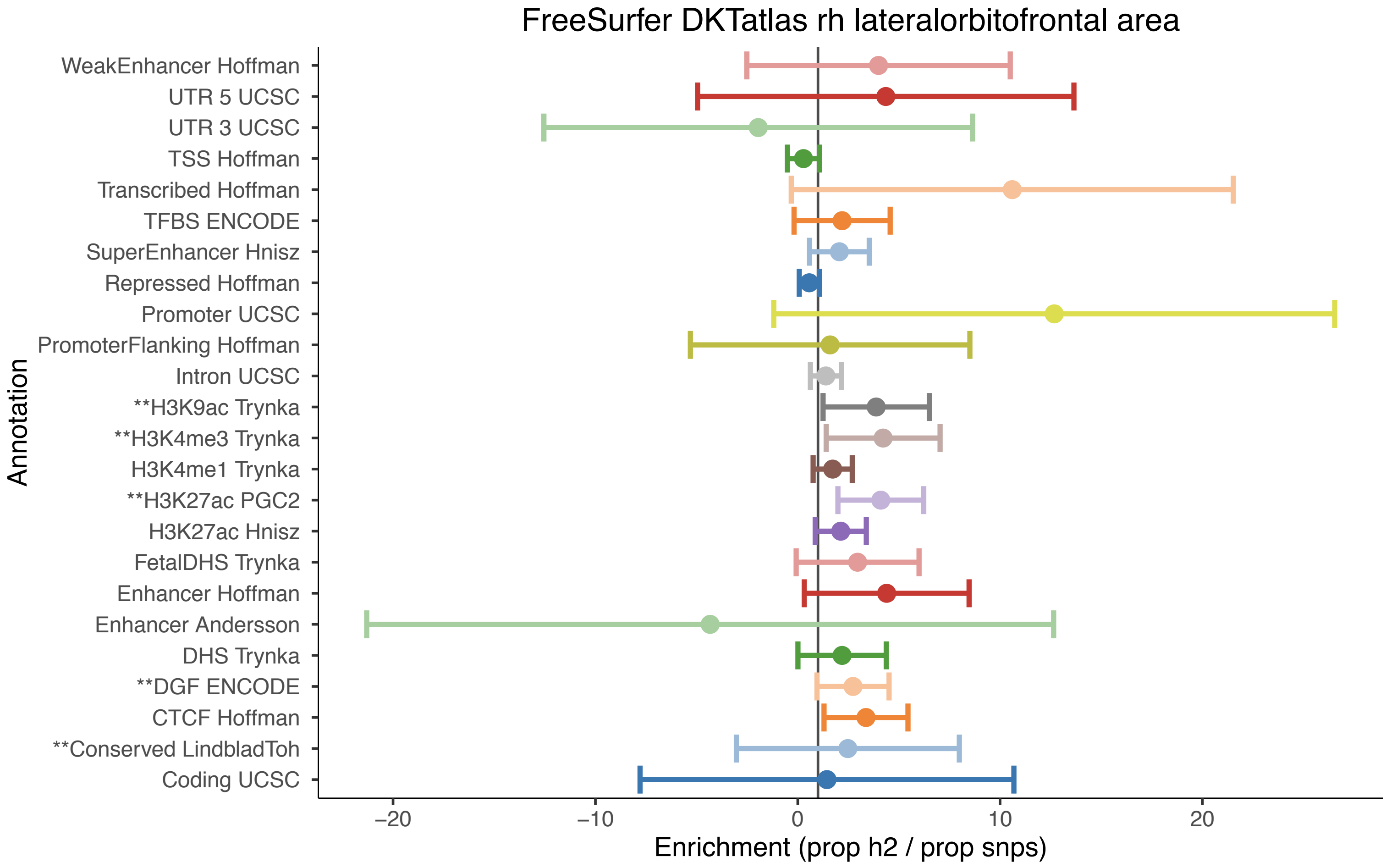

FreeSurfer DKTatlas rh lateralorbitofrontal area
WeakEnhancer Hoffman
UTR 5 UCSC
UTR 3 UCSC
TSS Hoffman
Transcribed Hoffman
TFBS ENCODE
SuperEnhancer Hnisz
Repressed Hoffman
Promoter UCSC
PromoterFlanking Hoffman
Intron UCSC
**H3K9ac Trynka
**H3K4me3 Trynka
H3K4me1 Trynka
**H3K27ac PGC2
H3K27ac Hnisz
FetalDHS Trynka
Enhancer Hoffman
Enhancer Andersson
DHS Trynka
**DGF ENCODE
CTCF Hoffman
**Conserved LindbladToh
Coding UCSC
Annotation
−20
−10
0
10
20
Enrichment (prop h2 / prop snps)

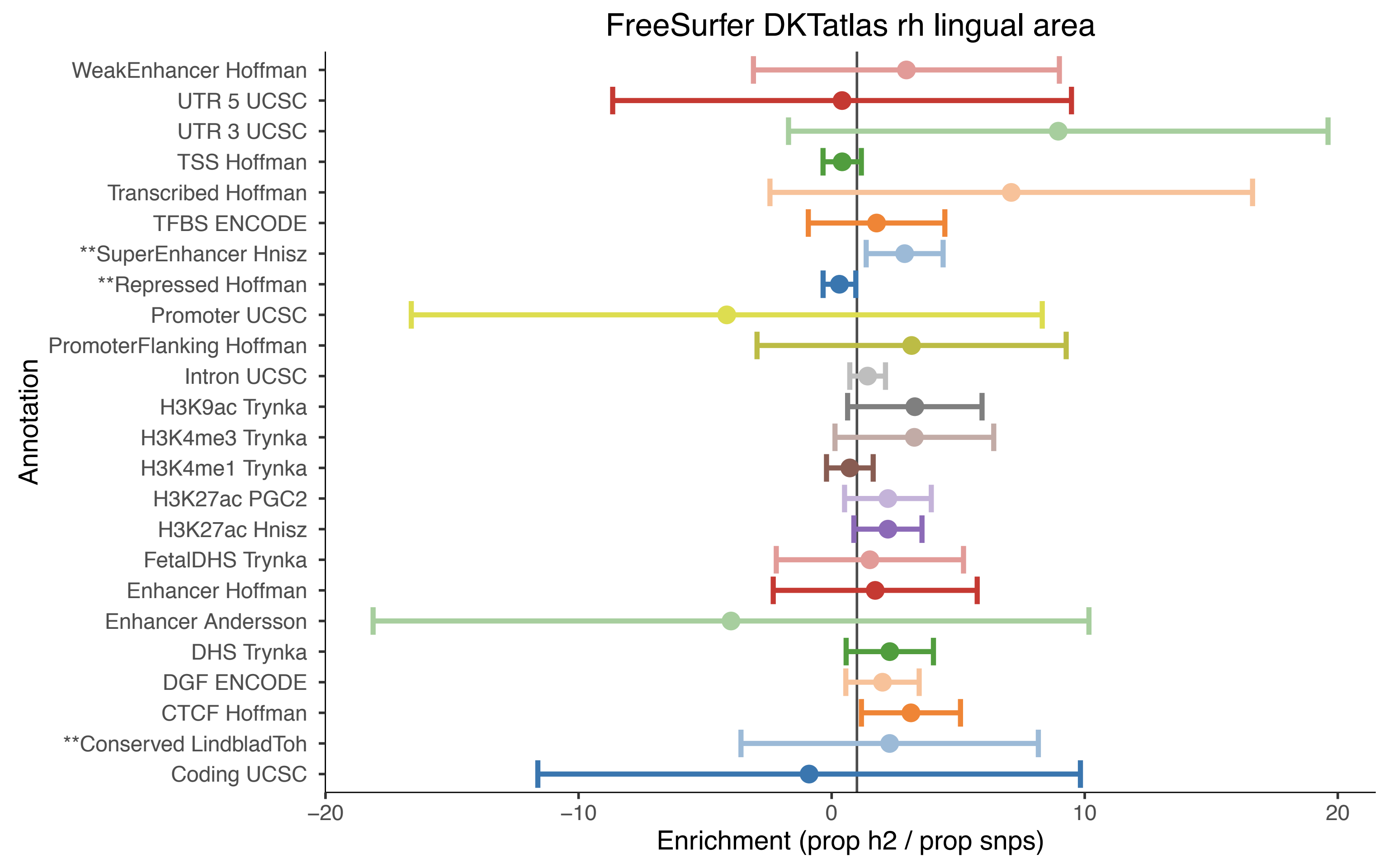

FreeSurfer DKTatlas rh lingual area
WeakEnhancer Hoffman
UTR 5 UCSC
UTR 3 UCSC
TSS Hoffman
Transcribed Hoffman
TFBS ENCODE
**SuperEnhancer Hnisz
**Repressed Hoffman
Promoter UCSC
PromoterFlanking Hoffman
Intron UCSC
H3K9ac Trynka
H3K4me3 Trynka
H3K4me1 Trynka
H3K27ac PGC2
H3K27ac Hnisz
FetalDHS Trynka
Enhancer Hoffman
Enhancer Andersson
DHS Trynka
DGF ENCODE
CTCF Hoffman
**Conserved LindbladToh
Coding UCSC
Annotation
−20
−10
0
10
20
Enrichment (prop h2 / prop snps)

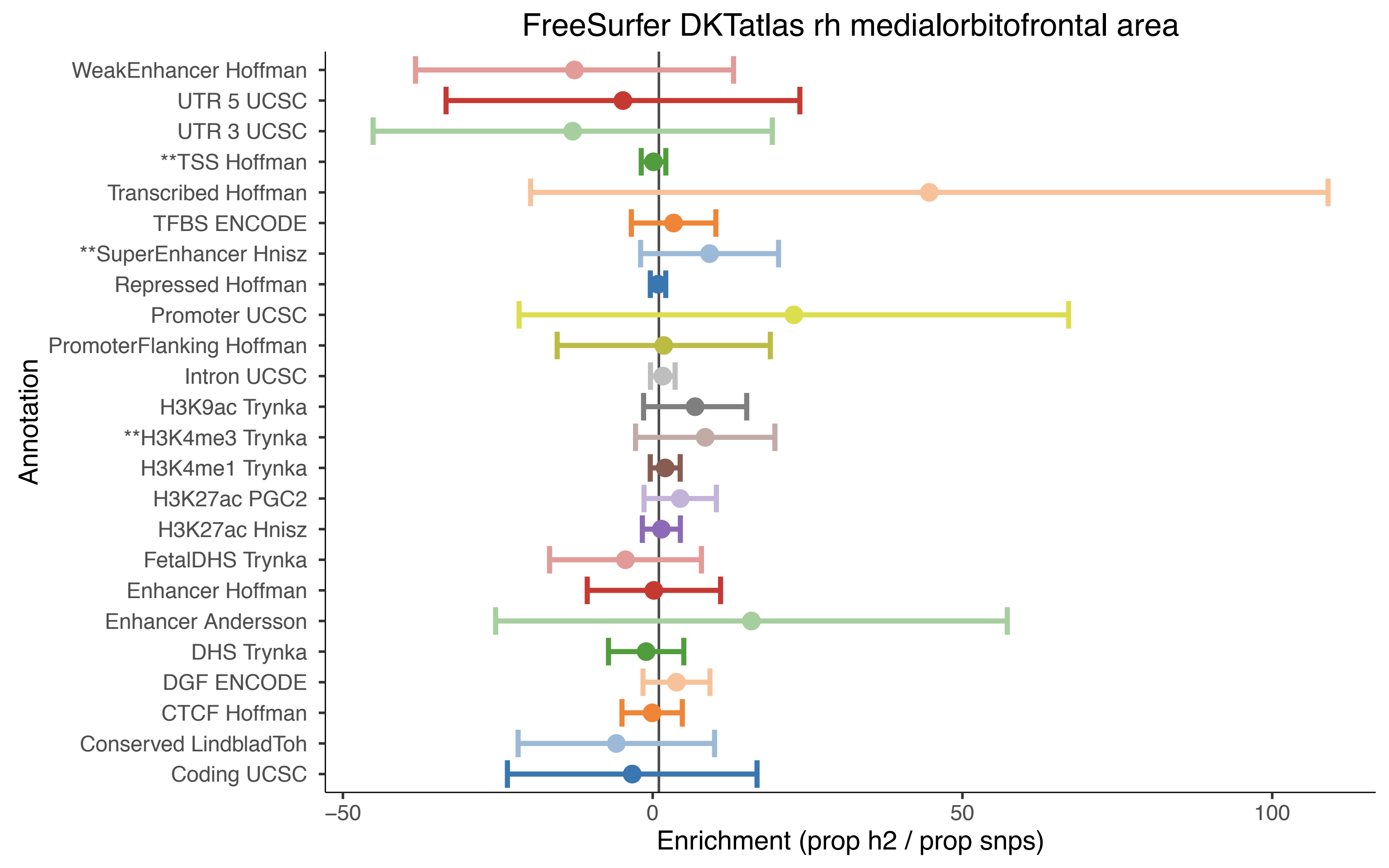
FreeSurfer DKTatlas rh medialorbitofrontal area
WeakEnhancer Hoffman
UTR 5 UCSC
UTR 3 UCSC
**TSS Hoffman
Transcribed Hoffman
TFBS ENCODE
**SuperEnhancer Hnisz
Repressed Hoffman
Promoter UCSC
PromoterFlanking Hoffman
Intron UCSC
H3K9ac Trynka
**H3K4me3 Trynka
H3K4me1 Trynka
H3K27ac PGC2
H3K27ac Hnisz
FetalDHS Trynka
Enhancer Hoffman
Enhancer Andersson
DHS Trynka
DGF ENCODE
CTCF Hoffman
Conserved LindbladToh
Coding UCSC
Annotation
−50
0
50
100
Enrichment (prop h2 / prop snps)

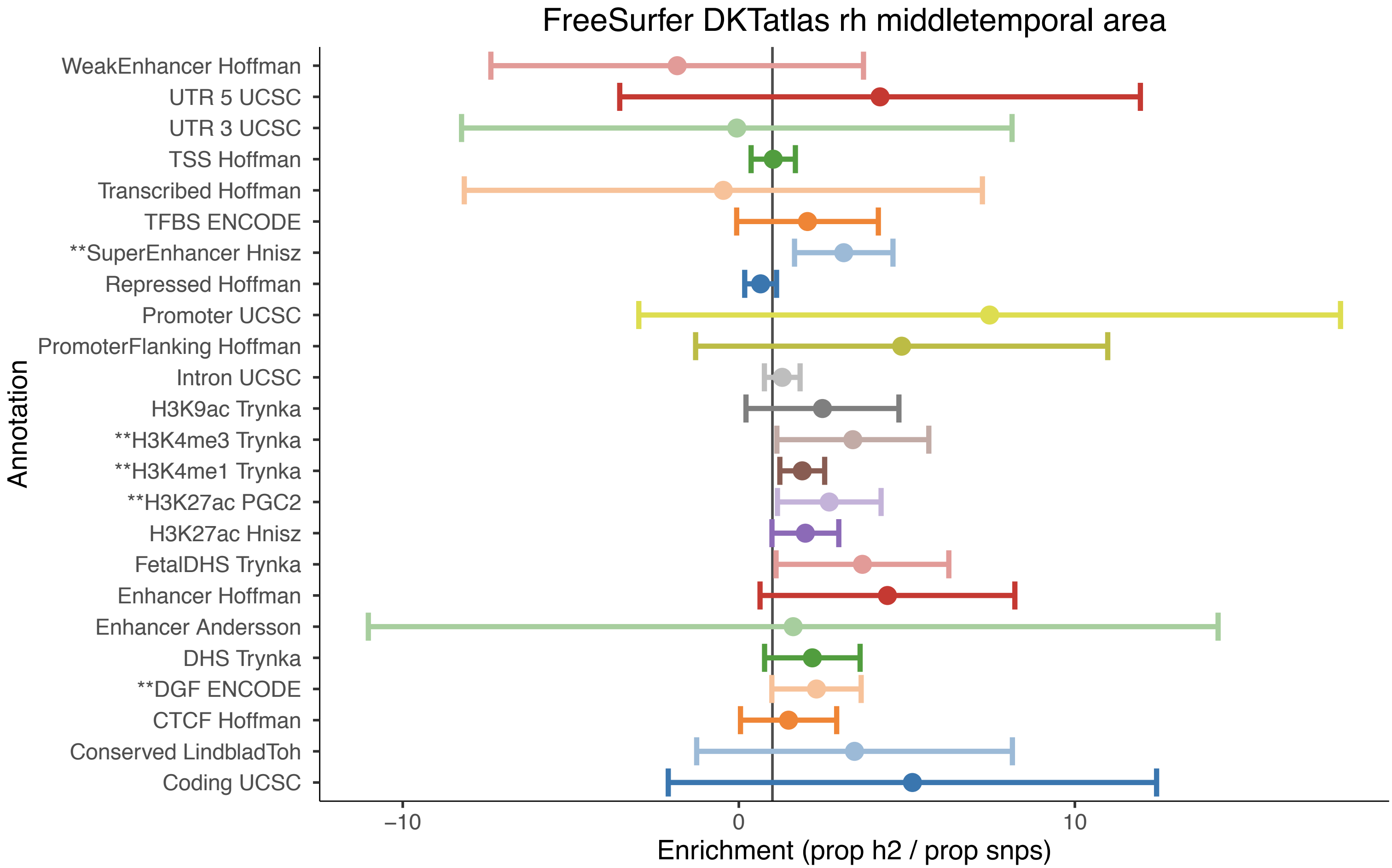
FreeSurfer DKTatlas rh middletemporal area
WeakEnhancer Hoffman
UTR 5 UCSC
UTR 3 UCSC
TSS Hoffman
Transcribed Hoffman
TFBS ENCODE
**SuperEnhancer Hnisz
Repressed Hoffman
Promoter UCSC
PromoterFlanking Hoffman
Intron UCSC
H3K9ac Trynka
**H3K4me3 Trynka
**H3K4me1 Trynka
**H3K27ac PGC2
H3K27ac Hnisz
FetalDHS Trynka
Enhancer Hoffman
Enhancer Andersson
DHS Trynka
**DGF ENCODE
CTCF Hoffman
Conserved LindbladToh
Coding UCSC
Annotation
−10
0
10
Enrichment (prop h2 / prop snps)

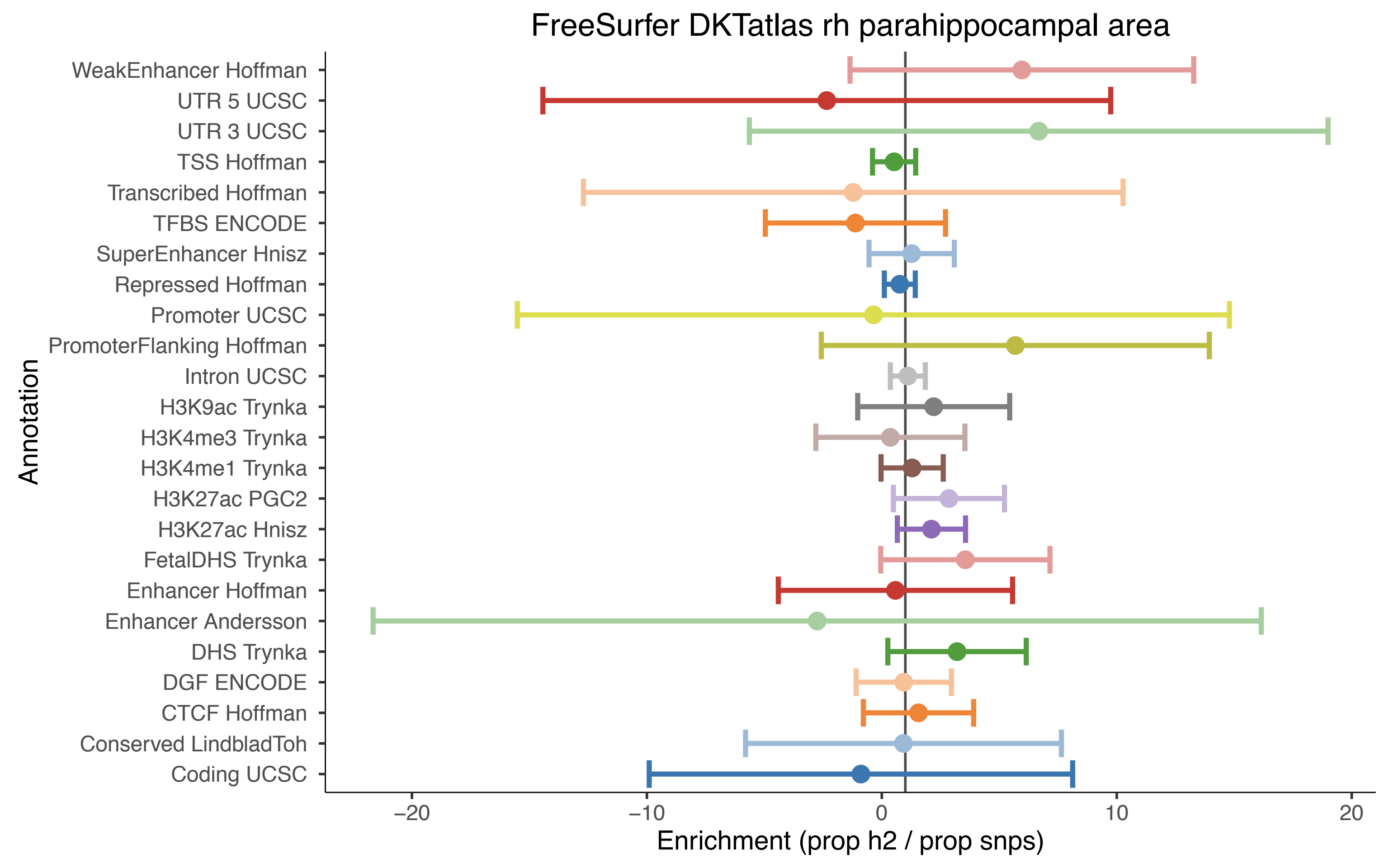
FreeSurfer DKTatlas rh parahippocampal area
WeakEnhancer Hoffman
UTR 5 UCSC
UTR 3 UCSC
TSS Hoffman
Transcribed Hoffman
TFBS ENCODE
SuperEnhancer Hnisz
Repressed Hoffman
Promoter UCSC
PromoterFlanking Hoffman
Intron UCSC
H3K9ac Trynka
H3K4me3 Trynka
H3K4me1 Trynka
H3K27ac PGC2
H3K27ac Hnisz
FetalDHS Trynka
Enhancer Hoffman
Enhancer Andersson
DHS Trynka
DGF ENCODE
CTCF Hoffman
Conserved LindbladToh
Coding UCSC
Annotation
−20
−10
0
10
20
Enrichment (prop h2 / prop snps)

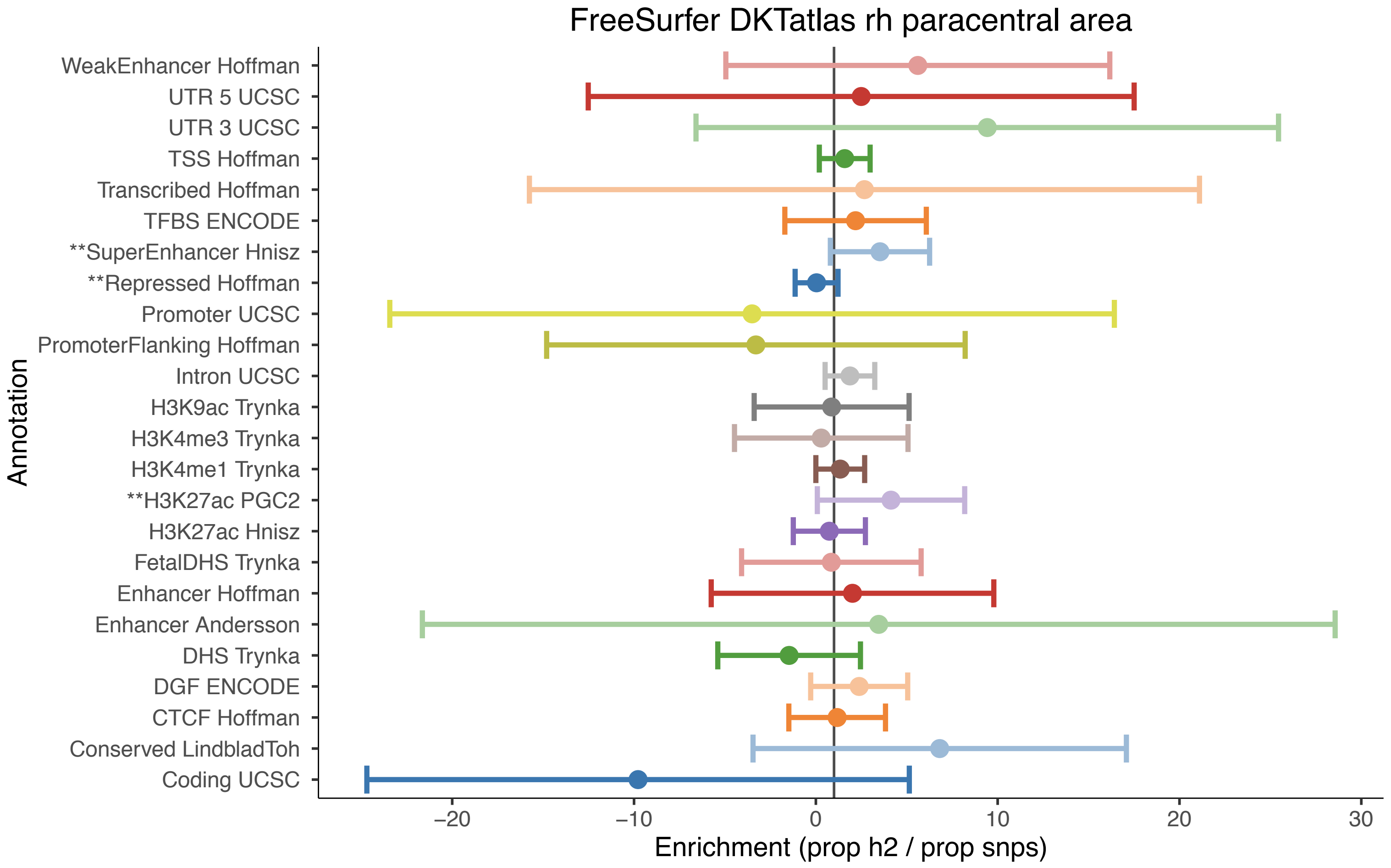
FreeSurfer DKTatlas rh paracentral area
WeakEnhancer Hoffman
UTR 5 UCSC
UTR 3 UCSC
TSS Hoffman
Transcribed Hoffman
TFBS ENCODE
**SuperEnhancer Hnisz
**Repressed Hoffman
Promoter UCSC
PromoterFlanking Hoffman
Intron UCSC
H3K9ac Trynka
H3K4me3 Trynka
H3K4me1 Trynka
**H3K27ac PGC2
H3K27ac Hnisz
FetalDHS Trynka
Enhancer Hoffman
Enhancer Andersson
DHS Trynka
DGF ENCODE
CTCF Hoffman
Conserved LindbladToh
Coding UCSC
Annotation
−20
−10
0
10
20
30
Enrichment (prop h2 / prop snps)

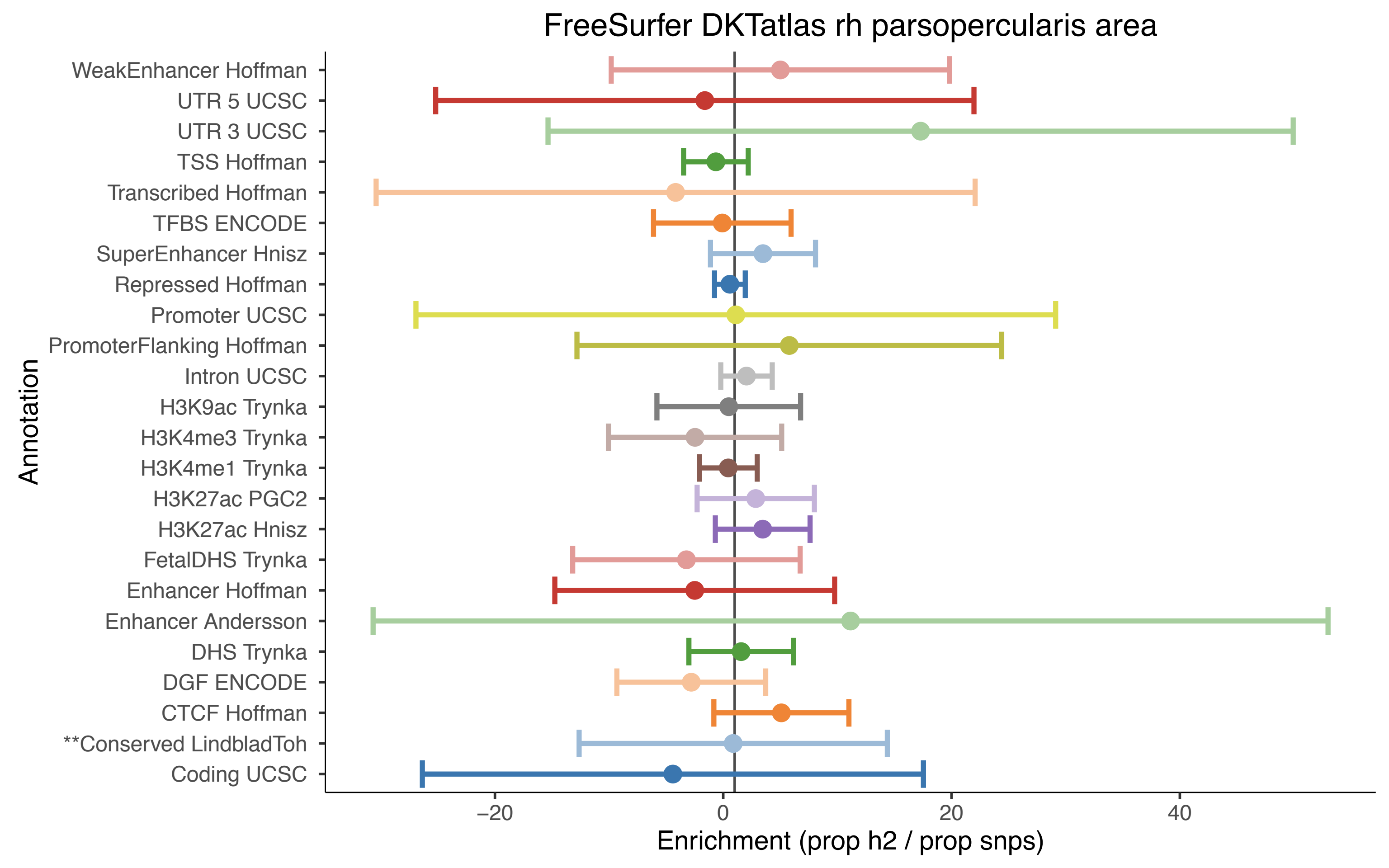
FreeSurfer DKTatlas rh parsopercularis area
WeakEnhancer Hoffman
UTR 5 UCSC
UTR 3 UCSC
TSS Hoffman
Transcribed Hoffman
TFBS ENCODE
SuperEnhancer Hnisz
Repressed Hoffman
Promoter UCSC
PromoterFlanking Hoffman
Intron UCSC
H3K9ac Trynka
H3K4me3 Trynka
H3K4me1 Trynka
H3K27ac PGC2
H3K27ac Hnisz
FetalDHS Trynka
Enhancer Hoffman
Enhancer Andersson
DHS Trynka
DGF ENCODE
CTCF Hoffman
**Conserved LindbladToh
Coding UCSC
Annotation
−20
0
20
40
Enrichment (prop h2 / prop snps)

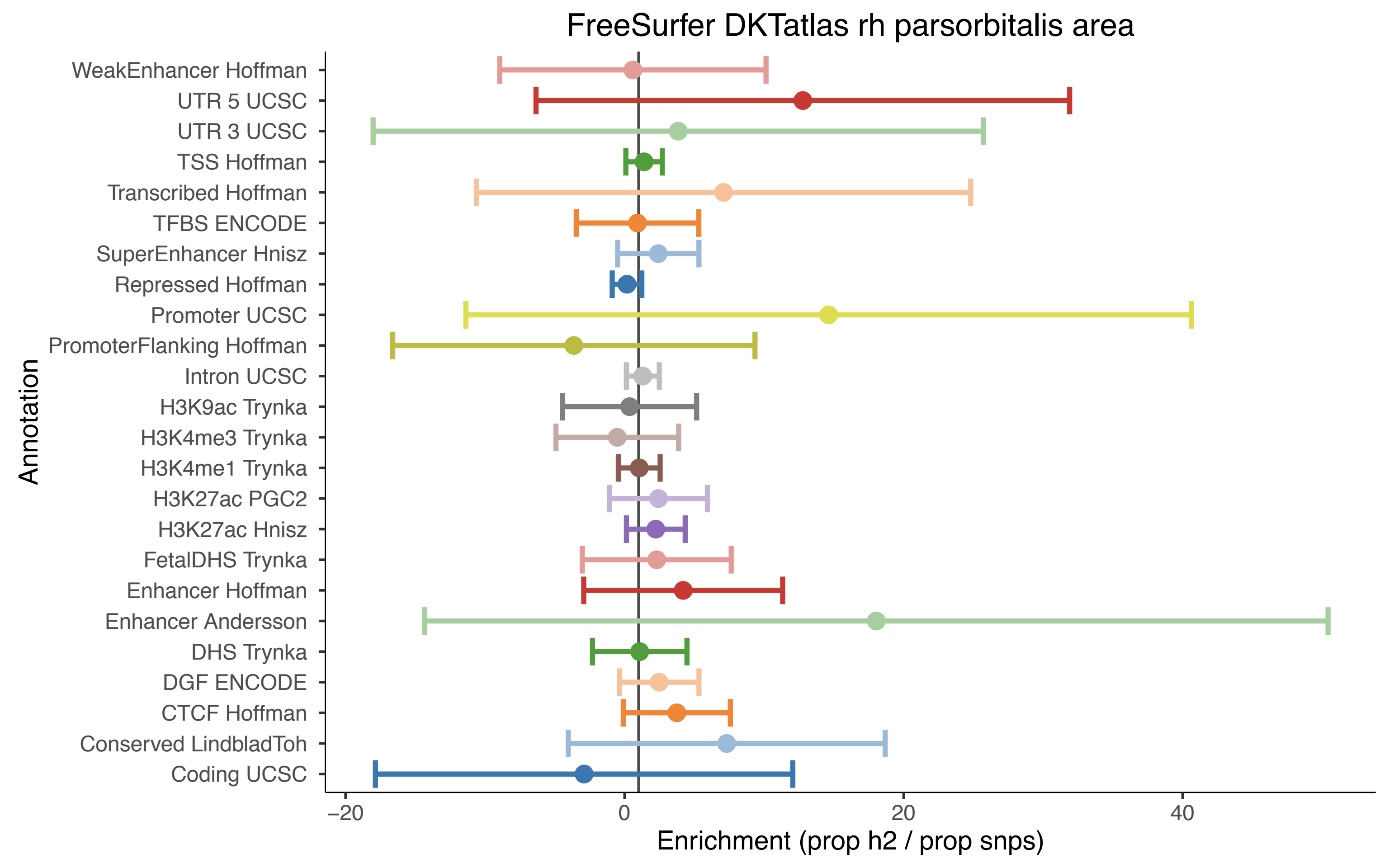

FreeSurfer DKTatlas rh parsorbitalis area
WeakEnhancer Hoffman
UTR 5 UCSC
UTR 3 UCSC
TSS Hoffman
Transcribed Hoffman
TFBS ENCODE
SuperEnhancer Hnisz
Repressed Hoffman
Promoter UCSC
PromoterFlanking Hoffman
Intron UCSC
H3K9ac Trynka
H3K4me3 Trynka
H3K4me1 Trynka
H3K27ac PGC2
H3K27ac Hnisz
FetalDHS Trynka
Enhancer Hoffman
Enhancer Andersson
DHS Trynka
DGF ENCODE
CTCF Hoffman
Conserved LindbladToh
Coding UCSC
Annotation
−20
0
20
40
Enrichment (prop h2 / prop snps)

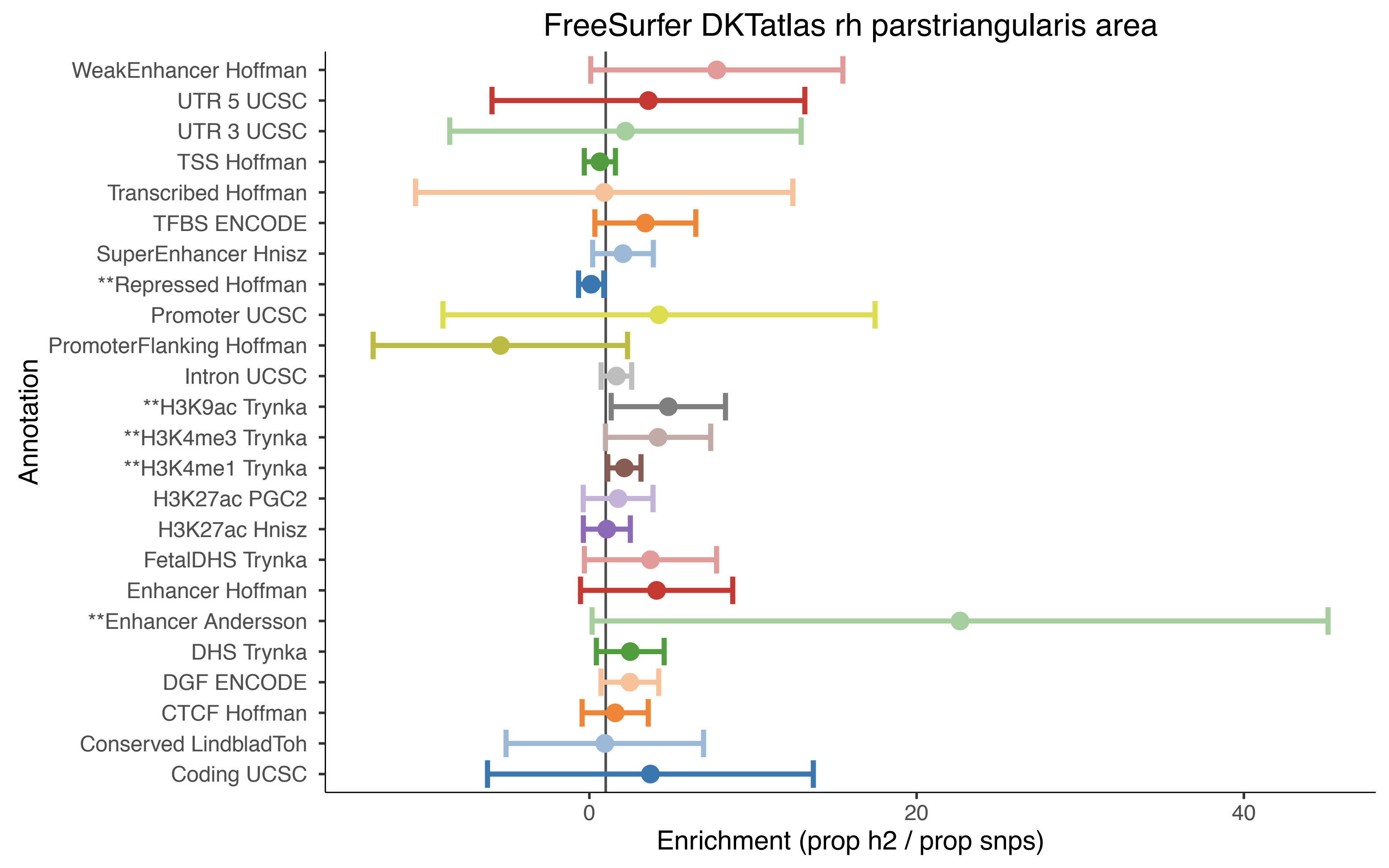
FreeSurfer DKTatlas rh parstriangularis area
WeakEnhancer Hoffman
UTR 5 UCSC
UTR 3 UCSC
TSS Hoffman
Transcribed Hoffman
TFBS ENCODE
SuperEnhancer Hnisz
**Repressed Hoffman
Promoter UCSC
PromoterFlanking Hoffman
Intron UCSC
**H3K9ac Trynka
**H3K4me3 Trynka
**H3K4me1 Trynka
H3K27ac PGC2
H3K27ac Hnisz
FetalDHS Trynka
Enhancer Hoffman
**Enhancer Andersson
DHS Trynka
DGF ENCODE
CTCF Hoffman
Conserved LindbladToh
Coding UCSC
Annotation
0
20
40
Enrichment (prop h2 / prop snps)

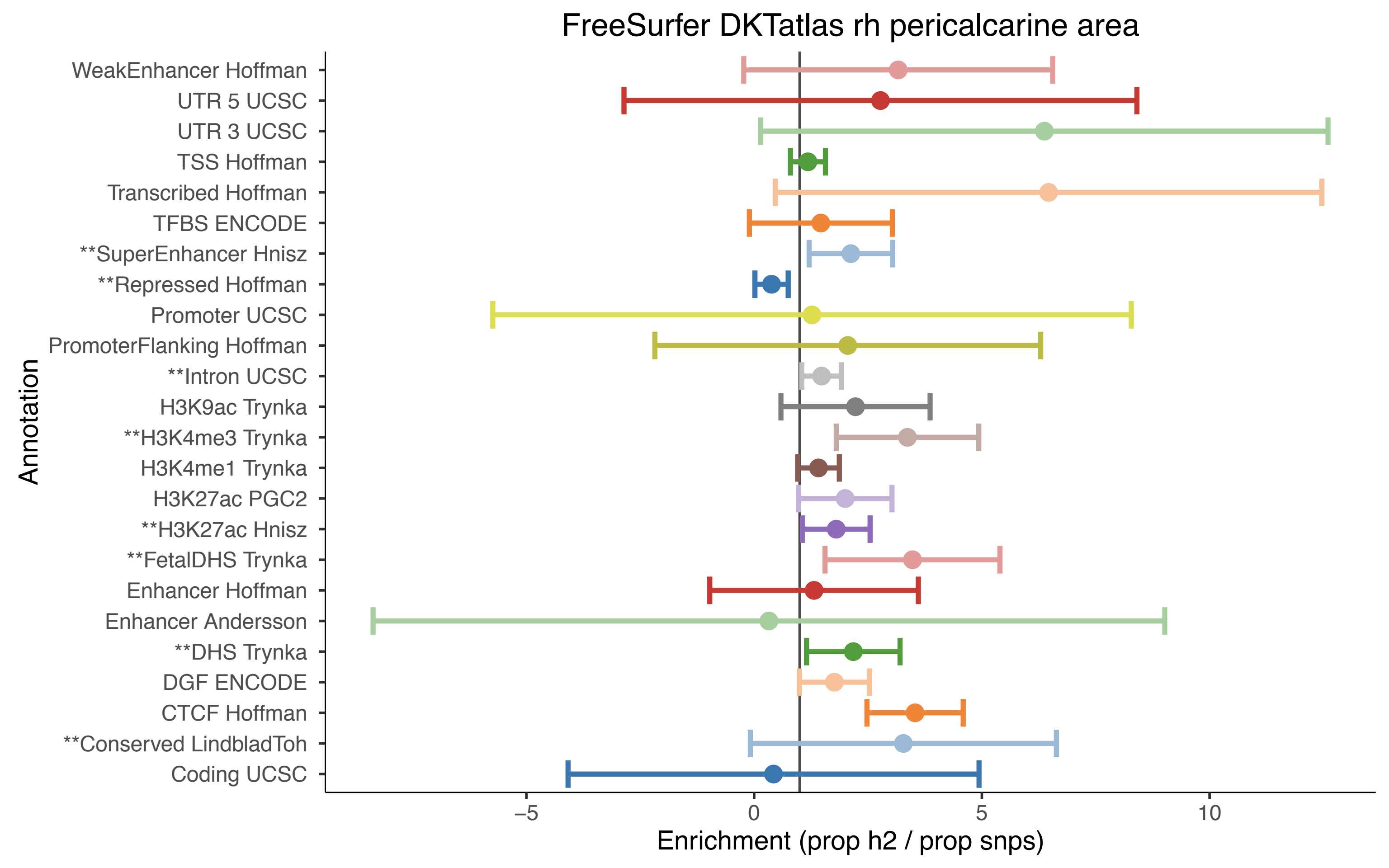
FreeSurfer DKTatlas rh pericalcarine area
WeakEnhancer Hoffman
UTR 5 UCSC
UTR 3 UCSC
TSS Hoffman
Transcribed Hoffman
TFBS ENCODE
**SuperEnhancer Hnisz
**Repressed Hoffman
Promoter UCSC
PromoterFlanking Hoffman
**Intron UCSC
H3K9ac Trynka
**H3K4me3 Trynka
H3K4me1 Trynka
H3K27ac PGC2
**H3K27ac Hnisz
**FetalDHS Trynka
Enhancer Hoffman
Enhancer Andersson
**DHS Trynka
DGF ENCODE
CTCF Hoffman
**Conserved LindbladToh
Coding UCSC
Annotation
−5
0
5
10
Enrichment (prop h2 / prop snps)

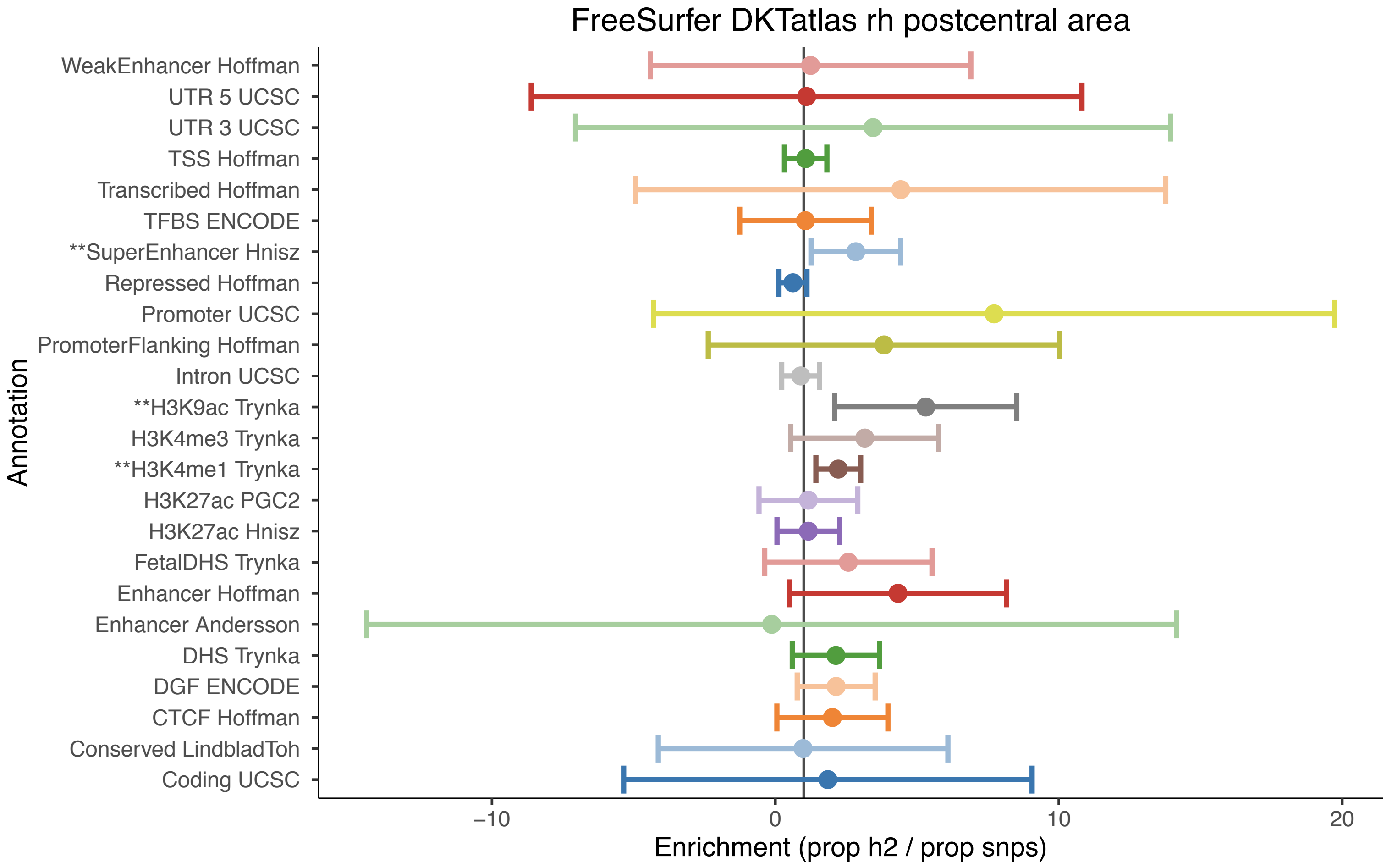
FreeSurfer DKTatlas rh postcentral area
WeakEnhancer Hoffman
UTR 5 UCSC
UTR 3 UCSC
TSS Hoffman
Transcribed Hoffman
TFBS ENCODE
**SuperEnhancer Hnisz
Repressed Hoffman
Promoter UCSC
PromoterFlanking Hoffman
Intron UCSC
**H3K9ac Trynka
H3K4me3 Trynka
**H3K4me1 Trynka
H3K27ac PGC2
H3K27ac Hnisz
FetalDHS Trynka
Enhancer Hoffman
Enhancer Andersson
DHS Trynka
DGF ENCODE
CTCF Hoffman
Conserved LindbladToh
Coding UCSC
Annotation
−10
0
10
20
Enrichment (prop h2 / prop snps)

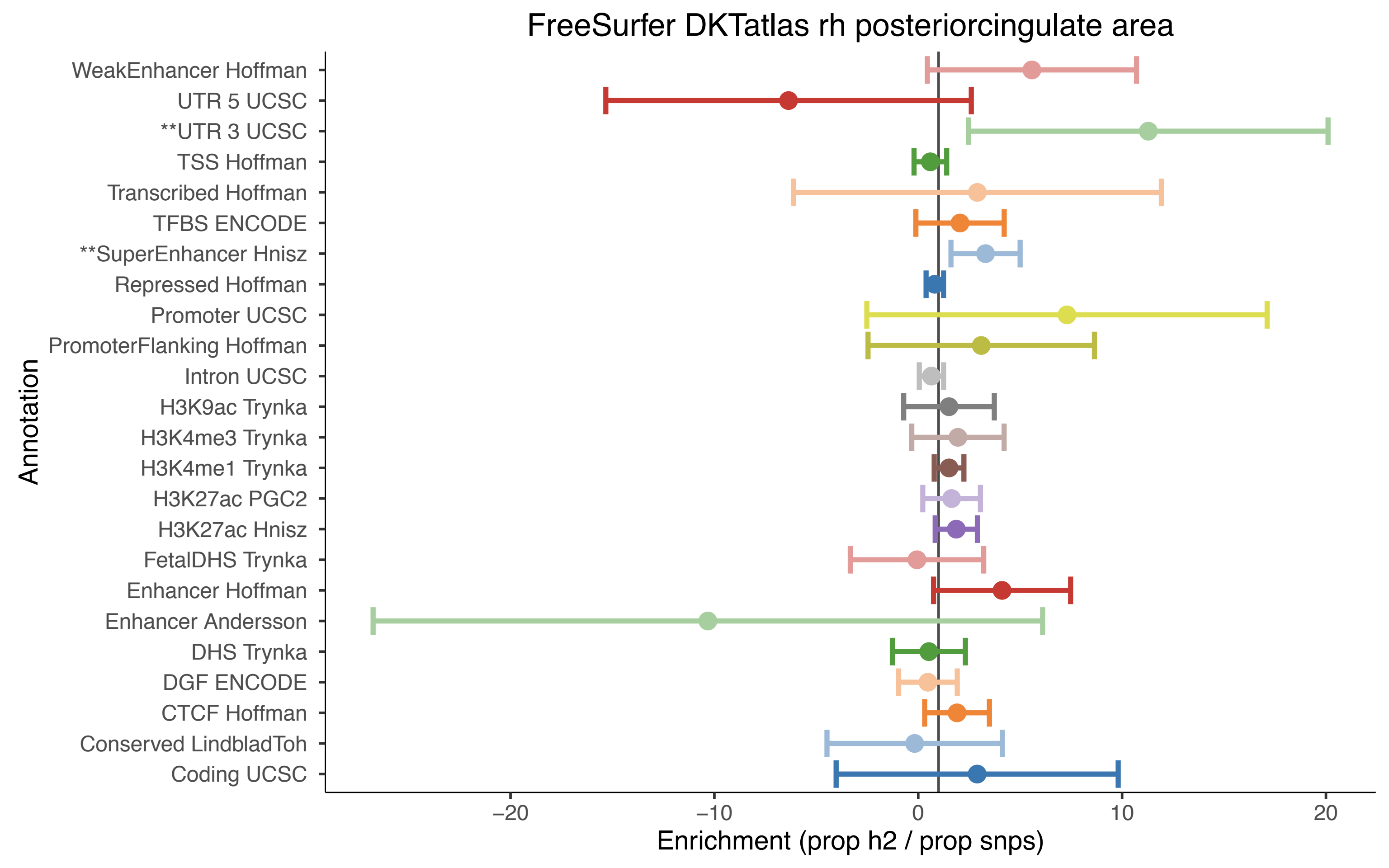

FreeSurfer DKTatlas rh posteriorcingulate area
WeakEnhancer Hoffman
UTR 5 UCSC
**UTR 3 UCSC
TSS Hoffman
Transcribed Hoffman
TFBS ENCODE
**SuperEnhancer Hnisz
Repressed Hoffman
Promoter UCSC
PromoterFlanking Hoffman
Intron UCSC
H3K9ac Trynka
H3K4me3 Trynka
H3K4me1 Trynka
H3K27ac PGC2
H3K27ac Hnisz
FetalDHS Trynka
Enhancer Hoffman
Enhancer Andersson
DHS Trynka
DGF ENCODE
CTCF Hoffman
Conserved LindbladToh
Coding UCSC
Annotation
−20
−10
0
10
20
Enrichment (prop h2 / prop snps)

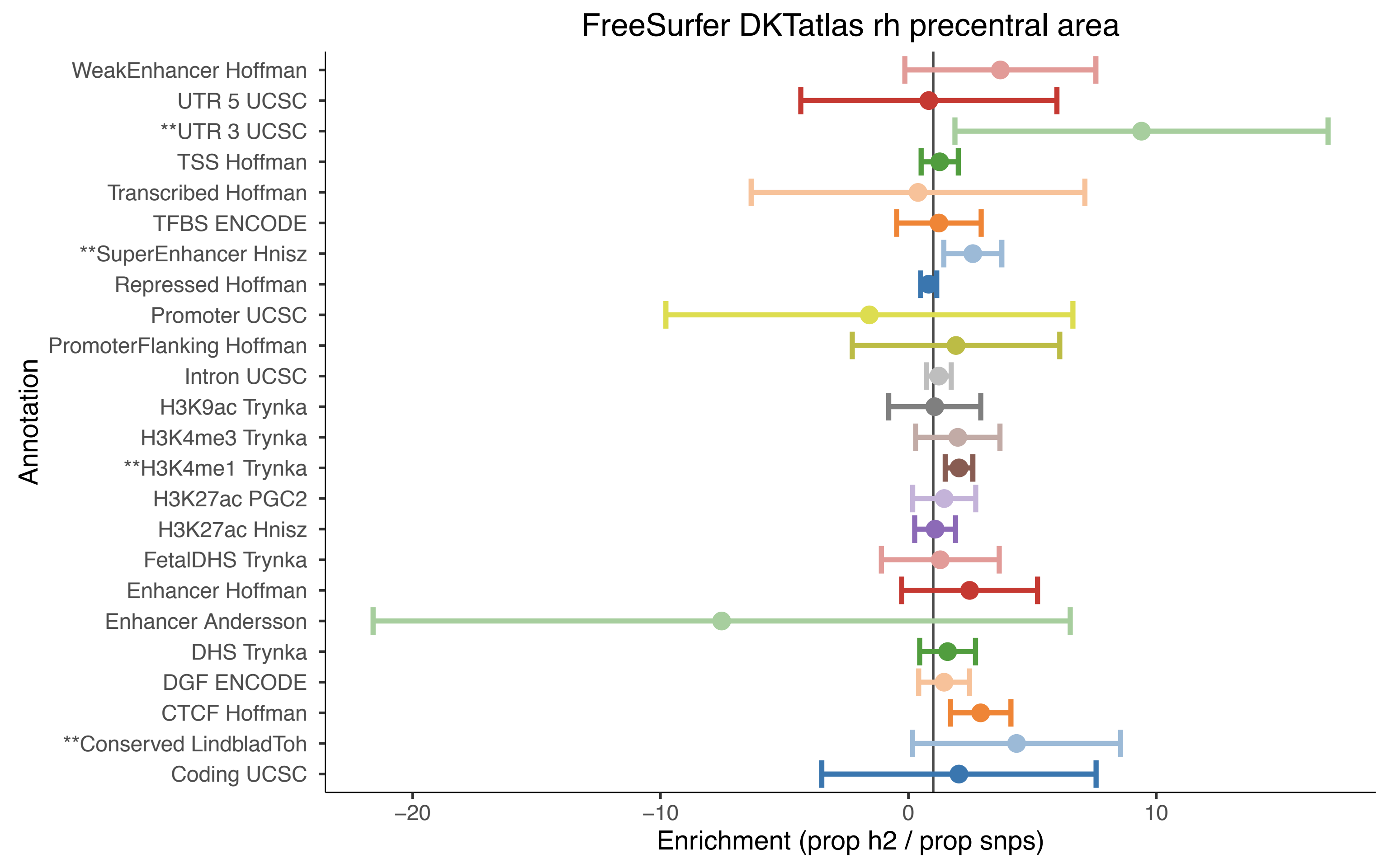
FreeSurfer DKTatlas rh precentral area
WeakEnhancer Hoffman
UTR 5 UCSC
**UTR 3 UCSC
TSS Hoffman
Transcribed Hoffman
TFBS ENCODE
**SuperEnhancer Hnisz
Repressed Hoffman
Promoter UCSC
PromoterFlanking Hoffman
Intron UCSC
H3K9ac Trynka
H3K4me3 Trynka
**H3K4me1 Trynka
H3K27ac PGC2
H3K27ac Hnisz
FetalDHS Trynka
Enhancer Hoffman
Enhancer Andersson
DHS Trynka
DGF ENCODE
CTCF Hoffman
**Conserved LindbladToh
Coding UCSC
Annotation
−20
−10
0
10
Enrichment (prop h2 / prop snps)

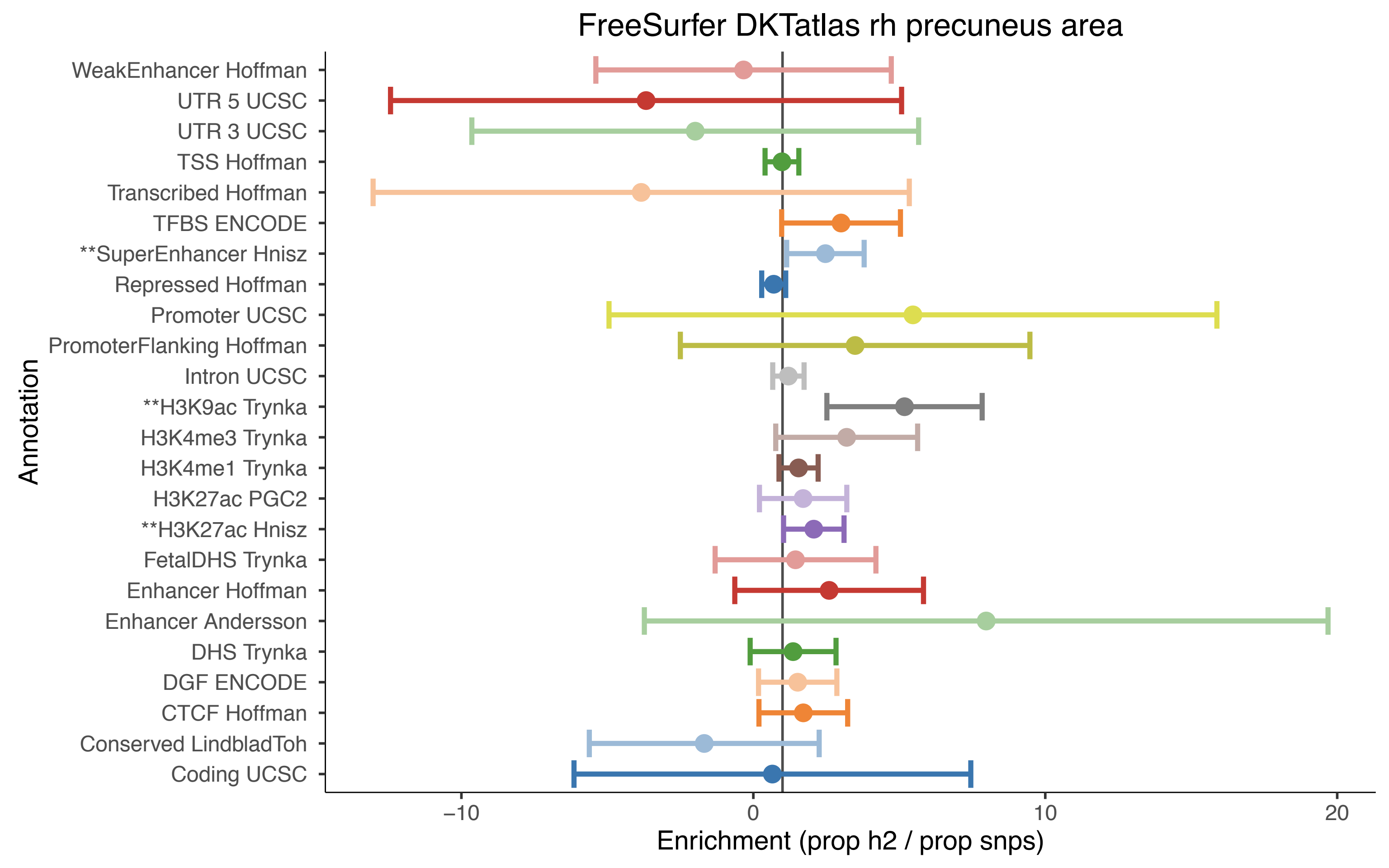
FreeSurfer DKTatlas rh precuneus area
Annotation
WeakEnhancer Hoffman
UTR 5 UCSC
UTR 3 UCSC
TSS Hoffman
Transcribed Hoffman
TFBS ENCODE
**SuperEnhancer Hnisz
Repressed Hoffman
Promoter UCSC
PromoterFlanking Hoffman
Intron UCSC
**H3K9ac Trynka
H3K4me3 Trynka
H3K4me1 Trynka
H3K27ac PGC2
**H3K27ac Hnisz
FetalDHS Trynka
Enhancer Hoffman
Enhancer Andersson
DHS Trynka
DGF ENCODE
CTCF Hoffman
Conserved LindbladToh
Coding UCSC
−10
0
10
20
Enrichment (prop h2 / prop snps)

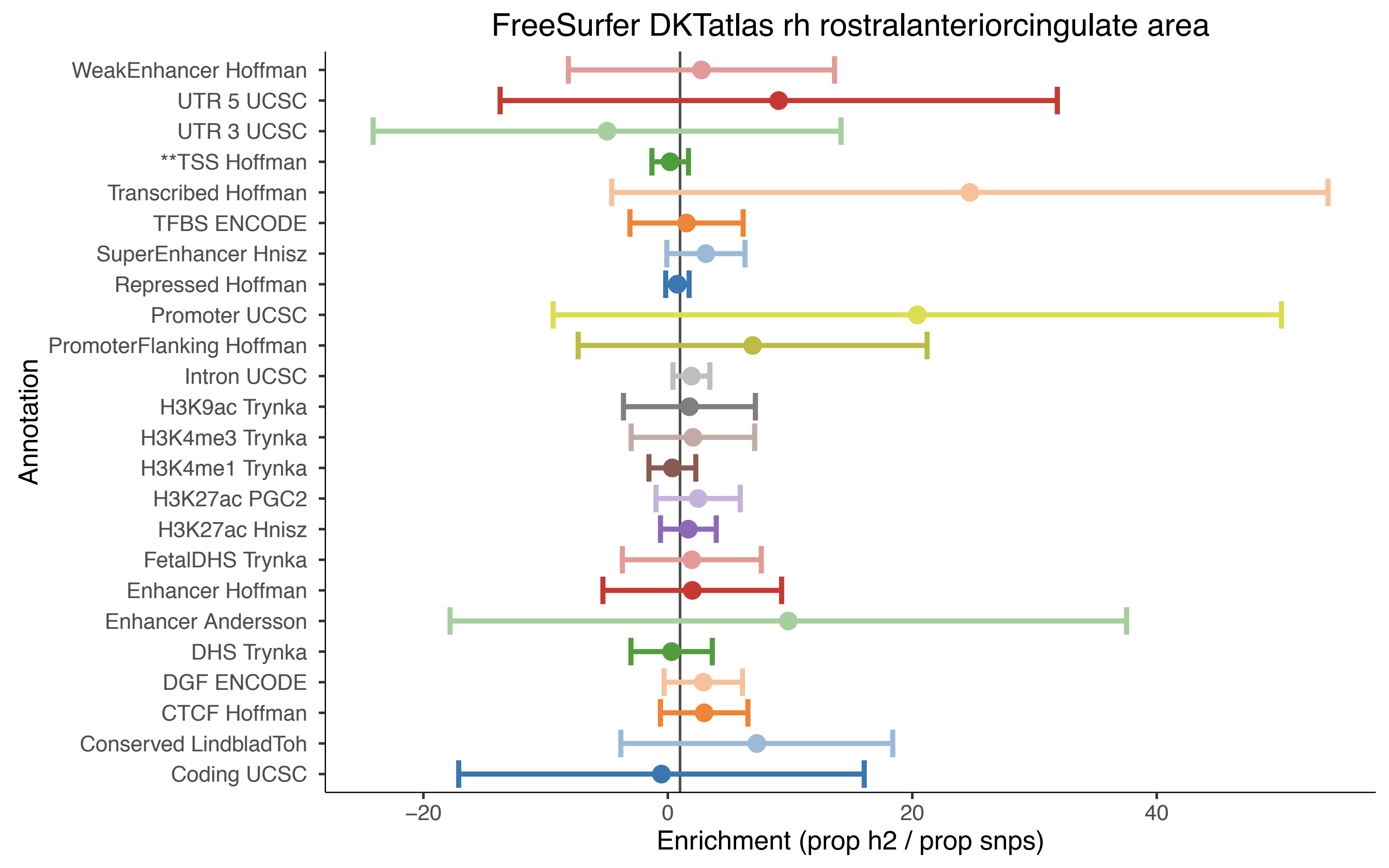
FreeSurfer DKTatlas rh rostralanteriorcingulate area
WeakEnhancer Hoffman
UTR 5 UCSC
UTR 3 UCSC
**TSS Hoffman
Transcribed Hoffman
TFBS ENCODE
SuperEnhancer Hnisz
Repressed Hoffman
Promoter UCSC
PromoterFlanking Hoffman
Intron UCSC
H3K9ac Trynka
H3K4me3 Trynka
H3K4me1 Trynka
H3K27ac PGC2
H3K27ac Hnisz
FetalDHS Trynka
Enhancer Hoffman
Enhancer Andersson
DHS Trynka
DGF ENCODE
CTCF Hoffman
Conserved LindbladToh
Coding UCSC
Annotation
−20
0
20
40
Enrichment (prop h2 / prop snps)

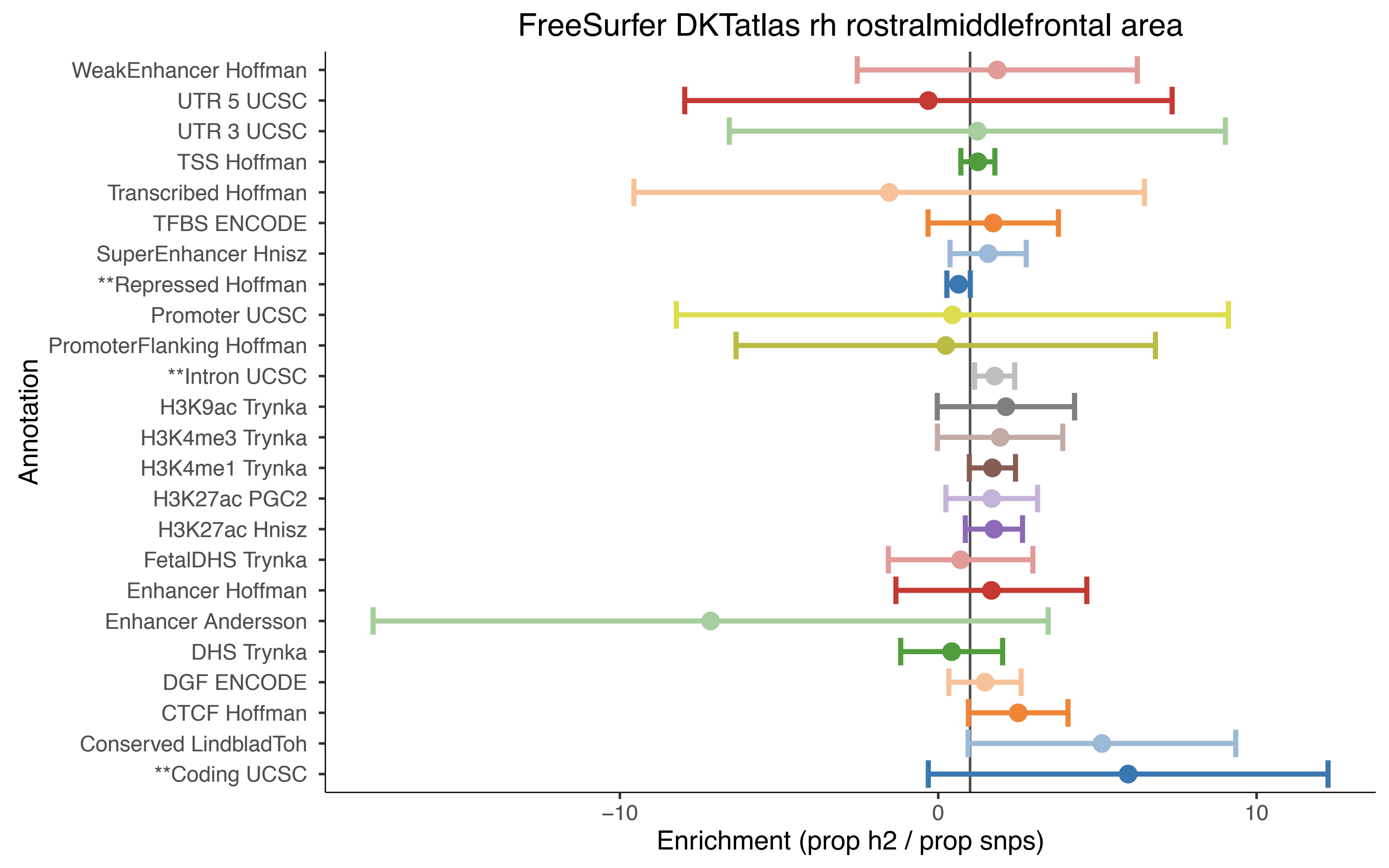
FreeSurfer DKTatlas rh rostralmiddlefrontal area
WeakEnhancer Hoffman
UTR 5 UCSC
UTR 3 UCSC
TSS Hoffman
Transcribed Hoffman
TFBS ENCODE
SuperEnhancer Hnisz
**Repressed Hoffman
Promoter UCSC
PromoterFlanking Hoffman
**Intron UCSC
H3K9ac Trynka
H3K4me3 Trynka
H3K4me1 Trynka
H3K27ac PGC2
H3K27ac Hnisz
FetalDHS Trynka
Enhancer Hoffman
Enhancer Andersson
DHS Trynka
DGF ENCODE
CTCF Hoffman
Conserved LindbladToh
**Coding UCSC
Annotation
−10
0
10
Enrichment (prop h2 / prop snps)

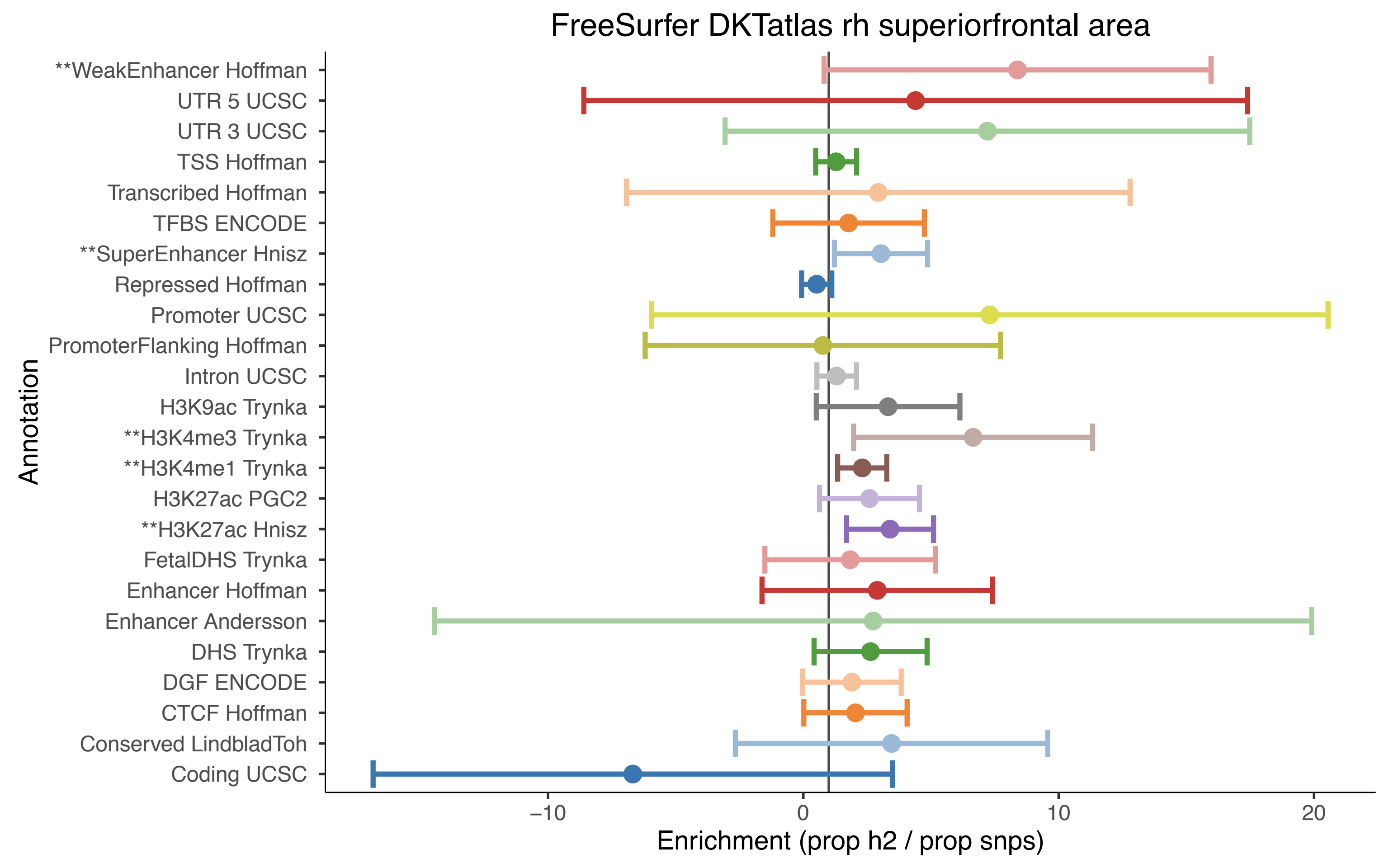
FreeSurfer DKTatlas rh superiorfrontal area
**WeakEnhancer Hoffman
UTR 5 UCSC
UTR 3 UCSC
TSS Hoffman
Transcribed Hoffman
TFBS ENCODE
**SuperEnhancer Hnisz
Repressed Hoffman
Promoter UCSC
PromoterFlanking Hoffman
Intron UCSC
H3K9ac Trynka
**H3K4me3 Trynka
**H3K4me1 Trynka
H3K27ac PGC2
**H3K27ac Hnisz
FetalDHS Trynka
Enhancer Hoffman
Enhancer Andersson
DHS Trynka
DGF ENCODE
CTCF Hoffman
Conserved LindbladToh
Coding UCSC
Annotation
−10
0
10
20
Enrichment (prop h2 / prop snps)

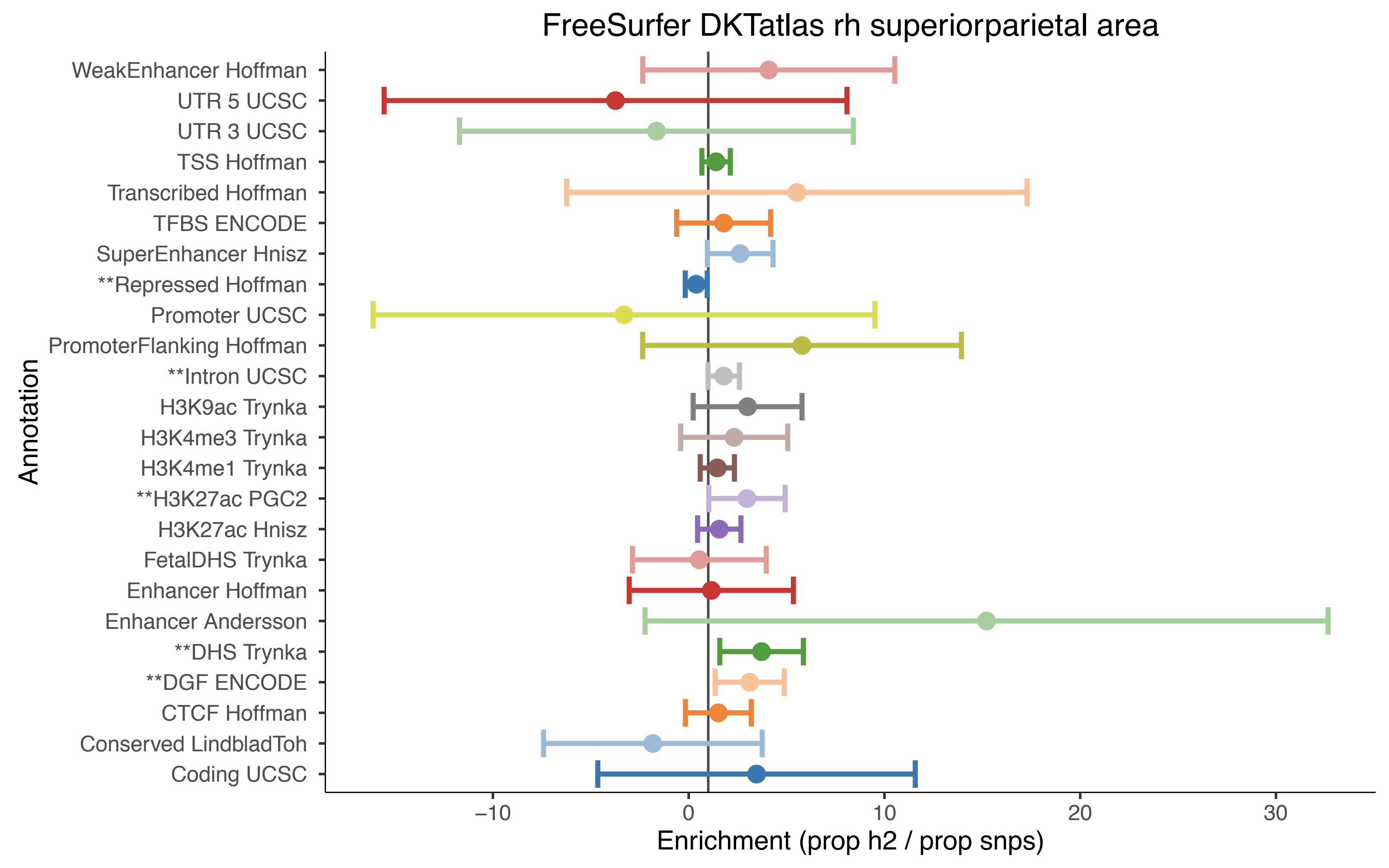

FreeSurfer DKTatlas rh superiorparietal area
WeakEnhancer Hoffman
UTR 5 UCSC
UTR 3 UCSC
TSS Hoffman
Transcribed Hoffman
TFBS ENCODE
SuperEnhancer Hnisz
**Repressed Hoffman
Promoter UCSC
PromoterFlanking Hoffman
**Intron UCSC
H3K9ac Trynka
H3K4me3 Trynka
H3K4me1 Trynka
**H3K27ac PGC2
H3K27ac Hnisz
FetalDHS Trynka
Enhancer Hoffman
Enhancer Andersson
**DHS Trynka
**DGF ENCODE
CTCF Hoffman
Conserved LindbladToh
Coding UCSC
Annotation
−10
0
10
20
30
Enrichment (prop h2 / prop snps)

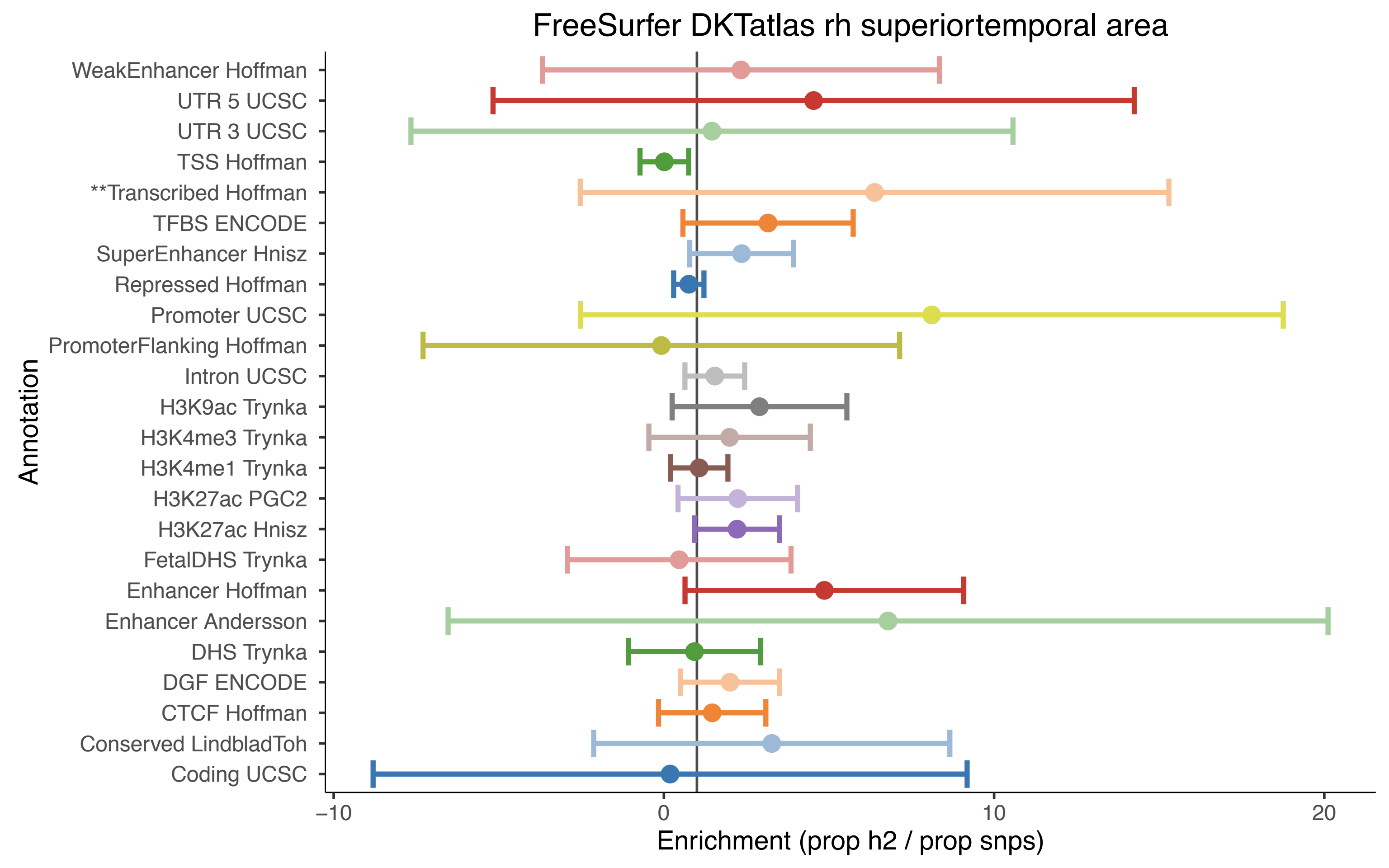

FreeSurfer DKTatlas rh superiortemporal area
WeakEnhancer Hoffman
UTR 5 UCSC
UTR 3 UCSC
TSS Hoffman
**Transcribed Hoffman
TFBS ENCODE
SuperEnhancer Hnisz
Repressed Hoffman
Promoter UCSC
PromoterFlanking Hoffman
Intron UCSC
H3K9ac Trynka
H3K4me3 Trynka
H3K4me1 Trynka
H3K27ac PGC2
H3K27ac Hnisz
FetalDHS Trynka
Enhancer Hoffman
Enhancer Andersson
DHS Trynka
DGF ENCODE
CTCF Hoffman
Conserved LindbladToh
Coding UCSC
Annotation
−10
0
10
20
Enrichment (prop h2 / prop snps)

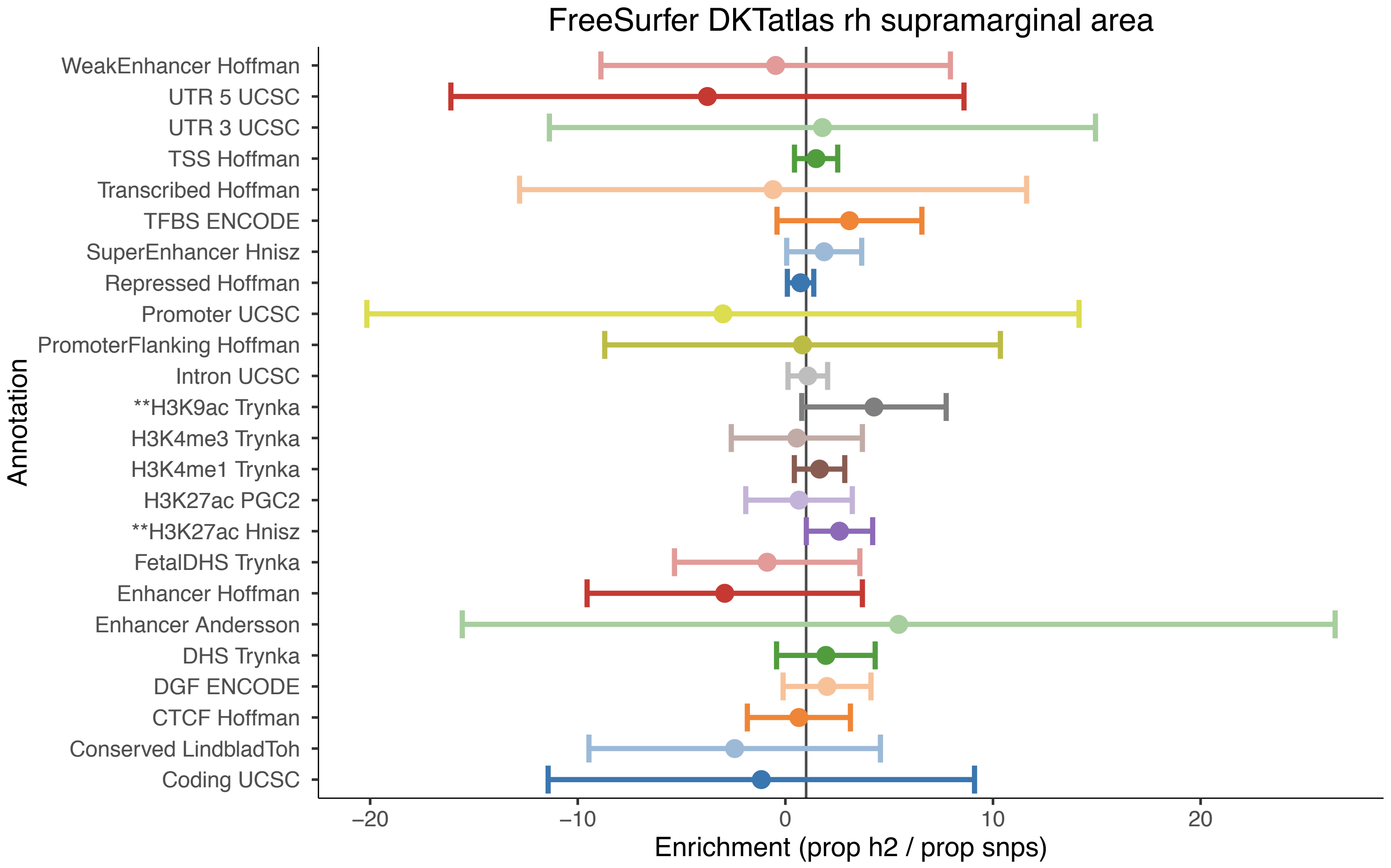
FreeSurfer DKTatlas rh supramarginal area
WeakEnhancer Hoffman
UTR 5 UCSC
UTR 3 UCSC
TSS Hoffman
Transcribed Hoffman
TFBS ENCODE
SuperEnhancer Hnisz
Repressed Hoffman
Promoter UCSC
PromoterFlanking Hoffman
Intron UCSC
**H3K9ac Trynka
H3K4me3 Trynka
H3K4me1 Trynka
H3K27ac PGC2
**H3K27ac Hnisz
FetalDHS Trynka
Enhancer Hoffman
Enhancer Andersson
DHS Trynka
DGF ENCODE
CTCF Hoffman
Conserved LindbladToh
Coding UCSC
Annotation
−20
−10
0
10
20
Enrichment (prop h2 / prop snps)

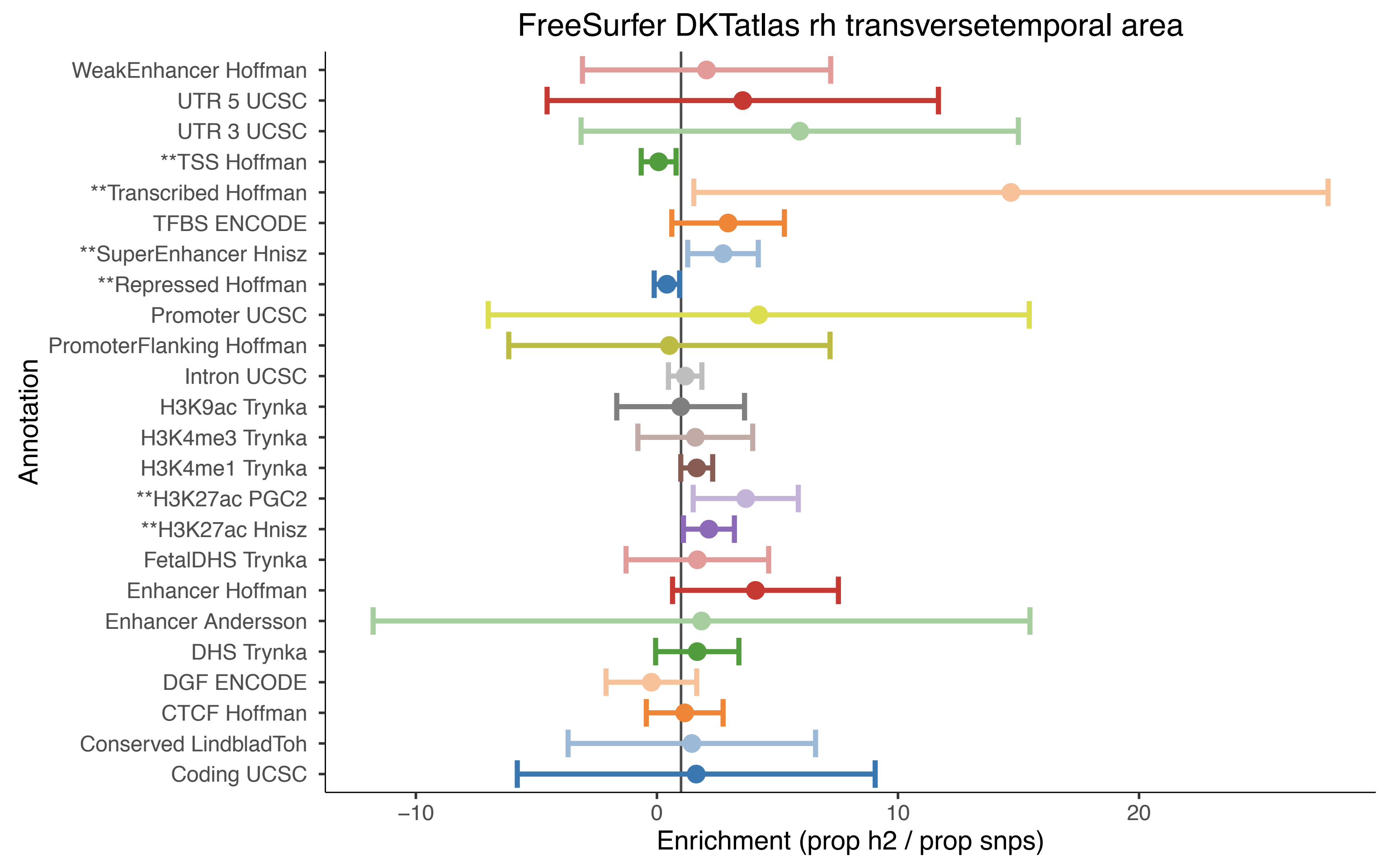

FreeSurfer DKTatlas rh transversetemporal area
Annotation
WeakEnhancer Hoffman
UTR 5 UCSC
UTR 3 UCSC
**TSS Hoffman
**Transcribed Hoffman
TFBS ENCODE
**SuperEnhancer Hnisz
**Repressed Hoffman
Promoter UCSC
PromoterFlanking Hoffman
Intron UCSC
H3K9ac Trynka
H3K4me3 Trynka
H3K4me1 Trynka
**H3K27ac PGC2
**H3K27ac Hnisz
FetalDHS Trynka
Enhancer Hoffman
Enhancer Andersson
DHS Trynka
DGF ENCODE
CTCF Hoffman
Conserved LindbladToh
Coding UCSC
−10
0
10
20
Enrichment (prop h2 / prop snps)

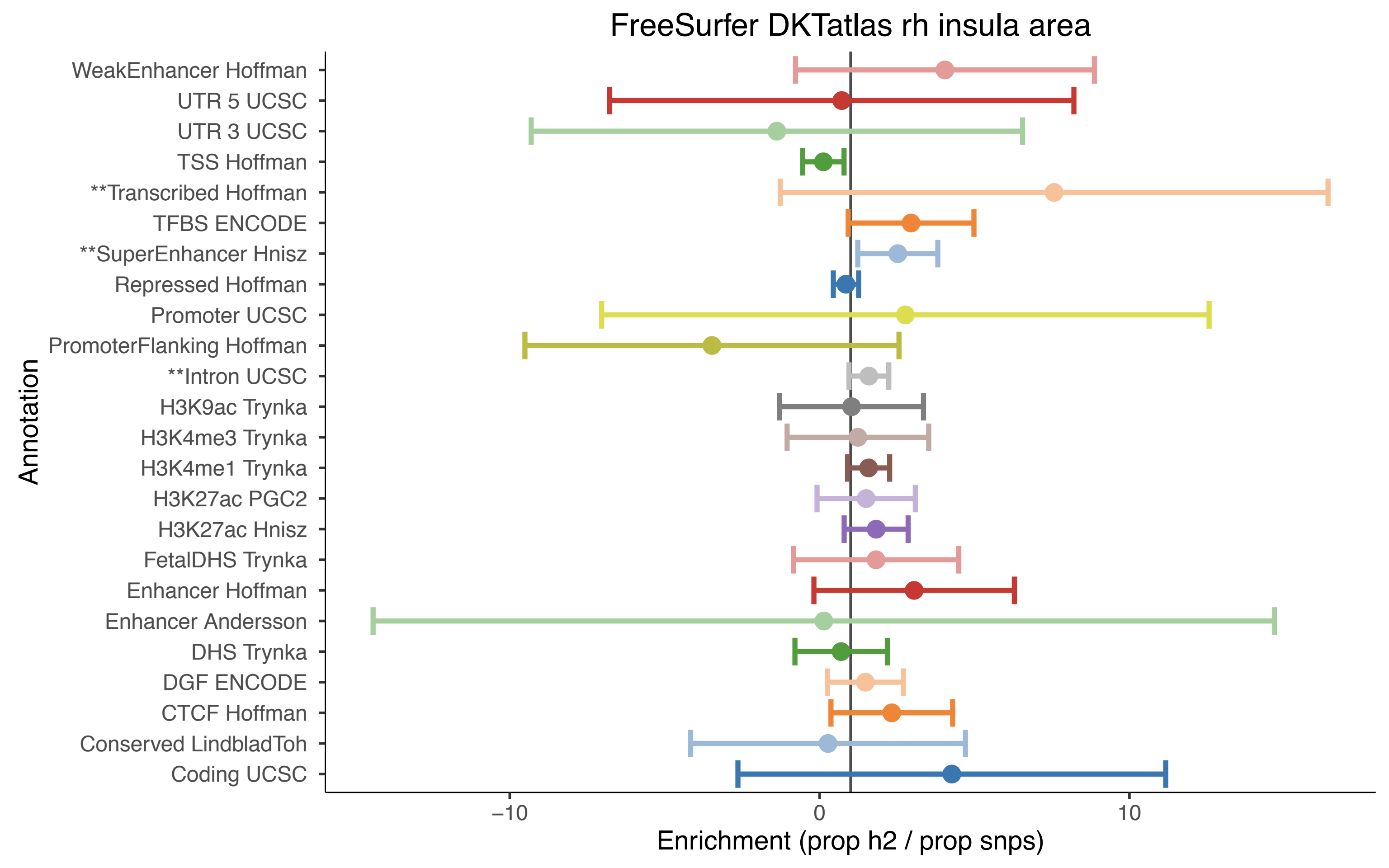

FreeSurfer DKTatlas rh insula area
WeakEnhancer Hoffman
UTR 5 UCSC
UTR 3 UCSC
TSS Hoffman
**Transcribed Hoffman
TFBS ENCODE
**SuperEnhancer Hnisz
Repressed Hoffman
Promoter UCSC
PromoterFlanking Hoffman
**Intron UCSC
H3K9ac Trynka
H3K4me3 Trynka
H3K4me1 Trynka
H3K27ac PGC2
H3K27ac Hnisz
FetalDHS Trynka
Enhancer Hoffman
Enhancer Andersson
DHS Trynka
DGF ENCODE
CTCF Hoffman
Conserved LindbladToh
Coding UCSC
Annotation
−10
0
10
Enrichment (prop h2 / prop snps)

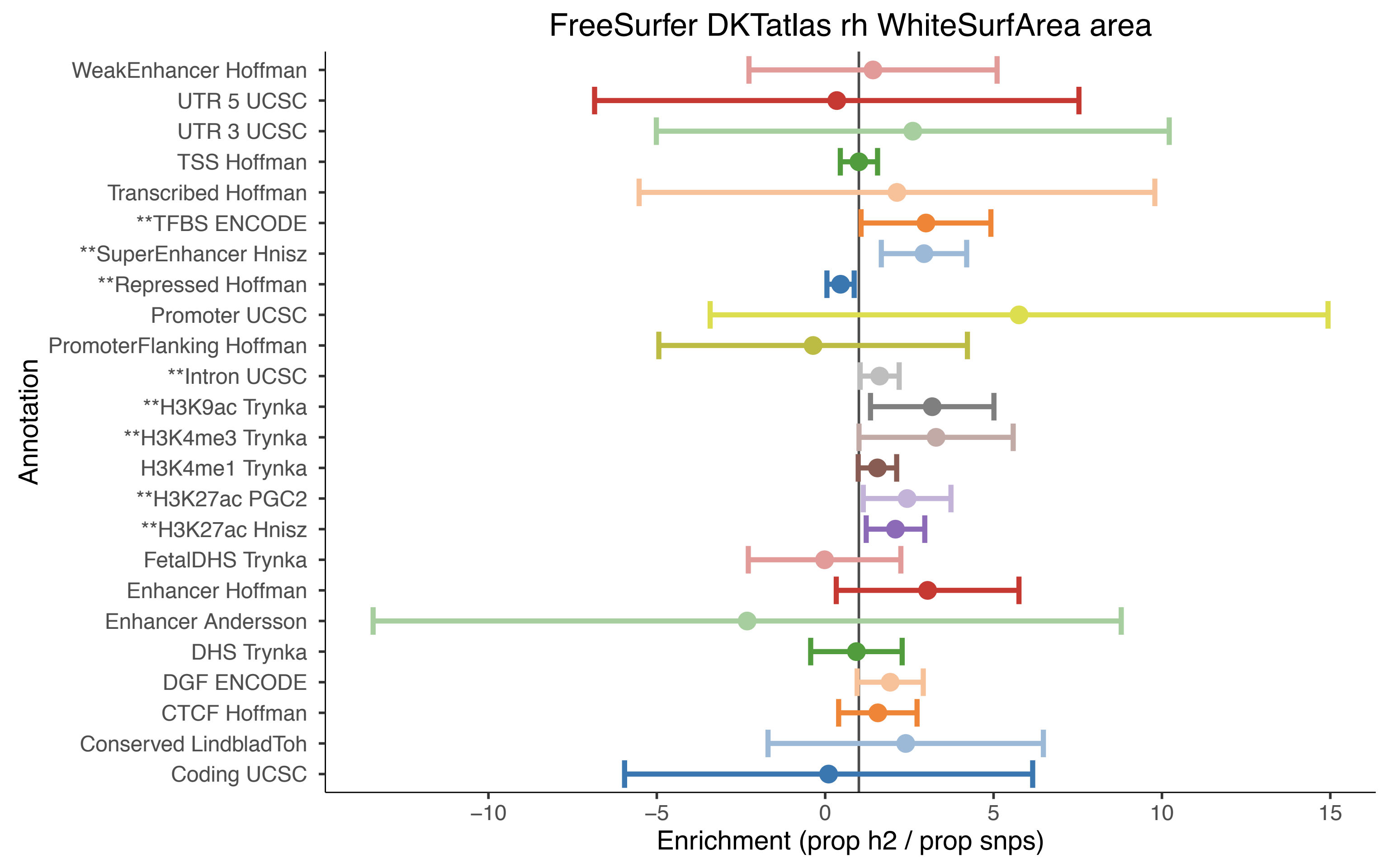

FreeSurfer DKTatlas rh WhiteSurfArea area
WeakEnhancer Hoffman
UTR 5 UCSC
UTR 3 UCSC
TSS Hoffman
Transcribed Hoffman
**TFBS ENCODE
**SuperEnhancer Hnisz
**Repressed Hoffman
Promoter UCSC
PromoterFlanking Hoffman
**Intron UCSC
**H3K9ac Trynka
**H3K4me3 Trynka
H3K4me1 Trynka
**H3K27ac PGC2
**H3K27ac Hnisz
FetalDHS Trynka
Enhancer Hoffman
Enhancer Andersson
DHS Trynka
DGF ENCODE
CTCF Hoffman
Conserved LindbladToh
Coding UCSC
Annotation
−10
−5
0
5
10
15
Enrichment (prop h2 / prop snps)

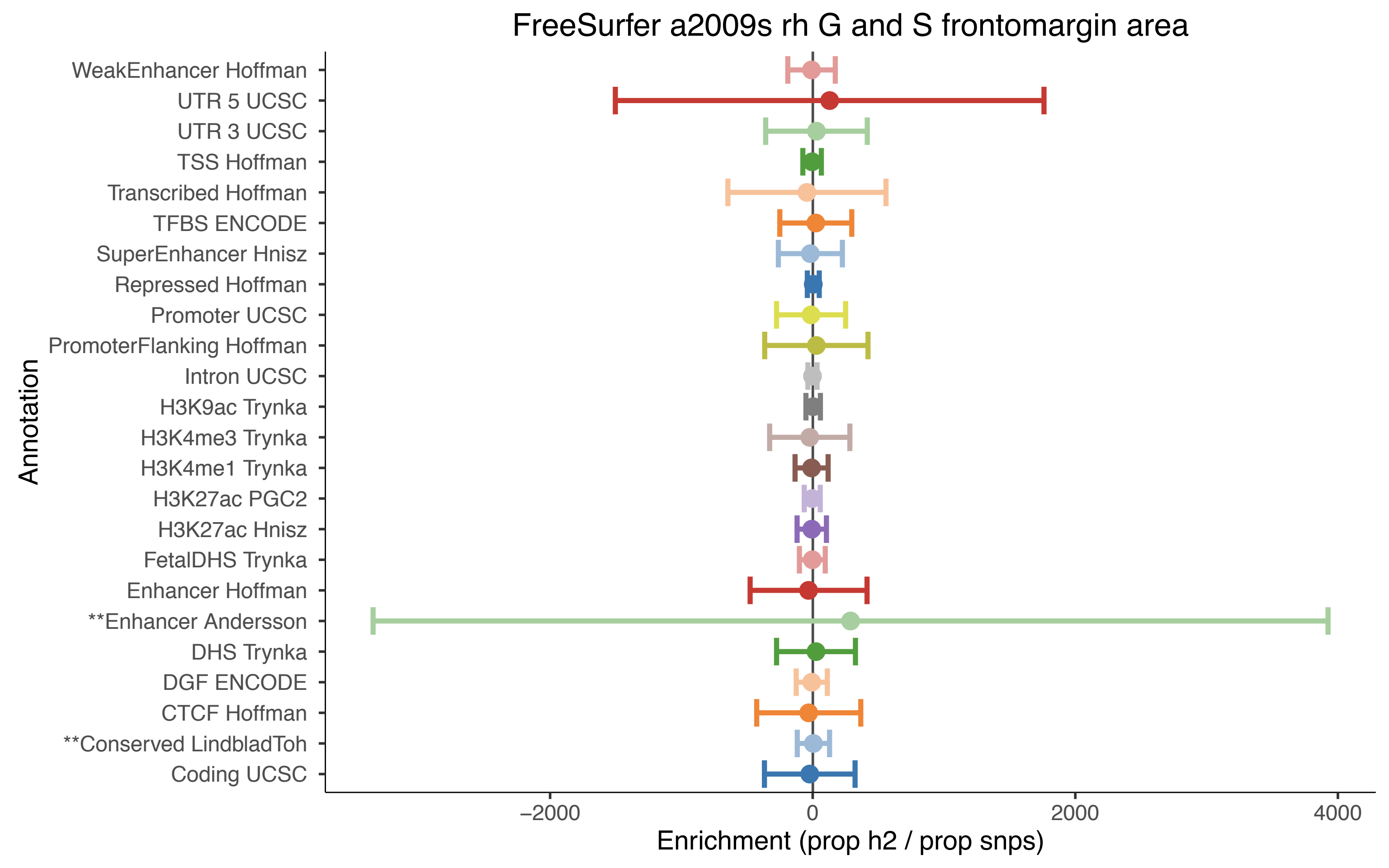

FreeSurfer a2009s rh G and S frontomargin area
Annotation
WeakEnhancer Hoffman
UTR 5 UCSC
UTR 3 UCSC
TSS Hoffman
Transcribed Hoffman
TFBS ENCODE
SuperEnhancer Hnisz
Repressed Hoffman
Promoter UCSC
PromoterFlanking Hoffman
Intron UCSC
H3K9ac Trynka
H3K4me3 Trynka
H3K4me1 Trynka
H3K27ac PGC2
H3K27ac Hnisz
FetalDHS Trynka
Enhancer Hoffman
**Enhancer Andersson
DHS Trynka
DGF ENCODE
CTCF Hoffman
**Conserved LindbladToh
Coding UCSC
−2000
0
2000
4000
Enrichment (prop h2 / prop snps)

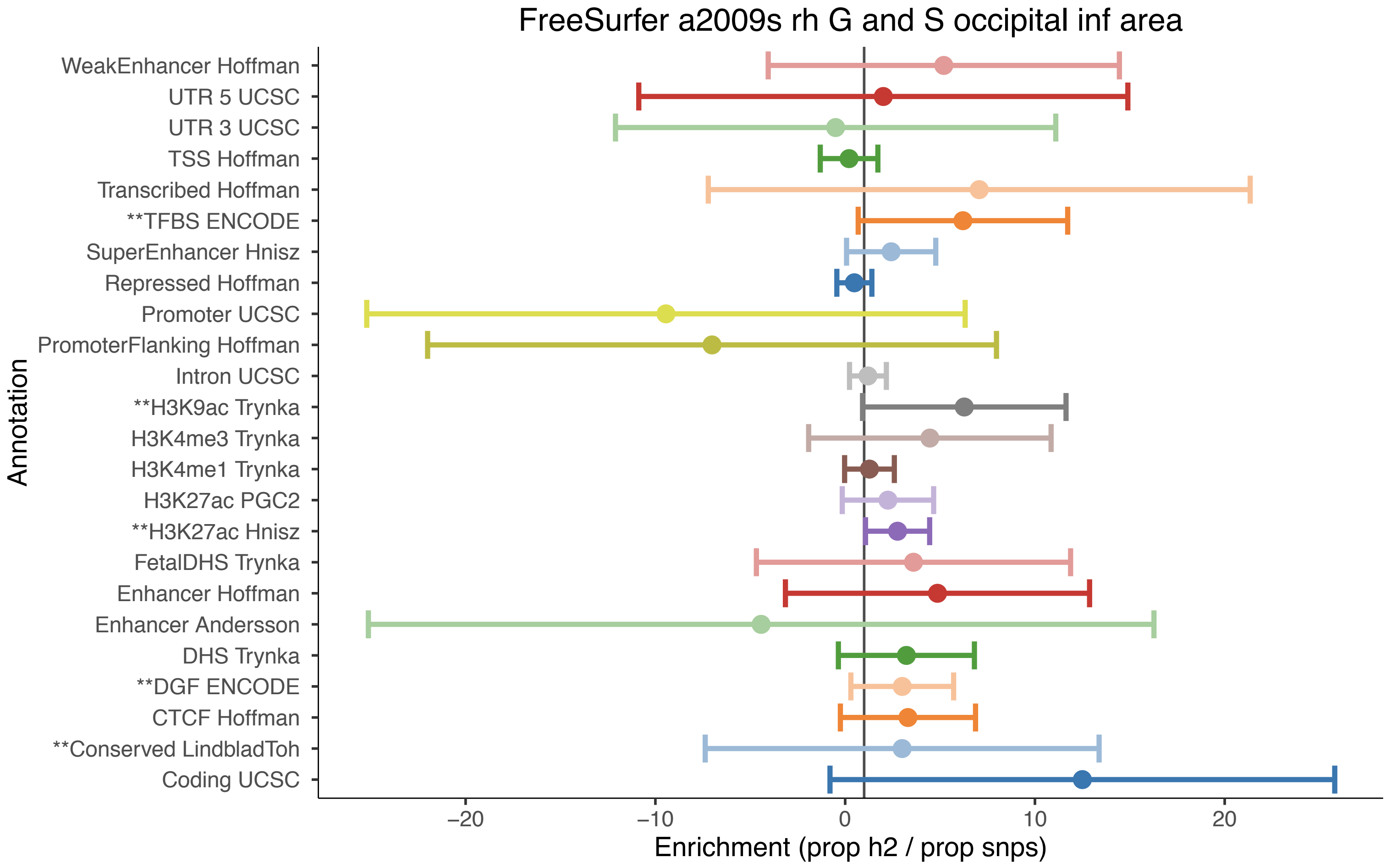

FreeSurfer a2009s rh G and S occipital inf area
WeakEnhancer Hoffman
UTR 5 UCSC
UTR 3 UCSC
TSS Hoffman
Transcribed Hoffman
**TFBS ENCODE
SuperEnhancer Hnisz
Repressed Hoffman
Promoter UCSC
PromoterFlanking Hoffman
Intron UCSC
**H3K9ac Trynka
H3K4me3 Trynka
H3K4me1 Trynka
H3K27ac PGC2
**H3K27ac Hnisz
FetalDHS Trynka
Enhancer Hoffman
Enhancer Andersson
DHS Trynka
**DGF ENCODE
CTCF Hoffman
**Conserved LindbladToh
Coding UCSC
Annotation
−20
−10
0
10
20
Enrichment (prop h2 / prop snps)

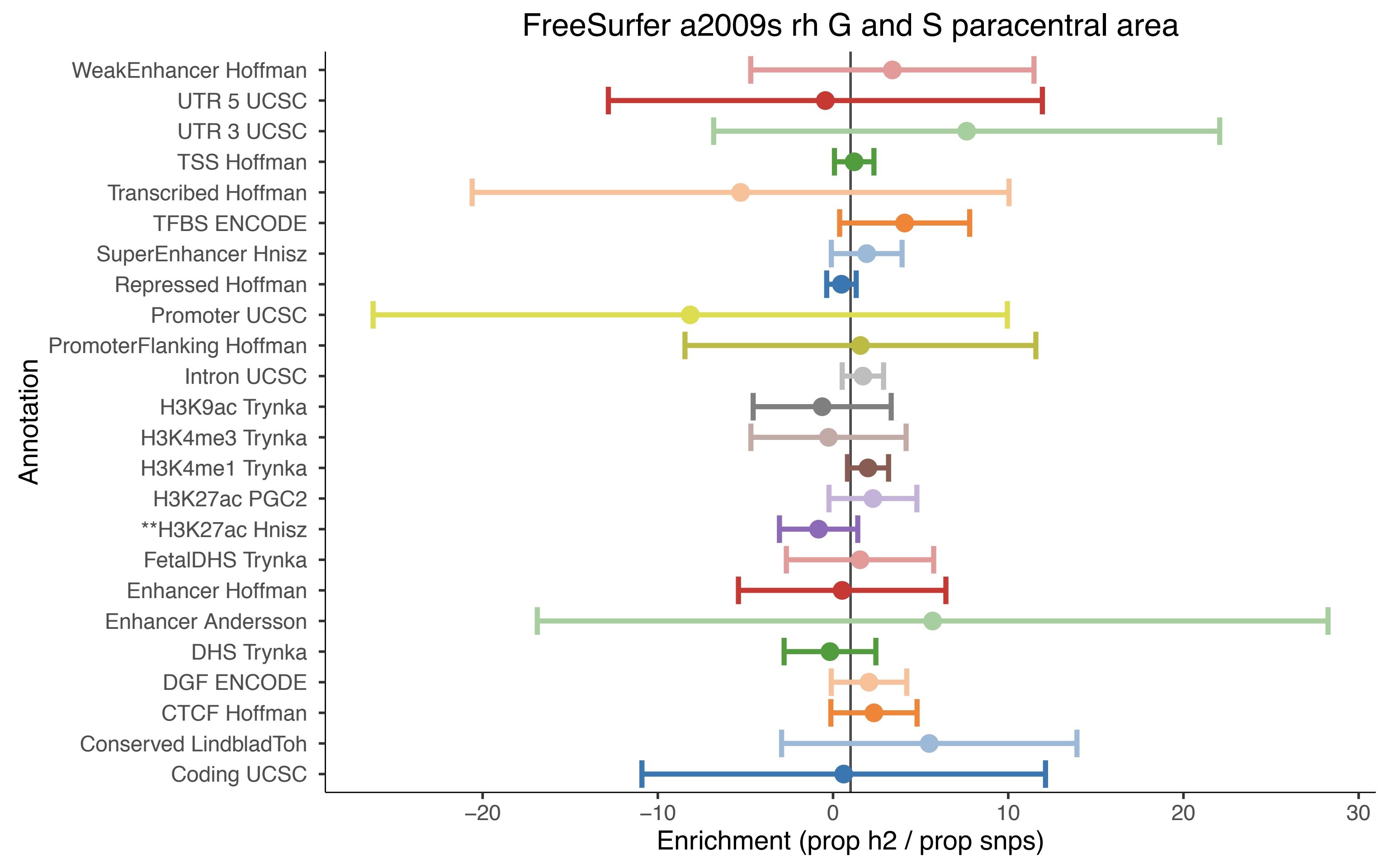
FreeSurfer a2009s rh G and S paracentral area
WeakEnhancer Hoffman
UTR 5 UCSC
UTR 3 UCSC
TSS Hoffman
Transcribed Hoffman
TFBS ENCODE
SuperEnhancer Hnisz
Repressed Hoffman
Promoter UCSC
PromoterFlanking Hoffman
Intron UCSC
H3K9ac Trynka
H3K4me3 Trynka
H3K4me1 Trynka
H3K27ac PGC2
**H3K27ac Hnisz
FetalDHS Trynka
Enhancer Hoffman
Enhancer Andersson
DHS Trynka
DGF ENCODE
CTCF Hoffman
Conserved LindbladToh
Coding UCSC
Annotation
−20
−10
0
10
20
30
Enrichment (prop h2 / prop snps)

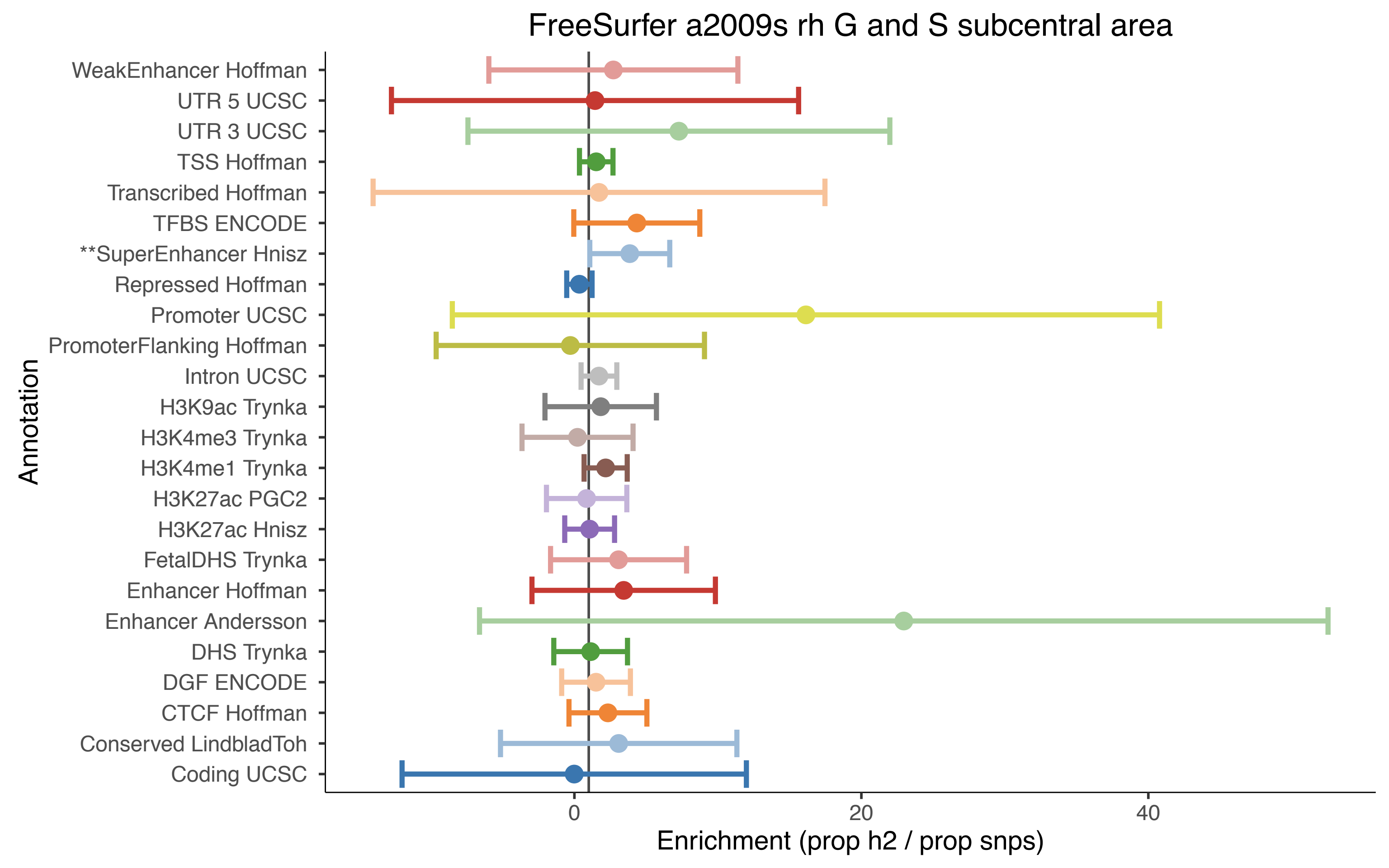
FreeSurfer a2009s rh G and S subcentral area
WeakEnhancer Hoffman
UTR 5 UCSC
UTR 3 UCSC
TSS Hoffman
Transcribed Hoffman
TFBS ENCODE
**SuperEnhancer Hnisz
Repressed Hoffman
Promoter UCSC
PromoterFlanking Hoffman
Intron UCSC
H3K9ac Trynka
H3K4me3 Trynka
H3K4me1 Trynka
H3K27ac PGC2
H3K27ac Hnisz
FetalDHS Trynka
Enhancer Hoffman
Enhancer Andersson
DHS Trynka
DGF ENCODE
CTCF Hoffman
Conserved LindbladToh
Coding UCSC
Annotation
0
20
40
Enrichment (prop h2 / prop snps)

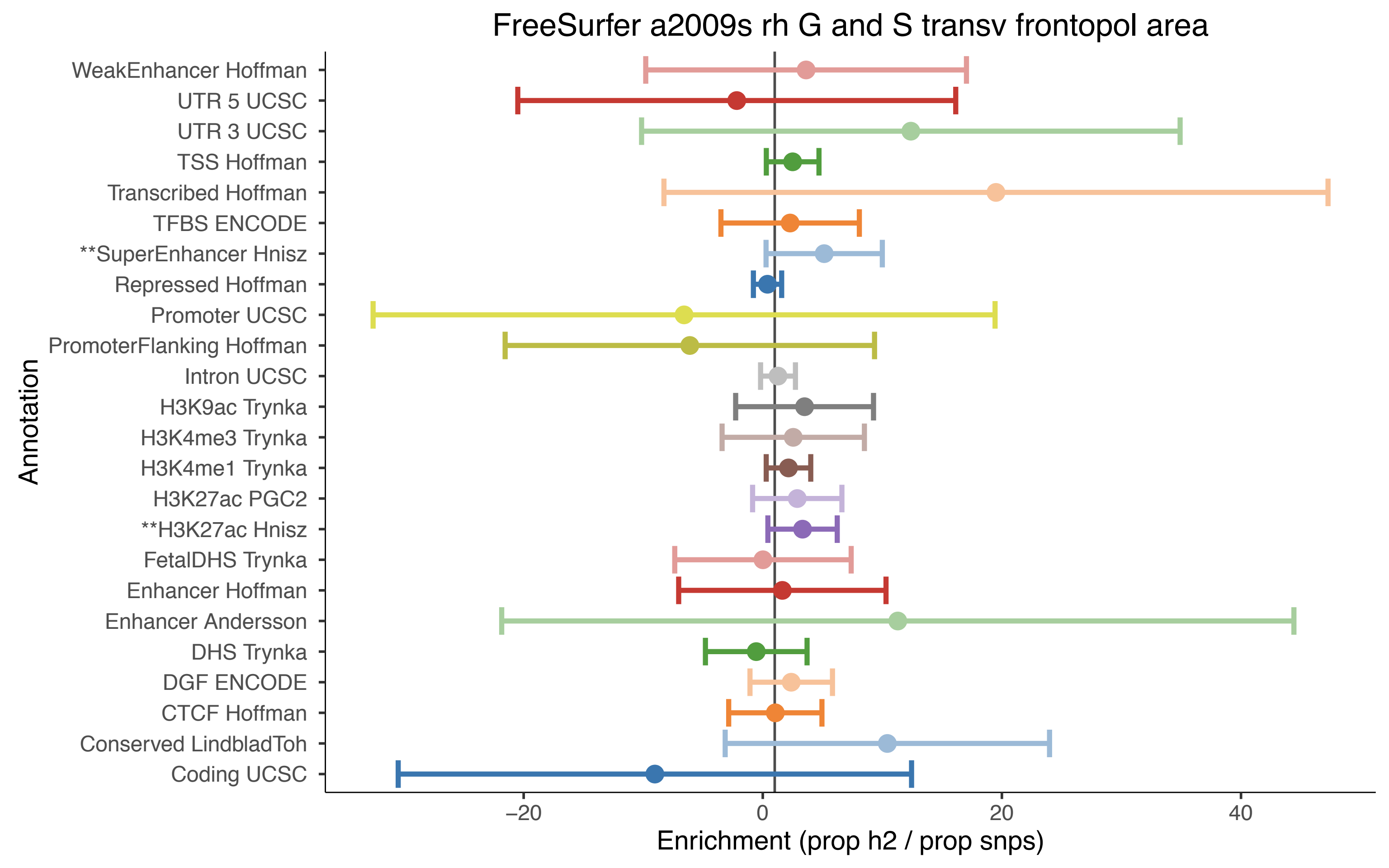
FreeSurfer a2009s rh G and S transv frontopol area
WeakEnhancer Hoffman
UTR 5 UCSC
UTR 3 UCSC
TSS Hoffman
Transcribed Hoffman
TFBS ENCODE
**SuperEnhancer Hnisz
Repressed Hoffman
Promoter UCSC
PromoterFlanking Hoffman
Intron UCSC
H3K9ac Trynka
H3K4me3 Trynka
H3K4me1 Trynka
H3K27ac PGC2
**H3K27ac Hnisz
FetalDHS Trynka
Enhancer Hoffman
Enhancer Andersson
DHS Trynka
DGF ENCODE
CTCF Hoffman
Conserved LindbladToh
Coding UCSC
Annotation
−20
0
20
40
Enrichment (prop h2 / prop snps)

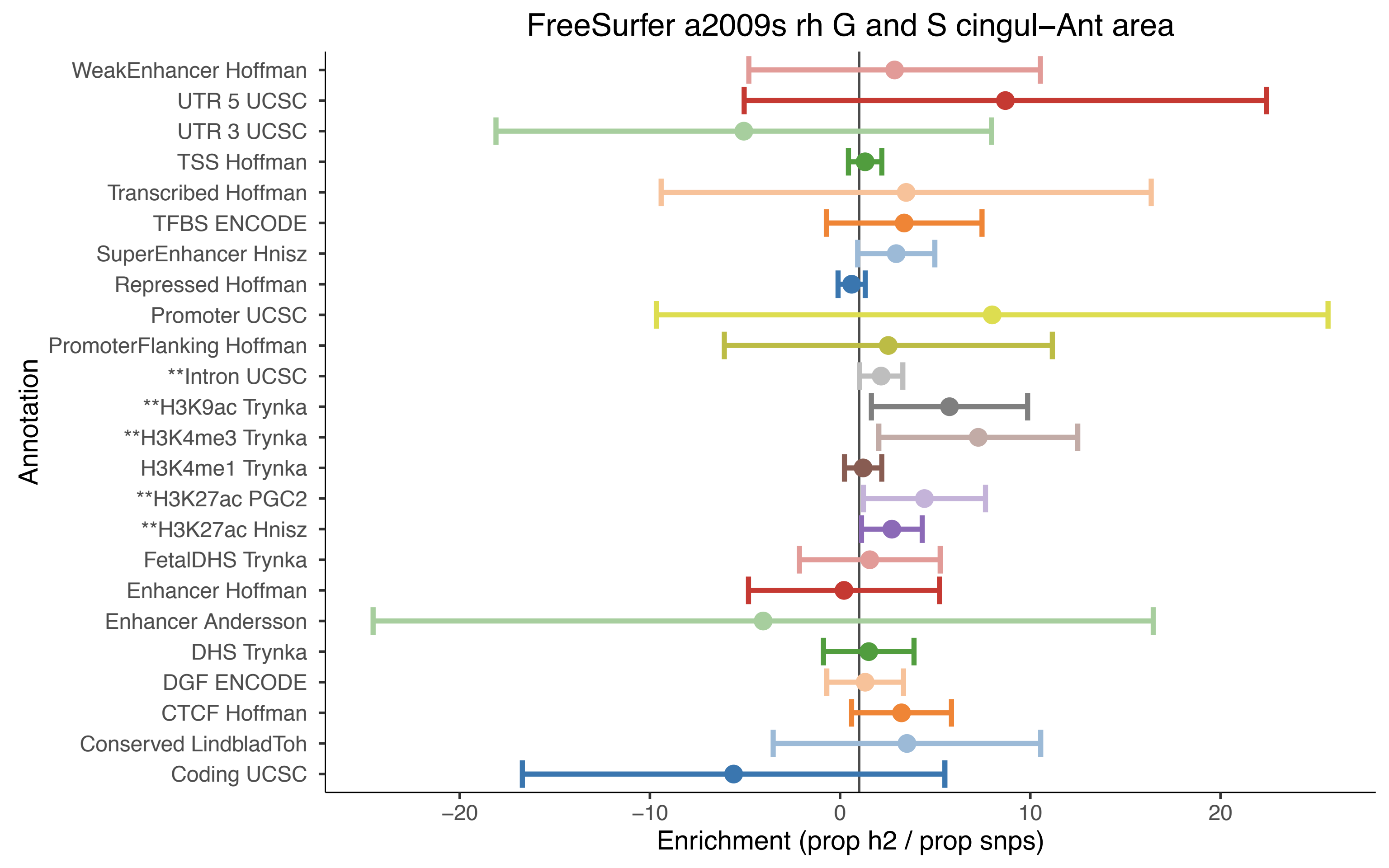

FreeSurfer a2009s rh G and S cingul−Ant area
WeakEnhancer Hoffman
UTR 5 UCSC
UTR 3 UCSC
TSS Hoffman
Transcribed Hoffman
TFBS ENCODE
SuperEnhancer Hnisz
Repressed Hoffman
Promoter UCSC
PromoterFlanking Hoffman
**Intron UCSC
**H3K9ac Trynka
**H3K4me3 Trynka
H3K4me1 Trynka
**H3K27ac PGC2
**H3K27ac Hnisz
FetalDHS Trynka
Enhancer Hoffman
Enhancer Andersson
DHS Trynka
DGF ENCODE
CTCF Hoffman
Conserved LindbladToh
Coding UCSC
Annotation
−20
−10
0
10
20
Enrichment (prop h2 / prop snps)

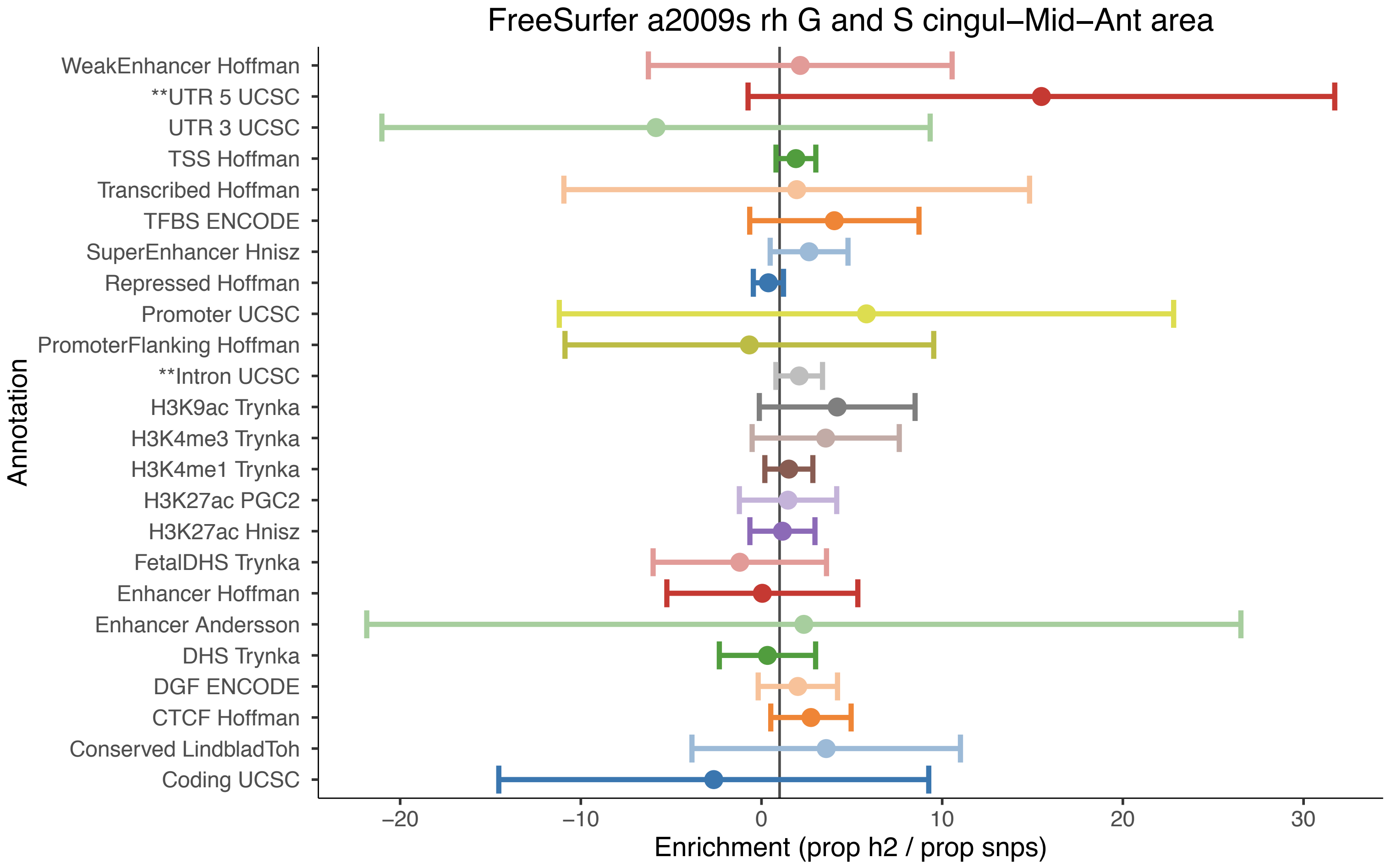
FreeSurfer a2009s rh G and S cingul−Mid−Ant area
WeakEnhancer Hoffman
**UTR 5 UCSC
UTR 3 UCSC
TSS Hoffman
Transcribed Hoffman
TFBS ENCODE
SuperEnhancer Hnisz
Repressed Hoffman
Promoter UCSC
PromoterFlanking Hoffman
**Intron UCSC
H3K9ac Trynka
H3K4me3 Trynka
H3K4me1 Trynka
H3K27ac PGC2
H3K27ac Hnisz
FetalDHS Trynka
Enhancer Hoffman
Enhancer Andersson
DHS Trynka
DGF ENCODE
CTCF Hoffman
Conserved LindbladToh
Coding UCSC
Annotation
−20
−10
0
10
20
30
Enrichment (prop h2 / prop snps)

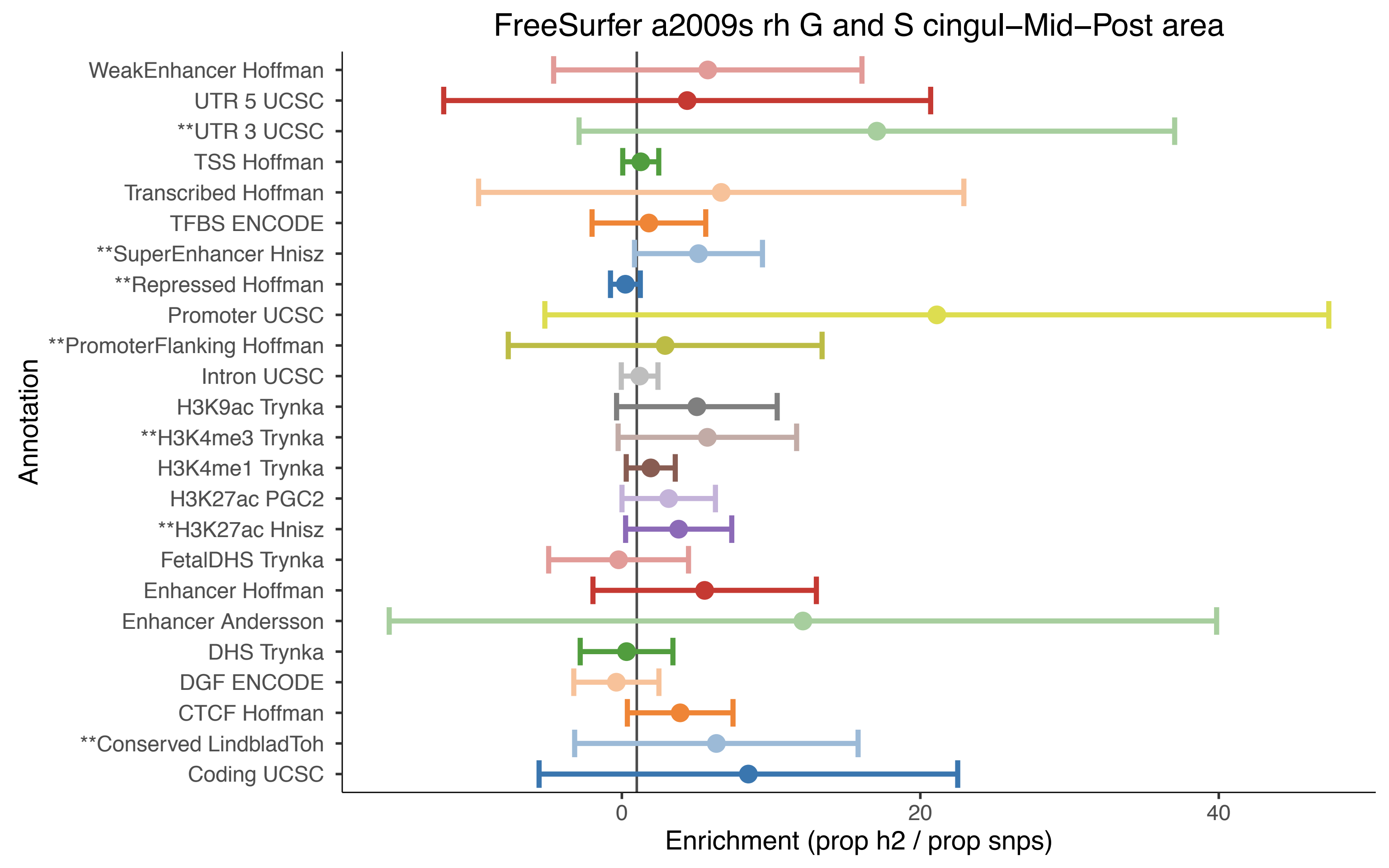
FreeSurfer a2009s rh G and S cingul−Mid−Post area
WeakEnhancer Hoffman
UTR 5 UCSC
**UTR 3 UCSC
TSS Hoffman
Transcribed Hoffman
TFBS ENCODE
**SuperEnhancer Hnisz
**Repressed Hoffman
Promoter UCSC
**PromoterFlanking Hoffman
Intron UCSC
H3K9ac Trynka
**H3K4me3 Trynka
H3K4me1 Trynka
H3K27ac PGC2
**H3K27ac Hnisz
FetalDHS Trynka
Enhancer Hoffman
Enhancer Andersson
DHS Trynka
DGF ENCODE
CTCF Hoffman
**Conserved LindbladToh
Coding UCSC
Annotation
0
20
40
Enrichment (prop h2 / prop snps)

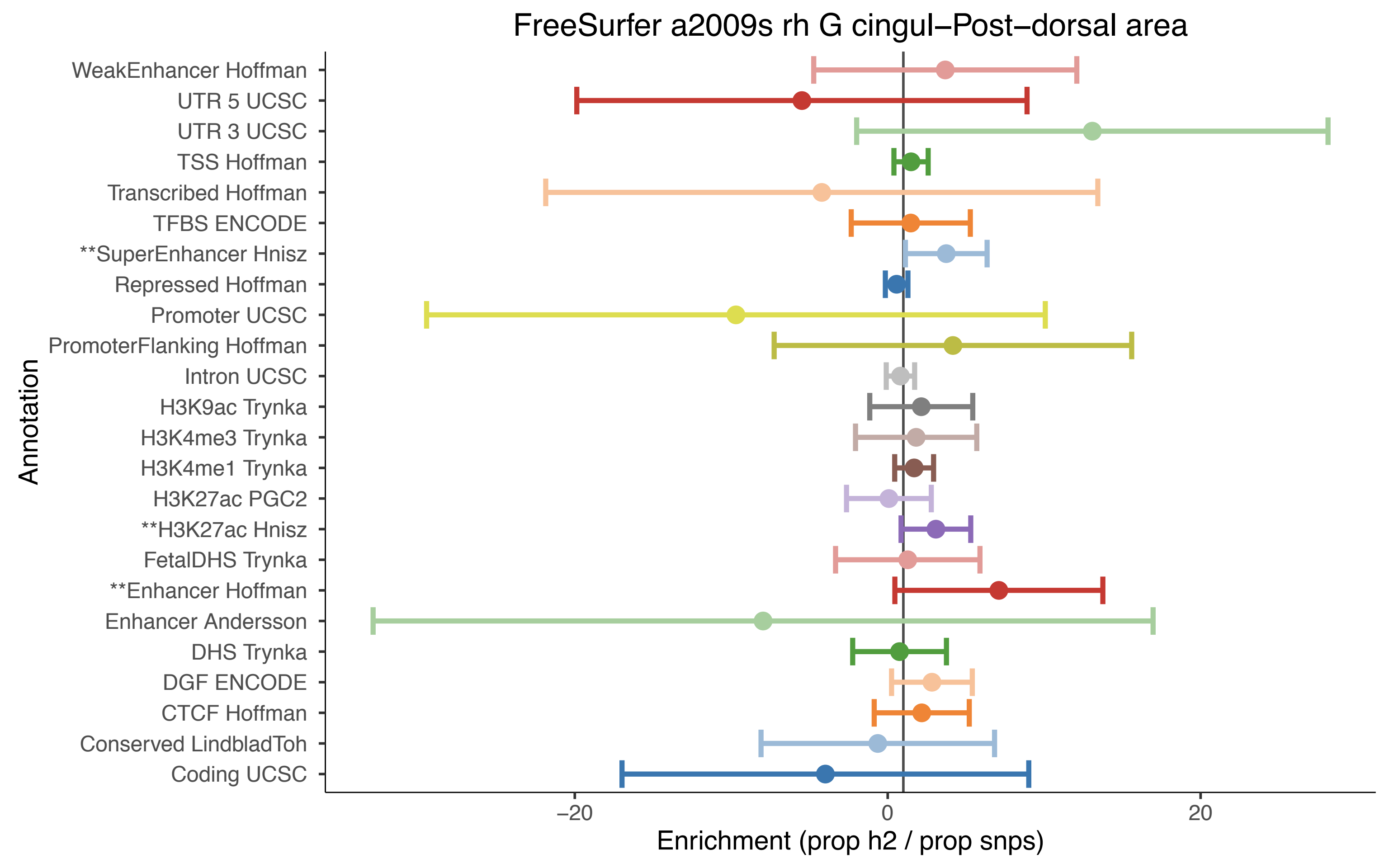

FreeSurfer a2009s rh G cingul−Post−dorsal area
WeakEnhancer Hoffman
UTR 5 UCSC
UTR 3 UCSC
TSS Hoffman
Transcribed Hoffman
TFBS ENCODE
**SuperEnhancer Hnisz
Repressed Hoffman
Promoter UCSC
PromoterFlanking Hoffman
Intron UCSC
H3K9ac Trynka
H3K4me3 Trynka
H3K4me1 Trynka
H3K27ac PGC2
**H3K27ac Hnisz
FetalDHS Trynka
**Enhancer Hoffman
Enhancer Andersson
DHS Trynka
DGF ENCODE
CTCF Hoffman
Conserved LindbladToh
Coding UCSC
Annotation
−20
0
20
Enrichment (prop h2 / prop snps)

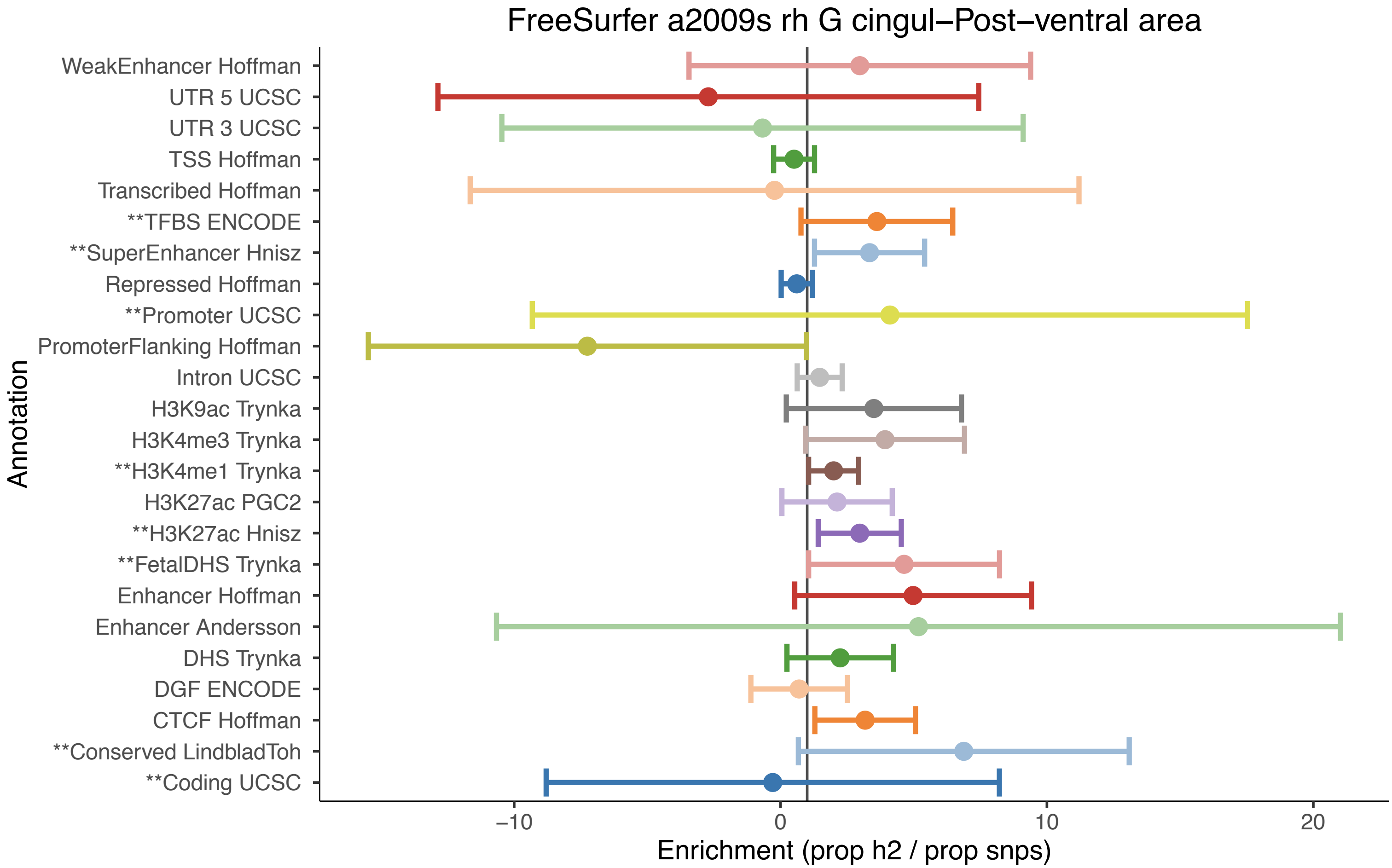
FreeSurfer a2009s rh G cingul−Post−ventral area
WeakEnhancer Hoffman
UTR 5 UCSC
UTR 3 UCSC
TSS Hoffman
Transcribed Hoffman
**TFBS ENCODE
**SuperEnhancer Hnisz
Repressed Hoffman
**Promoter UCSC
PromoterFlanking Hoffman
Intron UCSC
H3K9ac Trynka
H3K4me3 Trynka
**H3K4me1 Trynka
H3K27ac PGC2
**H3K27ac Hnisz
**FetalDHS Trynka
Enhancer Hoffman
Enhancer Andersson
DHS Trynka
DGF ENCODE
CTCF Hoffman
**Conserved LindbladToh
**Coding UCSC
Annotation
−10
0
10
20
Enrichment (prop h2 / prop snps)

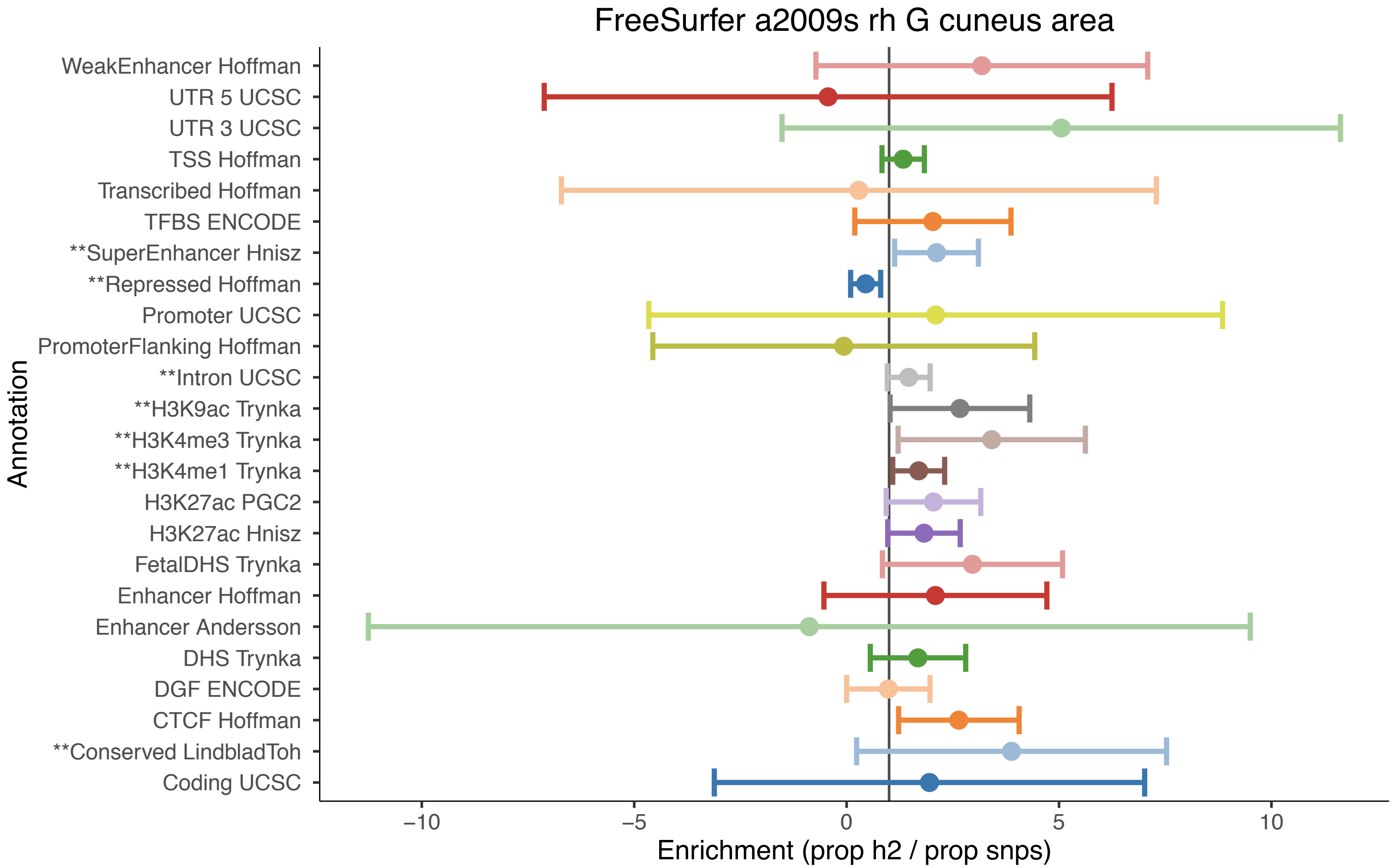
FreeSurfer a2009s rh G cuneus area
WeakEnhancer Hoffman
UTR 5 UCSC
UTR 3 UCSC
TSS Hoffman
Transcribed Hoffman
TFBS ENCODE
**SuperEnhancer Hnisz
**Repressed Hoffman
Promoter UCSC
PromoterFlanking Hoffman
**Intron UCSC
**H3K9ac Trynka
**H3K4me3 Trynka
**H3K4me1 Trynka
H3K27ac PGC2
H3K27ac Hnisz
FetalDHS Trynka
Enhancer Hoffman
Enhancer Andersson
DHS Trynka
DGF ENCODE
CTCF Hoffman
**Conserved LindbladToh
Coding UCSC
Annotation
−10
−5
0
5
10
Enrichment (prop h2 / prop snps)

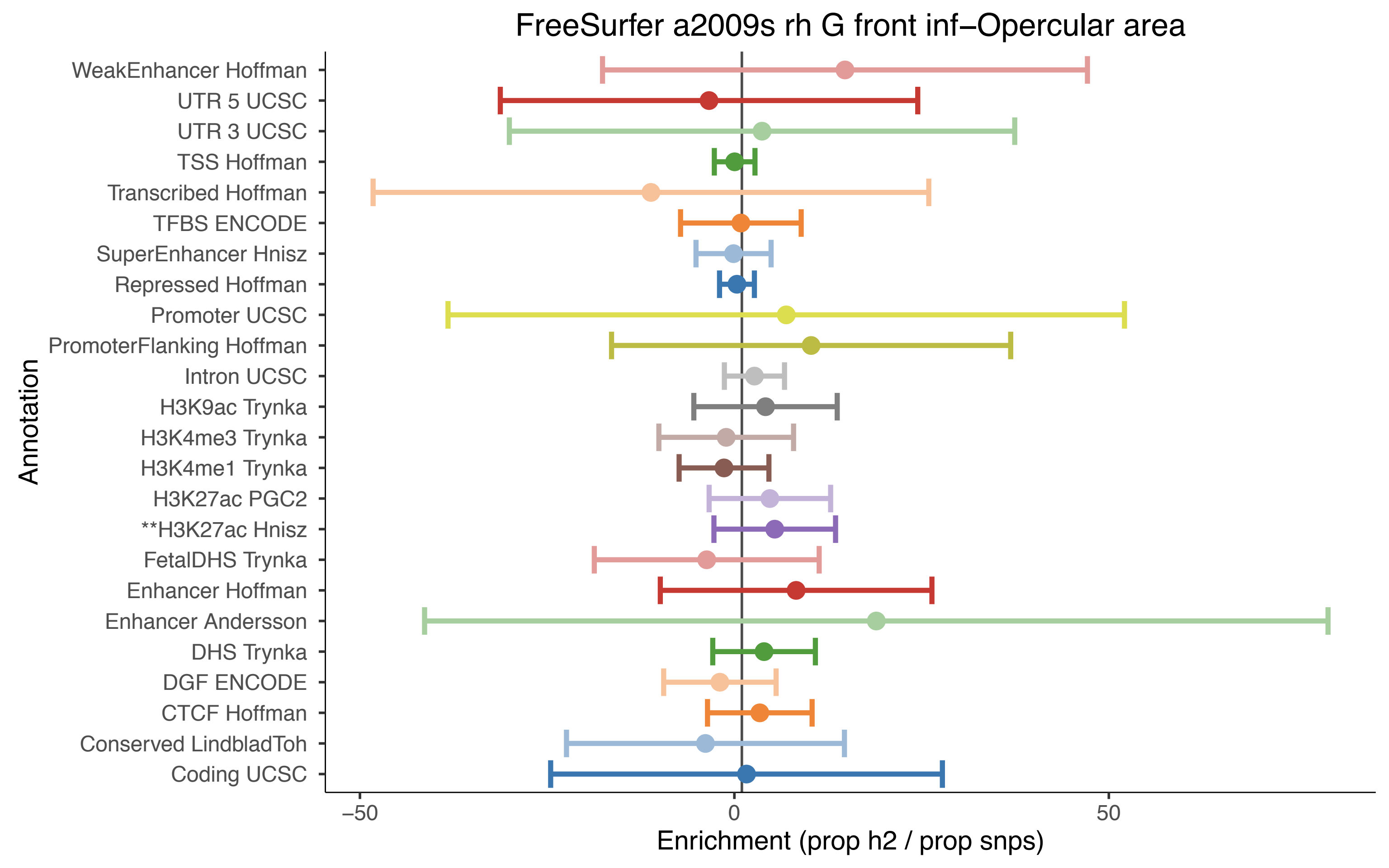
FreeSurfer a2009s rh G front inf−Opercular area
WeakEnhancer Hoffman
UTR 5 UCSC
UTR 3 UCSC
TSS Hoffman
Transcribed Hoffman
TFBS ENCODE
SuperEnhancer Hnisz
Repressed Hoffman
Promoter UCSC
PromoterFlanking Hoffman
Intron UCSC
H3K9ac Trynka
H3K4me3 Trynka
H3K4me1 Trynka
H3K27ac PGC2
**H3K27ac Hnisz
FetalDHS Trynka
Enhancer Hoffman
Enhancer Andersson
DHS Trynka
DGF ENCODE
CTCF Hoffman
Conserved LindbladToh
Coding UCSC
Annotation
−50
0
50
Enrichment (prop h2 / prop snps)

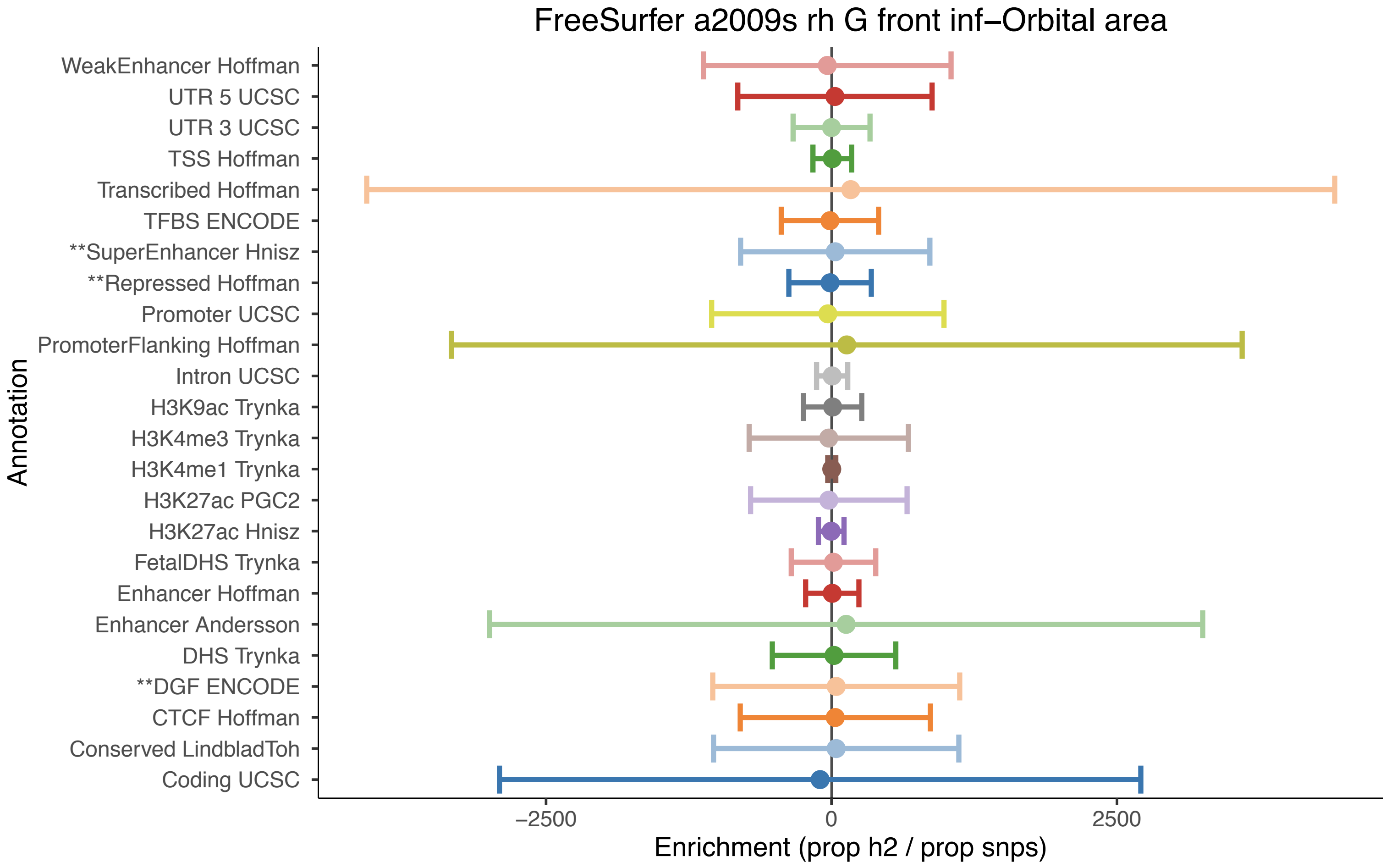
FreeSurfer a2009s rh G front inf−Orbital area
WeakEnhancer Hoffman
UTR 5 UCSC
UTR 3 UCSC
TSS Hoffman
Transcribed Hoffman
TFBS ENCODE
**SuperEnhancer Hnisz
**Repressed Hoffman
Promoter UCSC
PromoterFlanking Hoffman
Intron UCSC
H3K9ac Trynka
H3K4me3 Trynka
H3K4me1 Trynka
H3K27ac PGC2
H3K27ac Hnisz
FetalDHS Trynka
Enhancer Hoffman
Enhancer Andersson
DHS Trynka
**DGF ENCODE
CTCF Hoffman
Conserved LindbladToh
Coding UCSC
Annotation
−2500
0
2500
Enrichment (prop h2 / prop snps)

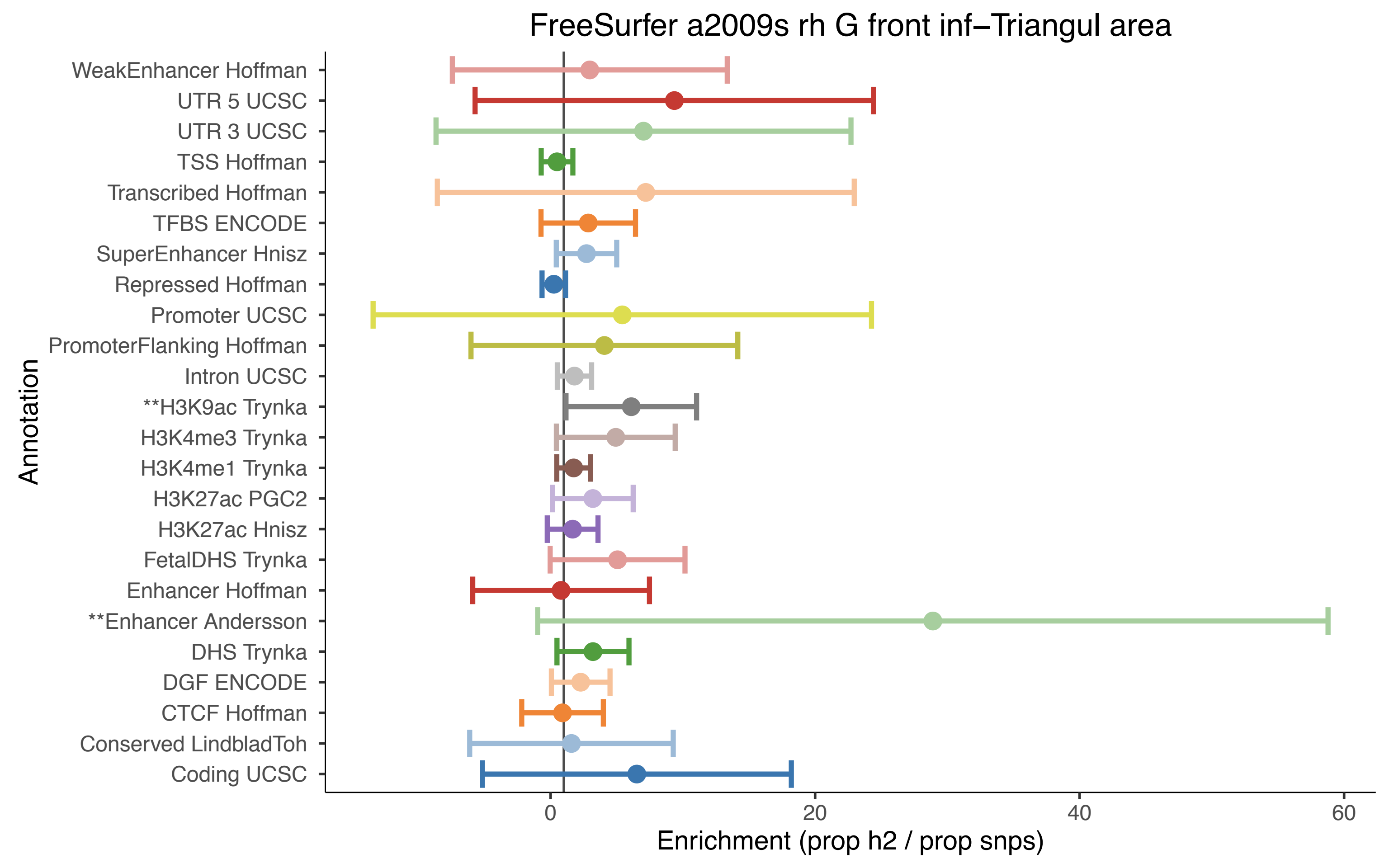
FreeSurfer a2009s rh G front inf−Triangul area
WeakEnhancer Hoffman
UTR 5 UCSC
UTR 3 UCSC
TSS Hoffman
Transcribed Hoffman
TFBS ENCODE
SuperEnhancer Hnisz
Repressed Hoffman
Promoter UCSC
PromoterFlanking Hoffman
Intron UCSC
**H3K9ac Trynka
H3K4me3 Trynka
H3K4me1 Trynka
H3K27ac PGC2
H3K27ac Hnisz
FetalDHS Trynka
Enhancer Hoffman
**Enhancer Andersson
DHS Trynka
DGF ENCODE
CTCF Hoffman
Conserved LindbladToh
Coding UCSC
Annotation
0
20
40
60
Enrichment (prop h2 / prop snps)

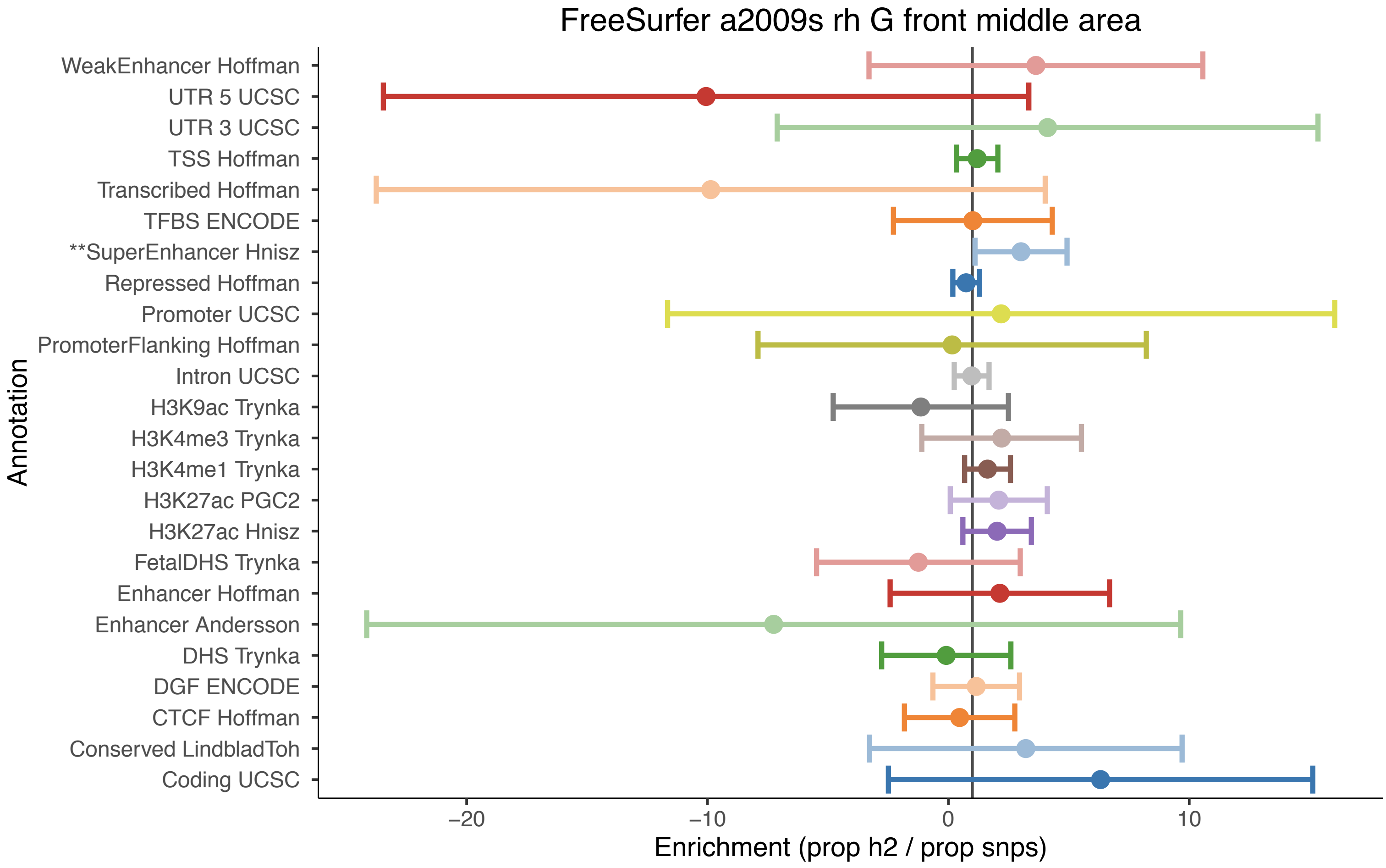
FreeSurfer a2009s rh G front middle area
WeakEnhancer Hoffman
UTR 5 UCSC
UTR 3 UCSC
TSS Hoffman
Transcribed Hoffman
TFBS ENCODE
**SuperEnhancer Hnisz
Repressed Hoffman
Promoter UCSC
PromoterFlanking Hoffman
Intron UCSC
H3K9ac Trynka
H3K4me3 Trynka
H3K4me1 Trynka
H3K27ac PGC2
H3K27ac Hnisz
FetalDHS Trynka
Enhancer Hoffman
Enhancer Andersson
DHS Trynka
DGF ENCODE
CTCF Hoffman
Conserved LindbladToh
Coding UCSC
Annotation
−20
−10
0
10
Enrichment (prop h2 / prop snps)

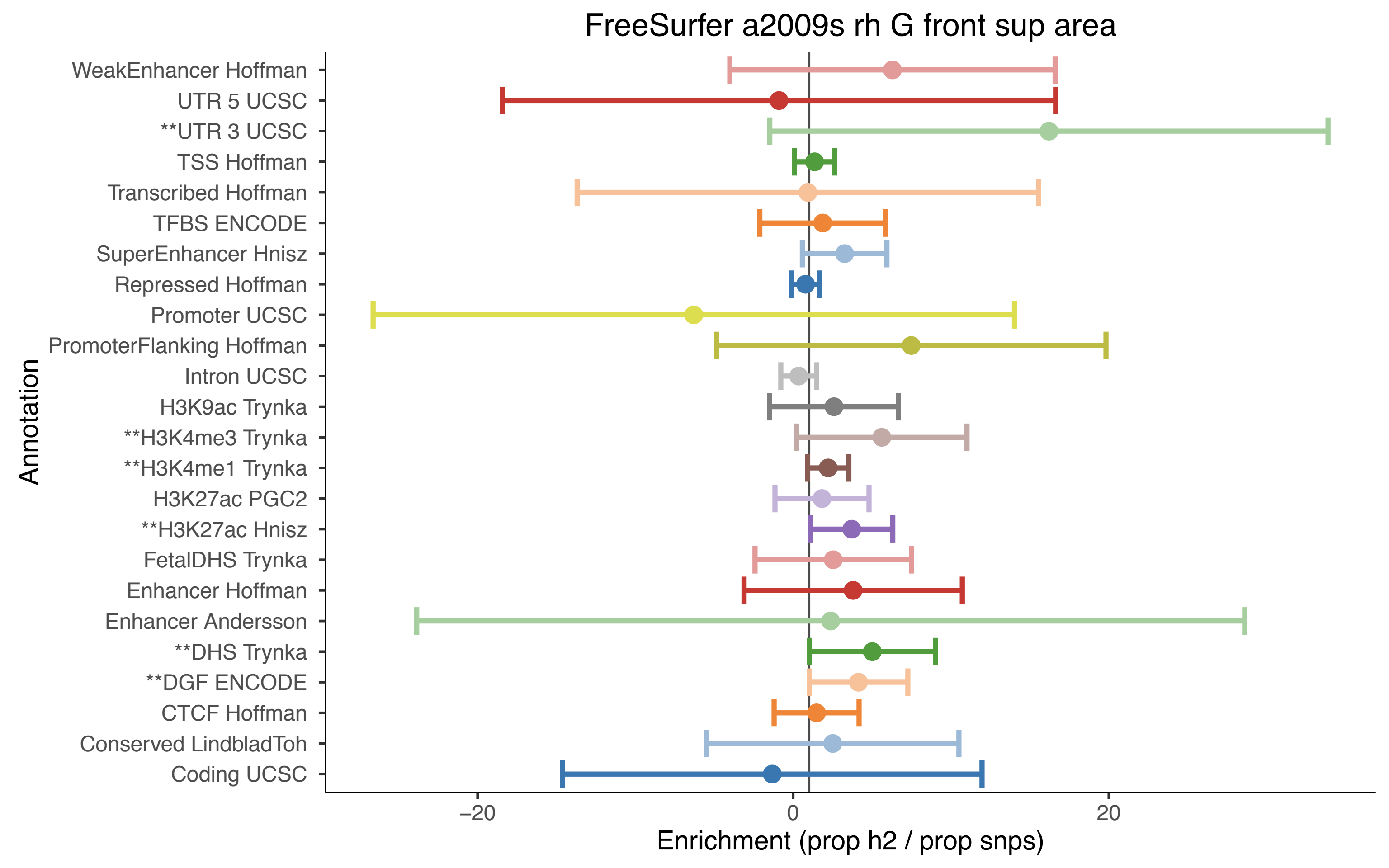

FreeSurfer a2009s rh G front sup area
WeakEnhancer Hoffman
UTR 5 UCSC
**UTR 3 UCSC
TSS Hoffman
Transcribed Hoffman
TFBS ENCODE
SuperEnhancer Hnisz
Repressed Hoffman
Promoter UCSC
PromoterFlanking Hoffman
Intron UCSC
H3K9ac Trynka
**H3K4me3 Trynka
**H3K4me1 Trynka
H3K27ac PGC2
**H3K27ac Hnisz
FetalDHS Trynka
Enhancer Hoffman
Enhancer Andersson
**DHS Trynka
**DGF ENCODE
CTCF Hoffman
Conserved LindbladToh
Coding UCSC
Annotation
−20
0
20
Enrichment (prop h2 / prop snps)

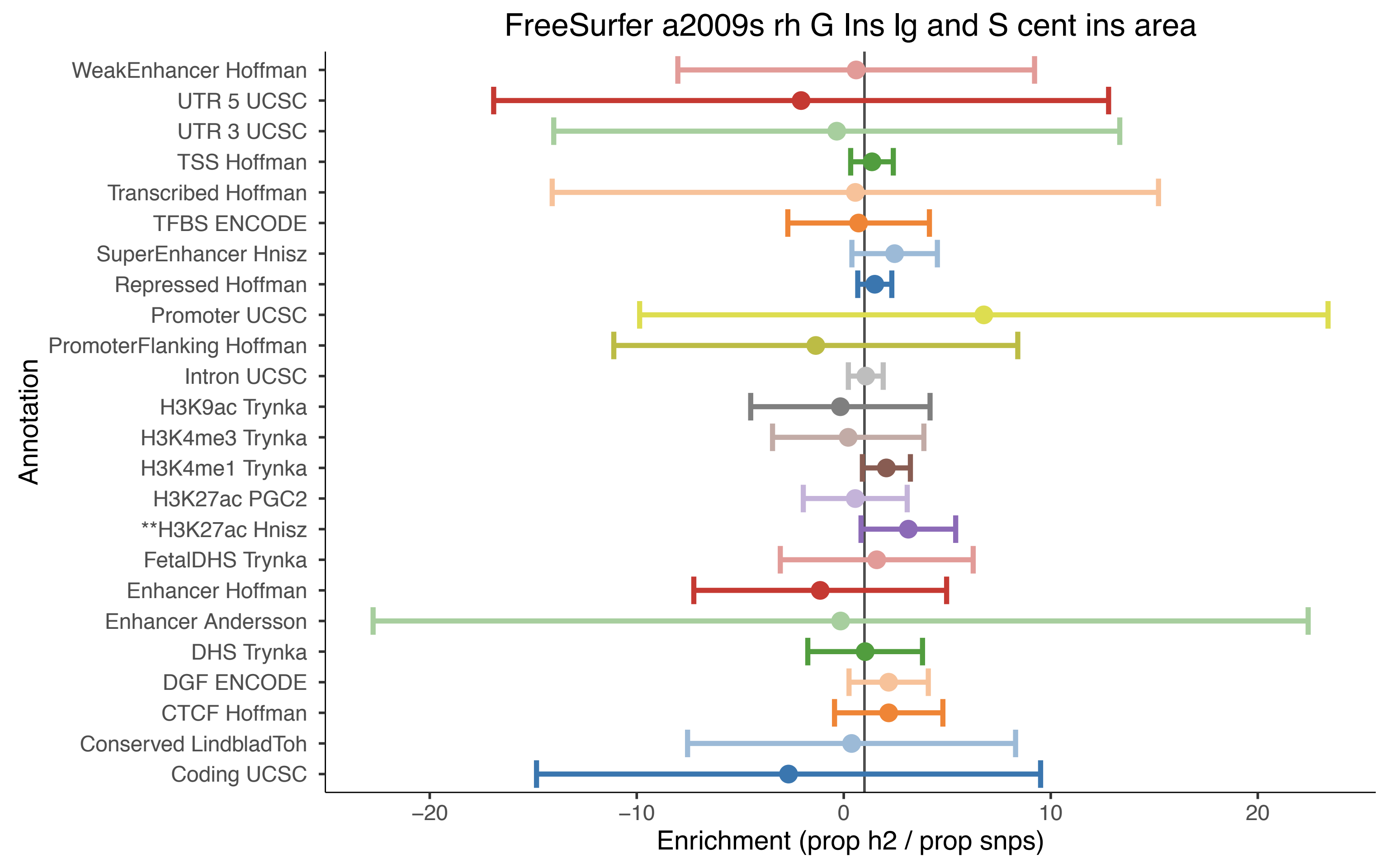
FreeSurfer a2009s rh G Ins lg and S cent ins area
WeakEnhancer Hoffman
UTR 5 UCSC
UTR 3 UCSC
TSS Hoffman
Transcribed Hoffman
TFBS ENCODE
SuperEnhancer Hnisz
Repressed Hoffman
Promoter UCSC
PromoterFlanking Hoffman
Intron UCSC
H3K9ac Trynka
H3K4me3 Trynka
H3K4me1 Trynka
H3K27ac PGC2
**H3K27ac Hnisz
FetalDHS Trynka
Enhancer Hoffman
Enhancer Andersson
DHS Trynka
DGF ENCODE
CTCF Hoffman
Conserved LindbladToh
Coding UCSC
Annotation
−20
−10
0
10
20
Enrichment (prop h2 / prop snps)

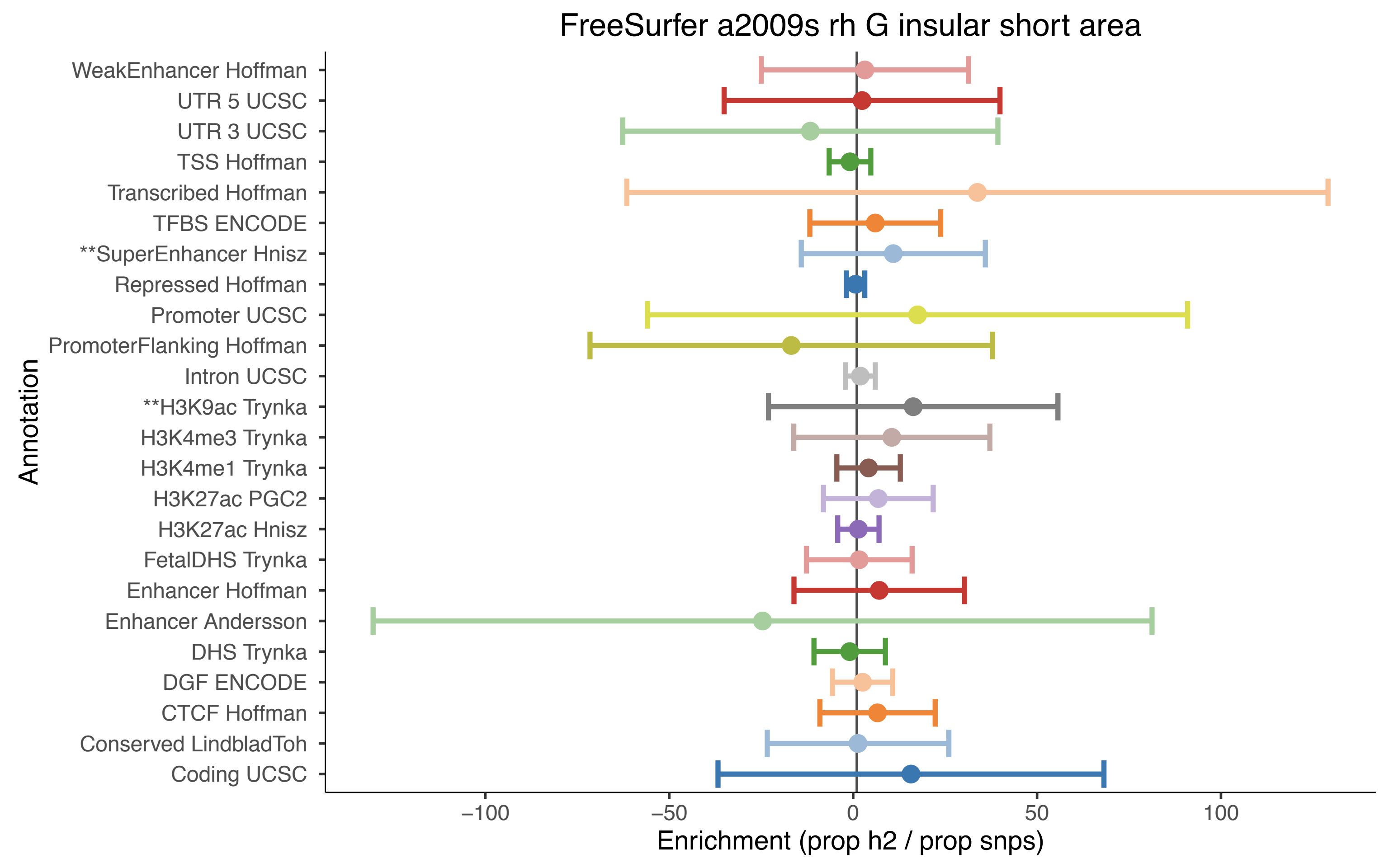

FreeSurfer a2009s rh G insular short area
WeakEnhancer Hoffman
UTR 5 UCSC
UTR 3 UCSC
TSS Hoffman
Transcribed Hoffman
TFBS ENCODE
**SuperEnhancer Hnisz
Repressed Hoffman
Promoter UCSC
PromoterFlanking Hoffman
Intron UCSC
**H3K9ac Trynka
H3K4me3 Trynka
H3K4me1 Trynka
H3K27ac PGC2
H3K27ac Hnisz
FetalDHS Trynka
Enhancer Hoffman
Enhancer Andersson
DHS Trynka
DGF ENCODE
CTCF Hoffman
Conserved LindbladToh
Coding UCSC
Annotation
−100
−50
0
50
100
Enrichment (prop h2 / prop snps)

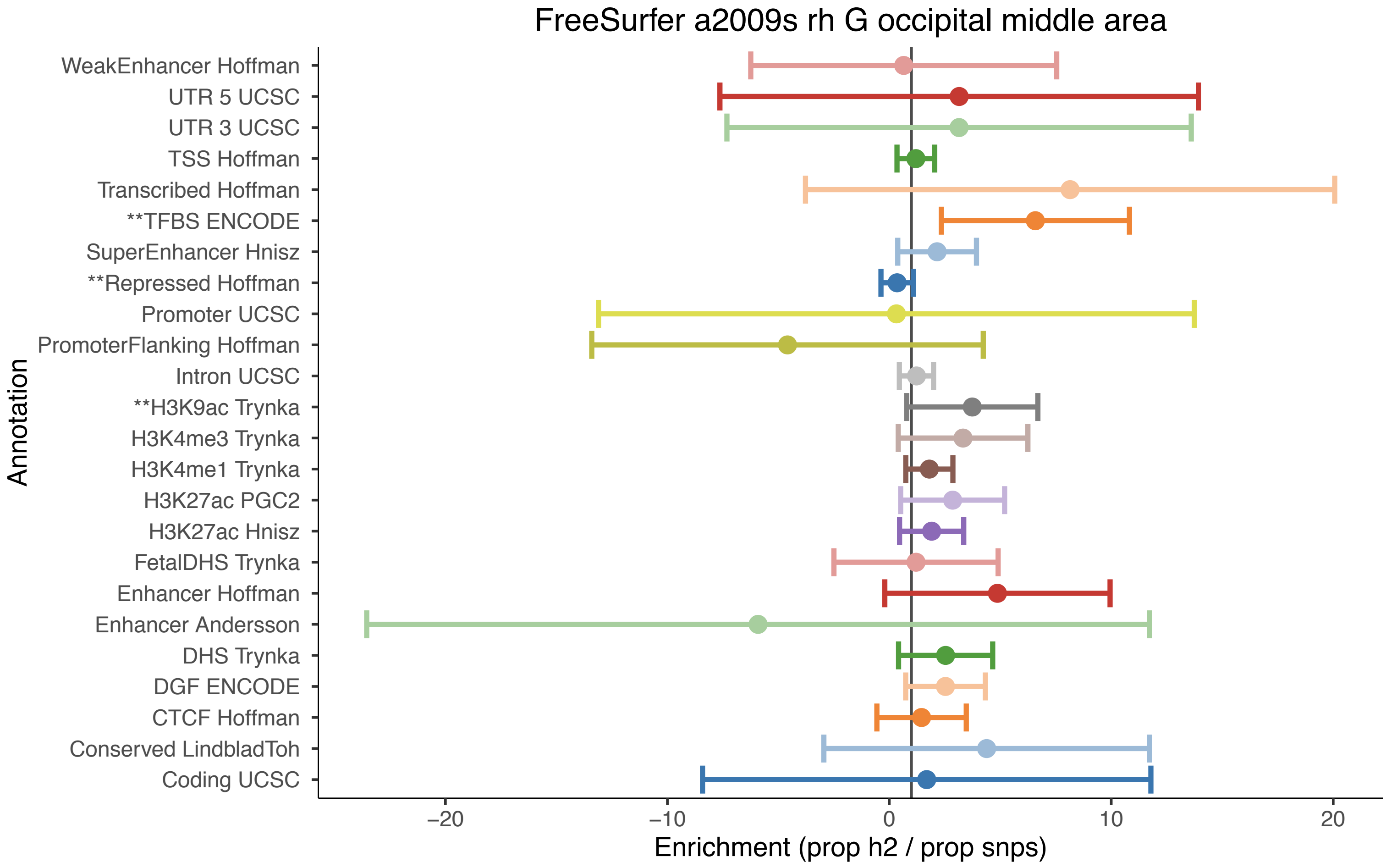
FreeSurfer a2009s rh G occipital middle area
WeakEnhancer Hoffman
UTR 5 UCSC
UTR 3 UCSC
TSS Hoffman
Transcribed Hoffman
**TFBS ENCODE
SuperEnhancer Hnisz
**Repressed Hoffman
Promoter UCSC
PromoterFlanking Hoffman
Intron UCSC
**H3K9ac Trynka
H3K4me3 Trynka
H3K4me1 Trynka
H3K27ac PGC2
H3K27ac Hnisz
FetalDHS Trynka
Enhancer Hoffman
Enhancer Andersson
DHS Trynka
DGF ENCODE
CTCF Hoffman
Conserved LindbladToh
Coding UCSC
Annotation
−20
−10
0
10
20
Enrichment (prop h2 / prop snps)

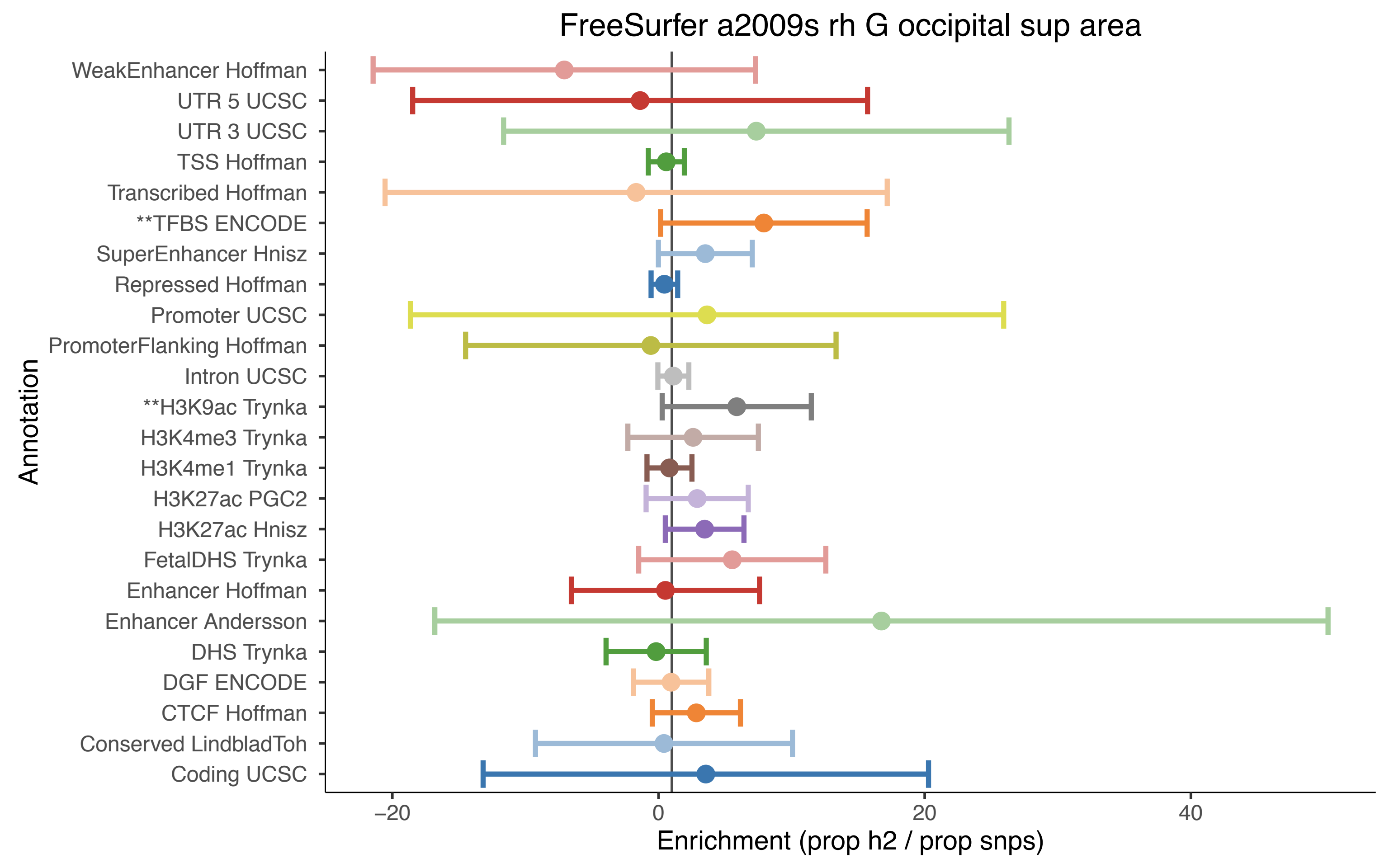
FreeSurfer a2009s rh G occipital sup area
WeakEnhancer Hoffman
UTR 5 UCSC
UTR 3 UCSC
TSS Hoffman
Transcribed Hoffman
**TFBS ENCODE
SuperEnhancer Hnisz
Repressed Hoffman
Promoter UCSC
PromoterFlanking Hoffman
Intron UCSC
**H3K9ac Trynka
H3K4me3 Trynka
H3K4me1 Trynka
H3K27ac PGC2
H3K27ac Hnisz
FetalDHS Trynka
Enhancer Hoffman
Enhancer Andersson
DHS Trynka
DGF ENCODE
CTCF Hoffman
Conserved LindbladToh
Coding UCSC
Annotation
−20
0
20
40
Enrichment (prop h2 / prop snps)

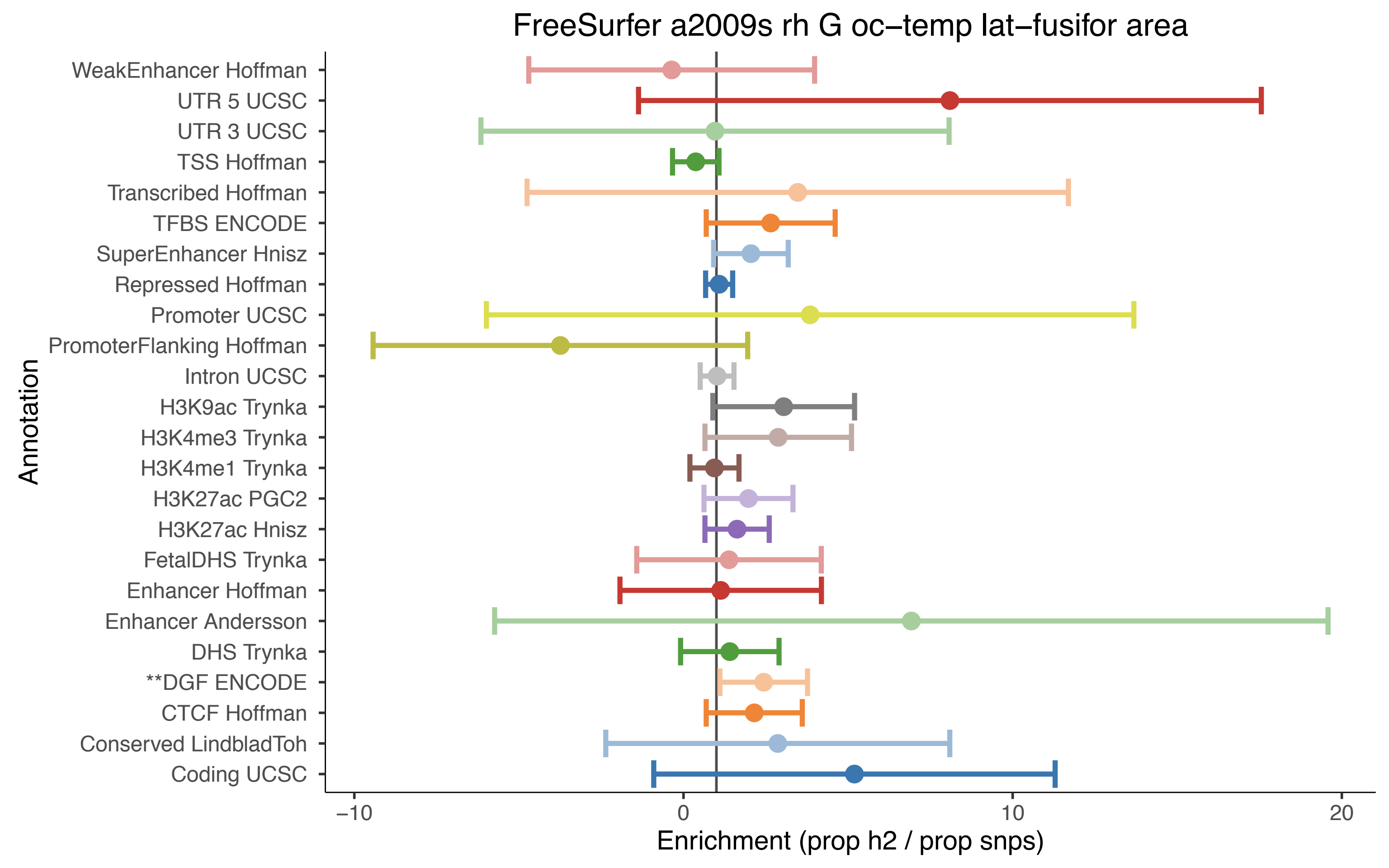

FreeSurfer a2009s rh G oc−temp lat−fusifor area
WeakEnhancer Hoffman
UTR 5 UCSC
UTR 3 UCSC
TSS Hoffman
Transcribed Hoffman
TFBS ENCODE
SuperEnhancer Hnisz
Repressed Hoffman
Promoter UCSC
PromoterFlanking Hoffman
Intron UCSC
H3K9ac Trynka
H3K4me3 Trynka
H3K4me1 Trynka
H3K27ac PGC2
H3K27ac Hnisz
FetalDHS Trynka
Enhancer Hoffman
Enhancer Andersson
DHS Trynka
**DGF ENCODE
CTCF Hoffman
Conserved LindbladToh
Coding UCSC
Annotation
−10
0
10
20
Enrichment (prop h2 / prop snps)

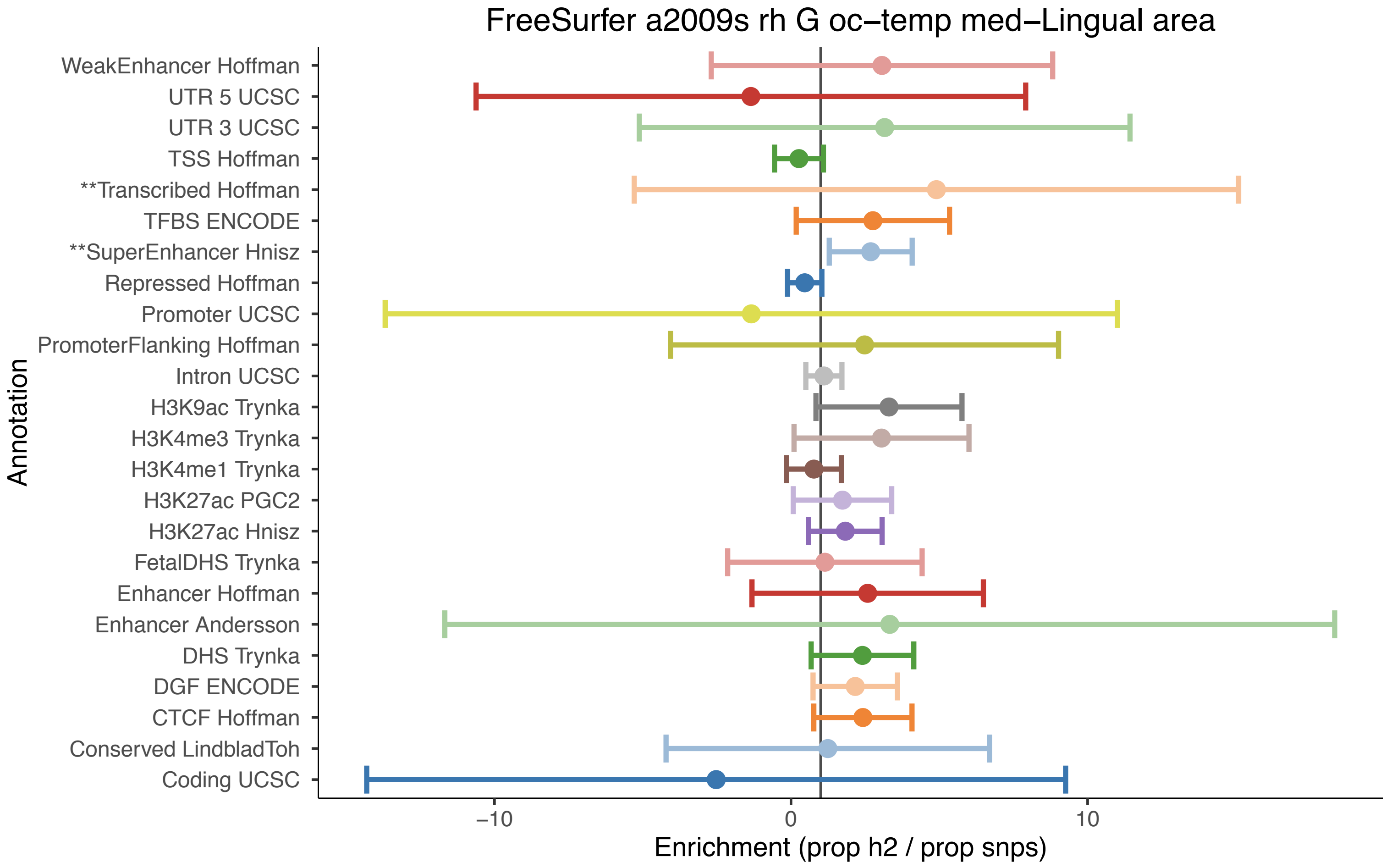

FreeSurfer a2009s rh G oc−temp med−Lingual area
WeakEnhancer Hoffman
UTR 5 UCSC
UTR 3 UCSC
TSS Hoffman
**Transcribed Hoffman
TFBS ENCODE
**SuperEnhancer Hnisz
Repressed Hoffman
Promoter UCSC
PromoterFlanking Hoffman
Intron UCSC
H3K9ac Trynka
H3K4me3 Trynka
H3K4me1 Trynka
H3K27ac PGC2
H3K27ac Hnisz
FetalDHS Trynka
Enhancer Hoffman
Enhancer Andersson
DHS Trynka
DGF ENCODE
CTCF Hoffman
Conserved LindbladToh
Coding UCSC
Annotation
−10
0
10
Enrichment (prop h2 / prop snps)

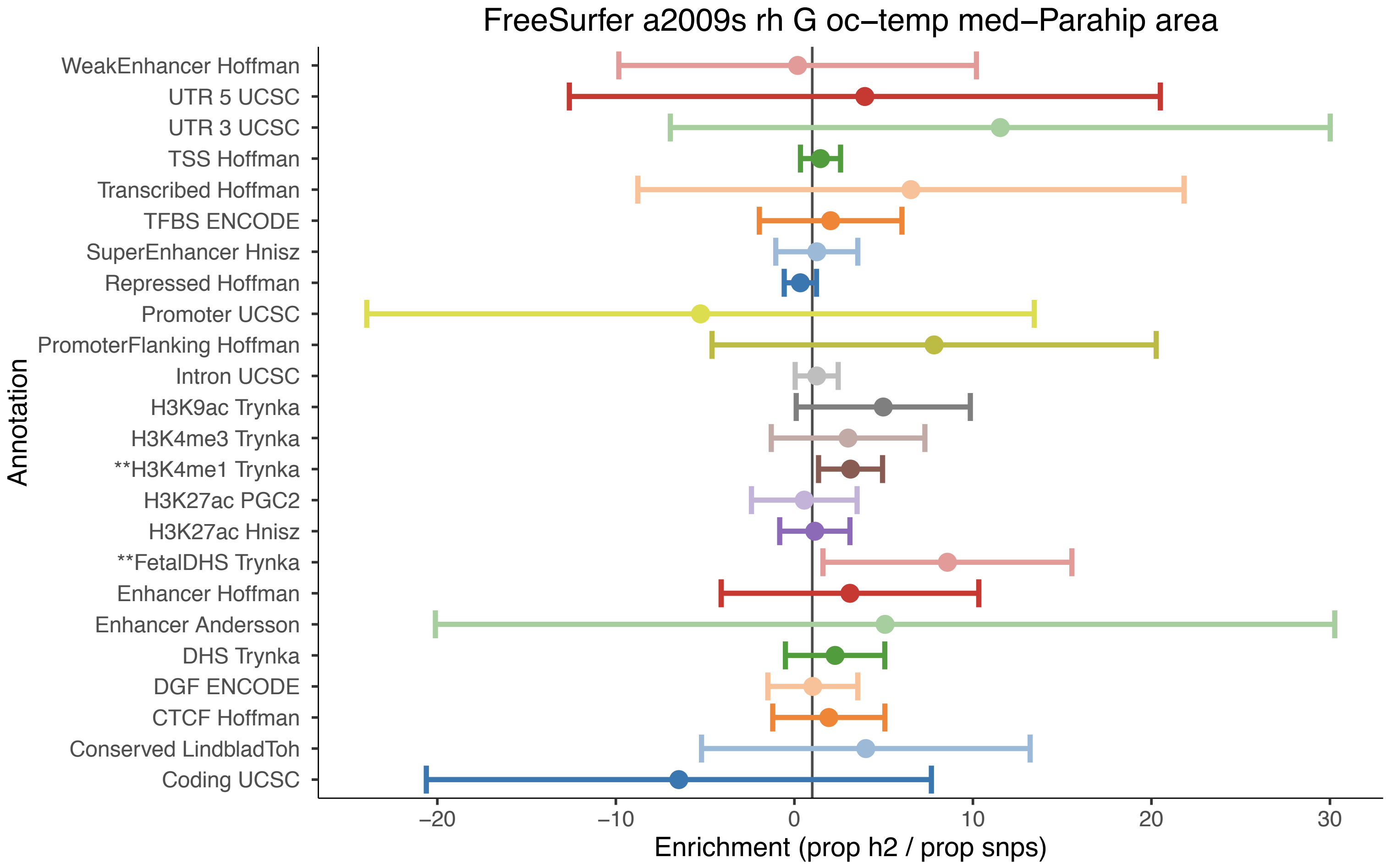
FreeSurfer a2009s rh G oc−temp med−Parahip area
WeakEnhancer Hoffman
UTR 5 UCSC
UTR 3 UCSC
TSS Hoffman
Transcribed Hoffman
TFBS ENCODE
SuperEnhancer Hnisz
Repressed Hoffman
Promoter UCSC
PromoterFlanking Hoffman
Intron UCSC
H3K9ac Trynka
H3K4me3 Trynka
**H3K4me1 Trynka
H3K27ac PGC2
H3K27ac Hnisz
**FetalDHS Trynka
Enhancer Hoffman
Enhancer Andersson
DHS Trynka
DGF ENCODE
CTCF Hoffman
Conserved LindbladToh
Coding UCSC
Annotation
−20
−10
0
10
20
30
Enrichment (prop h2 / prop snps)

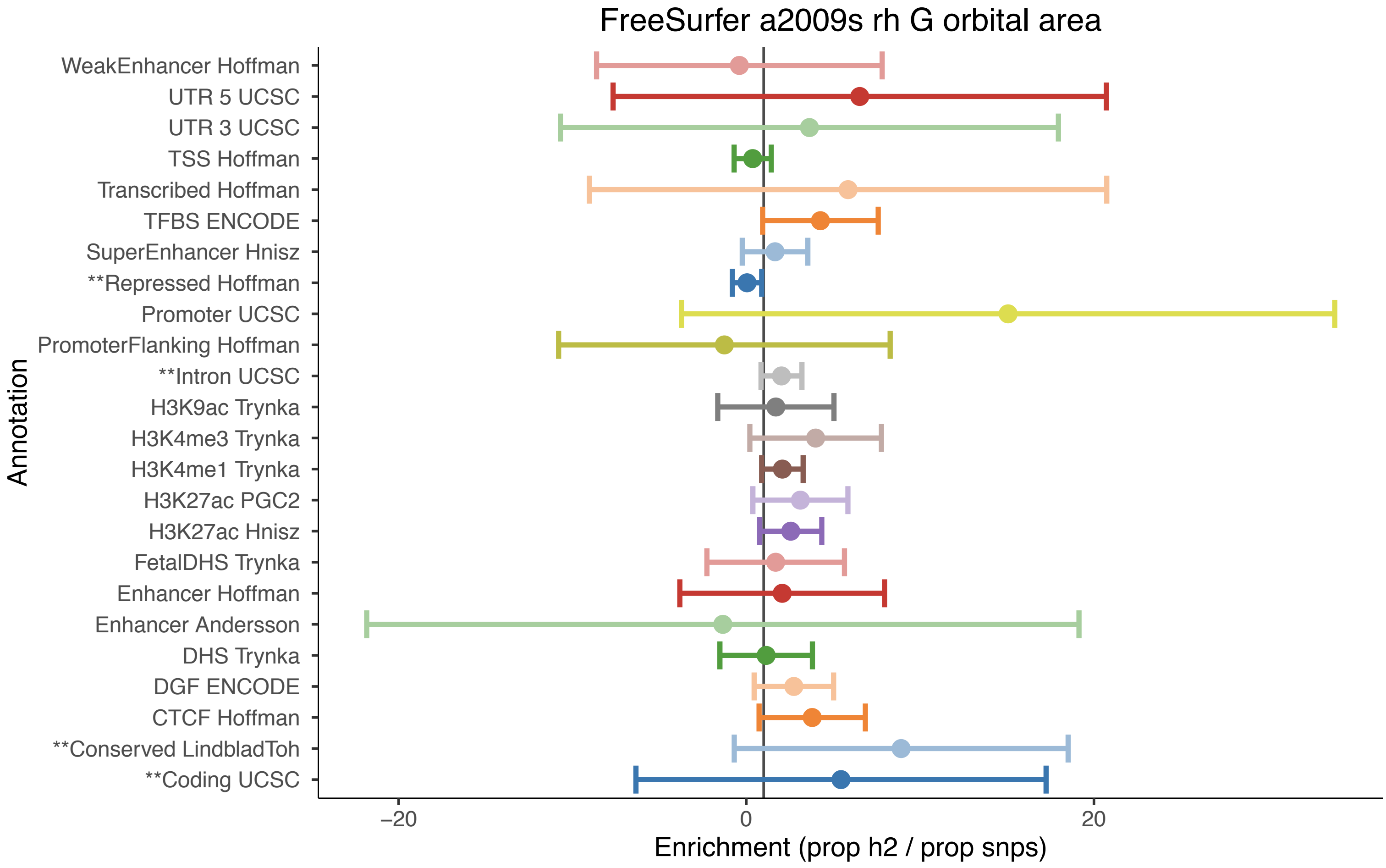

FreeSurfer a2009s rh G orbital area
WeakEnhancer Hoffman
UTR 5 UCSC
UTR 3 UCSC
TSS Hoffman
Transcribed Hoffman
TFBS ENCODE
SuperEnhancer Hnisz
**Repressed Hoffman
Promoter UCSC
PromoterFlanking Hoffman
**Intron UCSC
H3K9ac Trynka
H3K4me3 Trynka
H3K4me1 Trynka
H3K27ac PGC2
H3K27ac Hnisz
FetalDHS Trynka
Enhancer Hoffman
Enhancer Andersson
DHS Trynka
DGF ENCODE
CTCF Hoffman
**Conserved LindbladToh
**Coding UCSC
Annotation
−20
0
20
Enrichment (prop h2 / prop snps)

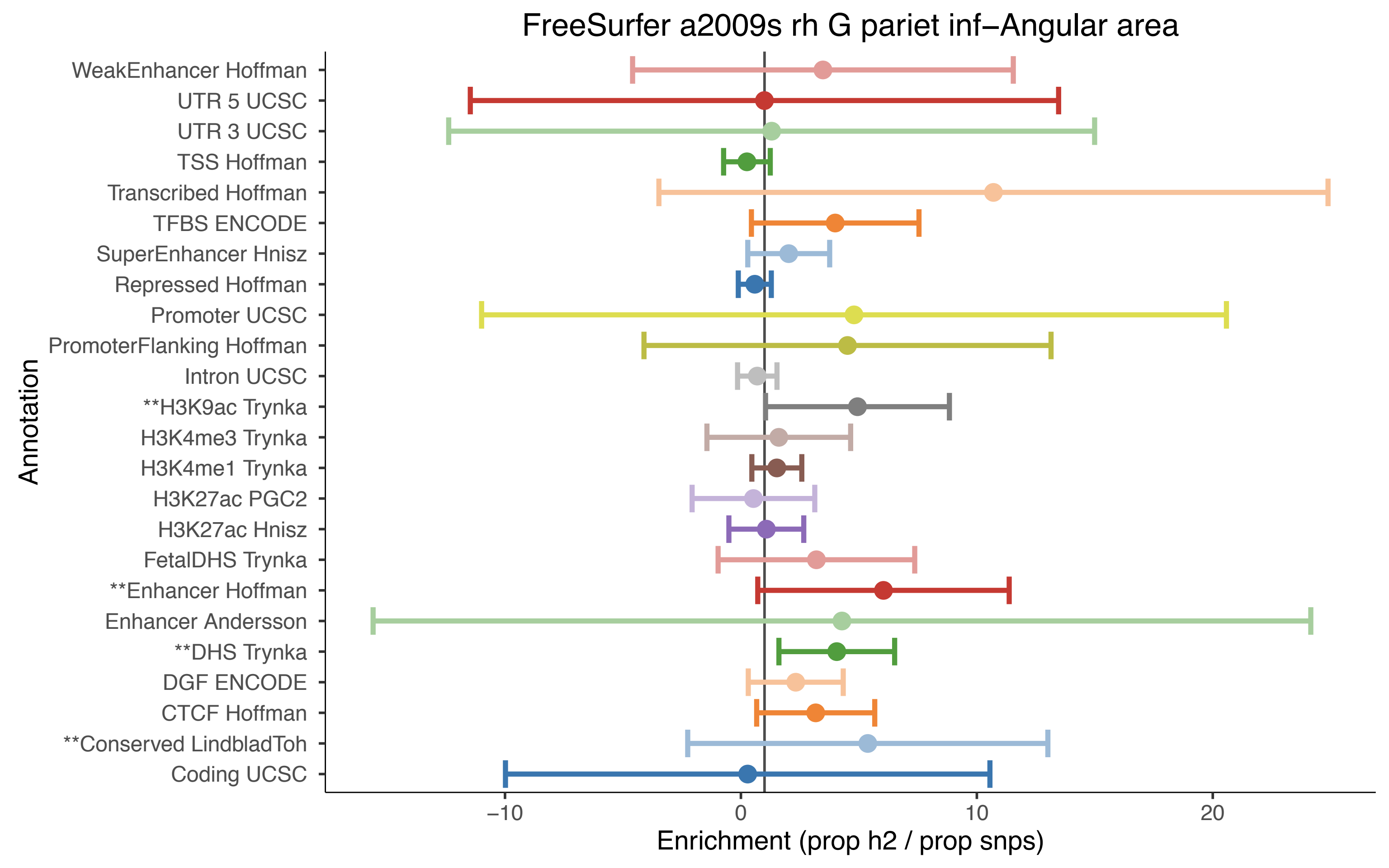

FreeSurfer a2009s rh G pariet inf−Angular area
WeakEnhancer Hoffman
UTR 5 UCSC
UTR 3 UCSC
TSS Hoffman
Transcribed Hoffman
TFBS ENCODE
SuperEnhancer Hnisz
Repressed Hoffman
Promoter UCSC
PromoterFlanking Hoffman
Intron UCSC
**H3K9ac Trynka
H3K4me3 Trynka
H3K4me1 Trynka
H3K27ac PGC2
H3K27ac Hnisz
FetalDHS Trynka
**Enhancer Hoffman
Enhancer Andersson
**DHS Trynka
DGF ENCODE
CTCF Hoffman
**Conserved LindbladToh
Coding UCSC
Annotation
−10
0
10
20
Enrichment (prop h2 / prop snps)

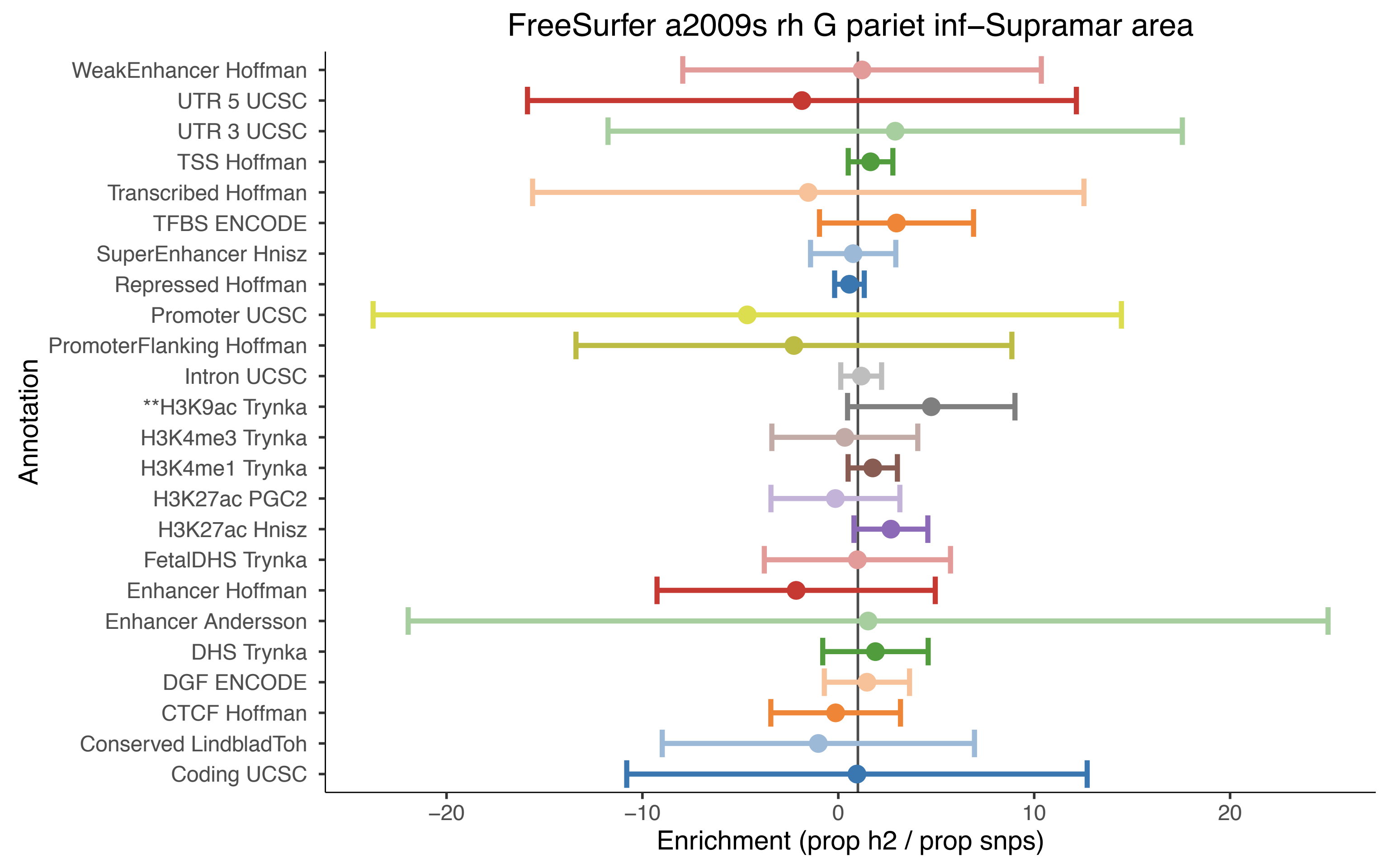
FreeSurfer a2009s rh G pariet inf−Supramar area
WeakEnhancer Hoffman
UTR 5 UCSC
UTR 3 UCSC
TSS Hoffman
Transcribed Hoffman
TFBS ENCODE
SuperEnhancer Hnisz
Repressed Hoffman
Promoter UCSC
PromoterFlanking Hoffman
Intron UCSC
**H3K9ac Trynka
H3K4me3 Trynka
H3K4me1 Trynka
H3K27ac PGC2
H3K27ac Hnisz
FetalDHS Trynka
Enhancer Hoffman
Enhancer Andersson
DHS Trynka
DGF ENCODE
CTCF Hoffman
Conserved LindbladToh
Coding UCSC
Annotation
−20
−10
0
10
20
Enrichment (prop h2 / prop snps)

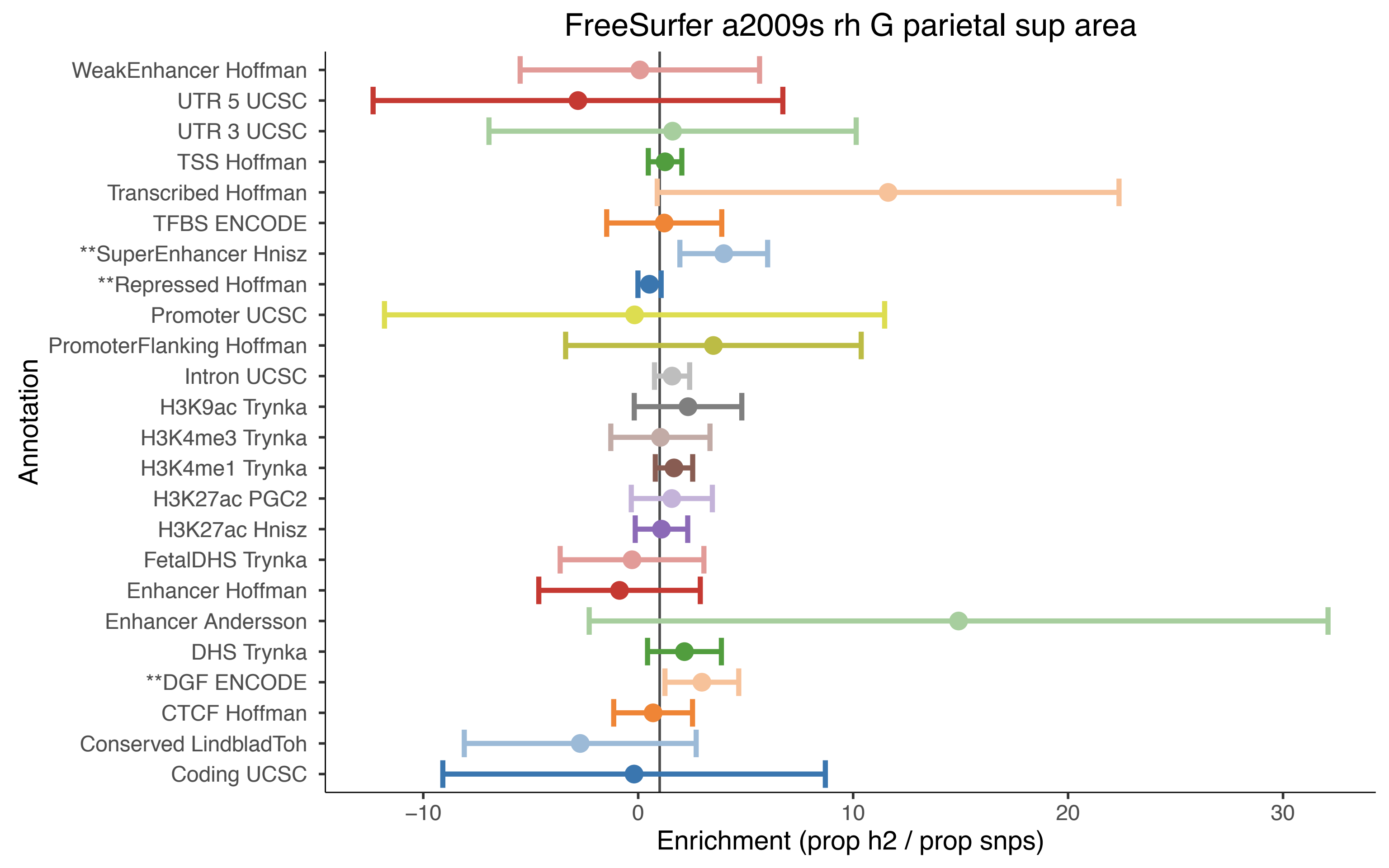
FreeSurfer a2009s rh G parietal sup area
WeakEnhancer Hoffman
UTR 5 UCSC
UTR 3 UCSC
TSS Hoffman
Transcribed Hoffman
TFBS ENCODE
**SuperEnhancer Hnisz
**Repressed Hoffman
Promoter UCSC
PromoterFlanking Hoffman
Intron UCSC
H3K9ac Trynka
H3K4me3 Trynka
H3K4me1 Trynka
H3K27ac PGC2
H3K27ac Hnisz
FetalDHS Trynka
Enhancer Hoffman
Enhancer Andersson
DHS Trynka
**DGF ENCODE
CTCF Hoffman
Conserved LindbladToh
Coding UCSC
Annotation
−10
0
10
20
30
Enrichment (prop h2 / prop snps)

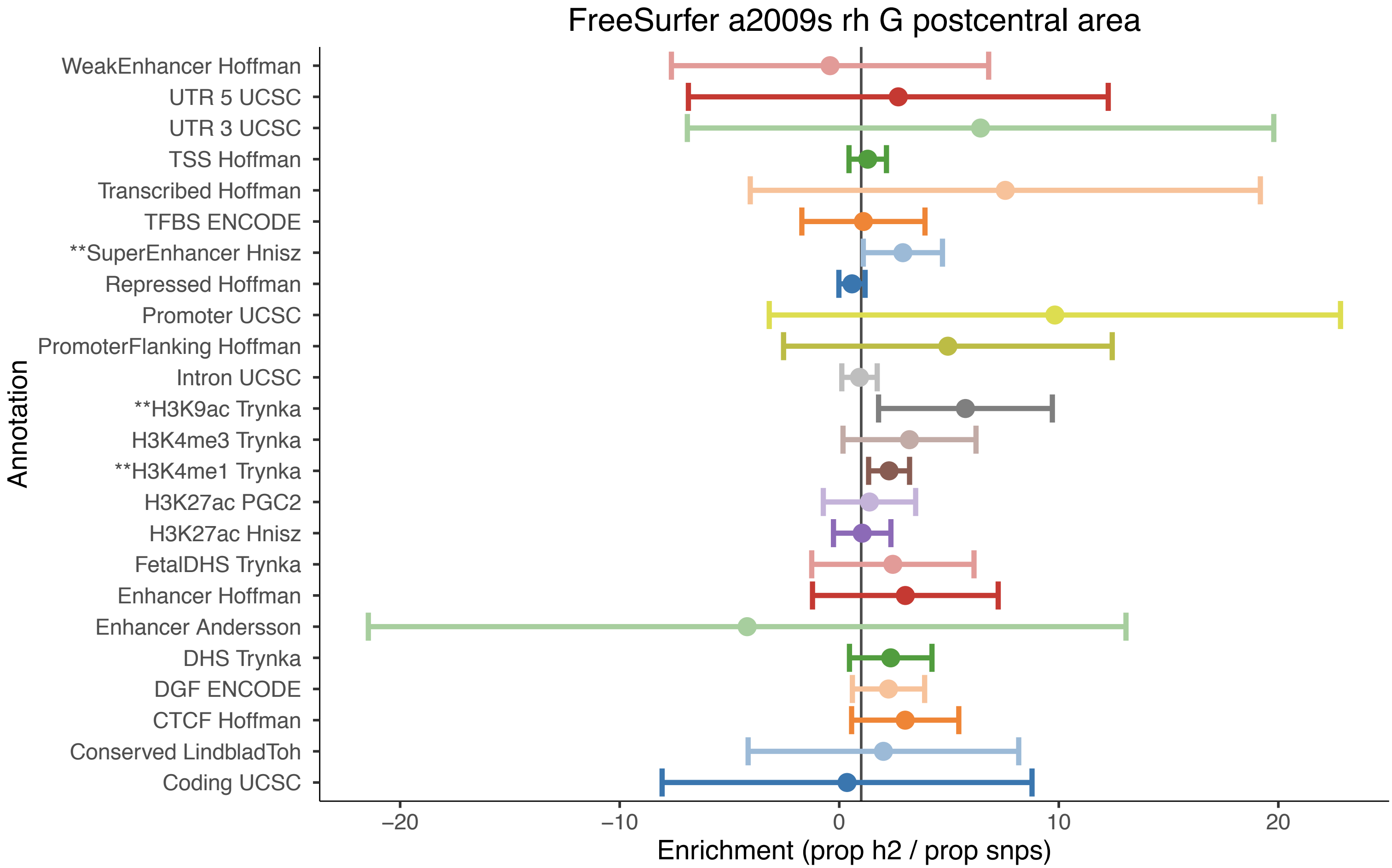
FreeSurfer a2009s rh G postcentral area
WeakEnhancer Hoffman
UTR 5 UCSC
UTR 3 UCSC
TSS Hoffman
Transcribed Hoffman
TFBS ENCODE
**SuperEnhancer Hnisz
Repressed Hoffman
Promoter UCSC
PromoterFlanking Hoffman
Intron UCSC
**H3K9ac Trynka
H3K4me3 Trynka
**H3K4me1 Trynka
H3K27ac PGC2
H3K27ac Hnisz
FetalDHS Trynka
Enhancer Hoffman
Enhancer Andersson
DHS Trynka
DGF ENCODE
CTCF Hoffman
Conserved LindbladToh
Coding UCSC
Annotation
−20
−10
0
10
20
Enrichment (prop h2 / prop snps)

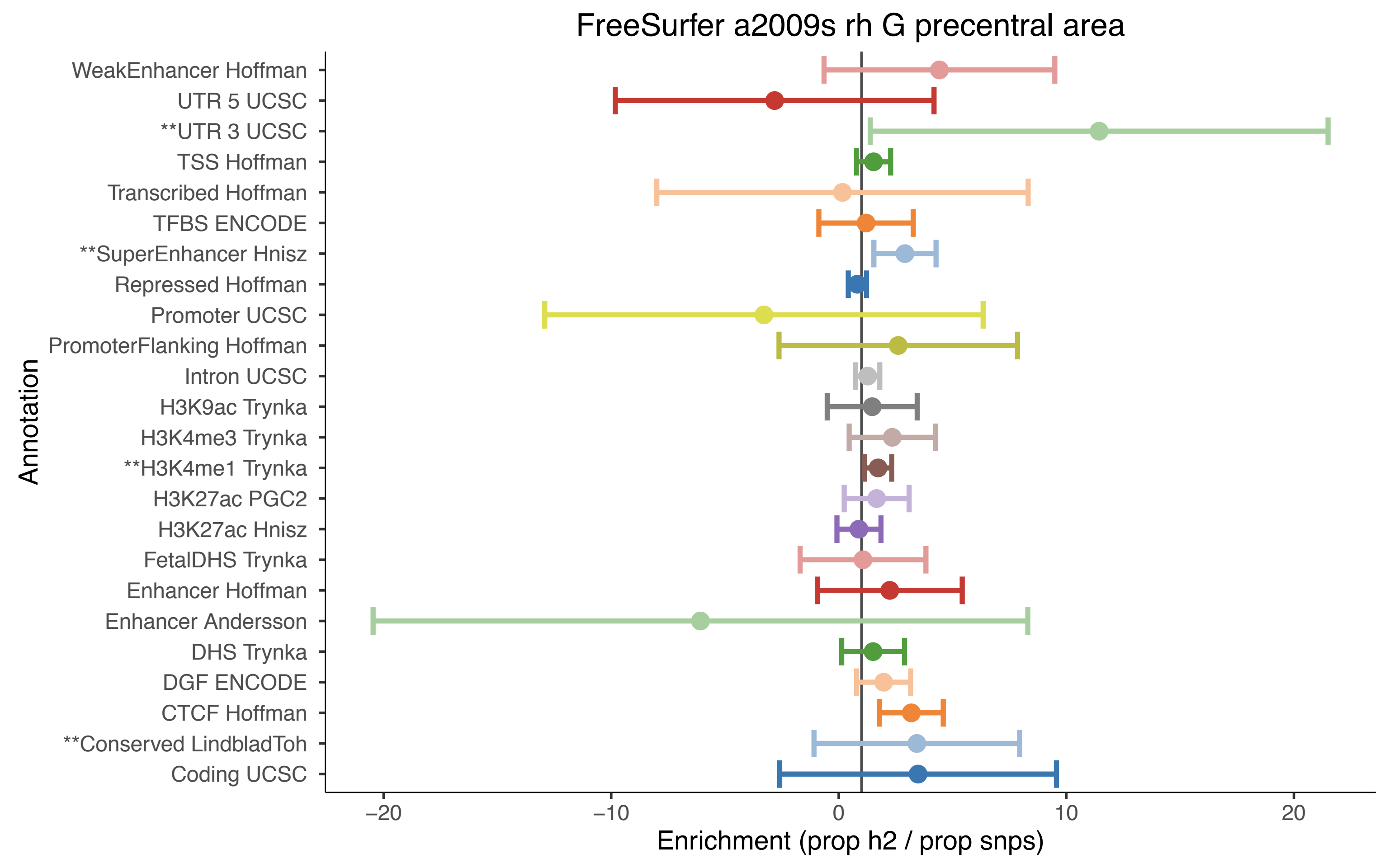

FreeSurfer a2009s rh G precentral area
WeakEnhancer Hoffman
UTR 5 UCSC
**UTR 3 UCSC
TSS Hoffman
Transcribed Hoffman
TFBS ENCODE
**SuperEnhancer Hnisz
Repressed Hoffman
Promoter UCSC
PromoterFlanking Hoffman
Intron UCSC
H3K9ac Trynka
H3K4me3 Trynka
**H3K4me1 Trynka
H3K27ac PGC2
H3K27ac Hnisz
FetalDHS Trynka
Enhancer Hoffman
Enhancer Andersson
DHS Trynka
DGF ENCODE
CTCF Hoffman
**Conserved LindbladToh
Coding UCSC
Annotation
−20
−10
0
10
20
Enrichment (prop h2 / prop snps)

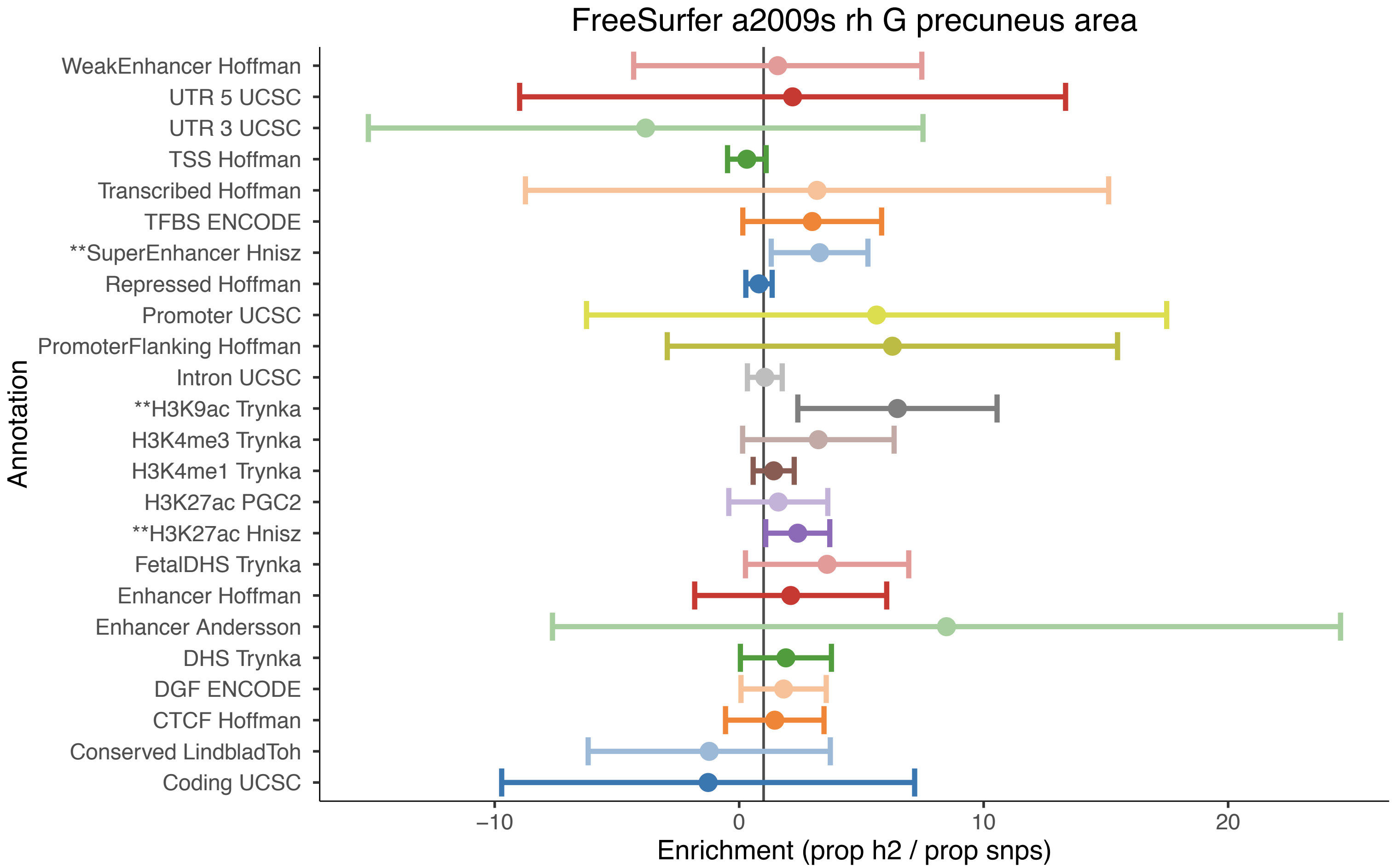

FreeSurfer a2009s rh G precuneus area
WeakEnhancer Hoffman
UTR 5 UCSC
UTR 3 UCSC
TSS Hoffman
Transcribed Hoffman
TFBS ENCODE
**SuperEnhancer Hnisz
Repressed Hoffman
Promoter UCSC
PromoterFlanking Hoffman
Intron UCSC
**H3K9ac Trynka
H3K4me3 Trynka
H3K4me1 Trynka
H3K27ac PGC2
**H3K27ac Hnisz
FetalDHS Trynka
Enhancer Hoffman
Enhancer Andersson
DHS Trynka
DGF ENCODE
CTCF Hoffman
Conserved LindbladToh
Coding UCSC
Annotation
−10
0
10
20
Enrichment (prop h2 / prop snps)

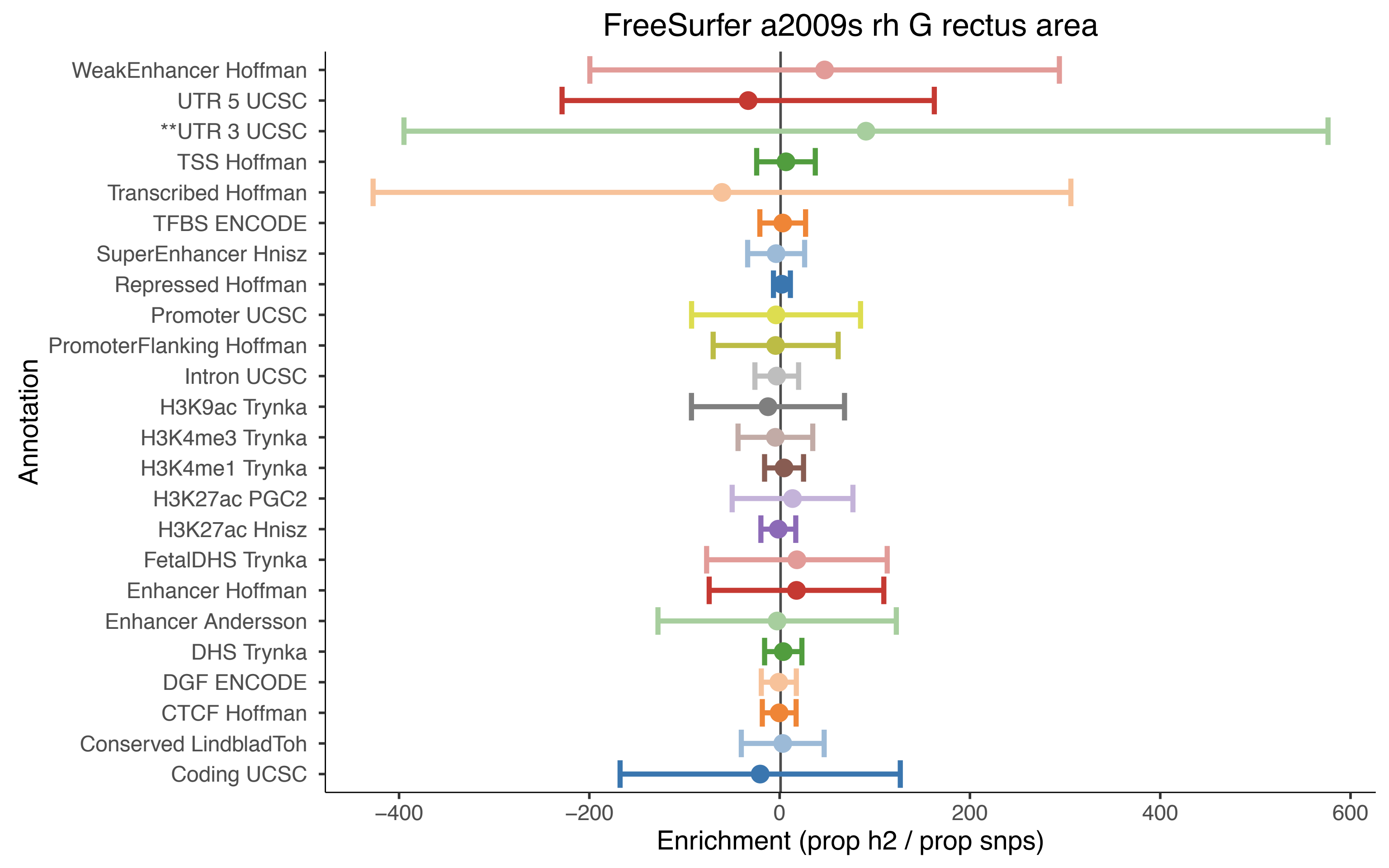

FreeSurfer a2009s rh G rectus area
WeakEnhancer Hoffman
UTR 5 UCSC
**UTR 3 UCSC
TSS Hoffman
Transcribed Hoffman
TFBS ENCODE
SuperEnhancer Hnisz
Repressed Hoffman
Promoter UCSC
PromoterFlanking Hoffman
Intron UCSC
H3K9ac Trynka
H3K4me3 Trynka
H3K4me1 Trynka
H3K27ac PGC2
H3K27ac Hnisz
FetalDHS Trynka
Enhancer Hoffman
Enhancer Andersson
DHS Trynka
DGF ENCODE
CTCF Hoffman
Conserved LindbladToh
Coding UCSC
Annotation
−400
−200
0
200
400
600
Enrichment (prop h2 / prop snps)

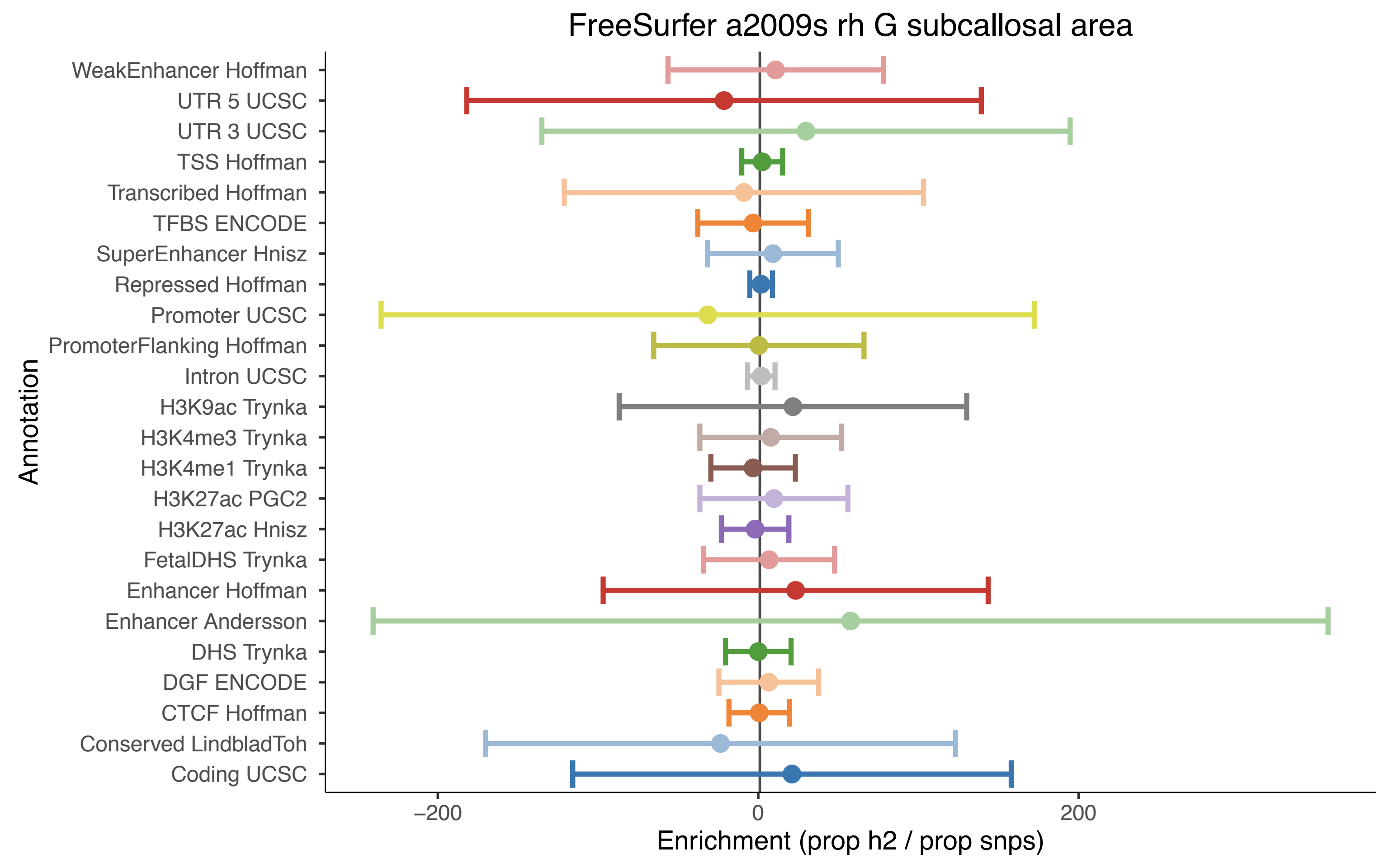

FreeSurfer a2009s rh G subcallosal area
WeakEnhancer Hoffman
UTR 5 UCSC
UTR 3 UCSC
TSS Hoffman
Transcribed Hoffman
TFBS ENCODE
SuperEnhancer Hnisz
Repressed Hoffman
Promoter UCSC
PromoterFlanking Hoffman
Intron UCSC
H3K9ac Trynka
H3K4me3 Trynka
H3K4me1 Trynka
H3K27ac PGC2
H3K27ac Hnisz
FetalDHS Trynka
Enhancer Hoffman
Enhancer Andersson
DHS Trynka
DGF ENCODE
CTCF Hoffman
Conserved LindbladToh
Coding UCSC
Annotation
−200
0
200
Enrichment (prop h2 / prop snps)

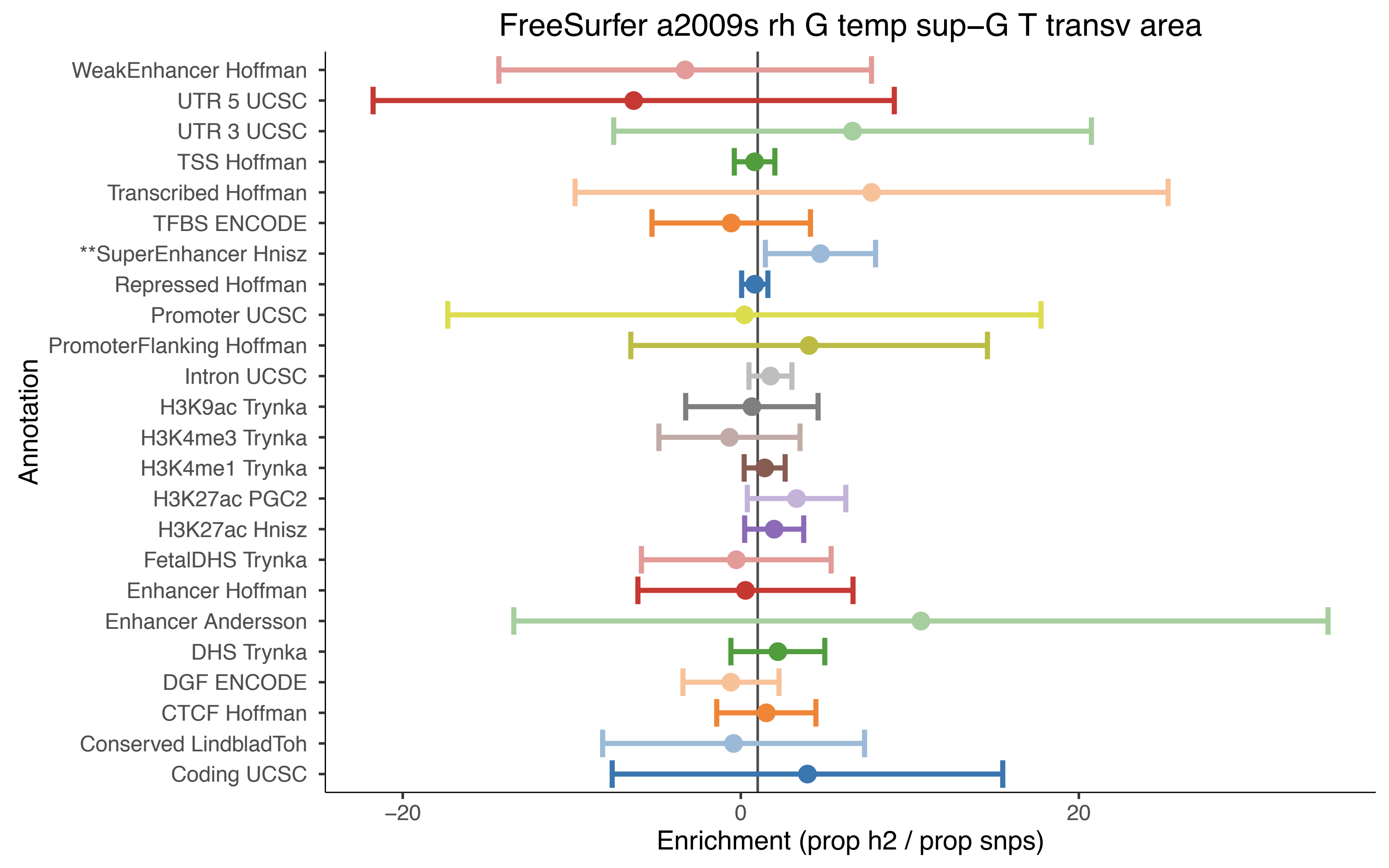

FreeSurfer a2009s rh G temp sup−G T transv area
WeakEnhancer Hoffman
UTR 5 UCSC
UTR 3 UCSC
TSS Hoffman
Transcribed Hoffman
TFBS ENCODE
**SuperEnhancer Hnisz
Repressed Hoffman
Promoter UCSC
PromoterFlanking Hoffman
Intron UCSC
H3K9ac Trynka
H3K4me3 Trynka
H3K4me1 Trynka
H3K27ac PGC2
H3K27ac Hnisz
FetalDHS Trynka
Enhancer Hoffman
Enhancer Andersson
DHS Trynka
DGF ENCODE
CTCF Hoffman
Conserved LindbladToh
Coding UCSC
Annotation
−20
0
20
Enrichment (prop h2 / prop snps)

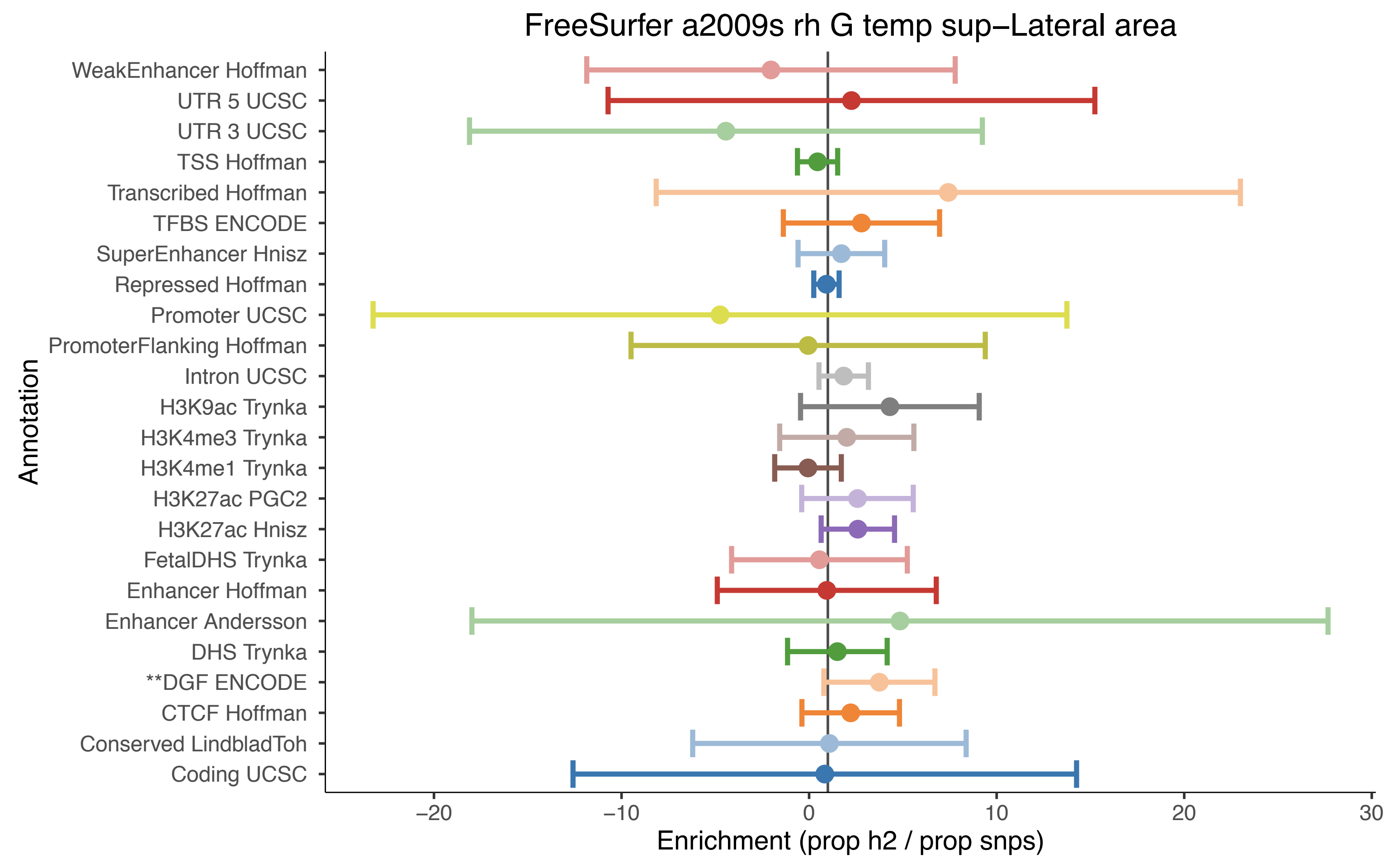
FreeSurfer a2009s rh G temp sup−Lateral area
WeakEnhancer Hoffman
UTR 5 UCSC
UTR 3 UCSC
TSS Hoffman
Transcribed Hoffman
TFBS ENCODE
SuperEnhancer Hnisz
Repressed Hoffman
Promoter UCSC
PromoterFlanking Hoffman
Intron UCSC
H3K9ac Trynka
H3K4me3 Trynka
H3K4me1 Trynka
H3K27ac PGC2
H3K27ac Hnisz
FetalDHS Trynka
Enhancer Hoffman
Enhancer Andersson
DHS Trynka
**DGF ENCODE
CTCF Hoffman
Conserved LindbladToh
Coding UCSC
Annotation
−20
−10
0
10
20
30
Enrichment (prop h2 / prop snps)

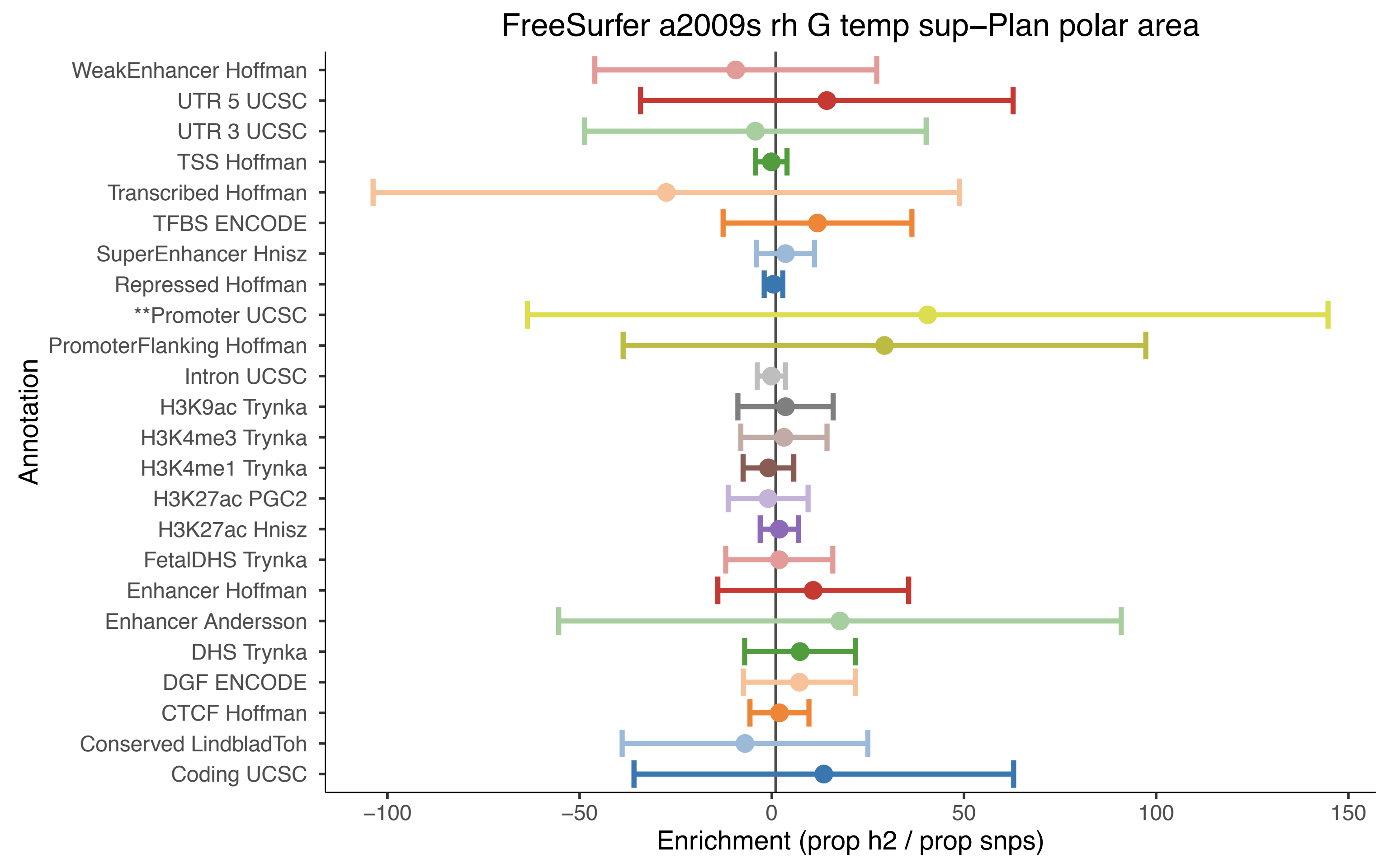
FreeSurfer a2009s rh G temp sup−Plan polar area
Annotation
WeakEnhancer Hoffman
UTR 5 UCSC
UTR 3 UCSC
TSS Hoffman
Transcribed Hoffman
TFBS ENCODE
SuperEnhancer Hnisz
Repressed Hoffman
**Promoter UCSC
PromoterFlanking Hoffman
Intron UCSC
H3K9ac Trynka
H3K4me3 Trynka
H3K4me1 Trynka
H3K27ac PGC2
H3K27ac Hnisz
FetalDHS Trynka
Enhancer Hoffman
Enhancer Andersson
DHS Trynka
DGF ENCODE
CTCF Hoffman
Conserved LindbladToh
Coding UCSC
−100
−50
0
50
100
150
Enrichment (prop h2 / prop snps)

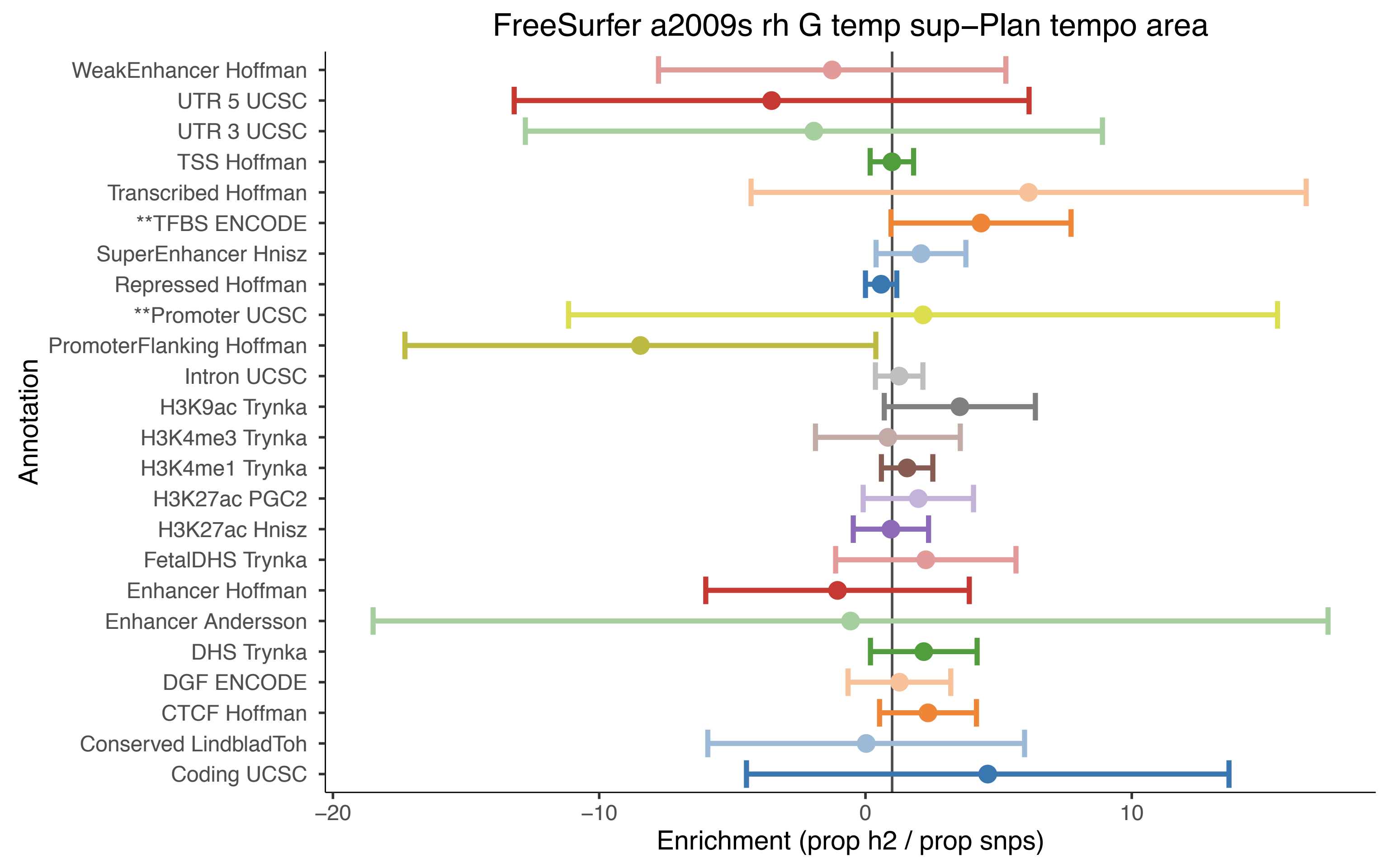
FreeSurfer a2009s rh G temp sup−Plan tempo area
WeakEnhancer Hoffman
UTR 5 UCSC
UTR 3 UCSC
TSS Hoffman
Transcribed Hoffman
**TFBS ENCODE
SuperEnhancer Hnisz
Repressed Hoffman
**Promoter UCSC
PromoterFlanking Hoffman
Intron UCSC
H3K9ac Trynka
H3K4me3 Trynka
H3K4me1 Trynka
H3K27ac PGC2
H3K27ac Hnisz
FetalDHS Trynka
Enhancer Hoffman
Enhancer Andersson
DHS Trynka
DGF ENCODE
CTCF Hoffman
Conserved LindbladToh
Coding UCSC
Annotation
−20
−10
0
10
Enrichment (prop h2 / prop snps)

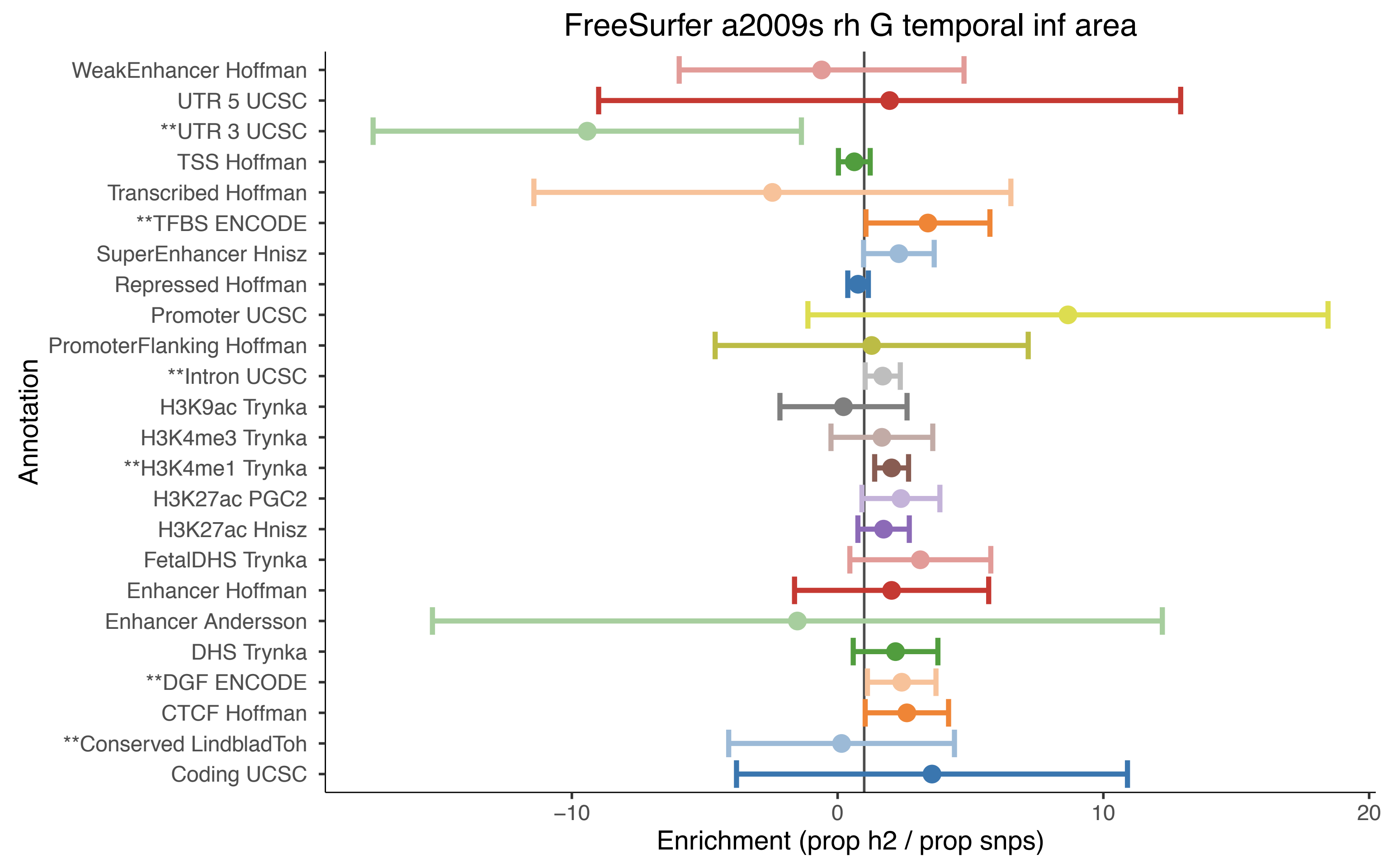
FreeSurfer a2009s rh G temporal inf area
WeakEnhancer Hoffman
UTR 5 UCSC
**UTR 3 UCSC
TSS Hoffman
Transcribed Hoffman
**TFBS ENCODE
SuperEnhancer Hnisz
Repressed Hoffman
Promoter UCSC
PromoterFlanking Hoffman
**Intron UCSC
H3K9ac Trynka
H3K4me3 Trynka
**H3K4me1 Trynka
H3K27ac PGC2
H3K27ac Hnisz
FetalDHS Trynka
Enhancer Hoffman
Enhancer Andersson
DHS Trynka
**DGF ENCODE
CTCF Hoffman
**Conserved LindbladToh
Coding UCSC
Annotation
−10
0
10
20
Enrichment (prop h2 / prop snps)

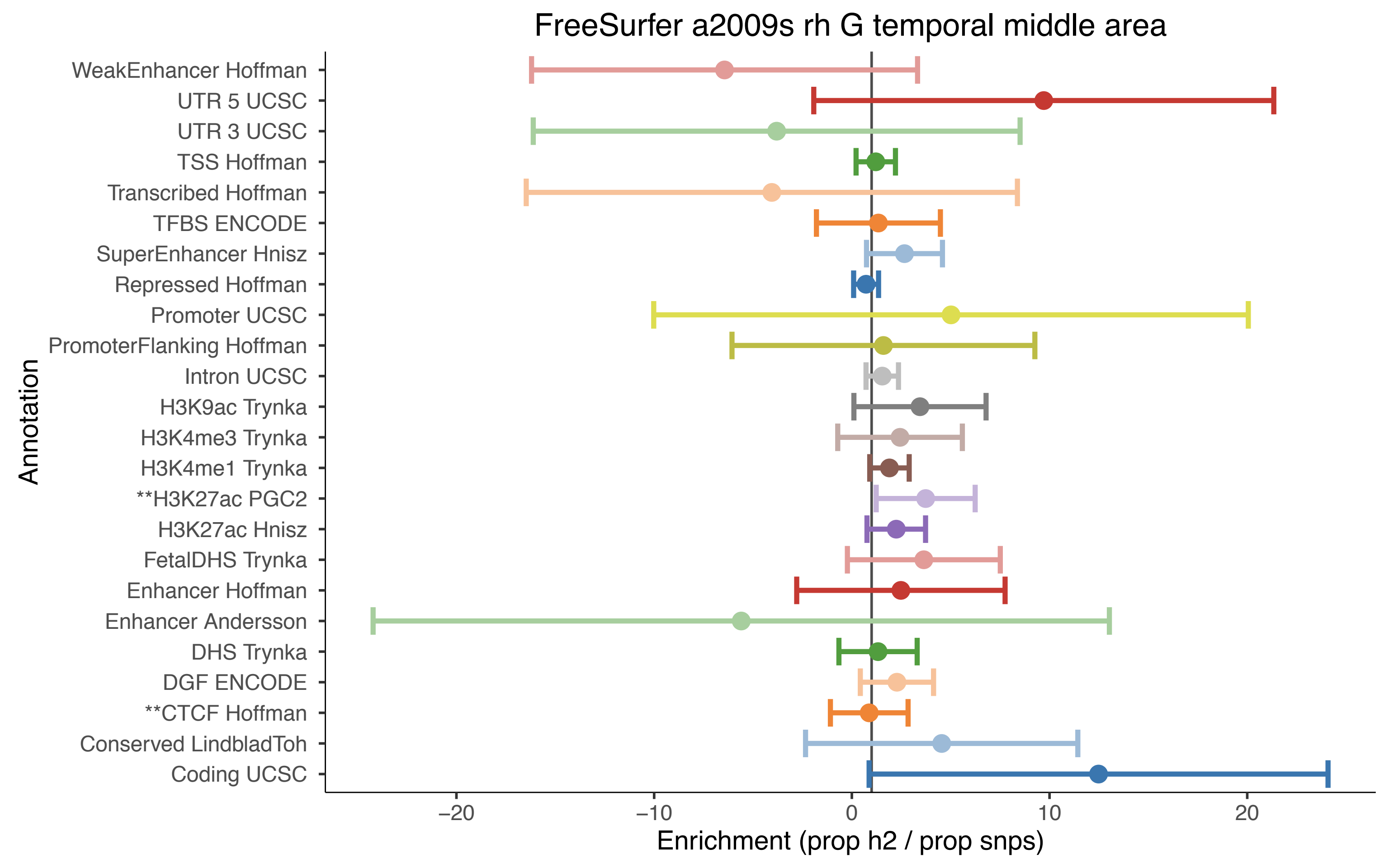
FreeSurfer a2009s rh G temporal middle area
WeakEnhancer Hoffman
UTR 5 UCSC
UTR 3 UCSC
TSS Hoffman
Transcribed Hoffman
TFBS ENCODE
SuperEnhancer Hnisz
Repressed Hoffman
Promoter UCSC
PromoterFlanking Hoffman
Intron UCSC
H3K9ac Trynka
H3K4me3 Trynka
H3K4me1 Trynka
**H3K27ac PGC2
H3K27ac Hnisz
FetalDHS Trynka
Enhancer Hoffman
Enhancer Andersson
DHS Trynka
DGF ENCODE
**CTCF Hoffman
Conserved LindbladToh
Coding UCSC
Annotation
−20
−10
0
10
20
Enrichment (prop h2 / prop snps)

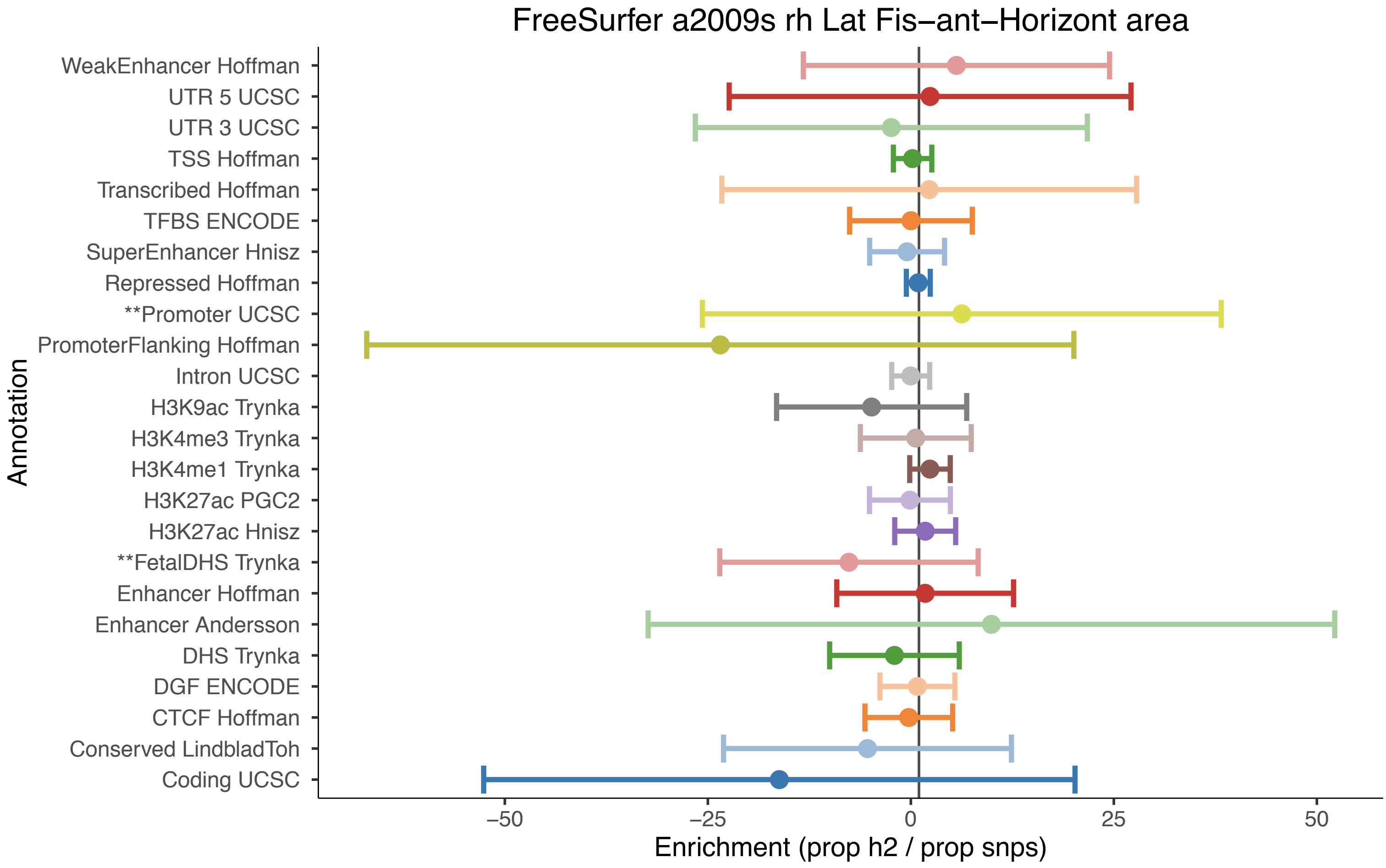
FreeSurfer a2009s rh Lat Fis−ant−Horizont area
WeakEnhancer Hoffman
UTR 5 UCSC
UTR 3 UCSC
TSS Hoffman
Transcribed Hoffman
TFBS ENCODE
SuperEnhancer Hnisz
Repressed Hoffman
**Promoter UCSC
PromoterFlanking Hoffman
Intron UCSC
H3K9ac Trynka
H3K4me3 Trynka
H3K4me1 Trynka
H3K27ac PGC2
H3K27ac Hnisz
**FetalDHS Trynka
Enhancer Hoffman
Enhancer Andersson
DHS Trynka
DGF ENCODE
CTCF Hoffman
Conserved LindbladToh
Coding UCSC
Annotation
−50
−25
0
25
50
Enrichment (prop h2 / prop snps)

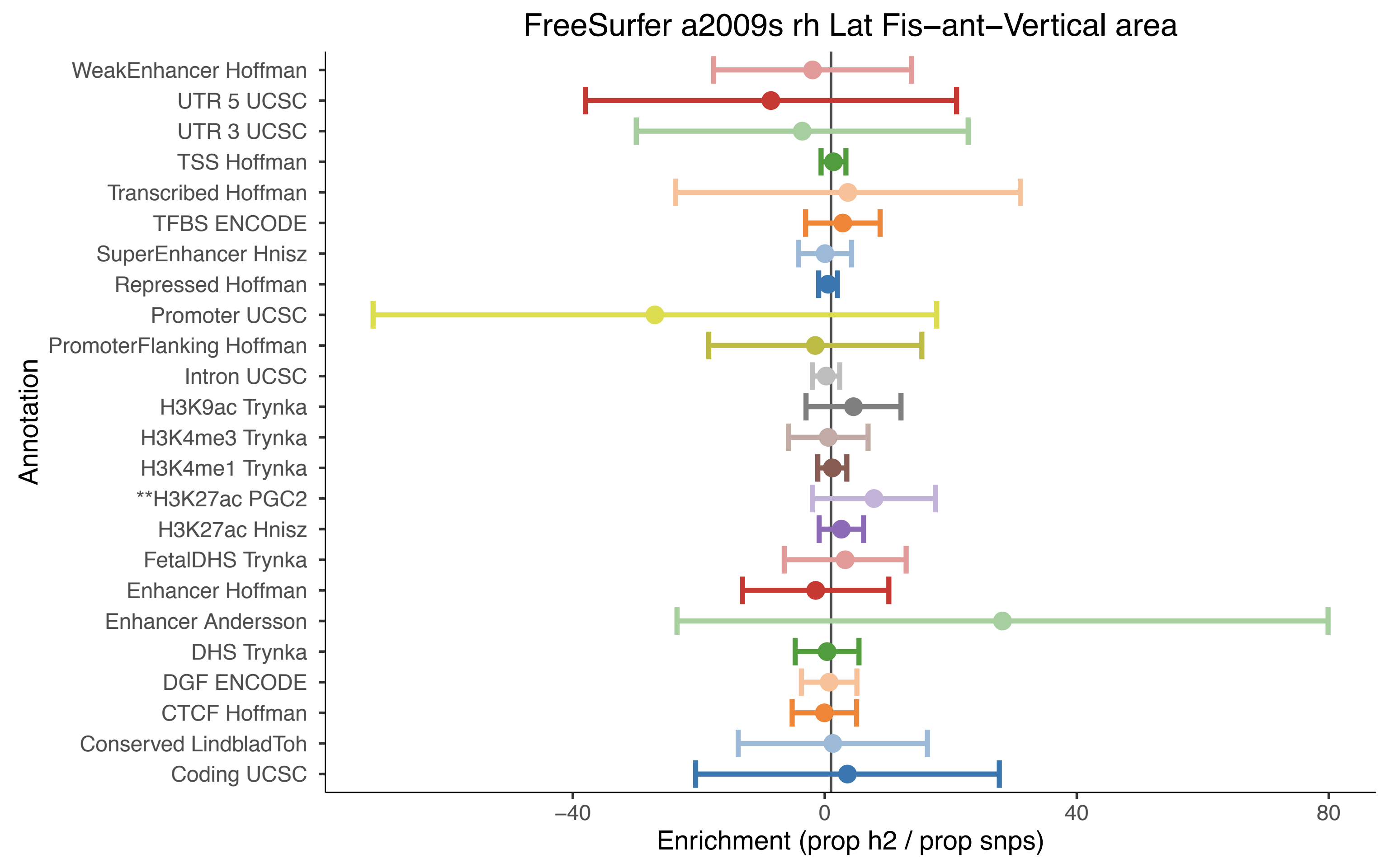

FreeSurfer a2009s rh Lat Fis−ant−Vertical area
WeakEnhancer Hoffman
UTR 5 UCSC
UTR 3 UCSC
TSS Hoffman
Transcribed Hoffman
TFBS ENCODE
SuperEnhancer Hnisz
Repressed Hoffman
Promoter UCSC
PromoterFlanking Hoffman
Intron UCSC
H3K9ac Trynka
H3K4me3 Trynka
H3K4me1 Trynka
**H3K27ac PGC2
H3K27ac Hnisz
FetalDHS Trynka
Enhancer Hoffman
Enhancer Andersson
DHS Trynka
DGF ENCODE
CTCF Hoffman
Conserved LindbladToh
Coding UCSC
Annotation
−40
0
40
80
Enrichment (prop h2 / prop snps)

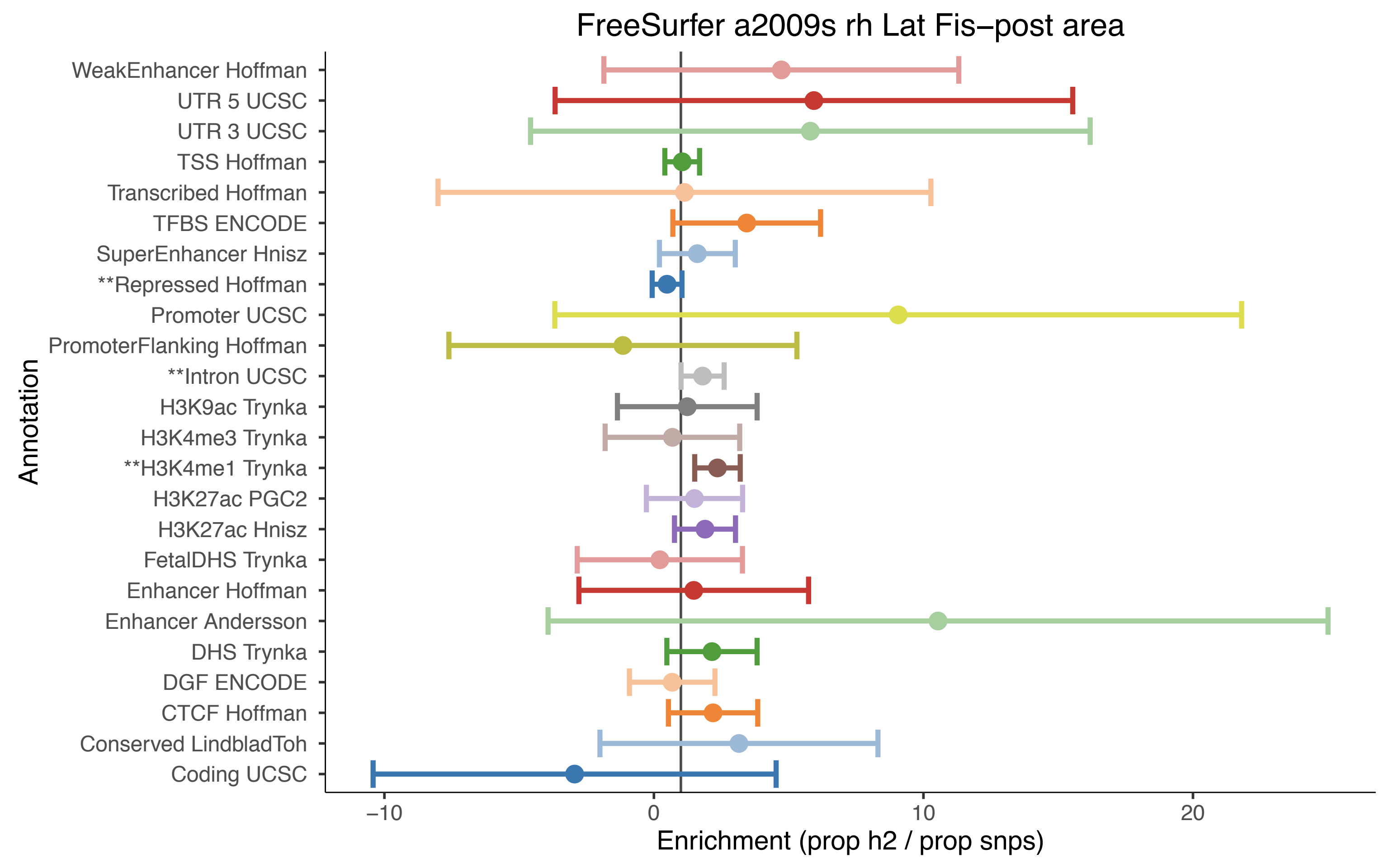
FreeSurfer a2009s rh Lat Fis−post area
WeakEnhancer Hoffman
UTR 5 UCSC
UTR 3 UCSC
TSS Hoffman
Transcribed Hoffman
TFBS ENCODE
SuperEnhancer Hnisz
**Repressed Hoffman
Promoter UCSC
PromoterFlanking Hoffman
**Intron UCSC
H3K9ac Trynka
H3K4me3 Trynka
**H3K4me1 Trynka
H3K27ac PGC2
H3K27ac Hnisz
FetalDHS Trynka
Enhancer Hoffman
Enhancer Andersson
DHS Trynka
DGF ENCODE
CTCF Hoffman
Conserved LindbladToh
Coding UCSC
Annotation
−10
0
10
20
Enrichment (prop h2 / prop snps)

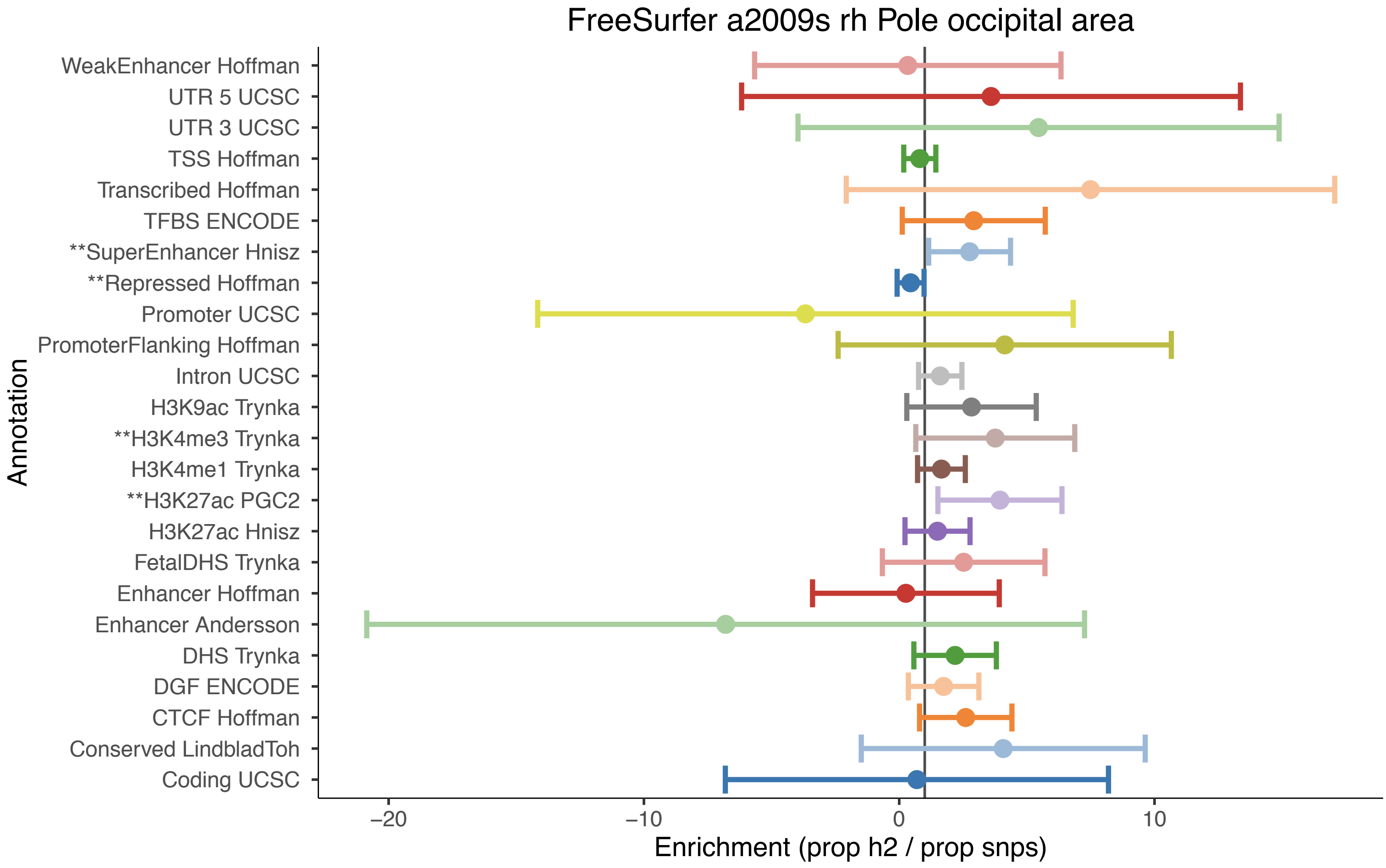
FreeSurfer a2009s rh Pole occipital area
WeakEnhancer Hoffman
UTR 5 UCSC
UTR 3 UCSC
TSS Hoffman
Transcribed Hoffman
TFBS ENCODE
**SuperEnhancer Hnisz
**Repressed Hoffman
Promoter UCSC
PromoterFlanking Hoffman
Intron UCSC
H3K9ac Trynka
**H3K4me3 Trynka
H3K4me1 Trynka
**H3K27ac PGC2
H3K27ac Hnisz
FetalDHS Trynka
Enhancer Hoffman
Enhancer Andersson
DHS Trynka
DGF ENCODE
CTCF Hoffman
Conserved LindbladToh
Coding UCSC
Annotation
−20
−10
0
10
Enrichment (prop h2 / prop snps)

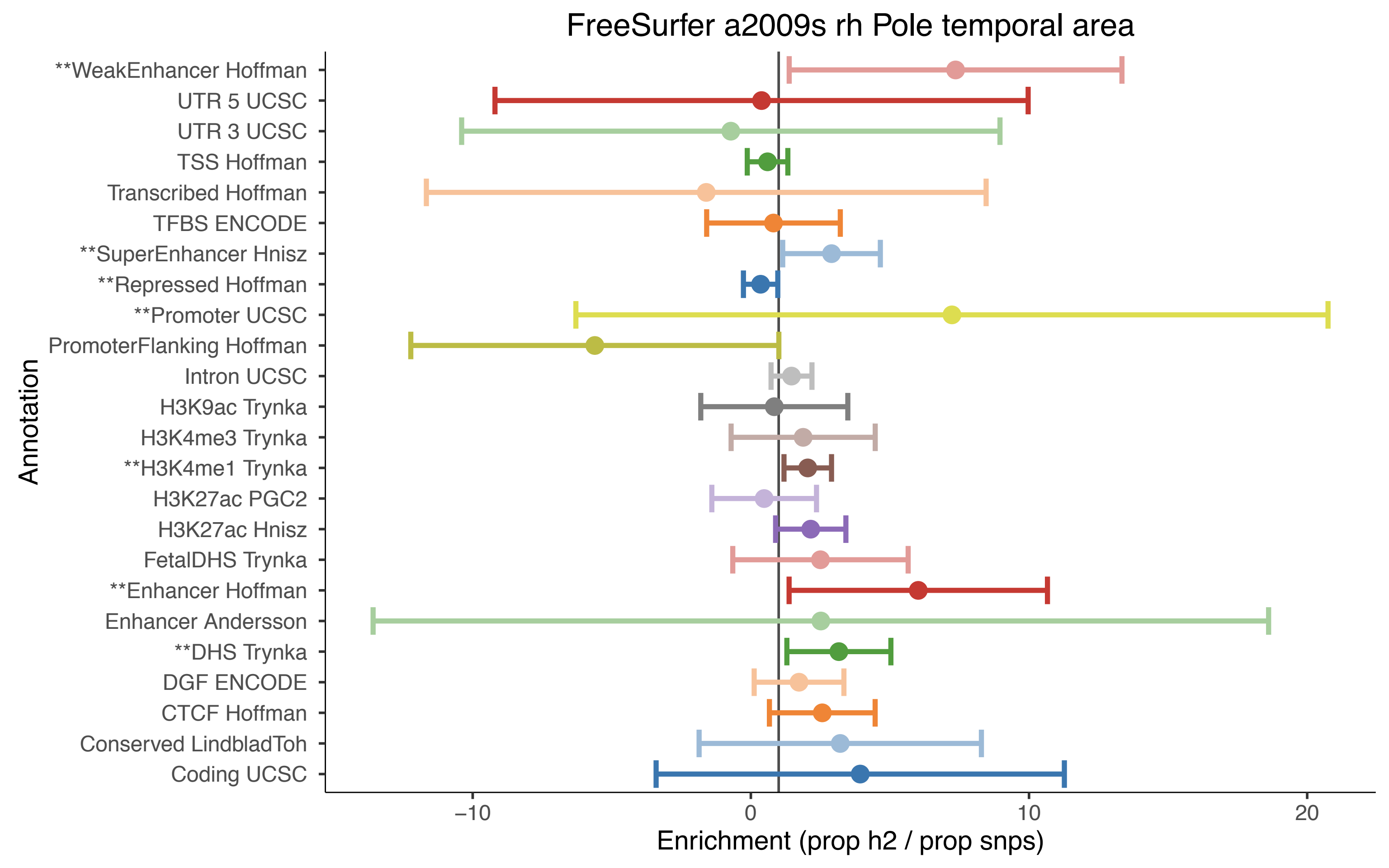

FreeSurfer a2009s rh Pole temporal area
Annotation
**WeakEnhancer Hoffman
UTR 5 UCSC
UTR 3 UCSC
TSS Hoffman
Transcribed Hoffman
TFBS ENCODE
**SuperEnhancer Hnisz
**Repressed Hoffman
**Promoter UCSC
PromoterFlanking Hoffman
Intron UCSC
H3K9ac Trynka
H3K4me3 Trynka
**H3K4me1 Trynka
H3K27ac PGC2
H3K27ac Hnisz
FetalDHS Trynka
**Enhancer Hoffman
Enhancer Andersson
**DHS Trynka
DGF ENCODE
CTCF Hoffman
Conserved LindbladToh
Coding UCSC
−10
0
10
20
Enrichment (prop h2 / prop snps)

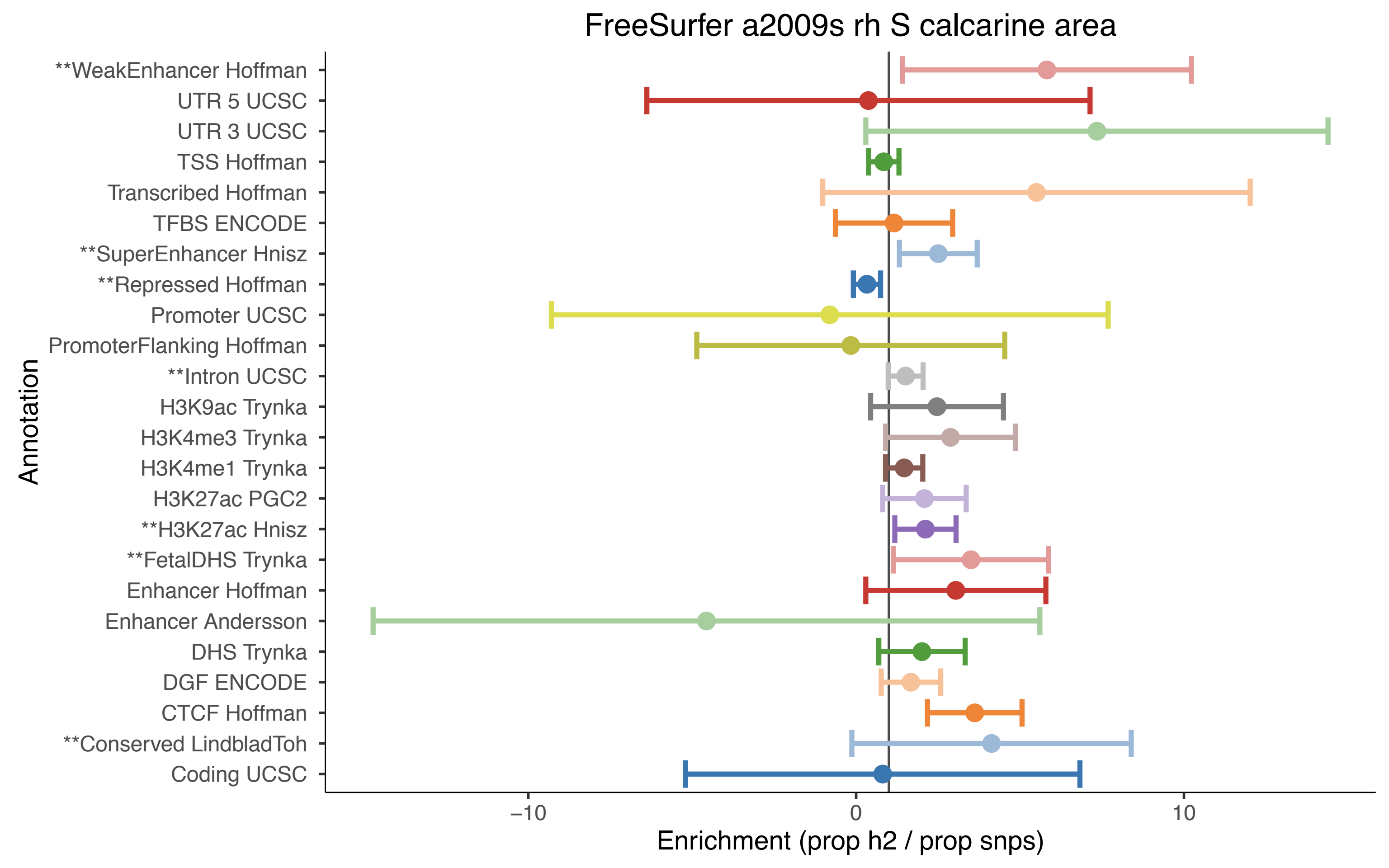

FreeSurfer a2009s rh S calcarine area
**WeakEnhancer Hoffman
UTR 5 UCSC
UTR 3 UCSC
TSS Hoffman
Transcribed Hoffman
TFBS ENCODE
**SuperEnhancer Hnisz
**Repressed Hoffman
Promoter UCSC
PromoterFlanking Hoffman
**Intron UCSC
H3K9ac Trynka
H3K4me3 Trynka
H3K4me1 Trynka
H3K27ac PGC2
**H3K27ac Hnisz
**FetalDHS Trynka
Enhancer Hoffman
Enhancer Andersson
DHS Trynka
DGF ENCODE
CTCF Hoffman
**Conserved LindbladToh
Coding UCSC
Annotation
−10
0
10
Enrichment (prop h2 / prop snps)

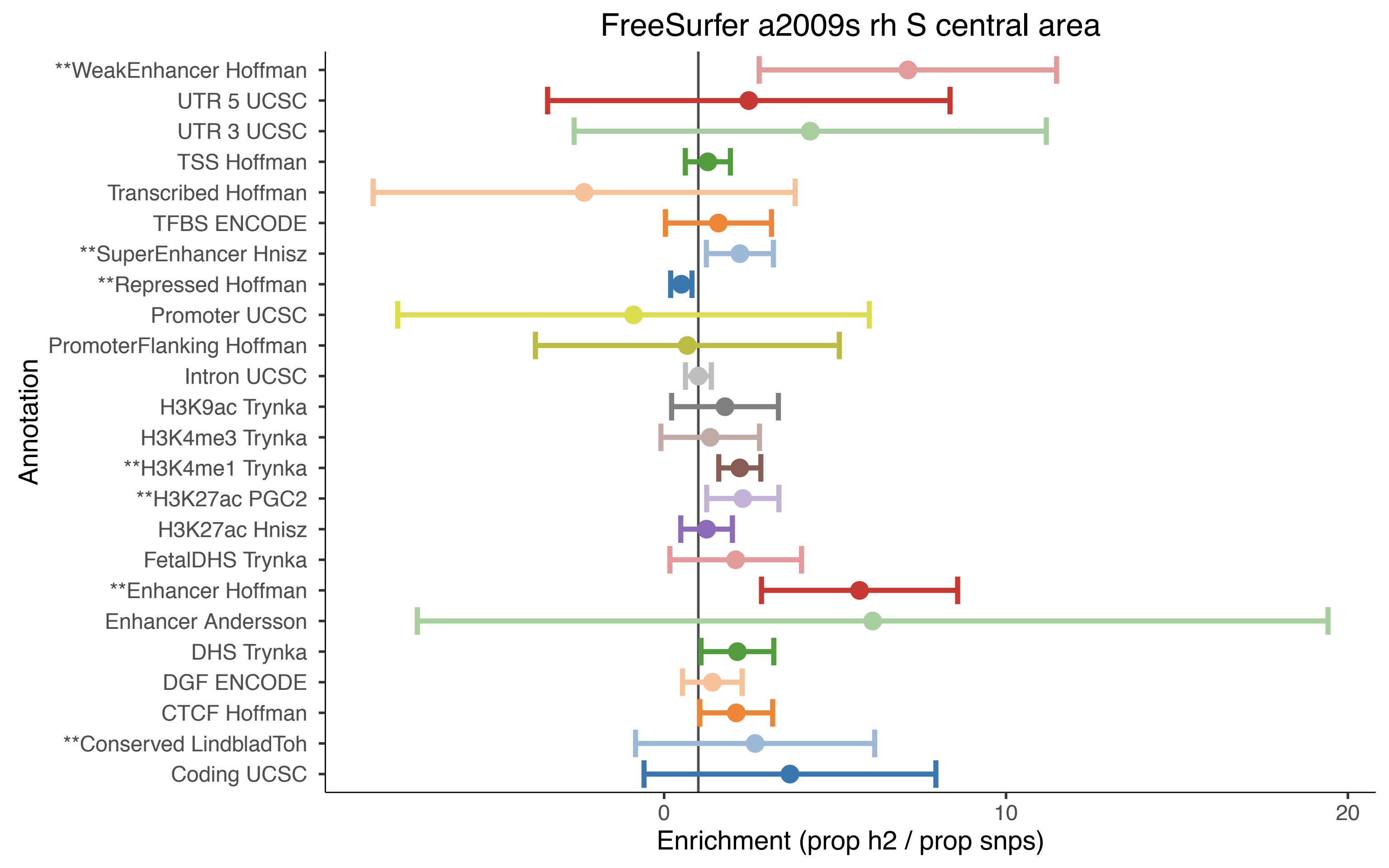
FreeSurfer a2009s rh S central area
**WeakEnhancer Hoffman
UTR 5 UCSC
UTR 3 UCSC
TSS Hoffman
Transcribed Hoffman
TFBS ENCODE
**SuperEnhancer Hnisz
**Repressed Hoffman
Promoter UCSC
PromoterFlanking Hoffman
Intron UCSC
H3K9ac Trynka
H3K4me3 Trynka
**H3K4me1 Trynka
**H3K27ac PGC2
H3K27ac Hnisz
FetalDHS Trynka
**Enhancer Hoffman
Enhancer Andersson
DHS Trynka
DGF ENCODE
CTCF Hoffman
**Conserved LindbladToh
Coding UCSC
Annotation
0
10
20
Enrichment (prop h2 / prop snps)

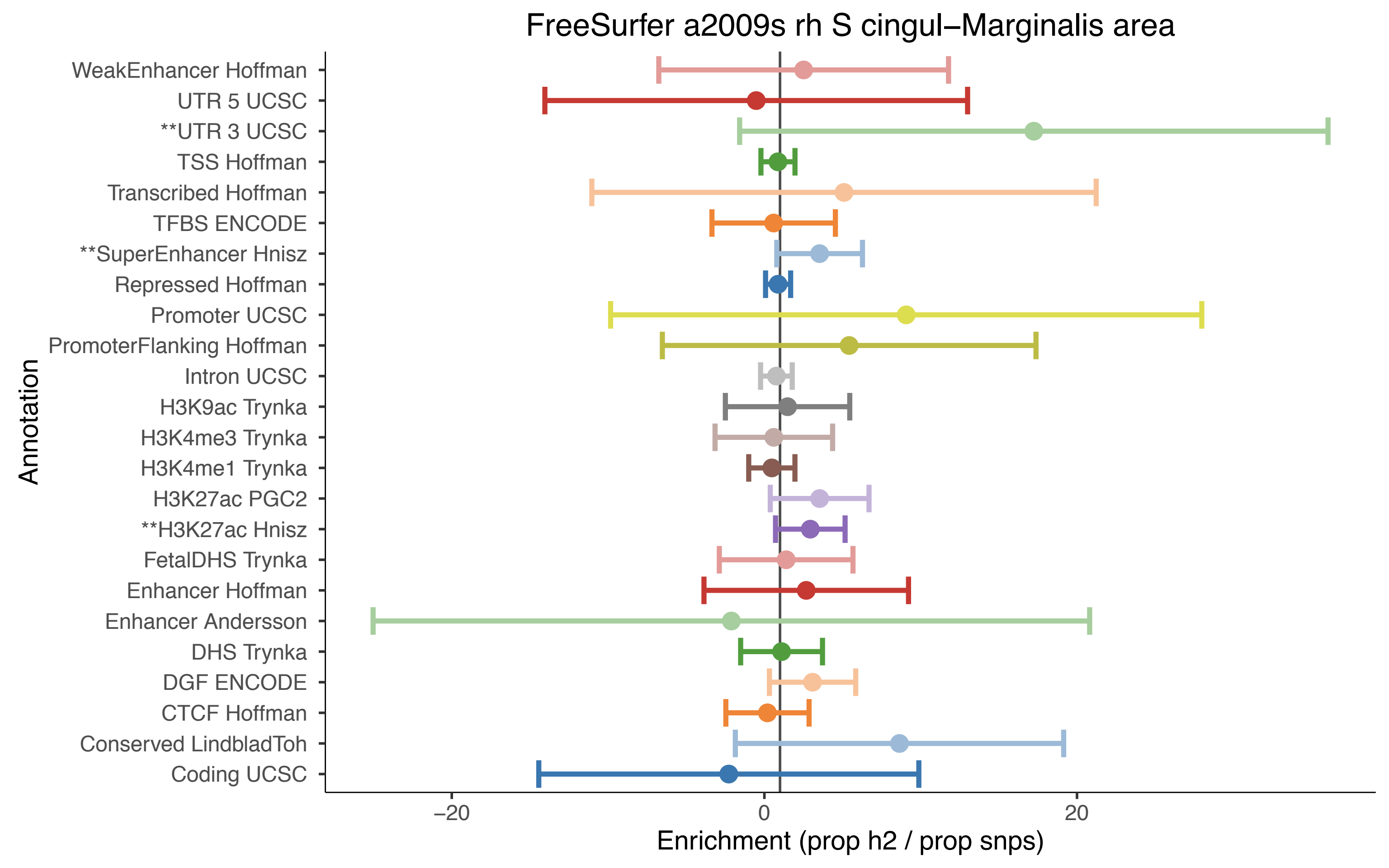
FreeSurfer a2009s rh S cingul–Marginalis area
WeakEnhancer Hoffman
UTR 5 UCSC
**UTR 3 UCSC
TSS Hoffman
Transcribed Hoffman
TFBS ENCODE
**SuperEnhancer Hnisz
Repressed Hoffman
Promoter UCSC
PromoterFlanking Hoffman
Intron UCSC
H3K9ac Trynka
H3K4me3 Trynka
H3K4me1 Trynka
H3K27ac PGC2
**H3K27ac Hnisz
FetalDHS Trynka
Enhancer Hoffman
Enhancer Andersson
DHS Trynka
DGF ENCODE
CTCF Hoffman
Conserved LindbladToh
Coding UCSC
Annotation
−20
0
20
Enrichment (prop h2 / prop snps)

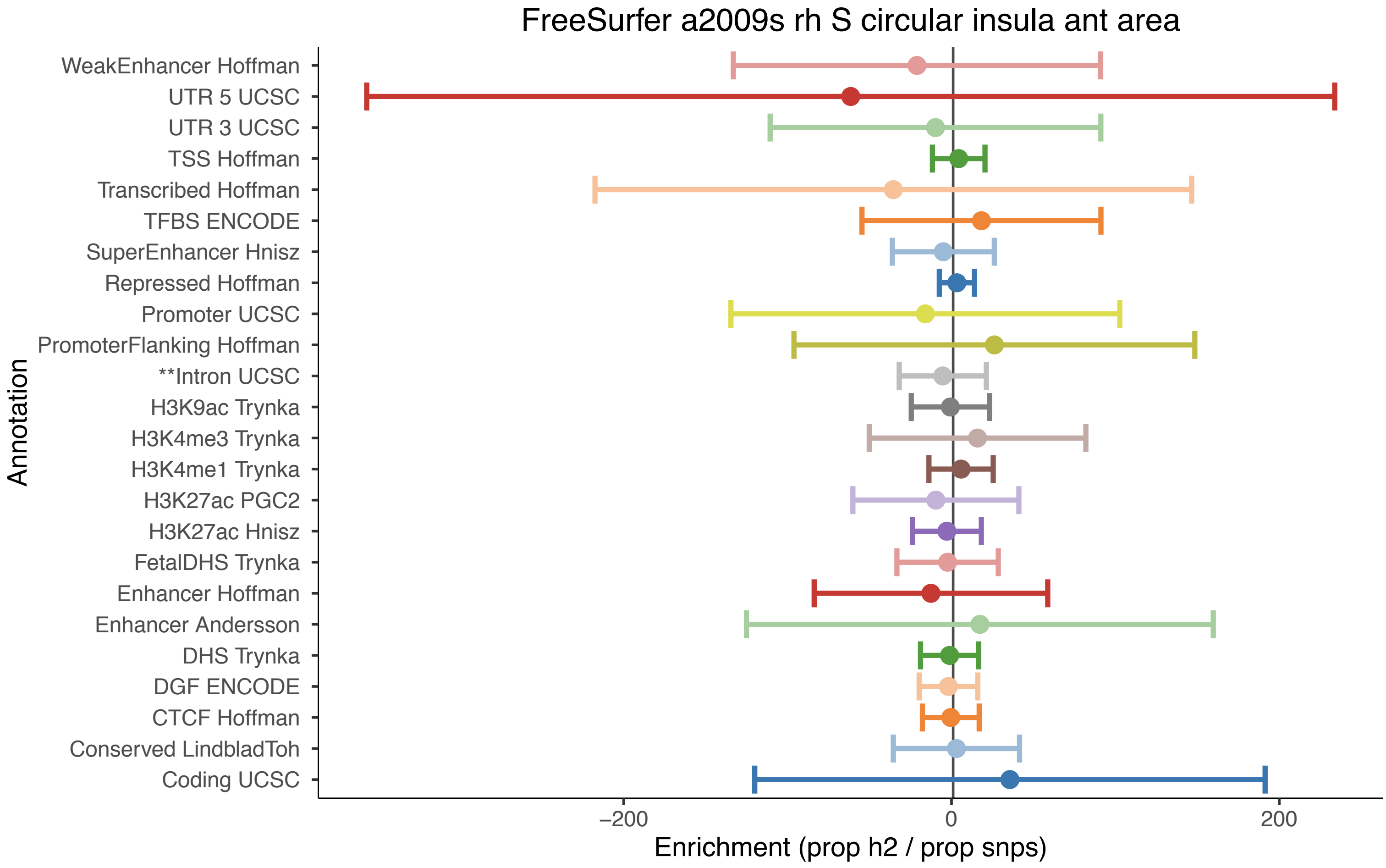
FreeSurfer a2009s rh S circular insula ant area
WeakEnhancer Hoffman
UTR 5 UCSC
UTR 3 UCSC
TSS Hoffman
Transcribed Hoffman
TFBS ENCODE
SuperEnhancer Hnisz
Repressed Hoffman
Promoter UCSC
PromoterFlanking Hoffman
**Intron UCSC
H3K9ac Trynka
H3K4me3 Trynka
H3K4me1 Trynka
H3K27ac PGC2
H3K27ac Hnisz
FetalDHS Trynka
Enhancer Hoffman
Enhancer Andersson
DHS Trynka
DGF ENCODE
CTCF Hoffman
Conserved LindbladToh
Coding UCSC
Annotation
−200
0
200
Enrichment (prop h2 / prop snps)

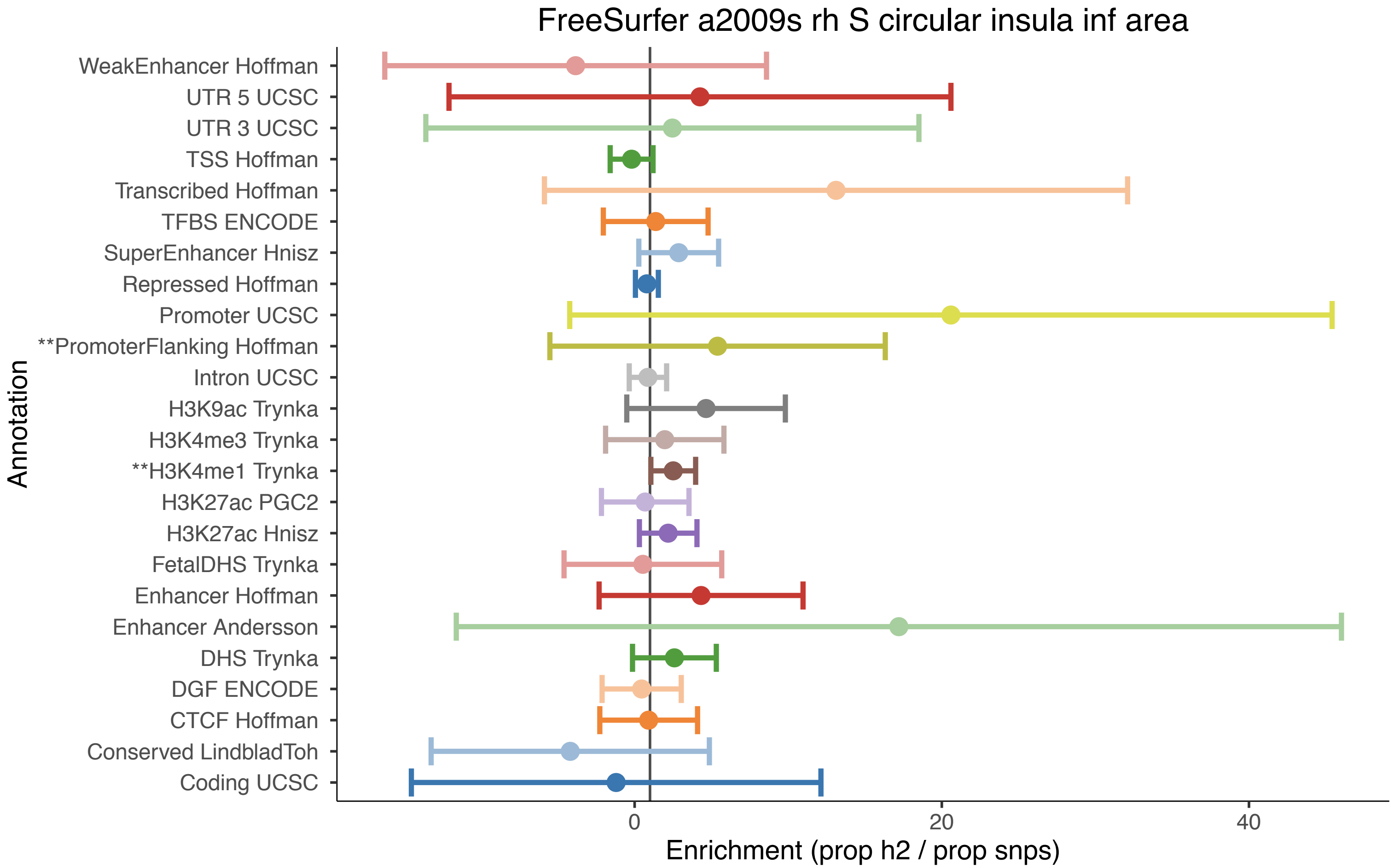

FreeSurfer a2009s rh S circular insula inf area
WeakEnhancer Hoffman
UTR 5 UCSC
UTR 3 UCSC
TSS Hoffman
Transcribed Hoffman
TFBS ENCODE
SuperEnhancer Hnisz
Repressed Hoffman
Promoter UCSC
**PromoterFlanking Hoffman
Intron UCSC
H3K9ac Trynka
H3K4me3 Trynka
**H3K4me1 Trynka
H3K27ac PGC2
H3K27ac Hnisz
FetalDHS Trynka
Enhancer Hoffman
Enhancer Andersson
DHS Trynka
DGF ENCODE
CTCF Hoffman
Conserved LindbladToh
Coding UCSC
Annotation
0
20
40
Enrichment (prop h2 / prop snps)

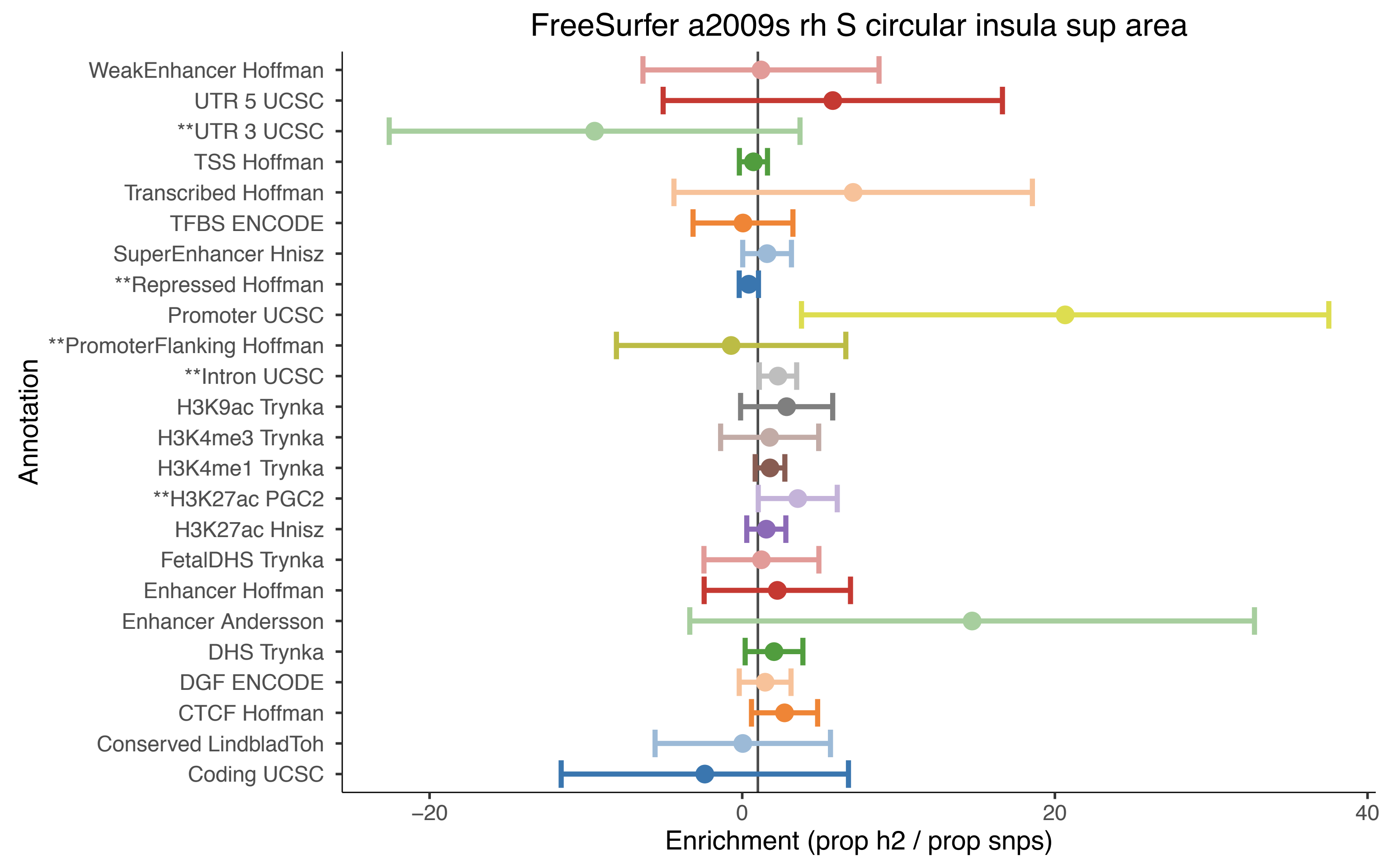

FreeSurfer a2009s rh S circular insula sup area
WeakEnhancer Hoffman
UTR 5 UCSC
**UTR 3 UCSC
TSS Hoffman
Transcribed Hoffman
TFBS ENCODE
SuperEnhancer Hnisz
**Repressed Hoffman
Promoter UCSC
**PromoterFlanking Hoffman
**Intron UCSC
H3K9ac Trynka
H3K4me3 Trynka
H3K4me1 Trynka
**H3K27ac PGC2
H3K27ac Hnisz
FetalDHS Trynka
Enhancer Hoffman
Enhancer Andersson
DHS Trynka
DGF ENCODE
CTCF Hoffman
Conserved LindbladToh
Coding UCSC
Annotation
−20
0
20
40
Enrichment (prop h2 / prop snps)

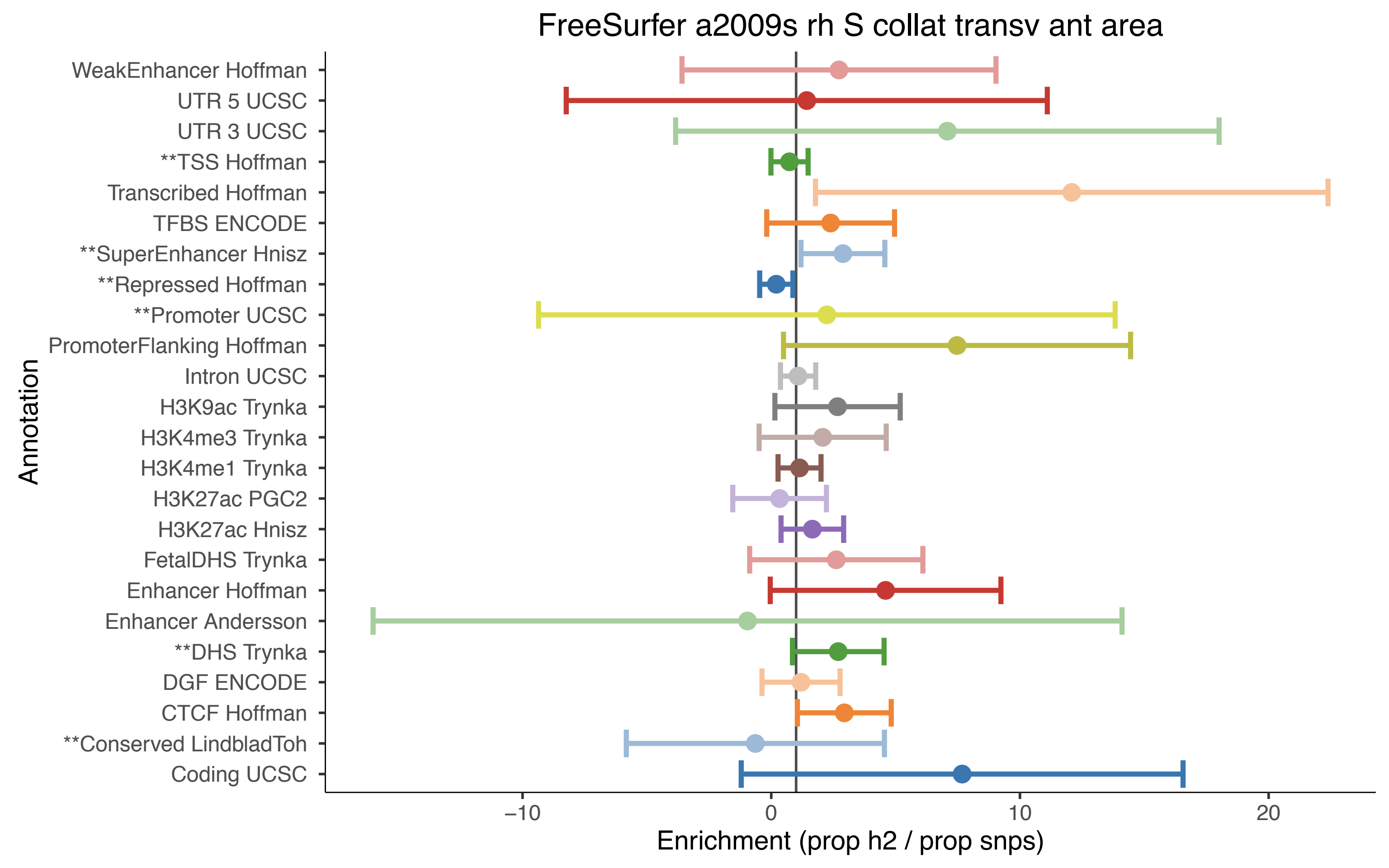

FreeSurfer a2009s rh S collat transv ant area
WeakEnhancer Hoffman
UTR 5 UCSC
UTR 3 UCSC
**TSS Hoffman
Transcribed Hoffman
TFBS ENCODE
**SuperEnhancer Hnisz
**Repressed Hoffman
**Promoter UCSC
PromoterFlanking Hoffman
Intron UCSC
H3K9ac Trynka
H3K4me3 Trynka
H3K4me1 Trynka
H3K27ac PGC2
H3K27ac Hnisz
FetalDHS Trynka
Enhancer Hoffman
Enhancer Andersson
**DHS Trynka
DGF ENCODE
CTCF Hoffman
**Conserved LindbladToh
Coding UCSC
Annotation
−10
0
10
20
Enrichment (prop h2 / prop snps)

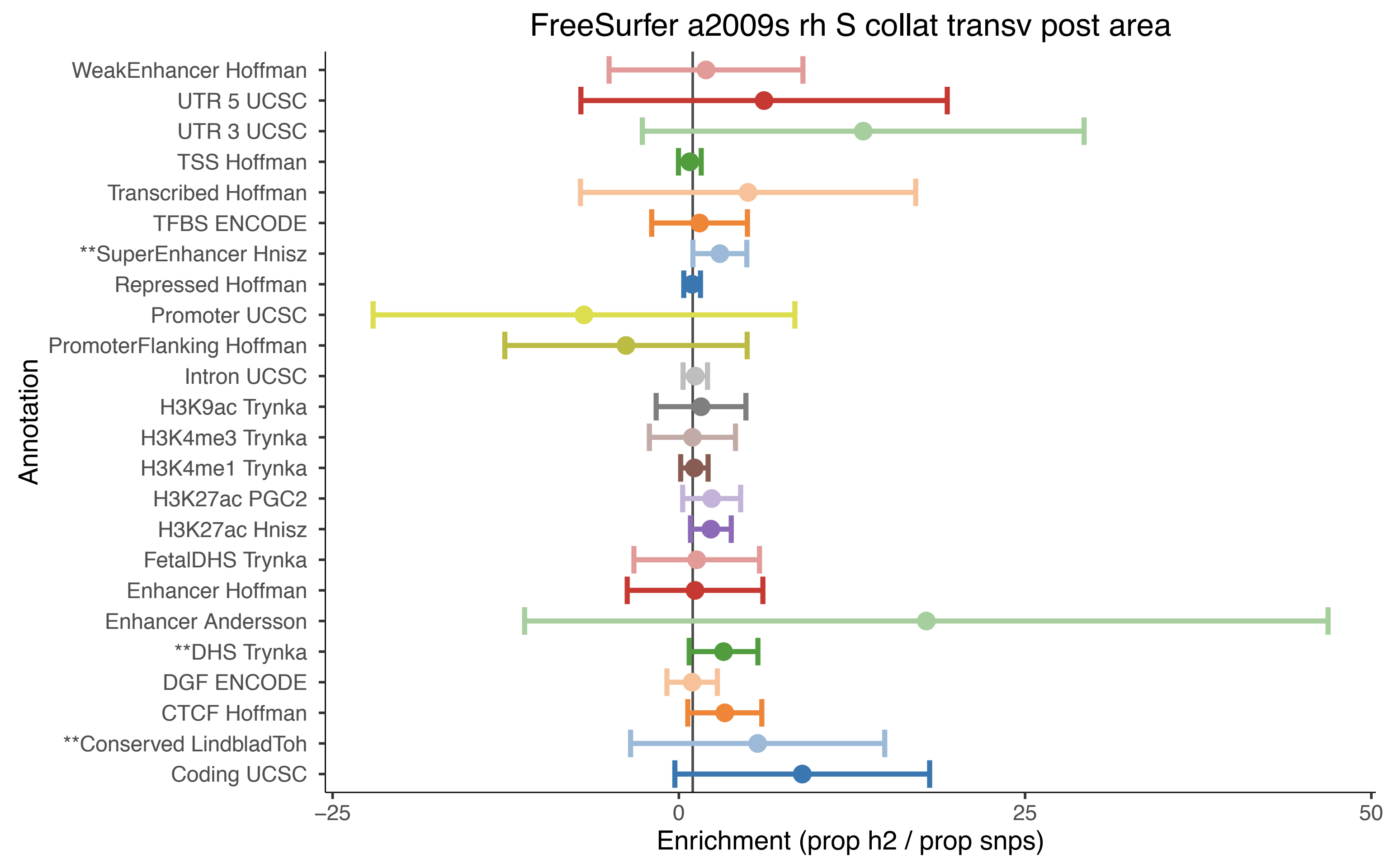

FreeSurfer a2009s rh S collat transv post area
WeakEnhancer Hoffman
UTR 5 UCSC
UTR 3 UCSC
TSS Hoffman
Transcribed Hoffman
TFBS ENCODE
**SuperEnhancer Hnisz
Repressed Hoffman
Promoter UCSC
PromoterFlanking Hoffman
Intron UCSC
H3K9ac Trynka
H3K4me3 Trynka
H3K4me1 Trynka
H3K27ac PGC2
H3K27ac Hnisz
FetalDHS Trynka
Enhancer Hoffman
Enhancer Andersson
**DHS Trynka
DGF ENCODE
CTCF Hoffman
**Conserved LindbladToh
Coding UCSC
Annotation
−25
0
25
50
Enrichment (prop h2 / prop snps)

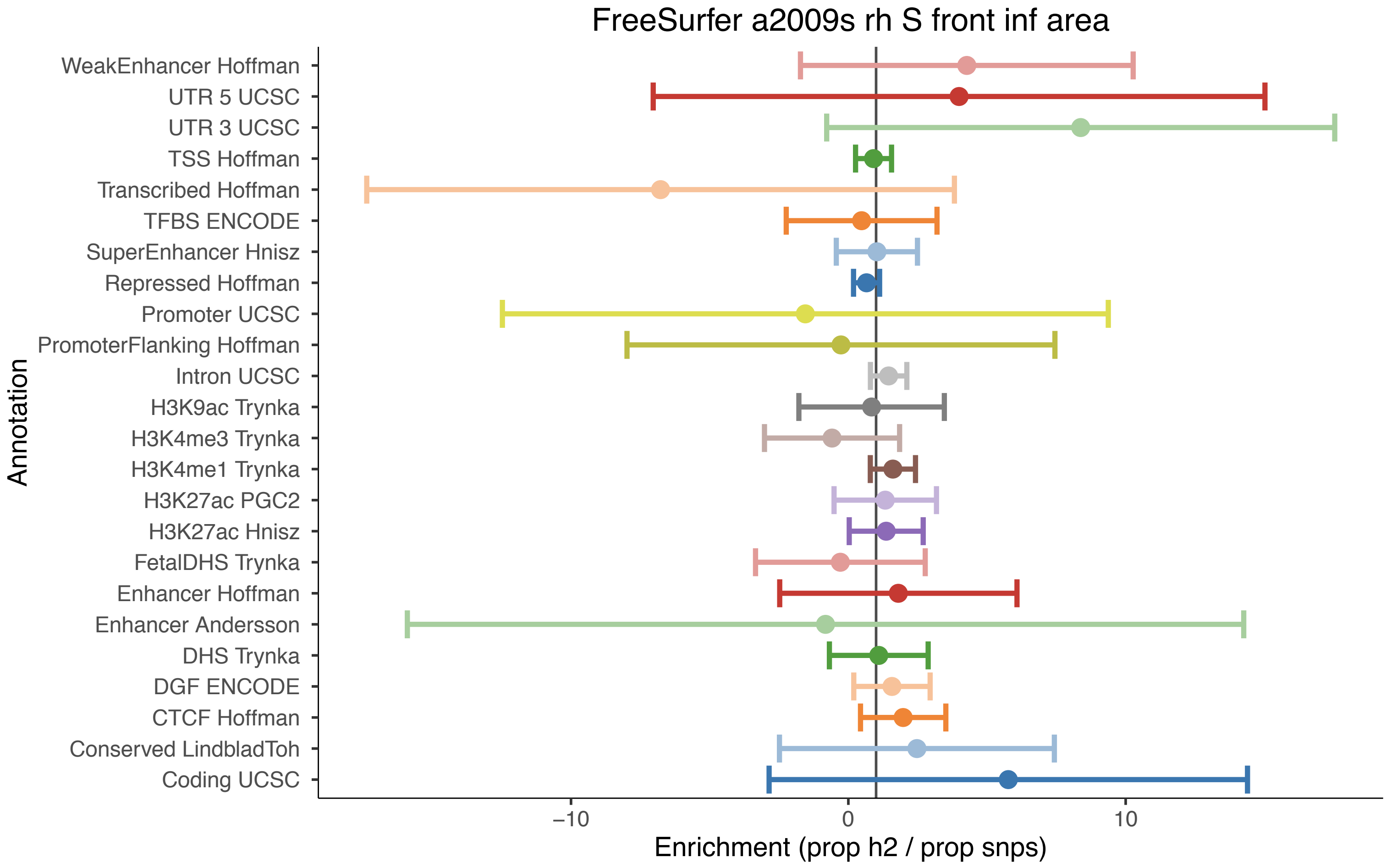
FreeSurfer a2009s rh S front inf area
WeakEnhancer Hoffman
UTR 5 UCSC
UTR 3 UCSC
TSS Hoffman
Transcribed Hoffman
TFBS ENCODE
SuperEnhancer Hnisz
Repressed Hoffman
Promoter UCSC
PromoterFlanking Hoffman
Intron UCSC
H3K9ac Trynka
H3K4me3 Trynka
H3K4me1 Trynka
H3K27ac PGC2
H3K27ac Hnisz
FetalDHS Trynka
Enhancer Hoffman
Enhancer Andersson
DHS Trynka
DGF ENCODE
CTCF Hoffman
Conserved LindbladToh
Coding UCSC
Annotation
−10
0
10
Enrichment (prop h2 / prop snps)

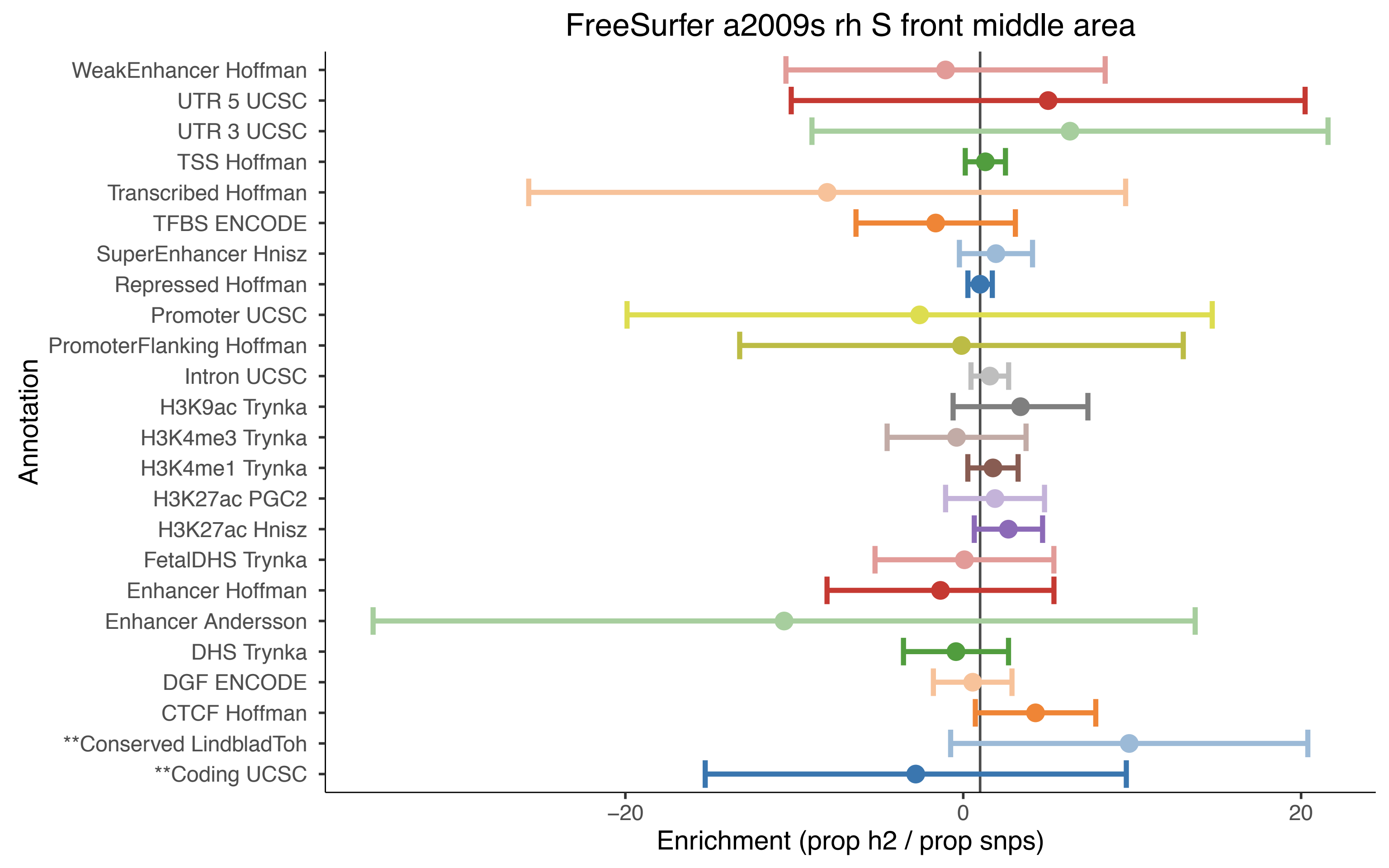

FreeSurfer a2009s rh S front middle area
WeakEnhancer Hoffman
UTR 5 UCSC
UTR 3 UCSC
TSS Hoffman
Transcribed Hoffman
TFBS ENCODE
SuperEnhancer Hnisz
Repressed Hoffman
Promoter UCSC
PromoterFlanking Hoffman
Intron UCSC
H3K9ac Trynka
H3K4me3 Trynka
H3K4me1 Trynka
H3K27ac PGC2
H3K27ac Hnisz
FetalDHS Trynka
Enhancer Hoffman
Enhancer Andersson
DHS Trynka
DGF ENCODE
CTCF Hoffman
**Conserved LindbladToh
**Coding UCSC
Annotation
−20
0
20
Enrichment (prop h2 / prop snps)

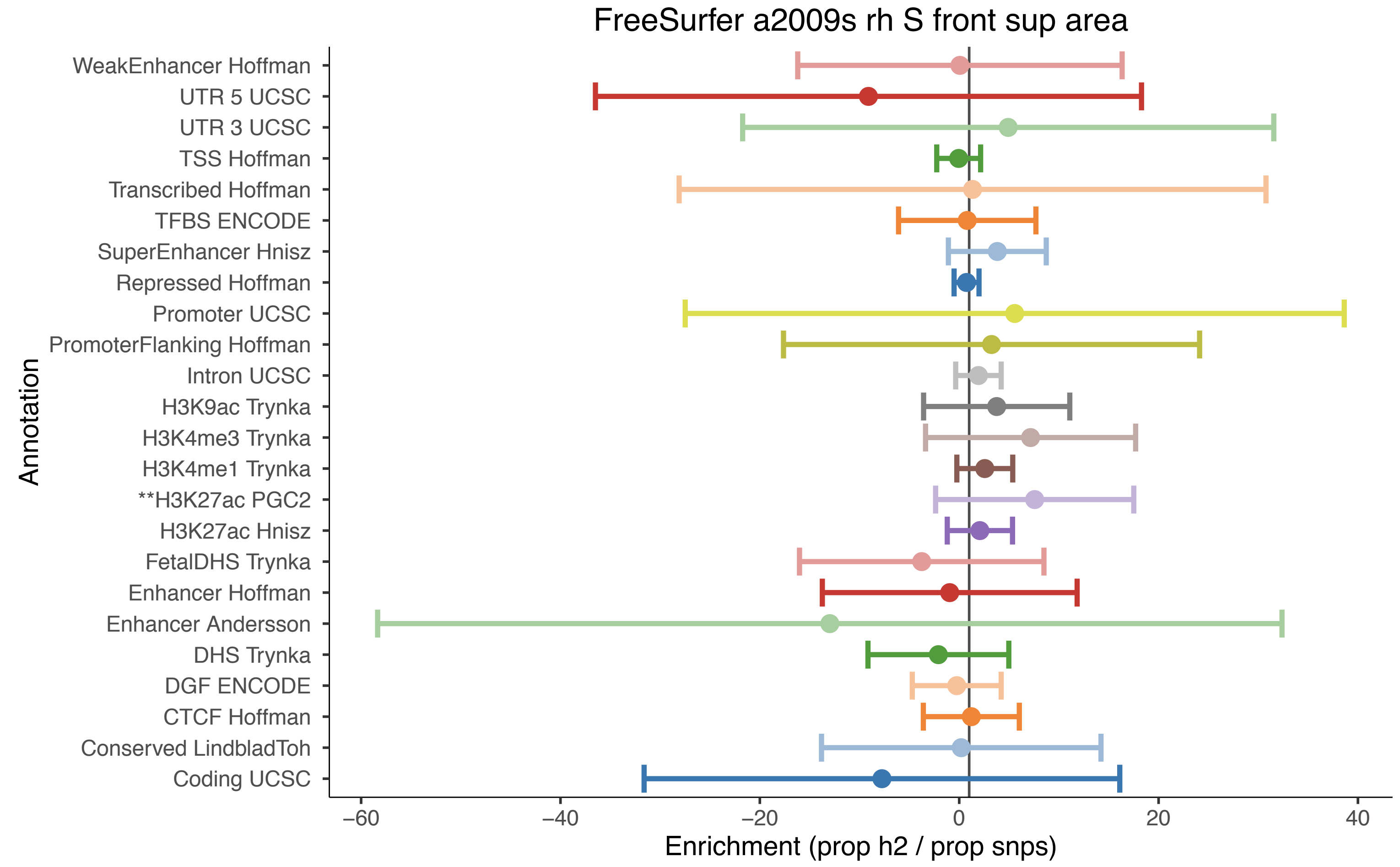
FreeSurfer a2009s rh S front sup area
WeakEnhancer Hoffman
UTR 5 UCSC
UTR 3 UCSC
TSS Hoffman
Transcribed Hoffman
TFBS ENCODE
SuperEnhancer Hnisz
Repressed Hoffman
Promoter UCSC
PromoterFlanking Hoffman
Intron UCSC
H3K9ac Trynka
H3K4me3 Trynka
H3K4me1 Trynka
**H3K27ac PGC2
H3K27ac Hnisz
FetalDHS Trynka
Enhancer Hoffman
Enhancer Andersson
DHS Trynka
DGF ENCODE
CTCF Hoffman
Conserved LindbladToh
Coding UCSC
Annotation
−60
−40
−20
0
20
40
Enrichment (prop h2 / prop snps)

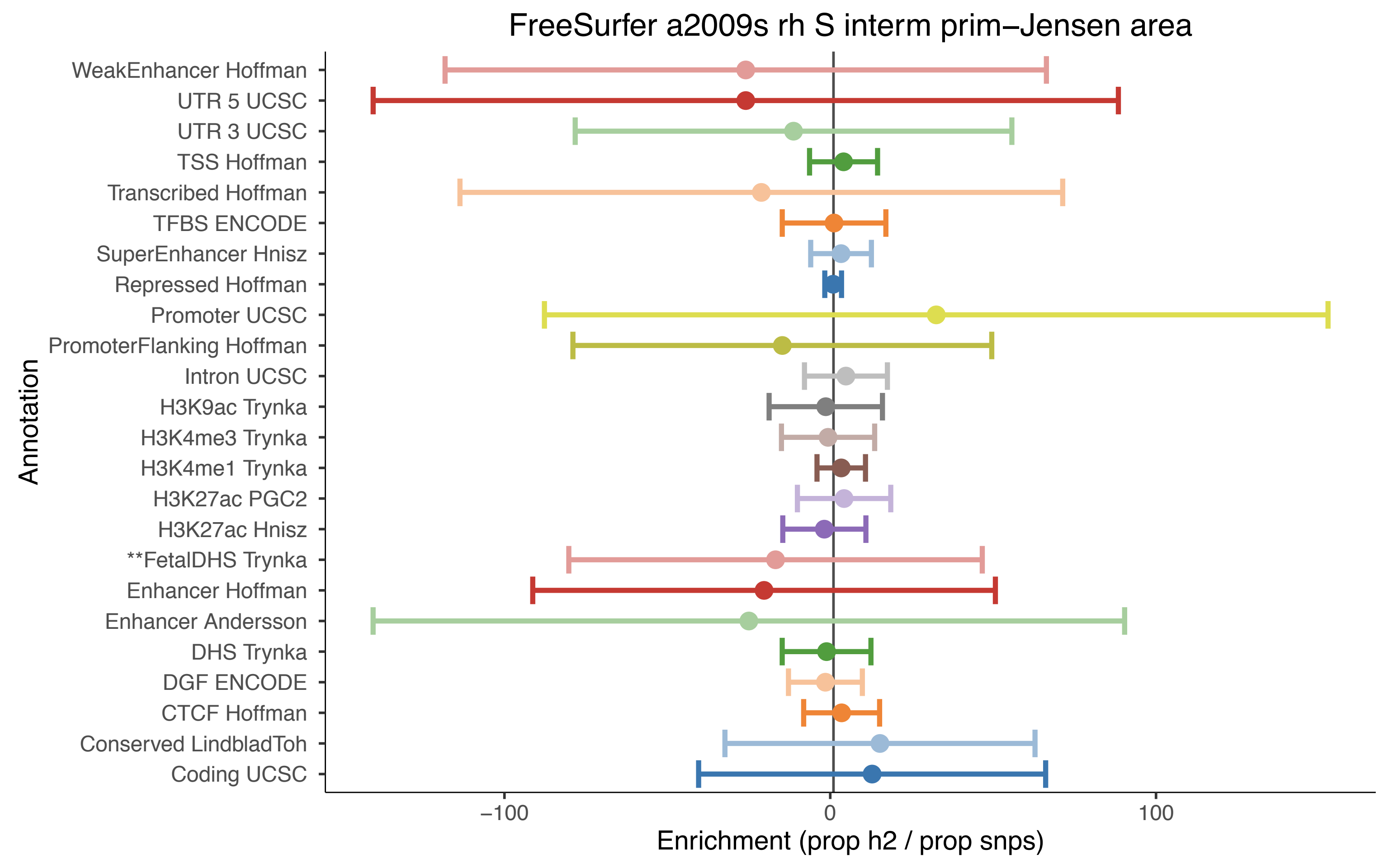
FreeSurfer a2009s rh S interm prim−Jensen area
WeakEnhancer Hoffman
UTR 5 UCSC
UTR 3 UCSC
TSS Hoffman
Transcribed Hoffman
TFBS ENCODE
SuperEnhancer Hnisz
Repressed Hoffman
Promoter UCSC
PromoterFlanking Hoffman
Intron UCSC
H3K9ac Trynka
H3K4me3 Trynka
H3K4me1 Trynka
H3K27ac PGC2
H3K27ac Hnisz
**FetalDHS Trynka
Enhancer Hoffman
Enhancer Andersson
DHS Trynka
DGF ENCODE
CTCF Hoffman
Conserved LindbladToh
Coding UCSC
Annotation
−100
0
100
Enrichment (prop h2 / prop snps)

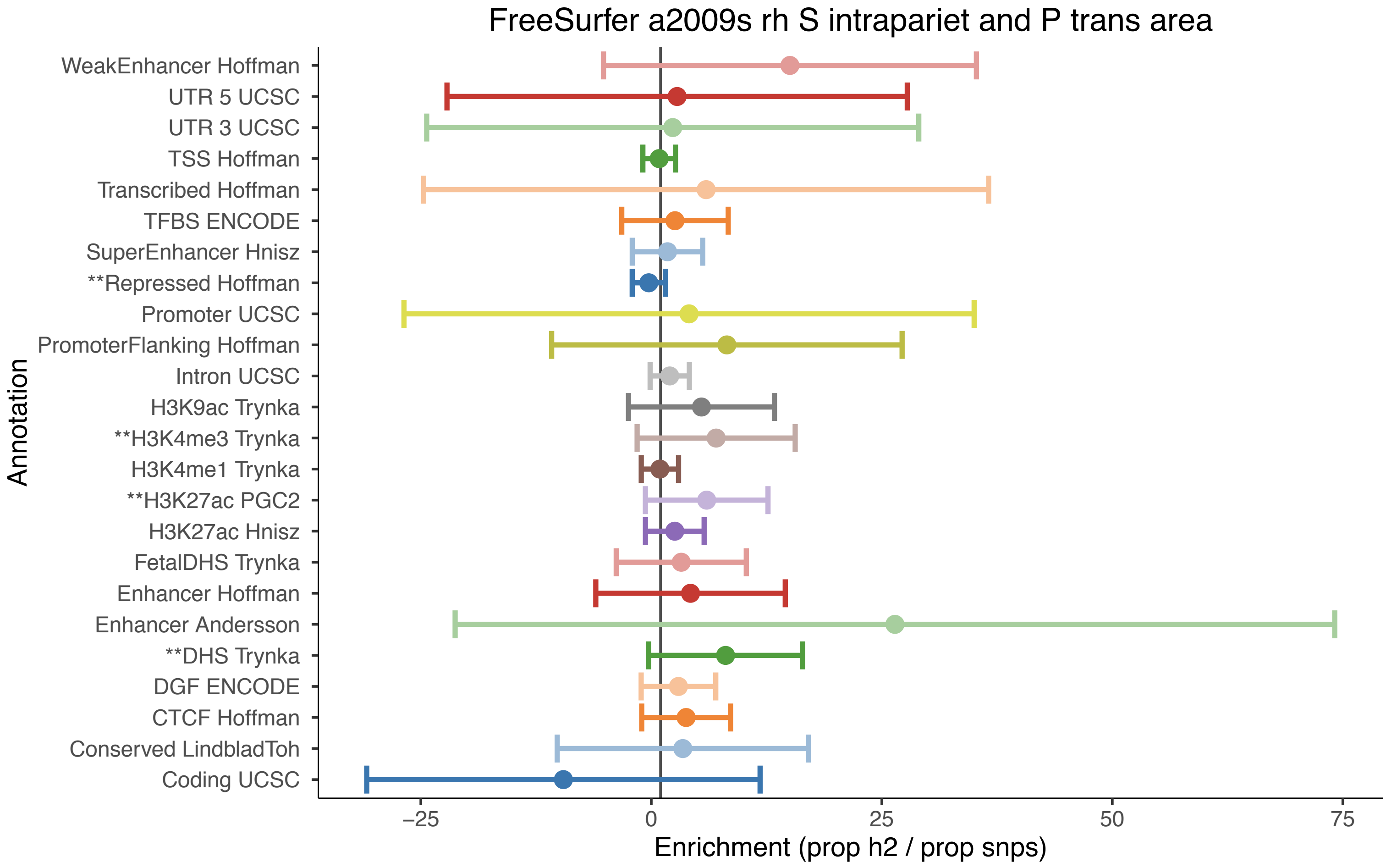

FreeSurfer a2009s rh S intrapariet and P trans area
WeakEnhancer Hoffman
UTR 5 UCSC
UTR 3 UCSC
TSS Hoffman
Transcribed Hoffman
TFBS ENCODE
SuperEnhancer Hnisz
**Repressed Hoffman
Promoter UCSC
PromoterFlanking Hoffman
Intron UCSC
H3K9ac Trynka
**H3K4me3 Trynka
H3K4me1 Trynka
**H3K27ac PGC2
H3K27ac Hnisz
FetalDHS Trynka
Enhancer Hoffman
Enhancer Andersson
**DHS Trynka
DGF ENCODE
CTCF Hoffman
Conserved LindbladToh
Coding UCSC
Annotation
−25
0
25
50
75
Enrichment (prop h2 / prop snps)

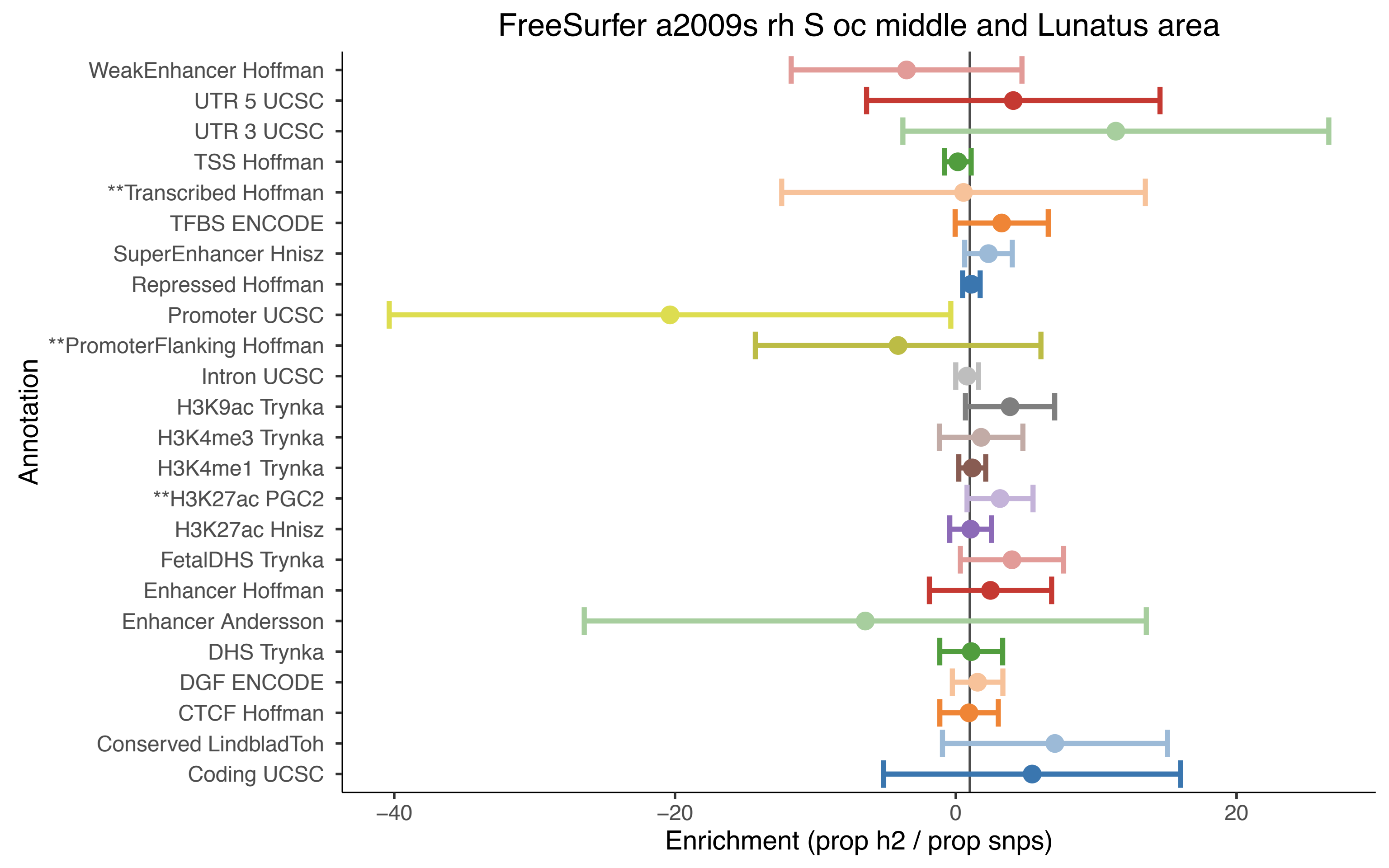
FreeSurfer a2009s rh S oc middle and Lunatus area
WeakEnhancer Hoffman
UTR 5 UCSC
UTR 3 UCSC
TSS Hoffman
**Transcribed Hoffman
TFBS ENCODE
SuperEnhancer Hnisz
Repressed Hoffman
Promoter UCSC
**PromoterFlanking Hoffman
Intron UCSC
H3K9ac Trynka
H3K4me3 Trynka
H3K4me1 Trynka
**H3K27ac PGC2
H3K27ac Hnisz
FetalDHS Trynka
Enhancer Hoffman
Enhancer Andersson
DHS Trynka
DGF ENCODE
CTCF Hoffman
Conserved LindbladToh
Coding UCSC
Annotation
−40
−20
0
20
Enrichment (prop h2 / prop snps)

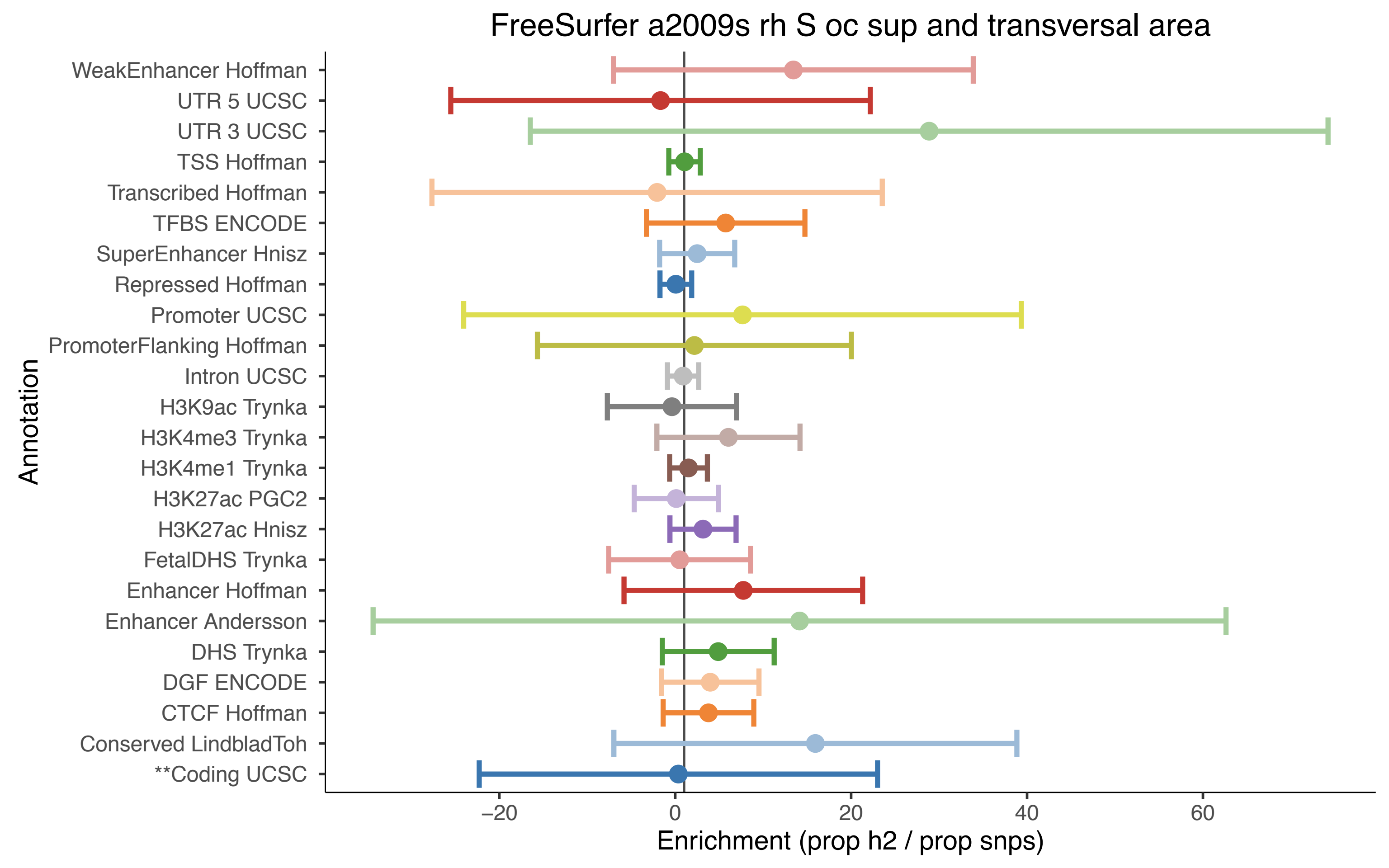

FreeSurfer a2009s rh S oc sup and transversal area
WeakEnhancer Hoffman
UTR 5 UCSC
UTR 3 UCSC
TSS Hoffman
Transcribed Hoffman
TFBS ENCODE
SuperEnhancer Hnisz
Repressed Hoffman
Promoter UCSC
PromoterFlanking Hoffman
Intron UCSC
H3K9ac Trynka
H3K4me3 Trynka
H3K4me1 Trynka
H3K27ac PGC2
H3K27ac Hnisz
FetalDHS Trynka
Enhancer Hoffman
Enhancer Andersson
DHS Trynka
DGF ENCODE
CTCF Hoffman
Conserved LindbladToh
**Coding UCSC
Annotation
−20
0
20
40
60
Enrichment (prop h2 / prop snps)

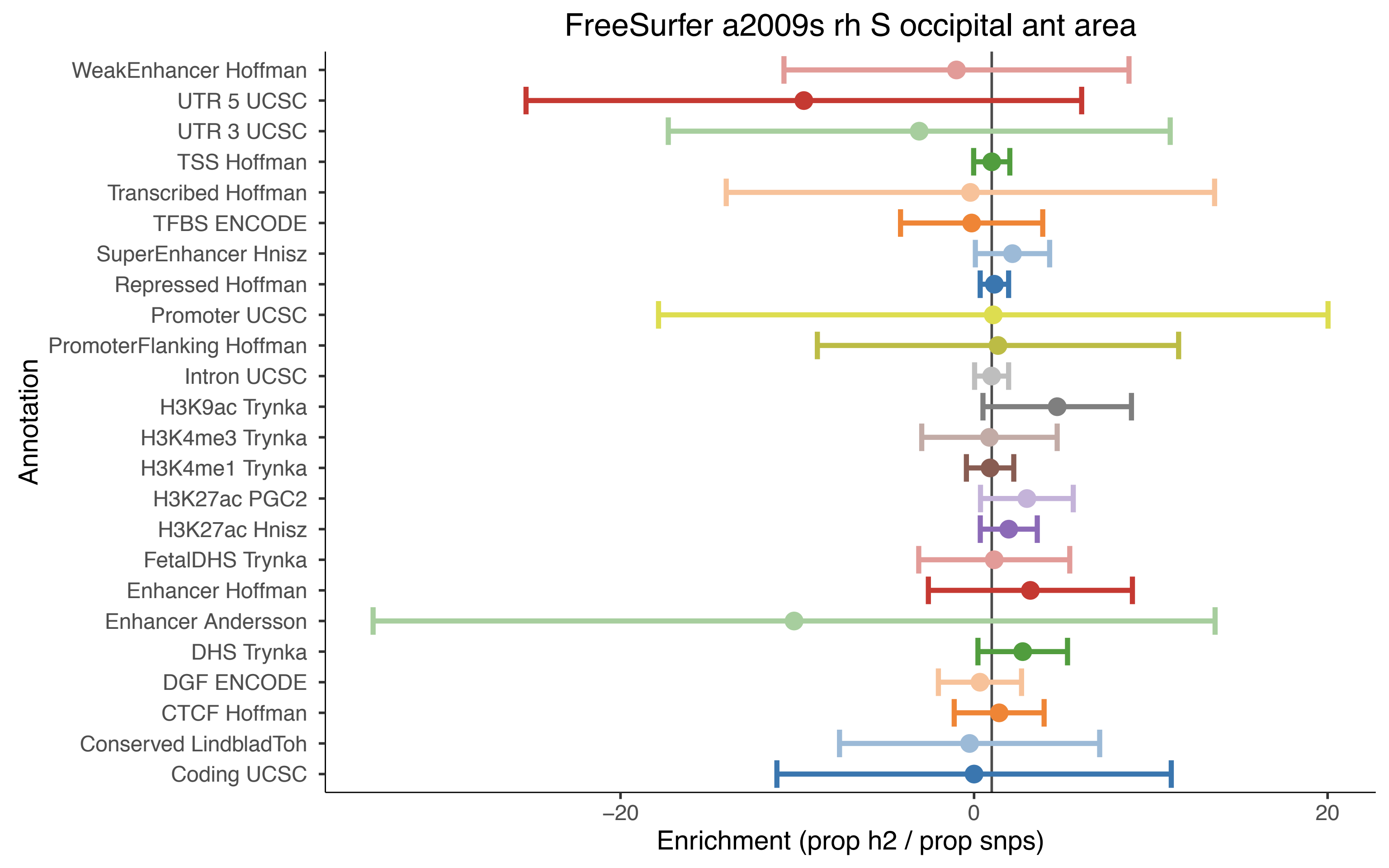
FreeSurfer a2009s rh S occipital ant area
WeakEnhancer Hoffman
UTR 5 UCSC
UTR 3 UCSC
TSS Hoffman
Transcribed Hoffman
TFBS ENCODE
SuperEnhancer Hnisz
Repressed Hoffman
Promoter UCSC
PromoterFlanking Hoffman
Intron UCSC
H3K9ac Trynka
H3K4me3 Trynka
H3K4me1 Trynka
H3K27ac PGC2
H3K27ac Hnisz
FetalDHS Trynka
Enhancer Hoffman
Enhancer Andersson
DHS Trynka
DGF ENCODE
CTCF Hoffman
Conserved LindbladToh
Coding UCSC
Annotation
−20
0
20
Enrichment (prop h2 / prop snps)

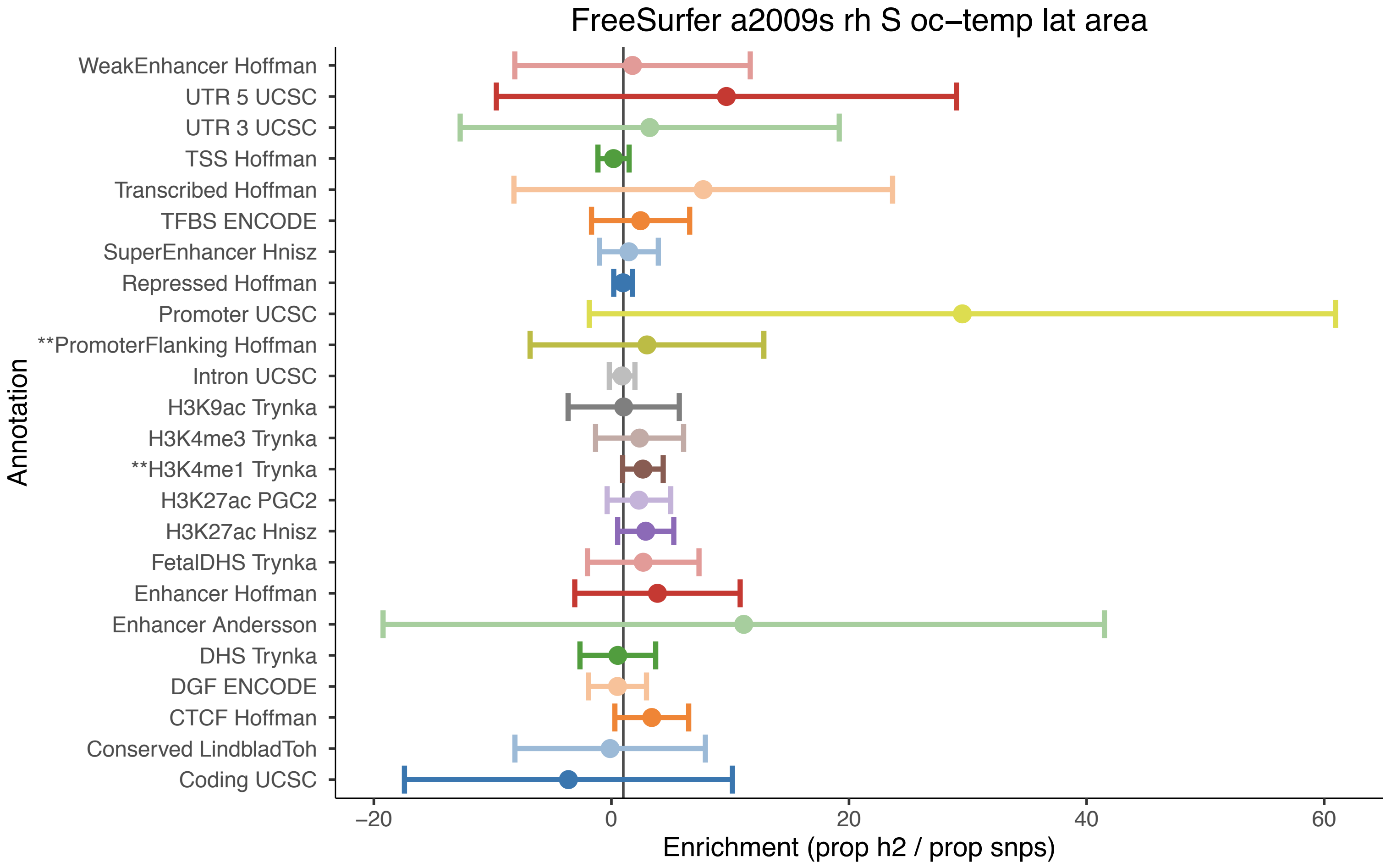

FreeSurfer a2009s rh S oc−temp lat area
WeakEnhancer Hoffman
UTR 5 UCSC
UTR 3 UCSC
TSS Hoffman
Transcribed Hoffman
TFBS ENCODE
SuperEnhancer Hnisz
Repressed Hoffman
Promoter UCSC
**PromoterFlanking Hoffman
Intron UCSC
H3K9ac Trynka
H3K4me3 Trynka
**H3K4me1 Trynka
H3K27ac PGC2
H3K27ac Hnisz
FetalDHS Trynka
Enhancer Hoffman
Enhancer Andersson
DHS Trynka
DGF ENCODE
CTCF Hoffman
Conserved LindbladToh
Coding UCSC
Annotation
−20
0
20
40
60
Enrichment (prop h2 / prop snps)

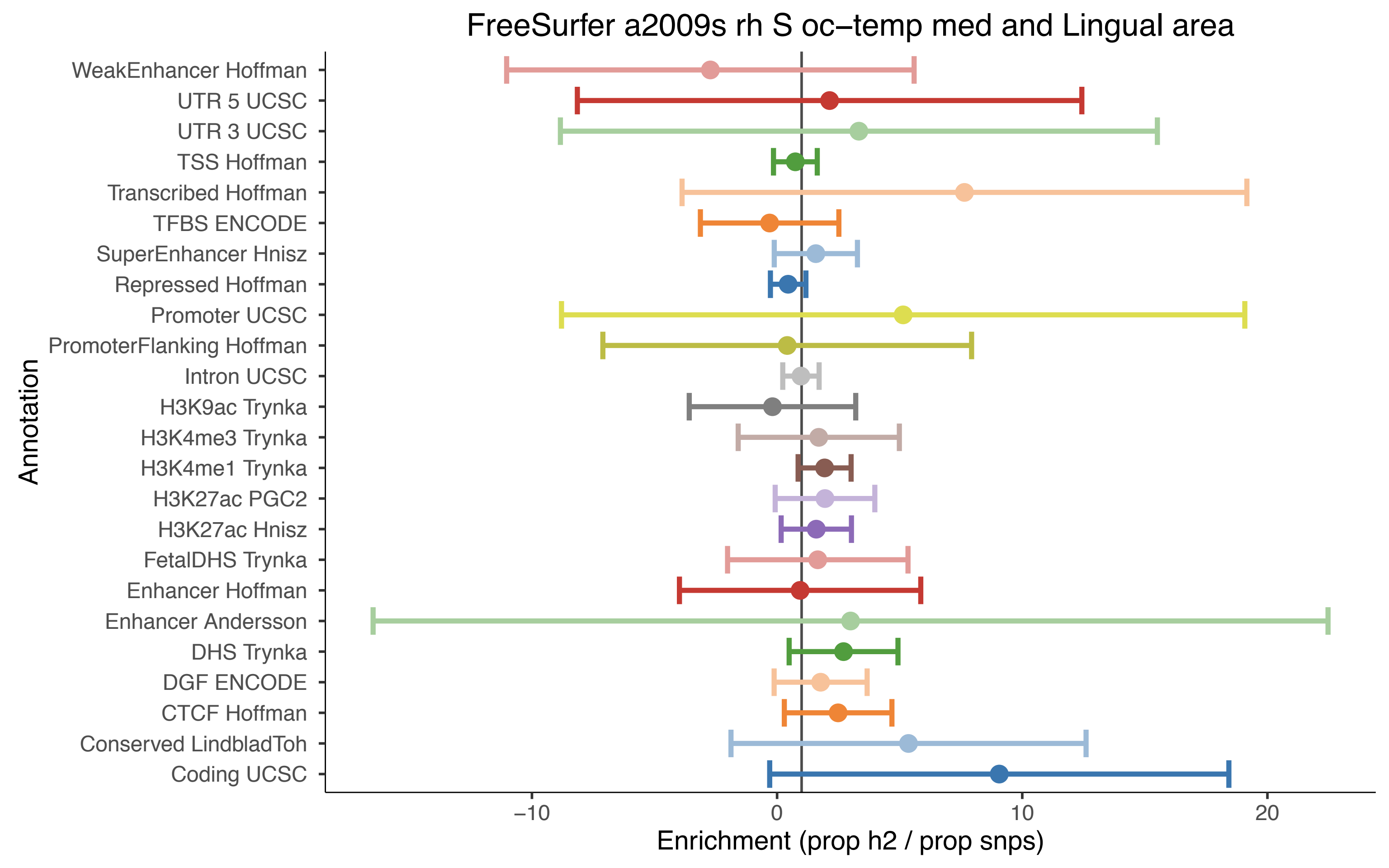
FreeSurfer a2009s rh S oc−temp med and Lingual area
WeakEnhancer Hoffman
UTR 5 UCSC
UTR 3 UCSC
TSS Hoffman
Transcribed Hoffman
TFBS ENCODE
SuperEnhancer Hnisz
Repressed Hoffman
Promoter UCSC
PromoterFlanking Hoffman
Intron UCSC
H3K9ac Trynka
H3K4me3 Trynka
H3K4me1 Trynka
H3K27ac PGC2
H3K27ac Hnisz
FetalDHS Trynka
Enhancer Hoffman
Enhancer Andersson
DHS Trynka
DGF ENCODE
CTCF Hoffman
Conserved LindbladToh
Coding UCSC
Annotation
−10
0
10
20
Enrichment (prop h2 / prop snps)

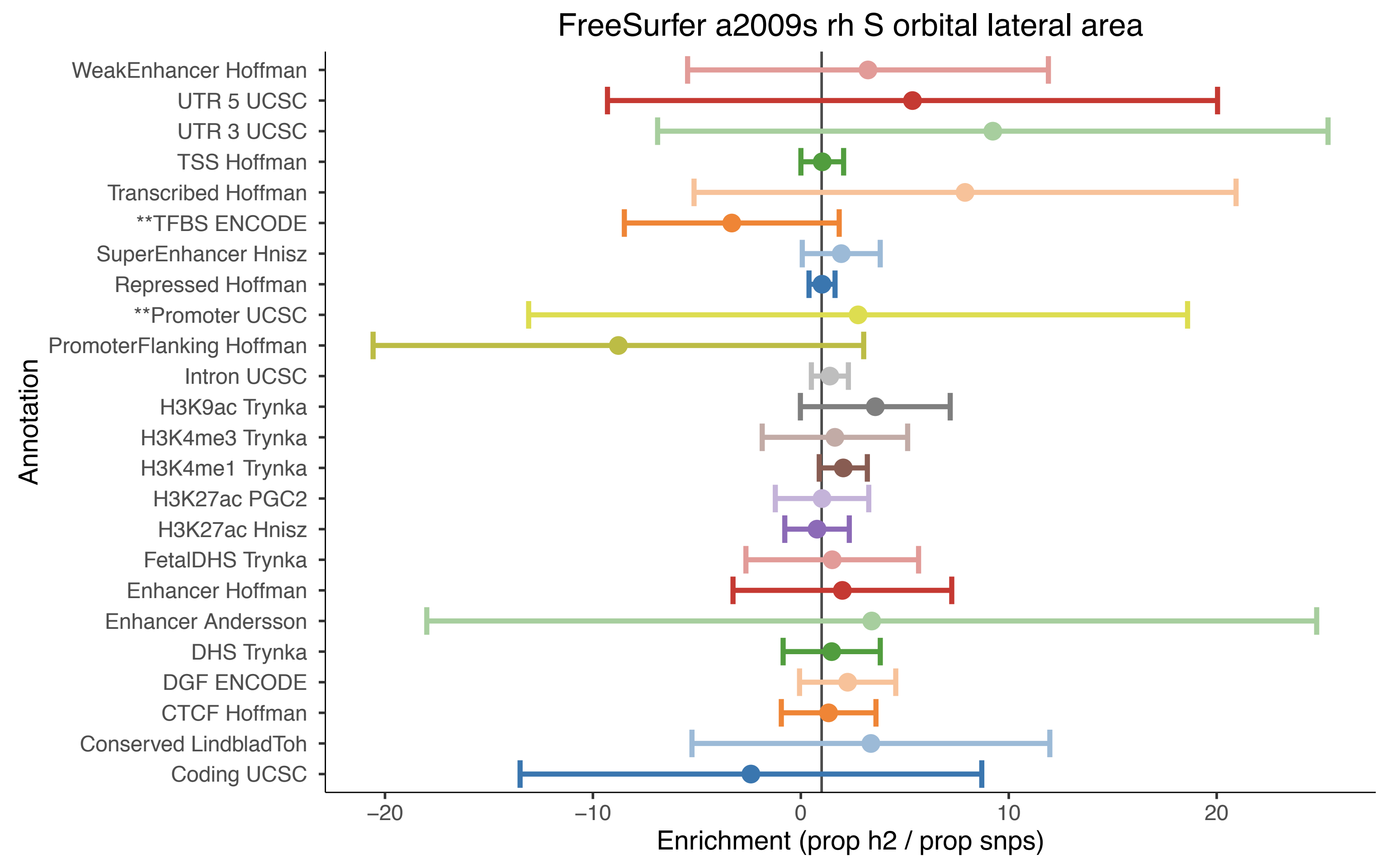

FreeSurfer a2009s rh S orbital lateral area
WeakEnhancer Hoffman
UTR 5 UCSC
UTR 3 UCSC
TSS Hoffman
Transcribed Hoffman
**TFBS ENCODE
SuperEnhancer Hnisz
Repressed Hoffman
**Promoter UCSC
PromoterFlanking Hoffman
Intron UCSC
H3K9ac Trynka
H3K4me3 Trynka
H3K4me1 Trynka
H3K27ac PGC2
H3K27ac Hnisz
FetalDHS Trynka
Enhancer Hoffman
Enhancer Andersson
DHS Trynka
DGF ENCODE
CTCF Hoffman
Conserved LindbladToh
Coding UCSC
Annotation
−20
−10
0
10
20
Enrichment (prop h2 / prop snps)

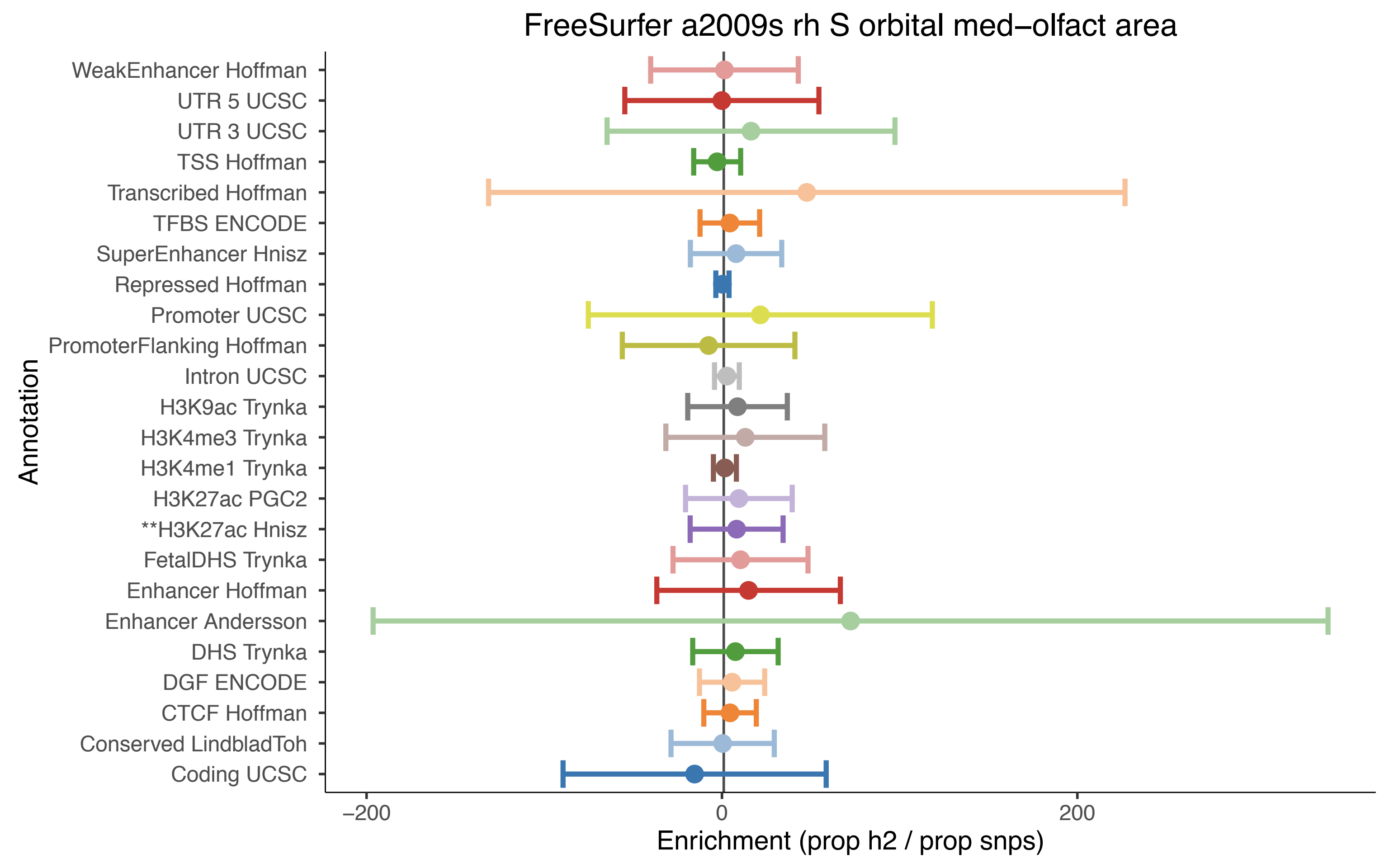

FreeSurfer a2009s rh S orbital med−olfact area
Annotation
WeakEnhancer Hoffman
UTR 5 UCSC
UTR 3 UCSC
TSS Hoffman
Transcribed Hoffman
TFBS ENCODE
SuperEnhancer Hnisz
Repressed Hoffman
Promoter UCSC
PromoterFlanking Hoffman
Intron UCSC
H3K9ac Trynka
H3K4me3 Trynka
H3K4me1 Trynka
H3K27ac PGC2
**H3K27ac Hnisz
FetalDHS Trynka
Enhancer Hoffman
Enhancer Andersson
DHS Trynka
DGF ENCODE
CTCF Hoffman
Conserved LindbladToh
Coding UCSC
−200
0
200
Enrichment (prop h2 / prop snps)

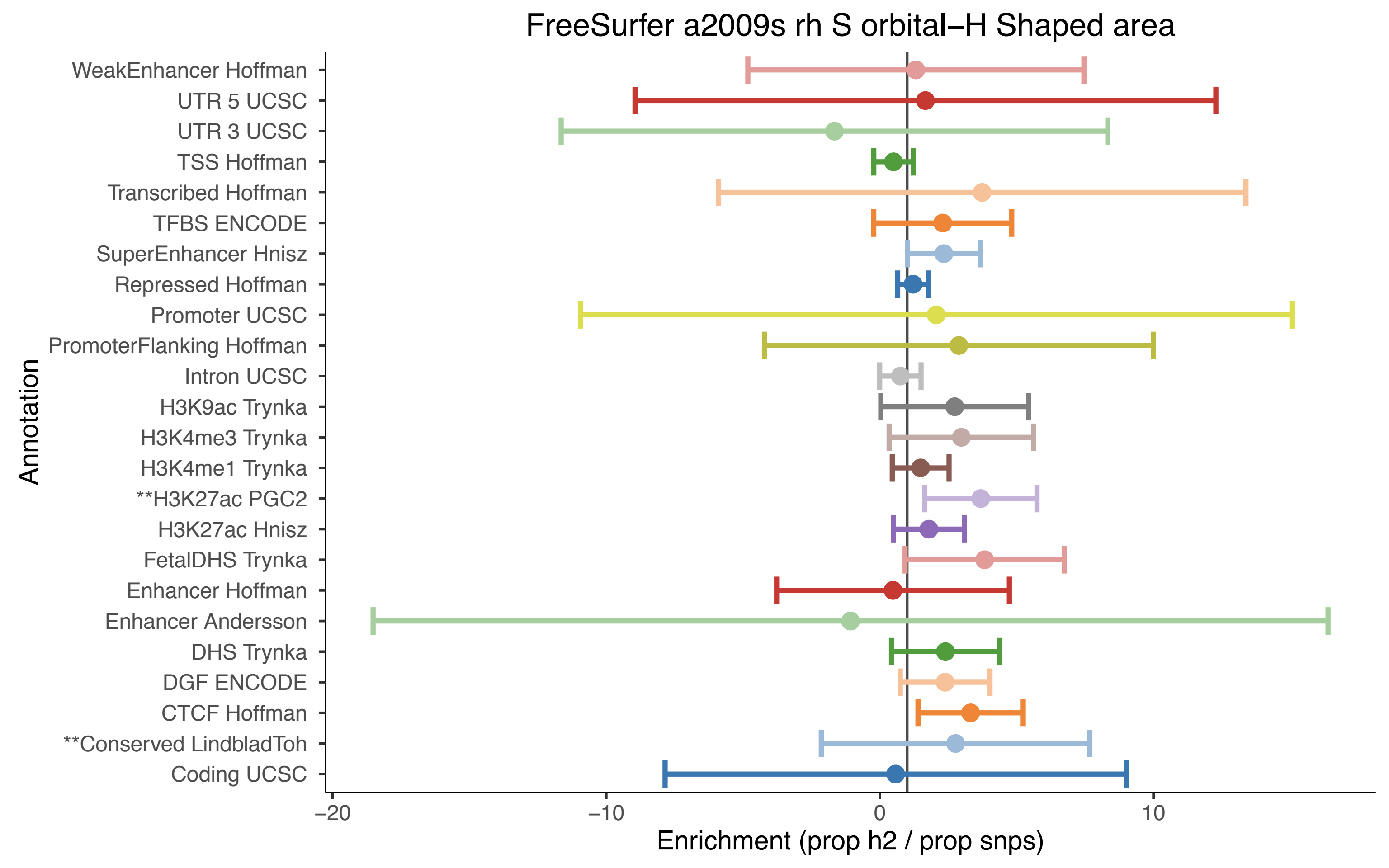
FreeSurfer a2009s rh S orbital–H Shaped area
WeakEnhancer Hoffman
UTR 5 UCSC
UTR 3 UCSC
TSS Hoffman
Transcribed Hoffman
TFBS ENCODE
SuperEnhancer Hnisz
Repressed Hoffman
Promoter UCSC
PromoterFlanking Hoffman
Intron UCSC
H3K9ac Trynka
H3K4me3 Trynka
H3K4me1 Trynka
**H3K27ac PGC2
H3K27ac Hnisz
FetalDHS Trynka
Enhancer Hoffman
Enhancer Andersson
DHS Trynka
DGF ENCODE
CTCF Hoffman
**Conserved LindbladToh
Coding UCSC
Annotation
−20
−10
0
10
Enrichment (prop h2 / prop snps)

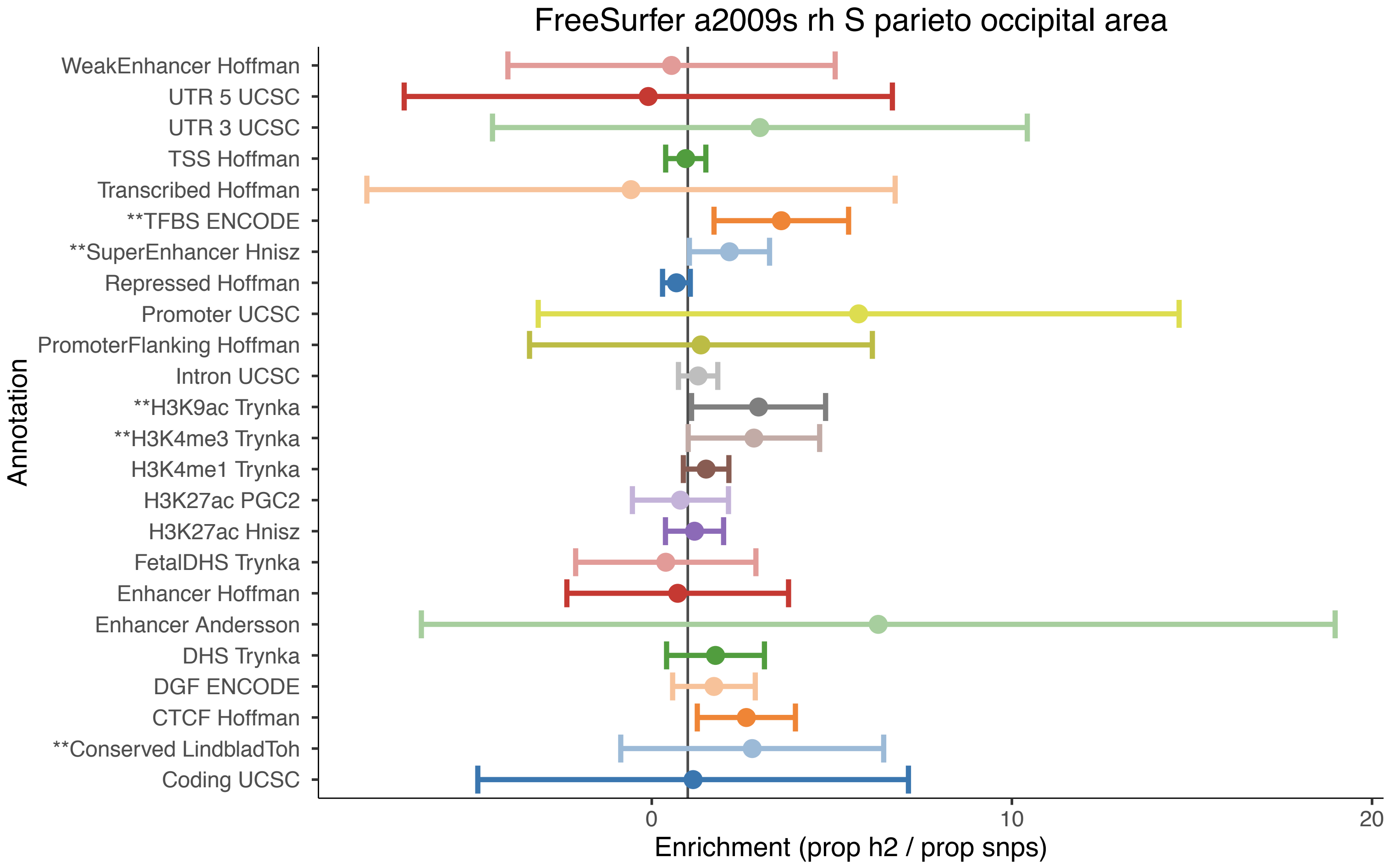

FreeSurfer a2009s rh S parieto occipital area
WeakEnhancer Hoffman
UTR 5 UCSC
UTR 3 UCSC
TSS Hoffman
Transcribed Hoffman
**TFBS ENCODE
**SuperEnhancer Hnisz
Repressed Hoffman
Promoter UCSC
PromoterFlanking Hoffman
Intron UCSC
**H3K9ac Trynka
**H3K4me3 Trynka
H3K4me1 Trynka
H3K27ac PGC2
H3K27ac Hnisz
FetalDHS Trynka
Enhancer Hoffman
Enhancer Andersson
DHS Trynka
DGF ENCODE
CTCF Hoffman
**Conserved LindbladToh
Coding UCSC
Annotation
0
10
20
Enrichment (prop h2 / prop snps)

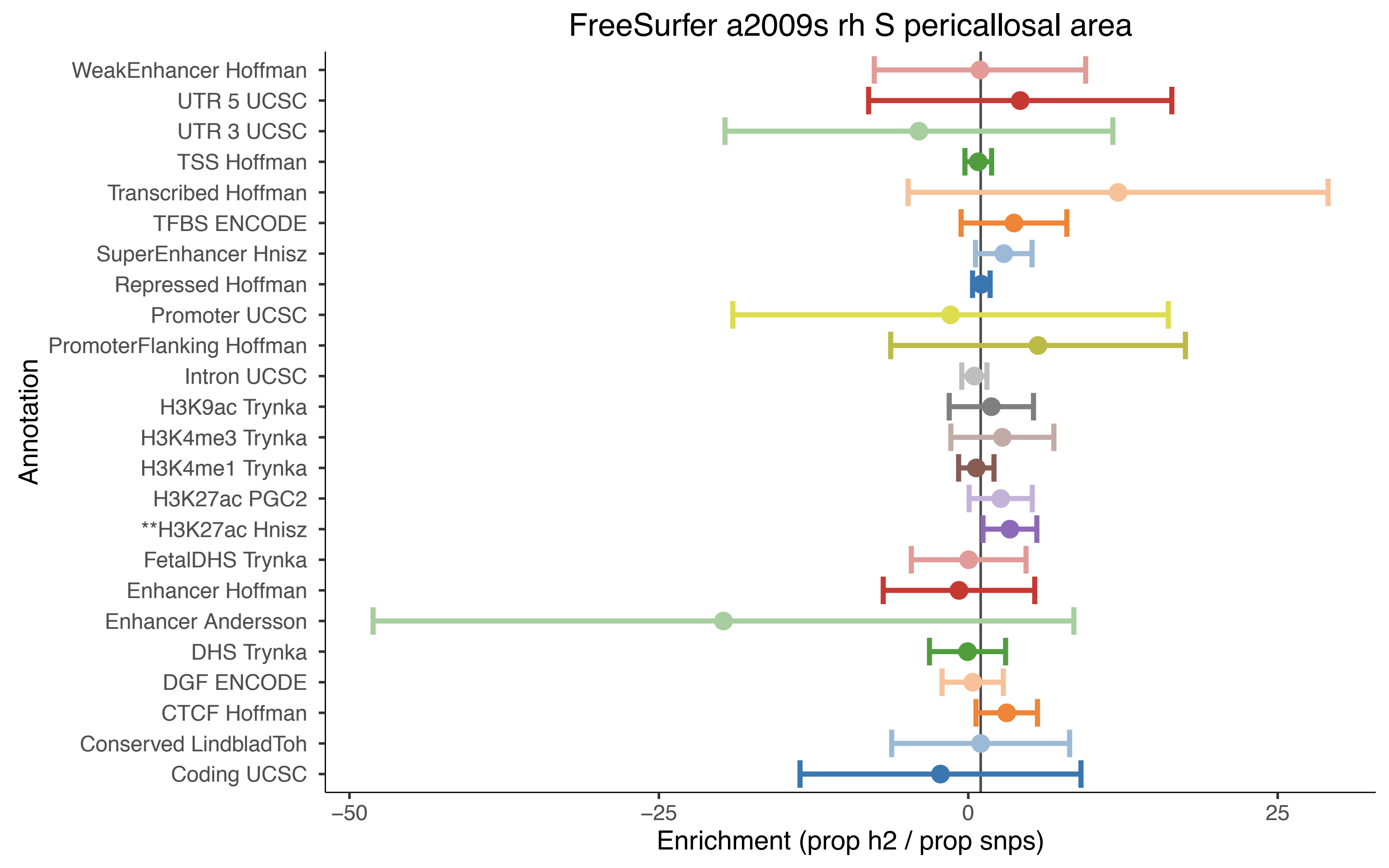
FreeSurfer a2009s rh S pericallosal area
WeakEnhancer Hoffman
UTR 5 UCSC
UTR 3 UCSC
TSS Hoffman
Transcribed Hoffman
TFBS ENCODE
SuperEnhancer Hnisz
Repressed Hoffman
Promoter UCSC
PromoterFlanking Hoffman
Intron UCSC
H3K9ac Trynka
H3K4me3 Trynka
H3K4me1 Trynka
H3K27ac PGC2
**H3K27ac Hnisz
FetalDHS Trynka
Enhancer Hoffman
Enhancer Andersson
DHS Trynka
DGF ENCODE
CTCF Hoffman
Conserved LindbladToh
Coding UCSC
Annotation
−50
−25
0
25
Enrichment (prop h2 / prop snps)

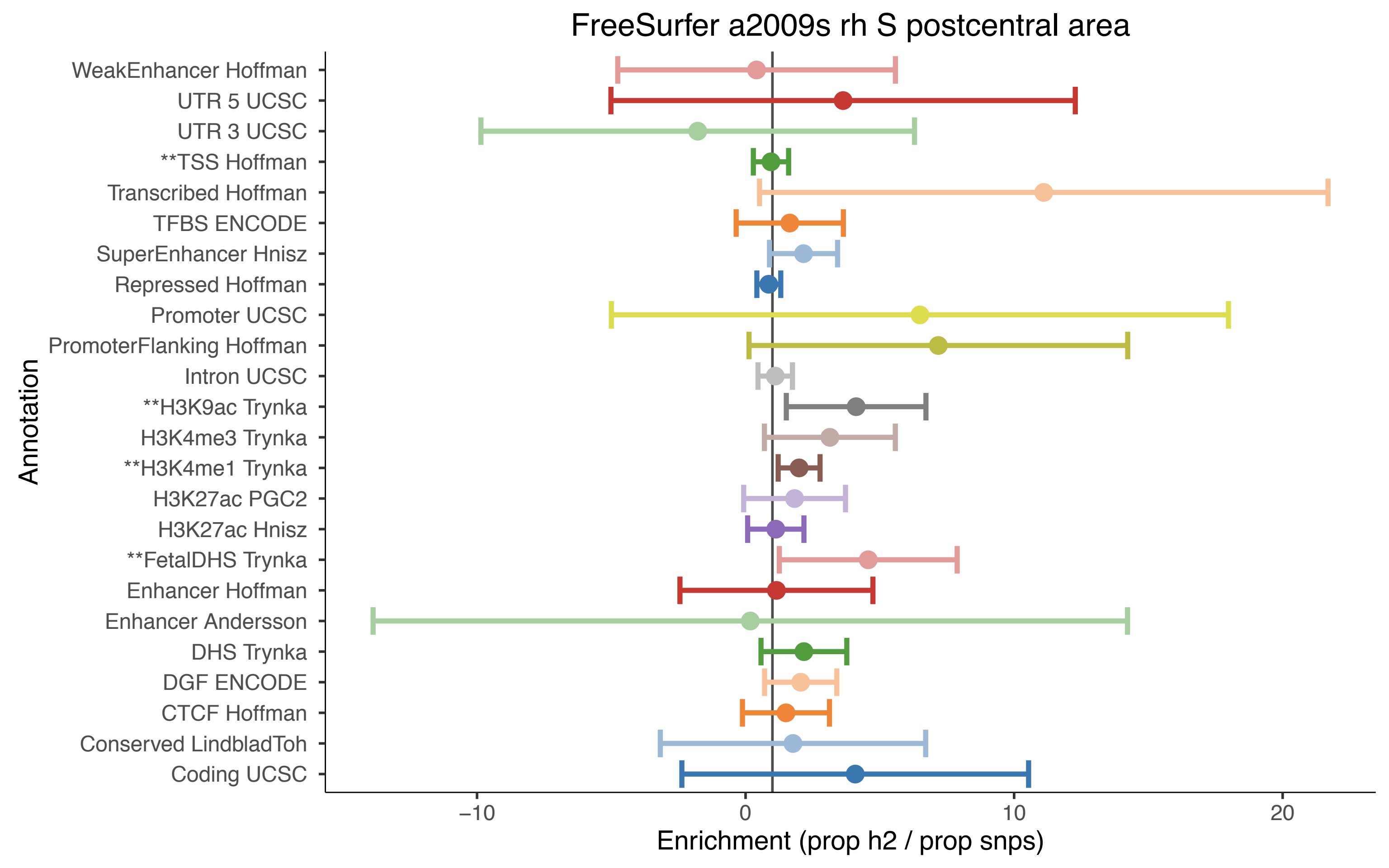
FreeSurfer a2009s rh S postcentral area
WeakEnhancer Hoffman
UTR 5 UCSC
UTR 3 UCSC
**TSS Hoffman
Transcribed Hoffman
TFBS ENCODE
SuperEnhancer Hnisz
Repressed Hoffman
Promoter UCSC
PromoterFlanking Hoffman
Intron UCSC
**H3K9ac Trynka
H3K4me3 Trynka
**H3K4me1 Trynka
H3K27ac PGC2
H3K27ac Hnisz
**FetalDHS Trynka
Enhancer Hoffman
Enhancer Andersson
DHS Trynka
DGF ENCODE
CTCF Hoffman
Conserved LindbladToh
Coding UCSC
Annotation
−10
0
10
20
Enrichment (prop h2 / prop snps)

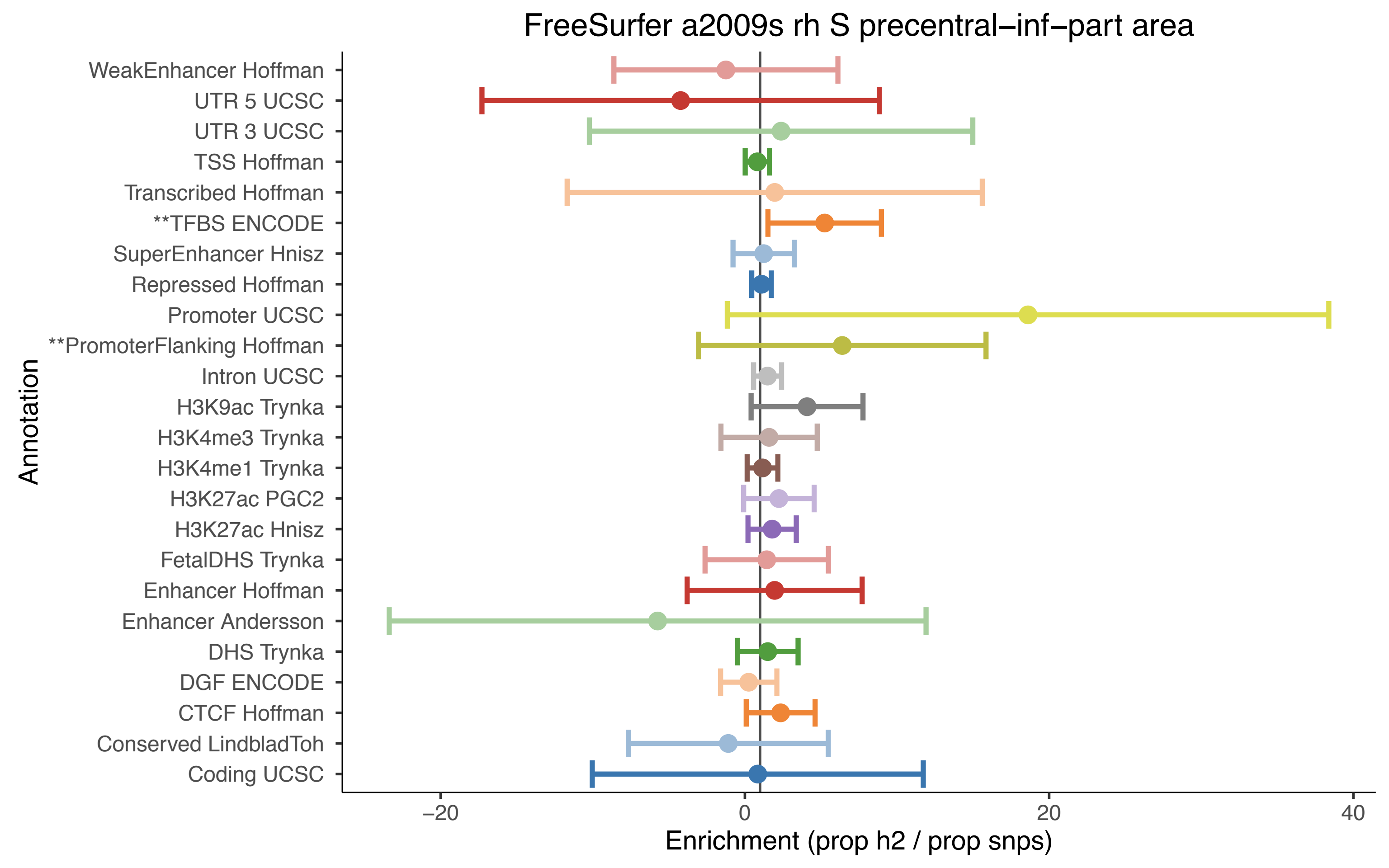

FreeSurfer a2009s rh S precentral−inf−part area
WeakEnhancer Hoffman
UTR 5 UCSC
UTR 3 UCSC
TSS Hoffman
Transcribed Hoffman
**TFBS ENCODE
SuperEnhancer Hnisz
Repressed Hoffman
Promoter UCSC
**PromoterFlanking Hoffman
Intron UCSC
H3K9ac Trynka
H3K4me3 Trynka
H3K4me1 Trynka
H3K27ac PGC2
H3K27ac Hnisz
FetalDHS Trynka
Enhancer Hoffman
Enhancer Andersson
DHS Trynka
DGF ENCODE
CTCF Hoffman
Conserved LindbladToh
Coding UCSC
Annotation
−20
0
20
40
Enrichment (prop h2 / prop snps)

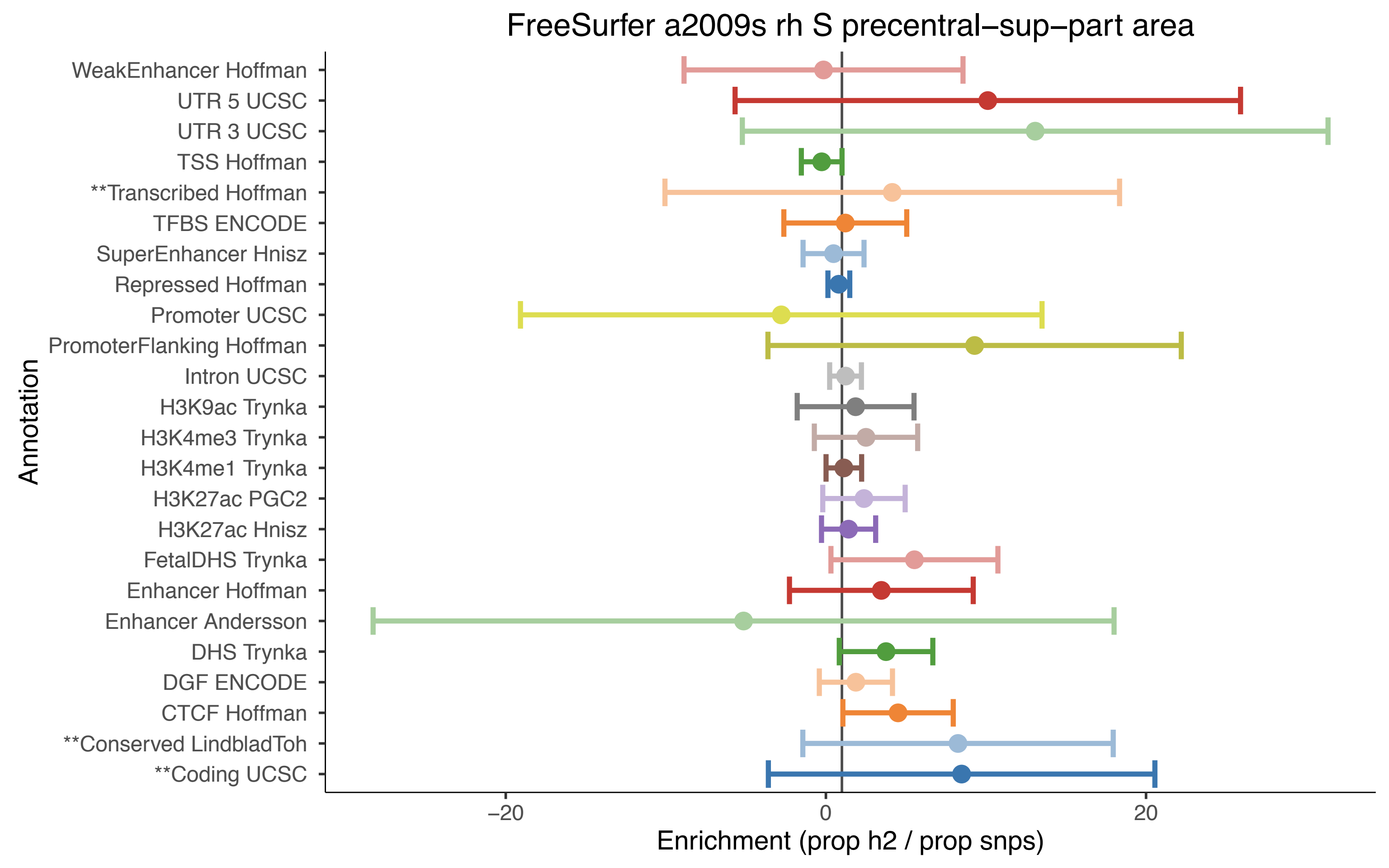

FreeSurfer a2009s rh S precentral−sup−part area
WeakEnhancer Hoffman
UTR 5 UCSC
UTR 3 UCSC
TSS Hoffman
**Transcribed Hoffman
TFBS ENCODE
SuperEnhancer Hnisz
Repressed Hoffman
Promoter UCSC
PromoterFlanking Hoffman
Intron UCSC
H3K9ac Trynka
H3K4me3 Trynka
H3K4me1 Trynka
H3K27ac PGC2
H3K27ac Hnisz
FetalDHS Trynka
Enhancer Hoffman
Enhancer Andersson
DHS Trynka
DGF ENCODE
CTCF Hoffman
**Conserved LindbladToh
**Coding UCSC
Annotation
−20
0
20
Enrichment (prop h2 / prop snps)

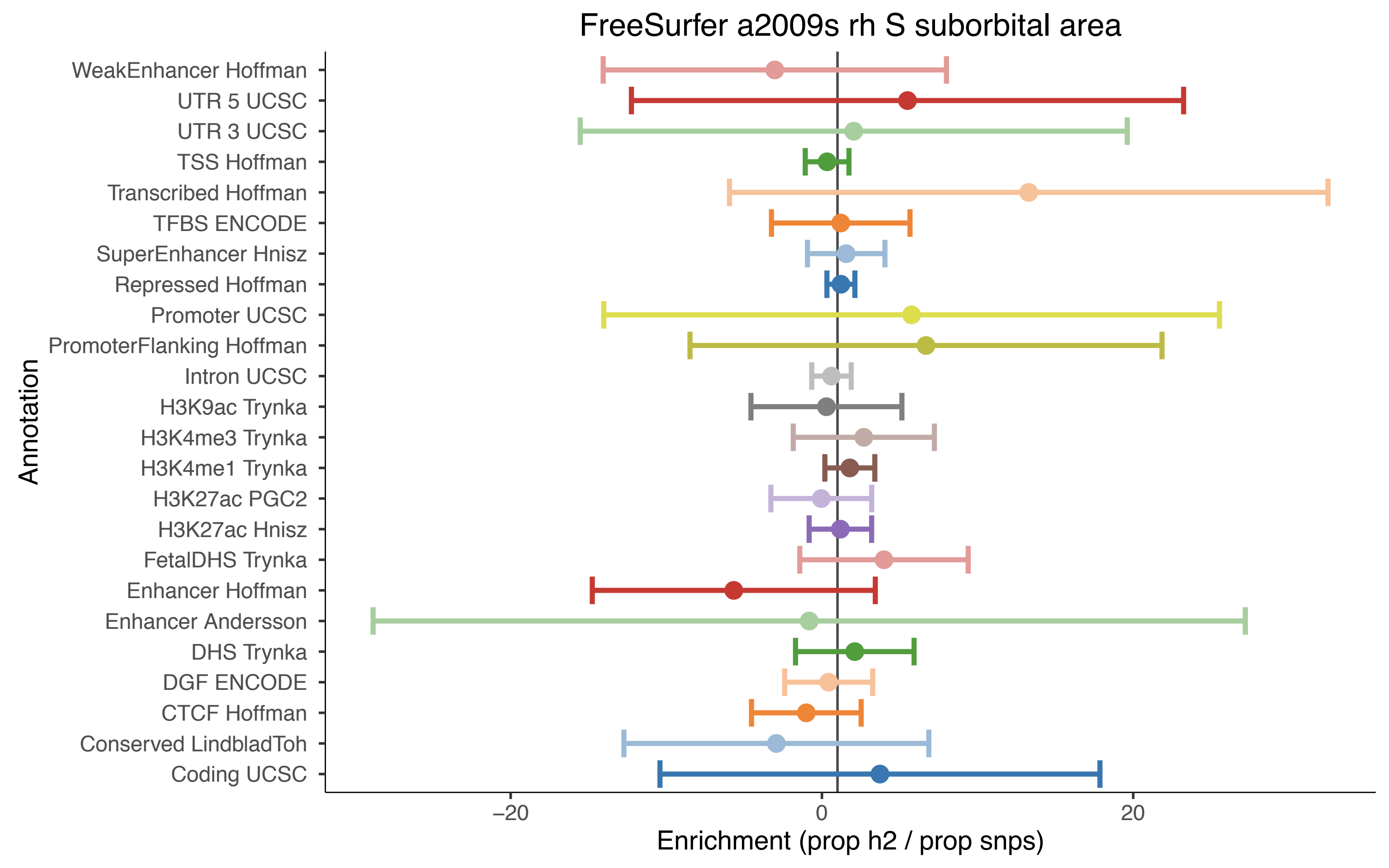

FreeSurfer a2009s rh S suborbital area
WeakEnhancer Hoffman
UTR 5 UCSC
UTR 3 UCSC
TSS Hoffman
Transcribed Hoffman
TFBS ENCODE
SuperEnhancer Hnisz
Repressed Hoffman
Promoter UCSC
PromoterFlanking Hoffman
Intron UCSC
H3K9ac Trynka
H3K4me3 Trynka
H3K4me1 Trynka
H3K27ac PGC2
H3K27ac Hnisz
FetalDHS Trynka
Enhancer Hoffman
Enhancer Andersson
DHS Trynka
DGF ENCODE
CTCF Hoffman
Conserved LindbladToh
Coding UCSC
Annotation
−20
0
20
Enrichment (prop h2 / prop snps)

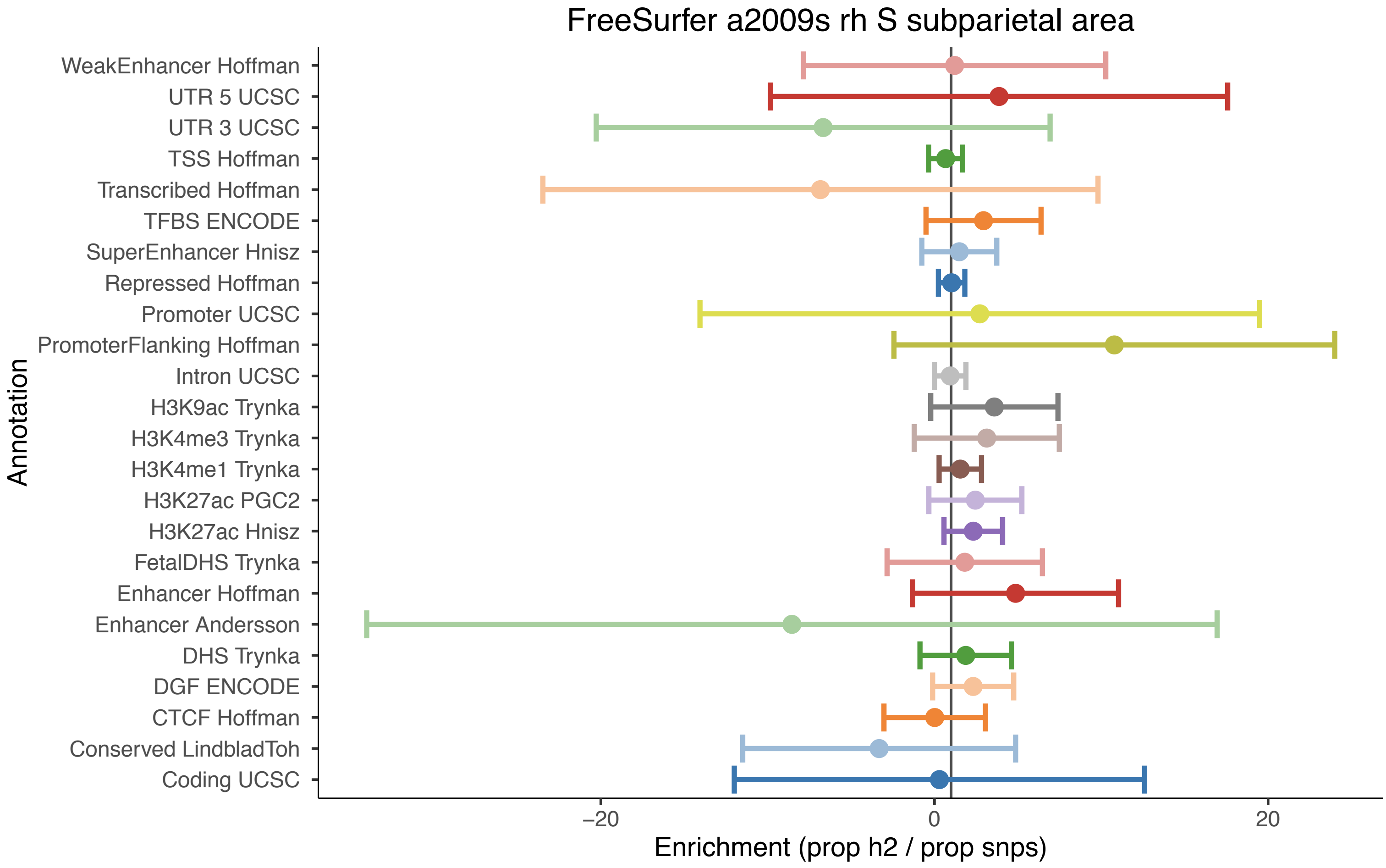
FreeSurfer a2009s rh S subparietal area
Annotation
WeakEnhancer Hoffman
UTR 5 UCSC
UTR 3 UCSC
TSS Hoffman
Transcribed Hoffman
TFBS ENCODE
SuperEnhancer Hnisz
Repressed Hoffman
Promoter UCSC
PromoterFlanking Hoffman
Intron UCSC
H3K9ac Trynka
H3K4me3 Trynka
H3K4me1 Trynka
H3K27ac PGC2
H3K27ac Hnisz
FetalDHS Trynka
Enhancer Hoffman
Enhancer Andersson
DHS Trynka
DGF ENCODE
CTCF Hoffman
Conserved LindbladToh
Coding UCSC
−20
0
20
Enrichment (prop h2 / prop snps)

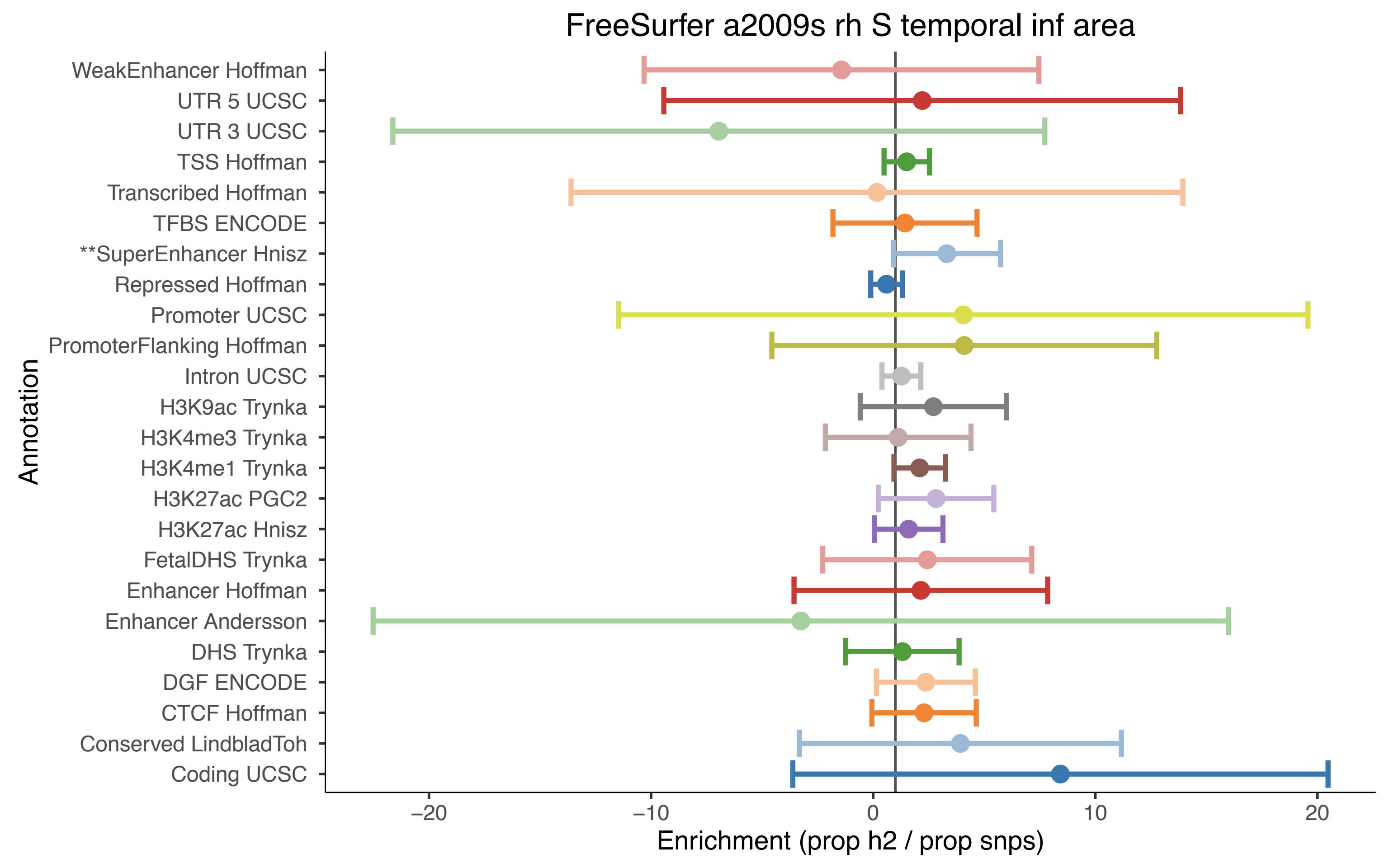
FreeSurfer a2009s rh S temporal inf area
WeakEnhancer Hoffman
UTR 5 UCSC
UTR 3 UCSC
TSS Hoffman
Transcribed Hoffman
TFBS ENCODE
**SuperEnhancer Hnisz
Repressed Hoffman
Promoter UCSC
PromoterFlanking Hoffman
Intron UCSC
H3K9ac Trynka
H3K4me3 Trynka
H3K4me1 Trynka
H3K27ac PGC2
H3K27ac Hnisz
FetalDHS Trynka
Enhancer Hoffman
Enhancer Andersson
DHS Trynka
DGF ENCODE
CTCF Hoffman
Conserved LindbladToh
Coding UCSC
Annotation
−20
−10
0
10
20
Enrichment (prop h2 / prop snps)

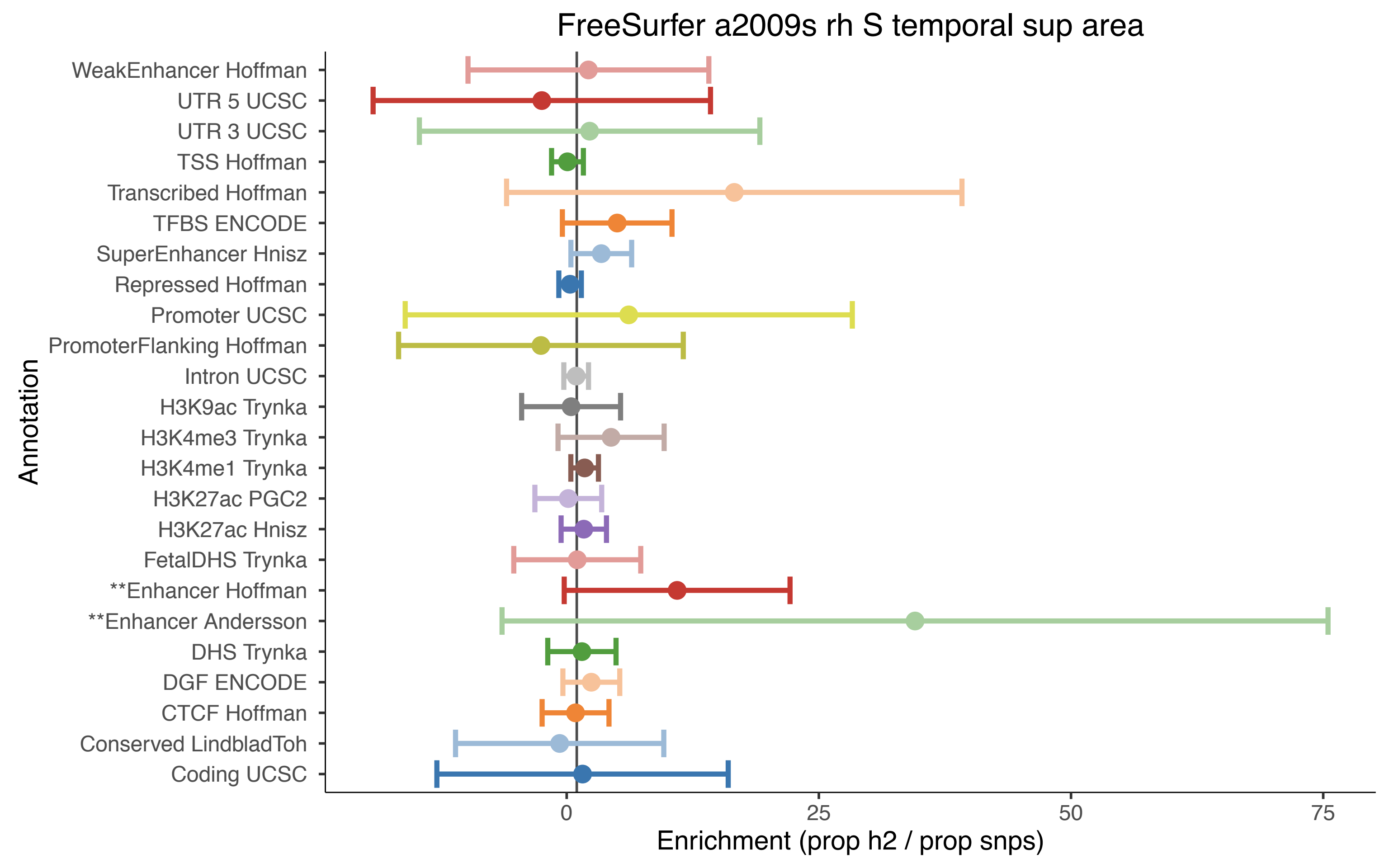
FreeSurfer a2009s rh S temporal sup area
WeakEnhancer Hoffman
UTR 5 UCSC
UTR 3 UCSC
TSS Hoffman
Transcribed Hoffman
TFBS ENCODE
SuperEnhancer Hnisz
Repressed Hoffman
Promoter UCSC
PromoterFlanking Hoffman
Intron UCSC
H3K9ac Trynka
H3K4me3 Trynka
H3K4me1 Trynka
H3K27ac PGC2
H3K27ac Hnisz
FetalDHS Trynka
**Enhancer Hoffman
**Enhancer Andersson
DHS Trynka
DGF ENCODE
CTCF Hoffman
Conserved LindbladToh
Coding UCSC
Annotation
0
25
50
75
Enrichment (prop h2 / prop snps)

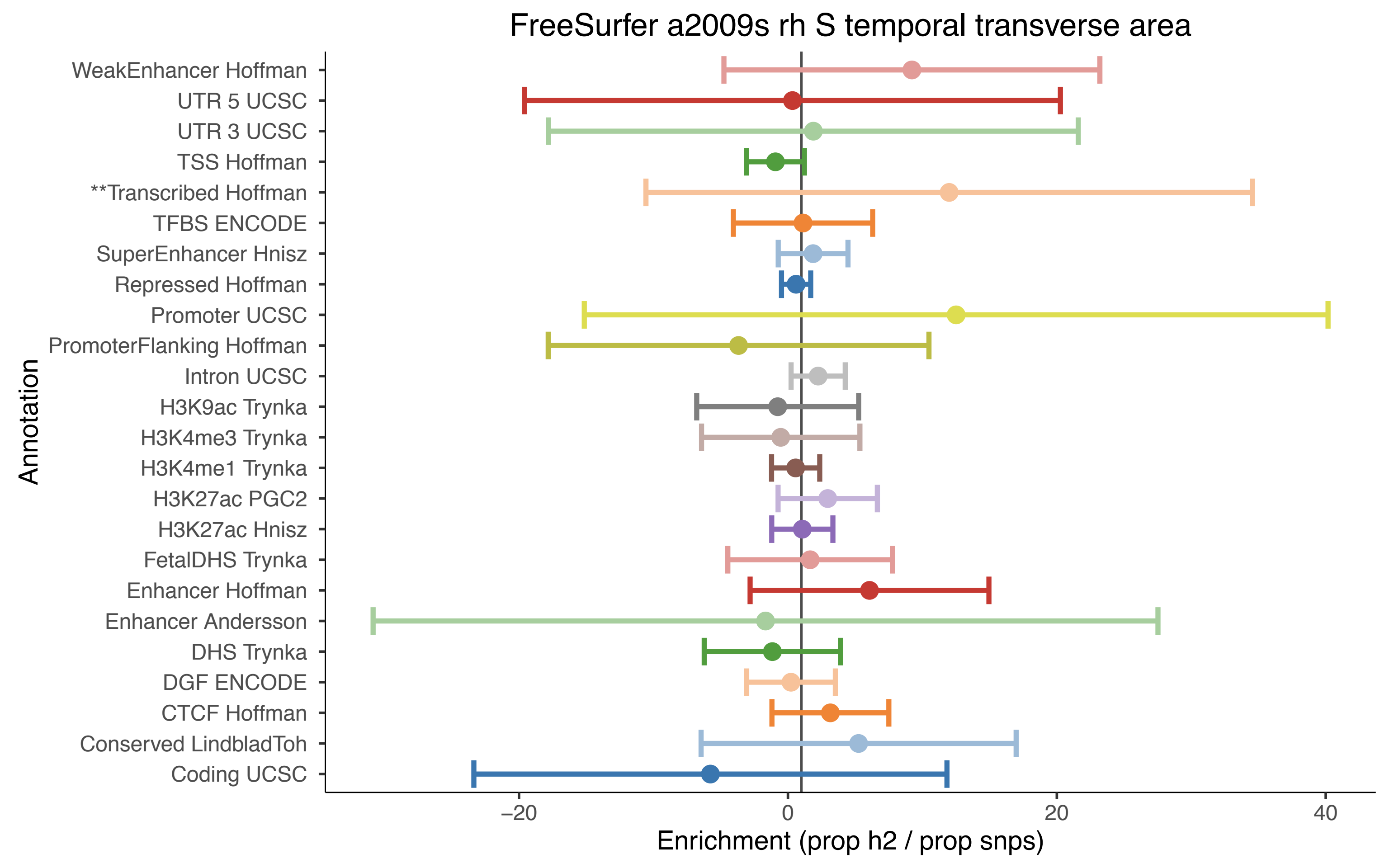
FreeSurfer a2009s rh S temporal transverse area
WeakEnhancer Hoffman
UTR 5 UCSC
UTR 3 UCSC
TSS Hoffman
**Transcribed Hoffman
TFBS ENCODE
SuperEnhancer Hnisz
Repressed Hoffman
Promoter UCSC
PromoterFlanking Hoffman
Intron UCSC
H3K9ac Trynka
H3K4me3 Trynka
H3K4me1 Trynka
H3K27ac PGC2
H3K27ac Hnisz
FetalDHS Trynka
Enhancer Hoffman
Enhancer Andersson
DHS Trynka
DGF ENCODE
CTCF Hoffman
Conserved LindbladToh
Coding UCSC
Annotation
−20
0
20
40
Enrichment (prop h2 / prop snps)

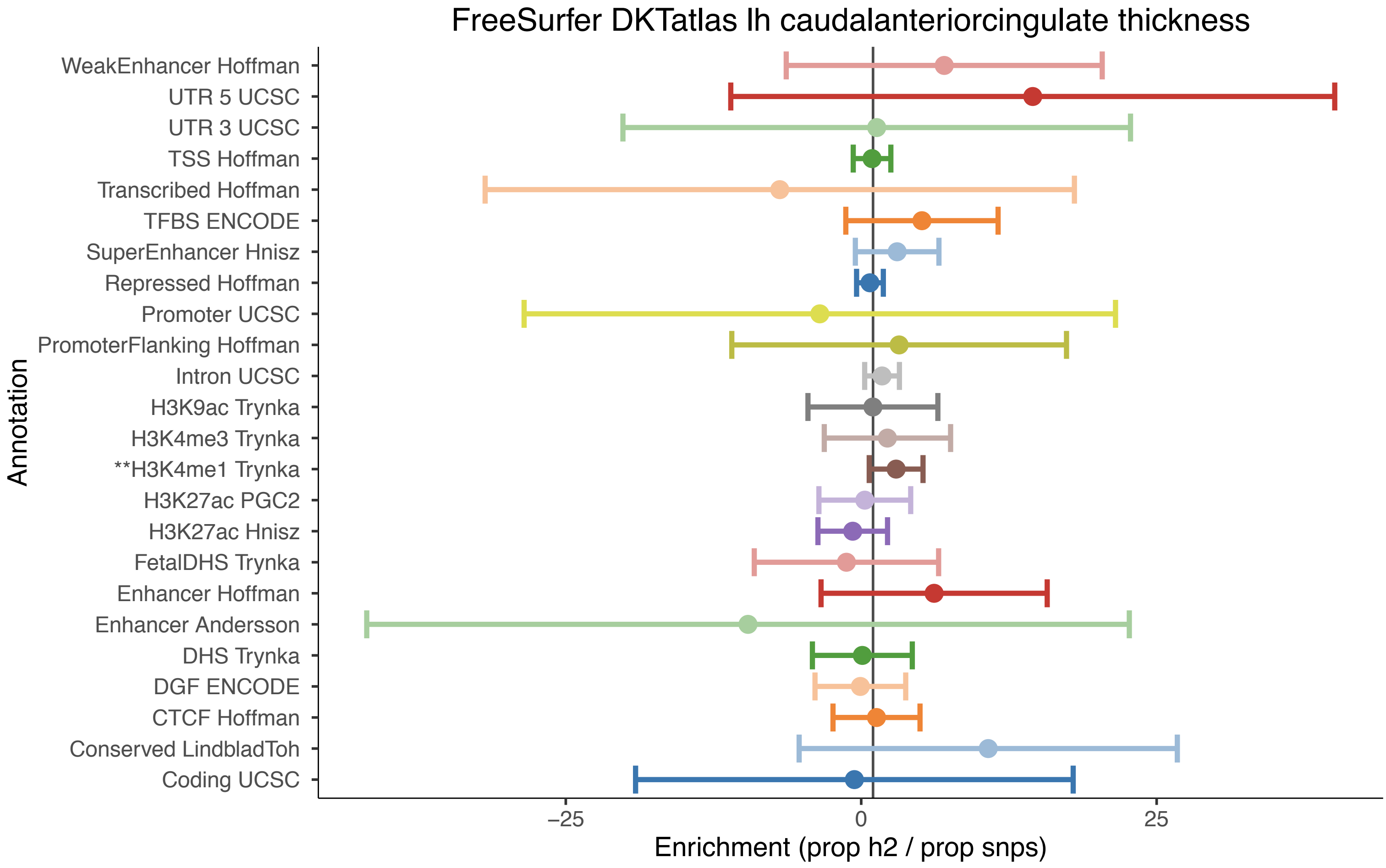

FreeSurfer DKTatlas lh caudalanteriorcingulate thickness
WeakEnhancer Hoffman
UTR 5 UCSC
UTR 3 UCSC
TSS Hoffman
Transcribed Hoffman
TFBS ENCODE
SuperEnhancer Hnisz
Repressed Hoffman
Promoter UCSC
PromoterFlanking Hoffman
Intron UCSC
H3K9ac Trynka
H3K4me3 Trynka
**H3K4me1 Trynka
H3K27ac PGC2
H3K27ac Hnisz
FetalDHS Trynka
Enhancer Hoffman
Enhancer Andersson
DHS Trynka
DGF ENCODE
CTCF Hoffman
Conserved LindbladToh
Coding UCSC
Annotation
−25
0
25
Enrichment (prop h2 / prop snps)

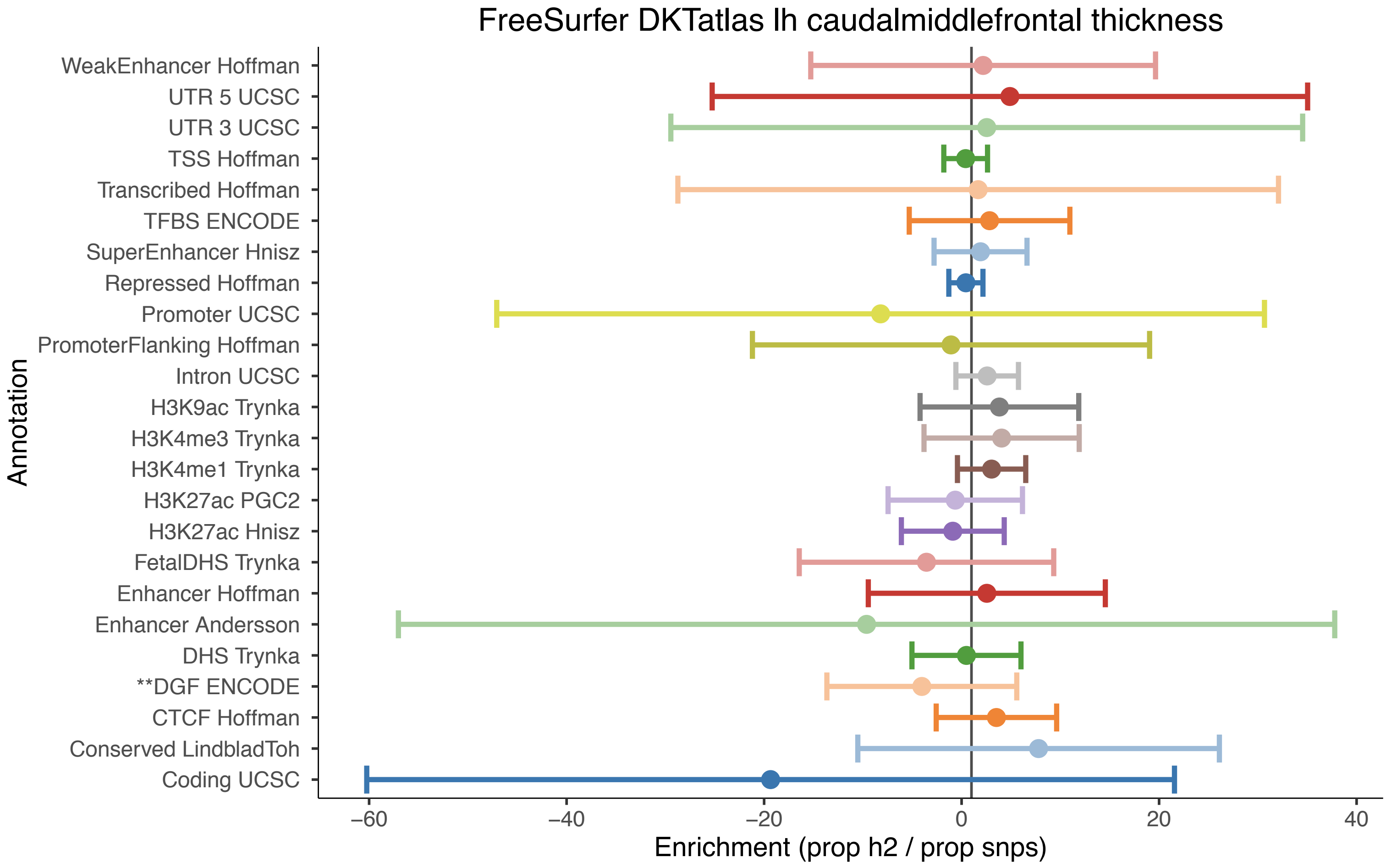

FreeSurfer DKTatlas lh caudalmiddlefrontal thickness
WeakEnhancer Hoffman
UTR 5 UCSC
UTR 3 UCSC
TSS Hoffman
Transcribed Hoffman
TFBS ENCODE
SuperEnhancer Hnisz
Repressed Hoffman
Promoter UCSC
PromoterFlanking Hoffman
Intron UCSC
H3K9ac Trynka
H3K4me3 Trynka
H3K4me1 Trynka
H3K27ac PGC2
H3K27ac Hnisz
FetalDHS Trynka
Enhancer Hoffman
Enhancer Andersson
DHS Trynka
**DGF ENCODE
CTCF Hoffman
Conserved LindbladToh
Coding UCSC
Annotation
−60
−40
−20
0
20
40
Enrichment (prop h2 / prop snps)

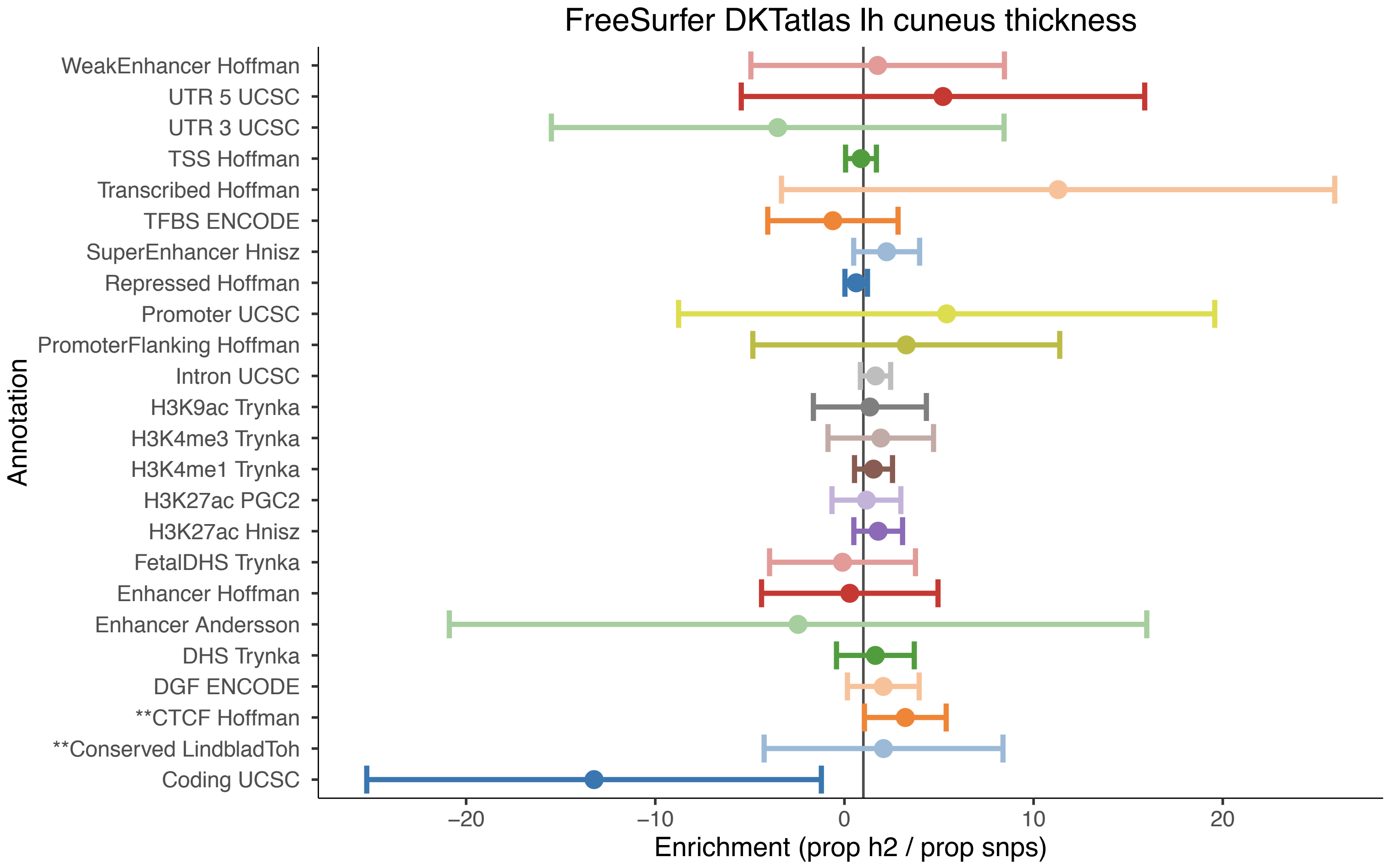

FreeSurfer DKTatlas lh cuneus thickness
WeakEnhancer Hoffman
UTR 5 UCSC
UTR 3 UCSC
TSS Hoffman
Transcribed Hoffman
TFBS ENCODE
SuperEnhancer Hnisz
Repressed Hoffman
Promoter UCSC
PromoterFlanking Hoffman
Intron UCSC
H3K9ac Trynka
H3K4me3 Trynka
H3K4me1 Trynka
H3K27ac PGC2
H3K27ac Hnisz
FetalDHS Trynka
Enhancer Hoffman
Enhancer Andersson
DHS Trynka
DGF ENCODE
**CTCF Hoffman
**Conserved LindbladToh
Coding UCSC
Annotation
−20
−10
0
10
20
Enrichment (prop h2 / prop snps)

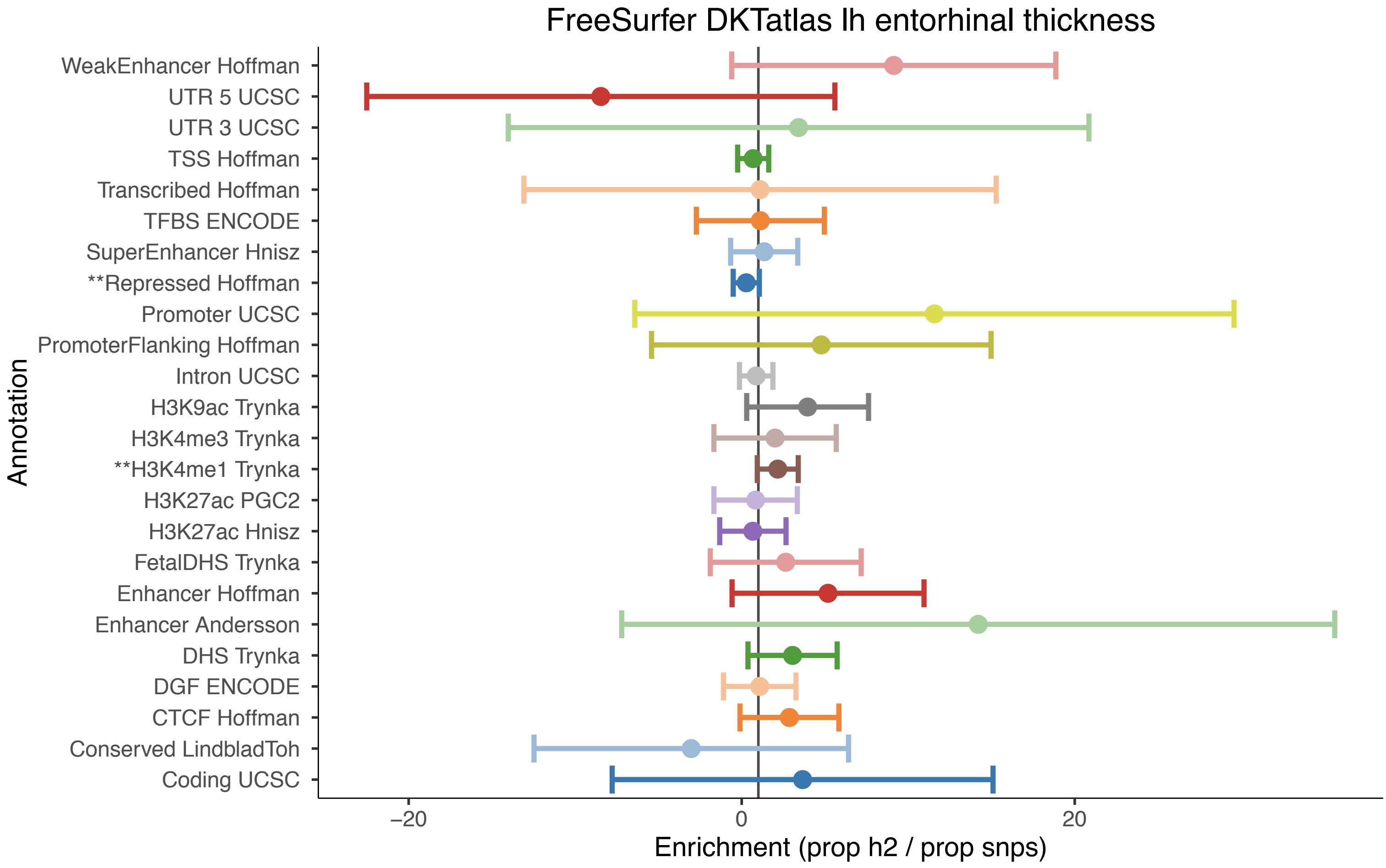

FreeSurfer DKTatlas lh entorhinal thickness
WeakEnhancer Hoffman
UTR 5 UCSC
UTR 3 UCSC
TSS Hoffman
Transcribed Hoffman
TFBS ENCODE
SuperEnhancer Hnisz
**Repressed Hoffman
Promoter UCSC
PromoterFlanking Hoffman
Intron UCSC
H3K9ac Trynka
H3K4me3 Trynka
**H3K4me1 Trynka
H3K27ac PGC2
H3K27ac Hnisz
FetalDHS Trynka
Enhancer Hoffman
Enhancer Andersson
DHS Trynka
DGF ENCODE
CTCF Hoffman
Conserved LindbladToh
Coding UCSC
Annotation
−20
0
20
Enrichment (prop h2 / prop snps)

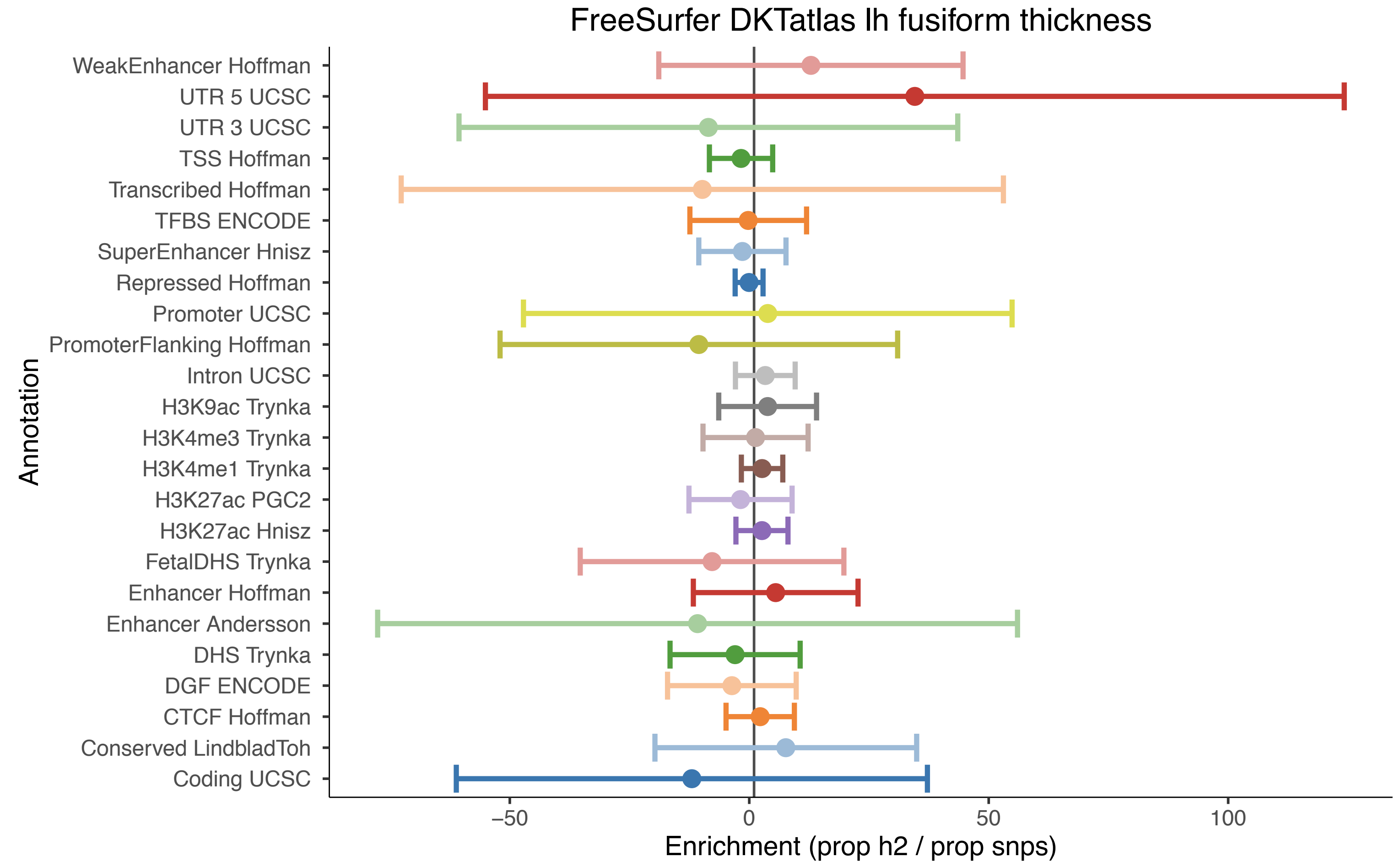
FreeSurfer DKTatlas lh fusiform thickness
Annotation
WeakEnhancer Hoffman
UTR 5 UCSC
UTR 3 UCSC
TSS Hoffman
Transcribed Hoffman
TFBS ENCODE
SuperEnhancer Hnisz
Repressed Hoffman
Promoter UCSC
PromoterFlanking Hoffman
Intron UCSC
H3K9ac Trynka
H3K4me3 Trynka
H3K4me1 Trynka
H3K27ac PGC2
H3K27ac Hnisz
FetalDHS Trynka
Enhancer Hoffman
Enhancer Andersson
DHS Trynka
DGF ENCODE
CTCF Hoffman
Conserved LindbladToh
Coding UCSC
−50
0
50
100
Enrichment (prop h2 / prop snps)

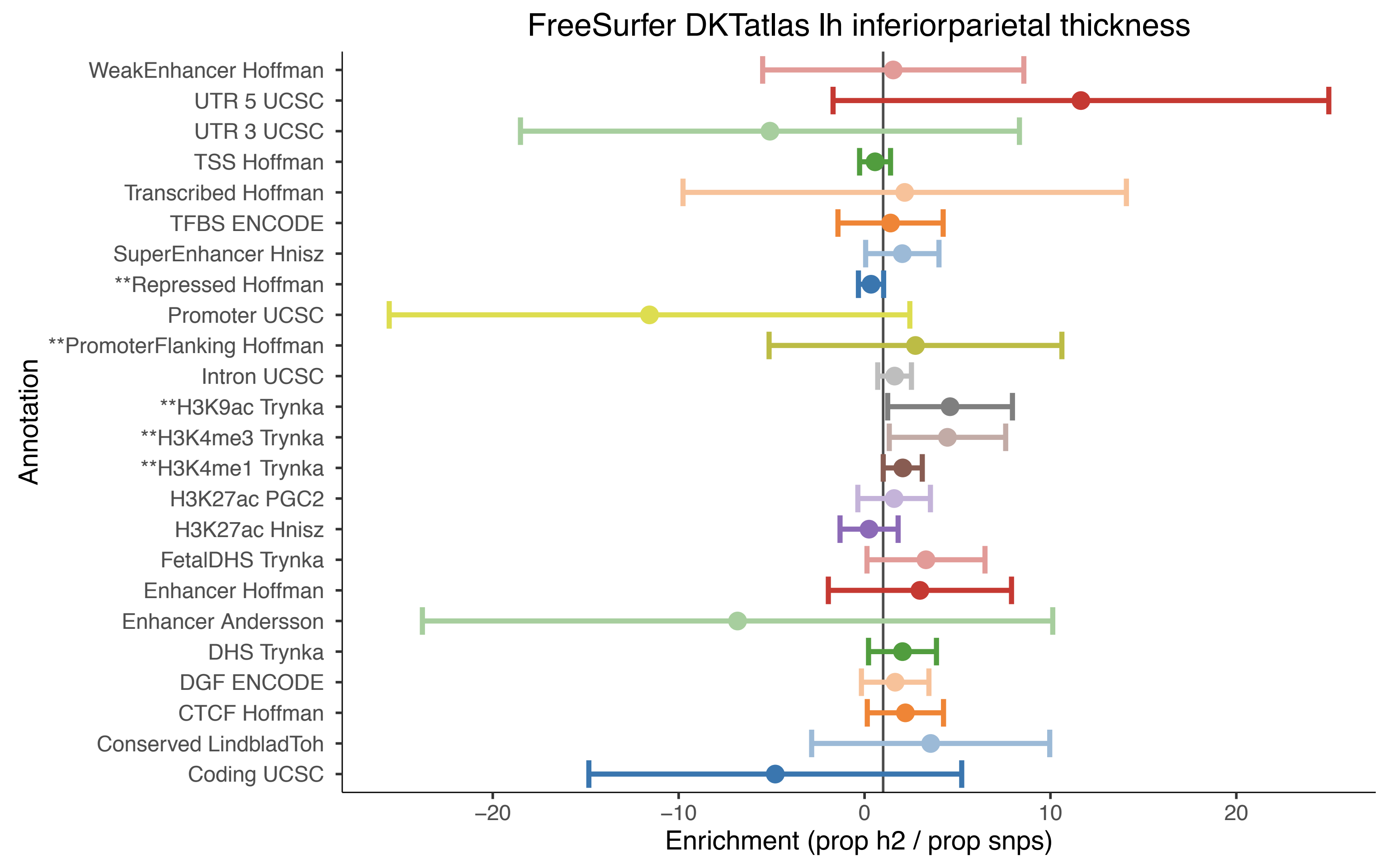

FreeSurfer DKTatlas lh inferiorparietal thickness
WeakEnhancer Hoffman
UTR 5 UCSC
UTR 3 UCSC
TSS Hoffman
Transcribed Hoffman
TFBS ENCODE
SuperEnhancer Hnisz
**Repressed Hoffman
Promoter UCSC
**PromoterFlanking Hoffman
Intron UCSC
**H3K9ac Trynka
**H3K4me3 Trynka
**H3K4me1 Trynka
H3K27ac PGC2
H3K27ac Hnisz
FetalDHS Trynka
Enhancer Hoffman
Enhancer Andersson
DHS Trynka
DGF ENCODE
CTCF Hoffman
Conserved LindbladToh
Coding UCSC
Annotation
−20
−10
0
10
20
Enrichment (prop h2 / prop snps)

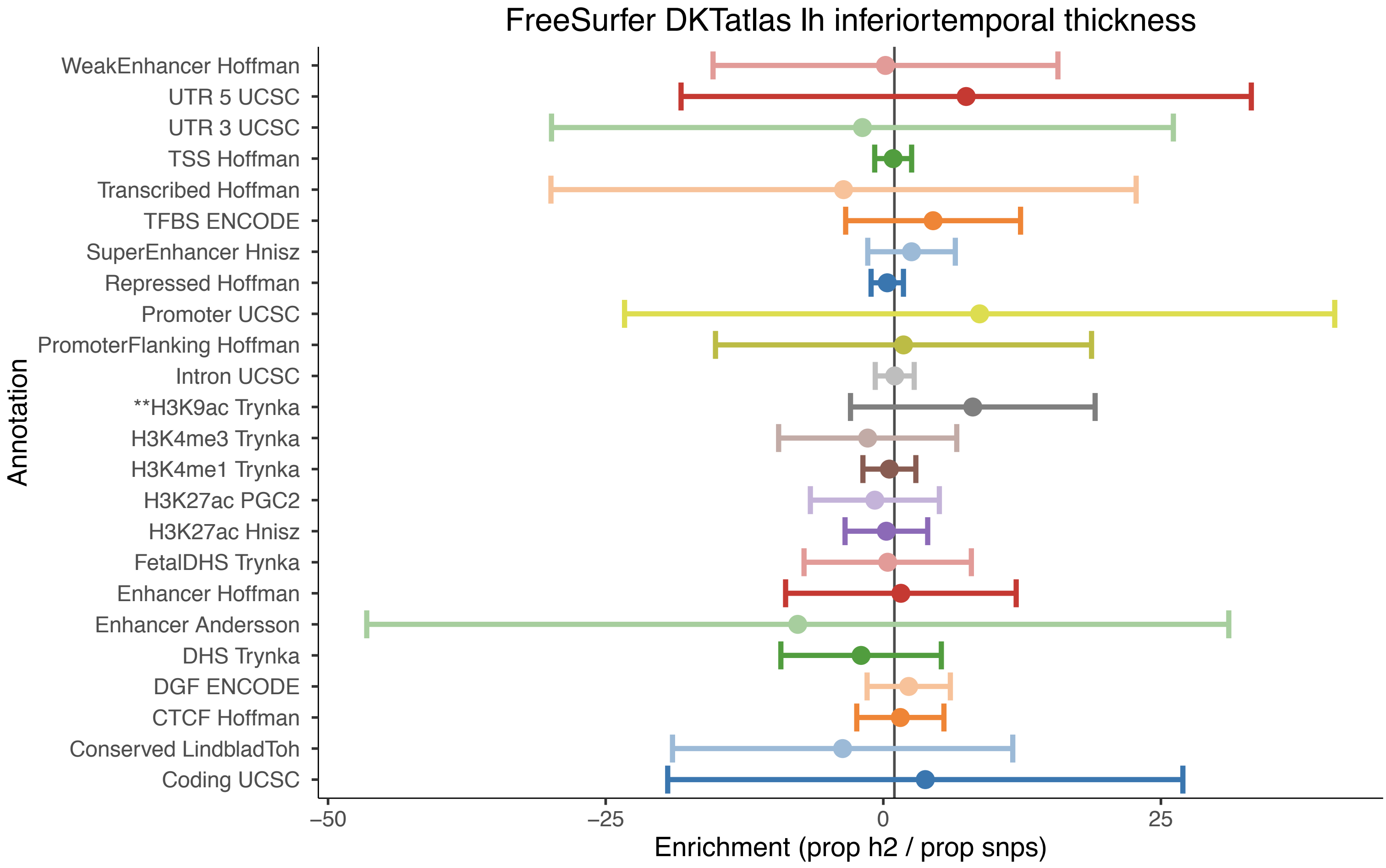

FreeSurfer DKTatlas lh inferiortemporal thickness
WeakEnhancer Hoffman
UTR 5 UCSC
UTR 3 UCSC
TSS Hoffman
Transcribed Hoffman
TFBS ENCODE
SuperEnhancer Hnisz
Repressed Hoffman
Promoter UCSC
PromoterFlanking Hoffman
Intron UCSC
**H3K9ac Trynka
H3K4me3 Trynka
H3K4me1 Trynka
H3K27ac PGC2
H3K27ac Hnisz
FetalDHS Trynka
Enhancer Hoffman
Enhancer Andersson
DHS Trynka
DGF ENCODE
CTCF Hoffman
Conserved LindbladToh
Coding UCSC
Annotation
−50
−25
0
25
Enrichment (prop h2 / prop snps)

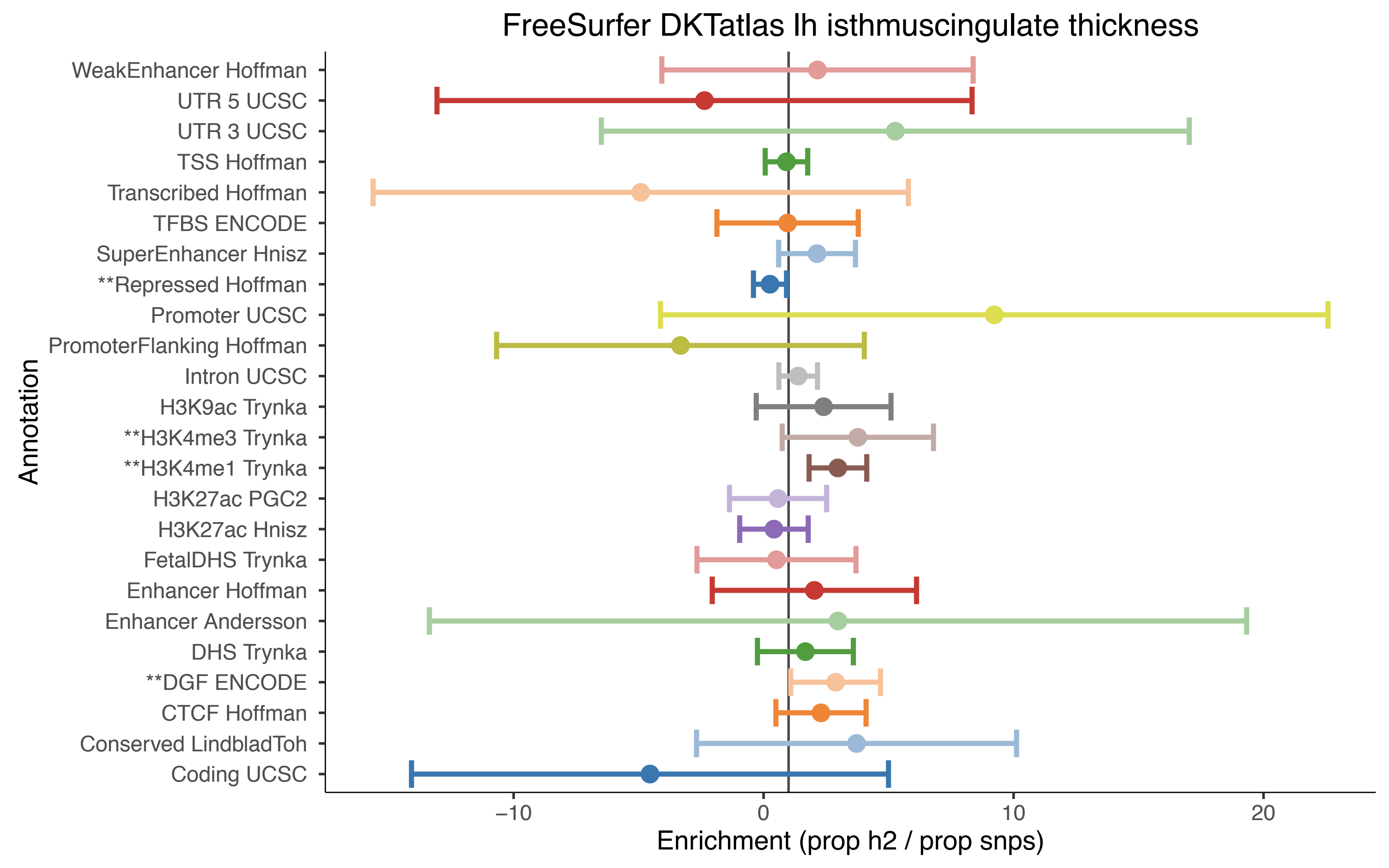

FreeSurfer DKTatlas lh isthmuscingulate thickness
WeakEnhancer Hoffman
UTR 5 UCSC
UTR 3 UCSC
TSS Hoffman
Transcribed Hoffman
TFBS ENCODE
SuperEnhancer Hnisz
**Repressed Hoffman
Promoter UCSC
PromoterFlanking Hoffman
Intron UCSC
H3K9ac Trynka
**H3K4me3 Trynka
**H3K4me1 Trynka
H3K27ac PGC2
H3K27ac Hnisz
FetalDHS Trynka
Enhancer Hoffman
Enhancer Andersson
DHS Trynka
**DGF ENCODE
CTCF Hoffman
Conserved LindbladToh
Coding UCSC
Annotation
−10
0
10
20
Enrichment (prop h2 / prop snps)

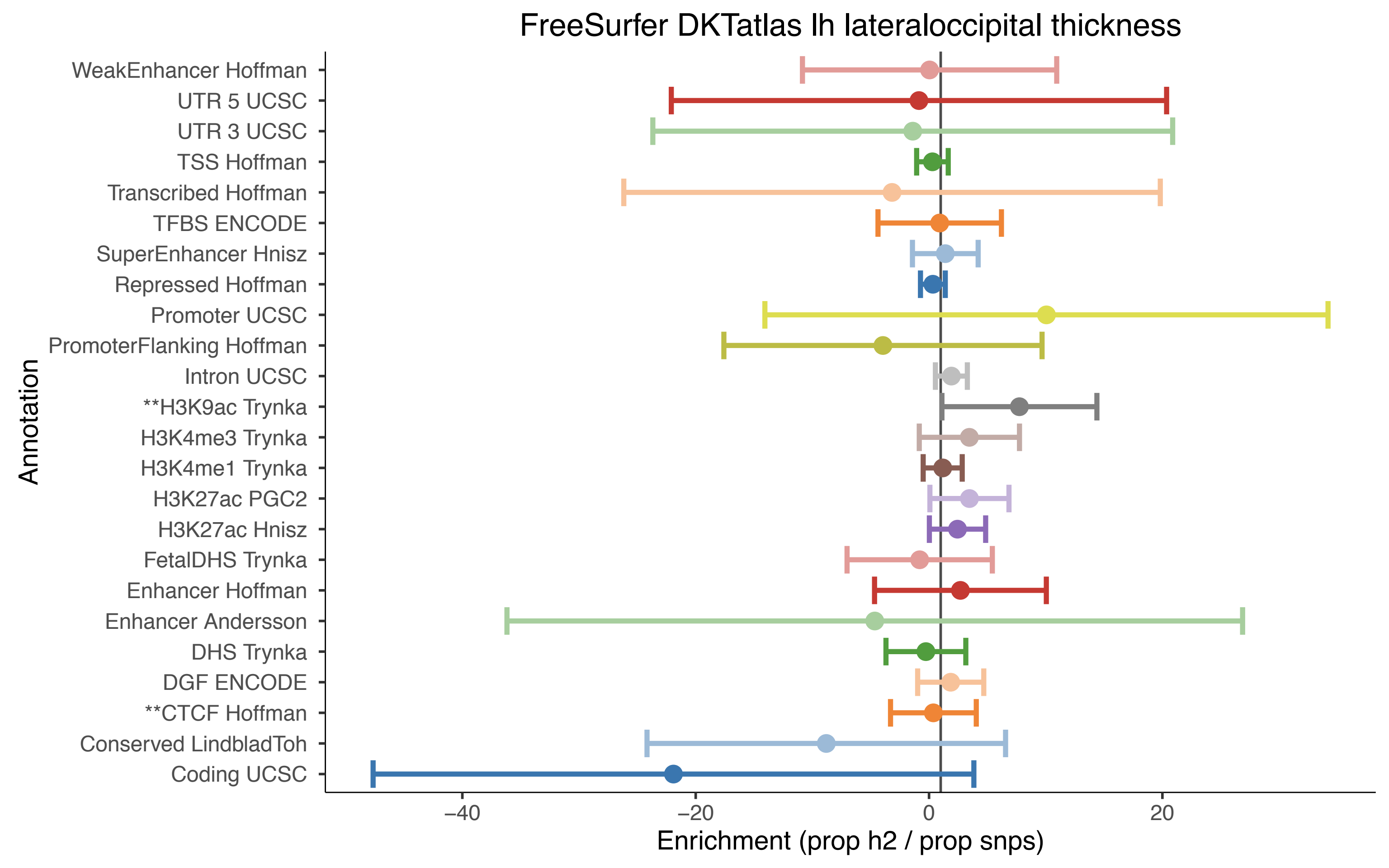
FreeSurfer DKTatlas lh lateraloccipital thickness
WeakEnhancer Hoffman
UTR 5 UCSC
UTR 3 UCSC
TSS Hoffman
Transcribed Hoffman
TFBS ENCODE
SuperEnhancer Hnisz
Repressed Hoffman
Promoter UCSC
PromoterFlanking Hoffman
Intron UCSC
**H3K9ac Trynka
H3K4me3 Trynka
H3K4me1 Trynka
H3K27ac PGC2
H3K27ac Hnisz
FetalDHS Trynka
Enhancer Hoffman
Enhancer Andersson
DHS Trynka
DGF ENCODE
**CTCF Hoffman
Conserved LindbladToh
Coding UCSC
Annotation
−40
−20
0
20
Enrichment (prop h2 / prop snps)

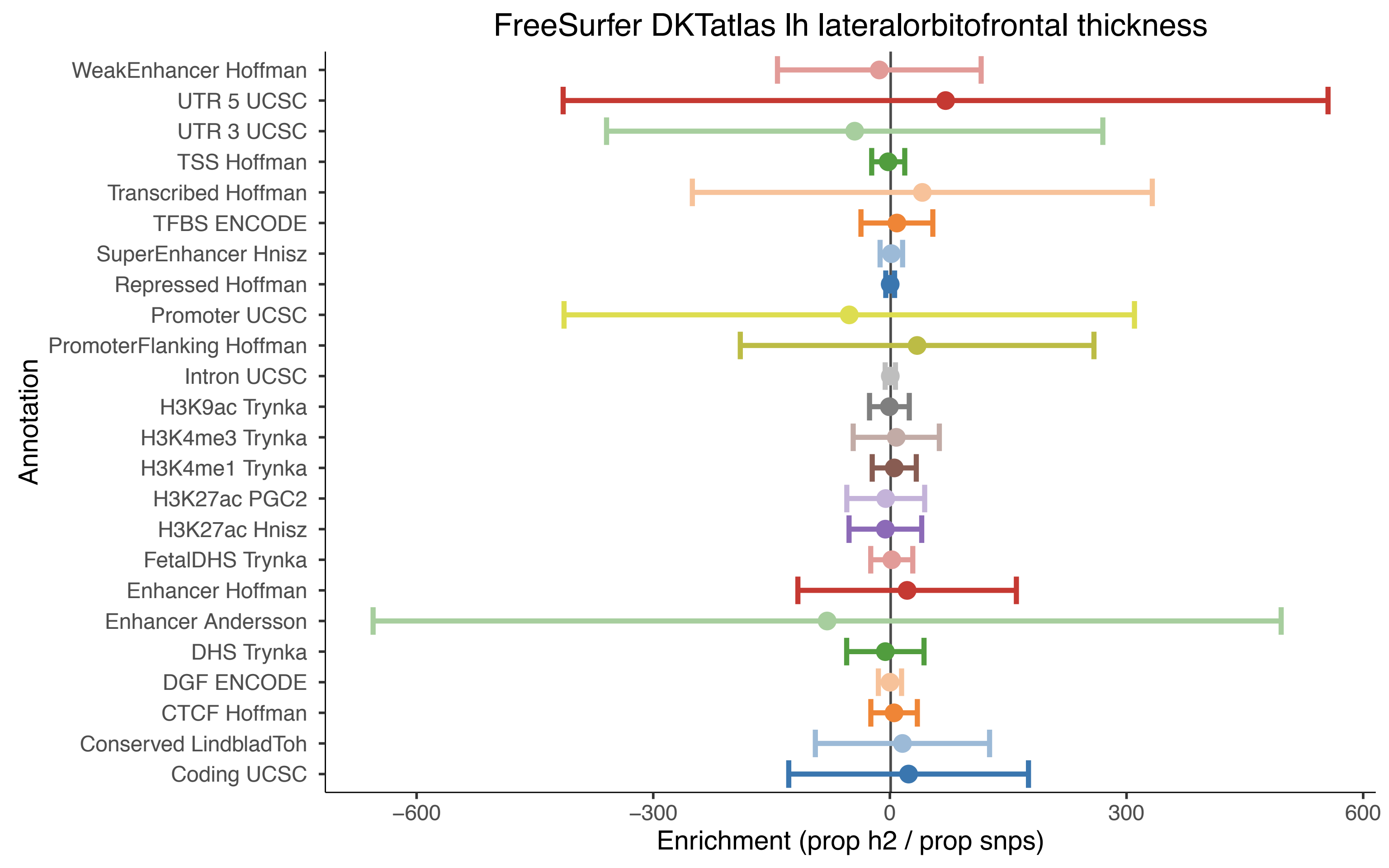

FreeSurfer DKTatlas lh lateralorbitofrontal thickness
WeakEnhancer Hoffman
UTR 5 UCSC
UTR 3 UCSC
TSS Hoffman
Transcribed Hoffman
TFBS ENCODE
SuperEnhancer Hnisz
Repressed Hoffman
Promoter UCSC
PromoterFlanking Hoffman
Intron UCSC
H3K9ac Trynka
H3K4me3 Trynka
H3K4me1 Trynka
H3K27ac PGC2
H3K27ac Hnisz
FetalDHS Trynka
Enhancer Hoffman
Enhancer Andersson
DHS Trynka
DGF ENCODE
CTCF Hoffman
Conserved LindbladToh
Coding UCSC
Annotation
−600
−300
0
300
600
Enrichment (prop h2 / prop snps)

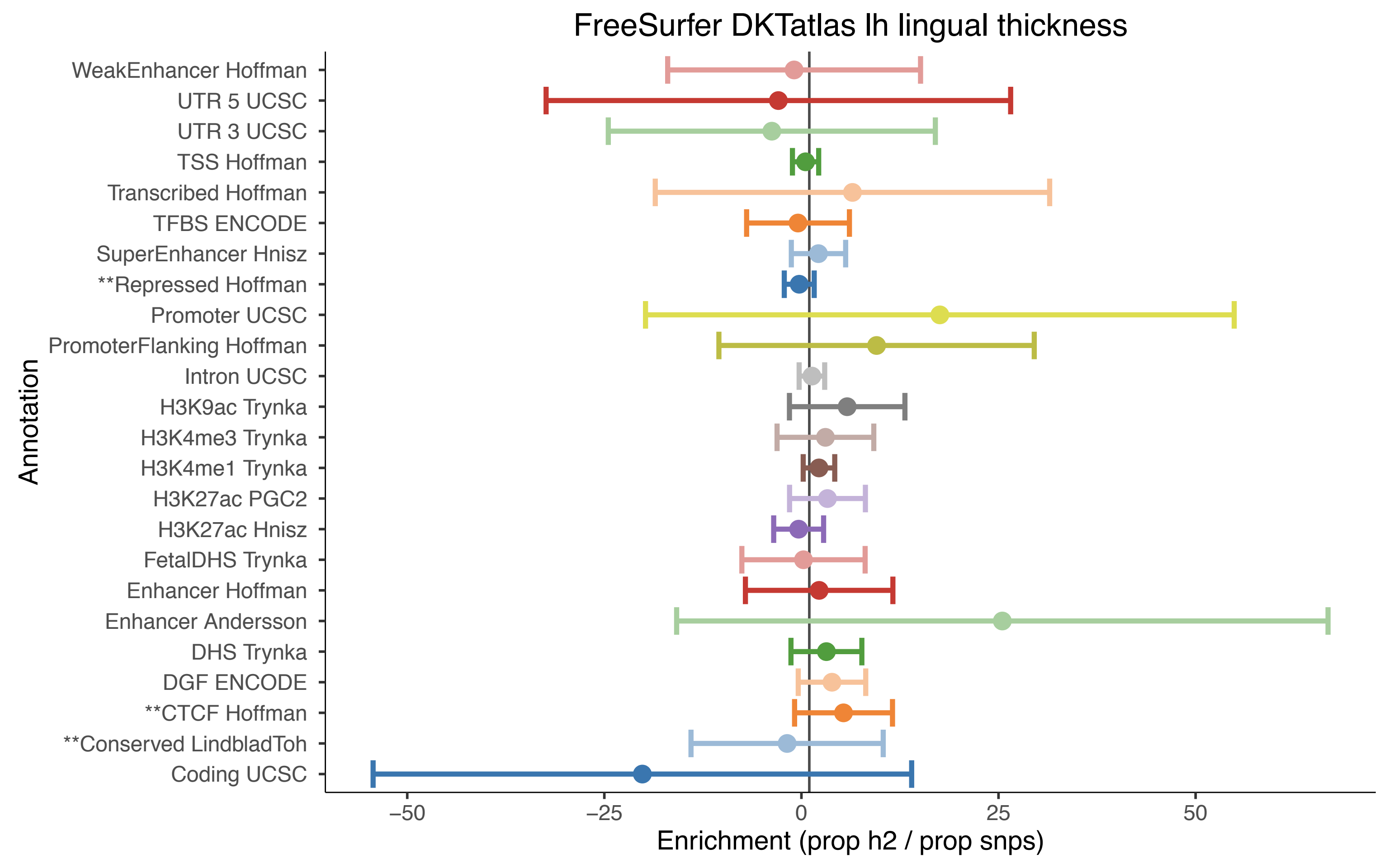

FreeSurfer DKTatlas lh lingual thickness
WeakEnhancer Hoffman
UTR 5 UCSC
UTR 3 UCSC
TSS Hoffman
Transcribed Hoffman
TFBS ENCODE
SuperEnhancer Hnisz
**Repressed Hoffman
Promoter UCSC
PromoterFlanking Hoffman
Intron UCSC
H3K9ac Trynka
H3K4me3 Trynka
H3K4me1 Trynka
H3K27ac PGC2
H3K27ac Hnisz
FetalDHS Trynka
Enhancer Hoffman
Enhancer Andersson
DHS Trynka
DGF ENCODE
**CTCF Hoffman
**Conserved LindbladToh
Coding UCSC
Annotation
−50
−25
0
25
50
Enrichment (prop h2 / prop snps)

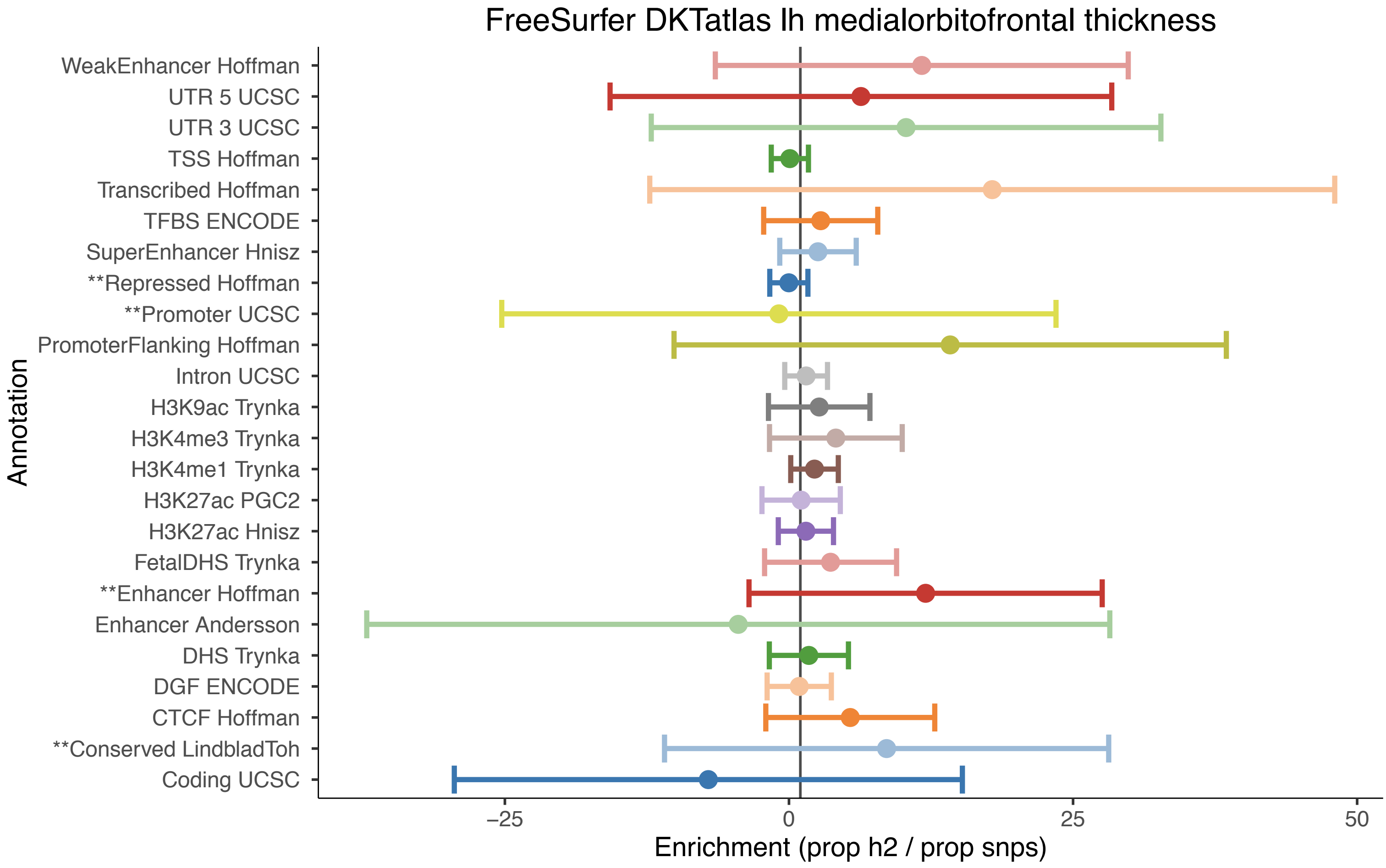

FreeSurfer DKTatlas lh medialorbitofrontal thickness
WeakEnhancer Hoffman
UTR 5 UCSC
UTR 3 UCSC
TSS Hoffman
Transcribed Hoffman
TFBS ENCODE
SuperEnhancer Hnisz
**Repressed Hoffman
**Promoter UCSC
PromoterFlanking Hoffman
Intron UCSC
H3K9ac Trynka
H3K4me3 Trynka
H3K4me1 Trynka
H3K27ac PGC2
H3K27ac Hnisz
FetalDHS Trynka
**Enhancer Hoffman
Enhancer Andersson
DHS Trynka
DGF ENCODE
CTCF Hoffman
**Conserved LindbladToh
Coding UCSC
Annotation
−25
0
25
50
Enrichment (prop h2 / prop snps)

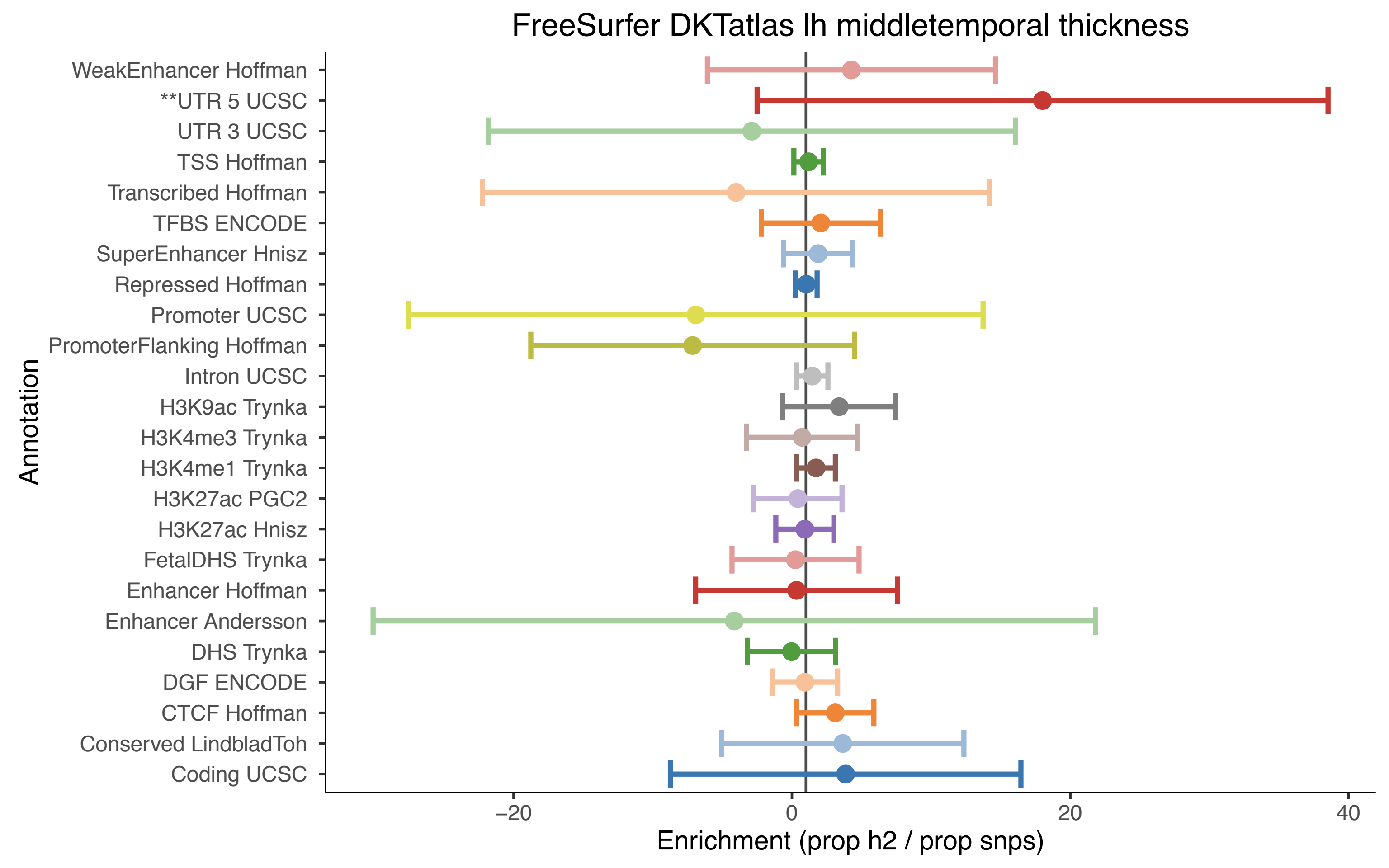
FreeSurfer DKTatlas lh middletemporal thickness
WeakEnhancer Hoffman
**UTR 5 UCSC
UTR 3 UCSC
TSS Hoffman
Transcribed Hoffman
TFBS ENCODE
SuperEnhancer Hnisz
Repressed Hoffman
Promoter UCSC
PromoterFlanking Hoffman
Intron UCSC
H3K9ac Trynka
H3K4me3 Trynka
H3K4me1 Trynka
H3K27ac PGC2
H3K27ac Hnisz
FetalDHS Trynka
Enhancer Hoffman
Enhancer Andersson
DHS Trynka
DGF ENCODE
CTCF Hoffman
Conserved LindbladToh
Coding UCSC
Annotation
−20
0
20
40
Enrichment (prop h2 / prop snps)

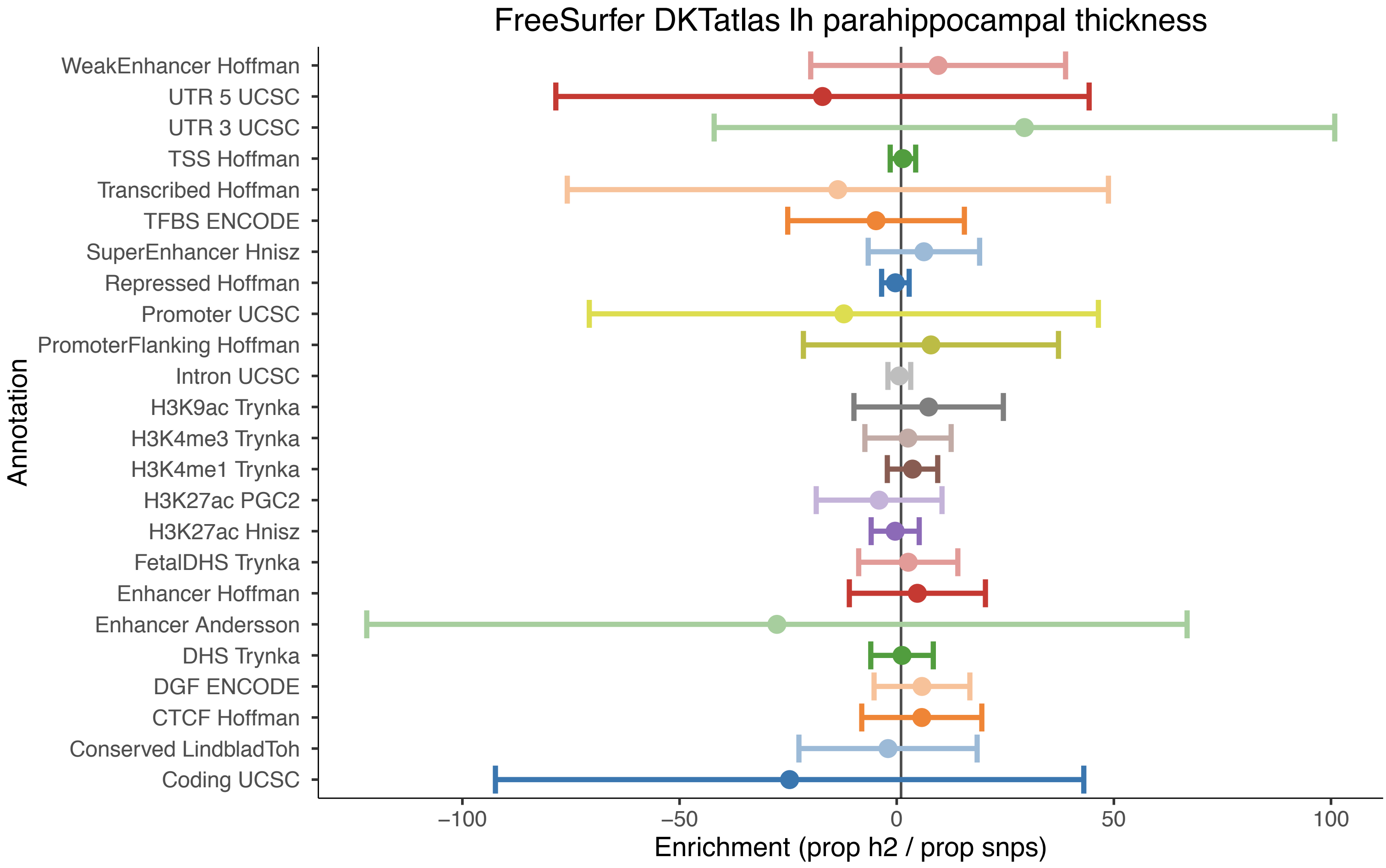
FreeSurfer DKTatlas lh parahippocampal thickness
Annotation
WeakEnhancer Hoffman
UTR 5 UCSC
UTR 3 UCSC
TSS Hoffman
Transcribed Hoffman
TFBS ENCODE
SuperEnhancer Hnisz
Repressed Hoffman
Promoter UCSC
PromoterFlanking Hoffman
Intron UCSC
H3K9ac Trynka
H3K4me3 Trynka
H3K4me1 Trynka
H3K27ac PGC2
H3K27ac Hnisz
FetalDHS Trynka
Enhancer Hoffman
Enhancer Andersson
DHS Trynka
DGF ENCODE
CTCF Hoffman
Conserved LindbladToh
Coding UCSC
−100
−50
0
50
100
Enrichment (prop h2 / prop snps)

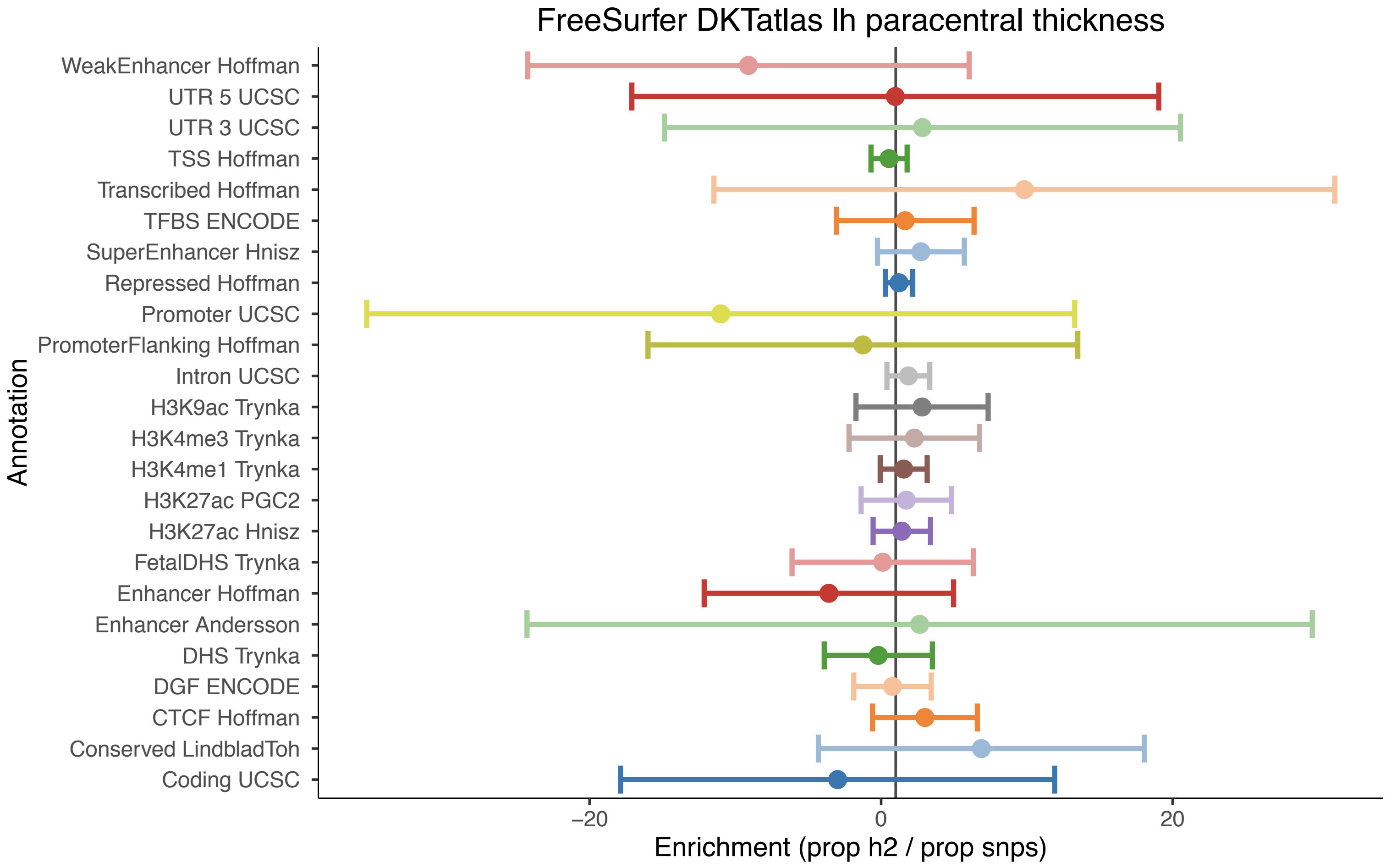
FreeSurfer DKTatlas lh paracentral thickness
WeakEnhancer Hoffman
UTR 5 UCSC
UTR 3 UCSC
TSS Hoffman
Transcribed Hoffman
TFBS ENCODE
SuperEnhancer Hnisz
Repressed Hoffman
Promoter UCSC
PromoterFlanking Hoffman
Intron UCSC
H3K9ac Trynka
H3K4me3 Trynka
H3K4me1 Trynka
H3K27ac PGC2
H3K27ac Hnisz
FetalDHS Trynka
Enhancer Hoffman
Enhancer Andersson
DHS Trynka
DGF ENCODE
CTCF Hoffman
Conserved LindbladToh
Coding UCSC
Annotation
−20
0
20
Enrichment (prop h2 / prop snps)

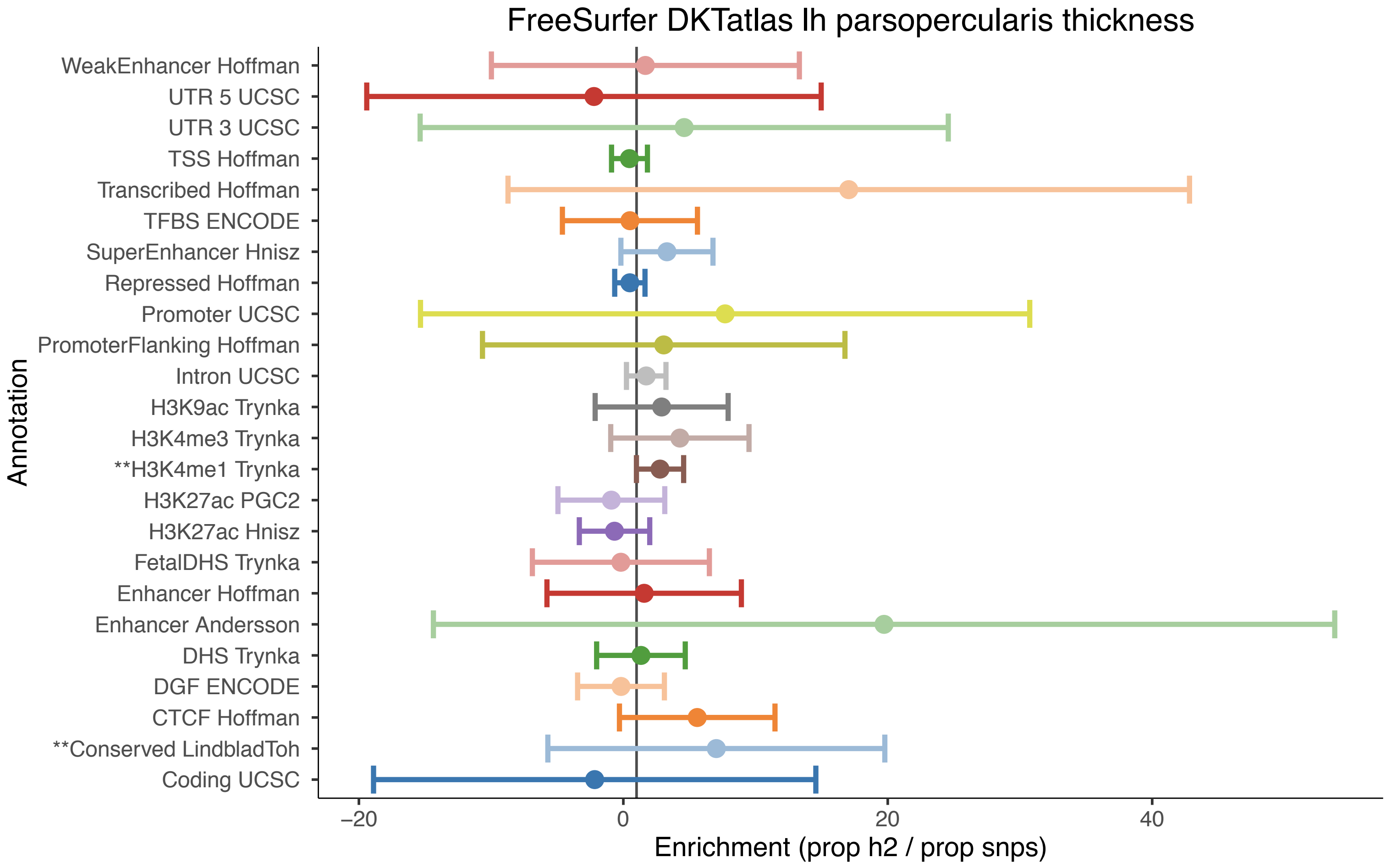

FreeSurfer DKTatlas lh parsopercularis thickness
WeakEnhancer Hoffman
UTR 5 UCSC
UTR 3 UCSC
TSS Hoffman
Transcribed Hoffman
TFBS ENCODE
SuperEnhancer Hnisz
Repressed Hoffman
Promoter UCSC
PromoterFlanking Hoffman
Intron UCSC
H3K9ac Trynka
H3K4me3 Trynka
**H3K4me1 Trynka
H3K27ac PGC2
H3K27ac Hnisz
FetalDHS Trynka
Enhancer Hoffman
Enhancer Andersson
DHS Trynka
DGF ENCODE
CTCF Hoffman
**Conserved LindbladToh
Coding UCSC
Annotation
−20
0
20
40
Enrichment (prop h2 / prop snps)

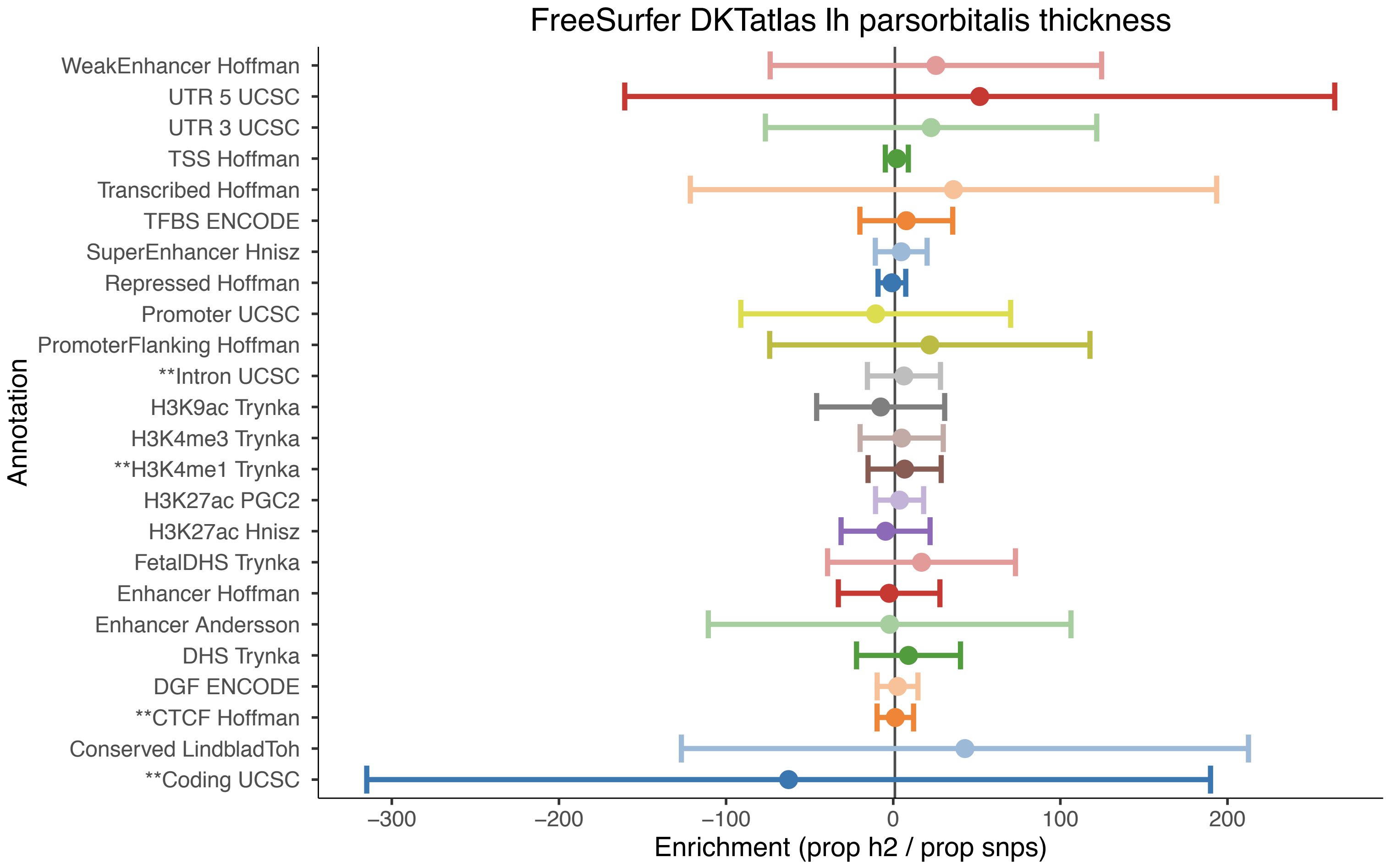

FreeSurfer DKTatlas lh parsorbitalis thickness
WeakEnhancer Hoffman
UTR 5 UCSC
UTR 3 UCSC
TSS Hoffman
Transcribed Hoffman
TFBS ENCODE
SuperEnhancer Hnisz
Repressed Hoffman
Promoter UCSC
PromoterFlanking Hoffman
**Intron UCSC
H3K9ac Trynka
H3K4me3 Trynka
**H3K4me1 Trynka
H3K27ac PGC2
H3K27ac Hnisz
FetalDHS Trynka
Enhancer Hoffman
Enhancer Andersson
DHS Trynka
DGF ENCODE
**CTCF Hoffman
Conserved LindbladToh
**Coding UCSC
Annotation
−300
−200
−100
0
100
200
Enrichment (prop h2 / prop snps)

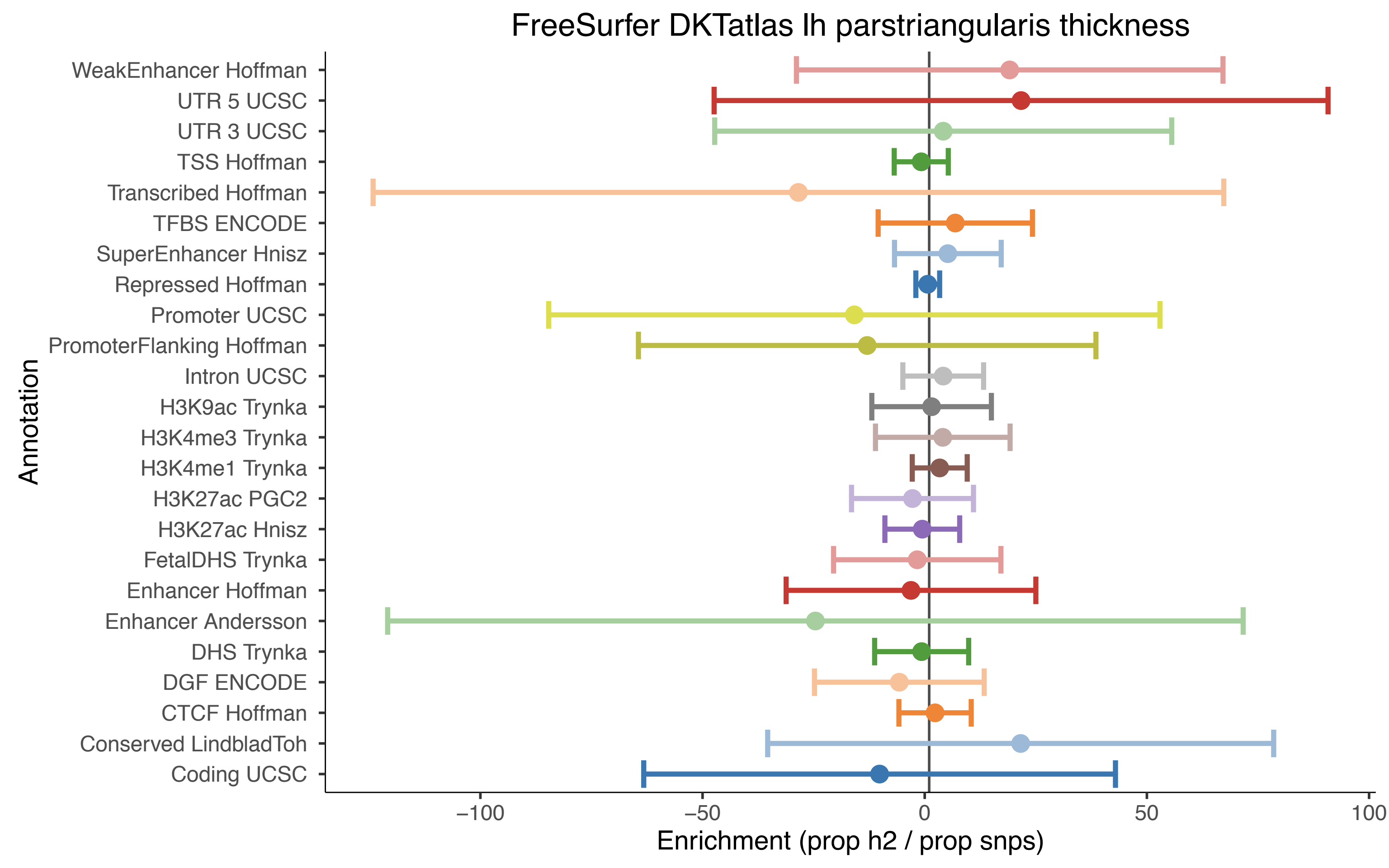

FreeSurfer DKTatlas lh parstriangularis thickness
WeakEnhancer Hoffman
UTR 5 UCSC
UTR 3 UCSC
TSS Hoffman
Transcribed Hoffman
TFBS ENCODE
SuperEnhancer Hnisz
Repressed Hoffman
Promoter UCSC
PromoterFlanking Hoffman
Intron UCSC
H3K9ac Trynka
H3K4me3 Trynka
H3K4me1 Trynka
H3K27ac PGC2
H3K27ac Hnisz
FetalDHS Trynka
Enhancer Hoffman
Enhancer Andersson
DHS Trynka
DGF ENCODE
CTCF Hoffman
Conserved LindbladToh
Coding UCSC
Annotation
−100
−50
0
50
100
Enrichment (prop h2 / prop snps)

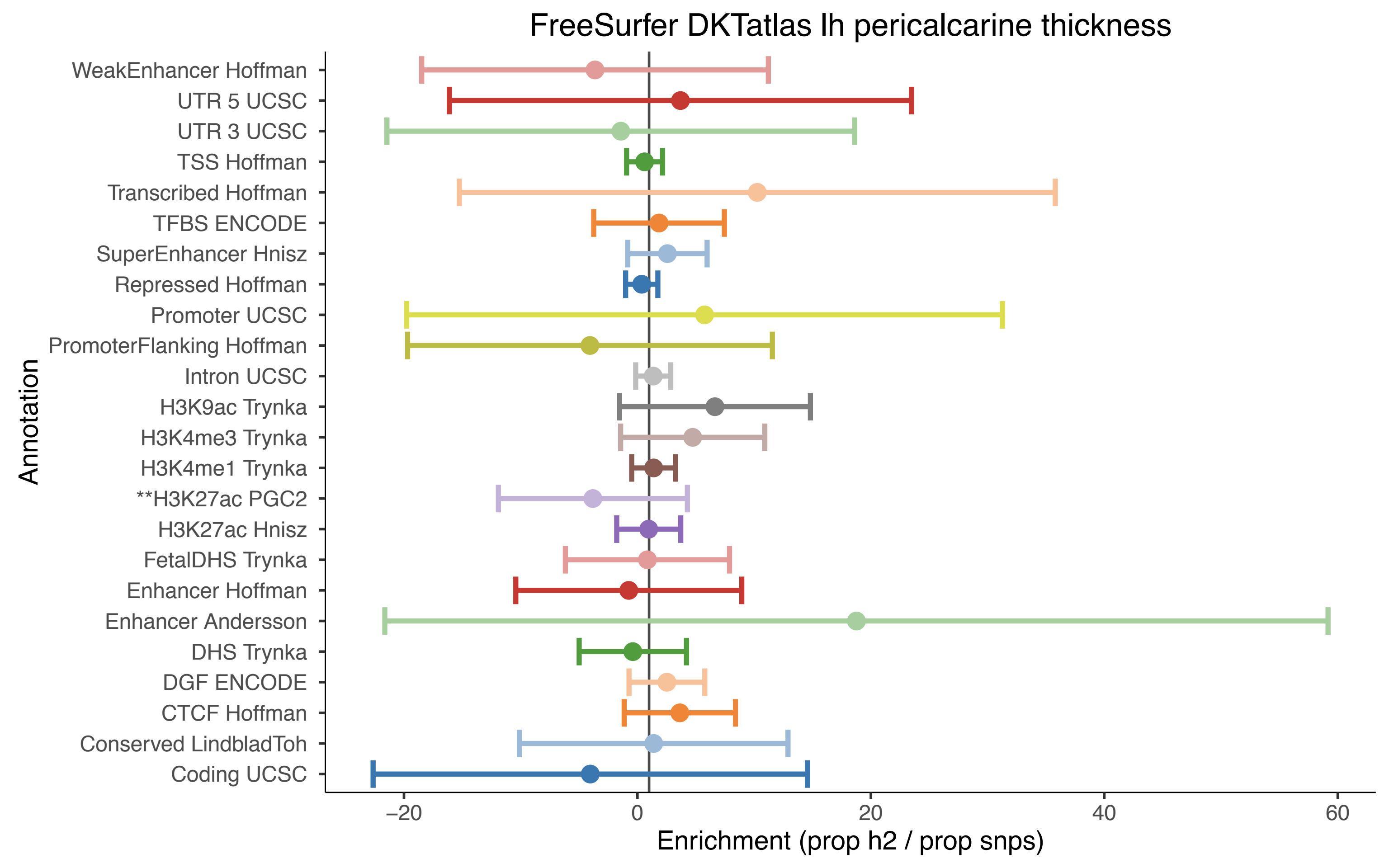
FreeSurfer DKTatlas lh pericalcarine thickness
Annotation
WeakEnhancer Hoffman
UTR 5 UCSC
UTR 3 UCSC
TSS Hoffman
Transcribed Hoffman
TFBS ENCODE
SuperEnhancer Hnisz
Repressed Hoffman
Promoter UCSC
PromoterFlanking Hoffman
Intron UCSC
H3K9ac Trynka
H3K4me3 Trynka
H3K4me1 Trynka
**H3K27ac PGC2
H3K27ac Hnisz
FetalDHS Trynka
Enhancer Hoffman
Enhancer Andersson
DHS Trynka
DGF ENCODE
CTCF Hoffman
Conserved LindbladToh
Coding UCSC
−20
0
20
40
60
Enrichment (prop h2 / prop snps)

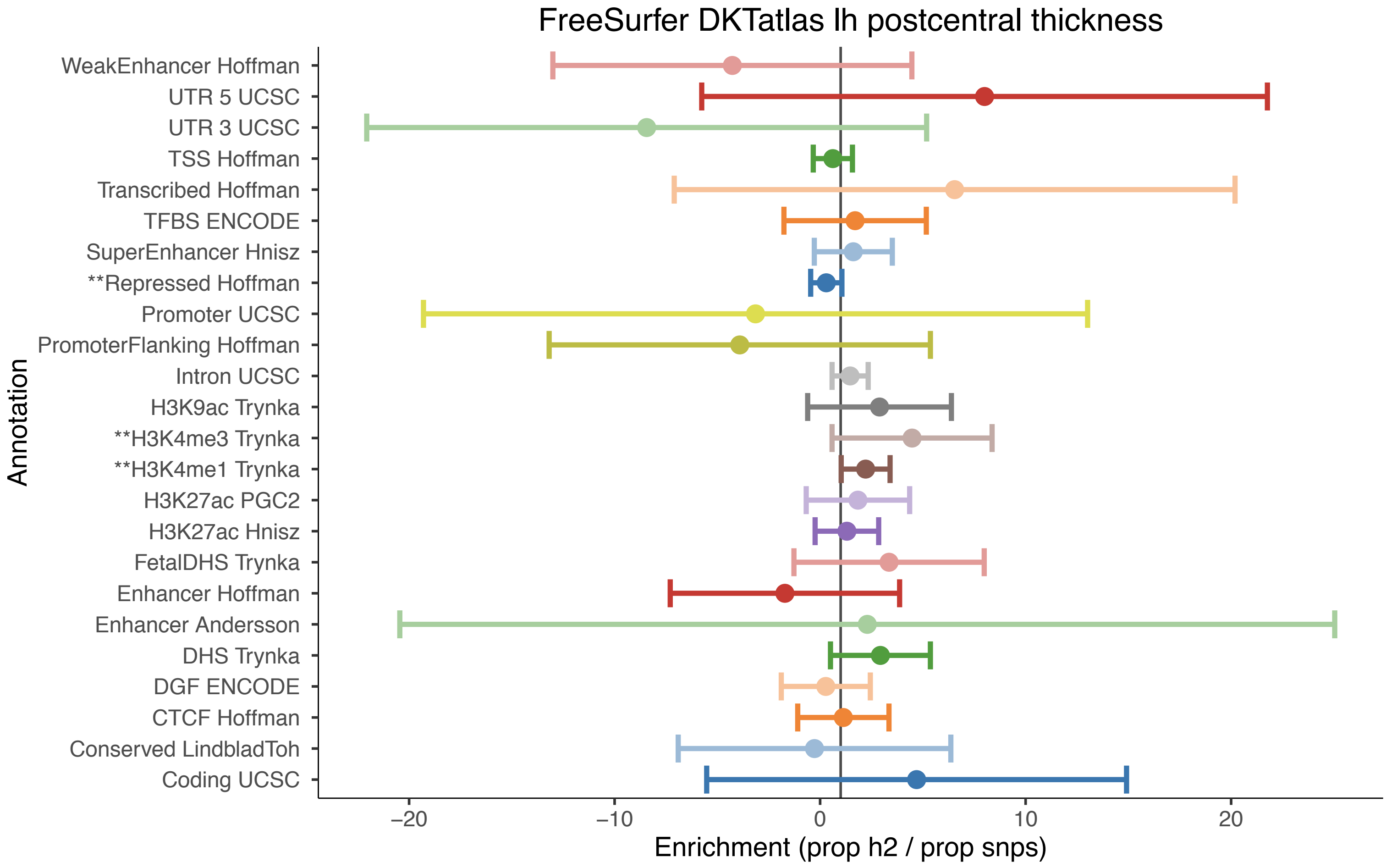
FreeSurfer DKTatlas lh postcentral thickness
WeakEnhancer Hoffman
UTR 5 UCSC
UTR 3 UCSC
TSS Hoffman
Transcribed Hoffman
TFBS ENCODE
SuperEnhancer Hnisz
**Repressed Hoffman
Promoter UCSC
PromoterFlanking Hoffman
Intron UCSC
H3K9ac Trynka
**H3K4me3 Trynka
**H3K4me1 Trynka
H3K27ac PGC2
H3K27ac Hnisz
FetalDHS Trynka
Enhancer Hoffman
Enhancer Andersson
DHS Trynka
DGF ENCODE
CTCF Hoffman
Conserved LindbladToh
Coding UCSC
Annotation
−20
−10
0
10
20
Enrichment (prop h2 / prop snps)

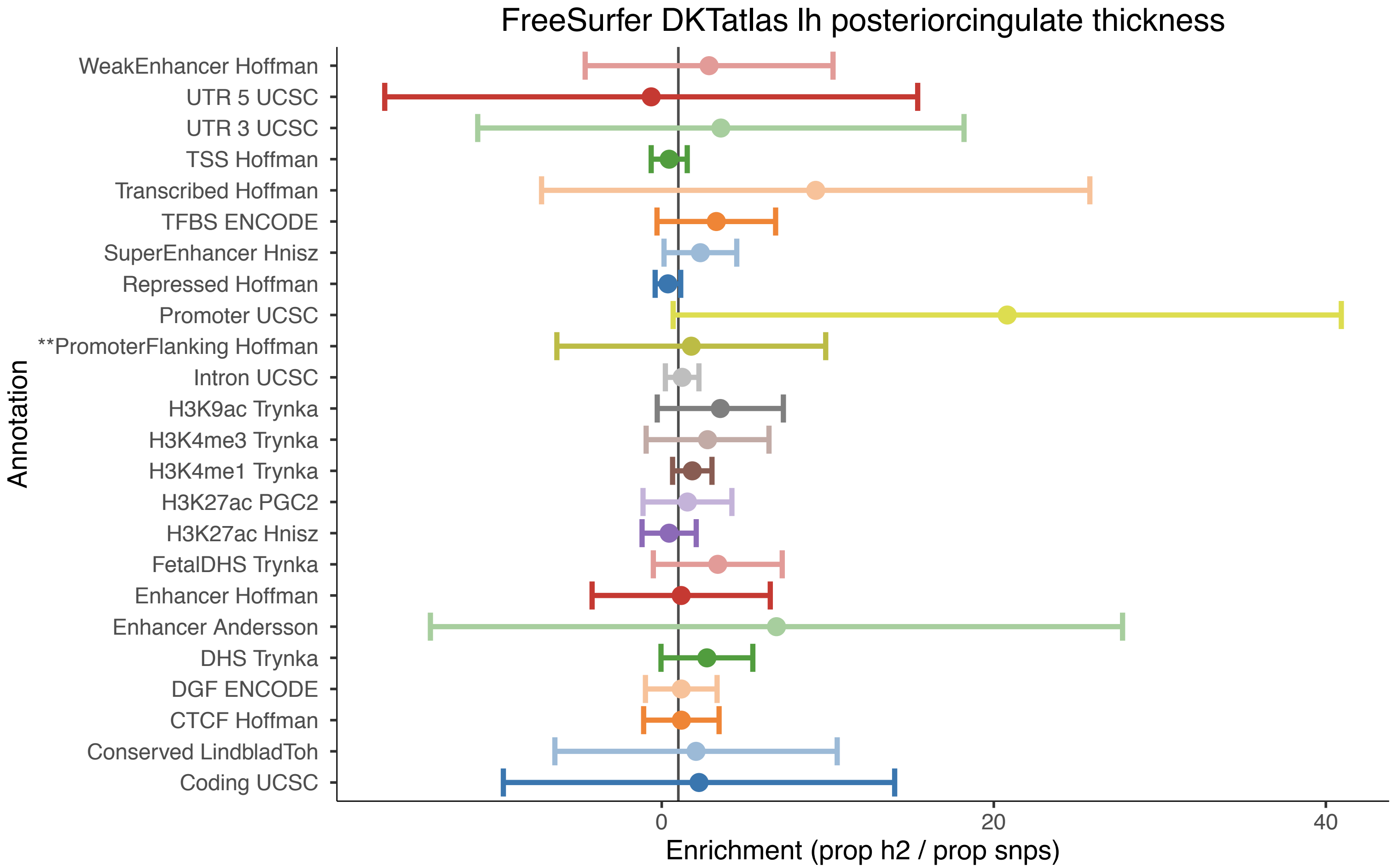
FreeSurfer DKTatlas lh posteriorcingulate thickness
WeakEnhancer Hoffman
UTR 5 UCSC
UTR 3 UCSC
TSS Hoffman
Transcribed Hoffman
TFBS ENCODE
SuperEnhancer Hnisz
Repressed Hoffman
Promoter UCSC
**PromoterFlanking Hoffman
Intron UCSC
H3K9ac Trynka
H3K4me3 Trynka
H3K4me1 Trynka
H3K27ac PGC2
H3K27ac Hnisz
FetalDHS Trynka
Enhancer Hoffman
Enhancer Andersson
DHS Trynka
DGF ENCODE
CTCF Hoffman
Conserved LindbladToh
Coding UCSC
Annotation
0
20
40
Enrichment (prop h2 / prop snps)

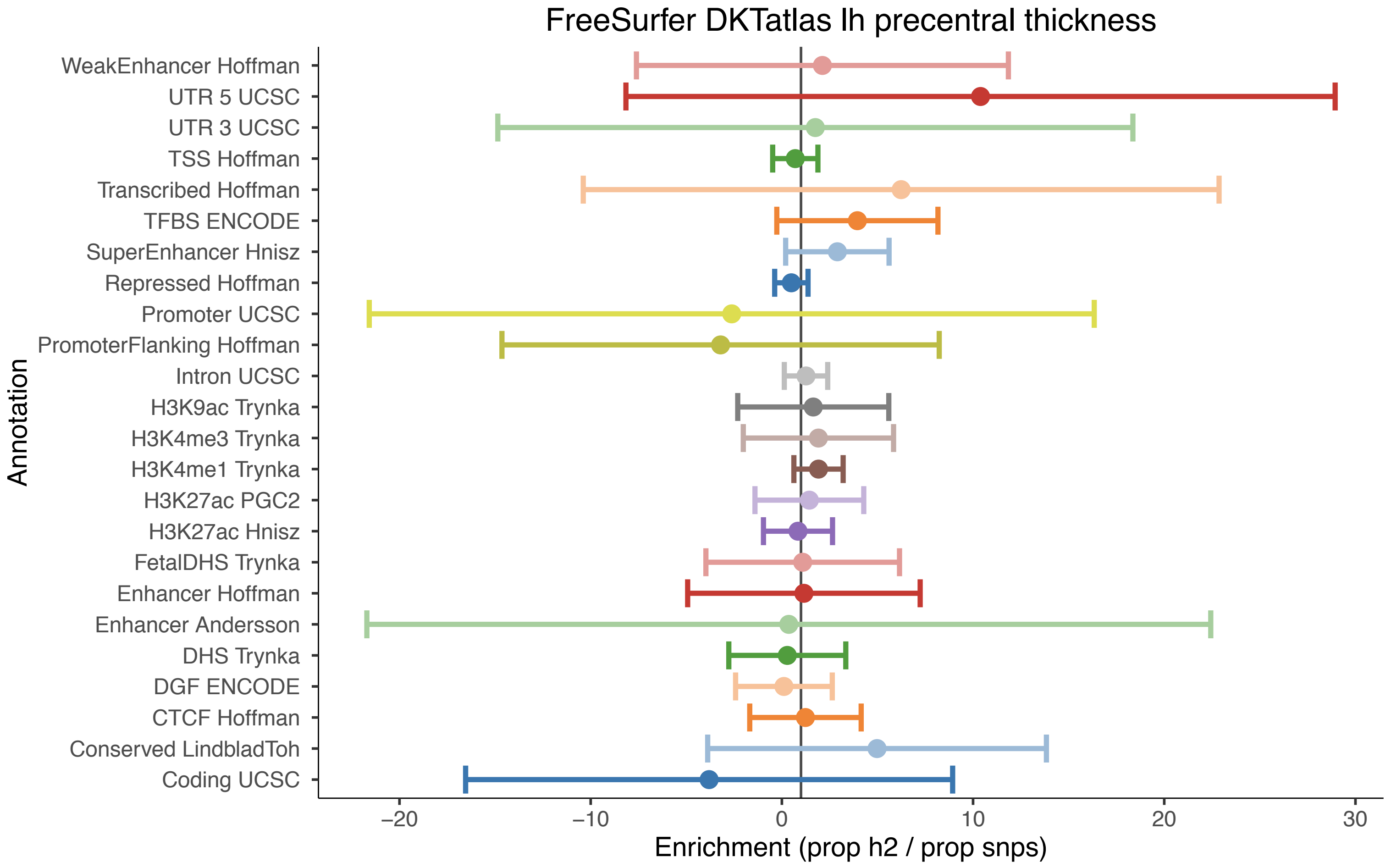
FreeSurfer DKTatlas lh precentral thickness
Annotation
WeakEnhancer Hoffman
UTR 5 UCSC
UTR 3 UCSC
TSS Hoffman
Transcribed Hoffman
TFBS ENCODE
SuperEnhancer Hnisz
Repressed Hoffman
Promoter UCSC
PromoterFlanking Hoffman
Intron UCSC
H3K9ac Trynka
H3K4me3 Trynka
H3K4me1 Trynka
H3K27ac PGC2
H3K27ac Hnisz
FetalDHS Trynka
Enhancer Hoffman
Enhancer Andersson
DHS Trynka
DGF ENCODE
CTCF Hoffman
Conserved LindbladToh
Coding UCSC
−20
−10
0
10
20
30
Enrichment (prop h2 / prop snps)

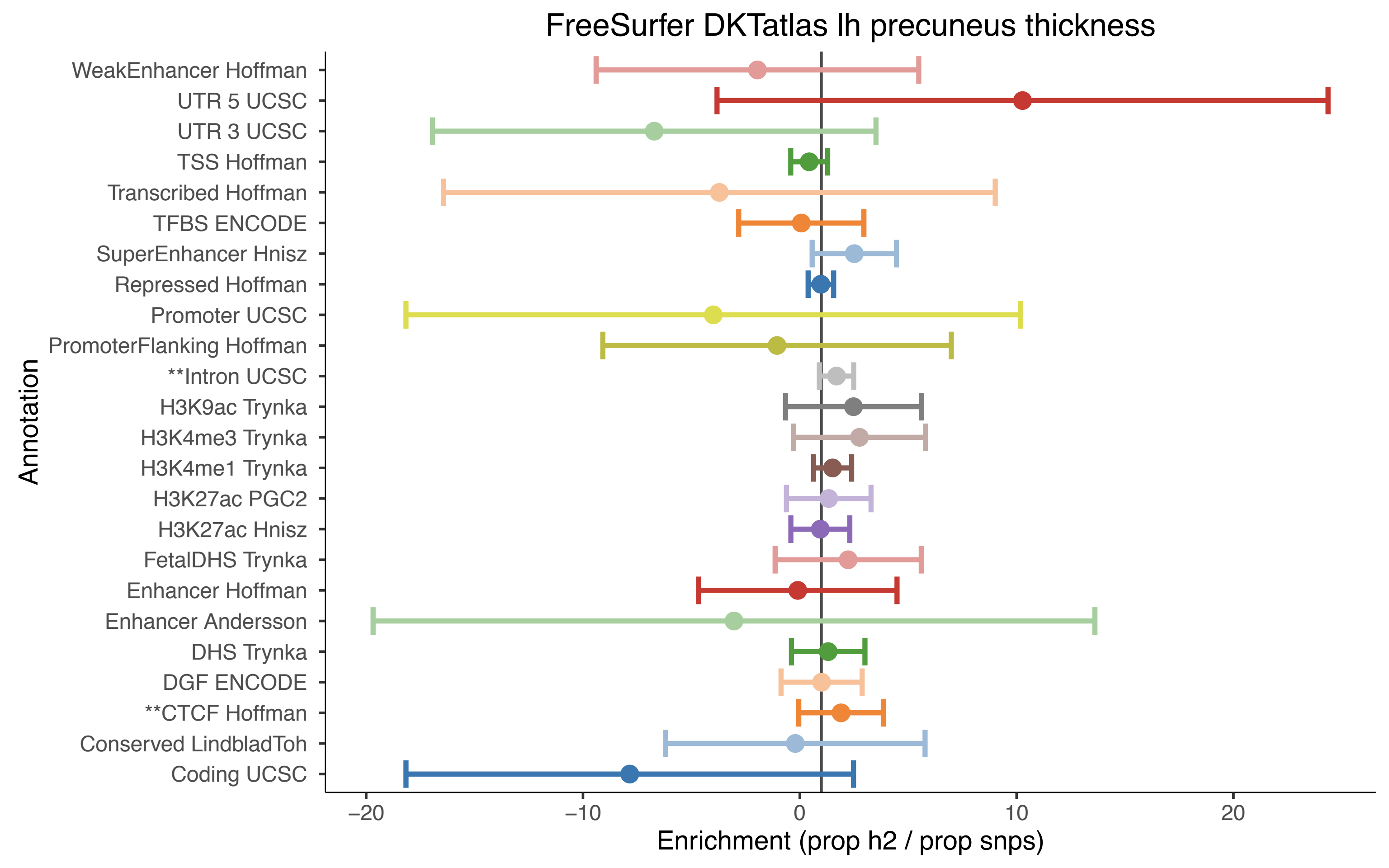
FreeSurfer DKTatlas lh precuneus thickness
WeakEnhancer Hoffman
UTR 5 UCSC
UTR 3 UCSC
TSS Hoffman
Transcribed Hoffman
TFBS ENCODE
SuperEnhancer Hnisz
Repressed Hoffman
Promoter UCSC
PromoterFlanking Hoffman
**Intron UCSC
H3K9ac Trynka
H3K4me3 Trynka
H3K4me1 Trynka
H3K27ac PGC2
H3K27ac Hnisz
FetalDHS Trynka
Enhancer Hoffman
Enhancer Andersson
DHS Trynka
DGF ENCODE
**CTCF Hoffman
Conserved LindbladToh
Coding UCSC
Annotation
−20
−10
0
10
20
Enrichment (prop h2 / prop snps)

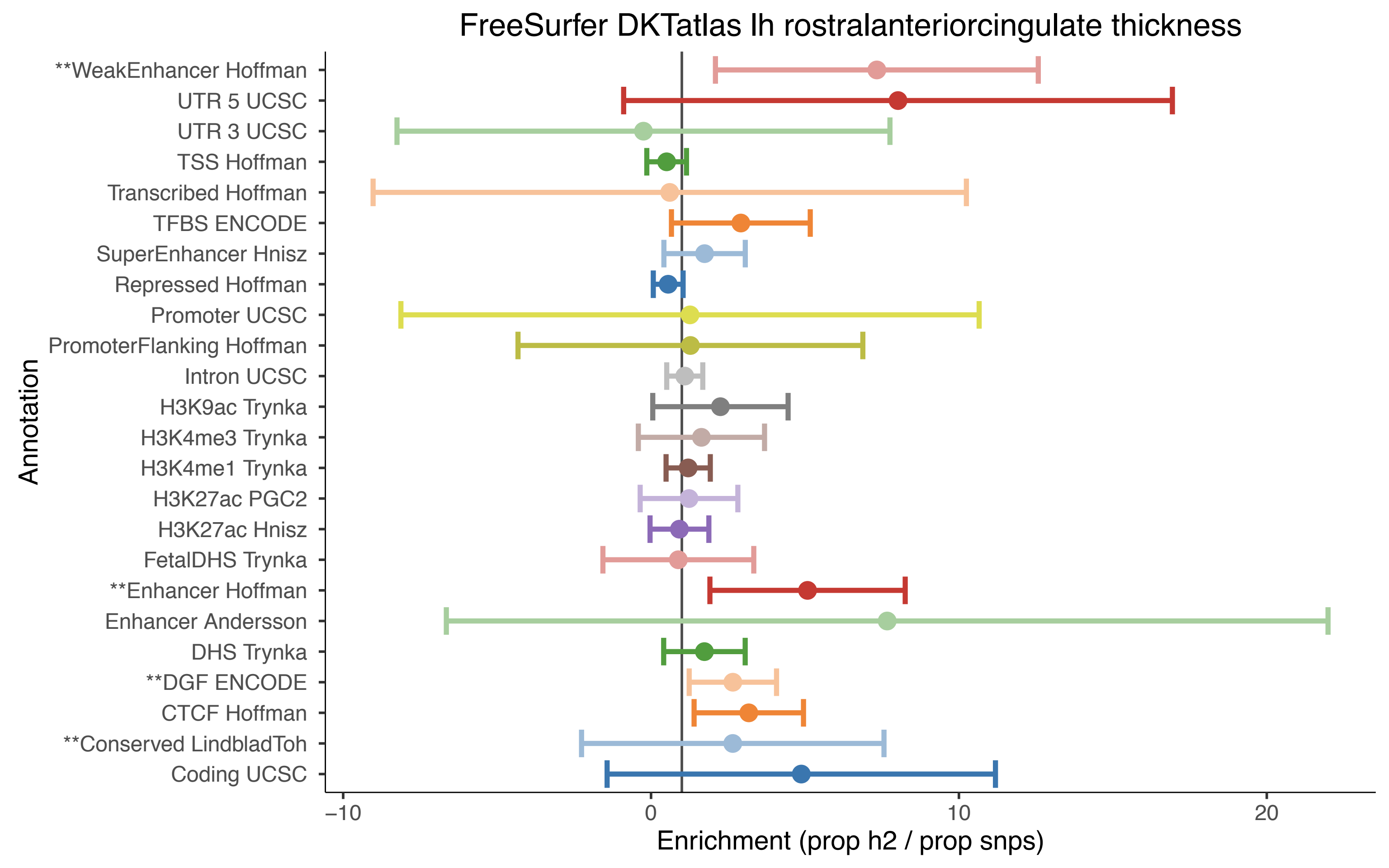
FreeSurfer DKTatlas lh rostralanteriorcingulate thickness
**WeakEnhancer Hoffman
UTR 5 UCSC
UTR 3 UCSC
TSS Hoffman
Transcribed Hoffman
TFBS ENCODE
SuperEnhancer Hnisz
Repressed Hoffman
Promoter UCSC
PromoterFlanking Hoffman
Intron UCSC
H3K9ac Trynka
H3K4me3 Trynka
H3K4me1 Trynka
H3K27ac PGC2
H3K27ac Hnisz
FetalDHS Trynka
**Enhancer Hoffman
Enhancer Andersson
DHS Trynka
**DGF ENCODE
CTCF Hoffman
**Conserved LindbladToh
Coding UCSC
Annotation
−10
0
10
20
Enrichment (prop h2 / prop snps)

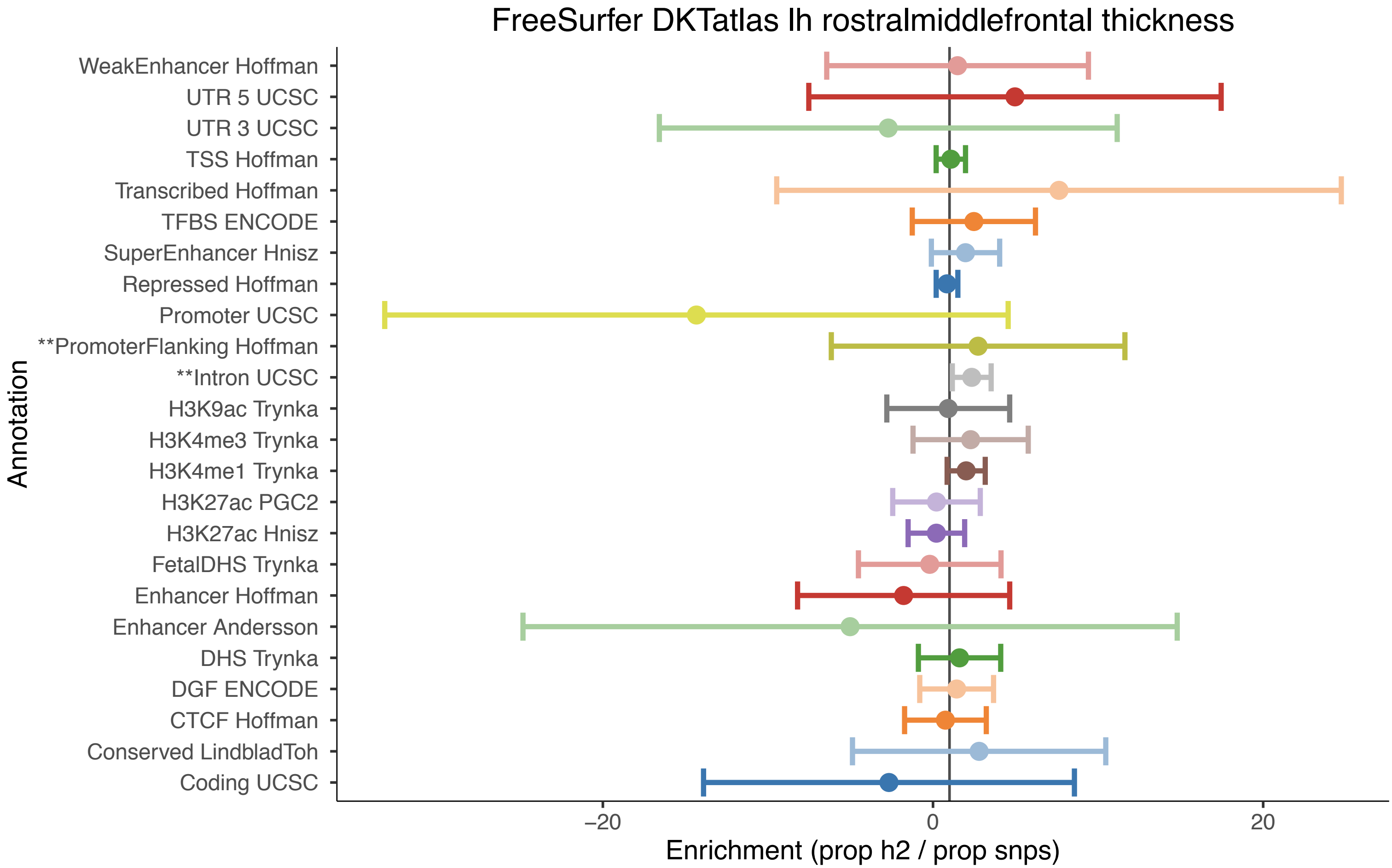

FreeSurfer DKTatlas lh rostralmiddlefrontal thickness
WeakEnhancer Hoffman
UTR 5 UCSC
UTR 3 UCSC
TSS Hoffman
Transcribed Hoffman
TFBS ENCODE
SuperEnhancer Hnisz
Repressed Hoffman
Promoter UCSC
**PromoterFlanking Hoffman
**Intron UCSC
H3K9ac Trynka
H3K4me3 Trynka
H3K4me1 Trynka
H3K27ac PGC2
H3K27ac Hnisz
FetalDHS Trynka
Enhancer Hoffman
Enhancer Andersson
DHS Trynka
DGF ENCODE
CTCF Hoffman
Conserved LindbladToh
Coding UCSC
Annotation
−20
0
20
Enrichment (prop h2 / prop snps)

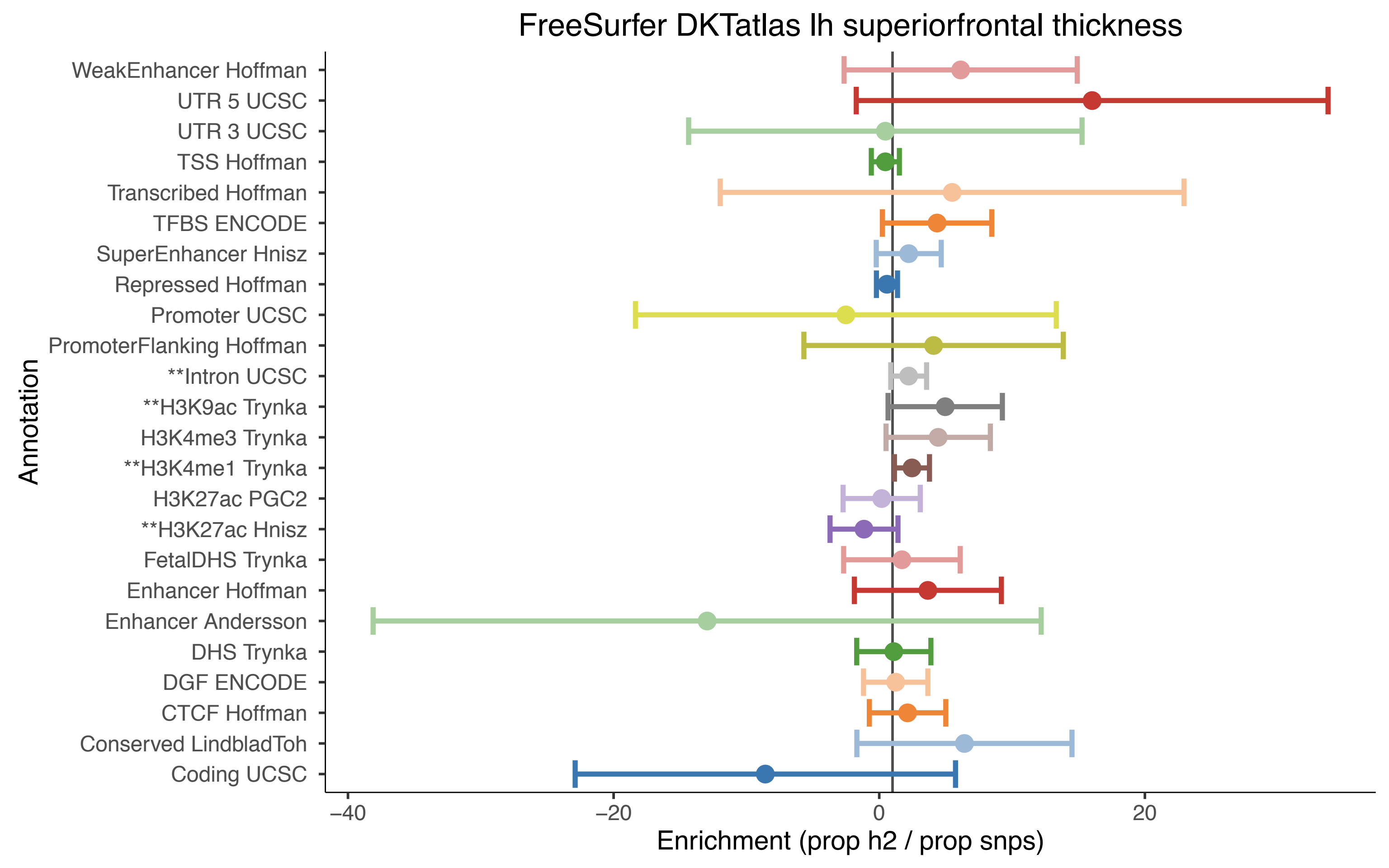
FreeSurfer DKTatlas lh superiorfrontal thickness
Annotation
WeakEnhancer Hoffman
UTR 5 UCSC
UTR 3 UCSC
TSS Hoffman
Transcribed Hoffman
TFBS ENCODE
SuperEnhancer Hnisz
Repressed Hoffman
Promoter UCSC
PromoterFlanking Hoffman
**Intron UCSC
**H3K9ac Trynka
H3K4me3 Trynka
**H3K4me1 Trynka
H3K27ac PGC2
**H3K27ac Hnisz
FetalDHS Trynka
Enhancer Hoffman
Enhancer Andersson
DHS Trynka
DGF ENCODE
CTCF Hoffman
Conserved LindbladToh
Coding UCSC
−40
−20
0
20
Enrichment (prop h2 / prop snps)

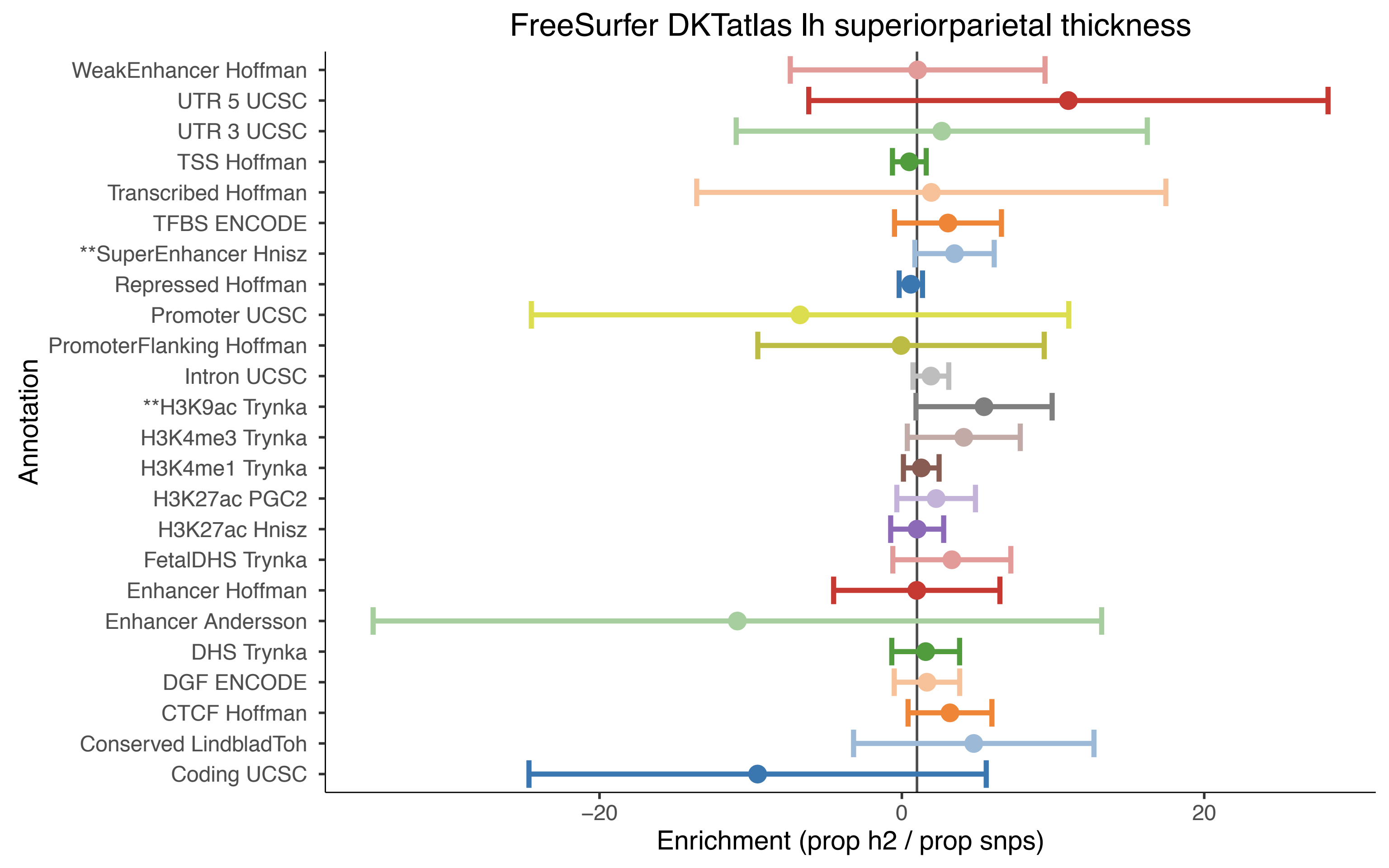
FreeSurfer DKTatlas lh superiorparietal thickness
WeakEnhancer Hoffman
UTR 5 UCSC
UTR 3 UCSC
TSS Hoffman
Transcribed Hoffman
TFBS ENCODE
**SuperEnhancer Hnisz
Repressed Hoffman
Promoter UCSC
PromoterFlanking Hoffman
Intron UCSC
**H3K9ac Trynka
H3K4me3 Trynka
H3K4me1 Trynka
H3K27ac PGC2
H3K27ac Hnisz
FetalDHS Trynka
Enhancer Hoffman
Enhancer Andersson
DHS Trynka
DGF ENCODE
CTCF Hoffman
Conserved LindbladToh
Coding UCSC
Annotation
−20
0
20
Enrichment (prop h2 / prop snps)

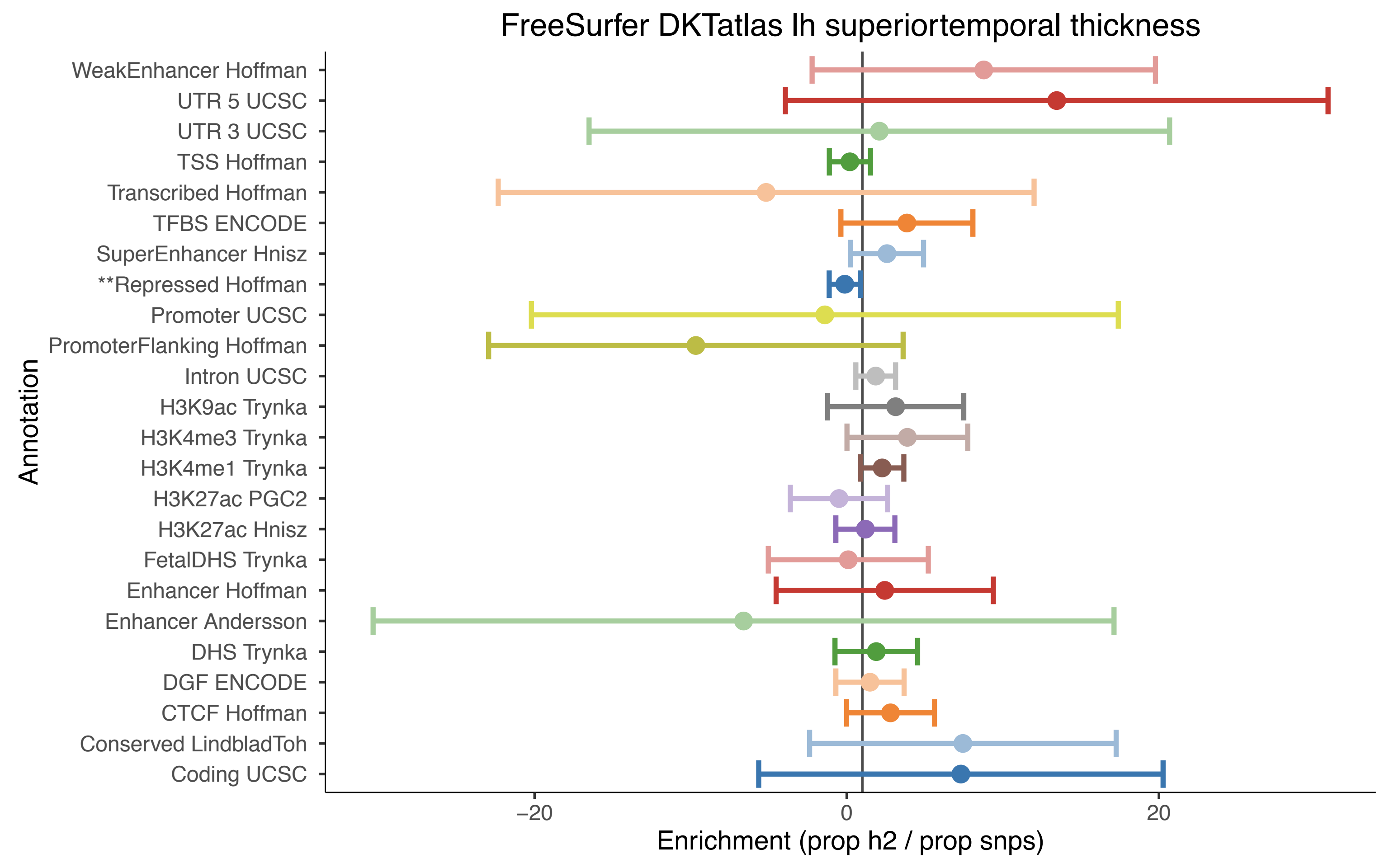

FreeSurfer DKTatlas lh superiortemporal thickness
WeakEnhancer Hoffman
UTR 5 UCSC
UTR 3 UCSC
TSS Hoffman
Transcribed Hoffman
TFBS ENCODE
SuperEnhancer Hnisz
**Repressed Hoffman
Promoter UCSC
PromoterFlanking Hoffman
Intron UCSC
H3K9ac Trynka
H3K4me3 Trynka
H3K4me1 Trynka
H3K27ac PGC2
H3K27ac Hnisz
FetalDHS Trynka
Enhancer Hoffman
Enhancer Andersson
DHS Trynka
DGF ENCODE
CTCF Hoffman
Conserved LindbladToh
Coding UCSC
Annotation
−20
0
20
Enrichment (prop h2 / prop snps)

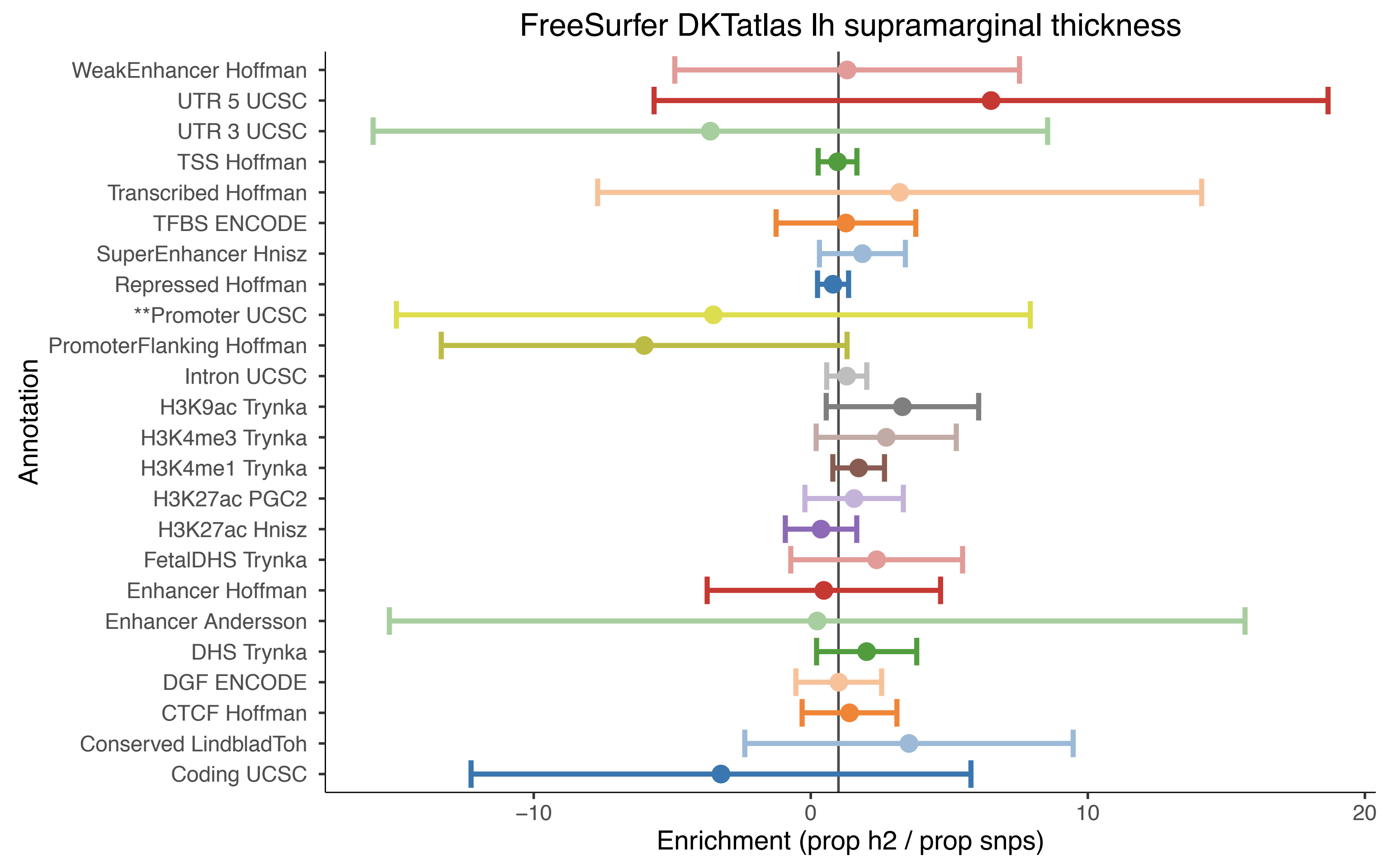

FreeSurfer DKTatlas lh supramarginal thickness
WeakEnhancer Hoffman
UTR 5 UCSC
UTR 3 UCSC
TSS Hoffman
Transcribed Hoffman
TFBS ENCODE
SuperEnhancer Hnisz
Repressed Hoffman
**Promoter UCSC
PromoterFlanking Hoffman
Intron UCSC
H3K9ac Trynka
H3K4me3 Trynka
H3K4me1 Trynka
H3K27ac PGC2
H3K27ac Hnisz
FetalDHS Trynka
Enhancer Hoffman
Enhancer Andersson
DHS Trynka
DGF ENCODE
CTCF Hoffman
Conserved LindbladToh
Coding UCSC
Annotation
−10
0
10
20
Enrichment (prop h2 / prop snps)

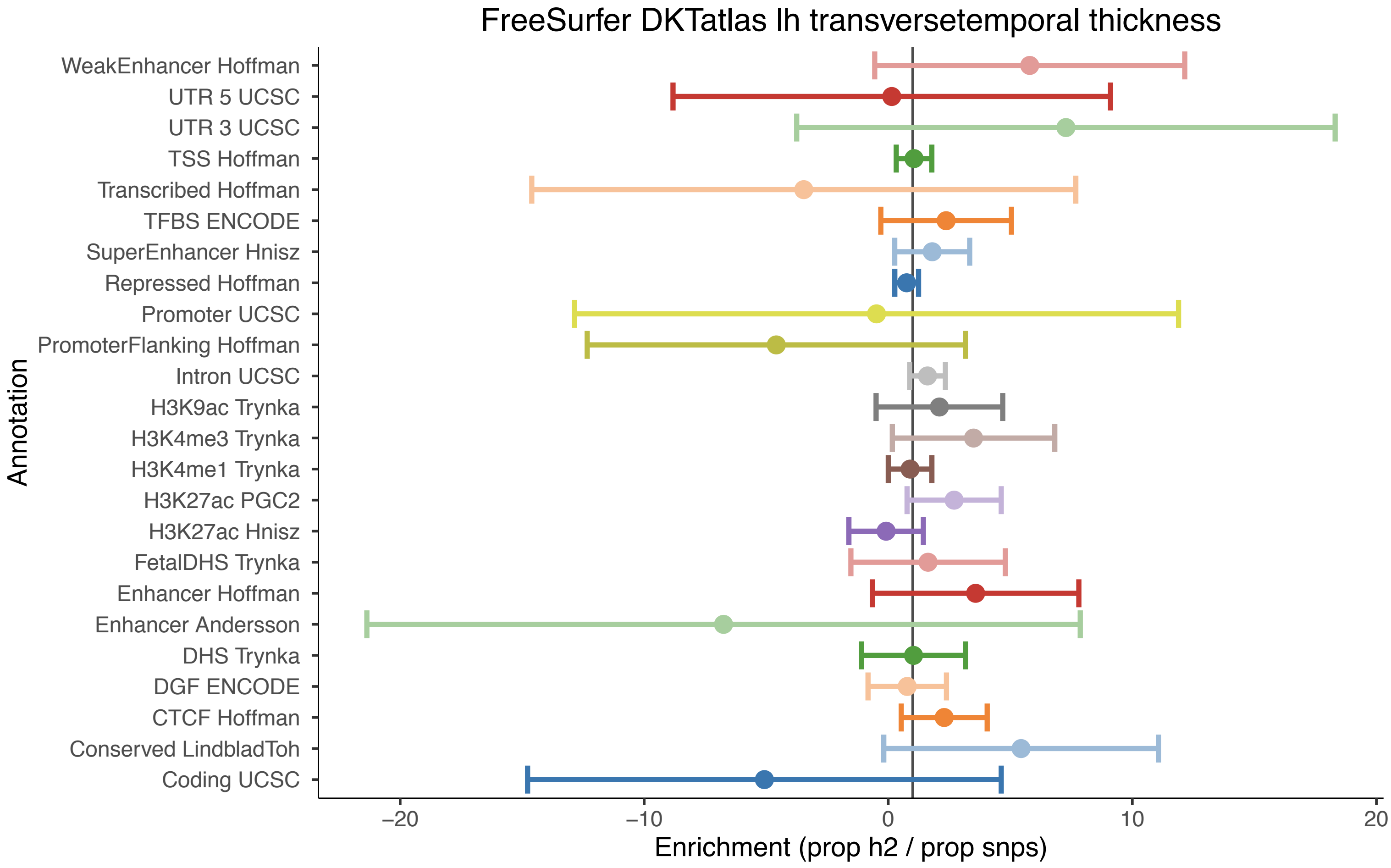

FreeSurfer DKTatlas lh transversetemporal thickness
WeakEnhancer Hoffman
UTR 5 UCSC
UTR 3 UCSC
TSS Hoffman
Transcribed Hoffman
TFBS ENCODE
SuperEnhancer Hnisz
Repressed Hoffman
Promoter UCSC
PromoterFlanking Hoffman
Intron UCSC
H3K9ac Trynka
H3K4me3 Trynka
H3K4me1 Trynka
H3K27ac PGC2
H3K27ac Hnisz
FetalDHS Trynka
Enhancer Hoffman
Enhancer Andersson
DHS Trynka
DGF ENCODE
CTCF Hoffman
Conserved LindbladToh
Coding UCSC
Annotation
−20
−10
0
10
20
Enrichment (prop h2 / prop snps)

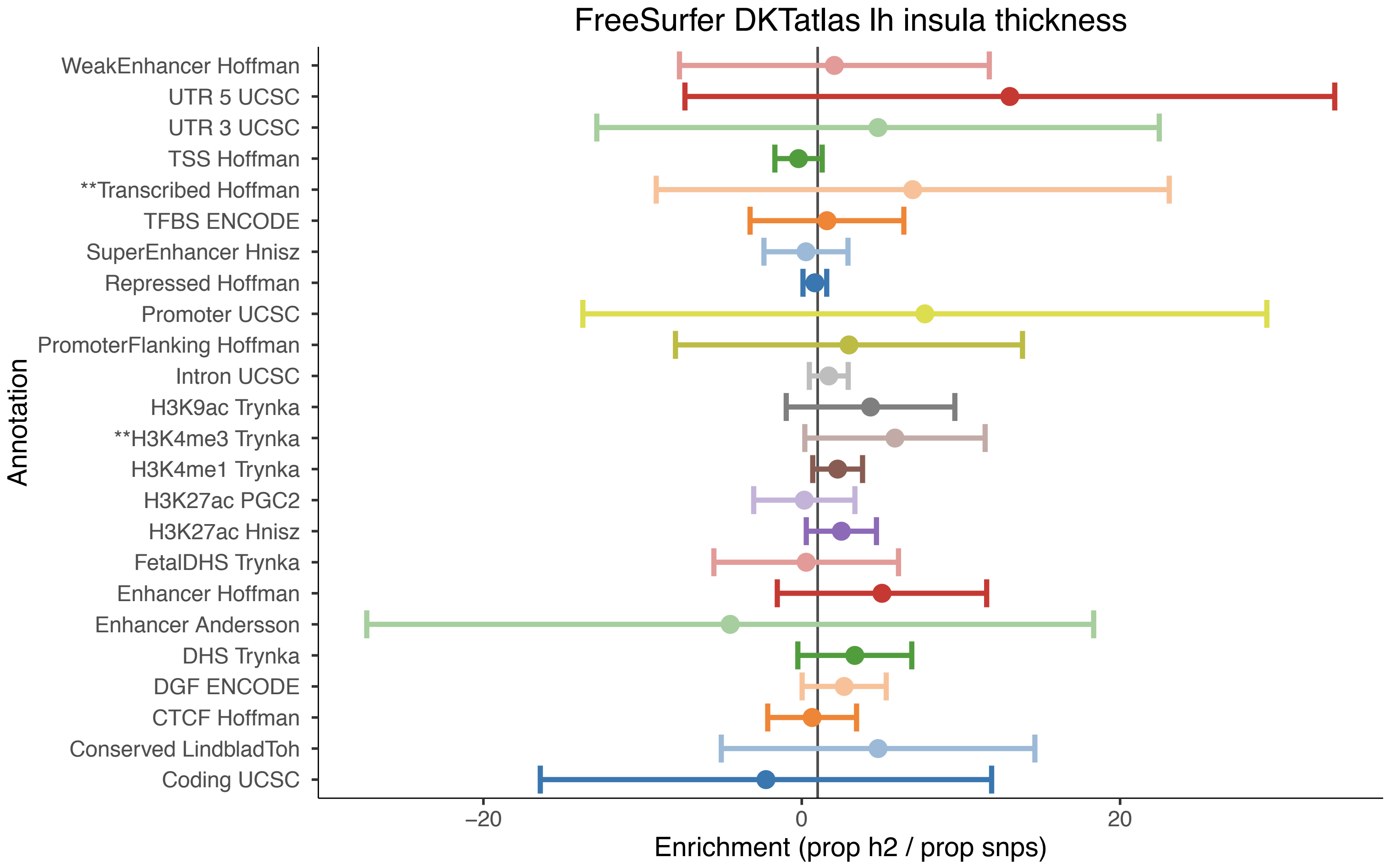

FreeSurfer DKTatlas lh insula thickness
WeakEnhancer Hoffman
UTR 5 UCSC
UTR 3 UCSC
TSS Hoffman
**Transcribed Hoffman
TFBS ENCODE
SuperEnhancer Hnisz
Repressed Hoffman
Promoter UCSC
PromoterFlanking Hoffman
Intron UCSC
H3K9ac Trynka
**H3K4me3 Trynka
H3K4me1 Trynka
H3K27ac PGC2
H3K27ac Hnisz
FetalDHS Trynka
Enhancer Hoffman
Enhancer Andersson
DHS Trynka
DGF ENCODE
CTCF Hoffman
Conserved LindbladToh
Coding UCSC
Annotation
−20
0
20
Enrichment (prop h2 / prop snps)

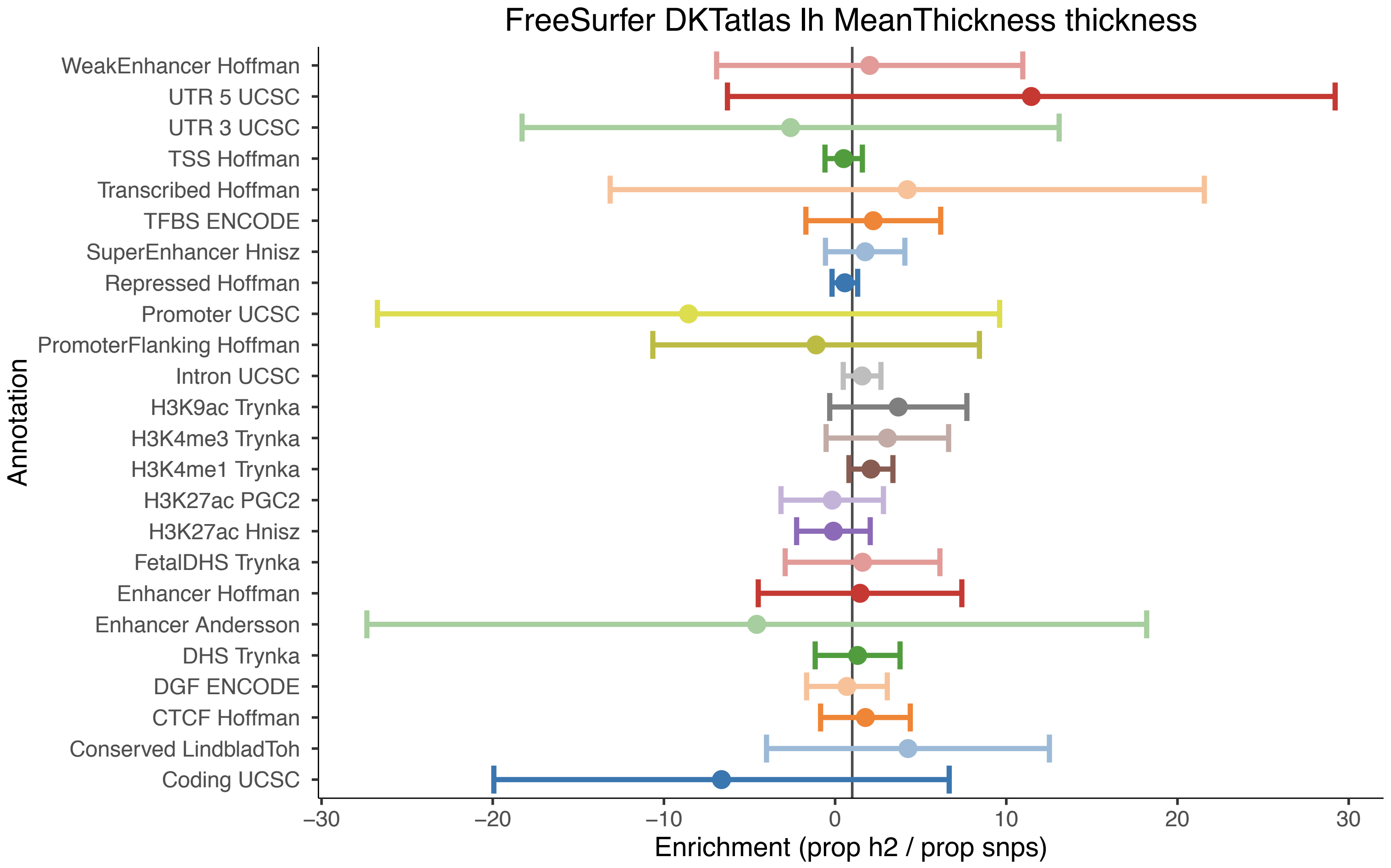

FreeSurfer DKTatlas lh MeanThickness thickness
WeakEnhancer Hoffman
UTR 5 UCSC
UTR 3 UCSC
TSS Hoffman
Transcribed Hoffman
TFBS ENCODE
SuperEnhancer Hnisz
Repressed Hoffman
Promoter UCSC
PromoterFlanking Hoffman
Intron UCSC
H3K9ac Trynka
H3K4me3 Trynka
H3K4me1 Trynka
H3K27ac PGC2
H3K27ac Hnisz
FetalDHS Trynka
Enhancer Hoffman
Enhancer Andersson
DHS Trynka
DGF ENCODE
CTCF Hoffman
Conserved LindbladToh
Coding UCSC
Annotation
−30
−20
−10
0
10
20
30
Enrichment (prop h2 / prop snps)

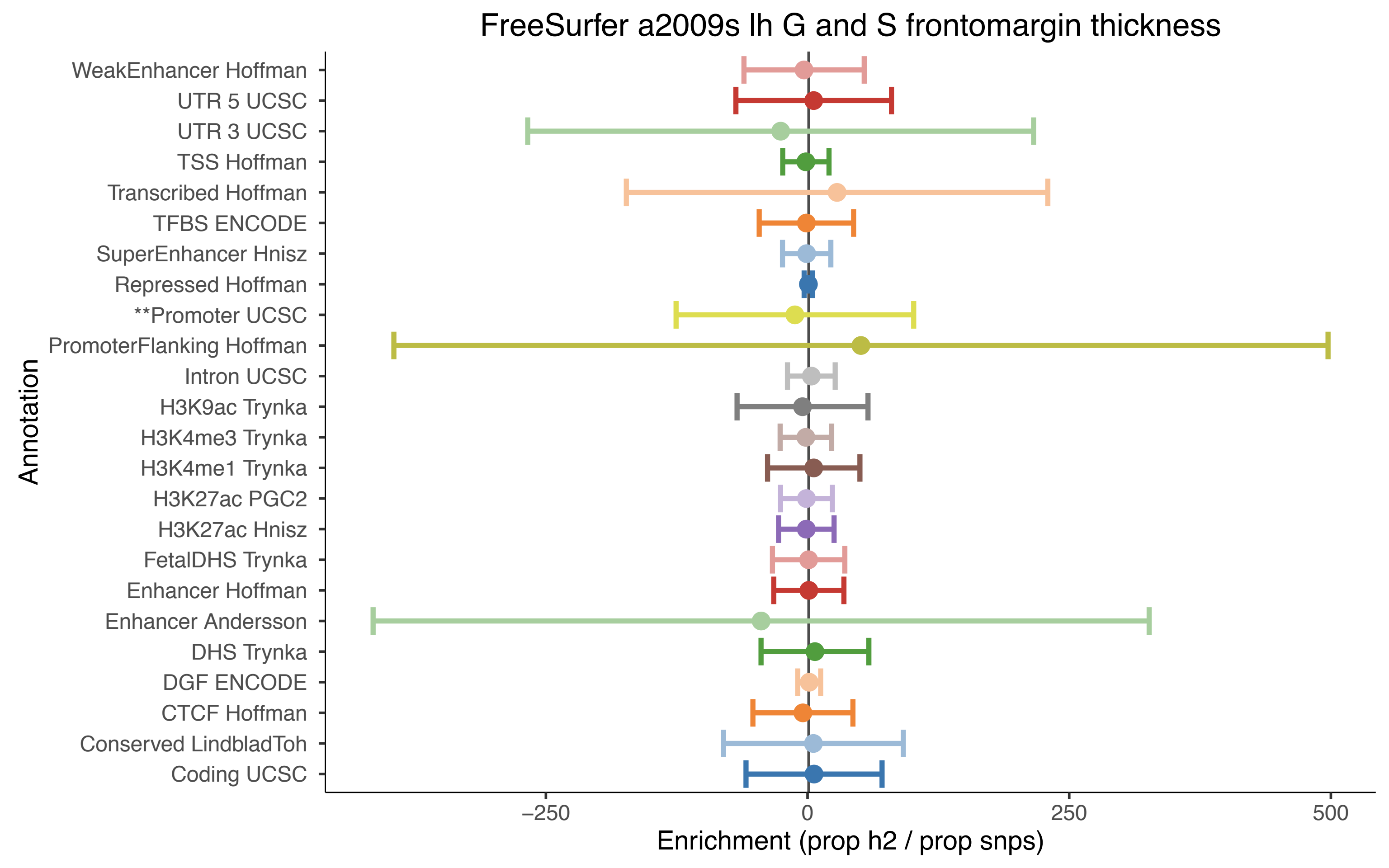
FreeSurfer a2009s lh G and S frontomargin thickness
WeakEnhancer Hoffman
UTR 5 UCSC
UTR 3 UCSC
TSS Hoffman
Transcribed Hoffman
TFBS ENCODE
SuperEnhancer Hnisz
Repressed Hoffman
**Promoter UCSC
PromoterFlanking Hoffman
Intron UCSC
H3K9ac Trynka
H3K4me3 Trynka
H3K4me1 Trynka
H3K27ac PGC2
H3K27ac Hnisz
FetalDHS Trynka
Enhancer Hoffman
Enhancer Andersson
DHS Trynka
DGF ENCODE
CTCF Hoffman
Conserved LindbladToh
Coding UCSC
Annotation
−250
0
250
500
Enrichment (prop h2 / prop snps)

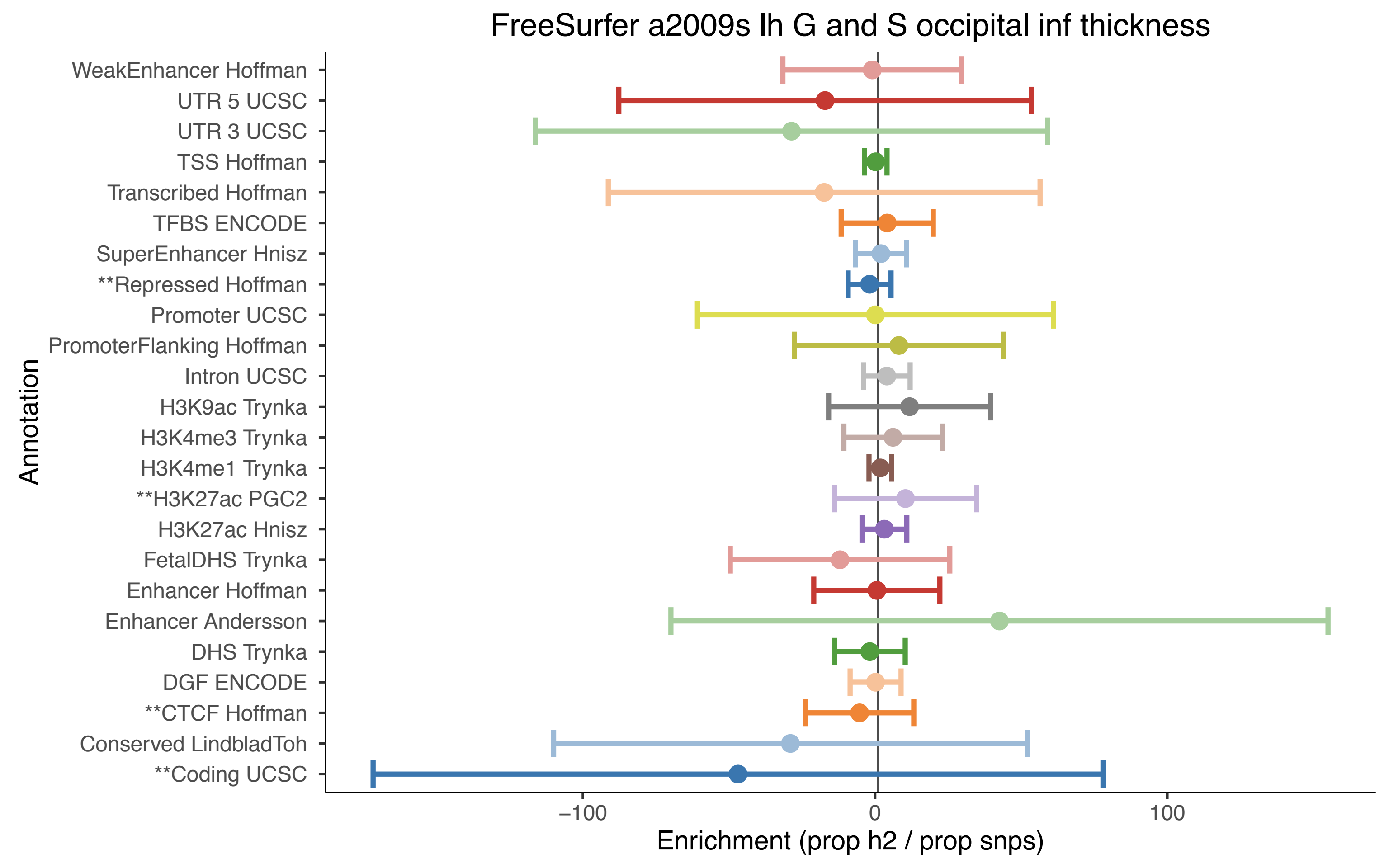

FreeSurfer a2009s lh G and S occipital inf thickness
Annotation
WeakEnhancer Hoffman
UTR 5 UCSC
UTR 3 UCSC
TSS Hoffman
Transcribed Hoffman
TFBS ENCODE
SuperEnhancer Hnisz
**Repressed Hoffman
Promoter UCSC
PromoterFlanking Hoffman
Intron UCSC
H3K9ac Trynka
H3K4me3 Trynka
H3K4me1 Trynka
**H3K27ac PGC2
H3K27ac Hnisz
FetalDHS Trynka
Enhancer Hoffman
Enhancer Andersson
DHS Trynka
DGF ENCODE
**CTCF Hoffman
Conserved LindbladToh
**Coding UCSC
−100
0
100
Enrichment (prop h2 / prop snps)

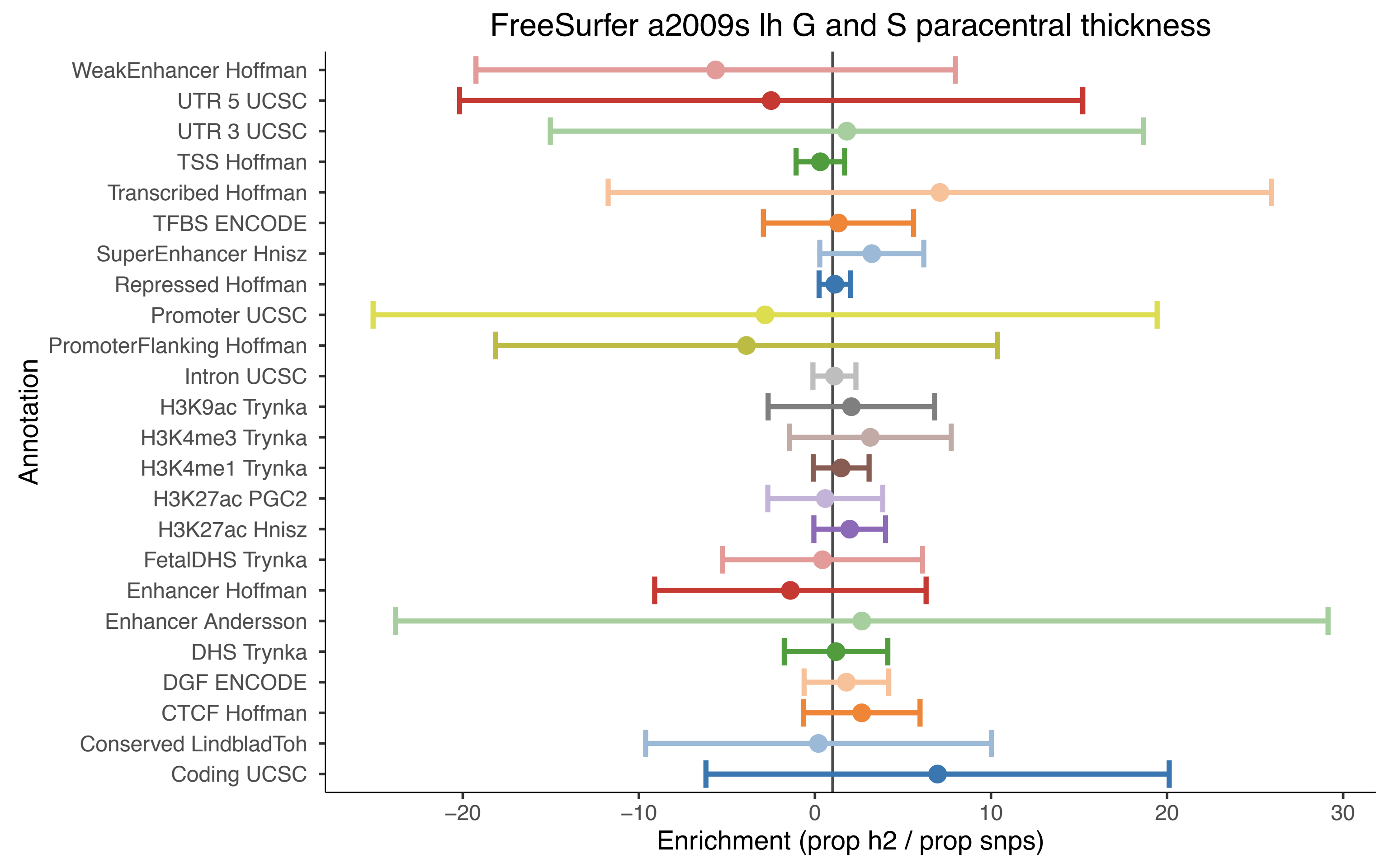

FreeSurfer a2009s lh G and S paracentral thickness
WeakEnhancer Hoffman
UTR 5 UCSC
UTR 3 UCSC
TSS Hoffman
Transcribed Hoffman
TFBS ENCODE
SuperEnhancer Hnisz
Repressed Hoffman
Promoter UCSC
PromoterFlanking Hoffman
Intron UCSC
H3K9ac Trynka
H3K4me3 Trynka
H3K4me1 Trynka
H3K27ac PGC2
H3K27ac Hnisz
FetalDHS Trynka
Enhancer Hoffman
Enhancer Andersson
DHS Trynka
DGF ENCODE
CTCF Hoffman
Conserved LindbladToh
Coding UCSC
Annotation
−20
−10
0
10
20
30
Enrichment (prop h2 / prop snps)

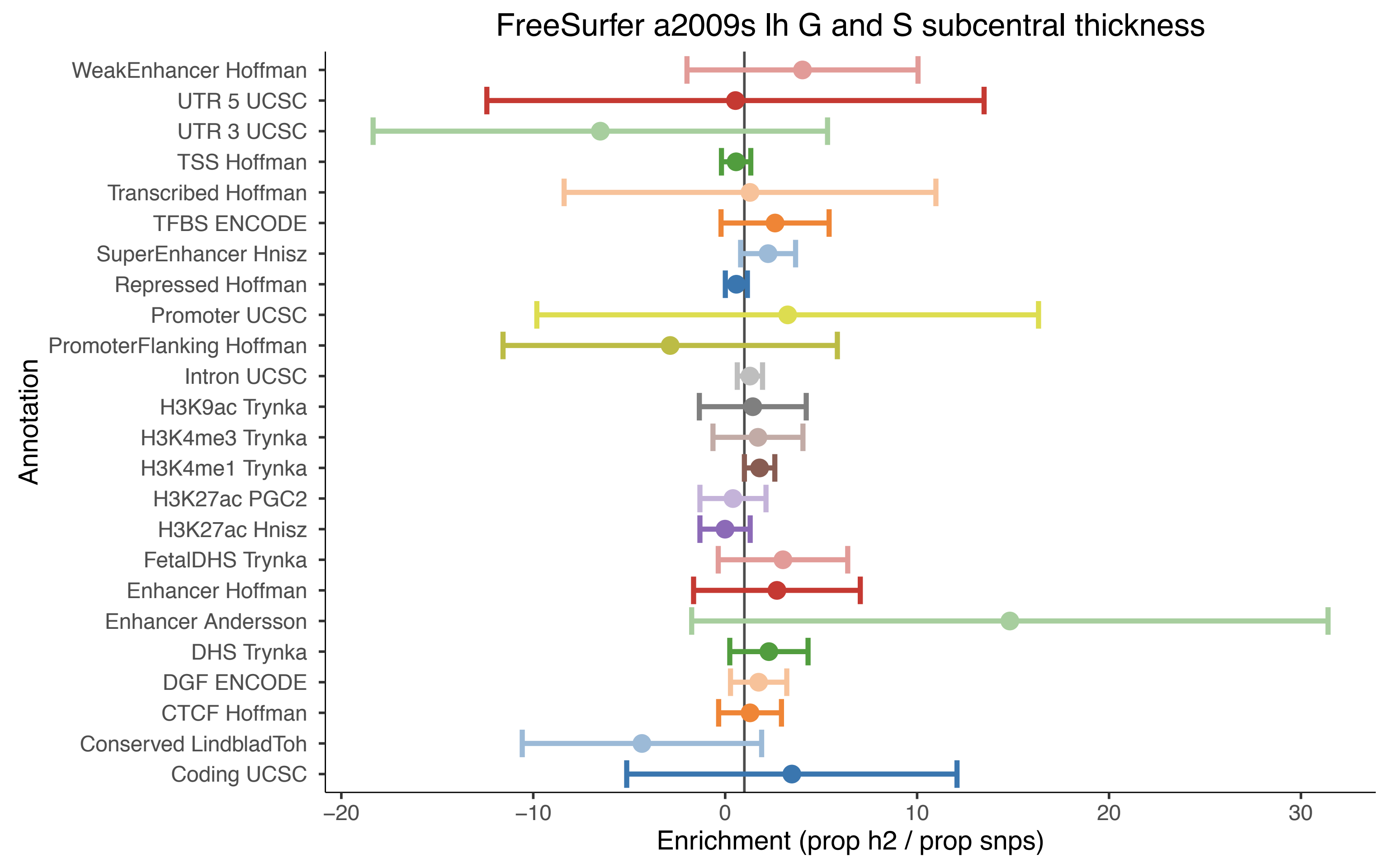
FreeSurfer a2009s lh G and S subcentral thickness
Annotation
WeakEnhancer Hoffman
UTR 5 UCSC
UTR 3 UCSC
TSS Hoffman
Transcribed Hoffman
TFBS ENCODE
SuperEnhancer Hnisz
Repressed Hoffman
Promoter UCSC
PromoterFlanking Hoffman
Intron UCSC
H3K9ac Trynka
H3K4me3 Trynka
H3K4me1 Trynka
H3K27ac PGC2
H3K27ac Hnisz
FetalDHS Trynka
Enhancer Hoffman
Enhancer Andersson
DHS Trynka
DGF ENCODE
CTCF Hoffman
Conserved LindbladToh
Coding UCSC
−20
−10
0
10
20
30
Enrichment (prop h2 / prop snps)

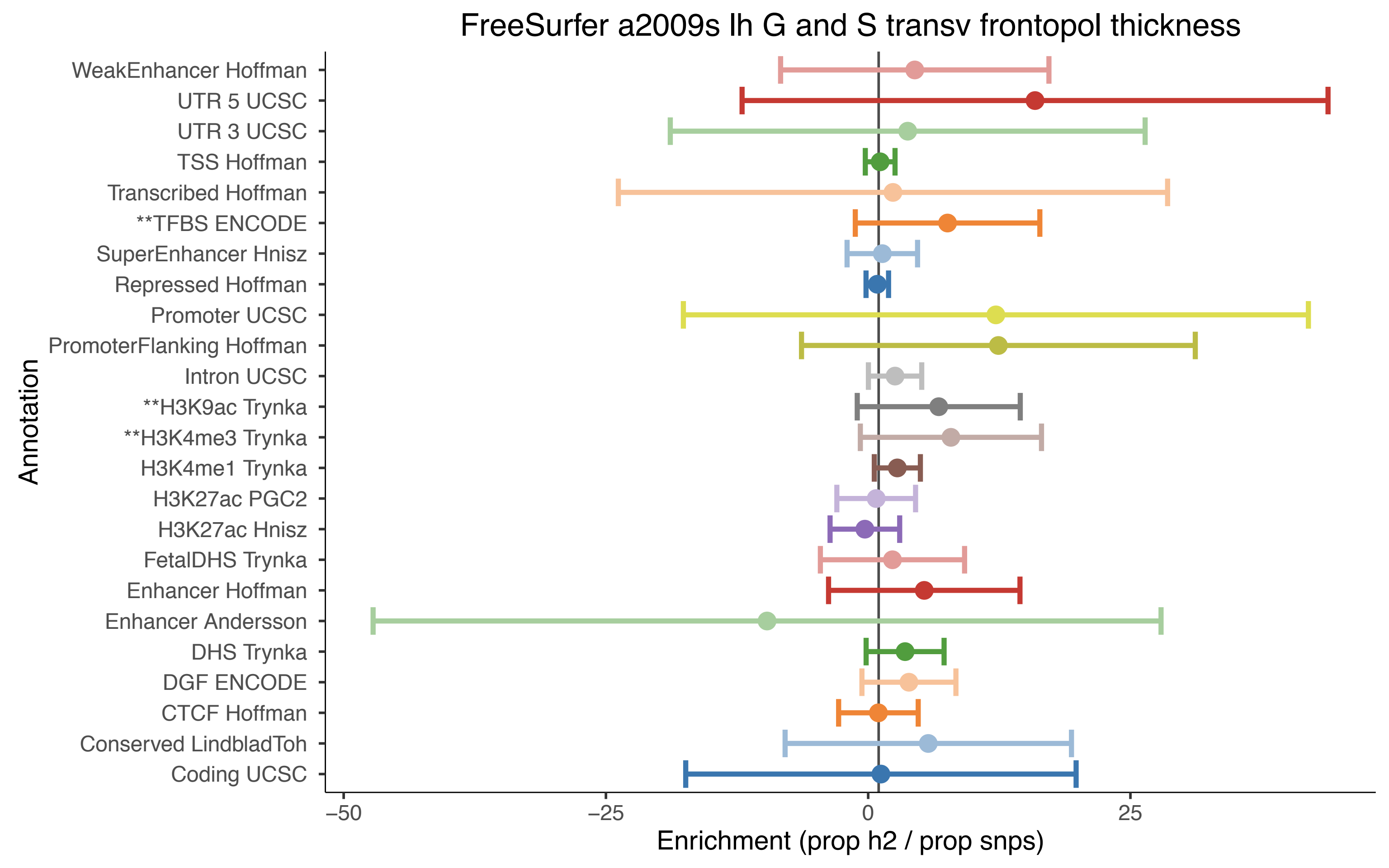
FreeSurfer a2009s lh G and S transv frontopol thickness
WeakEnhancer Hoffman
UTR 5 UCSC
UTR 3 UCSC
TSS Hoffman
Transcribed Hoffman
**TFBS ENCODE
SuperEnhancer Hnisz
Repressed Hoffman
Promoter UCSC
PromoterFlanking Hoffman
Intron UCSC
**H3K9ac Trynka
**H3K4me3 Trynka
H3K4me1 Trynka
H3K27ac PGC2
H3K27ac Hnisz
FetalDHS Trynka
Enhancer Hoffman
Enhancer Andersson
DHS Trynka
DGF ENCODE
CTCF Hoffman
Conserved LindbladToh
Coding UCSC
Annotation
−50
−25
0
25
Enrichment (prop h2 / prop snps)

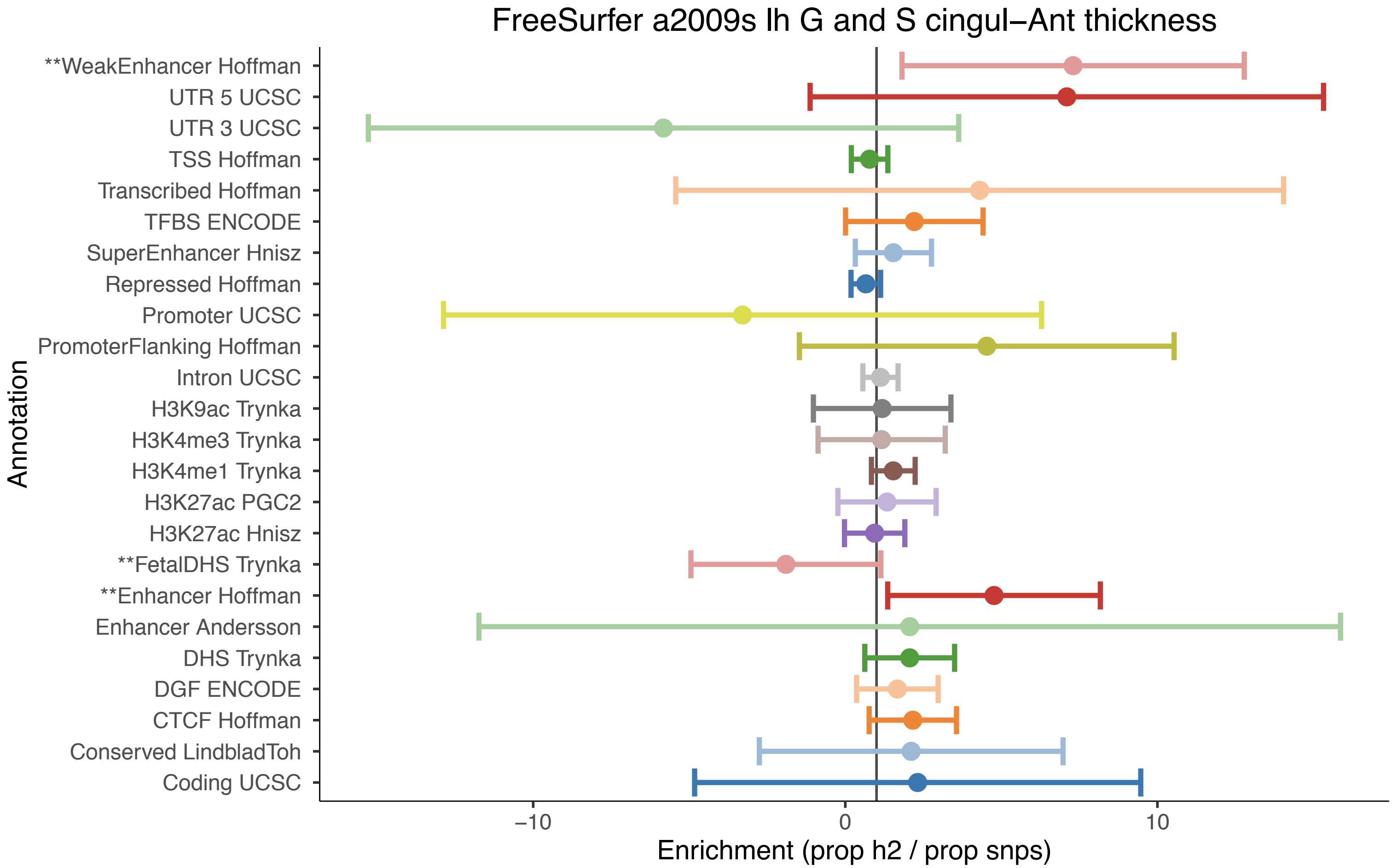
FreeSurfer a2009s lh G and S cingul−Ant thickness
**WeakEnhancer Hoffman
UTR 5 UCSC
UTR 3 UCSC
TSS Hoffman
Transcribed Hoffman
TFBS ENCODE
SuperEnhancer Hnisz
Repressed Hoffman
Promoter UCSC
PromoterFlanking Hoffman
Intron UCSC
H3K9ac Trynka
H3K4me3 Trynka
H3K4me1 Trynka
H3K27ac PGC2
H3K27ac Hnisz
**FetalDHS Trynka
**Enhancer Hoffman
Enhancer Andersson
DHS Trynka
DGF ENCODE
CTCF Hoffman
Conserved LindbladToh
Coding UCSC
Annotation
−10
0
10
Enrichment (prop h2 / prop snps)

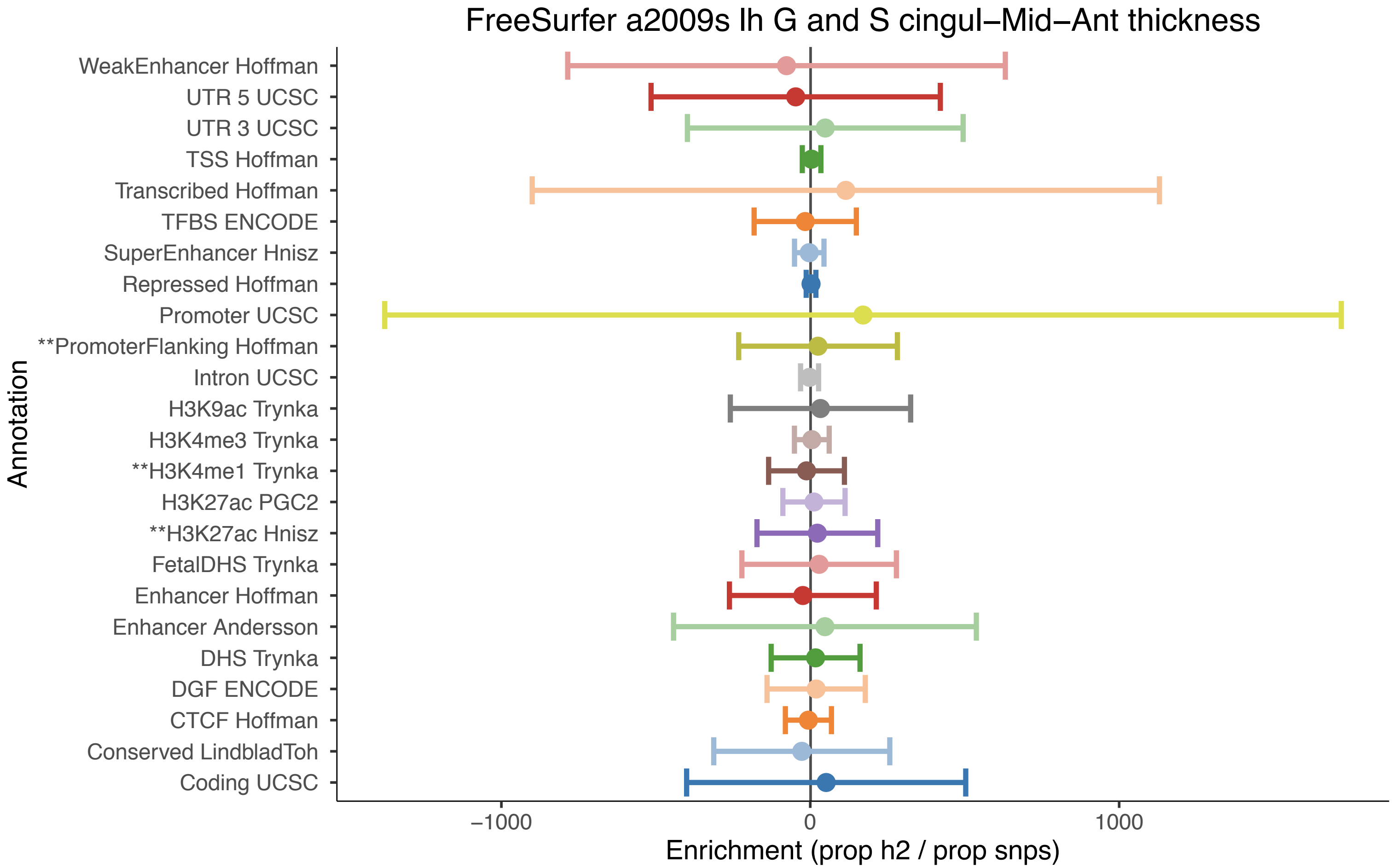
FreeSurfer a2009s lh G and S cingul−Mid−Ant thickness
WeakEnhancer Hoffman
UTR 5 UCSC
UTR 3 UCSC
TSS Hoffman
Transcribed Hoffman
TFBS ENCODE
SuperEnhancer Hnisz
Repressed Hoffman
Promoter UCSC
**PromoterFlanking Hoffman
Intron UCSC
H3K9ac Trynka
H3K4me3 Trynka
**H3K4me1 Trynka
H3K27ac PGC2
**H3K27ac Hnisz
FetalDHS Trynka
Enhancer Hoffman
Enhancer Andersson
DHS Trynka
DGF ENCODE
CTCF Hoffman
Conserved LindbladToh
Coding UCSC
Annotation
−1000
0
1000
Enrichment (prop h2 / prop snps)

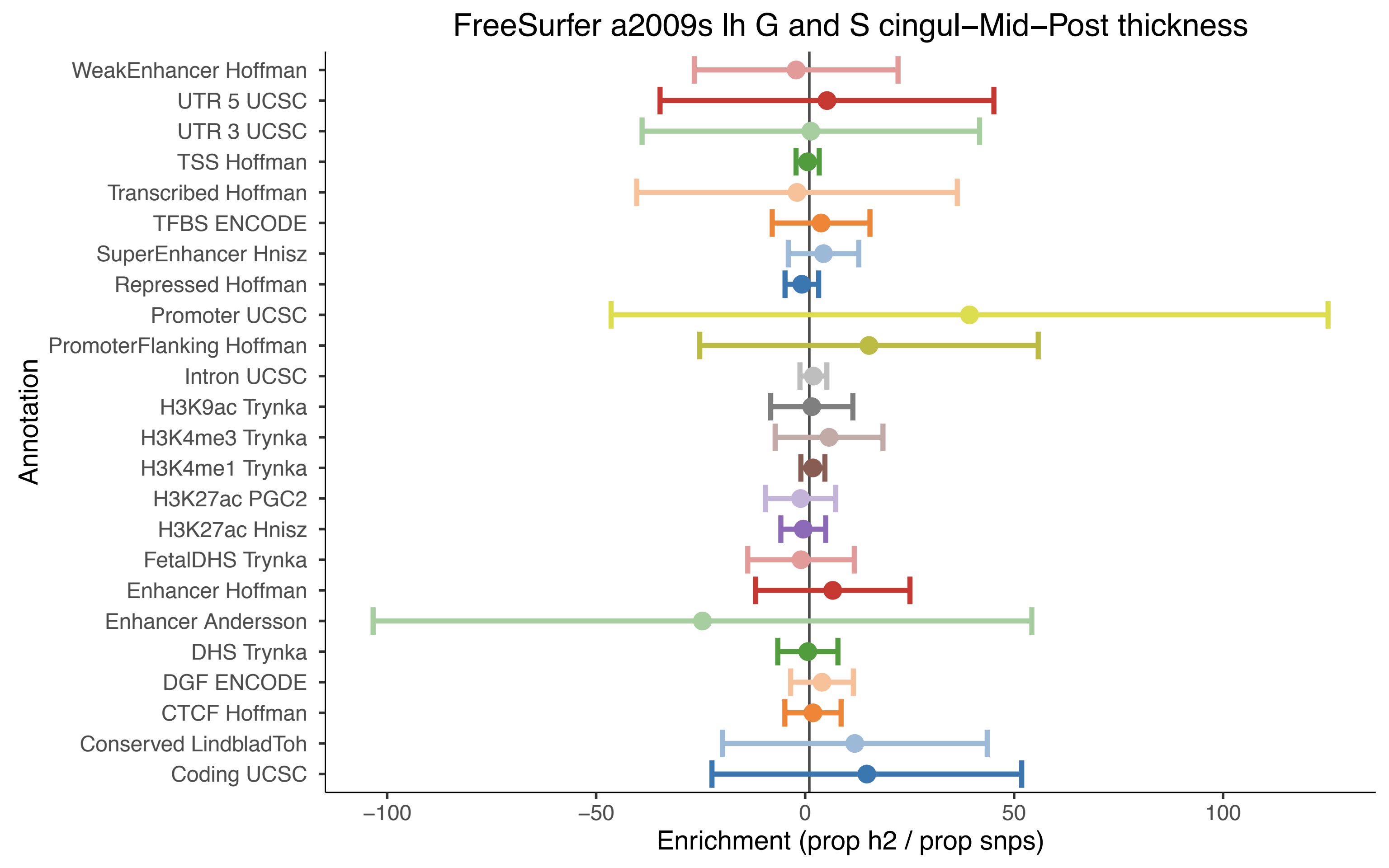
FreeSurfer a2009s lh G and S cingul−Mid−Post thickness
WeakEnhancer Hoffman
UTR 5 UCSC
UTR 3 UCSC
TSS Hoffman
Transcribed Hoffman
TFBS ENCODE
SuperEnhancer Hnisz
Repressed Hoffman
Promoter UCSC
PromoterFlanking Hoffman
Intron UCSC
H3K9ac Trynka
H3K4me3 Trynka
H3K4me1 Trynka
H3K27ac PGC2
H3K27ac Hnisz
FetalDHS Trynka
Enhancer Hoffman
Enhancer Andersson
DHS Trynka
DGF ENCODE
CTCF Hoffman
Conserved LindbladToh
Coding UCSC
Annotation
−100
−50
0
50
100
Enrichment (prop h2 / prop snps)

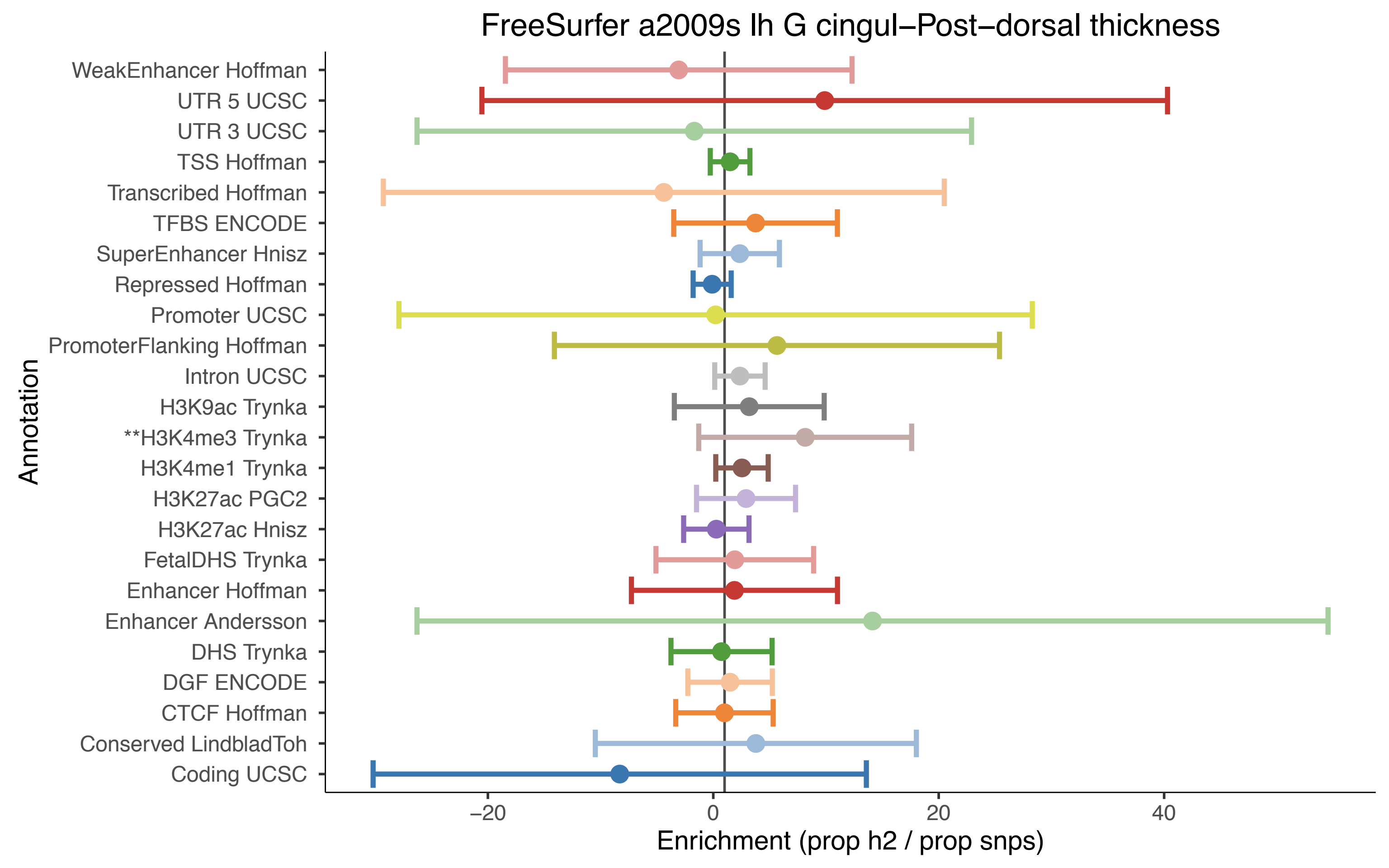

FreeSurfer a2009s lh G cingul−Post−dorsal thickness
WeakEnhancer Hoffman
UTR 5 UCSC
UTR 3 UCSC
TSS Hoffman
Transcribed Hoffman
TFBS ENCODE
SuperEnhancer Hnisz
Repressed Hoffman
Promoter UCSC
PromoterFlanking Hoffman
Intron UCSC
H3K9ac Trynka
**H3K4me3 Trynka
H3K4me1 Trynka
H3K27ac PGC2
H3K27ac Hnisz
FetalDHS Trynka
Enhancer Hoffman
Enhancer Andersson
DHS Trynka
DGF ENCODE
CTCF Hoffman
Conserved LindbladToh
Coding UCSC
Annotation
−20
0
20
40
Enrichment (prop h2 / prop snps)

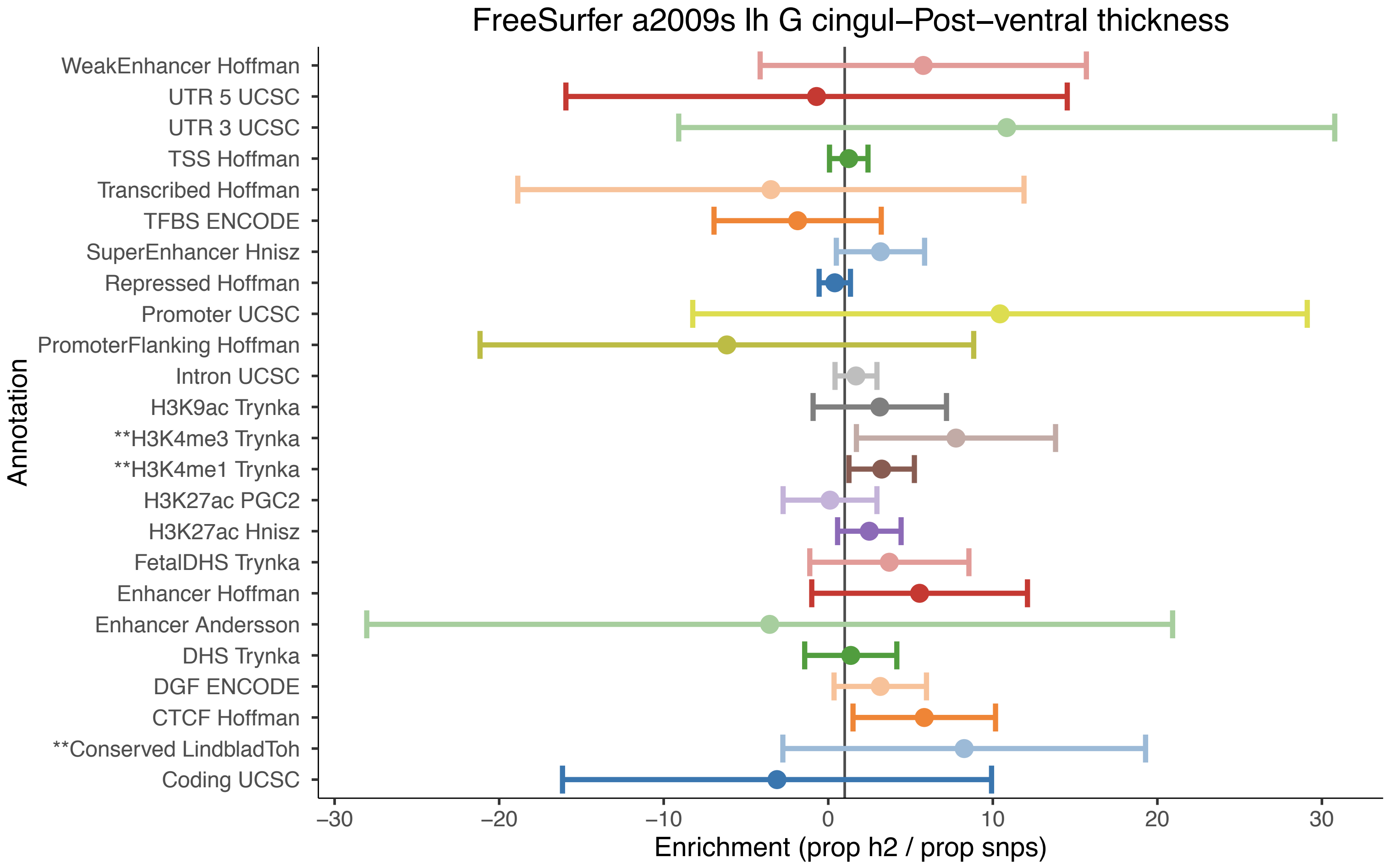

FreeSurfer a2009s lh G cingul−Post−ventral thickness
WeakEnhancer Hoffman
UTR 5 UCSC
UTR 3 UCSC
TSS Hoffman
Transcribed Hoffman
TFBS ENCODE
SuperEnhancer Hnisz
Repressed Hoffman
Promoter UCSC
PromoterFlanking Hoffman
Intron UCSC
H3K9ac Trynka
**H3K4me3 Trynka
**H3K4me1 Trynka
H3K27ac PGC2
H3K27ac Hnisz
FetalDHS Trynka
Enhancer Hoffman
Enhancer Andersson
DHS Trynka
DGF ENCODE
CTCF Hoffman
**Conserved LindbladToh
Coding UCSC
Annotation
−30
−20
−10
0
10
20
30
Enrichment (prop h2 / prop snps)

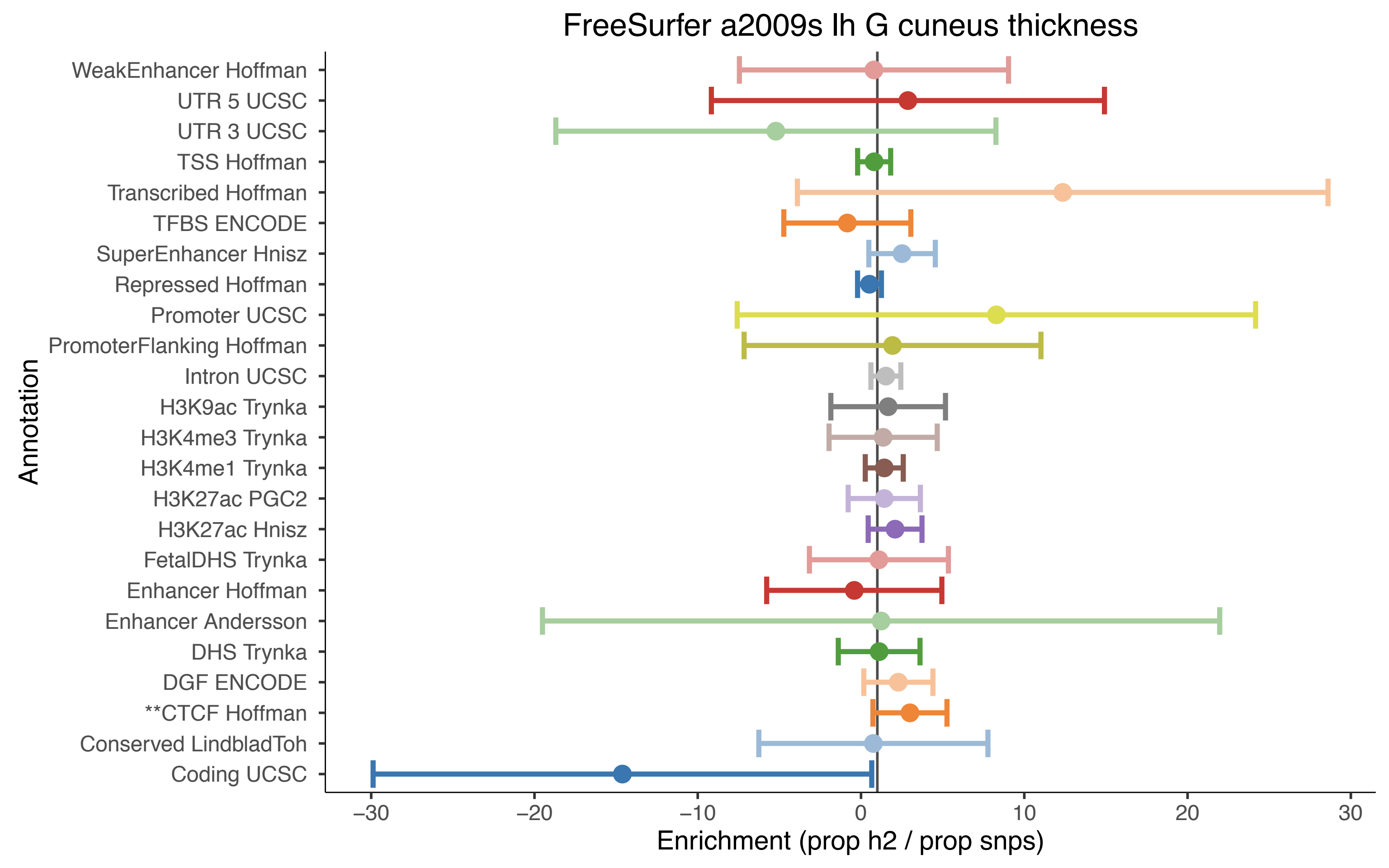
FreeSurfer a2009s lh G cuneus thickness
WeakEnhancer Hoffman
UTR 5 UCSC
UTR 3 UCSC
TSS Hoffman
Transcribed Hoffman
TFBS ENCODE
SuperEnhancer Hnisz
Repressed Hoffman
Promoter UCSC
PromoterFlanking Hoffman
Intron UCSC
H3K9ac Trynka
H3K4me3 Trynka
H3K4me1 Trynka
H3K27ac PGC2
H3K27ac Hnisz
FetalDHS Trynka
Enhancer Hoffman
Enhancer Andersson
DHS Trynka
DGF ENCODE
**CTCF Hoffman
Conserved LindbladToh
Coding UCSC
Annotation
−30
−20
−10
0
10
20
30
Enrichment (prop h2 / prop snps)

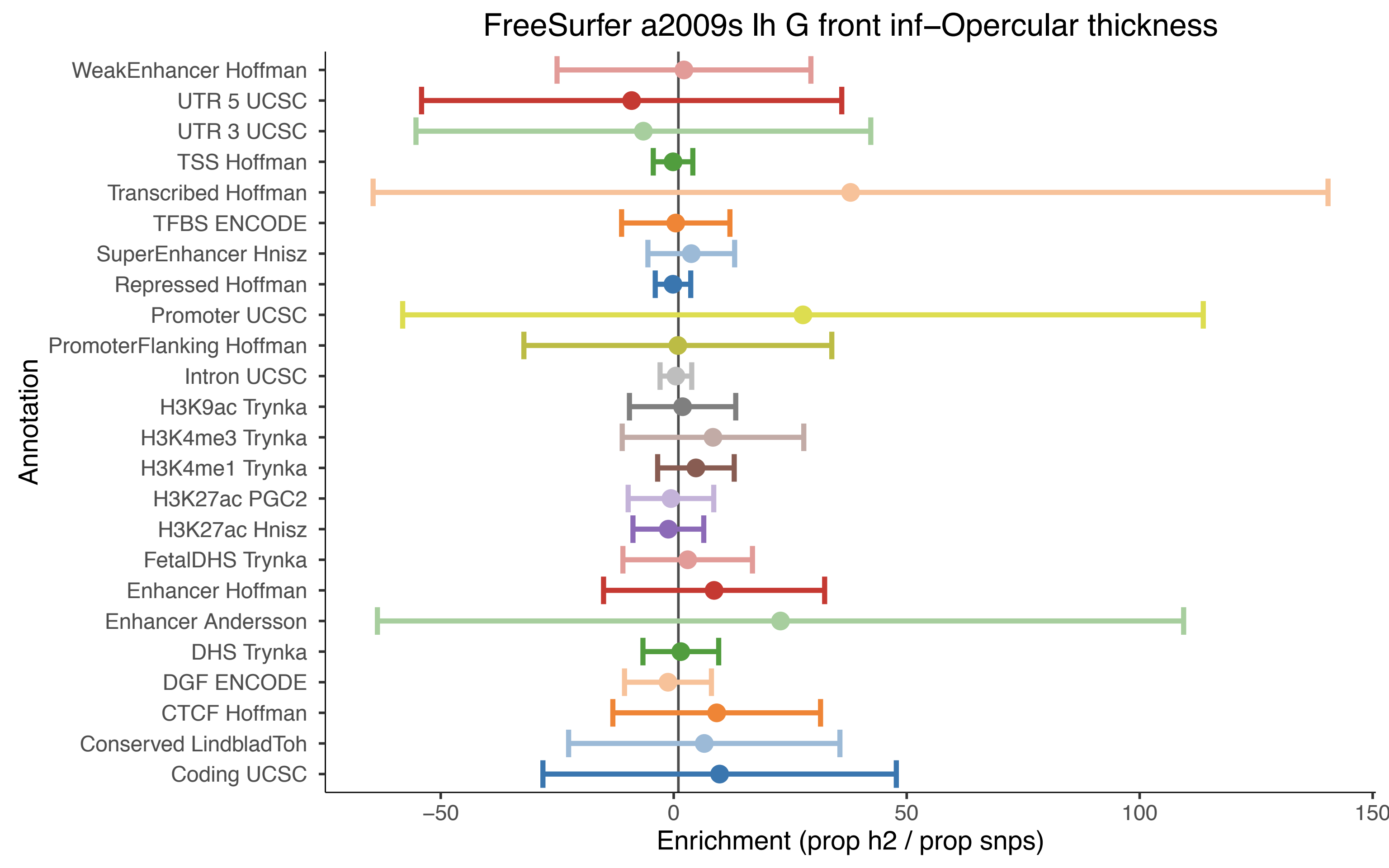
FreeSurfer a2009s lh G front inf−Opercular thickness
WeakEnhancer Hoffman
UTR 5 UCSC
UTR 3 UCSC
TSS Hoffman
Transcribed Hoffman
TFBS ENCODE
SuperEnhancer Hnisz
Repressed Hoffman
Promoter UCSC
PromoterFlanking Hoffman
Intron UCSC
H3K9ac Trynka
H3K4me3 Trynka
H3K4me1 Trynka
H3K27ac PGC2
H3K27ac Hnisz
FetalDHS Trynka
Enhancer Hoffman
Enhancer Andersson
DHS Trynka
DGF ENCODE
CTCF Hoffman
Conserved LindbladToh
Coding UCSC
Annotation
−50
0
50
100
150
Enrichment (prop h2 / prop snps)

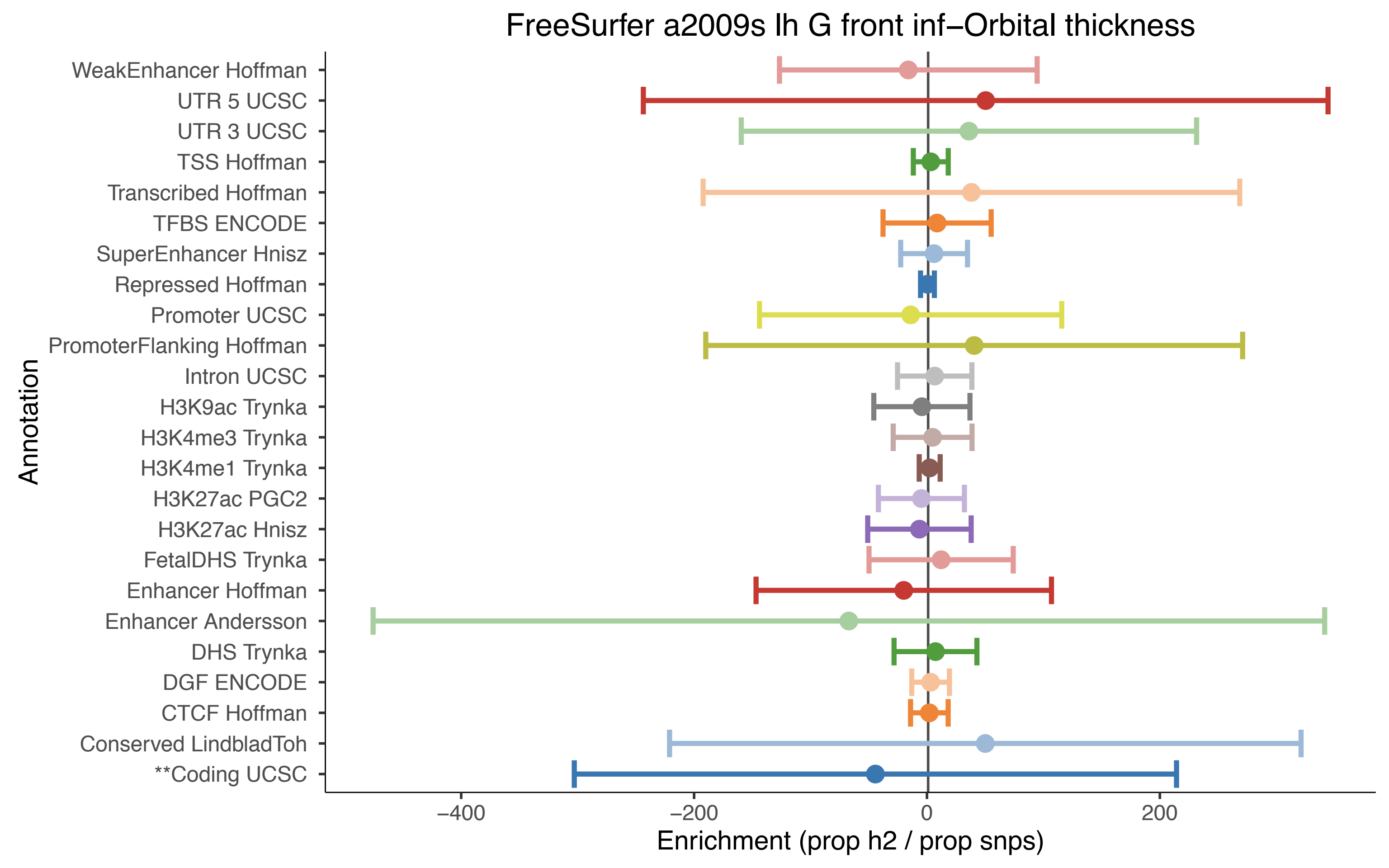
FreeSurfer a2009s lh G front inf−Orbital thickness
Annotation
WeakEnhancer Hoffman
UTR 5 UCSC
UTR 3 UCSC
TSS Hoffman
Transcribed Hoffman
TFBS ENCODE
SuperEnhancer Hnisz
Repressed Hoffman
Promoter UCSC
PromoterFlanking Hoffman
Intron UCSC
H3K9ac Trynka
H3K4me3 Trynka
H3K4me1 Trynka
H3K27ac PGC2
H3K27ac Hnisz
FetalDHS Trynka
Enhancer Hoffman
Enhancer Andersson
DHS Trynka
DGF ENCODE
CTCF Hoffman
Conserved LindbladToh
**Coding UCSC
−400
−200
0
200
Enrichment (prop h2 / prop snps)

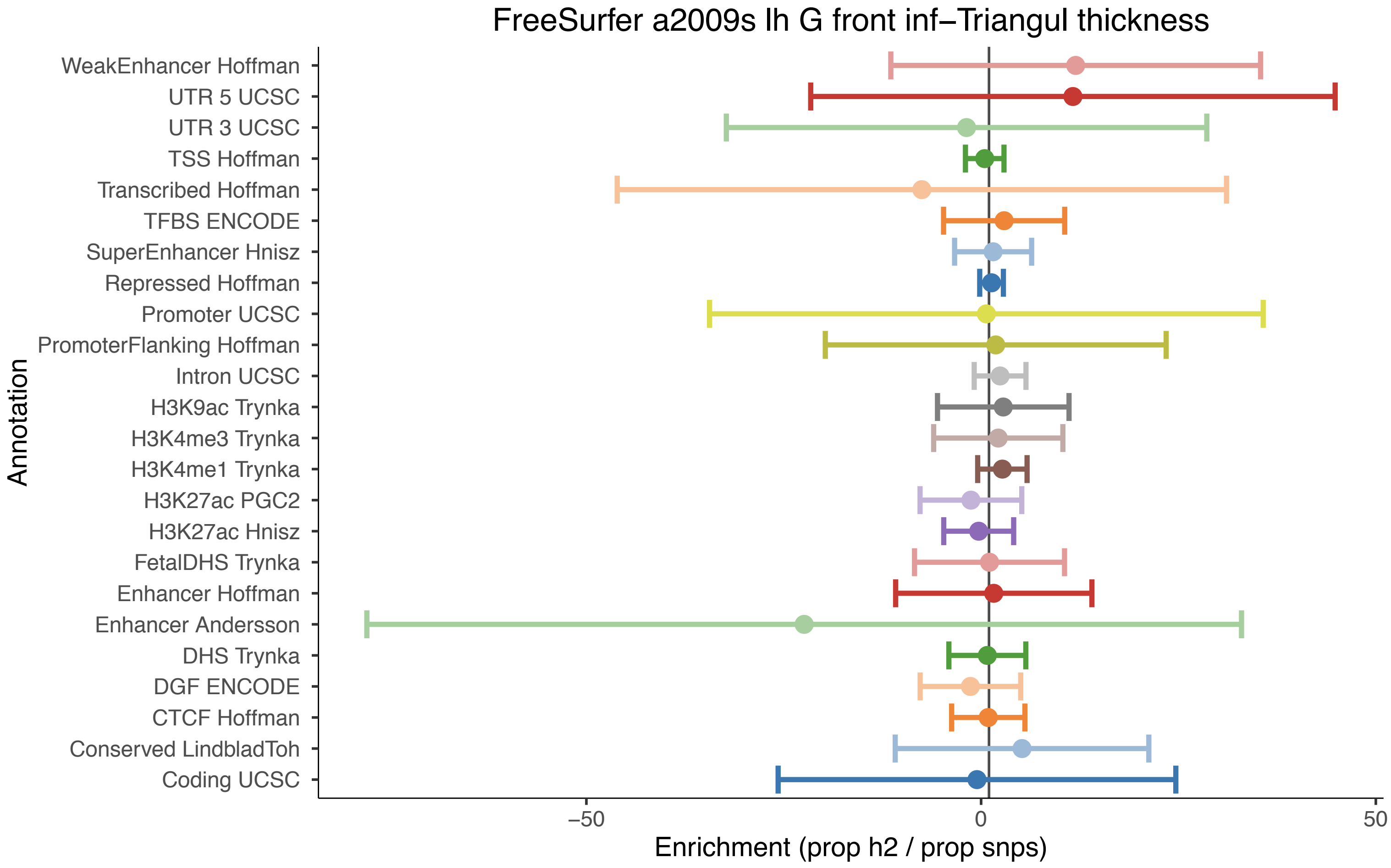
FreeSurfer a2009s lh G front inf−Triangul thickness
WeakEnhancer Hoffman
UTR 5 UCSC
UTR 3 UCSC
TSS Hoffman
Transcribed Hoffman
TFBS ENCODE
SuperEnhancer Hnisz
Repressed Hoffman
Promoter UCSC
PromoterFlanking Hoffman
Intron UCSC
H3K9ac Trynka
H3K4me3 Trynka
H3K4me1 Trynka
H3K27ac PGC2
H3K27ac Hnisz
FetalDHS Trynka
Enhancer Hoffman
Enhancer Andersson
DHS Trynka
DGF ENCODE
CTCF Hoffman
Conserved LindbladToh
Coding UCSC
Annotation
−50
0
50
Enrichment (prop h2 / prop snps)

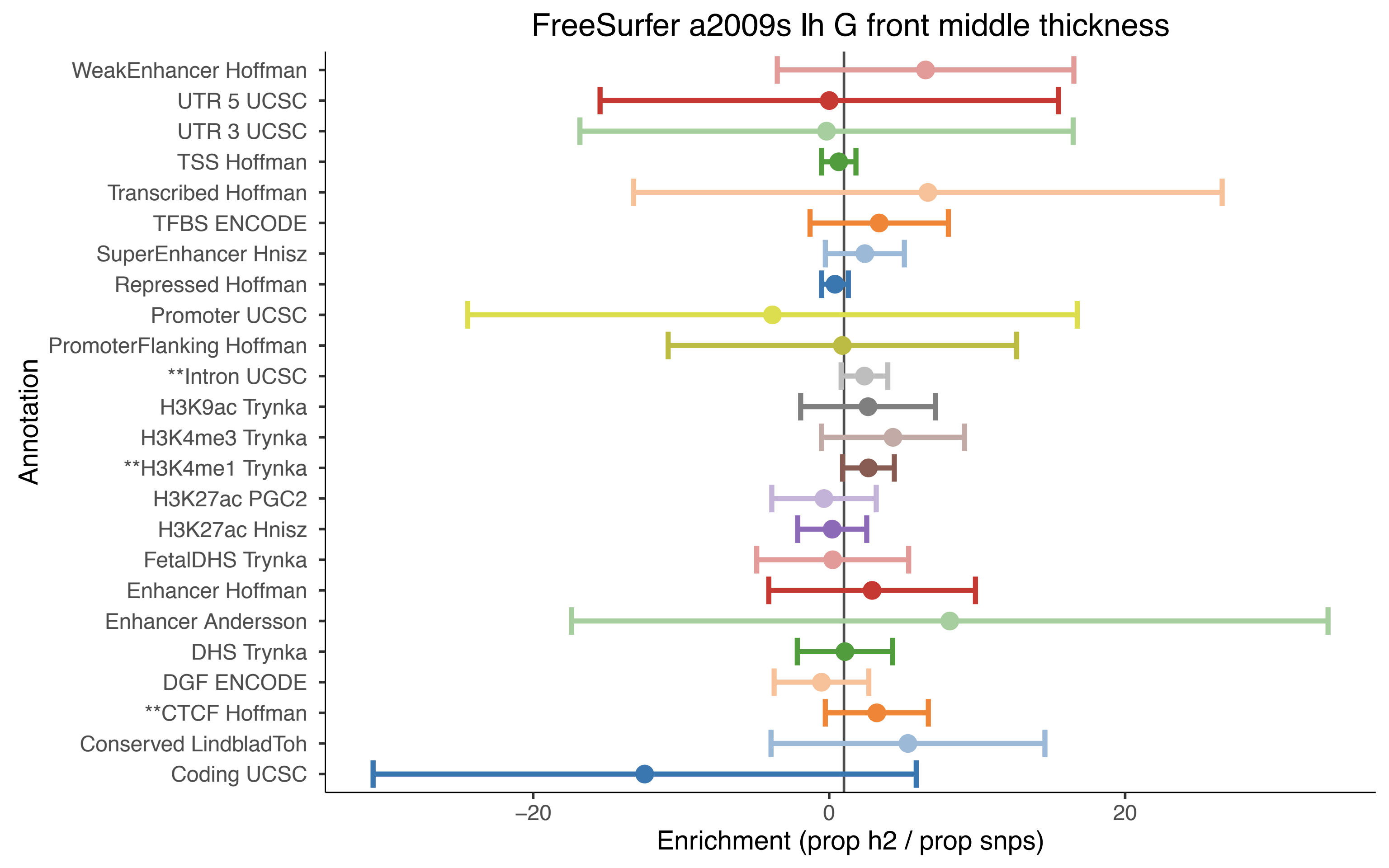
FreeSurfer a2009s lh G front middle thickness
WeakEnhancer Hoffman
UTR 5 UCSC
UTR 3 UCSC
TSS Hoffman
Transcribed Hoffman
TFBS ENCODE
SuperEnhancer Hnisz
Repressed Hoffman
Promoter UCSC
PromoterFlanking Hoffman
**Intron UCSC
H3K9ac Trynka
H3K4me3 Trynka
**H3K4me1 Trynka
H3K27ac PGC2
H3K27ac Hnisz
FetalDHS Trynka
Enhancer Hoffman
Enhancer Andersson
DHS Trynka
DGF ENCODE
**CTCF Hoffman
Conserved LindbladToh
Coding UCSC
Annotation
−20
0
20
Enrichment (prop h2 / prop snps)

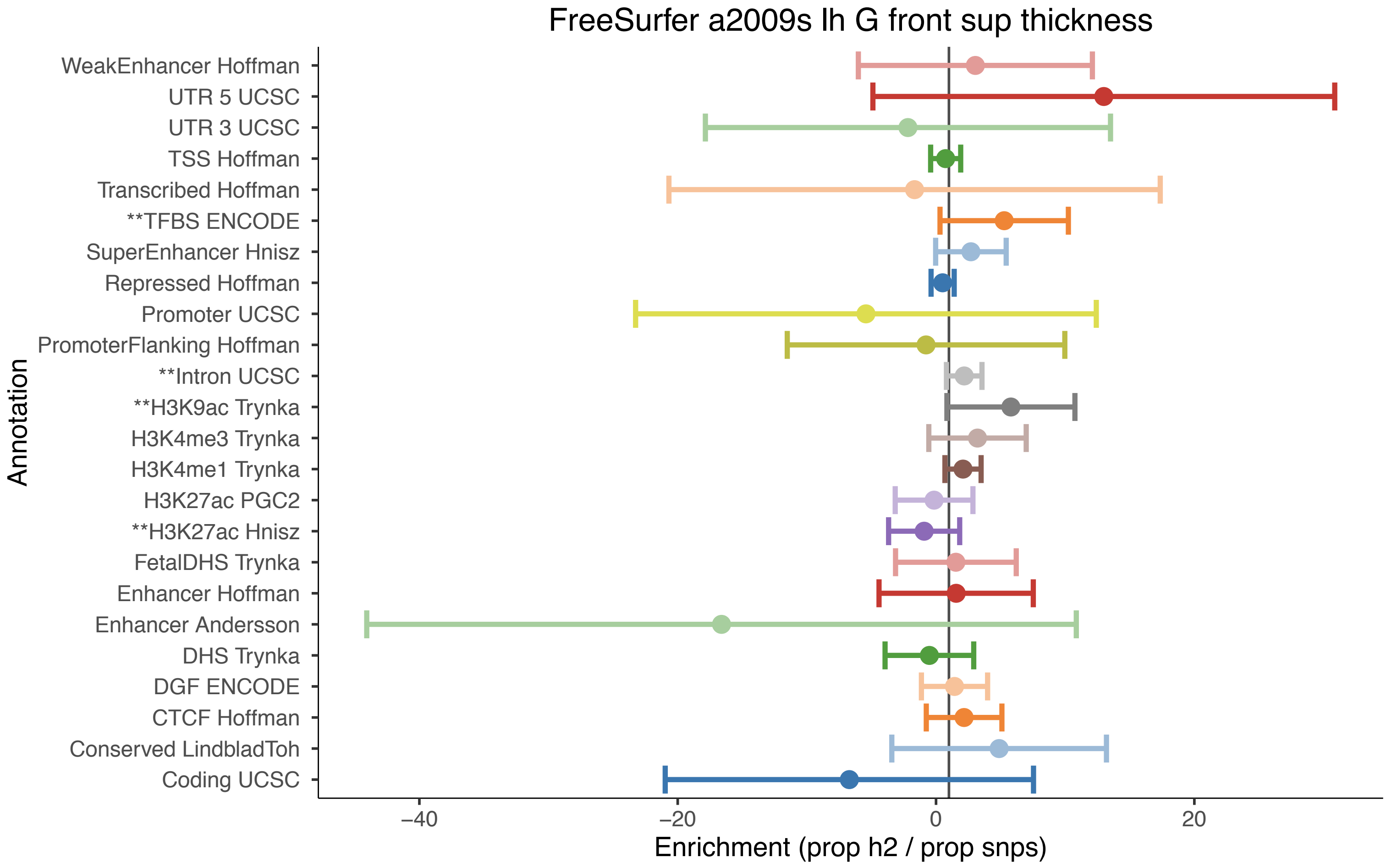
FreeSurfer a2009s lh G front sup thickness
WeakEnhancer Hoffman
UTR 5 UCSC
UTR 3 UCSC
TSS Hoffman
Transcribed Hoffman
**TFBS ENCODE
SuperEnhancer Hnisz
Repressed Hoffman
Promoter UCSC
PromoterFlanking Hoffman
**Intron UCSC
**H3K9ac Trynka
H3K4me3 Trynka
H3K4me1 Trynka
H3K27ac PGC2
**H3K27ac Hnisz
FetalDHS Trynka
Enhancer Hoffman
Enhancer Andersson
DHS Trynka
DGF ENCODE
CTCF Hoffman
Conserved LindbladToh
Coding UCSC
Annotation
−40
−20
0
20
Enrichment (prop h2 / prop snps)

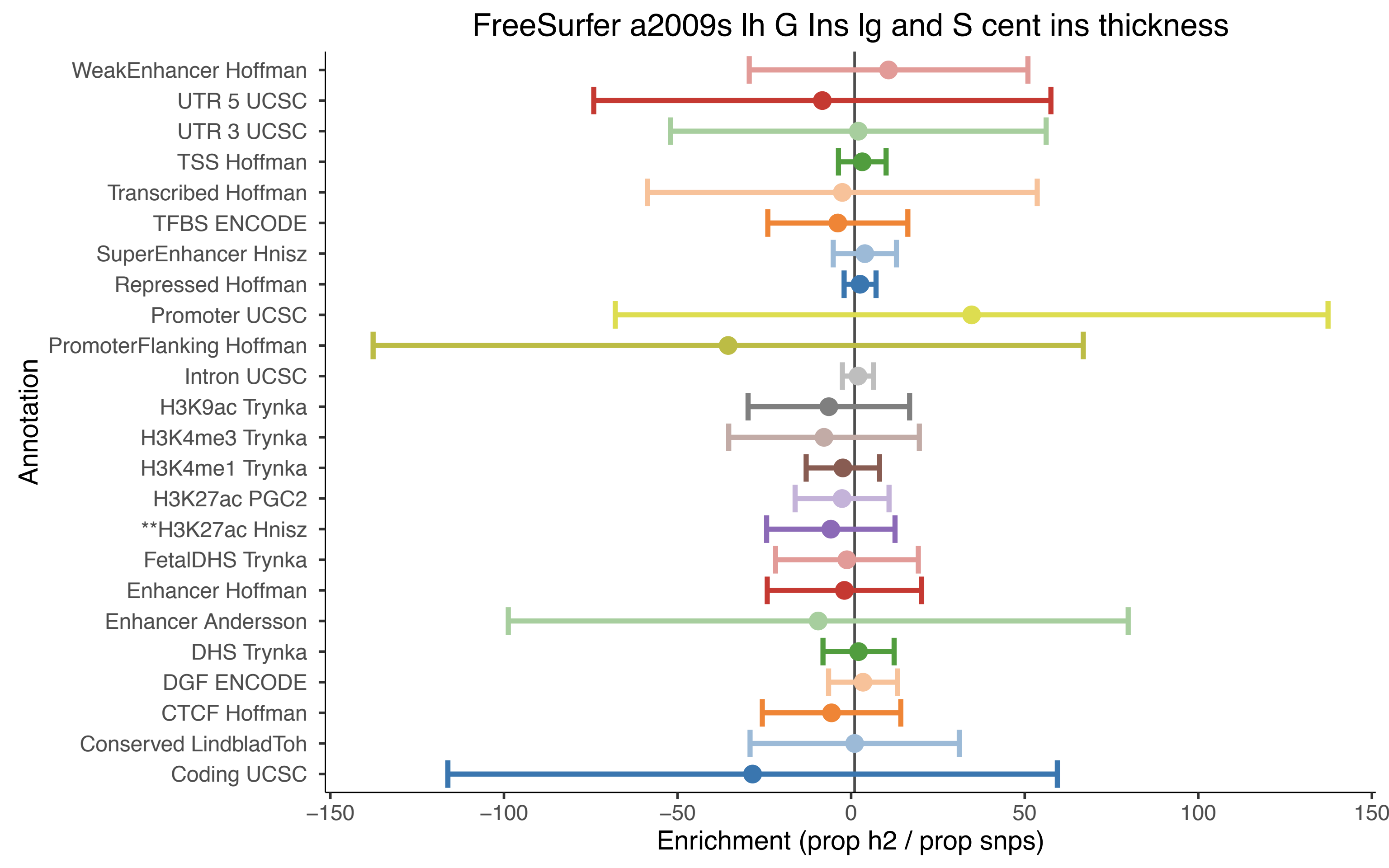

FreeSurfer a2009s lh G Ins lg and S cent ins thickness
WeakEnhancer Hoffman
UTR 5 UCSC
UTR 3 UCSC
TSS Hoffman
Transcribed Hoffman
TFBS ENCODE
SuperEnhancer Hnisz
Repressed Hoffman
Promoter UCSC
PromoterFlanking Hoffman
Intron UCSC
H3K9ac Trynka
H3K4me3 Trynka
H3K4me1 Trynka
H3K27ac PGC2
**H3K27ac Hnisz
FetalDHS Trynka
Enhancer Hoffman
Enhancer Andersson
DHS Trynka
DGF ENCODE
CTCF Hoffman
Conserved LindbladToh
Coding UCSC
Annotation
−150
−100
−50
0
50
100
150
Enrichment (prop h2 / prop snps)

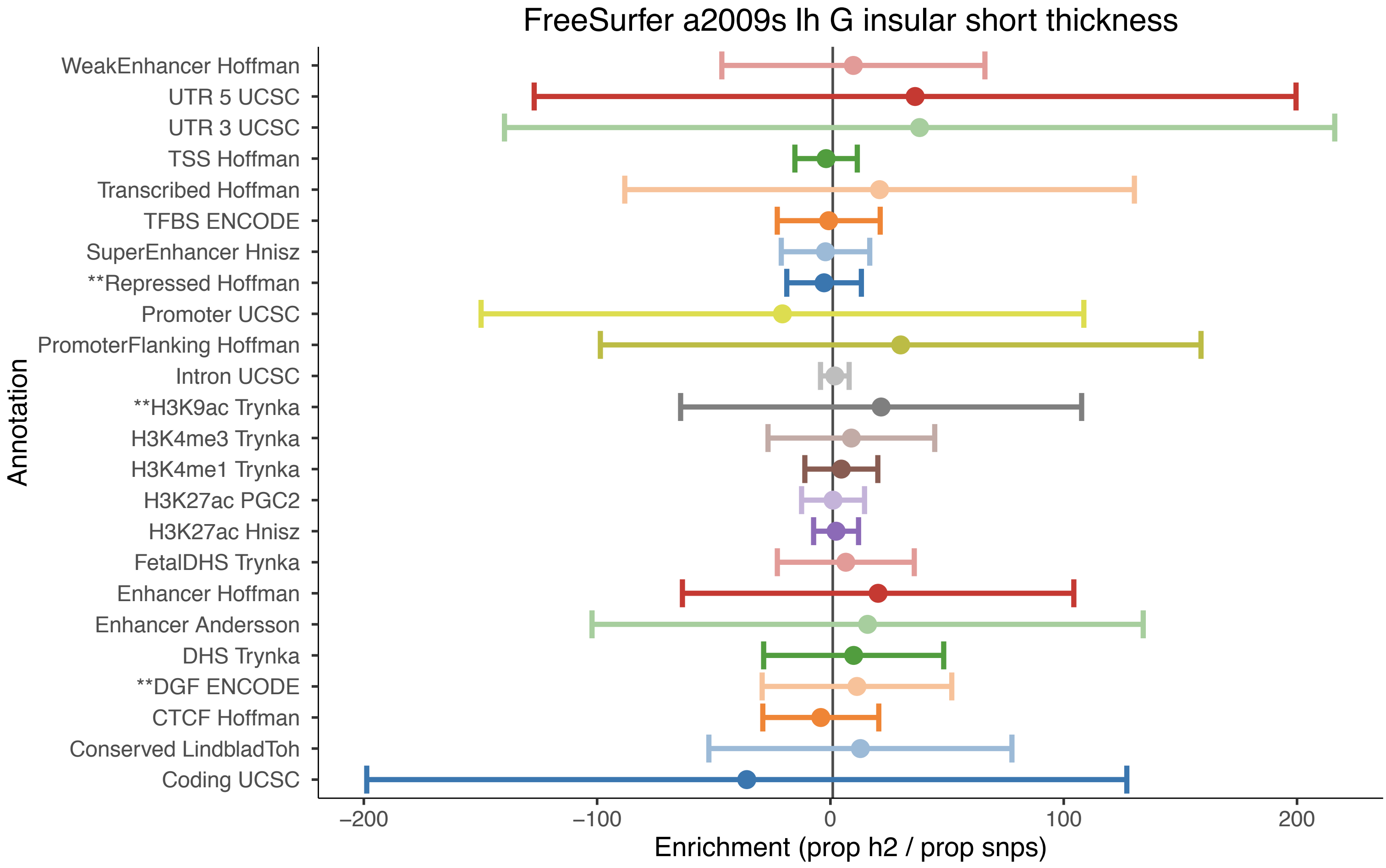

FreeSurfer a2009s lh G insular short thickness
WeakEnhancer Hoffman
UTR 5 UCSC
UTR 3 UCSC
TSS Hoffman
Transcribed Hoffman
TFBS ENCODE
SuperEnhancer Hnisz
**Repressed Hoffman
Promoter UCSC
PromoterFlanking Hoffman
Intron UCSC
**H3K9ac Trynka
H3K4me3 Trynka
H3K4me1 Trynka
H3K27ac PGC2
H3K27ac Hnisz
FetalDHS Trynka
Enhancer Hoffman
Enhancer Andersson
DHS Trynka
**DGF ENCODE
CTCF Hoffman
Conserved LindbladToh
Coding UCSC
Annotation
−200
−100
0
100
200
Enrichment (prop h2 / prop snps)

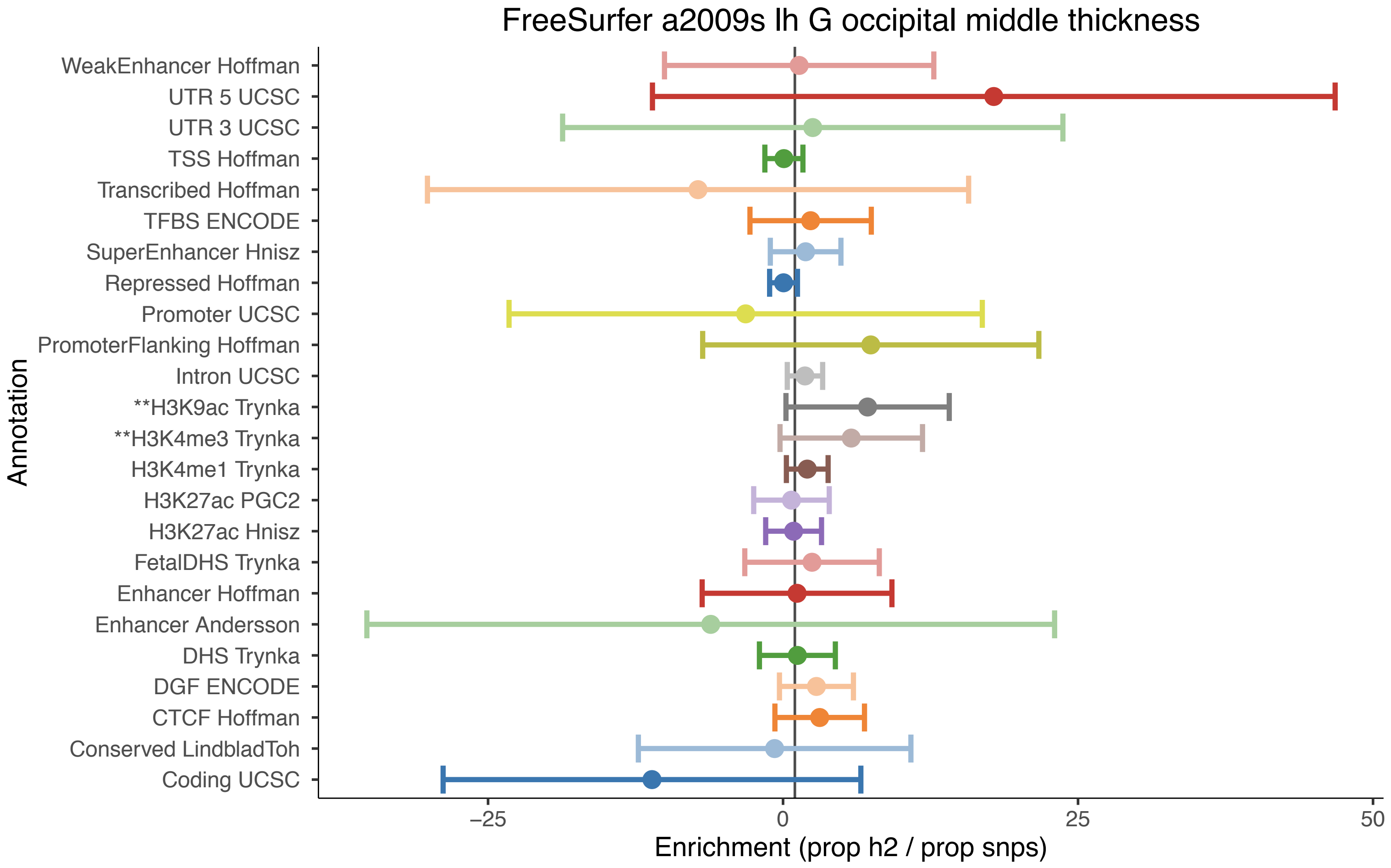
FreeSurfer a2009s lh G occipital middle thickness
WeakEnhancer Hoffman
UTR 5 UCSC
UTR 3 UCSC
TSS Hoffman
Transcribed Hoffman
TFBS ENCODE
SuperEnhancer Hnisz
Repressed Hoffman
Promoter UCSC
PromoterFlanking Hoffman
Intron UCSC
**H3K9ac Trynka
**H3K4me3 Trynka
H3K4me1 Trynka
H3K27ac PGC2
H3K27ac Hnisz
FetalDHS Trynka
Enhancer Hoffman
Enhancer Andersson
DHS Trynka
DGF ENCODE
CTCF Hoffman
Conserved LindbladToh
Coding UCSC
Annotation
−25
0
25
50
Enrichment (prop h2 / prop snps)

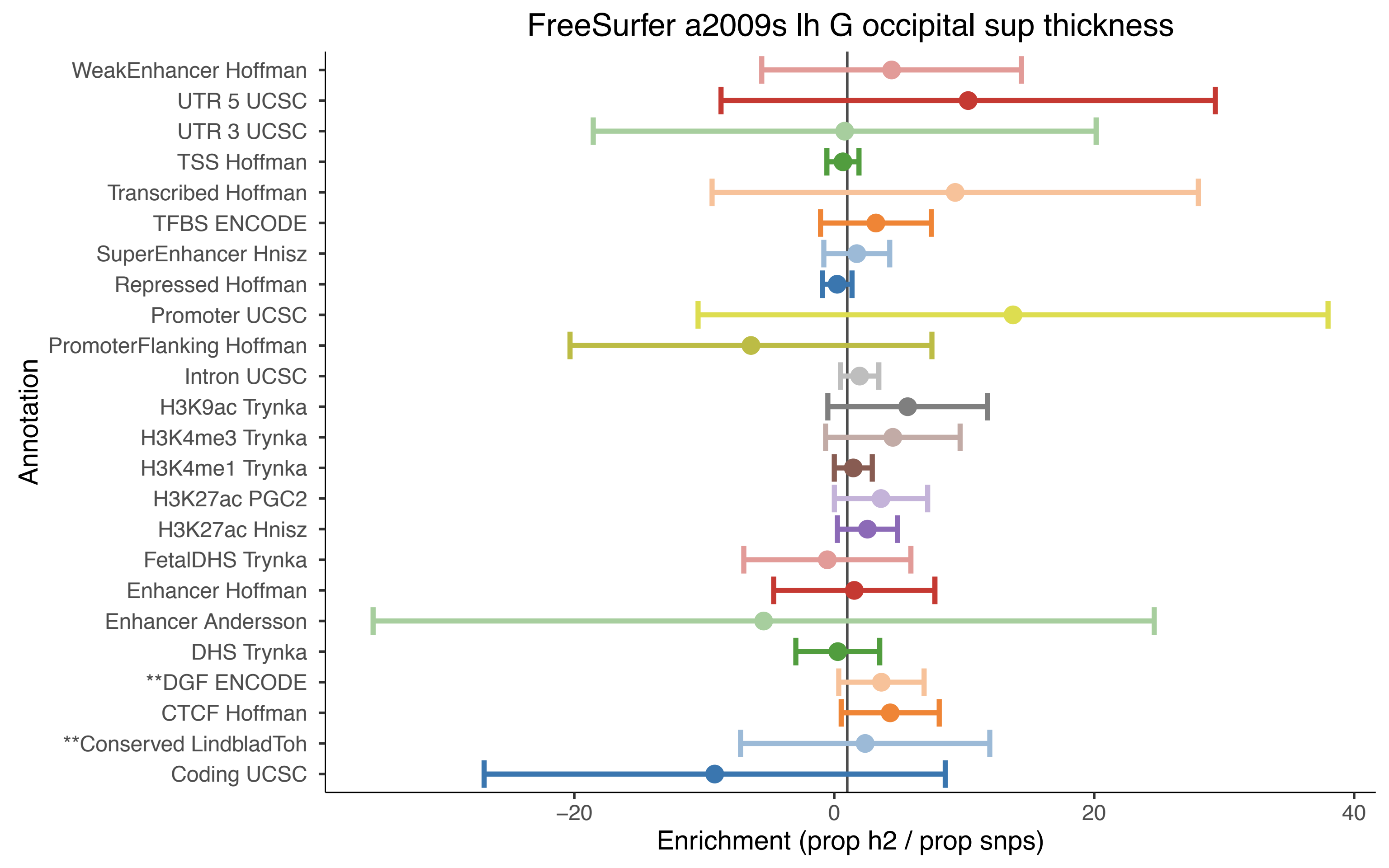
FreeSurfer a2009s lh G occipital sup thickness
WeakEnhancer Hoffman
UTR 5 UCSC
UTR 3 UCSC
TSS Hoffman
Transcribed Hoffman
TFBS ENCODE
SuperEnhancer Hnisz
Repressed Hoffman
Promoter UCSC
PromoterFlanking Hoffman
Intron UCSC
H3K9ac Trynka
H3K4me3 Trynka
H3K4me1 Trynka
H3K27ac PGC2
H3K27ac Hnisz
FetalDHS Trynka
Enhancer Hoffman
Enhancer Andersson
DHS Trynka
**DGF ENCODE
CTCF Hoffman
**Conserved LindbladToh
Coding UCSC
Annotation
−20
0
20
40
Enrichment (prop h2 / prop snps)

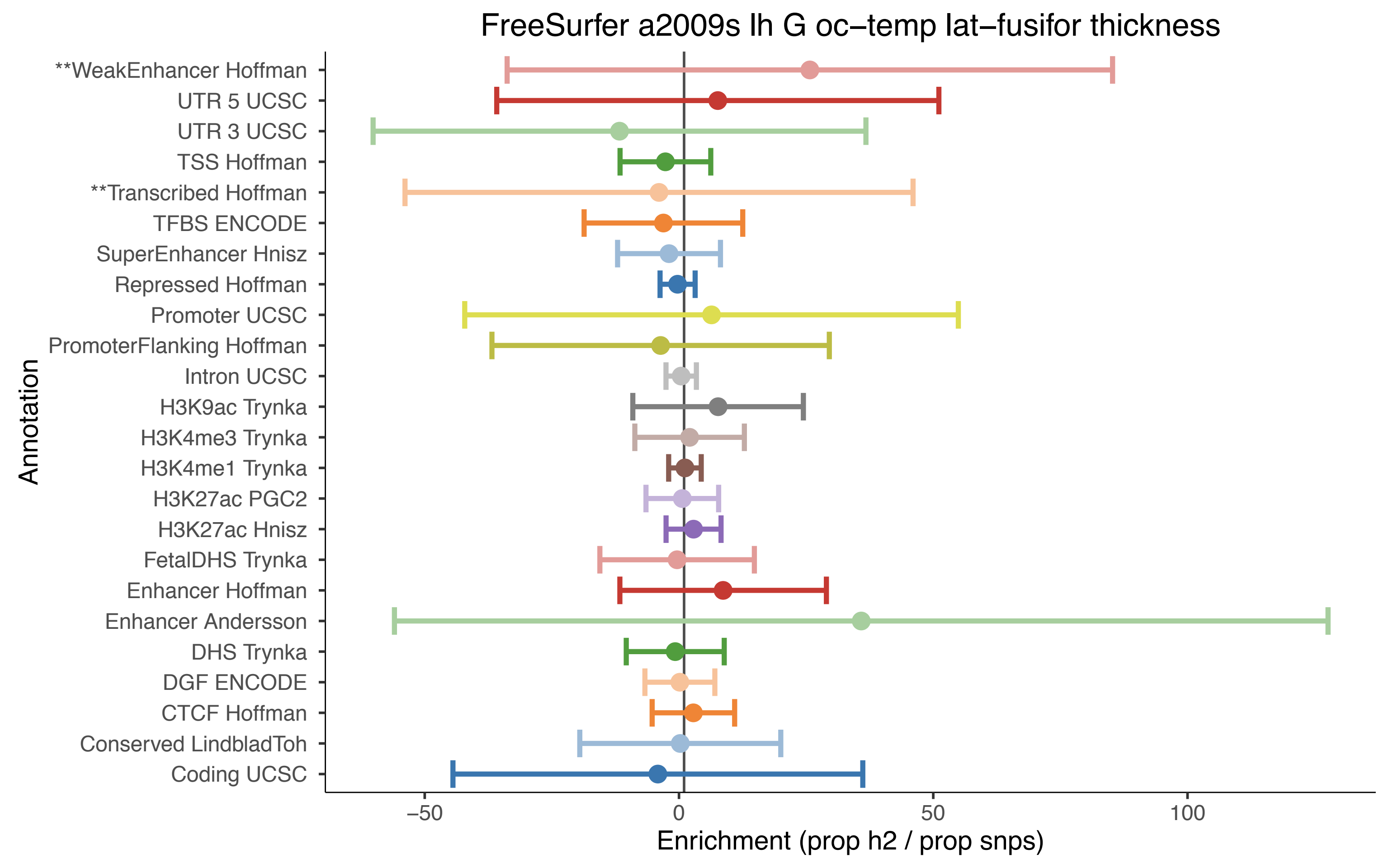
FreeSurfer a2009s lh G oc−temp lat−fusifor thickness
**WeakEnhancer Hoffman
UTR 5 UCSC
UTR 3 UCSC
TSS Hoffman
**Transcribed Hoffman
TFBS ENCODE
SuperEnhancer Hnisz
Repressed Hoffman
Promoter UCSC
PromoterFlanking Hoffman
Intron UCSC
H3K9ac Trynka
H3K4me3 Trynka
H3K4me1 Trynka
H3K27ac PGC2
H3K27ac Hnisz
FetalDHS Trynka
Enhancer Hoffman
Enhancer Andersson
DHS Trynka
DGF ENCODE
CTCF Hoffman
Conserved LindbladToh
Coding UCSC
Annotation
−50
0
50
100
Enrichment (prop h2 / prop snps)

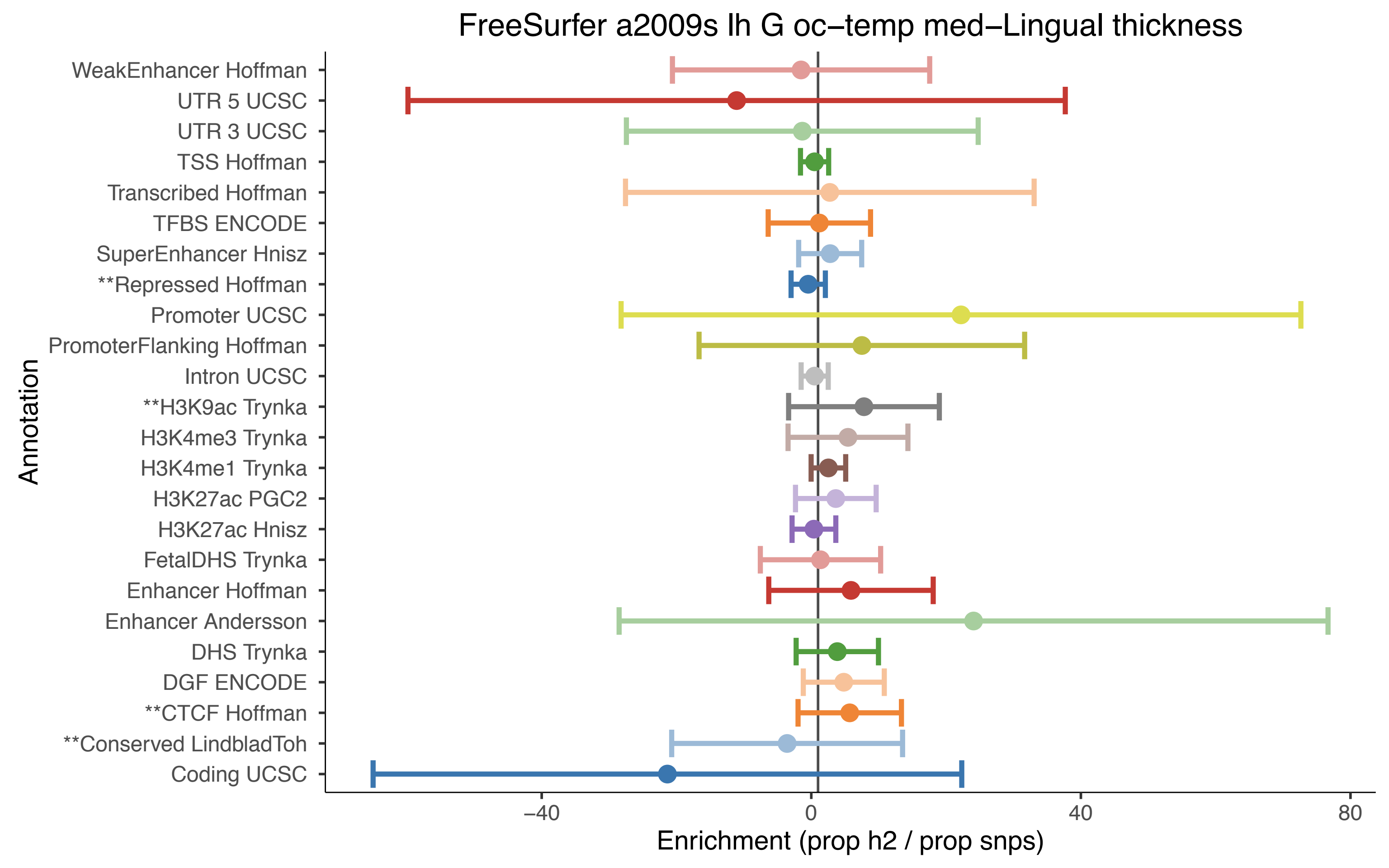
FreeSurfer a2009s lh G oc−temp med−Lingual thickness
WeakEnhancer Hoffman
UTR 5 UCSC
UTR 3 UCSC
TSS Hoffman
Transcribed Hoffman
TFBS ENCODE
SuperEnhancer Hnisz
**Repressed Hoffman
Promoter UCSC
PromoterFlanking Hoffman
Intron UCSC
**H3K9ac Trynka
H3K4me3 Trynka
H3K4me1 Trynka
H3K27ac PGC2
H3K27ac Hnisz
FetalDHS Trynka
Enhancer Hoffman
Enhancer Andersson
DHS Trynka
DGF ENCODE
**CTCF Hoffman
**Conserved LindbladToh
Coding UCSC
Annotation
−40
0
40
80
Enrichment (prop h2 / prop snps)

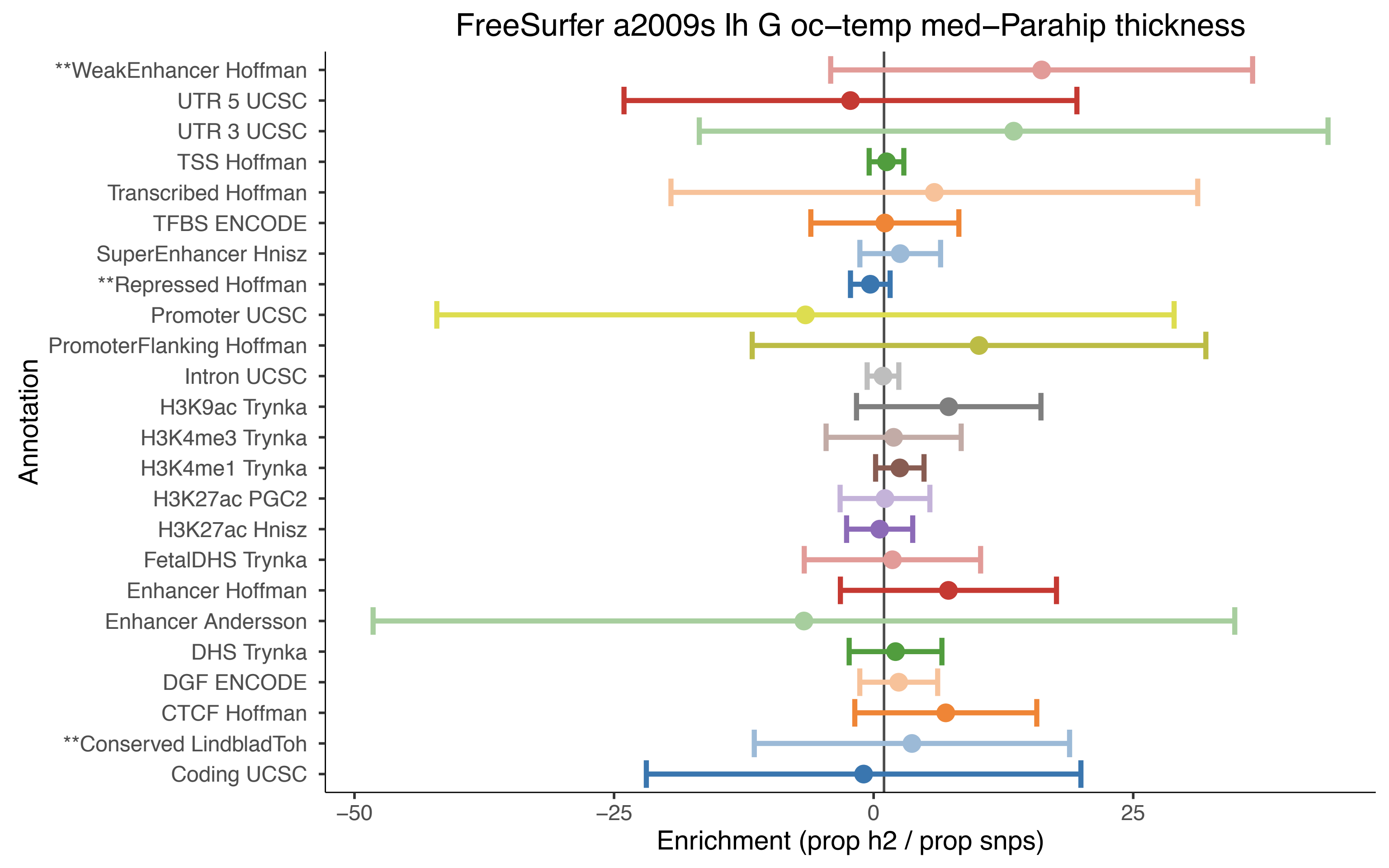
FreeSurfer a2009s lh G oc−temp med−Parahip thickness
Annotation
**WeakEnhancer Hoffman
UTR 5 UCSC
UTR 3 UCSC
TSS Hoffman
Transcribed Hoffman
TFBS ENCODE
SuperEnhancer Hnisz
**Repressed Hoffman
Promoter UCSC
PromoterFlanking Hoffman
Intron UCSC
H3K9ac Trynka
H3K4me3 Trynka
H3K4me1 Trynka
H3K27ac PGC2
H3K27ac Hnisz
FetalDHS Trynka
Enhancer Hoffman
Enhancer Andersson
DHS Trynka
DGF ENCODE
CTCF Hoffman
**Conserved LindbladToh
Coding UCSC
−50
−25
0
25
Enrichment (prop h2 / prop snps)

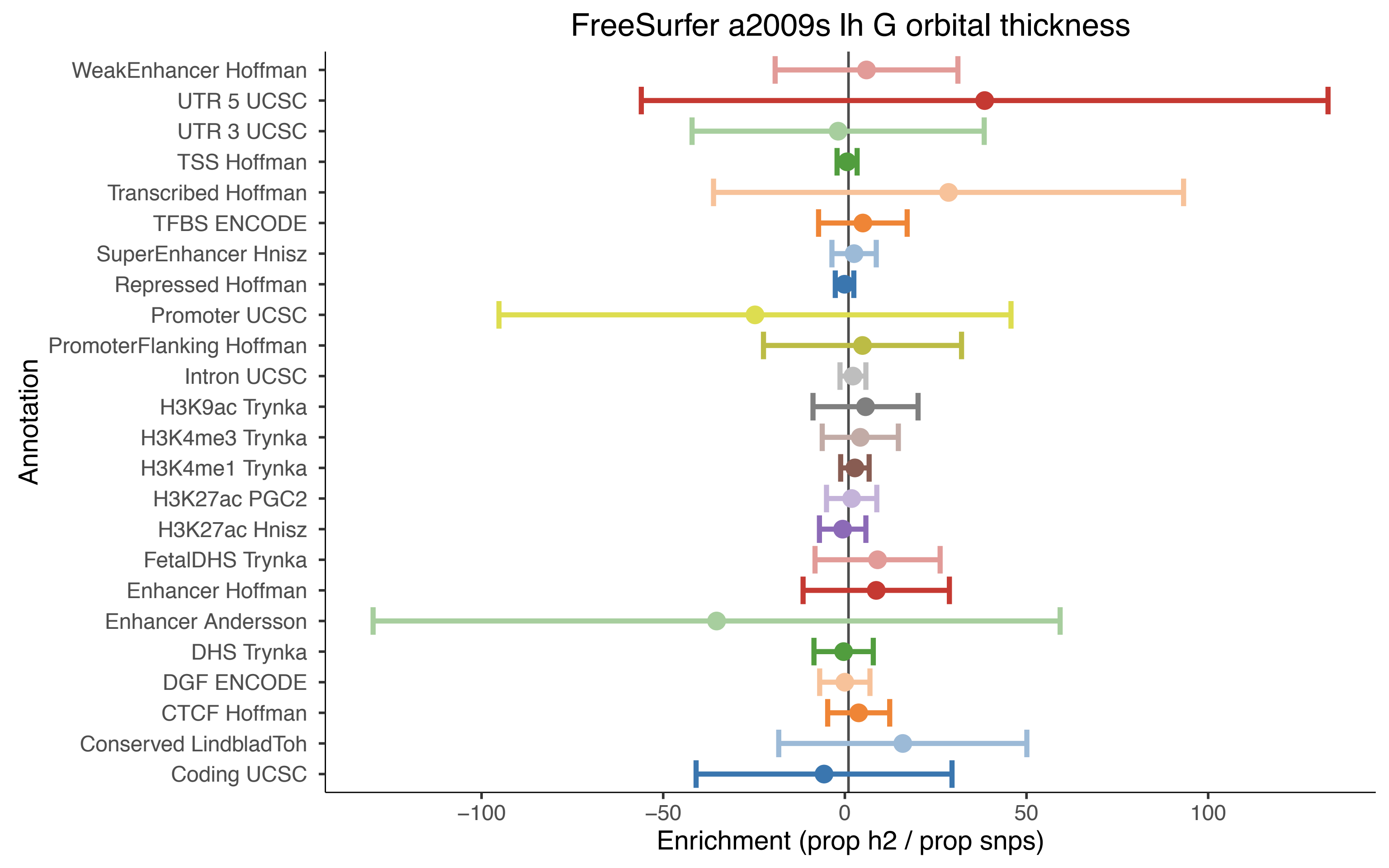
FreeSurfer a2009s lh G orbital thickness
WeakEnhancer Hoffman
UTR 5 UCSC
UTR 3 UCSC
TSS Hoffman
Transcribed Hoffman
TFBS ENCODE
SuperEnhancer Hnisz
Repressed Hoffman
Promoter UCSC
PromoterFlanking Hoffman
Intron UCSC
H3K9ac Trynka
H3K4me3 Trynka
H3K4me1 Trynka
H3K27ac PGC2
H3K27ac Hnisz
FetalDHS Trynka
Enhancer Hoffman
Enhancer Andersson
DHS Trynka
DGF ENCODE
CTCF Hoffman
Conserved LindbladToh
Coding UCSC
Annotation
−100
−50
0
50
100
Enrichment (prop h2 / prop snps)

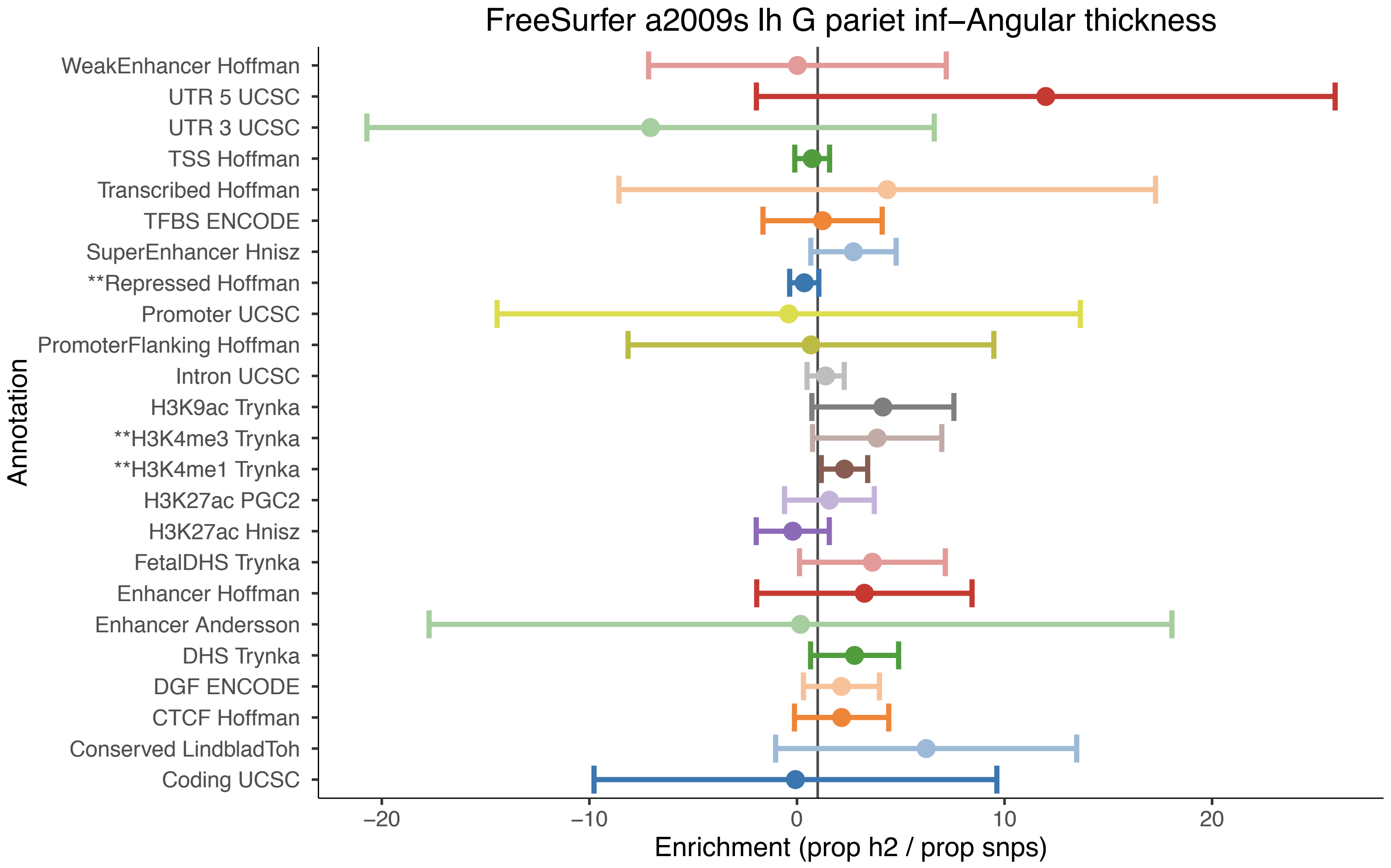
FreeSurfer a2009s lh G pariet inf−Angular thickness
WeakEnhancer Hoffman
UTR 5 UCSC
UTR 3 UCSC
TSS Hoffman
Transcribed Hoffman
TFBS ENCODE
SuperEnhancer Hnisz
**Repressed Hoffman
Promoter UCSC
PromoterFlanking Hoffman
Intron UCSC
H3K9ac Trynka
**H3K4me3 Trynka
**H3K4me1 Trynka
H3K27ac PGC2
H3K27ac Hnisz
FetalDHS Trynka
Enhancer Hoffman
Enhancer Andersson
DHS Trynka
DGF ENCODE
CTCF Hoffman
Conserved LindbladToh
Coding UCSC
Annotation
−20
−10
0
10
20
Enrichment (prop h2 / prop snps)

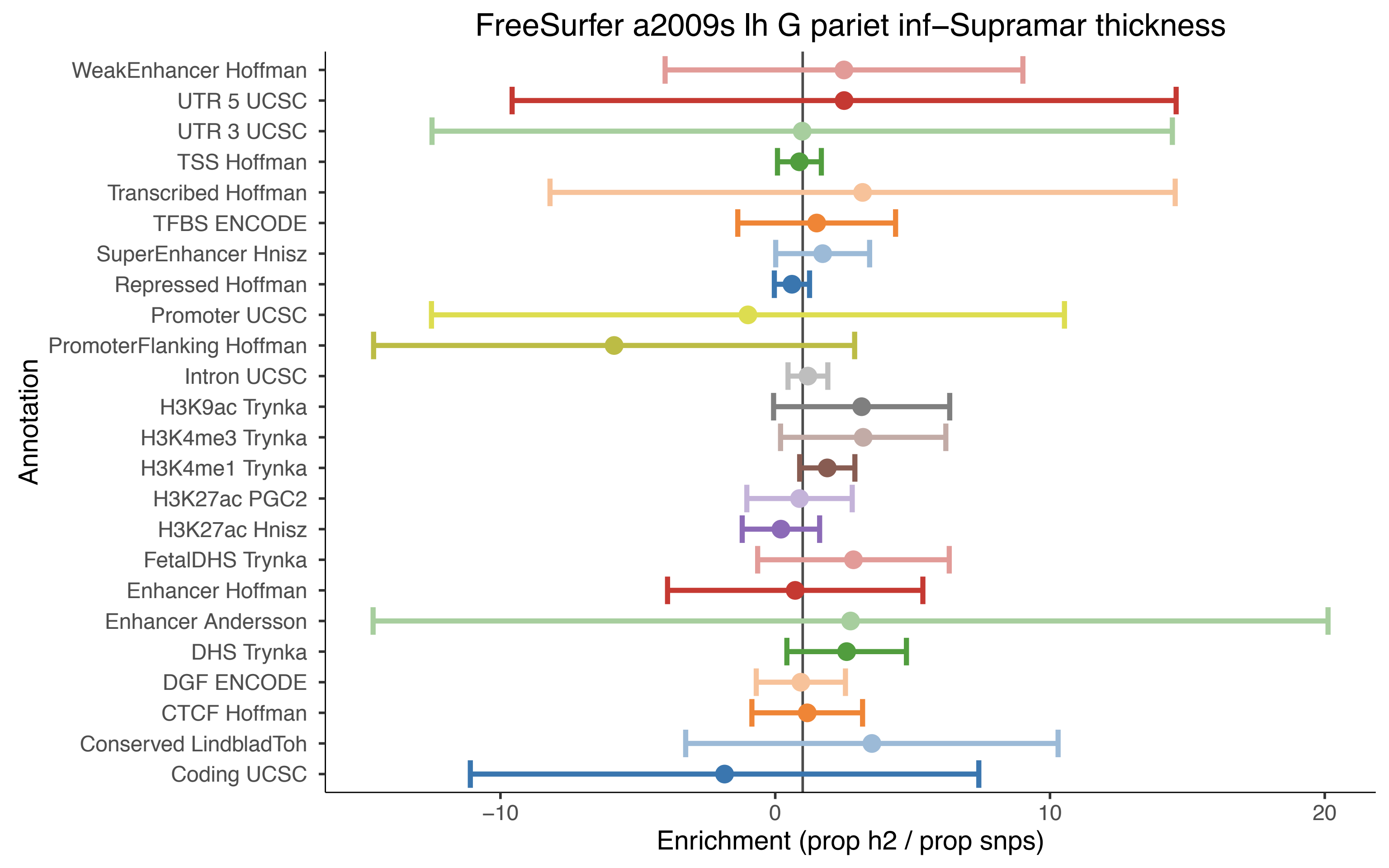

FreeSurfer a2009s lh G pariet inf−Supramar thickness
WeakEnhancer Hoffman
UTR 5 UCSC
UTR 3 UCSC
TSS Hoffman
Transcribed Hoffman
TFBS ENCODE
SuperEnhancer Hnisz
Repressed Hoffman
Promoter UCSC
PromoterFlanking Hoffman
Intron UCSC
H3K9ac Trynka
H3K4me3 Trynka
H3K4me1 Trynka
H3K27ac PGC2
H3K27ac Hnisz
FetalDHS Trynka
Enhancer Hoffman
Enhancer Andersson
DHS Trynka
DGF ENCODE
CTCF Hoffman
Conserved LindbladToh
Coding UCSC
Annotation
−10
0
10
20
Enrichment (prop h2 / prop snps)

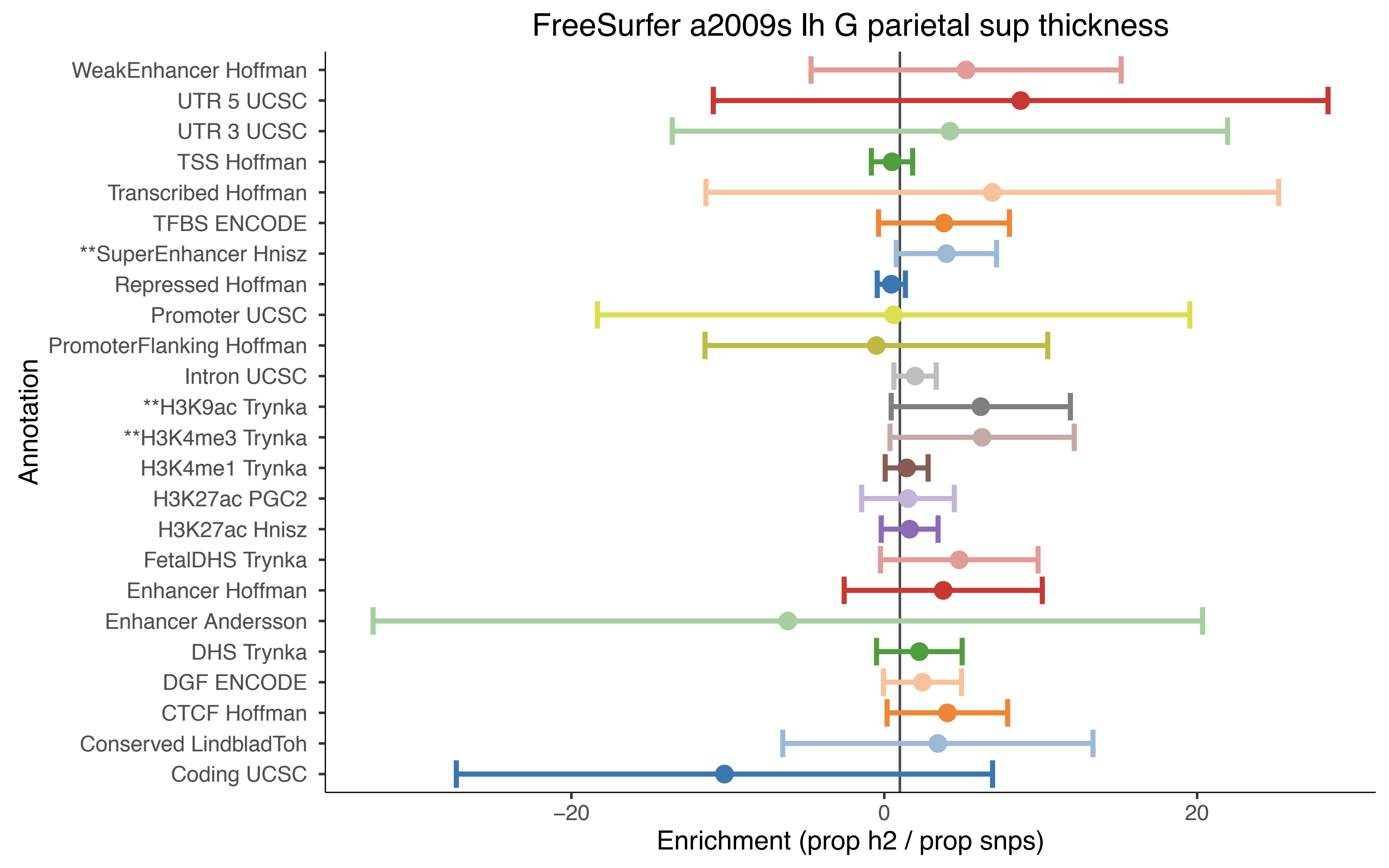
FreeSurfer a2009s lh G parietal sup thickness
WeakEnhancer Hoffman
UTR 5 UCSC
UTR 3 UCSC
TSS Hoffman
Transcribed Hoffman
TFBS ENCODE
**SuperEnhancer Hnisz
Repressed Hoffman
Promoter UCSC
PromoterFlanking Hoffman
Intron UCSC
**H3K9ac Trynka
**H3K4me3 Trynka
H3K4me1 Trynka
H3K27ac PGC2
H3K27ac Hnisz
FetalDHS Trynka
Enhancer Hoffman
Enhancer Andersson
DHS Trynka
DGF ENCODE
CTCF Hoffman
Conserved LindbladToh
Coding UCSC
Annotation
−20
0
20
Enrichment (prop h2 / prop snps)

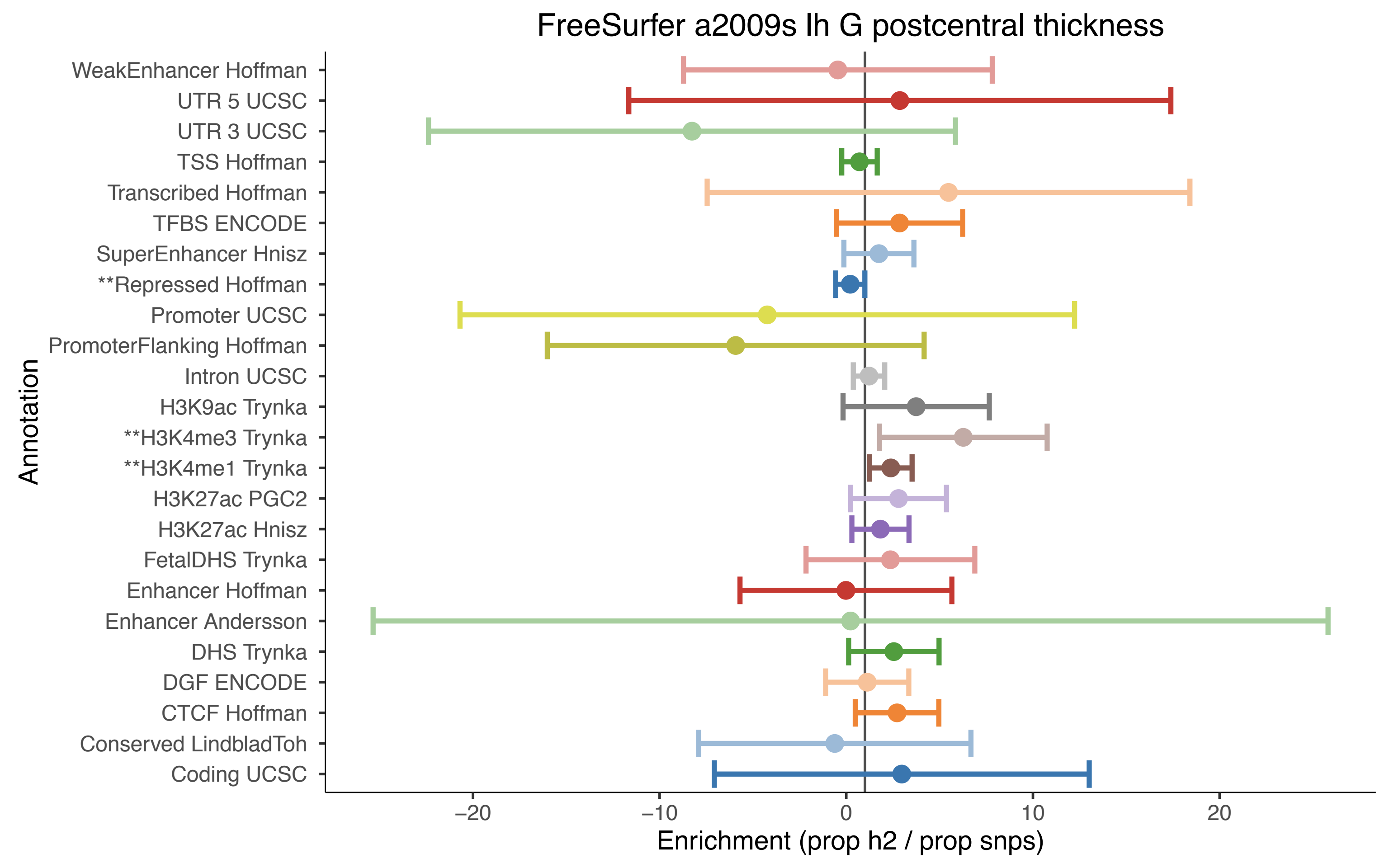
FreeSurfer a2009s lh G postcentral thickness
WeakEnhancer Hoffman
UTR 5 UCSC
UTR 3 UCSC
TSS Hoffman
Transcribed Hoffman
TFBS ENCODE
SuperEnhancer Hnisz
**Repressed Hoffman
Promoter UCSC
PromoterFlanking Hoffman
Intron UCSC
H3K9ac Trynka
**H3K4me3 Trynka
**H3K4me1 Trynka
H3K27ac PGC2
H3K27ac Hnisz
FetalDHS Trynka
Enhancer Hoffman
Enhancer Andersson
DHS Trynka
DGF ENCODE
CTCF Hoffman
Conserved LindbladToh
Coding UCSC
Annotation
−20
−10
0
10
20
Enrichment (prop h2 / prop snps)

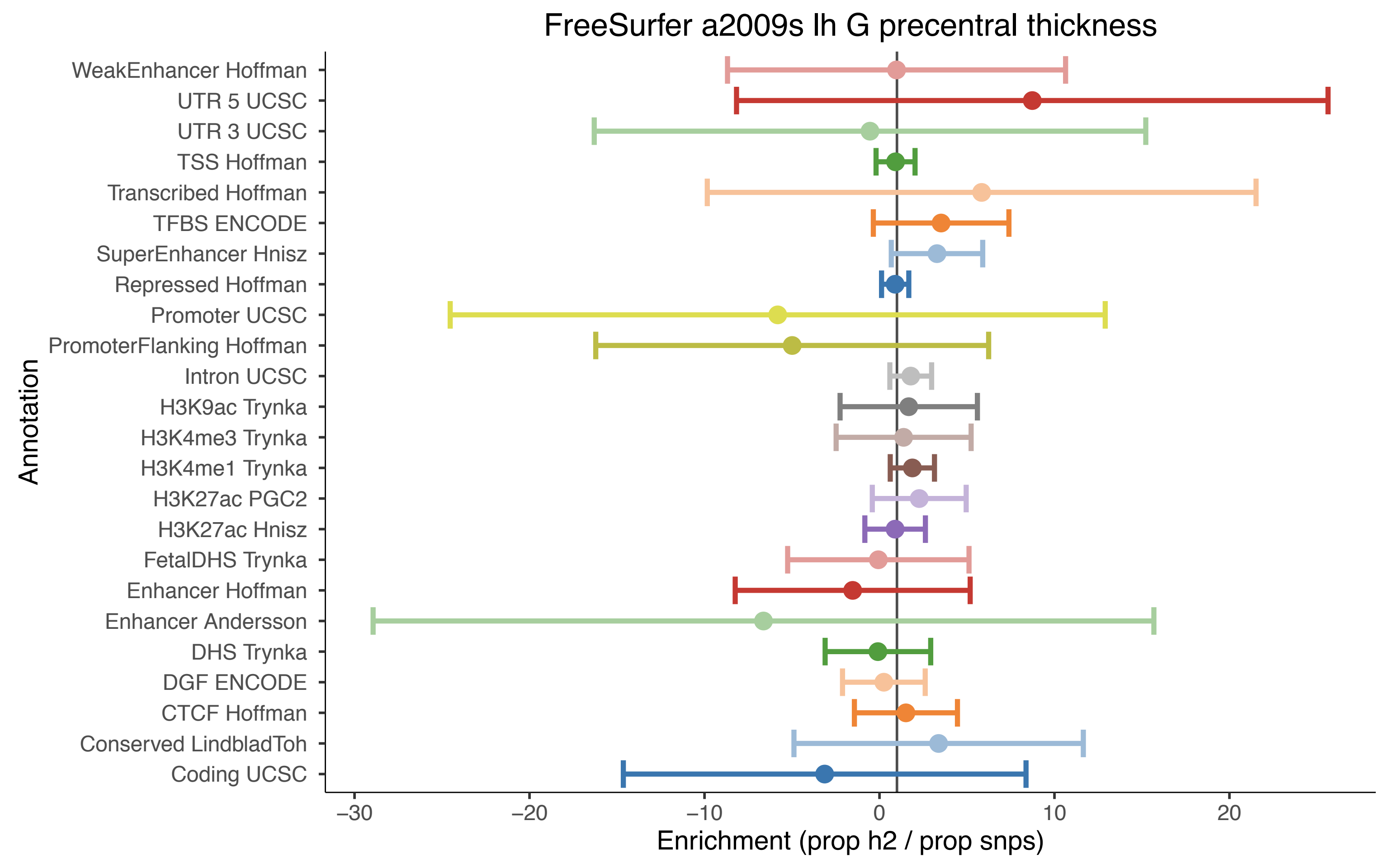

FreeSurfer a2009s lh G precentral thickness
WeakEnhancer Hoffman
UTR 5 UCSC
UTR 3 UCSC
TSS Hoffman
Transcribed Hoffman
TFBS ENCODE
SuperEnhancer Hnisz
Repressed Hoffman
Promoter UCSC
PromoterFlanking Hoffman
Intron UCSC
H3K9ac Trynka
H3K4me3 Trynka
H3K4me1 Trynka
H3K27ac PGC2
H3K27ac Hnisz
FetalDHS Trynka
Enhancer Hoffman
Enhancer Andersson
DHS Trynka
DGF ENCODE
CTCF Hoffman
Conserved LindbladToh
Coding UCSC
Annotation
−30
−20
−10
0
10
20
Enrichment (prop h2 / prop snps)

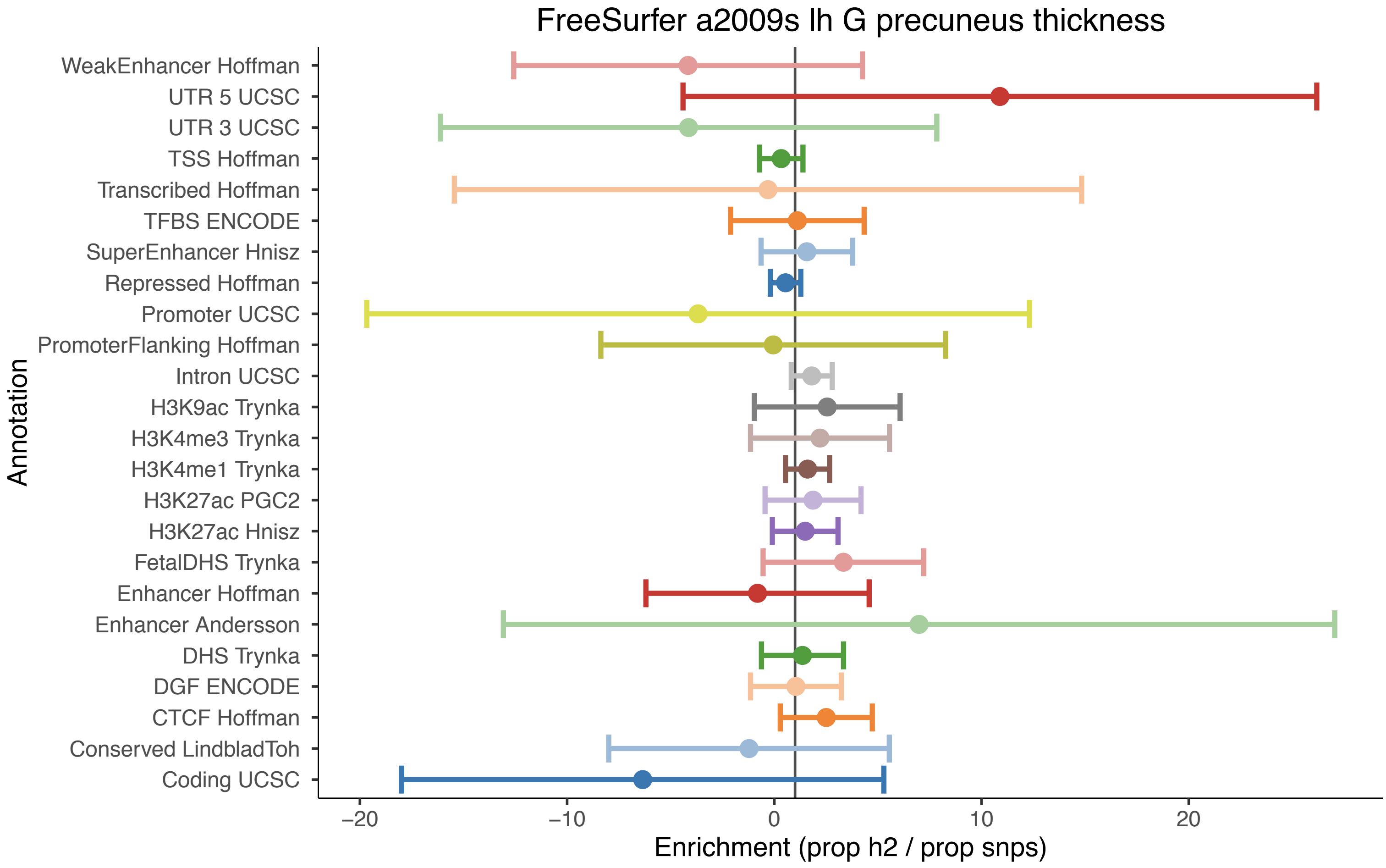
FreeSurfer a2009s lh G precuneus thickness
WeakEnhancer Hoffman
UTR 5 UCSC
UTR 3 UCSC
TSS Hoffman
Transcribed Hoffman
TFBS ENCODE
SuperEnhancer Hnisz
Repressed Hoffman
Promoter UCSC
PromoterFlanking Hoffman
Intron UCSC
H3K9ac Trynka
H3K4me3 Trynka
H3K4me1 Trynka
H3K27ac PGC2
H3K27ac Hnisz
FetalDHS Trynka
Enhancer Hoffman
Enhancer Andersson
DHS Trynka
DGF ENCODE
CTCF Hoffman
Conserved LindbladToh
Coding UCSC
Annotation
−20
−10
0
10
20
Enrichment (prop h2 / prop snps)

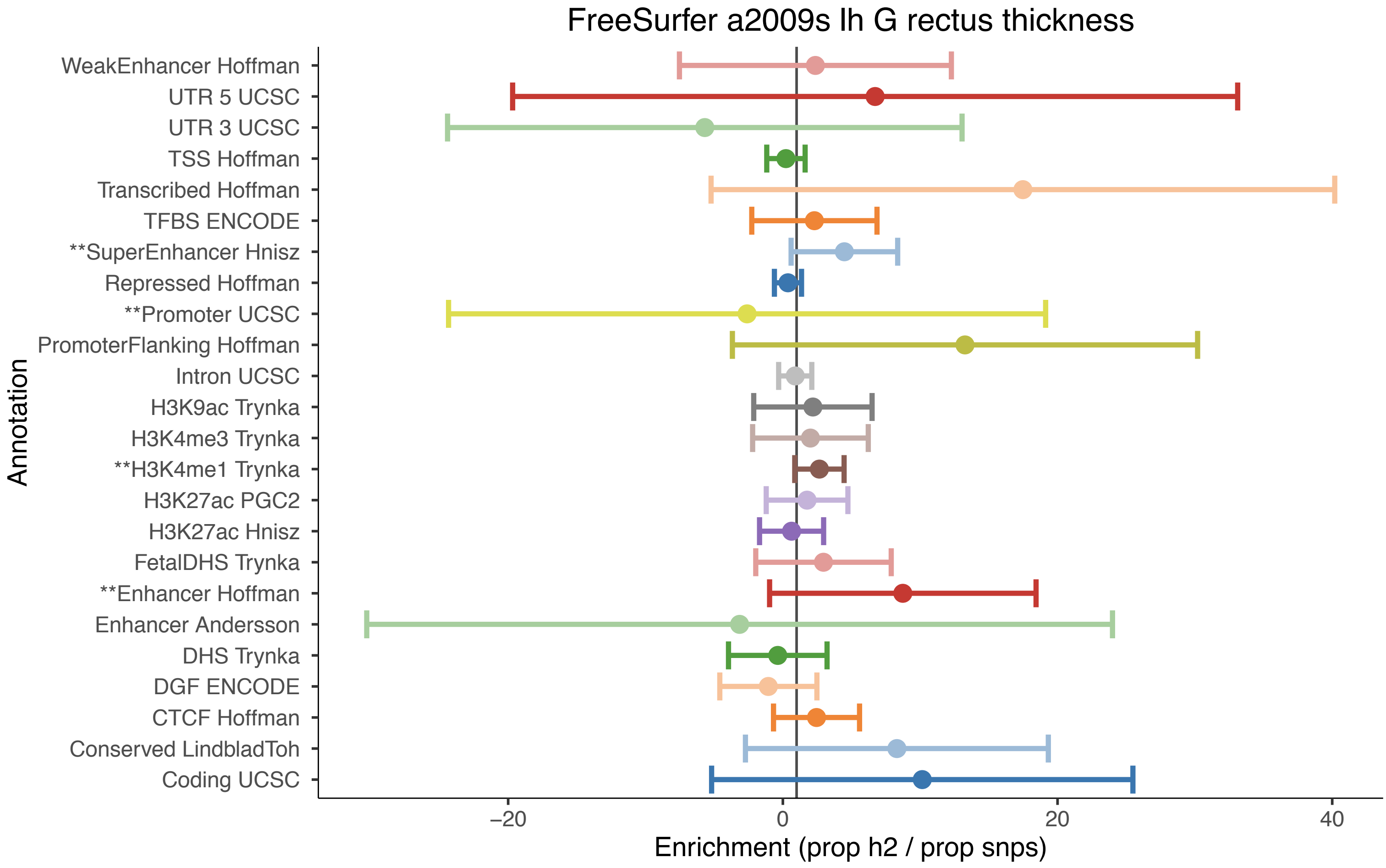
FreeSurfer a2009s lh G rectus thickness
WeakEnhancer Hoffman
UTR 5 UCSC
UTR 3 UCSC
TSS Hoffman
Transcribed Hoffman
TFBS ENCODE
**SuperEnhancer Hnisz
Repressed Hoffman
**Promoter UCSC
PromoterFlanking Hoffman
Intron UCSC
H3K9ac Trynka
H3K4me3 Trynka
**H3K4me1 Trynka
H3K27ac PGC2
H3K27ac Hnisz
FetalDHS Trynka
**Enhancer Hoffman
Enhancer Andersson
DHS Trynka
DGF ENCODE
CTCF Hoffman
Conserved LindbladToh
Coding UCSC
Annotation
−20
0
20
40
Enrichment (prop h2 / prop snps)

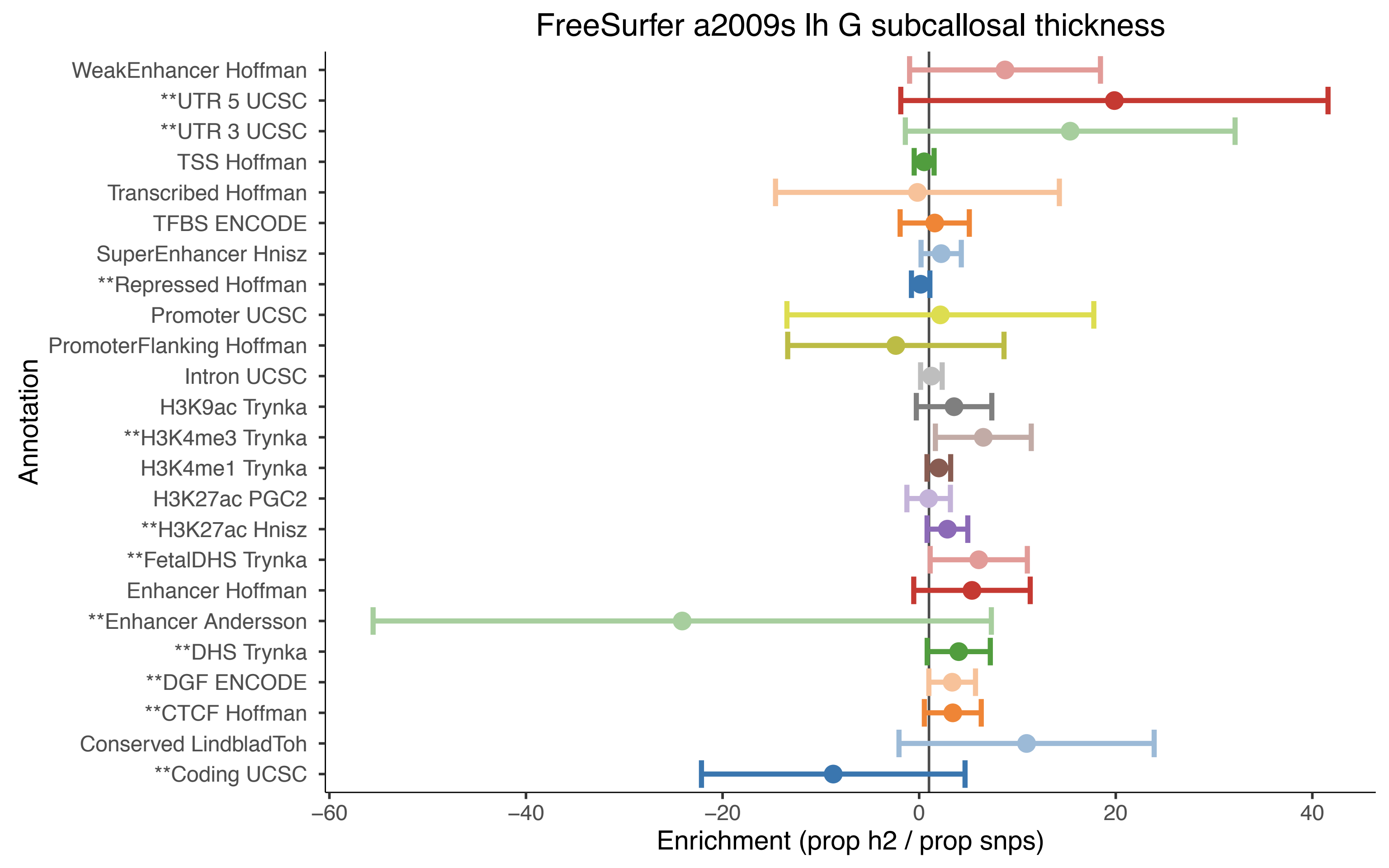
FreeSurfer a2009s lh G subcallosal thickness
WeakEnhancer Hoffman
**UTR 5 UCSC
**UTR 3 UCSC
TSS Hoffman
Transcribed Hoffman
TFBS ENCODE
SuperEnhancer Hnisz
**Repressed Hoffman
Promoter UCSC
PromoterFlanking Hoffman
Intron UCSC
H3K9ac Trynka
**H3K4me3 Trynka
H3K4me1 Trynka
H3K27ac PGC2
**H3K27ac Hnisz
**FetalDHS Trynka
Enhancer Hoffman
**Enhancer Andersson
**DHS Trynka
**DGF ENCODE
**CTCF Hoffman
Conserved LindbladToh
**Coding UCSC
Annotation
−60
−40
−20
0
20
40
Enrichment (prop h2 / prop snps)

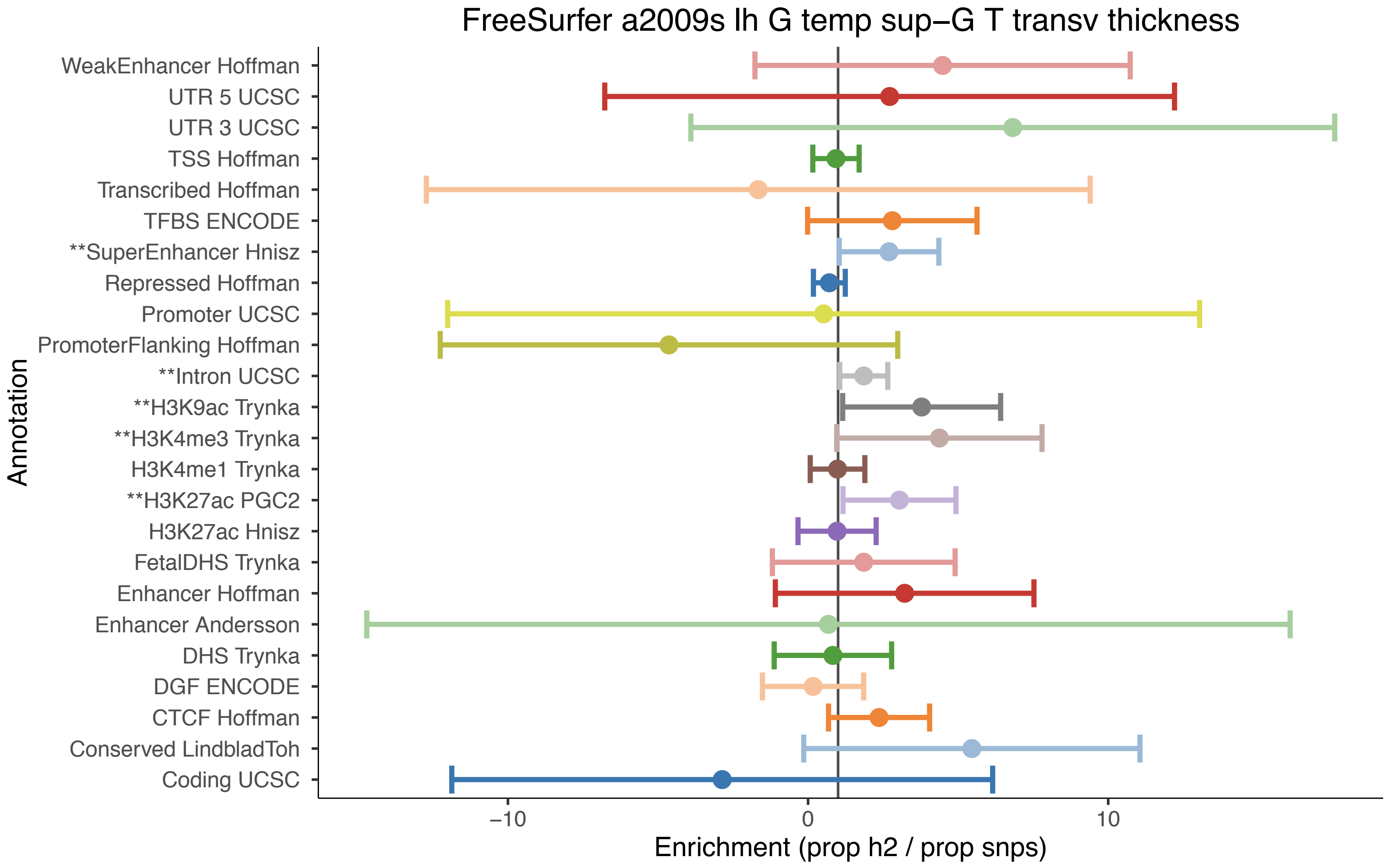
FreeSurfer a2009s lh G temp sup−G T transv thickness
WeakEnhancer Hoffman
UTR 5 UCSC
UTR 3 UCSC
TSS Hoffman
Transcribed Hoffman
TFBS ENCODE
**SuperEnhancer Hnisz
Repressed Hoffman
Promoter UCSC
PromoterFlanking Hoffman
**Intron UCSC
**H3K9ac Trynka
**H3K4me3 Trynka
H3K4me1 Trynka
**H3K27ac PGC2
H3K27ac Hnisz
FetalDHS Trynka
Enhancer Hoffman
Enhancer Andersson
DHS Trynka
DGF ENCODE
CTCF Hoffman
Conserved LindbladToh
Coding UCSC
Annotation
−10
0
10
Enrichment (prop h2 / prop snps)

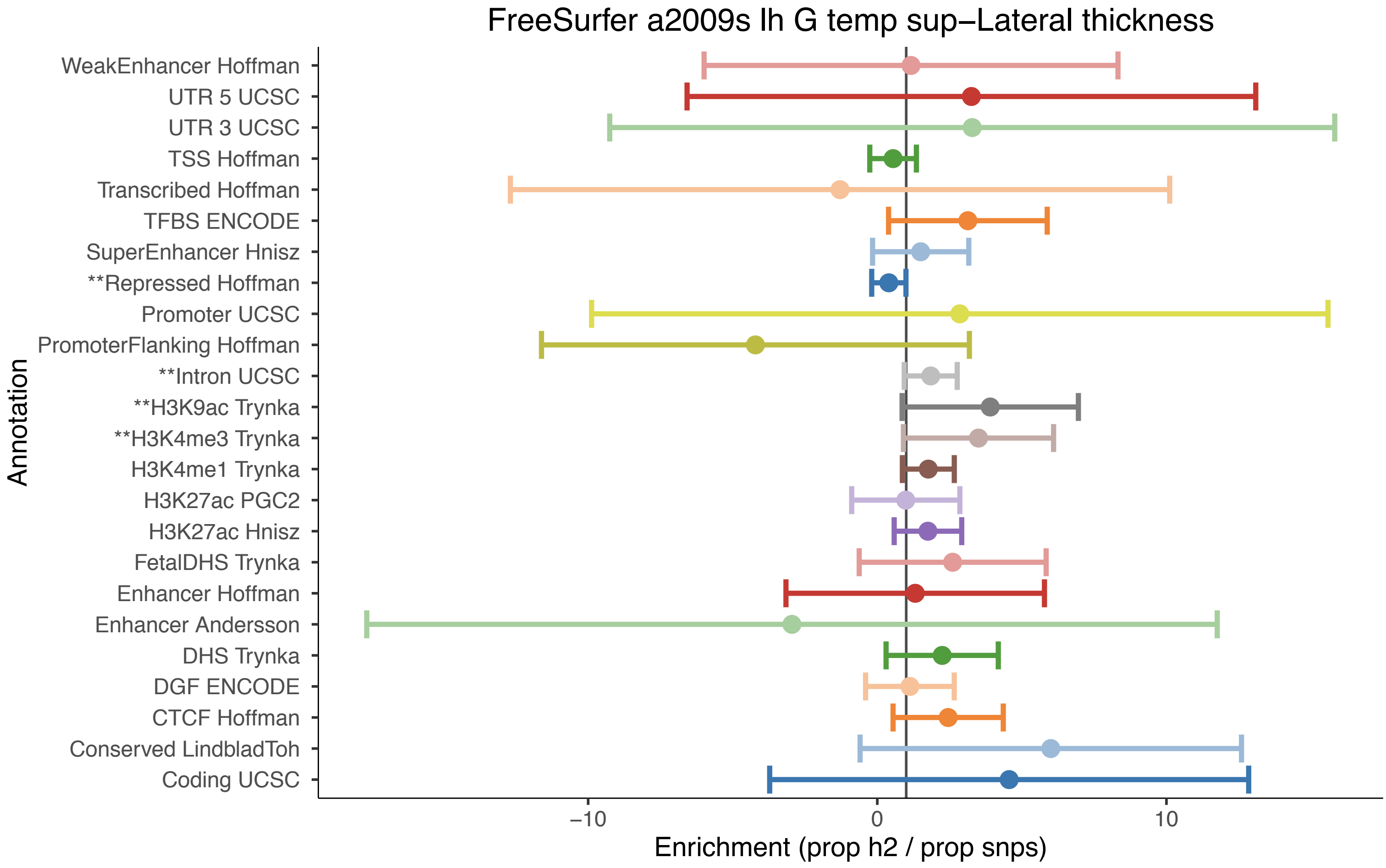

FreeSurfer a2009s lh G temp sup−Lateral thickness
WeakEnhancer Hoffman
UTR 5 UCSC
UTR 3 UCSC
TSS Hoffman
Transcribed Hoffman
TFBS ENCODE
SuperEnhancer Hnisz
**Repressed Hoffman
Promoter UCSC
PromoterFlanking Hoffman
**Intron UCSC
**H3K9ac Trynka
**H3K4me3 Trynka
H3K4me1 Trynka
H3K27ac PGC2
H3K27ac Hnisz
FetalDHS Trynka
Enhancer Hoffman
Enhancer Andersson
DHS Trynka
DGF ENCODE
CTCF Hoffman
Conserved LindbladToh
Coding UCSC
Annotation
−10
0
10
Enrichment (prop h2 / prop snps)

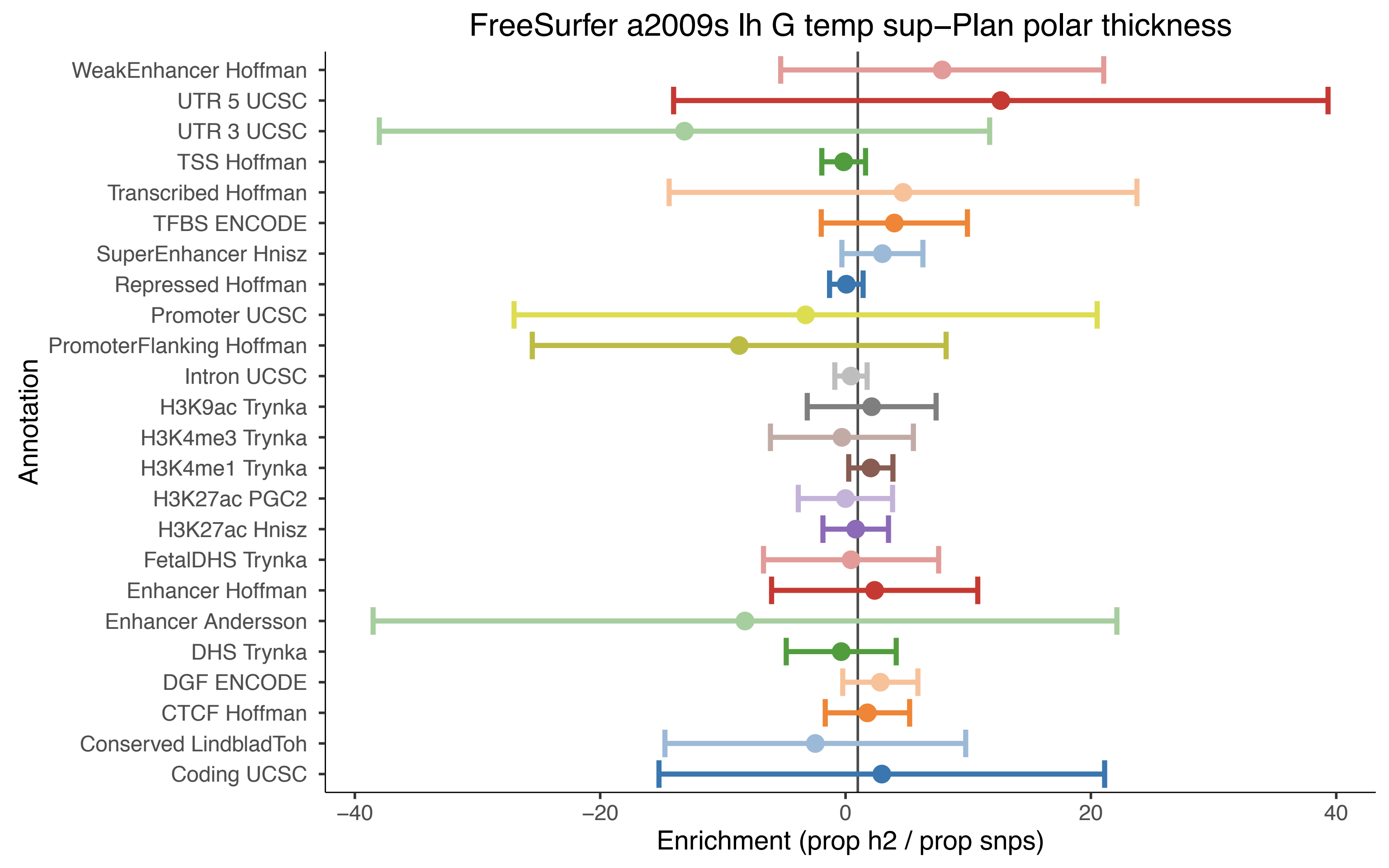
FreeSurfer a2009s lh G temp sup−Plan polar thickness
WeakEnhancer Hoffman
UTR 5 UCSC
UTR 3 UCSC
TSS Hoffman
Transcribed Hoffman
TFBS ENCODE
SuperEnhancer Hnisz
Repressed Hoffman
Promoter UCSC
PromoterFlanking Hoffman
Intron UCSC
H3K9ac Trynka
H3K4me3 Trynka
H3K4me1 Trynka
H3K27ac PGC2
H3K27ac Hnisz
FetalDHS Trynka
Enhancer Hoffman
Enhancer Andersson
DHS Trynka
DGF ENCODE
CTCF Hoffman
Conserved LindbladToh
Coding UCSC
Annotation
−40
−20
0
20
40
Enrichment (prop h2 / prop snps)

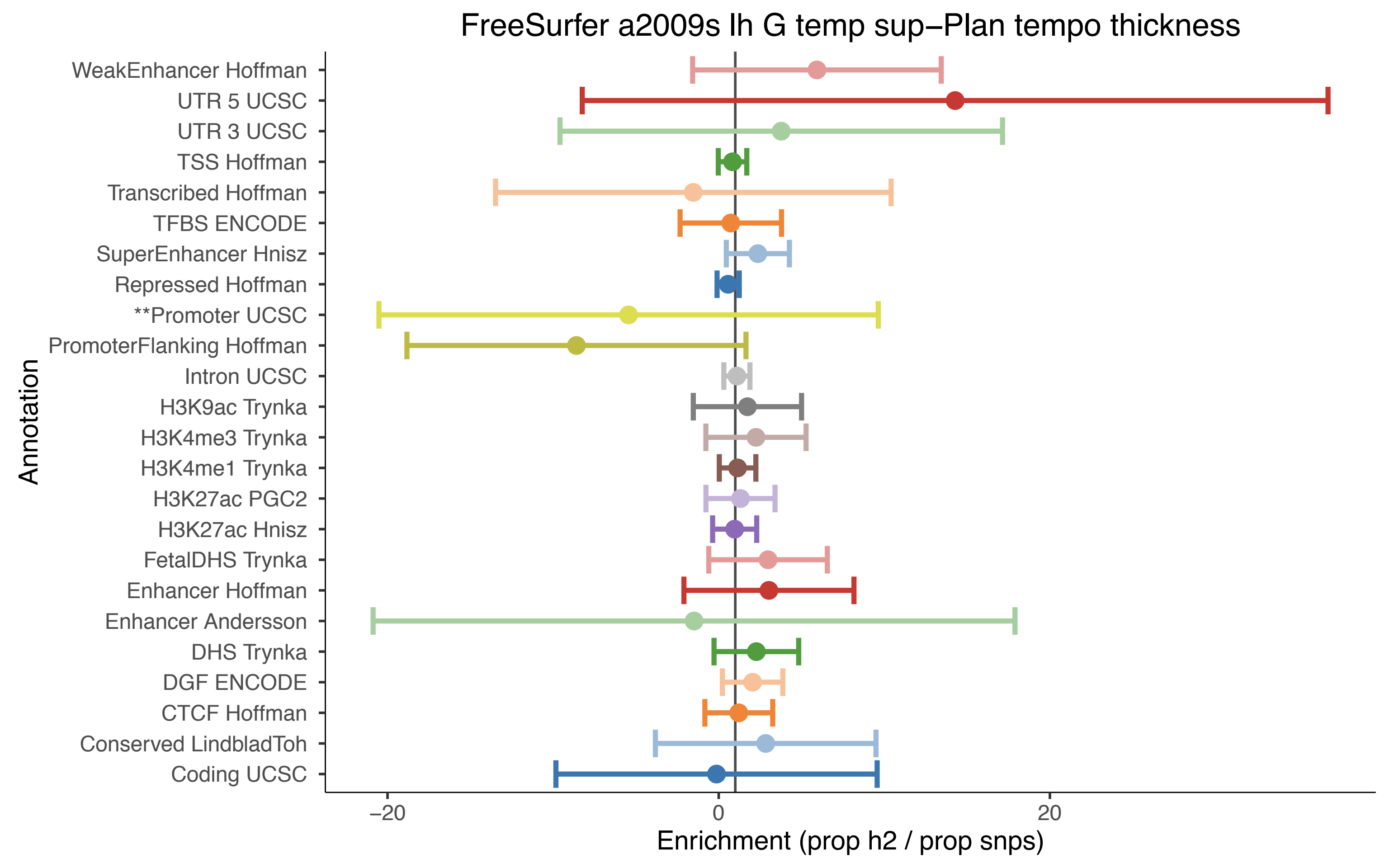

FreeSurfer a2009s lh G temp sup−Plan tempo thickness
WeakEnhancer Hoffman
UTR 5 UCSC
UTR 3 UCSC
TSS Hoffman
Transcribed Hoffman
TFBS ENCODE
SuperEnhancer Hnisz
Repressed Hoffman
**Promoter UCSC
PromoterFlanking Hoffman
Intron UCSC
H3K9ac Trynka
H3K4me3 Trynka
H3K4me1 Trynka
H3K27ac PGC2
H3K27ac Hnisz
FetalDHS Trynka
Enhancer Hoffman
Enhancer Andersson
DHS Trynka
DGF ENCODE
CTCF Hoffman
Conserved LindbladToh
Coding UCSC
Annotation
−20
0
20
Enrichment (prop h2 / prop snps)

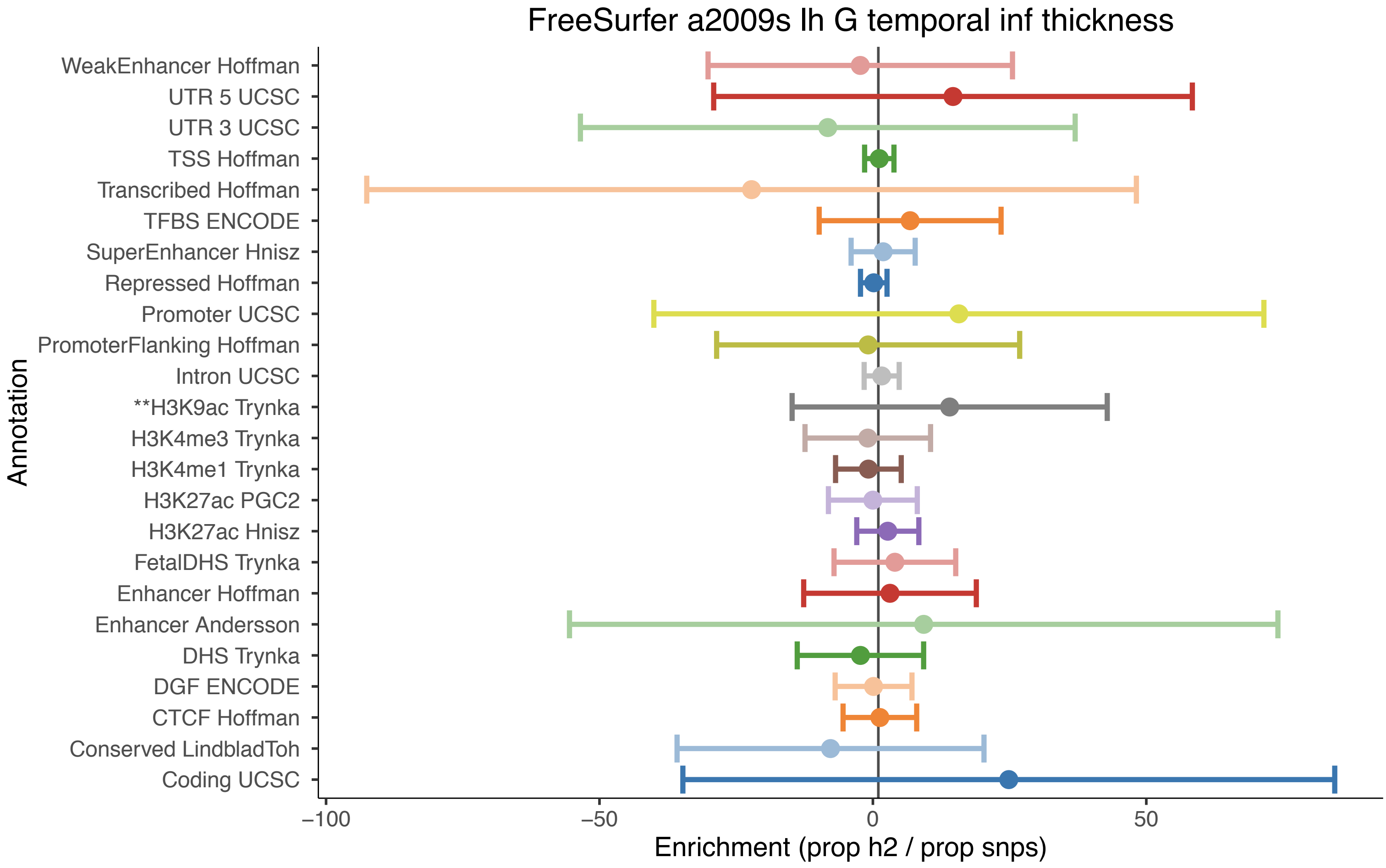
FreeSurfer a2009s lh G temporal inf thickness
WeakEnhancer Hoffman
UTR 5 UCSC
UTR 3 UCSC
TSS Hoffman
Transcribed Hoffman
TFBS ENCODE
SuperEnhancer Hnisz
Repressed Hoffman
Promoter UCSC
PromoterFlanking Hoffman
Intron UCSC
**H3K9ac Trynka
H3K4me3 Trynka
H3K4me1 Trynka
H3K27ac PGC2
H3K27ac Hnisz
FetalDHS Trynka
Enhancer Hoffman
Enhancer Andersson
DHS Trynka
DGF ENCODE
CTCF Hoffman
Conserved LindbladToh
Coding UCSC
Annotation
−100
−50
0
50
Enrichment (prop h2 / prop snps)

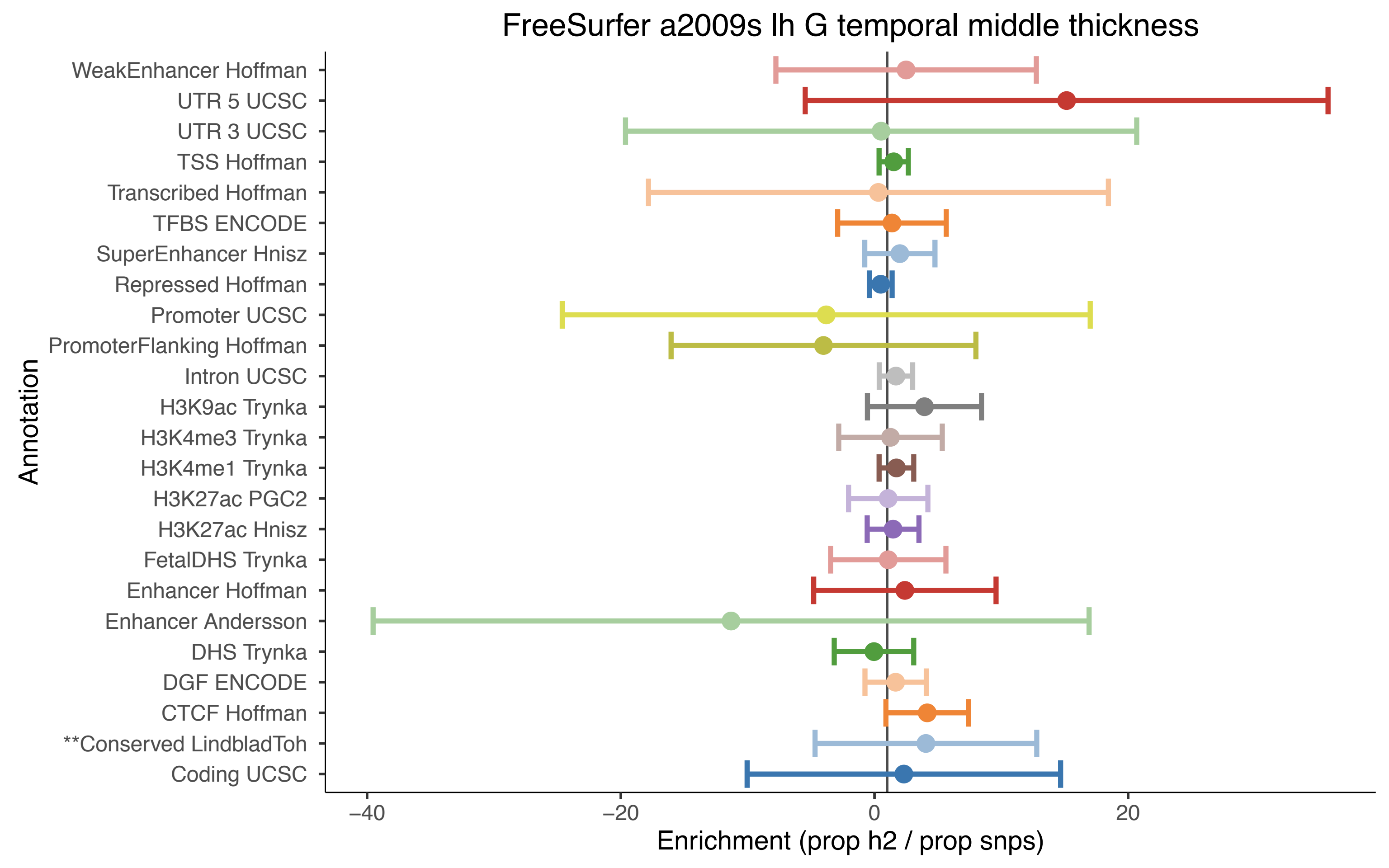

FreeSurfer a2009s lh G temporal middle thickness
WeakEnhancer Hoffman
UTR 5 UCSC
UTR 3 UCSC
TSS Hoffman
Transcribed Hoffman
TFBS ENCODE
SuperEnhancer Hnisz
Repressed Hoffman
Promoter UCSC
PromoterFlanking Hoffman
Intron UCSC
H3K9ac Trynka
H3K4me3 Trynka
H3K4me1 Trynka
H3K27ac PGC2
H3K27ac Hnisz
FetalDHS Trynka
Enhancer Hoffman
Enhancer Andersson
DHS Trynka
DGF ENCODE
CTCF Hoffman
**Conserved LindbladToh
Coding UCSC
Annotation
−40
−20
0
20
Enrichment (prop h2 / prop snps)

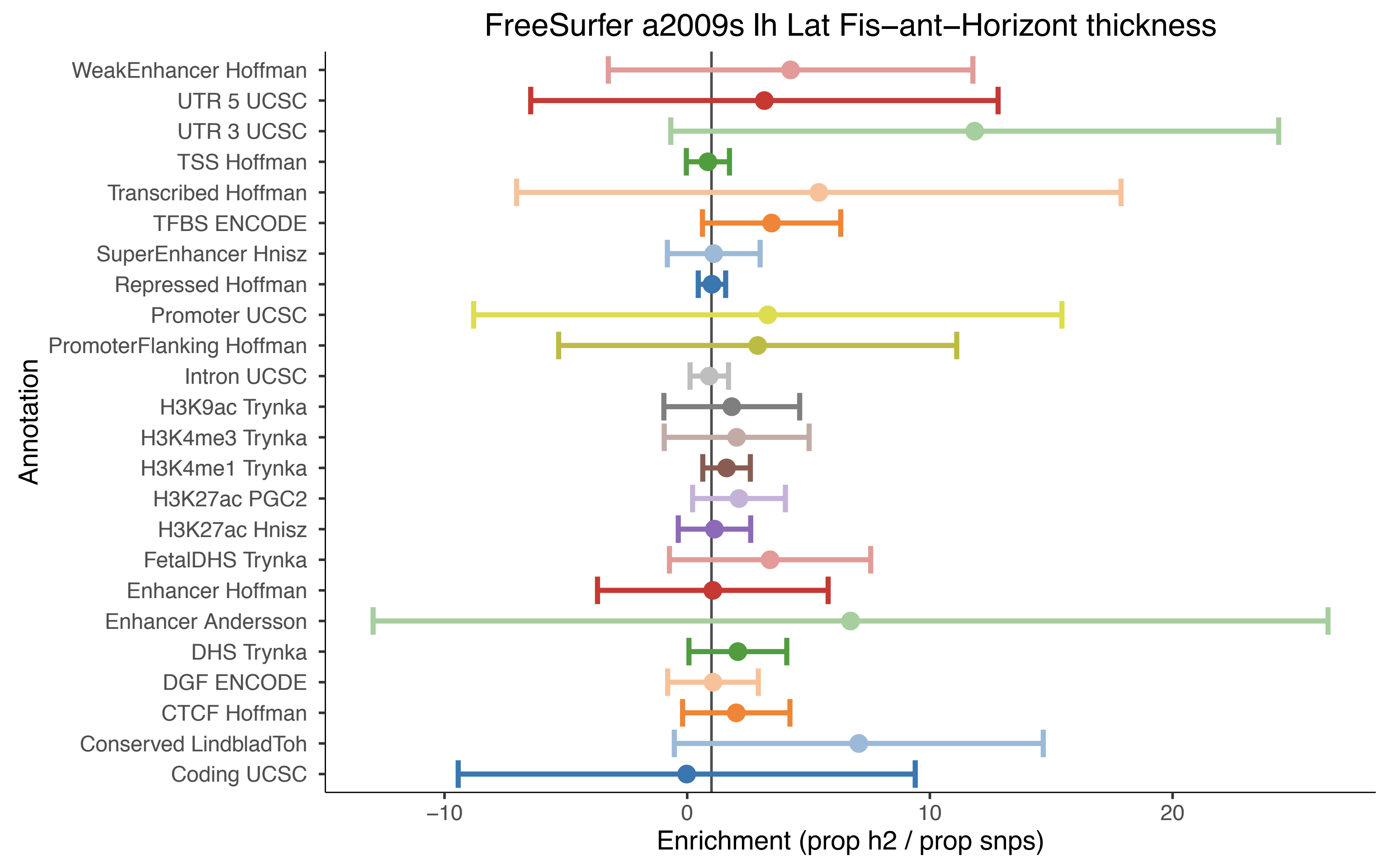
FreeSurfer a2009s lh Lat Fis−ant−Horizont thickness
WeakEnhancer Hoffman
UTR 5 UCSC
UTR 3 UCSC
TSS Hoffman
Transcribed Hoffman
TFBS ENCODE
SuperEnhancer Hnisz
Repressed Hoffman
Promoter UCSC
PromoterFlanking Hoffman
Intron UCSC
H3K9ac Trynka
H3K4me3 Trynka
H3K4me1 Trynka
H3K27ac PGC2
H3K27ac Hnisz
FetalDHS Trynka
Enhancer Hoffman
Enhancer Andersson
DHS Trynka
DGF ENCODE
CTCF Hoffman
Conserved LindbladToh
Coding UCSC
Annotation
−10
0
10
20
Enrichment (prop h2 / prop snps)

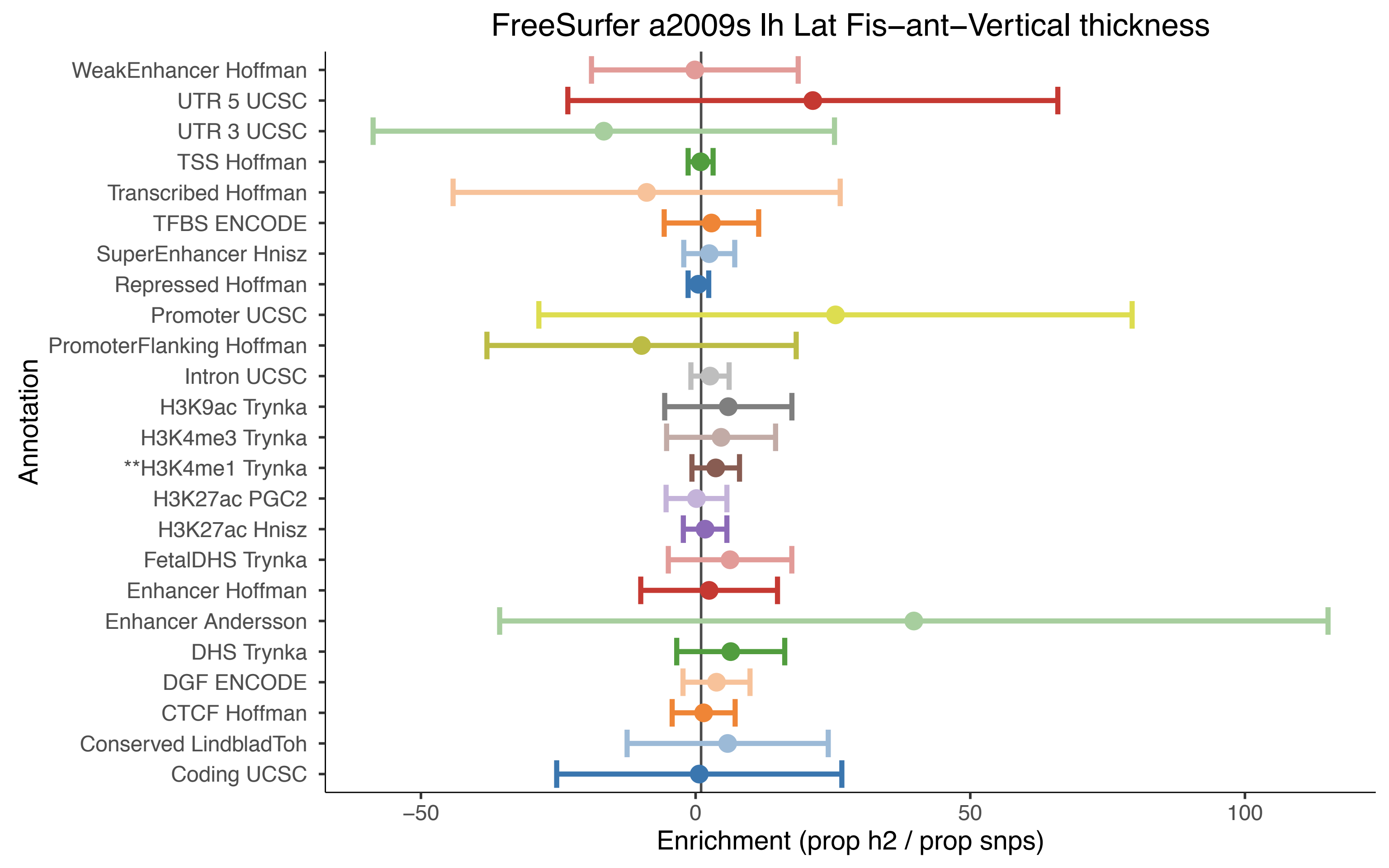
FreeSurfer a2009s lh Lat Fis−ant−Vertical thickness
Annotation
WeakEnhancer Hoffman
UTR 5 UCSC
UTR 3 UCSC
TSS Hoffman
Transcribed Hoffman
TFBS ENCODE
SuperEnhancer Hnisz
Repressed Hoffman
Promoter UCSC
PromoterFlanking Hoffman
Intron UCSC
H3K9ac Trynka
H3K4me3 Trynka
**H3K4me1 Trynka
H3K27ac PGC2
H3K27ac Hnisz
FetalDHS Trynka
Enhancer Hoffman
Enhancer Andersson
DHS Trynka
DGF ENCODE
CTCF Hoffman
Conserved LindbladToh
Coding UCSC
−50
0
50
100
Enrichment (prop h2 / prop snps)

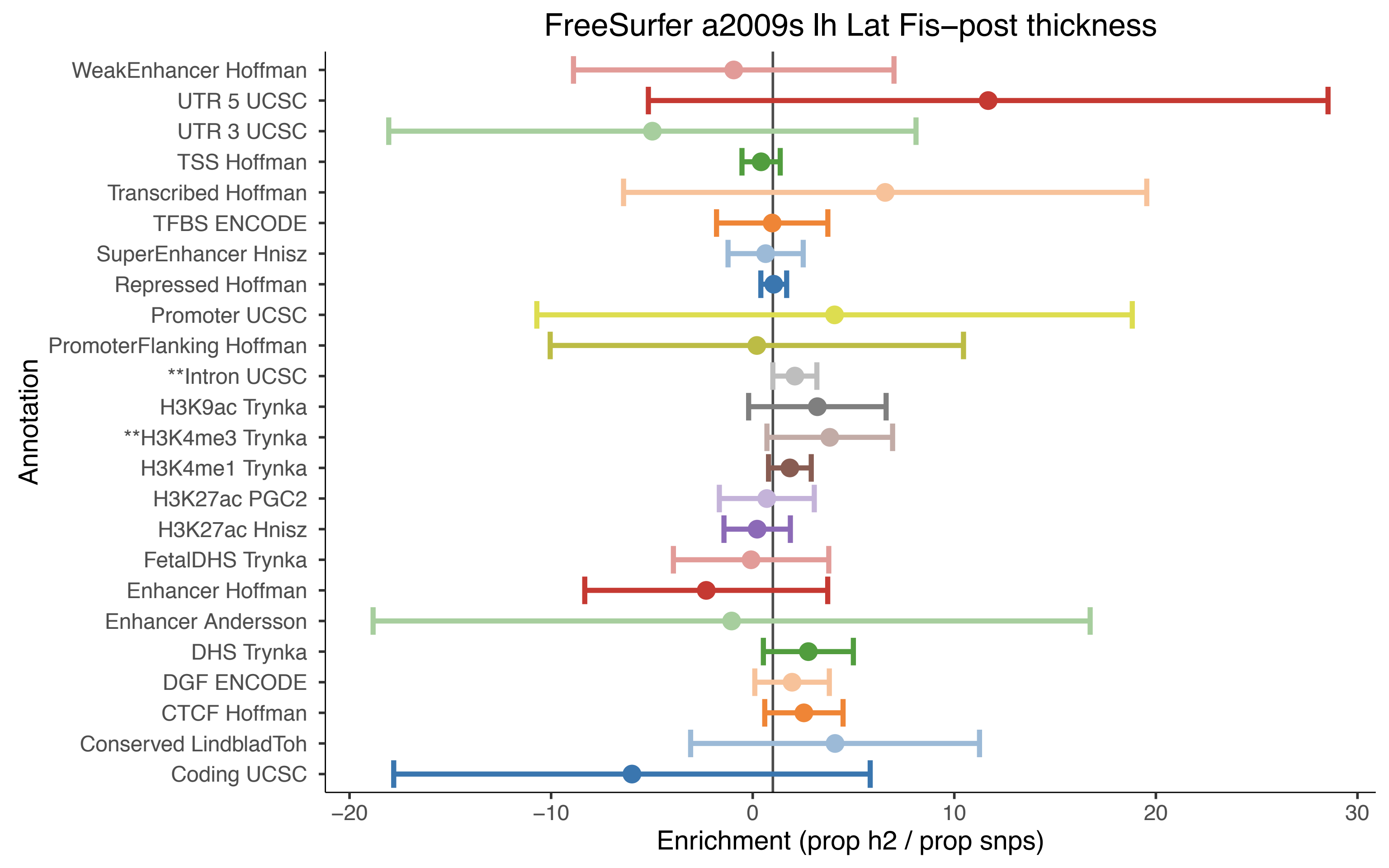
FreeSurfer a2009s lh Lat Fis−post thickness
WeakEnhancer Hoffman
UTR 5 UCSC
UTR 3 UCSC
TSS Hoffman
Transcribed Hoffman
TFBS ENCODE
SuperEnhancer Hnisz
Repressed Hoffman
Promoter UCSC
PromoterFlanking Hoffman
**Intron UCSC
H3K9ac Trynka
**H3K4me3 Trynka
H3K4me1 Trynka
H3K27ac PGC2
H3K27ac Hnisz
FetalDHS Trynka
Enhancer Hoffman
Enhancer Andersson
DHS Trynka
DGF ENCODE
CTCF Hoffman
Conserved LindbladToh
Coding UCSC
Annotation
−20
−10
0
10
20
30
Enrichment (prop h2 / prop snps)

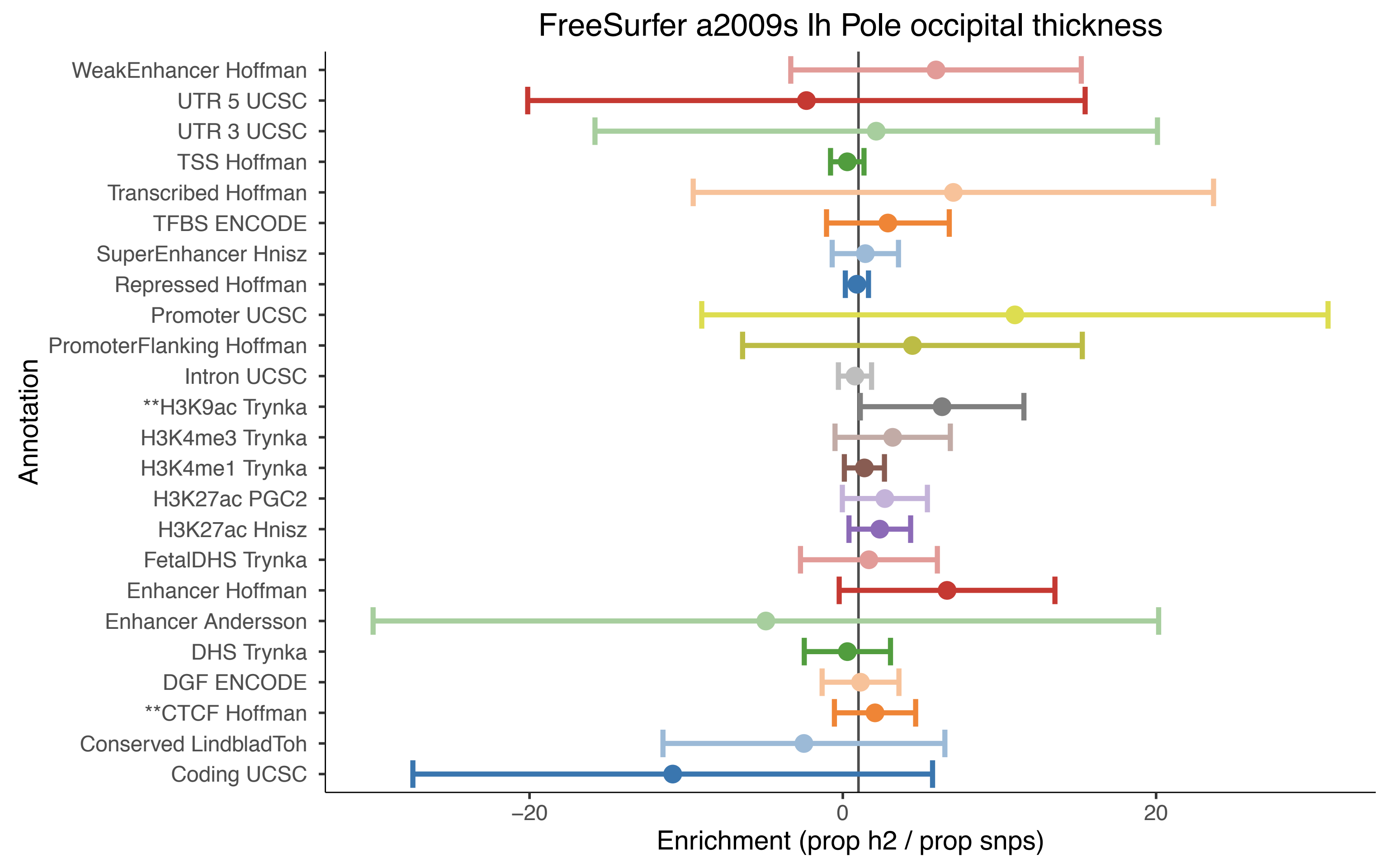

FreeSurfer a2009s lh Pole occipital thickness
WeakEnhancer Hoffman
UTR 5 UCSC
UTR 3 UCSC
TSS Hoffman
Transcribed Hoffman
TFBS ENCODE
SuperEnhancer Hnisz
Repressed Hoffman
Promoter UCSC
PromoterFlanking Hoffman
Intron UCSC
**H3K9ac Trynka
H3K4me3 Trynka
H3K4me1 Trynka
H3K27ac PGC2
H3K27ac Hnisz
FetalDHS Trynka
Enhancer Hoffman
Enhancer Andersson
DHS Trynka
DGF ENCODE
**CTCF Hoffman
Conserved LindbladToh
Coding UCSC
Annotation
−20
0
20
Enrichment (prop h2 / prop snps)

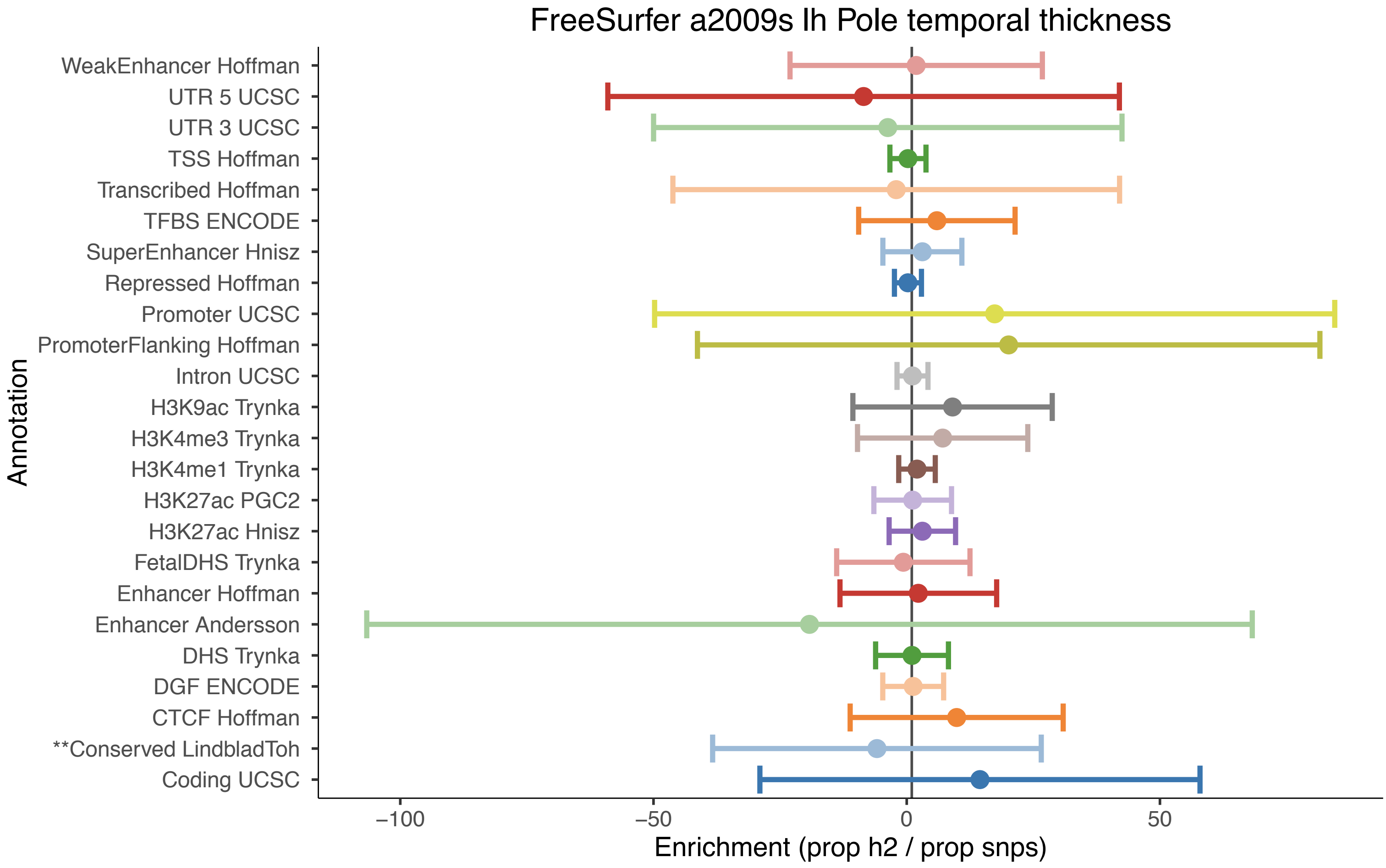
FreeSurfer a2009s lh Pole temporal thickness
WeakEnhancer Hoffman
UTR 5 UCSC
UTR 3 UCSC
TSS Hoffman
Transcribed Hoffman
TFBS ENCODE
SuperEnhancer Hnisz
Repressed Hoffman
Promoter UCSC
PromoterFlanking Hoffman
Intron UCSC
H3K9ac Trynka
H3K4me3 Trynka
H3K4me1 Trynka
H3K27ac PGC2
H3K27ac Hnisz
FetalDHS Trynka
Enhancer Hoffman
Enhancer Andersson
DHS Trynka
DGF ENCODE
CTCF Hoffman
**Conserved LindbladToh
Coding UCSC
Annotation
−100
−50
0
50
Enrichment (prop h2 / prop snps)

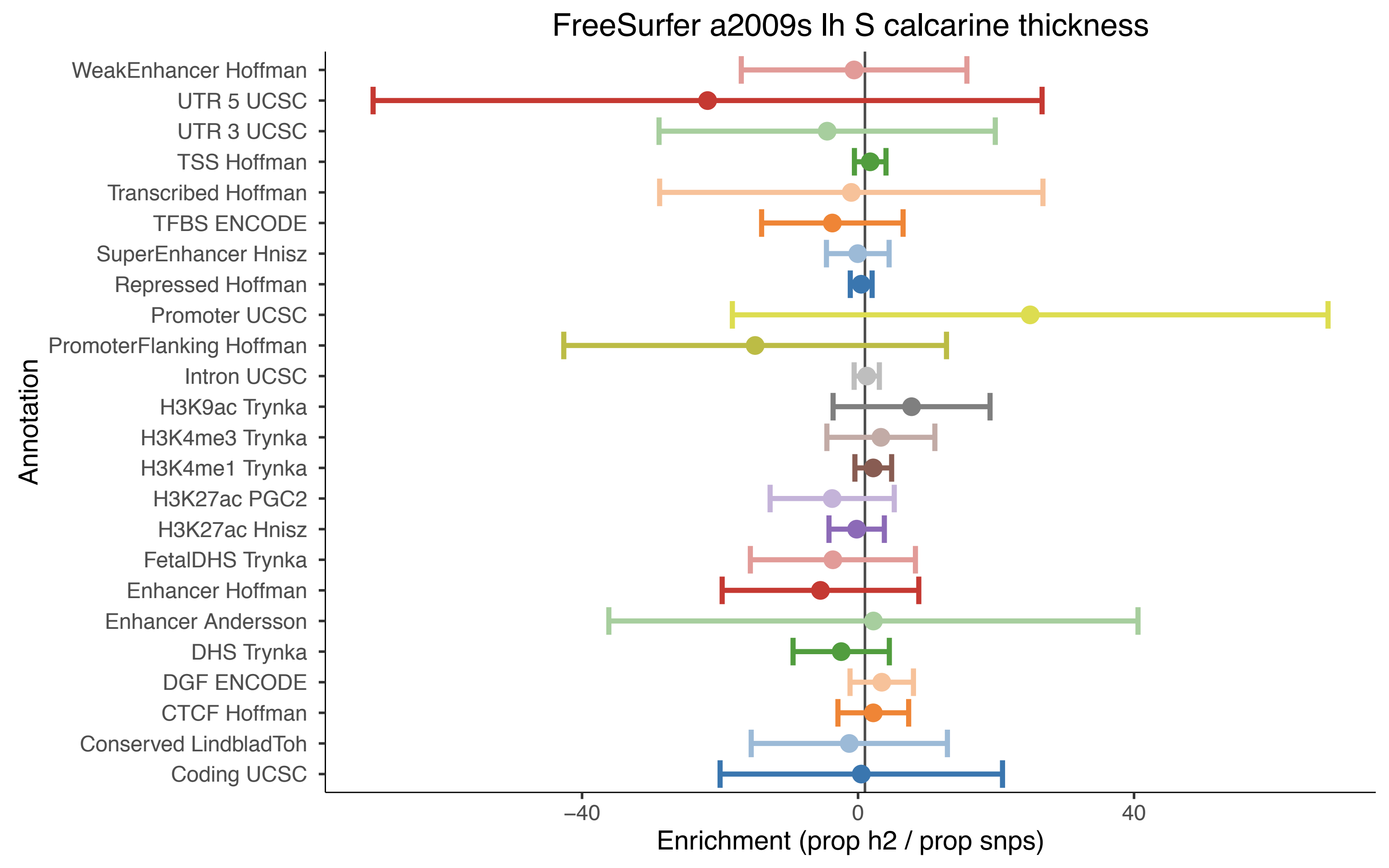

FreeSurfer a2009s lh S calcarine thickness
WeakEnhancer Hoffman
UTR 5 UCSC
UTR 3 UCSC
TSS Hoffman
Transcribed Hoffman
TFBS ENCODE
SuperEnhancer Hnisz
Repressed Hoffman
Promoter UCSC
PromoterFlanking Hoffman
Intron UCSC
H3K9ac Trynka
H3K4me3 Trynka
H3K4me1 Trynka
H3K27ac PGC2
H3K27ac Hnisz
FetalDHS Trynka
Enhancer Hoffman
Enhancer Andersson
DHS Trynka
DGF ENCODE
CTCF Hoffman
Conserved LindbladToh
Coding UCSC
Annotation
−40
0
40
Enrichment (prop h2 / prop snps)

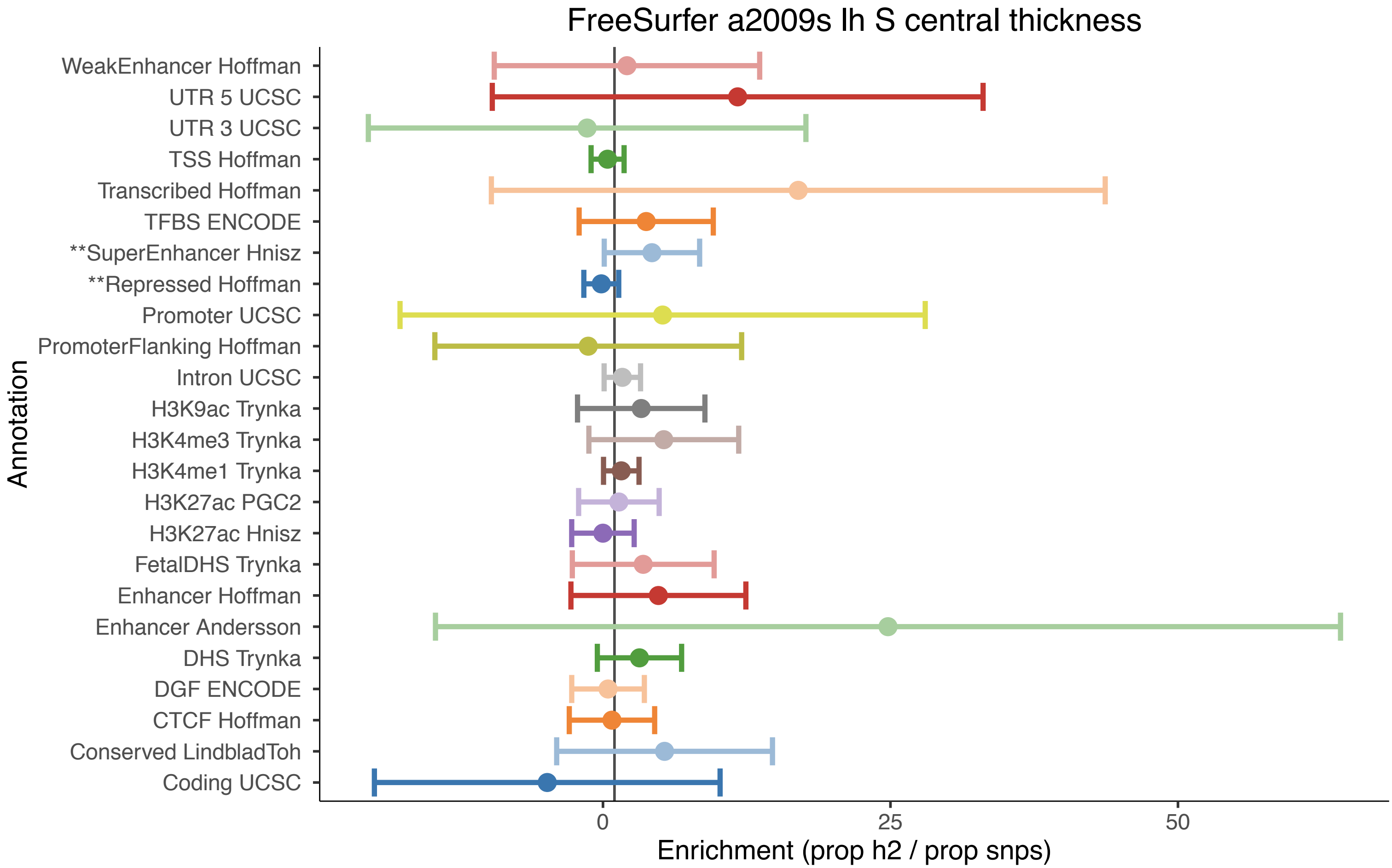
FreeSurfer a2009s lh S central thickness
WeakEnhancer Hoffman
UTR 5 UCSC
UTR 3 UCSC
TSS Hoffman
Transcribed Hoffman
TFBS ENCODE
**SuperEnhancer Hnisz
**Repressed Hoffman
Promoter UCSC
PromoterFlanking Hoffman
Intron UCSC
H3K9ac Trynka
H3K4me3 Trynka
H3K4me1 Trynka
H3K27ac PGC2
H3K27ac Hnisz
FetalDHS Trynka
Enhancer Hoffman
Enhancer Andersson
DHS Trynka
DGF ENCODE
CTCF Hoffman
Conserved LindbladToh
Coding UCSC
Annotation
0
25
50
Enrichment (prop h2 / prop snps)

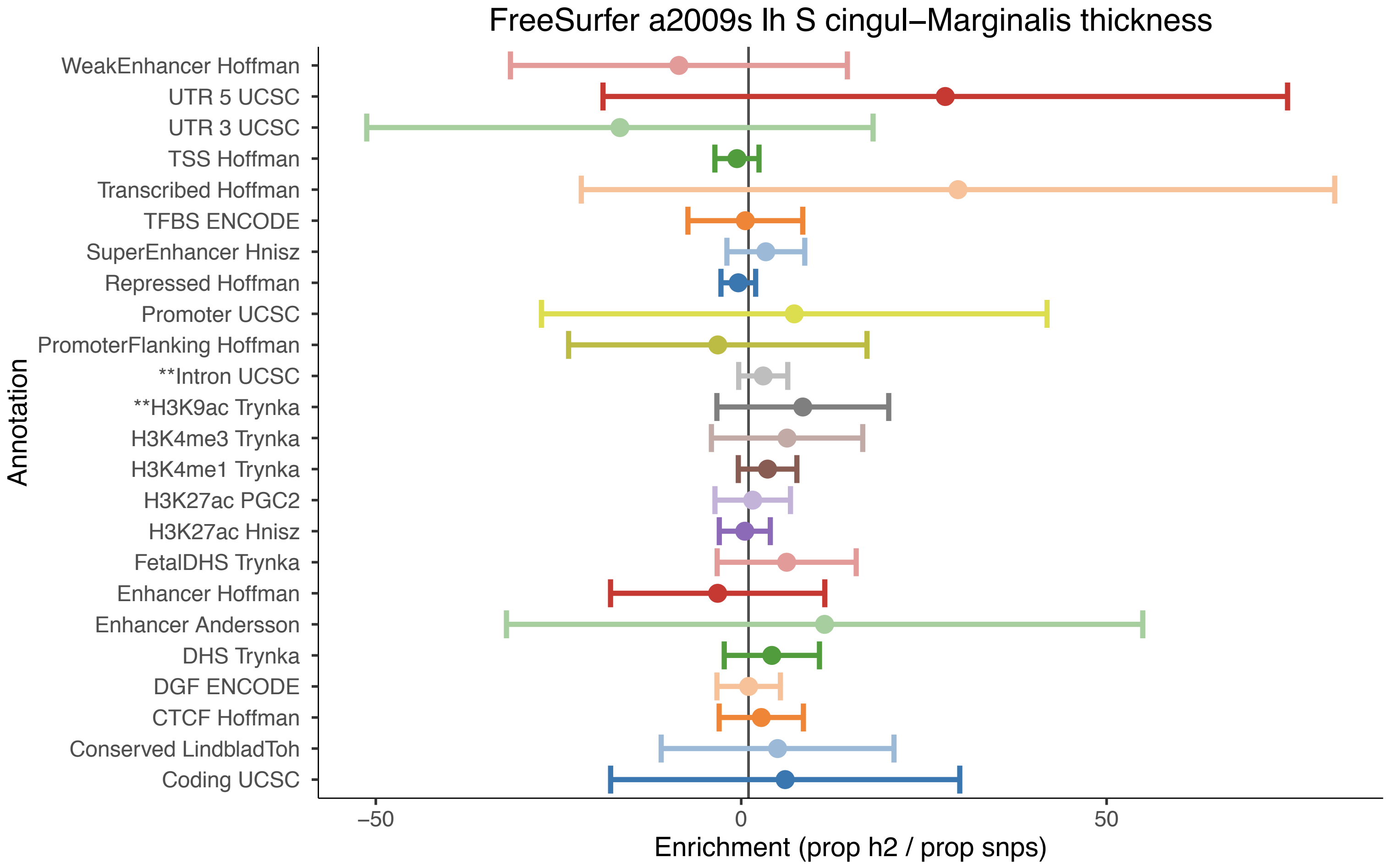

FreeSurfer a2009s lh S cingul−Marginalis thickness
WeakEnhancer Hoffman
UTR 5 UCSC
UTR 3 UCSC
TSS Hoffman
Transcribed Hoffman
TFBS ENCODE
SuperEnhancer Hnisz
Repressed Hoffman
Promoter UCSC
PromoterFlanking Hoffman
**Intron UCSC
**H3K9ac Trynka
H3K4me3 Trynka
H3K4me1 Trynka
H3K27ac PGC2
H3K27ac Hnisz
FetalDHS Trynka
Enhancer Hoffman
Enhancer Andersson
DHS Trynka
DGF ENCODE
CTCF Hoffman
Conserved LindbladToh
Coding UCSC
Annotation
−50
0
50
Enrichment (prop h2 / prop snps)

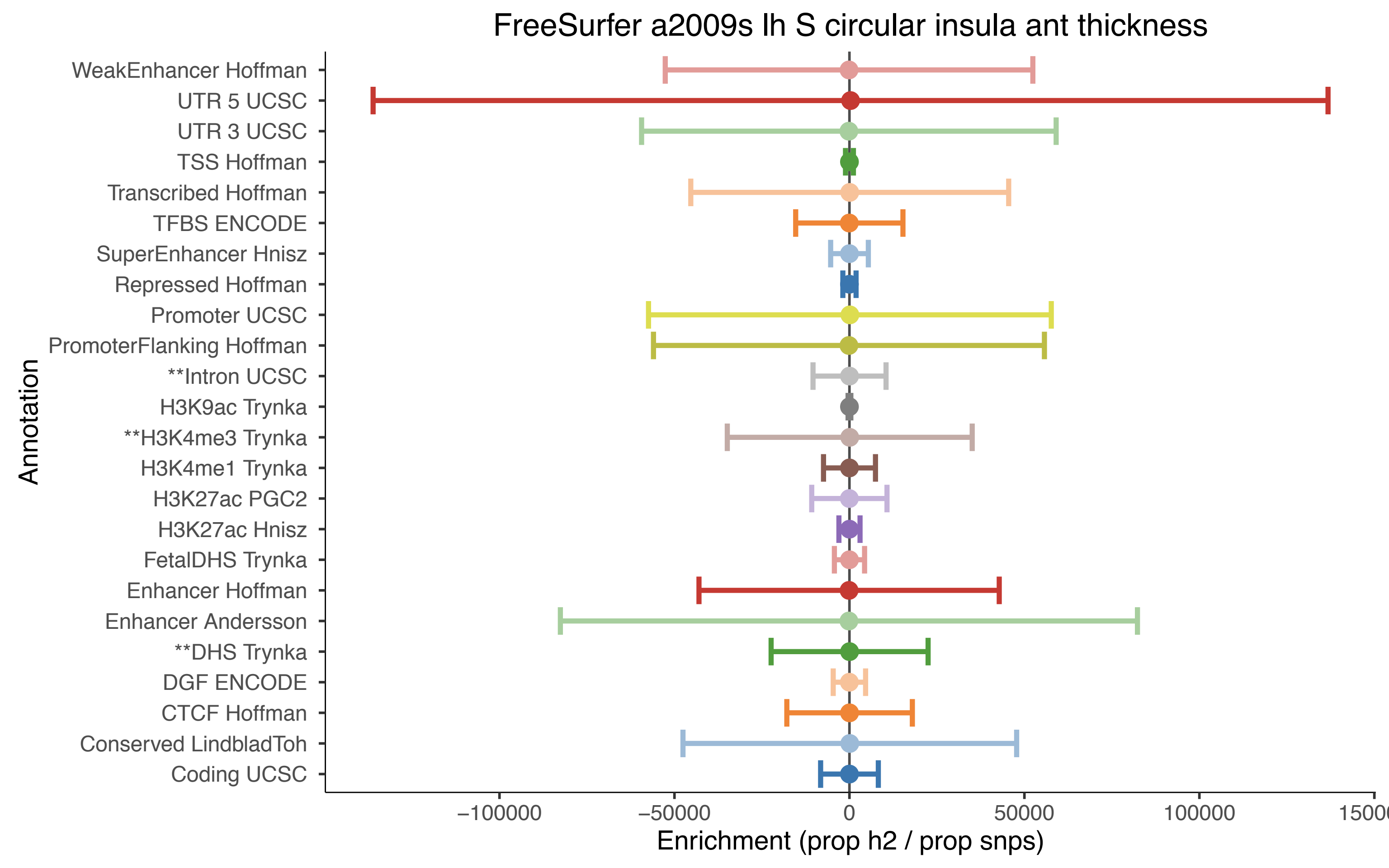

FreeSurfer a2009s lh S circular insula ant thickness
WeakEnhancer Hoffman
UTR 5 UCSC
UTR 3 UCSC
TSS Hoffman
Transcribed Hoffman
TFBS ENCODE
SuperEnhancer Hnisz
Repressed Hoffman
Promoter UCSC
PromoterFlanking Hoffman
**Intron UCSC
H3K9ac Trynka
**H3K4me3 Trynka
H3K4me1 Trynka
H3K27ac PGC2
H3K27ac Hnisz
FetalDHS Trynka
Enhancer Hoffman
Enhancer Andersson
**DHS Trynka
DGF ENCODE
CTCF Hoffman
Conserved LindbladToh
Coding UCSC
Annotation
−100000
−50000
0
50000
100000
Enrichment (prop h2 / prop snps)

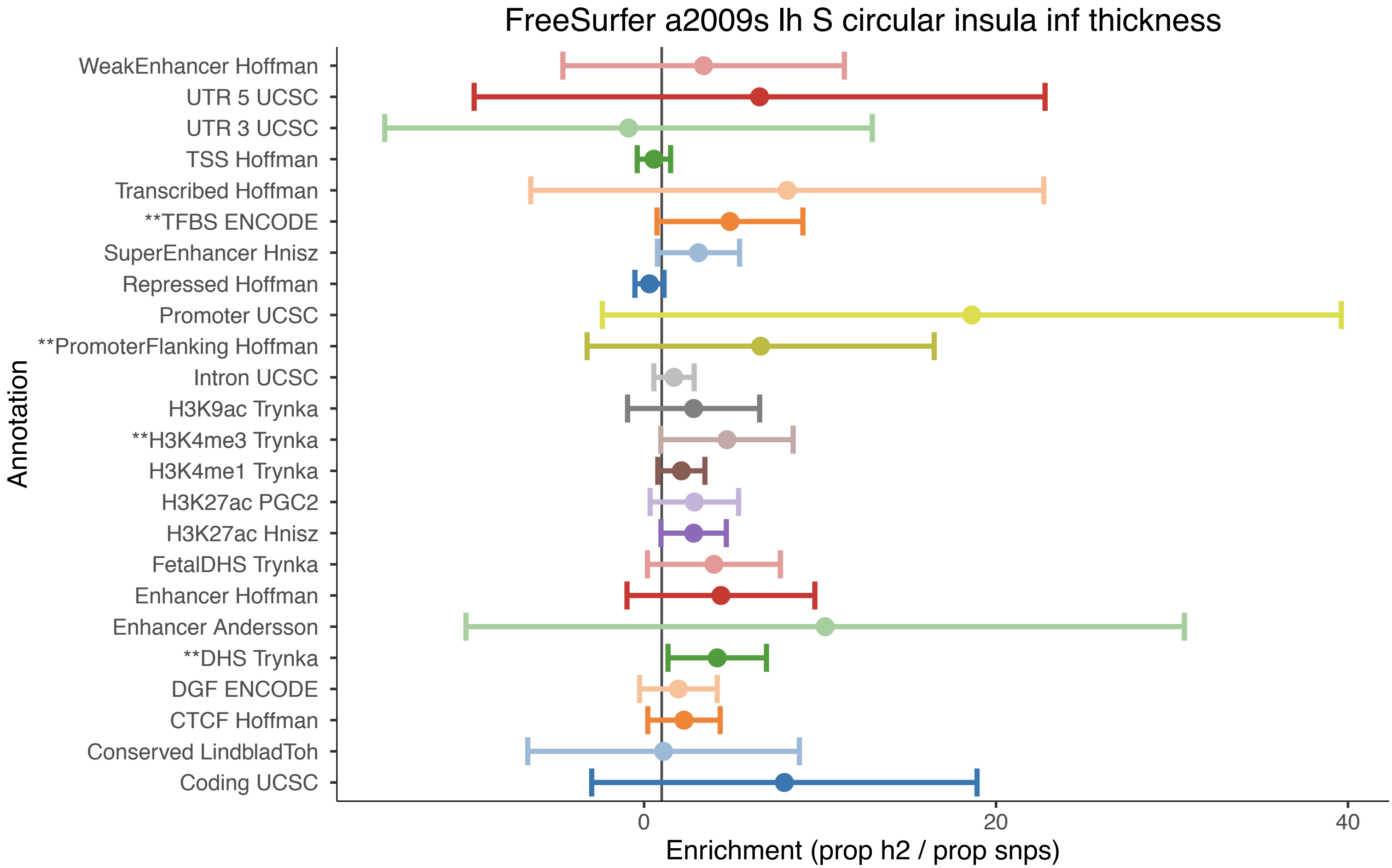

FreeSurfer a2009s lh S circular insula inf thickness
WeakEnhancer Hoffman
UTR 5 UCSC
UTR 3 UCSC
TSS Hoffman
Transcribed Hoffman
**TFBS ENCODE
SuperEnhancer Hnisz
Repressed Hoffman
Promoter UCSC
**PromoterFlanking Hoffman
Intron UCSC
H3K9ac Trynka
**H3K4me3 Trynka
H3K4me1 Trynka
H3K27ac PGC2
H3K27ac Hnisz
FetalDHS Trynka
Enhancer Hoffman
Enhancer Andersson
**DHS Trynka
DGF ENCODE
CTCF Hoffman
Conserved LindbladToh
Coding UCSC
Annotation
0
20
40
Enrichment (prop h2 / prop snps)

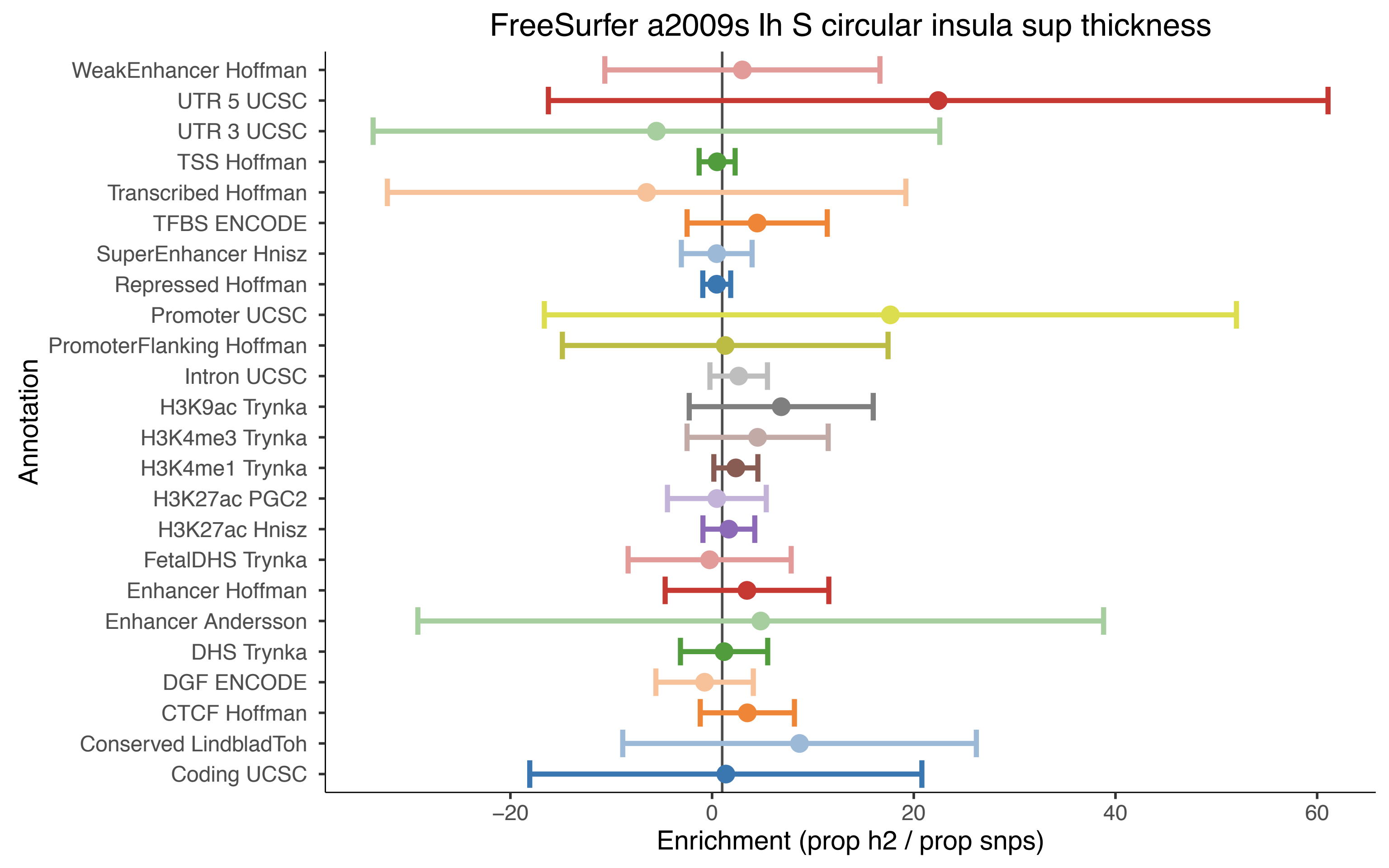
FreeSurfer a2009s lh S circular insula sup thickness
WeakEnhancer Hoffman
UTR 5 UCSC
UTR 3 UCSC
TSS Hoffman
Transcribed Hoffman
TFBS ENCODE
SuperEnhancer Hnisz
Repressed Hoffman
Promoter UCSC
PromoterFlanking Hoffman
Intron UCSC
H3K9ac Trynka
H3K4me3 Trynka
H3K4me1 Trynka
H3K27ac PGC2
H3K27ac Hnisz
FetalDHS Trynka
Enhancer Hoffman
Enhancer Andersson
DHS Trynka
DGF ENCODE
CTCF Hoffman
Conserved LindbladToh
Coding UCSC
Annotation
−20
0
20
40
60
Enrichment (prop h2 / prop snps)

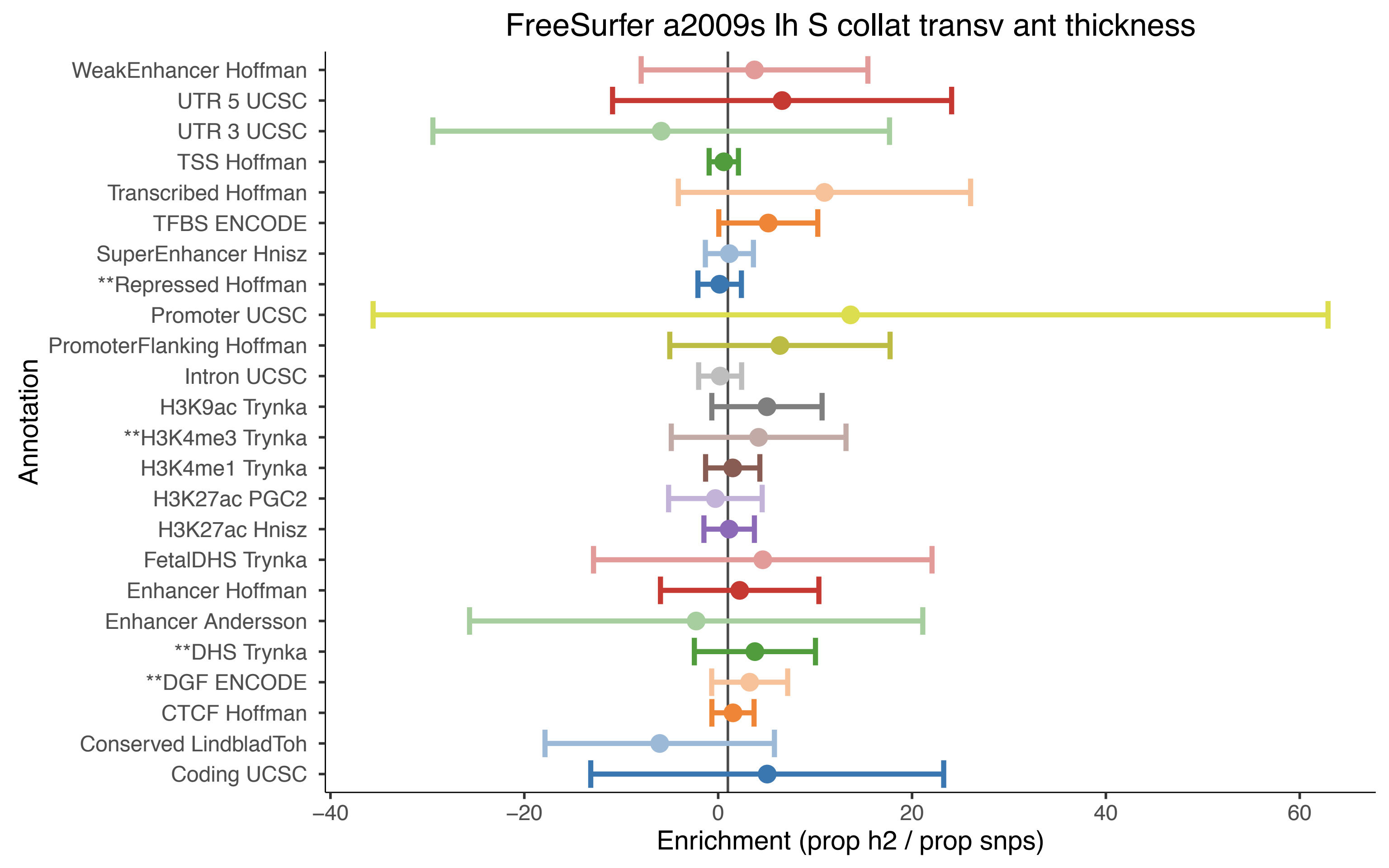

FreeSurfer a2009s lh S collat transv ant thickness
WeakEnhancer Hoffman
UTR 5 UCSC
UTR 3 UCSC
TSS Hoffman
Transcribed Hoffman
TFBS ENCODE
SuperEnhancer Hnisz
**Repressed Hoffman
Promoter UCSC
PromoterFlanking Hoffman
Intron UCSC
H3K9ac Trynka
**H3K4me3 Trynka
H3K4me1 Trynka
H3K27ac PGC2
H3K27ac Hnisz
FetalDHS Trynka
Enhancer Hoffman
Enhancer Andersson
**DHS Trynka
**DGF ENCODE
CTCF Hoffman
Conserved LindbladToh
Coding UCSC
Annotation
−40
−20
0
20
40
60
Enrichment (prop h2 / prop snps)

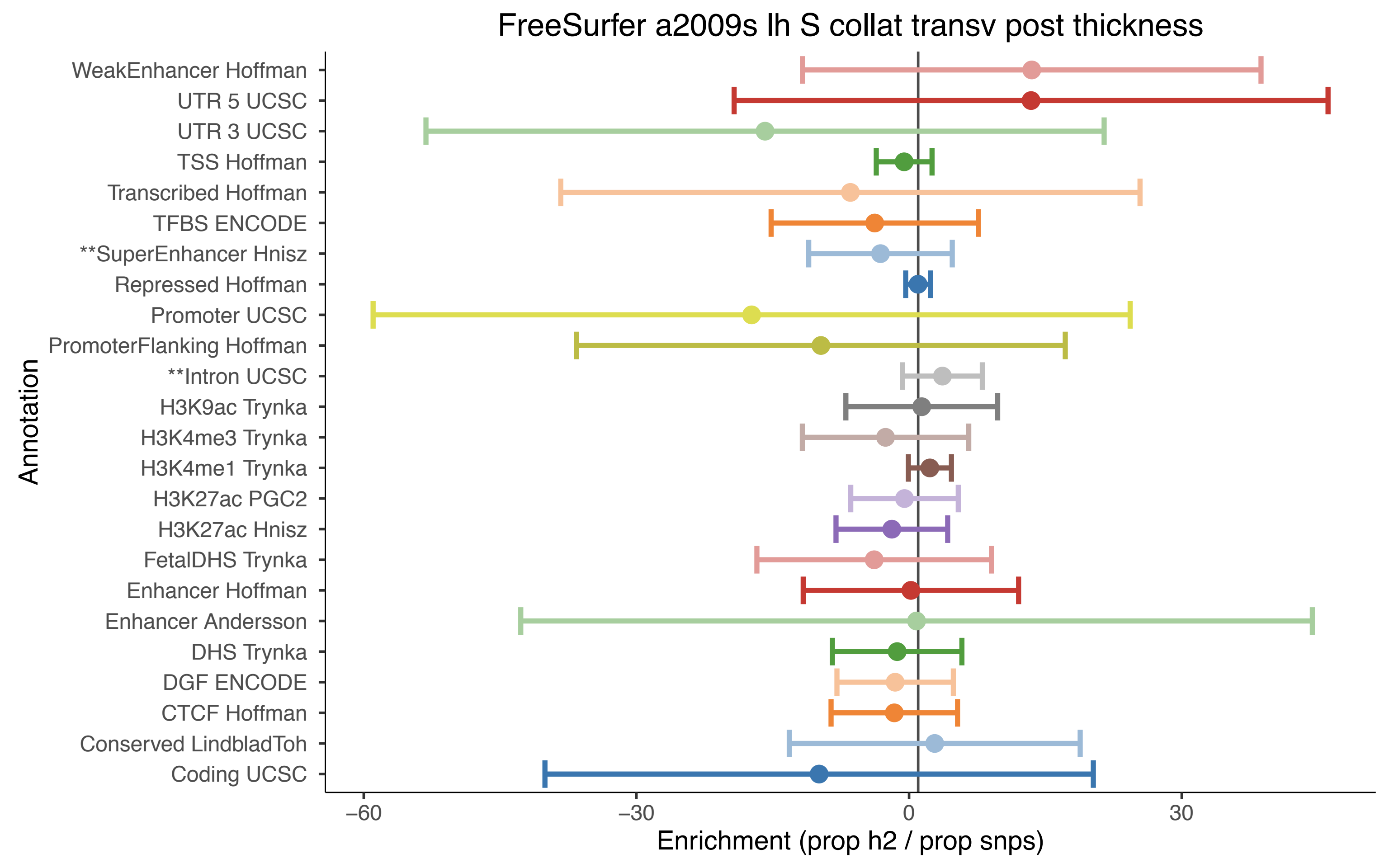

FreeSurfer a2009s lh S collat transv post thickness
WeakEnhancer Hoffman
UTR 5 UCSC
UTR 3 UCSC
TSS Hoffman
Transcribed Hoffman
TFBS ENCODE
**SuperEnhancer Hnisz
Repressed Hoffman
Promoter UCSC
PromoterFlanking Hoffman
**Intron UCSC
H3K9ac Trynka
H3K4me3 Trynka
H3K4me1 Trynka
H3K27ac PGC2
H3K27ac Hnisz
FetalDHS Trynka
Enhancer Hoffman
Enhancer Andersson
DHS Trynka
DGF ENCODE
CTCF Hoffman
Conserved LindbladToh
Coding UCSC
Annotation
−60
−30
0
30
Enrichment (prop h2 / prop snps)

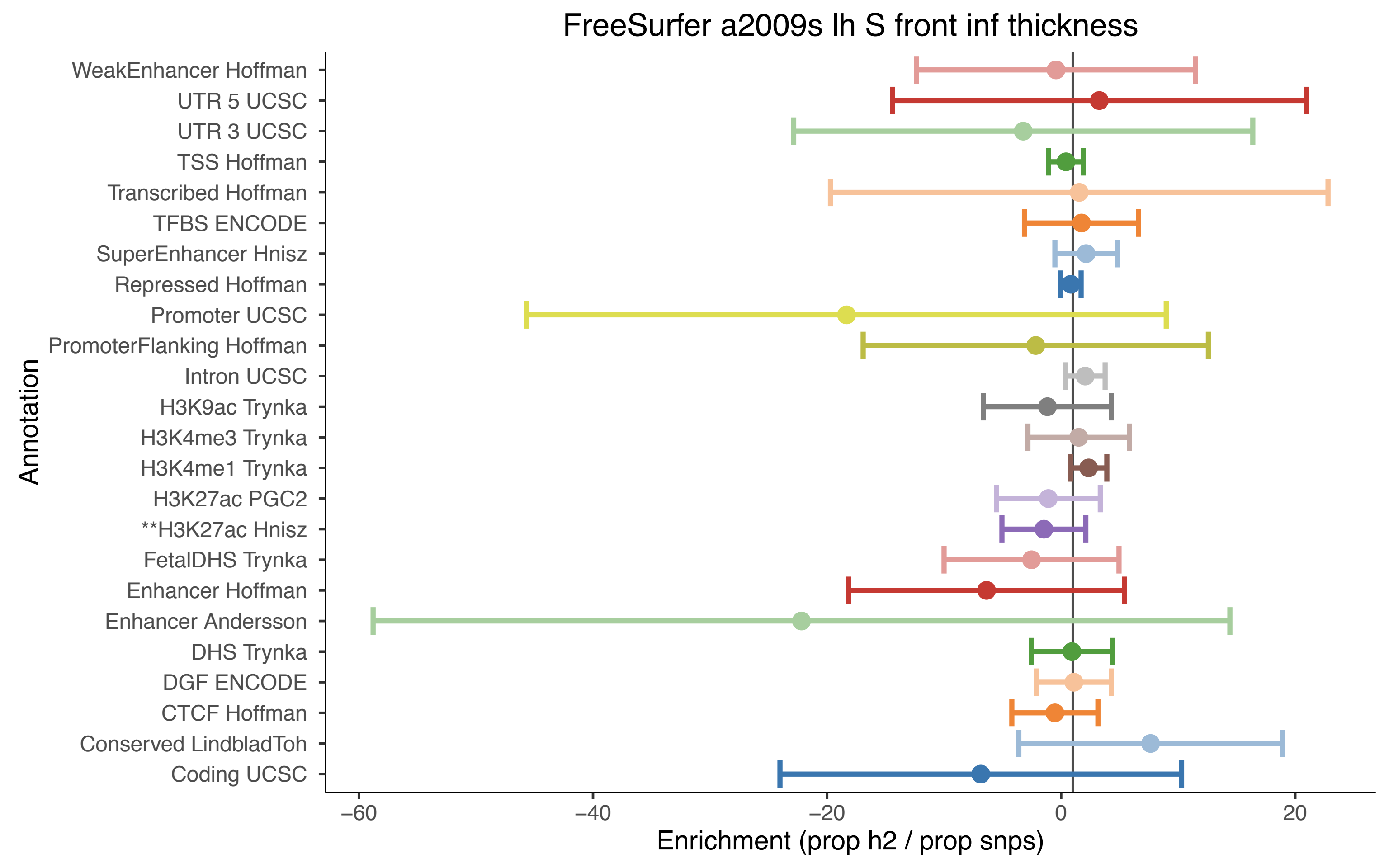

FreeSurfer a2009s lh S front inf thickness
WeakEnhancer Hoffman
UTR 5 UCSC
UTR 3 UCSC
TSS Hoffman
Transcribed Hoffman
TFBS ENCODE
SuperEnhancer Hnisz
Repressed Hoffman
Promoter UCSC
PromoterFlanking Hoffman
Intron UCSC
H3K9ac Trynka
H3K4me3 Trynka
H3K4me1 Trynka
H3K27ac PGC2
**H3K27ac Hnisz
FetalDHS Trynka
Enhancer Hoffman
Enhancer Andersson
DHS Trynka
DGF ENCODE
CTCF Hoffman
Conserved LindbladToh
Coding UCSC
Annotation
−60
−40
−20
0
20
Enrichment (prop h2 / prop snps)

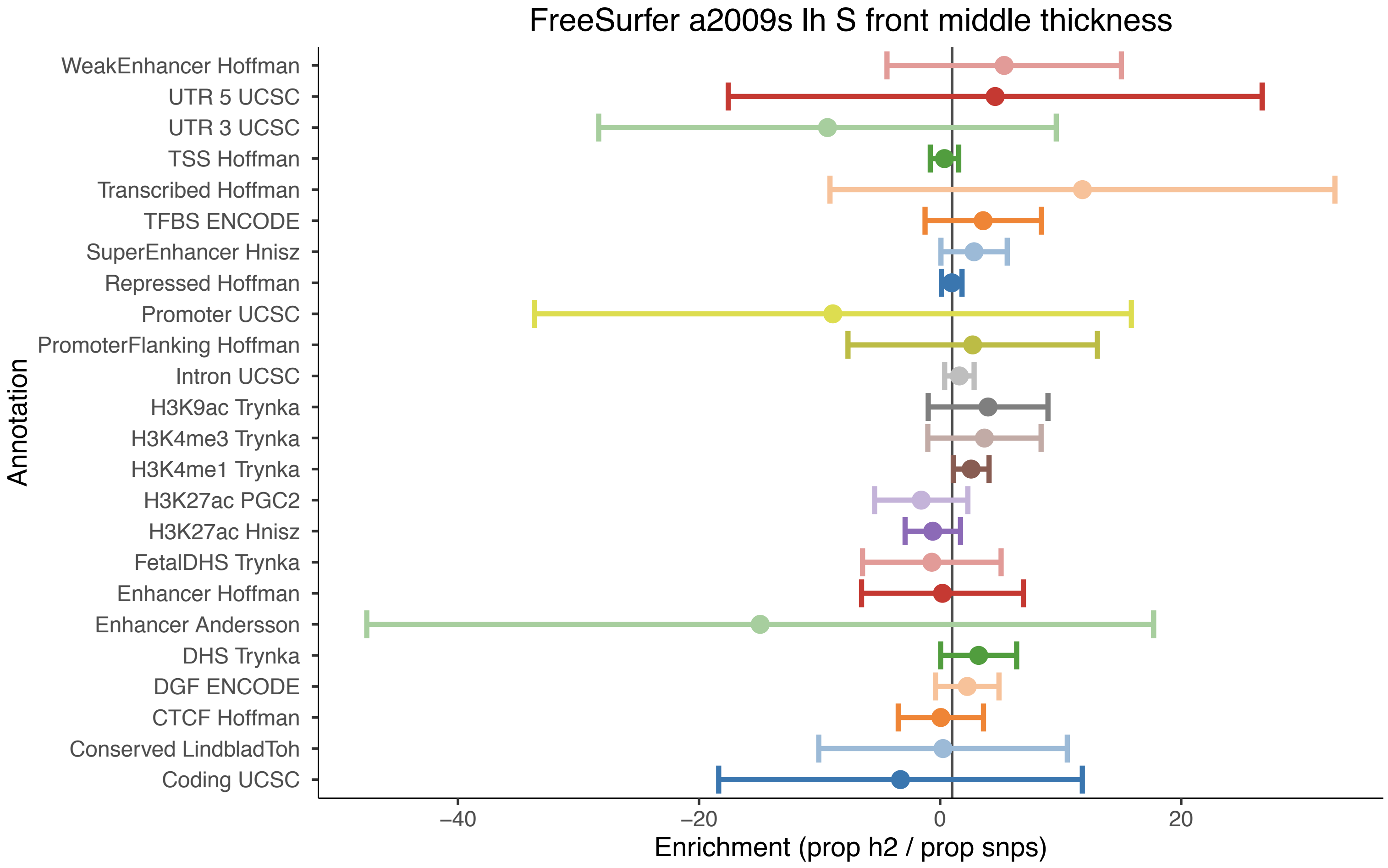
FreeSurfer a2009s lh S front middle thickness
WeakEnhancer Hoffman
UTR 5 UCSC
UTR 3 UCSC
TSS Hoffman
Transcribed Hoffman
TFBS ENCODE
SuperEnhancer Hnisz
Repressed Hoffman
Promoter UCSC
PromoterFlanking Hoffman
Intron UCSC
H3K9ac Trynka
H3K4me3 Trynka
H3K4me1 Trynka
H3K27ac PGC2
H3K27ac Hnisz
FetalDHS Trynka
Enhancer Hoffman
Enhancer Andersson
DHS Trynka
DGF ENCODE
CTCF Hoffman
Conserved LindbladToh
Coding UCSC
Annotation
−40
−20
0
20
Enrichment (prop h2 / prop snps)

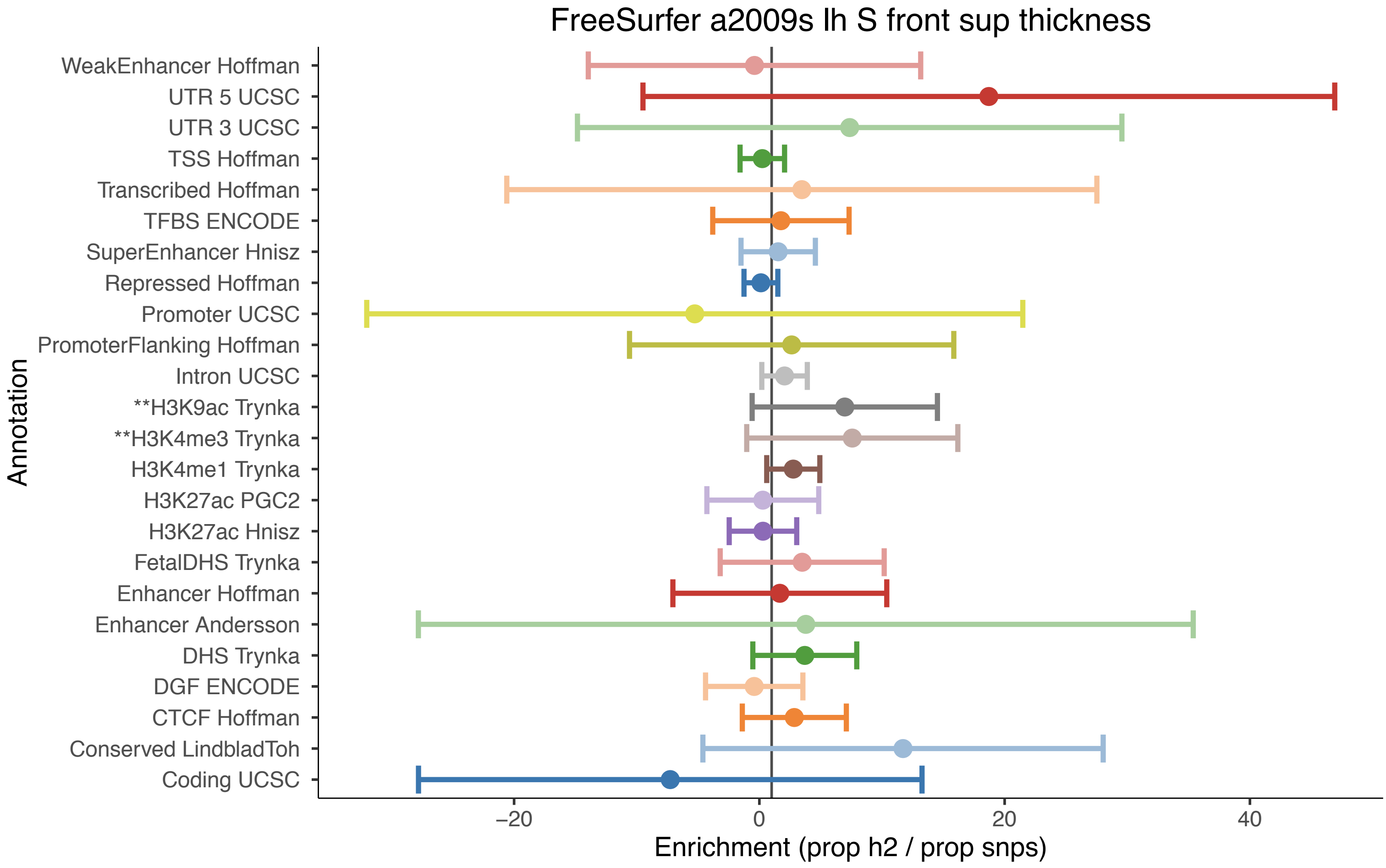
FreeSurfer a2009s lh S front sup thickness
WeakEnhancer Hoffman
UTR 5 UCSC
UTR 3 UCSC
TSS Hoffman
Transcribed Hoffman
TFBS ENCODE
SuperEnhancer Hnisz
Repressed Hoffman
Promoter UCSC
PromoterFlanking Hoffman
Intron UCSC
**H3K9ac Trynka
**H3K4me3 Trynka
H3K4me1 Trynka
H3K27ac PGC2
H3K27ac Hnisz
FetalDHS Trynka
Enhancer Hoffman
Enhancer Andersson
DHS Trynka
DGF ENCODE
CTCF Hoffman
Conserved LindbladToh
Coding UCSC
Annotation
−20
0
20
40
Enrichment (prop h2 / prop snps)

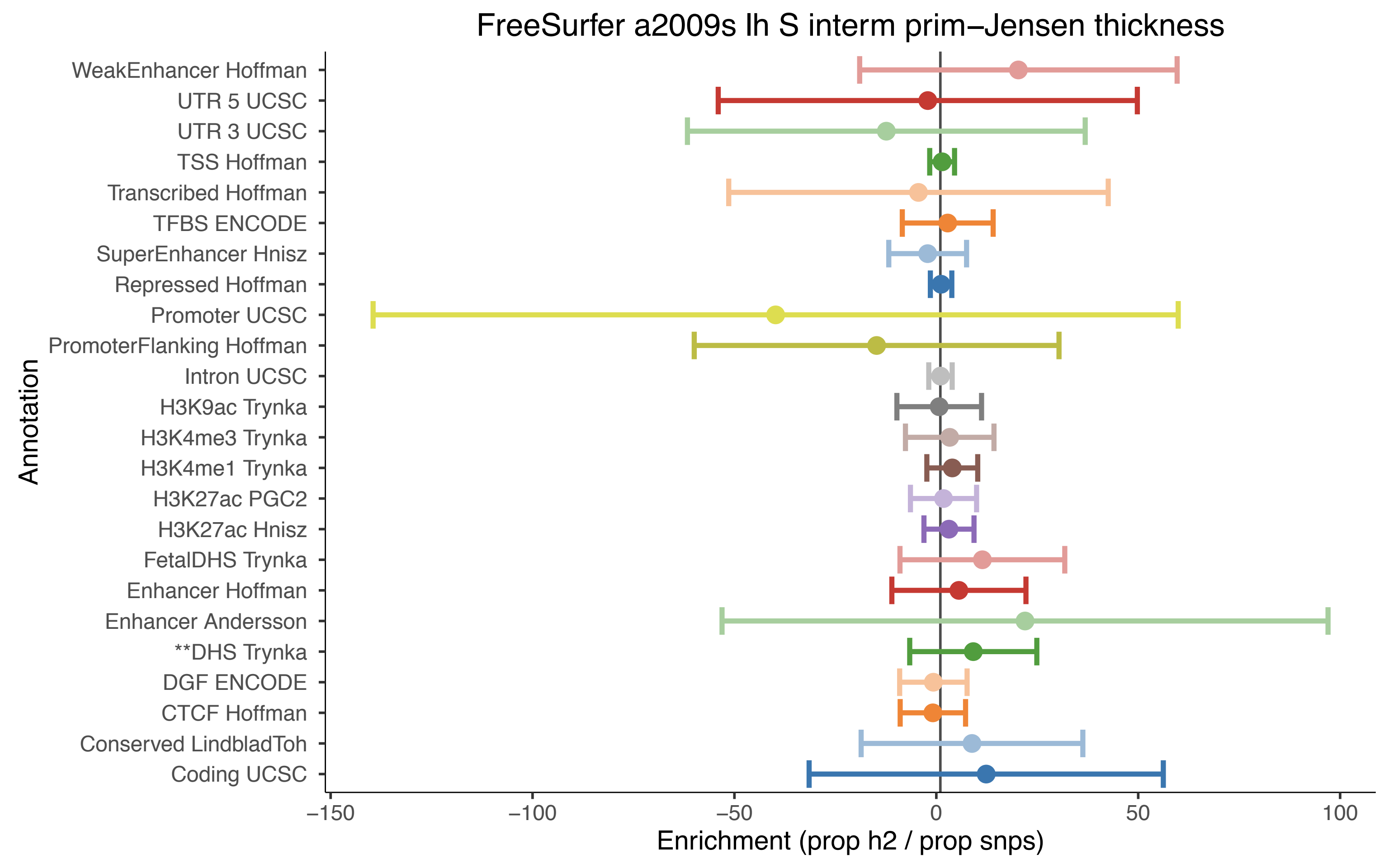
FreeSurfer a2009s lh S interm prim−Jensen thickness
Annotation
WeakEnhancer Hoffman
UTR 5 UCSC
UTR 3 UCSC
TSS Hoffman
Transcribed Hoffman
TFBS ENCODE
SuperEnhancer Hnisz
Repressed Hoffman
Promoter UCSC
PromoterFlanking Hoffman
Intron UCSC
H3K9ac Trynka
H3K4me3 Trynka
H3K4me1 Trynka
H3K27ac PGC2
H3K27ac Hnisz
FetalDHS Trynka
Enhancer Hoffman
Enhancer Andersson
**DHS Trynka
DGF ENCODE
CTCF Hoffman
Conserved LindbladToh
Coding UCSC
−150
−100
−50
0
50
100
Enrichment (prop h2 / prop snps)

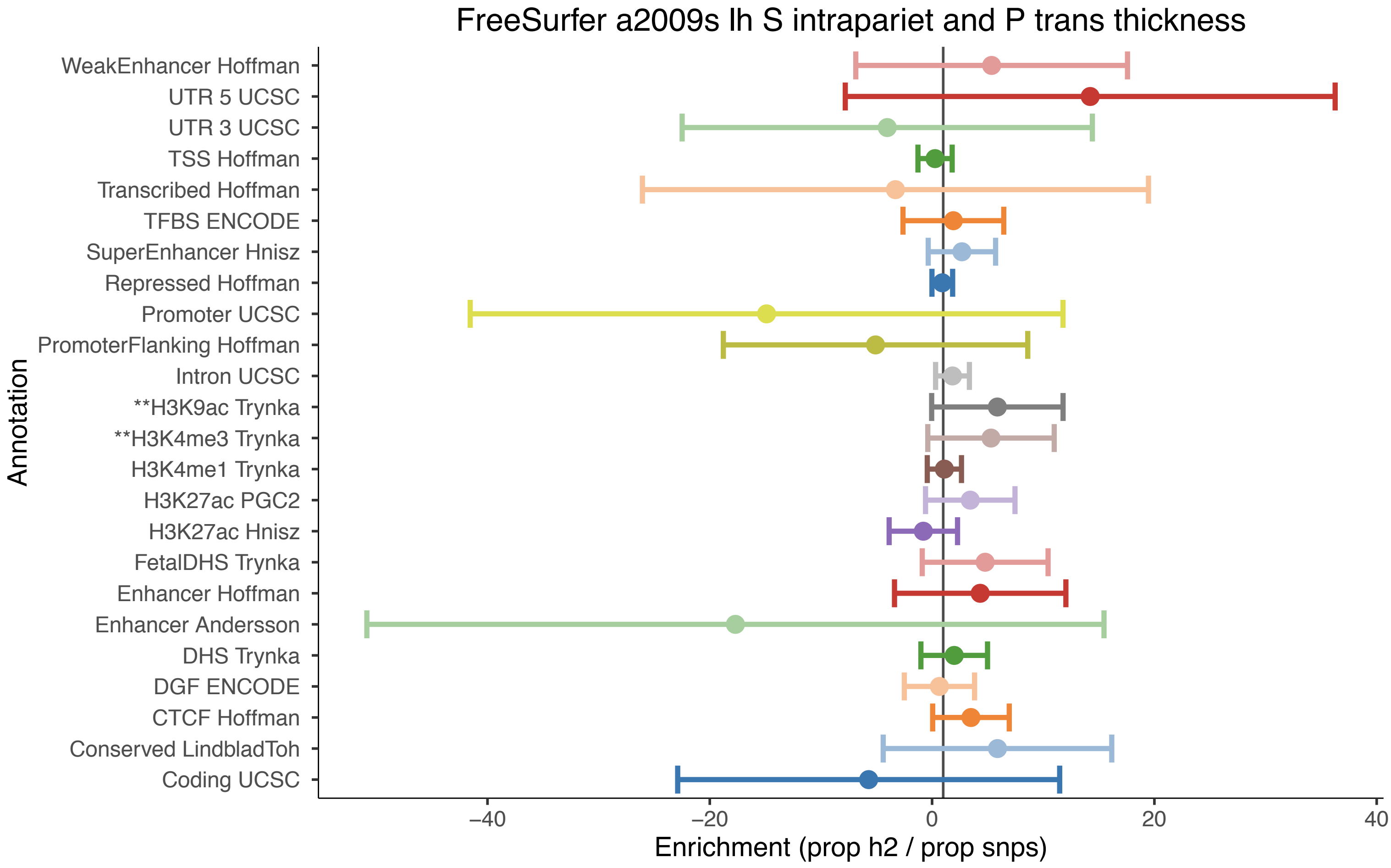
FreeSurfer a2009s lh S intrapariet and P trans thickness
Annotation
WeakEnhancer Hoffman
UTR 5 UCSC
UTR 3 UCSC
TSS Hoffman
Transcribed Hoffman
TFBS ENCODE
SuperEnhancer Hnisz
Repressed Hoffman
Promoter UCSC
PromoterFlanking Hoffman
Intron UCSC
**H3K9ac Trynka
**H3K4me3 Trynka
H3K4me1 Trynka
H3K27ac PGC2
H3K27ac Hnisz
FetalDHS Trynka
Enhancer Hoffman
Enhancer Andersson
DHS Trynka
DGF ENCODE
CTCF Hoffman
Conserved LindbladToh
Coding UCSC
−40
−20
0
20
40
Enrichment (prop h2 / prop snps)

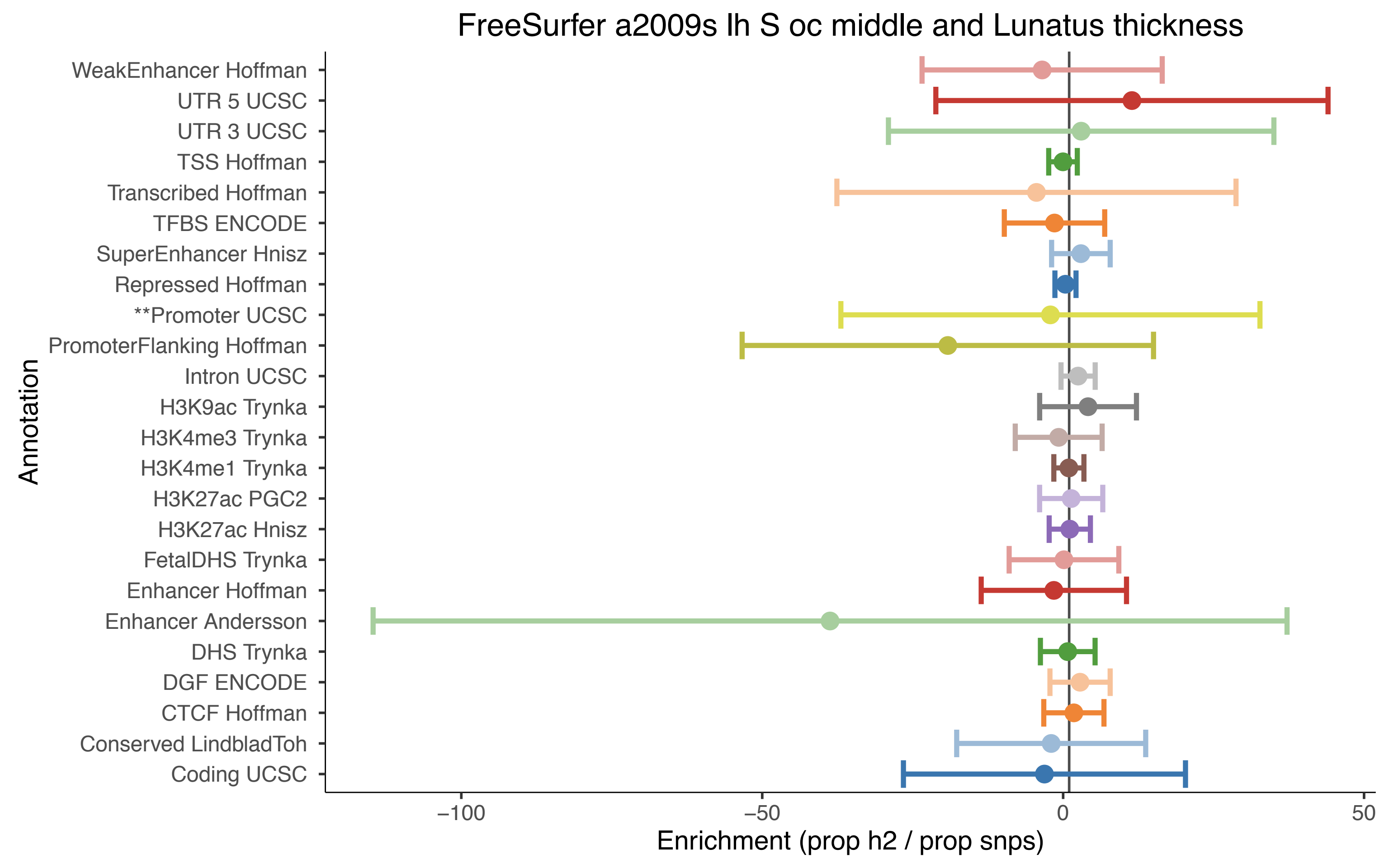

FreeSurfer a2009s lh S oc middle and Lunatus thickness
WeakEnhancer Hoffman
UTR 5 UCSC
UTR 3 UCSC
TSS Hoffman
Transcribed Hoffman
TFBS ENCODE
SuperEnhancer Hnisz
Repressed Hoffman
**Promoter UCSC
PromoterFlanking Hoffman
Intron UCSC
H3K9ac Trynka
H3K4me3 Trynka
H3K4me1 Trynka
H3K27ac PGC2
H3K27ac Hnisz
FetalDHS Trynka
Enhancer Hoffman
Enhancer Andersson
DHS Trynka
DGF ENCODE
CTCF Hoffman
Conserved LindbladToh
Coding UCSC
Annotation
−100
−50
0
50
Enrichment (prop h2 / prop snps)

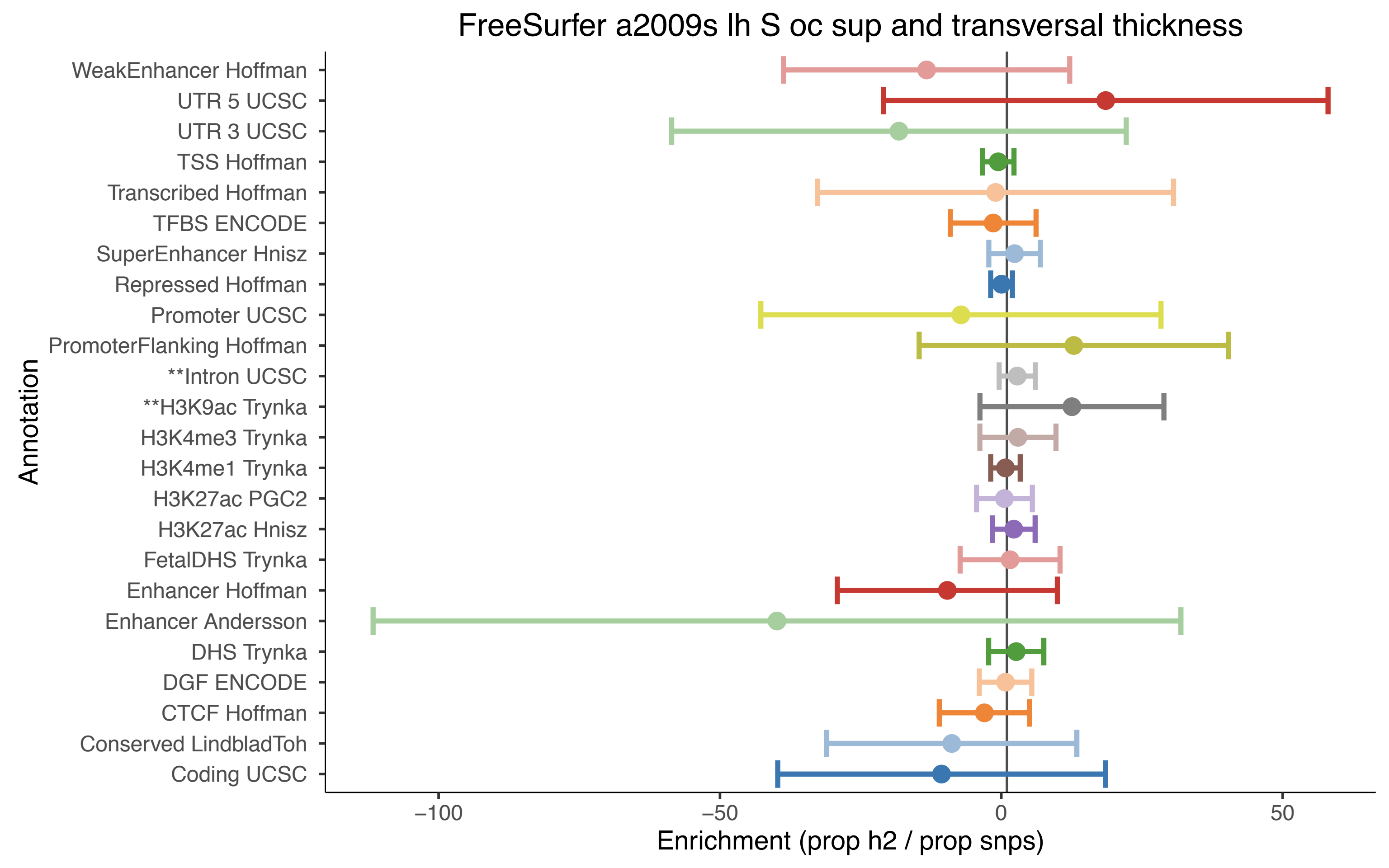
FreeSurfer a2009s lh S oc sup and transversal thickness
WeakEnhancer Hoffman
UTR 5 UCSC
UTR 3 UCSC
TSS Hoffman
Transcribed Hoffman
TFBS ENCODE
SuperEnhancer Hnisz
Repressed Hoffman
Promoter UCSC
PromoterFlanking Hoffman
**Intron UCSC
**H3K9ac Trynka
H3K4me3 Trynka
H3K4me1 Trynka
H3K27ac PGC2
H3K27ac Hnisz
FetalDHS Trynka
Enhancer Hoffman
Enhancer Andersson
DHS Trynka
DGF ENCODE
CTCF Hoffman
Conserved LindbladToh
Coding UCSC
Annotation
−100
−50
0
50
Enrichment (prop h2 / prop snps)

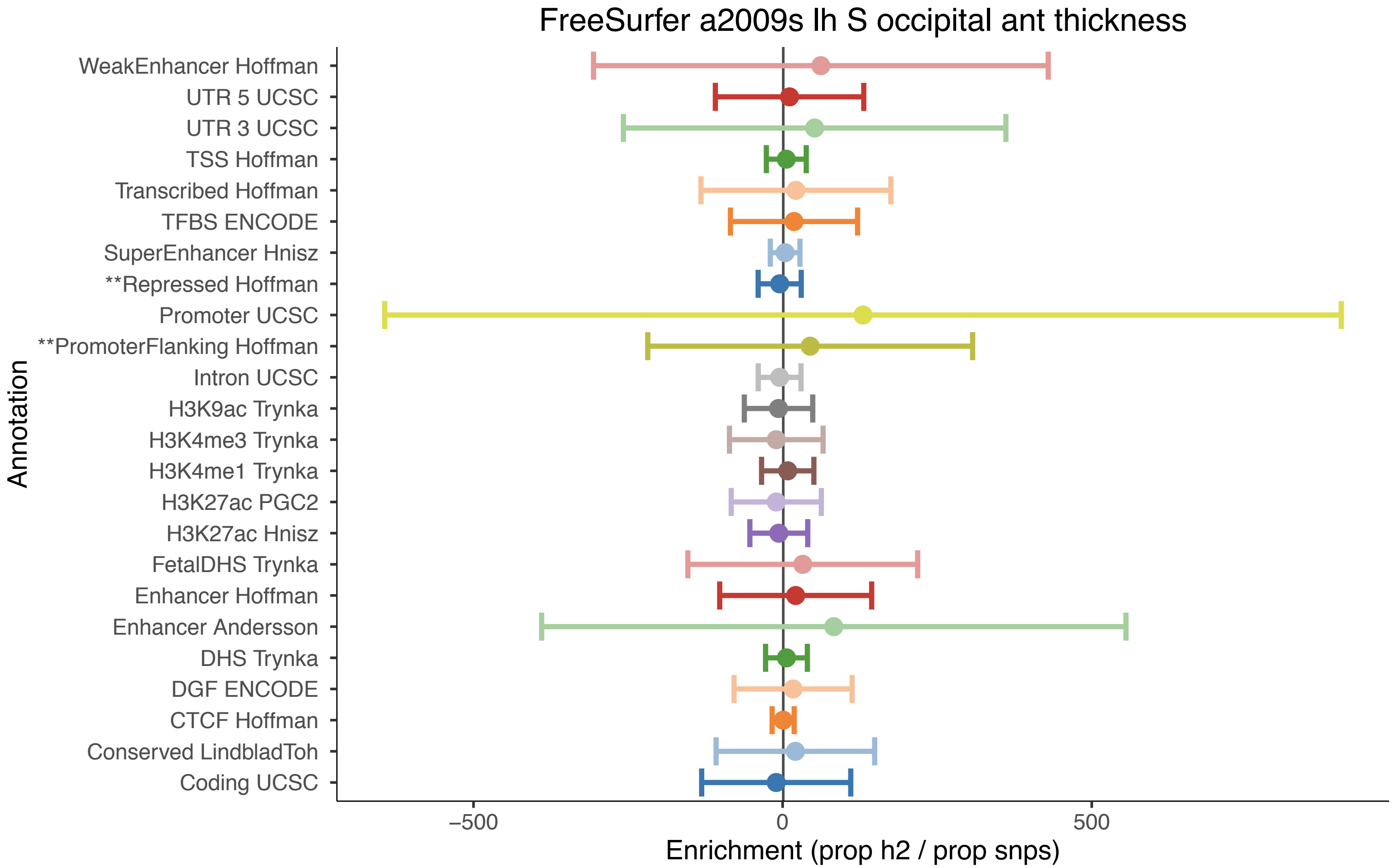
FreeSurfer a2009s lh S occipital ant thickness
WeakEnhancer Hoffman
UTR 5 UCSC
UTR 3 UCSC
TSS Hoffman
Transcribed Hoffman
TFBS ENCODE
SuperEnhancer Hnisz
**Repressed Hoffman
Promoter UCSC
**PromoterFlanking Hoffman
Intron UCSC
H3K9ac Trynka
H3K4me3 Trynka
H3K4me1 Trynka
H3K27ac PGC2
H3K27ac Hnisz
FetalDHS Trynka
Enhancer Hoffman
Enhancer Andersson
DHS Trynka
DGF ENCODE
CTCF Hoffman
Conserved LindbladToh
Coding UCSC
Annotation
−500
0
500
Enrichment (prop h2 / prop snps)

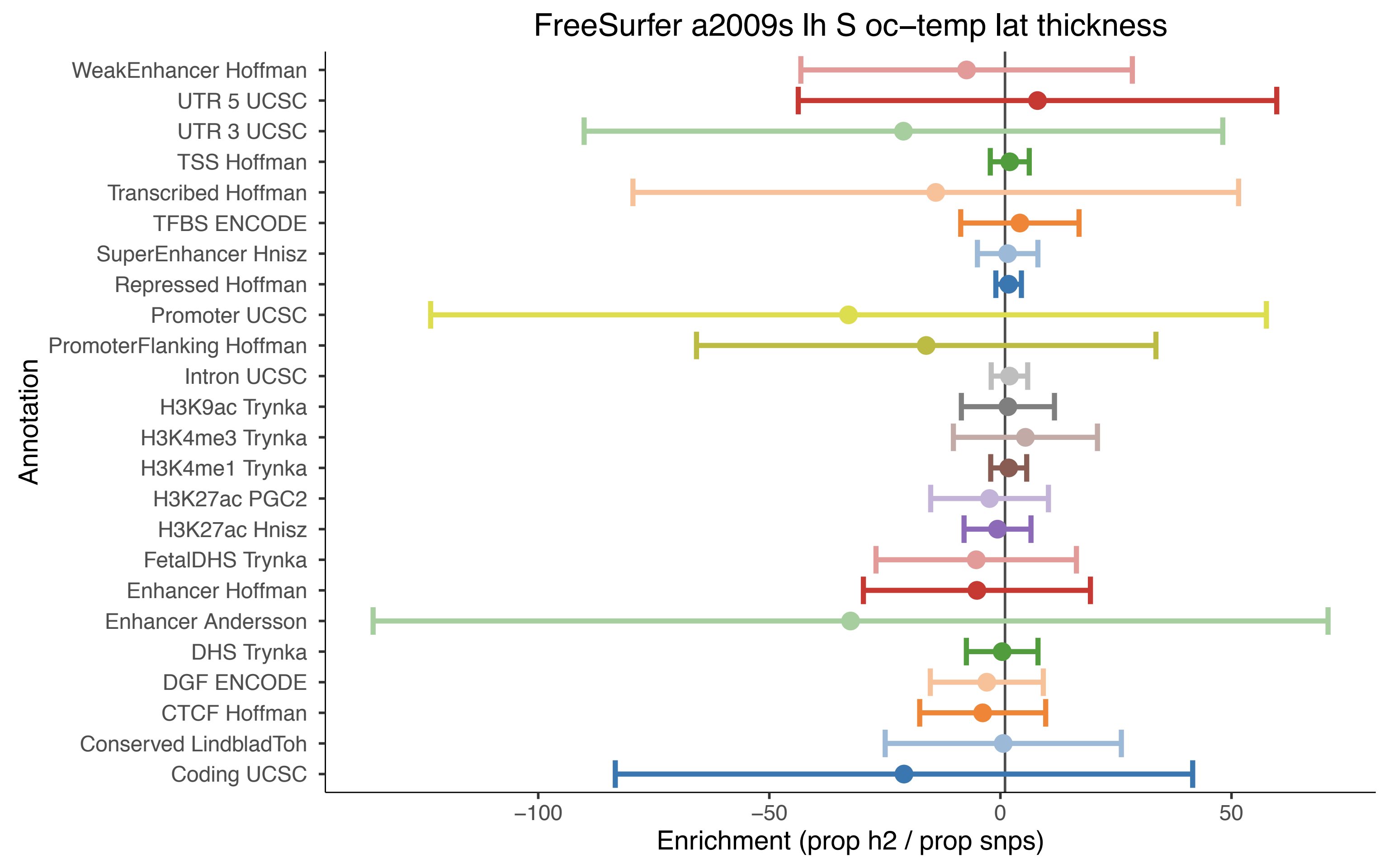

FreeSurfer a2009s lh S oc−temp lat thickness
WeakEnhancer Hoffman
UTR 5 UCSC
UTR 3 UCSC
TSS Hoffman
Transcribed Hoffman
TFBS ENCODE
SuperEnhancer Hnisz
Repressed Hoffman
Promoter UCSC
PromoterFlanking Hoffman
Intron UCSC
H3K9ac Trynka
H3K4me3 Trynka
H3K4me1 Trynka
H3K27ac PGC2
H3K27ac Hnisz
FetalDHS Trynka
Enhancer Hoffman
Enhancer Andersson
DHS Trynka
DGF ENCODE
CTCF Hoffman
Conserved LindbladToh
Coding UCSC
Annotation
−100
−50
0
50
Enrichment (prop h2 / prop snps)

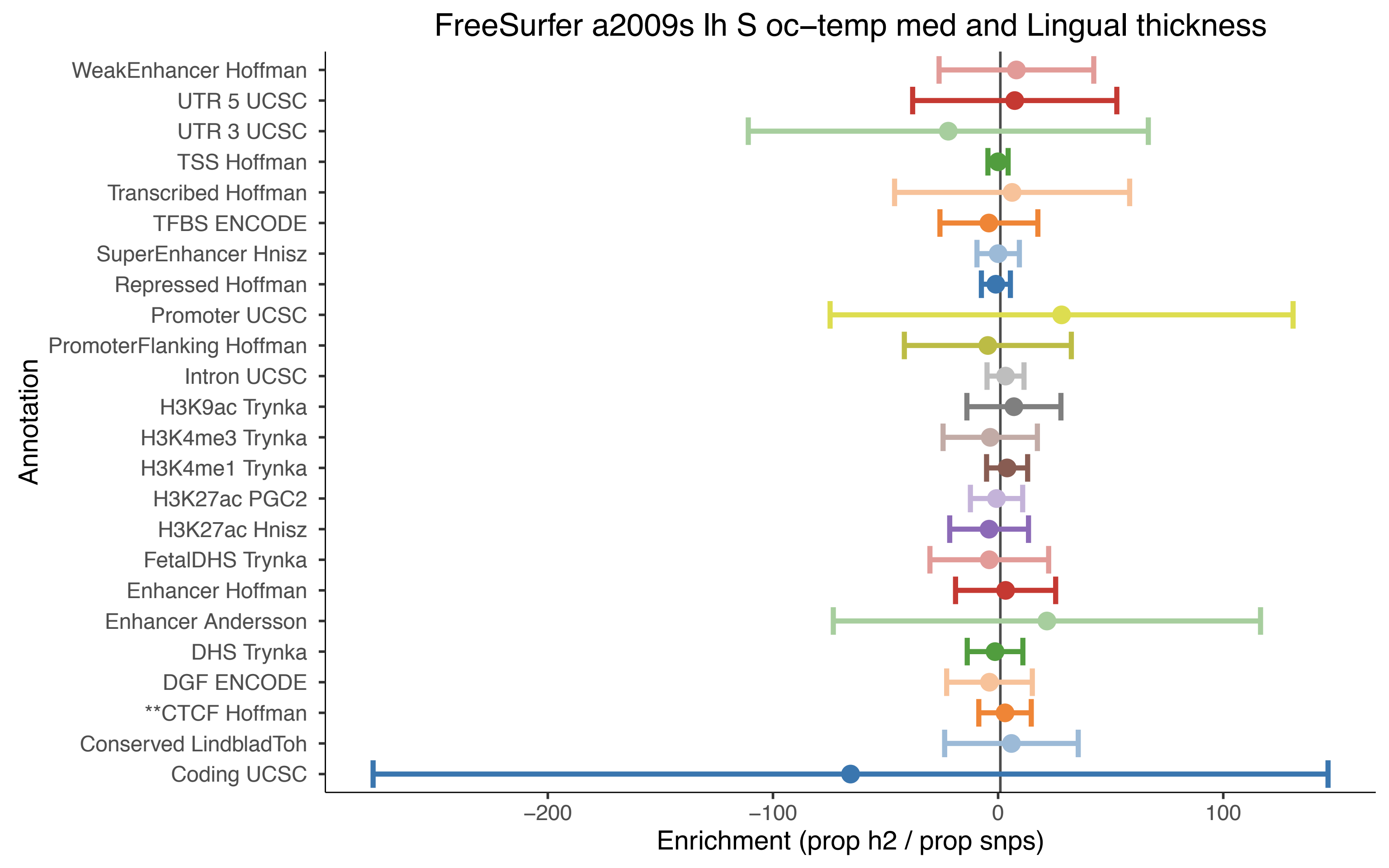

FreeSurfer a2009s lh S oc–temp med and Lingual thickness
WeakEnhancer Hoffman
UTR 5 UCSC
UTR 3 UCSC
TSS Hoffman
Transcribed Hoffman
TFBS ENCODE
SuperEnhancer Hnisz
Repressed Hoffman
Promoter UCSC
PromoterFlanking Hoffman
Intron UCSC
H3K9ac Trynka
H3K4me3 Trynka
H3K4me1 Trynka
H3K27ac PGC2
H3K27ac Hnisz
FetalDHS Trynka
Enhancer Hoffman
Enhancer Andersson
DHS Trynka
DGF ENCODE
**CTCF Hoffman
Conserved LindbladToh
Coding UCSC
Annotation
−200
−100
0
100
Enrichment (prop h2 / prop snps)

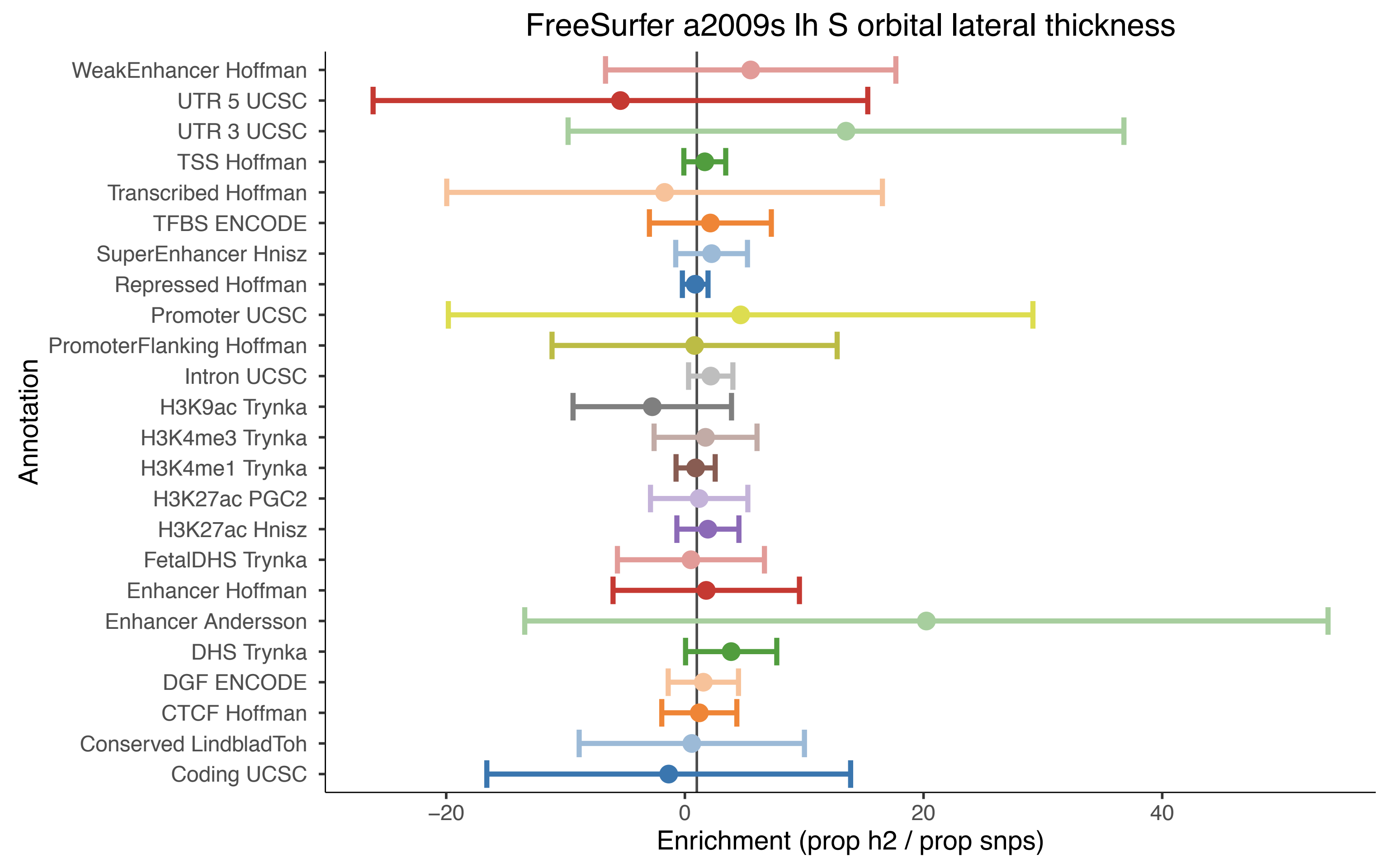
FreeSurfer a2009s lh S orbital lateral thickness
WeakEnhancer Hoffman
UTR 5 UCSC
UTR 3 UCSC
TSS Hoffman
Transcribed Hoffman
TFBS ENCODE
SuperEnhancer Hnisz
Repressed Hoffman
Promoter UCSC
PromoterFlanking Hoffman
Intron UCSC
H3K9ac Trynka
H3K4me3 Trynka
H3K4me1 Trynka
H3K27ac PGC2
H3K27ac Hnisz
FetalDHS Trynka
Enhancer Hoffman
Enhancer Andersson
DHS Trynka
DGF ENCODE
CTCF Hoffman
Conserved LindbladToh
Coding UCSC
Annotation
−20
0
20
40
Enrichment (prop h2 / prop snps)

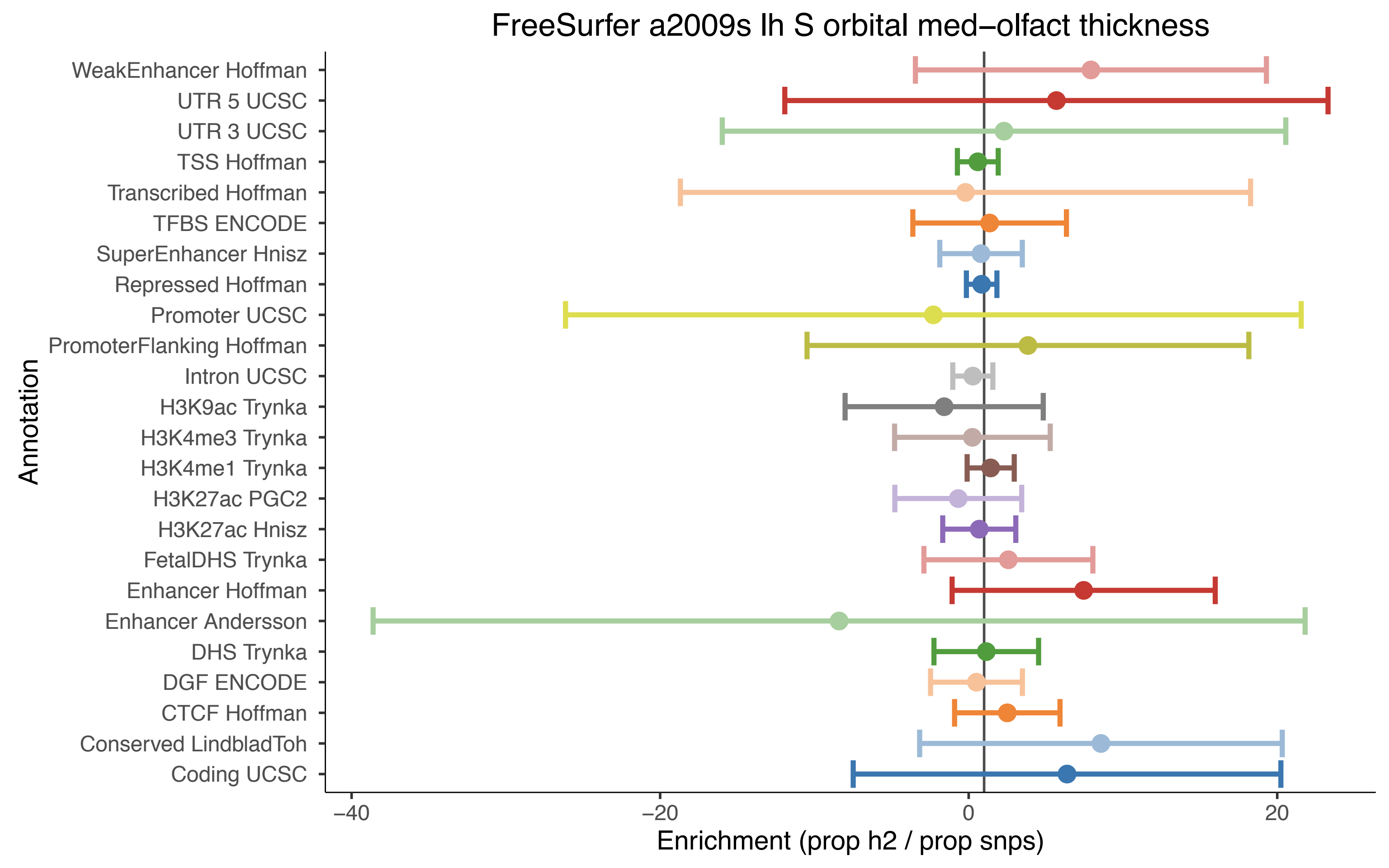
FreeSurfer a2009s lh S orbital med−olfact thickness
Annotation
WeakEnhancer Hoffman
UTR 5 UCSC
UTR 3 UCSC
TSS Hoffman
Transcribed Hoffman
TFBS ENCODE
SuperEnhancer Hnisz
Repressed Hoffman
Promoter UCSC
PromoterFlanking Hoffman
Intron UCSC
H3K9ac Trynka
H3K4me3 Trynka
H3K4me1 Trynka
H3K27ac PGC2
H3K27ac Hnisz
FetalDHS Trynka
Enhancer Hoffman
Enhancer Andersson
DHS Trynka
DGF ENCODE
CTCF Hoffman
Conserved LindbladToh
Coding UCSC
−40
−20
0
20
Enrichment (prop h2 / prop snps)

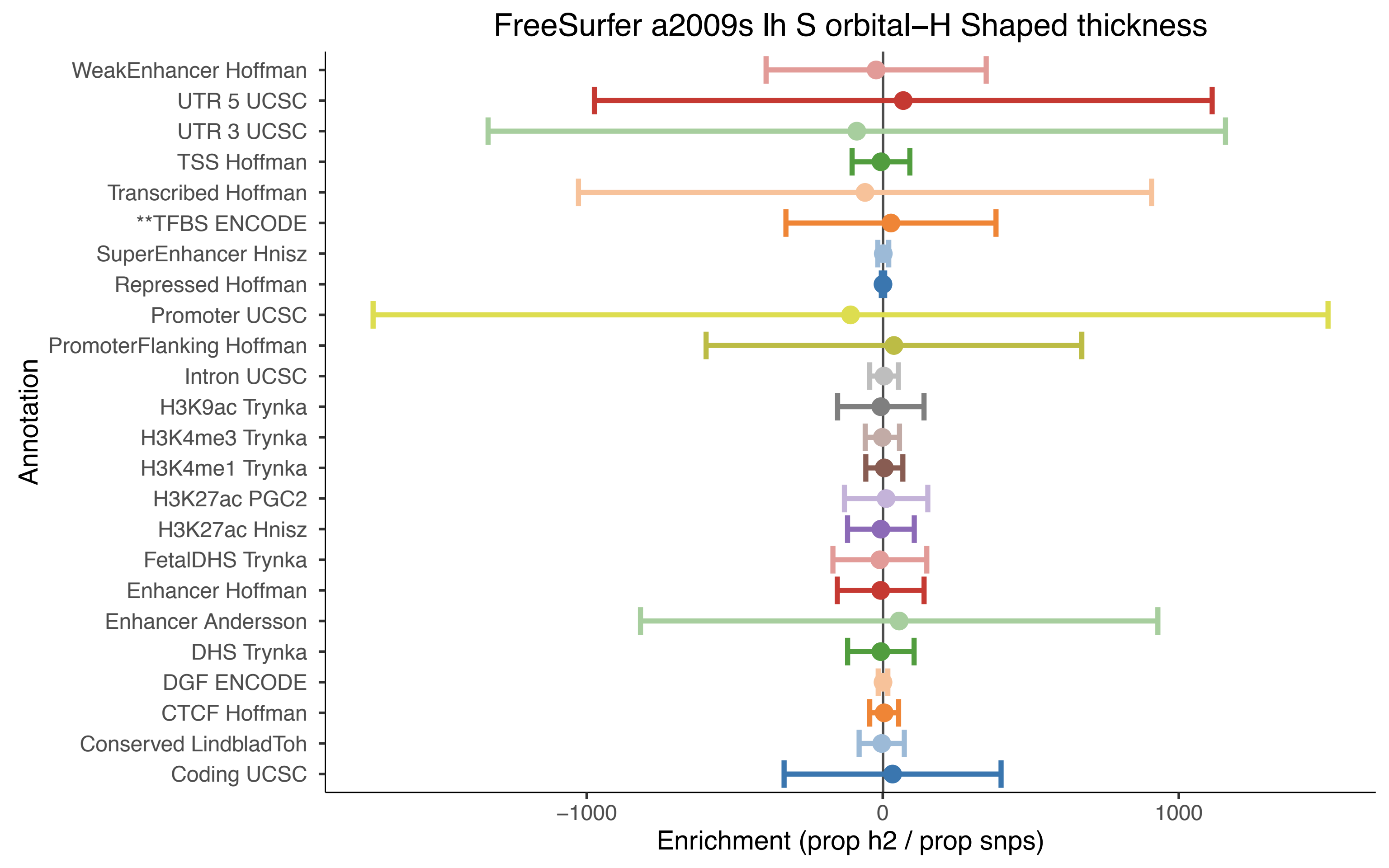
FreeSurfer a2009s lh S orbital−H Shaped thickness
WeakEnhancer Hoffman
UTR 5 UCSC
UTR 3 UCSC
TSS Hoffman
Transcribed Hoffman
**TFBS ENCODE
SuperEnhancer Hnisz
Repressed Hoffman
Promoter UCSC
PromoterFlanking Hoffman
Intron UCSC
H3K9ac Trynka
H3K4me3 Trynka
H3K4me1 Trynka
H3K27ac PGC2
H3K27ac Hnisz
FetalDHS Trynka
Enhancer Hoffman
Enhancer Andersson
DHS Trynka
DGF ENCODE
CTCF Hoffman
Conserved LindbladToh
Coding UCSC
Annotation
−1000
0
1000
Enrichment (prop h2 / prop snps)

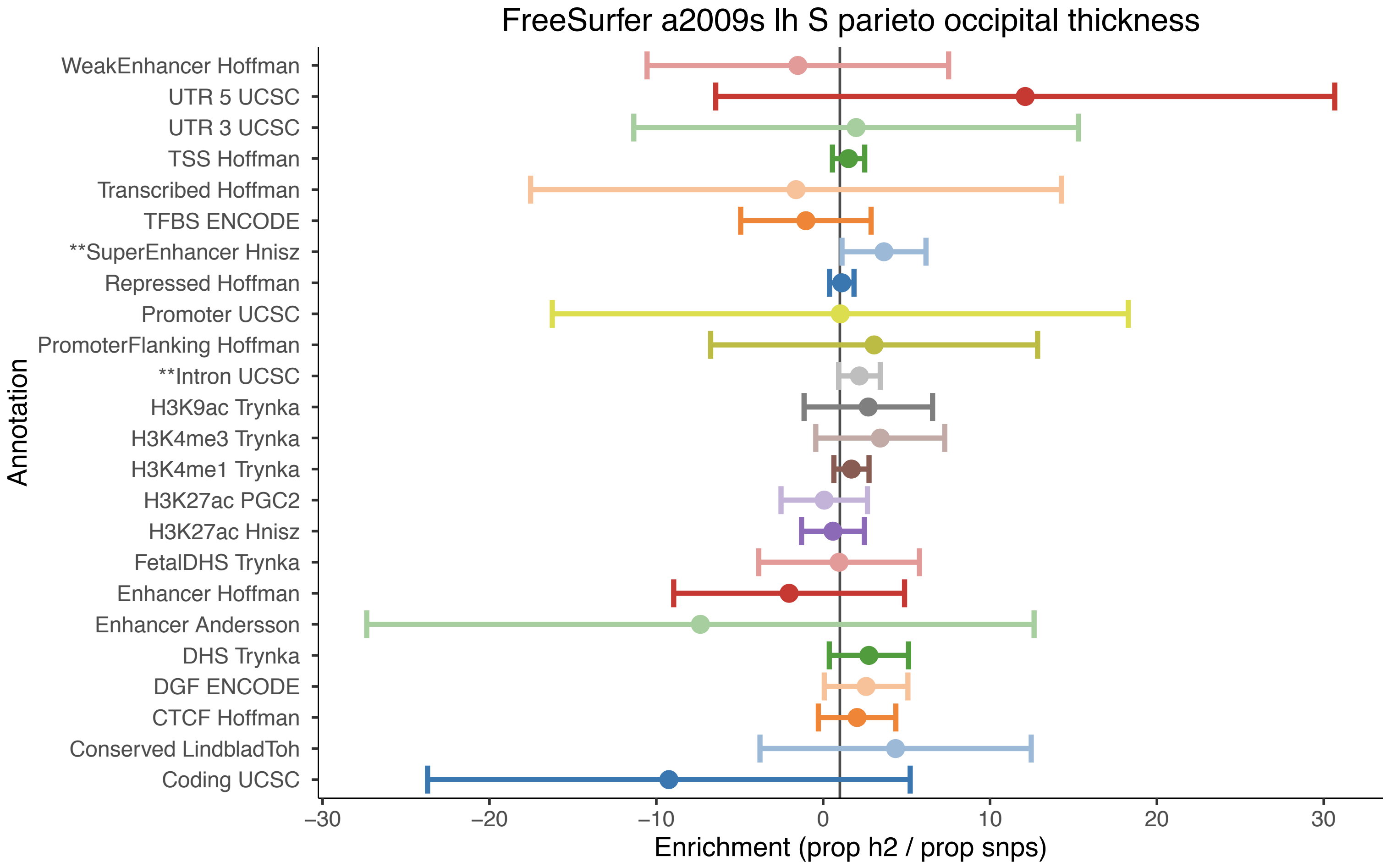
FreeSurfer a2009s lh S parieto occipital thickness
Annotation
WeakEnhancer Hoffman
UTR 5 UCSC
UTR 3 UCSC
TSS Hoffman
Transcribed Hoffman
TFBS ENCODE
**SuperEnhancer Hnisz
Repressed Hoffman
Promoter UCSC
PromoterFlanking Hoffman
**Intron UCSC
H3K9ac Trynka
H3K4me3 Trynka
H3K4me1 Trynka
H3K27ac PGC2
H3K27ac Hnisz
FetalDHS Trynka
Enhancer Hoffman
Enhancer Andersson
DHS Trynka
DGF ENCODE
CTCF Hoffman
Conserved LindbladToh
Coding UCSC
−30
−20
−10
0
10
20
30
Enrichment (prop h2 / prop snps)

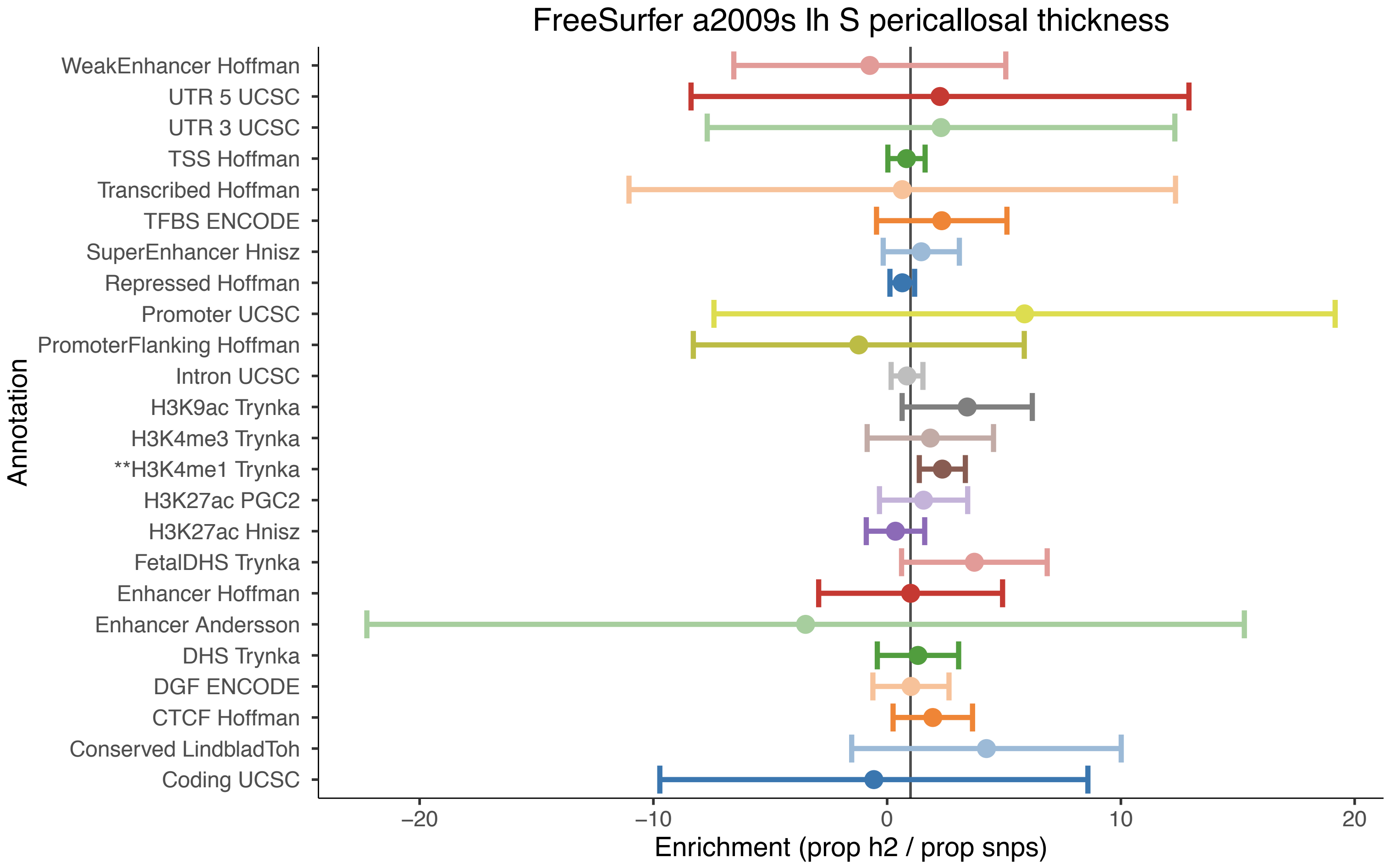

FreeSurfer a2009s lh S pericallosal thickness
WeakEnhancer Hoffman
UTR 5 UCSC
UTR 3 UCSC
TSS Hoffman
Transcribed Hoffman
TFBS ENCODE
SuperEnhancer Hnisz
Repressed Hoffman
Promoter UCSC
PromoterFlanking Hoffman
Intron UCSC
H3K9ac Trynka
H3K4me3 Trynka
**H3K4me1 Trynka
H3K27ac PGC2
H3K27ac Hnisz
FetalDHS Trynka
Enhancer Hoffman
Enhancer Andersson
DHS Trynka
DGF ENCODE
CTCF Hoffman
Conserved LindbladToh
Coding UCSC
Annotation
−20
−10
0
10
20
Enrichment (prop h2 / prop snps)

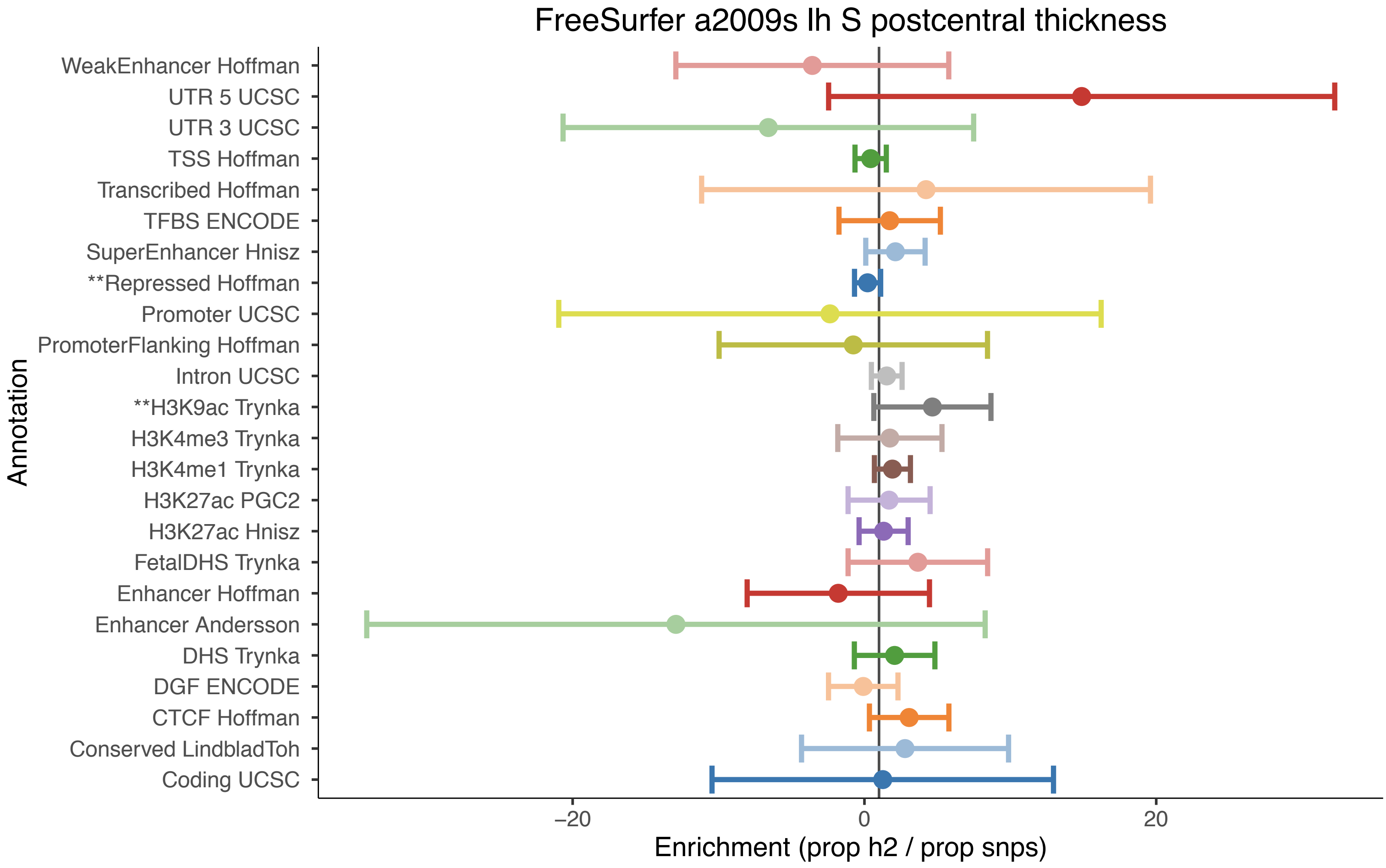

FreeSurfer a2009s lh S postcentral thickness
WeakEnhancer Hoffman
UTR 5 UCSC
UTR 3 UCSC
TSS Hoffman
Transcribed Hoffman
TFBS ENCODE
SuperEnhancer Hnisz
**Repressed Hoffman
Promoter UCSC
PromoterFlanking Hoffman
Intron UCSC
**H3K9ac Trynka
H3K4me3 Trynka
H3K4me1 Trynka
H3K27ac PGC2
H3K27ac Hnisz
FetalDHS Trynka
Enhancer Hoffman
Enhancer Andersson
DHS Trynka
DGF ENCODE
CTCF Hoffman
Conserved LindbladToh
Coding UCSC
Annotation
−20
0
20
Enrichment (prop h2 / prop snps)

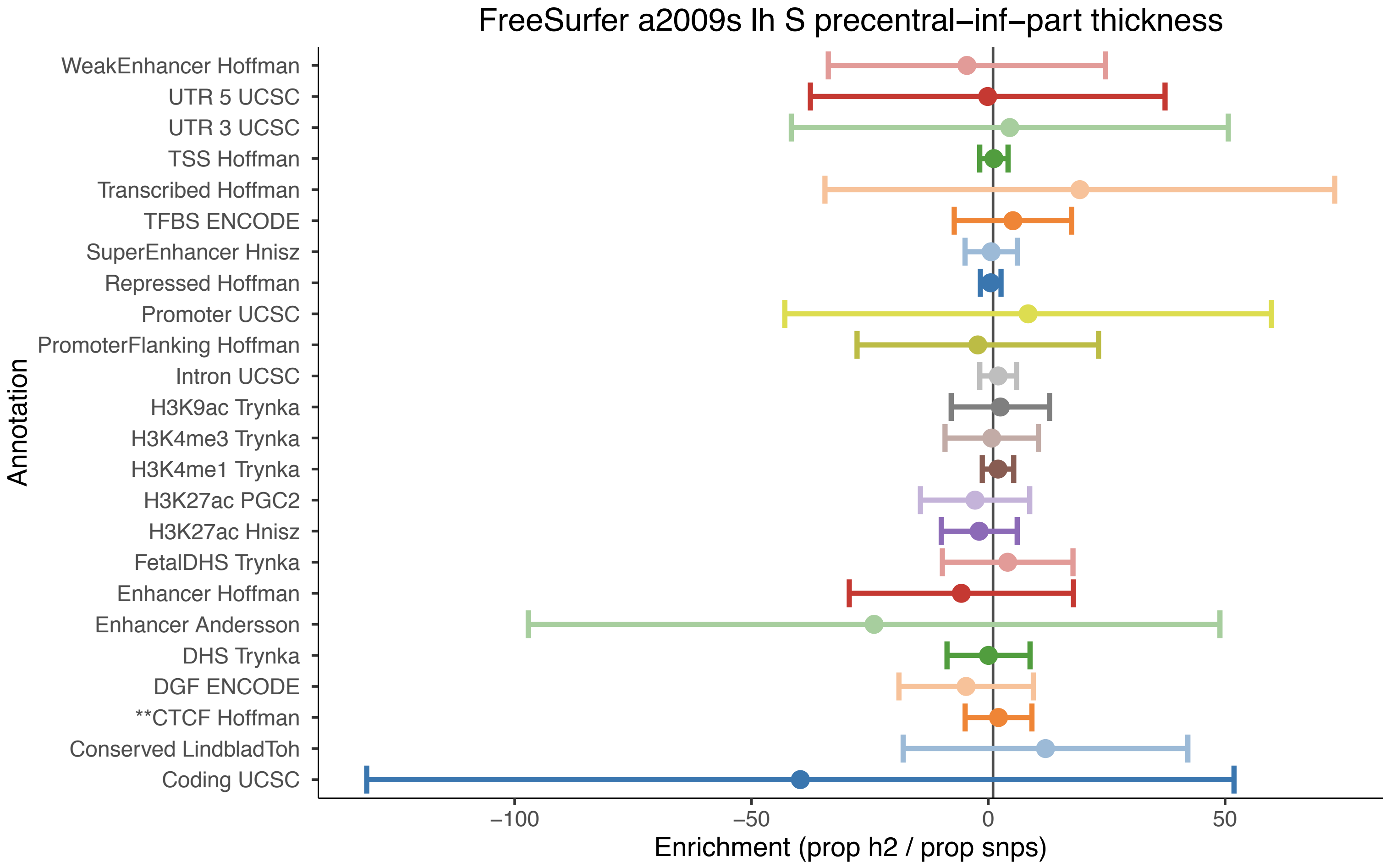
FreeSurfer a2009s lh S precentral−inf−part thickness
WeakEnhancer Hoffman
UTR 5 UCSC
UTR 3 UCSC
TSS Hoffman
Transcribed Hoffman
TFBS ENCODE
SuperEnhancer Hnisz
Repressed Hoffman
Promoter UCSC
PromoterFlanking Hoffman
Intron UCSC
H3K9ac Trynka
H3K4me3 Trynka
H3K4me1 Trynka
H3K27ac PGC2
H3K27ac Hnisz
FetalDHS Trynka
Enhancer Hoffman
Enhancer Andersson
DHS Trynka
DGF ENCODE
**CTCF Hoffman
Conserved LindbladToh
Coding UCSC
Annotation
−100
−50
0
50
Enrichment (prop h2 / prop snps)

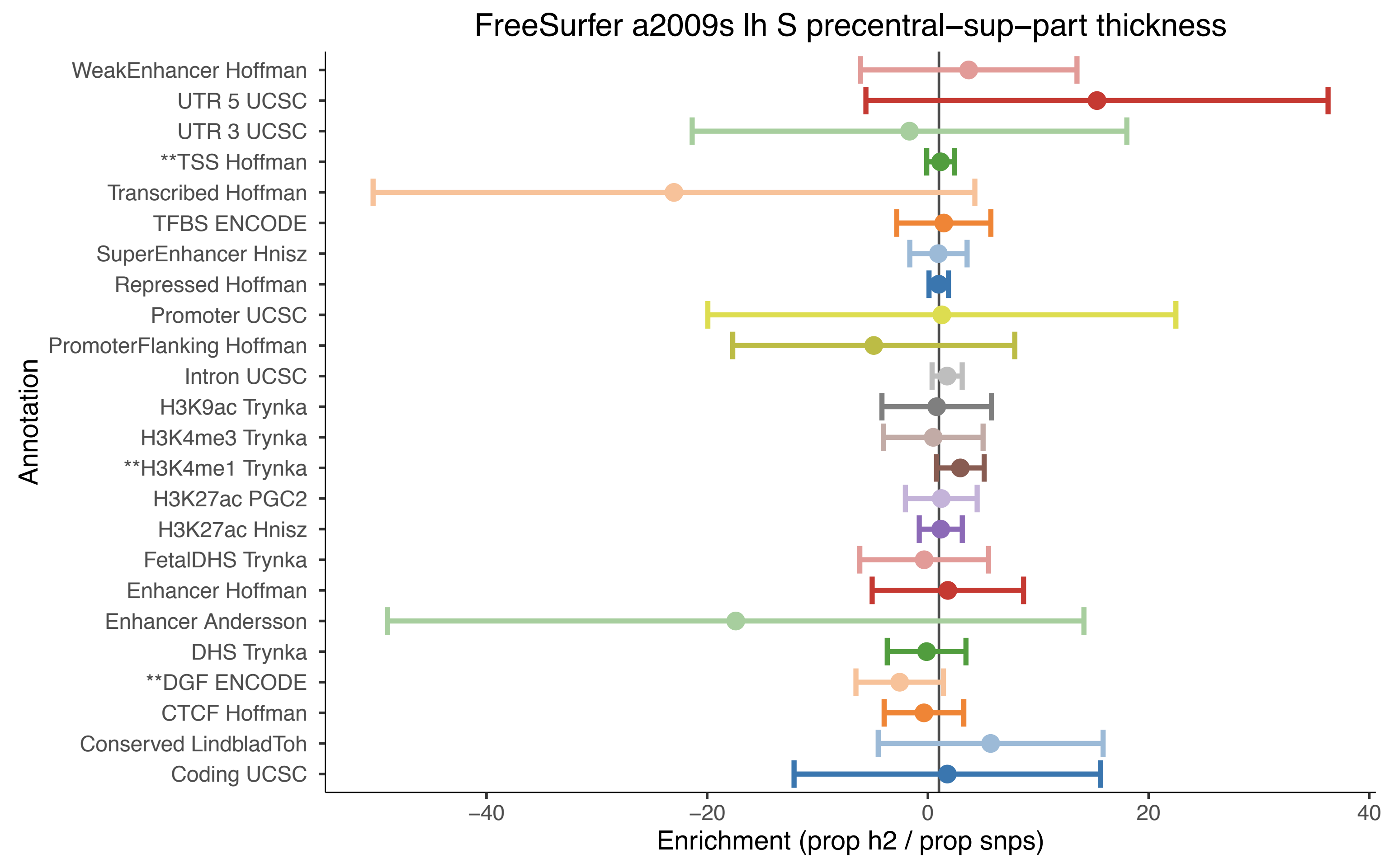

FreeSurfer a2009s lh S precentral−sup−part thickness
WeakEnhancer Hoffman
UTR 5 UCSC
UTR 3 UCSC
**TSS Hoffman
Transcribed Hoffman
TFBS ENCODE
SuperEnhancer Hnisz
Repressed Hoffman
Promoter UCSC
PromoterFlanking Hoffman
Intron UCSC
H3K9ac Trynka
H3K4me3 Trynka
**H3K4me1 Trynka
H3K27ac PGC2
H3K27ac Hnisz
FetalDHS Trynka
Enhancer Hoffman
Enhancer Andersson
DHS Trynka
**DGF ENCODE
CTCF Hoffman
Conserved LindbladToh
Coding UCSC
Annotation
−40
−20
0
20
40
Enrichment (prop h2 / prop snps)

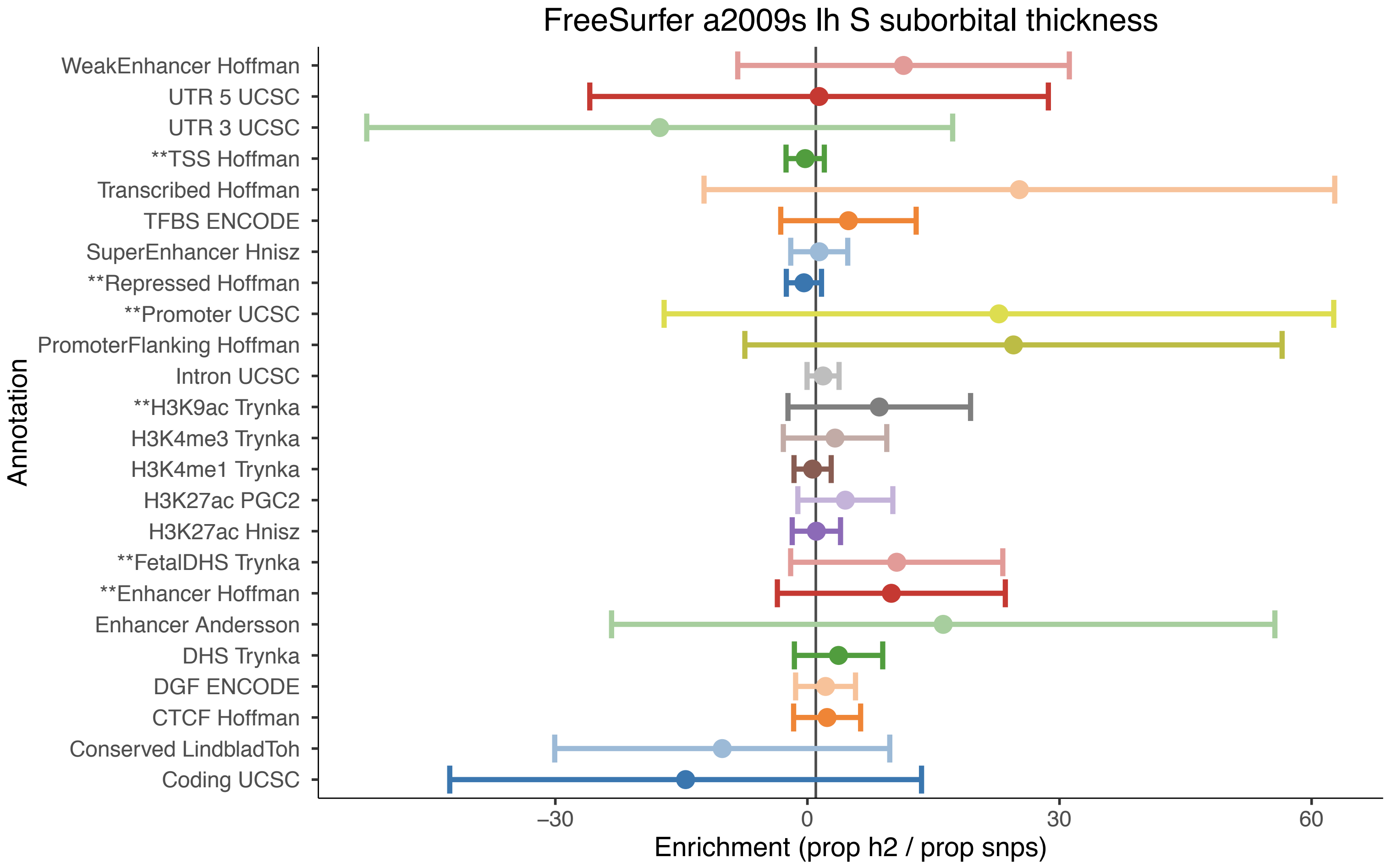
FreeSurfer a2009s lh S suborbital thickness
WeakEnhancer Hoffman
UTR 5 UCSC
UTR 3 UCSC
**TSS Hoffman
Transcribed Hoffman
TFBS ENCODE
SuperEnhancer Hnisz
**Repressed Hoffman
**Promoter UCSC
PromoterFlanking Hoffman
Intron UCSC
**H3K9ac Trynka
H3K4me3 Trynka
H3K4me1 Trynka
H3K27ac PGC2
H3K27ac Hnisz
**FetalDHS Trynka
**Enhancer Hoffman
Enhancer Andersson
DHS Trynka
DGF ENCODE
CTCF Hoffman
Conserved LindbladToh
Coding UCSC
Annotation
−30
0
30
60
Enrichment (prop h2 / prop snps)

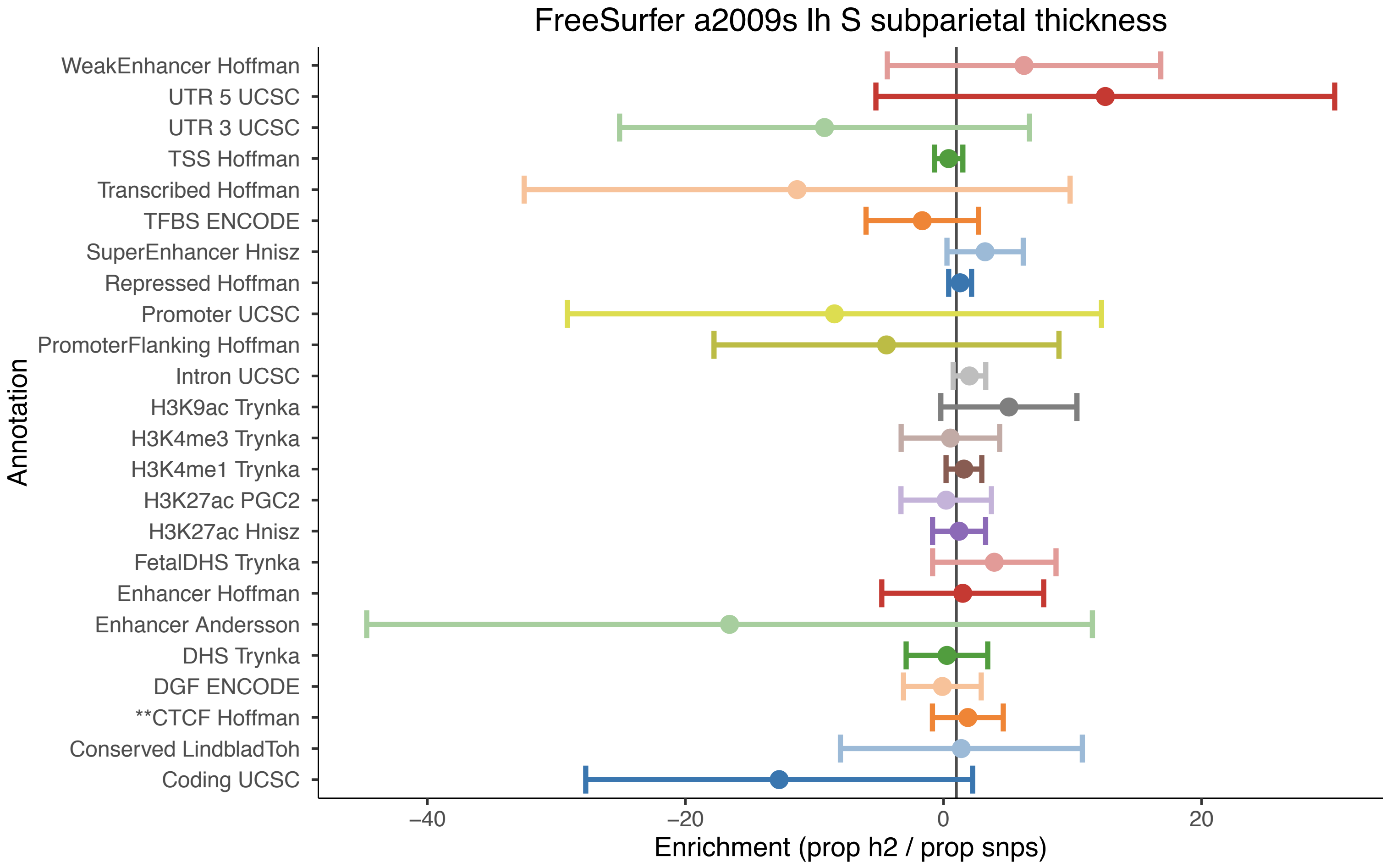
FreeSurfer a2009s lh S subparietal thickness
WeakEnhancer Hoffman
UTR 5 UCSC
UTR 3 UCSC
TSS Hoffman
Transcribed Hoffman
TFBS ENCODE
SuperEnhancer Hnisz
Repressed Hoffman
Promoter UCSC
PromoterFlanking Hoffman
Intron UCSC
H3K9ac Trynka
H3K4me3 Trynka
H3K4me1 Trynka
H3K27ac PGC2
H3K27ac Hnisz
FetalDHS Trynka
Enhancer Hoffman
Enhancer Andersson
DHS Trynka
DGF ENCODE
**CTCF Hoffman
Conserved LindbladToh
Coding UCSC
Annotation
−40
−20
0
20
Enrichment (prop h2 / prop snps)

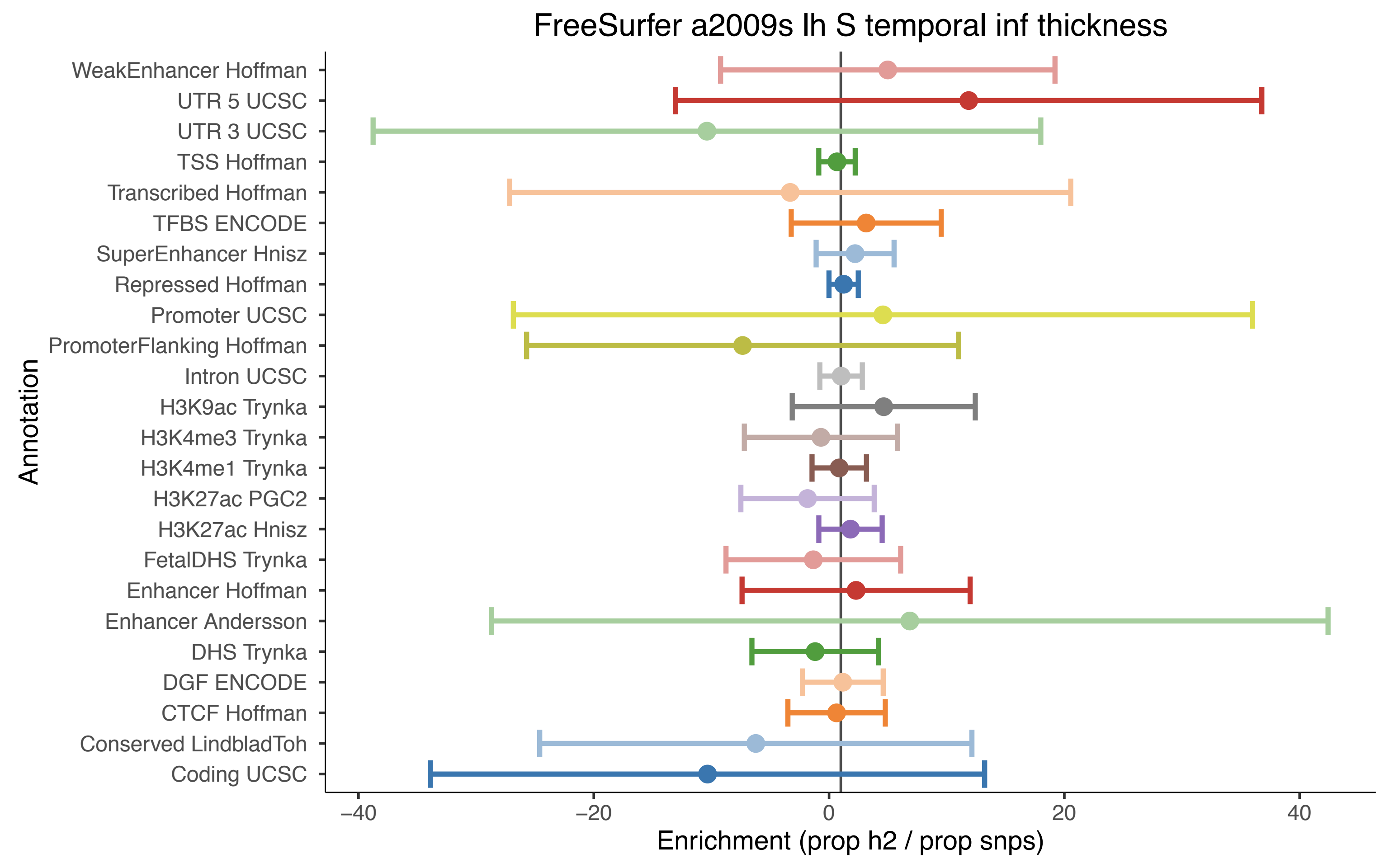
FreeSurfer a2009s lh S temporal inf thickness
WeakEnhancer Hoffman
UTR 5 UCSC
UTR 3 UCSC
TSS Hoffman
Transcribed Hoffman
TFBS ENCODE
SuperEnhancer Hnisz
Repressed Hoffman
Promoter UCSC
PromoterFlanking Hoffman
Intron UCSC
H3K9ac Trynka
H3K4me3 Trynka
H3K4me1 Trynka
H3K27ac PGC2
H3K27ac Hnisz
FetalDHS Trynka
Enhancer Hoffman
Enhancer Andersson
DHS Trynka
DGF ENCODE
CTCF Hoffman
Conserved LindbladToh
Coding UCSC
Annotation
−40
−20
0
20
40
Enrichment (prop h2 / prop snps)

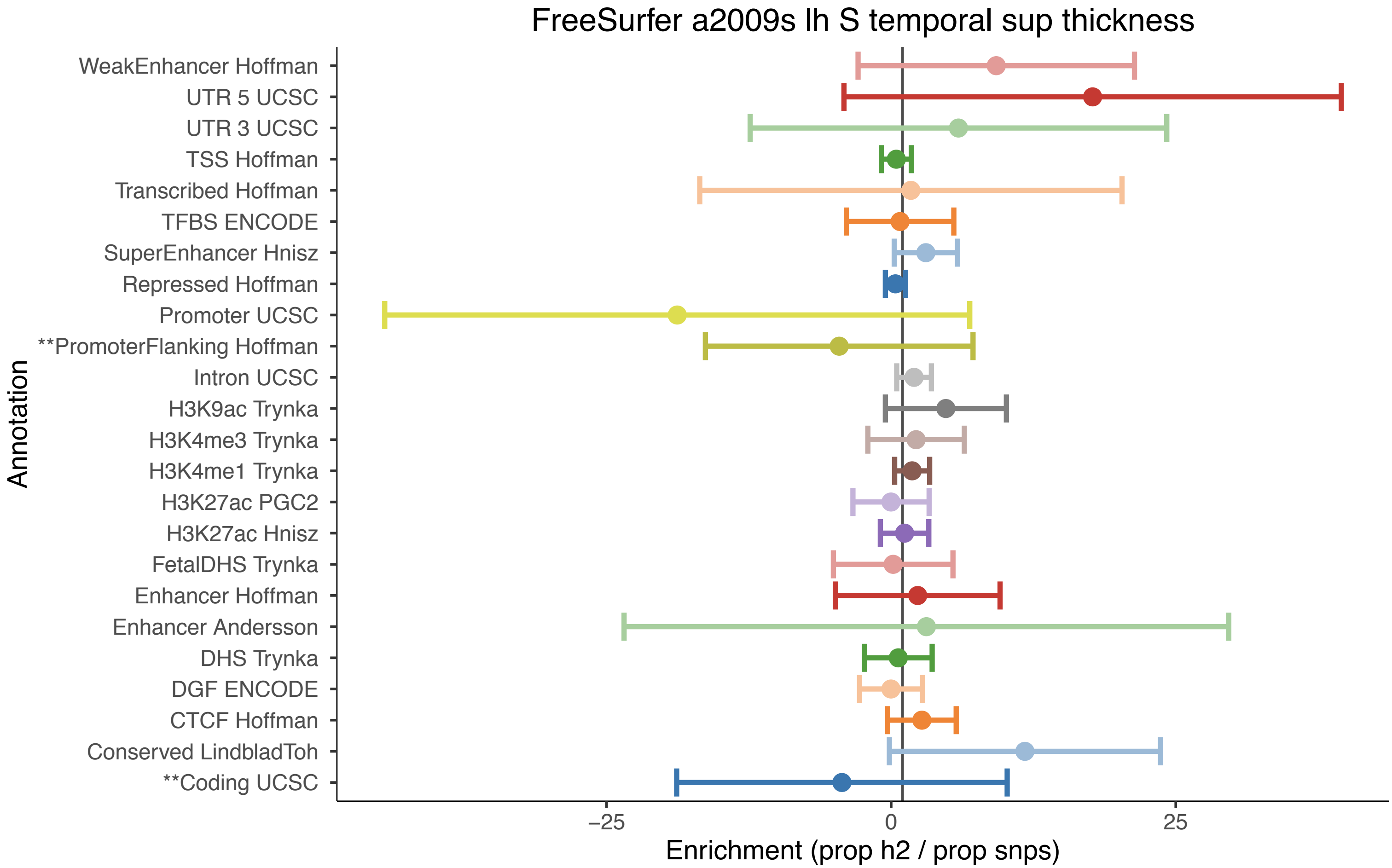

FreeSurfer a2009s lh S temporal sup thickness
WeakEnhancer Hoffman
UTR 5 UCSC
UTR 3 UCSC
TSS Hoffman
Transcribed Hoffman
TFBS ENCODE
SuperEnhancer Hnisz
Repressed Hoffman
Promoter UCSC
**PromoterFlanking Hoffman
Intron UCSC
H3K9ac Trynka
H3K4me3 Trynka
H3K4me1 Trynka
H3K27ac PGC2
H3K27ac Hnisz
FetalDHS Trynka
Enhancer Hoffman
Enhancer Andersson
DHS Trynka
DGF ENCODE
CTCF Hoffman
Conserved LindbladToh
**Coding UCSC
Annotation
−25
0
25
Enrichment (prop h2 / prop snps)

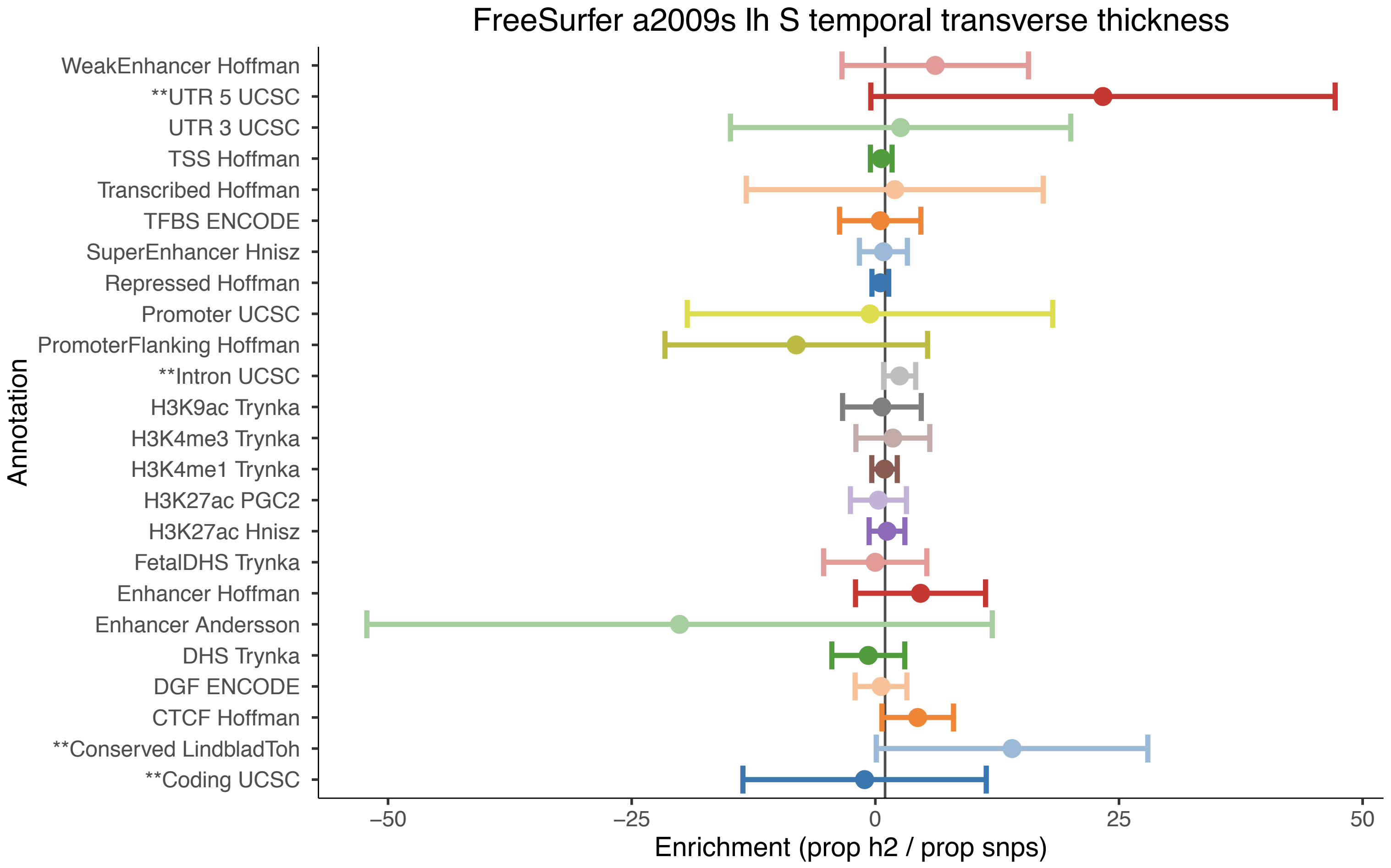

FreeSurfer a2009s lh S temporal transverse thickness
Annotation
WeakEnhancer Hoffman
**UTR 5 UCSC
UTR 3 UCSC
TSS Hoffman
Transcribed Hoffman
TFBS ENCODE
SuperEnhancer Hnisz
Repressed Hoffman
Promoter UCSC
PromoterFlanking Hoffman
**Intron UCSC
H3K9ac Trynka
H3K4me3 Trynka
H3K4me1 Trynka
H3K27ac PGC2
H3K27ac Hnisz
FetalDHS Trynka
Enhancer Hoffman
Enhancer Andersson
DHS Trynka
DGF ENCODE
CTCF Hoffman
**Conserved LindbladToh
**Coding UCSC
−50
−25
0
25
50
Enrichment (prop h2 / prop snps)

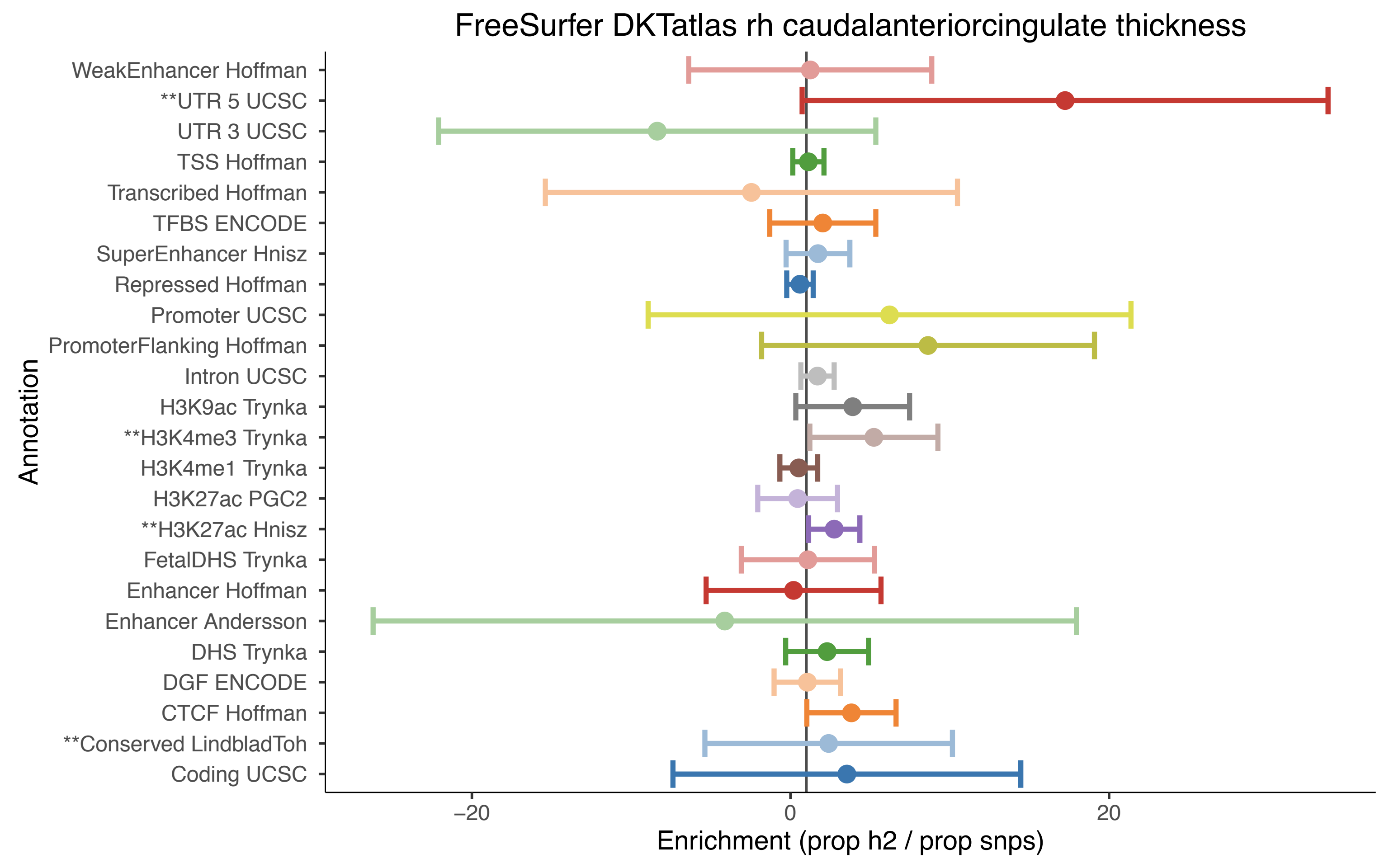
FreeSurfer DKTatlas rh caudalanteriorcingulate thickness
WeakEnhancer Hoffman
**UTR 5 UCSC
UTR 3 UCSC
TSS Hoffman
Transcribed Hoffman
TFBS ENCODE
SuperEnhancer Hnisz
Repressed Hoffman
Promoter UCSC
PromoterFlanking Hoffman
Intron UCSC
H3K9ac Trynka
**H3K4me3 Trynka
H3K4me1 Trynka
H3K27ac PGC2
**H3K27ac Hnisz
FetalDHS Trynka
Enhancer Hoffman
Enhancer Andersson
DHS Trynka
DGF ENCODE
CTCF Hoffman
**Conserved LindbladToh
Coding UCSC
Annotation
−20
0
20
Enrichment (prop h2 / prop snps)

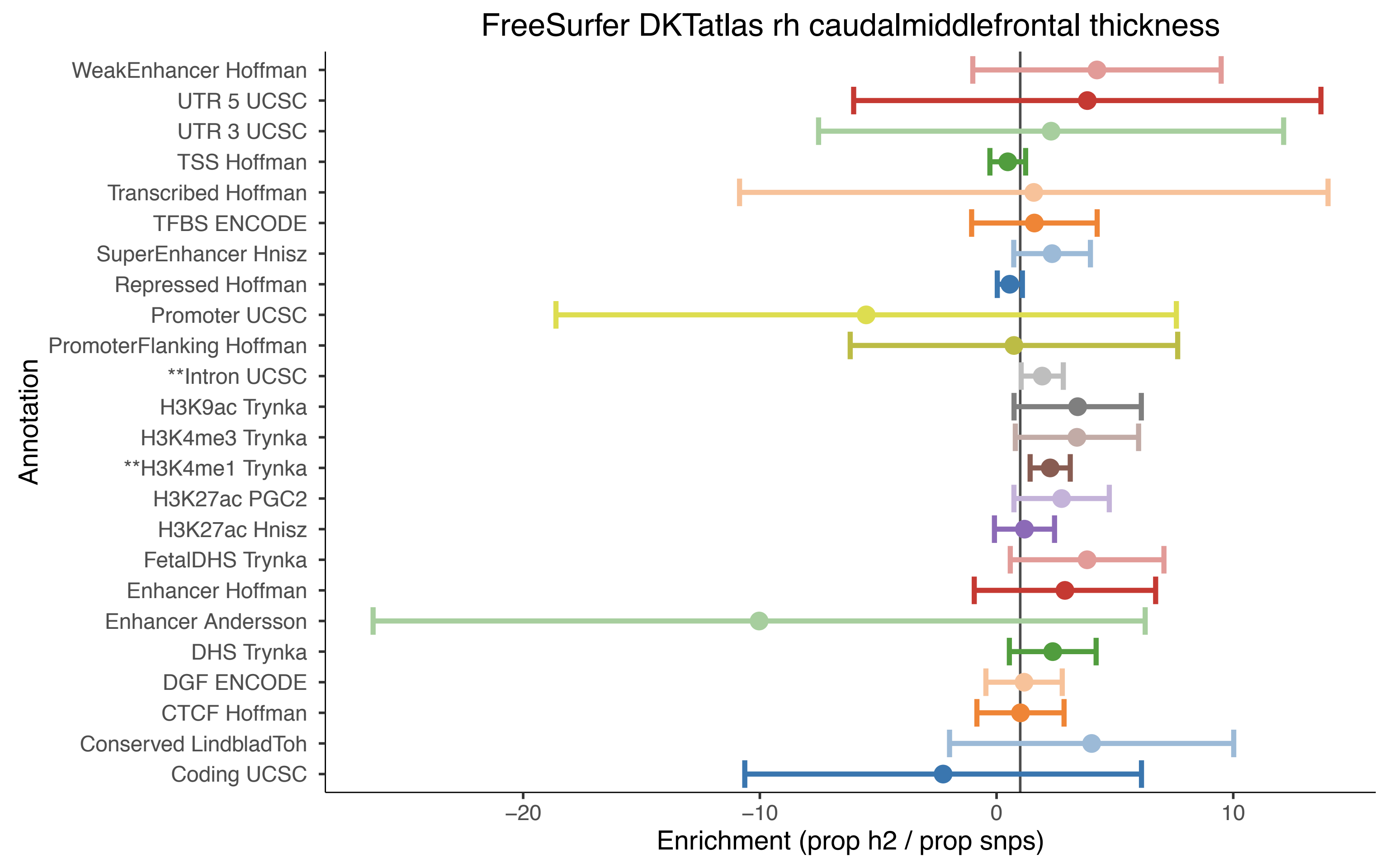

FreeSurfer DKTatlas rh caudalmiddlefrontal thickness
WeakEnhancer Hoffman
UTR 5 UCSC
UTR 3 UCSC
TSS Hoffman
Transcribed Hoffman
TFBS ENCODE
SuperEnhancer Hnisz
Repressed Hoffman
Promoter UCSC
PromoterFlanking Hoffman
**Intron UCSC
H3K9ac Trynka
H3K4me3 Trynka
**H3K4me1 Trynka
H3K27ac PGC2
H3K27ac Hnisz
FetalDHS Trynka
Enhancer Hoffman
Enhancer Andersson
DHS Trynka
DGF ENCODE
CTCF Hoffman
Conserved LindbladToh
Coding UCSC
Annotation
−20
−10
0
10
Enrichment (prop h2 / prop snps)

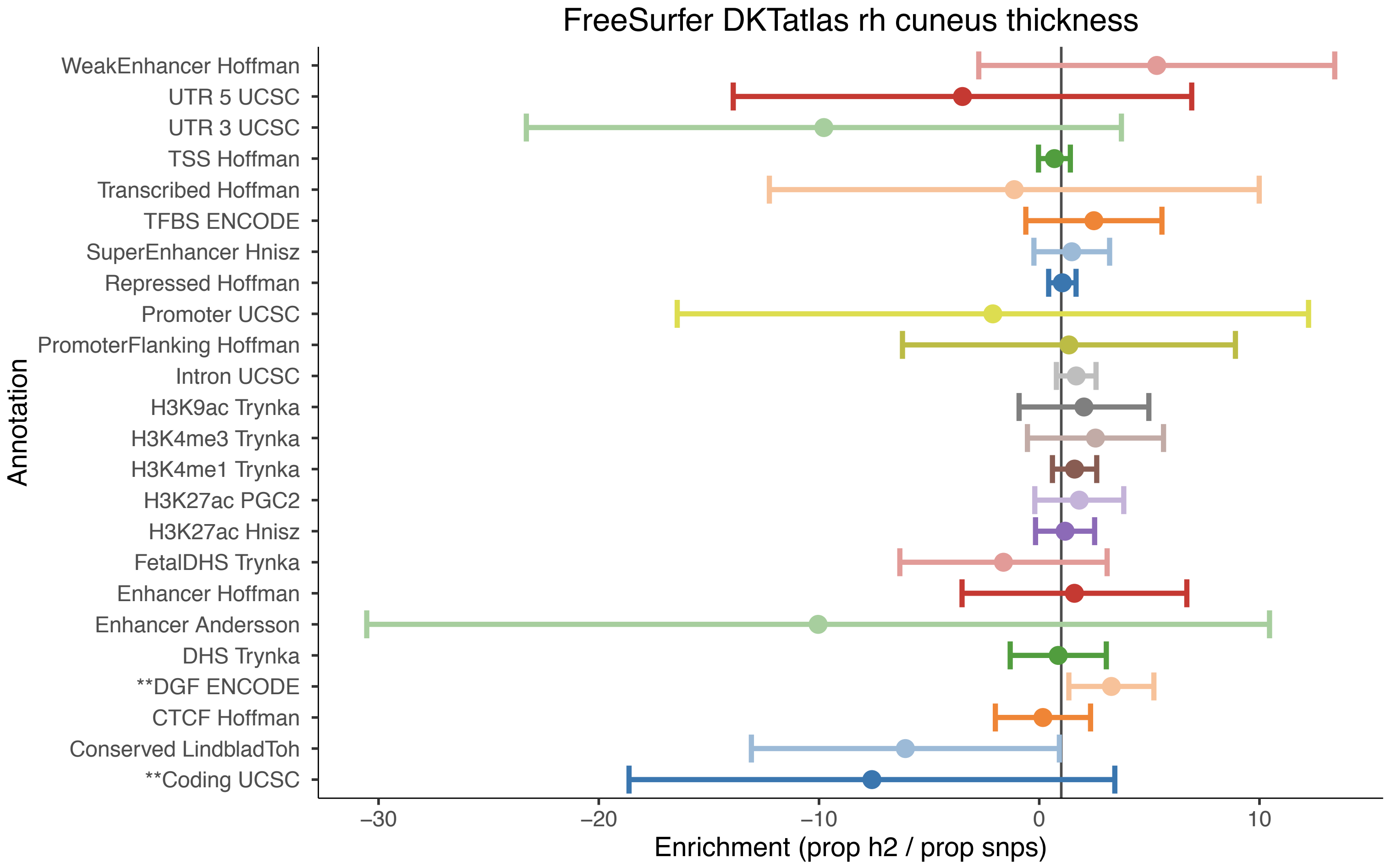
FreeSurfer DKTatlas rh cuneus thickness
WeakEnhancer Hoffman
UTR 5 UCSC
UTR 3 UCSC
TSS Hoffman
Transcribed Hoffman
TFBS ENCODE
SuperEnhancer Hnisz
Repressed Hoffman
Promoter UCSC
PromoterFlanking Hoffman
Intron UCSC
H3K9ac Trynka
H3K4me3 Trynka
H3K4me1 Trynka
H3K27ac PGC2
H3K27ac Hnisz
FetalDHS Trynka
Enhancer Hoffman
Enhancer Andersson
DHS Trynka
**DGF ENCODE
CTCF Hoffman
Conserved LindbladToh
**Coding UCSC
Annotation
−30
−20
−10
0
10
Enrichment (prop h2 / prop snps)

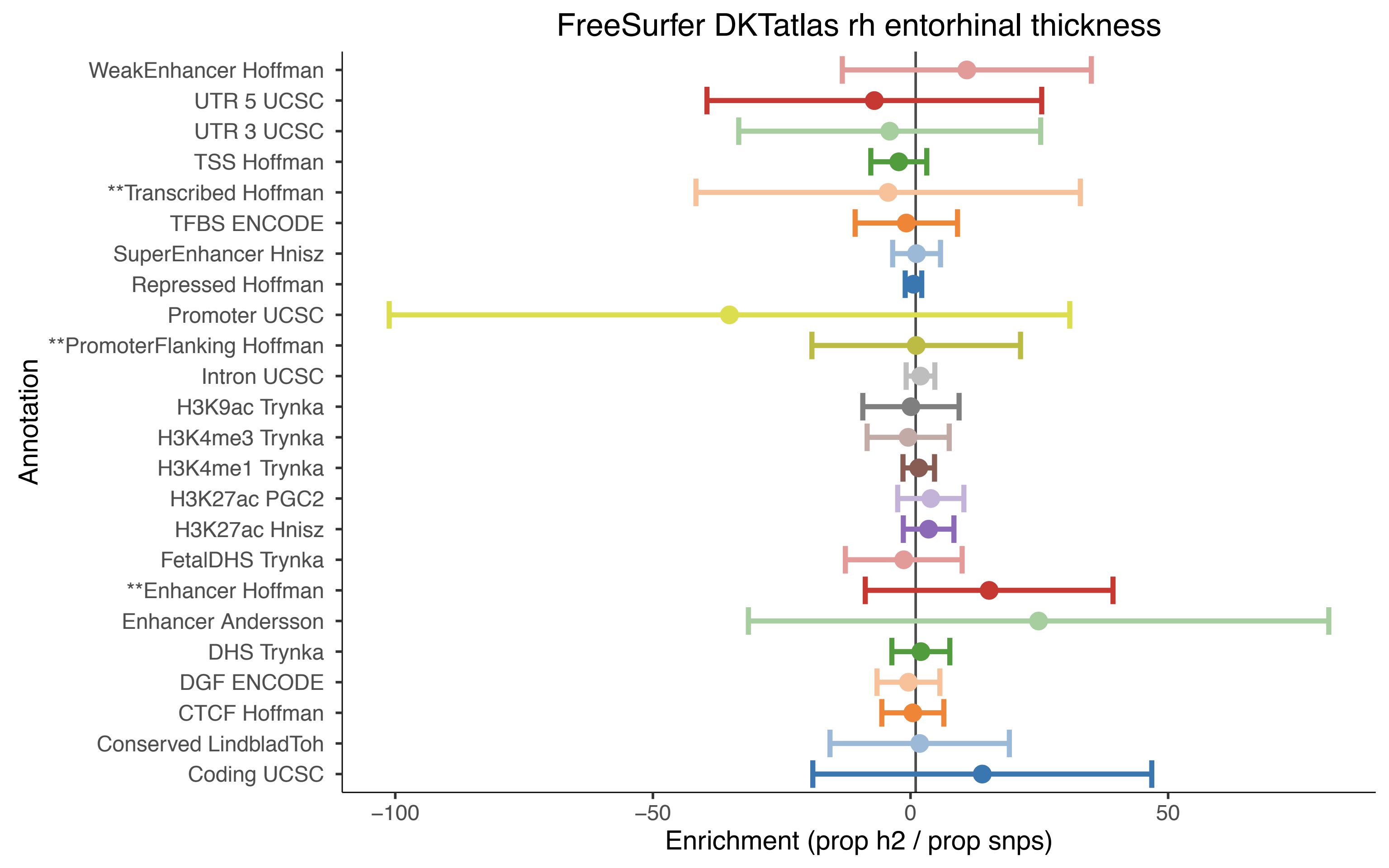

FreeSurfer DKTatlas rh entorhinal thickness
WeakEnhancer Hoffman
UTR 5 UCSC
UTR 3 UCSC
TSS Hoffman
**Transcribed Hoffman
TFBS ENCODE
SuperEnhancer Hnisz
Repressed Hoffman
Promoter UCSC
**PromoterFlanking Hoffman
Intron UCSC
H3K9ac Trynka
H3K4me3 Trynka
H3K4me1 Trynka
H3K27ac PGC2
H3K27ac Hnisz
FetalDHS Trynka
**Enhancer Hoffman
Enhancer Andersson
DHS Trynka
DGF ENCODE
CTCF Hoffman
Conserved LindbladToh
Coding UCSC
Annotation
−100
−50
0
50
Enrichment (prop h2 / prop snps)

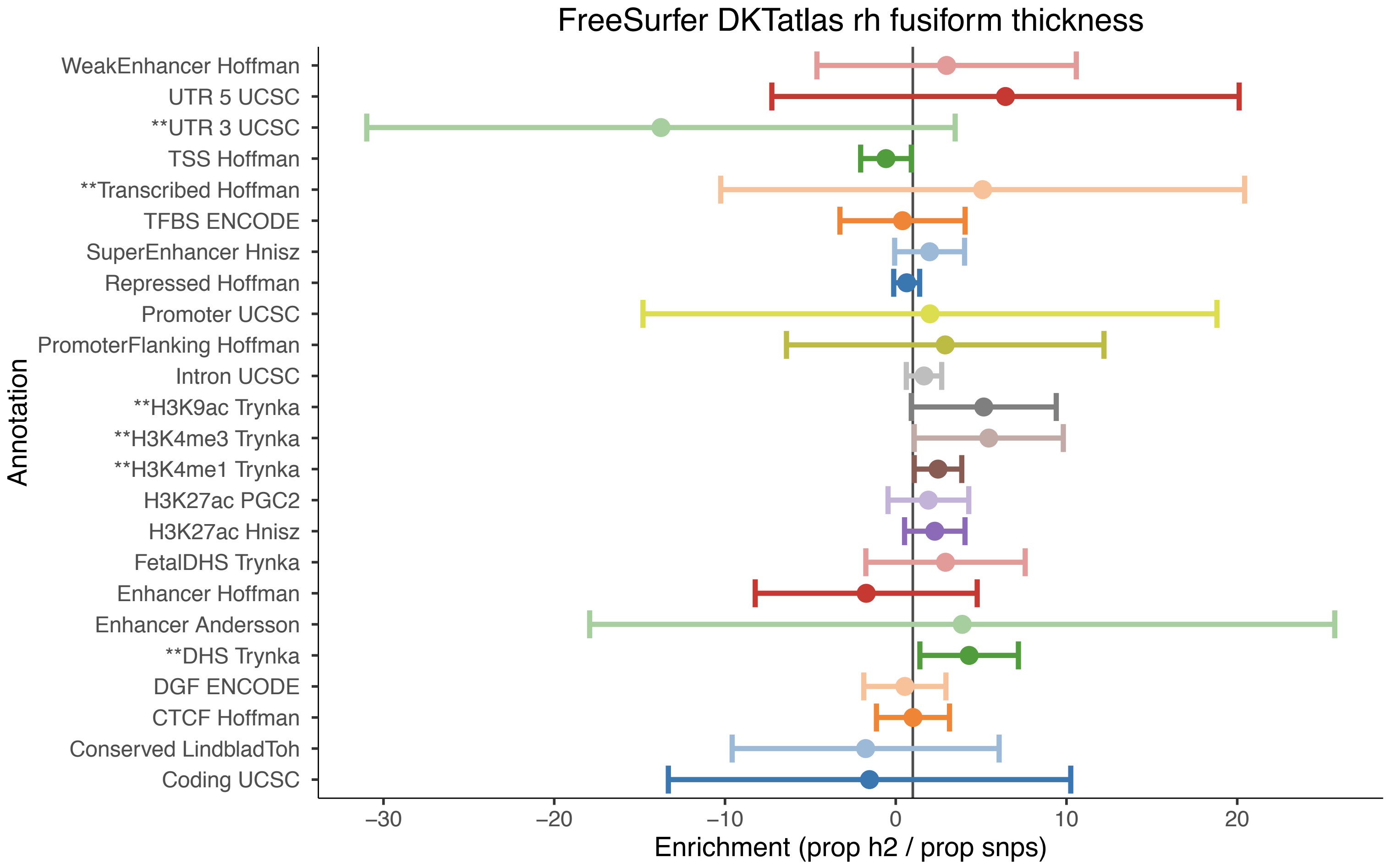
FreeSurfer DKTatlas rh fusiform thickness
WeakEnhancer Hoffman
UTR 5 UCSC
**UTR 3 UCSC
TSS Hoffman
**Transcribed Hoffman
TFBS ENCODE
SuperEnhancer Hnisz
Repressed Hoffman
Promoter UCSC
PromoterFlanking Hoffman
Intron UCSC
**H3K9ac Trynka
**H3K4me3 Trynka
**H3K4me1 Trynka
H3K27ac PGC2
H3K27ac Hnisz
FetalDHS Trynka
Enhancer Hoffman
Enhancer Andersson
**DHS Trynka
DGF ENCODE
CTCF Hoffman
Conserved LindbladToh
Coding UCSC
Annotation
−30
−20
−10
0
10
20
Enrichment (prop h2 / prop snps)

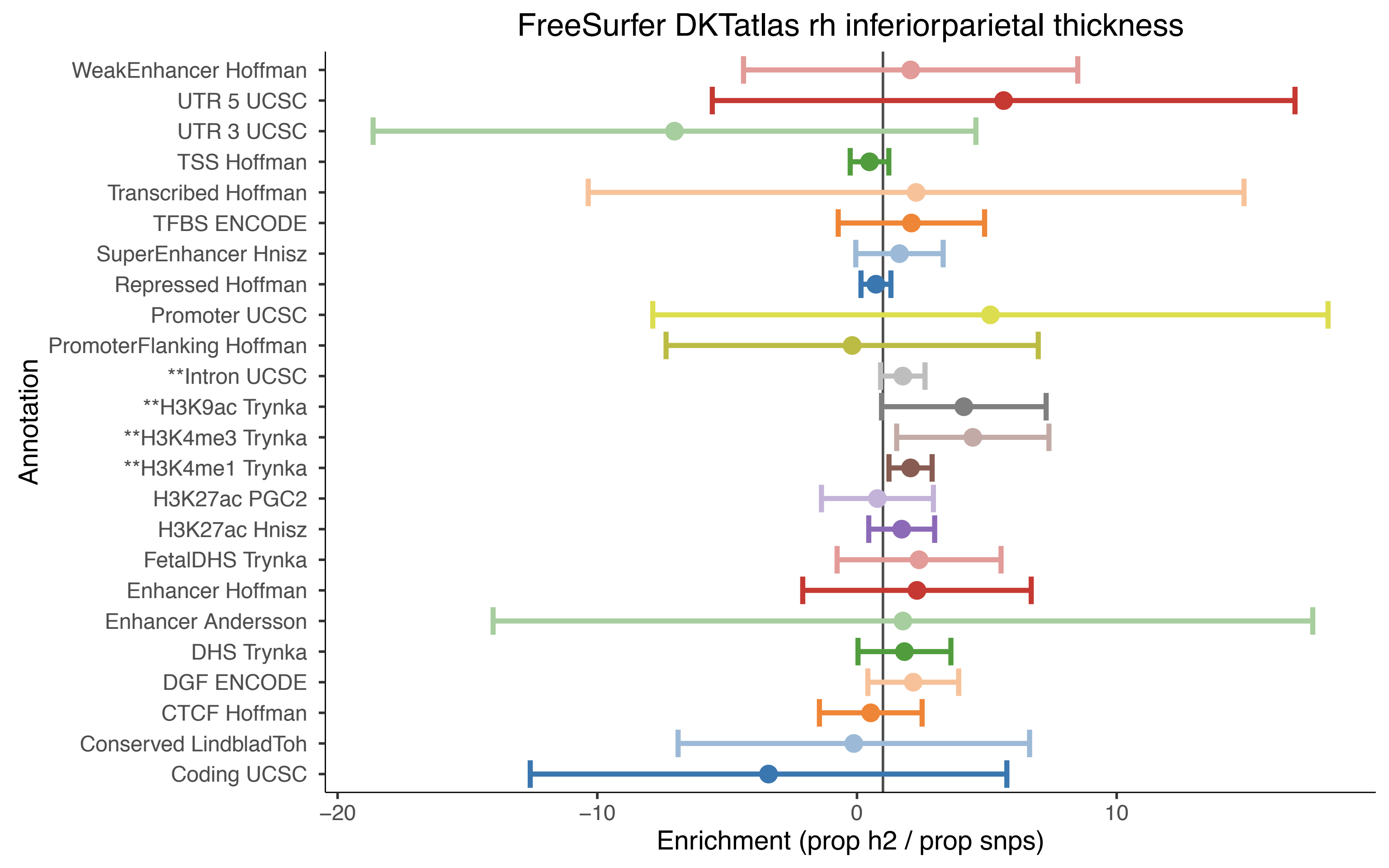

FreeSurfer DKTatlas rh inferiorparietal thickness
WeakEnhancer Hoffman
UTR 5 UCSC
UTR 3 UCSC
TSS Hoffman
Transcribed Hoffman
TFBS ENCODE
SuperEnhancer Hnisz
Repressed Hoffman
Promoter UCSC
PromoterFlanking Hoffman
**Intron UCSC
**H3K9ac Trynka
**H3K4me3 Trynka
**H3K4me1 Trynka
H3K27ac PGC2
H3K27ac Hnisz
FetalDHS Trynka
Enhancer Hoffman
Enhancer Andersson
DHS Trynka
DGF ENCODE
CTCF Hoffman
Conserved LindbladToh
Coding UCSC
Annotation
−20
−10
0
10
Enrichment (prop h2 / prop snps)

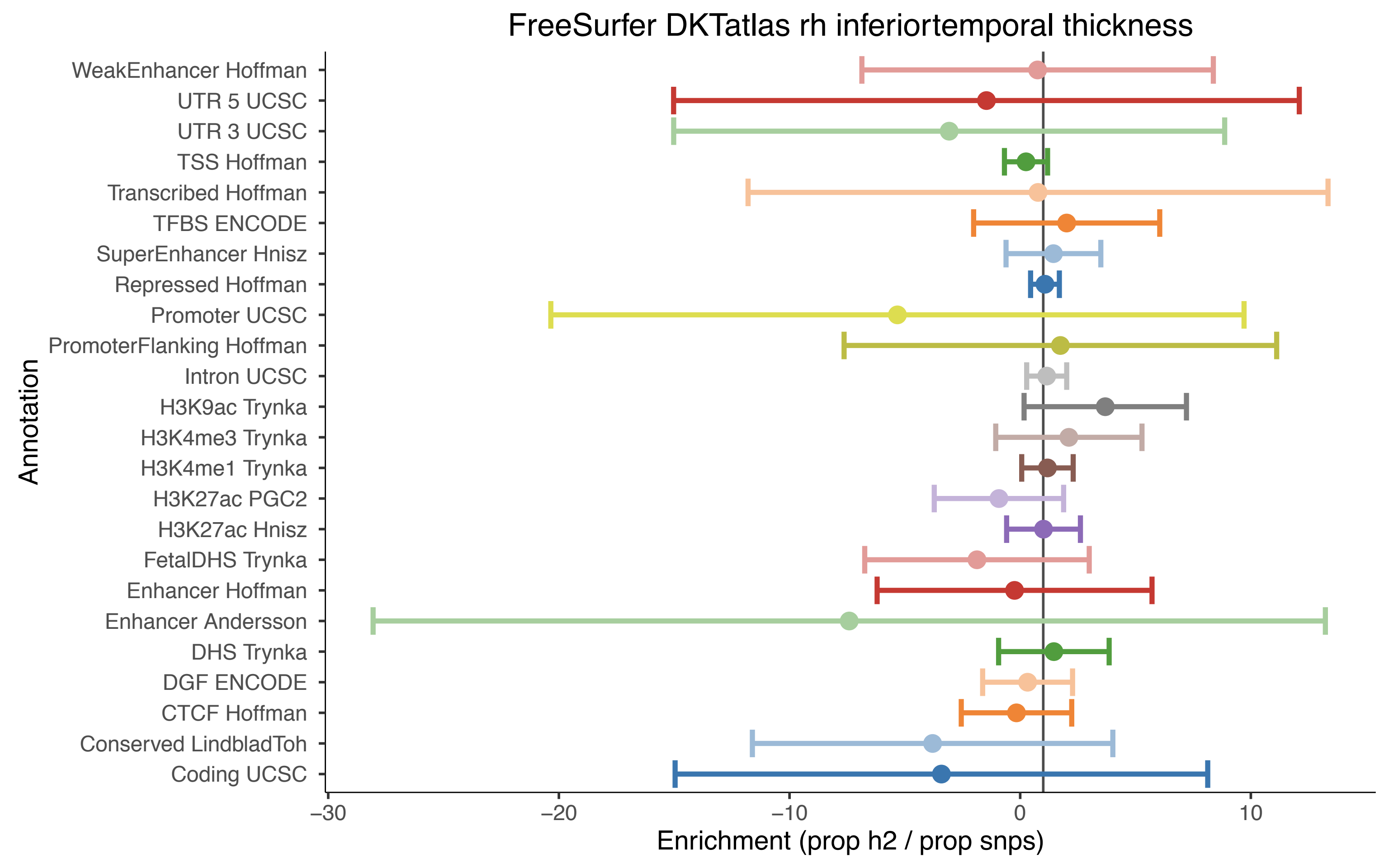
FreeSurfer DKTatlas rh inferiortemporal thickness
Annotation
WeakEnhancer Hoffman
UTR 5 UCSC
UTR 3 UCSC
TSS Hoffman
Transcribed Hoffman
TFBS ENCODE
SuperEnhancer Hnisz
Repressed Hoffman
Promoter UCSC
PromoterFlanking Hoffman
Intron UCSC
H3K9ac Trynka
H3K4me3 Trynka
H3K4me1 Trynka
H3K27ac PGC2
H3K27ac Hnisz
FetalDHS Trynka
Enhancer Hoffman
Enhancer Andersson
DHS Trynka
DGF ENCODE
CTCF Hoffman
Conserved LindbladToh
Coding UCSC
−30
−20
−10
0
10
Enrichment (prop h2 / prop snps)

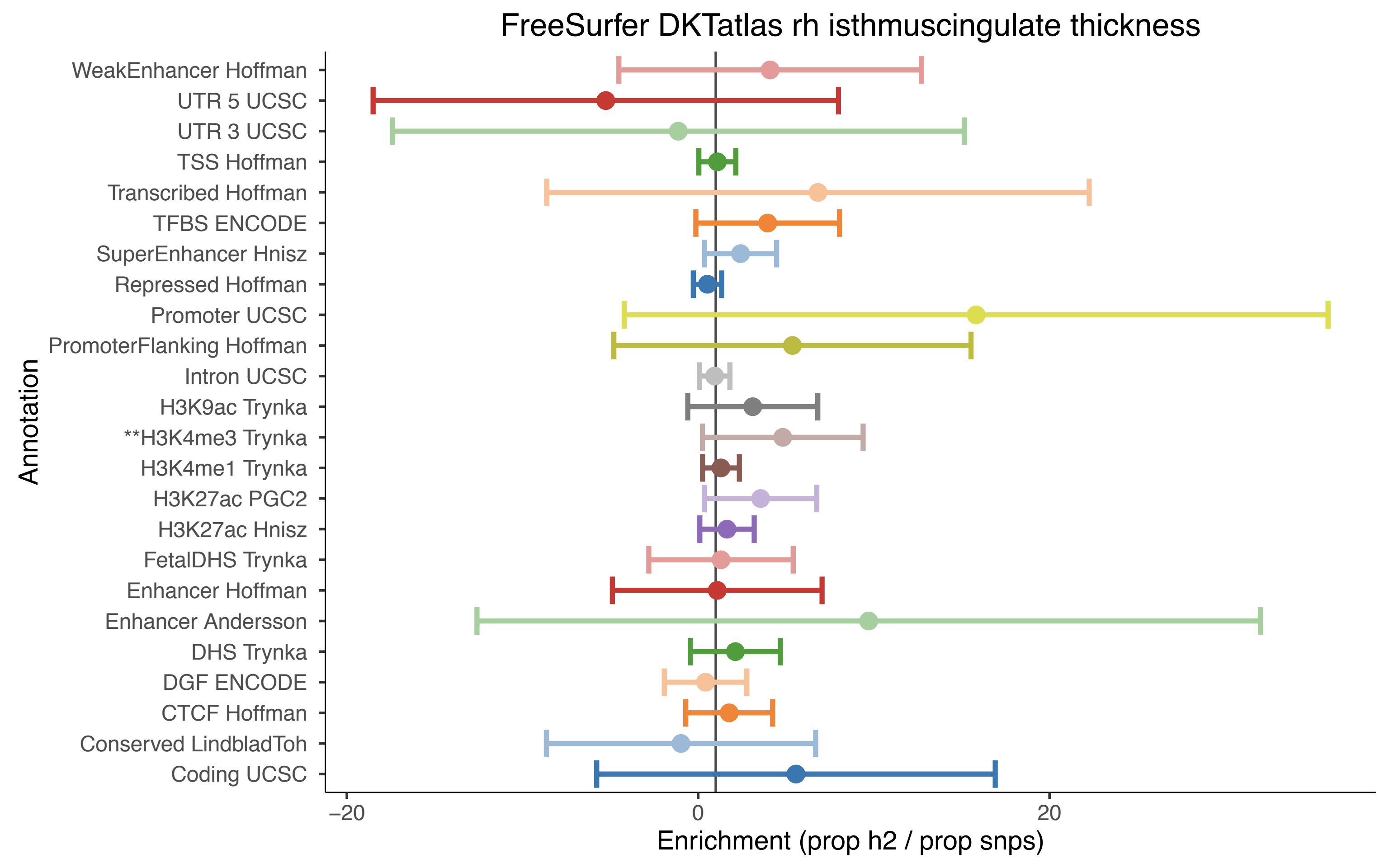
FreeSurfer DKTatlas rh isthmuscingulate thickness
WeakEnhancer Hoffman
UTR 5 UCSC
UTR 3 UCSC
TSS Hoffman
Transcribed Hoffman
TFBS ENCODE
SuperEnhancer Hnisz
Repressed Hoffman
Promoter UCSC
PromoterFlanking Hoffman
Intron UCSC
H3K9ac Trynka
**H3K4me3 Trynka
H3K4me1 Trynka
H3K27ac PGC2
H3K27ac Hnisz
FetalDHS Trynka
Enhancer Hoffman
Enhancer Andersson
DHS Trynka
DGF ENCODE
CTCF Hoffman
Conserved LindbladToh
Coding UCSC
Annotation
−20
0
20
Enrichment (prop h2 / prop snps)

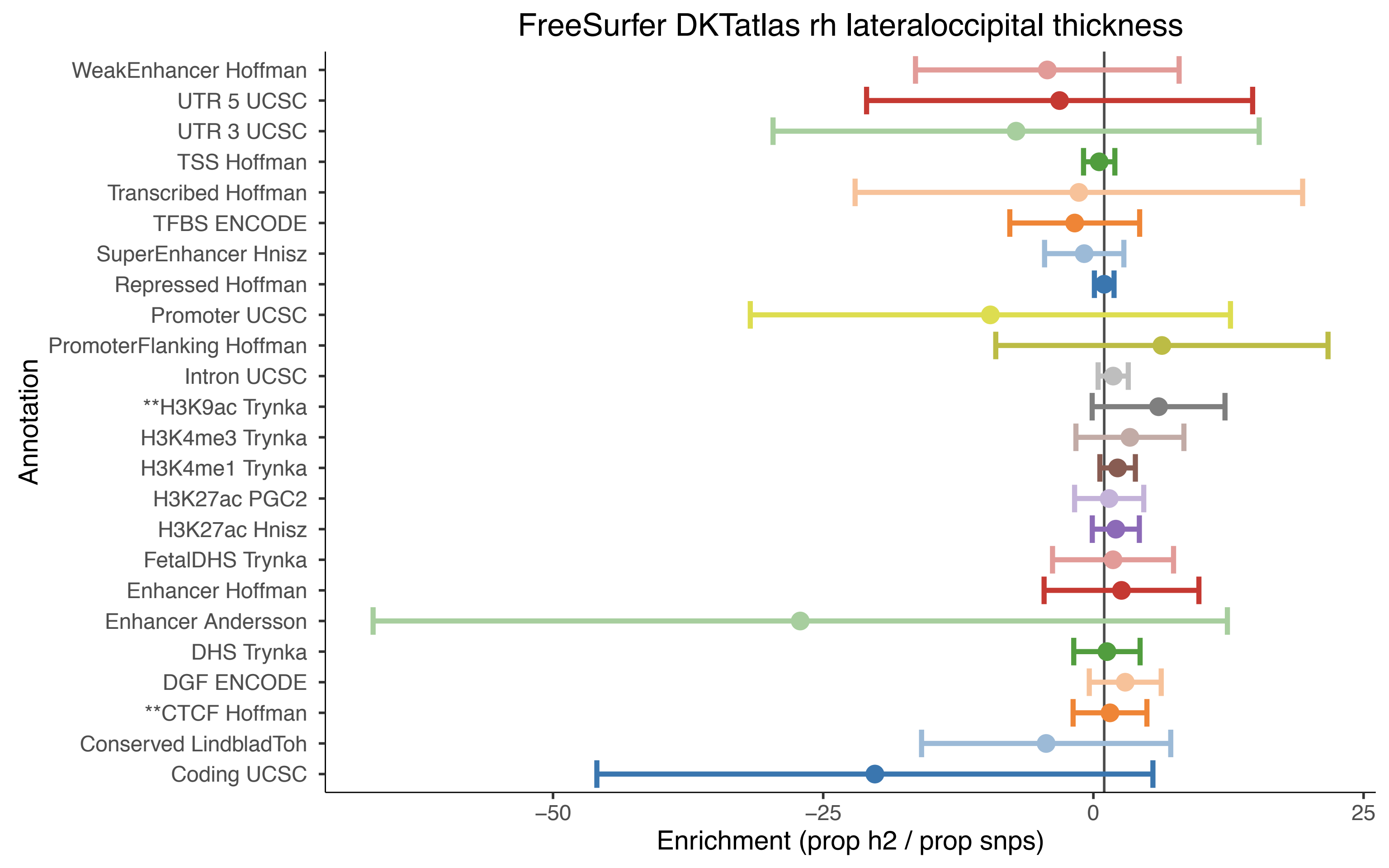
FreeSurfer DKTatlas rh lateraloccipital thickness
WeakEnhancer Hoffman
UTR 5 UCSC
UTR 3 UCSC
TSS Hoffman
Transcribed Hoffman
TFBS ENCODE
SuperEnhancer Hnisz
Repressed Hoffman
Promoter UCSC
PromoterFlanking Hoffman
Intron UCSC
**H3K9ac Trynka
H3K4me3 Trynka
H3K4me1 Trynka
H3K27ac PGC2
H3K27ac Hnisz
FetalDHS Trynka
Enhancer Hoffman
Enhancer Andersson
DHS Trynka
DGF ENCODE
**CTCF Hoffman
Conserved LindbladToh
Coding UCSC
Annotation
−50
−25
0
25
Enrichment (prop h2 / prop snps)

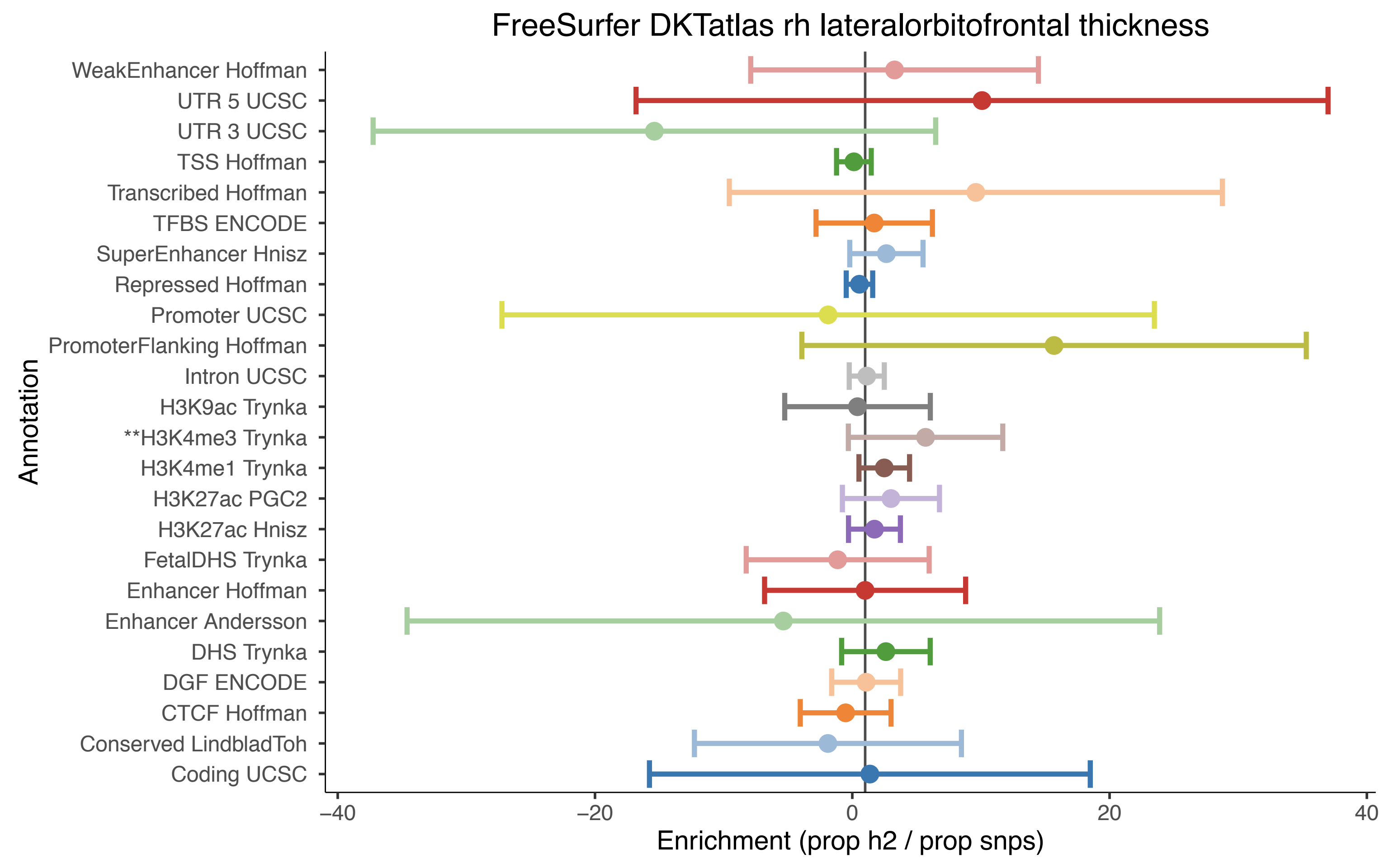
FreeSurfer DKTatlas rh lateralorbitofrontal thickness
WeakEnhancer Hoffman
UTR 5 UCSC
UTR 3 UCSC
TSS Hoffman
Transcribed Hoffman
TFBS ENCODE
SuperEnhancer Hnisz
Repressed Hoffman
Promoter UCSC
PromoterFlanking Hoffman
Intron UCSC
H3K9ac Trynka
**H3K4me3 Trynka
H3K4me1 Trynka
H3K27ac PGC2
H3K27ac Hnisz
FetalDHS Trynka
Enhancer Hoffman
Enhancer Andersson
DHS Trynka
DGF ENCODE
CTCF Hoffman
Conserved LindbladToh
Coding UCSC
Annotation
−40
−20
0
20
40
Enrichment (prop h2 / prop snps)

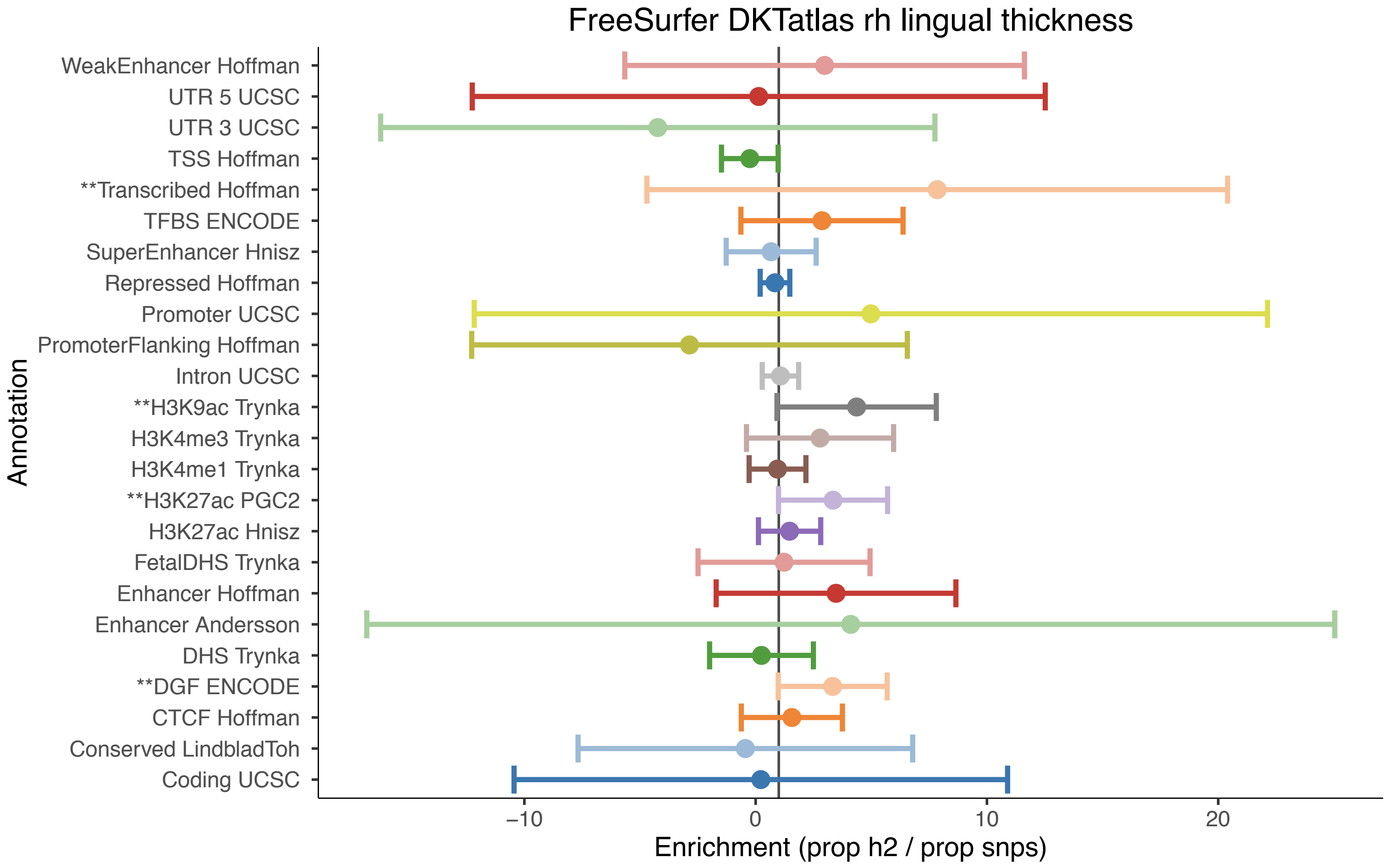
FreeSurfer DKTatlas rh lingual thickness
WeakEnhancer Hoffman
UTR 5 UCSC
UTR 3 UCSC
TSS Hoffman
**Transcribed Hoffman
TFBS ENCODE
SuperEnhancer Hnisz
Repressed Hoffman
Promoter UCSC
PromoterFlanking Hoffman
Intron UCSC
**H3K9ac Trynka
H3K4me3 Trynka
H3K4me1 Trynka
**H3K27ac PGC2
H3K27ac Hnisz
FetalDHS Trynka
Enhancer Hoffman
Enhancer Andersson
DHS Trynka
**DGF ENCODE
CTCF Hoffman
Conserved LindbladToh
Coding UCSC
Annotation
−10
0
10
20
Enrichment (prop h2 / prop snps)

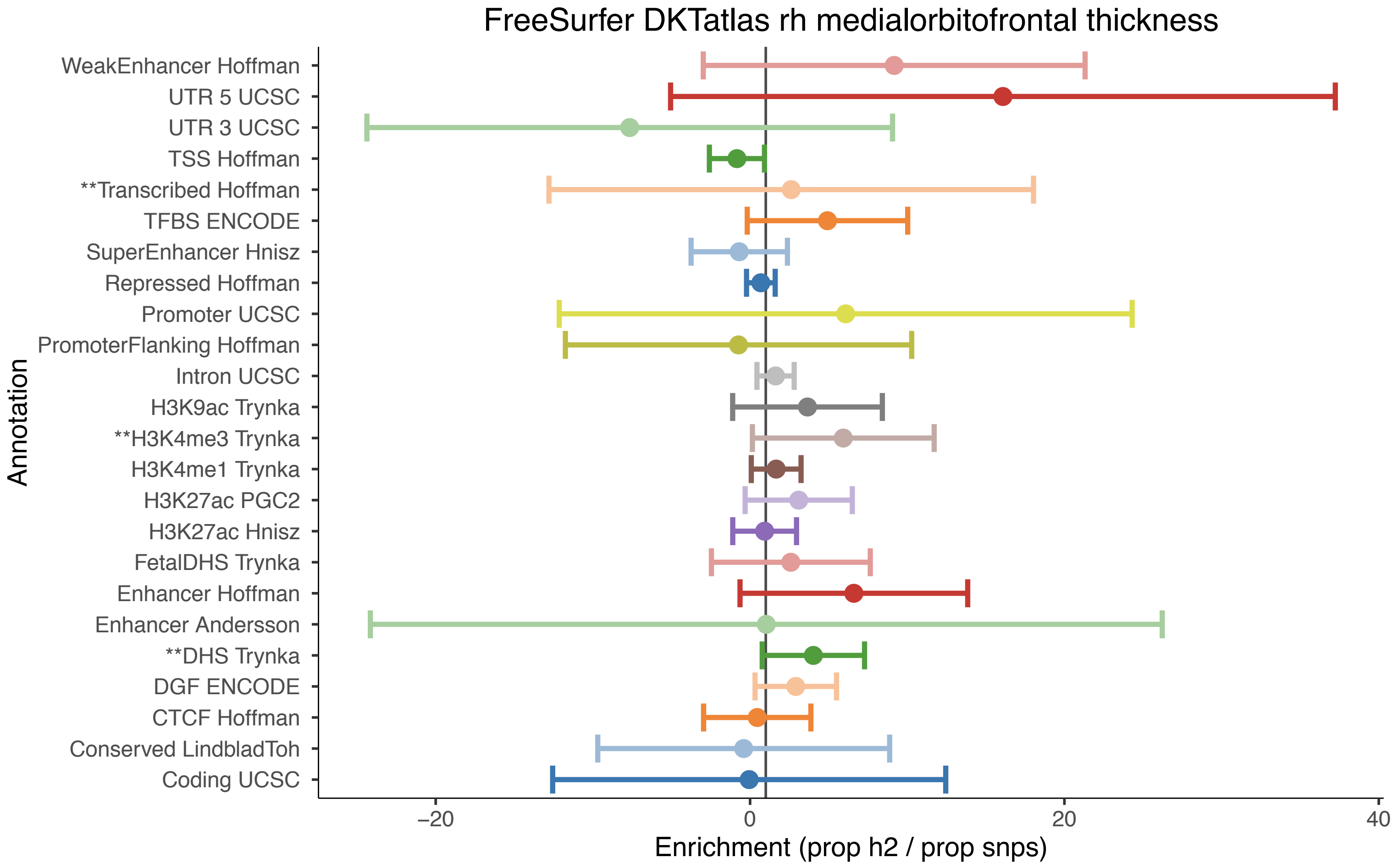
FreeSurfer DKTatlas rh medialorbitofrontal thickness
WeakEnhancer Hoffman
UTR 5 UCSC
UTR 3 UCSC
TSS Hoffman
**Transcribed Hoffman
TFBS ENCODE
SuperEnhancer Hnisz
Repressed Hoffman
Promoter UCSC
PromoterFlanking Hoffman
Intron UCSC
H3K9ac Trynka
**H3K4me3 Trynka
H3K4me1 Trynka
H3K27ac PGC2
H3K27ac Hnisz
FetalDHS Trynka
Enhancer Hoffman
Enhancer Andersson
**DHS Trynka
DGF ENCODE
CTCF Hoffman
Conserved LindbladToh
Coding UCSC
Annotation
−20
0
20
40
Enrichment (prop h2 / prop snps)

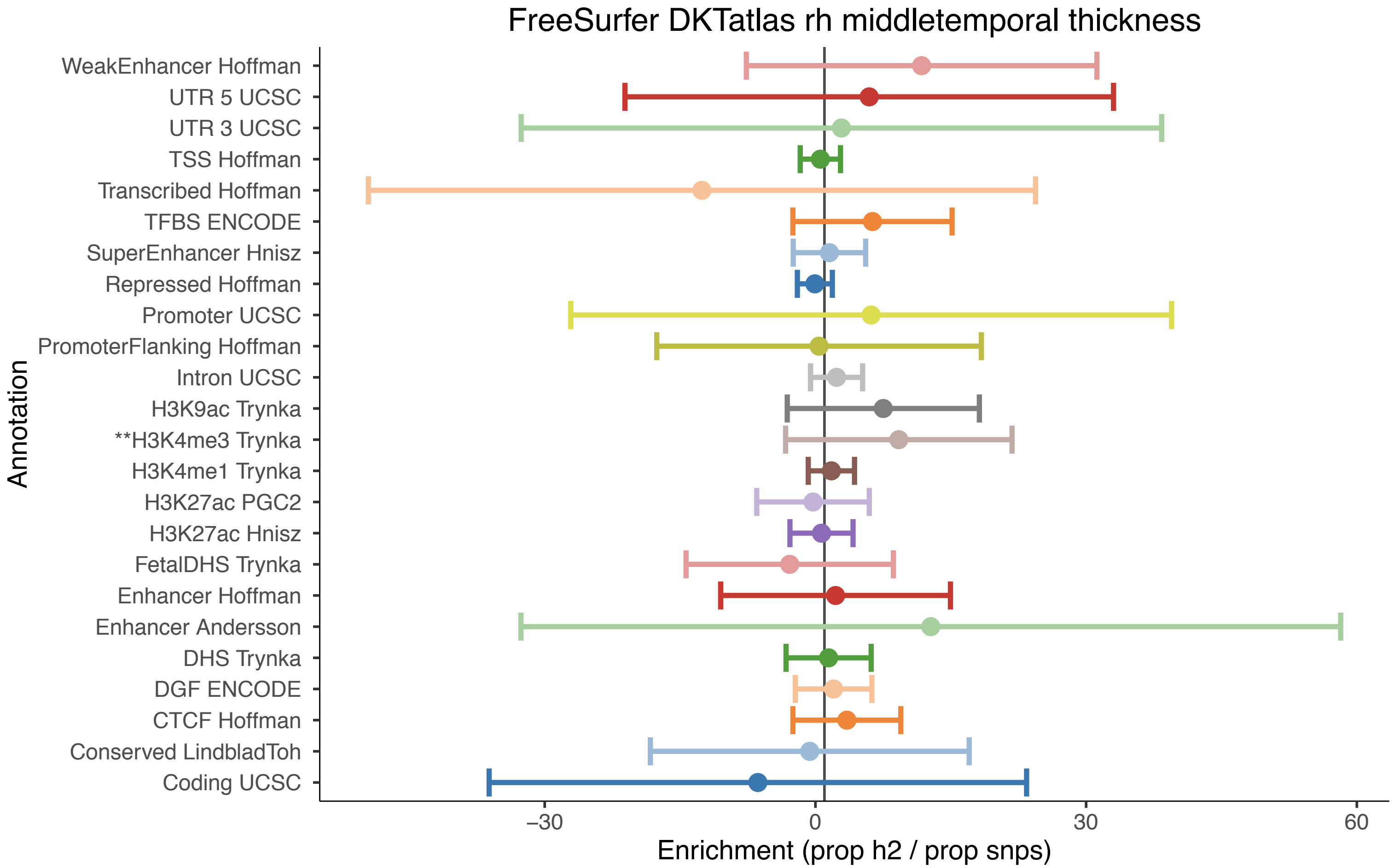
FreeSurfer DKTatlas rh middletemporal thickness
WeakEnhancer Hoffman
UTR 5 UCSC
UTR 3 UCSC
TSS Hoffman
Transcribed Hoffman
TFBS ENCODE
SuperEnhancer Hnisz
Repressed Hoffman
Promoter UCSC
PromoterFlanking Hoffman
Intron UCSC
H3K9ac Trynka
**H3K4me3 Trynka
H3K4me1 Trynka
H3K27ac PGC2
H3K27ac Hnisz
FetalDHS Trynka
Enhancer Hoffman
Enhancer Andersson
DHS Trynka
DGF ENCODE
CTCF Hoffman
Conserved LindbladToh
Coding UCSC
Annotation
−30
0
30
60
Enrichment (prop h2 / prop snps)

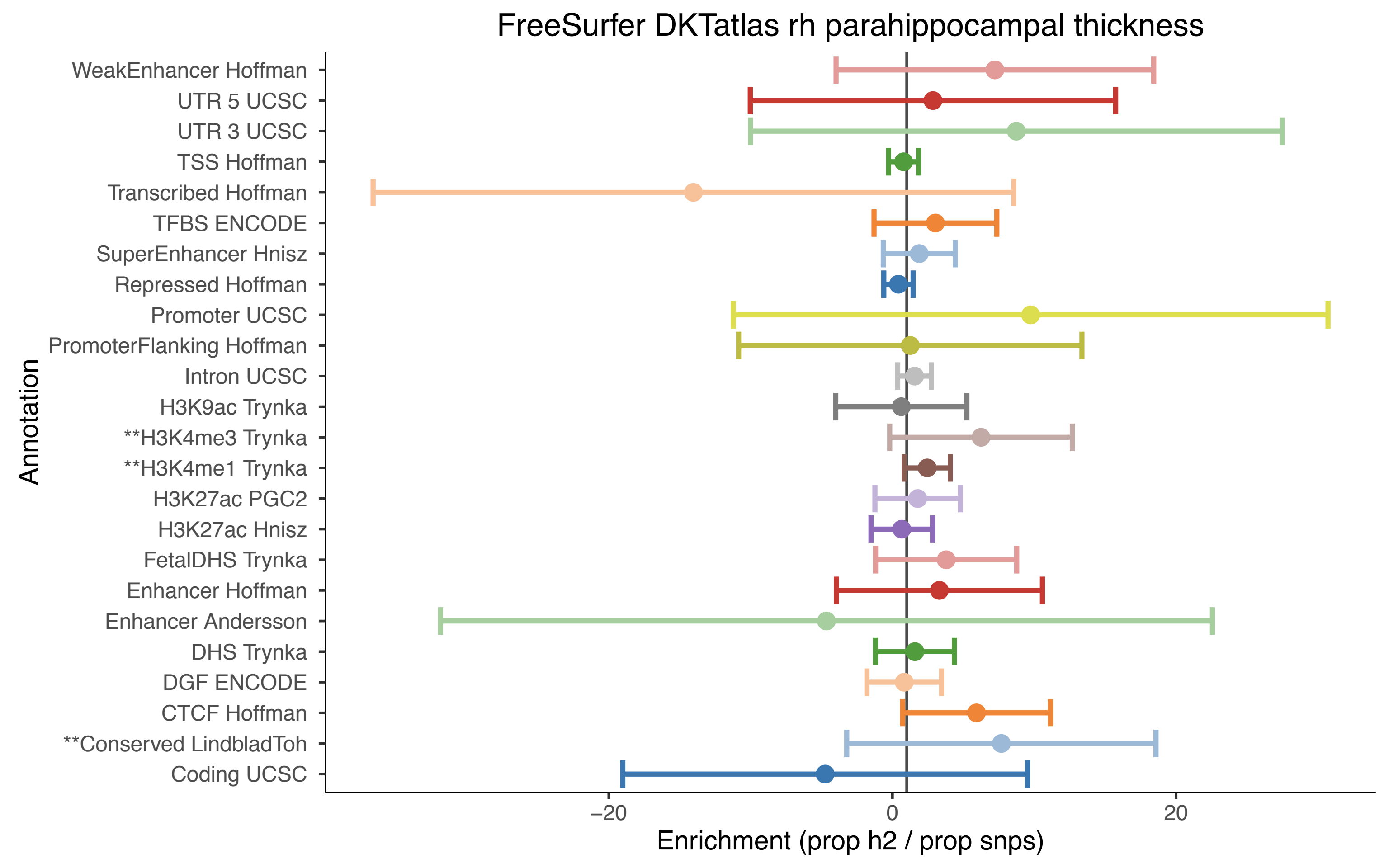

FreeSurfer DKTatlas rh parahippocampal thickness
WeakEnhancer Hoffman
UTR 5 UCSC
UTR 3 UCSC
TSS Hoffman
Transcribed Hoffman
TFBS ENCODE
SuperEnhancer Hnisz
Repressed Hoffman
Promoter UCSC
PromoterFlanking Hoffman
Intron UCSC
H3K9ac Trynka
**H3K4me3 Trynka
**H3K4me1 Trynka
H3K27ac PGC2
H3K27ac Hnisz
FetalDHS Trynka
Enhancer Hoffman
Enhancer Andersson
DHS Trynka
DGF ENCODE
CTCF Hoffman
**Conserved LindbladToh
Coding UCSC
Annotation
−20
0
20
Enrichment (prop h2 / prop snps)

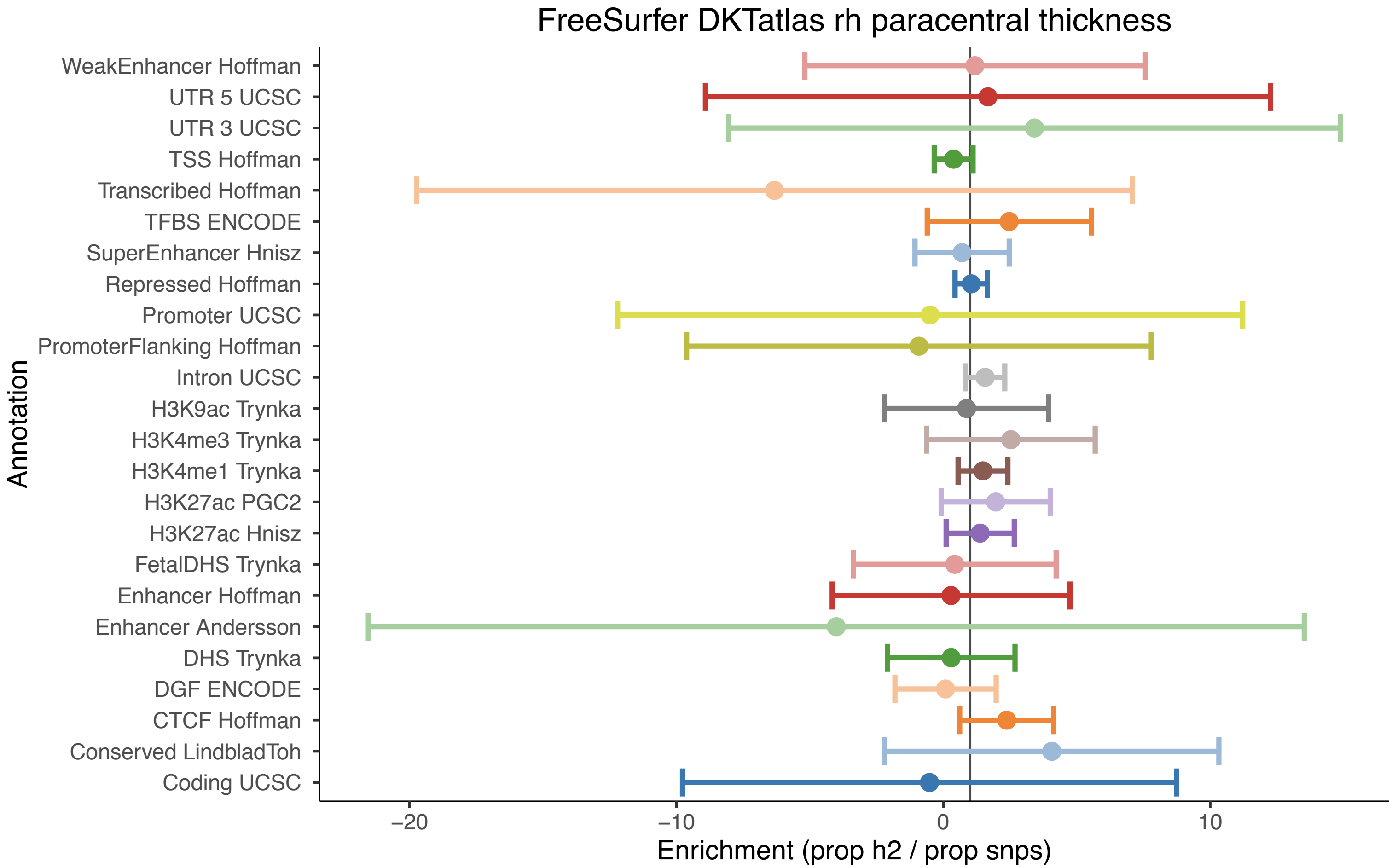
FreeSurfer DKTatlas rh paracentral thickness
WeakEnhancer Hoffman
UTR 5 UCSC
UTR 3 UCSC
TSS Hoffman
Transcribed Hoffman
TFBS ENCODE
SuperEnhancer Hnisz
Repressed Hoffman
Promoter UCSC
PromoterFlanking Hoffman
Intron UCSC
H3K9ac Trynka
H3K4me3 Trynka
H3K4me1 Trynka
H3K27ac PGC2
H3K27ac Hnisz
FetalDHS Trynka
Enhancer Hoffman
Enhancer Andersson
DHS Trynka
DGF ENCODE
CTCF Hoffman
Conserved LindbladToh
Coding UCSC
Annotation
−20
−10
0
10
Enrichment (prop h2 / prop snps)

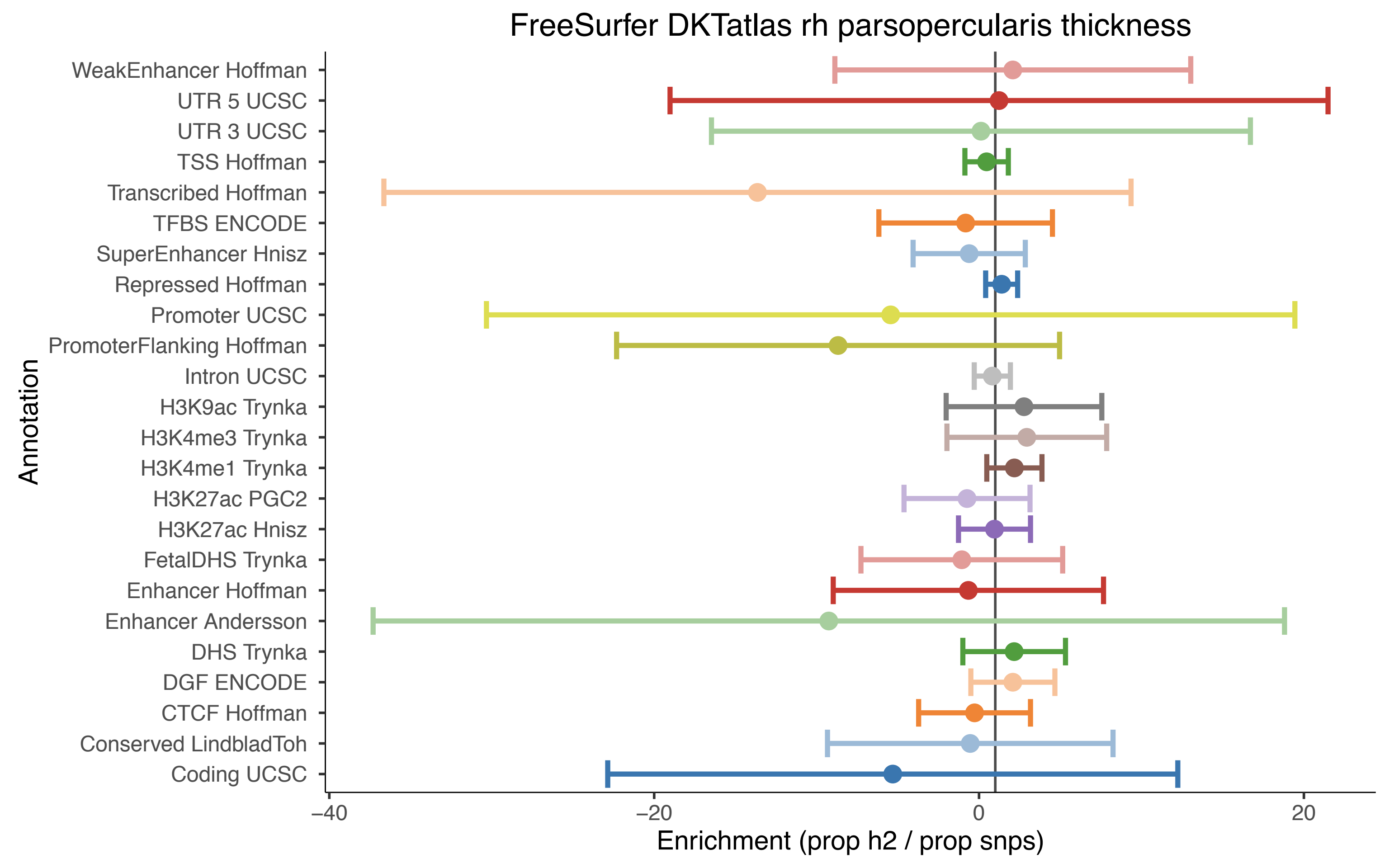

FreeSurfer DKTatlas rh parsopercularis thickness
Annotation
WeakEnhancer Hoffman
UTR 5 UCSC
UTR 3 UCSC
TSS Hoffman
Transcribed Hoffman
TFBS ENCODE
SuperEnhancer Hnisz
Repressed Hoffman
Promoter UCSC
PromoterFlanking Hoffman
Intron UCSC
H3K9ac Trynka
H3K4me3 Trynka
H3K4me1 Trynka
H3K27ac PGC2
H3K27ac Hnisz
FetalDHS Trynka
Enhancer Hoffman
Enhancer Andersson
DHS Trynka
DGF ENCODE
CTCF Hoffman
Conserved LindbladToh
Coding UCSC
−40
−20
0
20
Enrichment (prop h2 / prop snps)

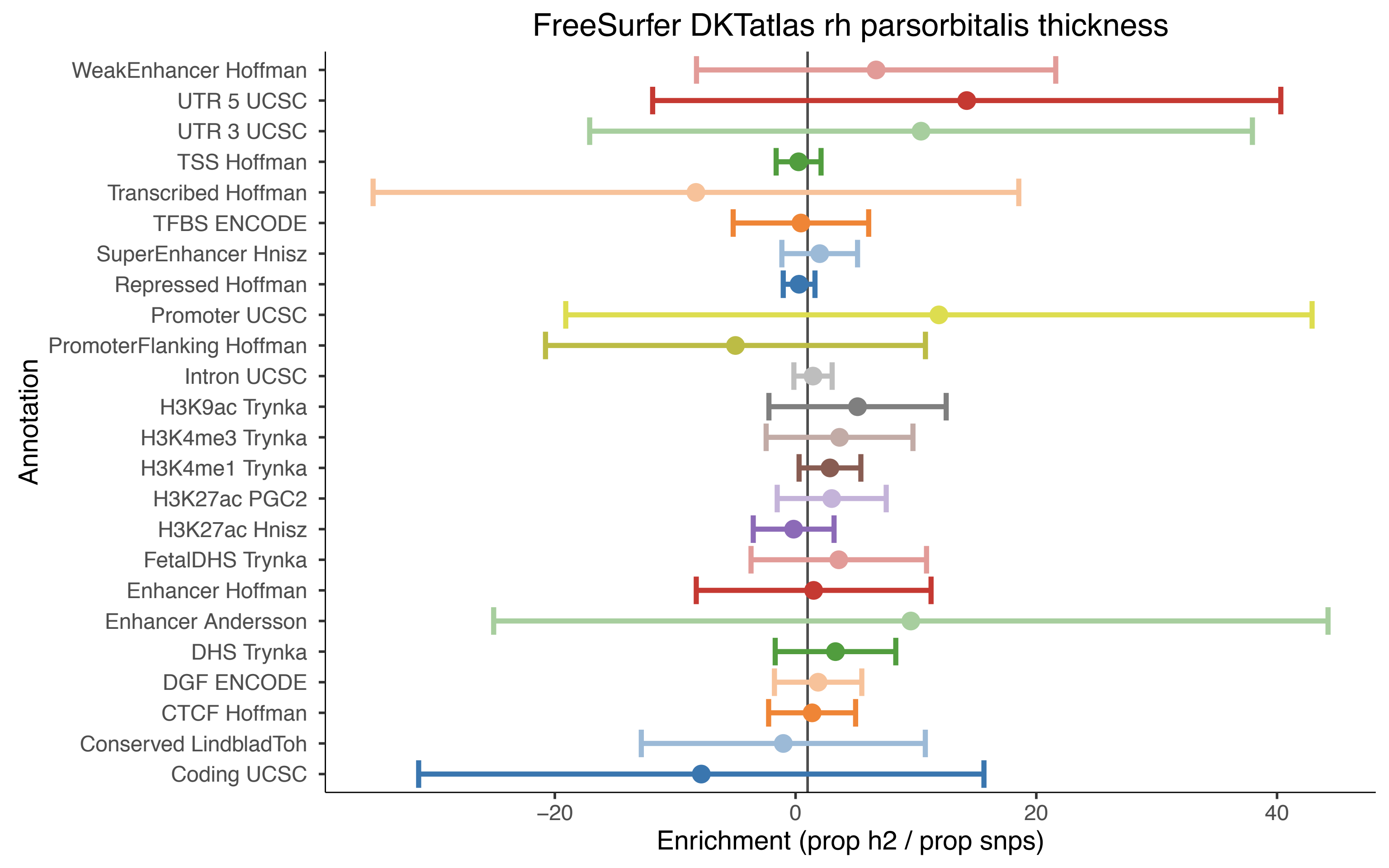

FreeSurfer DKTatlas rh parsorbitalis thickness
WeakEnhancer Hoffman
UTR 5 UCSC
UTR 3 UCSC
TSS Hoffman
Transcribed Hoffman
TFBS ENCODE
SuperEnhancer Hnisz
Repressed Hoffman
Promoter UCSC
PromoterFlanking Hoffman
Intron UCSC
H3K9ac Trynka
H3K4me3 Trynka
H3K4me1 Trynka
H3K27ac PGC2
H3K27ac Hnisz
FetalDHS Trynka
Enhancer Hoffman
Enhancer Andersson
DHS Trynka
DGF ENCODE
CTCF Hoffman
Conserved LindbladToh
Coding UCSC
Annotation
−20
0
20
40
Enrichment (prop h2 / prop snps)

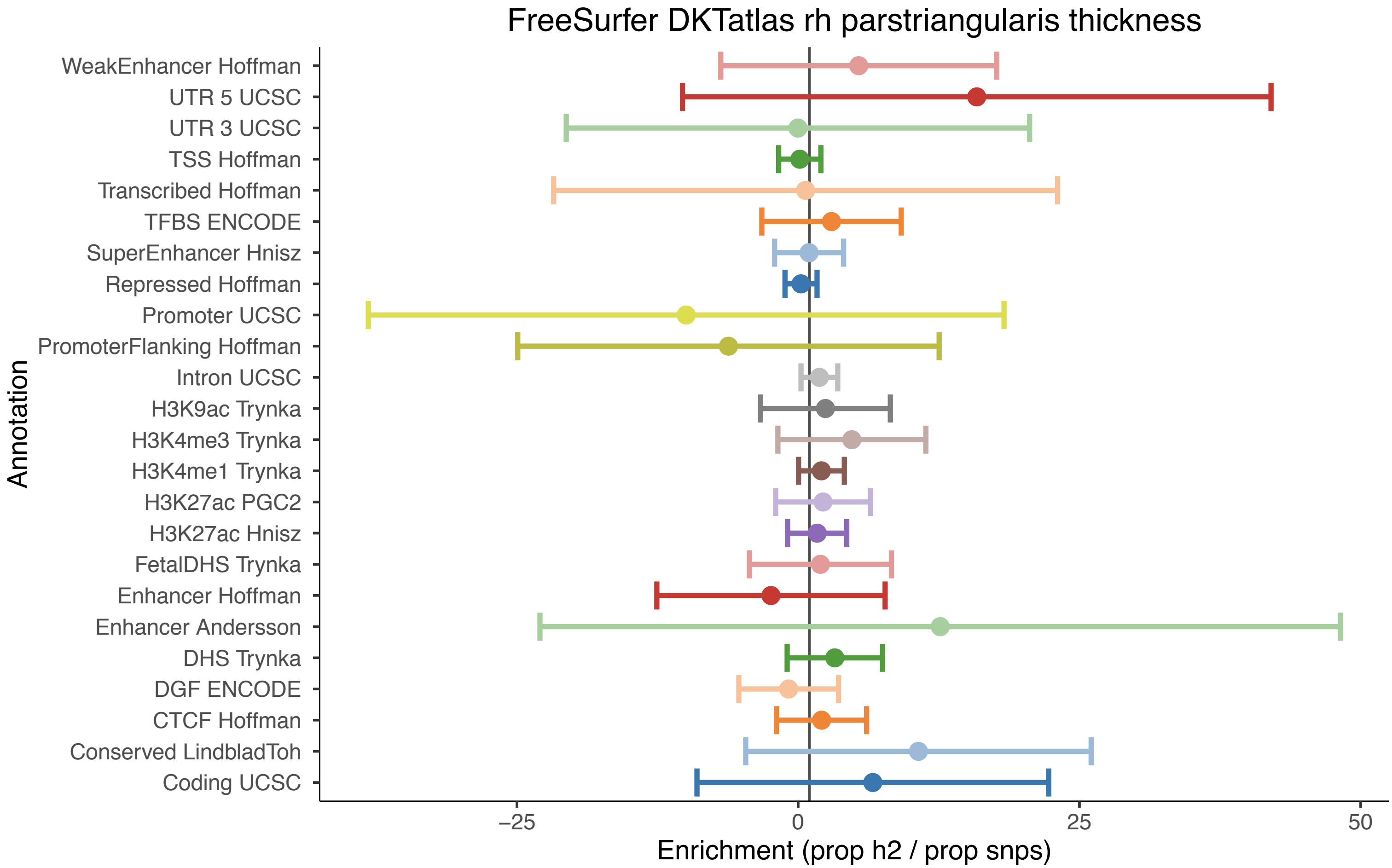

FreeSurfer DKTatlas rh parstriangularis thickness
WeakEnhancer Hoffman
UTR 5 UCSC
UTR 3 UCSC
TSS Hoffman
Transcribed Hoffman
TFBS ENCODE
SuperEnhancer Hnisz
Repressed Hoffman
Promoter UCSC
PromoterFlanking Hoffman
Intron UCSC
H3K9ac Trynka
H3K4me3 Trynka
H3K4me1 Trynka
H3K27ac PGC2
H3K27ac Hnisz
FetalDHS Trynka
Enhancer Hoffman
Enhancer Andersson
DHS Trynka
DGF ENCODE
CTCF Hoffman
Conserved LindbladToh
Coding UCSC
Annotation
−25
0
25
50
Enrichment (prop h2 / prop snps)

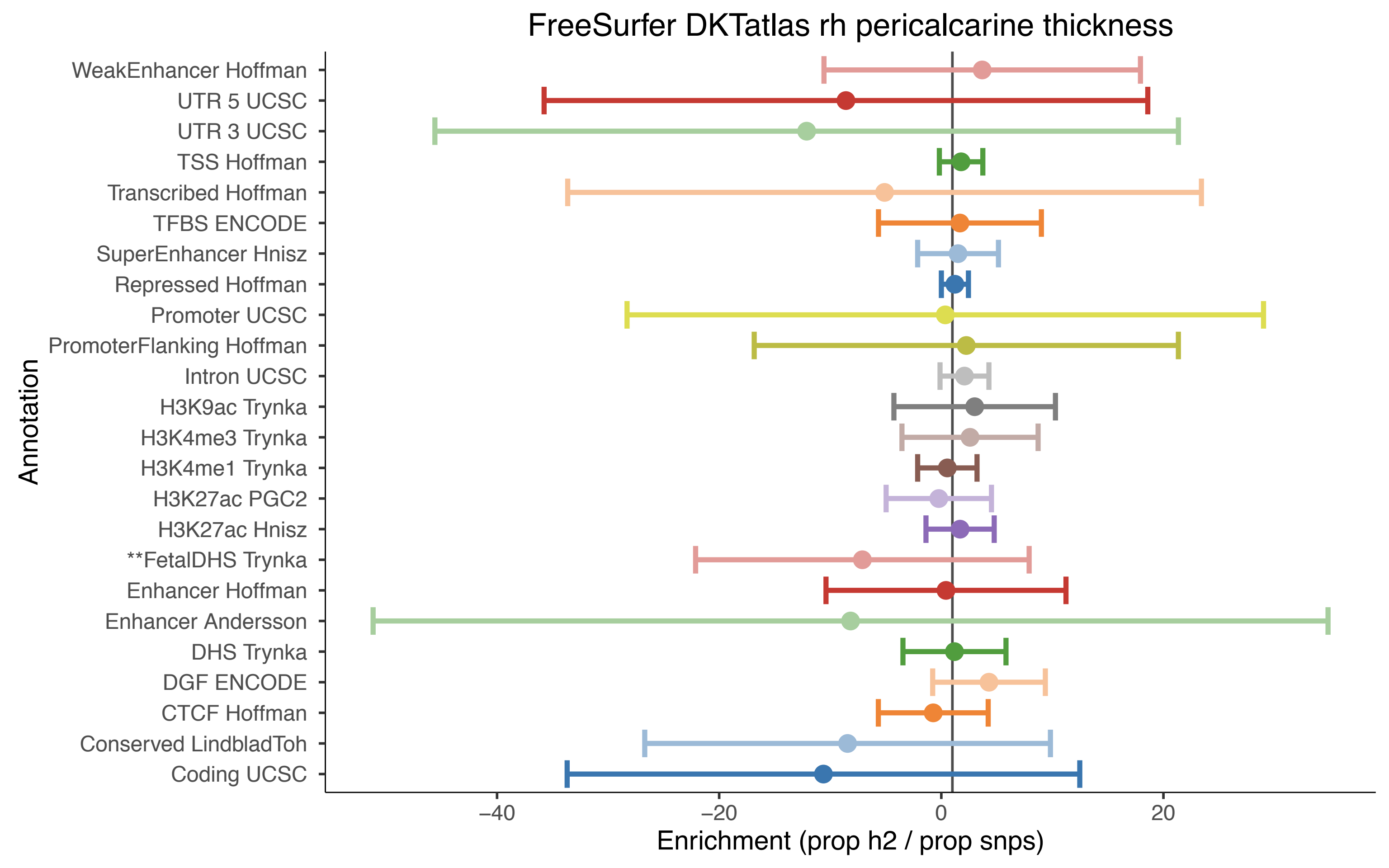
FreeSurfer DKTatlas rh pericalcarine thickness
WeakEnhancer Hoffman
UTR 5 UCSC
UTR 3 UCSC
TSS Hoffman
Transcribed Hoffman
TFBS ENCODE
SuperEnhancer Hnisz
Repressed Hoffman
Promoter UCSC
PromoterFlanking Hoffman
Intron UCSC
H3K9ac Trynka
H3K4me3 Trynka
H3K4me1 Trynka
H3K27ac PGC2
H3K27ac Hnisz
**FetalDHS Trynka
Enhancer Hoffman
Enhancer Andersson
DHS Trynka
DGF ENCODE
CTCF Hoffman
Conserved LindbladToh
Coding UCSC
Annotation
−40
−20
0
20
Enrichment (prop h2 / prop snps)

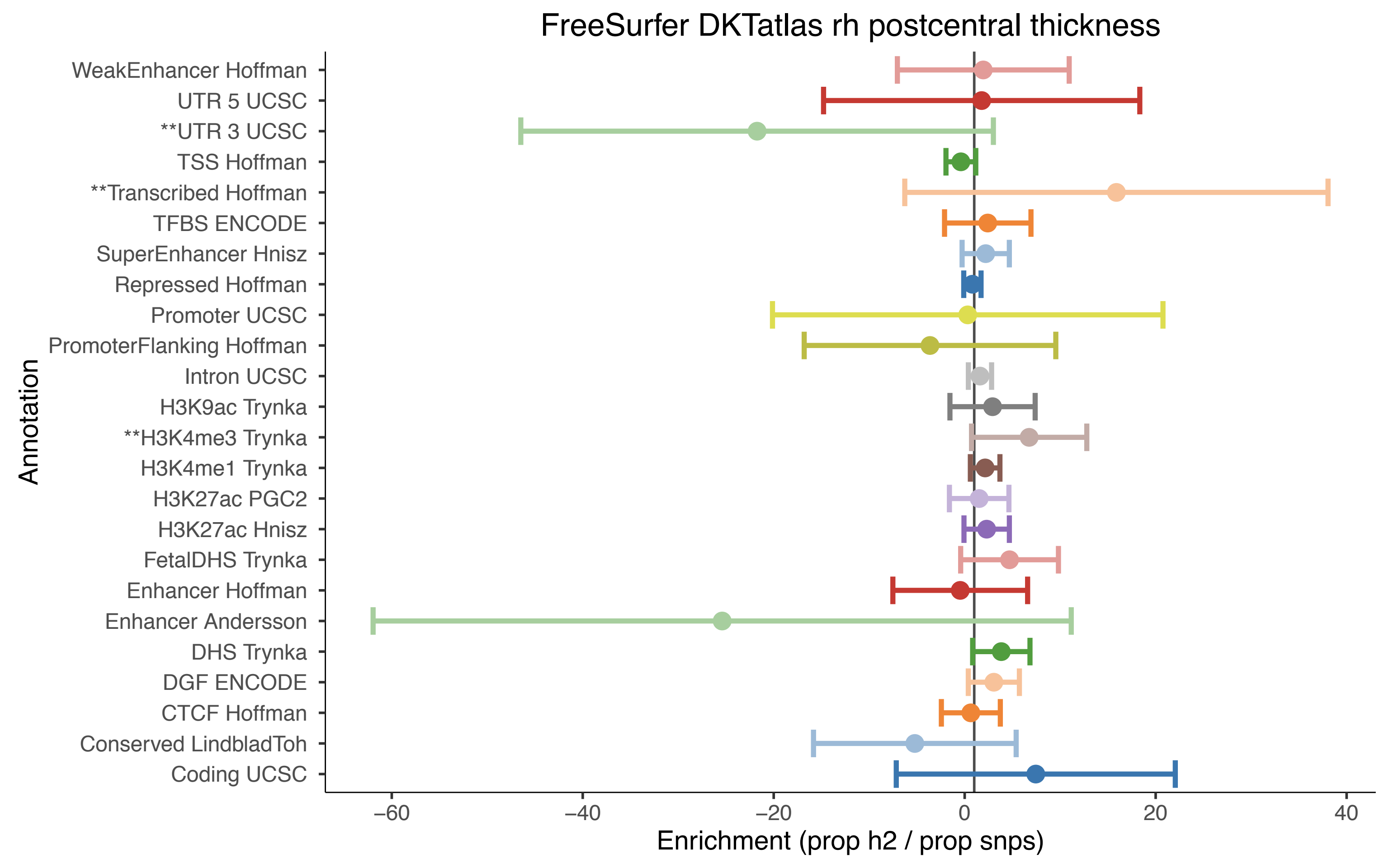

FreeSurfer DKTatlas rh postcentral thickness
WeakEnhancer Hoffman
UTR 5 UCSC
**UTR 3 UCSC
TSS Hoffman
**Transcribed Hoffman
TFBS ENCODE
SuperEnhancer Hnisz
Repressed Hoffman
Promoter UCSC
PromoterFlanking Hoffman
Intron UCSC
H3K9ac Trynka
**H3K4me3 Trynka
H3K4me1 Trynka
H3K27ac PGC2
H3K27ac Hnisz
FetalDHS Trynka
Enhancer Hoffman
Enhancer Andersson
DHS Trynka
DGF ENCODE
CTCF Hoffman
Conserved LindbladToh
Coding UCSC
Annotation
−60
−40
−20
0
20
40
Enrichment (prop h2 / prop snps)

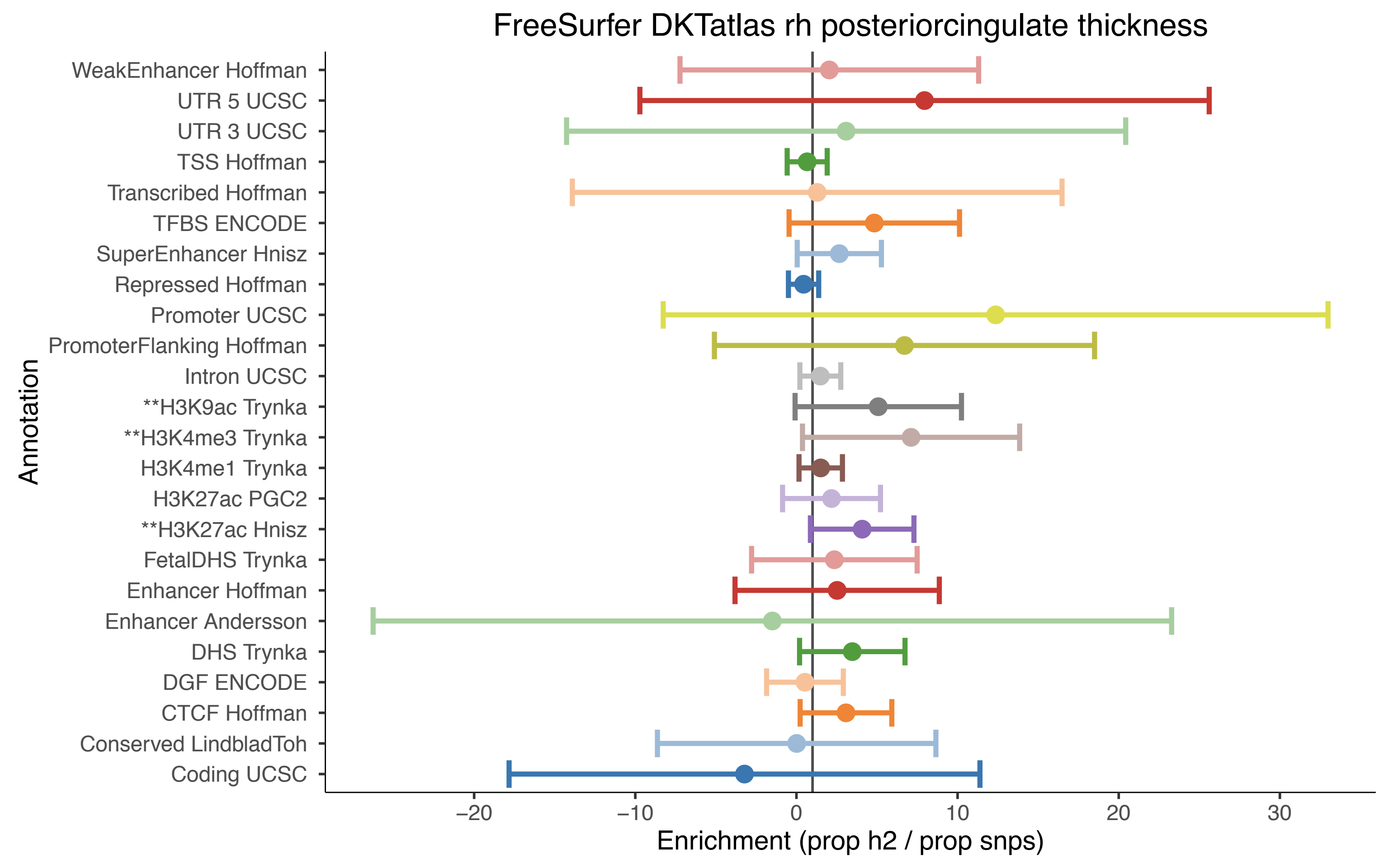
FreeSurfer DKTatlas rh posteriorcingulate thickness
WeakEnhancer Hoffman
UTR 5 UCSC
UTR 3 UCSC
TSS Hoffman
Transcribed Hoffman
TFBS ENCODE
SuperEnhancer Hnisz
Repressed Hoffman
Promoter UCSC
PromoterFlanking Hoffman
Intron UCSC
**H3K9ac Trynka
**H3K4me3 Trynka
H3K4me1 Trynka
H3K27ac PGC2
**H3K27ac Hnisz
FetalDHS Trynka
Enhancer Hoffman
Enhancer Andersson
DHS Trynka
DGF ENCODE
CTCF Hoffman
Conserved LindbladToh
Coding UCSC
Annotation
−20
−10
0
10
20
30
Enrichment (prop h2 / prop snps)

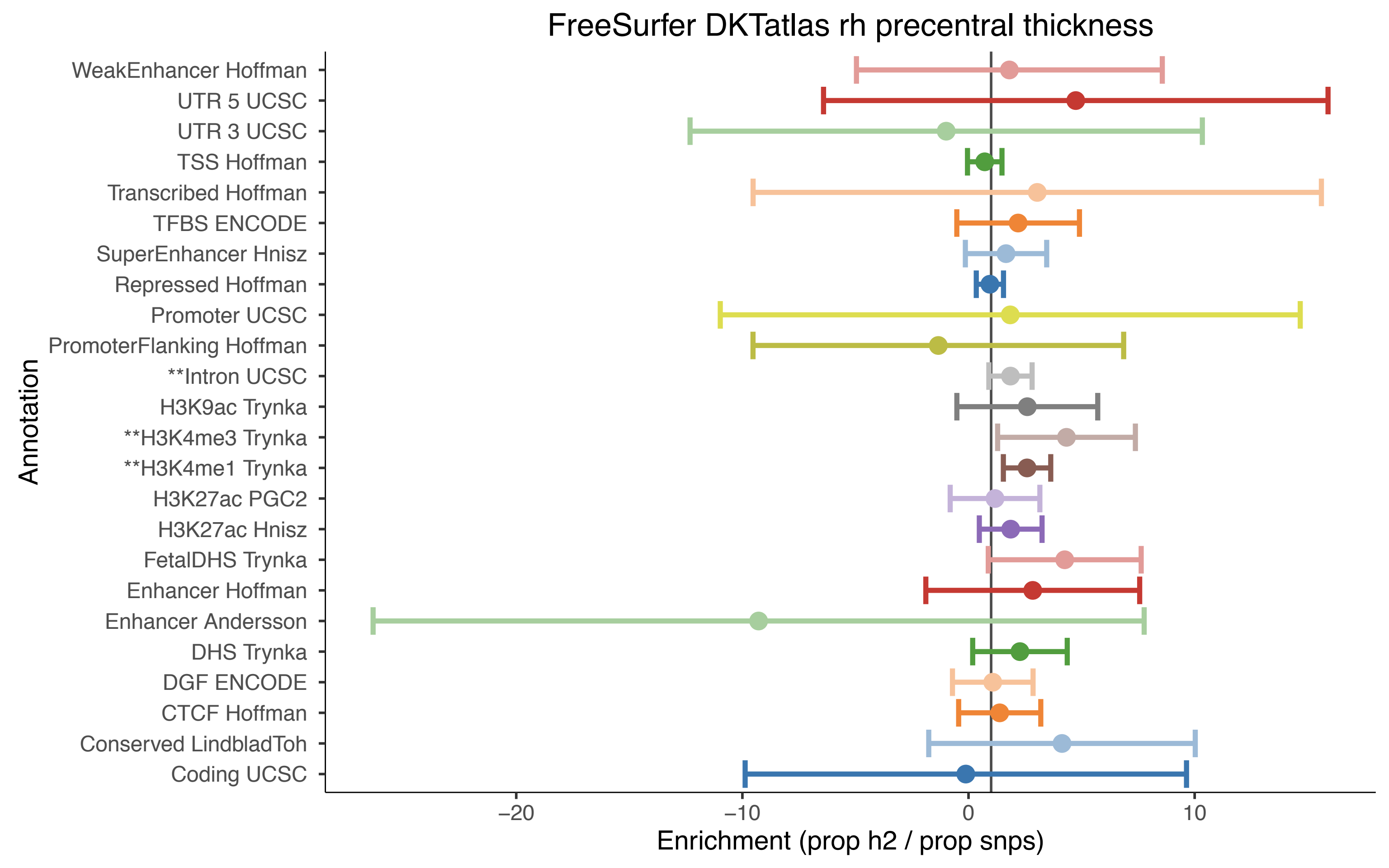
FreeSurfer DKTatlas rh precentral thickness
WeakEnhancer Hoffman
UTR 5 UCSC
UTR 3 UCSC
TSS Hoffman
Transcribed Hoffman
TFBS ENCODE
SuperEnhancer Hnisz
Repressed Hoffman
Promoter UCSC
PromoterFlanking Hoffman
**Intron UCSC
H3K9ac Trynka
**H3K4me3 Trynka
**H3K4me1 Trynka
H3K27ac PGC2
H3K27ac Hnisz
FetalDHS Trynka
Enhancer Hoffman
Enhancer Andersson
DHS Trynka
DGF ENCODE
CTCF Hoffman
Conserved LindbladToh
Coding UCSC
Annotation
−20
−10
0
10
Enrichment (prop h2 / prop snps)

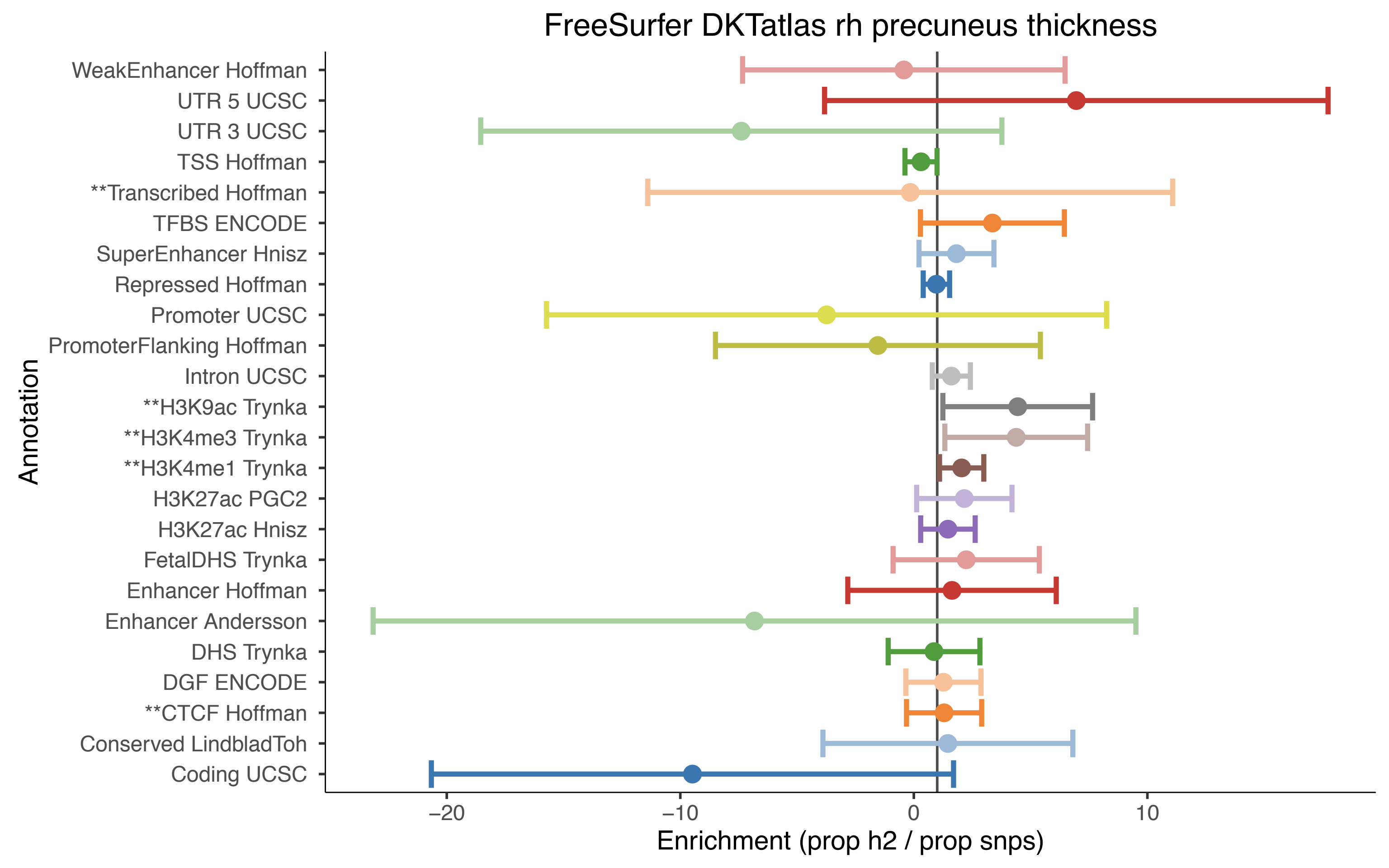

FreeSurfer DKTatlas rh precuneus thickness
WeakEnhancer Hoffman
UTR 5 UCSC
UTR 3 UCSC
TSS Hoffman
**Transcribed Hoffman
TFBS ENCODE
SuperEnhancer Hnisz
Repressed Hoffman
Promoter UCSC
PromoterFlanking Hoffman
Intron UCSC
**H3K9ac Trynka
**H3K4me3 Trynka
**H3K4me1 Trynka
H3K27ac PGC2
H3K27ac Hnisz
FetalDHS Trynka
Enhancer Hoffman
Enhancer Andersson
DHS Trynka
DGF ENCODE
**CTCF Hoffman
Conserved LindbladToh
Coding UCSC
Annotation
−20
−10
0
10
Enrichment (prop h2 / prop snps)

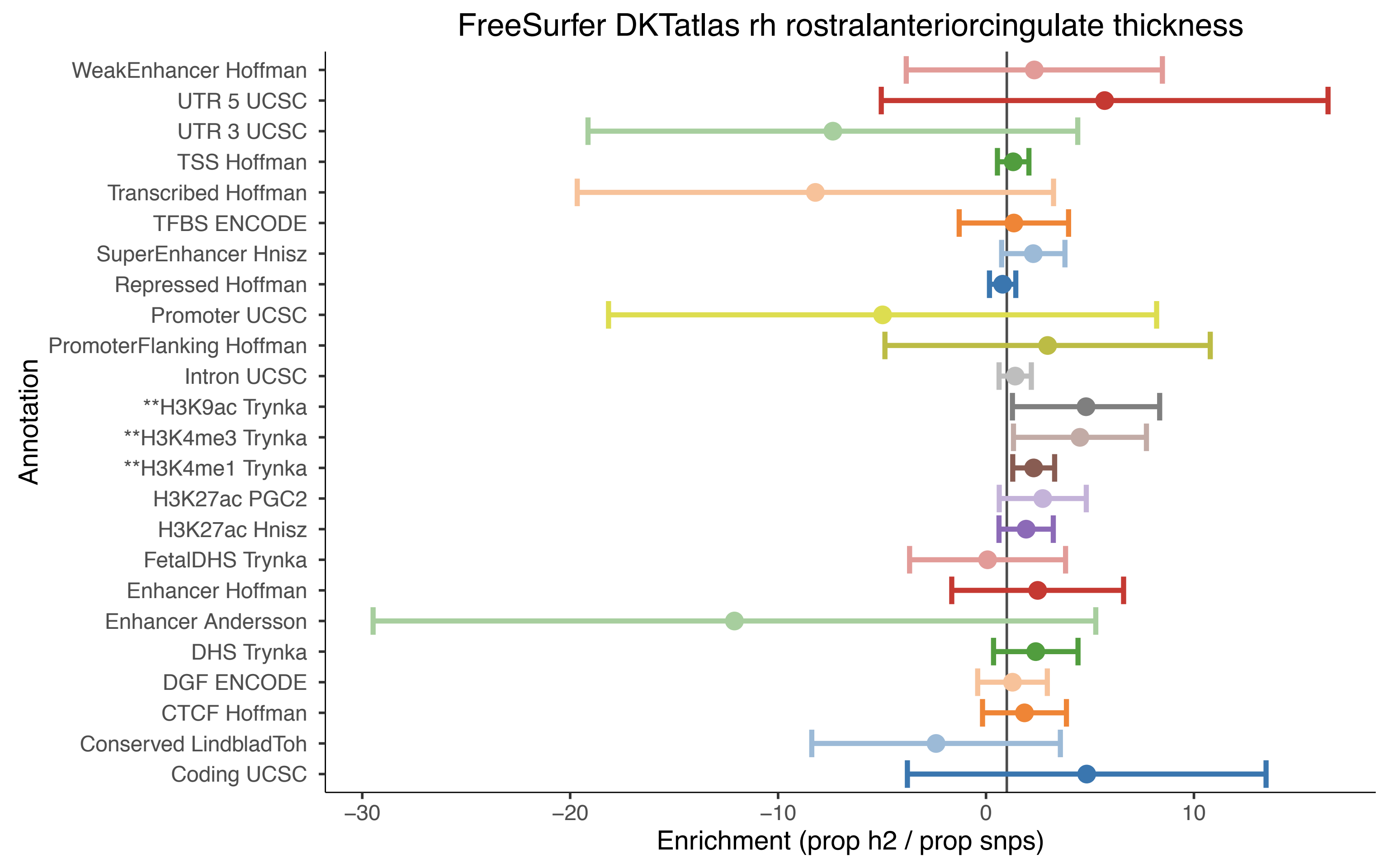

FreeSurfer DKTatlas rh rostralanteriorcingulate thickness
WeakEnhancer Hoffman
UTR 5 UCSC
UTR 3 UCSC
TSS Hoffman
Transcribed Hoffman
TFBS ENCODE
SuperEnhancer Hnisz
Repressed Hoffman
Promoter UCSC
PromoterFlanking Hoffman
Intron UCSC
**H3K9ac Trynka
**H3K4me3 Trynka
**H3K4me1 Trynka
H3K27ac PGC2
H3K27ac Hnisz
FetalDHS Trynka
Enhancer Hoffman
Enhancer Andersson
DHS Trynka
DGF ENCODE
CTCF Hoffman
Conserved LindbladToh
Coding UCSC
Annotation
−30
−20
−10
0
10
Enrichment (prop h2 / prop snps)

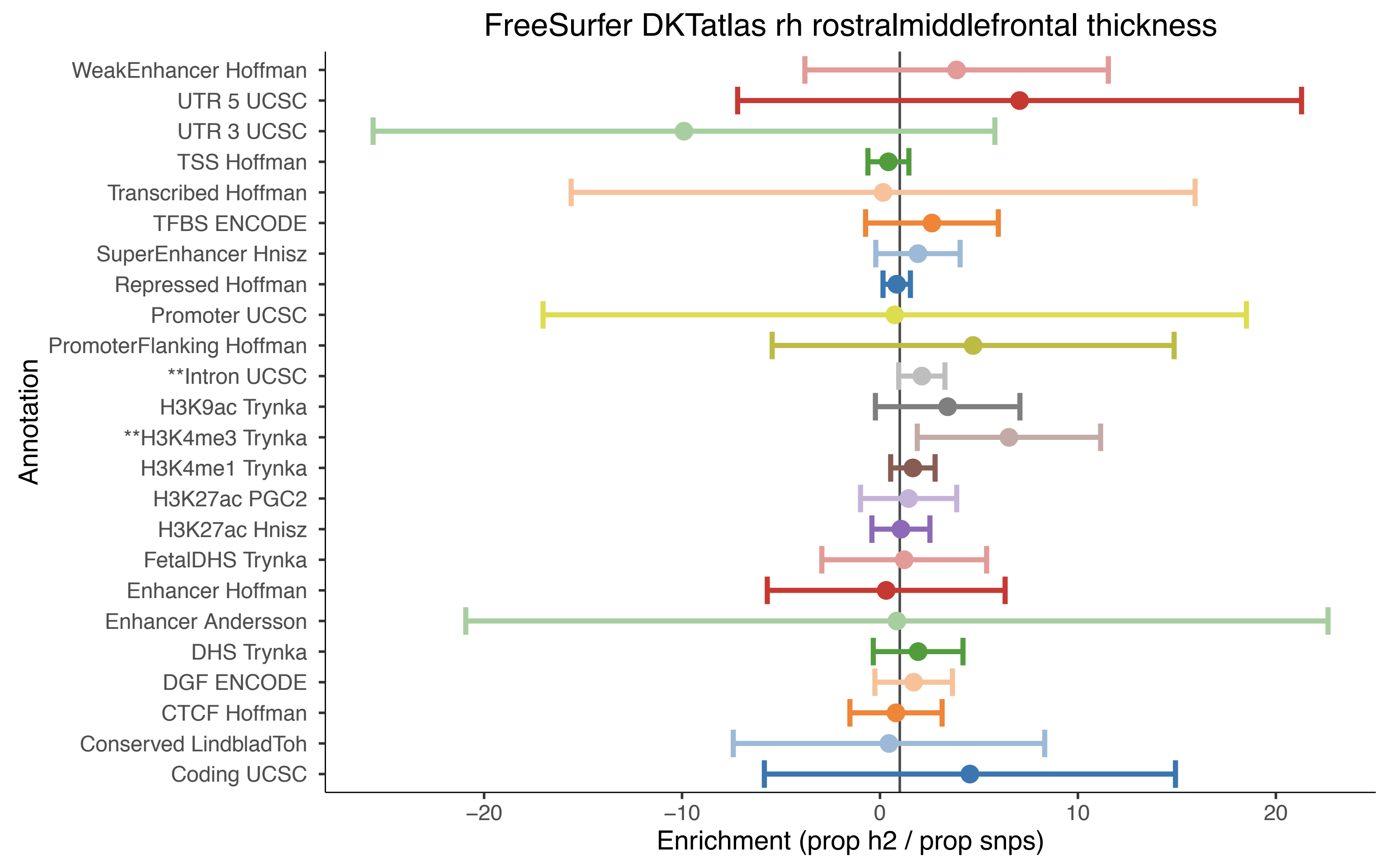

FreeSurfer DKTatlas rh rostralmiddlefrontal thickness
Annotation
WeakEnhancer Hoffman
UTR 5 UCSC
UTR 3 UCSC
TSS Hoffman
Transcribed Hoffman
TFBS ENCODE
SuperEnhancer Hnisz
Repressed Hoffman
Promoter UCSC
PromoterFlanking Hoffman
**Intron UCSC
H3K9ac Trynka
**H3K4me3 Trynka
H3K4me1 Trynka
H3K27ac PGC2
H3K27ac Hnisz
FetalDHS Trynka
Enhancer Hoffman
Enhancer Andersson
DHS Trynka
DGF ENCODE
CTCF Hoffman
Conserved LindbladToh
Coding UCSC
−20
−10
0
10
20
Enrichment (prop h2 / prop snps)

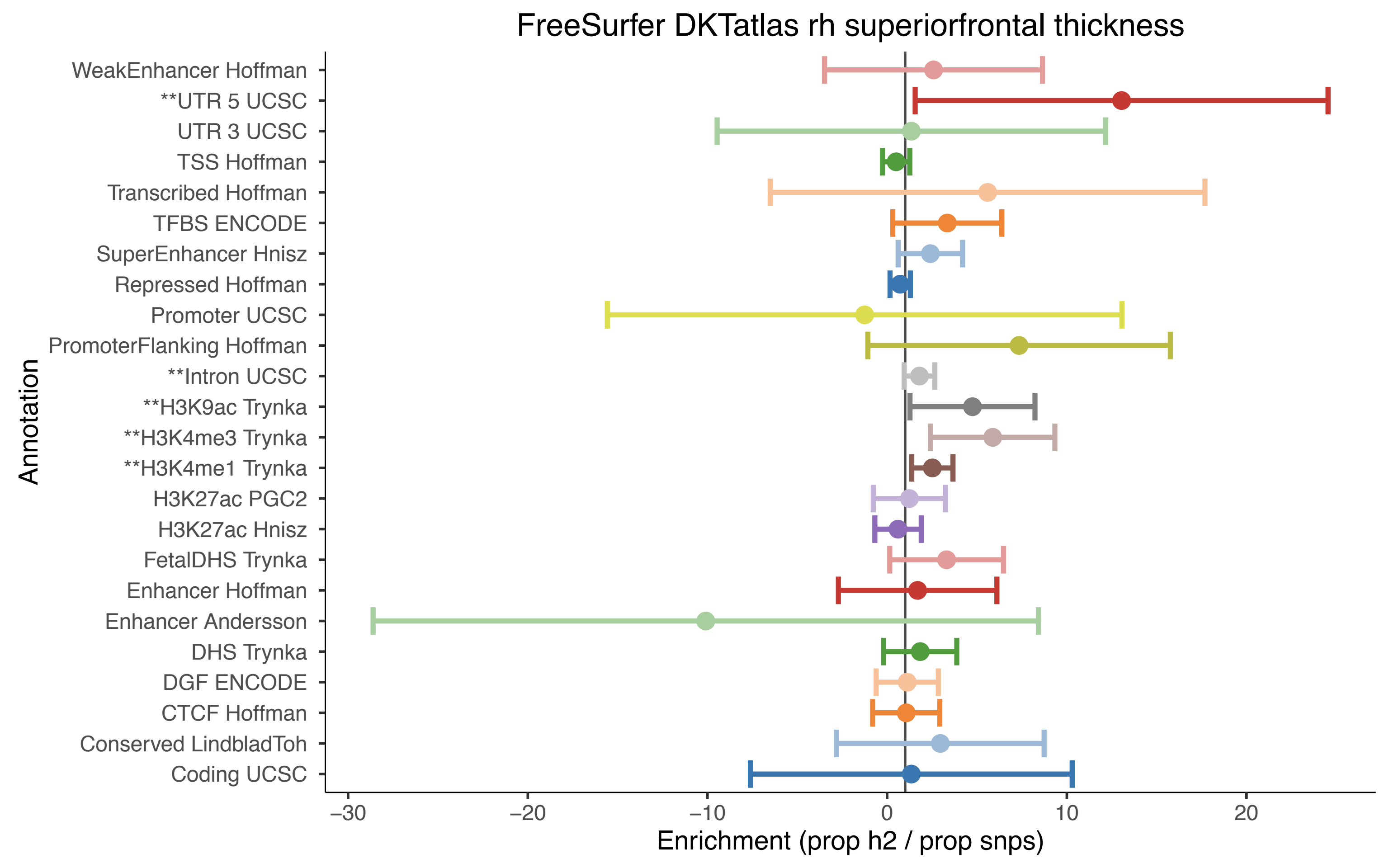
FreeSurfer DKTatlas rh superiorfrontal thickness
WeakEnhancer Hoffman
**UTR 5 UCSC
UTR 3 UCSC
TSS Hoffman
Transcribed Hoffman
TFBS ENCODE
SuperEnhancer Hnisz
Repressed Hoffman
Promoter UCSC
PromoterFlanking Hoffman
**Intron UCSC
**H3K9ac Trynka
**H3K4me3 Trynka
**H3K4me1 Trynka
H3K27ac PGC2
H3K27ac Hnisz
FetalDHS Trynka
Enhancer Hoffman
Enhancer Andersson
DHS Trynka
DGF ENCODE
CTCF Hoffman
Conserved LindbladToh
Coding UCSC
Annotation
−30
−20
−10
0
10
20
Enrichment (prop h2 / prop snps)

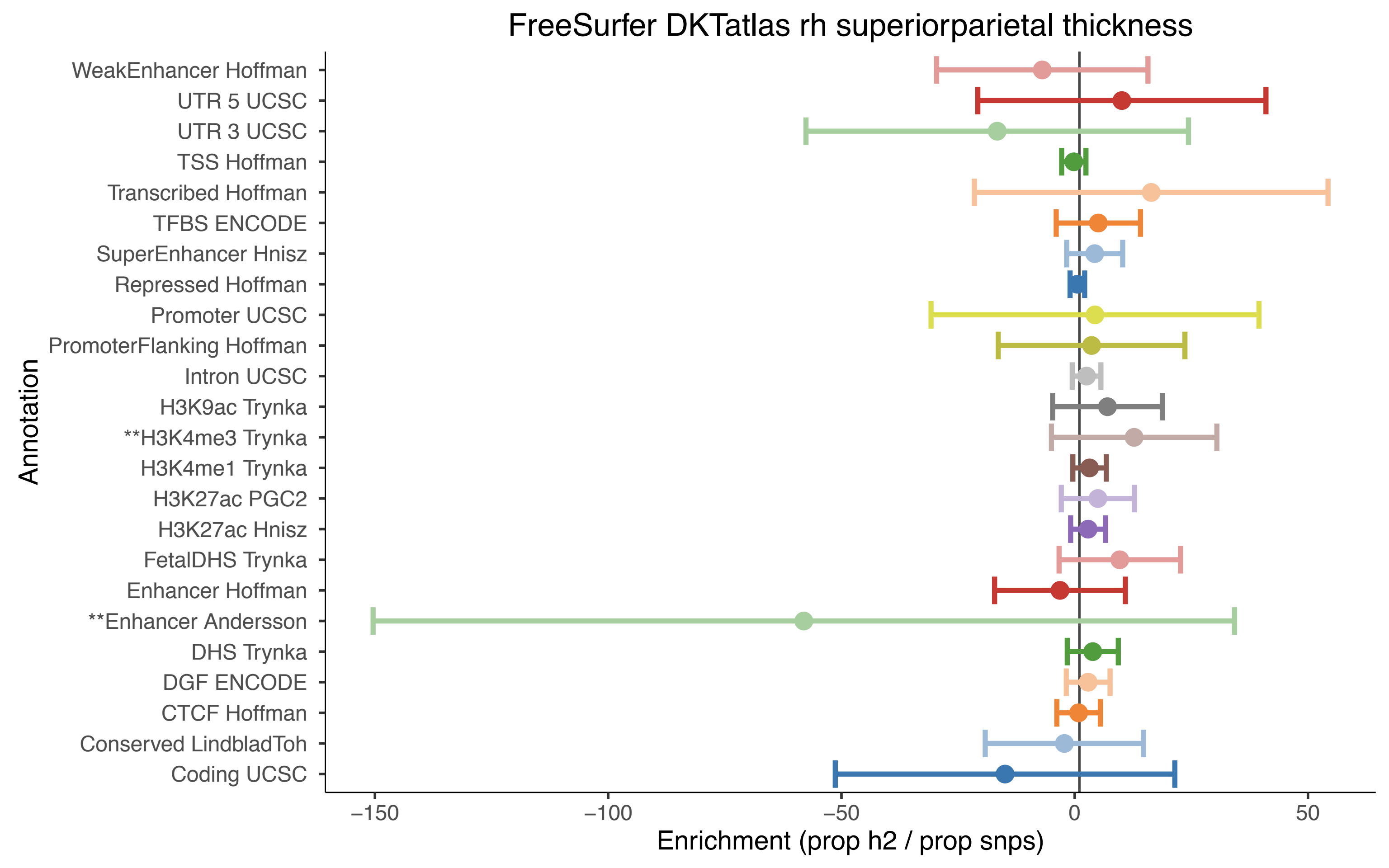
FreeSurfer DKTatlas rh superiorparietal thickness
WeakEnhancer Hoffman
UTR 5 UCSC
UTR 3 UCSC
TSS Hoffman
Transcribed Hoffman
TFBS ENCODE
SuperEnhancer Hnisz
Repressed Hoffman
Promoter UCSC
PromoterFlanking Hoffman
Intron UCSC
H3K9ac Trynka
**H3K4me3 Trynka
H3K4me1 Trynka
H3K27ac PGC2
H3K27ac Hnisz
FetalDHS Trynka
Enhancer Hoffman
**Enhancer Andersson
DHS Trynka
DGF ENCODE
CTCF Hoffman
Conserved LindbladToh
Coding UCSC
Annotation
−150
−100
−50
0
50
Enrichment (prop h2 / prop snps)

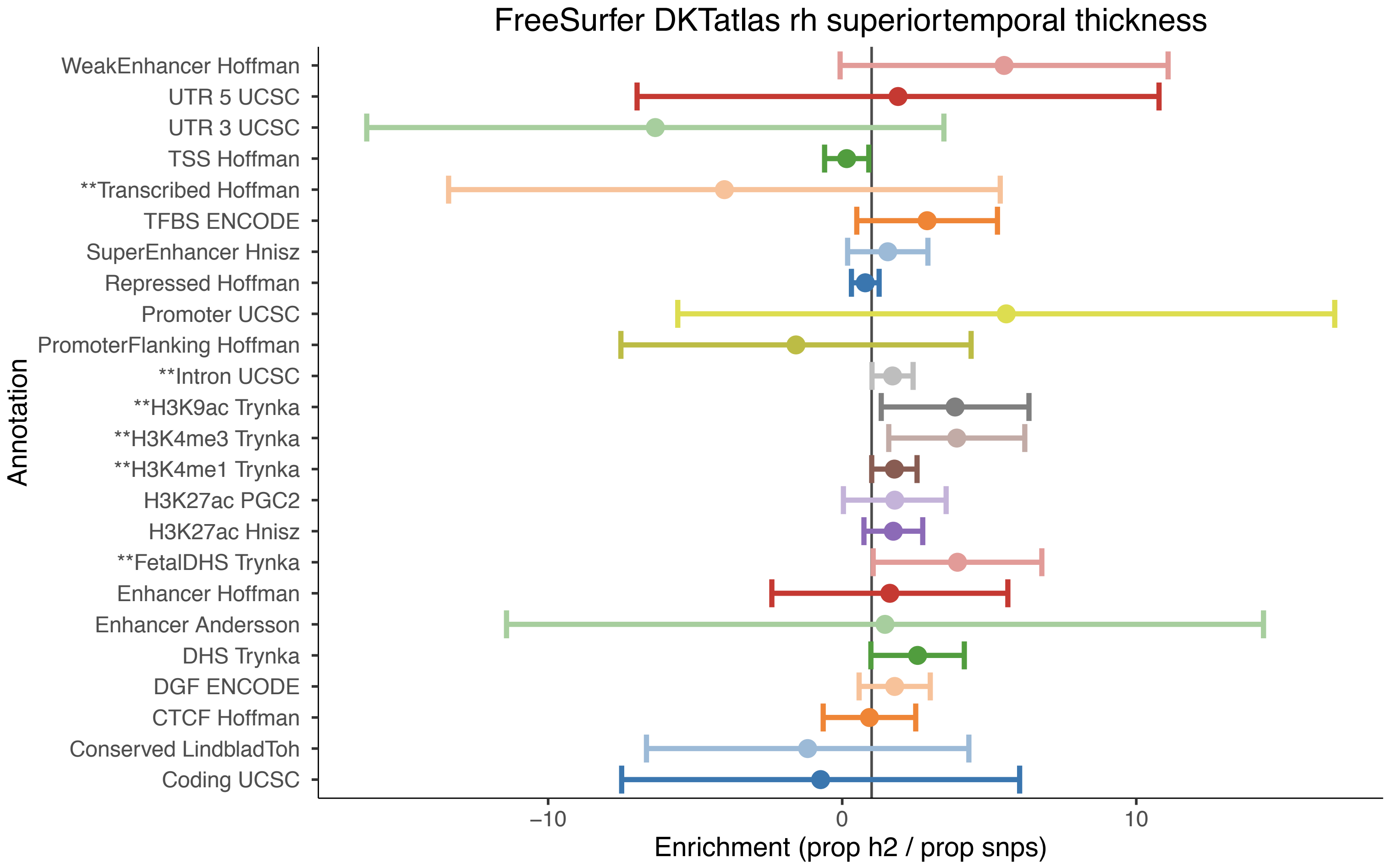

FreeSurfer DKTatlas rh superiortemporal thickness
WeakEnhancer Hoffman
UTR 5 UCSC
UTR 3 UCSC
TSS Hoffman
**Transcribed Hoffman
TFBS ENCODE
SuperEnhancer Hnisz
Repressed Hoffman
Promoter UCSC
PromoterFlanking Hoffman
**Intron UCSC
**H3K9ac Trynka
**H3K4me3 Trynka
**H3K4me1 Trynka
H3K27ac PGC2
H3K27ac Hnisz
**FetalDHS Trynka
Enhancer Hoffman
Enhancer Andersson
DHS Trynka
DGF ENCODE
CTCF Hoffman
Conserved LindbladToh
Coding UCSC
Annotation
−10
0
10
Enrichment (prop h2 / prop snps)

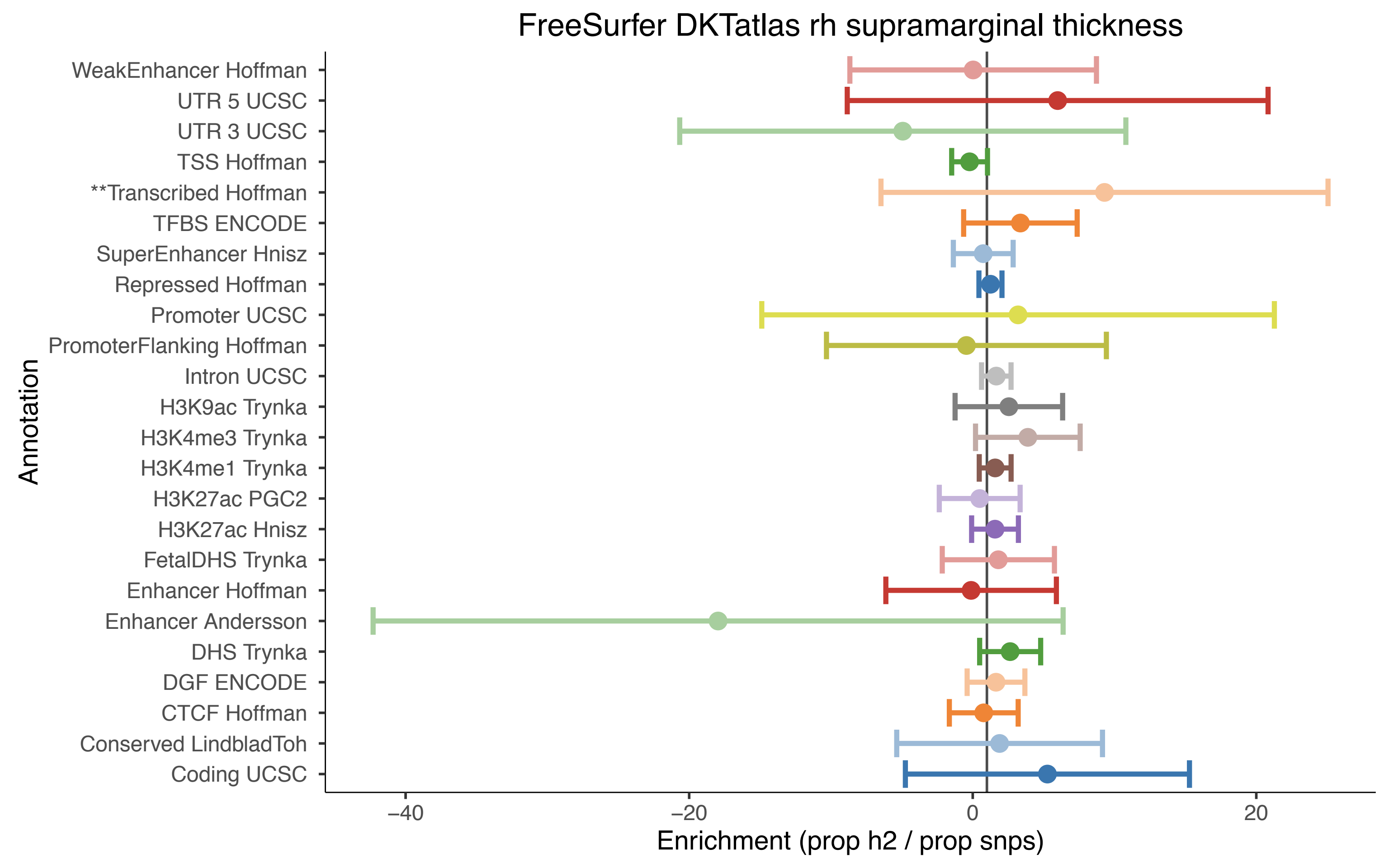

FreeSurfer DKTatlas rh supramarginal thickness
WeakEnhancer Hoffman
UTR 5 UCSC
UTR 3 UCSC
TSS Hoffman
**Transcribed Hoffman
TFBS ENCODE
SuperEnhancer Hnisz
Repressed Hoffman
Promoter UCSC
PromoterFlanking Hoffman
Intron UCSC
H3K9ac Trynka
H3K4me3 Trynka
H3K4me1 Trynka
H3K27ac PGC2
H3K27ac Hnisz
FetalDHS Trynka
Enhancer Hoffman
Enhancer Andersson
DHS Trynka
DGF ENCODE
CTCF Hoffman
Conserved LindbladToh
Coding UCSC
Annotation
−40
−20
0
20
Enrichment (prop h2 / prop snps)

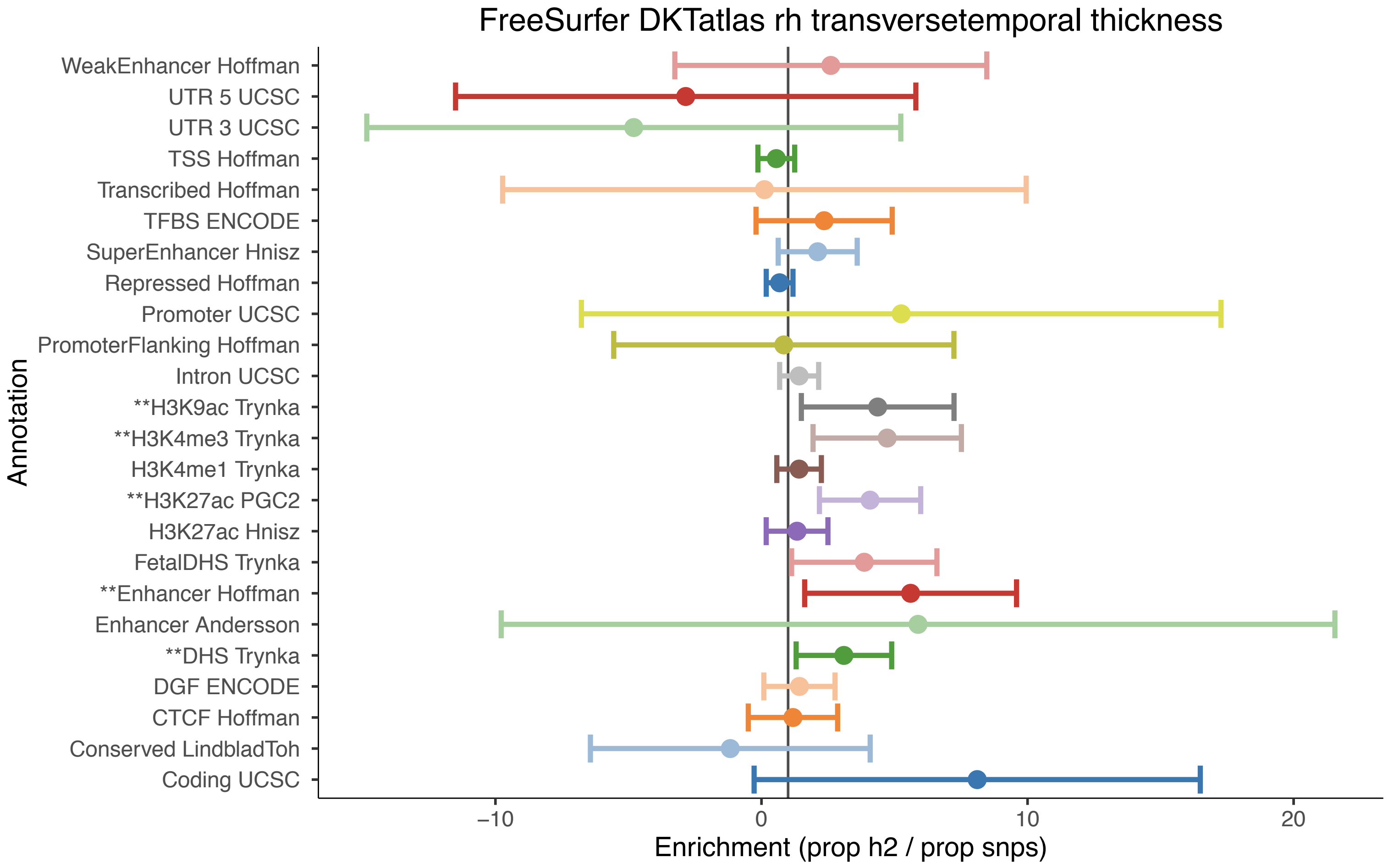

FreeSurfer DKTatlas rh transversetemporal thickness
WeakEnhancer Hoffman
UTR 5 UCSC
UTR 3 UCSC
TSS Hoffman
Transcribed Hoffman
TFBS ENCODE
SuperEnhancer Hnisz
Repressed Hoffman
Promoter UCSC
PromoterFlanking Hoffman
Intron UCSC
**H3K9ac Trynka
**H3K4me3 Trynka
H3K4me1 Trynka
**H3K27ac PGC2
H3K27ac Hnisz
FetalDHS Trynka
**Enhancer Hoffman
Enhancer Andersson
**DHS Trynka
DGF ENCODE
CTCF Hoffman
Conserved LindbladToh
Coding UCSC
Annotation
−10
0
10
20
Enrichment (prop h2 / prop snps)

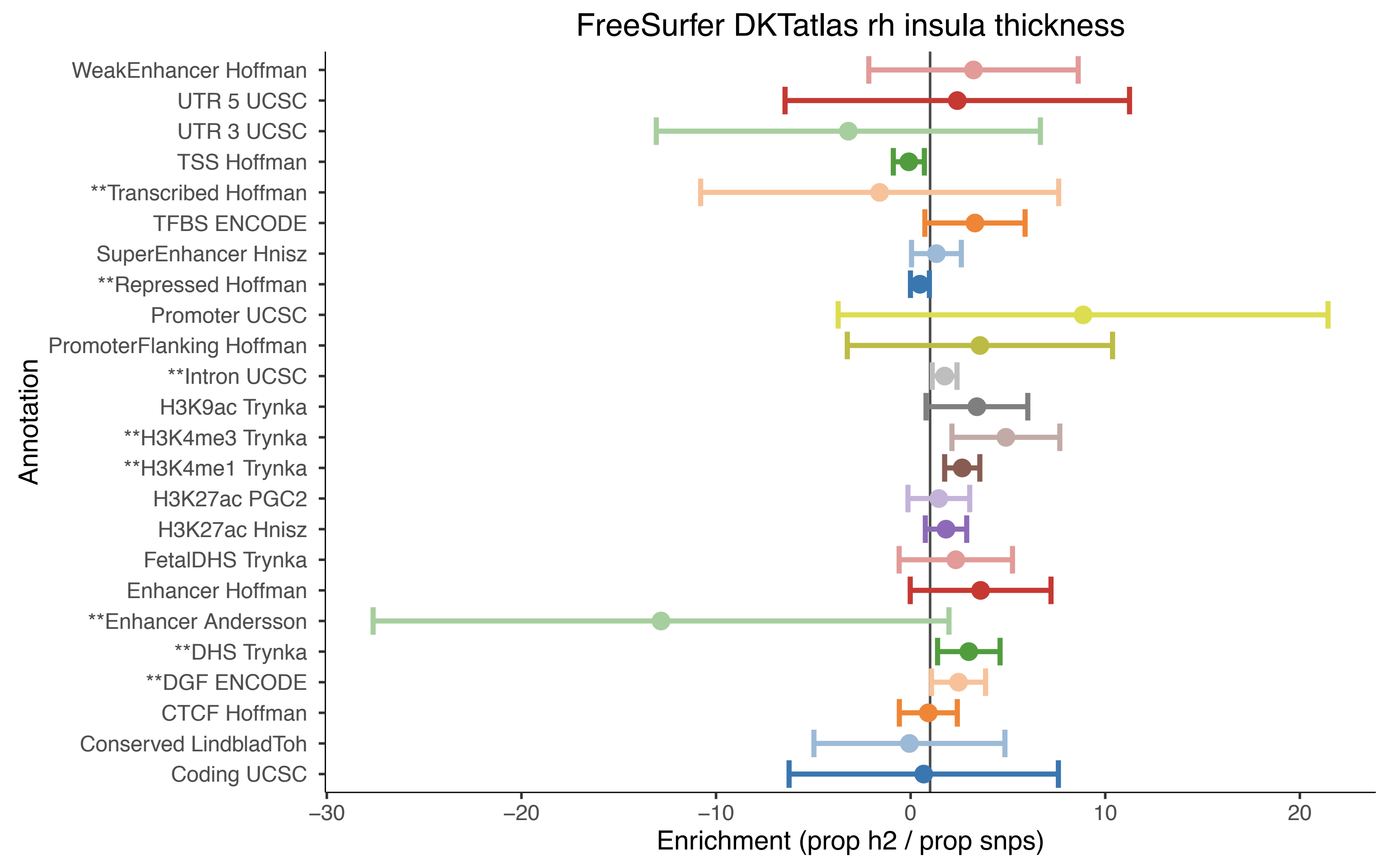

FreeSurfer DKTatlas rh insula thickness
WeakEnhancer Hoffman
UTR 5 UCSC
UTR 3 UCSC
TSS Hoffman
**Transcribed Hoffman
TFBS ENCODE
SuperEnhancer Hnisz
**Repressed Hoffman
Promoter UCSC
PromoterFlanking Hoffman
**Intron UCSC
H3K9ac Trynka
**H3K4me3 Trynka
**H3K4me1 Trynka
H3K27ac PGC2
H3K27ac Hnisz
FetalDHS Trynka
Enhancer Hoffman
**Enhancer Andersson
**DHS Trynka
**DGF ENCODE
CTCF Hoffman
Conserved LindbladToh
Coding UCSC
Annotation
−30
−20
−10
0
10
20
Enrichment (prop h2 / prop snps)

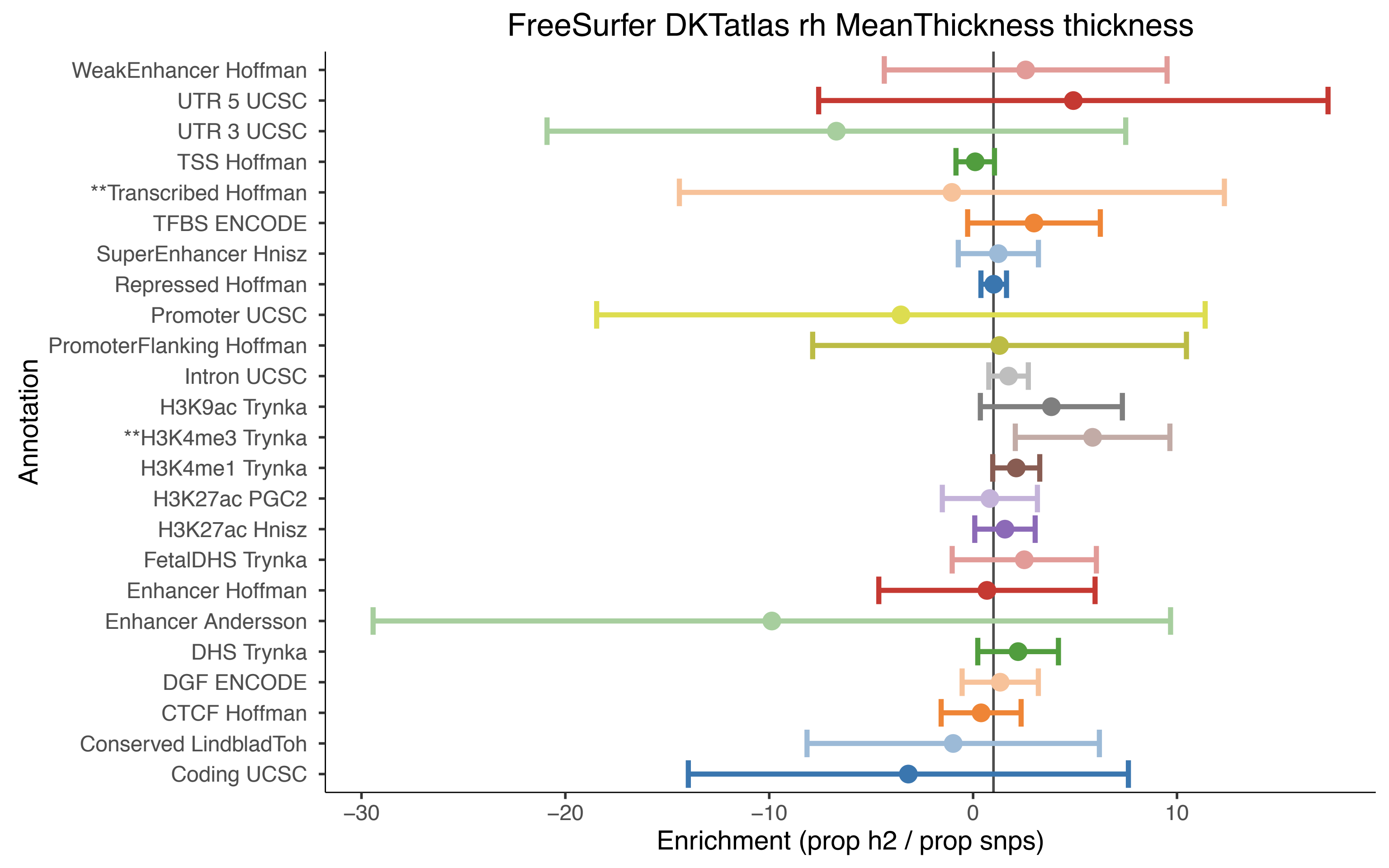
FreeSurfer DKTatlas rh MeanThickness thickness
WeakEnhancer Hoffman
UTR 5 UCSC
UTR 3 UCSC
TSS Hoffman
**Transcribed Hoffman
TFBS ENCODE
SuperEnhancer Hnisz
Repressed Hoffman
Promoter UCSC
PromoterFlanking Hoffman
Intron UCSC
H3K9ac Trynka
**H3K4me3 Trynka
H3K4me1 Trynka
H3K27ac PGC2
H3K27ac Hnisz
FetalDHS Trynka
Enhancer Hoffman
Enhancer Andersson
DHS Trynka
DGF ENCODE
CTCF Hoffman
Conserved LindbladToh
Coding UCSC
Annotation
−30
−20
−10
0
10
Enrichment (prop h2 / prop snps)

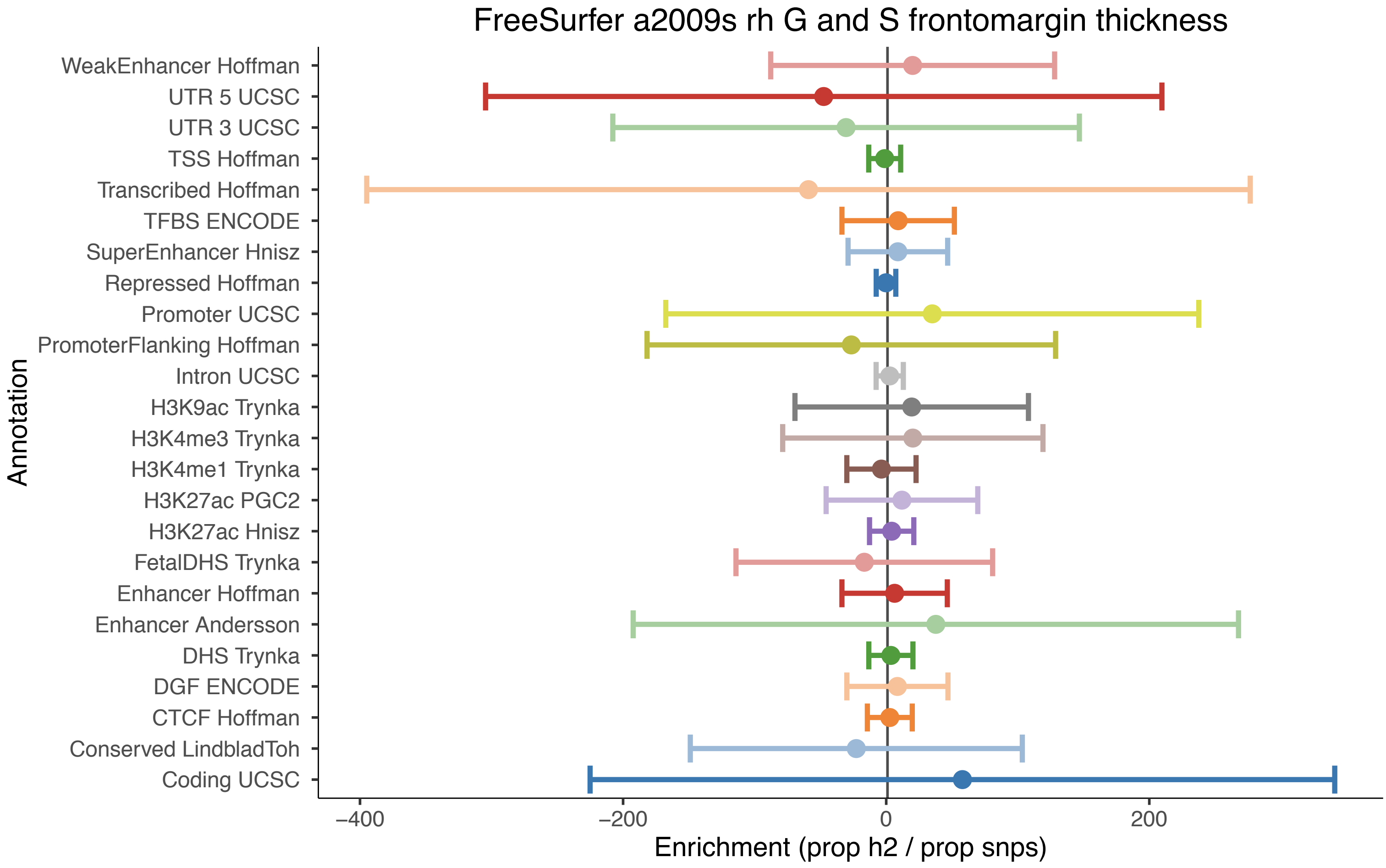

FreeSurfer a2009s rh G and S frontomargin thickness
WeakEnhancer Hoffman
UTR 5 UCSC
UTR 3 UCSC
TSS Hoffman
Transcribed Hoffman
TFBS ENCODE
SuperEnhancer Hnisz
Repressed Hoffman
Promoter UCSC
PromoterFlanking Hoffman
Intron UCSC
H3K9ac Trynka
H3K4me3 Trynka
H3K4me1 Trynka
H3K27ac PGC2
H3K27ac Hnisz
FetalDHS Trynka
Enhancer Hoffman
Enhancer Andersson
DHS Trynka
DGF ENCODE
CTCF Hoffman
Conserved LindbladToh
Coding UCSC
Annotation
−400
−200
0
200
Enrichment (prop h2 / prop snps)

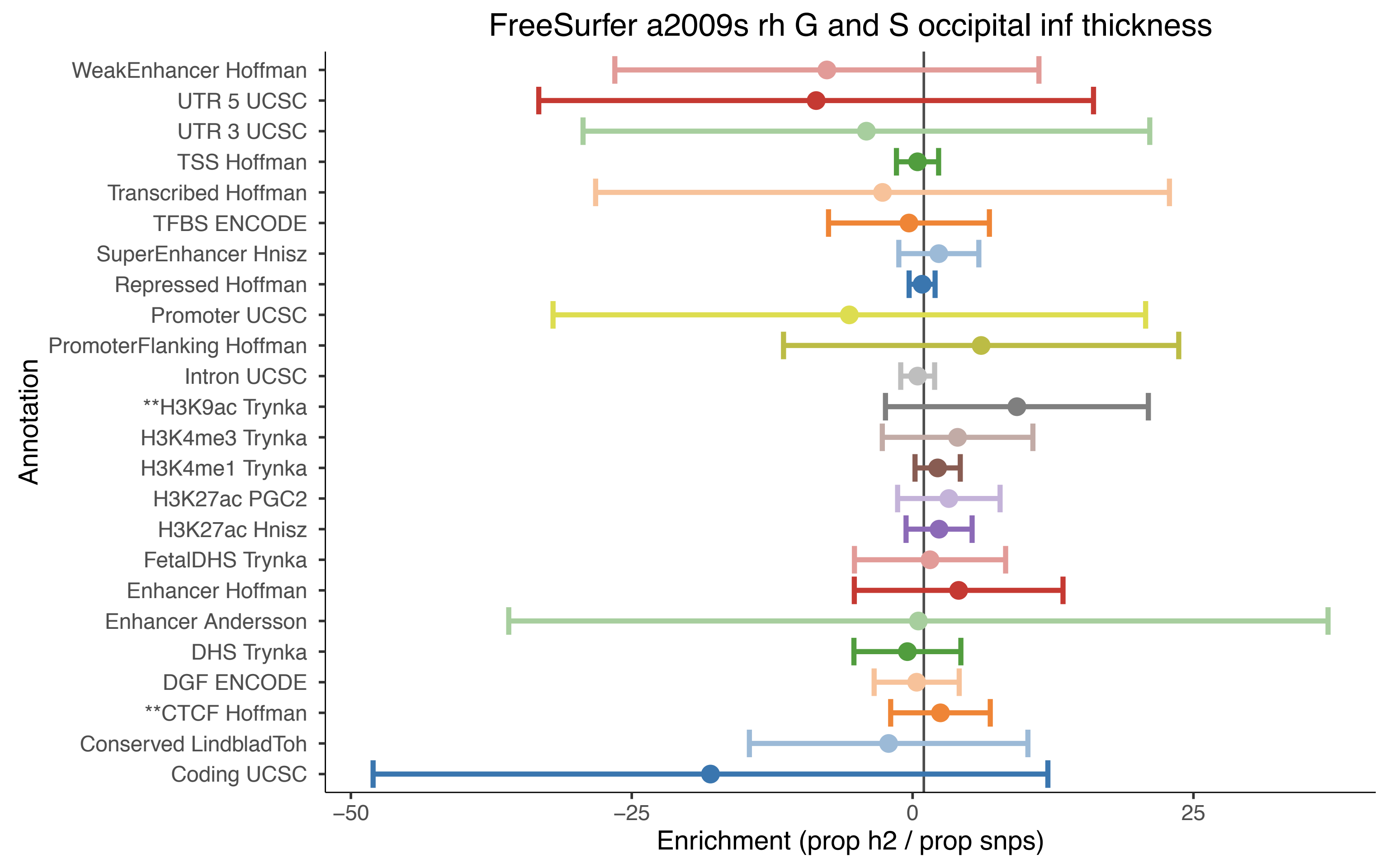

FreeSurfer a2009s rh G and S occipital inf thickness
WeakEnhancer Hoffman
UTR 5 UCSC
UTR 3 UCSC
TSS Hoffman
Transcribed Hoffman
TFBS ENCODE
SuperEnhancer Hnisz
Repressed Hoffman
Promoter UCSC
PromoterFlanking Hoffman
Intron UCSC
**H3K9ac Trynka
H3K4me3 Trynka
H3K4me1 Trynka
H3K27ac PGC2
H3K27ac Hnisz
FetalDHS Trynka
Enhancer Hoffman
Enhancer Andersson
DHS Trynka
DGF ENCODE
**CTCF Hoffman
Conserved LindbladToh
Coding UCSC
Annotation
−50
−25
0
25
Enrichment (prop h2 / prop snps)

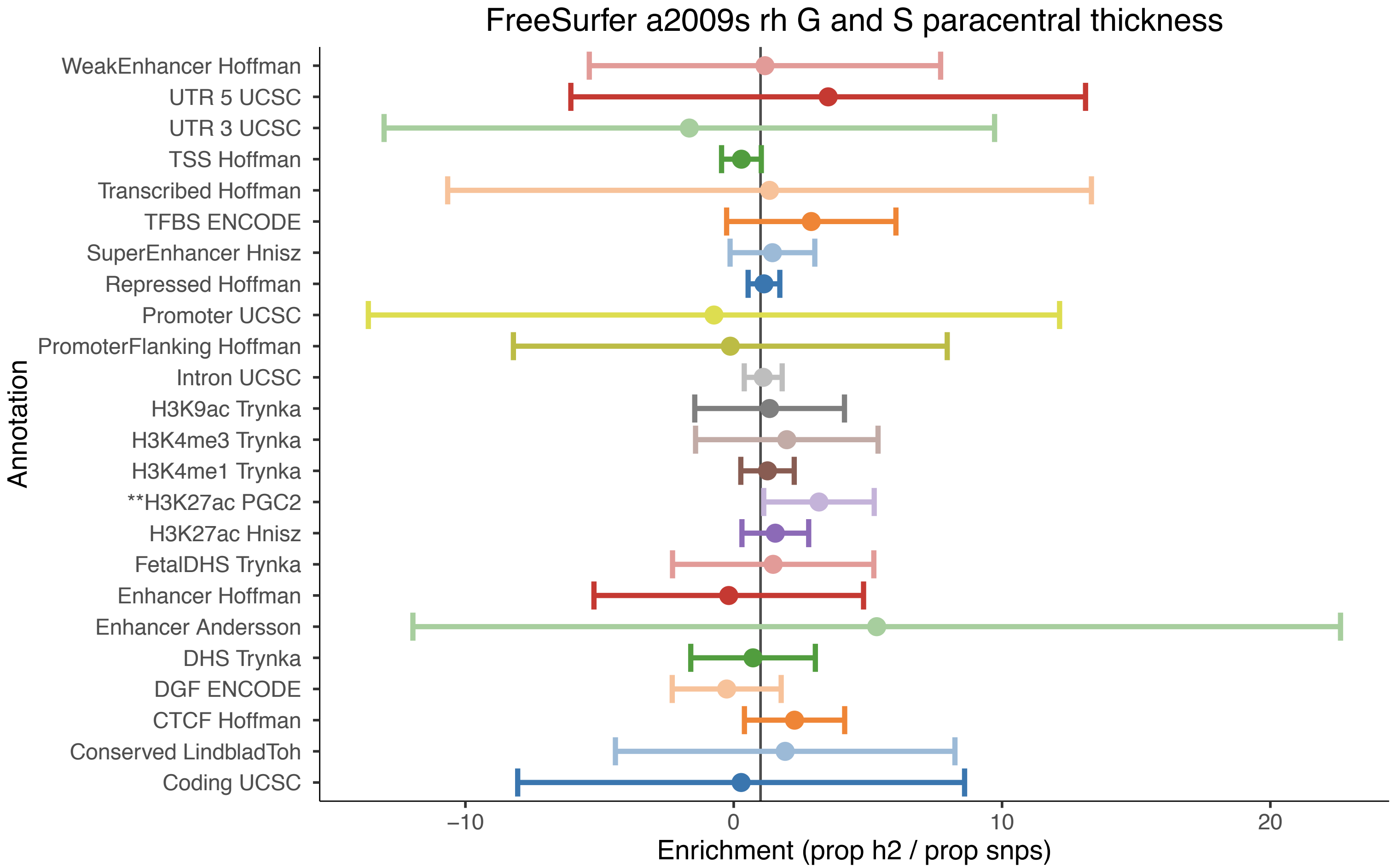
FreeSurfer a2009s rh G and S paracentral thickness
Annotation
WeakEnhancer Hoffman
UTR 5 UCSC
UTR 3 UCSC
TSS Hoffman
Transcribed Hoffman
TFBS ENCODE
SuperEnhancer Hnisz
Repressed Hoffman
Promoter UCSC
PromoterFlanking Hoffman
Intron UCSC
H3K9ac Trynka
H3K4me3 Trynka
H3K4me1 Trynka
**H3K27ac PGC2
H3K27ac Hnisz
FetalDHS Trynka
Enhancer Hoffman
Enhancer Andersson
DHS Trynka
DGF ENCODE
CTCF Hoffman
Conserved LindbladToh
Coding UCSC
−10
0
10
20
Enrichment (prop h2 / prop snps)

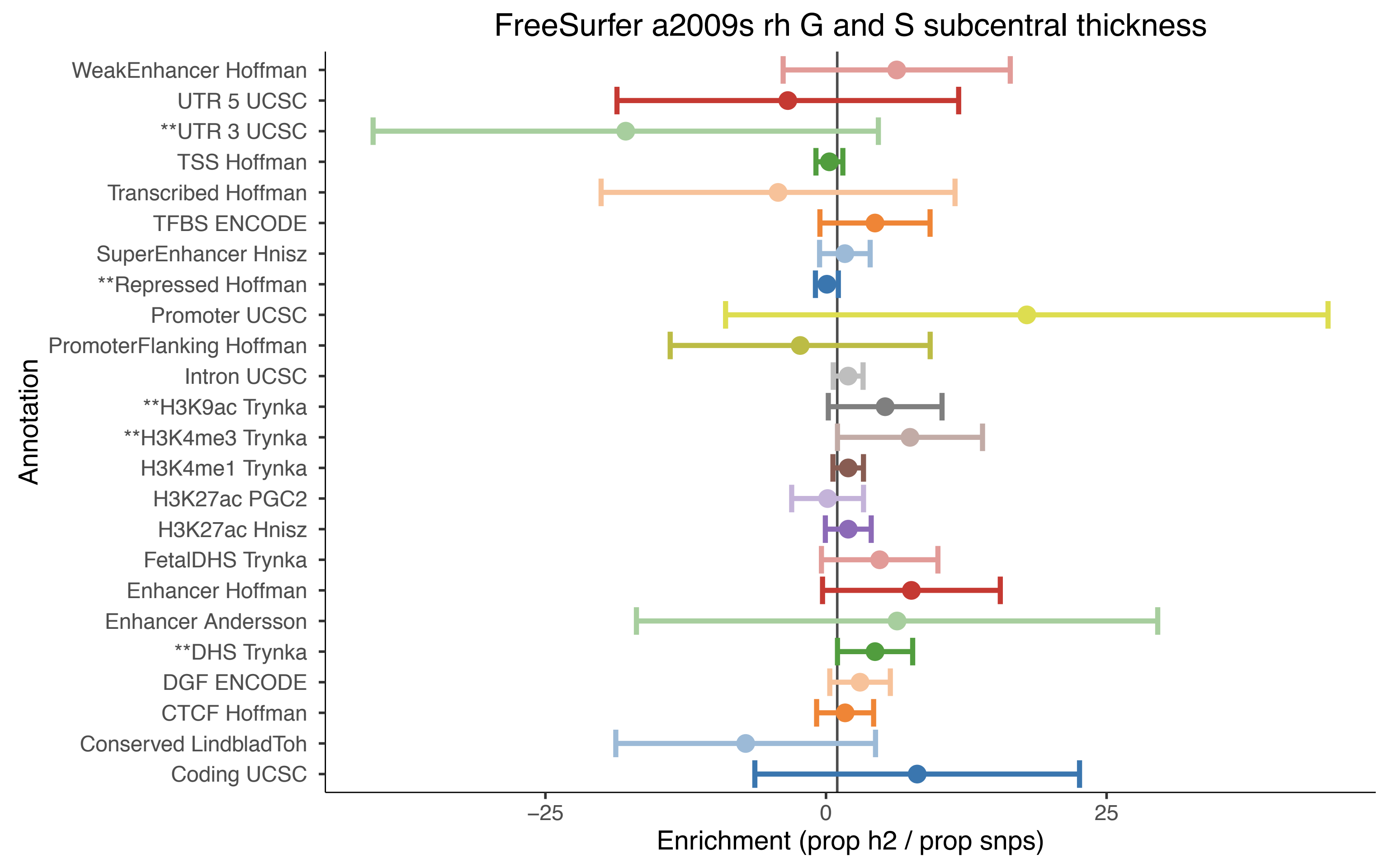
FreeSurfer a2009s rh G and S subcentral thickness
WeakEnhancer Hoffman
UTR 5 UCSC
**UTR 3 UCSC
TSS Hoffman
Transcribed Hoffman
TFBS ENCODE
SuperEnhancer Hnisz
**Repressed Hoffman
Promoter UCSC
PromoterFlanking Hoffman
Intron UCSC
**H3K9ac Trynka
**H3K4me3 Trynka
H3K4me1 Trynka
H3K27ac PGC2
H3K27ac Hnisz
FetalDHS Trynka
Enhancer Hoffman
Enhancer Andersson
**DHS Trynka
DGF ENCODE
CTCF Hoffman
Conserved LindbladToh
Coding UCSC
Annotation
−25
0
25
Enrichment (prop h2 / prop snps)

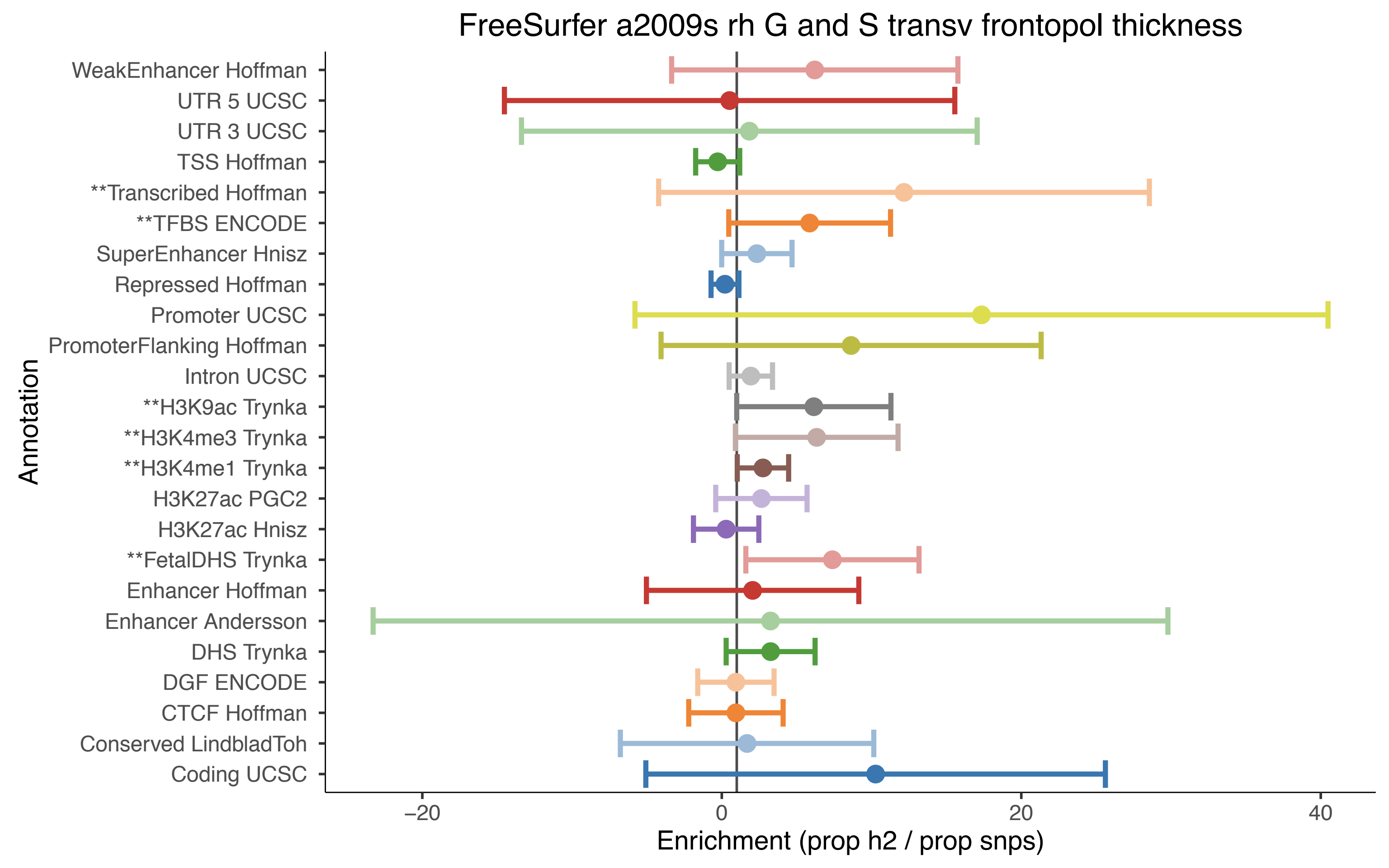
FreeSurfer a2009s rh G and S transv frontopol thickness
WeakEnhancer Hoffman
UTR 5 UCSC
UTR 3 UCSC
TSS Hoffman
**Transcribed Hoffman
**TFBS ENCODE
SuperEnhancer Hnisz
Repressed Hoffman
Promoter UCSC
PromoterFlanking Hoffman
Intron UCSC
**H3K9ac Trynka
**H3K4me3 Trynka
**H3K4me1 Trynka
H3K27ac PGC2
H3K27ac Hnisz
**FetalDHS Trynka
Enhancer Hoffman
Enhancer Andersson
DHS Trynka
DGF ENCODE
CTCF Hoffman
Conserved LindbladToh
Coding UCSC
Annotation
−20
0
20
40
Enrichment (prop h2 / prop snps)

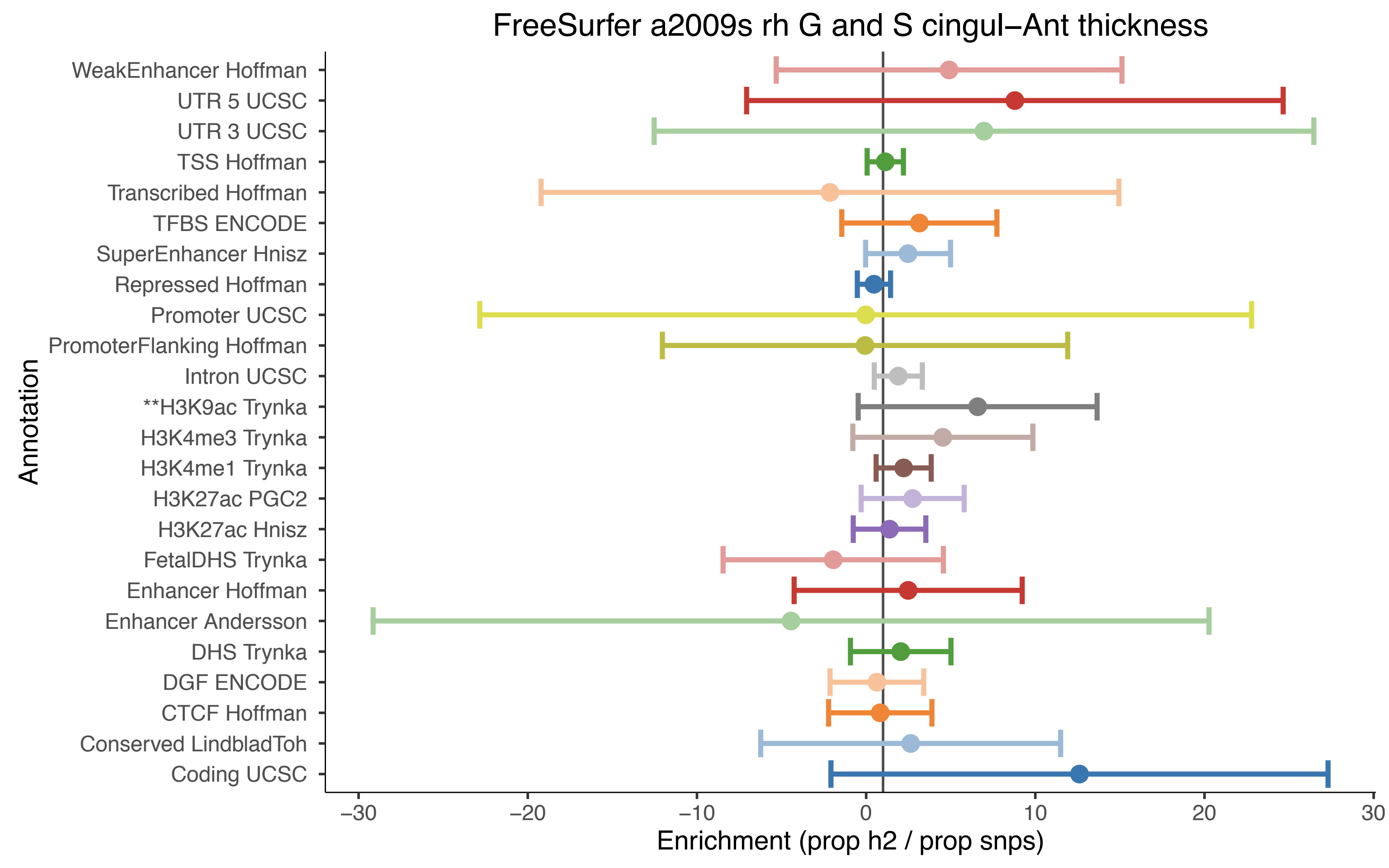
FreeSurfer a2009s rh G and S cingul−Ant thickness
WeakEnhancer Hoffman
UTR 5 UCSC
UTR 3 UCSC
TSS Hoffman
Transcribed Hoffman
TFBS ENCODE
SuperEnhancer Hnisz
Repressed Hoffman
Promoter UCSC
PromoterFlanking Hoffman
Intron UCSC
**H3K9ac Trynka
H3K4me3 Trynka
H3K4me1 Trynka
H3K27ac PGC2
H3K27ac Hnisz
FetalDHS Trynka
Enhancer Hoffman
Enhancer Andersson
DHS Trynka
DGF ENCODE
CTCF Hoffman
Conserved LindbladToh
Coding UCSC
Annotation
−30
−20
−10
0
10
20
30
Enrichment (prop h2 / prop snps)

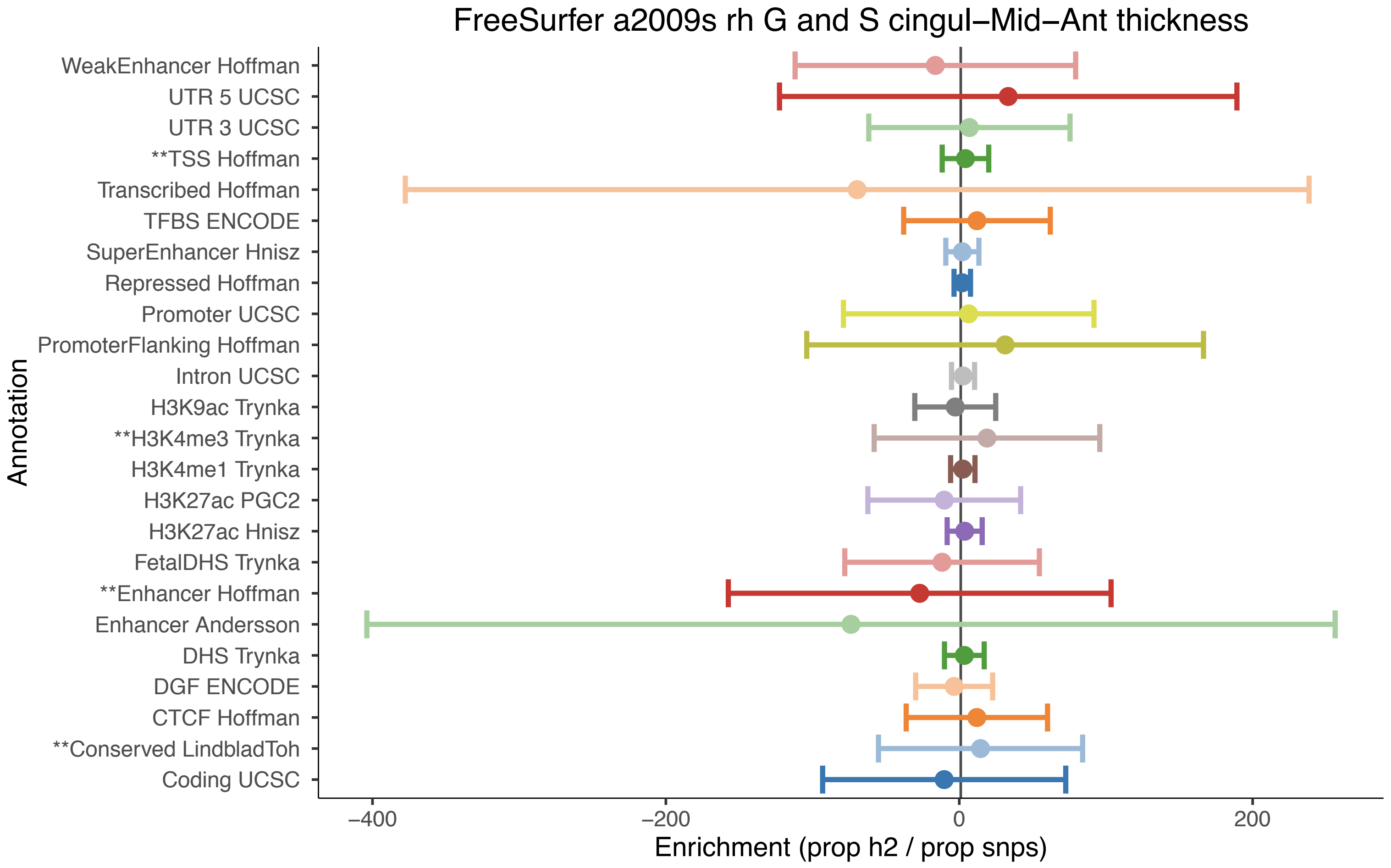
FreeSurfer a2009s rh G and S cingul−Mid−Ant thickness
WeakEnhancer Hoffman
UTR 5 UCSC
UTR 3 UCSC
**TSS Hoffman
Transcribed Hoffman
TFBS ENCODE
SuperEnhancer Hnisz
Repressed Hoffman
Promoter UCSC
PromoterFlanking Hoffman
Intron UCSC
H3K9ac Trynka
**H3K4me3 Trynka
H3K4me1 Trynka
H3K27ac PGC2
H3K27ac Hnisz
FetalDHS Trynka
**Enhancer Hoffman
Enhancer Andersson
DHS Trynka
DGF ENCODE
CTCF Hoffman
**Conserved LindbladToh
Coding UCSC
Annotation
−400
−200
0
200
Enrichment (prop h2 / prop snps)

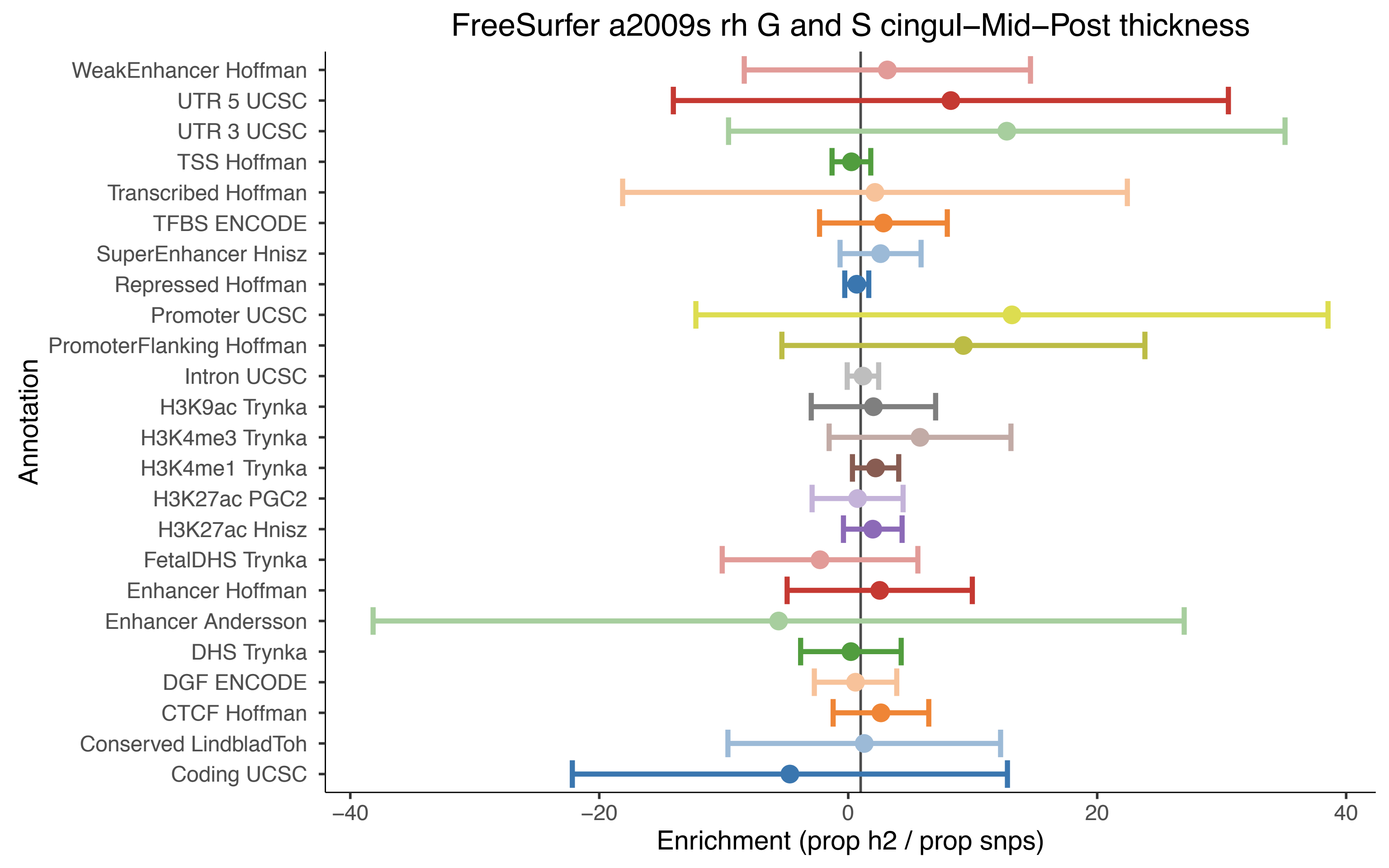

FreeSurfer a2009s rh G and S cingul−Mid−Post thickness
WeakEnhancer Hoffman
UTR 5 UCSC
UTR 3 UCSC
TSS Hoffman
Transcribed Hoffman
TFBS ENCODE
SuperEnhancer Hnisz
Repressed Hoffman
Promoter UCSC
PromoterFlanking Hoffman
Intron UCSC
H3K9ac Trynka
H3K4me3 Trynka
H3K4me1 Trynka
H3K27ac PGC2
H3K27ac Hnisz
FetalDHS Trynka
Enhancer Hoffman
Enhancer Andersson
DHS Trynka
DGF ENCODE
CTCF Hoffman
Conserved LindbladToh
Coding UCSC
Annotation
−40
−20
0
20
40
Enrichment (prop h2 / prop snps)

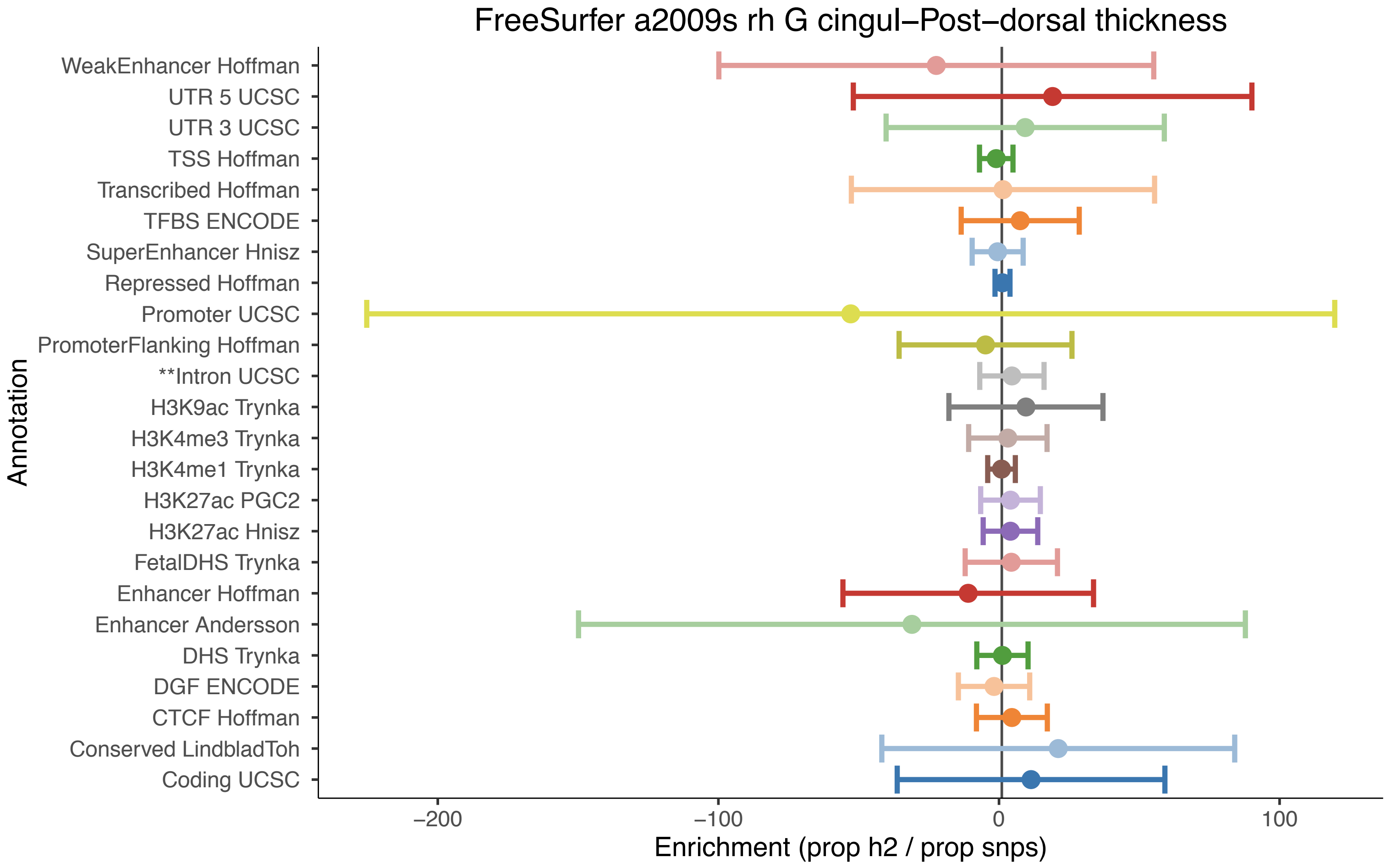

FreeSurfer a2009s rh G cingul−Post−dorsal thickness
WeakEnhancer Hoffman
UTR 5 UCSC
UTR 3 UCSC
TSS Hoffman
Transcribed Hoffman
TFBS ENCODE
SuperEnhancer Hnisz
Repressed Hoffman
Promoter UCSC
PromoterFlanking Hoffman
**Intron UCSC
H3K9ac Trynka
H3K4me3 Trynka
H3K4me1 Trynka
H3K27ac PGC2
H3K27ac Hnisz
FetalDHS Trynka
Enhancer Hoffman
Enhancer Andersson
DHS Trynka
DGF ENCODE
CTCF Hoffman
Conserved LindbladToh
Coding UCSC
Annotation
−200
−100
0
100
Enrichment (prop h2 / prop snps)

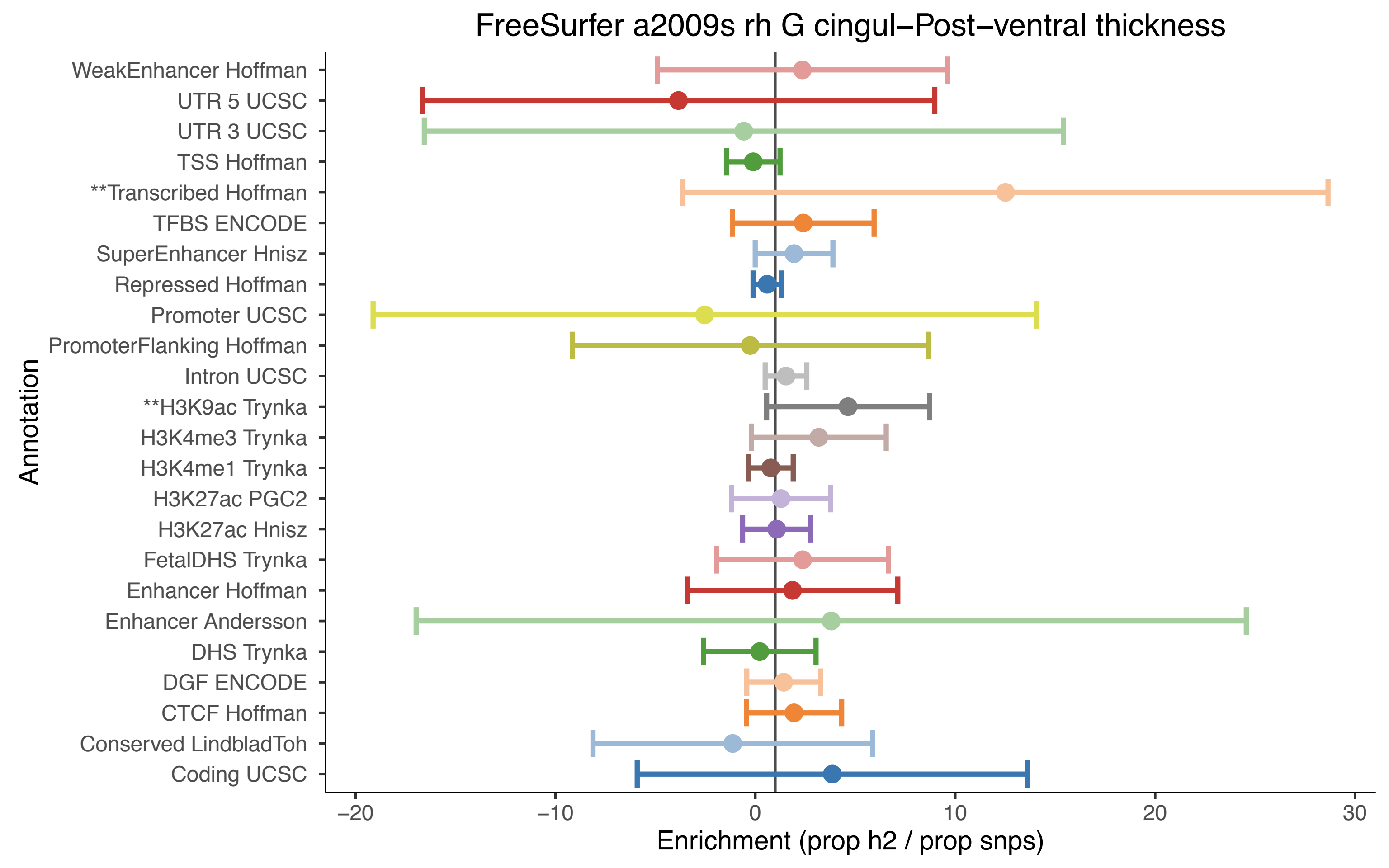

FreeSurfer a2009s rh G cingul−Post−ventral thickness
WeakEnhancer Hoffman
UTR 5 UCSC
UTR 3 UCSC
TSS Hoffman
**Transcribed Hoffman
TFBS ENCODE
SuperEnhancer Hnisz
Repressed Hoffman
Promoter UCSC
PromoterFlanking Hoffman
Intron UCSC
**H3K9ac Trynka
H3K4me3 Trynka
H3K4me1 Trynka
H3K27ac PGC2
H3K27ac Hnisz
FetalDHS Trynka
Enhancer Hoffman
Enhancer Andersson
DHS Trynka
DGF ENCODE
CTCF Hoffman
Conserved LindbladToh
Coding UCSC
Annotation
−20
−10
0
10
20
30
Enrichment (prop h2 / prop snps)

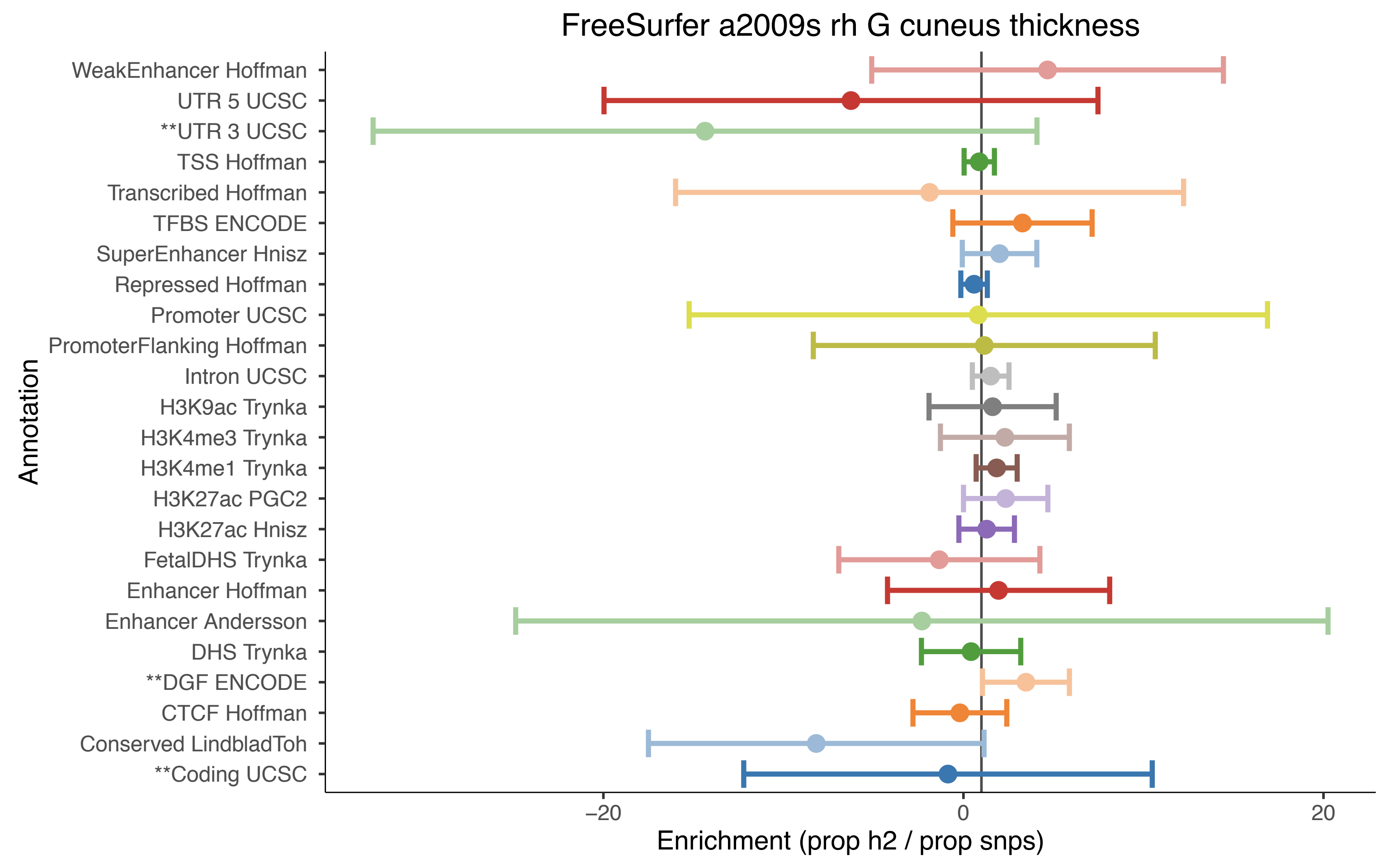

FreeSurfer a2009s rh G cuneus thickness
WeakEnhancer Hoffman
UTR 5 UCSC
**UTR 3 UCSC
TSS Hoffman
Transcribed Hoffman
TFBS ENCODE
SuperEnhancer Hnisz
Repressed Hoffman
Promoter UCSC
PromoterFlanking Hoffman
Intron UCSC
H3K9ac Trynka
H3K4me3 Trynka
H3K4me1 Trynka
H3K27ac PGC2
H3K27ac Hnisz
FetalDHS Trynka
Enhancer Hoffman
Enhancer Andersson
DHS Trynka
**DGF ENCODE
CTCF Hoffman
Conserved LindbladToh
**Coding UCSC
Annotation
−20
0
20
Enrichment (prop h2 / prop snps)

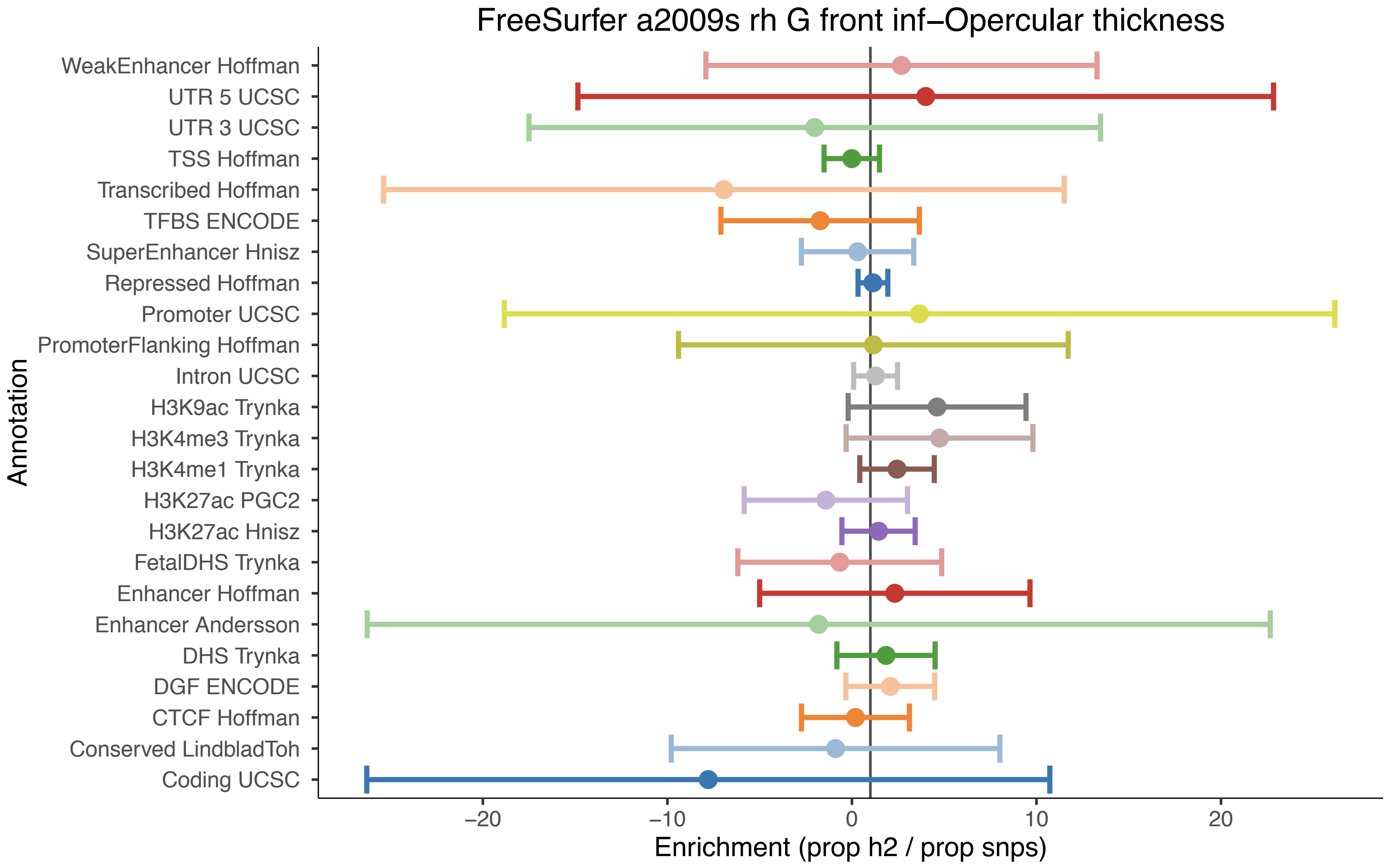

FreeSurfer a2009s rh G front inf−Opercular thickness
WeakEnhancer Hoffman
UTR 5 UCSC
UTR 3 UCSC
TSS Hoffman
Transcribed Hoffman
TFBS ENCODE
SuperEnhancer Hnisz
Repressed Hoffman
Promoter UCSC
PromoterFlanking Hoffman
Intron UCSC
H3K9ac Trynka
H3K4me3 Trynka
H3K4me1 Trynka
H3K27ac PGC2
H3K27ac Hnisz
FetalDHS Trynka
Enhancer Hoffman
Enhancer Andersson
DHS Trynka
DGF ENCODE
CTCF Hoffman
Conserved LindbladToh
Coding UCSC
Annotation
−20
−10
0
10
20
Enrichment (prop h2 / prop snps)

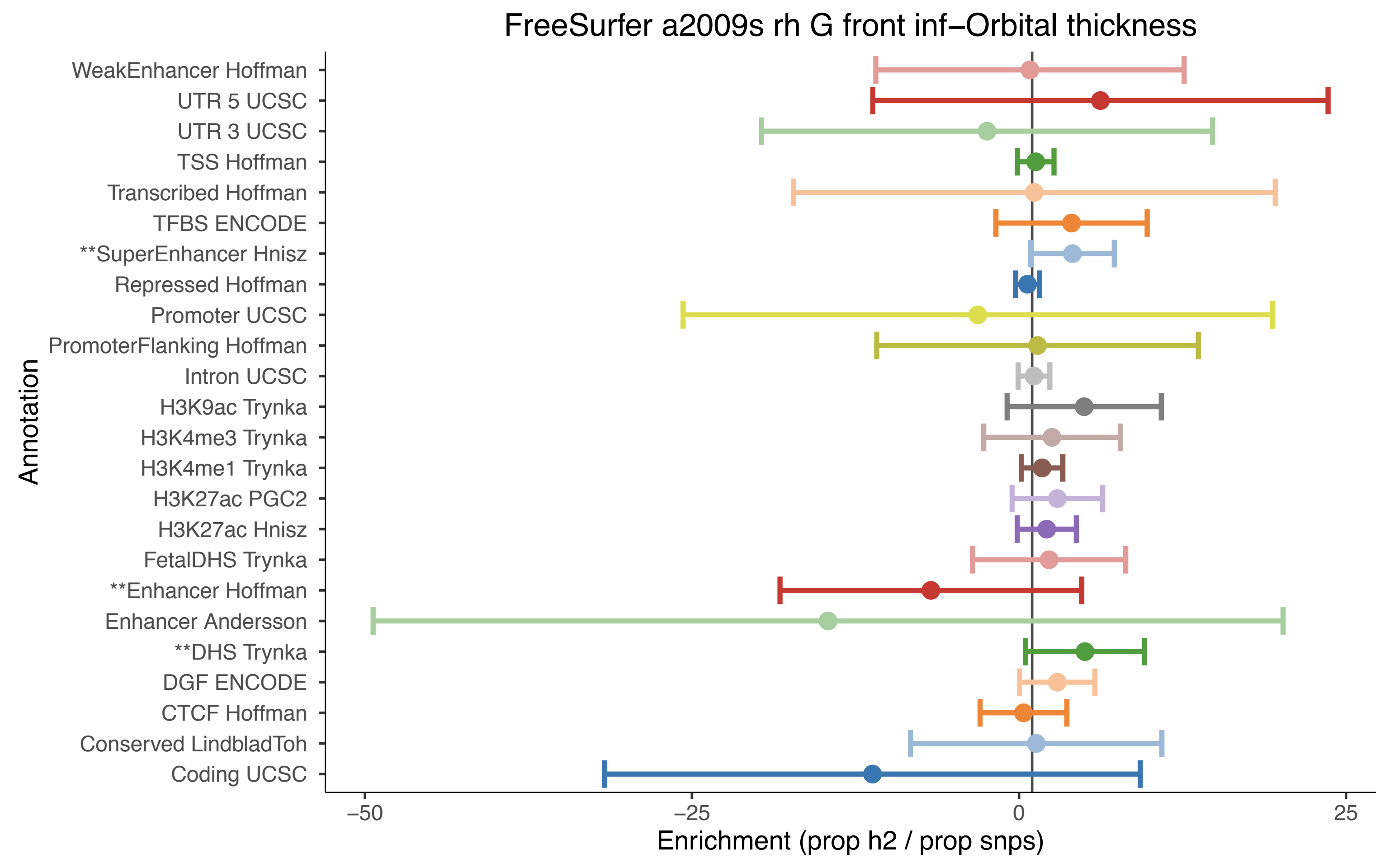

FreeSurfer a2009s rh G front inf−Orbital thickness
WeakEnhancer Hoffman
UTR 5 UCSC
UTR 3 UCSC
TSS Hoffman
Transcribed Hoffman
TFBS ENCODE
**SuperEnhancer Hnisz
Repressed Hoffman
Promoter UCSC
PromoterFlanking Hoffman
Intron UCSC
H3K9ac Trynka
H3K4me3 Trynka
H3K4me1 Trynka
H3K27ac PGC2
H3K27ac Hnisz
FetalDHS Trynka
**Enhancer Hoffman
Enhancer Andersson
**DHS Trynka
DGF ENCODE
CTCF Hoffman
Conserved LindbladToh
Coding UCSC
Annotation
−50
−25
0
25
Enrichment (prop h2 / prop snps)

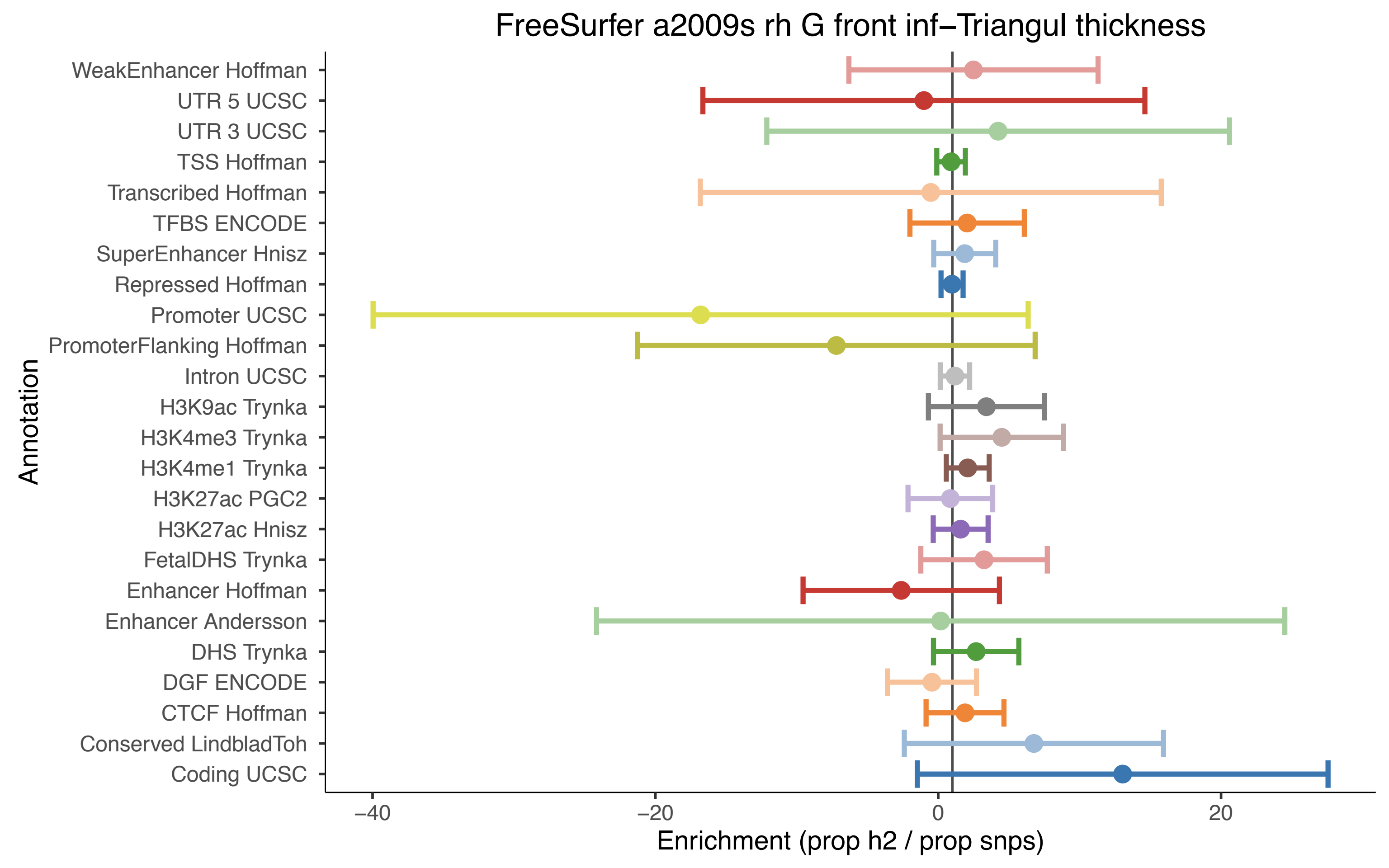

FreeSurfer a2009s rh G front inf−Triangul thickness
WeakEnhancer Hoffman
UTR 5 UCSC
UTR 3 UCSC
TSS Hoffman
Transcribed Hoffman
TFBS ENCODE
SuperEnhancer Hnisz
Repressed Hoffman
Promoter UCSC
PromoterFlanking Hoffman
Intron UCSC
H3K9ac Trynka
H3K4me3 Trynka
H3K4me1 Trynka
H3K27ac PGC2
H3K27ac Hnisz
FetalDHS Trynka
Enhancer Hoffman
Enhancer Andersson
DHS Trynka
DGF ENCODE
CTCF Hoffman
Conserved LindbladToh
Coding UCSC
Annotation
−40
−20
0
20
Enrichment (prop h2 / prop snps)

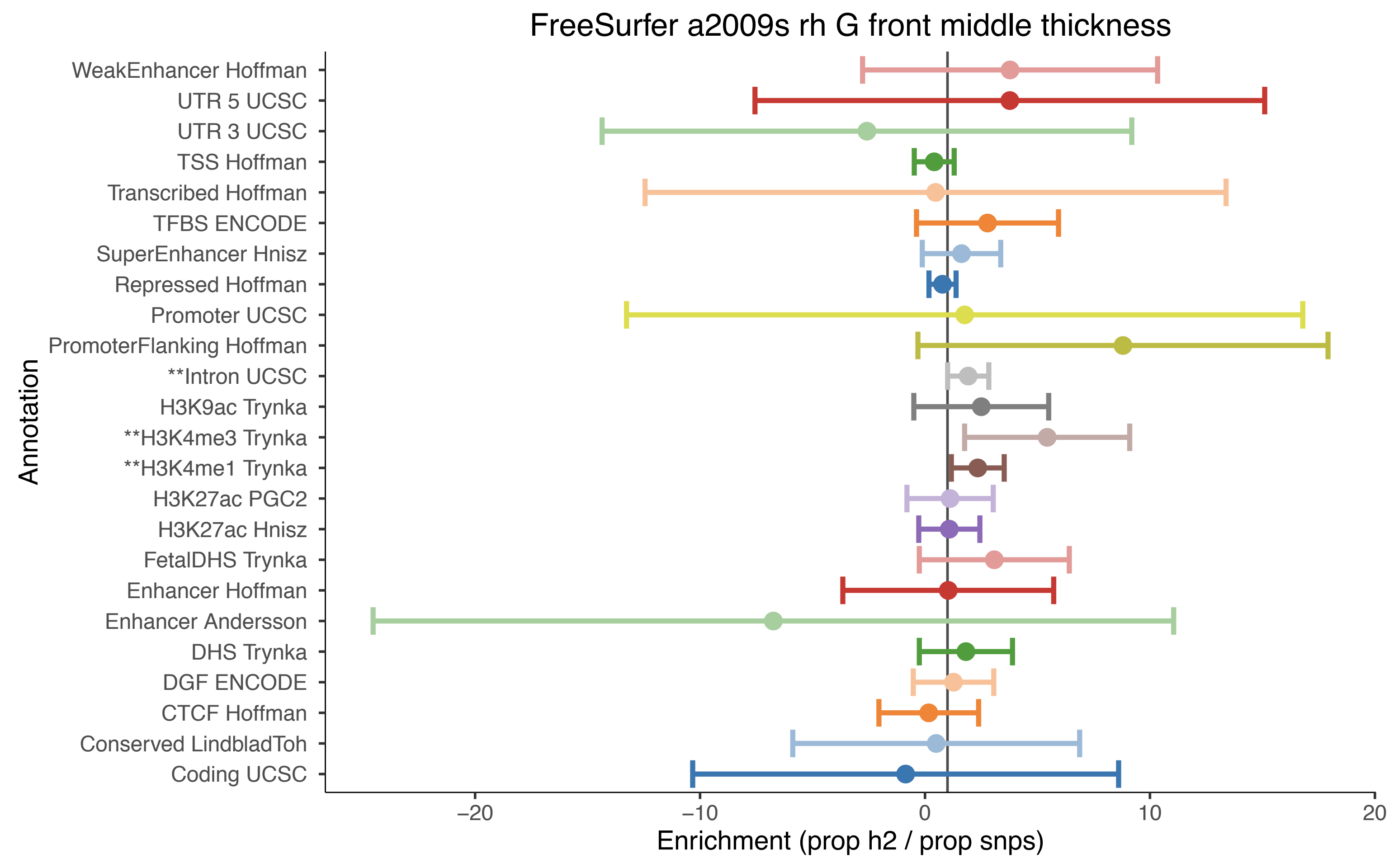

FreeSurfer a2009s rh G front middle thickness
WeakEnhancer Hoffman
UTR 5 UCSC
UTR 3 UCSC
TSS Hoffman
Transcribed Hoffman
TFBS ENCODE
SuperEnhancer Hnisz
Repressed Hoffman
Promoter UCSC
PromoterFlanking Hoffman
**Intron UCSC
H3K9ac Trynka
**H3K4me3 Trynka
**H3K4me1 Trynka
H3K27ac PGC2
H3K27ac Hnisz
FetalDHS Trynka
Enhancer Hoffman
Enhancer Andersson
DHS Trynka
DGF ENCODE
CTCF Hoffman
Conserved LindbladToh
Coding UCSC
Annotation
−20
−10
0
10
20
Enrichment (prop h2 / prop snps)

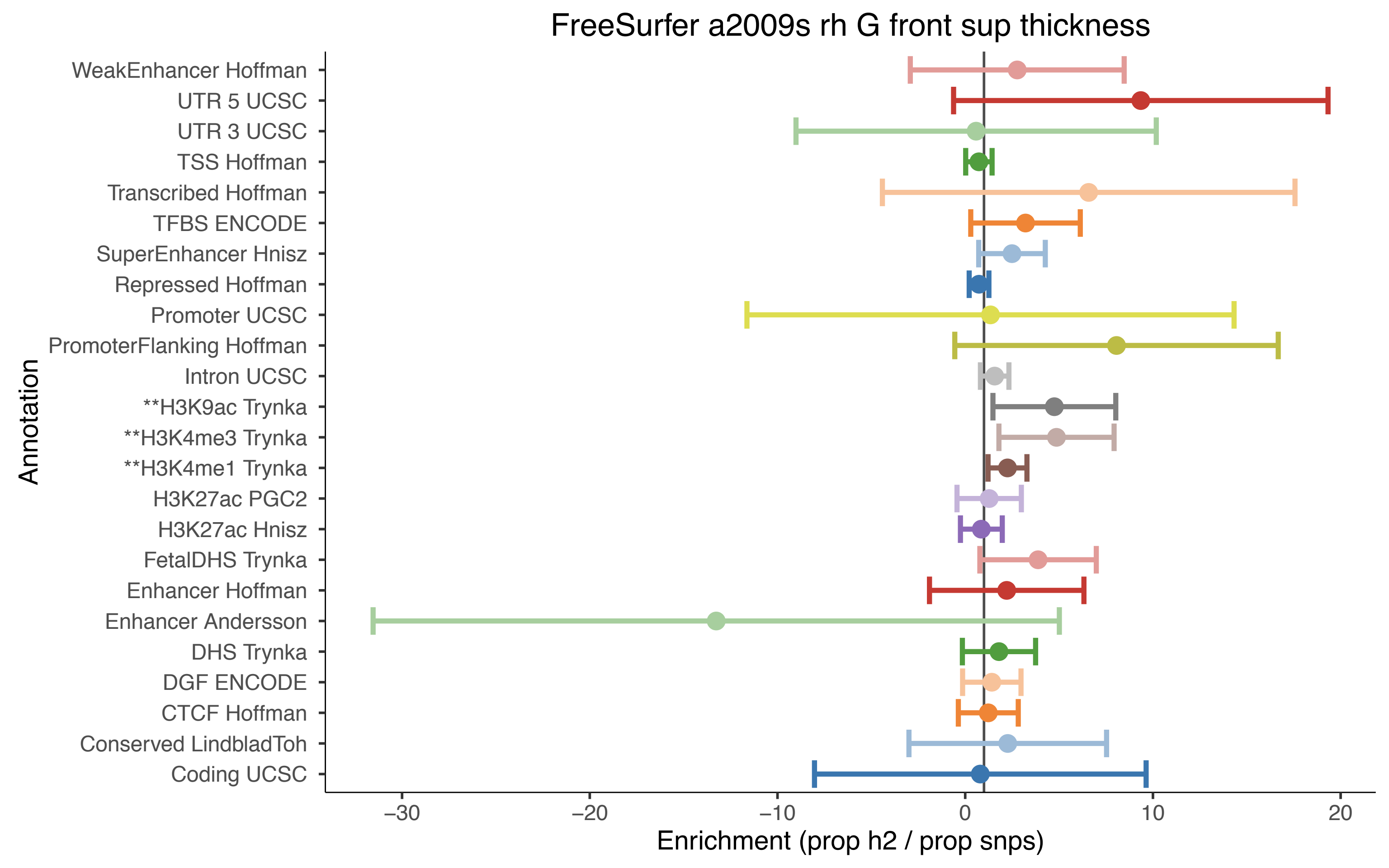
FreeSurfer a2009s rh G front sup thickness
Annotation
WeakEnhancer Hoffman
UTR 5 UCSC
UTR 3 UCSC
TSS Hoffman
Transcribed Hoffman
TFBS ENCODE
SuperEnhancer Hnisz
Repressed Hoffman
Promoter UCSC
PromoterFlanking Hoffman
Intron UCSC
**H3K9ac Trynka
**H3K4me3 Trynka
**H3K4me1 Trynka
H3K27ac PGC2
H3K27ac Hnisz
FetalDHS Trynka
Enhancer Hoffman
Enhancer Andersson
DHS Trynka
DGF ENCODE
CTCF Hoffman
Conserved LindbladToh
Coding UCSC
−30
−20
−10
0
10
20
Enrichment (prop h2 / prop snps)

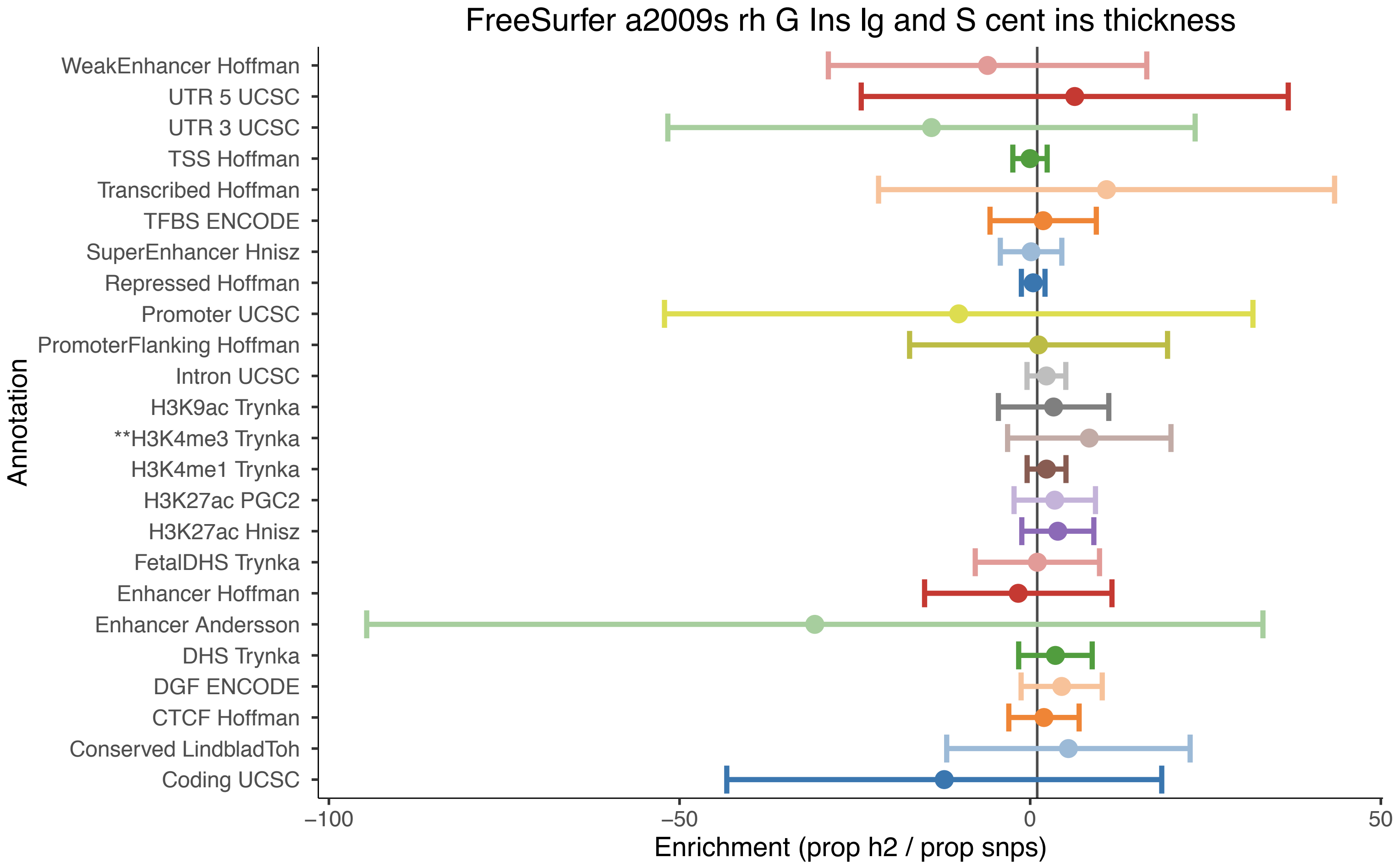

FreeSurfer a2009s rh G Ins lg and S cent ins thickness
WeakEnhancer Hoffman
UTR 5 UCSC
UTR 3 UCSC
TSS Hoffman
Transcribed Hoffman
TFBS ENCODE
SuperEnhancer Hnisz
Repressed Hoffman
Promoter UCSC
PromoterFlanking Hoffman
Intron UCSC
H3K9ac Trynka
**H3K4me3 Trynka
H3K4me1 Trynka
H3K27ac PGC2
H3K27ac Hnisz
FetalDHS Trynka
Enhancer Hoffman
Enhancer Andersson
DHS Trynka
DGF ENCODE
CTCF Hoffman
Conserved LindbladToh
Coding UCSC
Annotation
−100
−50
0
50
Enrichment (prop h2 / prop snps)

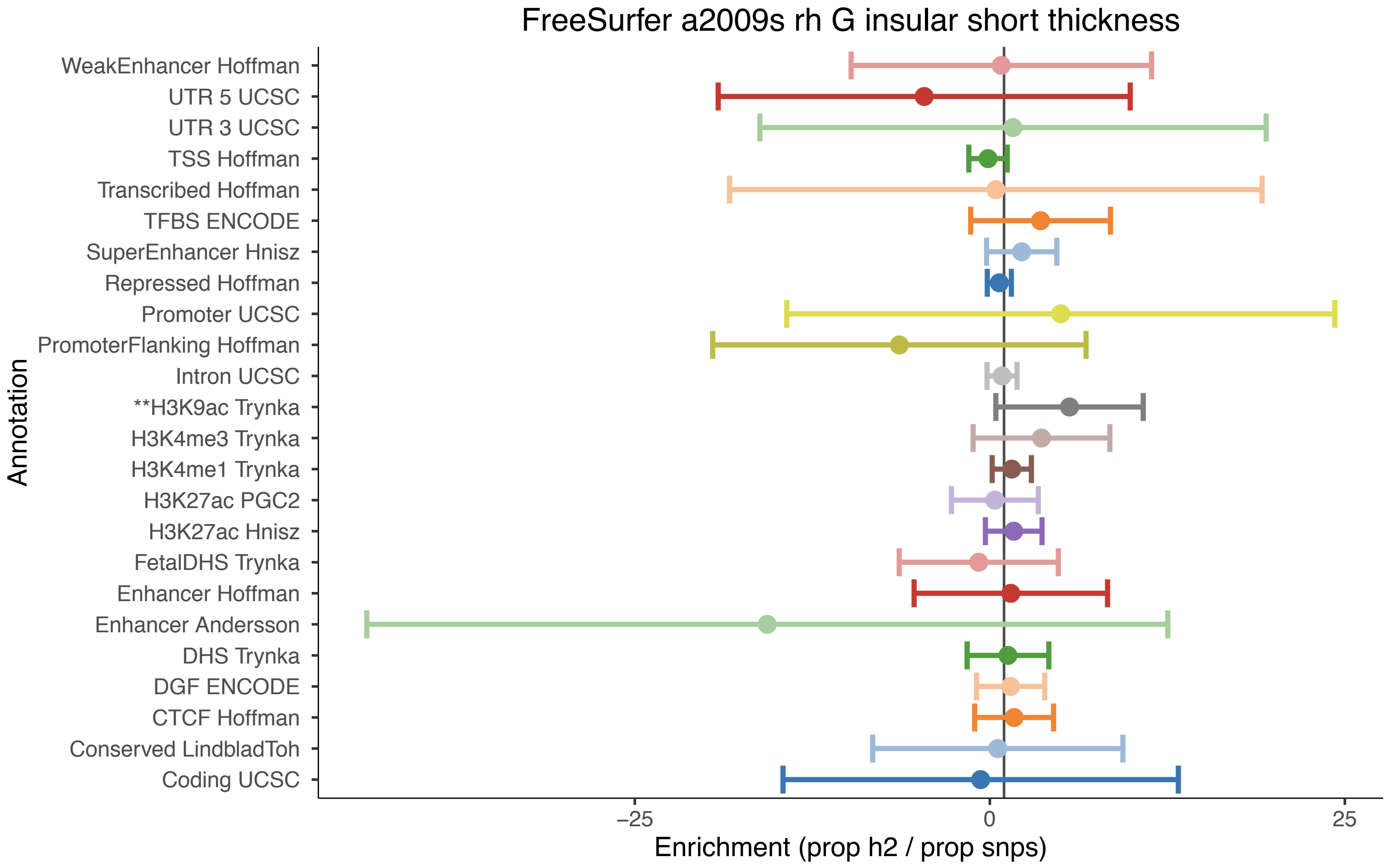
FreeSurfer a2009s rh G insular short thickness
WeakEnhancer Hoffman
UTR 5 UCSC
UTR 3 UCSC
TSS Hoffman
Transcribed Hoffman
TFBS ENCODE
SuperEnhancer Hnisz
Repressed Hoffman
Promoter UCSC
PromoterFlanking Hoffman
Intron UCSC
**H3K9ac Trynka
H3K4me3 Trynka
H3K4me1 Trynka
H3K27ac PGC2
H3K27ac Hnisz
FetalDHS Trynka
Enhancer Hoffman
Enhancer Andersson
DHS Trynka
DGF ENCODE
CTCF Hoffman
Conserved LindbladToh
Coding UCSC
Annotation
−25
0
25
Enrichment (prop h2 / prop snps)

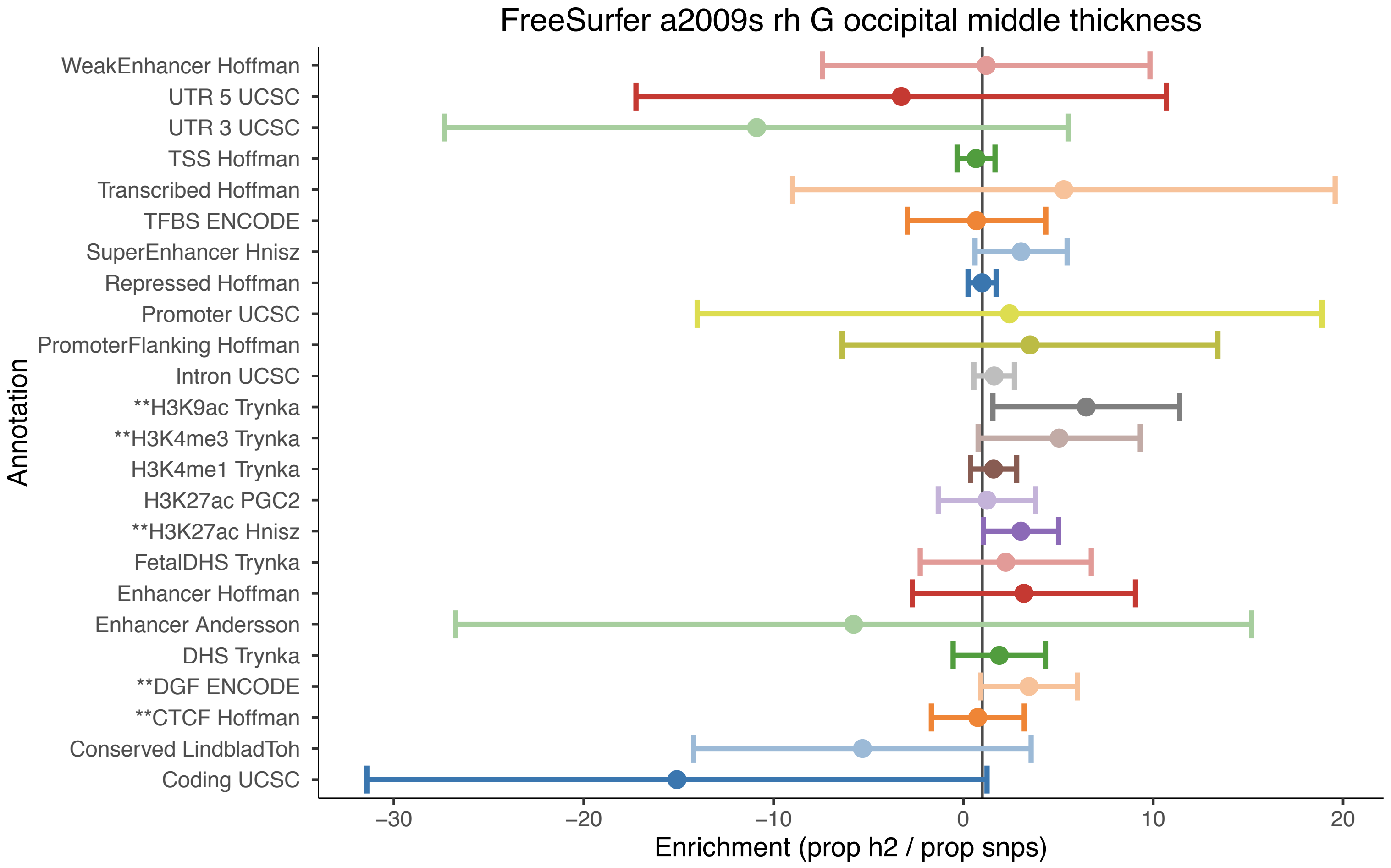
FreeSurfer a2009s rh G occipital middle thickness
WeakEnhancer Hoffman
UTR 5 UCSC
UTR 3 UCSC
TSS Hoffman
Transcribed Hoffman
TFBS ENCODE
SuperEnhancer Hnisz
Repressed Hoffman
Promoter UCSC
PromoterFlanking Hoffman
Intron UCSC
**H3K9ac Trynka
**H3K4me3 Trynka
H3K4me1 Trynka
H3K27ac PGC2
**H3K27ac Hnisz
FetalDHS Trynka
Enhancer Hoffman
Enhancer Andersson
DHS Trynka
**DGF ENCODE
**CTCF Hoffman
Conserved LindbladToh
Coding UCSC
Annotation
−30
−20
−10
0
10
20
Enrichment (prop h2 / prop snps)

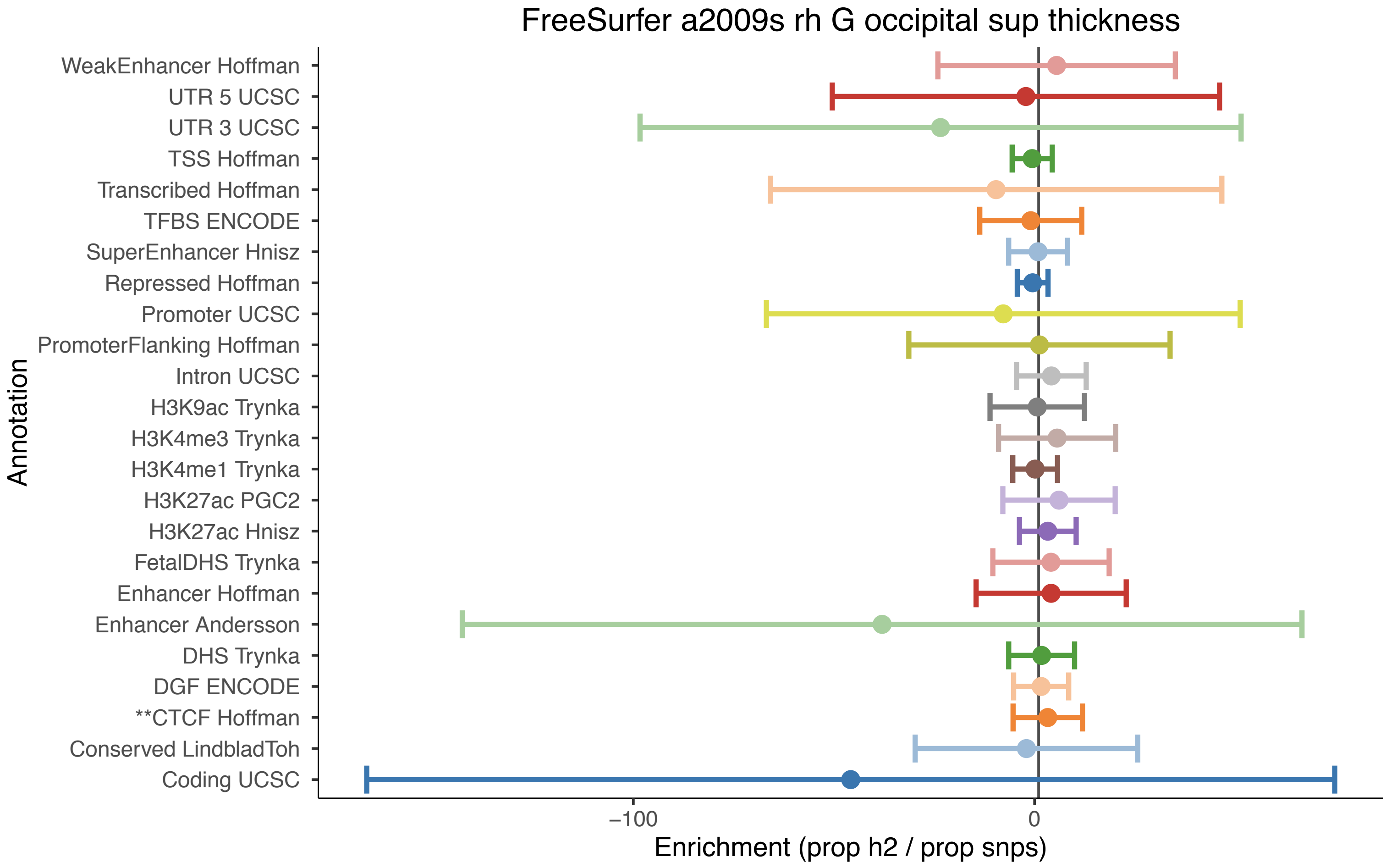

FreeSurfer a2009s rh G occipital sup thickness
WeakEnhancer Hoffman
UTR 5 UCSC
UTR 3 UCSC
TSS Hoffman
Transcribed Hoffman
TFBS ENCODE
SuperEnhancer Hnisz
Repressed Hoffman
Promoter UCSC
PromoterFlanking Hoffman
Intron UCSC
H3K9ac Trynka
H3K4me3 Trynka
H3K4me1 Trynka
H3K27ac PGC2
H3K27ac Hnisz
FetalDHS Trynka
Enhancer Hoffman
Enhancer Andersson
DHS Trynka
DGF ENCODE
**CTCF Hoffman
Conserved LindbladToh
Coding UCSC
Annotation
−100
0
Enrichment (prop h2 / prop snps)

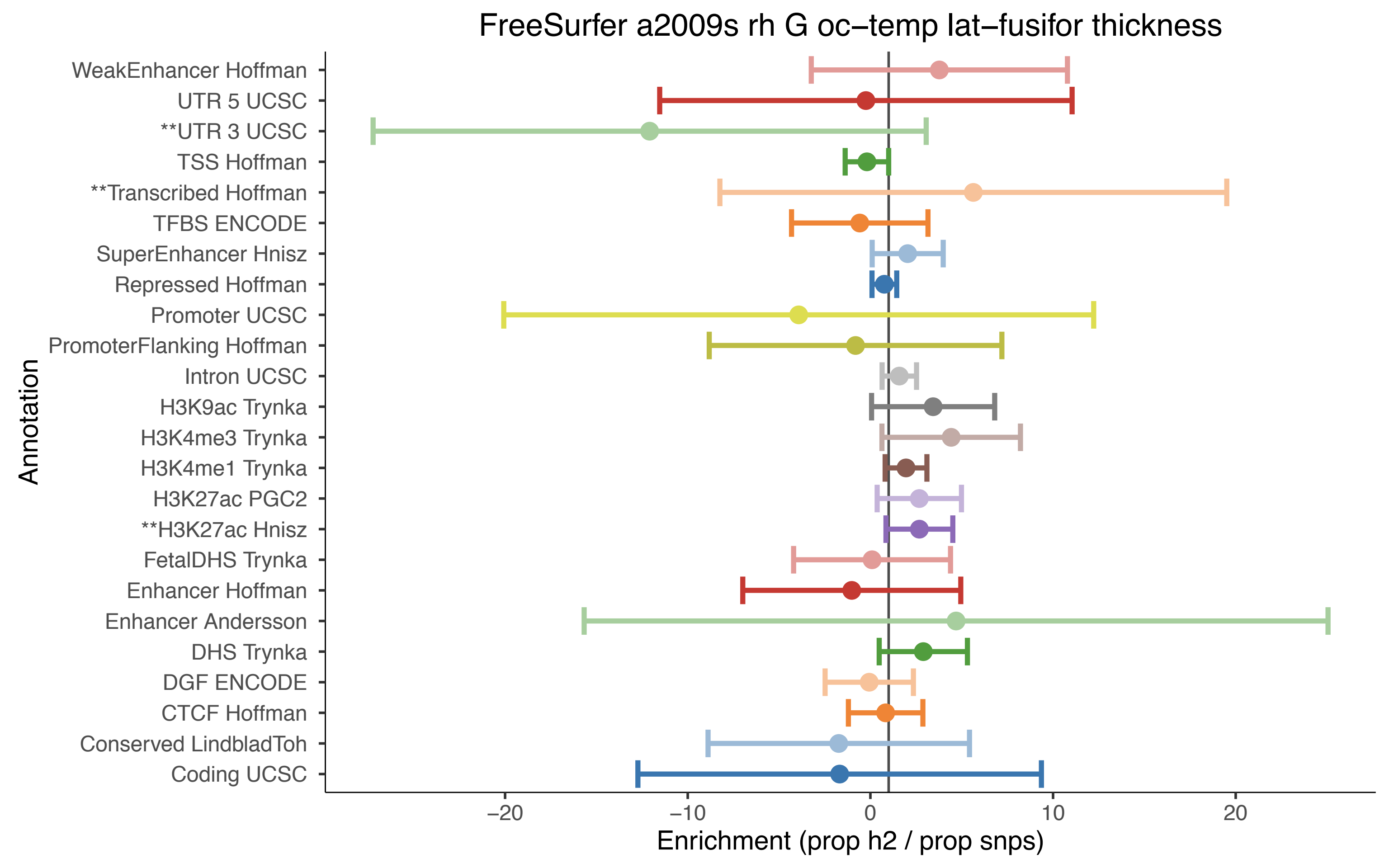

FreeSurfer a2009s rh G oc−temp lat−fusifor thickness
WeakEnhancer Hoffman
UTR 5 UCSC
**UTR 3 UCSC
TSS Hoffman
**Transcribed Hoffman
TFBS ENCODE
SuperEnhancer Hnisz
Repressed Hoffman
Promoter UCSC
PromoterFlanking Hoffman
Intron UCSC
H3K9ac Trynka
H3K4me3 Trynka
H3K4me1 Trynka
H3K27ac PGC2
**H3K27ac Hnisz
FetalDHS Trynka
Enhancer Hoffman
Enhancer Andersson
DHS Trynka
DGF ENCODE
CTCF Hoffman
Conserved LindbladToh
Coding UCSC
Annotation
−20
−10
0
10
20
Enrichment (prop h2 / prop snps)

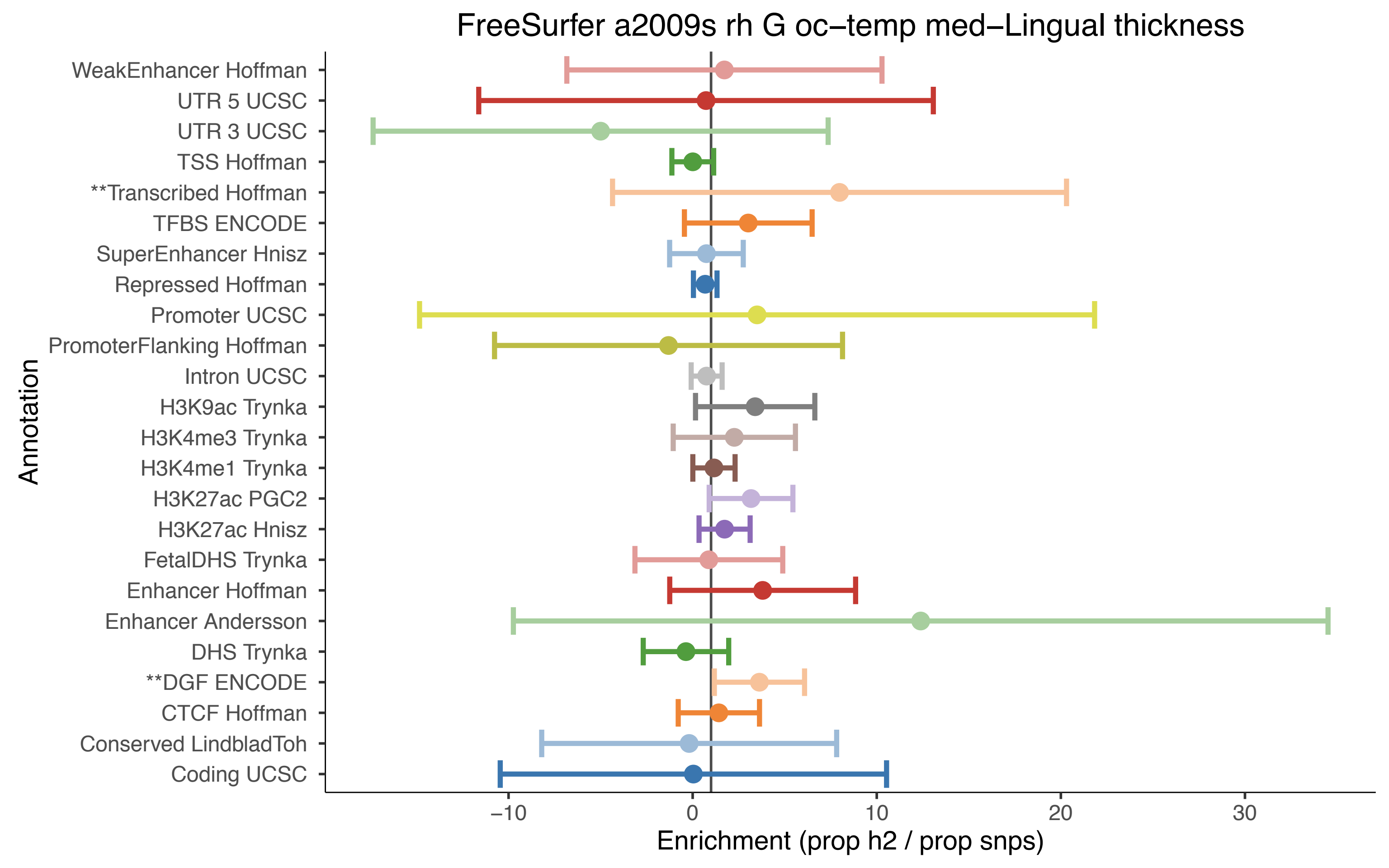

FreeSurfer a2009s rh G oc−temp med−Lingual thickness
WeakEnhancer Hoffman
UTR 5 UCSC
UTR 3 UCSC
TSS Hoffman
**Transcribed Hoffman
TFBS ENCODE
SuperEnhancer Hnisz
Repressed Hoffman
Promoter UCSC
PromoterFlanking Hoffman
Intron UCSC
H3K9ac Trynka
H3K4me3 Trynka
H3K4me1 Trynka
H3K27ac PGC2
H3K27ac Hnisz
FetalDHS Trynka
Enhancer Hoffman
Enhancer Andersson
DHS Trynka
**DGF ENCODE
CTCF Hoffman
Conserved LindbladToh
Coding UCSC
Annotation
−10
0
10
20
30
Enrichment (prop h2 / prop snps)

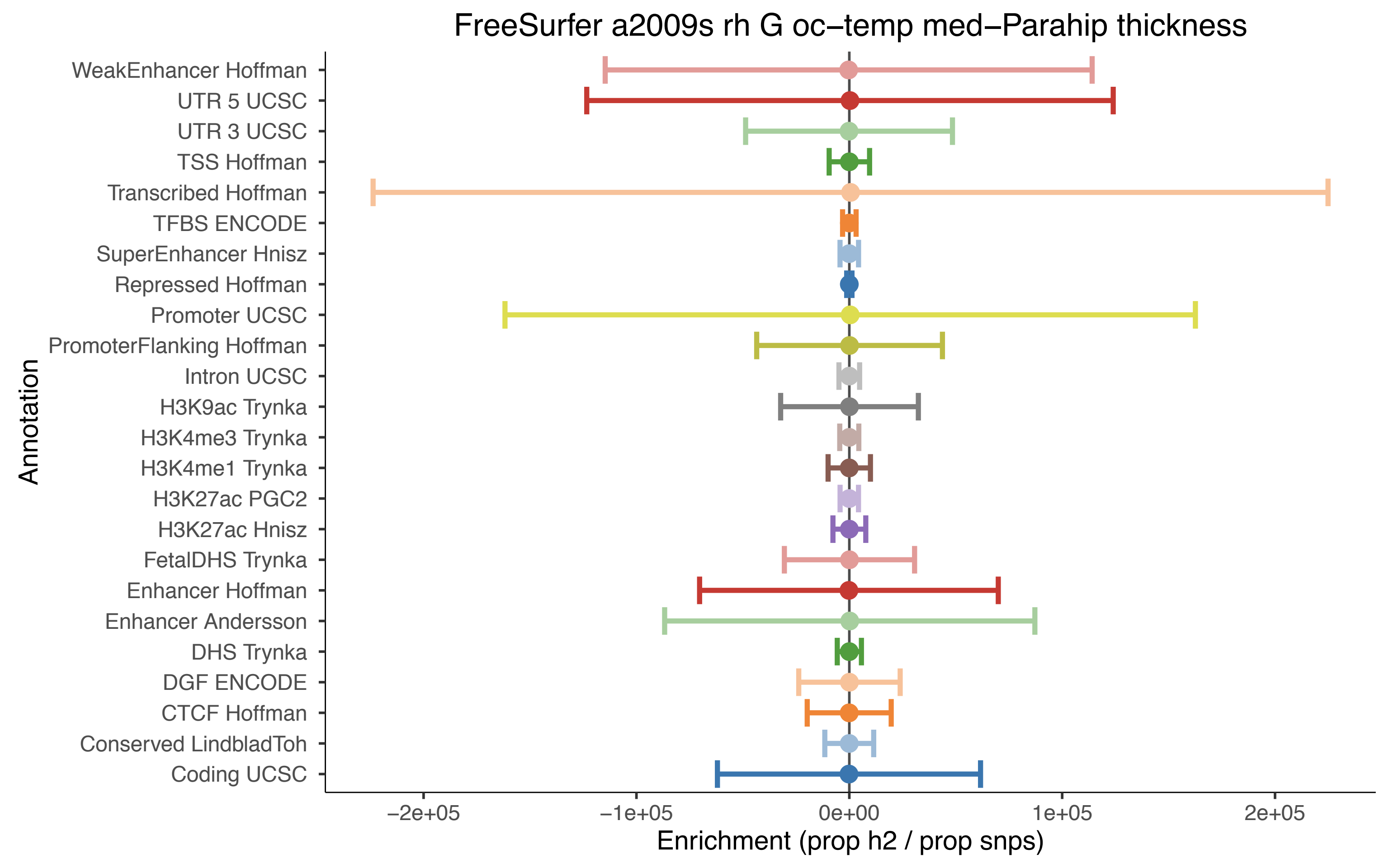

FreeSurfer a2009s rh G oc−temp med−Parahip thickness
WeakEnhancer Hoffman
UTR 5 UCSC
UTR 3 UCSC
TSS Hoffman
Transcribed Hoffman
TFBS ENCODE
SuperEnhancer Hnisz
Repressed Hoffman
Promoter UCSC
PromoterFlanking Hoffman
Intron UCSC
H3K9ac Trynka
H3K4me3 Trynka
H3K4me1 Trynka
H3K27ac PGC2
H3K27ac Hnisz
FetalDHS Trynka
Enhancer Hoffman
Enhancer Andersson
DHS Trynka
DGF ENCODE
CTCF Hoffman
Conserved LindbladToh
Coding UCSC
Annotation
−2e+05
−1e+05
0e+00
1e+05
2e+05
Enrichment (prop h2 / prop snps)

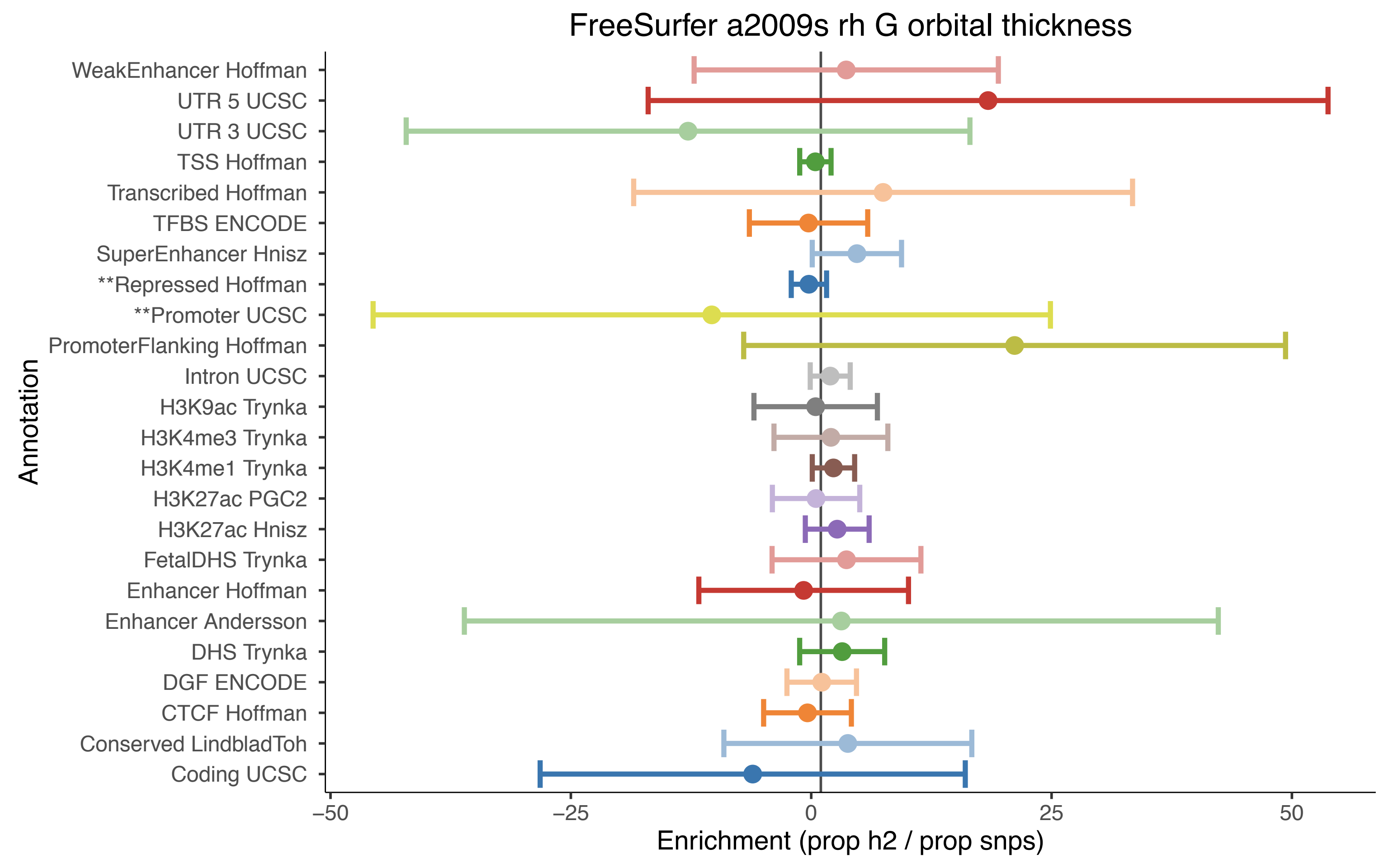
FreeSurfer a2009s rh G orbital thickness
WeakEnhancer Hoffman
UTR 5 UCSC
UTR 3 UCSC
TSS Hoffman
Transcribed Hoffman
TFBS ENCODE
SuperEnhancer Hnisz
**Repressed Hoffman
**Promoter UCSC
PromoterFlanking Hoffman
Intron UCSC
H3K9ac Trynka
H3K4me3 Trynka
H3K4me1 Trynka
H3K27ac PGC2
H3K27ac Hnisz
FetalDHS Trynka
Enhancer Hoffman
Enhancer Andersson
DHS Trynka
DGF ENCODE
CTCF Hoffman
Conserved LindbladToh
Coding UCSC
Annotation
−50
−25
0
25
50
Enrichment (prop h2 / prop snps)

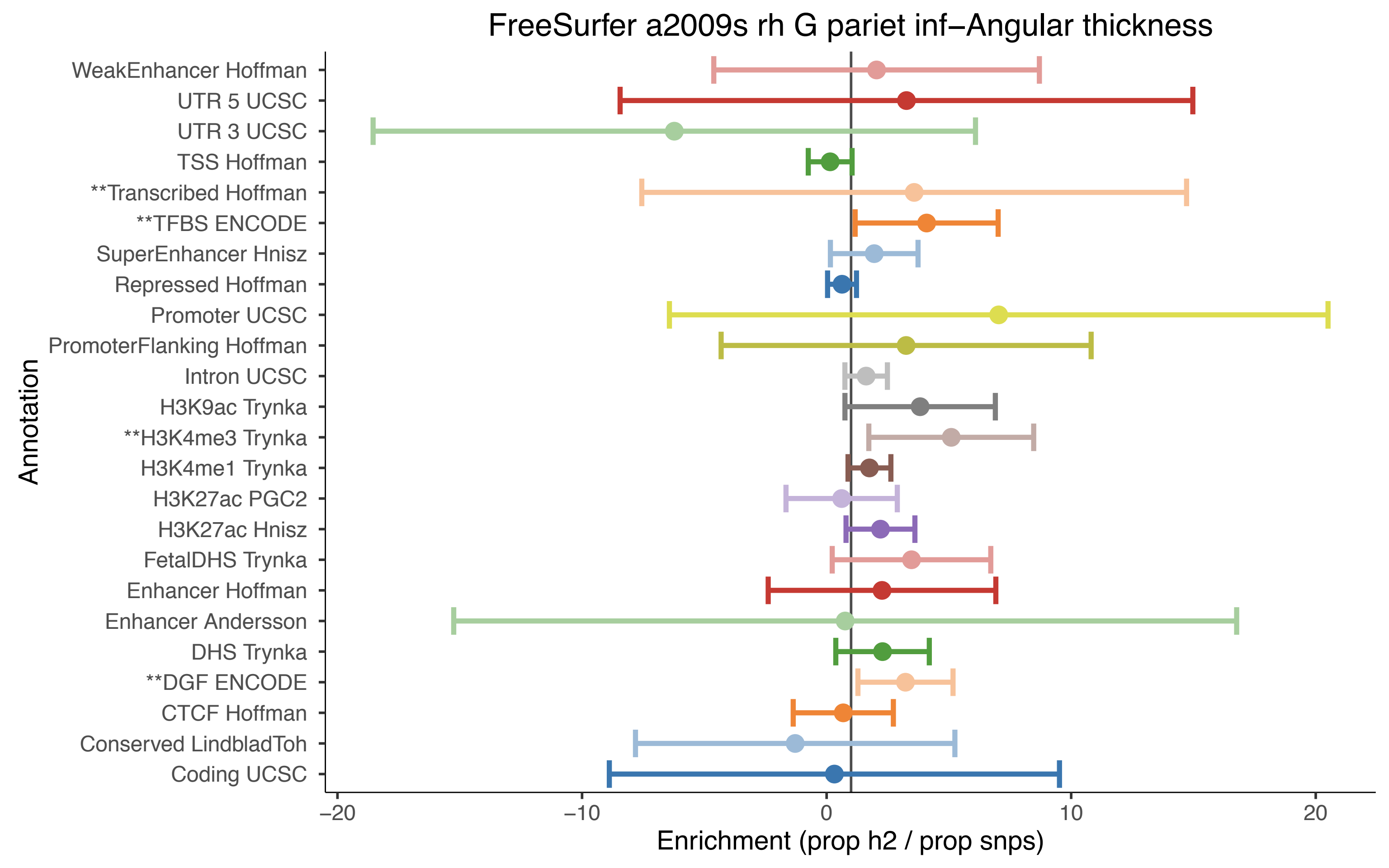
FreeSurfer a2009s rh G pariet inf−Angular thickness
WeakEnhancer Hoffman
UTR 5 UCSC
UTR 3 UCSC
TSS Hoffman
**Transcribed Hoffman
**TFBS ENCODE
SuperEnhancer Hnisz
Repressed Hoffman
Promoter UCSC
PromoterFlanking Hoffman
Intron UCSC
H3K9ac Trynka
**H3K4me3 Trynka
H3K4me1 Trynka
H3K27ac PGC2
H3K27ac Hnisz
FetalDHS Trynka
Enhancer Hoffman
Enhancer Andersson
DHS Trynka
**DGF ENCODE
CTCF Hoffman
Conserved LindbladToh
Coding UCSC
Annotation
−20
−10
0
10
20
Enrichment (prop h2 / prop snps)

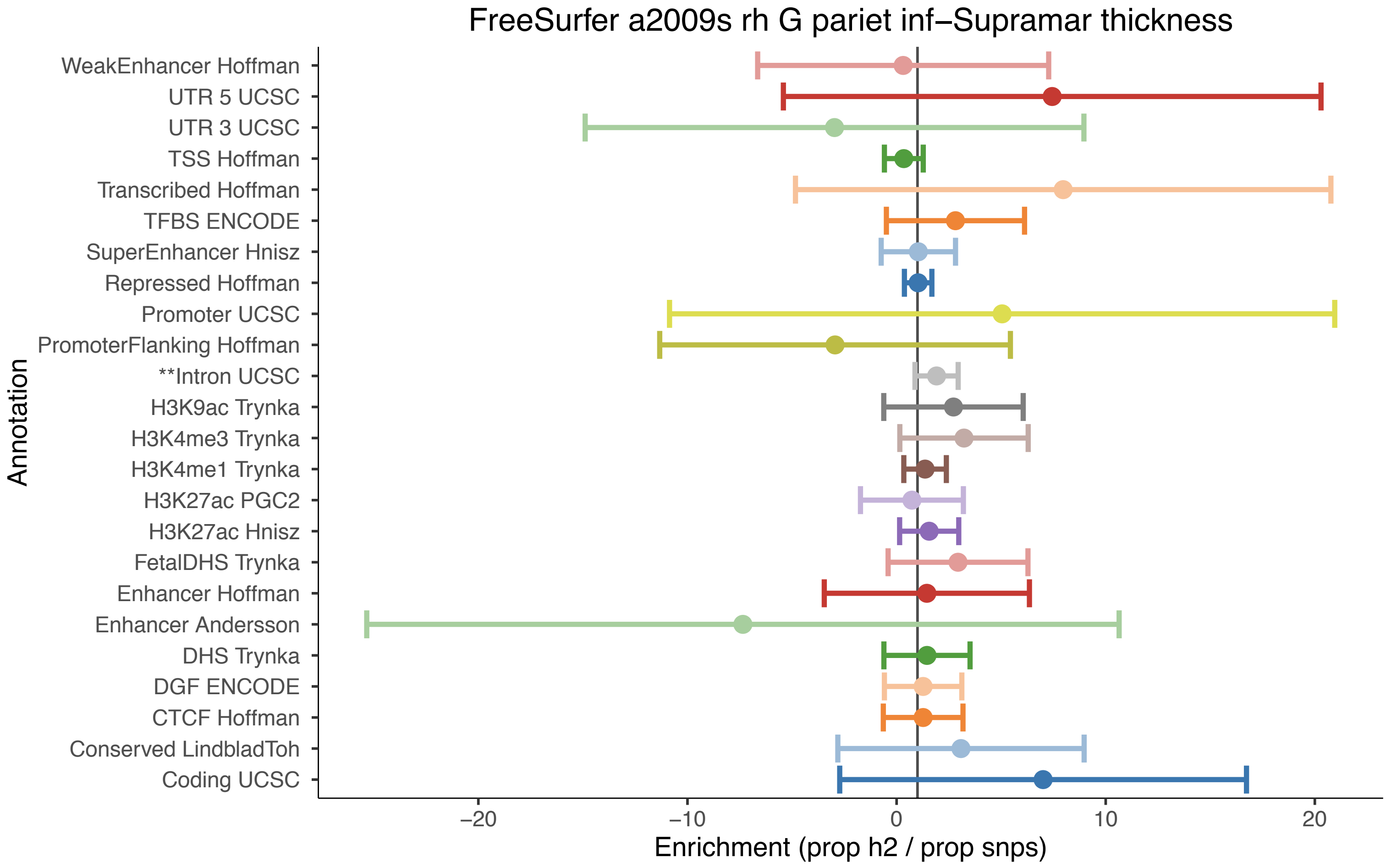

FreeSurfer a2009s rh G pariet inf−Supramar thickness
WeakEnhancer Hoffman
UTR 5 UCSC
UTR 3 UCSC
TSS Hoffman
Transcribed Hoffman
TFBS ENCODE
SuperEnhancer Hnisz
Repressed Hoffman
Promoter UCSC
PromoterFlanking Hoffman
**Intron UCSC
H3K9ac Trynka
H3K4me3 Trynka
H3K4me1 Trynka
H3K27ac PGC2
H3K27ac Hnisz
FetalDHS Trynka
Enhancer Hoffman
Enhancer Andersson
DHS Trynka
DGF ENCODE
CTCF Hoffman
Conserved LindbladToh
Coding UCSC
Annotation
−20
−10
0
10
20
Enrichment (prop h2 / prop snps)

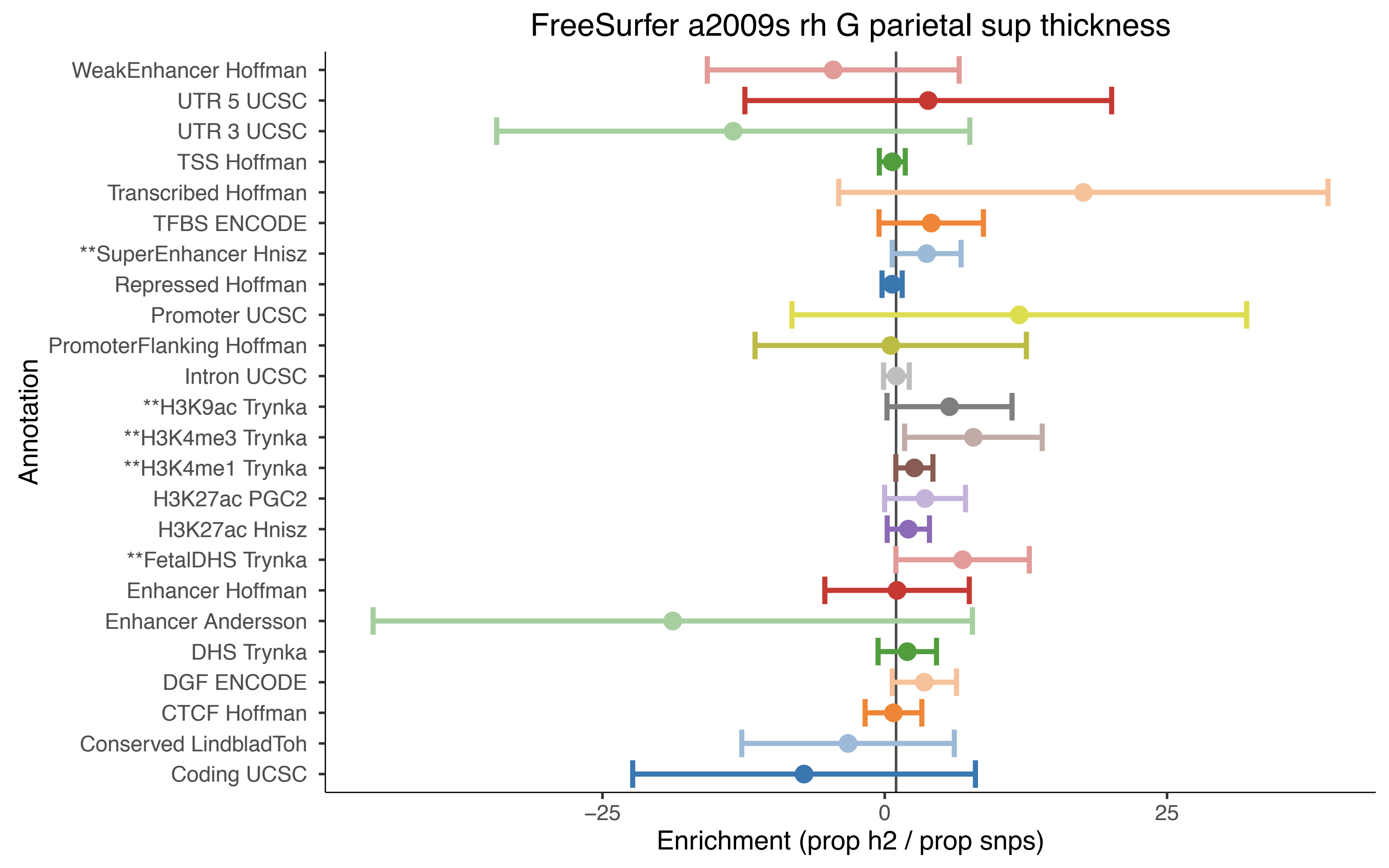
FreeSurfer a2009s rh G parietal sup thickness
WeakEnhancer Hoffman
UTR 5 UCSC
UTR 3 UCSC
TSS Hoffman
Transcribed Hoffman
TFBS ENCODE
**SuperEnhancer Hnisz
Repressed Hoffman
Promoter UCSC
PromoterFlanking Hoffman
Intron UCSC
**H3K9ac Trynka
**H3K4me3 Trynka
**H3K4me1 Trynka
H3K27ac PGC2
H3K27ac Hnisz
**FetalDHS Trynka
Enhancer Hoffman
Enhancer Andersson
DHS Trynka
DGF ENCODE
CTCF Hoffman
Conserved LindbladToh
Coding UCSC
Annotation
−25
0
25
Enrichment (prop h2 / prop snps)

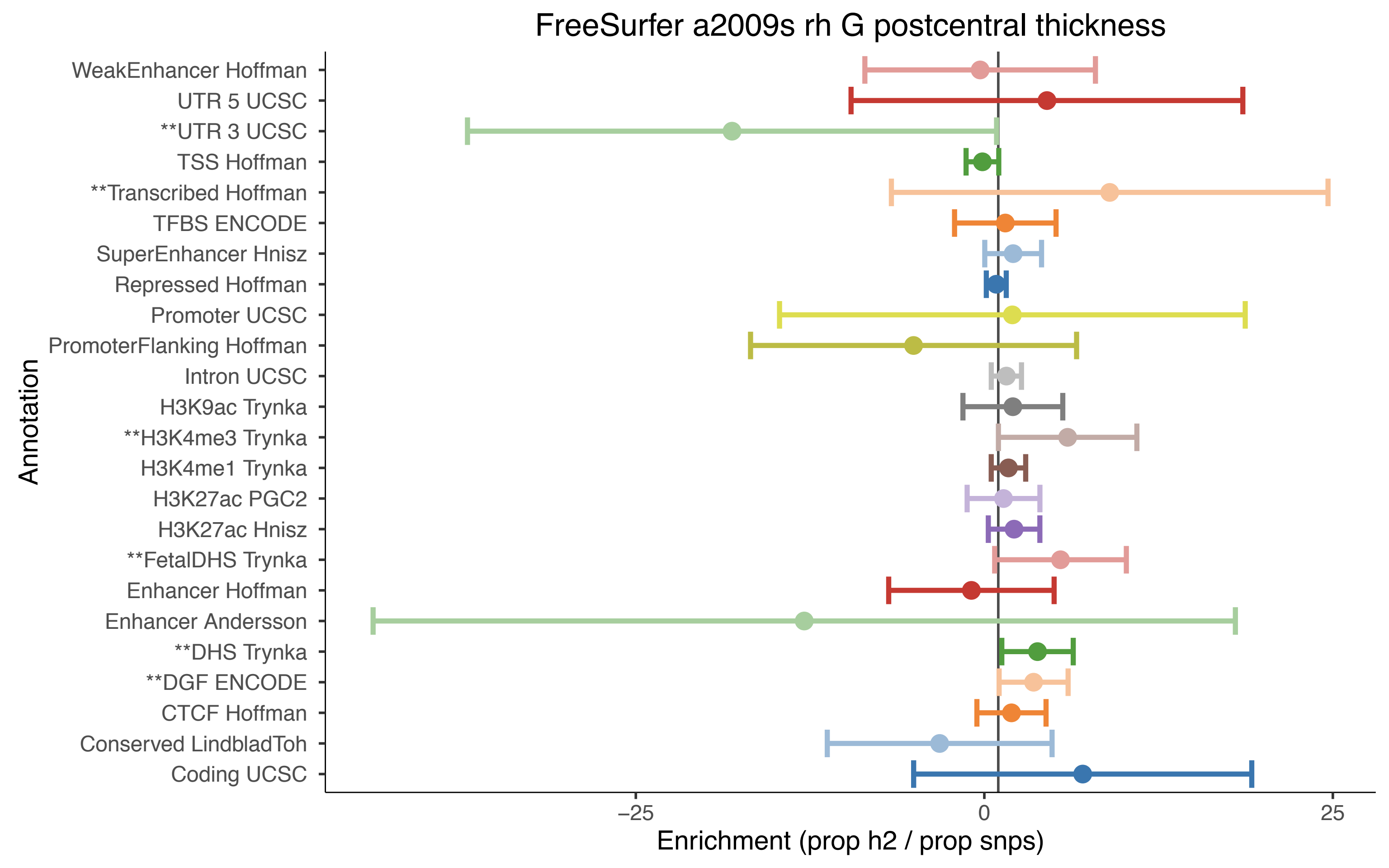
FreeSurfer a2009s rh G postcentral thickness
Annotation
WeakEnhancer Hoffman
UTR 5 UCSC
**UTR 3 UCSC
TSS Hoffman
**Transcribed Hoffman
TFBS ENCODE
SuperEnhancer Hnisz
Repressed Hoffman
Promoter UCSC
PromoterFlanking Hoffman
Intron UCSC
H3K9ac Trynka
**H3K4me3 Trynka
H3K4me1 Trynka
H3K27ac PGC2
H3K27ac Hnisz
**FetalDHS Trynka
Enhancer Hoffman
Enhancer Andersson
**DHS Trynka
**DGF ENCODE
CTCF Hoffman
Conserved LindbladToh
Coding UCSC
−25
0
25
Enrichment (prop h2 / prop snps)

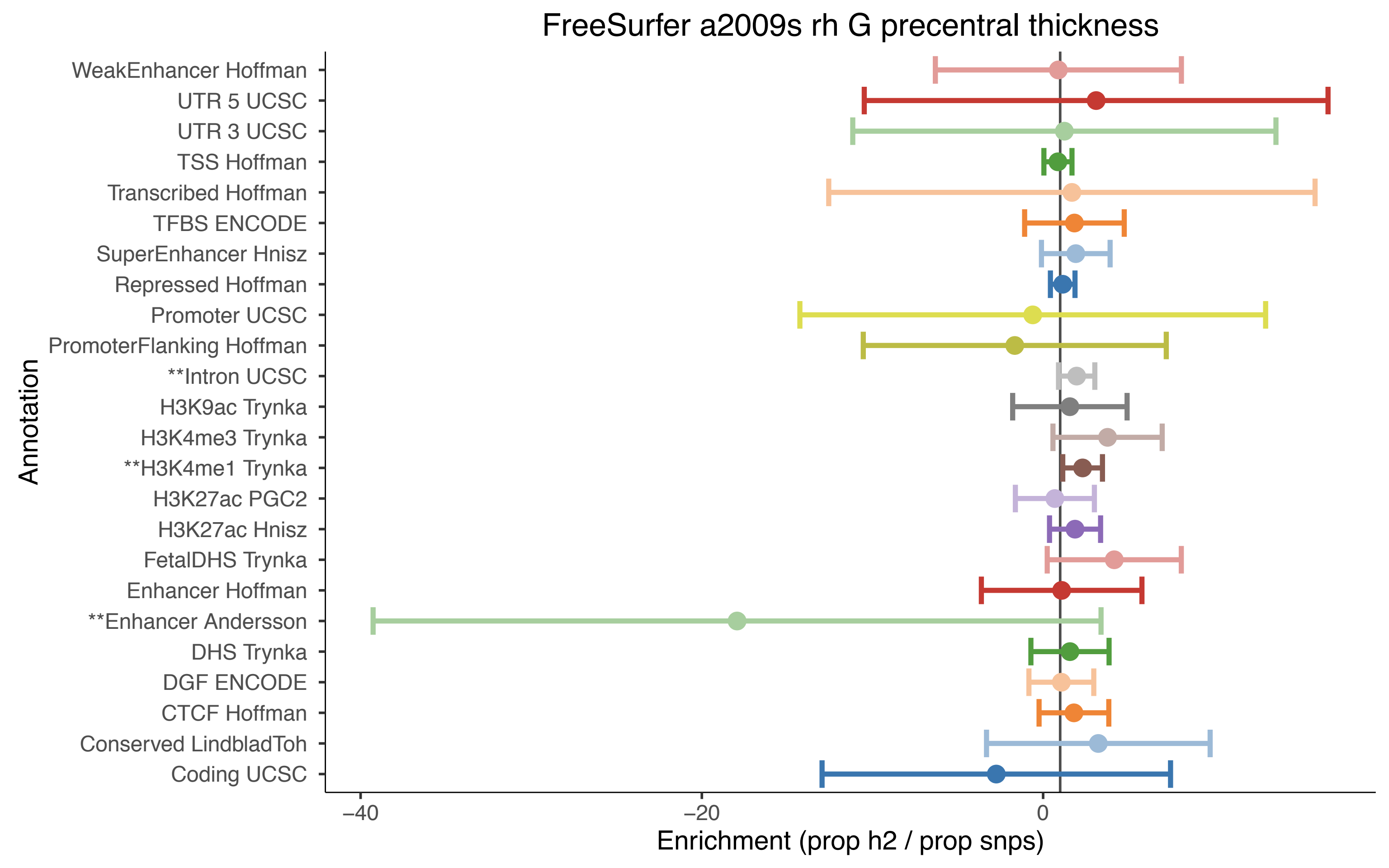
FreeSurfer a2009s rh G precentral thickness
WeakEnhancer Hoffman
UTR 5 UCSC
UTR 3 UCSC
TSS Hoffman
Transcribed Hoffman
TFBS ENCODE
SuperEnhancer Hnisz
Repressed Hoffman
Promoter UCSC
PromoterFlanking Hoffman
**Intron UCSC
H3K9ac Trynka
H3K4me3 Trynka
**H3K4me1 Trynka
H3K27ac PGC2
H3K27ac Hnisz
FetalDHS Trynka
Enhancer Hoffman
**Enhancer Andersson
DHS Trynka
DGF ENCODE
CTCF Hoffman
Conserved LindbladToh
Coding UCSC
Annotation
−40
−20
0
Enrichment (prop h2 / prop snps)

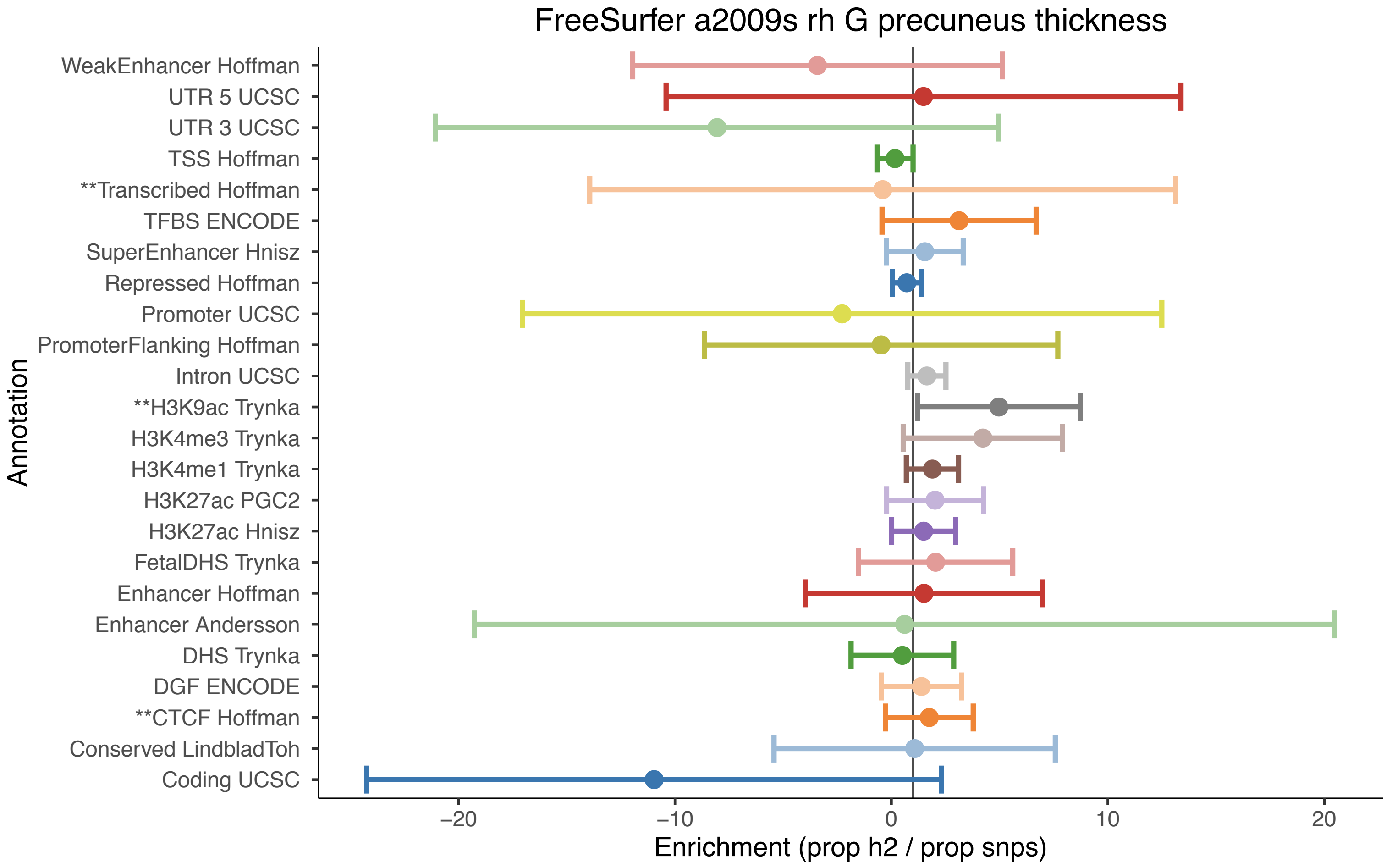

FreeSurfer a2009s rh G precuneus thickness
WeakEnhancer Hoffman
UTR 5 UCSC
UTR 3 UCSC
TSS Hoffman
**Transcribed Hoffman
TFBS ENCODE
SuperEnhancer Hnisz
Repressed Hoffman
Promoter UCSC
PromoterFlanking Hoffman
Intron UCSC
**H3K9ac Trynka
H3K4me3 Trynka
H3K4me1 Trynka
H3K27ac PGC2
H3K27ac Hnisz
FetalDHS Trynka
Enhancer Hoffman
Enhancer Andersson
DHS Trynka
DGF ENCODE
**CTCF Hoffman
Conserved LindbladToh
Coding UCSC
Annotation
−20
−10
0
10
20
Enrichment (prop h2 / prop snps)

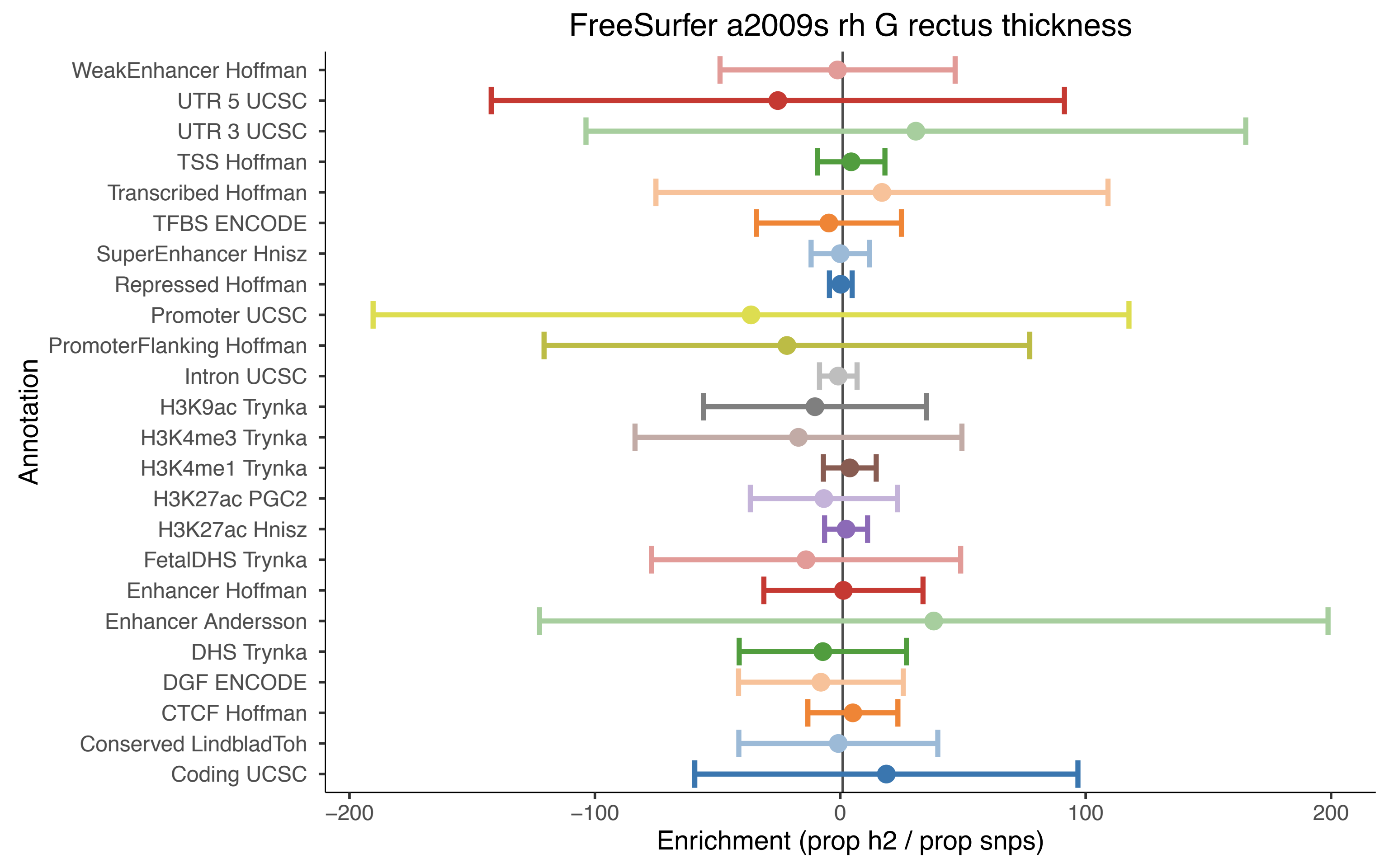

FreeSurfer a2009s rh G rectus thickness
Annotation
WeakEnhancer Hoffman
UTR 5 UCSC
UTR 3 UCSC
TSS Hoffman
Transcribed Hoffman
TFBS ENCODE
SuperEnhancer Hnisz
Repressed Hoffman
Promoter UCSC
PromoterFlanking Hoffman
Intron UCSC
H3K9ac Trynka
H3K4me3 Trynka
H3K4me1 Trynka
H3K27ac PGC2
H3K27ac Hnisz
FetalDHS Trynka
Enhancer Hoffman
Enhancer Andersson
DHS Trynka
DGF ENCODE
CTCF Hoffman
Conserved LindbladToh
Coding UCSC
−200
−100
0
100
200
Enrichment (prop h2 / prop snps)

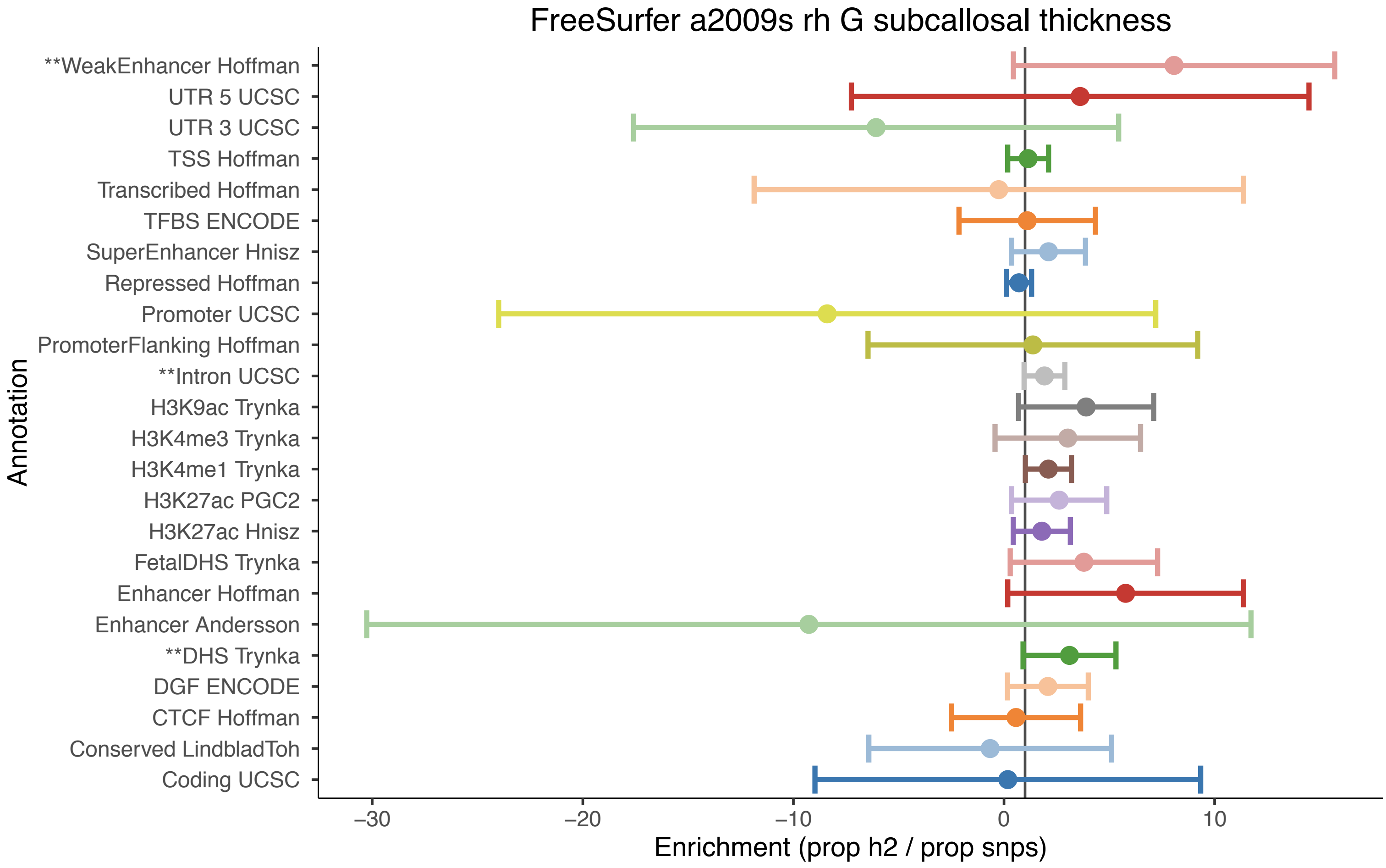
FreeSurfer a2009s rh G subcallosal thickness
**WeakEnhancer Hoffman
UTR 5 UCSC
UTR 3 UCSC
TSS Hoffman
Transcribed Hoffman
TFBS ENCODE
SuperEnhancer Hnisz
Repressed Hoffman
Promoter UCSC
PromoterFlanking Hoffman
**Intron UCSC
H3K9ac Trynka
H3K4me3 Trynka
H3K4me1 Trynka
H3K27ac PGC2
H3K27ac Hnisz
FetalDHS Trynka
Enhancer Hoffman
Enhancer Andersson
**DHS Trynka
DGF ENCODE
CTCF Hoffman
Conserved LindbladToh
Coding UCSC
Annotation
−30
−20
−10
0
10
Enrichment (prop h2 / prop snps)

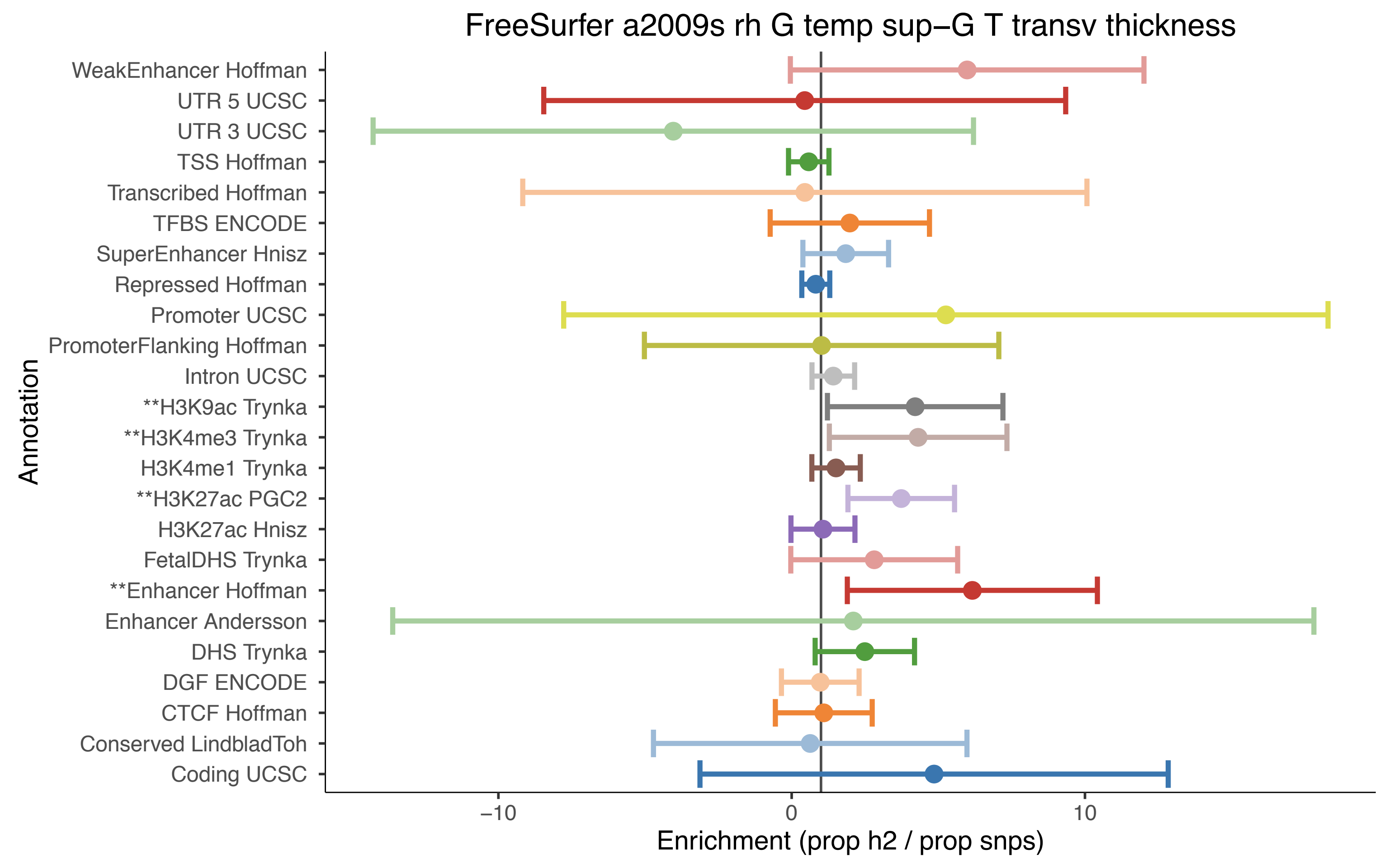
FreeSurfer a2009s rh G temp sup−G T transv thickness
Annotation
WeakEnhancer Hoffman
UTR 5 UCSC
UTR 3 UCSC
TSS Hoffman
Transcribed Hoffman
TFBS ENCODE
SuperEnhancer Hnisz
Repressed Hoffman
Promoter UCSC
PromoterFlanking Hoffman
Intron UCSC
**H3K9ac Trynka
**H3K4me3 Trynka
H3K4me1 Trynka
**H3K27ac PGC2
H3K27ac Hnisz
FetalDHS Trynka
**Enhancer Hoffman
Enhancer Andersson
DHS Trynka
DGF ENCODE
CTCF Hoffman
Conserved LindbladToh
Coding UCSC
−10
0
10
Enrichment (prop h2 / prop snps)

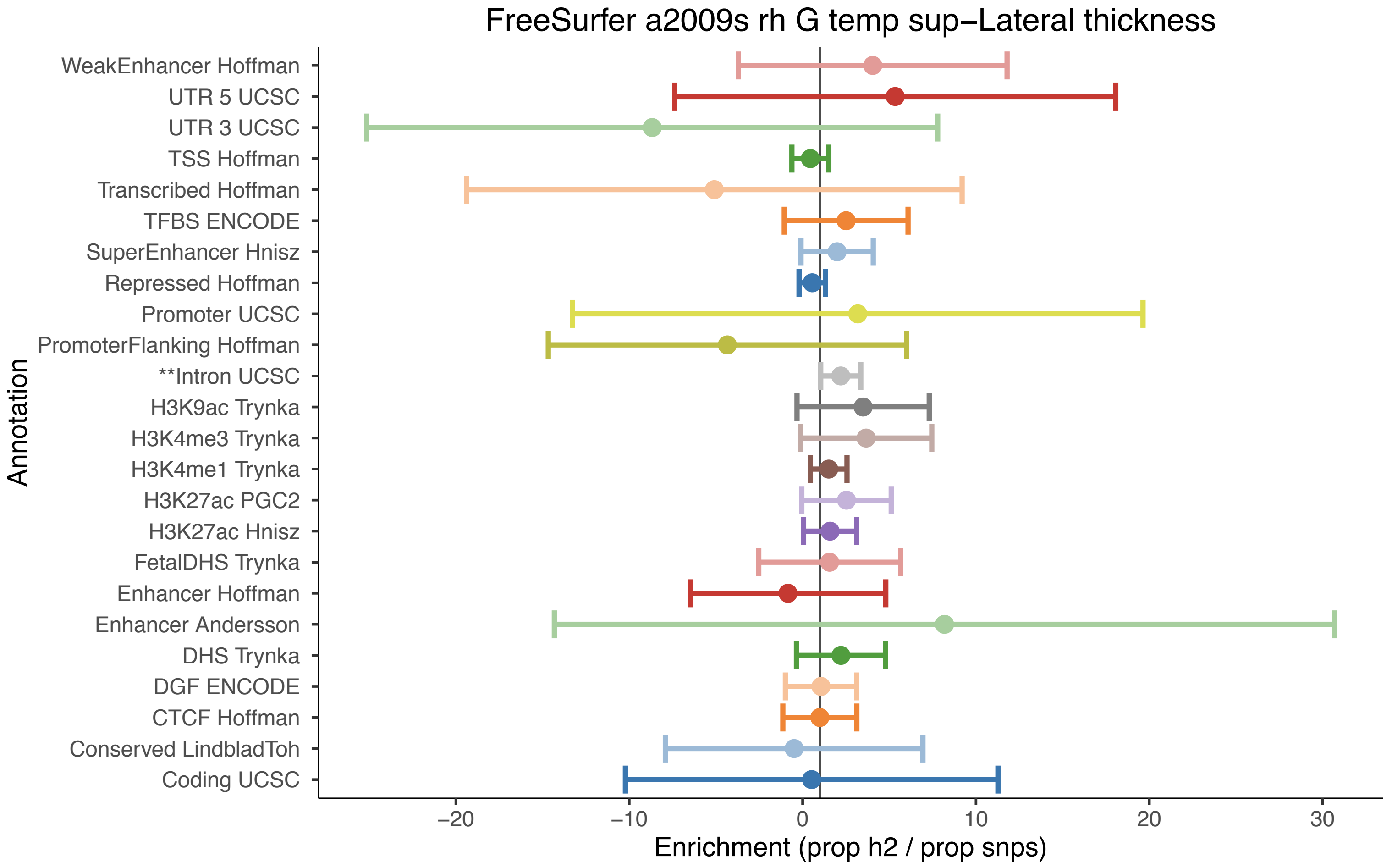

FreeSurfer a2009s rh G temp sup−Lateral thickness
WeakEnhancer Hoffman
UTR 5 UCSC
UTR 3 UCSC
TSS Hoffman
Transcribed Hoffman
TFBS ENCODE
SuperEnhancer Hnisz
Repressed Hoffman
Promoter UCSC
PromoterFlanking Hoffman
**Intron UCSC
H3K9ac Trynka
H3K4me3 Trynka
H3K4me1 Trynka
H3K27ac PGC2
H3K27ac Hnisz
FetalDHS Trynka
Enhancer Hoffman
Enhancer Andersson
DHS Trynka
DGF ENCODE
CTCF Hoffman
Conserved LindbladToh
Coding UCSC
Annotation
−20
−10
0
10
20
30
Enrichment (prop h2 / prop snps)

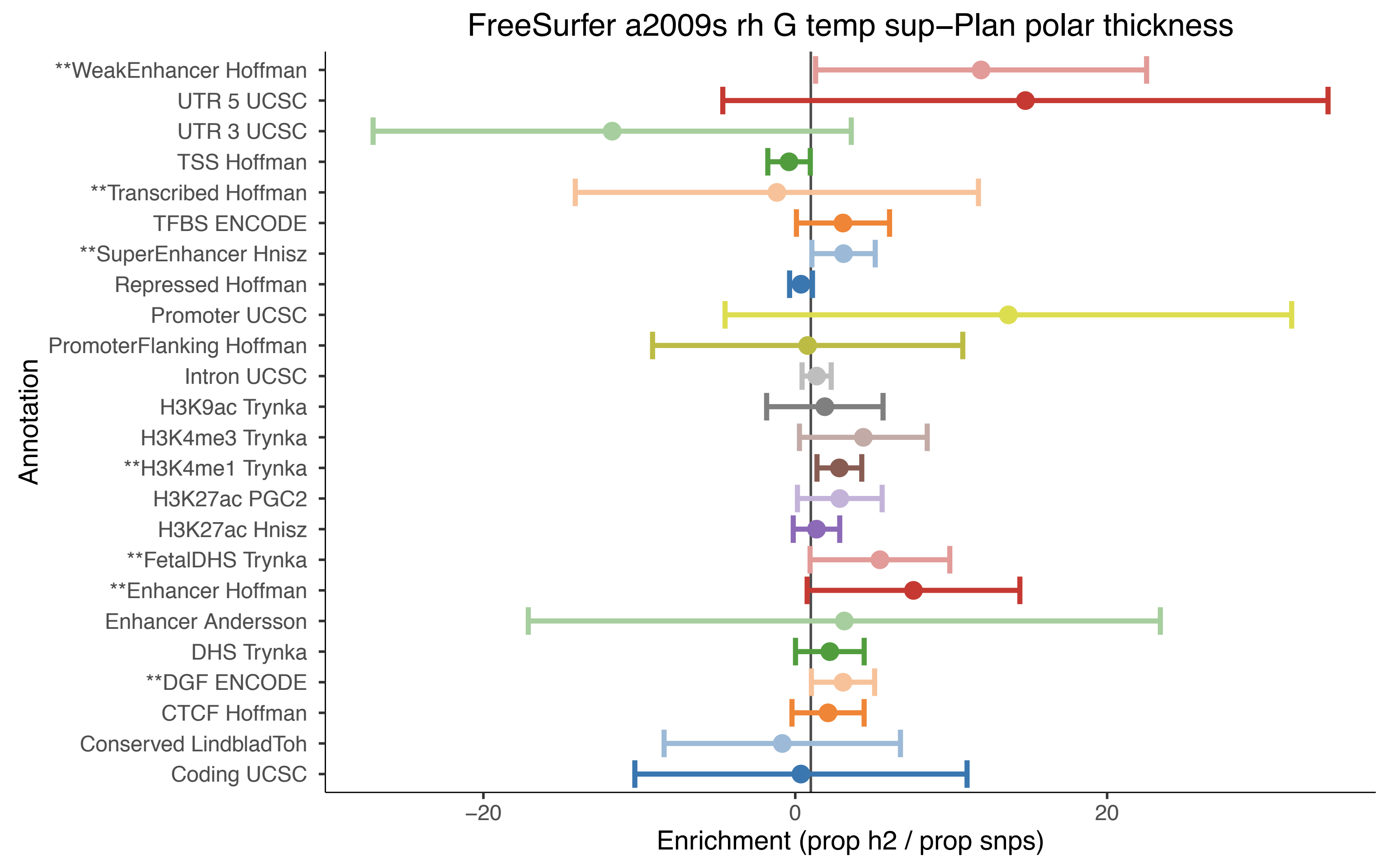
FreeSurfer a2009s rh G temp sup−Plan polar thickness
**WeakEnhancer Hoffman
UTR 5 UCSC
UTR 3 UCSC
TSS Hoffman
**Transcribed Hoffman
TFBS ENCODE
**SuperEnhancer Hnisz
Repressed Hoffman
Promoter UCSC
PromoterFlanking Hoffman
Intron UCSC
H3K9ac Trynka
H3K4me3 Trynka
**H3K4me1 Trynka
H3K27ac PGC2
H3K27ac Hnisz
**FetalDHS Trynka
**Enhancer Hoffman
Enhancer Andersson
DHS Trynka
**DGF ENCODE
CTCF Hoffman
Conserved LindbladToh
Coding UCSC
Annotation
−20
0
20
Enrichment (prop h2 / prop snps)

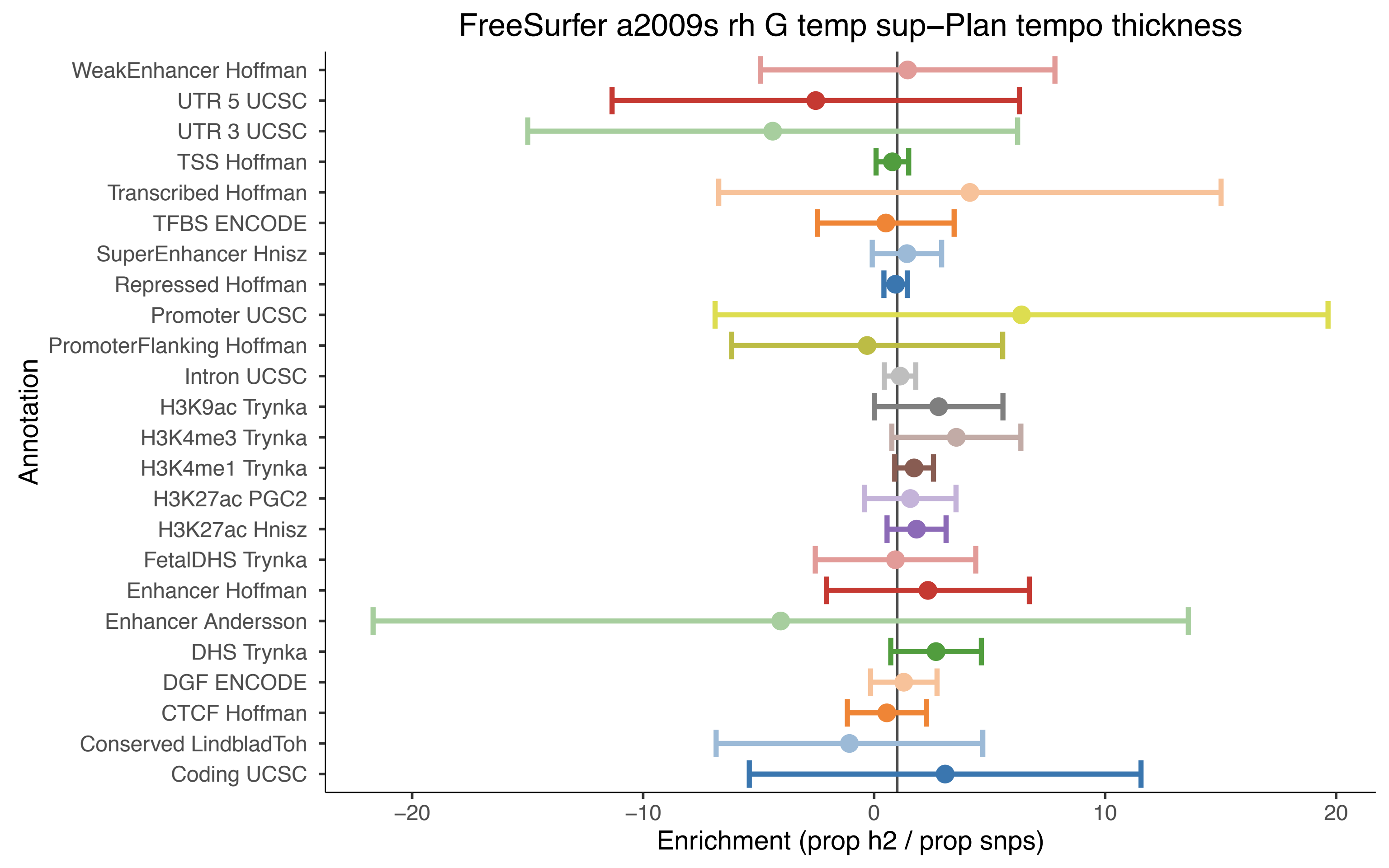
FreeSurfer a2009s rh G temp sup−Plan tempo thickness
WeakEnhancer Hoffman
UTR 5 UCSC
UTR 3 UCSC
TSS Hoffman
Transcribed Hoffman
TFBS ENCODE
SuperEnhancer Hnisz
Repressed Hoffman
Promoter UCSC
PromoterFlanking Hoffman
Intron UCSC
H3K9ac Trynka
H3K4me3 Trynka
H3K4me1 Trynka
H3K27ac PGC2
H3K27ac Hnisz
FetalDHS Trynka
Enhancer Hoffman
Enhancer Andersson
DHS Trynka
DGF ENCODE
CTCF Hoffman
Conserved LindbladToh
Coding UCSC
Annotation
−20
−10
0
10
20
Enrichment (prop h2 / prop snps)

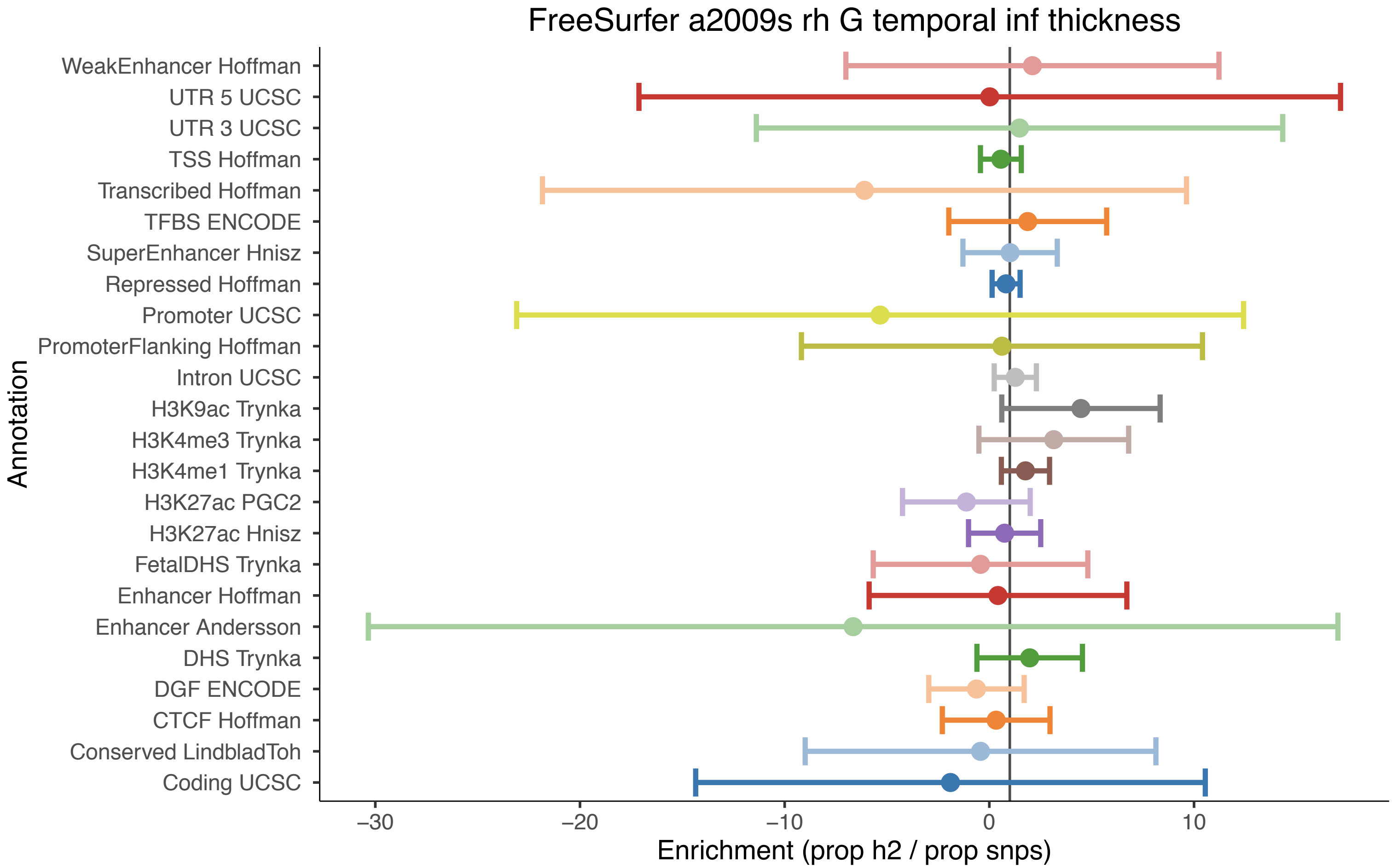

FreeSurfer a2009s rh G temporal inf thickness
WeakEnhancer Hoffman
UTR 5 UCSC
UTR 3 UCSC
TSS Hoffman
Transcribed Hoffman
TFBS ENCODE
SuperEnhancer Hnisz
Repressed Hoffman
Promoter UCSC
PromoterFlanking Hoffman
Intron UCSC
H3K9ac Trynka
H3K4me3 Trynka
H3K4me1 Trynka
H3K27ac PGC2
H3K27ac Hnisz
FetalDHS Trynka
Enhancer Hoffman
Enhancer Andersson
DHS Trynka
DGF ENCODE
CTCF Hoffman
Conserved LindbladToh
Coding UCSC
Annotation
−30
−20
−10
0
10
Enrichment (prop h2 / prop snps)

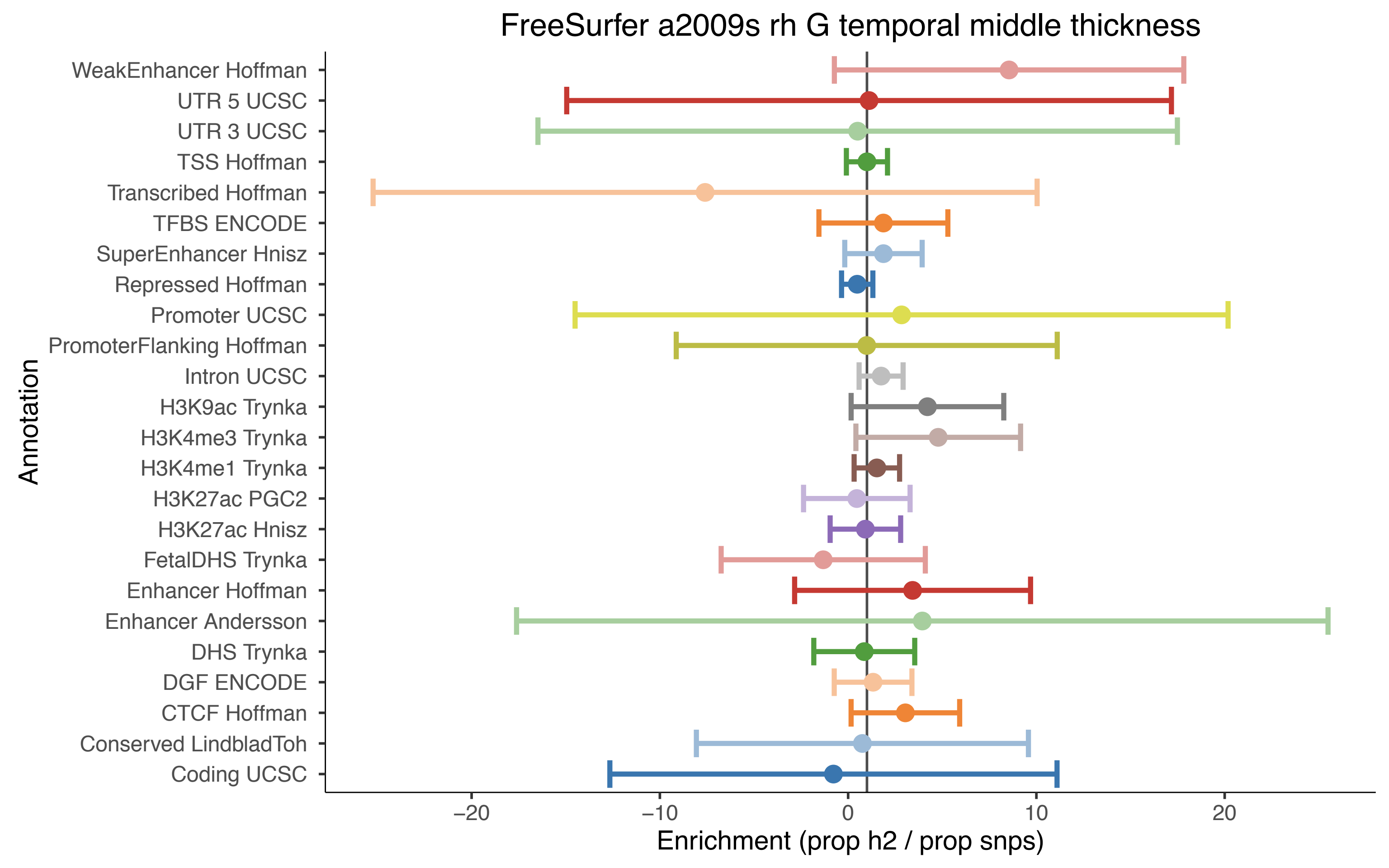
FreeSurfer a2009s rh G temporal middle thickness
WeakEnhancer Hoffman
UTR 5 UCSC
UTR 3 UCSC
TSS Hoffman
Transcribed Hoffman
TFBS ENCODE
SuperEnhancer Hnisz
Repressed Hoffman
Promoter UCSC
PromoterFlanking Hoffman
Intron UCSC
H3K9ac Trynka
H3K4me3 Trynka
H3K4me1 Trynka
H3K27ac PGC2
H3K27ac Hnisz
FetalDHS Trynka
Enhancer Hoffman
Enhancer Andersson
DHS Trynka
DGF ENCODE
CTCF Hoffman
Conserved LindbladToh
Coding UCSC
Annotation
−20
−10
0
10
20
Enrichment (prop h2 / prop snps)

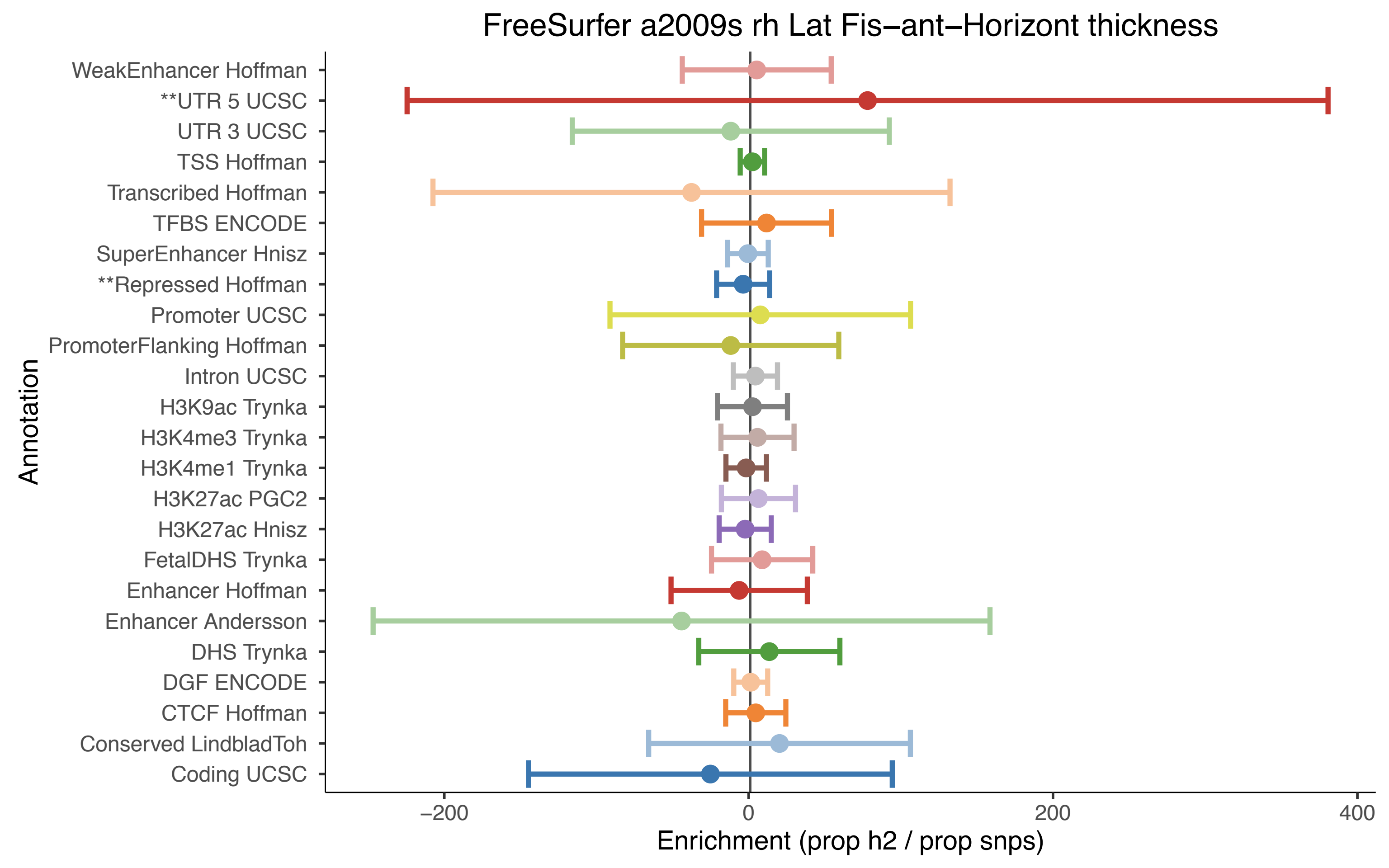

FreeSurfer a2009s rh Lat Fis−ant−Horizont thickness
WeakEnhancer Hoffman
**UTR 5 UCSC
UTR 3 UCSC
TSS Hoffman
Transcribed Hoffman
TFBS ENCODE
SuperEnhancer Hnisz
**Repressed Hoffman
Promoter UCSC
PromoterFlanking Hoffman
Intron UCSC
H3K9ac Trynka
H3K4me3 Trynka
H3K4me1 Trynka
H3K27ac PGC2
H3K27ac Hnisz
FetalDHS Trynka
Enhancer Hoffman
Enhancer Andersson
DHS Trynka
DGF ENCODE
CTCF Hoffman
Conserved LindbladToh
Coding UCSC
Annotation
−200
0
200
400
Enrichment (prop h2 / prop snps)

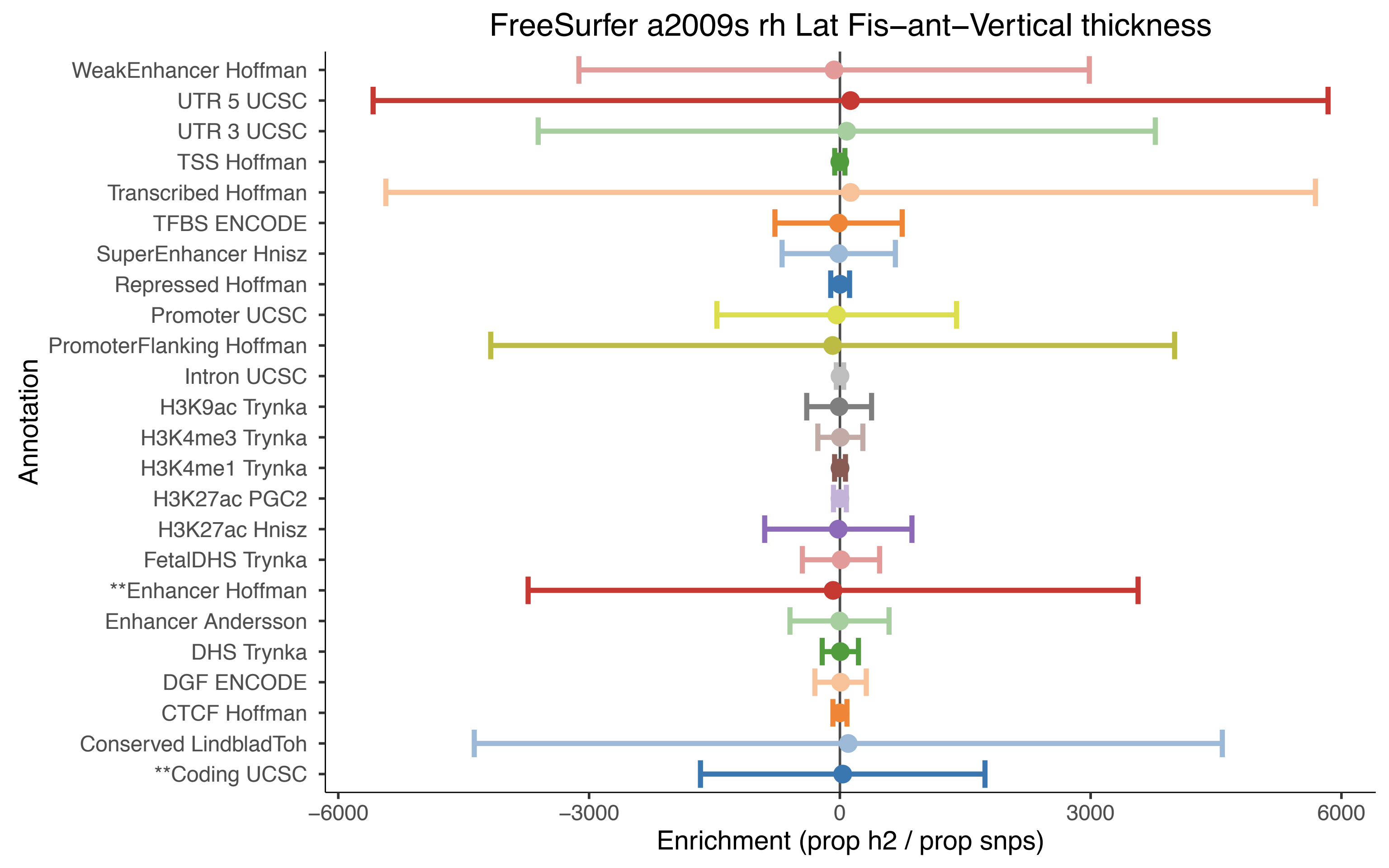

FreeSurfer a2009s rh Lat Fis−ant−Vertical thickness
WeakEnhancer Hoffman
UTR 5 UCSC
UTR 3 UCSC
TSS Hoffman
Transcribed Hoffman
TFBS ENCODE
SuperEnhancer Hnisz
Repressed Hoffman
Promoter UCSC
PromoterFlanking Hoffman
Intron UCSC
H3K9ac Trynka
H3K4me3 Trynka
H3K4me1 Trynka
H3K27ac PGC2
H3K27ac Hnisz
FetalDHS Trynka
**Enhancer Hoffman
Enhancer Andersson
DHS Trynka
DGF ENCODE
CTCF Hoffman
Conserved LindbladToh
**Coding UCSC
Annotation
−6000
−3000
0
3000
6000
Enrichment (prop h2 / prop snps)

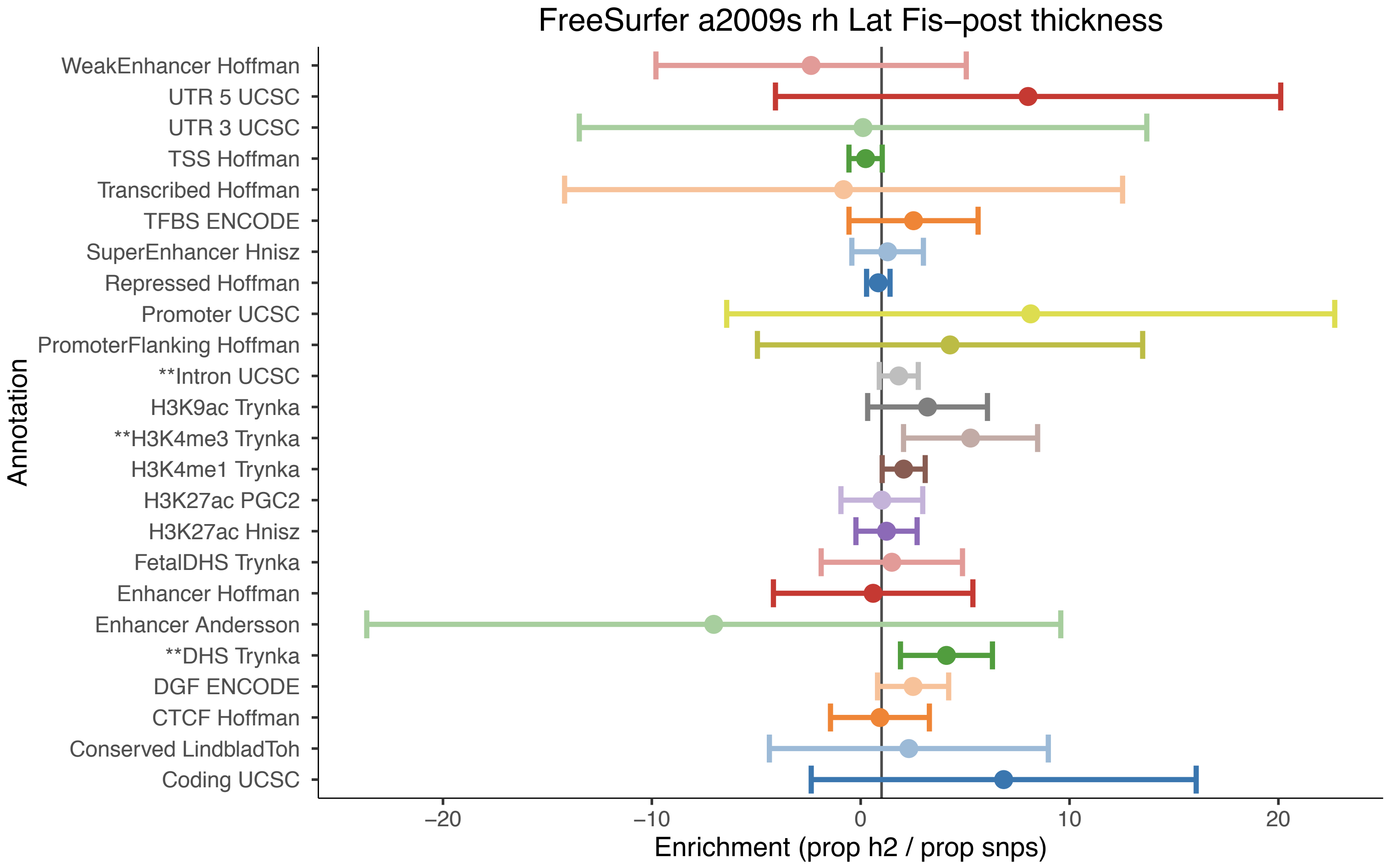

FreeSurfer a2009s rh Lat Fis−post thickness
WeakEnhancer Hoffman
UTR 5 UCSC
UTR 3 UCSC
TSS Hoffman
Transcribed Hoffman
TFBS ENCODE
SuperEnhancer Hnisz
Repressed Hoffman
Promoter UCSC
PromoterFlanking Hoffman
**Intron UCSC
H3K9ac Trynka
**H3K4me3 Trynka
H3K4me1 Trynka
H3K27ac PGC2
H3K27ac Hnisz
FetalDHS Trynka
Enhancer Hoffman
Enhancer Andersson
**DHS Trynka
DGF ENCODE
CTCF Hoffman
Conserved LindbladToh
Coding UCSC
Annotation
−20
−10
0
10
20
Enrichment (prop h2 / prop snps)

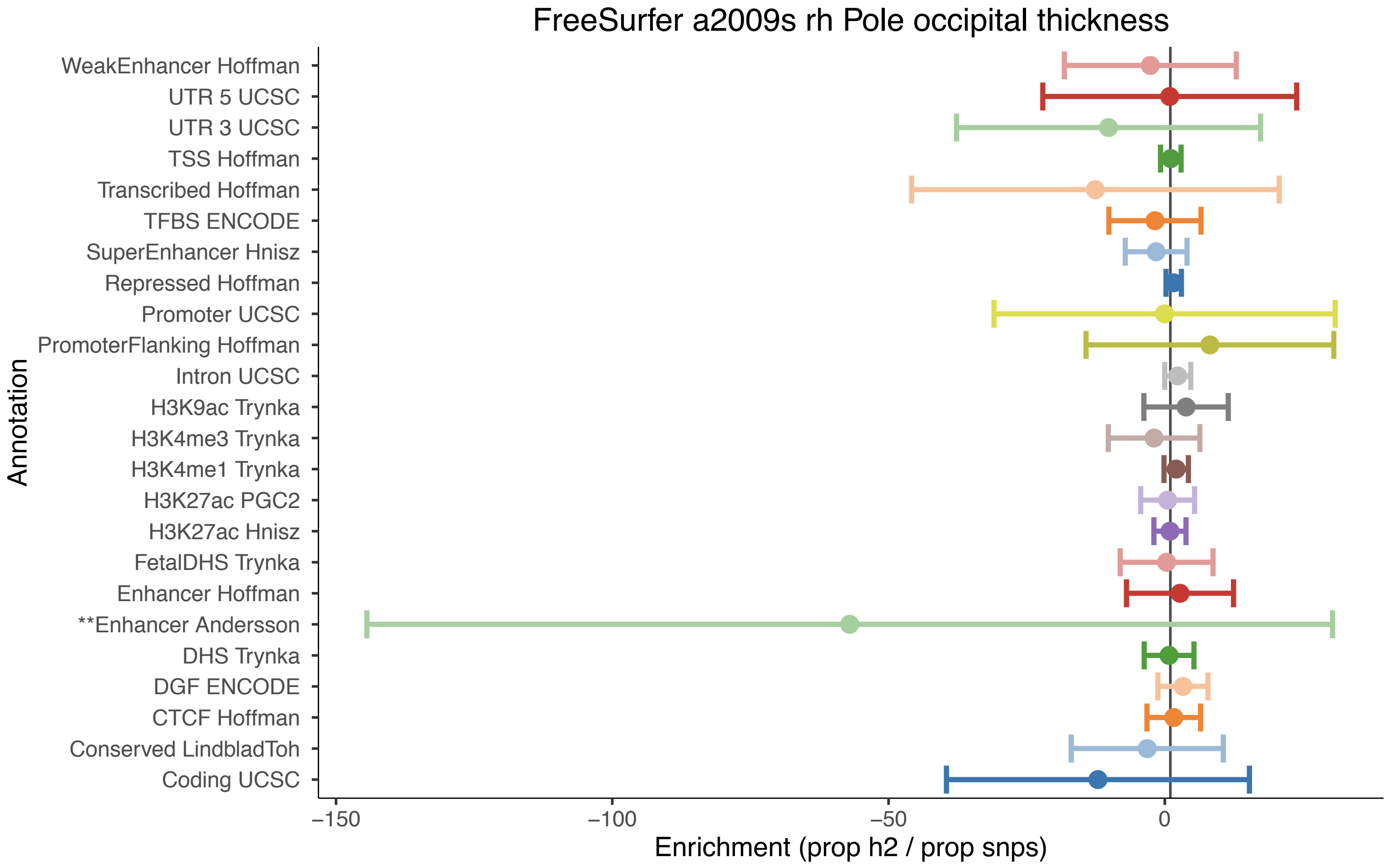

FreeSurfer a2009s rh Pole occipital thickness
WeakEnhancer Hoffman
UTR 5 UCSC
UTR 3 UCSC
TSS Hoffman
Transcribed Hoffman
TFBS ENCODE
SuperEnhancer Hnisz
Repressed Hoffman
Promoter UCSC
PromoterFlanking Hoffman
Intron UCSC
H3K9ac Trynka
H3K4me3 Trynka
H3K4me1 Trynka
H3K27ac PGC2
H3K27ac Hnisz
FetalDHS Trynka
Enhancer Hoffman
**Enhancer Andersson
DHS Trynka
DGF ENCODE
CTCF Hoffman
Conserved LindbladToh
Coding UCSC
Annotation
−150
−100
−50
0
Enrichment (prop h2 / prop snps)

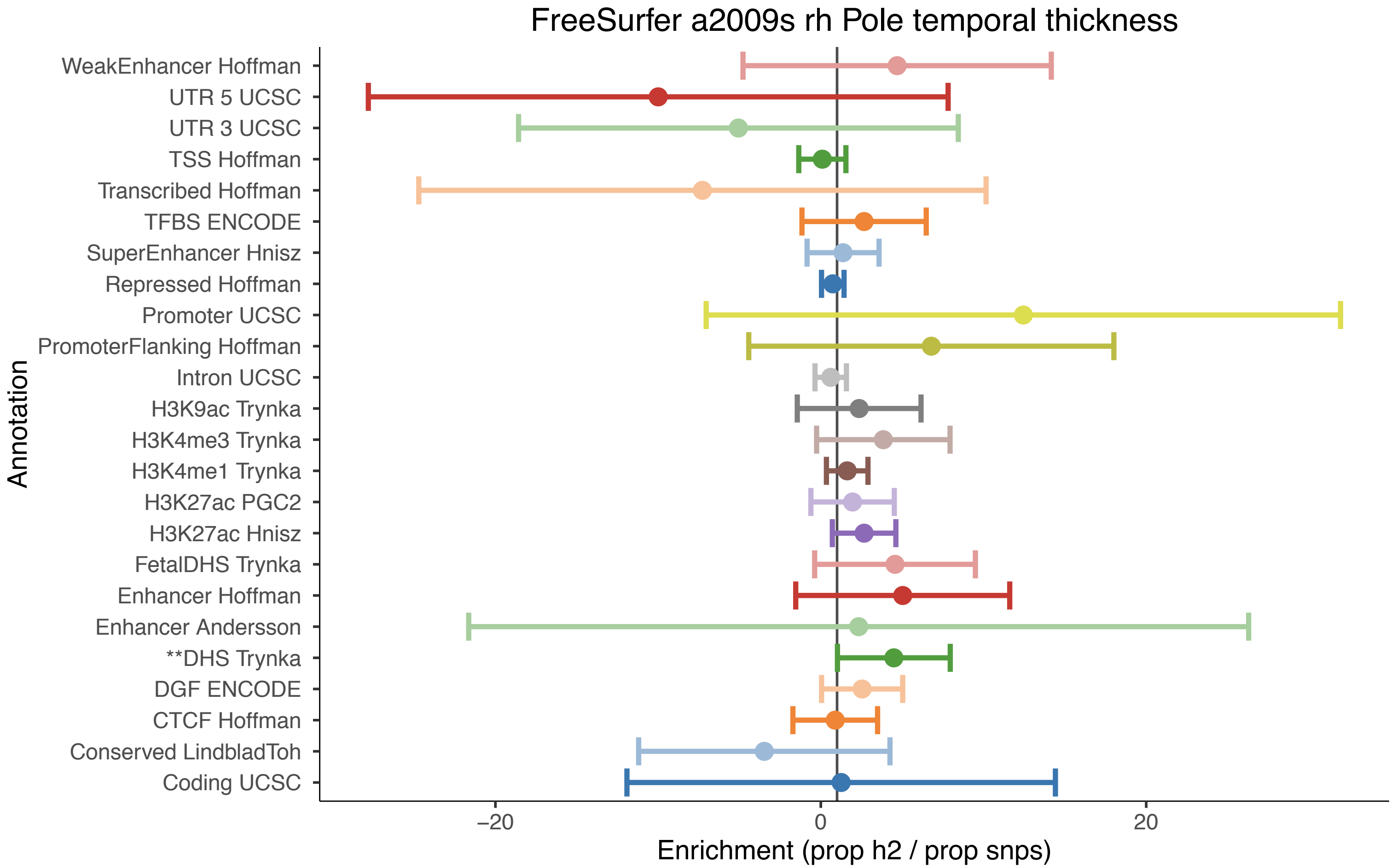

FreeSurfer a2009s rh Pole temporal thickness
WeakEnhancer Hoffman
UTR 5 UCSC
UTR 3 UCSC
TSS Hoffman
Transcribed Hoffman
TFBS ENCODE
SuperEnhancer Hnisz
Repressed Hoffman
Promoter UCSC
PromoterFlanking Hoffman
Intron UCSC
H3K9ac Trynka
H3K4me3 Trynka
H3K4me1 Trynka
H3K27ac PGC2
H3K27ac Hnisz
FetalDHS Trynka
Enhancer Hoffman
Enhancer Andersson
**DHS Trynka
DGF ENCODE
CTCF Hoffman
Conserved LindbladToh
Coding UCSC
Annotation
−20
0
20
Enrichment (prop h2 / prop snps)

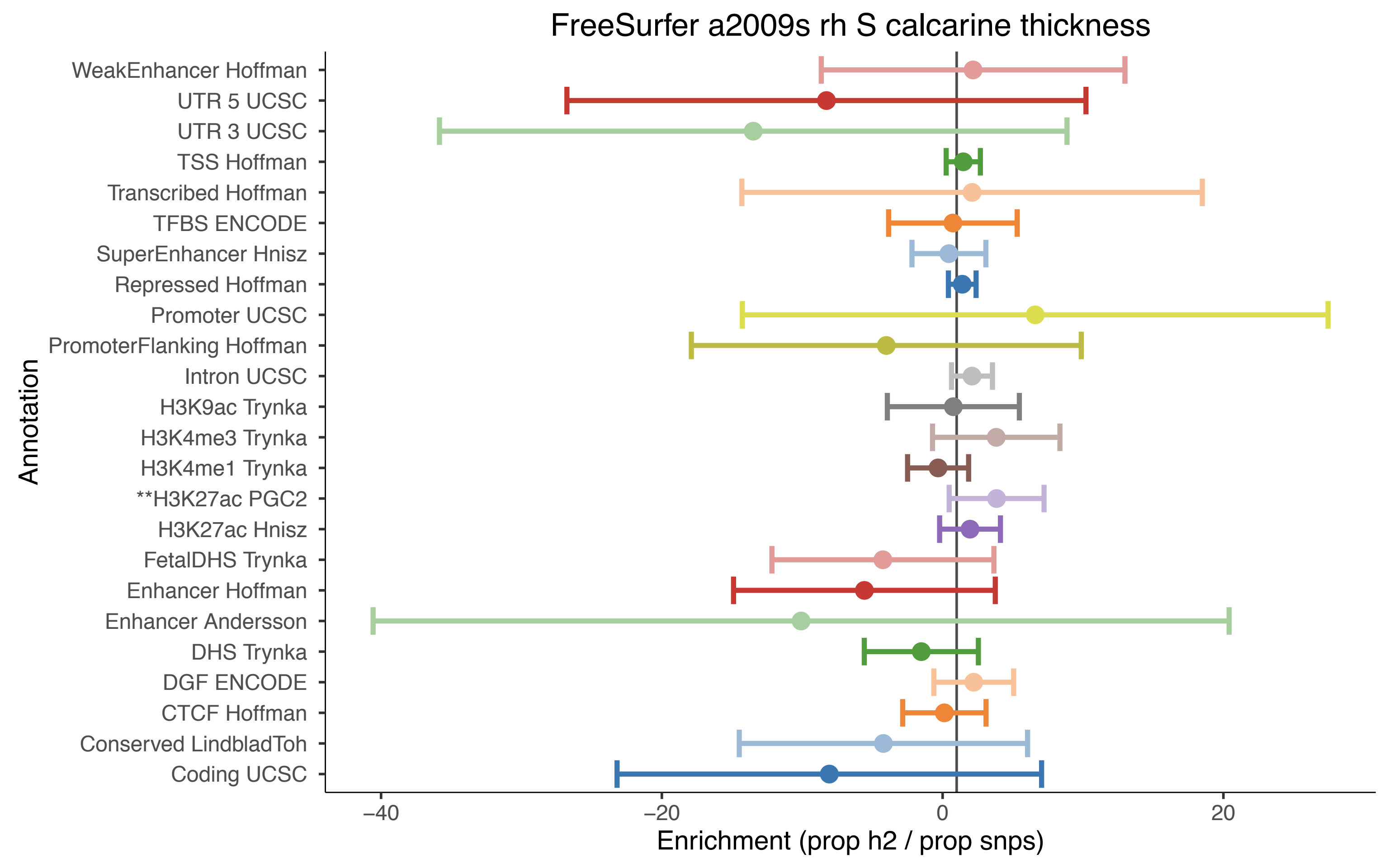

FreeSurfer a2009s rh S calcarine thickness
WeakEnhancer Hoffman
UTR 5 UCSC
UTR 3 UCSC
TSS Hoffman
Transcribed Hoffman
TFBS ENCODE
SuperEnhancer Hnisz
Repressed Hoffman
Promoter UCSC
PromoterFlanking Hoffman
Intron UCSC
H3K9ac Trynka
H3K4me3 Trynka
H3K4me1 Trynka
**H3K27ac PGC2
H3K27ac Hnisz
FetalDHS Trynka
Enhancer Hoffman
Enhancer Andersson
DHS Trynka
DGF ENCODE
CTCF Hoffman
Conserved LindbladToh
Coding UCSC
Annotation
−40
−20
0
20
Enrichment (prop h2 / prop snps)

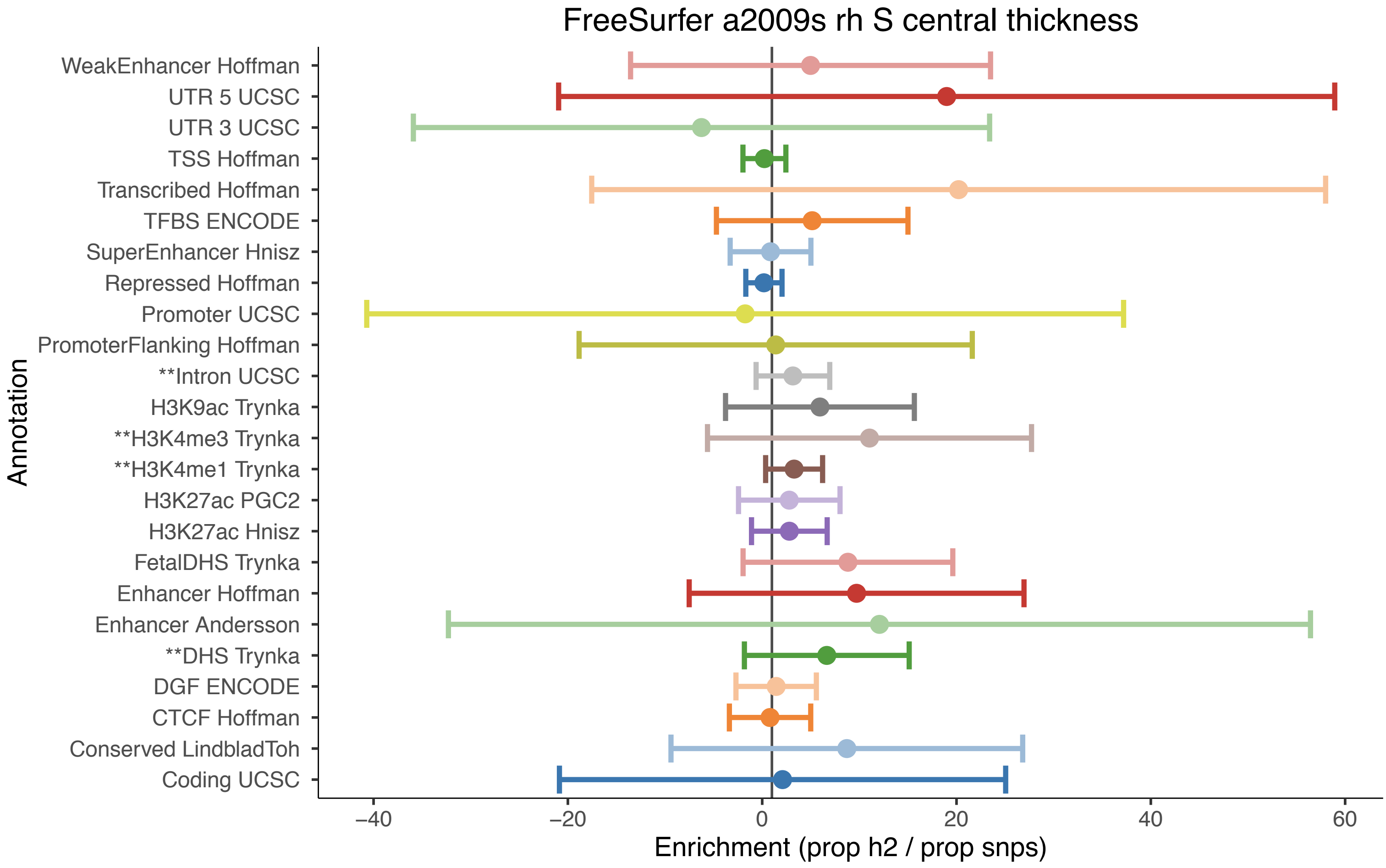

FreeSurfer a2009s rh S central thickness
WeakEnhancer Hoffman
UTR 5 UCSC
UTR 3 UCSC
TSS Hoffman
Transcribed Hoffman
TFBS ENCODE
SuperEnhancer Hnisz
Repressed Hoffman
Promoter UCSC
PromoterFlanking Hoffman
**Intron UCSC
H3K9ac Trynka
**H3K4me3 Trynka
**H3K4me1 Trynka
H3K27ac PGC2
H3K27ac Hnisz
FetalDHS Trynka
Enhancer Hoffman
Enhancer Andersson
**DHS Trynka
DGF ENCODE
CTCF Hoffman
Conserved LindbladToh
Coding UCSC
Annotation
−40
−20
0
20
40
60
Enrichment (prop h2 / prop snps)

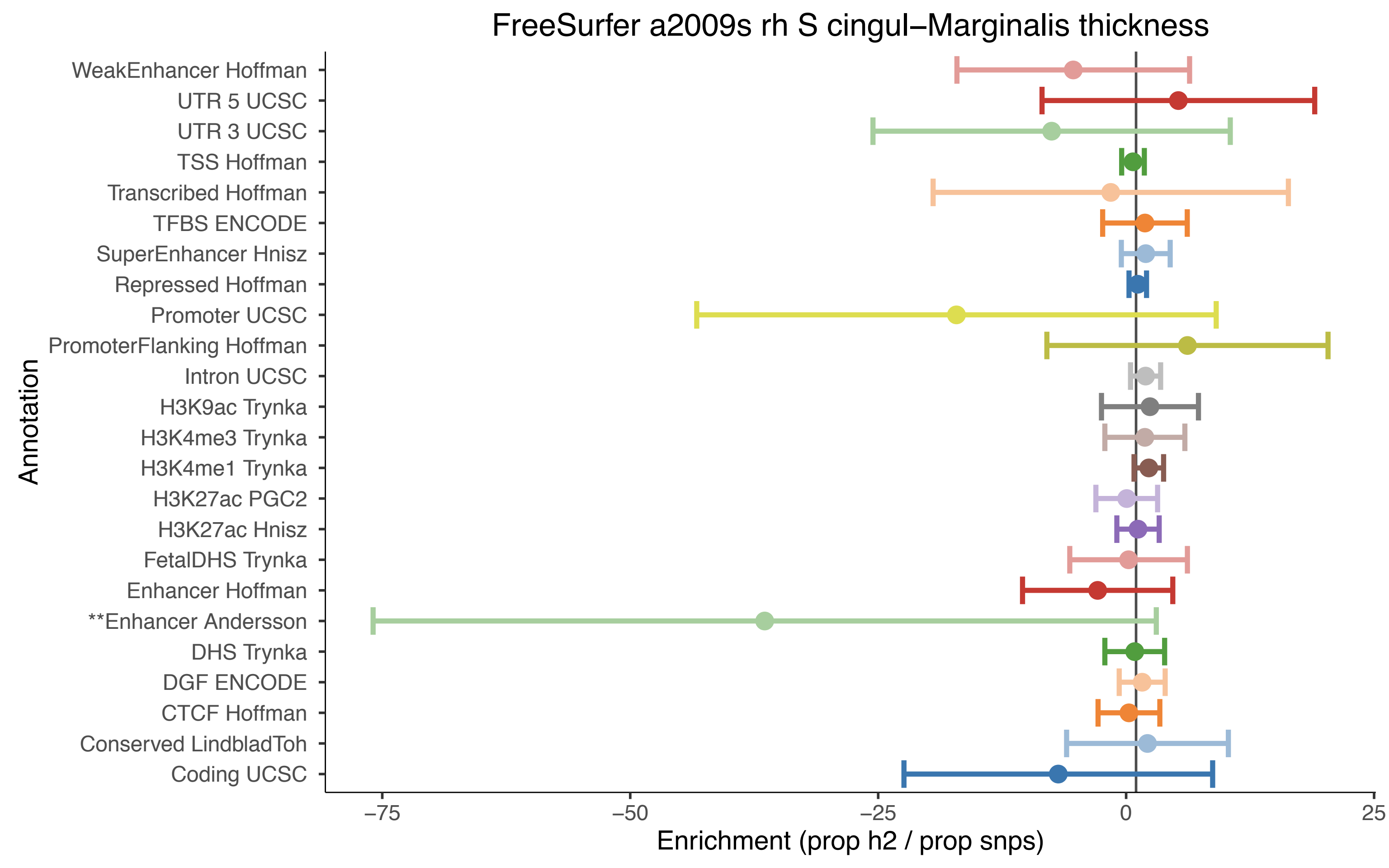
FreeSurfer a2009s rh S cingul−Marginalis thickness
Annotation
WeakEnhancer Hoffman
UTR 5 UCSC
UTR 3 UCSC
TSS Hoffman
Transcribed Hoffman
TFBS ENCODE
SuperEnhancer Hnisz
Repressed Hoffman
Promoter UCSC
PromoterFlanking Hoffman
Intron UCSC
H3K9ac Trynka
H3K4me3 Trynka
H3K4me1 Trynka
H3K27ac PGC2
H3K27ac Hnisz
FetalDHS Trynka
Enhancer Hoffman
**Enhancer Andersson
DHS Trynka
DGF ENCODE
CTCF Hoffman
Conserved LindbladToh
Coding UCSC
−75
−50
−25
0
25
Enrichment (prop h2 / prop snps)

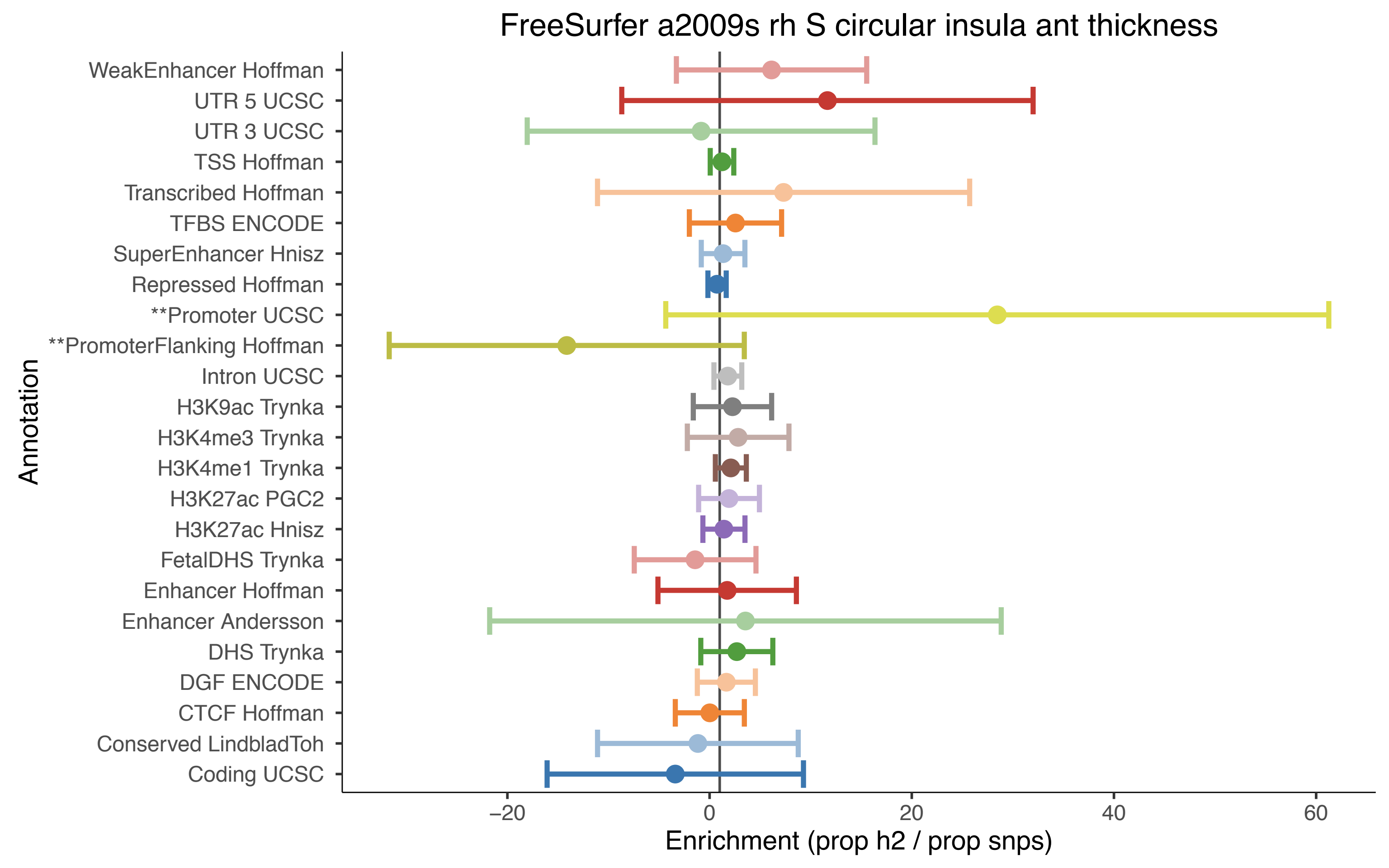
FreeSurfer a2009s rh S circular insula ant thickness
WeakEnhancer Hoffman
UTR 5 UCSC
UTR 3 UCSC
TSS Hoffman
Transcribed Hoffman
TFBS ENCODE
SuperEnhancer Hnisz
Repressed Hoffman
**Promoter UCSC
**PromoterFlanking Hoffman
Intron UCSC
H3K9ac Trynka
H3K4me3 Trynka
H3K4me1 Trynka
H3K27ac PGC2
H3K27ac Hnisz
FetalDHS Trynka
Enhancer Hoffman
Enhancer Andersson
DHS Trynka
DGF ENCODE
CTCF Hoffman
Conserved LindbladToh
Coding UCSC
Annotation
−20
0
20
40
60
Enrichment (prop h2 / prop snps)

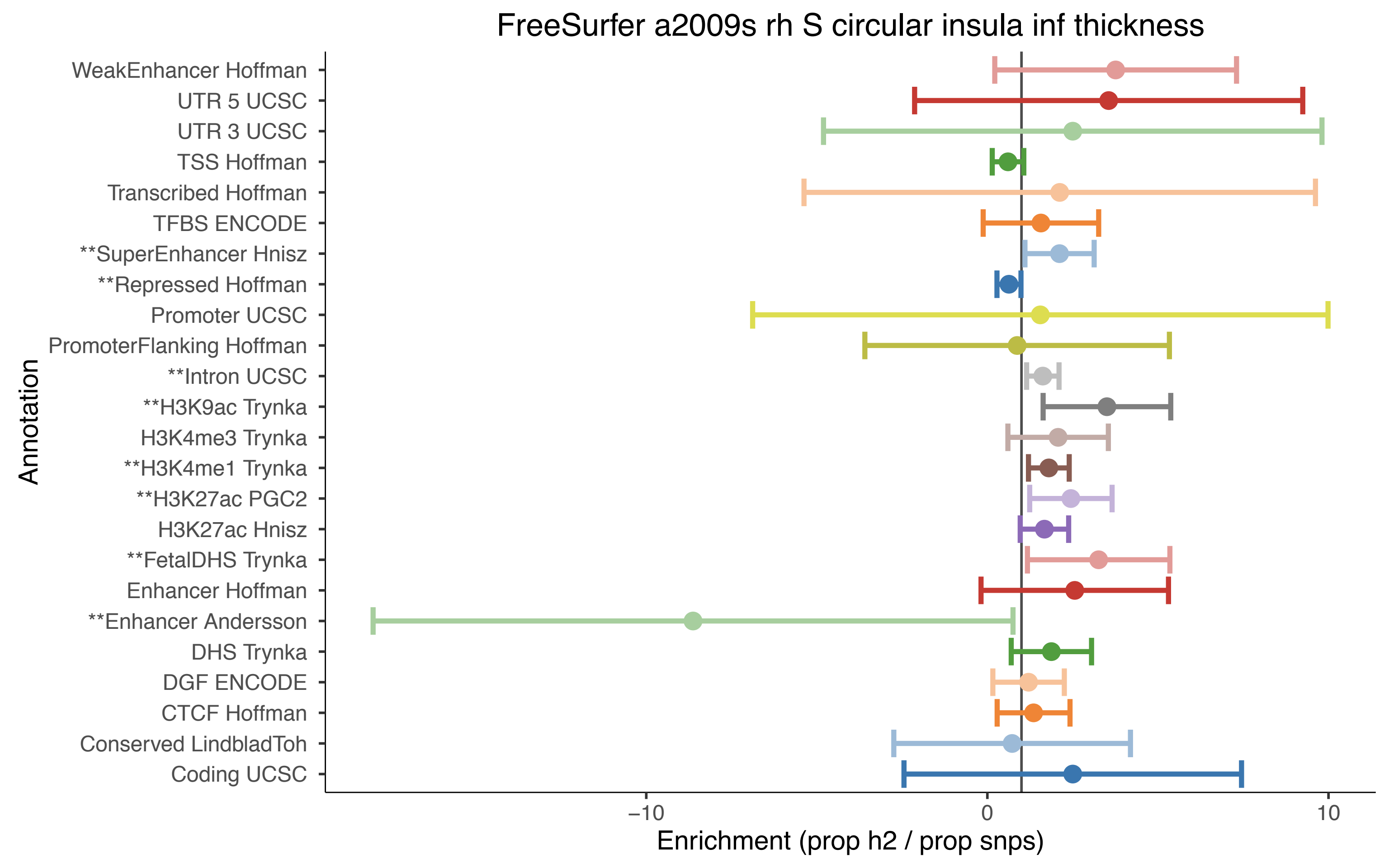

FreeSurfer a2009s rh S circular insula inf thickness
WeakEnhancer Hoffman
UTR 5 UCSC
UTR 3 UCSC
TSS Hoffman
Transcribed Hoffman
TFBS ENCODE
**SuperEnhancer Hnisz
**Repressed Hoffman
Promoter UCSC
PromoterFlanking Hoffman
**Intron UCSC
**H3K9ac Trynka
H3K4me3 Trynka
**H3K4me1 Trynka
**H3K27ac PGC2
H3K27ac Hnisz
**FetalDHS Trynka
Enhancer Hoffman
**Enhancer Andersson
DHS Trynka
DGF ENCODE
CTCF Hoffman
Conserved LindbladToh
Coding UCSC
Annotation
−10
0
10
Enrichment (prop h2 / prop snps)

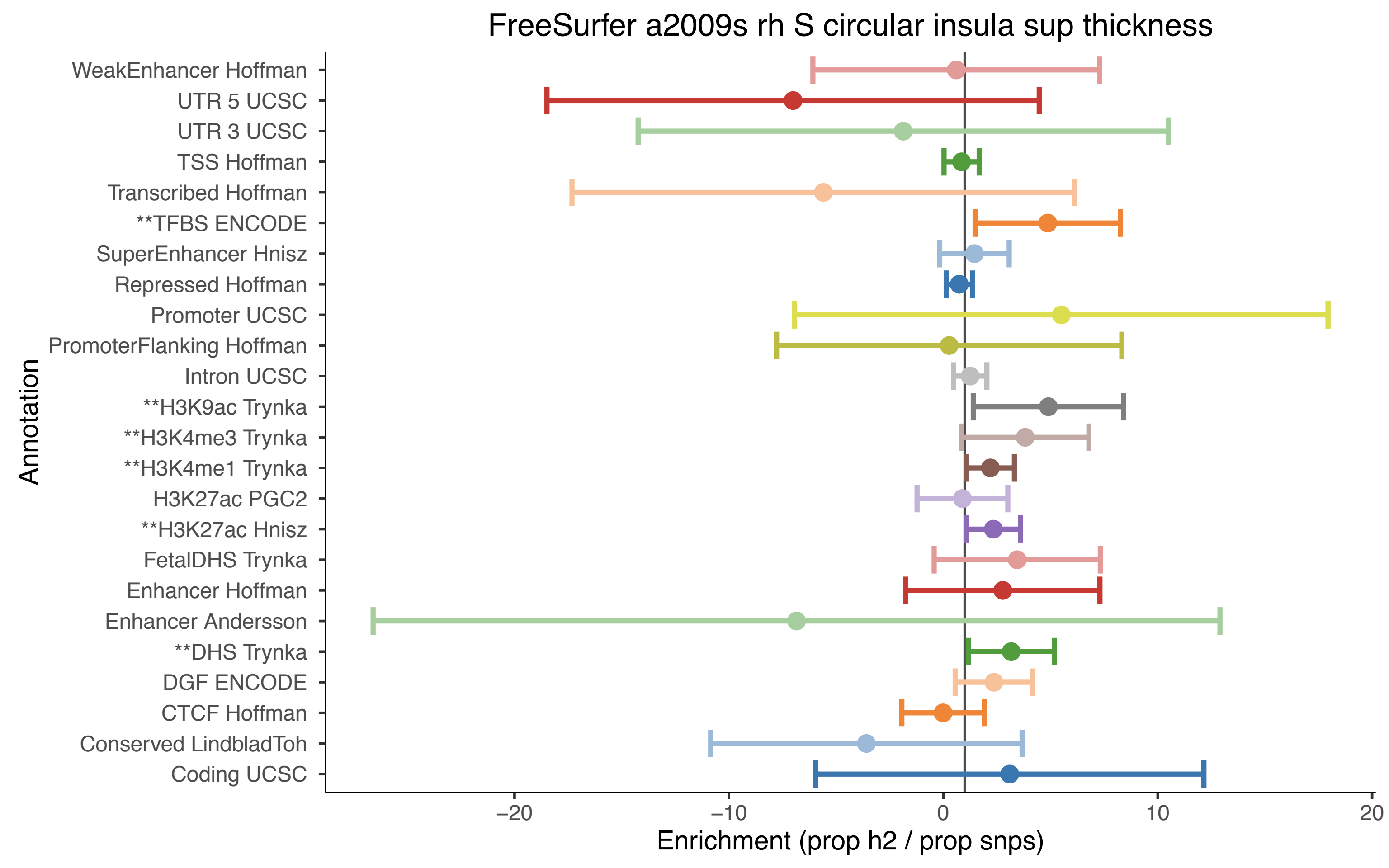

FreeSurfer a2009s rh S circular insula sup thickness
WeakEnhancer Hoffman
UTR 5 UCSC
UTR 3 UCSC
TSS Hoffman
Transcribed Hoffman
**TFBS ENCODE
SuperEnhancer Hnisz
Repressed Hoffman
Promoter UCSC
PromoterFlanking Hoffman
Intron UCSC
**H3K9ac Trynka
**H3K4me3 Trynka
**H3K4me1 Trynka
H3K27ac PGC2
**H3K27ac Hnisz
FetalDHS Trynka
Enhancer Hoffman
Enhancer Andersson
**DHS Trynka
DGF ENCODE
CTCF Hoffman
Conserved LindbladToh
Coding UCSC
Annotation
−20
−10
0
10
20
Enrichment (prop h2 / prop snps)

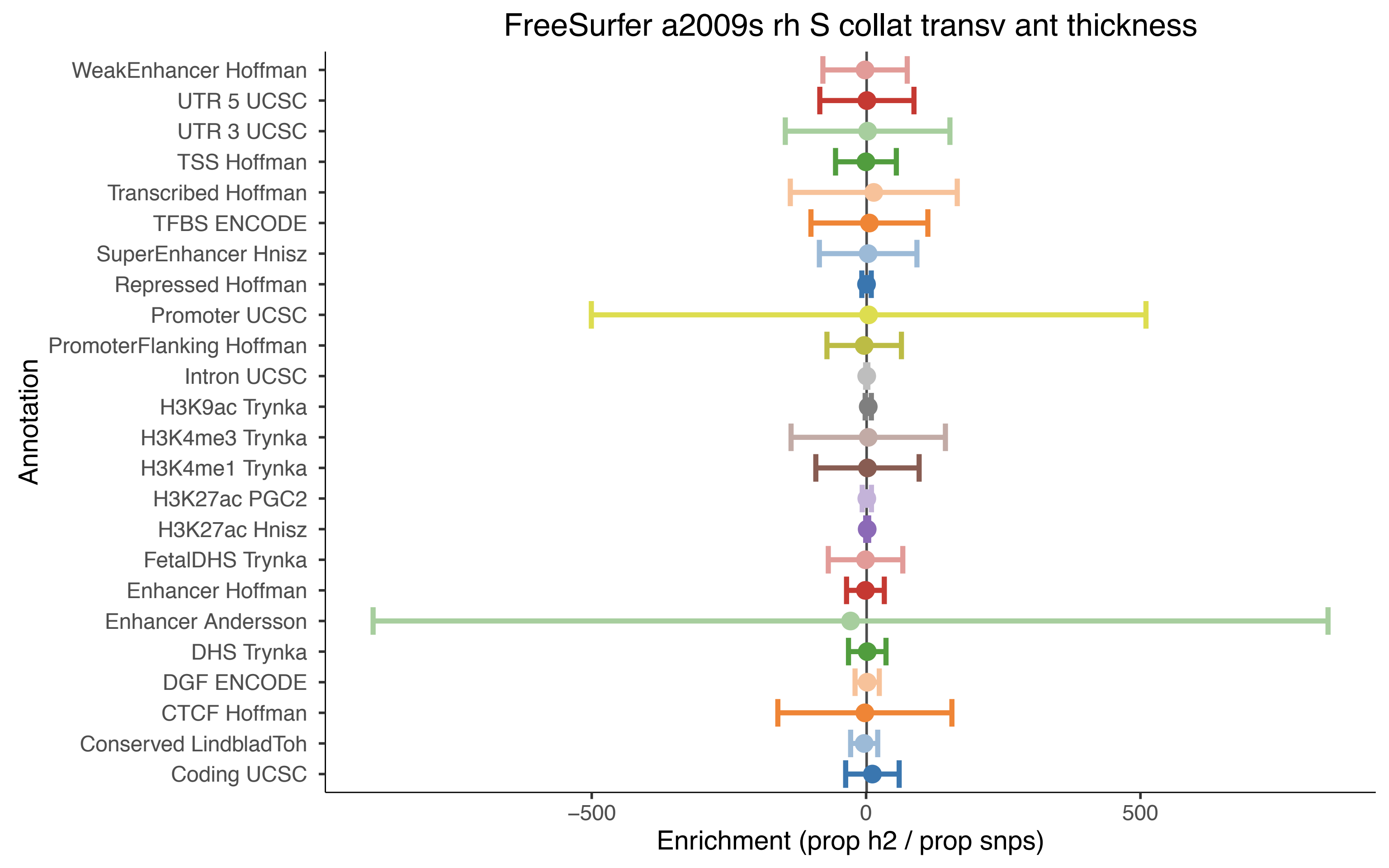
FreeSurfer a2009s rh S collat transv ant thickness
WeakEnhancer Hoffman
UTR 5 UCSC
UTR 3 UCSC
TSS Hoffman
Transcribed Hoffman
TFBS ENCODE
SuperEnhancer Hnisz
Repressed Hoffman
Promoter UCSC
PromoterFlanking Hoffman
Intron UCSC
H3K9ac Trynka
H3K4me3 Trynka
H3K4me1 Trynka
H3K27ac PGC2
H3K27ac Hnisz
FetalDHS Trynka
Enhancer Hoffman
Enhancer Andersson
DHS Trynka
DGF ENCODE
CTCF Hoffman
Conserved LindbladToh
Coding UCSC
Annotation
−500
0
500
Enrichment (prop h2 / prop snps)

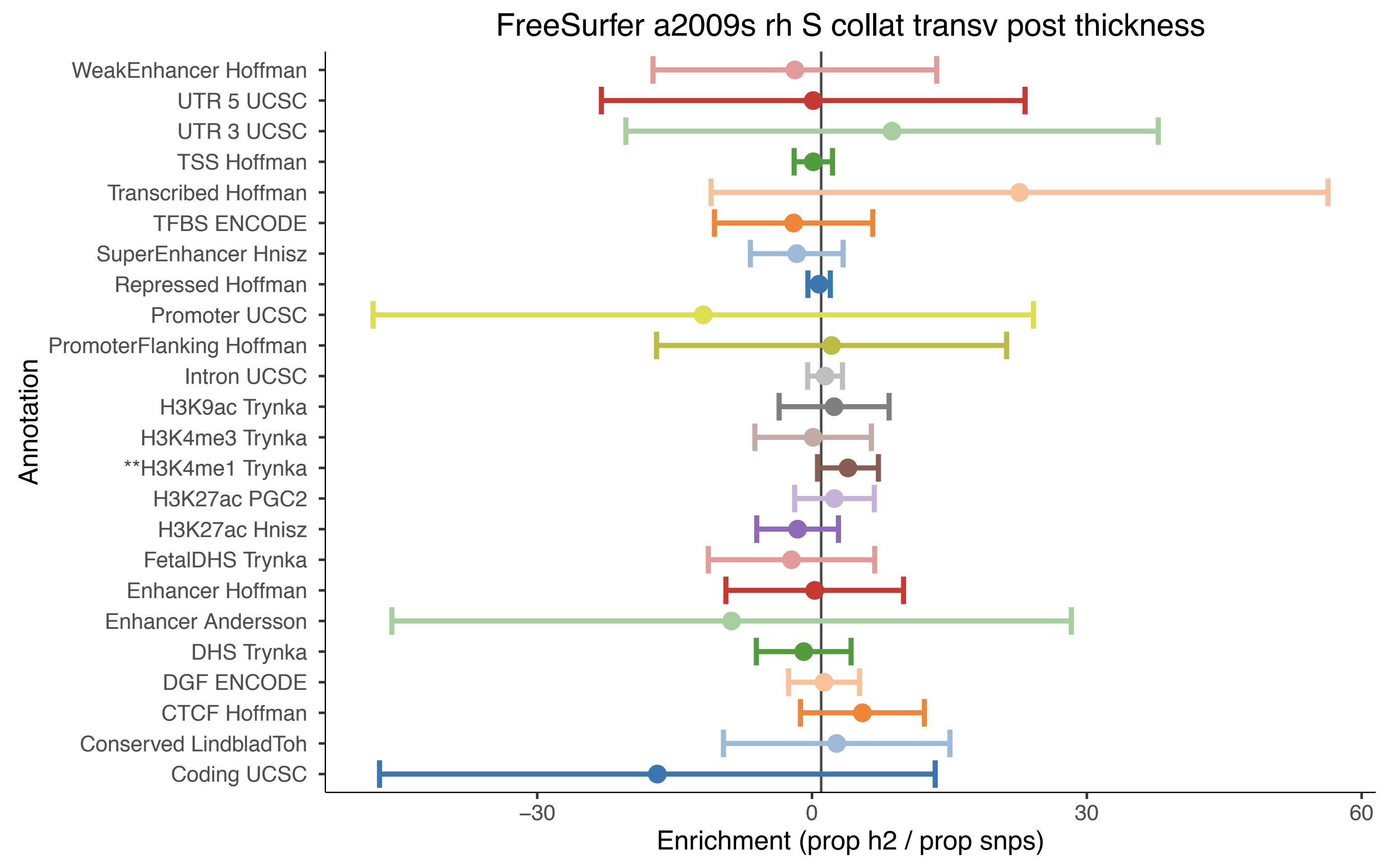

FreeSurfer a2009s rh S collat transv post thickness
WeakEnhancer Hoffman
UTR 5 UCSC
UTR 3 UCSC
TSS Hoffman
Transcribed Hoffman
TFBS ENCODE
SuperEnhancer Hnisz
Repressed Hoffman
Promoter UCSC
PromoterFlanking Hoffman
Intron UCSC
H3K9ac Trynka
H3K4me3 Trynka
**H3K4me1 Trynka
H3K27ac PGC2
H3K27ac Hnisz
FetalDHS Trynka
Enhancer Hoffman
Enhancer Andersson
DHS Trynka
DGF ENCODE
CTCF Hoffman
Conserved LindbladToh
Coding UCSC
Annotation
−30
0
30
60
Enrichment (prop h2 / prop snps)

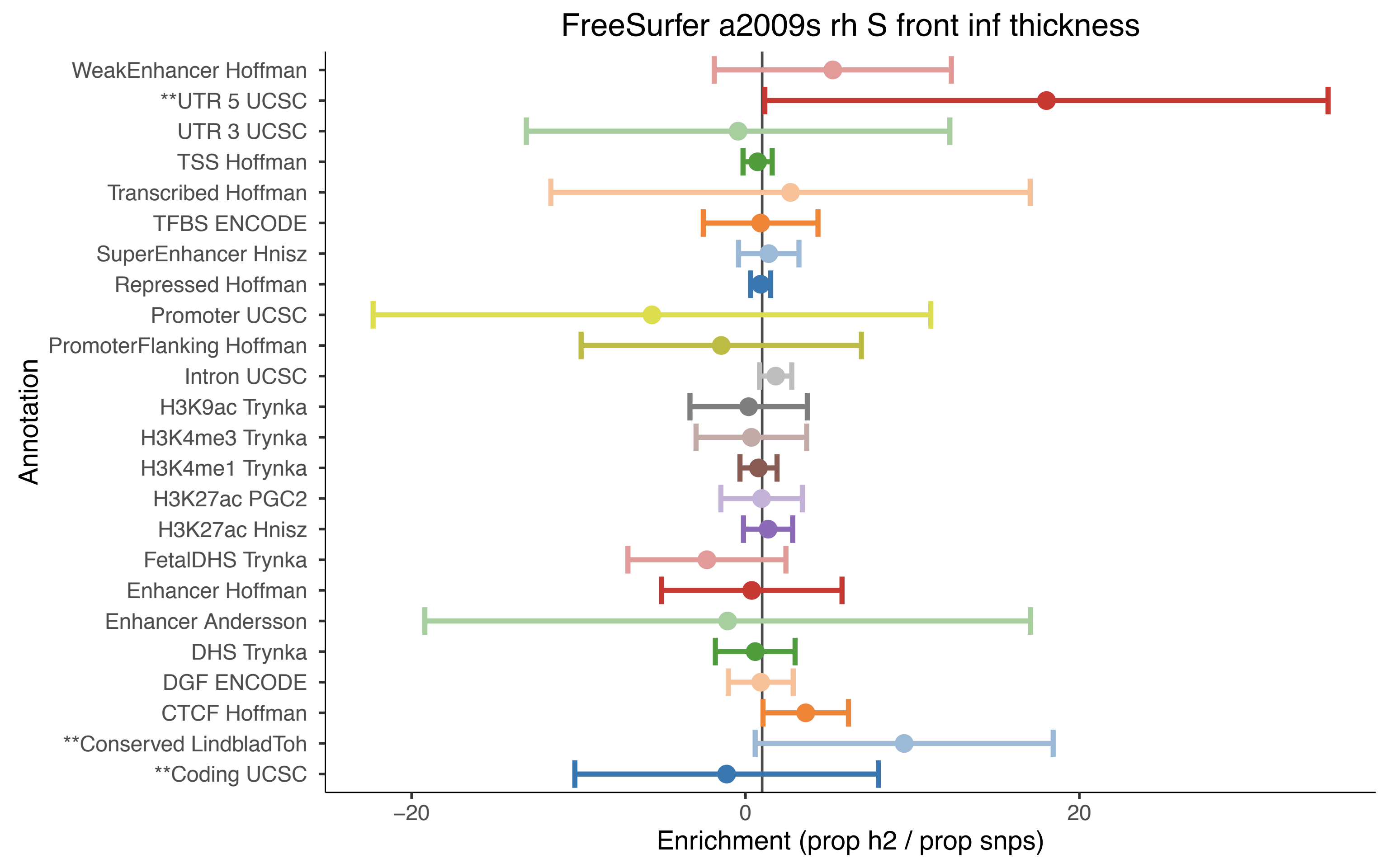
FreeSurfer a2009s rh S front inf thickness
Annotation
WeakEnhancer Hoffman
**UTR 5 UCSC
UTR 3 UCSC
TSS Hoffman
Transcribed Hoffman
TFBS ENCODE
SuperEnhancer Hnisz
Repressed Hoffman
Promoter UCSC
PromoterFlanking Hoffman
Intron UCSC
H3K9ac Trynka
H3K4me3 Trynka
H3K4me1 Trynka
H3K27ac PGC2
H3K27ac Hnisz
FetalDHS Trynka
Enhancer Hoffman
Enhancer Andersson
DHS Trynka
DGF ENCODE
CTCF Hoffman
**Conserved LindbladToh
**Coding UCSC
−20
0
20
Enrichment (prop h2 / prop snps)

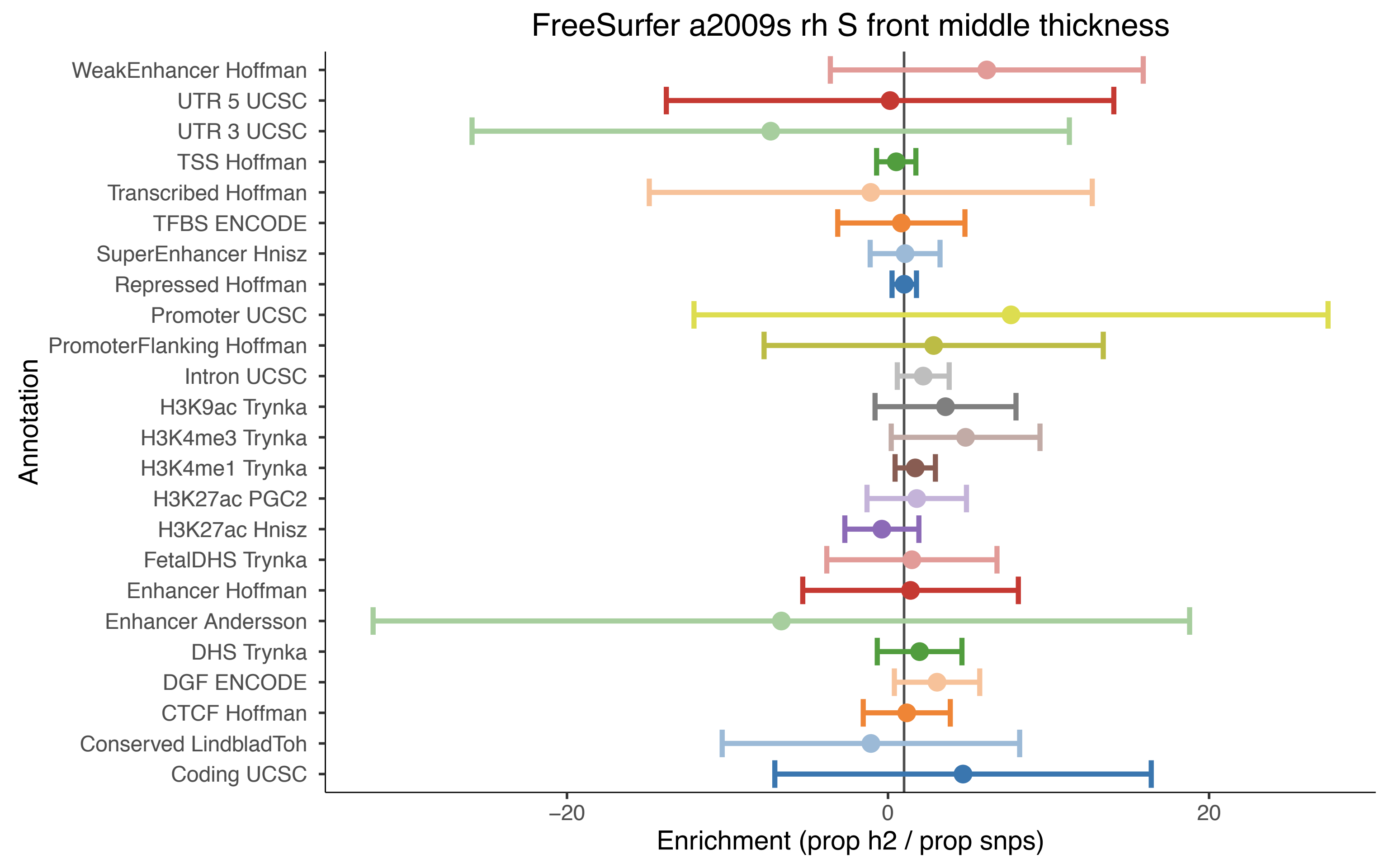

FreeSurfer a2009s rh S front middle thickness
WeakEnhancer Hoffman
UTR 5 UCSC
UTR 3 UCSC
TSS Hoffman
Transcribed Hoffman
TFBS ENCODE
SuperEnhancer Hnisz
Repressed Hoffman
Promoter UCSC
PromoterFlanking Hoffman
Intron UCSC
H3K9ac Trynka
H3K4me3 Trynka
H3K4me1 Trynka
H3K27ac PGC2
H3K27ac Hnisz
FetalDHS Trynka
Enhancer Hoffman
Enhancer Andersson
DHS Trynka
DGF ENCODE
CTCF Hoffman
Conserved LindbladToh
Coding UCSC
Annotation
−20
0
20
Enrichment (prop h2 / prop snps)

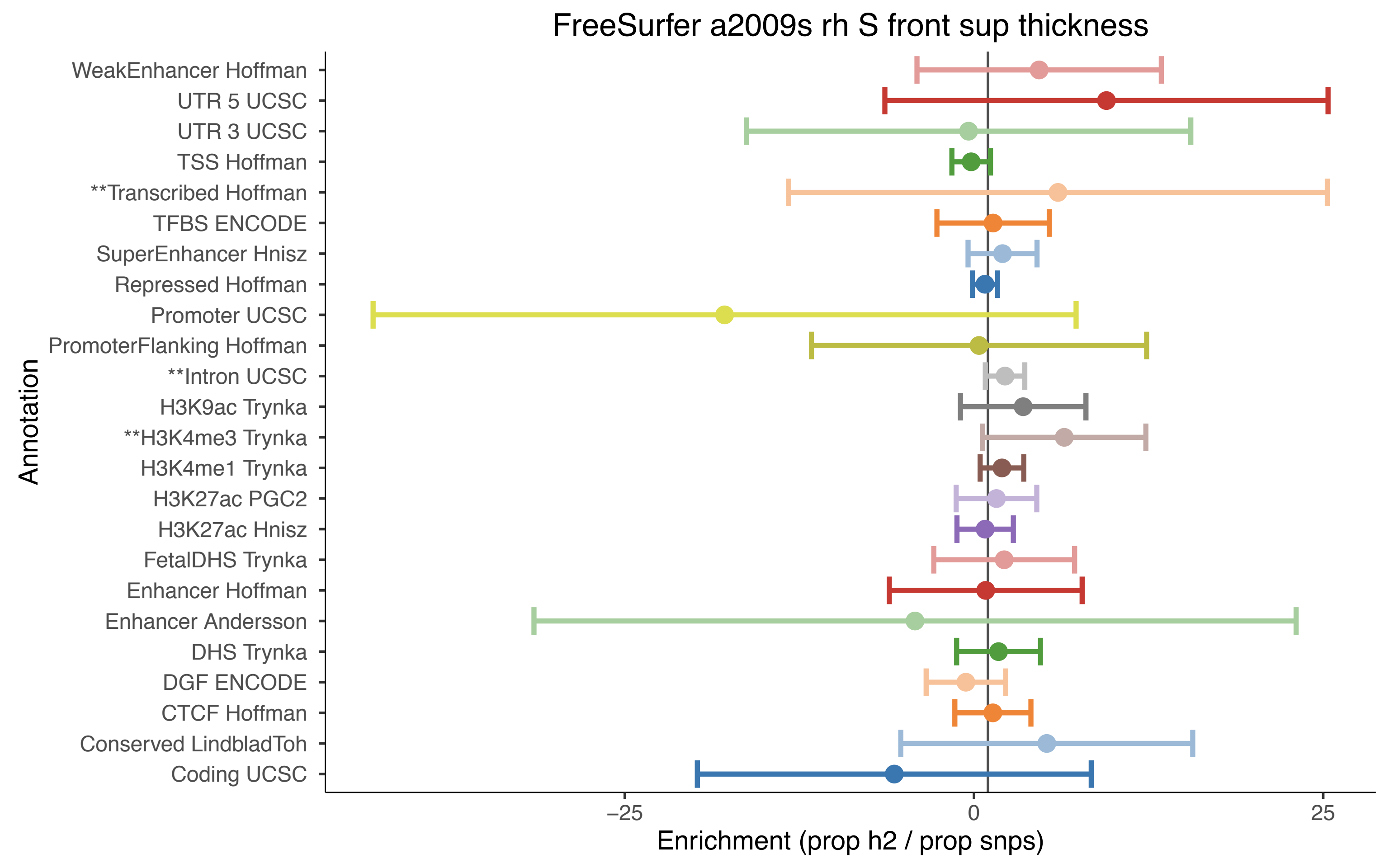

FreeSurfer a2009s rh S front sup thickness
WeakEnhancer Hoffman
UTR 5 UCSC
UTR 3 UCSC
TSS Hoffman
**Transcribed Hoffman
TFBS ENCODE
SuperEnhancer Hnisz
Repressed Hoffman
Promoter UCSC
PromoterFlanking Hoffman
**Intron UCSC
H3K9ac Trynka
**H3K4me3 Trynka
H3K4me1 Trynka
H3K27ac PGC2
H3K27ac Hnisz
FetalDHS Trynka
Enhancer Hoffman
Enhancer Andersson
DHS Trynka
DGF ENCODE
CTCF Hoffman
Conserved LindbladToh
Coding UCSC
Annotation
−25
0
25
Enrichment (prop h2 / prop snps)

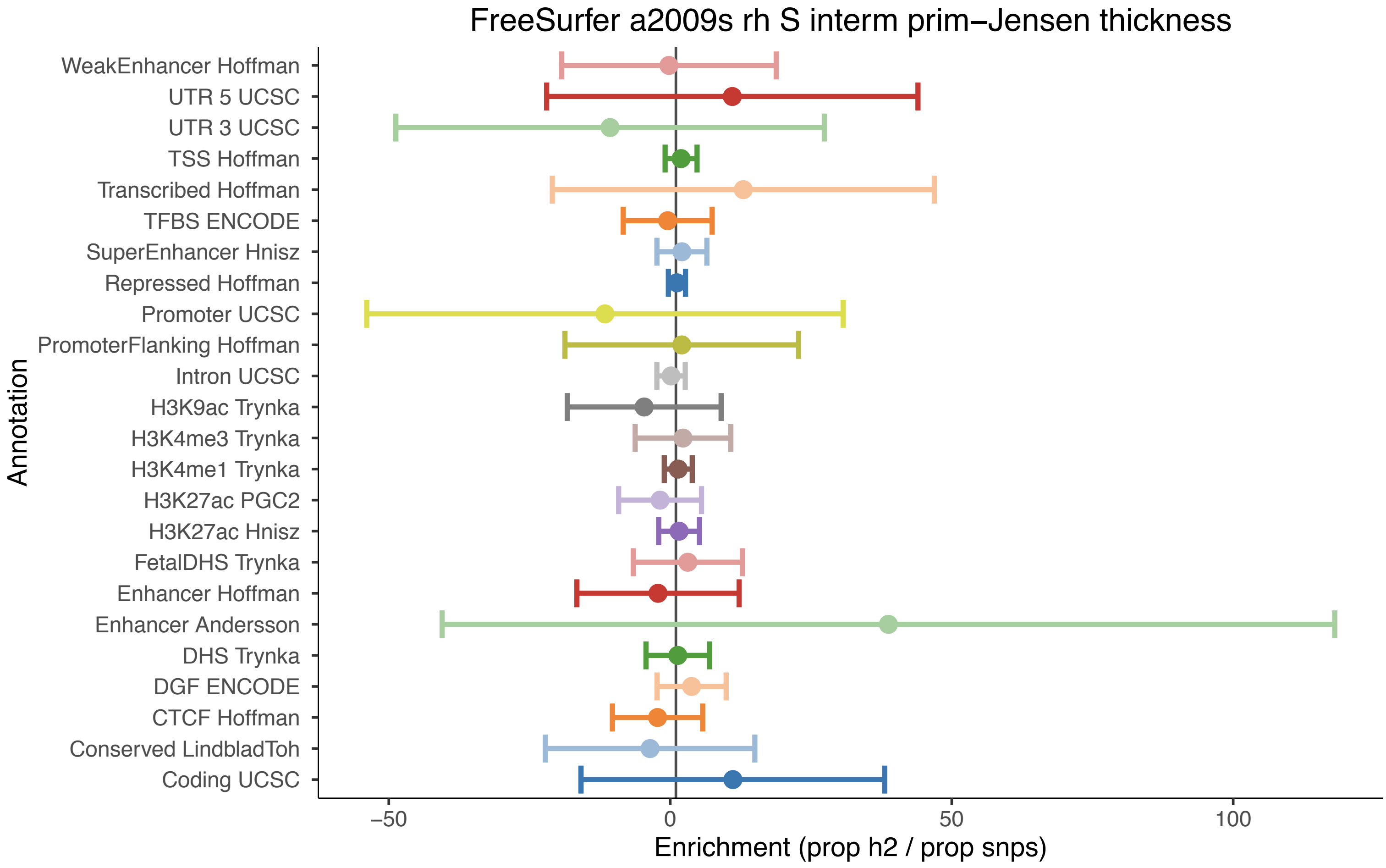

FreeSurfer a2009s rh S interm prim−Jensen thickness
Annotation
WeakEnhancer Hoffman
UTR 5 UCSC
UTR 3 UCSC
TSS Hoffman
Transcribed Hoffman
TFBS ENCODE
SuperEnhancer Hnisz
Repressed Hoffman
Promoter UCSC
PromoterFlanking Hoffman
Intron UCSC
H3K9ac Trynka
H3K4me3 Trynka
H3K4me1 Trynka
H3K27ac PGC2
H3K27ac Hnisz
FetalDHS Trynka
Enhancer Hoffman
Enhancer Andersson
DHS Trynka
DGF ENCODE
CTCF Hoffman
Conserved LindbladToh
Coding UCSC
−50
0
50
100
Enrichment (prop h2 / prop snps)

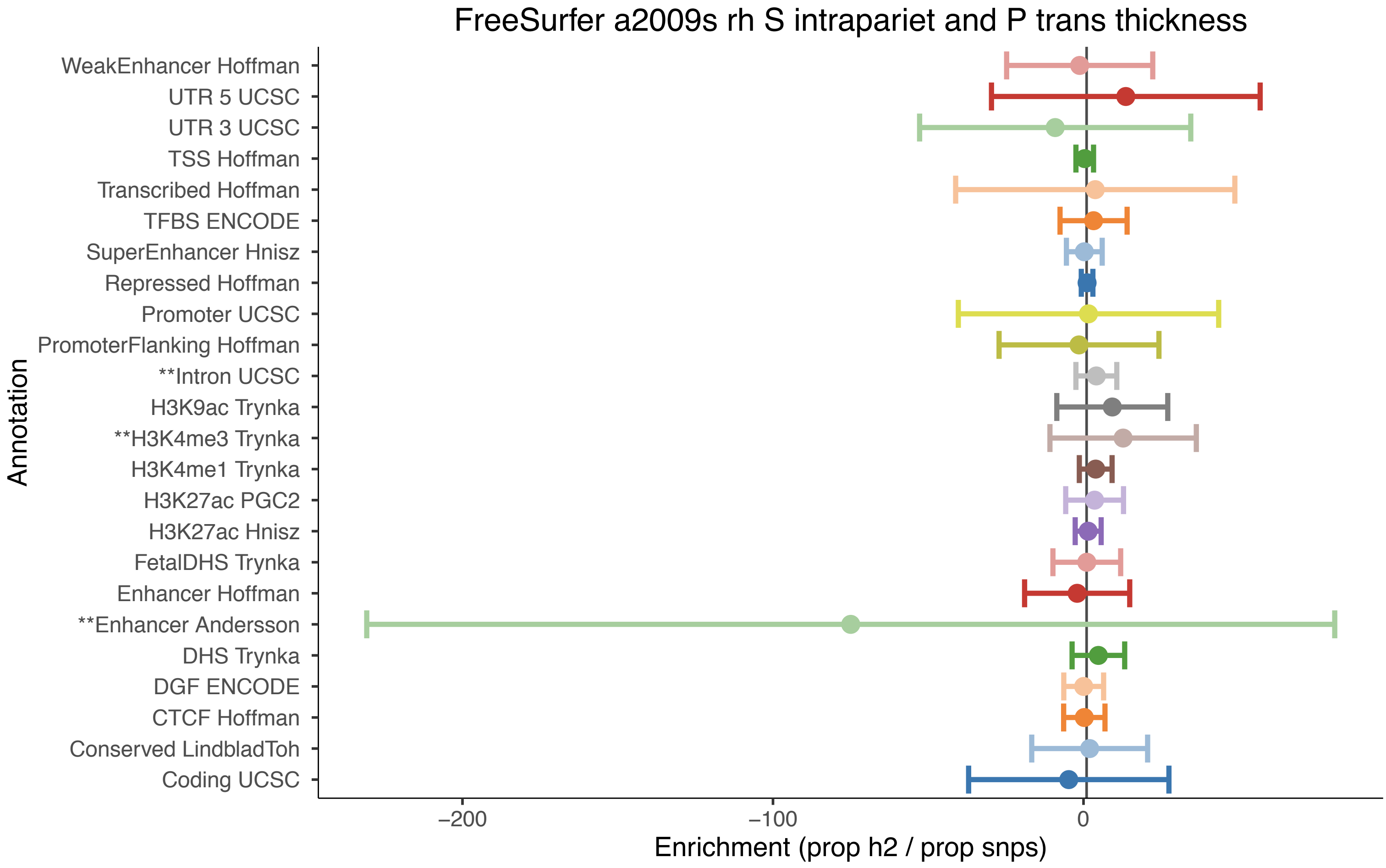

FreeSurfer a2009s rh S intrapariet and P trans thickness
Annotation
WeakEnhancer Hoffman
UTR 5 UCSC
UTR 3 UCSC
TSS Hoffman
Transcribed Hoffman
TFBS ENCODE
SuperEnhancer Hnisz
Repressed Hoffman
Promoter UCSC
PromoterFlanking Hoffman
**Intron UCSC
H3K9ac Trynka
**H3K4me3 Trynka
H3K4me1 Trynka
H3K27ac PGC2
H3K27ac Hnisz
FetalDHS Trynka
Enhancer Hoffman
**Enhancer Andersson
DHS Trynka
DGF ENCODE
CTCF Hoffman
Conserved LindbladToh
Coding UCSC
−200
−100
0
Enrichment (prop h2 / prop snps)

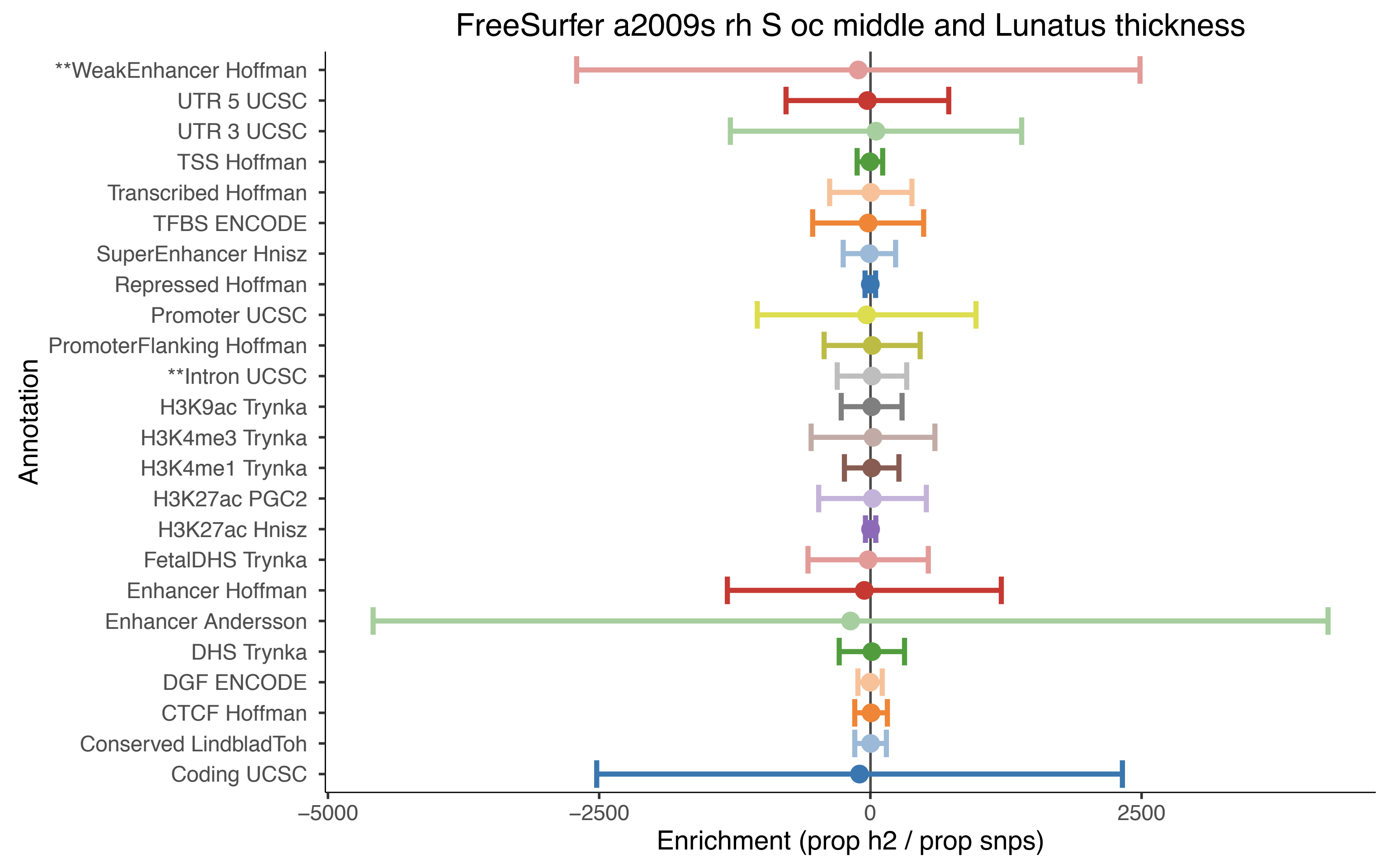
FreeSurfer a2009s rh S oc middle and Lunatus thickness
**WeakEnhancer Hoffman
UTR 5 UCSC
UTR 3 UCSC
TSS Hoffman
Transcribed Hoffman
TFBS ENCODE
SuperEnhancer Hnisz
Repressed Hoffman
Promoter UCSC
PromoterFlanking Hoffman
**Intron UCSC
H3K9ac Trynka
H3K4me3 Trynka
H3K4me1 Trynka
H3K27ac PGC2
H3K27ac Hnisz
FetalDHS Trynka
Enhancer Hoffman
Enhancer Andersson
DHS Trynka
DGF ENCODE
CTCF Hoffman
Conserved LindbladToh
Coding UCSC
Annotation
−5000
−2500
0
2500
Enrichment (prop h2 / prop snps)

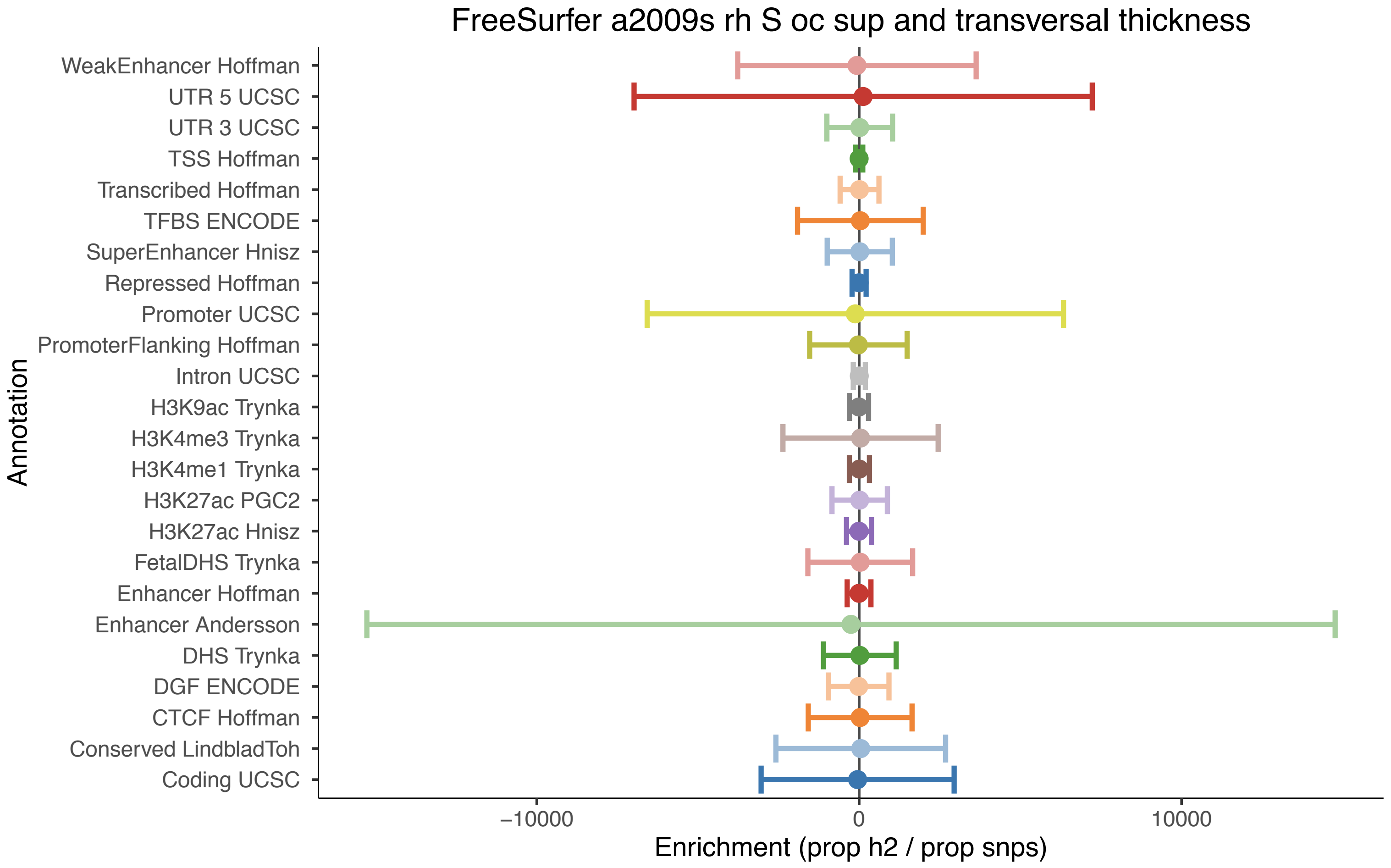
FreeSurfer a2009s rh S oc sup and transversal thickness
Annotation
WeakEnhancer Hoffman
UTR 5 UCSC
UTR 3 UCSC
TSS Hoffman
Transcribed Hoffman
TFBS ENCODE
SuperEnhancer Hnisz
Repressed Hoffman
Promoter UCSC
PromoterFlanking Hoffman
Intron UCSC
H3K9ac Trynka
H3K4me3 Trynka
H3K4me1 Trynka
H3K27ac PGC2
H3K27ac Hnisz
FetalDHS Trynka
Enhancer Hoffman
Enhancer Andersson
DHS Trynka
DGF ENCODE
CTCF Hoffman
Conserved LindbladToh
Coding UCSC
−10000
0
10000
Enrichment (prop h2 / prop snps)

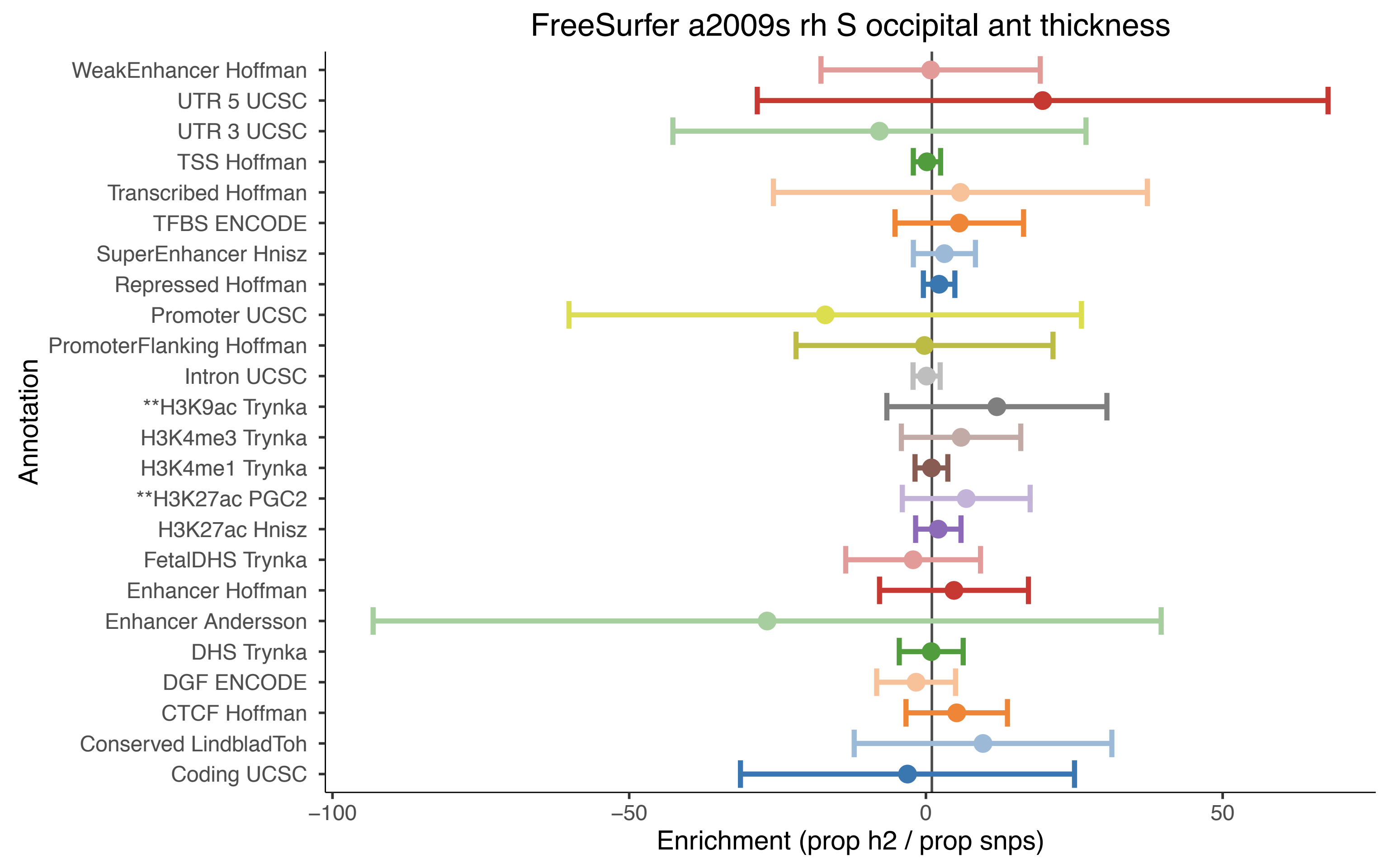
FreeSurfer a2009s rh S occipital ant thickness
WeakEnhancer Hoffman
UTR 5 UCSC
UTR 3 UCSC
TSS Hoffman
Transcribed Hoffman
TFBS ENCODE
SuperEnhancer Hnisz
Repressed Hoffman
Promoter UCSC
PromoterFlanking Hoffman
Intron UCSC
**H3K9ac Trynka
H3K4me3 Trynka
H3K4me1 Trynka
**H3K27ac PGC2
H3K27ac Hnisz
FetalDHS Trynka
Enhancer Hoffman
Enhancer Andersson
DHS Trynka
DGF ENCODE
CTCF Hoffman
Conserved LindbladToh
Coding UCSC
Annotation
−100
−50
0
50
Enrichment (prop h2 / prop snps)

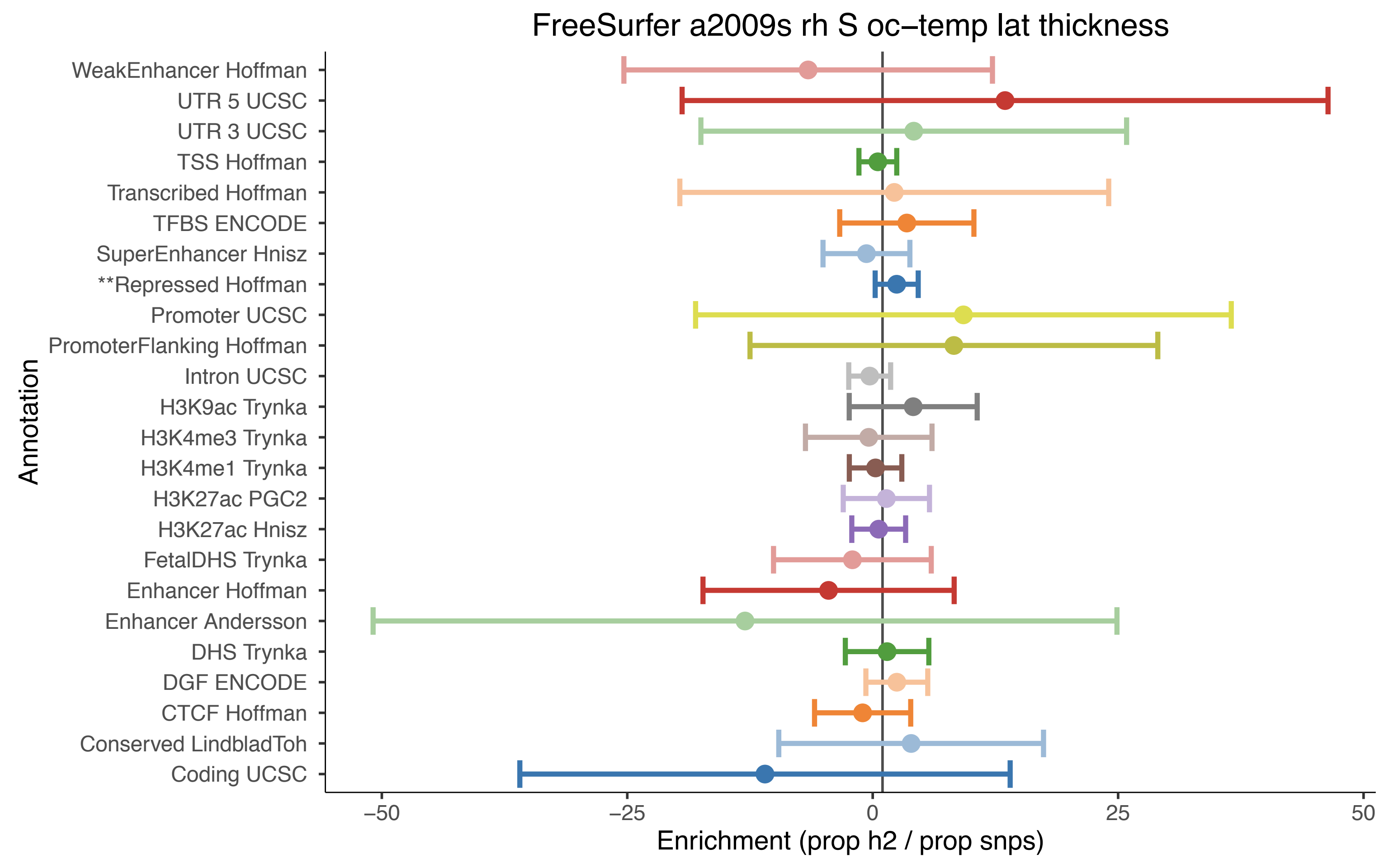
FreeSurfer a2009s rh S oc−temp lat thickness
WeakEnhancer Hoffman
UTR 5 UCSC
UTR 3 UCSC
TSS Hoffman
Transcribed Hoffman
TFBS ENCODE
SuperEnhancer Hnisz
**Repressed Hoffman
Promoter UCSC
PromoterFlanking Hoffman
Intron UCSC
H3K9ac Trynka
H3K4me3 Trynka
H3K4me1 Trynka
H3K27ac PGC2
H3K27ac Hnisz
FetalDHS Trynka
Enhancer Hoffman
Enhancer Andersson
DHS Trynka
DGF ENCODE
CTCF Hoffman
Conserved LindbladToh
Coding UCSC
Annotation
−50
−25
0
25
50
Enrichment (prop h2 / prop snps)

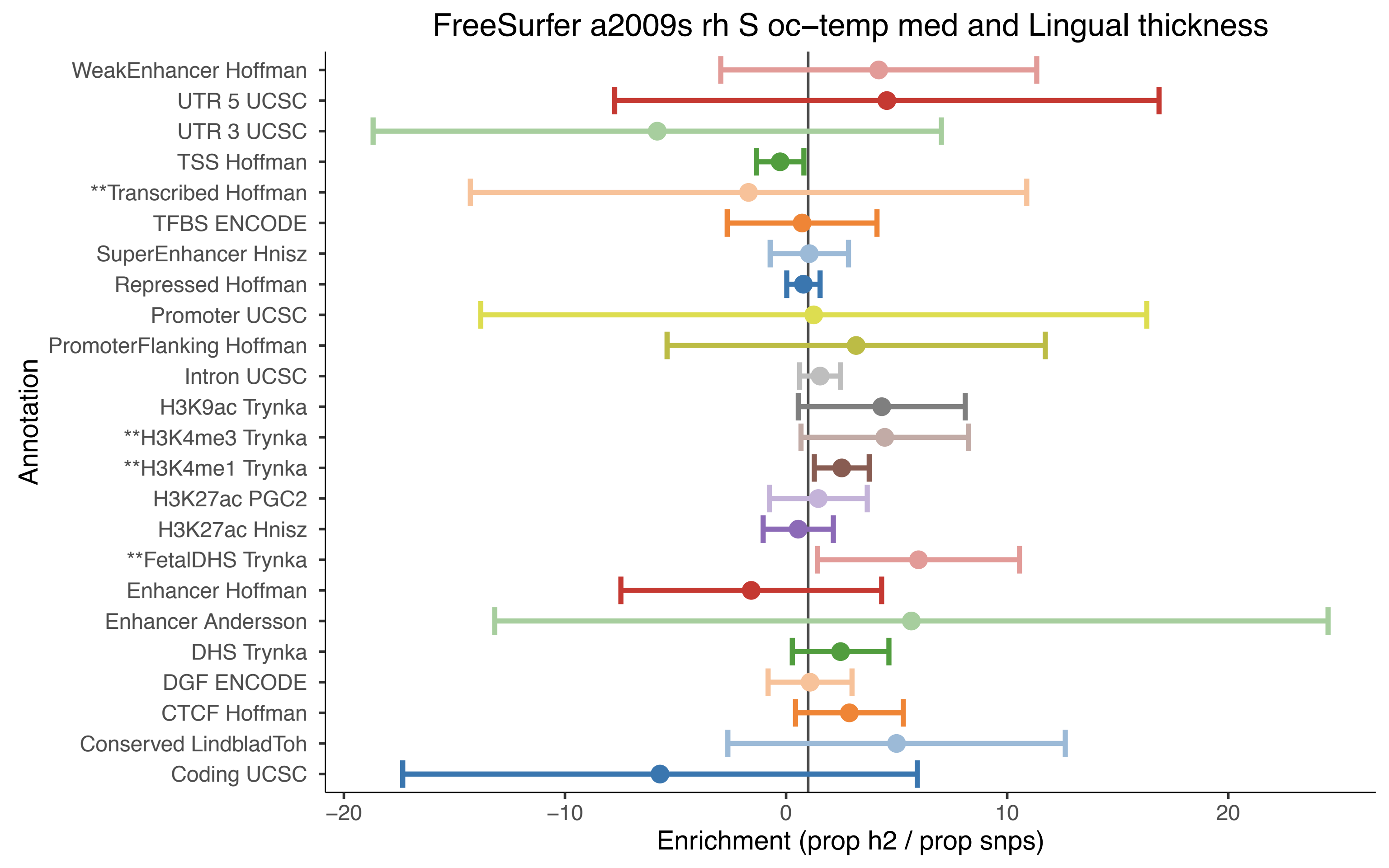
FreeSurfer a2009s rh S oc–temp med and Lingual thickness
WeakEnhancer Hoffman
UTR 5 UCSC
UTR 3 UCSC
TSS Hoffman
**Transcribed Hoffman
TFBS ENCODE
SuperEnhancer Hnisz
Repressed Hoffman
Promoter UCSC
PromoterFlanking Hoffman
Intron UCSC
H3K9ac Trynka
**H3K4me3 Trynka
**H3K4me1 Trynka
H3K27ac PGC2
H3K27ac Hnisz
**FetalDHS Trynka
Enhancer Hoffman
Enhancer Andersson
DHS Trynka
DGF ENCODE
CTCF Hoffman
Conserved LindbladToh
Coding UCSC
Annotation
−20
−10
0
10
20
Enrichment (prop h2 / prop snps)

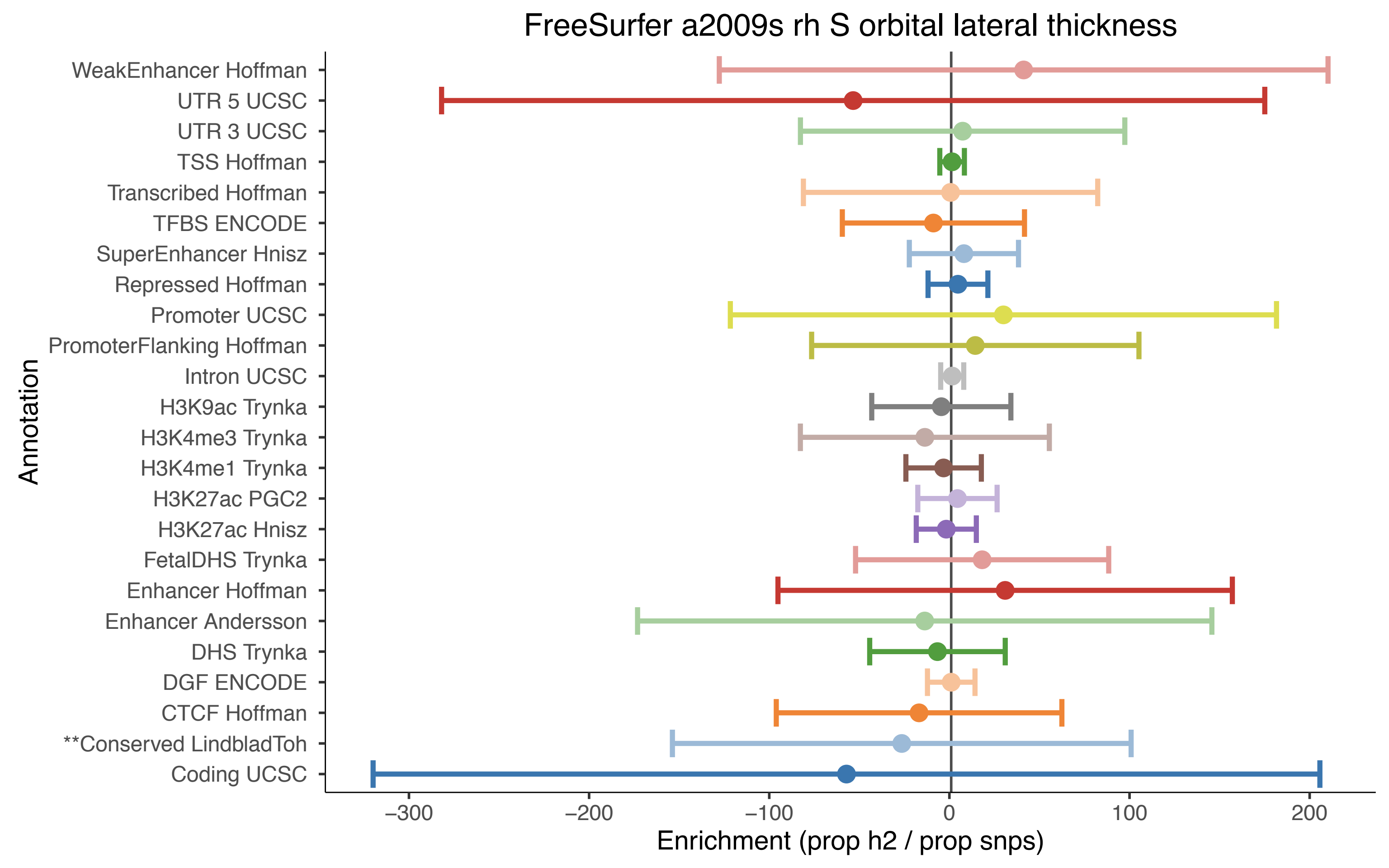

FreeSurfer a2009s rh S orbital lateral thickness
WeakEnhancer Hoffman
UTR 5 UCSC
UTR 3 UCSC
TSS Hoffman
Transcribed Hoffman
TFBS ENCODE
SuperEnhancer Hnisz
Repressed Hoffman
Promoter UCSC
PromoterFlanking Hoffman
Intron UCSC
H3K9ac Trynka
H3K4me3 Trynka
H3K4me1 Trynka
H3K27ac PGC2
H3K27ac Hnisz
FetalDHS Trynka
Enhancer Hoffman
Enhancer Andersson
DHS Trynka
DGF ENCODE
CTCF Hoffman
**Conserved LindbladToh
Coding UCSC
Annotation
−300
−200
−100
0
100
200
Enrichment (prop h2 / prop snps)

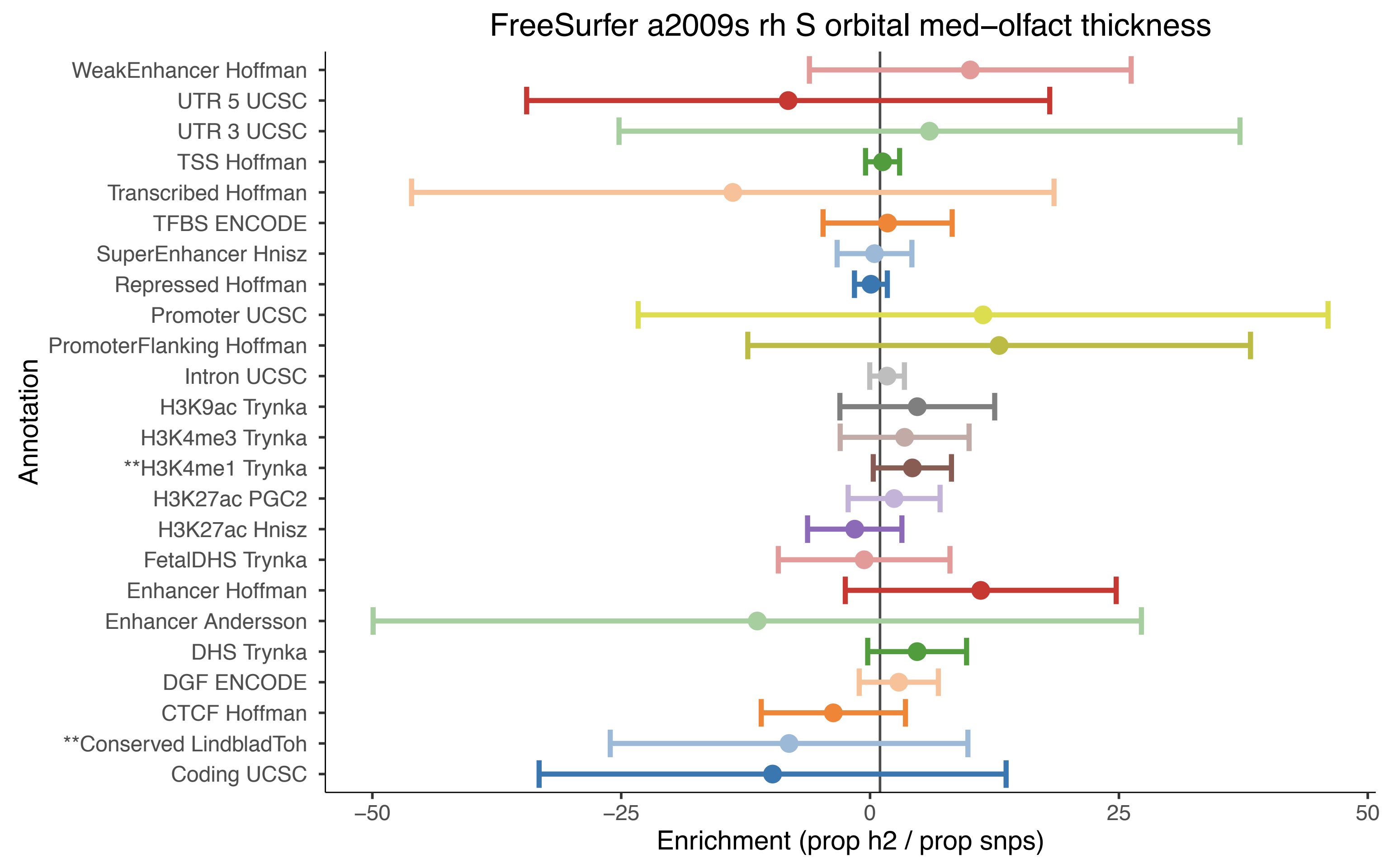

FreeSurfer a2009s rh S orbital med−olfact thickness
WeakEnhancer Hoffman
UTR 5 UCSC
UTR 3 UCSC
TSS Hoffman
Transcribed Hoffman
TFBS ENCODE
SuperEnhancer Hnisz
Repressed Hoffman
Promoter UCSC
PromoterFlanking Hoffman
Intron UCSC
H3K9ac Trynka
H3K4me3 Trynka
**H3K4me1 Trynka
H3K27ac PGC2
H3K27ac Hnisz
FetalDHS Trynka
Enhancer Hoffman
Enhancer Andersson
DHS Trynka
DGF ENCODE
CTCF Hoffman
**Conserved LindbladToh
Coding UCSC
Annotation
−50
−25
0
25
50
Enrichment (prop h2 / prop snps)

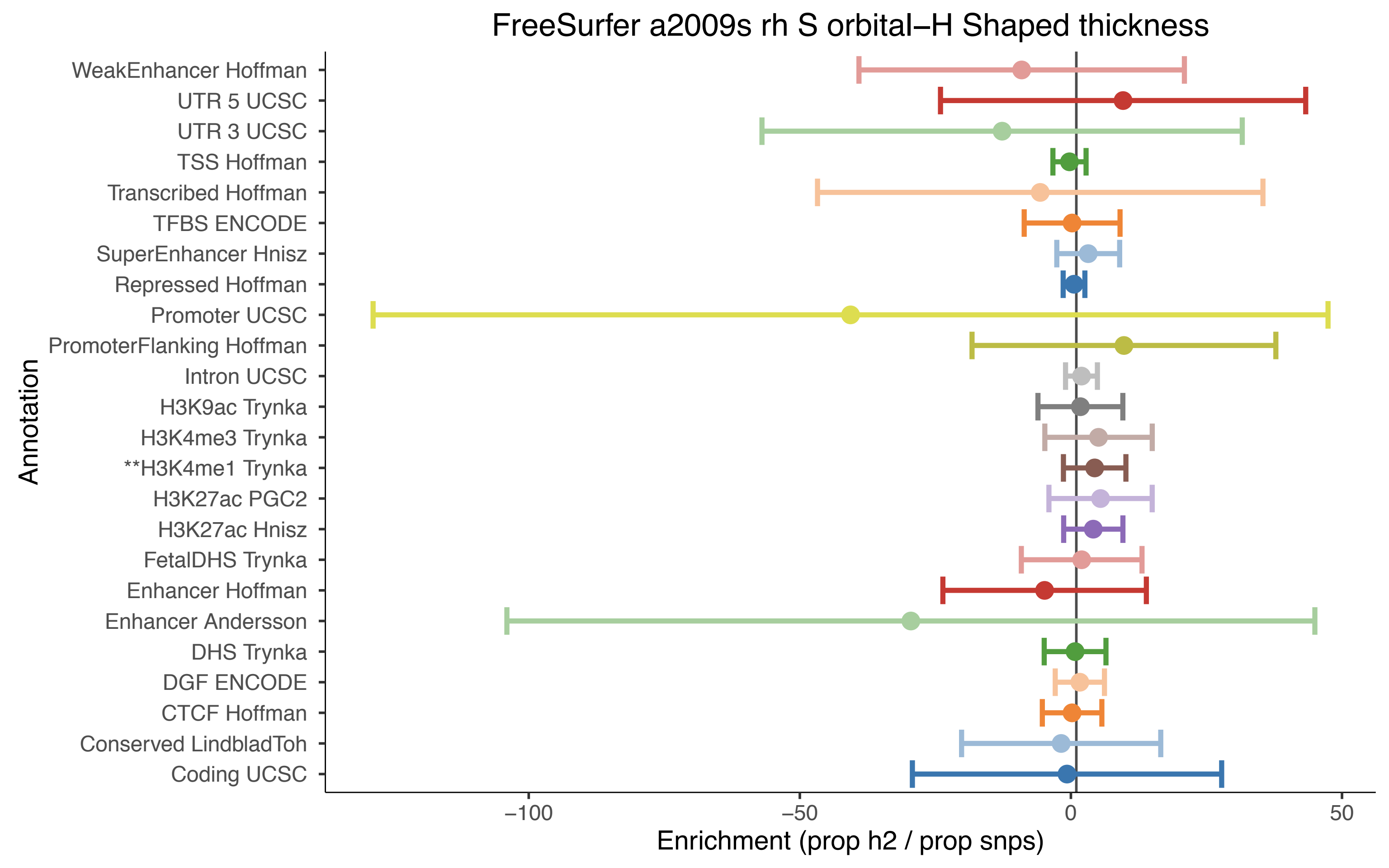

FreeSurfer a2009s rh S orbital–H Shaped thickness
WeakEnhancer Hoffman
UTR 5 UCSC
UTR 3 UCSC
TSS Hoffman
Transcribed Hoffman
TFBS ENCODE
SuperEnhancer Hnisz
Repressed Hoffman
Promoter UCSC
PromoterFlanking Hoffman
Intron UCSC
H3K9ac Trynka
H3K4me3 Trynka
**H3K4me1 Trynka
H3K27ac PGC2
H3K27ac Hnisz
FetalDHS Trynka
Enhancer Hoffman
Enhancer Andersson
DHS Trynka
DGF ENCODE
CTCF Hoffman
Conserved LindbladToh
Coding UCSC
Annotation
−100
−50
0
50
Enrichment (prop h2 / prop snps)

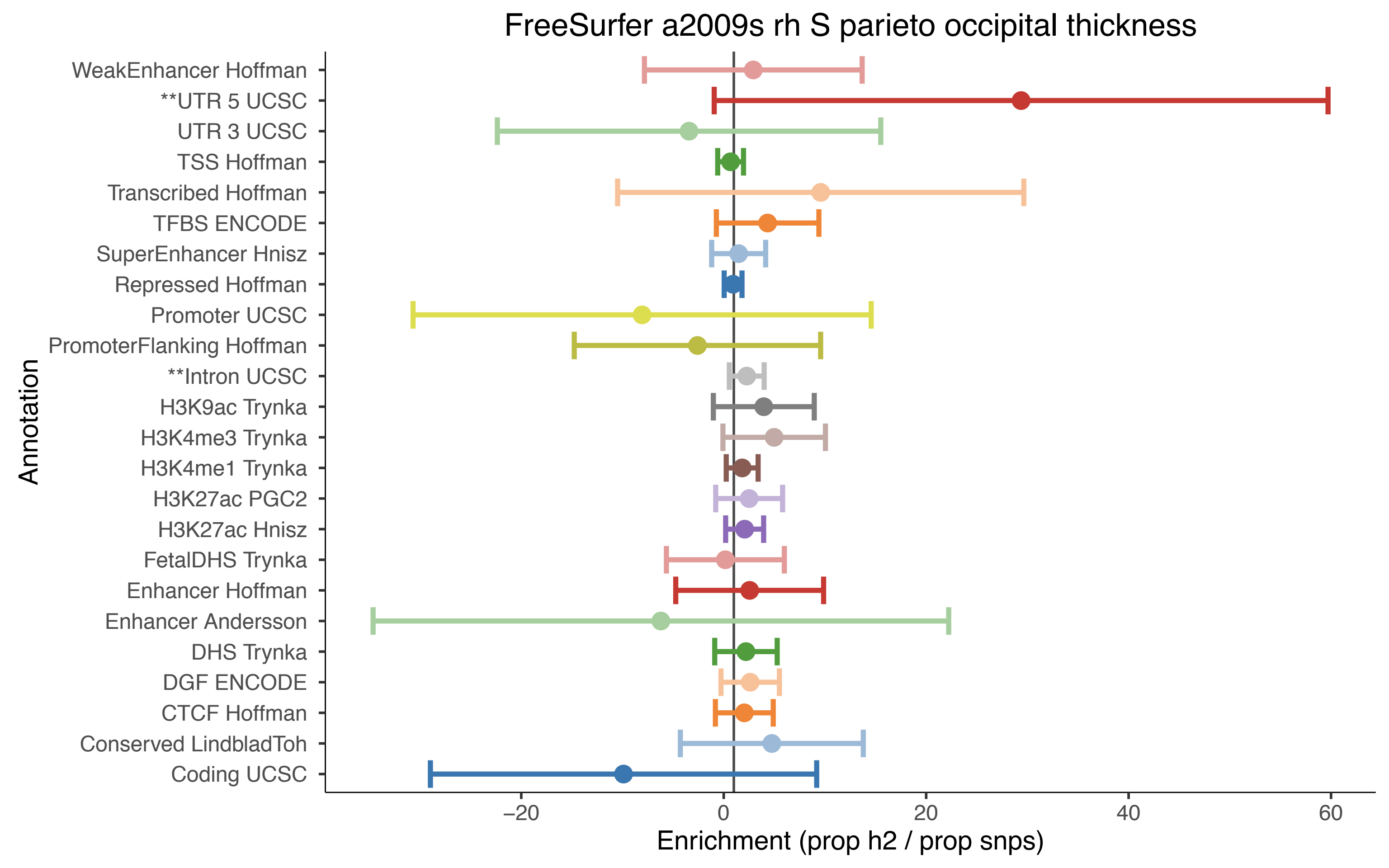

FreeSurfer a2009s rh S parieto occipital thickness
WeakEnhancer Hoffman
**UTR 5 UCSC
UTR 3 UCSC
TSS Hoffman
Transcribed Hoffman
TFBS ENCODE
SuperEnhancer Hnisz
Repressed Hoffman
Promoter UCSC
PromoterFlanking Hoffman
**Intron UCSC
H3K9ac Trynka
H3K4me3 Trynka
H3K4me1 Trynka
H3K27ac PGC2
H3K27ac Hnisz
FetalDHS Trynka
Enhancer Hoffman
Enhancer Andersson
DHS Trynka
DGF ENCODE
CTCF Hoffman
Conserved LindbladToh
Coding UCSC
Annotation
−20
0
20
40
60
Enrichment (prop h2 / prop snps)

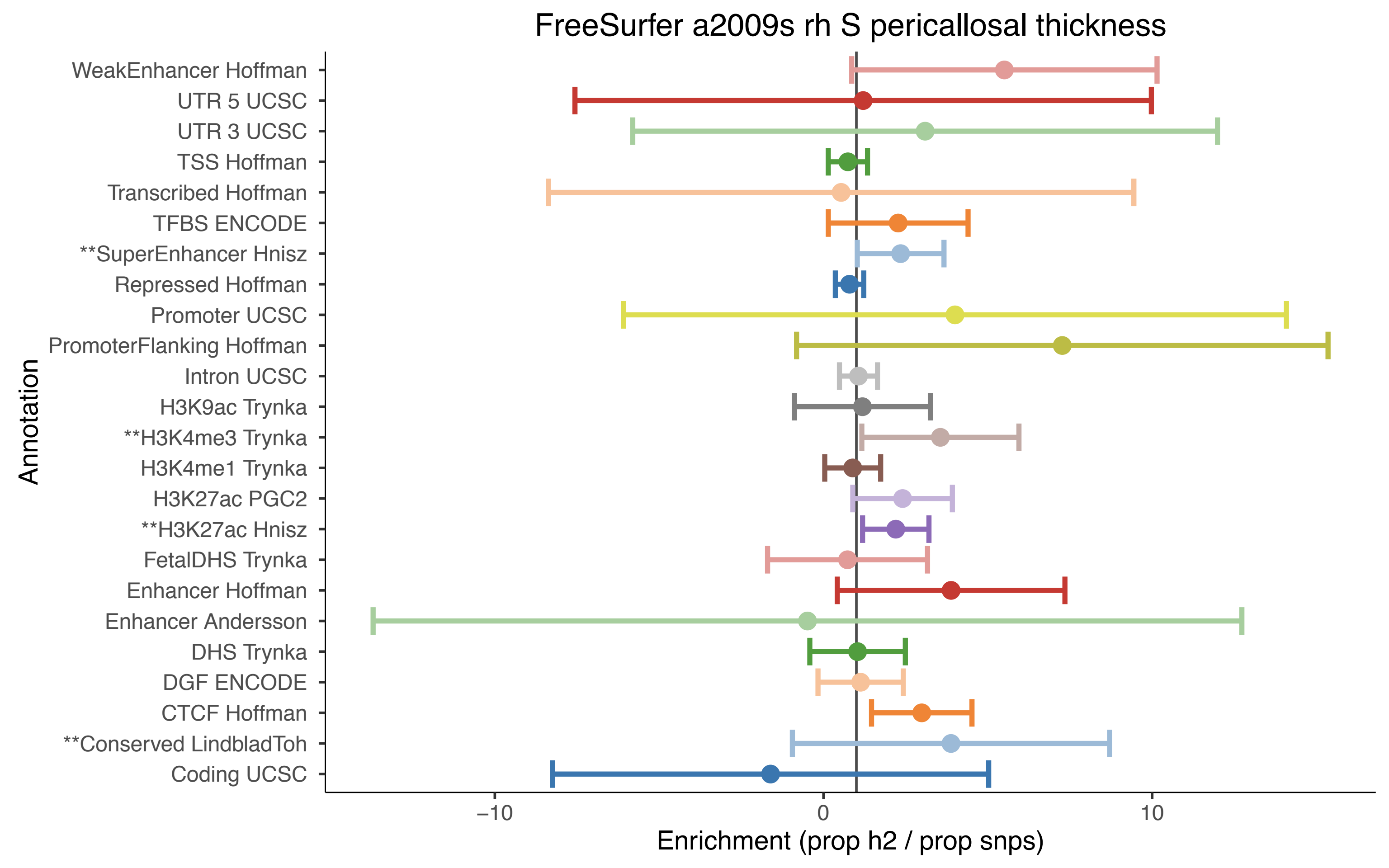
FreeSurfer a2009s rh S pericallosal thickness
Annotation
WeakEnhancer Hoffman
UTR 5 UCSC
UTR 3 UCSC
TSS Hoffman
Transcribed Hoffman
TFBS ENCODE
**SuperEnhancer Hnisz
Repressed Hoffman
Promoter UCSC
PromoterFlanking Hoffman
Intron UCSC
H3K9ac Trynka
**H3K4me3 Trynka
H3K4me1 Trynka
H3K27ac PGC2
**H3K27ac Hnisz
FetalDHS Trynka
Enhancer Hoffman
Enhancer Andersson
DHS Trynka
DGF ENCODE
CTCF Hoffman
**Conserved LindbladToh
Coding UCSC
−10
0
10
Enrichment (prop h2 / prop snps)

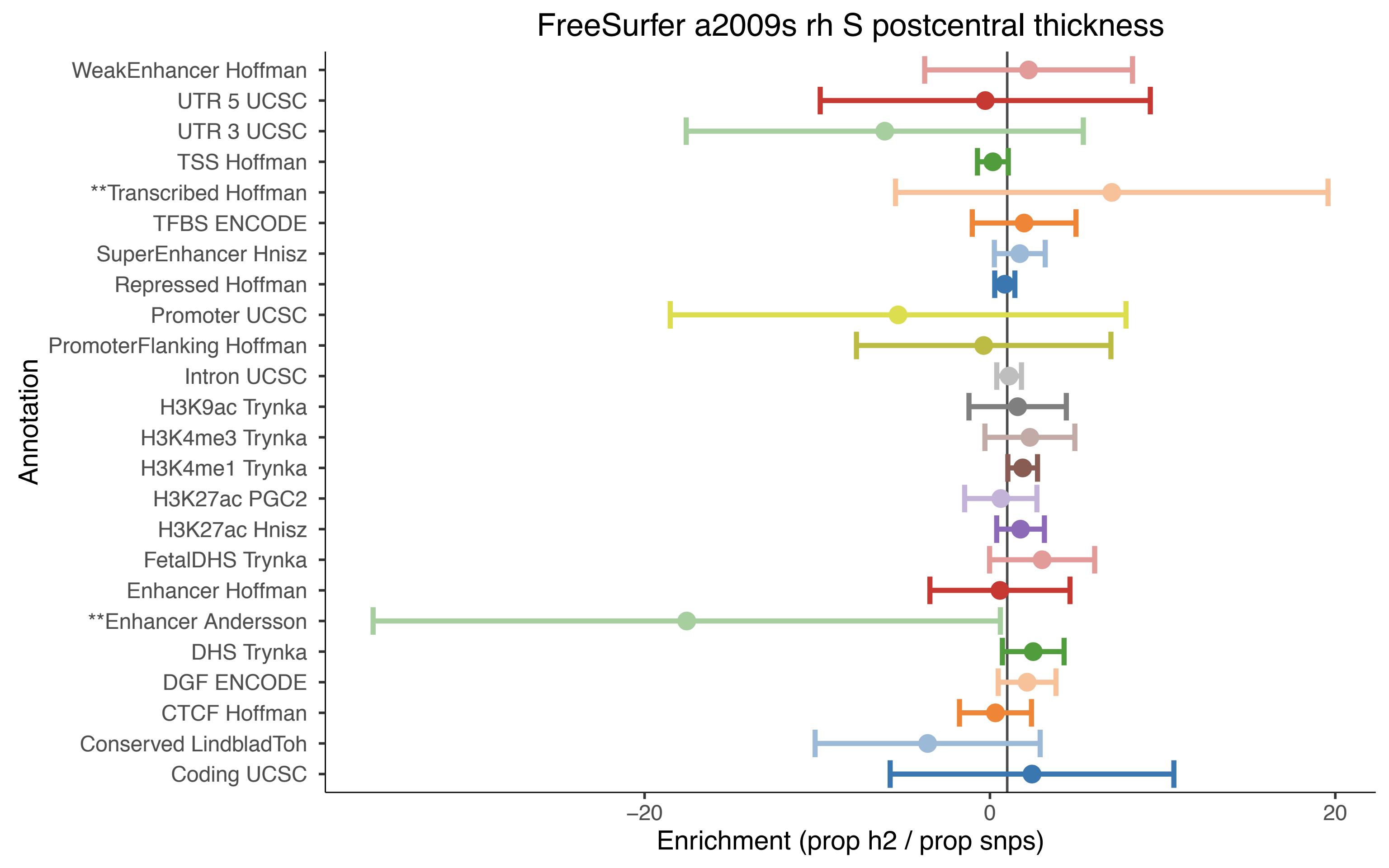

FreeSurfer a2009s rh S postcentral thickness
WeakEnhancer Hoffman
UTR 5 UCSC
UTR 3 UCSC
TSS Hoffman
**Transcribed Hoffman
TFBS ENCODE
SuperEnhancer Hnisz
Repressed Hoffman
Promoter UCSC
PromoterFlanking Hoffman
Intron UCSC
H3K9ac Trynka
H3K4me3 Trynka
H3K4me1 Trynka
H3K27ac PGC2
H3K27ac Hnisz
FetalDHS Trynka
Enhancer Hoffman
**Enhancer Andersson
DHS Trynka
DGF ENCODE
CTCF Hoffman
Conserved LindbladToh
Coding UCSC
Annotation
−20
0
20
Enrichment (prop h2 / prop snps)

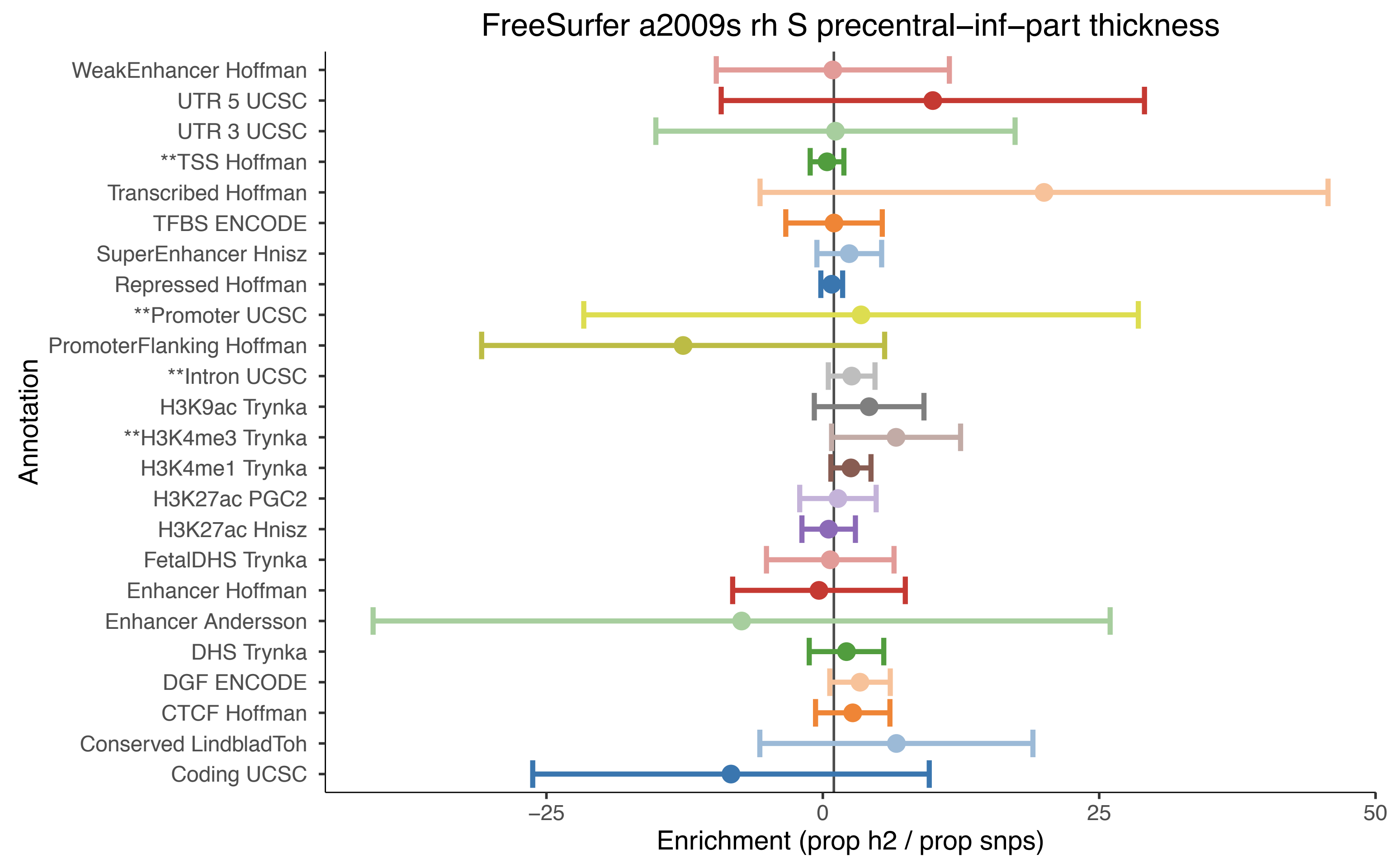
FreeSurfer a2009s rh S precentral−inf−part thickness
WeakEnhancer Hoffman
UTR 5 UCSC
UTR 3 UCSC
**TSS Hoffman
Transcribed Hoffman
TFBS ENCODE
SuperEnhancer Hnisz
Repressed Hoffman
**Promoter UCSC
PromoterFlanking Hoffman
**Intron UCSC
H3K9ac Trynka
**H3K4me3 Trynka
H3K4me1 Trynka
H3K27ac PGC2
H3K27ac Hnisz
FetalDHS Trynka
Enhancer Hoffman
Enhancer Andersson
DHS Trynka
DGF ENCODE
CTCF Hoffman
Conserved LindbladToh
Coding UCSC
Annotation
−25
0
25
50
Enrichment (prop h2 / prop snps)

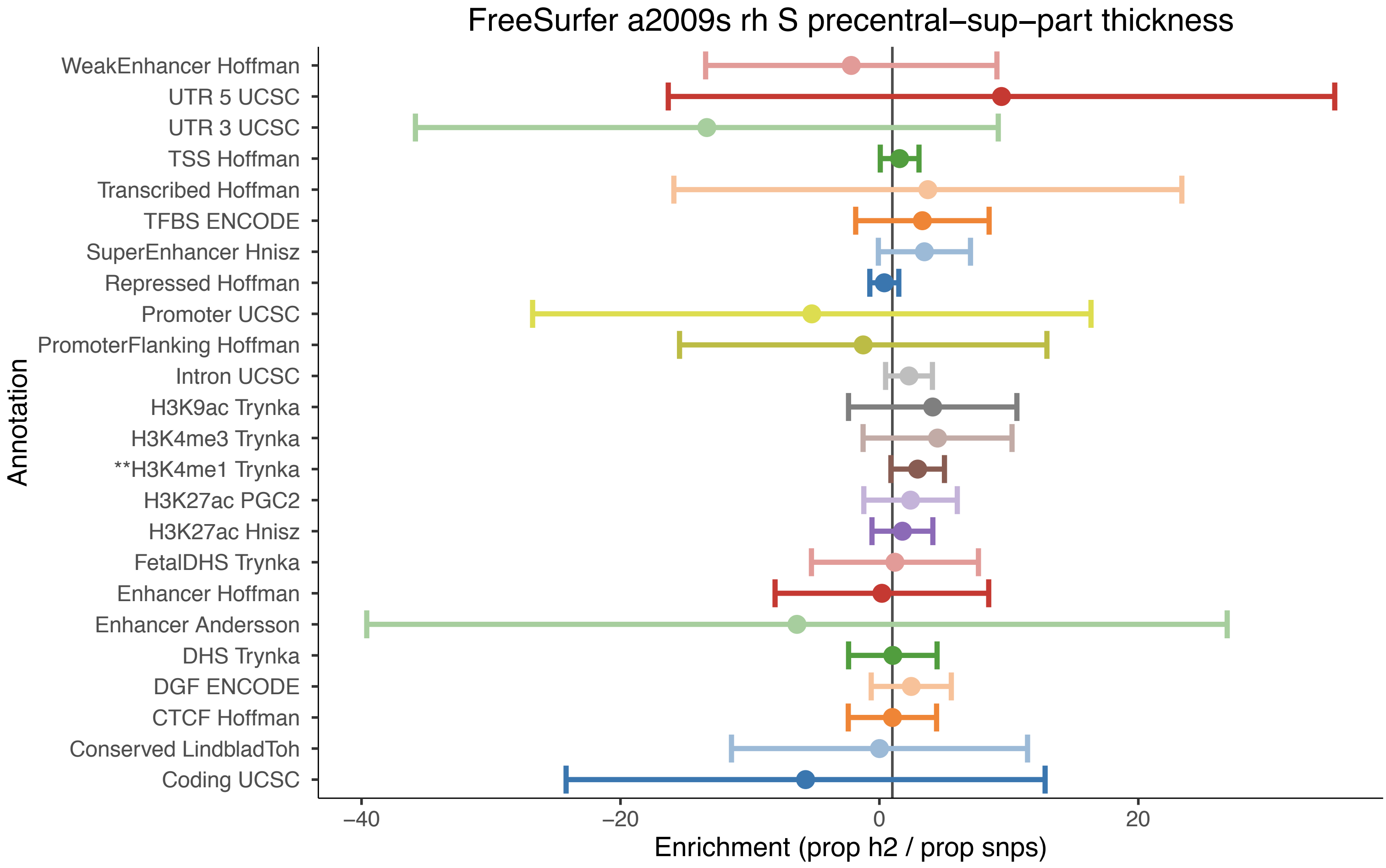
FreeSurfer a2009s rh S precentral−sup−part thickness
WeakEnhancer Hoffman
UTR 5 UCSC
UTR 3 UCSC
TSS Hoffman
Transcribed Hoffman
TFBS ENCODE
SuperEnhancer Hnisz
Repressed Hoffman
Promoter UCSC
PromoterFlanking Hoffman
Intron UCSC
H3K9ac Trynka
H3K4me3 Trynka
**H3K4me1 Trynka
H3K27ac PGC2
H3K27ac Hnisz
FetalDHS Trynka
Enhancer Hoffman
Enhancer Andersson
DHS Trynka
DGF ENCODE
CTCF Hoffman
Conserved LindbladToh
Coding UCSC
Annotation
−40
−20
0
20
Enrichment (prop h2 / prop snps)

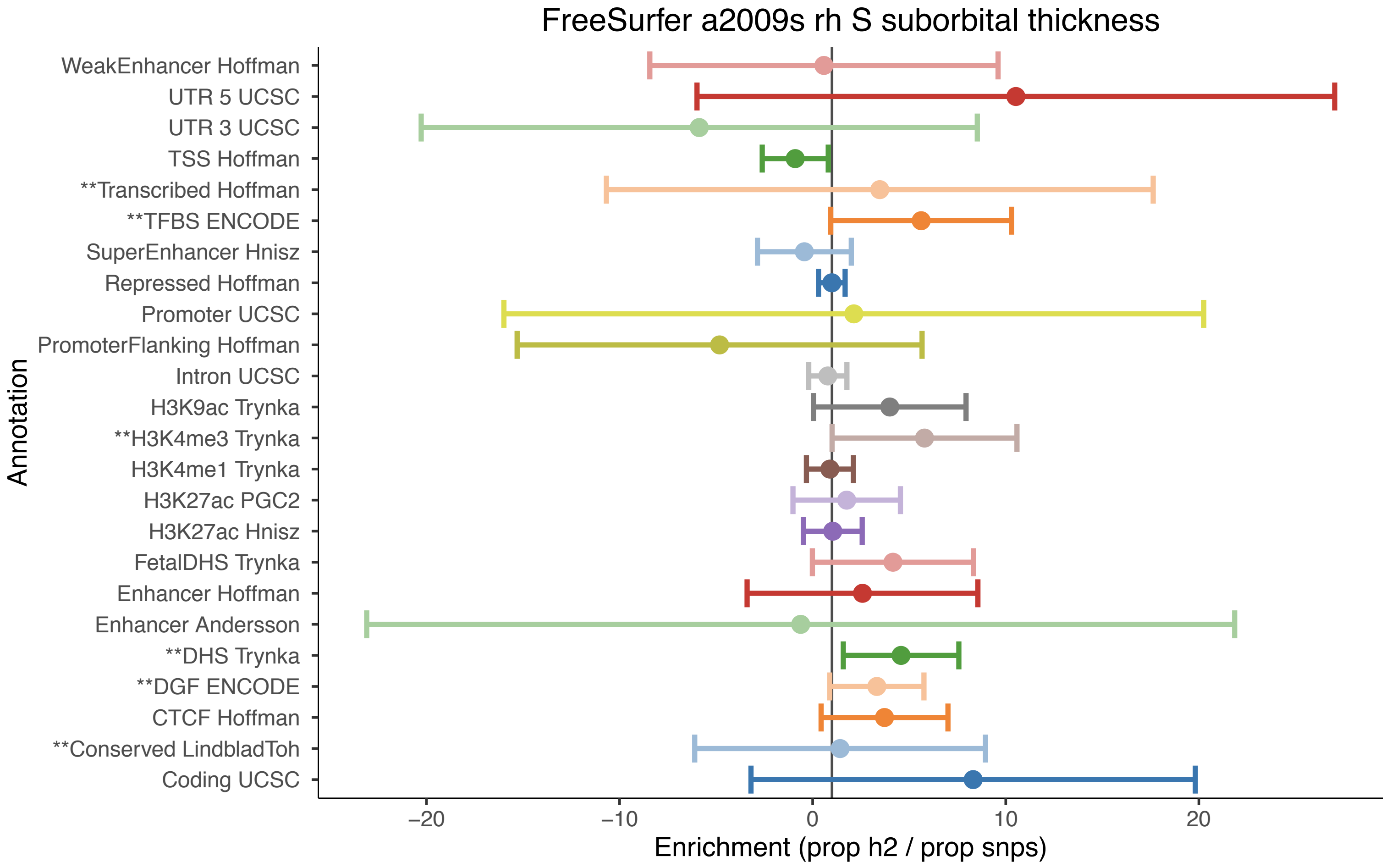

FreeSurfer a2009s rh S suborbital thickness
WeakEnhancer Hoffman
UTR 5 UCSC
UTR 3 UCSC
TSS Hoffman
**Transcribed Hoffman
**TFBS ENCODE
SuperEnhancer Hnisz
Repressed Hoffman
Promoter UCSC
PromoterFlanking Hoffman
Intron UCSC
H3K9ac Trynka
**H3K4me3 Trynka
H3K4me1 Trynka
H3K27ac PGC2
H3K27ac Hnisz
FetalDHS Trynka
Enhancer Hoffman
Enhancer Andersson
**DHS Trynka
**DGF ENCODE
CTCF Hoffman
**Conserved LindbladToh
Coding UCSC
Annotation
−20
−10
0
10
20
Enrichment (prop h2 / prop snps)

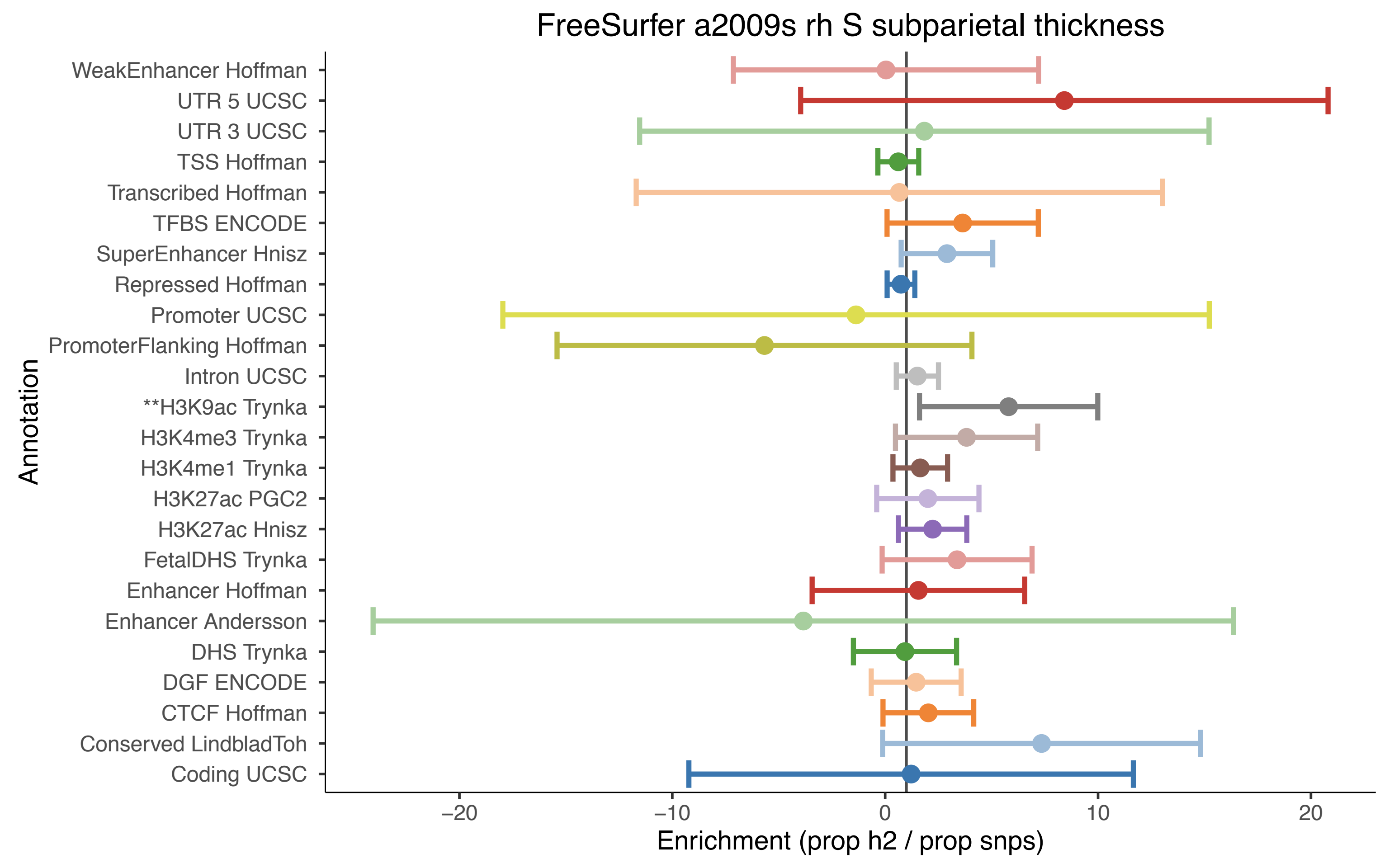
FreeSurfer a2009s rh S subparietal thickness
WeakEnhancer Hoffman
UTR 5 UCSC
UTR 3 UCSC
TSS Hoffman
Transcribed Hoffman
TFBS ENCODE
SuperEnhancer Hnisz
Repressed Hoffman
Promoter UCSC
PromoterFlanking Hoffman
Intron UCSC
**H3K9ac Trynka
H3K4me3 Trynka
H3K4me1 Trynka
H3K27ac PGC2
H3K27ac Hnisz
FetalDHS Trynka
Enhancer Hoffman
Enhancer Andersson
DHS Trynka
DGF ENCODE
CTCF Hoffman
Conserved LindbladToh
Coding UCSC
Annotation
−20
−10
0
10
20
Enrichment (prop h2 / prop snps)

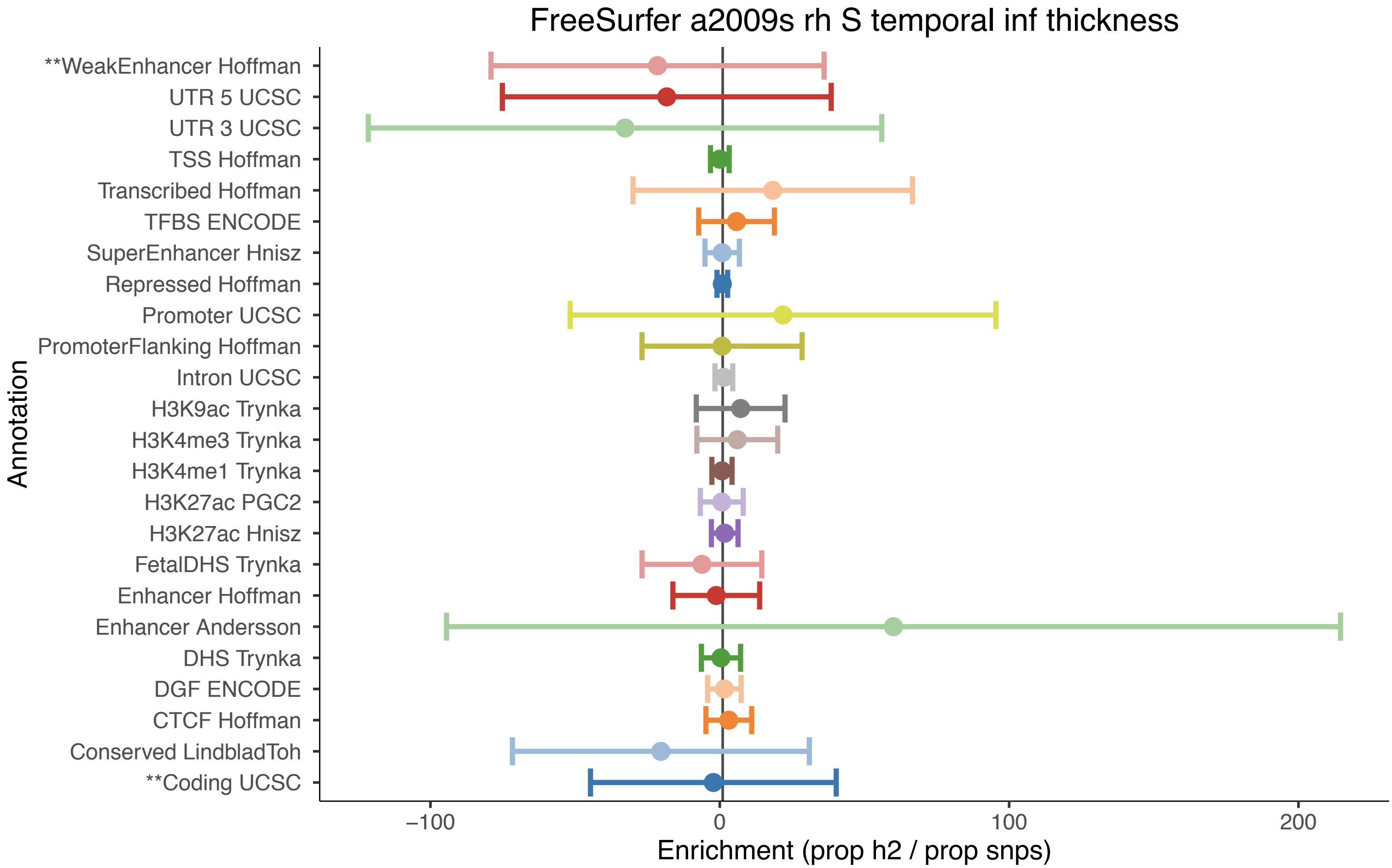
FreeSurfer a2009s rh S temporal inf thickness
**WeakEnhancer Hoffman
UTR 5 UCSC
UTR 3 UCSC
TSS Hoffman
Transcribed Hoffman
TFBS ENCODE
SuperEnhancer Hnisz
Repressed Hoffman
Promoter UCSC
PromoterFlanking Hoffman
Intron UCSC
H3K9ac Trynka
H3K4me3 Trynka
H3K4me1 Trynka
H3K27ac PGC2
H3K27ac Hnisz
FetalDHS Trynka
Enhancer Hoffman
Enhancer Andersson
DHS Trynka
DGF ENCODE
CTCF Hoffman
Conserved LindbladToh
**Coding UCSC
Annotation
−100
0
100
200
Enrichment (prop h2 / prop snps)

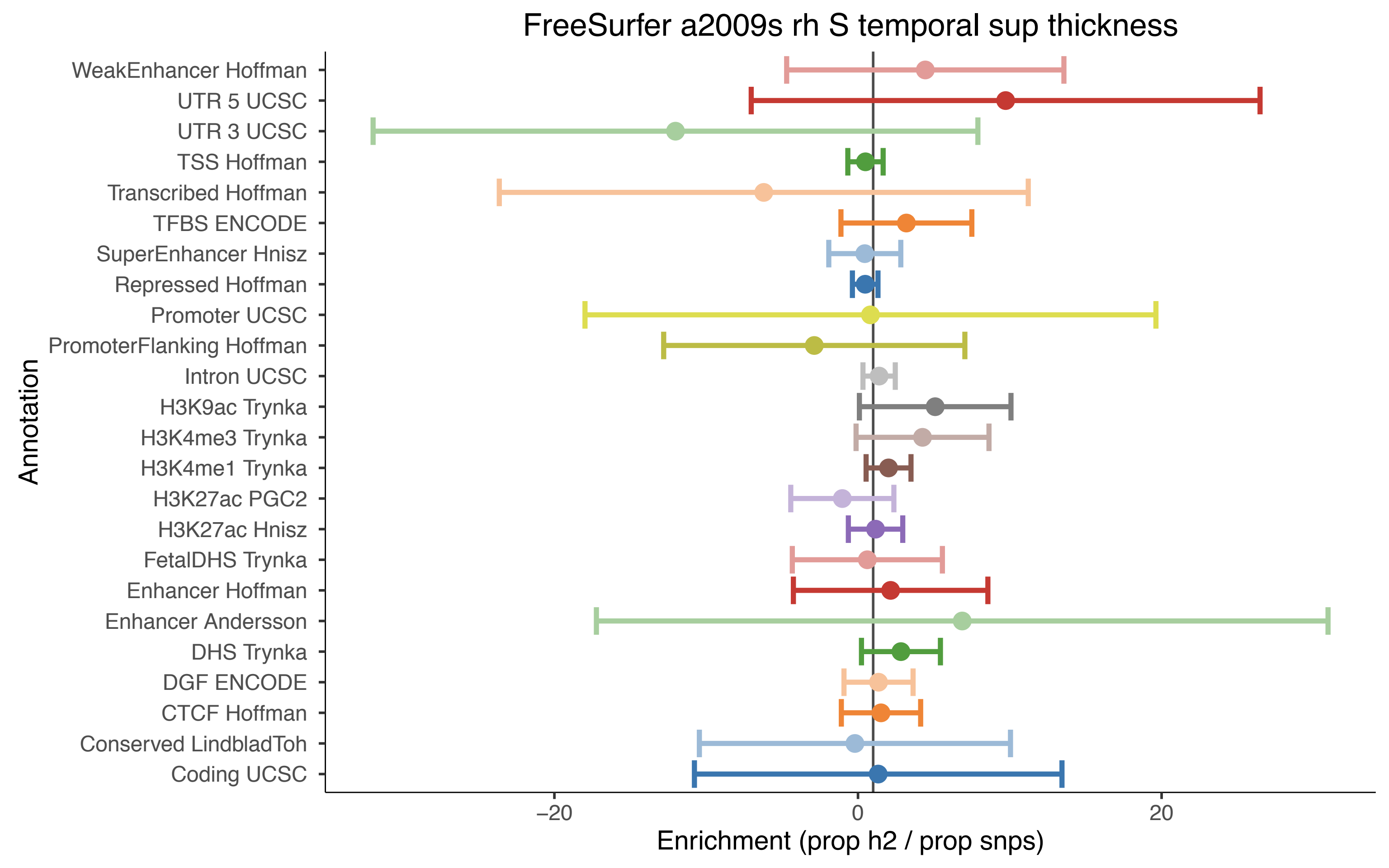

FreeSurfer a2009s rh S temporal sup thickness
WeakEnhancer Hoffman
UTR 5 UCSC
UTR 3 UCSC
TSS Hoffman
Transcribed Hoffman
TFBS ENCODE
SuperEnhancer Hnisz
Repressed Hoffman
Promoter UCSC
PromoterFlanking Hoffman
Intron UCSC
H3K9ac Trynka
H3K4me3 Trynka
H3K4me1 Trynka
H3K27ac PGC2
H3K27ac Hnisz
FetalDHS Trynka
Enhancer Hoffman
Enhancer Andersson
DHS Trynka
DGF ENCODE
CTCF Hoffman
Conserved LindbladToh
Coding UCSC
Annotation
−20
0
20
Enrichment (prop h2 / prop snps)

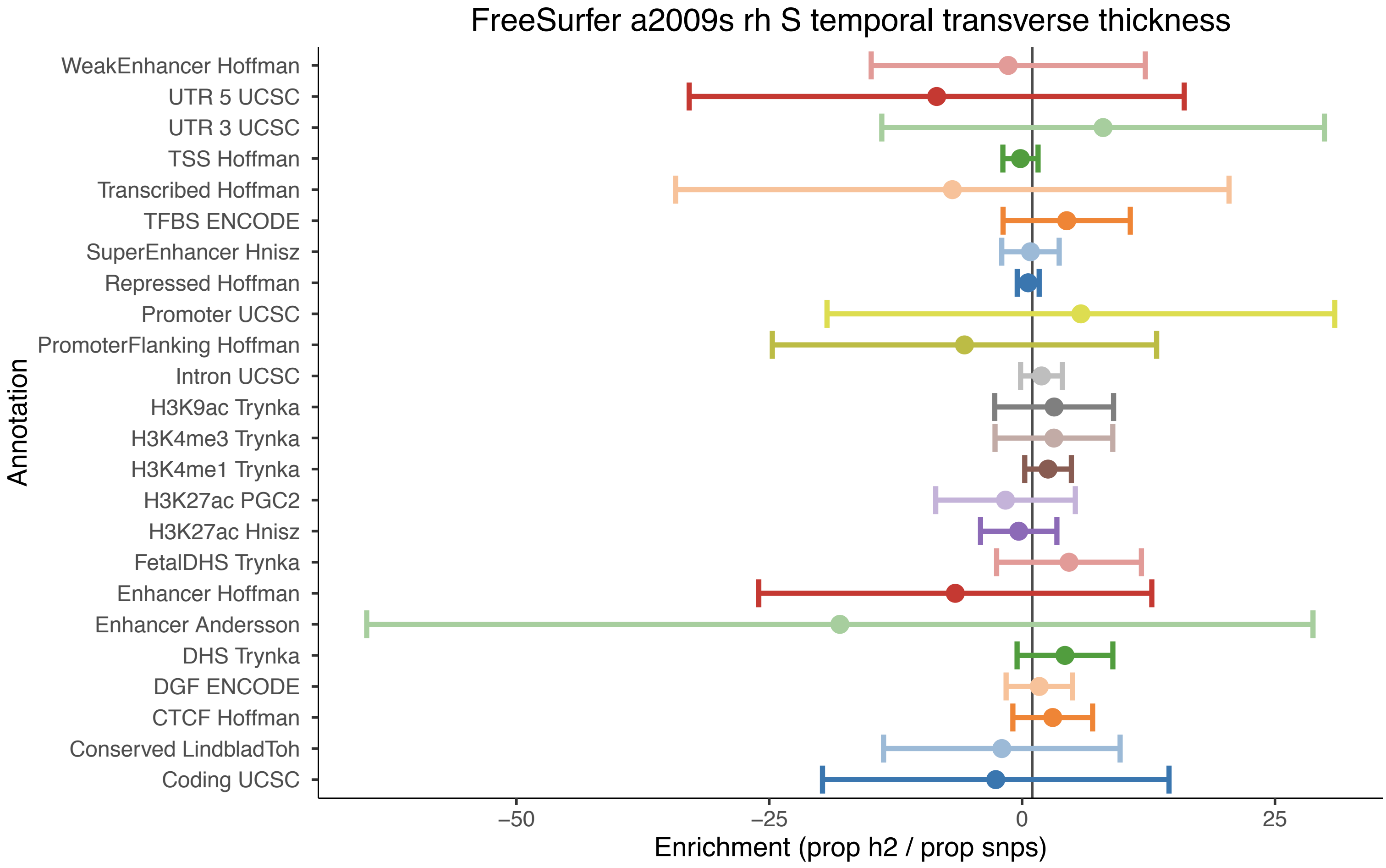
FreeSurfer a2009s rh S temporal transverse thickness
Annotation
WeakEnhancer Hoffman
UTR 5 UCSC
UTR 3 UCSC
TSS Hoffman
Transcribed Hoffman
TFBS ENCODE
SuperEnhancer Hnisz
Repressed Hoffman
Promoter UCSC
PromoterFlanking Hoffman
Intron UCSC
H3K9ac Trynka
H3K4me3 Trynka
H3K4me1 Trynka
H3K27ac PGC2
H3K27ac Hnisz
FetalDHS Trynka
Enhancer Hoffman
Enhancer Andersson
DHS Trynka
DGF ENCODE
CTCF Hoffman
Conserved LindbladToh
Coding UCSC
−50
−25
0
25
Enrichment (prop h2 / prop snps)

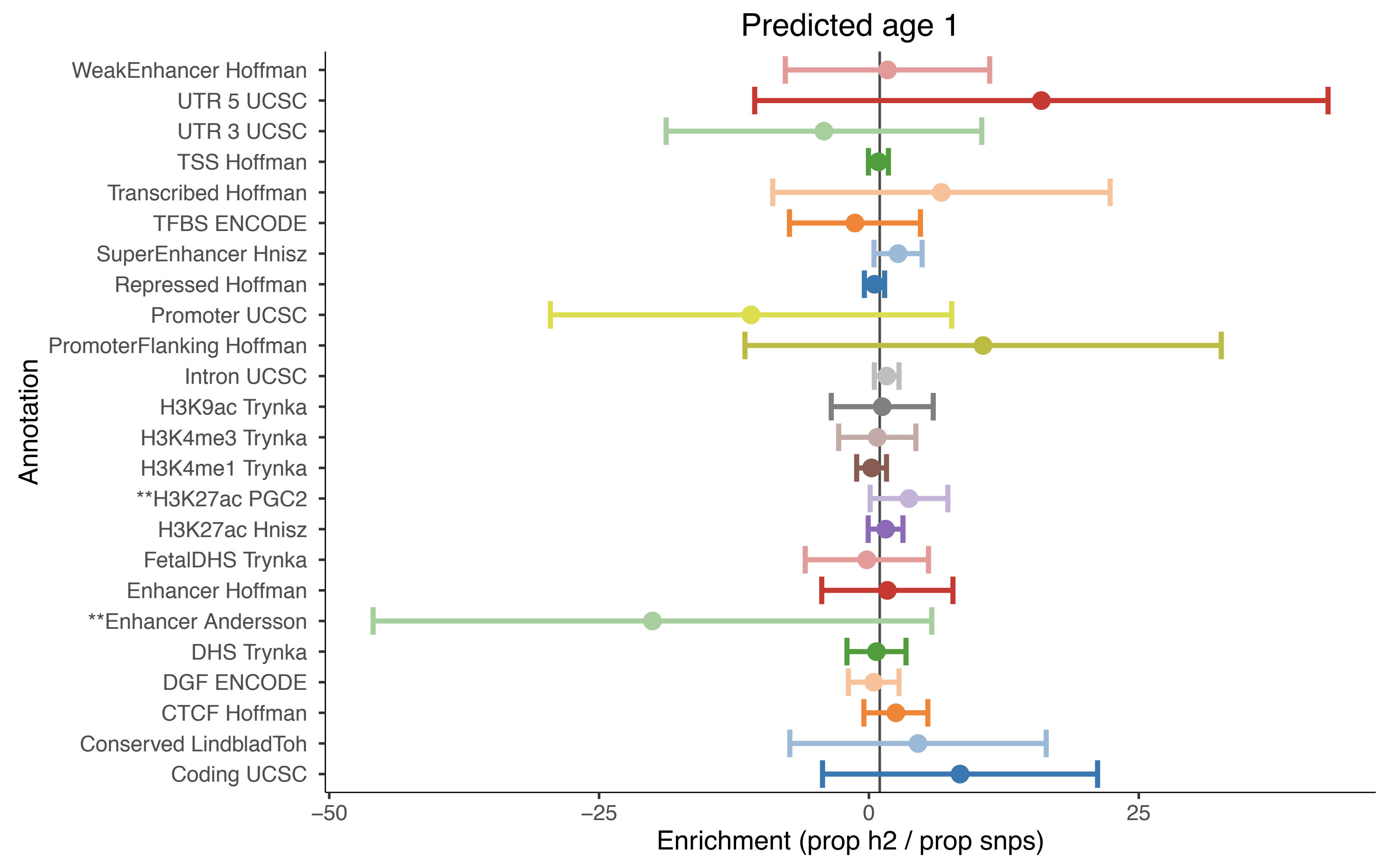

Predicted age 1
WeakEnhancer Hoffman
UTR 5 UCSC
UTR 3 UCSC
TSS Hoffman
Transcribed Hoffman
TFBS ENCODE
SuperEnhancer Hnisz
Repressed Hoffman
Promoter UCSC
PromoterFlanking Hoffman
Intron UCSC
H3K9ac Trynka
H3K4me3 Trynka
H3K4me1 Trynka
**H3K27ac PGC2
H3K27ac Hnisz
FetalDHS Trynka
Enhancer Hoffman
**Enhancer Andersson
DHS Trynka
DGF ENCODE
CTCF Hoffman
Conserved LindbladToh
Coding UCSC
Annotation
−50
−25
0
25
Enrichment (prop h2 / prop snps)

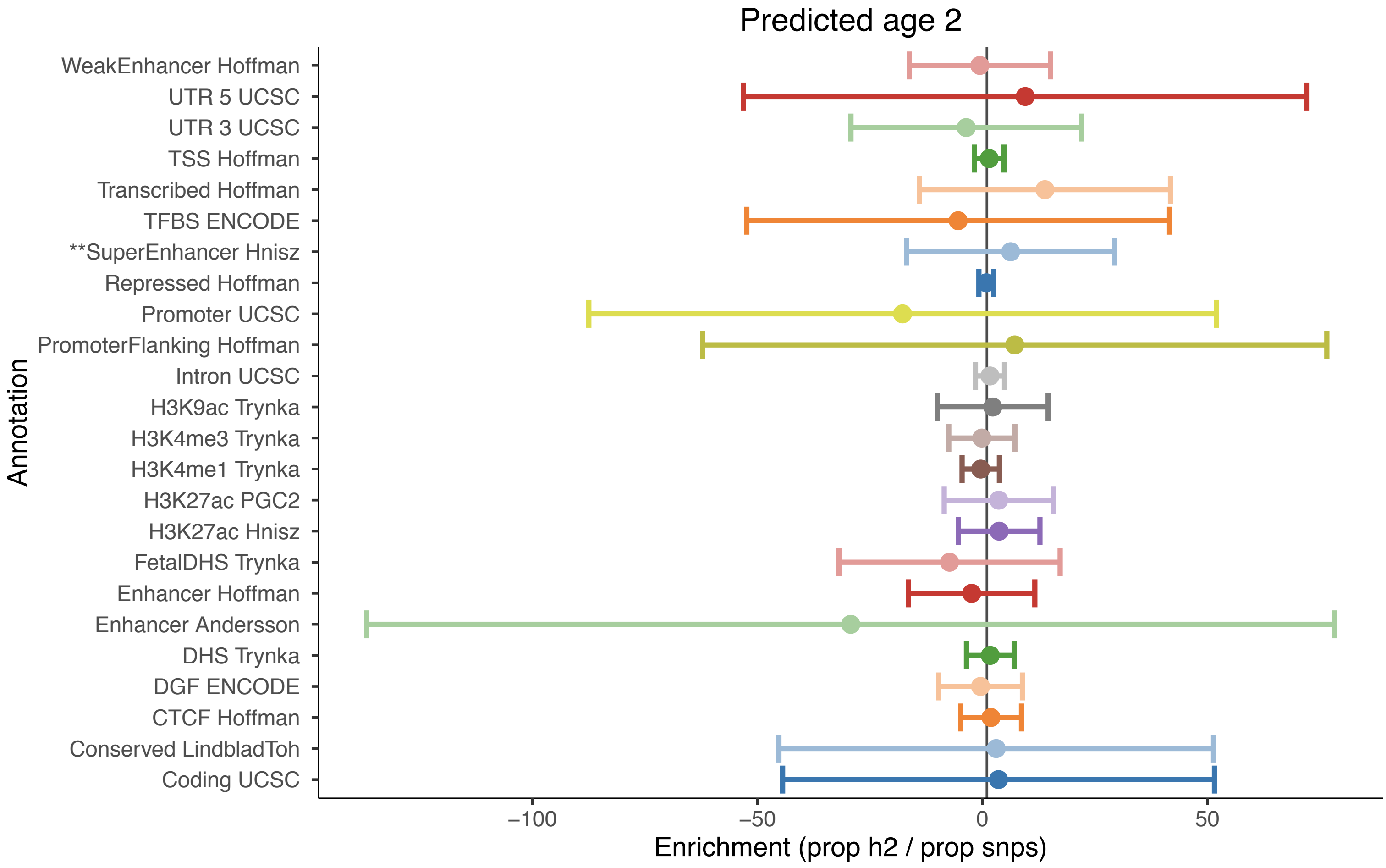
Predicted age 2
WeakEnhancer Hoffman
UTR 5 UCSC
UTR 3 UCSC
TSS Hoffman
Transcribed Hoffman
TFBS ENCODE
**SuperEnhancer Hnisz
Repressed Hoffman
Promoter UCSC
PromoterFlanking Hoffman
Intron UCSC
H3K9ac Trynka
H3K4me3 Trynka
H3K4me1 Trynka
H3K27ac PGC2
H3K27ac Hnisz
FetalDHS Trynka
Enhancer Hoffman
Enhancer Andersson
DHS Trynka
DGF ENCODE
CTCF Hoffman
Conserved LindbladToh
Coding UCSC
Annotation
−100
−50
0
50
Enrichment (prop h2 / prop snps)

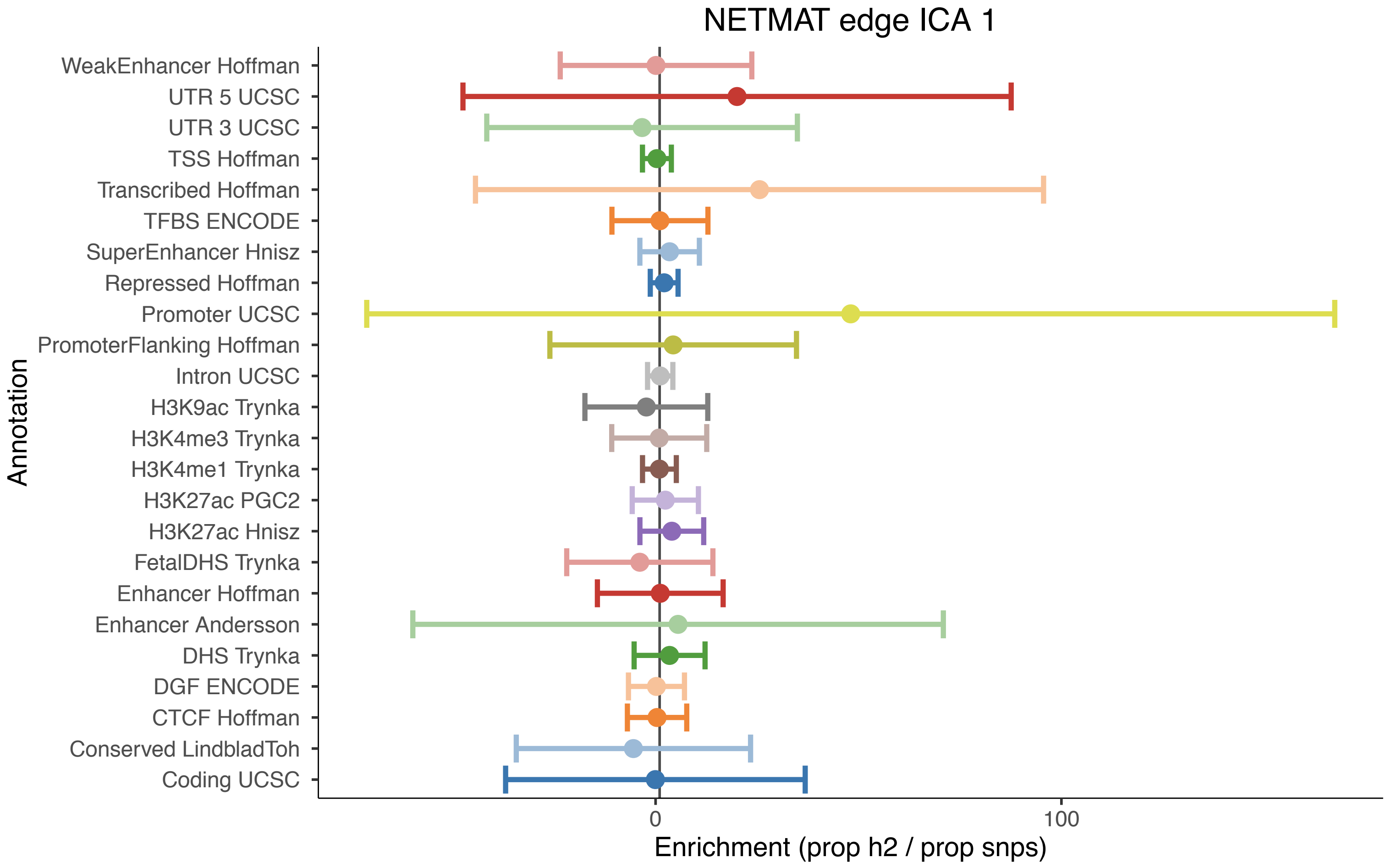

NETMAT edge ICA 1
WeakEnhancer Hoffman
UTR 5 UCSC
UTR 3 UCSC
TSS Hoffman
Transcribed Hoffman
TFBS ENCODE
SuperEnhancer Hnisz
Repressed Hoffman
Promoter UCSC
PromoterFlanking Hoffman
Intron UCSC
H3K9ac Trynka
H3K4me3 Trynka
H3K4me1 Trynka
H3K27ac PGC2
H3K27ac Hnisz
FetalDHS Trynka
Enhancer Hoffman
Enhancer Andersson
DHS Trynka
DGF ENCODE
CTCF Hoffman
Conserved LindbladToh
Coding UCSC
Annotation
0
100
Enrichment (prop h2 / prop snps)

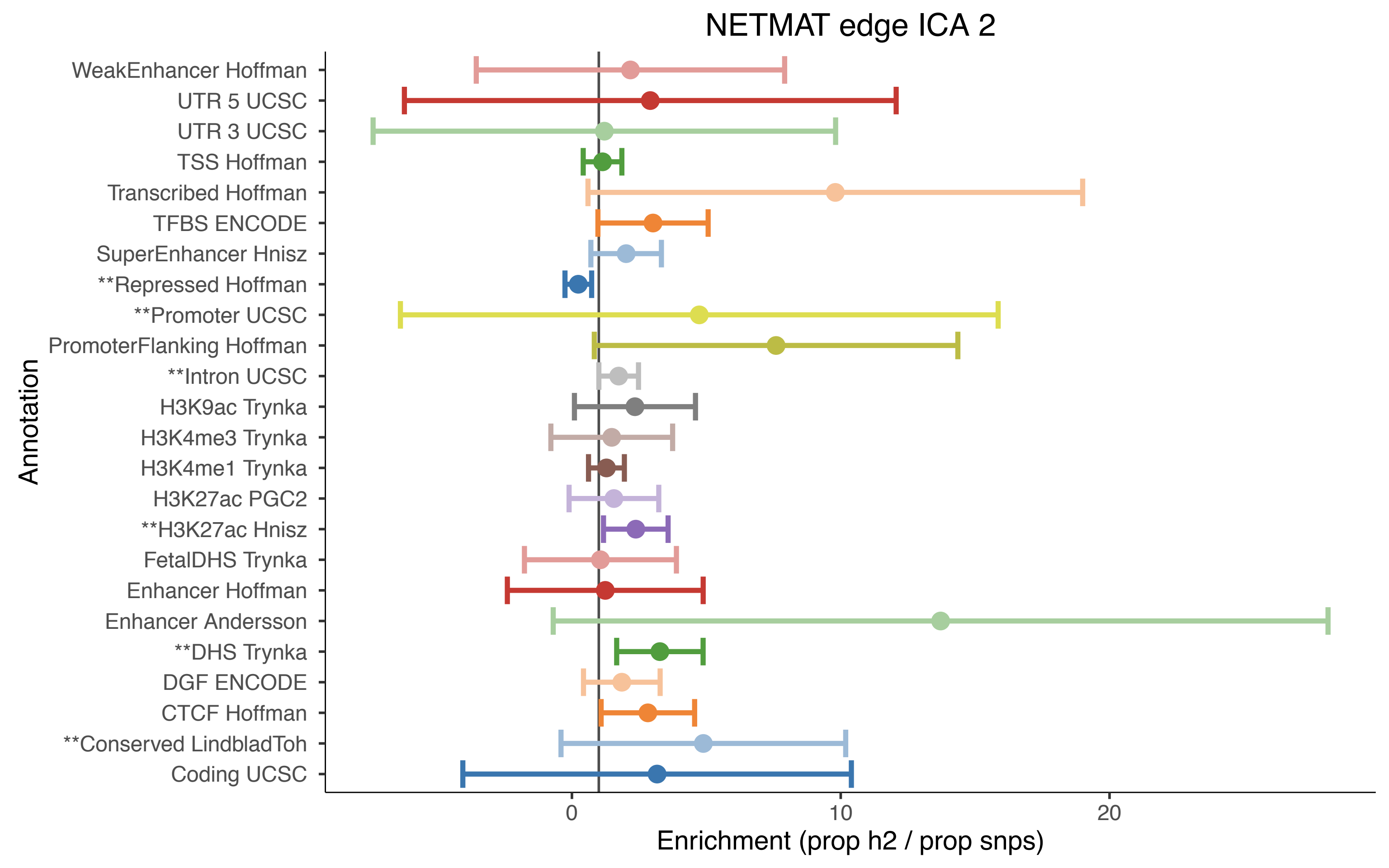
NETMAT edge ICA 2
WeakEnhancer Hoffman
UTR 5 UCSC
UTR 3 UCSC
TSS Hoffman
Transcribed Hoffman
TFBS ENCODE
SuperEnhancer Hnisz
**Repressed Hoffman
**Promoter UCSC
PromoterFlanking Hoffman
**Intron UCSC
H3K9ac Trynka
H3K4me3 Trynka
H3K4me1 Trynka
H3K27ac PGC2
**H3K27ac Hnisz
FetalDHS Trynka
Enhancer Hoffman
Enhancer Andersson
**DHS Trynka
DGF ENCODE
CTCF Hoffman
**Conserved LindbladToh
Coding UCSC
Annotation
0
10
20
Enrichment (prop h2 / prop snps)

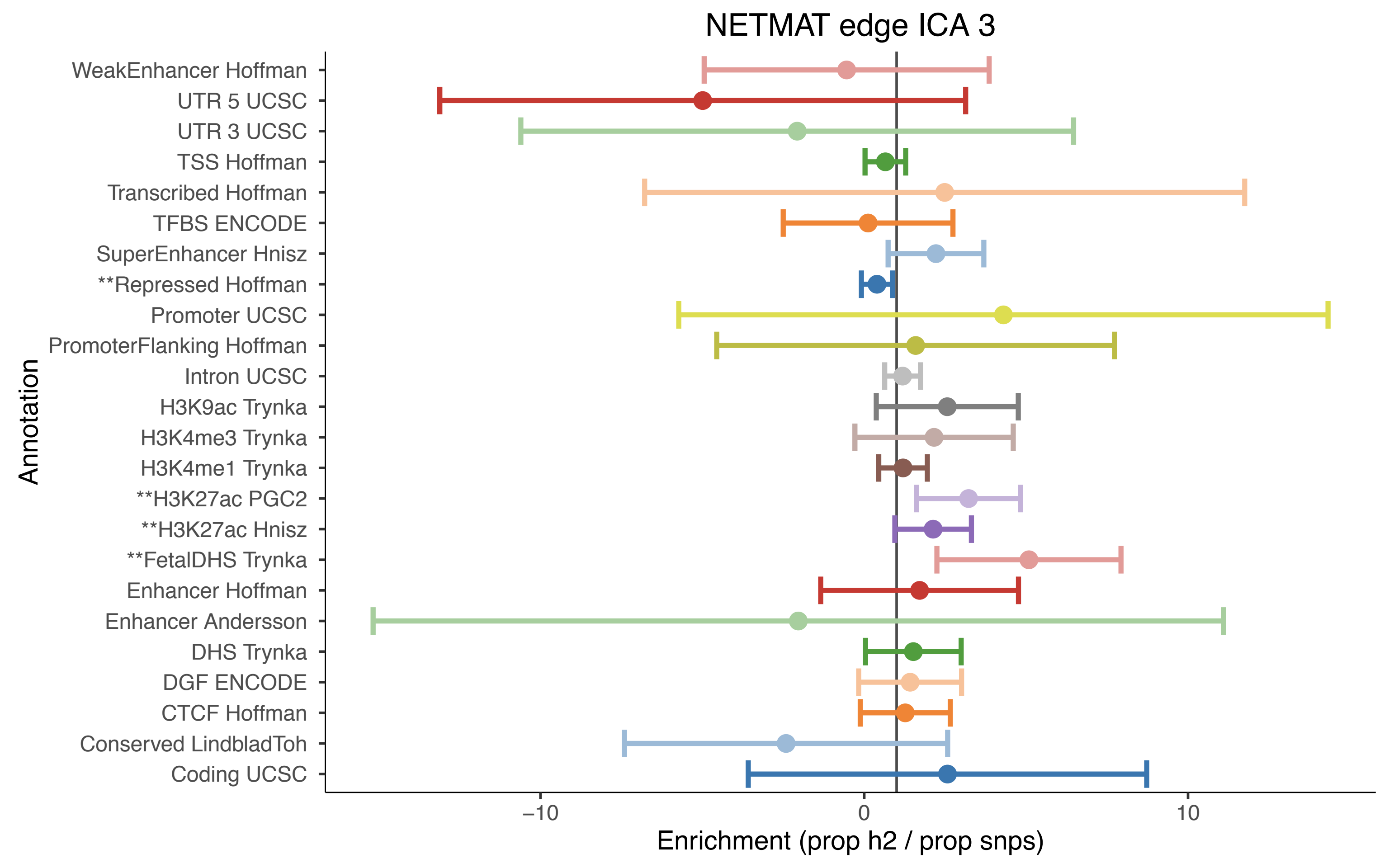
NETMAT edge ICA 3
WeakEnhancer Hoffman
UTR 5 UCSC
UTR 3 UCSC
TSS Hoffman
Transcribed Hoffman
TFBS ENCODE
SuperEnhancer Hnisz
**Repressed Hoffman
Promoter UCSC
PromoterFlanking Hoffman
Intron UCSC
H3K9ac Trynka
H3K4me3 Trynka
H3K4me1 Trynka
**H3K27ac PGC2
**H3K27ac Hnisz
**FetalDHS Trynka
Enhancer Hoffman
Enhancer Andersson
DHS Trynka
DGF ENCODE
CTCF Hoffman
Conserved LindbladToh
Coding UCSC
Annotation
−10
0
10
Enrichment (prop h2 / prop snps)

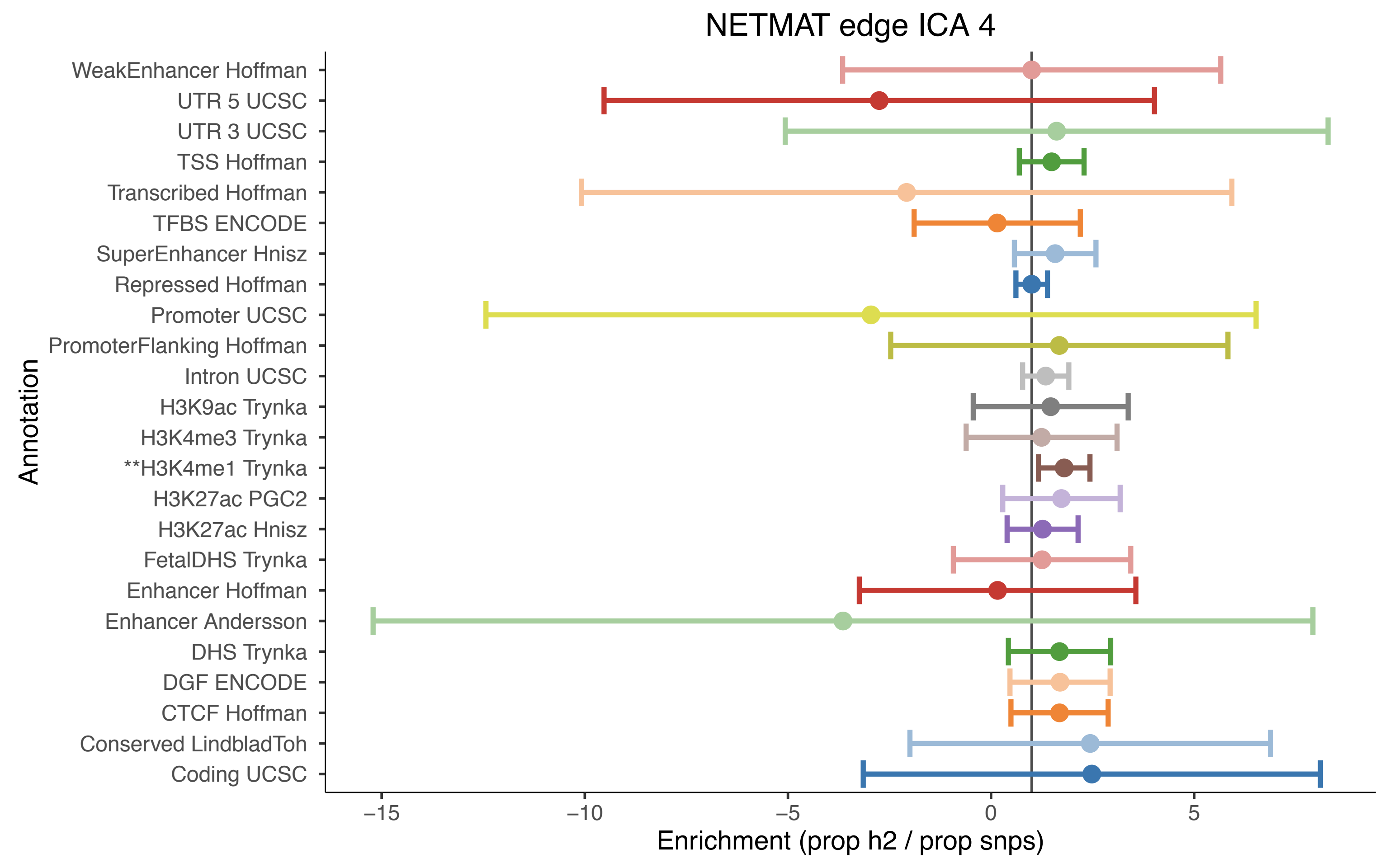
NETMAT edge ICA 4
WeakEnhancer Hoffman
UTR 5 UCSC
UTR 3 UCSC
TSS Hoffman
Transcribed Hoffman
TFBS ENCODE
SuperEnhancer Hnisz
Repressed Hoffman
Promoter UCSC
PromoterFlanking Hoffman
Intron UCSC
H3K9ac Trynka
H3K4me3 Trynka
**H3K4me1 Trynka
H3K27ac PGC2
H3K27ac Hnisz
FetalDHS Trynka
Enhancer Hoffman
Enhancer Andersson
DHS Trynka
DGF ENCODE
CTCF Hoffman
Conserved LindbladToh
Coding UCSC
Annotation
−15
−10
−5
0
5
Enrichment (prop h2 / prop snps)

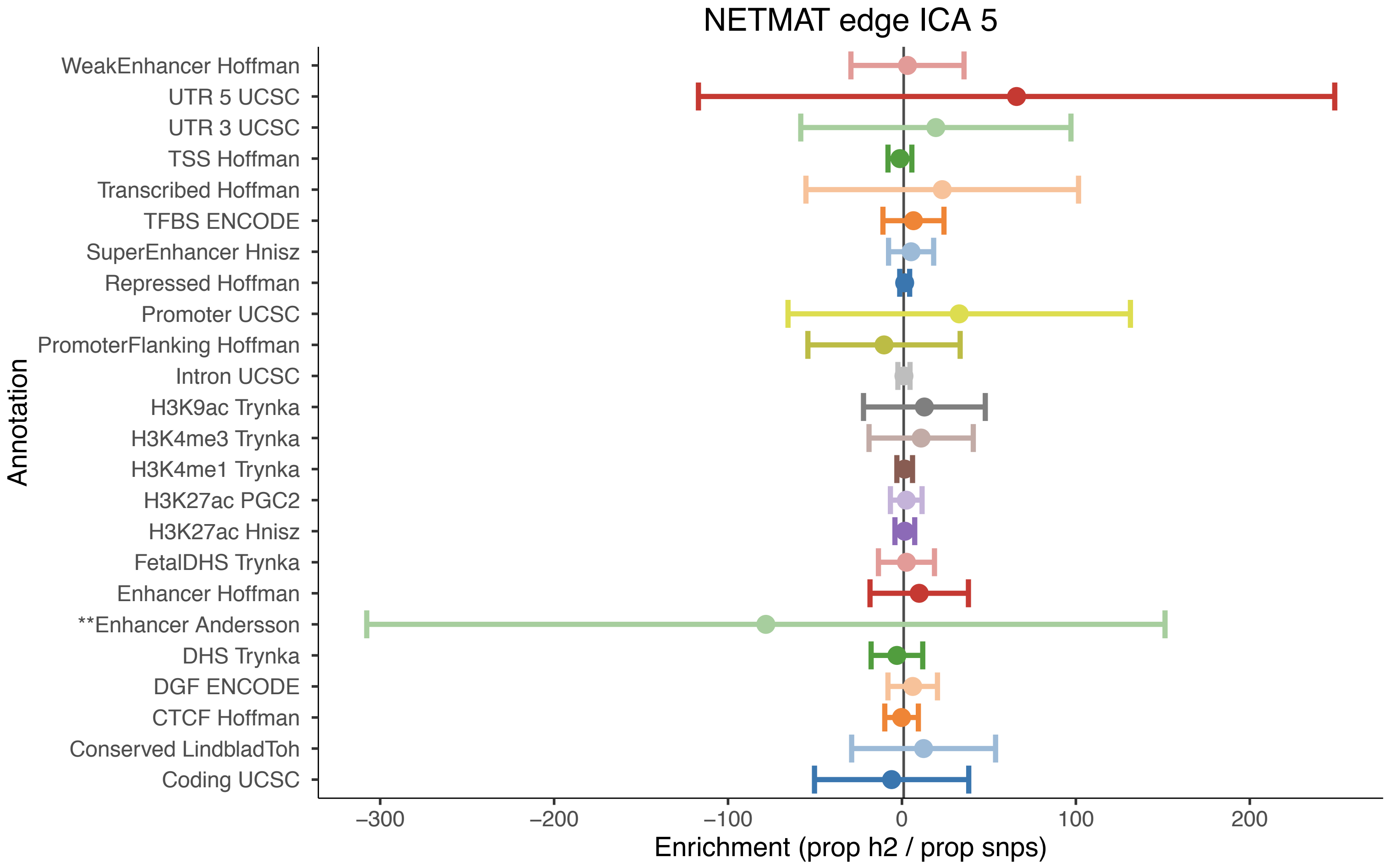
NETMAT edge ICA 5
WeakEnhancer Hoffman
UTR 5 UCSC
UTR 3 UCSC
TSS Hoffman
Transcribed Hoffman
TFBS ENCODE
SuperEnhancer Hnisz
Repressed Hoffman
Promoter UCSC
PromoterFlanking Hoffman
Intron UCSC
H3K9ac Trynka
H3K4me3 Trynka
H3K4me1 Trynka
H3K27ac PGC2
H3K27ac Hnisz
FetalDHS Trynka
Enhancer Hoffman
**Enhancer Andersson
DHS Trynka
DGF ENCODE
CTCF Hoffman
Conserved LindbladToh
Coding UCSC
Annotation
−300
−200
−100
0
100
200
Enrichment (prop h2 / prop snps)

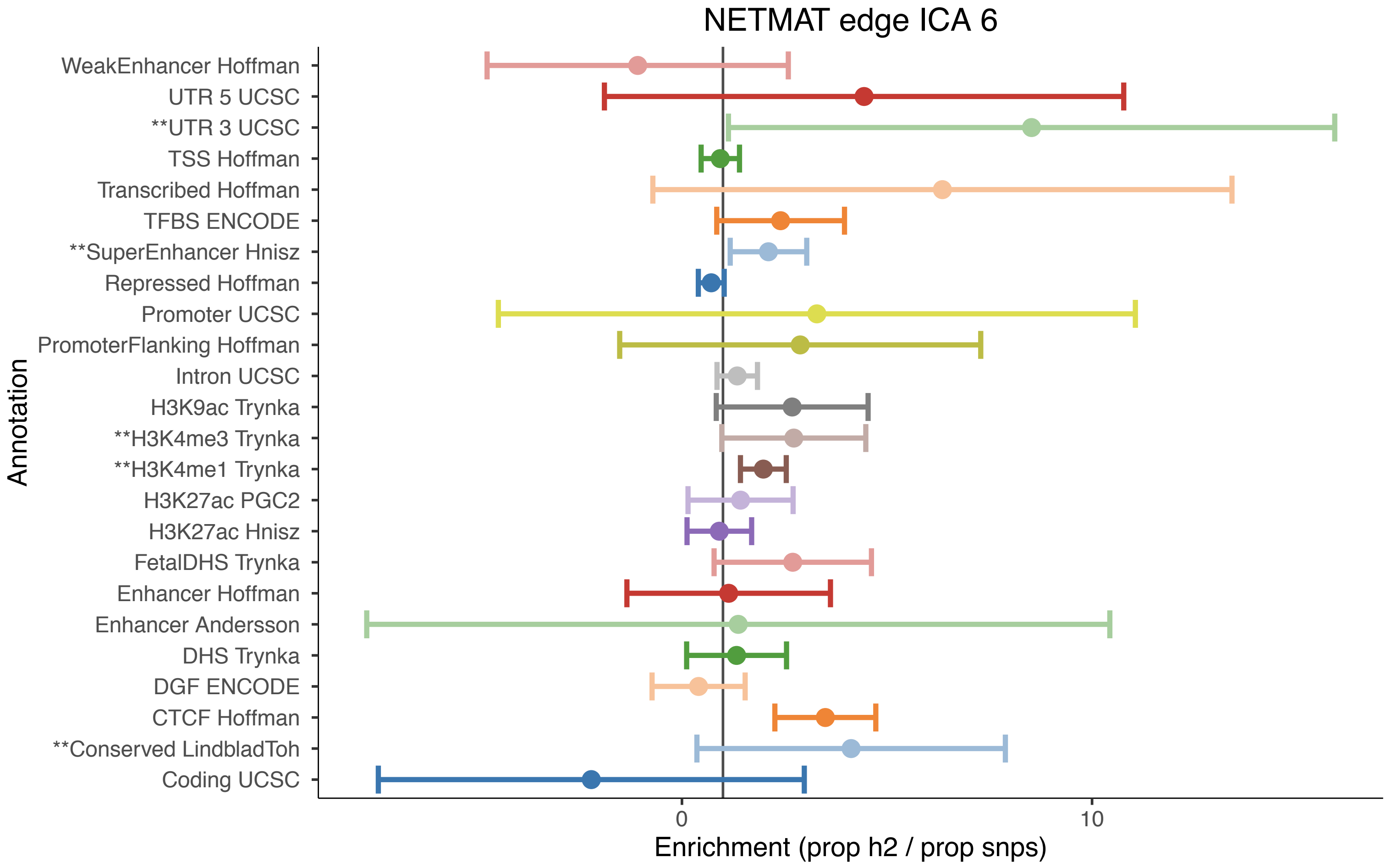

NETMAT edge ICA 6
WeakEnhancer Hoffman
UTR 5 UCSC
**UTR 3 UCSC
TSS Hoffman
Transcribed Hoffman
TFBS ENCODE
**SuperEnhancer Hnisz
Repressed Hoffman
Promoter UCSC
PromoterFlanking Hoffman
Intron UCSC
H3K9ac Trynka
**H3K4me3 Trynka
**H3K4me1 Trynka
H3K27ac PGC2
H3K27ac Hnisz
FetalDHS Trynka
Enhancer Hoffman
Enhancer Andersson
DHS Trynka
DGF ENCODE
CTCF Hoffman
**Conserved LindbladToh
Coding UCSC
Annotation
0
10
Enrichment (prop h2 / prop snps)
