# Supplementary material for: Genome-wide association studies of brain imaging phenotypes in UK Biobank
Source: Nature. 2018 Oct 10;562(7726):210–6. doi: 10.1038/s41586-018-0571-7 (PMC6786974; doi:10.1038/s41586-018-0571-7)
Supplement: Supplementary file 3 — This file contains Supplementary Figures S1-S22. [file Figure-S6.pdf › Figure-S6.pdf]

IDP GWAS Manhattan Plots

List of Figures

1 T1 SIENAX peripheral grey normalised volume . . . 1
2 T1 SIENAX peripheral grey unnormalised volume . . . 2
3 T1 SIENAX CSF normalised volume . . . 3
4 T1 SIENAX CSF unnormalised volume . . . 4
5 T1 SIENAX grey normalised volume . . . 5
6 T1 SIENAX grey unnormalised volume . . . 6
7 T1 SIENAX white normalised volume . . . 7
8 T1 SIENAX white unnormalised volume . . . 8
9 T1 SIENAX brain-normalised volume . . . 9
10 T1 SIENAX brain-unnormalised volume . . . 10
11 T1 FIRST left thalamus volume . . . 11
12 T1 FIRST right thalamus volume . . . 12
13 T1 FIRST left caudate volume . . . 13
14 T1 FIRST right caudate volume . . . 14
15 T1 FIRST left putamen volume . . . 15
16 T1 FIRST right putamen volume . . . 16
17 T1 FIRST left pallidum volume . . . 17
18 T1 FIRST right pallidum volume . . . 18
19 T1 FIRST left hippocampus volume . . . 19
20 T1 FIRST right hippocampus volume . . . 20
21 T1 FIRST left amygdala volume . . . 21
22 T1 FIRST right amygdala volume . . . 22
23 T1 FIRST left accumbens volume . . . 23
24 T1 FIRST right accumbens volume . . . 24
25 T1 FIRST brain stem+4th ventricle volume . . . 25
26 T1 FAST ROIs L frontal pole . . . 26
27 T1 FAST ROIs R frontal pole . . . 27
28 T1 FAST ROIs L insular cortex . . . 28
29 T1 FAST ROIs R insular cortex . . . 29
30 T1 FAST ROIs L sup front gyrus . . . 30
31 T1 FAST ROIs R sup front gyrus . . . 31
32 T1 FAST ROIs L mid front gyrus . . . 32
33 T1 FAST ROIs R mid front gyrus . . . 33
34 T1 FAST ROIs L inf front gyrus parstri . . . 34
35 T1 FAST ROIs R inf front gyrus parstri . . . 35
36 T1 FAST ROIs L inf front gyrus parsop . . . 36
37 T1 FAST ROIs R inf front gyrus parsop . . . 37
38 T1 FAST ROIs L precentral gyrus . . . 38
39 T1 FAST ROIs R precentral gyrus . . . 39
40 T1 FAST ROIs L temporal pole . . . 40
41 T1 FAST ROIs R temporal pole . . . 41
42 T1 FAST ROIs L sup temp gyrus ant . . . 42
43 T1 FAST ROIs R sup temp gyrus ant . . . 43
44 T1 FAST ROIs L sup temp gyrus post . . . 44
45 T1 FAST ROIs R sup temp gyrus post . . . 45
46 T1 FAST ROIs L mid temp gyrus ant . . . 46
47 T1 FAST ROIs R mid temp gyrus ant . . . 47
48 T1 FAST ROIs L mid temp gyrus post . . . 48

49 T1 FAST ROIs R mid temp gyrus post . . . 49
50 T1 FAST ROIs L mid temp gyrus tempocc . . . 50
51 T1 FAST ROIs R mid temp gyrus tempocc . . . 51
52 T1 FAST ROIs L inf temp gyrus ant . . . 52
53 T1 FAST ROIs R inf temp gyrus ant . . . 53
54 T1 FAST ROIs L inf temp gyrus post . . . 54
55 T1 FAST ROIs R inf temp gyrus post . . . 55
56 T1 FAST ROIs L inf temp gyrus tempocc . . . 56
57 T1 FAST ROIs R inf temp gyrus tempocc . . . 57
58 T1 FAST ROIs L postcent gyrus . . . 58
59 T1 FAST ROIs R postcent gyrus . . . 59
60 T1 FAST ROIs L sup parietal lobule . . . 60
61 T1 FAST ROIs R sup parietal lobule . . . 61
62 T1 FAST ROIs L supramarg gyrus ant . . . 62
63 T1 FAST ROIs R supramarg gyrus ant . . . 63
64 T1 FAST ROIs L supramarg gyrus post . . . 64
65 T1 FAST ROIs R supramarg gyrus post . . . 65
66 T1 FAST ROIs L angular gyrus . . . 66
67 T1 FAST ROIs R angular gyrus . . . 67
68 T1 FAST ROIs L latocc cortex sup . . . 68
69 T1 FAST ROIs R latocc cortex sup . . . 69
70 T1 FAST ROIs L latocc cortex inf . . . 70
71 T1 FAST ROIs R latocc cortex inf . . . 71
72 T1 FAST ROIs L intracalc cortex . . . 72
73 T1 FAST ROIs R intracalc cortex . . . 73
74 T1 FAST ROIs L front med cortex . . . 74
75 T1 FAST ROIs R front med cortex . . . 75
76 T1 FAST ROIs L juxtapos lobule cortex . . . 76
77 T1 FAST ROIs R juxtapos lobule cortex . . . 77
78 T1 FAST ROIs L subcallosal cortex . . . 78
79 T1 FAST ROIs R subcallosal cortex . . . 79
80 T1 FAST ROIs L paracing gyrus . . . 80
81 T1 FAST ROIs R paracing gyrus . . . 81
82 T1 FAST ROIs L cing gyrus ant . . . 82
83 T1 FAST ROIs R cing gyrus ant . . . 83
84 T1 FAST ROIs L cing gyrus post . . . 84
85 T1 FAST ROIs R cing gyrus post . . . 85
86 T1 FAST ROIs L precun cortex . . . 86
87 T1 FAST ROIs R precun cortex . . . 87
88 T1 FAST ROIs L cuneal cortex . . . 88
89 T1 FAST ROIs R cuneal cortex . . . 89
90 T1 FAST ROIs L front orb cortex . . . 90
91 T1 FAST ROIs R front orb cortex . . . 91
92 T1 FAST ROIs L parahipp gyrus ant . . . 92
93 T1 FAST ROIs R parahipp gyrus ant . . . 93
94 T1 FAST ROIs L parahipp gyrus post . . . 94
95 T1 FAST ROIs R parahipp gyrus post . . . 95
96 T1 FAST ROIs L lingual gyrus . . . 96
97 T1 FAST ROIs R lingual gyrus . . . 97
98 T1 FAST ROIs L temp fusif cortex ant . . . 98
99 T1 FAST ROIs R temp fusif cortex ant . . . 99
100 T1 FAST ROIs L temp fusif cortex post . . . 100
101 T1 FAST ROIs R temp fusif cortex post . . . 101
102 T1 FAST ROIs L temp occ fusif cortex . . . 102
103 T1 FAST ROIs R temp occ fusif cortex . . . 103
104 T1 FAST ROIs L occ fusif gyrus . . . 104
105 T1 FAST ROIs R occ fusif gyrus . . . 105
106 T1 FAST ROIs L front operc cortex . . . 106
107 T1 FAST ROIs R front operc cortex . . . 107
108 T1 FAST ROIs L cent operc cortex . . . 108

109 T1 FAST ROIs R cent operc cortex 109
110 T1 FAST ROIs L parietal operc cortex 110
111 T1 FAST ROIs R parietal operc cortex 111
112 T1 FAST ROIs L planum polare 112
113 T1 FAST ROIs R planum polare 113
114 T1 FAST ROIs L heschl gyrus 114
115 T1 FAST ROIs R heschl gyrus 115
116 T1 FAST ROIs L planum temporale 116
117 T1 FAST ROIs R planum temporale 117
118 T1 FAST ROIs L supracalc cortex 118
119 T1 FAST ROIs R supracalc cortex 119
120 T1 FAST ROIs L occ pole 120
121 T1 FAST ROIs R occ pole 121
122 T1 FAST ROIs L thalamus 122
123 T1 FAST ROIs R thalamus 123
124 T1 FAST ROIs L caudate 124
125 T1 FAST ROIs R caudate 125
126 T1 FAST ROIs L putamen 126
127 T1 FAST ROIs R putamen 127
128 T1 FAST ROIs L pallidum 128
129 T1 FAST ROIs R pallidum 129
130 T1 FAST ROIs L hippocampus 130
131 T1 FAST ROIs R hippocampus 131
132 T1 FAST ROIs L amygdala 132
133 T1 FAST ROIs R amygdala 133
134 T1 FAST ROIs L ventral striatum 134
135 T1 FAST ROIs R ventral striatum 135
136 T1 FAST ROIs brain stem 136
137 T1 FAST ROIs L cerebellum I-IV 137
138 T1 FAST ROIs R cerebellum I-IV 138
139 T1 FAST ROIs L cerebellum V 139
140 T1 FAST ROIs R cerebellum V 140
141 T1 FAST ROIs L cerebellum VI 141
142 T1 FAST ROIs V cerebellum VI 142
143 T1 FAST ROIs R cerebellum VI 143
144 T1 FAST ROIs L cerebellum crus I 144
145 T1 FAST ROIs V cerebellum crus I 145
146 T1 FAST ROIs R cerebellum crus I 146
147 T1 FAST ROIs L cerebellum crus II 147
148 T1 FAST ROIs V cerebellum crus II 148
149 T1 FAST ROIs R cerebellum crus II 149
150 T1 FAST ROIs L cerebellum VIIb 150
151 T1 FAST ROIs V cerebellum VIIb 151
152 T1 FAST ROIs R cerebellum VIIb 152
153 T1 FAST ROIs L cerebellum VIIIa 153
154 T1 FAST ROIs V cerebellum VIIIa 154
155 T1 FAST ROIs R cerebellum VIIIa 155
156 T1 FAST ROIs L cerebellum VIIIb 156
157 T1 FAST ROIs V cerebellum VIIIb 157
158 T1 FAST ROIs R cerebellum VIIIb 158
159 T1 FAST ROIs L cerebellum IX 159
160 T1 FAST ROIs V cerebellum IX 160
161 T1 FAST ROIs R cerebellum IX 161
162 T1 FAST ROIs L cerebellum X 162
163 T1 FAST ROIs V cerebellum X 163
164 T1 FAST ROIs R cerebellum X 164
165 T2 FLAIR BIANCA WMH volume 165
166 SWI T2star left thalamus 166
167 SWI T2star right thalamus 167
168 SWI T2star left caudate 168

169 SWI T2star right caudate . . . 169
170 SWI T2star left putamen . . . 170
171 SWI T2star right putamen . . . 171
172 SWI T2star left pallidum . . . 172
173 SWI T2star right pallidum . . . 173
174 SWI T2star left hippocampus . . . 174
175 SWI T2star right hippocampus . . . 175
176 SWI T2star left amygdala . . . 176
177 SWI T2star right amygdala . . . 177
178 SWI T2star left accumbens . . . 178
179 SWI T2star right accumbens . . . 179
180 tfMRI median BOLD shapes . . . 180
181 tfMRI 90th-percentile BOLD shapes . . . 181
182 tfMRI median zstat shapes . . . 182
183 tfMRI 90th-percentile zstat shapes . . . 183
184 tfMRI median BOLD faces . . . 184
185 tfMRI 90th-percentile BOLD faces . . . 185
186 tfMRI median zstat faces . . . 186
187 tfMRI 90th-percentile zstat faces . . . 187
188 tfMRI median BOLD faces-shapes . . . 188
189 tfMRI 90th-percentile BOLD faces-shapes . . . 189
190 tfMRI median zstat faces-shapes . . . 190
191 tfMRI 90th-percentile zstat faces-shapes . . . 191
192 tfMRI median BOLD faces-shapes amygdala . . . 192
193 tfMRI 90th-percentile BOLD faces-shapes amygdala . . . 193
194 tfMRI median zstat faces-shapes amygdala . . . 194
195 tfMRI 90th-percentile zstat faces-shapes amygdala . . . 195
196 dMRI TBSS FA Middle cerebellar peduncle . . . 196
197 dMRI TBSS FA Pontine crossing tract . . . 197
198 dMRI TBSS FA Genu of corpus callosum . . . 198
199 dMRI TBSS FA Body of corpus callosum . . . 199
200 dMRI TBSS FA Splenium of corpus callosum . . . 200
201 dMRI TBSS FA Fornix . . . 201
202 dMRI TBSS FA Corticospinal tract R . . . 202
203 dMRI TBSS FA Corticospinal tract L . . . 203
204 dMRI TBSS FA Medial lemniscus R . . . 204
205 dMRI TBSS FA Medial lemniscus L . . . 205
206 dMRI TBSS FA Inferior cerebellar peduncle R . . . 206
207 dMRI TBSS FA Inferior cerebellar peduncle L . . . 207
208 dMRI TBSS FA Superior cerebellar peduncle R . . . 208
209 dMRI TBSS FA Superior cerebellar peduncle L . . . 209
210 dMRI TBSS FA Cerebral peduncle R . . . 210
211 dMRI TBSS FA Cerebral peduncle L . . . 211
212 dMRI TBSS FA Anterior limb of internal capsule R . . . 212
213 dMRI TBSS FA Anterior limb of internal capsule L . . . 213
214 dMRI TBSS FA Posterior limb of internal capsule R . . . 214
215 dMRI TBSS FA Posterior limb of internal capsule L . . . 215
216 dMRI TBSS FA Retrolenticular part of internal capsule R . . . 216
217 dMRI TBSS FA Retrolenticular part of internal capsule L . . . 217
218 dMRI TBSS FA Anterior corona radiata R . . . 218
219 dMRI TBSS FA Anterior corona radiata L . . . 219
220 dMRI TBSS FA Superior corona radiata R . . . 220
221 dMRI TBSS FA Superior corona radiata L . . . 221
222 dMRI TBSS FA Posterior corona radiata R . . . 222
223 dMRI TBSS FA Posterior corona radiata L . . . 223
224 dMRI TBSS FA Posterior thalamic radiation R . . . 224
225 dMRI TBSS FA Posterior thalamic radiation L . . . 225
226 dMRI TBSS FA Sagittal stratum R . . . 226
227 dMRI TBSS FA Sagittal stratum L . . . 227
228 dMRI TBSS FA External capsule R . . . 228

229 dMRI TBSS FA External capsule L 229
230 dMRI TBSS FA Cingulum cingulate gyrus R 230
231 dMRI TBSS FA Cingulum cingulate gyrus L 231
232 dMRI TBSS FA Cingulum hippocampus R 232
233 dMRI TBSS FA Cingulum hippocampus L 233
234 dMRI TBSS FA Fornix cres+Stria terminalis R 234
235 dMRI TBSS FA Fornix cres+Stria terminalis L 235
236 dMRI TBSS FA Superior longitudinal fasciculus R 236
237 dMRI TBSS FA Superior longitudinal fasciculus L 237
238 dMRI TBSS FA Superior fronto-occipital fasciculus R 238
239 dMRI TBSS FA Superior fronto-occipital fasciculus L 239
240 dMRI TBSS FA Uncinate fasciculus R 240
241 dMRI TBSS FA Uncinate fasciculus L 241
242 dMRI TBSS FA Tapetum R 242
243 dMRI TBSS FA Tapetum L 243
244 dMRI TBSS MD Middle cerebellar peduncle 244
245 dMRI TBSS MD Pontine crossing tract 245
246 dMRI TBSS MD Genu of corpus callosum 246
247 dMRI TBSS MD Body of corpus callosum 247
248 dMRI TBSS MD Splenium of corpus callosum 248
249 dMRI TBSS MD Fornix 249
250 dMRI TBSS MD Corticospinal tract R 250
251 dMRI TBSS MD Corticospinal tract L 251
252 dMRI TBSS MD Medial lemniscus R 252
253 dMRI TBSS MD Medial lemniscus L 253
254 dMRI TBSS MD Inferior cerebellar peduncle R 254
255 dMRI TBSS MD Inferior cerebellar peduncle L 255
256 dMRI TBSS MD Superior cerebellar peduncle R 256
257 dMRI TBSS MD Superior cerebellar peduncle L 257
258 dMRI TBSS MD Cerebral peduncle R 258
259 dMRI TBSS MD Cerebral peduncle L 259
260 dMRI TBSS MD Anterior limb of internal capsule R 260
261 dMRI TBSS MD Anterior limb of internal capsule L 261
262 dMRI TBSS MD Posterior limb of internal capsule R 262
263 dMRI TBSS MD Posterior limb of internal capsule L 263
264 dMRI TBSS MD Retrolenticular part of internal capsule R 264
265 dMRI TBSS MD Retrolenticular part of internal capsule L 265
266 dMRI TBSS MD Anterior corona radiata R 266
267 dMRI TBSS MD Anterior corona radiata L 267
268 dMRI TBSS MD Superior corona radiata R 268
269 dMRI TBSS MD Superior corona radiata L 269
270 dMRI TBSS MD Posterior corona radiata R 270
271 dMRI TBSS MD Posterior corona radiata L 271
272 dMRI TBSS MD Posterior thalamic radiation R 272
273 dMRI TBSS MD Posterior thalamic radiation L 273
274 dMRI TBSS MD Sagittal stratum R 274
275 dMRI TBSS MD Sagittal stratum L 275
276 dMRI TBSS MD External capsule R 276
277 dMRI TBSS MD External capsule L 277
278 dMRI TBSS MD Cingulum cingulate gyrus R 278
279 dMRI TBSS MD Cingulum cingulate gyrus L 279
280 dMRI TBSS MD Cingulum hippocampus R 280
281 dMRI TBSS MD Cingulum hippocampus L 281
282 dMRI TBSS MD Fornix cres+Stria terminalis R 282
283 dMRI TBSS MD Fornix cres+Stria terminalis L 283
284 dMRI TBSS MD Superior longitudinal fasciculus R 284
285 dMRI TBSS MD Superior longitudinal fasciculus L 285
286 dMRI TBSS MD Superior fronto-occipital fasciculus R 286
287 dMRI TBSS MD Superior fronto-occipital fasciculus L 287
288 dMRI TBSS MD Uncinate fasciculus R 288

289 dMRI TBSS MD Uncinate fasciculus L . . . 289
290 dMRI TBSS MD Tapetum R . . . 290
291 dMRI TBSS MD Tapetum L . . . 291
292 dMRI TBSS MO Middle cerebellar peduncle . . . 292
293 dMRI TBSS MO Pontine crossing tract . . . 293
294 dMRI TBSS MO Genu of corpus callosum . . . 294
295 dMRI TBSS MO Body of corpus callosum . . . 295
296 dMRI TBSS MO Splenium of corpus callosum . . . 296
297 dMRI TBSS MO Fornix . . . 297
298 dMRI TBSS MO Corticospinal tract R . . . 298
299 dMRI TBSS MO Corticospinal tract L . . . 299
300 dMRI TBSS MO Medial lemniscus R . . . 300
301 dMRI TBSS MO Medial lemniscus L . . . 301
302 dMRI TBSS MO Inferior cerebellar peduncle R . . . 302
303 dMRI TBSS MO Inferior cerebellar peduncle L . . . 303
304 dMRI TBSS MO Superior cerebellar peduncle R . . . 304
305 dMRI TBSS MO Superior cerebellar peduncle L . . . 305
306 dMRI TBSS MO Cerebral peduncle R . . . 306
307 dMRI TBSS MO Cerebral peduncle L . . . 307
308 dMRI TBSS MO Anterior limb of internal capsule R . . . 308
309 dMRI TBSS MO Anterior limb of internal capsule L . . . 309
310 dMRI TBSS MO Posterior limb of internal capsule R . . . 310
311 dMRI TBSS MO Posterior limb of internal capsule L . . . 311
312 dMRI TBSS MO Retrolenticular part of internal capsule R . . . 312
313 dMRI TBSS MO Retrolenticular part of internal capsule L . . . 313
314 dMRI TBSS MO Anterior corona radiata R . . . 314
315 dMRI TBSS MO Anterior corona radiata L . . . 315
316 dMRI TBSS MO Superior corona radiata R . . . 316
317 dMRI TBSS MO Superior corona radiata L . . . 317
318 dMRI TBSS MO Posterior corona radiata R . . . 318
319 dMRI TBSS MO Posterior corona radiata L . . . 319
320 dMRI TBSS MO Posterior thalamic radiation R . . . 320
321 dMRI TBSS MO Posterior thalamic radiation L . . . 321
322 dMRI TBSS MO Sagittal stratum R . . . 322
323 dMRI TBSS MO Sagittal stratum L . . . 323
324 dMRI TBSS MO External capsule R . . . 324
325 dMRI TBSS MO External capsule L . . . 325
326 dMRI TBSS MO Cingulum cingulate gyrus R . . . 326
327 dMRI TBSS MO Cingulum cingulate gyrus L . . . 327
328 dMRI TBSS MO Cingulum hippocampus R . . . 328
329 dMRI TBSS MO Cingulum hippocampus L . . . 329
330 dMRI TBSS MO Fornix cres+Stria terminalis R . . . 330
331 dMRI TBSS MO Fornix cres+Stria terminalis L . . . 331
332 dMRI TBSS MO Superior longitudinal fasciculus R . . . 332
333 dMRI TBSS MO Superior longitudinal fasciculus L . . . 333
334 dMRI TBSS MO Superior fronto-occipital fasciculus R . . . 334
335 dMRI TBSS MO Superior fronto-occipital fasciculus L . . . 335
336 dMRI TBSS MO Uncinate fasciculus R . . . 336
337 dMRI TBSS MO Uncinate fasciculus L . . . 337
338 dMRI TBSS MO Tapetum R . . . 338
339 dMRI TBSS MO Tapetum L . . . 339
340 dMRI TBSS L1 Middle cerebellar peduncle . . . 340
341 dMRI TBSS L1 Pontine crossing tract . . . 341
342 dMRI TBSS L1 Genu of corpus callosum . . . 342
343 dMRI TBSS L1 Body of corpus callosum . . . 343
344 dMRI TBSS L1 Splenium of corpus callosum . . . 344
345 dMRI TBSS L1 Fornix . . . 345
346 dMRI TBSS L1 Corticospinal tract R . . . 346
347 dMRI TBSS L1 Corticospinal tract L . . . 347
348 dMRI TBSS L1 Medial lemniscus R . . . 348

349 dMRI TBSS L1 Medial lemniscus L 349
350 dMRI TBSS L1 Inferior cerebellar peduncle R 350
351 dMRI TBSS L1 Inferior cerebellar peduncle L 351
352 dMRI TBSS L1 Superior cerebellar peduncle R 352
353 dMRI TBSS L1 Superior cerebellar peduncle L 353
354 dMRI TBSS L1 Cerebral peduncle R 354
355 dMRI TBSS L1 Cerebral peduncle L 355
356 dMRI TBSS L1 Anterior limb of internal capsule R 356
357 dMRI TBSS L1 Anterior limb of internal capsule L 357
358 dMRI TBSS L1 Posterior limb of internal capsule R 358
359 dMRI TBSS L1 Posterior limb of internal capsule L 359
360 dMRI TBSS L1 Retrolenticular part of internal capsule R 360
361 dMRI TBSS L1 Retrolenticular part of internal capsule L 361
362 dMRI TBSS L1 Anterior corona radiata R 362
363 dMRI TBSS L1 Anterior corona radiata L 363
364 dMRI TBSS L1 Superior corona radiata R 364
365 dMRI TBSS L1 Superior corona radiata L 365
366 dMRI TBSS L1 Posterior corona radiata R 366
367 dMRI TBSS L1 Posterior corona radiata L 367
368 dMRI TBSS L1 Posterior thalamic radiation R 368
369 dMRI TBSS L1 Posterior thalamic radiation L 369
370 dMRI TBSS L1 Sagittal stratum R 370
371 dMRI TBSS L1 Sagittal stratum L 371
372 dMRI TBSS L1 External capsule R 372
373 dMRI TBSS L1 External capsule L 373
374 dMRI TBSS L1 Cingulum cingulate gyrus R 374
375 dMRI TBSS L1 Cingulum cingulate gyrus L 375
376 dMRI TBSS L1 Cingulum hippocampus R 376
377 dMRI TBSS L1 Cingulum hippocampus L 377
378 dMRI TBSS L1 Fornix cres+Stria terminalis R 378
379 dMRI TBSS L1 Fornix cres+Stria terminalis L 379
380 dMRI TBSS L1 Superior longitudinal fasciculus R 380
381 dMRI TBSS L1 Superior longitudinal fasciculus L 381
382 dMRI TBSS L1 Superior fronto-occipital fasciculus R 382
383 dMRI TBSS L1 Superior fronto-occipital fasciculus L 383
384 dMRI TBSS L1 Uncinate fasciculus R 384
385 dMRI TBSS L1 Uncinate fasciculus L 385
386 dMRI TBSS L1 Tapetum R 386
387 dMRI TBSS L1 Tapetum L 387
388 dMRI TBSS L2 Middle cerebellar peduncle 388
389 dMRI TBSS L2 Pontine crossing tract 389
390 dMRI TBSS L2 Genu of corpus callosum 390
391 dMRI TBSS L2 Body of corpus callosum 391
392 dMRI TBSS L2 Splenium of corpus callosum 392
393 dMRI TBSS L2 Fornix 393
394 dMRI TBSS L2 Corticospinal tract R 394
395 dMRI TBSS L2 Corticospinal tract L 395
396 dMRI TBSS L2 Medial lemniscus R 396
397 dMRI TBSS L2 Medial lemniscus L 397
398 dMRI TBSS L2 Inferior cerebellar peduncle R 398
399 dMRI TBSS L2 Inferior cerebellar peduncle L 399
400 dMRI TBSS L2 Superior cerebellar peduncle R 400
401 dMRI TBSS L2 Superior cerebellar peduncle L 401
402 dMRI TBSS L2 Cerebral peduncle R 402
403 dMRI TBSS L2 Cerebral peduncle L 403
404 dMRI TBSS L2 Anterior limb of internal capsule R 404
405 dMRI TBSS L2 Anterior limb of internal capsule L 405
406 dMRI TBSS L2 Posterior limb of internal capsule R 406
407 dMRI TBSS L2 Posterior limb of internal capsule L 407
408 dMRI TBSS L2 Retrolenticular part of internal capsule R 408

409 dMRI TBSS L2 Retrolenticular part of internal capsule L 409
410 dMRI TBSS L2 Anterior corona radiata R 410
411 dMRI TBSS L2 Anterior corona radiata L 411
412 dMRI TBSS L2 Superior corona radiata R 412
413 dMRI TBSS L2 Superior corona radiata L 413
414 dMRI TBSS L2 Posterior corona radiata R 414
415 dMRI TBSS L2 Posterior corona radiata L 415
416 dMRI TBSS L2 Posterior thalamic radiation R 416
417 dMRI TBSS L2 Posterior thalamic radiation L 417
418 dMRI TBSS L2 Sagittal stratum R 418
419 dMRI TBSS L2 Sagittal stratum L 419
420 dMRI TBSS L2 External capsule R 420
421 dMRI TBSS L2 External capsule L 421
422 dMRI TBSS L2 Cingulum cingulate gyrus R 422
423 dMRI TBSS L2 Cingulum cingulate gyrus L 423
424 dMRI TBSS L2 Cingulum hippocampus R 424
425 dMRI TBSS L2 Cingulum hippocampus L 425
426 dMRI TBSS L2 Fornix cres+Stria terminalis R 426
427 dMRI TBSS L2 Fornix cres+Stria terminalis L 427
428 dMRI TBSS L2 Superior longitudinal fasciculus R 428
429 dMRI TBSS L2 Superior longitudinal fasciculus L 429
430 dMRI TBSS L2 Superior fronto-occipital fasciculus R 430
431 dMRI TBSS L2 Superior fronto-occipital fasciculus L 431
432 dMRI TBSS L2 Uncinate fasciculus R 432
433 dMRI TBSS L2 Uncinate fasciculus L 433
434 dMRI TBSS L2 Tapetum R 434
435 dMRI TBSS L2 Tapetum L 435
436 dMRI TBSS L3 Middle cerebellar peduncle 436
437 dMRI TBSS L3 Pontine crossing tract 437
438 dMRI TBSS L3 Genu of corpus callosum 438
439 dMRI TBSS L3 Body of corpus callosum 439
440 dMRI TBSS L3 Splenium of corpus callosum 440
441 dMRI TBSS L3 Fornix 441
442 dMRI TBSS L3 Corticospinal tract R 442
443 dMRI TBSS L3 Corticospinal tract L 443
444 dMRI TBSS L3 Medial lemniscus R 444
445 dMRI TBSS L3 Medial lemniscus L 445
446 dMRI TBSS L3 Inferior cerebellar peduncle R 446
447 dMRI TBSS L3 Inferior cerebellar peduncle L 447
448 dMRI TBSS L3 Superior cerebellar peduncle R 448
449 dMRI TBSS L3 Superior cerebellar peduncle L 449
450 dMRI TBSS L3 Cerebral peduncle R 450
451 dMRI TBSS L3 Cerebral peduncle L 451
452 dMRI TBSS L3 Anterior limb of internal capsule R 452
453 dMRI TBSS L3 Anterior limb of internal capsule L 453
454 dMRI TBSS L3 Posterior limb of internal capsule R 454
455 dMRI TBSS L3 Posterior limb of internal capsule L 455
456 dMRI TBSS L3 Retrolenticular part of internal capsule R 456
457 dMRI TBSS L3 Retrolenticular part of internal capsule L 457
458 dMRI TBSS L3 Anterior corona radiata R 458
459 dMRI TBSS L3 Anterior corona radiata L 459
460 dMRI TBSS L3 Superior corona radiata R 460
461 dMRI TBSS L3 Superior corona radiata L 461
462 dMRI TBSS L3 Posterior corona radiata R 462
463 dMRI TBSS L3 Posterior corona radiata L 463
464 dMRI TBSS L3 Posterior thalamic radiation R 464
465 dMRI TBSS L3 Posterior thalamic radiation L 465
466 dMRI TBSS L3 Sagittal stratum R 466
467 dMRI TBSS L3 Sagittal stratum L 467
468 dMRI TBSS L3 External capsule R 468

469 dMRI TBSS L3 External capsule L . . . 469
470 dMRI TBSS L3 Cingulum cingulate gyrus R . . . 470
471 dMRI TBSS L3 Cingulum cingulate gyrus L . . . 471
472 dMRI TBSS L3 Cingulum hippocampus R . . . 472
473 dMRI TBSS L3 Cingulum hippocampus L . . . 473
474 dMRI TBSS L3 Fornix cres+Stria terminalis R . . . 474
475 dMRI TBSS L3 Fornix cres+Stria terminalis L . . . 475
476 dMRI TBSS L3 Superior longitudinal fasciculus R . . . 476
477 dMRI TBSS L3 Superior longitudinal fasciculus L . . . 477
478 dMRI TBSS L3 Superior fronto-occipital fasciculus R . . . 478
479 dMRI TBSS L3 Superior fronto-occipital fasciculus L . . . 479
480 dMRI TBSS L3 Uncinate fasciculus R . . . 480
481 dMRI TBSS L3 Uncinate fasciculus L . . . 481
482 dMRI TBSS L3 Tapetum R . . . 482
483 dMRI TBSS L3 Tapetum L . . . 483
484 dMRI TBSS ICVF Middle cerebellar peduncle . . . 484
485 dMRI TBSS ICVF Pontine crossing tract . . . 485
486 dMRI TBSS ICVF Genu of corpus callosum . . . 486
487 dMRI TBSS ICVF Body of corpus callosum . . . 487
488 dMRI TBSS ICVF Splenium of corpus callosum . . . 488
489 dMRI TBSS ICVF Fornix . . . 489
490 dMRI TBSS ICVF Corticospinal tract R . . . 490
491 dMRI TBSS ICVF Corticospinal tract L . . . 491
492 dMRI TBSS ICVF Medial lemniscus R . . . 492
493 dMRI TBSS ICVF Medial lemniscus L . . . 493
494 dMRI TBSS ICVF Inferior cerebellar peduncle R . . . 494
495 dMRI TBSS ICVF Inferior cerebellar peduncle L . . . 495
496 dMRI TBSS ICVF Superior cerebellar peduncle R . . . 496
497 dMRI TBSS ICVF Superior cerebellar peduncle L . . . 497
498 dMRI TBSS ICVF Cerebral peduncle R . . . 498
499 dMRI TBSS ICVF Cerebral peduncle L . . . 499
500 dMRI TBSS ICVF Anterior limb of internal capsule R . . . 500
501 dMRI TBSS ICVF Anterior limb of internal capsule L . . . 501
502 dMRI TBSS ICVF Posterior limb of internal capsule R . . . 502
503 dMRI TBSS ICVF Posterior limb of internal capsule L . . . 503
504 dMRI TBSS ICVF Retrolenticular part of internal capsule R . . . 504
505 dMRI TBSS ICVF Retrolenticular part of internal capsule L . . . 505
506 dMRI TBSS ICVF Anterior corona radiata R . . . 506
507 dMRI TBSS ICVF Anterior corona radiata L . . . 507
508 dMRI TBSS ICVF Superior corona radiata R . . . 508
509 dMRI TBSS ICVF Superior corona radiata L . . . 509
510 dMRI TBSS ICVF Posterior corona radiata R . . . 510
511 dMRI TBSS ICVF Posterior corona radiata L . . . 511
512 dMRI TBSS ICVF Posterior thalamic radiation R . . . 512
513 dMRI TBSS ICVF Posterior thalamic radiation L . . . 513
514 dMRI TBSS ICVF Sagittal stratum R . . . 514
515 dMRI TBSS ICVF Sagittal stratum L . . . 515
516 dMRI TBSS ICVF External capsule R . . . 516
517 dMRI TBSS ICVF External capsule L . . . 517
518 dMRI TBSS ICVF Cingulum cingulate gyrus R . . . 518
519 dMRI TBSS ICVF Cingulum cingulate gyrus L . . . 519
520 dMRI TBSS ICVF Cingulum hippocampus R . . . 520
521 dMRI TBSS ICVF Cingulum hippocampus L . . . 521
522 dMRI TBSS ICVF Fornix cres+Stria terminalis R . . . 522
523 dMRI TBSS ICVF Fornix cres+Stria terminalis L . . . 523
524 dMRI TBSS ICVF Superior longitudinal fasciculus R . . . 524
525 dMRI TBSS ICVF Superior longitudinal fasciculus L . . . 525
526 dMRI TBSS ICVF Superior fronto-occipital fasciculus R . . . 526
527 dMRI TBSS ICVF Superior fronto-occipital fasciculus L . . . 527
528 dMRI TBSS ICVF Uncinate fasciculus R . . . 528

529 dMRI TBSS ICVF Uncinate fasciculus L 529
530 dMRI TBSS ICVF Tapetum R 530
531 dMRI TBSS ICVF Tapetum L 531
532 dMRI TBSS OD Middle cerebellar peduncle 532
533 dMRI TBSS OD Pontine crossing tract 533
534 dMRI TBSS OD Genu of corpus callosum 534
535 dMRI TBSS OD Body of corpus callosum 535
536 dMRI TBSS OD Splenium of corpus callosum 536
537 dMRI TBSS OD Fornix 537
538 dMRI TBSS OD Corticospinal tract R 538
539 dMRI TBSS OD Corticospinal tract L 539
540 dMRI TBSS OD Medial lemniscus R 540
541 dMRI TBSS OD Medial lemniscus L 541
542 dMRI TBSS OD Inferior cerebellar peduncle R 542
543 dMRI TBSS OD Inferior cerebellar peduncle L 543
544 dMRI TBSS OD Superior cerebellar peduncle R 544
545 dMRI TBSS OD Superior cerebellar peduncle L 545
546 dMRI TBSS OD Cerebral peduncle R 546
547 dMRI TBSS OD Cerebral peduncle L 547
548 dMRI TBSS OD Anterior limb of internal capsule R 548
549 dMRI TBSS OD Anterior limb of internal capsule L 549
550 dMRI TBSS OD Posterior limb of internal capsule R 550
551 dMRI TBSS OD Posterior limb of internal capsule L 551
552 dMRI TBSS OD Retrolenticular part of internal capsule R 552
553 dMRI TBSS OD Retrolenticular part of internal capsule L 553
554 dMRI TBSS OD Anterior corona radiata R 554
555 dMRI TBSS OD Anterior corona radiata L 555
556 dMRI TBSS OD Superior corona radiata R 556
557 dMRI TBSS OD Superior corona radiata L 557
558 dMRI TBSS OD Posterior corona radiata R 558
559 dMRI TBSS OD Posterior corona radiata L 559
560 dMRI TBSS OD Posterior thalamic radiation R 560
561 dMRI TBSS OD Posterior thalamic radiation L 561
562 dMRI TBSS OD Sagittal stratum R 562
563 dMRI TBSS OD Sagittal stratum L 563
564 dMRI TBSS OD External capsule R 564
565 dMRI TBSS OD External capsule L 565
566 dMRI TBSS OD Cingulum cingulate gyrus R 566
567 dMRI TBSS OD Cingulum cingulate gyrus L 567
568 dMRI TBSS OD Cingulum hippocampus R 568
569 dMRI TBSS OD Cingulum hippocampus L 569
570 dMRI TBSS OD Fornix cres+Stria terminalis R 570
571 dMRI TBSS OD Fornix cres+Stria terminalis L 571
572 dMRI TBSS OD Superior longitudinal fasciculus R 572
573 dMRI TBSS OD Superior longitudinal fasciculus L 573
574 dMRI TBSS OD Superior fronto-occipital fasciculus R 574
575 dMRI TBSS OD Superior fronto-occipital fasciculus L 575
576 dMRI TBSS OD Uncinate fasciculus R 576
577 dMRI TBSS OD Uncinate fasciculus L 577
578 dMRI TBSS OD Tapetum R 578
579 dMRI TBSS OD Tapetum L 579
580 dMRI TBSS ISOVF Middle cerebellar peduncle 580
581 dMRI TBSS ISOVF Pontine crossing tract 581
582 dMRI TBSS ISOVF Genu of corpus callosum 582
583 dMRI TBSS ISOVF Body of corpus callosum 583
584 dMRI TBSS ISOVF Splenium of corpus callosum 584
585 dMRI TBSS ISOVF Fornix 585
586 dMRI TBSS ISOVF Corticospinal tract R 586
587 dMRI TBSS ISOVF Corticospinal tract L 587
588 dMRI TBSS ISOVF Medial lemniscus R 588

589 dMRI TBSS ISOVF Medial lemniscus L . . . 589
590 dMRI TBSS ISOVF Inferior cerebellar peduncle R . . . 590
591 dMRI TBSS ISOVF Inferior cerebellar peduncle L . . . 591
592 dMRI TBSS ISOVF Superior cerebellar peduncle R . . . 592
593 dMRI TBSS ISOVF Superior cerebellar peduncle L . . . 593
594 dMRI TBSS ISOVF Cerebral peduncle R . . . 594
595 dMRI TBSS ISOVF Cerebral peduncle L . . . 595
596 dMRI TBSS ISOVF Anterior limb of internal capsule R . . . 596
597 dMRI TBSS ISOVF Anterior limb of internal capsule L . . . 597
598 dMRI TBSS ISOVF Posterior limb of internal capsule R . . . 598
599 dMRI TBSS ISOVF Posterior limb of internal capsule L . . . 599
600 dMRI TBSS ISOVF Retrolenticular part of internal capsule R . . . 600
601 dMRI TBSS ISOVF Retrolenticular part of internal capsule L . . . 601
602 dMRI TBSS ISOVF Anterior corona radiata R . . . 602
603 dMRI TBSS ISOVF Anterior corona radiata L . . . 603
604 dMRI TBSS ISOVF Superior corona radiata R . . . 604
605 dMRI TBSS ISOVF Superior corona radiata L . . . 605
606 dMRI TBSS ISOVF Posterior corona radiata R . . . 606
607 dMRI TBSS ISOVF Posterior corona radiata L . . . 607
608 dMRI TBSS ISOVF Posterior thalamic radiation R . . . 608
609 dMRI TBSS ISOVF Posterior thalamic radiation L . . . 609
610 dMRI TBSS ISOVF Sagittal stratum R . . . 610
611 dMRI TBSS ISOVF Sagittal stratum L . . . 611
612 dMRI TBSS ISOVF External capsule R . . . 612
613 dMRI TBSS ISOVF External capsule L . . . 613
614 dMRI TBSS ISOVF Cingulum cingulate gyrus R . . . 614
615 dMRI TBSS ISOVF Cingulum cingulate gyrus L . . . 615
616 dMRI TBSS ISOVF Cingulum hippocampus R . . . 616
617 dMRI TBSS ISOVF Cingulum hippocampus L . . . 617
618 dMRI TBSS ISOVF Fornix cres+Stria terminalis R . . . 618
619 dMRI TBSS ISOVF Fornix cres+Stria terminalis L . . . 619
620 dMRI TBSS ISOVF Superior longitudinal fasciculus R . . . 620
621 dMRI TBSS ISOVF Superior longitudinal fasciculus L . . . 621
622 dMRI TBSS ISOVF Superior fronto-occipital fasciculus R . . . 622
623 dMRI TBSS ISOVF Superior fronto-occipital fasciculus L . . . 623
624 dMRI TBSS ISOVF Uncinate fasciculus R . . . 624
625 dMRI TBSS ISOVF Uncinate fasciculus L . . . 625
626 dMRI TBSS ISOVF Tapetum R . . . 626
627 dMRI TBSS ISOVF Tapetum L . . . 627
628 dMRI ProbtrackX FA ar l . . . 628
629 dMRI ProbtrackX FA ar r . . . 629
630 dMRI ProbtrackX FA atr l . . . 630
631 dMRI ProbtrackX FA atr r . . . 631
632 dMRI ProbtrackX FA cgc l . . . 632
633 dMRI ProbtrackX FA cgc r . . . 633
634 dMRI ProbtrackX FA cgh l . . . 634
635 dMRI ProbtrackX FA cgh r . . . 635
636 dMRI ProbtrackX FA cst l . . . 636
637 dMRI ProbtrackX FA cst r . . . 637
638 dMRI ProbtrackX FA fma . . . 638
639 dMRI ProbtrackX FA fmi . . . 639
640 dMRI ProbtrackX FA ifo l . . . 640
641 dMRI ProbtrackX FA ifo r . . . 641
642 dMRI ProbtrackX FA ilf l . . . 642
643 dMRI ProbtrackX FA ilf r . . . 643
644 dMRI ProbtrackX FA mcp . . . 644
645 dMRI ProbtrackX FA ml l . . . 645
646 dMRI ProbtrackX FA ml r . . . 646
647 dMRI ProbtrackX FA ptr l . . . 647
648 dMRI ProbtrackX FA ptr r . . . 648

649 dMRI ProbtrackX FA slf l 649
650 dMRI ProbtrackX FA slf r 650
651 dMRI ProbtrackX FA str l 651
652 dMRI ProbtrackX FA str r 652
653 dMRI ProbtrackX FA unc l 653
654 dMRI ProbtrackX FA unc r 654
655 dMRI ProbtrackX MD ar l 655
656 dMRI ProbtrackX MD ar r 656
657 dMRI ProbtrackX MD atr l 657
658 dMRI ProbtrackX MD atr r 658
659 dMRI ProbtrackX MD cgc l 659
660 dMRI ProbtrackX MD cgc r 660
661 dMRI ProbtrackX MD cgh l 661
662 dMRI ProbtrackX MD cgh r 662
663 dMRI ProbtrackX MD cst l 663
664 dMRI ProbtrackX MD cst r 664
665 dMRI ProbtrackX MD fma 665
666 dMRI ProbtrackX MD fmi 666
667 dMRI ProbtrackX MD ifo l 667
668 dMRI ProbtrackX MD ifo r 668
669 dMRI ProbtrackX MD ilf l 669
670 dMRI ProbtrackX MD ilf r 670
671 dMRI ProbtrackX MD mcp 671
672 dMRI ProbtrackX MD ml l 672
673 dMRI ProbtrackX MD ml r 673
674 dMRI ProbtrackX MD ptr l 674
675 dMRI ProbtrackX MD ptr r 675
676 dMRI ProbtrackX MD slf l 676
677 dMRI ProbtrackX MD slf r 677
678 dMRI ProbtrackX MD str l 678
679 dMRI ProbtrackX MD str r 679
680 dMRI ProbtrackX MD unc l 680
681 dMRI ProbtrackX MD unc r 681
682 dMRI ProbtrackX MO ar l 682
683 dMRI ProbtrackX MO ar r 683
684 dMRI ProbtrackX MO atr l 684
685 dMRI ProbtrackX MO atr r 685
686 dMRI ProbtrackX MO cgc l 686
687 dMRI ProbtrackX MO cgc r 687
688 dMRI ProbtrackX MO cgh l 688
689 dMRI ProbtrackX MO cgh r 689
690 dMRI ProbtrackX MO cst l 690
691 dMRI ProbtrackX MO cst r 691
692 dMRI ProbtrackX MO fma 692
693 dMRI ProbtrackX MO fmi 693
694 dMRI ProbtrackX MO ifo l 694
695 dMRI ProbtrackX MO ifo r 695
696 dMRI ProbtrackX MO ilf l 696
697 dMRI ProbtrackX MO ilf r 697
698 dMRI ProbtrackX MO mcp 698
699 dMRI ProbtrackX MO ml l 699
700 dMRI ProbtrackX MO ml r 700
701 dMRI ProbtrackX MO ptr l 701
702 dMRI ProbtrackX MO ptr r 702
703 dMRI ProbtrackX MO slf l 703
704 dMRI ProbtrackX MO slf r 704
705 dMRI ProbtrackX MO str l 705
706 dMRI ProbtrackX MO str r 706
707 dMRI ProbtrackX MO unc l 707
708 dMRI ProbtrackX MO unc r 708

709 dMRI ProbtrackX L1 ar l . . . 709
710 dMRI ProbtrackX L1 ar r . . . 710
711 dMRI ProbtrackX L1 atr l . . . 711
712 dMRI ProbtrackX L1 atr r . . . 712
713 dMRI ProbtrackX L1 cgc l . . . 713
714 dMRI ProbtrackX L1 cgc r . . . 714
715 dMRI ProbtrackX L1 cgh l . . . 715
716 dMRI ProbtrackX L1 cgh r . . . 716
717 dMRI ProbtrackX L1 cst l . . . 717
718 dMRI ProbtrackX L1 cst r . . . 718
719 dMRI ProbtrackX L1 fma . . . 719
720 dMRI ProbtrackX L1 fmi . . . 720
721 dMRI ProbtrackX L1 ifo l . . . 721
722 dMRI ProbtrackX L1 ifo r . . . 722
723 dMRI ProbtrackX L1 ilf l . . . 723
724 dMRI ProbtrackX L1 ilf r . . . 724
725 dMRI ProbtrackX L1 mcp . . . 725
726 dMRI ProbtrackX L1 ml l . . . 726
727 dMRI ProbtrackX L1 ml r . . . 727
728 dMRI ProbtrackX L1 ptr l . . . 728
729 dMRI ProbtrackX L1 ptr r . . . 729
730 dMRI ProbtrackX L1 slf l . . . 730
731 dMRI ProbtrackX L1 slf r . . . 731
732 dMRI ProbtrackX L1 str l . . . 732
733 dMRI ProbtrackX L1 str r . . . 733
734 dMRI ProbtrackX L1 unc l . . . 734
735 dMRI ProbtrackX L1 unc r . . . 735
736 dMRI ProbtrackX L2 ar l . . . 736
737 dMRI ProbtrackX L2 ar r . . . 737
738 dMRI ProbtrackX L2 atr l . . . 738
739 dMRI ProbtrackX L2 atr r . . . 739
740 dMRI ProbtrackX L2 cgc l . . . 740
741 dMRI ProbtrackX L2 cgc r . . . 741
742 dMRI ProbtrackX L2 cgh l . . . 742
743 dMRI ProbtrackX L2 cgh r . . . 743
744 dMRI ProbtrackX L2 cst l . . . 744
745 dMRI ProbtrackX L2 cst r . . . 745
746 dMRI ProbtrackX L2 fma . . . 746
747 dMRI ProbtrackX L2 fmi . . . 747
748 dMRI ProbtrackX L2 ifo l . . . 748
749 dMRI ProbtrackX L2 ifo r . . . 749
750 dMRI ProbtrackX L2 ilf l . . . 750
751 dMRI ProbtrackX L2 ilf r . . . 751
752 dMRI ProbtrackX L2 mcp . . . 752
753 dMRI ProbtrackX L2 ml l . . . 753
754 dMRI ProbtrackX L2 ml r . . . 754
755 dMRI ProbtrackX L2 ptr l . . . 755
756 dMRI ProbtrackX L2 ptr r . . . 756
757 dMRI ProbtrackX L2 slf l . . . 757
758 dMRI ProbtrackX L2 slf r . . . 758
759 dMRI ProbtrackX L2 str l . . . 759
760 dMRI ProbtrackX L2 str r . . . 760
761 dMRI ProbtrackX L2 unc l . . . 761
762 dMRI ProbtrackX L2 unc r . . . 762
763 dMRI ProbtrackX L3 ar l . . . 763
764 dMRI ProbtrackX L3 ar r . . . 764
765 dMRI ProbtrackX L3 atr l . . . 765
766 dMRI ProbtrackX L3 atr r . . . 766
767 dMRI ProbtrackX L3 cgc l . . . 767
768 dMRI ProbtrackX L3 cgc r . . . 768

769 dMRI ProbtrackX L3 cgh l . . . 769
770 dMRI ProbtrackX L3 cgh r . . . 770
771 dMRI ProbtrackX L3 cst l . . . 771
772 dMRI ProbtrackX L3 cst r . . . 772
773 dMRI ProbtrackX L3 fma . . . 773
774 dMRI ProbtrackX L3 fmi . . . 774
775 dMRI ProbtrackX L3 ifo l . . . 775
776 dMRI ProbtrackX L3 ifo r . . . 776
777 dMRI ProbtrackX L3 ilf l . . . 777
778 dMRI ProbtrackX L3 ilf r . . . 778
779 dMRI ProbtrackX L3 mcp . . . 779
780 dMRI ProbtrackX L3 ml l . . . 780
781 dMRI ProbtrackX L3 ml r . . . 781
782 dMRI ProbtrackX L3 ptr l . . . 782
783 dMRI ProbtrackX L3 ptr r . . . 783
784 dMRI ProbtrackX L3 slf l . . . 784
785 dMRI ProbtrackX L3 slf r . . . 785
786 dMRI ProbtrackX L3 str l . . . 786
787 dMRI ProbtrackX L3 str r . . . 787
788 dMRI ProbtrackX L3 unc l . . . 788
789 dMRI ProbtrackX L3 unc r . . . 789
790 dMRI ProbtrackX ICVF ar l . . . 790
791 dMRI ProbtrackX ICVF ar r . . . 791
792 dMRI ProbtrackX ICVF atr l . . . 792
793 dMRI ProbtrackX ICVF atr r . . . 793
794 dMRI ProbtrackX ICVF cgc l . . . 794
795 dMRI ProbtrackX ICVF cgc r . . . 795
796 dMRI ProbtrackX ICVF cgh l . . . 796
797 dMRI ProbtrackX ICVF cgh r . . . 797
798 dMRI ProbtrackX ICVF cst l . . . 798
799 dMRI ProbtrackX ICVF cst r . . . 799
800 dMRI ProbtrackX ICVF fma . . . 800
801 dMRI ProbtrackX ICVF fmi . . . 801
802 dMRI ProbtrackX ICVF ifo l . . . 802
803 dMRI ProbtrackX ICVF ifo r . . . 803
804 dMRI ProbtrackX ICVF ilf l . . . 804
805 dMRI ProbtrackX ICVF ilf r . . . 805
806 dMRI ProbtrackX ICVF mcp . . . 806
807 dMRI ProbtrackX ICVF ml l . . . 807
808 dMRI ProbtrackX ICVF ml r . . . 808
809 dMRI ProbtrackX ICVF ptr l . . . 809
810 dMRI ProbtrackX ICVF ptr r . . . 810
811 dMRI ProbtrackX ICVF slf l . . . 811
812 dMRI ProbtrackX ICVF slf r . . . 812
813 dMRI ProbtrackX ICVF str l . . . 813
814 dMRI ProbtrackX ICVF str r . . . 814
815 dMRI ProbtrackX ICVF unc l . . . 815
816 dMRI ProbtrackX ICVF unc r . . . 816
817 dMRI ProbtrackX OD ar l . . . 817
818 dMRI ProbtrackX OD ar r . . . 818
819 dMRI ProbtrackX OD atr l . . . 819
820 dMRI ProbtrackX OD atr r . . . 820
821 dMRI ProbtrackX OD cgc l . . . 821
822 dMRI ProbtrackX OD cgc r . . . 822
823 dMRI ProbtrackX OD cgh l . . . 823
824 dMRI ProbtrackX OD cgh r . . . 824
825 dMRI ProbtrackX OD cst l . . . 825
826 dMRI ProbtrackX OD cst r . . . 826
827 dMRI ProbtrackX OD fma . . . 827
828 dMRI ProbtrackX OD fmi . . . 828

829 dMRI ProbtrackX OD ifo l . . . 829
830 dMRI ProbtrackX OD ifo r . . . 830
831 dMRI ProbtrackX OD ilf l . . . 831
832 dMRI ProbtrackX OD ilf r . . . 832
833 dMRI ProbtrackX OD mcp . . . 833
834 dMRI ProbtrackX OD ml l . . . 834
835 dMRI ProbtrackX OD ml r . . . 835
836 dMRI ProbtrackX OD ptr l . . . 836
837 dMRI ProbtrackX OD ptr r . . . 837
838 dMRI ProbtrackX OD slf l . . . 838
839 dMRI ProbtrackX OD slf r . . . 839
840 dMRI ProbtrackX OD str l . . . 840
841 dMRI ProbtrackX OD str r . . . 841
842 dMRI ProbtrackX OD unc l . . . 842
843 dMRI ProbtrackX OD unc r . . . 843
844 dMRI ProbtrackX ISOVF ar l . . . 844
845 dMRI ProbtrackX ISOVF ar r . . . 845
846 dMRI ProbtrackX ISOVF atr l . . . 846
847 dMRI ProbtrackX ISOVF atr r . . . 847
848 dMRI ProbtrackX ISOVF cgc l . . . 848
849 dMRI ProbtrackX ISOVF cgc r . . . 849
850 dMRI ProbtrackX ISOVF cgh l . . . 850
851 dMRI ProbtrackX ISOVF cgh r . . . 851
852 dMRI ProbtrackX ISOVF cst l . . . 852
853 dMRI ProbtrackX ISOVF cst r . . . 853
854 dMRI ProbtrackX ISOVF fma . . . 854
855 dMRI ProbtrackX ISOVF fmi . . . 855
856 dMRI ProbtrackX ISOVF ifo l . . . 856
857 dMRI ProbtrackX ISOVF ifo r . . . 857
858 dMRI ProbtrackX ISOVF ilf l . . . 858
859 dMRI ProbtrackX ISOVF ilf r . . . 859
860 dMRI ProbtrackX ISOVF mcp . . . 860
861 dMRI ProbtrackX ISOVF ml l . . . 861
862 dMRI ProbtrackX ISOVF ml r . . . 862
863 dMRI ProbtrackX ISOVF ptr l . . . 863
864 dMRI ProbtrackX ISOVF ptr r . . . 864
865 dMRI ProbtrackX ISOVF slf l . . . 865
866 dMRI ProbtrackX ISOVF slf r . . . 866
867 dMRI ProbtrackX ISOVF str l . . . 867
868 dMRI ProbtrackX ISOVF str r . . . 868
869 dMRI ProbtrackX ISOVF unc l . . . 869
870 dMRI ProbtrackX ISOVF unc r . . . 870
871 NETMAT amplitudes (25) 01 . . . 871
872 NETMAT amplitudes (25) 02 . . . 872
873 NETMAT amplitudes (25) 03 . . . 873
874 NETMAT amplitudes (25) 04 . . . 874
875 NETMAT amplitudes (25) 05 . . . 875
876 NETMAT amplitudes (25) 06 . . . 876
877 NETMAT amplitudes (25) 07 . . . 877
878 NETMAT amplitudes (25) 08 . . . 878
879 NETMAT amplitudes (25) 09 . . . 879
880 NETMAT amplitudes (25) 10 . . . 880
881 NETMAT amplitudes (25) 11 . . . 881
882 NETMAT amplitudes (25) 12 . . . 882
883 NETMAT amplitudes (25) 13 . . . 883
884 NETMAT amplitudes (25) 14 . . . 884
885 NETMAT amplitudes (25) 15 . . . 885
886 NETMAT amplitudes (25) 16 . . . 886
887 NETMAT amplitudes (25) 17 . . . 887
888 NETMAT amplitudes (25) 18 . . . 888

889 NETMAT amplitudes (25) 19 889
890 NETMAT amplitudes (25) 20 890
891 NETMAT amplitudes (25) 21 891
892 NETMAT amplitudes (100) 01 892
893 NETMAT amplitudes (100) 02 893
894 NETMAT amplitudes (100) 03 894
895 NETMAT amplitudes (100) 04 895
896 NETMAT amplitudes (100) 05 896
897 NETMAT amplitudes (100) 06 897
898 NETMAT amplitudes (100) 07 898
899 NETMAT amplitudes (100) 08 899
900 NETMAT amplitudes (100) 09 900
901 NETMAT amplitudes (100) 10 901
902 NETMAT amplitudes (100) 11 902
903 NETMAT amplitudes (100) 12 903
904 NETMAT amplitudes (100) 13 904
905 NETMAT amplitudes (100) 14 905
906 NETMAT amplitudes (100) 15 906
907 NETMAT amplitudes (100) 16 907
908 NETMAT amplitudes (100) 17 908
909 NETMAT amplitudes (100) 18 909
910 NETMAT amplitudes (100) 19 910
911 NETMAT amplitudes (100) 20 911
912 NETMAT amplitudes (100) 21 912
913 NETMAT amplitudes (100) 22 913
914 NETMAT amplitudes (100) 23 914
915 NETMAT amplitudes (100) 24 915
916 NETMAT amplitudes (100) 25 916
917 NETMAT amplitudes (100) 26 917
918 NETMAT amplitudes (100) 27 918
919 NETMAT amplitudes (100) 28 919
920 NETMAT amplitudes (100) 29 920
921 NETMAT amplitudes (100) 30 921
922 NETMAT amplitudes (100) 31 922
923 NETMAT amplitudes (100) 32 923
924 NETMAT amplitudes (100) 33 924
925 NETMAT amplitudes (100) 34 925
926 NETMAT amplitudes (100) 35 926
927 NETMAT amplitudes (100) 36 927
928 NETMAT amplitudes (100) 37 928
929 NETMAT amplitudes (100) 38 929
930 NETMAT amplitudes (100) 39 930
931 NETMAT amplitudes (100) 40 931
932 NETMAT amplitudes (100) 41 932
933 NETMAT amplitudes (100) 42 933
934 NETMAT amplitudes (100) 43 934
935 NETMAT amplitudes (100) 44 935
936 NETMAT amplitudes (100) 45 936
937 NETMAT amplitudes (100) 46 937
938 NETMAT amplitudes (100) 47 938
939 NETMAT amplitudes (100) 48 939
940 NETMAT amplitudes (100) 49 940
941 NETMAT amplitudes (100) 50 941
942 NETMAT amplitudes (100) 51 942
943 NETMAT amplitudes (100) 52 943
944 NETMAT amplitudes (100) 53 944
945 NETMAT amplitudes (100) 54 945
946 NETMAT amplitudes (100) 55 946
947 T1 FIRST left plus right thalamus volume 947
948 T1 FIRST left plus right caudate volume 948

949 T1 FIRST left plus right putamen volume . . . 949
950 T1 FIRST left plus right pallidum volume . . . 950
951 T1 FIRST left plus right hippocampus volume . . . 951
952 T1 FIRST left plus right amygdala volume . . . 952
953 T1 FIRST left plus right accumbens volume . . . 953
954 SWI T2star left plus right thalamus . . . 954
955 SWI T2star left plus right caudate . . . 955
956 SWI T2star left plus right putamen . . . 956
957 SWI T2star left plus right pallidum . . . 957
958 SWI T2star left plus right hippocampus . . . 958
959 SWI T2star left plus right amygdala . . . 959
960 SWI T2star left plus right accumbens . . . 960
961 FreeSurfer volume Left-Lateral-Ventricle . . . 961
962 FreeSurfer volume Left-Inf-Lat-Vent . . . 962
963 FreeSurfer volume Left-Cerebellum-White-Matter . . . 963
964 FreeSurfer volume Left-Cerebellum-Cortex . . . 964
965 FreeSurfer volume Left-Thalamus-Proper . . . 965
966 FreeSurfer volume Left-Caudate . . . 966
967 FreeSurfer volume Left-Putamen . . . 967
968 FreeSurfer volume Left-Pallidum . . . 968
969 FreeSurfer volume 3rd-Ventricle . . . 969
970 FreeSurfer volume 4th-Ventricle . . . 970
971 FreeSurfer volume Brain-Stem . . . 971
972 FreeSurfer volume Left-Hippocampus . . . 972
973 FreeSurfer volume Left-Amygdala . . . 973
974 FreeSurfer volume CSF . . . 974
975 FreeSurfer volume Left-Accumbens-area . . . 975
976 FreeSurfer volume Left-VentralDC . . . 976
977 FreeSurfer volume Left-vessel . . . 977
978 FreeSurfer volume Left-choroid-plexus . . . 978
979 FreeSurfer volume Right-Lateral-Ventricle . . . 979
980 FreeSurfer volume Right-Inf-Lat-Vent . . . 980
981 FreeSurfer volume Right-Cerebellum-White-Matter . . . 981
982 FreeSurfer volume Right-Cerebellum-Cortex . . . 982
983 FreeSurfer volume Right-Thalamus-Proper . . . 983
984 FreeSurfer volume Right-Caudate . . . 984
985 FreeSurfer volume Right-Putamen . . . 985
986 FreeSurfer volume Right-Pallidum . . . 986
987 FreeSurfer volume Right-Hippocampus . . . 987
988 FreeSurfer volume Right-Amygdala . . . 988
989 FreeSurfer volume Right-Accumbens-area . . . 989
990 FreeSurfer volume Right-VentralDC . . . 990
991 FreeSurfer volume Right-vessel . . . 991
992 FreeSurfer volume Right-choroid-plexus . . . 992
993 FreeSurfer volume Optic-Chiasm . . . 993
994 FreeSurfer volume CC Posterior . . . 994
995 FreeSurfer volume CC Mid Posterior . . . 995
996 FreeSurfer volume CC Central . . . 996
997 FreeSurfer volume CC Mid Anterior . . . 997
998 FreeSurfer volume CC Anterior . . . 998
999 FreeSurfer volume BrainSegVol . . . 999
1000 FreeSurfer volume BrainSegVolNotVent . . . 1000
1001 FreeSurfer volume BrainSegVolNotVentSurf . . . 1001
1002 FreeSurfer volume lhCortexVol . . . 1002
1003 FreeSurfer volume rhCortexVol . . . 1003
1004 FreeSurfer volume CortexVol . . . 1004
1005 FreeSurfer volume lhCerebralWhiteMatterVol . . . 1005
1006 FreeSurfer volume rhCerebralWhiteMatterVol . . . 1006
1007 FreeSurfer volume CerebralWhiteMatterVol . . . 1007
1008 FreeSurfer volume SubCortGrayVol . . . 1008

1009 FreeSurfer volume TotalGrayVol . . . 1009
1010 FreeSurfer volume SupraTentorialVol . . . 1010
1011 FreeSurfer volume SupraTentorialVolNotVent . . . 1011
1012 FreeSurfer volume SupraTentorialVolNotVentVox . . . 1012
1013 FreeSurfer volume MaskVol . . . 1013
1014 FreeSurfer volume BrainSegVol-to-eTIV . . . 1014
1015 FreeSurfer volume MaskVol-to-eTIV . . . 1015
1016 FreeSurfer volume lhSurfaceHoles . . . 1016
1017 FreeSurfer volume rhSurfaceHoles . . . 1017
1018 FreeSurfer volume SurfaceHoles . . . 1018
1019 FreeSurfer volume EstimatedTotalIntraCranialVol . . . 1019
1020 FreeSurfer DKTatlas lh caudalanteriorcingulate area . . . 1020
1021 FreeSurfer DKTatlas lh caudalmiddlefrontal area . . . 1021
1022 FreeSurfer DKTatlas lh cuneus area . . . 1022
1023 FreeSurfer DKTatlas lh entorhinal area . . . 1023
1024 FreeSurfer DKTatlas lh fusiform area . . . 1024
1025 FreeSurfer DKTatlas lh inferiorparietal area . . . 1025
1026 FreeSurfer DKTatlas lh inferiortemporal area . . . 1026
1027 FreeSurfer DKTatlas lh isthmuscingulate area . . . 1027
1028 FreeSurfer DKTatlas lh lateraloccipital area . . . 1028
1029 FreeSurfer DKTatlas lh lateralorbitofrontal area . . . 1029
1030 FreeSurfer DKTatlas lh lingual area . . . 1030
1031 FreeSurfer DKTatlas lh medialorbitofrontal area . . . 1031
1032 FreeSurfer DKTatlas lh middletemporal area . . . 1032
1033 FreeSurfer DKTatlas lh parahippocampal area . . . 1033
1034 FreeSurfer DKTatlas lh paracentral area . . . 1034
1035 FreeSurfer DKTatlas lh parsopercularis area . . . 1035
1036 FreeSurfer DKTatlas lh parsorbitalis area . . . 1036
1037 FreeSurfer DKTatlas lh parstriangularis area . . . 1037
1038 FreeSurfer DKTatlas lh pericalcarine area . . . 1038
1039 FreeSurfer DKTatlas lh postcentral area . . . 1039
1040 FreeSurfer DKTatlas lh posteriorcingulate area . . . 1040
1041 FreeSurfer DKTatlas lh precentral area . . . 1041
1042 FreeSurfer DKTatlas lh precuneus area . . . 1042
1043 FreeSurfer DKTatlas lh rostralanteriorcingulate area . . . 1043
1044 FreeSurfer DKTatlas lh rostralmiddlefrontal area . . . 1044
1045 FreeSurfer DKTatlas lh superiorfrontal area . . . 1045
1046 FreeSurfer DKTatlas lh superiorparietal area . . . 1046
1047 FreeSurfer DKTatlas lh superiortemporal area . . . 1047
1048 FreeSurfer DKTatlas lh supramarginal area . . . 1048
1049 FreeSurfer DKTatlas lh transversetemporal area . . . 1049
1050 FreeSurfer DKTatlas lh insula area . . . 1050
1051 FreeSurfer DKTatlas lh WhiteSurfArea area . . . 1051
1052 FreeSurfer a2009s lh G and S frontomargin area . . . 1052
1053 FreeSurfer a2009s lh G and S occipital inf area . . . 1053
1054 FreeSurfer a2009s lh G and S paracentral area . . . 1054
1055 FreeSurfer a2009s lh G and S subcentral area . . . 1055
1056 FreeSurfer a2009s lh G and S transv frontopol area . . . 1056
1057 FreeSurfer a2009s lh G and S cingul-Ant area . . . 1057
1058 FreeSurfer a2009s lh G and S cingul-Mid-Ant area . . . 1058
1059 FreeSurfer a2009s lh G and S cingul-Mid-Post area . . . 1059
1060 FreeSurfer a2009s lh G cingul-Post-dorsal area . . . 1060
1061 FreeSurfer a2009s lh G cingul-Post-ventral area . . . 1061
1062 FreeSurfer a2009s lh G cuneus area . . . 1062
1063 FreeSurfer a2009s lh G front inf-Opercular area . . . 1063
1064 FreeSurfer a2009s lh G front inf-Orbital area . . . 1064
1065 FreeSurfer a2009s lh G front inf-Triangul area . . . 1065
1066 FreeSurfer a2009s lh G front middle area . . . 1066
1067 FreeSurfer a2009s lh G front sup area . . . 1067
1068 FreeSurfer a2009s lh G Ins lg and S cent ins area . . . 1068

1069 FreeSurfer a2009s lh G insular short area . . . 1069
1070 FreeSurfer a2009s lh G occipital middle area . . . 1070
1071 FreeSurfer a2009s lh G occipital sup area . . . 1071
1072 FreeSurfer a2009s lh G oc-temp lat-fusifor area . . . 1072
1073 FreeSurfer a2009s lh G oc-temp med-Lingual area . . . 1073
1074 FreeSurfer a2009s lh G oc-temp med-Parahip area . . . 1074
1075 FreeSurfer a2009s lh G orbital area . . . 1075
1076 FreeSurfer a2009s lh G pariet inf-Angular area . . . 1076
1077 FreeSurfer a2009s lh G pariet inf-Supramar area . . . 1077
1078 FreeSurfer a2009s lh G parietal sup area . . . 1078
1079 FreeSurfer a2009s lh G postcentral area . . . 1079
1080 FreeSurfer a2009s lh G precentral area . . . 1080
1081 FreeSurfer a2009s lh G precuneus area . . . 1081
1082 FreeSurfer a2009s lh G rectus area . . . 1082
1083 FreeSurfer a2009s lh G subcallosal area . . . 1083
1084 FreeSurfer a2009s lh G temp sup-G T transv area . . . 1084
1085 FreeSurfer a2009s lh G temp sup-Lateral area . . . 1085
1086 FreeSurfer a2009s lh G temp sup-Plan polar area . . . 1086
1087 FreeSurfer a2009s lh G temp sup-Plan tempo area . . . 1087
1088 FreeSurfer a2009s lh G temporal inf area . . . 1088
1089 FreeSurfer a2009s lh G temporal middle area . . . 1089
1090 FreeSurfer a2009s lh Lat Fis-ant-Horizont area . . . 1090
1091 FreeSurfer a2009s lh Lat Fis-ant-Vertical area . . . 1091
1092 FreeSurfer a2009s lh Lat Fis-post area . . . 1092
1093 FreeSurfer a2009s lh Pole occipital area . . . 1093
1094 FreeSurfer a2009s lh Pole temporal area . . . 1094
1095 FreeSurfer a2009s lh S calcarine area . . . 1095
1096 FreeSurfer a2009s lh S central area . . . 1096
1097 FreeSurfer a2009s lh S cingul-Marginalis area . . . 1097
1098 FreeSurfer a2009s lh S circular insula ant area . . . 1098
1099 FreeSurfer a2009s lh S circular insula inf area . . . 1099
1100 FreeSurfer a2009s lh S circular insula sup area . . . 1100
1101 FreeSurfer a2009s lh S collat transv ant area . . . 1101
1102 FreeSurfer a2009s lh S collat transv post area . . . 1102
1103 FreeSurfer a2009s lh S front inf area . . . 1103
1104 FreeSurfer a2009s lh S front middle area . . . 1104
1105 FreeSurfer a2009s lh S front sup area . . . 1105
1106 FreeSurfer a2009s lh S interm prim-Jensen area . . . 1106
1107 FreeSurfer a2009s lh S intrapariet and P trans area . . . 1107
1108 FreeSurfer a2009s lh S oc middle and Lunatus area . . . 1108
1109 FreeSurfer a2009s lh S oc sup and transversal area . . . 1109
1110 FreeSurfer a2009s lh S occipital ant area . . . 1110
1111 FreeSurfer a2009s lh S oc-temp lat area . . . 1111
1112 FreeSurfer a2009s lh S oc-temp med and Lingual area . . . 1112
1113 FreeSurfer a2009s lh S orbital lateral area . . . 1113
1114 FreeSurfer a2009s lh S orbital med-olfact area . . . 1114
1115 FreeSurfer a2009s lh S orbital-H Shaped area . . . 1115
1116 FreeSurfer a2009s lh S parieto occipital area . . . 1116
1117 FreeSurfer a2009s lh S pericallosal area . . . 1117
1118 FreeSurfer a2009s lh S postcentral area . . . 1118
1119 FreeSurfer a2009s lh S precentral-inf-part area . . . 1119
1120 FreeSurfer a2009s lh S precentral-sup-part area . . . 1120
1121 FreeSurfer a2009s lh S suborbital area . . . 1121
1122 FreeSurfer a2009s lh S subparietal area . . . 1122
1123 FreeSurfer a2009s lh S temporal inf area . . . 1123
1124 FreeSurfer a2009s lh S temporal sup area . . . 1124
1125 FreeSurfer a2009s lh S temporal transverse area . . . 1125
1126 FreeSurfer DKTatlas rh caudalanteriorcingulate area . . . 1126
1127 FreeSurfer DKTatlas rh caudalmiddlefrontal area . . . 1127
1128 FreeSurfer DKTatlas rh cuneus area . . . 1128

1129 FreeSurfer DKTatlas rh entorhinal area . . . 1129
1130 FreeSurfer DKTatlas rh fusiform area . . . 1130
1131 FreeSurfer DKTatlas rh inferiorparietal area . . . 1131
1132 FreeSurfer DKTatlas rh inferiortemporal area . . . 1132
1133 FreeSurfer DKTatlas rh isthmuscingulate area . . . 1133
1134 FreeSurfer DKTatlas rh lateraloccipital area . . . 1134
1135 FreeSurfer DKTatlas rh lateralorbitofrontal area . . . 1135
1136 FreeSurfer DKTatlas rh lingual area . . . 1136
1137 FreeSurfer DKTatlas rh medialorbitofrontal area . . . 1137
1138 FreeSurfer DKTatlas rh middletemporal area . . . 1138
1139 FreeSurfer DKTatlas rh parahippocampal area . . . 1139
1140 FreeSurfer DKTatlas rh paracentral area . . . 1140
1141 FreeSurfer DKTatlas rh parsopercularis area . . . 1141
1142 FreeSurfer DKTatlas rh parsorbitalis area . . . 1142
1143 FreeSurfer DKTatlas rh parstriangularis area . . . 1143
1144 FreeSurfer DKTatlas rh pericalcarine area . . . 1144
1145 FreeSurfer DKTatlas rh postcentral area . . . 1145
1146 FreeSurfer DKTatlas rh posteriorcingulate area . . . 1146
1147 FreeSurfer DKTatlas rh precentral area . . . 1147
1148 FreeSurfer DKTatlas rh precuneus area . . . 1148
1149 FreeSurfer DKTatlas rh rostralanteriorcingulate area . . . 1149
1150 FreeSurfer DKTatlas rh rostralmiddlefrontal area . . . 1150
1151 FreeSurfer DKTatlas rh superiorfrontal area . . . 1151
1152 FreeSurfer DKTatlas rh superiorparietal area . . . 1152
1153 FreeSurfer DKTatlas rh superiortemporal area . . . 1153
1154 FreeSurfer DKTatlas rh supramarginal area . . . 1154
1155 FreeSurfer DKTatlas rh transversetemporal area . . . 1155
1156 FreeSurfer DKTatlas rh insula area . . . 1156
1157 FreeSurfer DKTatlas rh WhiteSurfArea area . . . 1157
1158 FreeSurfer a2009s rh G and S frontomargin area . . . 1158
1159 FreeSurfer a2009s rh G and S occipital inf area . . . 1159
1160 FreeSurfer a2009s rh G and S paracentral area . . . 1160
1161 FreeSurfer a2009s rh G and S subcentral area . . . 1161
1162 FreeSurfer a2009s rh G and S transv frontopol area . . . 1162
1163 FreeSurfer a2009s rh G and S cingul-Ant area . . . 1163
1164 FreeSurfer a2009s rh G and S cingul-Mid-Ant area . . . 1164
1165 FreeSurfer a2009s rh G and S cingul-Mid-Post area . . . 1165
1166 FreeSurfer a2009s rh G cingul-Post-dorsal area . . . 1166
1167 FreeSurfer a2009s rh G cingul-Post-ventral area . . . 1167
1168 FreeSurfer a2009s rh G cuneus area . . . 1168
1169 FreeSurfer a2009s rh G front inf-Opercular area . . . 1169
1170 FreeSurfer a2009s rh G front inf-Orbital area . . . 1170
1171 FreeSurfer a2009s rh G front inf-Triangul area . . . 1171
1172 FreeSurfer a2009s rh G front middle area . . . 1172
1173 FreeSurfer a2009s rh G front sup area . . . 1173
1174 FreeSurfer a2009s rh G Ins lg and S cent ins area . . . 1174
1175 FreeSurfer a2009s rh G insular short area . . . 1175
1176 FreeSurfer a2009s rh G occipital middle area . . . 1176
1177 FreeSurfer a2009s rh G occipital sup area . . . 1177
1178 FreeSurfer a2009s rh G oc-temp lat-fusifor area . . . 1178
1179 FreeSurfer a2009s rh G oc-temp med-Lingual area . . . 1179
1180 FreeSurfer a2009s rh G oc-temp med-Parahip area . . . 1180
1181 FreeSurfer a2009s rh G orbital area . . . 1181
1182 FreeSurfer a2009s rh G pariet inf-Angular area . . . 1182
1183 FreeSurfer a2009s rh G pariet inf-Supramar area . . . 1183
1184 FreeSurfer a2009s rh G parietal sup area . . . 1184
1185 FreeSurfer a2009s rh G postcentral area . . . 1185
1186 FreeSurfer a2009s rh G precentral area . . . 1186
1187 FreeSurfer a2009s rh G precuneus area . . . 1187
1188 FreeSurfer a2009s rh G rectus area . . . 1188

1189 FreeSurfer a2009s rh G subcallosal area . . . 1189
1190 FreeSurfer a2009s rh G temp sup-G T transv area . . . 1190
1191 FreeSurfer a2009s rh G temp sup-Lateral area . . . 1191
1192 FreeSurfer a2009s rh G temp sup-Plan polar area . . . 1192
1193 FreeSurfer a2009s rh G temp sup-Plan tempo area . . . 1193
1194 FreeSurfer a2009s rh G temporal inf area . . . 1194
1195 FreeSurfer a2009s rh G temporal middle area . . . 1195
1196 FreeSurfer a2009s rh Lat Fis-ant-Horizont area . . . 1196
1197 FreeSurfer a2009s rh Lat Fis-ant-Vertical area . . . 1197
1198 FreeSurfer a2009s rh Lat Fis-post area . . . 1198
1199 FreeSurfer a2009s rh Pole occipital area . . . 1199
1200 FreeSurfer a2009s rh Pole temporal area . . . 1200
1201 FreeSurfer a2009s rh S calcarine area . . . 1201
1202 FreeSurfer a2009s rh S central area . . . 1202
1203 FreeSurfer a2009s rh S cingul-Marginalis area . . . 1203
1204 FreeSurfer a2009s rh S circular insula ant area . . . 1204
1205 FreeSurfer a2009s rh S circular insula inf area . . . 1205
1206 FreeSurfer a2009s rh S circular insula sup area . . . 1206
1207 FreeSurfer a2009s rh S collat transv ant area . . . 1207
1208 FreeSurfer a2009s rh S collat transv post area . . . 1208
1209 FreeSurfer a2009s rh S front inf area . . . 1209
1210 FreeSurfer a2009s rh S front middle area . . . 1210
1211 FreeSurfer a2009s rh S front sup area . . . 1211
1212 FreeSurfer a2009s rh S interm prim-Jensen area . . . 1212
1213 FreeSurfer a2009s rh S intrapariet and P trans area . . . 1213
1214 FreeSurfer a2009s rh S oc middle and Lunatus area . . . 1214
1215 FreeSurfer a2009s rh S oc sup and transversal area . . . 1215
1216 FreeSurfer a2009s rh S occipital ant area . . . 1216
1217 FreeSurfer a2009s rh S oc-temp lat area . . . 1217
1218 FreeSurfer a2009s rh S oc-temp med and Lingual area . . . 1218
1219 FreeSurfer a2009s rh S orbital lateral area . . . 1219
1220 FreeSurfer a2009s rh S orbital med-olfact area . . . 1220
1221 FreeSurfer a2009s rh S orbital-H Shaped area . . . 1221
1222 FreeSurfer a2009s rh S parieto occipital area . . . 1222
1223 FreeSurfer a2009s rh S pericallosal area . . . 1223
1224 FreeSurfer a2009s rh S postcentral area . . . 1224
1225 FreeSurfer a2009s rh S precentral-inf-part area . . . 1225
1226 FreeSurfer a2009s rh S precentral-sup-part area . . . 1226
1227 FreeSurfer a2009s rh S suborbital area . . . 1227
1228 FreeSurfer a2009s rh S subparietal area . . . 1228
1229 FreeSurfer a2009s rh S temporal inf area . . . 1229
1230 FreeSurfer a2009s rh S temporal sup area . . . 1230
1231 FreeSurfer a2009s rh S temporal transverse area . . . 1231
1232 FreeSurfer DKTatlas lh caudalanteriorcingulate thickness . . . 1232
1233 FreeSurfer DKTatlas lh caudalmiddlefrontal thickness . . . 1233
1234 FreeSurfer DKTatlas lh cuneus thickness . . . 1234
1235 FreeSurfer DKTatlas lh entorhinal thickness . . . 1235
1236 FreeSurfer DKTatlas lh fusiform thickness . . . 1236
1237 FreeSurfer DKTatlas lh inferiorparietal thickness . . . 1237
1238 FreeSurfer DKTatlas lh inferiortemporal thickness . . . 1238
1239 FreeSurfer DKTatlas lh isthmuscingulate thickness . . . 1239
1240 FreeSurfer DKTatlas lh lateraloccipital thickness . . . 1240
1241 FreeSurfer DKTatlas lh lateralorbitofrontal thickness . . . 1241
1242 FreeSurfer DKTatlas lh lingual thickness . . . 1242
1243 FreeSurfer DKTatlas lh medialorbitofrontal thickness . . . 1243
1244 FreeSurfer DKTatlas lh middletemporal thickness . . . 1244
1245 FreeSurfer DKTatlas lh parahippocampal thickness . . . 1245
1246 FreeSurfer DKTatlas lh paracentral thickness . . . 1246
1247 FreeSurfer DKTatlas lh parsopercularis thickness . . . 1247
1248 FreeSurfer DKTatlas lh parsorbitalis thickness . . . 1248

1249 FreeSurfer DKTatlas lh parstriangularis thickness . . . 1249
1250 FreeSurfer DKTatlas lh pericalcarine thickness . . . 1250
1251 FreeSurfer DKTatlas lh postcentral thickness . . . 1251
1252 FreeSurfer DKTatlas lh posteriorcingulate thickness . . . 1252
1253 FreeSurfer DKTatlas lh precentral thickness . . . 1253
1254 FreeSurfer DKTatlas lh precuneus thickness . . . 1254
1255 FreeSurfer DKTatlas lh rostralanteriorcingulate thickness . . . 1255
1256 FreeSurfer DKTatlas lh rostralmiddlefrontal thickness . . . 1256
1257 FreeSurfer DKTatlas lh superiorfrontal thickness . . . 1257
1258 FreeSurfer DKTatlas lh superiorparietal thickness . . . 1258
1259 FreeSurfer DKTatlas lh superiortemporal thickness . . . 1259
1260 FreeSurfer DKTatlas lh supramarginal thickness . . . 1260
1261 FreeSurfer DKTatlas lh transversetemporal thickness . . . 1261
1262 FreeSurfer DKTatlas lh insula thickness . . . 1262
1263 FreeSurfer DKTatlas lh MeanThickness thickness . . . 1263
1264 FreeSurfer a2009s lh G and S frontomargin thickness . . . 1264
1265 FreeSurfer a2009s lh G and S occipital inf thickness . . . 1265
1266 FreeSurfer a2009s lh G and S paracentral thickness . . . 1266
1267 FreeSurfer a2009s lh G and S subcentral thickness . . . 1267
1268 FreeSurfer a2009s lh G and S transv frontopol thickness . . . 1268
1269 FreeSurfer a2009s lh G and S cingul-Ant thickness . . . 1269
1270 FreeSurfer a2009s lh G and S cingul-Mid-Ant thickness . . . 1270
1271 FreeSurfer a2009s lh G and S cingul-Mid-Post thickness . . . 1271
1272 FreeSurfer a2009s lh G cingul-Post-dorsal thickness . . . 1272
1273 FreeSurfer a2009s lh G cingul-Post-ventral thickness . . . 1273
1274 FreeSurfer a2009s lh G cuneus thickness . . . 1274
1275 FreeSurfer a2009s lh G front inf-Opercular thickness . . . 1275
1276 FreeSurfer a2009s lh G front inf-Orbital thickness . . . 1276
1277 FreeSurfer a2009s lh G front inf-Triangul thickness . . . 1277
1278 FreeSurfer a2009s lh G front middle thickness . . . 1278
1279 FreeSurfer a2009s lh G front sup thickness . . . 1279
1280 FreeSurfer a2009s lh G Ins lg and S cent ins thickness . . . 1280
1281 FreeSurfer a2009s lh G insular short thickness . . . 1281
1282 FreeSurfer a2009s lh G occipital middle thickness . . . 1282
1283 FreeSurfer a2009s lh G occipital sup thickness . . . 1283
1284 FreeSurfer a2009s lh G oc-temp lat-fusifor thickness . . . 1284
1285 FreeSurfer a2009s lh G oc-temp med-Lingual thickness . . . 1285
1286 FreeSurfer a2009s lh G oc-temp med-Parahip thickness . . . 1286
1287 FreeSurfer a2009s lh G orbital thickness . . . 1287
1288 FreeSurfer a2009s lh G pariet inf-Angular thickness . . . 1288
1289 FreeSurfer a2009s lh G pariet inf-Supramar thickness . . . 1289
1290 FreeSurfer a2009s lh G parietal sup thickness . . . 1290
1291 FreeSurfer a2009s lh G postcentral thickness . . . 1291
1292 FreeSurfer a2009s lh G precentral thickness . . . 1292
1293 FreeSurfer a2009s lh G precuneus thickness . . . 1293
1294 FreeSurfer a2009s lh G rectus thickness . . . 1294
1295 FreeSurfer a2009s lh G subcallosal thickness . . . 1295
1296 FreeSurfer a2009s lh G temp sup-G T transv thickness . . . 1296
1297 FreeSurfer a2009s lh G temp sup-Lateral thickness . . . 1297
1298 FreeSurfer a2009s lh G temp sup-Plan polar thickness . . . 1298
1299 FreeSurfer a2009s lh G temp sup-Plan tempo thickness . . . 1299
1300 FreeSurfer a2009s lh G temporal inf thickness . . . 1300
1301 FreeSurfer a2009s lh G temporal middle thickness . . . 1301
1302 FreeSurfer a2009s lh Lat Fis-ant-Horizont thickness . . . 1302
1303 FreeSurfer a2009s lh Lat Fis-ant-Vertical thickness . . . 1303
1304 FreeSurfer a2009s lh Lat Fis-post thickness . . . 1304
1305 FreeSurfer a2009s lh Pole occipital thickness . . . 1305
1306 FreeSurfer a2009s lh Pole temporal thickness . . . 1306
1307 FreeSurfer a2009s lh S calcarine thickness . . . 1307
1308 FreeSurfer a2009s lh S central thickness . . . 1308

1309 FreeSurfer a2009s lh S cingul-Marginalis thickness . . . 1309
1310 FreeSurfer a2009s lh S circular insula ant thickness . . . 1310
1311 FreeSurfer a2009s lh S circular insula inf thickness . . . 1311
1312 FreeSurfer a2009s lh S circular insula sup thickness . . . 1312
1313 FreeSurfer a2009s lh S collat transv ant thickness . . . 1313
1314 FreeSurfer a2009s lh S collat transv post thickness . . . 1314
1315 FreeSurfer a2009s lh S front inf thickness . . . 1315
1316 FreeSurfer a2009s lh S front middle thickness . . . 1316
1317 FreeSurfer a2009s lh S front sup thickness . . . 1317
1318 FreeSurfer a2009s lh S interm prim-Jensen thickness . . . 1318
1319 FreeSurfer a2009s lh S intrapariet and P trans thickness . . . 1319
1320 FreeSurfer a2009s lh S oc middle and Lunatus thickness . . . 1320
1321 FreeSurfer a2009s lh S oc sup and transversal thickness . . . 1321
1322 FreeSurfer a2009s lh S occipital ant thickness . . . 1322
1323 FreeSurfer a2009s lh S oc-temp lat thickness . . . 1323
1324 FreeSurfer a2009s lh S oc-temp med and Lingual thickness . . . 1324
1325 FreeSurfer a2009s lh S orbital lateral thickness . . . 1325
1326 FreeSurfer a2009s lh S orbital med-olfact thickness . . . 1326
1327 FreeSurfer a2009s lh S orbital-H Shaped thickness . . . 1327
1328 FreeSurfer a2009s lh S parieto occipital thickness . . . 1328
1329 FreeSurfer a2009s lh S pericallosal thickness . . . 1329
1330 FreeSurfer a2009s lh S postcentral thickness . . . 1330
1331 FreeSurfer a2009s lh S precentral-inf-part thickness . . . 1331
1332 FreeSurfer a2009s lh S precentral-sup-part thickness . . . 1332
1333 FreeSurfer a2009s lh S suborbital thickness . . . 1333
1334 FreeSurfer a2009s lh S subparietal thickness . . . 1334
1335 FreeSurfer a2009s lh S temporal inf thickness . . . 1335
1336 FreeSurfer a2009s lh S temporal sup thickness . . . 1336
1337 FreeSurfer a2009s lh S temporal transverse thickness . . . 1337
1338 FreeSurfer DKTatlas rh caudalanteriorcingulate thickness . . . 1338
1339 FreeSurfer DKTatlas rh caudalmiddlefrontal thickness . . . 1339
1340 FreeSurfer DKTatlas rh cuneus thickness . . . 1340
1341 FreeSurfer DKTatlas rh entorhinal thickness . . . 1341
1342 FreeSurfer DKTatlas rh fusiform thickness . . . 1342
1343 FreeSurfer DKTatlas rh inferiorparietal thickness . . . 1343
1344 FreeSurfer DKTatlas rh inferiortemporal thickness . . . 1344
1345 FreeSurfer DKTatlas rh isthmuscingulate thickness . . . 1345
1346 FreeSurfer DKTatlas rh lateraloccipital thickness . . . 1346
1347 FreeSurfer DKTatlas rh lateralorbitofrontal thickness . . . 1347
1348 FreeSurfer DKTatlas rh lingual thickness . . . 1348
1349 FreeSurfer DKTatlas rh medialorbitofrontal thickness . . . 1349
1350 FreeSurfer DKTatlas rh middletemporal thickness . . . 1350
1351 FreeSurfer DKTatlas rh parahippocampal thickness . . . 1351
1352 FreeSurfer DKTatlas rh paracentral thickness . . . 1352
1353 FreeSurfer DKTatlas rh parsopercularis thickness . . . 1353
1354 FreeSurfer DKTatlas rh parsorbitalis thickness . . . 1354
1355 FreeSurfer DKTatlas rh parstriangularis thickness . . . 1355
1356 FreeSurfer DKTatlas rh pericalcarine thickness . . . 1356
1357 FreeSurfer DKTatlas rh postcentral thickness . . . 1357
1358 FreeSurfer DKTatlas rh posteriorcingulate thickness . . . 1358
1359 FreeSurfer DKTatlas rh precentral thickness . . . 1359
1360 FreeSurfer DKTatlas rh precuneus thickness . . . 1360
1361 FreeSurfer DKTatlas rh rostralanteriorcingulate thickness . . . 1361
1362 FreeSurfer DKTatlas rh rostralmiddlefrontal thickness . . . 1362
1363 FreeSurfer DKTatlas rh superiorfrontal thickness . . . 1363
1364 FreeSurfer DKTatlas rh superiorparietal thickness . . . 1364
1365 FreeSurfer DKTatlas rh superiortemporal thickness . . . 1365
1366 FreeSurfer DKTatlas rh supramarginal thickness . . . 1366
1367 FreeSurfer DKTatlas rh transversetemporal thickness . . . 1367
1368 FreeSurfer DKTatlas rh insula thickness . . . 1368

1369 FreeSurfer DKTatlas rh MeanThickness thickness . . . 1369
1370 FreeSurfer a2009s rh G and S frontomargin thickness . . . 1370
1371 FreeSurfer a2009s rh G and S occipital inf thickness . . . 1371
1372 FreeSurfer a2009s rh G and S paracentral thickness . . . 1372
1373 FreeSurfer a2009s rh G and S subcentral thickness . . . 1373
1374 FreeSurfer a2009s rh G and S transv frontopol thickness . . . 1374
1375 FreeSurfer a2009s rh G and S cingul-Ant thickness . . . 1375
1376 FreeSurfer a2009s rh G and S cingul-Mid-Ant thickness . . . 1376
1377 FreeSurfer a2009s rh G and S cingul-Mid-Post thickness . . . 1377
1378 FreeSurfer a2009s rh G cingul-Post-dorsal thickness . . . 1378
1379 FreeSurfer a2009s rh G cingul-Post-ventral thickness . . . 1379
1380 FreeSurfer a2009s rh G cuneus thickness . . . 1380
1381 FreeSurfer a2009s rh G front inf-Opercular thickness . . . 1381
1382 FreeSurfer a2009s rh G front inf-Orbital thickness . . . 1382
1383 FreeSurfer a2009s rh G front inf-Triangul thickness . . . 1383
1384 FreeSurfer a2009s rh G front middle thickness . . . 1384
1385 FreeSurfer a2009s rh G front sup thickness . . . 1385
1386 FreeSurfer a2009s rh G Ins lg and S cent ins thickness . . . 1386
1387 FreeSurfer a2009s rh G insular short thickness . . . 1387
1388 FreeSurfer a2009s rh G occipital middle thickness . . . 1388
1389 FreeSurfer a2009s rh G occipital sup thickness . . . 1389
1390 FreeSurfer a2009s rh G oc-temp lat-fusifor thickness . . . 1390
1391 FreeSurfer a2009s rh G oc-temp med-Lingual thickness . . . 1391
1392 FreeSurfer a2009s rh G oc-temp med-Parahip thickness . . . 1392
1393 FreeSurfer a2009s rh G orbital thickness . . . 1393
1394 FreeSurfer a2009s rh G pariet inf-Angular thickness . . . 1394
1395 FreeSurfer a2009s rh G pariet inf-Supramar thickness . . . 1395
1396 FreeSurfer a2009s rh G parietal sup thickness . . . 1396
1397 FreeSurfer a2009s rh G postcentral thickness . . . 1397
1398 FreeSurfer a2009s rh G precentral thickness . . . 1398
1399 FreeSurfer a2009s rh G precuneus thickness . . . 1399
1400 FreeSurfer a2009s rh G rectus thickness . . . 1400
1401 FreeSurfer a2009s rh G subcallosal thickness . . . 1401
1402 FreeSurfer a2009s rh G temp sup-G T transv thickness . . . 1402
1403 FreeSurfer a2009s rh G temp sup-Lateral thickness . . . 1403
1404 FreeSurfer a2009s rh G temp sup-Plan polar thickness . . . 1404
1405 FreeSurfer a2009s rh G temp sup-Plan tempo thickness . . . 1405
1406 FreeSurfer a2009s rh G temporal inf thickness . . . 1406
1407 FreeSurfer a2009s rh G temporal middle thickness . . . 1407
1408 FreeSurfer a2009s rh Lat Fis-ant-Horizont thickness . . . 1408
1409 FreeSurfer a2009s rh Lat Fis-ant-Vertical thickness . . . 1409
1410 FreeSurfer a2009s rh Lat Fis-post thickness . . . 1410
1411 FreeSurfer a2009s rh Pole occipital thickness . . . 1411
1412 FreeSurfer a2009s rh Pole temporal thickness . . . 1412
1413 FreeSurfer a2009s rh S calcarine thickness . . . 1413
1414 FreeSurfer a2009s rh S central thickness . . . 1414
1415 FreeSurfer a2009s rh S cingul-Marginalis thickness . . . 1415
1416 FreeSurfer a2009s rh S circular insula ant thickness . . . 1416
1417 FreeSurfer a2009s rh S circular insula inf thickness . . . 1417
1418 FreeSurfer a2009s rh S circular insula sup thickness . . . 1418
1419 FreeSurfer a2009s rh S collat transv ant thickness . . . 1419
1420 FreeSurfer a2009s rh S collat transv post thickness . . . 1420
1421 FreeSurfer a2009s rh S front inf thickness . . . 1421
1422 FreeSurfer a2009s rh S front middle thickness . . . 1422
1423 FreeSurfer a2009s rh S front sup thickness . . . 1423
1424 FreeSurfer a2009s rh S interm prim-Jensen thickness . . . 1424
1425 FreeSurfer a2009s rh S intrapariet and P trans thickness . . . 1425
1426 FreeSurfer a2009s rh S oc middle and Lunatus thickness . . . 1426
1427 FreeSurfer a2009s rh S oc sup and transversal thickness . . . 1427
1428 FreeSurfer a2009s rh S occipital ant thickness . . . 1428

1429 FreeSurfer a2009s rh S oc-temp lat thickness . . . 1429
1430 FreeSurfer a2009s rh S oc-temp med and Lingual thickness . . . 1430
1431 FreeSurfer a2009s rh S orbital lateral thickness . . . 1431
1432 FreeSurfer a2009s rh S orbital med-olfact thickness . . . 1432
1433 FreeSurfer a2009s rh S orbital-H Shaped thickness . . . 1433
1434 FreeSurfer a2009s rh S parieto occipital thickness . . . 1434
1435 FreeSurfer a2009s rh S pericallosal thickness . . . 1435
1436 FreeSurfer a2009s rh S postcentral thickness . . . 1436
1437 FreeSurfer a2009s rh S precentral-inf-part thickness . . . 1437
1438 FreeSurfer a2009s rh S precentral-sup-part thickness . . . 1438
1439 FreeSurfer a2009s rh S suborbital thickness . . . 1439
1440 FreeSurfer a2009s rh S subparietal thickness . . . 1440
1441 FreeSurfer a2009s rh S temporal inf thickness . . . 1441
1442 FreeSurfer a2009s rh S temporal sup thickness . . . 1442
1443 FreeSurfer a2009s rh S temporal transverse thickness . . . 1443
1444 NETMAT edge ICA 1 . . . 1444
1445 NETMAT edge ICA 2 . . . 1445
1446 NETMAT edge ICA 3 . . . 1446
1447 NETMAT edge ICA 4 . . . 1447
1448 NETMAT edge ICA 5 . . . 1448
1449 NETMAT edge ICA 6 . . . 1449

Figure 1: T1 SIENAX peripheral grey normalised volume

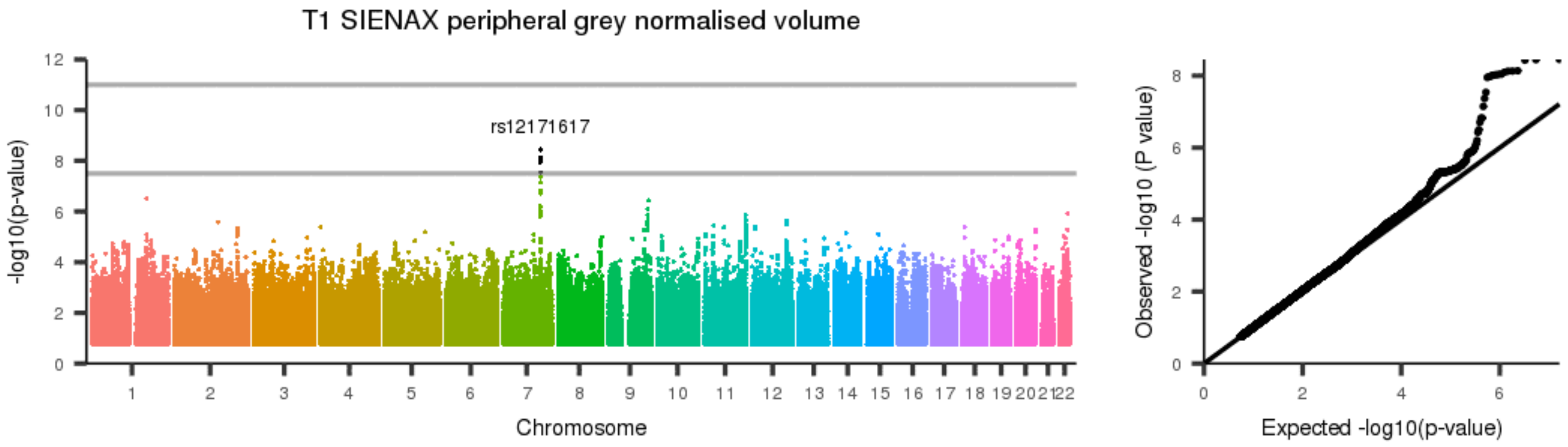

back to list of plots

Figure 2: T1 SIENAX peripheral grey unnormalised volume

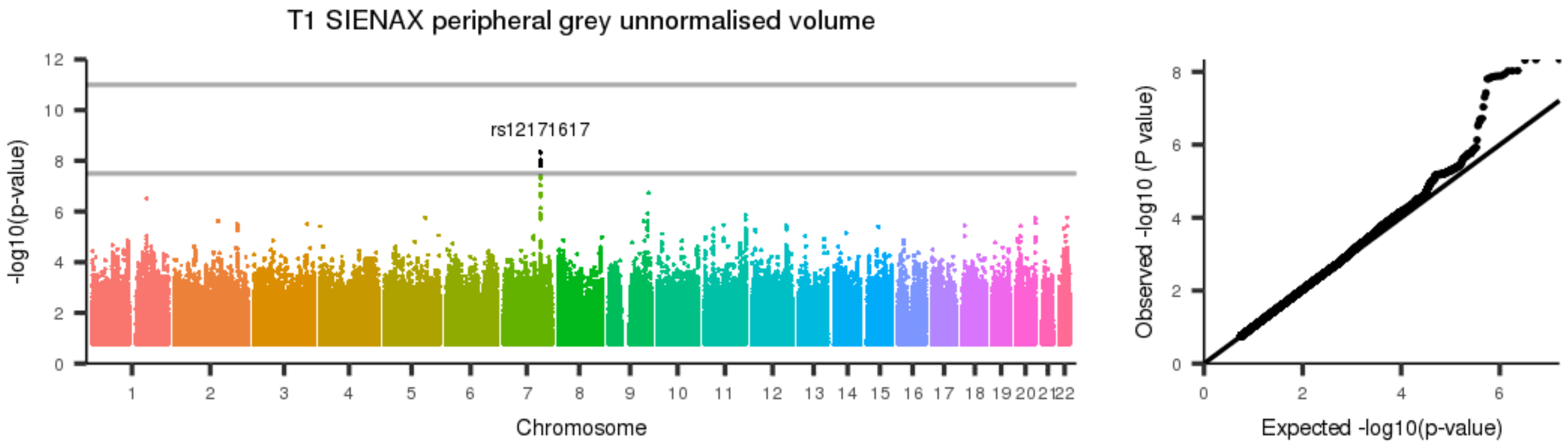

back to list of plots

Figure 3: T1 SIENAX CSF normalised volume

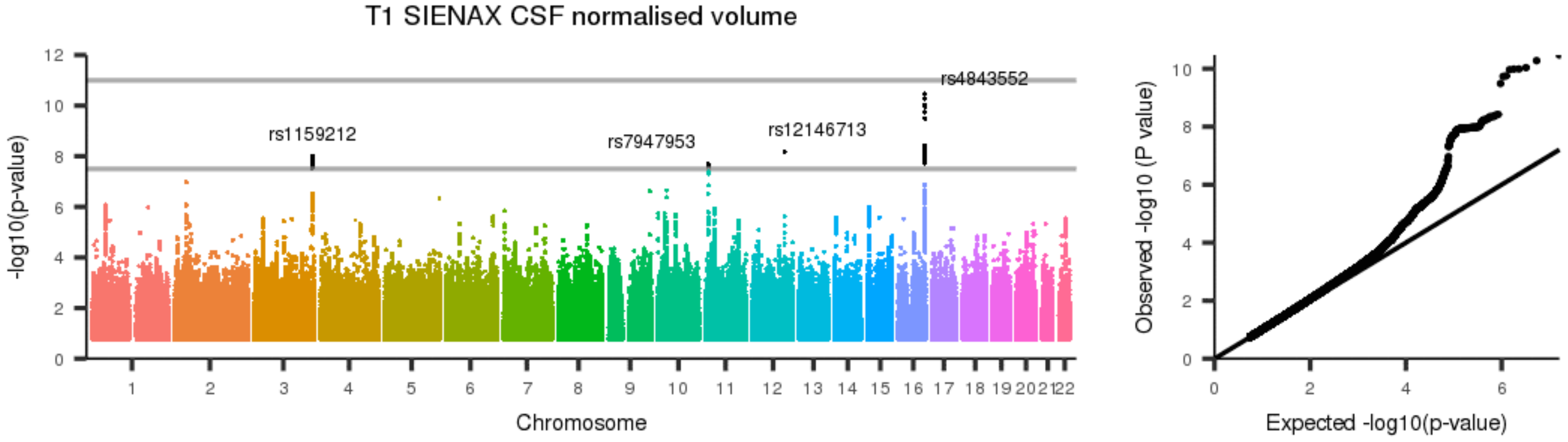

back to list of plots

Figure 4: T1 SIENAX CSF unnormalised volume

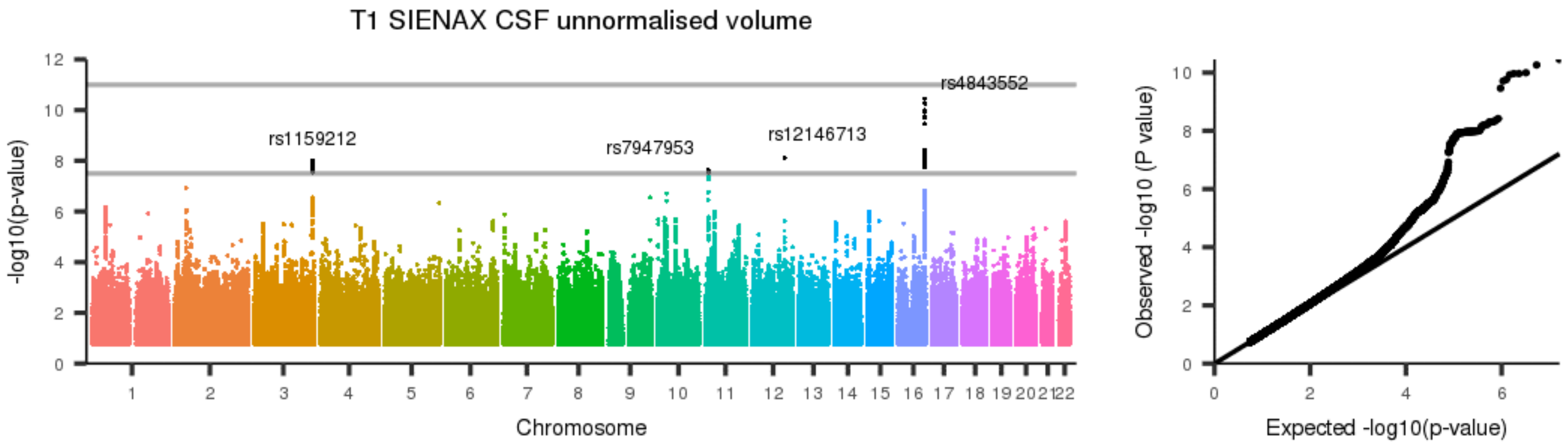

back to list of plots

Figure 5: T1 SIENAX grey normalised volume

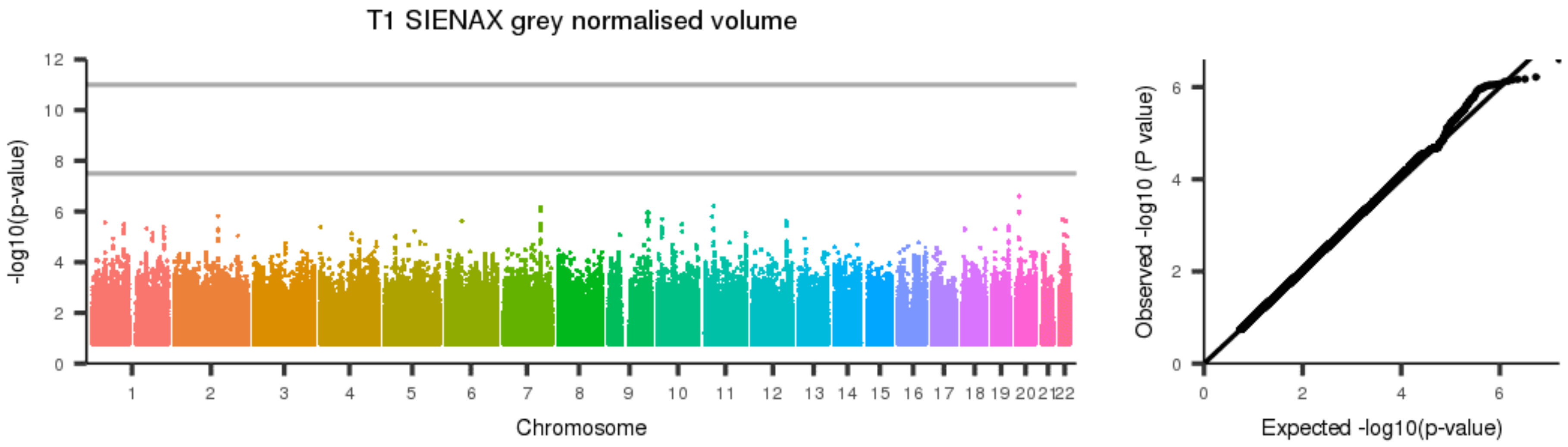

back to list of plots

Figure 6: T1 SIENAX grey unnormalised volume

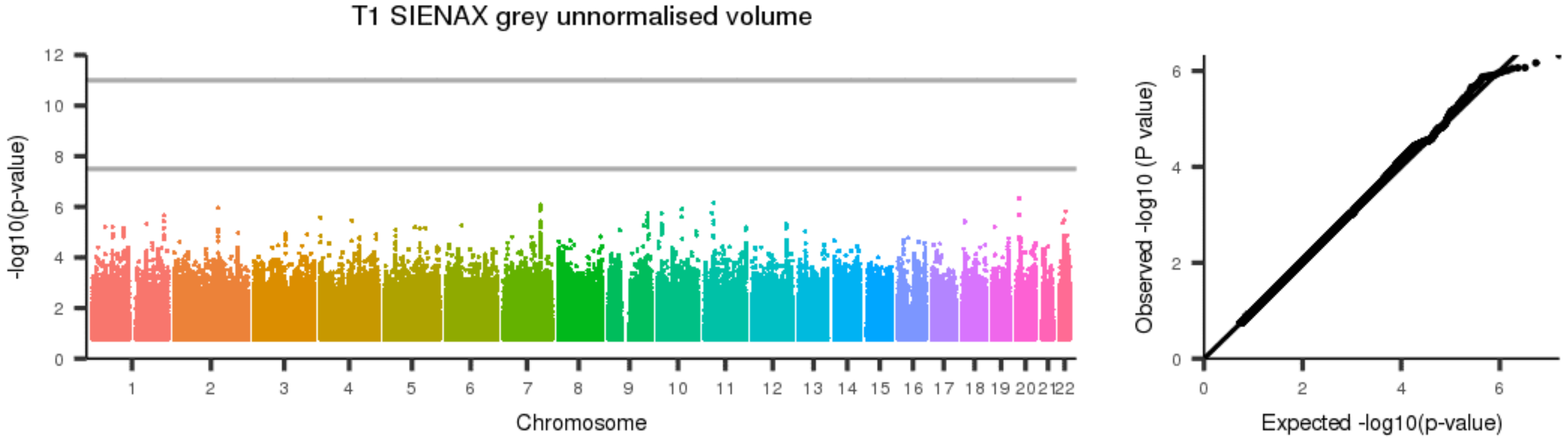

back to list of plots

Figure 7: T1 SIENAX white normalised volume

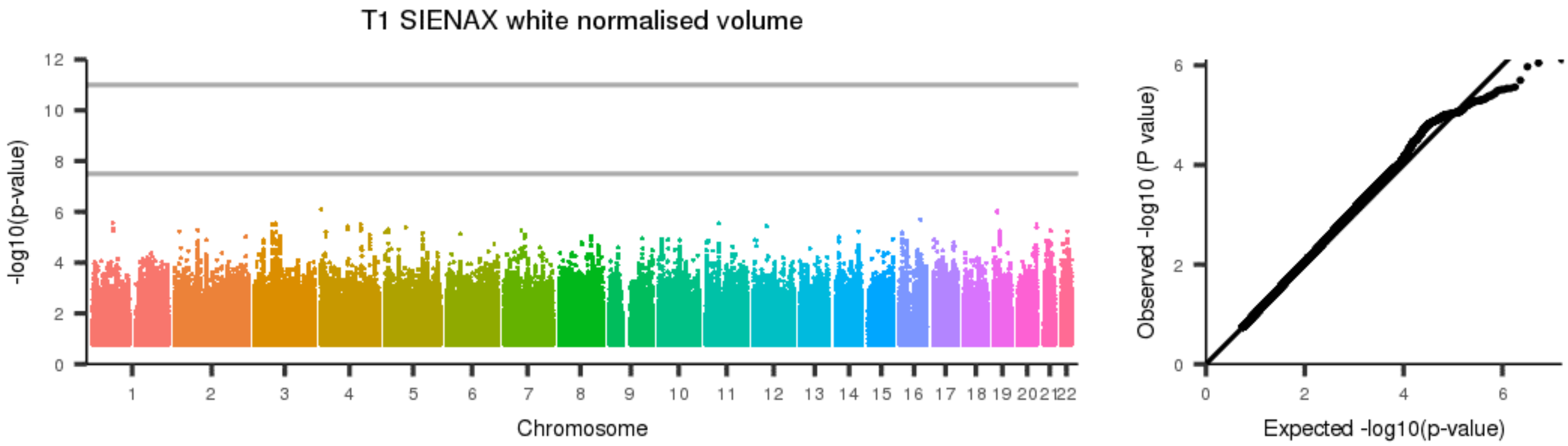

back to list of plots

Figure 8: T1 SIENAX white unnormalised volume

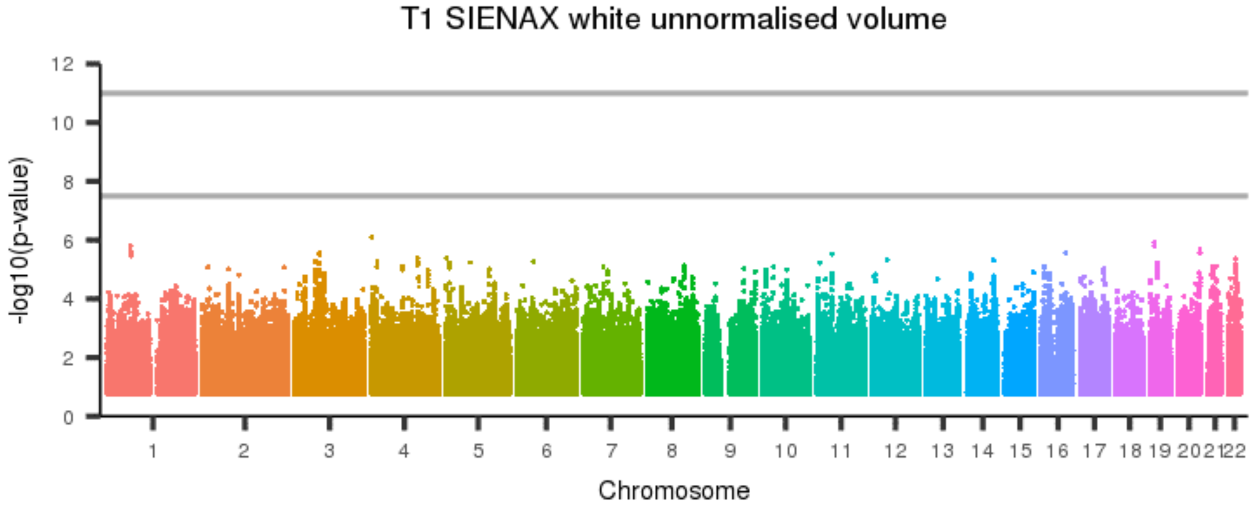

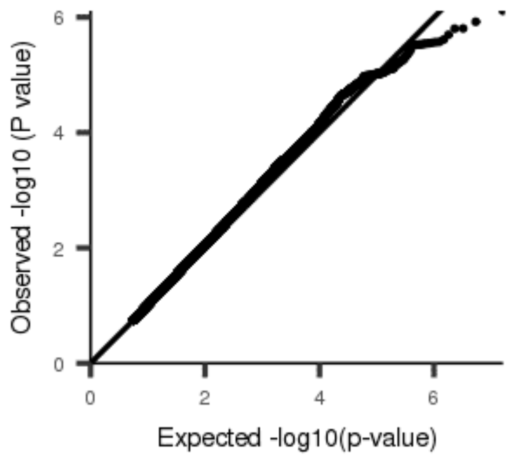

back to list of plots

Figure 9: T1 SIENAX brain-normalised volume

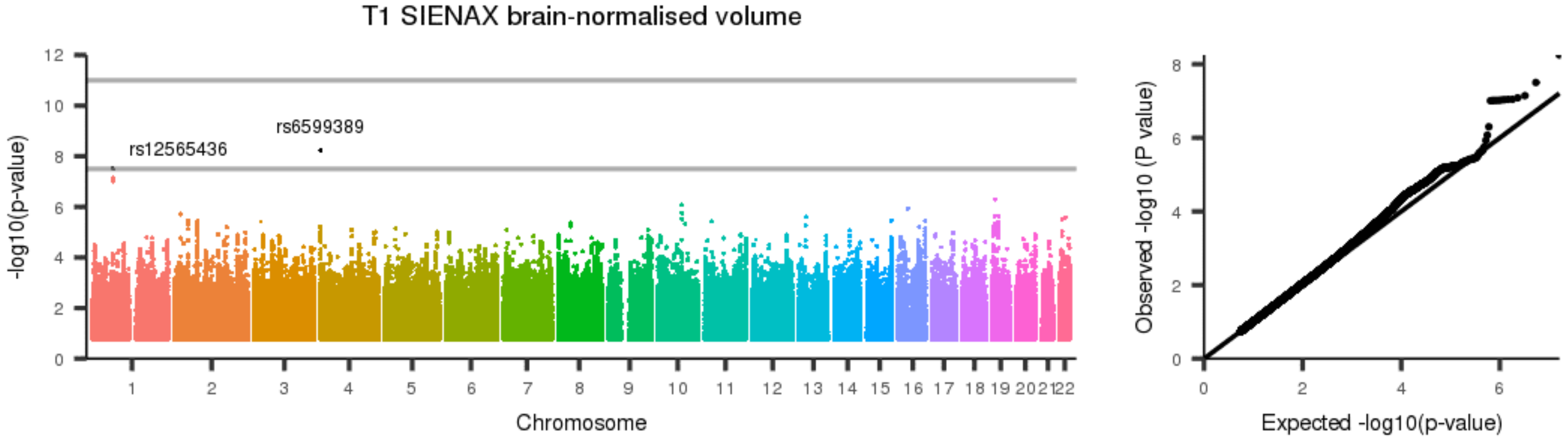

back to list of plots

Figure 10: T1 SIENAX brain-unnormalised volume

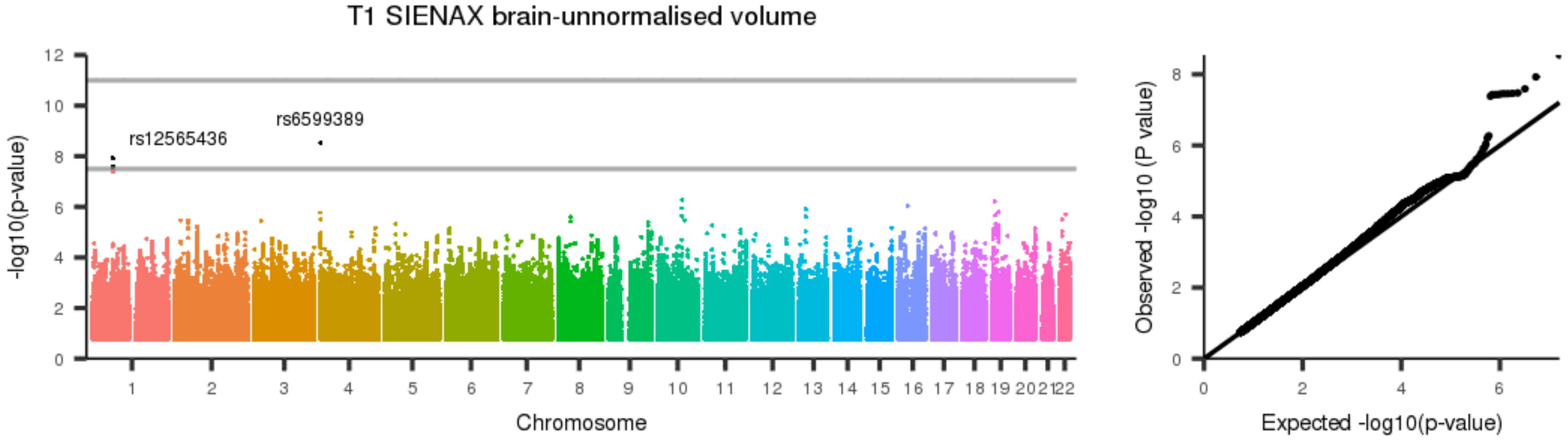

back to list of plots

Figure 11: T1 FIRST left thalamus volume

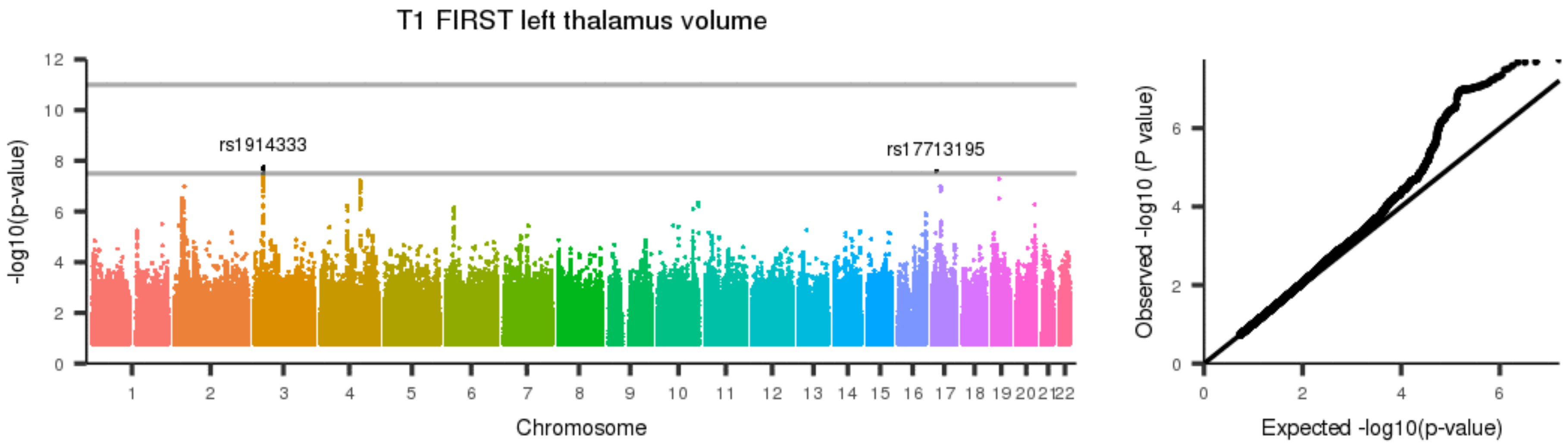

back to list of plots

Figure 12: T1 FIRST right thalamus volume

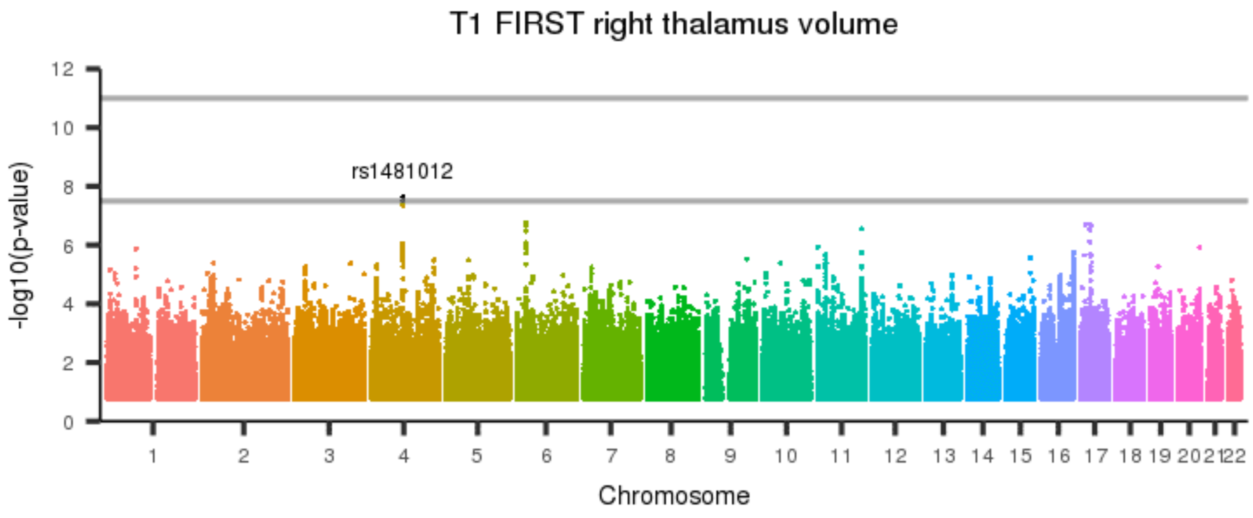

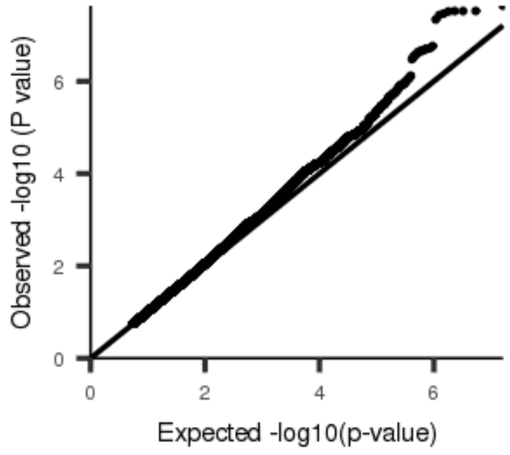

back to list of plots

Figure 13: T1 FIRST left caudate volume

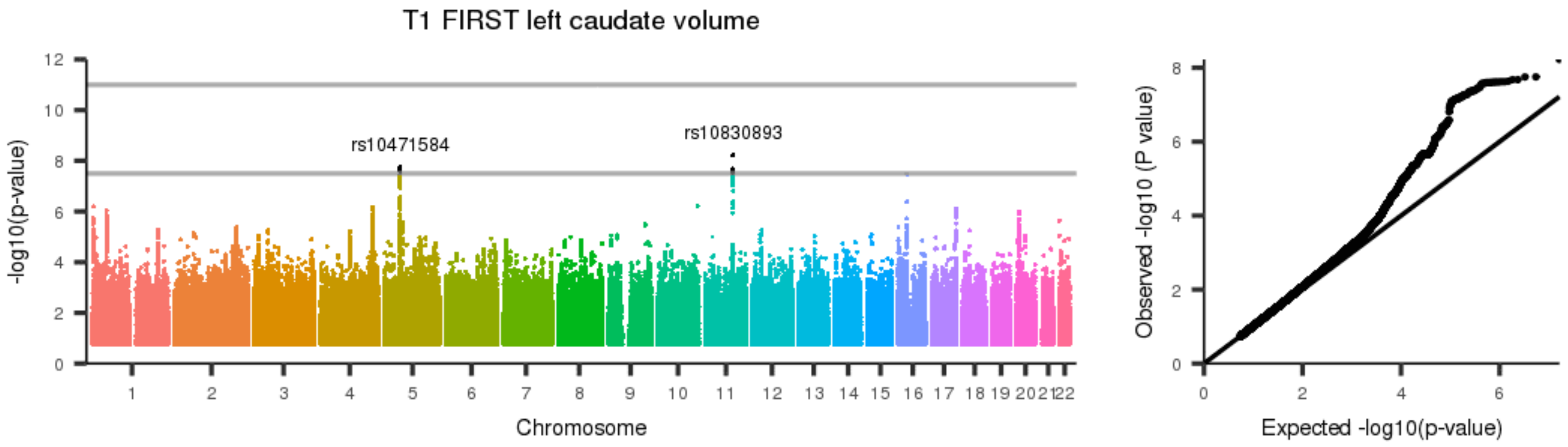

back to list of plots

Figure 14: T1 FIRST right caudate volume

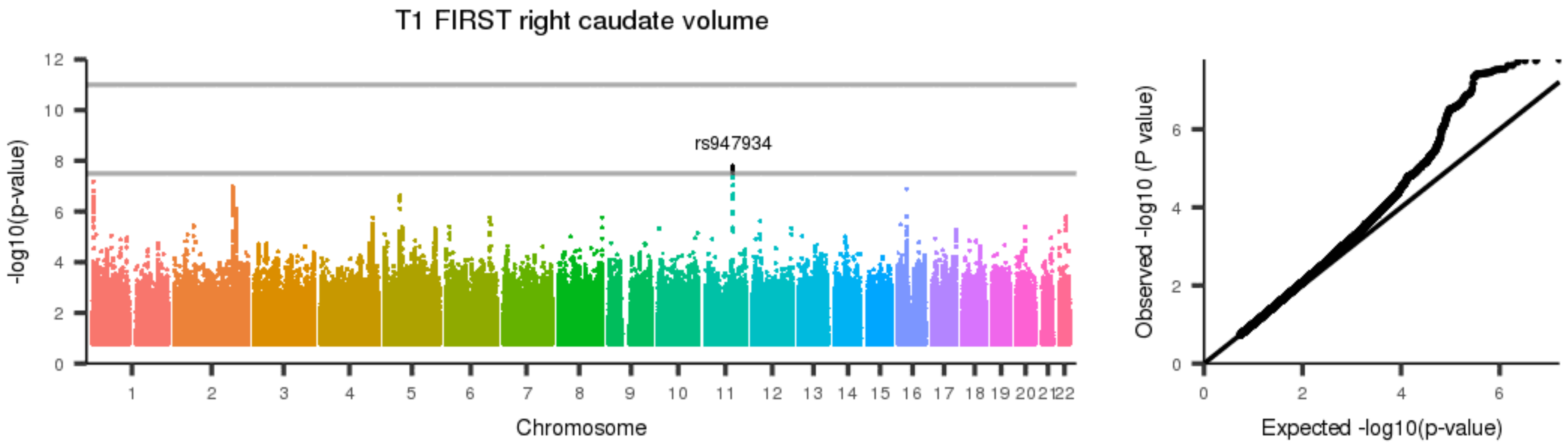

back to list of plots

Figure 15: T1 FIRST left putamen volume

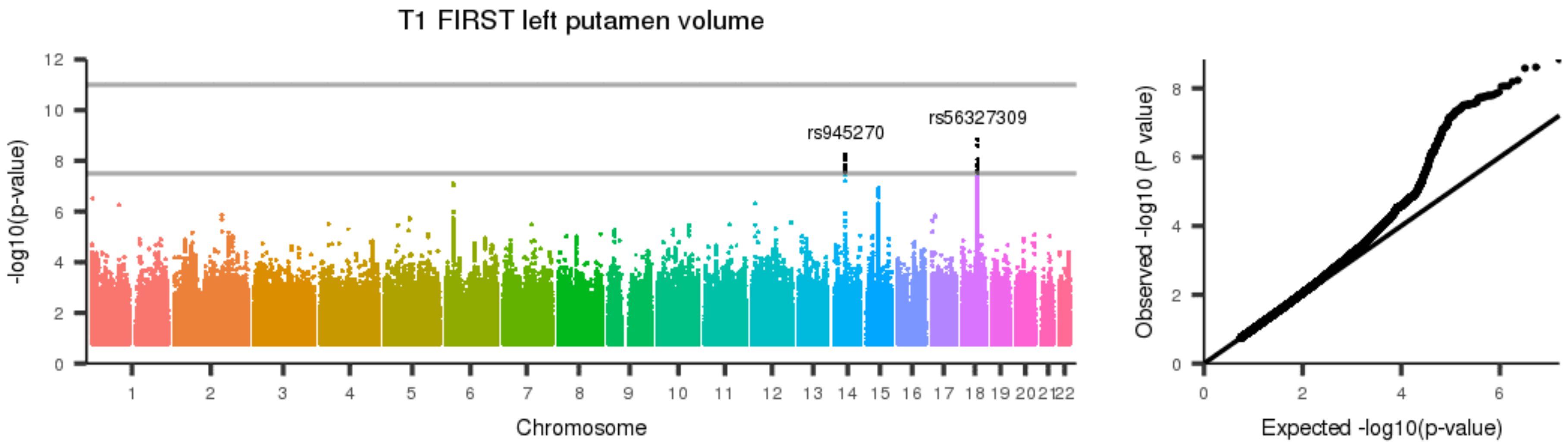

back to list of plots

Figure 16: T1 FIRST right putamen volume

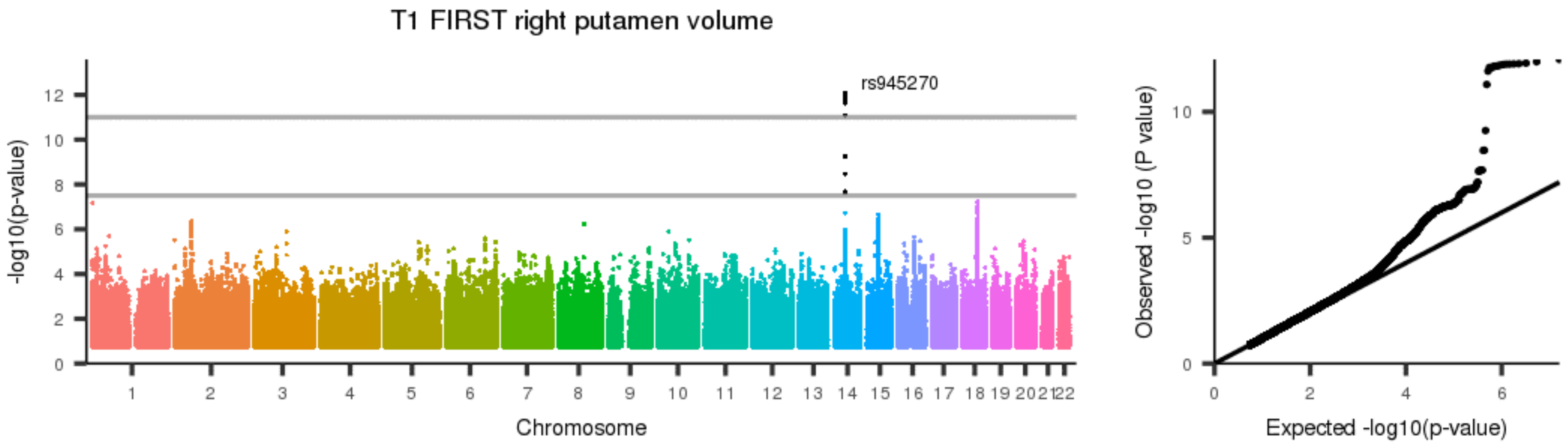

back to list of plots

Figure 17: T1 FIRST left pallidum volume

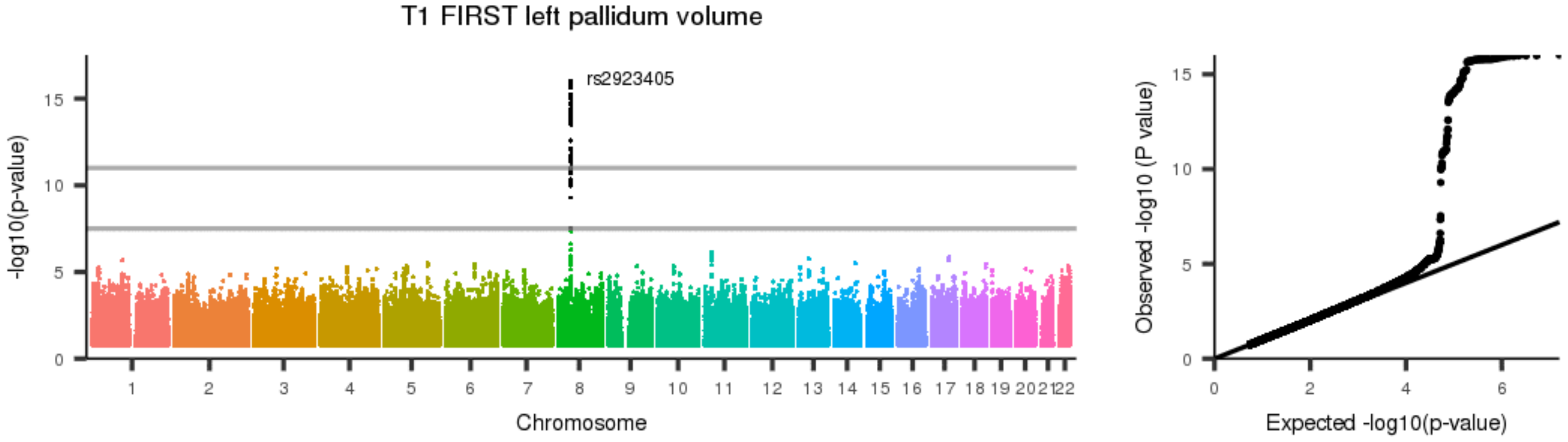

back to list of plots

Figure 18: T1 FIRST right pallidum volume

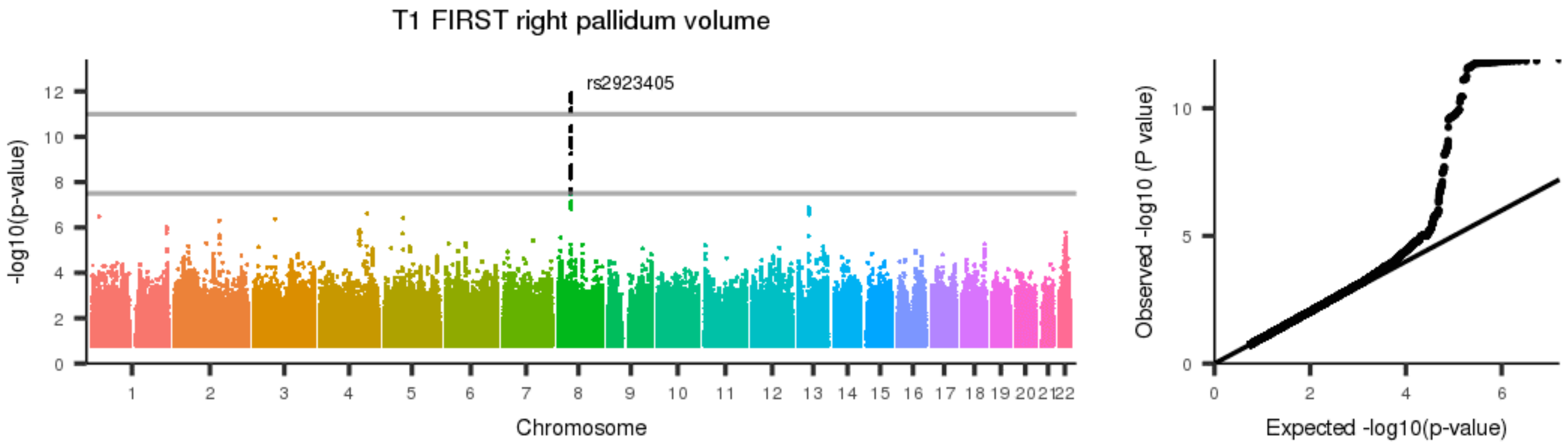

back to list of plots

Figure 19: T1 FIRST left hippocampus volume

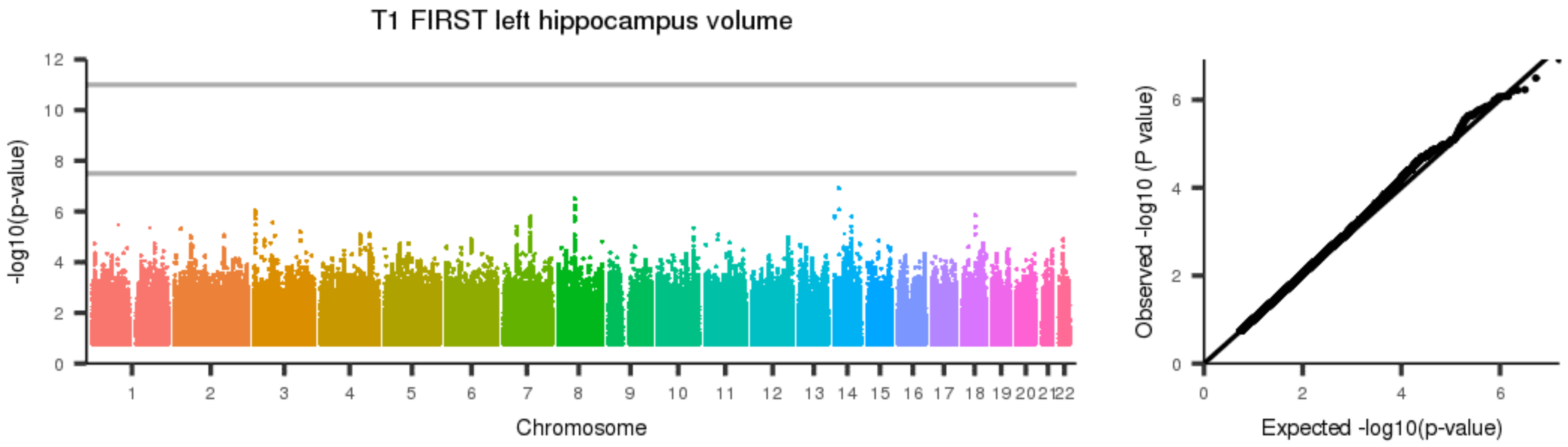

back to list of plots

Figure 20: T1 FIRST right hippocampus volume

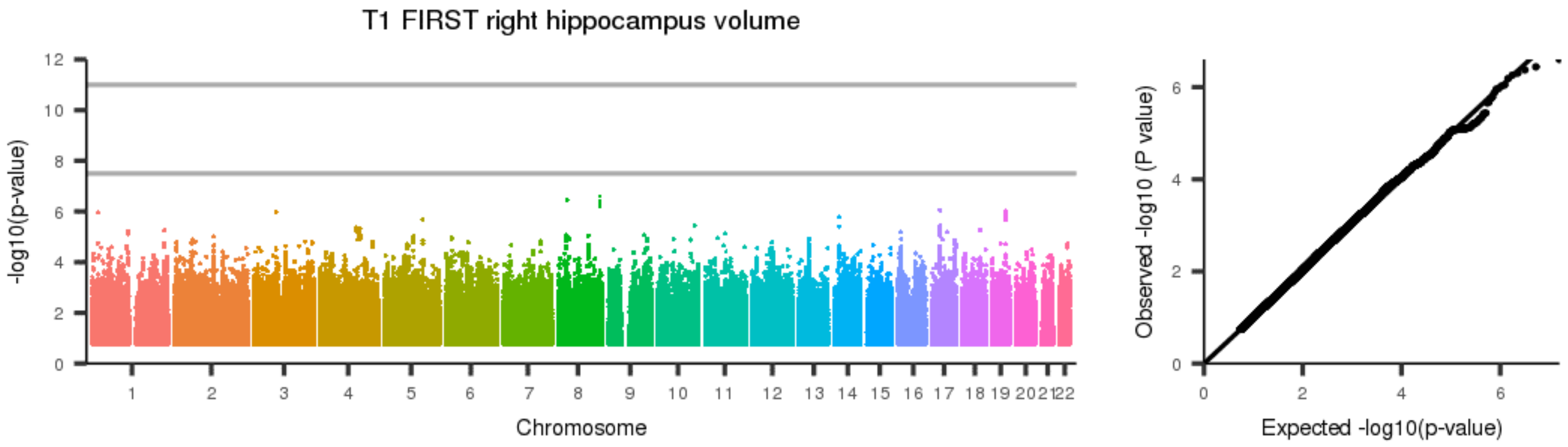

back to list of plots

Figure 21: T1 FIRST left amygdala volume

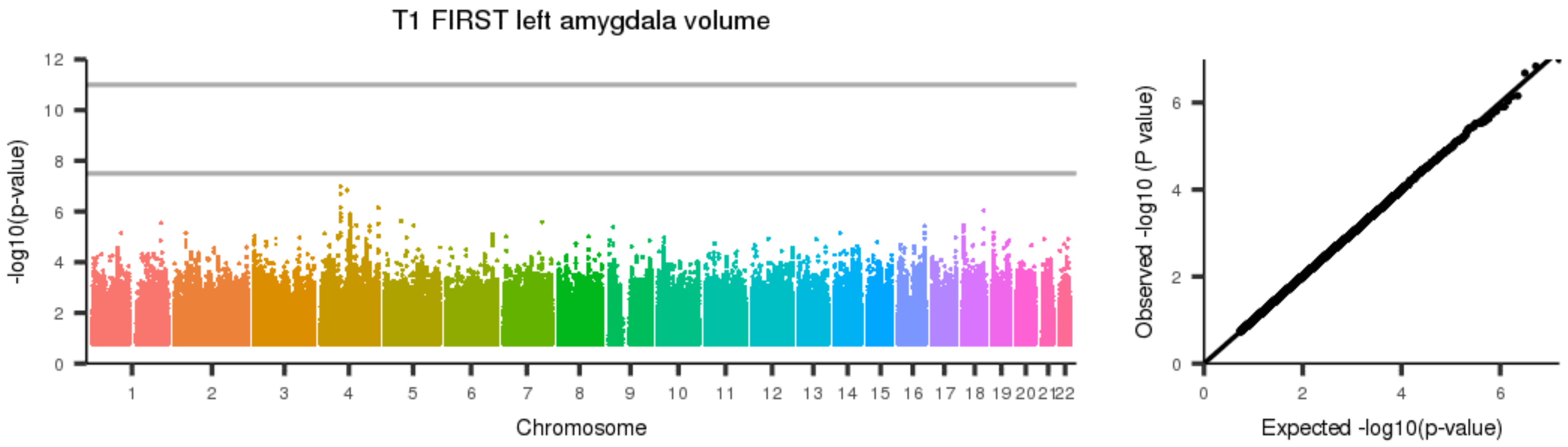

back to list of plots

Figure 22: T1 FIRST right amygdala volume

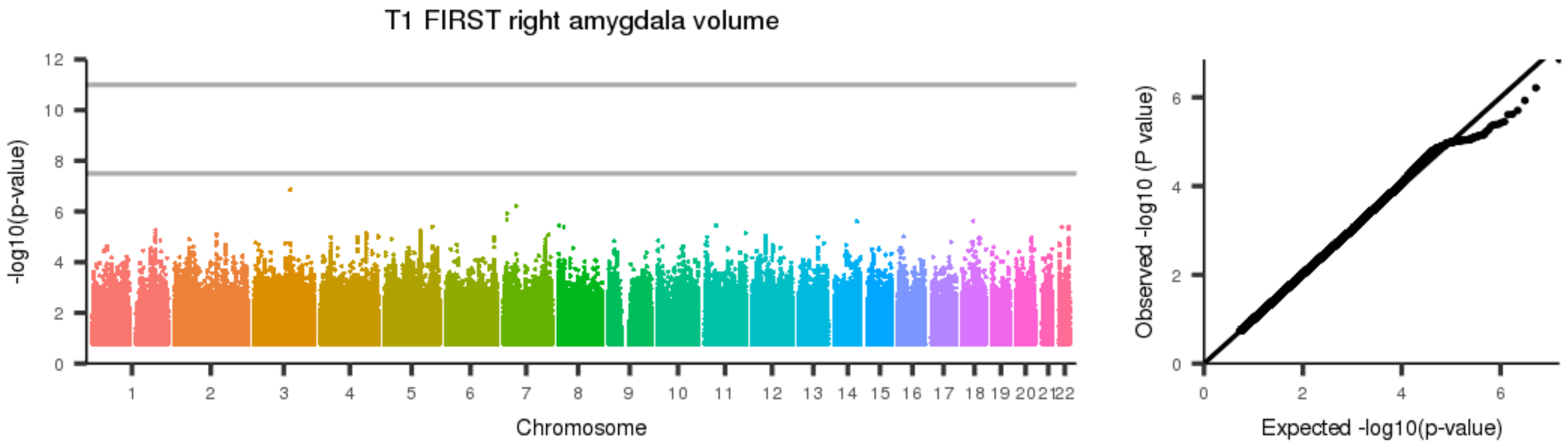

back to list of plots

Figure 23: T1 FIRST left accumbens volume

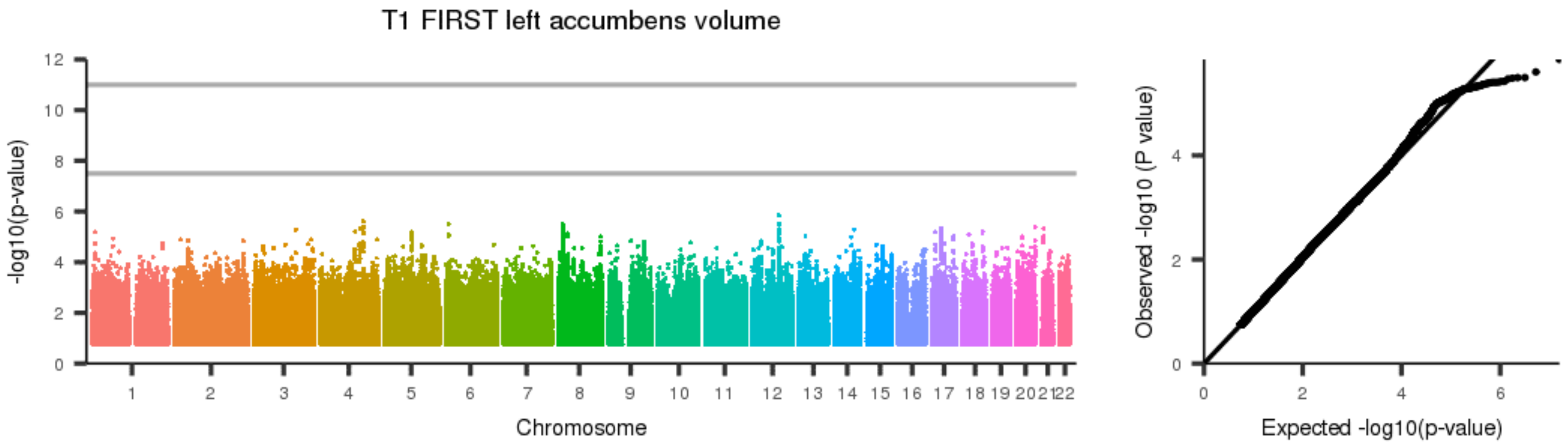

back to list of plots

Figure 24: T1 FIRST right accumbens volume

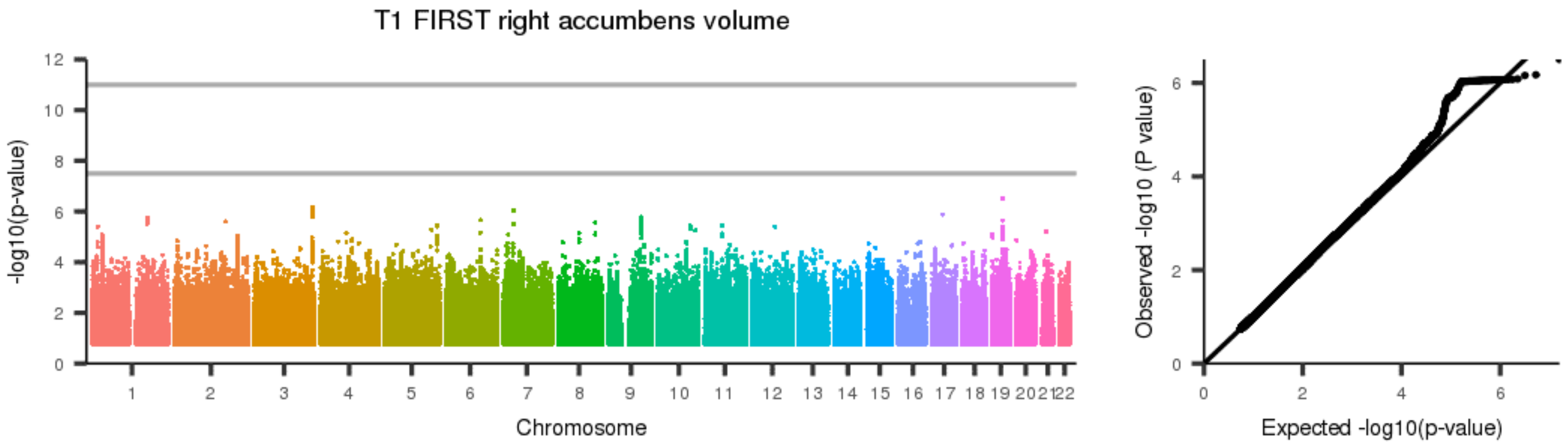

back to list of plots

Figure 25: T1 FIRST brain stem+4th ventricle volume

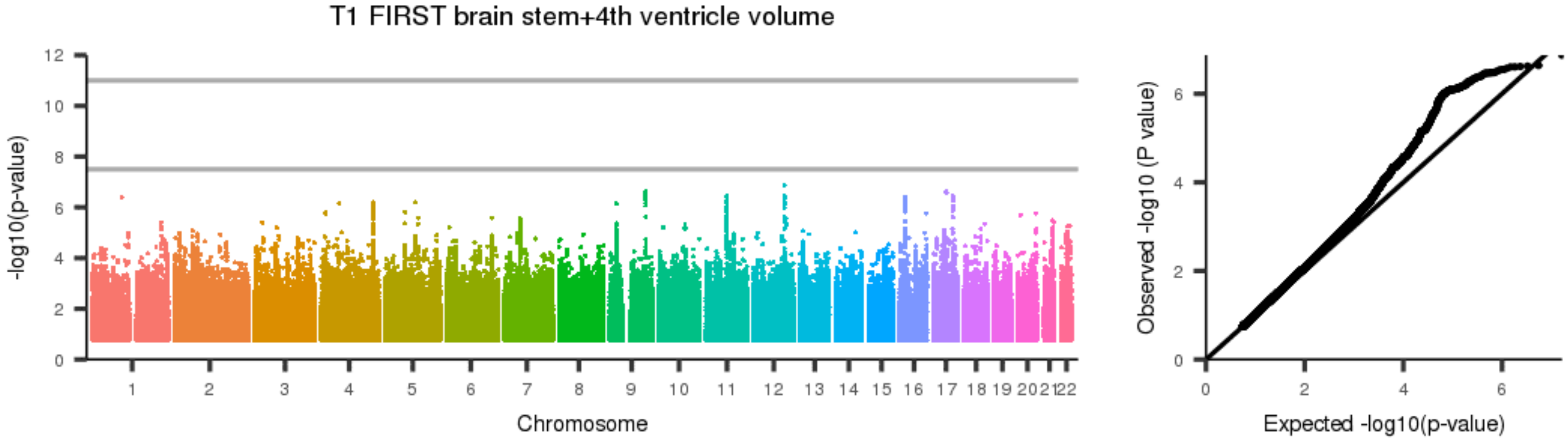

back to list of plots

Figure 26: T1 FAST ROIs L frontal pole

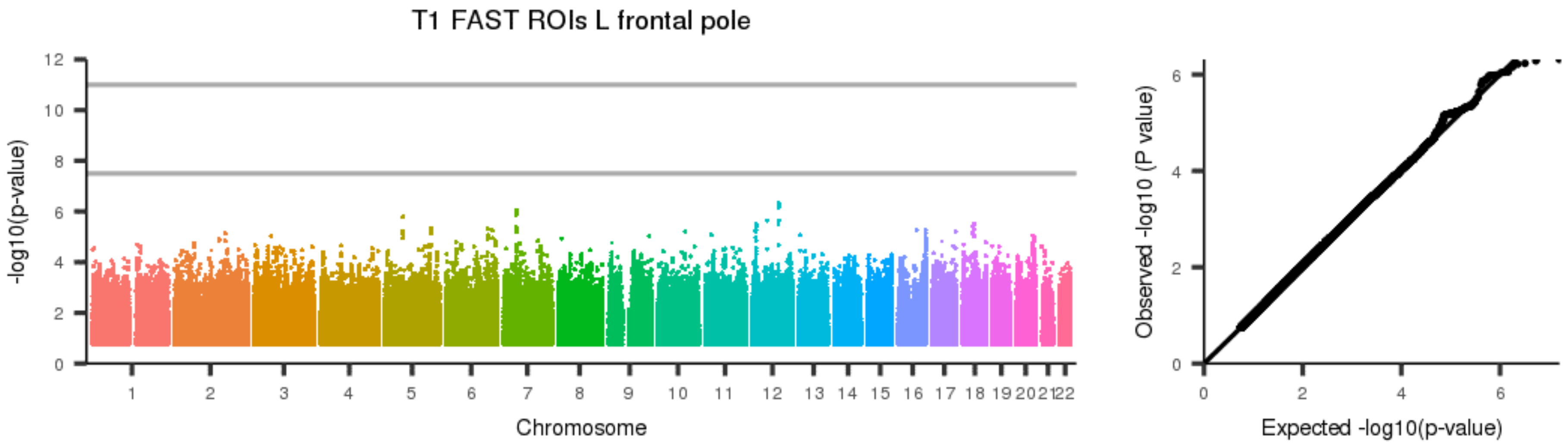

back to list of plots

Figure 27: T1 FAST ROIs R frontal pole

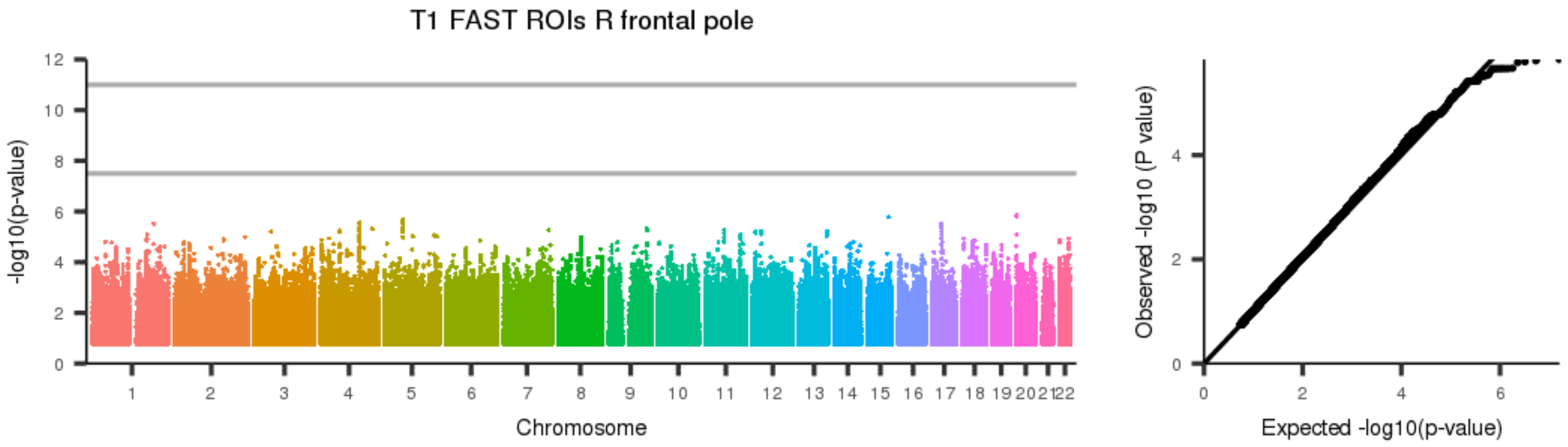

back to list of plots

Figure 28: T1 FAST ROIs L insular cortex

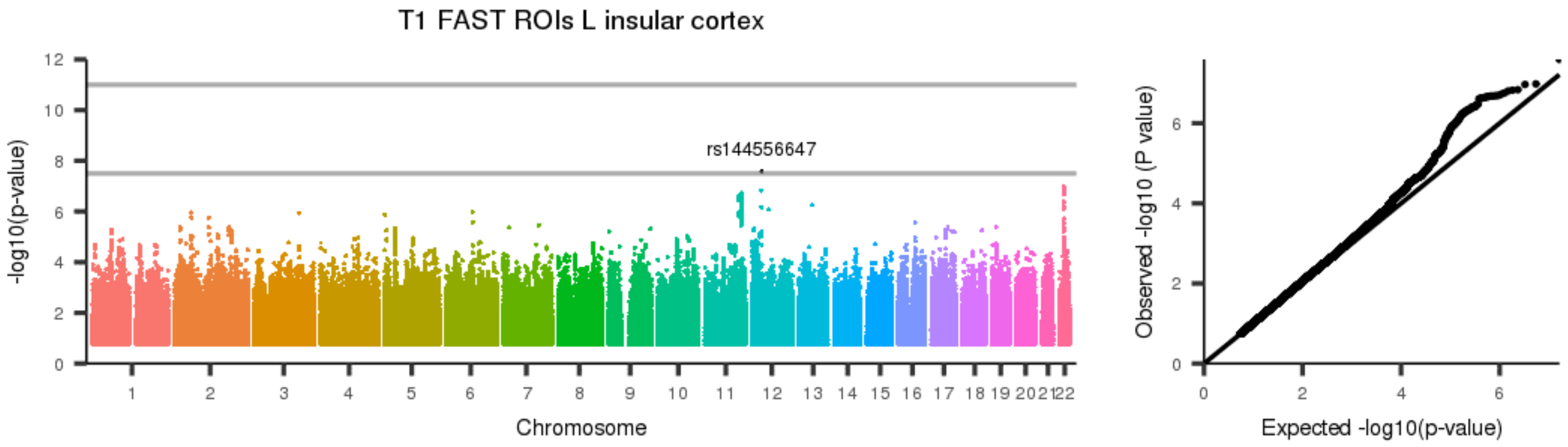

back to list of plots

Figure 29: T1 FAST ROIs R insular cortex

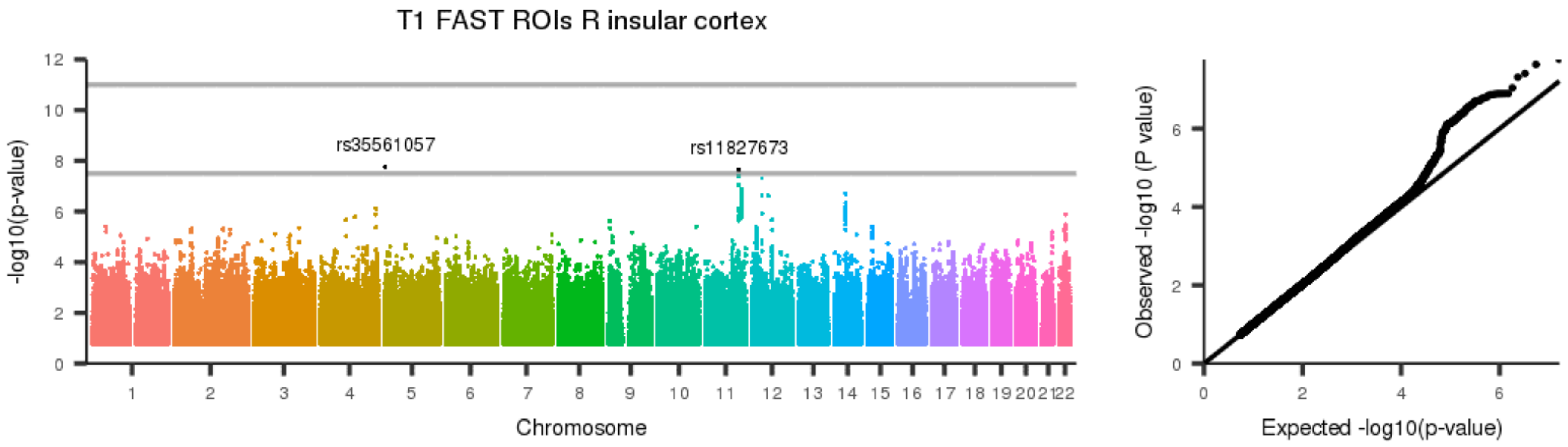

back to list of plots

Figure 30: T1 FAST ROIs L sup front gyrus

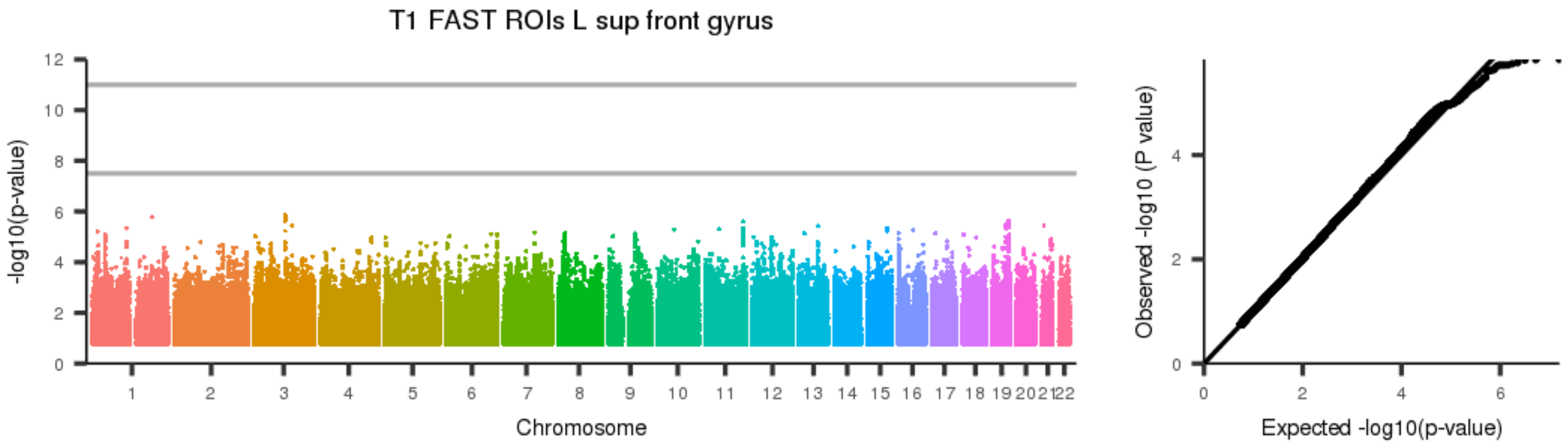

back to list of plots

Figure 31: T1 FAST ROIs R sup front gyrus

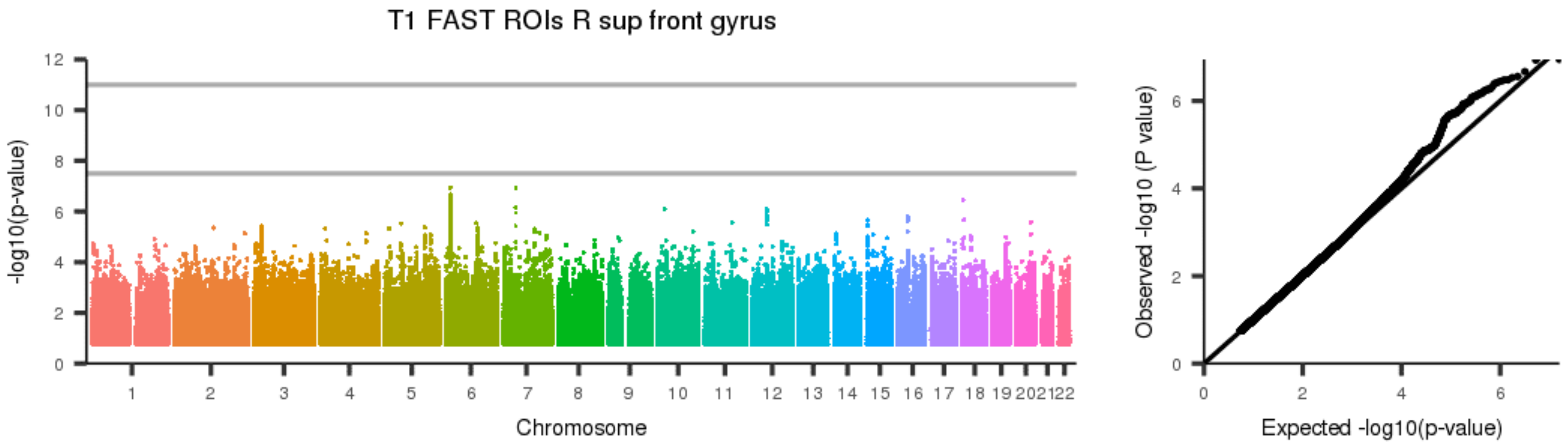

back to list of plots

Figure 32: T1 FAST ROIs L mid front gyrus

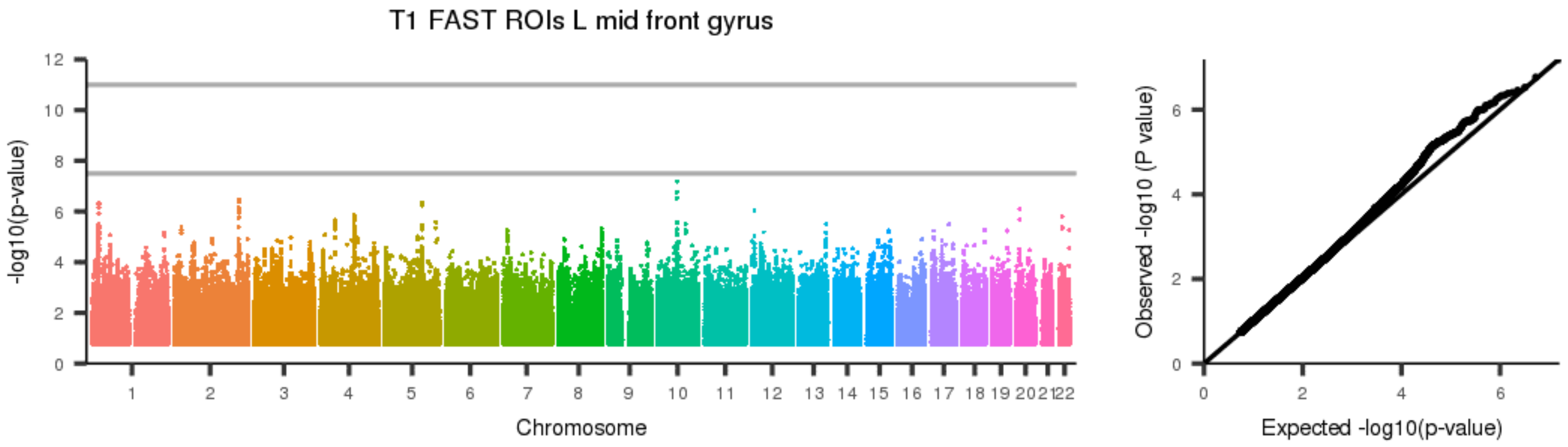

back to list of plots

Figure 33: T1 FAST ROIs R mid front gyrus

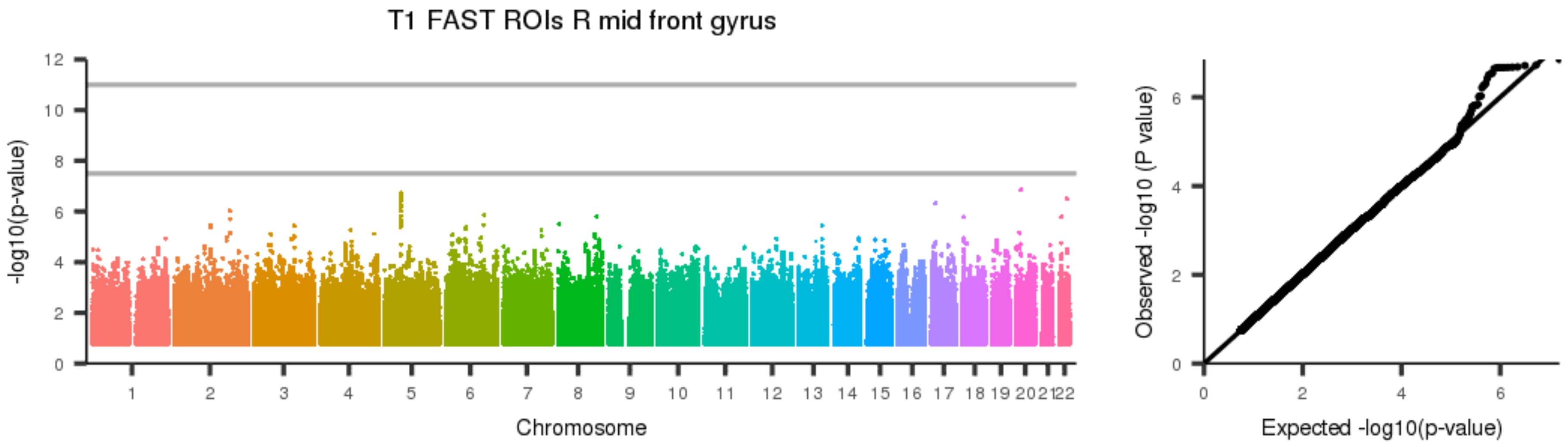

back to list of plots

Figure 34: T1 FAST ROIs L inf front gyrus parstri

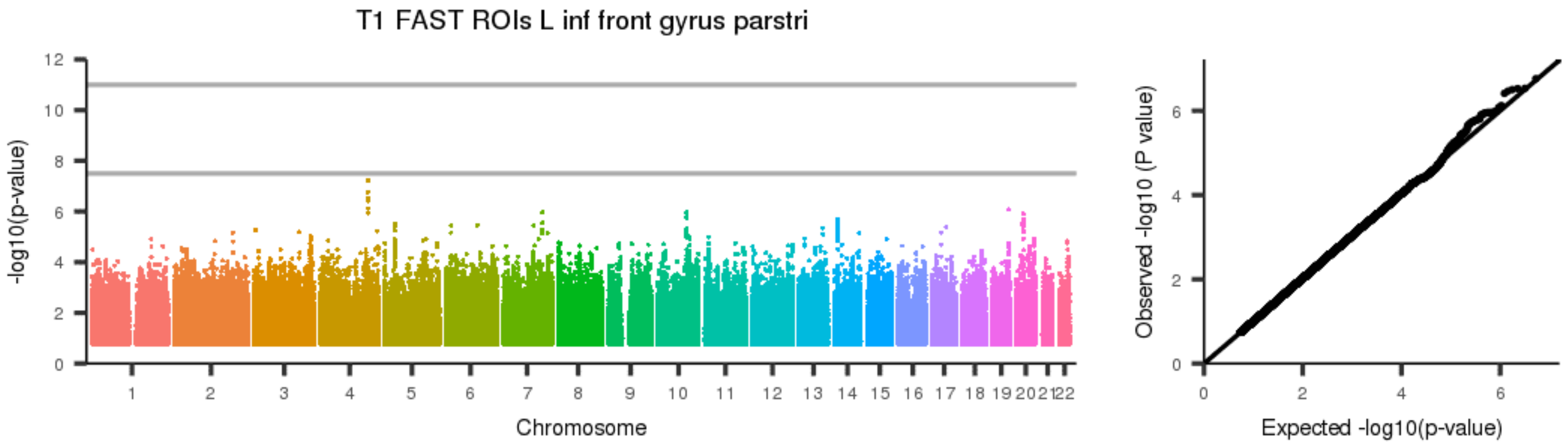

back to list of plots

Figure 35: T1 FAST ROIs R inf front gyrus parstri

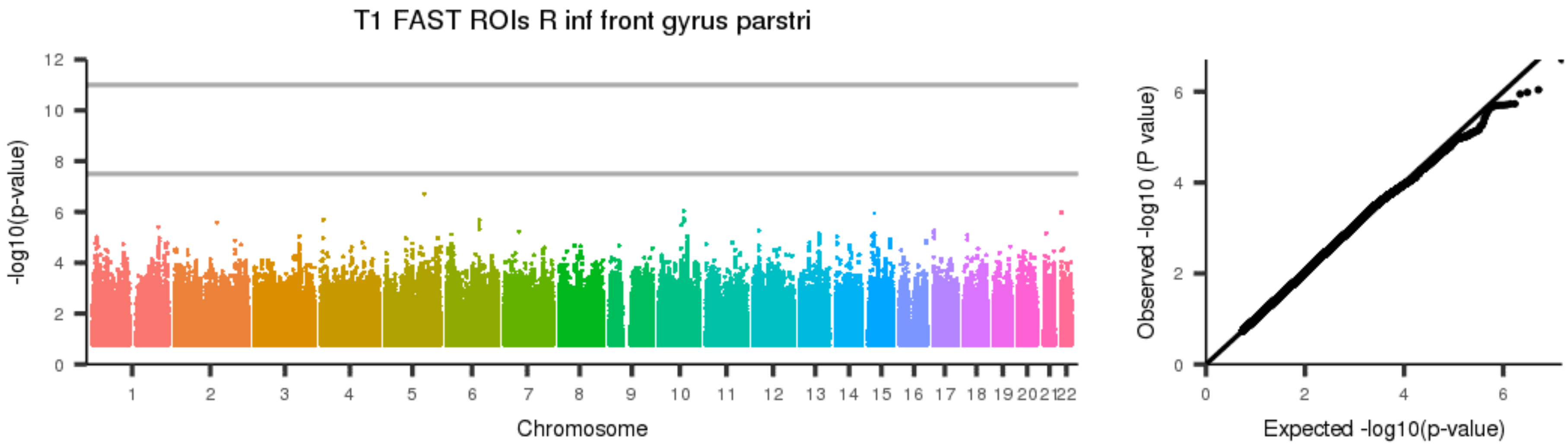

back to list of plots

Figure 36: T1 FAST ROIs L inf front gyrus parsop

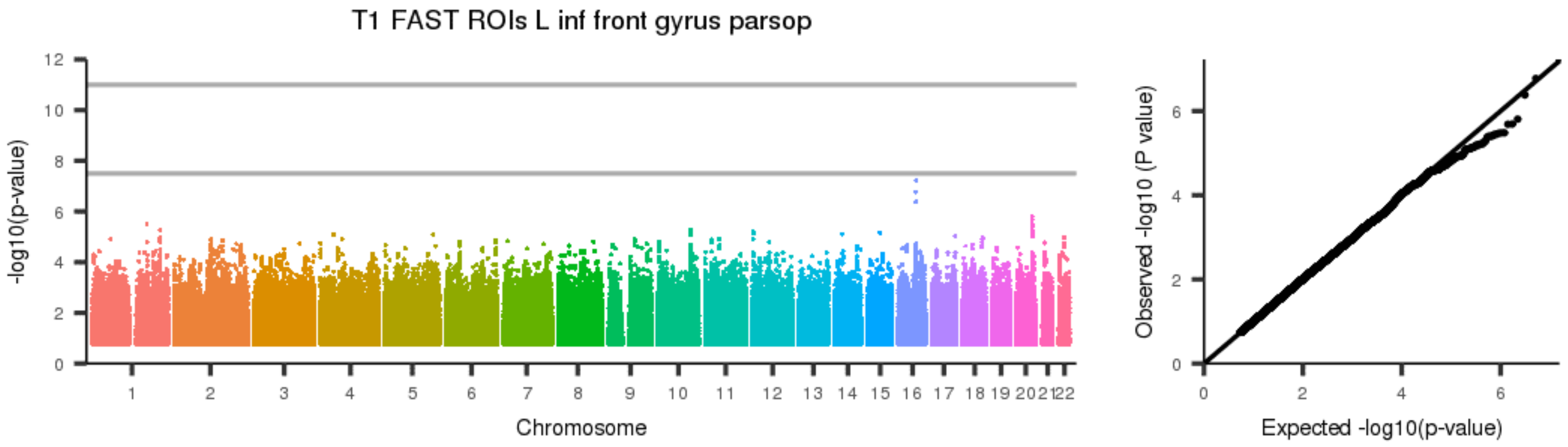

back to list of plots

Figure 37: T1 FAST ROIs R inf front gyrus parsop

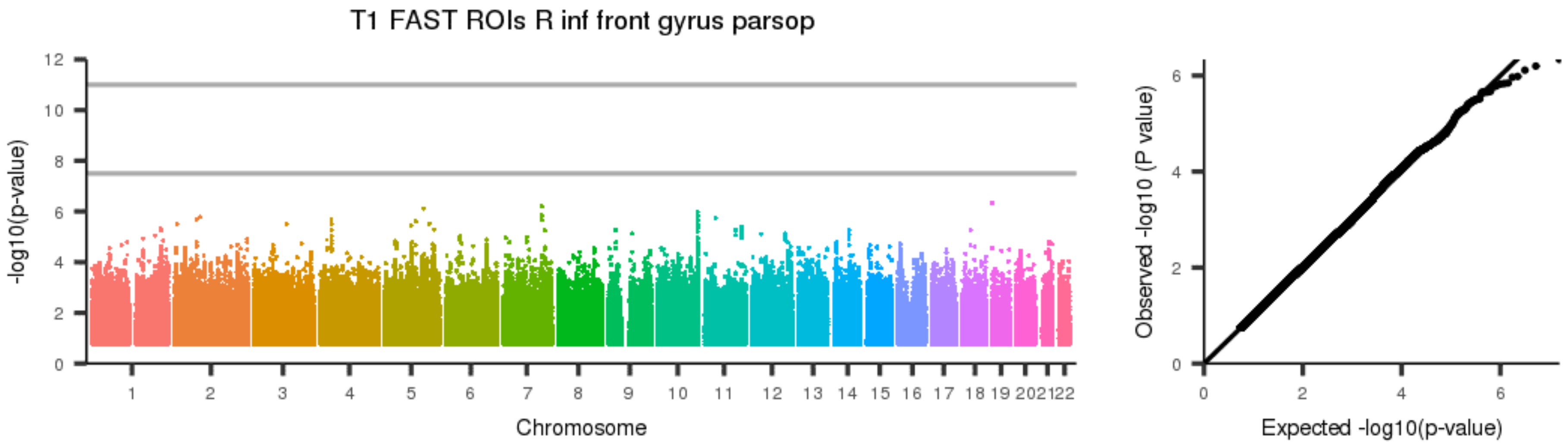

back to list of plots

Figure 38: T1 FAST ROIs L precentral gyrus

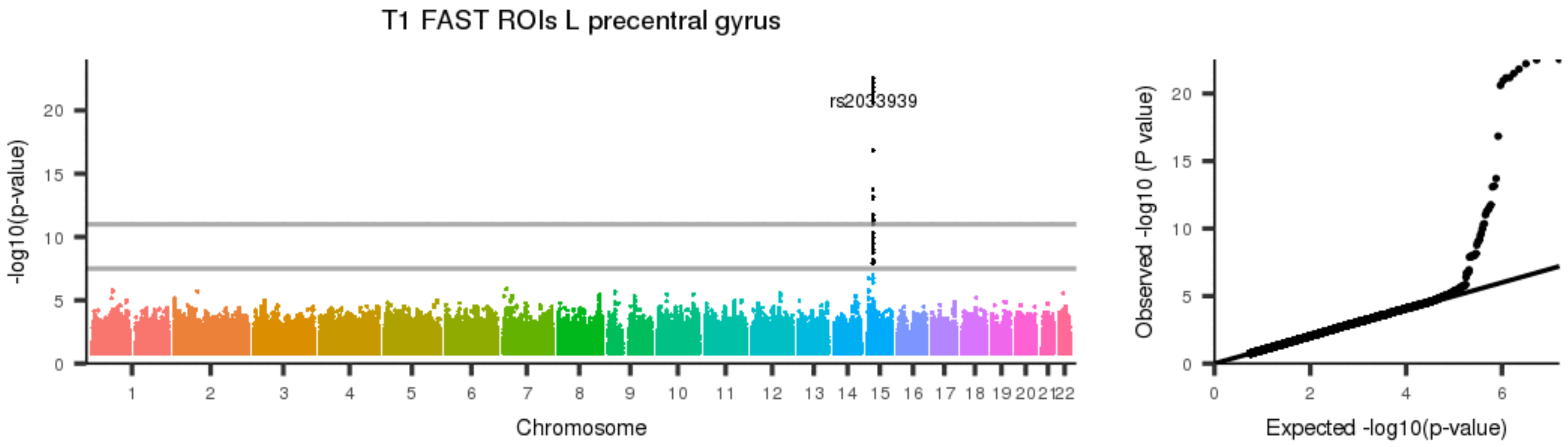

back to list of plots

Figure 39: T1 FAST ROIs R precentral gyrus

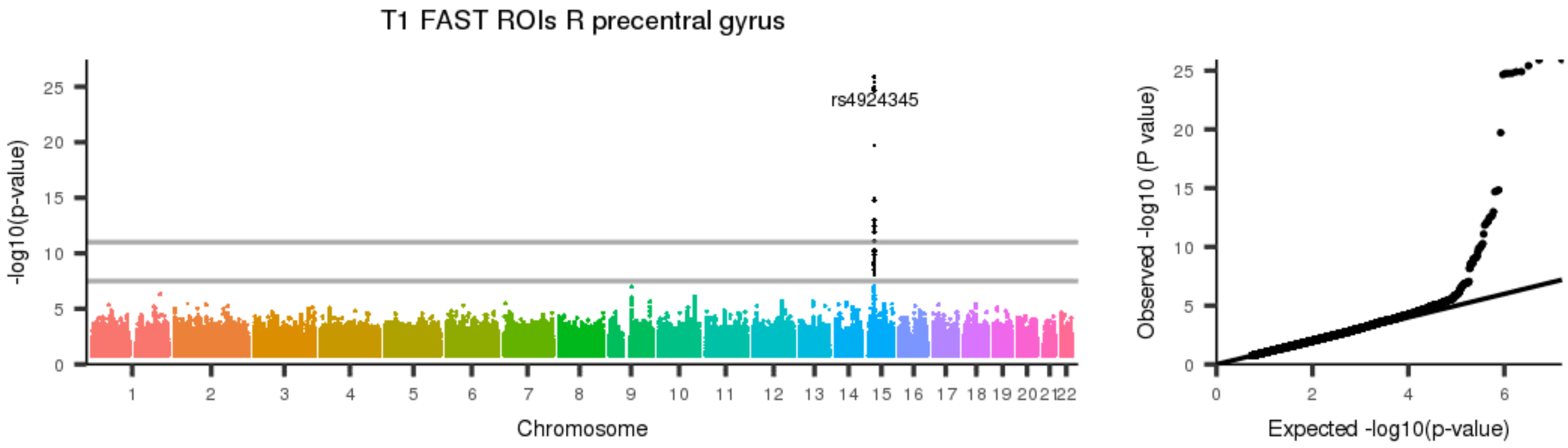

back to list of plots

Figure 40: T1 FAST ROIs L temporal pole

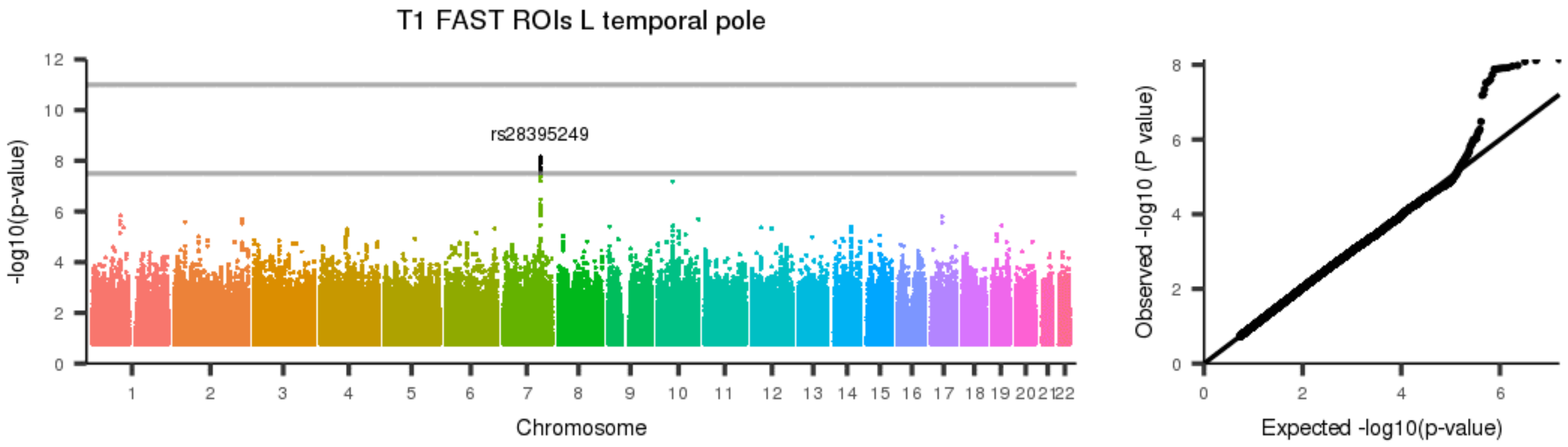

back to list of plots

Figure 41: T1 FAST ROIs R temporal pole

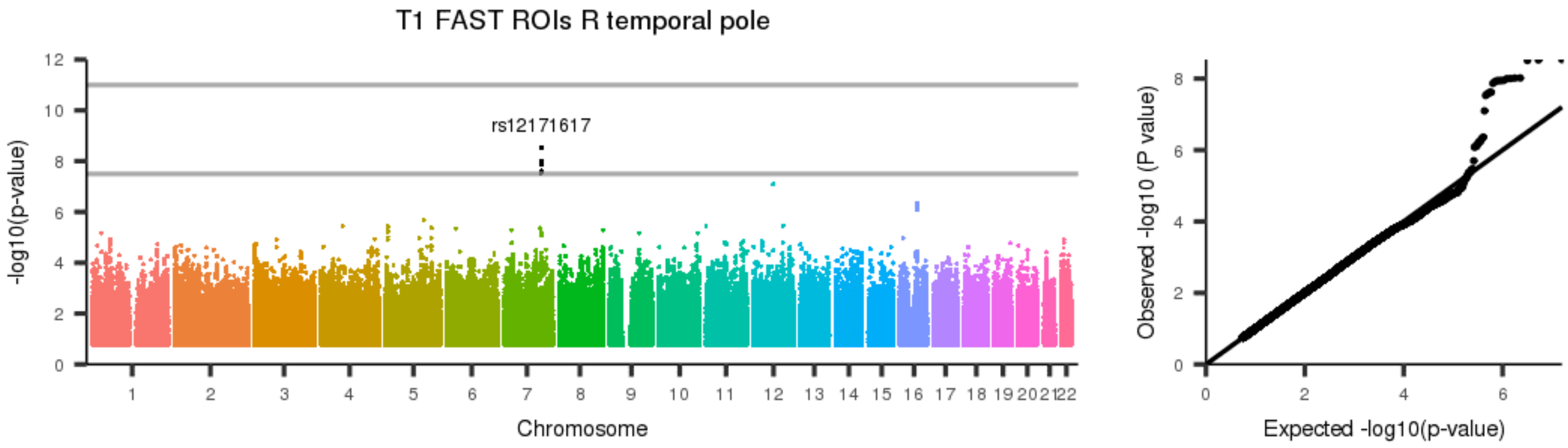

back to list of plots

Figure 42: T1 FAST ROIs L sup temp gyrus ant

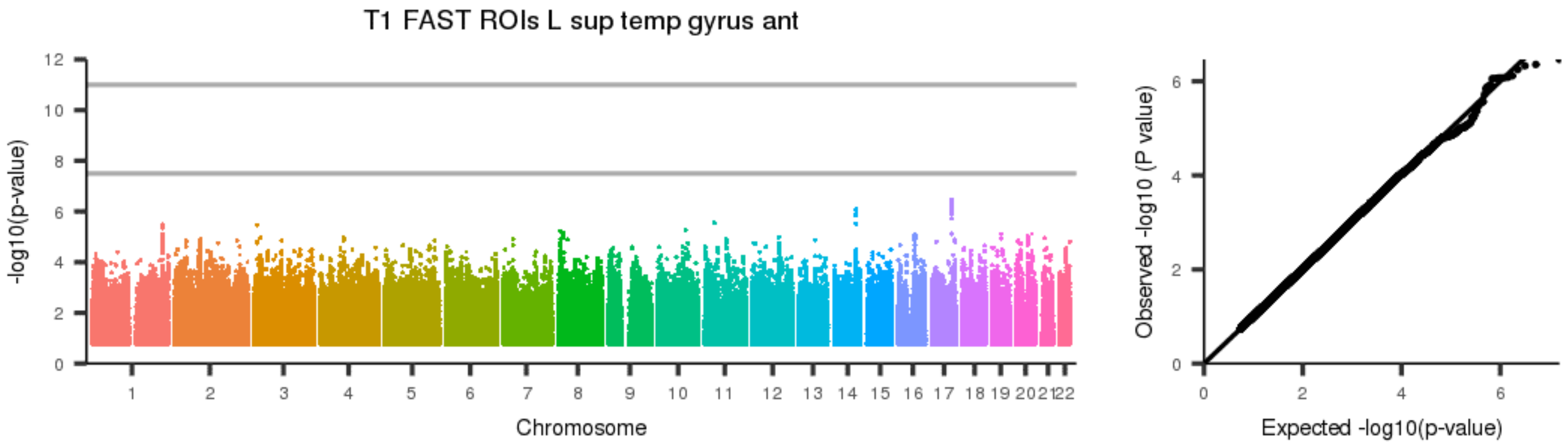

back to list of plots

Figure 43: T1 FAST ROIs R sup temp gyrus ant

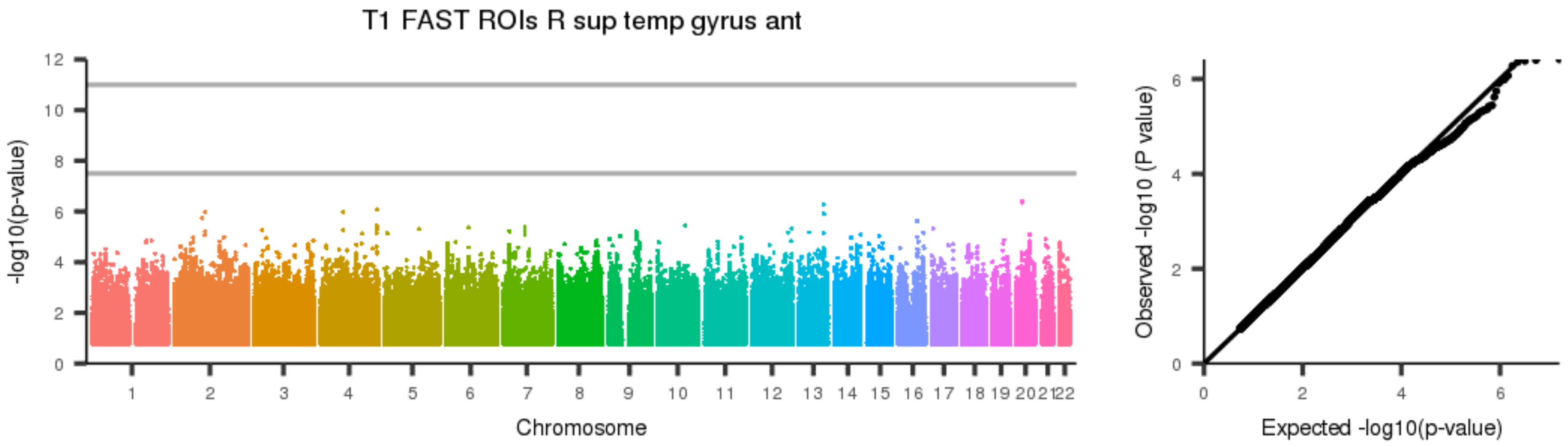

back to list of plots

Figure 44: T1 FAST ROIs L sup temp gyrus post

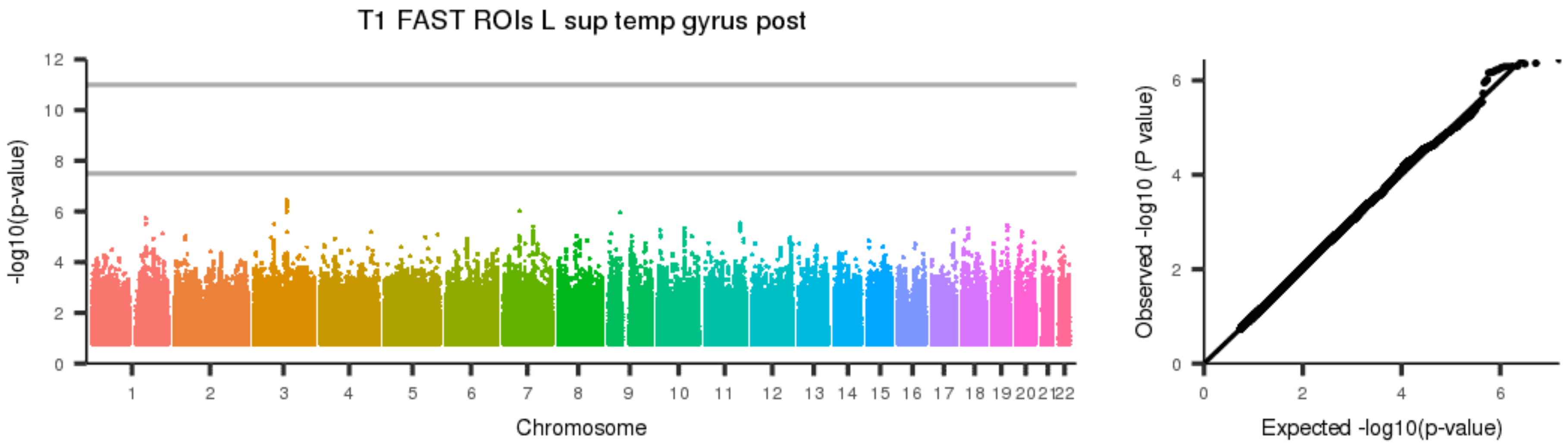

back to list of plots

Figure 45: T1 FAST ROIs R sup temp gyrus post

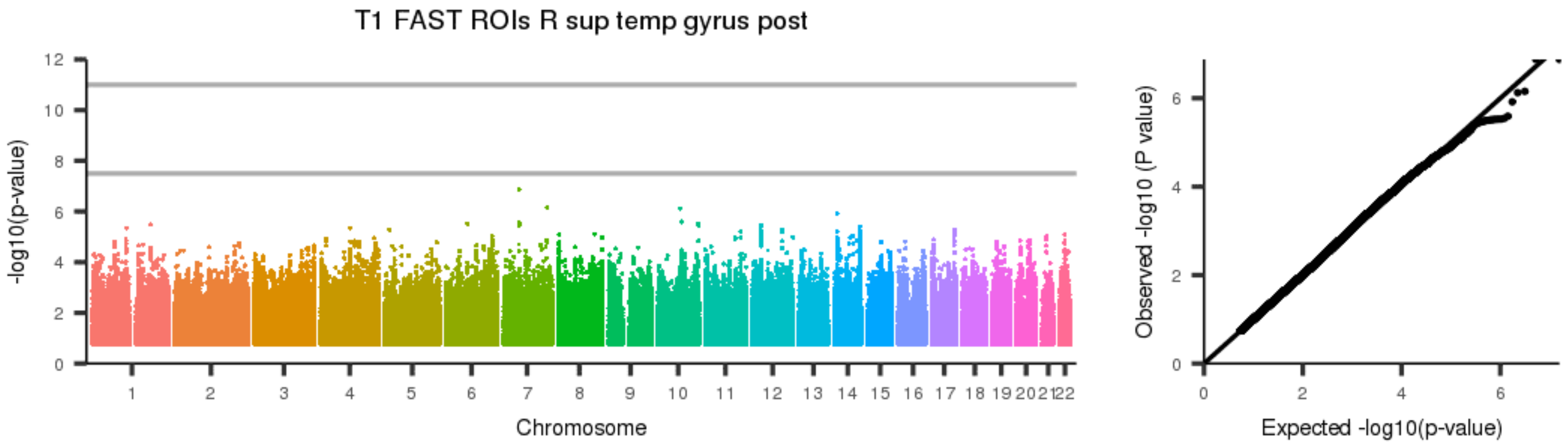

back to list of plots

Figure 46: T1 FAST ROIs L mid temp gyrus ant

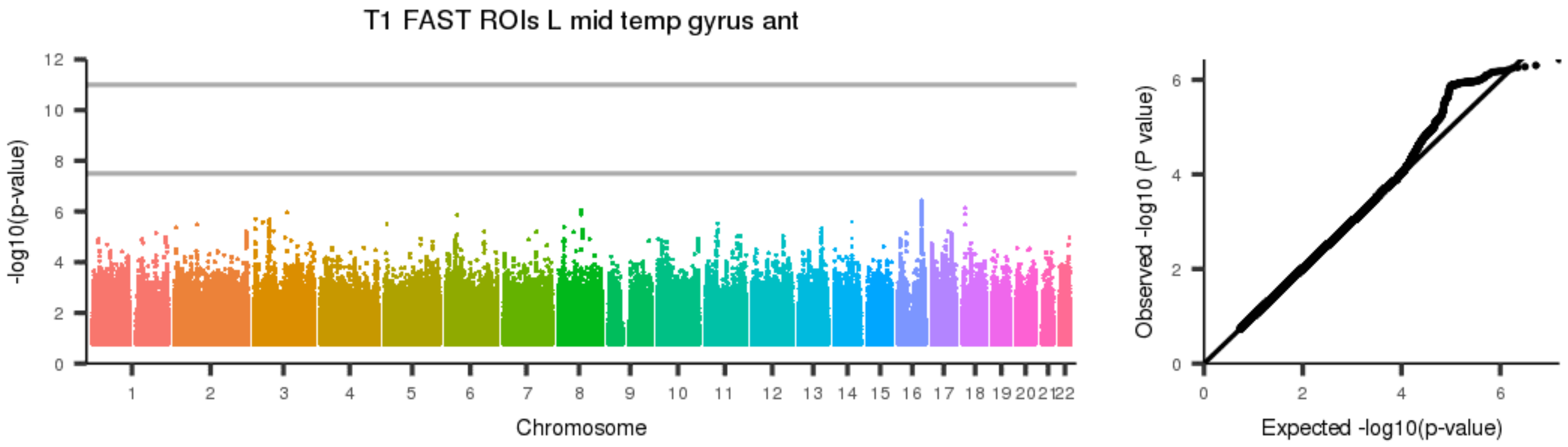

back to list of plots

Figure 47: T1 FAST ROIs R mid temp gyrus ant

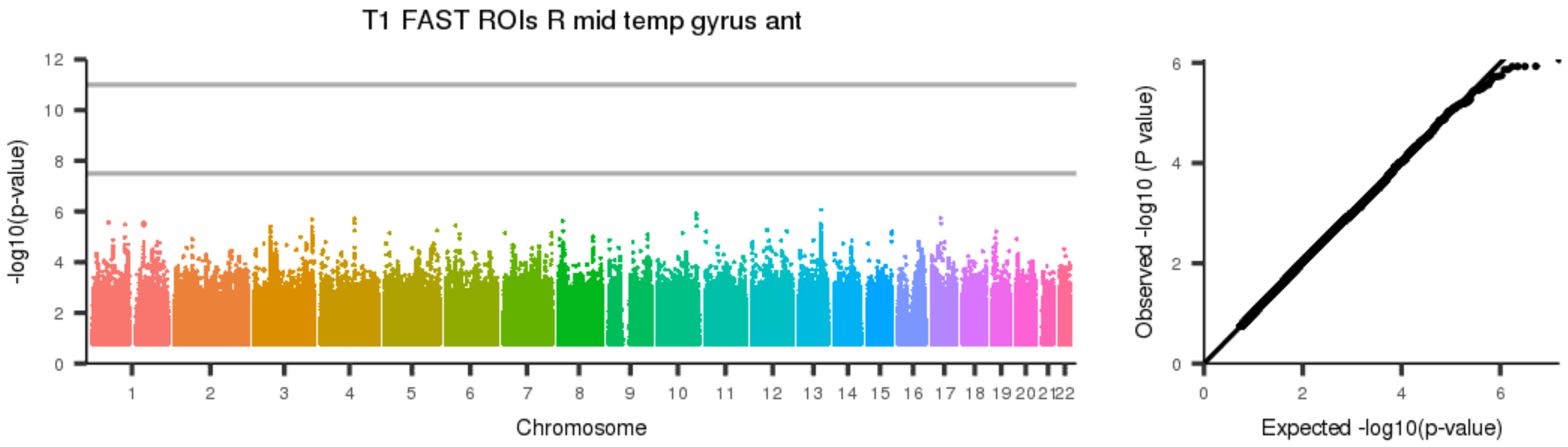

back to list of plots

Figure 48: T1 FAST ROIs L mid temp gyrus post

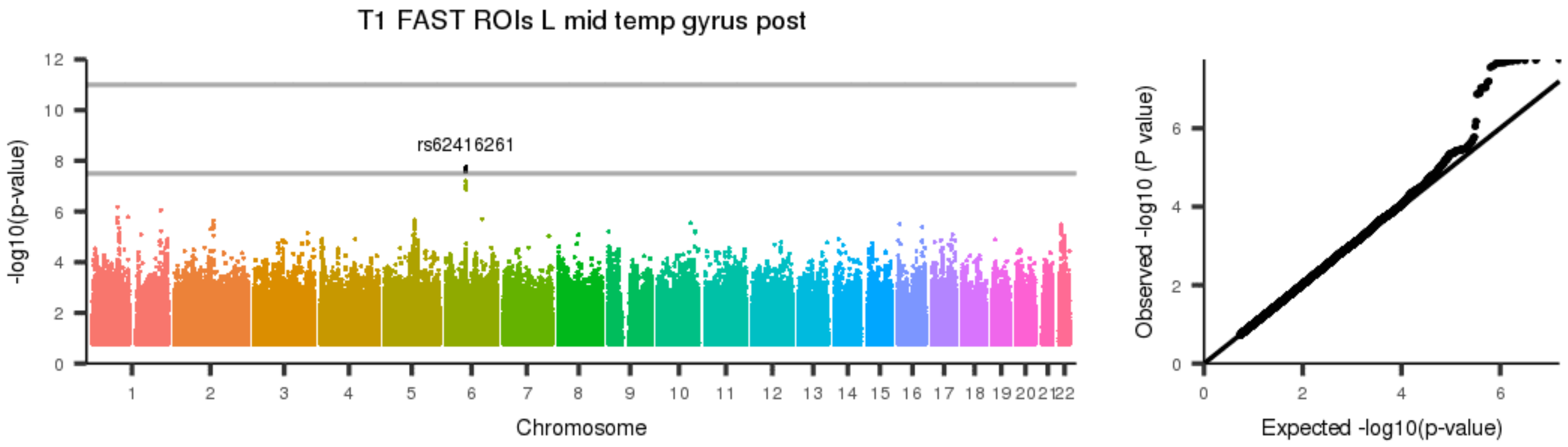

back to list of plots

Figure 49: T1 FAST ROIs R mid temp gyrus post

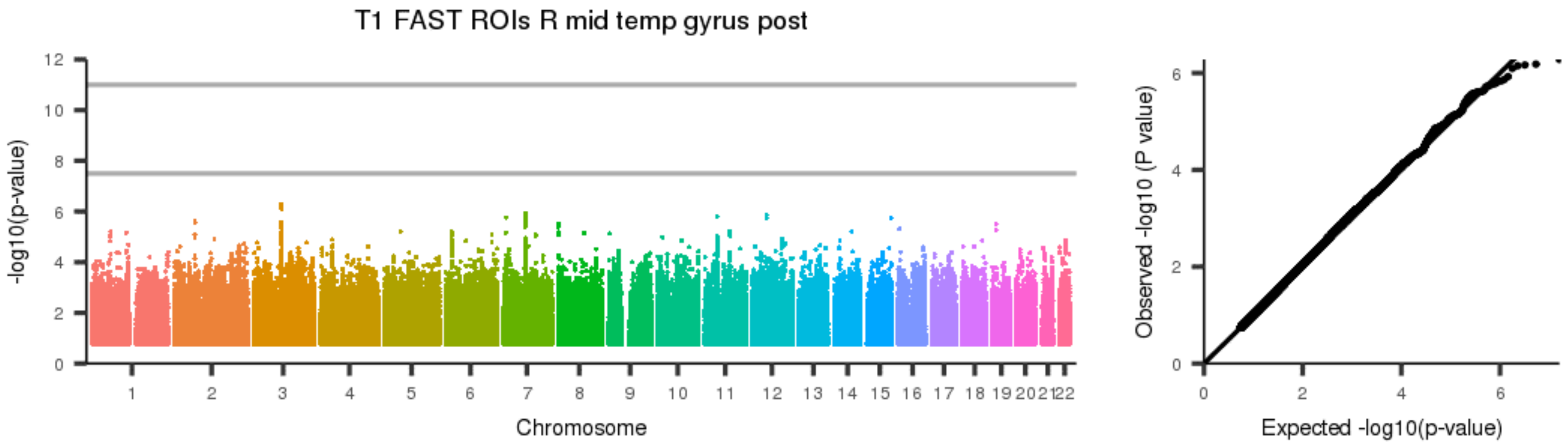

back to list of plots

Figure 50: T1 FAST ROIs L mid temp gyrus tempocc

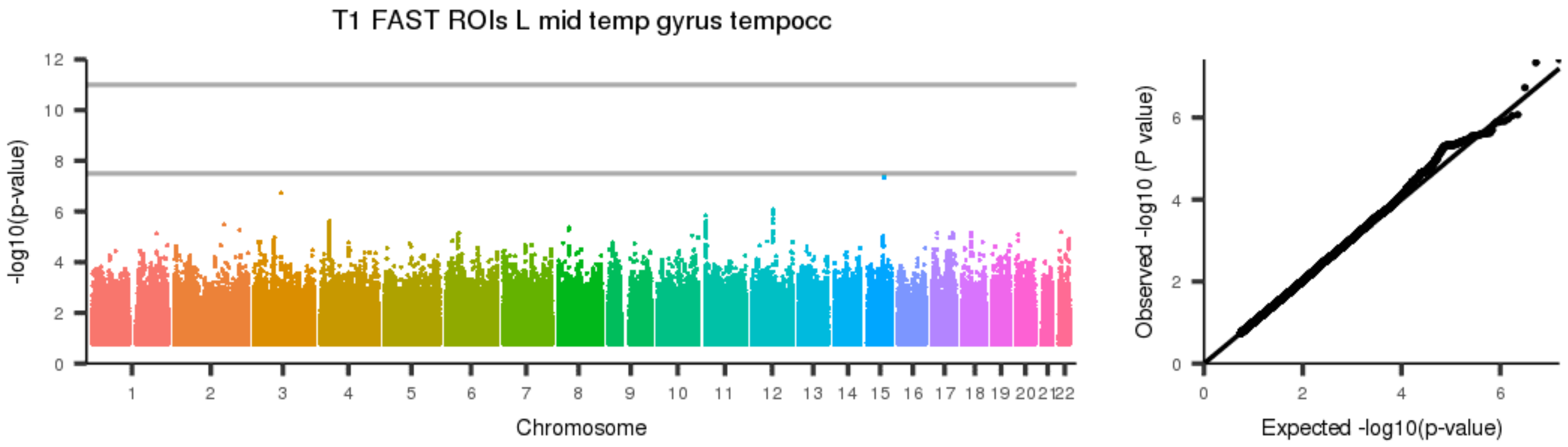

back to list of plots

Figure 51: T1 FAST ROIs R mid temp gyrus tempocc

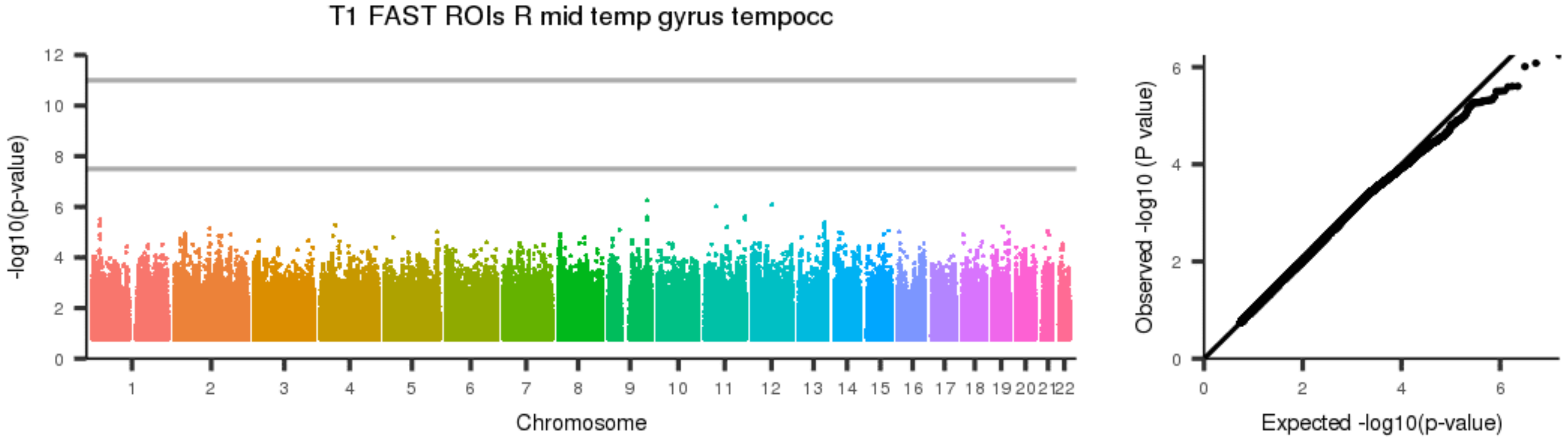

back to list of plots

Figure 52: T1 FAST ROIs L inf temp gyrus ant

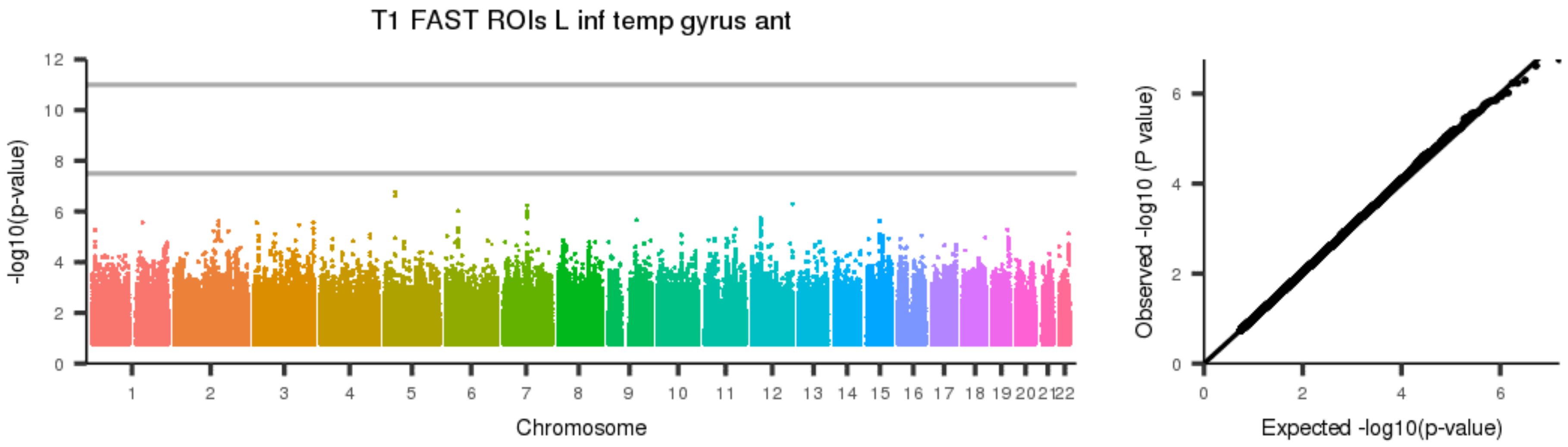

back to list of plots

Figure 53: T1 FAST ROIs R inf temp gyrus ant

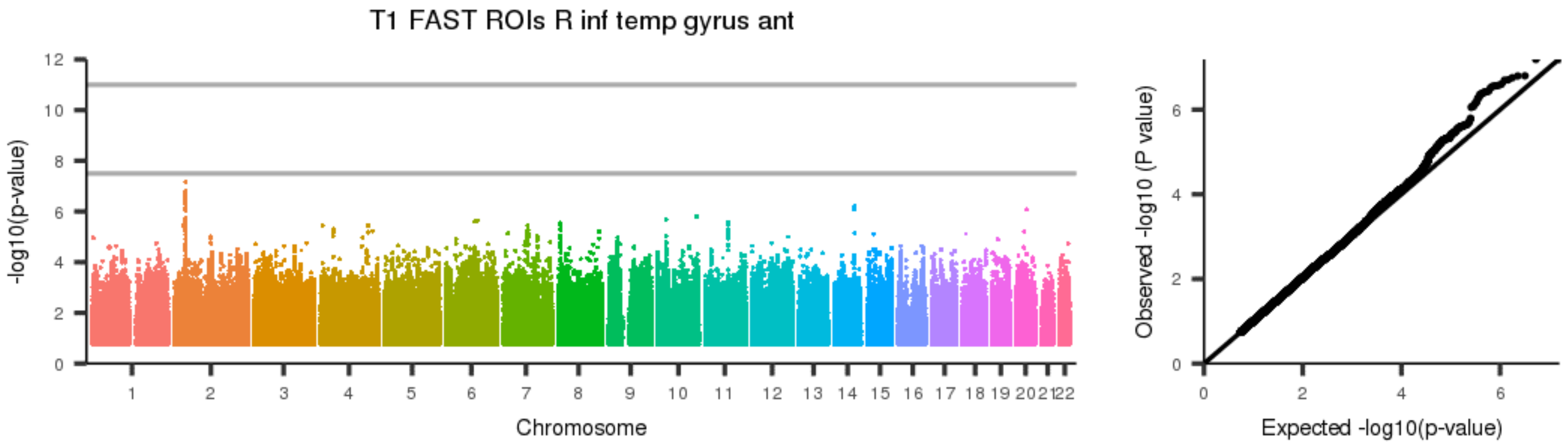

back to list of plots

Figure 54: T1 FAST ROIs L inf temp gyrus post

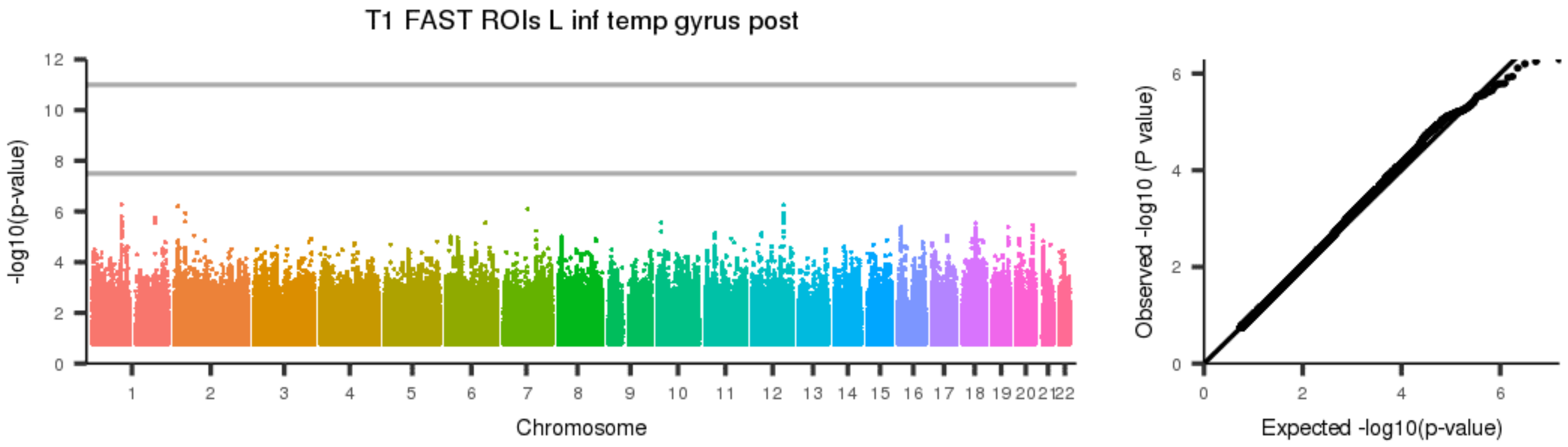

back to list of plots

Figure 55: T1 FAST ROIs R inf temp gyrus post

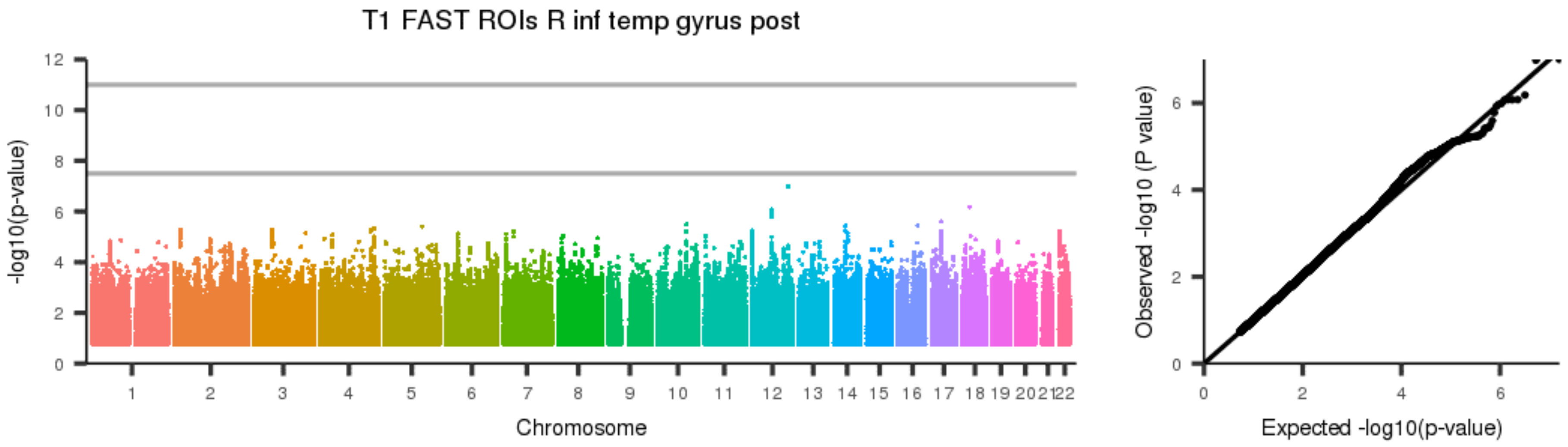

back to list of plots

Figure 56: T1 FAST ROIs L inf temp gyrus tempocc

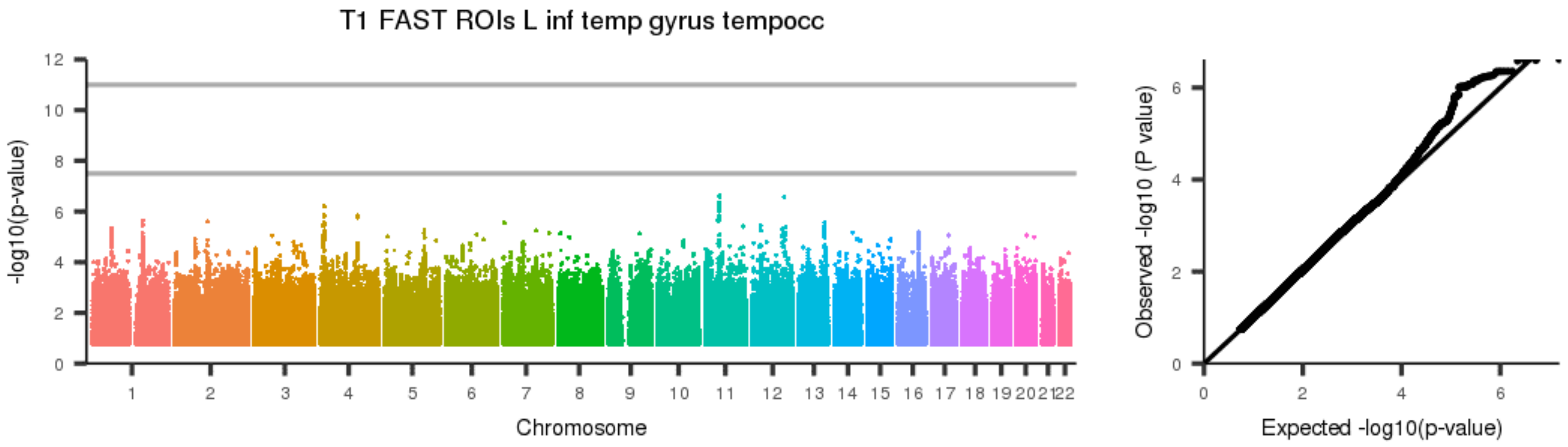

back to list of plots

Figure 57: T1 FAST ROIs R inf temp gyrus tempocc

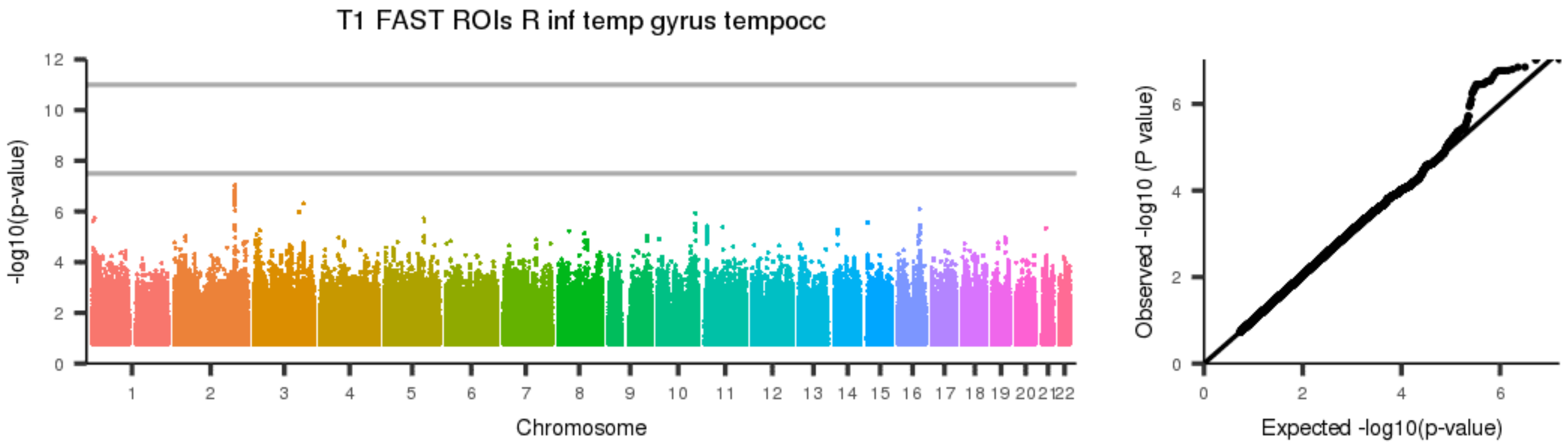

back to list of plots

Figure 58: T1 FAST ROIs L postcent gyrus

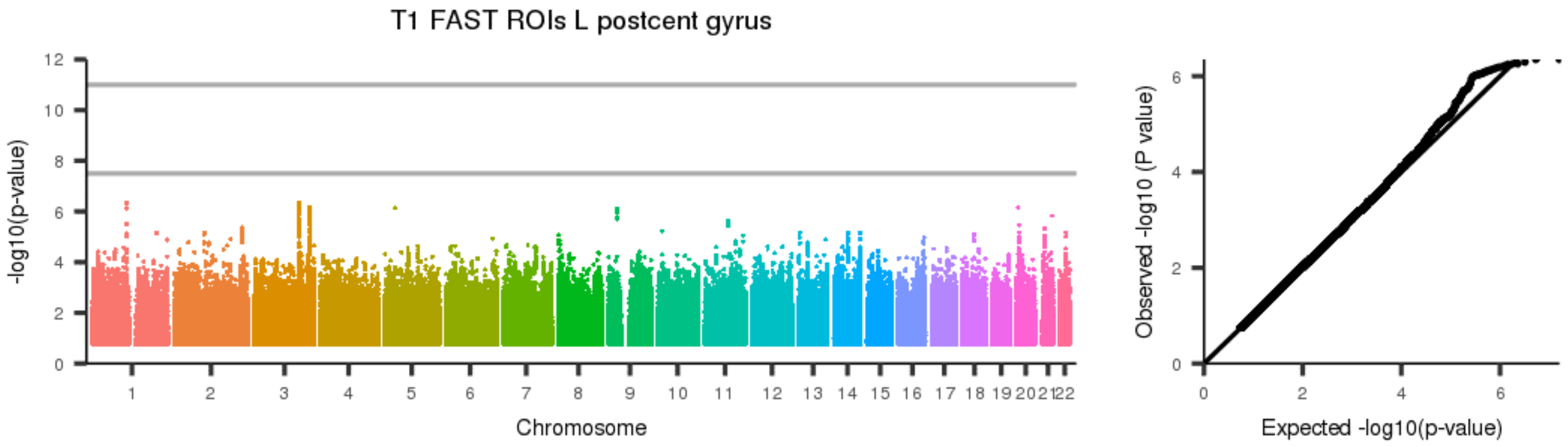

back to list of plots

Figure 59: T1 FAST ROIs R postcent gyrus

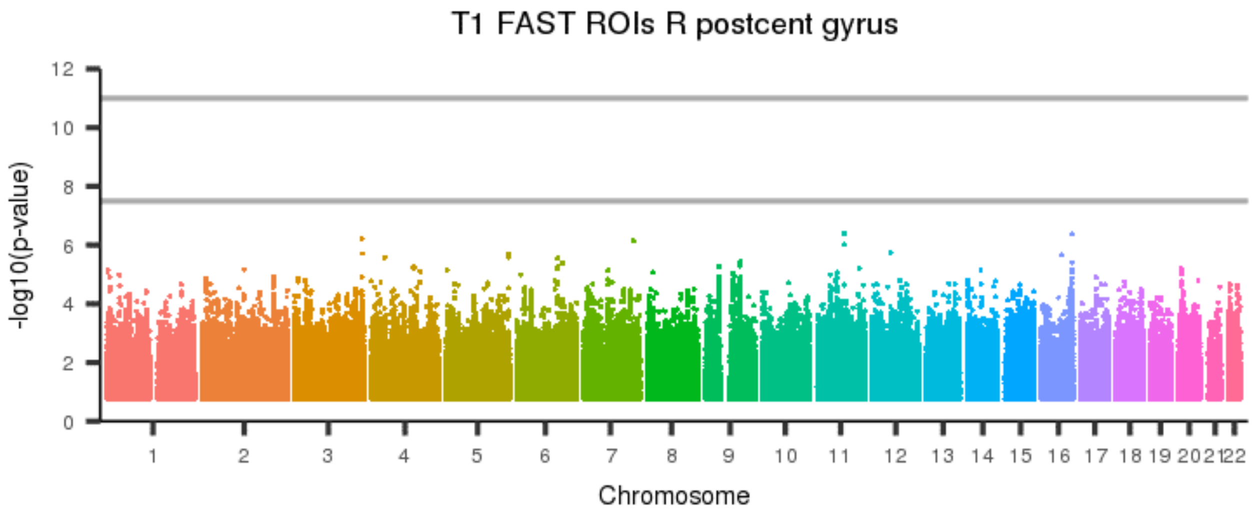

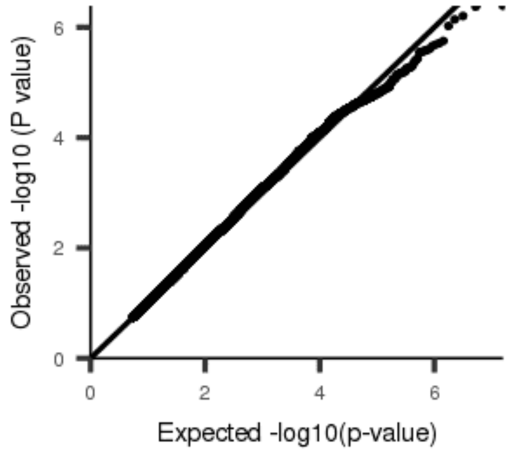

back to list of plots

Figure 60: T1 FAST ROIs L sup parietal lobule

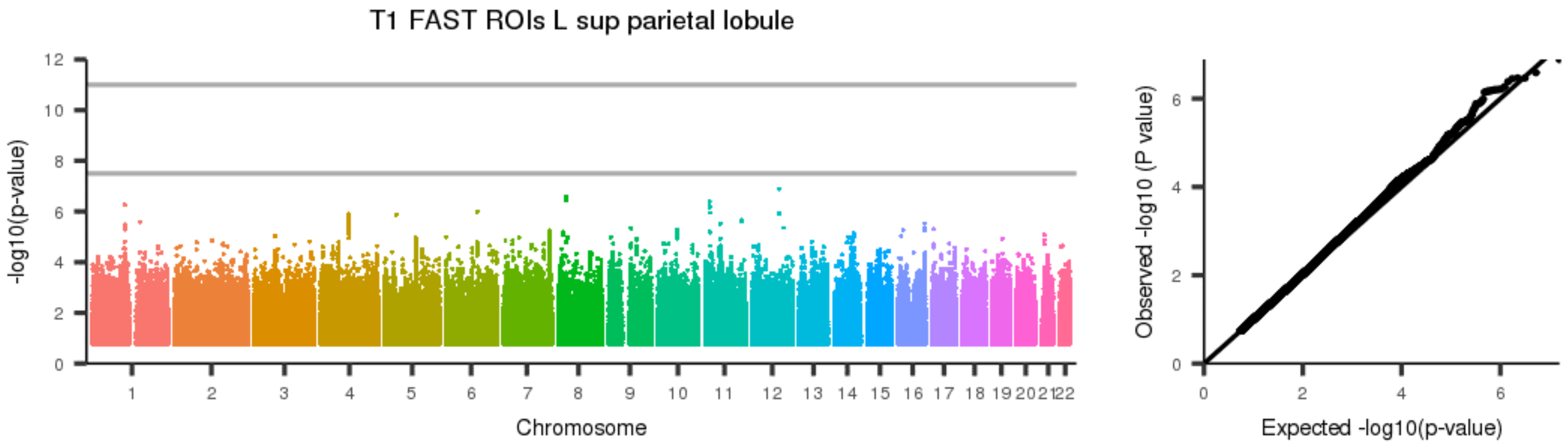

back to list of plots

Figure 61: T1 FAST ROIs R sup parietal lobule

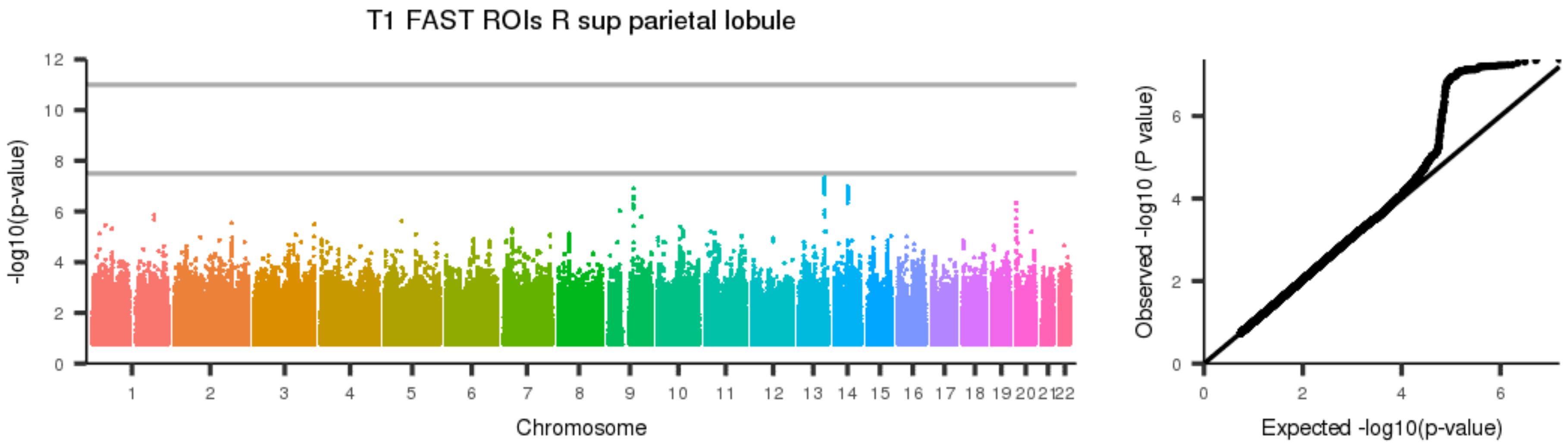

back to list of plots

Figure 62: T1 FAST ROIs L supramarg gyrus ant

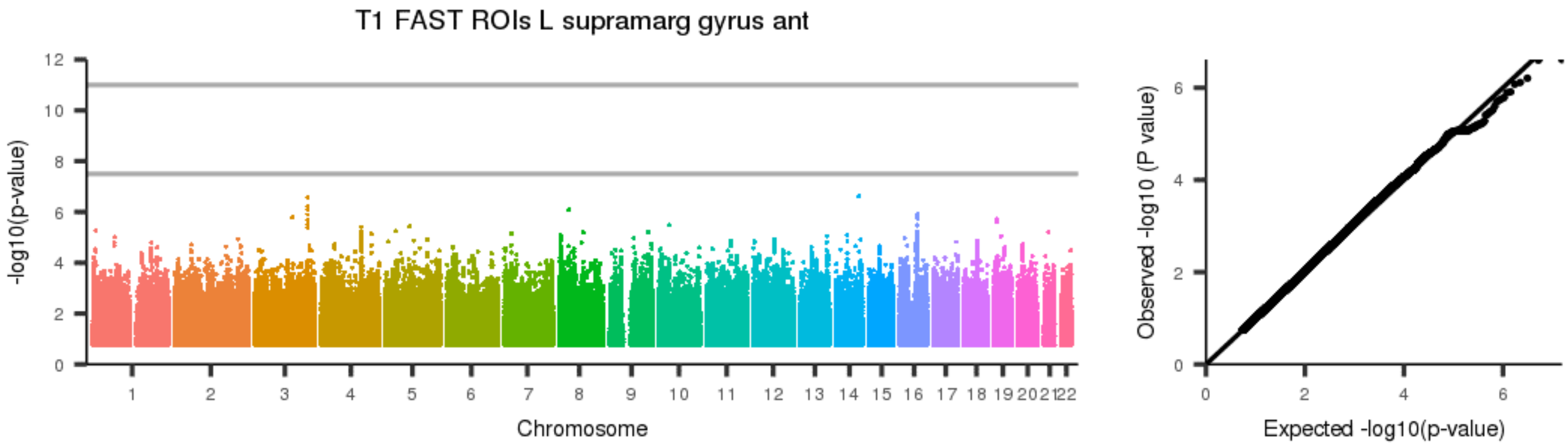

back to list of plots

Figure 63: T1 FAST ROIs R supramarg gyrus ant

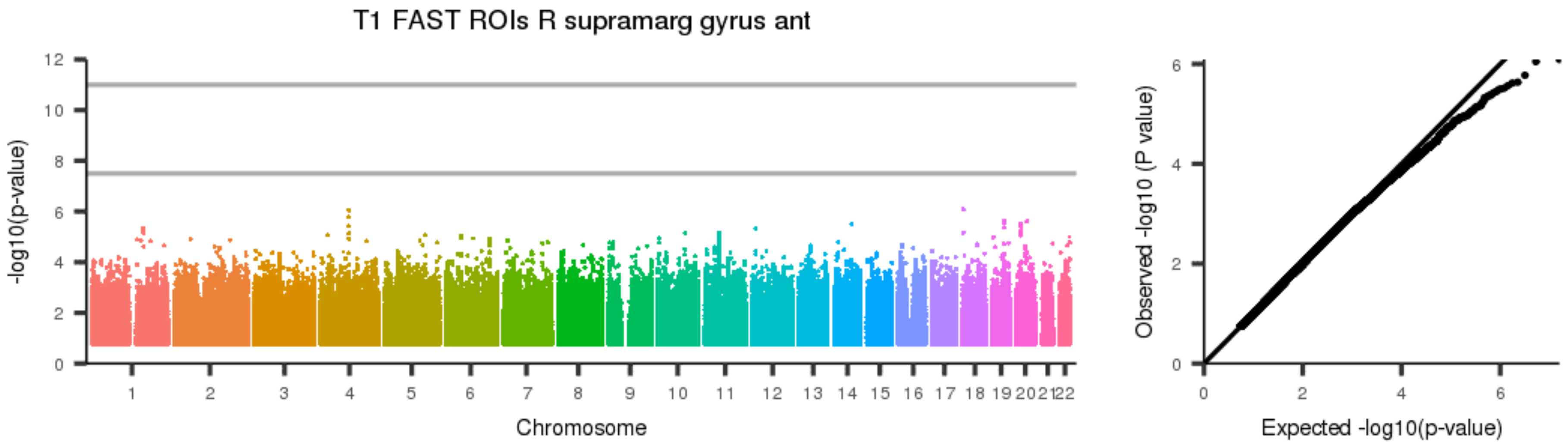

back to list of plots

Figure 64: T1 FAST ROIs L supramarg gyrus post

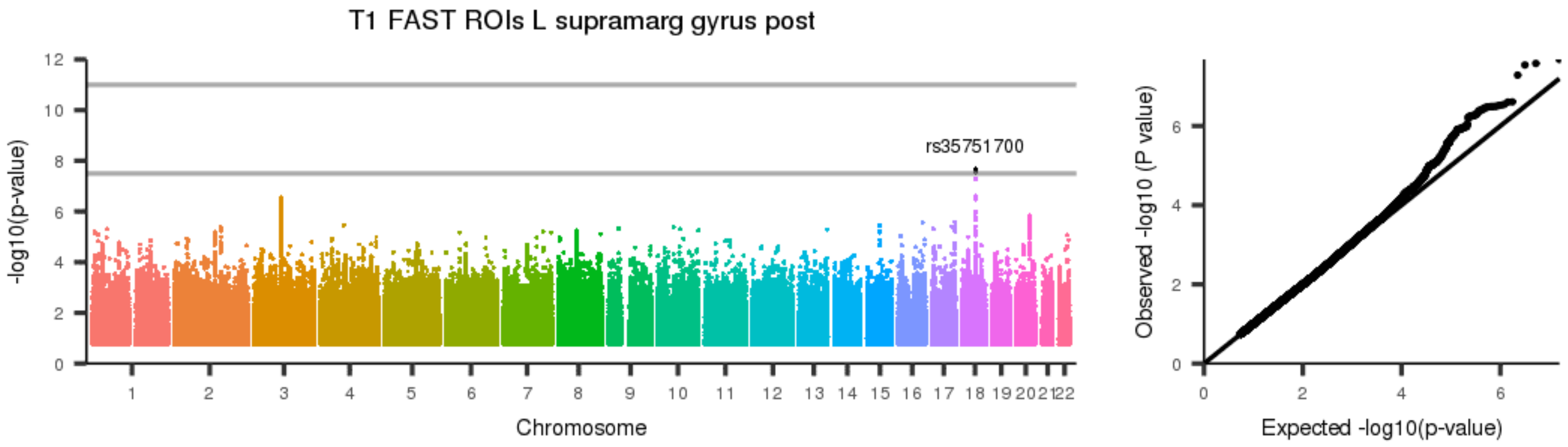

back to list of plots

Figure 65: T1 FAST ROIs R supramarg gyrus post

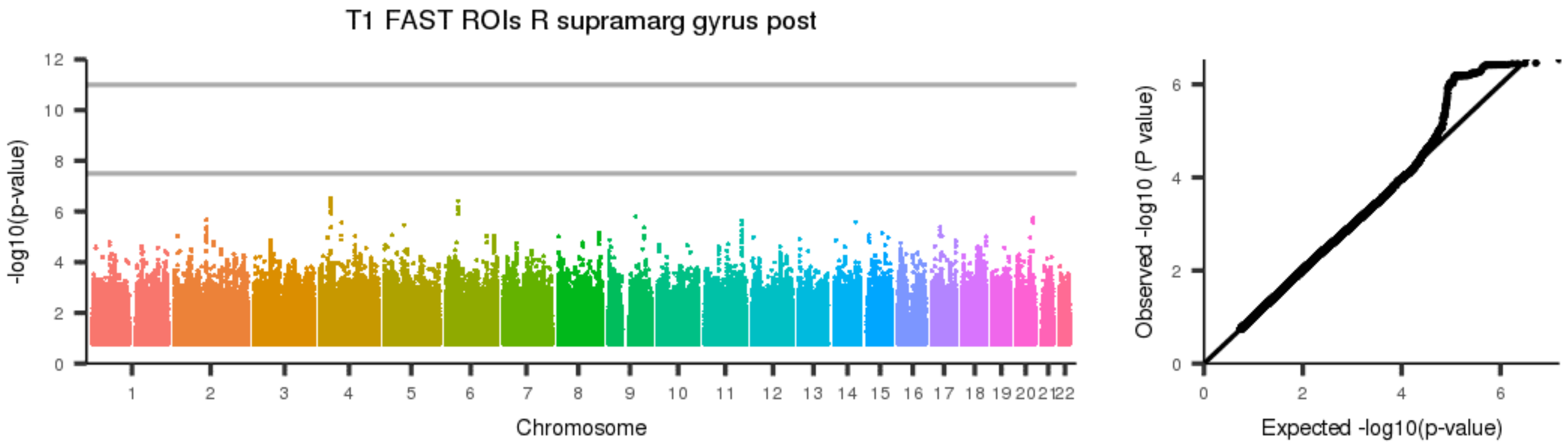

back to list of plots

Figure 66: T1 FAST ROIs L angular gyrus

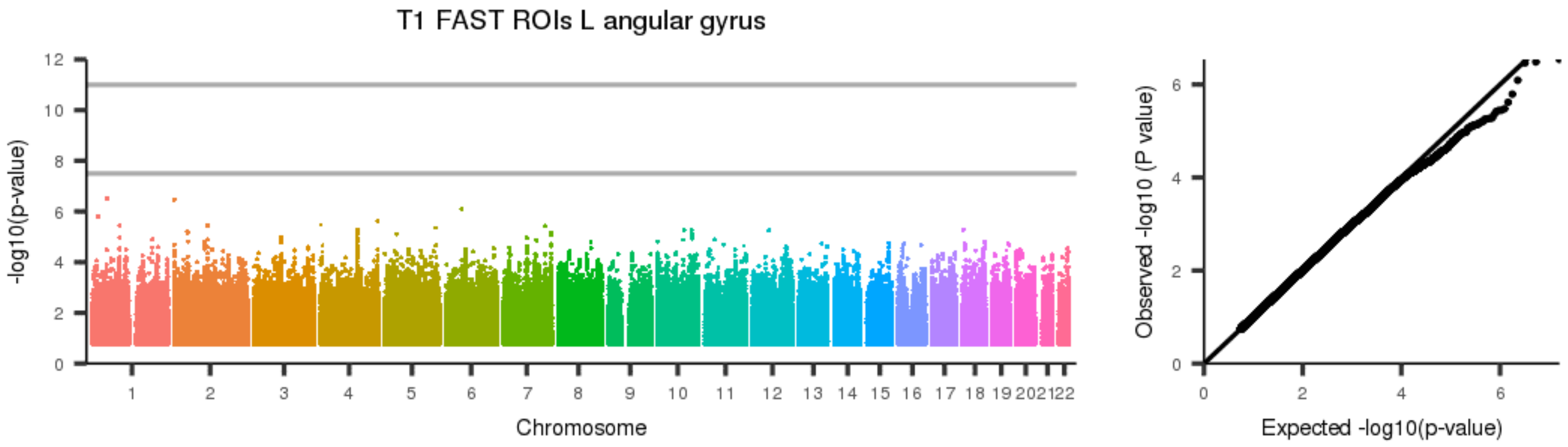

back to list of plots

Figure 67: T1 FAST ROIs R angular gyrus

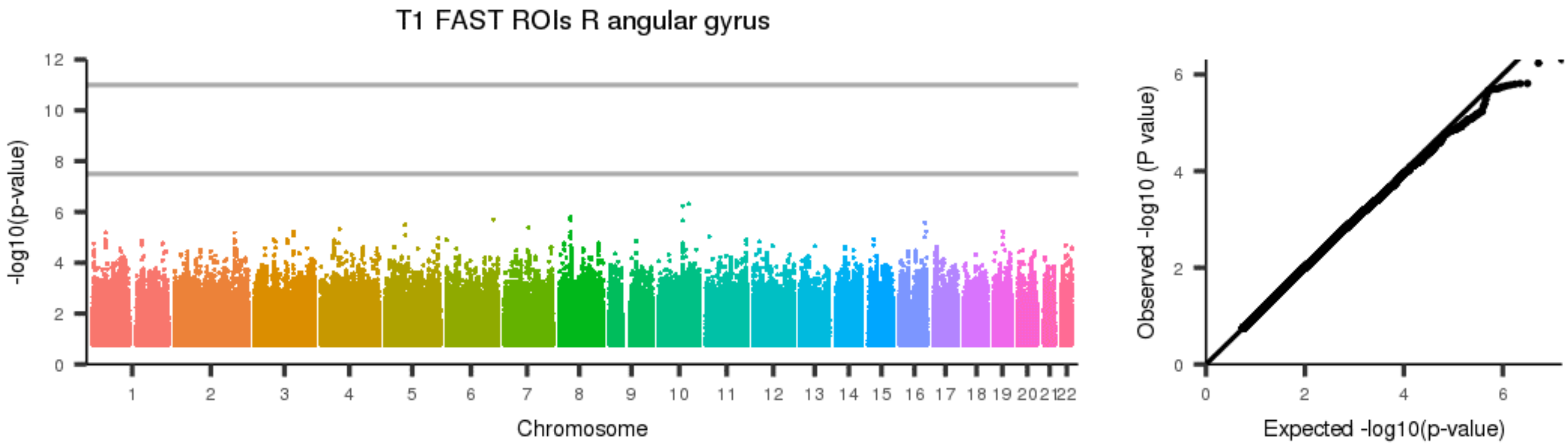

back to list of plots

Figure 68: T1 FAST ROIs L latocc cortex sup

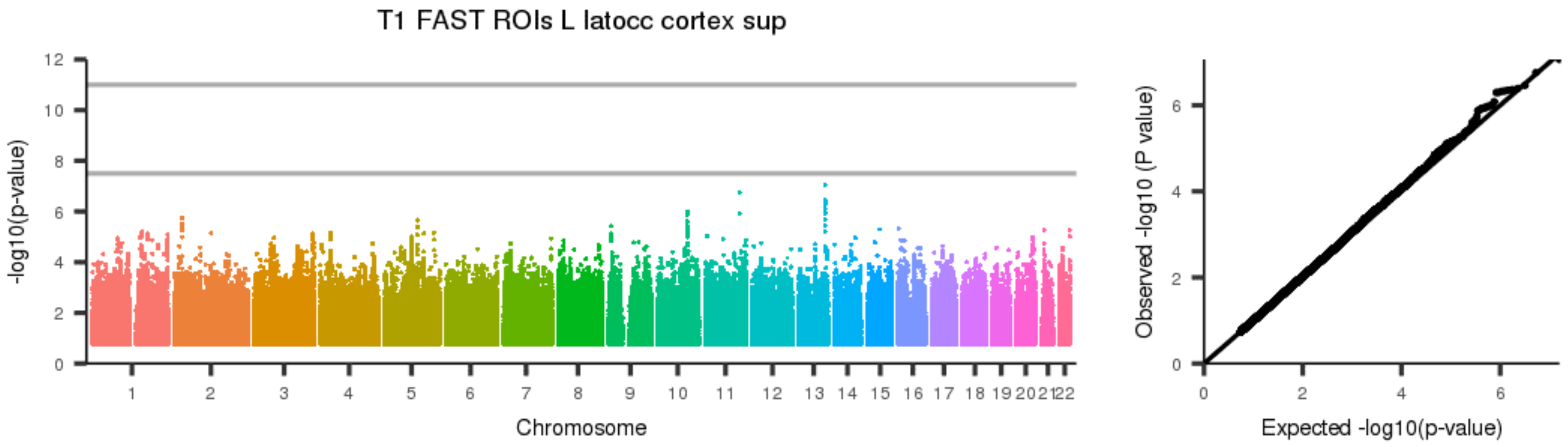

back to list of plots

Figure 69: T1 FAST ROIs R latocc cortex sup

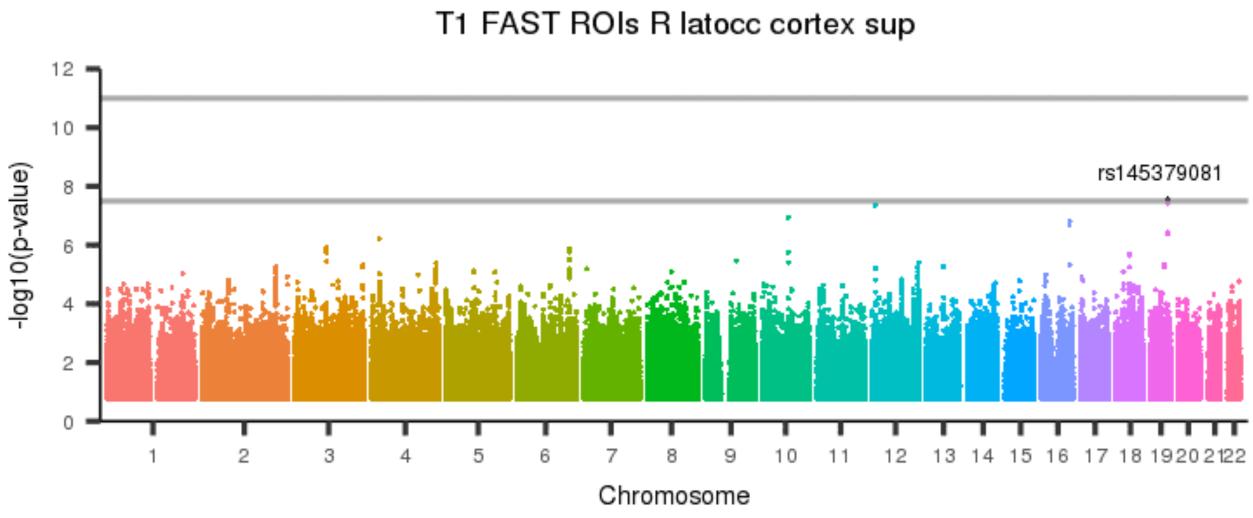

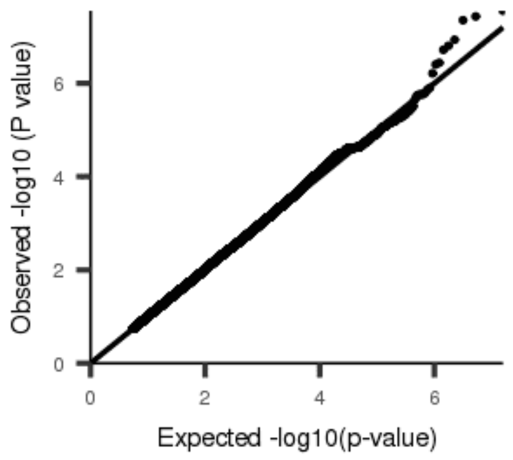

back to list of plots

Figure 70: T1 FAST ROIs L latocc cortex inf

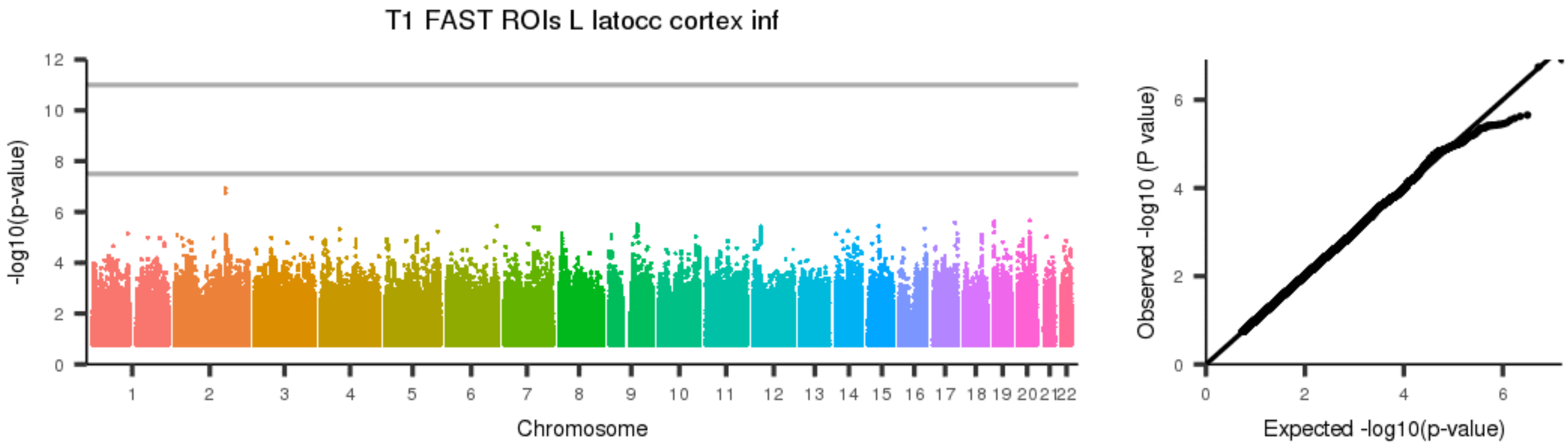

back to list of plots

Figure 71: T1 FAST ROIs R latocc cortex inf

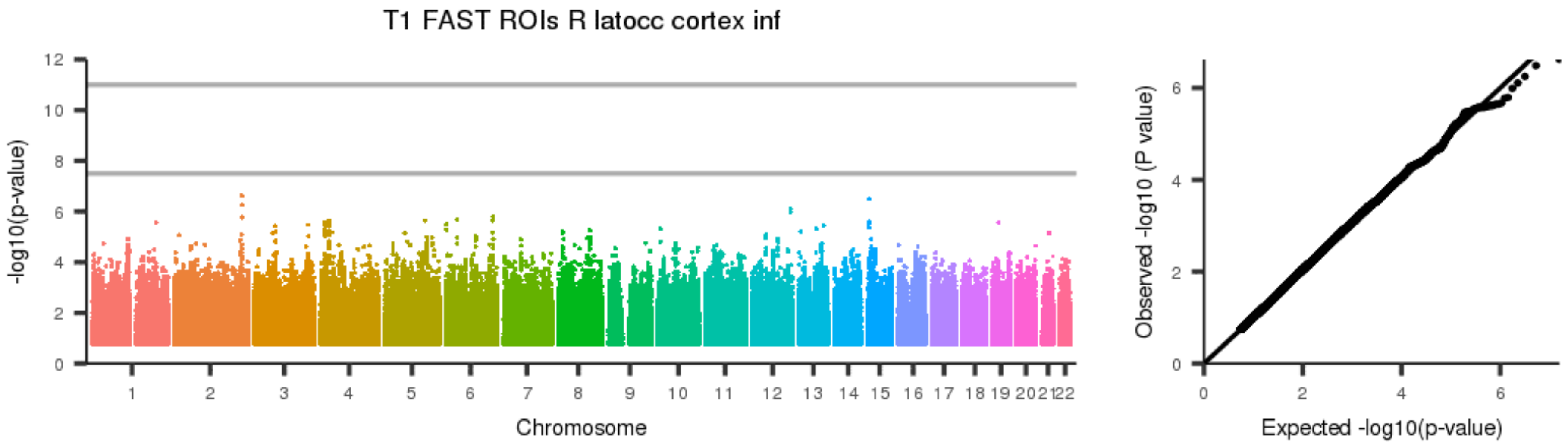

back to list of plots

Figure 72: T1 FAST ROIs L intracalc cortex

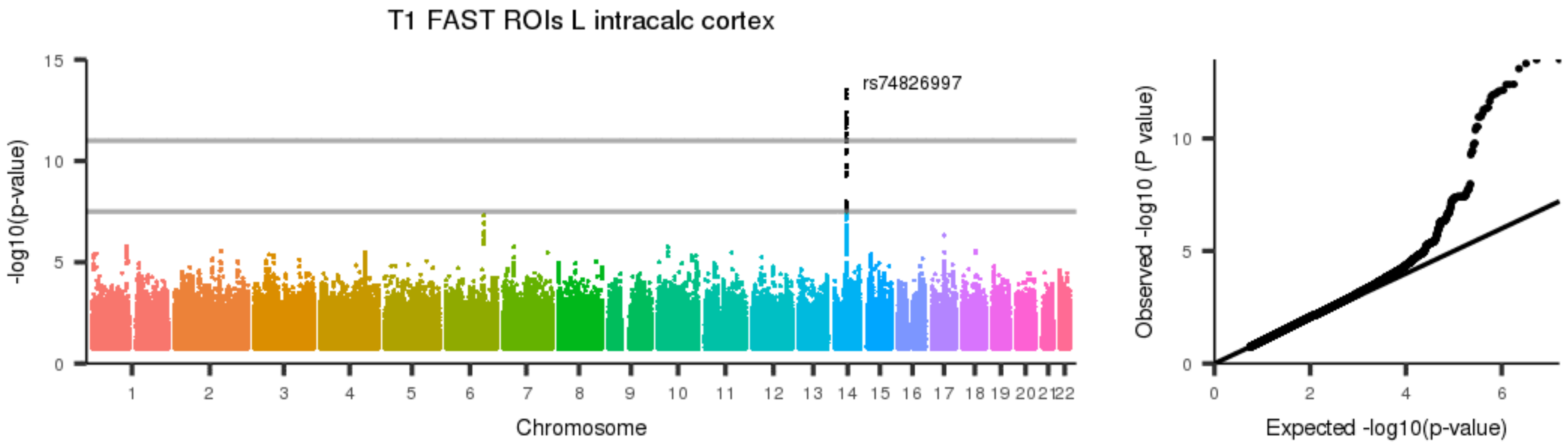

back to list of plots

Figure 73: T1 FAST ROIs R intracalc cortex

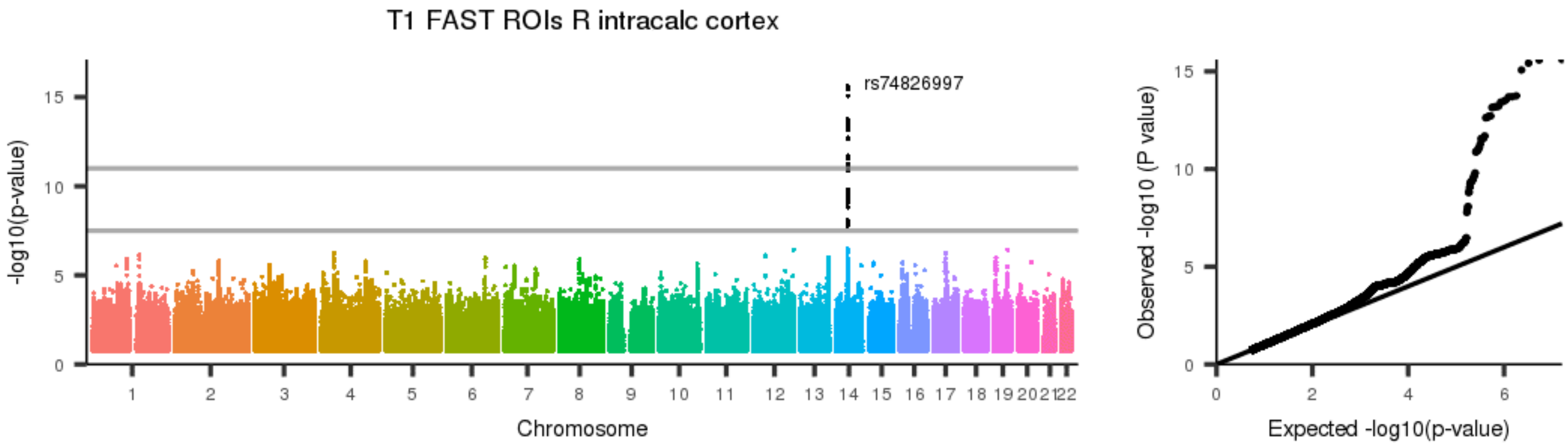

back to list of plots

Figure 74: T1 FAST ROIs L front med cortex

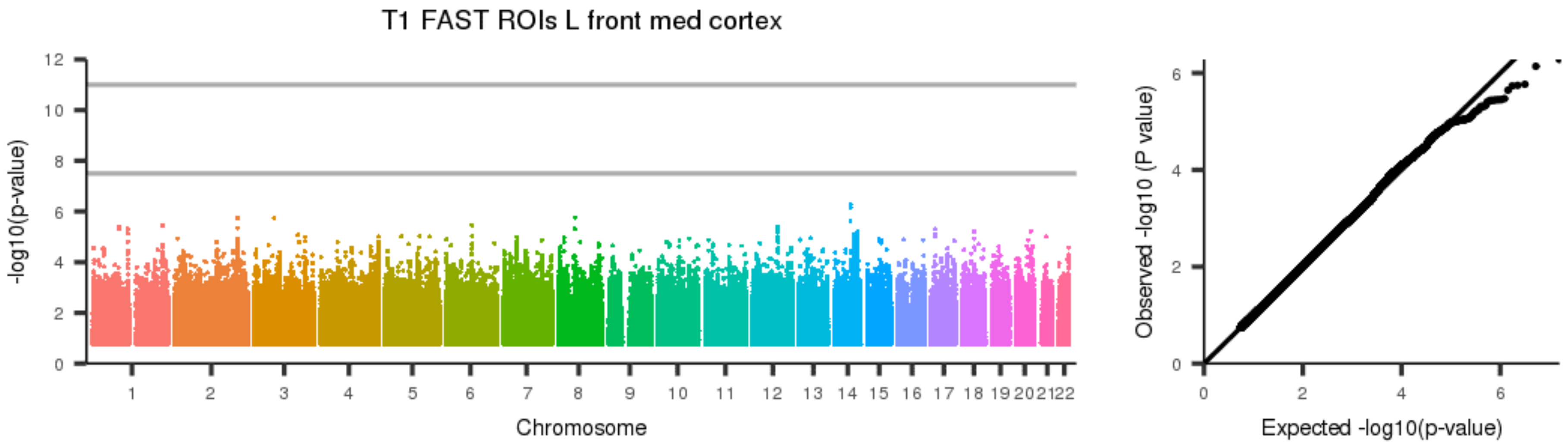

back to list of plots

Figure 75: T1 FAST ROIs R front med cortex

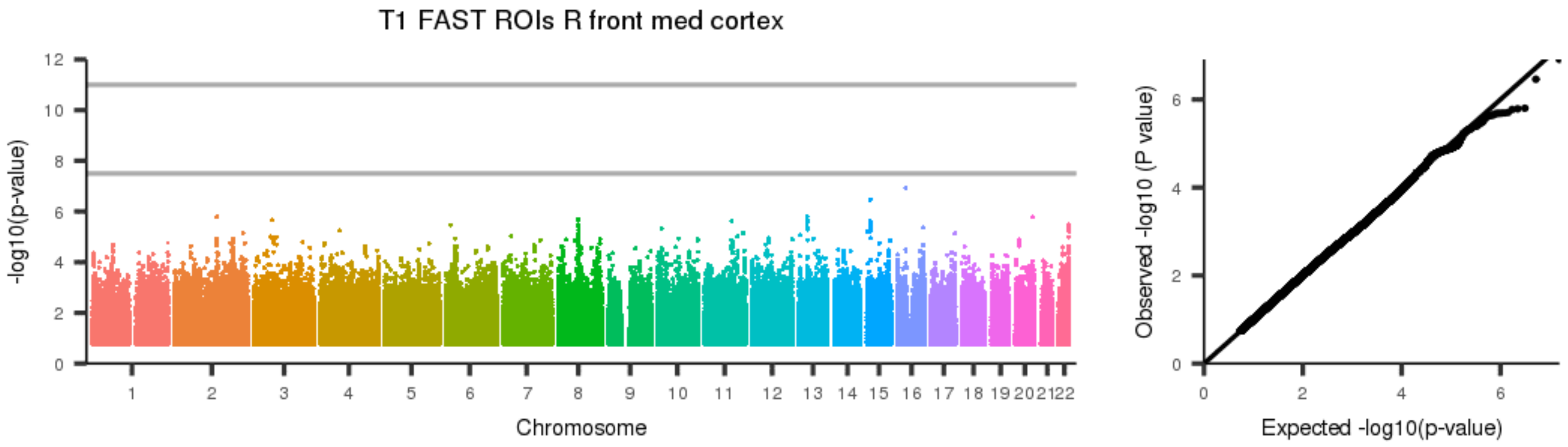

back to list of plots

Figure 76: T1 FAST ROIs L juxtapos lobule cortex

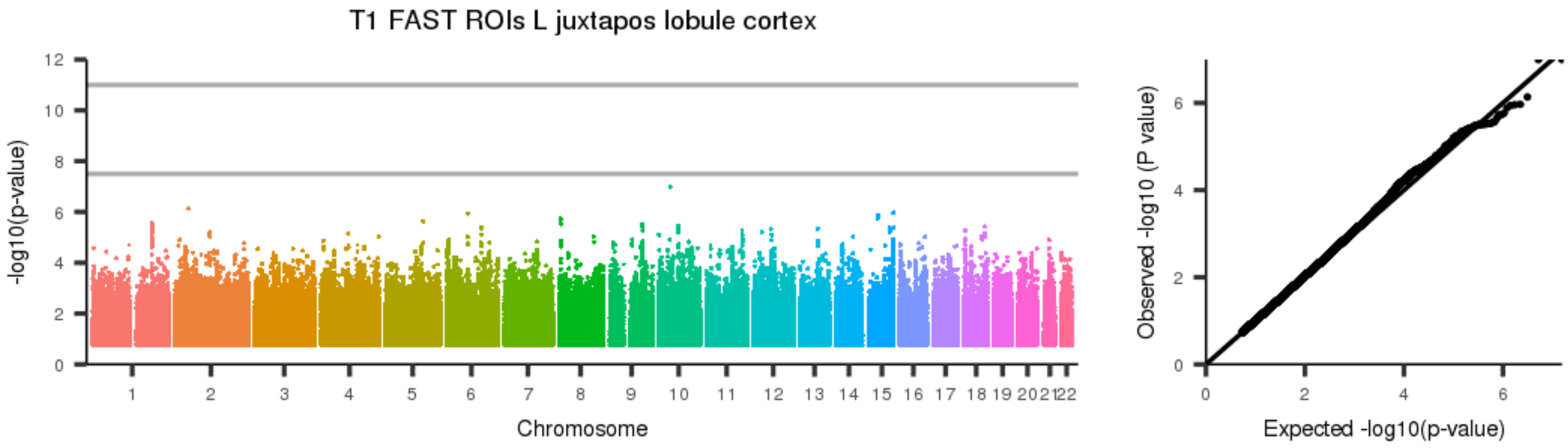

back to list of plots

Figure 77: T1 FAST ROIs R juxtapos lobule cortex

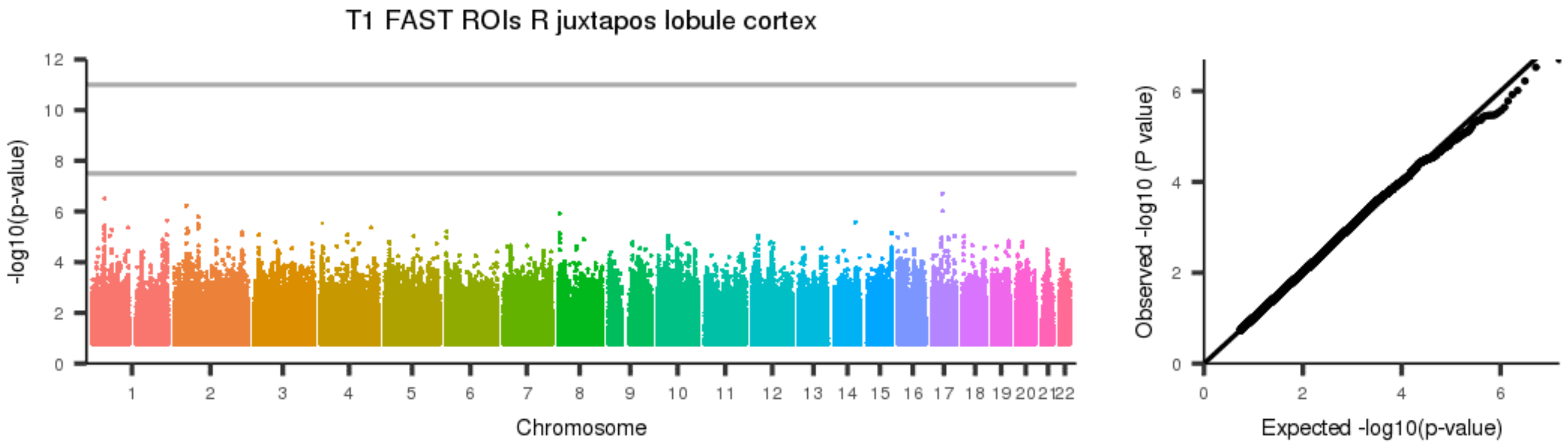

back to list of plots

Figure 78: T1 FAST ROIs L subcallosal cortex

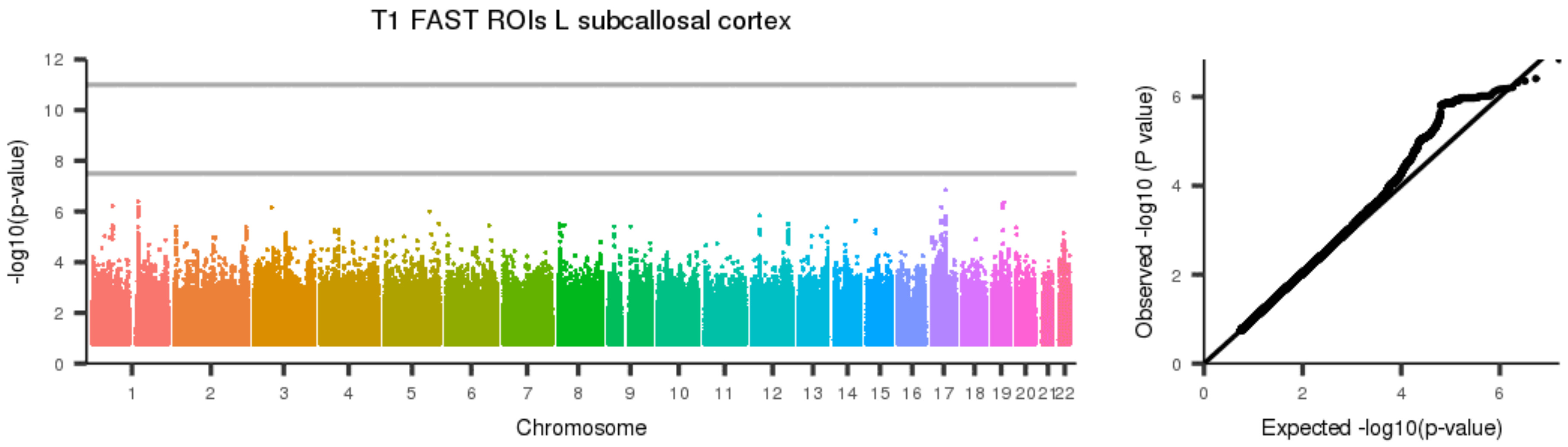

back to list of plots

Figure 79: T1 FAST ROIs R subcallosal cortex

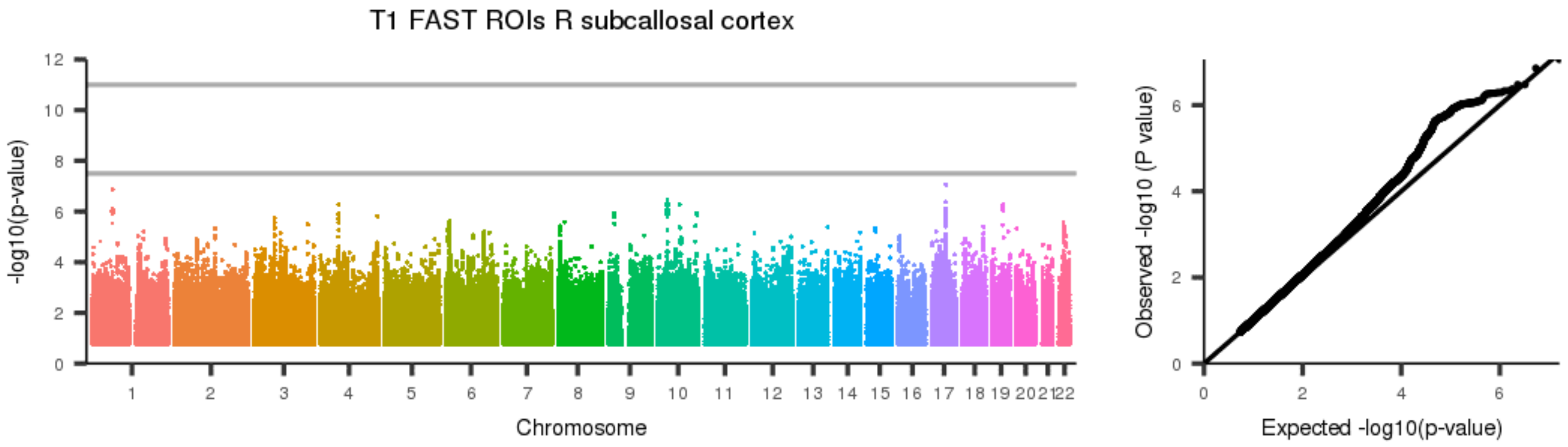

back to list of plots

Figure 80: T1 FAST ROIs L paracing gyrus

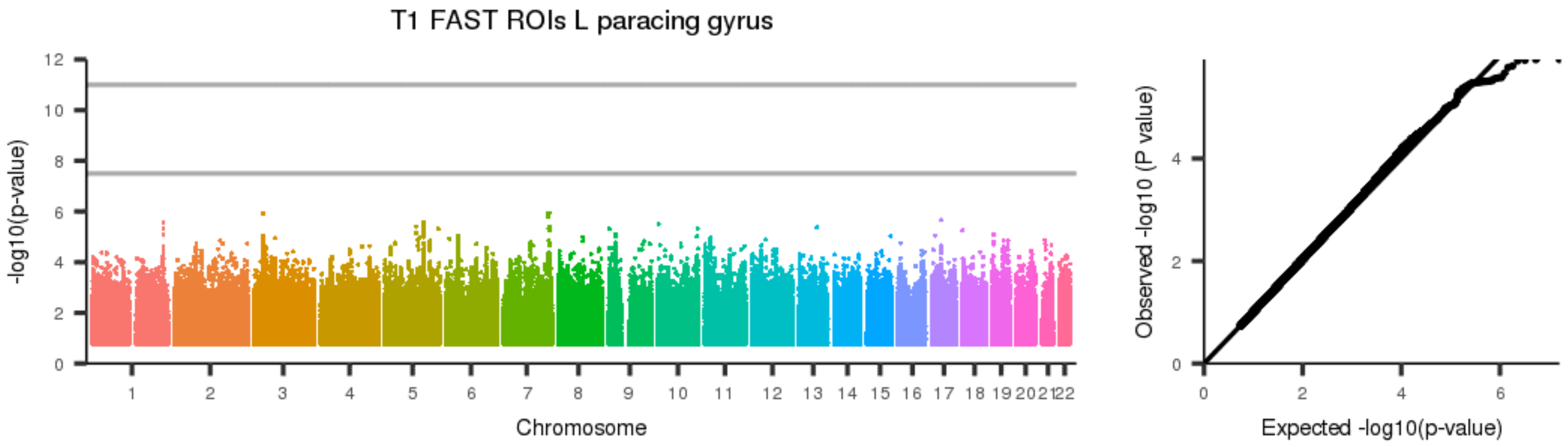

back to list of plots

Figure 81: T1 FAST ROIs R paracing gyrus

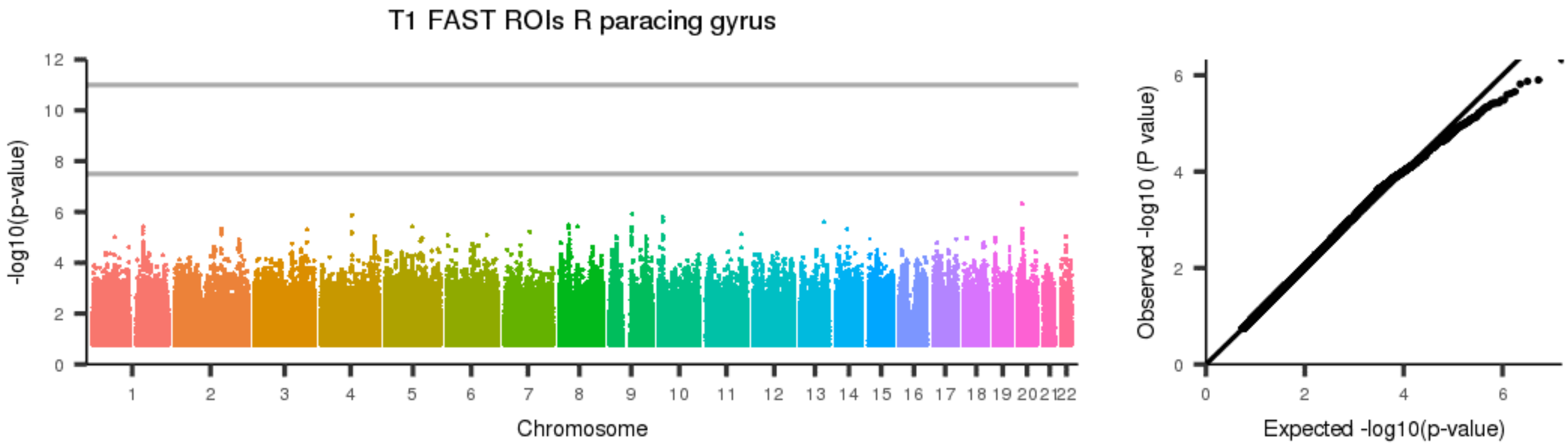

back to list of plots

Figure 82: T1 FAST ROIs L cing gyrus ant

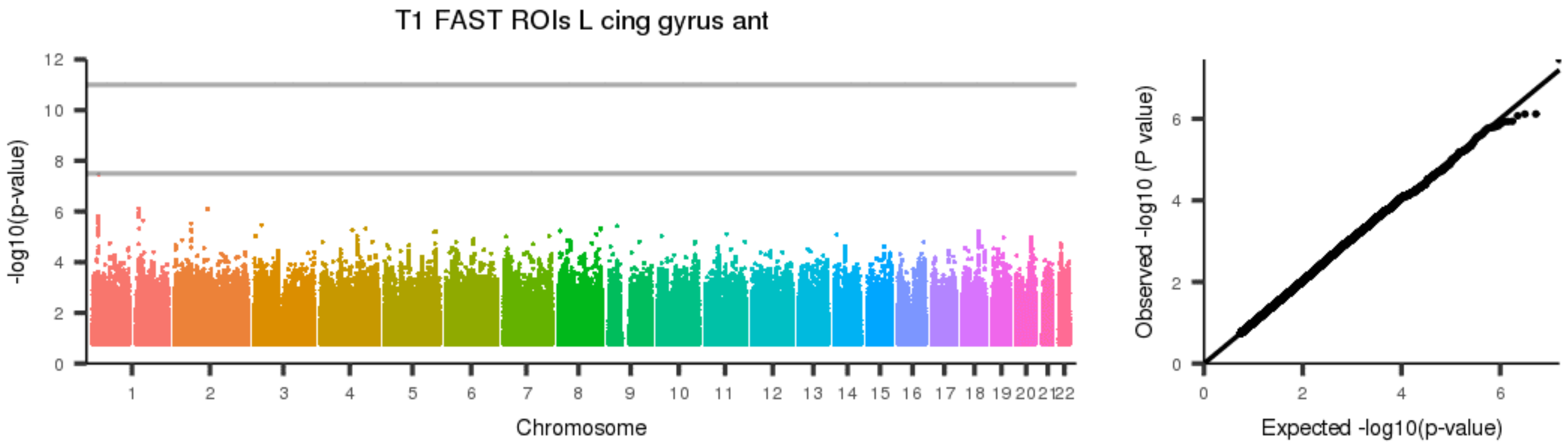

back to list of plots

Figure 83: T1 FAST ROIs R cing gyrus ant

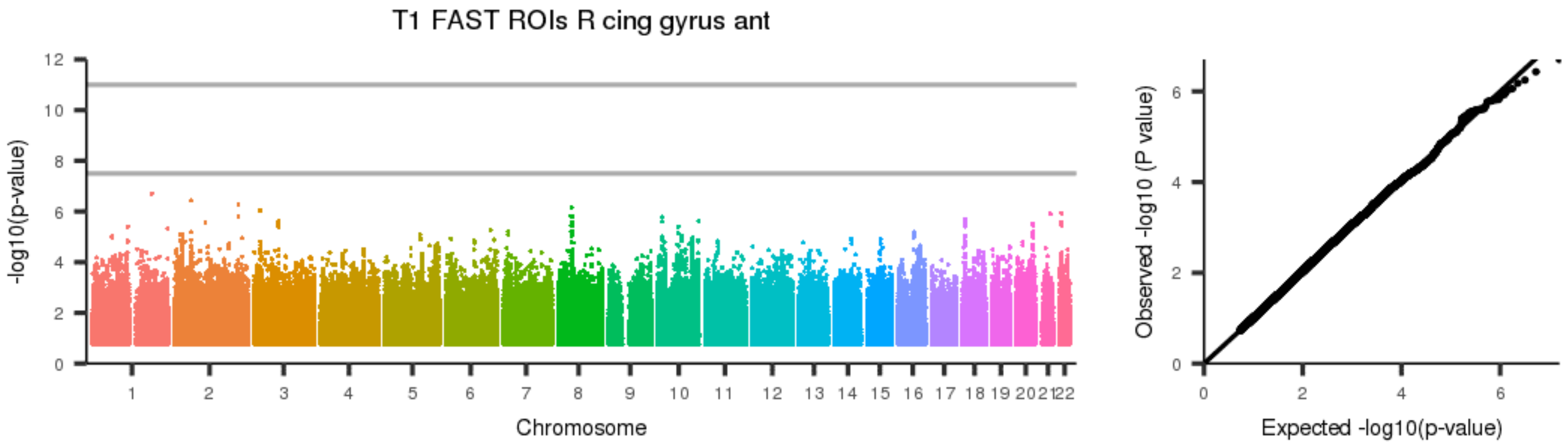

back to list of plots

Figure 84: T1 FAST ROIs L cing gyrus post

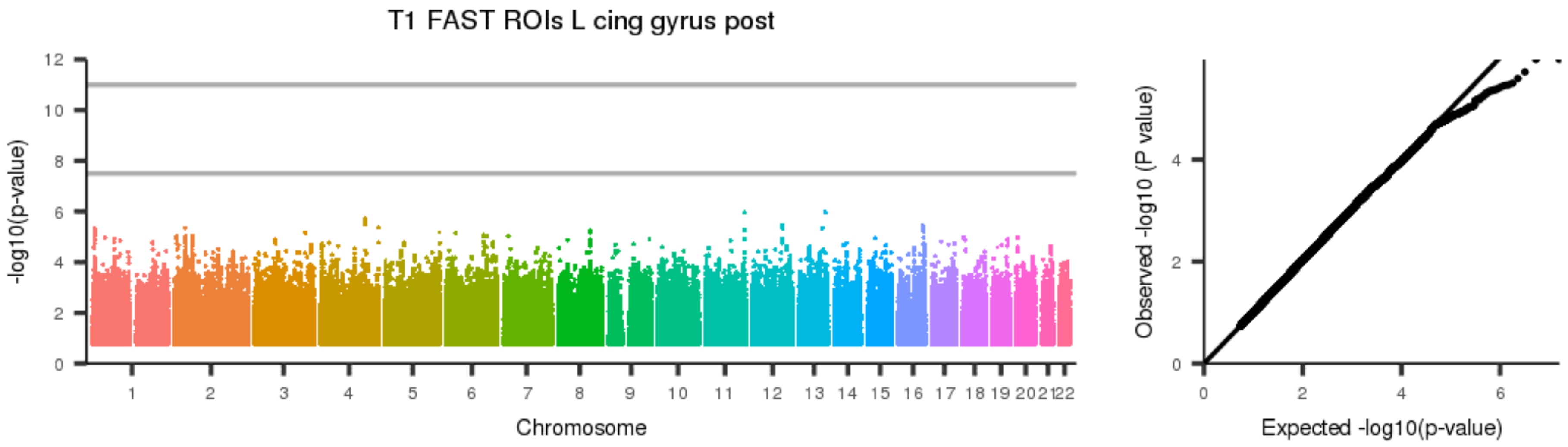

back to list of plots

Figure 85: T1 FAST ROIs R cing gyrus post

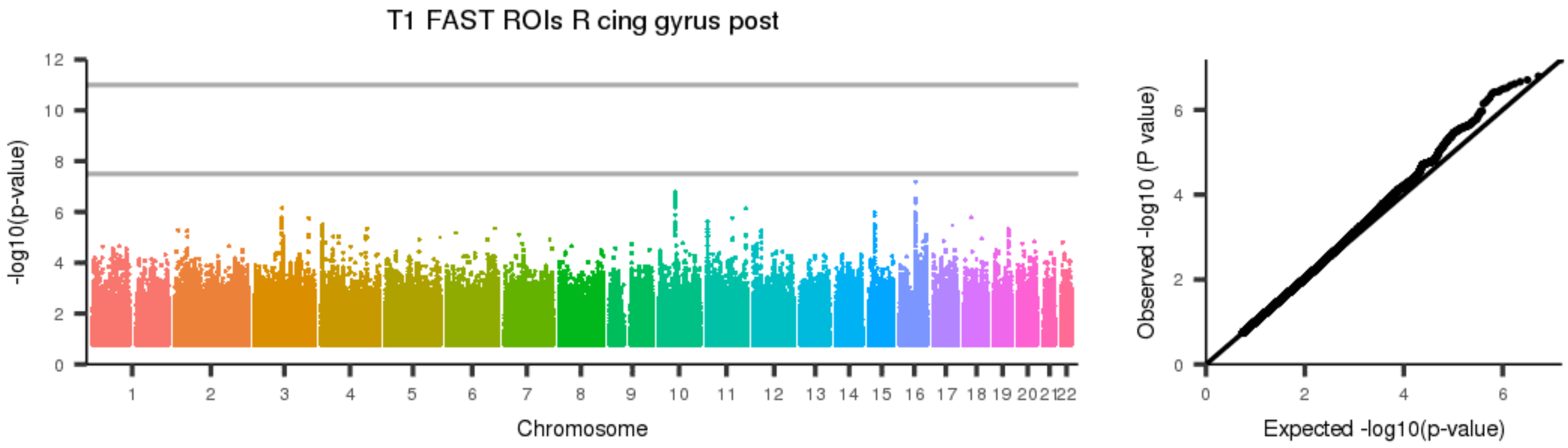

back to list of plots

Figure 86: T1 FAST ROIs L precun cortex

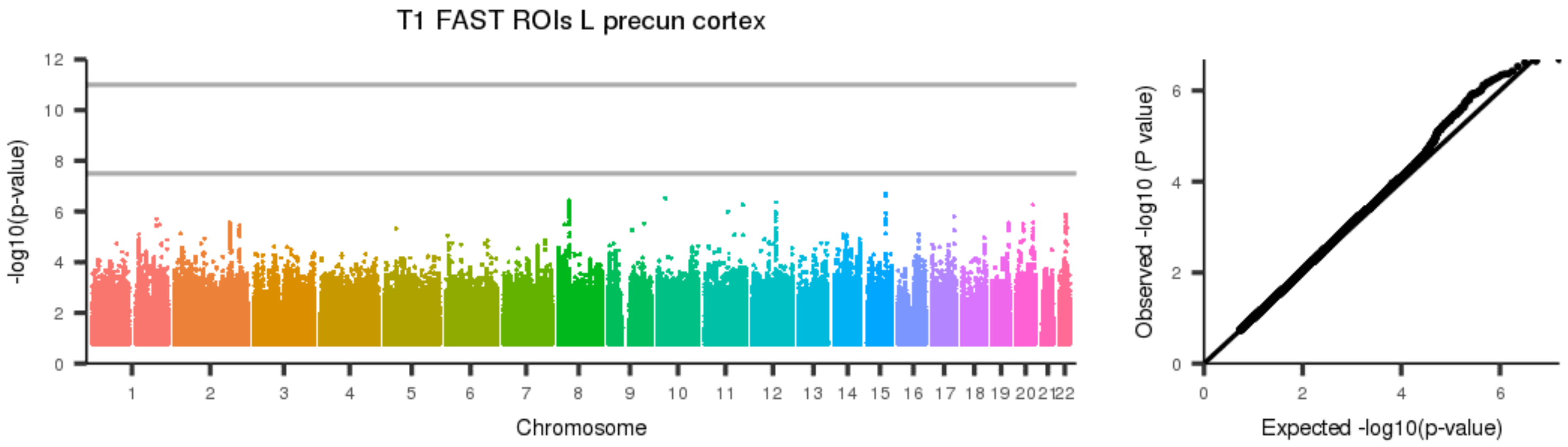

back to list of plots

Figure 87: T1 FAST ROIs R precun cortex

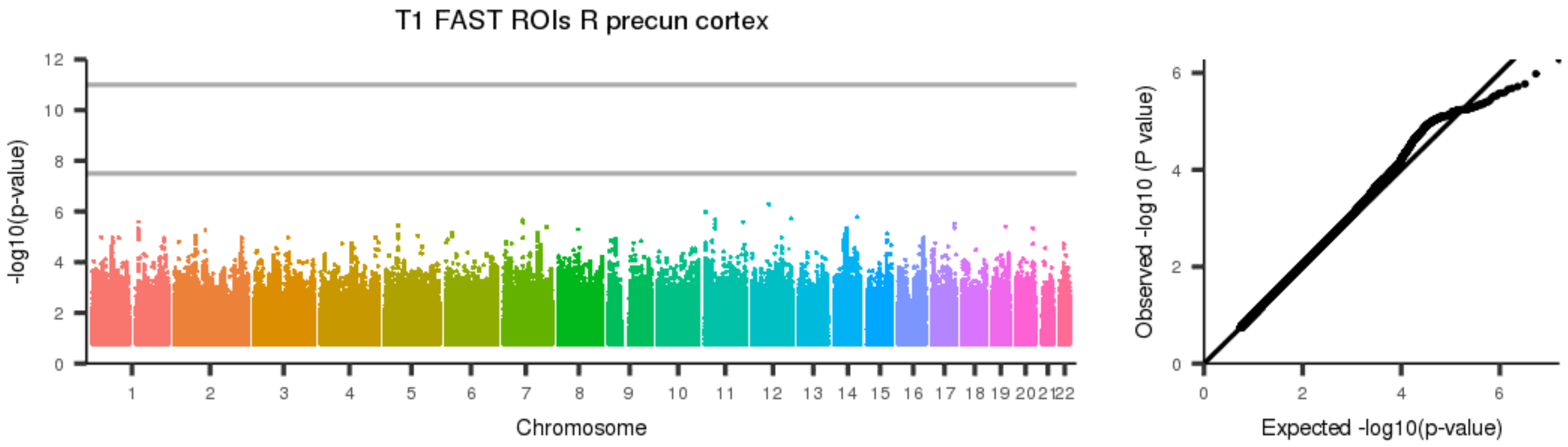

back to list of plots

Figure 88: T1 FAST ROIs L cuneal cortex

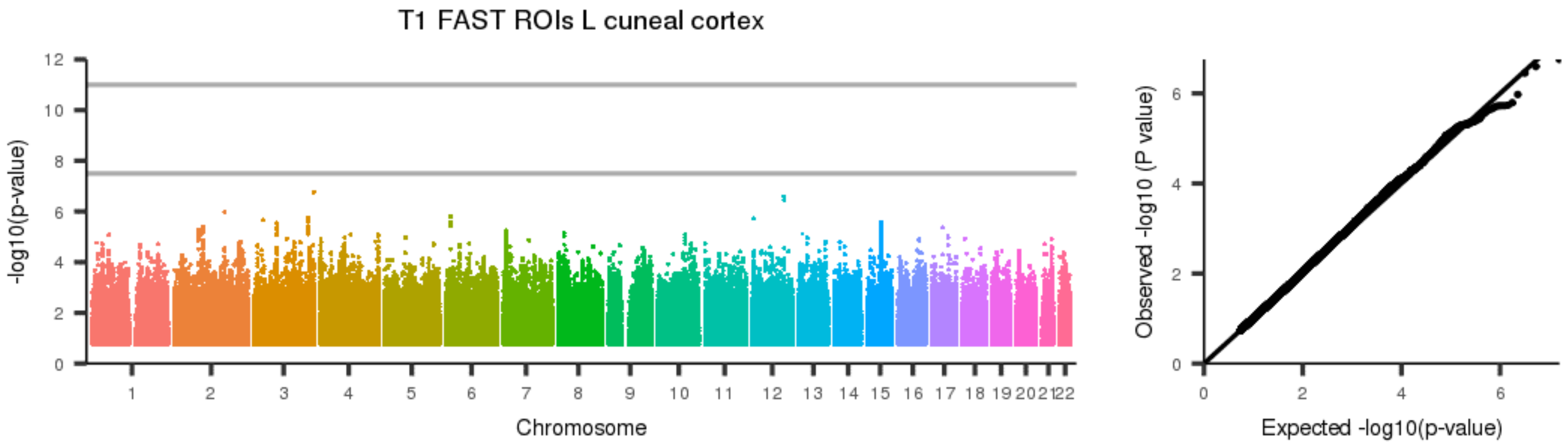

back to list of plots

Figure 89: T1 FAST ROIs R cuneal cortex

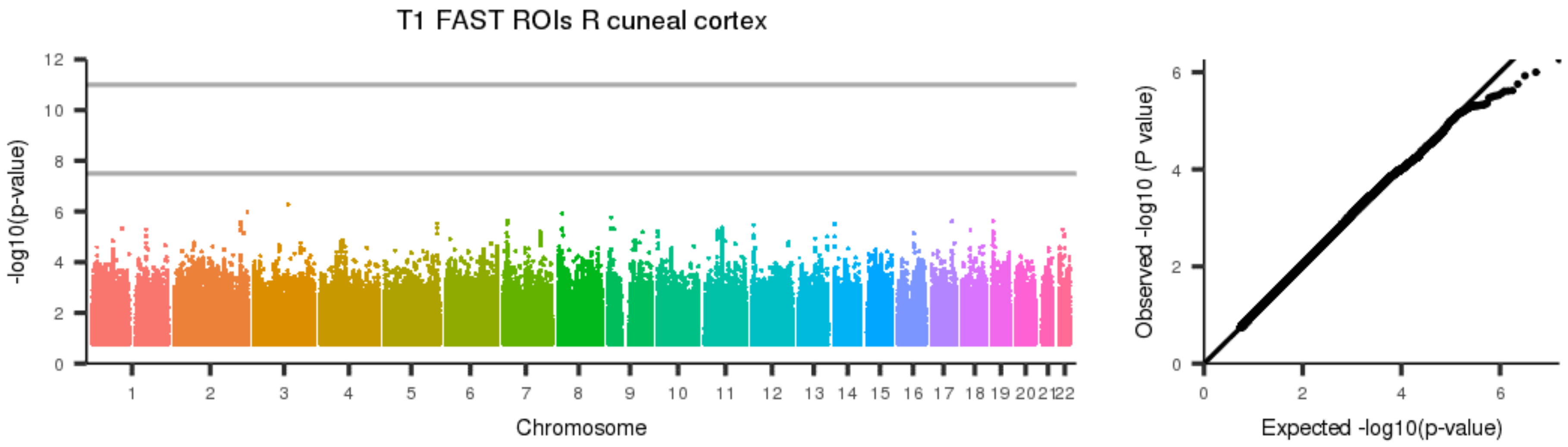

back to list of plots

Figure 90: T1 FAST ROIs L front orb cortex

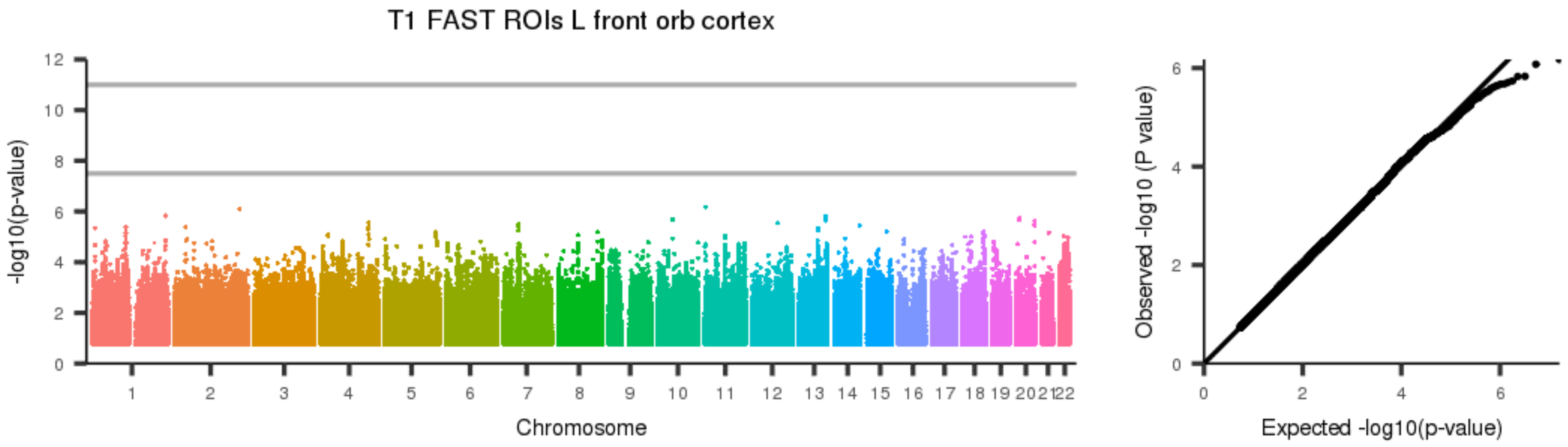

back to list of plots

Figure 91: T1 FAST ROIs R front orb cortex

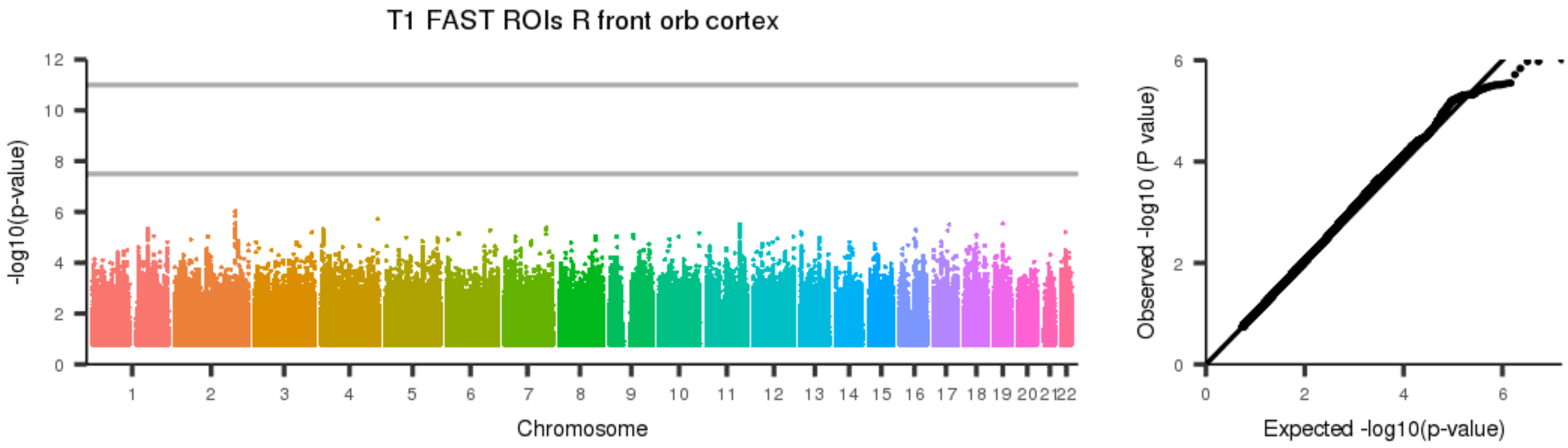

back to list of plots

Figure 92: T1 FAST ROIs L parahipp gyrus ant

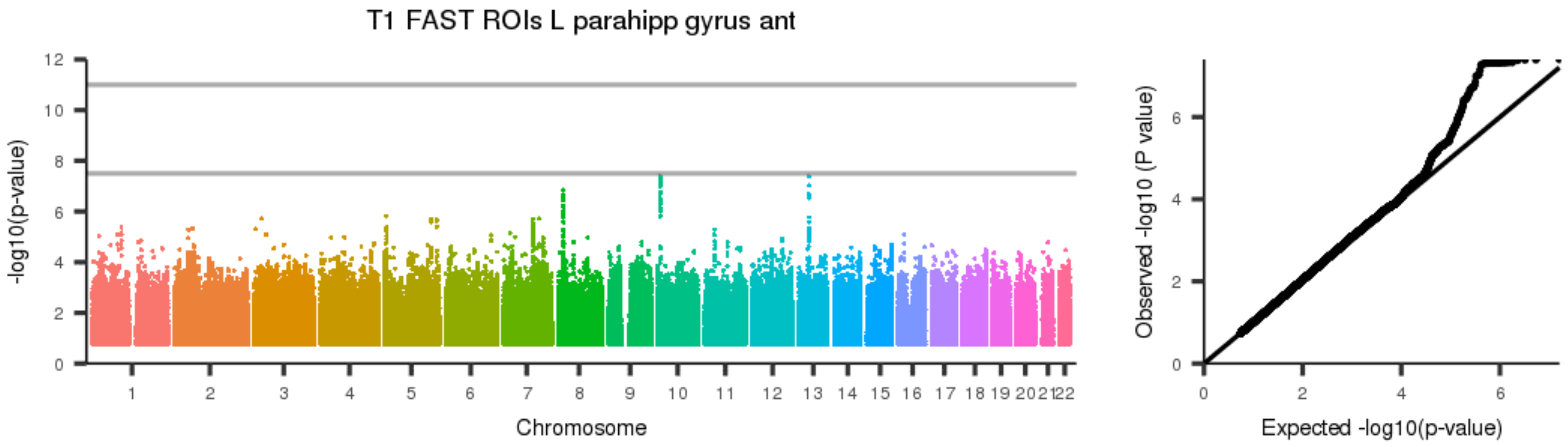

back to list of plots

Figure 93: T1 FAST ROIs R parahipp gyrus ant

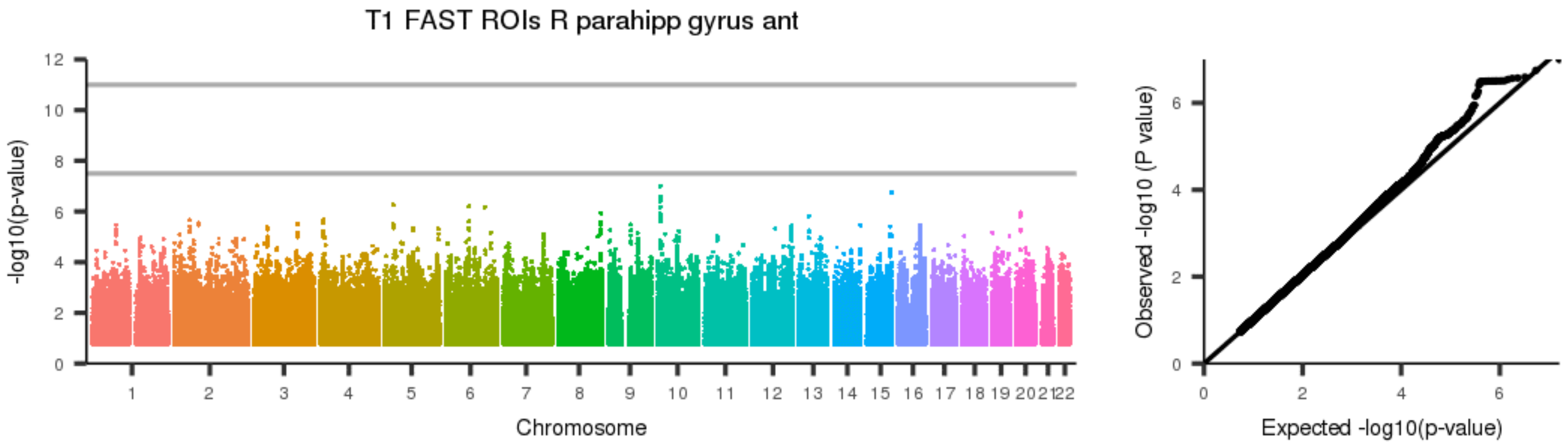

back to list of plots

Figure 94: T1 FAST ROIs L parahipp gyrus post

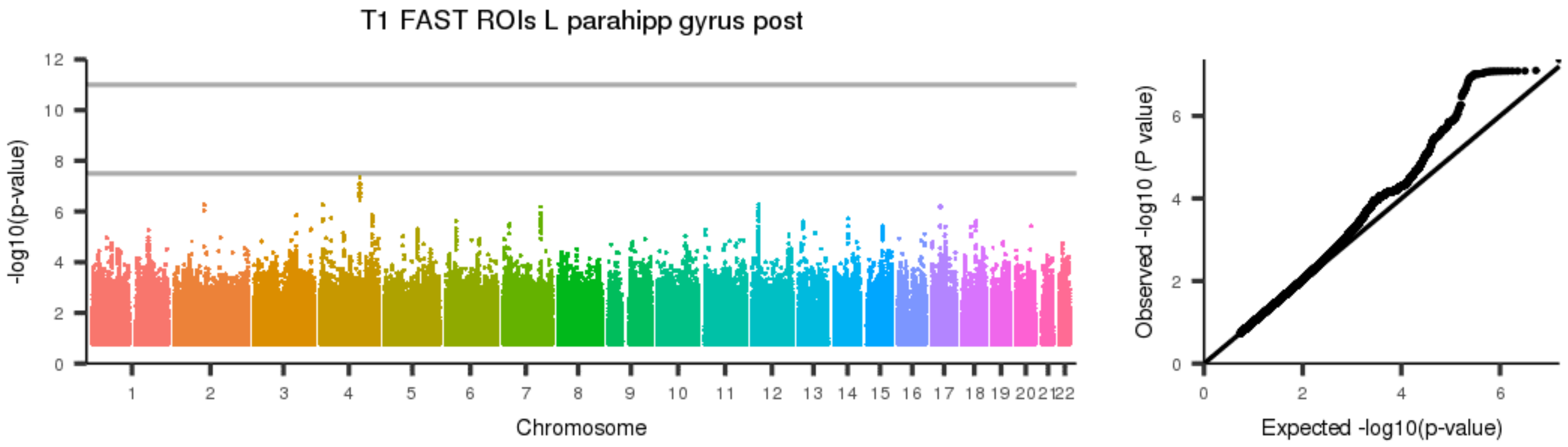

back to list of plots

Figure 95: T1 FAST ROIs R parahipp gyrus post

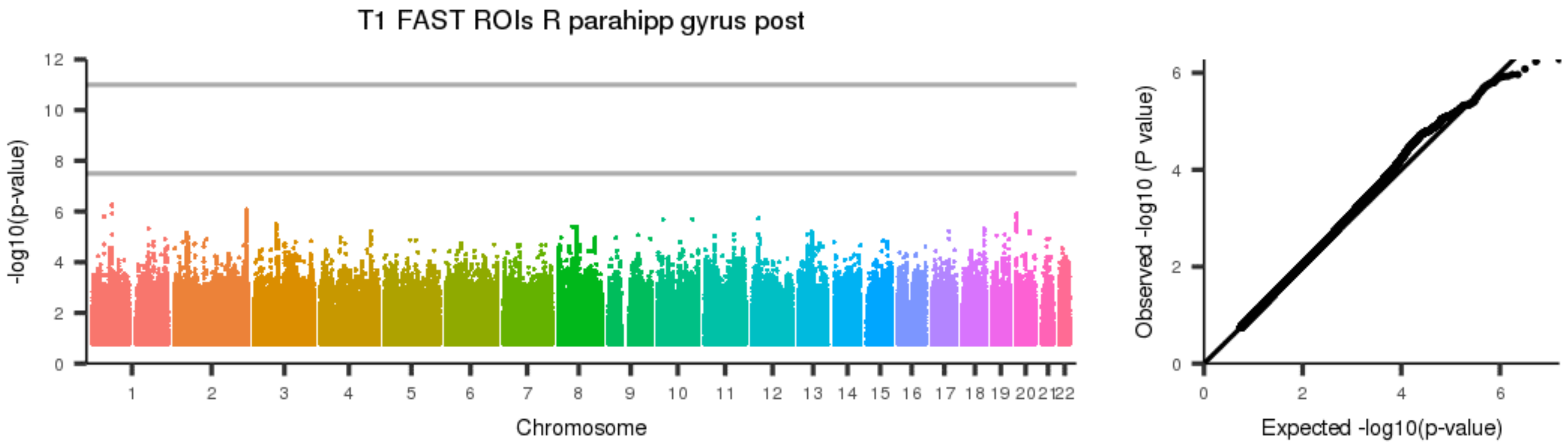

back to list of plots

Figure 96: T1 FAST ROIs L lingual gyrus

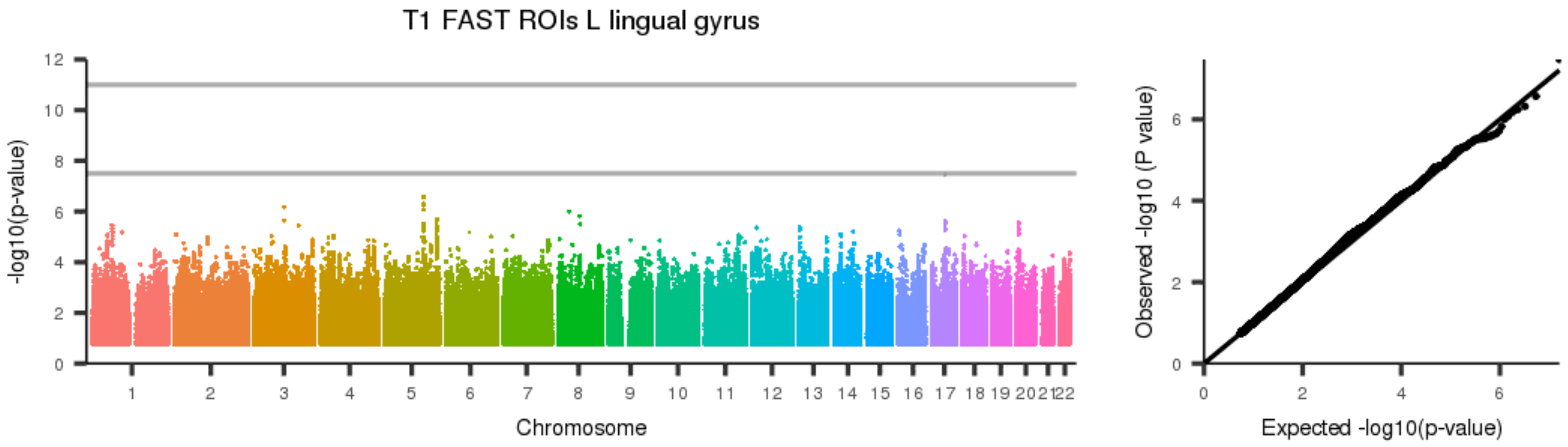

back to list of plots

Figure 97: T1 FAST ROIs R lingual gyrus

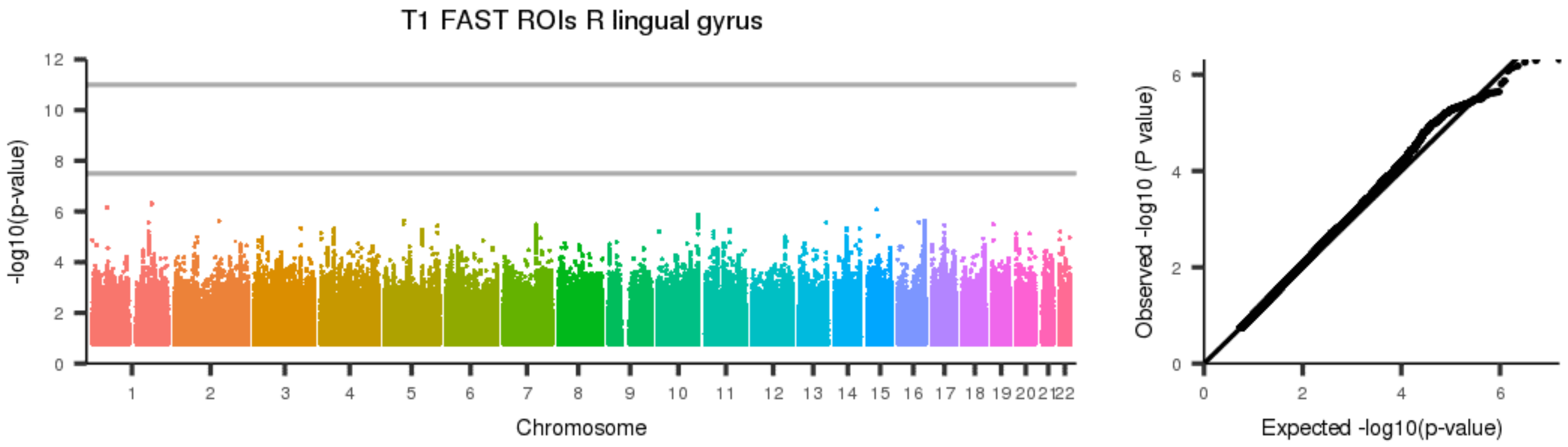

back to list of plots

Figure 98: T1 FAST ROIs L temp fusif cortex ant

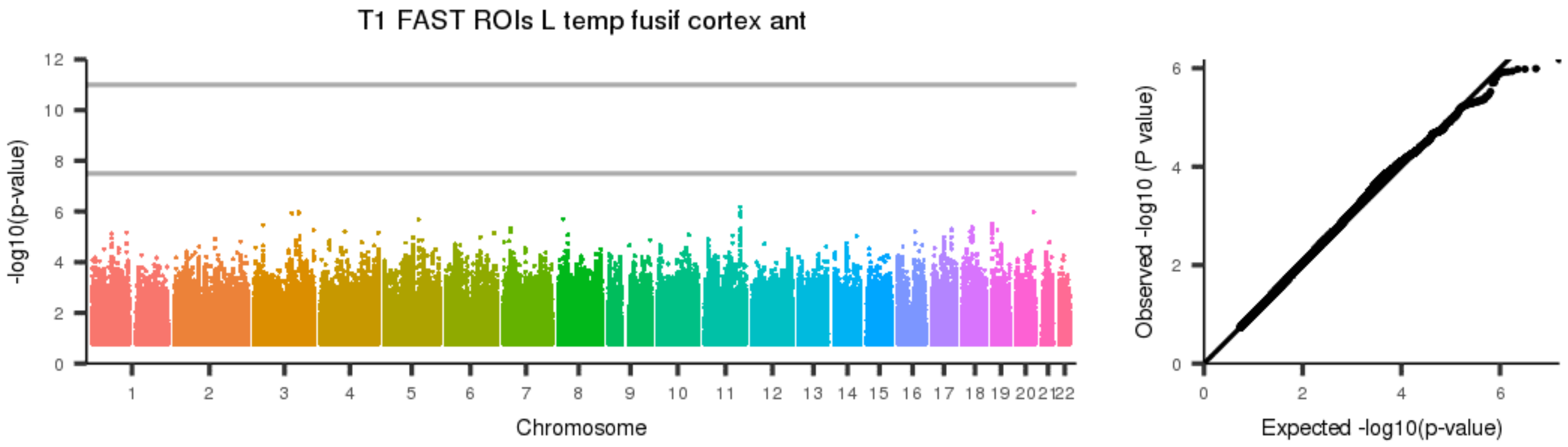

back to list of plots

Figure 99: T1 FAST ROIs R temp fusif cortex ant

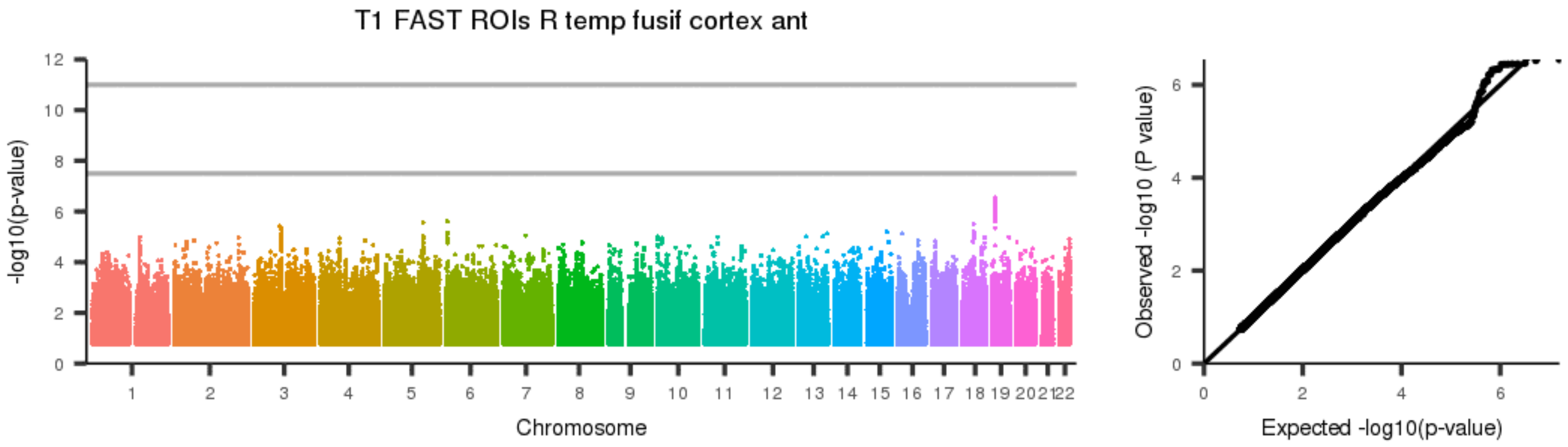

back to list of plots

Figure 100: T1 FAST ROIs L temp fusif cortex post

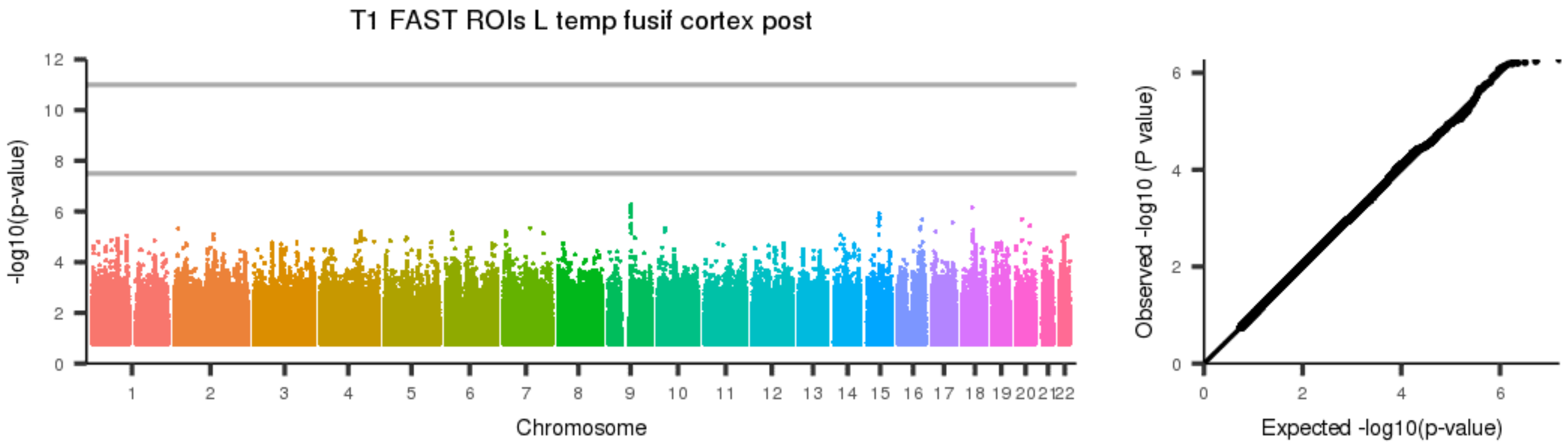

back to list of plots

Figure 101: T1 FAST ROIs R temp fusif cortex post

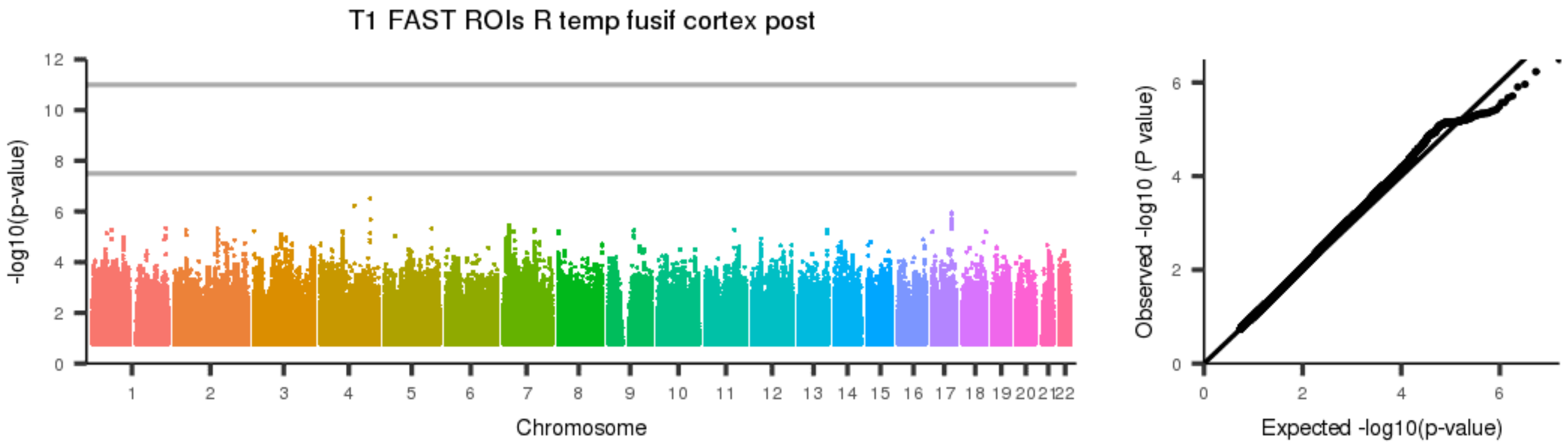

back to list of plots

Figure 102: T1 FAST ROIs L temp occ fusif cortex

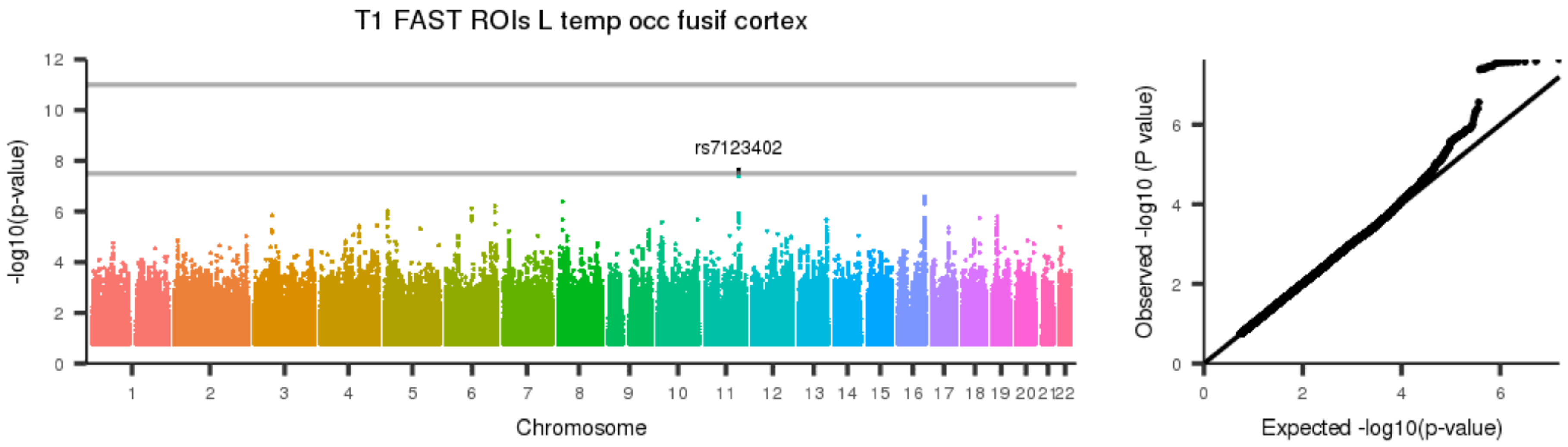

back to list of plots

Figure 103: T1 FAST ROIs R temp occ fusif cortex

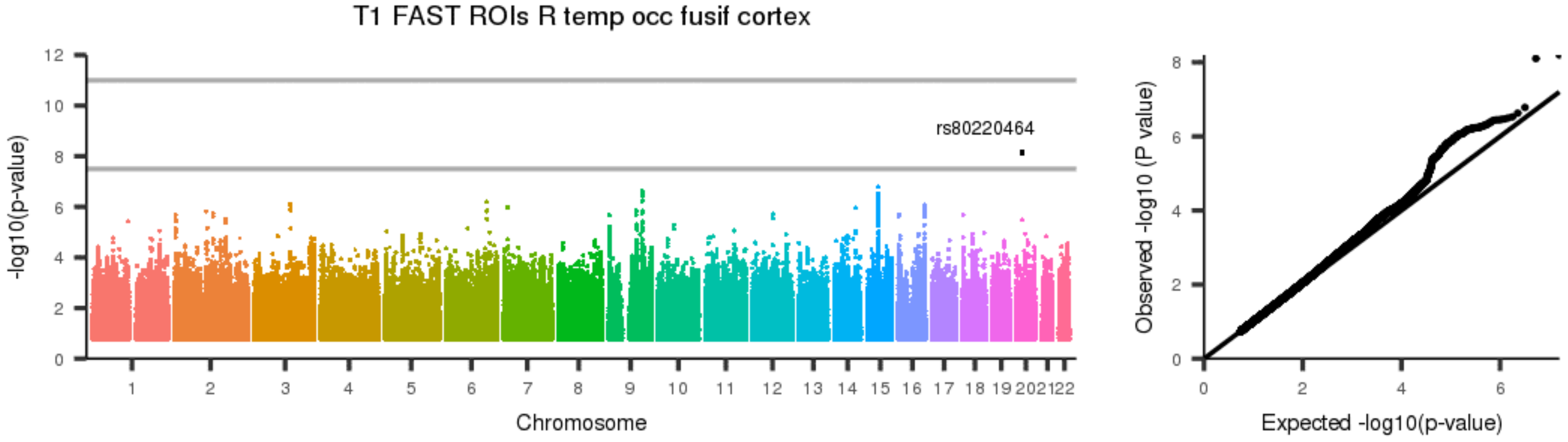

back to list of plots

Figure 104: T1 FAST ROIs L occ fusif gyrus

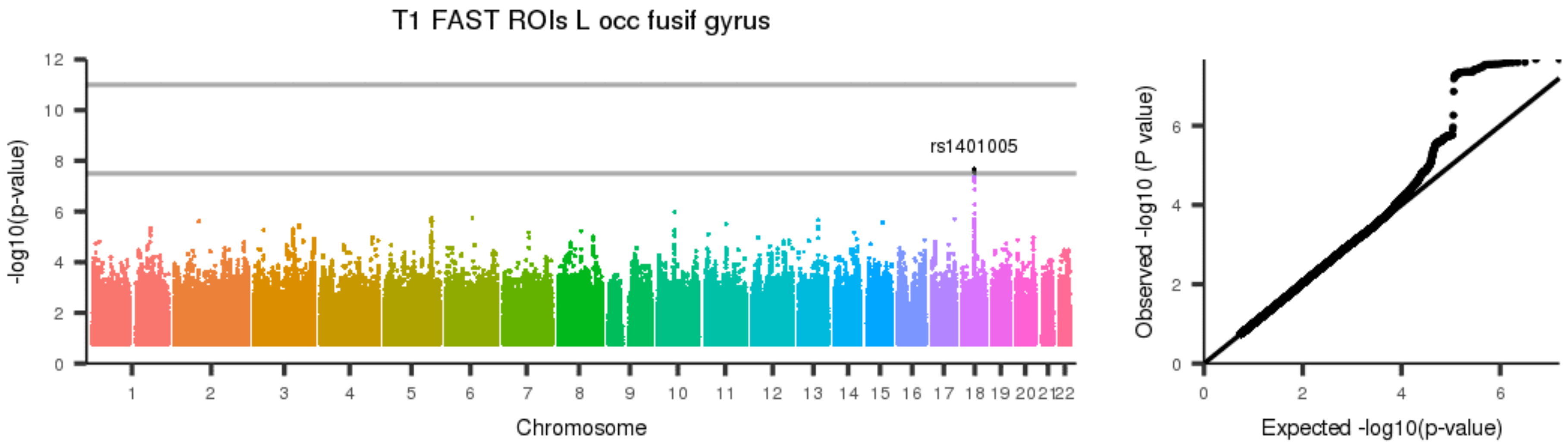

back to list of plots

Figure 105: T1 FAST ROIs R occ fusif gyrus

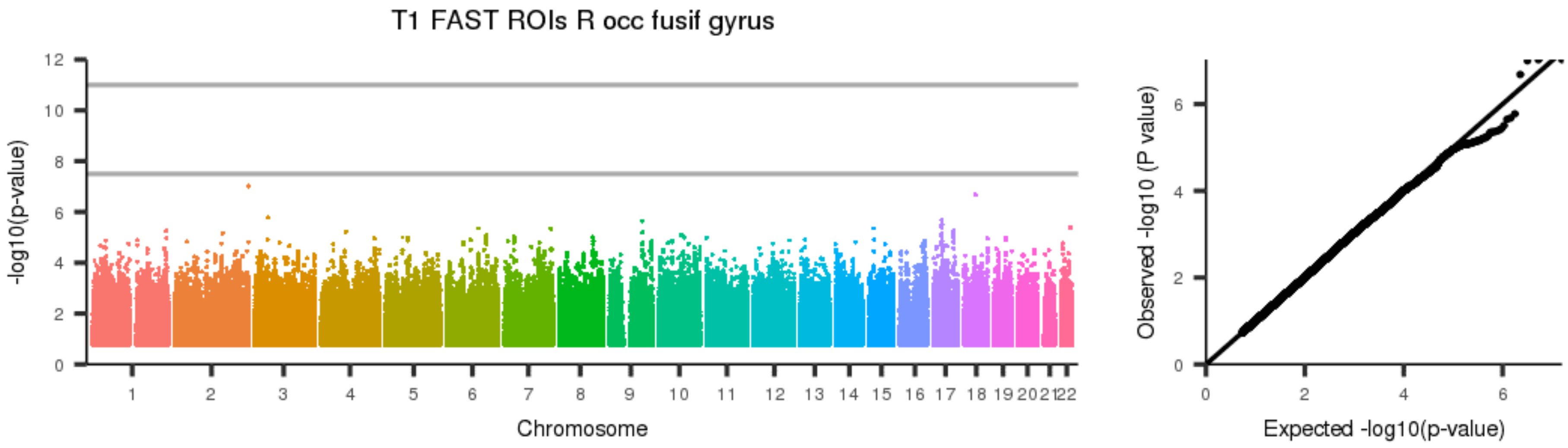

back to list of plots

Figure 106: T1 FAST ROIs L front operc cortex

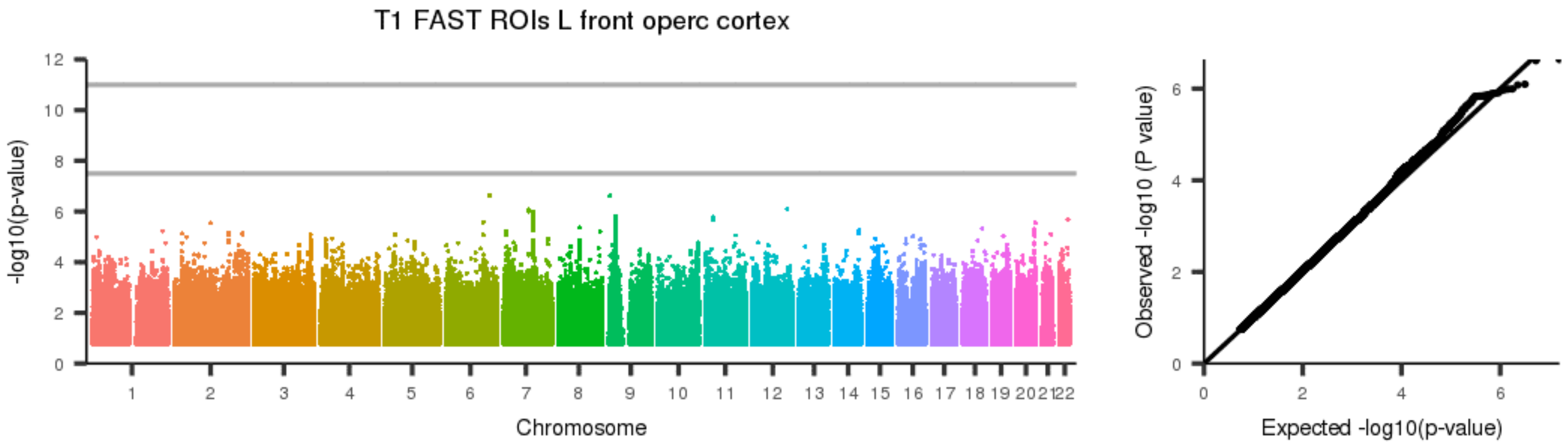

back to list of plots

Figure 107: T1 FAST ROIs R front operc cortex

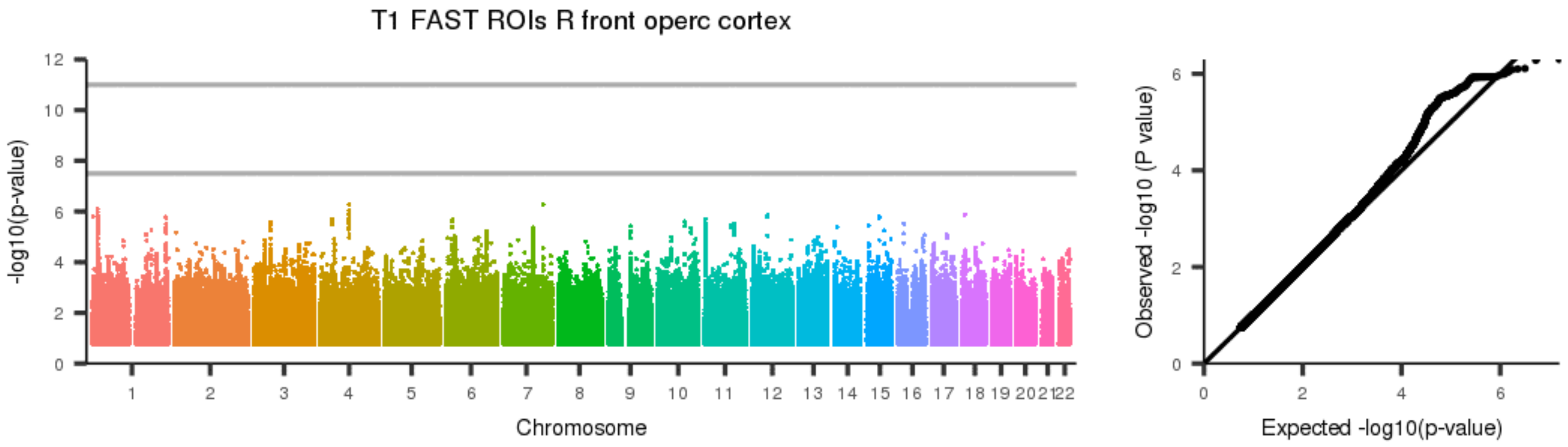

back to list of plots

Figure 108: T1 FAST ROIs L cent operc cortex

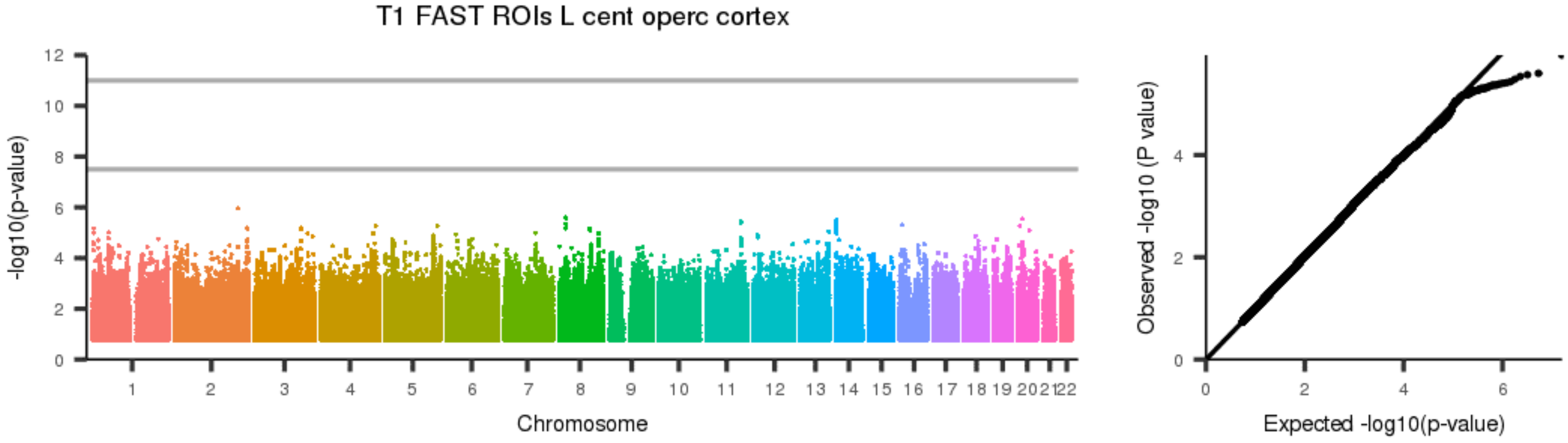

back to list of plots

Figure 109: T1 FAST ROIs R cent operc cortex

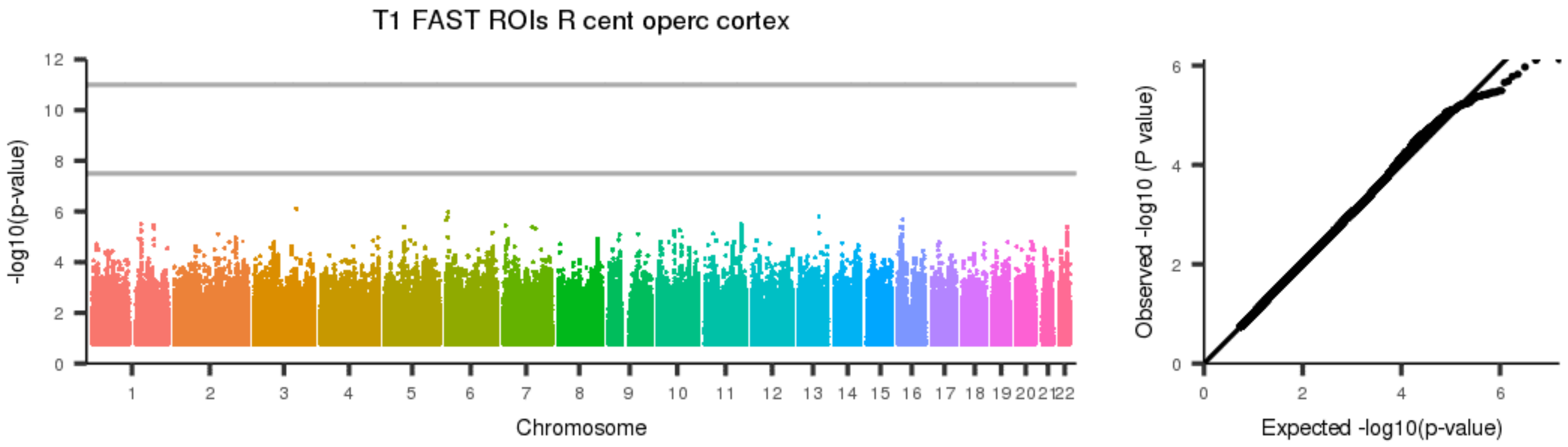

back to list of plots

Figure 110: T1 FAST ROIs L parietal operc cortex

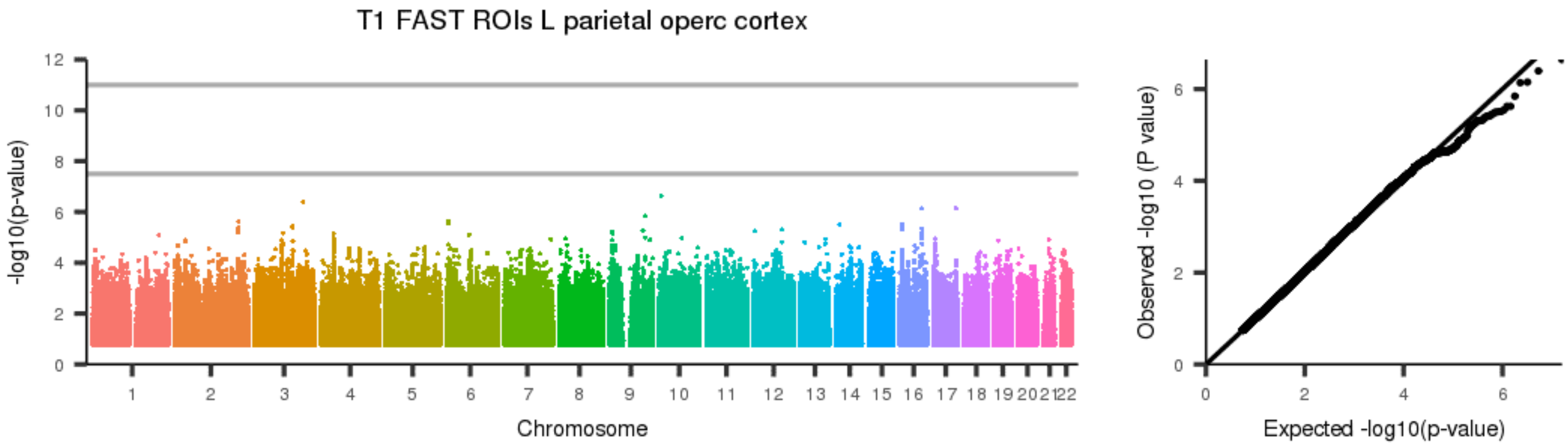

back to list of plots

Figure 111: T1 FAST ROIs R parietal operc cortex

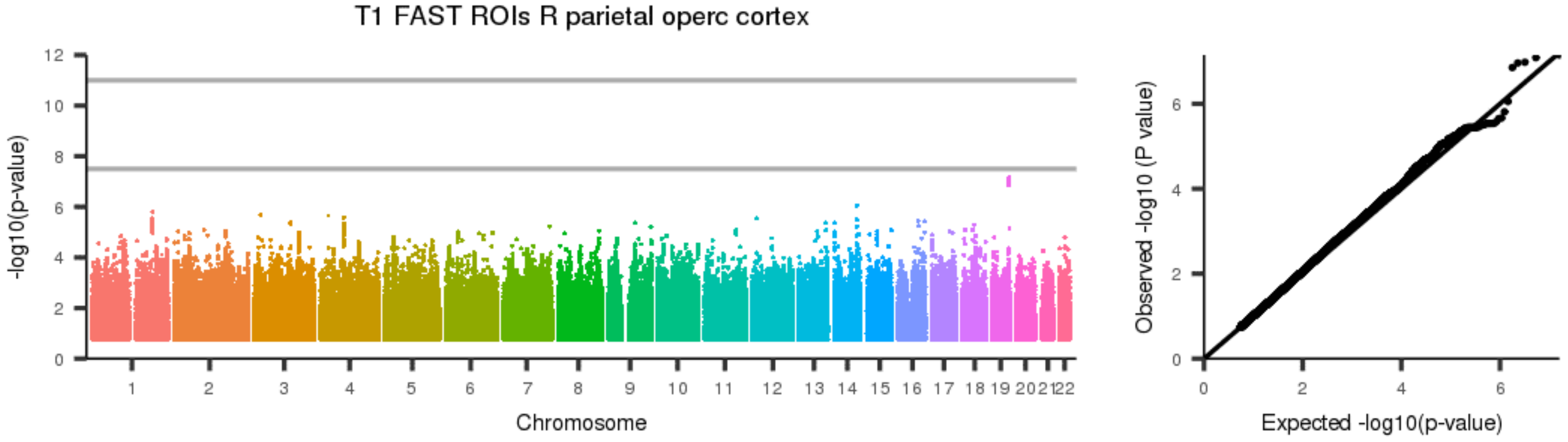

back to list of plots

Figure 112: T1 FAST ROIs L planum polare

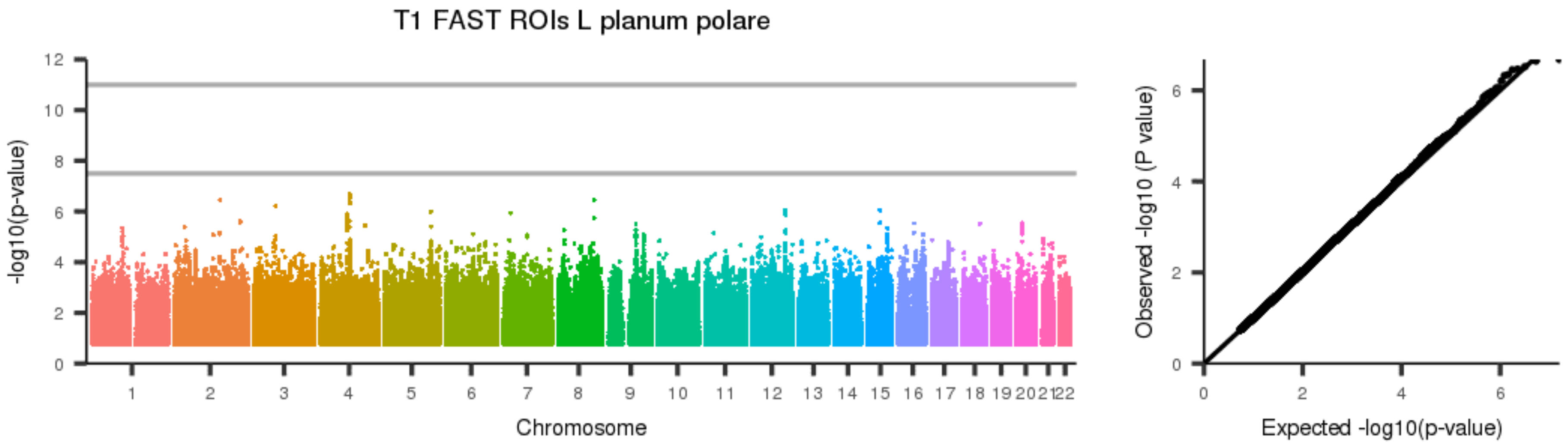

back to list of plots

Figure 113: T1 FAST ROIs R planum polare

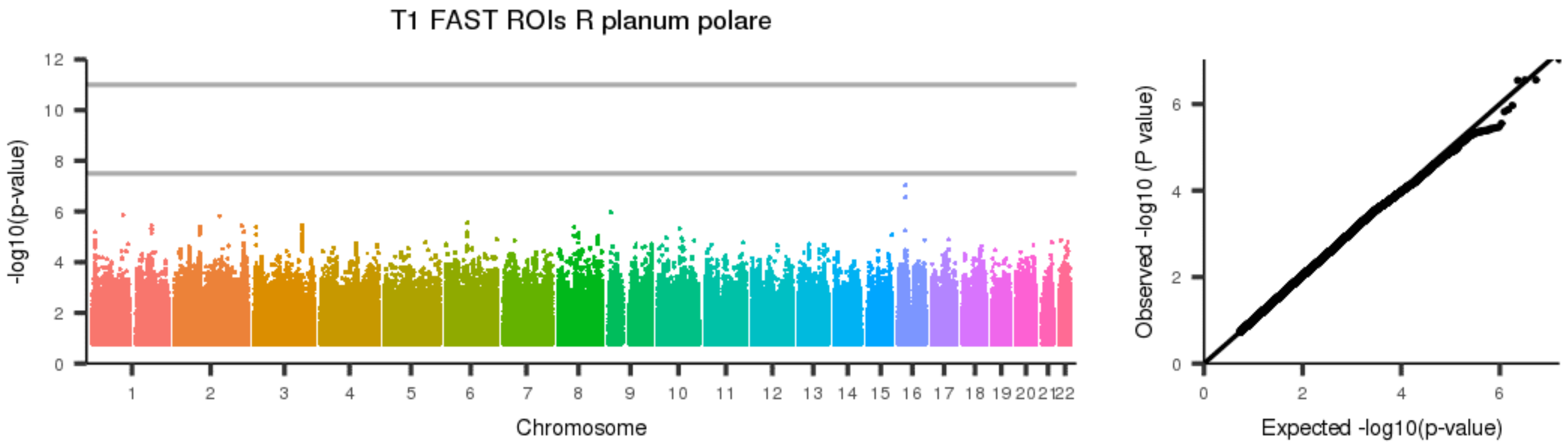

back to list of plots

Figure 114: T1 FAST ROIs L heschl gyrus

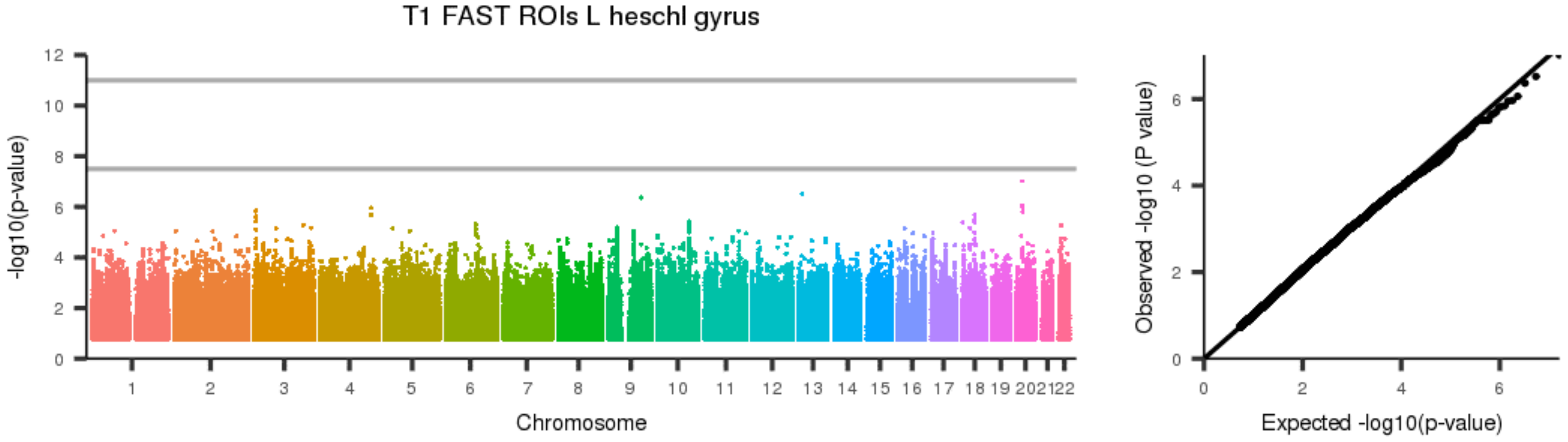

back to list of plots

Figure 115: T1 FAST ROIs R heschl gyrus

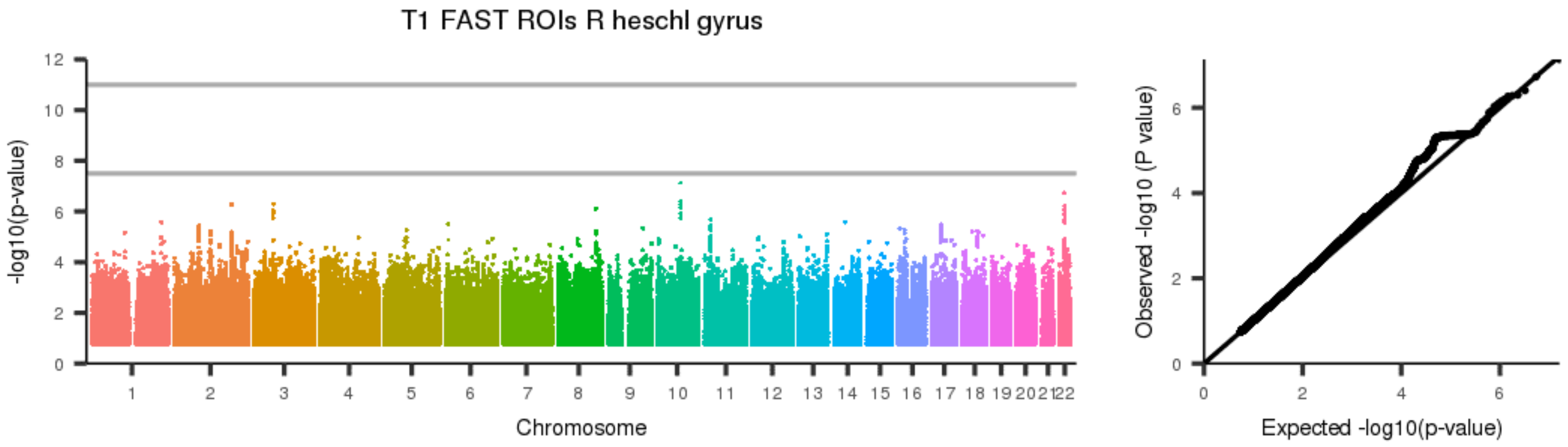

back to list of plots

Figure 116: T1 FAST ROIs L planum temporale

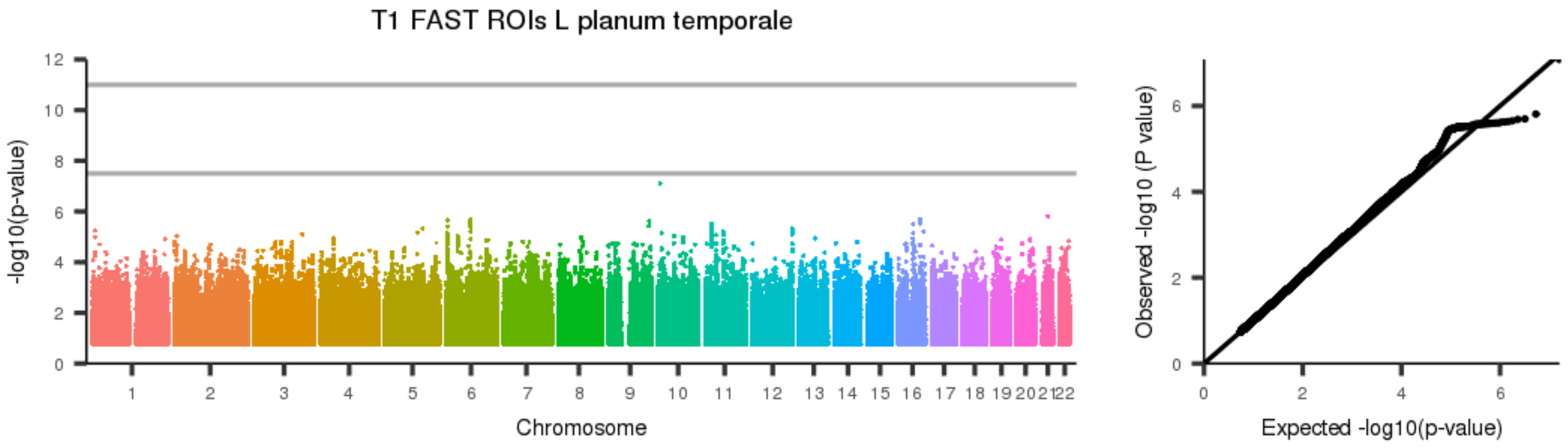

back to list of plots

Figure 117: T1 FAST ROIs R planum temporale

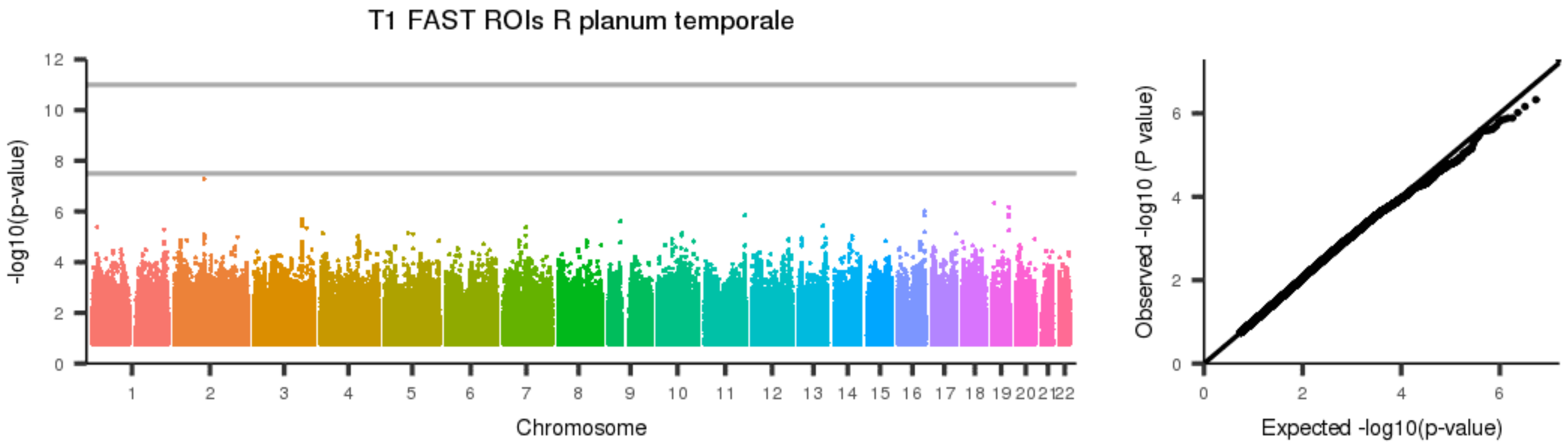

back to list of plots

Figure 118: T1 FAST ROIs L supracalc cortex

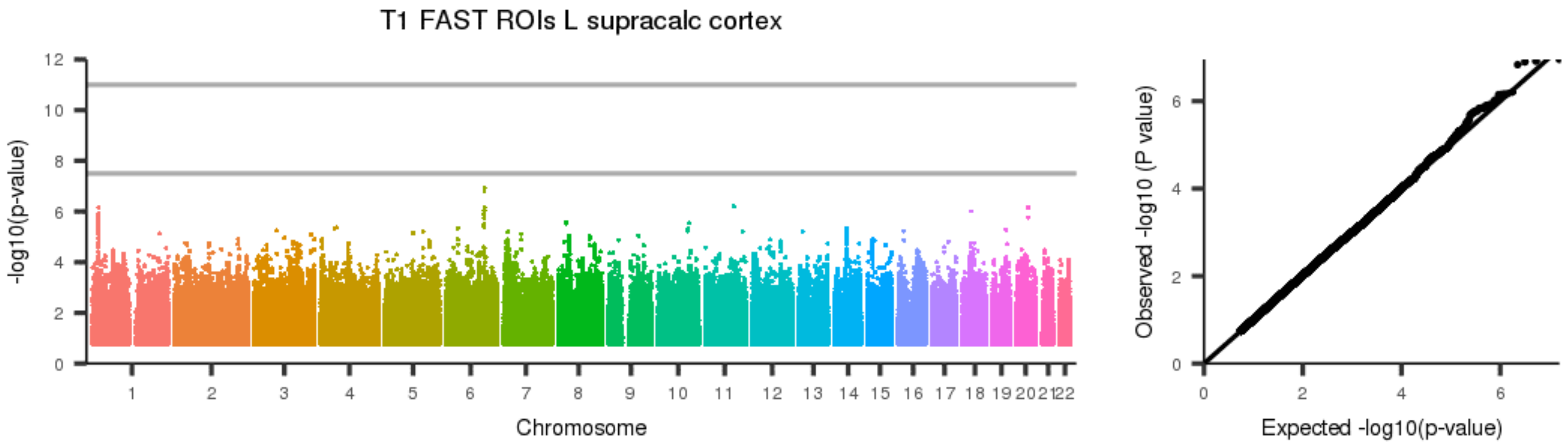

back to list of plots

Figure 119: T1 FAST ROIs R supracalc cortex

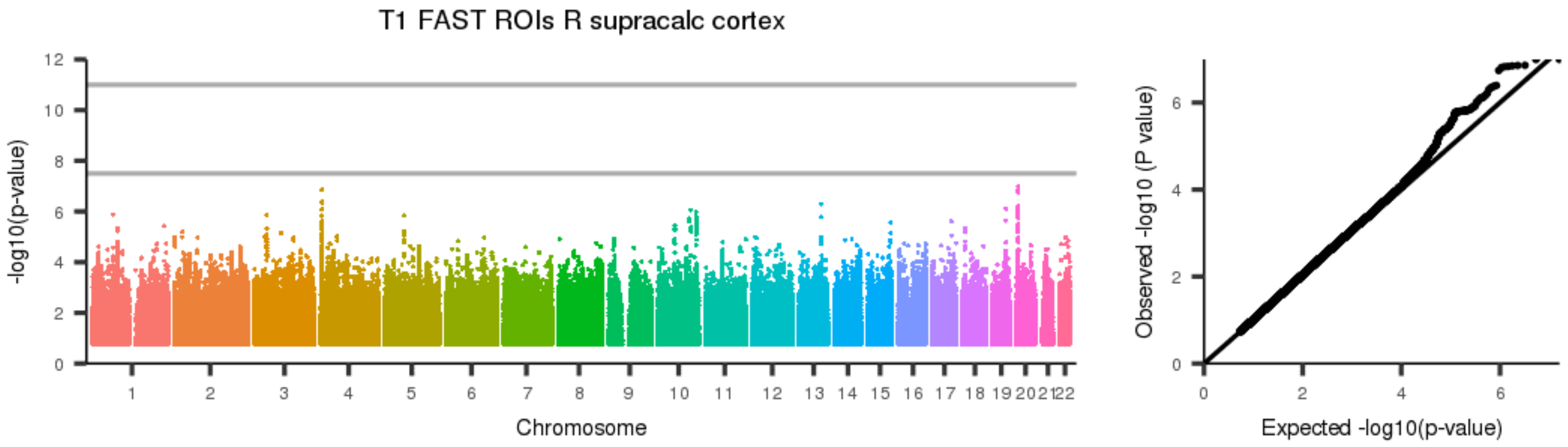

back to list of plots

Figure 120: T1 FAST ROIs L occ pole

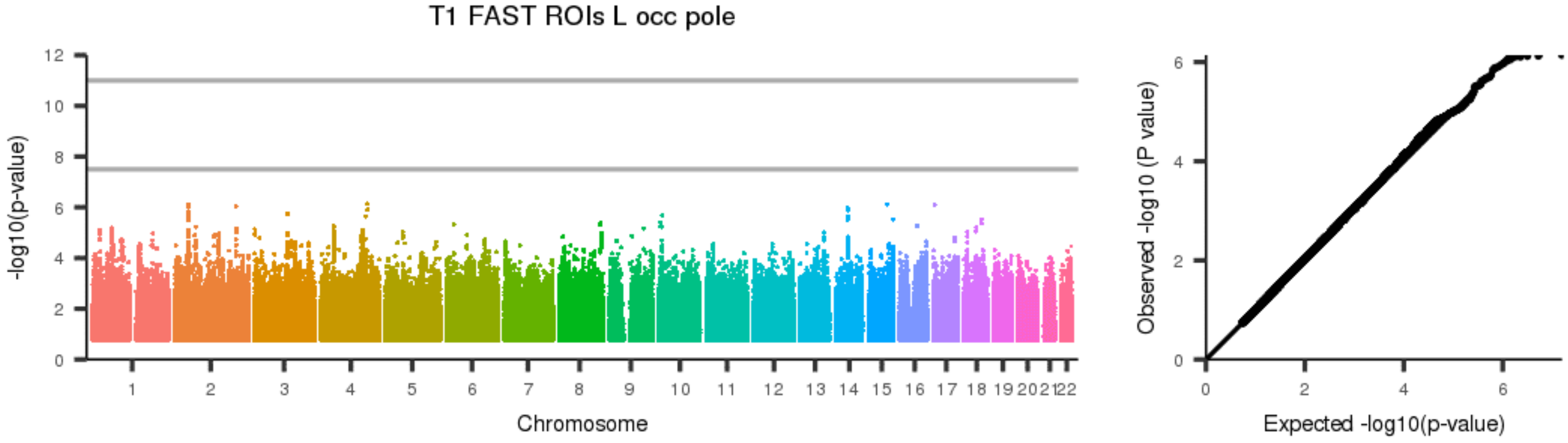

back to list of plots

Figure 121: T1 FAST ROIs R occ pole

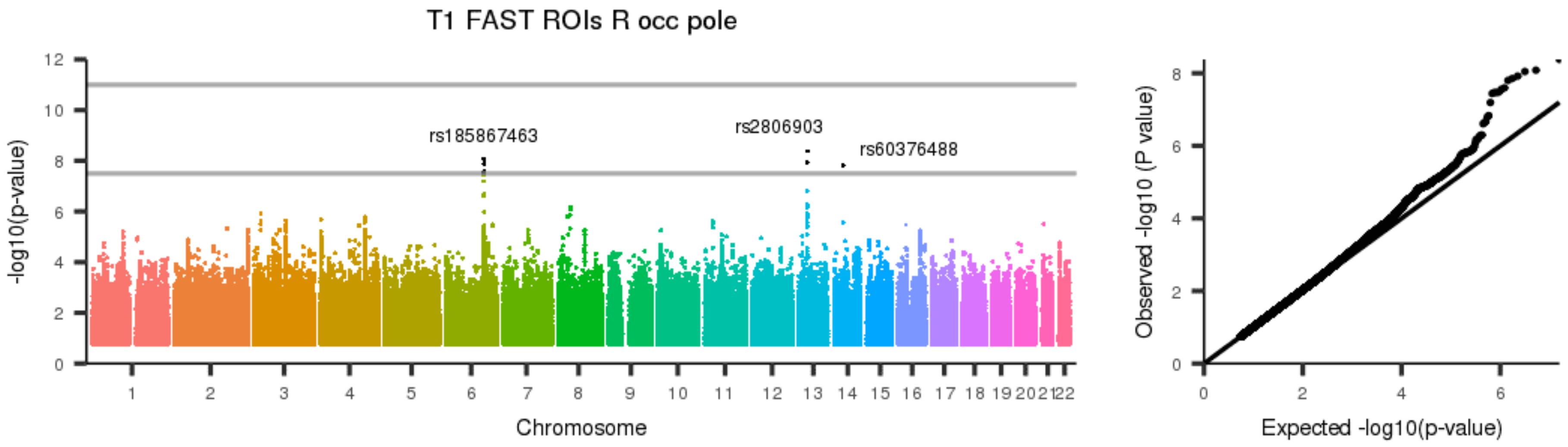

back to list of plots

Figure 122: T1 FAST ROIs L thalamus

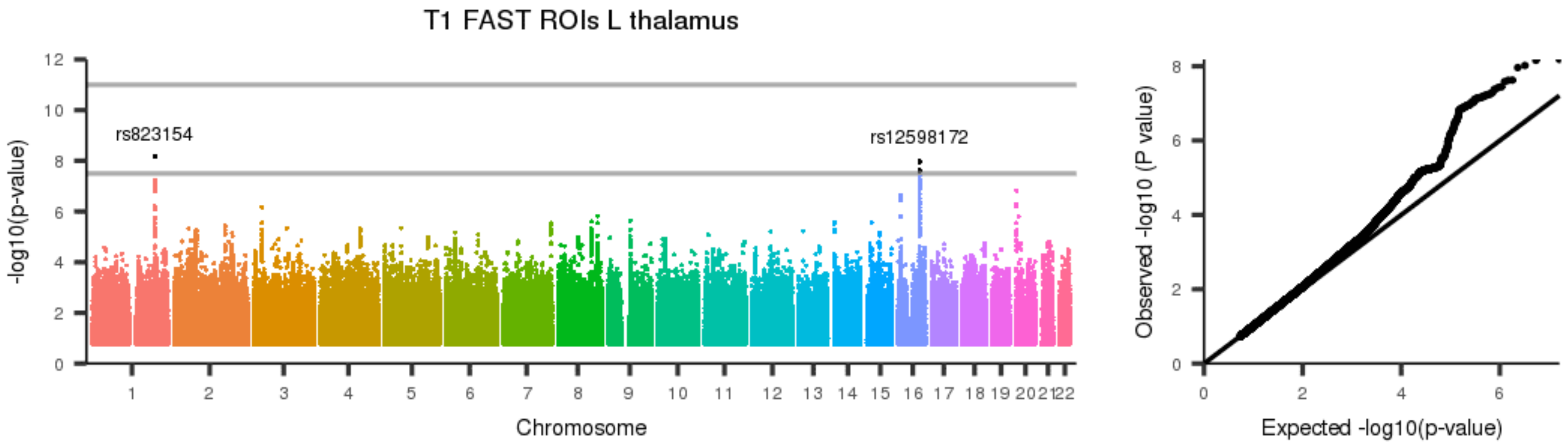

back to list of plots

Figure 123: T1 FAST ROIs R thalamus

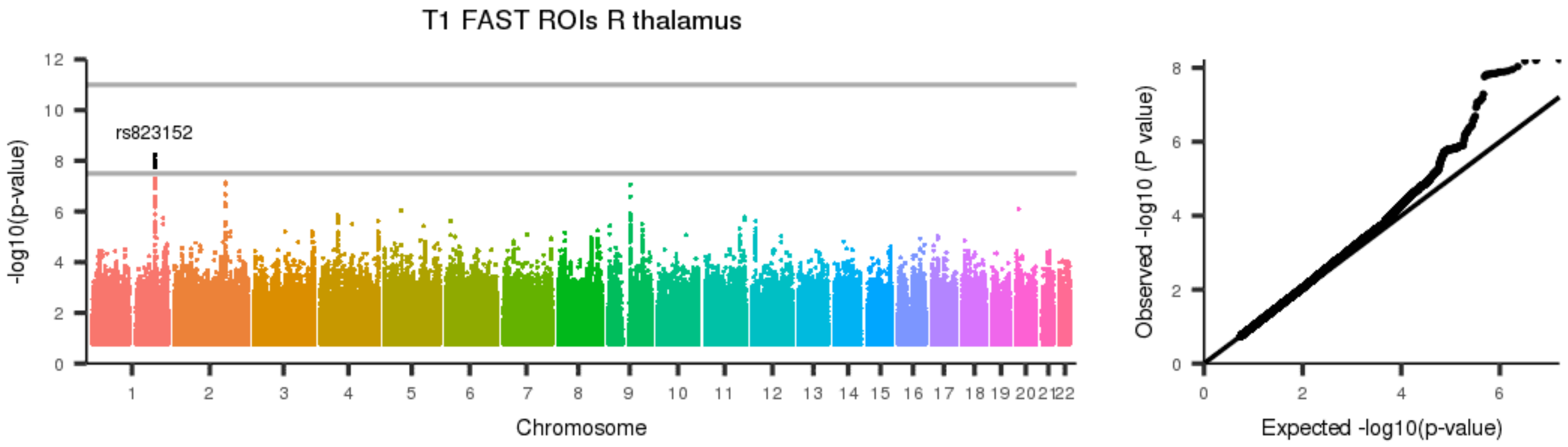

back to list of plots

Figure 124: T1 FAST ROIs L caudate

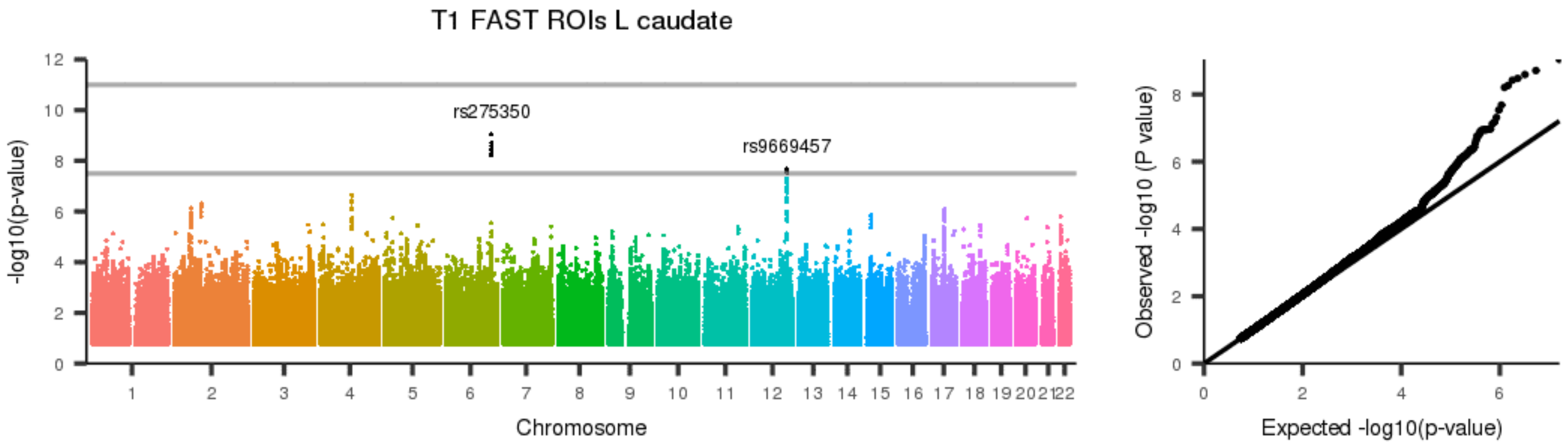

back to list of plots

Figure 125: T1 FAST ROIs R caudate

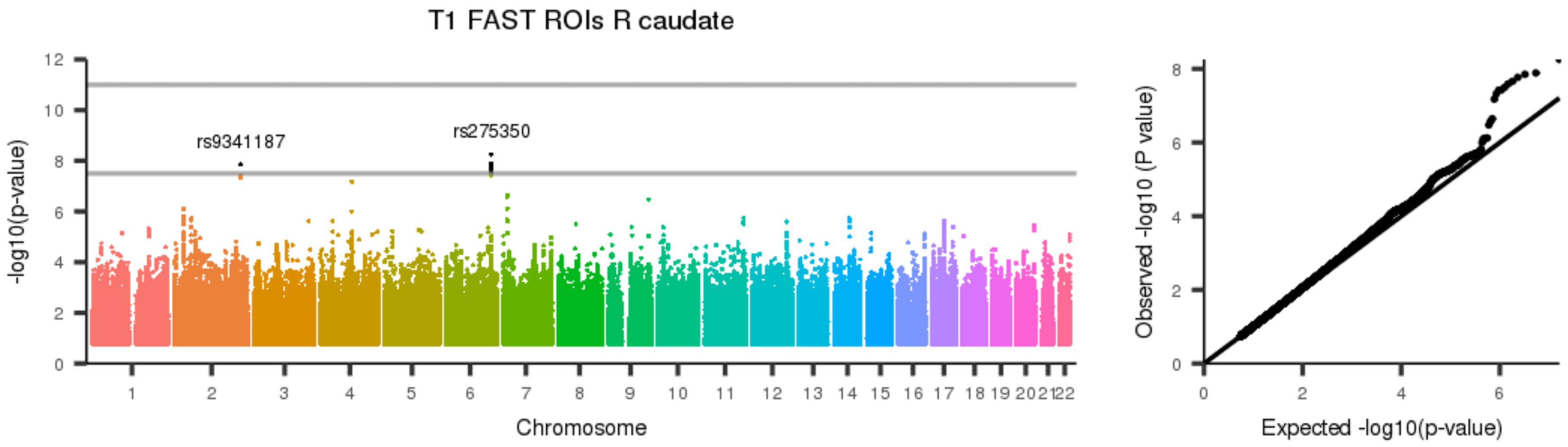

back to list of plots

Figure 126: T1 FAST ROIs L putamen

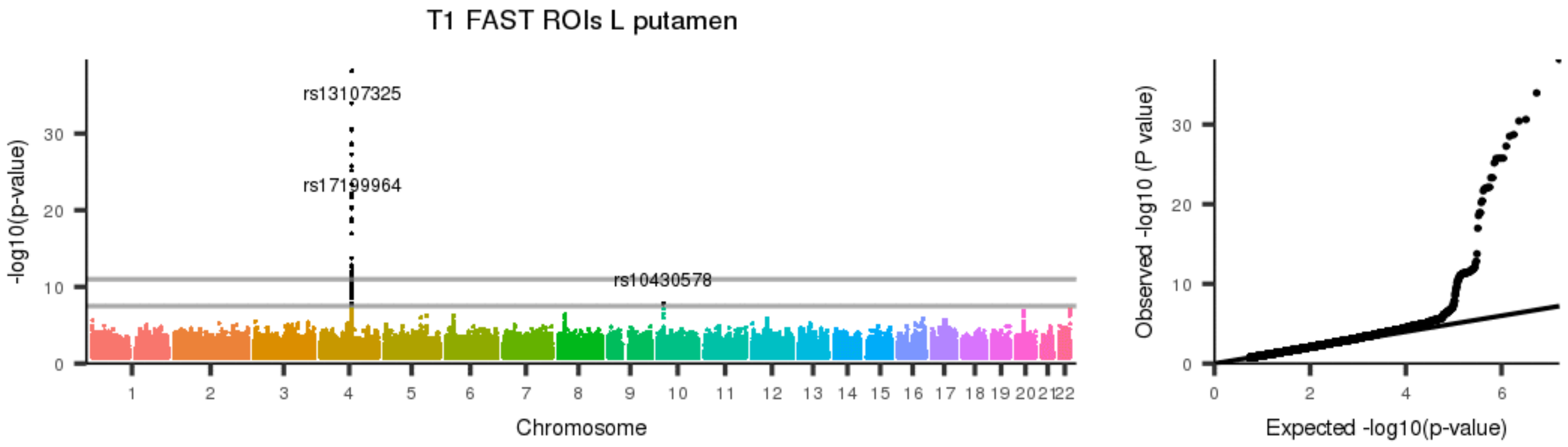

back to list of plots

Figure 127: T1 FAST ROIs R putamen

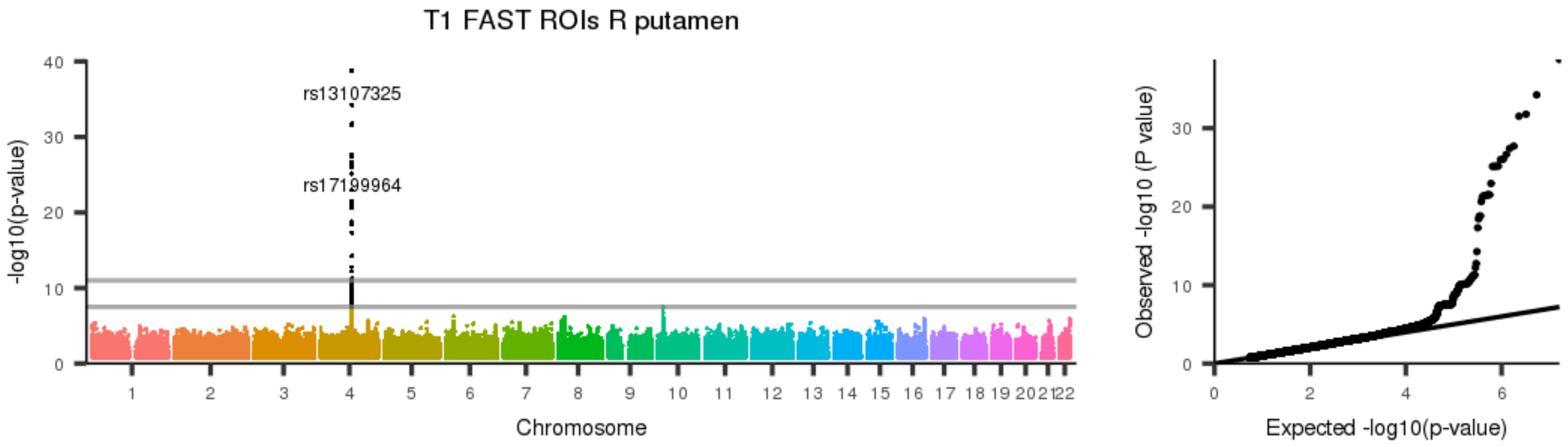

back to list of plots

Figure 128: T1 FAST ROIs L pallidum

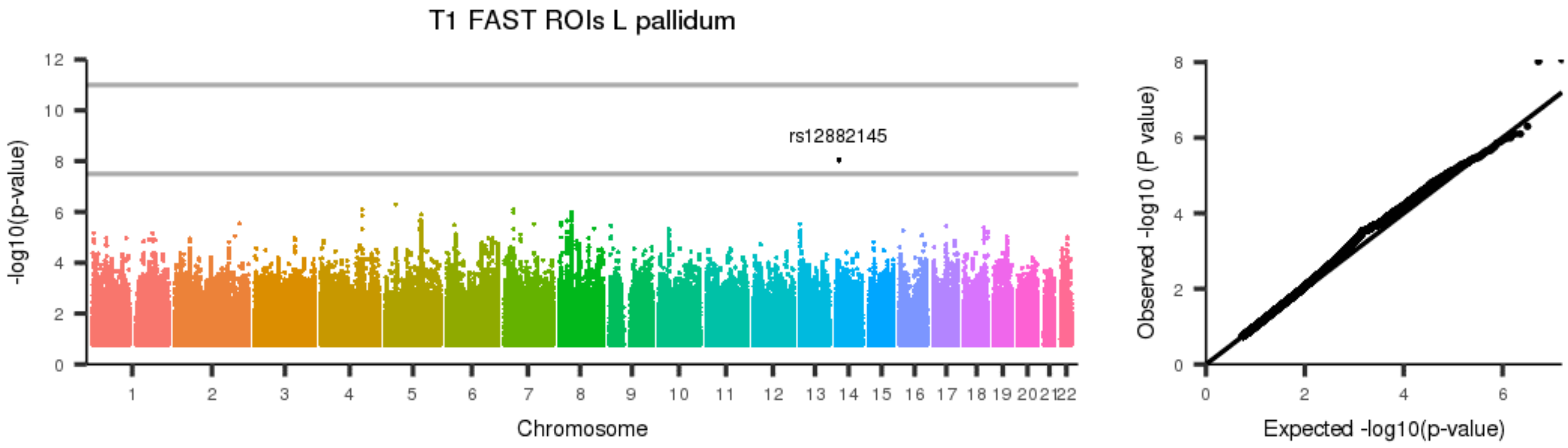

back to list of plots

Figure 129: T1 FAST ROIs R pallidum

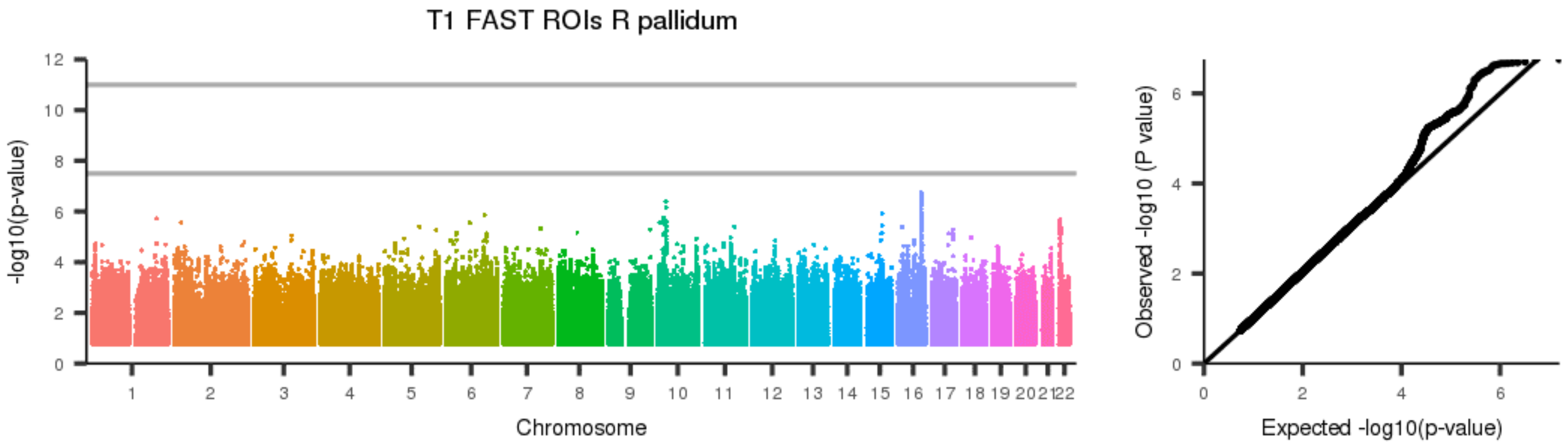

back to list of plots

Figure 130: T1 FAST ROIs L hippocampus

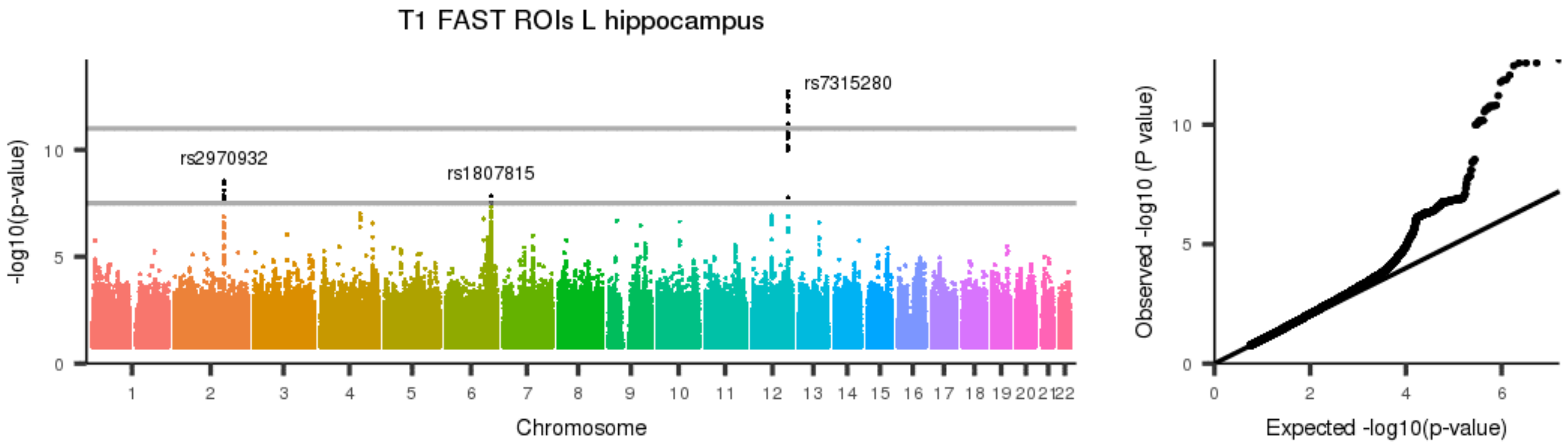

back to list of plots

Figure 131: T1 FAST ROIs R hippocampus

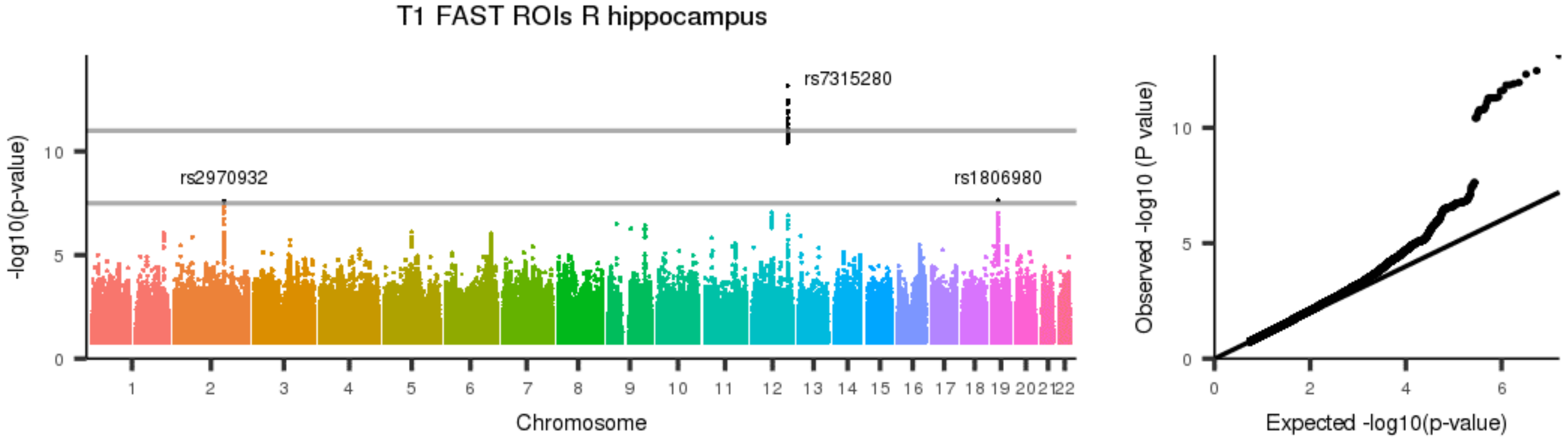

back to list of plots

Figure 132: T1 FAST ROIs L amygdala

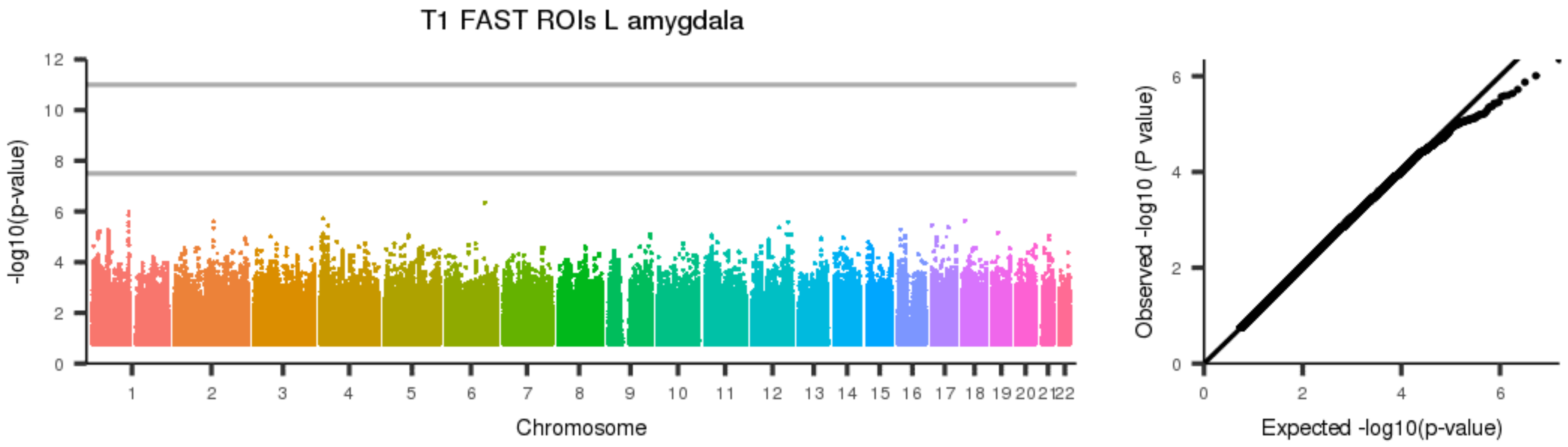

back to list of plots

Figure 133: T1 FAST ROIs R amygdala

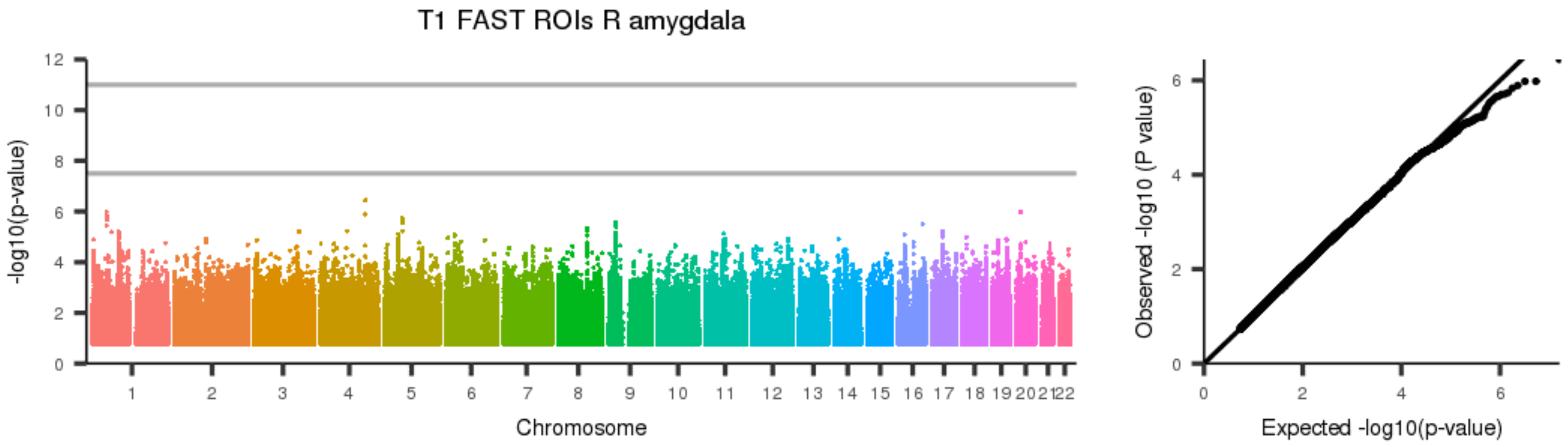

back to list of plots

Figure 134: T1 FAST ROIs L ventral striatum

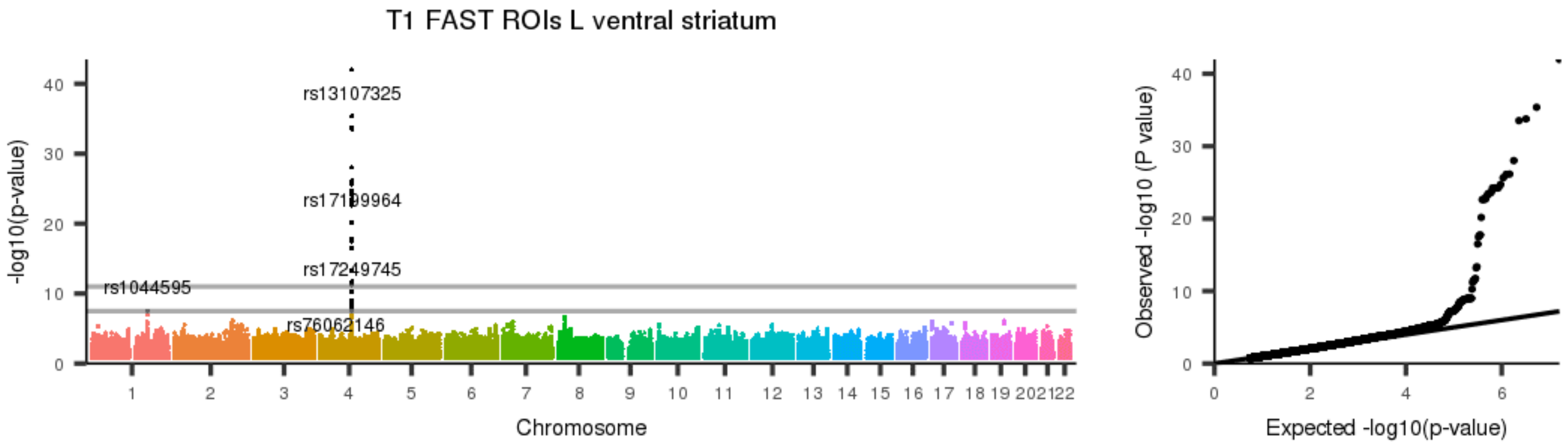

back to list of plots

Figure 135: T1 FAST ROIs R ventral striatum

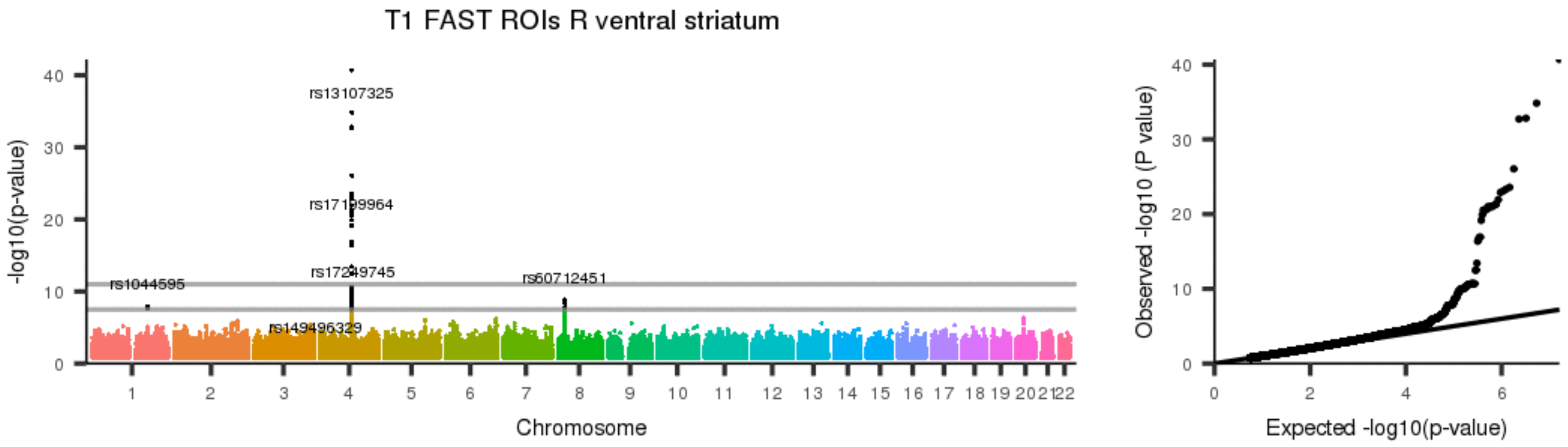

back to list of plots

Figure 136: T1 FAST ROIs brain stem

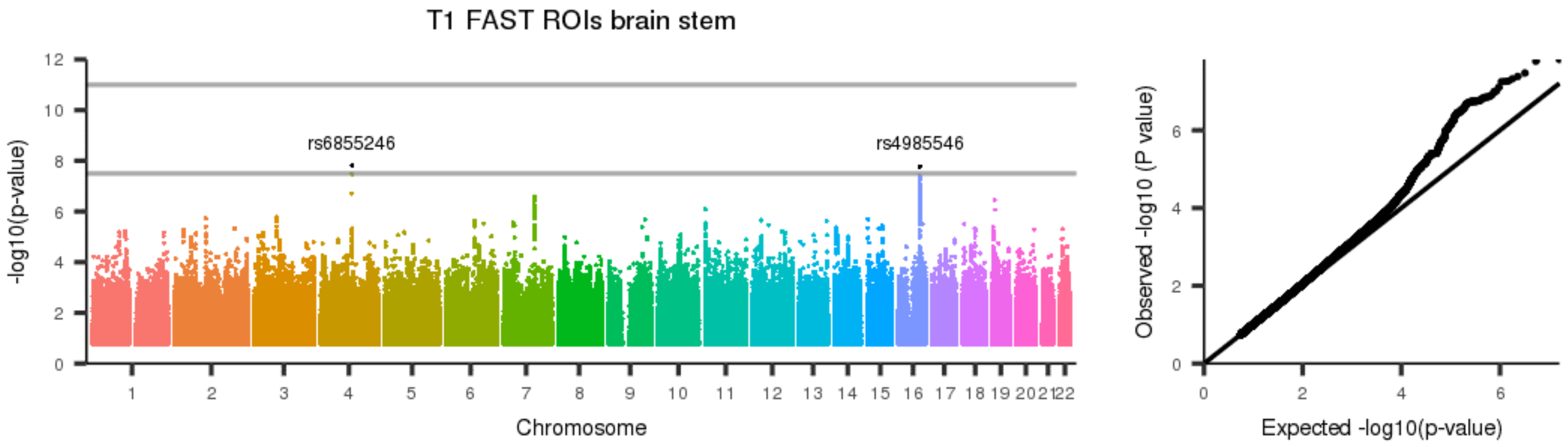

back to list of plots

Figure 137: T1 FAST ROIs L cerebellum I-IV

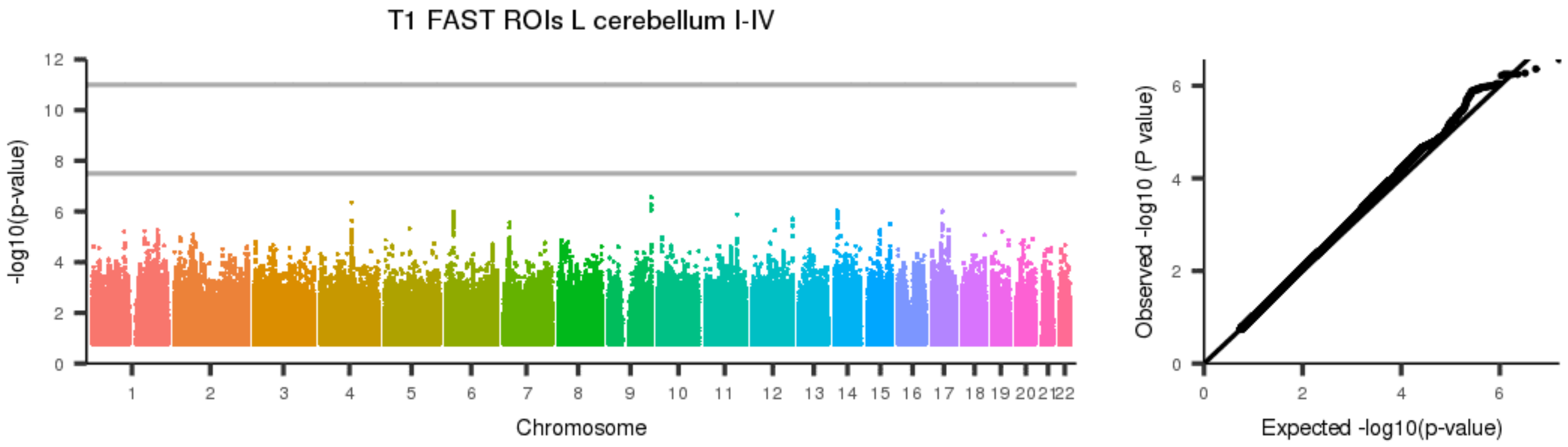

back to list of plots

Figure 138: T1 FAST ROIs R cerebellum I-IV

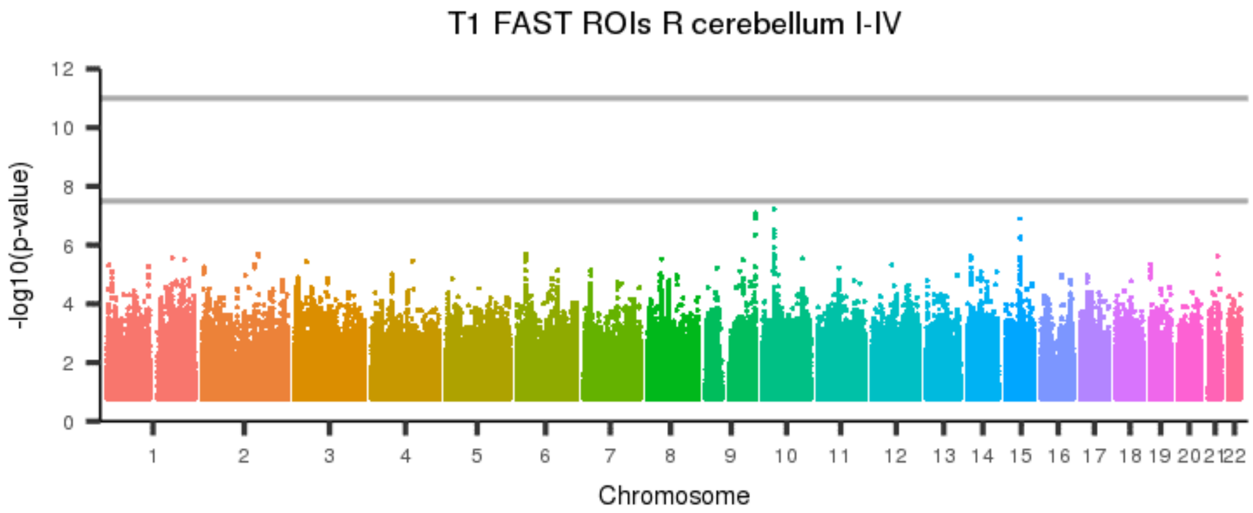

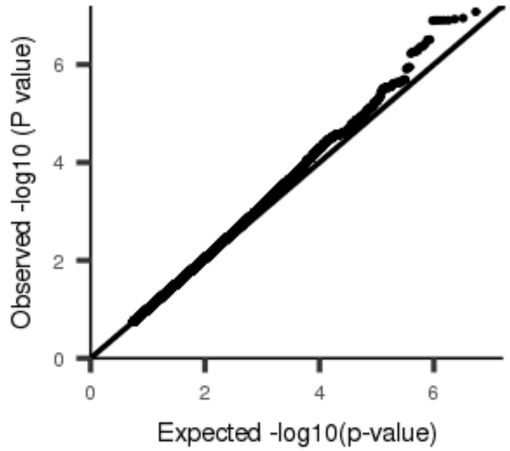

back to list of plots

Figure 139: T1 FAST ROIs L cerebellum V

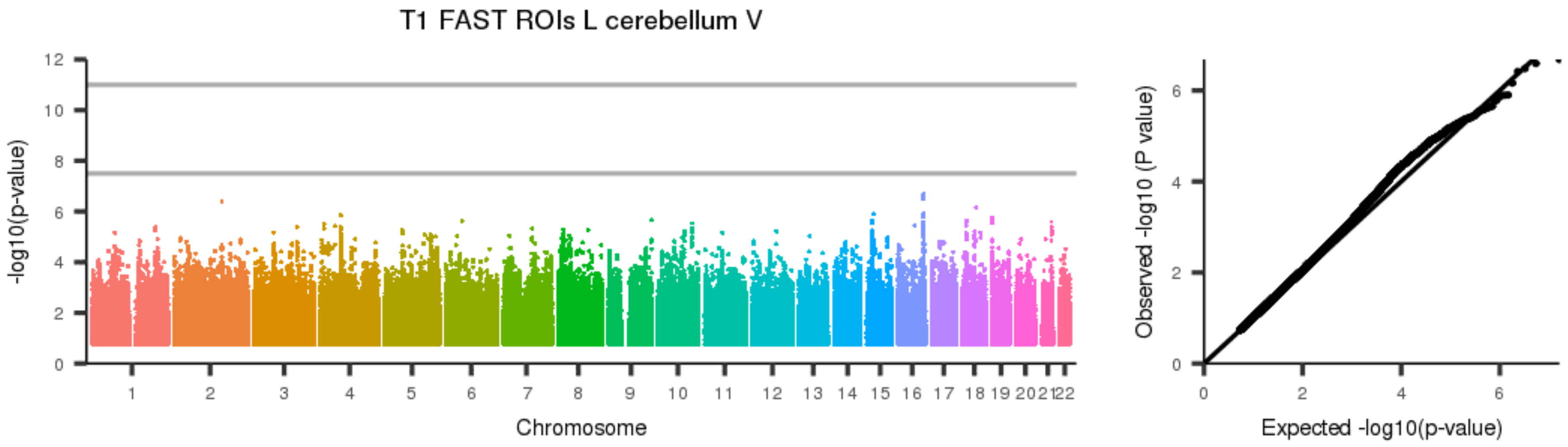

back to list of plots

Figure 140: T1 FAST ROIs R cerebellum V

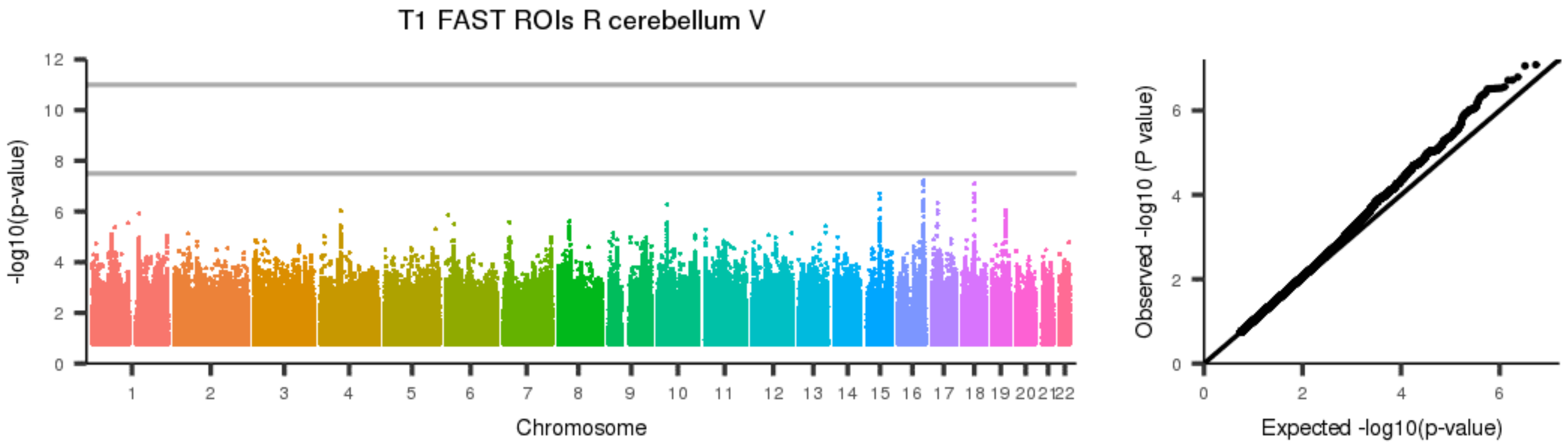

back to list of plots

Figure 141: T1 FAST ROIs L cerebellum VI

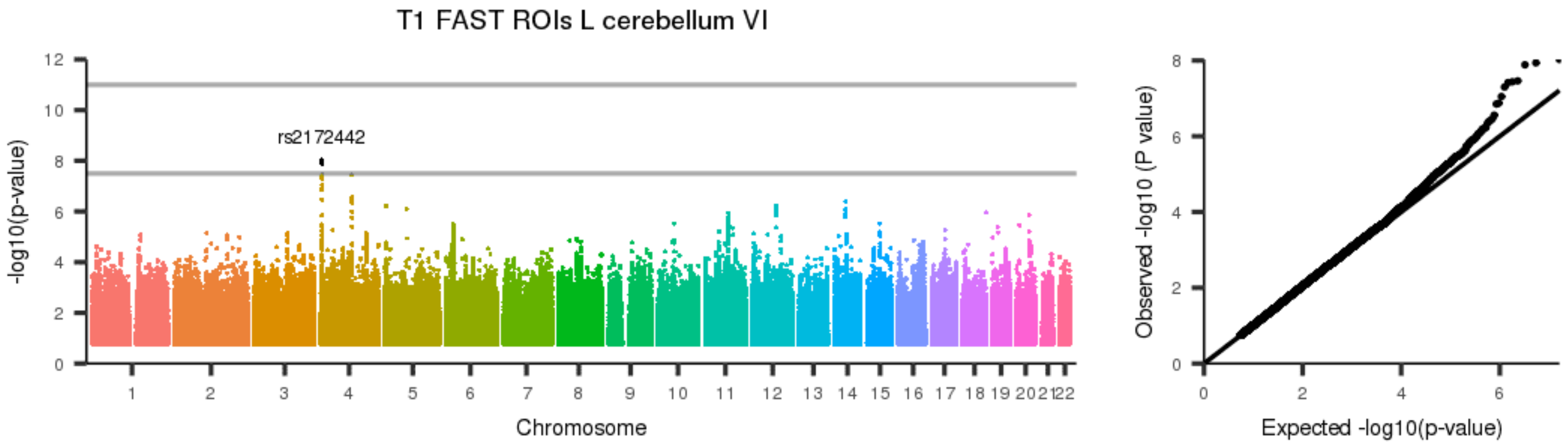

back to list of plots

Figure 142: T1 FAST ROIs V cerebellum VI

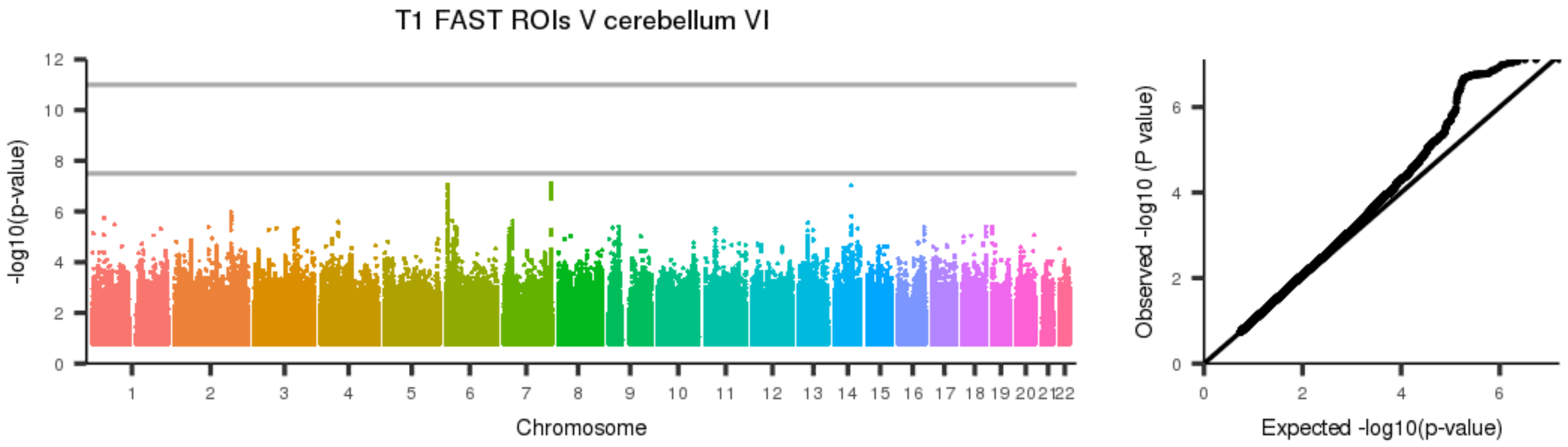

back to list of plots

Figure 143: T1 FAST ROIs R cerebellum VI

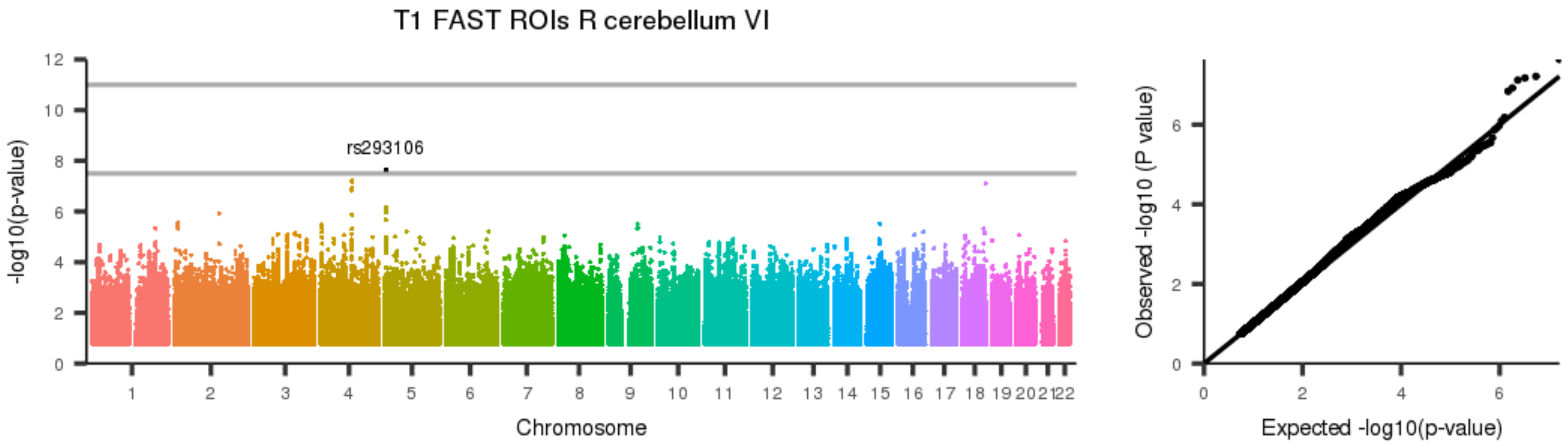

back to list of plots

Figure 144: T1 FAST ROIs L cerebellum crus I

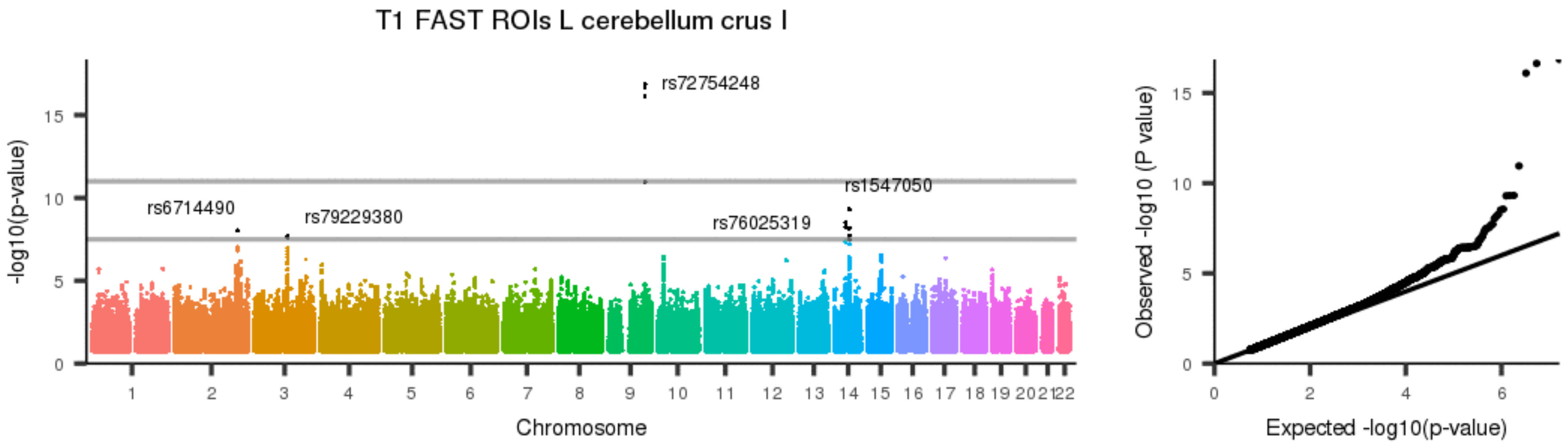

back to list of plots

Figure 145: T1 FAST ROIs V cerebellum crus I

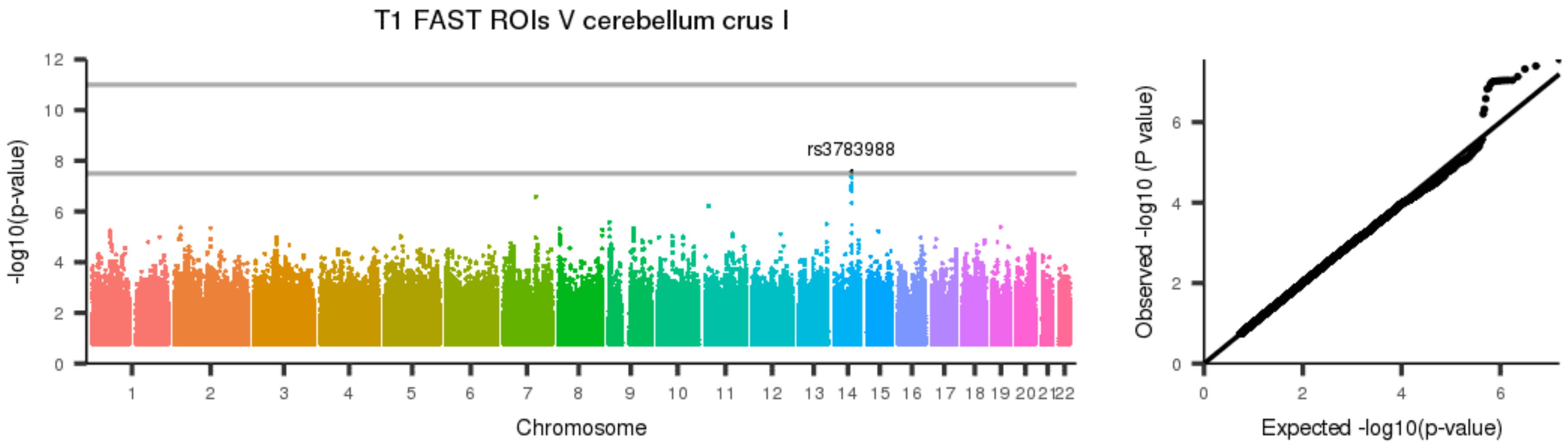

back to list of plots

Figure 146: T1 FAST ROIs R cerebellum crus I

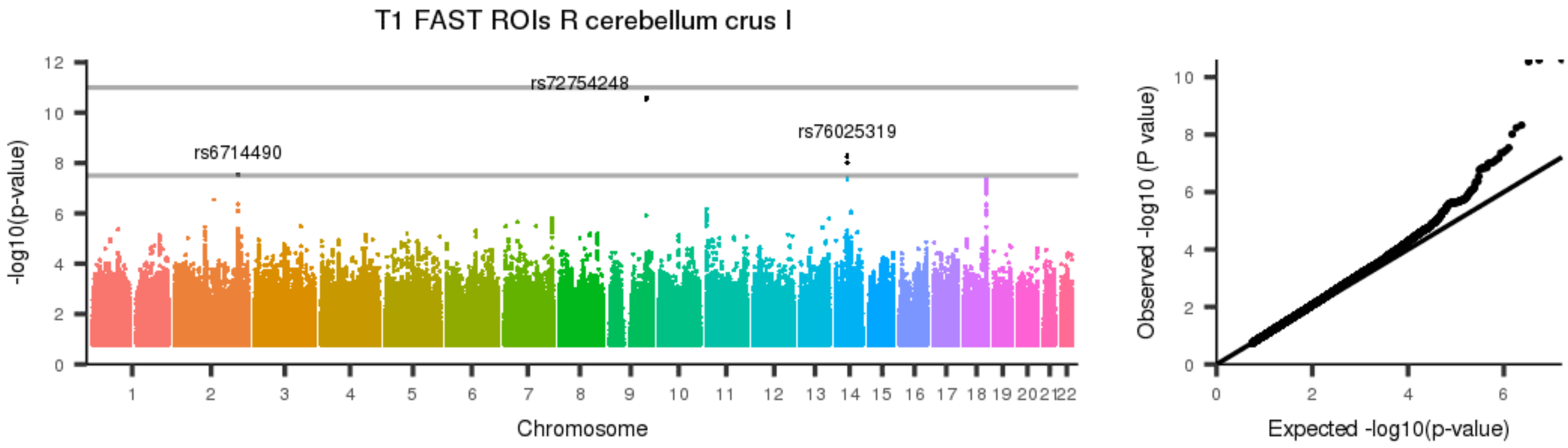

back to list of plots

Figure 147: T1 FAST ROIs L cerebellum crus II

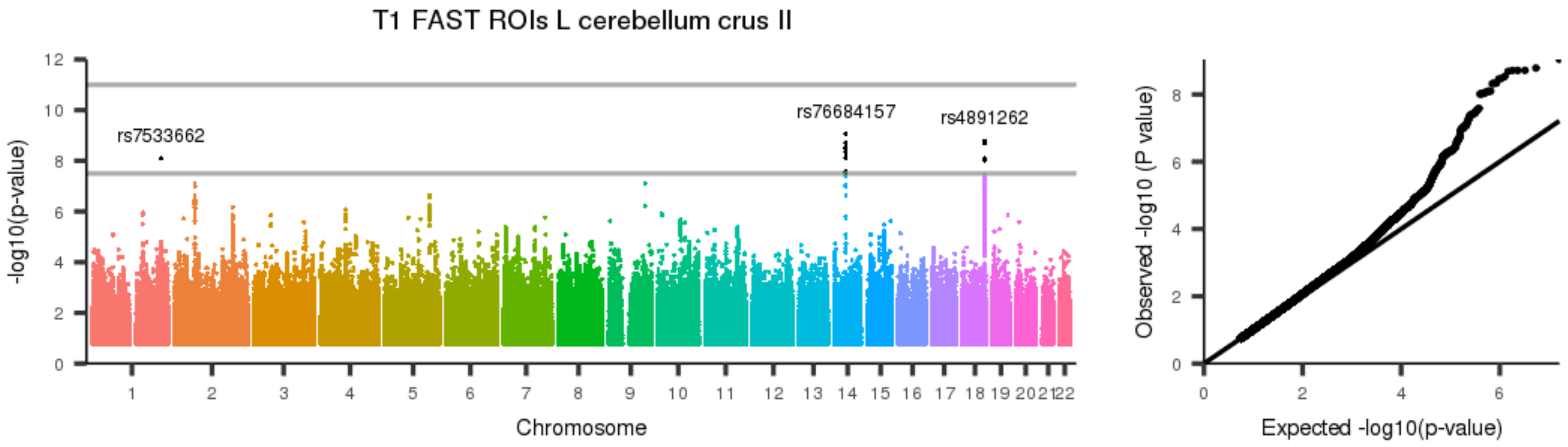

back to list of plots

Figure 148: T1 FAST ROIs V cerebellum crus II

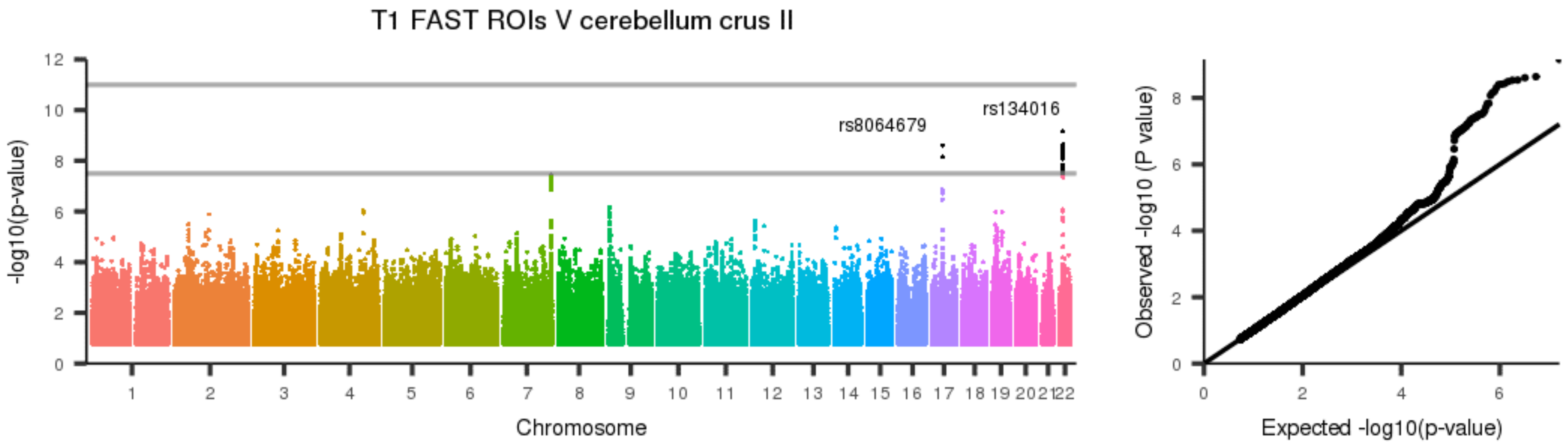

back to list of plots

Figure 149: T1 FAST ROIs R cerebellum crus II

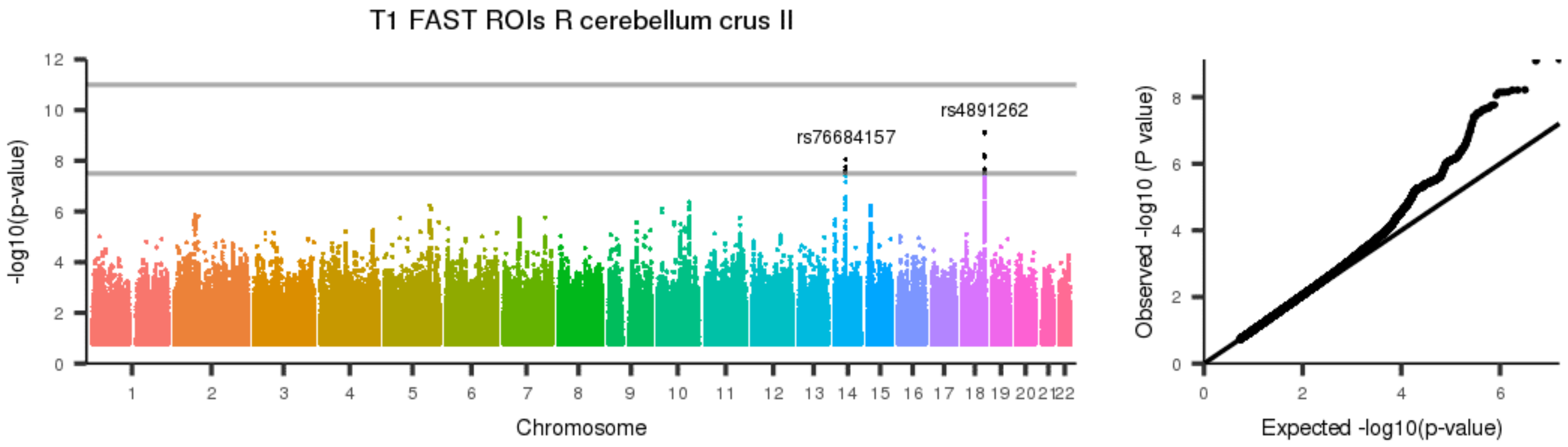

back to list of plots

Figure 150: T1 FAST ROIs L cerebellum VIIb

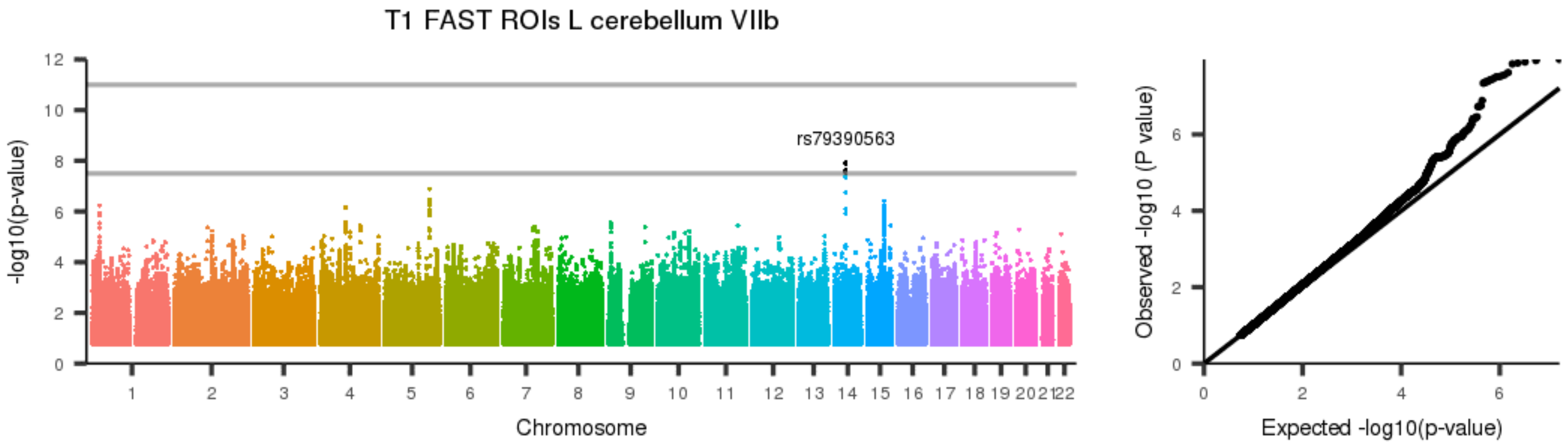

back to list of plots

Figure 151: T1 FAST ROIs V cerebellum VIIb

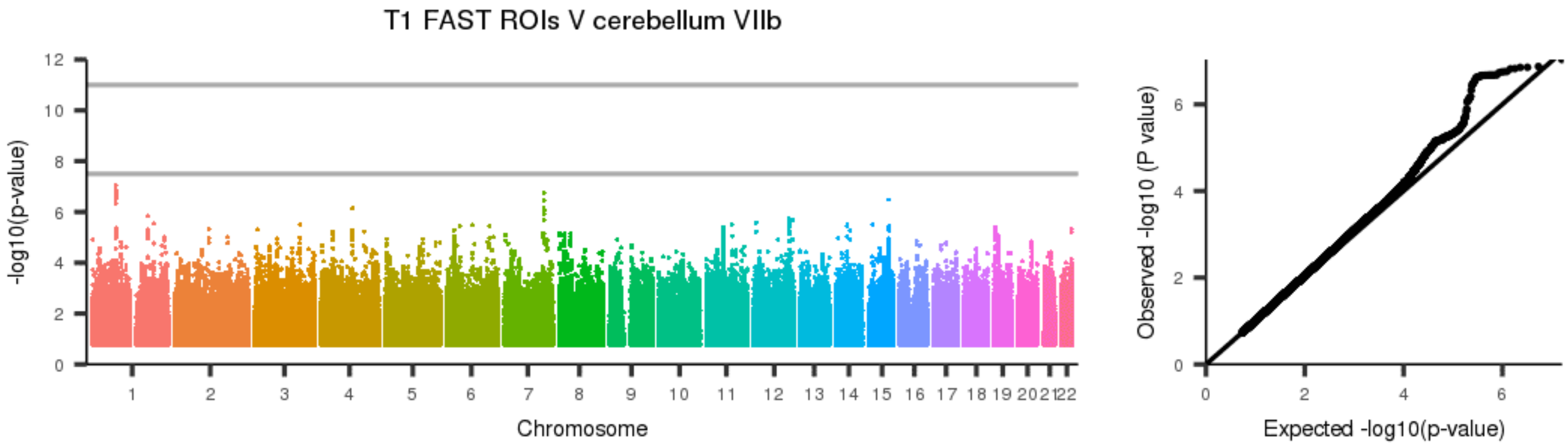

back to list of plots

Figure 152: T1 FAST ROIs R cerebellum VIIb

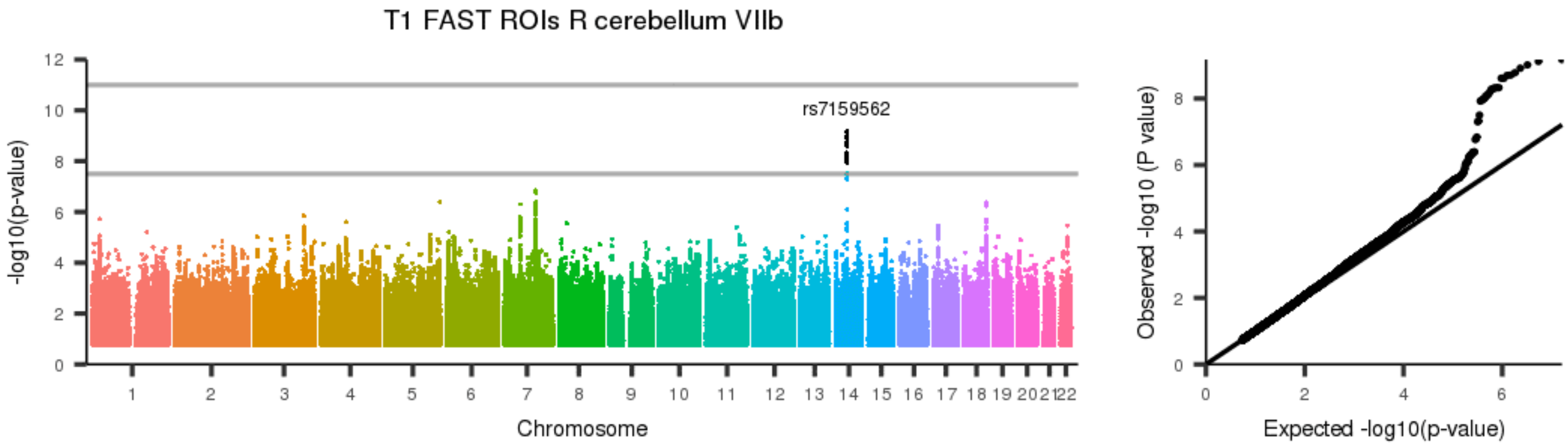

back to list of plots

Figure 153: T1 FAST ROIs L cerebellum VIIIa

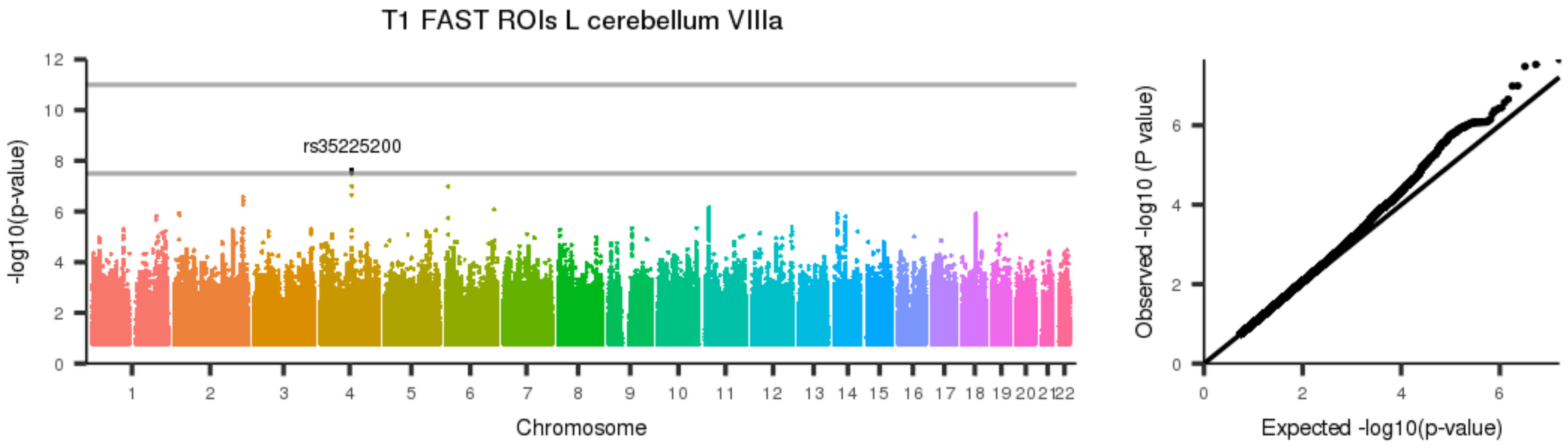

back to list of plots

Figure 154: T1 FAST ROIs V cerebellum VIIIa

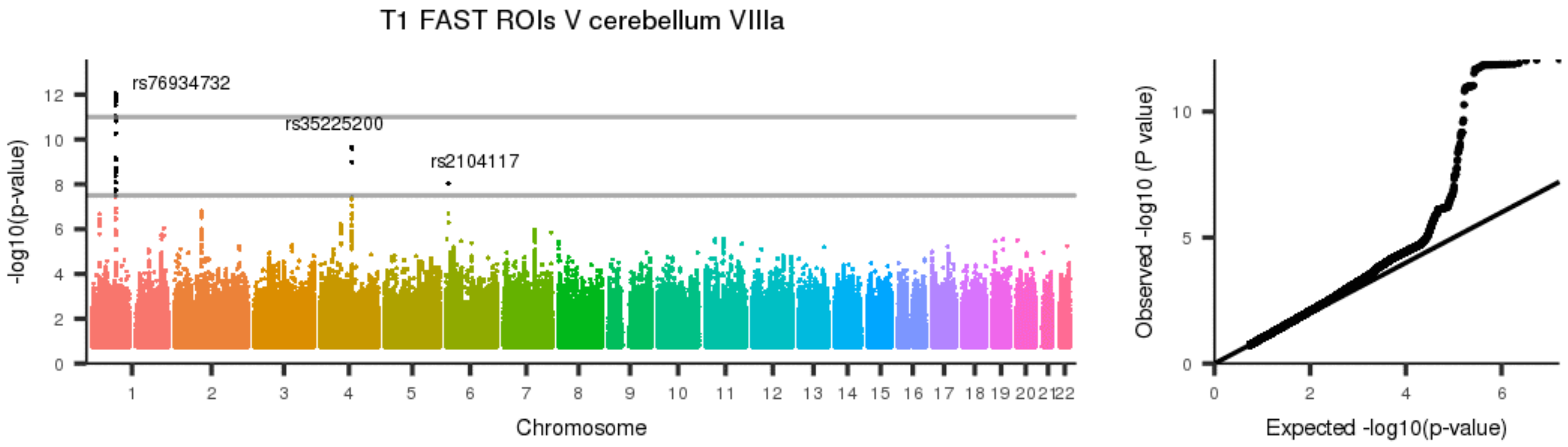

back to list of plots

Figure 155: T1 FAST ROIs R cerebellum VIIIa

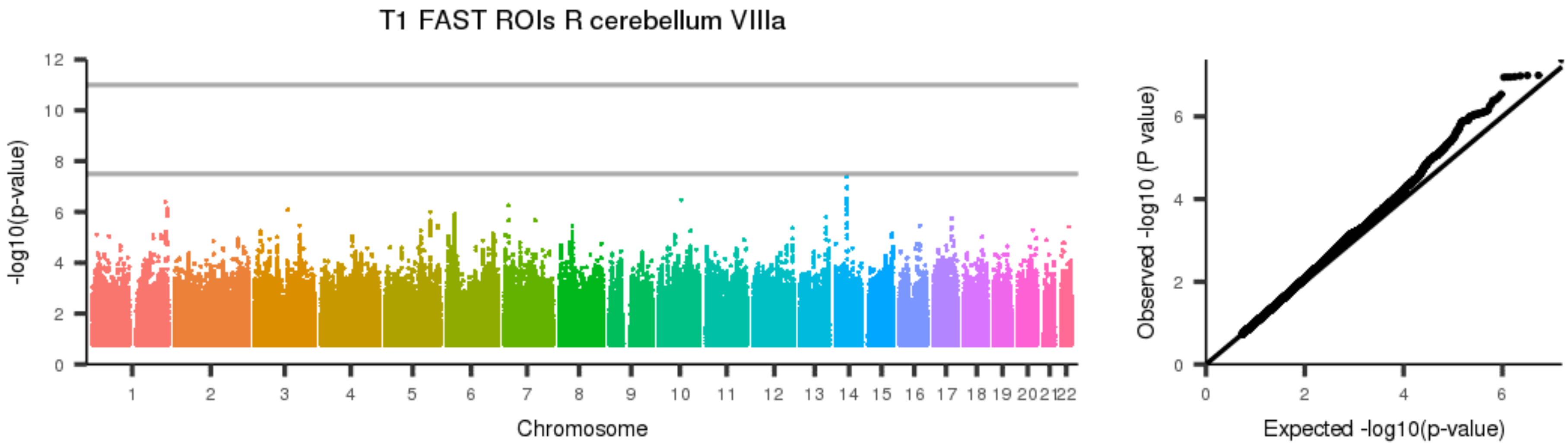

back to list of plots

Figure 156: T1 FAST ROIs L cerebellum VIIIb

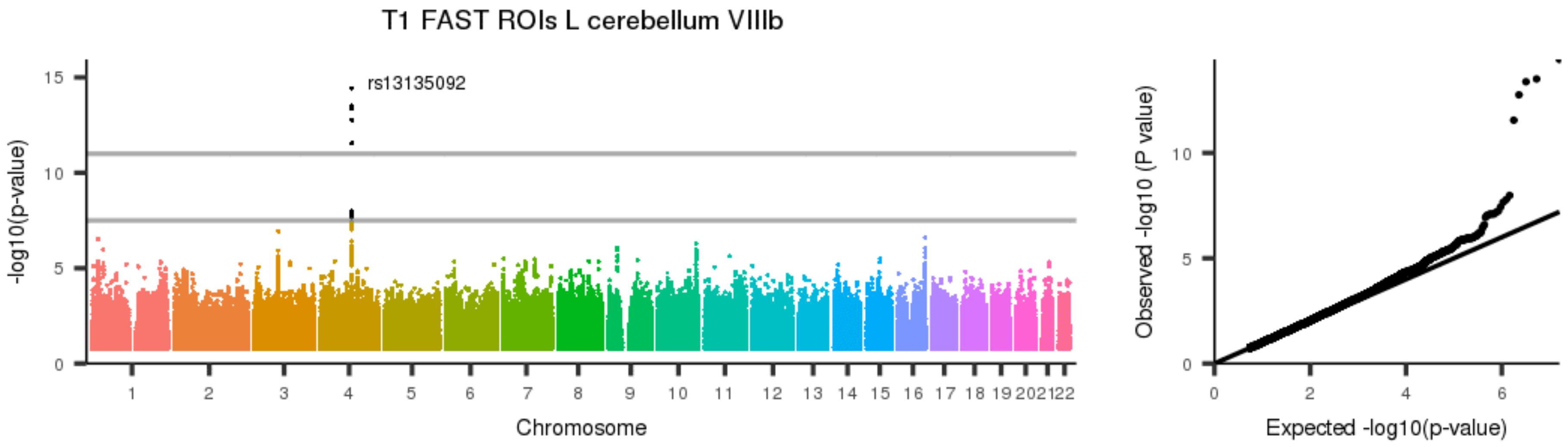

back to list of plots

Figure 157: T1 FAST ROIs V cerebellum VIIIb

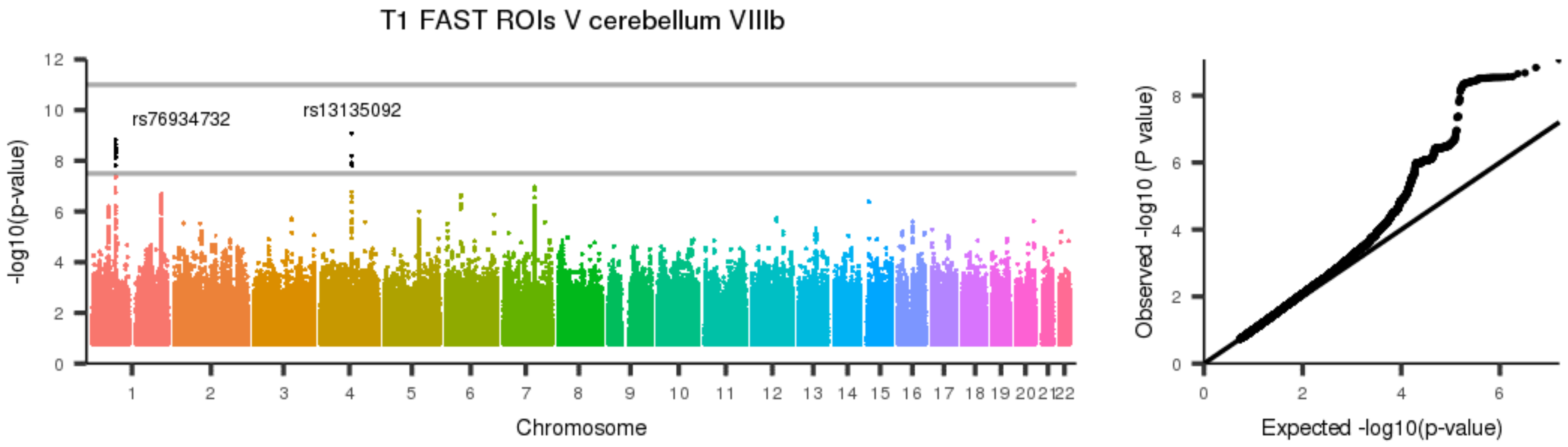

back to list of plots

Figure 158: T1 FAST ROIs R cerebellum VIIIb

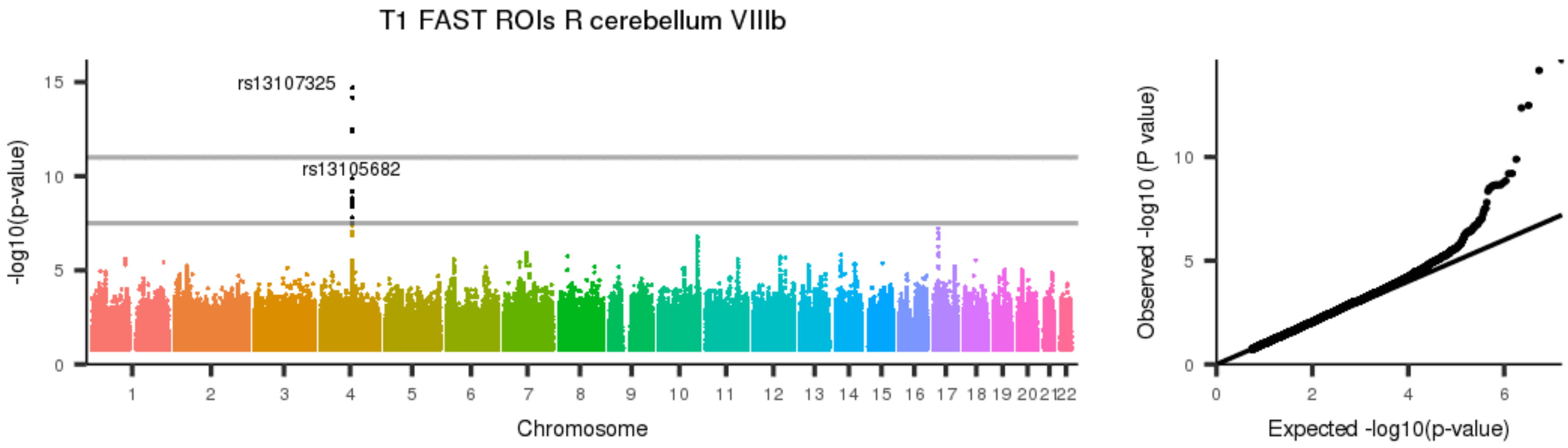

back to list of plots

Figure 159: T1 FAST ROIs L cerebellum IX

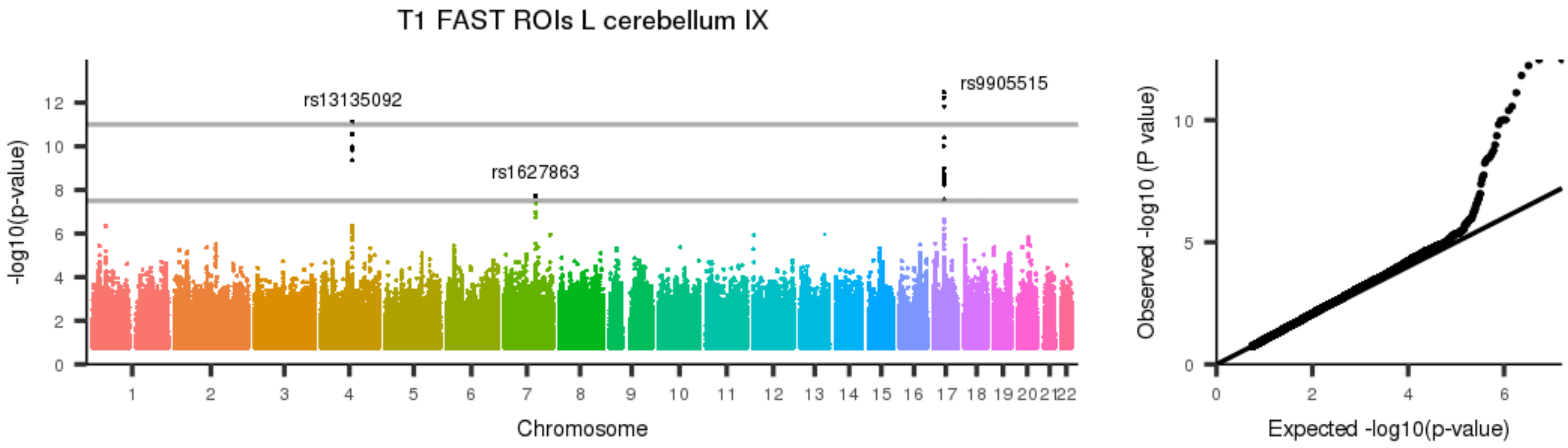

back to list of plots

Figure 160: T1 FAST ROIs V cerebellum IX

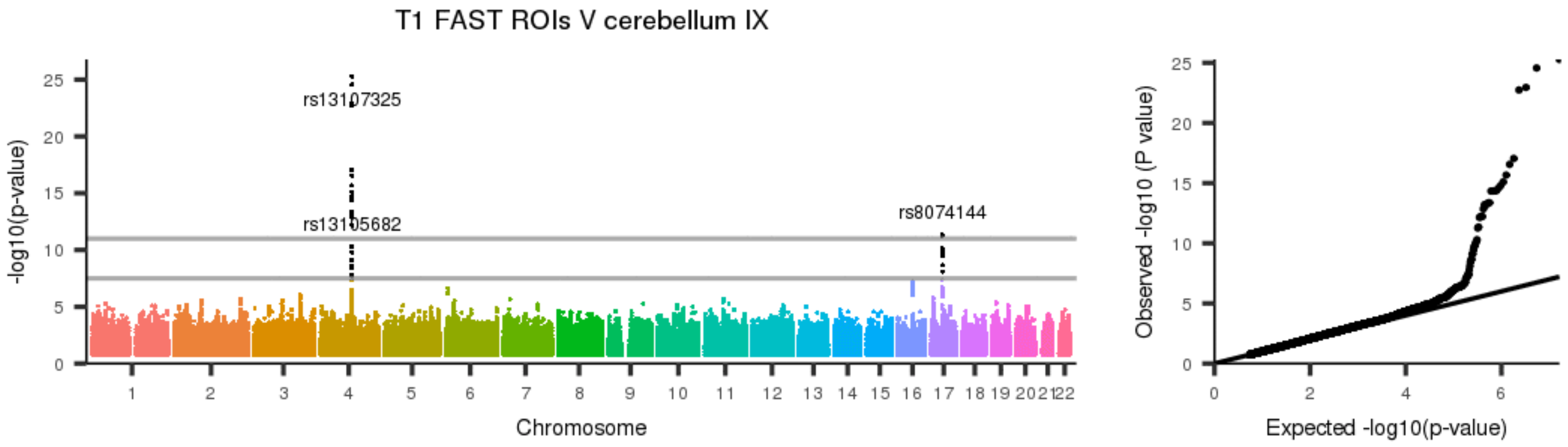

back to list of plots

Figure 161: T1 FAST ROIs R cerebellum IX

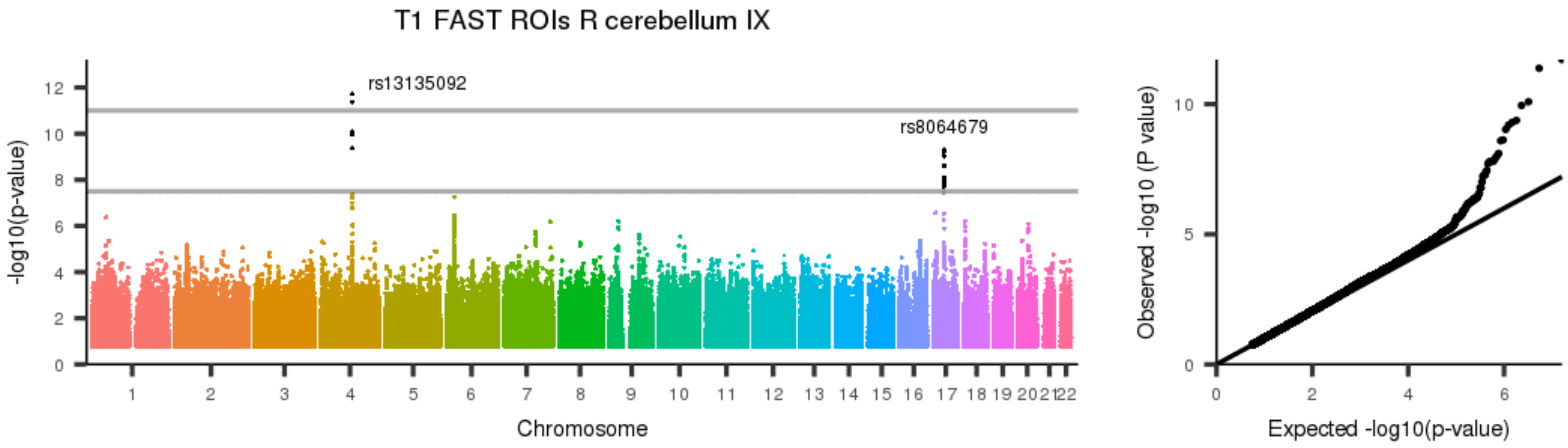

back to list of plots

Figure 162: T1 FAST ROIs L cerebellum X

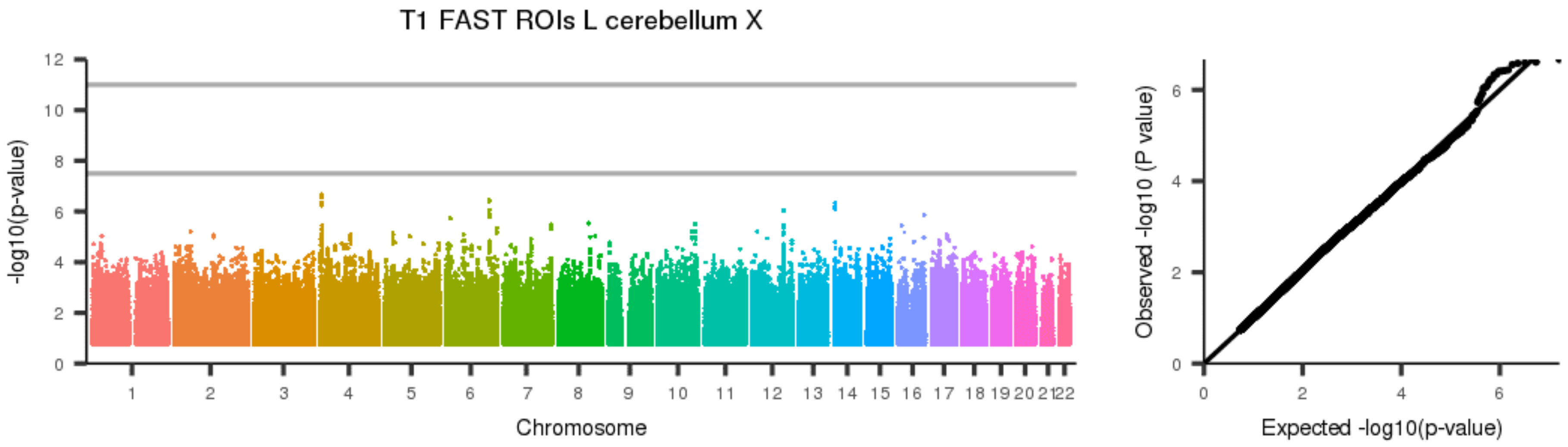

back to list of plots

Figure 163: T1 FAST ROIs V cerebellum X

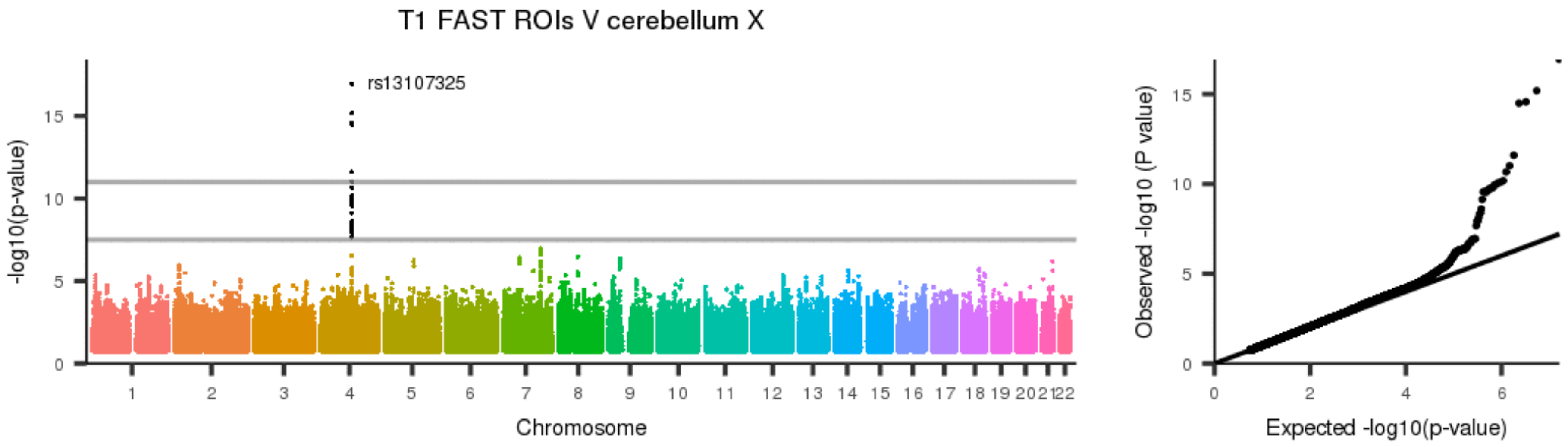

back to list of plots

Figure 164: T1 FAST ROIs R cerebellum X

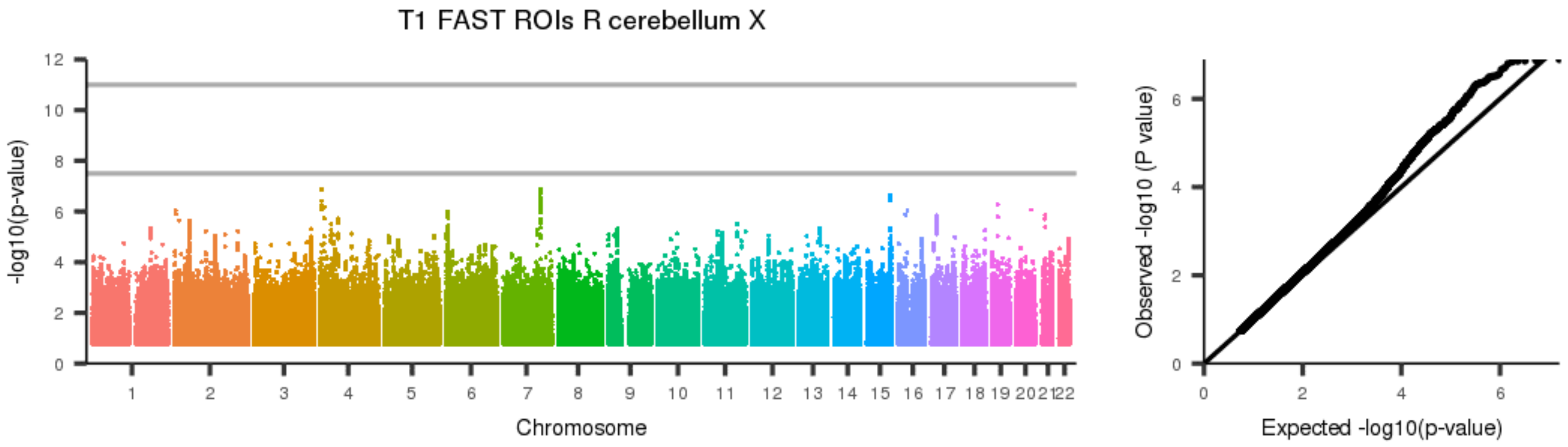

back to list of plots

Figure 165: T2 FLAIR BIANCA WMH volume

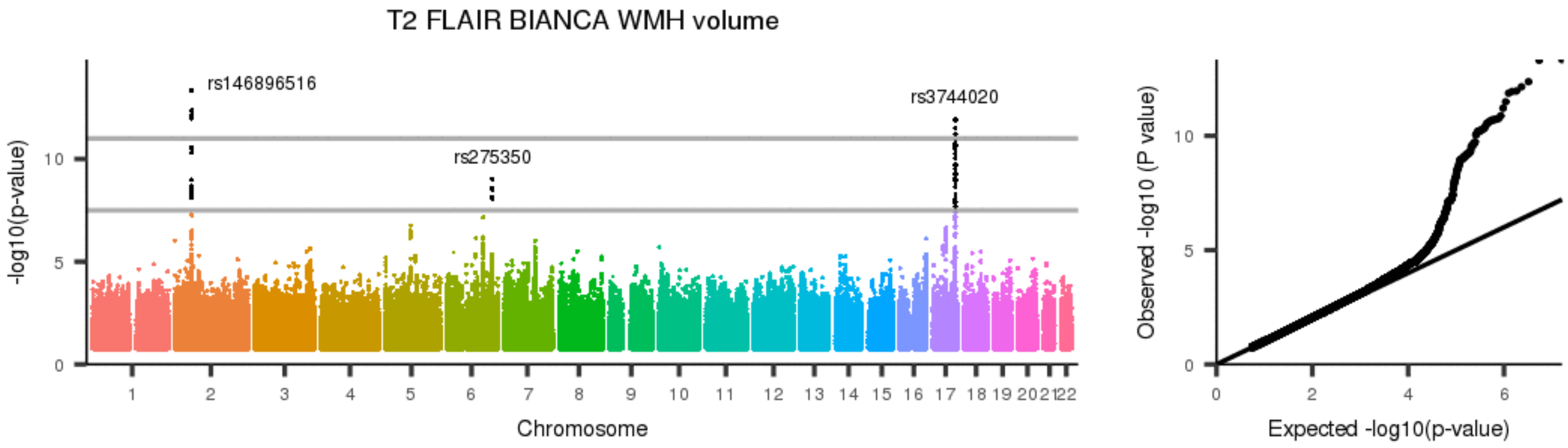

back to list of plots

Figure 166: SWI T2star left thalamus

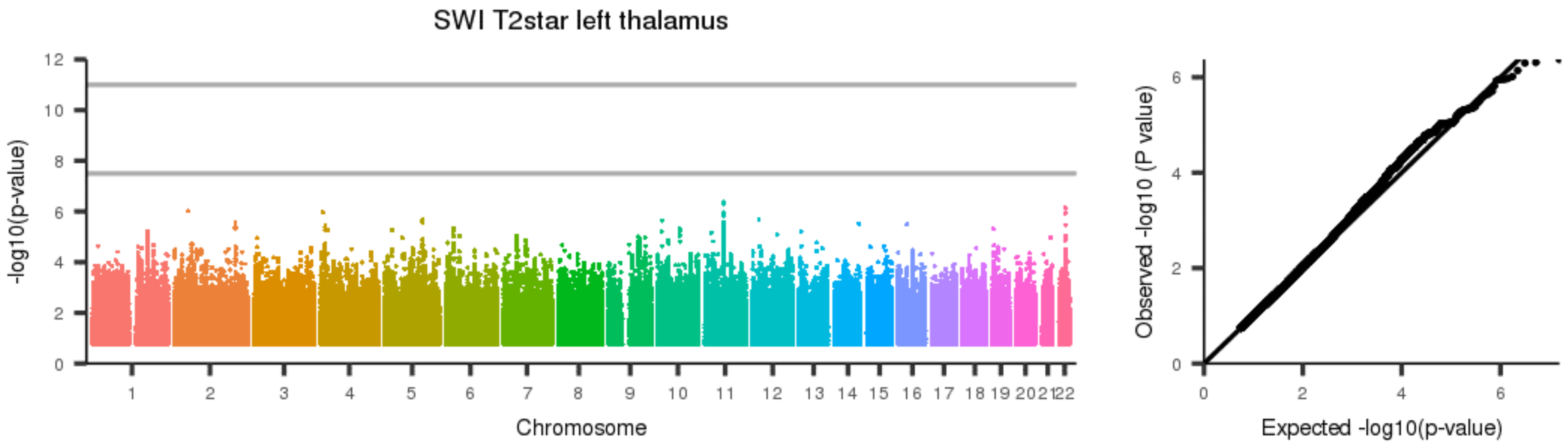

back to list of plots

Figure 167: SWI T2star right thalamus

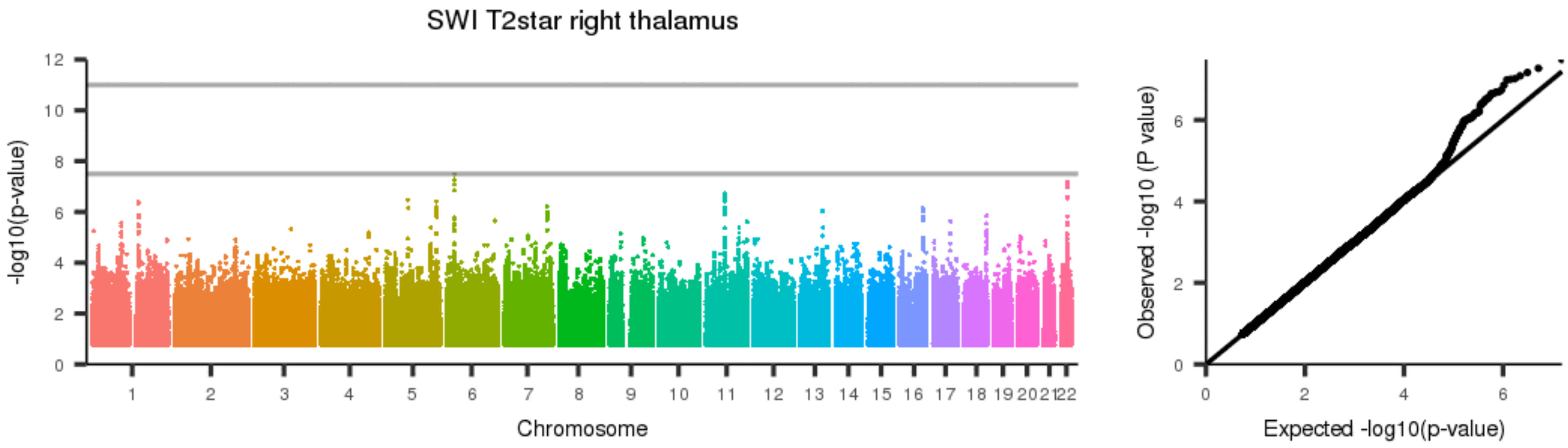

back to list of plots

Figure 168: SWI T2star left caudate

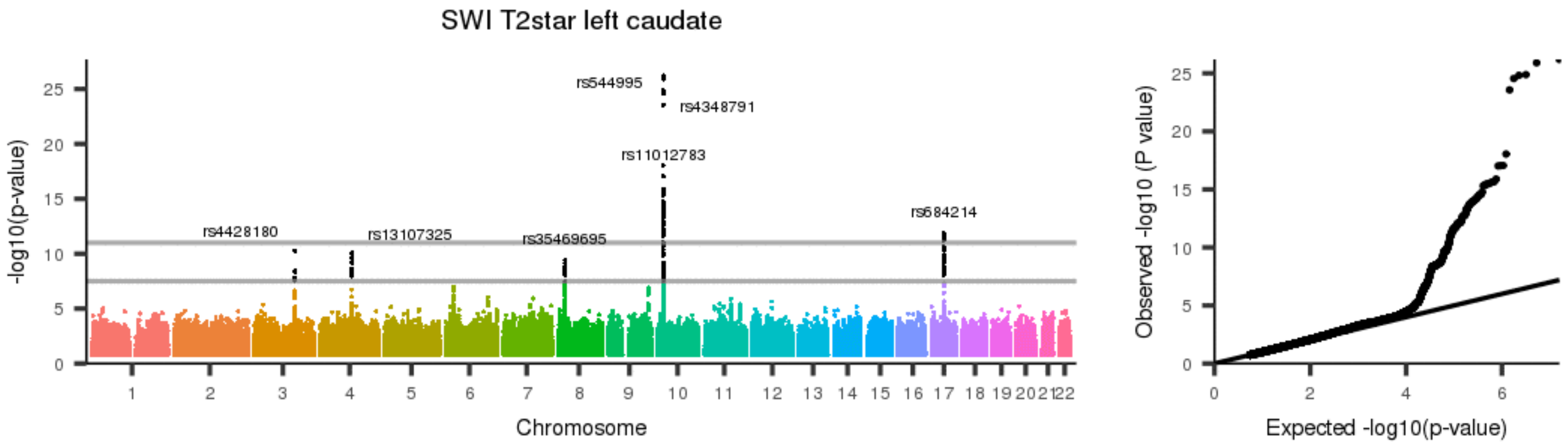

back to list of plots

Figure 169: SWI T2star right caudate

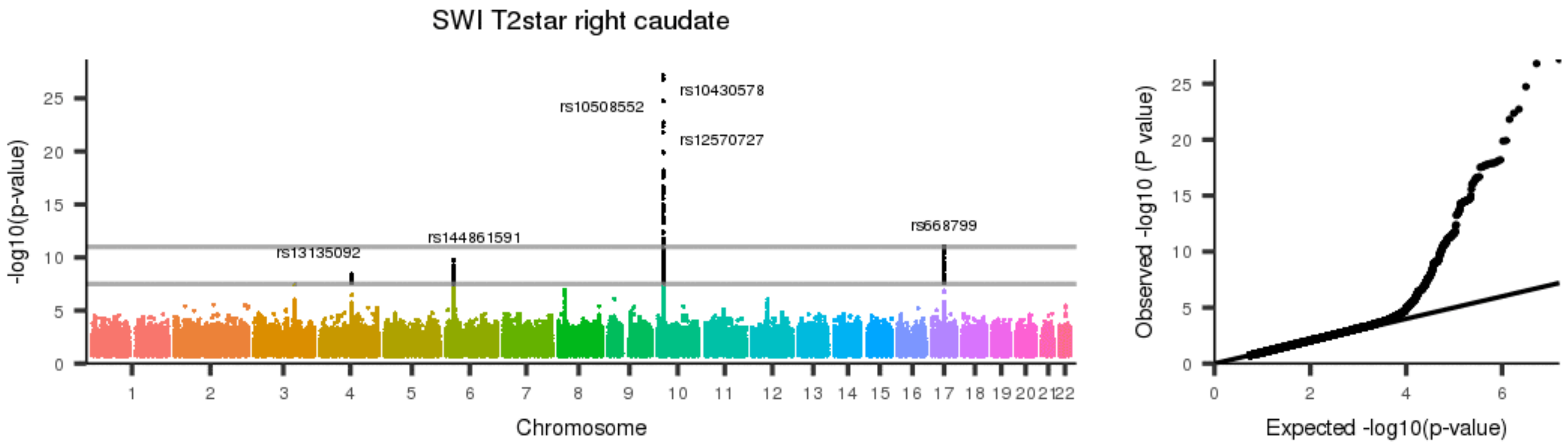

back to list of plots

Figure 170: SWI T2star left putamen

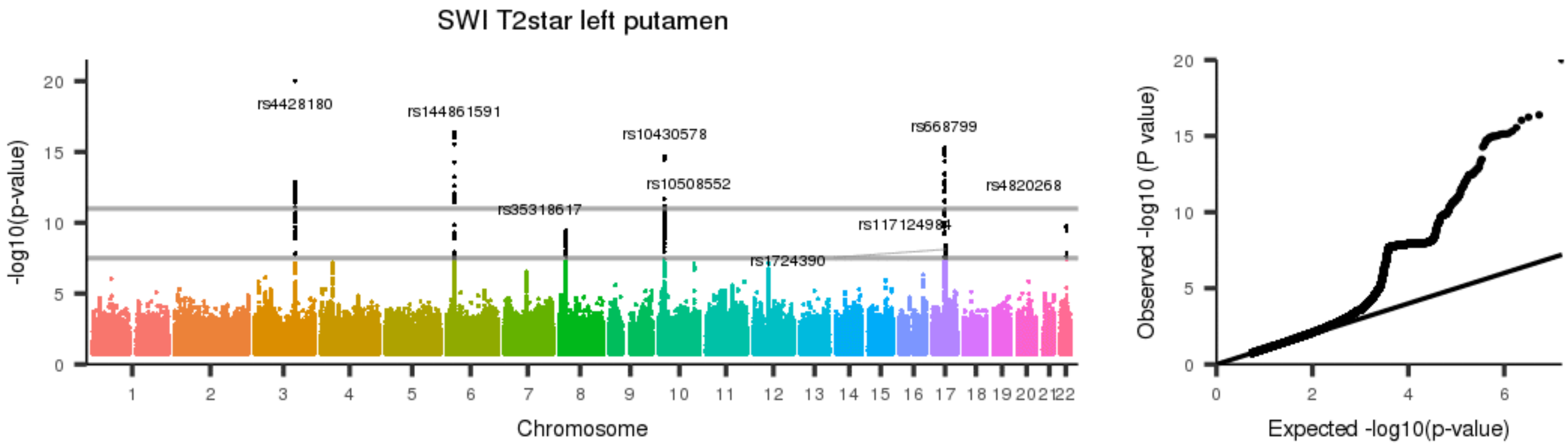

back to list of plots

Figure 171: SWI T2star right putamen

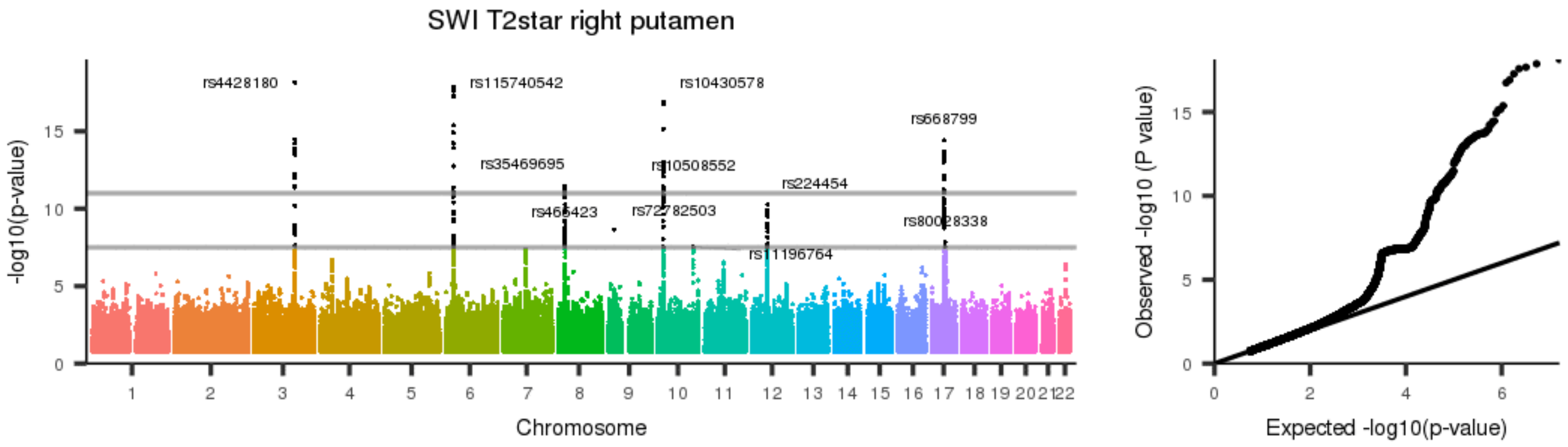

back to list of plots

Figure 172: SWI T2star left pallidum

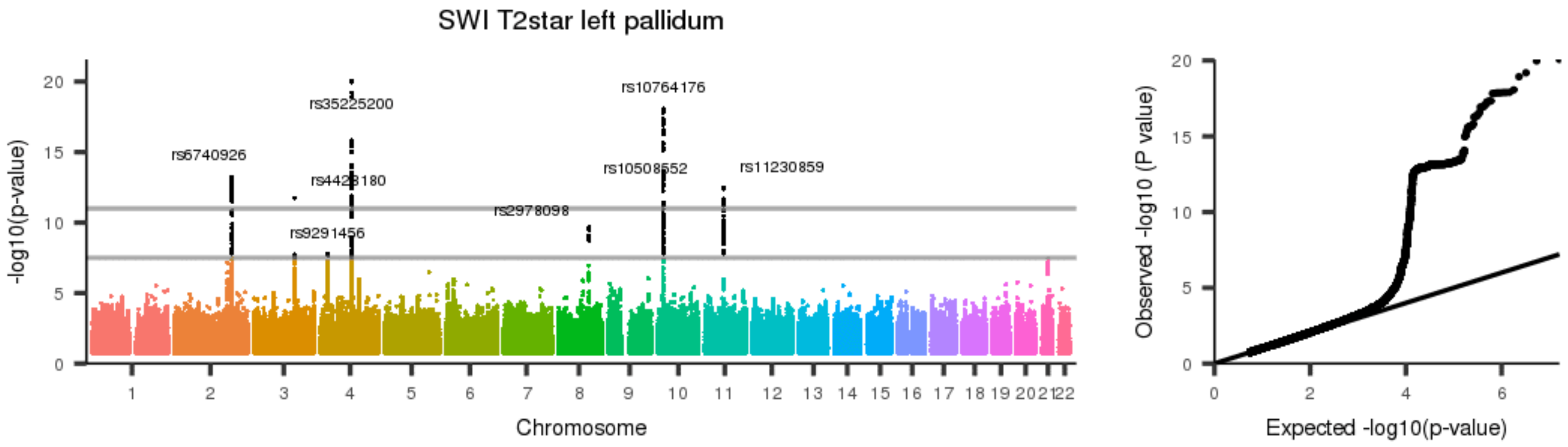

back to list of plots

Figure 173: SWI T2star right pallidum

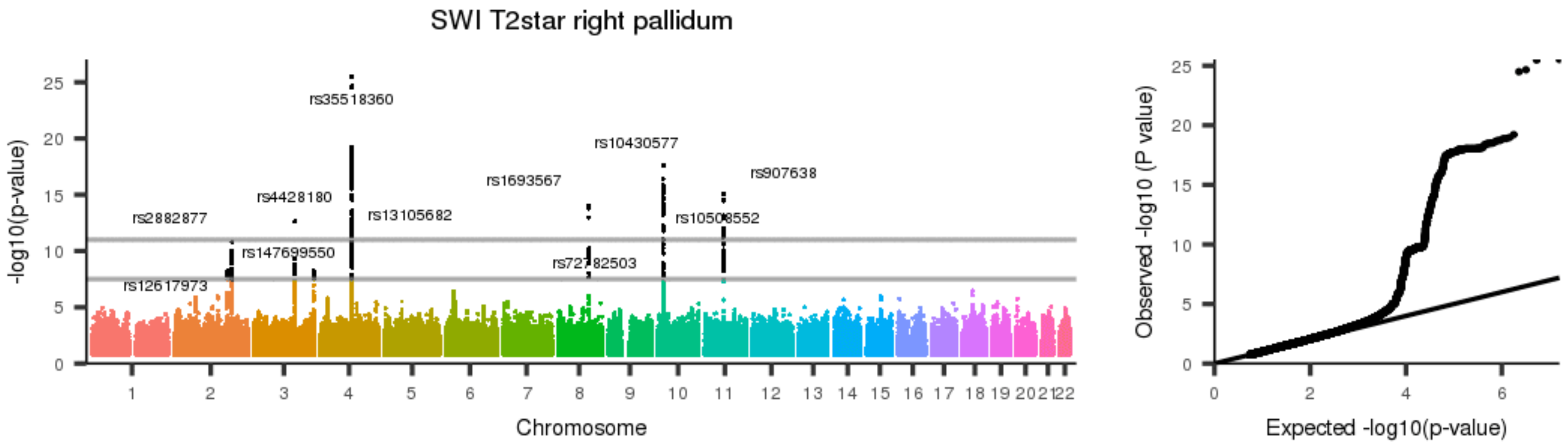

back to list of plots

Figure 174: SWI T2star left hippocampus

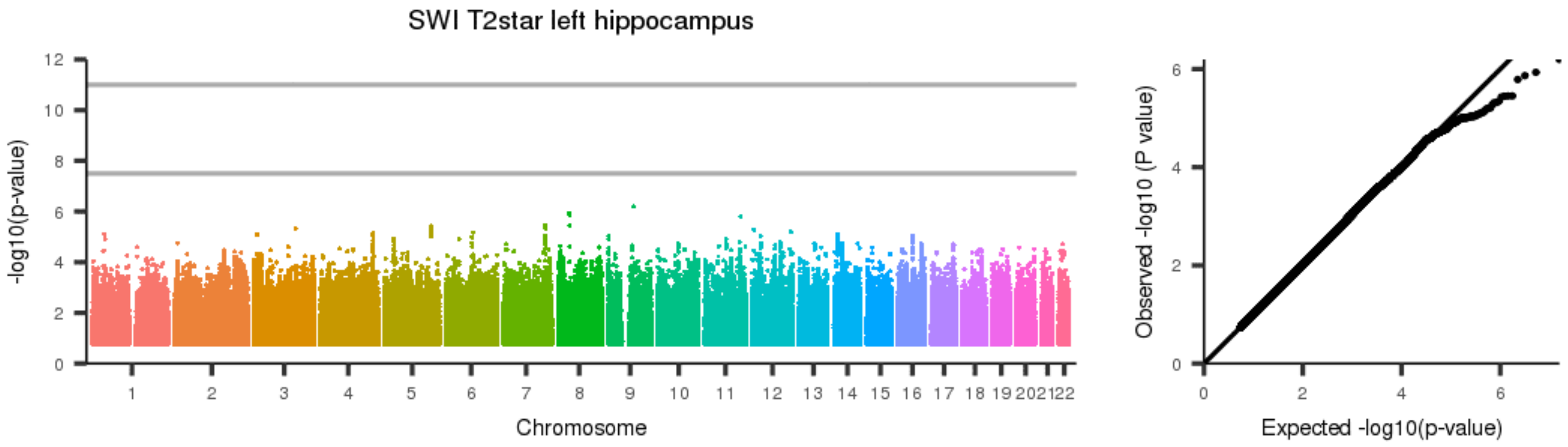

back to list of plots

Figure 175: SWI T2star right hippocampus

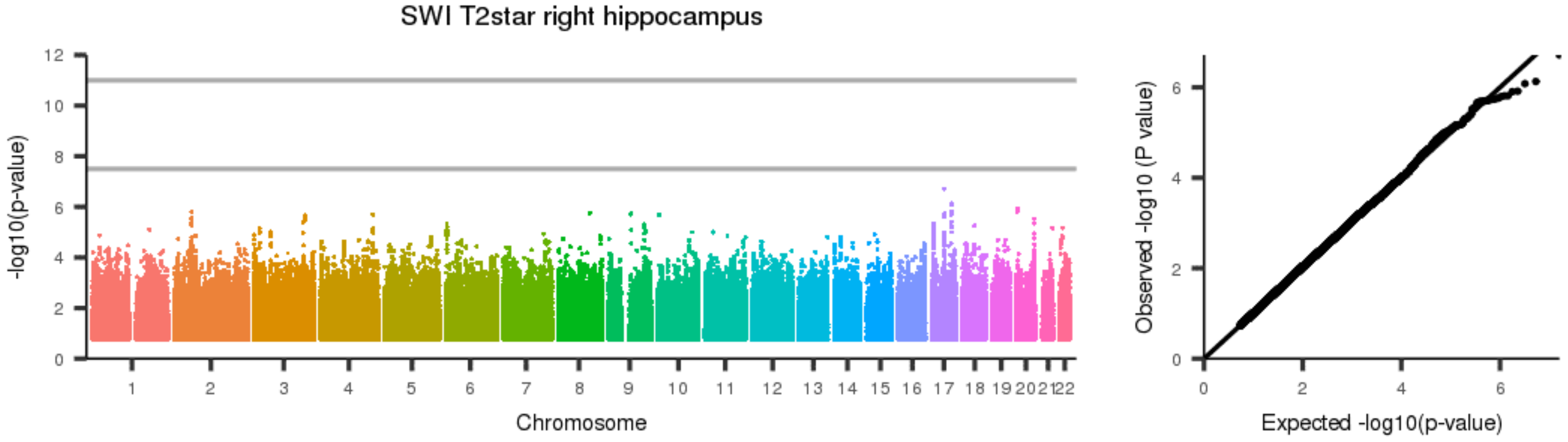

back to list of plots

Figure 176: SWI T2star left amygdala

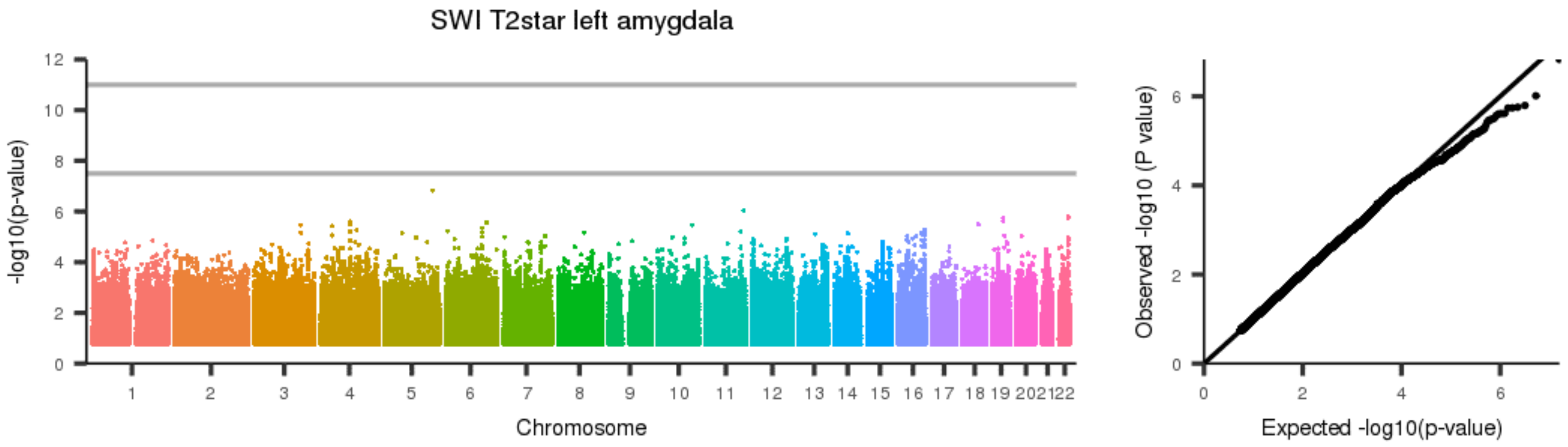

back to list of plots

Figure 177: SWI T2star right amygdala

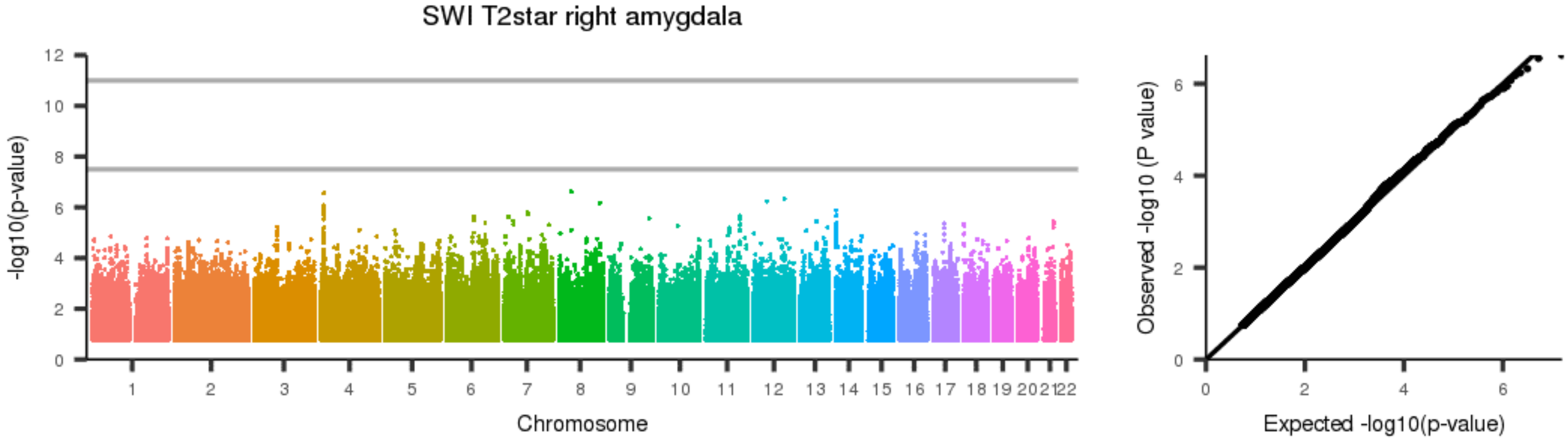

back to list of plots

Figure 178: SWI T2star left accumbens

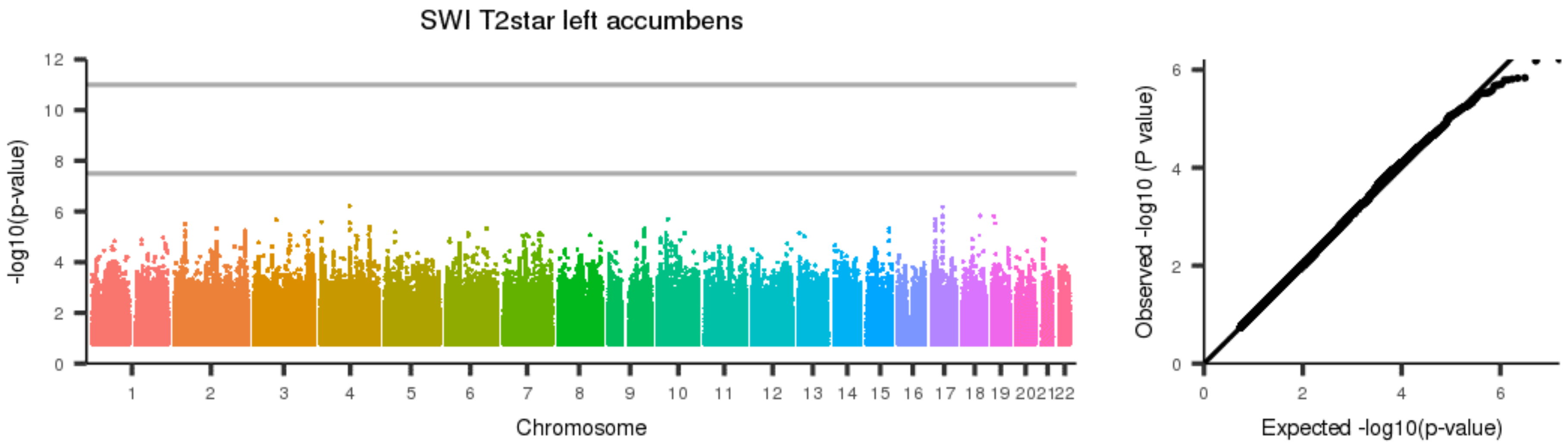

back to list of plots

Figure 179: SWI T2star right accumbens

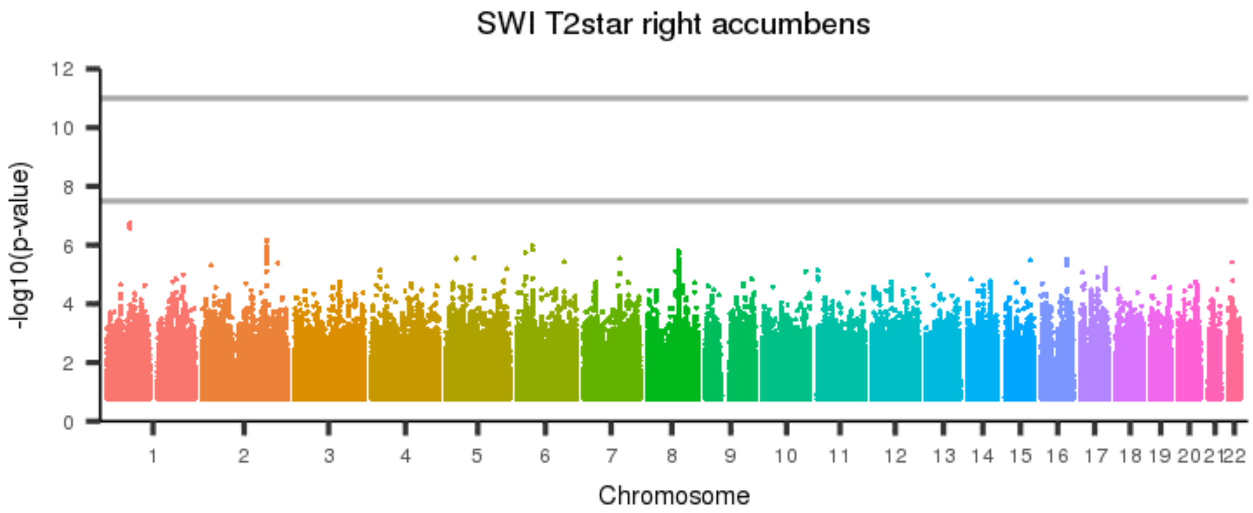

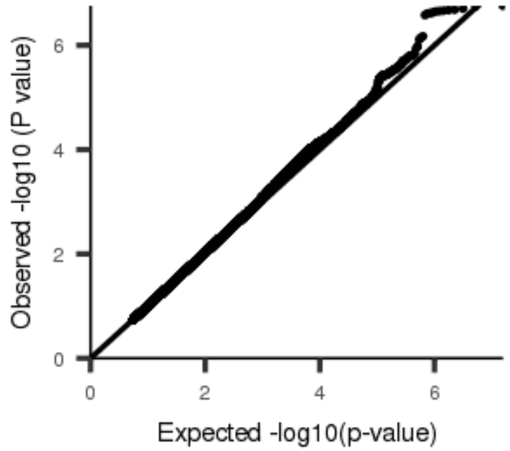

back to list of plots

Figure 180: tfMRI median BOLD shapes

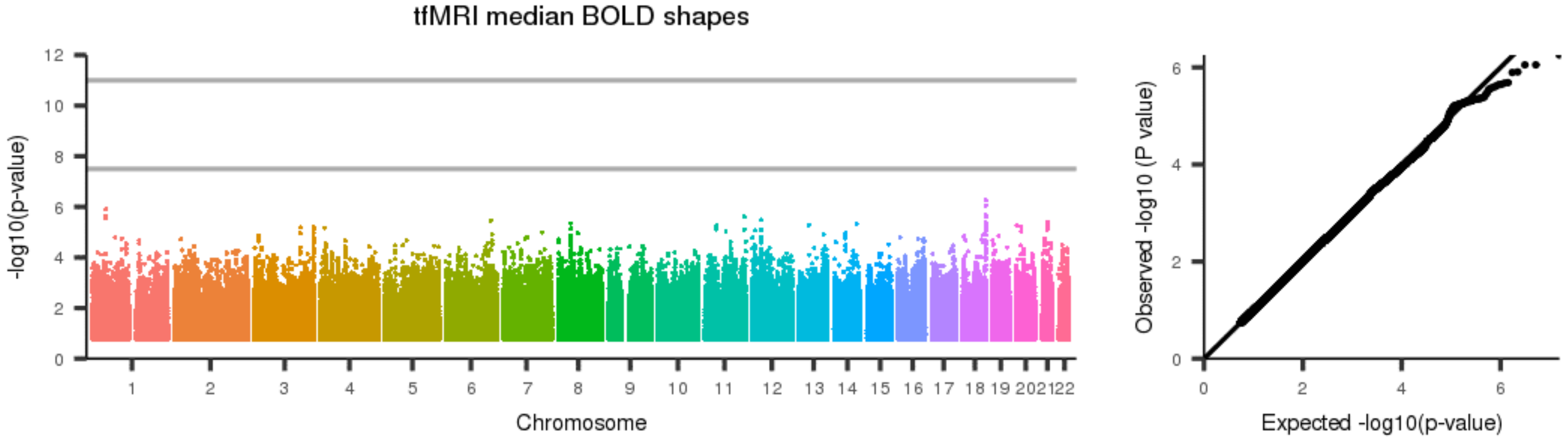

back to list of plots

Figure 181: tfMRI 90th-percentile BOLD shapes

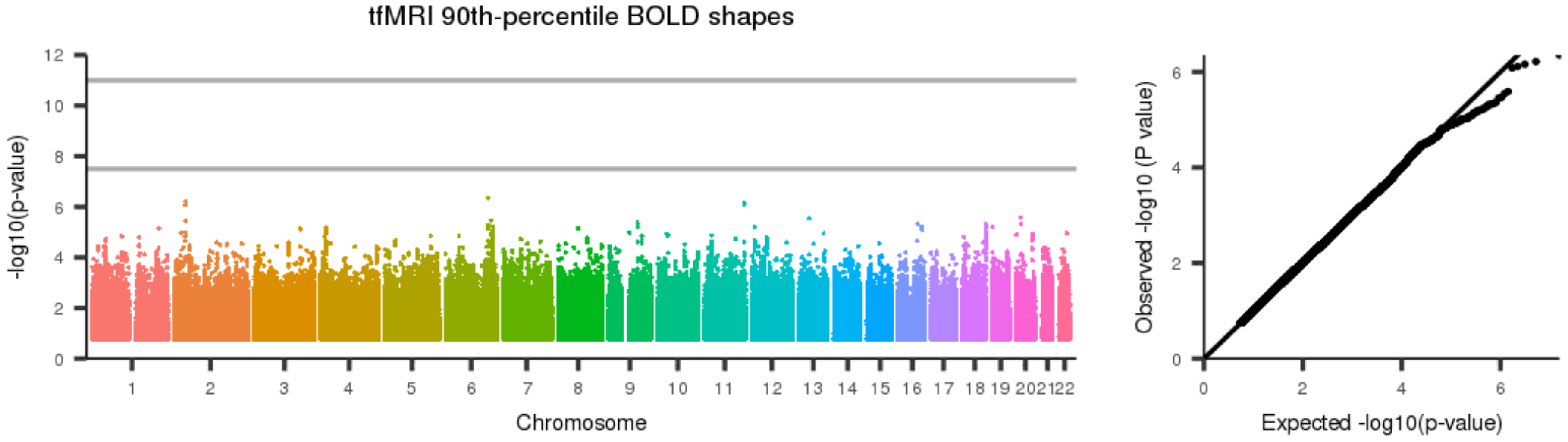

back to list of plots

Figure 182: tfMRI median zstat shapes

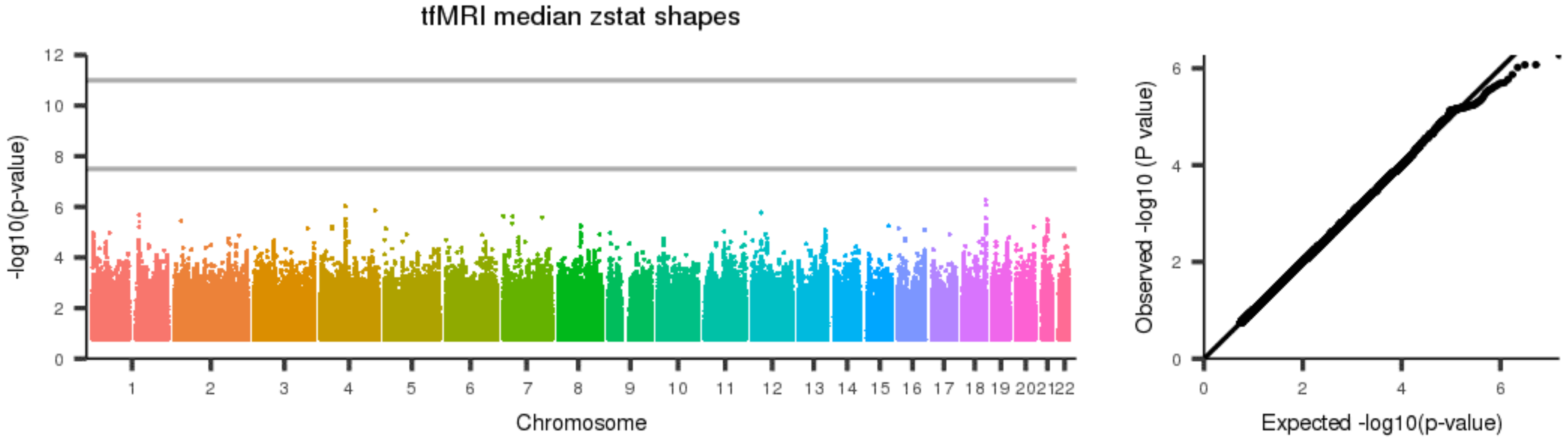

back to list of plots

Figure 183: tfMRI 90th-percentile zstat shapes

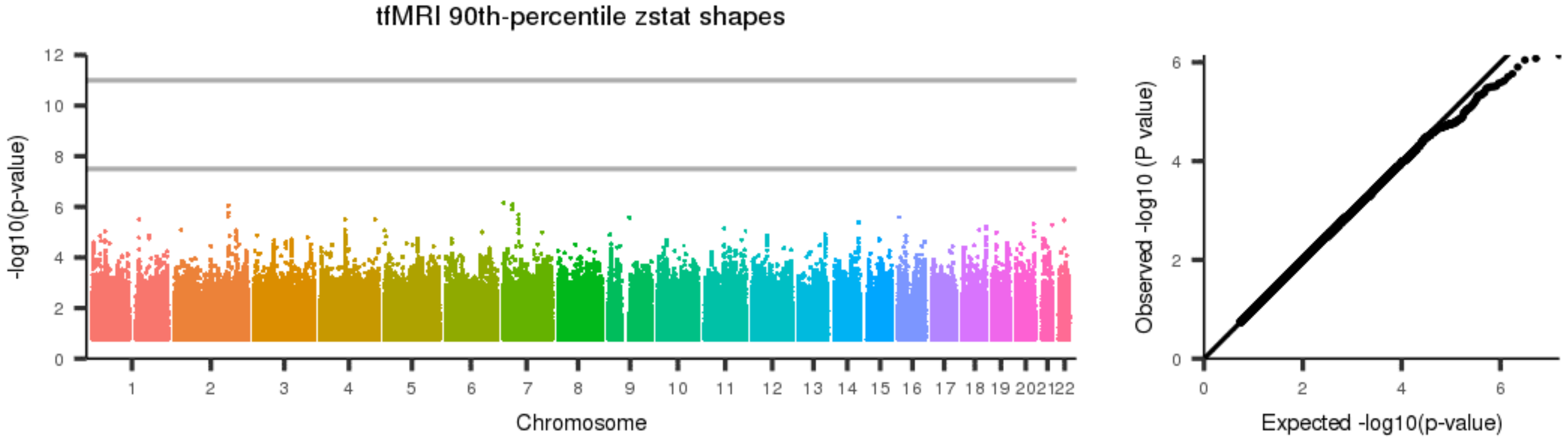

back to list of plots

Figure 184: tfMRI median BOLD faces

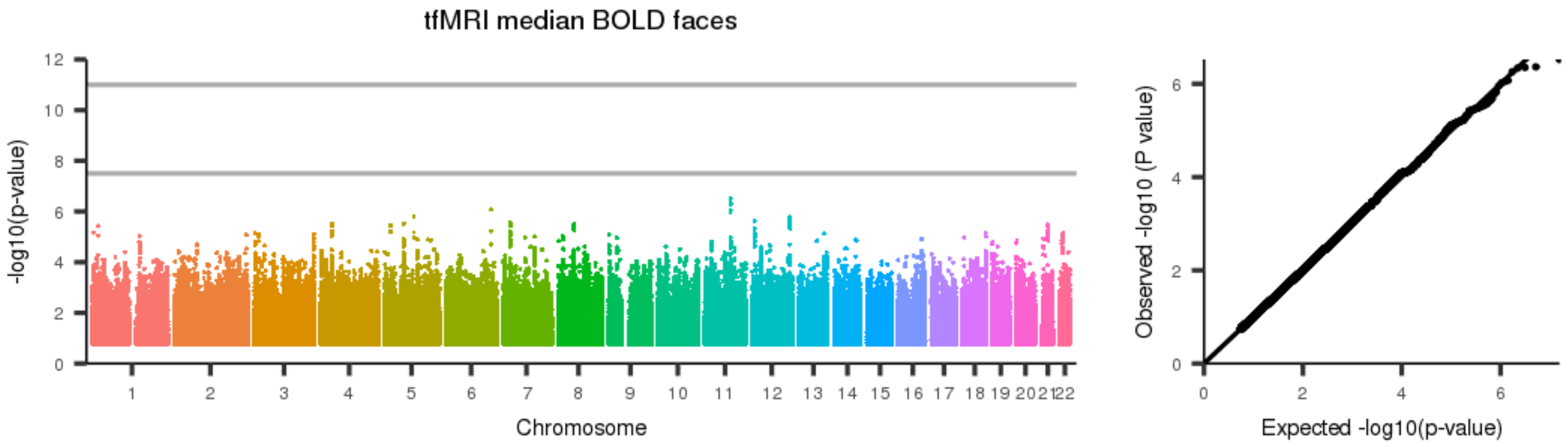

back to list of plots

Figure 185: tfMRI 90th-percentile BOLD faces

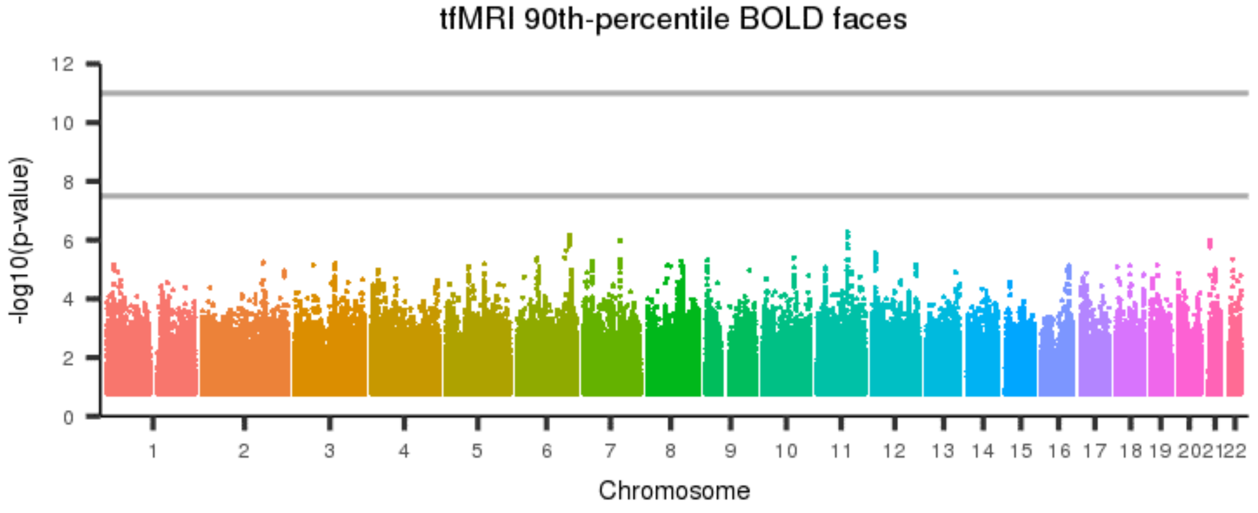

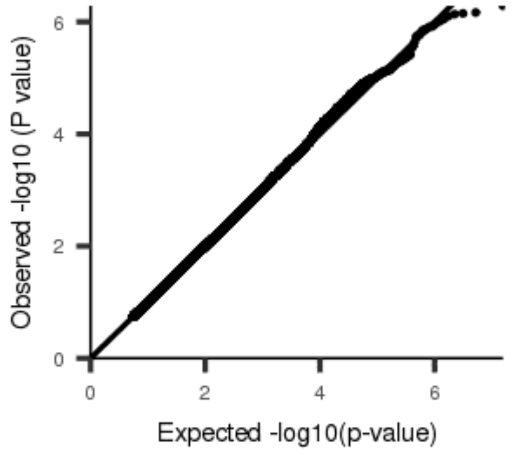

back to list of plots

Figure 186: tfMRI median zstat faces

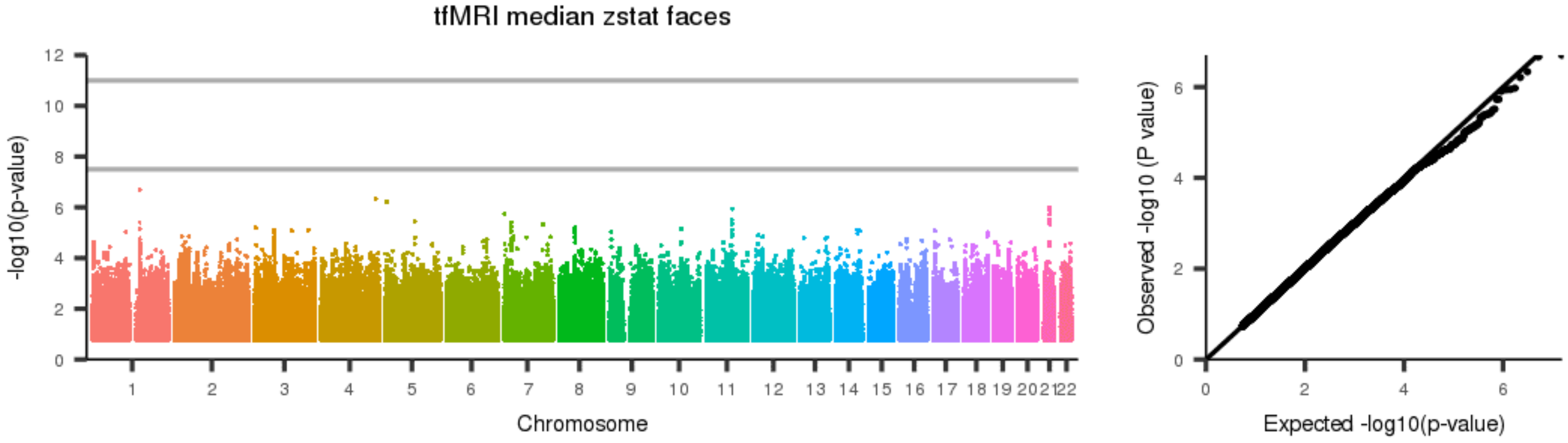

back to list of plots

Figure 187: tfMRI 90th-percentile zstat faces

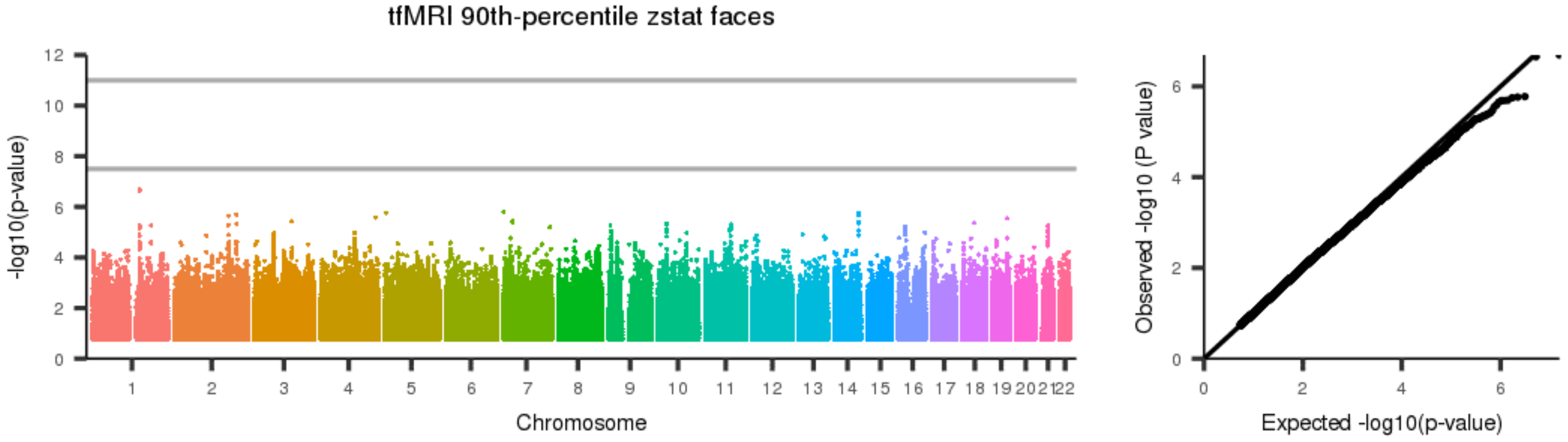

back to list of plots

Figure 188: tfMRI median BOLD faces-shapes

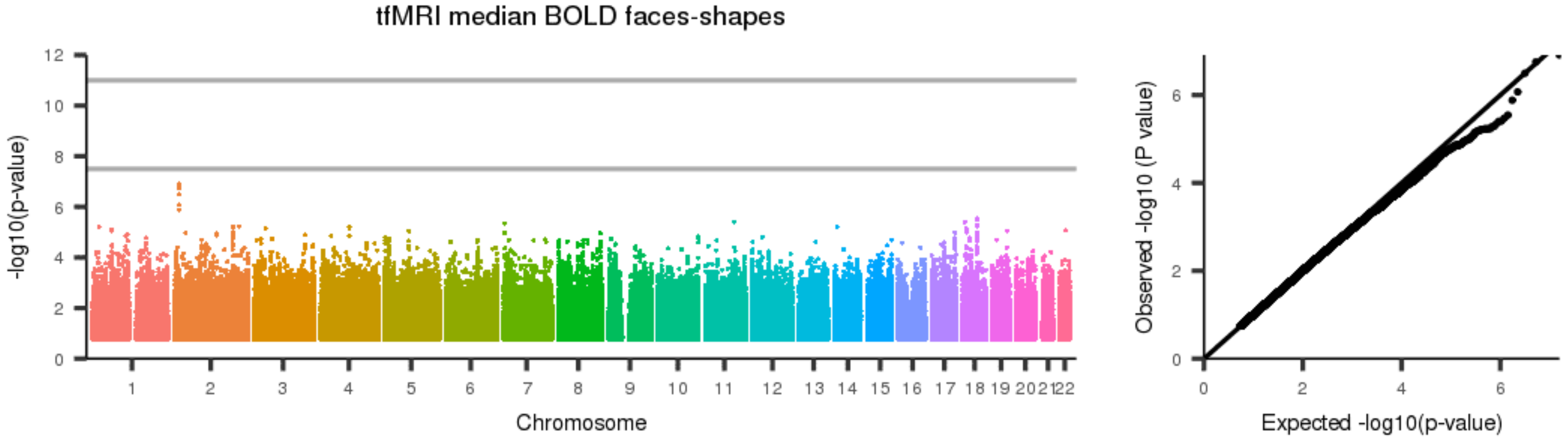

back to list of plots

Figure 189: tfMRI 90th-percentile BOLD faces-shapes

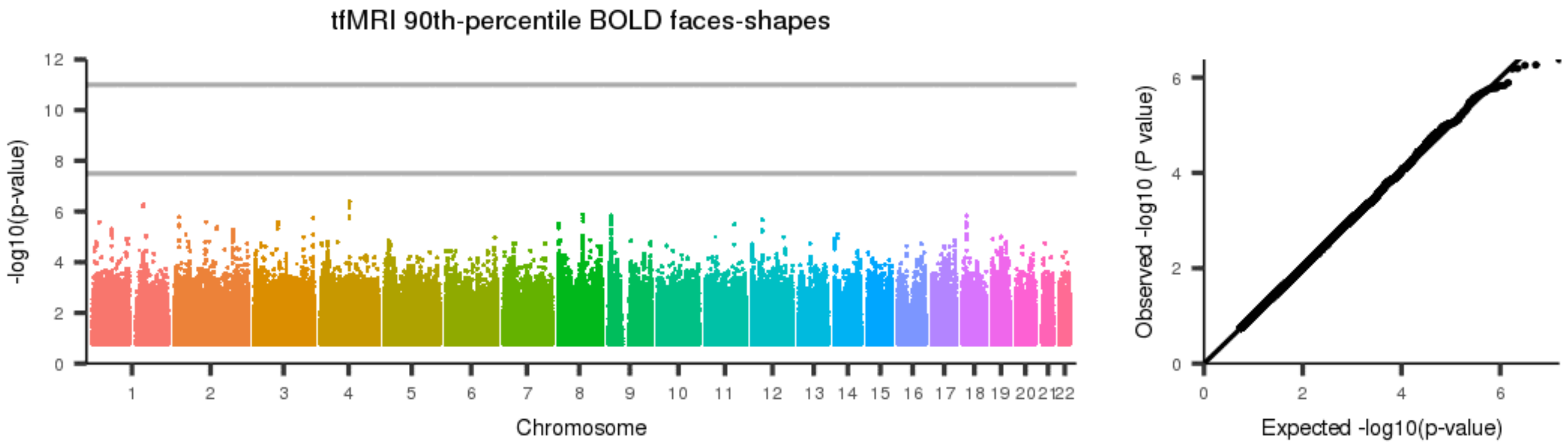

back to list of plots

Figure 190: tfMRI median zstat faces-shapes

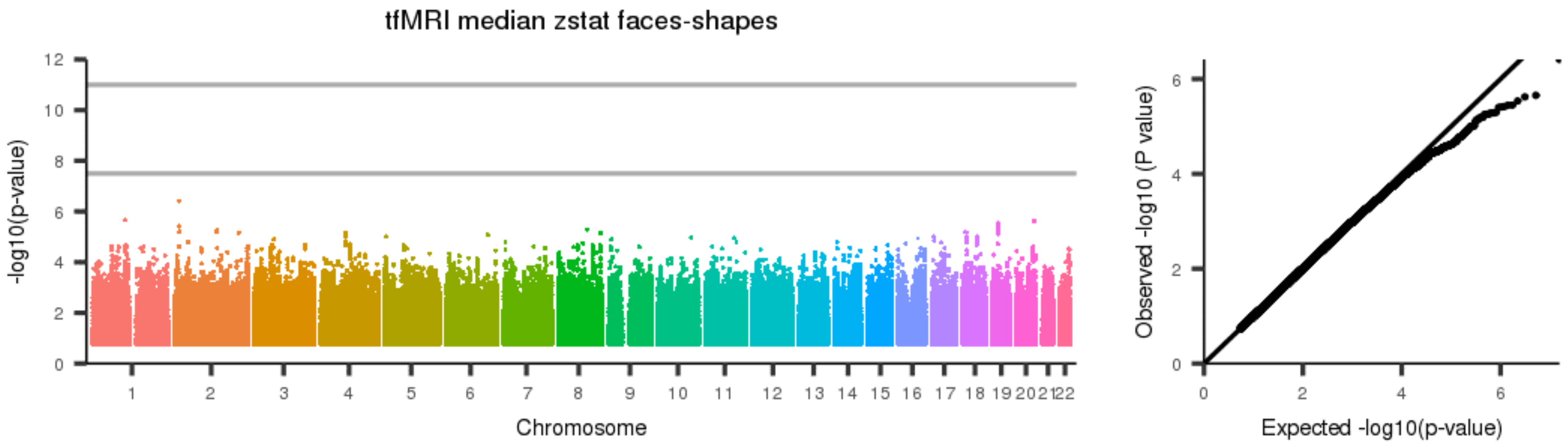

back to list of plots

Figure 191: tfMRI 90th-percentile zstat faces-shapes

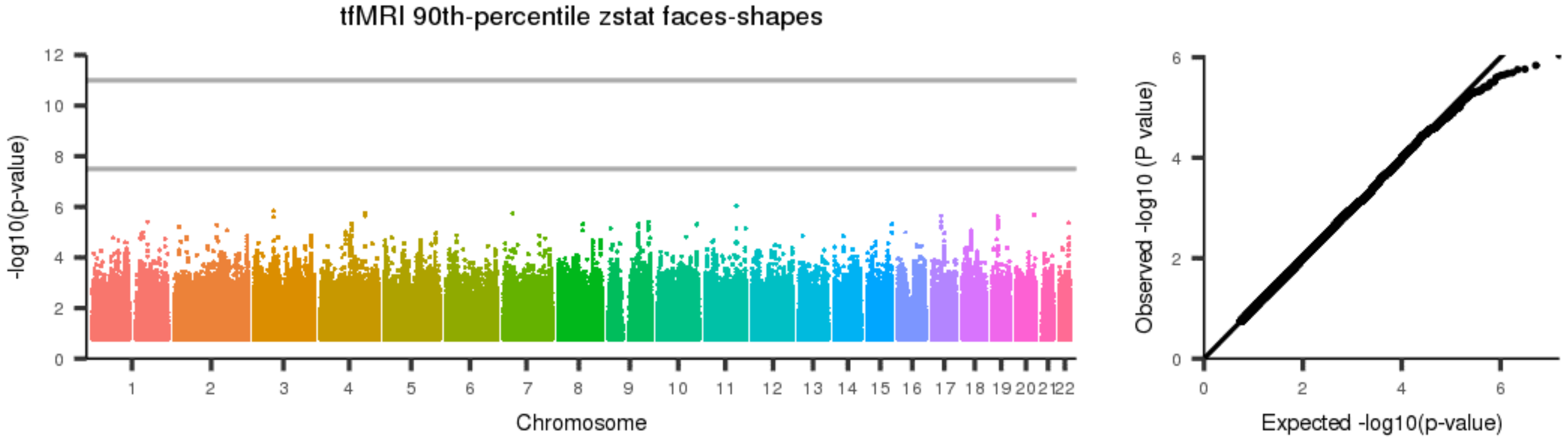

back to list of plots

Figure 192: tfMRI median BOLD faces-shapes amygdala

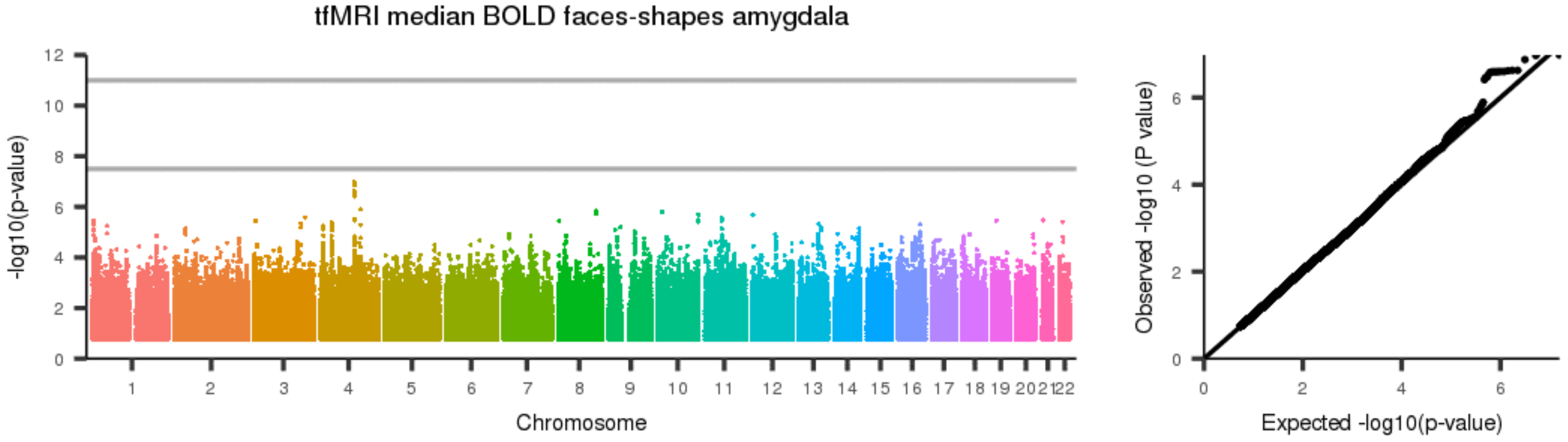

back to list of plots

Figure 193: tfMRI 90th-percentile BOLD faces-shapes amygdala

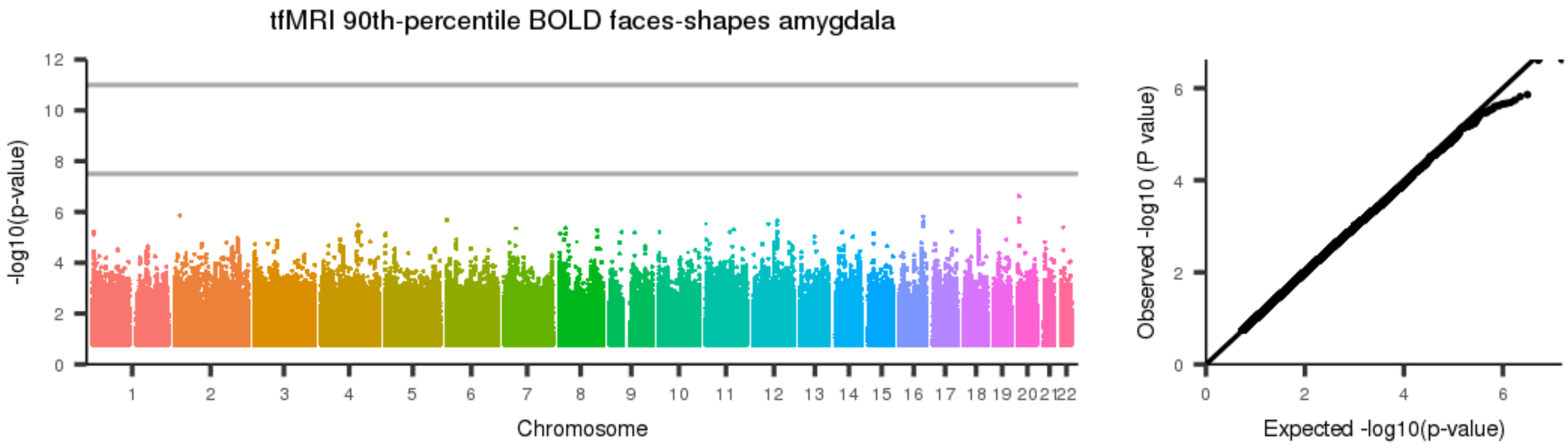

back to list of plots

Figure 194: tfMRI median zstat faces-shapes amygdala

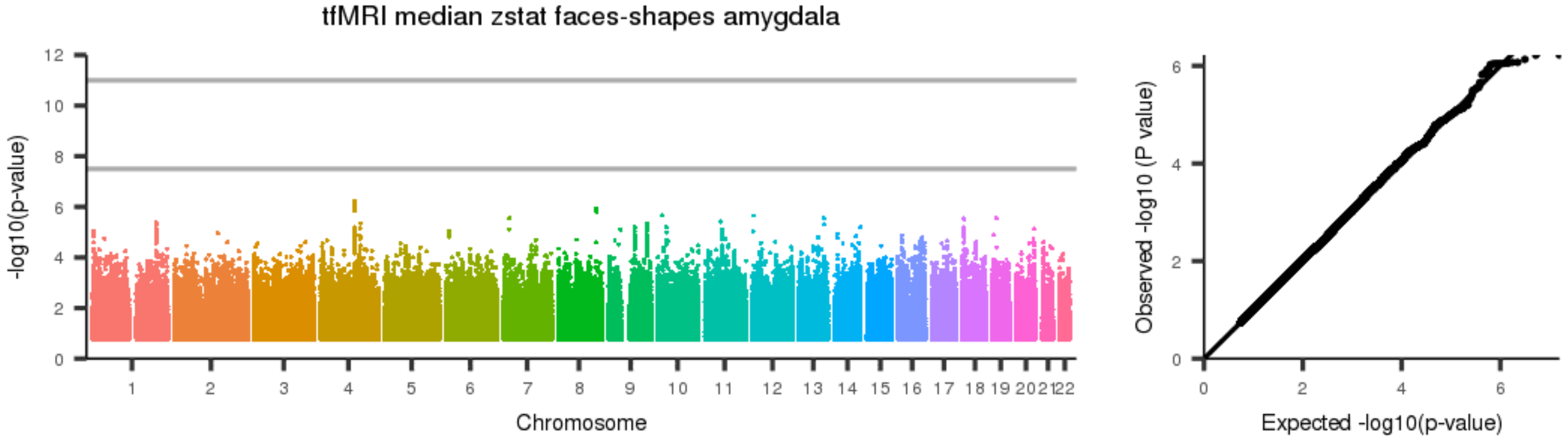

back to list of plots

Figure 195: tfMRI 90th-percentile zstat faces-shapes amygdala

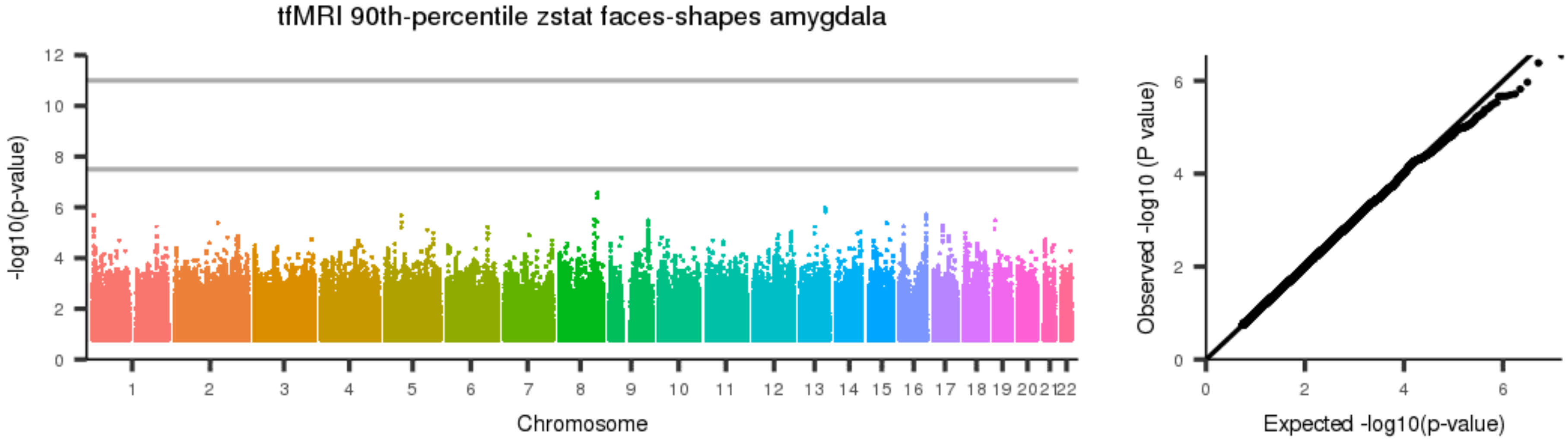

back to list of plots

Figure 196: dMRI TBSS FA Middle cerebellar peduncle

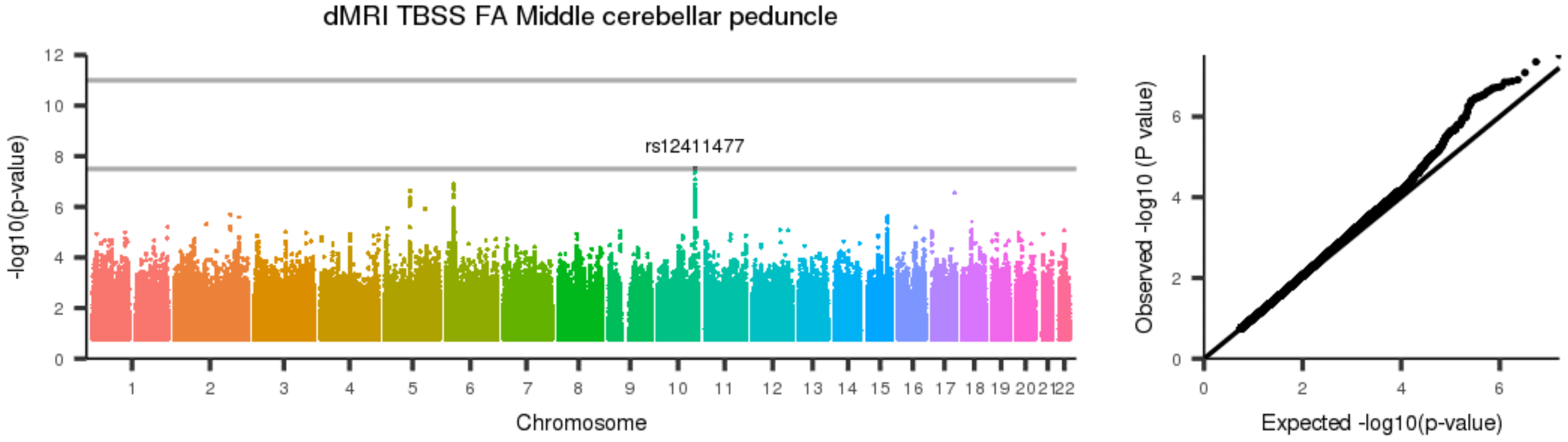

back to list of plots

Figure 197: dMRI TBSS FA Pontine crossing tract

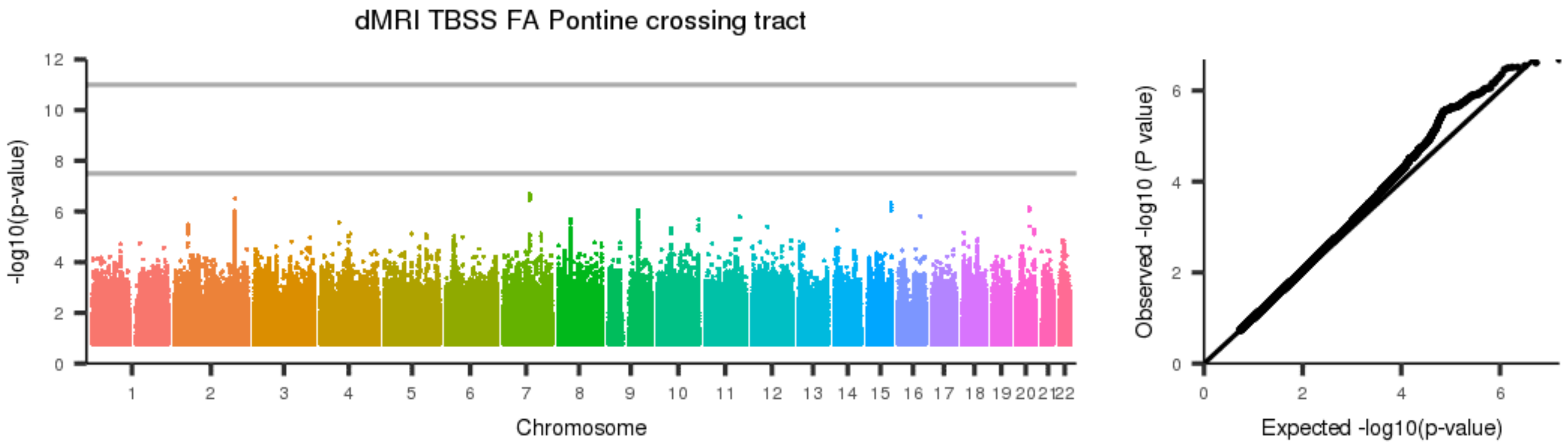

back to list of plots

Figure 198: dMRI TBSS FA Genu of corpus callosum

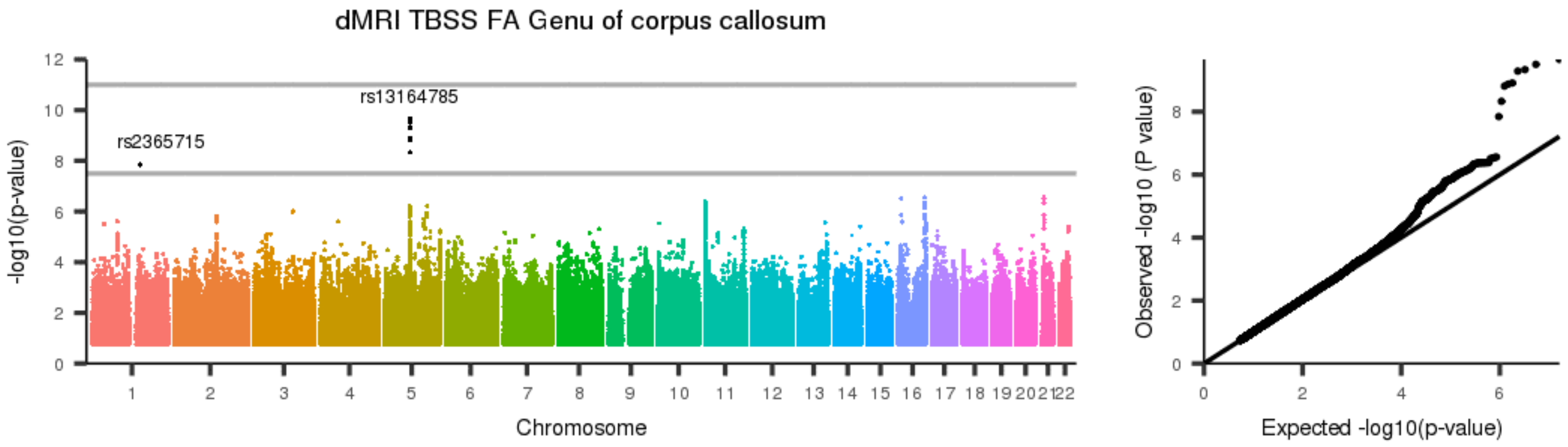

back to list of plots

Figure 199: dMRI TBSS FA Body of corpus callosum

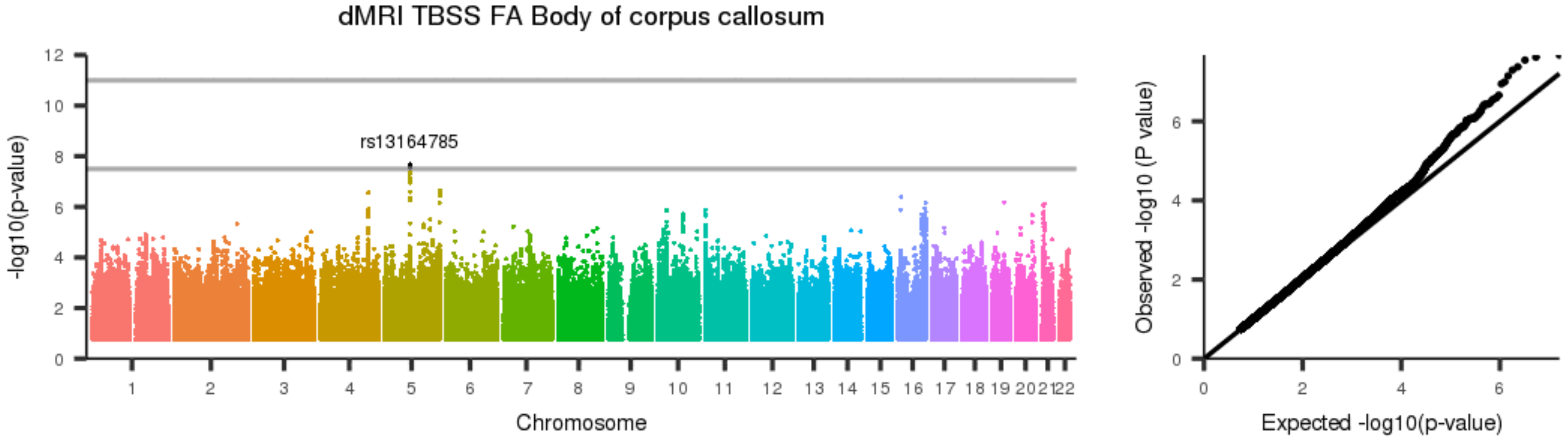

back to list of plots

Figure 200: dMRI TBSS FA Splenium of corpus callosum

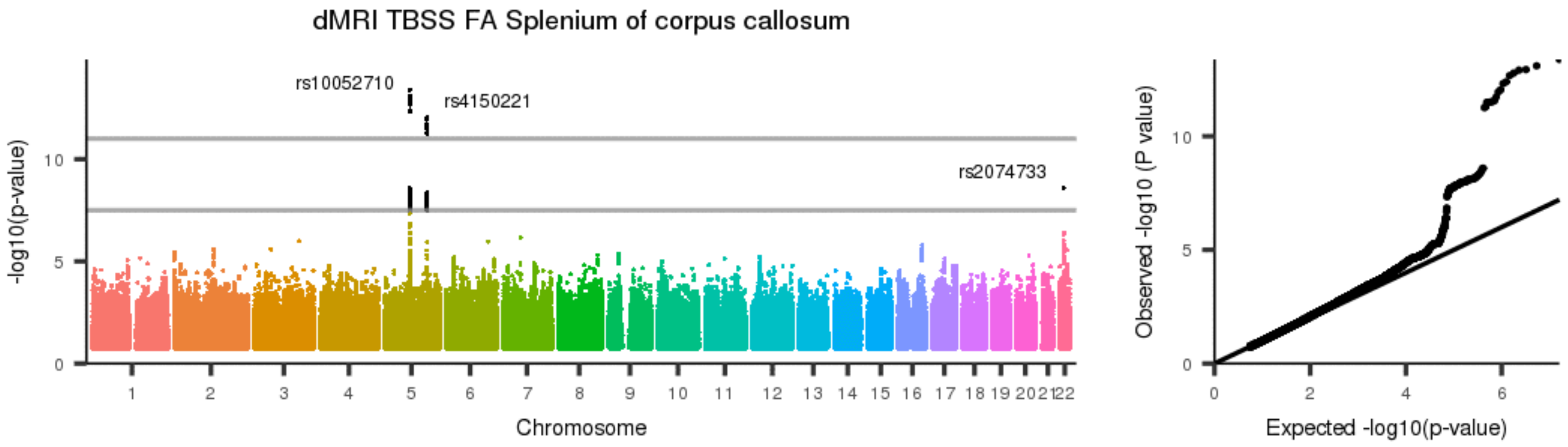

back to list of plots

Figure 201: dMRI TBSS FA Fornix

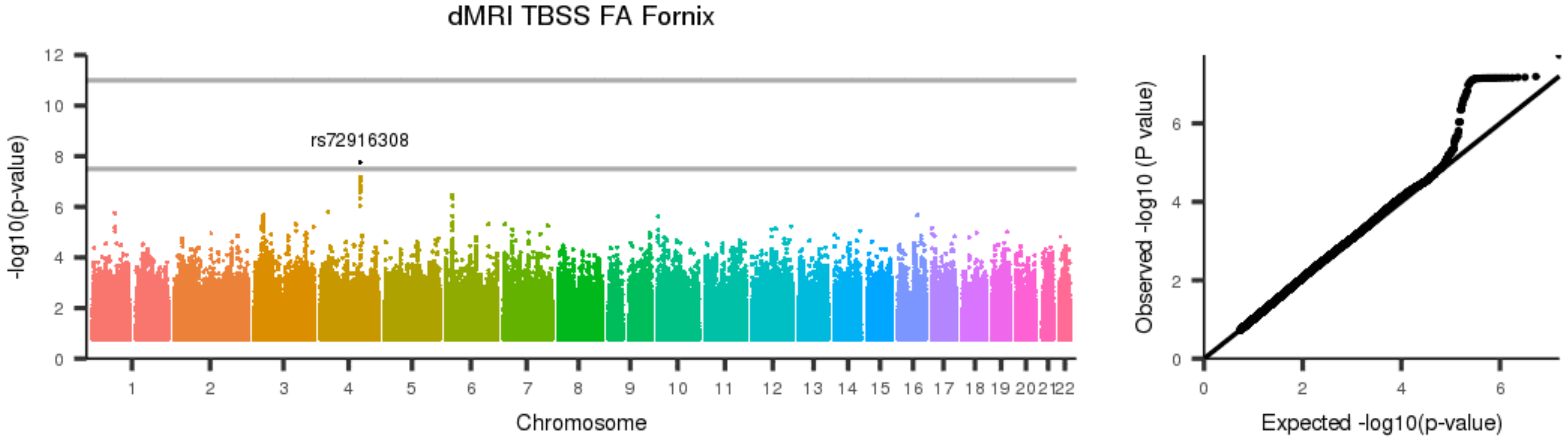

back to list of plots

Figure 202: dMRI TBSS FA Corticospinal tract R

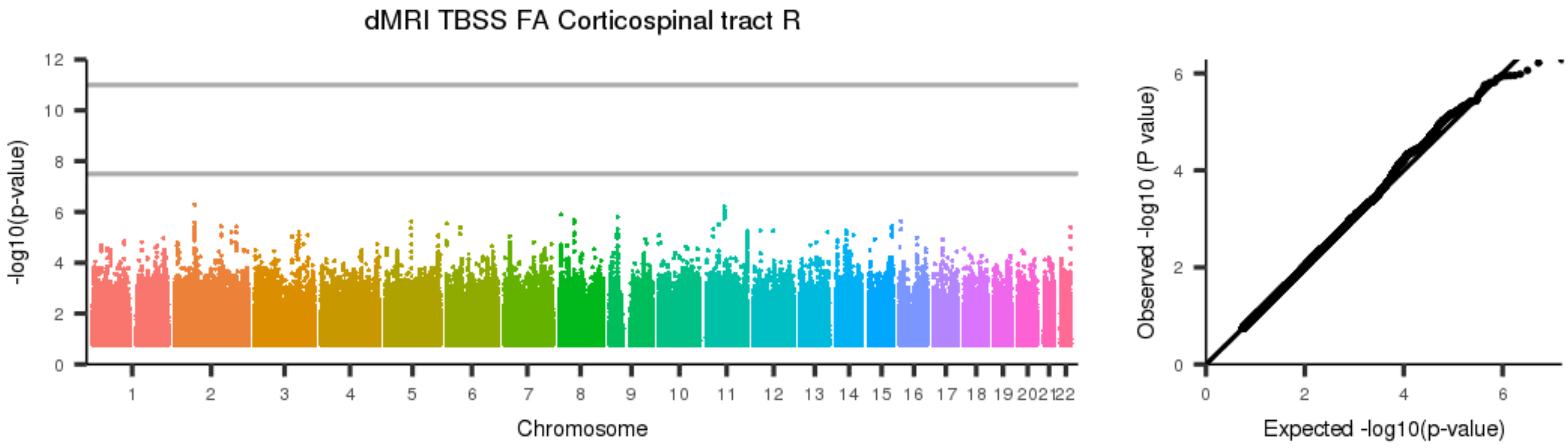

back to list of plots

Figure 203: dMRI TBSS FA Corticospinal tract L

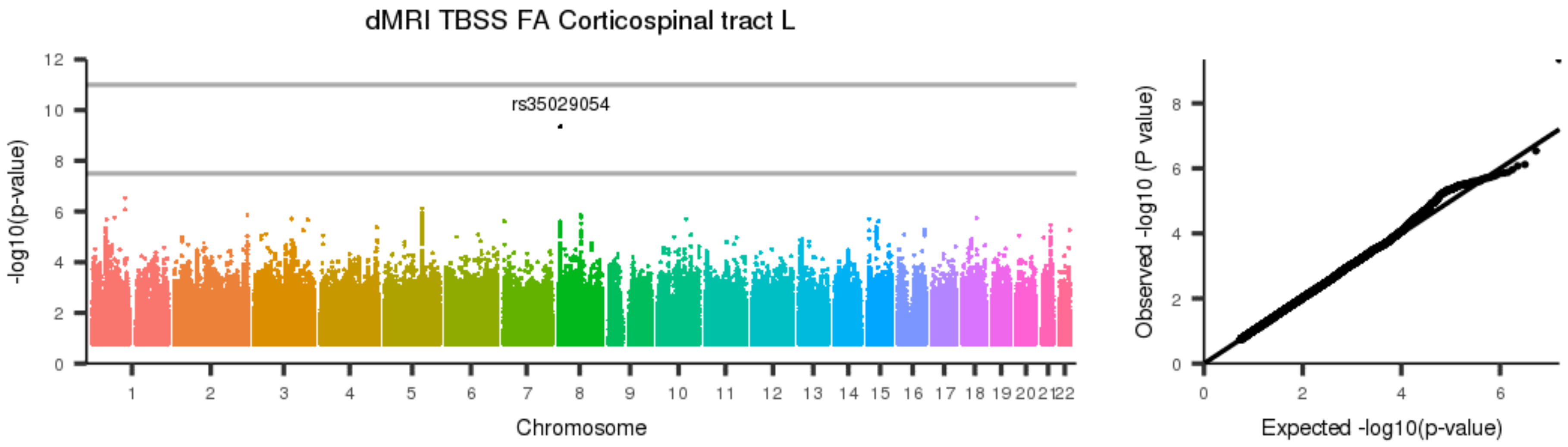

back to list of plots

Figure 204: dMRI TBSS FA Medial lemniscus R

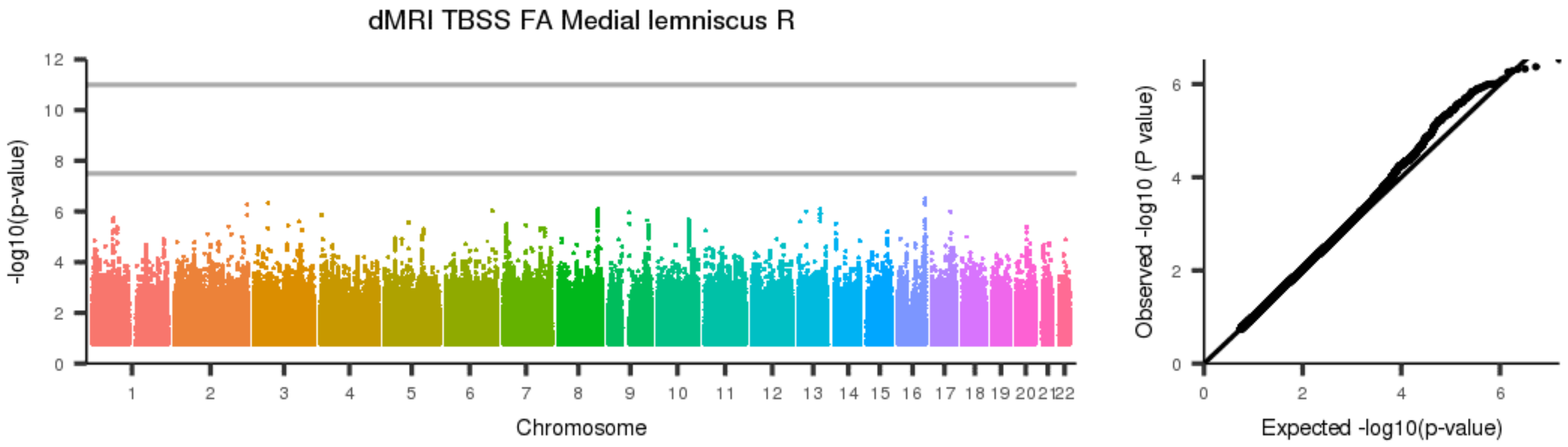

back to list of plots

Figure 205: dMRI TBSS FA Medial lemniscus L

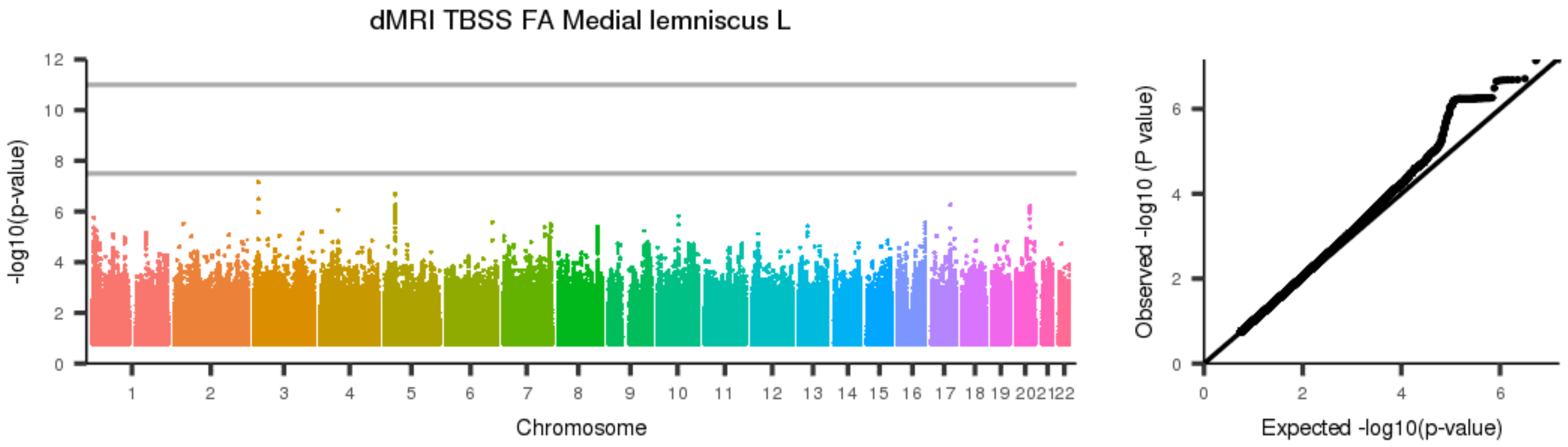

back to list of plots

Figure 206: dMRI TBSS FA Inferior cerebellar peduncle R

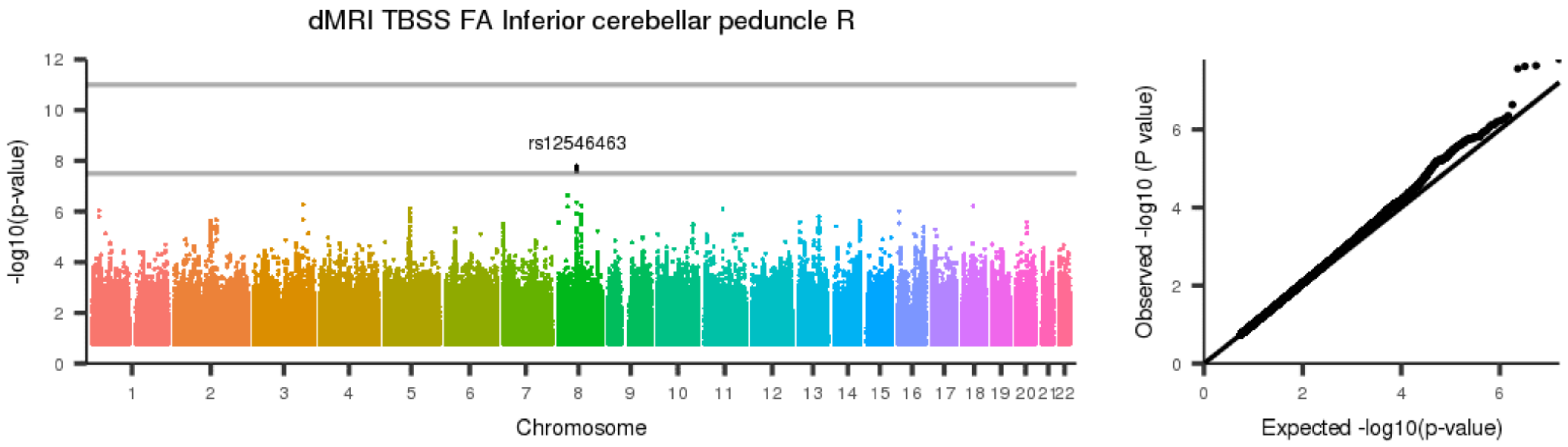

back to list of plots

Figure 207: dMRI TBSS FA Inferior cerebellar peduncle L

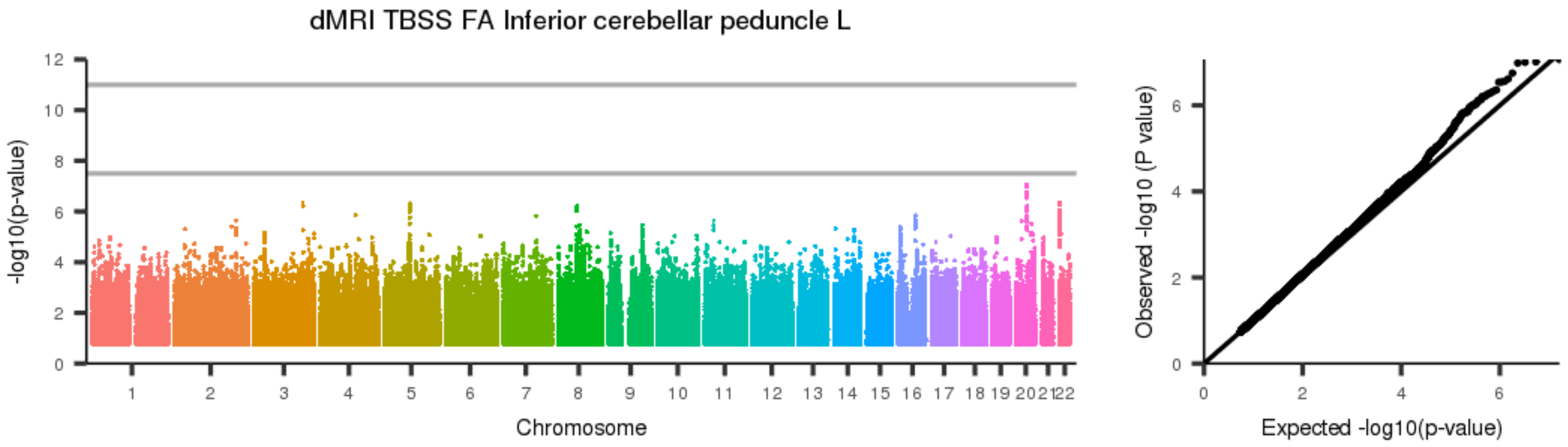

back to list of plots

Figure 208: dMRI TBSS FA Superior cerebellar peduncle R

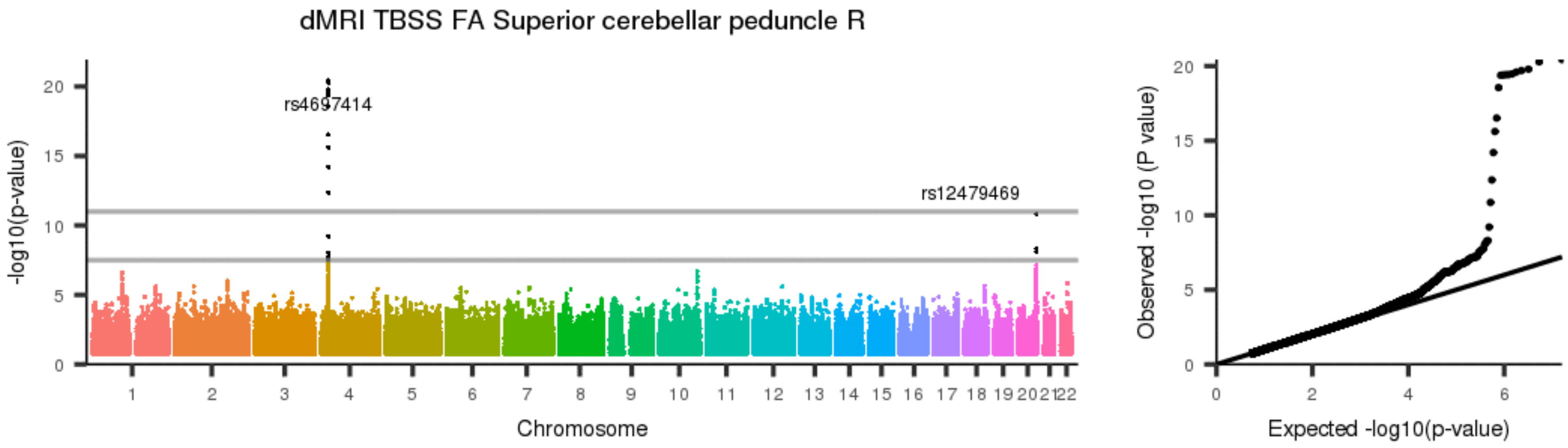

back to list of plots

Figure 209: dMRI TBSS FA Superior cerebellar peduncle L

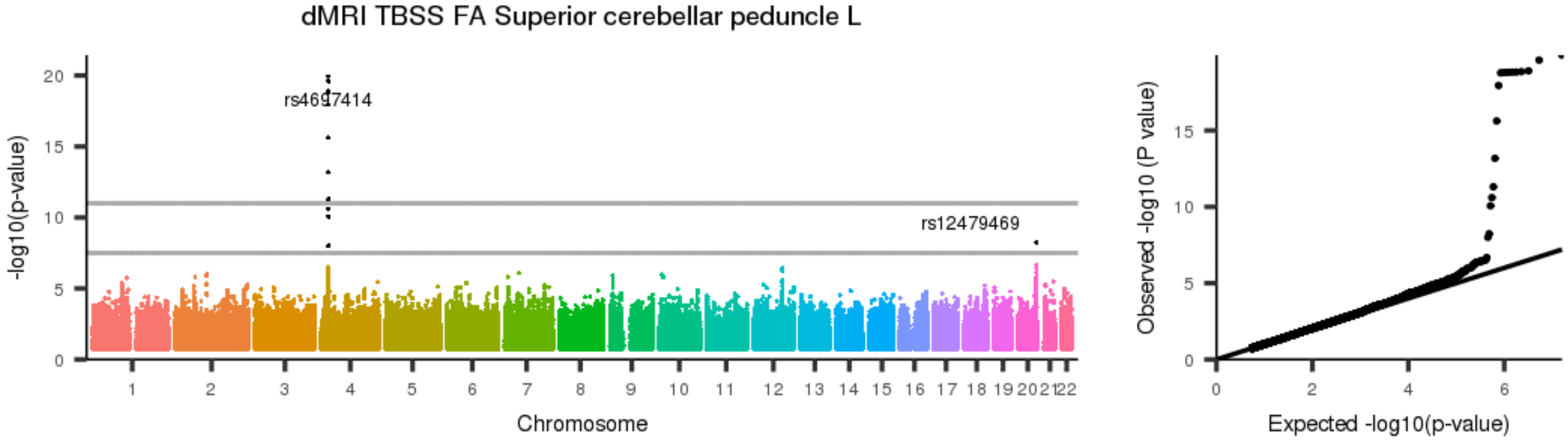

back to list of plots

Figure 210: dMRI TBSS FA Cerebral peduncle R

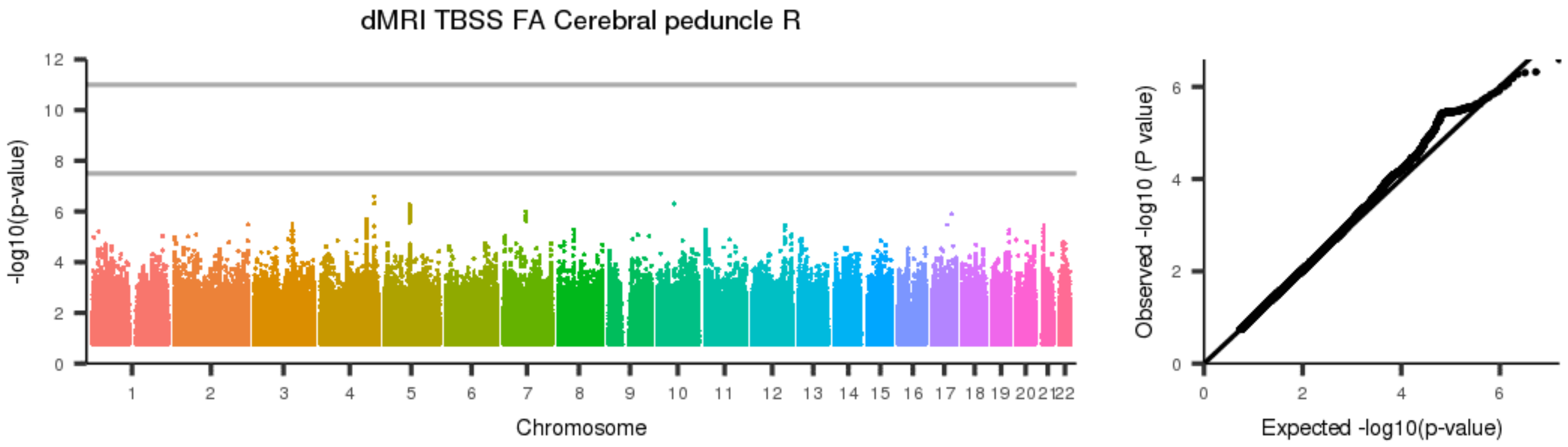

back to list of plots

Figure 211: dMRI TBSS FA Cerebral peduncle L

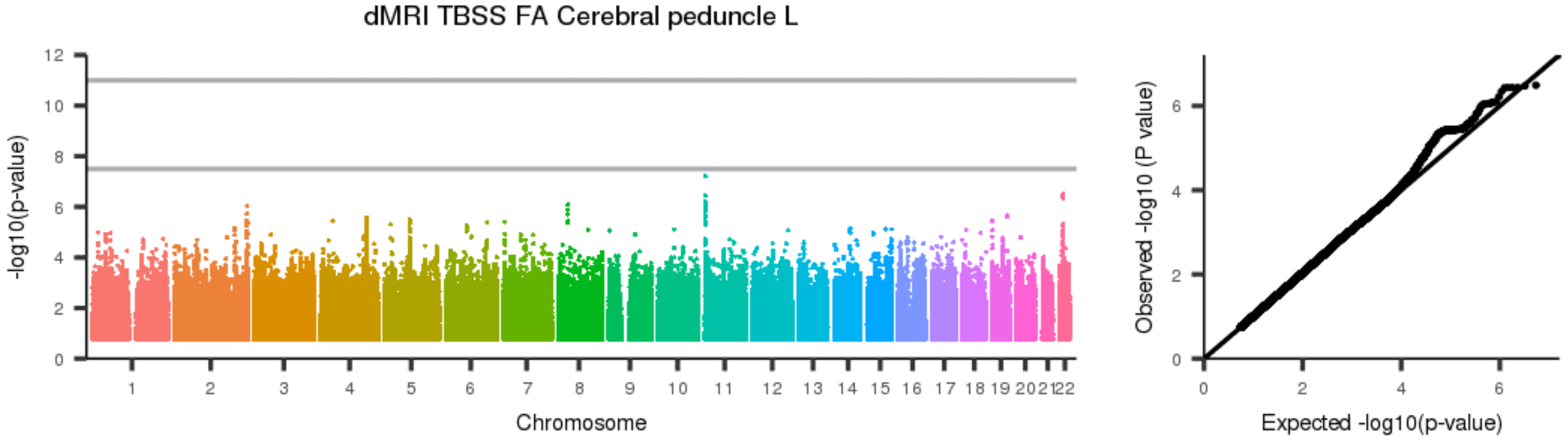

back to list of plots

Figure 212: dMRI TBSS FA Anterior limb of internal capsule R

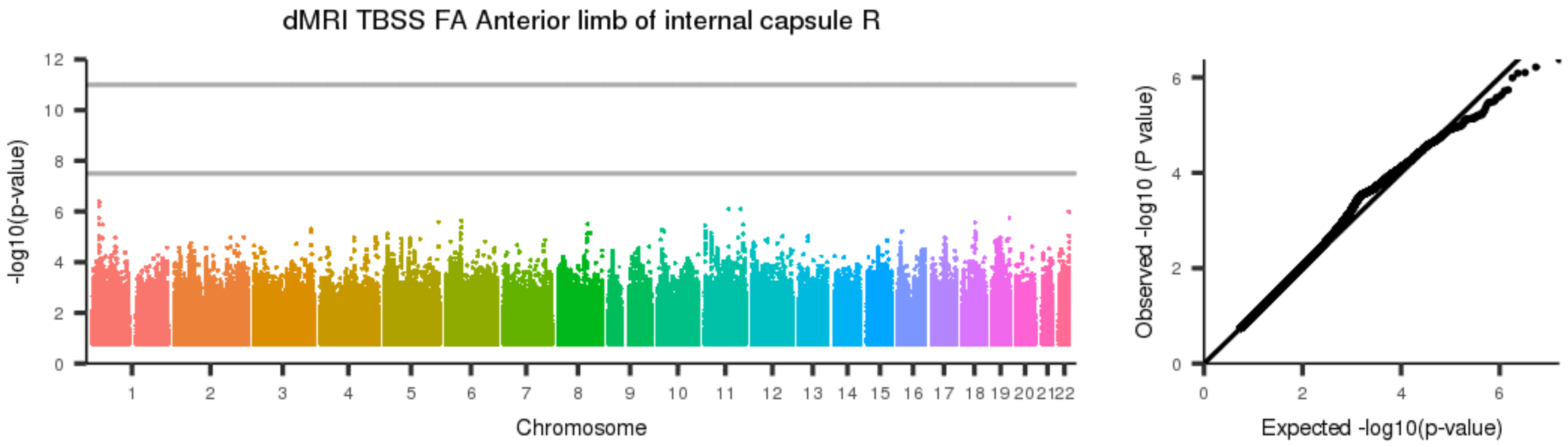

back to list of plots

Figure 213: dMRI TBSS FA Anterior limb of internal capsule L

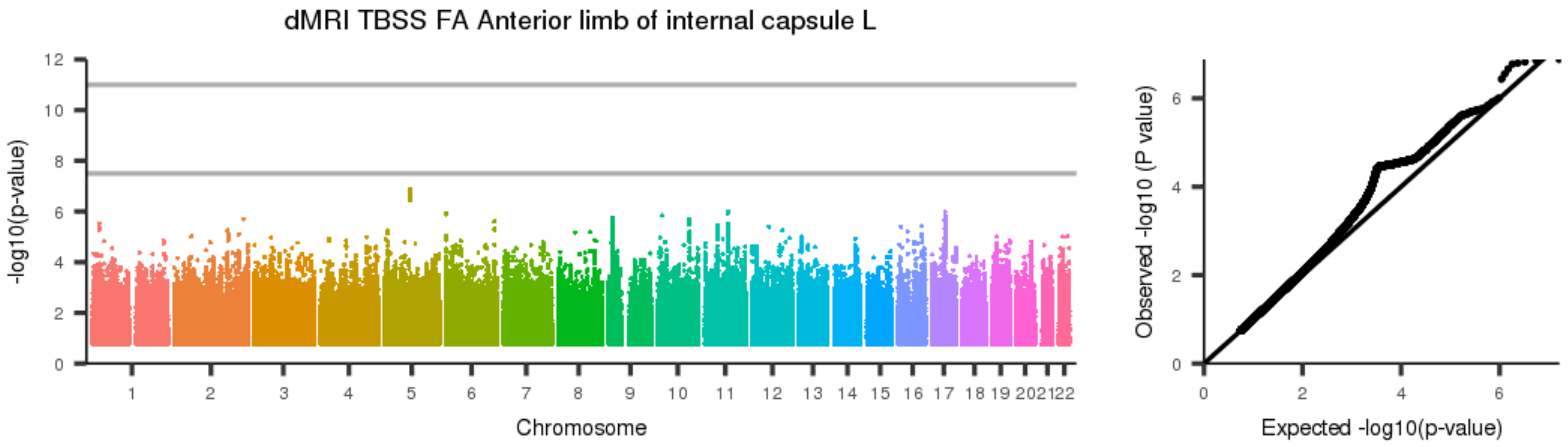

back to list of plots

Figure 214: dMRI TBSS FA Posterior limb of internal capsule R

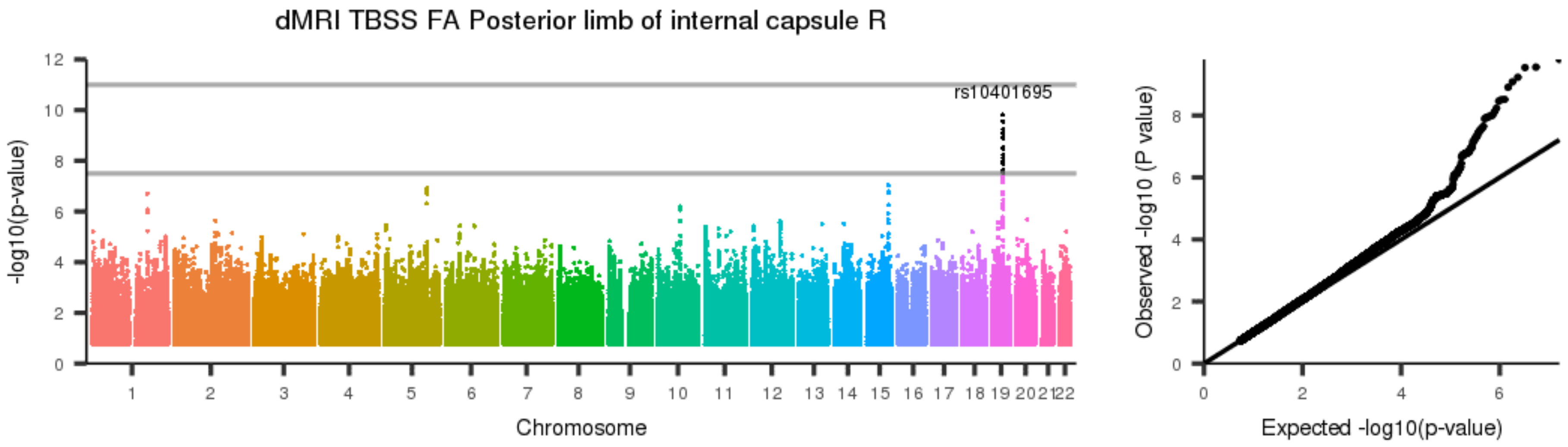

back to list of plots

Figure 215: dMRI TBSS FA Posterior limb of internal capsule L

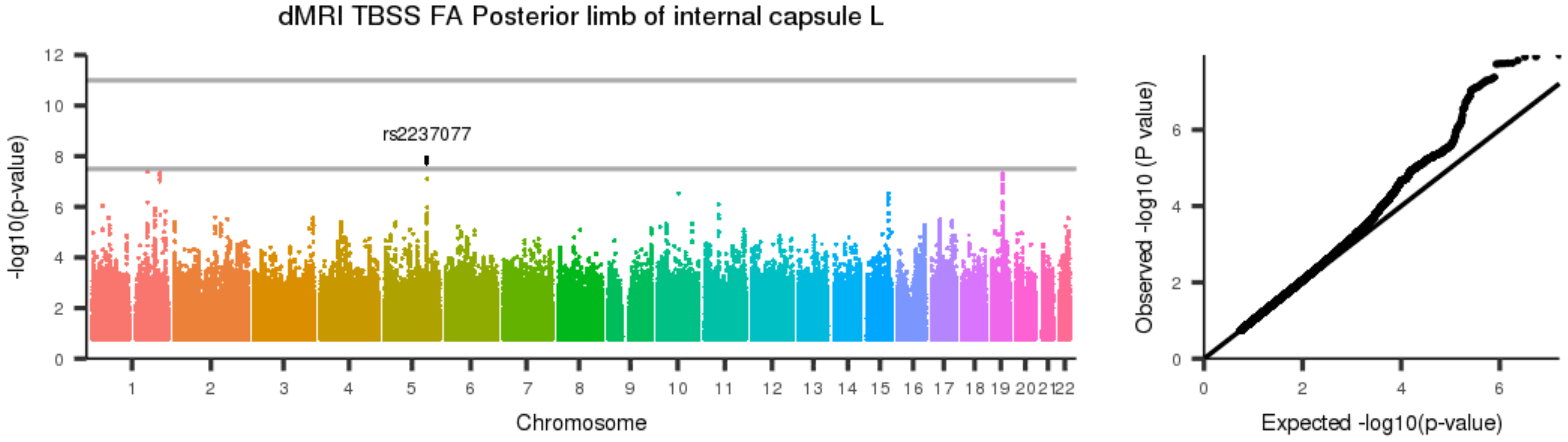

back to list of plots

Figure 216: dMRI TBSS FA Retrolenticular part of internal capsule R

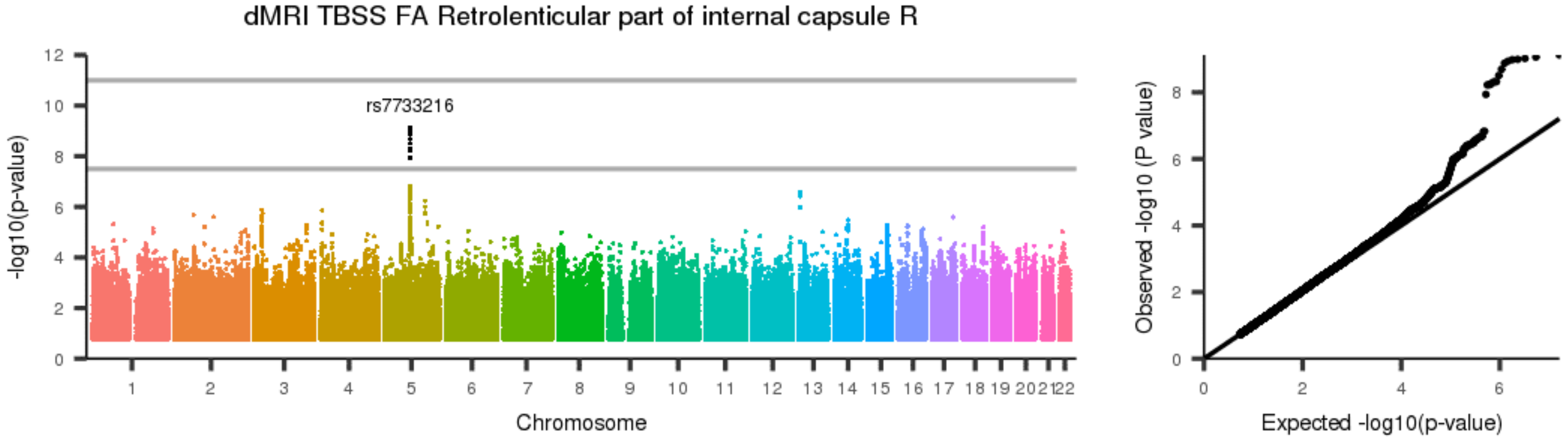

back to list of plots

Figure 217: dMRI TBSS FA Retrolenticular part of internal capsule L

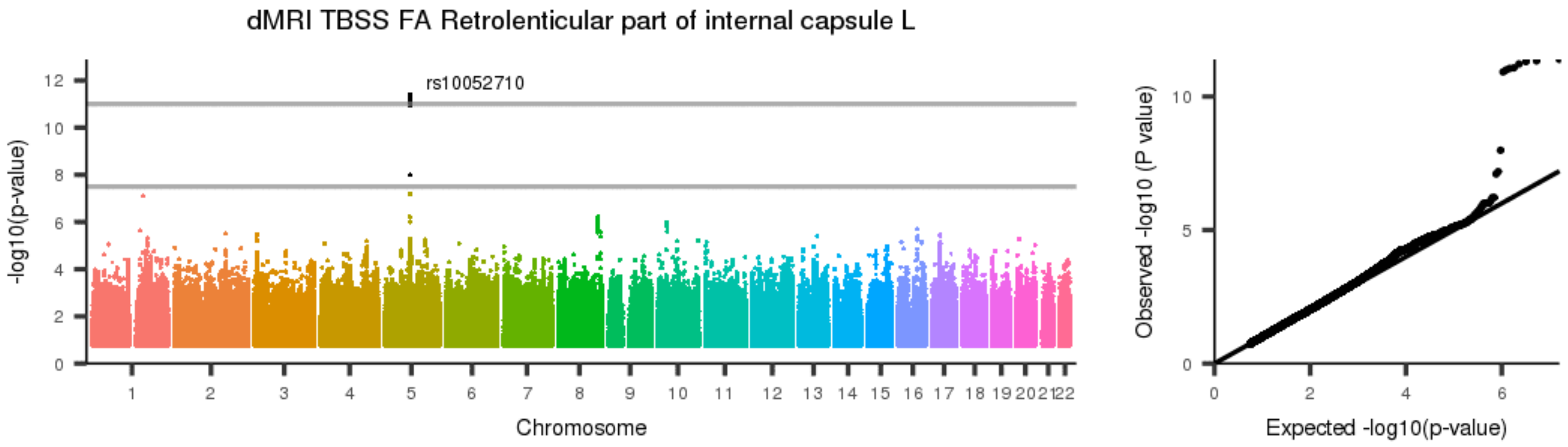

back to list of plots

Figure 218: dMRI TBSS FA Anterior corona radiata R

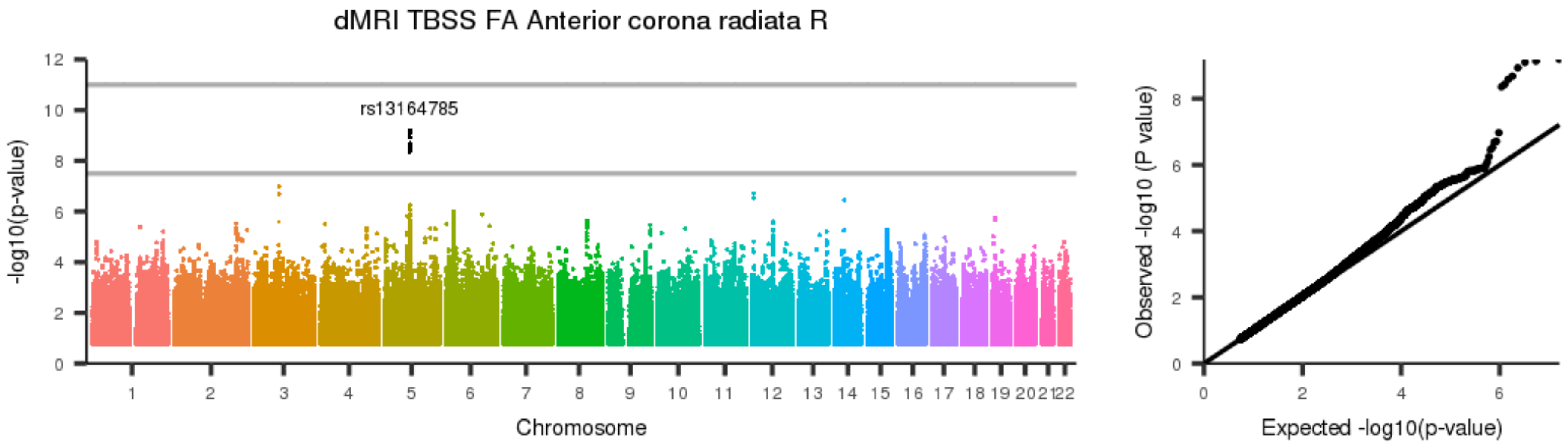

back to list of plots

Figure 219: dMRI TBSS FA Anterior corona radiata L

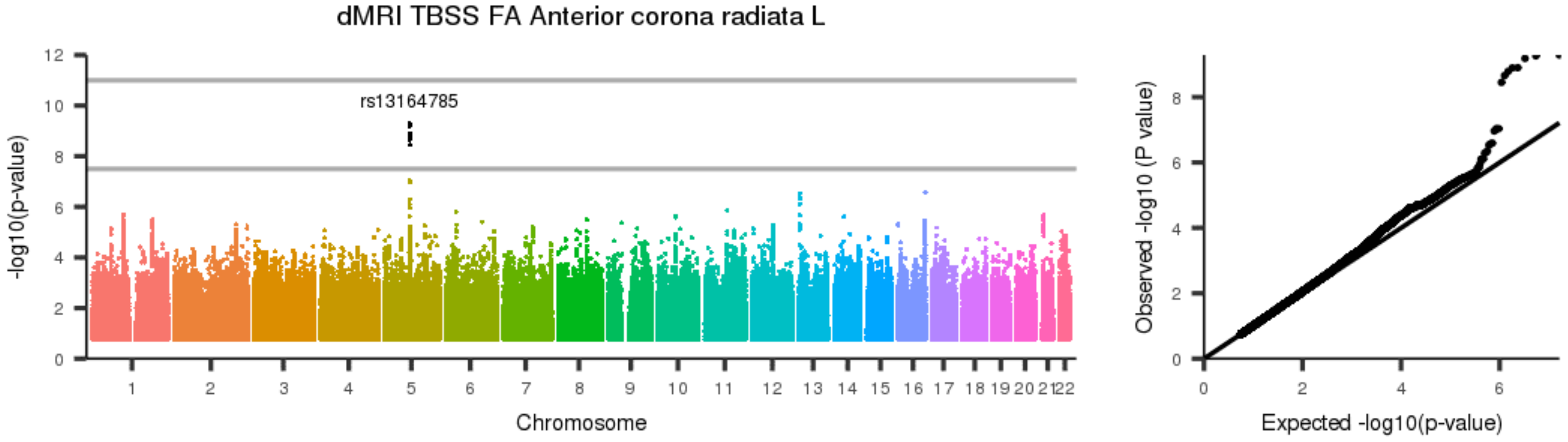

back to list of plots

Figure 220: dMRI TBSS FA Superior corona radiata R

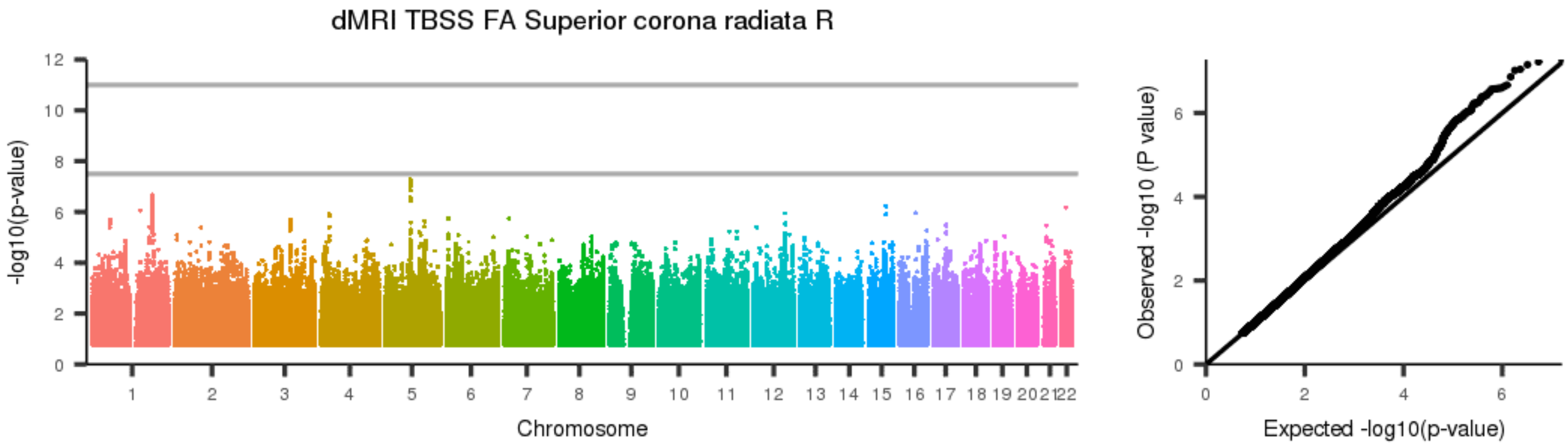

back to list of plots

Figure 221: dMRI TBSS FA Superior corona radiata L

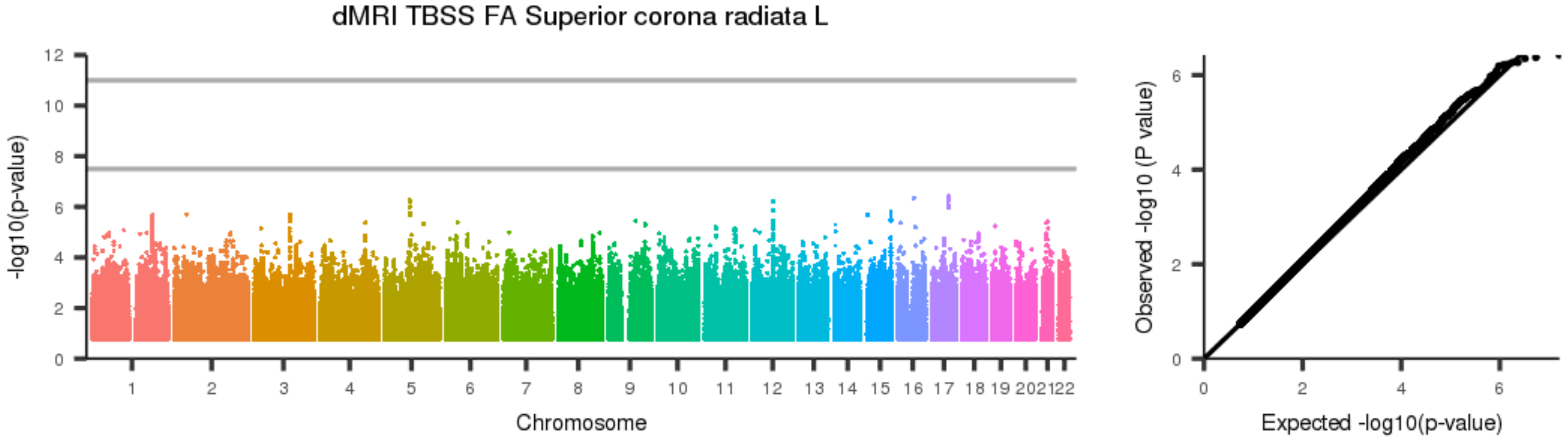

back to list of plots

Figure 222: dMRI TBSS FA Posterior corona radiata R

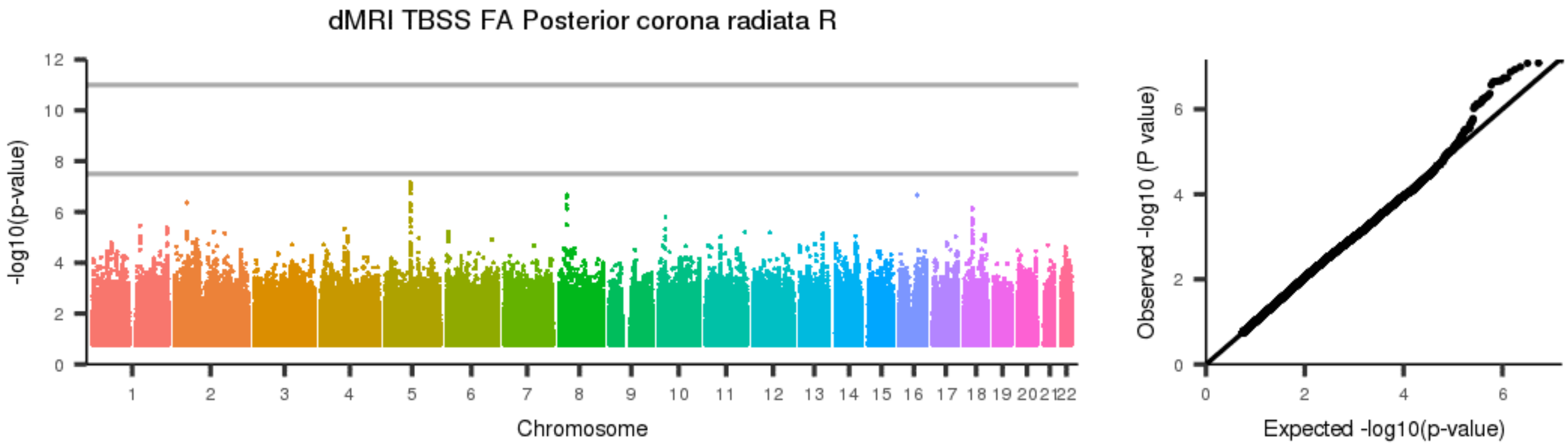

back to list of plots

Figure 223: dMRI TBSS FA Posterior corona radiata L

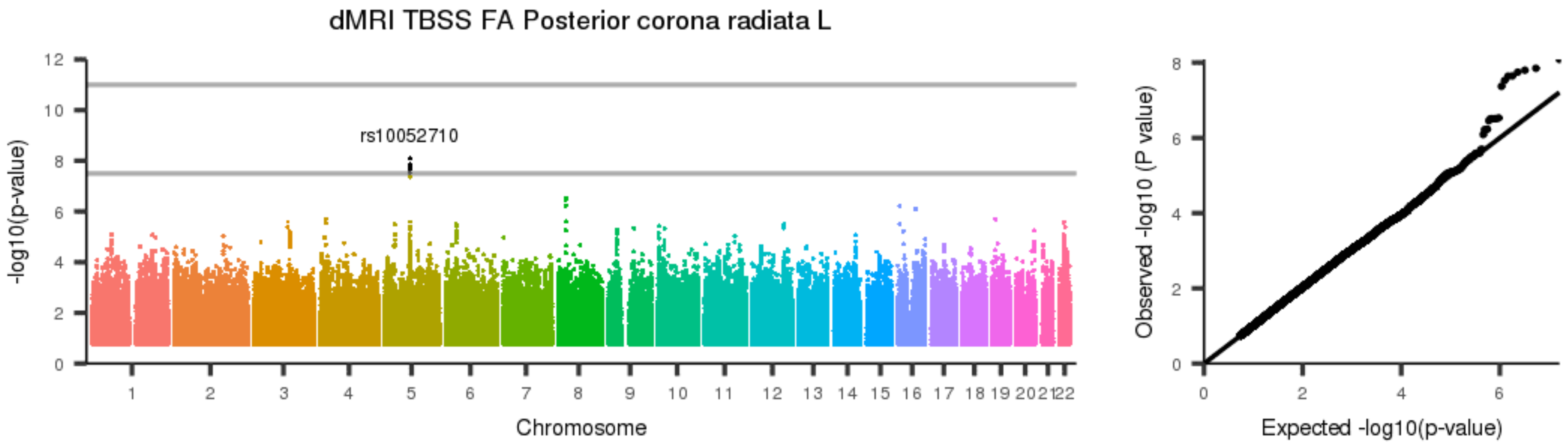

back to list of plots

Figure 224: dMRI TBSS FA Posterior thalamic radiation R

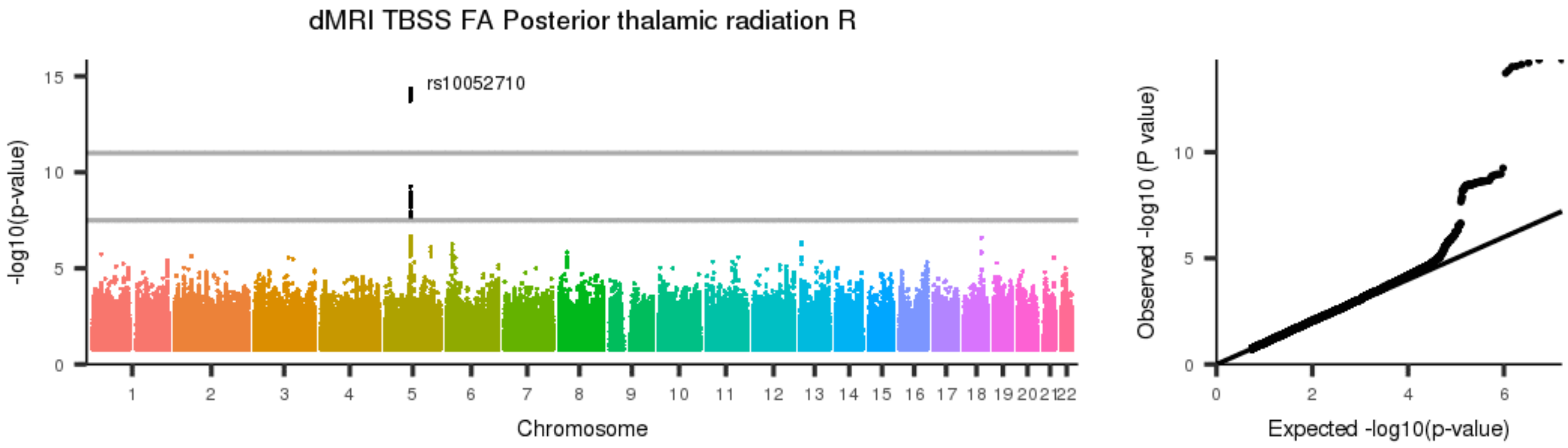

back to list of plots

Figure 225: dMRI TBSS FA Posterior thalamic radiation L

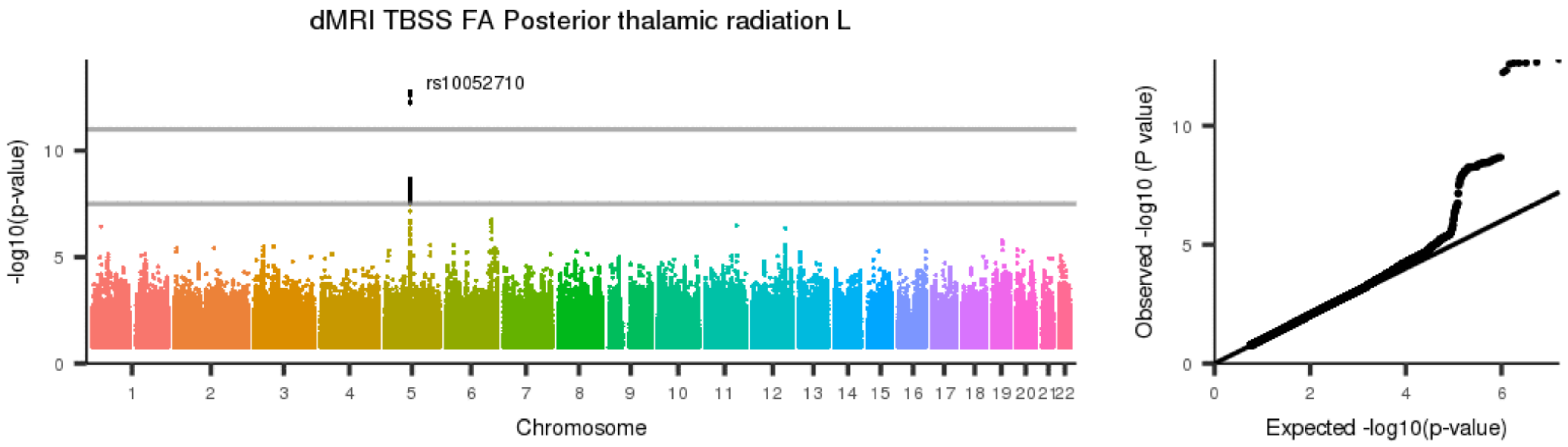

back to list of plots

Figure 226: dMRI TBSS FA Sagittal stratum R

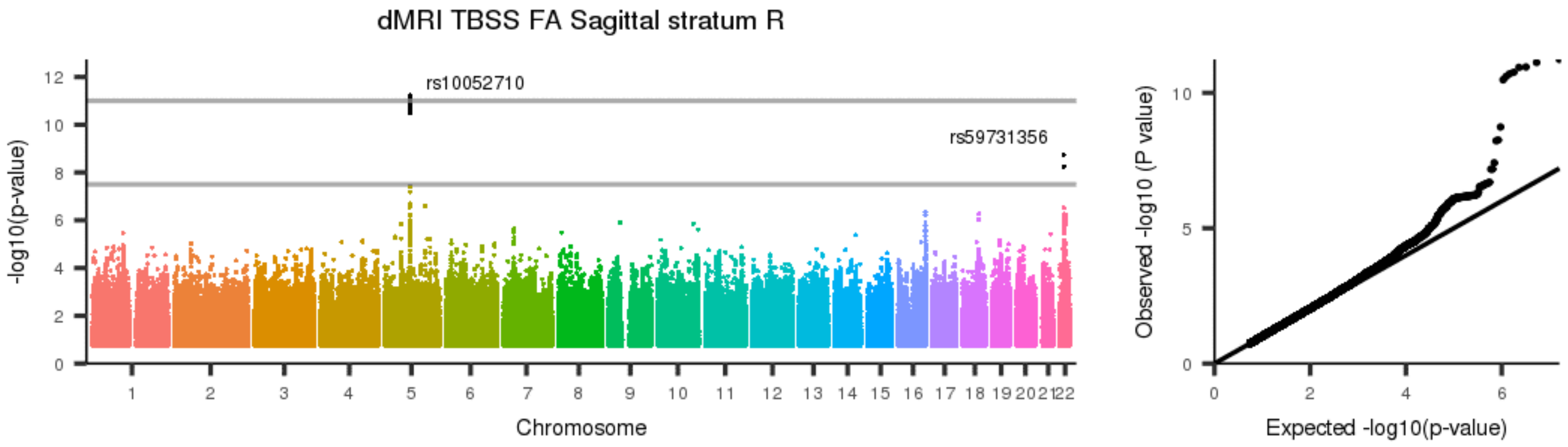

back to list of plots

Figure 227: dMRI TBSS FA Sagittal stratum L

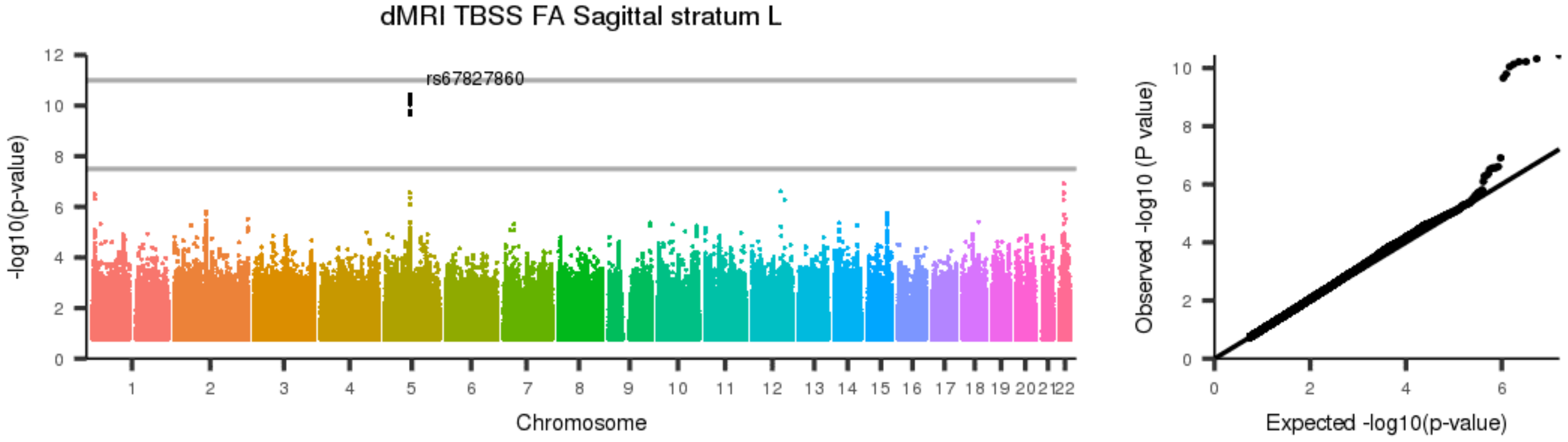

back to list of plots

Figure 228: dMRI TBSS FA External capsule R

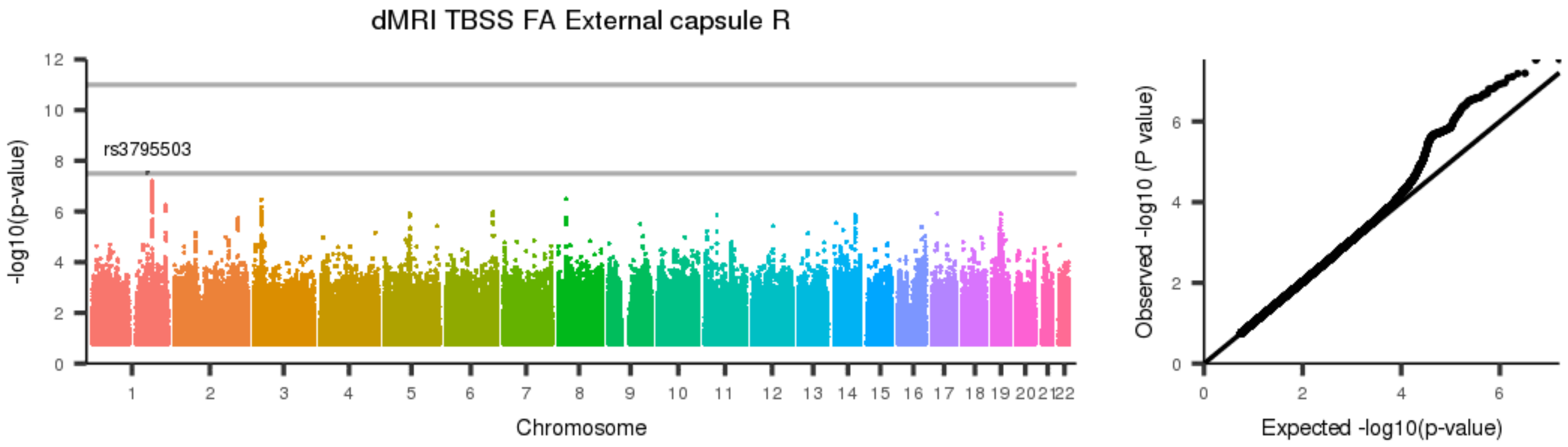

back to list of plots

Figure 229: dMRI TBSS FA External capsule L

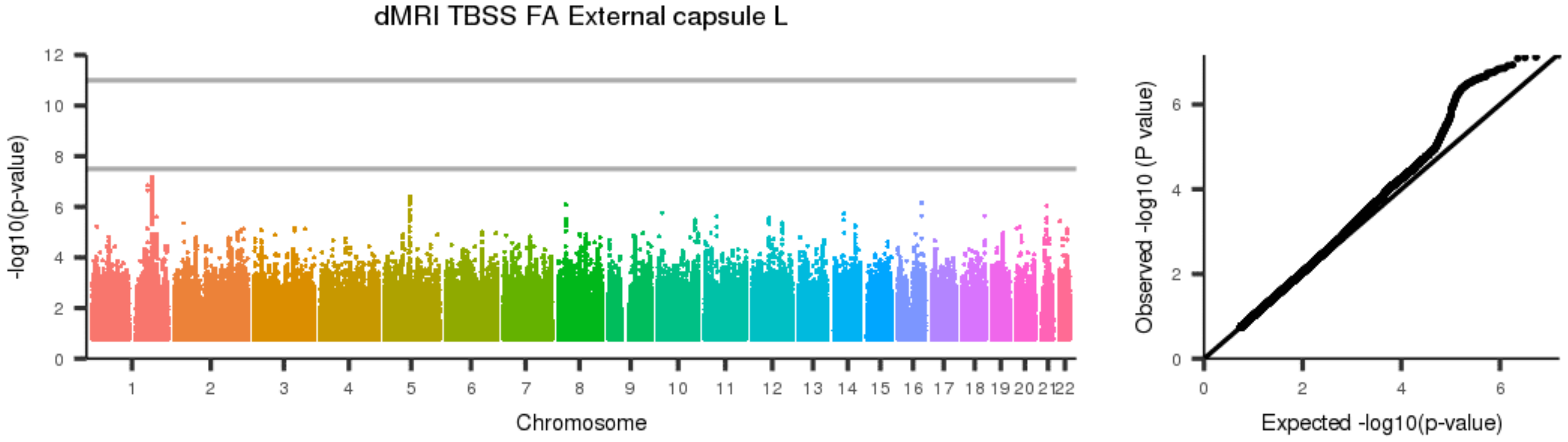

back to list of plots

Figure 230: dMRI TBSS FA Cingulum cingulate gyrus R

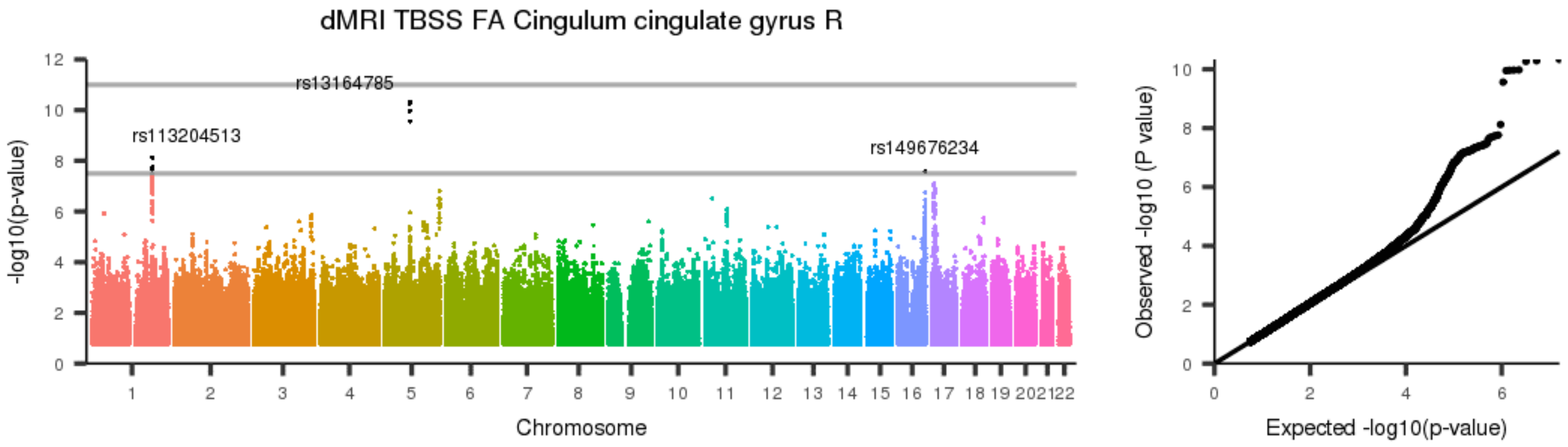

back to list of plots

Figure 231: dMRI TBSS FA Cingulum cingulate gyrus L

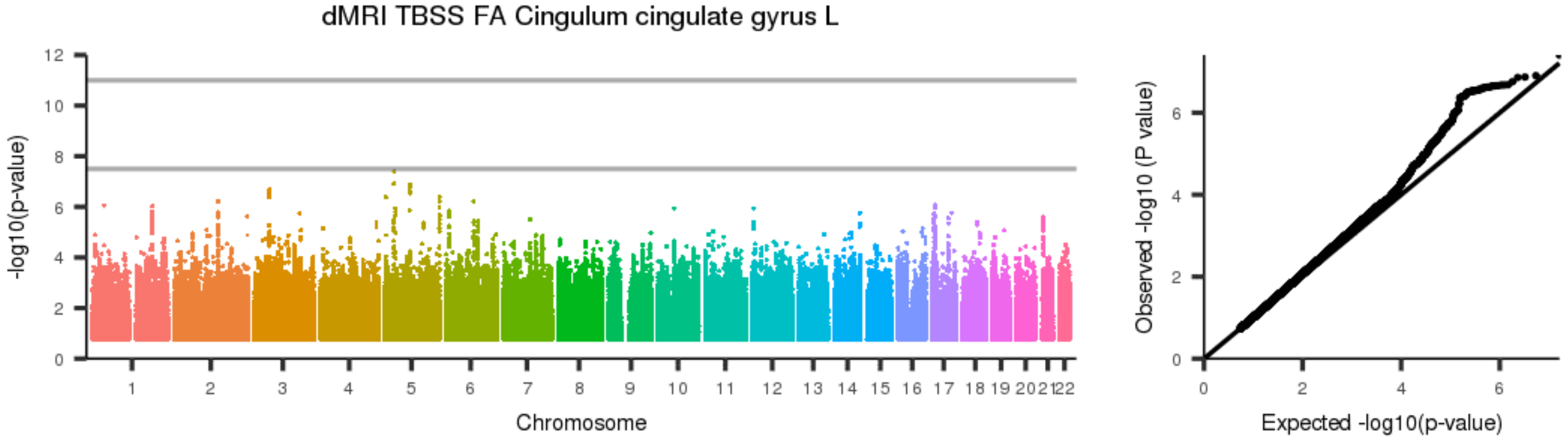

back to list of plots

Figure 232: dMRI TBSS FA Cingulum hippocampus R

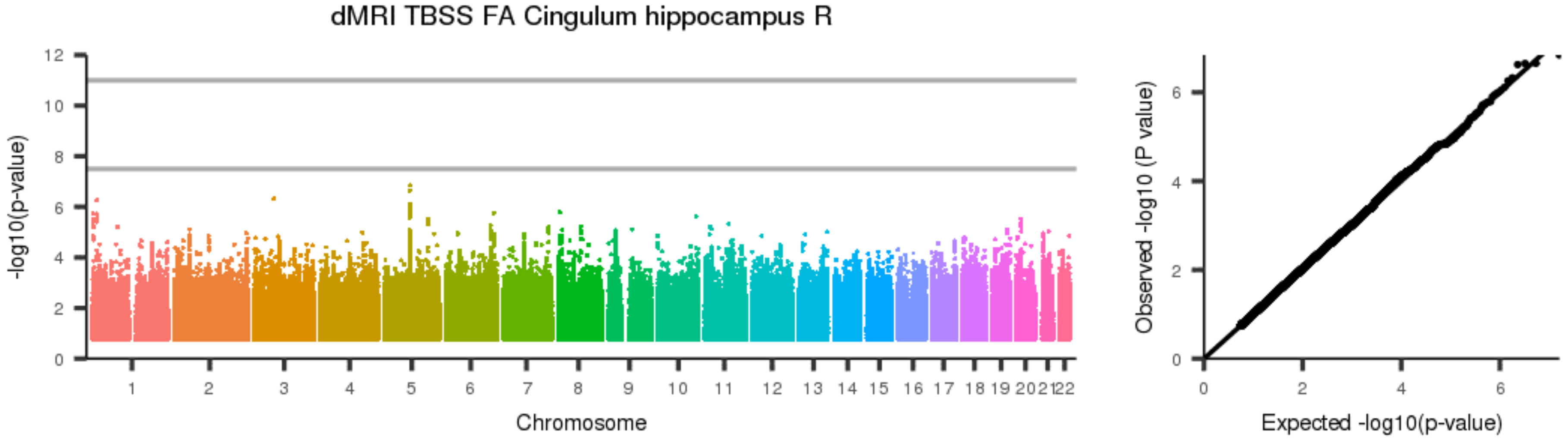

back to list of plots

Figure 233: dMRI TBSS FA Cingulum hippocampus L

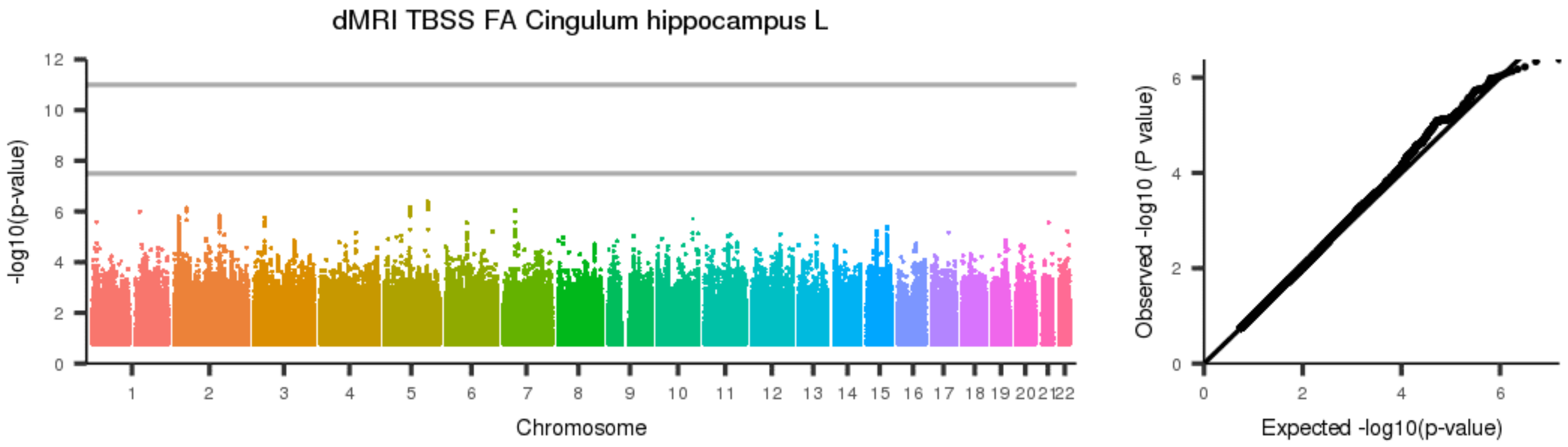

back to list of plots

Figure 234: dMRI TBSS FA Fornix cres+Stria terminalis R

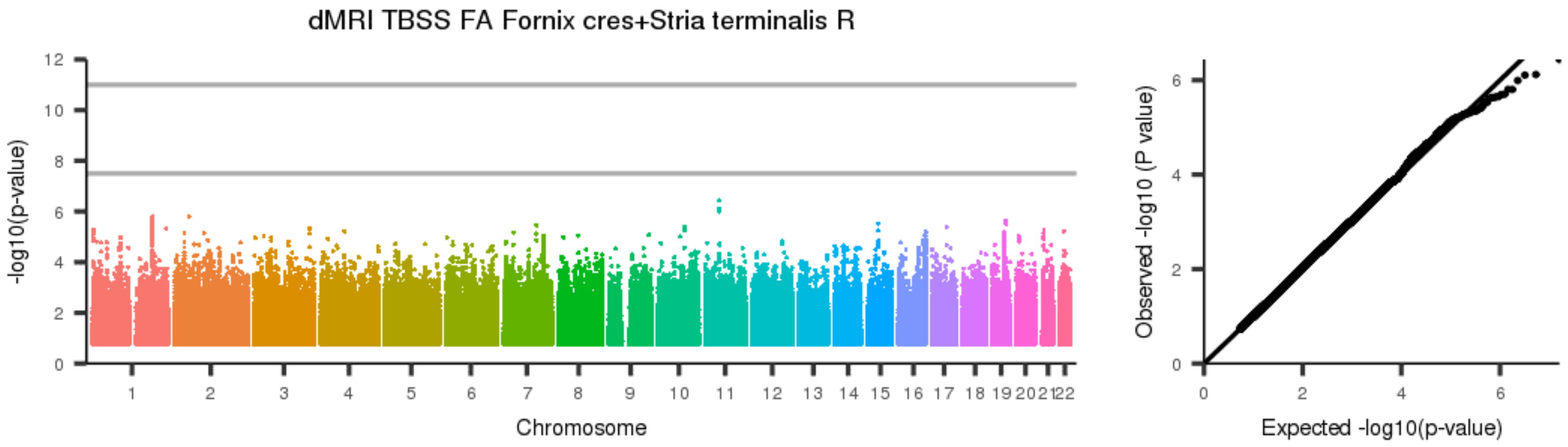

back to list of plots

Figure 235: dMRI TBSS FA Fornix cres+Stria terminalis L

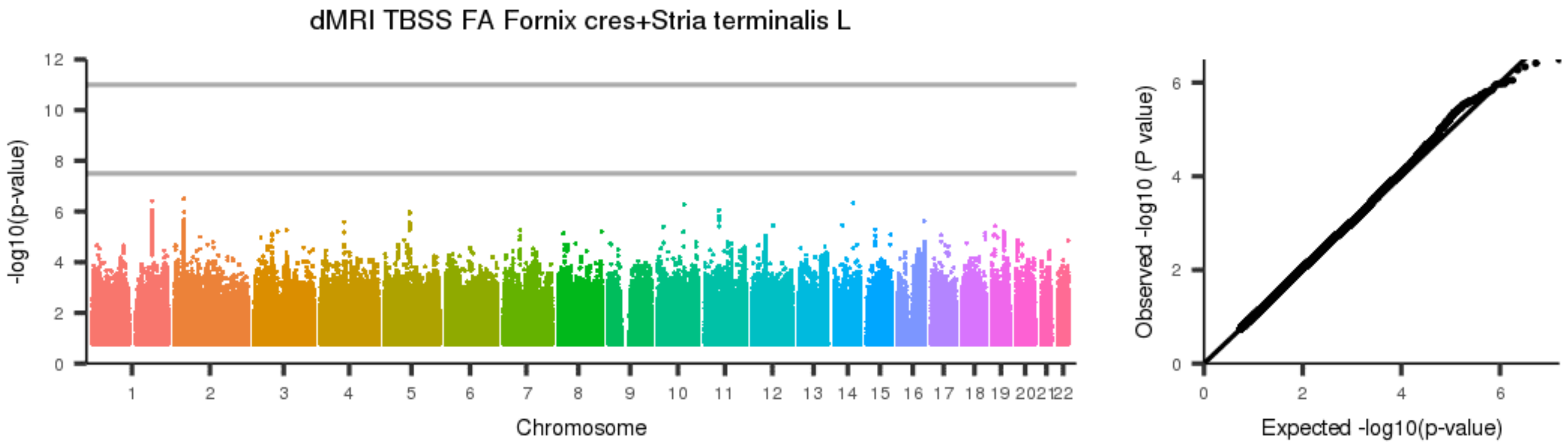

back to list of plots

Figure 236: dMRI TBSS FA Superior longitudinal fasciculus R

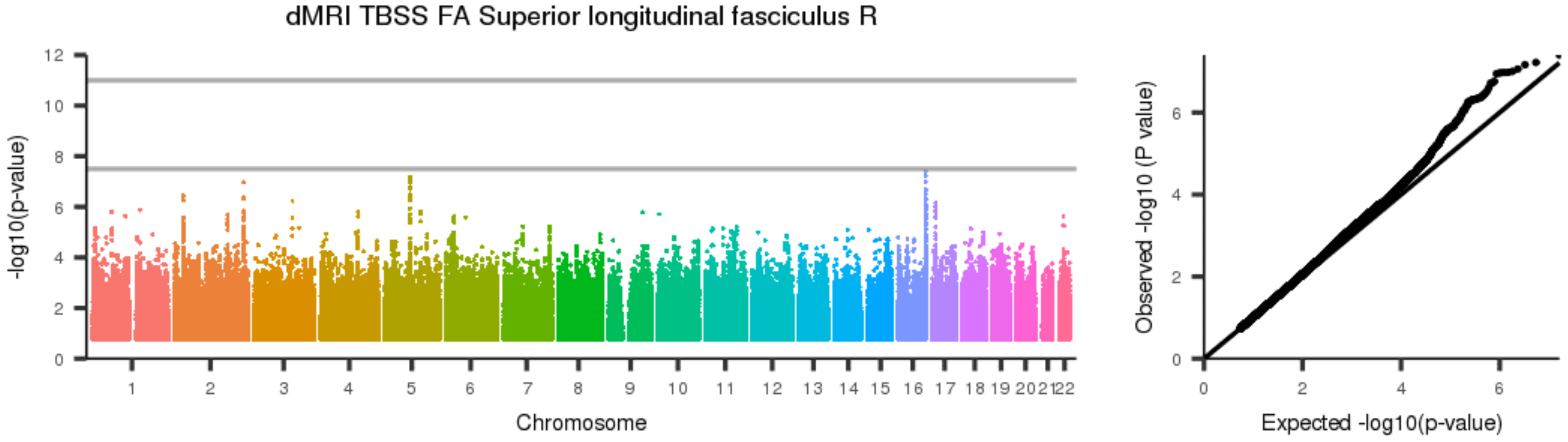

back to list of plots

Figure 237: dMRI TBSS FA Superior longitudinal fasciculus L

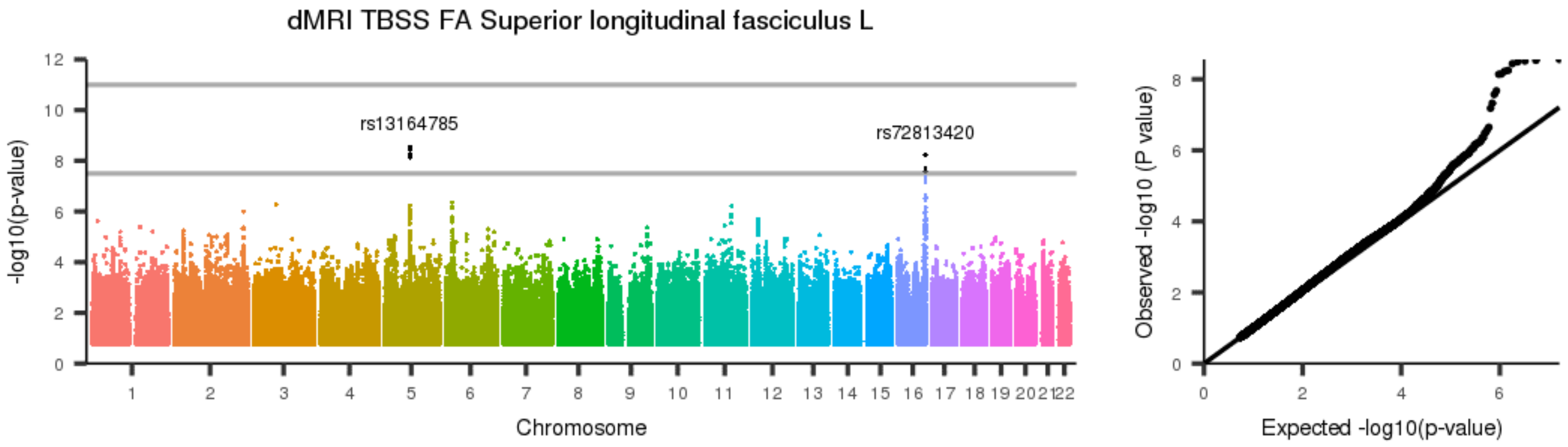

back to list of plots

Figure 238: dMRI TBSS FA Superior fronto-occipital fasciculus R

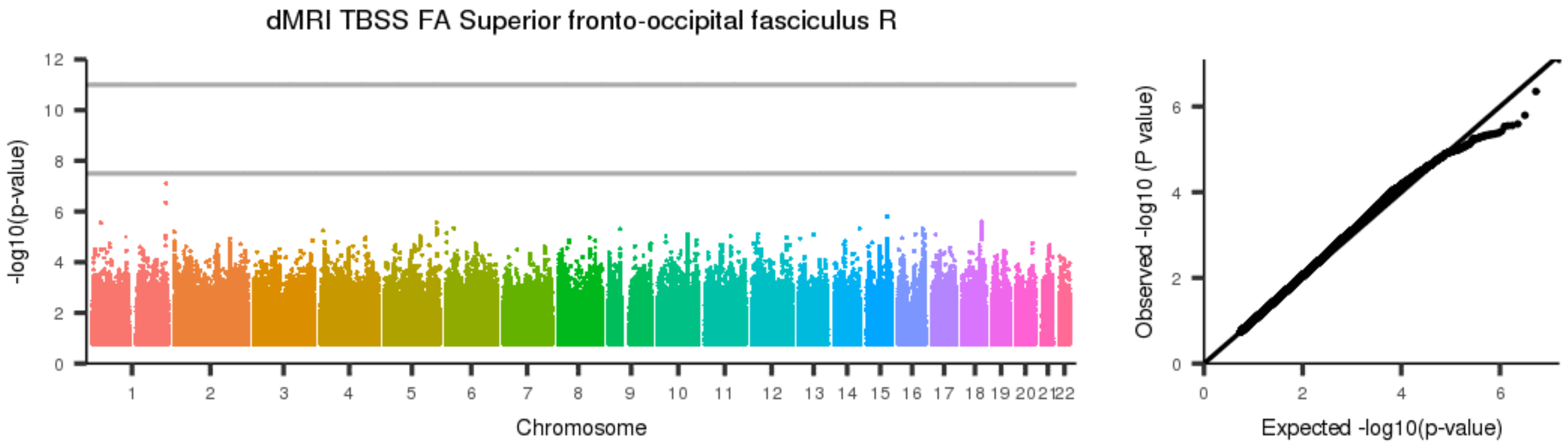

back to list of plots

Figure 239: dMRI TBSS FA Superior fronto-occipital fasciculus L

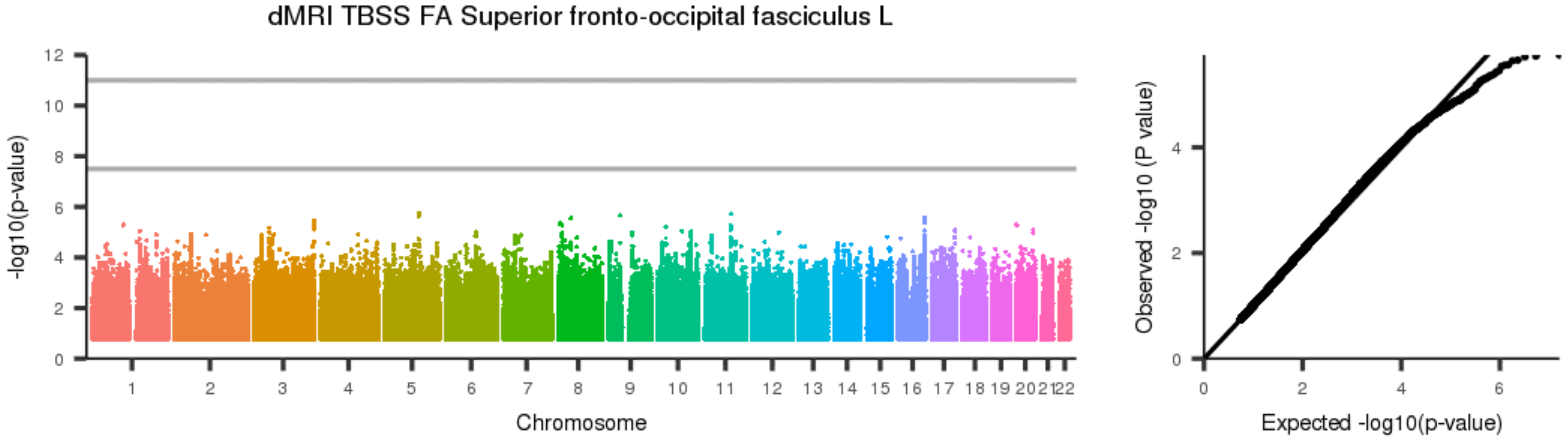

back to list of plots

Figure 240: dMRI TBSS FA Uncinate fasciculus R

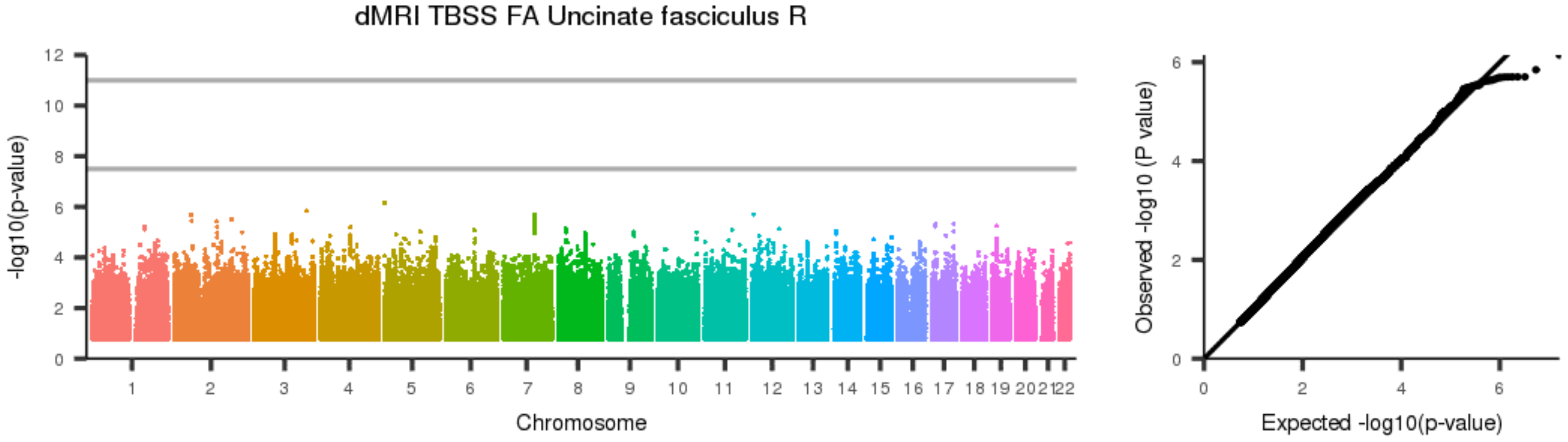

back to list of plots

Figure 241: dMRI TBSS FA Uncinate fasciculus L

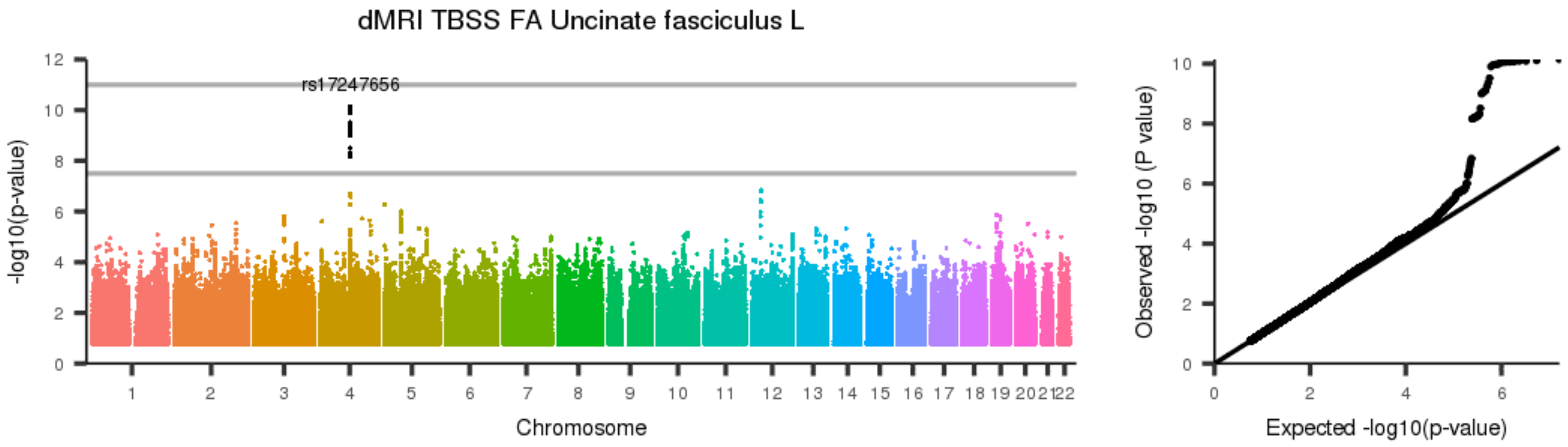

back to list of plots

Figure 242: dMRI TBSS FA Tapetum R

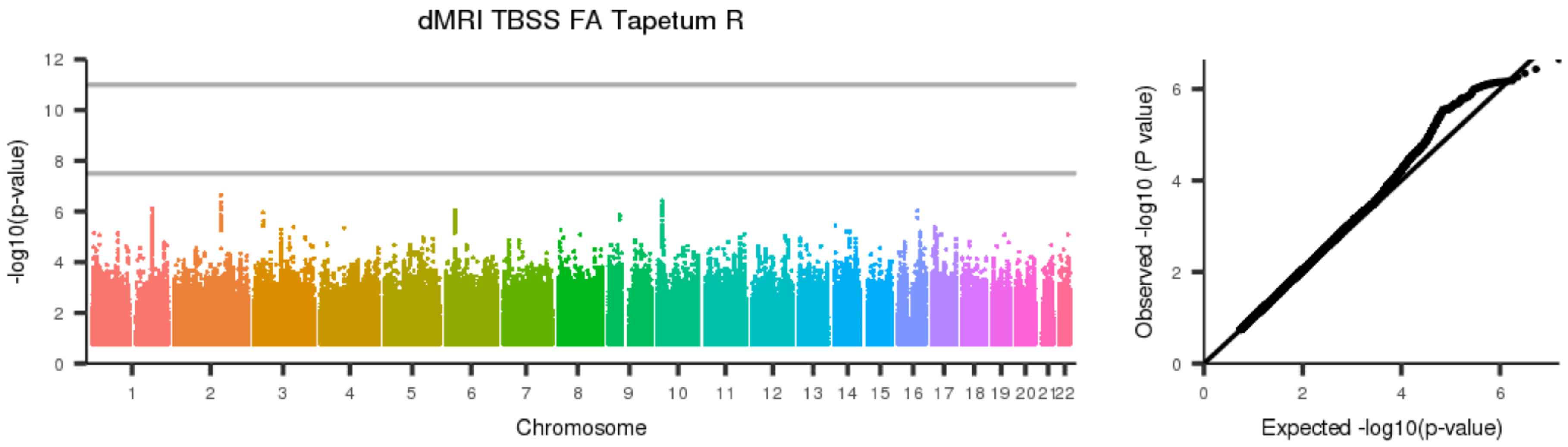

back to list of plots

Figure 243: dMRI TBSS FA Tapetum L

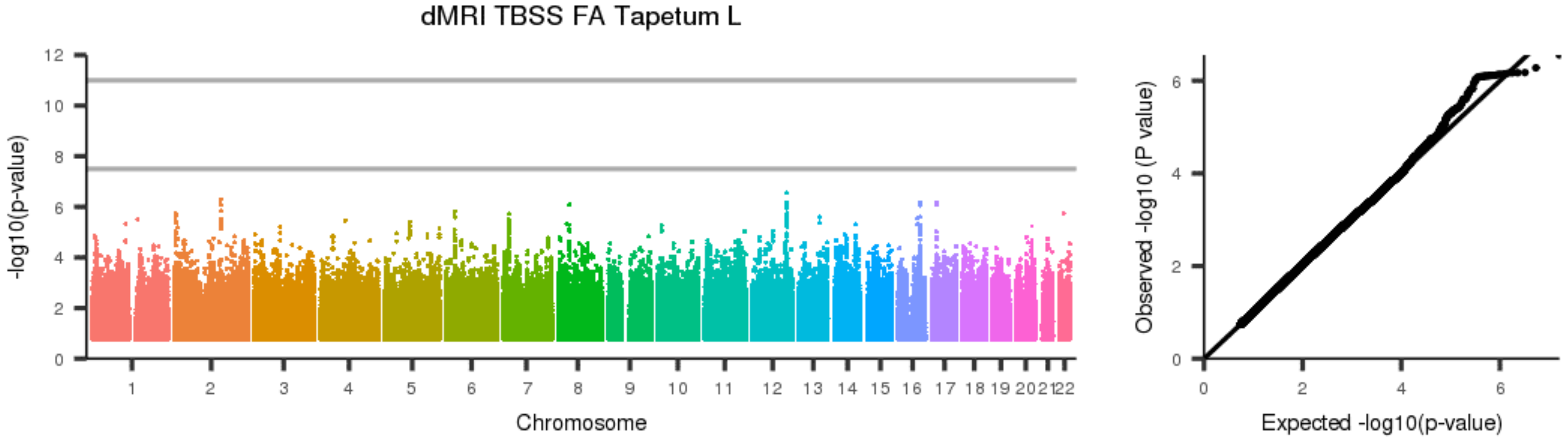

back to list of plots

Figure 244: dMRI TBSS MD Middle cerebellar peduncle

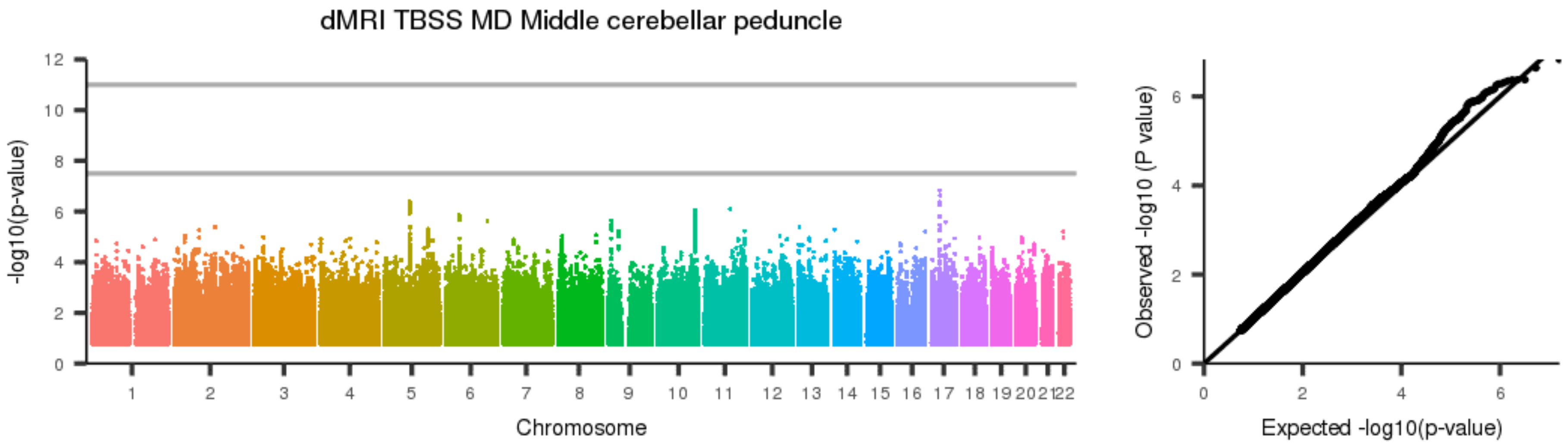

back to list of plots

Figure 245: dMRI TBSS MD Pontine crossing tract

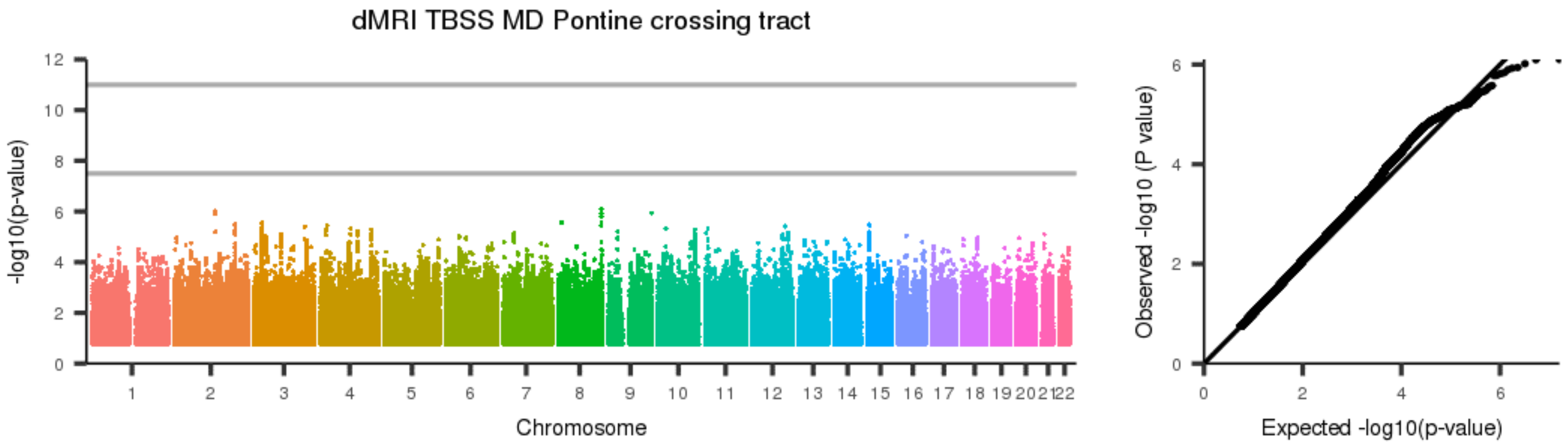

back to list of plots

Figure 246: dMRI TBSS MD Genu of corpus callosum

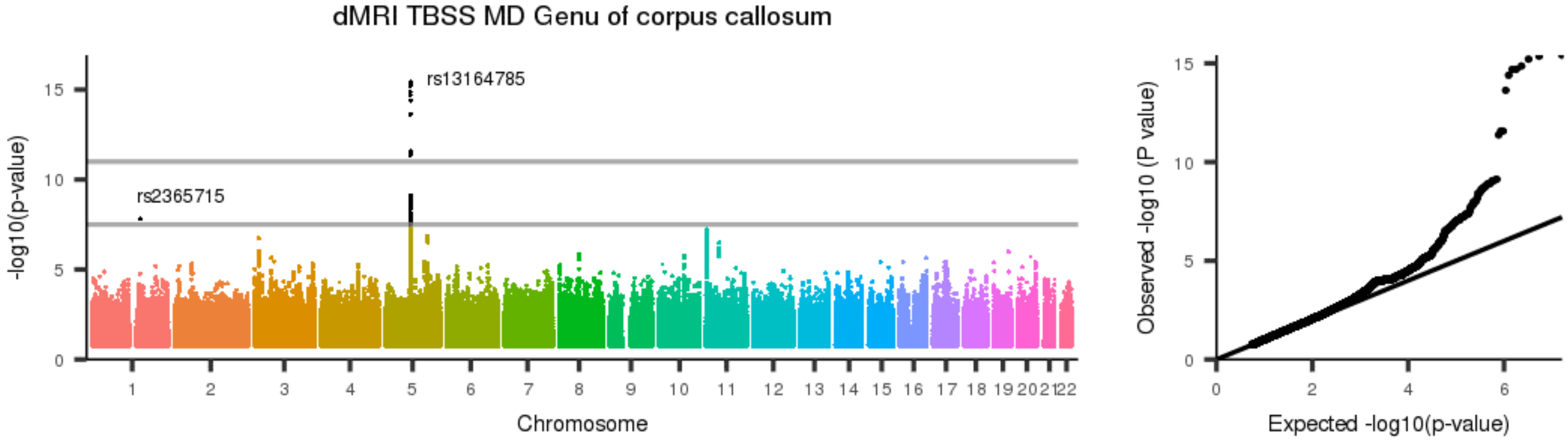

back to list of plots

Figure 247: dMRI TBSS MD Body of corpus callosum

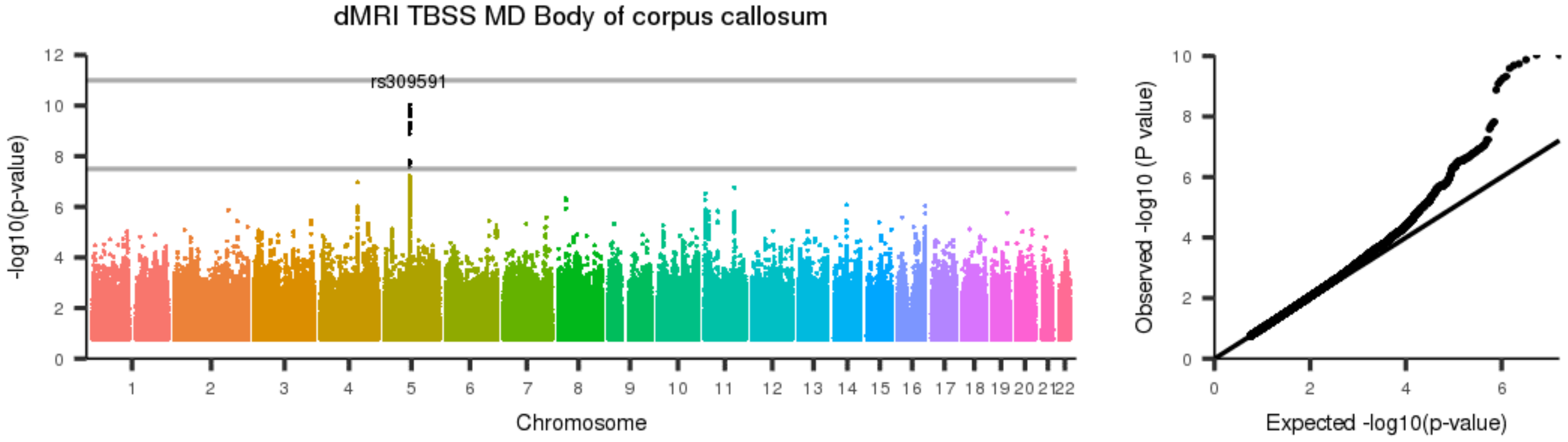

back to list of plots

Figure 248: dMRI TBSS MD Splenium of corpus callosum

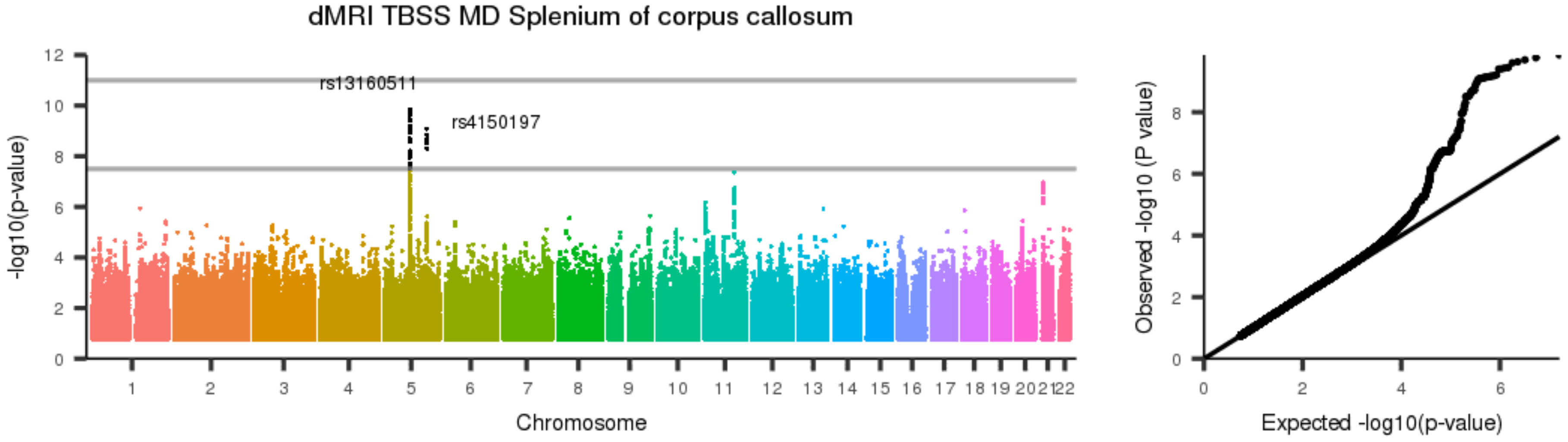

back to list of plots

Figure 249: dMRI TBSS MD Fornix

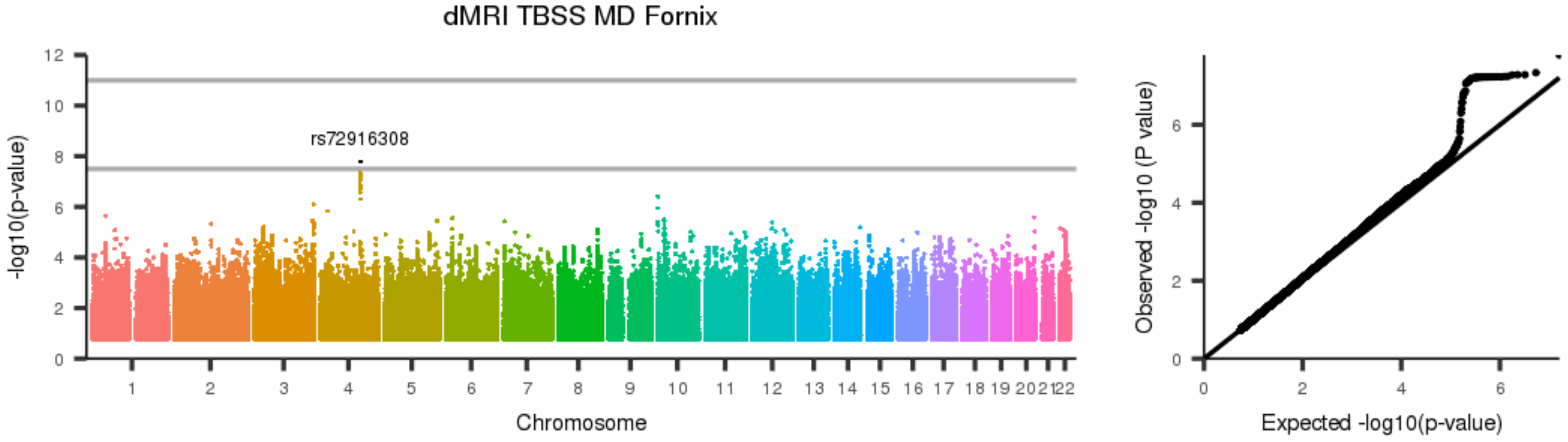

back to list of plots

Figure 250: dMRI TBSS MD Corticospinal tract R

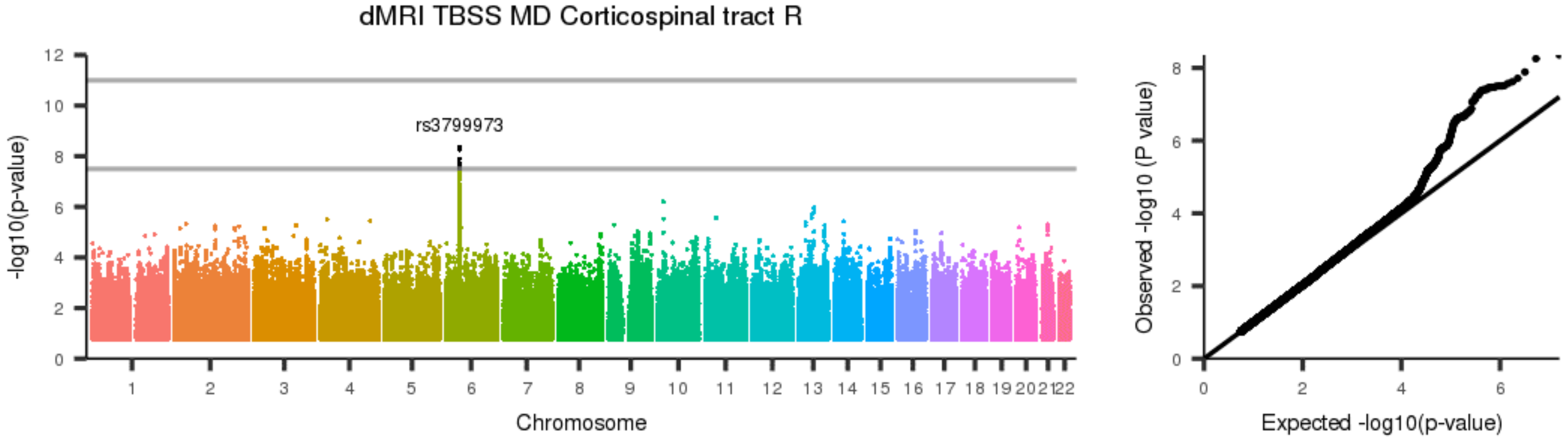

back to list of plots

Figure 251: dMRI TBSS MD Corticospinal tract L

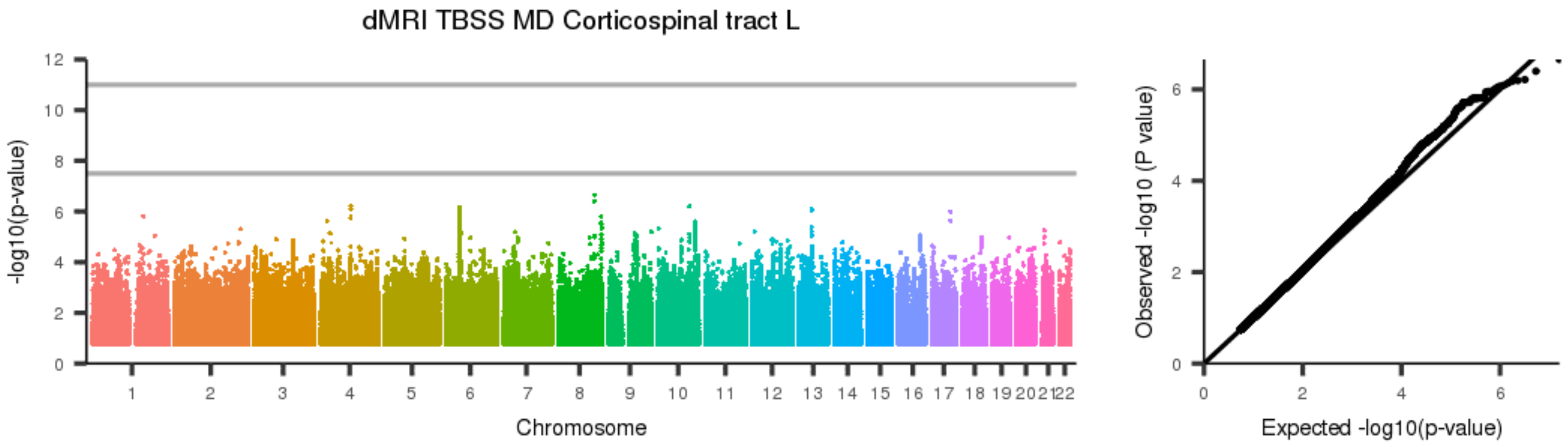

back to list of plots

Figure 252: dMRI TBSS MD Medial lemniscus R

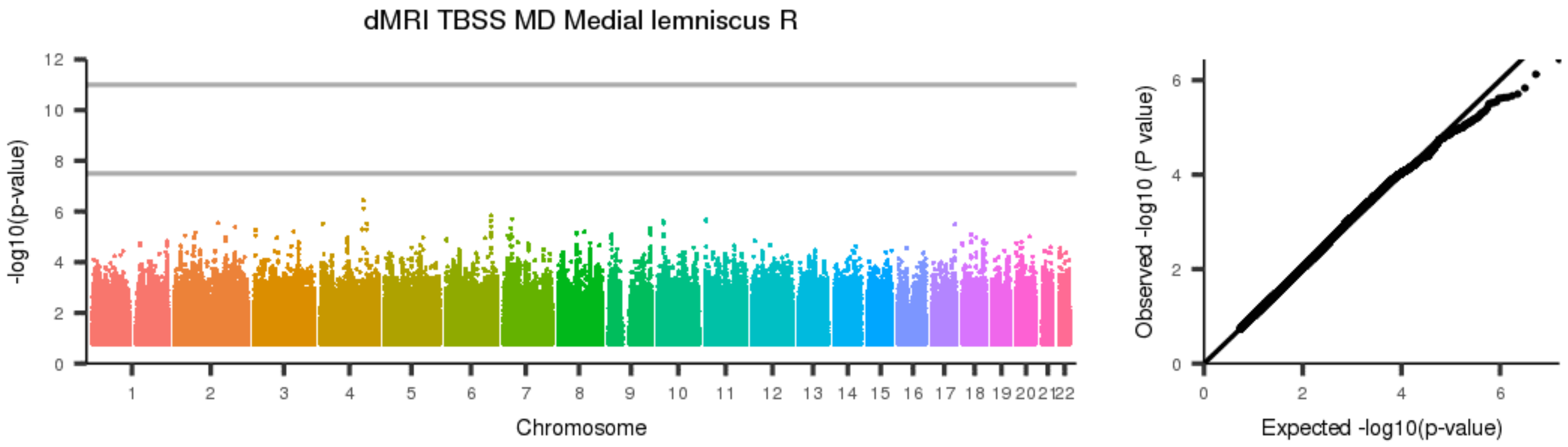

back to list of plots

Figure 253: dMRI TBSS MD Medial lemniscus L

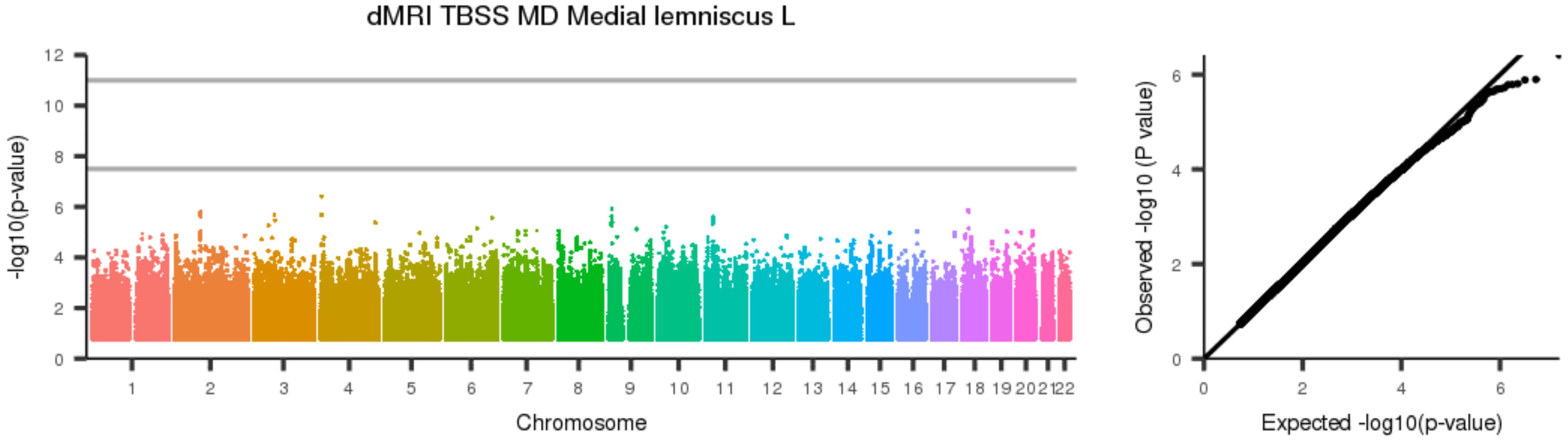

back to list of plots

Figure 254: dMRI TBSS MD Inferior cerebellar peduncle R

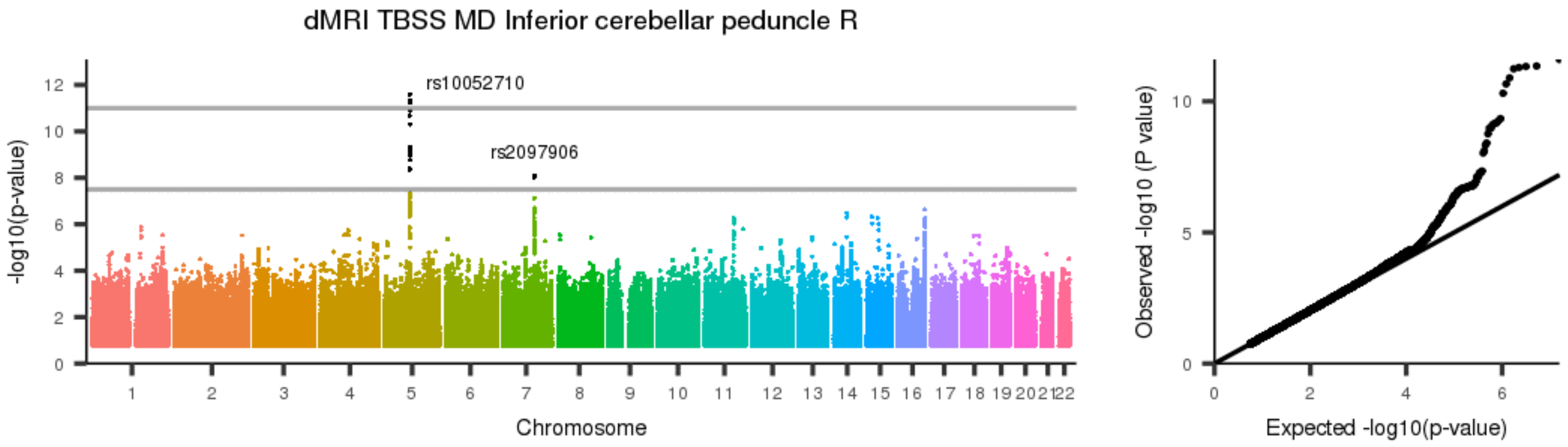

back to list of plots

Figure 255: dMRI TBSS MD Inferior cerebellar peduncle L

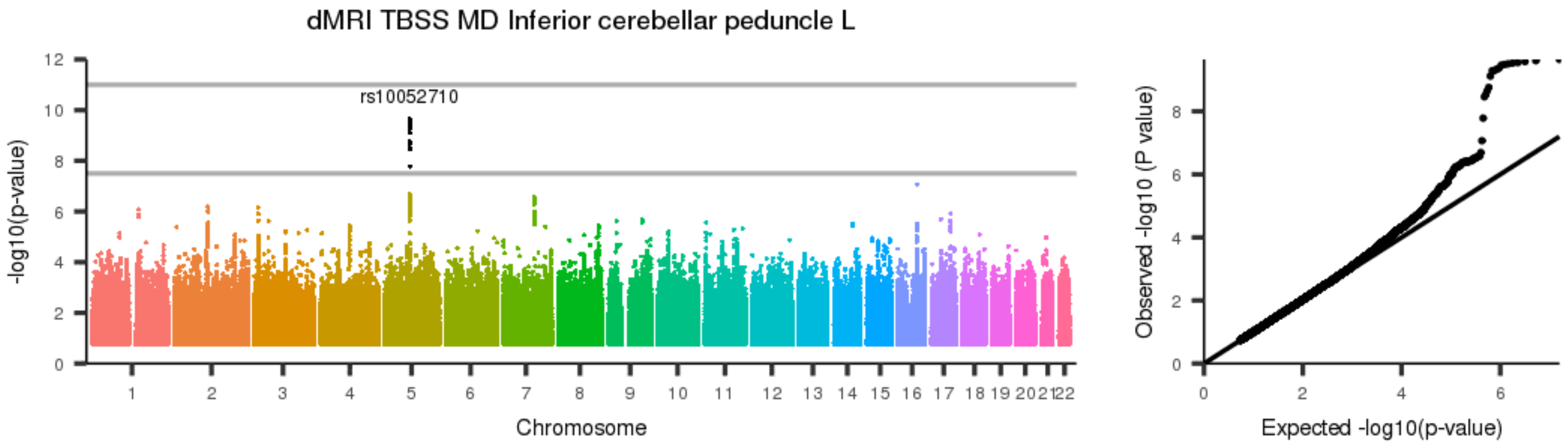

back to list of plots

Figure 256: dMRI TBSS MD Superior cerebellar peduncle R

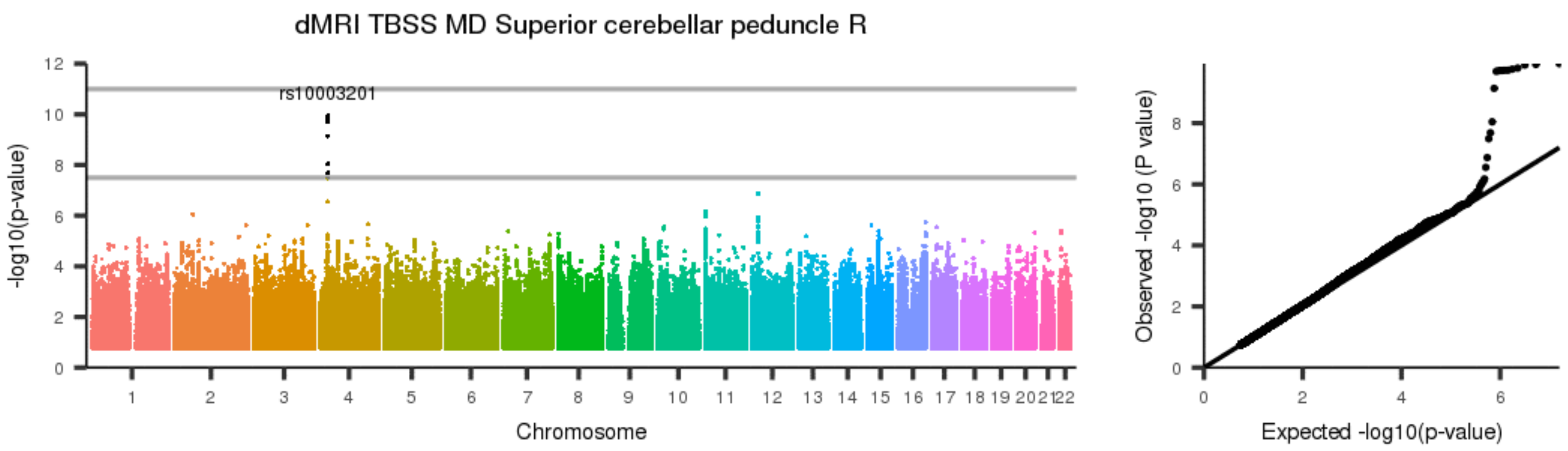

back to list of plots

Figure 257: dMRI TBSS MD Superior cerebellar peduncle L

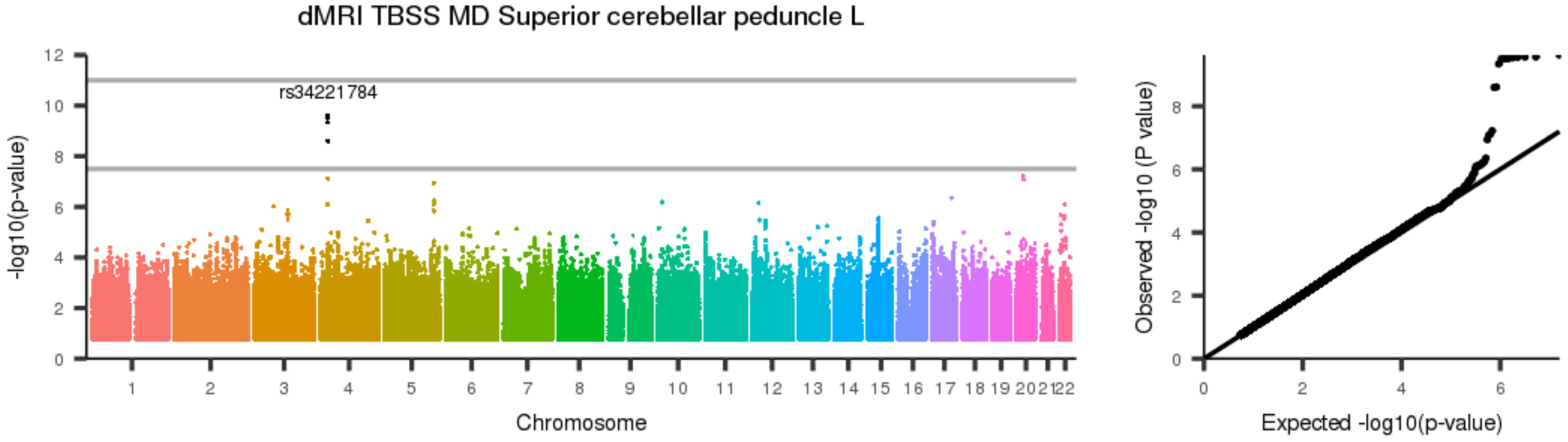

back to list of plots

Figure 258: dMRI TBSS MD Cerebral peduncle R

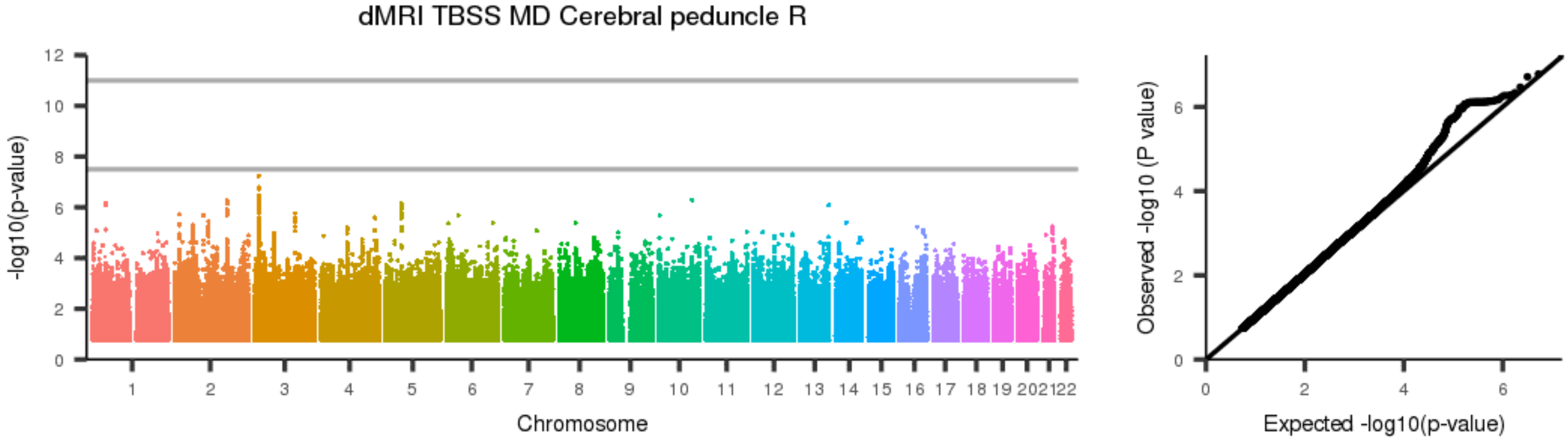

back to list of plots

Figure 259: dMRI TBSS MD Cerebral peduncle L

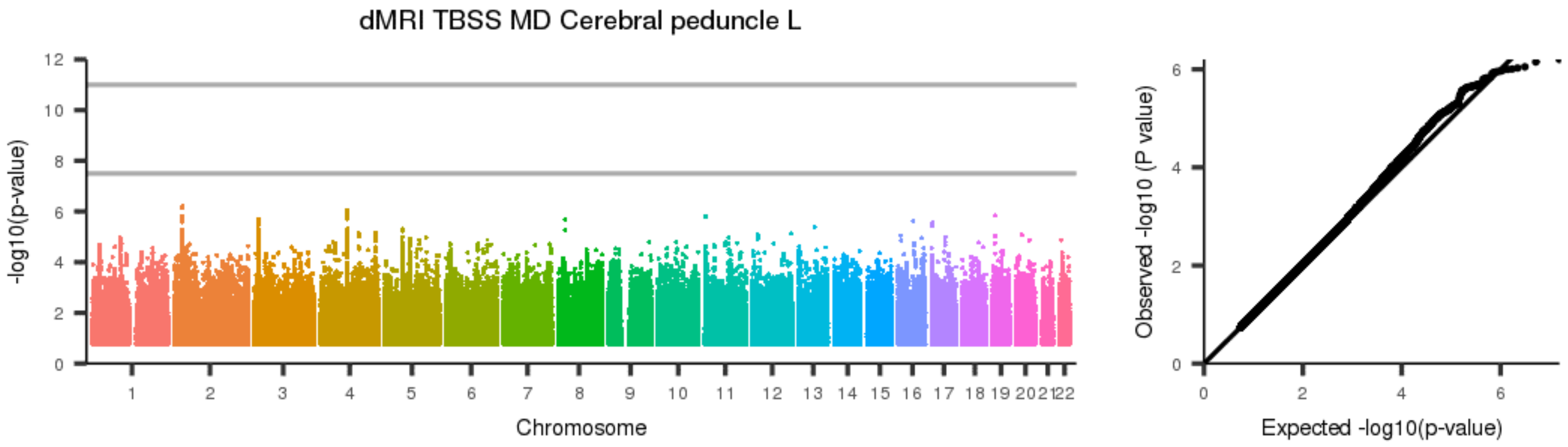

back to list of plots

Figure 260: dMRI TBSS MD Anterior limb of internal capsule R

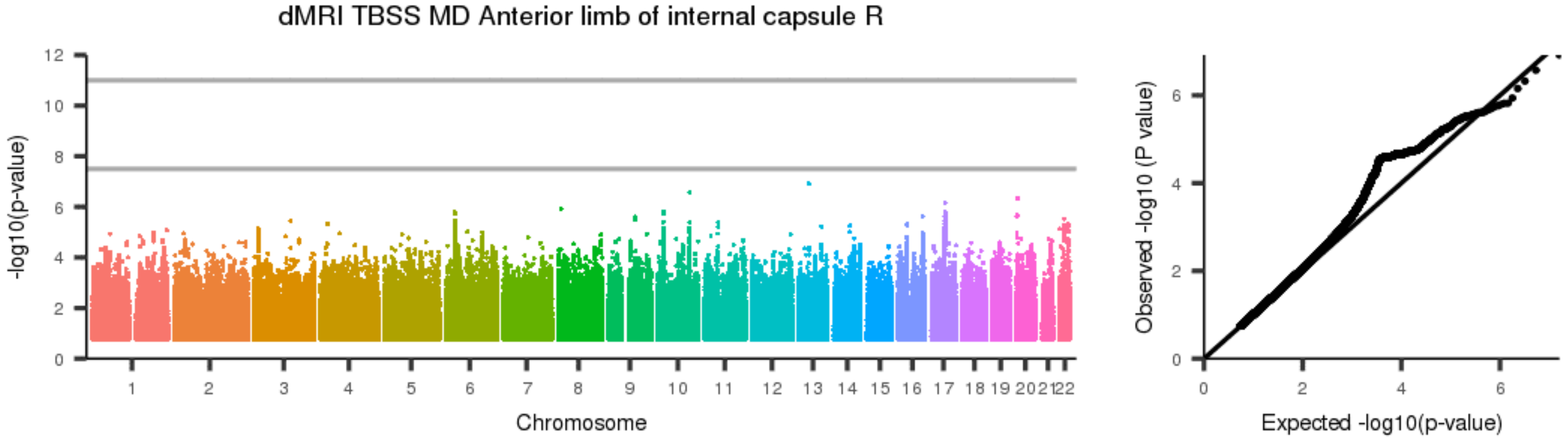

back to list of plots

Figure 261: dMRI TBSS MD Anterior limb of internal capsule L

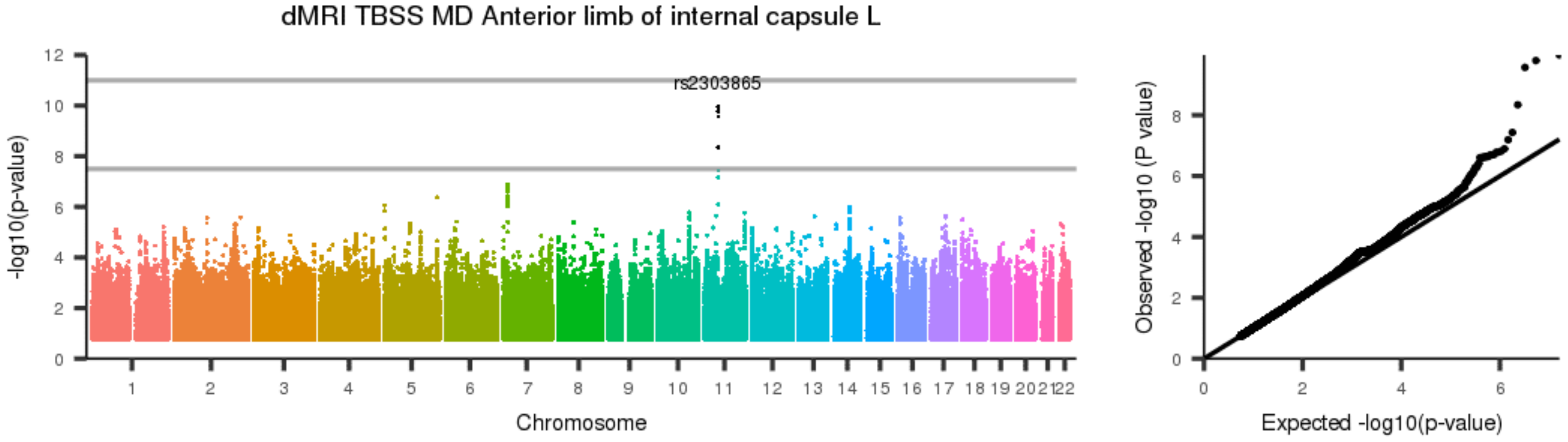

back to list of plots

Figure 262: dMRI TBSS MD Posterior limb of internal capsule R

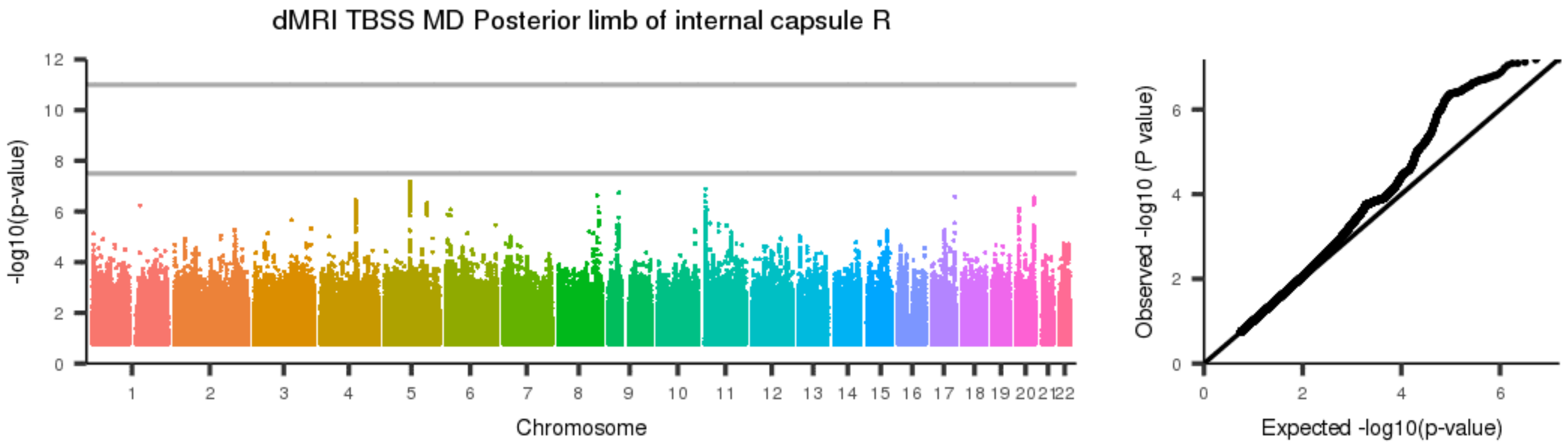

back to list of plots

Figure 263: dMRI TBSS MD Posterior limb of internal capsule L

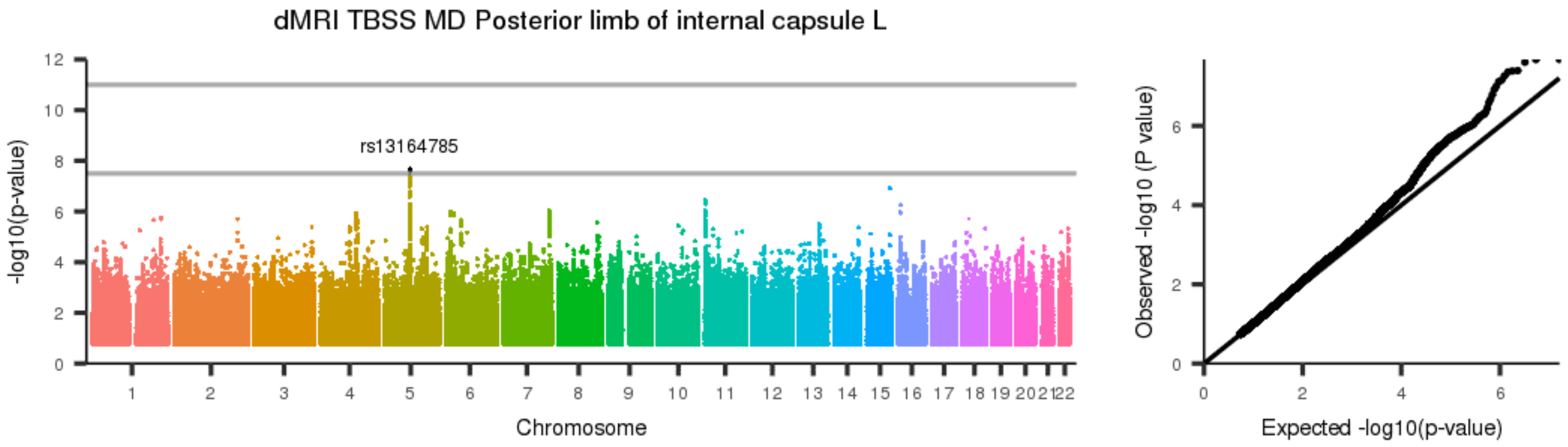

back to list of plots

Figure 264: dMRI TBSS MD Retrolenticular part of internal capsule R

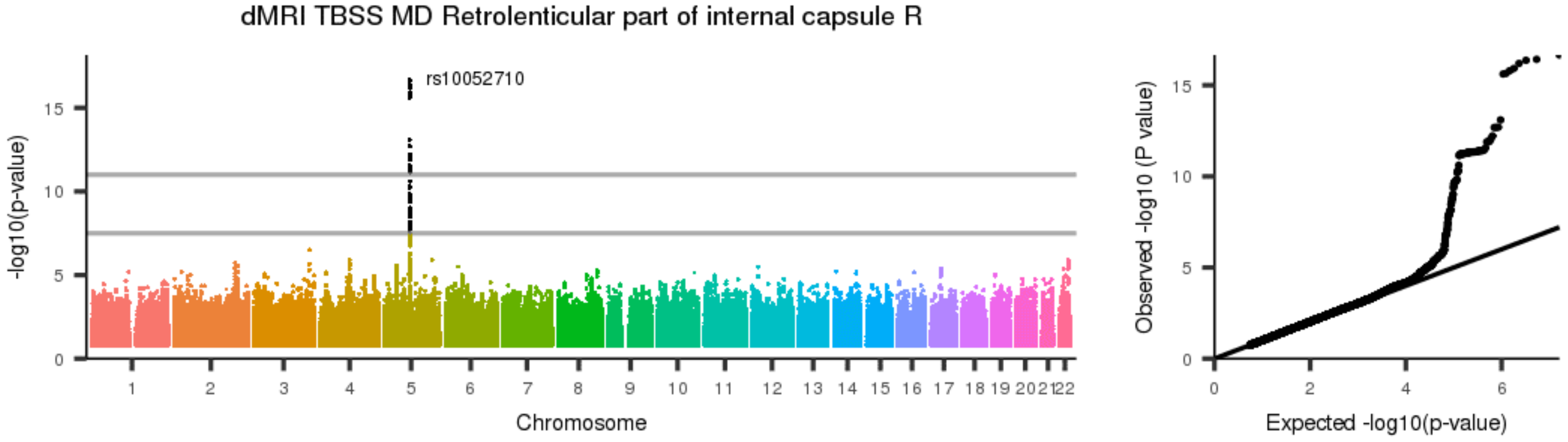

back to list of plots

Figure 265: dMRI TBSS MD Retrolenticular part of internal capsule L

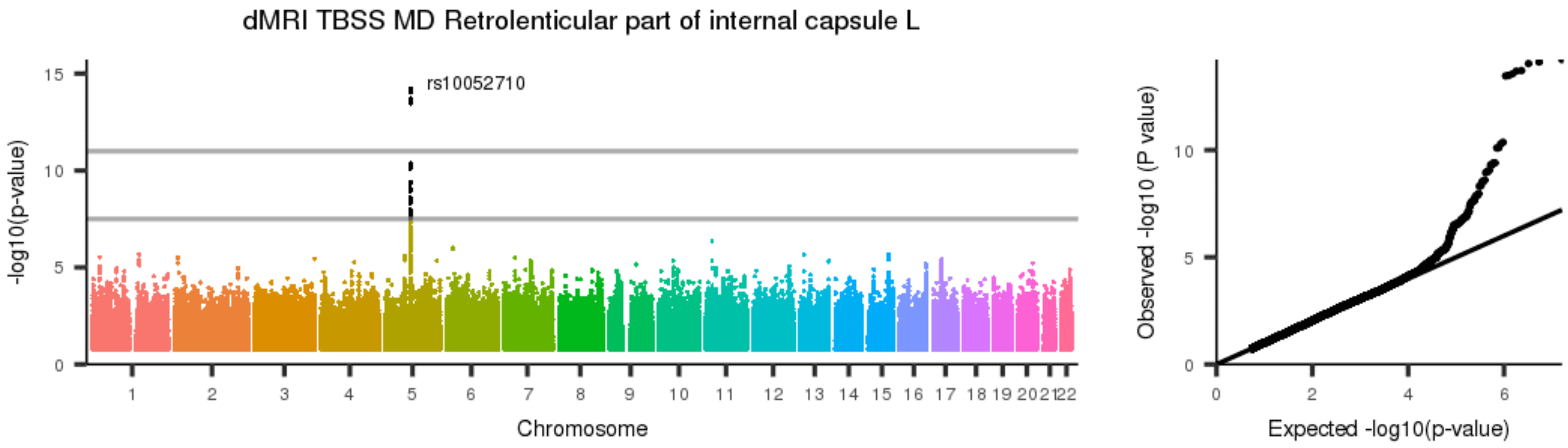

back to list of plots

Figure 266: dMRI TBSS MD Anterior corona radiata R

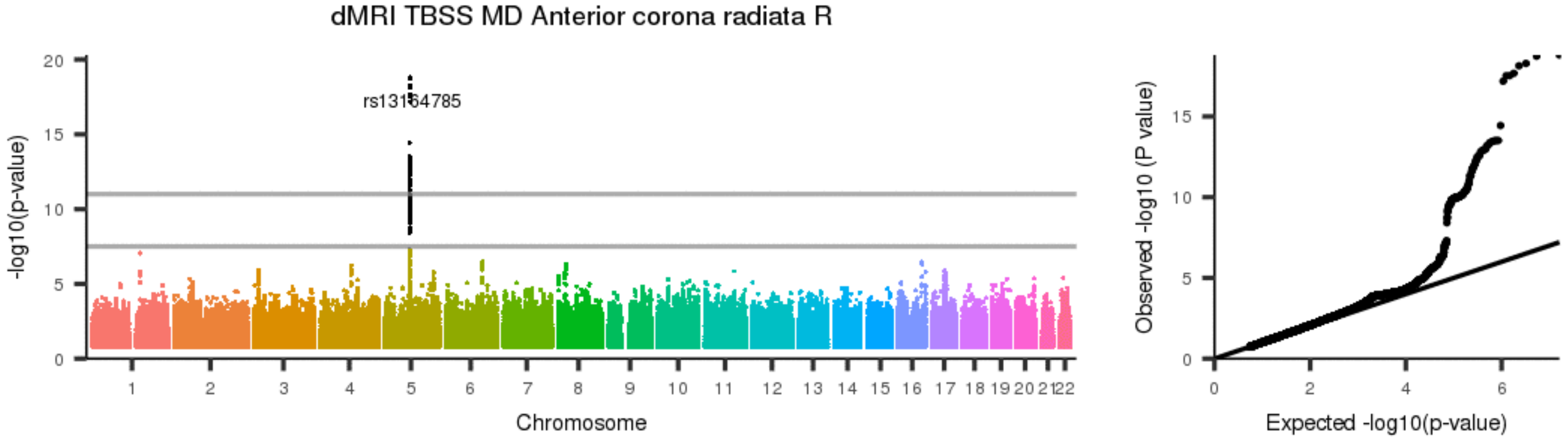

back to list of plots

Figure 267: dMRI TBSS MD Anterior corona radiata L

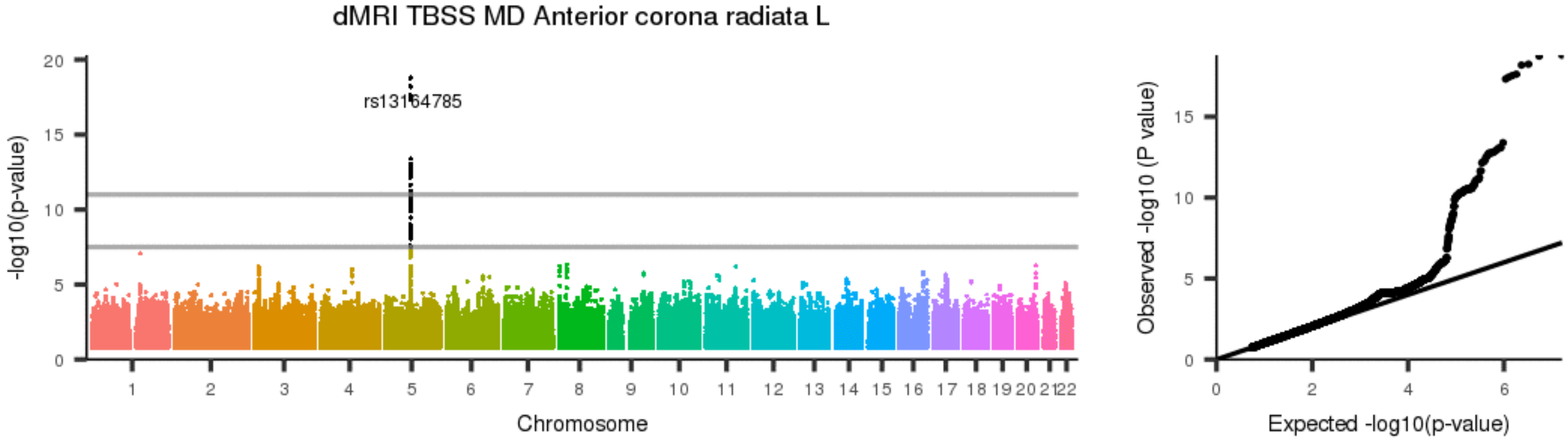

back to list of plots

Figure 268: dMRI TBSS MD Superior corona radiata R

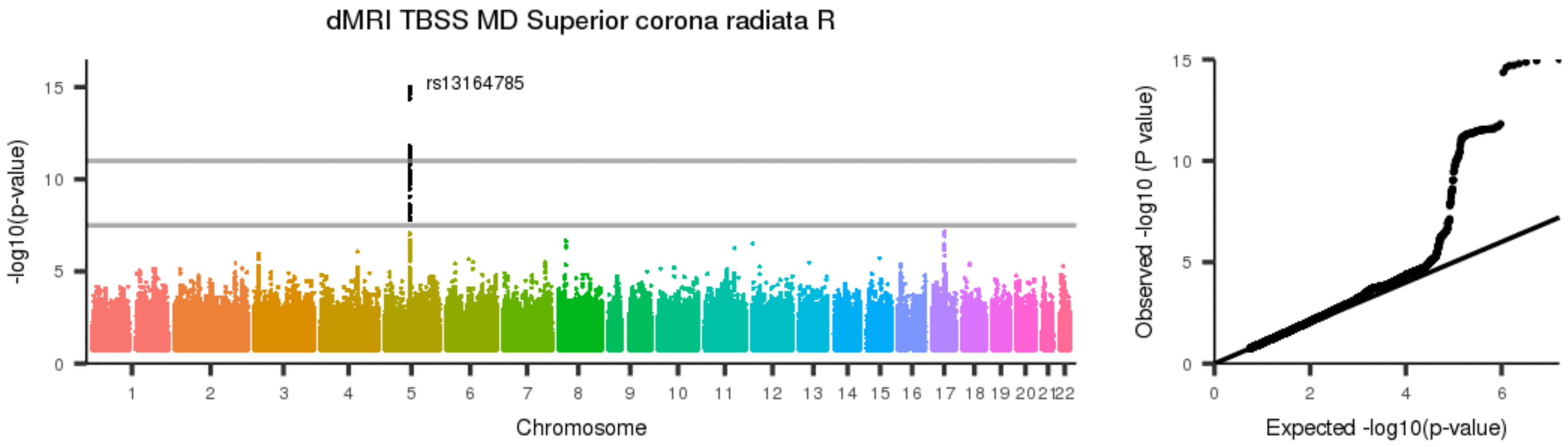

back to list of plots

Figure 269: dMRI TBSS MD Superior corona radiata L

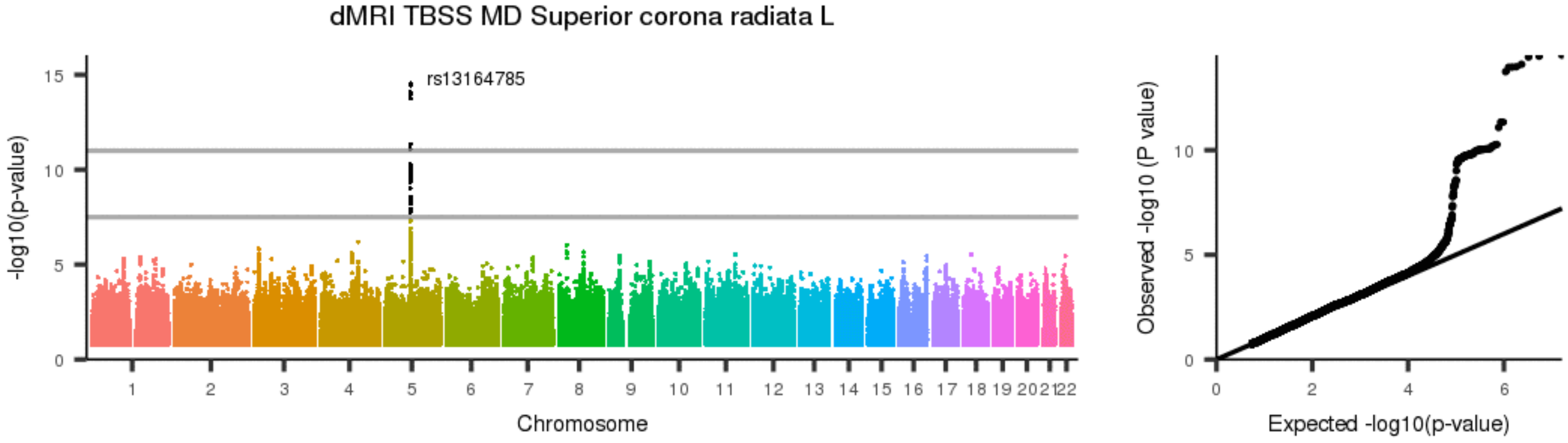

back to list of plots

Figure 270: dMRI TBSS MD Posterior corona radiata R

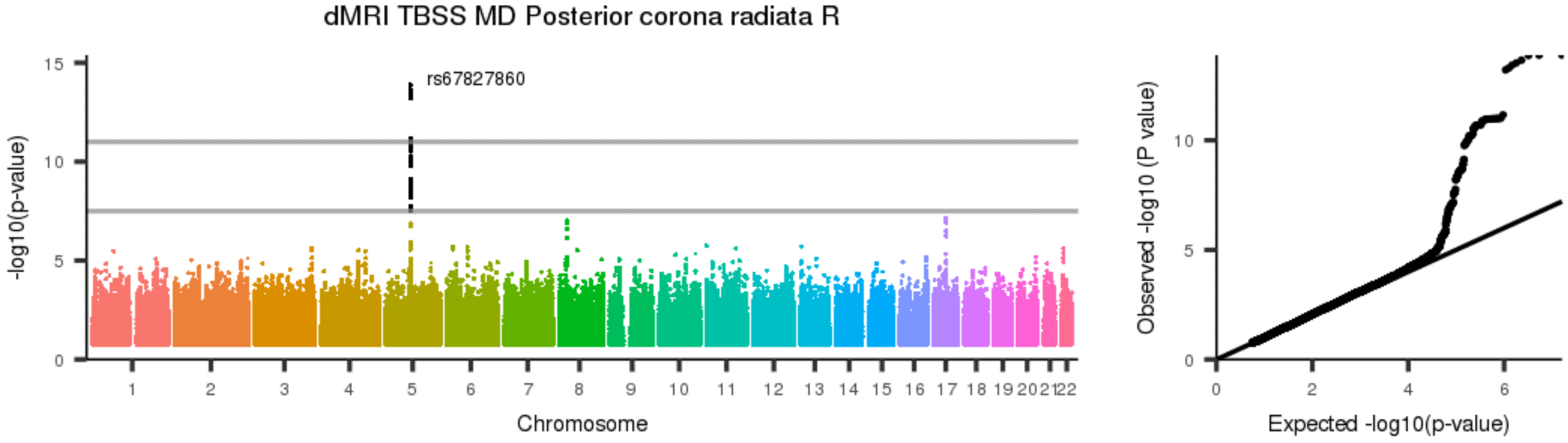

back to list of plots

Figure 271: dMRI TBSS MD Posterior corona radiata L

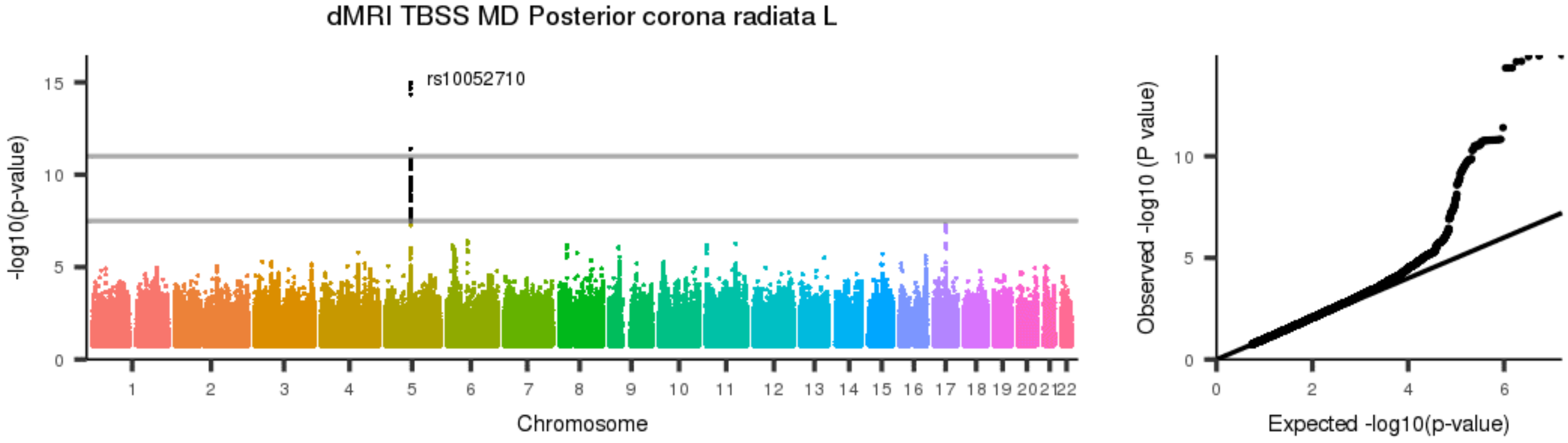

back to list of plots

Figure 272: dMRI TBSS MD Posterior thalamic radiation R

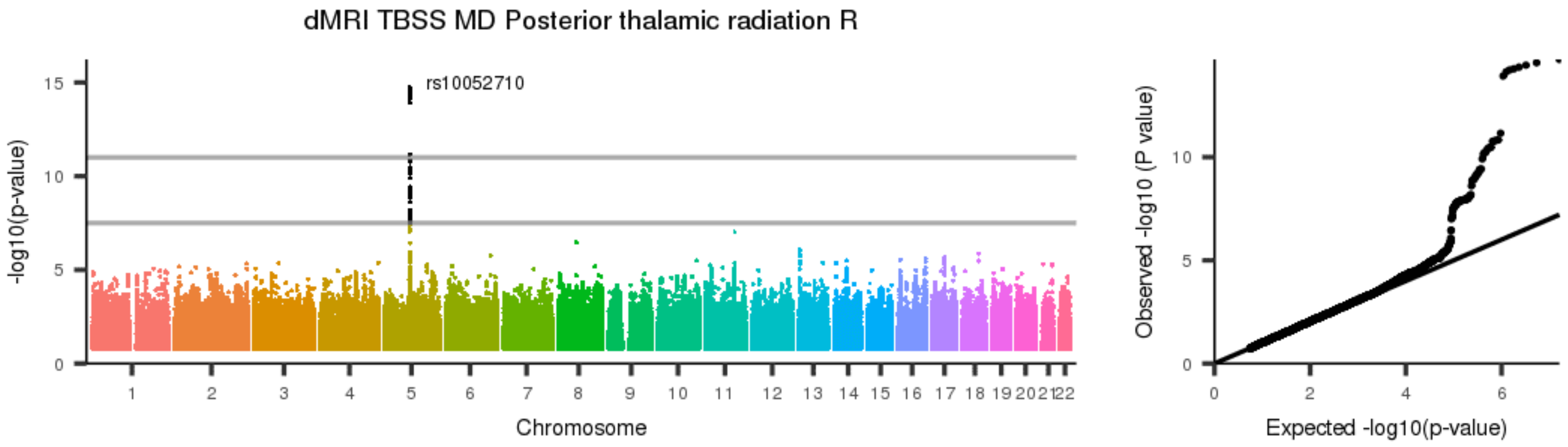

back to list of plots

Figure 273: dMRI TBSS MD Posterior thalamic radiation L

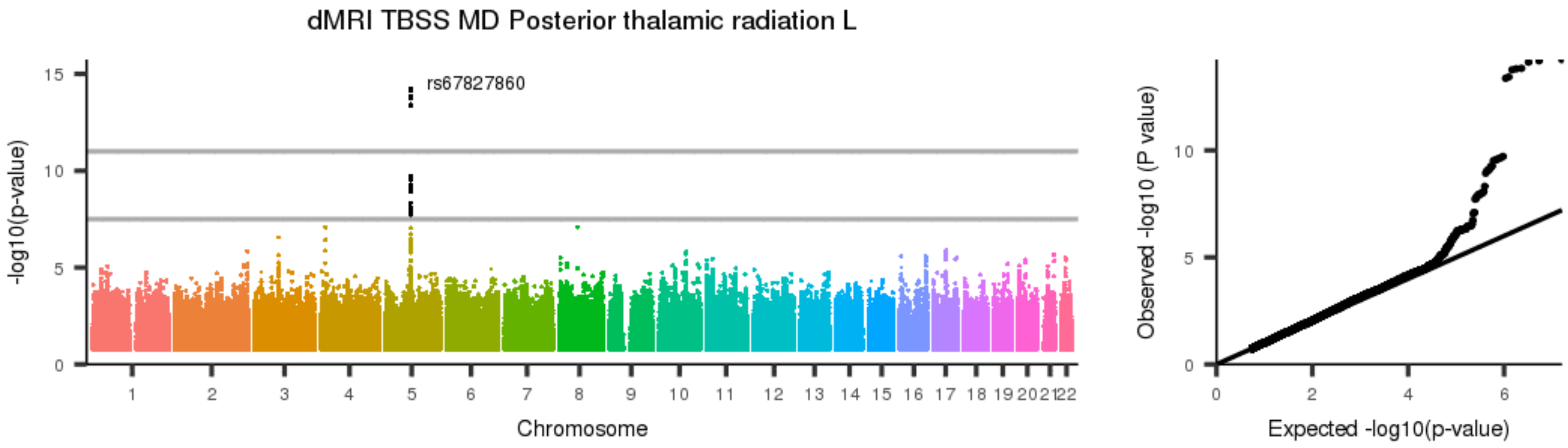

back to list of plots

Figure 274: dMRI TBSS MD Sagittal stratum R

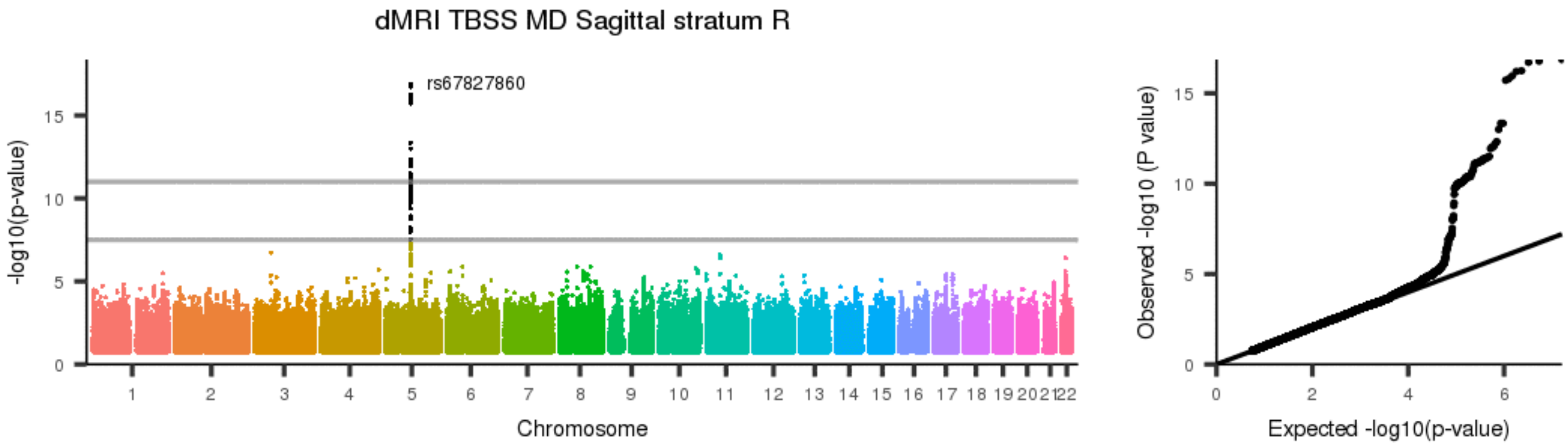

back to list of plots

Figure 275: dMRI TBSS MD Sagittal stratum L

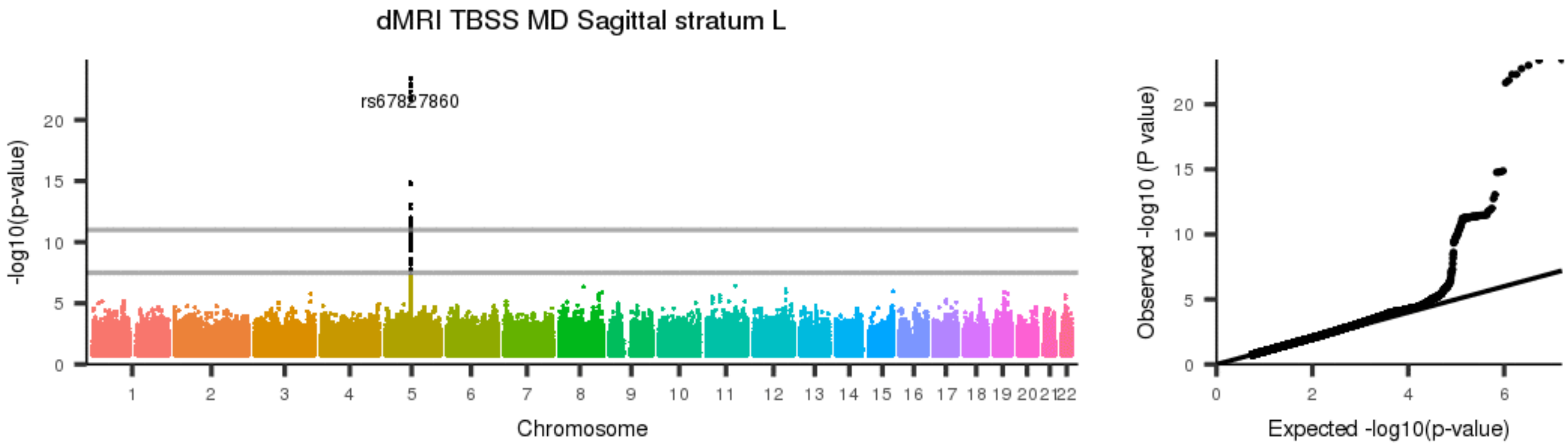

back to list of plots

Figure 276: dMRI TBSS MD External capsule R

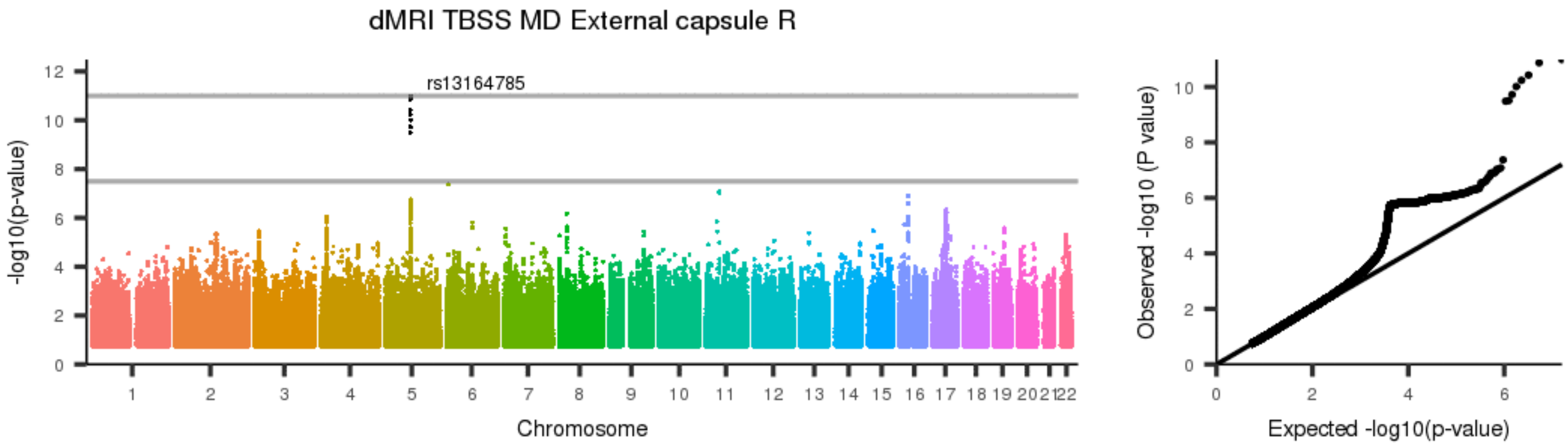

back to list of plots

Figure 277: dMRI TBSS MD External capsule L

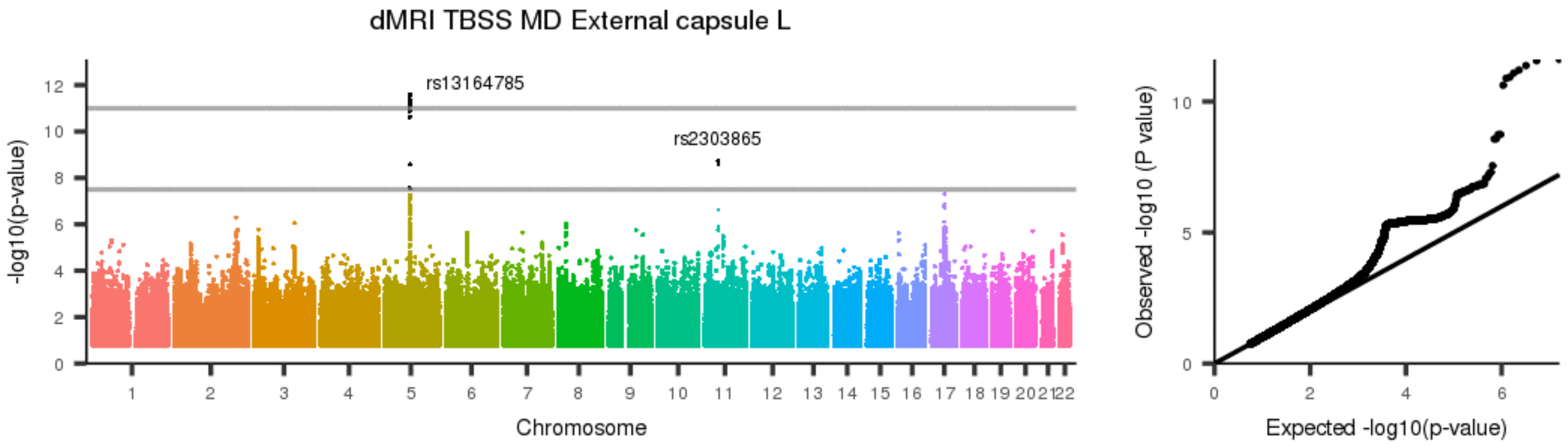

back to list of plots

Figure 278: dMRI TBSS MD Cingulum cingulate gyrus R

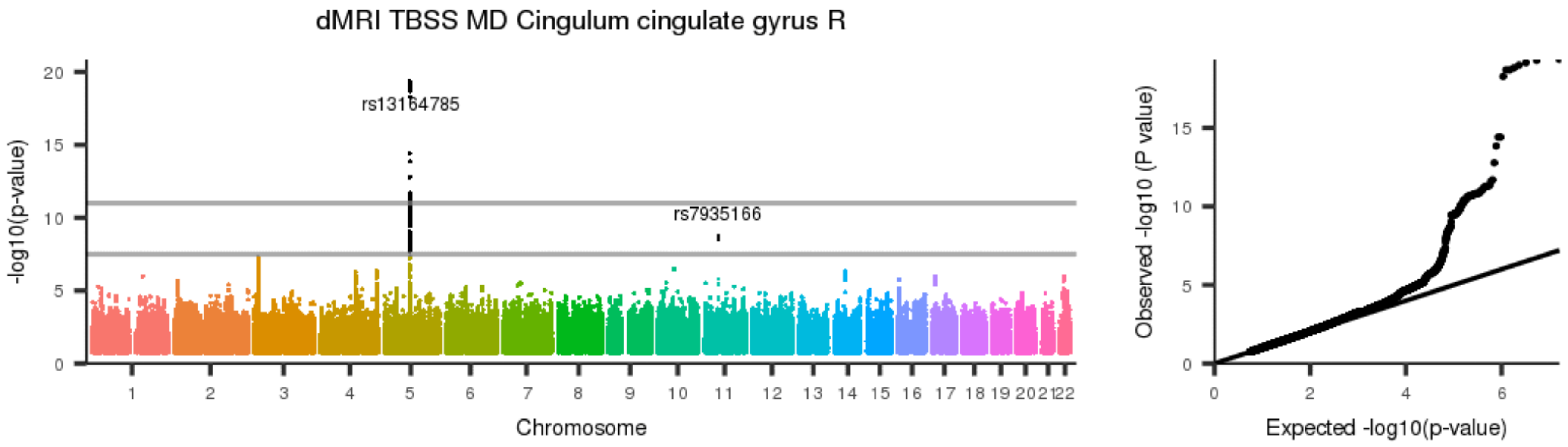

back to list of plots

Figure 279: dMRI TBSS MD Cingulum cingulate gyrus L

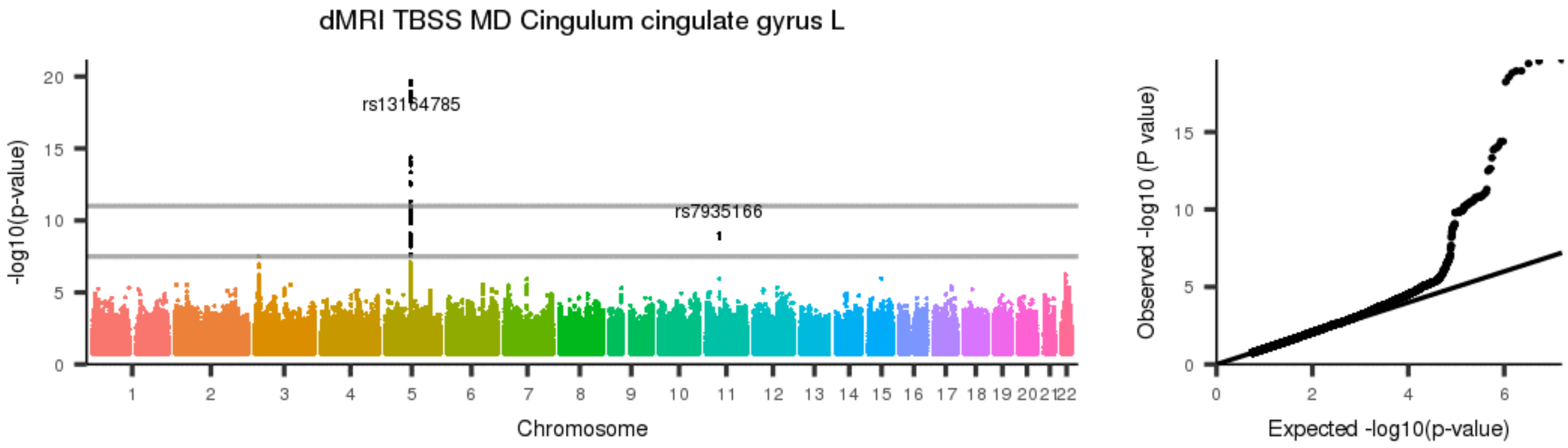

back to list of plots

Figure 280: dMRI TBSS MD Cingulum hippocampus R

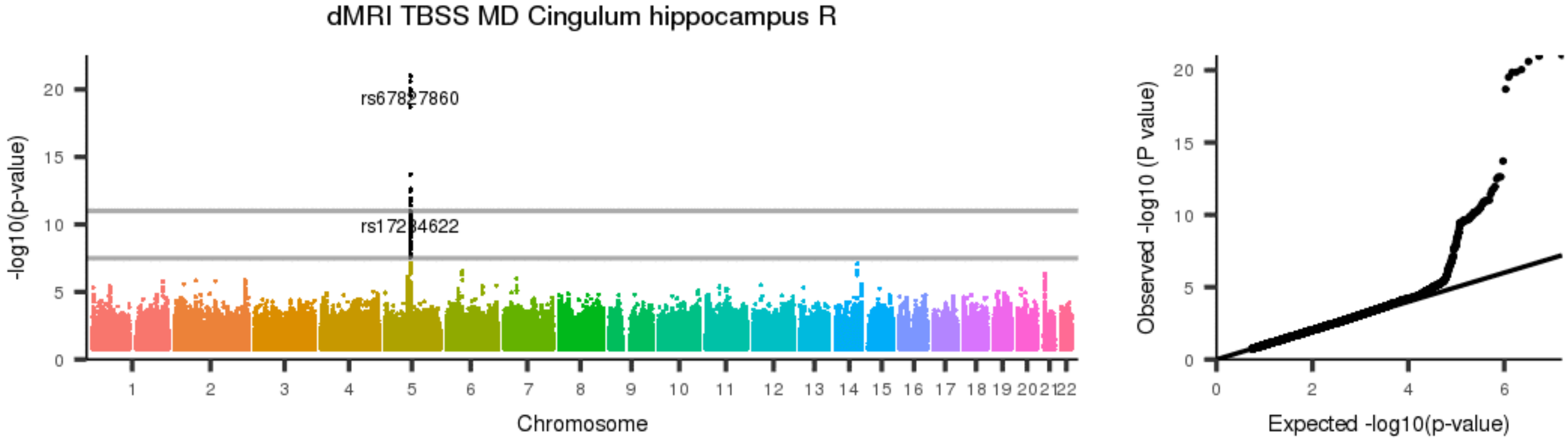

back to list of plots

Figure 281: dMRI TBSS MD Cingulum hippocampus L

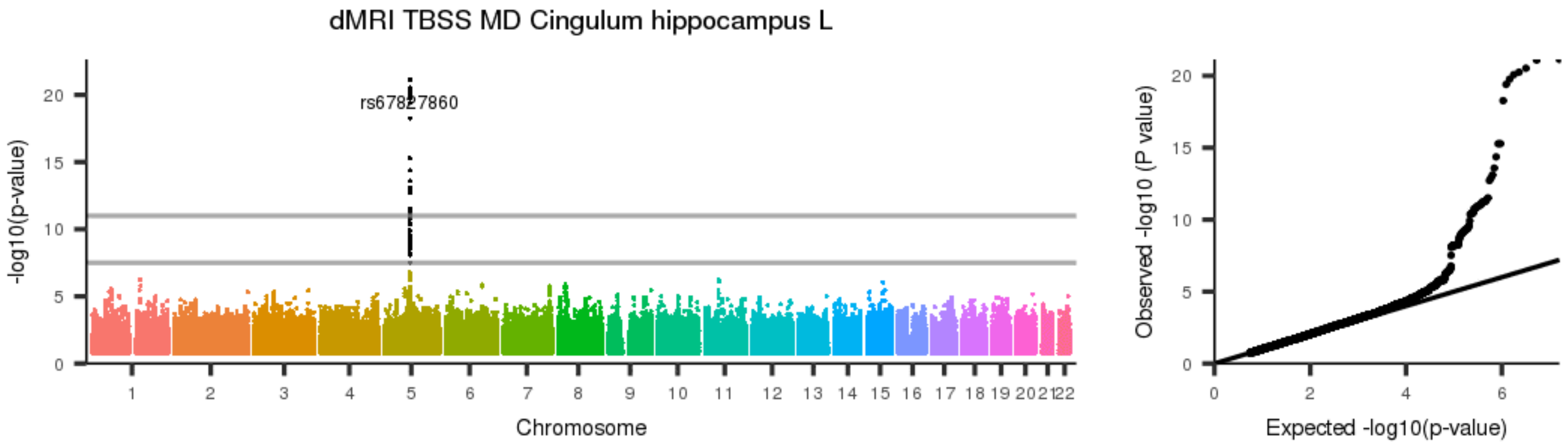

back to list of plots

Figure 282: dMRI TBSS MD Fornix cres+Stria terminalis R

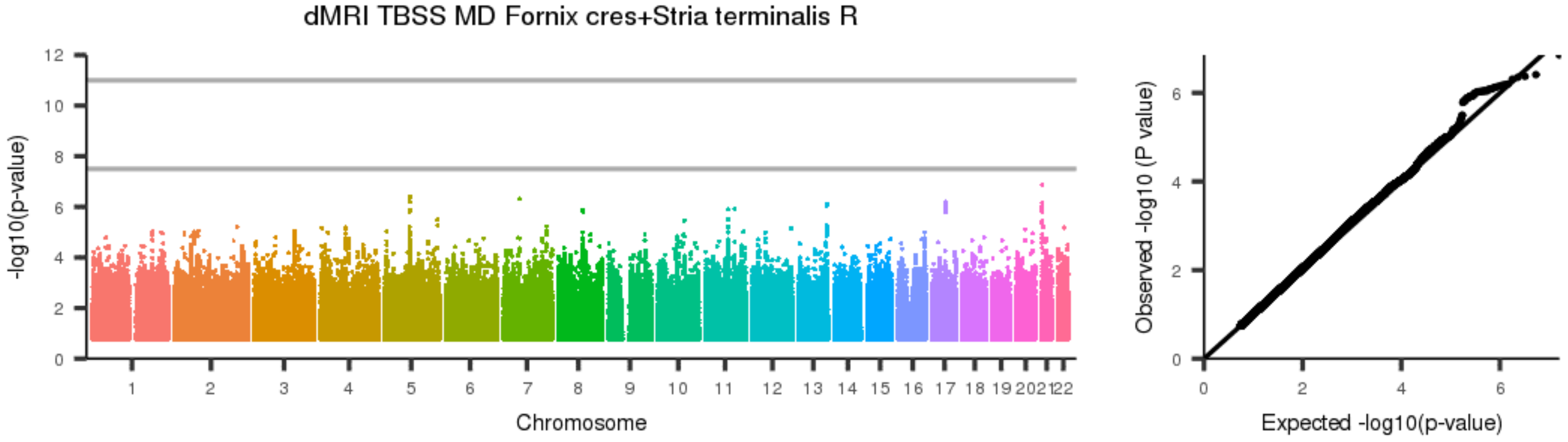

back to list of plots

Figure 283: dMRI TBSS MD Fornix cres+Stria terminalis L

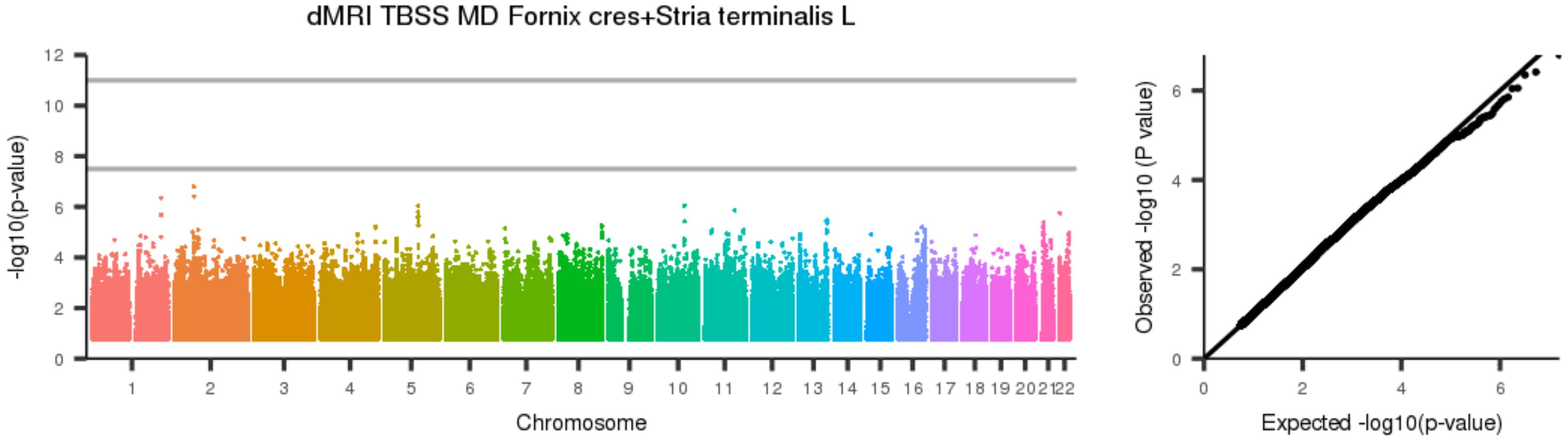

back to list of plots

Figure 284: dMRI TBSS MD Superior longitudinal fasciculus R

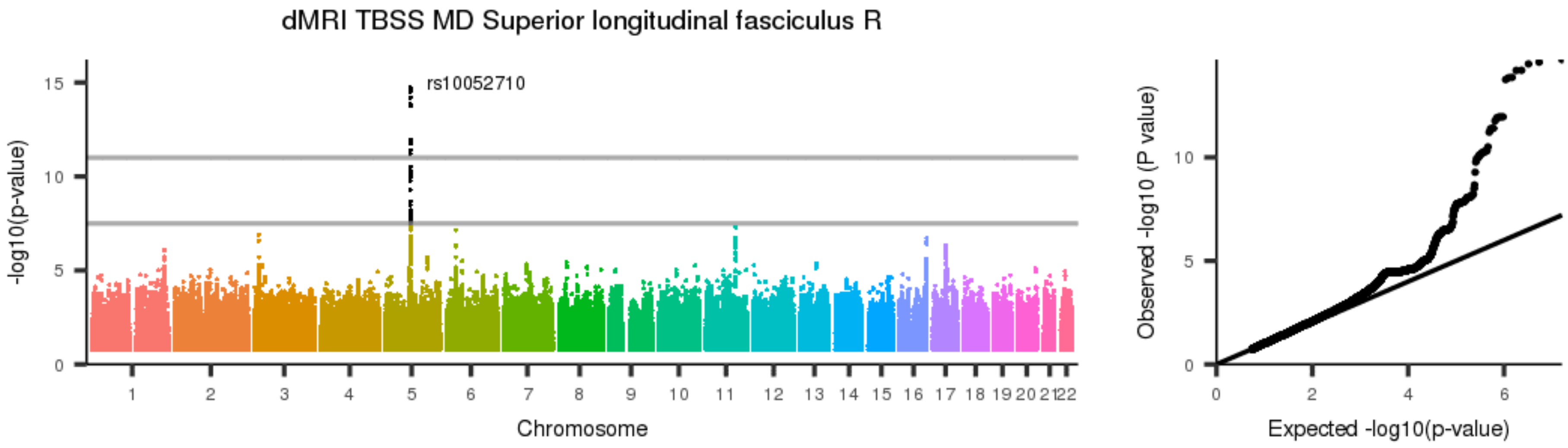

back to list of plots

Figure 285: dMRI TBSS MD Superior longitudinal fasciculus L

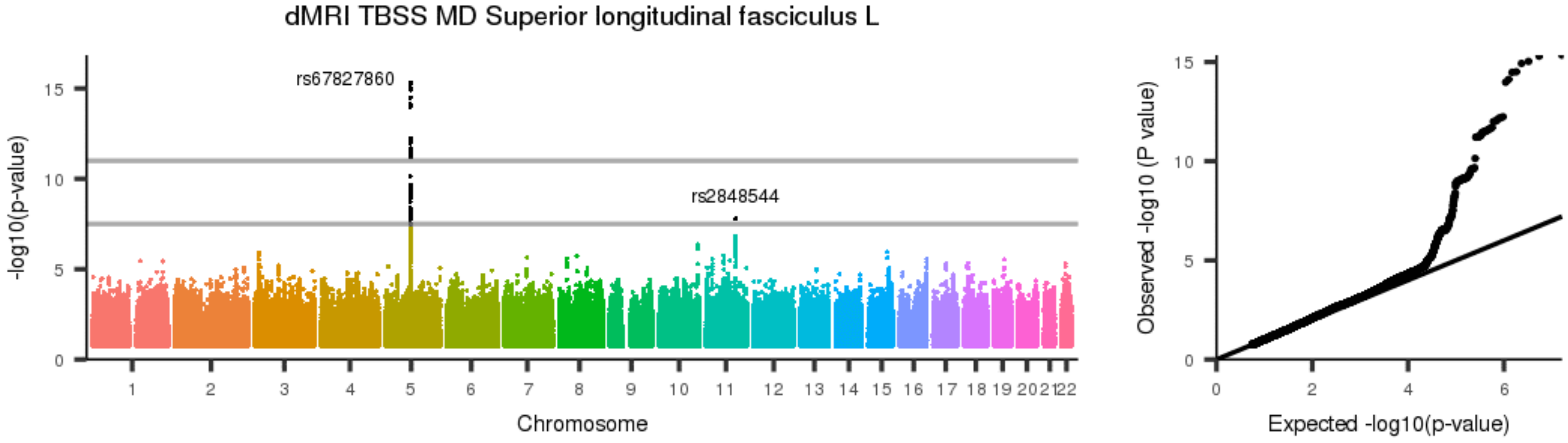

back to list of plots

Figure 286: dMRI TBSS MD Superior fronto-occipital fasciculus R

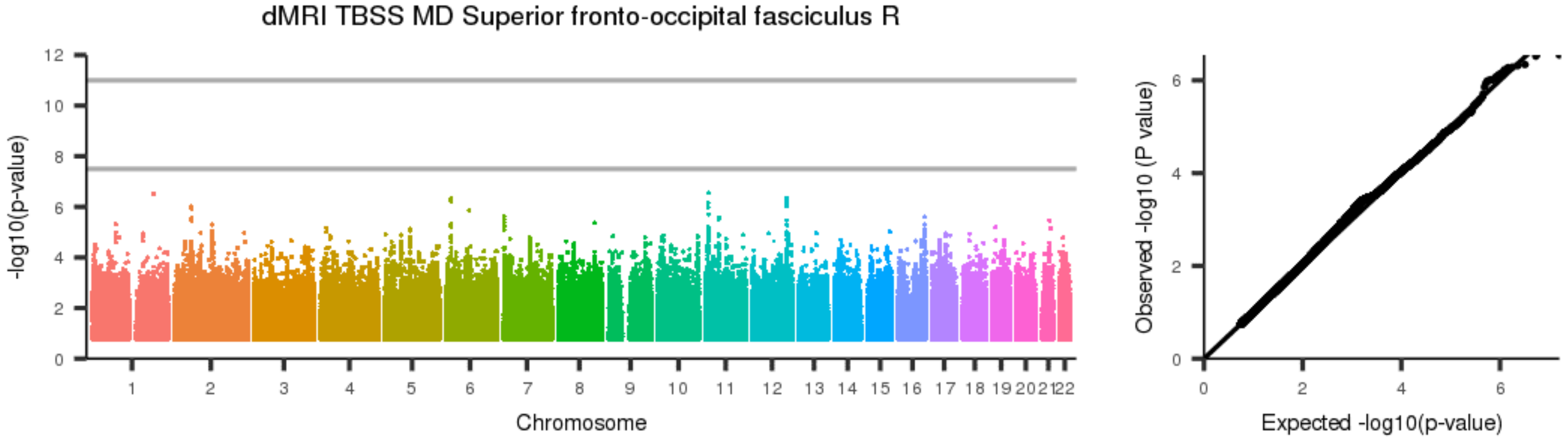

back to list of plots

Figure 287: dMRI TBSS MD Superior fronto-occipital fasciculus L

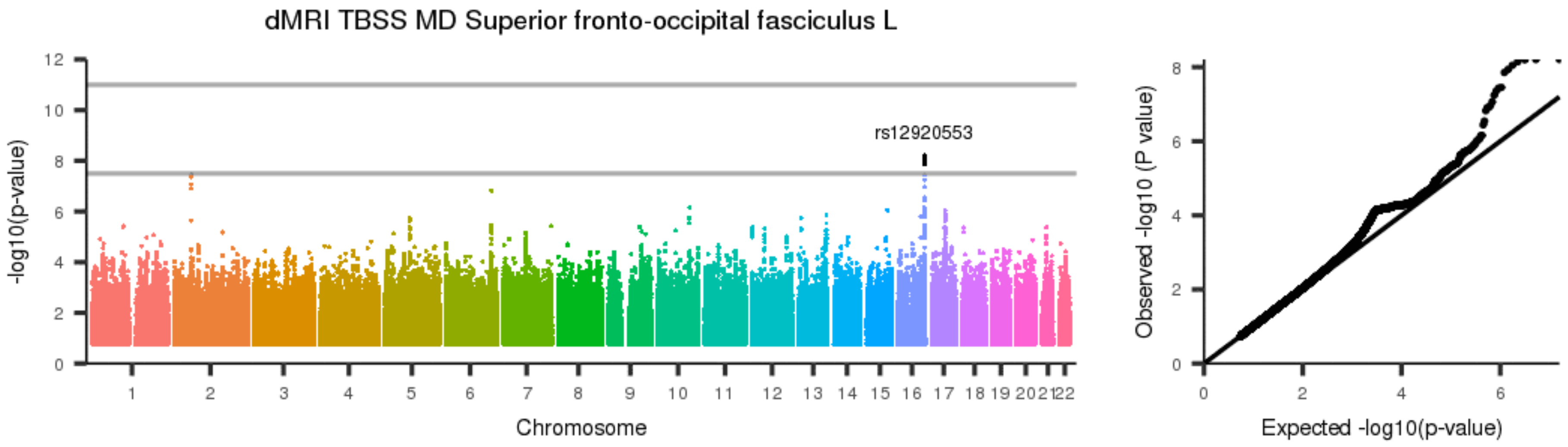

back to list of plots

Figure 288: dMRI TBSS MD Uncinate fasciculus R

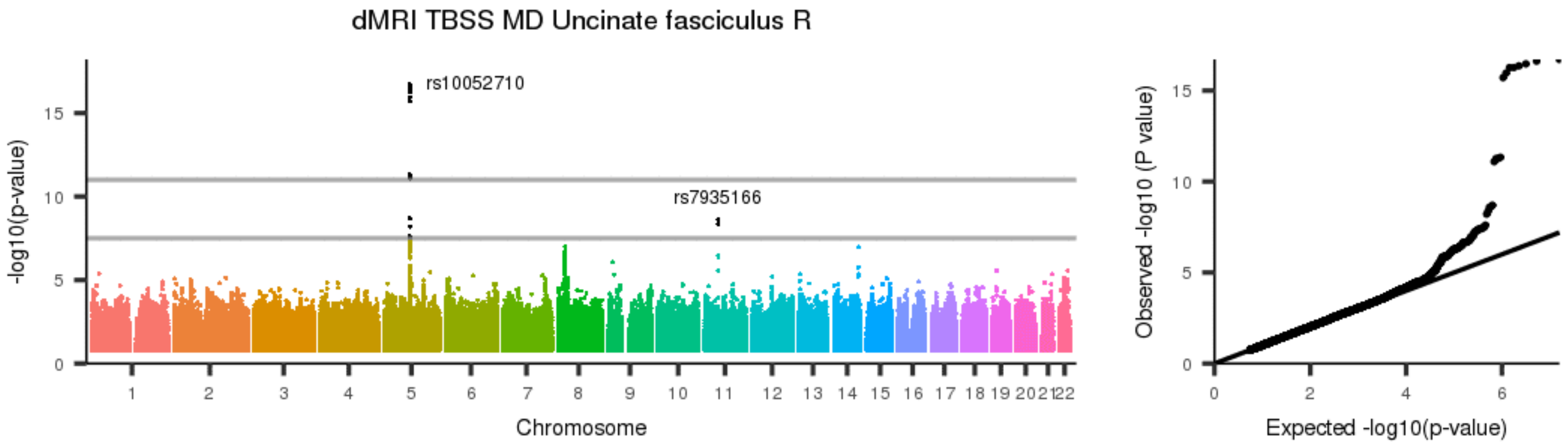

back to list of plots

Figure 289: dMRI TBSS MD Uncinate fasciculus L

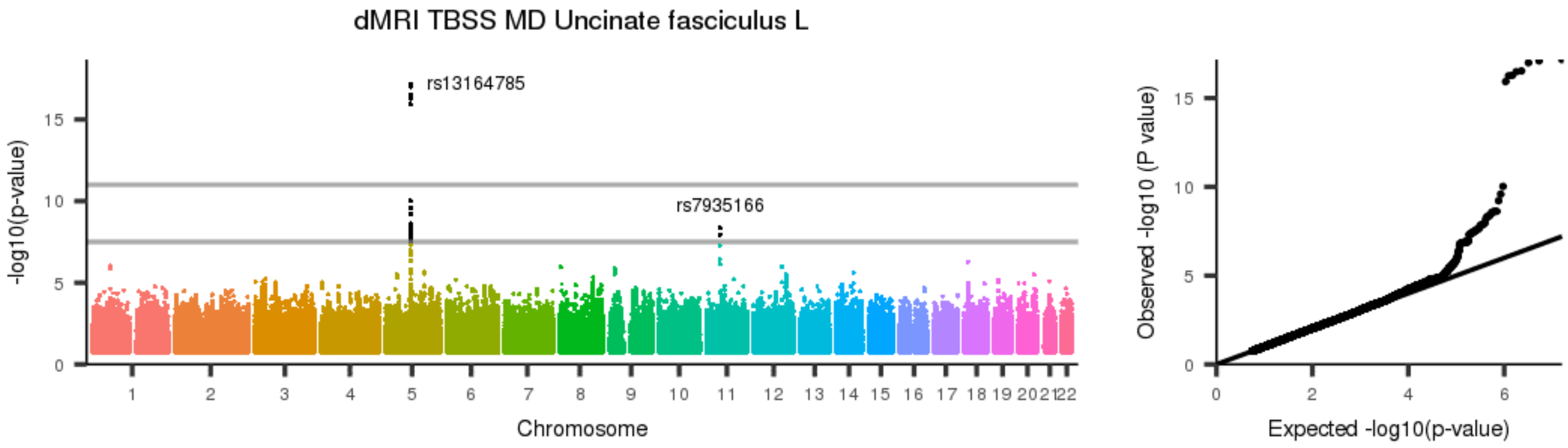

back to list of plots

Figure 290: dMRI TBSS MD Tapetum R

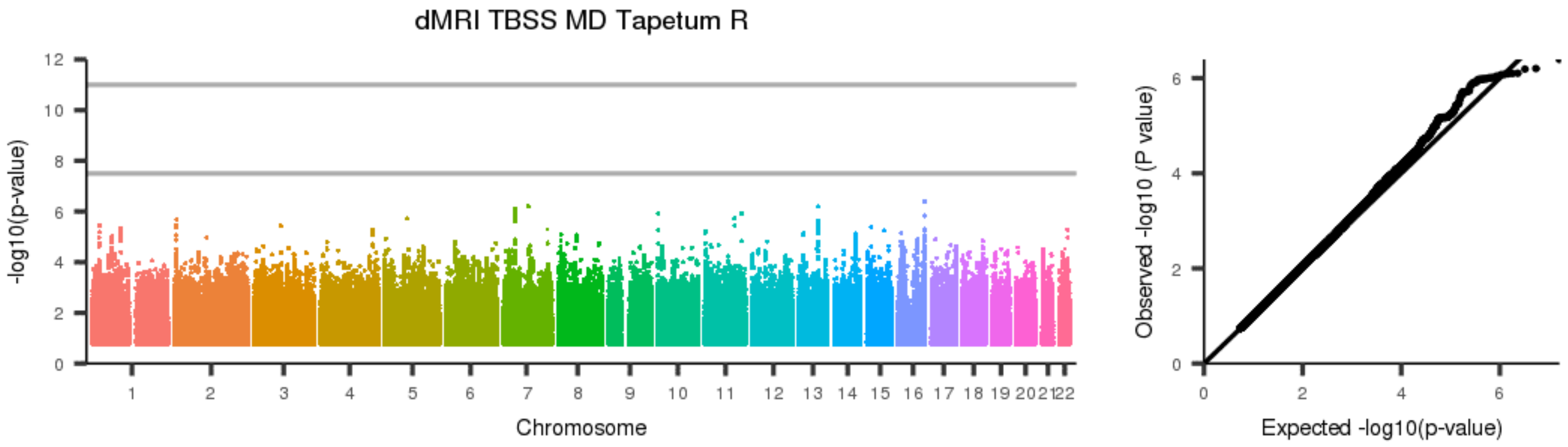

back to list of plots

Figure 291: dMRI TBSS MD Tapetum L

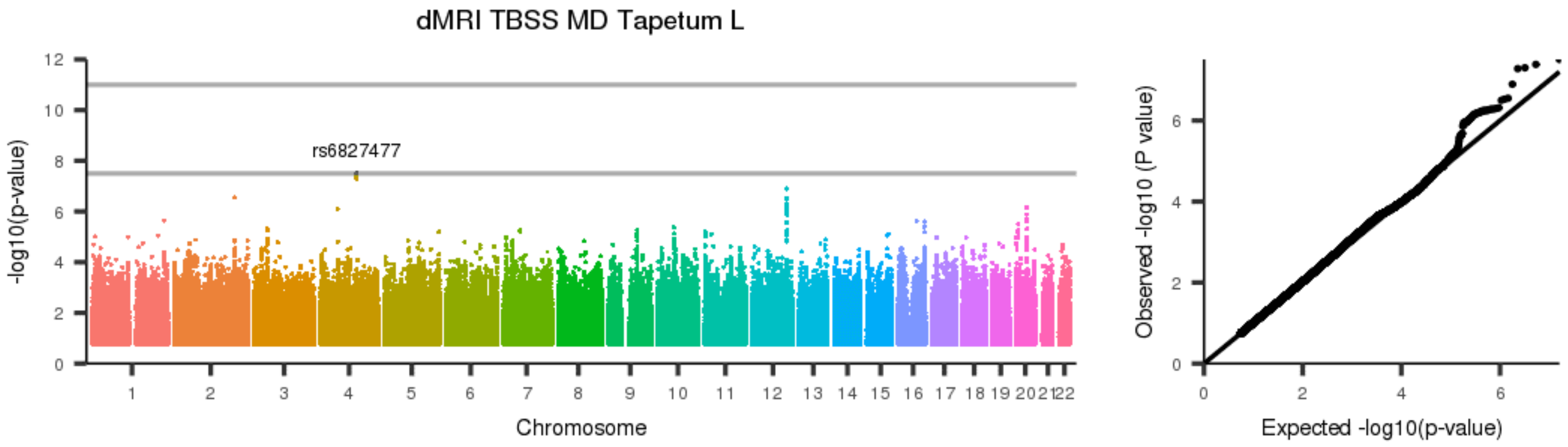

back to list of plots

Figure 292: dMRI TBSS MO Middle cerebellar peduncle

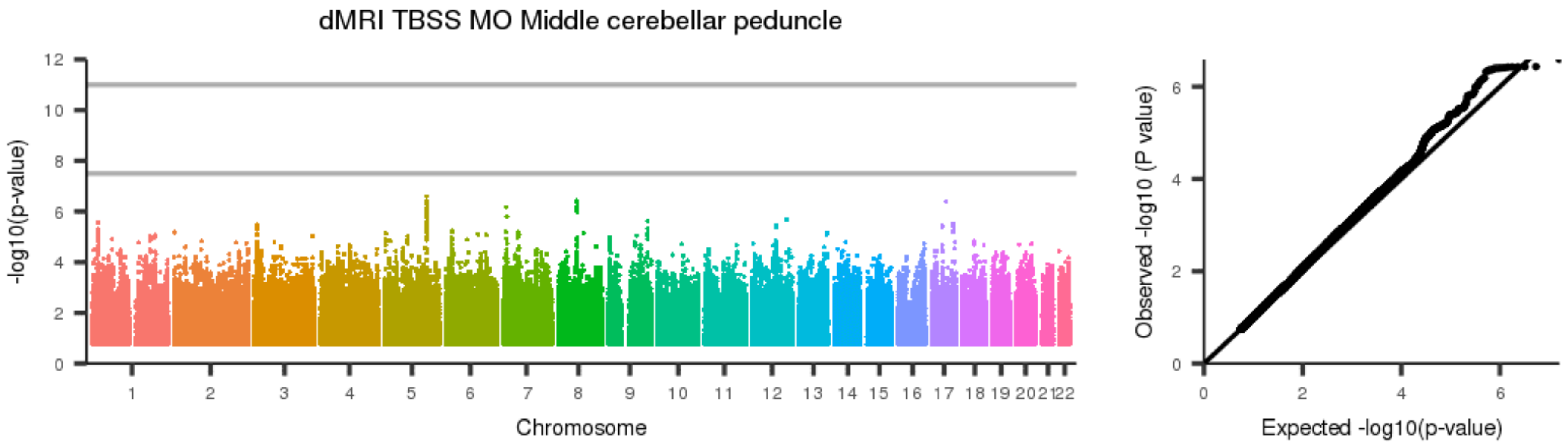

back to list of plots

Figure 293: dMRI TBSS MO Pontine crossing tract

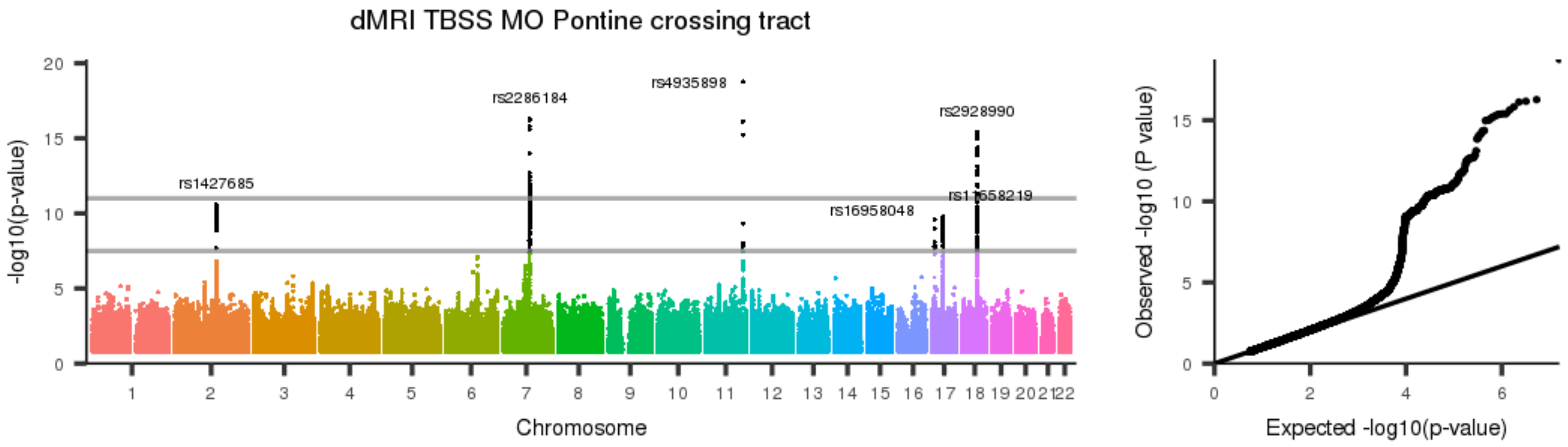

back to list of plots

Figure 294: dMRI TBSS MO Genu of corpus callosum

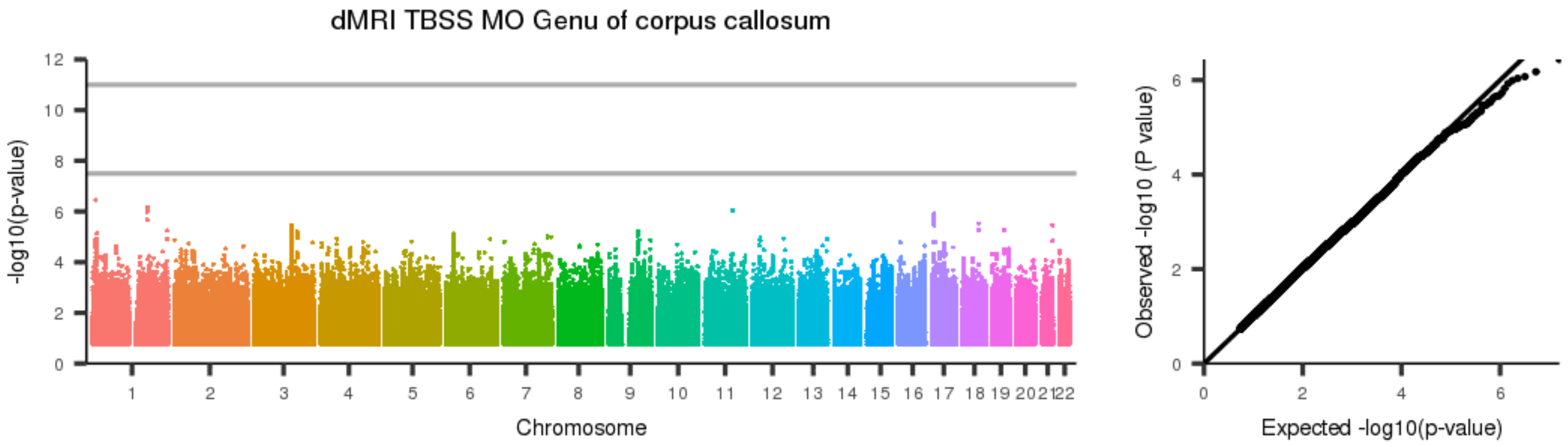

back to list of plots

Figure 295: dMRI TBSS MO Body of corpus callosum

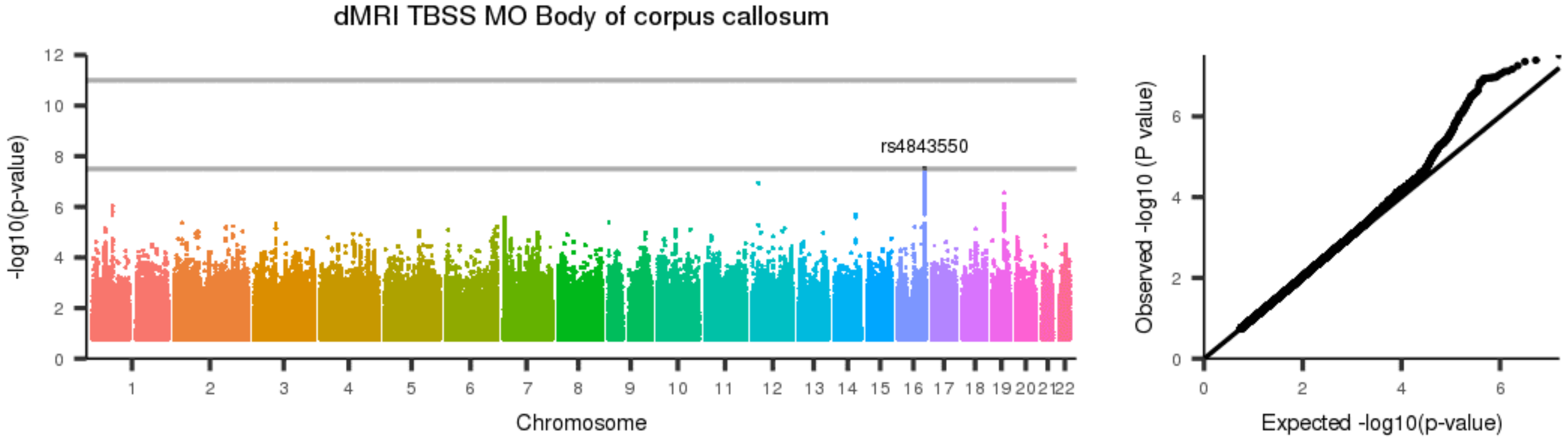

back to list of plots

Figure 296: dMRI TBSS MO Splenium of corpus callosum

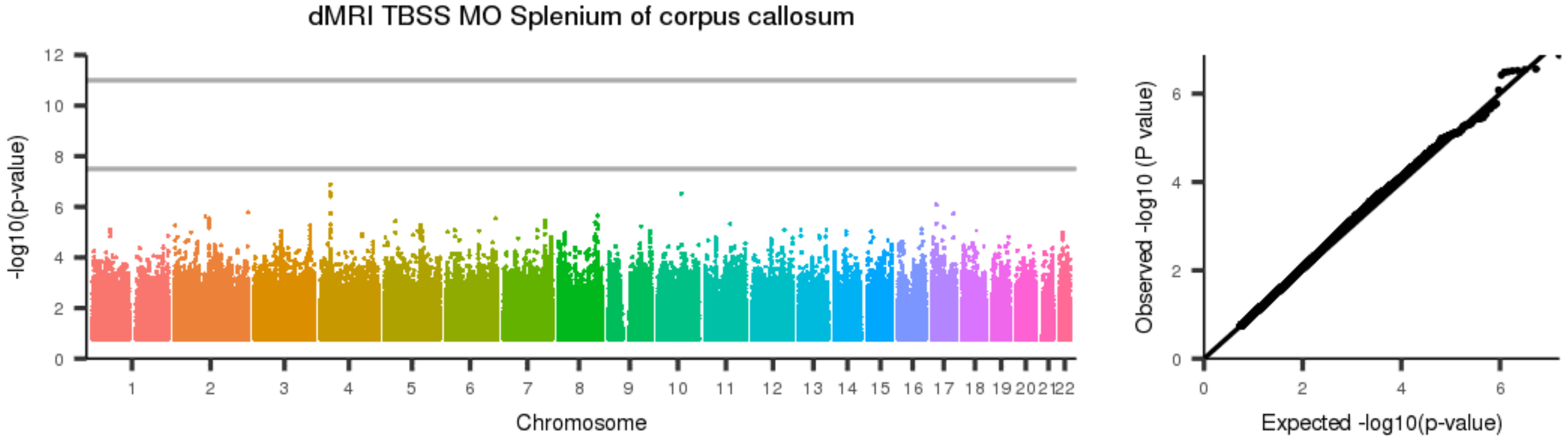

back to list of plots

Figure 297: dMRI TBSS MO Fornix

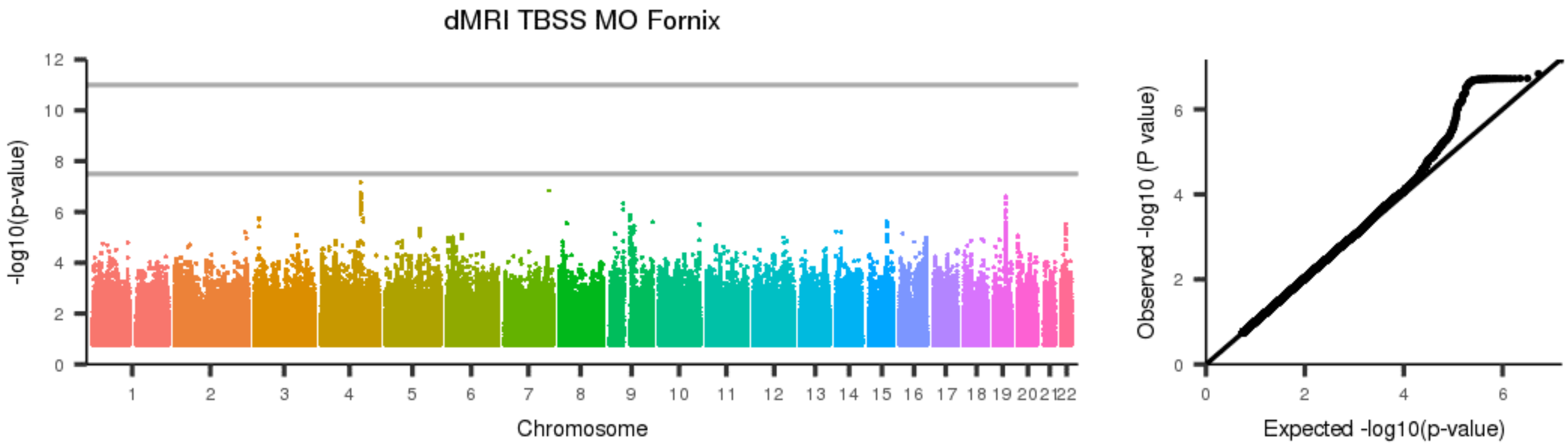

back to list of plots

Figure 298: dMRI TBSS MO Corticospinal tract R

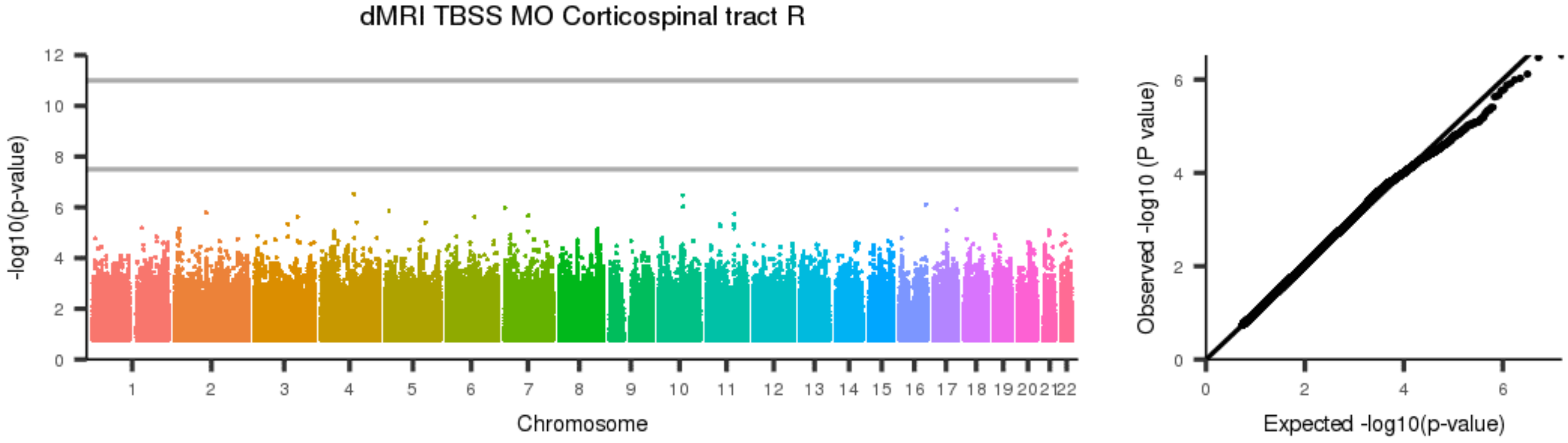

back to list of plots

Figure 299: dMRI TBSS MO Corticospinal tract L

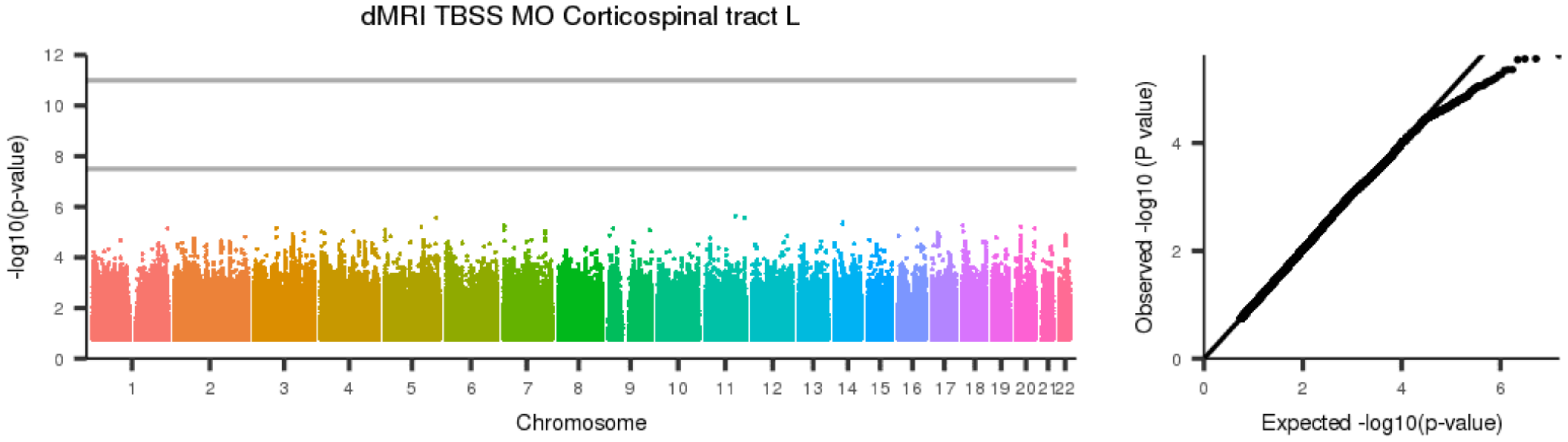

back to list of plots

Figure 300: dMRI TBSS MO Medial lemniscus R

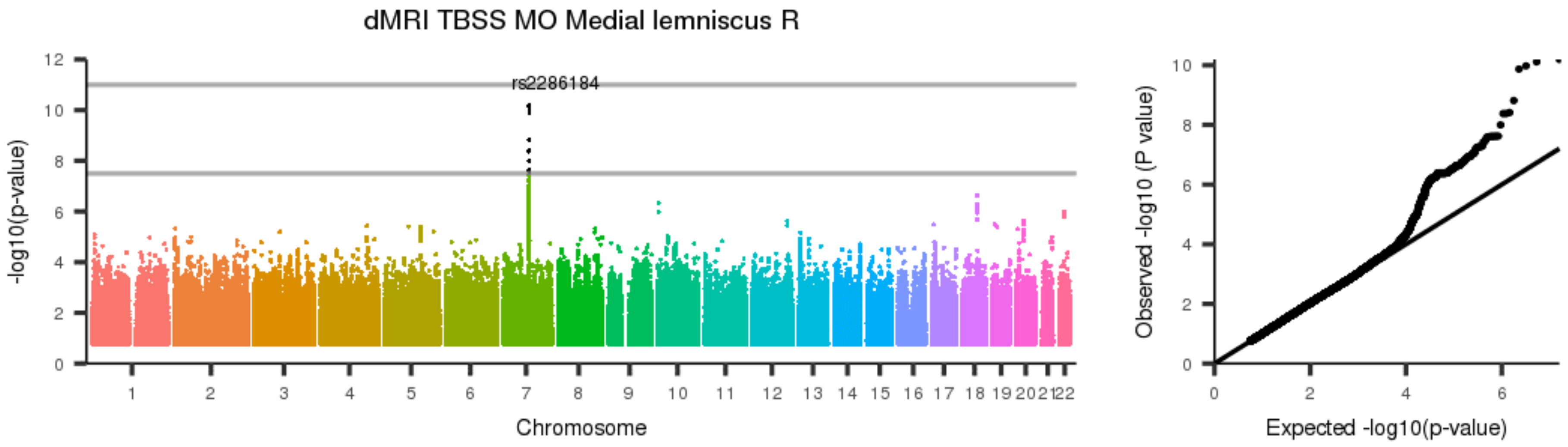

back to list of plots

Figure 301: dMRI TBSS MO Medial lemniscus L

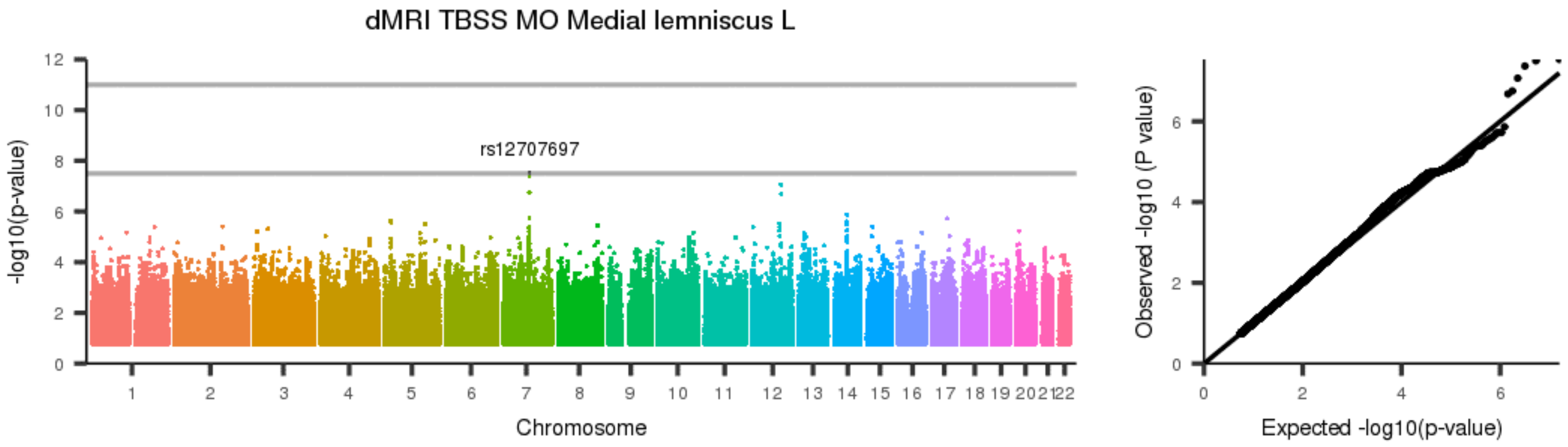

back to list of plots

Figure 302: dMRI TBSS MO Inferior cerebellar peduncle R

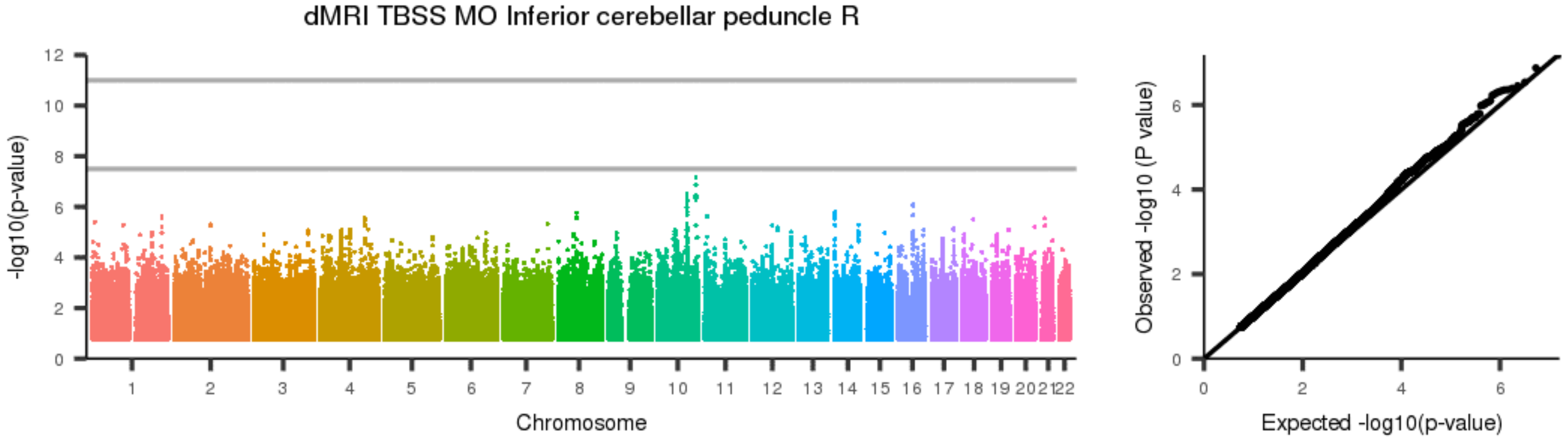

back to list of plots

Figure 303: dMRI TBSS MO Inferior cerebellar peduncle L

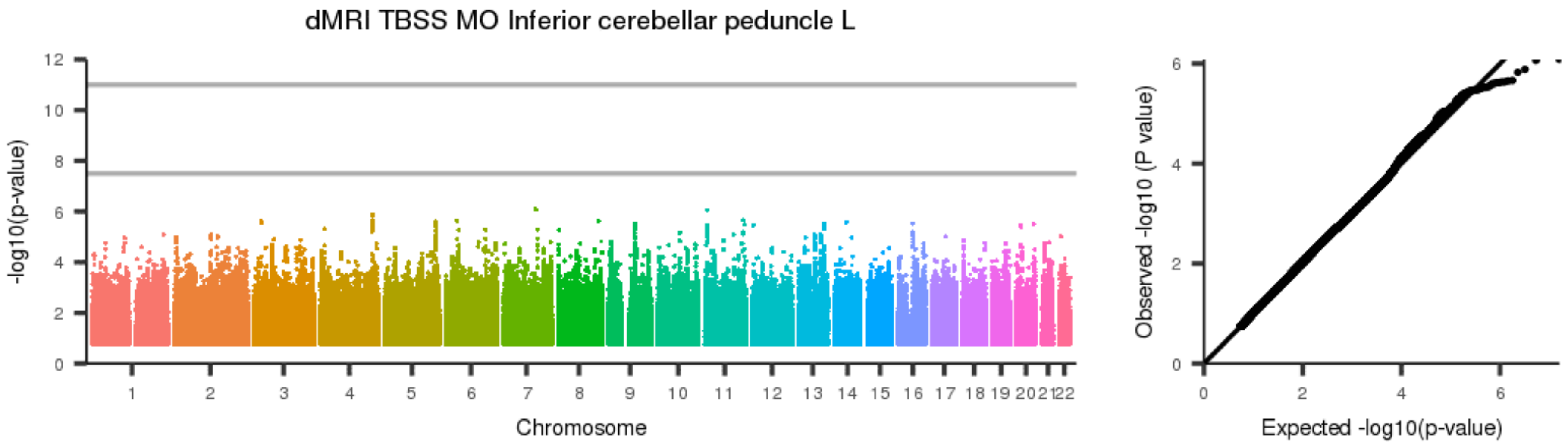

back to list of plots

Figure 304: dMRI TBSS MO Superior cerebellar peduncle R

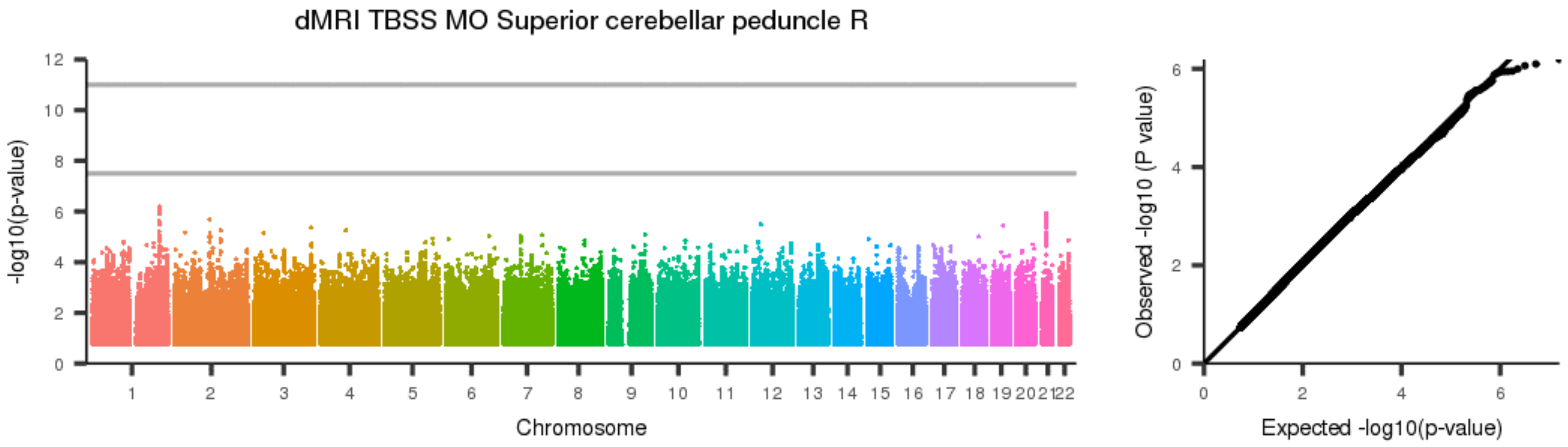

back to list of plots

Figure 305: dMRI TBSS MO Superior cerebellar peduncle L

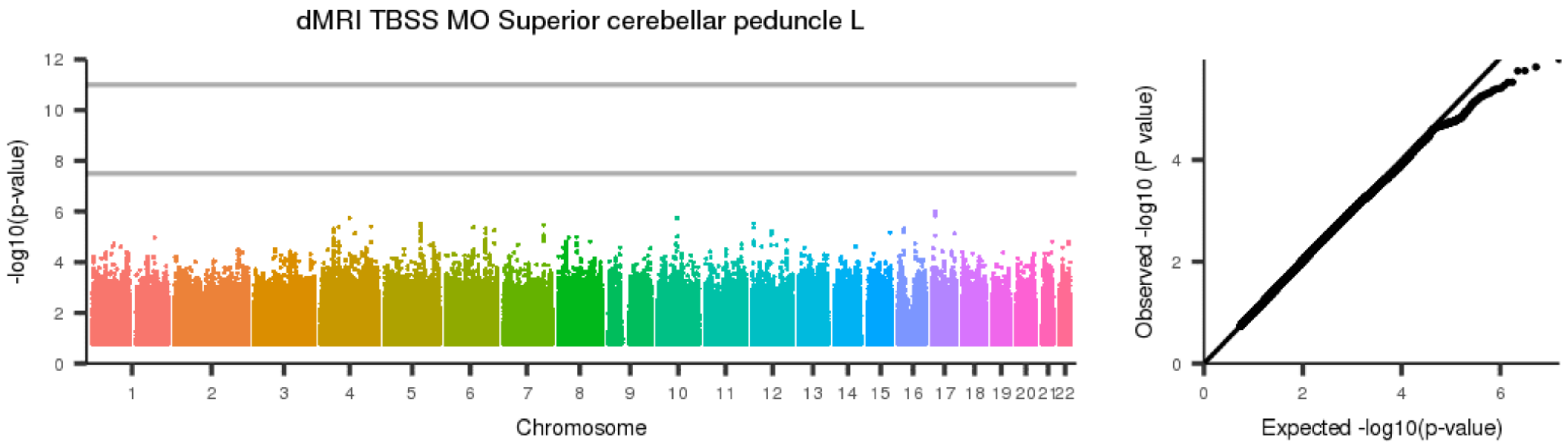

back to list of plots

Figure 306: dMRI TBSS MO Cerebral peduncle R

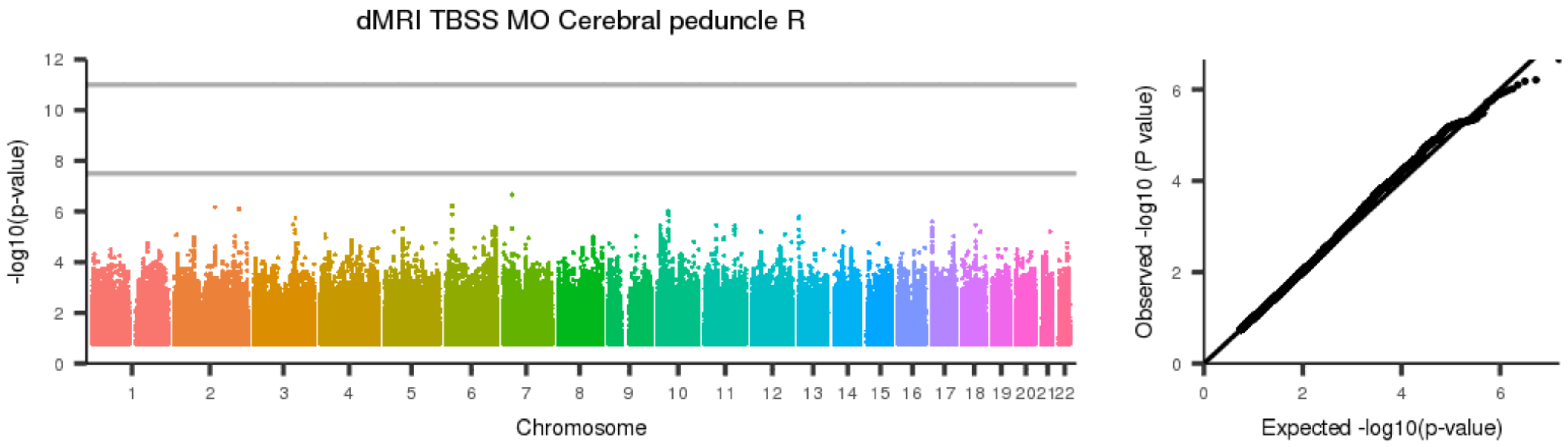

back to list of plots

Figure 307: dMRI TBSS MO Cerebral peduncle L

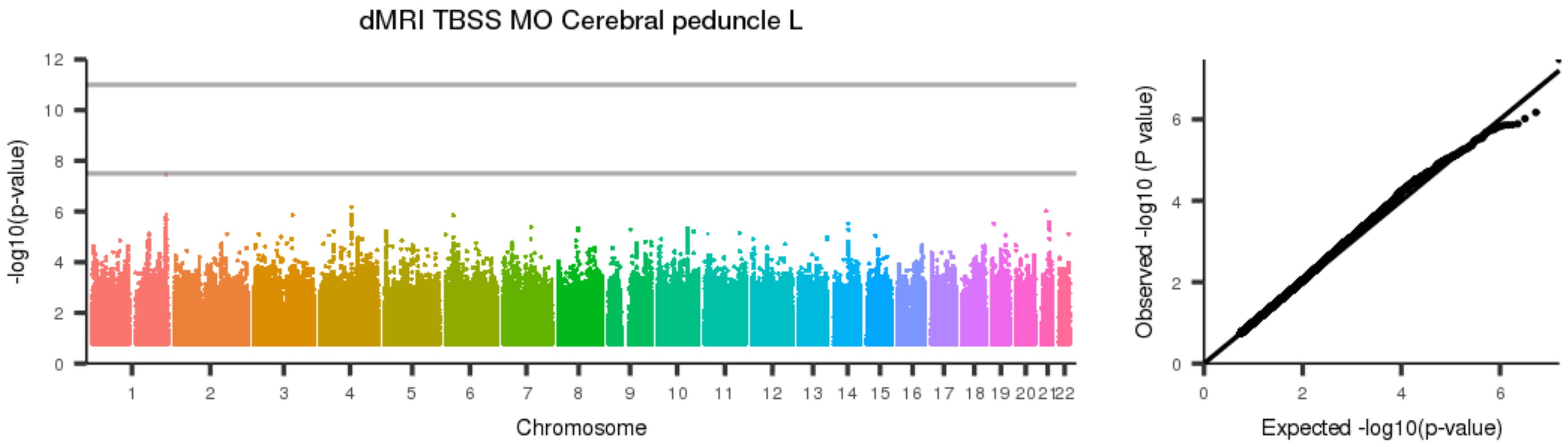

back to list of plots

Figure 308: dMRI TBSS MO Anterior limb of internal capsule R

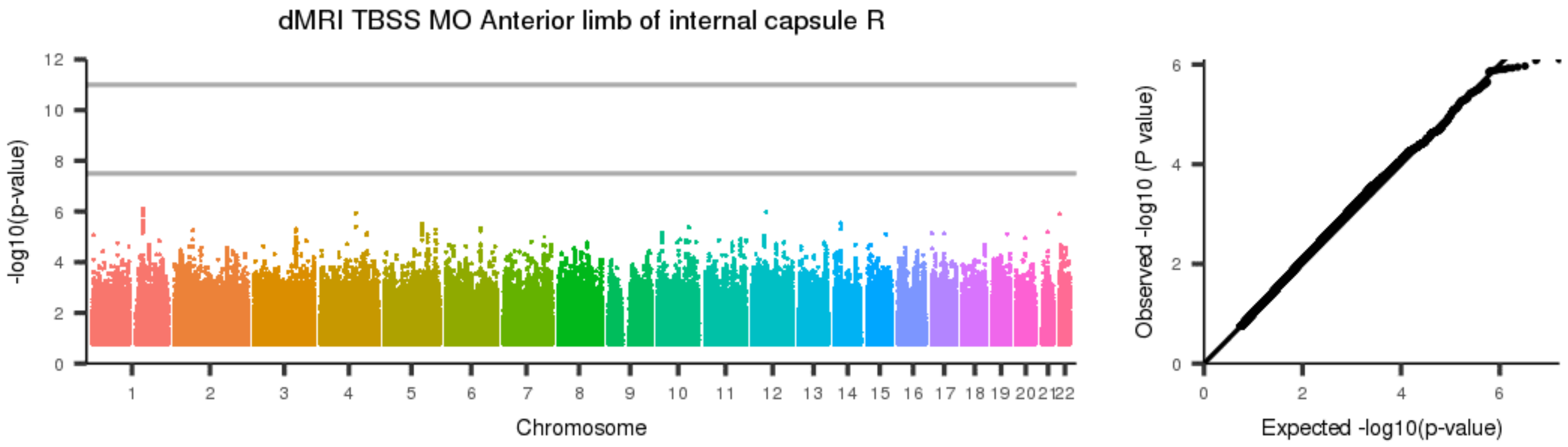

back to list of plots

Figure 309: dMRI TBSS MO Anterior limb of internal capsule L

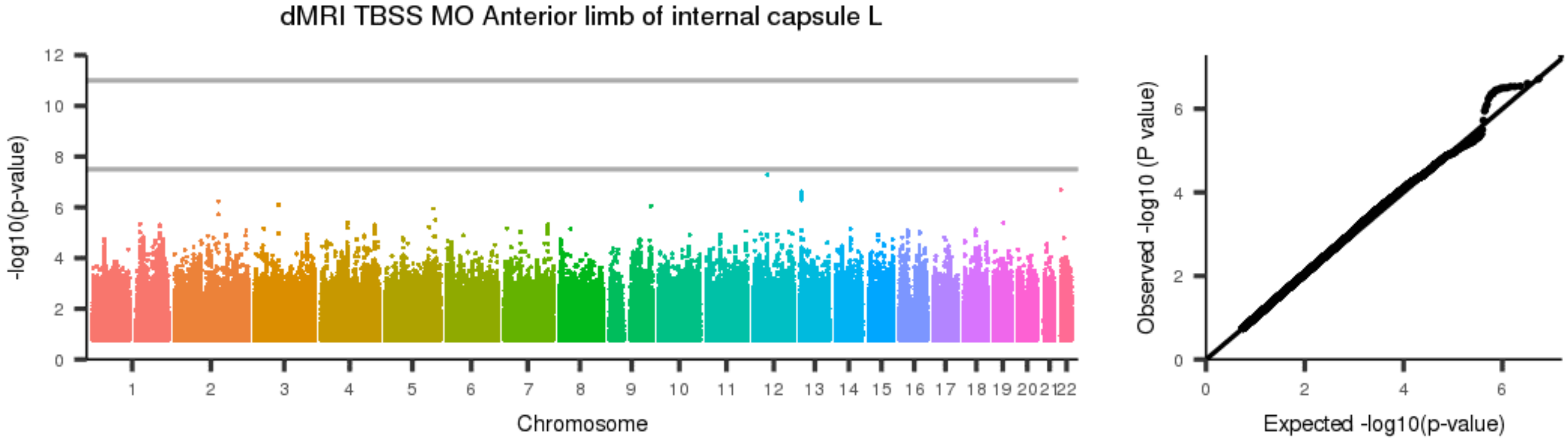

back to list of plots

Figure 310: dMRI TBSS MO Posterior limb of internal capsule R

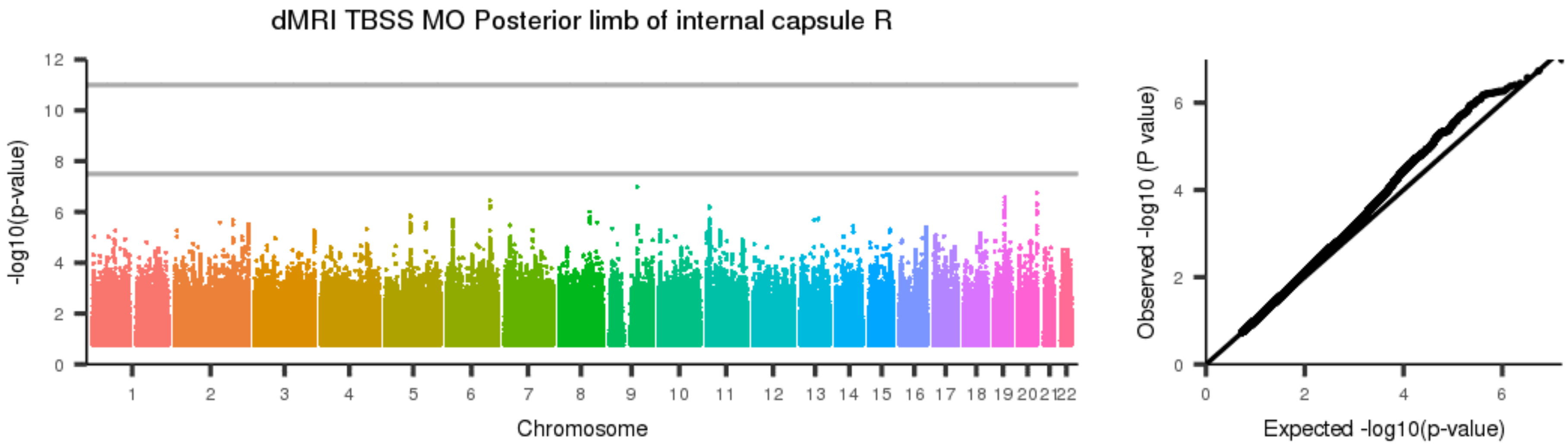

back to list of plots

Figure 311: dMRI TBSS MO Posterior limb of internal capsule L

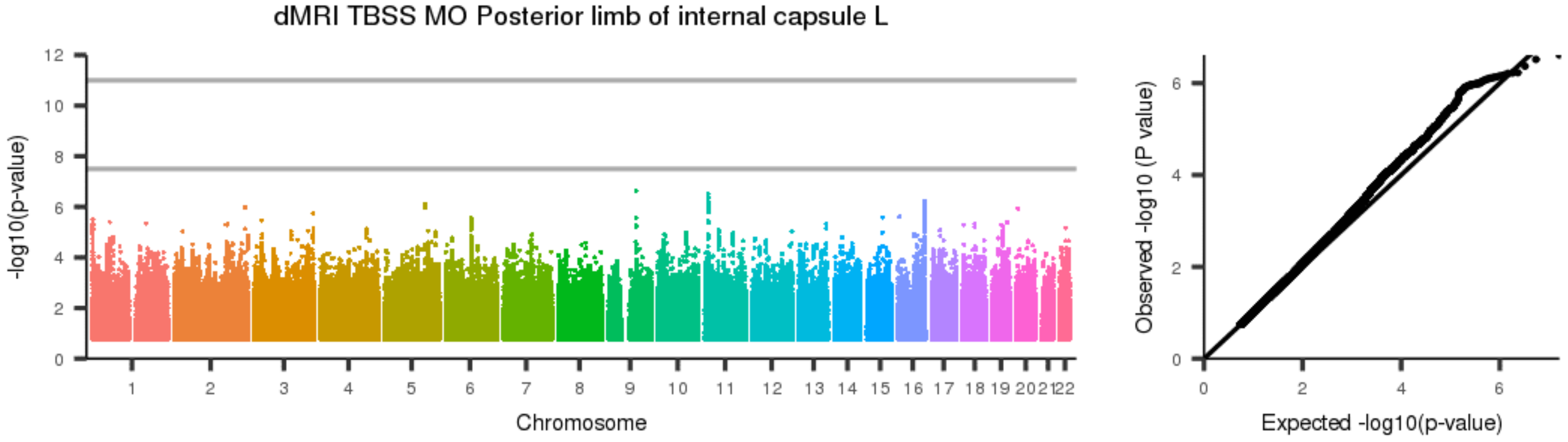

back to list of plots

Figure 312: dMRI TBSS MO Retrolenticular part of internal capsule R

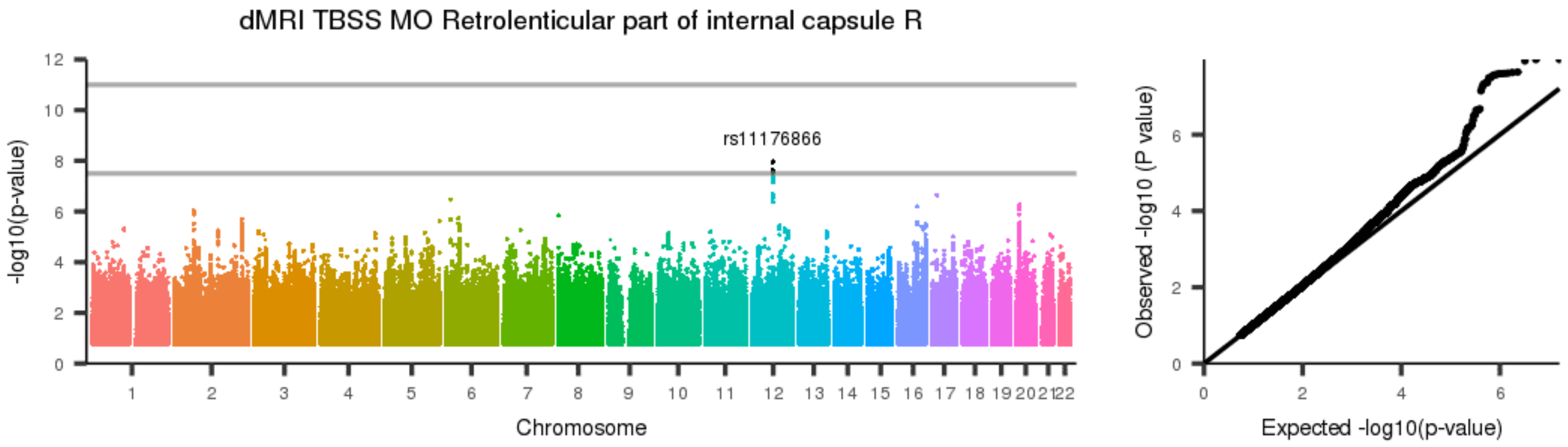

back to list of plots

Figure 313: dMRI TBSS MO Retrolenticular part of internal capsule L

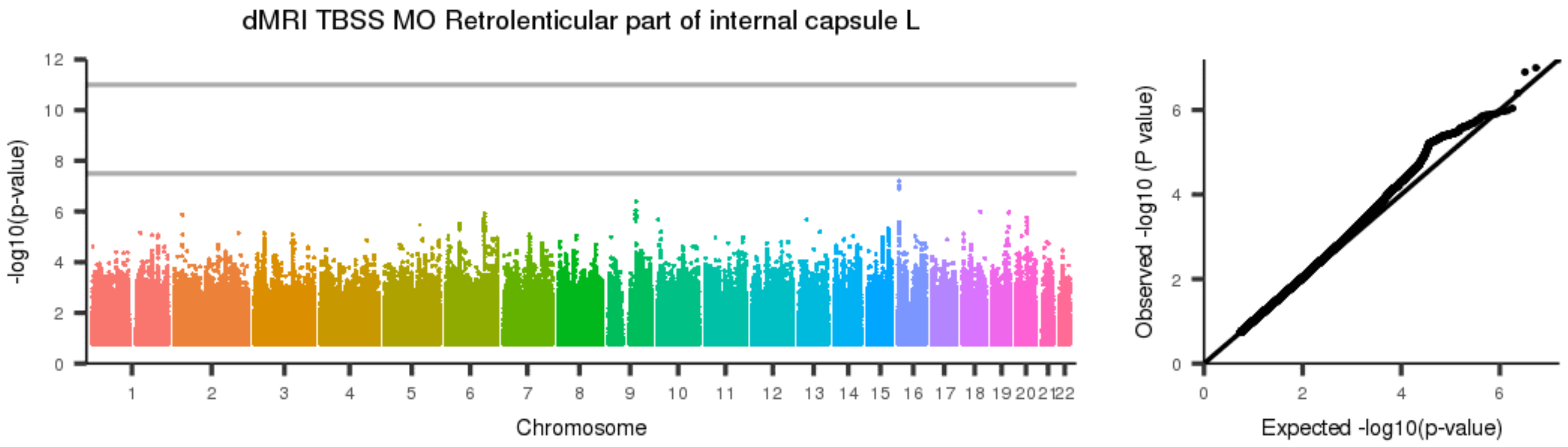

back to list of plots

Figure 314: dMRI TBSS MO Anterior corona radiata R

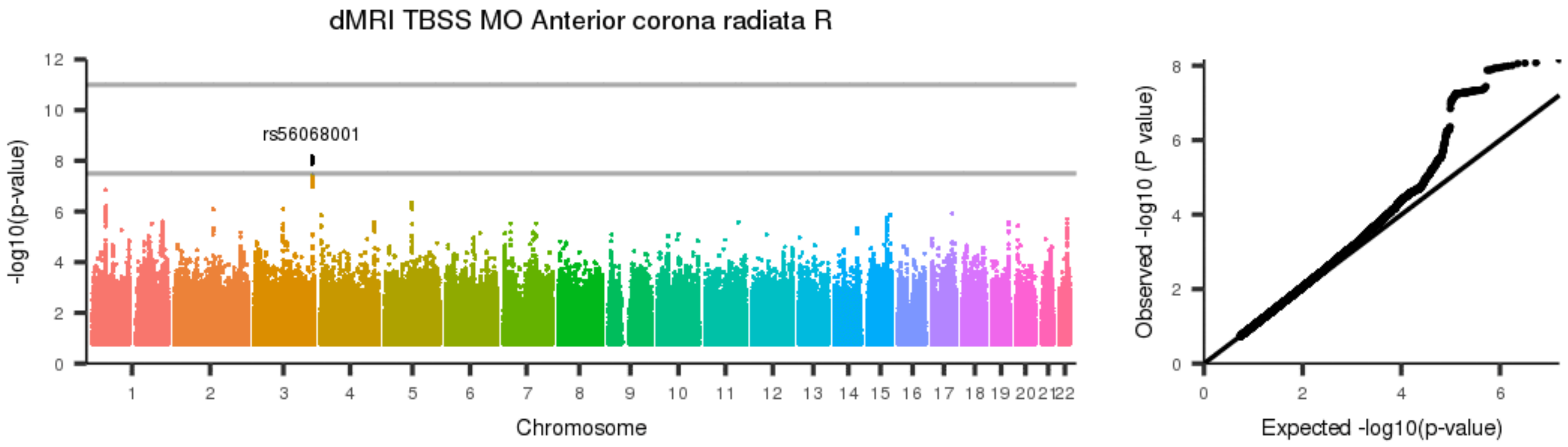

back to list of plots

Figure 315: dMRI TBSS MO Anterior corona radiata L

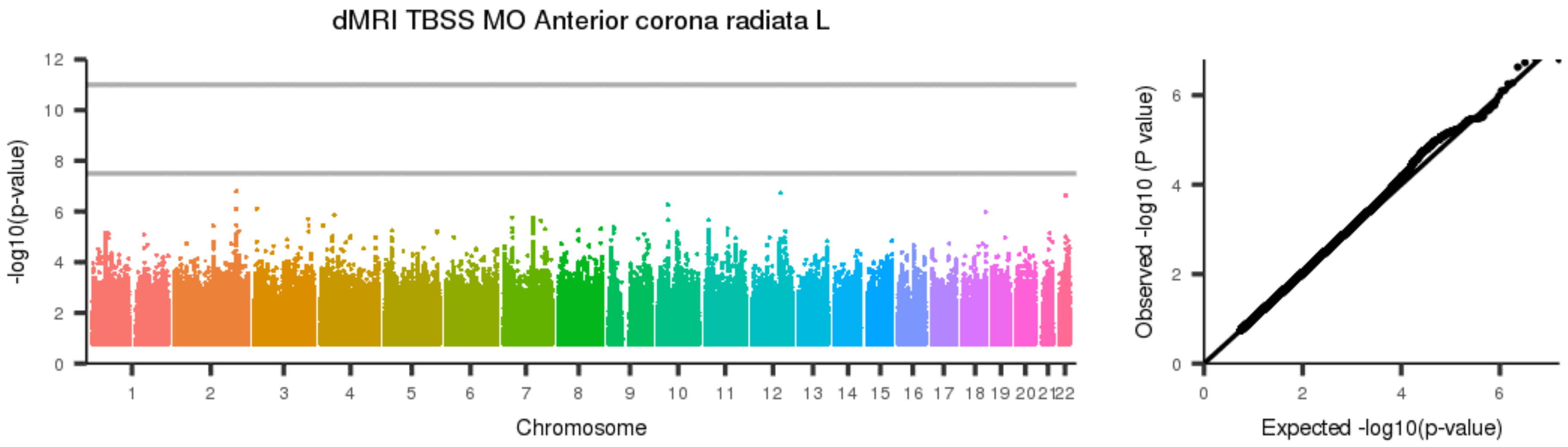

back to list of plots

Figure 316: dMRI TBSS MO Superior corona radiata R

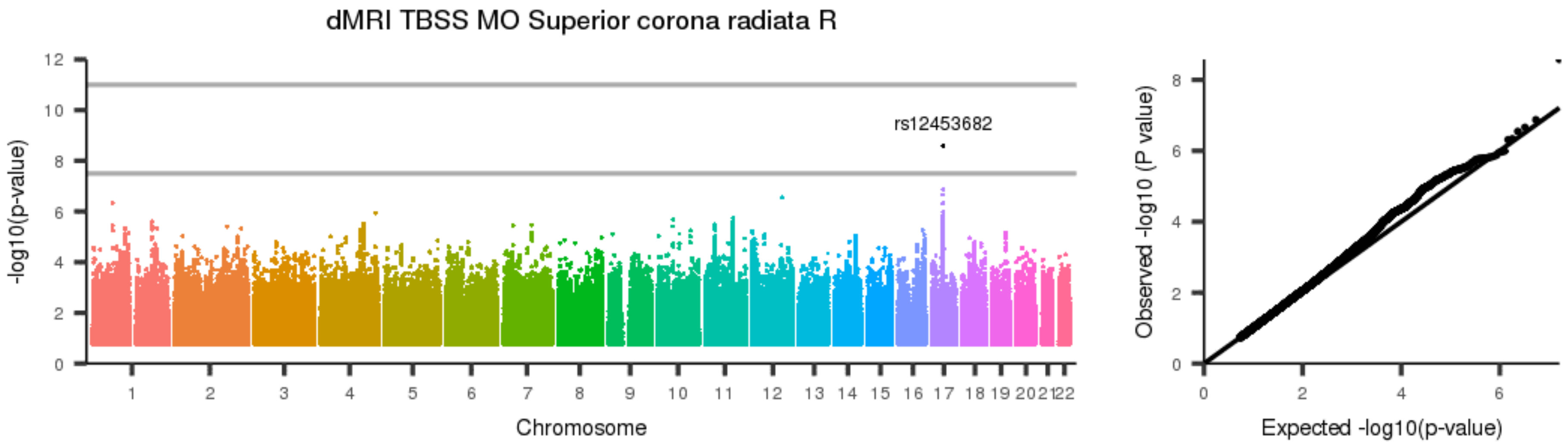

back to list of plots

Figure 317: dMRI TBSS MO Superior corona radiata L

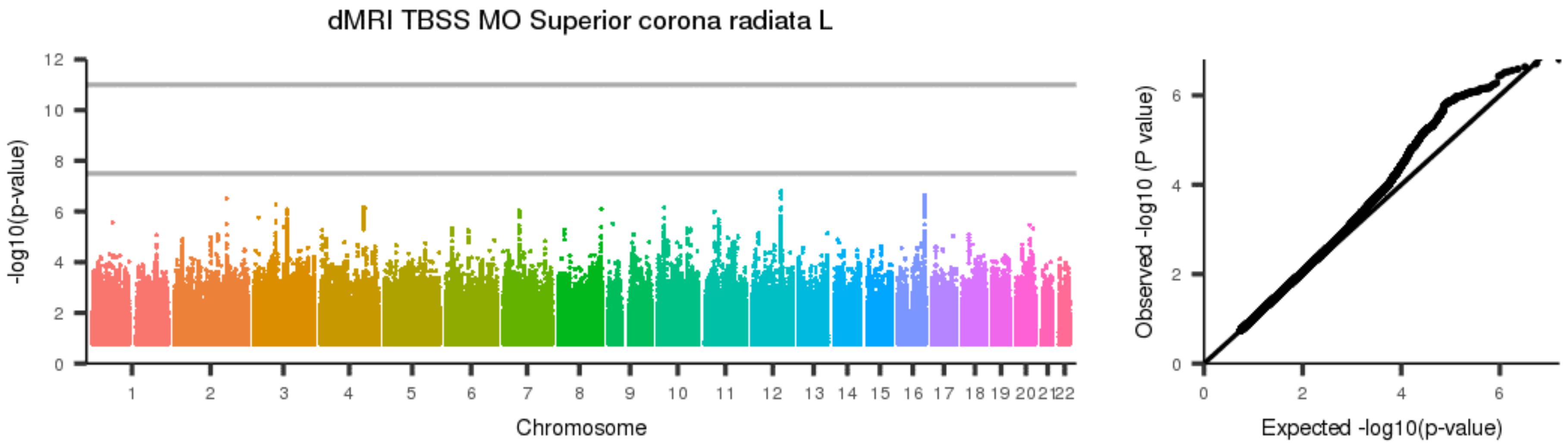

back to list of plots

Figure 318: dMRI TBSS MO Posterior corona radiata R

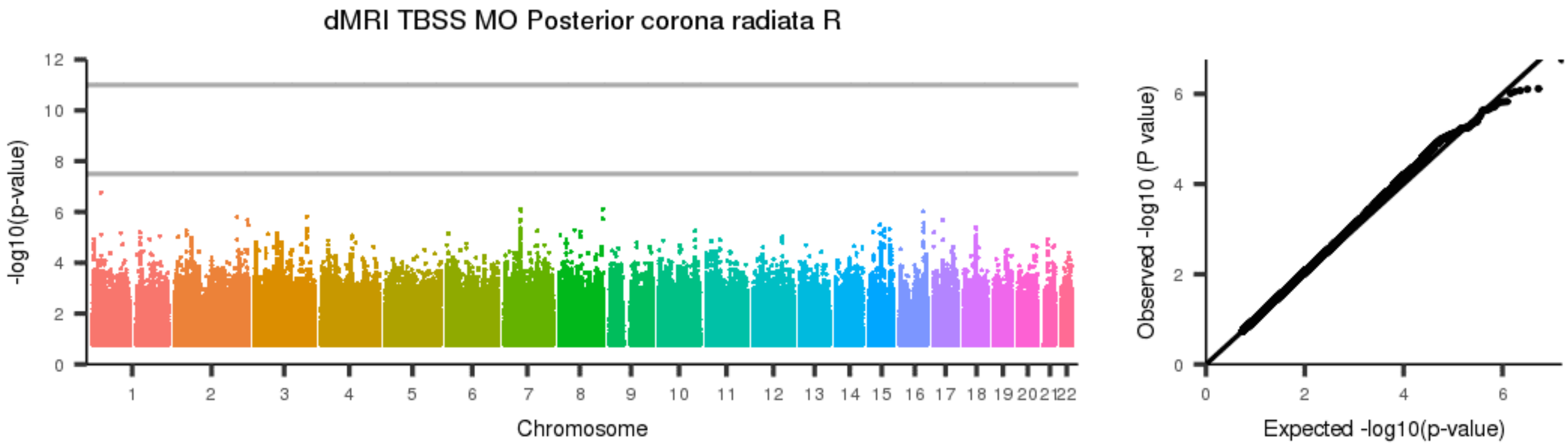

back to list of plots

Figure 319: dMRI TBSS MO Posterior corona radiata L

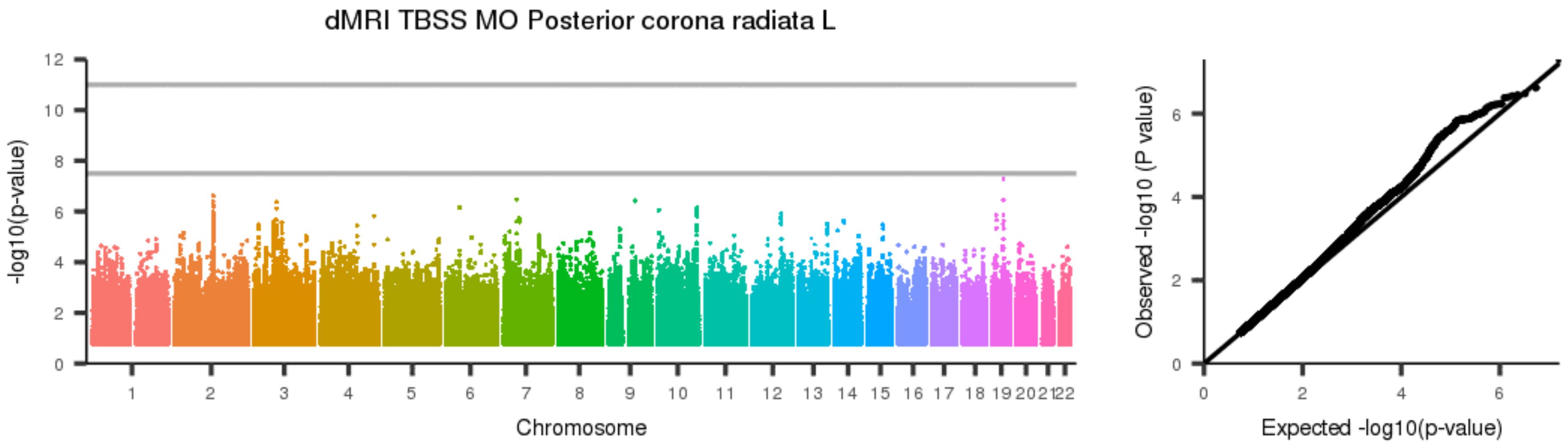

back to list of plots

Figure 320: dMRI TBSS MO Posterior thalamic radiation R

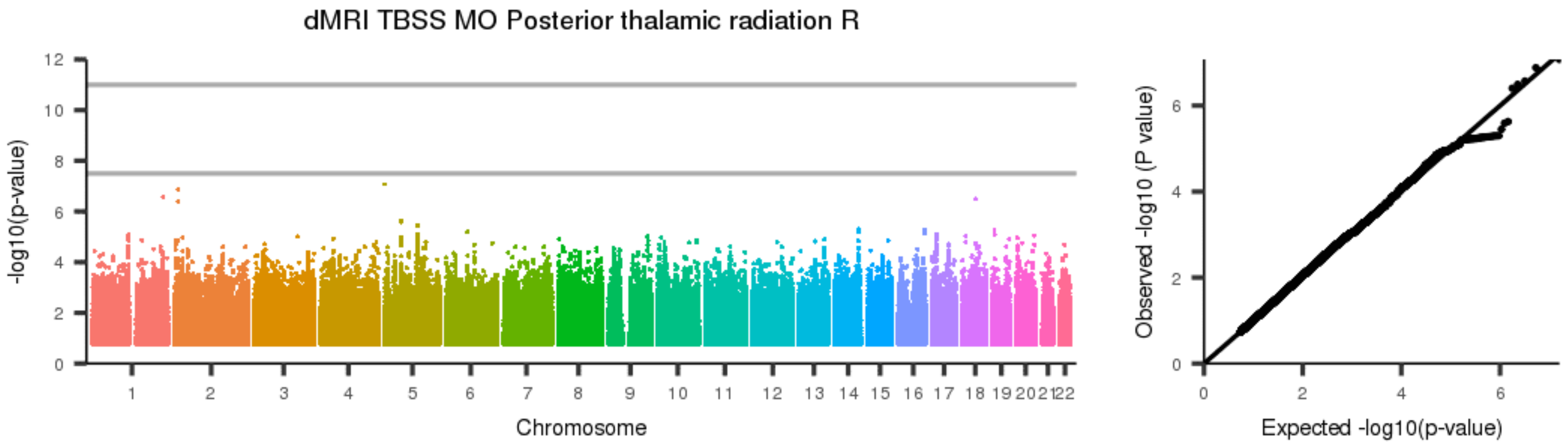

back to list of plots

Figure 321: dMRI TBSS MO Posterior thalamic radiation L

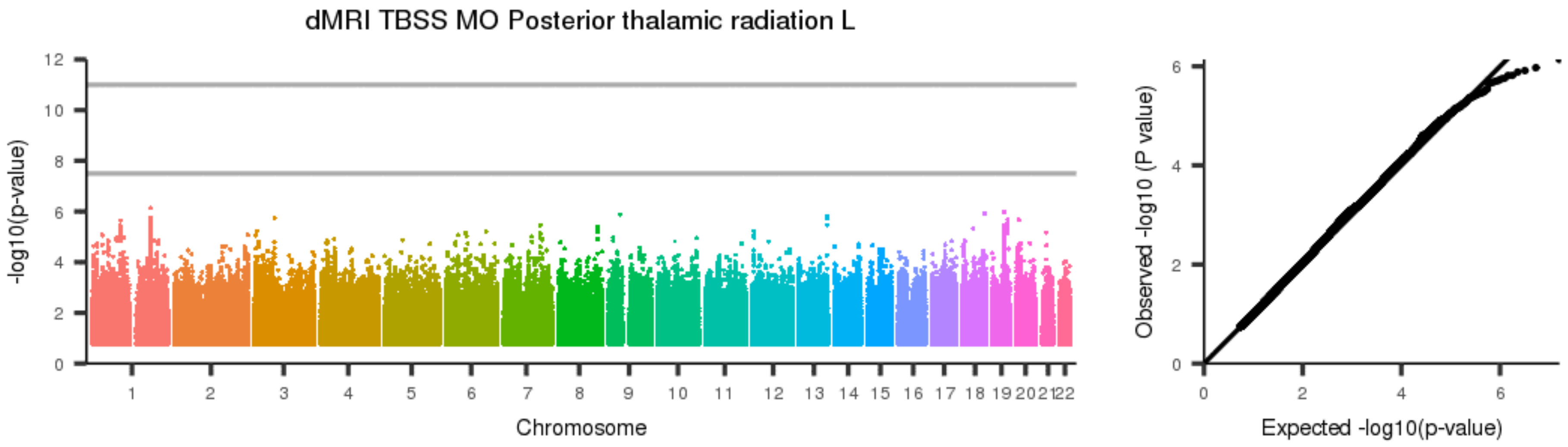

back to list of plots

Figure 322: dMRI TBSS MO Sagittal stratum R

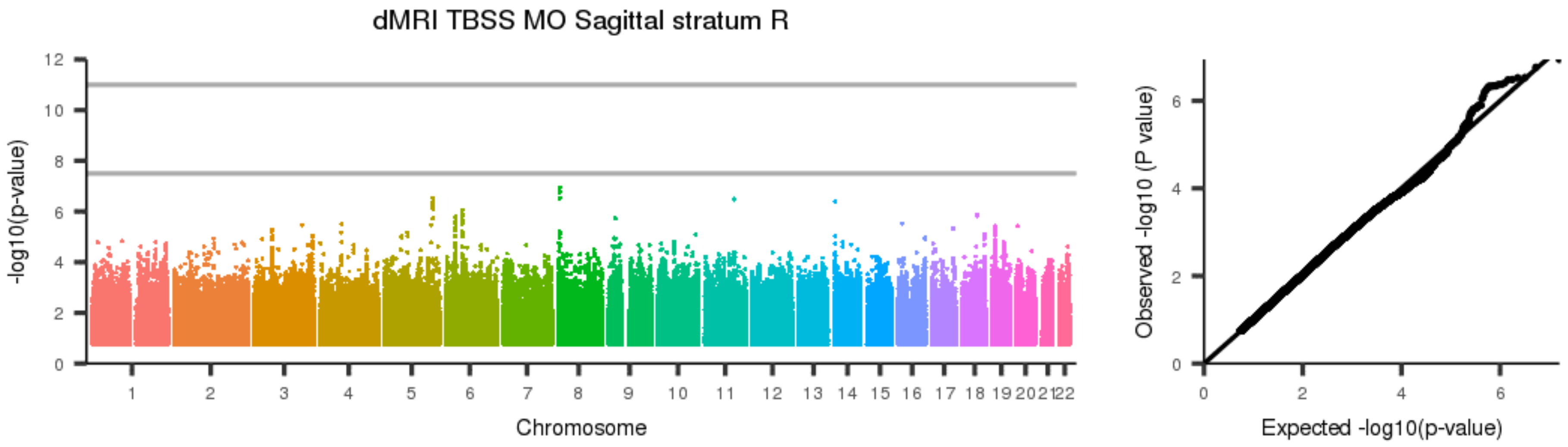

back to list of plots

Figure 323: dMRI TBSS MO Sagittal stratum L

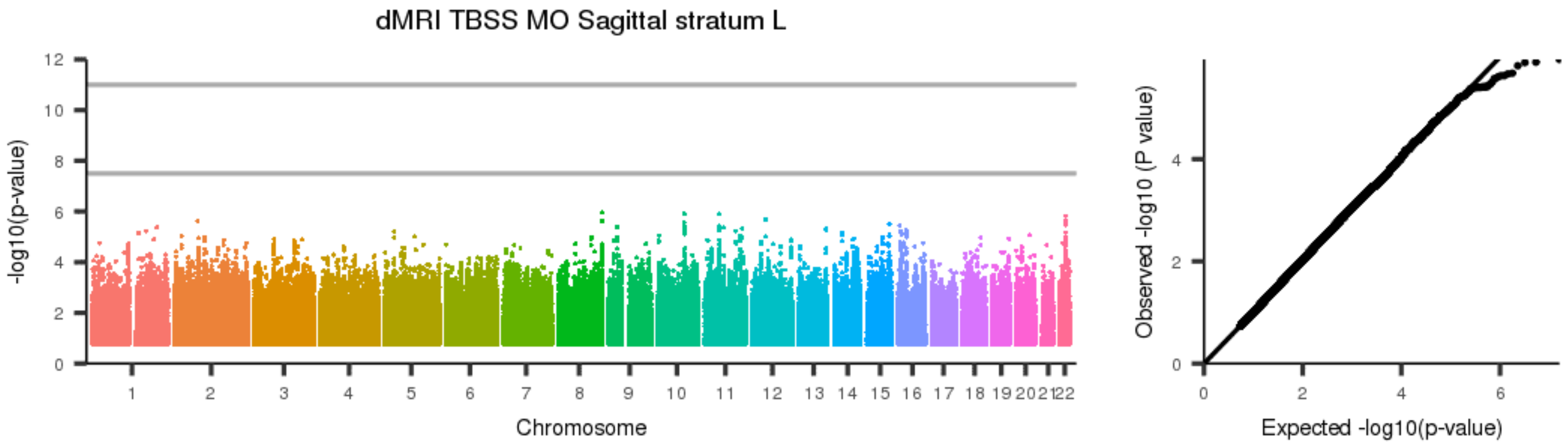

back to list of plots

Figure 324: dMRI TBSS MO External capsule R

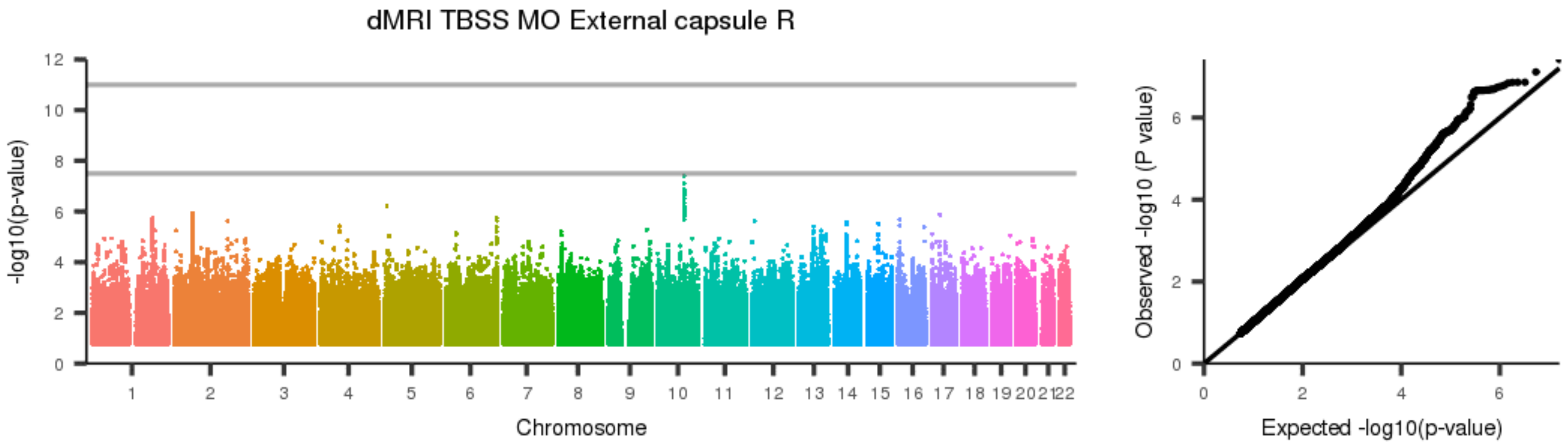

back to list of plots

Figure 325: dMRI TBSS MO External capsule L

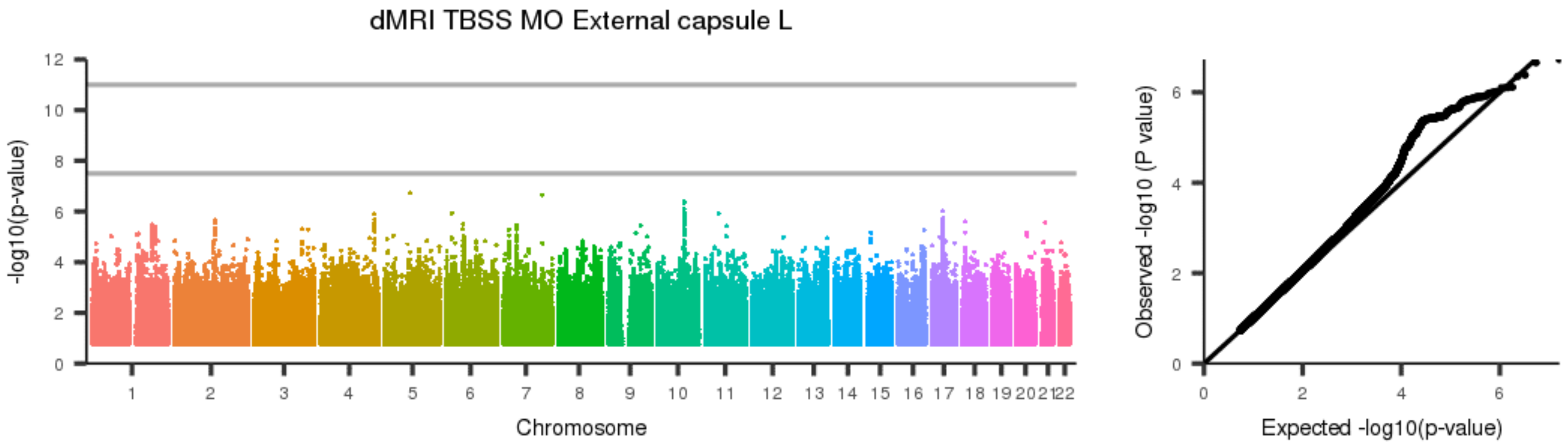

back to list of plots

Figure 326: dMRI TBSS MO Cingulum cingulate gyrus R

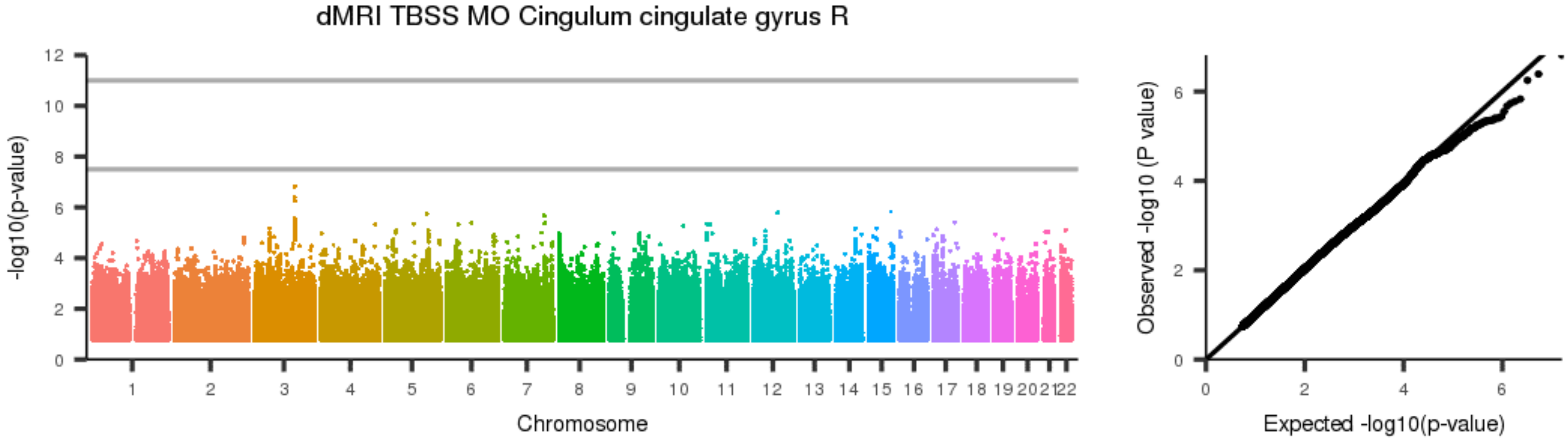

back to list of plots

Figure 327: dMRI TBSS MO Cingulum cingulate gyrus L

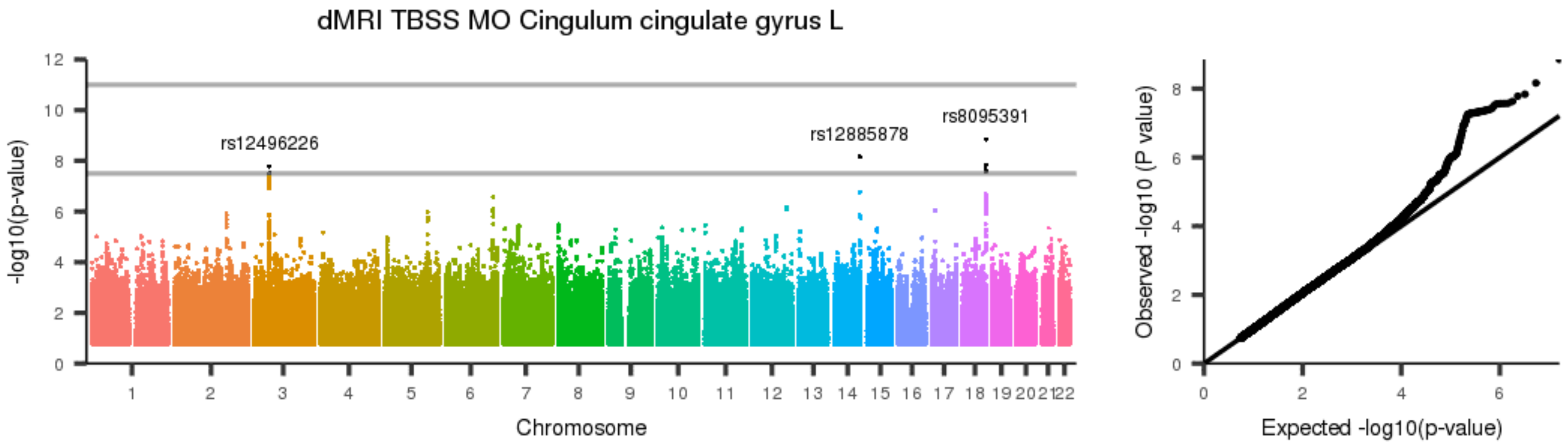

back to list of plots

Figure 328: dMRI TBSS MO Cingulum hippocampus R

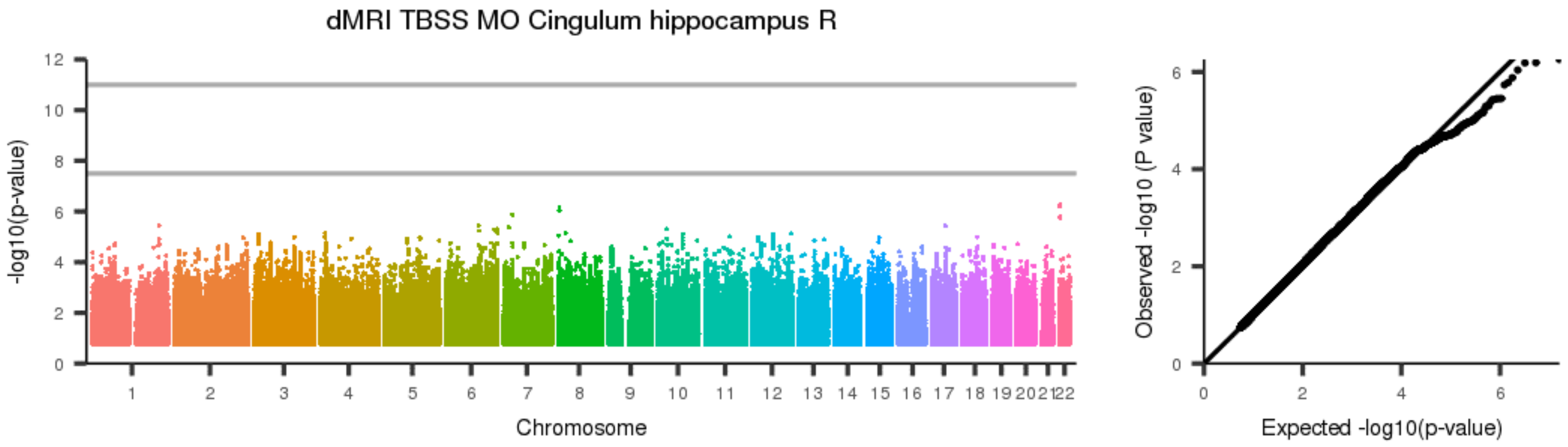

back to list of plots

Figure 329: dMRI TBSS MO Cingulum hippocampus L

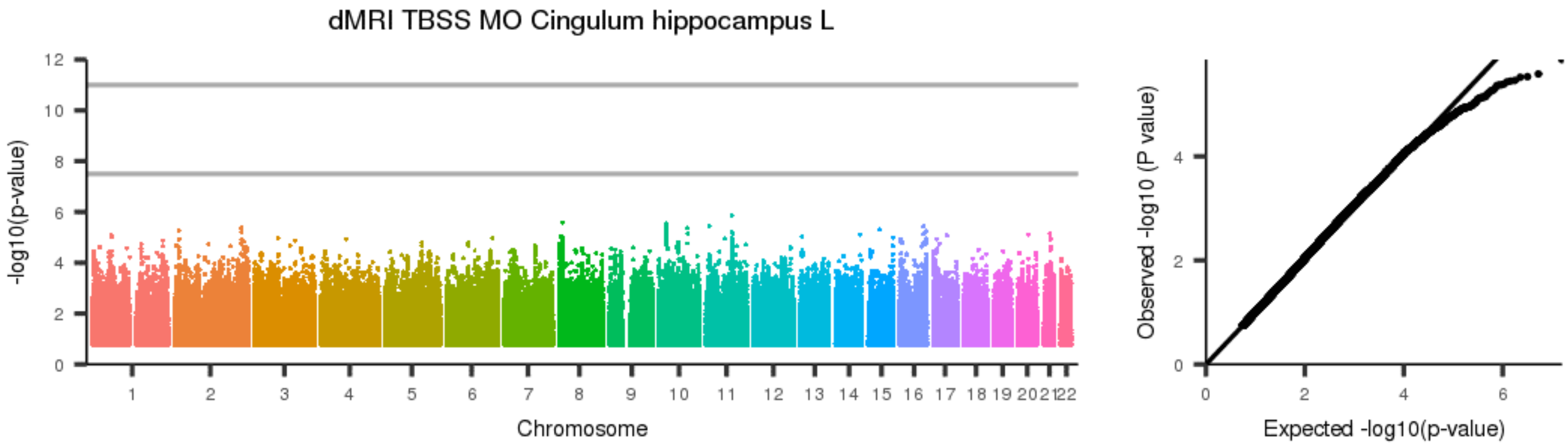

back to list of plots

Figure 330: dMRI TBSS MO Fornix cres+Stria terminalis R

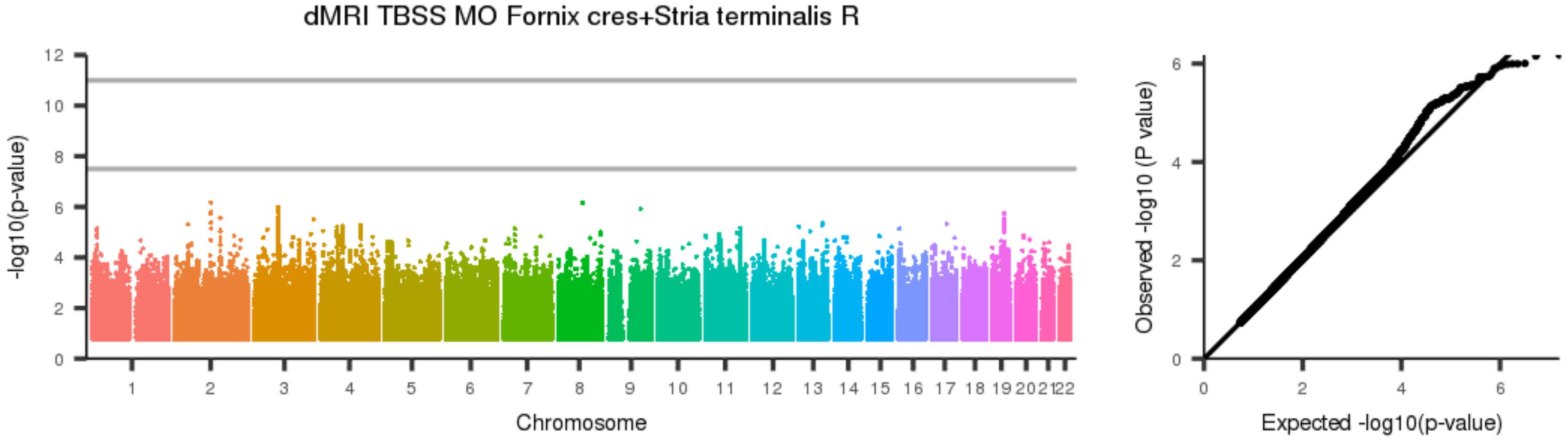

back to list of plots

Figure 331: dMRI TBSS MO Fornix cres+Stria terminalis L

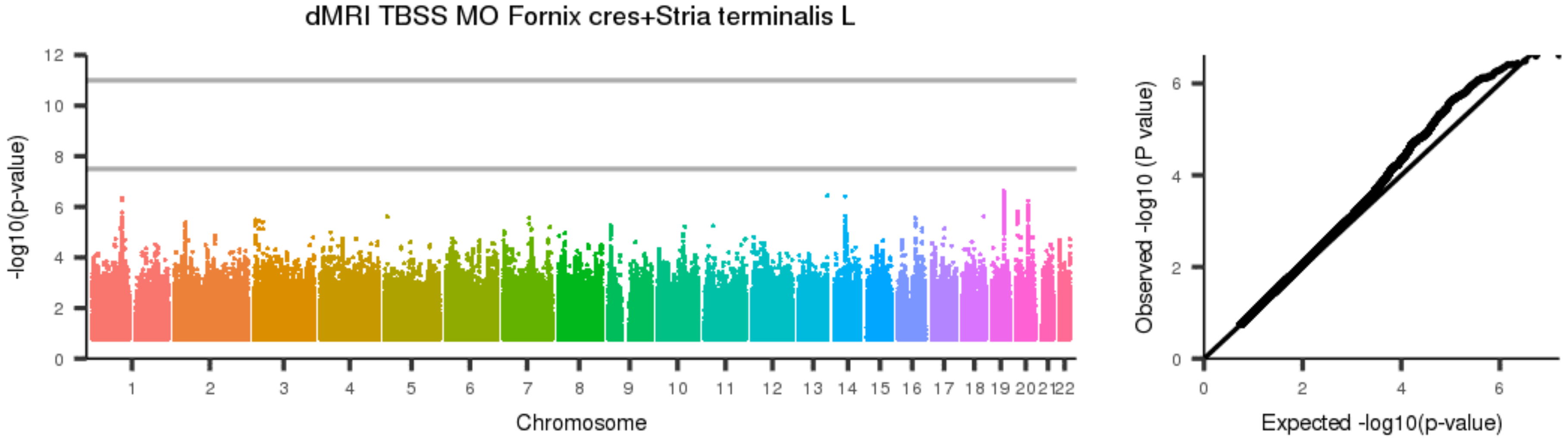

back to list of plots

Figure 332: dMRI TBSS MO Superior longitudinal fasciculus R

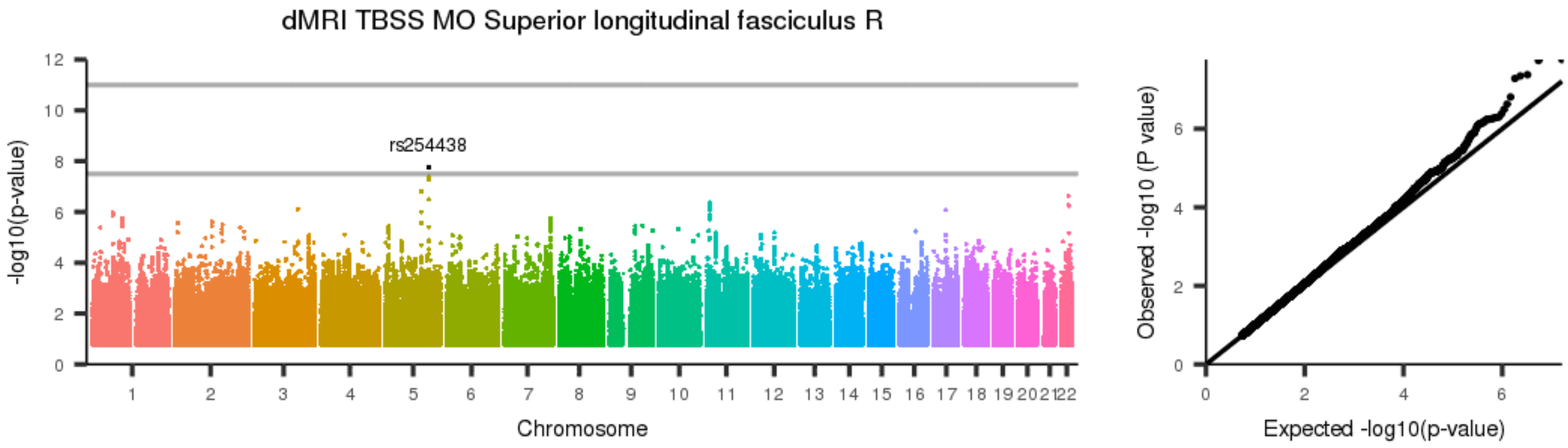

back to list of plots

Figure 333: dMRI TBSS MO Superior longitudinal fasciculus L

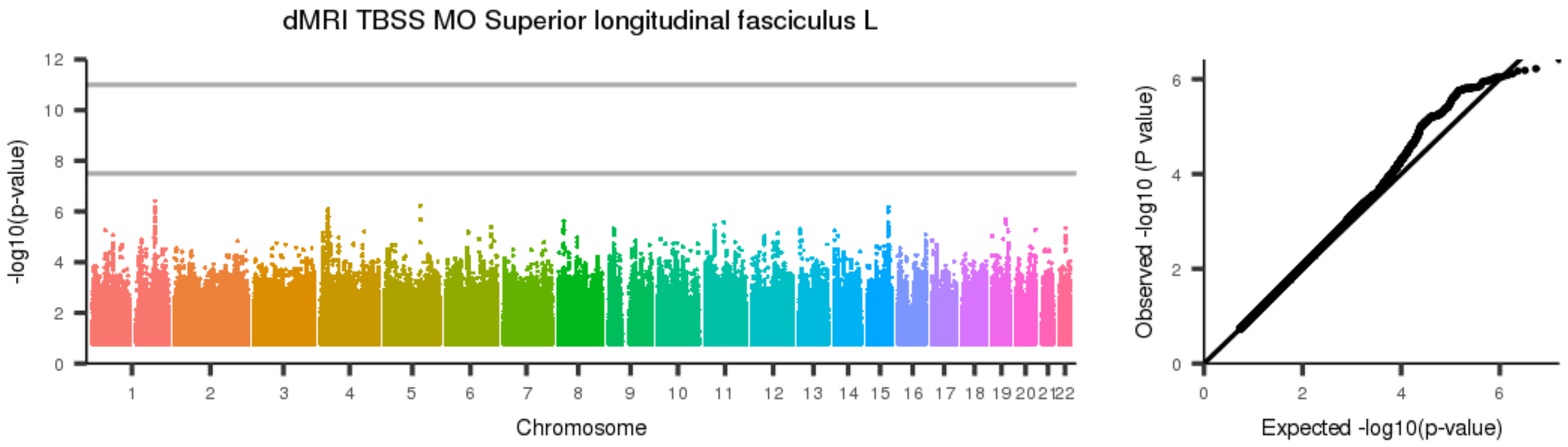

back to list of plots

Figure 334: dMRI TBSS MO Superior fronto-occipital fasciculus R

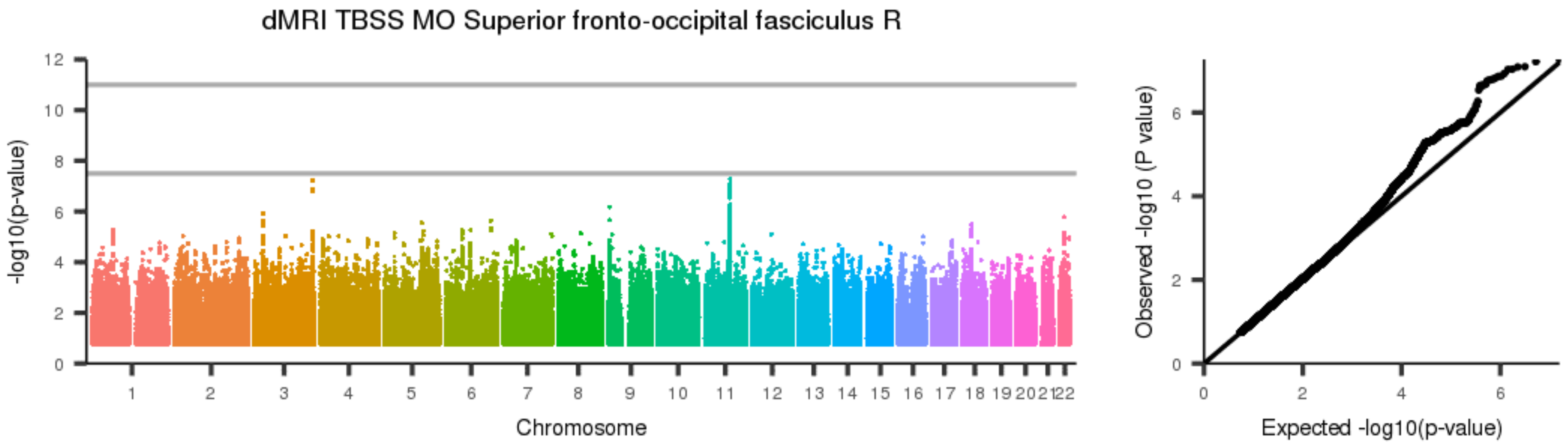

back to list of plots

Figure 335: dMRI TBSS MO Superior fronto-occipital fasciculus L

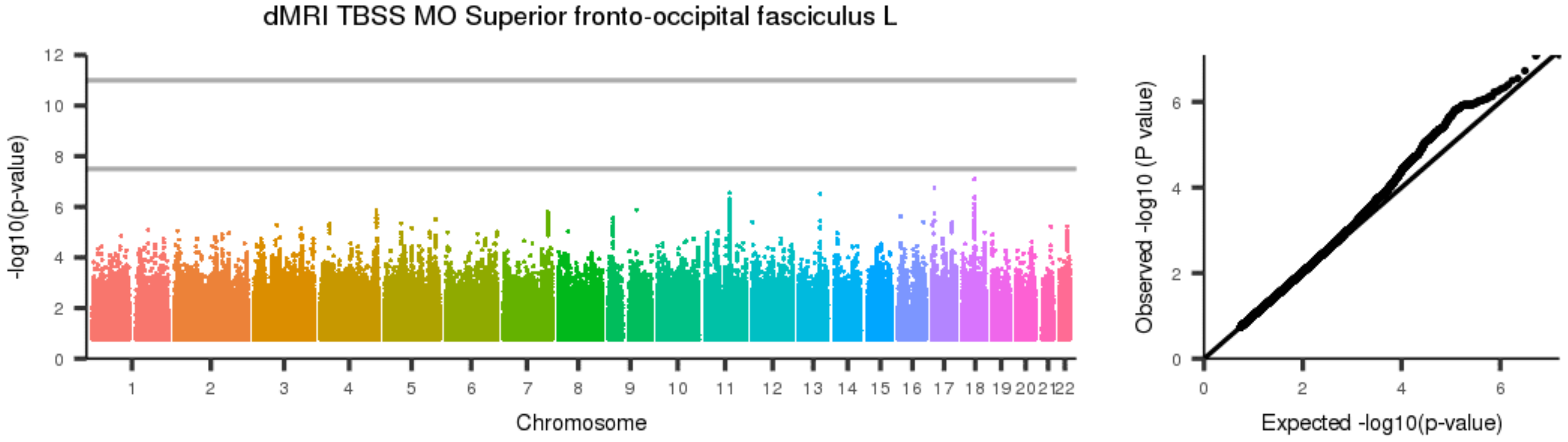

back to list of plots

Figure 336: dMRI TBSS MO Uncinate fasciculus R

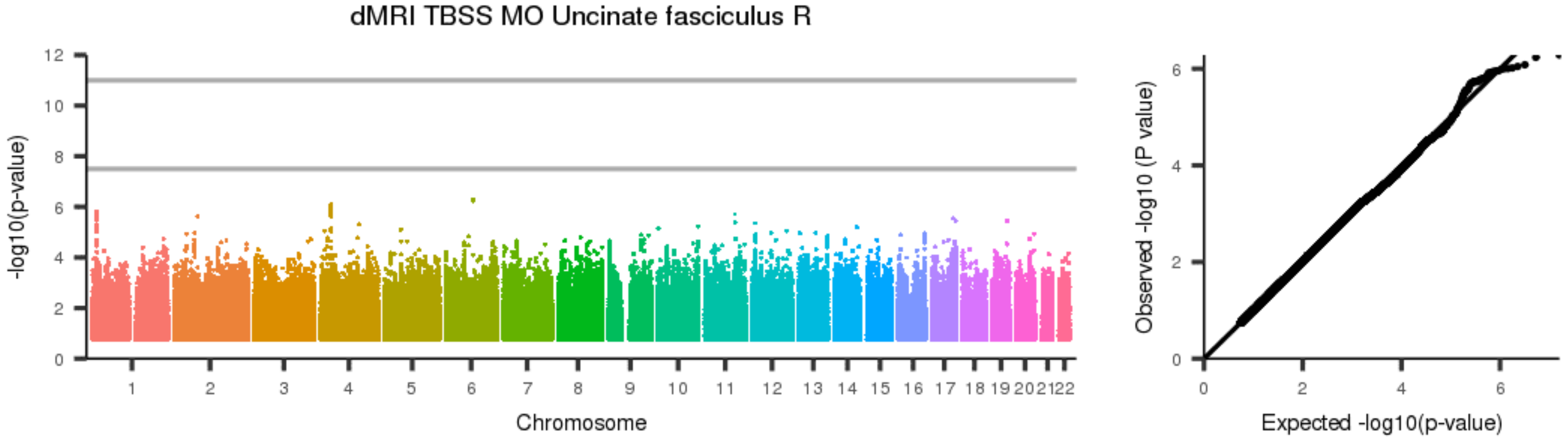

back to list of plots

Figure 337: dMRI TBSS MO Uncinate fasciculus L

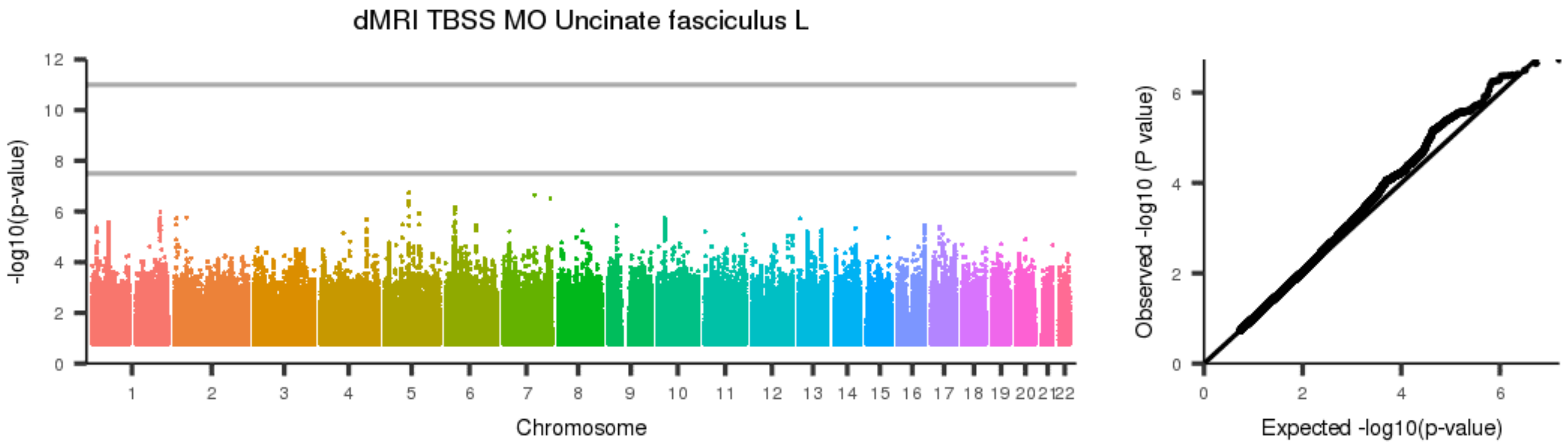

back to list of plots

Figure 338: dMRI TBSS MO Tapetum R

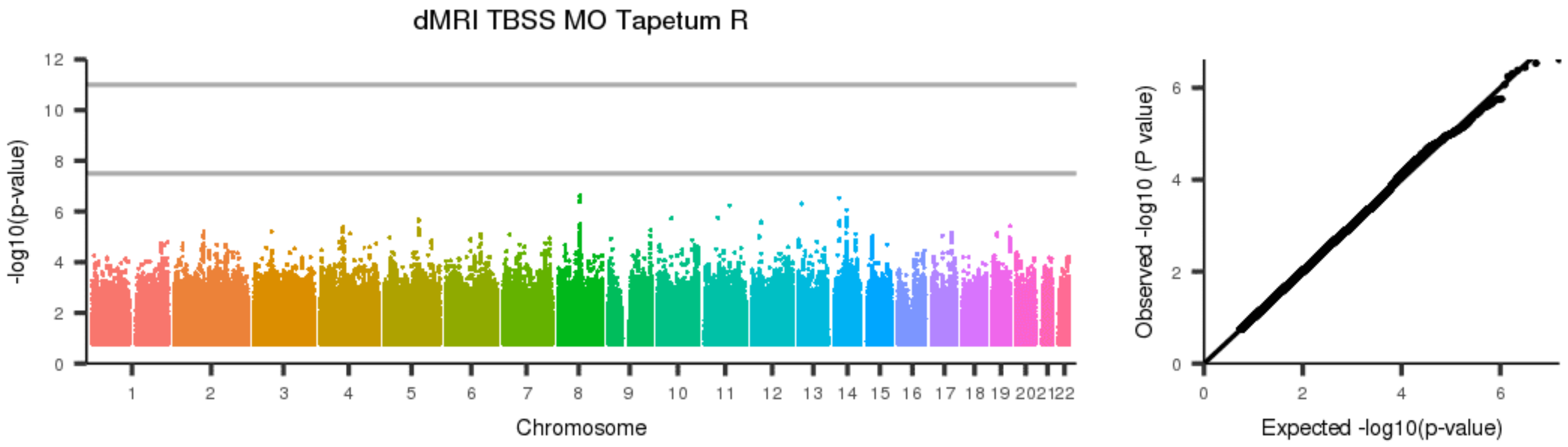

back to list of plots

Figure 339: dMRI TBSS MO Tapetum L

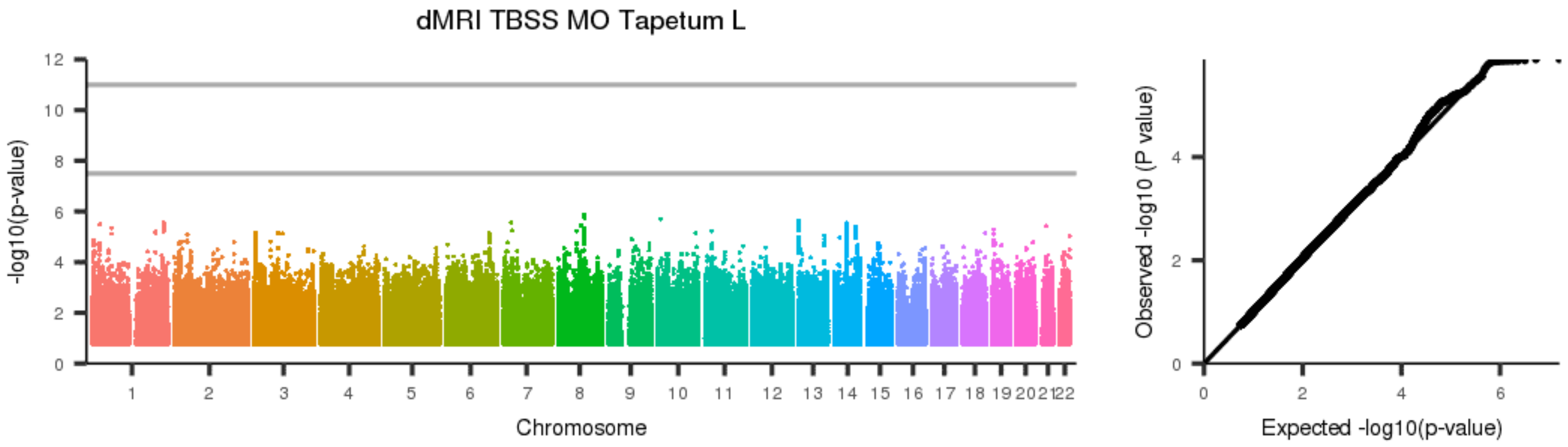

back to list of plots

Figure 340: dMRI TBSS L1 Middle cerebellar peduncle

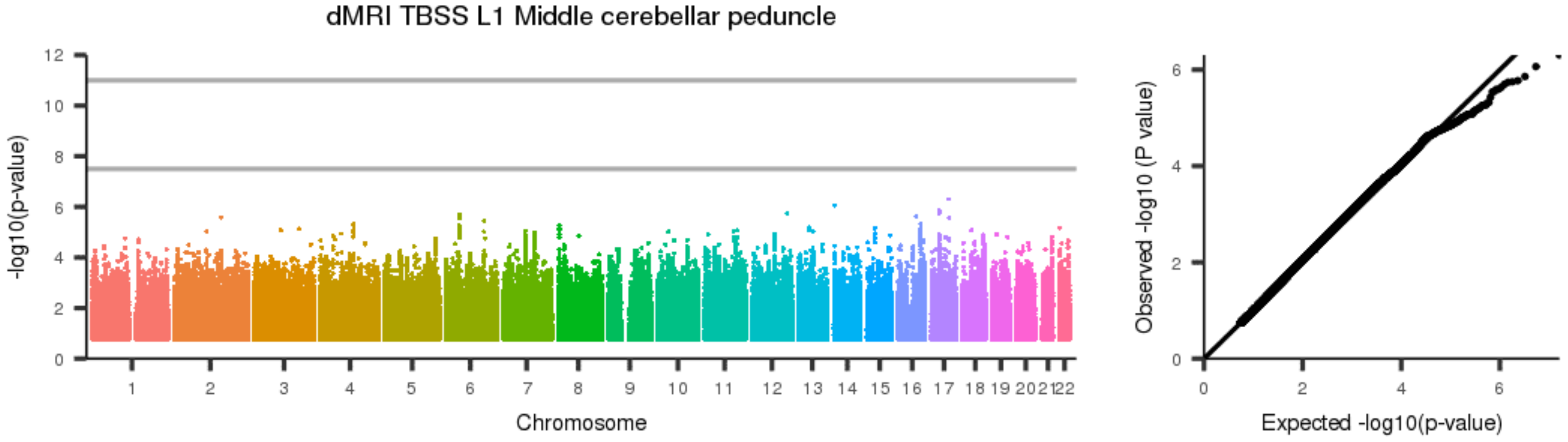

back to list of plots

Figure 341: dMRI TBSS L1 Pontine crossing tract

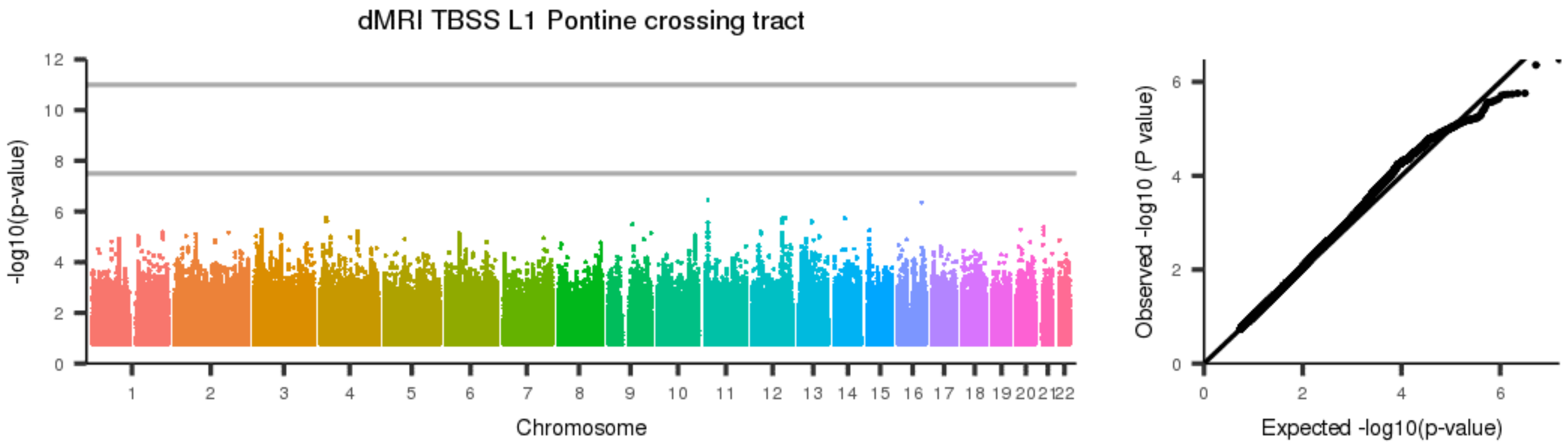

back to list of plots

Figure 342: dMRI TBSS L1 Genu of corpus callosum

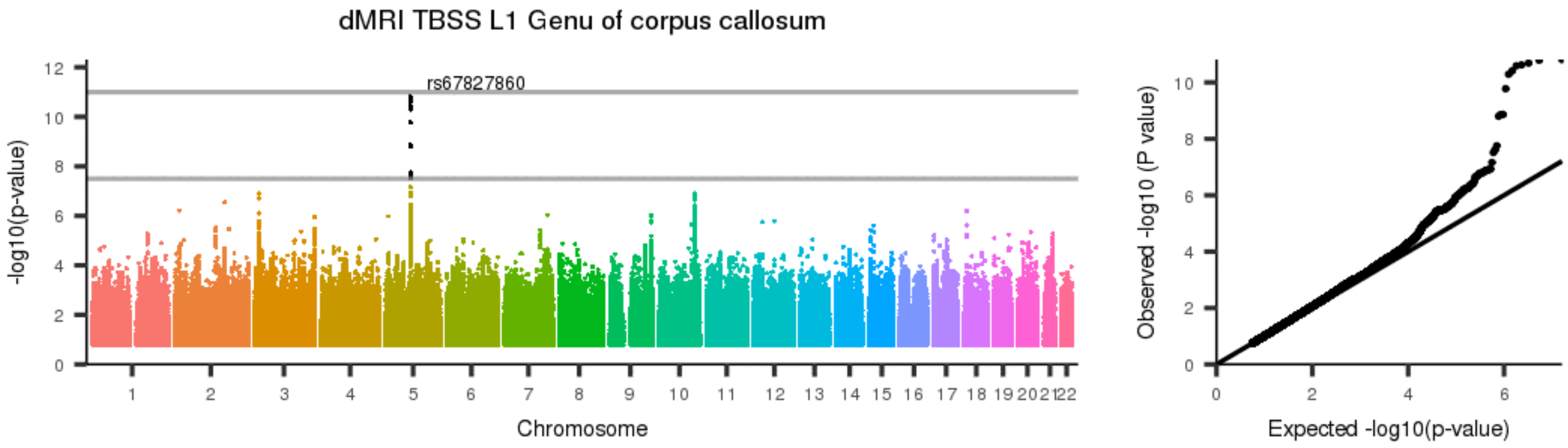

back to list of plots

Figure 343: dMRI TBSS L1 Body of corpus callosum

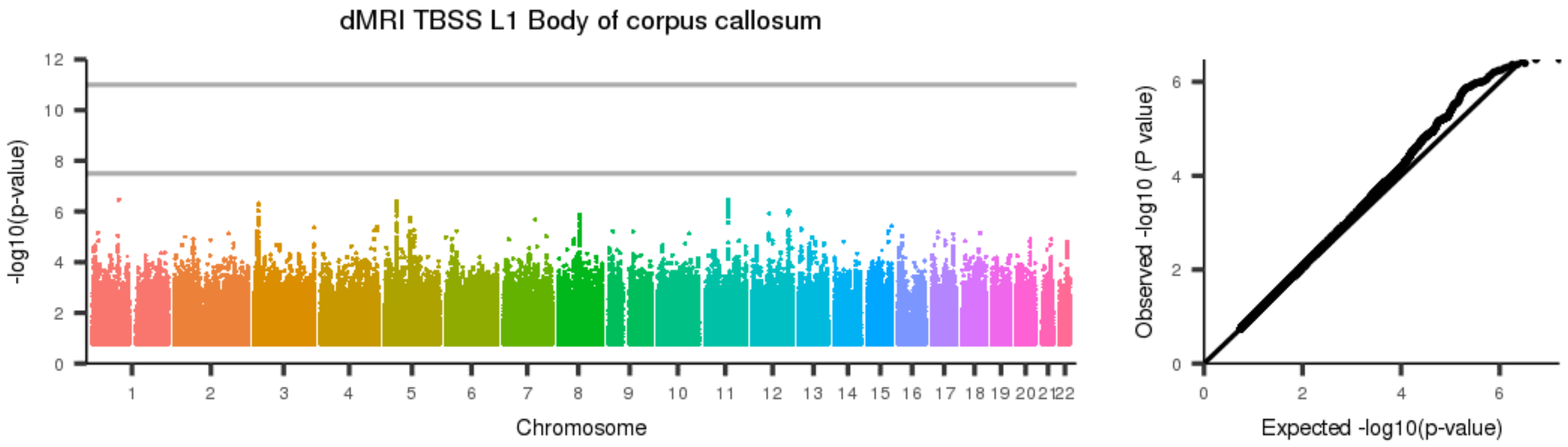

back to list of plots

Figure 344: dMRI TBSS L1 Splenium of corpus callosum

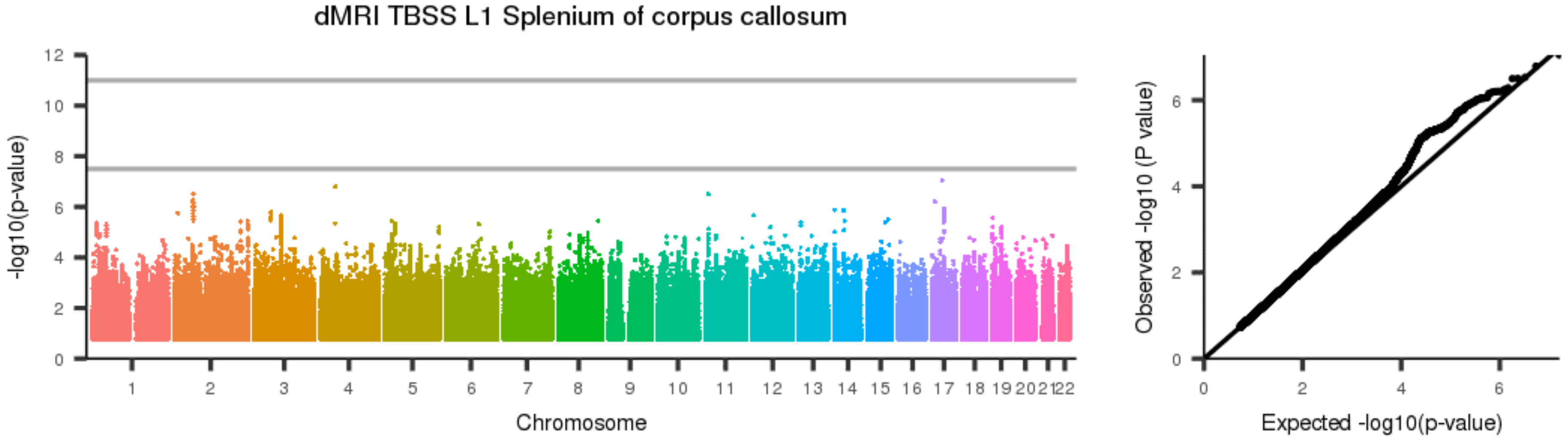

back to list of plots

Figure 345: dMRI TBSS L1 Fornix

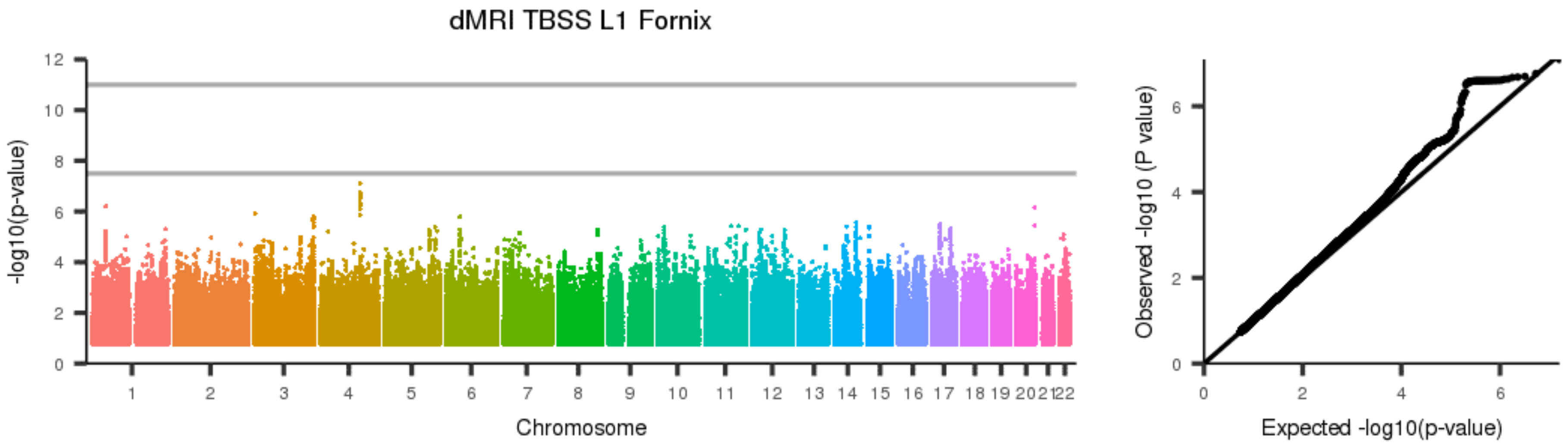

back to list of plots

Figure 346: dMRI TBSS L1 Corticospinal tract R

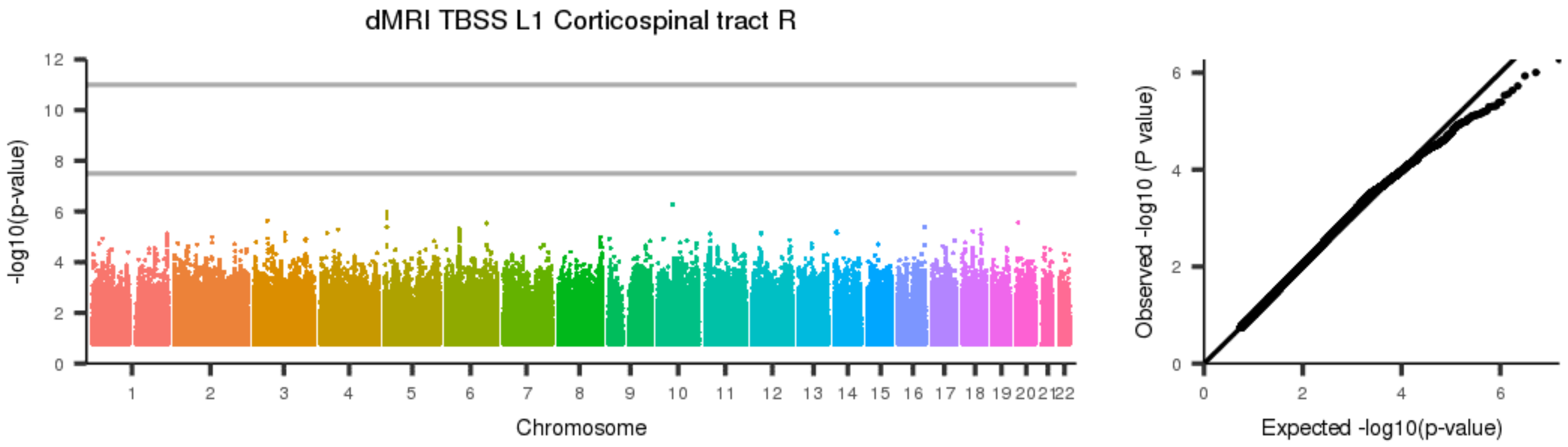

back to list of plots

Figure 347: dMRI TBSS L1 Corticospinal tract L

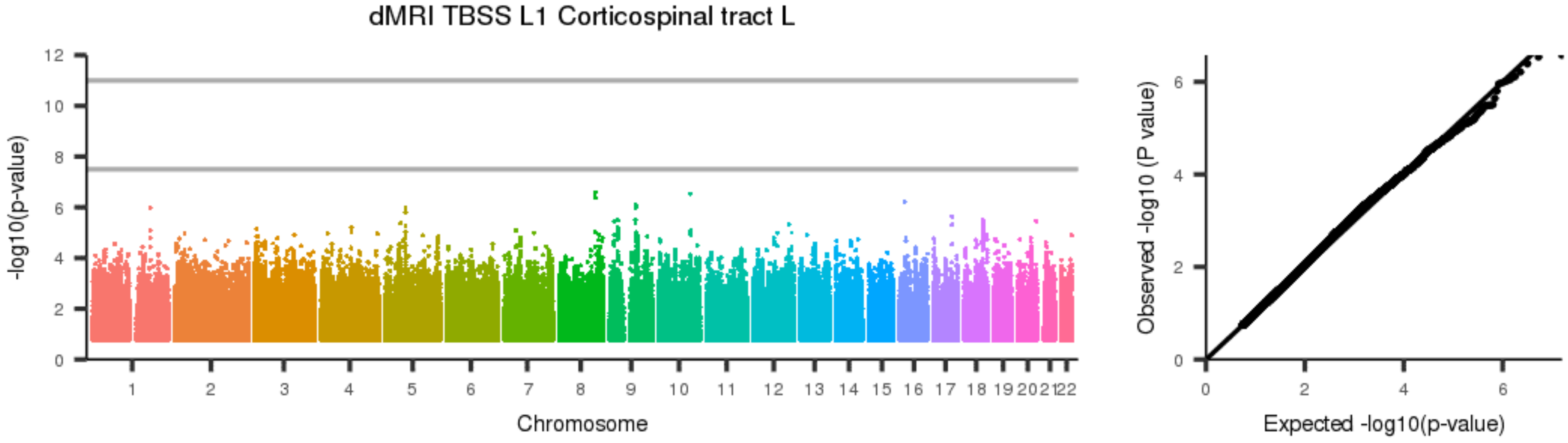

back to list of plots

Figure 348: dMRI TBSS L1 Medial lemniscus R

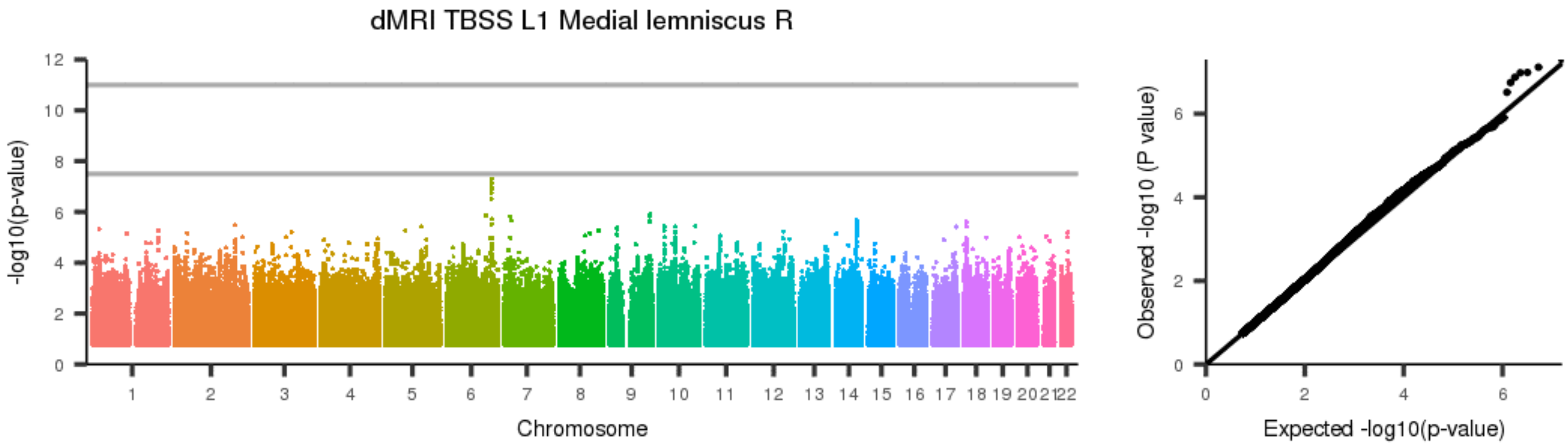

back to list of plots

Figure 349: dMRI TBSS L1 Medial lemniscus L

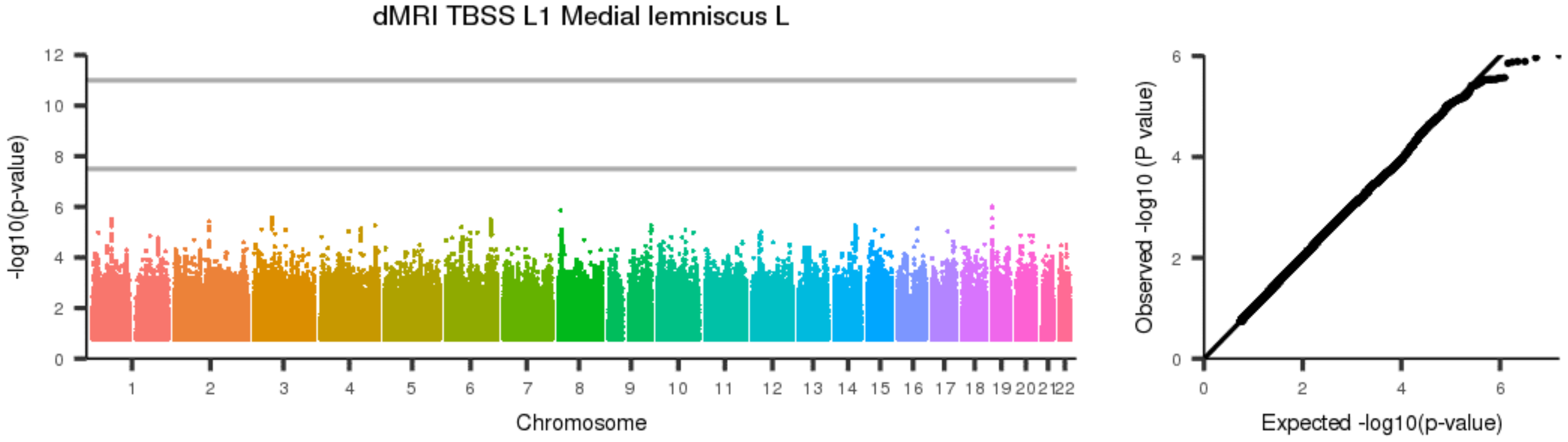

back to list of plots

Figure 350: dMRI TBSS L1 Inferior cerebellar peduncle R

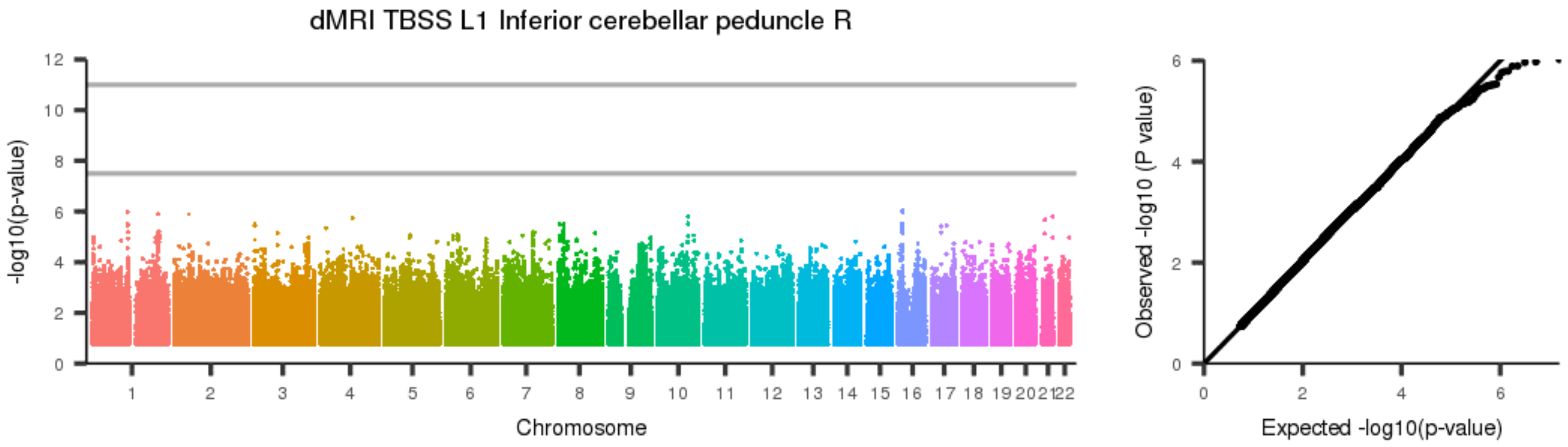

back to list of plots

Figure 351: dMRI TBSS L1 Inferior cerebellar peduncle L

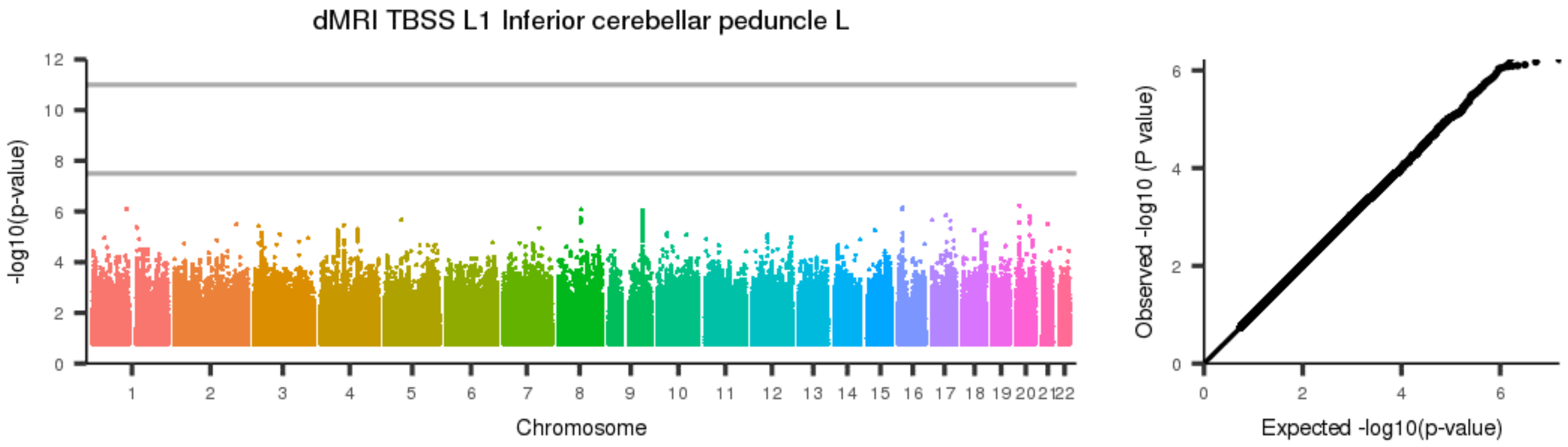

back to list of plots

Figure 352: dMRI TBSS L1 Superior cerebellar peduncle R

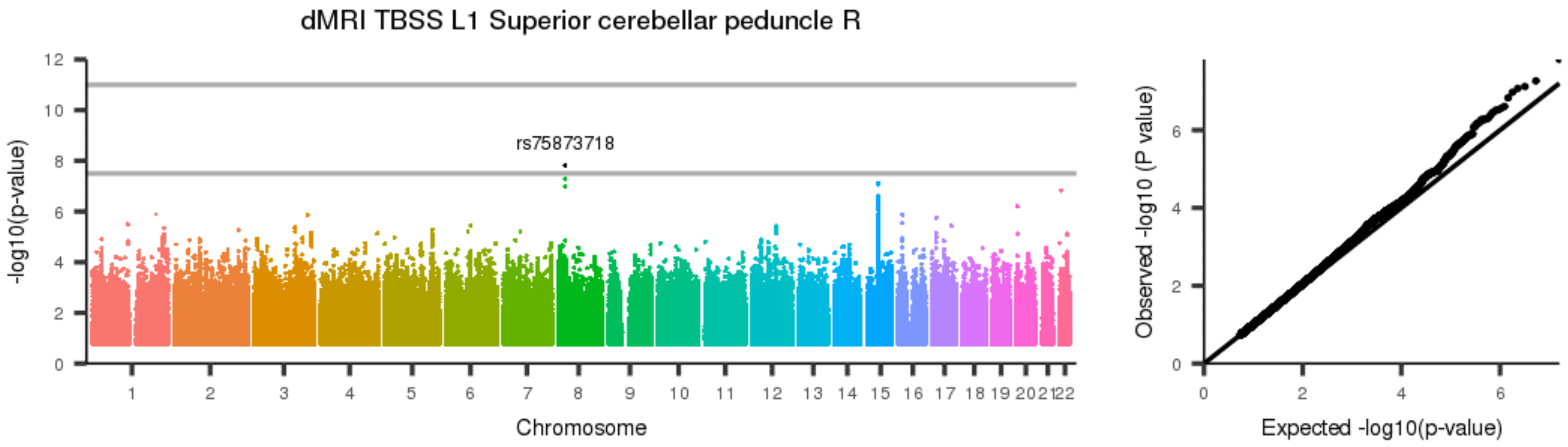

back to list of plots

Figure 353: dMRI TBSS L1 Superior cerebellar peduncle L

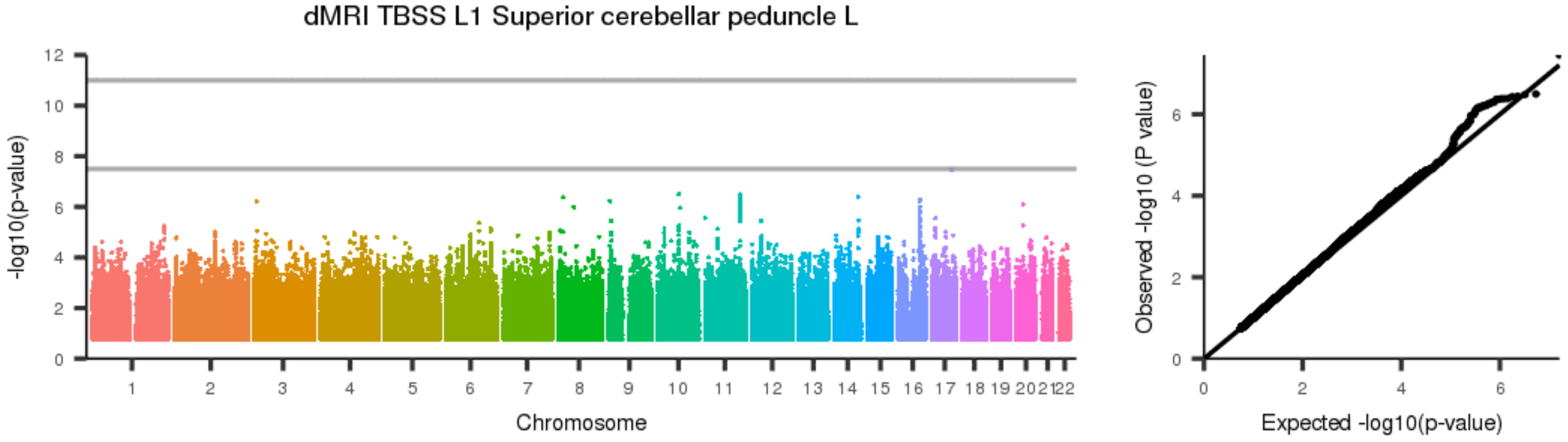

back to list of plots

Figure 354: dMRI TBSS L1 Cerebral peduncle R

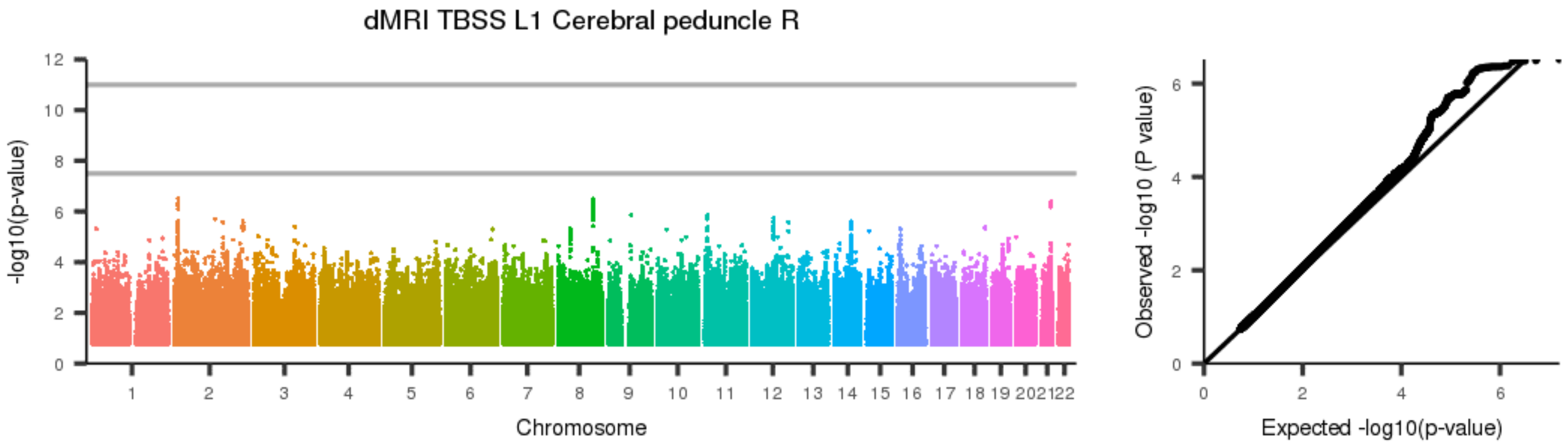

back to list of plots

Figure 355: dMRI TBSS L1 Cerebral peduncle L

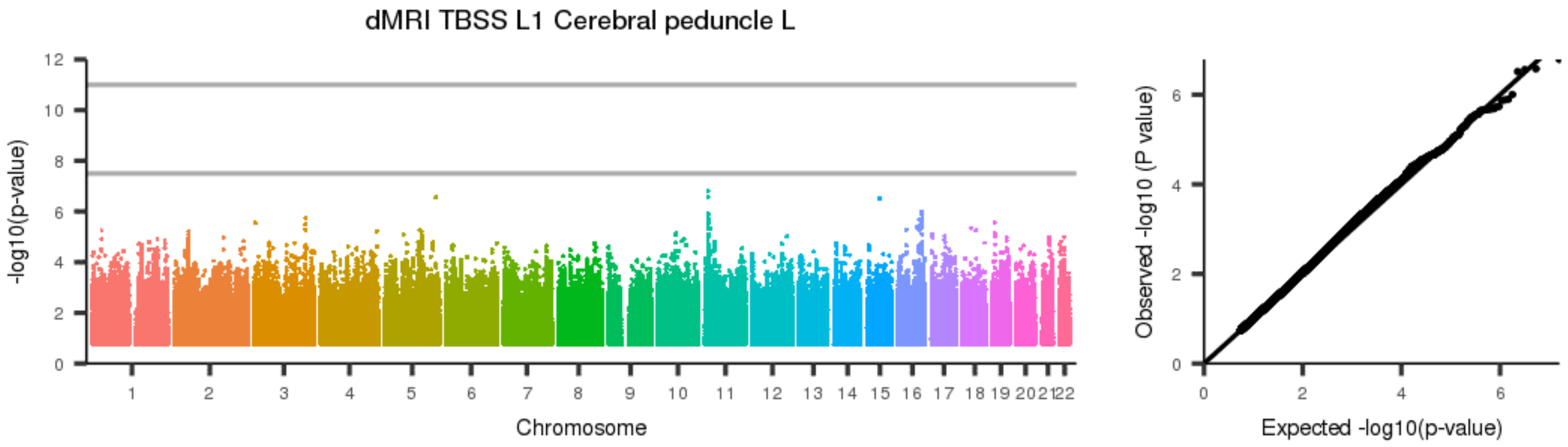

back to list of plots

Figure 356: dMRI TBSS L1 Anterior limb of internal capsule R

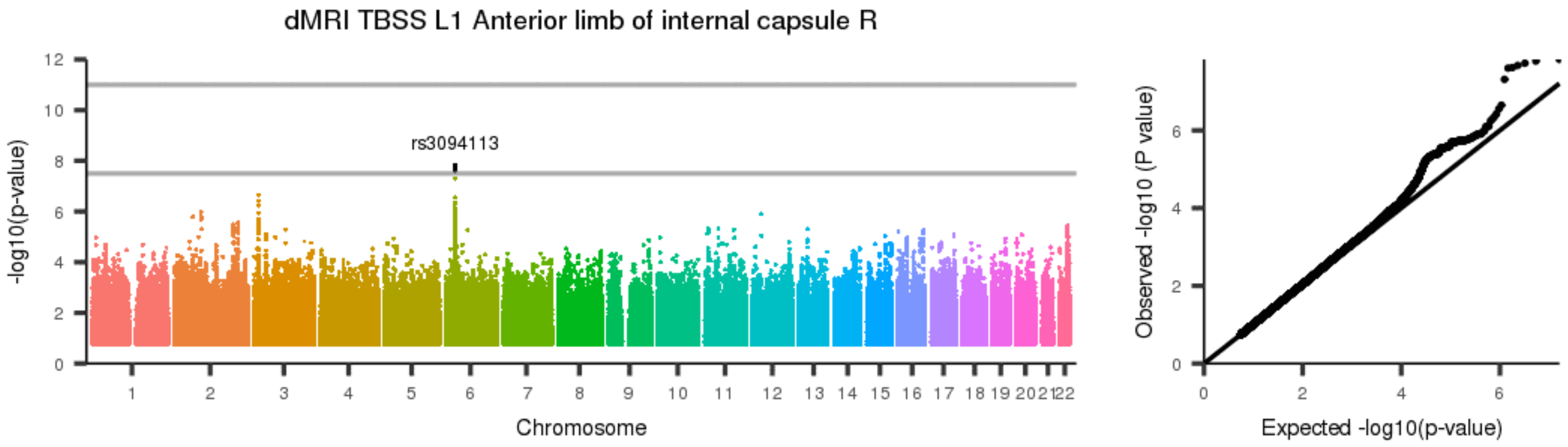

back to list of plots

Figure 357: dMRI TBSS L1 Anterior limb of internal capsule L

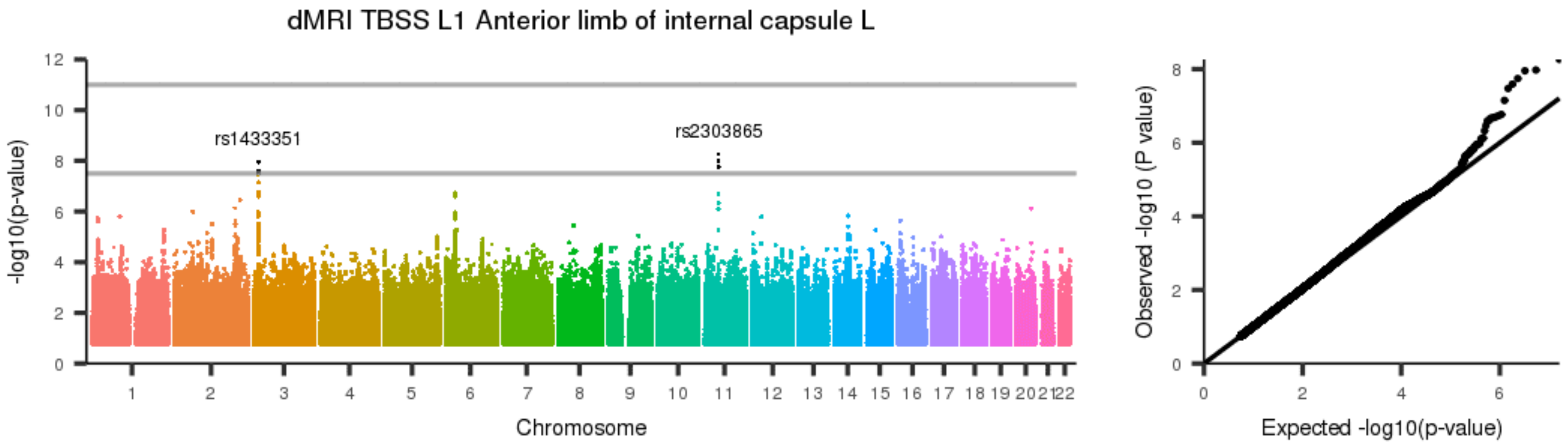

back to list of plots

Figure 358: dMRI TBSS L1 Posterior limb of internal capsule R

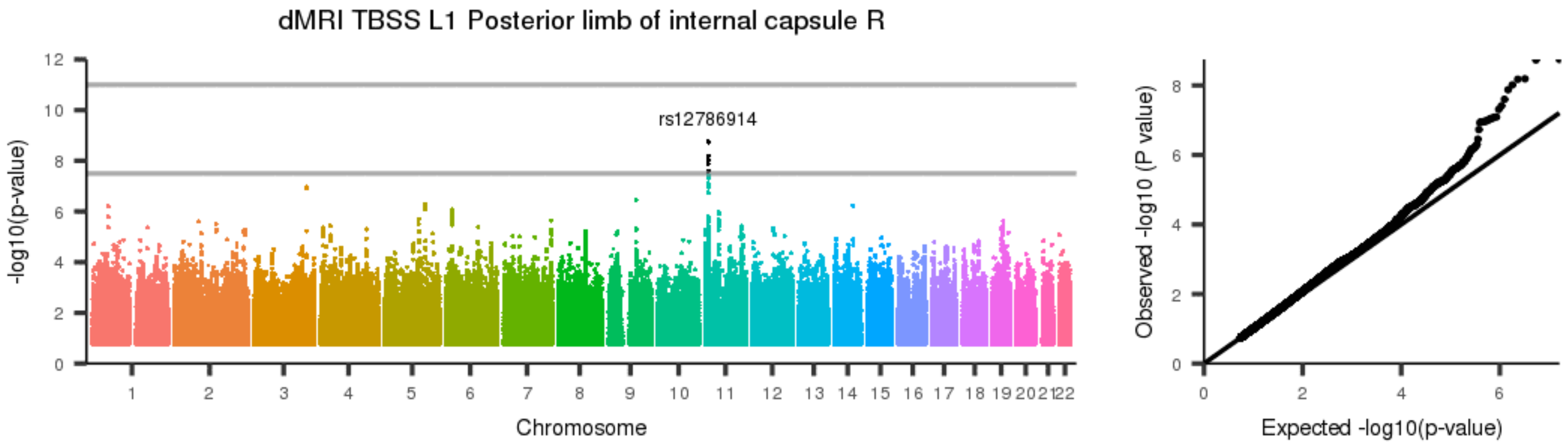

back to list of plots

Figure 359: dMRI TBSS L1 Posterior limb of internal capsule L

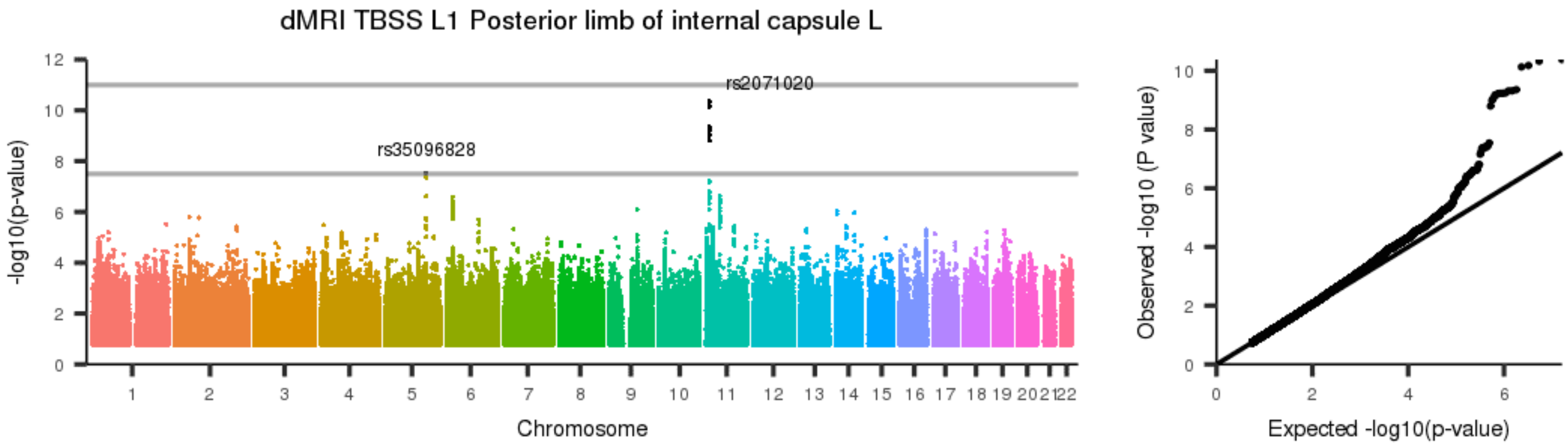

back to list of plots

Figure 360: dMRI TBSS L1 Retrolenticular part of internal capsule R

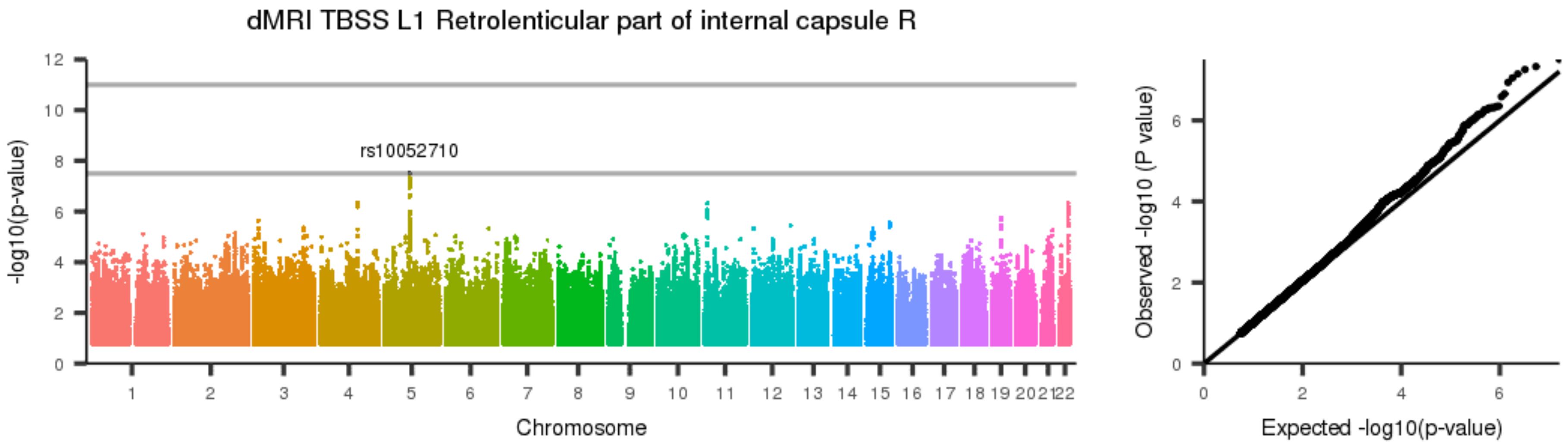

back to list of plots

Figure 361: dMRI TBSS L1 Retrolenticular part of internal capsule L

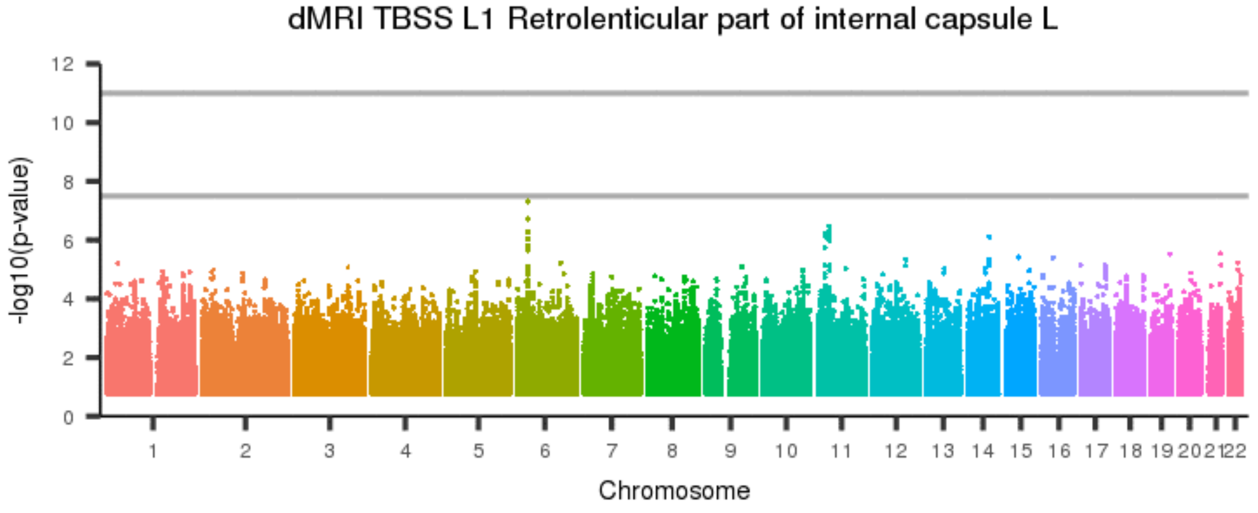

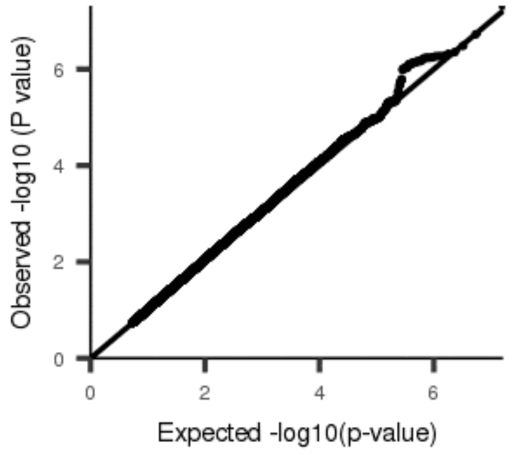

back to list of plots

Figure 362: dMRI TBSS L1 Anterior corona radiata R

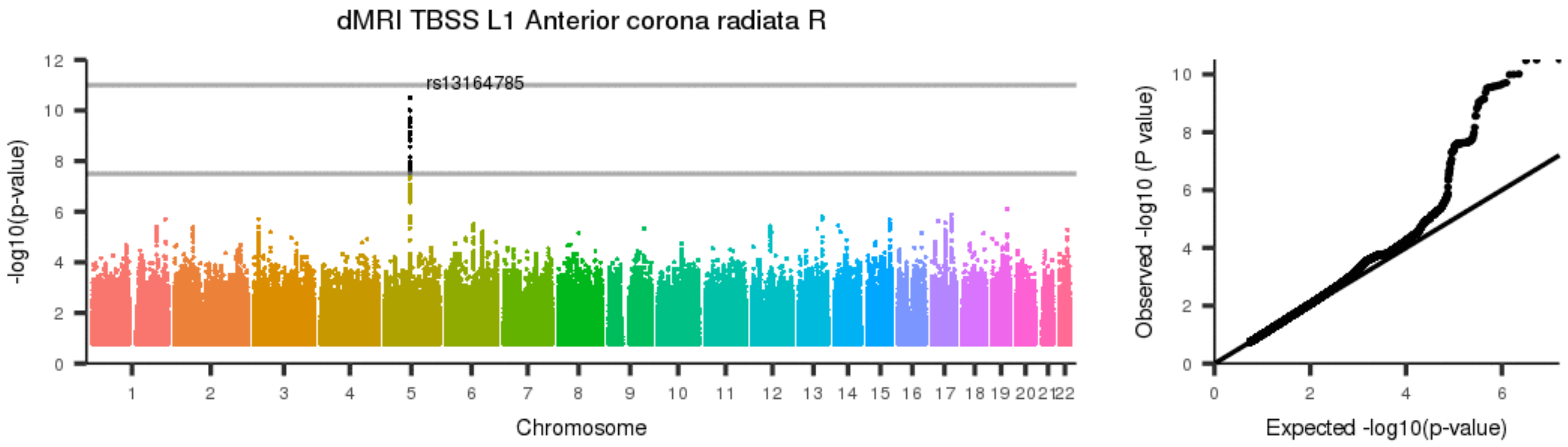

back to list of plots

Figure 363: dMRI TBSS L1 Anterior corona radiata L

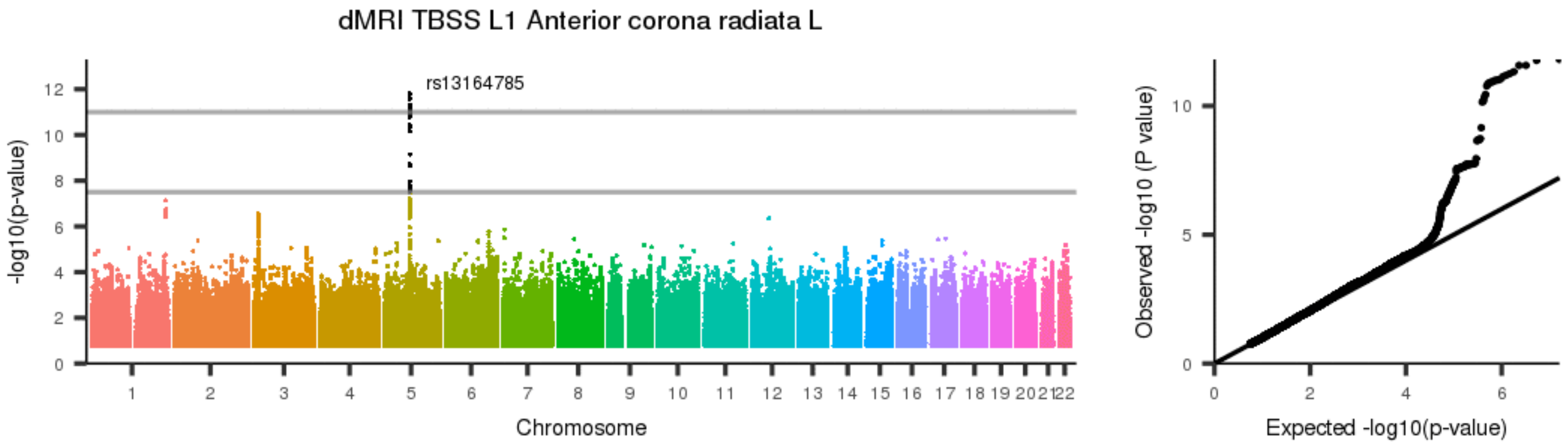

back to list of plots

Figure 364: dMRI TBSS L1 Superior corona radiata R

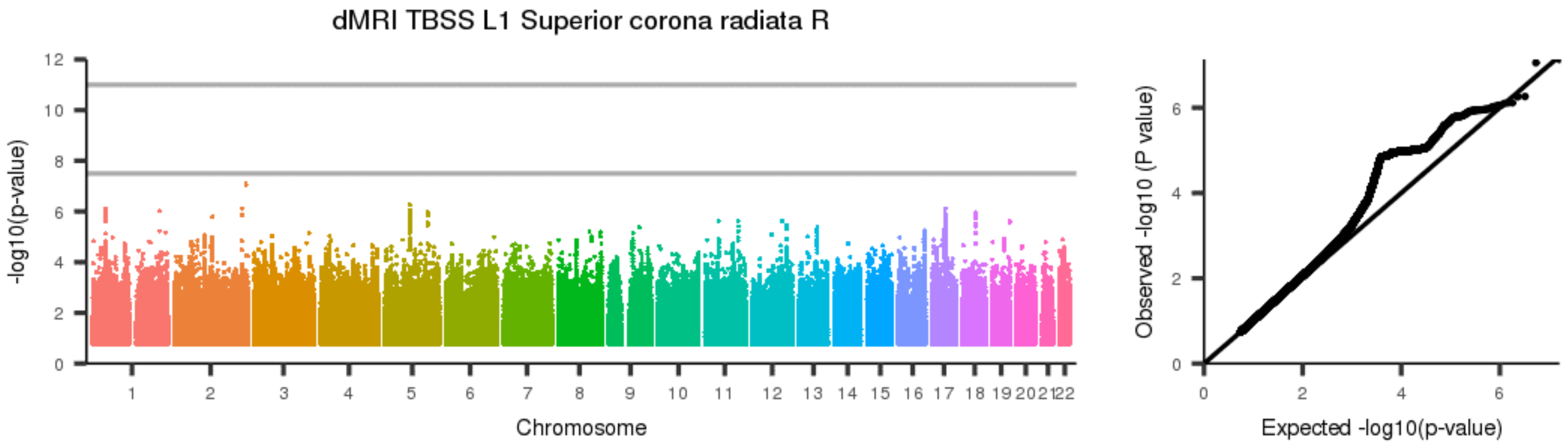

back to list of plots

Figure 365: dMRI TBSS L1 Superior corona radiata L

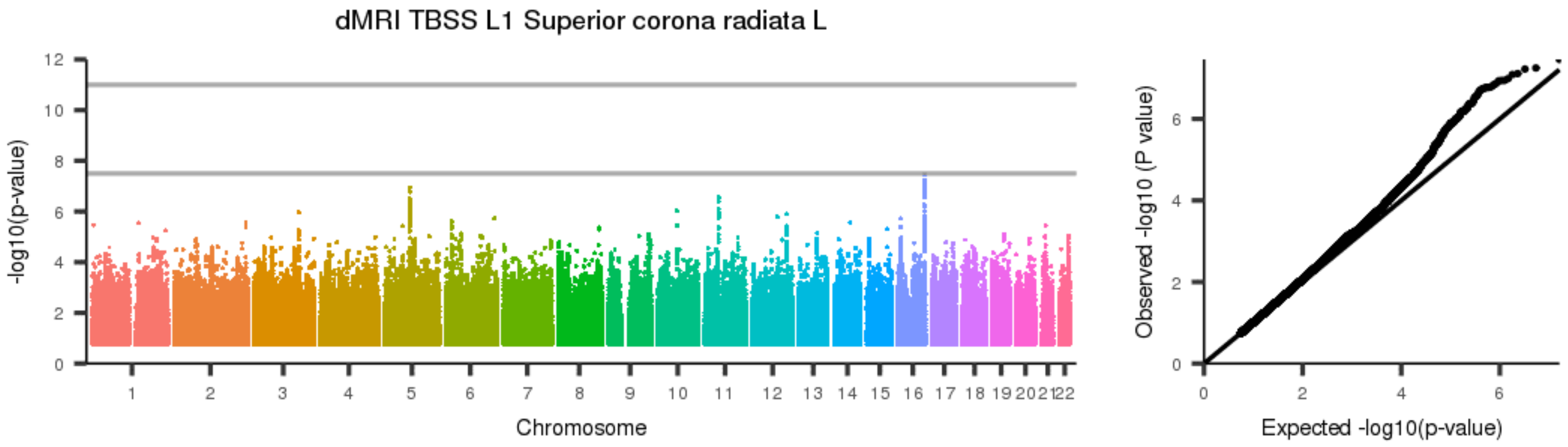

back to list of plots

Figure 366: dMRI TBSS L1 Posterior corona radiata R

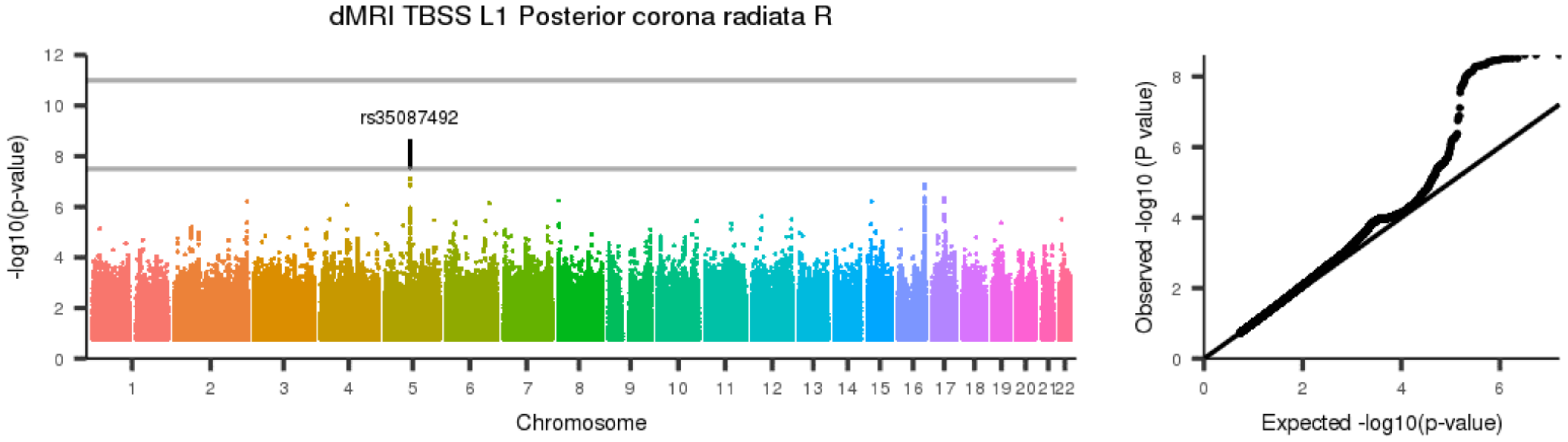

back to list of plots

Figure 367: dMRI TBSS L1 Posterior corona radiata L

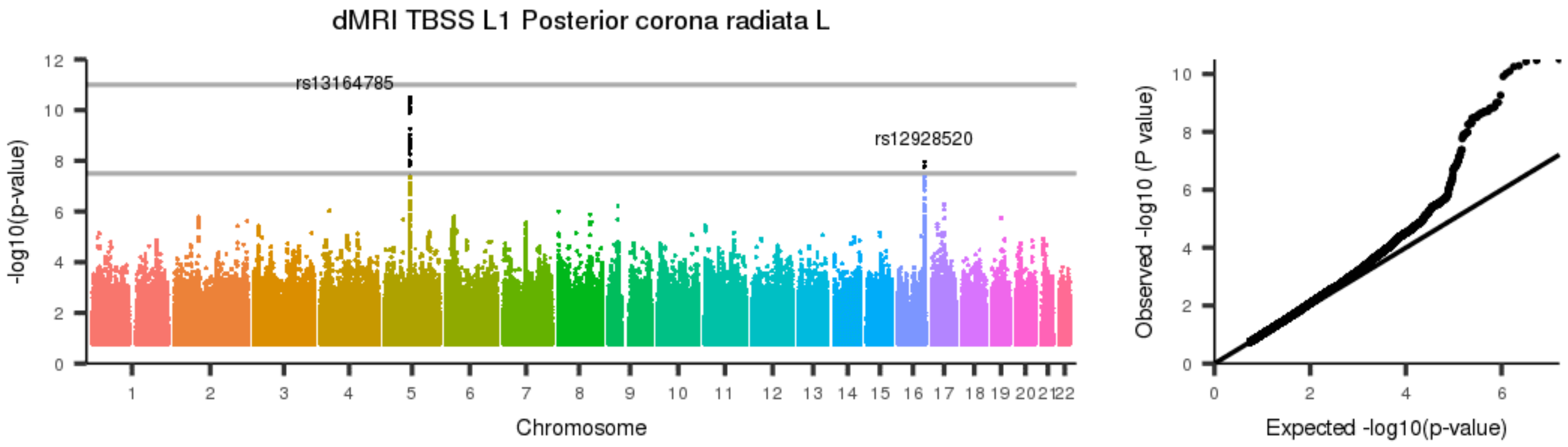

back to list of plots

Figure 368: dMRI TBSS L1 Posterior thalamic radiation R

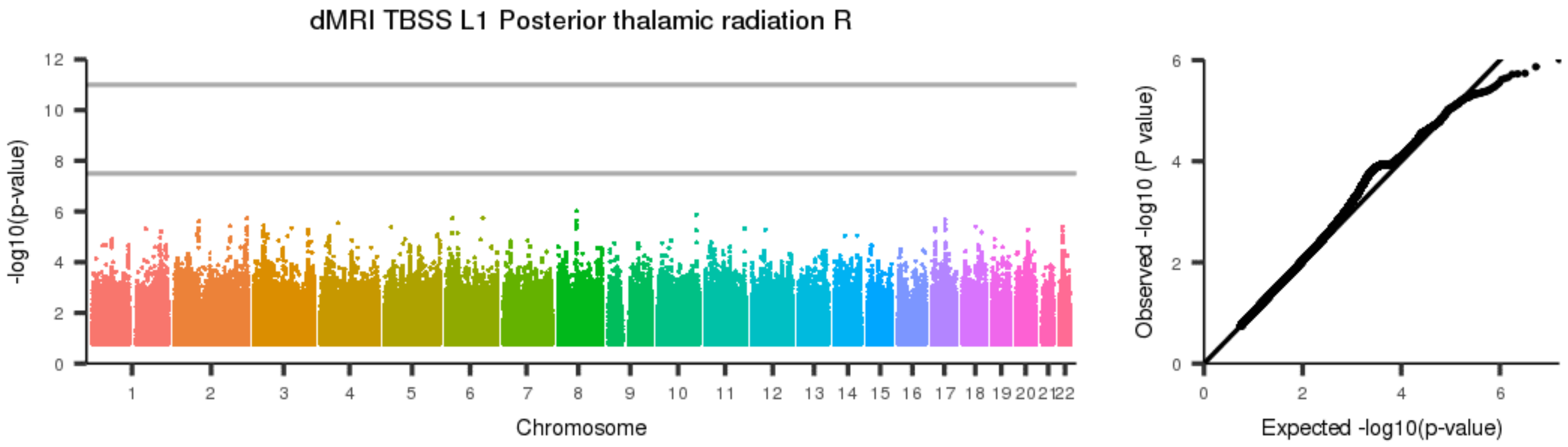

back to list of plots

Figure 369: dMRI TBSS L1 Posterior thalamic radiation L

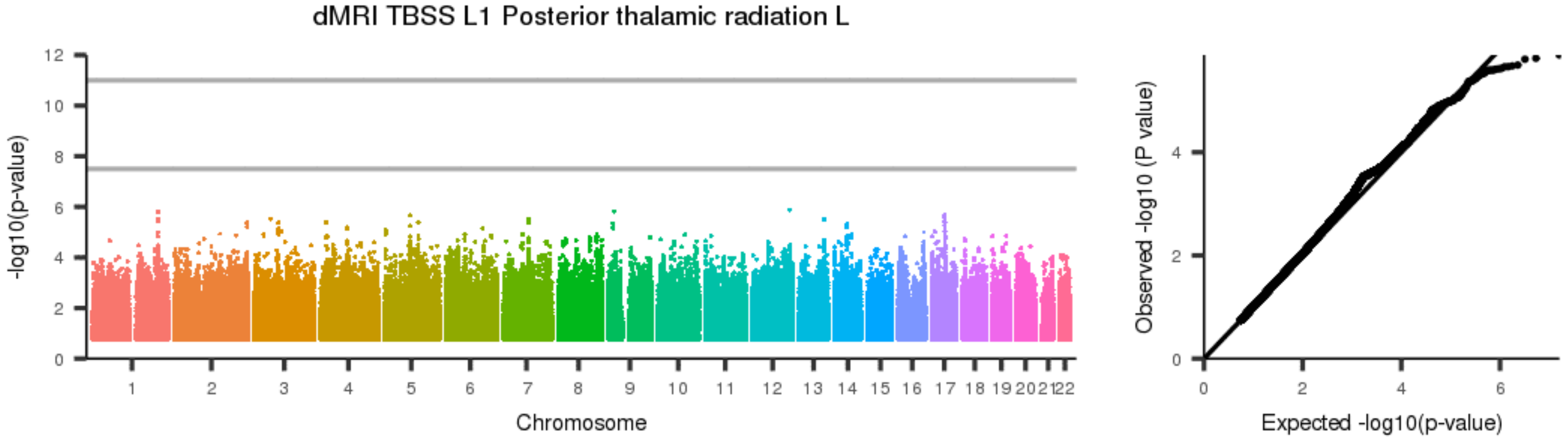

back to list of plots

Figure 370: dMRI TBSS L1 Sagittal stratum R

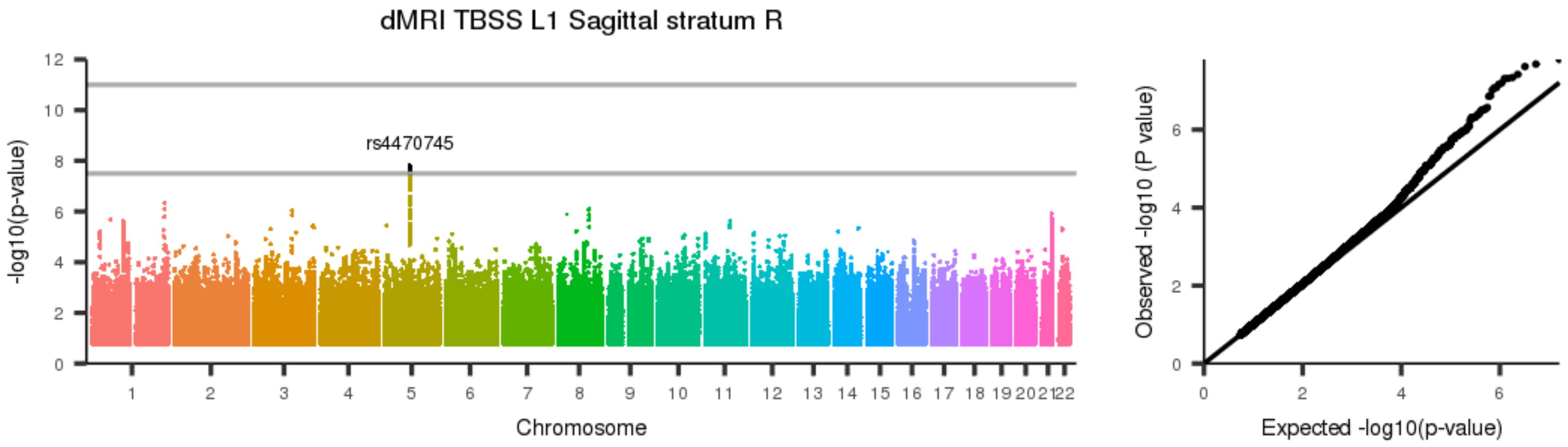

back to list of plots

Figure 371: dMRI TBSS L1 Sagittal stratum L

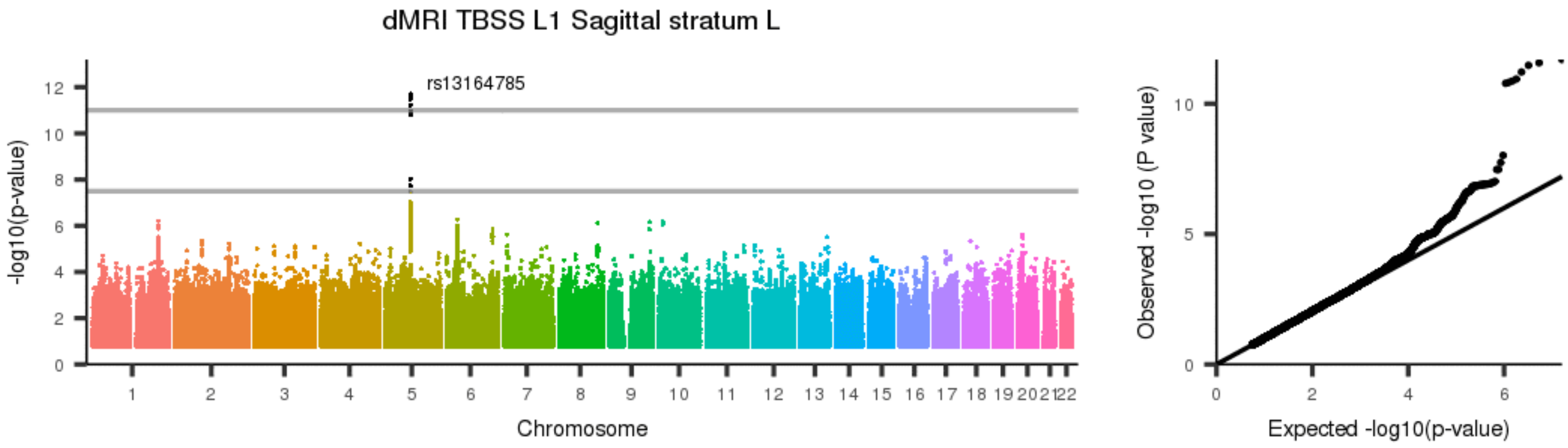

back to list of plots

Figure 372: dMRI TBSS L1 External capsule R

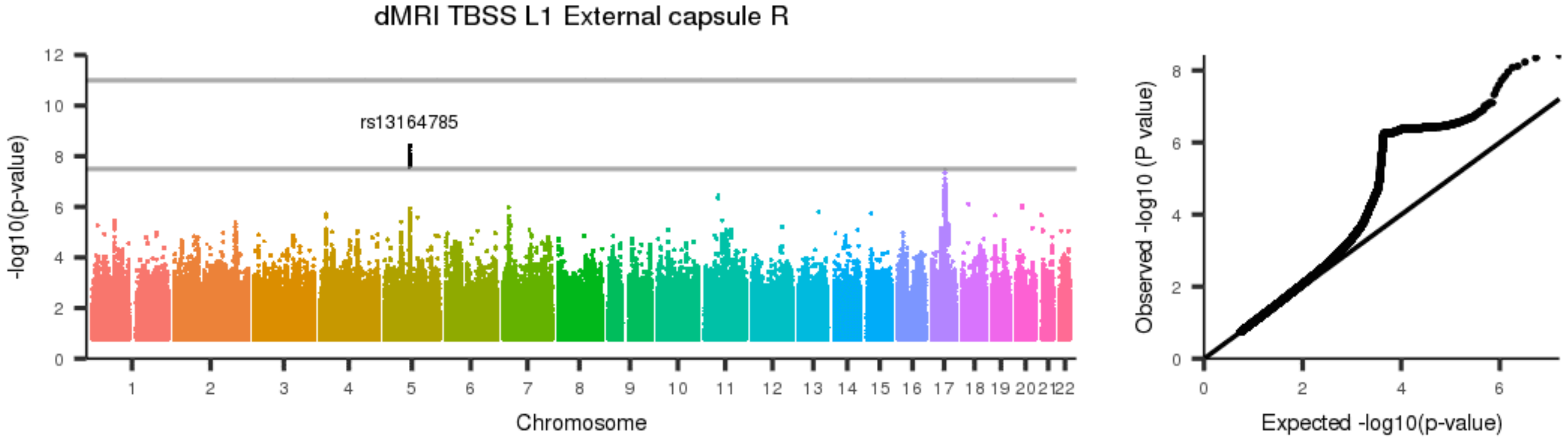

back to list of plots

Figure 373: dMRI TBSS L1 External capsule L

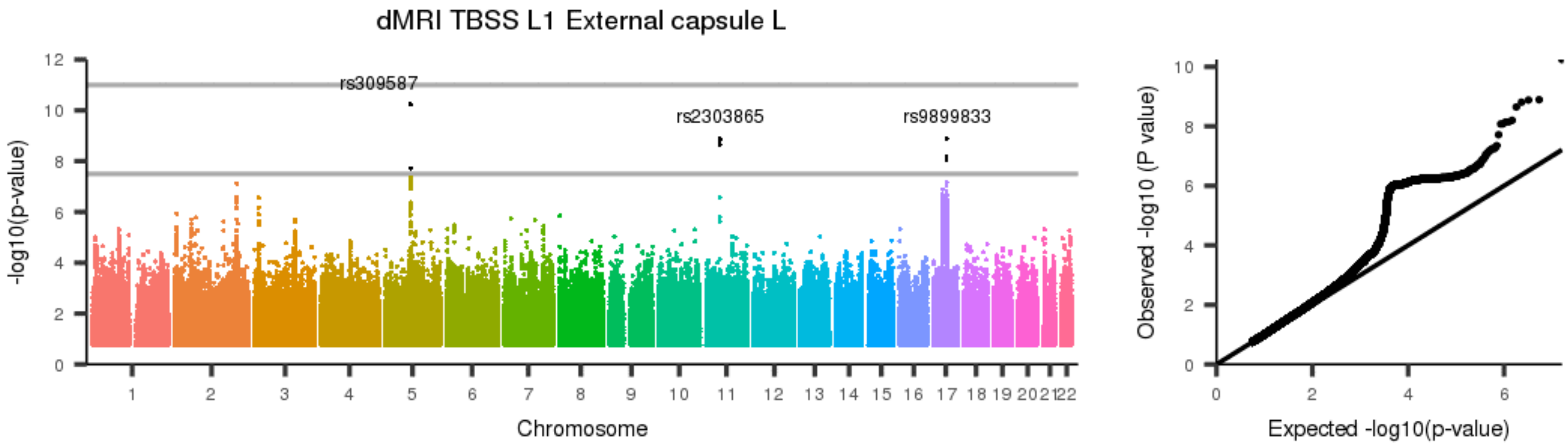

back to list of plots

Figure 374: dMRI TBSS L1 Cingulum cingulate gyrus R

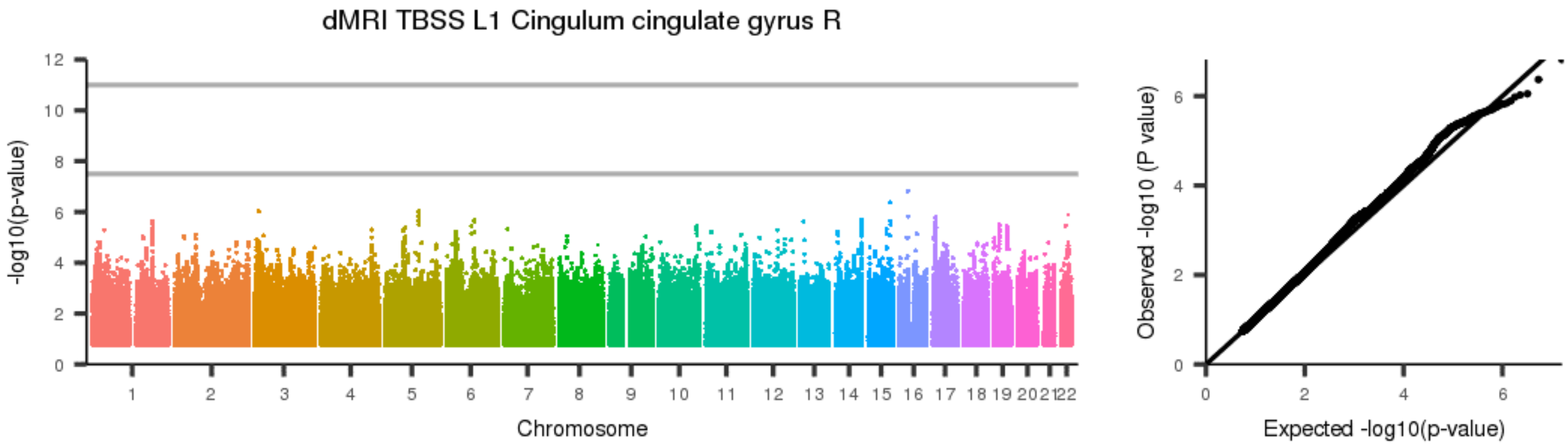

back to list of plots

Figure 375: dMRI TBSS L1 Cingulum cingulate gyrus L

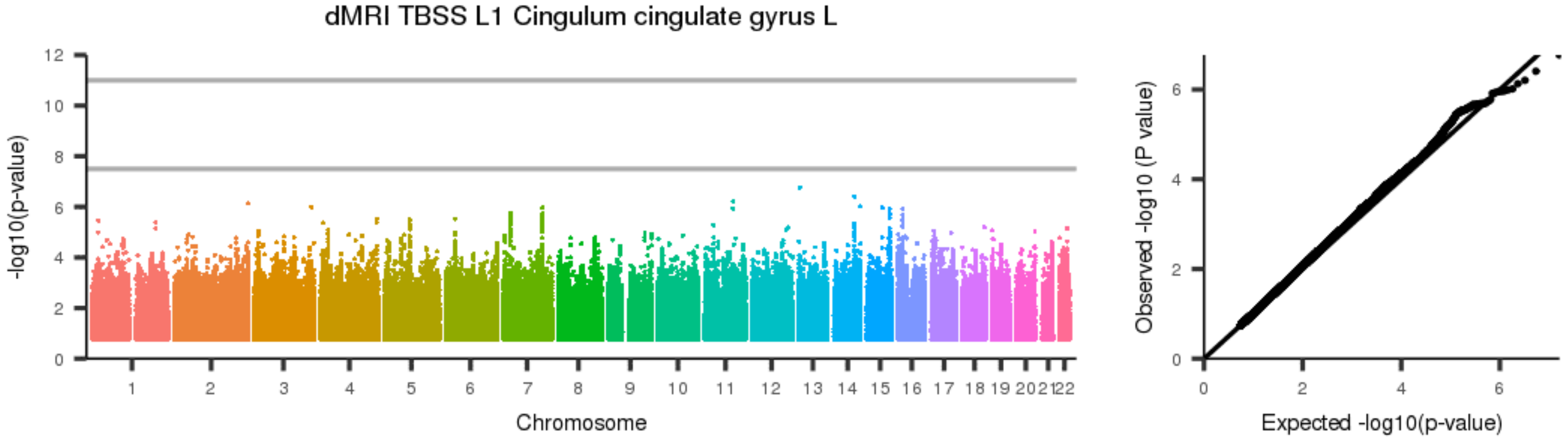

back to list of plots

Figure 376: dMRI TBSS L1 Cingulum hippocampus R

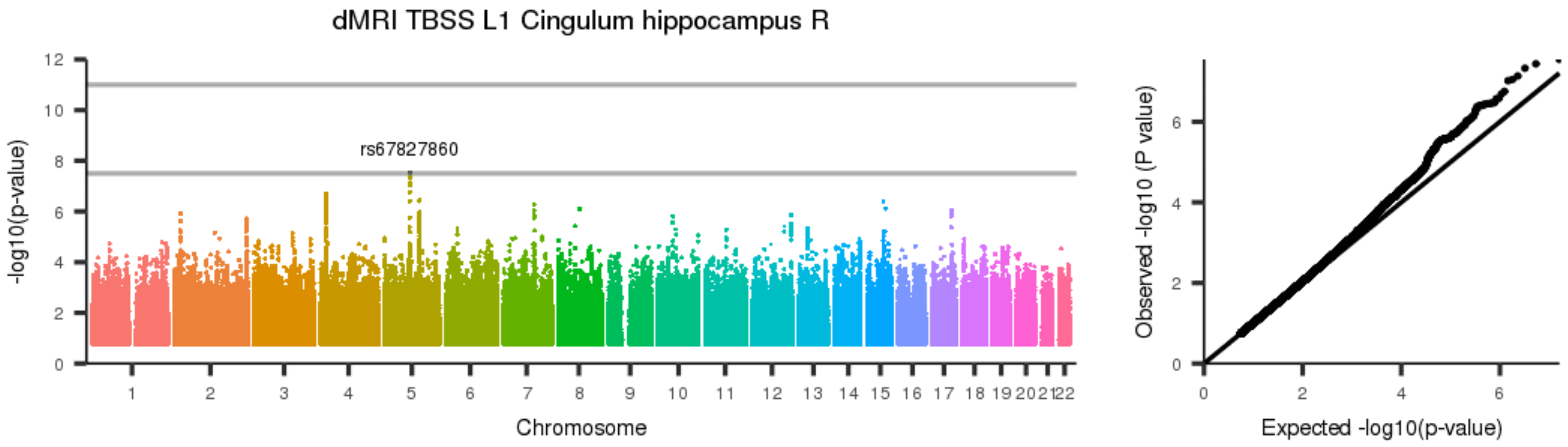

back to list of plots

Figure 377: dMRI TBSS L1 Cingulum hippocampus L

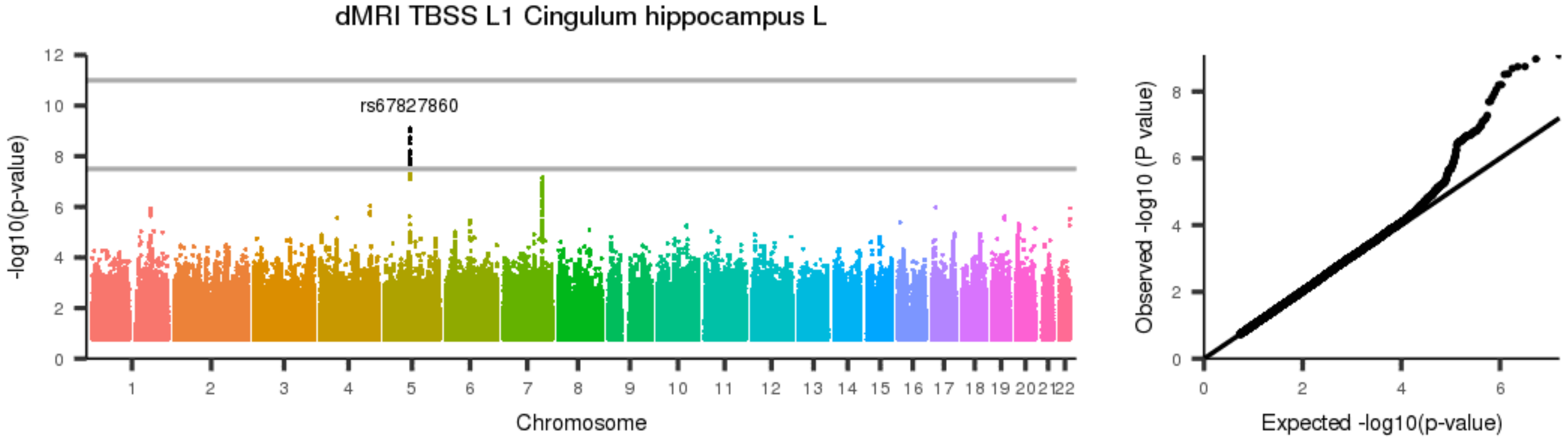

back to list of plots

Figure 378: dMRI TBSS L1 Fornix cres+Stria terminalis R

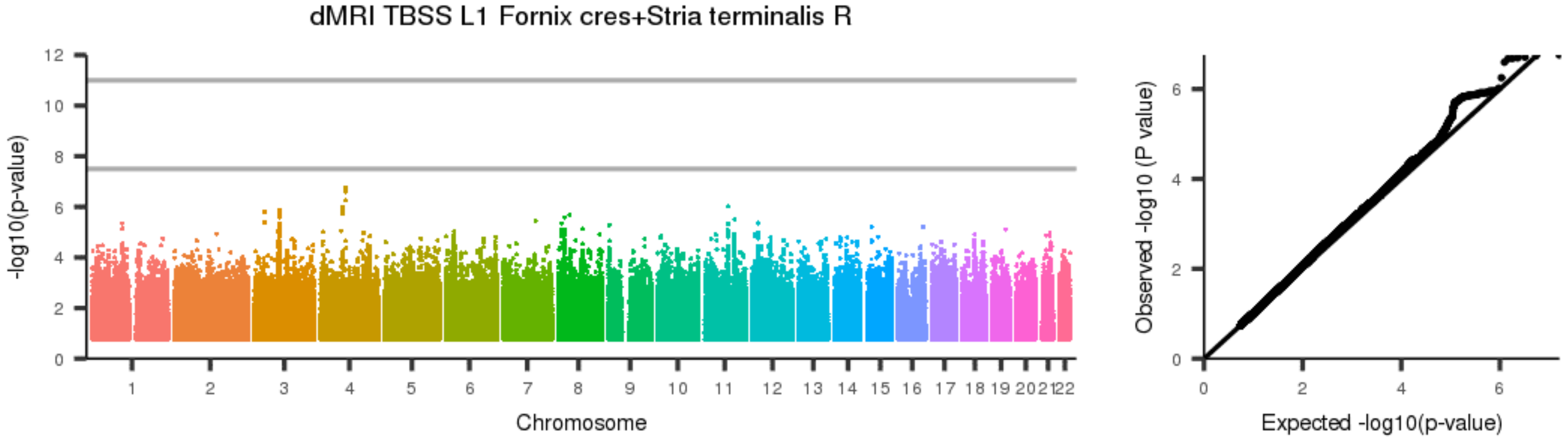

back to list of plots

Figure 379: dMRI TBSS L1 Fornix cres+Stria terminalis L

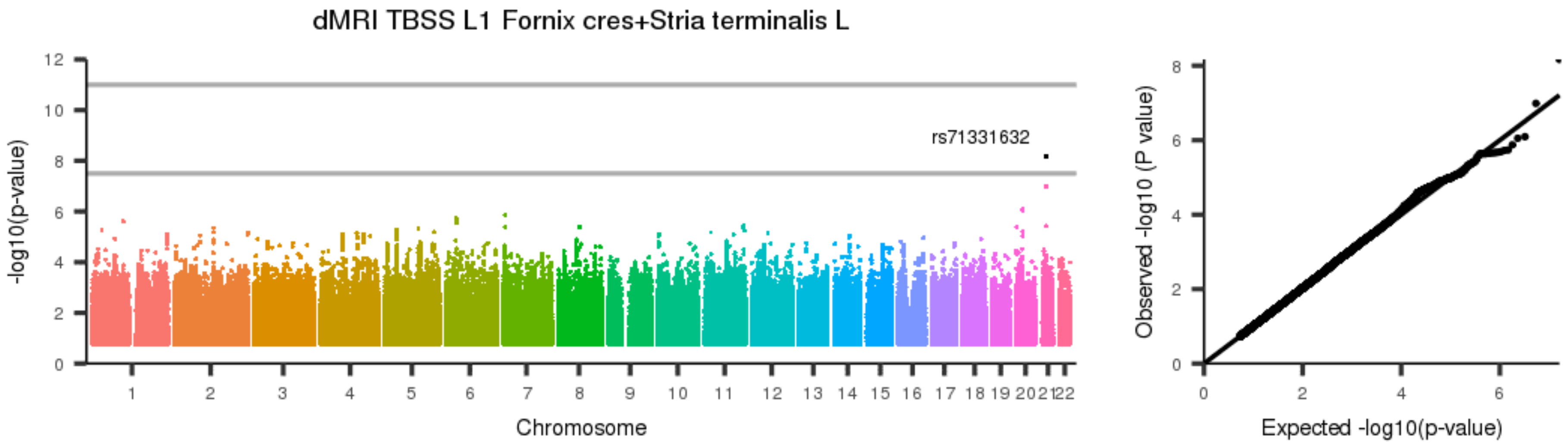

back to list of plots

Figure 380: dMRI TBSS L1 Superior longitudinal fasciculus R

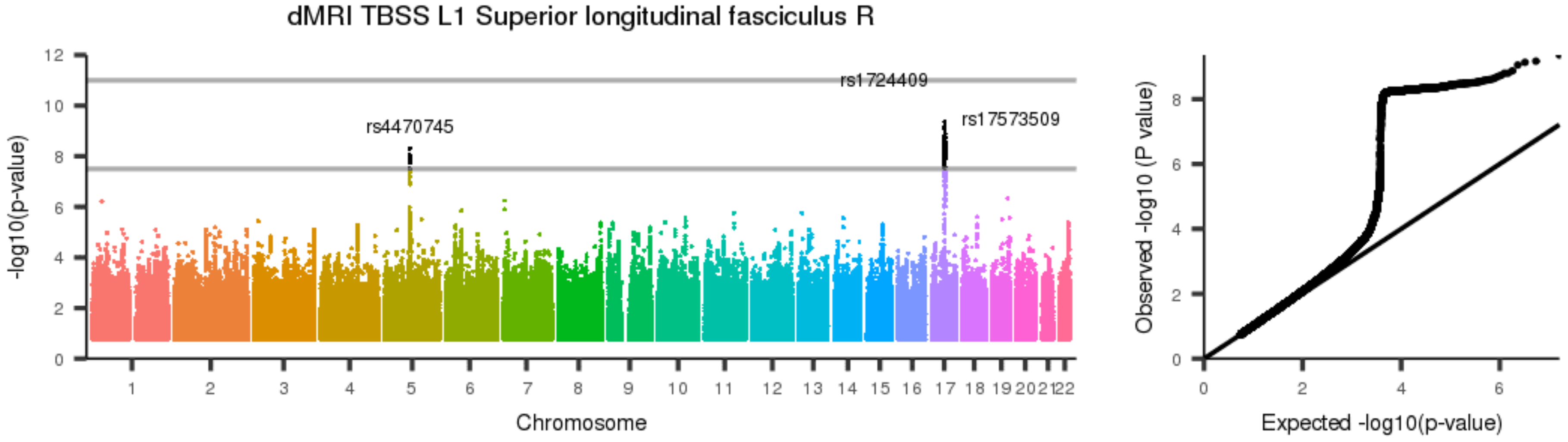

back to list of plots

Figure 381: dMRI TBSS L1 Superior longitudinal fasciculus L

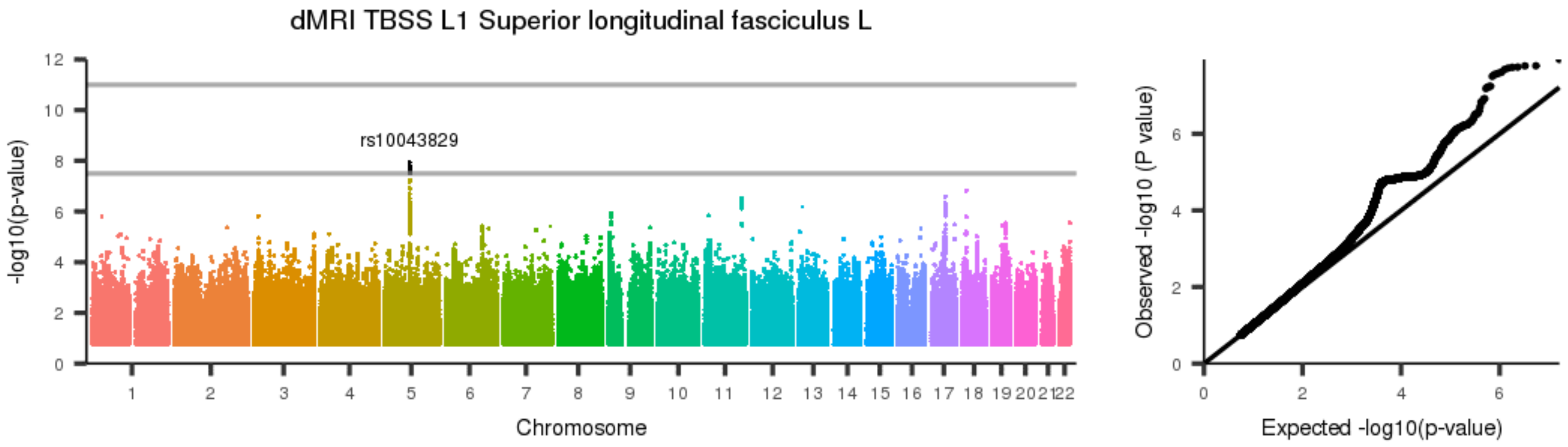

back to list of plots

Figure 382: dMRI TBSS L1 Superior fronto-occipital fasciculus R

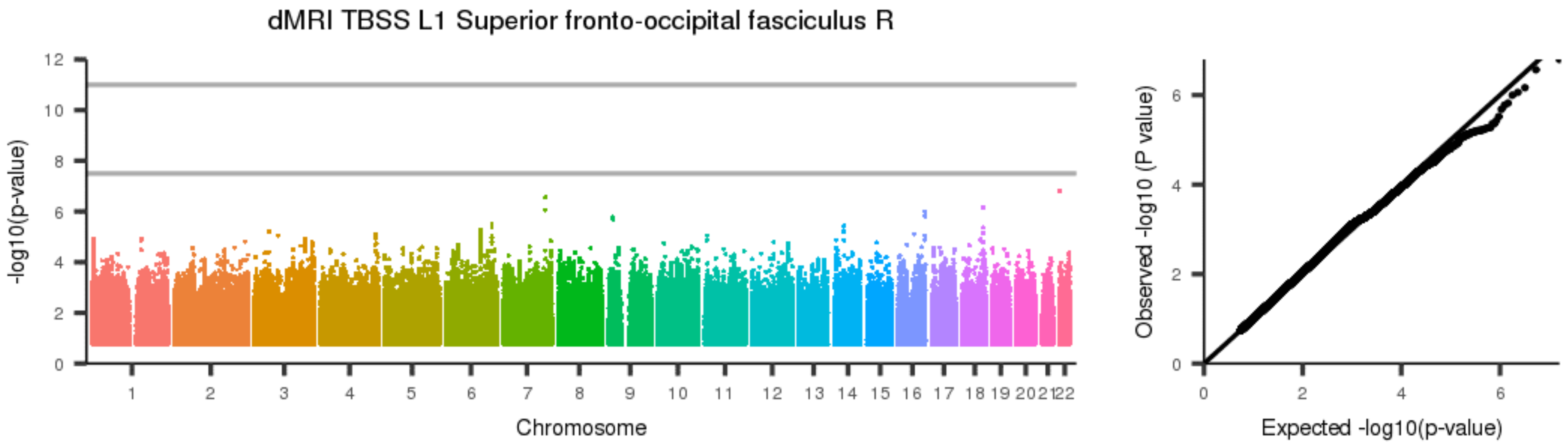

back to list of plots

Figure 383: dMRI TBSS L1 Superior fronto-occipital fasciculus L

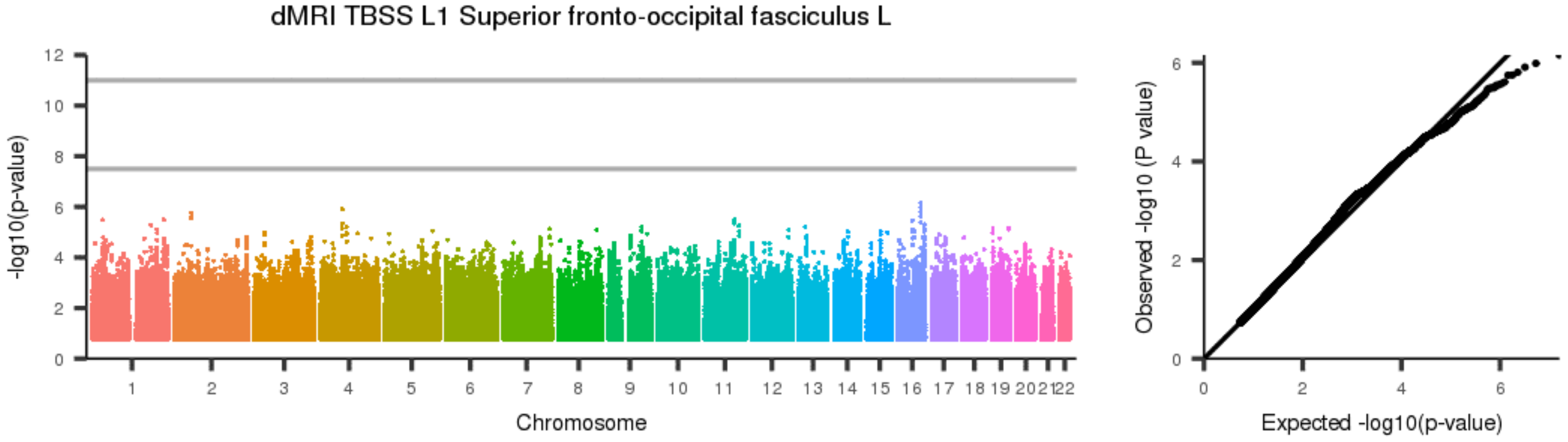

back to list of plots

Figure 384: dMRI TBSS L1 Uncinate fasciculus R

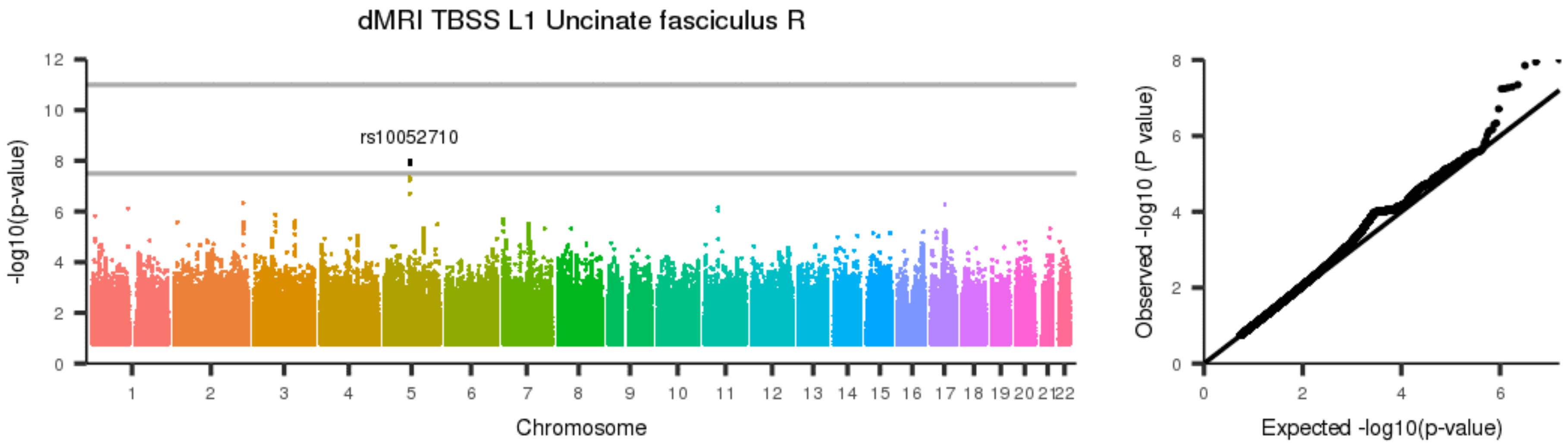

back to list of plots

Figure 385: dMRI TBSS L1 Uncinate fasciculus L

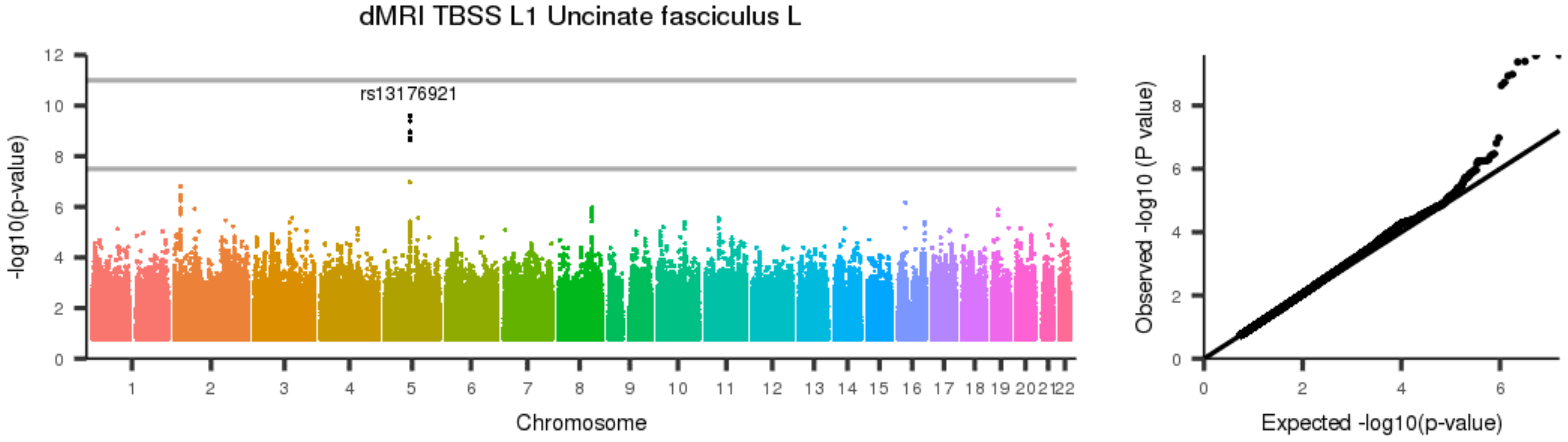

back to list of plots

Figure 386: dMRI TBSS L1 Tapetum R

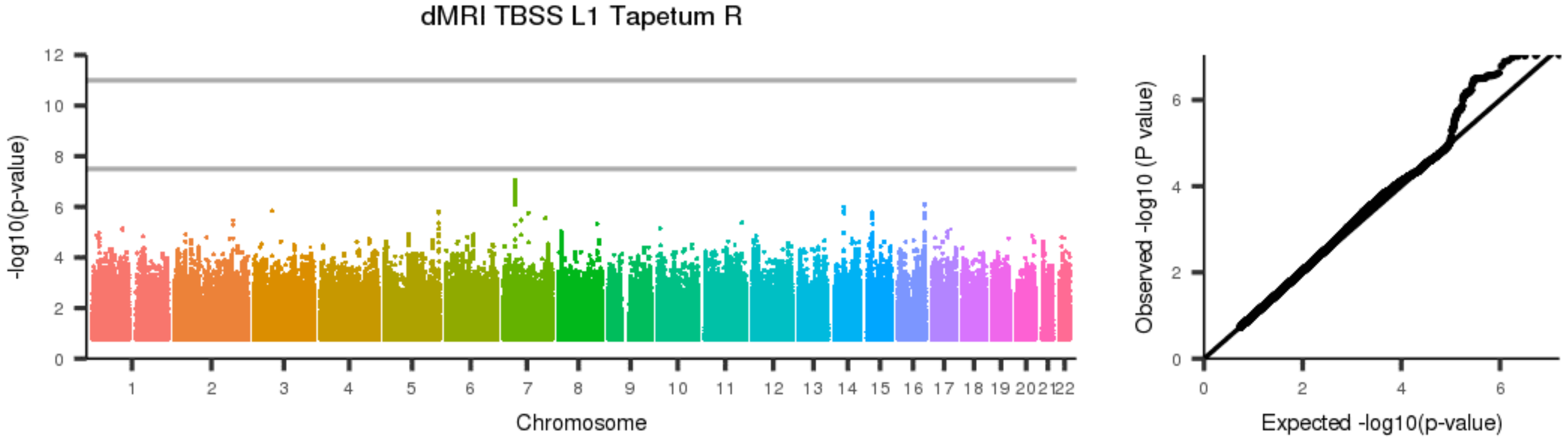

back to list of plots

Figure 387: dMRI TBSS L1 Tapetum L

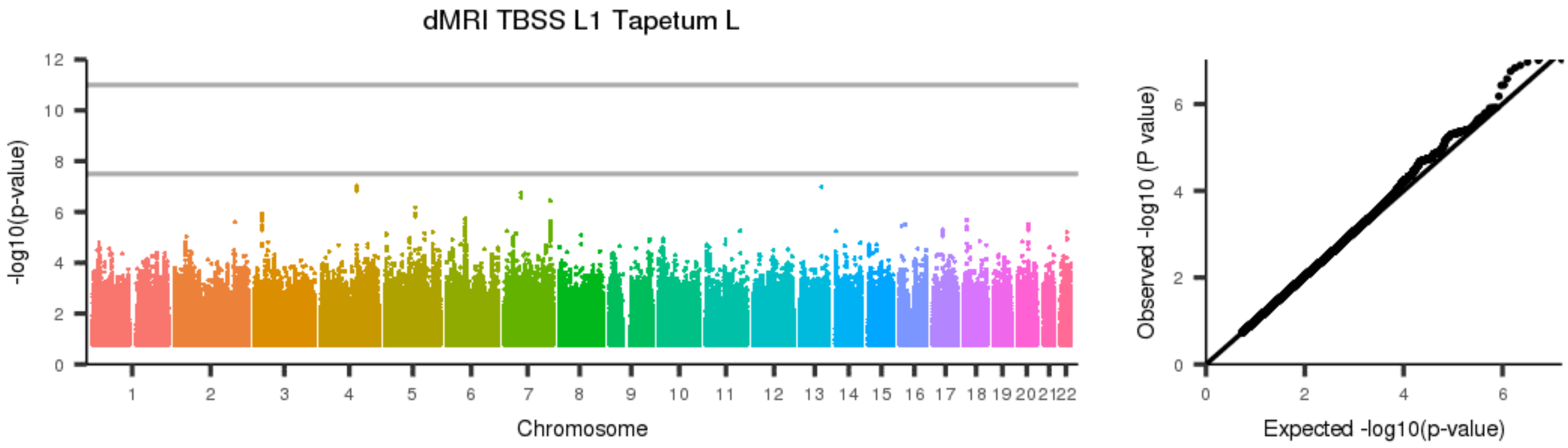

back to list of plots

Figure 388: dMRI TBSS L2 Middle cerebellar peduncle

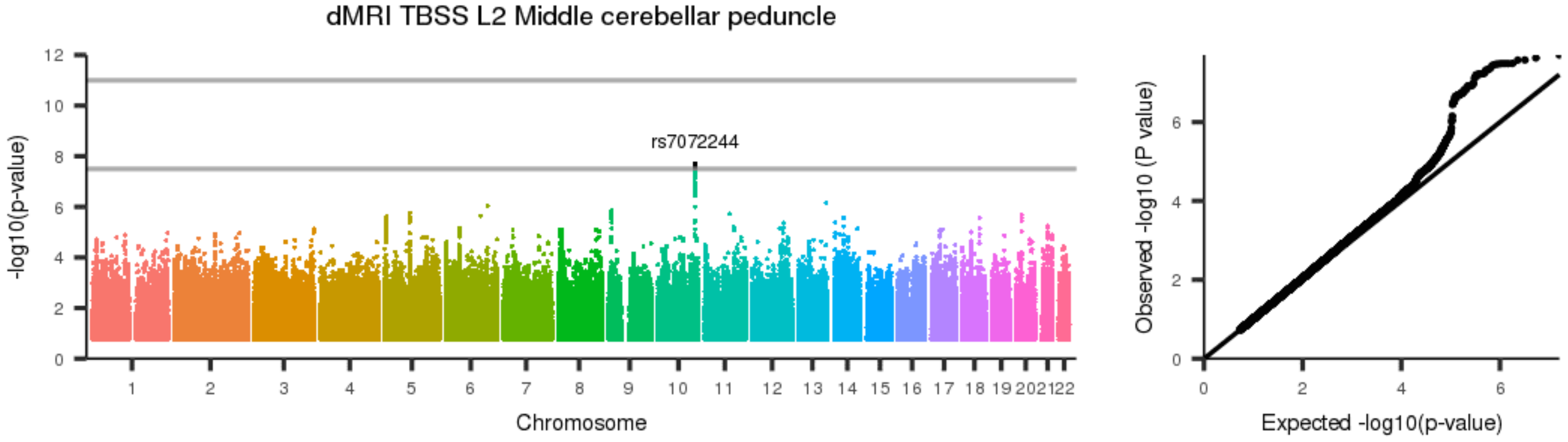

back to list of plots

Figure 389: dMRI TBSS L2 Pontine crossing tract

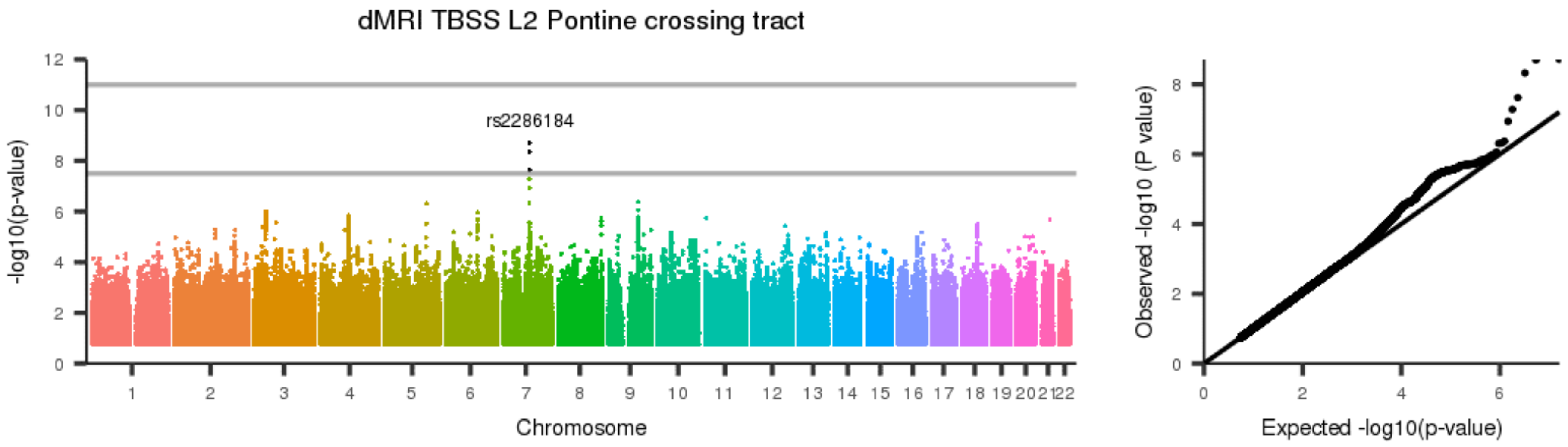

back to list of plots

Figure 390: dMRI TBSS L2 Genu of corpus callosum

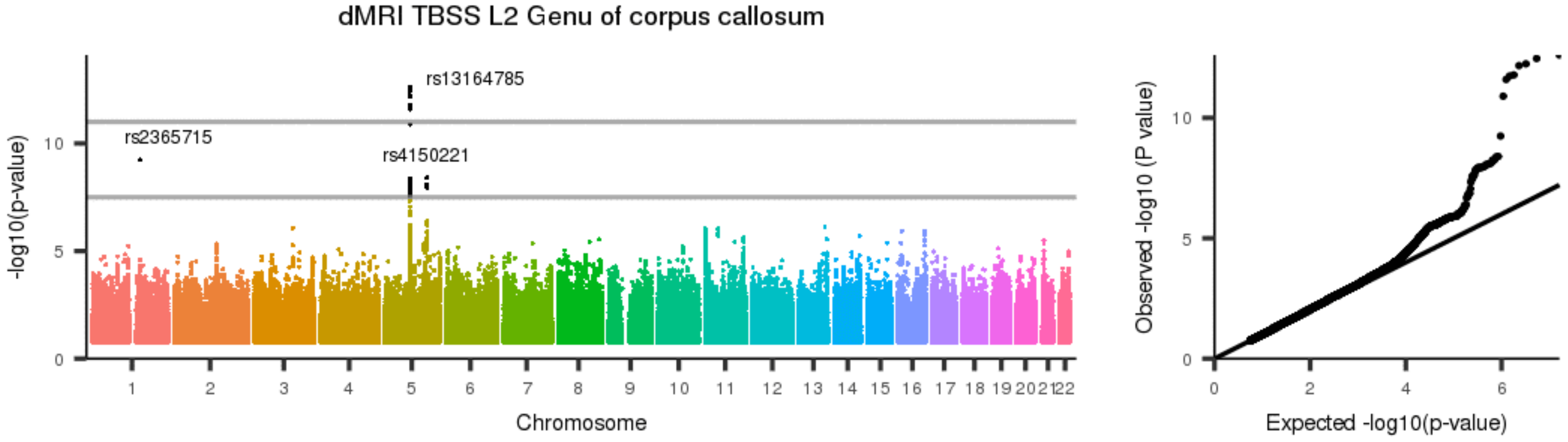

back to list of plots

Figure 391: dMRI TBSS L2 Body of corpus callosum

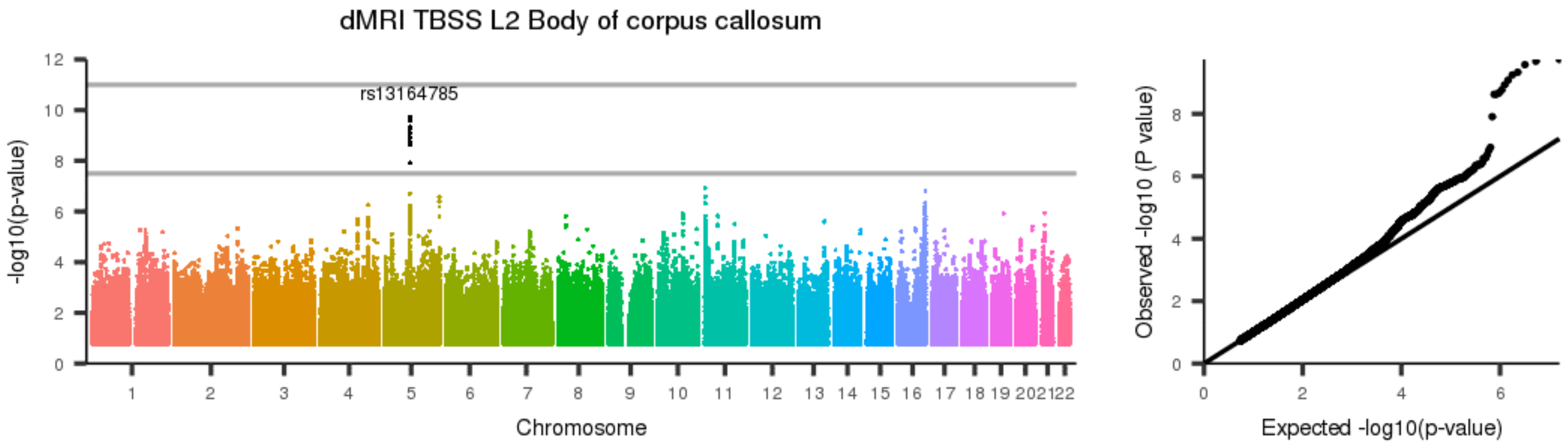

back to list of plots

Figure 392: dMRI TBSS L2 Splenium of corpus callosum

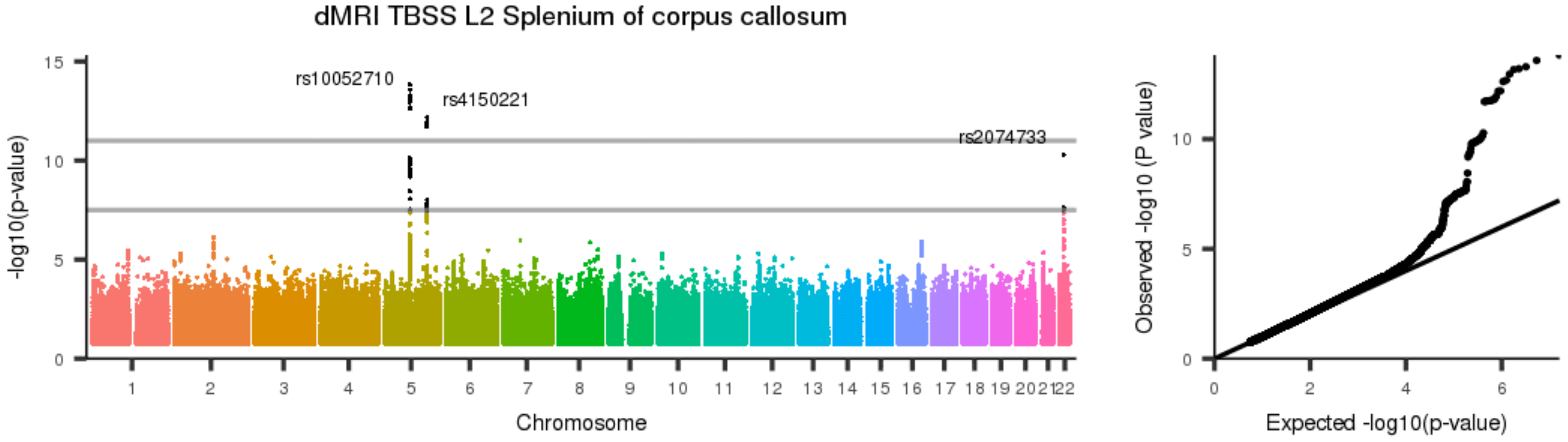

back to list of plots

Figure 393: dMRI TBSS L2 Fornix

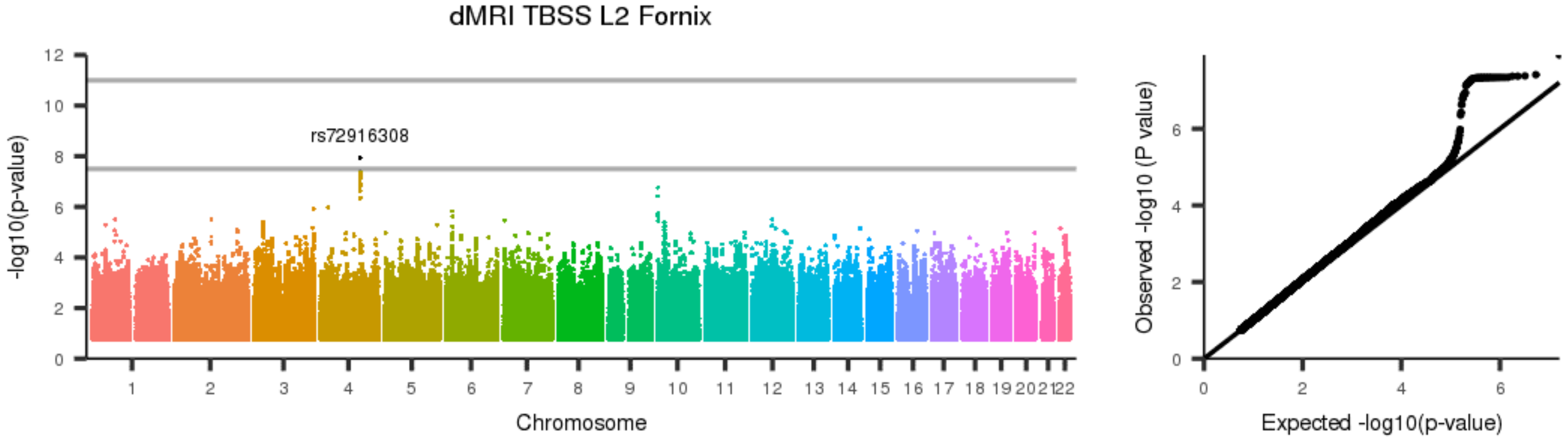

back to list of plots

Figure 394: dMRI TBSS L2 Corticospinal tract R

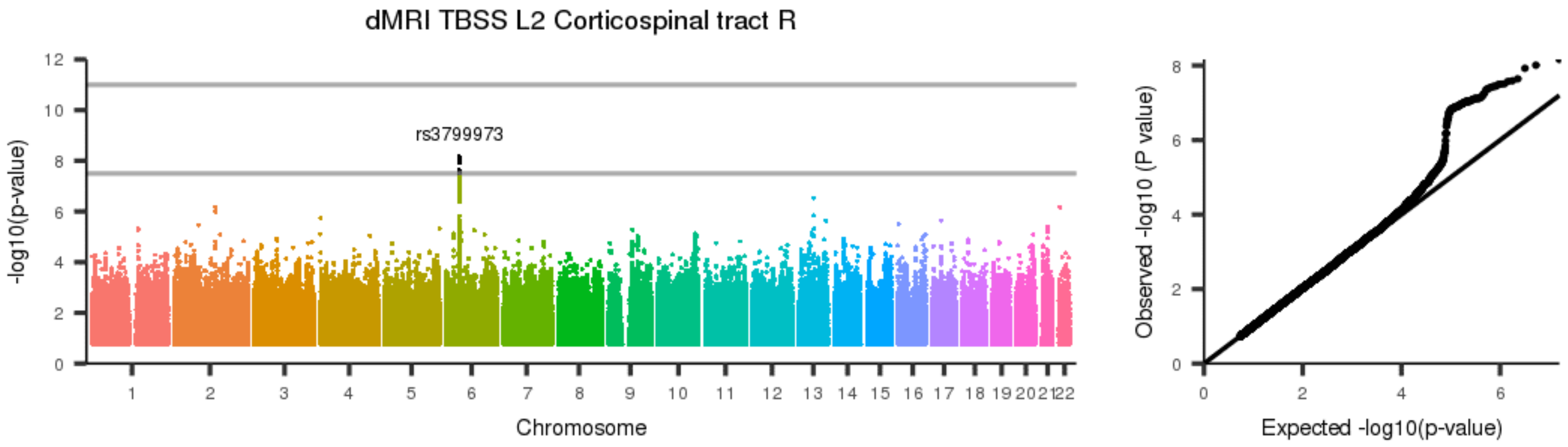

back to list of plots

Figure 395: dMRI TBSS L2 Corticospinal tract L

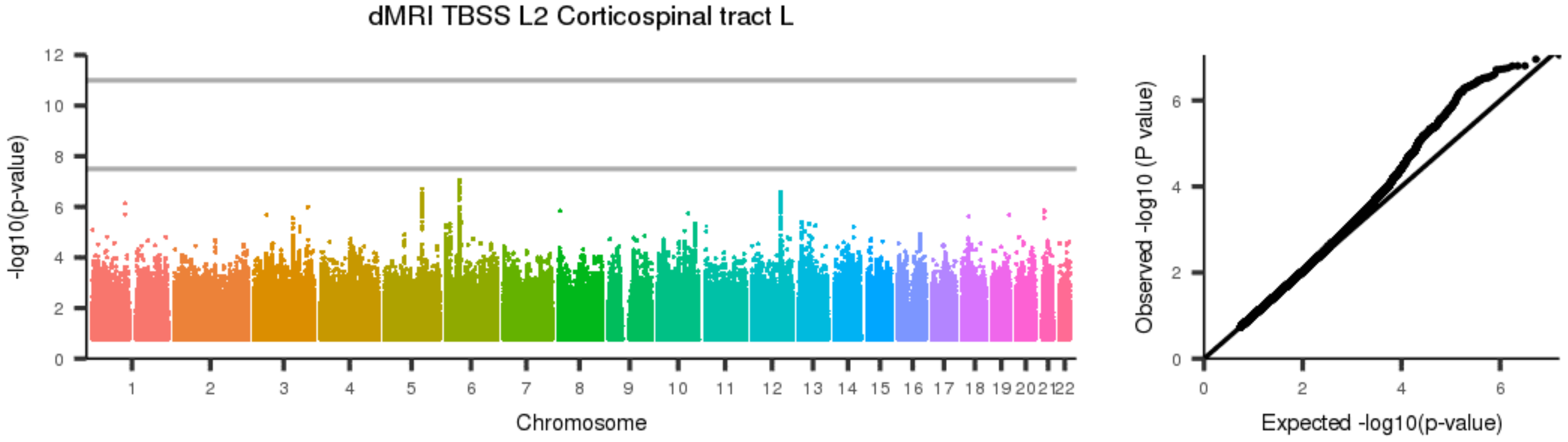

back to list of plots

Figure 396: dMRI TBSS L2 Medial lemniscus R

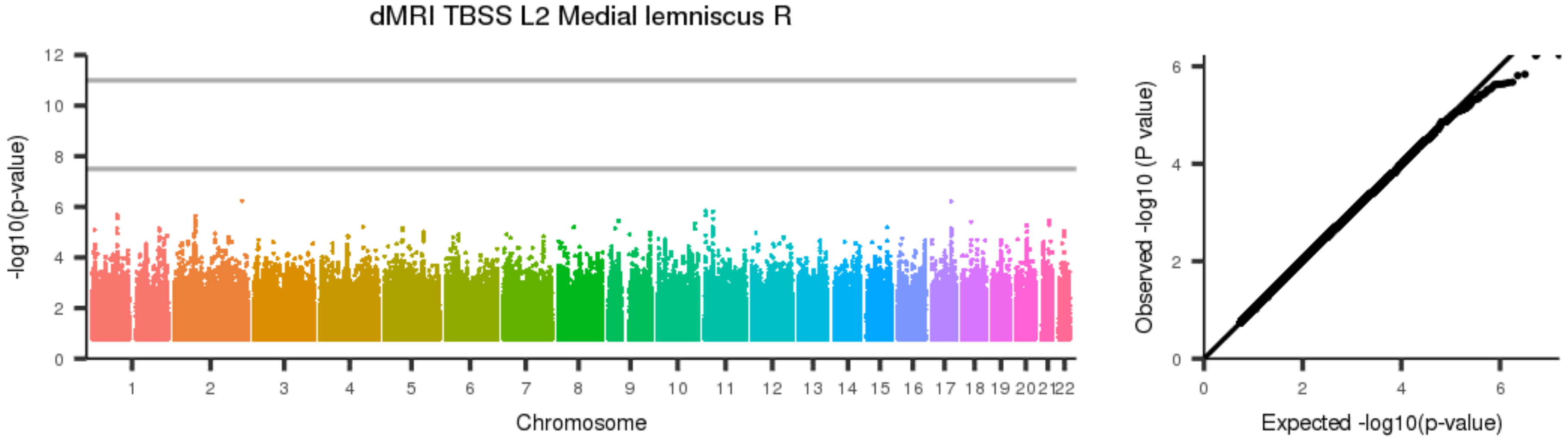

back to list of plots

Figure 397: dMRI TBSS L2 Medial lemniscus L

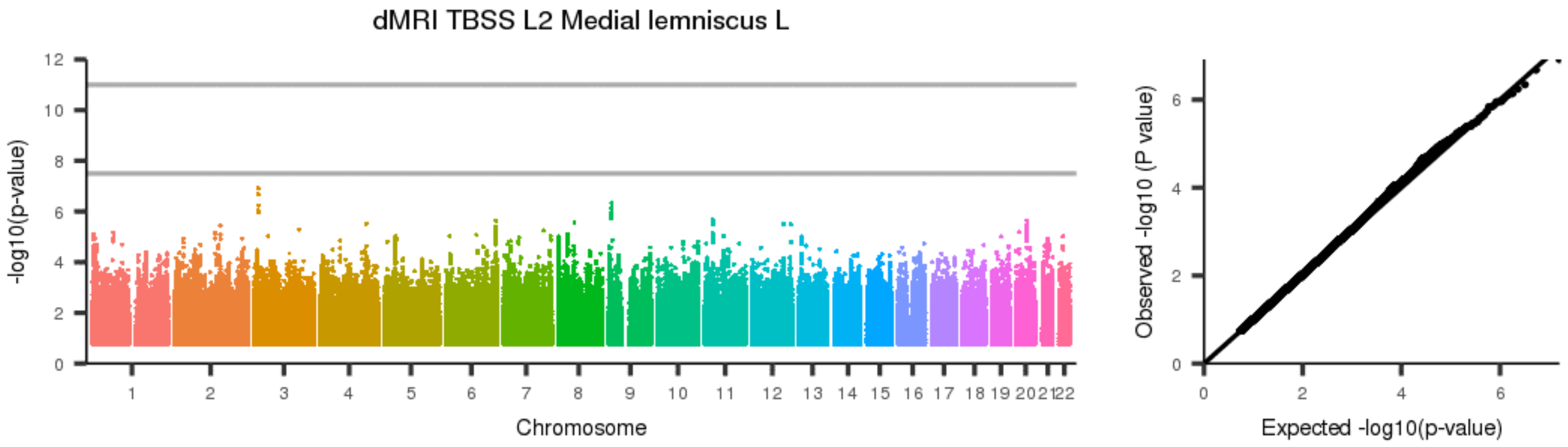

back to list of plots

Figure 398: dMRI TBSS L2 Inferior cerebellar peduncle R

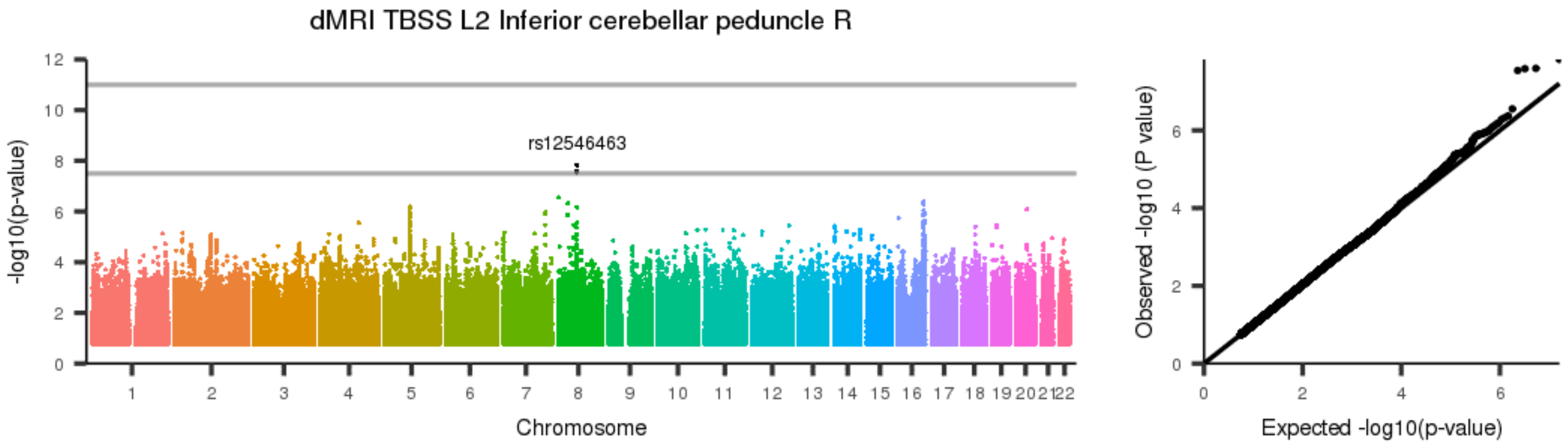

back to list of plots

Figure 399: dMRI TBSS L2 Inferior cerebellar peduncle L

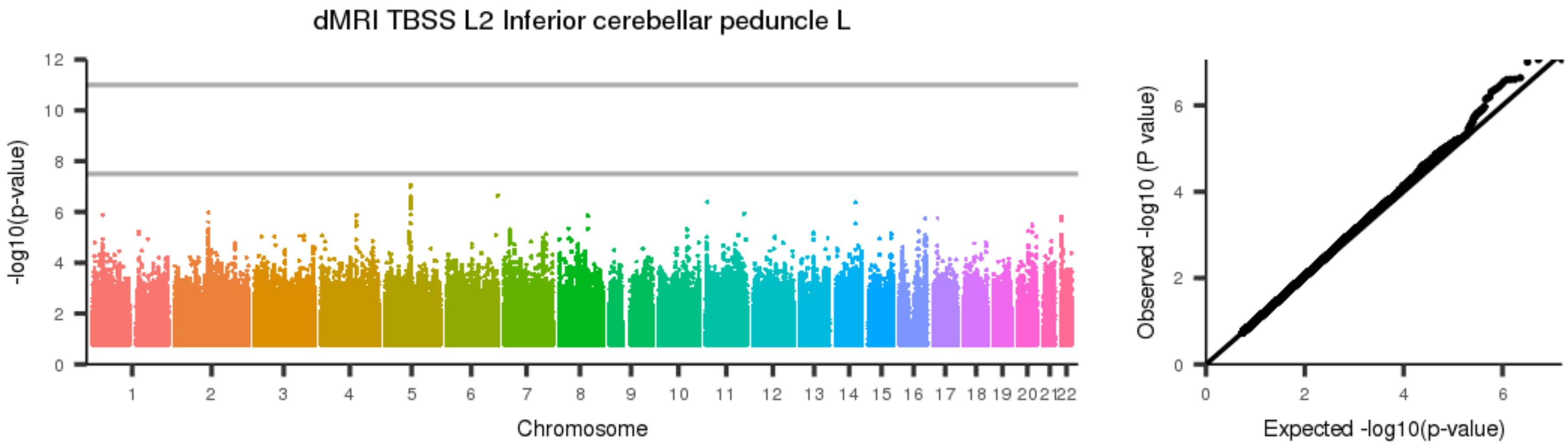

back to list of plots

Figure 400: dMRI TBSS L2 Superior cerebellar peduncle R

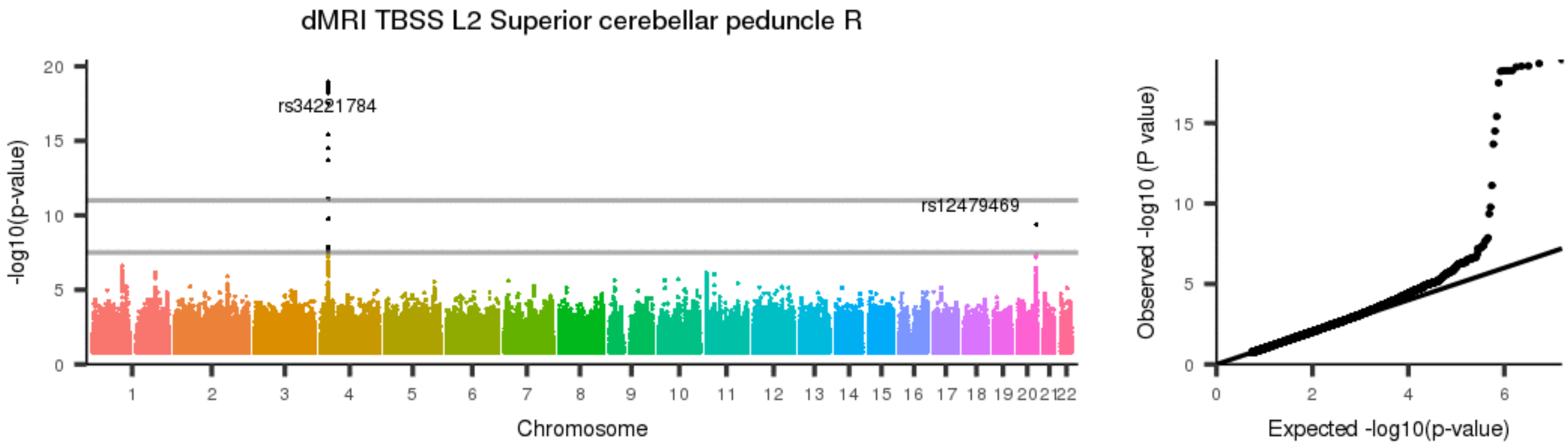

back to list of plots

Figure 401: dMRI TBSS L2 Superior cerebellar peduncle L

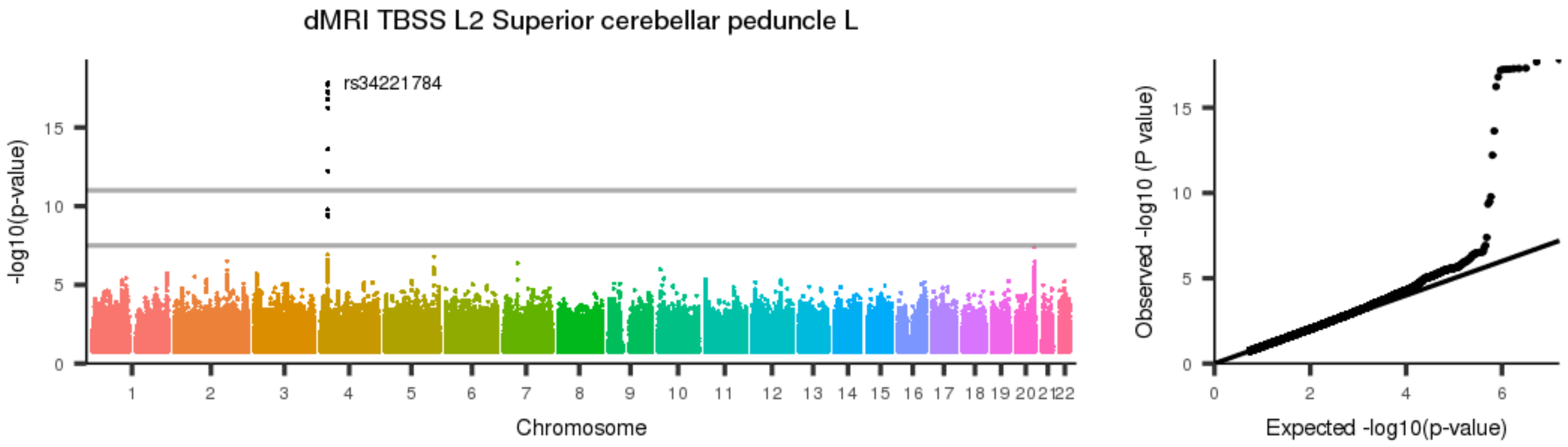

back to list of plots

Figure 402: dMRI TBSS L2 Cerebral peduncle R

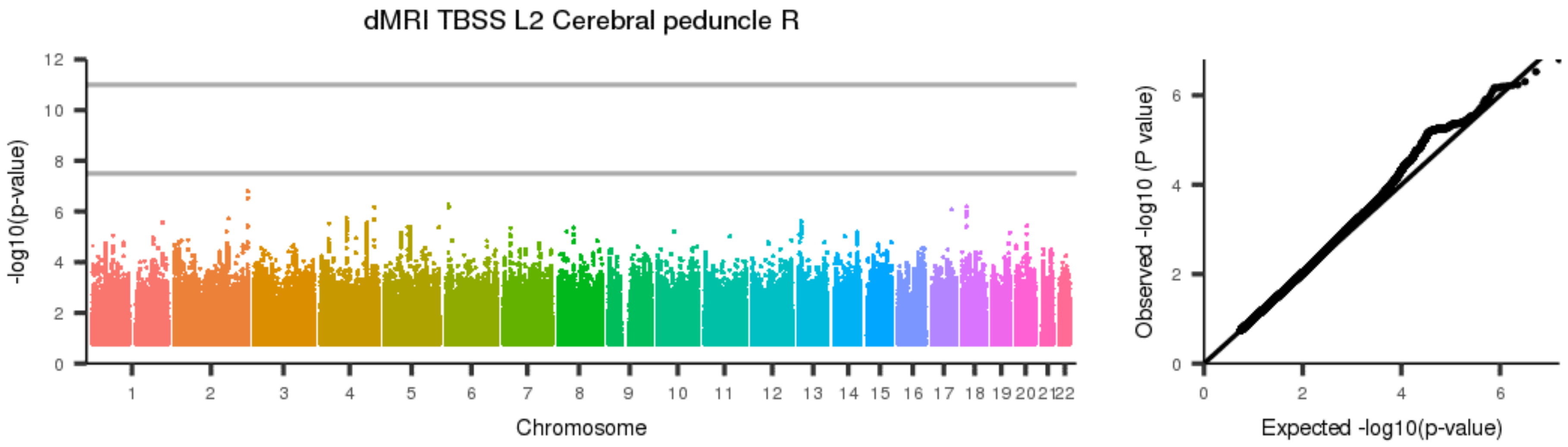

back to list of plots

Figure 403: dMRI TBSS L2 Cerebral peduncle L

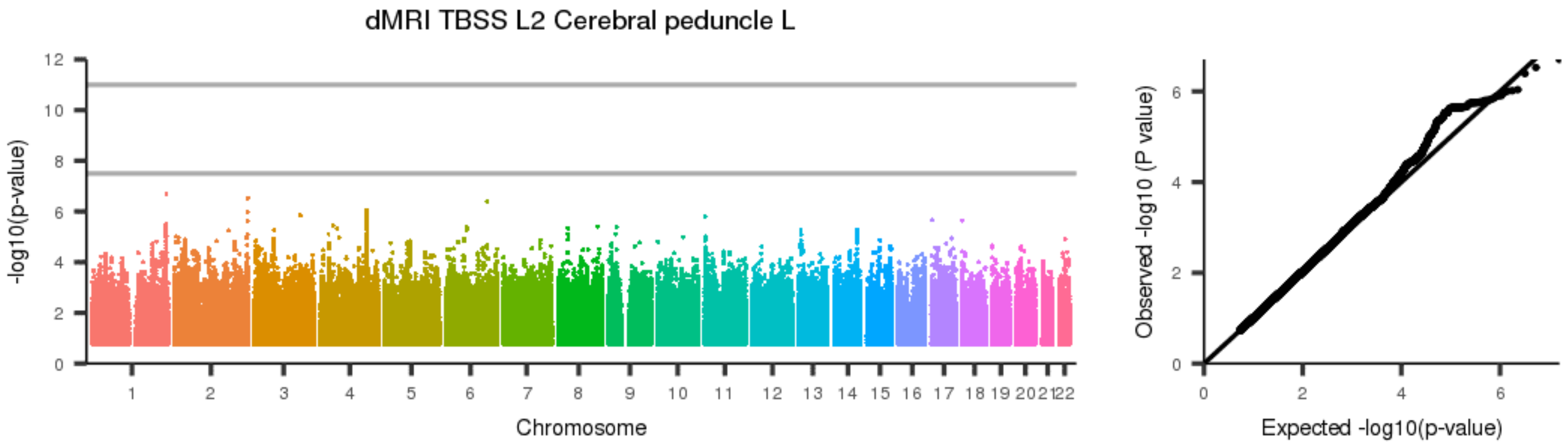

back to list of plots

Figure 404: dMRI TBSS L2 Anterior limb of internal capsule R

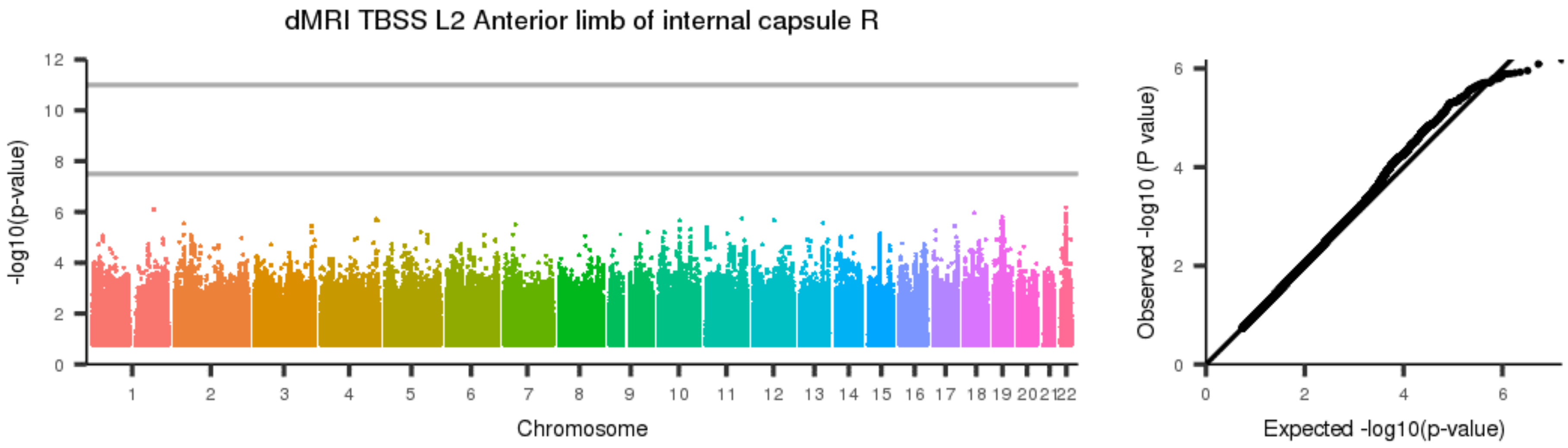

back to list of plots

Figure 405: dMRI TBSS L2 Anterior limb of internal capsule L

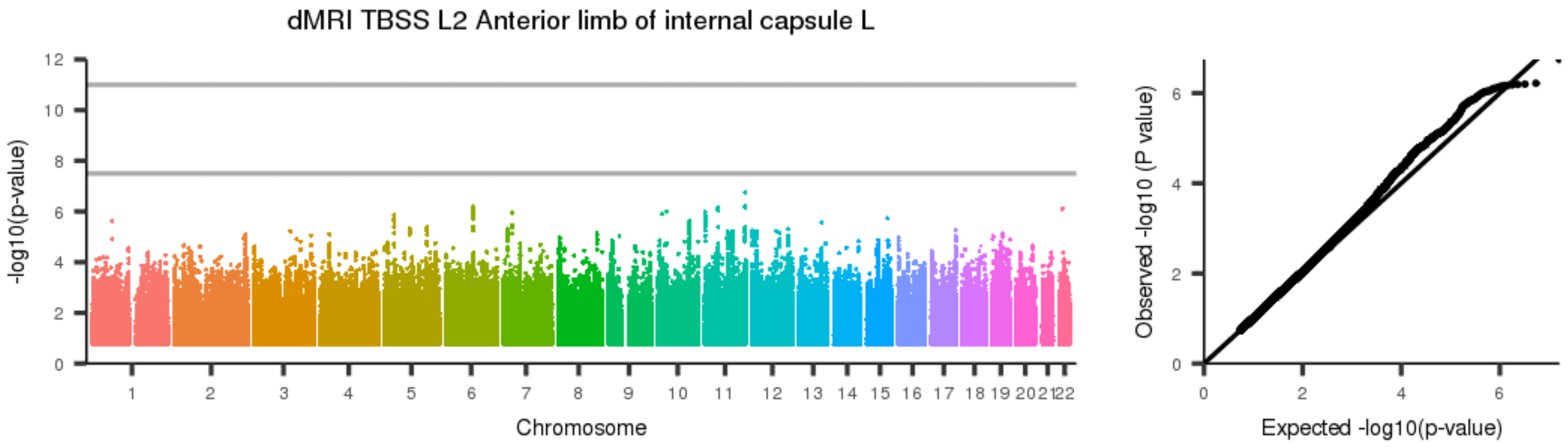

back to list of plots

Figure 406: dMRI TBSS L2 Posterior limb of internal capsule R

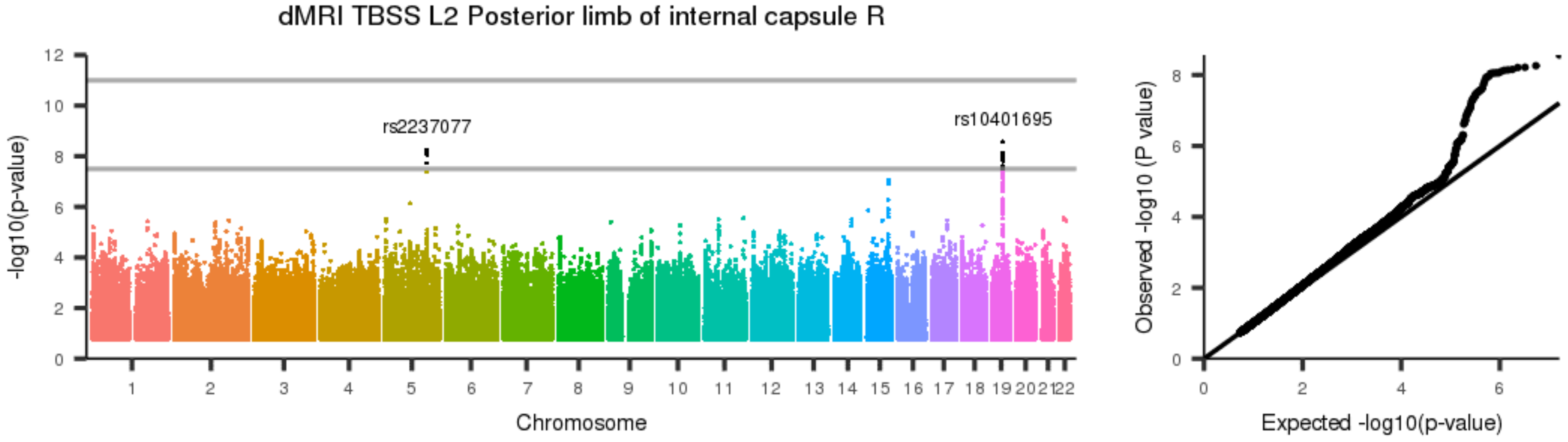

back to list of plots

Figure 407: dMRI TBSS L2 Posterior limb of internal capsule L

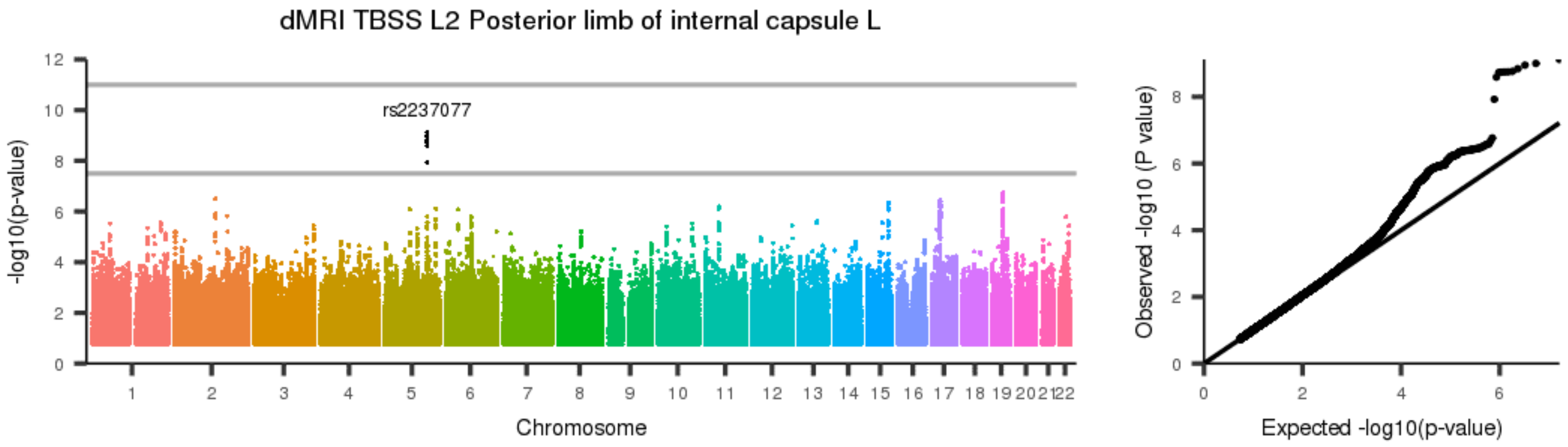

back to list of plots

Figure 408: dMRI TBSS L2 Retrolenticular part of internal capsule R

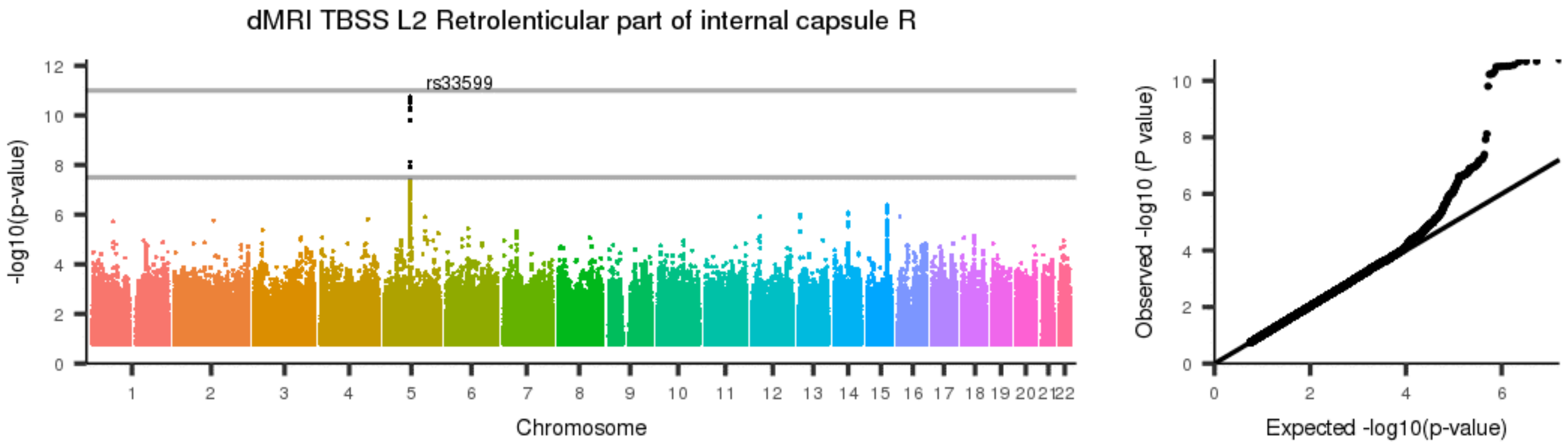

back to list of plots

Figure 409: dMRI TBSS L2 Retrolenticular part of internal capsule L

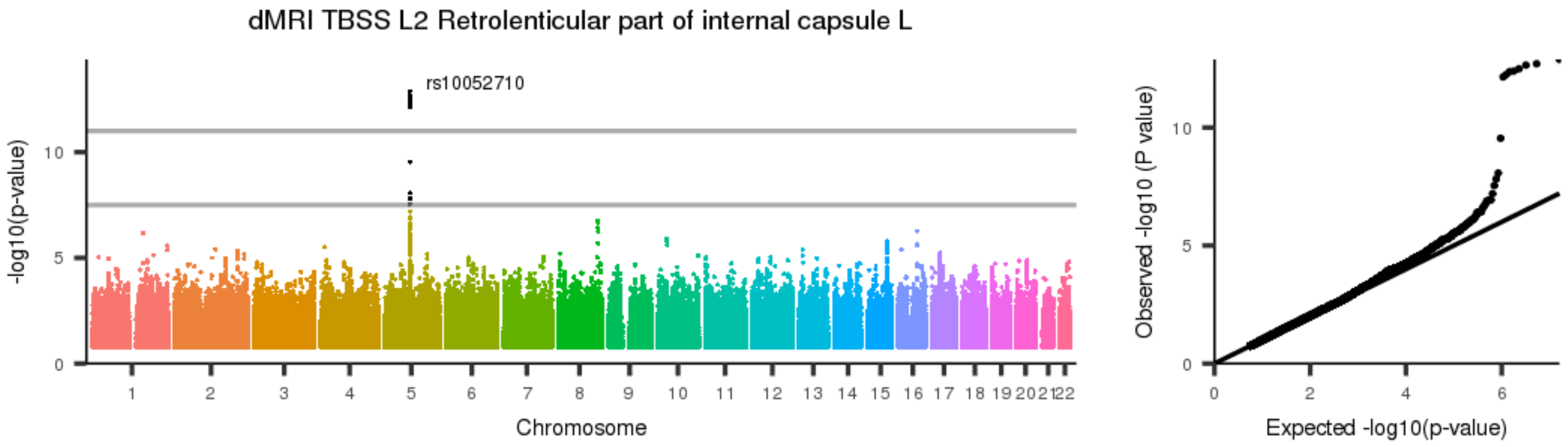

back to list of plots

Figure 410: dMRI TBSS L2 Anterior corona radiata R

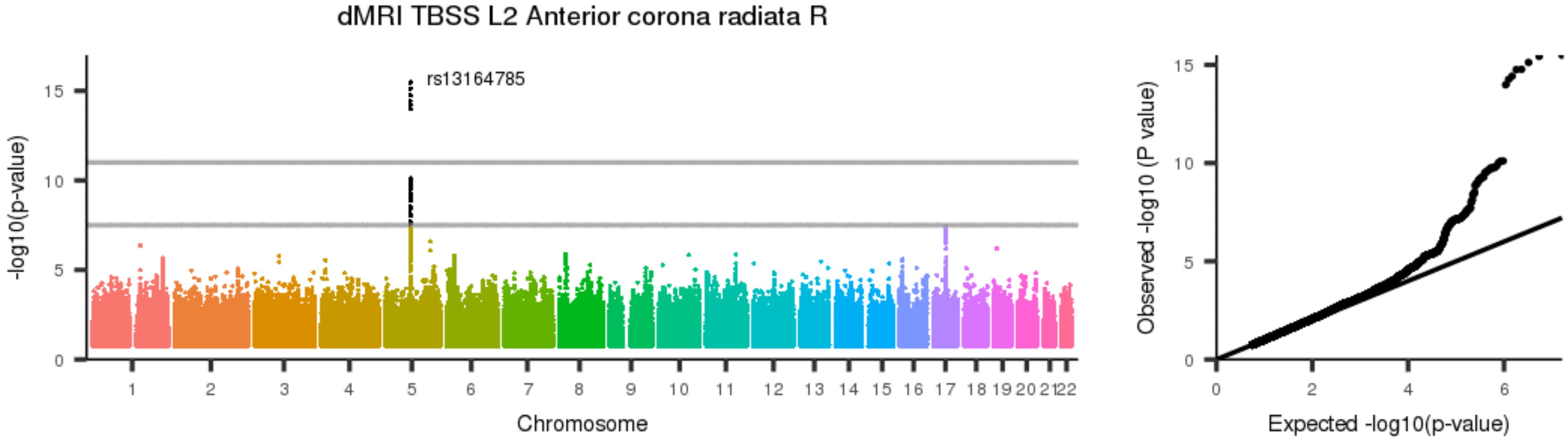

back to list of plots

Figure 411: dMRI TBSS L2 Anterior corona radiata L

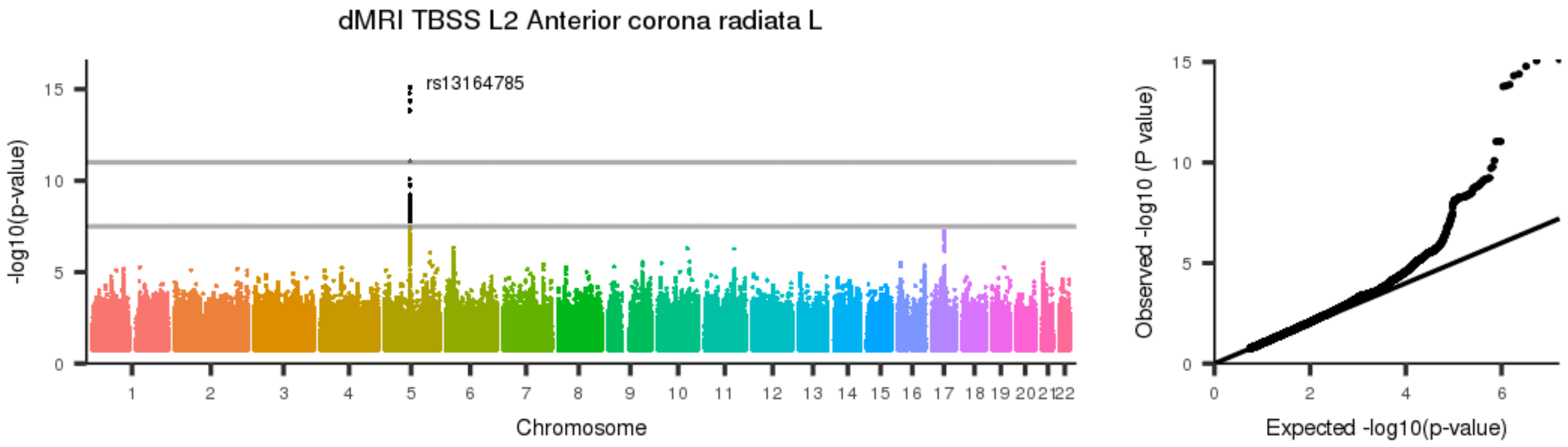

back to list of plots

Figure 412: dMRI TBSS L2 Superior corona radiata R

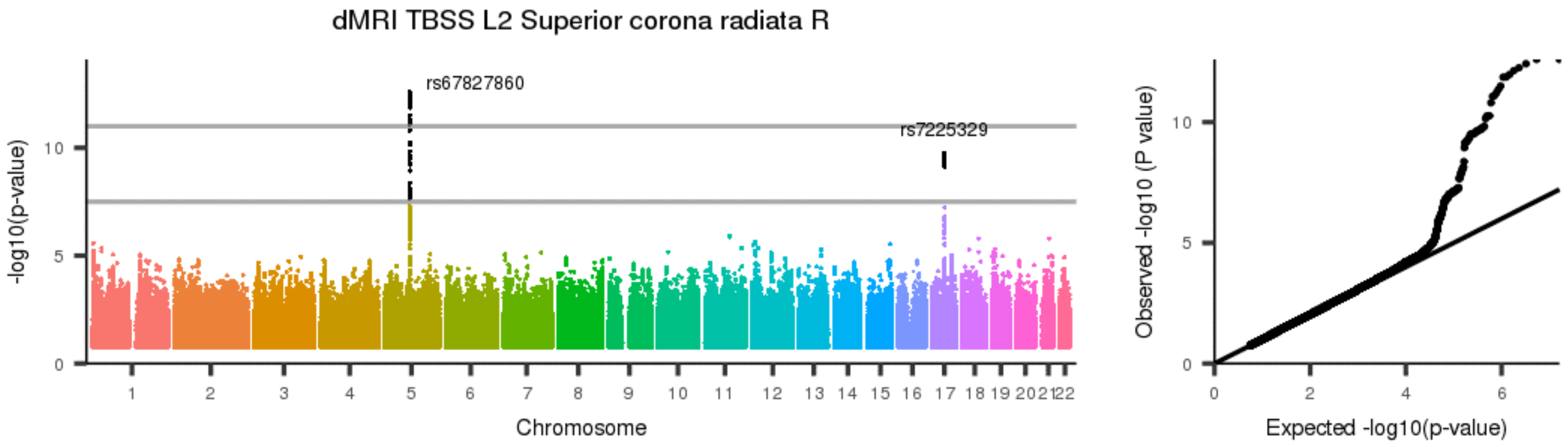

back to list of plots

Figure 413: dMRI TBSS L2 Superior corona radiata L

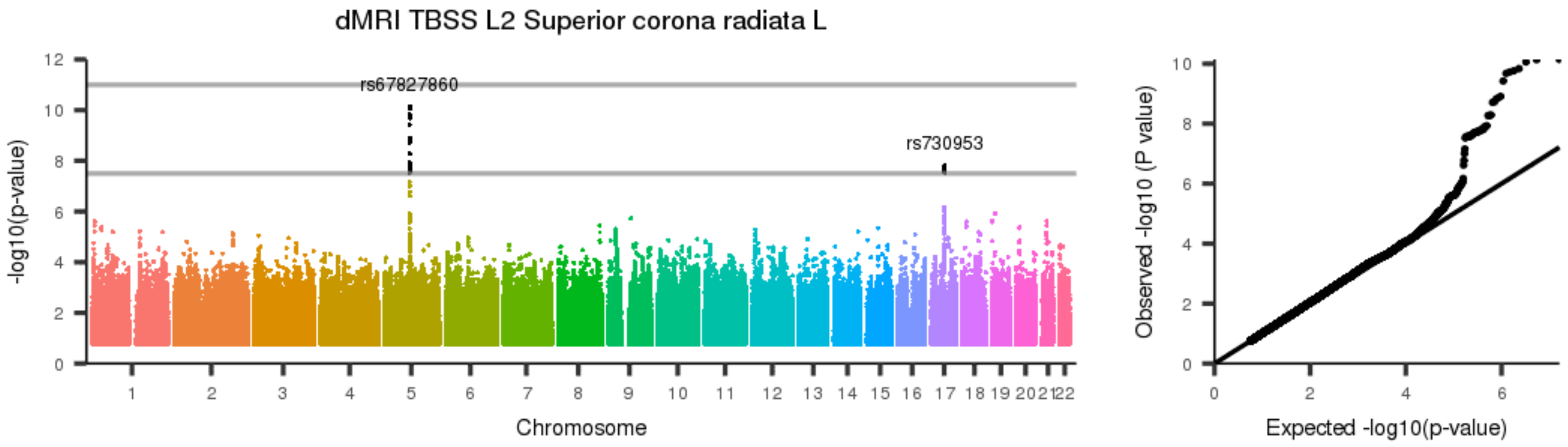

back to list of plots

Figure 414: dMRI TBSS L2 Posterior corona radiata R

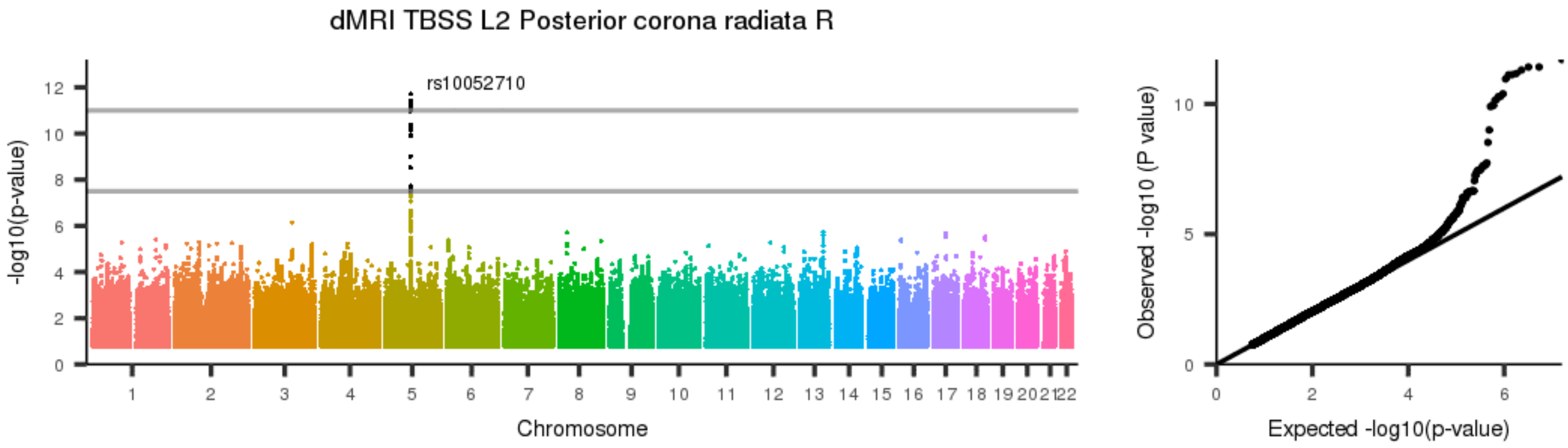

back to list of plots

Figure 415: dMRI TBSS L2 Posterior corona radiata L

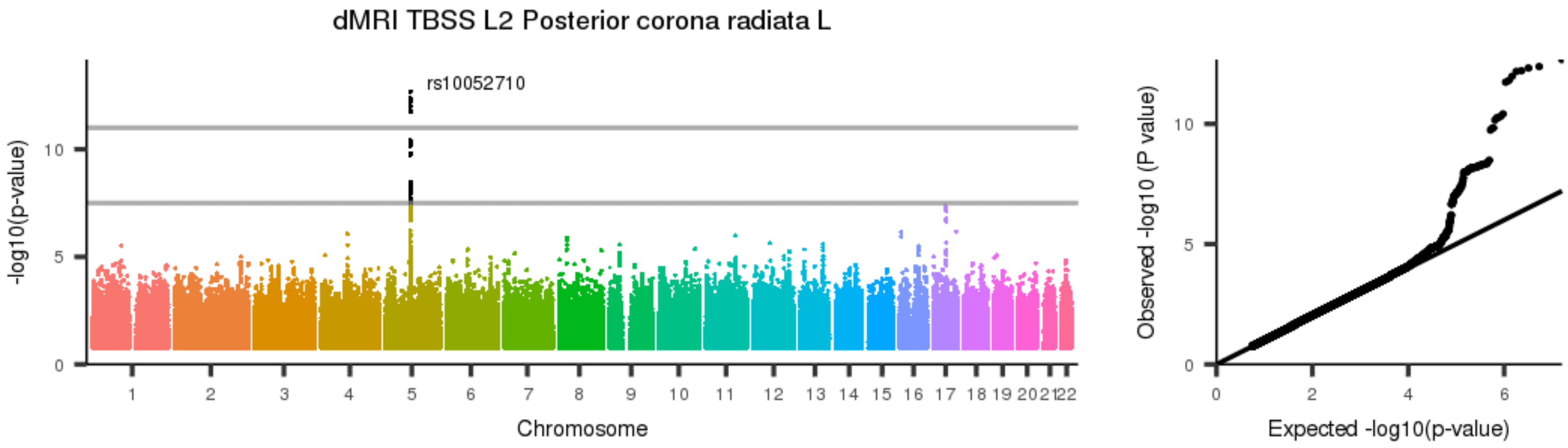

back to list of plots

Figure 416: dMRI TBSS L2 Posterior thalamic radiation R

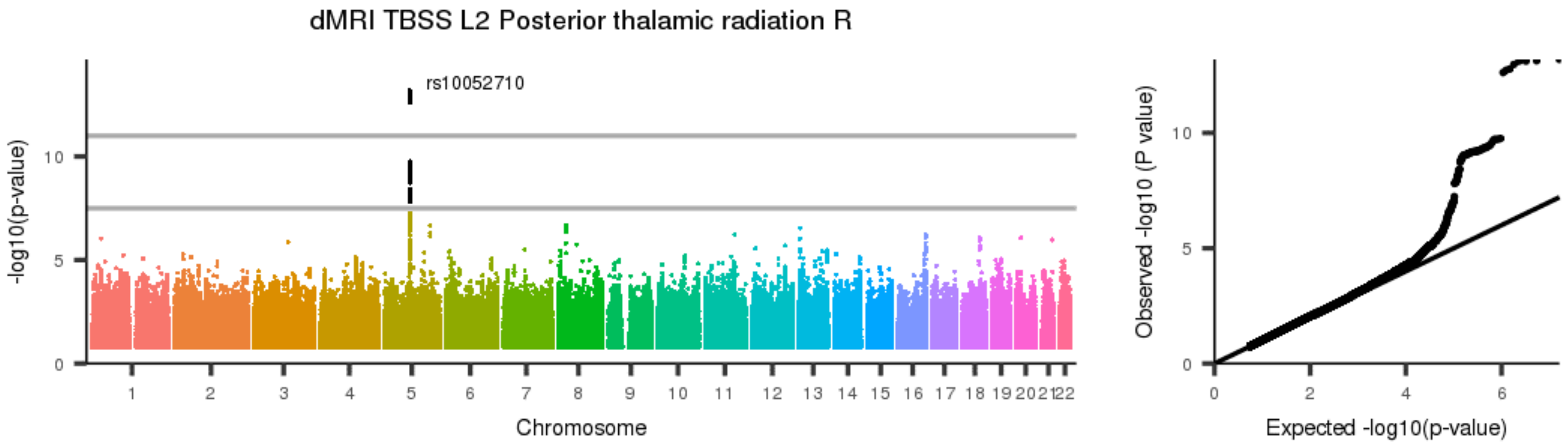

back to list of plots

Figure 417: dMRI TBSS L2 Posterior thalamic radiation L

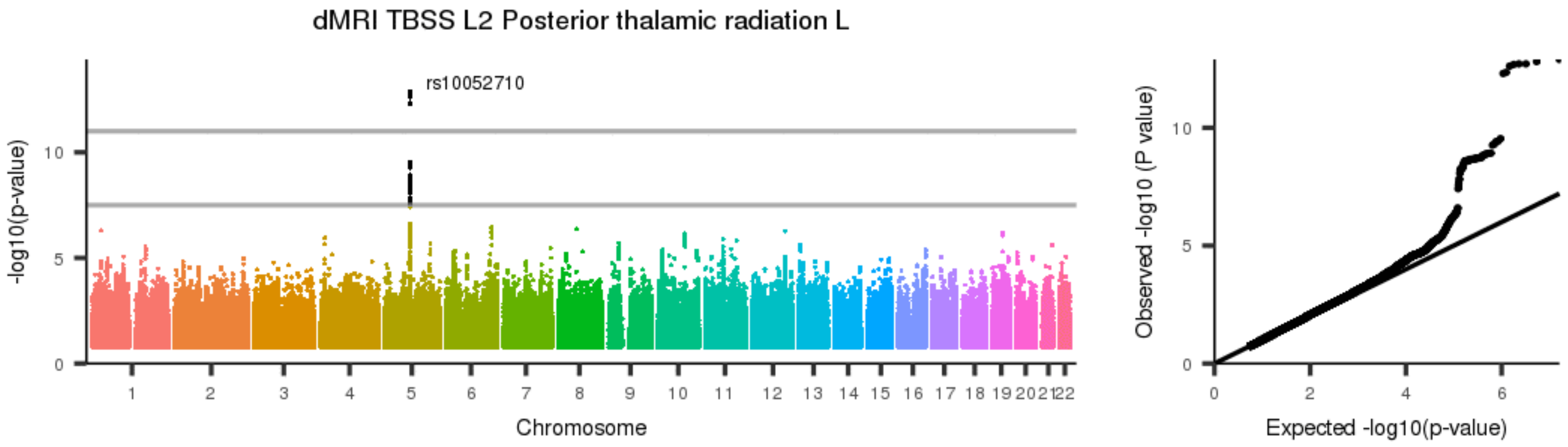

back to list of plots

Figure 418: dMRI TBSS L2 Sagittal stratum R

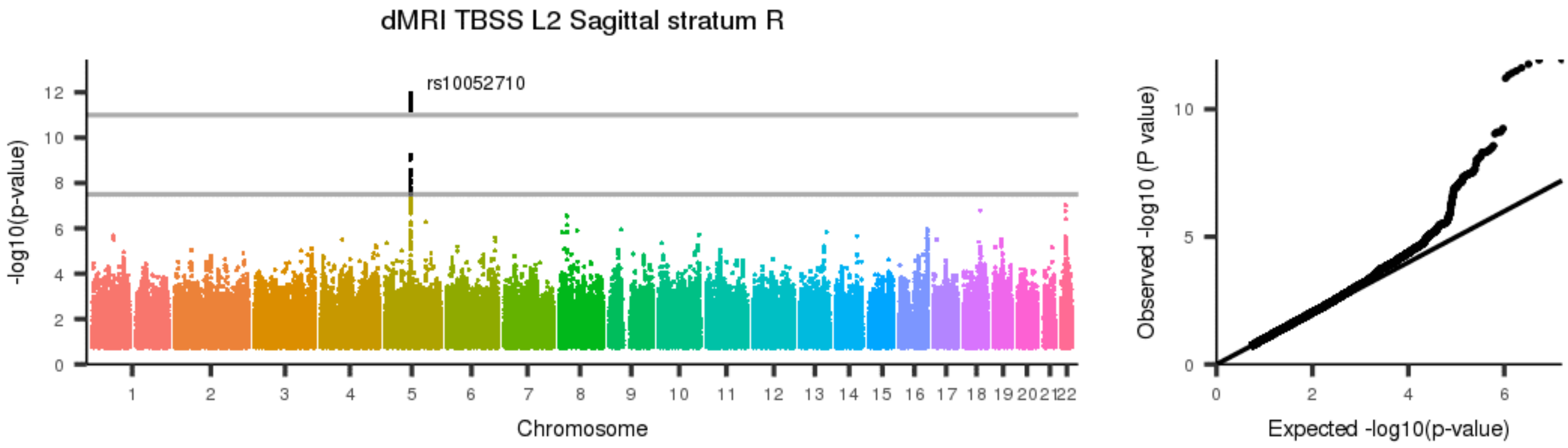

back to list of plots

Figure 419: dMRI TBSS L2 Sagittal stratum L

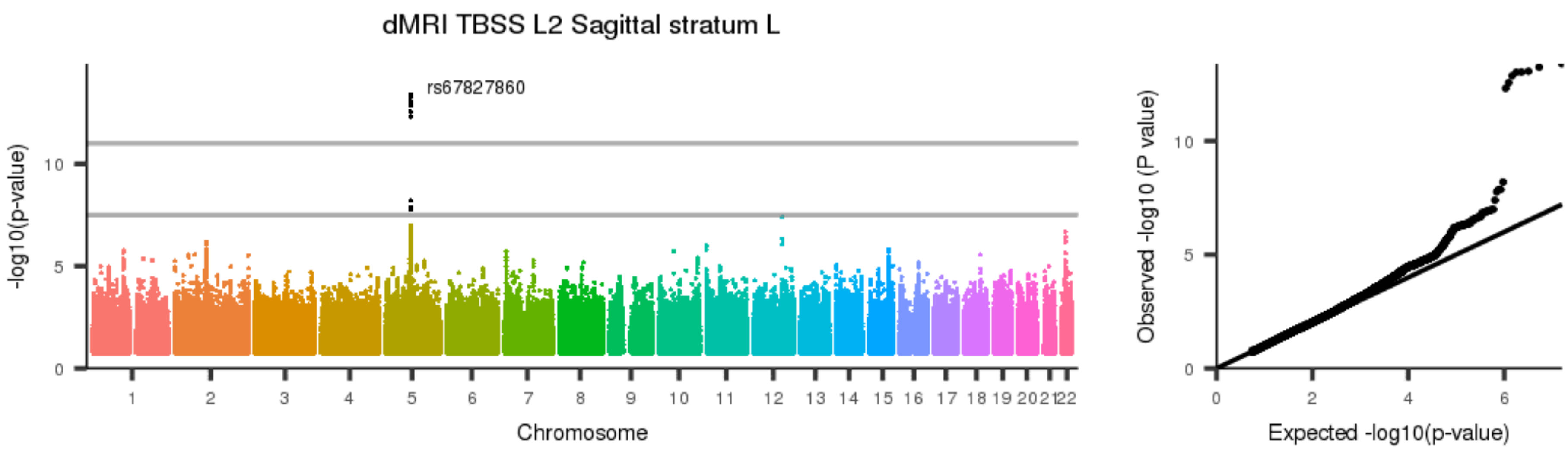

back to list of plots

Figure 420: dMRI TBSS L2 External capsule R

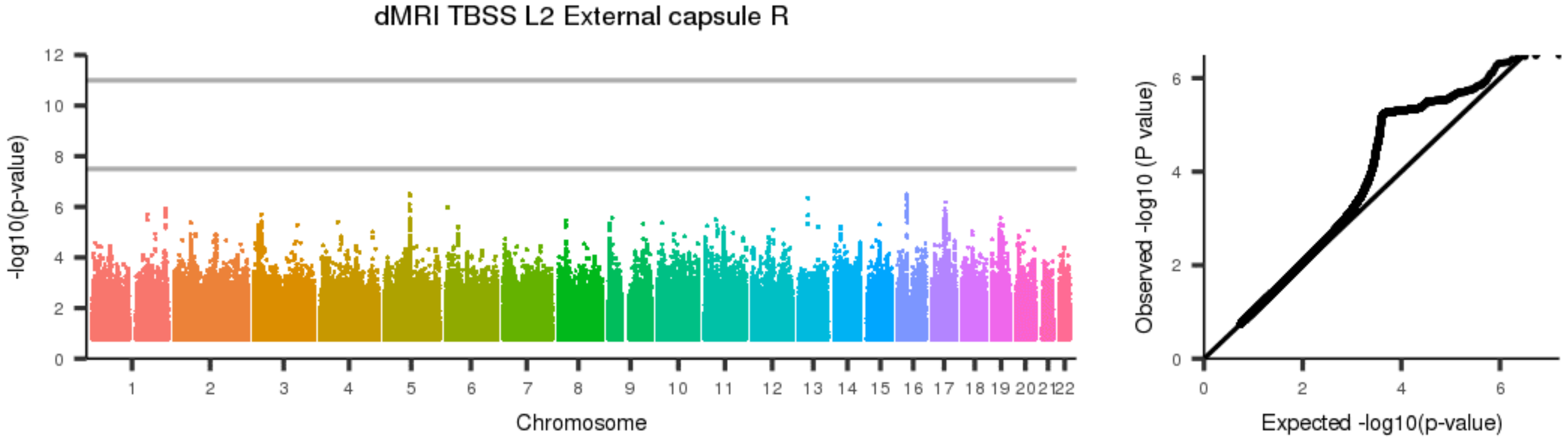

back to list of plots

Figure 421: dMRI TBSS L2 External capsule L

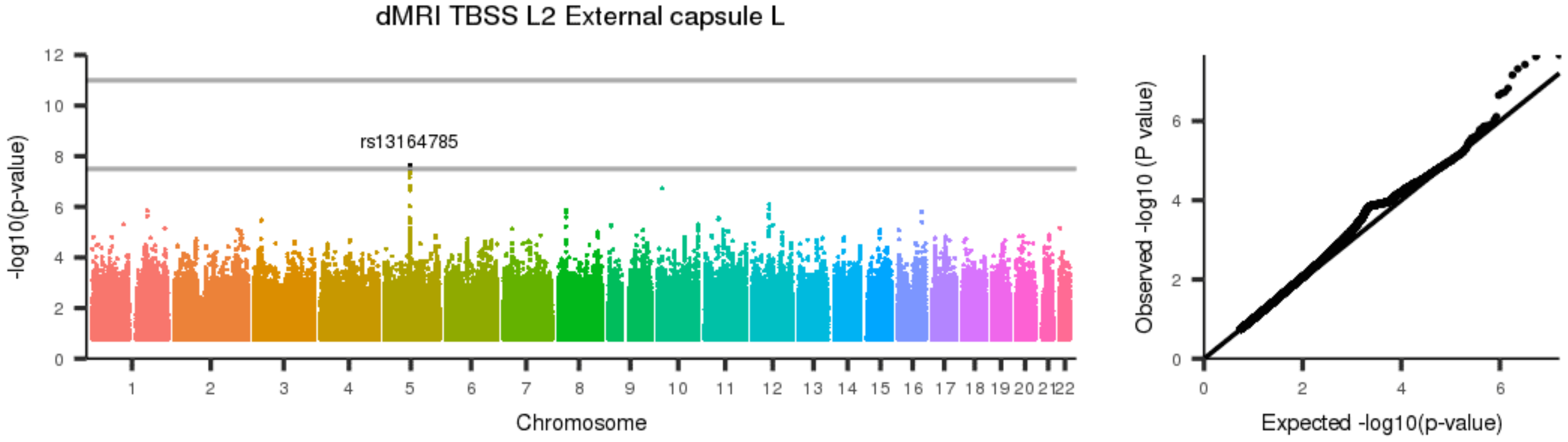

back to list of plots

Figure 422: dMRI TBSS L2 Cingulum cingulate gyrus R

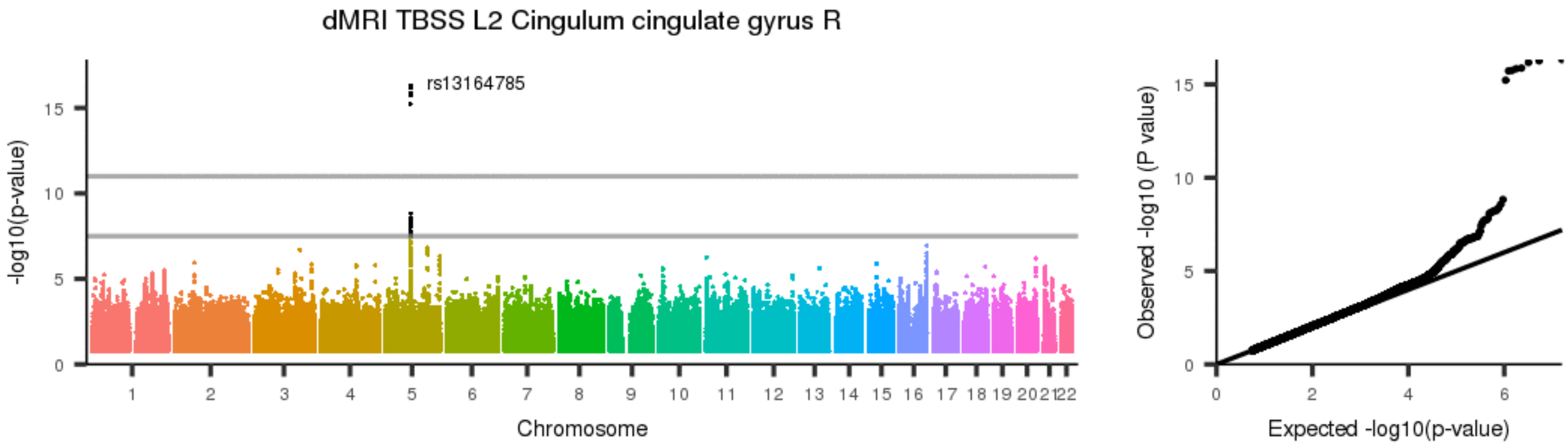

back to list of plots

Figure 423: dMRI TBSS L2 Cingulum cingulate gyrus L

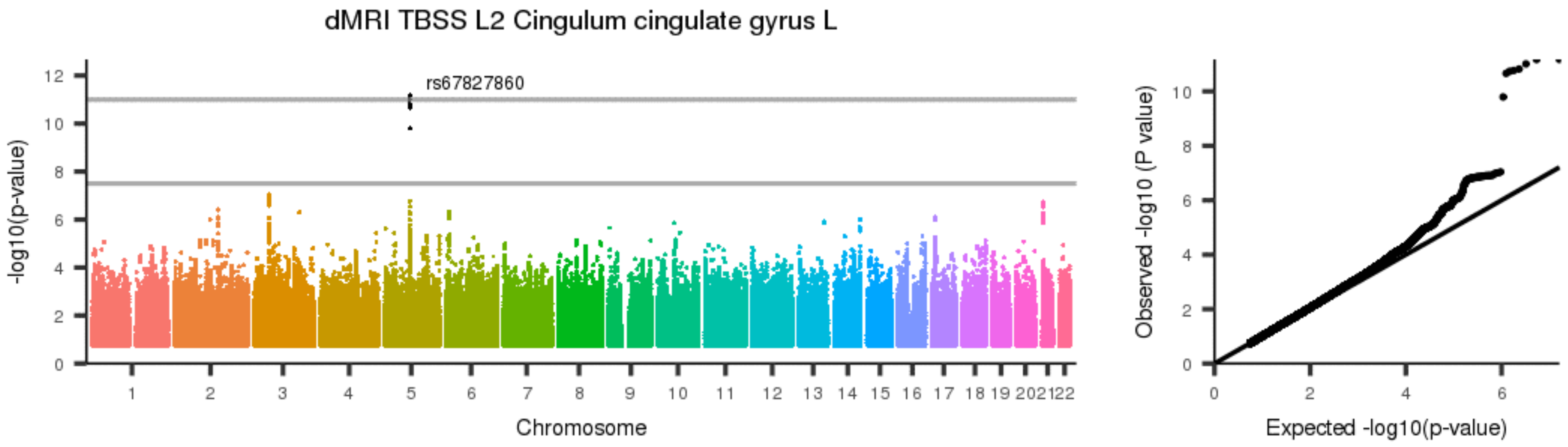

back to list of plots

Figure 424: dMRI TBSS L2 Cingulum hippocampus R

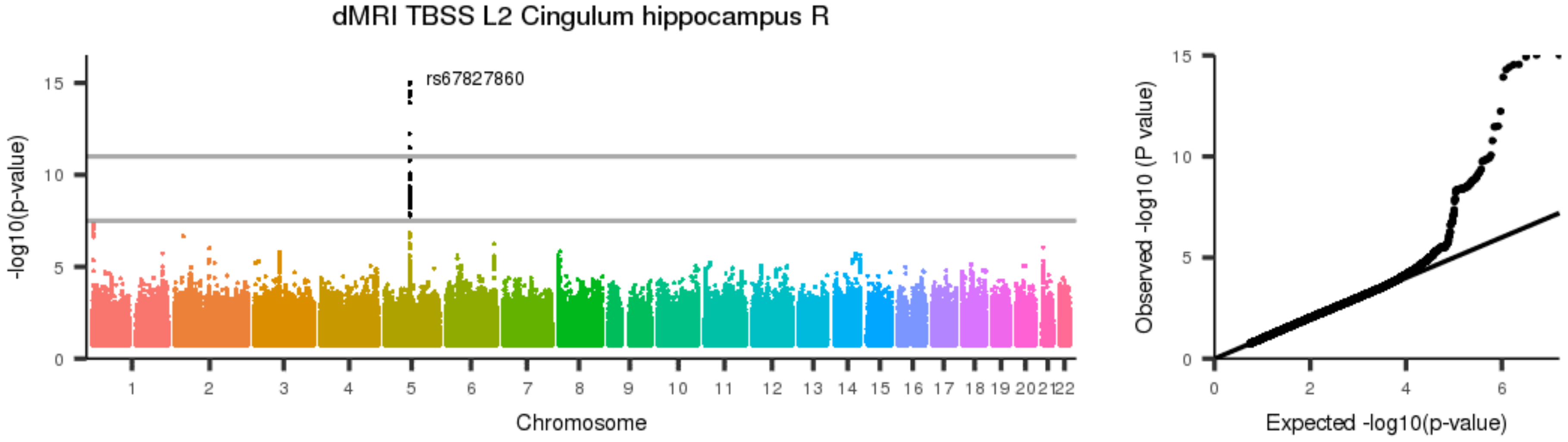

back to list of plots

Figure 425: dMRI TBSS L2 Cingulum hippocampus L

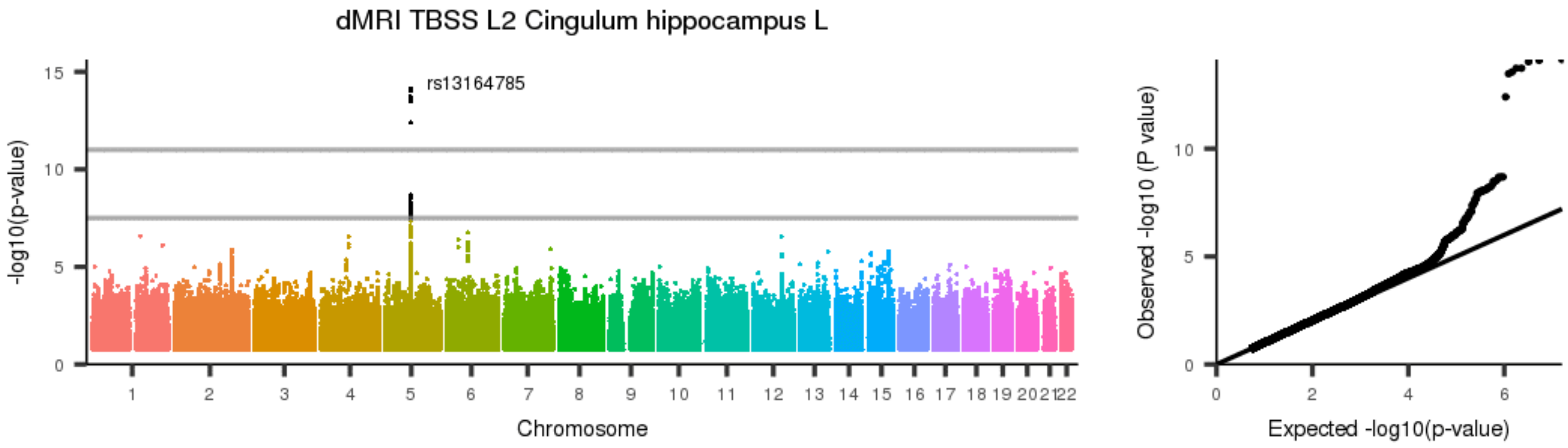

back to list of plots

Figure 426: dMRI TBSS L2 Fornix cres+Stria terminalis R

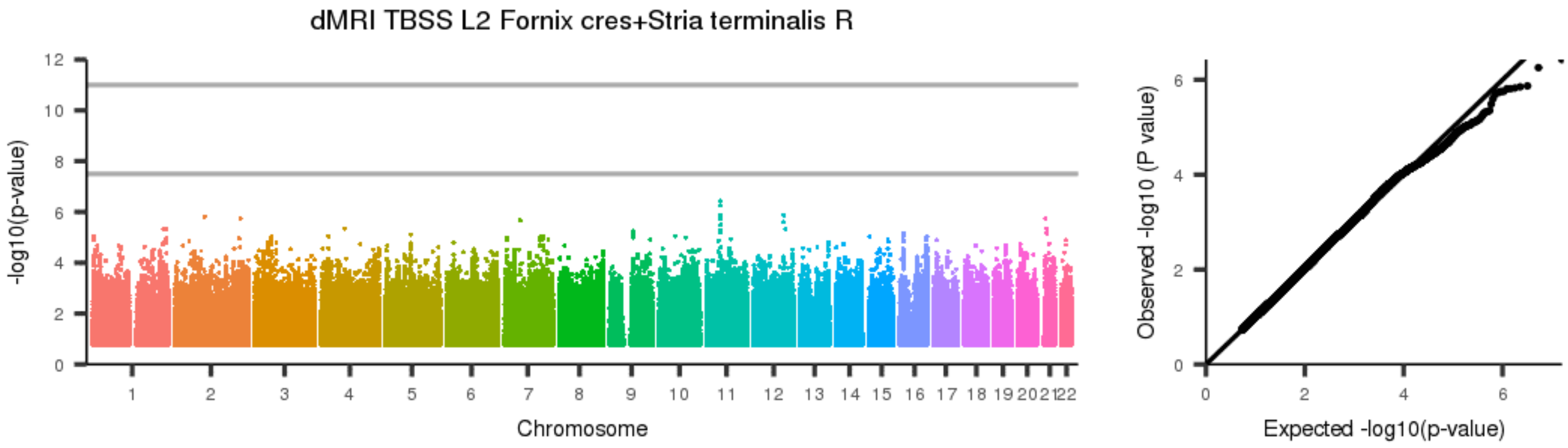

back to list of plots

Figure 427: dMRI TBSS L2 Fornix cres+Stria terminalis L

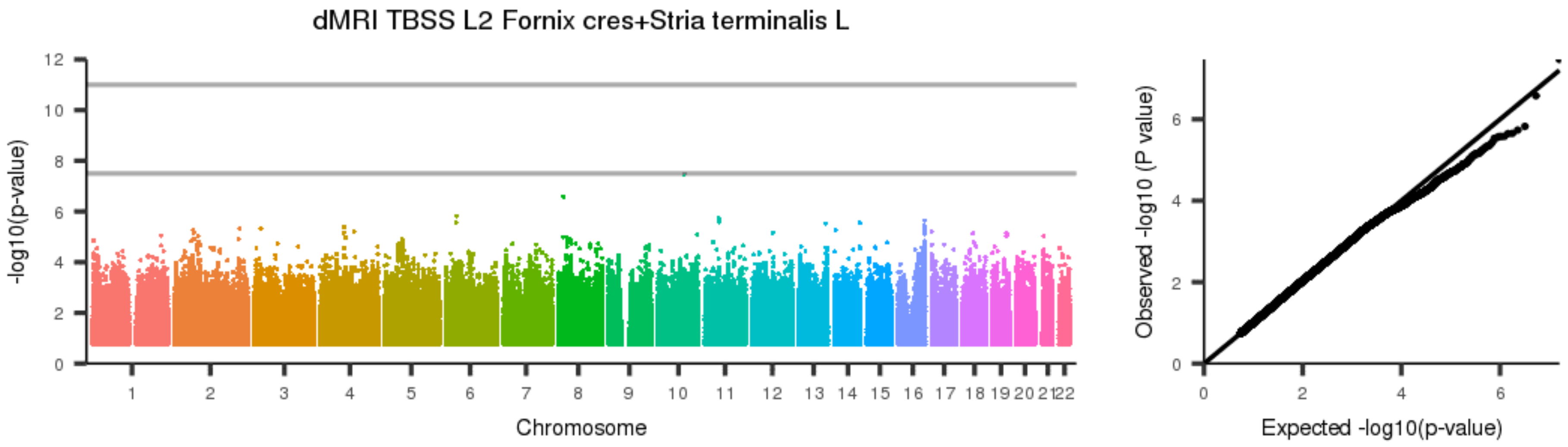

back to list of plots

Figure 428: dMRI TBSS L2 Superior longitudinal fasciculus R

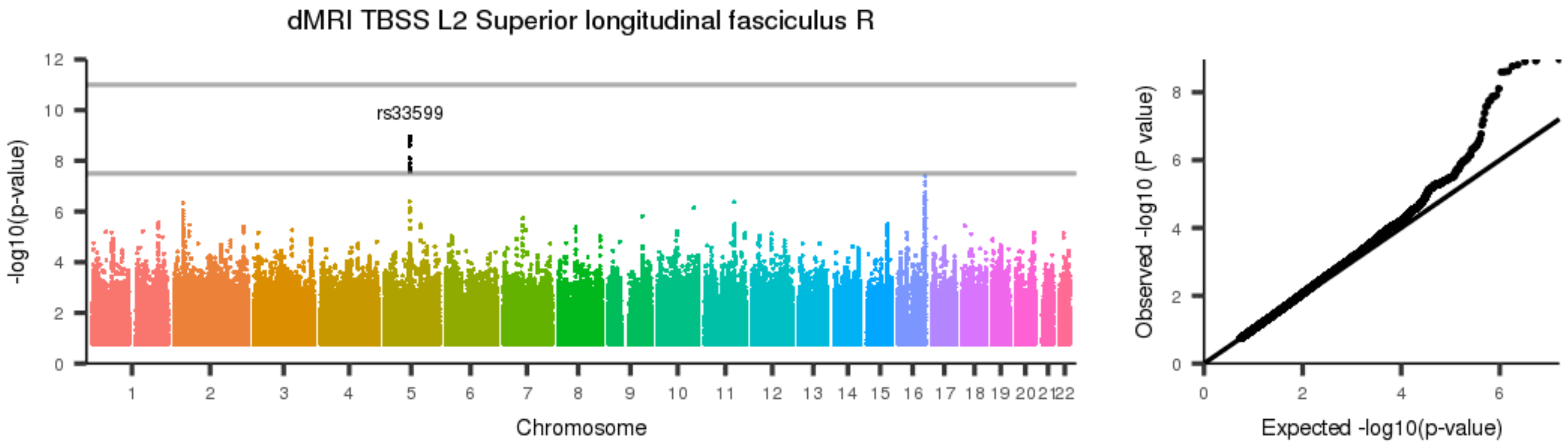

back to list of plots

Figure 429: dMRI TBSS L2 Superior longitudinal fasciculus L

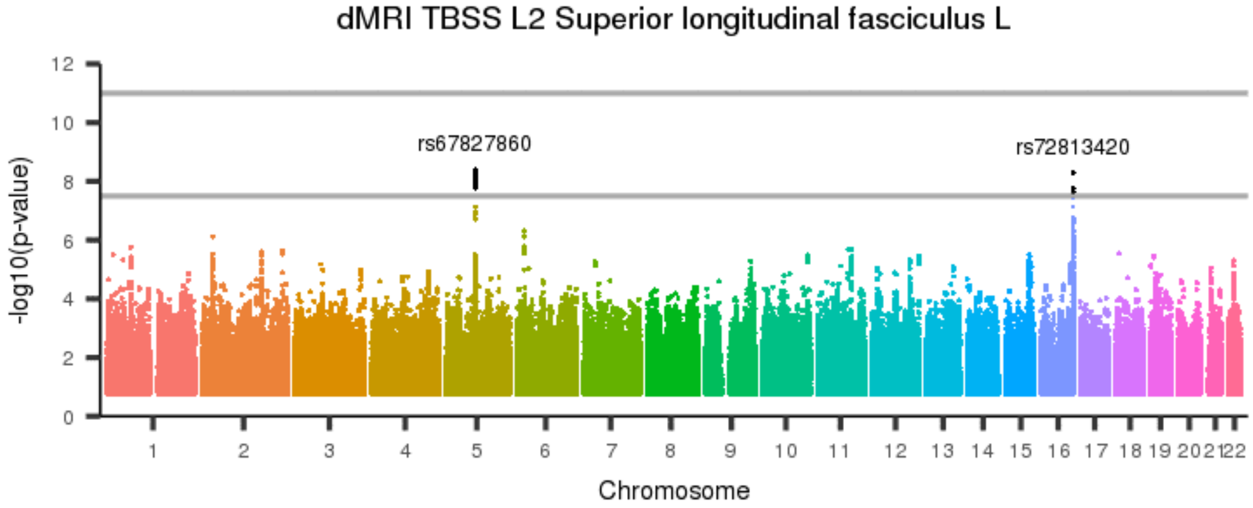

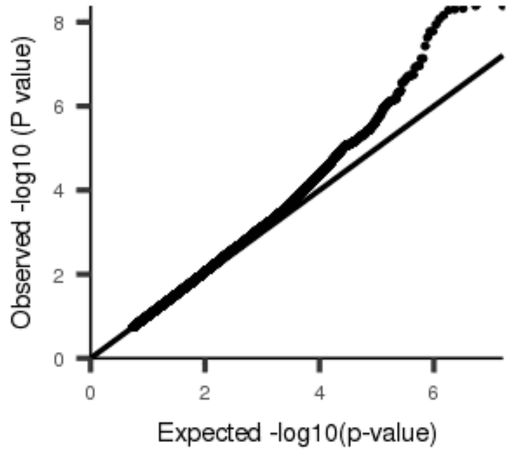

back to list of plots

Figure 430: dMRI TBSS L2 Superior fronto-occipital fasciculus R

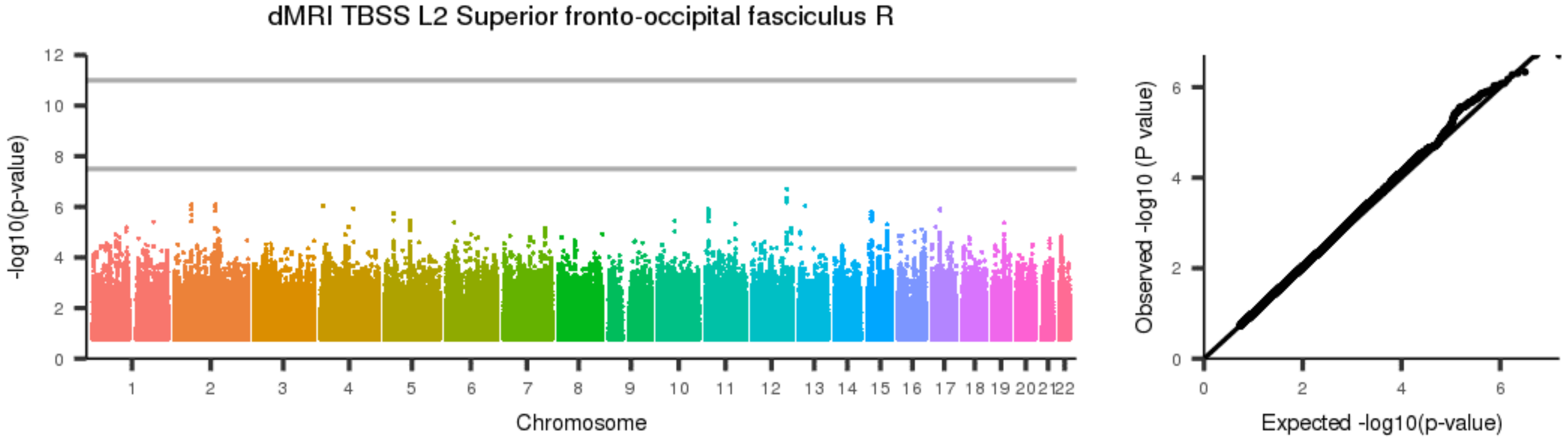

back to list of plots

Figure 431: dMRI TBSS L2 Superior fronto-occipital fasciculus L

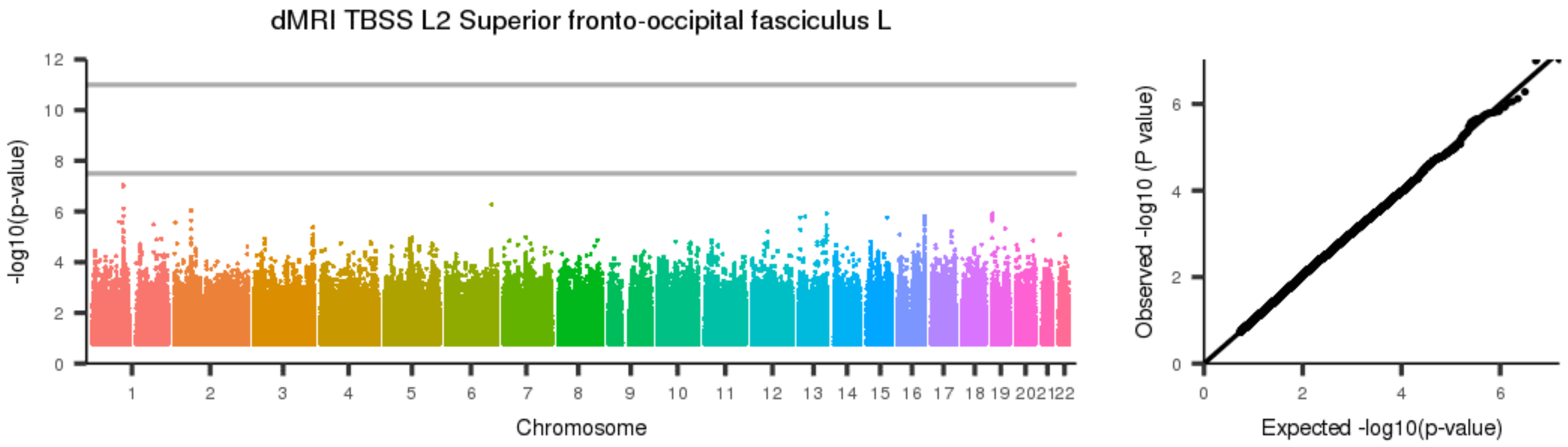

back to list of plots

Figure 432: dMRI TBSS L2 Uncinate fasciculus R

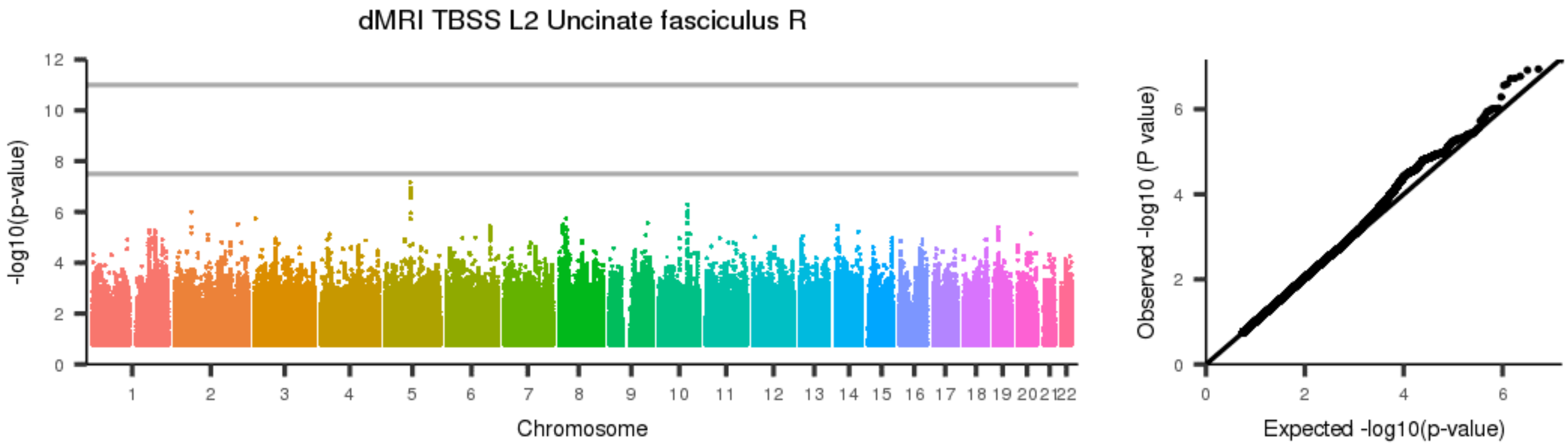

back to list of plots

Figure 433: dMRI TBSS L2 Uncinate fasciculus L

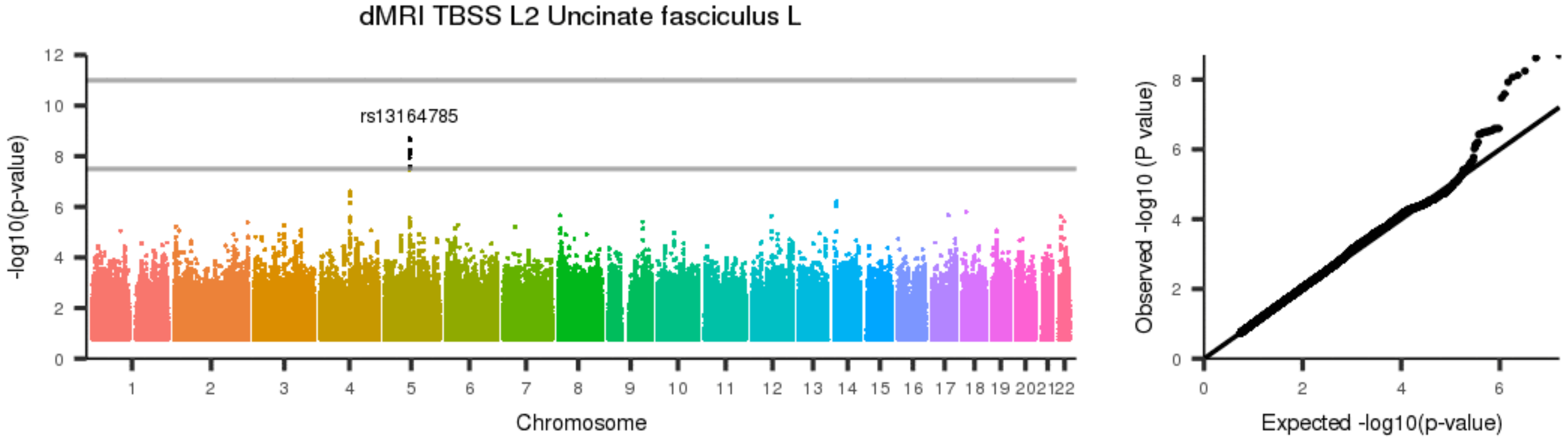

back to list of plots

Figure 434: dMRI TBSS L2 Tapetum R

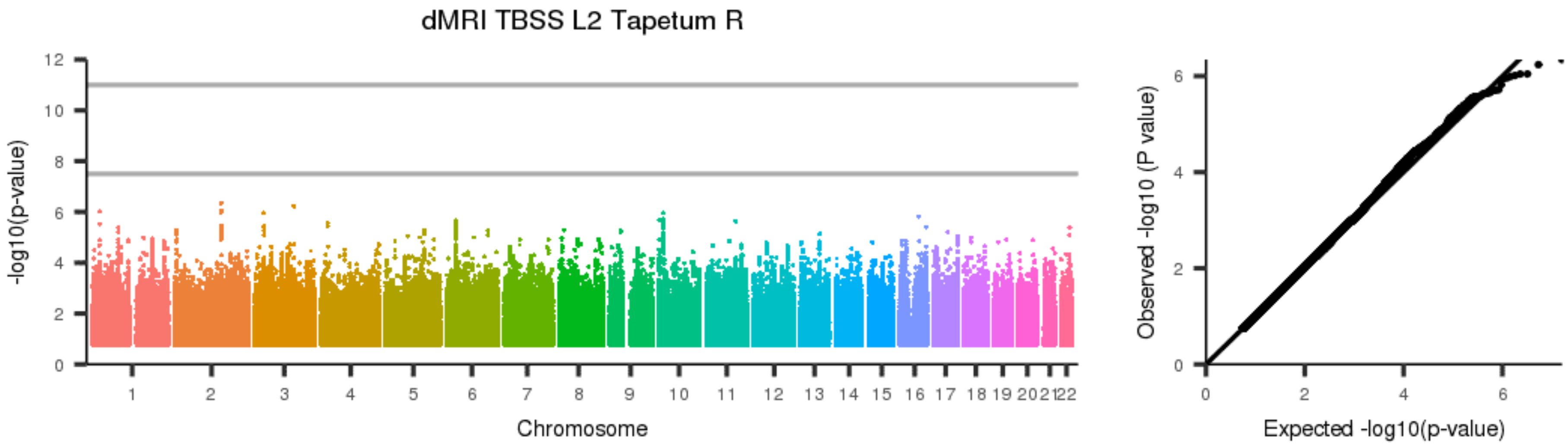

back to list of plots

Figure 435: dMRI TBSS L2 Tapetum L

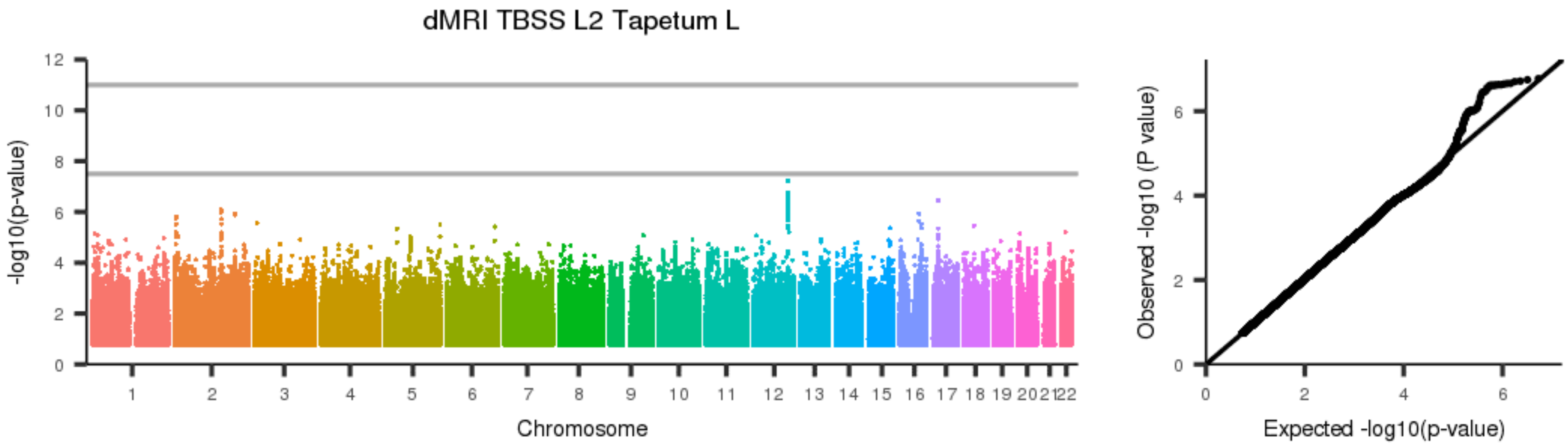

back to list of plots

Figure 436: dMRI TBSS L3 Middle cerebellar peduncle

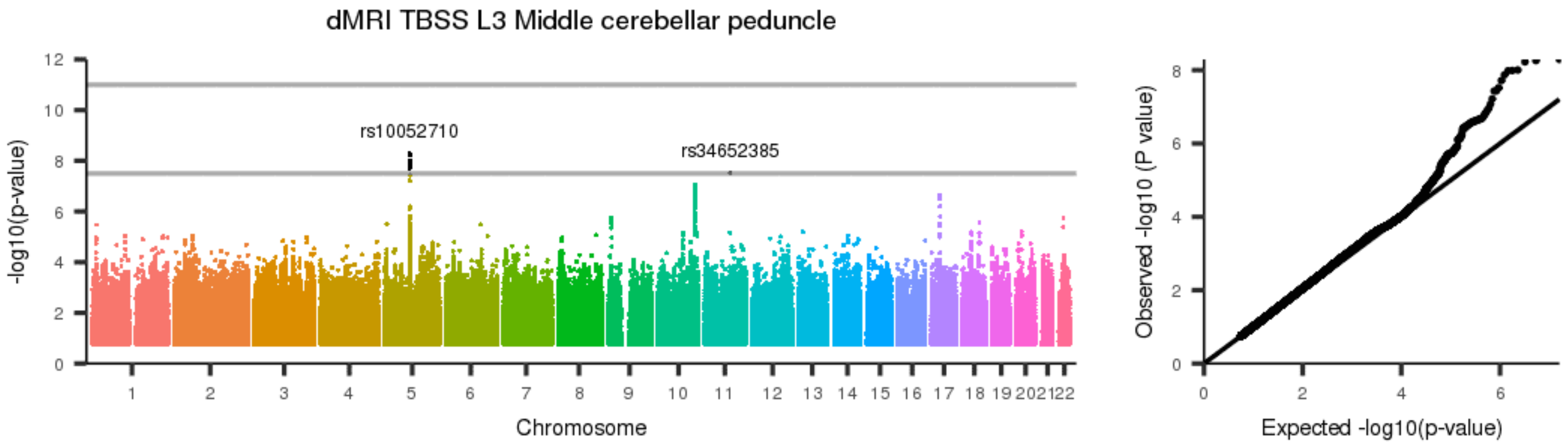

back to list of plots

Figure 437: dMRI TBSS L3 Pontine crossing tract

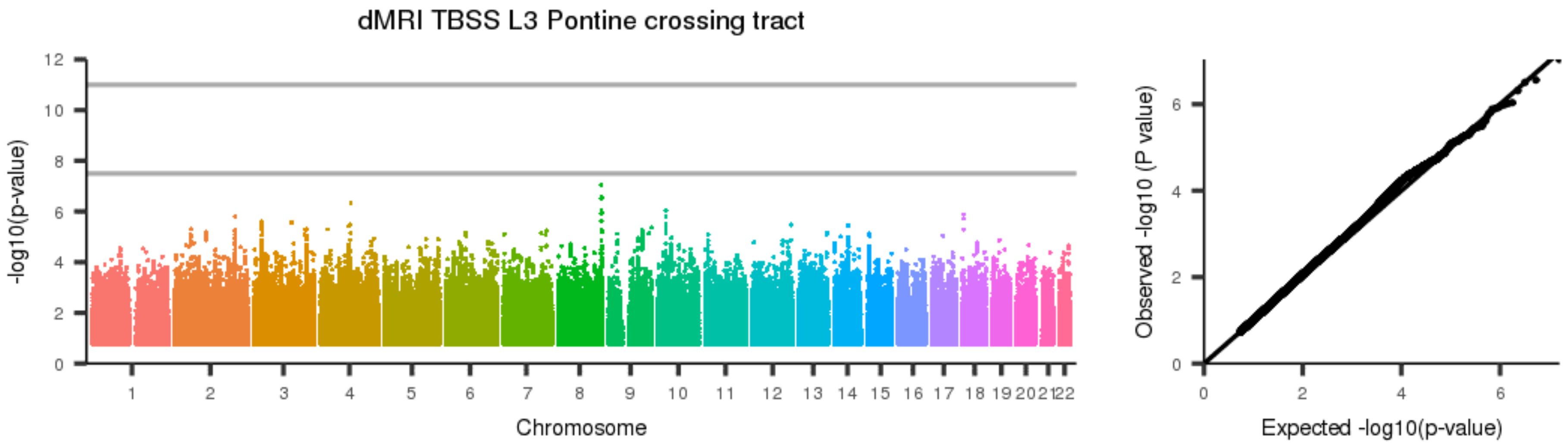

back to list of plots

Figure 438: dMRI TBSS L3 Genu of corpus callosum

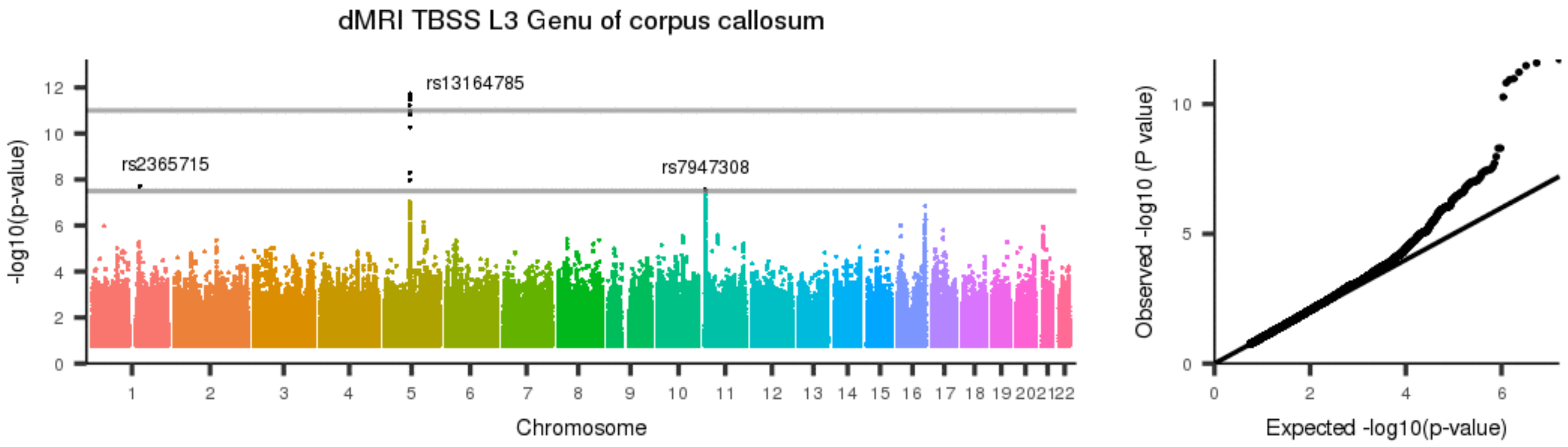

back to list of plots

Figure 439: dMRI TBSS L3 Body of corpus callosum

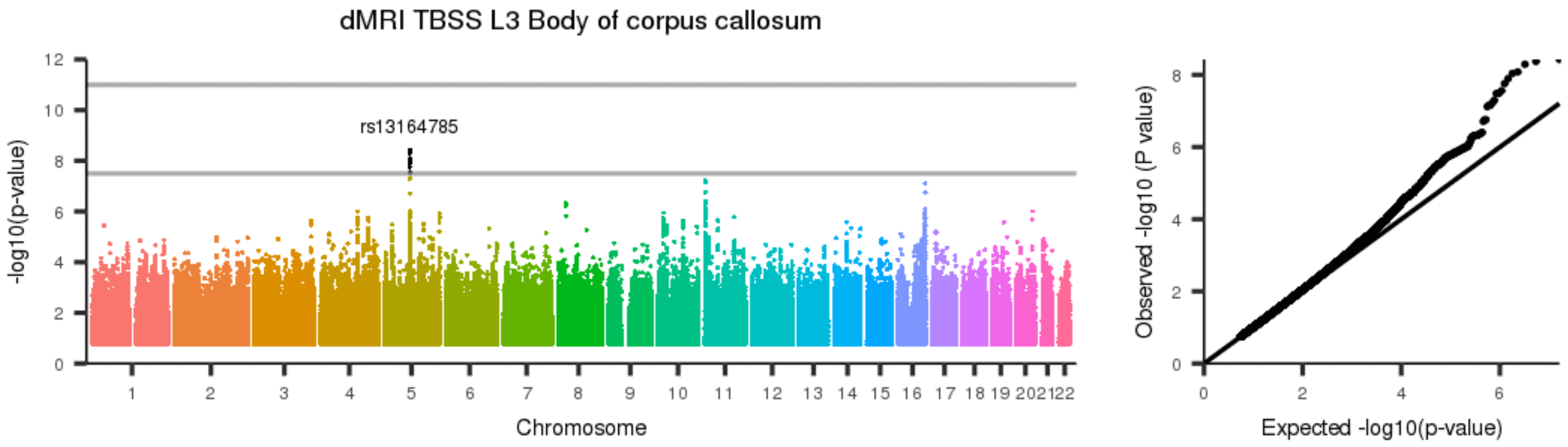

back to list of plots

Figure 440: dMRI TBSS L3 Splenium of corpus callosum

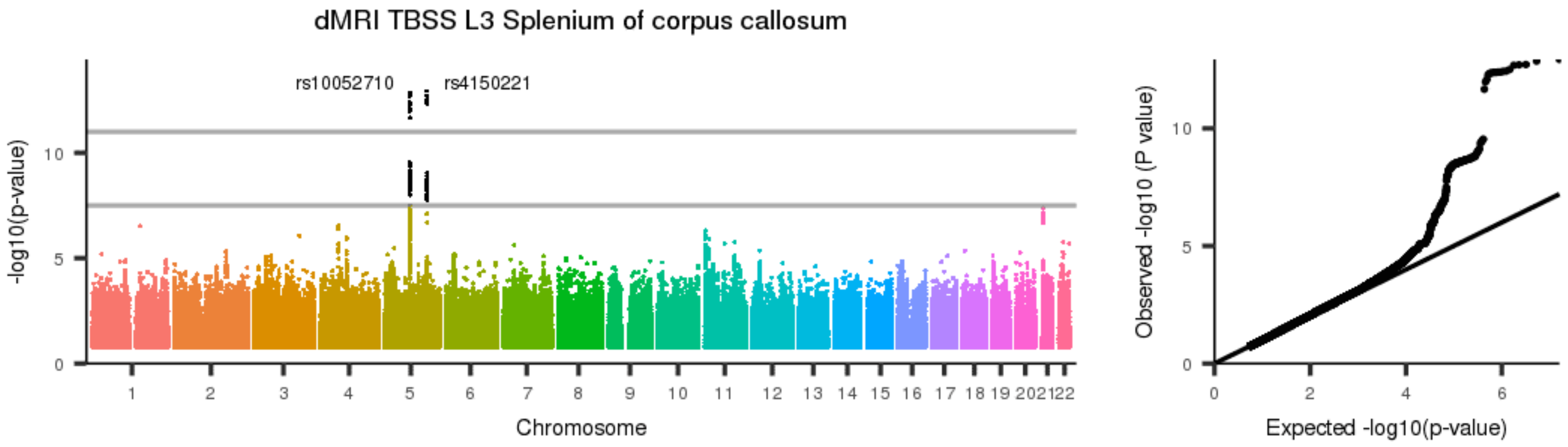

back to list of plots

Figure 441: dMRI TBSS L3 Fornix

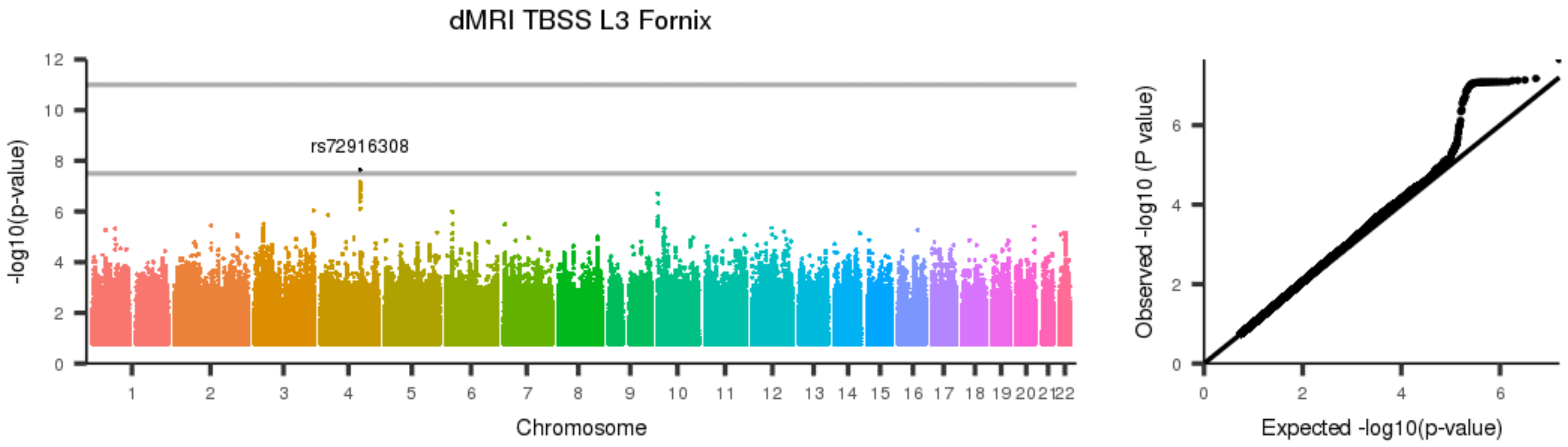

back to list of plots

Figure 442: dMRI TBSS L3 Corticospinal tract R

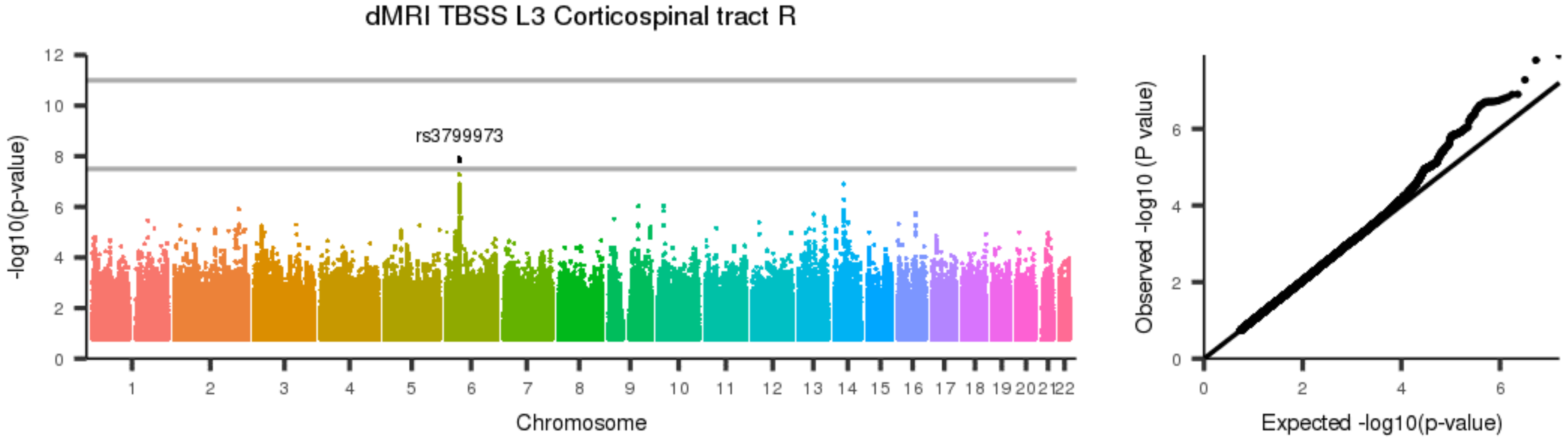

back to list of plots

Figure 443: dMRI TBSS L3 Corticospinal tract L

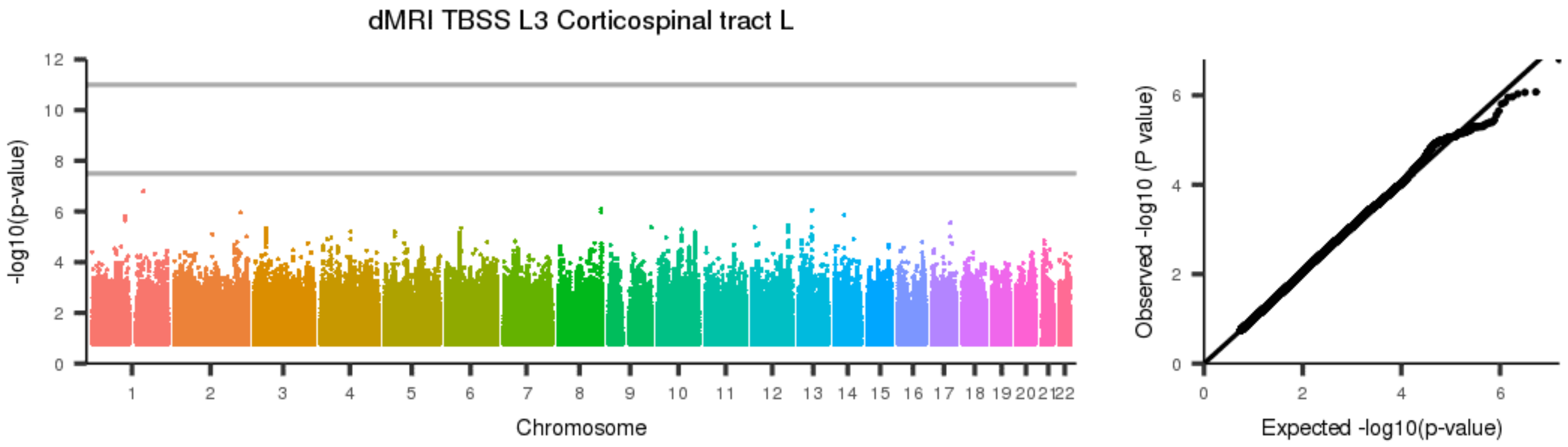

back to list of plots

Figure 444: dMRI TBSS L3 Medial lemniscus R

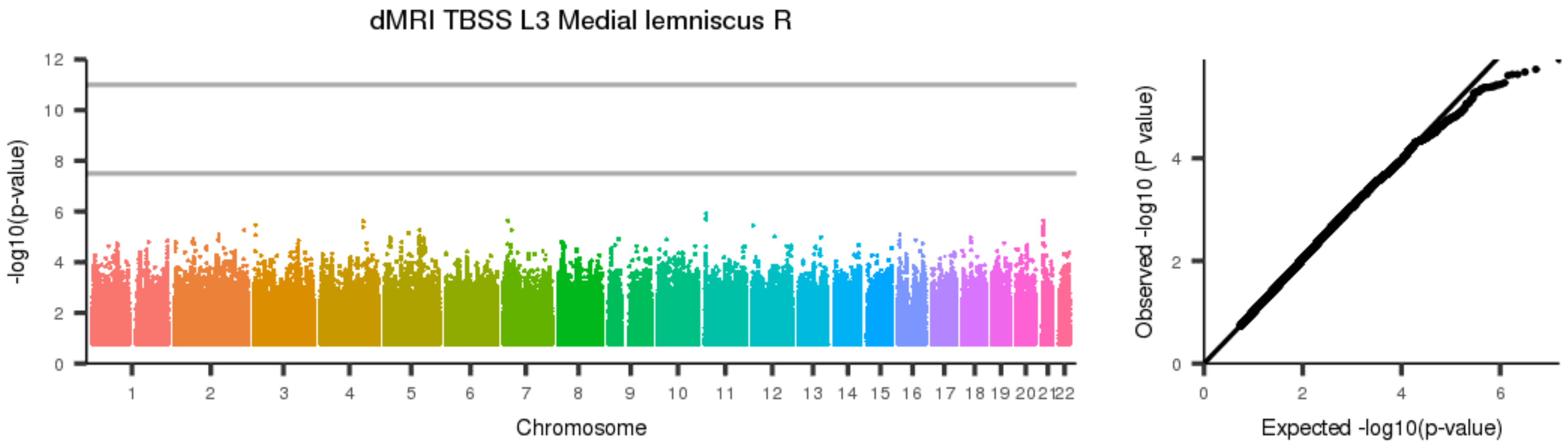

back to list of plots

Figure 445: dMRI TBSS L3 Medial lemniscus L

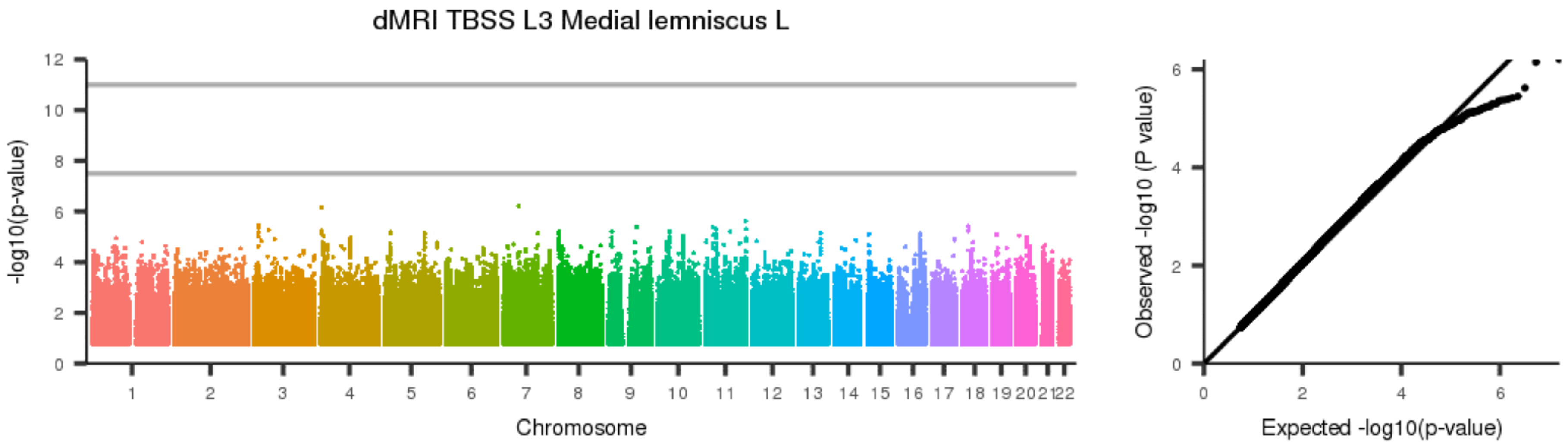

back to list of plots

Figure 446: dMRI TBSS L3 Inferior cerebellar peduncle R

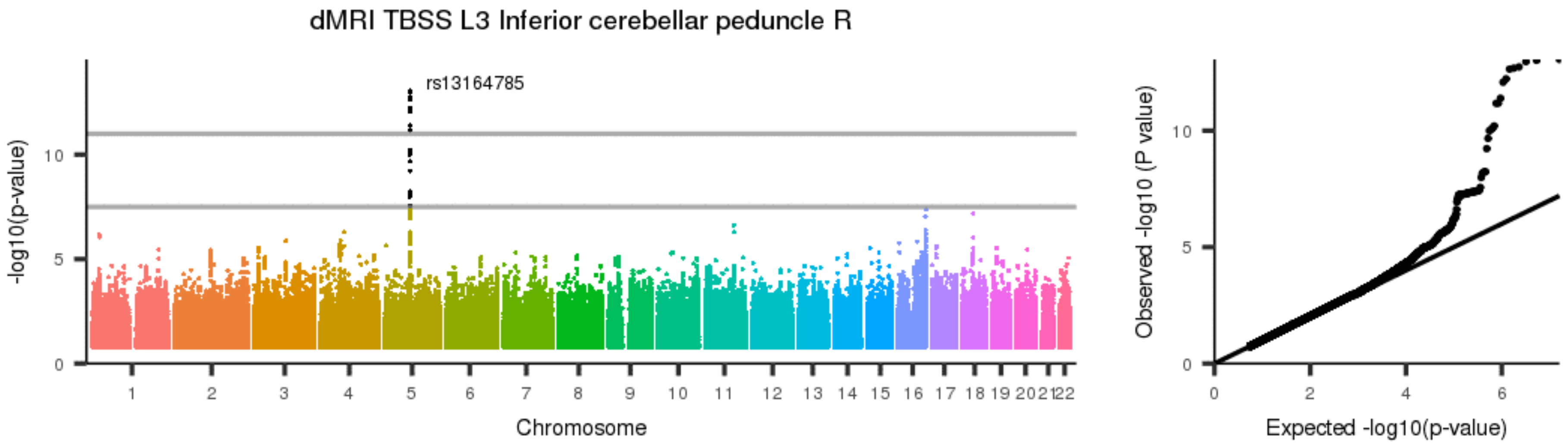

back to list of plots

Figure 447: dMRI TBSS L3 Inferior cerebellar peduncle L

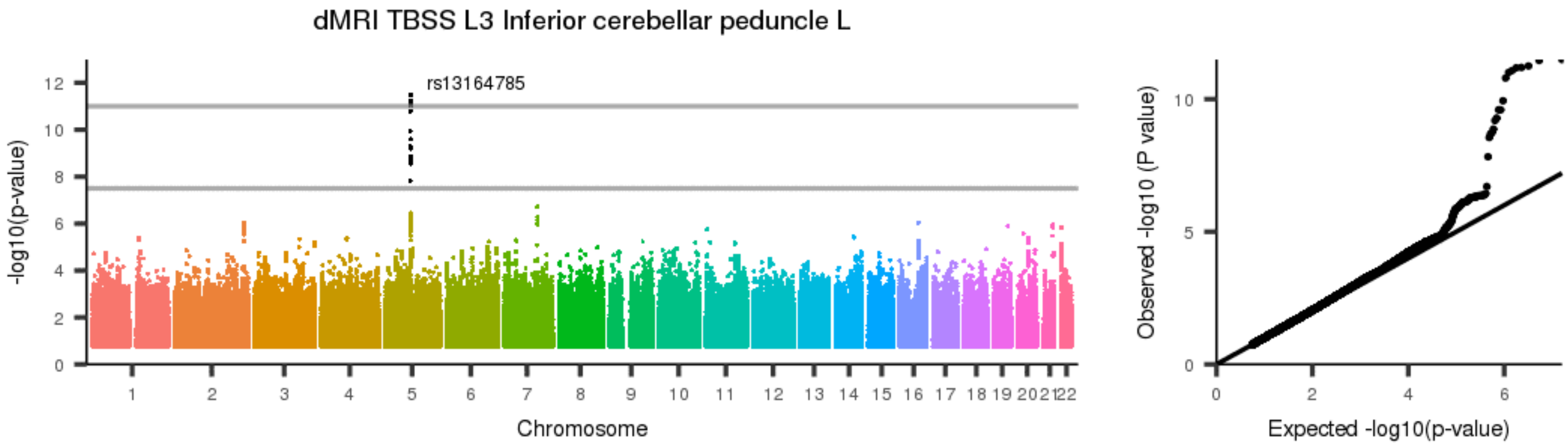

back to list of plots

Figure 448: dMRI TBSS L3 Superior cerebellar peduncle R

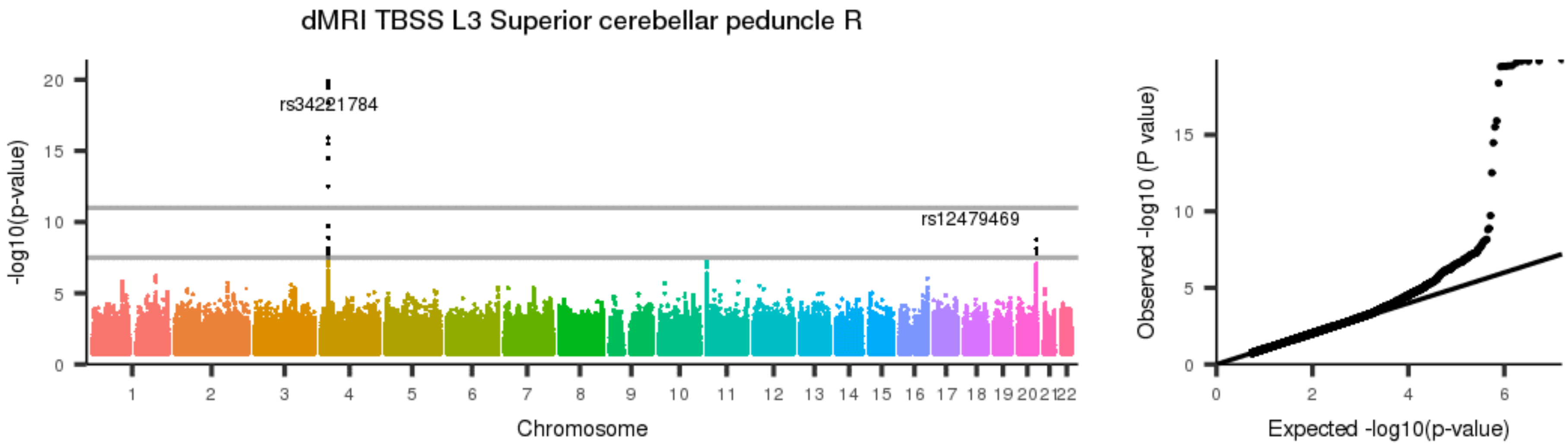

back to list of plots

Figure 449: dMRI TBSS L3 Superior cerebellar peduncle L

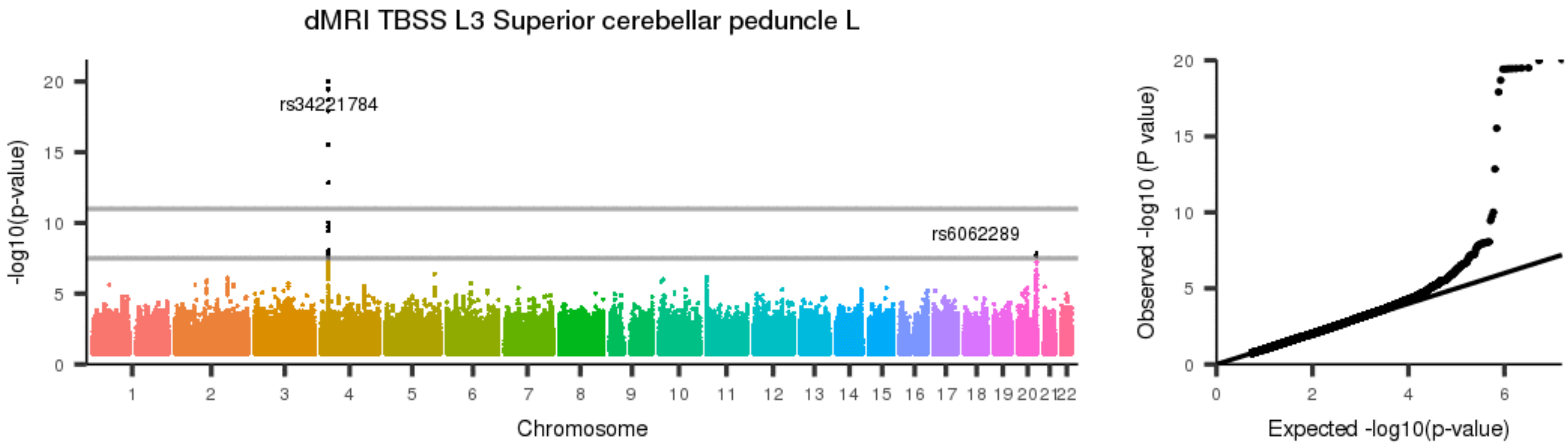

back to list of plots

Figure 450: dMRI TBSS L3 Cerebral peduncle R

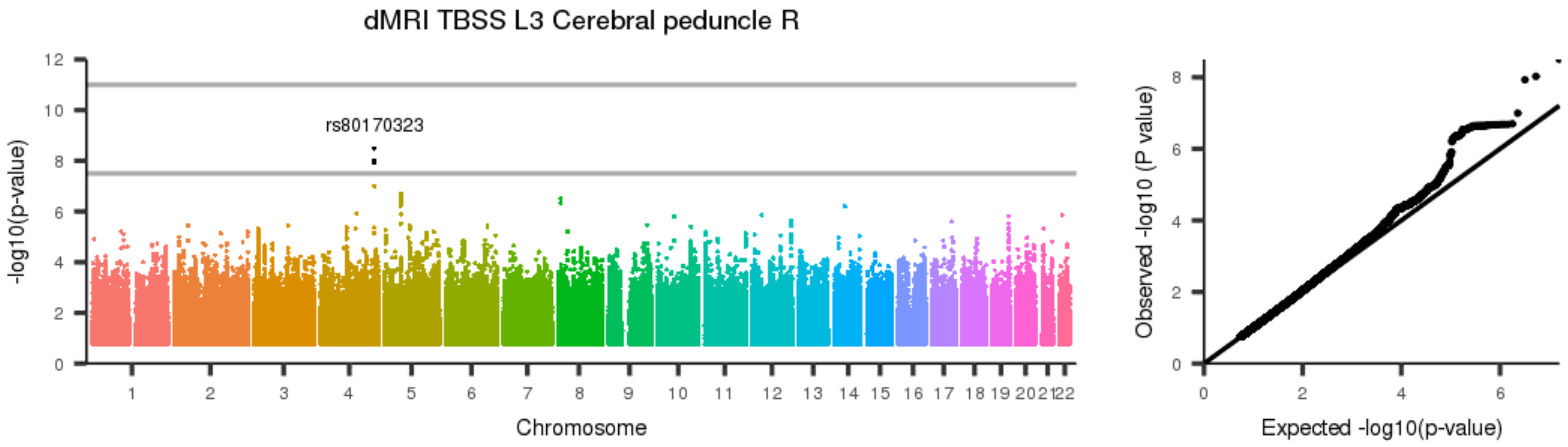

back to list of plots

Figure 451: dMRI TBSS L3 Cerebral peduncle L

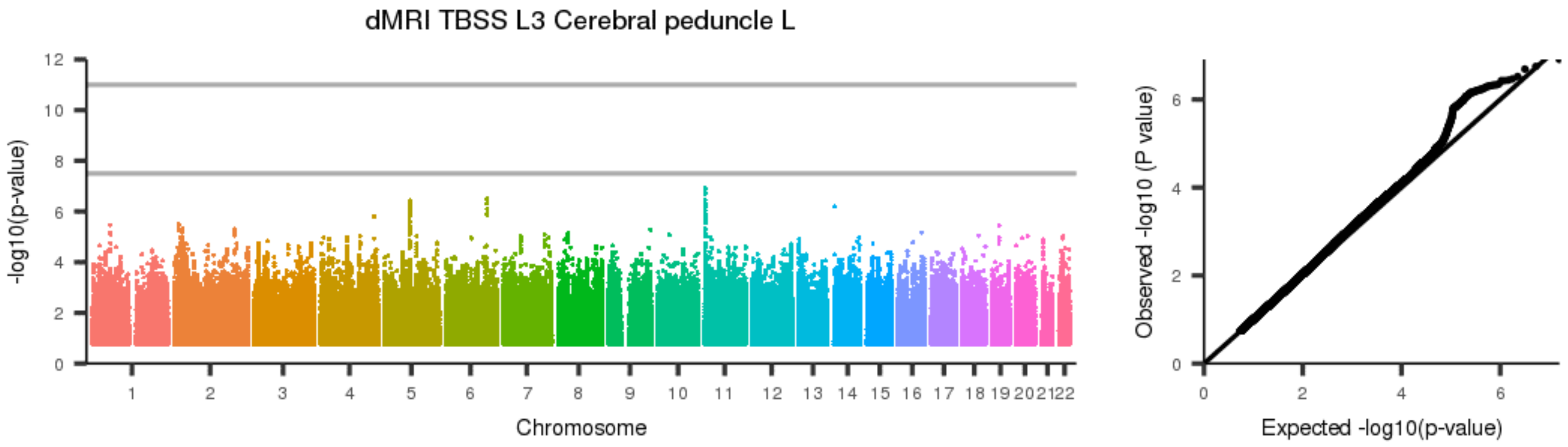

back to list of plots

Figure 452: dMRI TBSS L3 Anterior limb of internal capsule R

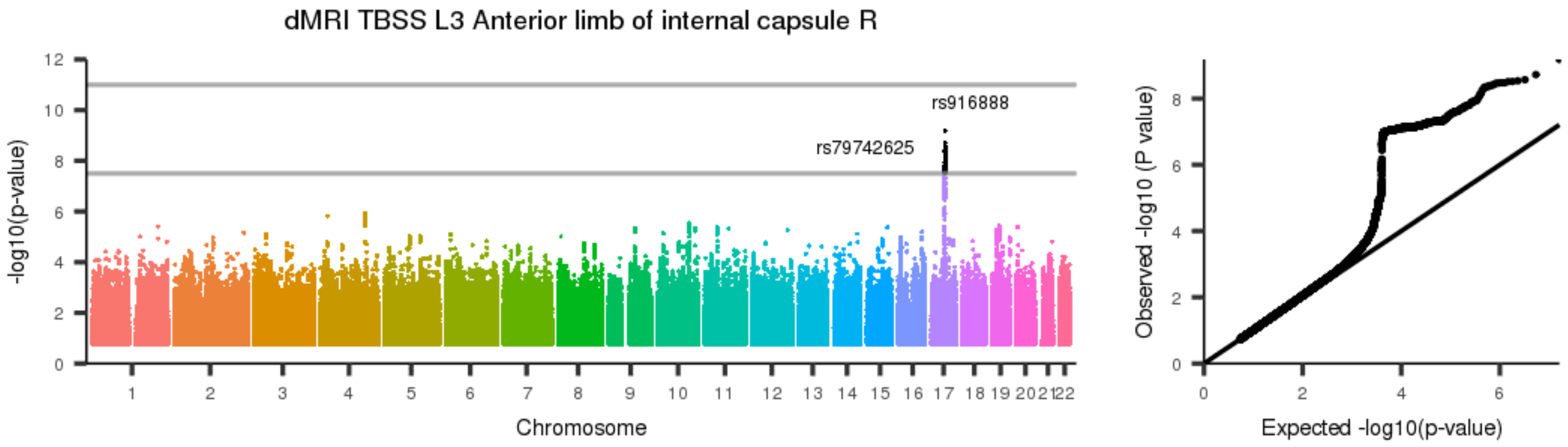

back to list of plots

Figure 453: dMRI TBSS L3 Anterior limb of internal capsule L

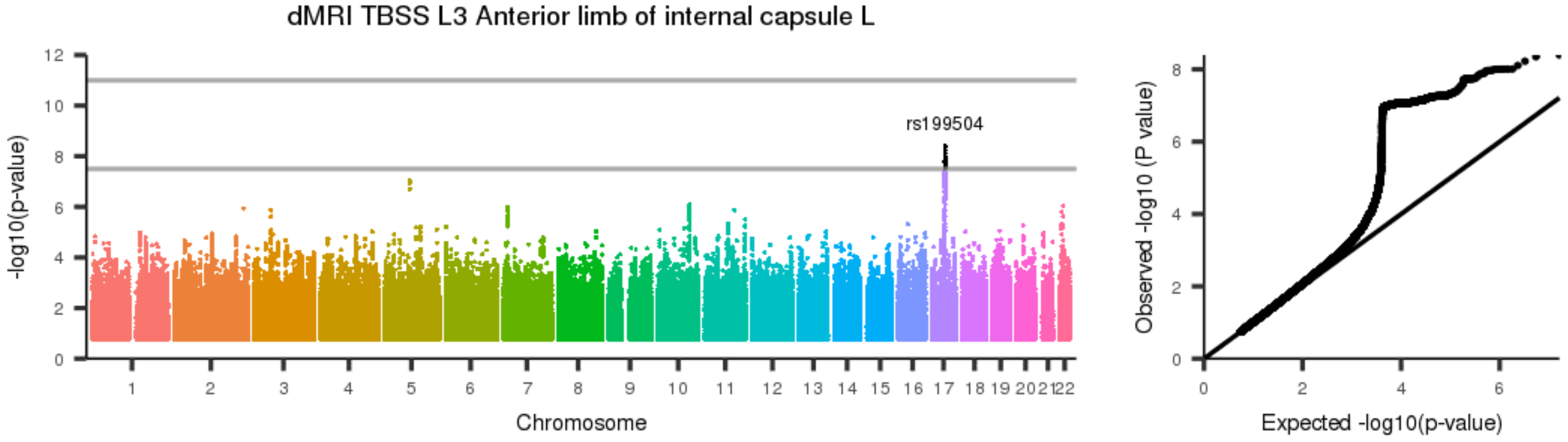

back to list of plots

Figure 454: dMRI TBSS L3 Posterior limb of internal capsule R

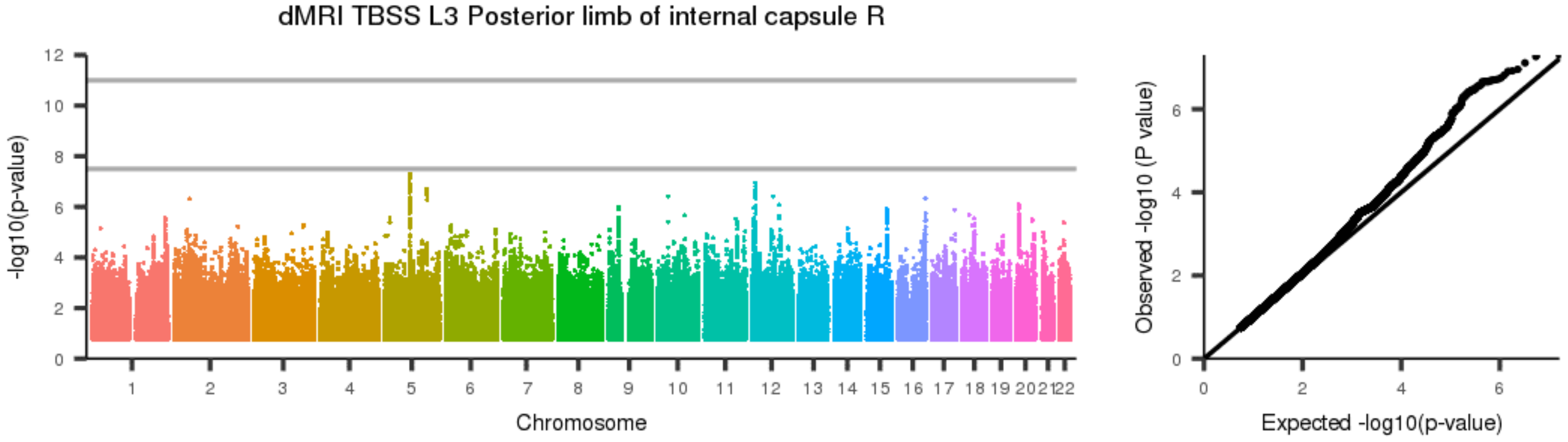

back to list of plots

Figure 455: dMRI TBSS L3 Posterior limb of internal capsule L

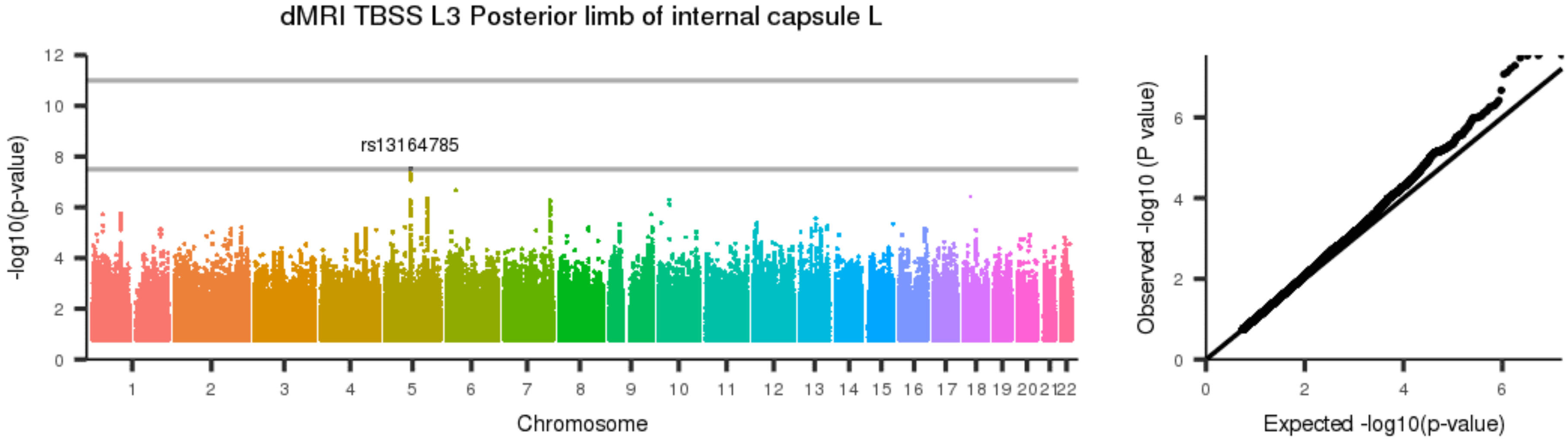

back to list of plots

Figure 456: dMRI TBSS L3 Retrolenticular part of internal capsule R

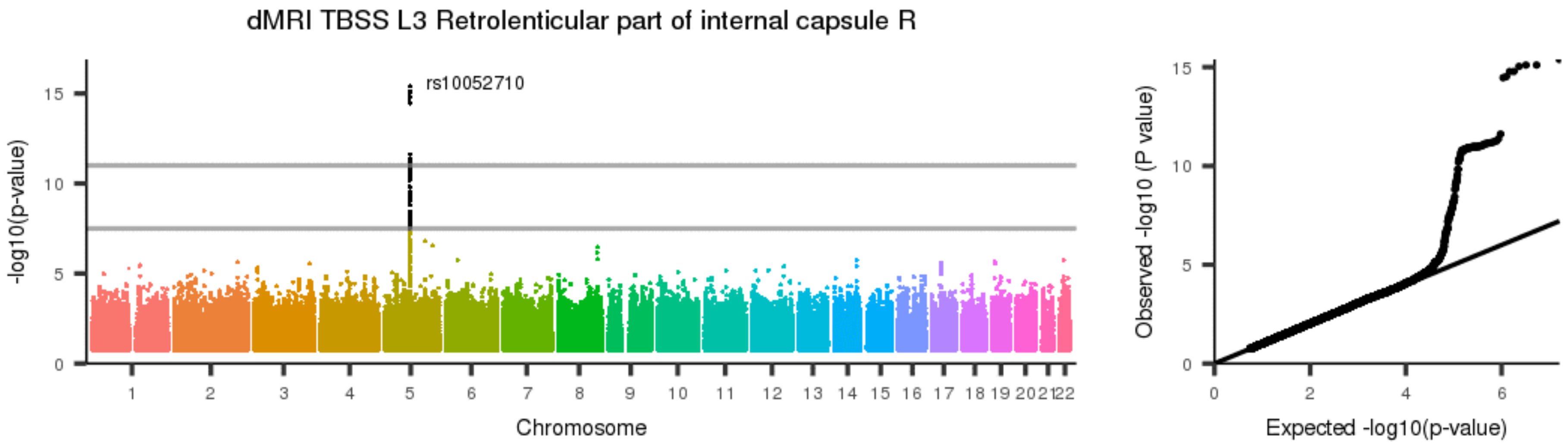

back to list of plots

Figure 457: dMRI TBSS L3 Retrolenticular part of internal capsule L

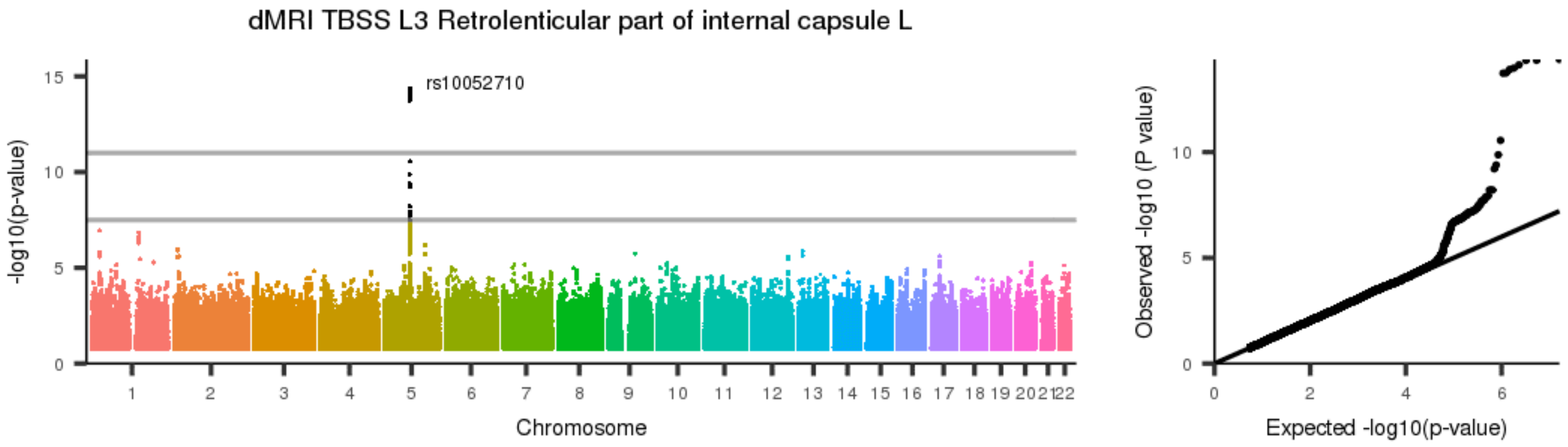

back to list of plots

Figure 458: dMRI TBSS L3 Anterior corona radiata R

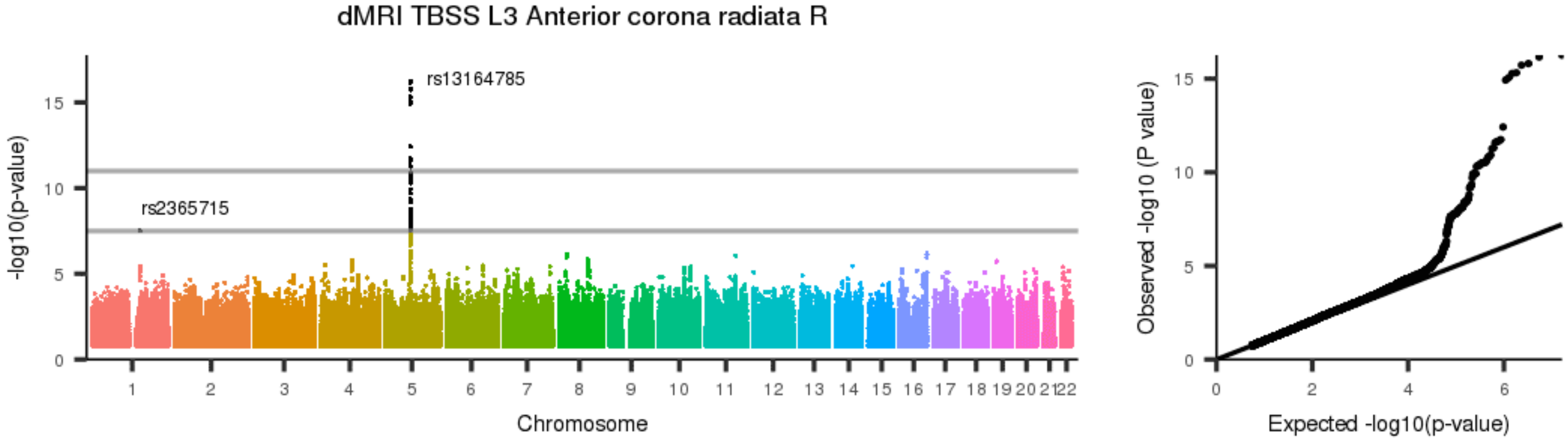

back to list of plots

Figure 459: dMRI TBSS L3 Anterior corona radiata L

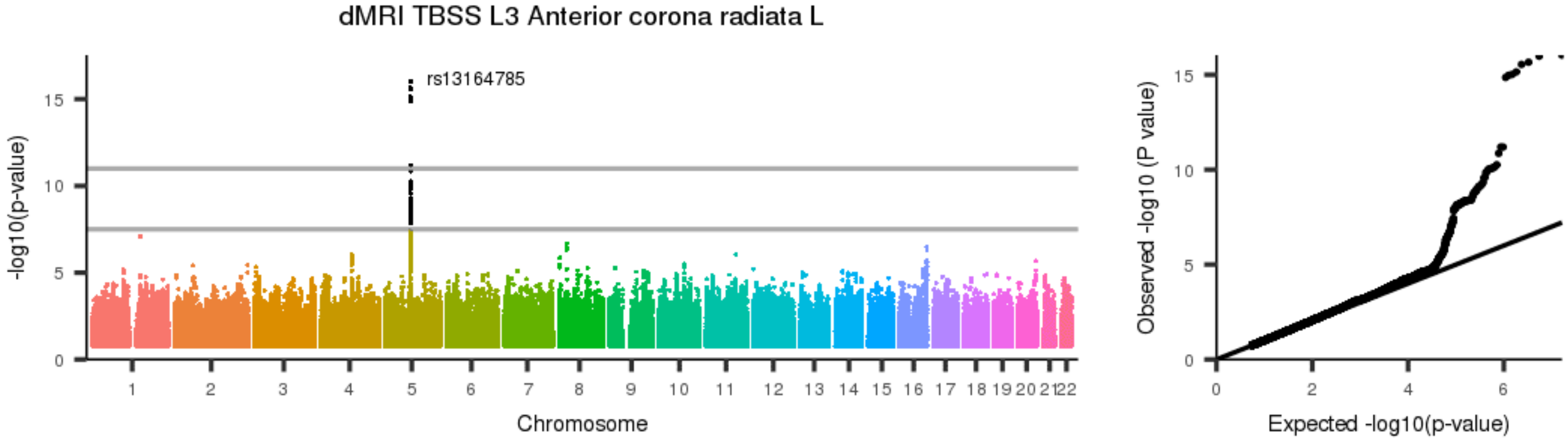

back to list of plots

Figure 460: dMRI TBSS L3 Superior corona radiata R

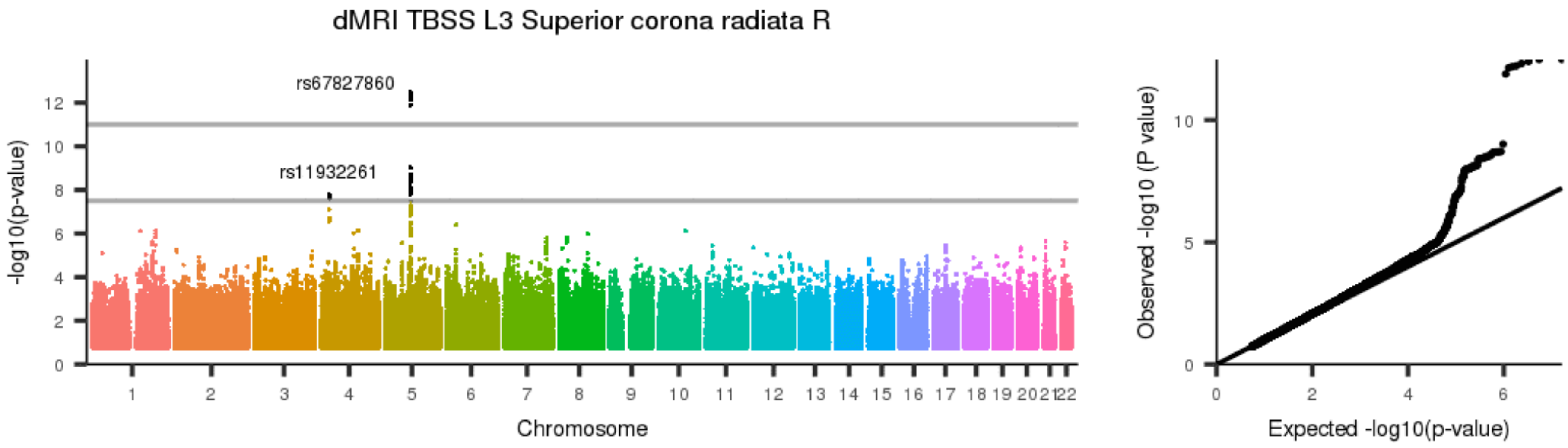

back to list of plots

Figure 461: dMRI TBSS L3 Superior corona radiata L

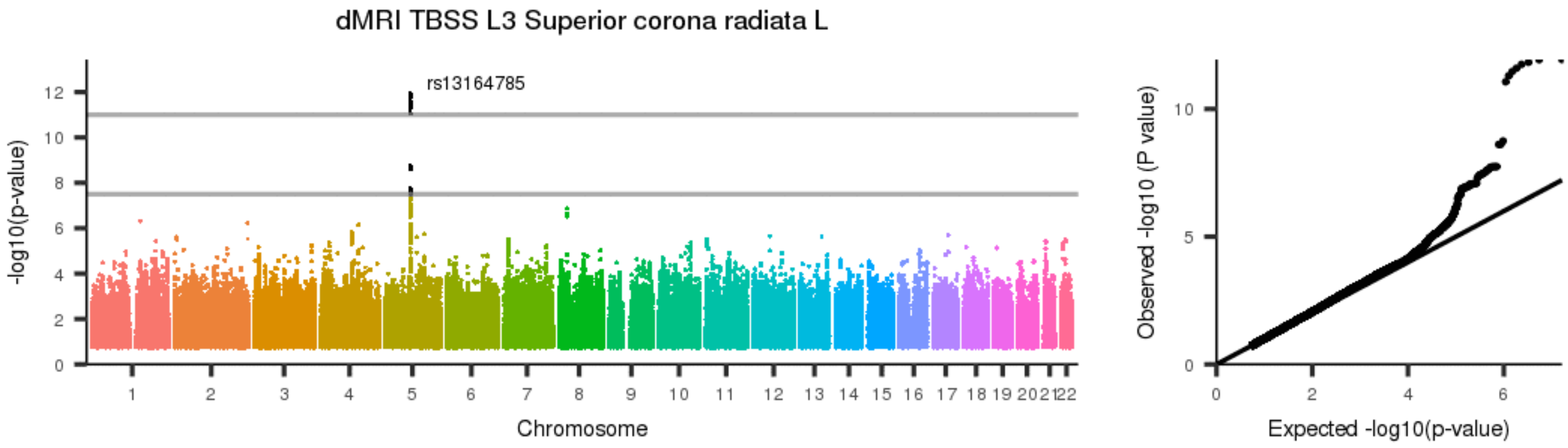

back to list of plots

Figure 462: dMRI TBSS L3 Posterior corona radiata R

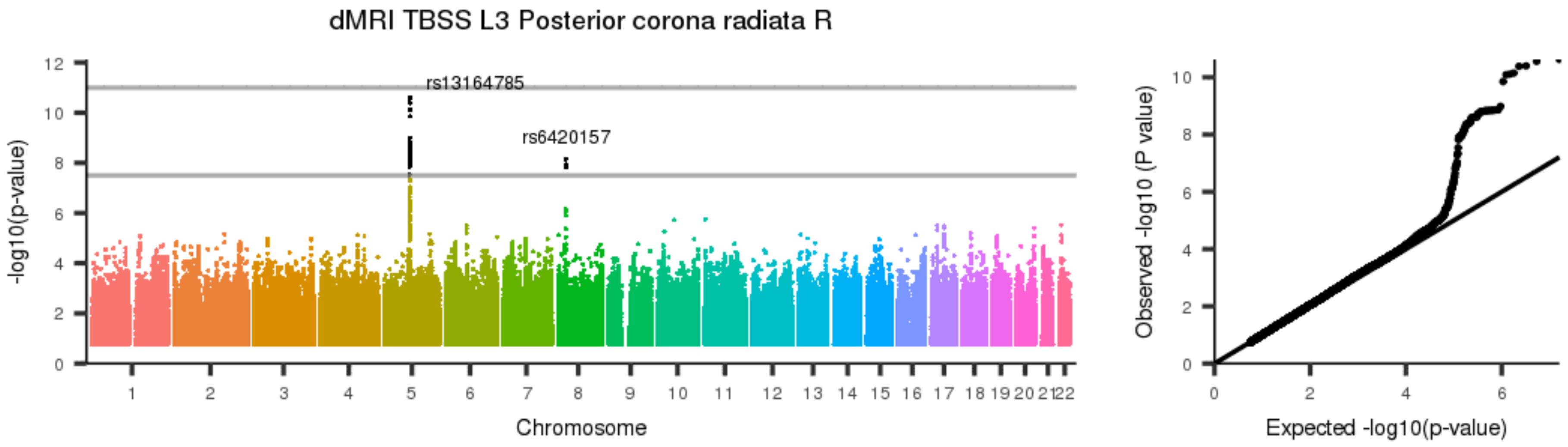

back to list of plots

Figure 463: dMRI TBSS L3 Posterior corona radiata L

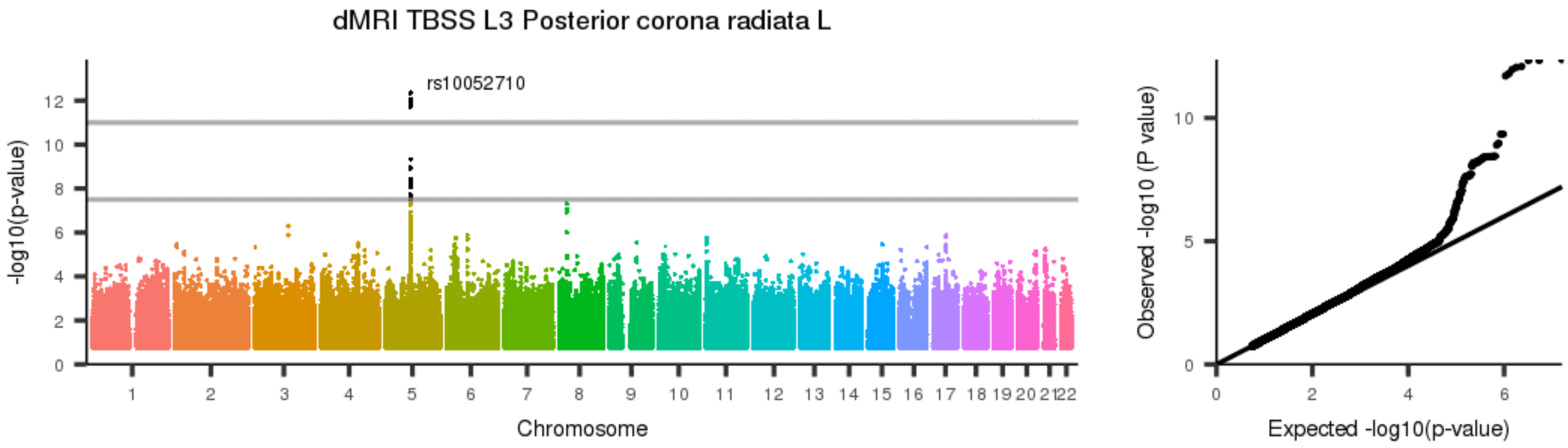

back to list of plots

Figure 464: dMRI TBSS L3 Posterior thalamic radiation R

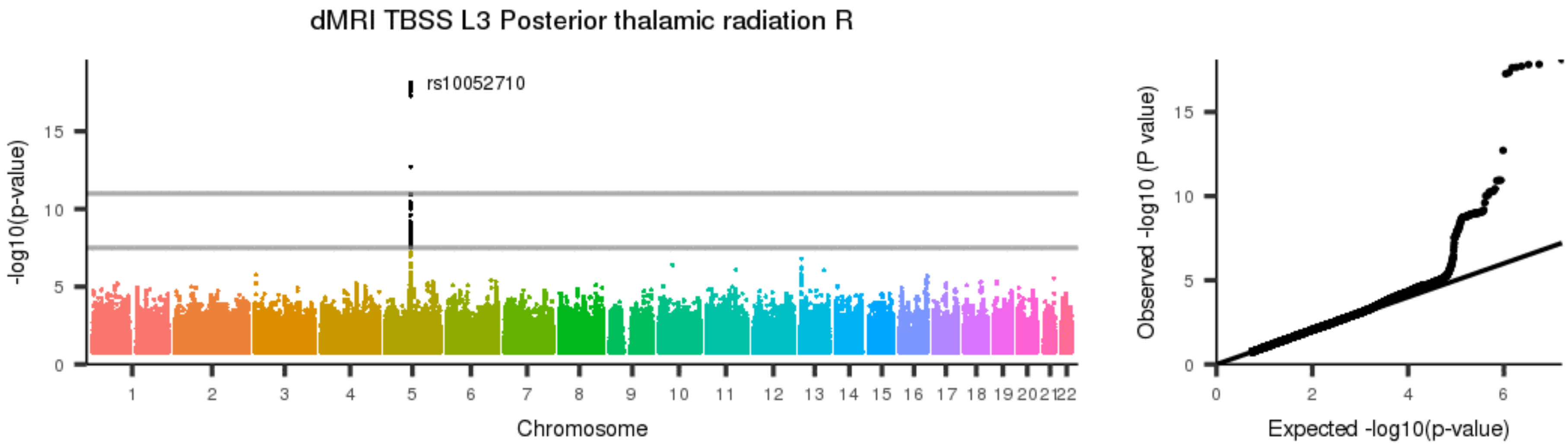

back to list of plots

Figure 465: dMRI TBSS L3 Posterior thalamic radiation L

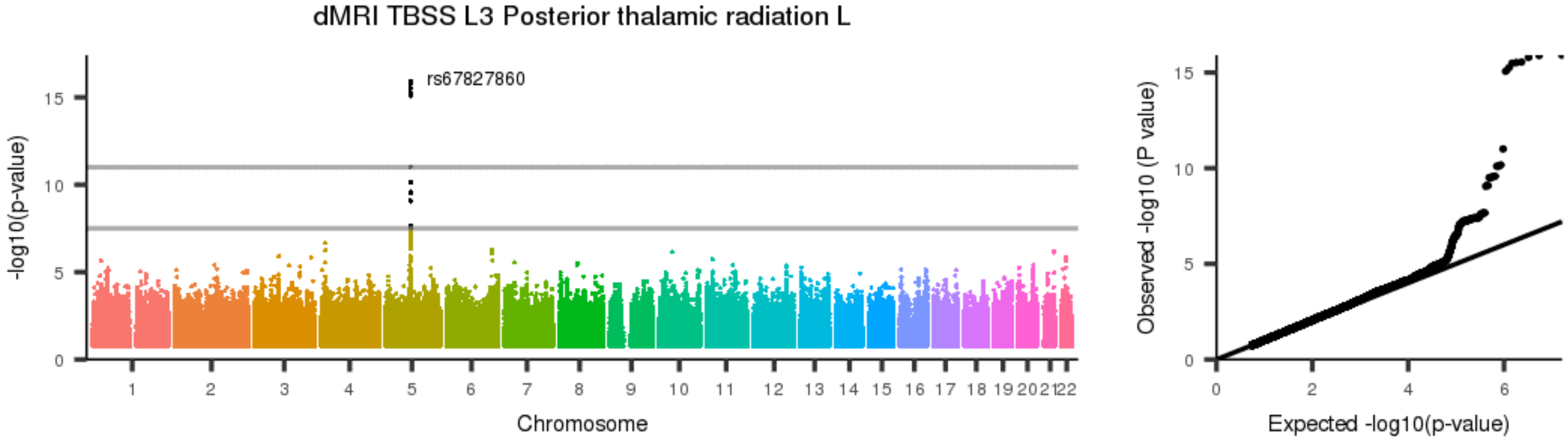

back to list of plots

Figure 466: dMRI TBSS L3 Sagittal stratum R

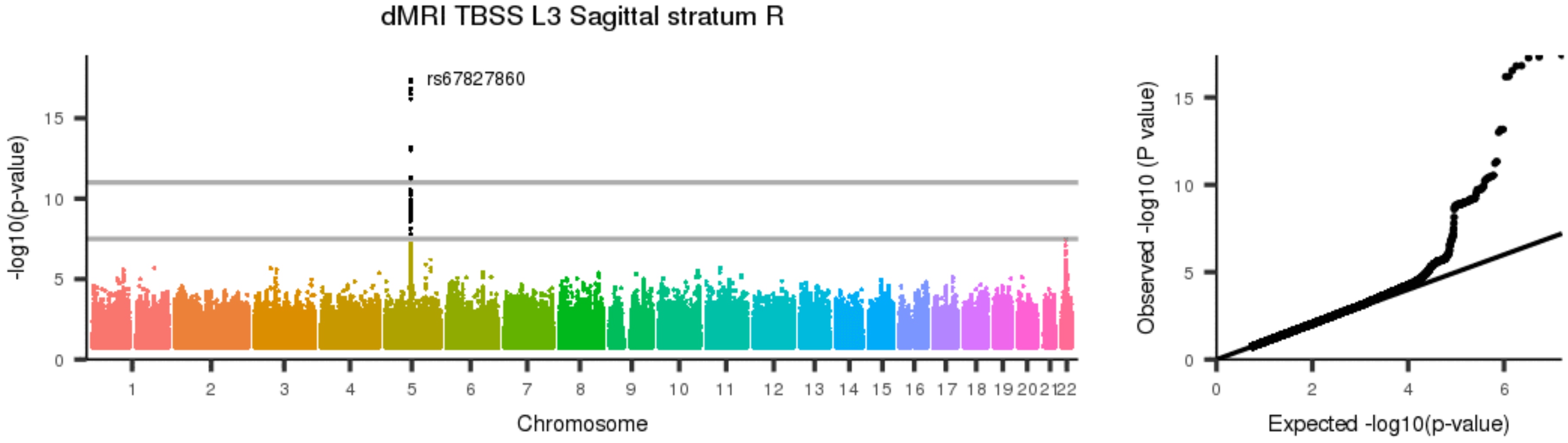

back to list of plots

Figure 467: dMRI TBSS L3 Sagittal stratum L

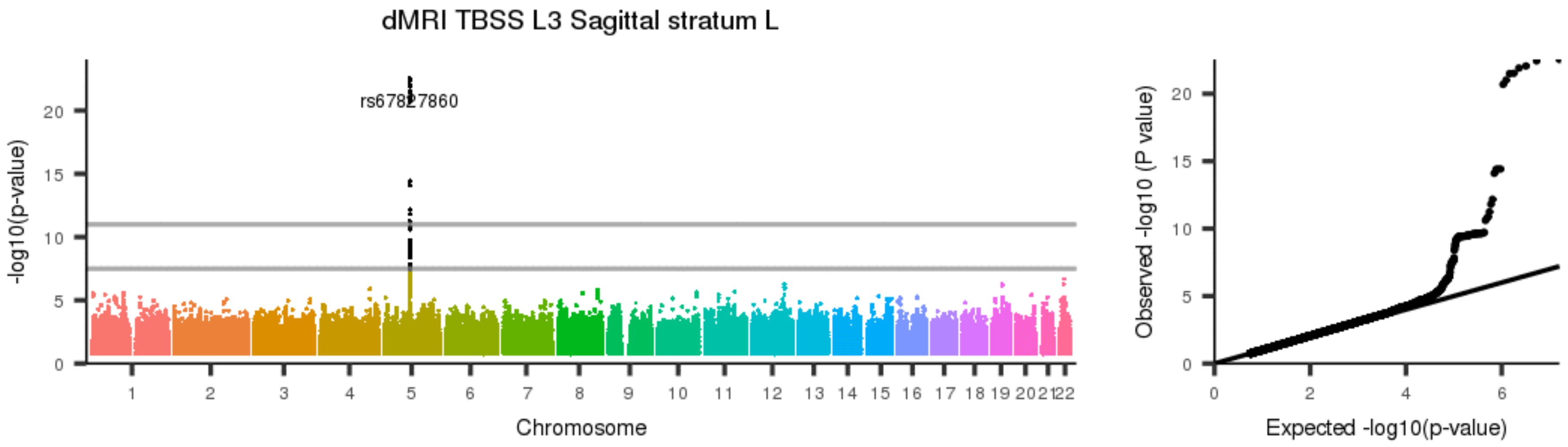

back to list of plots

Figure 468: dMRI TBSS L3 External capsule R

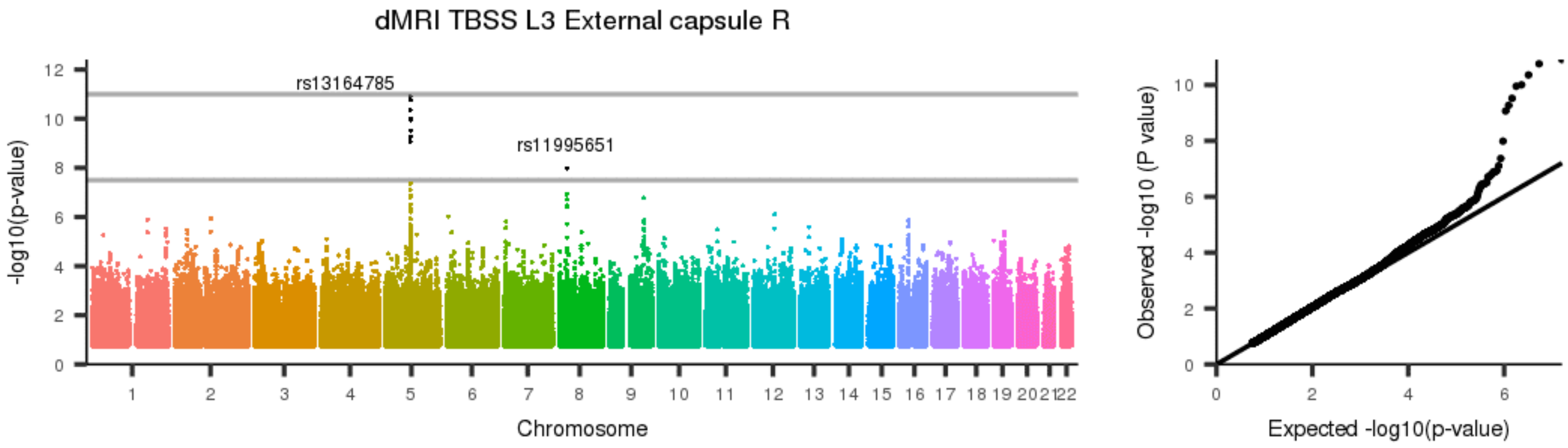

back to list of plots

Figure 469: dMRI TBSS L3 External capsule L

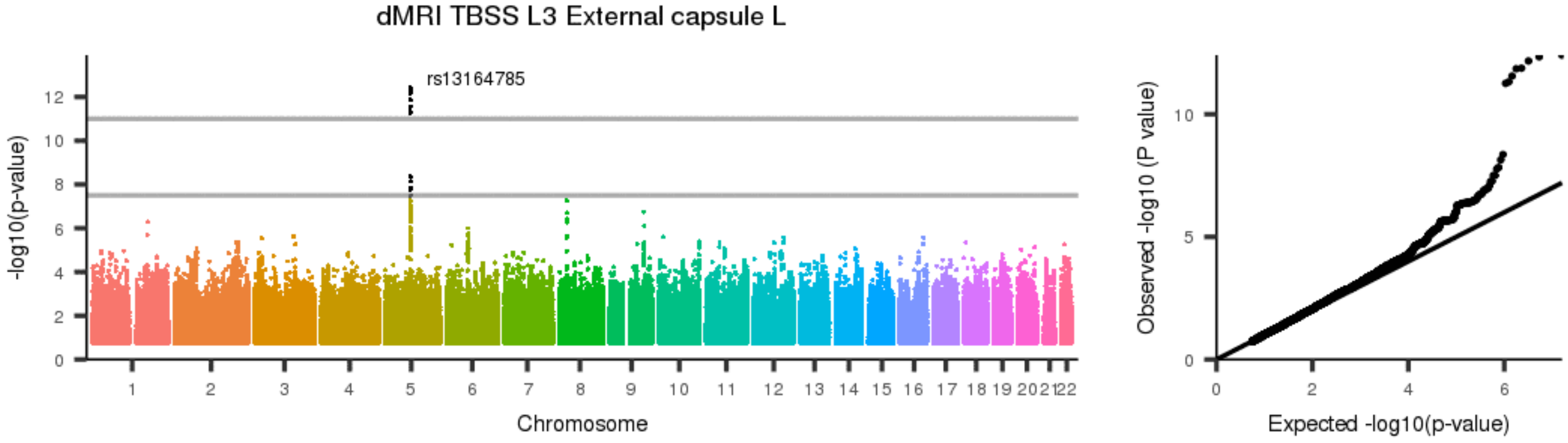

back to list of plots

Figure 470: dMRI TBSS L3 Cingulum cingulate gyrus R

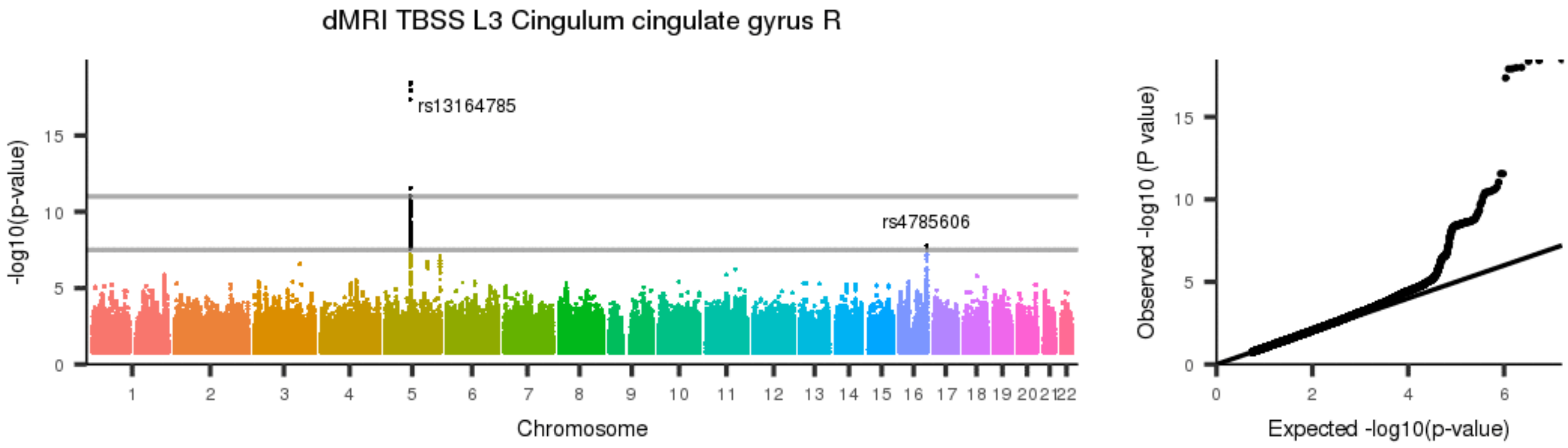

back to list of plots

Figure 471: dMRI TBSS L3 Cingulum cingulate gyrus L

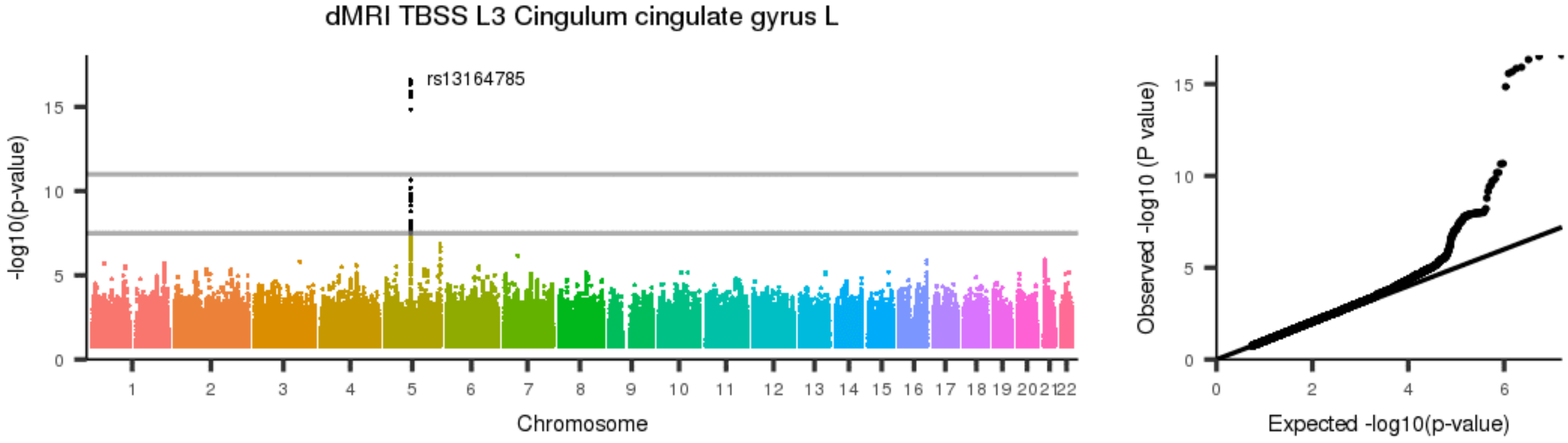

back to list of plots

Figure 472: dMRI TBSS L3 Cingulum hippocampus R

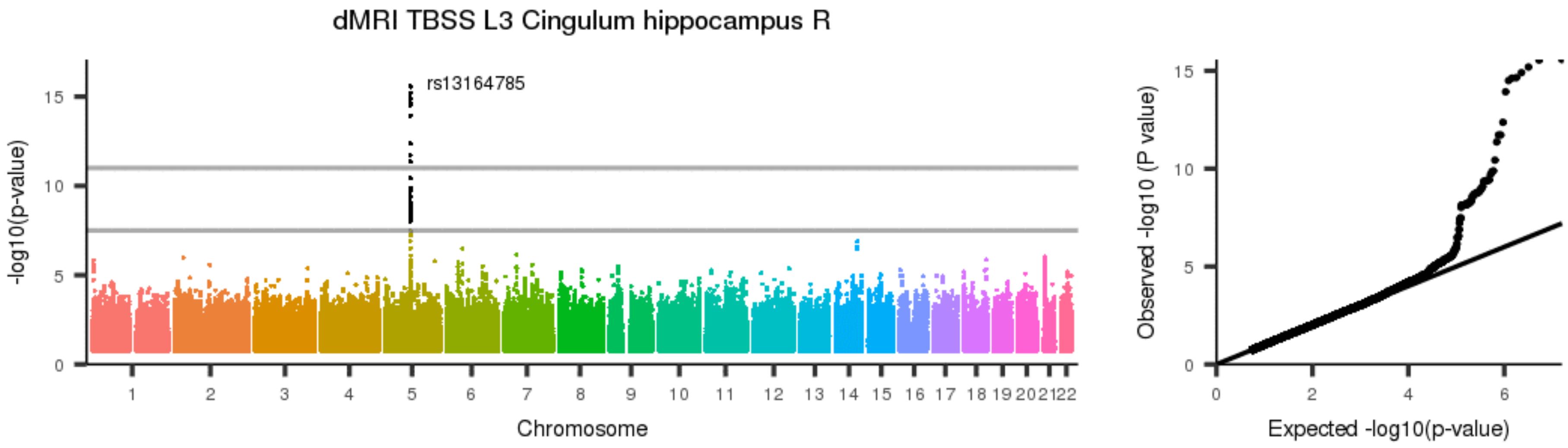

back to list of plots

Figure 473: dMRI TBSS L3 Cingulum hippocampus L

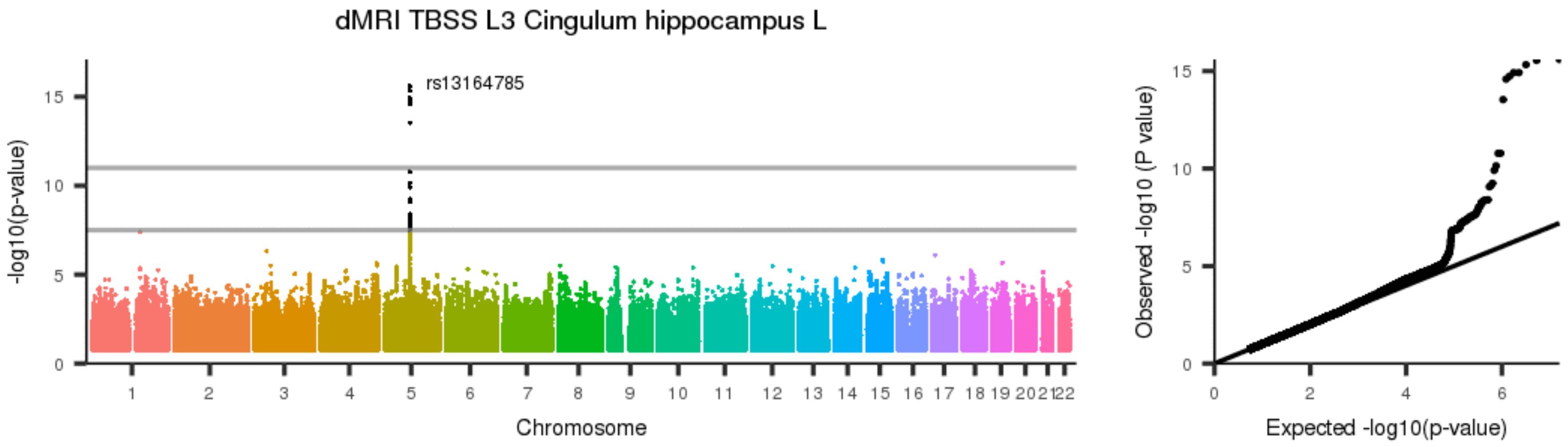

back to list of plots

Figure 474: dMRI TBSS L3 Fornix cres+Stria terminalis R

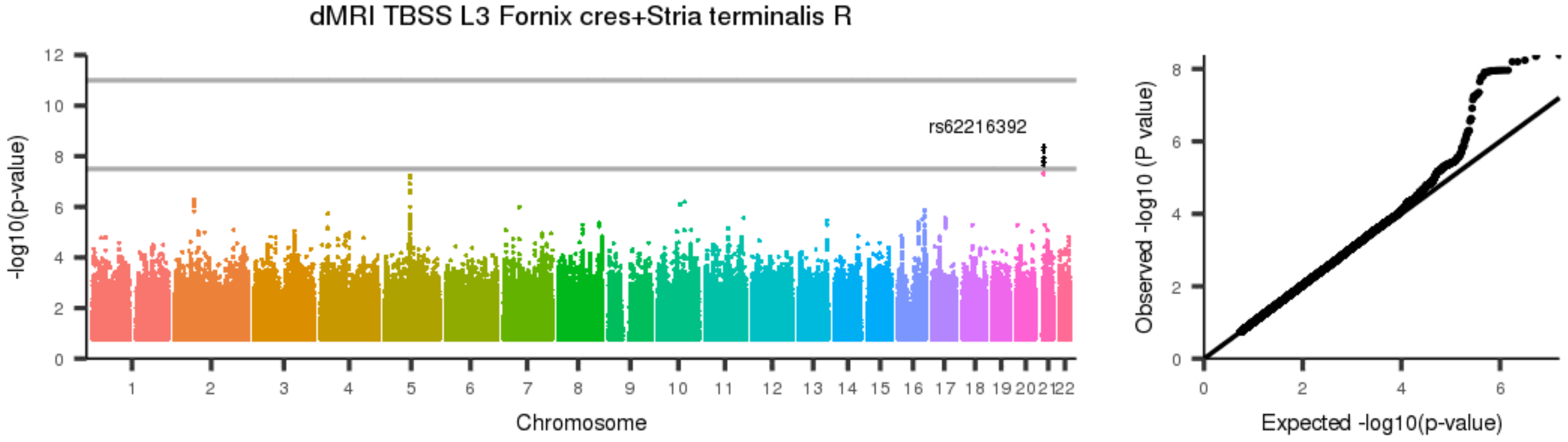

back to list of plots

Figure 475: dMRI TBSS L3 Fornix cres+Stria terminalis L

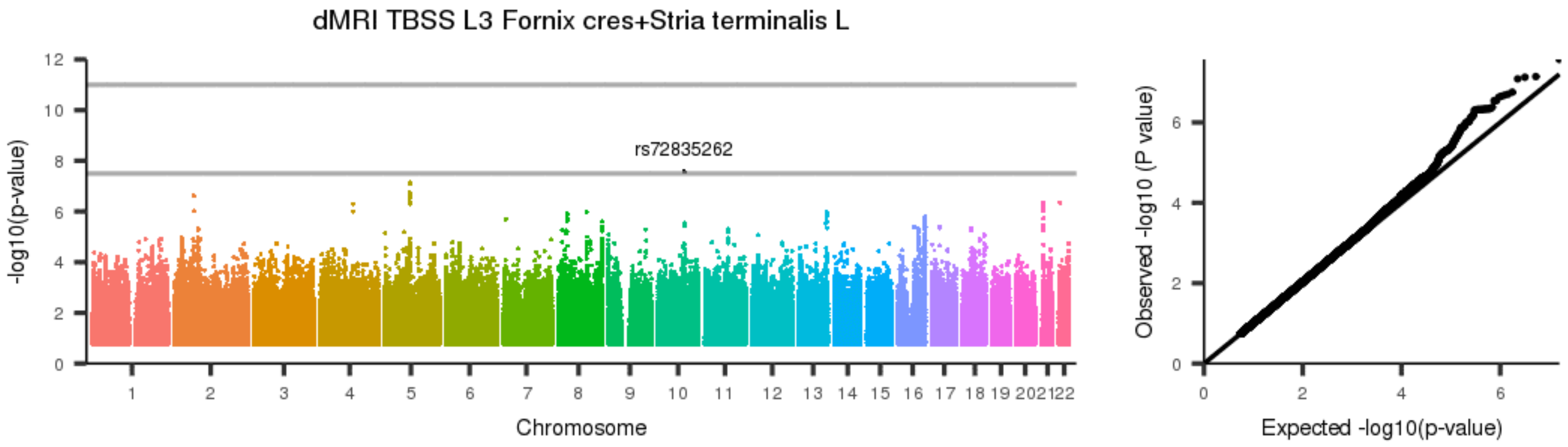

back to list of plots

Figure 476: dMRI TBSS L3 Superior longitudinal fasciculus R

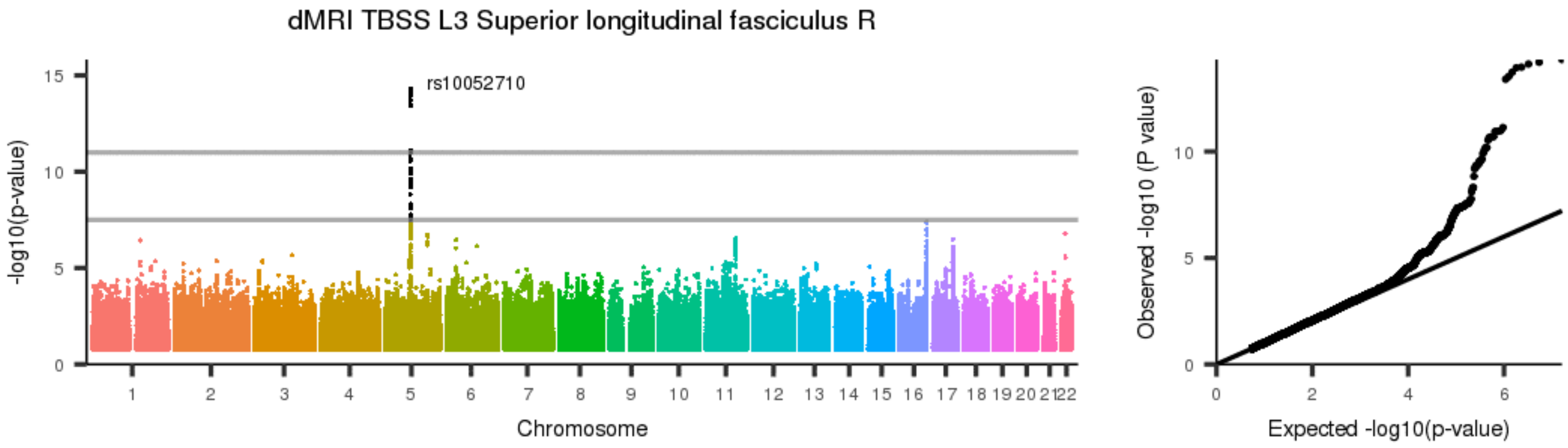

back to list of plots

Figure 477: dMRI TBSS L3 Superior longitudinal fasciculus L

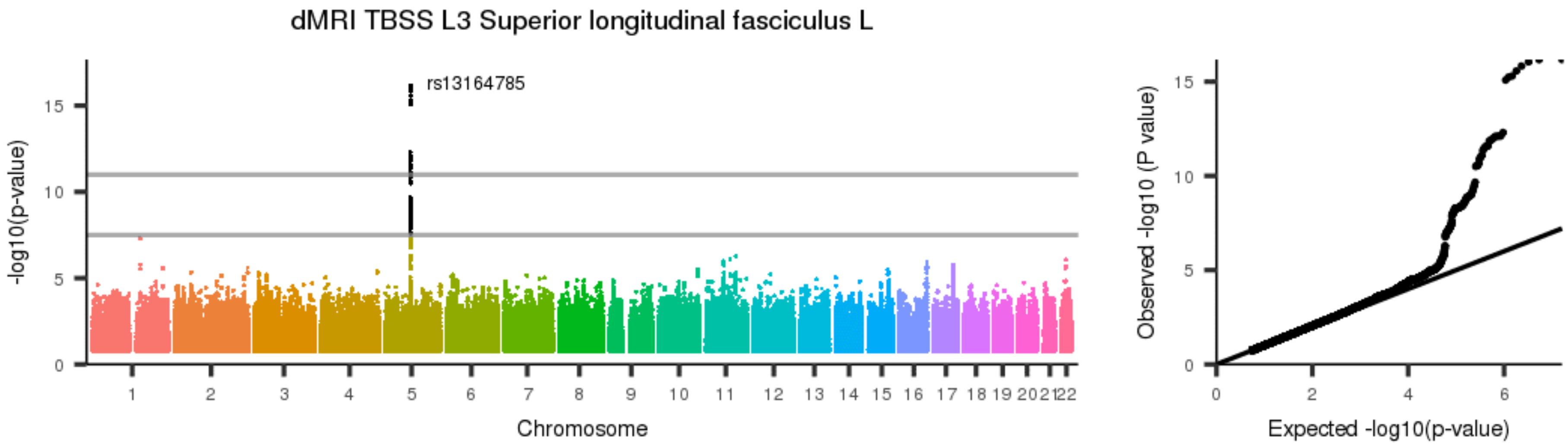

back to list of plots

Figure 478: dMRI TBSS L3 Superior fronto-occipital fasciculus R

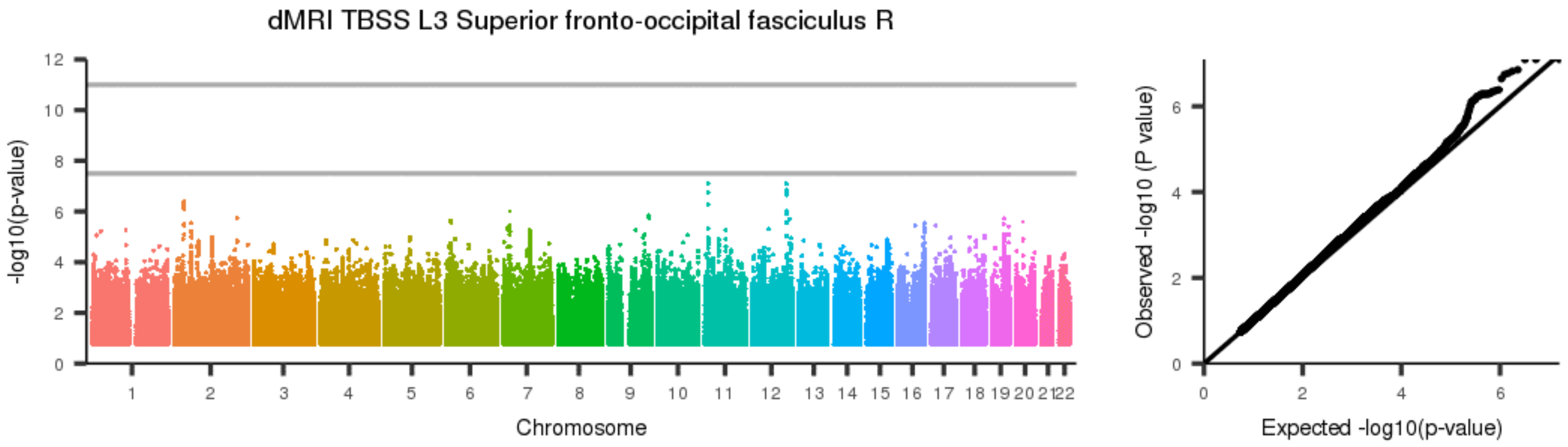

back to list of plots

Figure 479: dMRI TBSS L3 Superior fronto-occipital fasciculus L

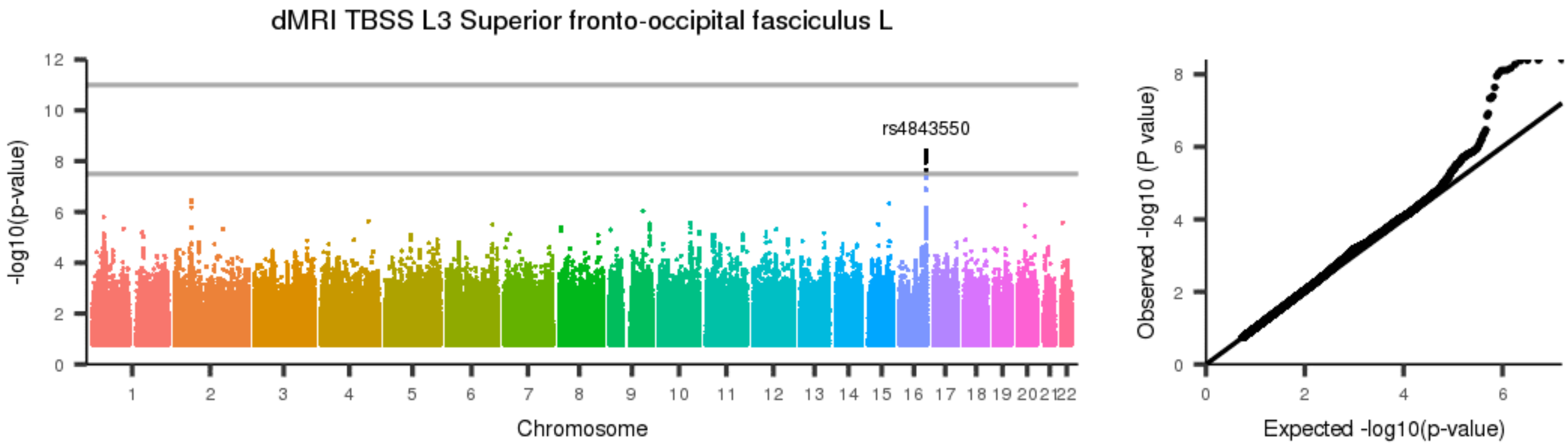

back to list of plots

Figure 480: dMRI TBSS L3 Uncinate fasciculus R

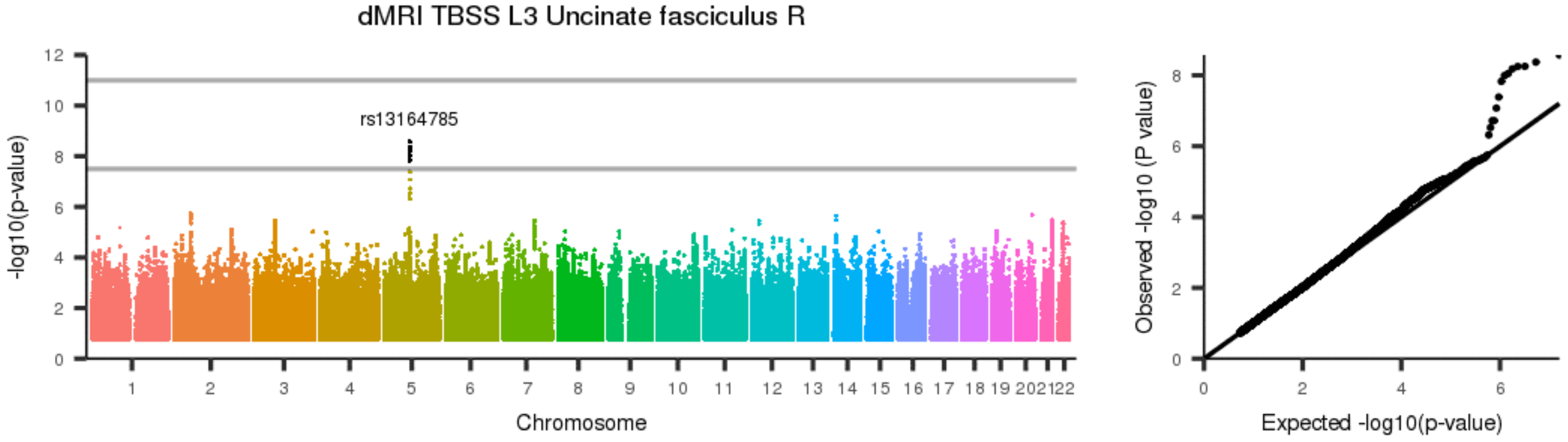

back to list of plots

Figure 481: dMRI TBSS L3 Uncinate fasciculus L

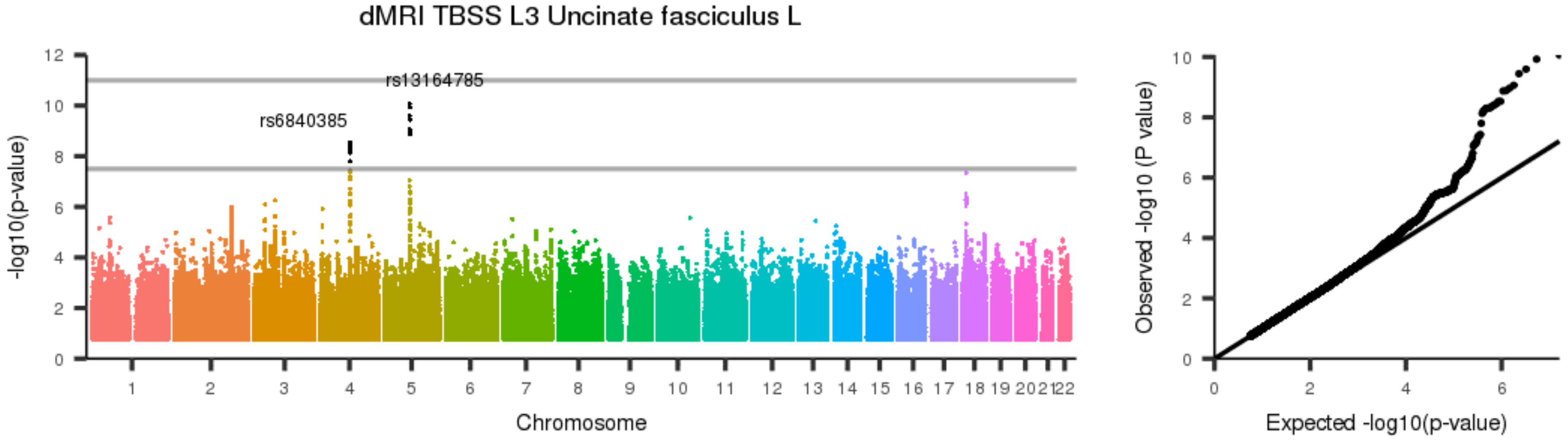

back to list of plots

Figure 482: dMRI TBSS L3 Tapetum R

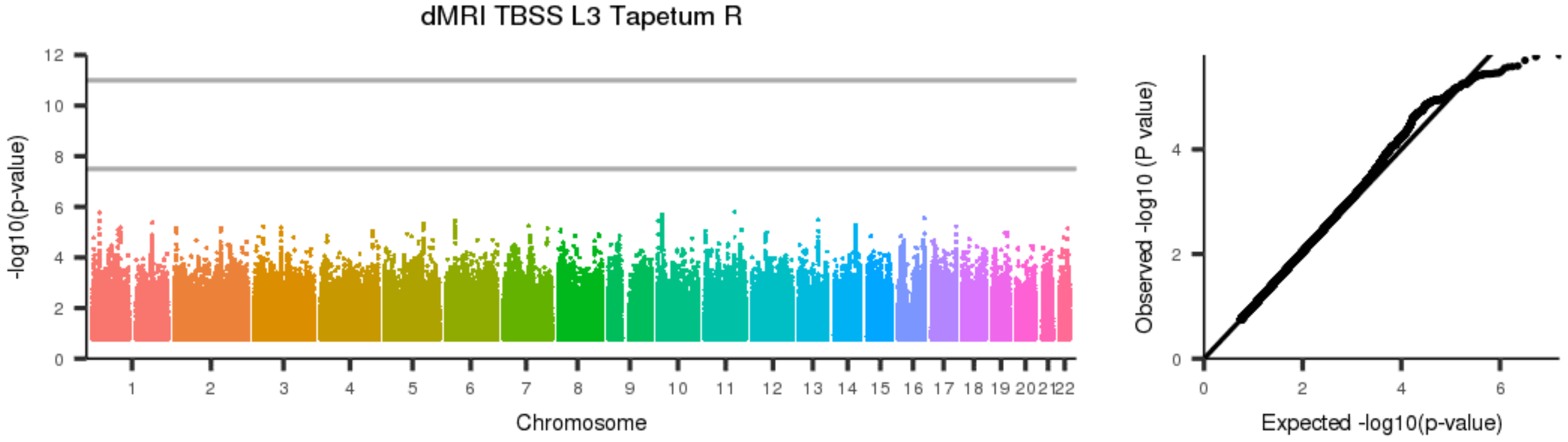

back to list of plots

Figure 483: dMRI TBSS L3 Tapetum L

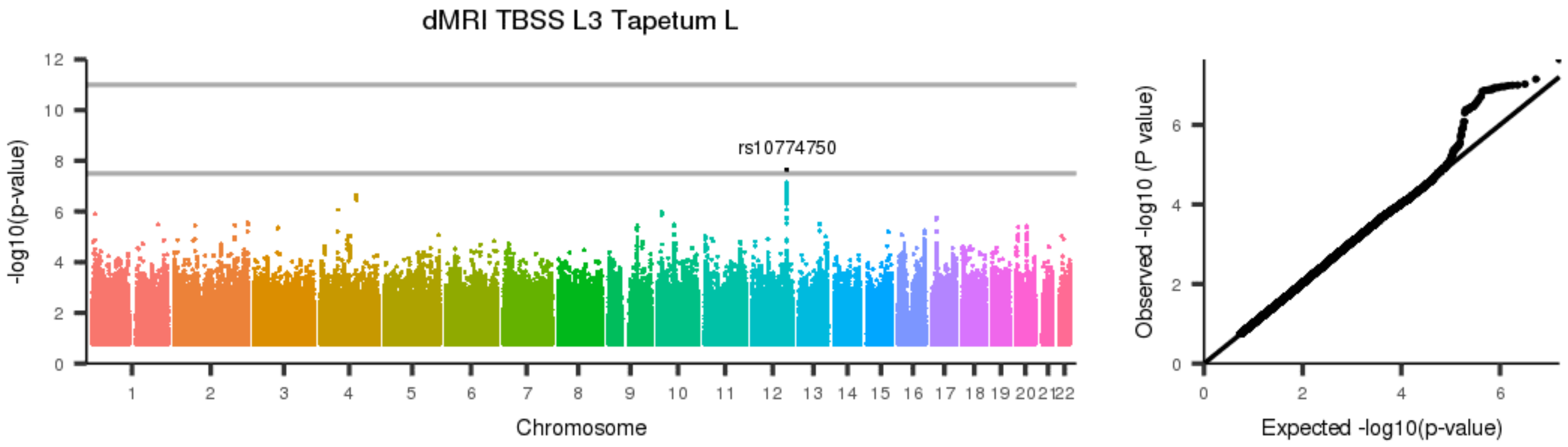

back to list of plots

Figure 484: dMRI TBSS ICVF Middle cerebellar peduncle

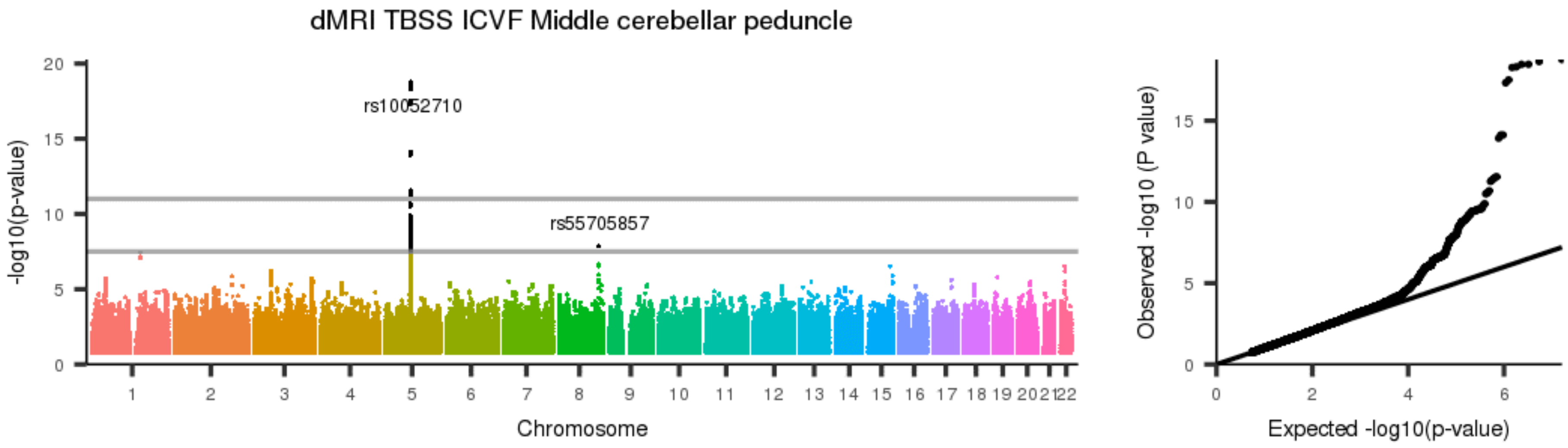

back to list of plots

Figure 485: dMRI TBSS ICVF Pontine crossing tract

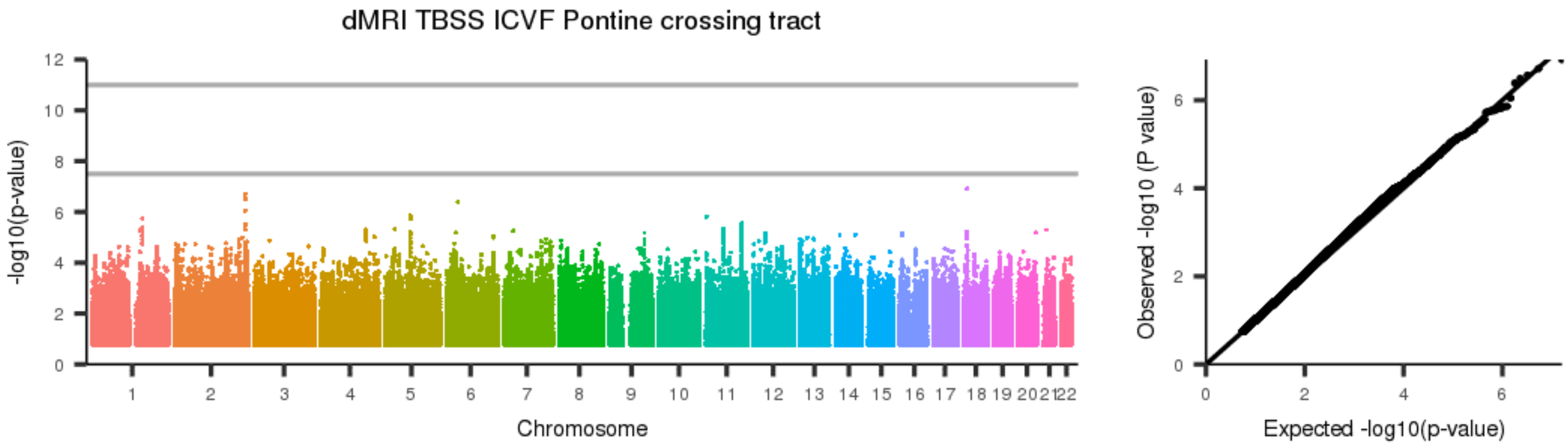

back to list of plots

Figure 486: dMRI TBSS ICVF Genu of corpus callosum

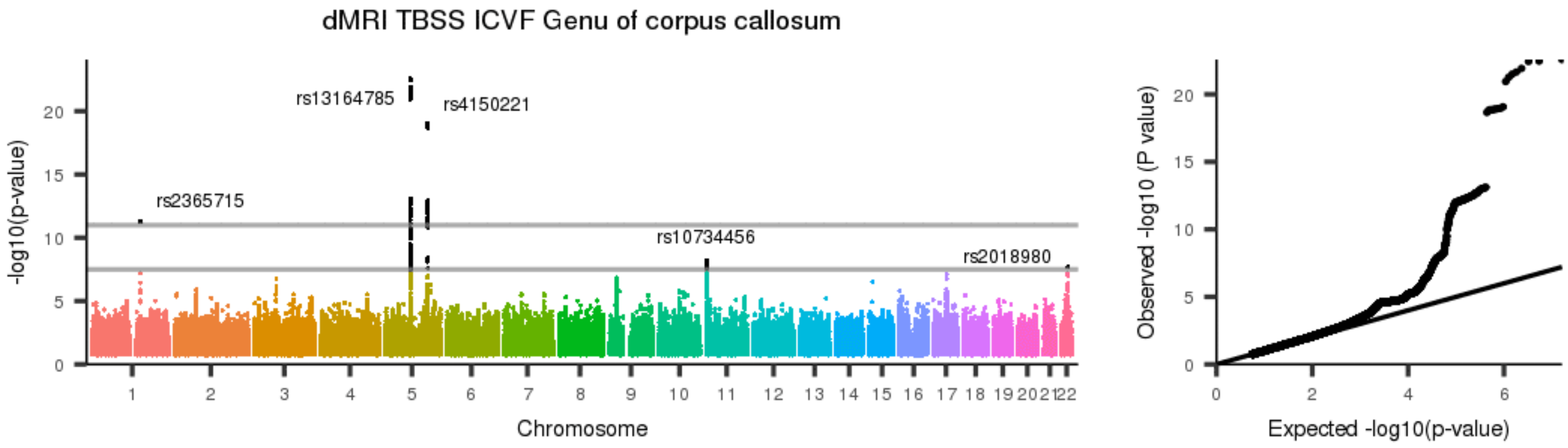

back to list of plots

Figure 487: dMRI TBSS ICVF Body of corpus callosum

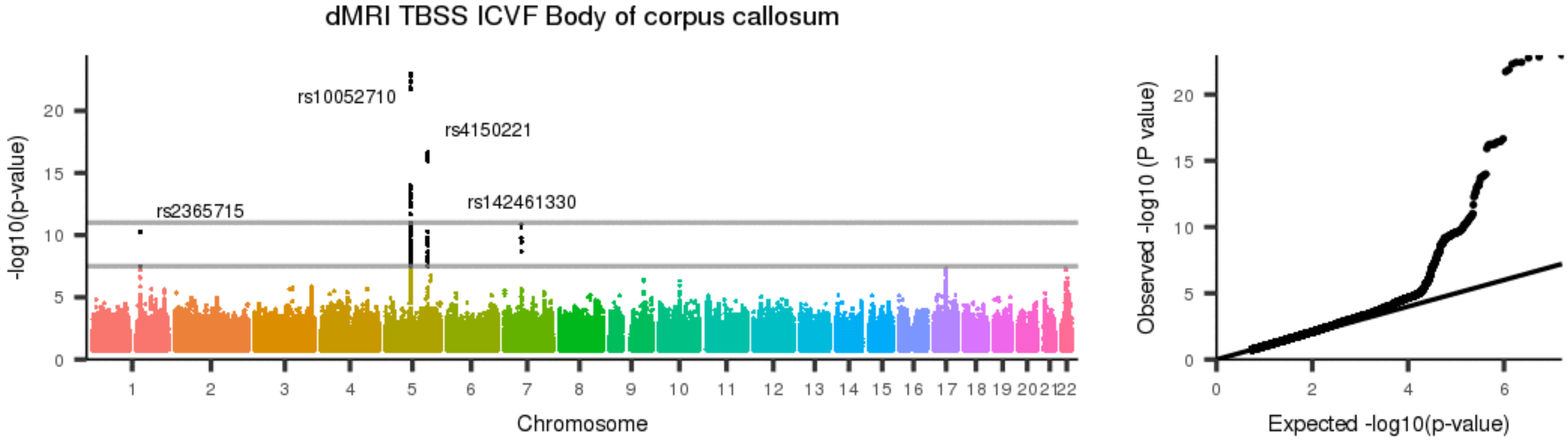

back to list of plots

Figure 488: dMRI TBSS ICVF Splenium of corpus callosum

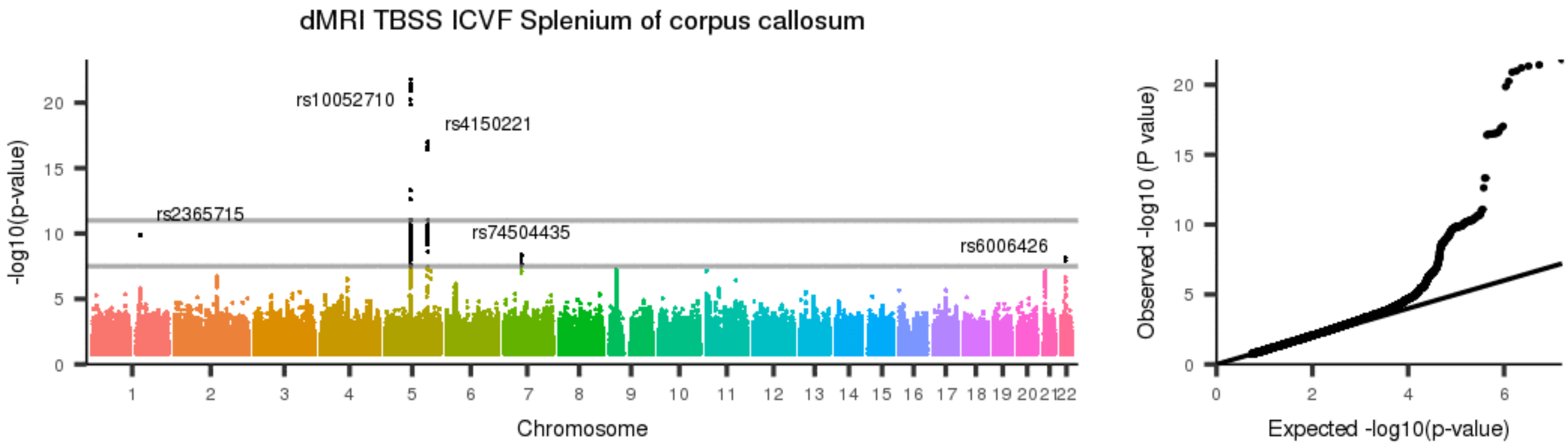

back to list of plots

Figure 489: dMRI TBSS ICVF Fornix

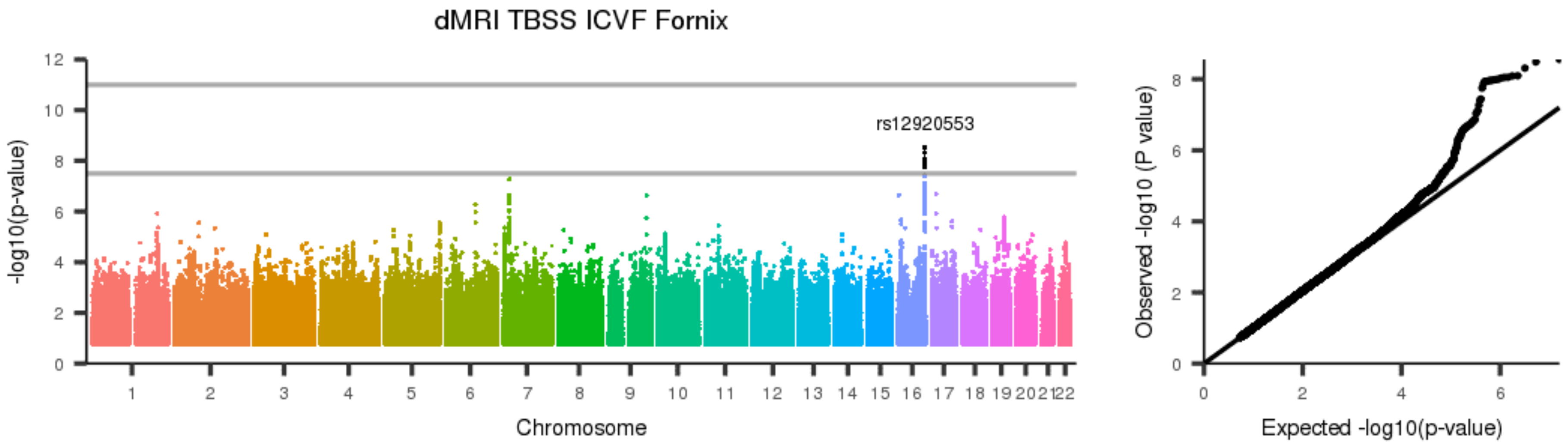

back to list of plots

Figure 490: dMRI TBSS ICVF Corticospinal tract R

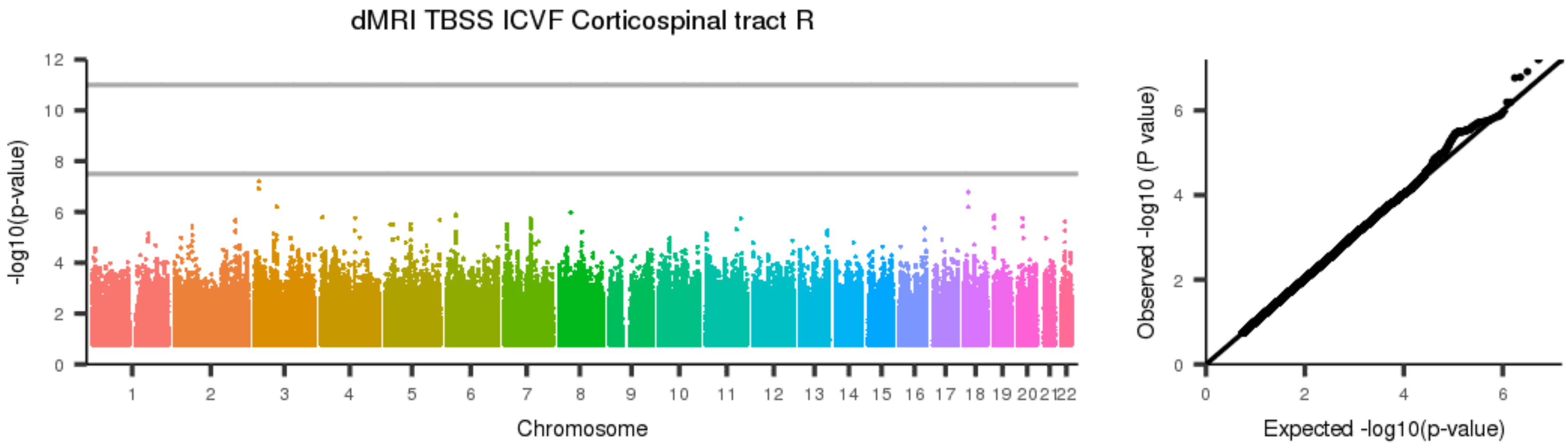

back to list of plots

Figure 491: dMRI TBSS ICVF Corticospinal tract L

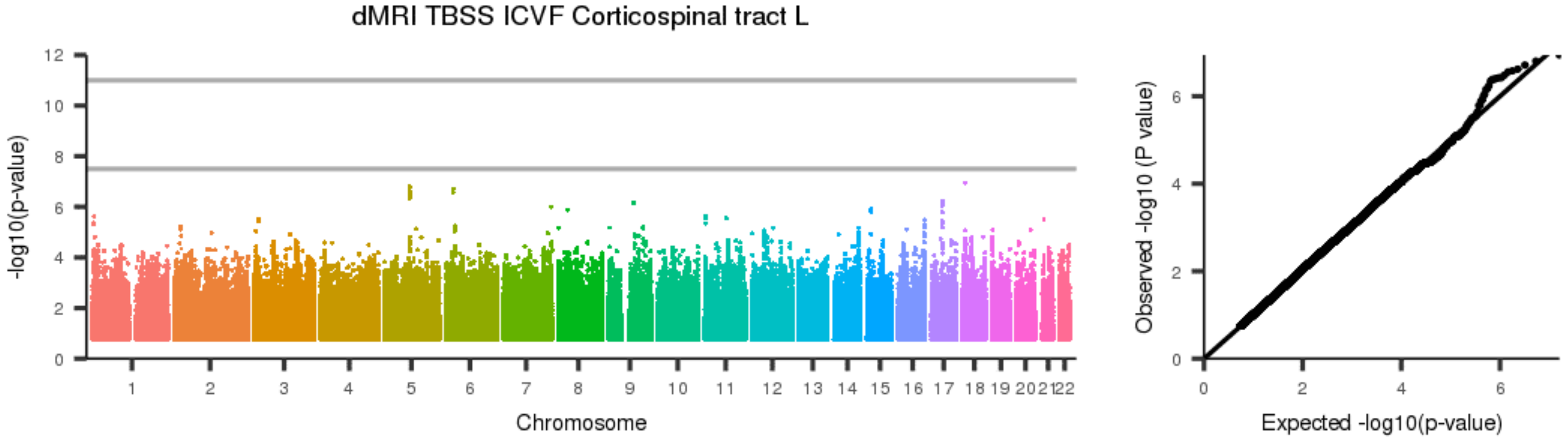

back to list of plots

Figure 492: dMRI TBSS ICVF Medial lemniscus R

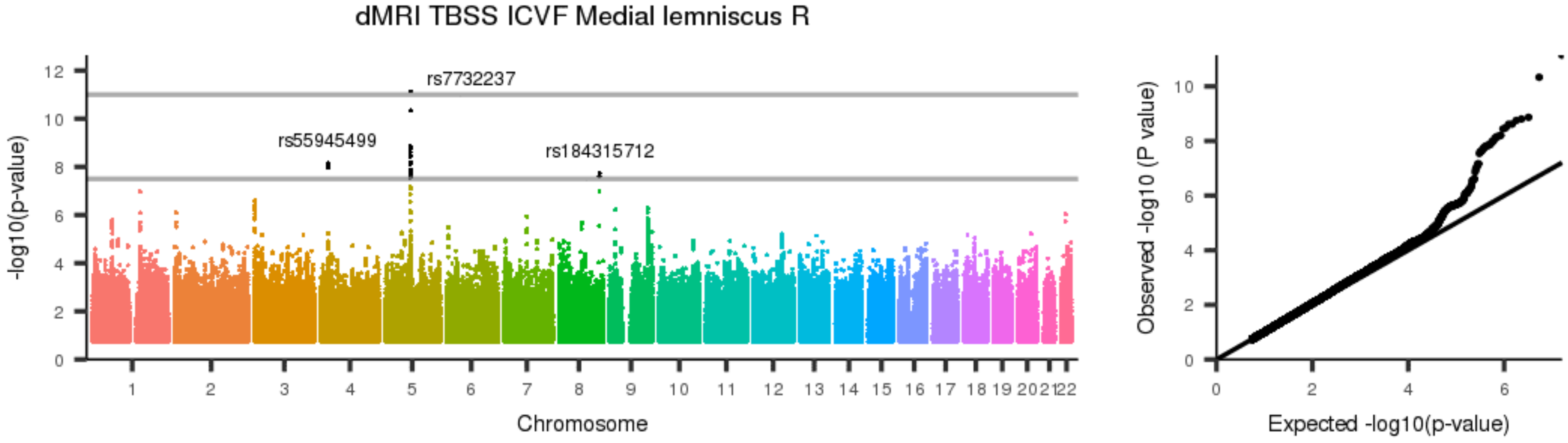

back to list of plots

Figure 493: dMRI TBSS ICVF Medial lemniscus L

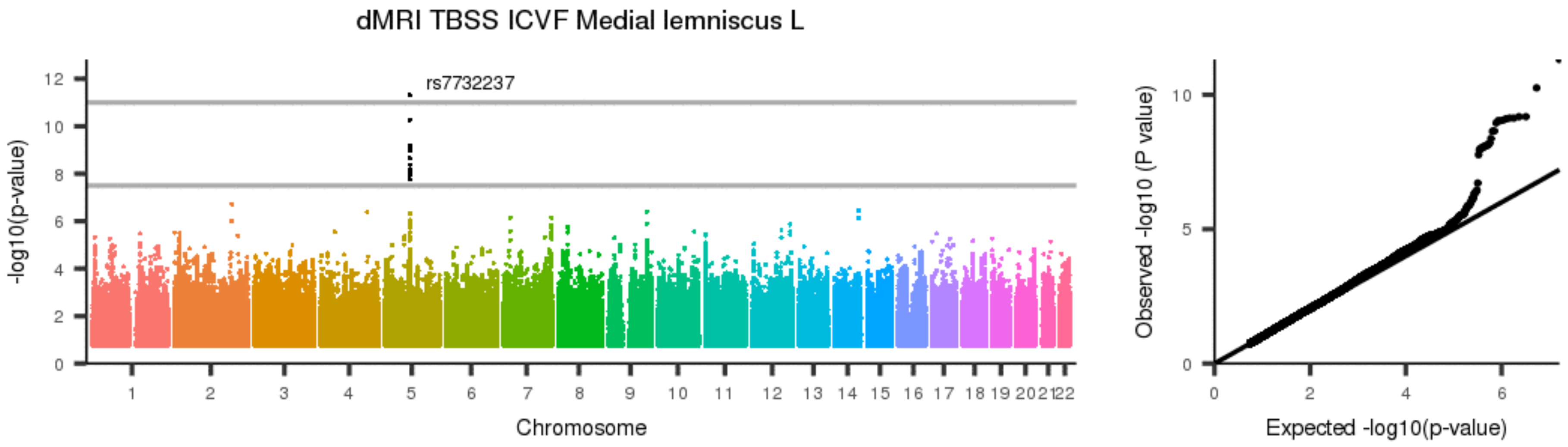

back to list of plots

Figure 494: dMRI TBSS ICVF Inferior cerebellar peduncle R

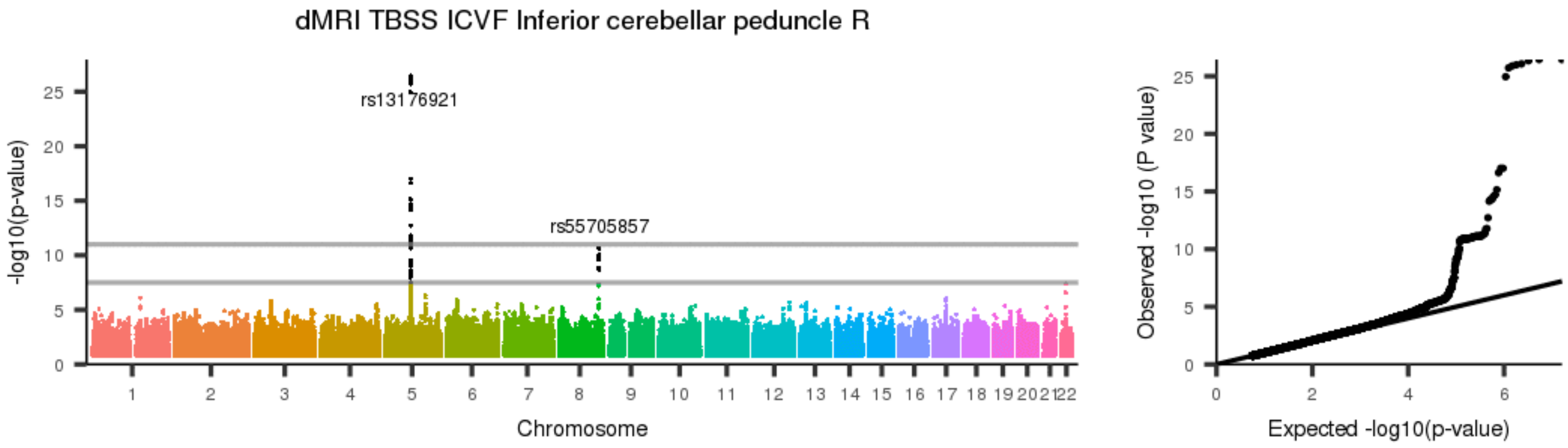

back to list of plots

Figure 495: dMRI TBSS ICVF Inferior cerebellar peduncle L

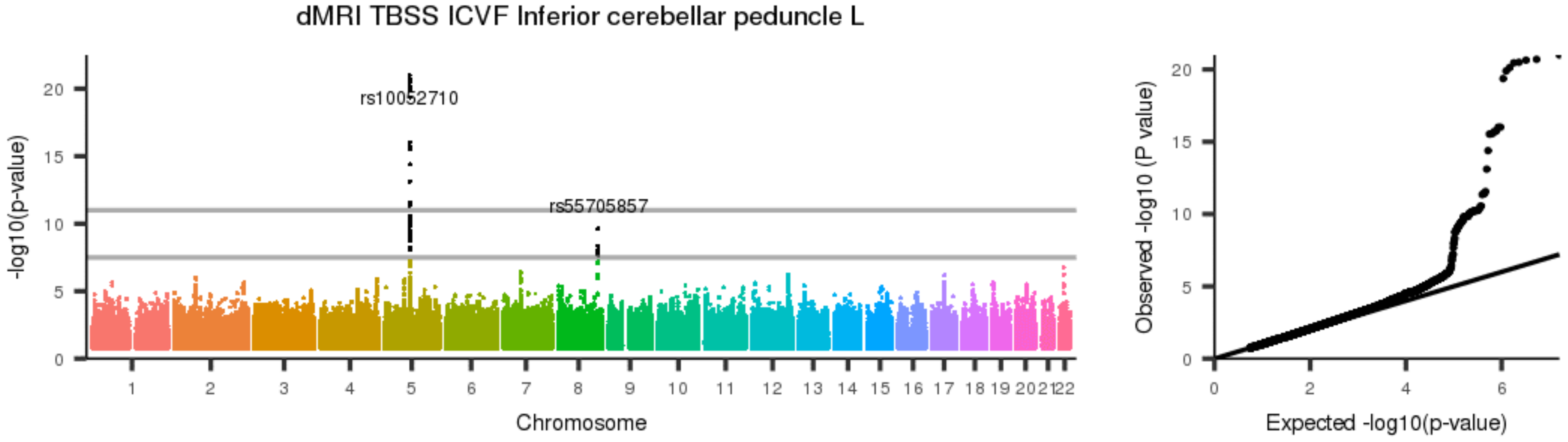

back to list of plots

Figure 496: dMRI TBSS ICVF Superior cerebellar peduncle R

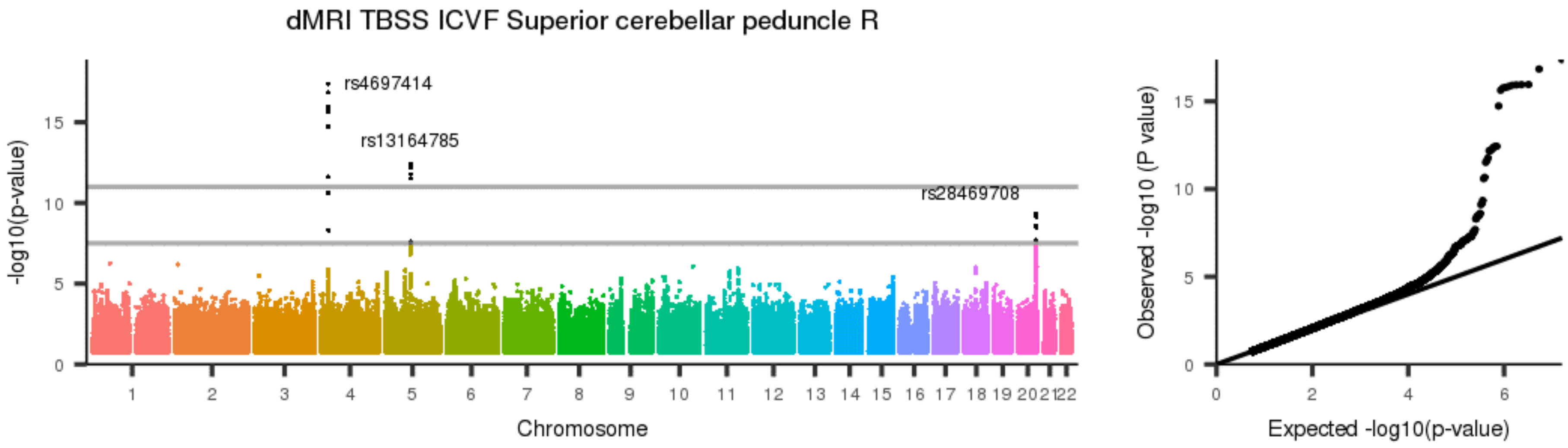

back to list of plots

Figure 497: dMRI TBSS ICVF Superior cerebellar peduncle L

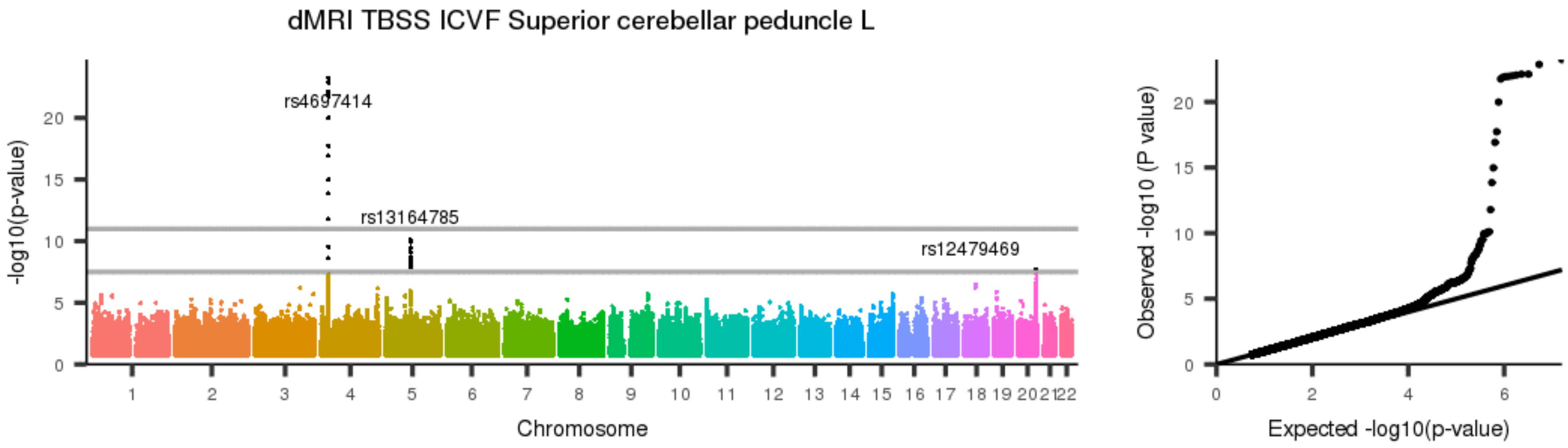

back to list of plots

Figure 498: dMRI TBSS ICVF Cerebral peduncle R

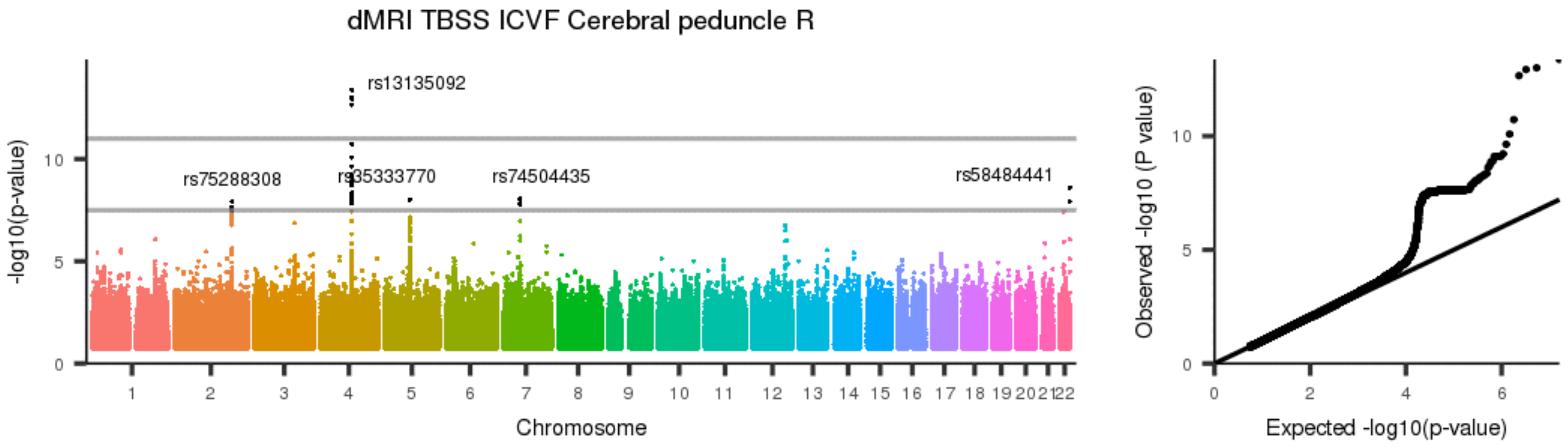

back to list of plots

Figure 499: dMRI TBSS ICVF Cerebral peduncle L

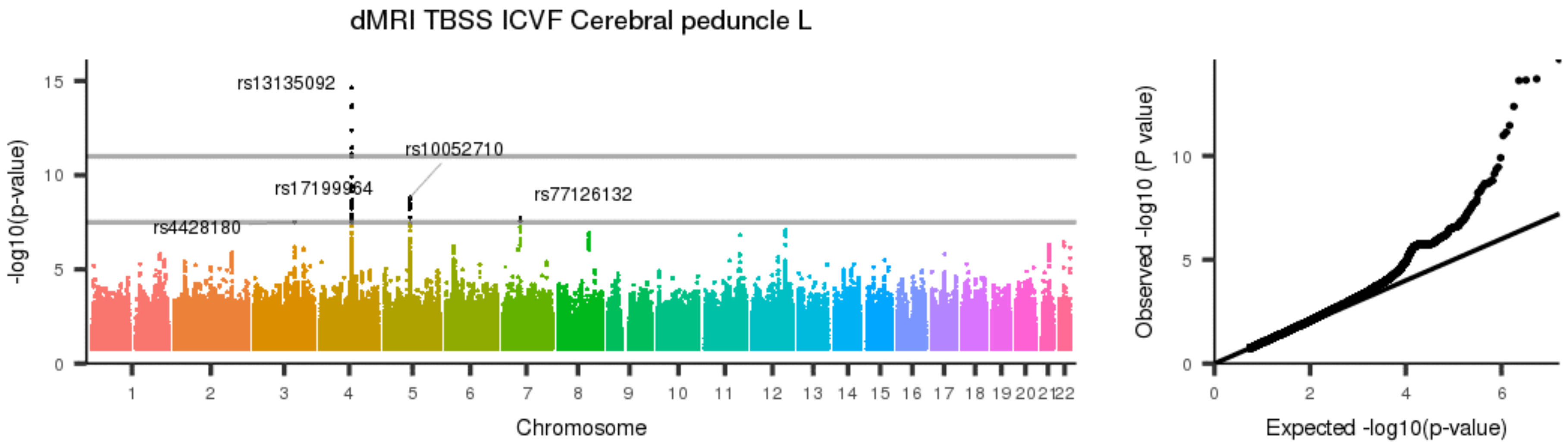

back to list of plots

Figure 500: dMRI TBSS ICVF Anterior limb of internal capsule R

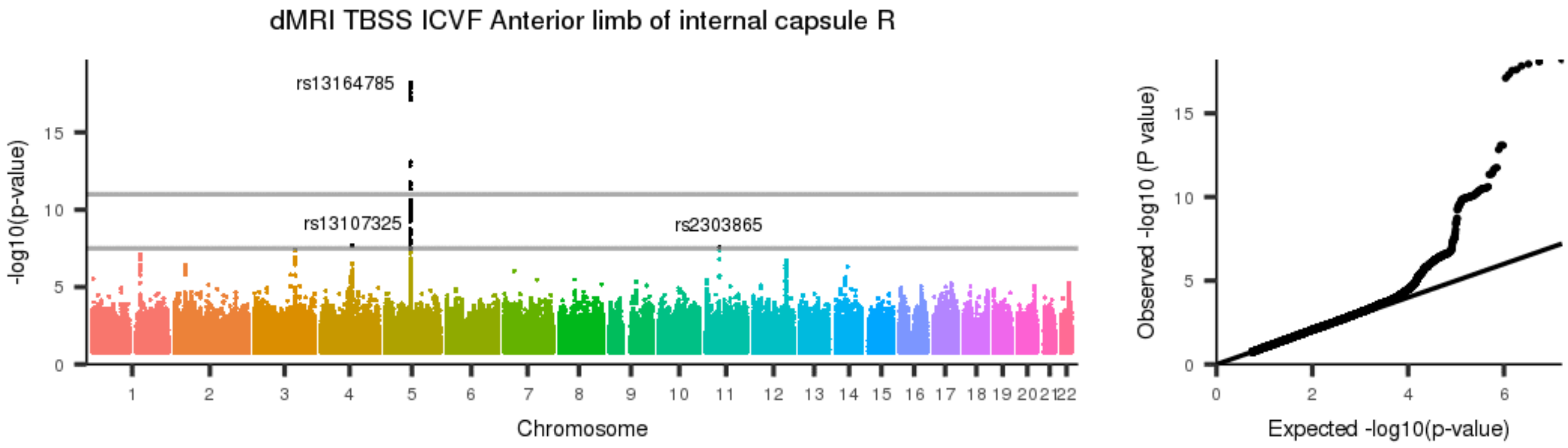

back to list of plots

Figure 501: dMRI TBSS ICVF Anterior limb of internal capsule L

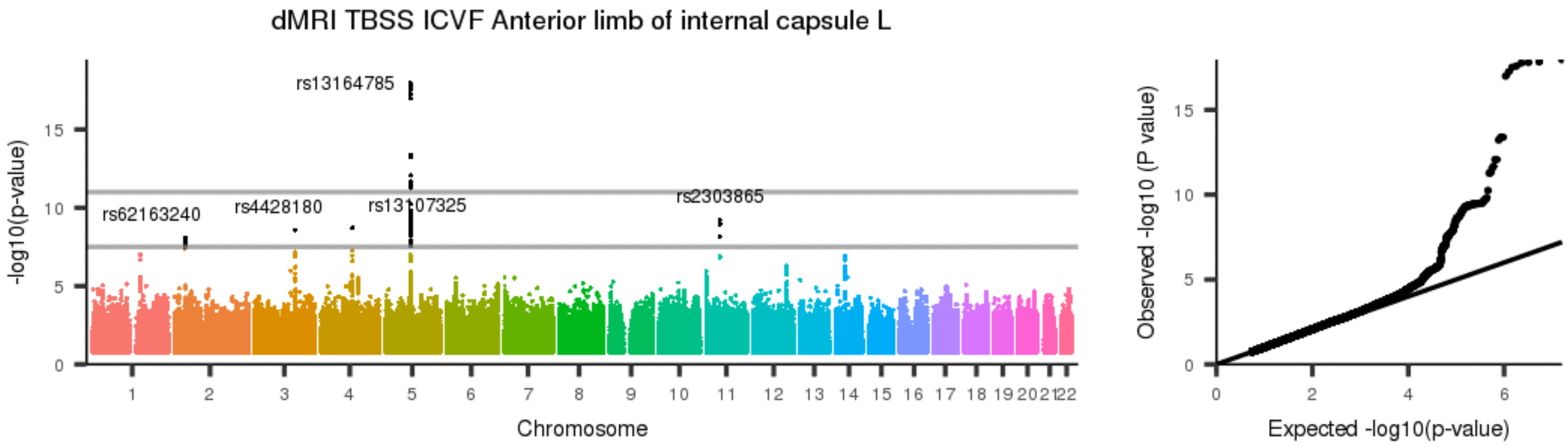

back to list of plots

Figure 502: dMRI TBSS ICVF Posterior limb of internal capsule R

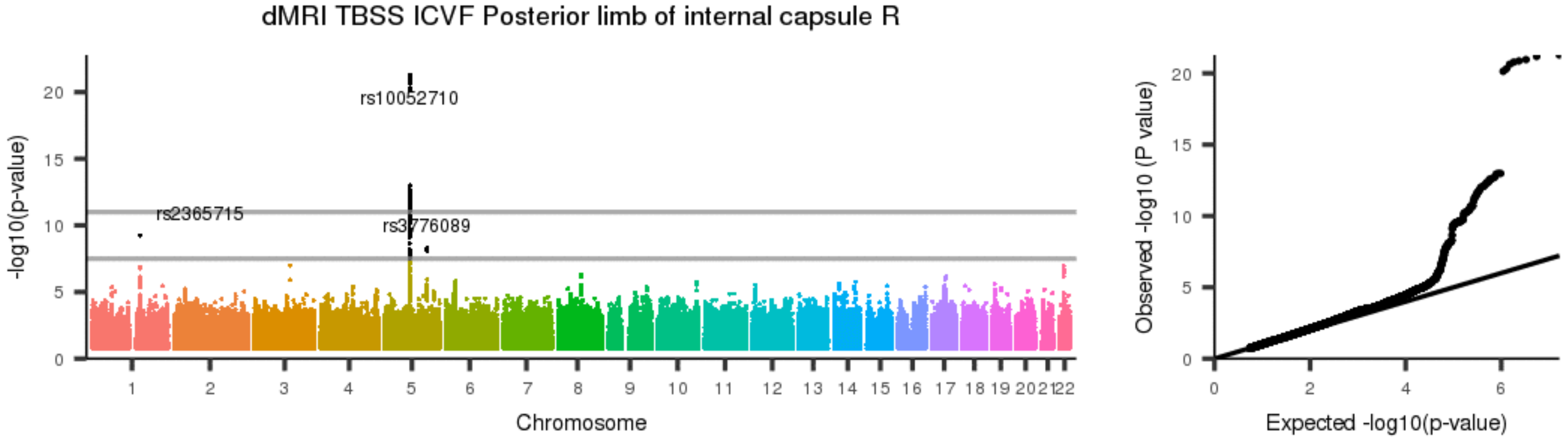

back to list of plots

Figure 503: dMRI TBSS ICVF Posterior limb of internal capsule L

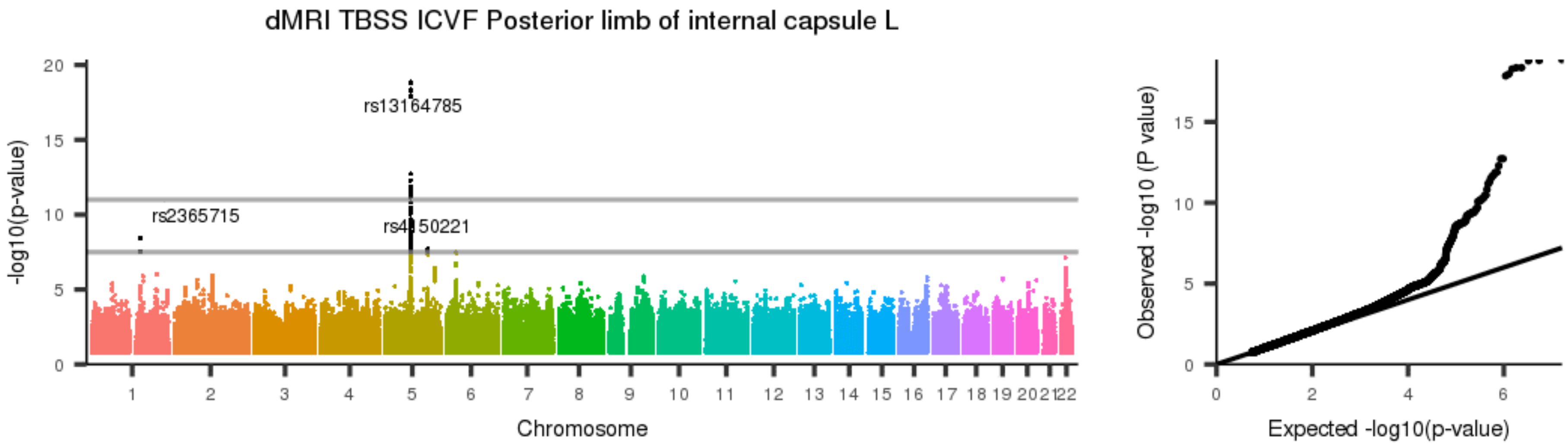

back to list of plots

Figure 504: dMRI TBSS ICVF Retrolenticular part of internal capsule R

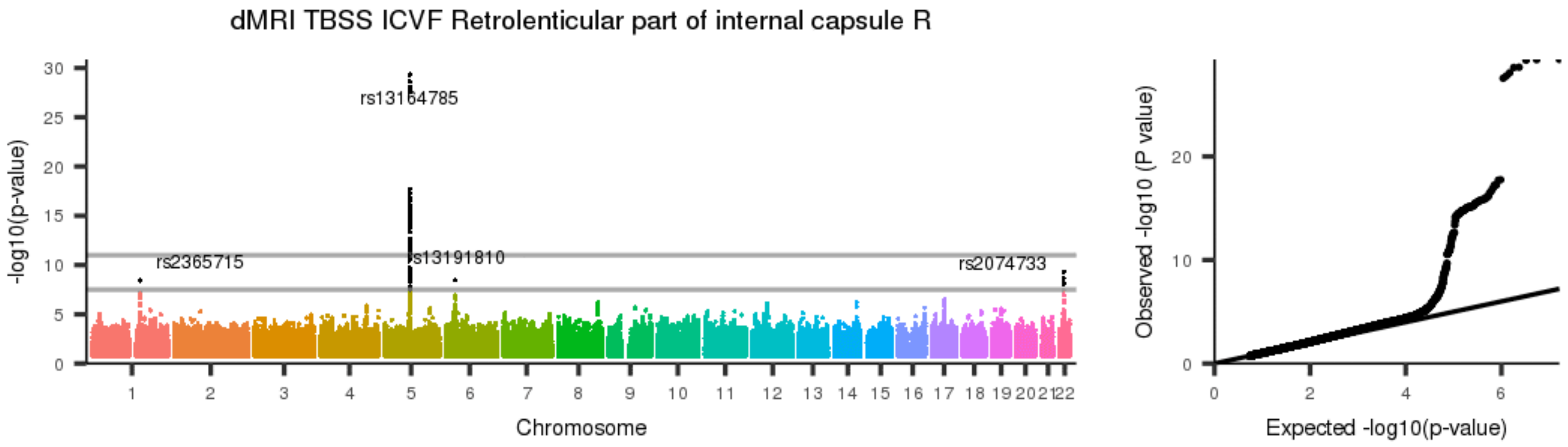

back to list of plots

Figure 505: dMRI TBSS ICVF Retrolenticular part of internal capsule L

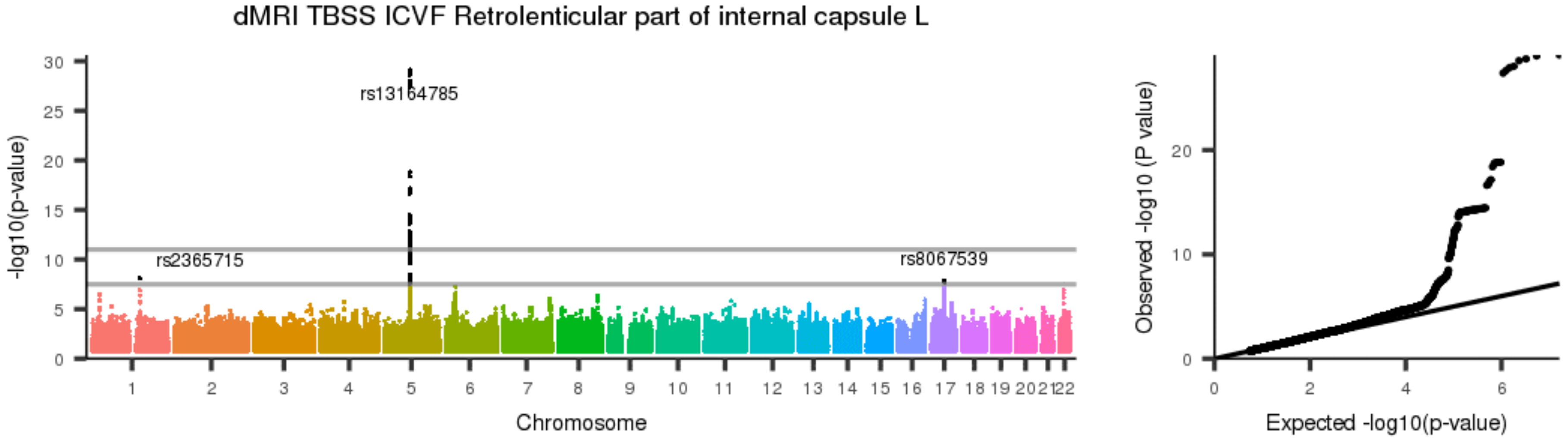

back to list of plots

Figure 506: dMRI TBSS ICVF Anterior corona radiata R

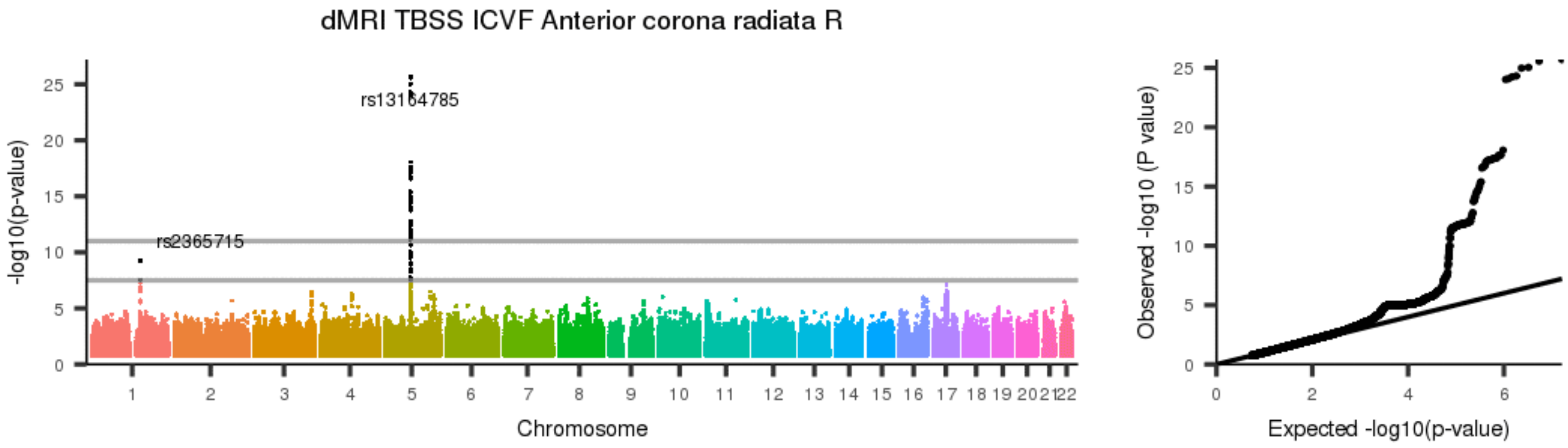

back to list of plots

Figure 507: dMRI TBSS ICVF Anterior corona radiata L

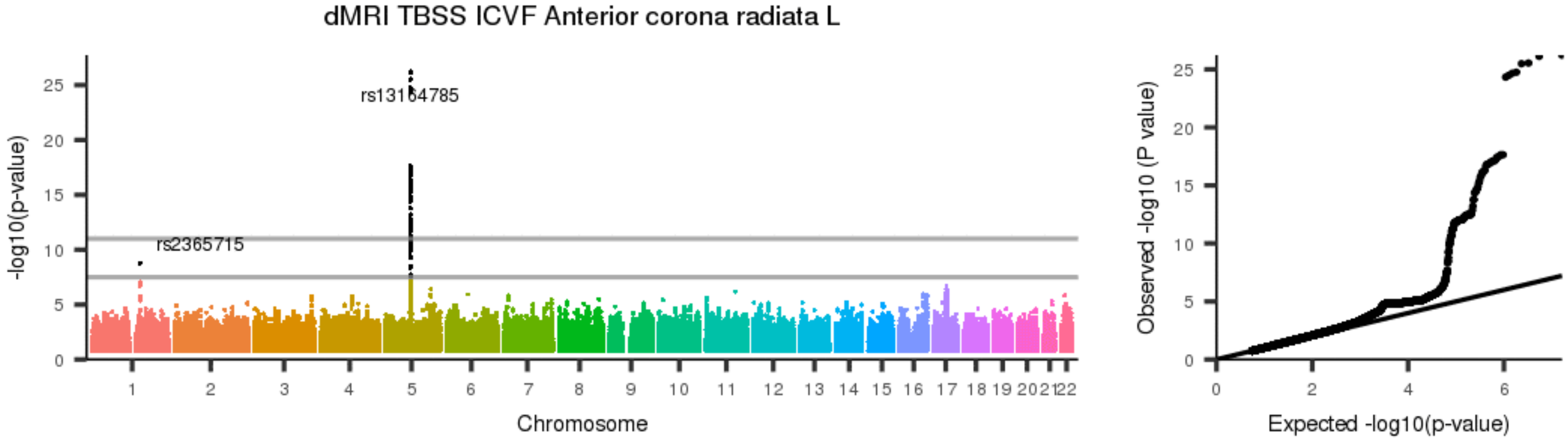

back to list of plots

Figure 508: dMRI TBSS ICVF Superior corona radiata R

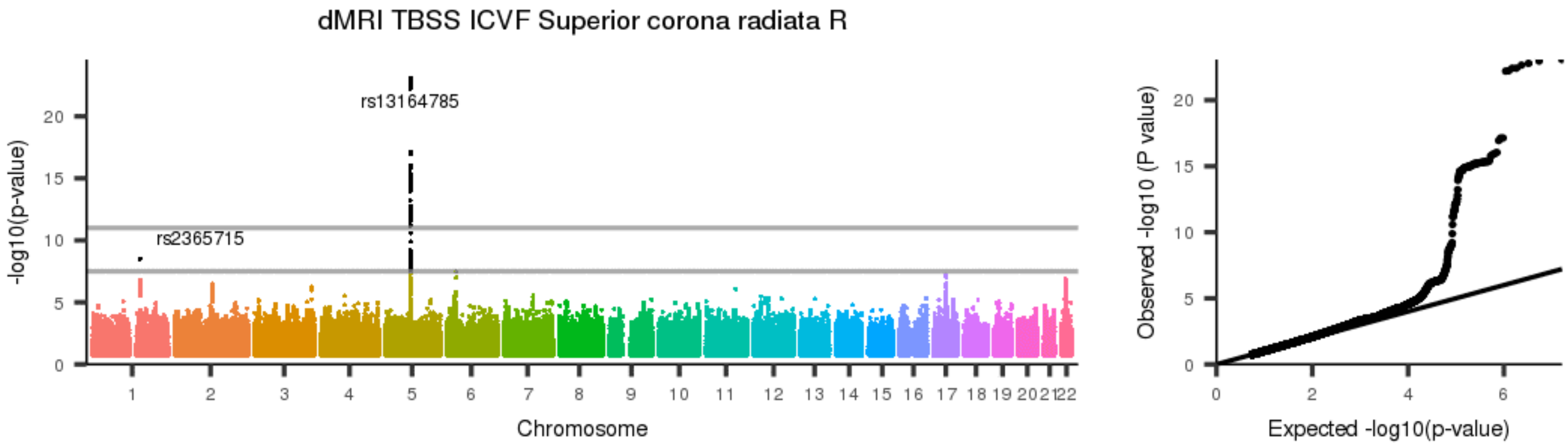

back to list of plots

Figure 509: dMRI TBSS ICVF Superior corona radiata L

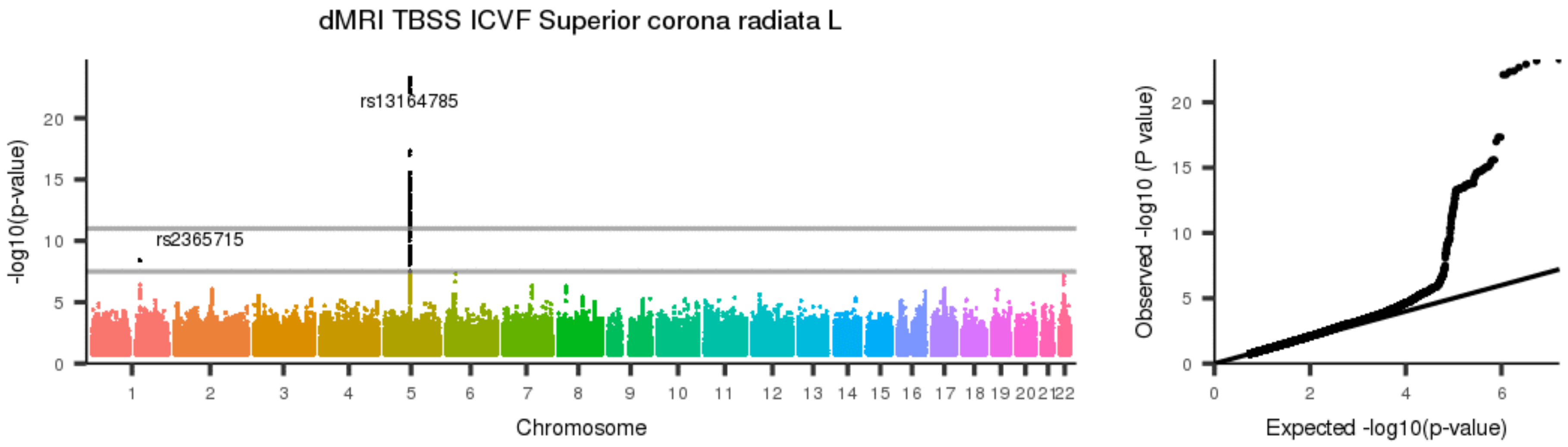

back to list of plots

Figure 510: dMRI TBSS ICVF Posterior corona radiata R

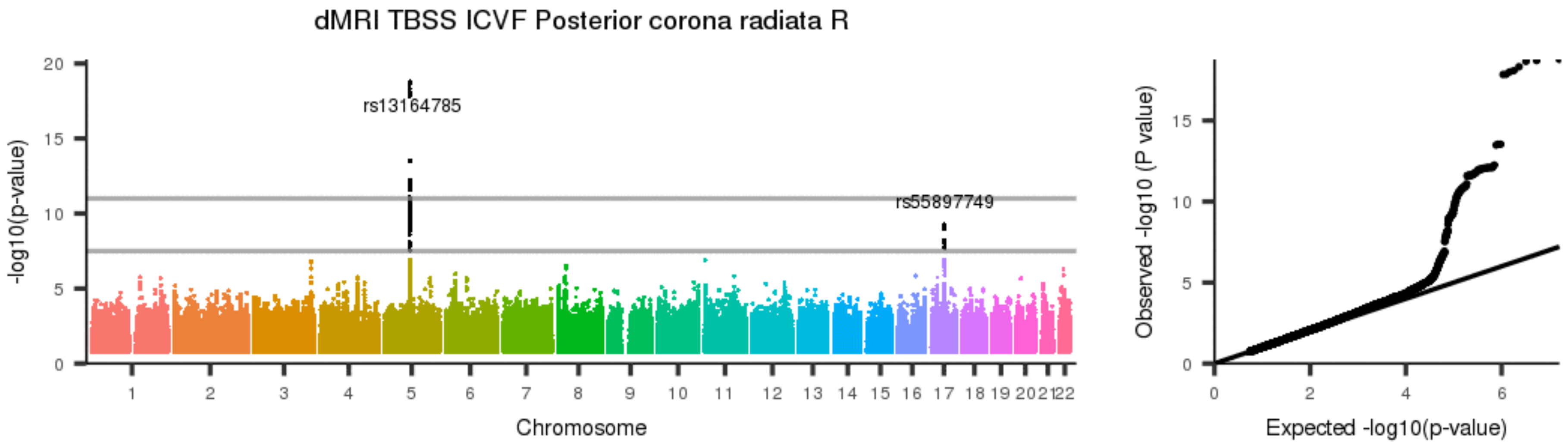

back to list of plots

Figure 511: dMRI TBSS ICVF Posterior corona radiata L

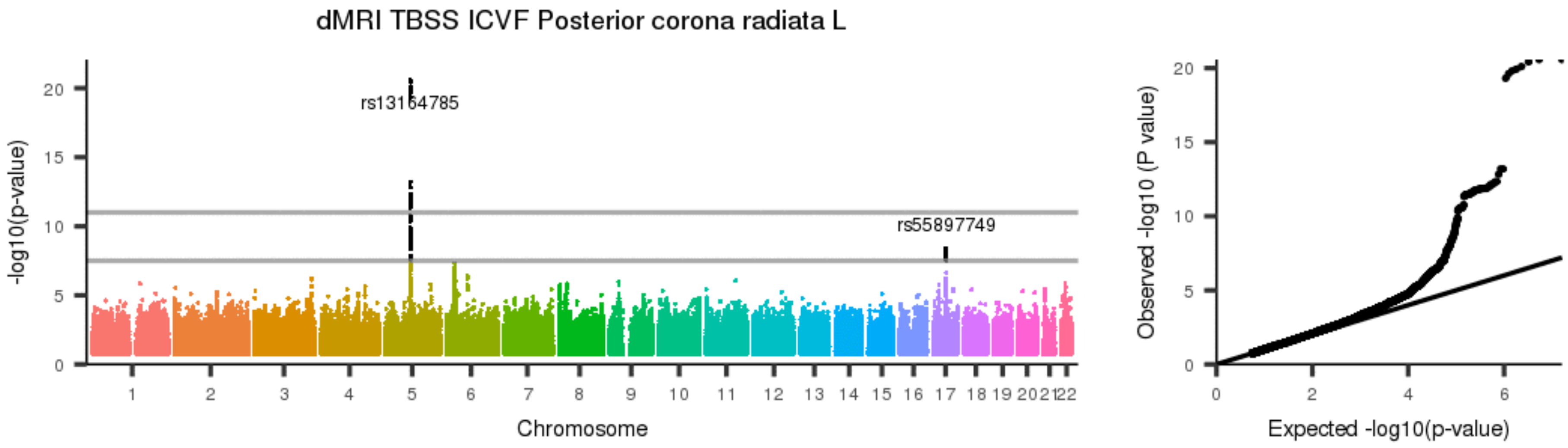

back to list of plots

Figure 512: dMRI TBSS ICVF Posterior thalamic radiation R

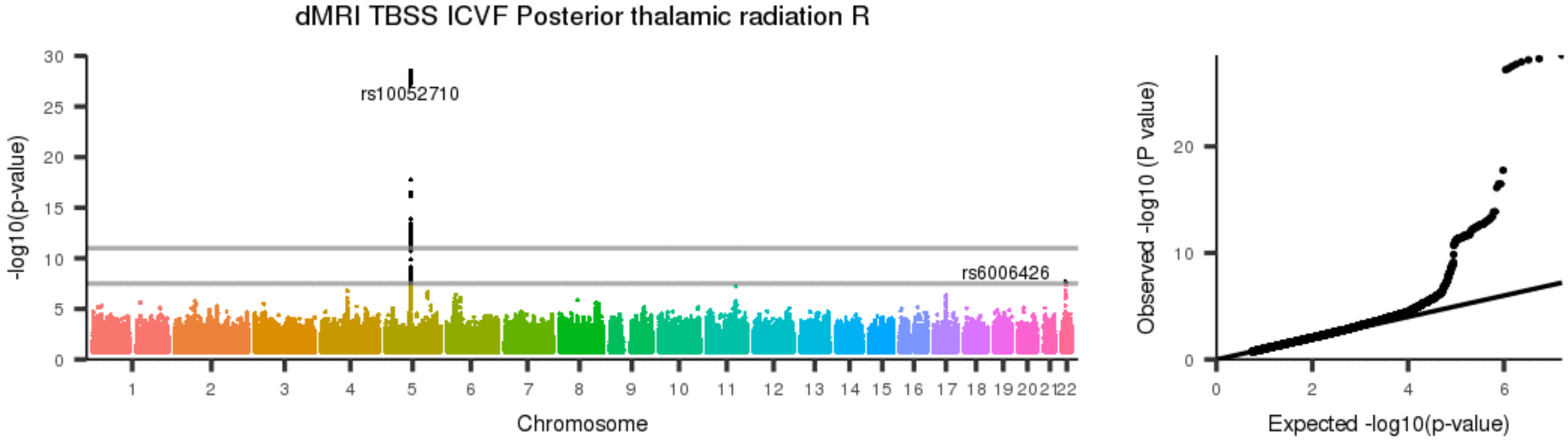

back to list of plots

Figure 513: dMRI TBSS ICVF Posterior thalamic radiation L

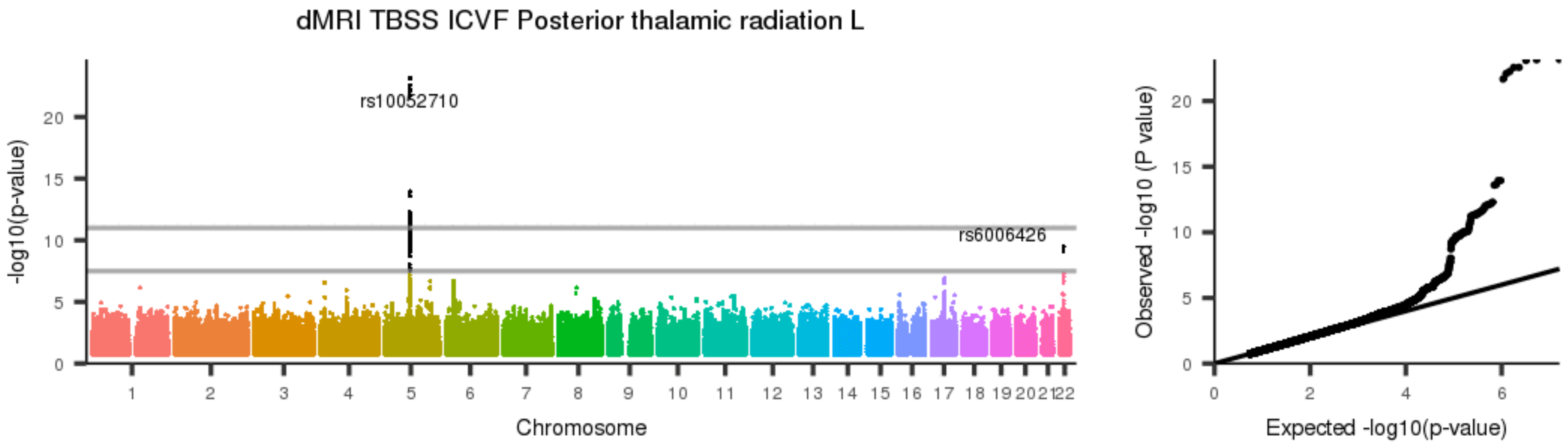

back to list of plots

Figure 514: dMRI TBSS ICVF Sagittal stratum R

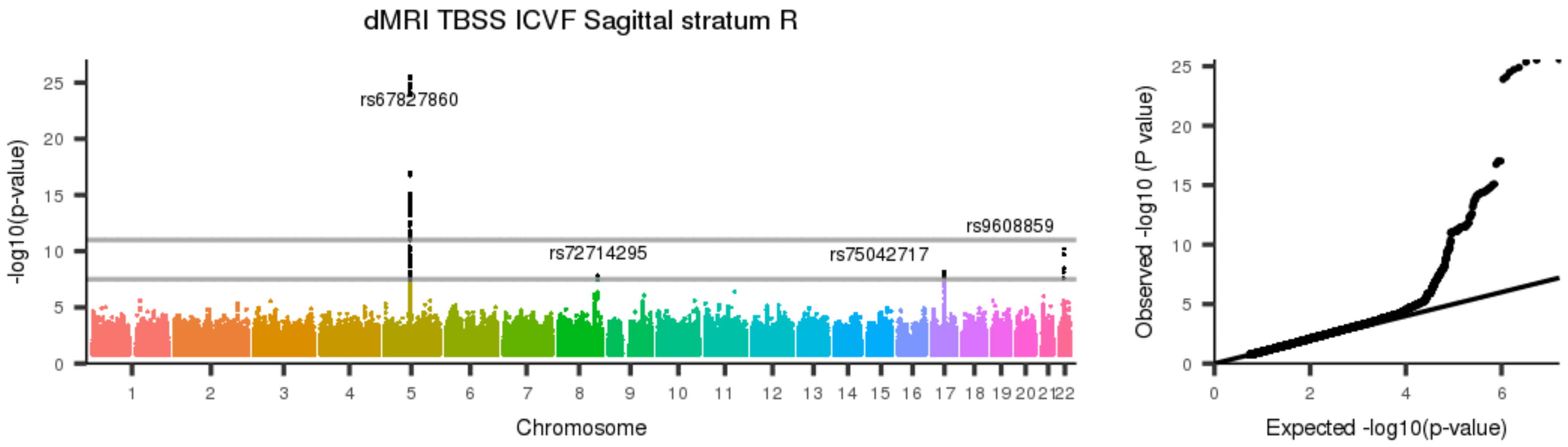

back to list of plots

Figure 515: dMRI TBSS ICVF Sagittal stratum L

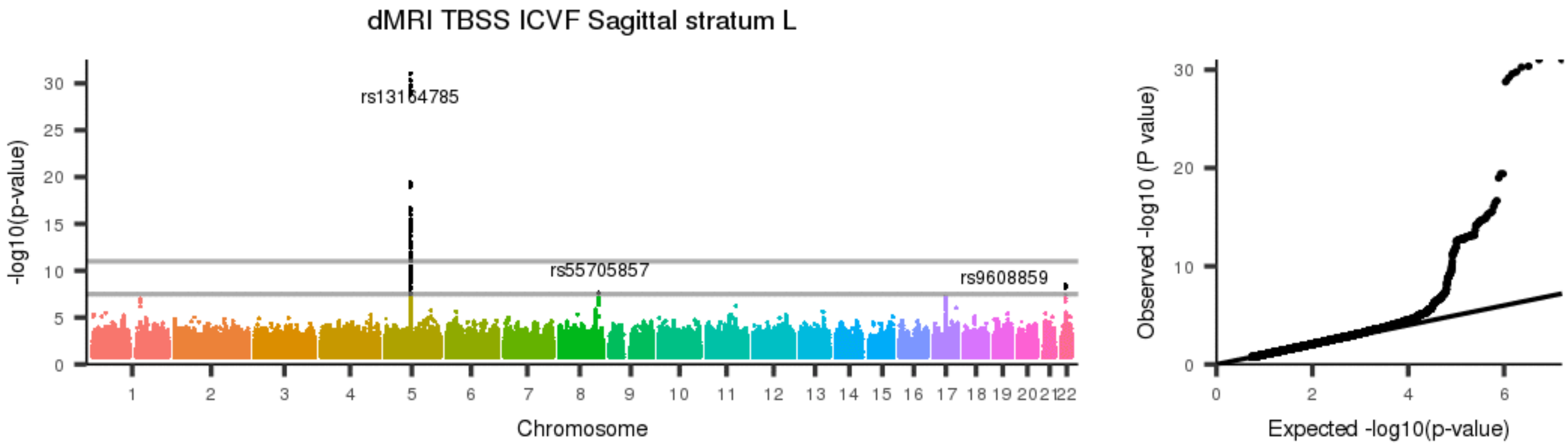

back to list of plots

Figure 516: dMRI TBSS ICVF External capsule R

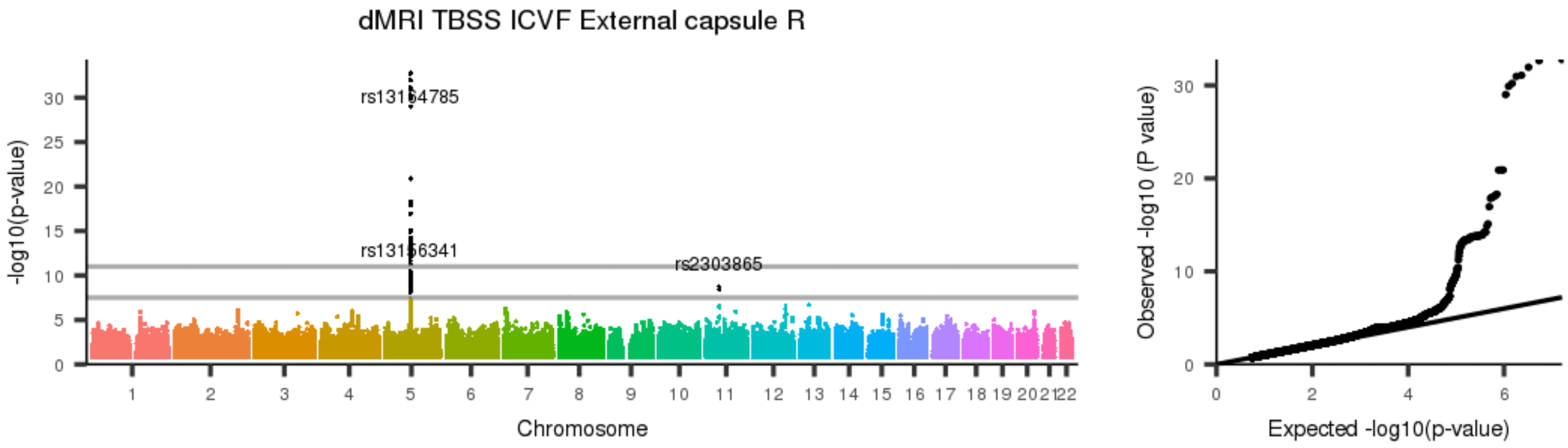

back to list of plots

Figure 517: dMRI TBSS ICVF External capsule L

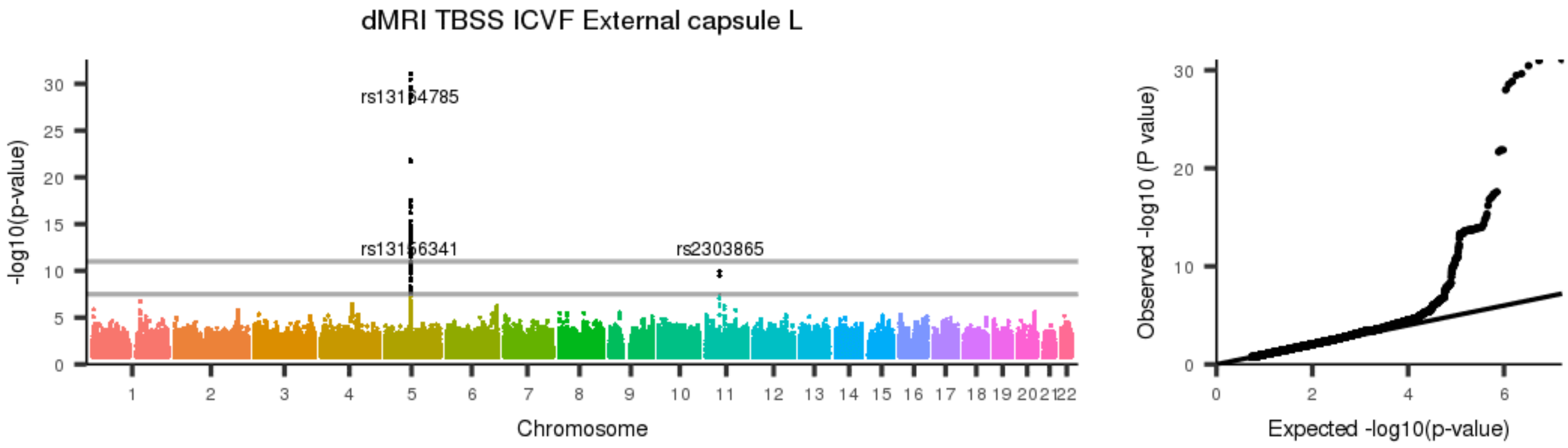

back to list of plots

Figure 518: dMRI TBSS ICVF Cingulum cingulate gyrus R

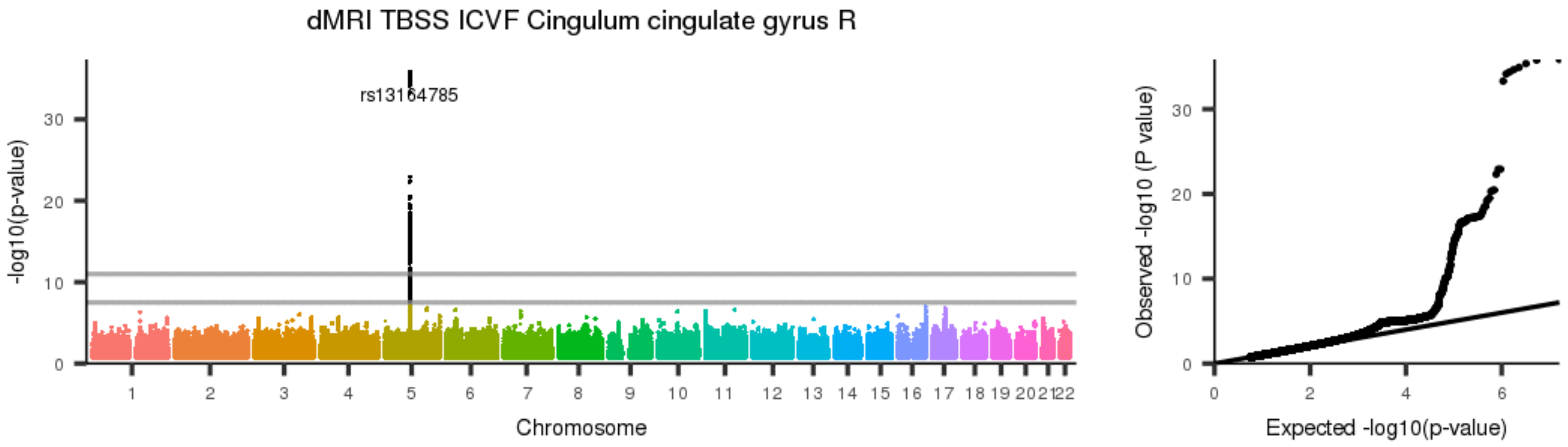

back to list of plots

Figure 519: dMRI TBSS ICVF Cingulum cingulate gyrus L

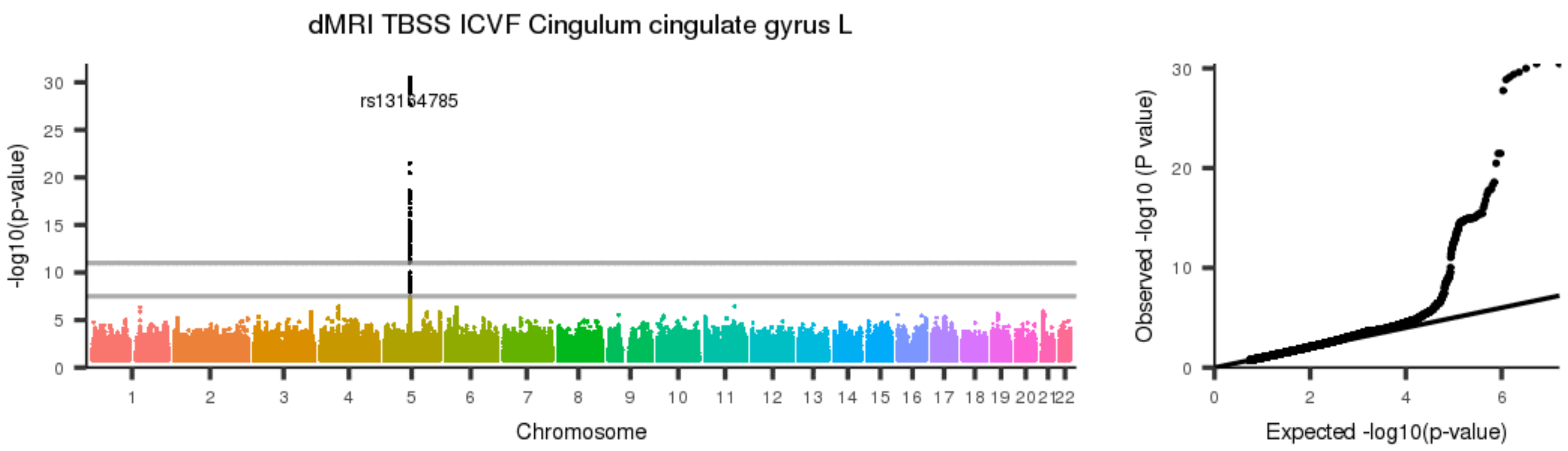

back to list of plots

Figure 520: dMRI TBSS ICVF Cingulum hippocampus R

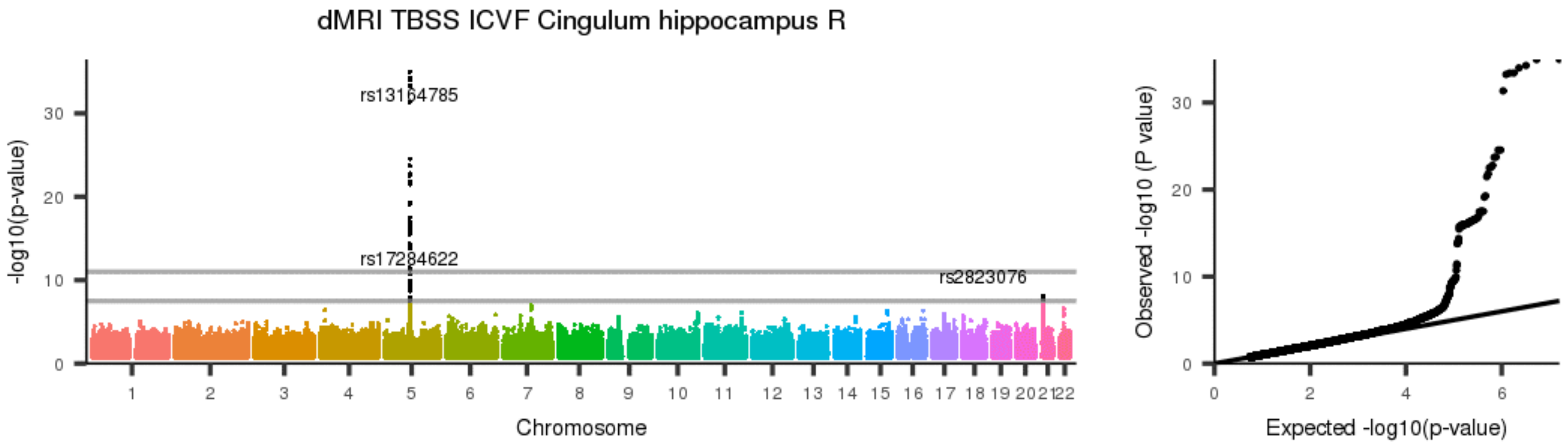

back to list of plots

Figure 521: dMRI TBSS ICVF Cingulum hippocampus L

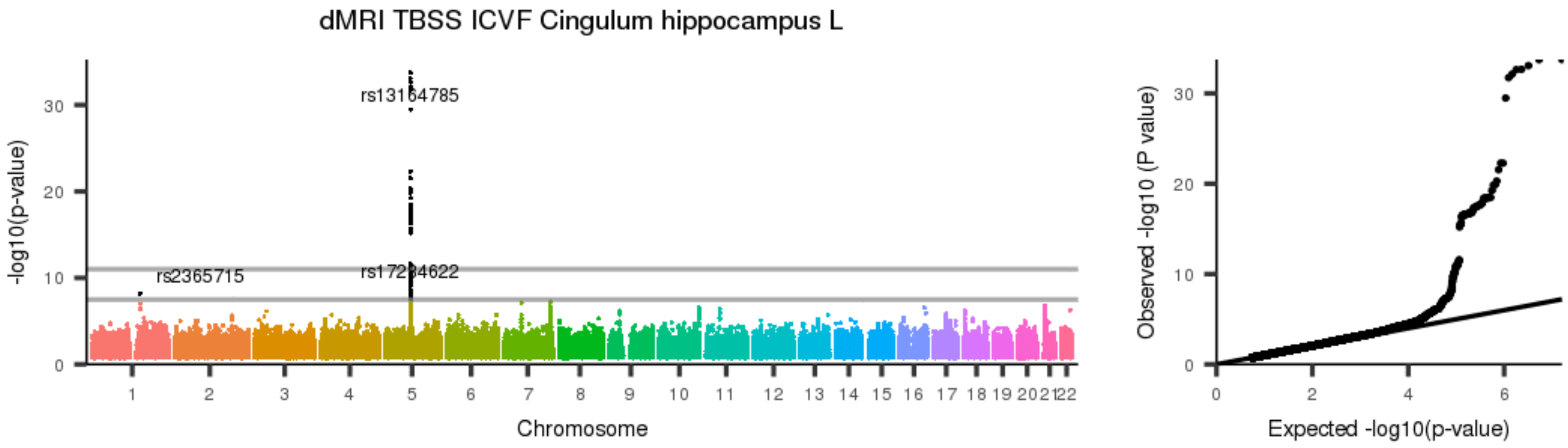

back to list of plots

Figure 522: dMRI TBSS ICVF Fornix cres+Stria terminalis R

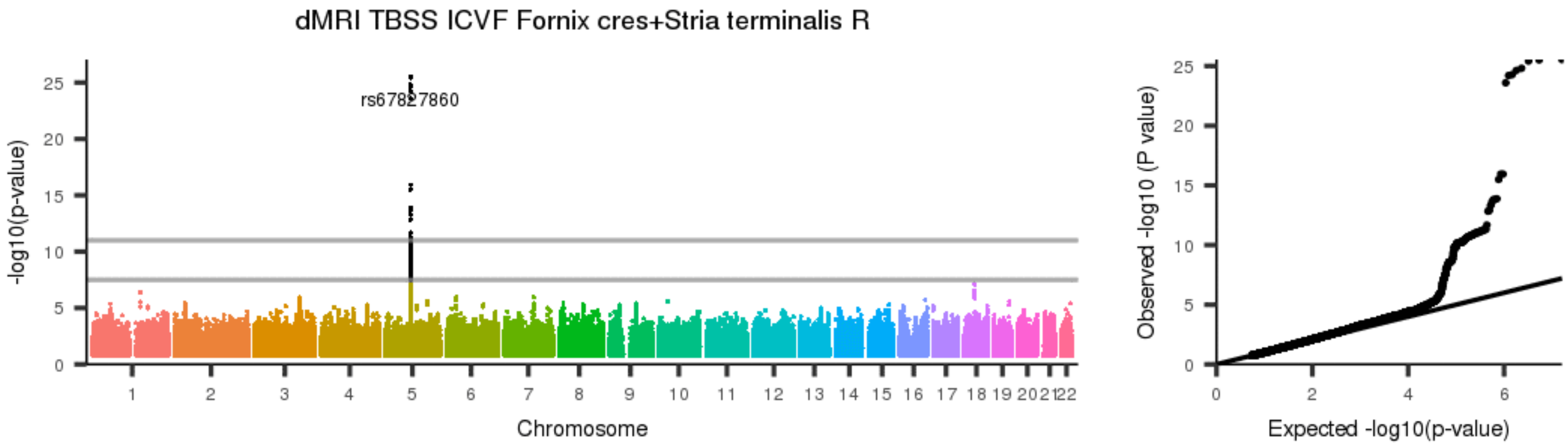

back to list of plots

Figure 523: dMRI TBSS ICVF Fornix cres+Stria terminalis L

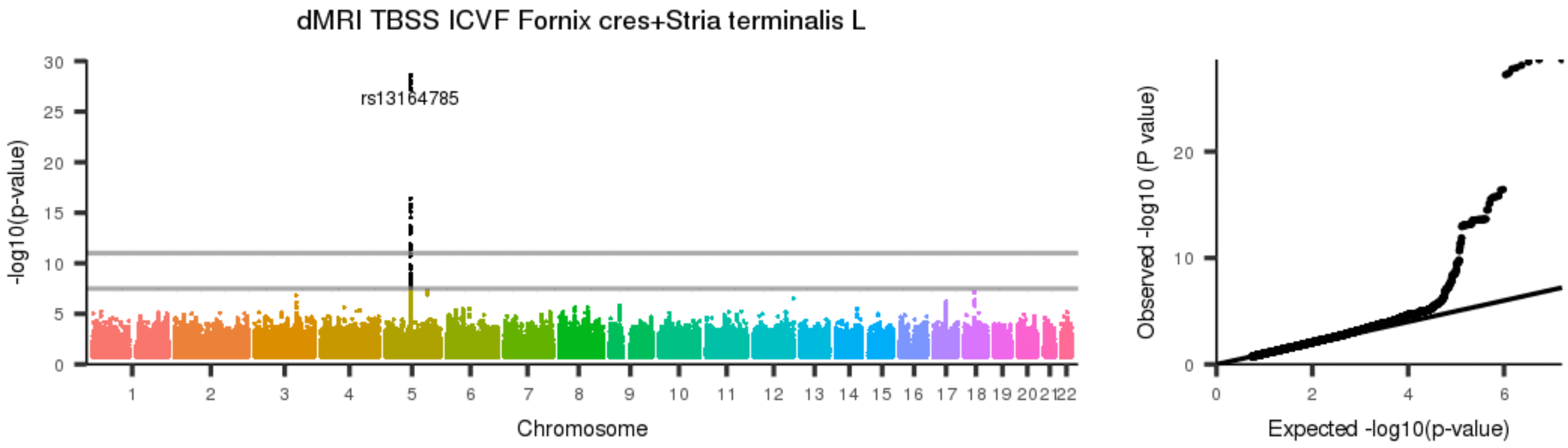

back to list of plots

Figure 524: dMRI TBSS ICVF Superior longitudinal fasciculus R

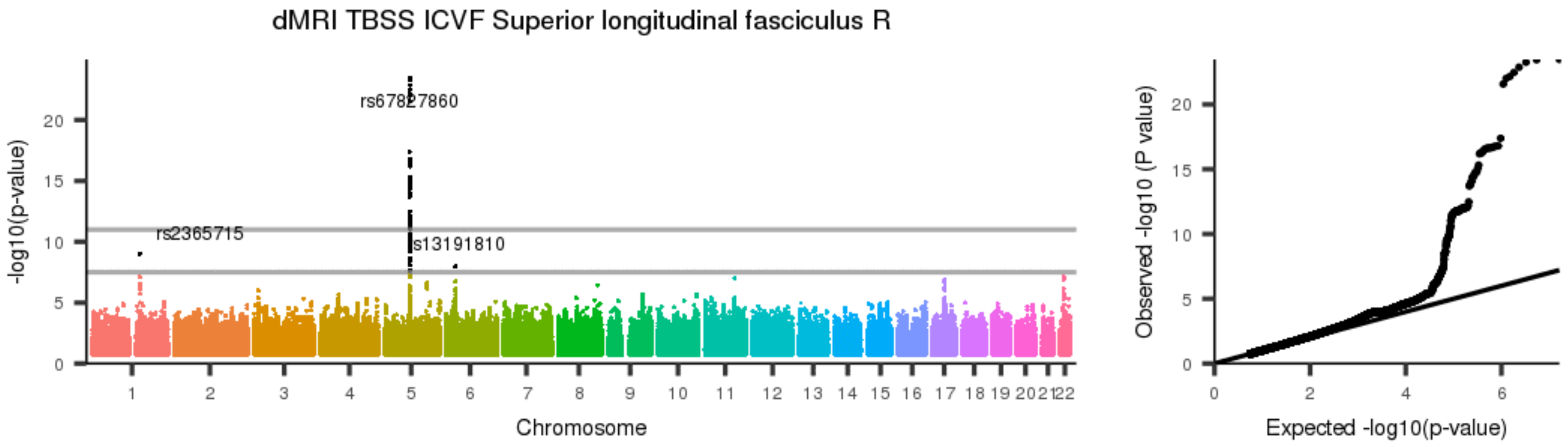

back to list of plots

Figure 525: dMRI TBSS ICVF Superior longitudinal fasciculus L

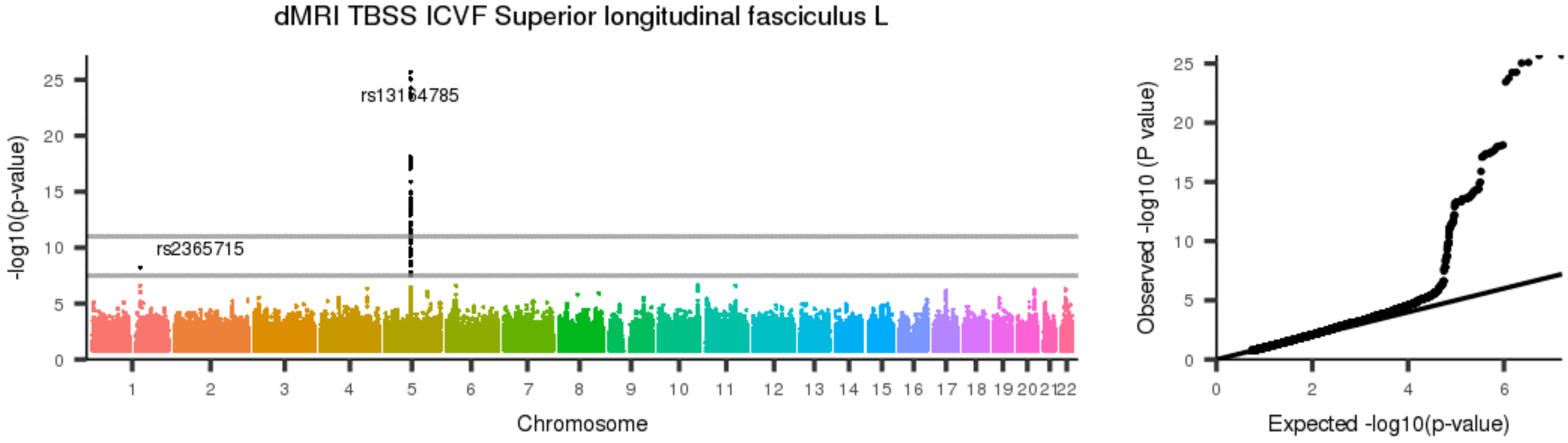

back to list of plots

Figure 526: dMRI TBSS ICVF Superior fronto-occipital fasciculus R

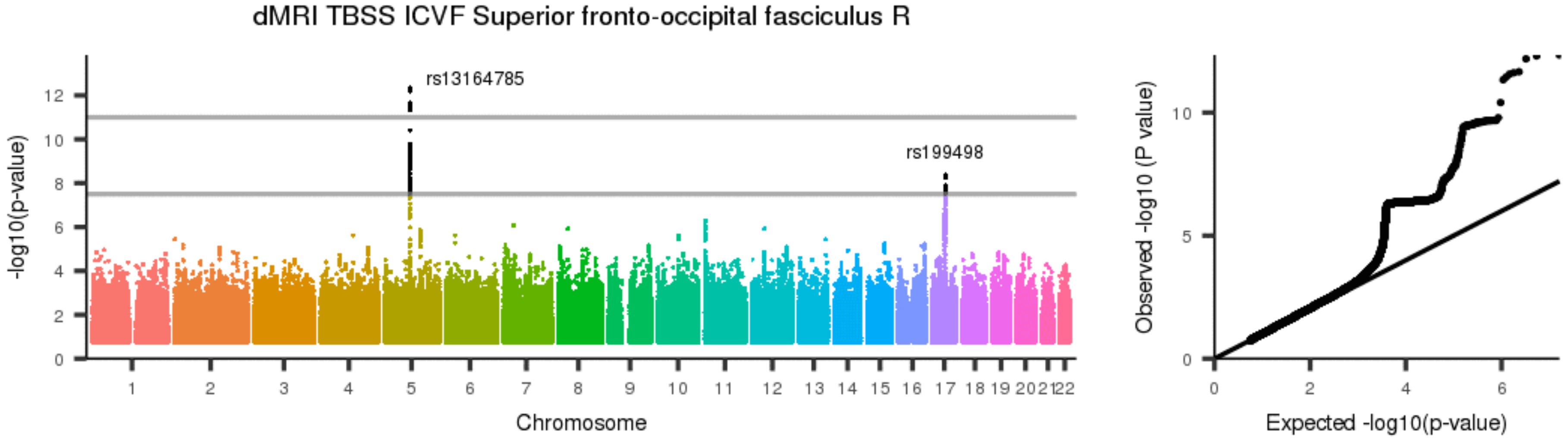

back to list of plots

Figure 527: dMRI TBSS ICVF Superior fronto-occipital fasciculus L

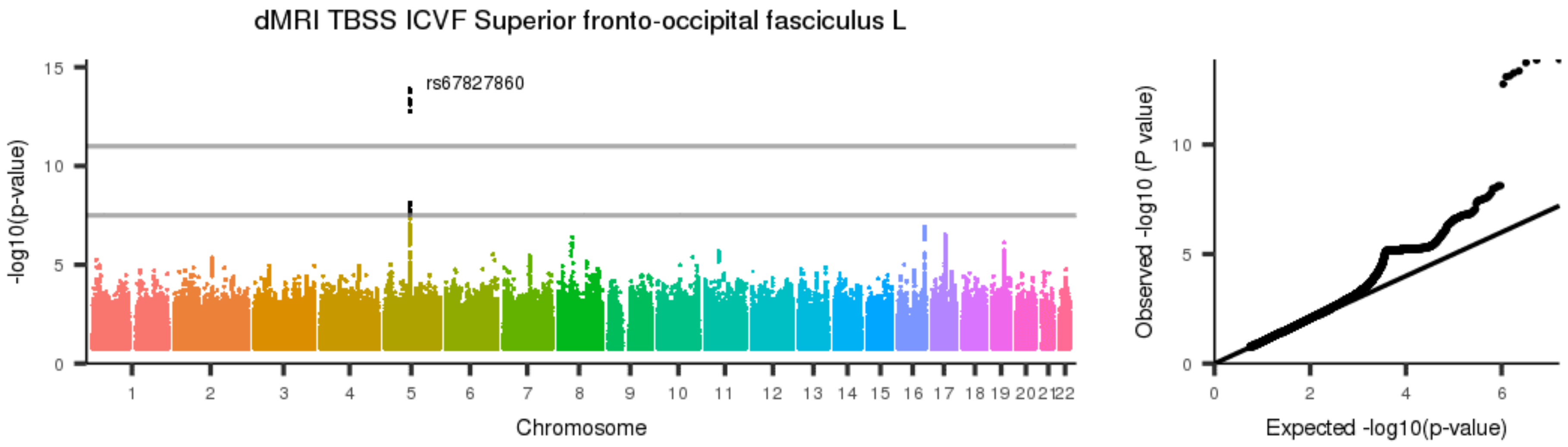

back to list of plots

Figure 528: dMRI TBSS ICVF Uncinate fasciculus R

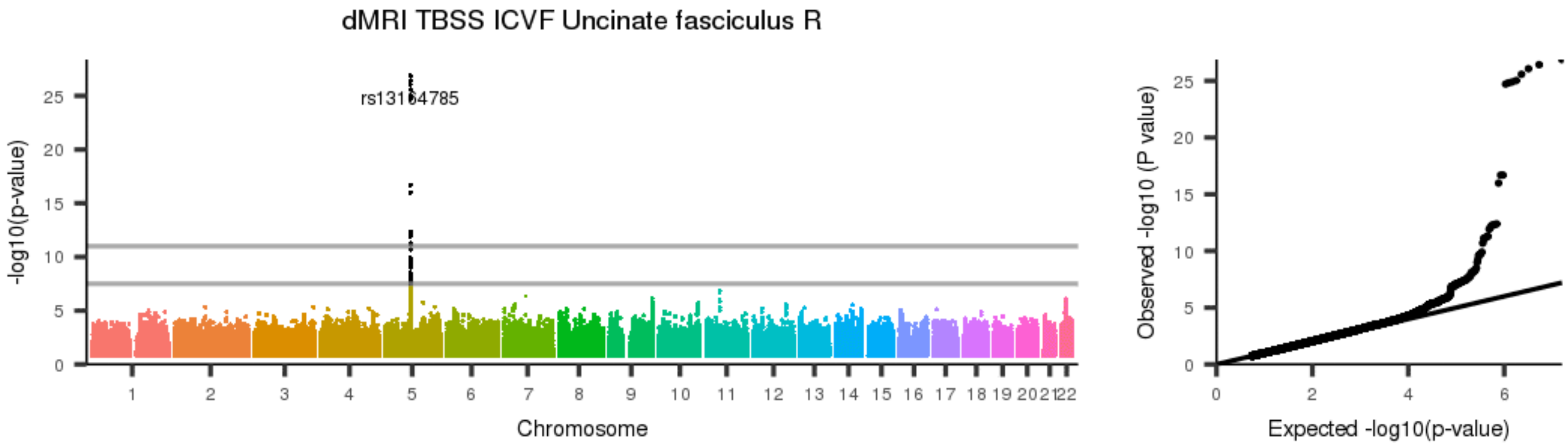

back to list of plots

Figure 529: dMRI TBSS ICVF Uncinate fasciculus L

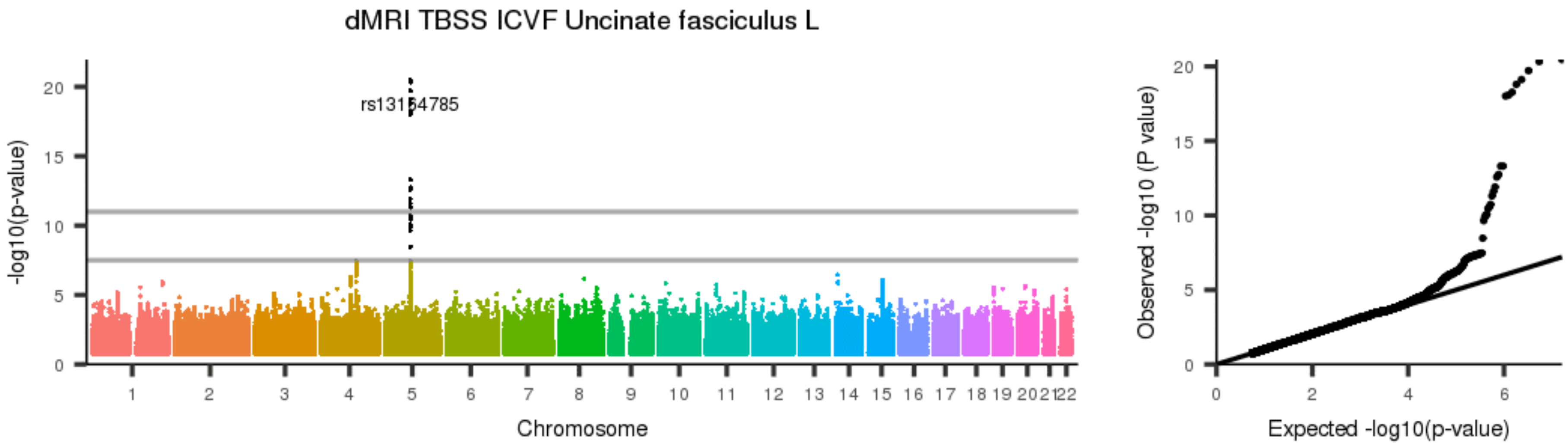

back to list of plots

Figure 530: dMRI TBSS ICVF Tapetum R

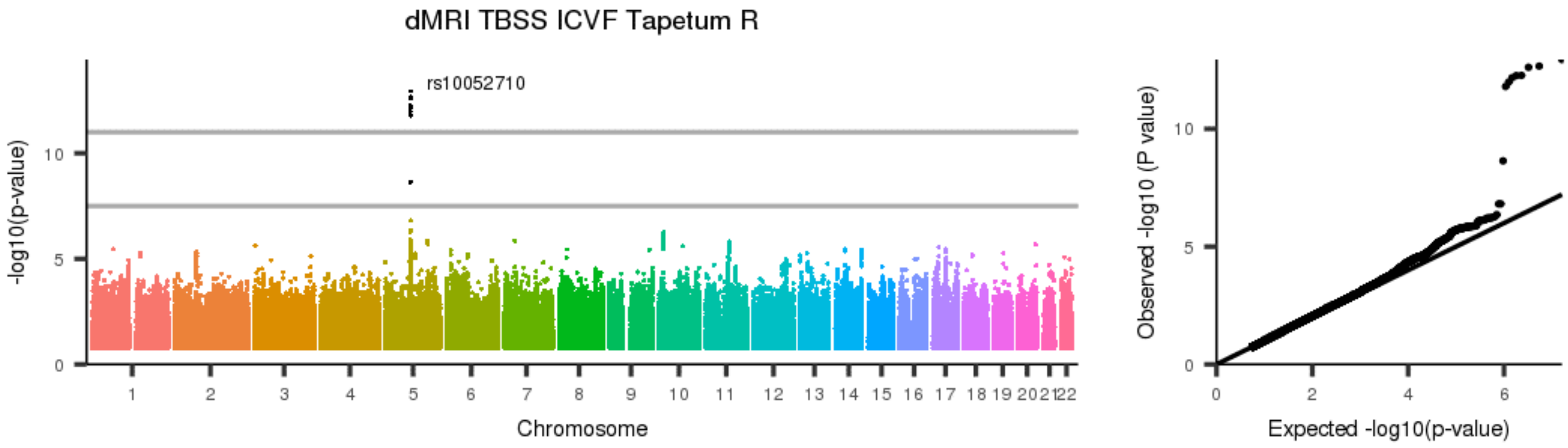

back to list of plots

Figure 531: dMRI TBSS ICVF Tapetum L

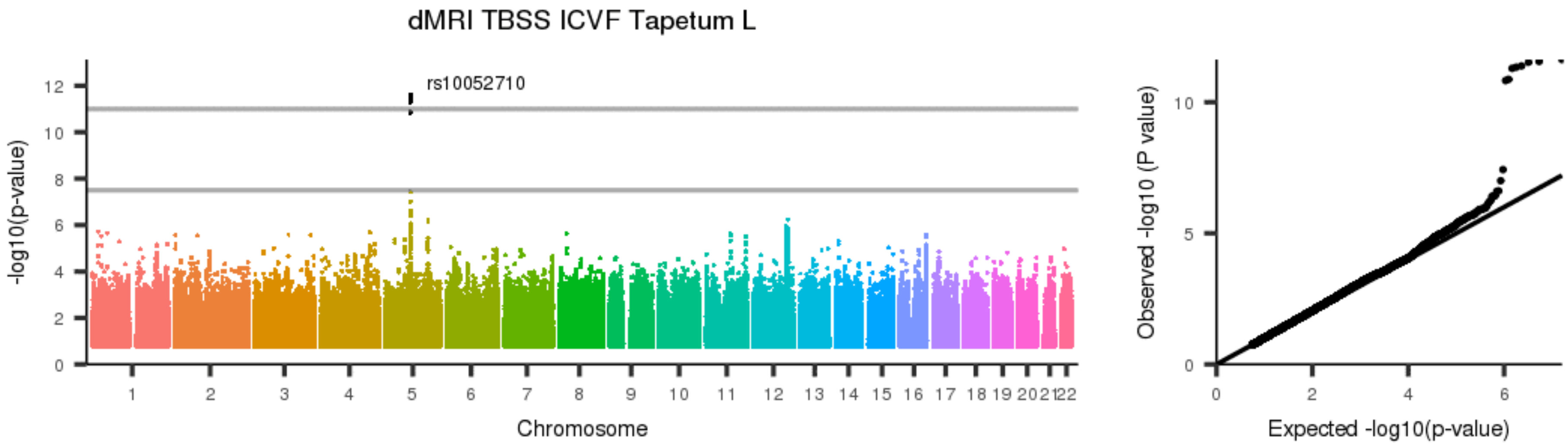

back to list of plots

Figure 532: dMRI TBSS OD Middle cerebellar peduncle

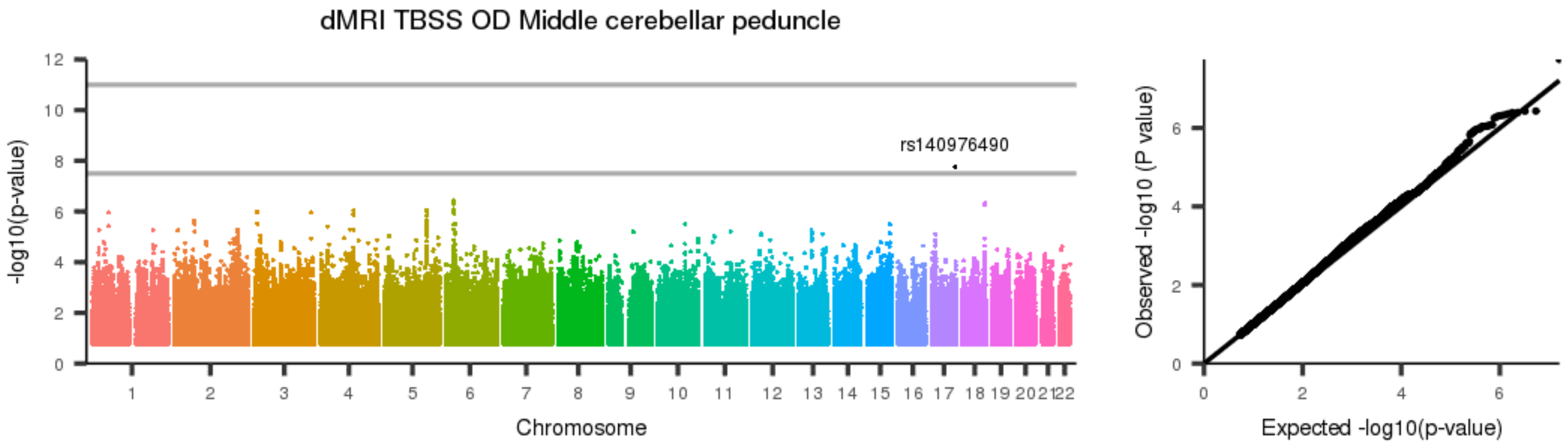

back to list of plots

Figure 533: dMRI TBSS OD Pontine crossing tract

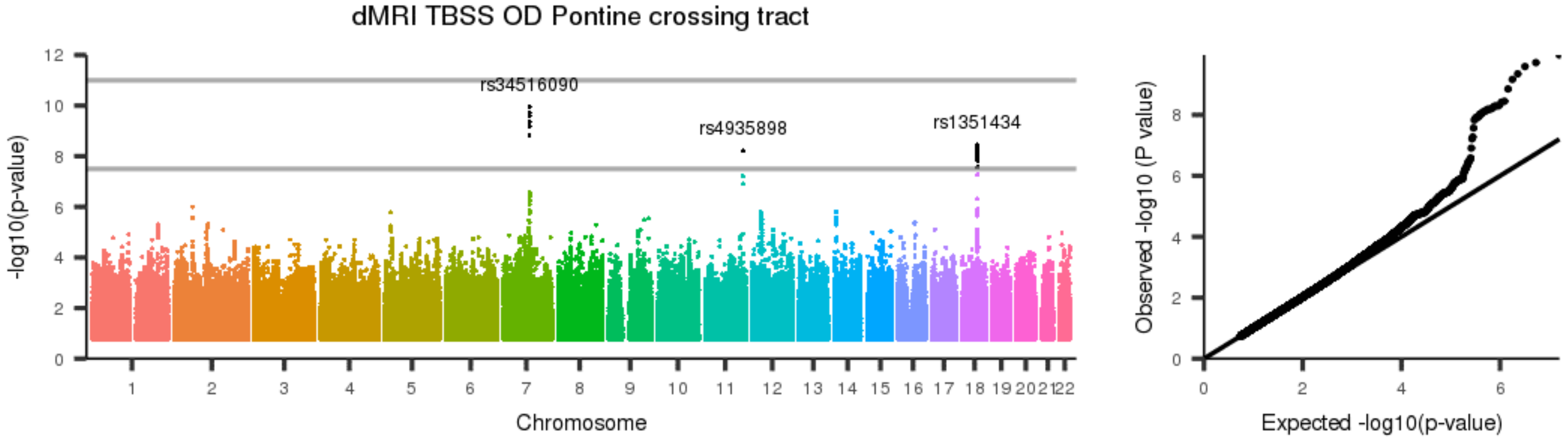

back to list of plots

Figure 534: dMRI TBSS OD Genu of corpus callosum

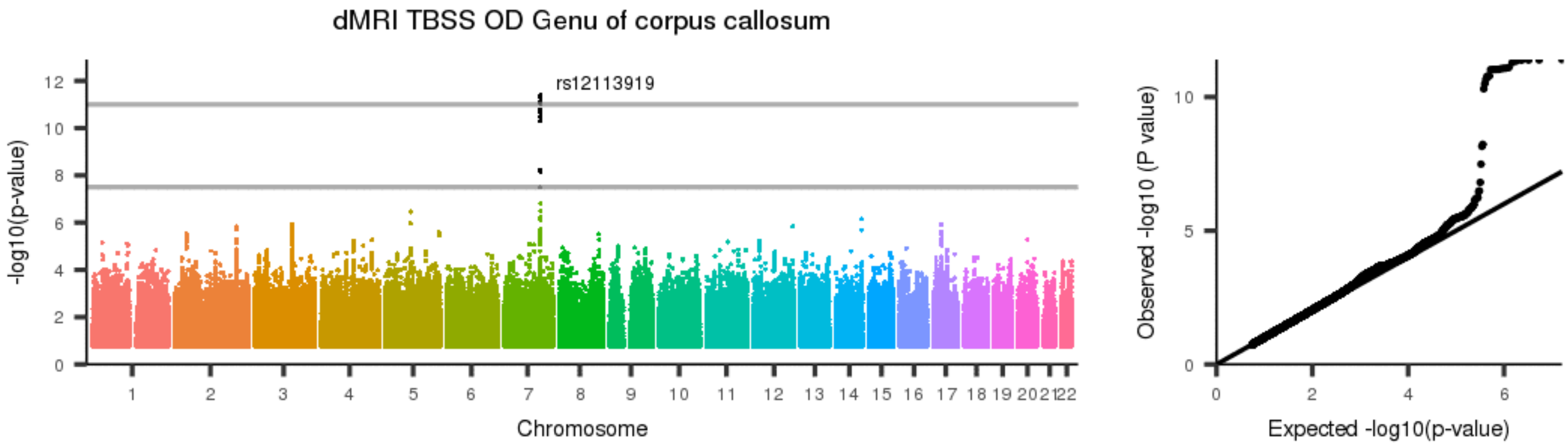

back to list of plots

Figure 535: dMRI TBSS OD Body of corpus callosum

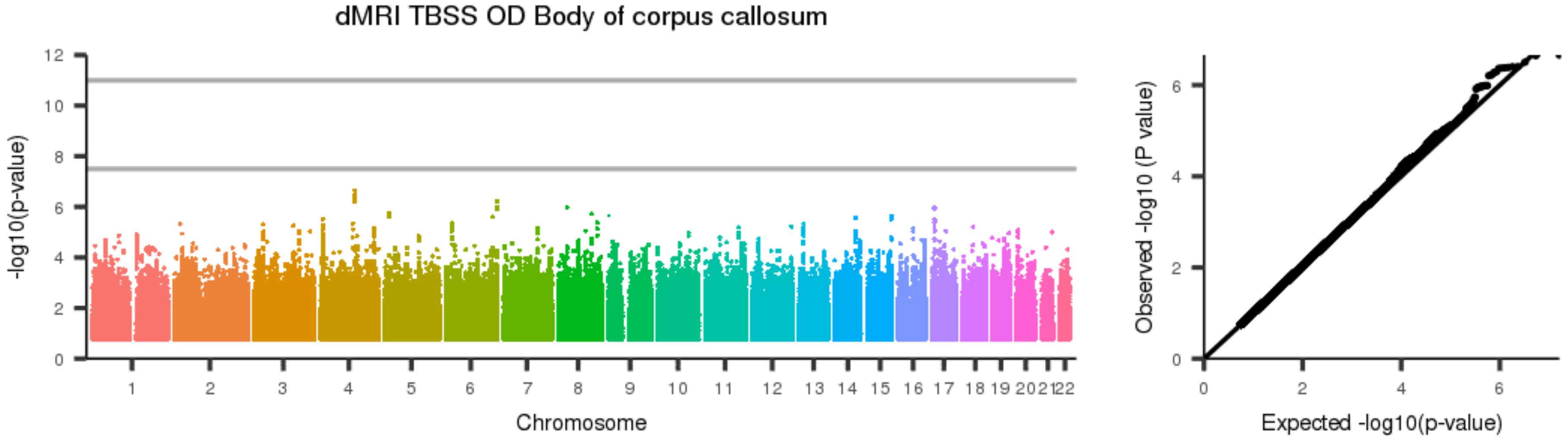

back to list of plots

Figure 536: dMRI TBSS OD Splenium of corpus callosum

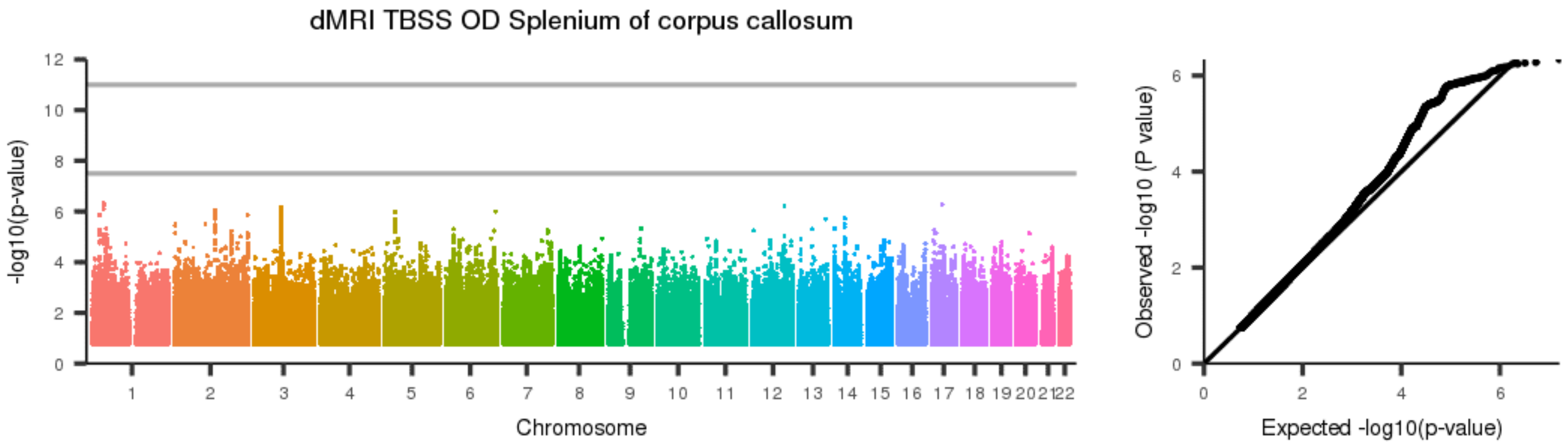

back to list of plots

Figure 537: dMRI TBSS OD Fornix

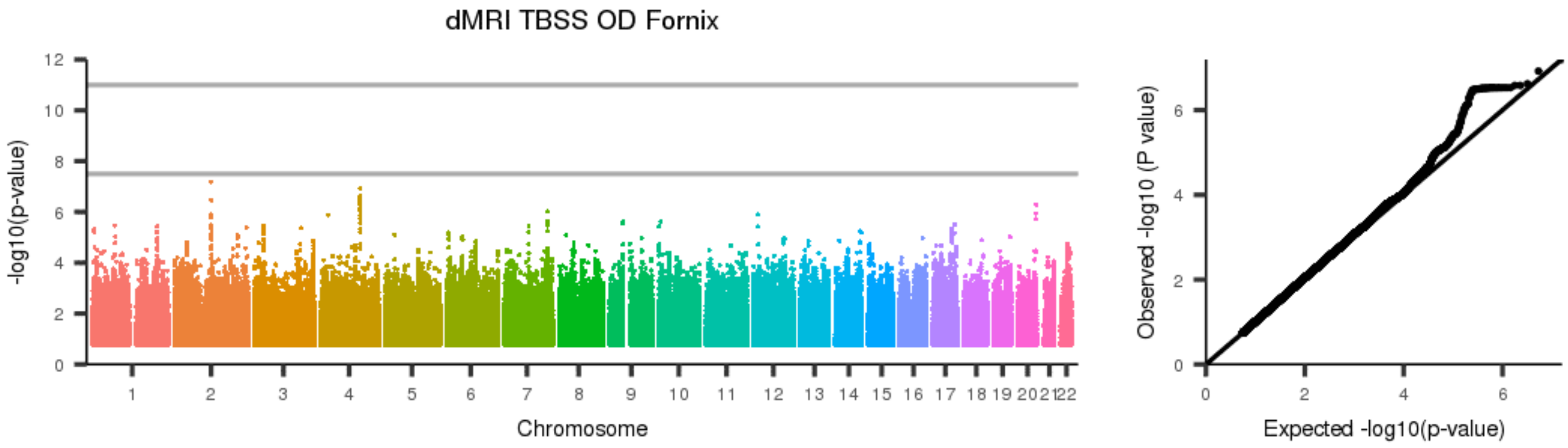

back to list of plots

Figure 538: dMRI TBSS OD Corticospinal tract R

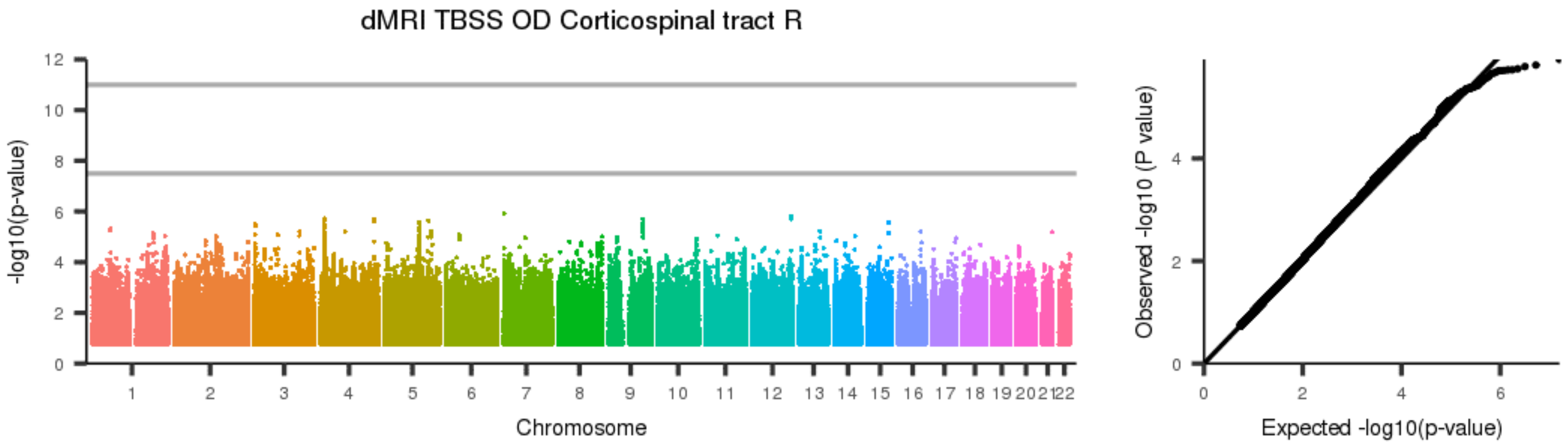

back to list of plots

Figure 539: dMRI TBSS OD Corticospinal tract L

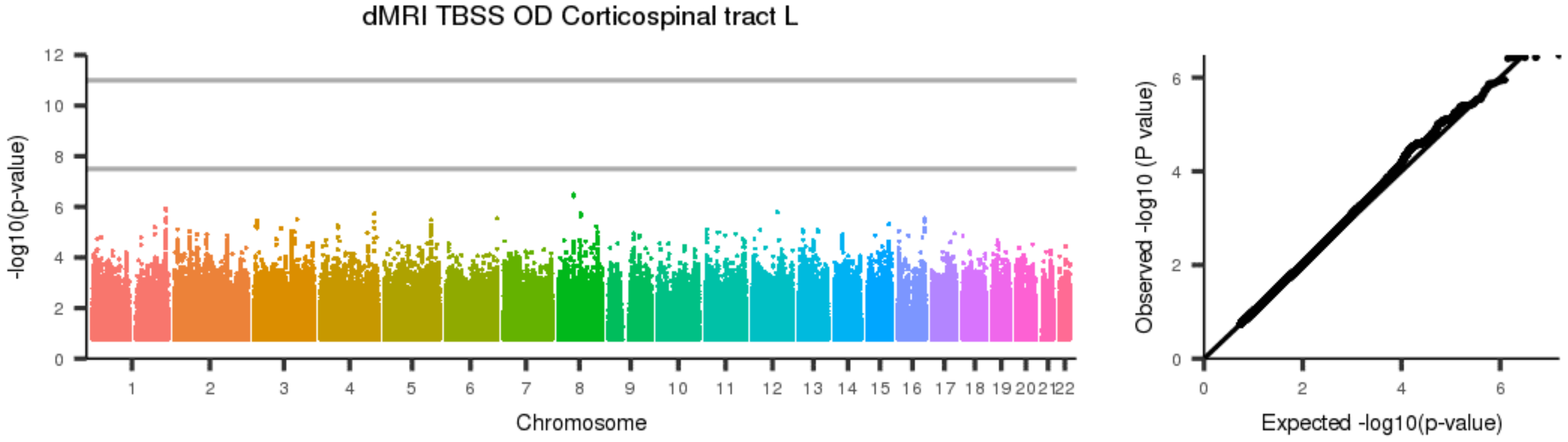

back to list of plots

Figure 540: dMRI TBSS OD Medial lemniscus R

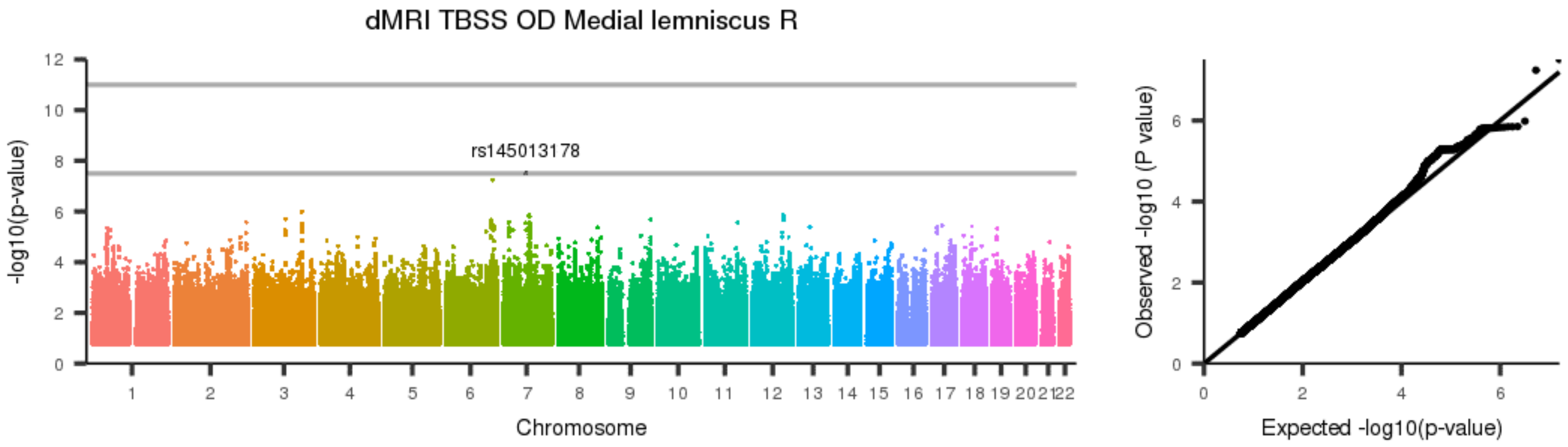

back to list of plots

Figure 541: dMRI TBSS OD Medial lemniscus L

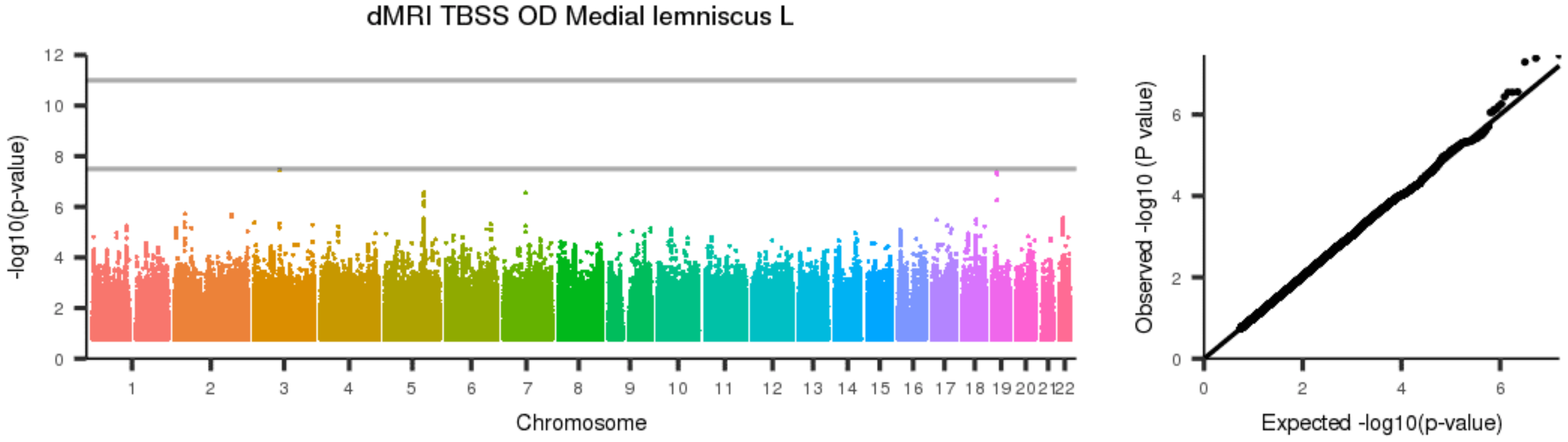

back to list of plots

Figure 542: dMRI TBSS OD Inferior cerebellar peduncle R

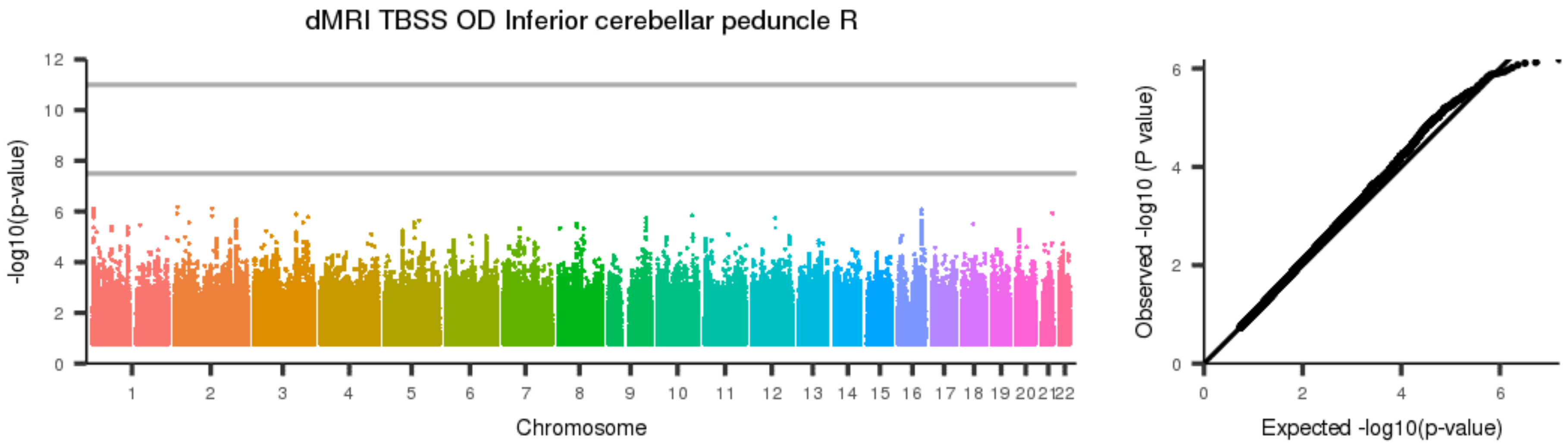

back to list of plots

Figure 543: dMRI TBSS OD Inferior cerebellar peduncle L

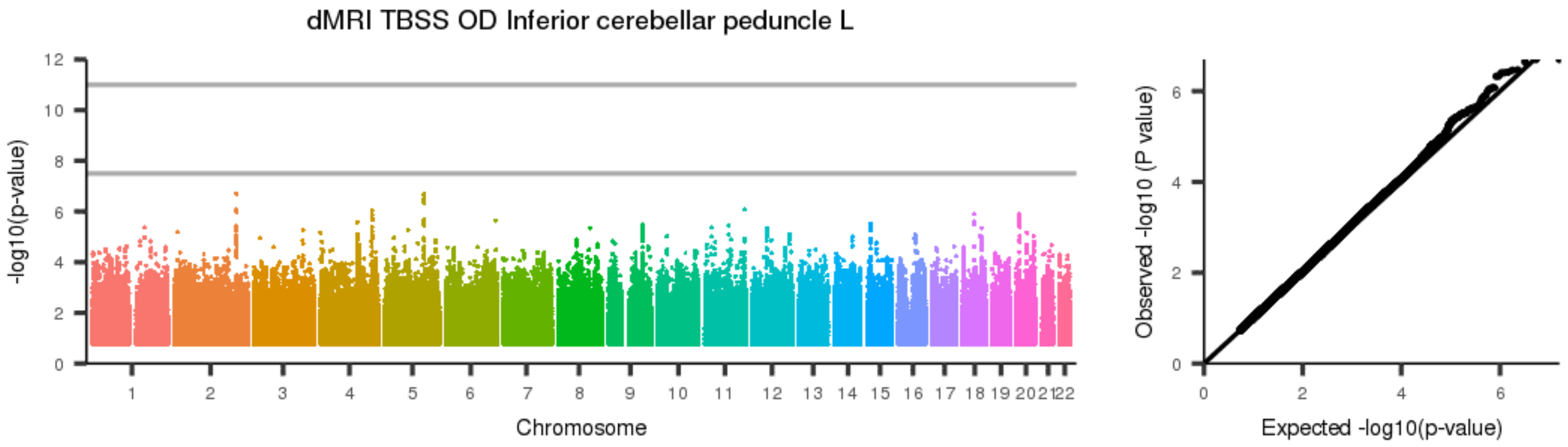

back to list of plots

Figure 544: dMRI TBSS OD Superior cerebellar peduncle R

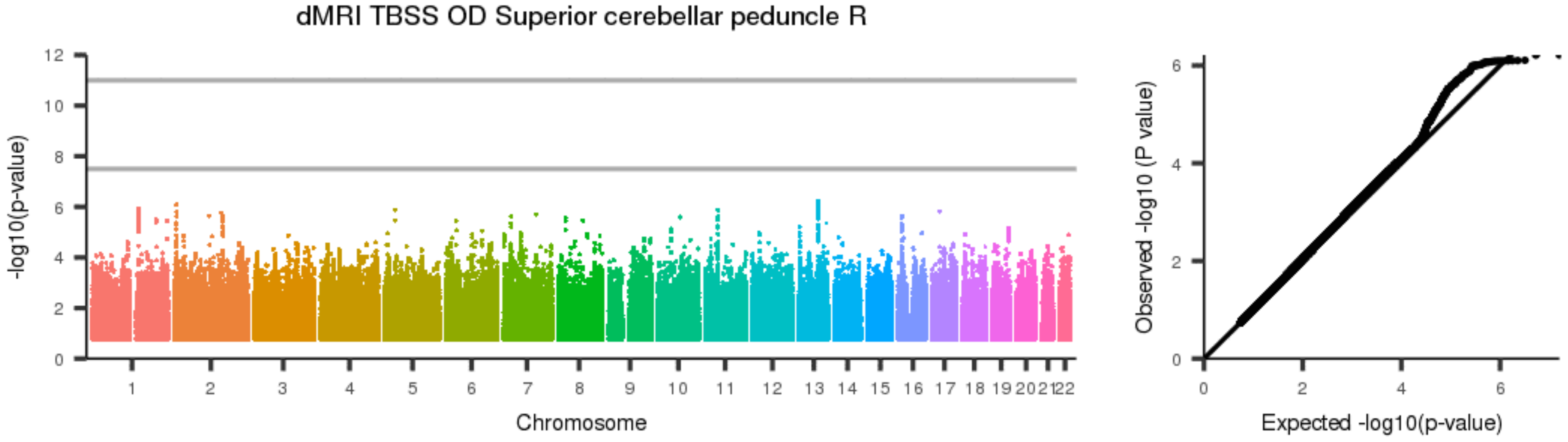

back to list of plots

Figure 545: dMRI TBSS OD Superior cerebellar peduncle L

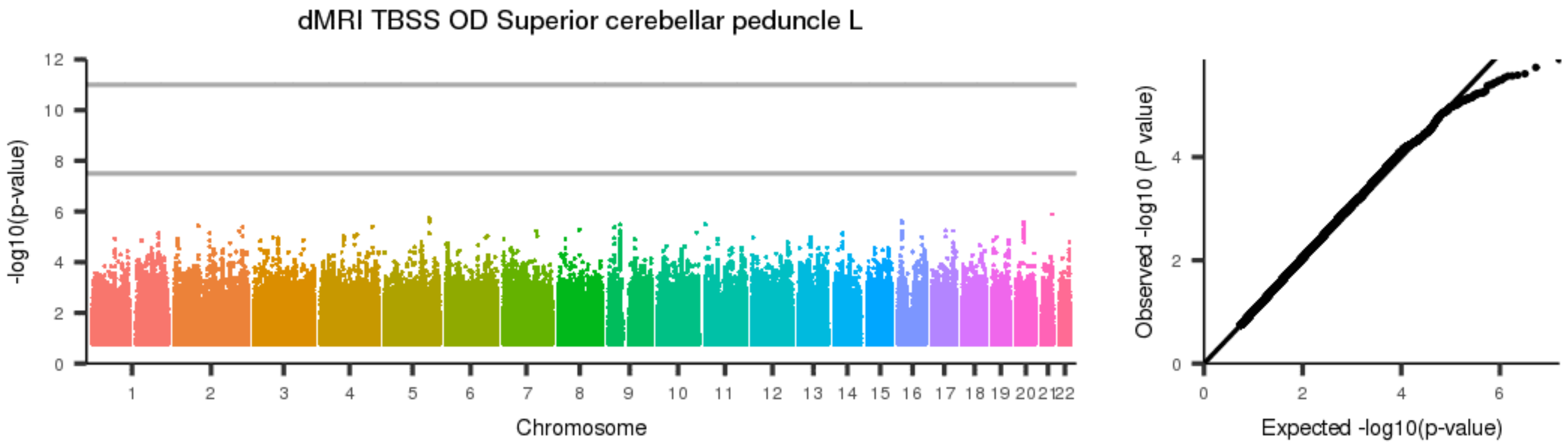

back to list of plots

Figure 546: dMRI TBSS OD Cerebral peduncle R

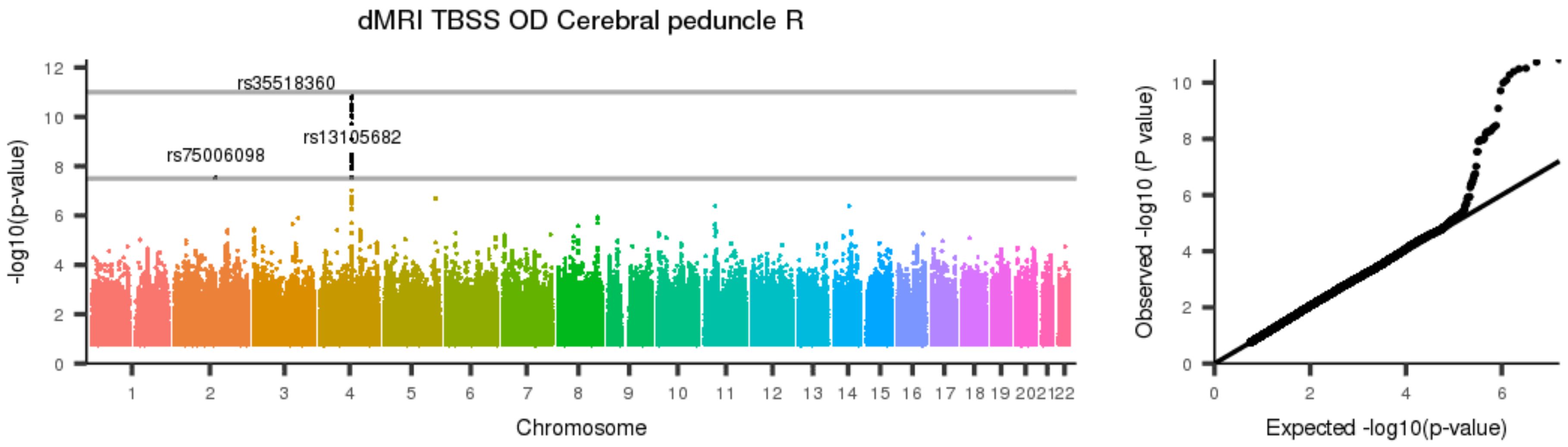

back to list of plots

Figure 547: dMRI TBSS OD Cerebral peduncle L

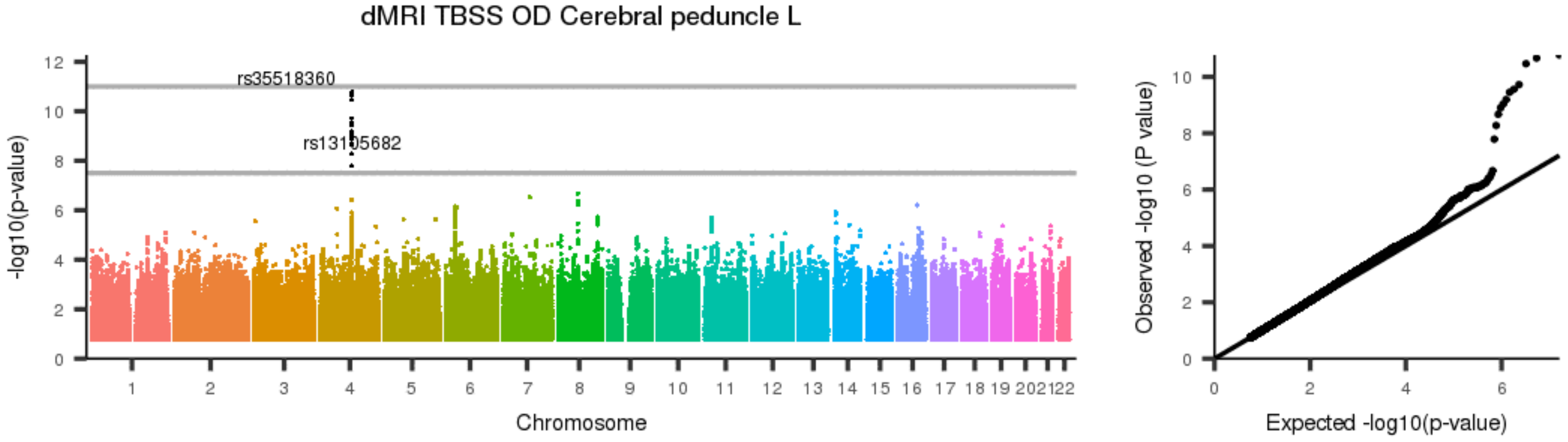

back to list of plots

Figure 548: dMRI TBSS OD Anterior limb of internal capsule R

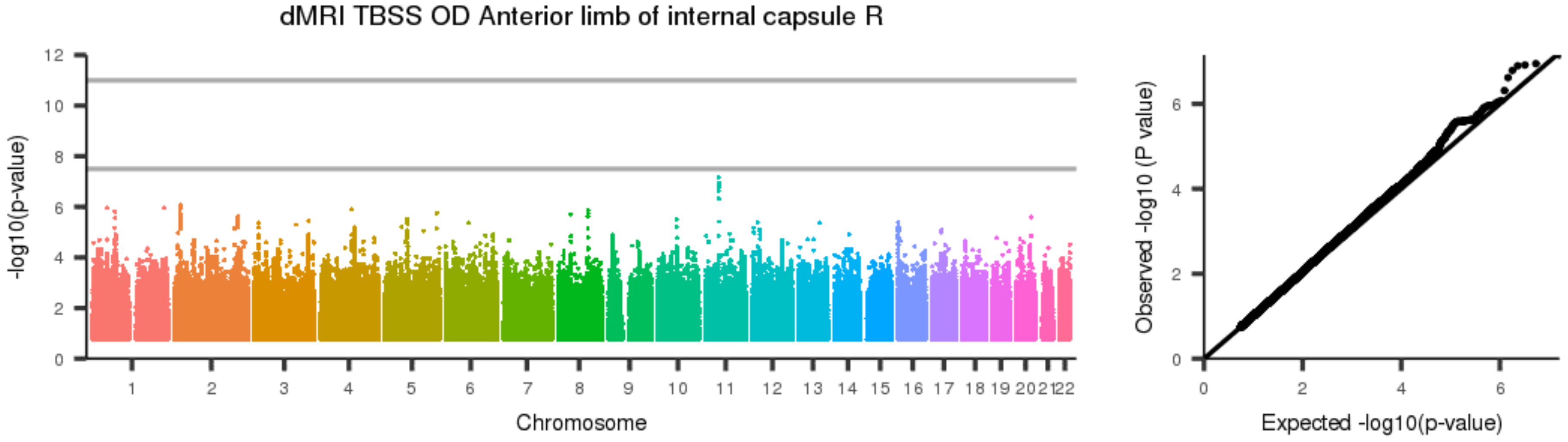

back to list of plots

Figure 549: dMRI TBSS OD Anterior limb of internal capsule L

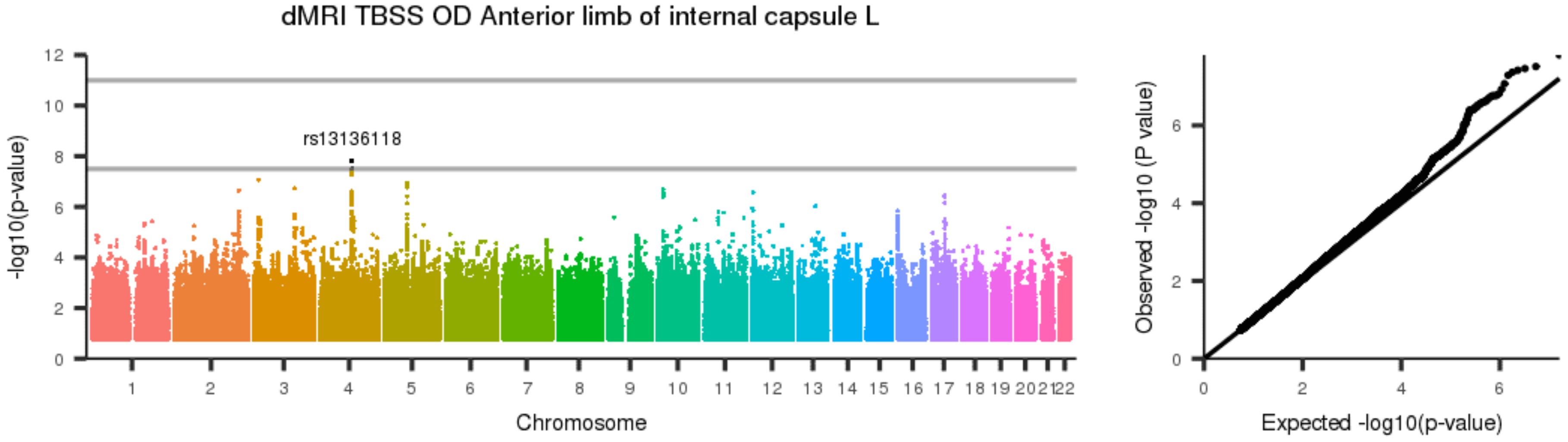

back to list of plots

Figure 550: dMRI TBSS OD Posterior limb of internal capsule R

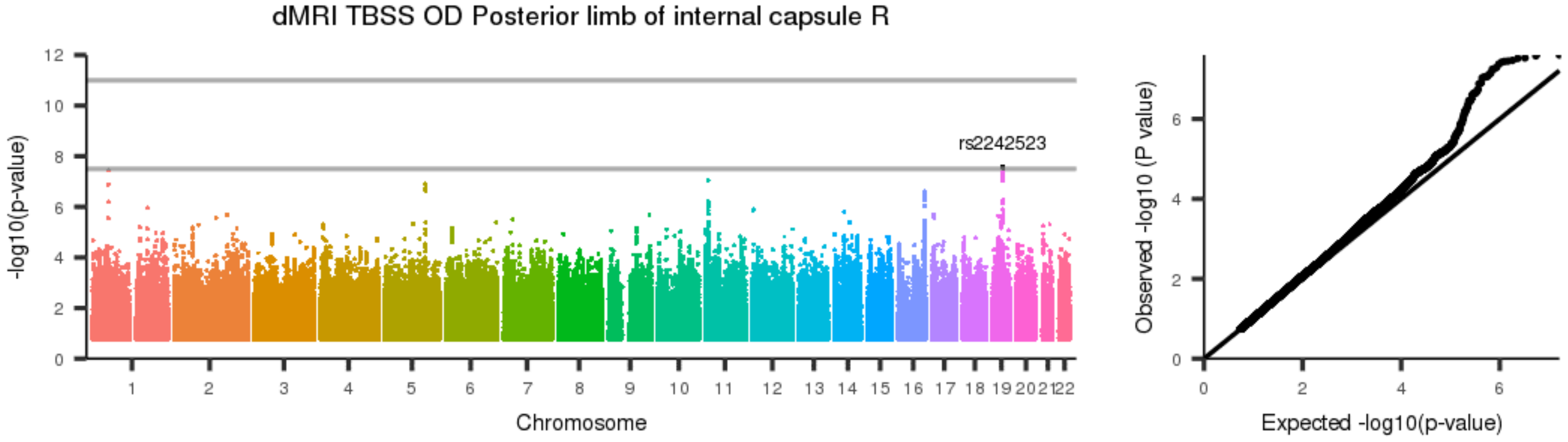

back to list of plots

Figure 551: dMRI TBSS OD Posterior limb of internal capsule L

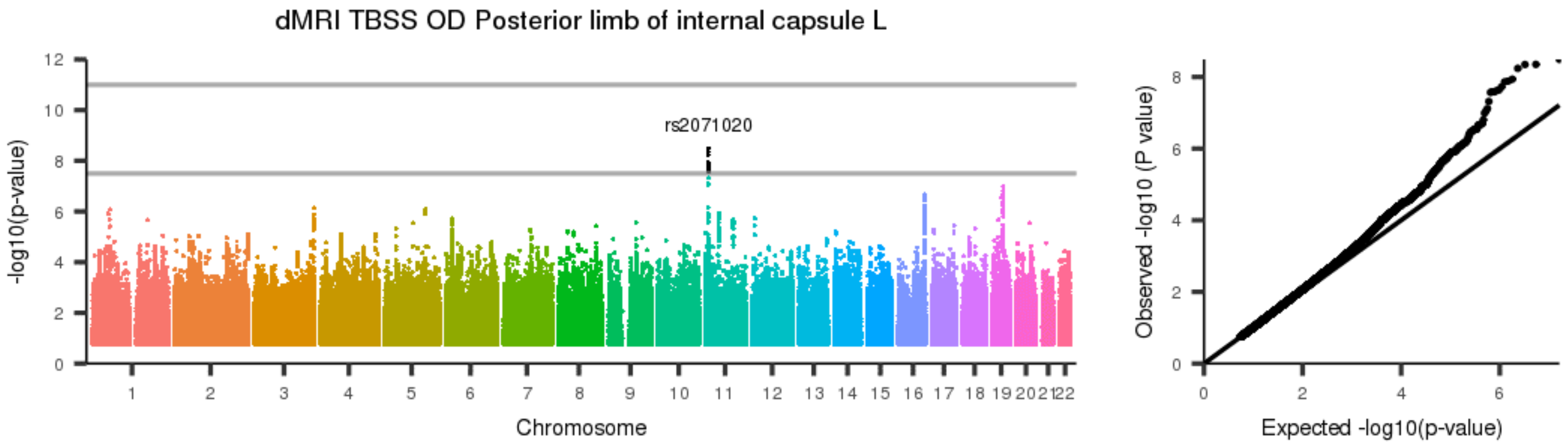

back to list of plots

Figure 552: dMRI TBSS OD Retrolenticular part of internal capsule R

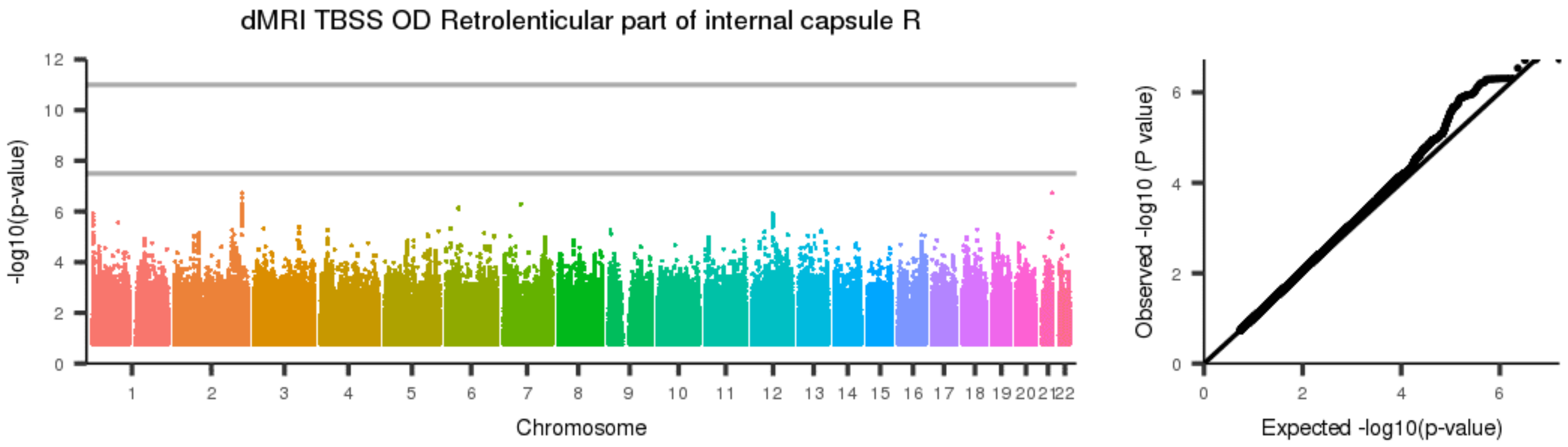

back to list of plots

Figure 553: dMRI TBSS OD Retrolenticular part of internal capsule L

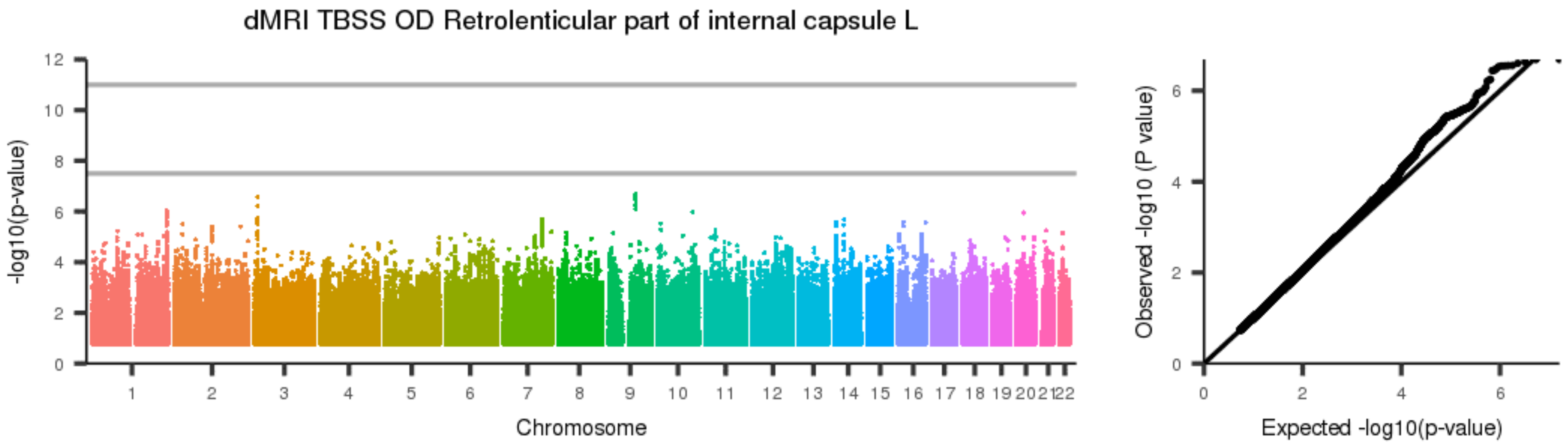

back to list of plots

Figure 554: dMRI TBSS OD Anterior corona radiata R

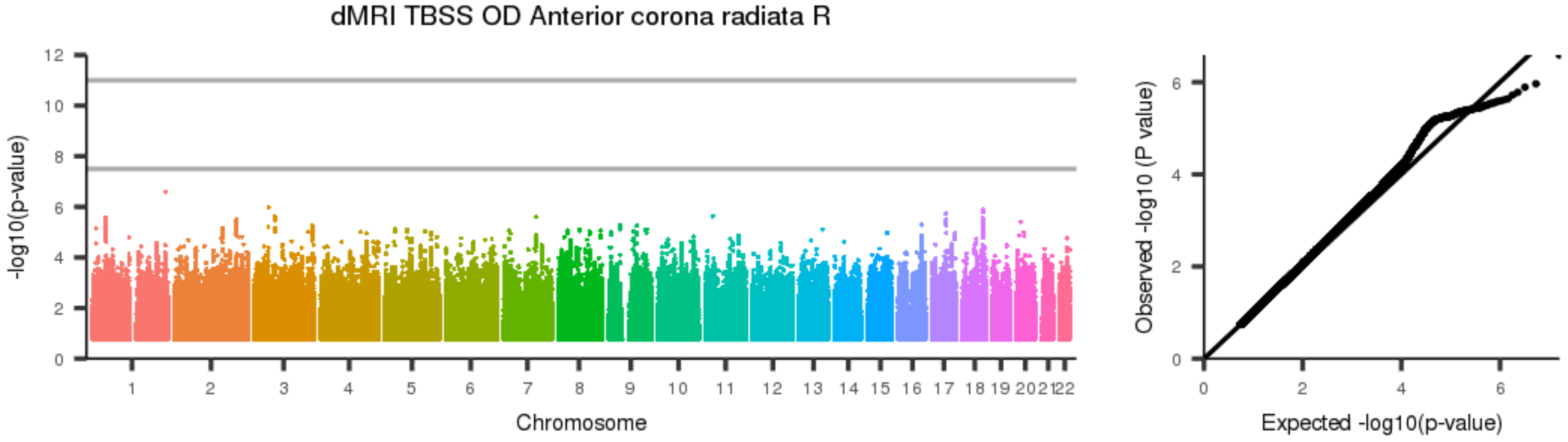

back to list of plots

Figure 555: dMRI TBSS OD Anterior corona radiata L

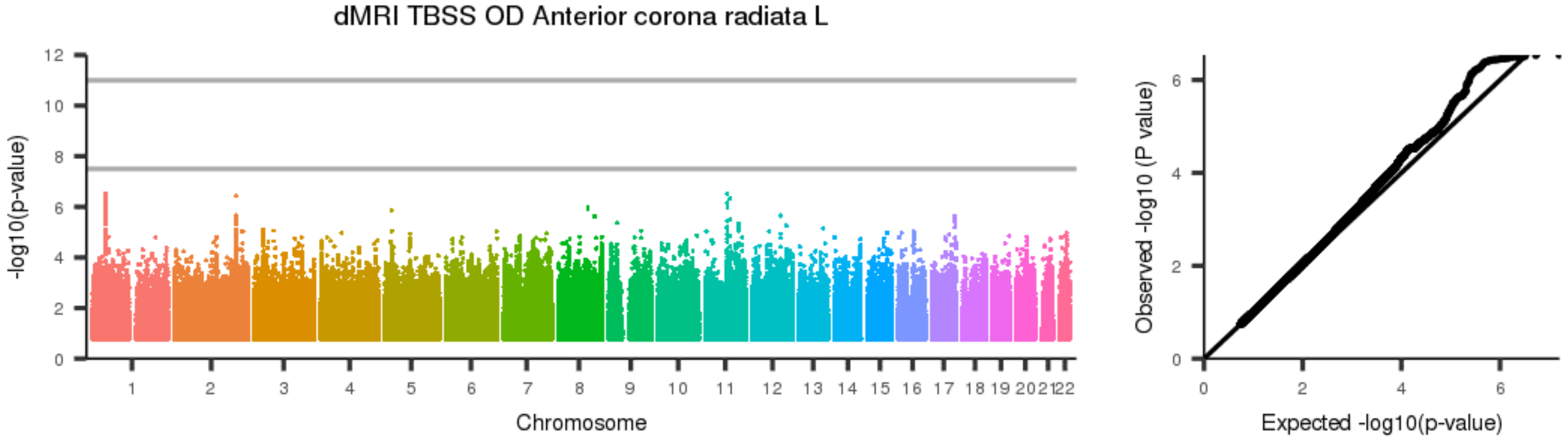

back to list of plots

Figure 556: dMRI TBSS OD Superior corona radiata R

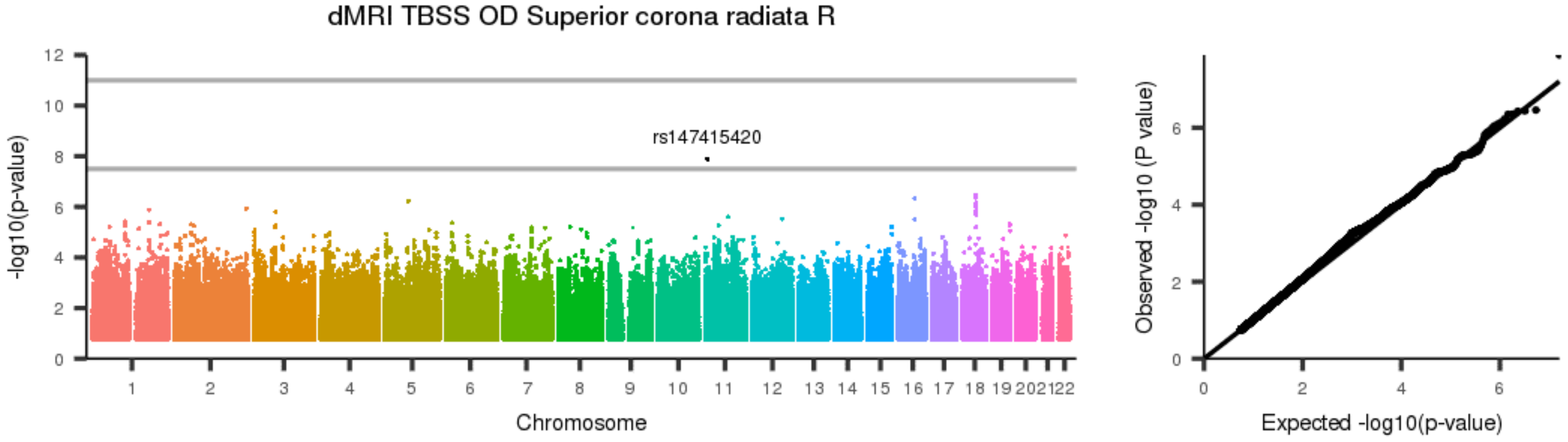

back to list of plots

Figure 557: dMRI TBSS OD Superior corona radiata L

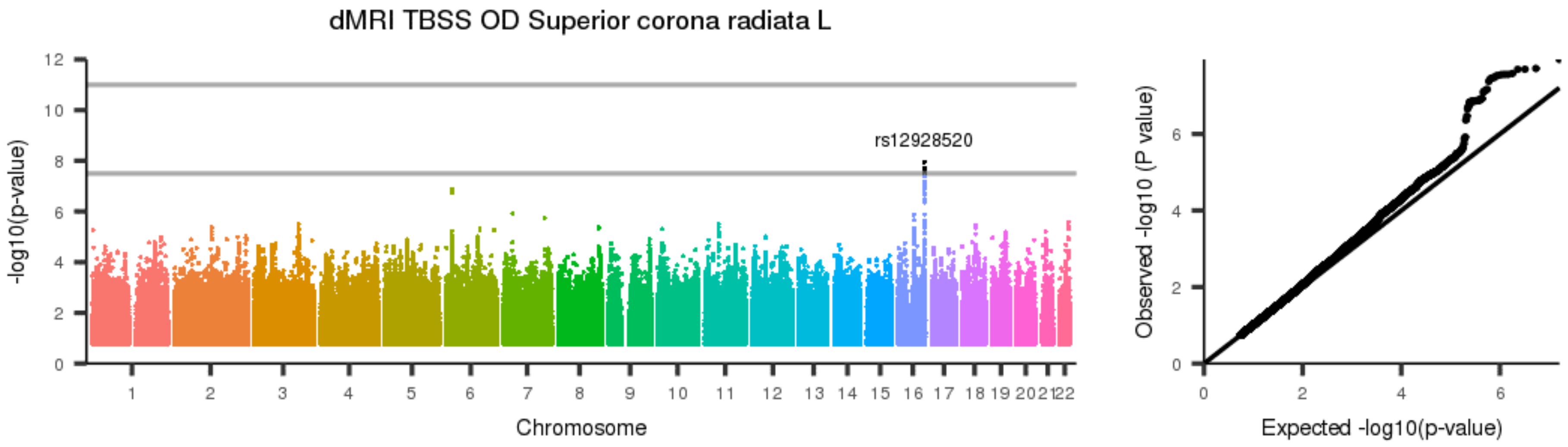

back to list of plots

Figure 558: dMRI TBSS OD Posterior corona radiata R

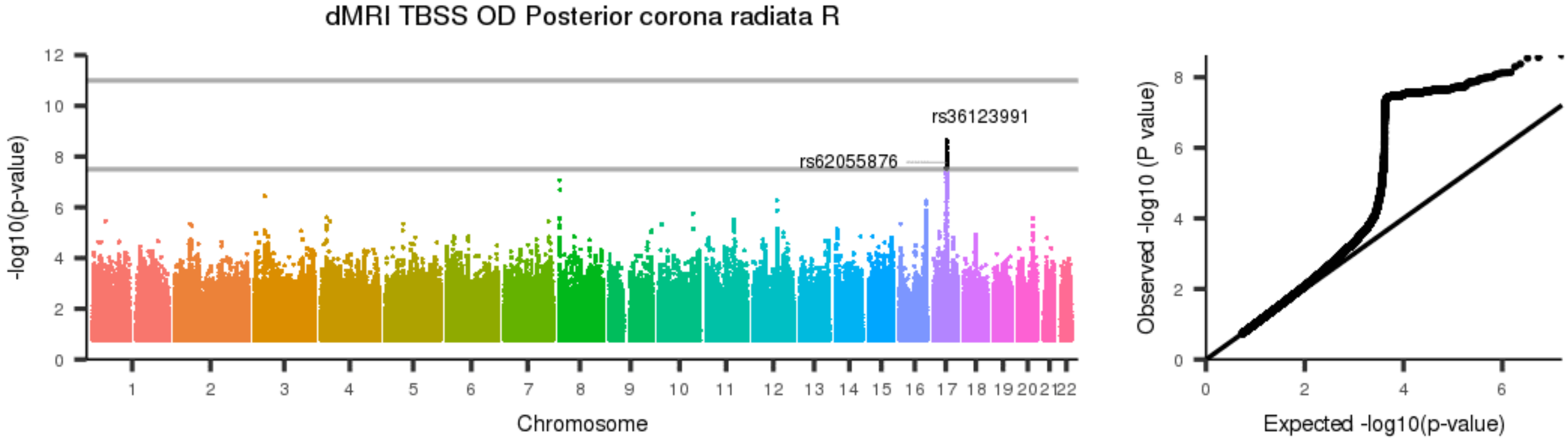

back to list of plots

Figure 559: dMRI TBSS OD Posterior corona radiata L

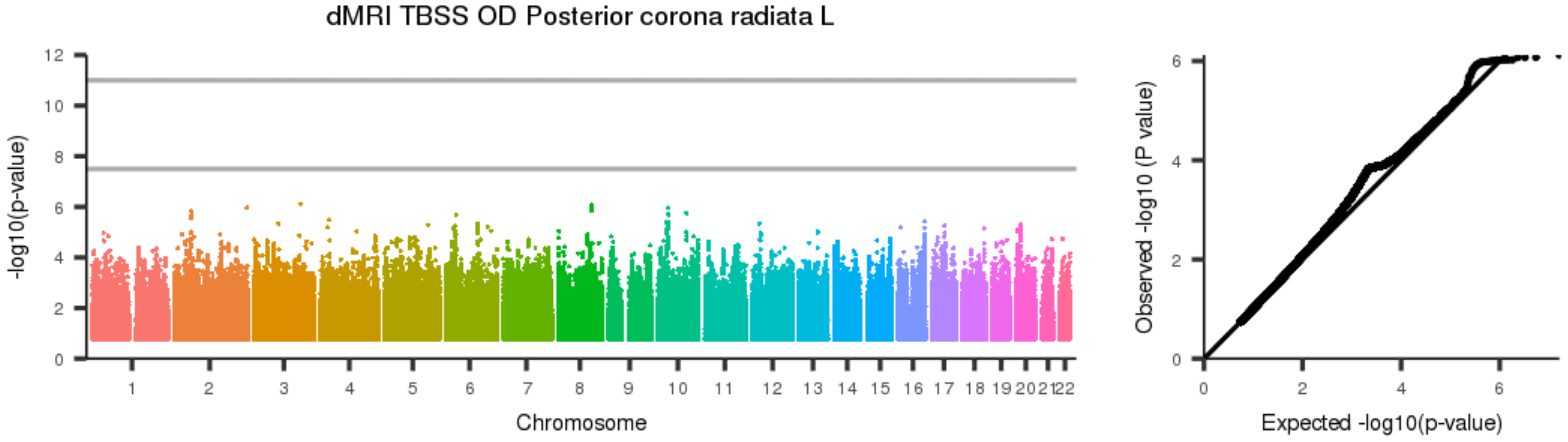

back to list of plots

Figure 560: dMRI TBSS OD Posterior thalamic radiation R

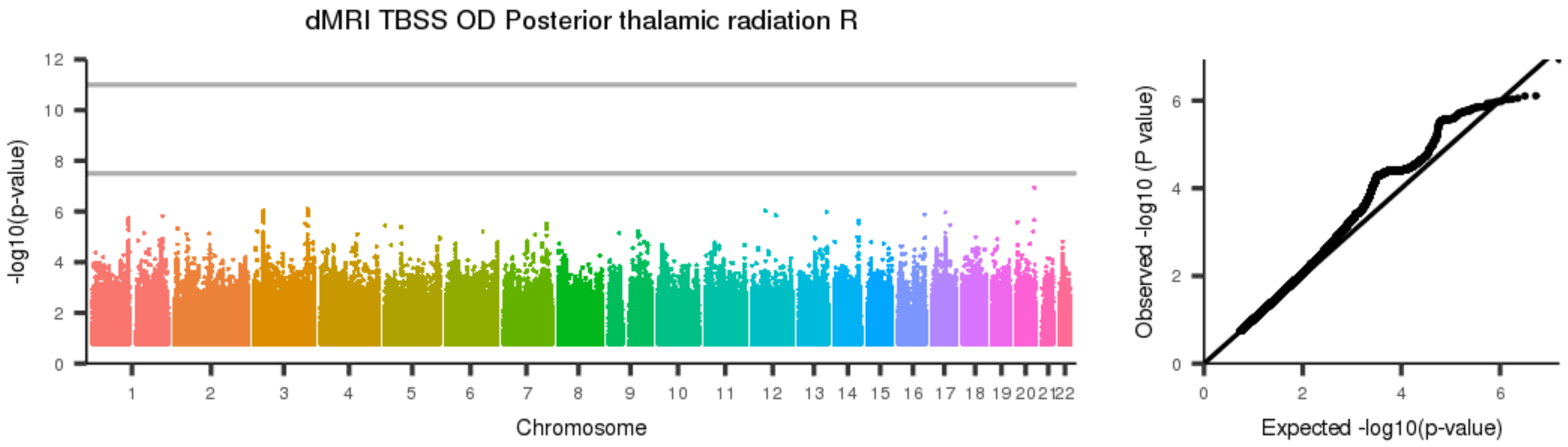

back to list of plots

Figure 561: dMRI TBSS OD Posterior thalamic radiation L

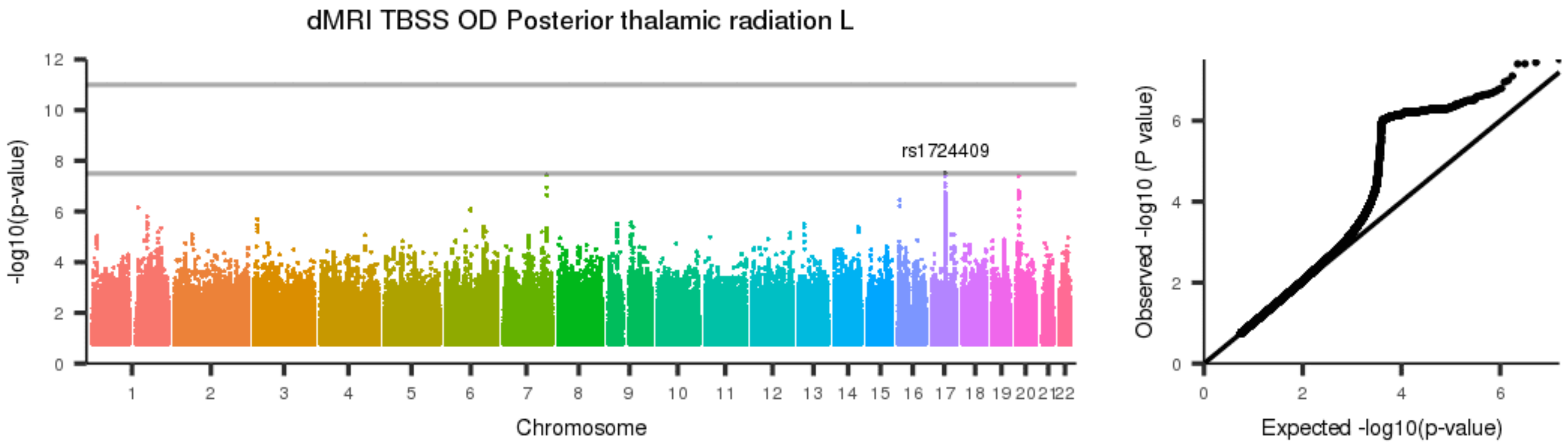

back to list of plots

Figure 562: dMRI TBSS OD Sagittal stratum R

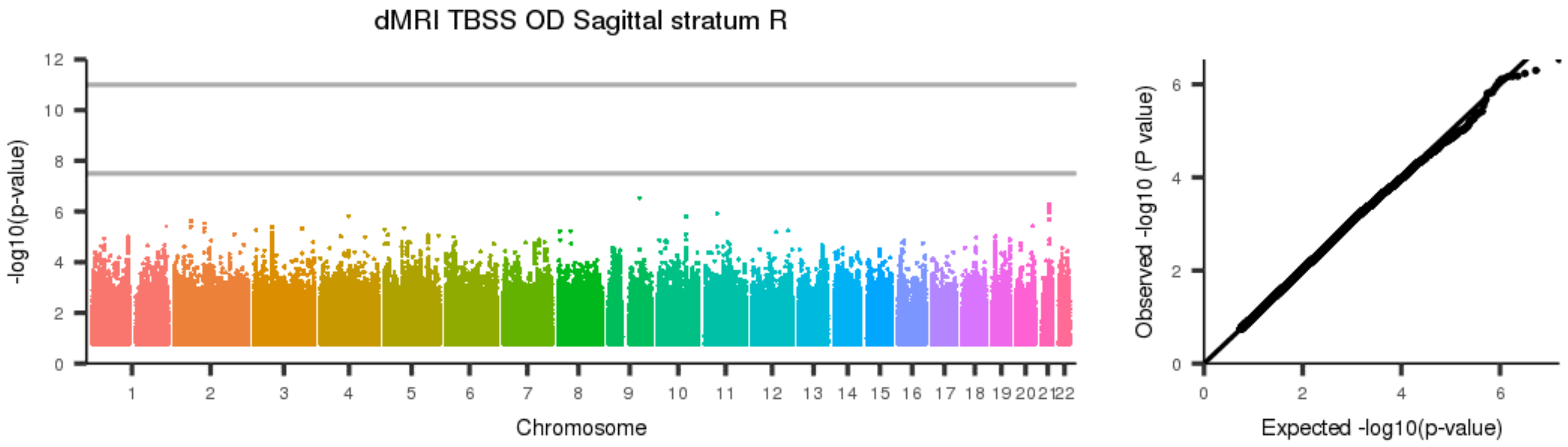

back to list of plots

Figure 563: dMRI TBSS OD Sagittal stratum L

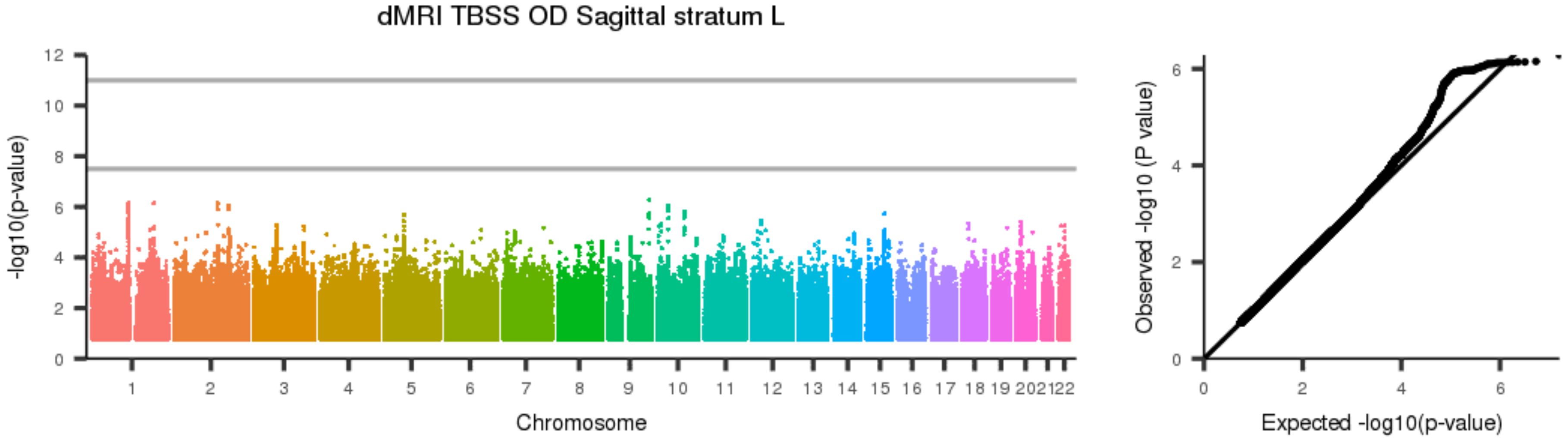

back to list of plots

Figure 564: dMRI TBSS OD External capsule R

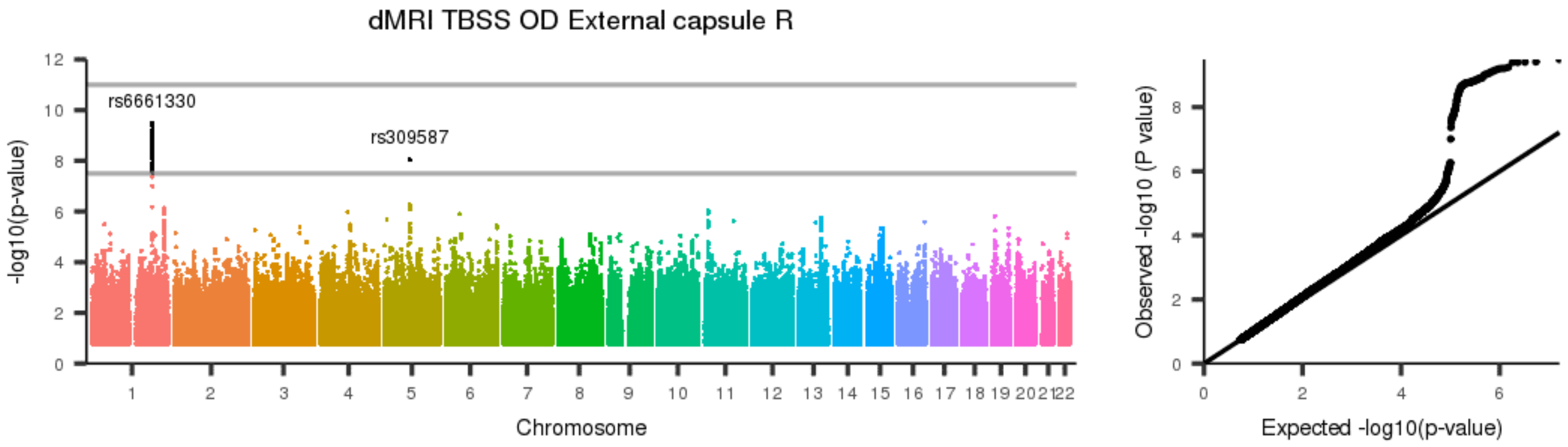

back to list of plots

Figure 565: dMRI TBSS OD External capsule L

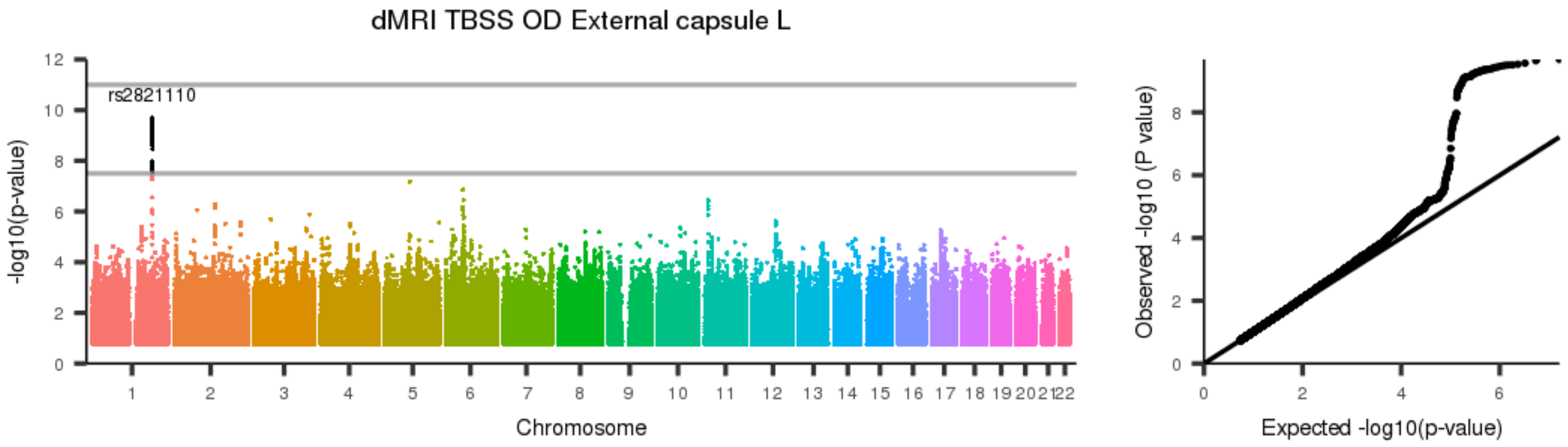

back to list of plots

Figure 566: dMRI TBSS OD Cingulum cingulate gyrus R

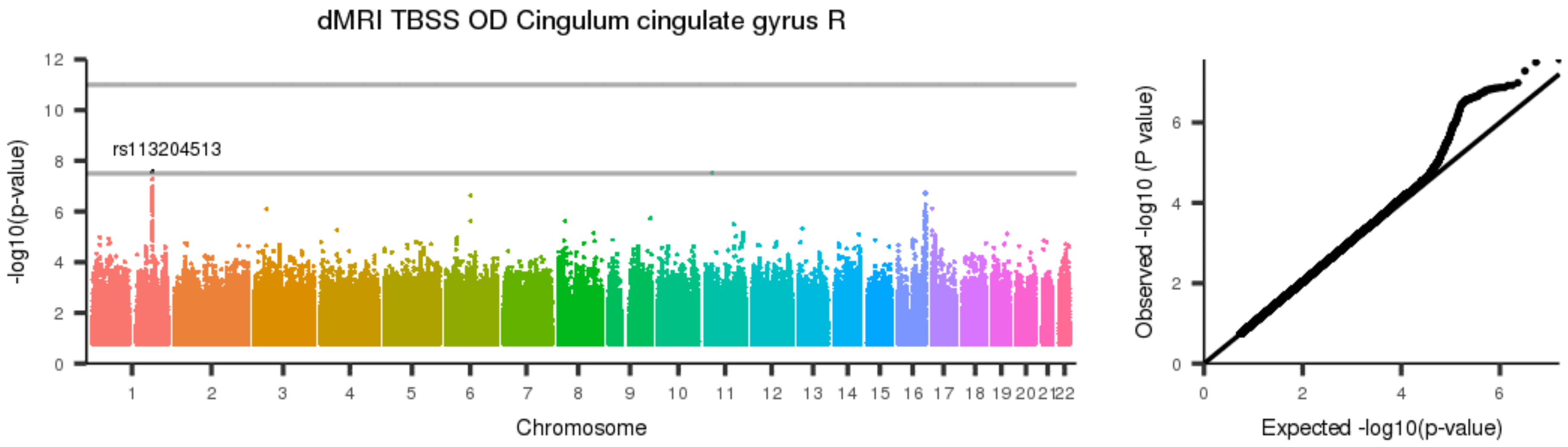

back to list of plots

Figure 567: dMRI TBSS OD Cingulum cingulate gyrus L

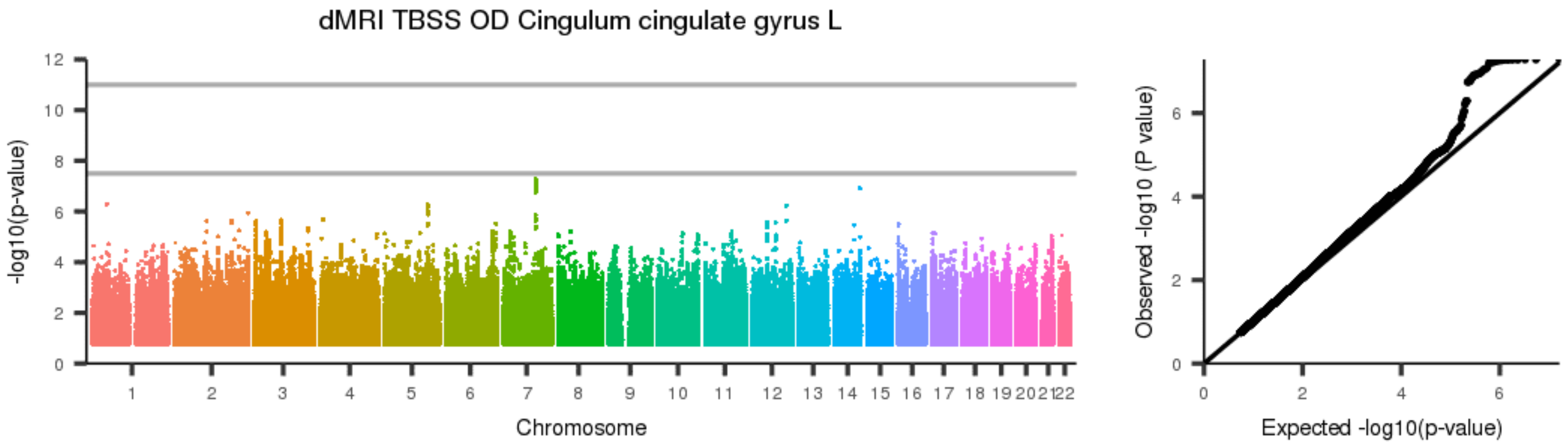

back to list of plots

Figure 568: dMRI TBSS OD Cingulum hippocampus R

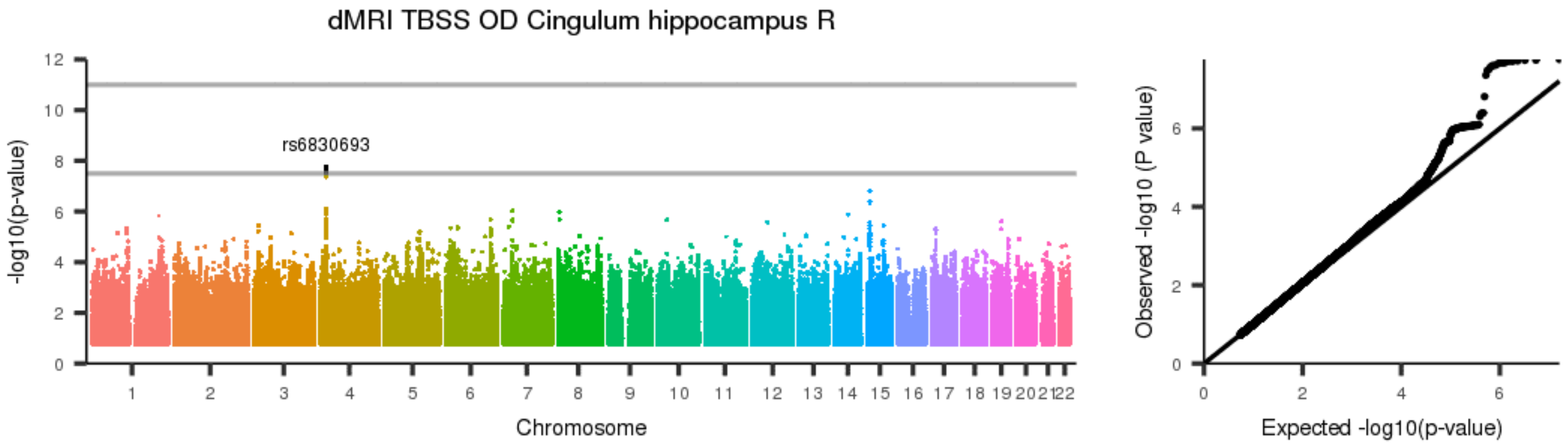

back to list of plots

Figure 569: dMRI TBSS OD Cingulum hippocampus L

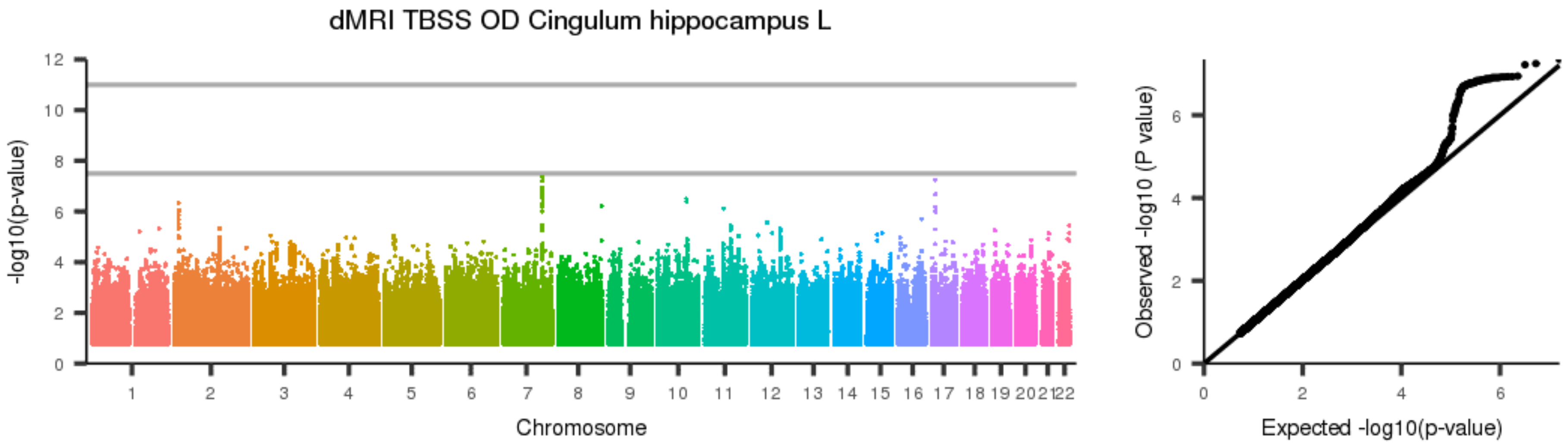

back to list of plots

Figure 570: dMRI TBSS OD Fornix cres+Stria terminalis R

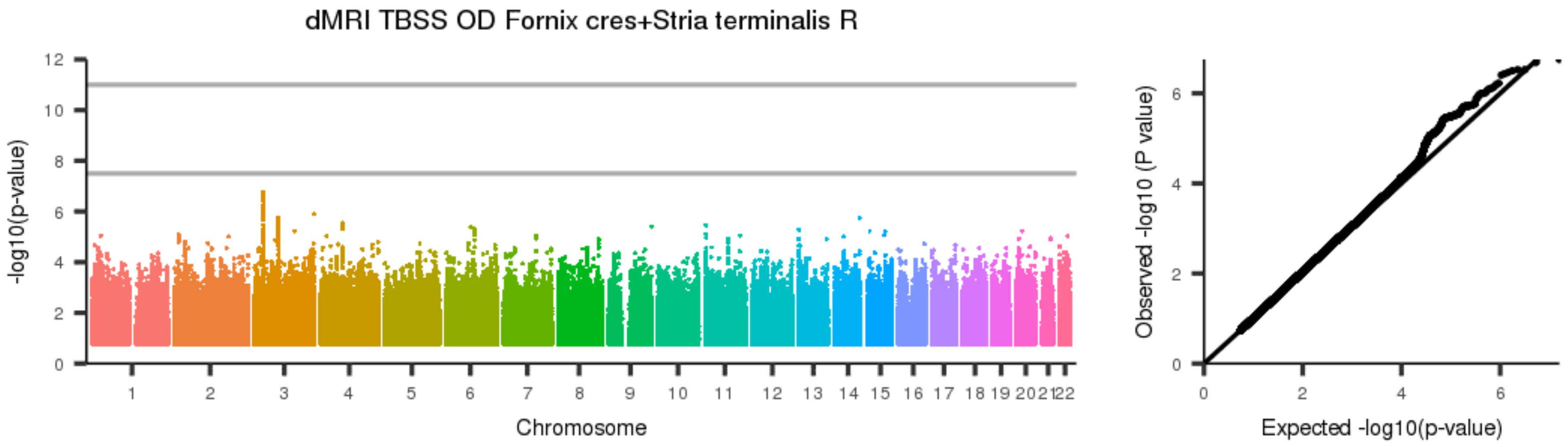

back to list of plots

Figure 571: dMRI TBSS OD Fornix cres+Stria terminalis L

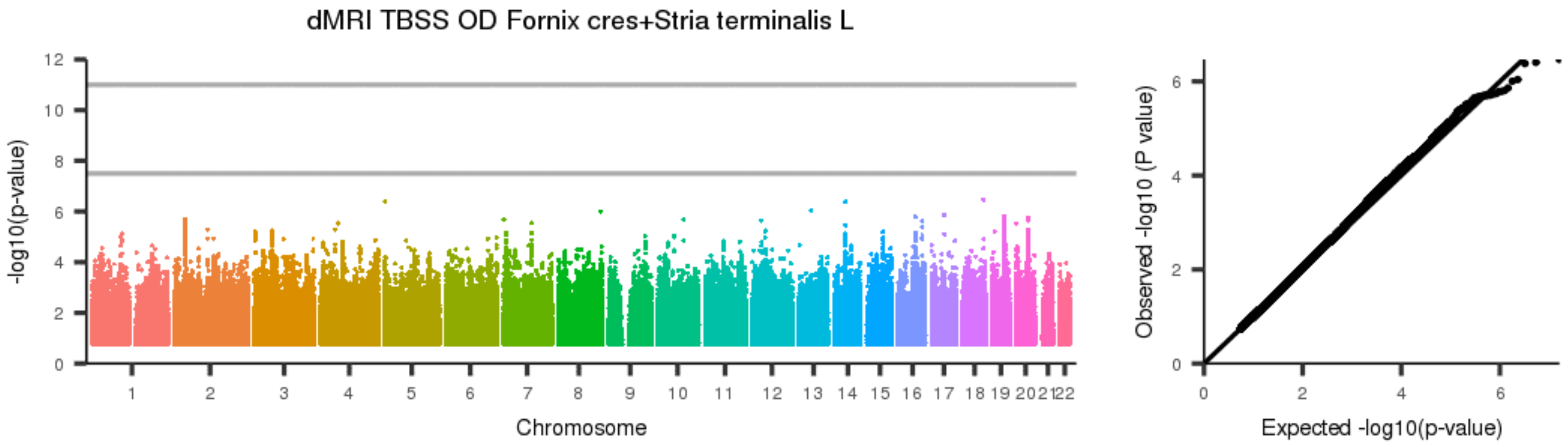

back to list of plots

Figure 572: dMRI TBSS OD Superior longitudinal fasciculus R

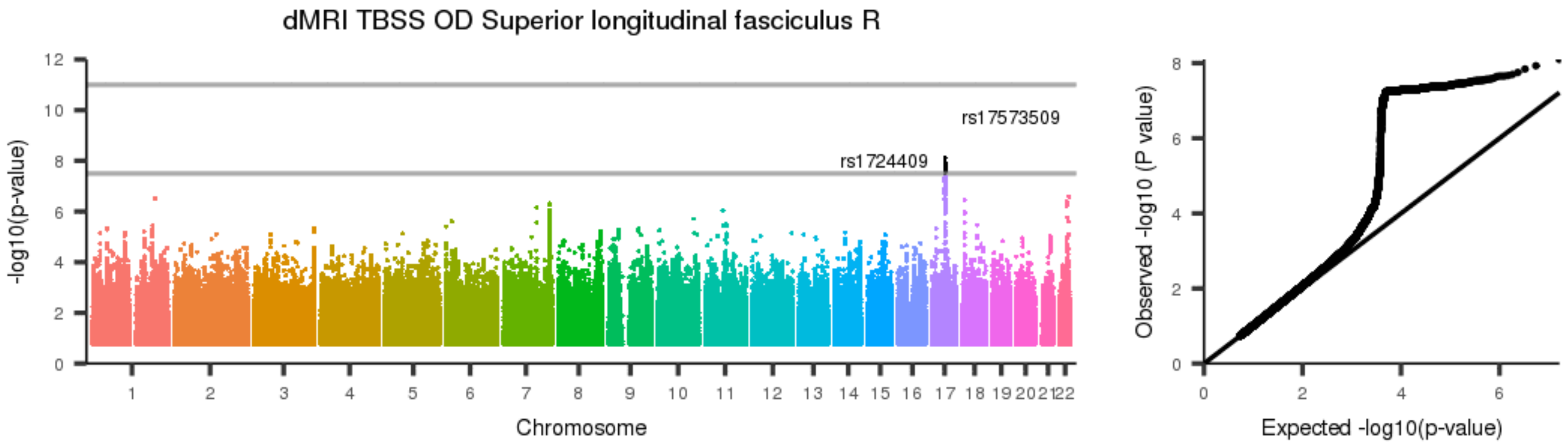

back to list of plots

Figure 573: dMRI TBSS OD Superior longitudinal fasciculus L

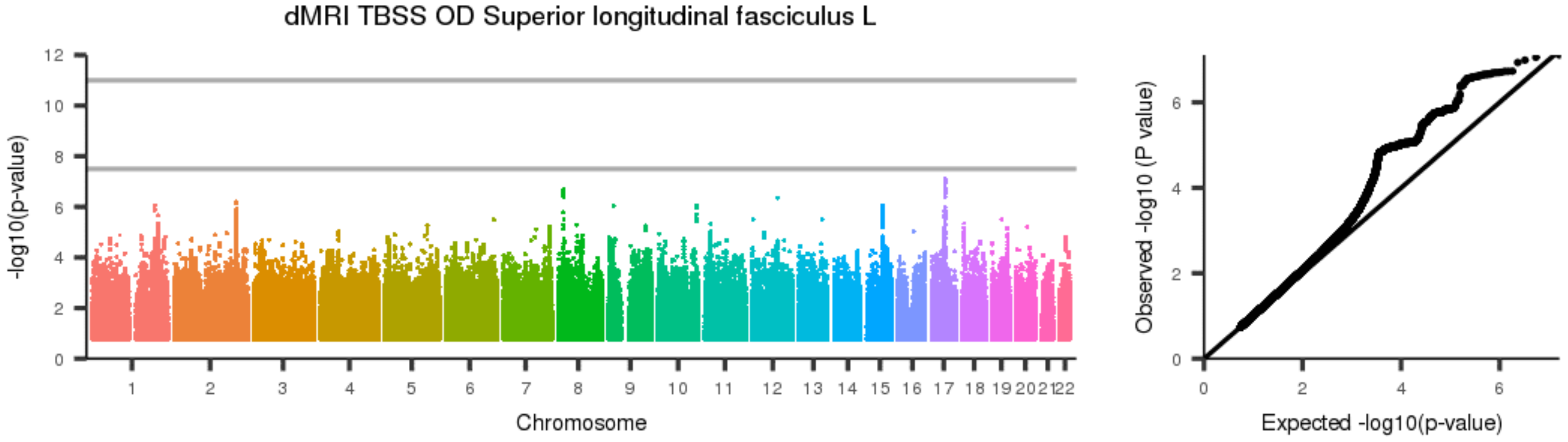

back to list of plots

Figure 574: dMRI TBSS OD Superior fronto-occipital fasciculus R

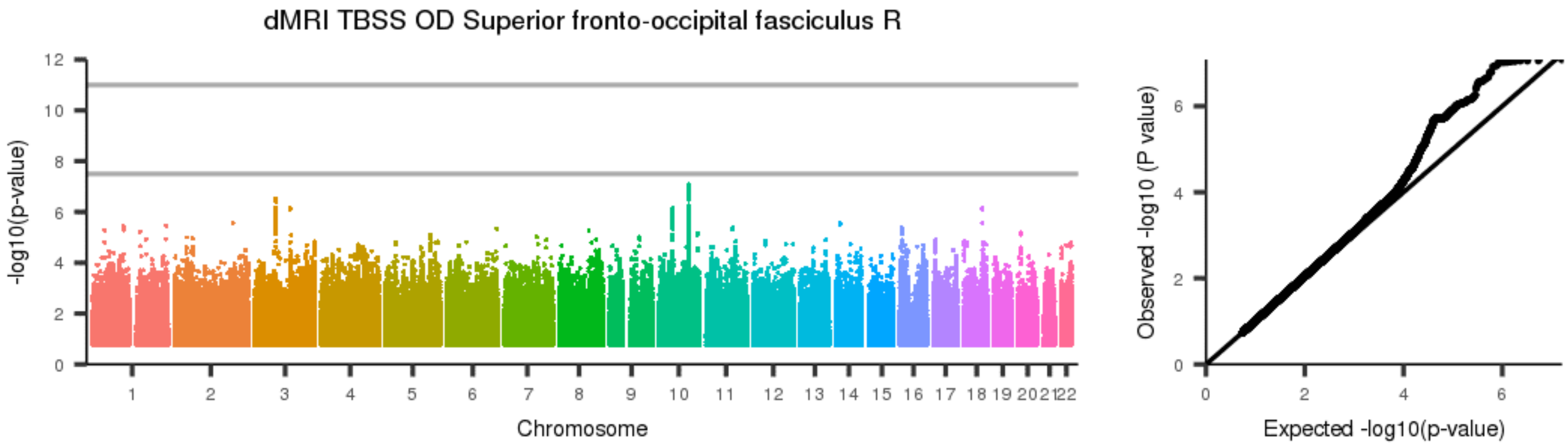

back to list of plots

Figure 575: dMRI TBSS OD Superior fronto-occipital fasciculus L

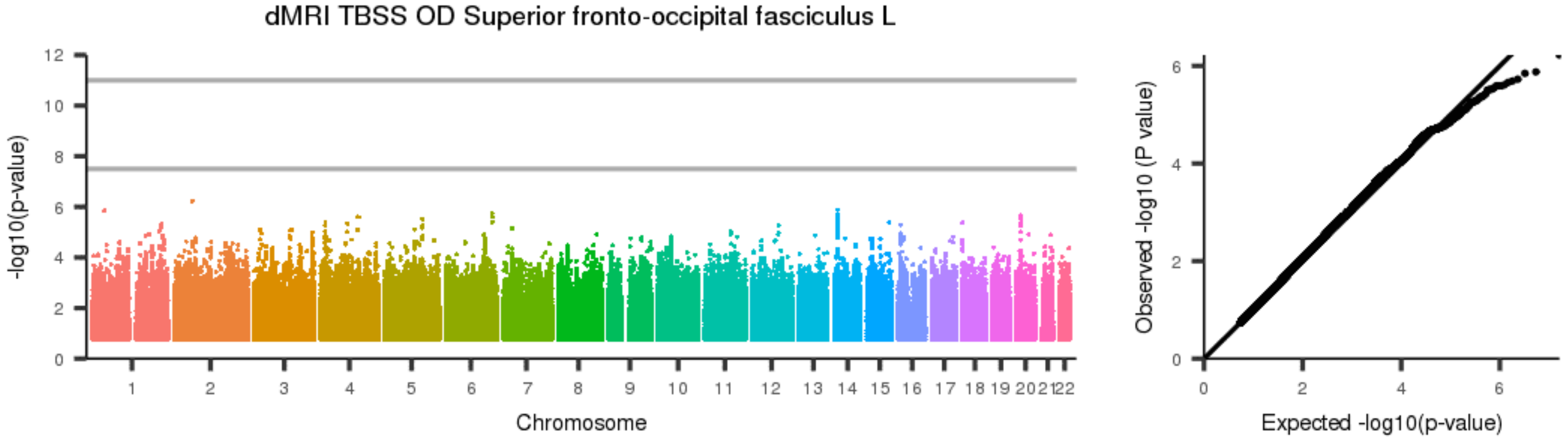

back to list of plots

Figure 576: dMRI TBSS OD Uncinate fasciculus R

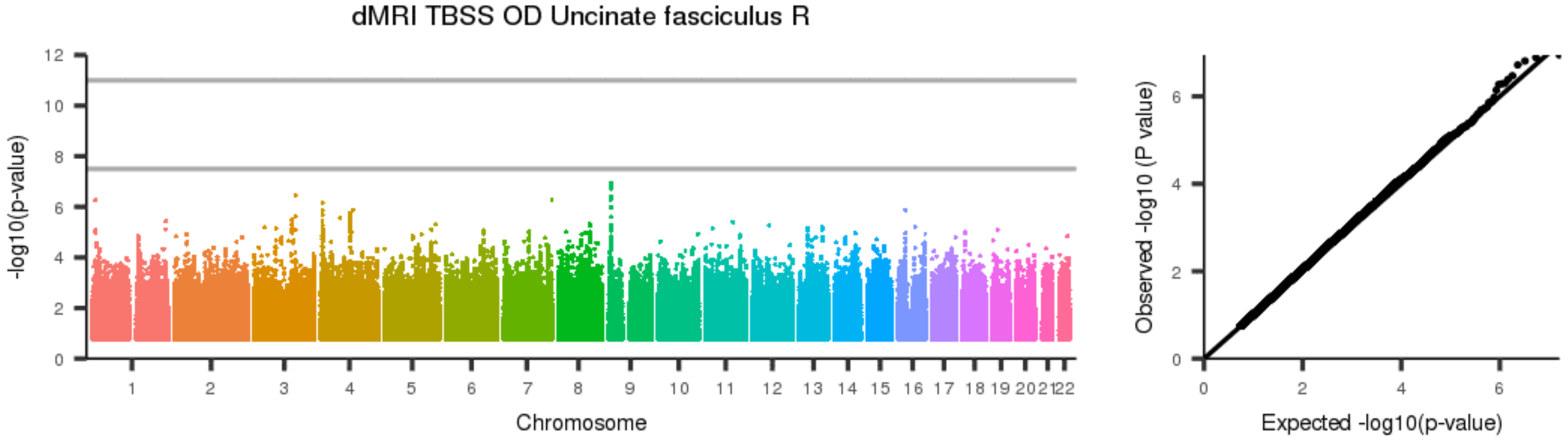

back to list of plots

Figure 577: dMRI TBSS OD Uncinate fasciculus L

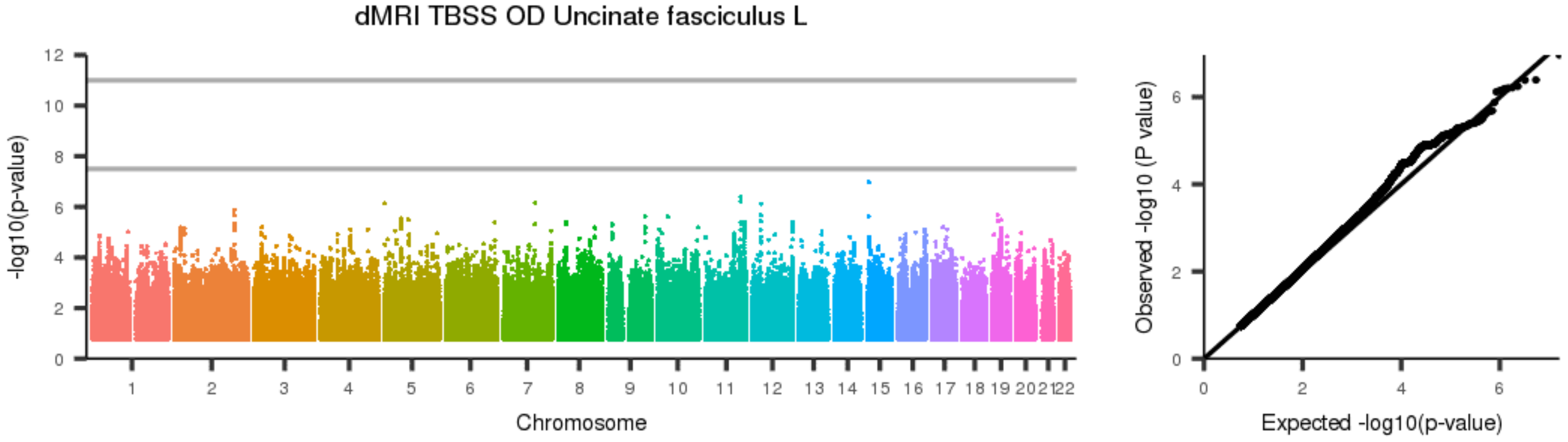

back to list of plots

Figure 578: dMRI TBSS OD Tapetum R

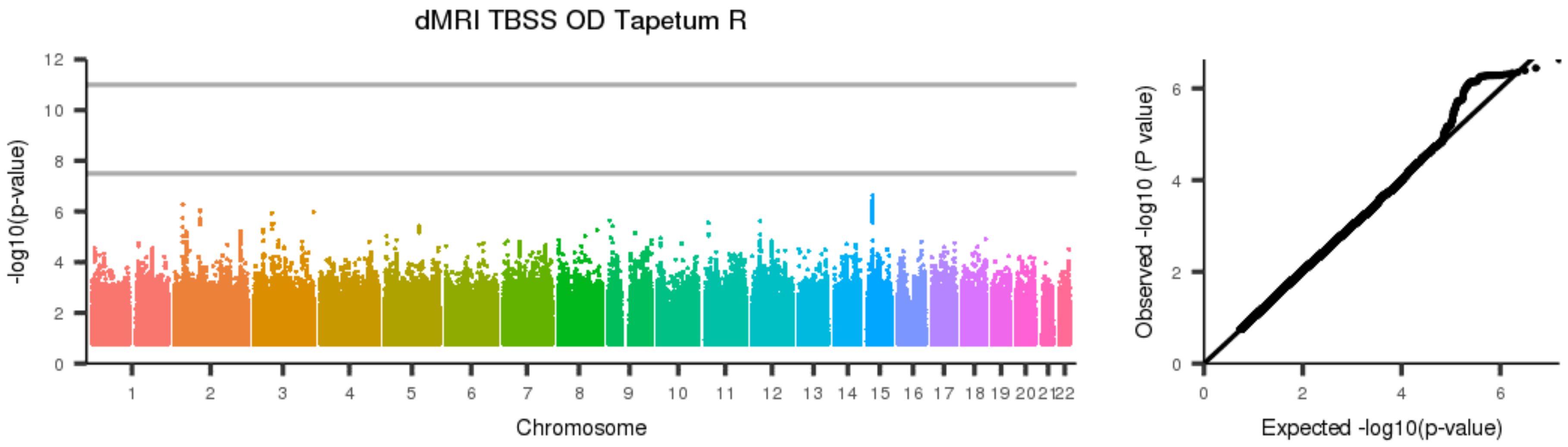

back to list of plots

Figure 579: dMRI TBSS OD Tapetum L

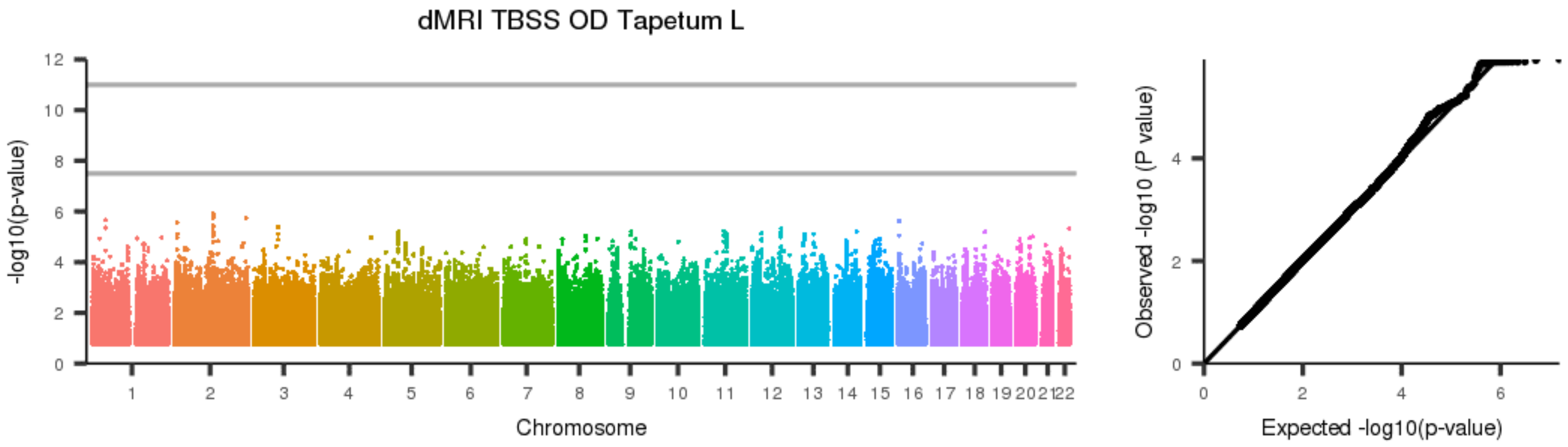

back to list of plots

Figure 580: dMRI TBSS ISOVF Middle cerebellar peduncle

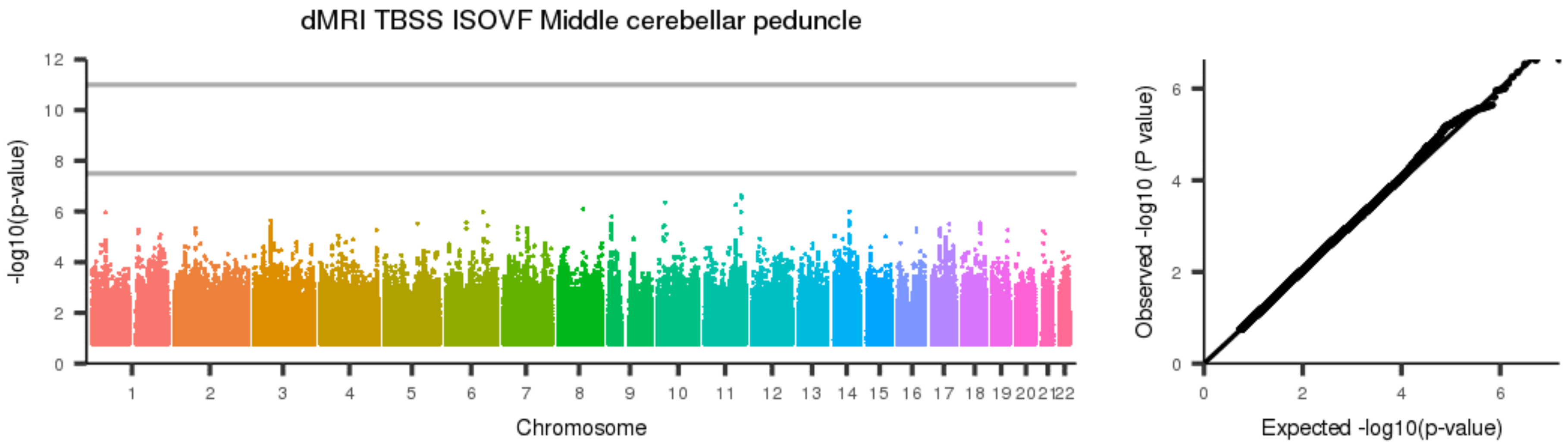

back to list of plots

Figure 581: dMRI TBSS ISOVF Pontine crossing tract

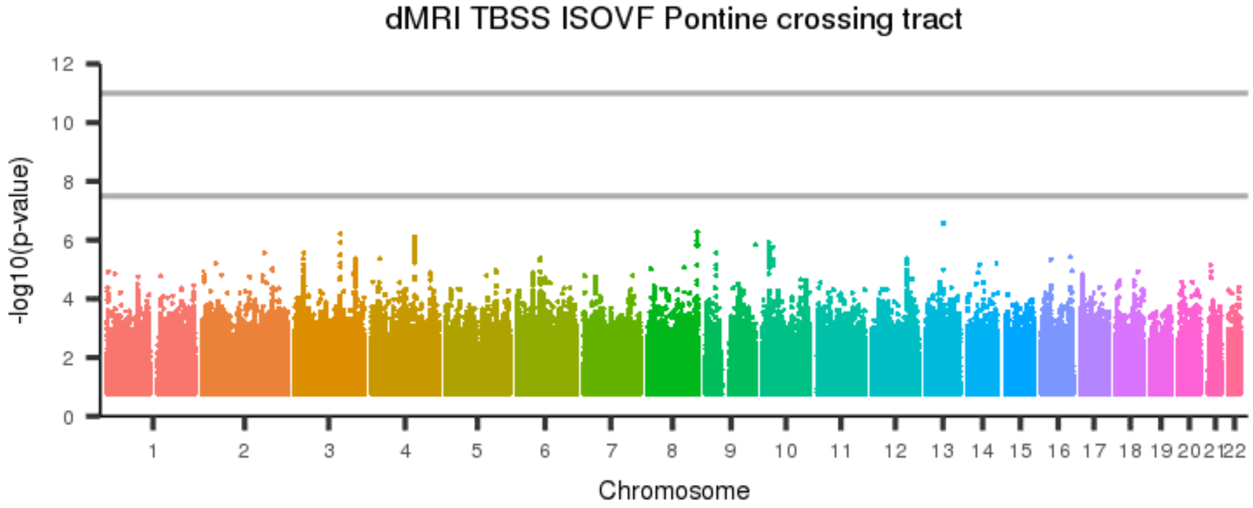

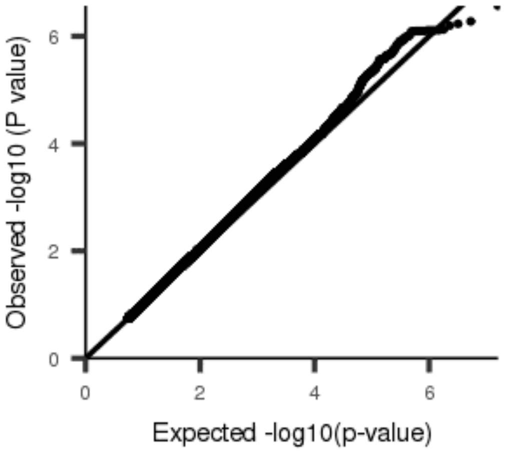

back to list of plots

Figure 582: dMRI TBSS ISOVF Genu of corpus callosum

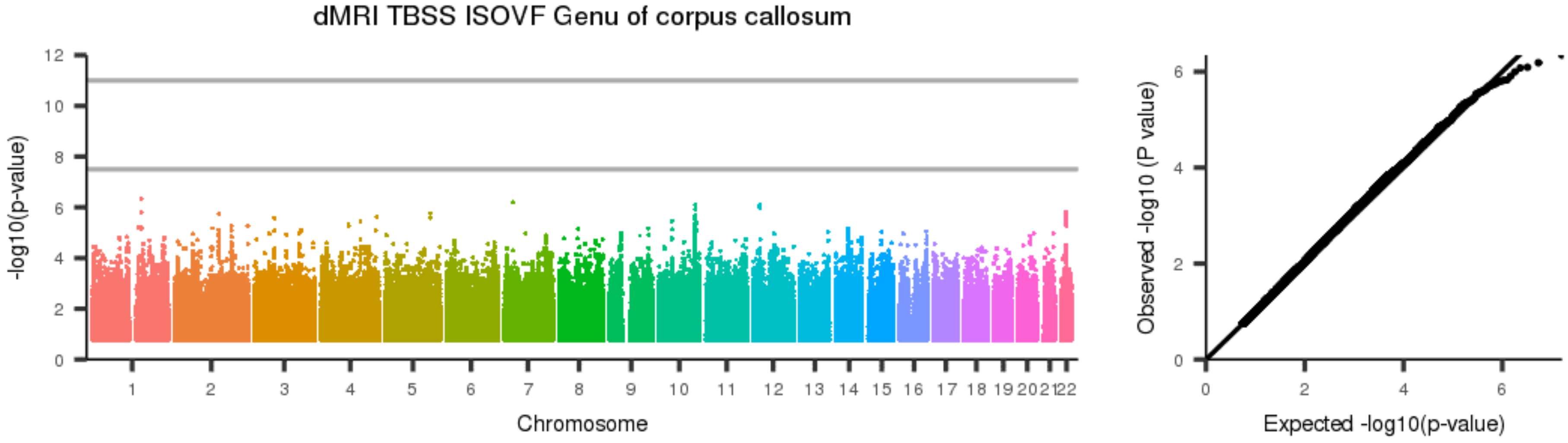

back to list of plots

Figure 583: dMRI TBSS ISOVF Body of corpus callosum

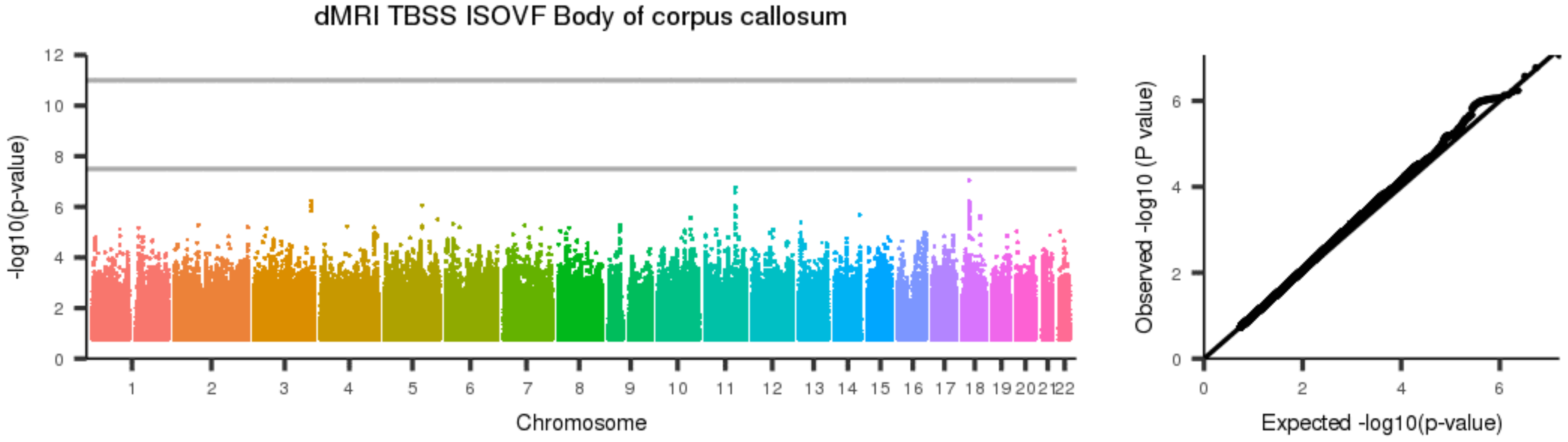

back to list of plots

Figure 584: dMRI TBSS ISOVF Splenium of corpus callosum

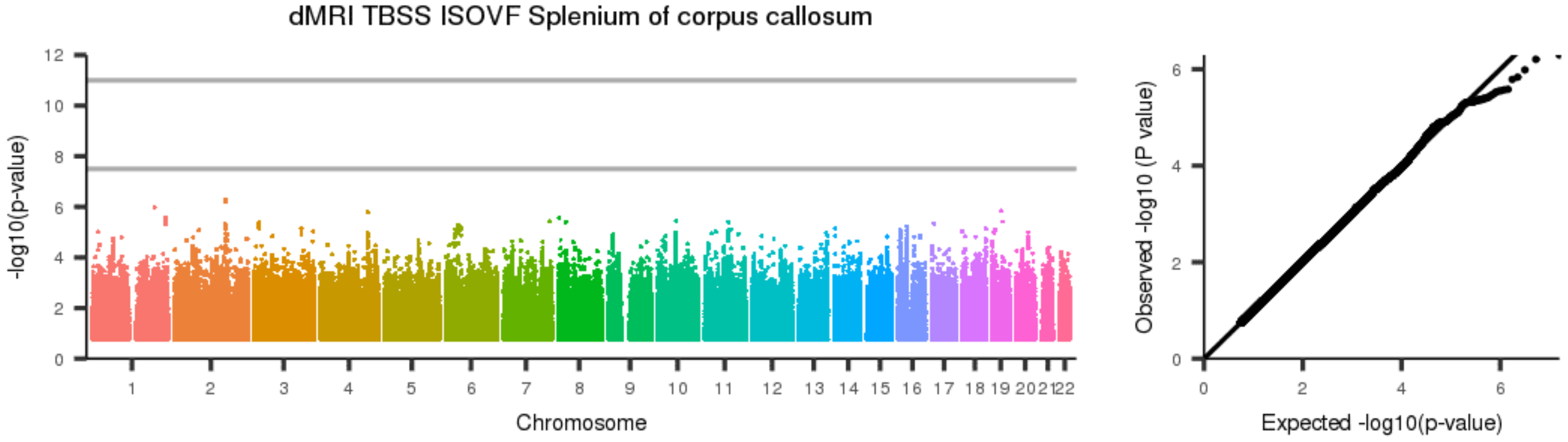

back to list of plots

Figure 585: dMRI TBSS ISOVF Fornix

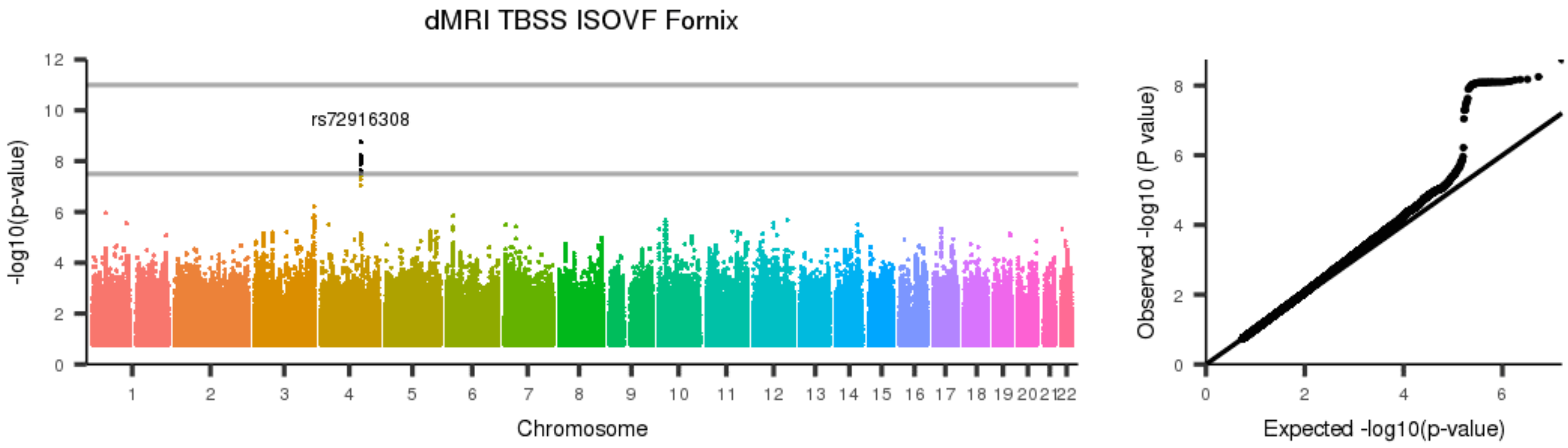

back to list of plots

Figure 586: dMRI TBSS ISOVF Corticospinal tract R

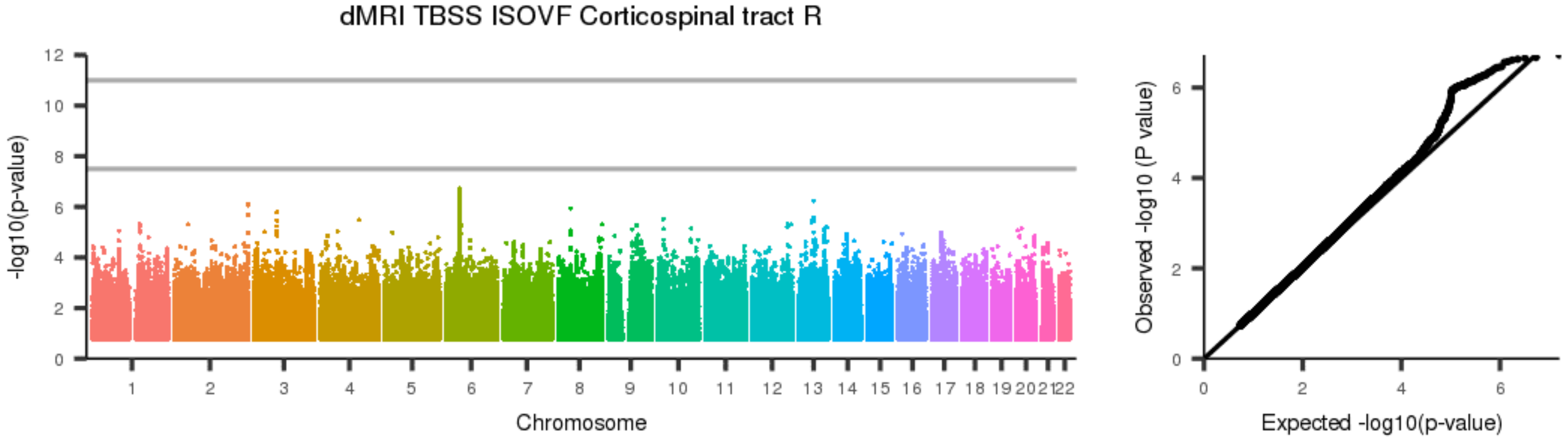

back to list of plots

Figure 587: dMRI TBSS ISOVF Corticospinal tract L

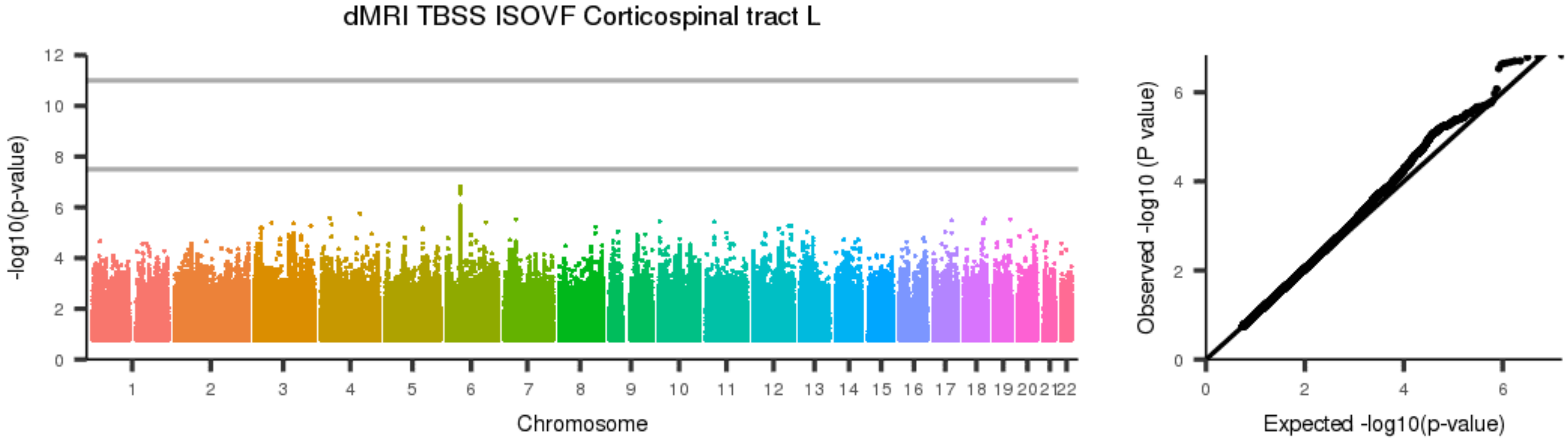

back to list of plots

Figure 588: dMRI TBSS ISOVF Medial lemniscus R

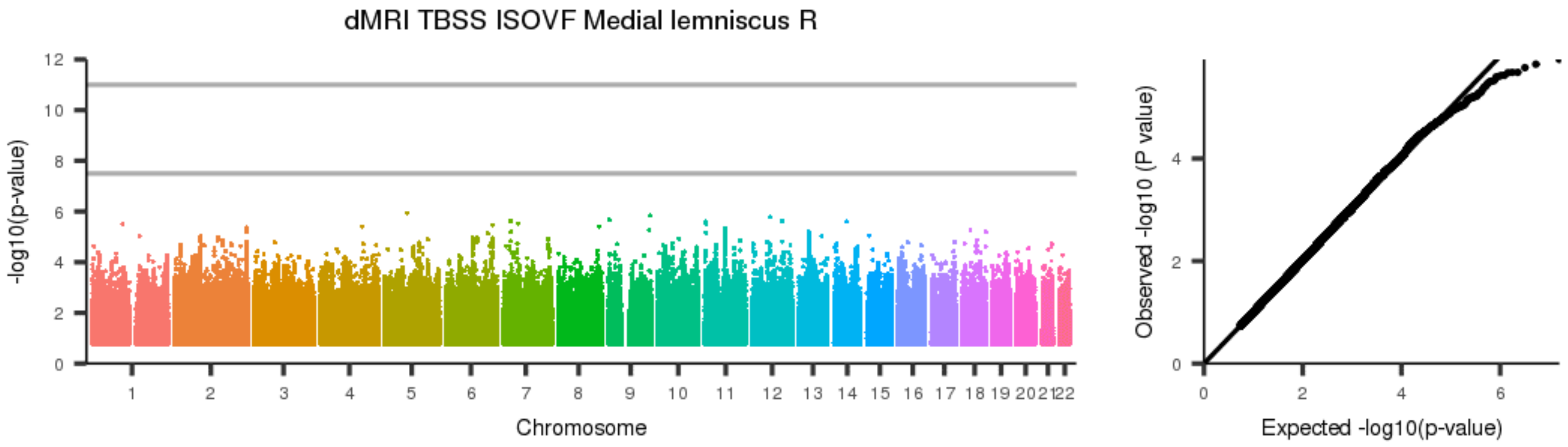

back to list of plots

Figure 589: dMRI TBSS ISOVF Medial lemniscus L

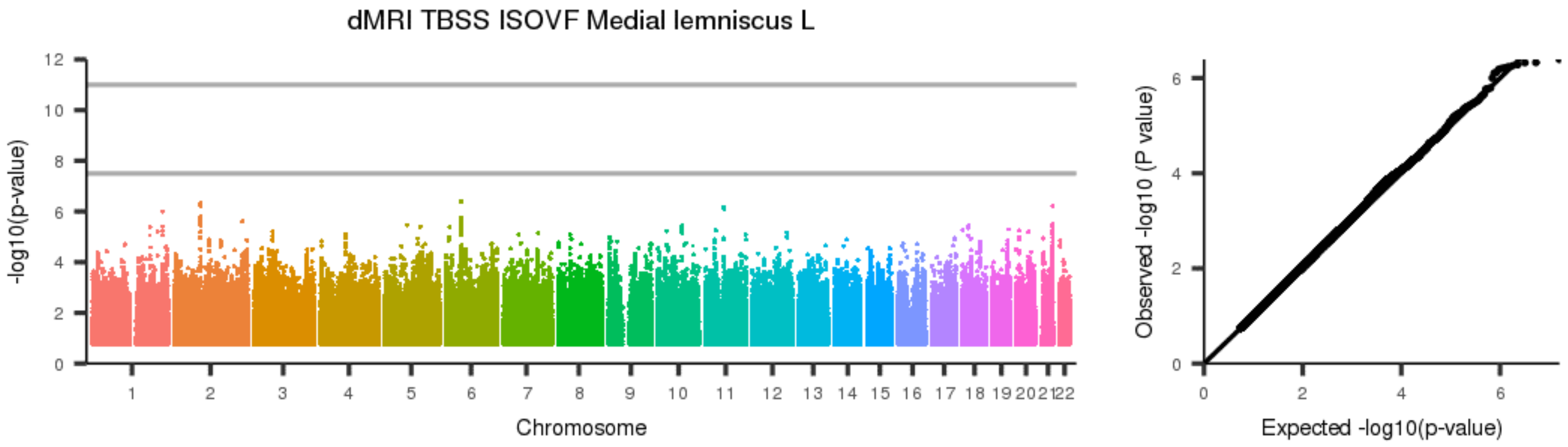

back to list of plots

Figure 590: dMRI TBSS ISOVF Inferior cerebellar peduncle R

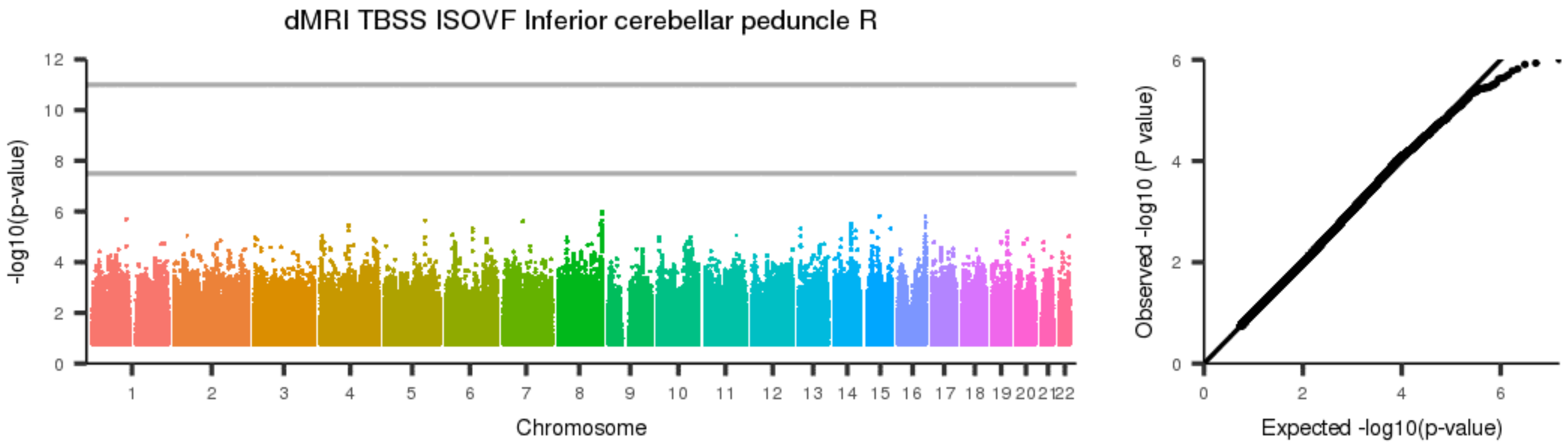

back to list of plots

Figure 591: dMRI TBSS ISOVF Inferior cerebellar peduncle L

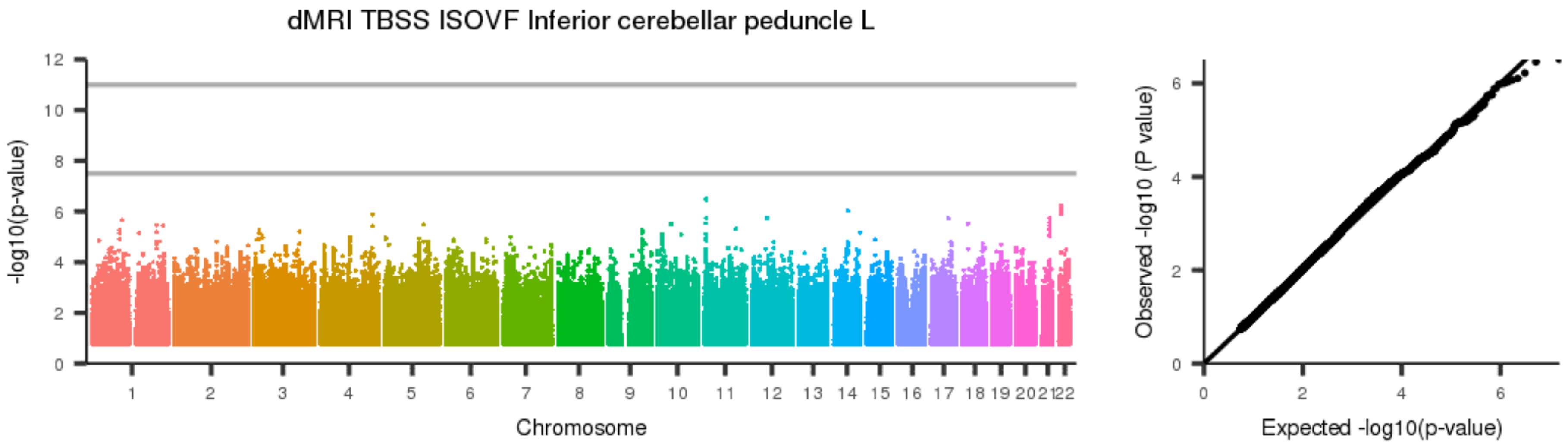

back to list of plots

Figure 592: dMRI TBSS ISOVF Superior cerebellar peduncle R

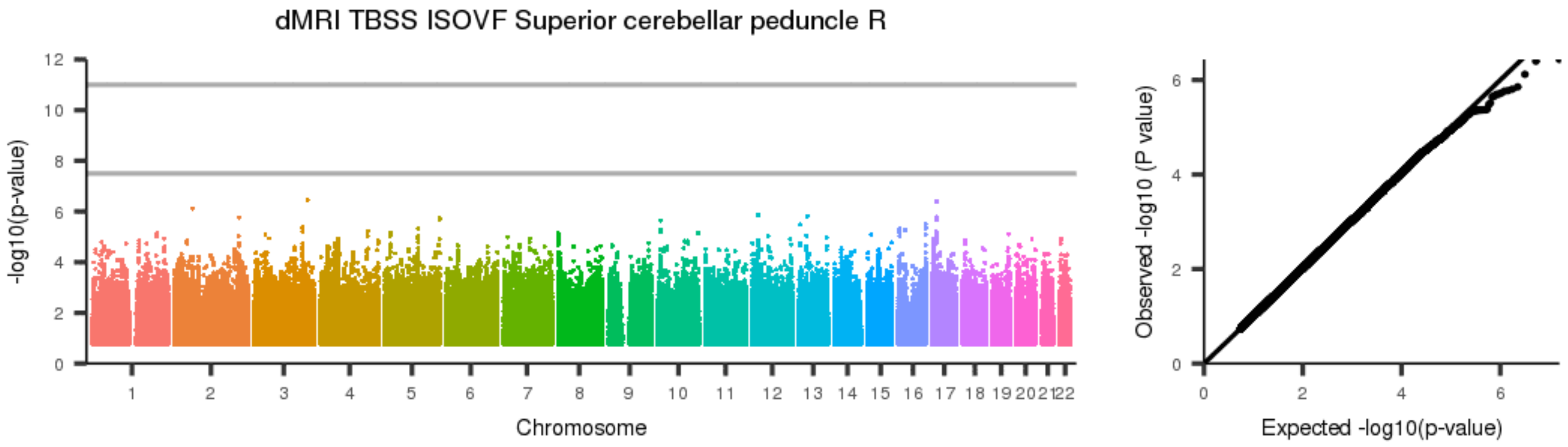

back to list of plots

Figure 593: dMRI TBSS ISOVF Superior cerebellar peduncle L

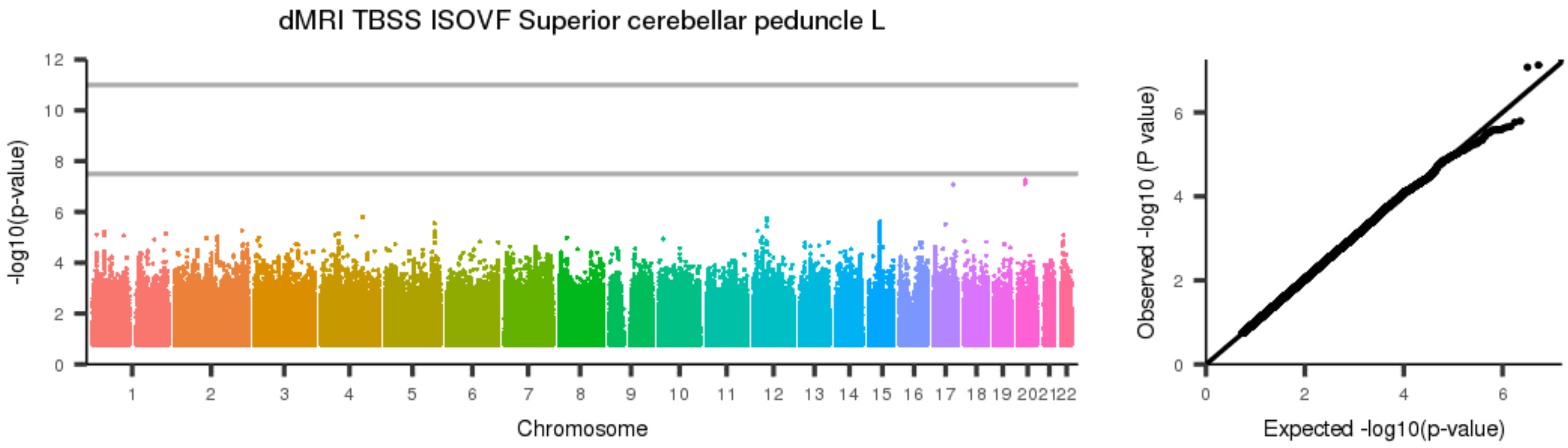

back to list of plots

Figure 594: dMRI TBSS ISOVF Cerebral peduncle R

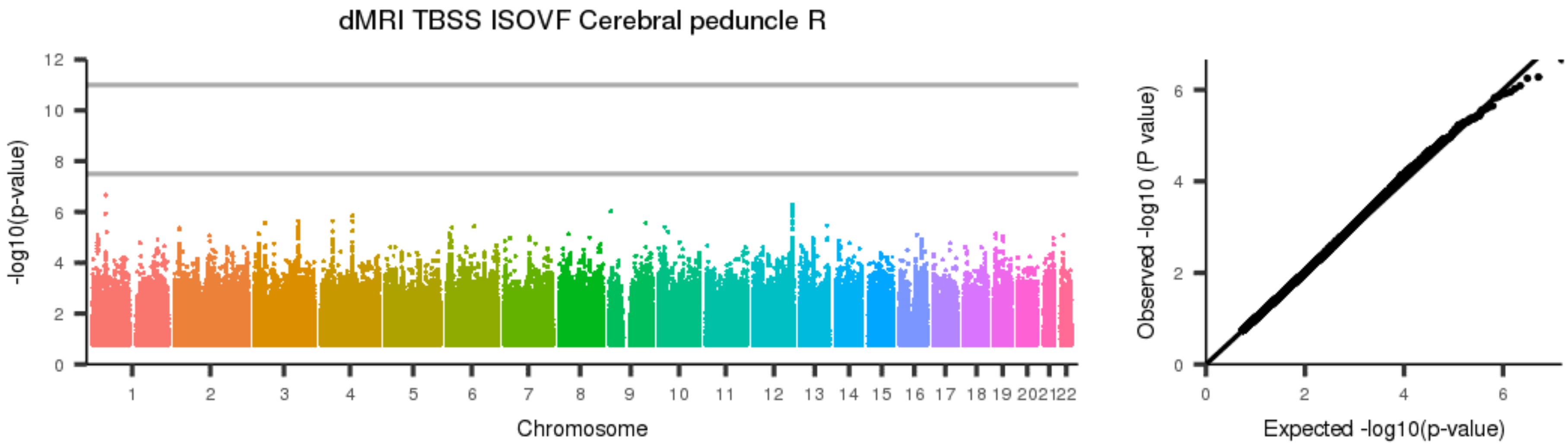

back to list of plots

Figure 595: dMRI TBSS ISOVF Cerebral peduncle L

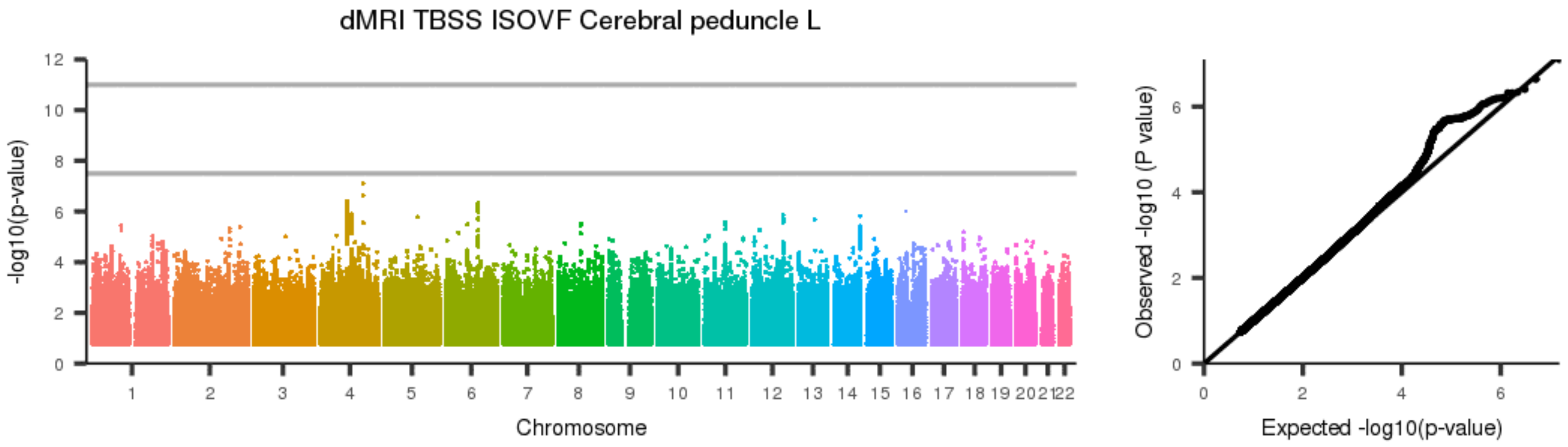

back to list of plots

Figure 596: dMRI TBSS ISOVF Anterior limb of internal capsule R

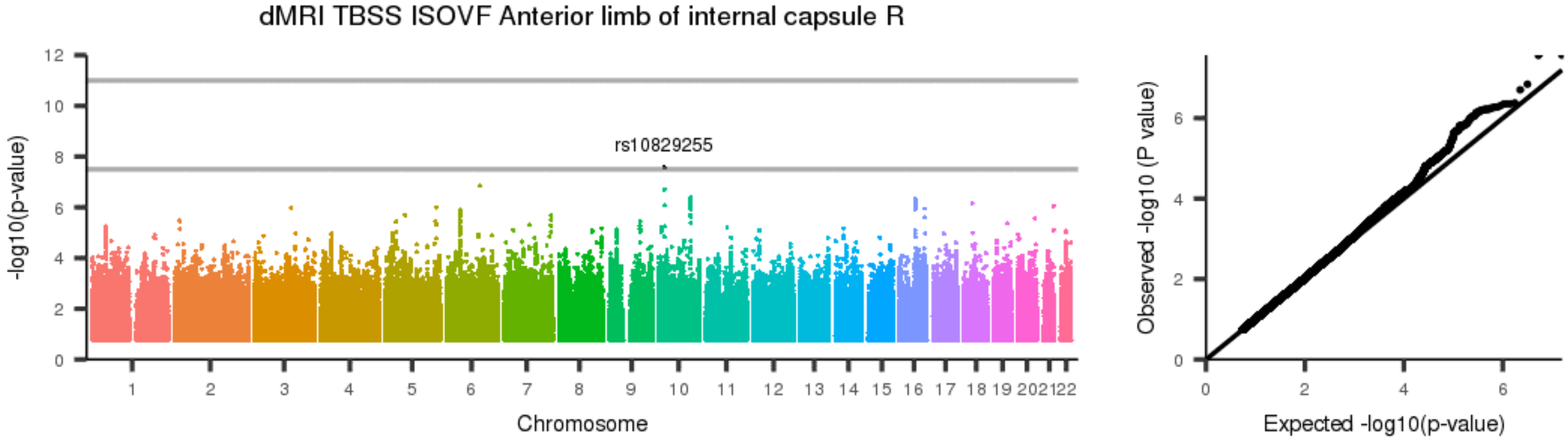

back to list of plots

Figure 597: dMRI TBSS ISOVF Anterior limb of internal capsule L

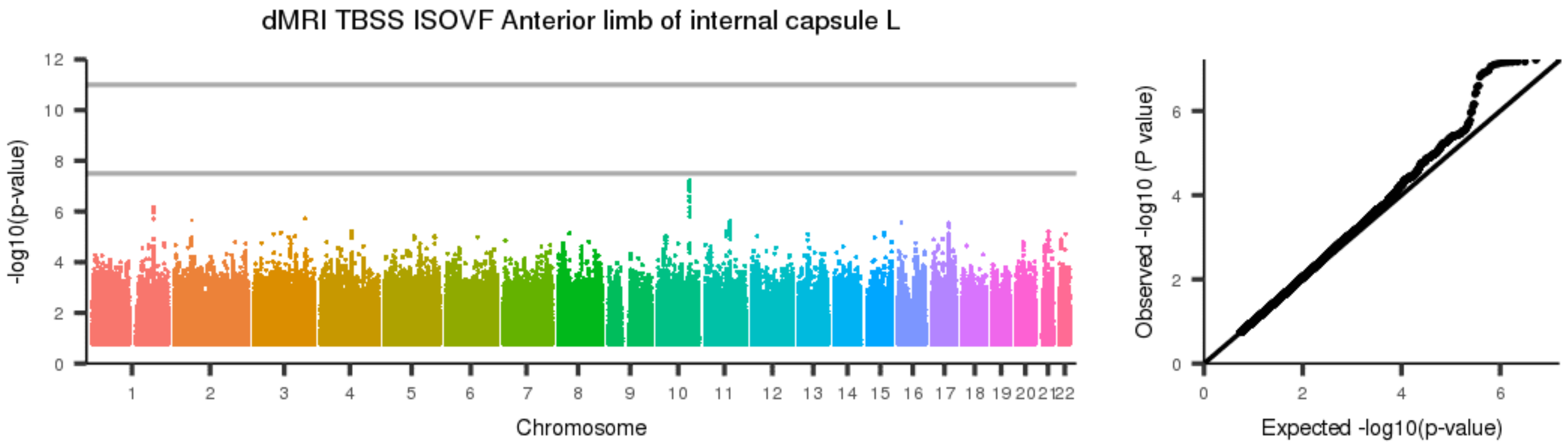

back to list of plots

Figure 598: dMRI TBSS ISOVF Posterior limb of internal capsule R

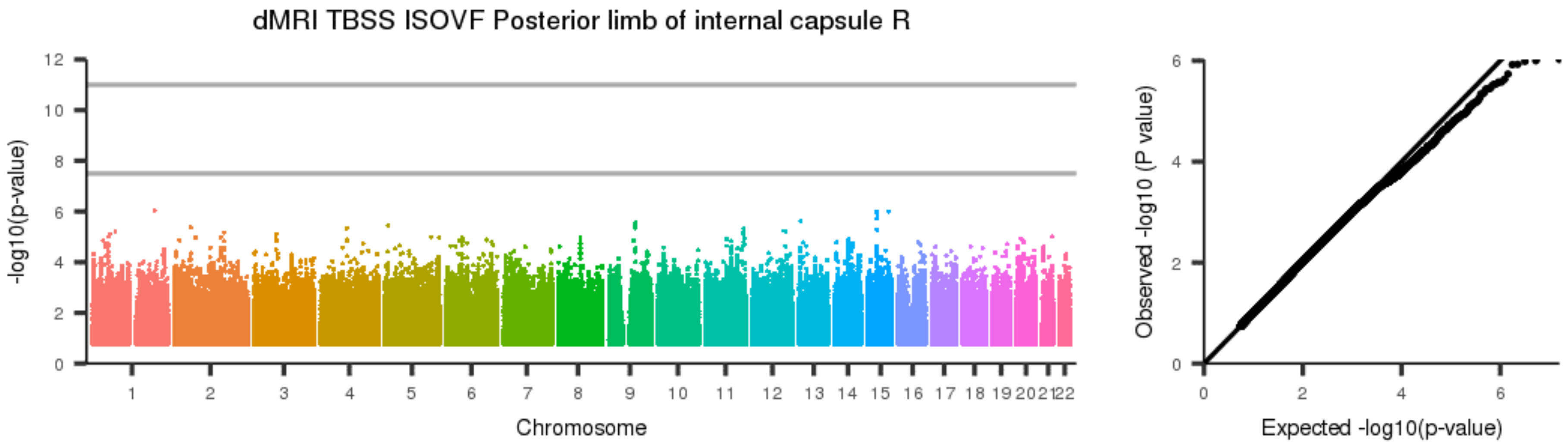

back to list of plots

Figure 599: dMRI TBSS ISOVF Posterior limb of internal capsule L

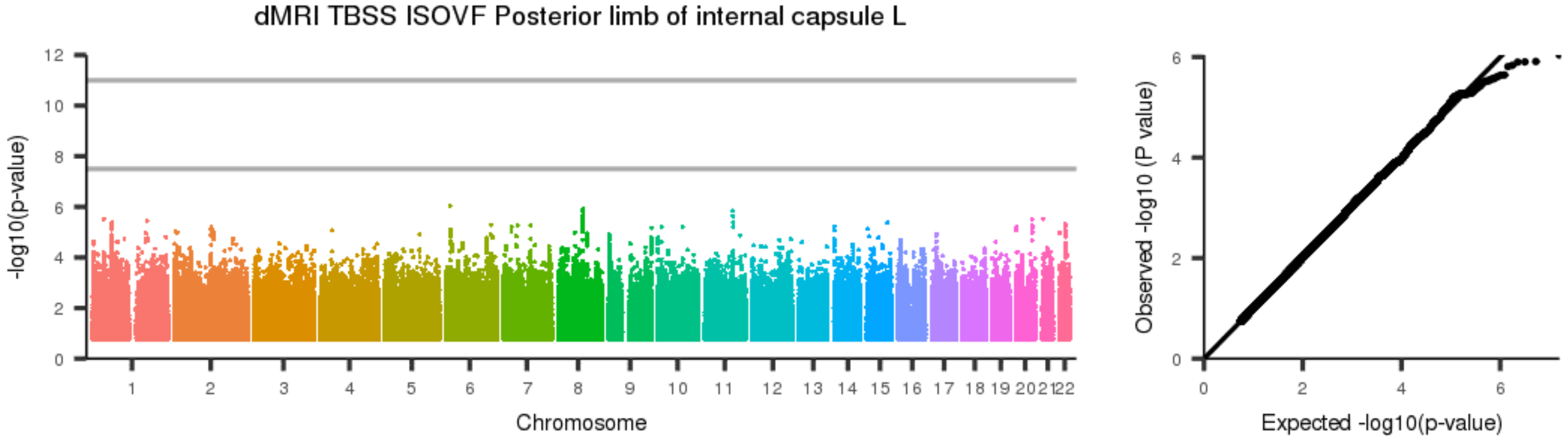

back to list of plots

Figure 600: dMRI TBSS ISOVF Retrolenticular part of internal capsule R

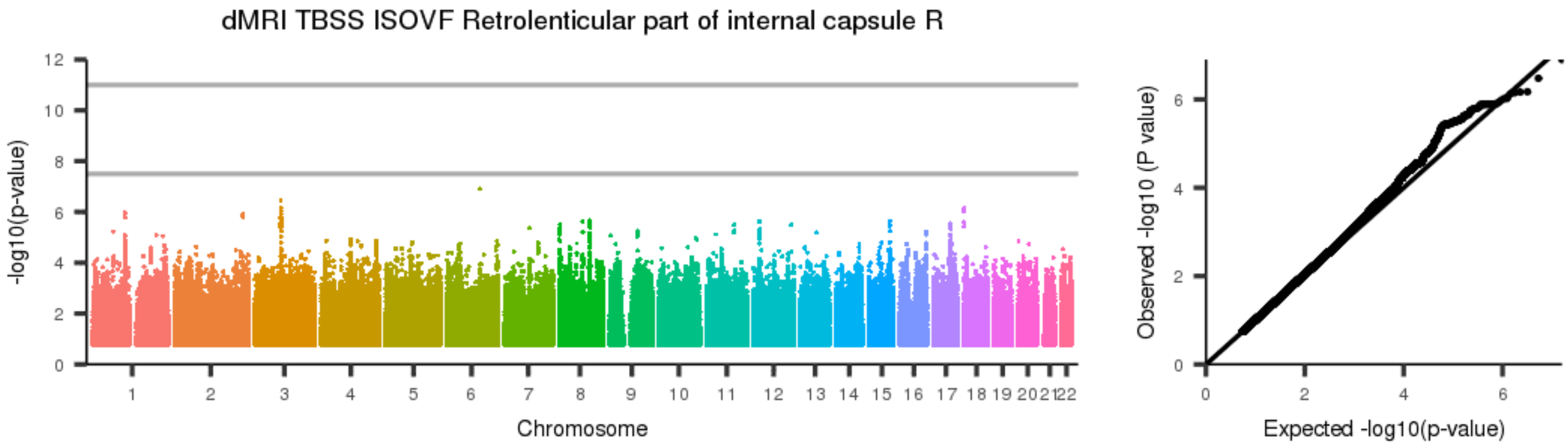

back to list of plots

Figure 601: dMRI TBSS ISOVF Retrolenticular part of internal capsule L

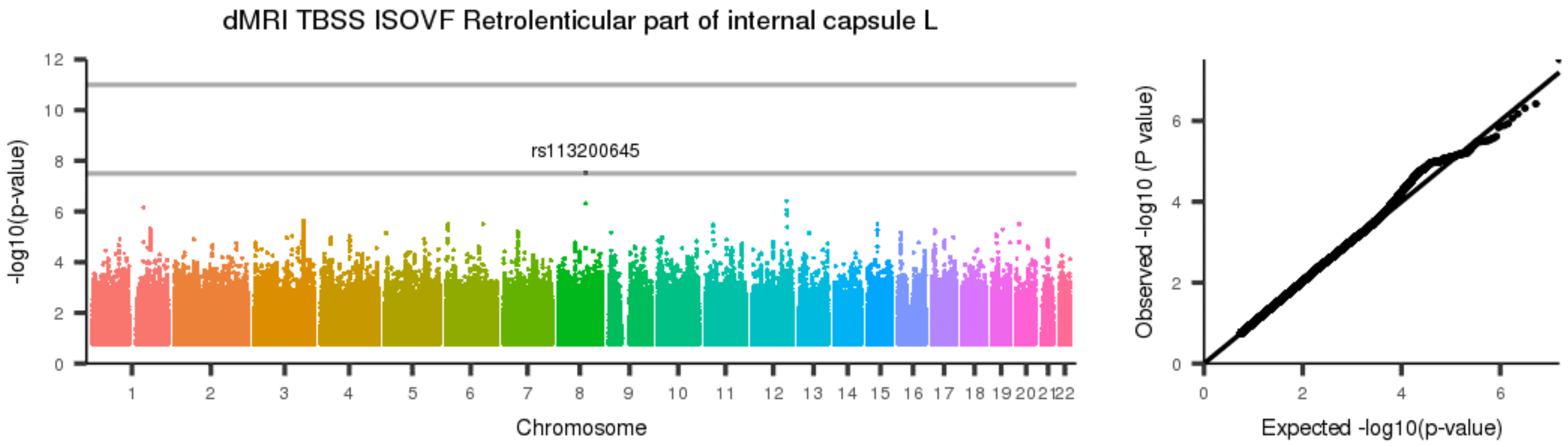

back to list of plots

Figure 602: dMRI TBSS ISOVF Anterior corona radiata R

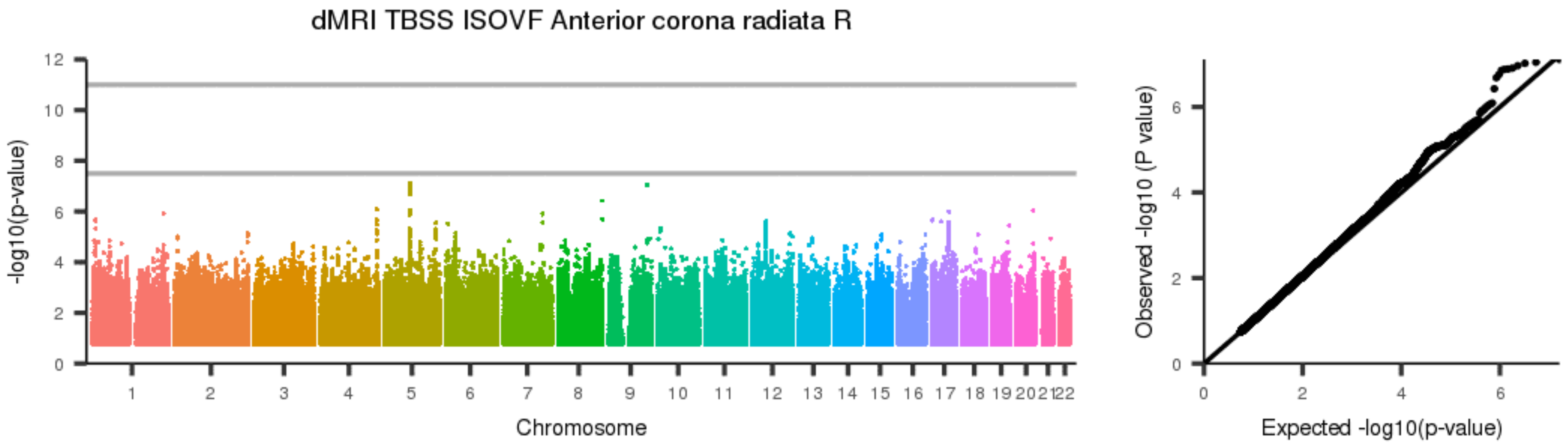

back to list of plots

Figure 603: dMRI TBSS ISOVF Anterior corona radiata L

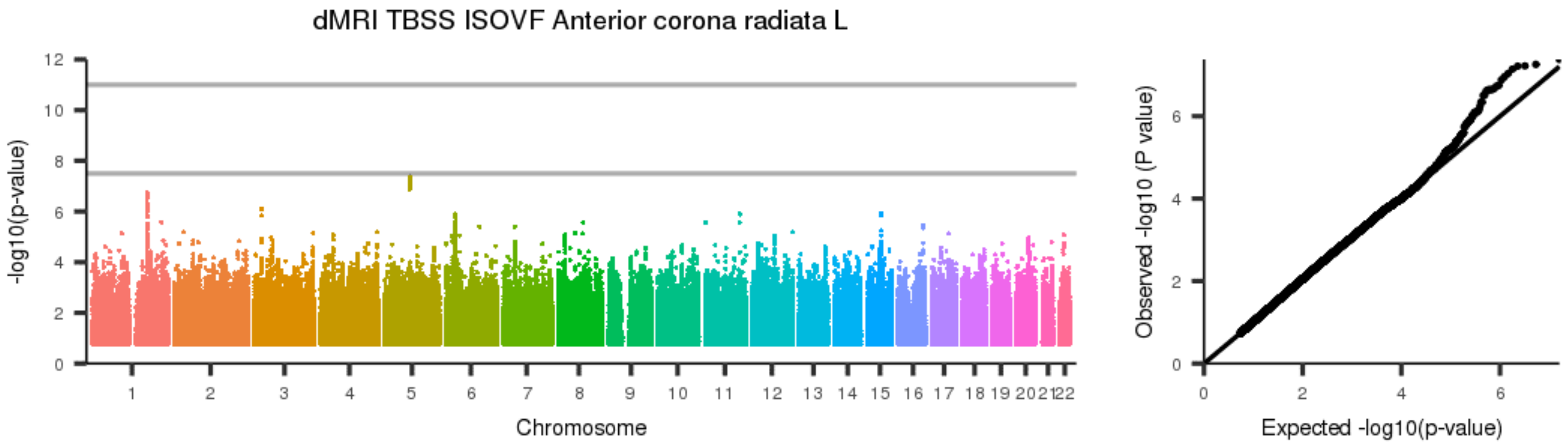

back to list of plots

Figure 604: dMRI TBSS ISOVF Superior corona radiata R

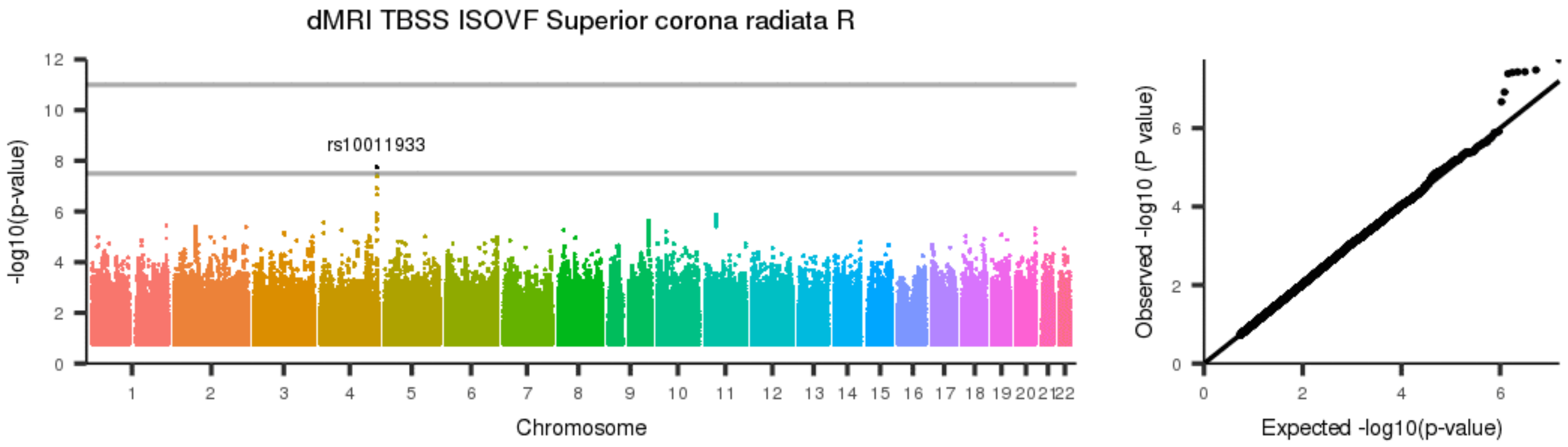

back to list of plots

Figure 605: dMRI TBSS ISOVF Superior corona radiata L

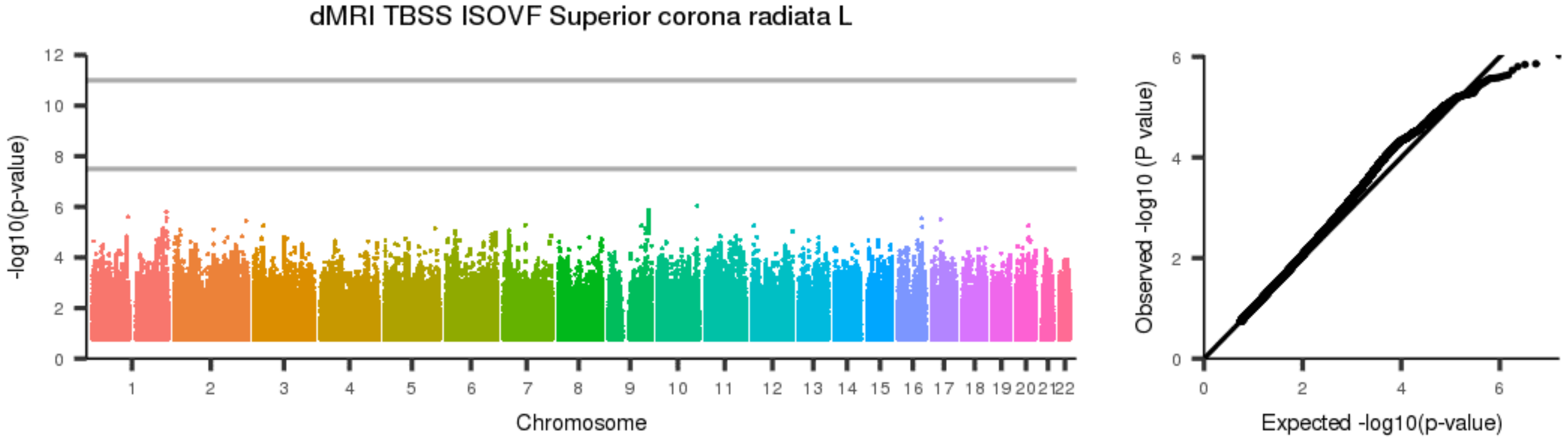

back to list of plots

Figure 606: dMRI TBSS ISOVF Posterior corona radiata R

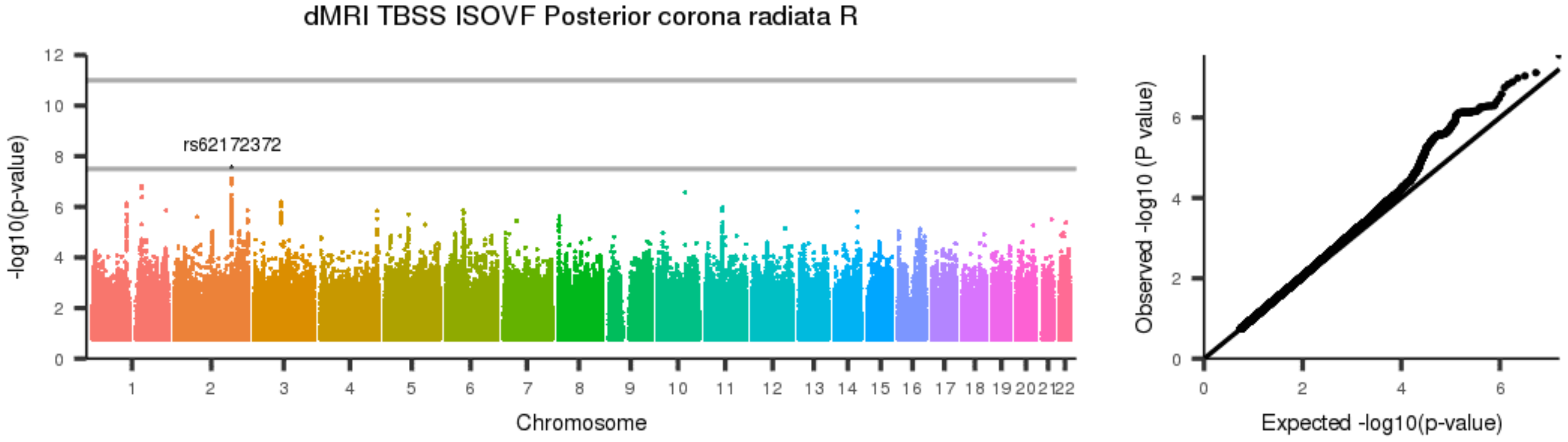

back to list of plots

Figure 607: dMRI TBSS ISOVF Posterior corona radiata L

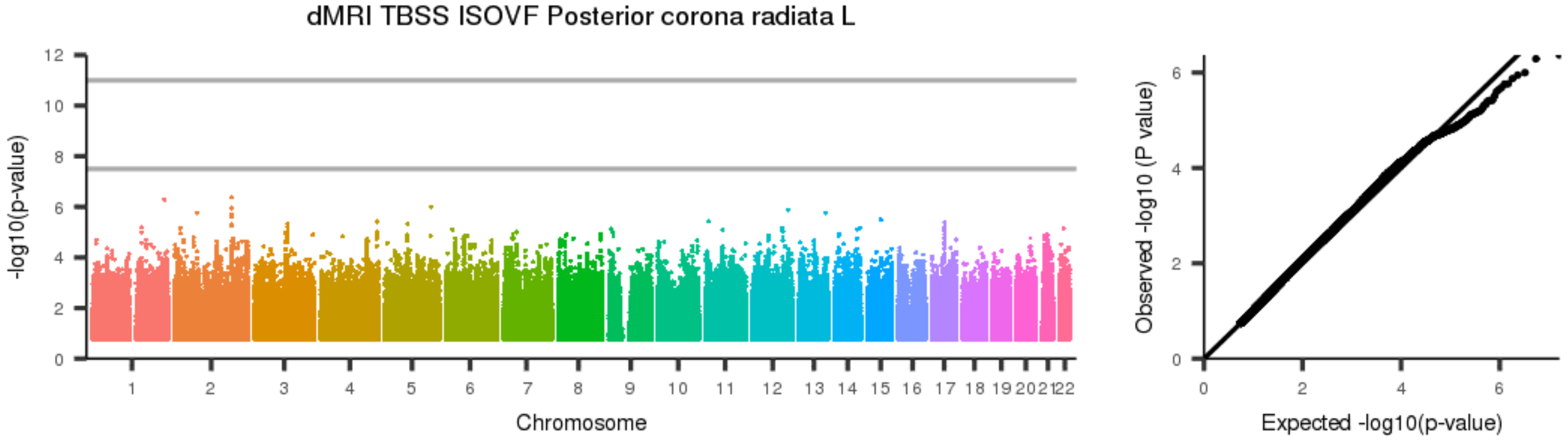

back to list of plots

Figure 608: dMRI TBSS ISOVF Posterior thalamic radiation R

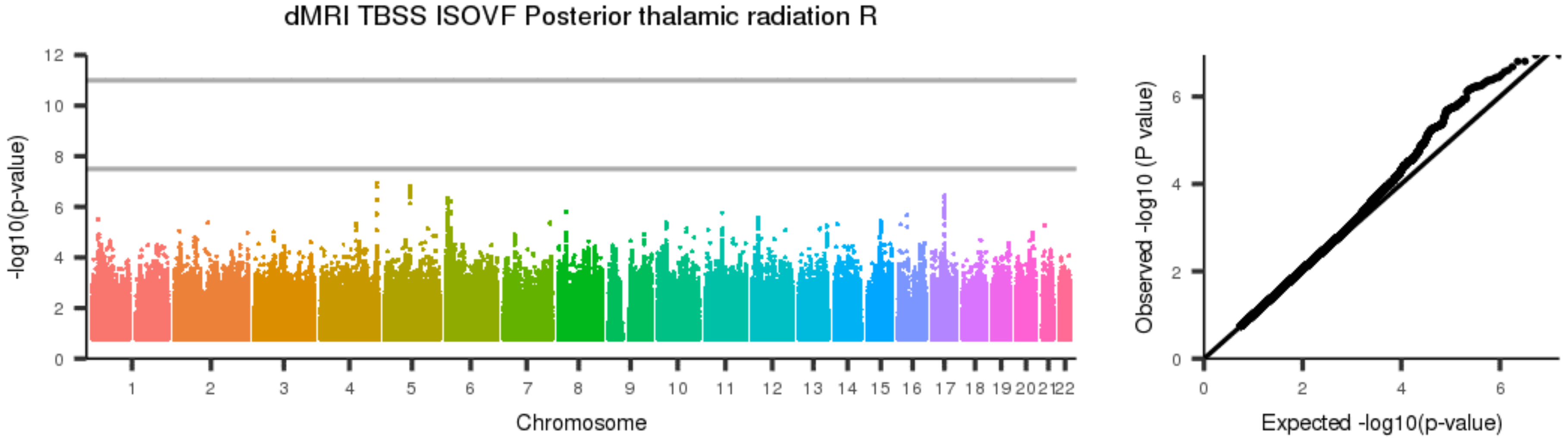

back to list of plots

Figure 609: dMRI TBSS ISOVF Posterior thalamic radiation L

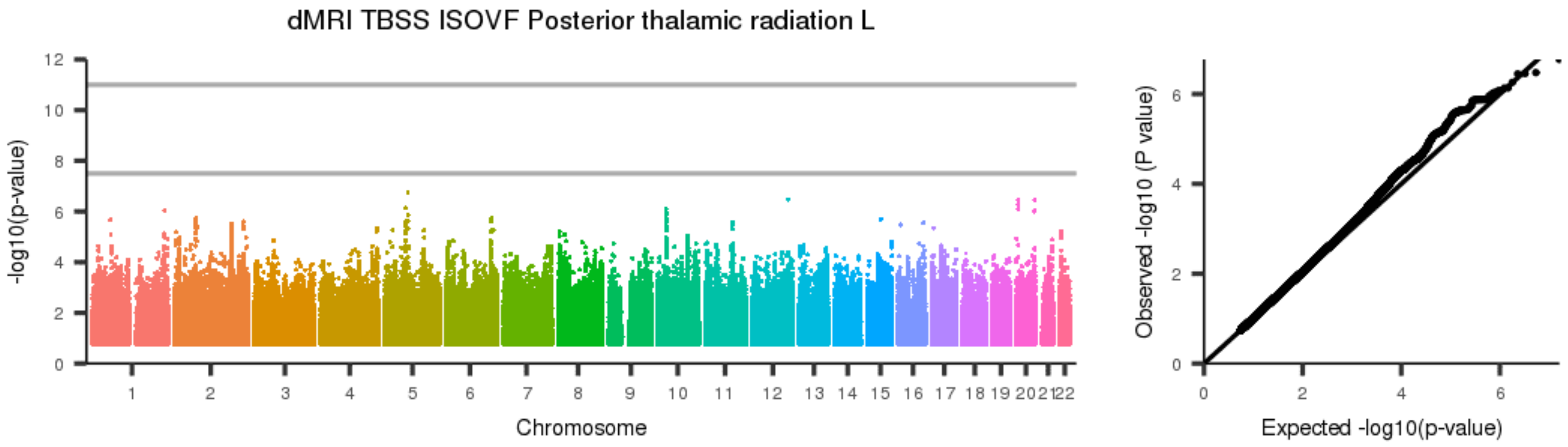

back to list of plots

Figure 610: dMRI TBSS ISOVF Sagittal stratum R

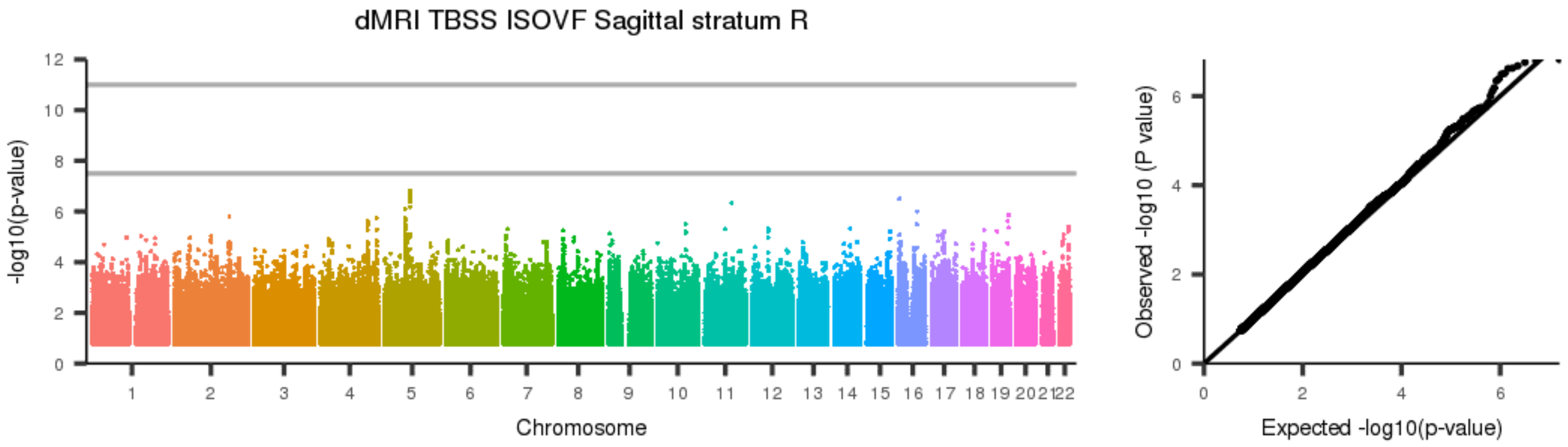

back to list of plots

Figure 611: dMRI TBSS ISOVF Sagittal stratum L

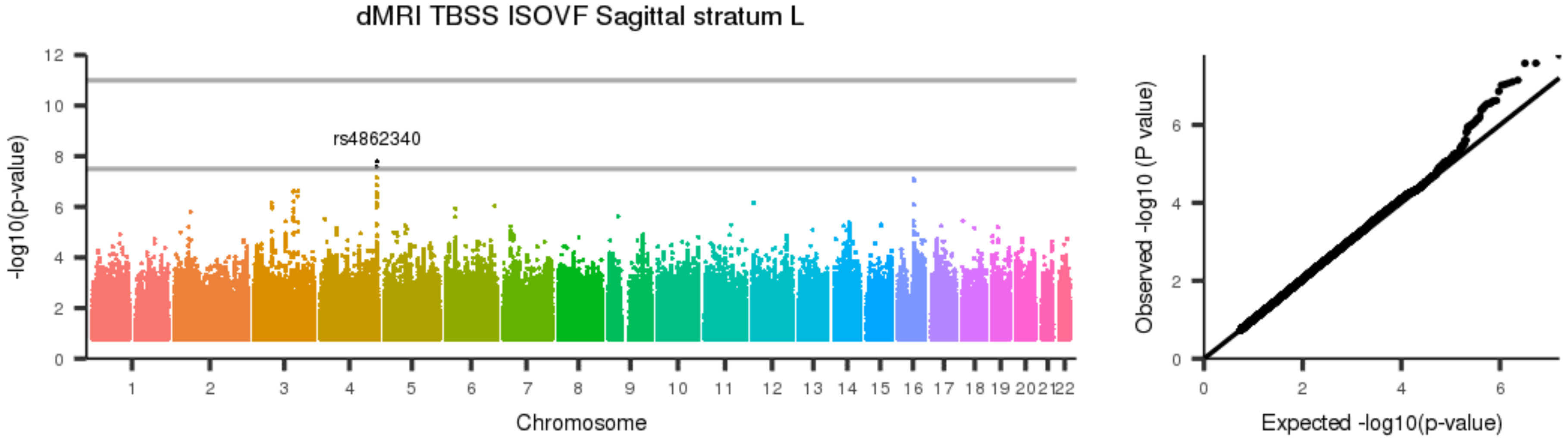

back to list of plots

Figure 612: dMRI TBSS ISOVF External capsule R

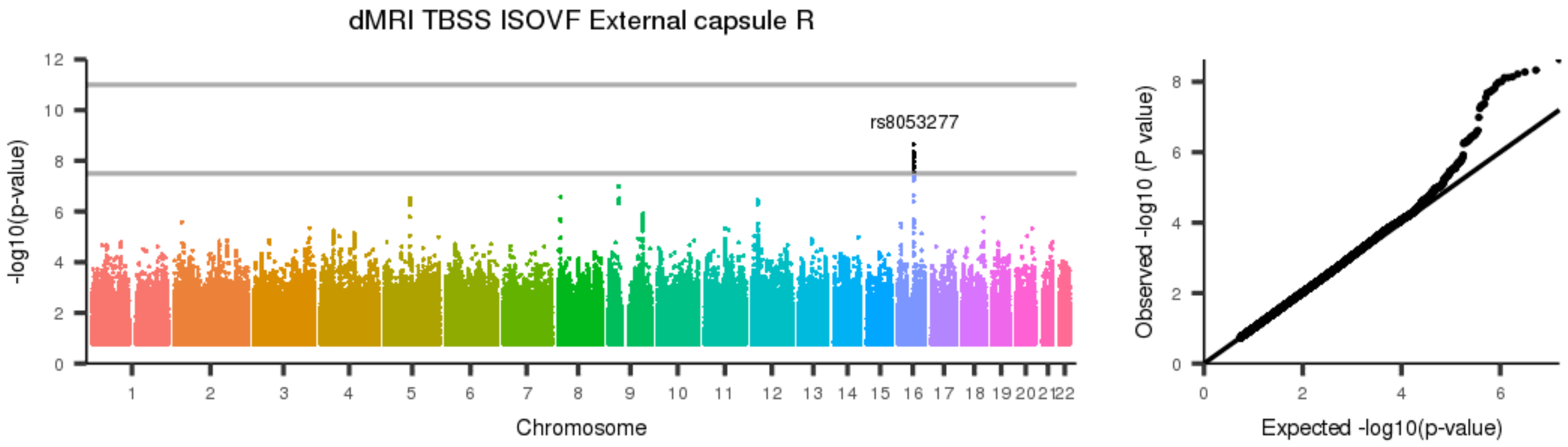

back to list of plots

Figure 613: dMRI TBSS ISOVF External capsule L

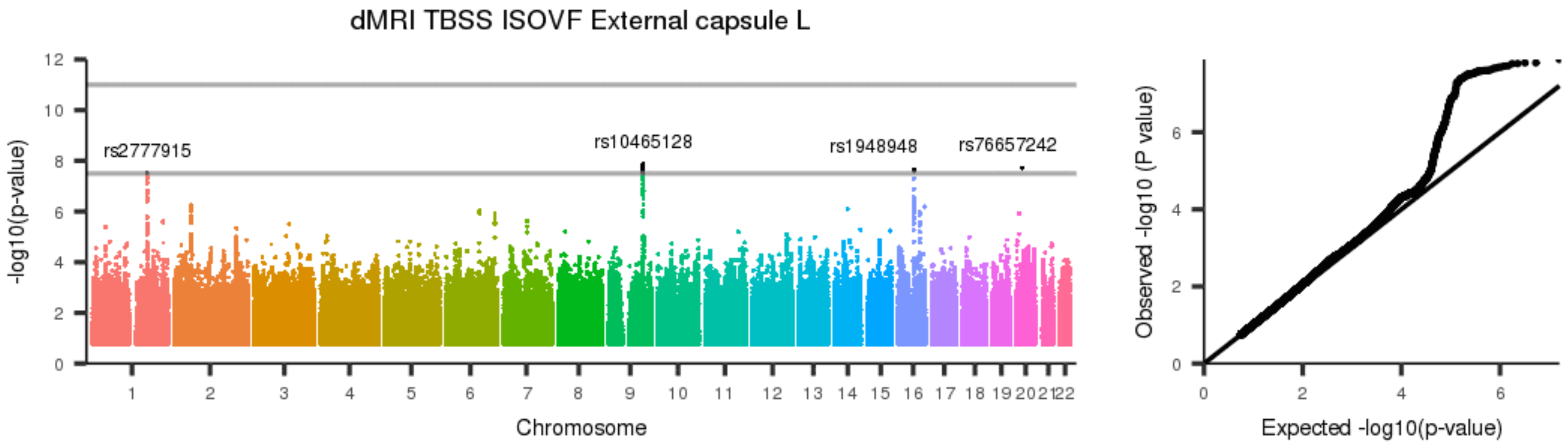

back to list of plots

Figure 614: dMRI TBSS ISOVF Cingulum cingulate gyrus R

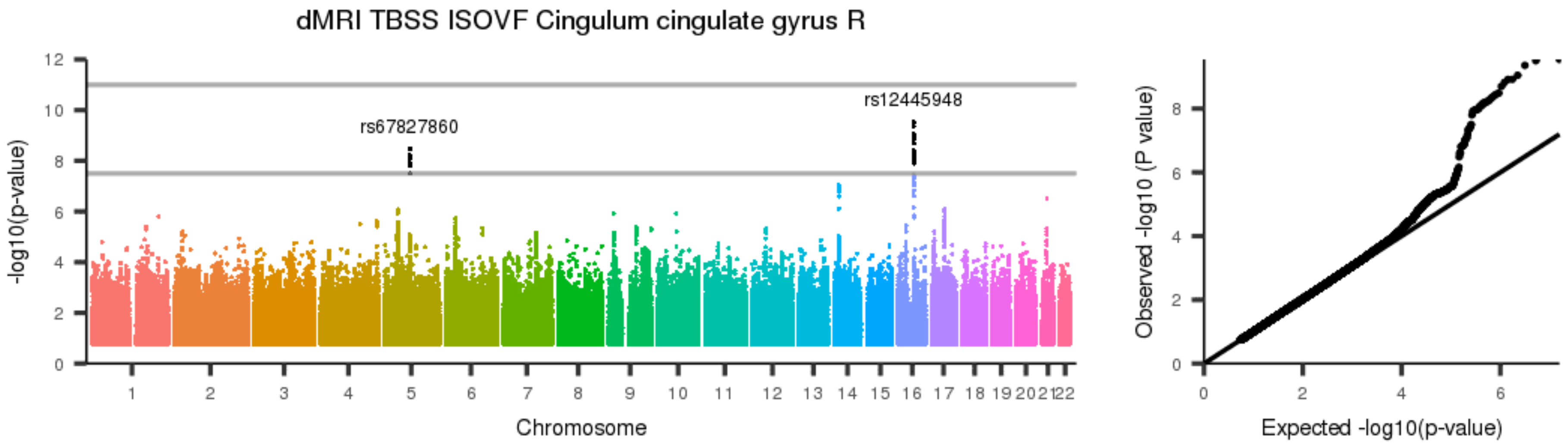

back to list of plots

Figure 615: dMRI TBSS ISOVF Cingulum cingulate gyrus L

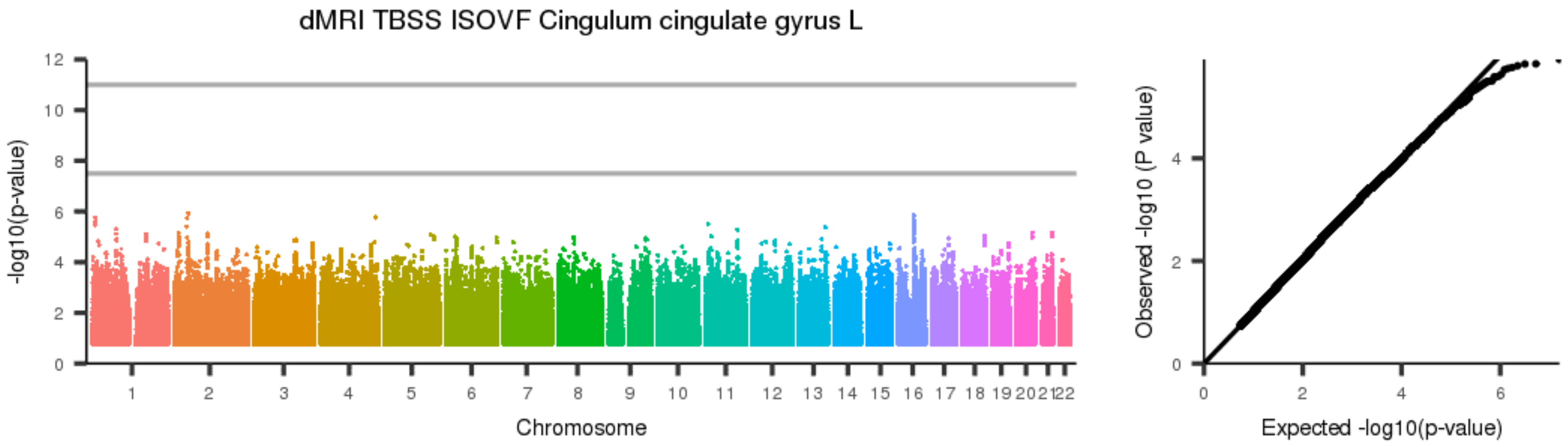

back to list of plots

Figure 616: dMRI TBSS ISOVF Cingulum hippocampus R

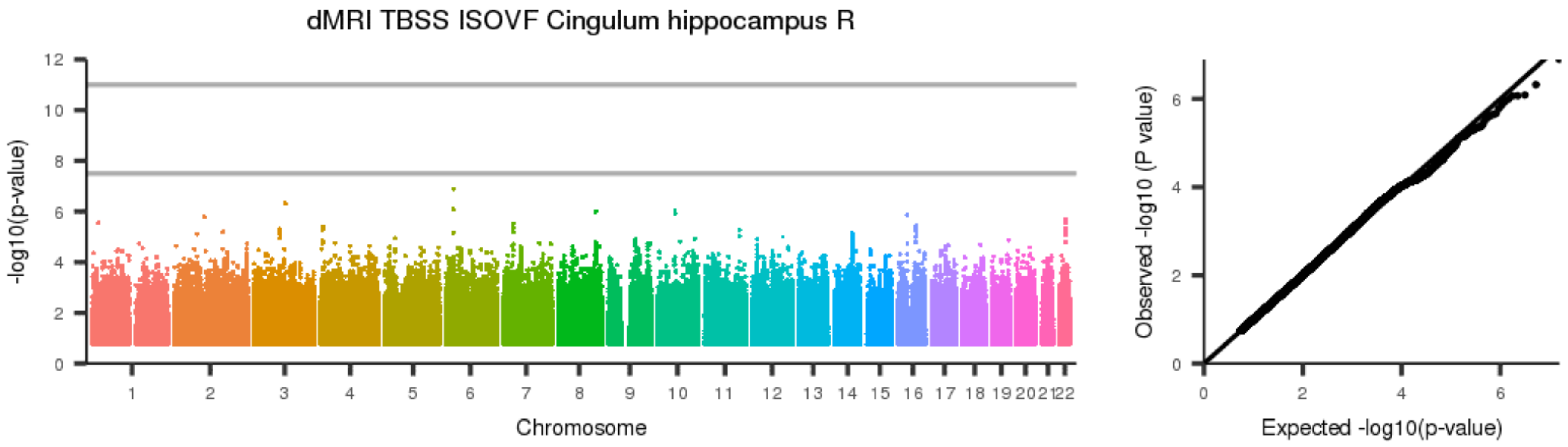

back to list of plots

Figure 617: dMRI TBSS ISOVF Cingulum hippocampus L

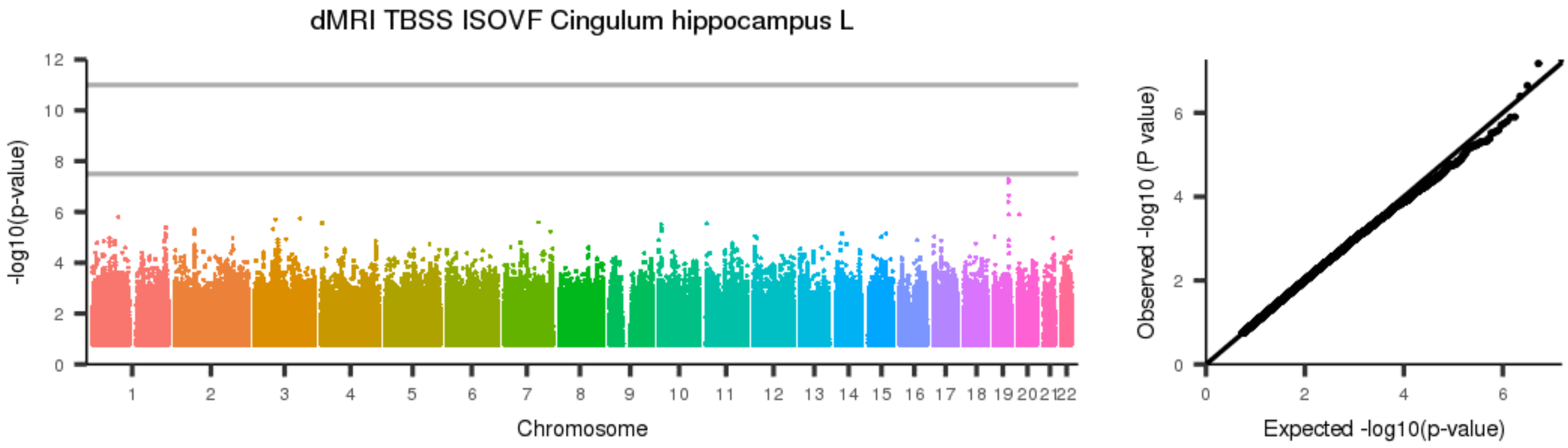

back to list of plots

Figure 618: dMRI TBSS ISOVF Fornix cres+Stria terminalis R

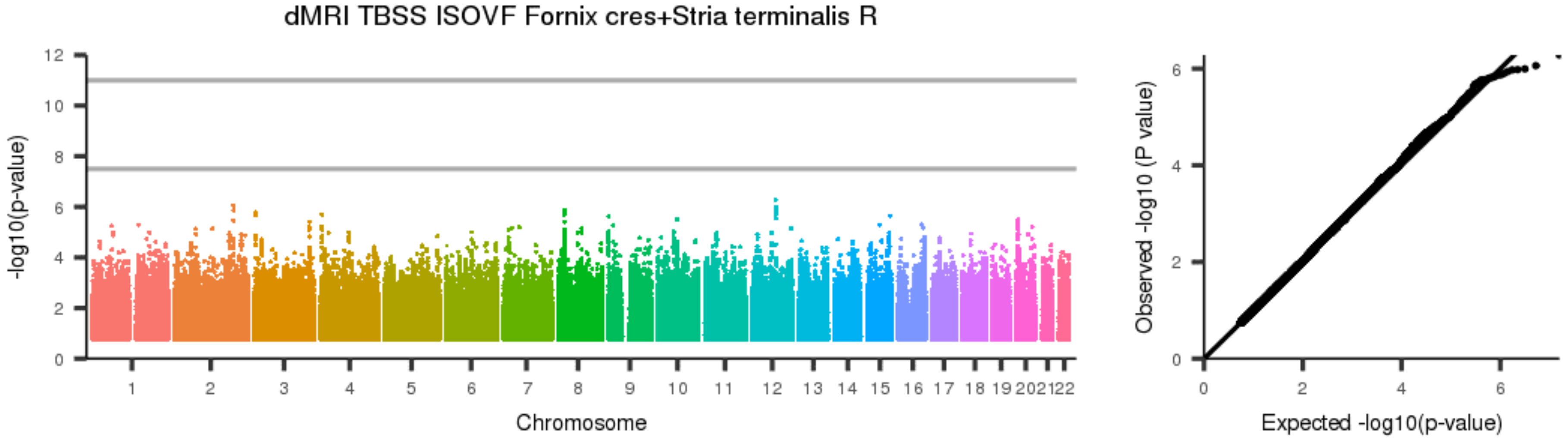

back to list of plots

Figure 619: dMRI TBSS ISOVF Fornix cres+Stria terminalis L

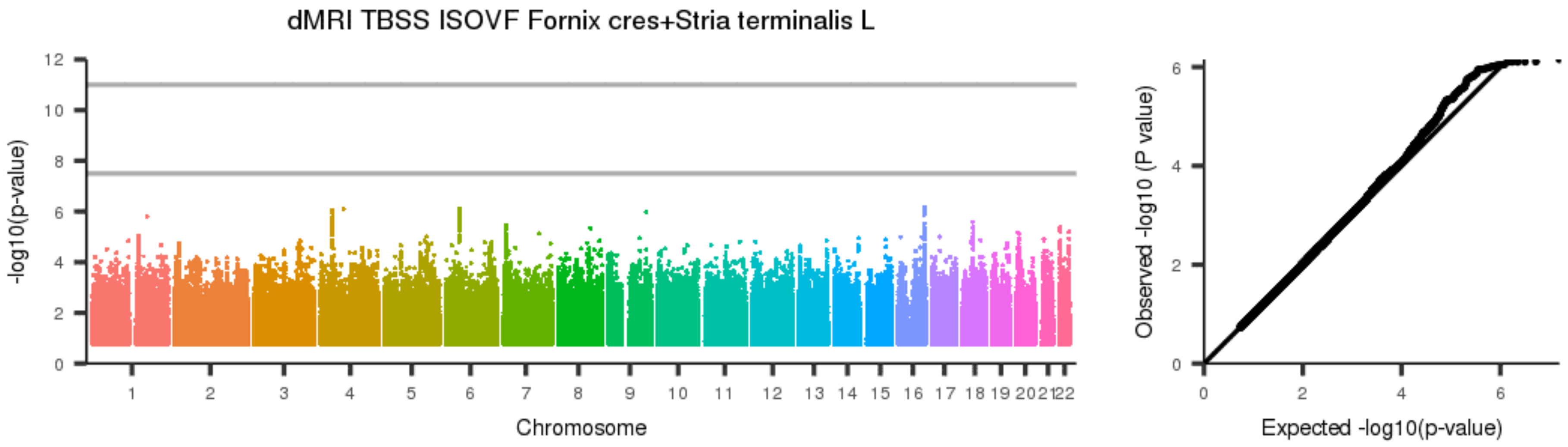

back to list of plots

Figure 620: dMRI TBSS ISOVF Superior longitudinal fasciculus R

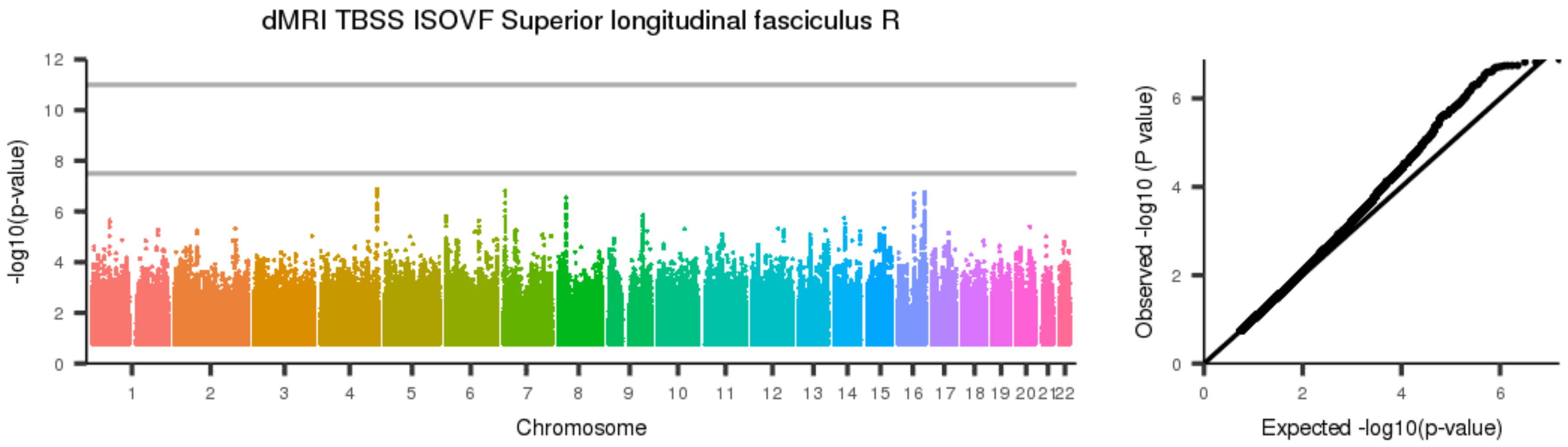

back to list of plots

Figure 621: dMRI TBSS ISOVF Superior longitudinal fasciculus L

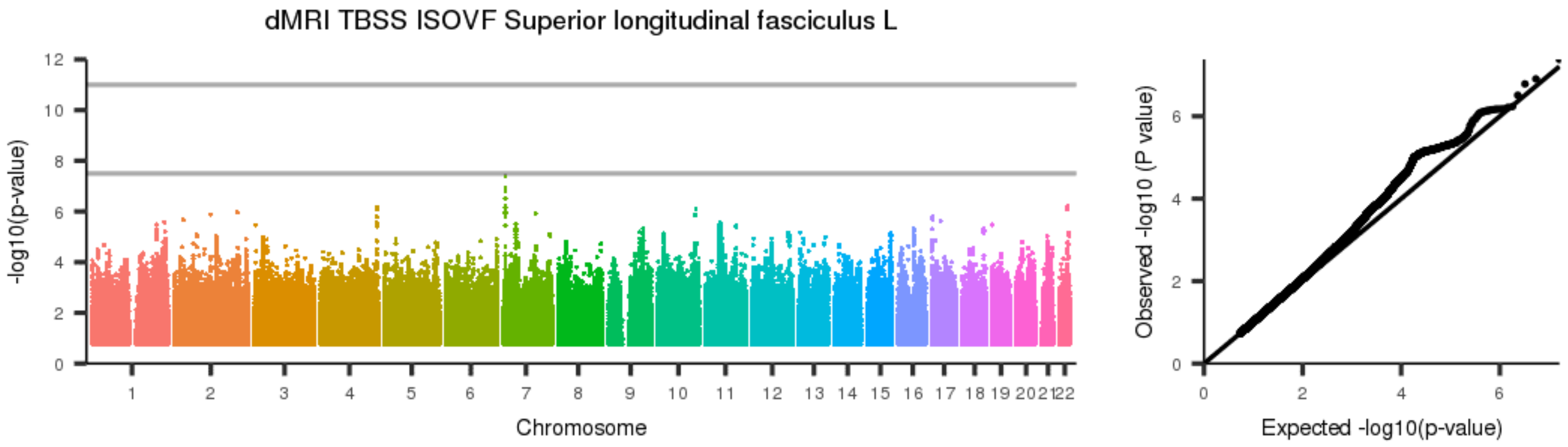

back to list of plots

Figure 622: dMRI TBSS ISOVF Superior fronto-occipital fasciculus R

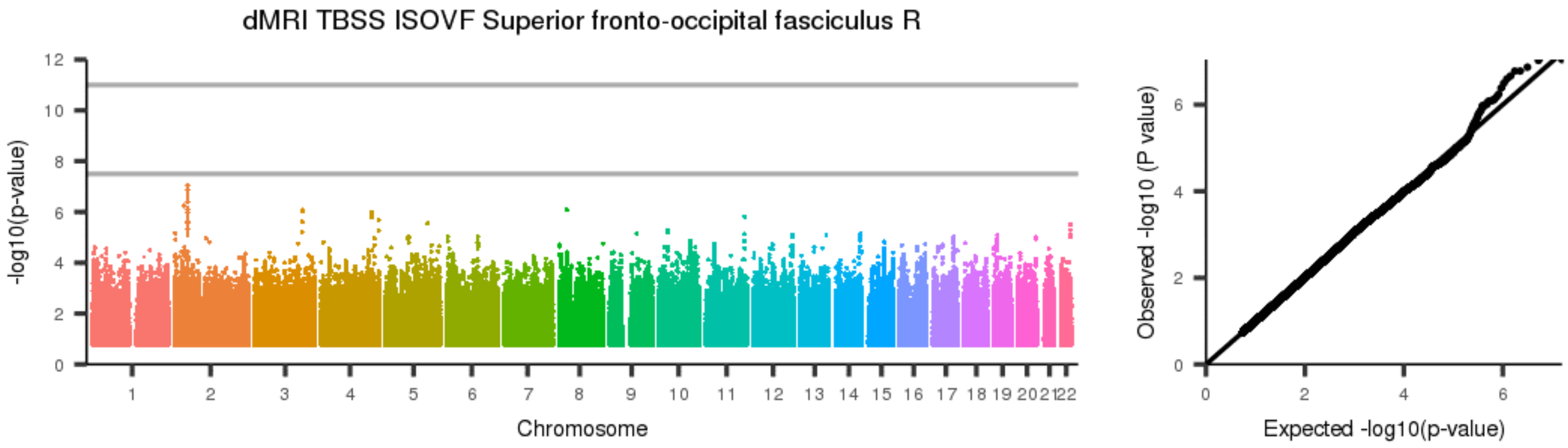

back to list of plots

Figure 623: dMRI TBSS ISOVF Superior fronto-occipital fasciculus L

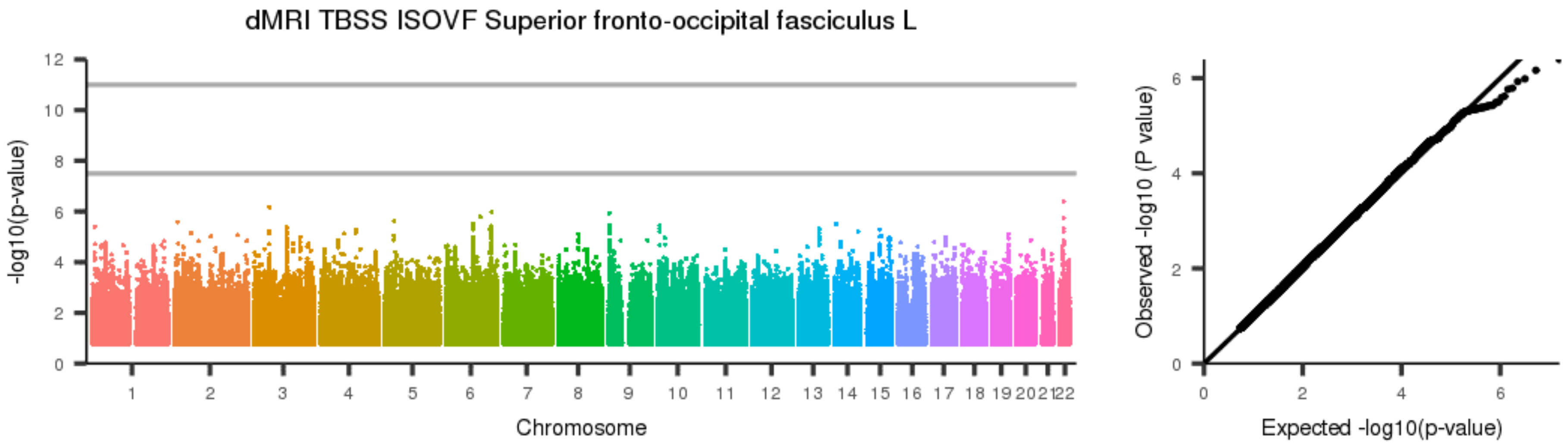

back to list of plots

Figure 624: dMRI TBSS ISOVF Uncinate fasciculus R

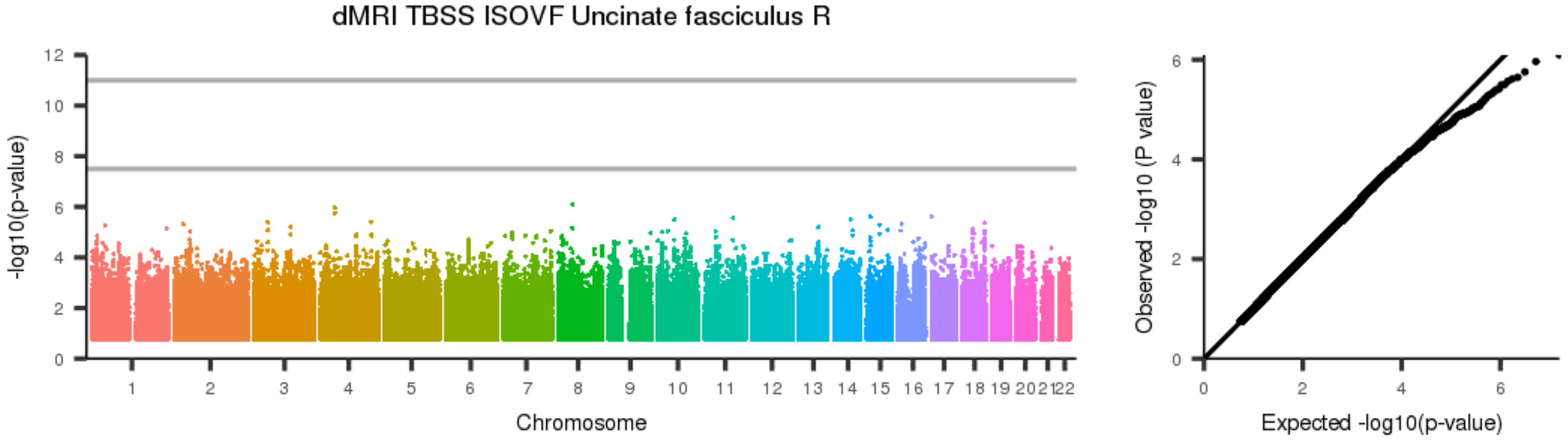

back to list of plots

Figure 625: dMRI TBSS ISOVF Uncinate fasciculus L

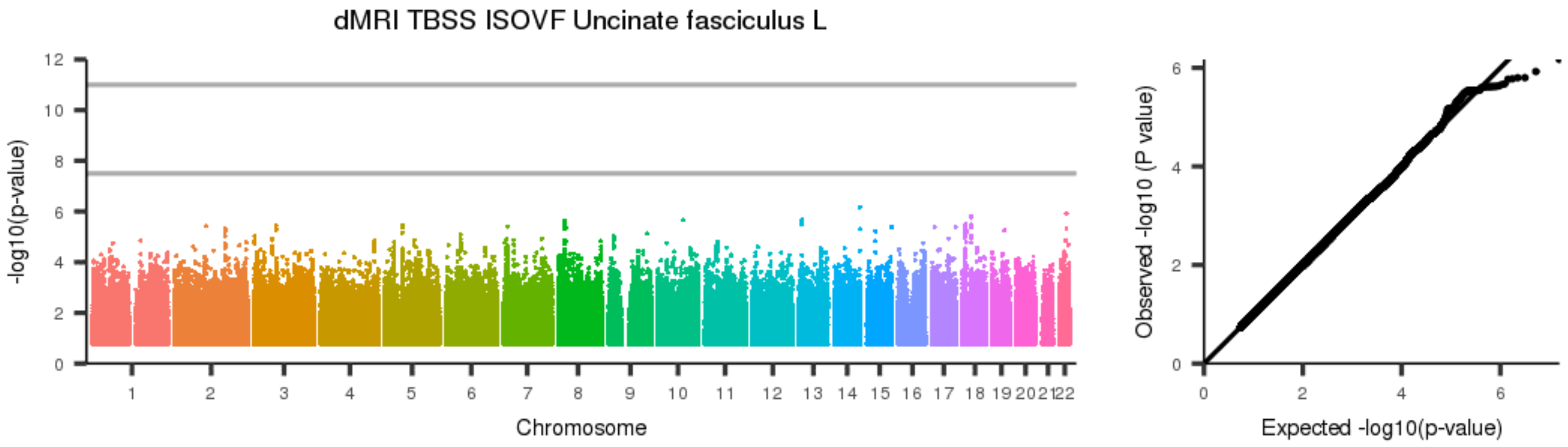

back to list of plots

Figure 626: dMRI TBSS ISOVF Tapetum R

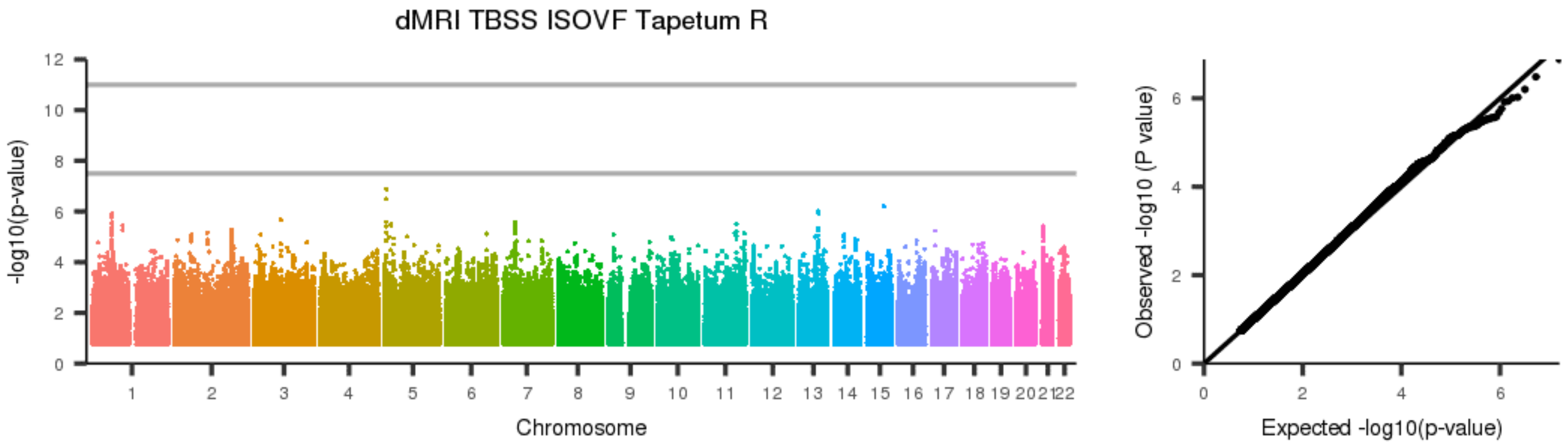

back to list of plots

Figure 627: dMRI TBSS ISOVF Tapetum L

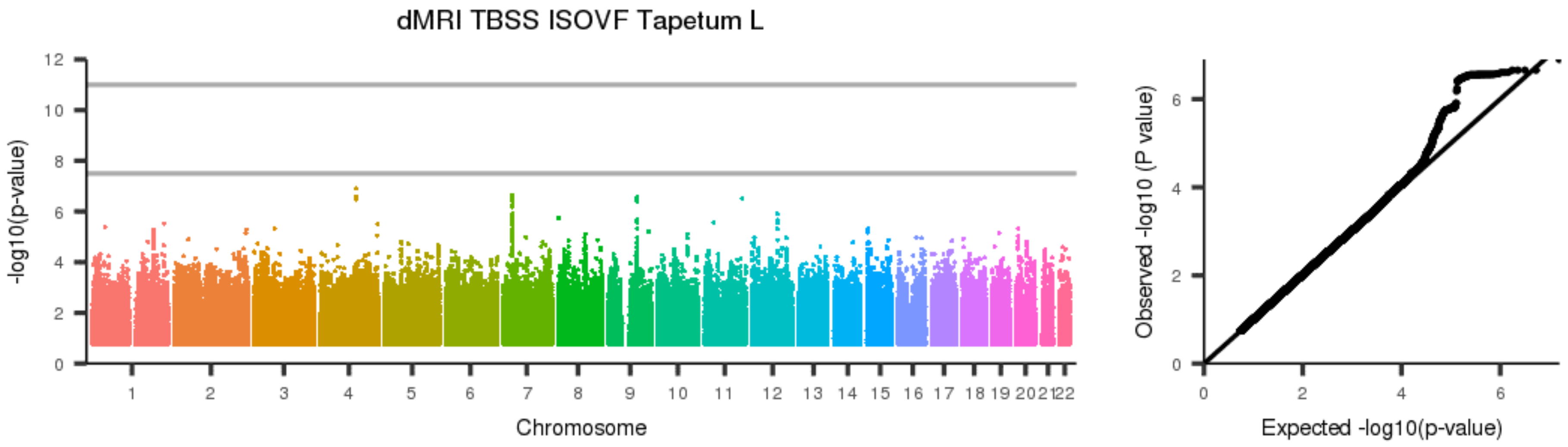

back to list of plots

Figure 628: dMRI ProbtrackX FA ar l

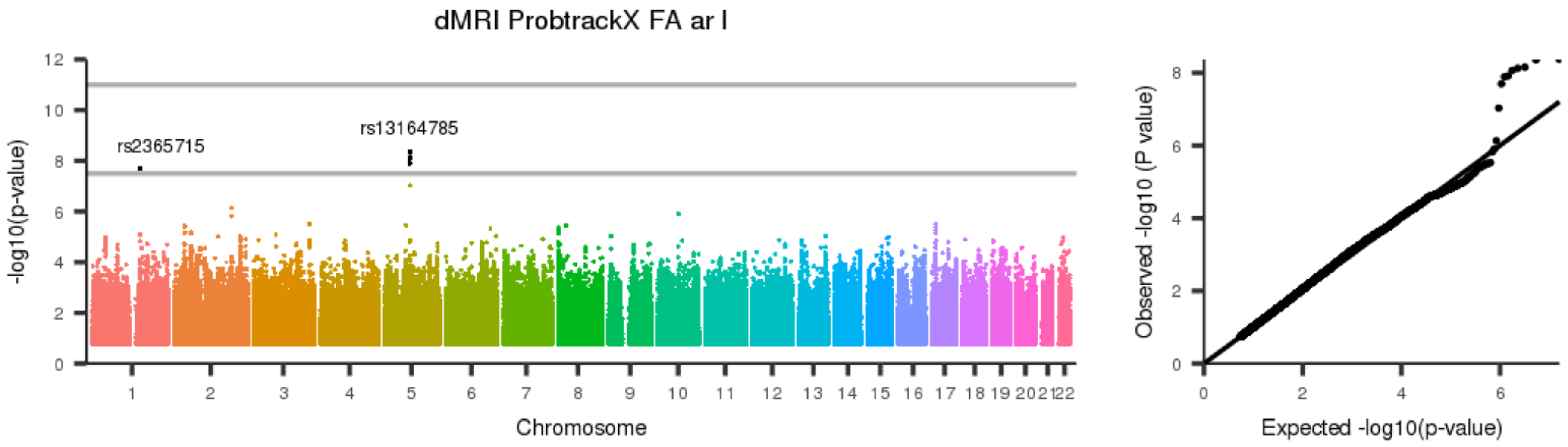

back to list of plots

Figure 629: dMRI ProbtrackX FA ar r

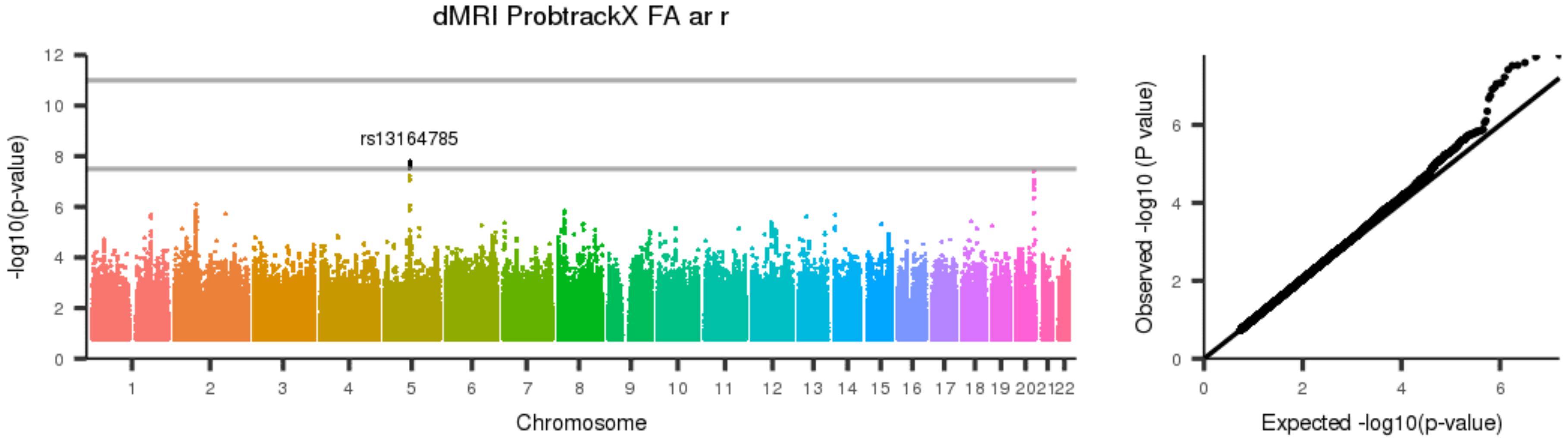

back to list of plots

Figure 630: dMRI ProbtrackX FA atr l

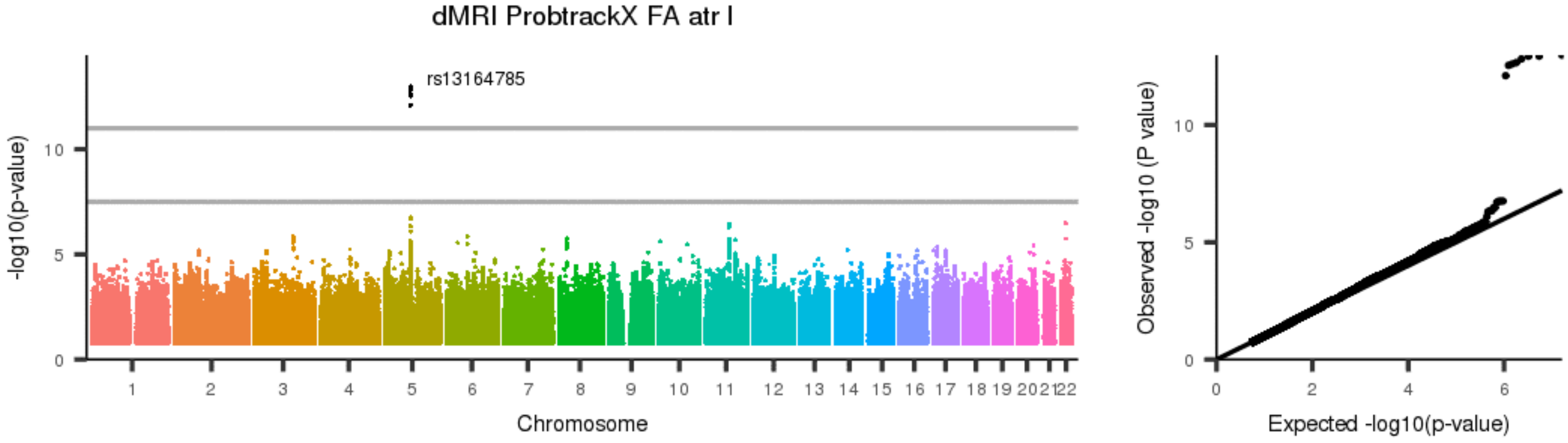

back to list of plots

Figure 631: dMRI ProbtrackX FA atr r

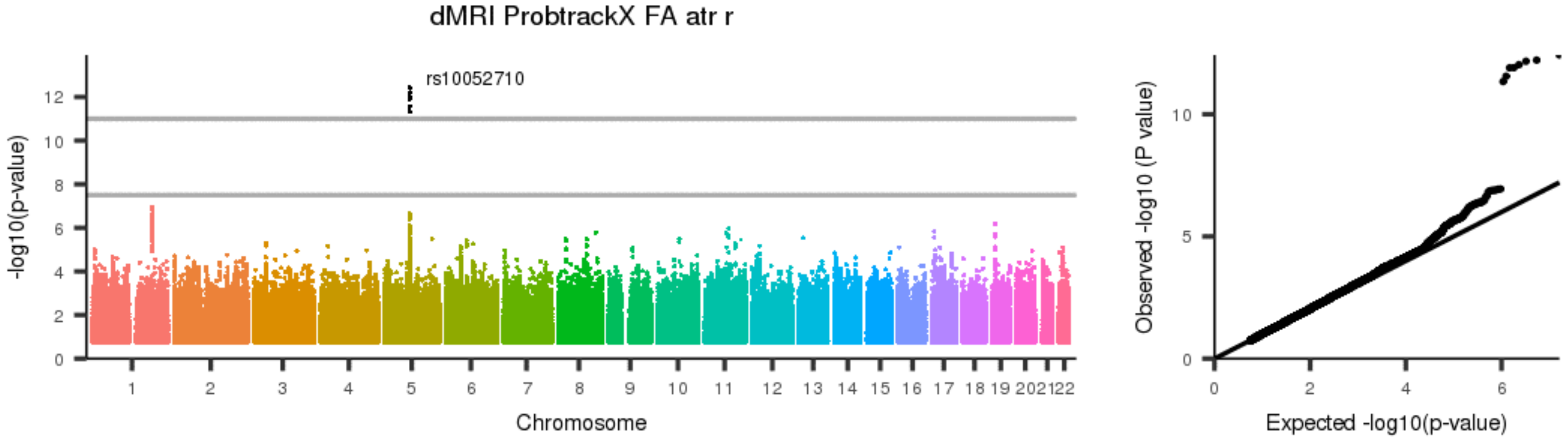

back to list of plots

Figure 632: dMRI ProbtrackX FA cgc l

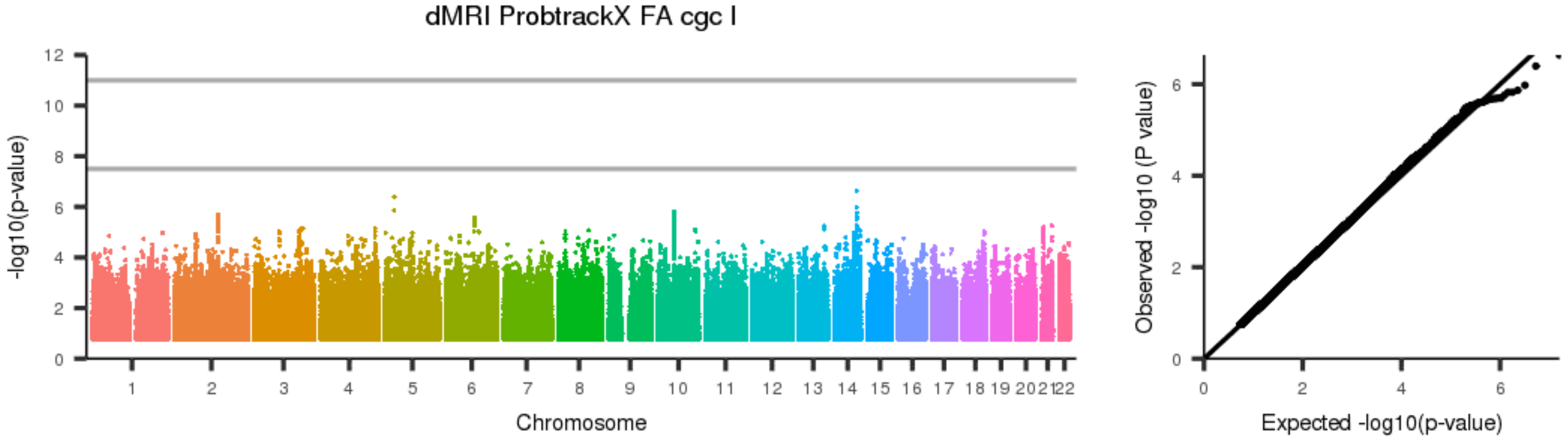

back to list of plots

Figure 633: dMRI ProbtrackX FA cgc r

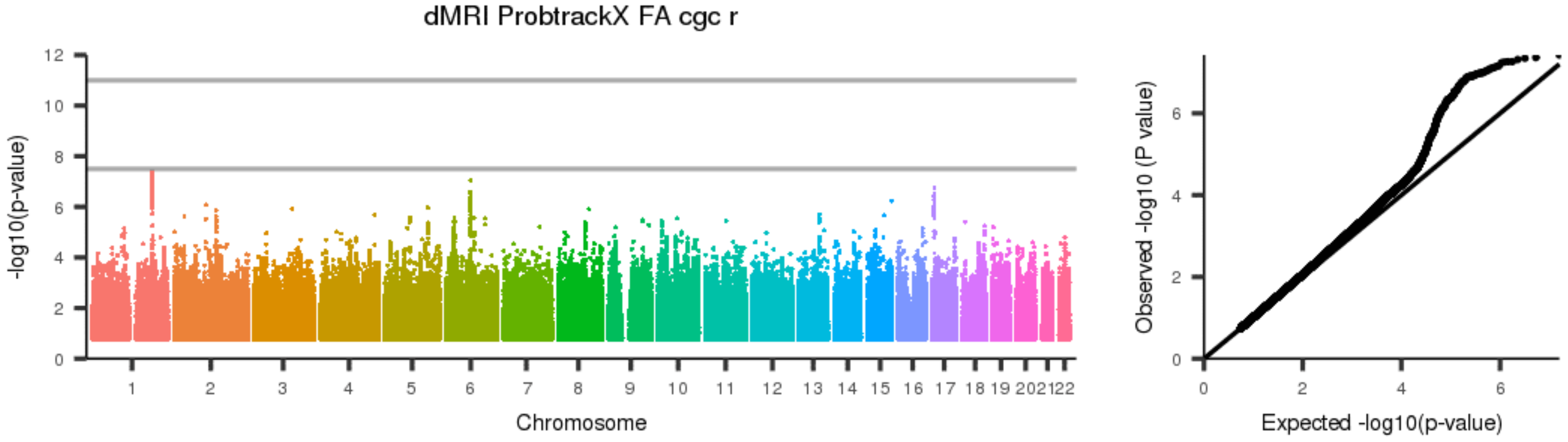

back to list of plots

Figure 634: dMRI ProbtrackX FA cgh l

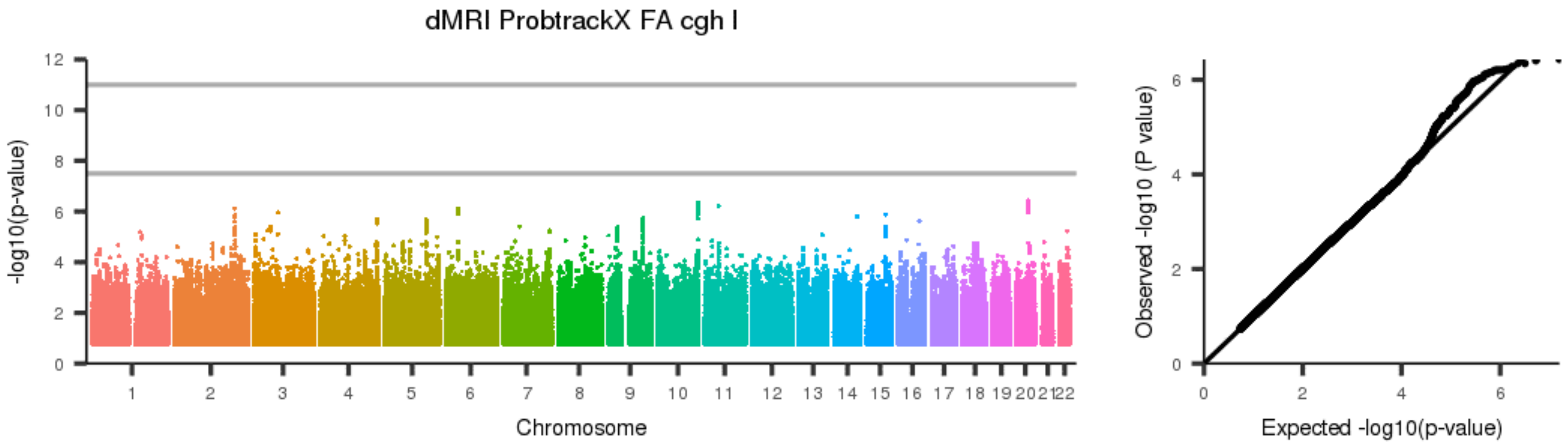

back to list of plots

Figure 635: dMRI ProbtrackX FA cgh r

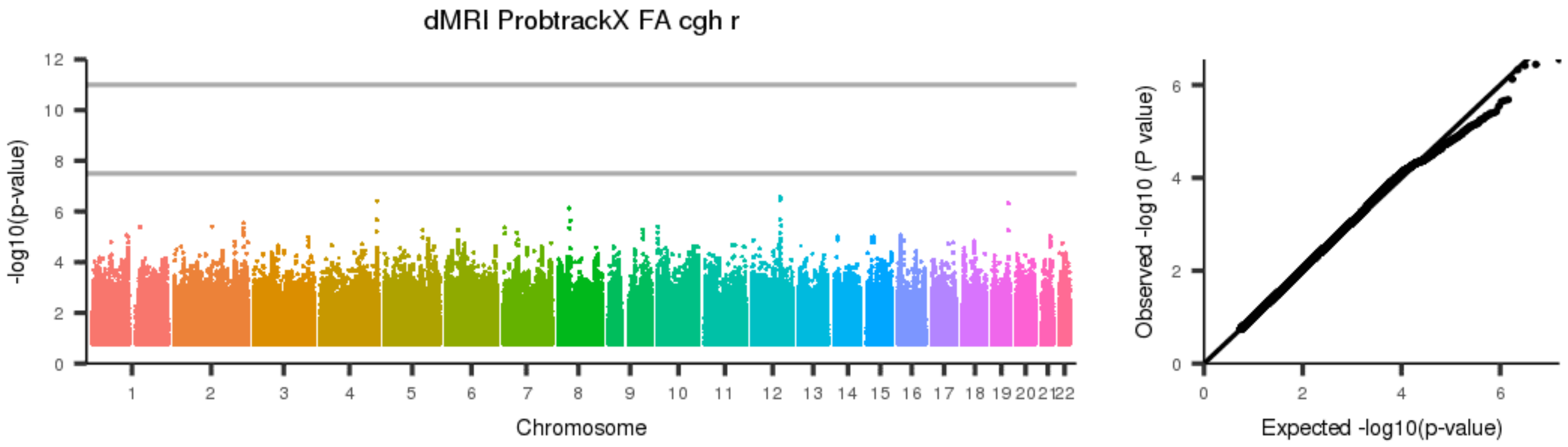

back to list of plots

Figure 636: dMRI ProbtrackX FA cst l

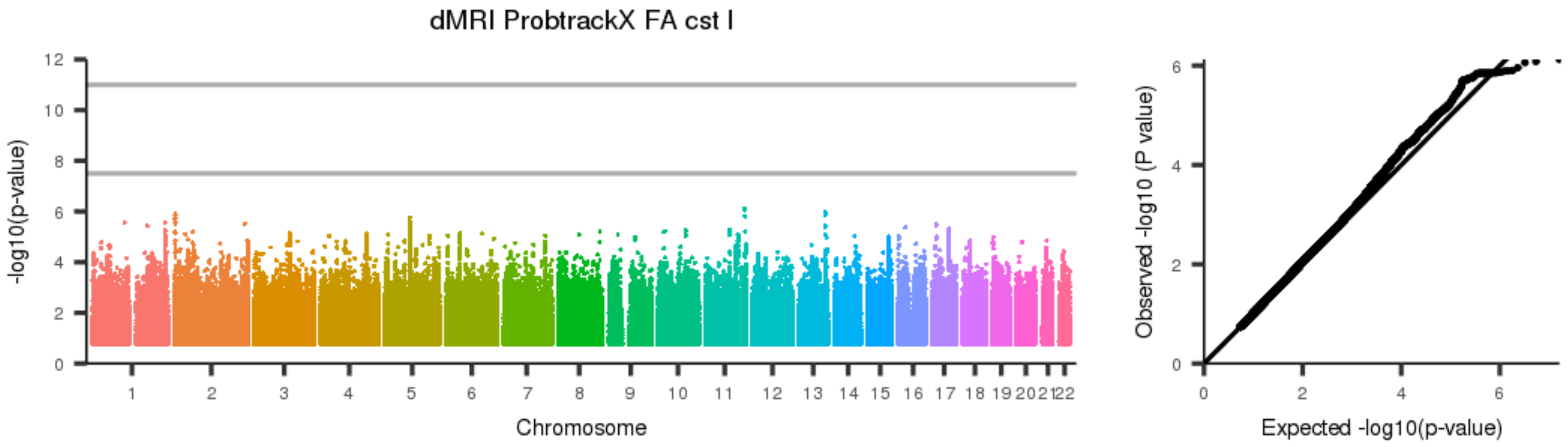

back to list of plots

Figure 637: dMRI ProbtrackX FA cst r

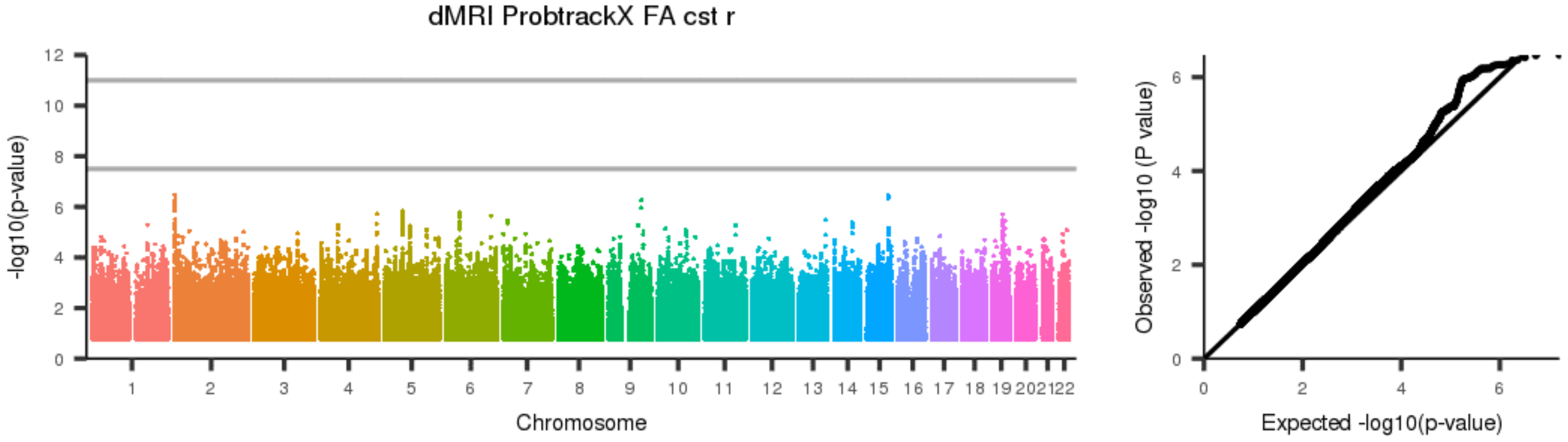

back to list of plots

Figure 638: dMRI ProbtrackX FA fma

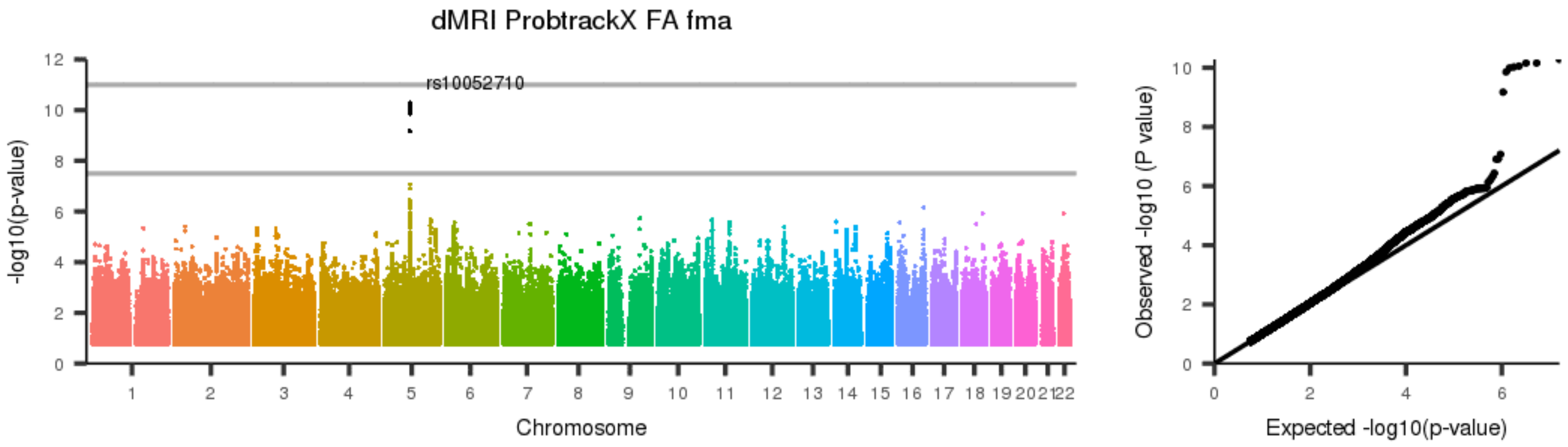

back to list of plots

Figure 639: dMRI ProbtrackX FA fmi

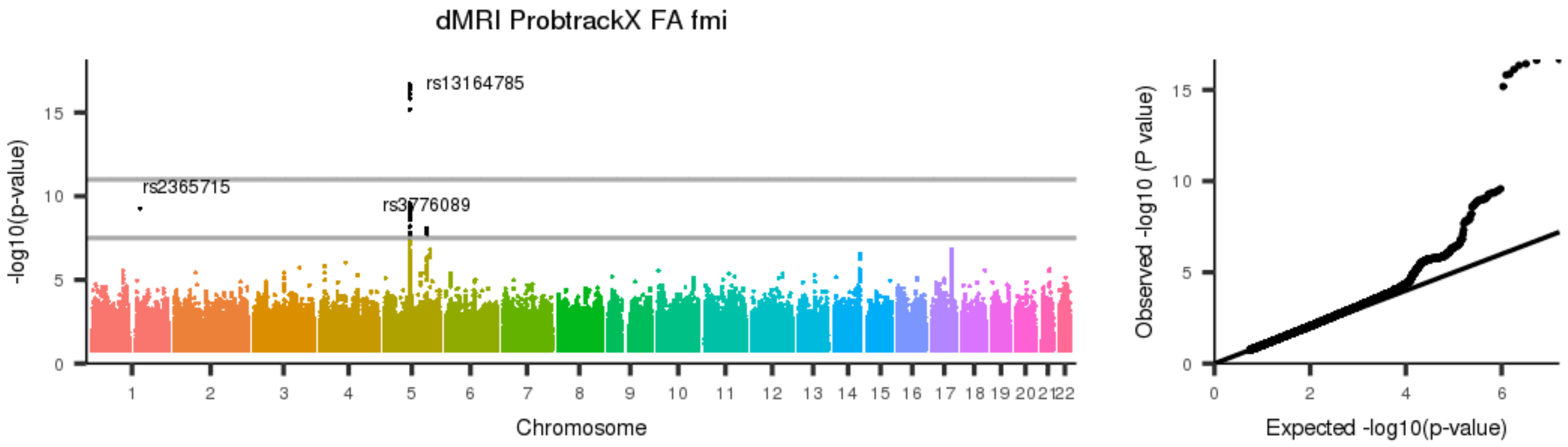

back to list of plots

Figure 640: dMRI ProbtrackX FA ifo l

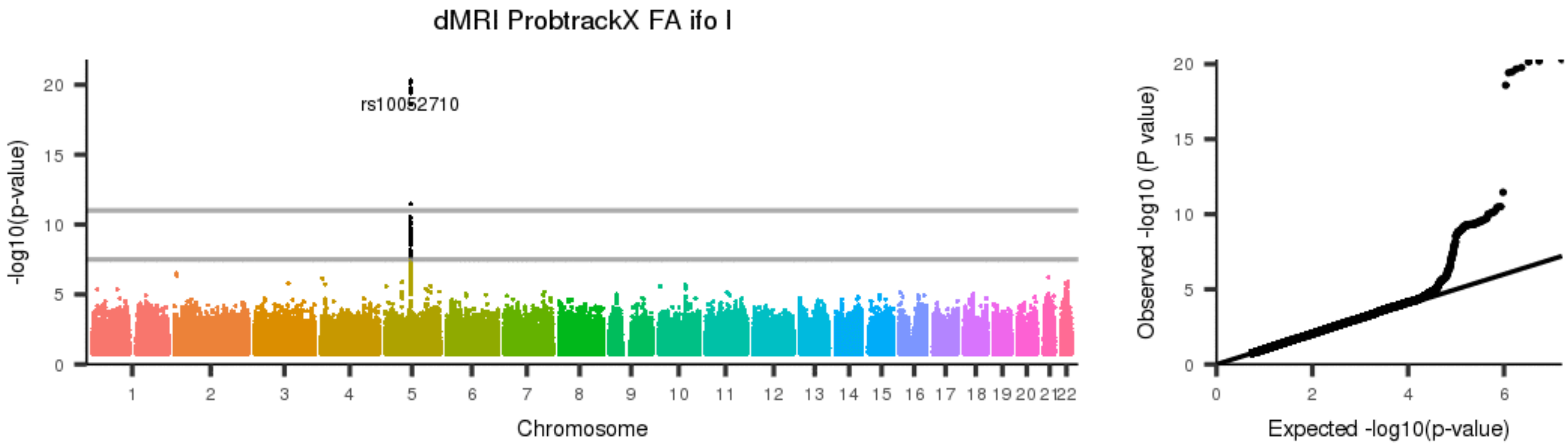

back to list of plots

Figure 641: dMRI ProbtrackX FA ifo r

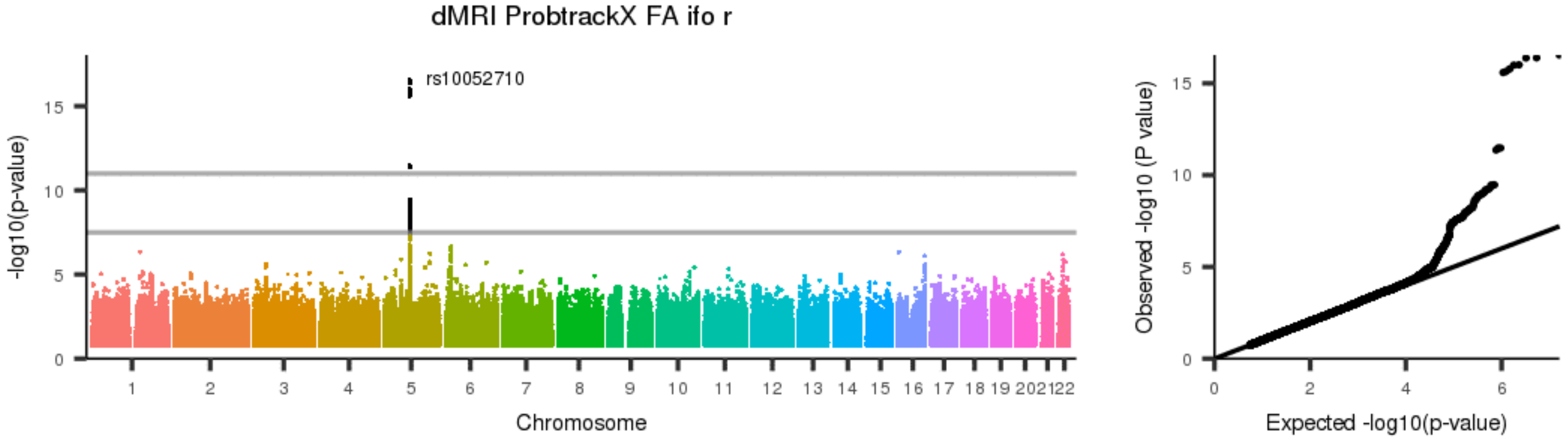

back to list of plots

Figure 642: dMRI ProbtrackX FA ilf l

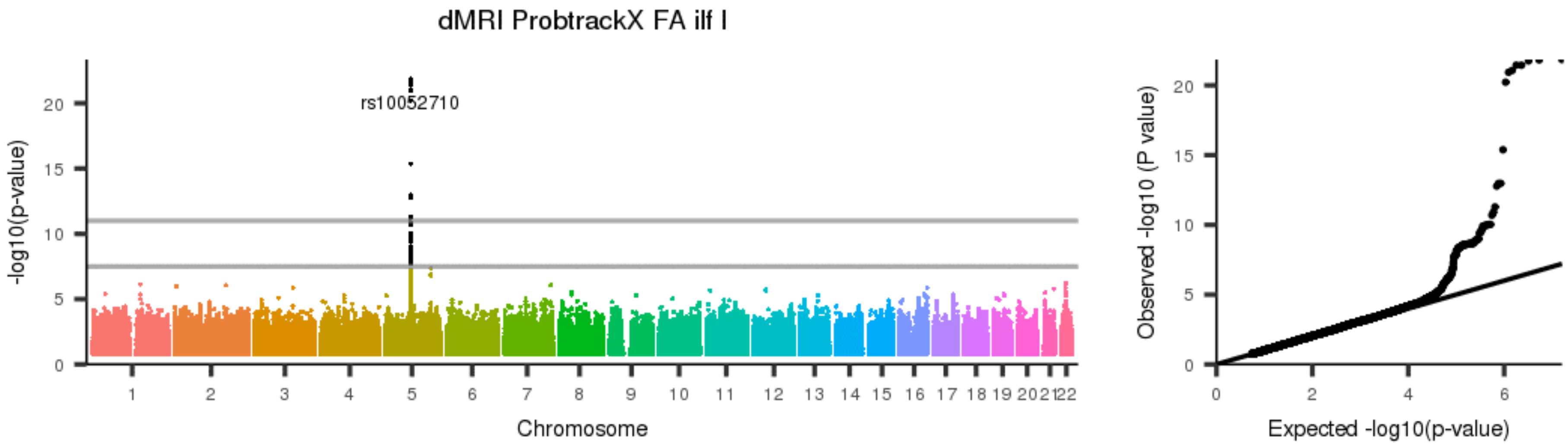

back to list of plots

Figure 643: dMRI ProbtrackX FA ilf r

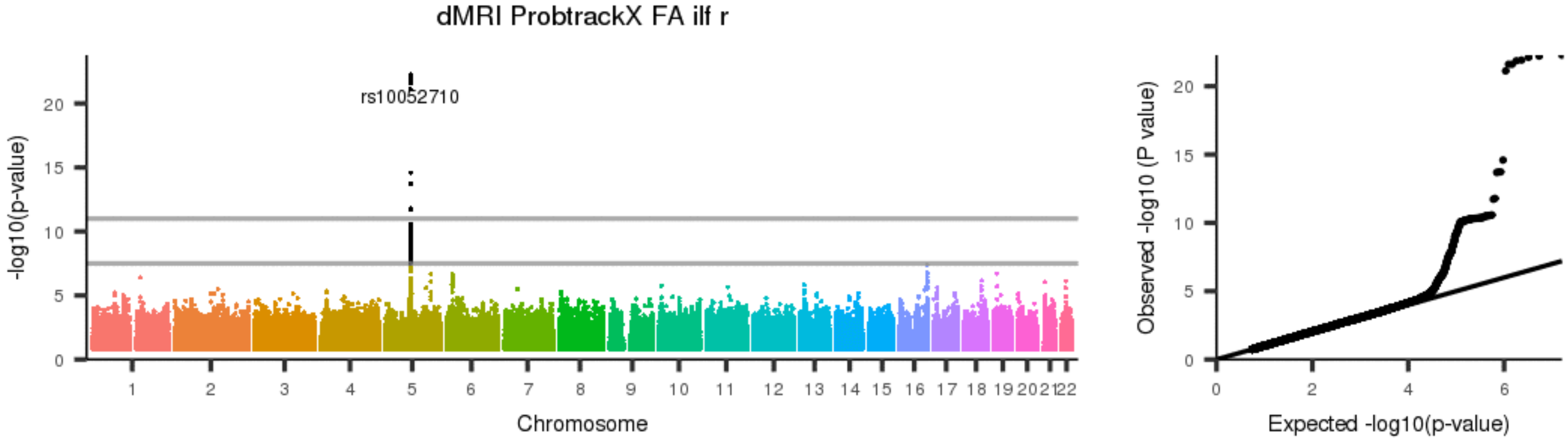

back to list of plots

Figure 644: dMRI ProbtrackX FA mcp

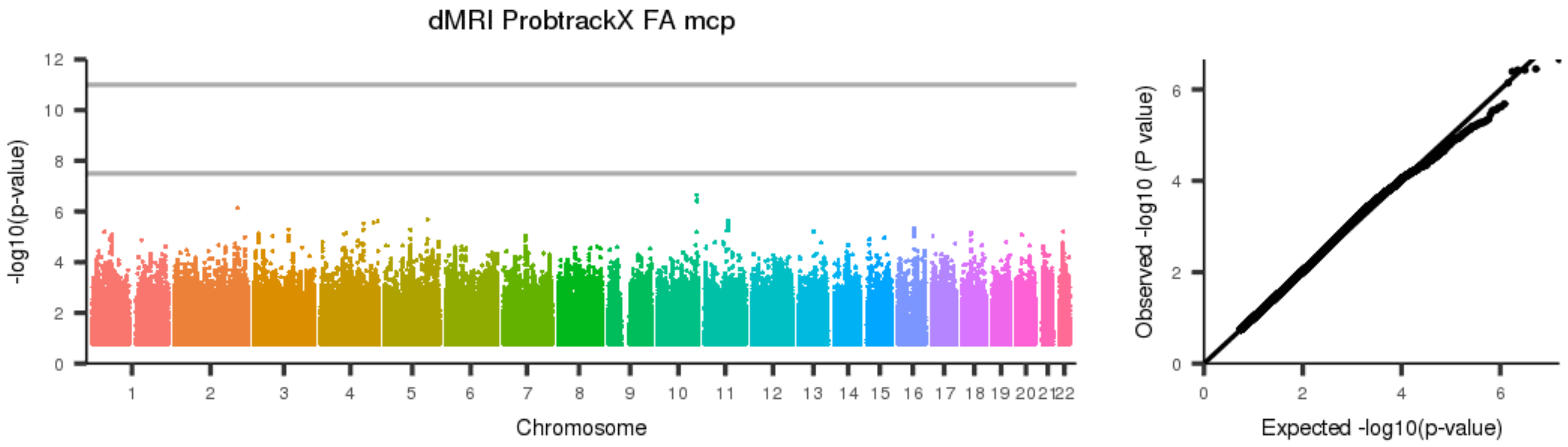

back to list of plots

Figure 645: dMRI ProbtrackX FA ml l

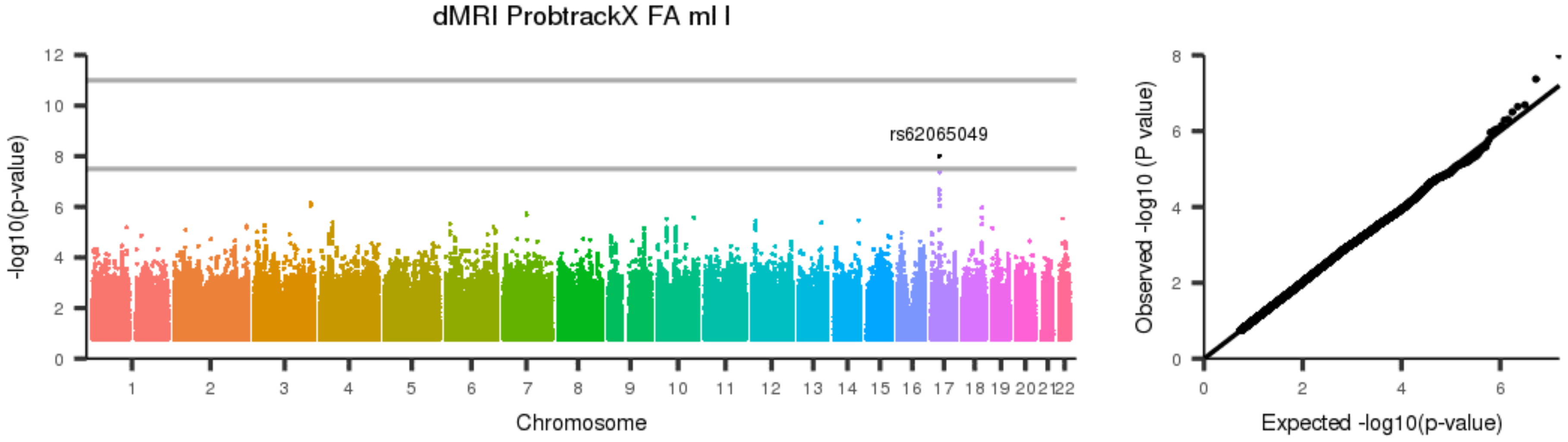

back to list of plots

Figure 646: dMRI ProbtrackX FA ml r

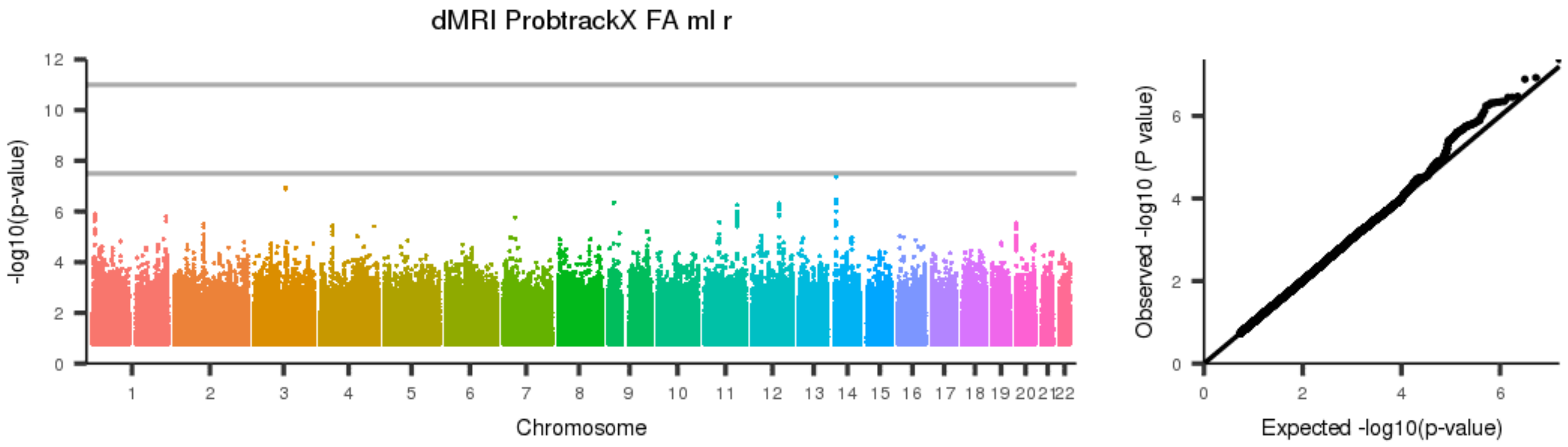

back to list of plots

Figure 647: dMRI ProbtrackX FA ptr l

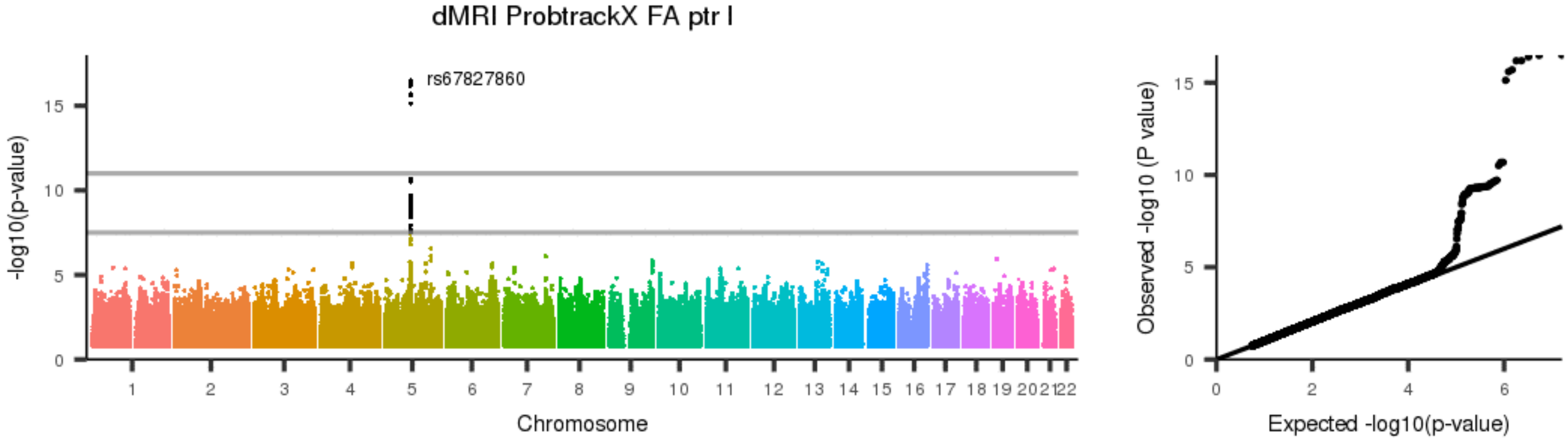

back to list of plots

Figure 648: dMRI ProbtrackX FA ptr r

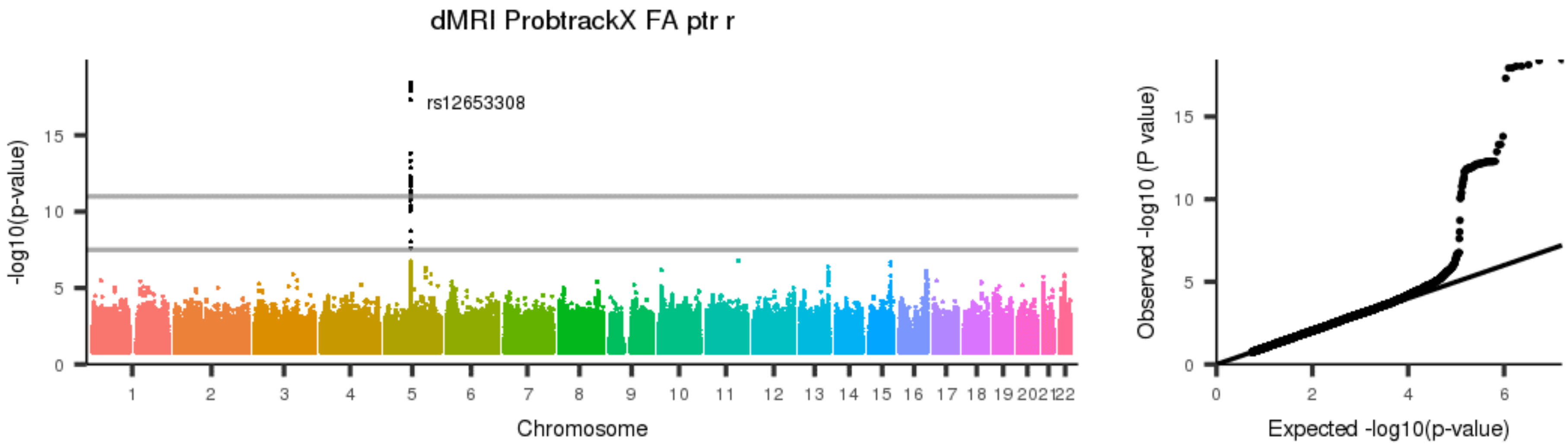

back to list of plots

Figure 649: dMRI ProbtrackX FA slf I

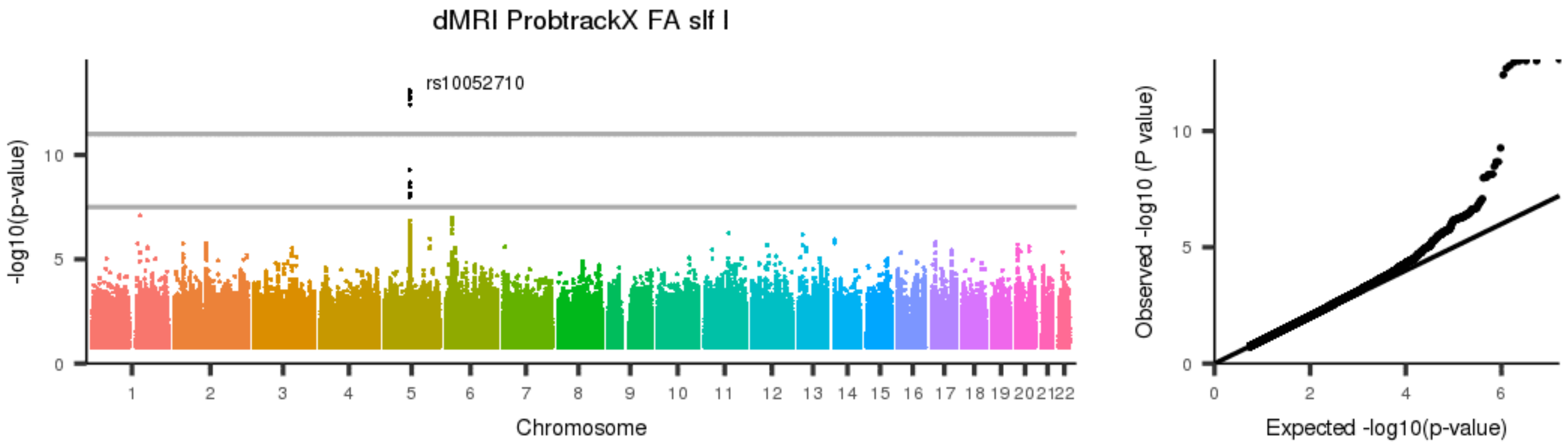

back to list of plots

Figure 650: dMRI ProbtrackX FA slf r

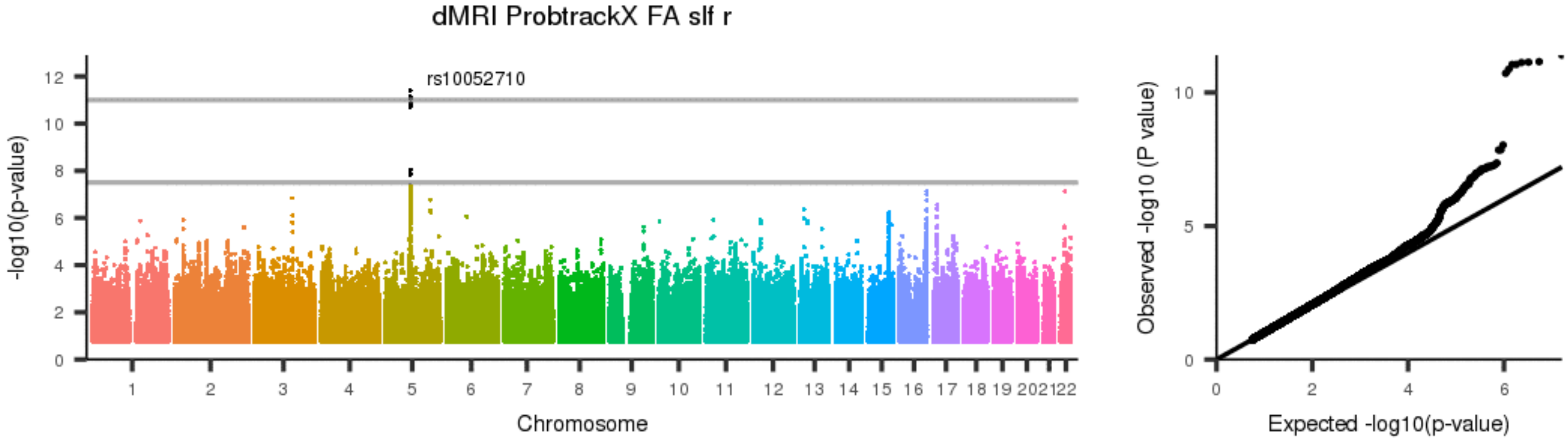

back to list of plots

Figure 651: dMRI ProbtrackX FA str l

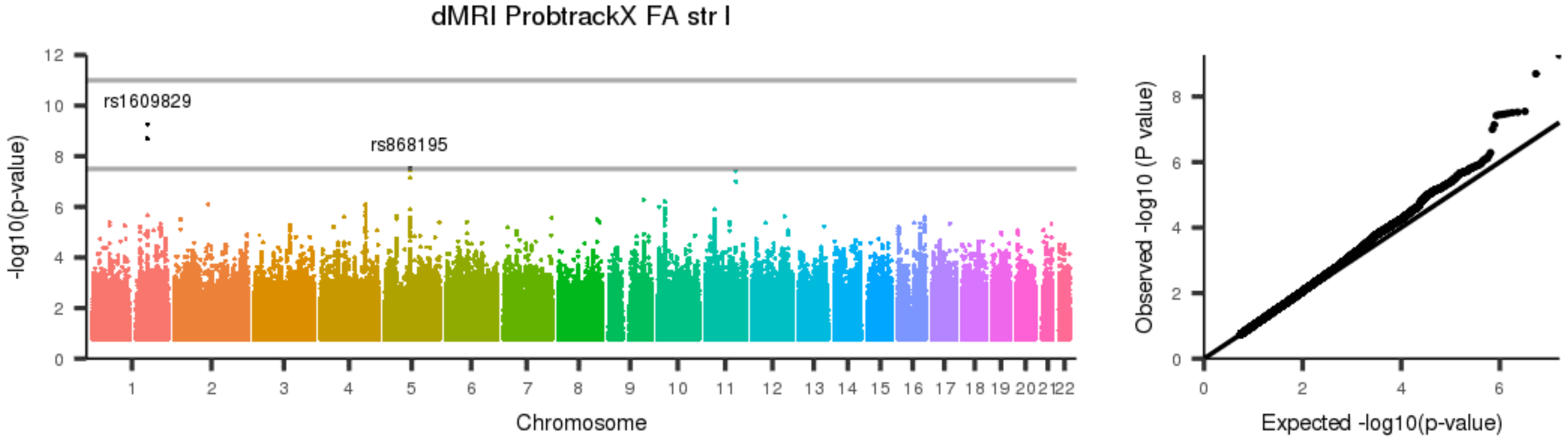

back to list of plots

Figure 652: dMRI ProbtrackX FA str r

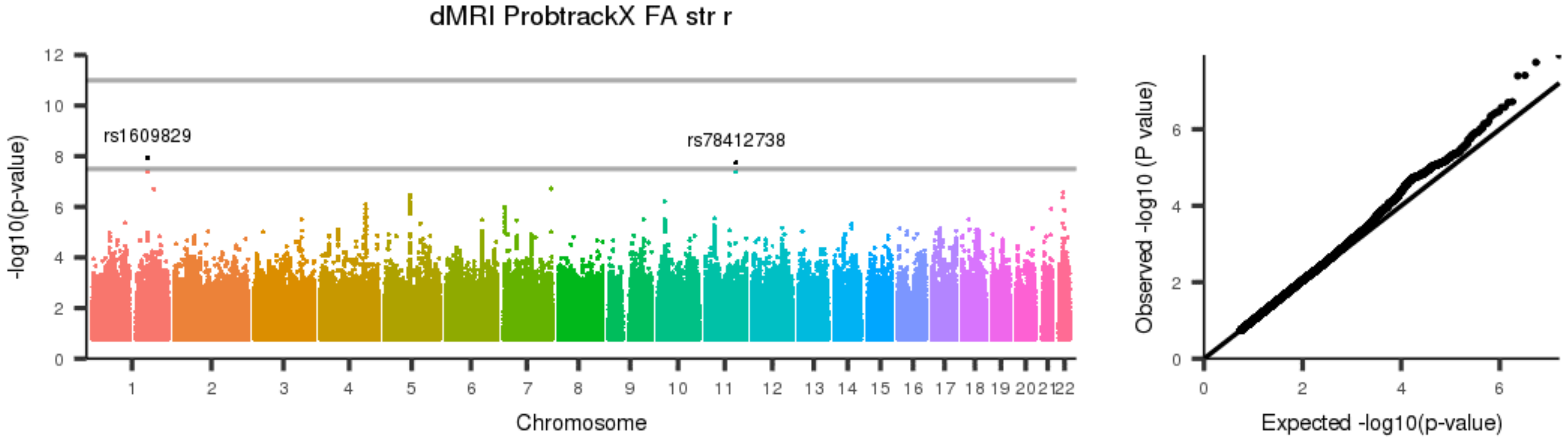

back to list of plots

Figure 653: dMRI ProbtrackX FA unc l

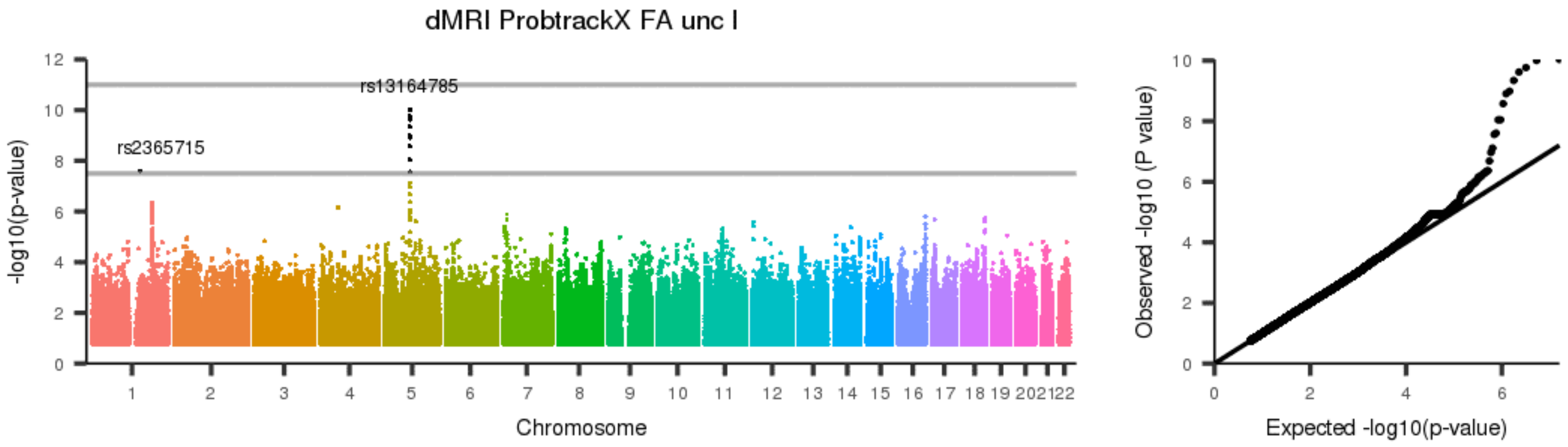

back to list of plots

Figure 654: dMRI ProbtrackX FA unc r

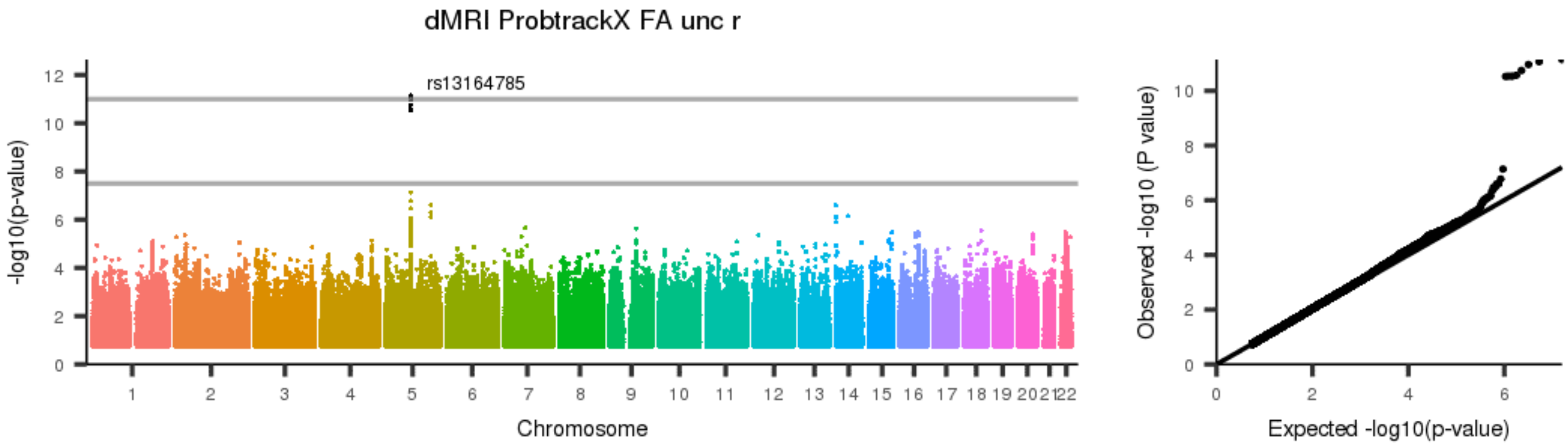

back to list of plots

Figure 655: dMRI ProbtrackX MD ar l

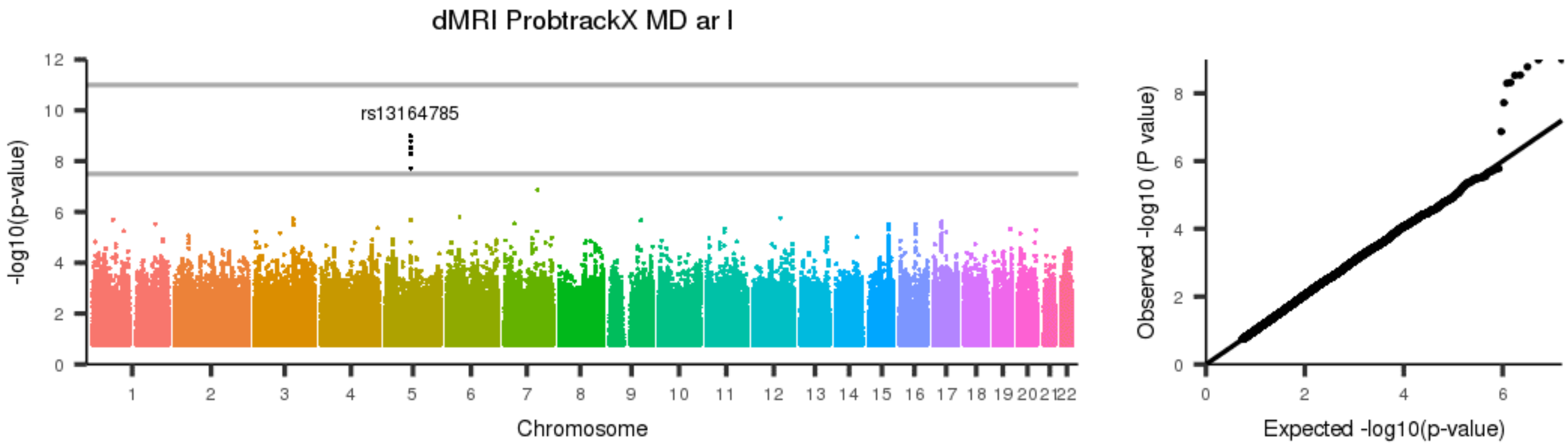

back to list of plots

Figure 656: dMRI ProbtrackX MD ar r

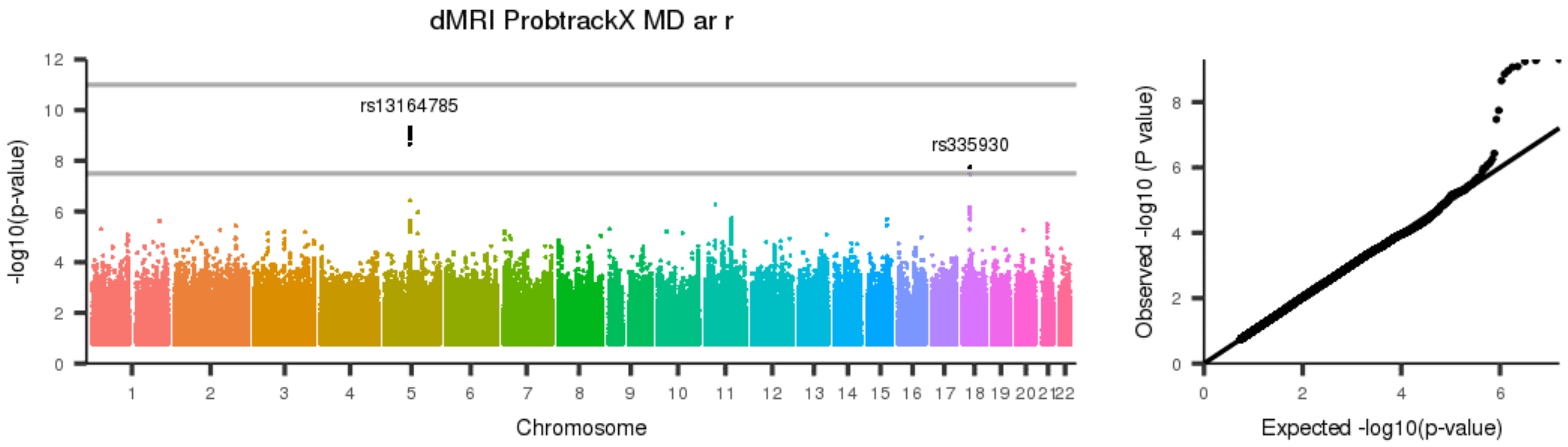

back to list of plots

Figure 657: dMRI ProbtrackX MD atr l

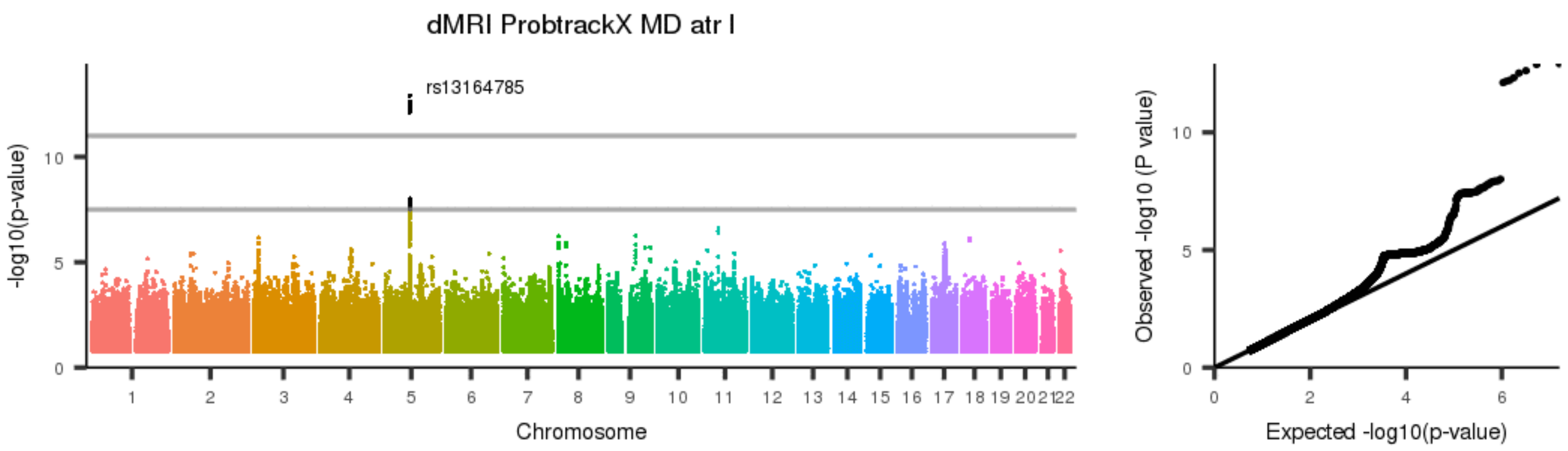

back to list of plots

Figure 658: dMRI ProbtrackX MD atr r

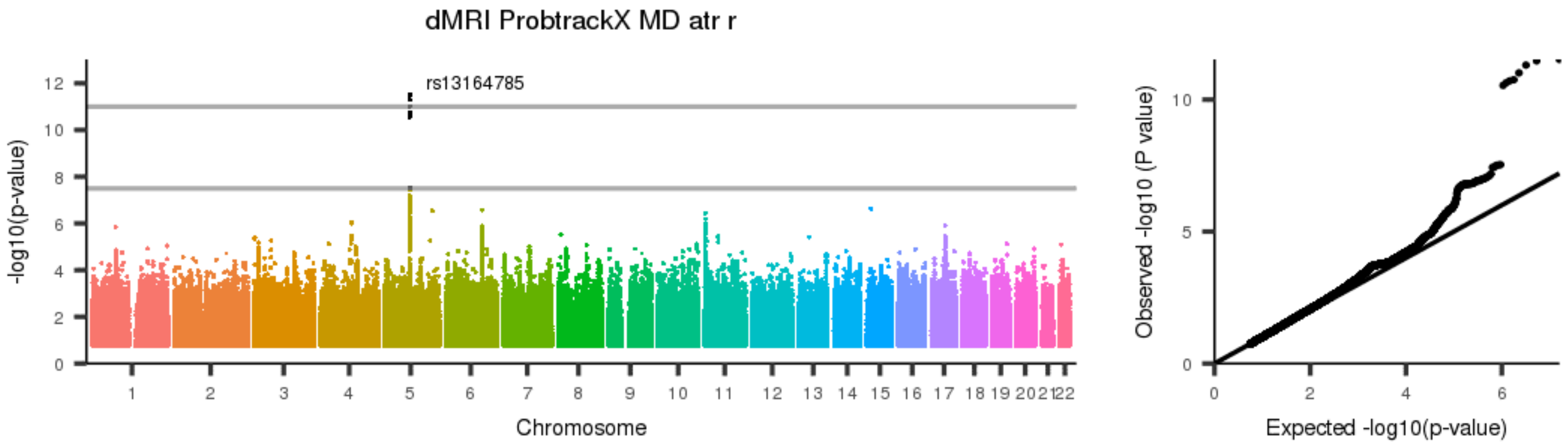

back to list of plots

Figure 659: dMRI ProbtrackX MD cgc l

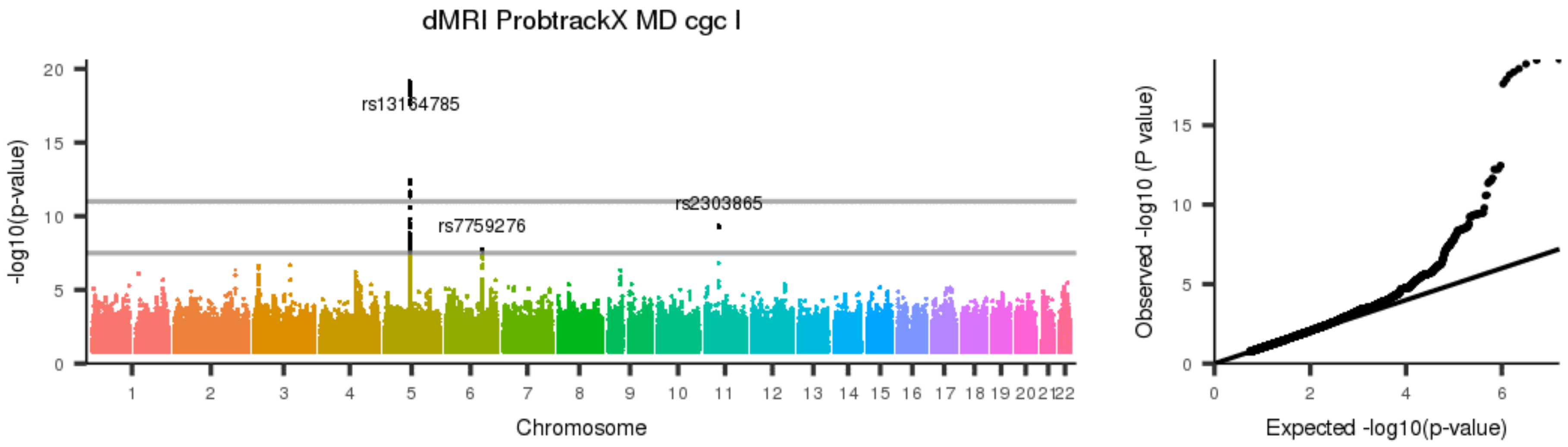

back to list of plots

Figure 660: dMRI ProbtrackX MD cgc r

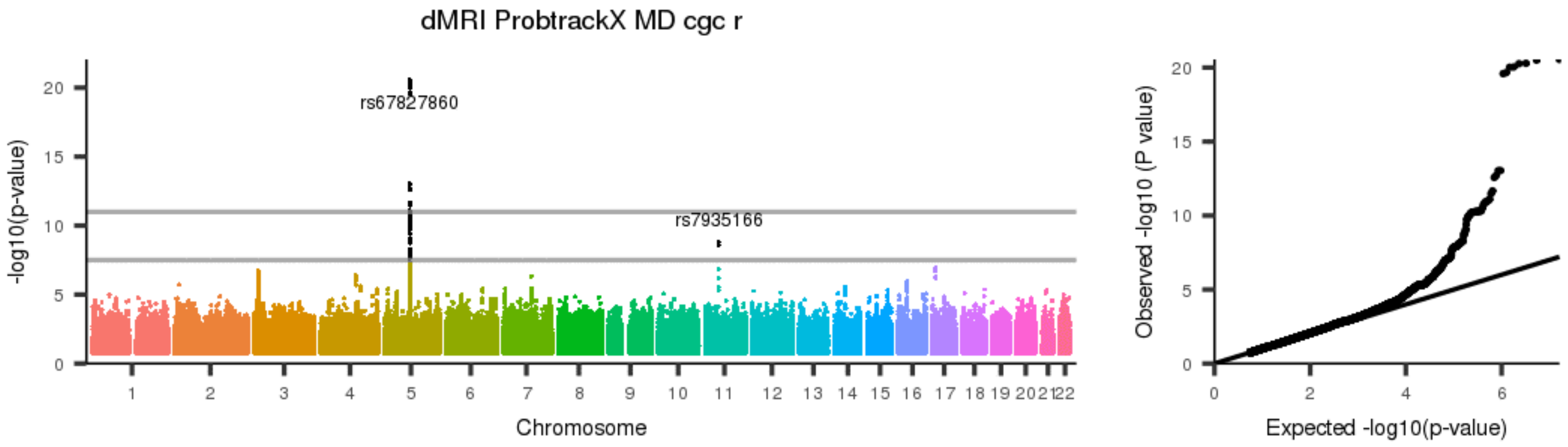

back to list of plots

Figure 661: dMRI ProbtrackX MD cgh l

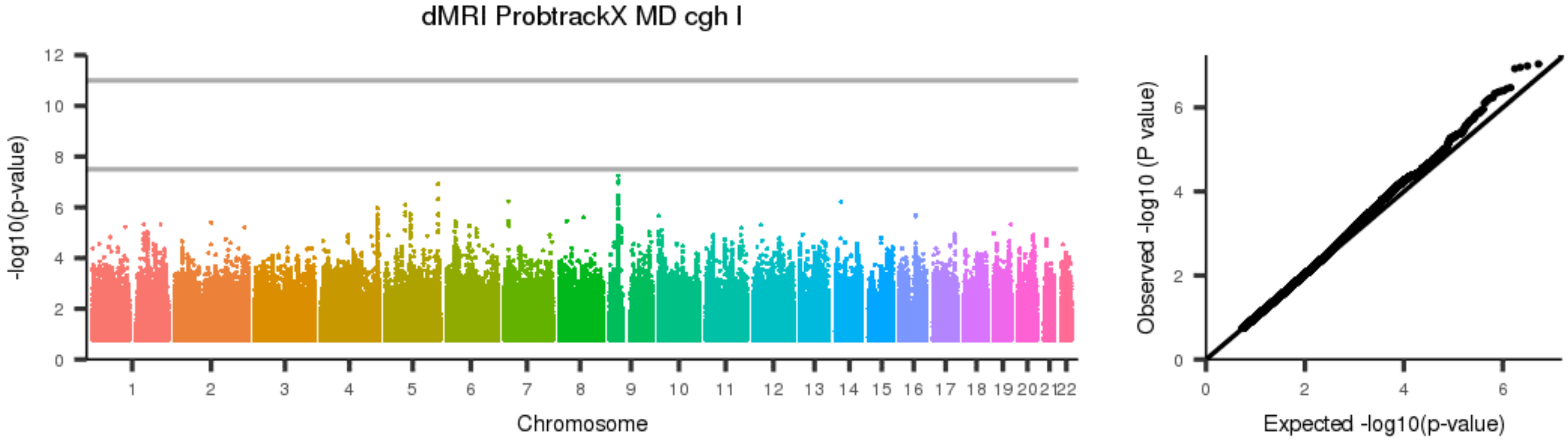

back to list of plots

Figure 662: dMRI ProbtrackX MD cgh r

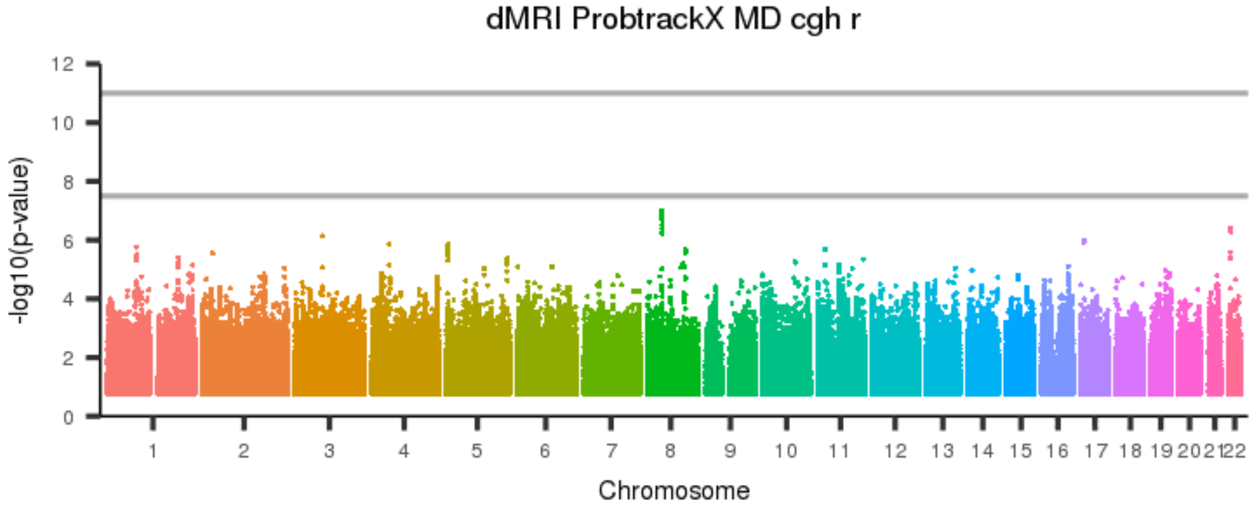

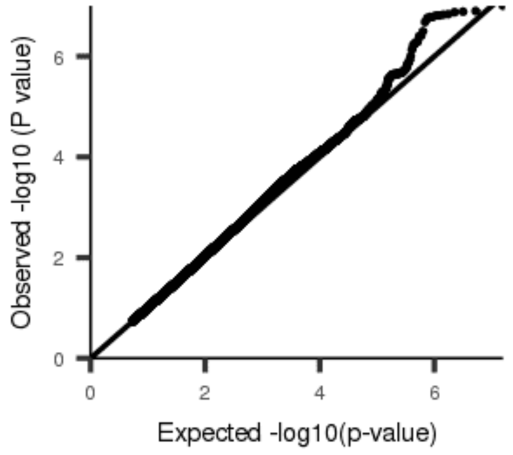

back to list of plots

Figure 663: dMRI ProbtrackX MD cst l

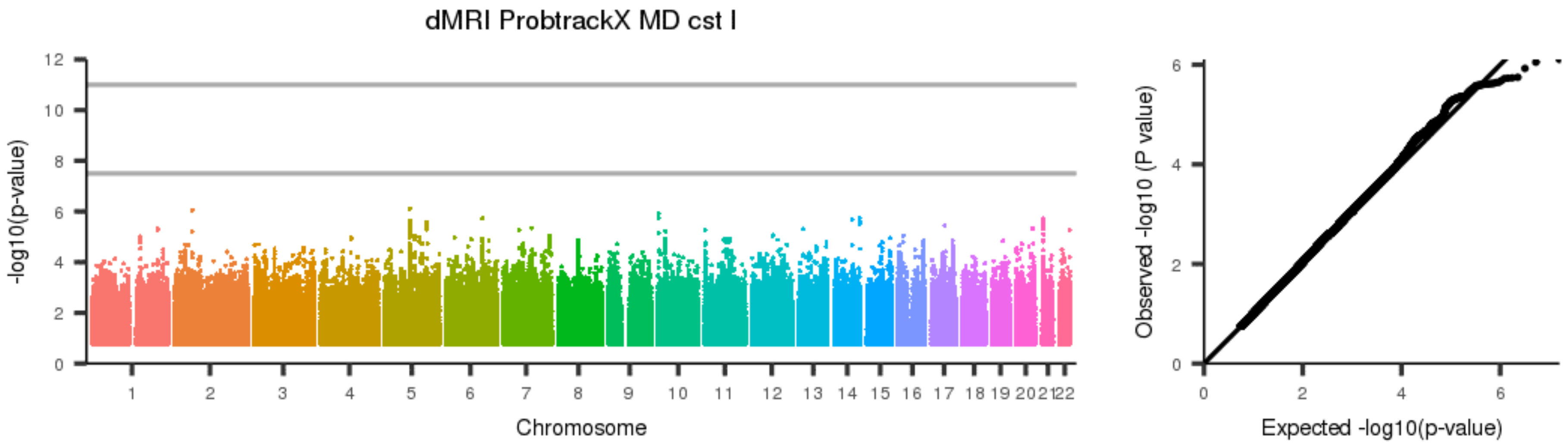

back to list of plots

Figure 664: dMRI ProbtrackX MD cst r

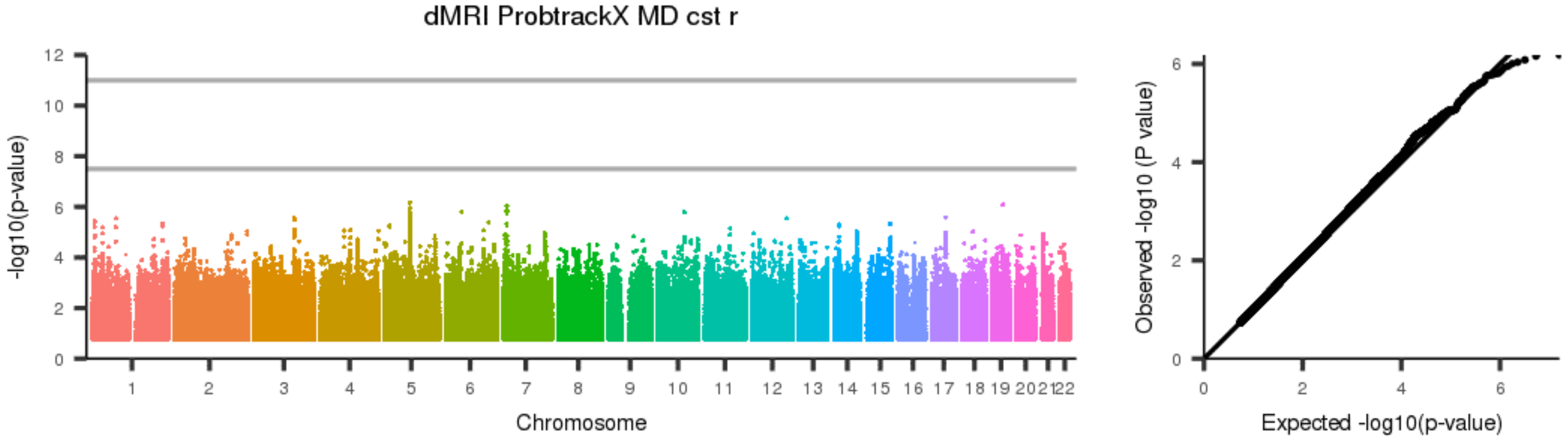

back to list of plots

Figure 665: dMRI ProbtrackX MD fma

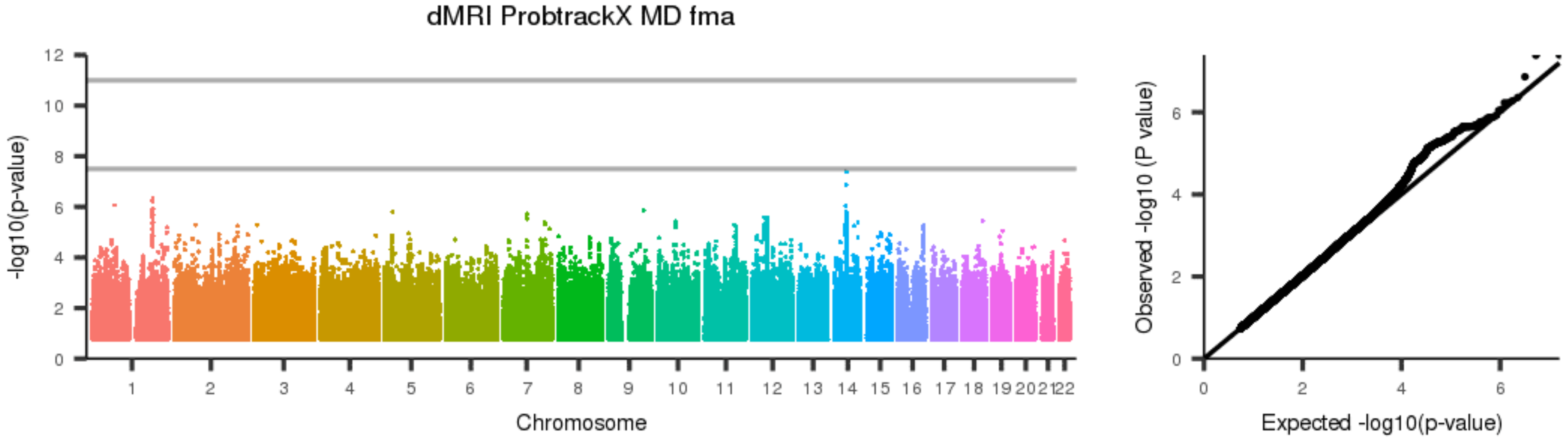

back to list of plots

Figure 666: dMRI ProbtrackX MD fmi

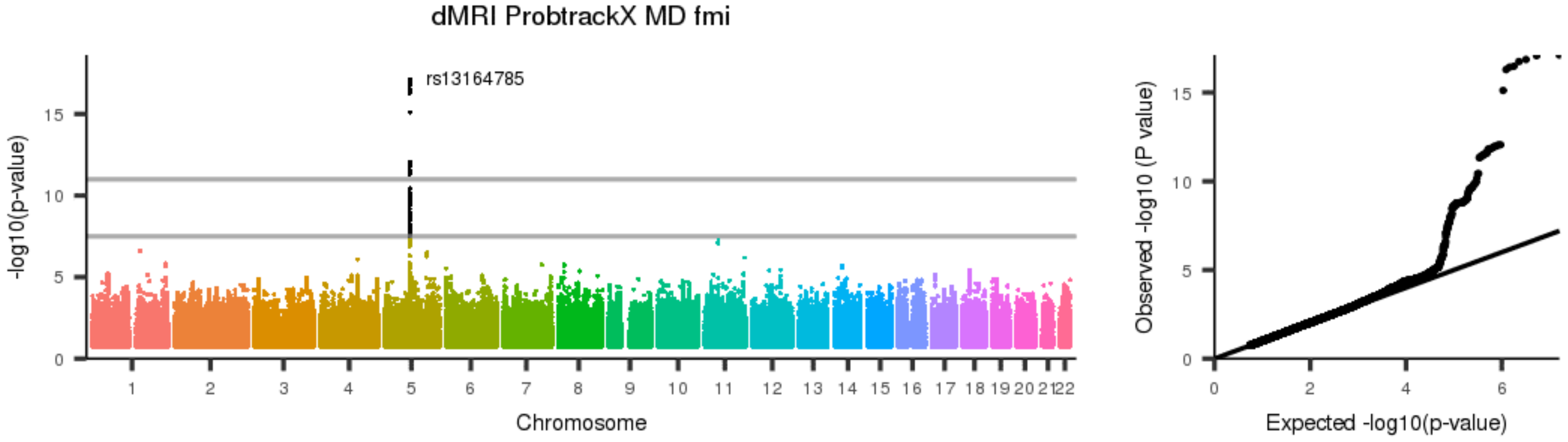

back to list of plots

Figure 667: dMRI ProbtrackX MD ifo l

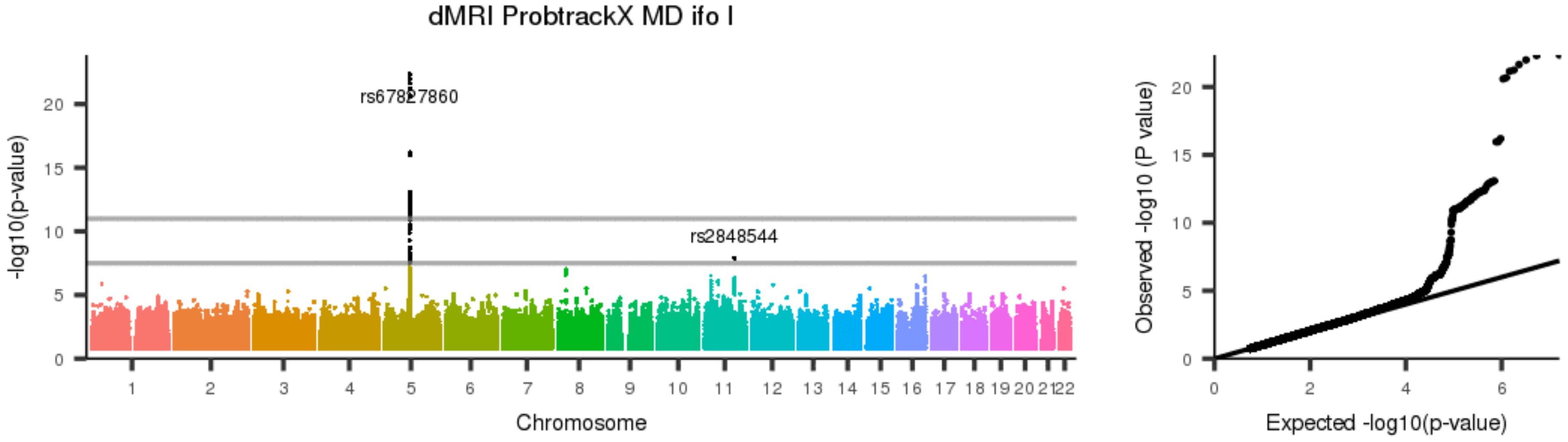

back to list of plots

Figure 668: dMRI ProbtrackX MD ifo r

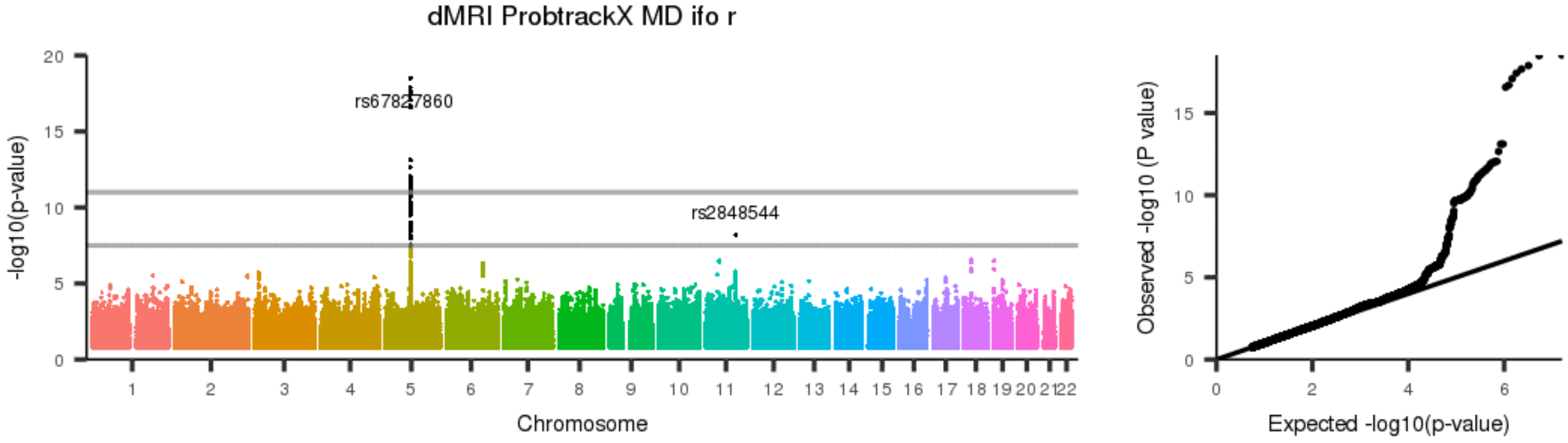

back to list of plots

Figure 669: dMRI ProbtrackX MD ilf l

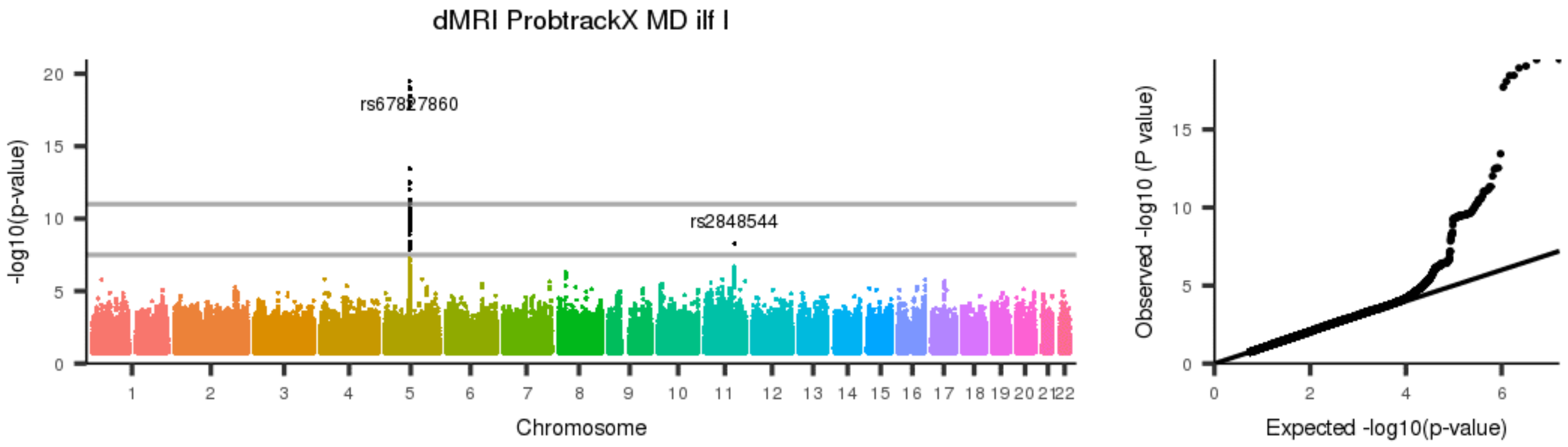

back to list of plots

Figure 670: dMRI ProbtrackX MD ilf r

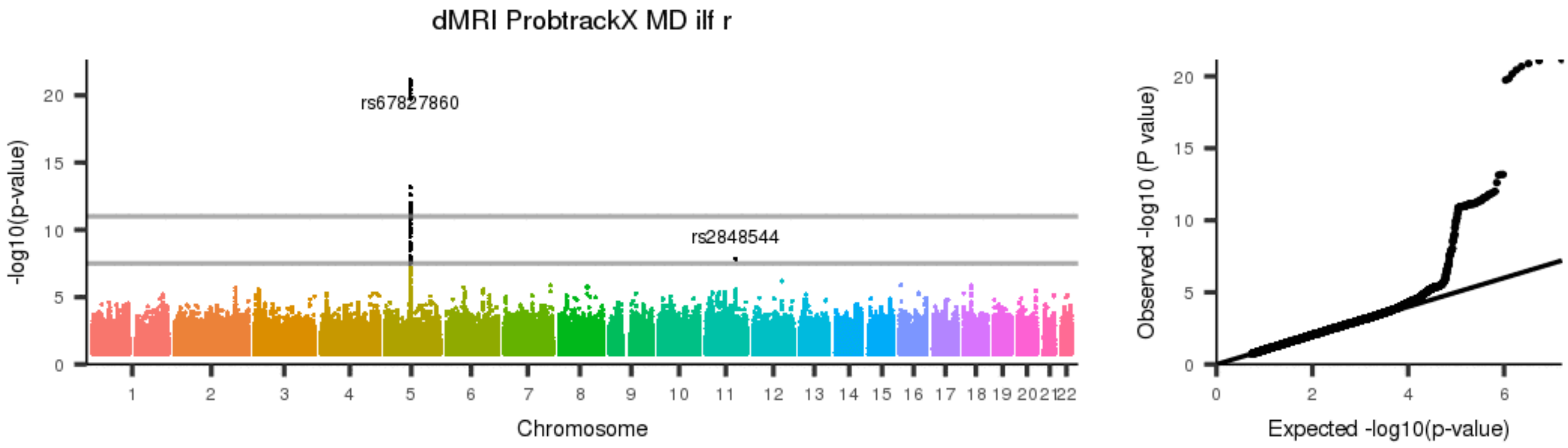

back to list of plots

Figure 671: dMRI ProbtrackX MD mcp

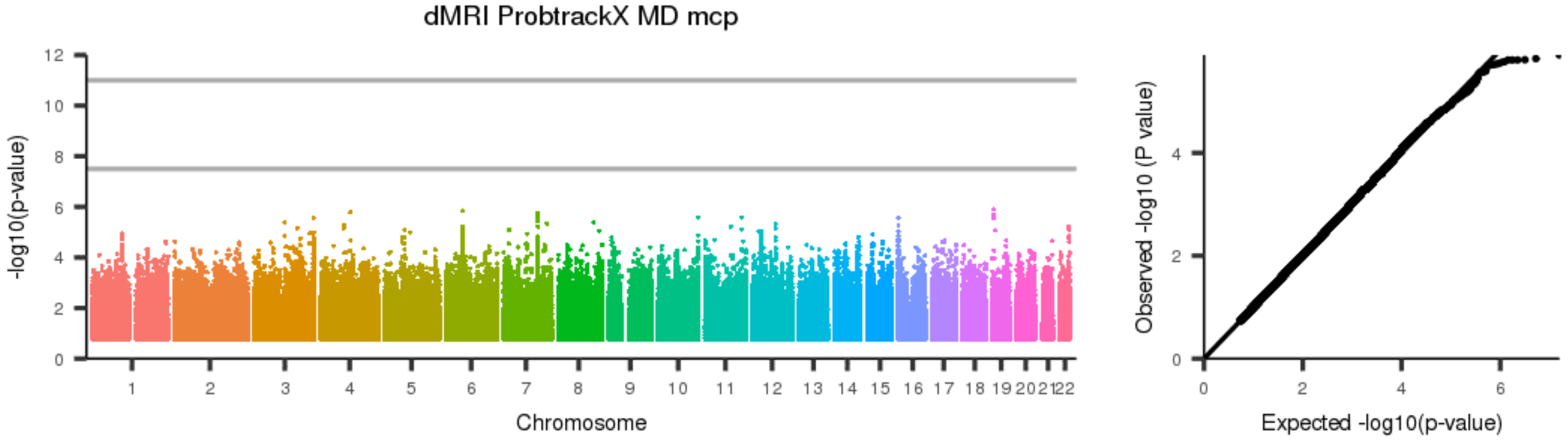

back to list of plots

Figure 672: dMRI ProbtrackX MD ml l

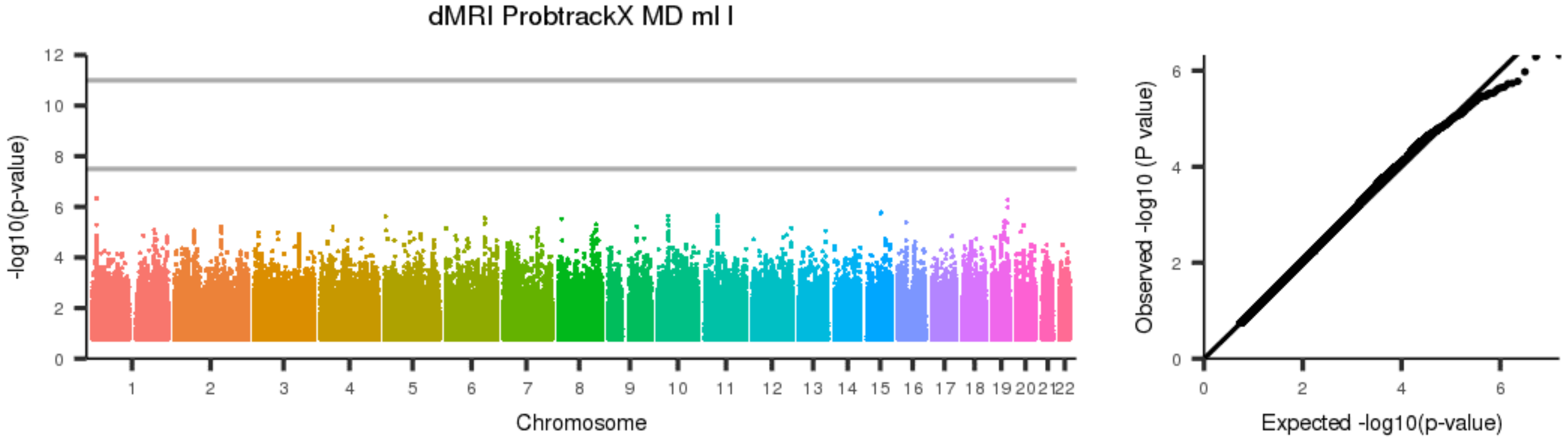

back to list of plots

Figure 673: dMRI ProbtrackX MD ml r

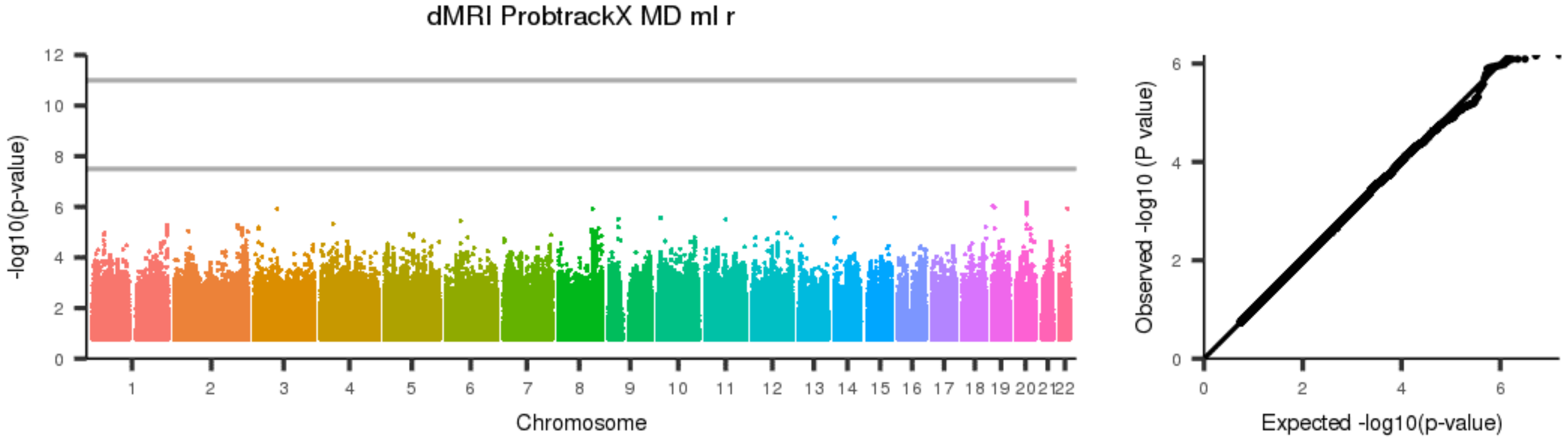

back to list of plots

Figure 674: dMRI ProbtrackX MD ptr l

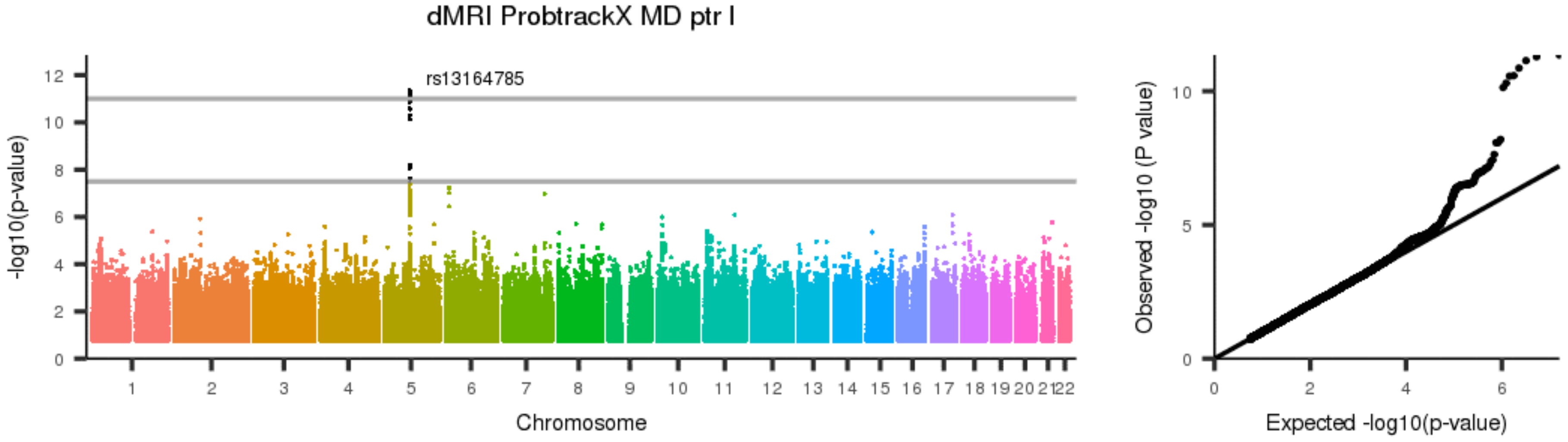

back to list of plots

Figure 675: dMRI ProbtrackX MD ptr r

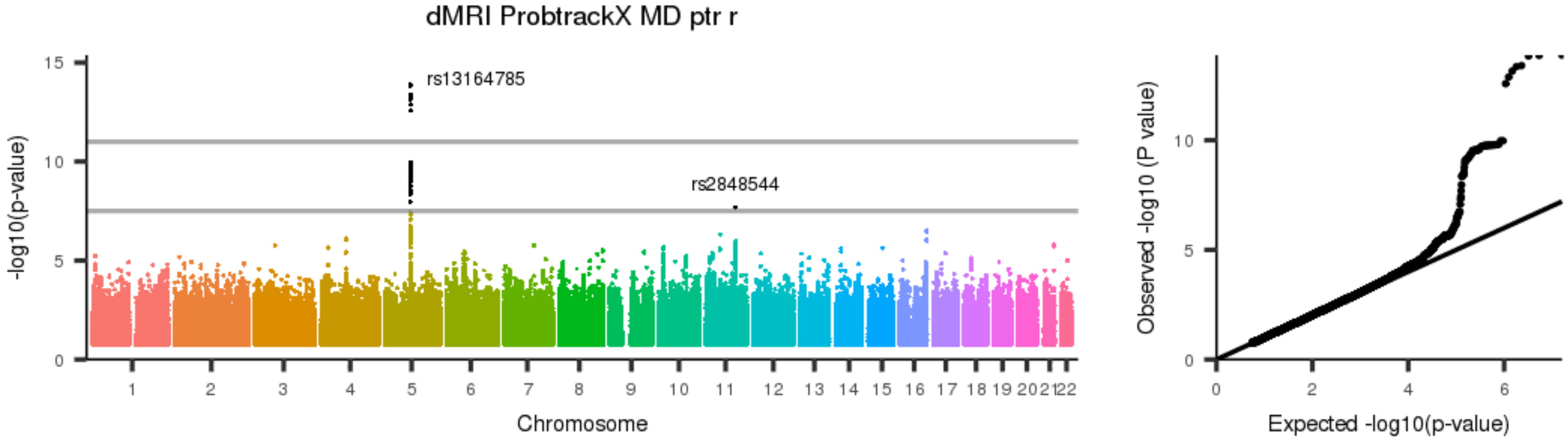

back to list of plots

Figure 676: dMRI ProbtrackX MD slf I

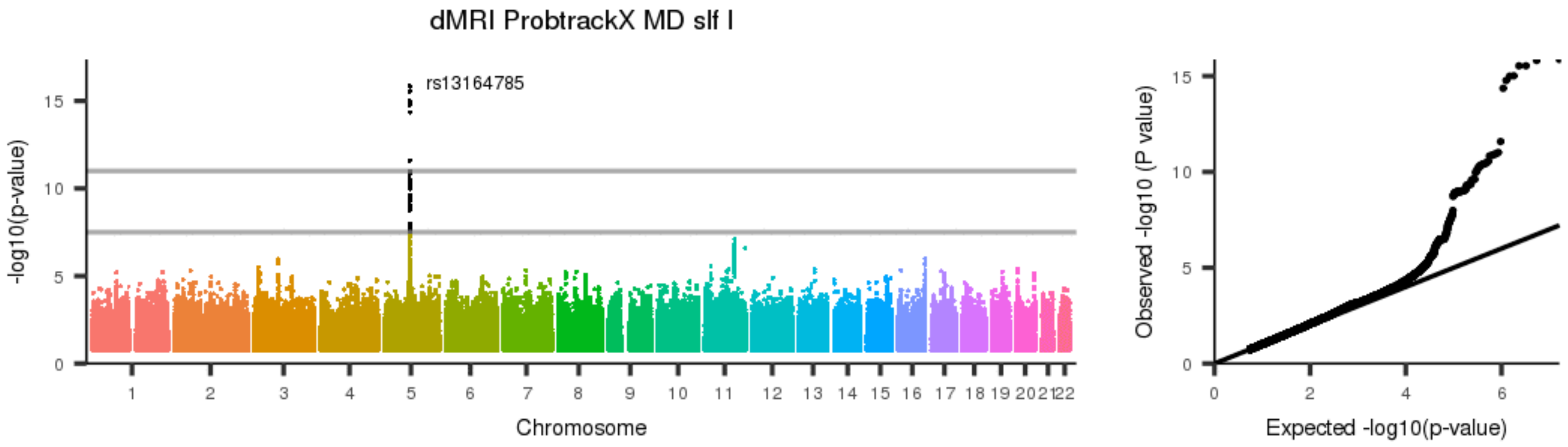

back to list of plots

Figure 677: dMRI ProbtrackX MD slf r

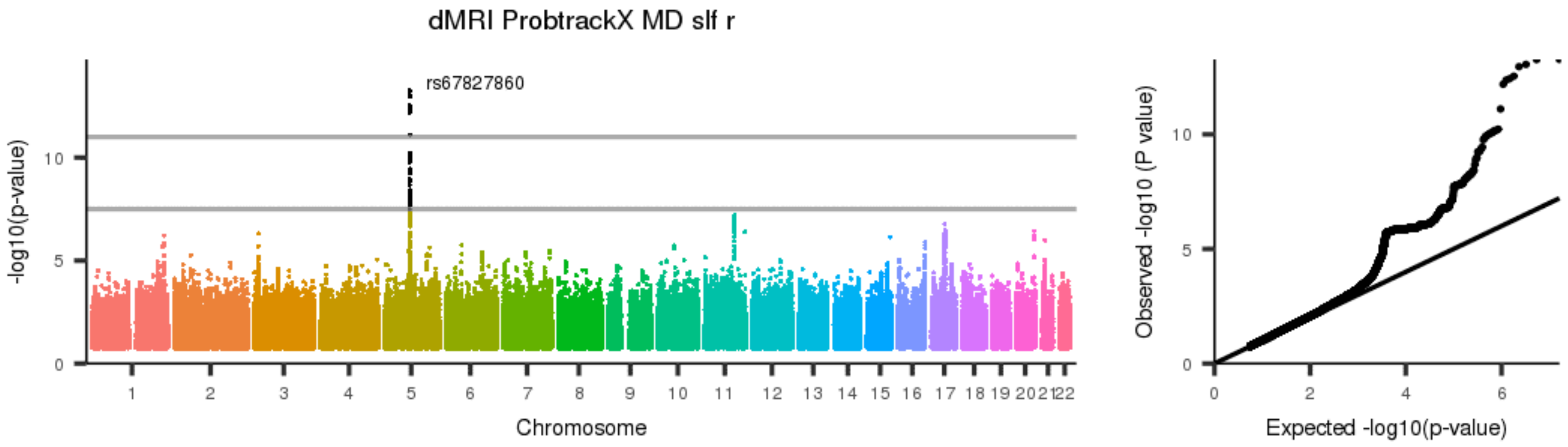

back to list of plots

Figure 678: dMRI ProbtrackX MD str l

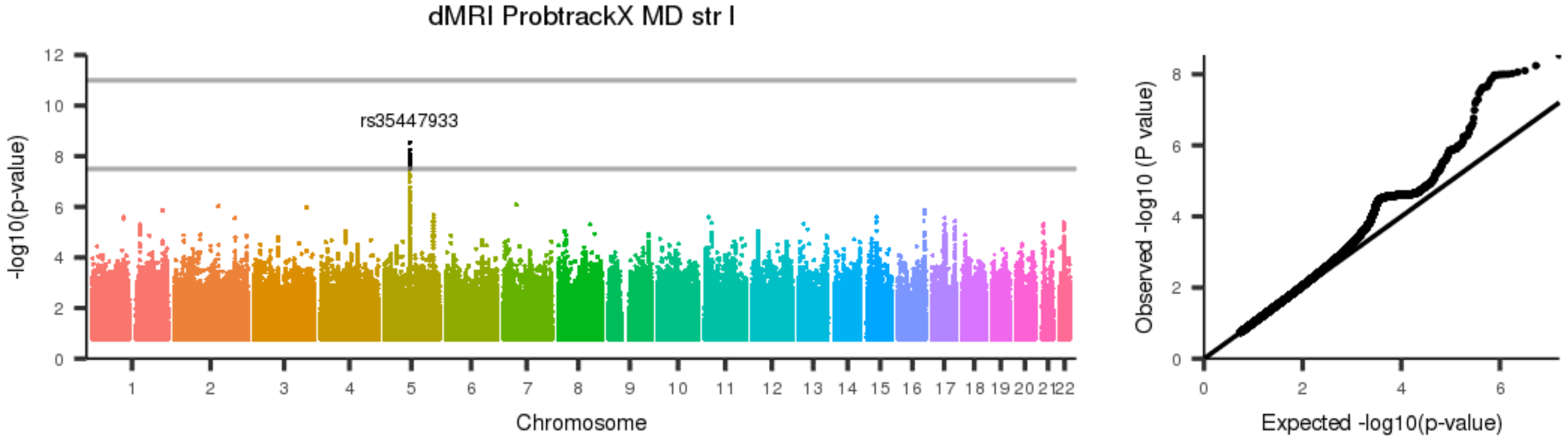

back to list of plots

Figure 679: dMRI ProbtrackX MD str r

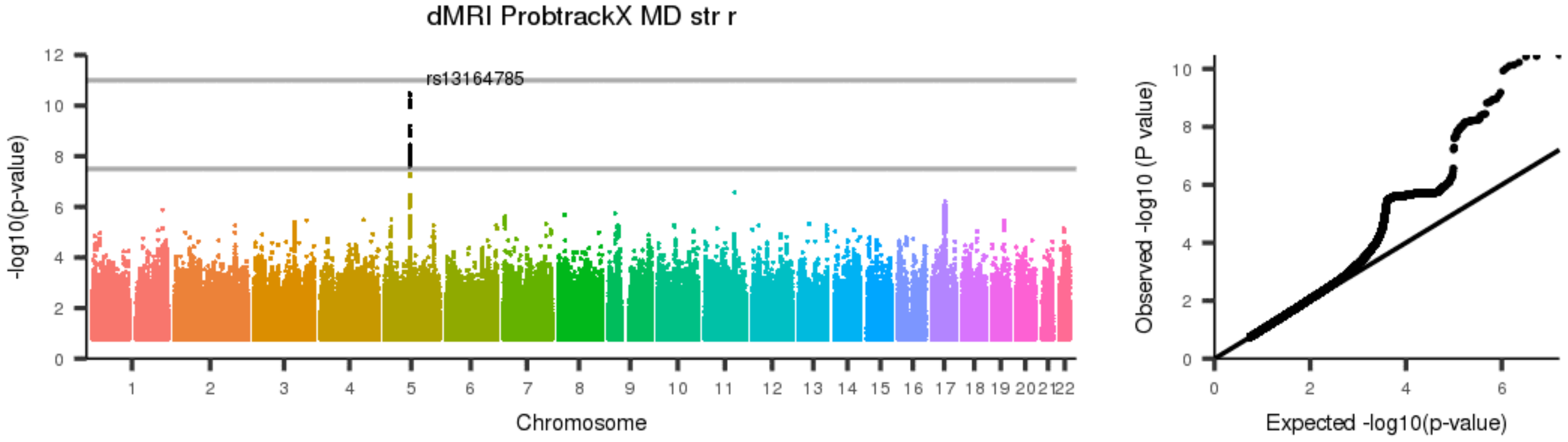

back to list of plots

Figure 680: dMRI ProbtrackX MD unc l

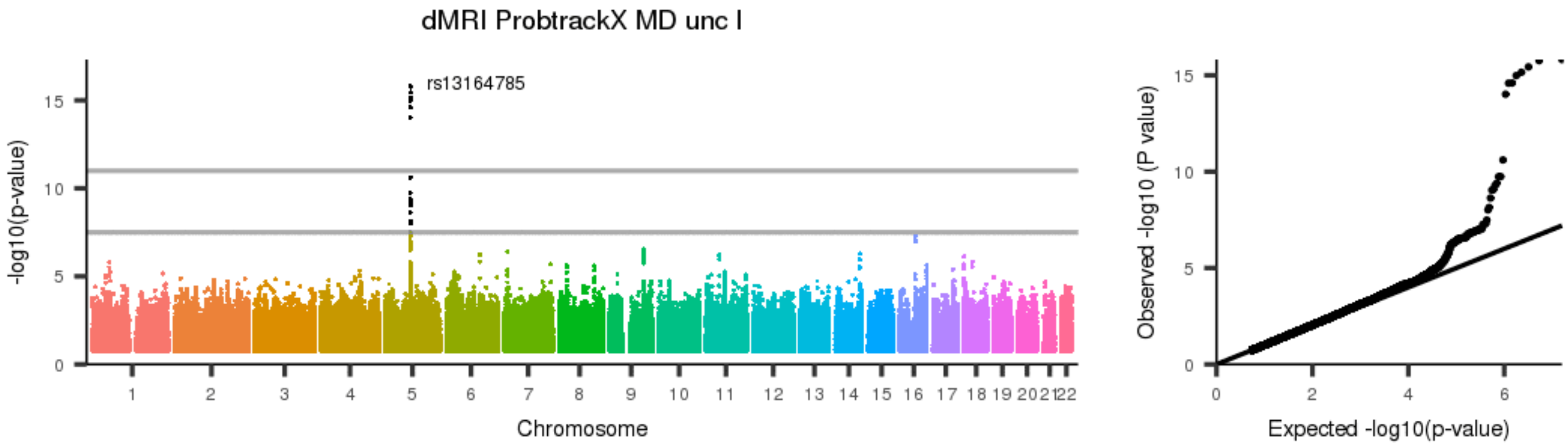

back to list of plots

Figure 681: dMRI ProbtrackX MD unc r

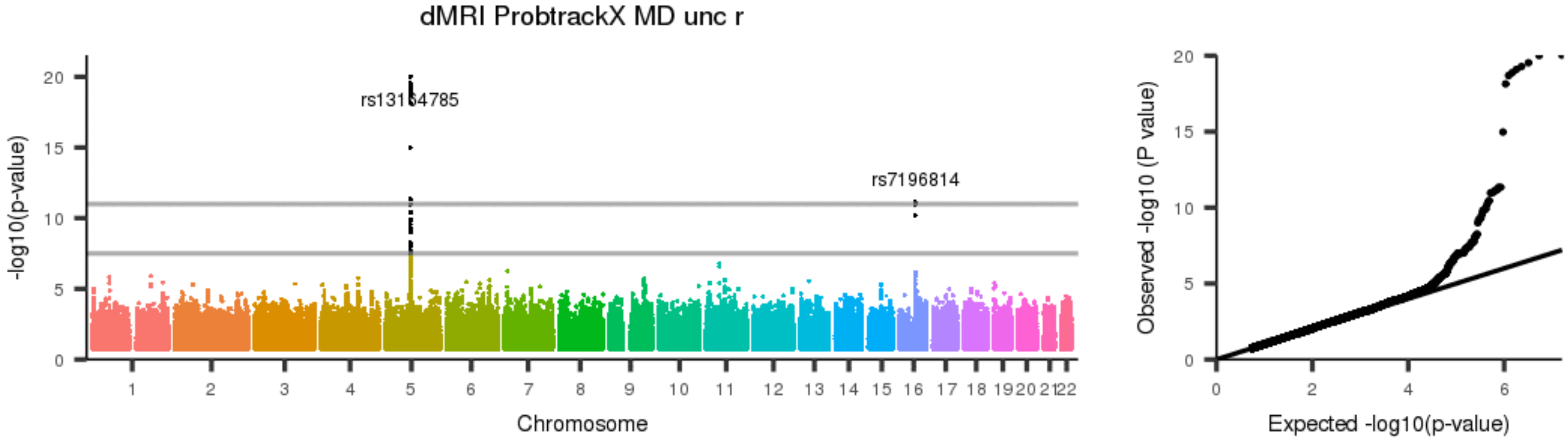

back to list of plots

Figure 682: dMRI ProbtrackX MO ar l

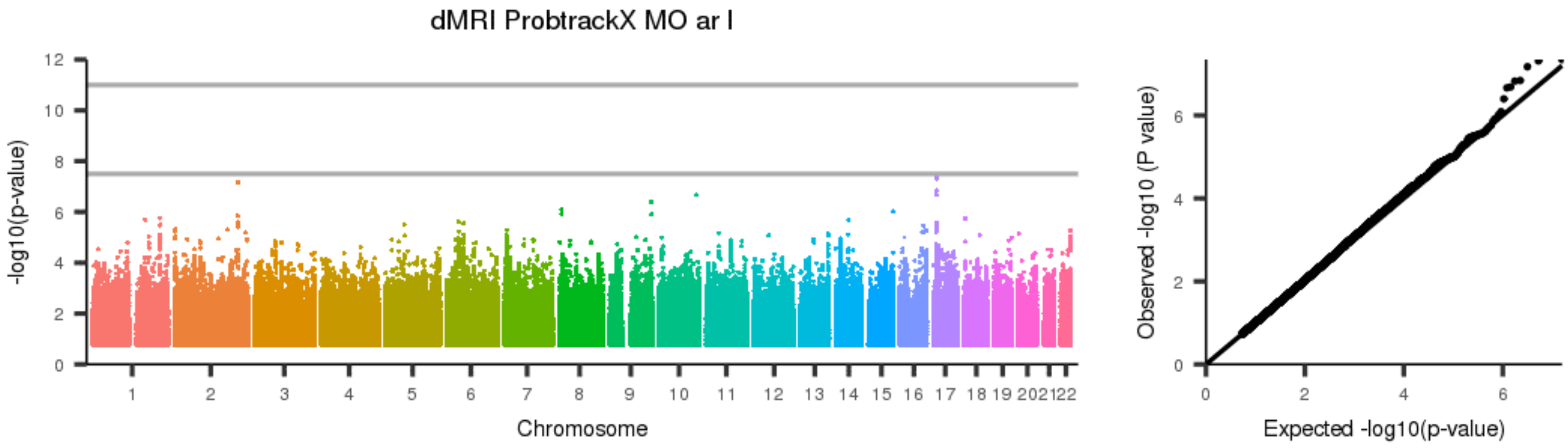

back to list of plots

Figure 683: dMRI ProbtrackX MO ar r

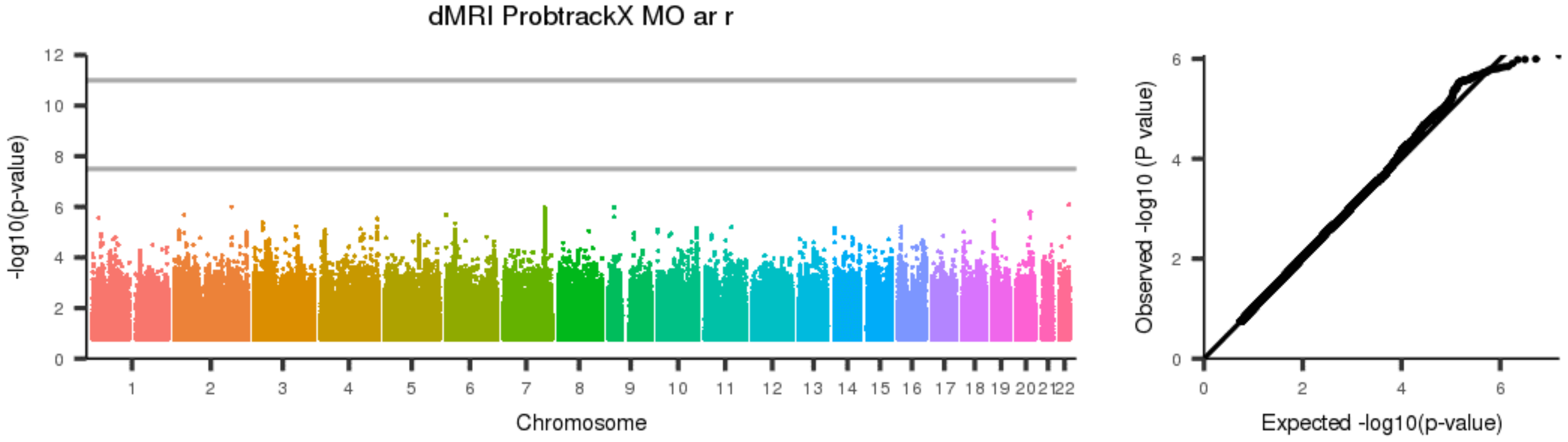

back to list of plots

Figure 684: dMRI ProbtrackX MO atr l

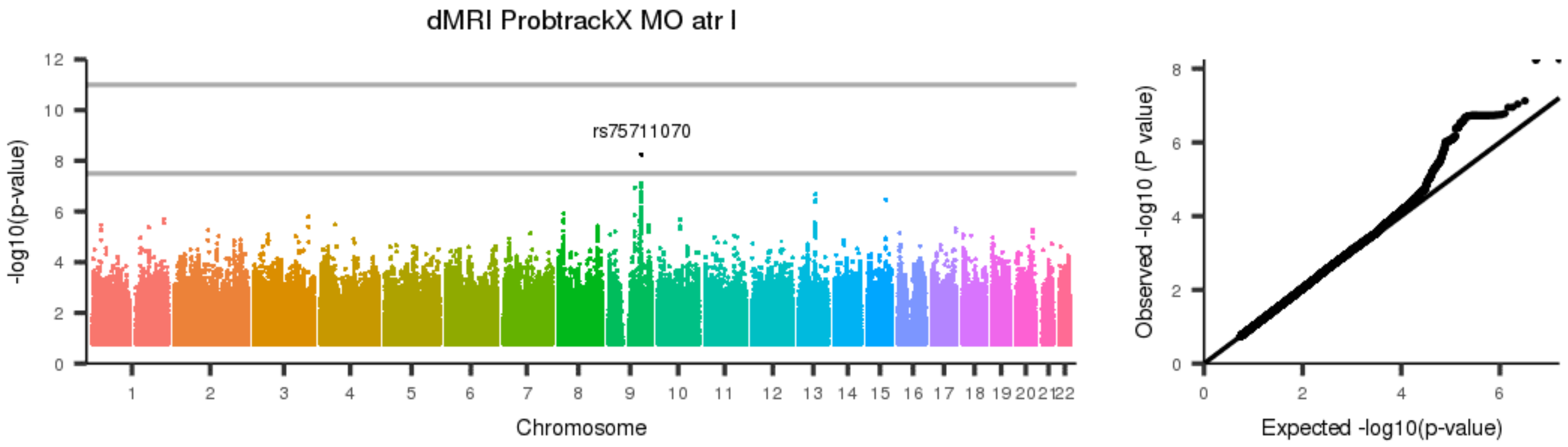

back to list of plots

Figure 685: dMRI ProbtrackX MO atr r

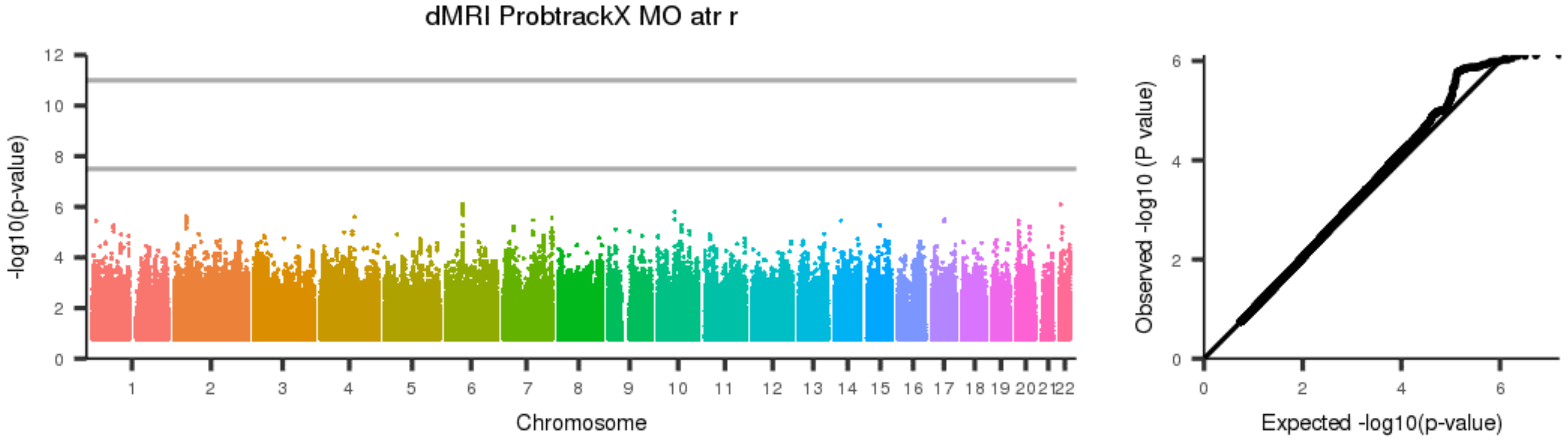

back to list of plots

Figure 686: dMRI ProbtrackX MO cgc l

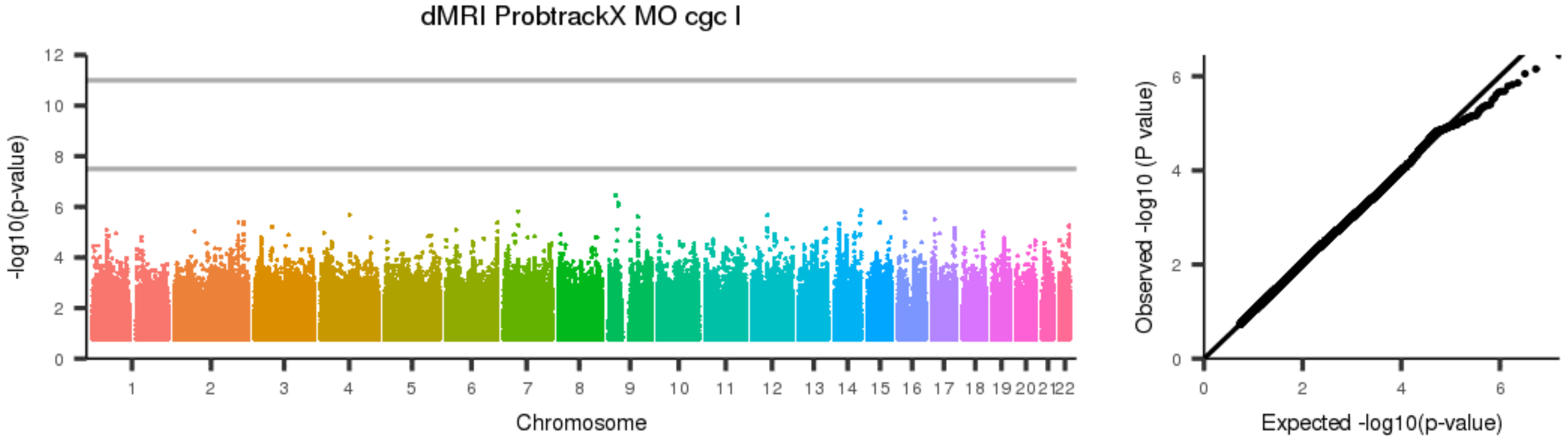

back to list of plots

Figure 687: dMRI ProbtrackX MO cgc r

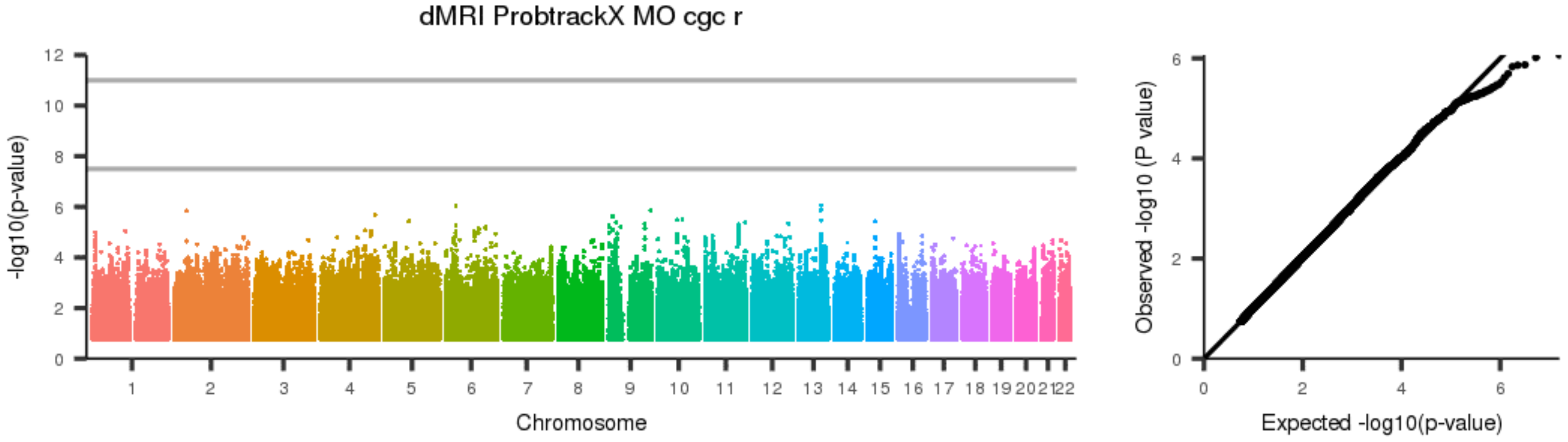

back to list of plots

Figure 688: dMRI ProbtrackX MO cgh l

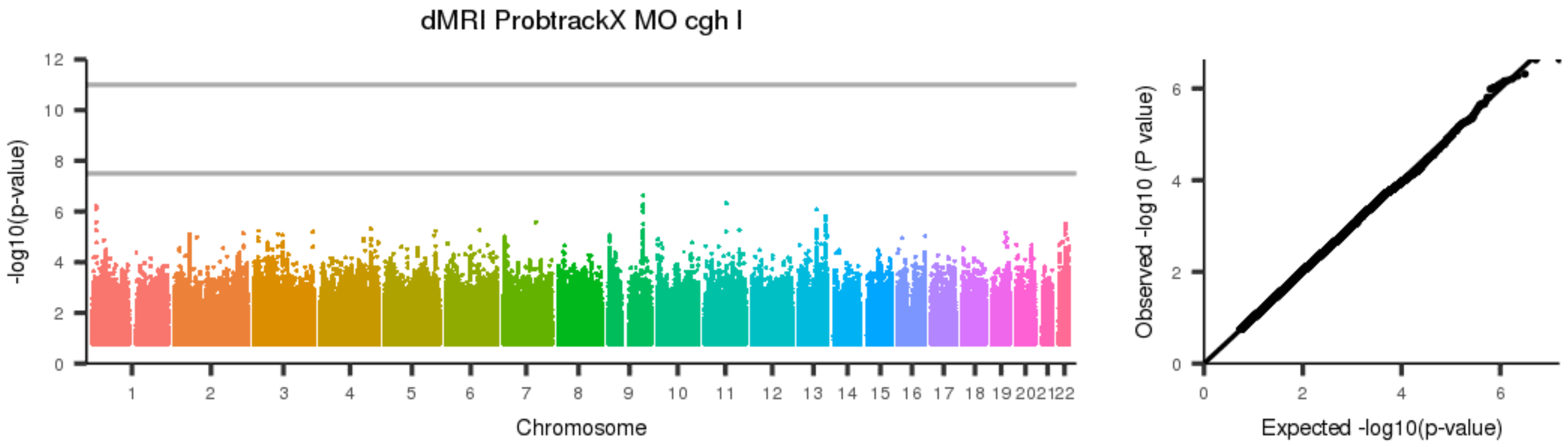

back to list of plots

Figure 689: dMRI ProbtrackX MO cgh r

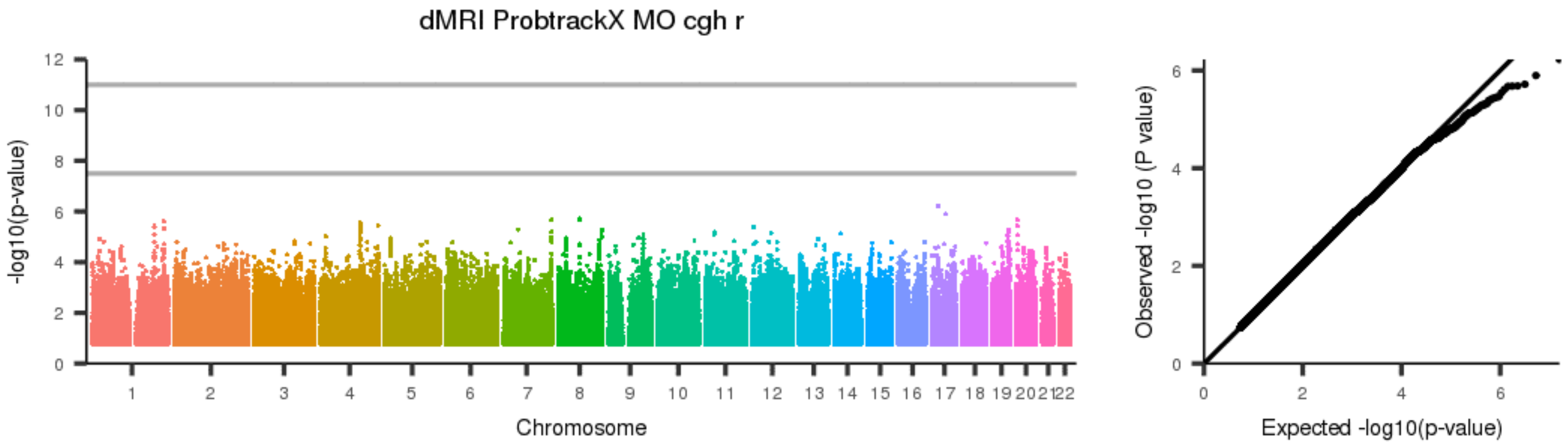

back to list of plots

Figure 690: dMRI ProbtrackX MO cst l

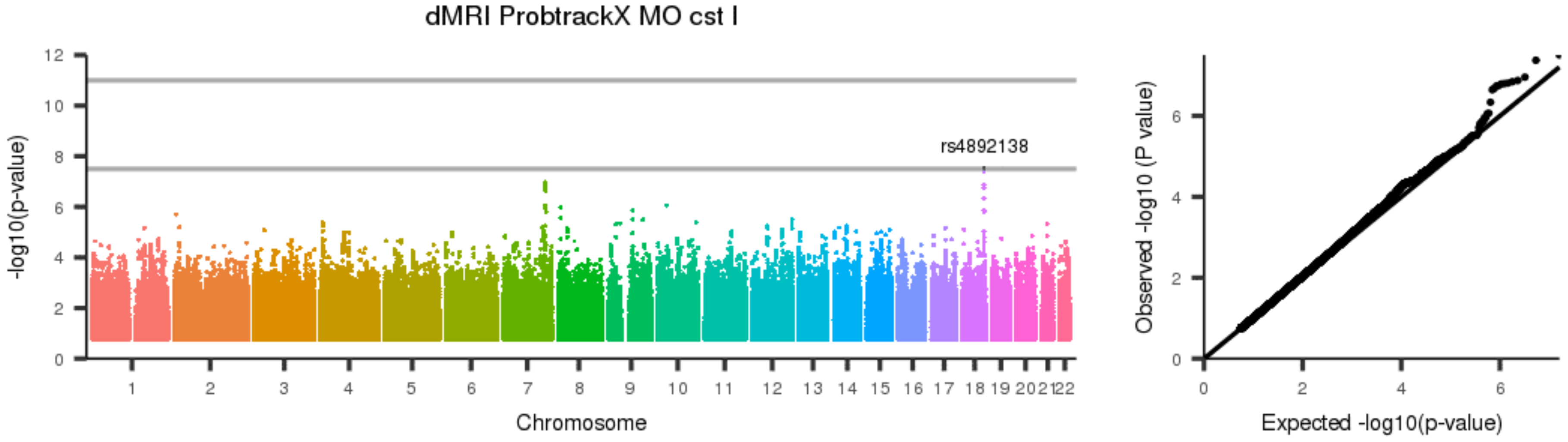

back to list of plots

Figure 691: dMRI ProbtrackX MO cst r

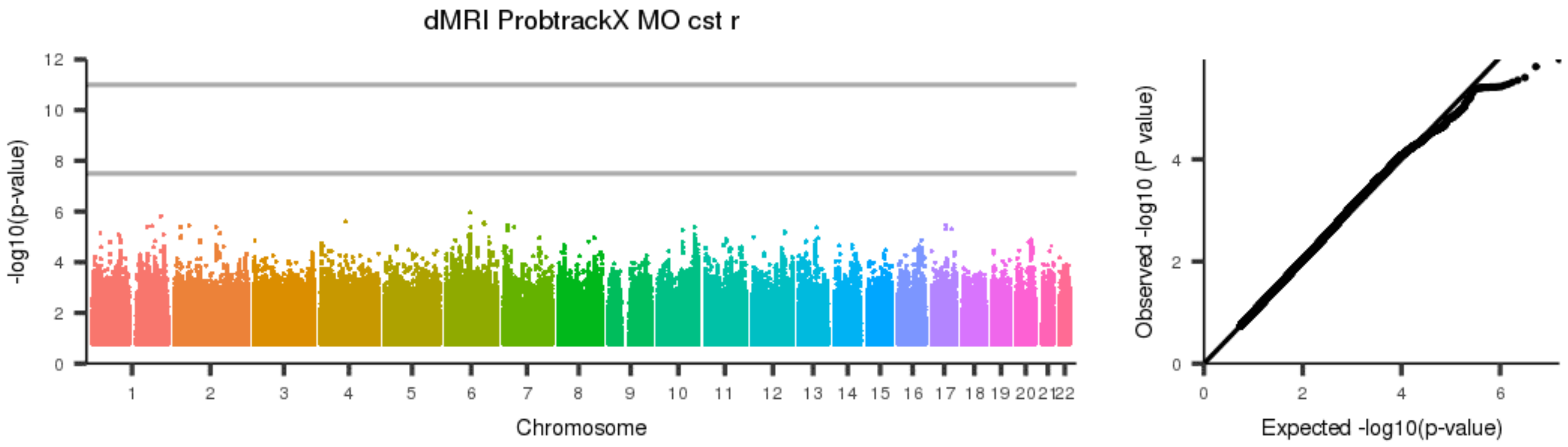

back to list of plots

Figure 692: dMRI ProbtrackX MO fma

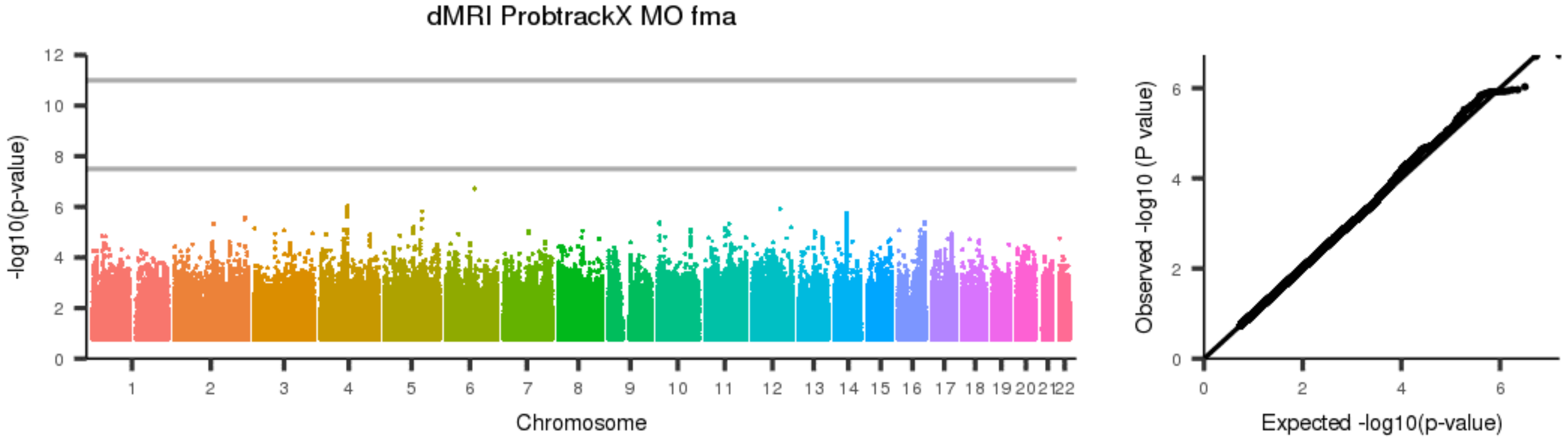

back to list of plots

Figure 693: dMRI ProbtrackX MO fmi

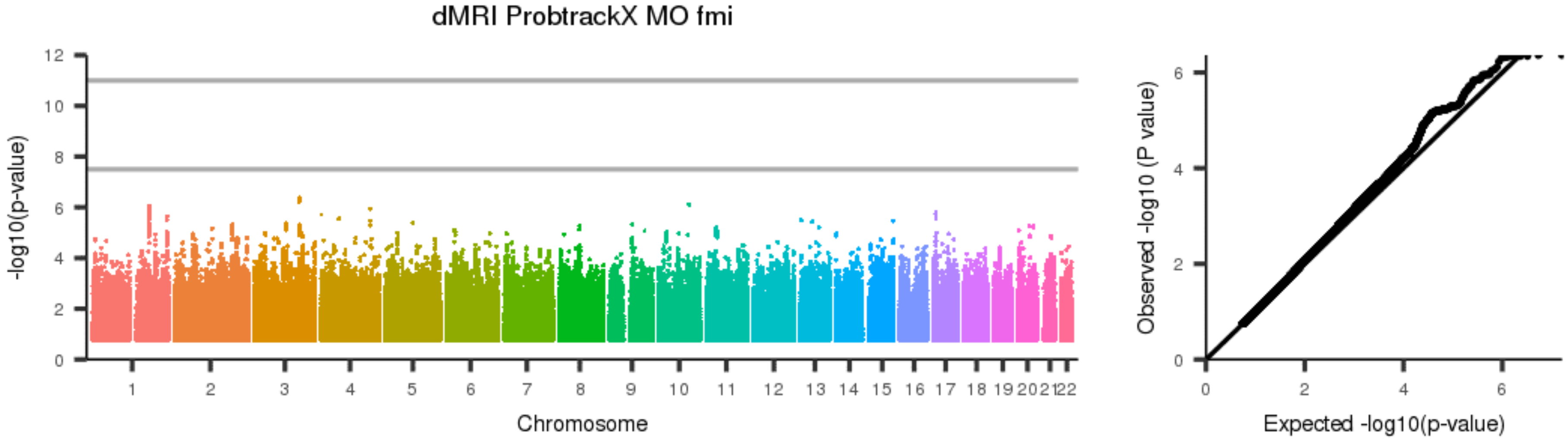

back to list of plots

Figure 694: dMRI ProbtrackX MO ifo l

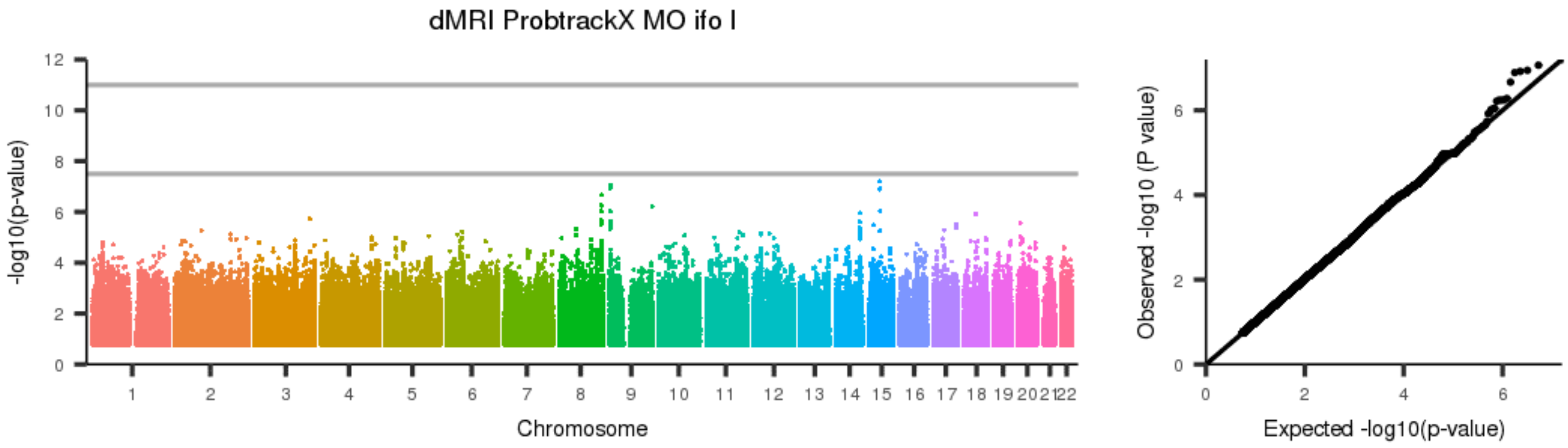

back to list of plots

Figure 695: dMRI ProbtrackX MO ifo r

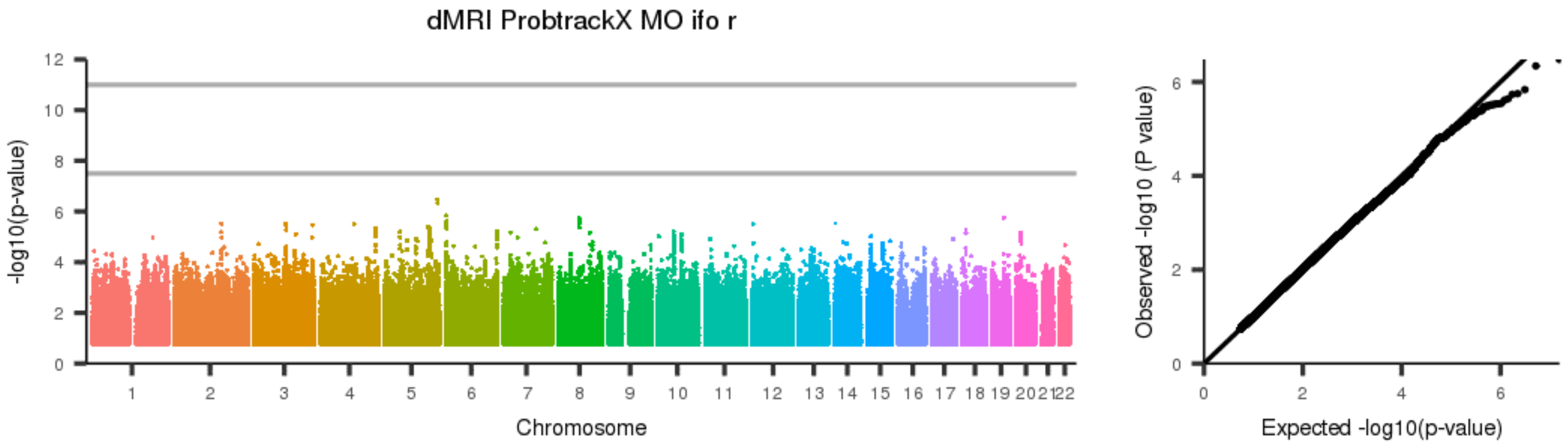

back to list of plots

Figure 696: dMRI ProbtrackX MO ilf l

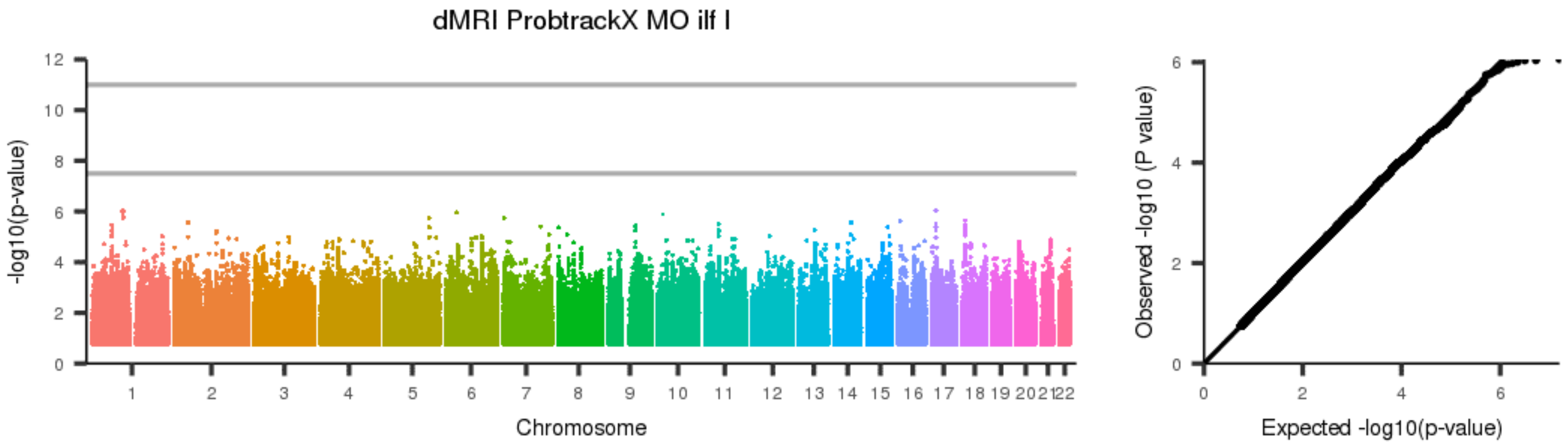

back to list of plots

Figure 697: dMRI ProbtrackX MO ilf r

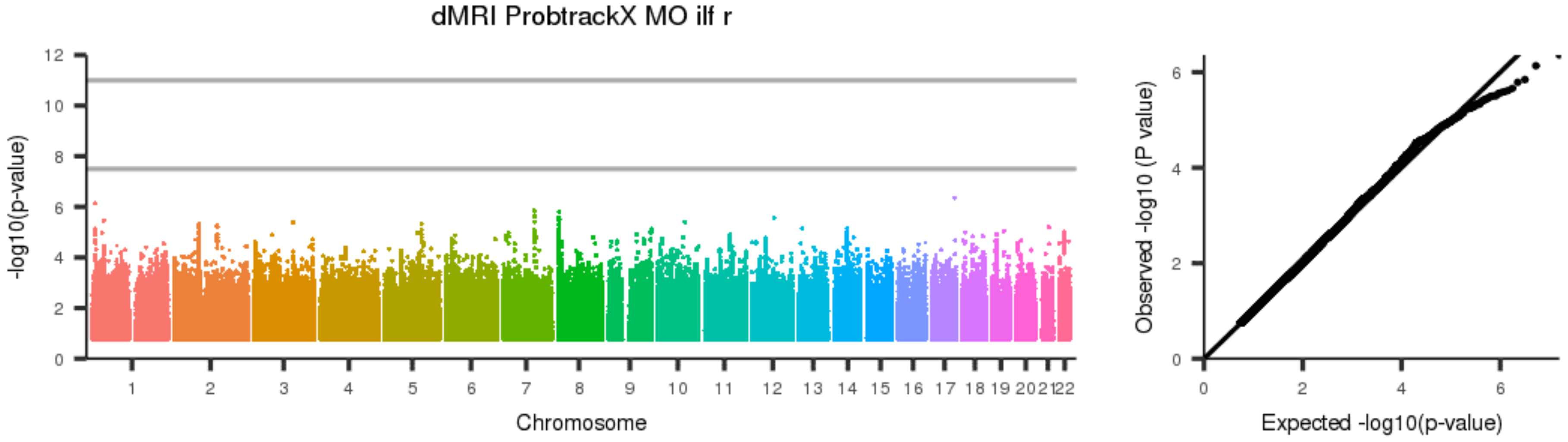

back to list of plots

Figure 698: dMRI ProbtrackX MO mcp

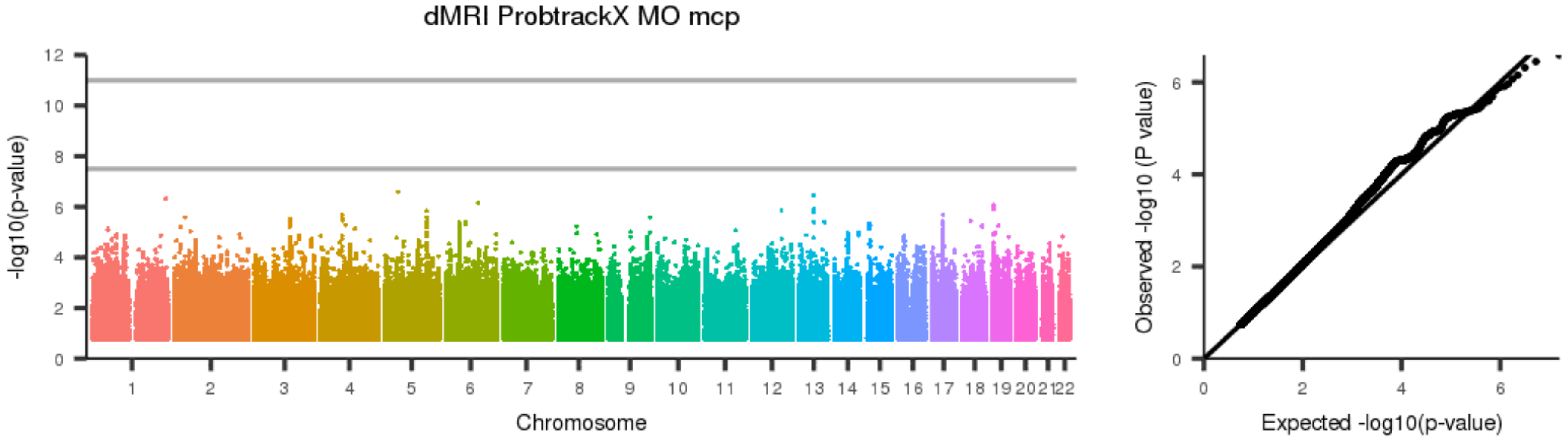

back to list of plots

Figure 699: dMRI ProbtrackX MO ml l

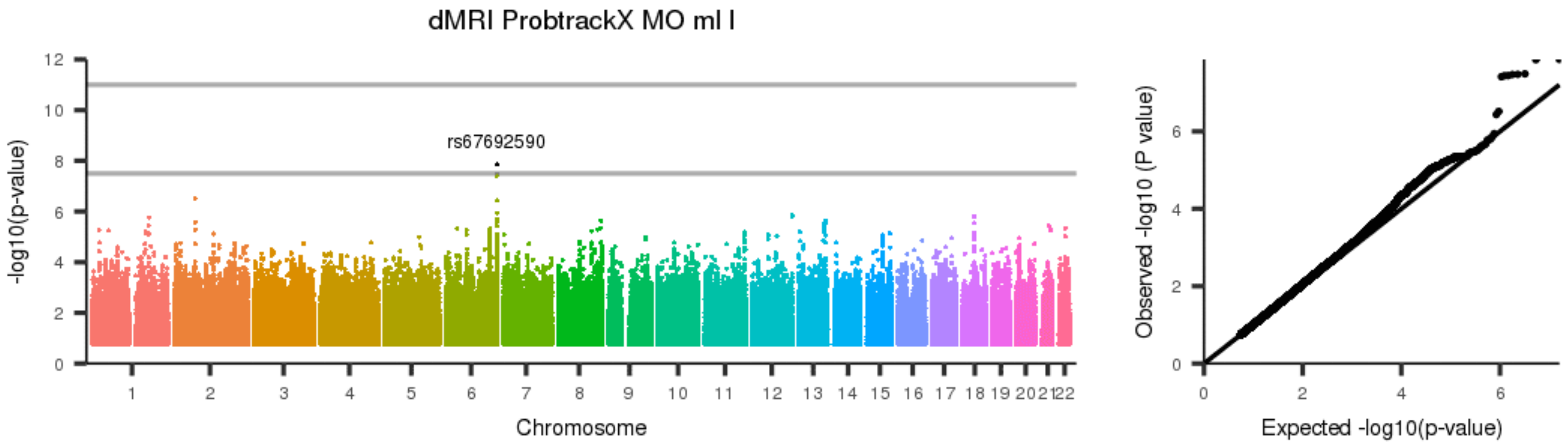

back to list of plots

Figure 700: dMRI ProbtrackX MO ml r

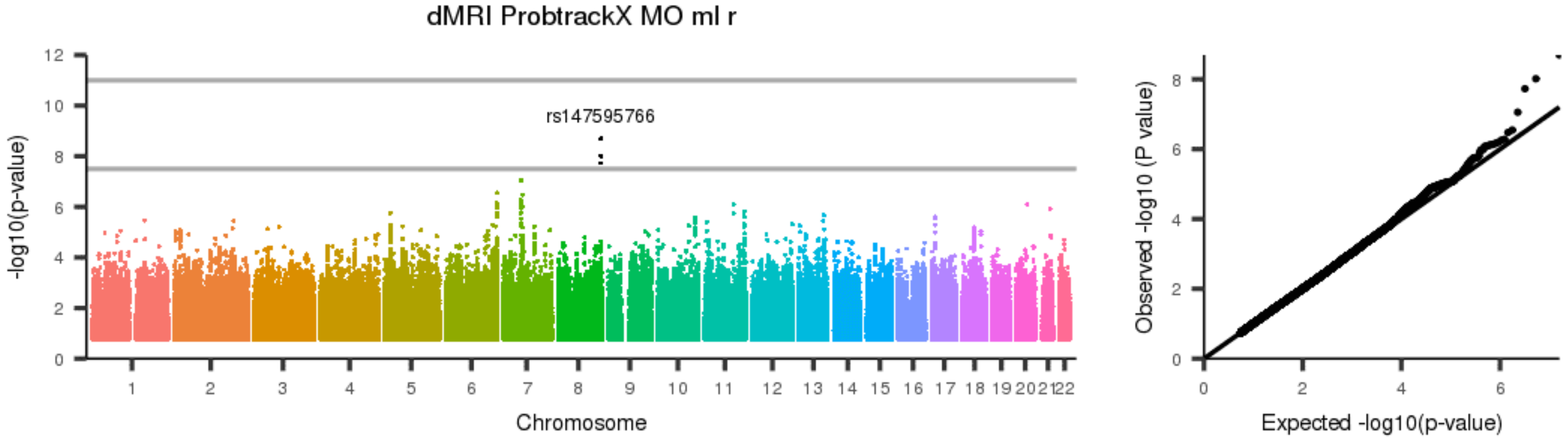

back to list of plots

Figure 701: dMRI ProbtrackX MO ptr l

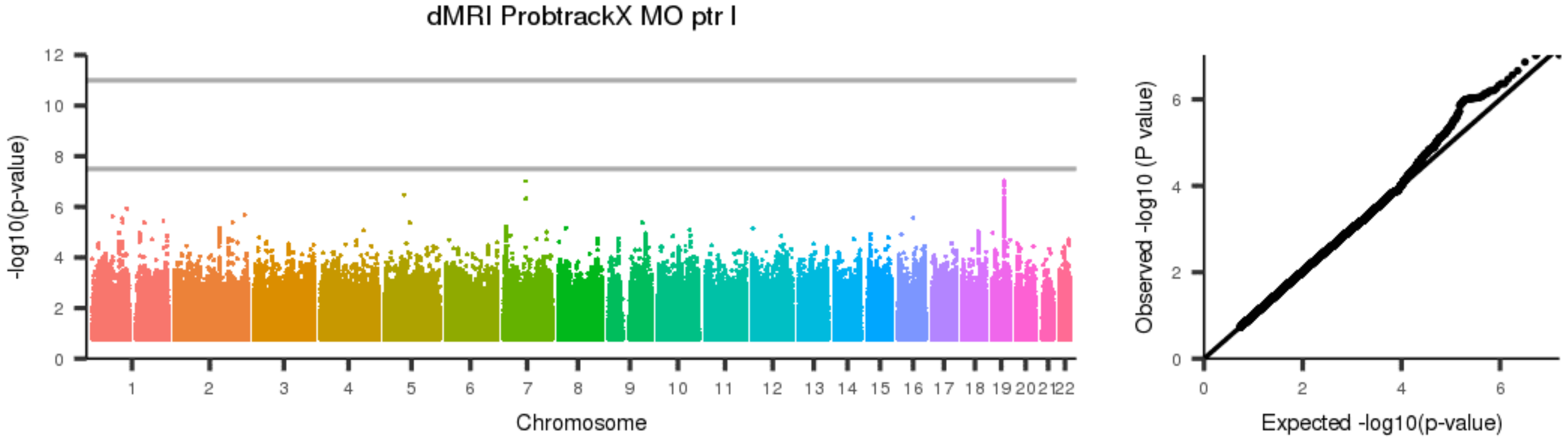

back to list of plots

Figure 702: dMRI ProbtrackX MO ptr r

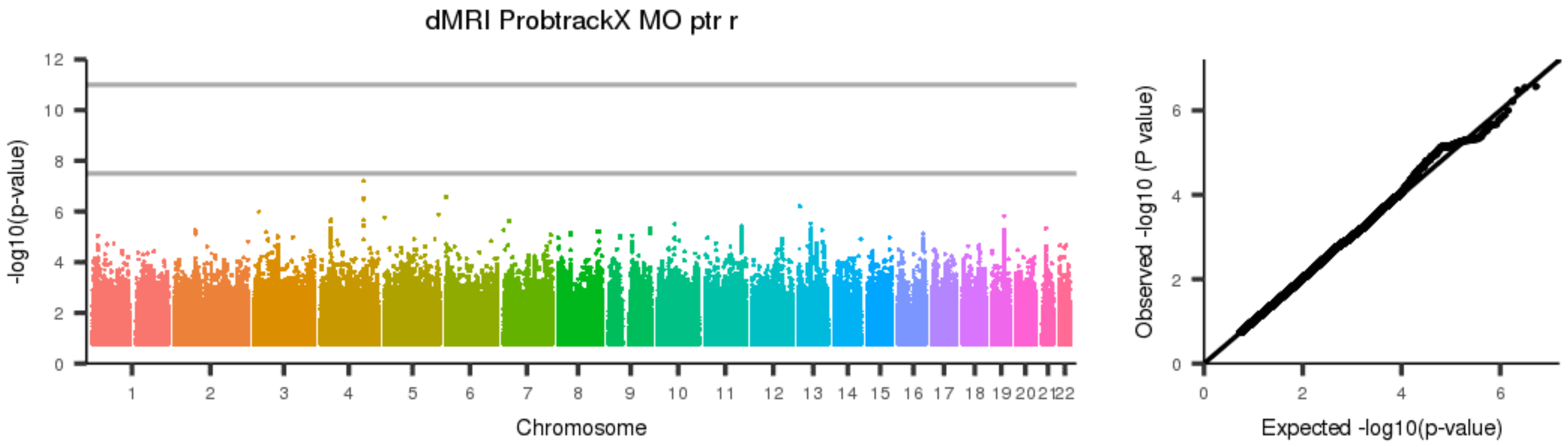

back to list of plots

Figure 703: dMRI ProbtrackX MO slf I

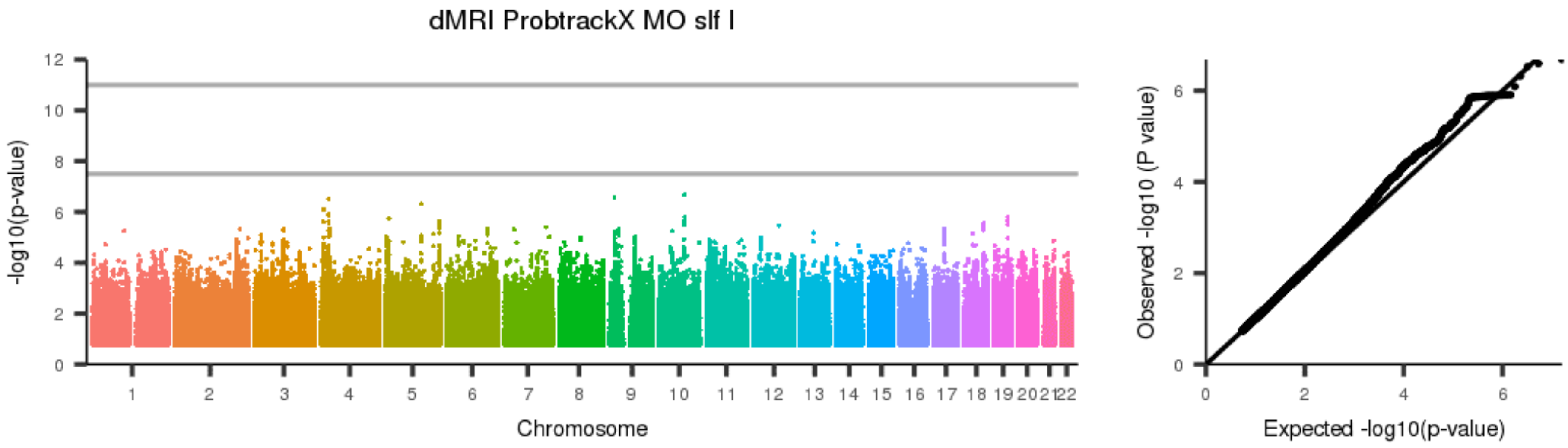

back to list of plots

Figure 704: dMRI ProbtrackX MO slf r

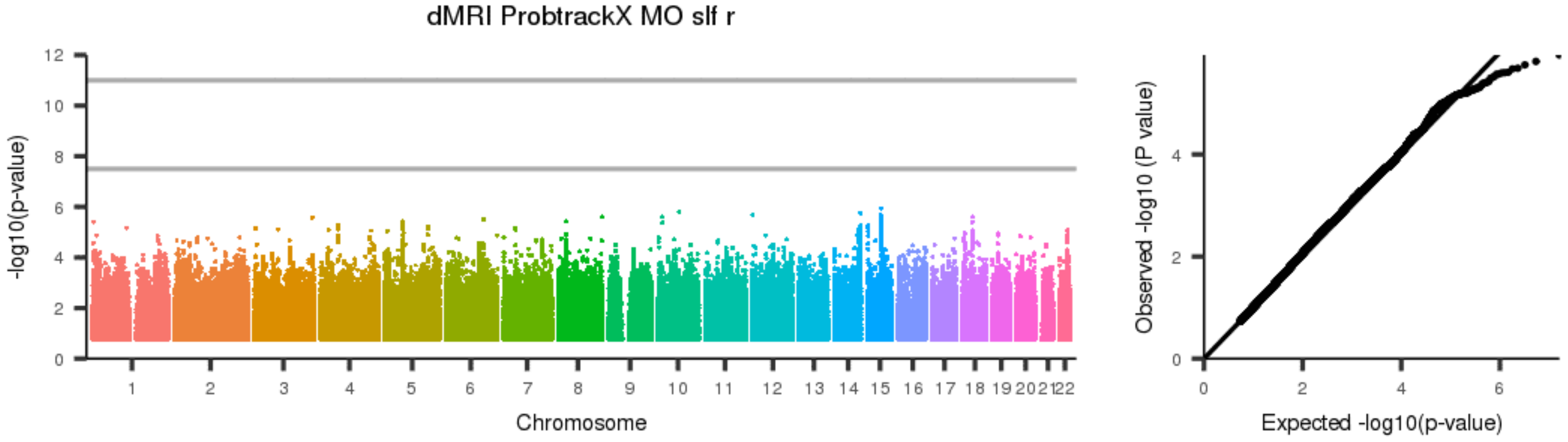

back to list of plots

Figure 705: dMRI ProbtrackX MO str l

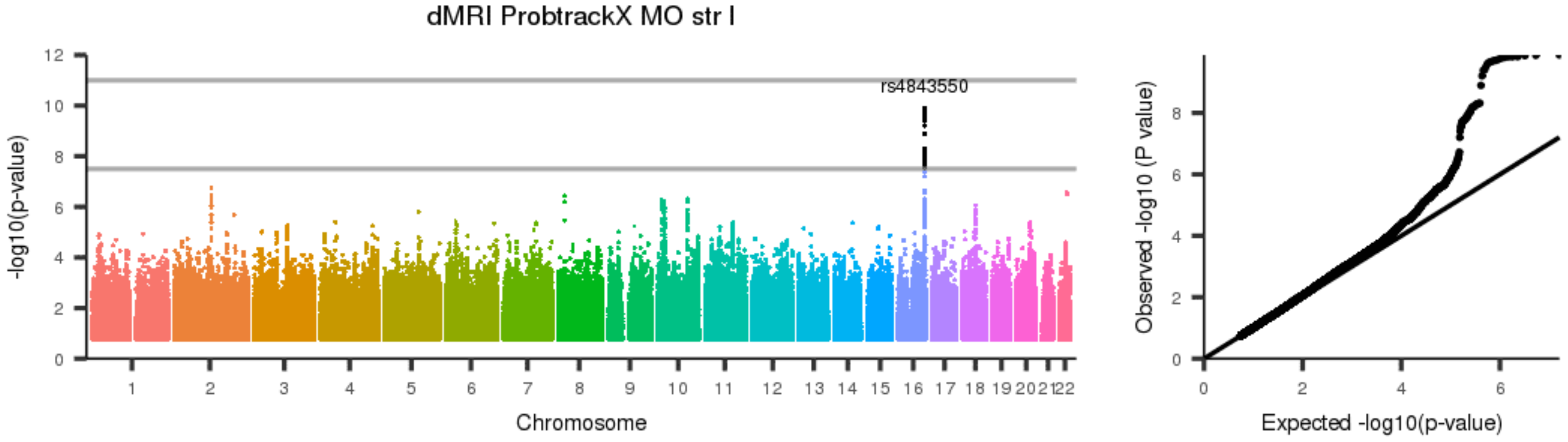

back to list of plots

Figure 706: dMRI ProbtrackX MO str r

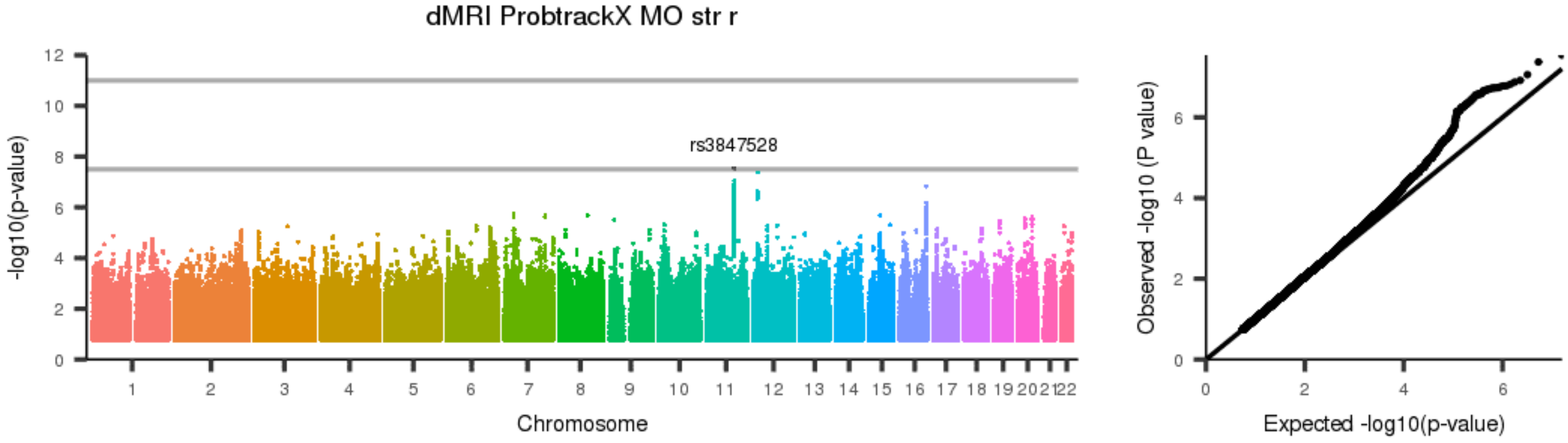

back to list of plots

Figure 707: dMRI ProbtrackX MO unc l

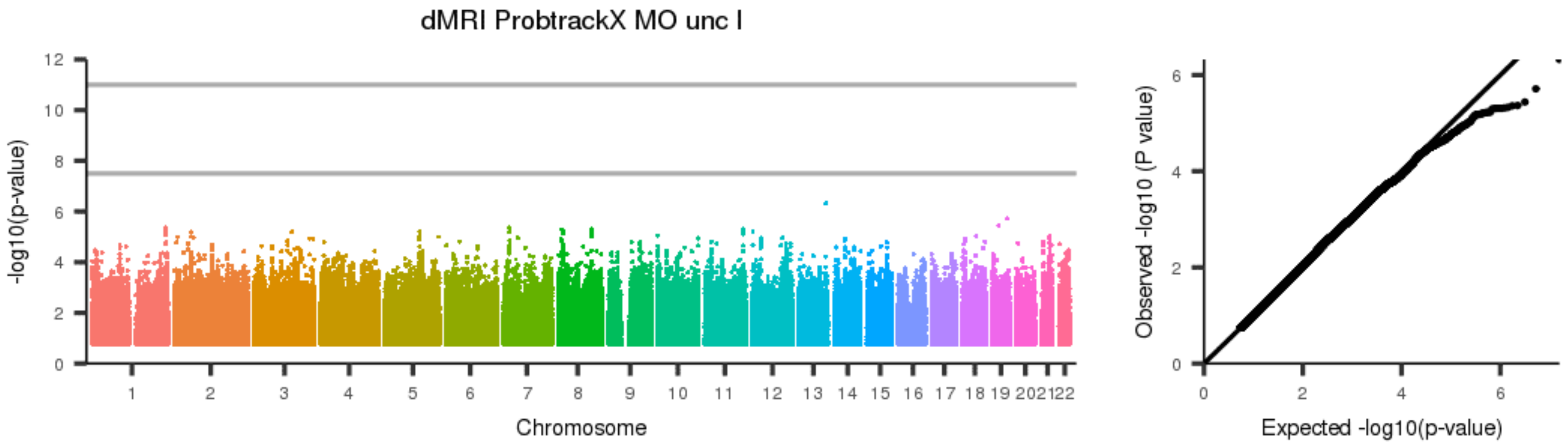

back to list of plots

Figure 708: dMRI ProbtrackX MO unc r

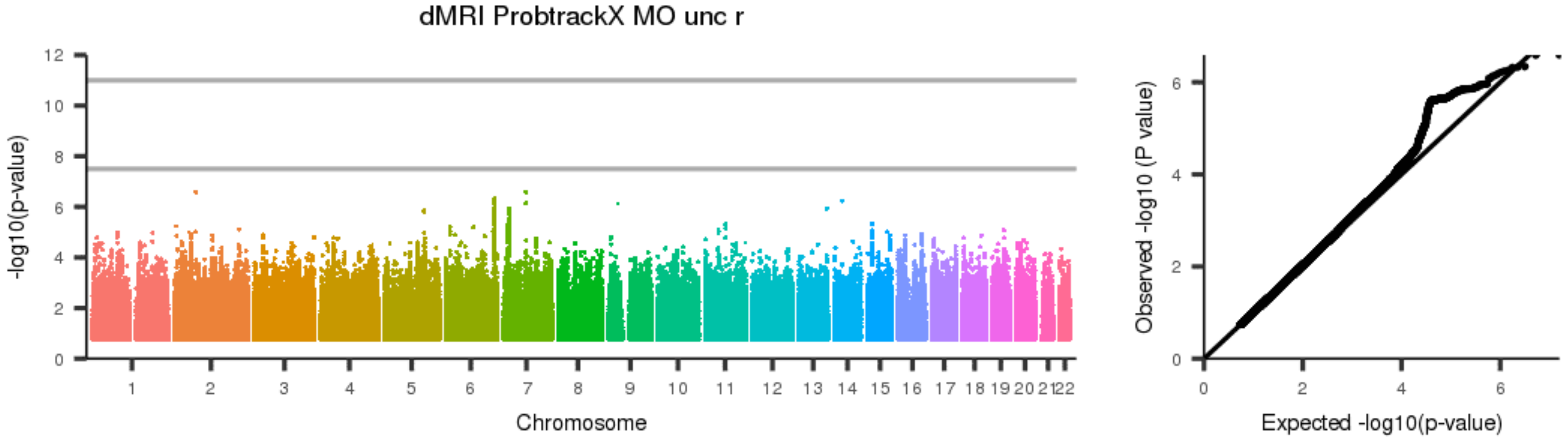

back to list of plots

Figure 709: dMRI ProbtrackX L1 ar l

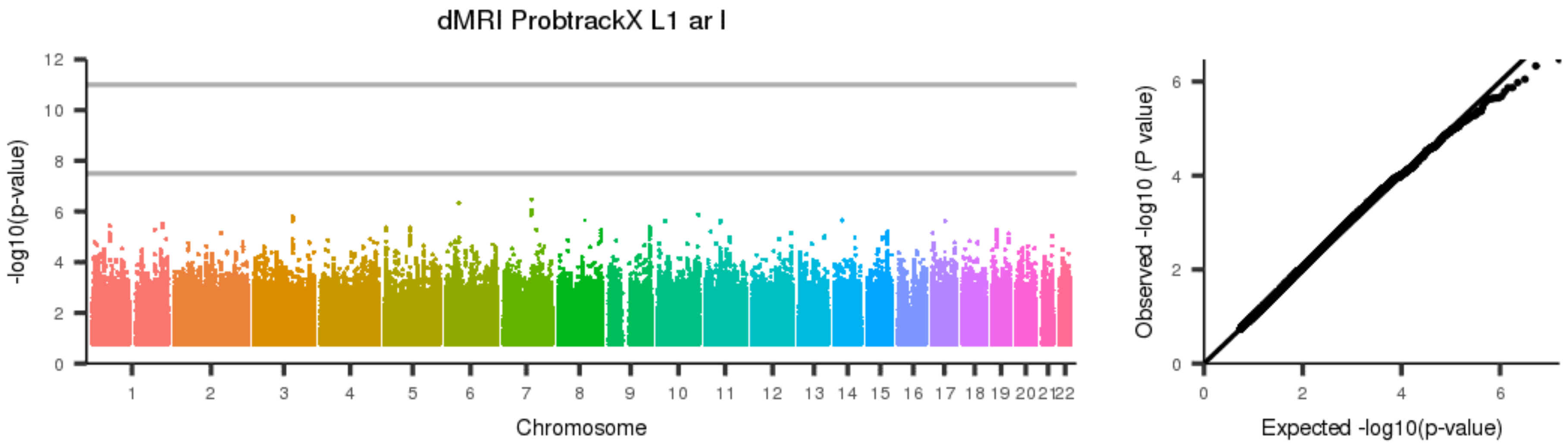

back to list of plots

Figure 710: dMRI ProbtrackX L1 ar r

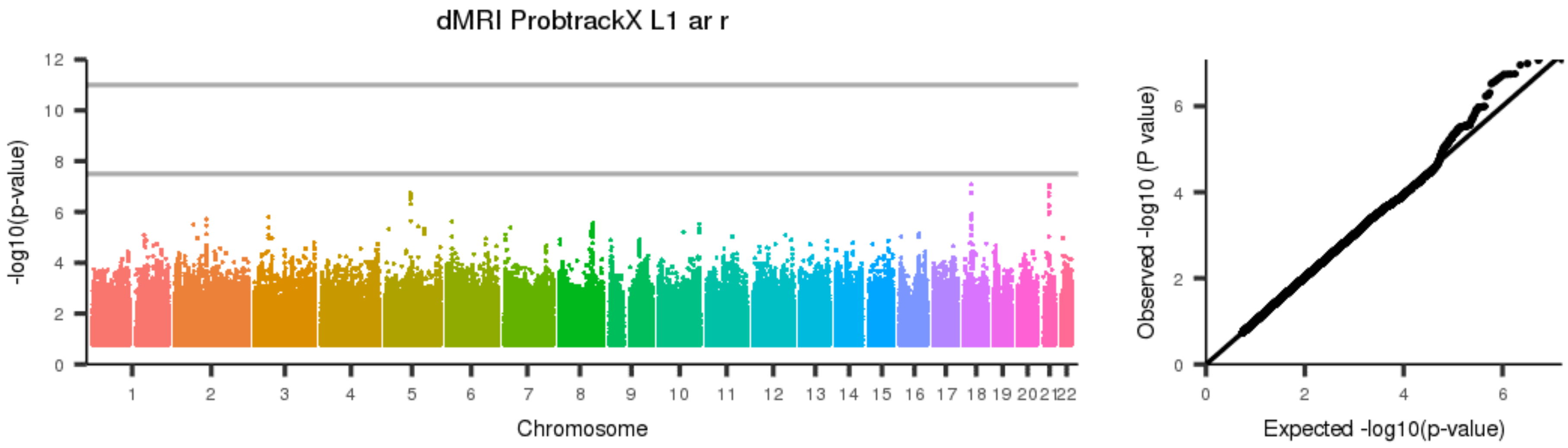

back to list of plots

Figure 711: dMRI ProbtrackX L1 atr l

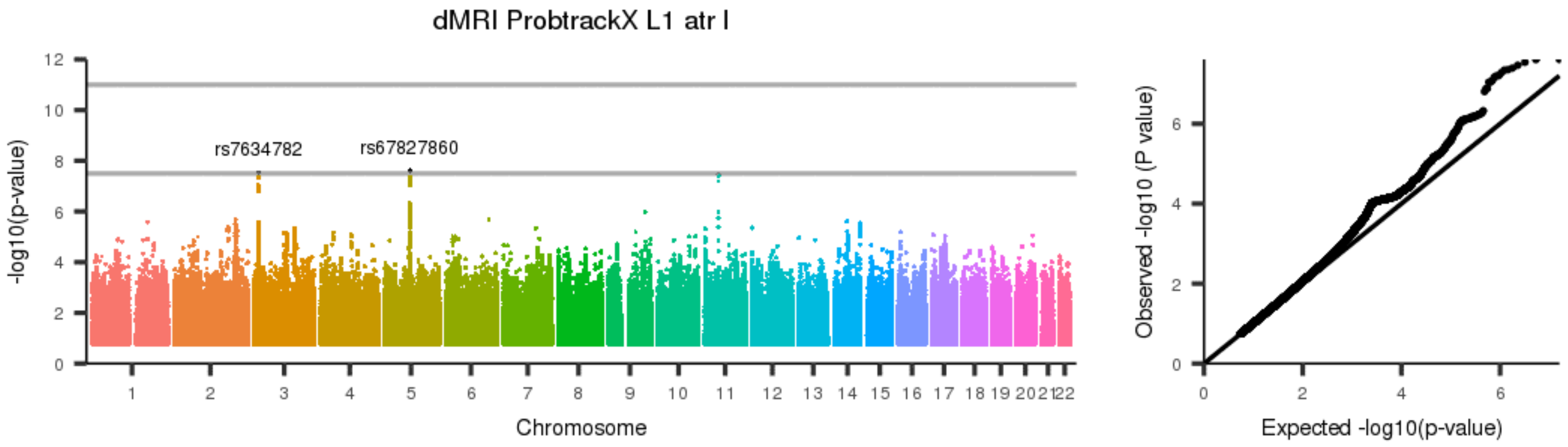

back to list of plots

Figure 712: dMRI ProbtrackX L1 atr r

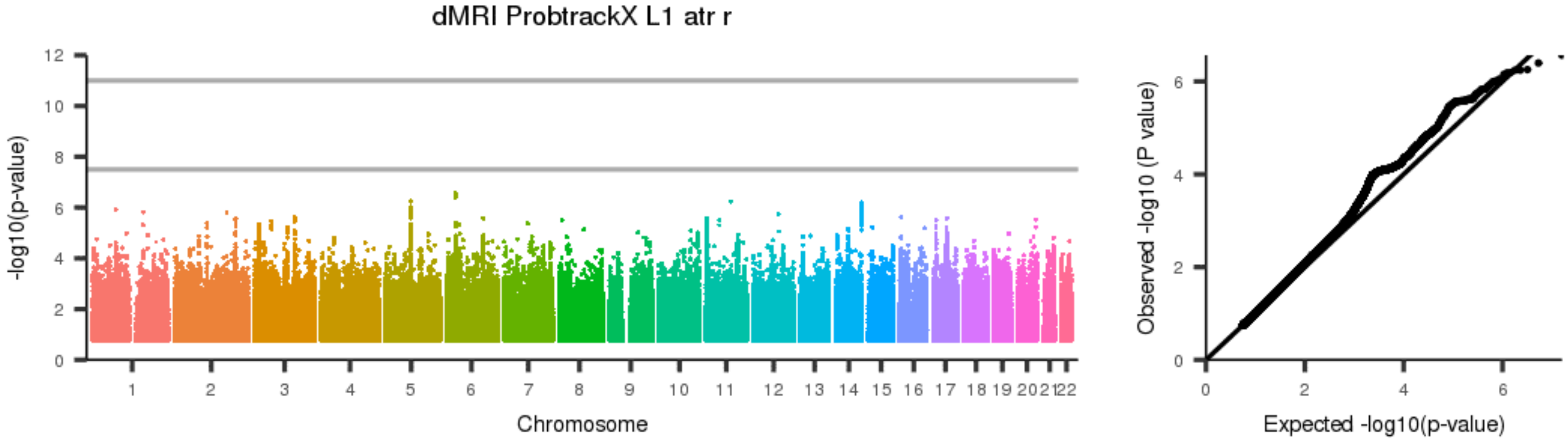

back to list of plots

Figure 713: dMRI ProbtrackX L1 cgc l

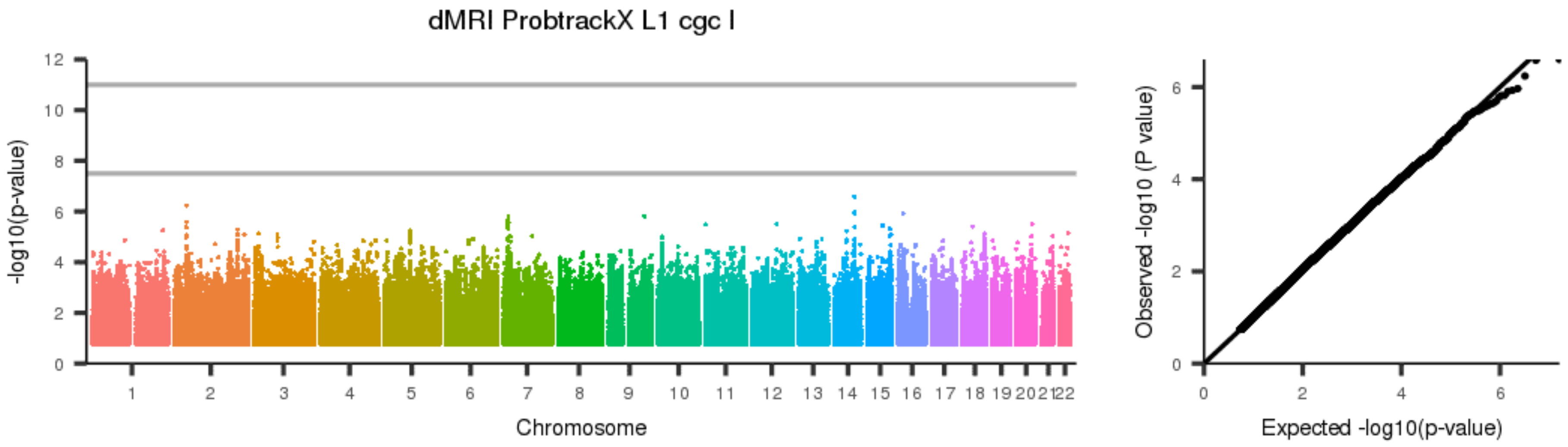

back to list of plots

Figure 714: dMRI ProbtrackX L1 cgc r

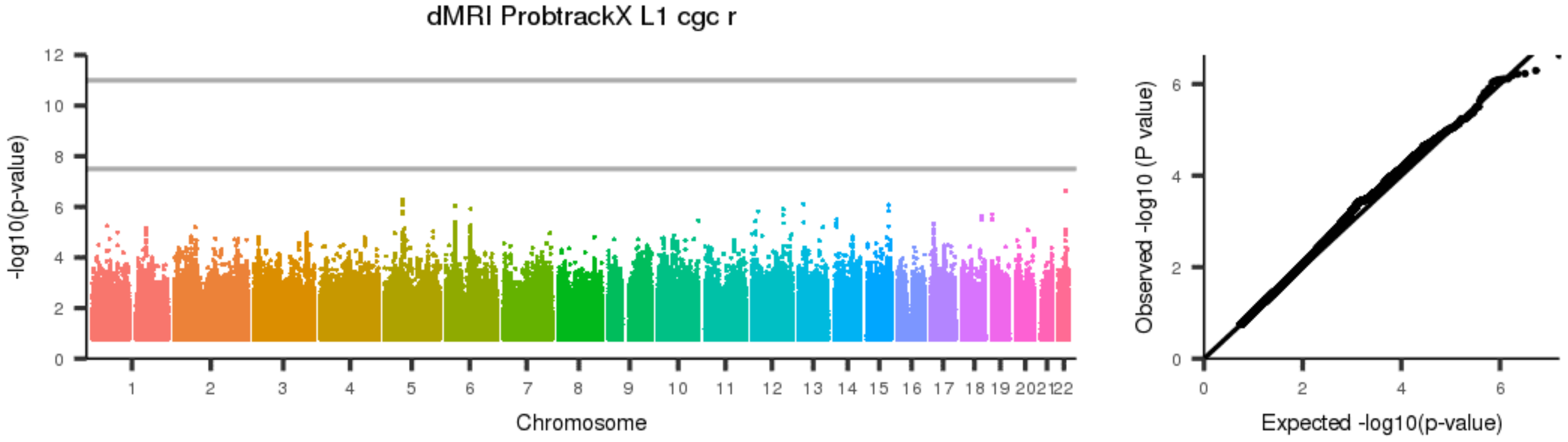

back to list of plots

Figure 715: dMRI ProbtrackX L1 cgh l

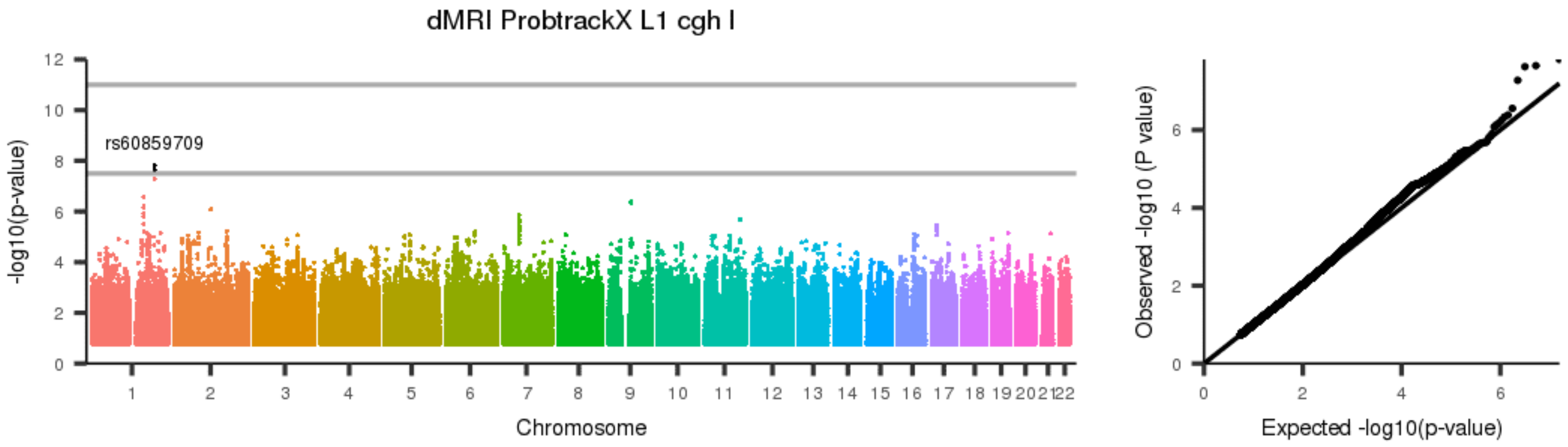

back to list of plots

Figure 716: dMRI ProbtrackX L1 cgh r

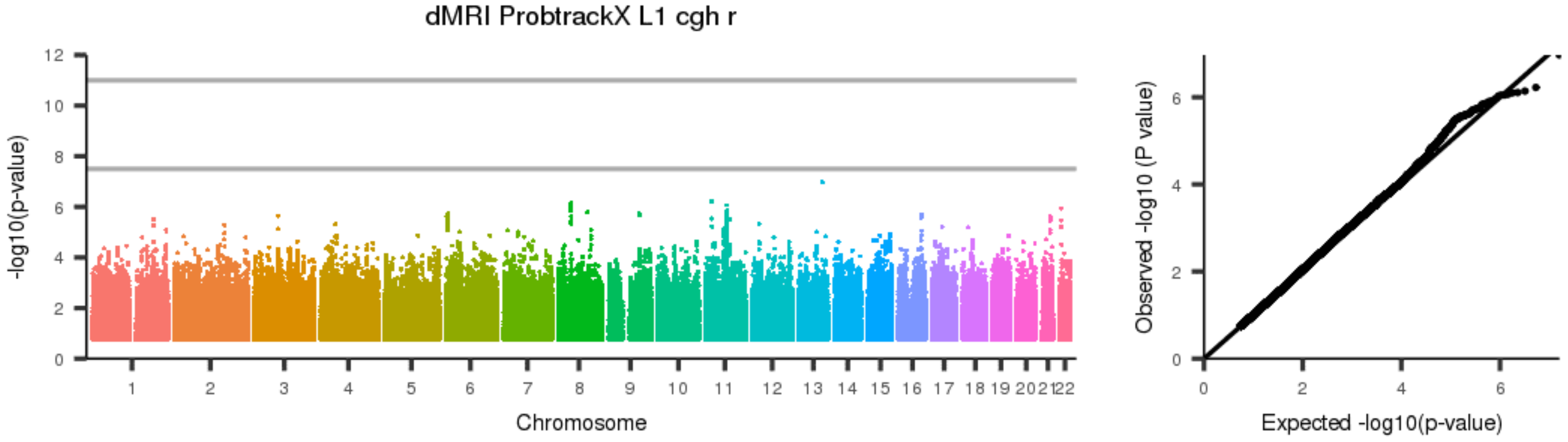

back to list of plots

Figure 717: dMRI ProbtrackX L1 cst l

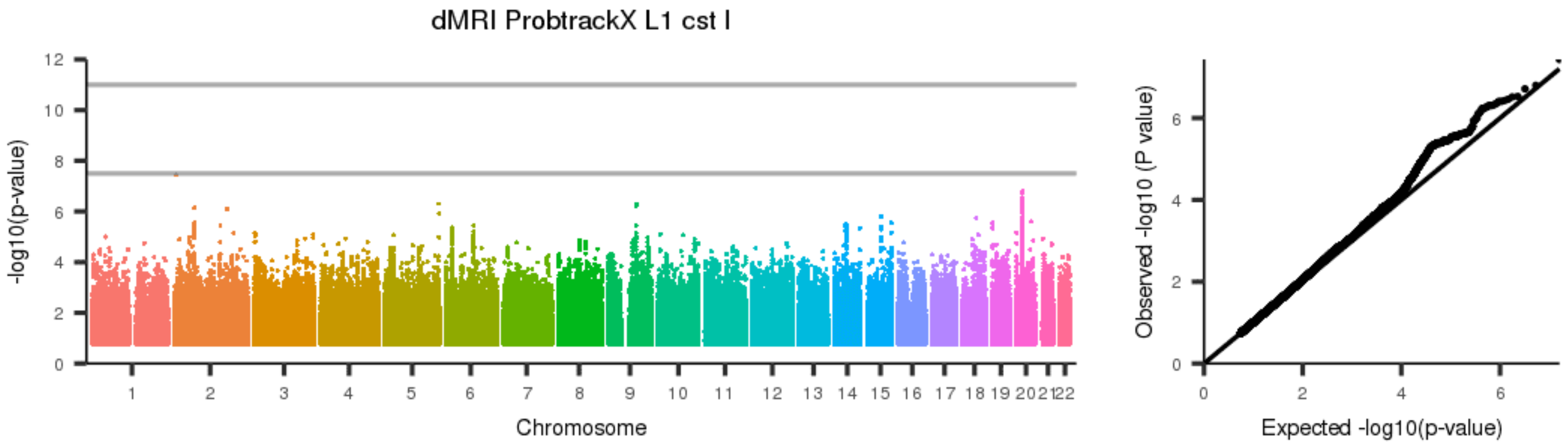

back to list of plots

Figure 718: dMRI ProbtrackX L1 cst r

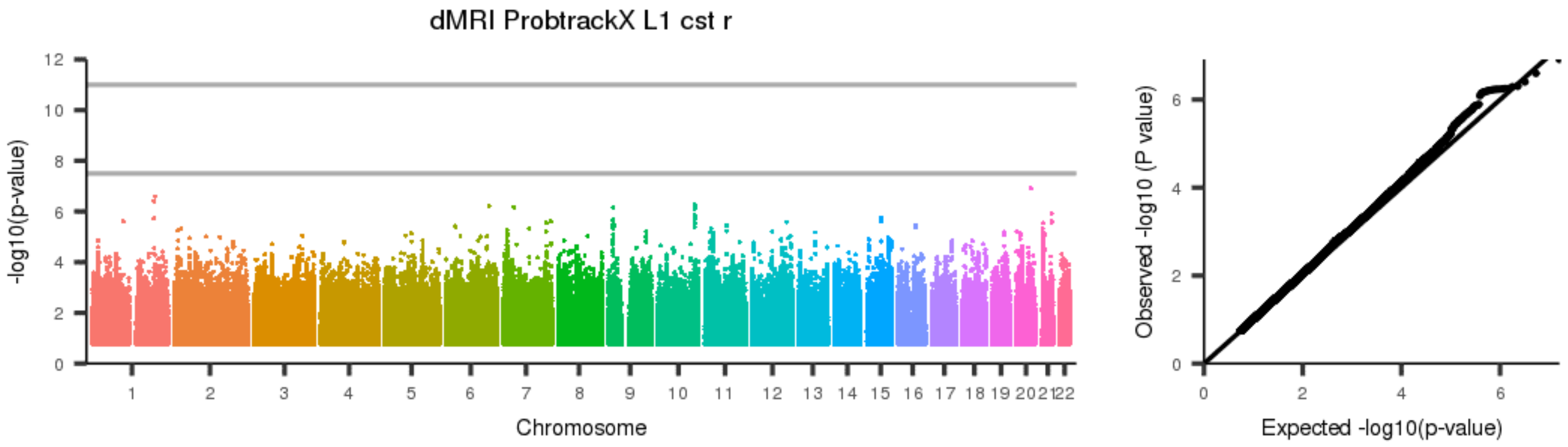

back to list of plots

Figure 719: dMRI ProbtrackX L1 fma

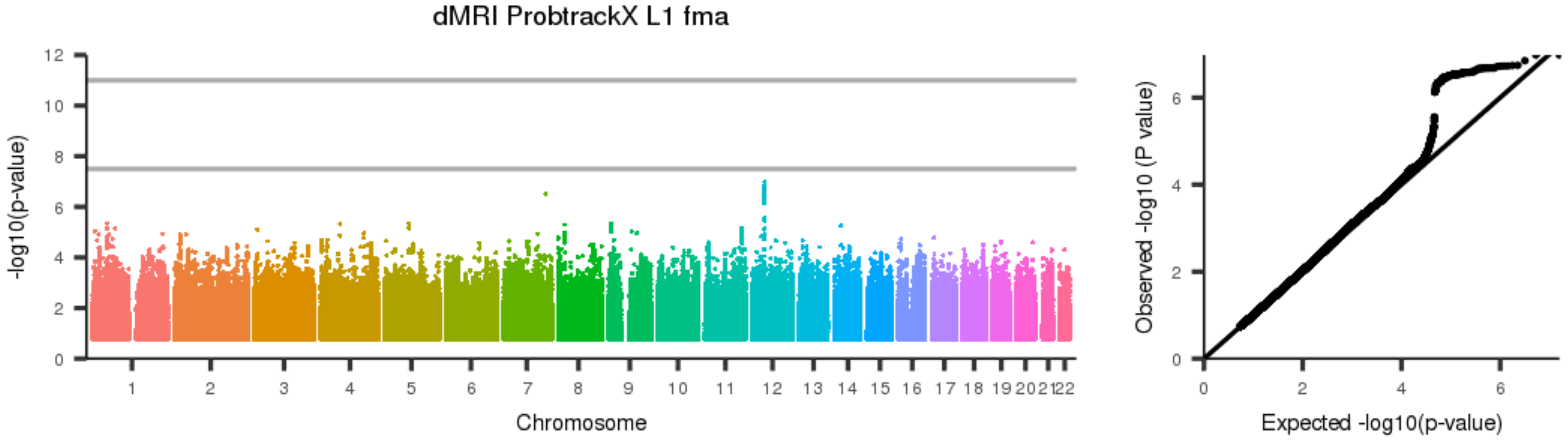

back to list of plots

Figure 720: dMRI ProbtrackX L1 fmi

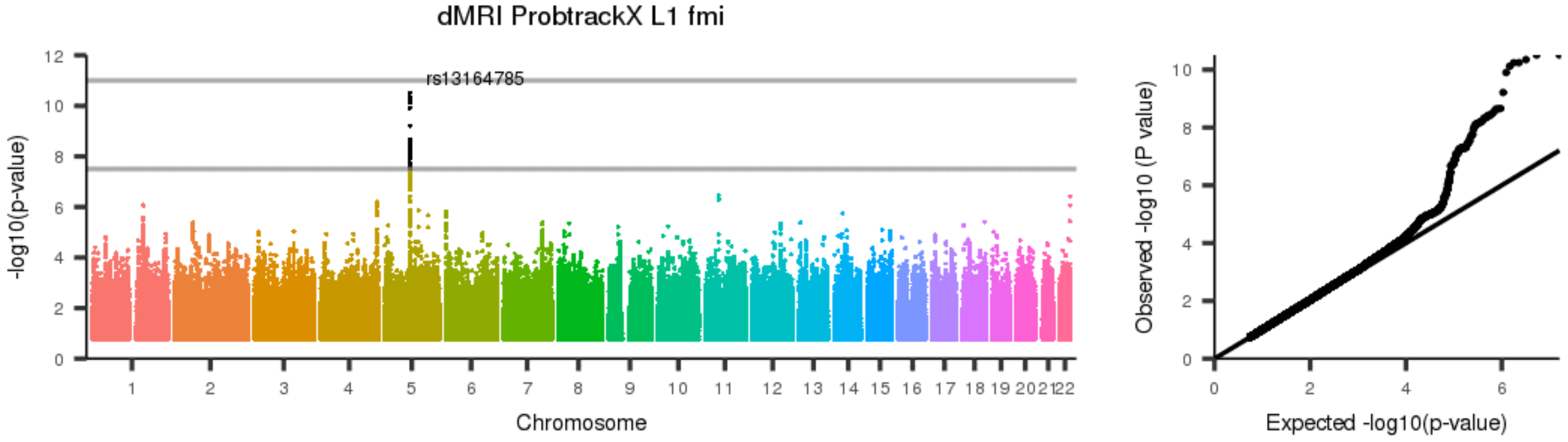

back to list of plots

Figure 721: dMRI ProbtrackX L1 ifo l

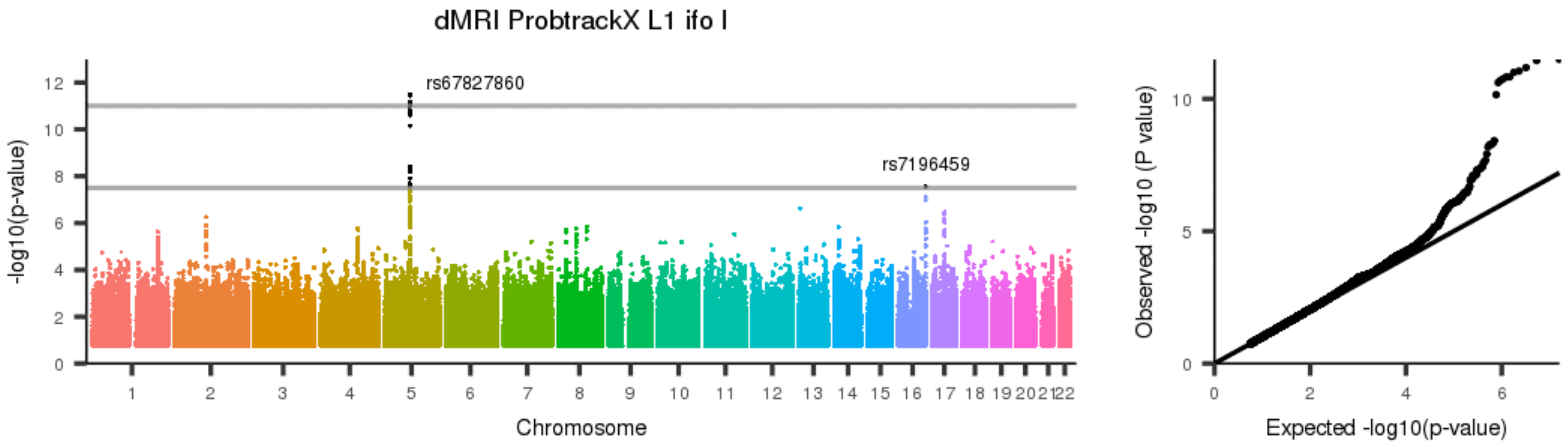

back to list of plots

Figure 722: dMRI ProbtrackX L1 ifo r

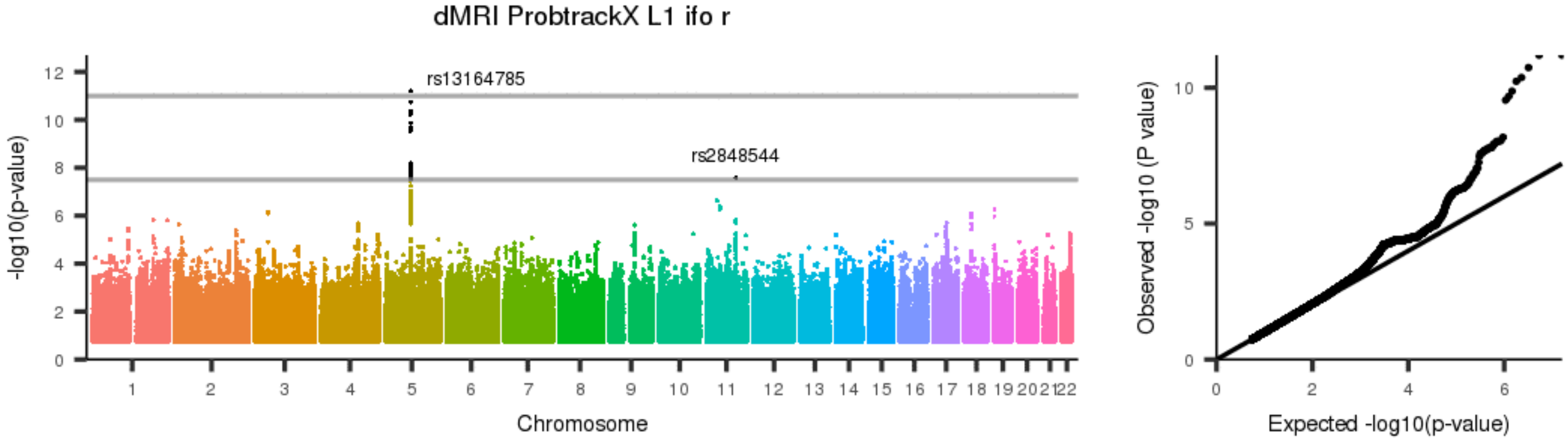

back to list of plots

Figure 723: dMRI ProbtrackX L1 ilf l

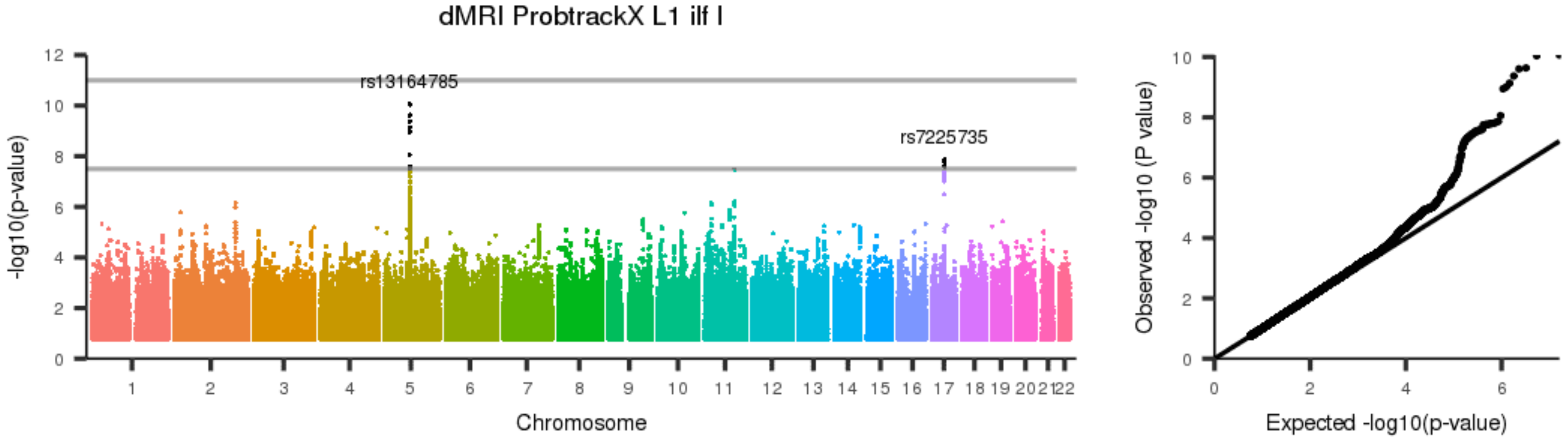

back to list of plots

Figure 724: dMRI ProbtrackX L1 ilf r

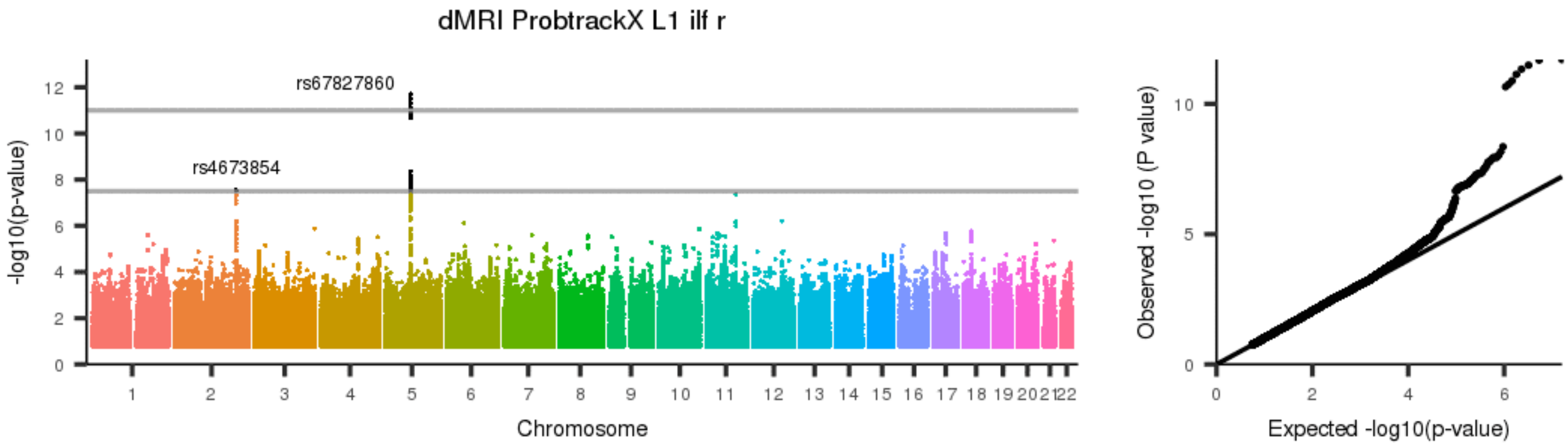

back to list of plots

Figure 725: dMRI ProbtrackX L1 mcp

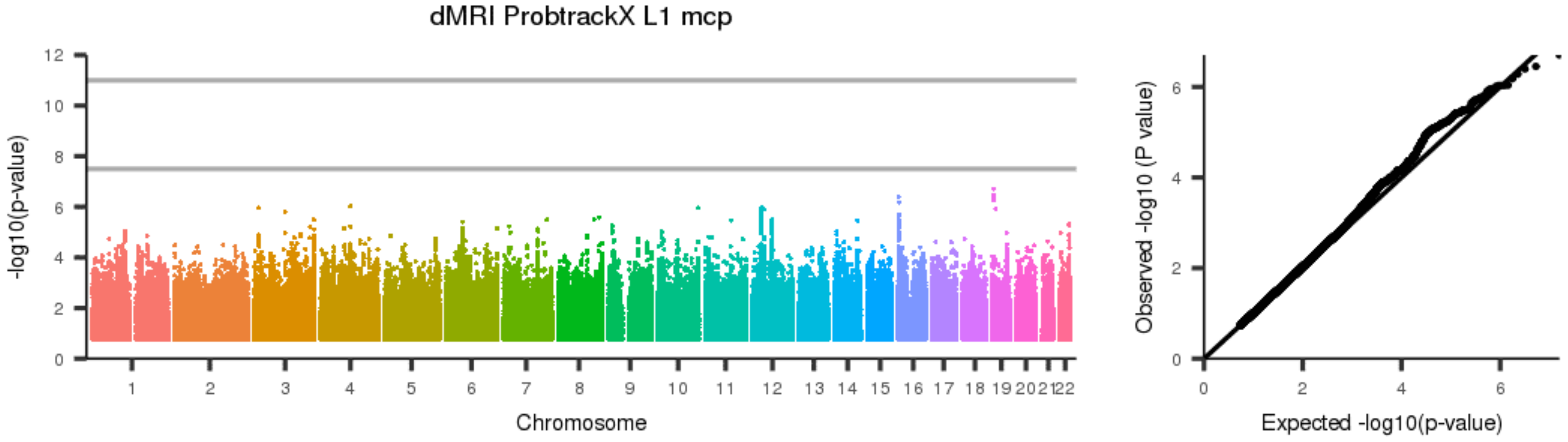

back to list of plots

Figure 726: dMRI ProbtrackX L1 ml l

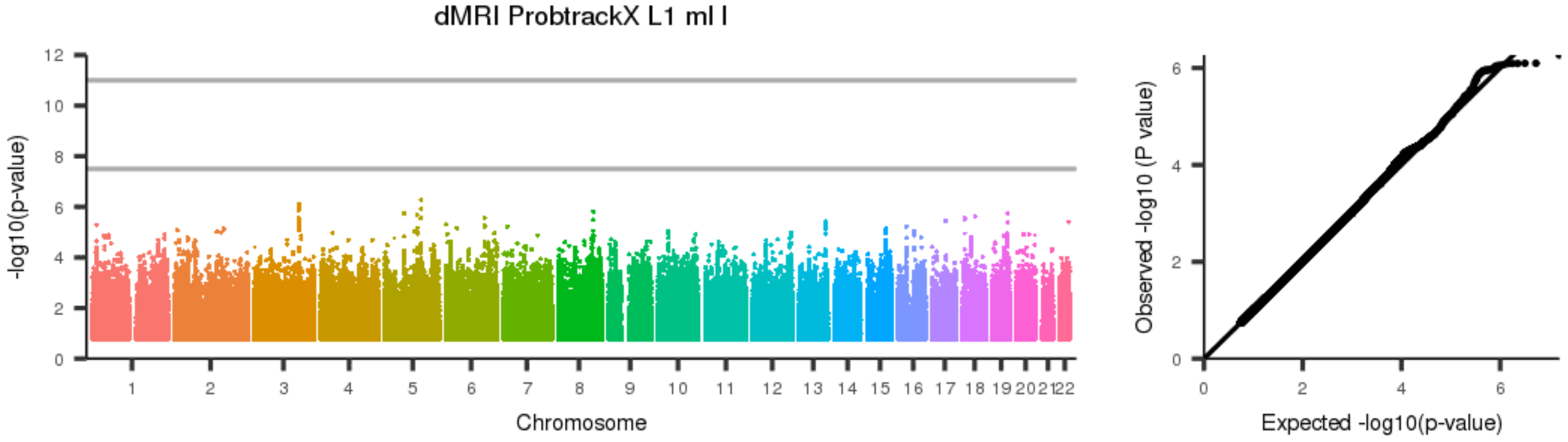

back to list of plots

Figure 727: dMRI ProbtrackX L1 ml r

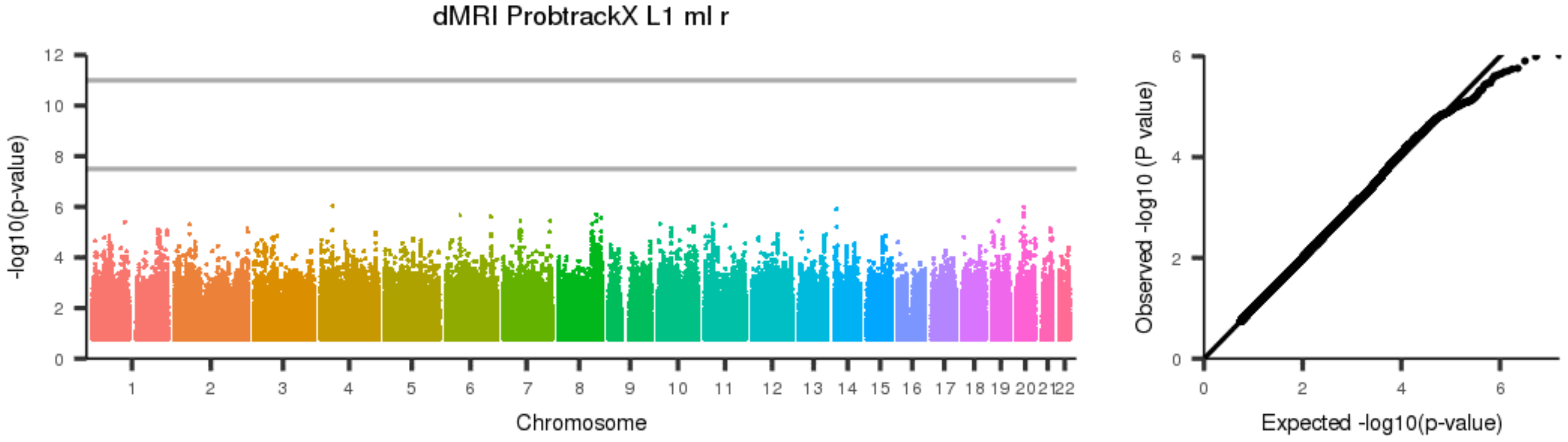

back to list of plots

Figure 728: dMRI ProbtrackX L1 ptr l

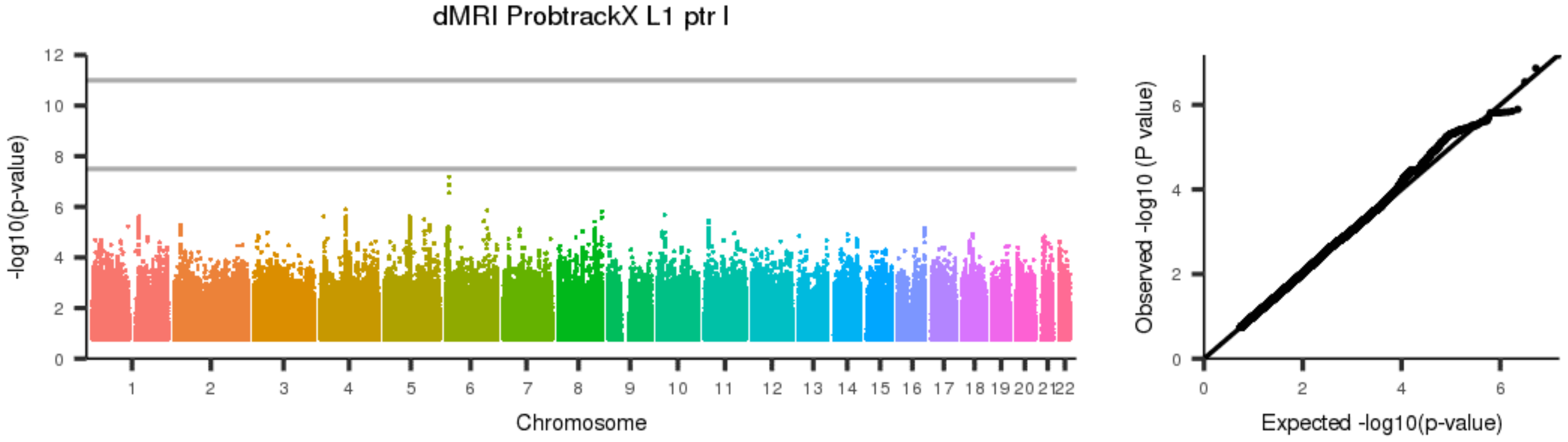

back to list of plots

Figure 729: dMRI ProbtrackX L1 ptr r

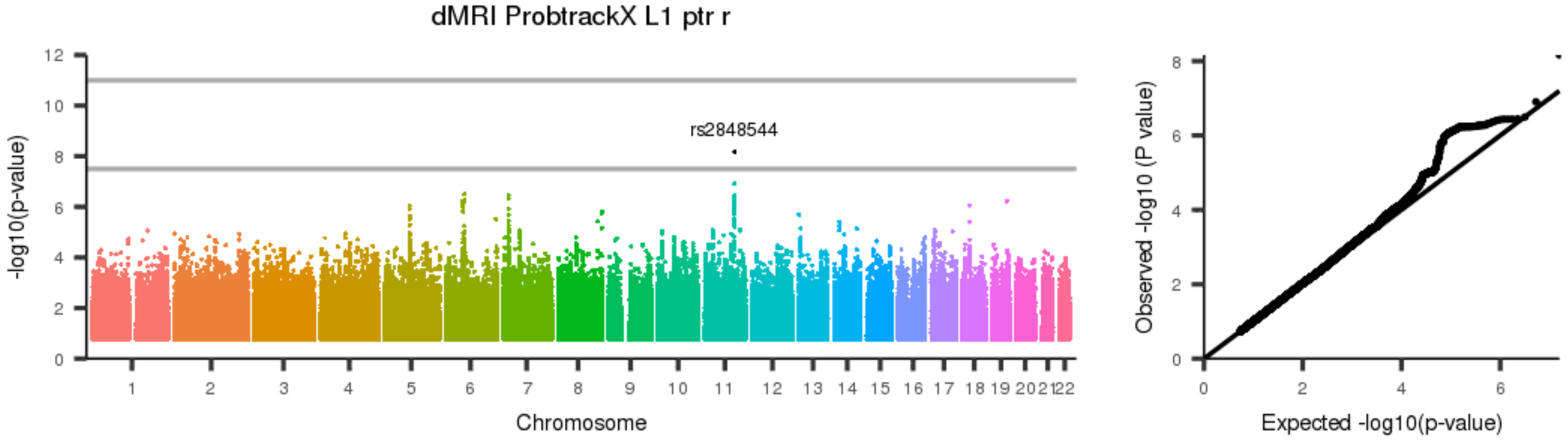

back to list of plots

Figure 730: dMRI ProbtrackX L1 slf I

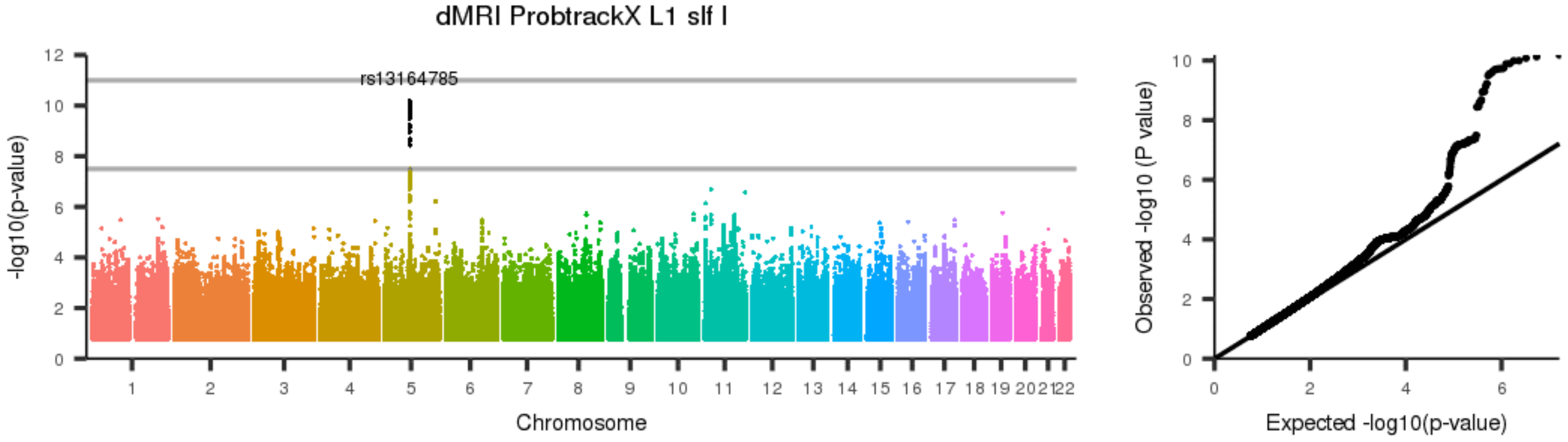

back to list of plots

Figure 731: dMRI ProbtrackX L1 slf r

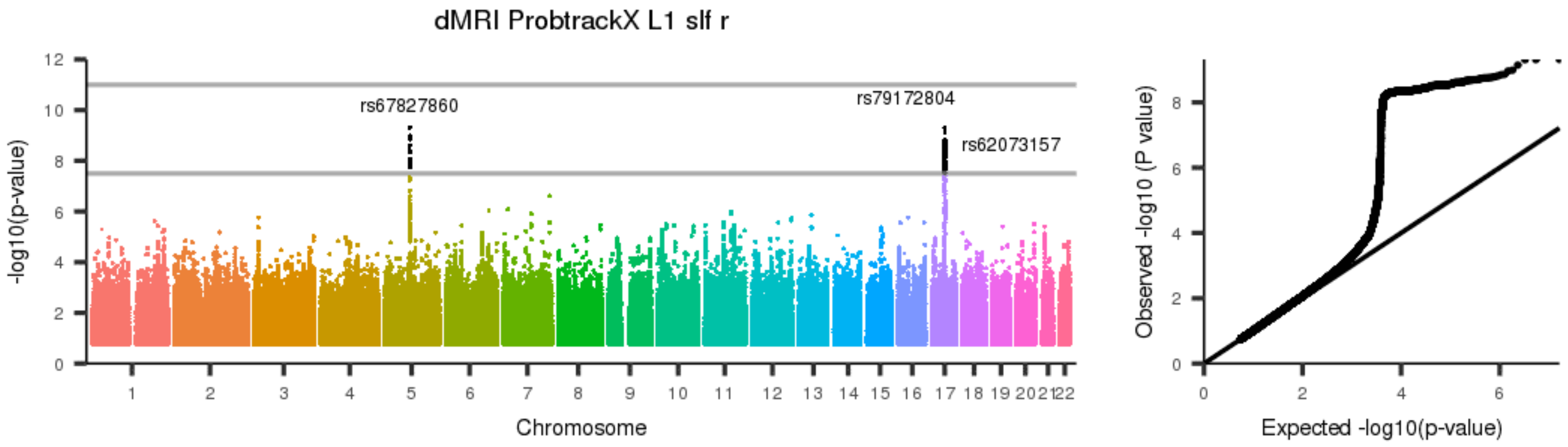

back to list of plots

Figure 732: dMRI ProbtrackX L1 str l

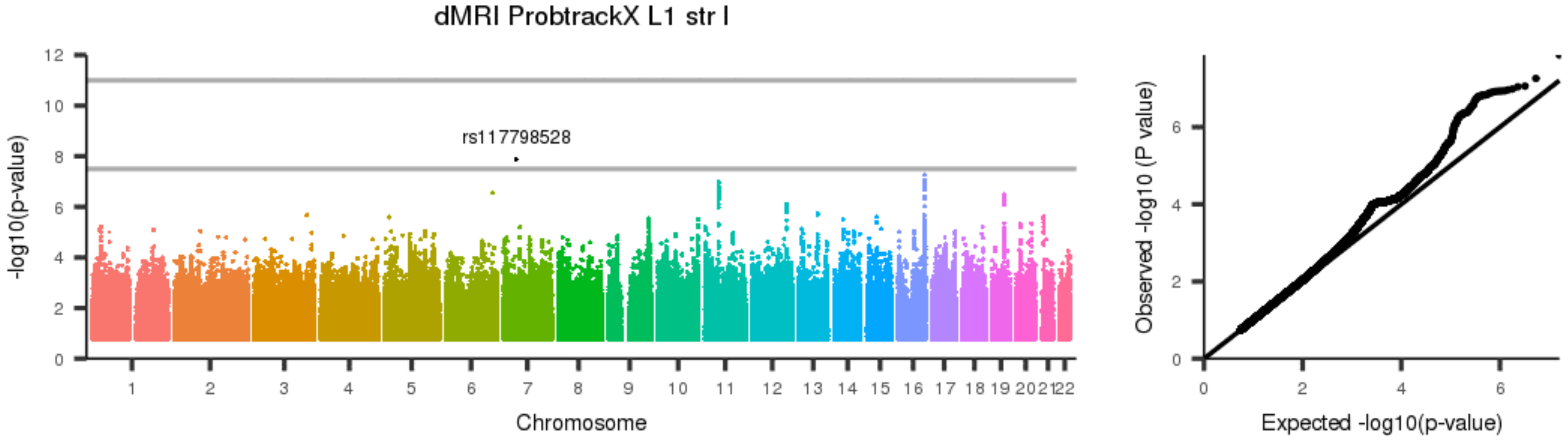

back to list of plots

Figure 733: dMRI ProbtrackX L1 str r

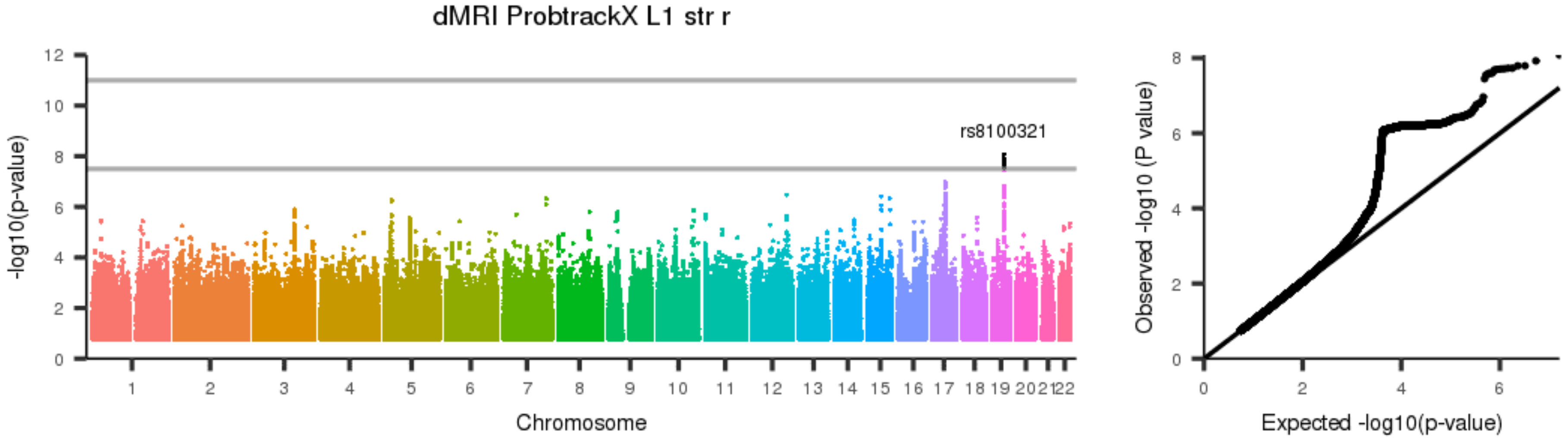

back to list of plots

Figure 734: dMRI ProbtrackX L1 unc l

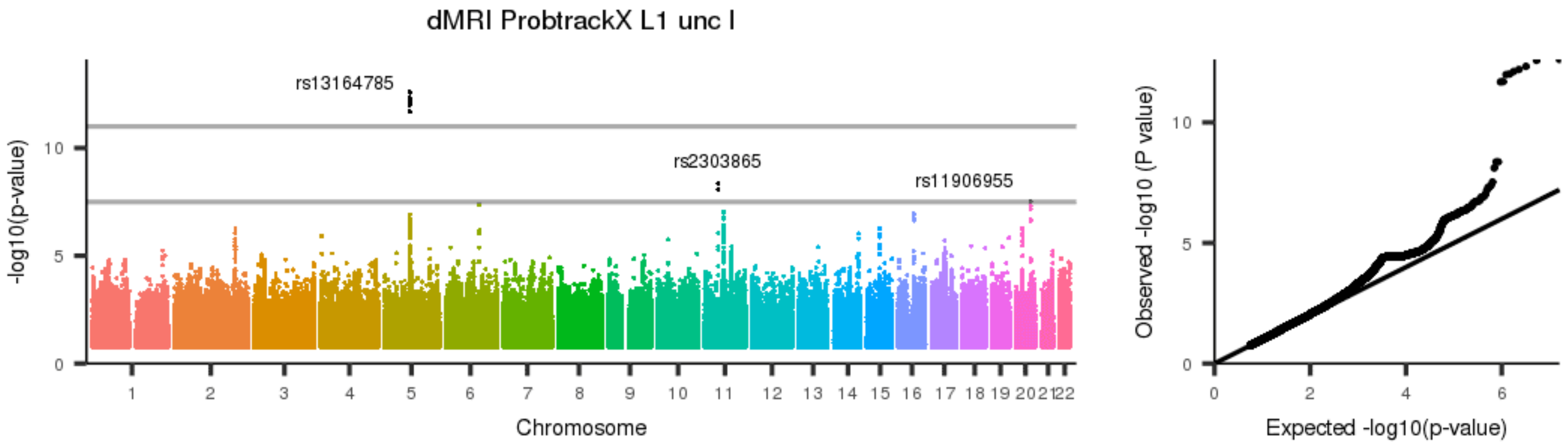

back to list of plots

Figure 735: dMRI ProbtrackX L1 unc r

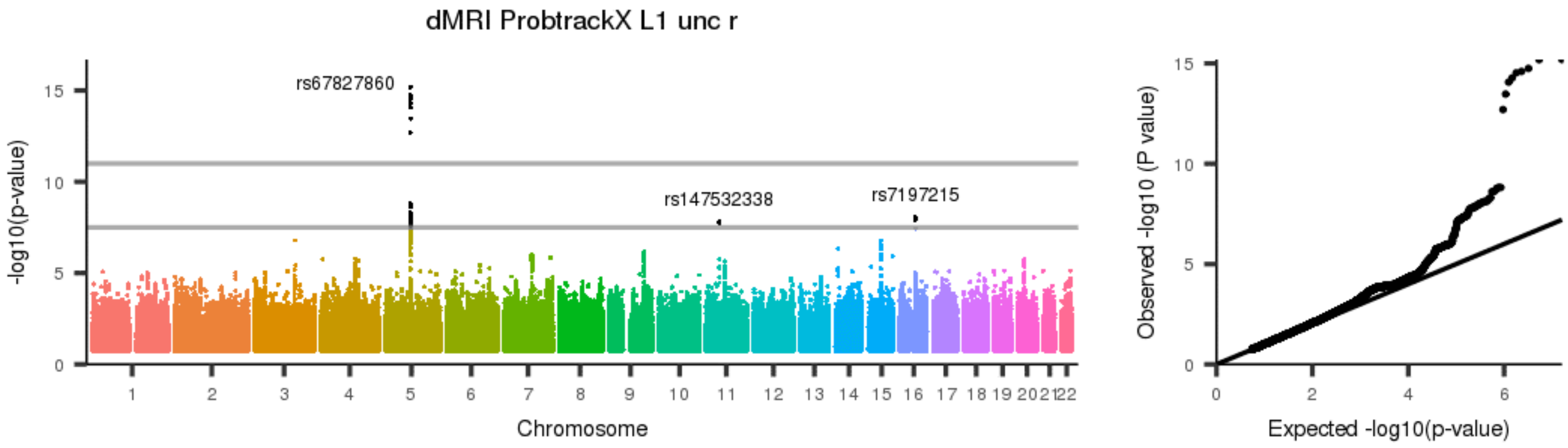

back to list of plots

Figure 736: dMRI ProbtrackX L2 ar l

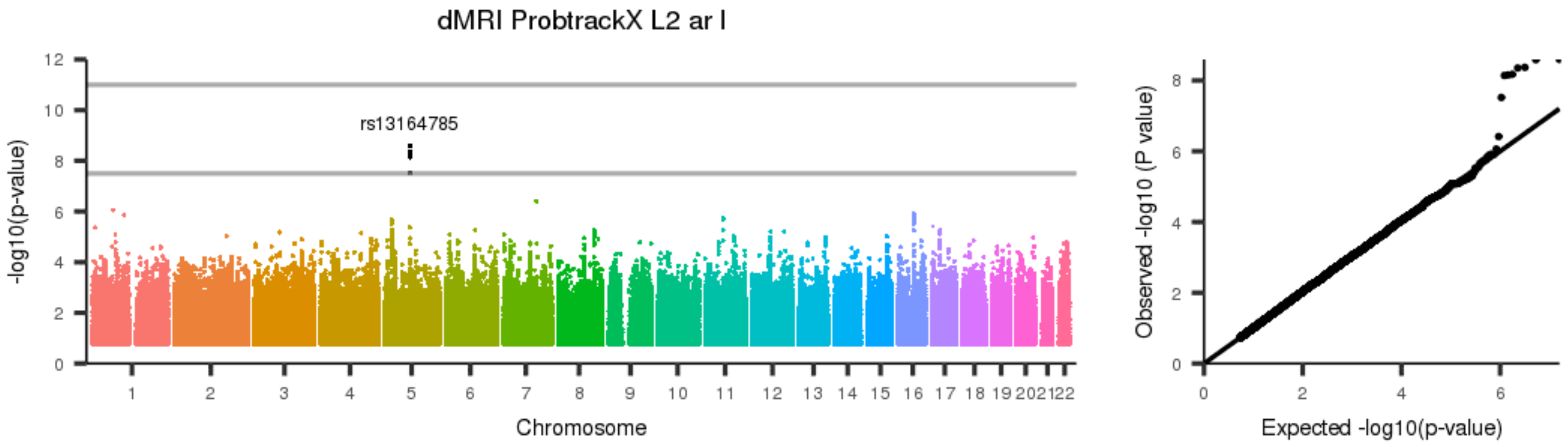

back to list of plots

Figure 737: dMRI ProbtrackX L2 ar r

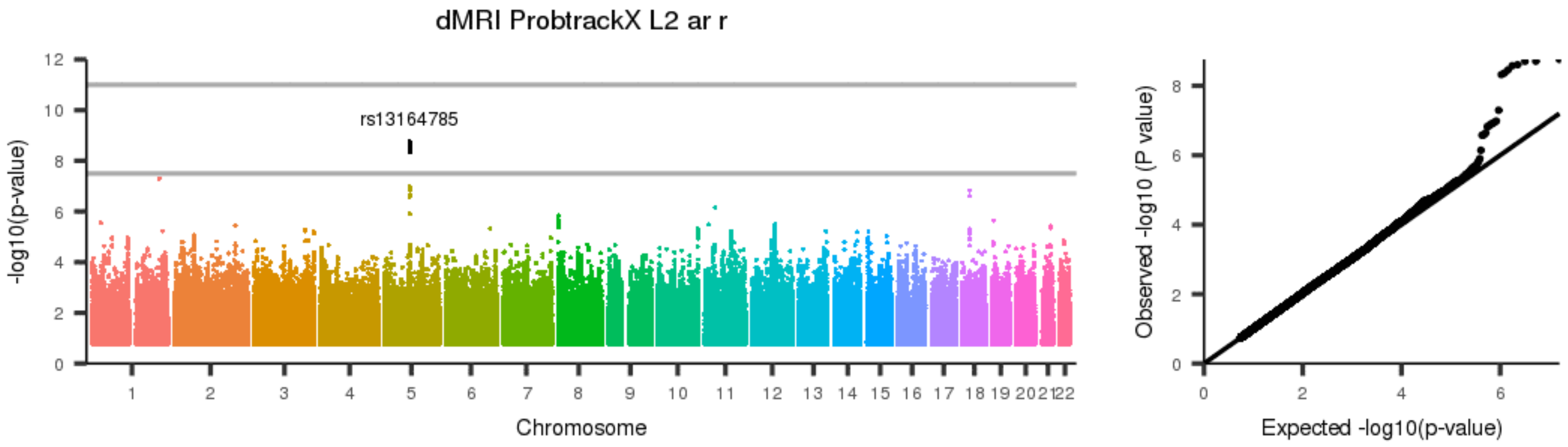

back to list of plots

Figure 738: dMRI ProbtrackX L2 atr l

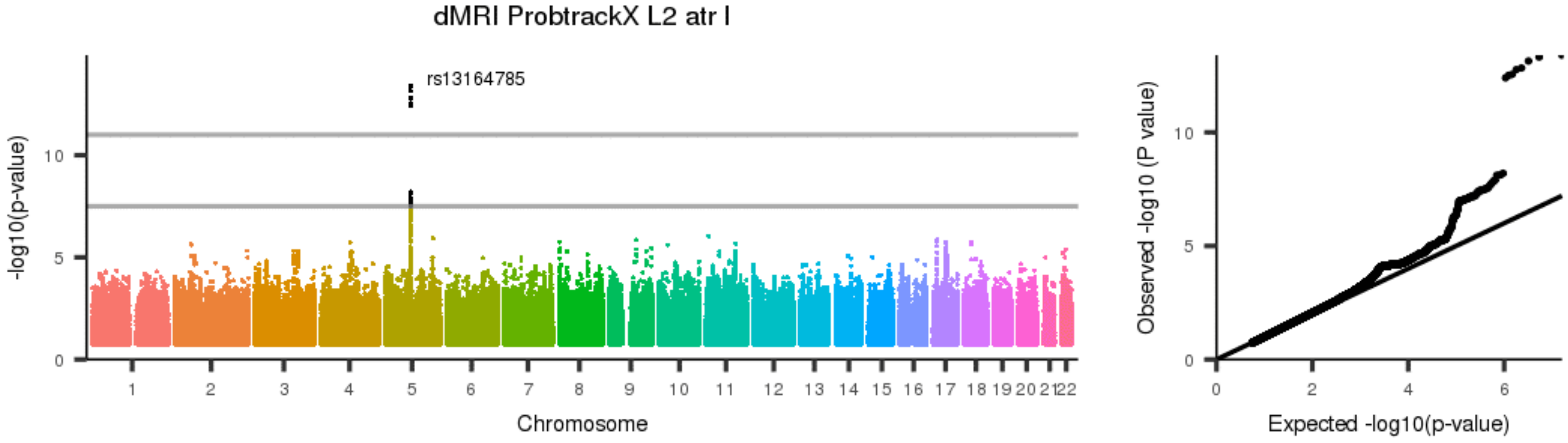

back to list of plots

Figure 739: dMRI ProbtrackX L2 atr r

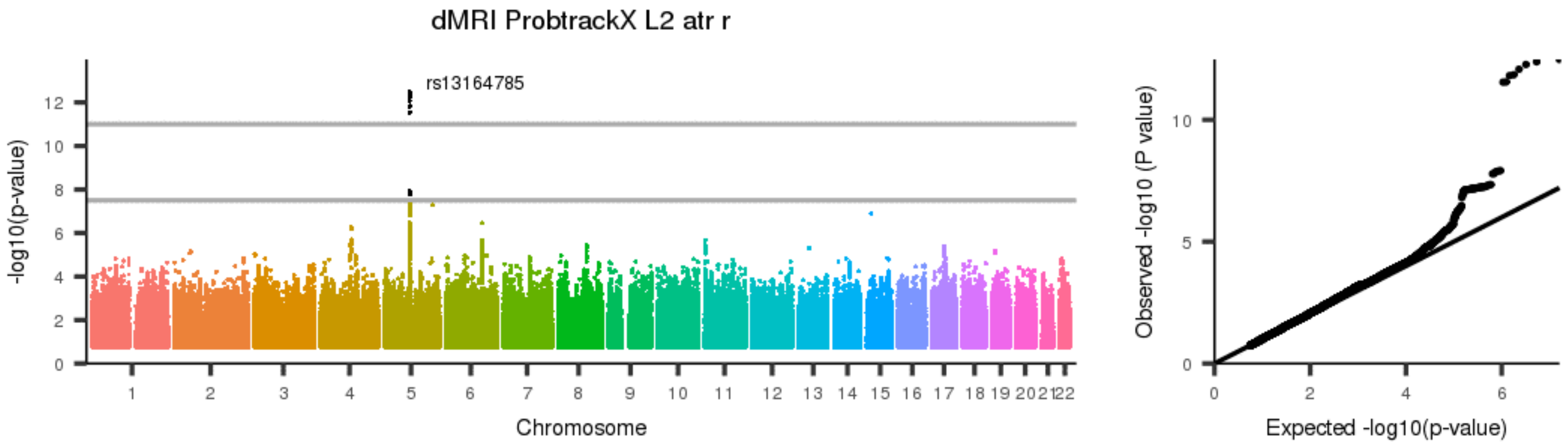

back to list of plots

Figure 740: dMRI ProbtrackX L2 cgc l

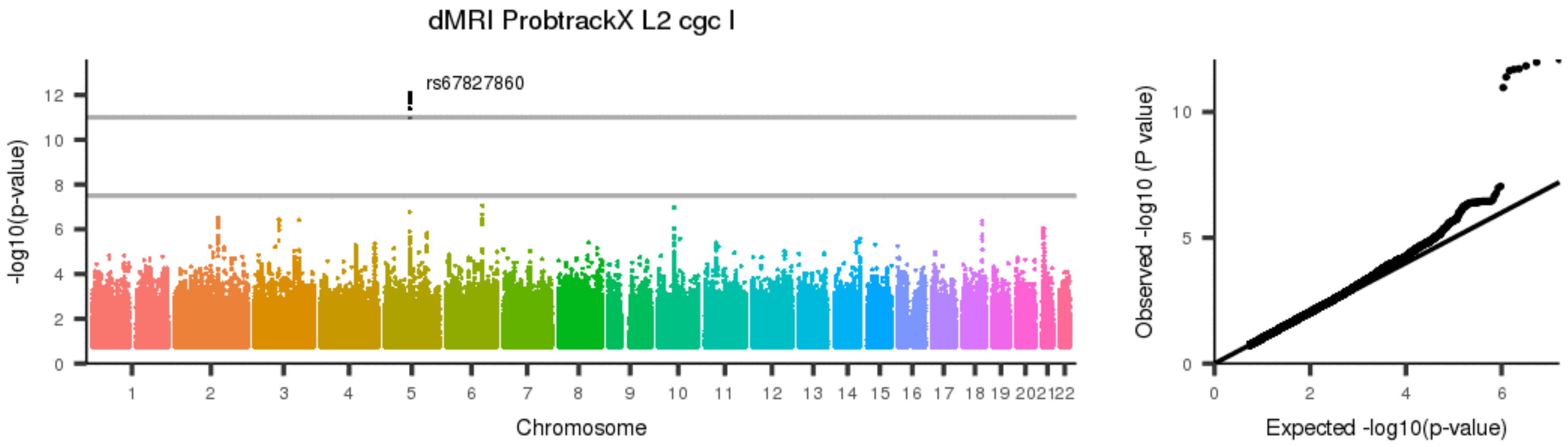

back to list of plots

Figure 741: dMRI ProbtrackX L2 cgc r

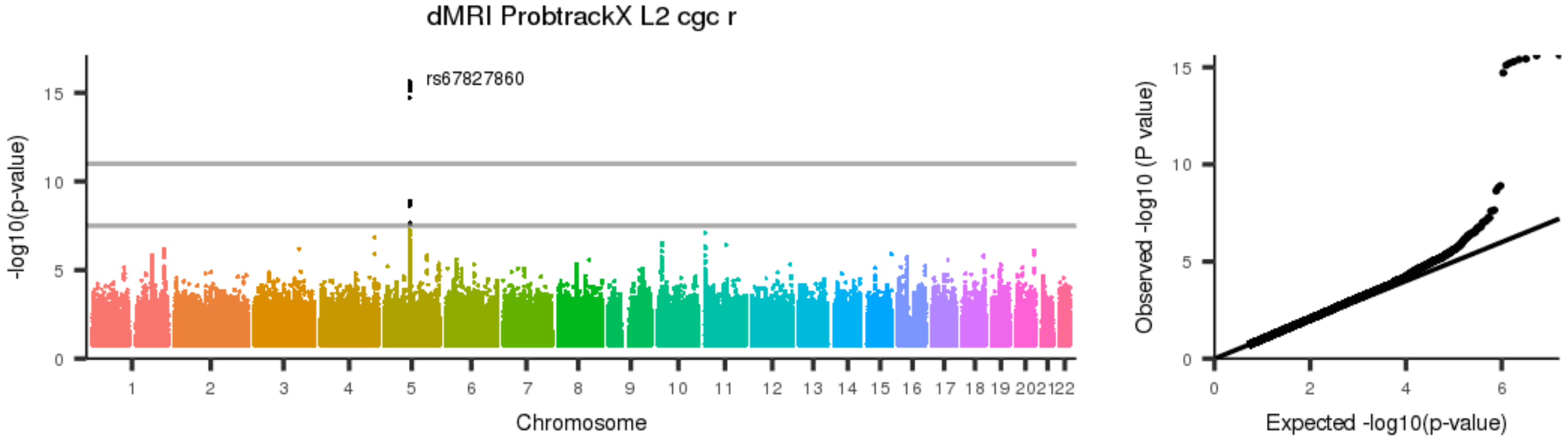

back to list of plots

Figure 742: dMRI ProbtrackX L2 cgh l

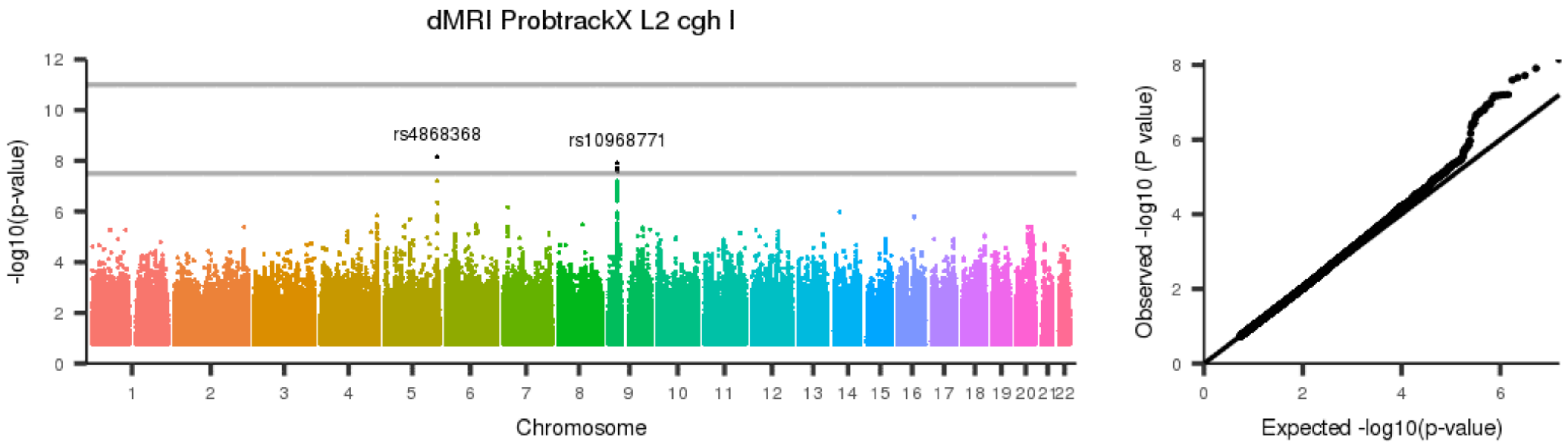

back to list of plots

Figure 743: dMRI ProbtrackX L2 cgh r

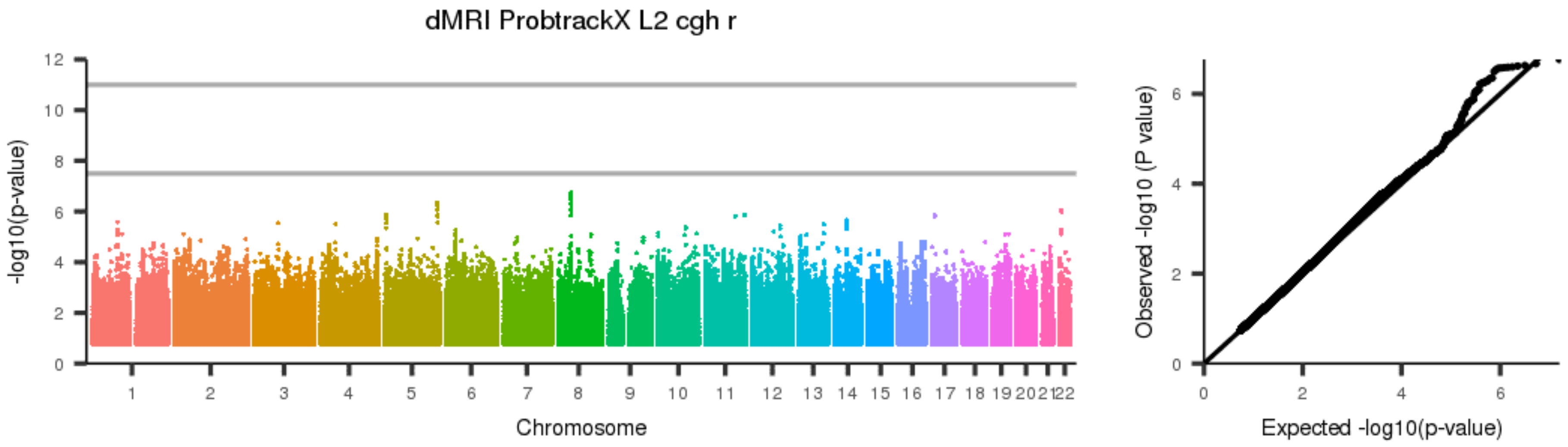

back to list of plots

Figure 744: dMRI ProbtrackX L2 cst l

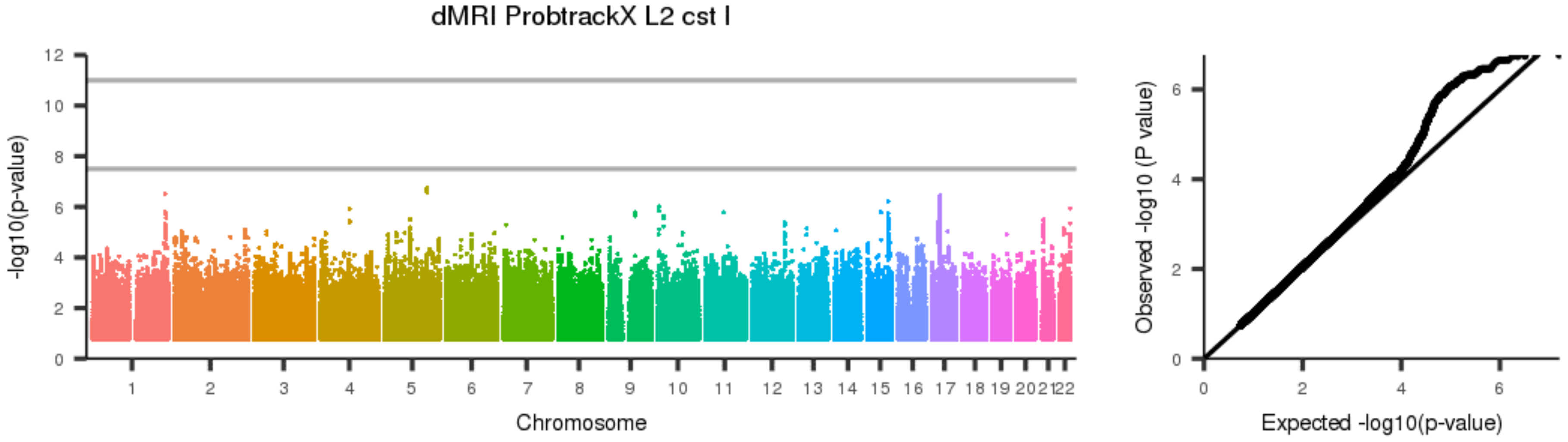

back to list of plots

Figure 745: dMRI ProbtrackX L2 cst r

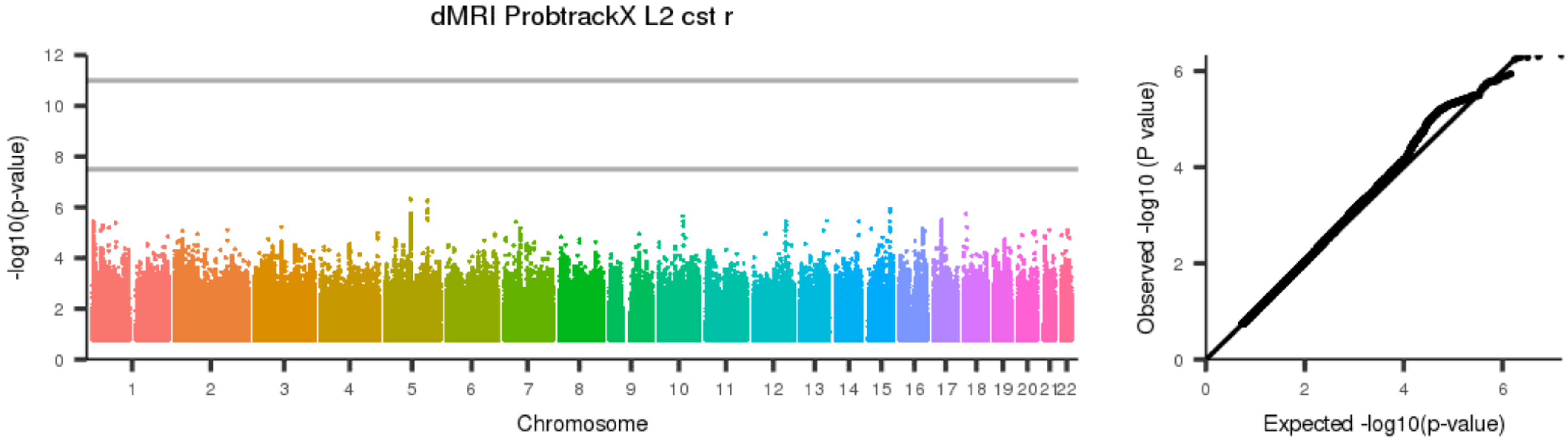

back to list of plots

Figure 746: dMRI ProbtrackX L2 fma

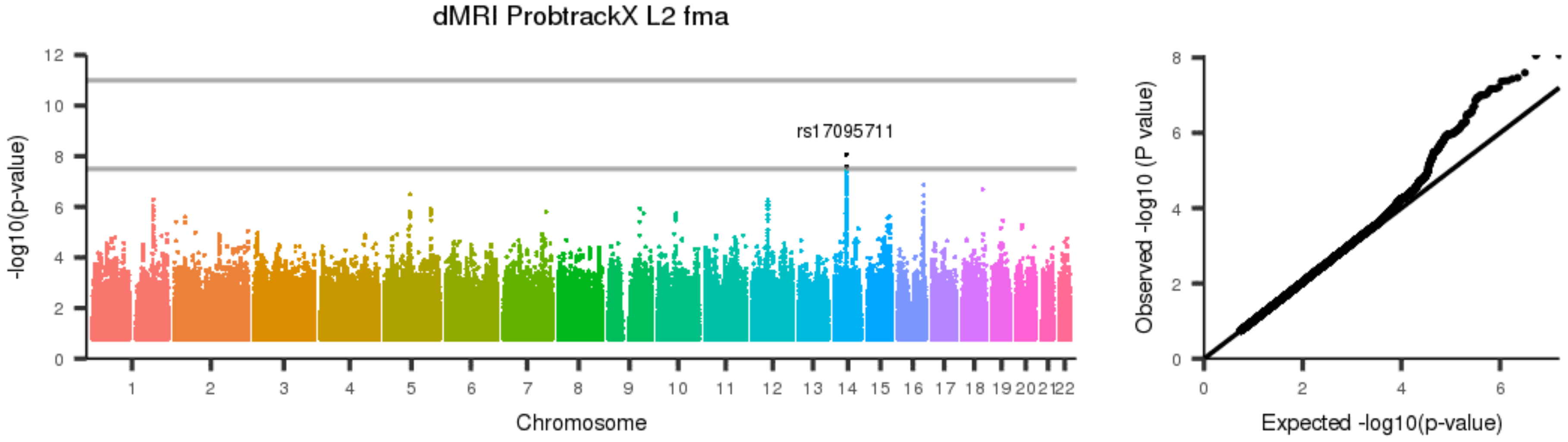

back to list of plots

Figure 747: dMRI ProbtrackX L2 fmi

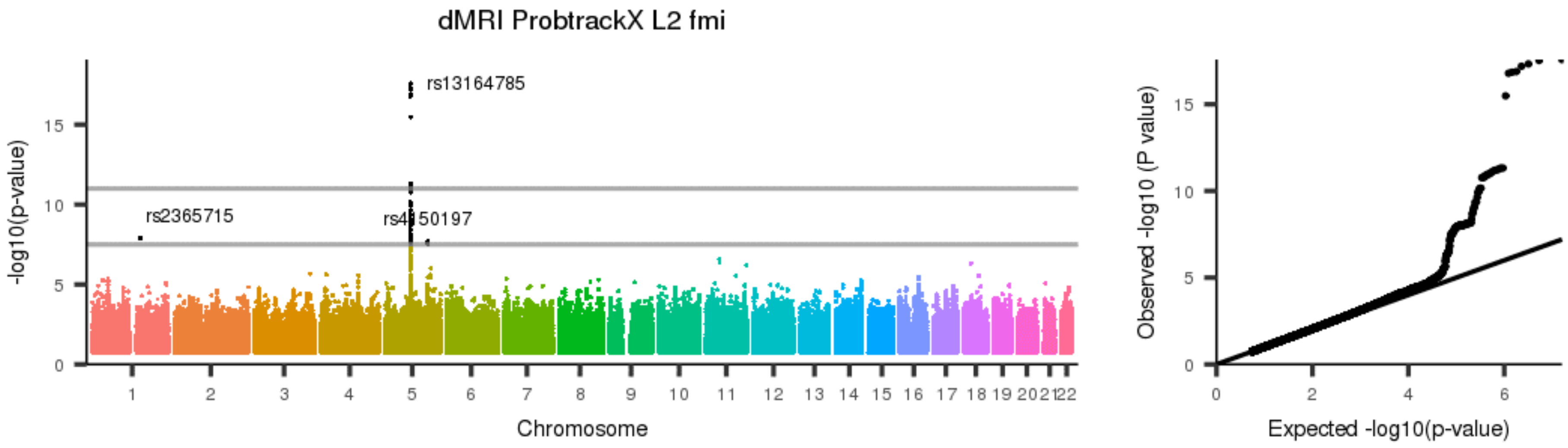

back to list of plots

Figure 748: dMRI ProbtrackX L2 ifo l

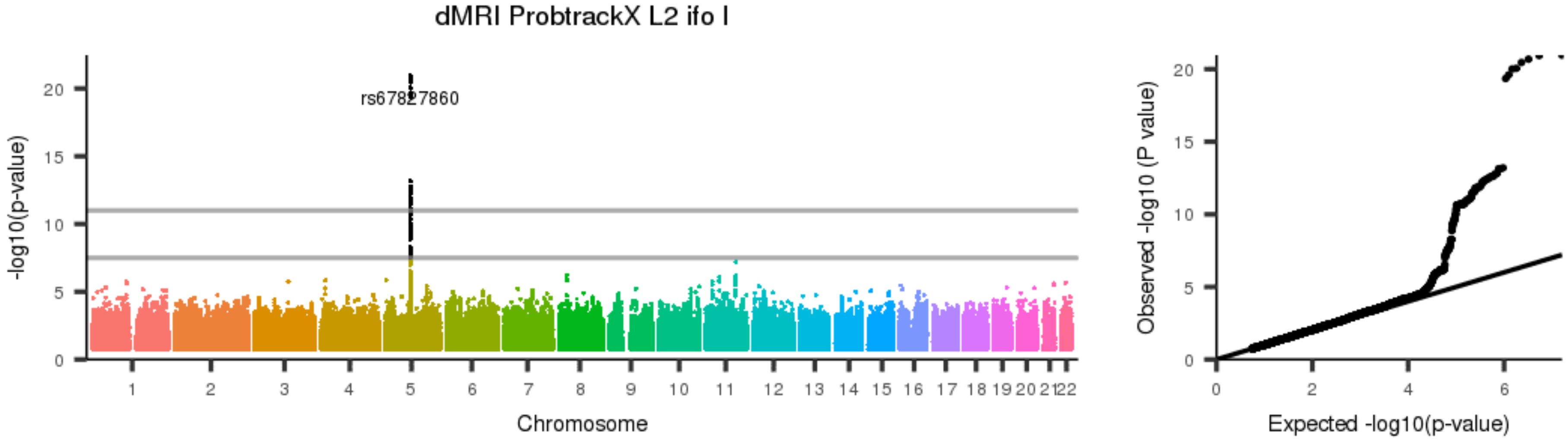

back to list of plots

Figure 749: dMRI ProbtrackX L2 ifo r

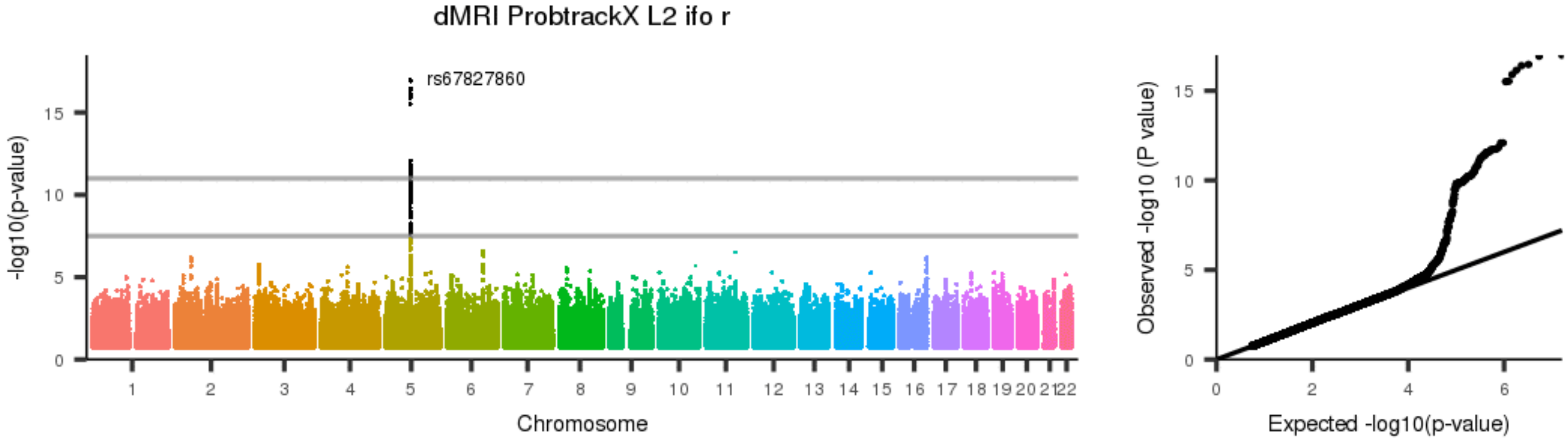

back to list of plots

Figure 750: dMRI ProbtrackX L2 ilf l

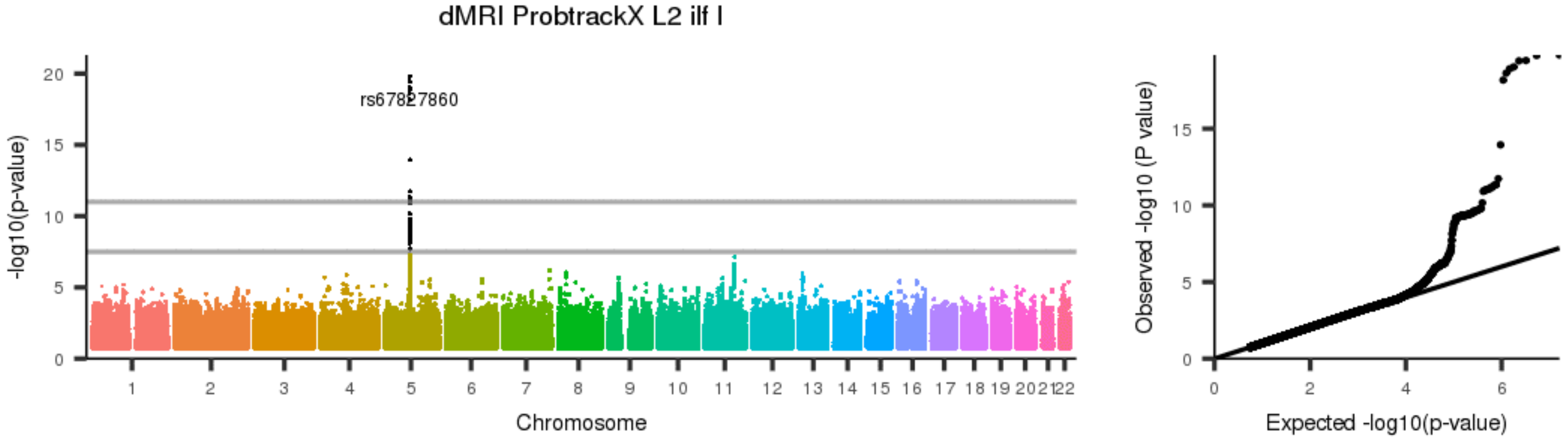

back to list of plots

Figure 751: dMRI ProbtrackX L2 ilf r

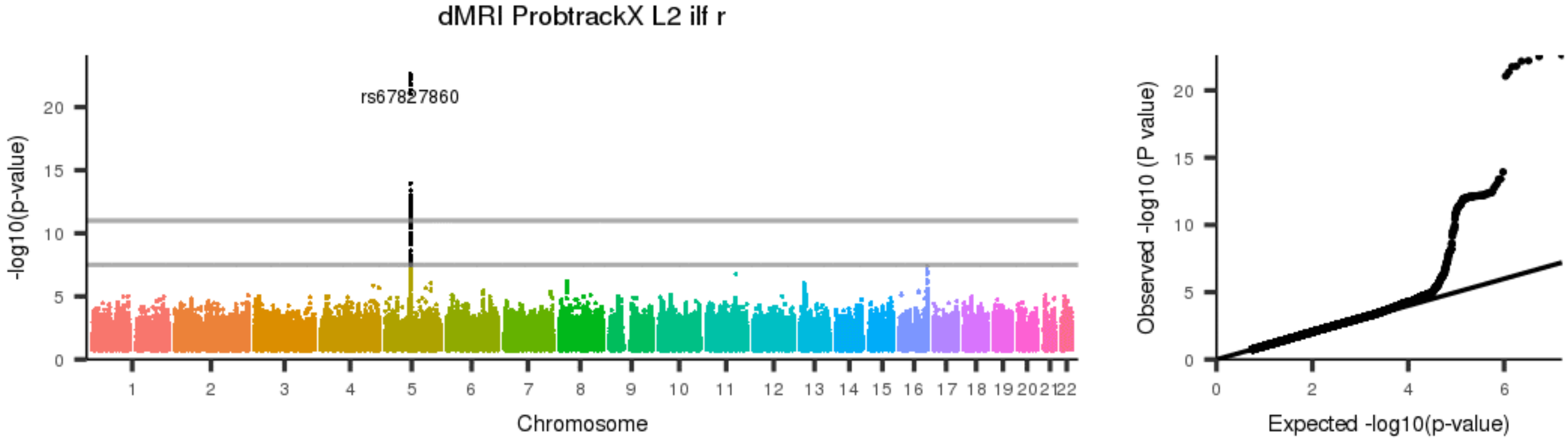

back to list of plots

Figure 752: dMRI ProbtrackX L2 mcp

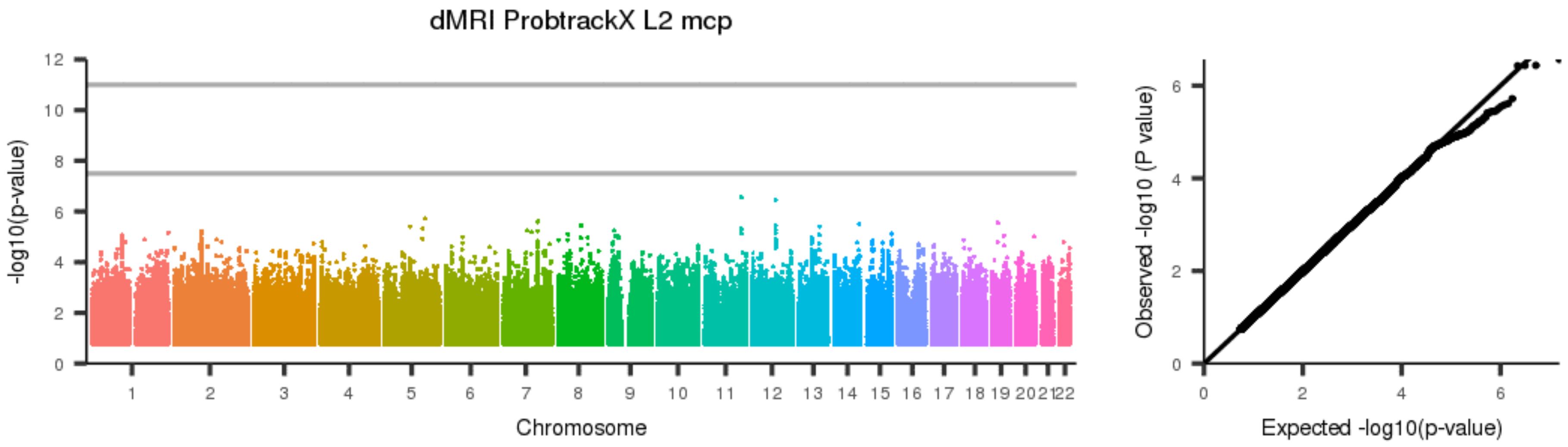

back to list of plots

Figure 753: dMRI ProbtrackX L2 ml l

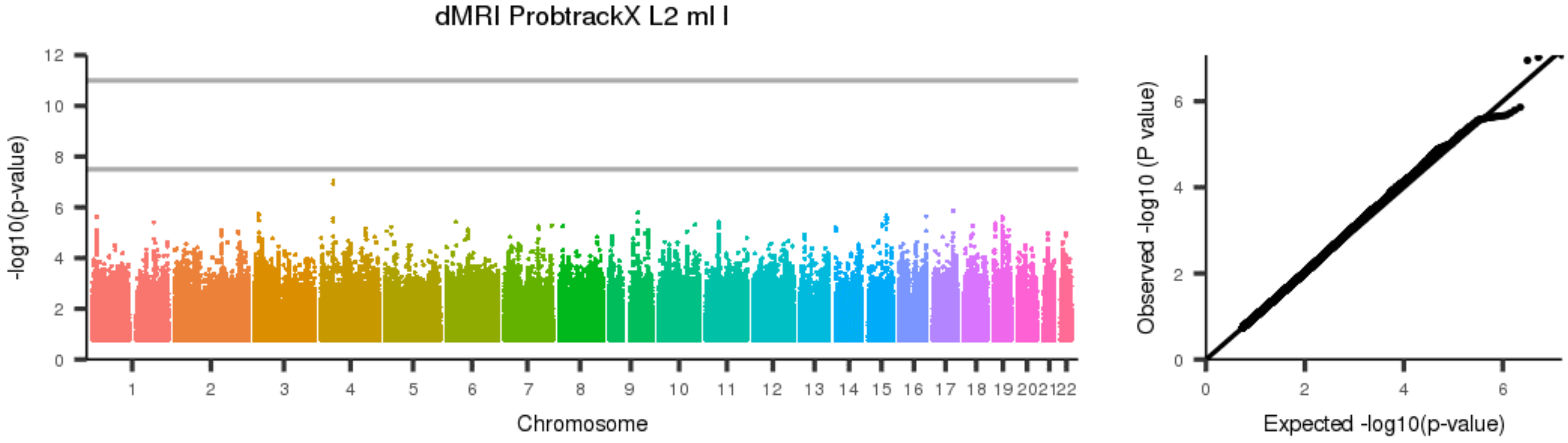

back to list of plots

Figure 754: dMRI ProbtrackX L2 ml r

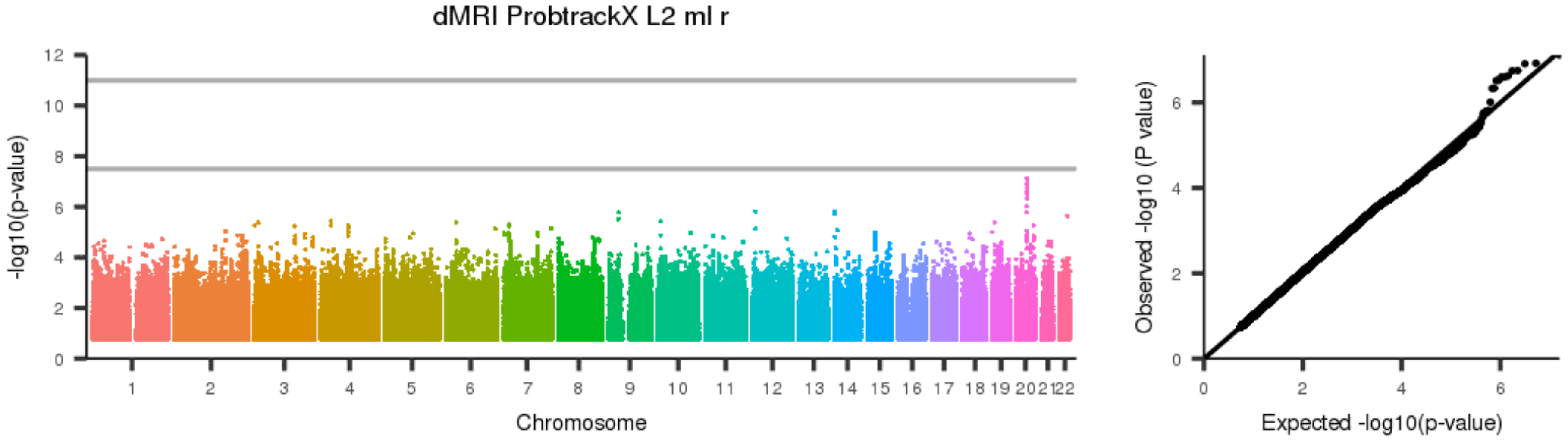

back to list of plots

Figure 755: dMRI ProbtrackX L2 ptr l

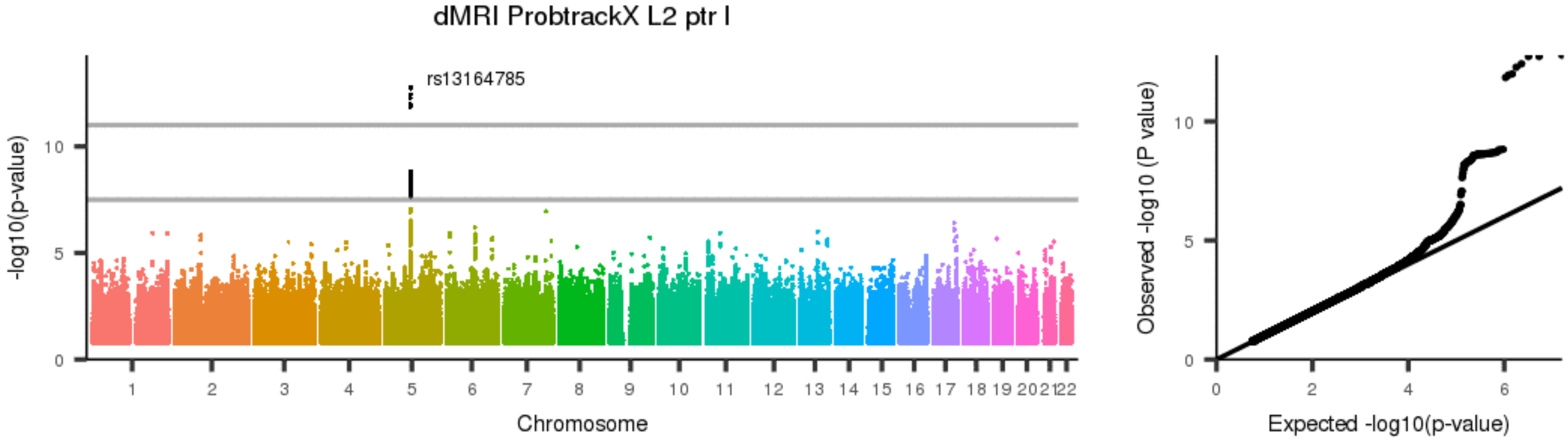

back to list of plots

Figure 756: dMRI ProbtrackX L2 ptr r

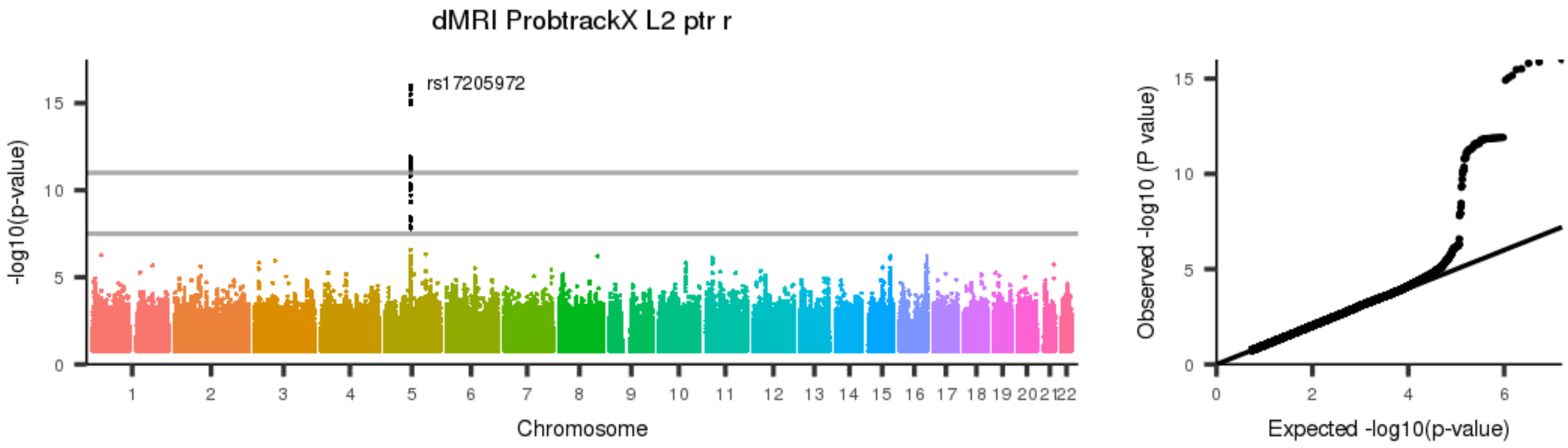

back to list of plots

Figure 757: dMRI ProbtrackX L2 slf I

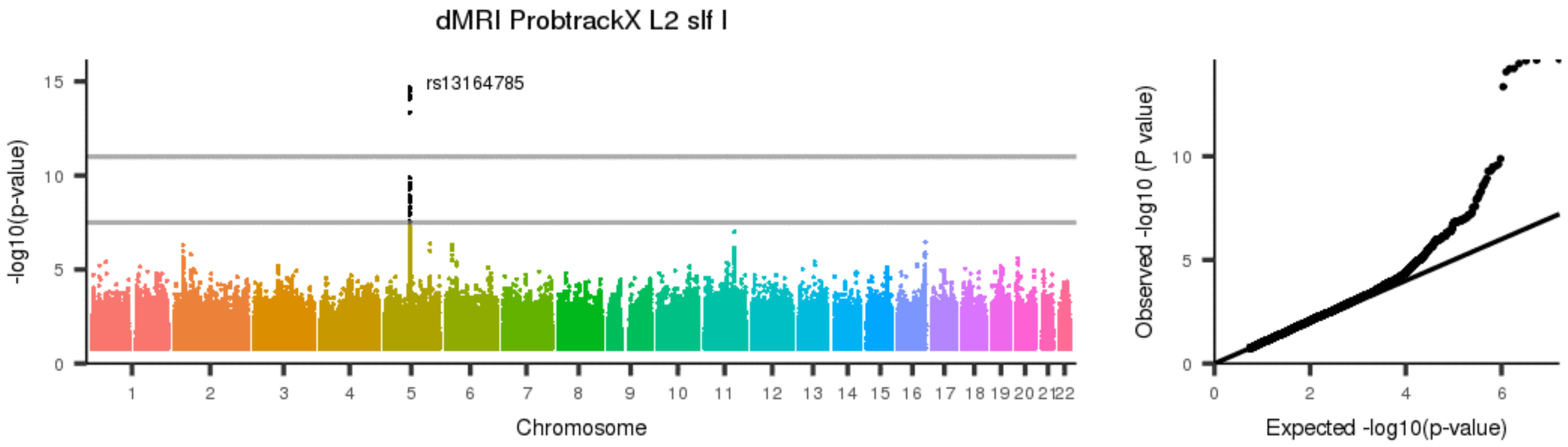

back to list of plots

Figure 758: dMRI ProbtrackX L2 slf r

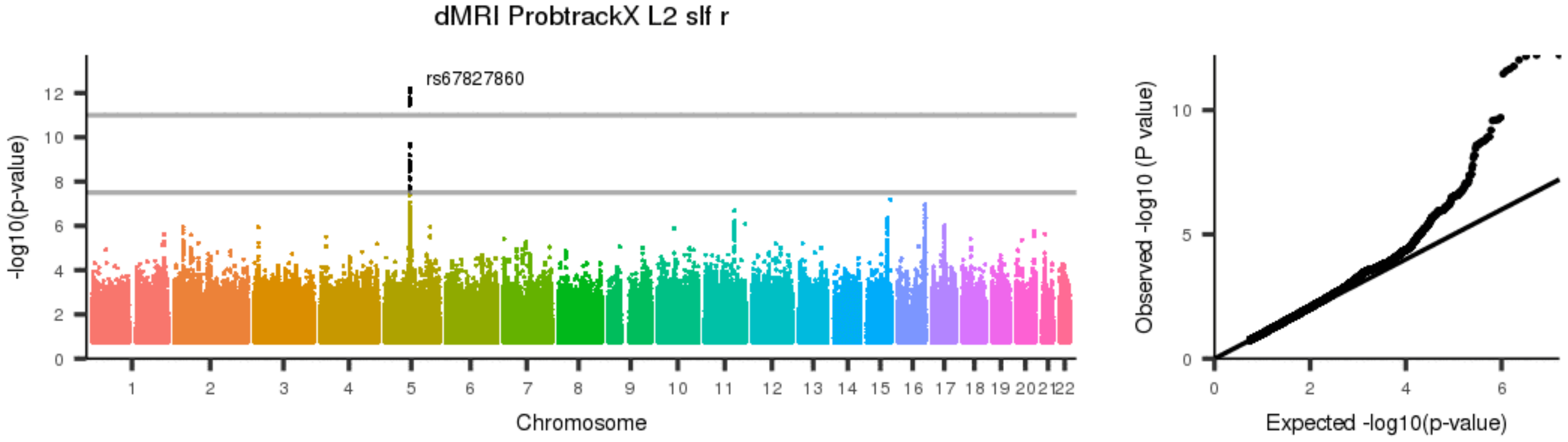

back to list of plots

Figure 759: dMRI ProbtrackX L2 str l

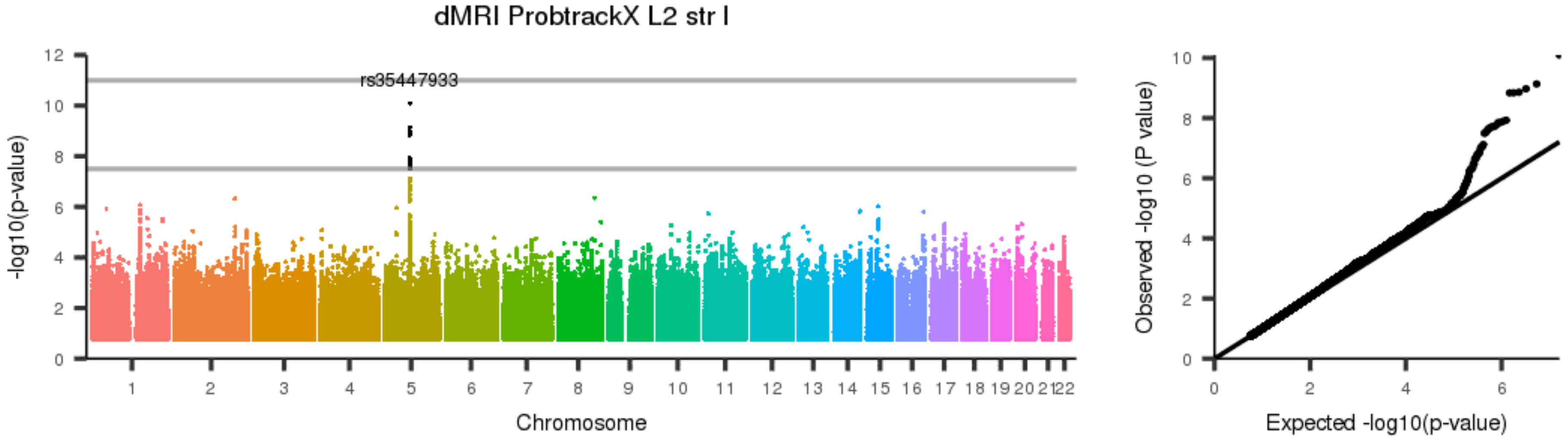

back to list of plots

Figure 760: dMRI ProbtrackX L2 str r

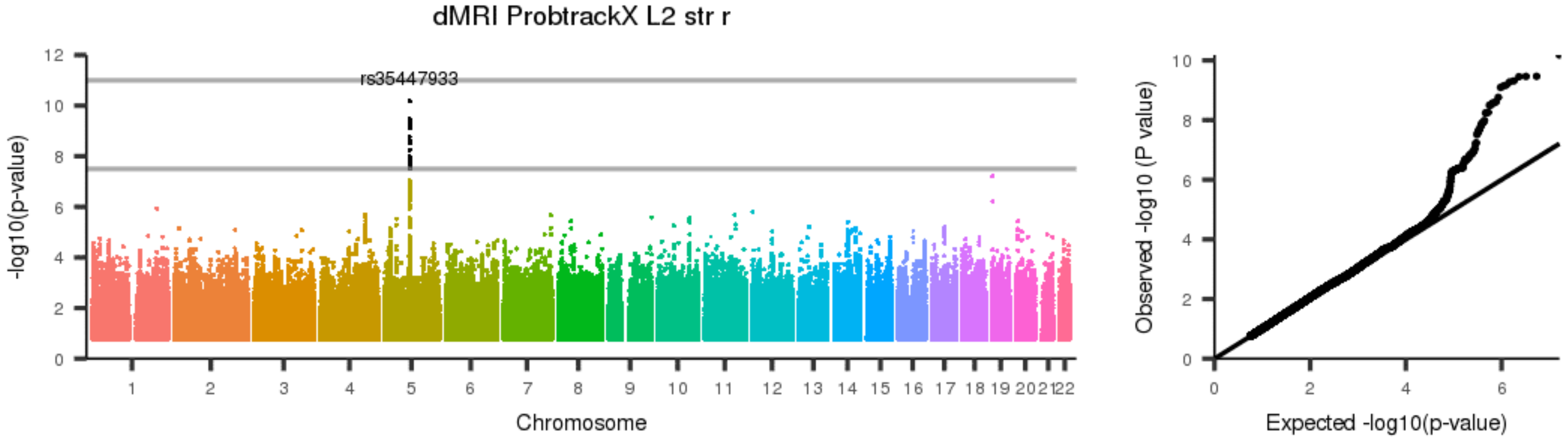

back to list of plots

Figure 761: dMRI ProbtrackX L2 unc l

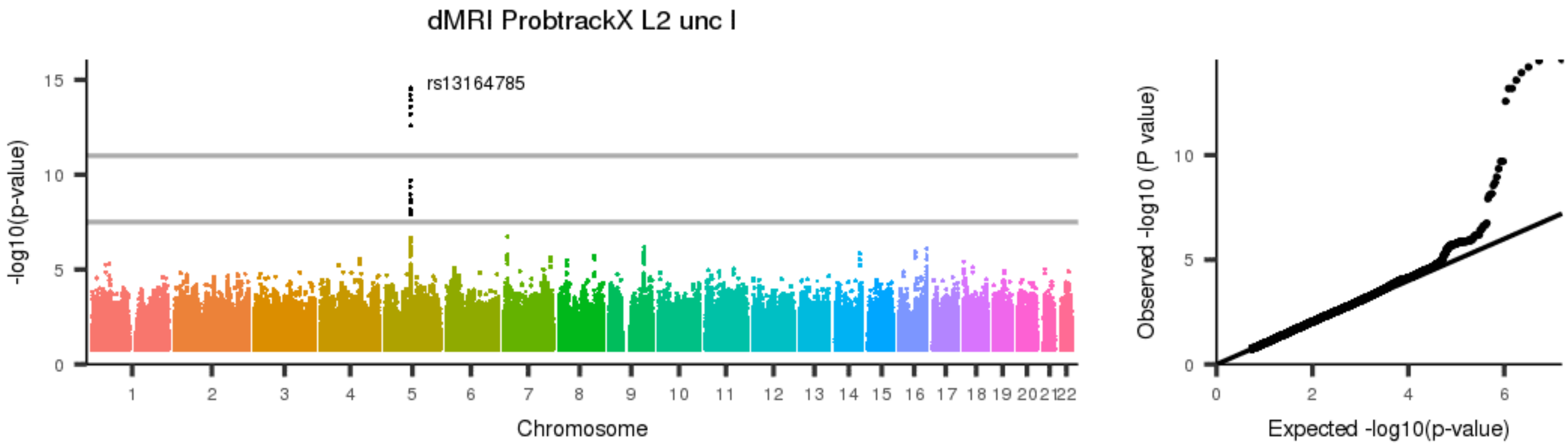

back to list of plots

Figure 762: dMRI ProbtrackX L2 unc r

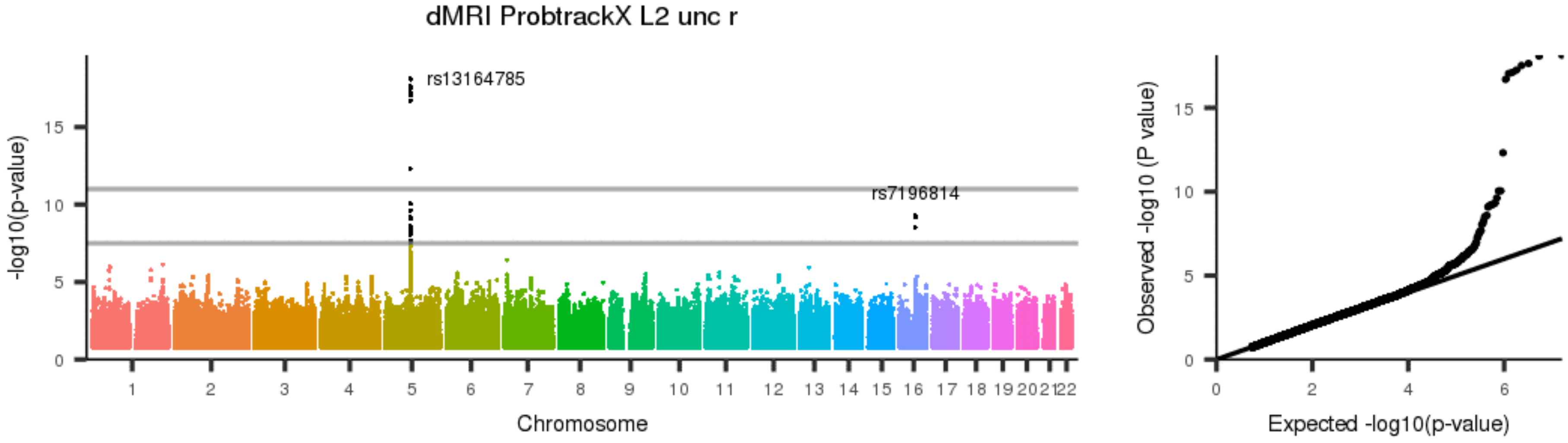

back to list of plots

Figure 763: dMRI ProbtrackX L3 ar l

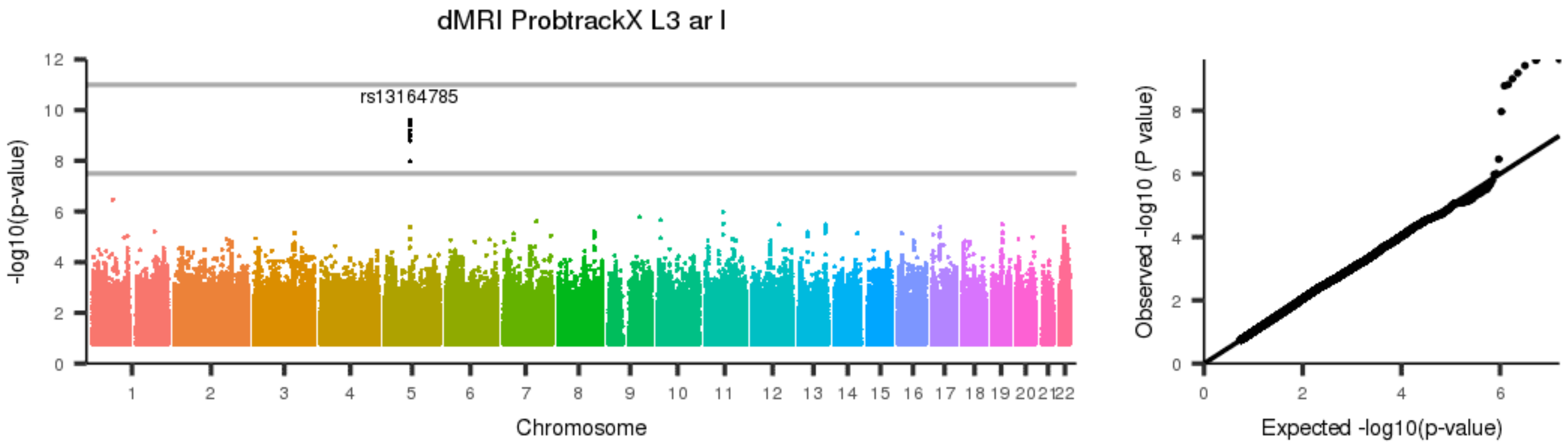

back to list of plots

Figure 764: dMRI ProbtrackX L3 ar r

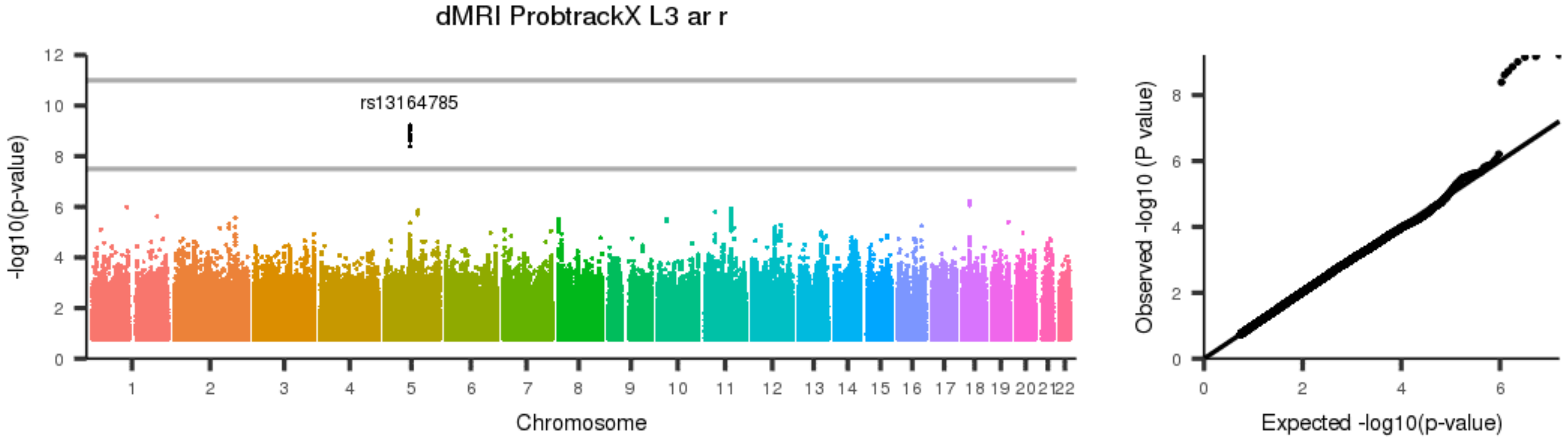

back to list of plots

Figure 765: dMRI ProbtrackX L3 atr l

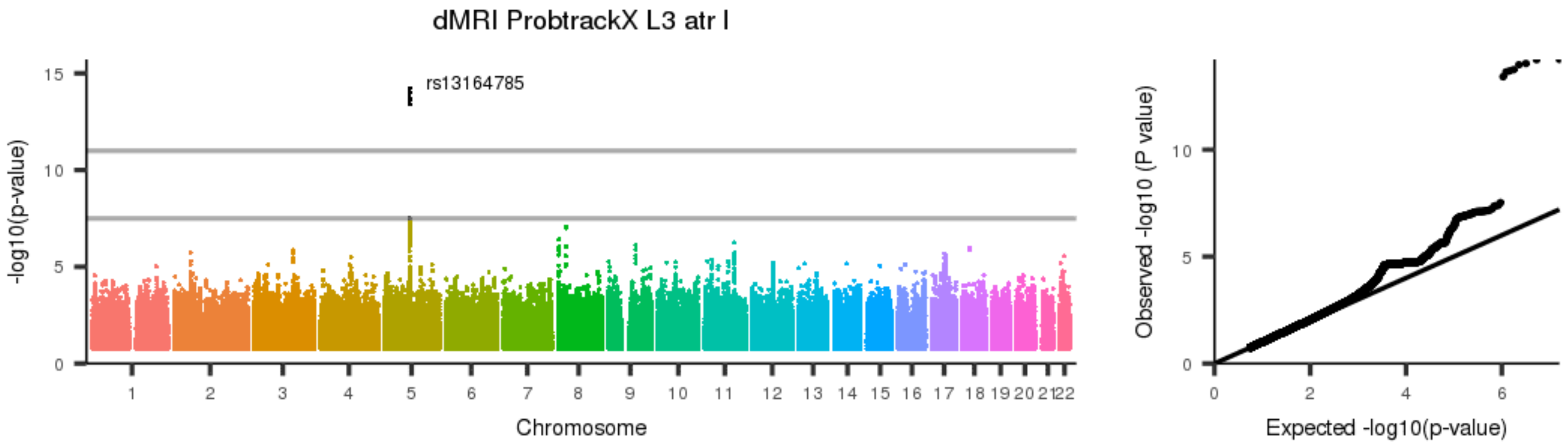

back to list of plots

Figure 766: dMRI ProbtrackX L3 atr r

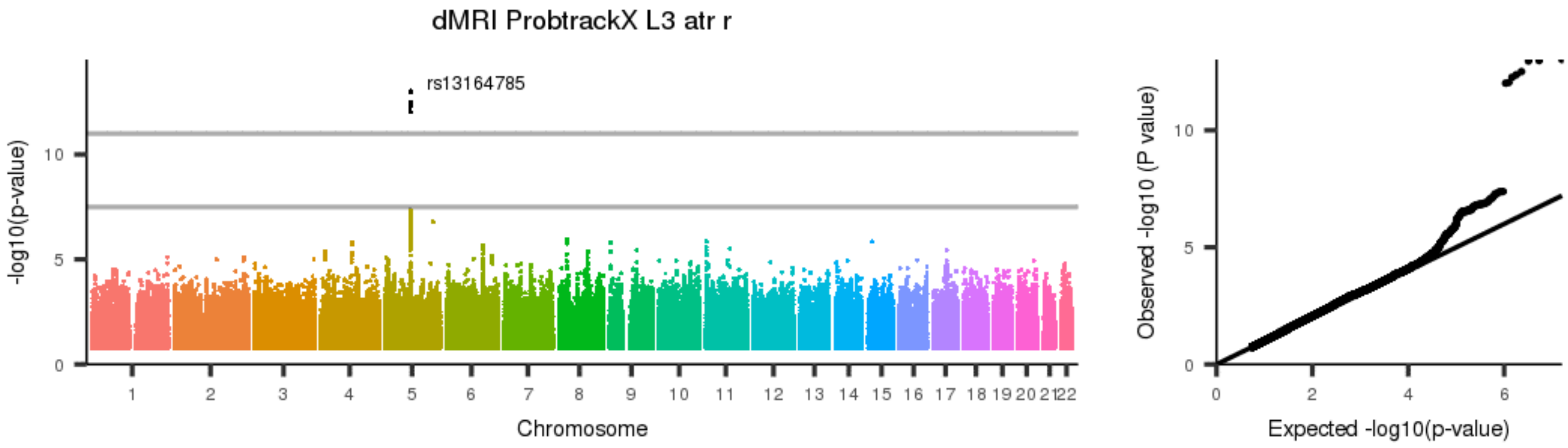

back to list of plots

Figure 767: dMRI ProbtrackX L3 cgc l

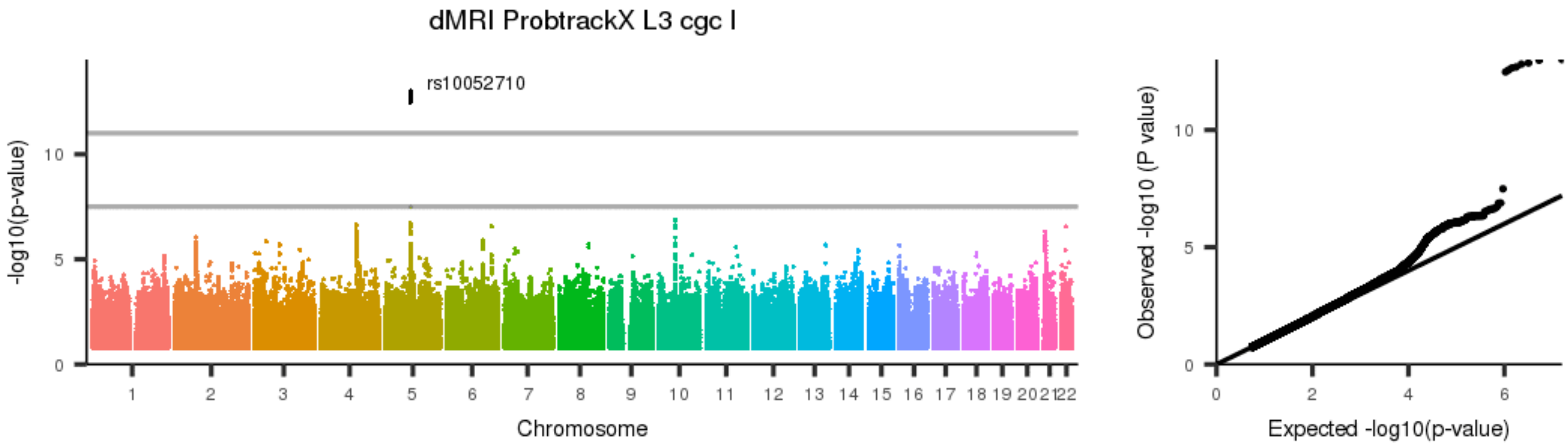

back to list of plots

Figure 768: dMRI ProbtrackX L3 cgc r

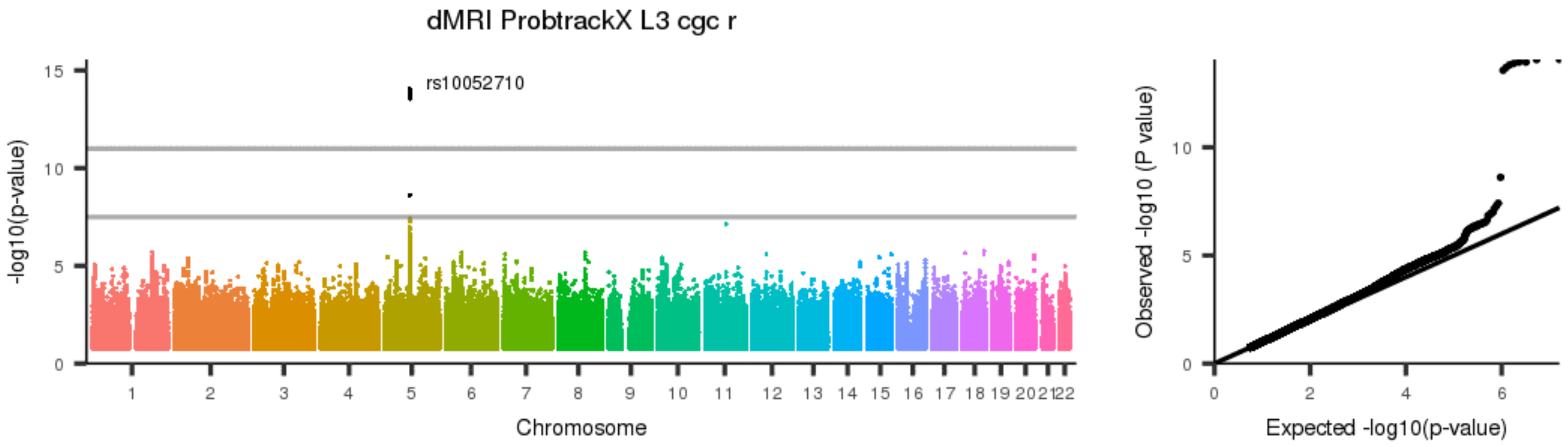

back to list of plots

Figure 769: dMRI ProbtrackX L3 cgh l

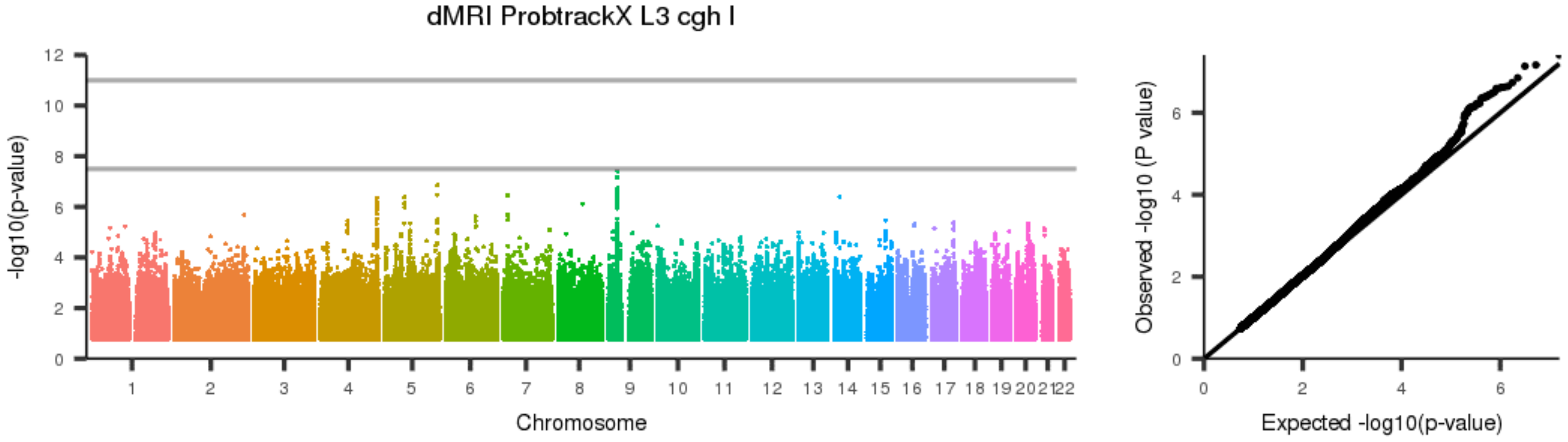

back to list of plots

Figure 770: dMRI ProbtrackX L3 cgh r

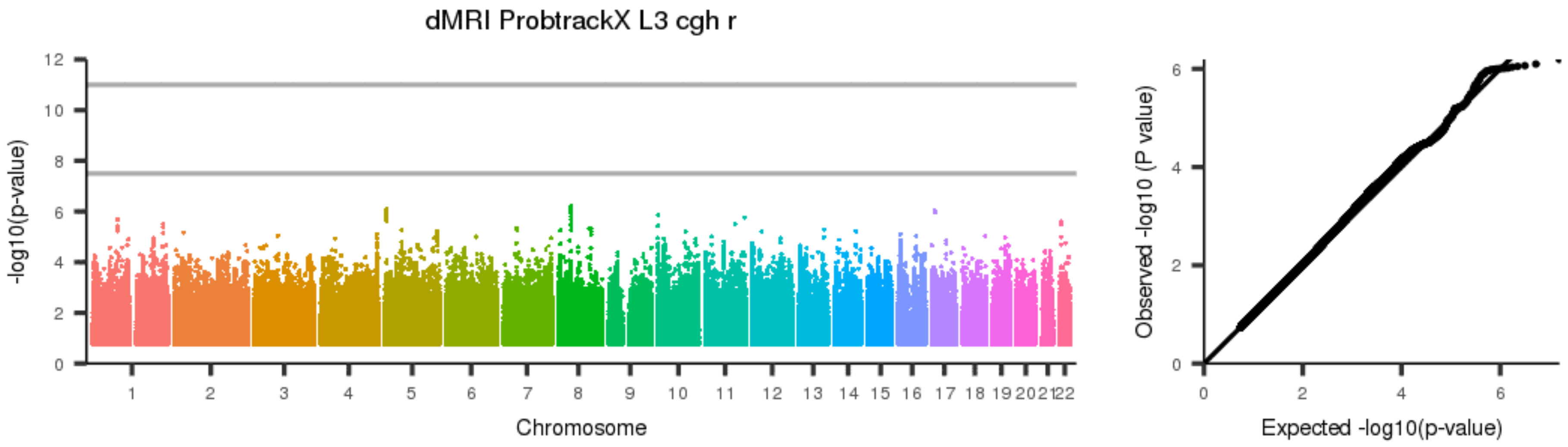

back to list of plots

Figure 771: dMRI ProbtrackX L3 cst l

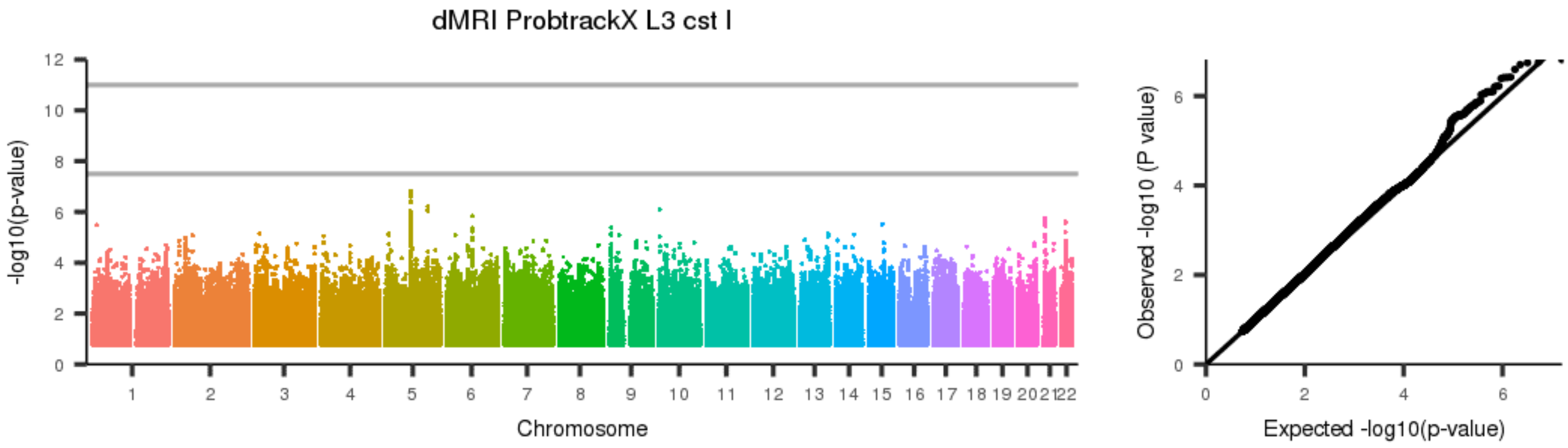

back to list of plots

Figure 772: dMRI ProbtrackX L3 cst r

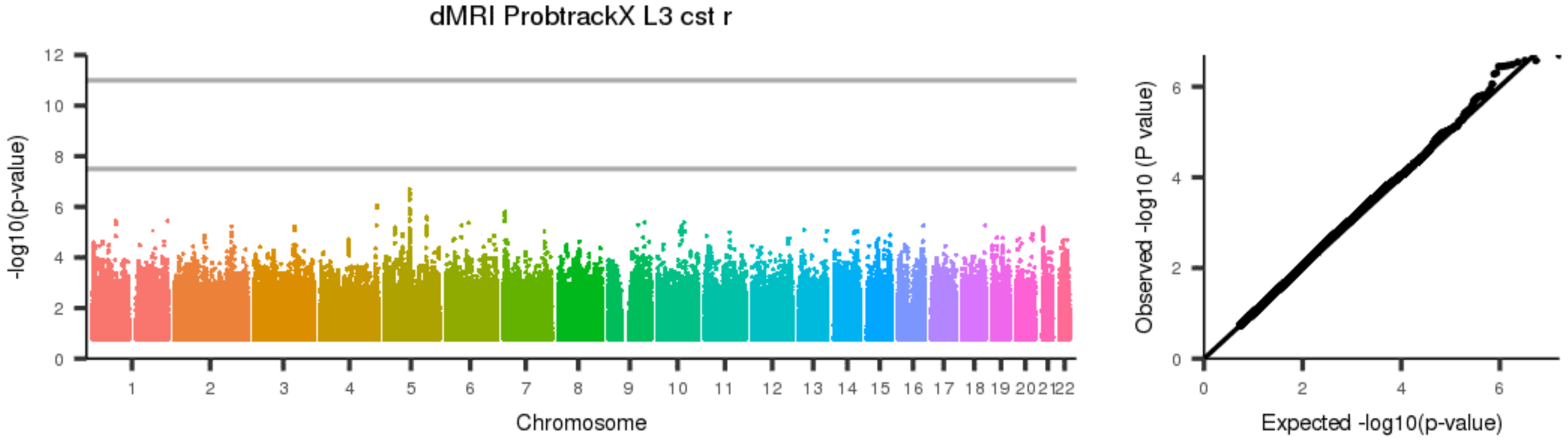

back to list of plots

Figure 773: dMRI ProbtrackX L3 fma

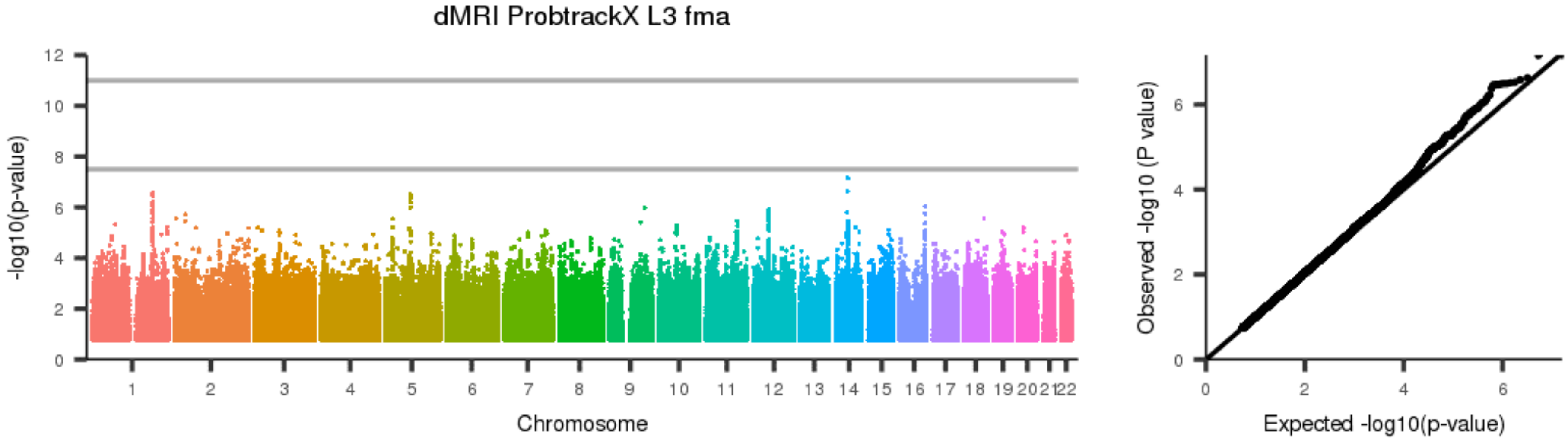

back to list of plots

Figure 774: dMRI ProbtrackX L3 fmi

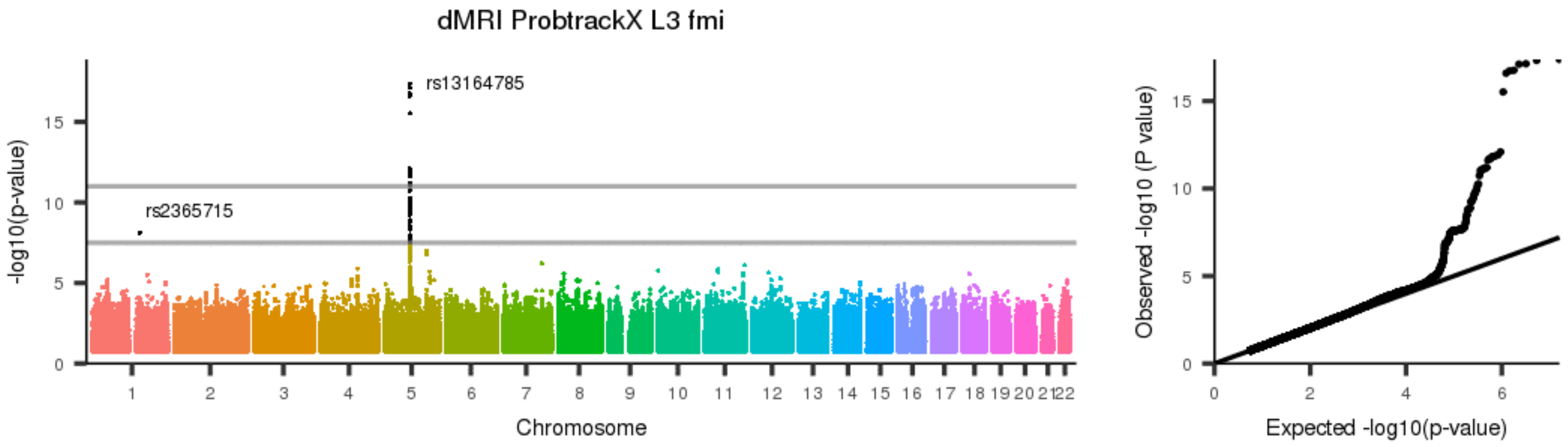

back to list of plots

Figure 775: dMRI ProbtrackX L3 ifo l

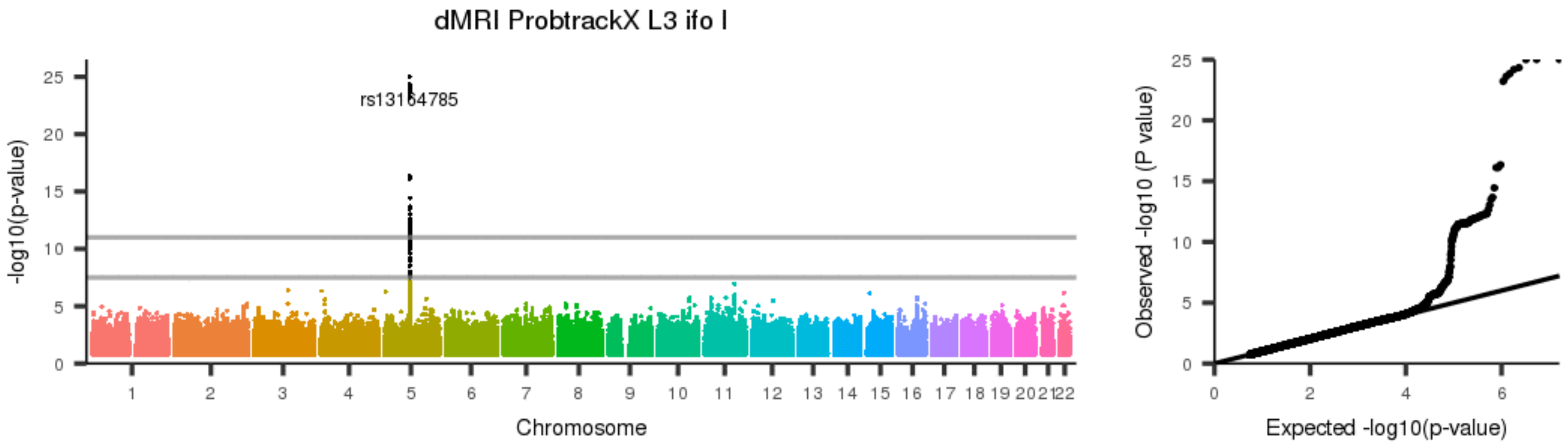

back to list of plots

Figure 776: dMRI ProbtrackX L3 ifo r

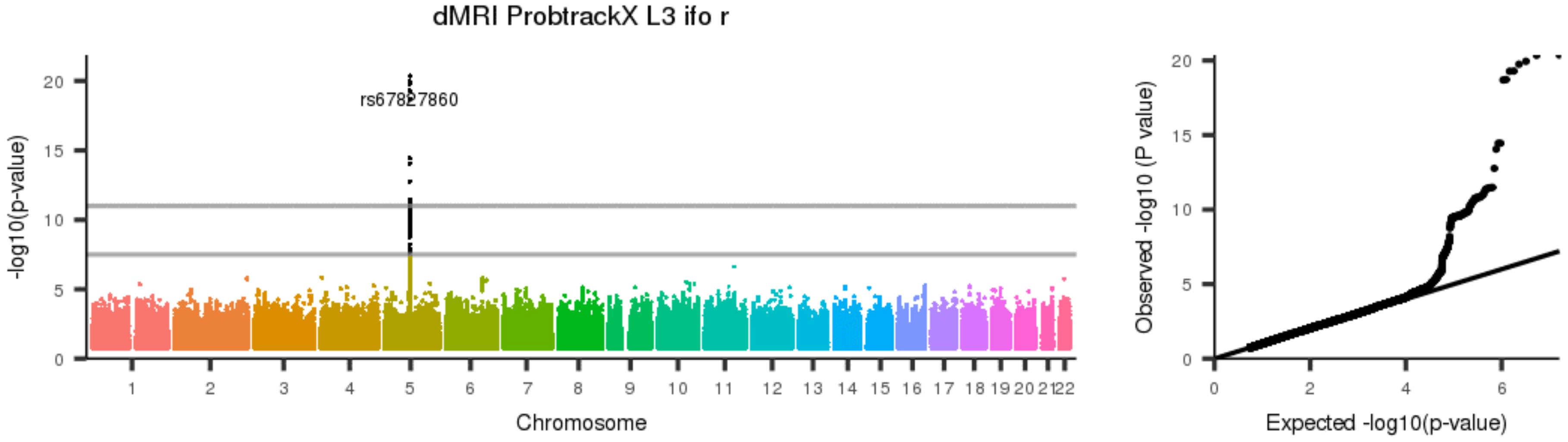

back to list of plots

Figure 777: dMRI ProbtrackX L3 ilf l

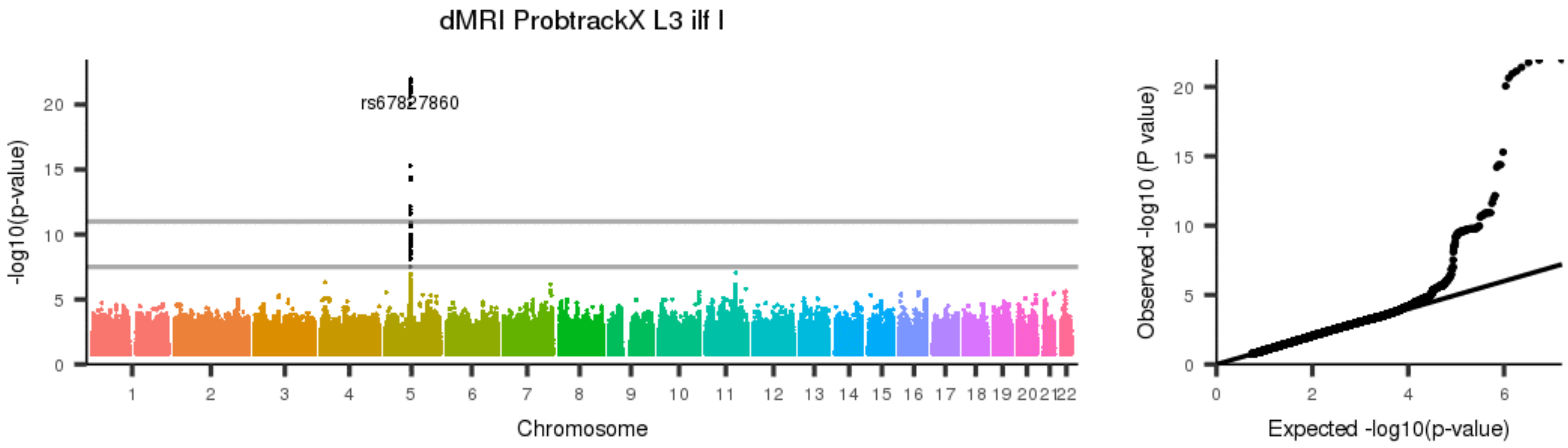

back to list of plots

Figure 778: dMRI ProbtrackX L3 ilf r

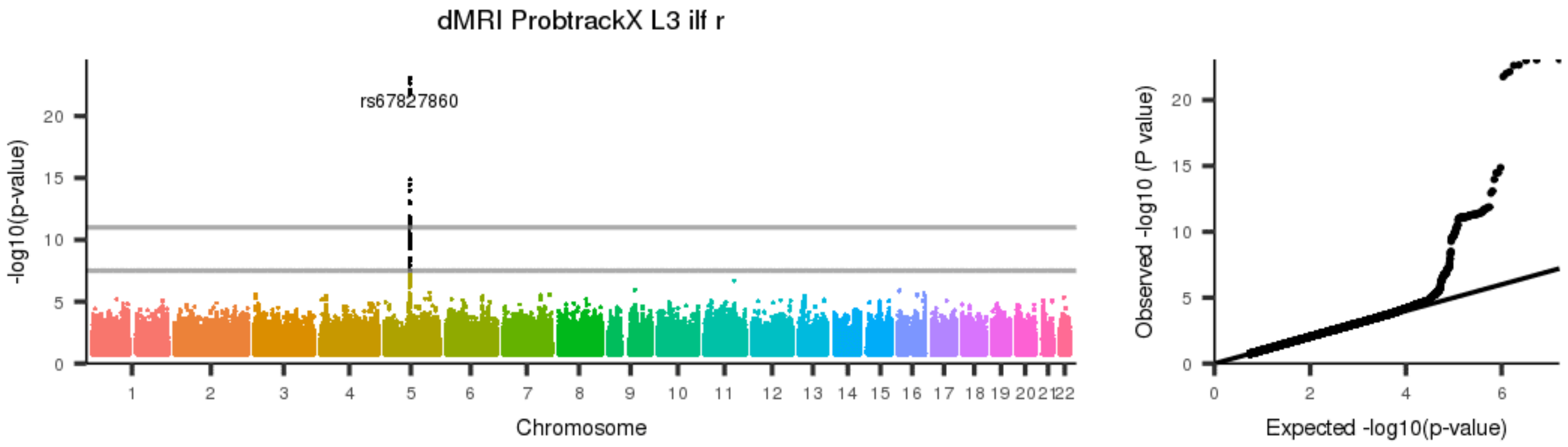

back to list of plots

Figure 779: dMRI ProbtrackX L3 mcp

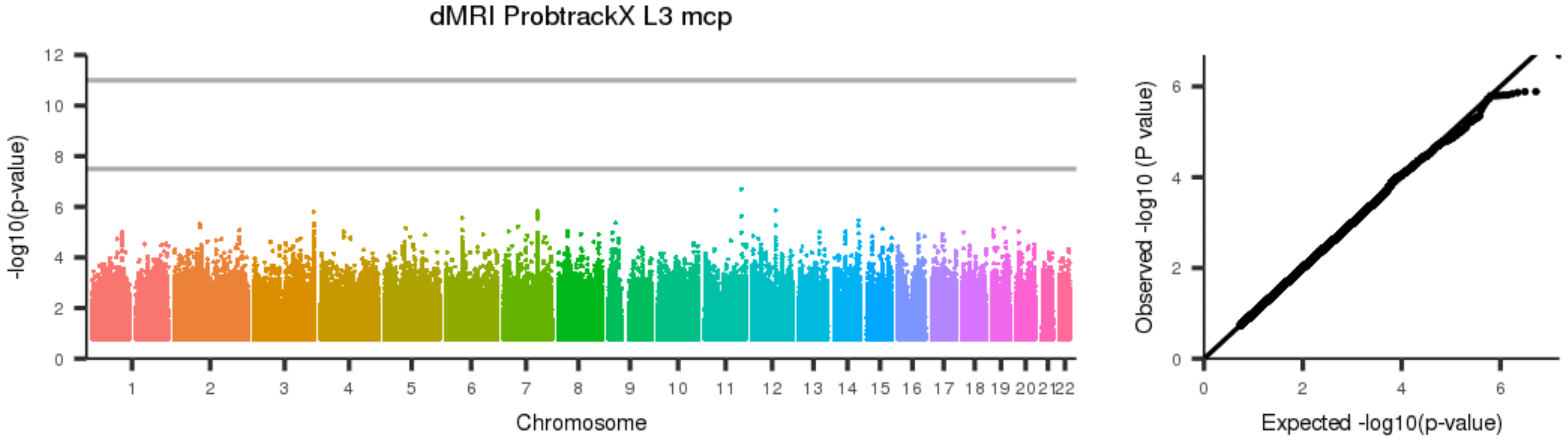

back to list of plots

Figure 780: dMRI ProbtrackX L3 ml l

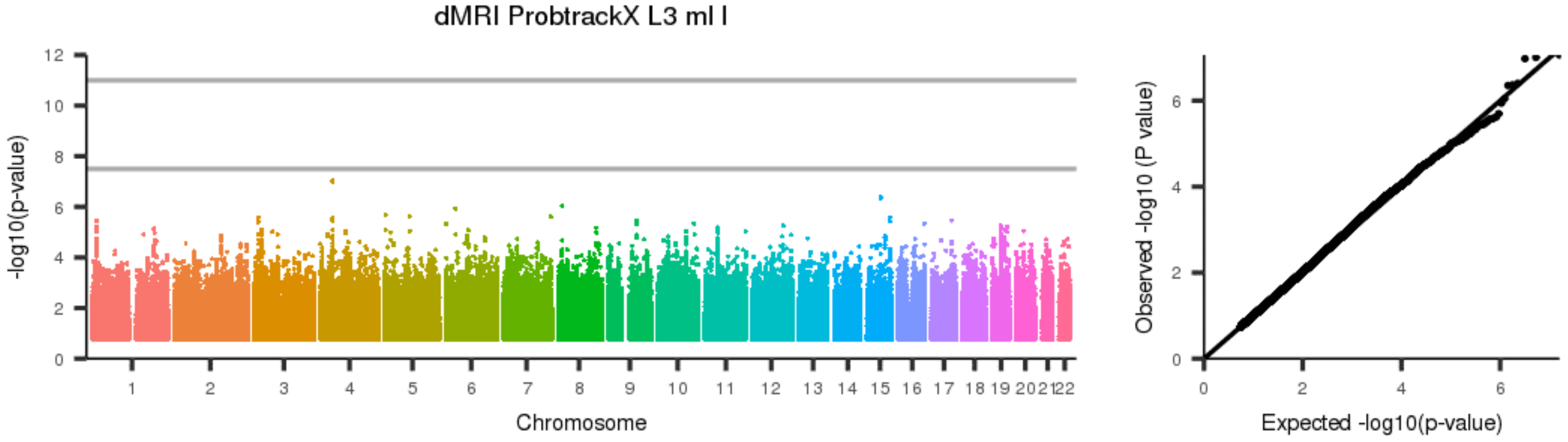

back to list of plots

Figure 781: dMRI ProbtrackX L3 ml r

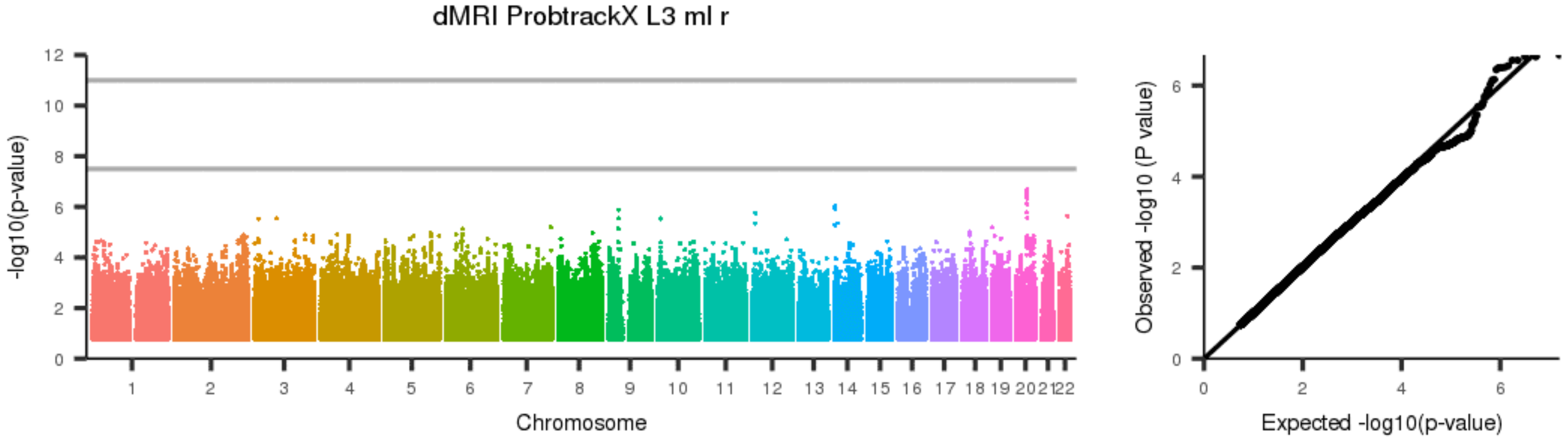

back to list of plots

Figure 782: dMRI ProbtrackX L3 ptr l

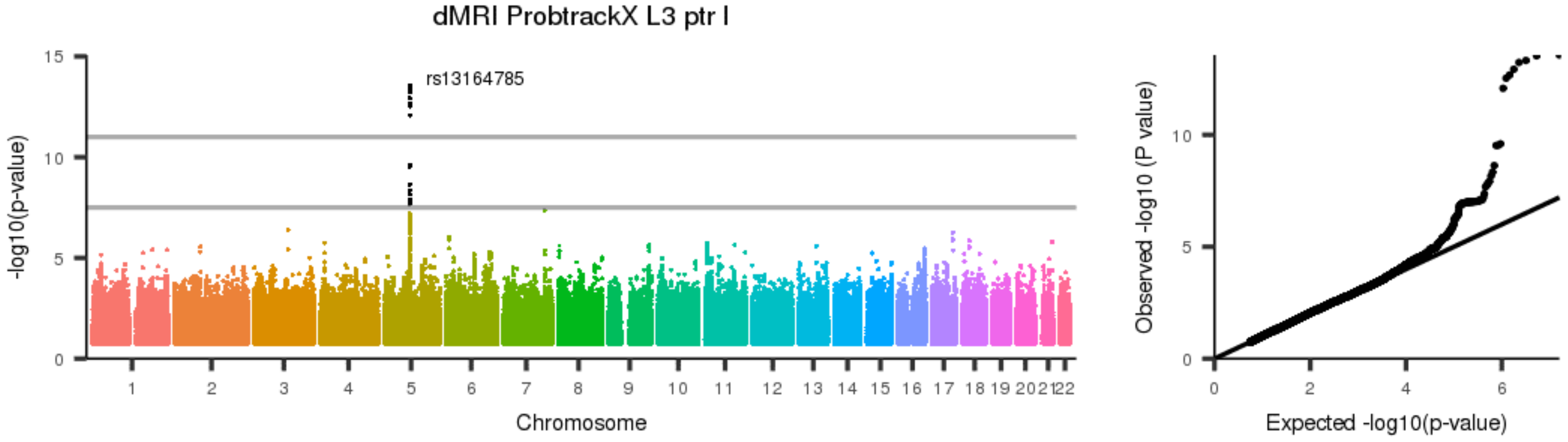

back to list of plots

Figure 783: dMRI ProbtrackX L3 ptr r

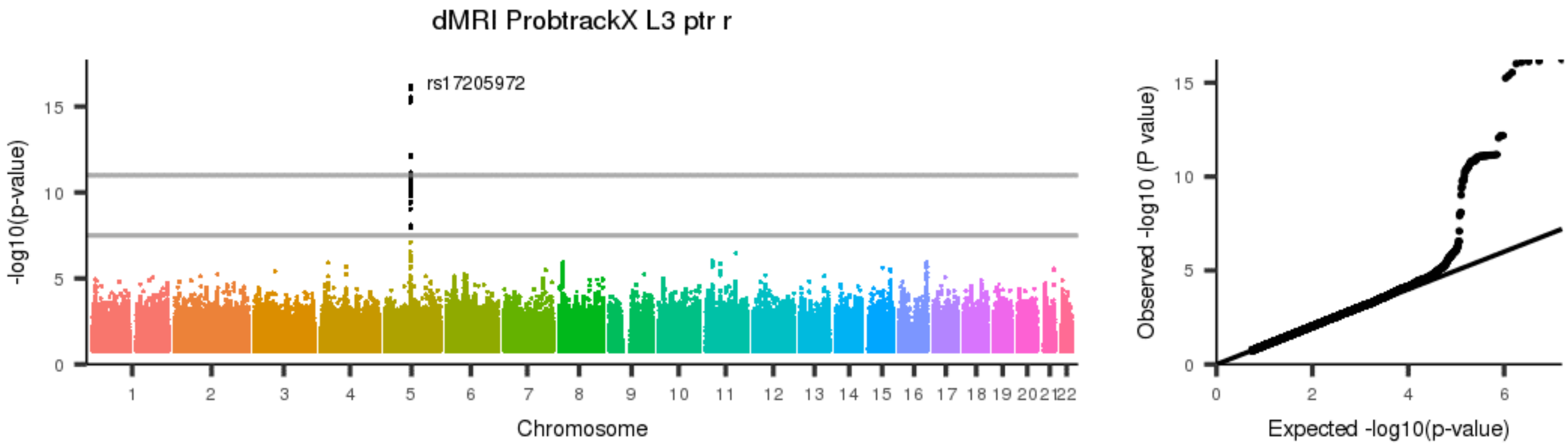

back to list of plots

Figure 784: dMRI ProbtrackX L3 slf l

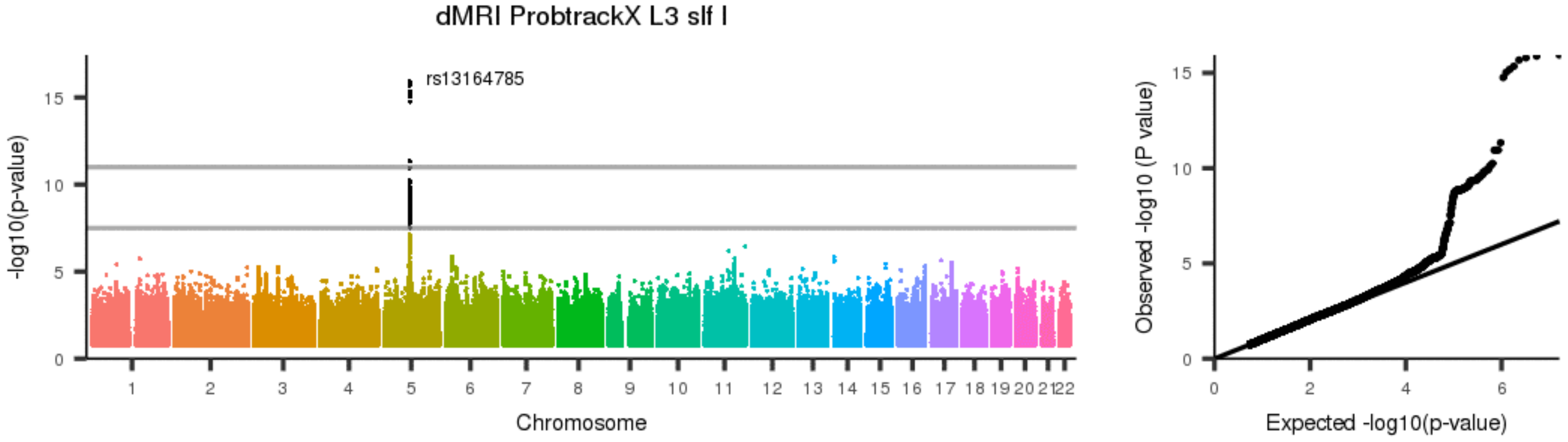

back to list of plots

Figure 785: dMRI ProbtrackX L3 slf r

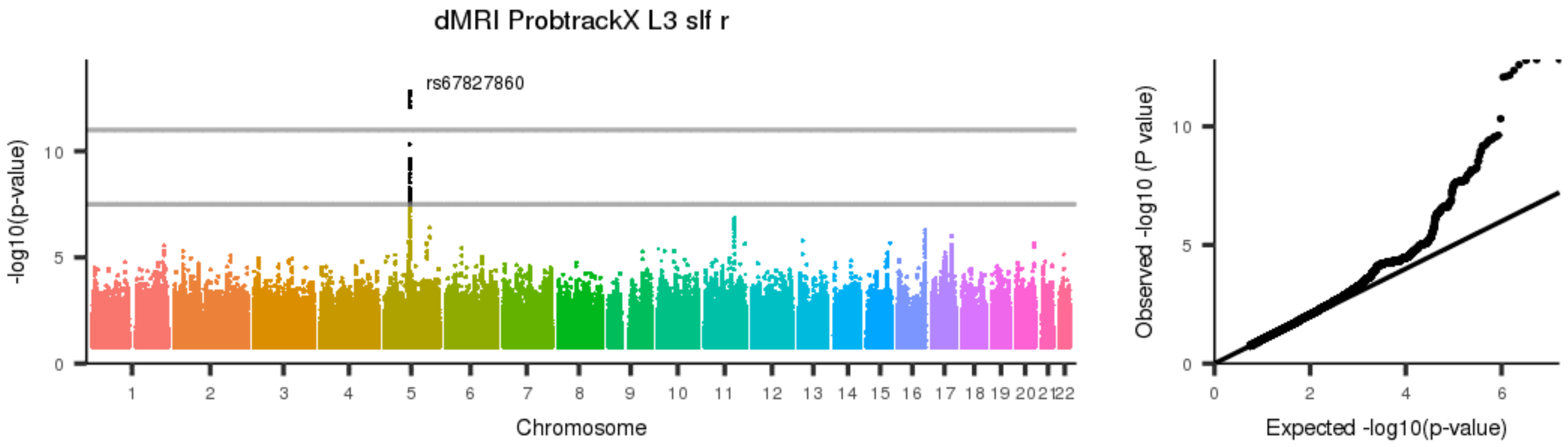

back to list of plots

Figure 786: dMRI ProbtrackX L3 str l

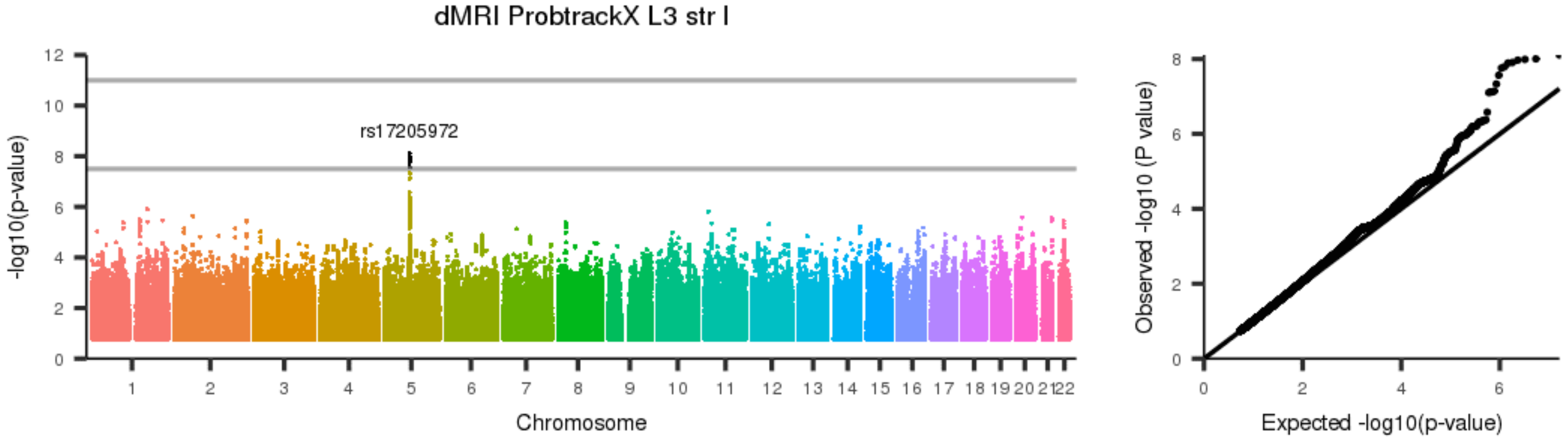

back to list of plots

Figure 787: dMRI ProbtrackX L3 str r

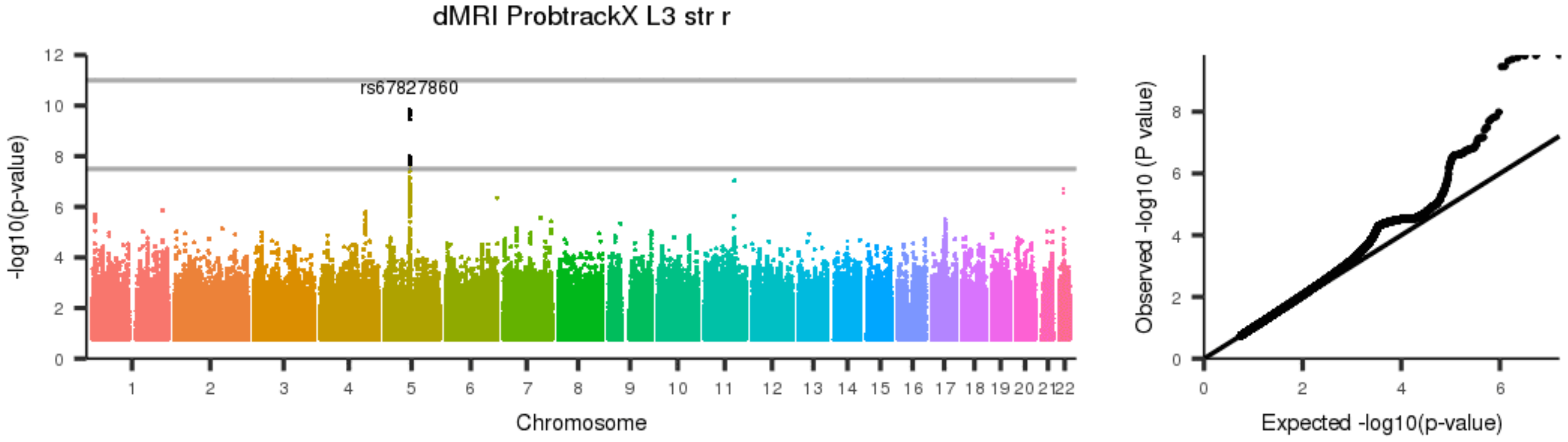

back to list of plots

Figure 788: dMRI ProbtrackX L3 unc l

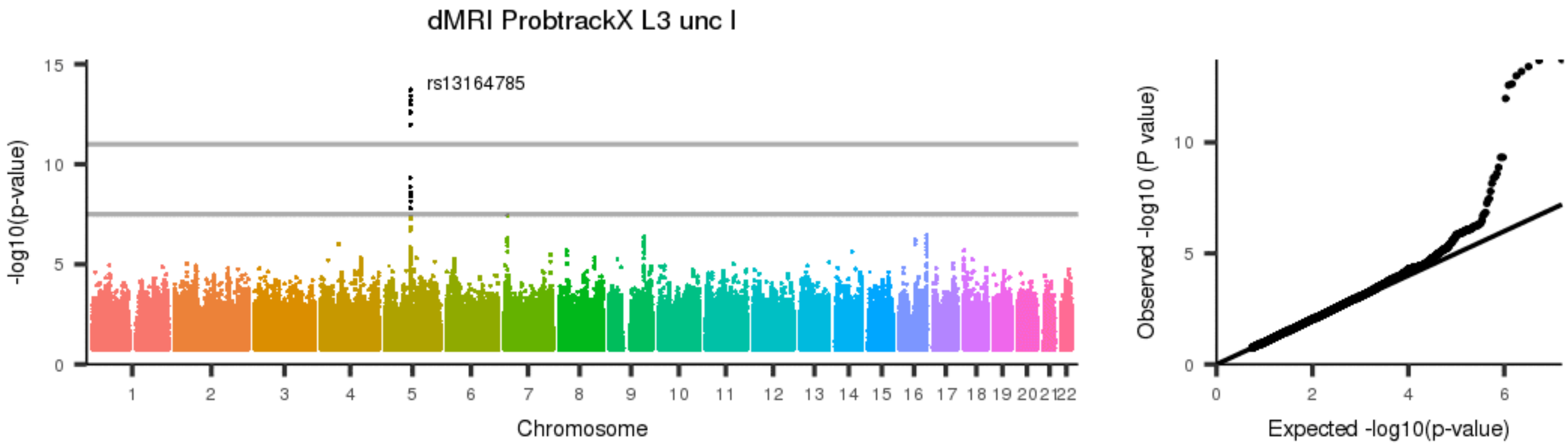

back to list of plots

Figure 789: dMRI ProbtrackX L3 unc r

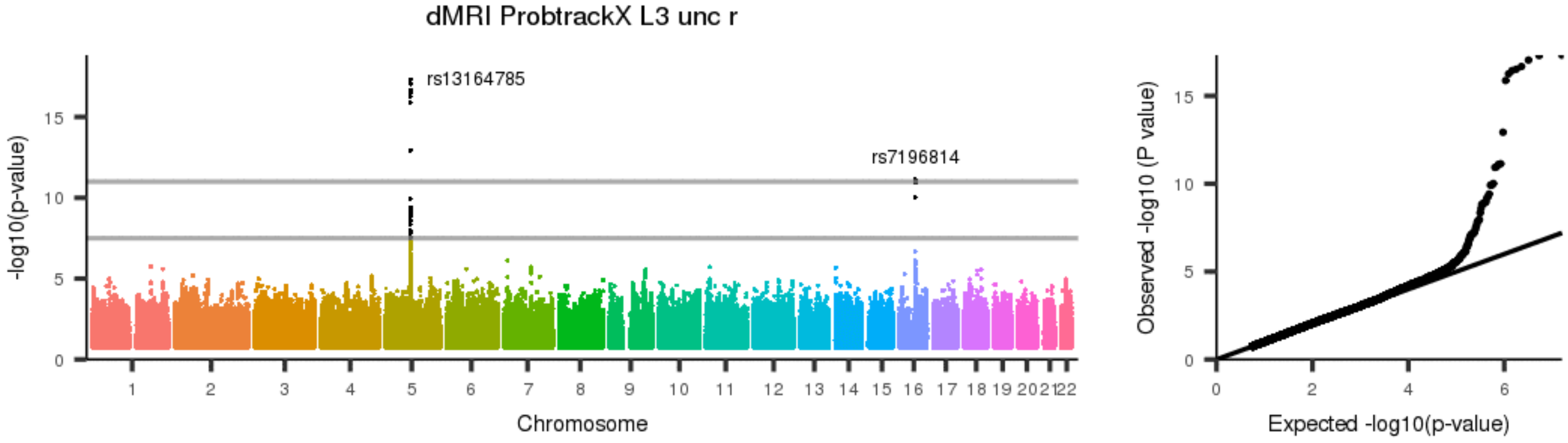

back to list of plots

Figure 790: dMRI ProbtrackX ICVF ar l

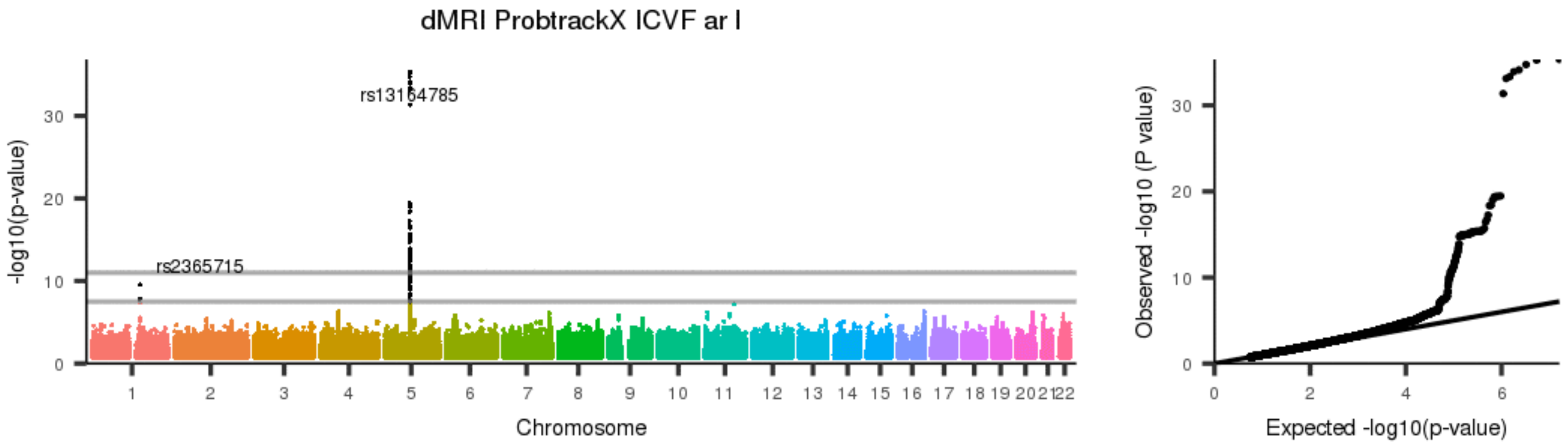

back to list of plots

Figure 791: dMRI ProbtrackX ICVF ar r

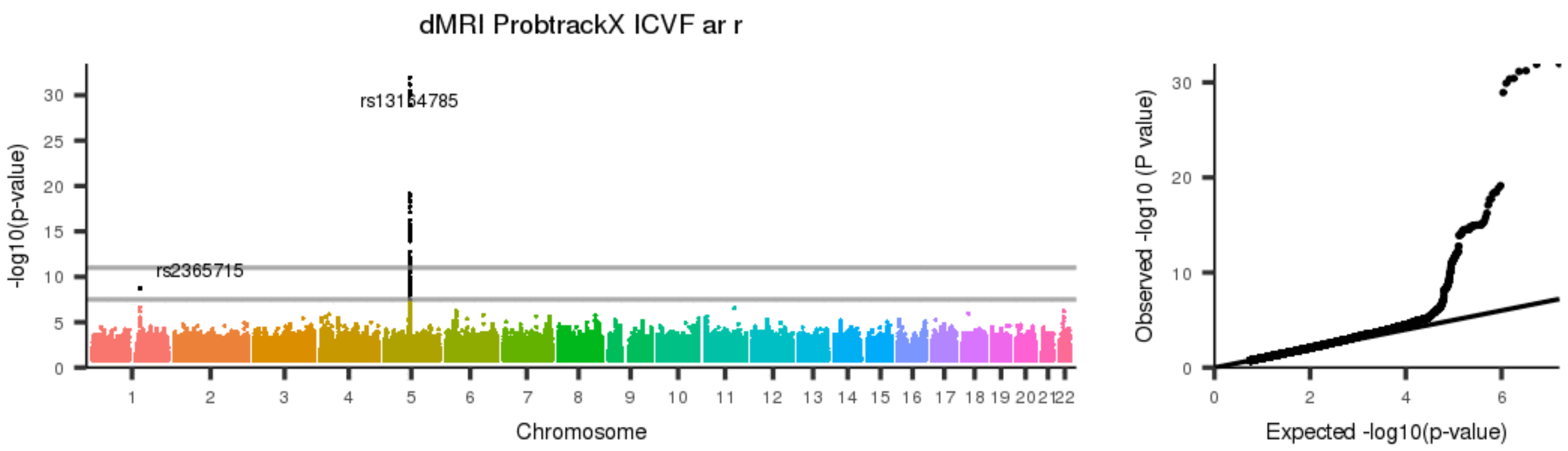

back to list of plots

Figure 792: dMRI ProbtrackX ICVF atr l

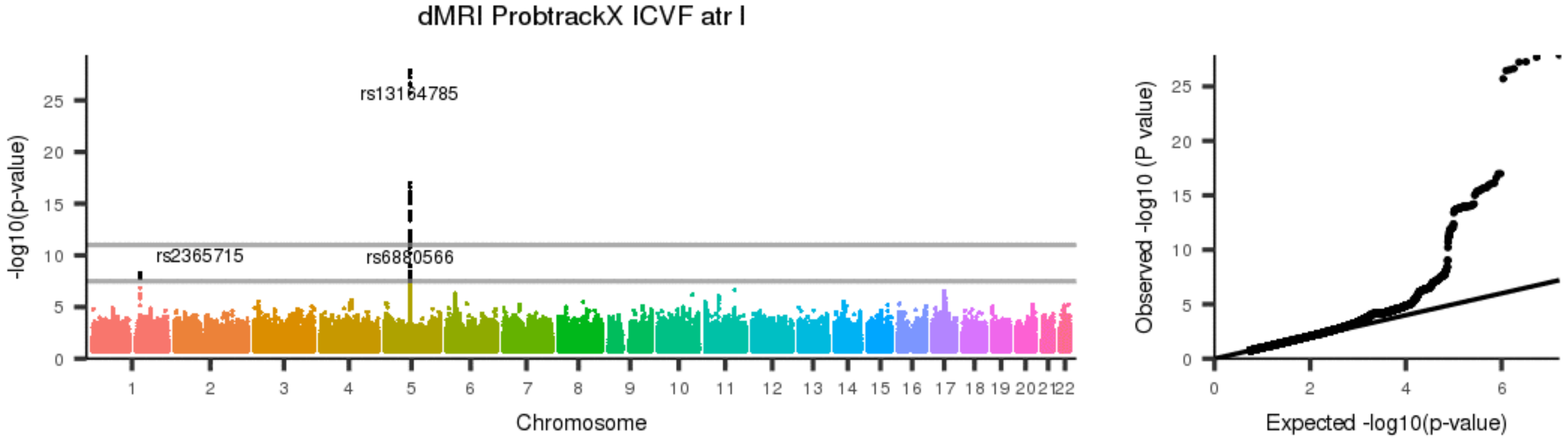

back to list of plots

Figure 793: dMRI ProbtrackX ICVF atr r

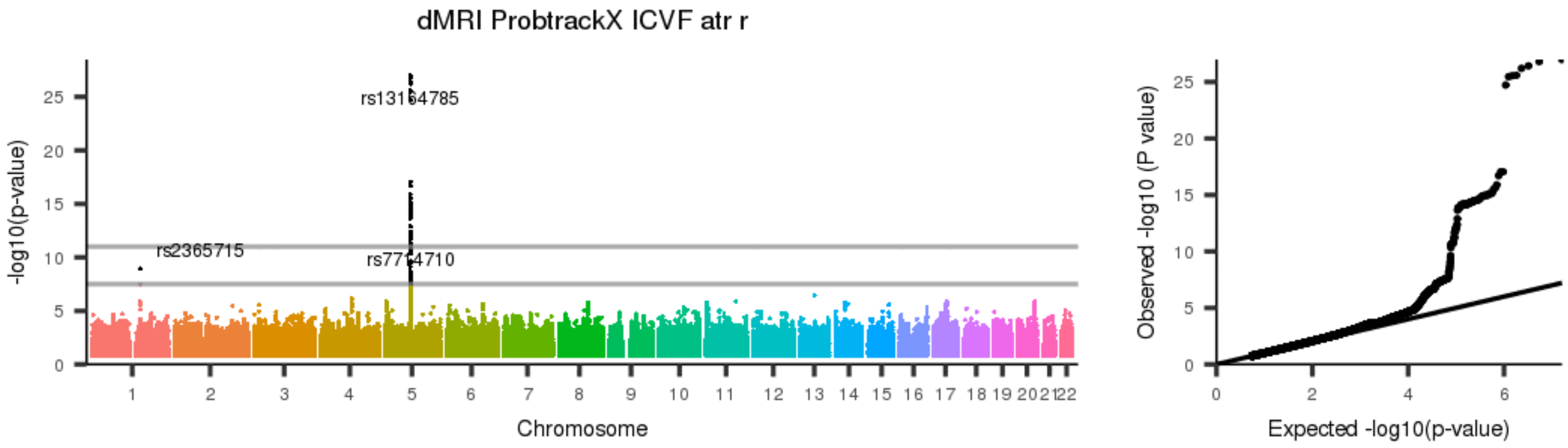

back to list of plots

Figure 794: dMRI ProbtrackX ICVF cgc l

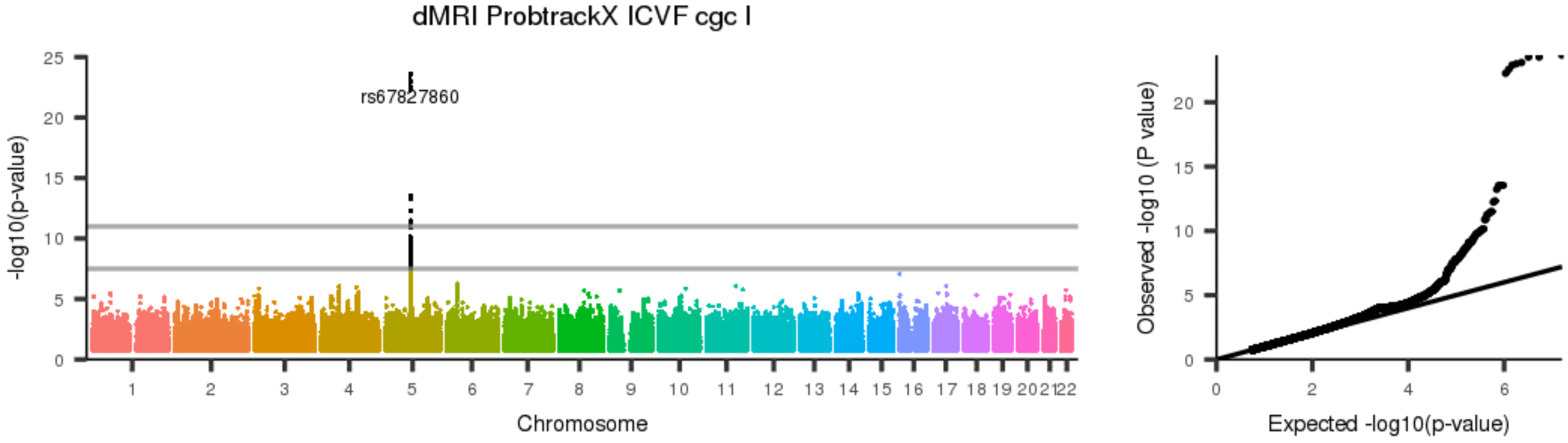

back to list of plots

Figure 795: dMRI ProbtrackX ICVF cgc r

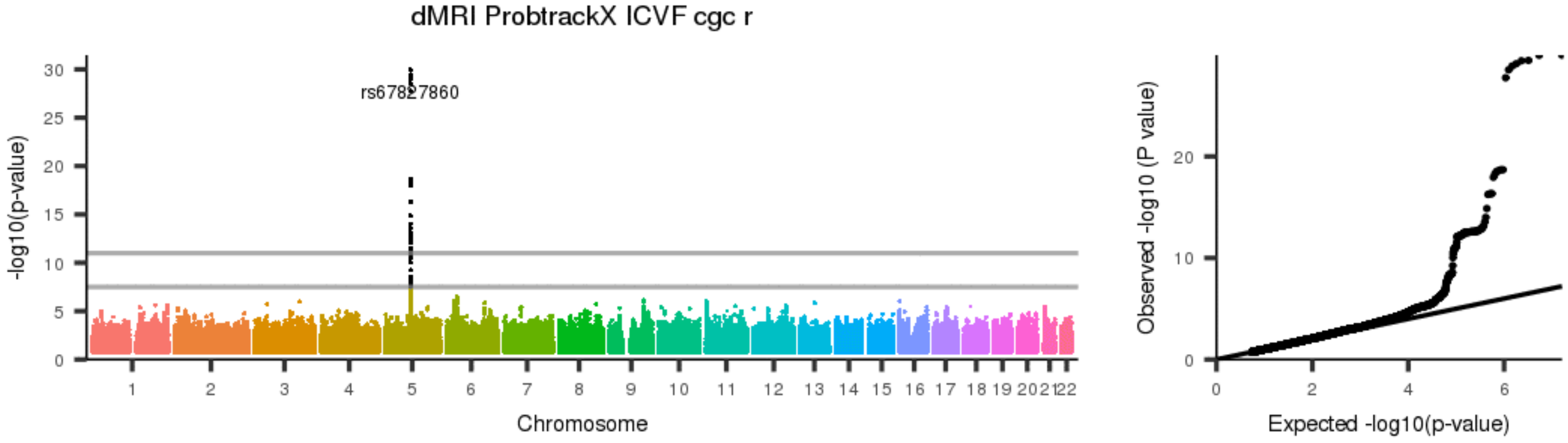

back to list of plots

Figure 796: dMRI ProbtrackX ICVF cgh l

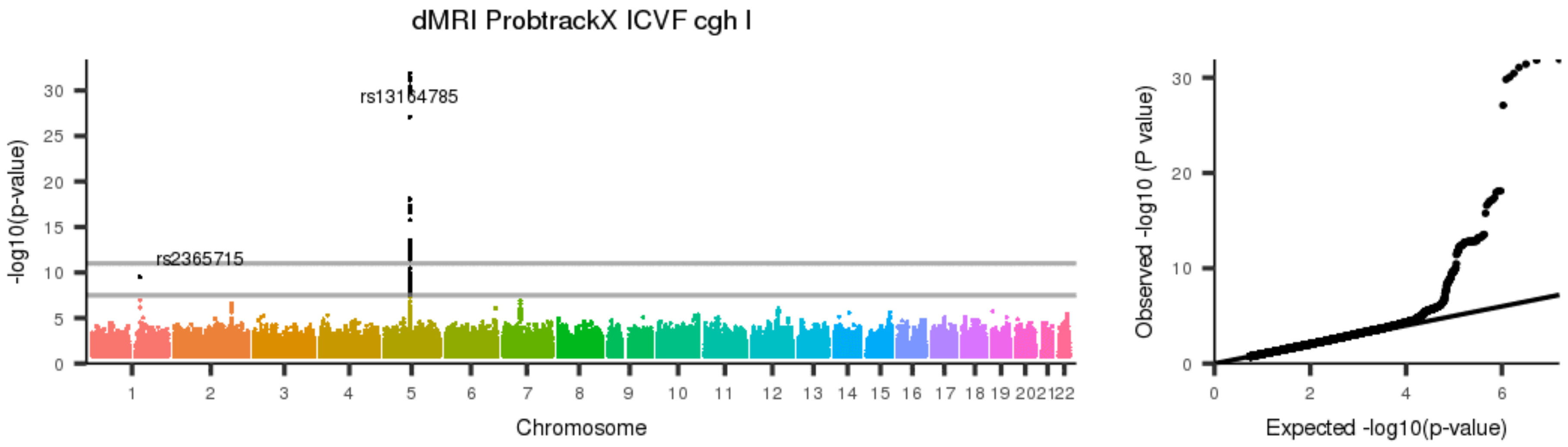

back to list of plots

Figure 797: dMRI ProbtrackX ICVF cgh r

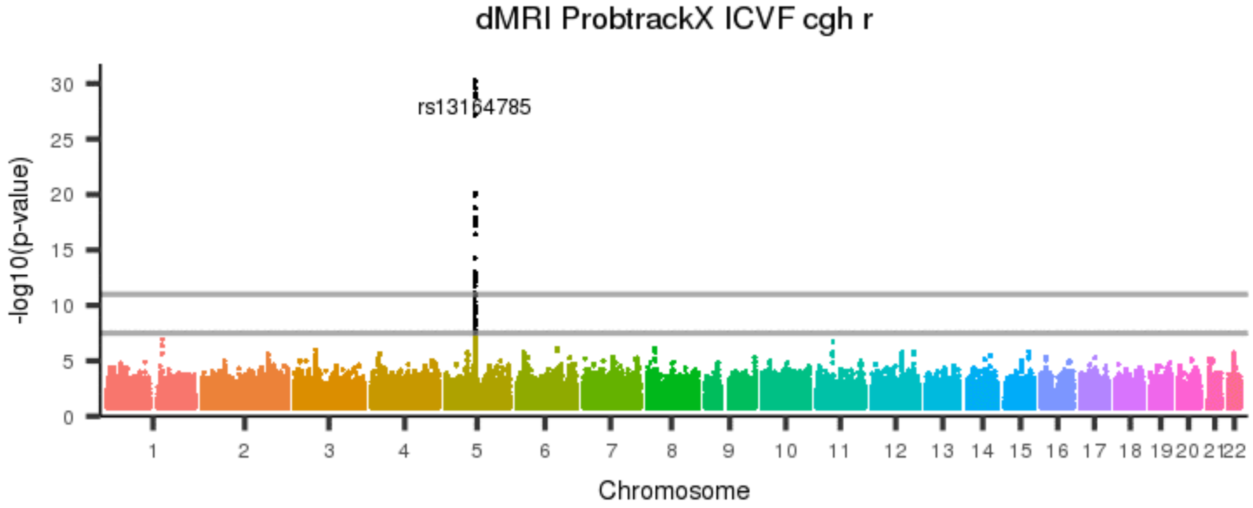

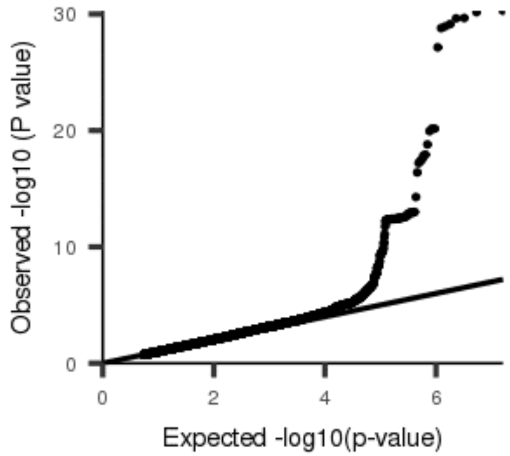

back to list of plots

Figure 798: dMRI ProbtrackX ICVF cst l

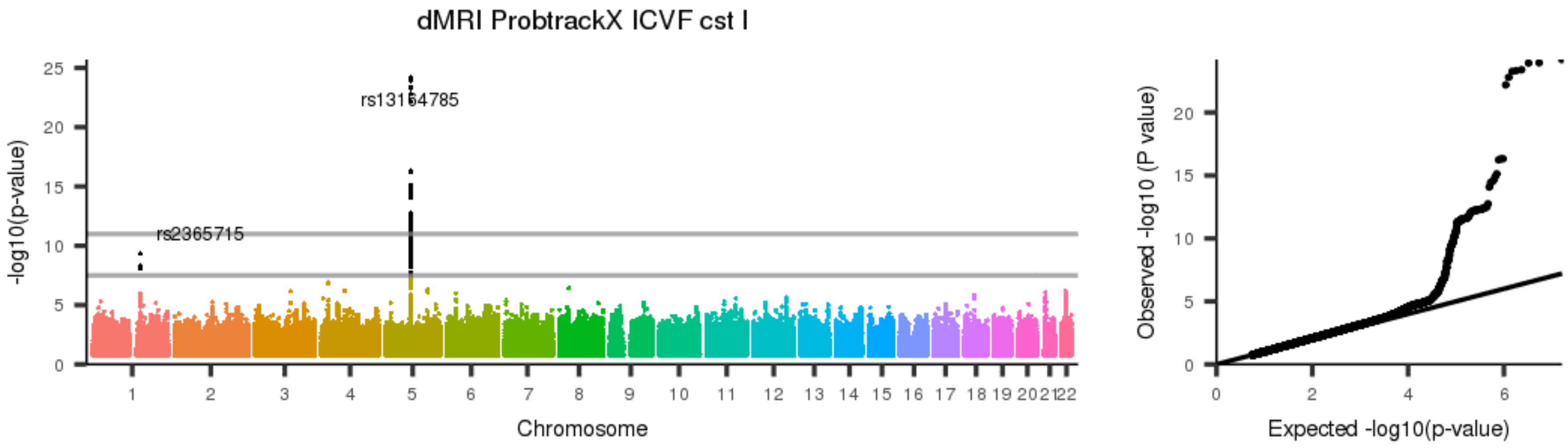

back to list of plots

Figure 799: dMRI ProbtrackX ICVF cst r

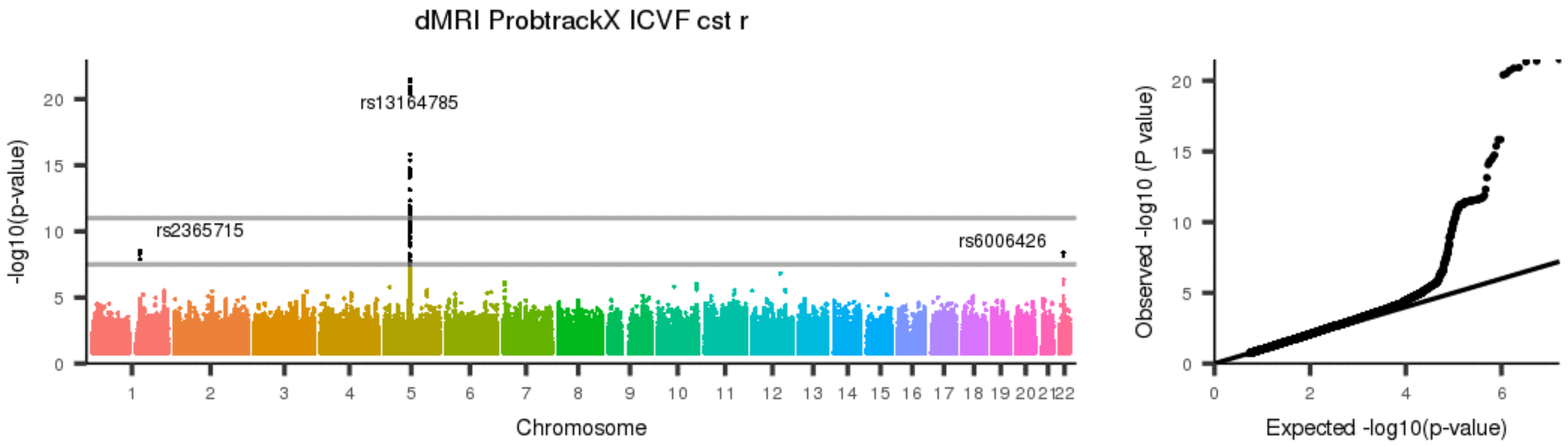

back to list of plots

Figure 800: dMRI ProbtrackX ICVF fma

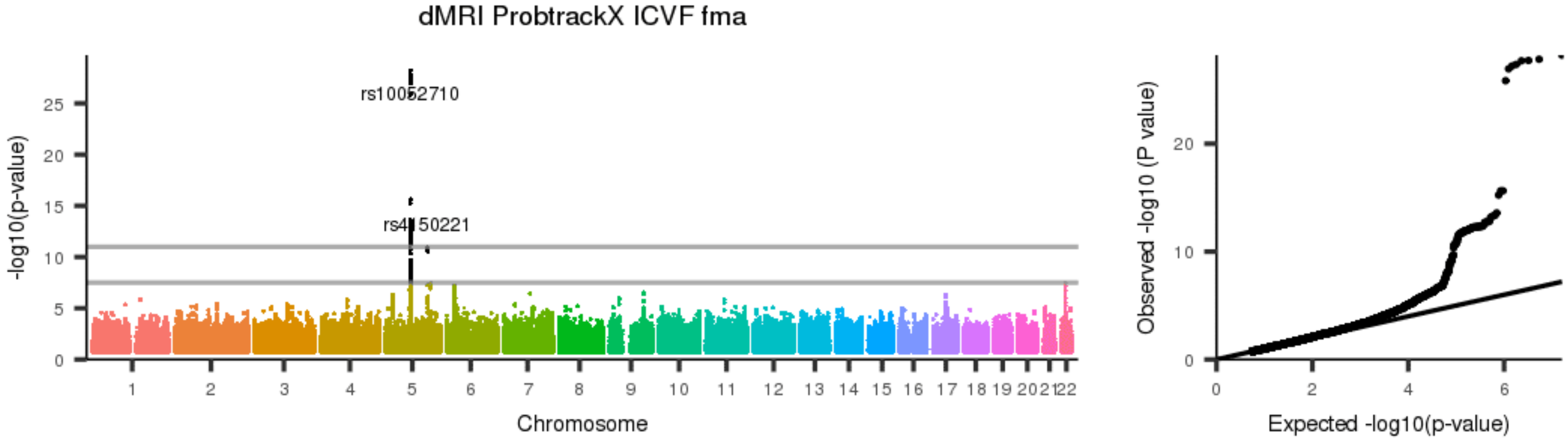

back to list of plots

Figure 801: dMRI ProbtrackX ICVF fmi

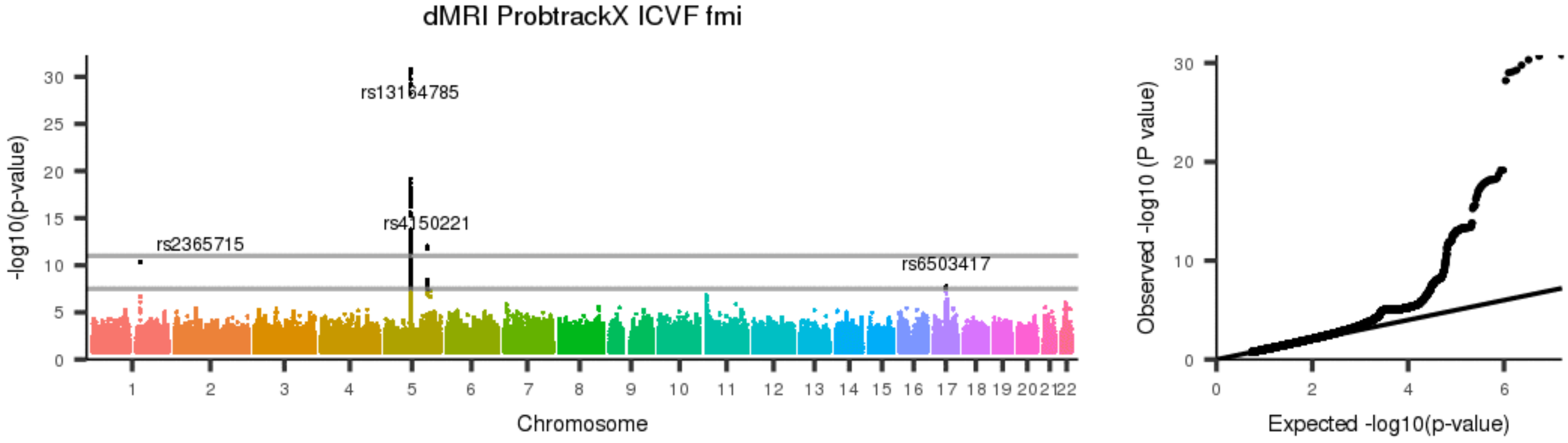

back to list of plots

Figure 802: dMRI ProbtrackX ICVF ifo l

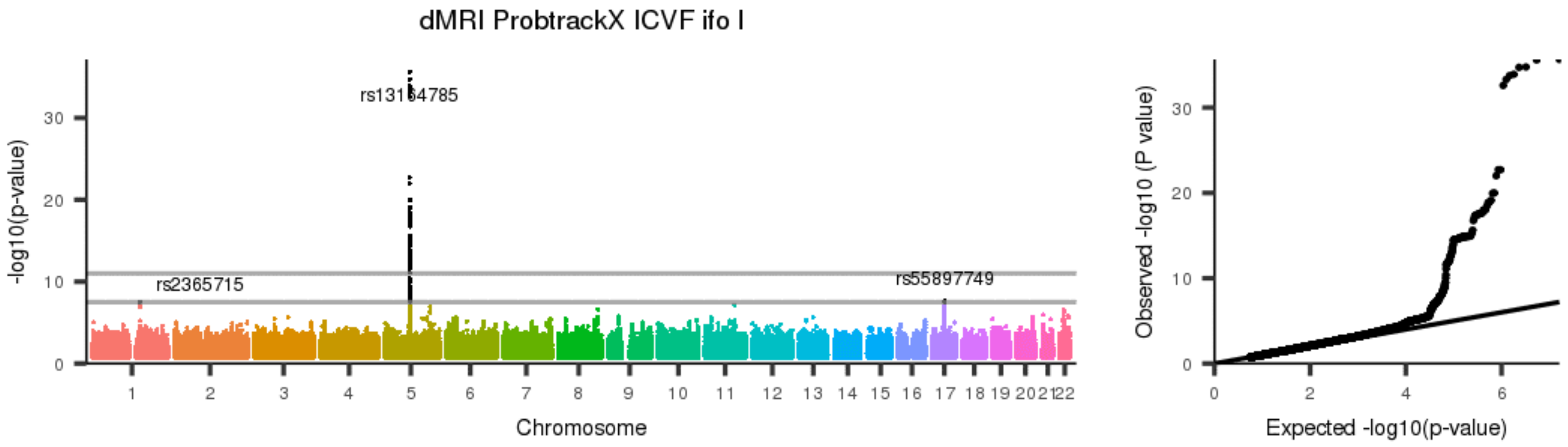

back to list of plots

Figure 803: dMRI ProbtrackX ICVF ifo r

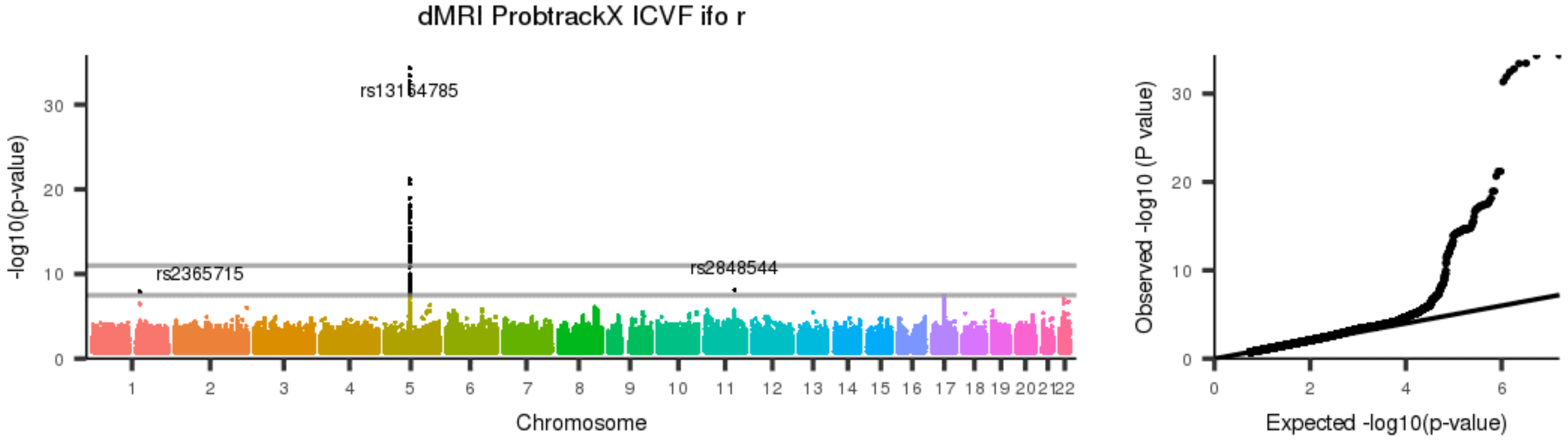

back to list of plots

Figure 804: dMRI ProbtrackX ICVF ilf l

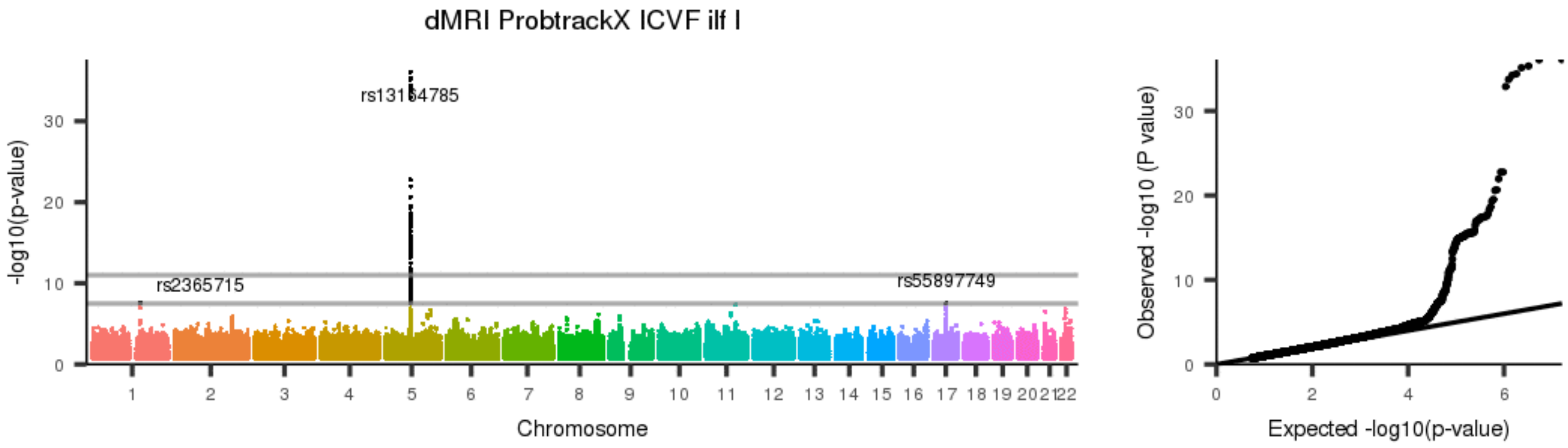

back to list of plots

Figure 805: dMRI ProbtrackX ICVF ilf r

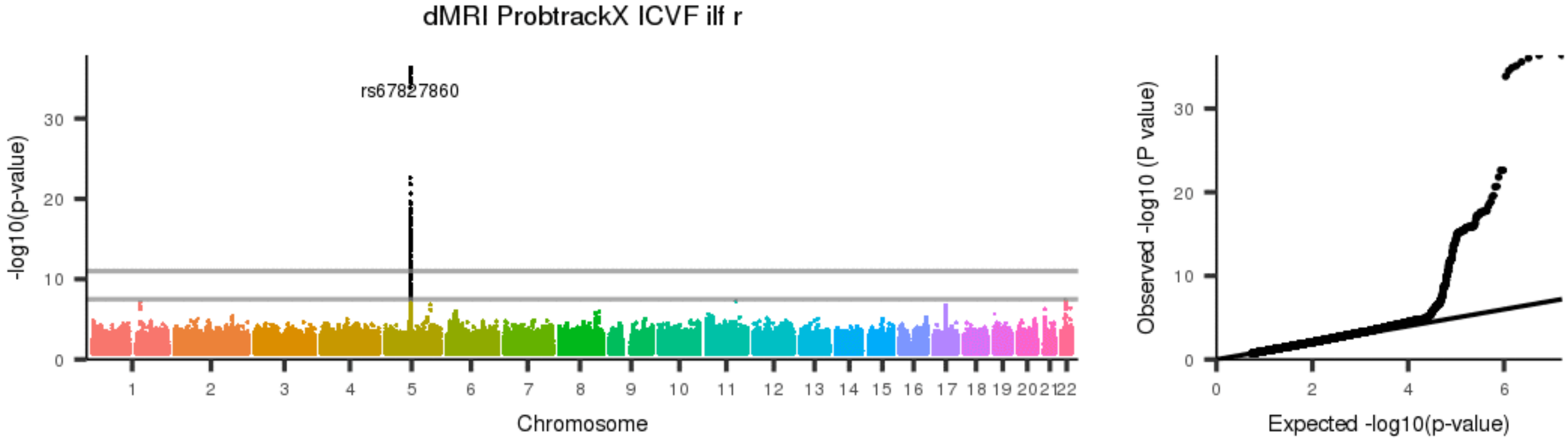

back to list of plots

Figure 806: dMRI ProbtrackX ICVF mcp

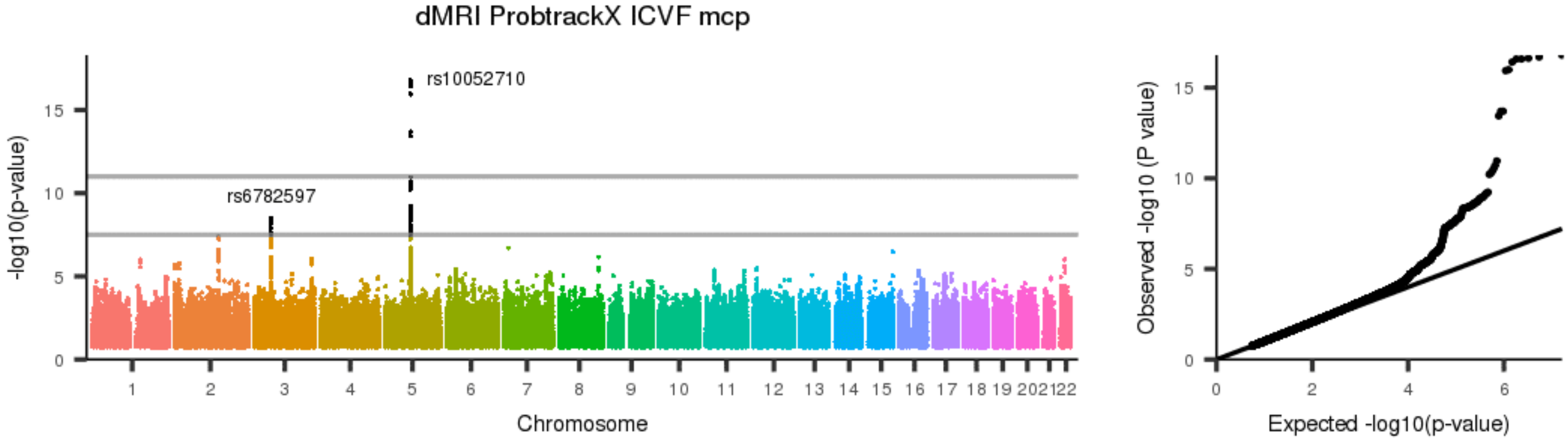

back to list of plots

Figure 807: dMRI ProbtrackX ICVF ml l

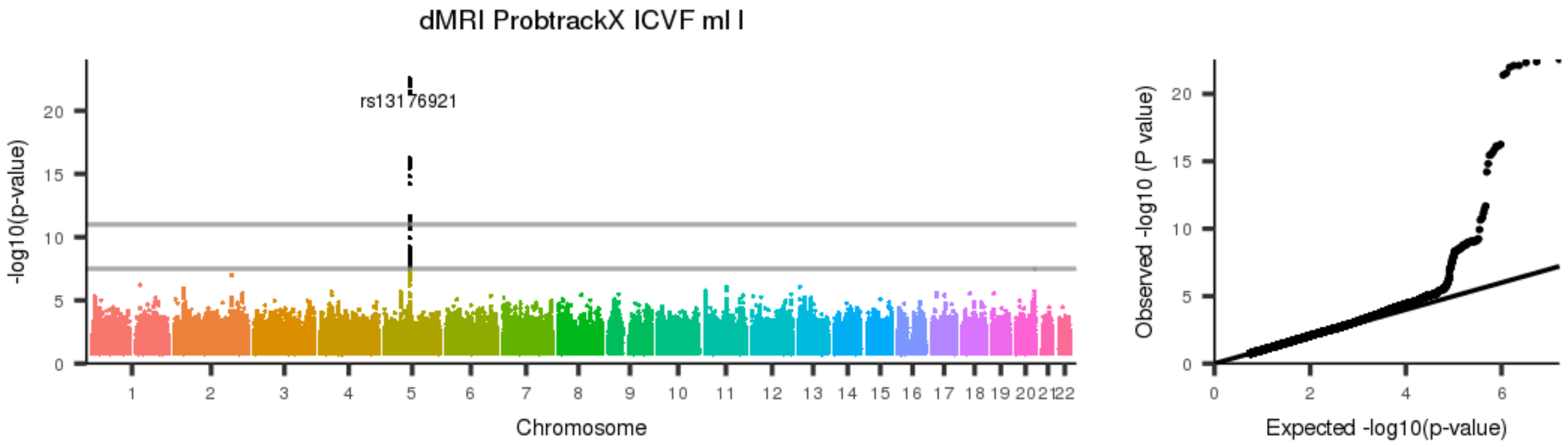

back to list of plots

Figure 808: dMRI ProbtrackX ICVF ml r

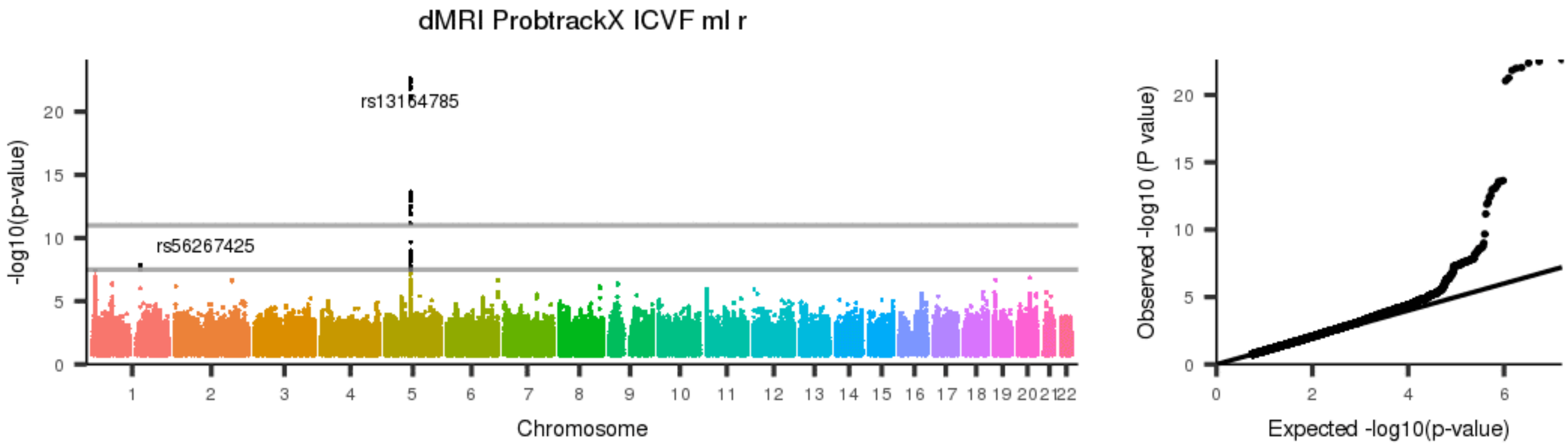

back to list of plots

Figure 809: dMRI ProbtrackX ICVF ptr l

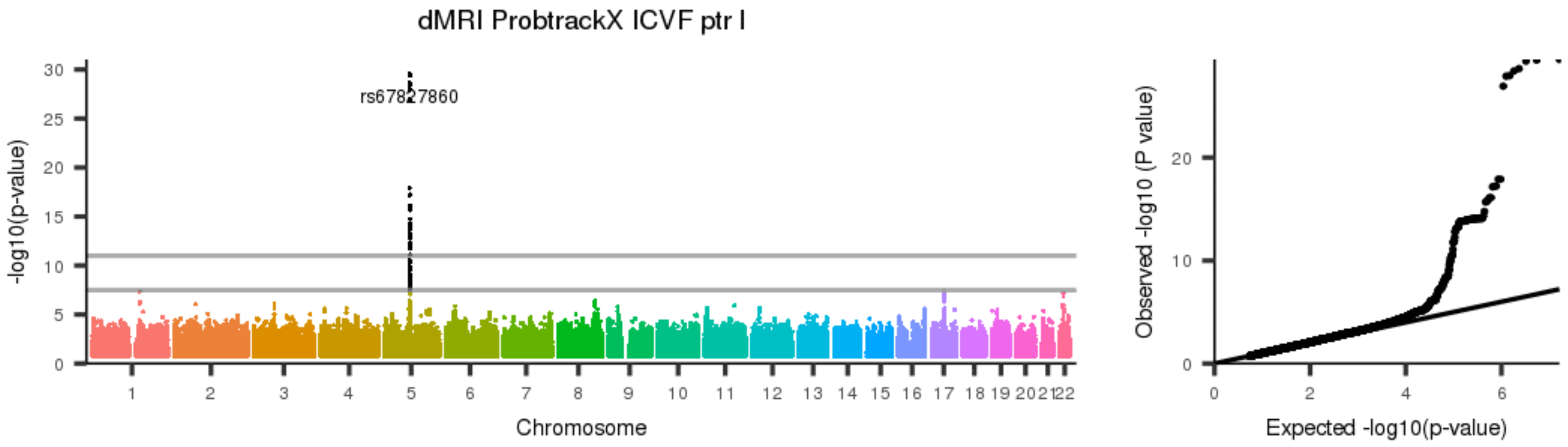

back to list of plots

Figure 810: dMRI ProbtrackX ICVF ptr r

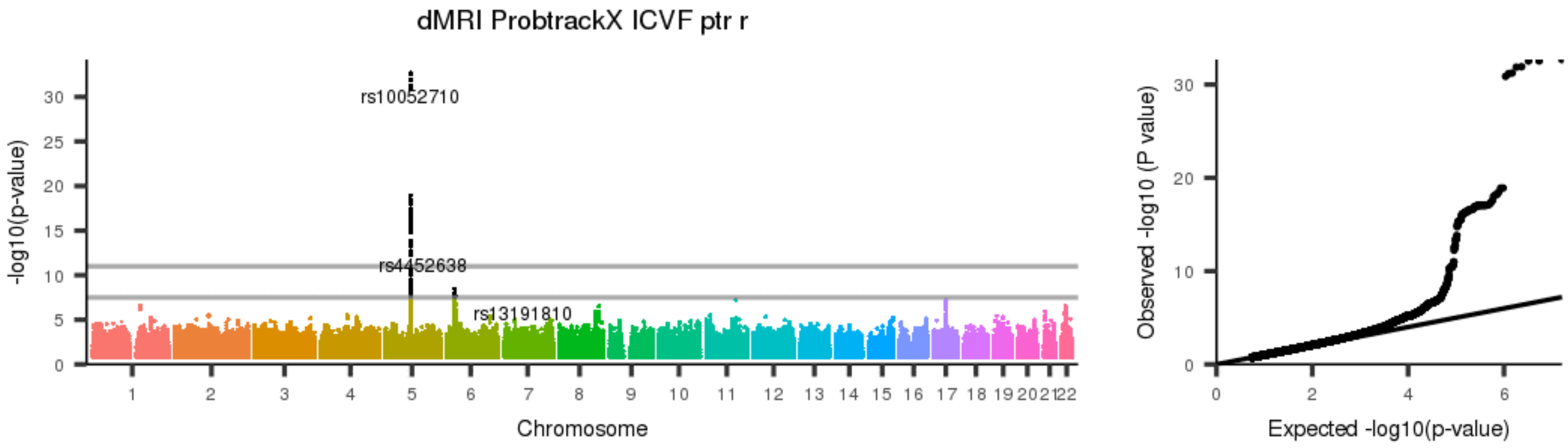

back to list of plots

Figure 811: dMRI ProbtrackX ICVF slf I

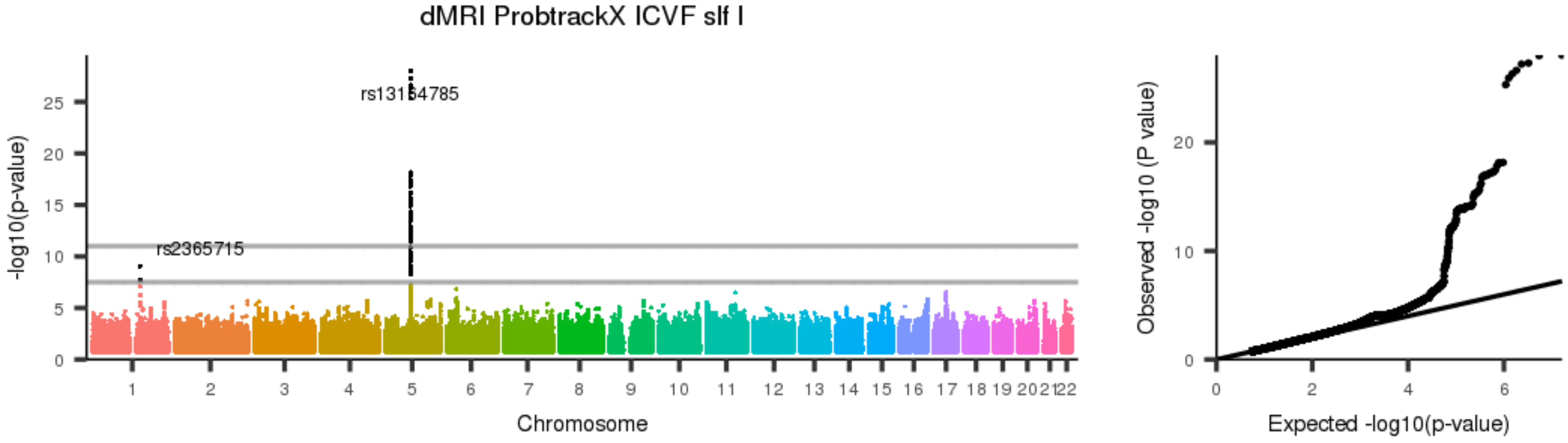

back to list of plots

Figure 812: dMRI ProbtrackX ICVF slf r

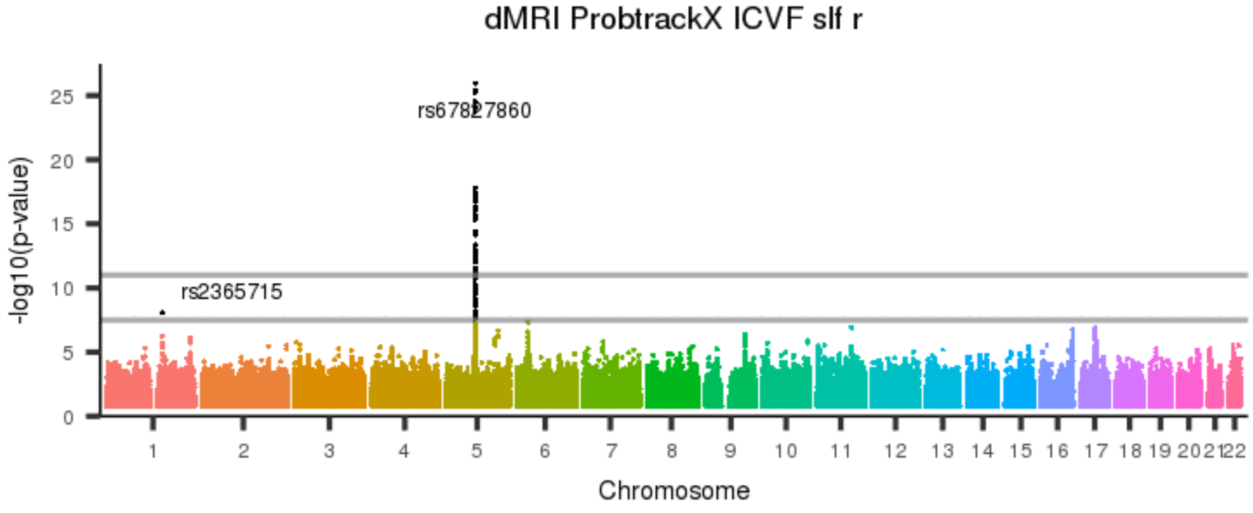

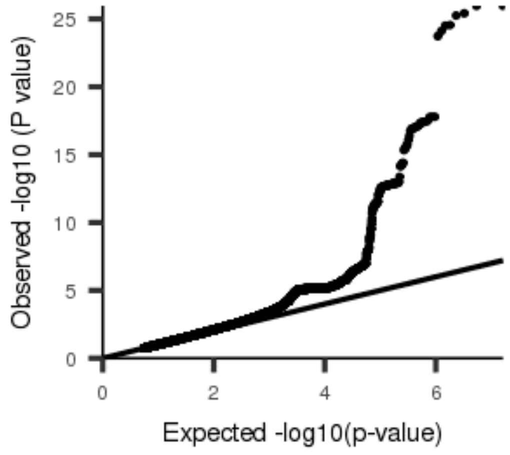

back to list of plots

Figure 813: dMRI ProbtrackX ICVF str l

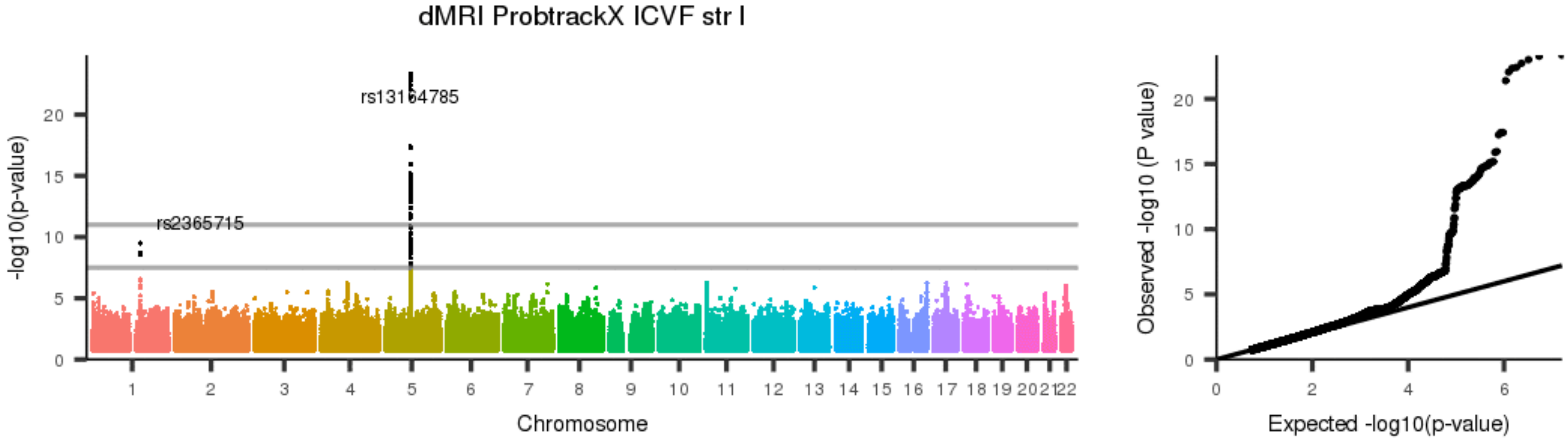

back to list of plots

Figure 814: dMRI ProbtrackX ICVF str r

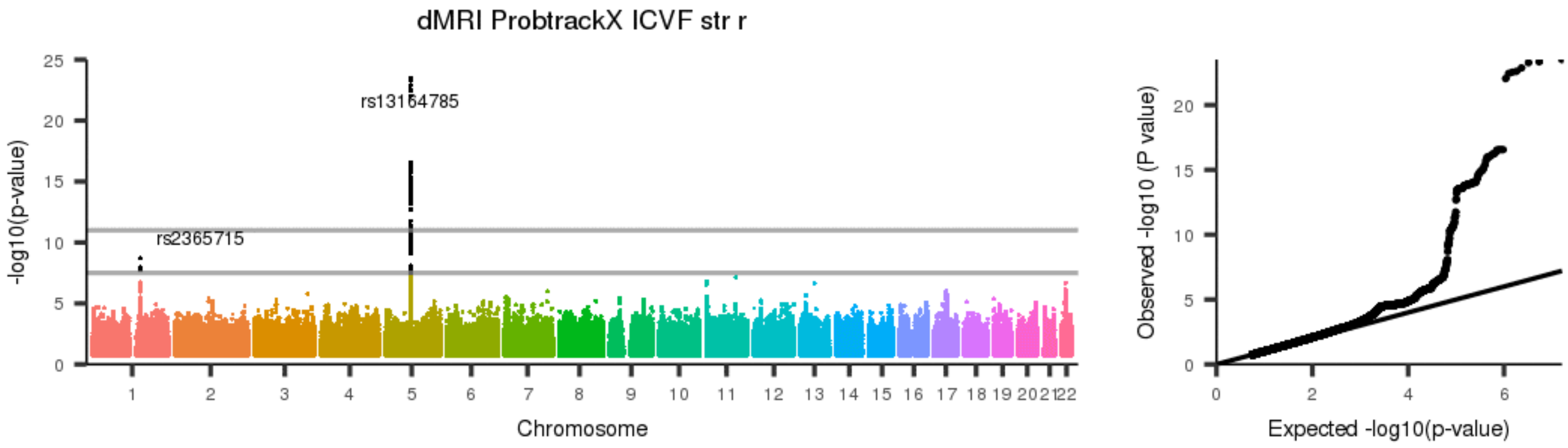

back to list of plots

Figure 815: dMRI ProbtrackX ICVF unc l

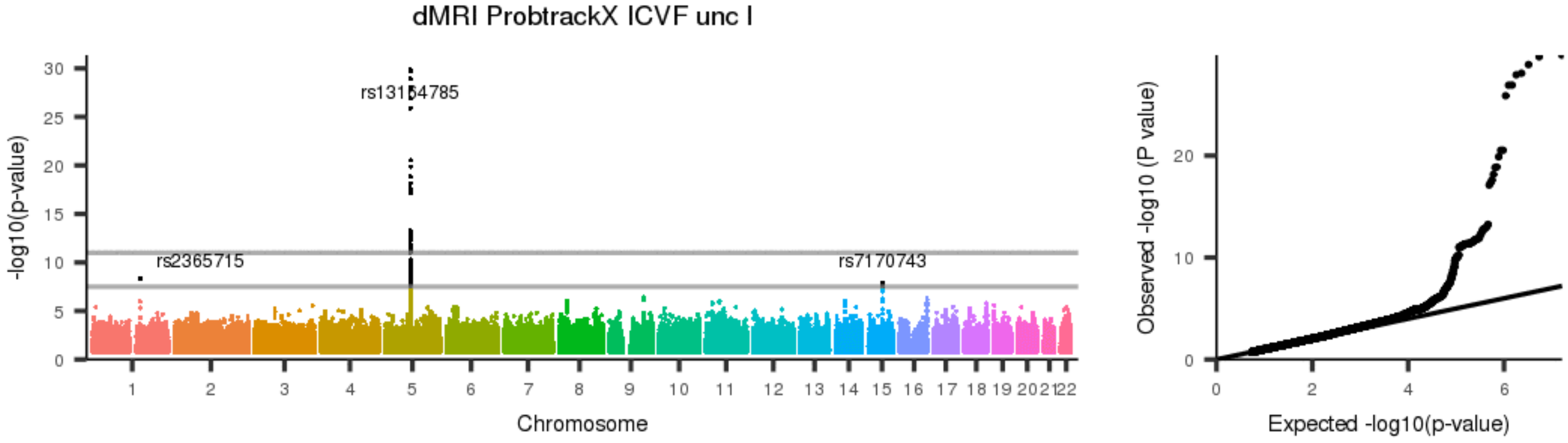

back to list of plots

Figure 816: dMRI ProbtrackX ICVF unc r

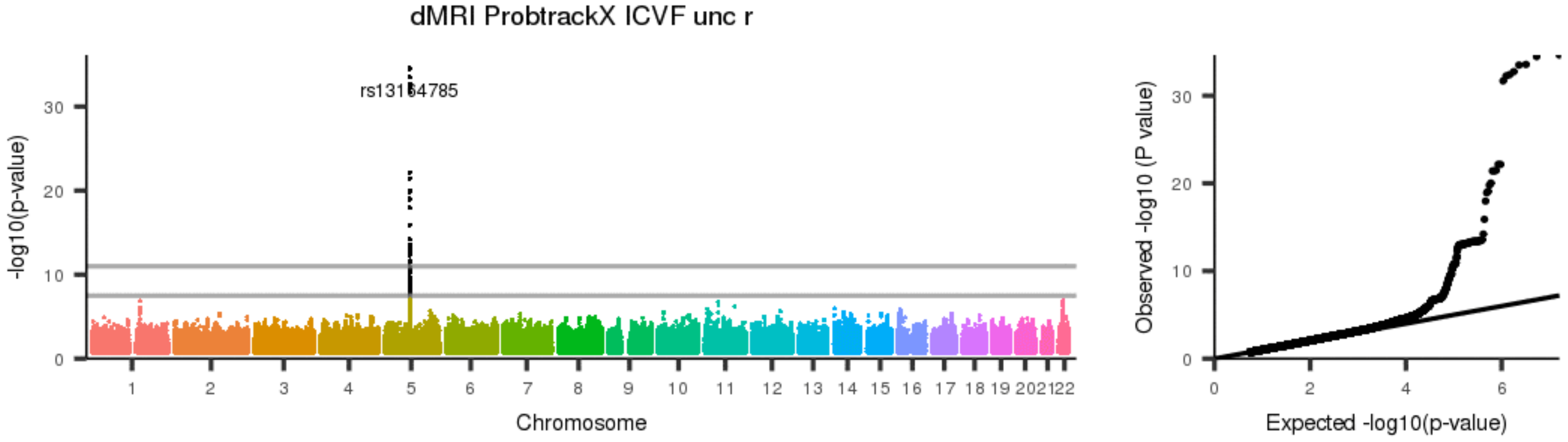

back to list of plots

Figure 817: dMRI ProbtrackX OD ar l

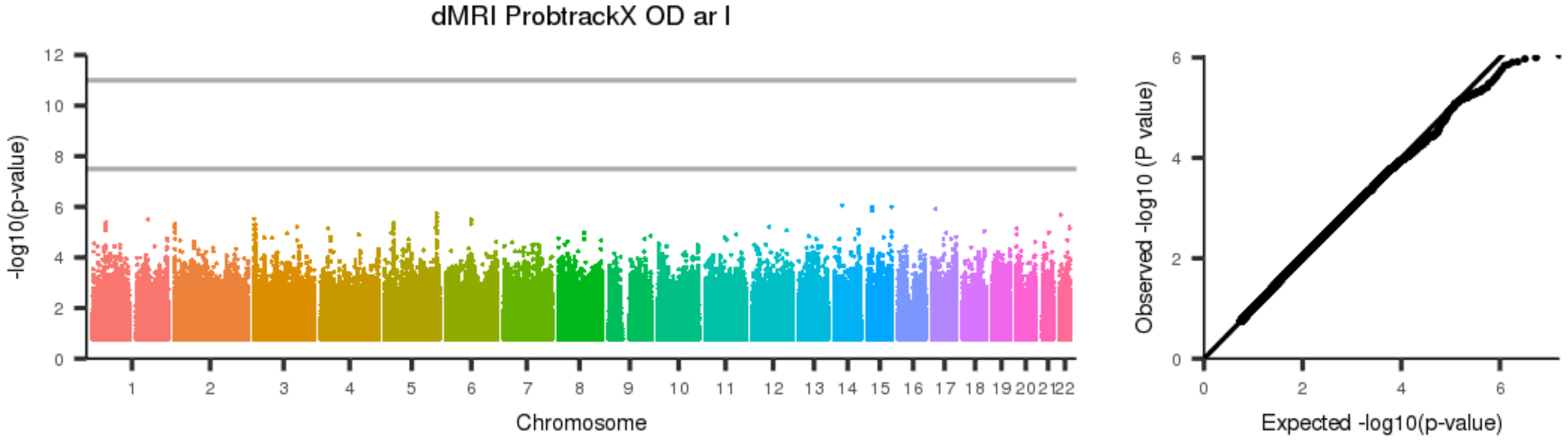

back to list of plots

Figure 818: dMRI ProbtrackX OD ar r

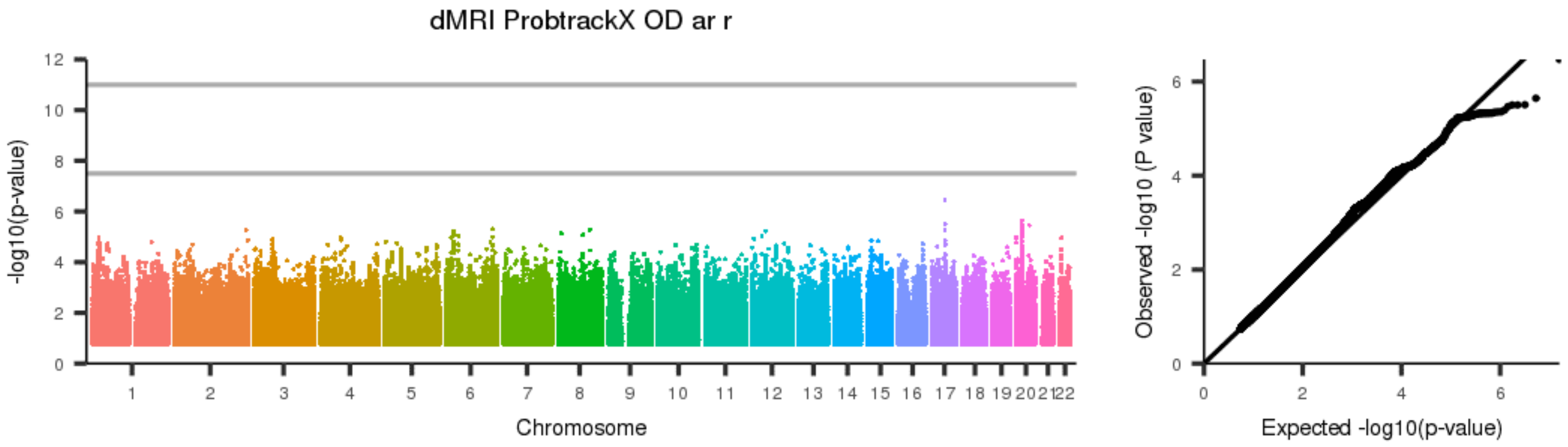

back to list of plots

Figure 819: dMRI ProbtrackX OD atr l

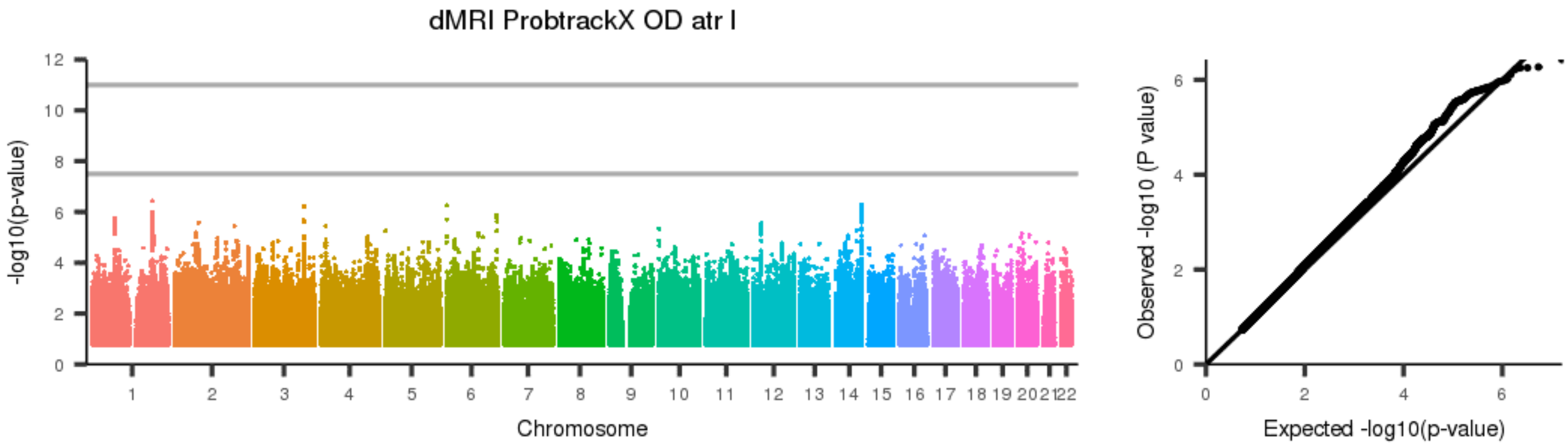

back to list of plots

Figure 820: dMRI ProbtrackX OD atr r

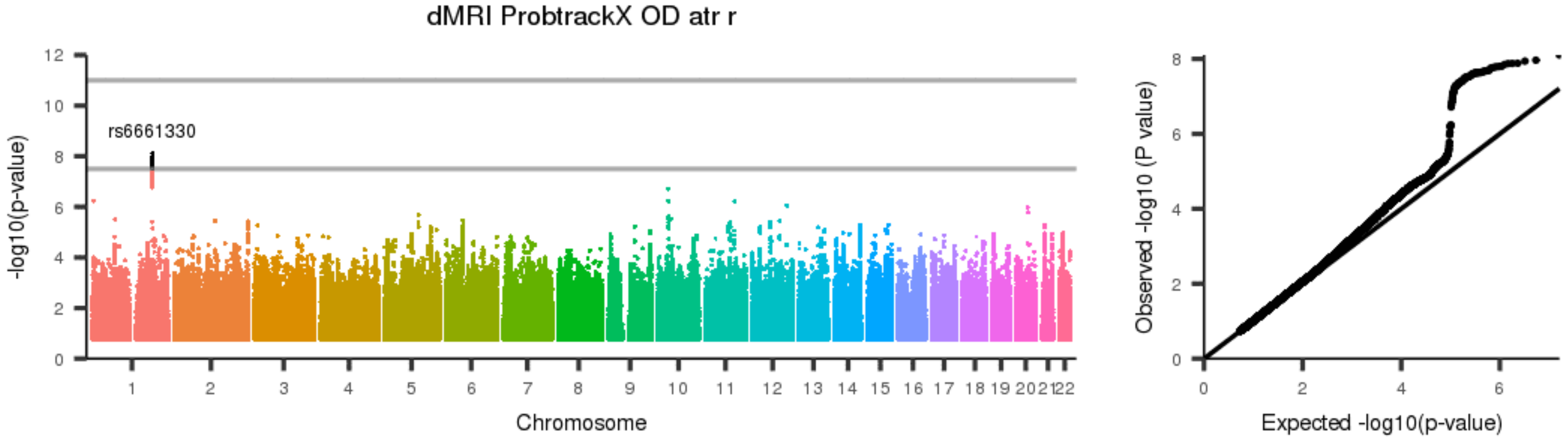

back to list of plots

Figure 821: dMRI ProbtrackX OD cgc l

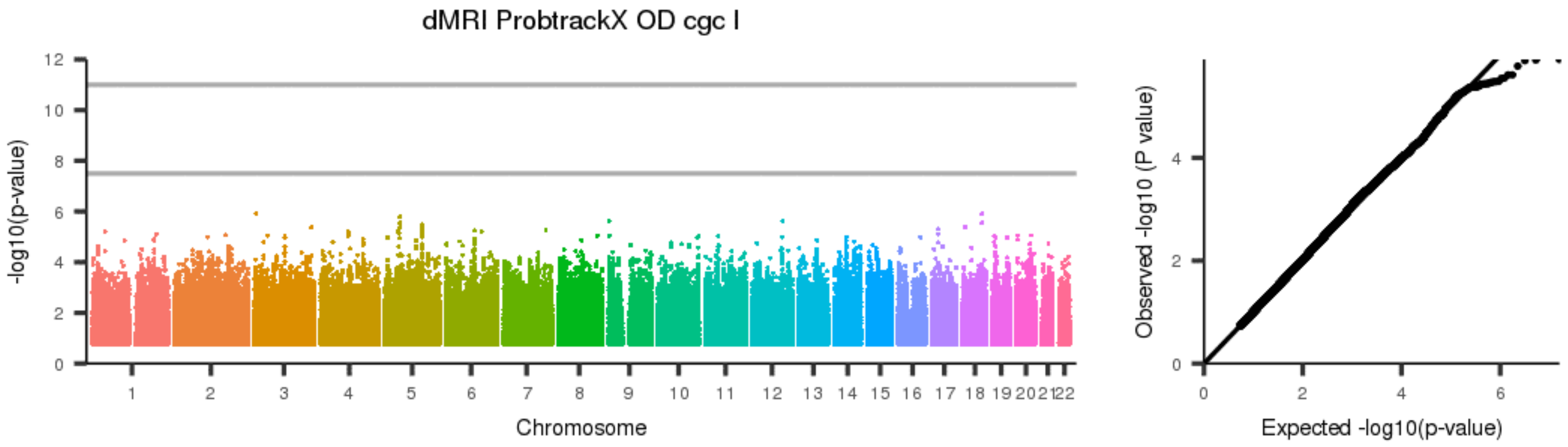

back to list of plots

Figure 822: dMRI ProbtrackX OD cgc r

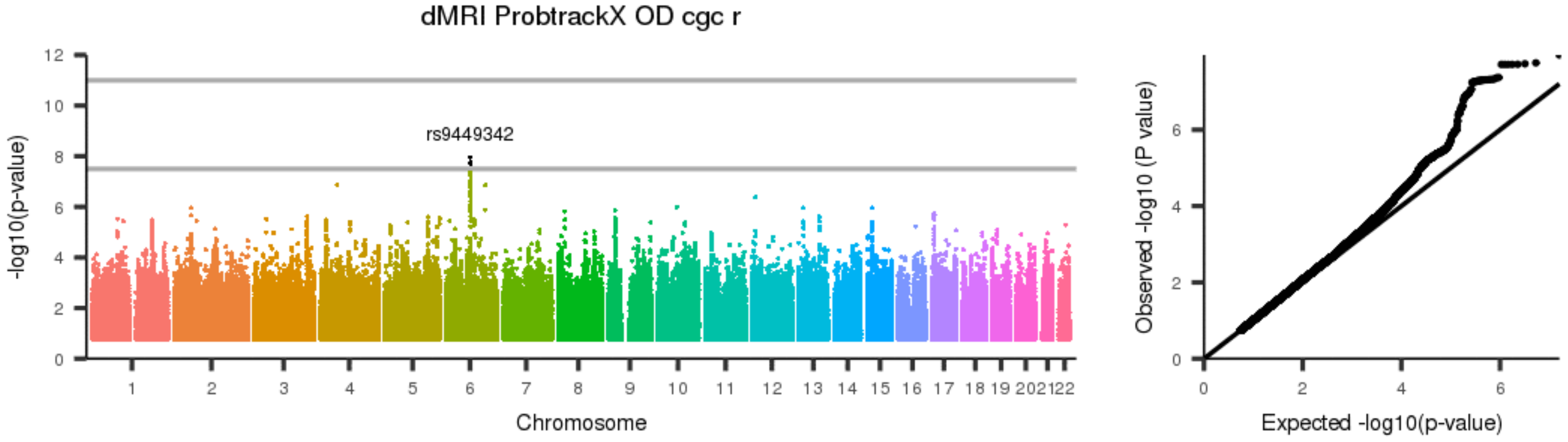

back to list of plots

Figure 823: dMRI ProbtrackX OD cgh l

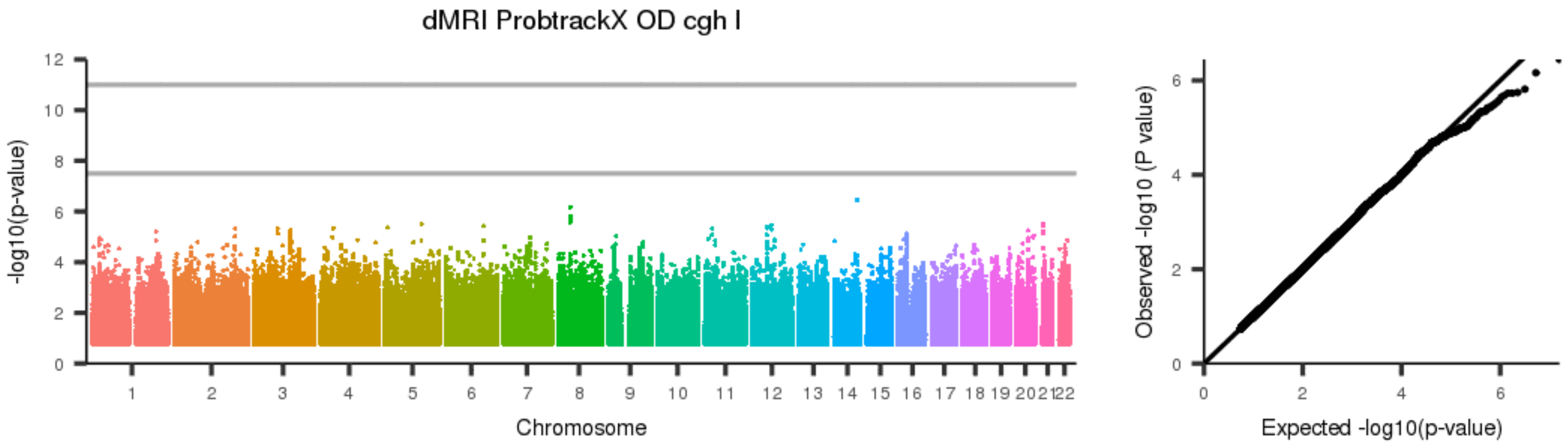

back to list of plots

Figure 824: dMRI ProbtrackX OD cgh r

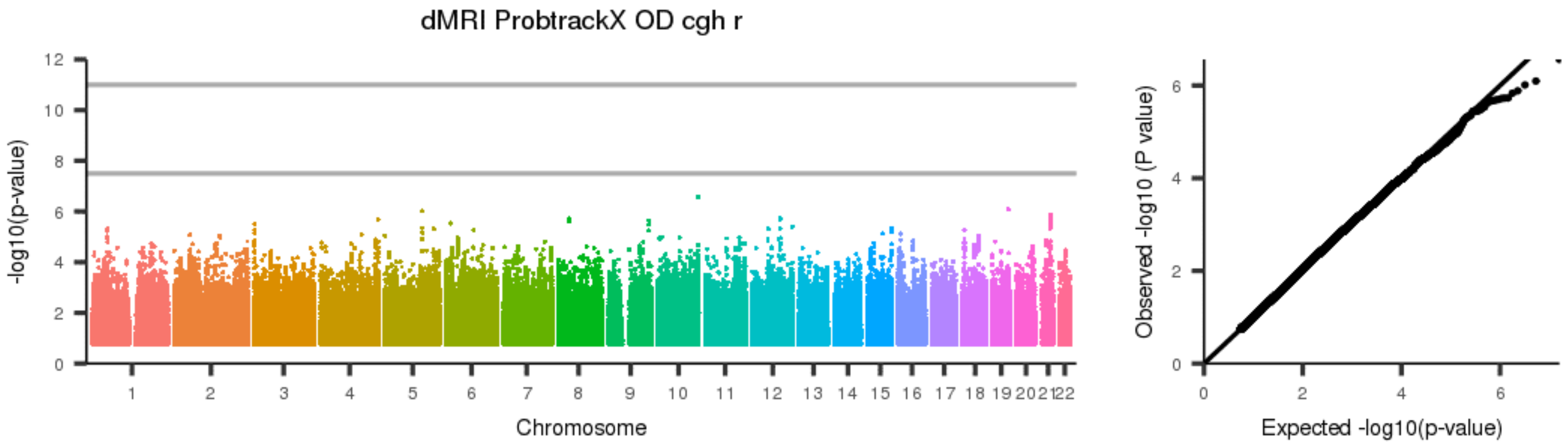

back to list of plots

Figure 825: dMRI ProbtrackX OD cst l

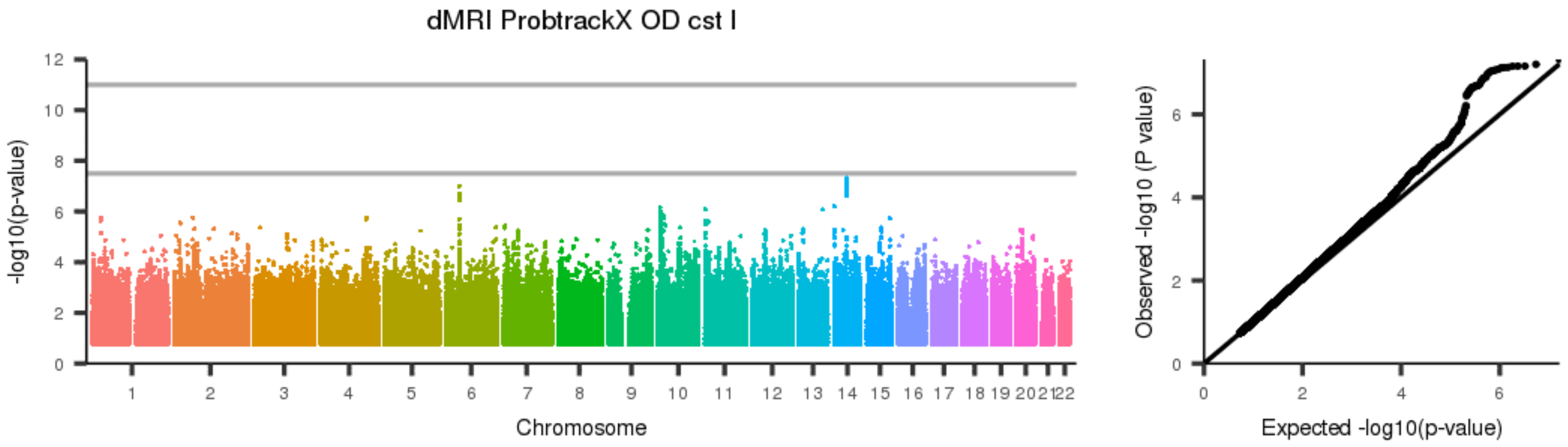

back to list of plots

Figure 826: dMRI ProbtrackX OD cst r

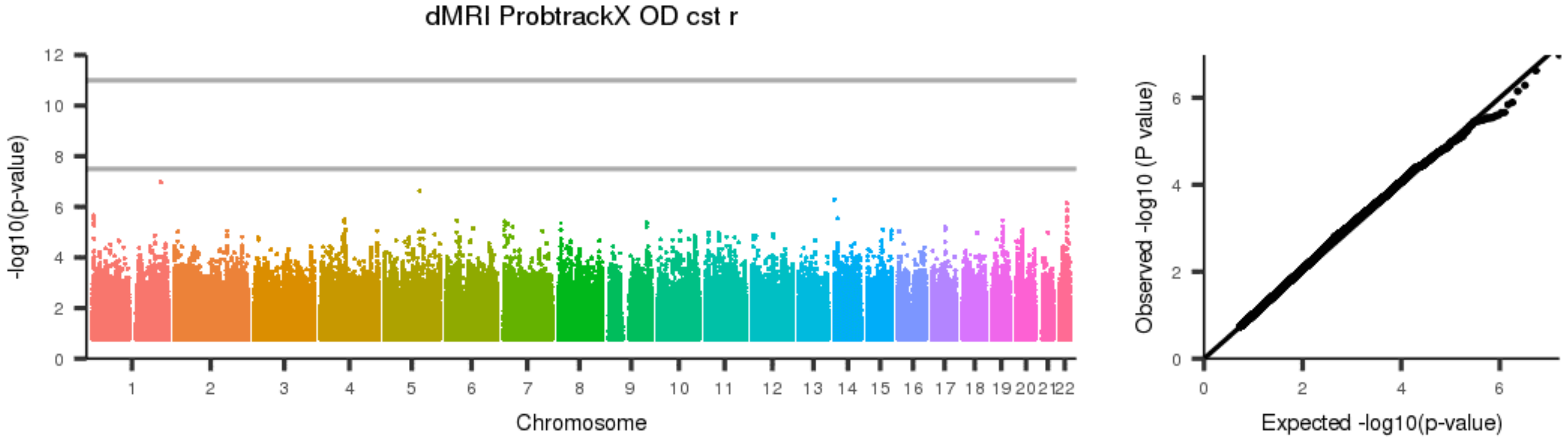

back to list of plots

Figure 827: dMRI ProbtrackX OD fma

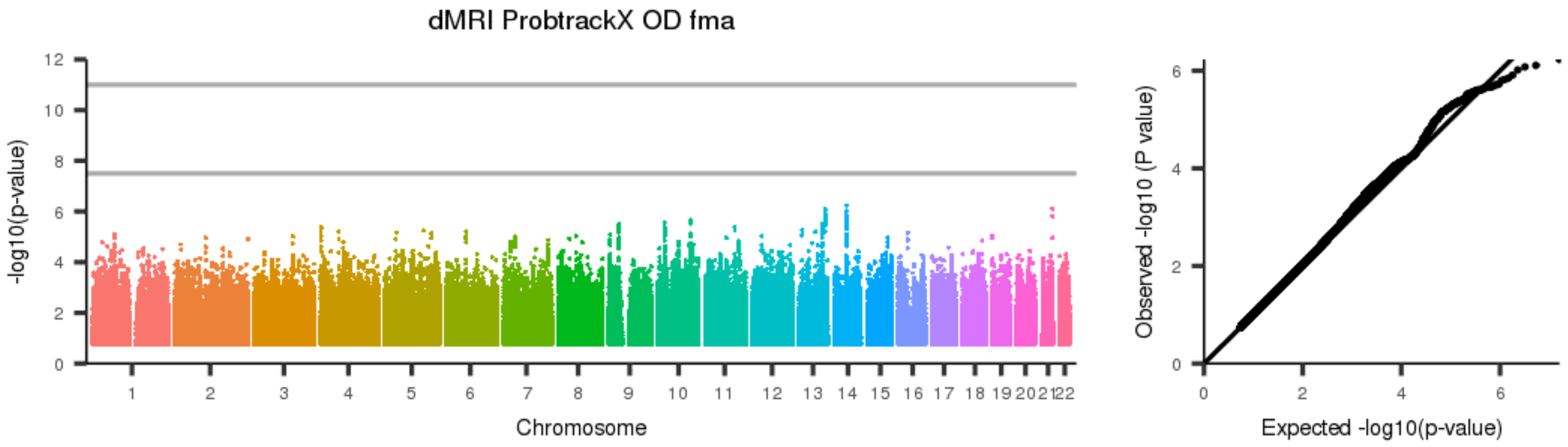

back to list of plots

Figure 828: dMRI ProbtrackX OD fmi

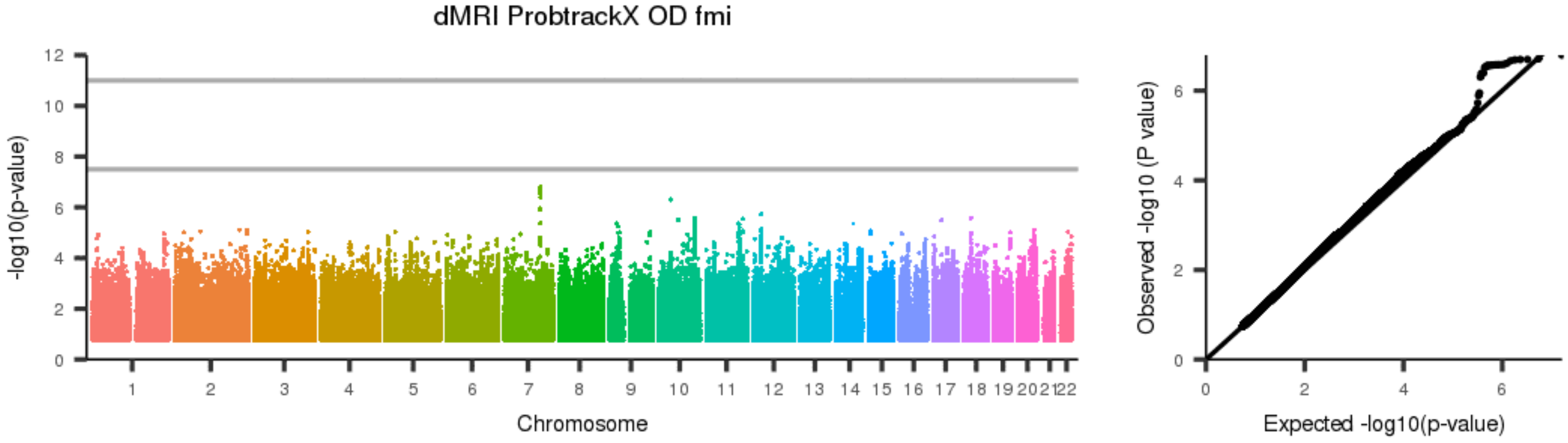

back to list of plots

Figure 829: dMRI ProbtrackX OD ifo l

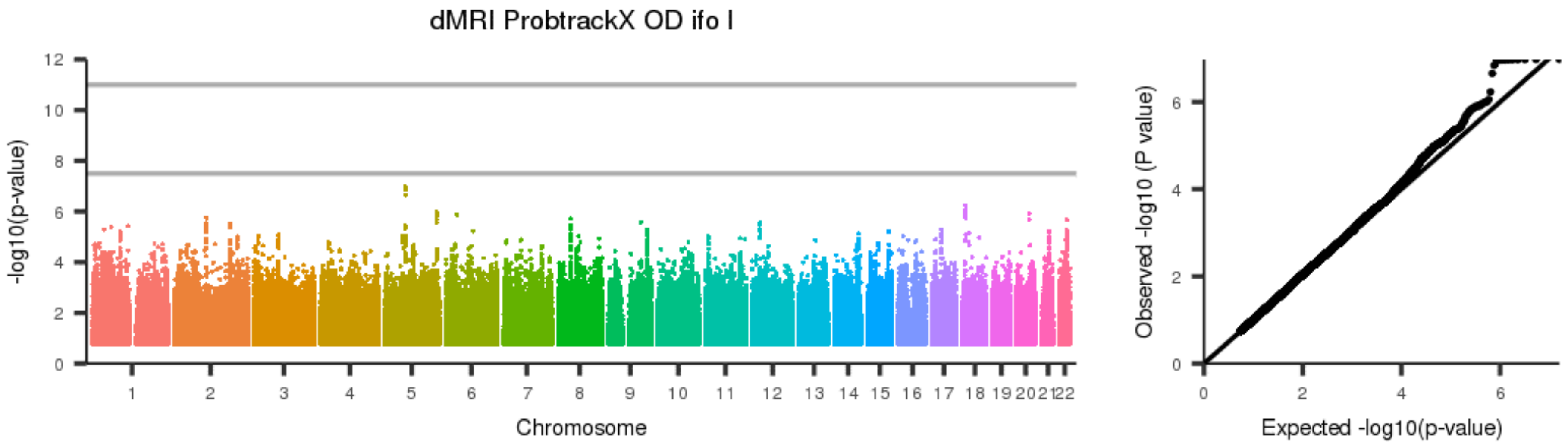

back to list of plots

Figure 830: dMRI ProbtrackX OD ifo r

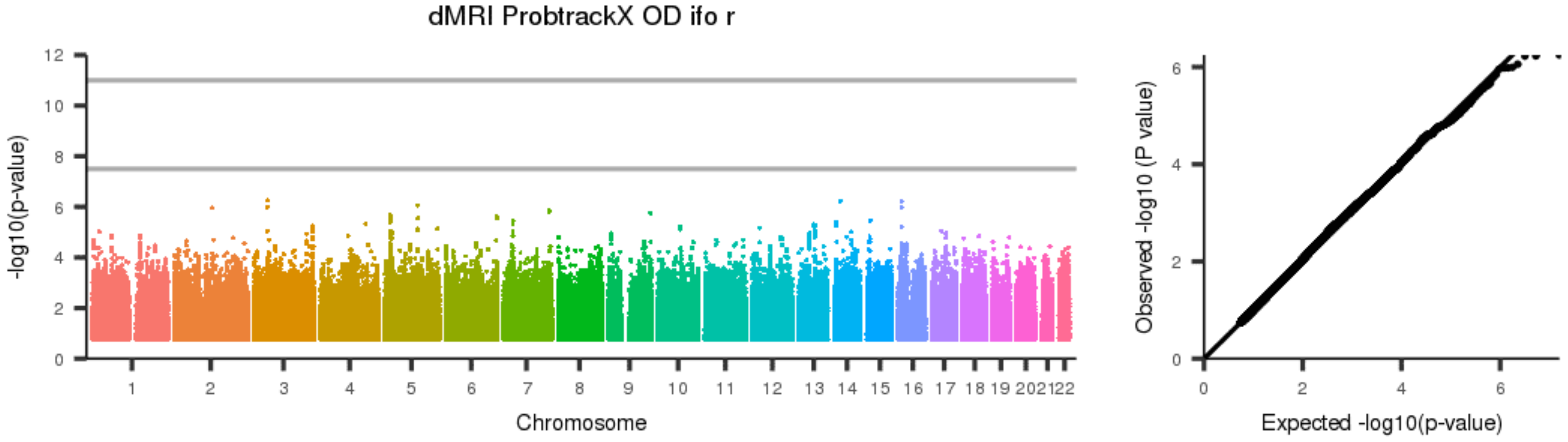

back to list of plots

Figure 831: dMRI ProbtrackX OD ilf l

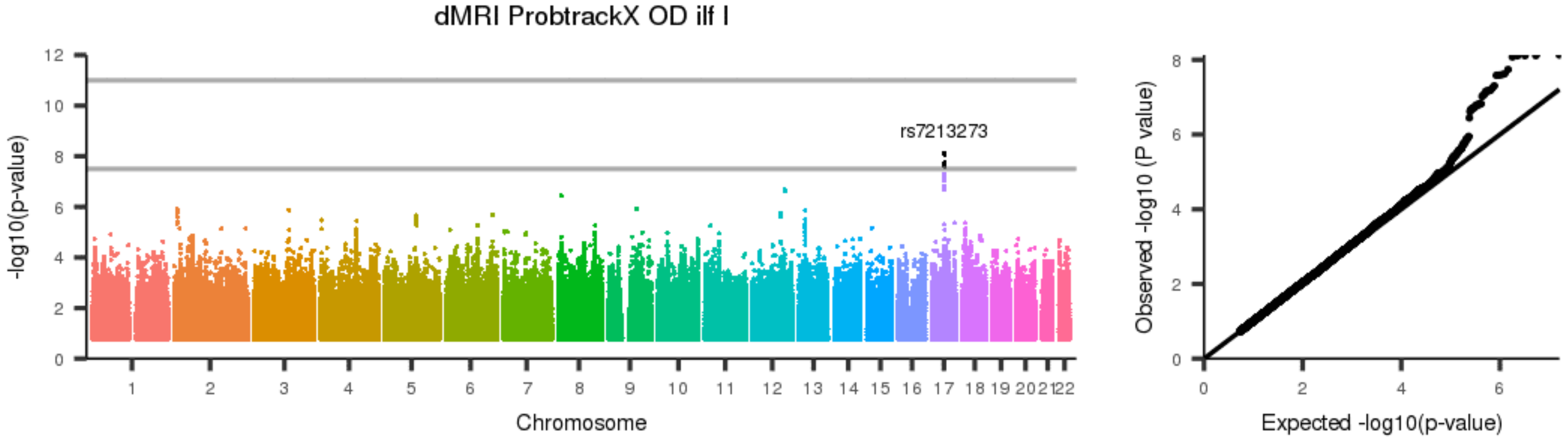

back to list of plots

Figure 832: dMRI ProbtrackX OD ilf r

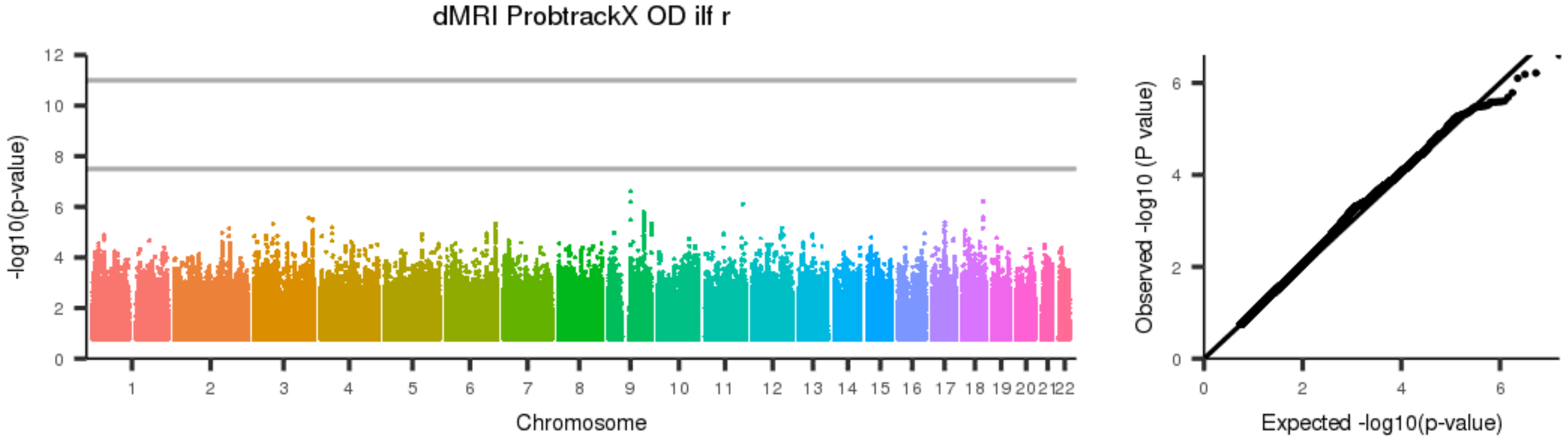

back to list of plots

Figure 833: dMRI ProbtrackX OD mcp

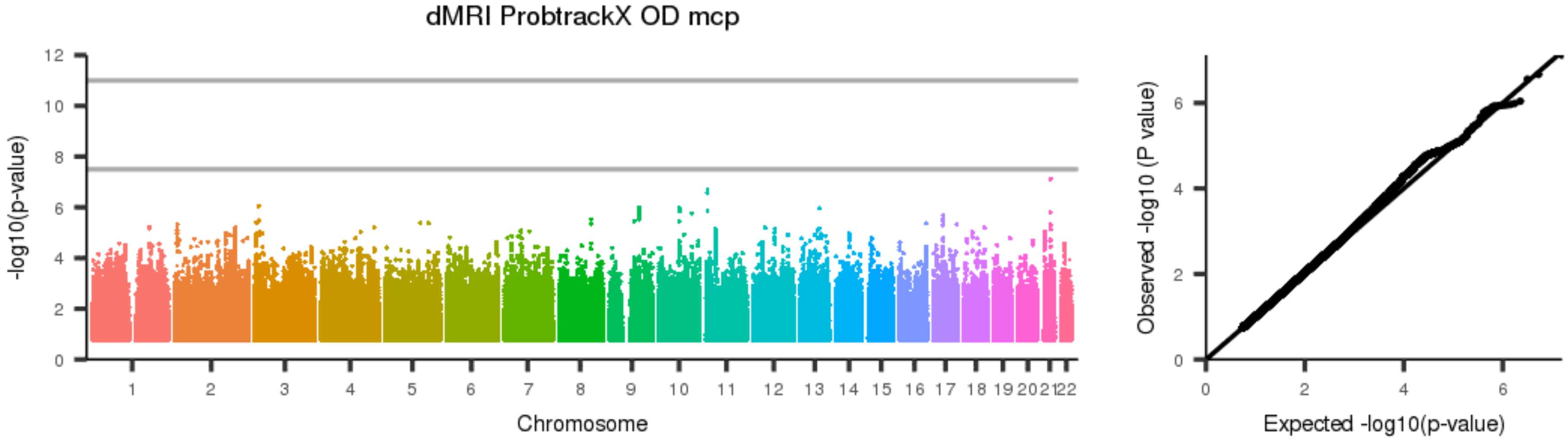

back to list of plots

Figure 834: dMRI ProbtrackX OD ml l

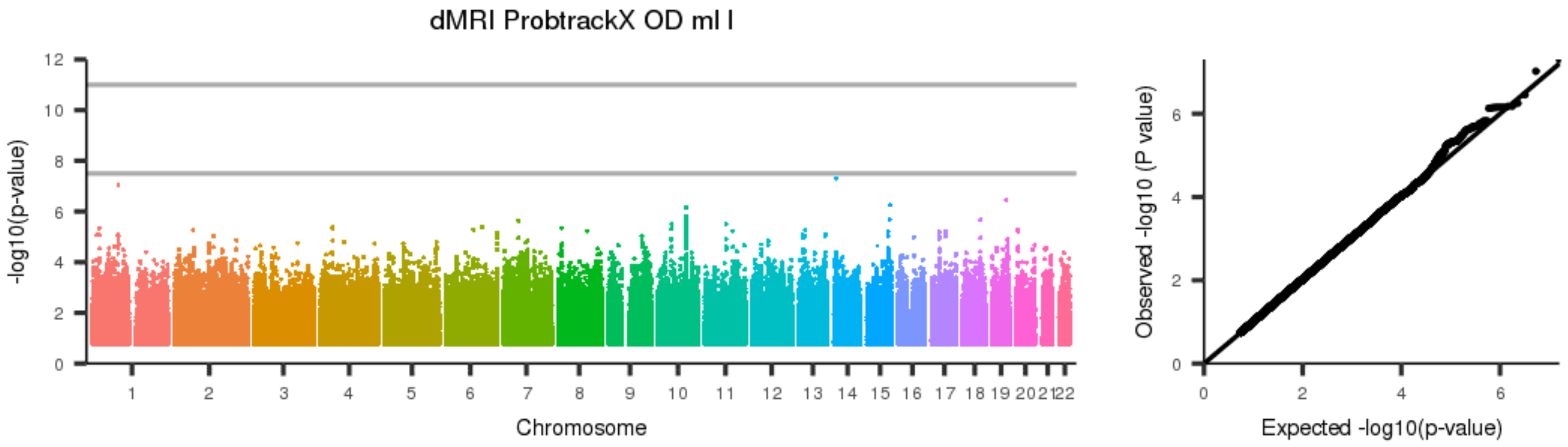

back to list of plots

Figure 835: dMRI ProbtrackX OD ml r

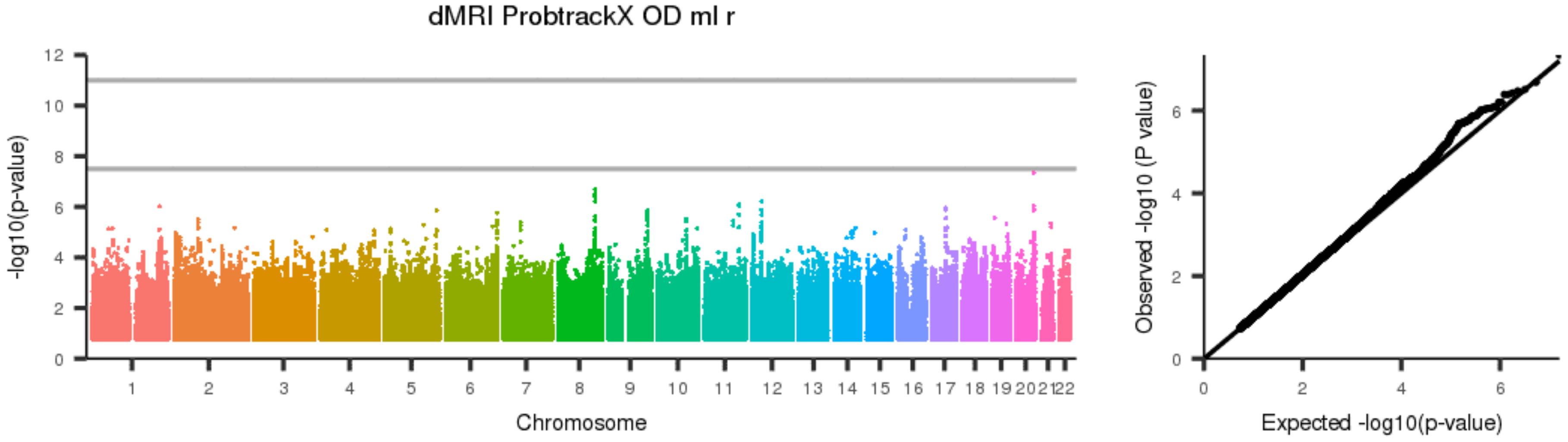

back to list of plots

Figure 836: dMRI ProbtrackX OD ptr l

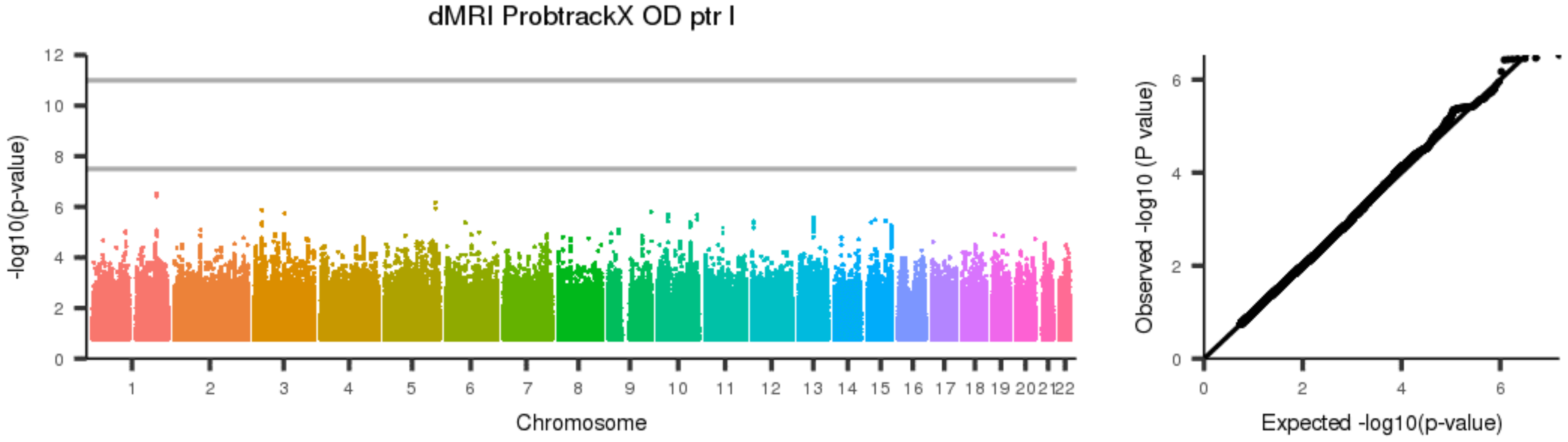

back to list of plots

Figure 837: dMRI ProbtrackX OD ptr r

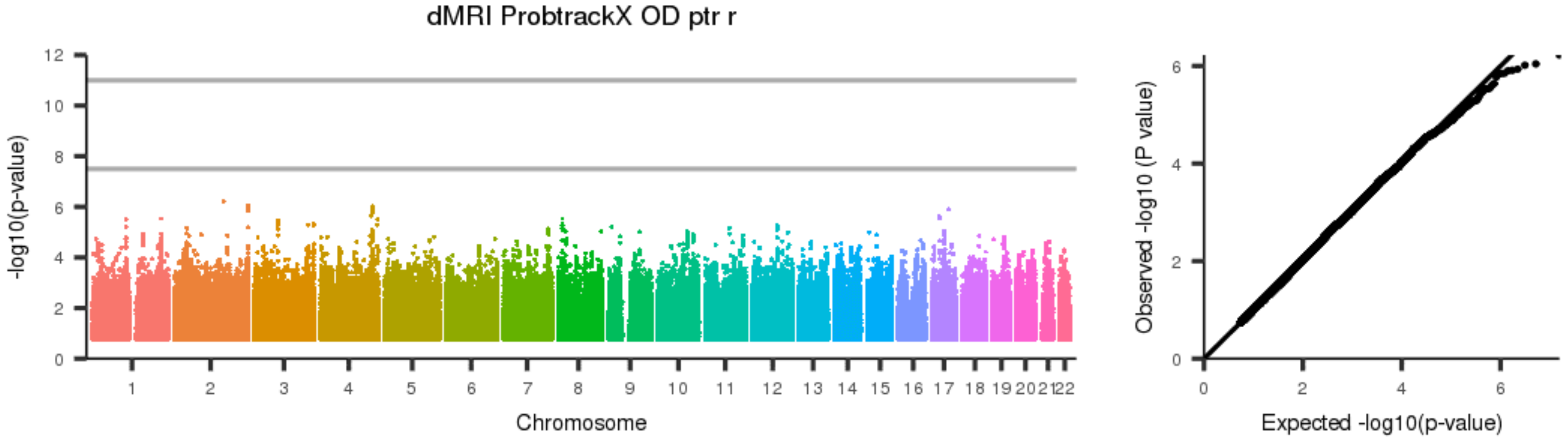

back to list of plots

Figure 838: dMRI ProbtrackX OD slf I

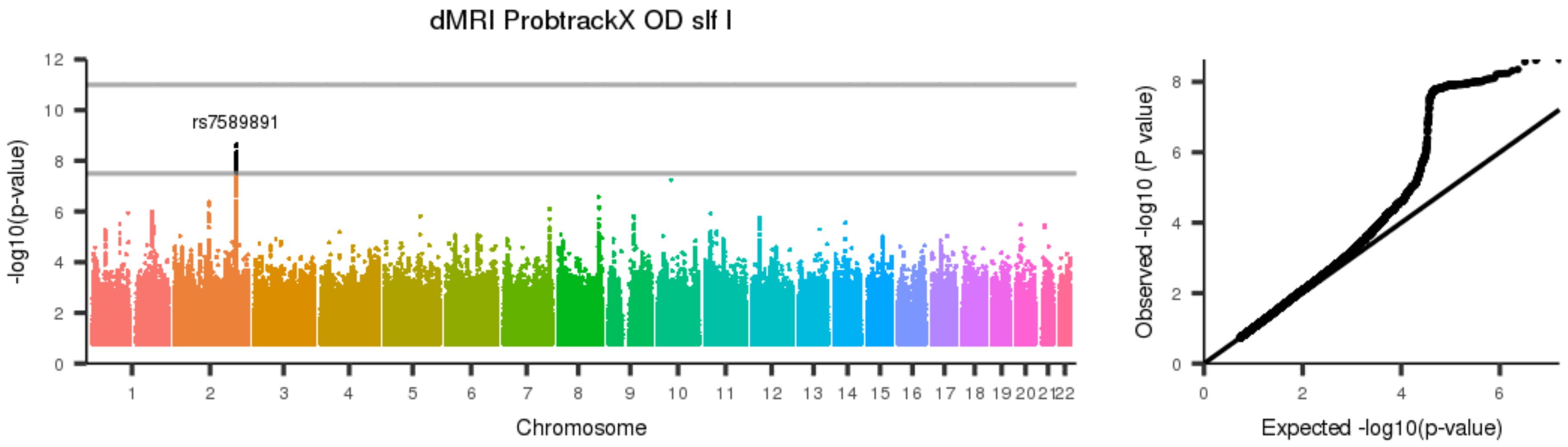

back to list of plots

Figure 839: dMRI ProbtrackX OD slf r

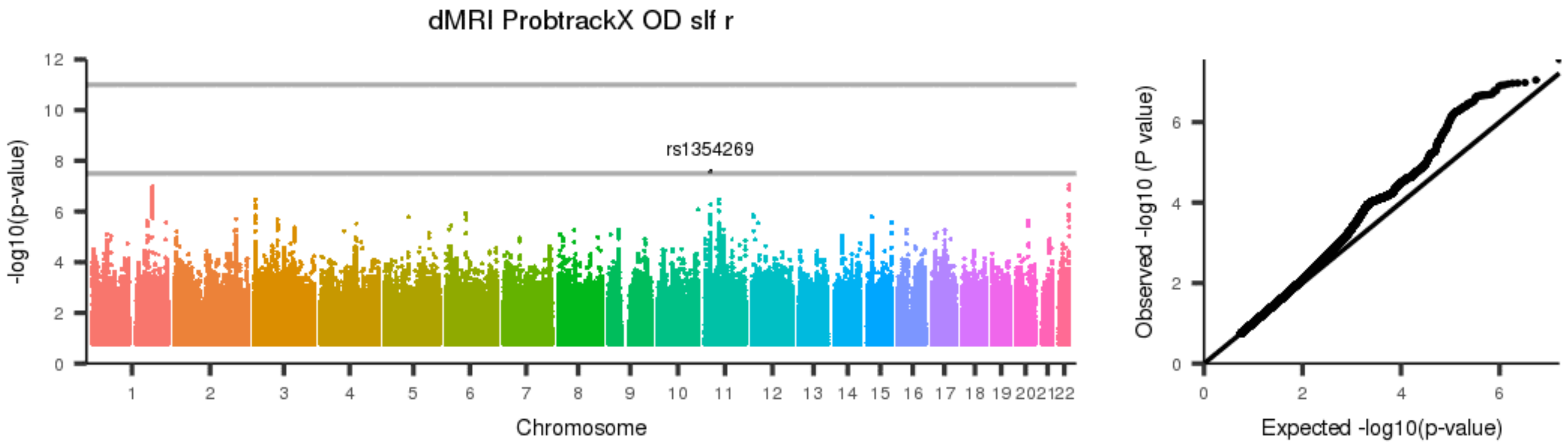

back to list of plots

Figure 840: dMRI ProbtrackX OD str l

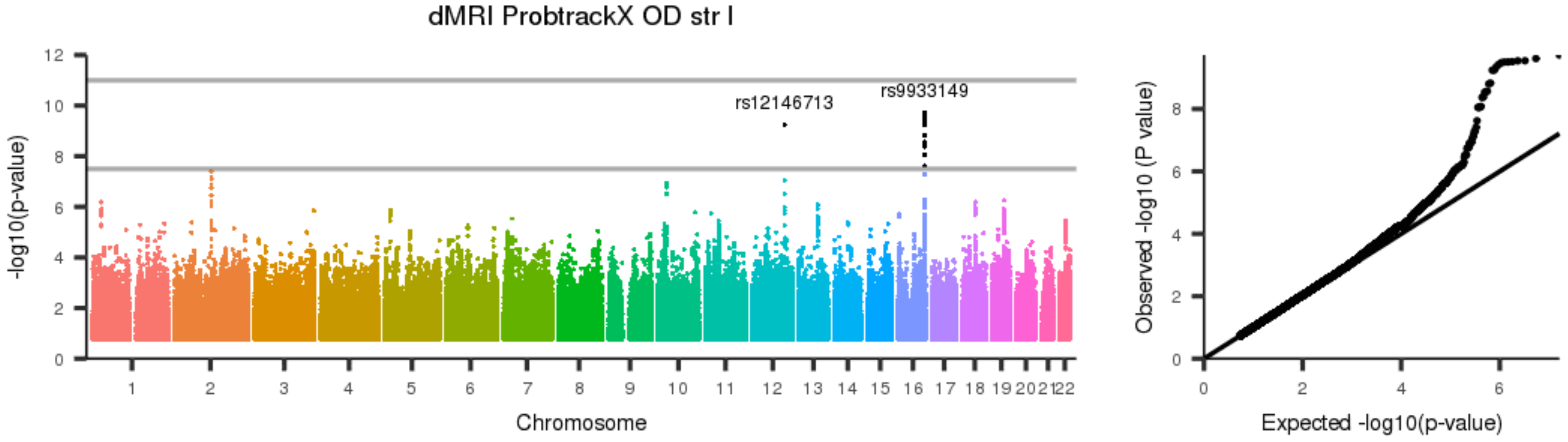

back to list of plots

Figure 841: dMRI ProbtrackX OD str r

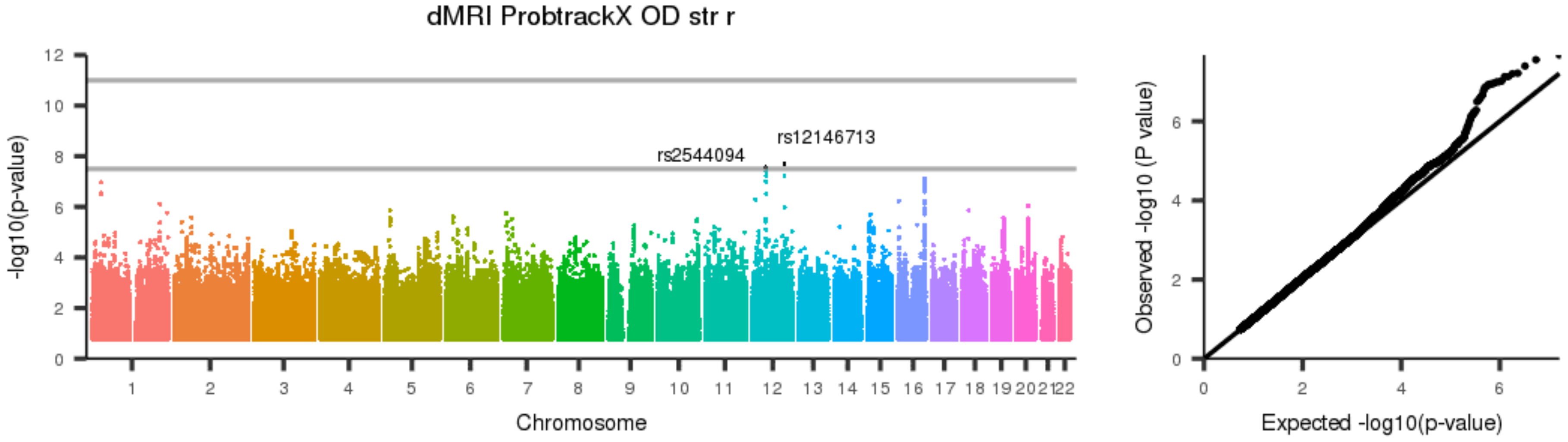

back to list of plots

Figure 842: dMRI ProbtrackX OD unc l

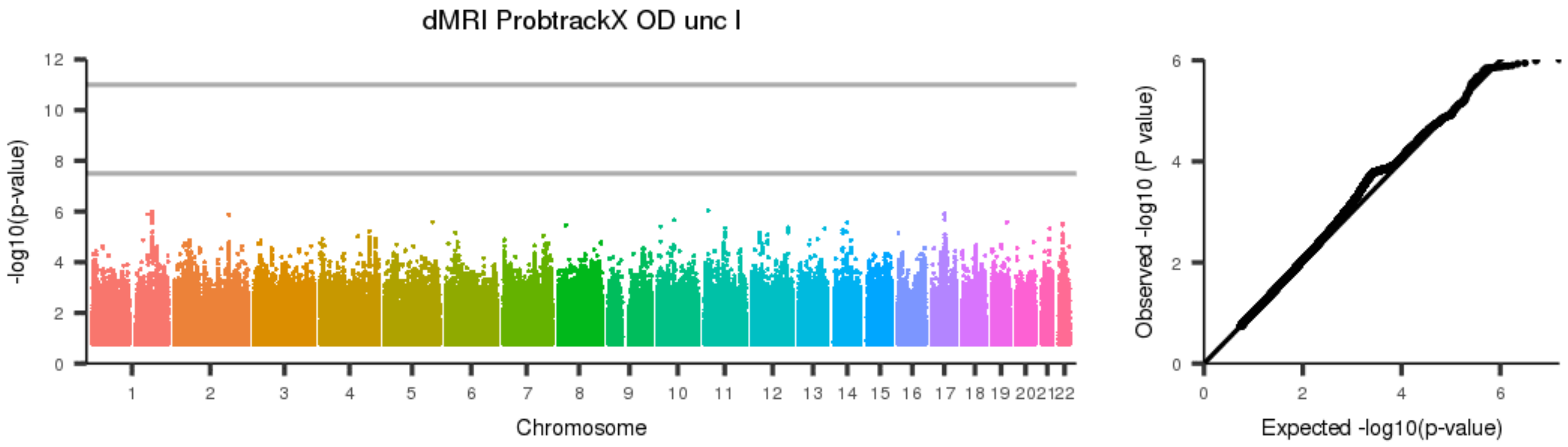

back to list of plots

Figure 843: dMRI ProbtrackX OD unc r

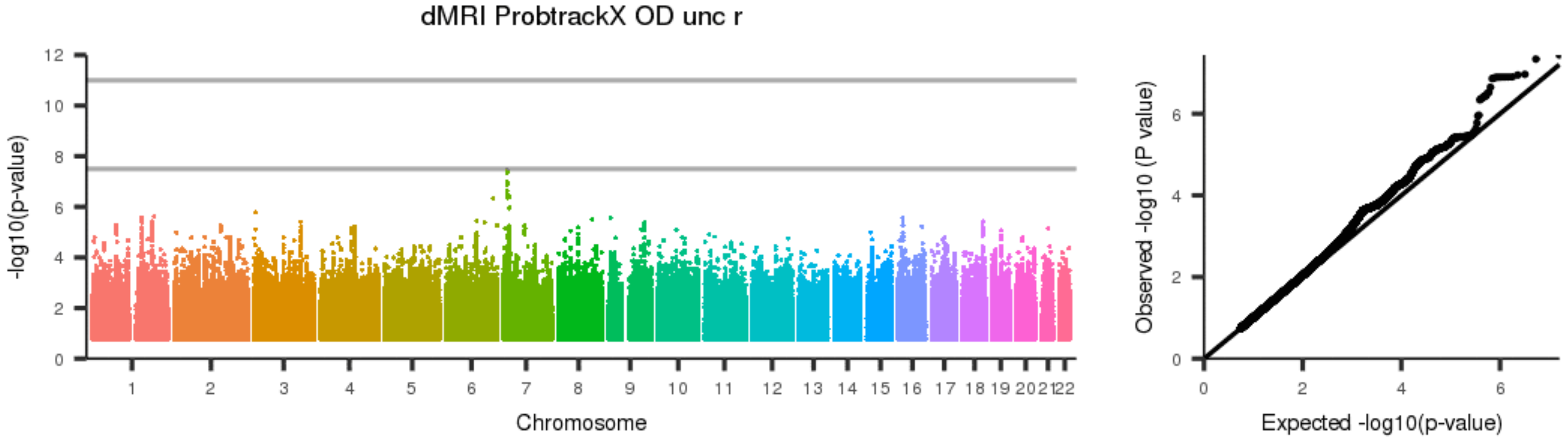

back to list of plots

Figure 844: dMRI ProbtrackX ISOVF ar l

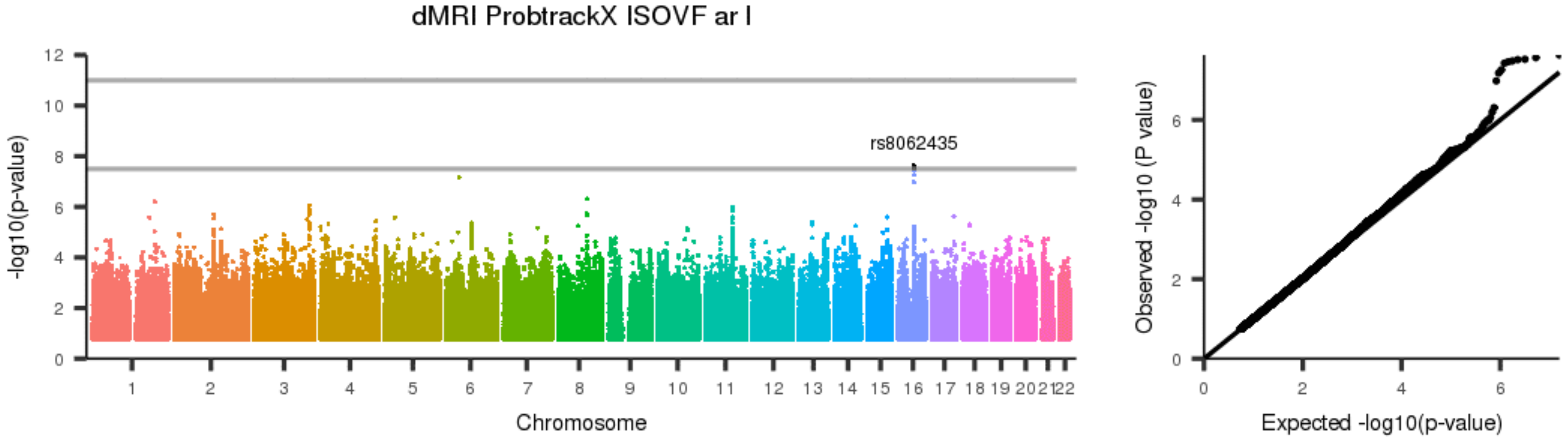

back to list of plots

Figure 845: dMRI ProbtrackX ISOVF ar r

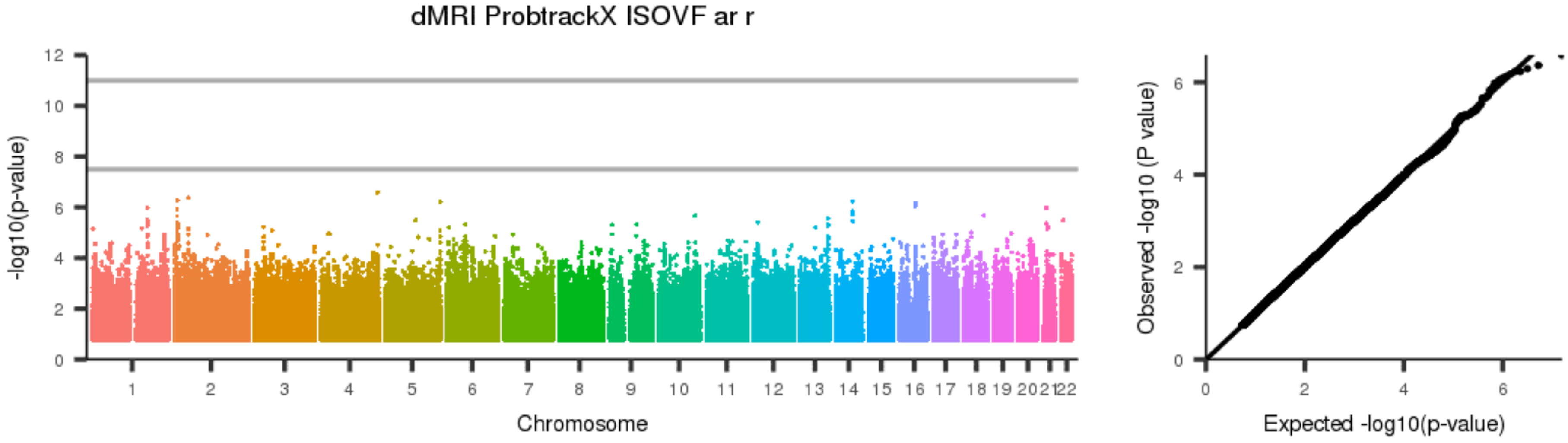

back to list of plots

Figure 846: dMRI ProbtrackX ISOVF atr l

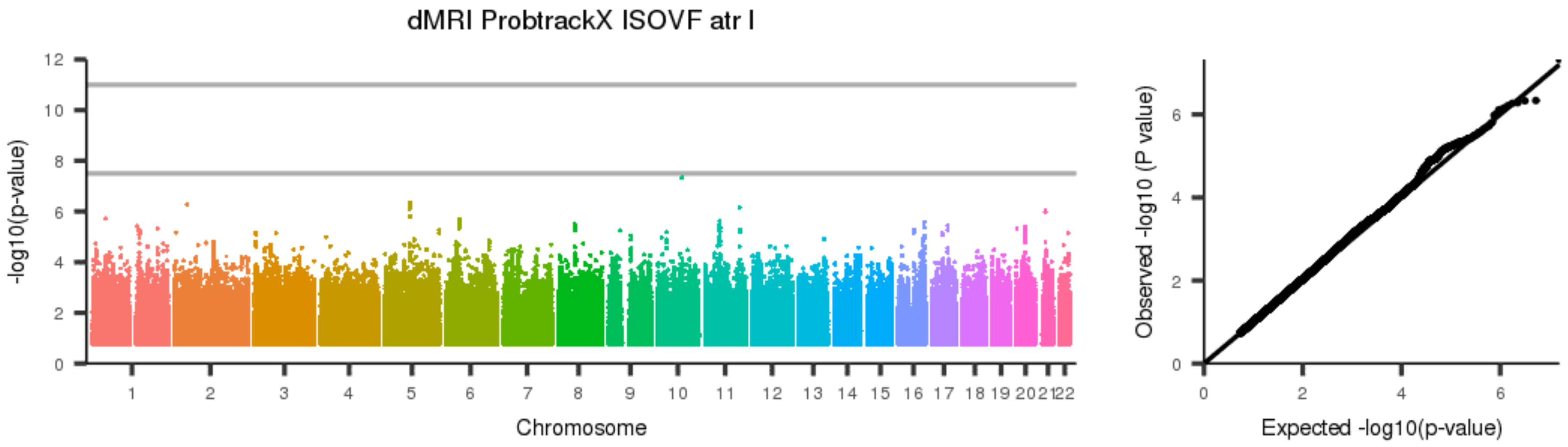

back to list of plots

Figure 847: dMRI ProbtrackX ISOVF atr r

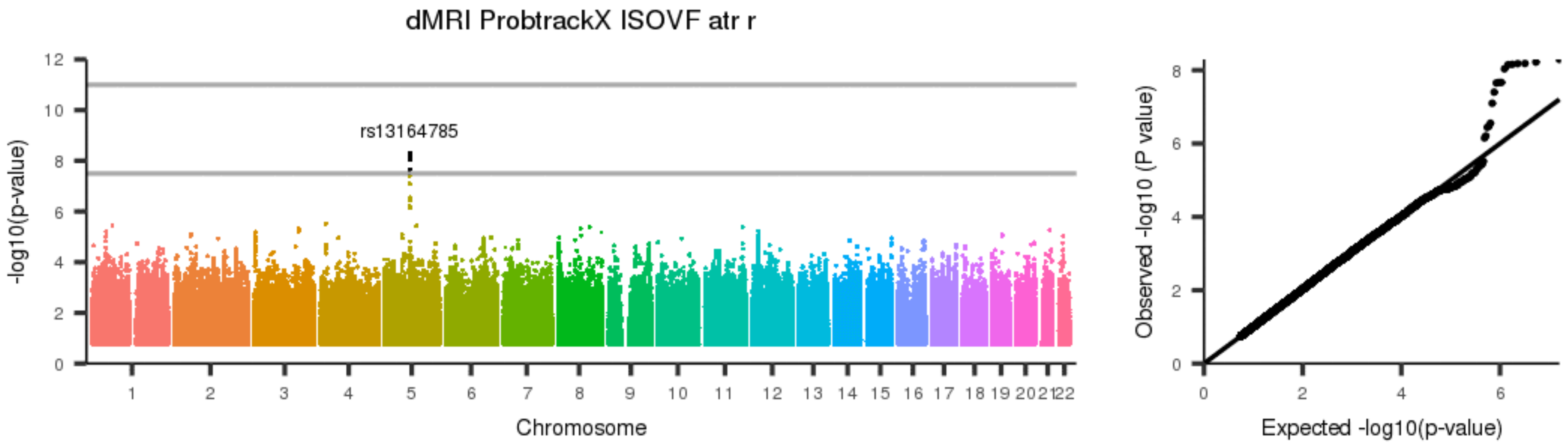

back to list of plots

Figure 848: dMRI ProbtrackX ISOVF cgc l

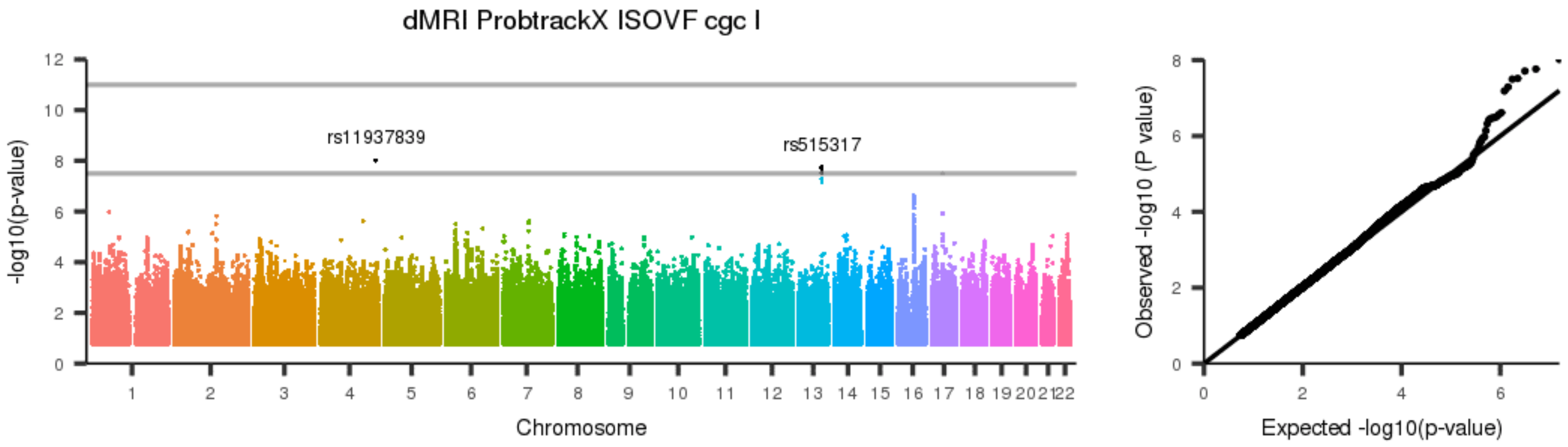

back to list of plots

Figure 849: dMRI ProbtrackX ISOVF cgc r

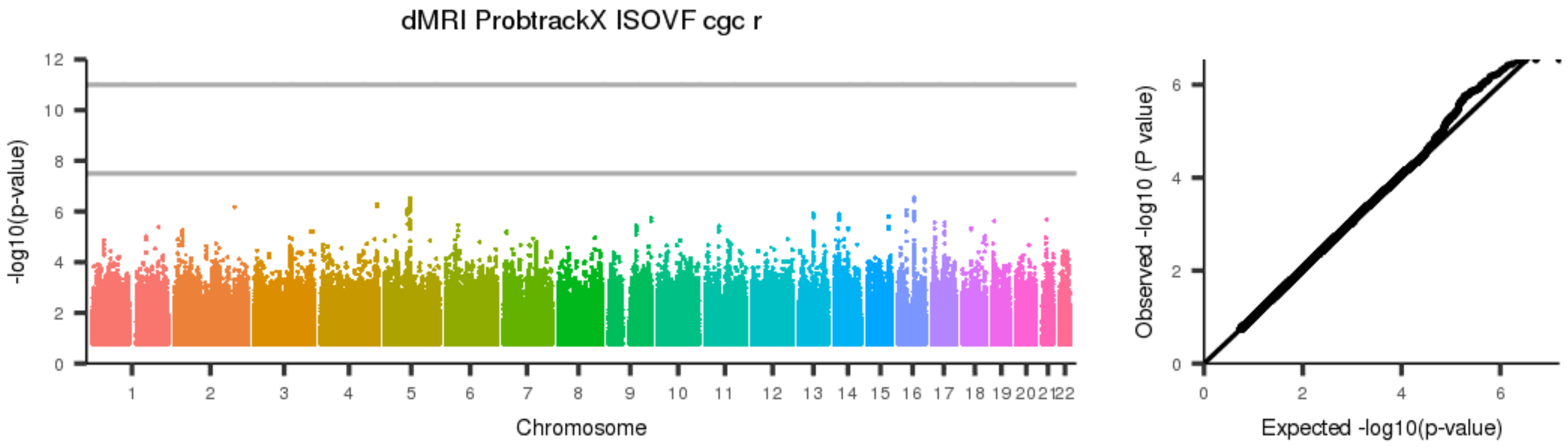

back to list of plots

Figure 850: dMRI ProbtrackX ISOVF cgh l

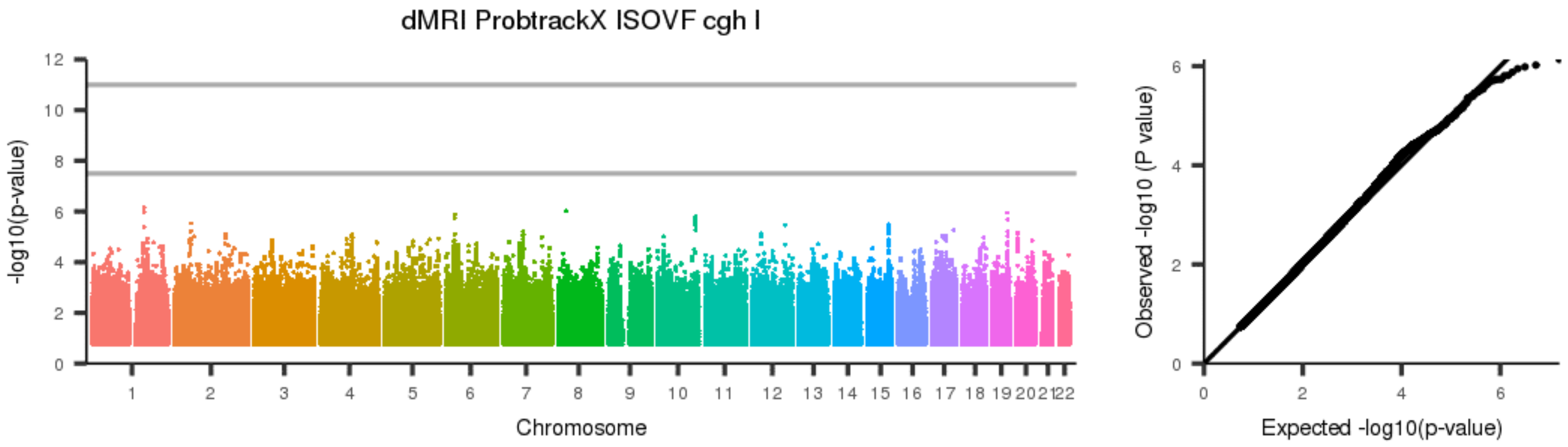

back to list of plots

Figure 851: dMRI ProbtrackX ISOVF cgh r

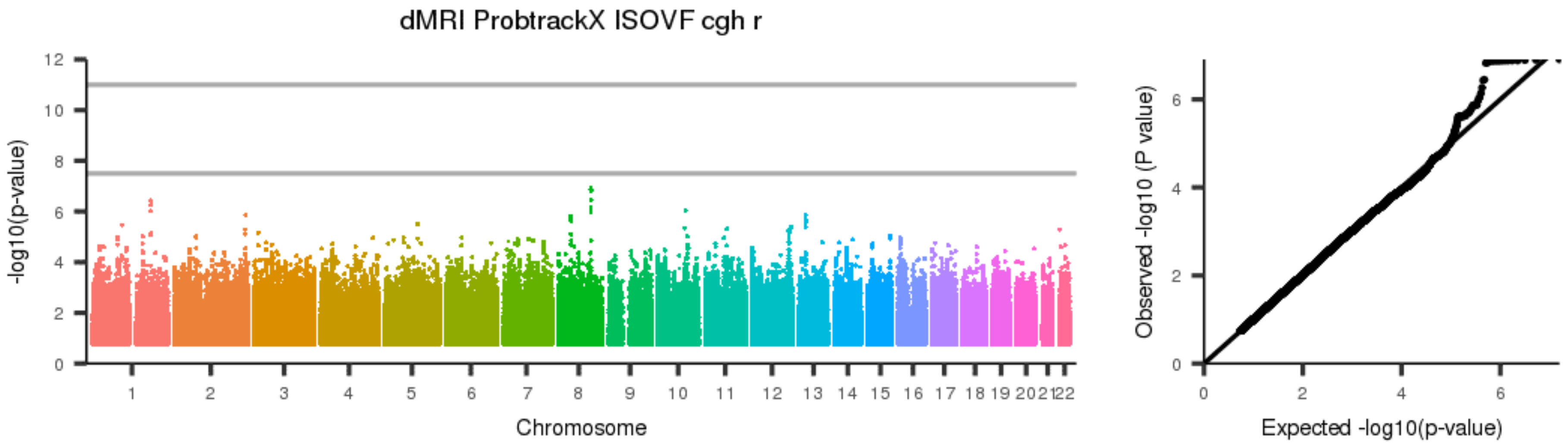

back to list of plots

Figure 852: dMRI ProbtrackX ISOVF cst l

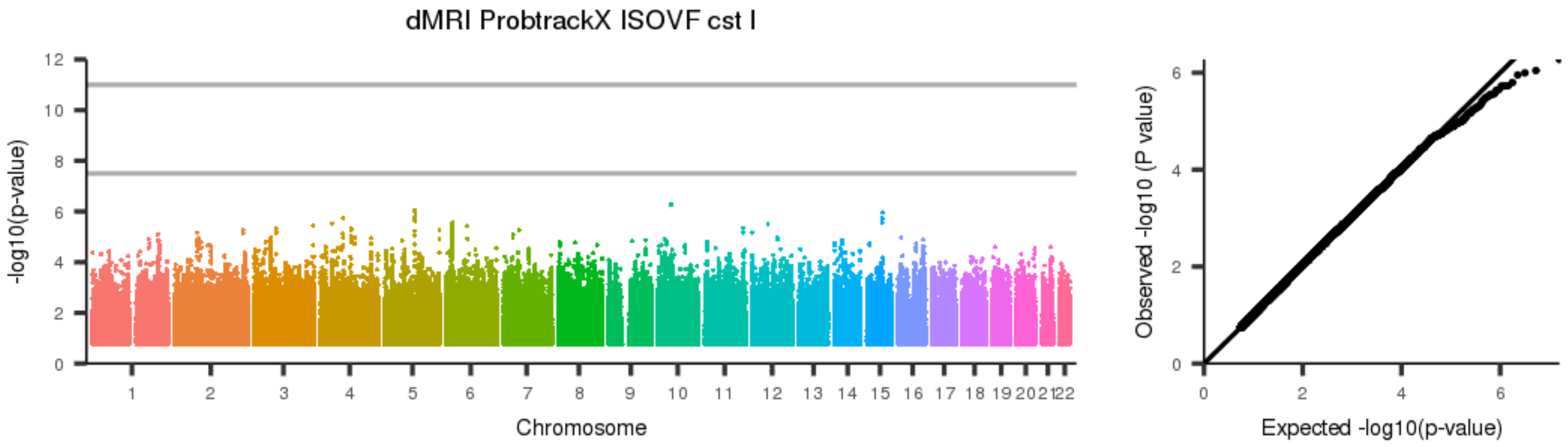

back to list of plots

Figure 853: dMRI ProbtrackX ISOVF cst r

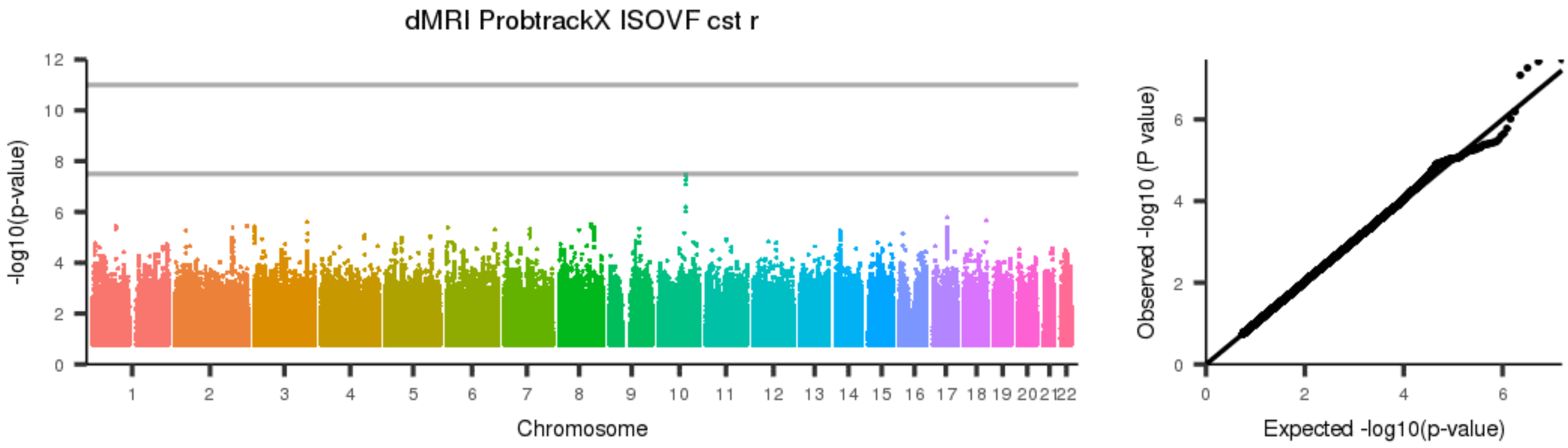

back to list of plots

Figure 854: dMRI ProbtrackX ISOVF fma

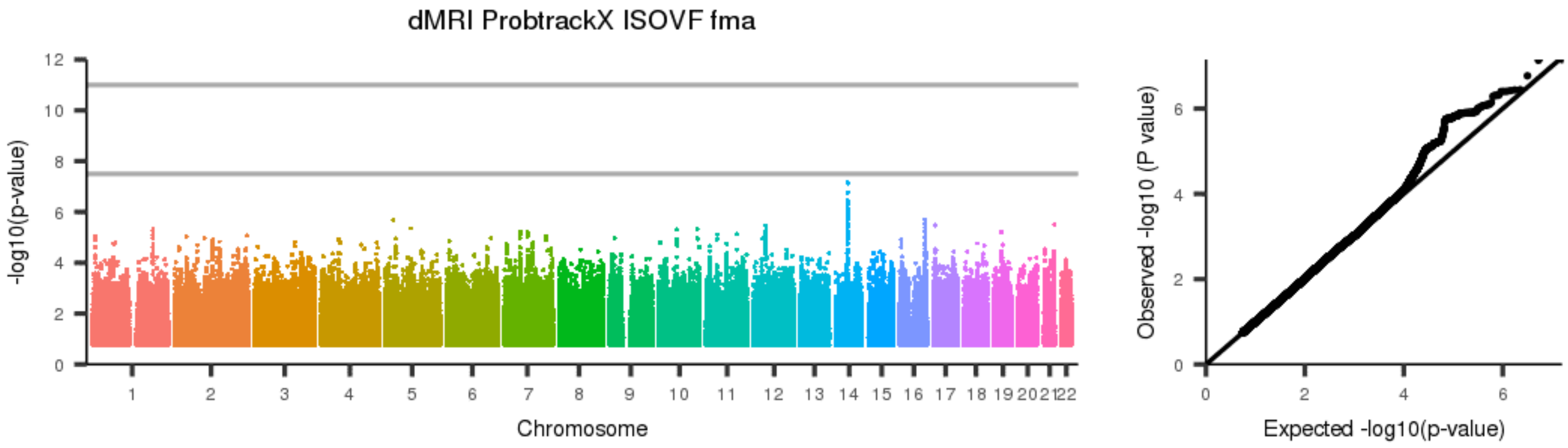

back to list of plots

Figure 855: dMRI ProbtrackX ISOVF fmi

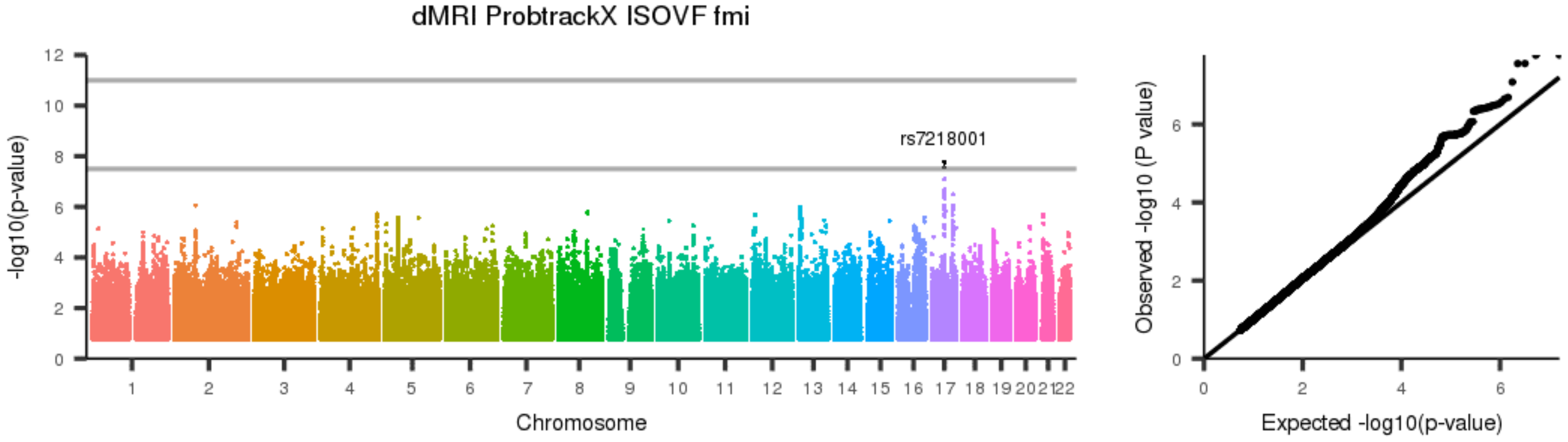

back to list of plots

Figure 856: dMRI ProbtrackX ISOVF ifo l

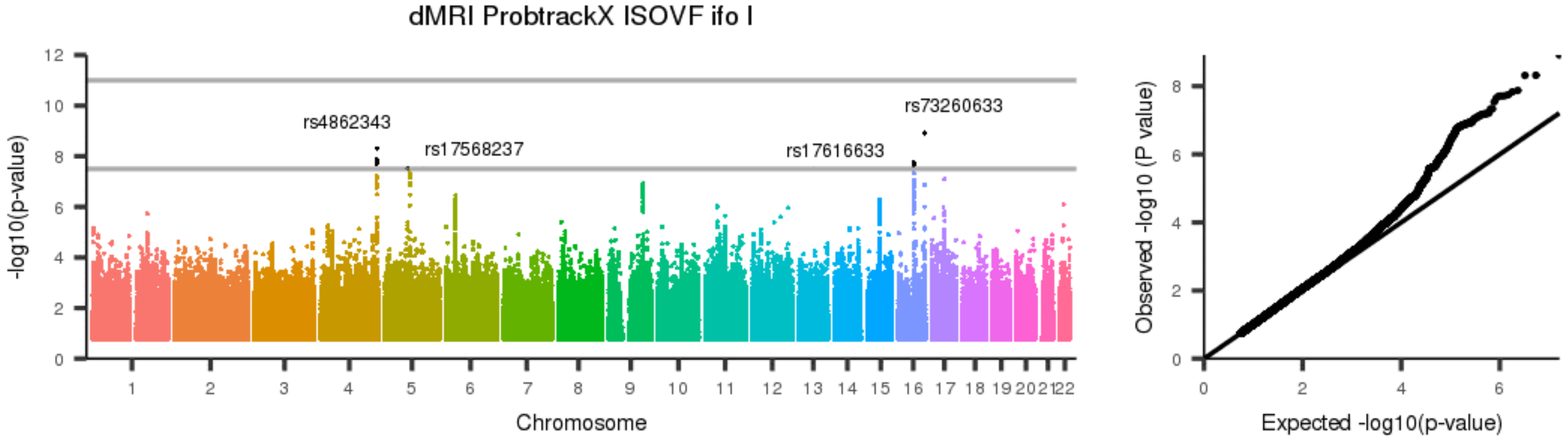

back to list of plots

Figure 857: dMRI ProbtrackX ISOVF ifo r

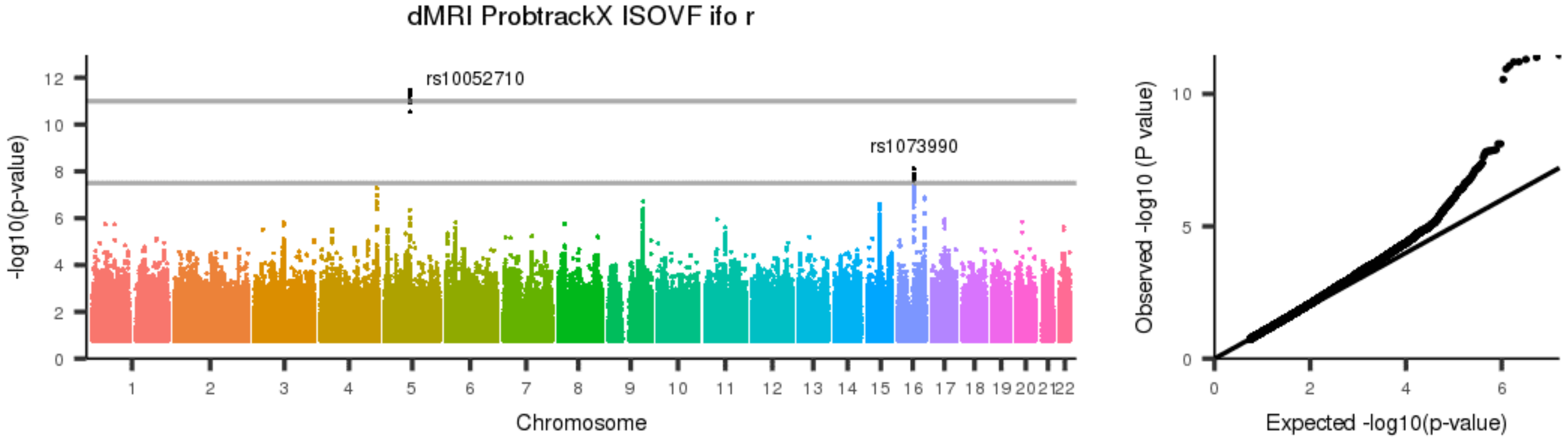

back to list of plots

Figure 858: dMRI ProbtrackX ISOVF ilf l

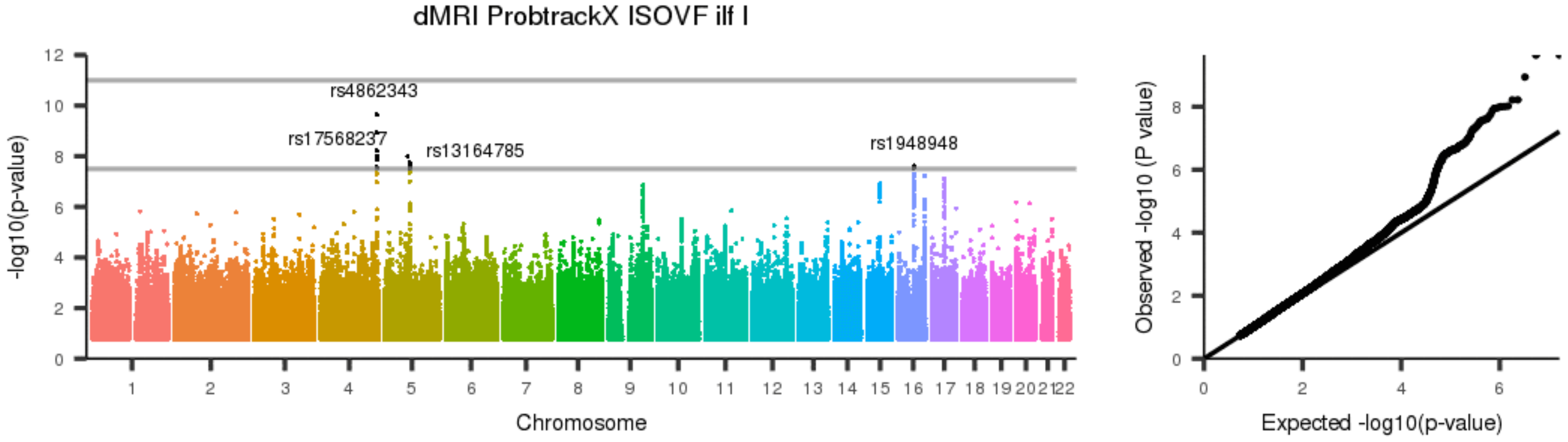

back to list of plots

Figure 859: dMRI ProbtrackX ISOVF ilf r

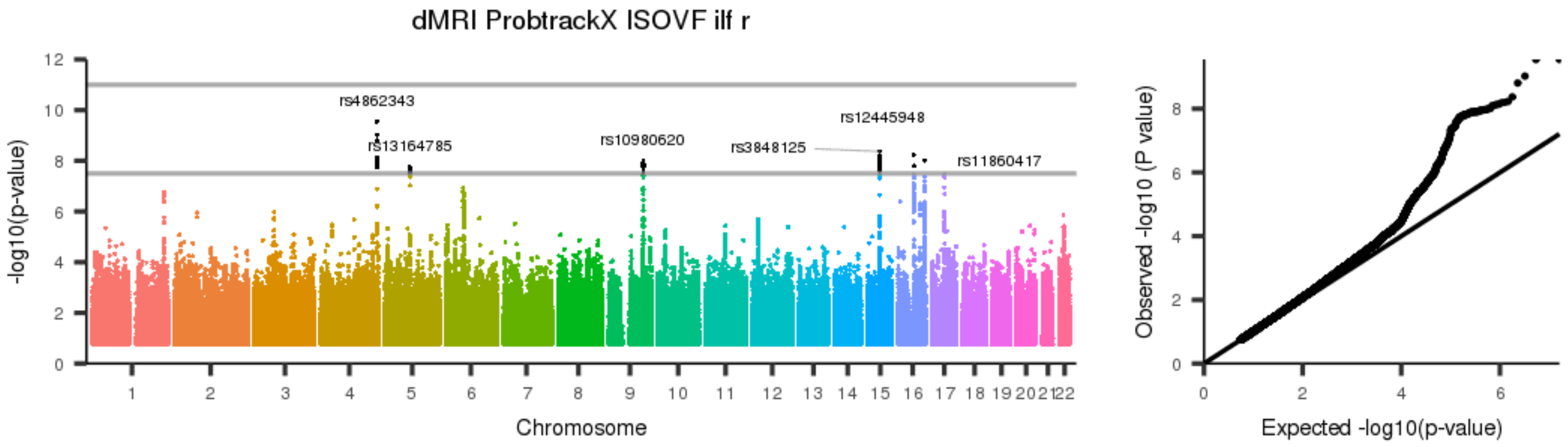

back to list of plots

Figure 860: dMRI ProbtrackX ISOVF mcp

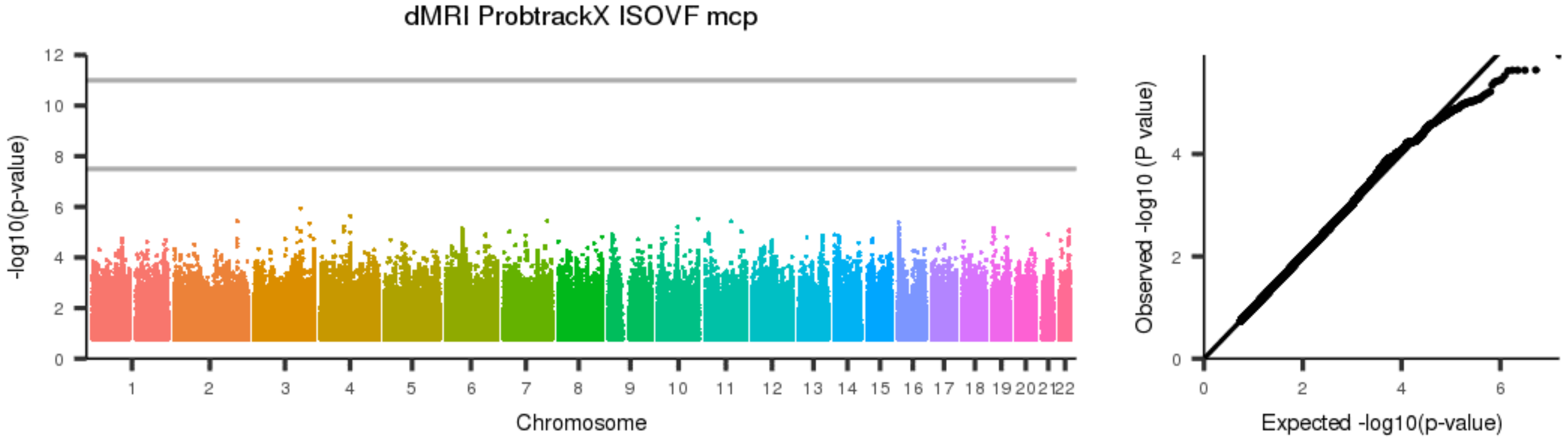

back to list of plots

Figure 861: dMRI ProbtrackX ISOVF ml l

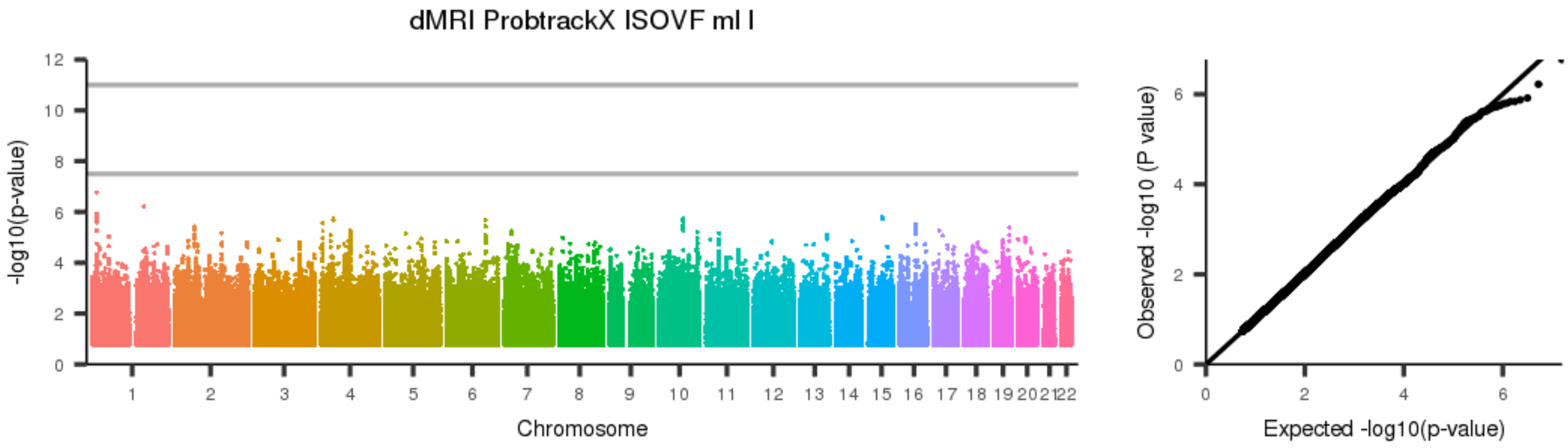

back to list of plots

Figure 862: dMRI ProbtrackX ISOVF ml r

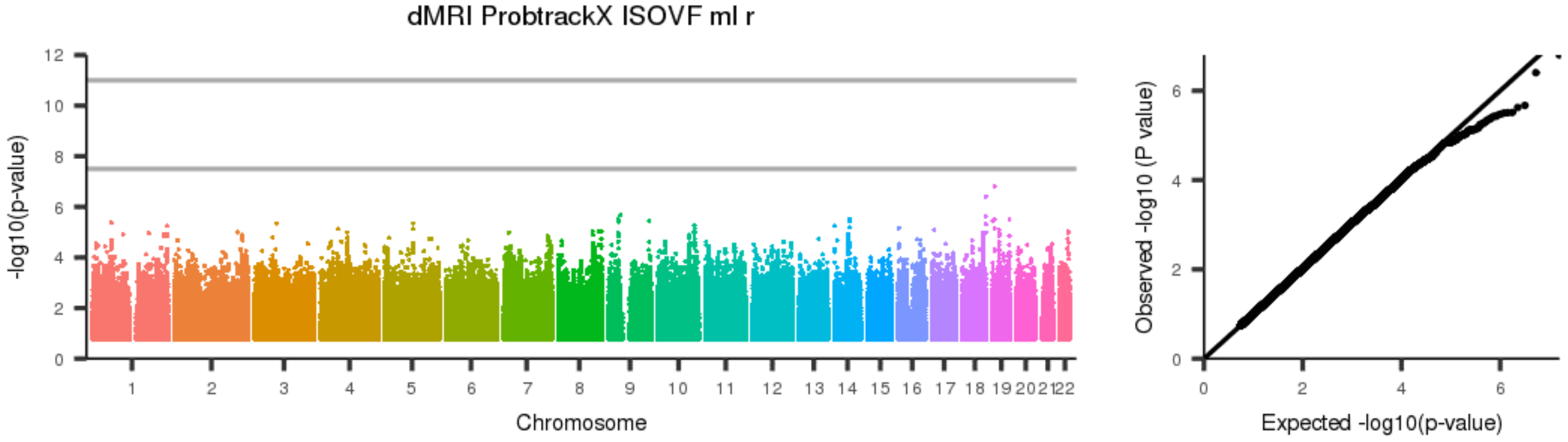

back to list of plots

Figure 863: dMRI ProbtrackX ISOVF ptr l

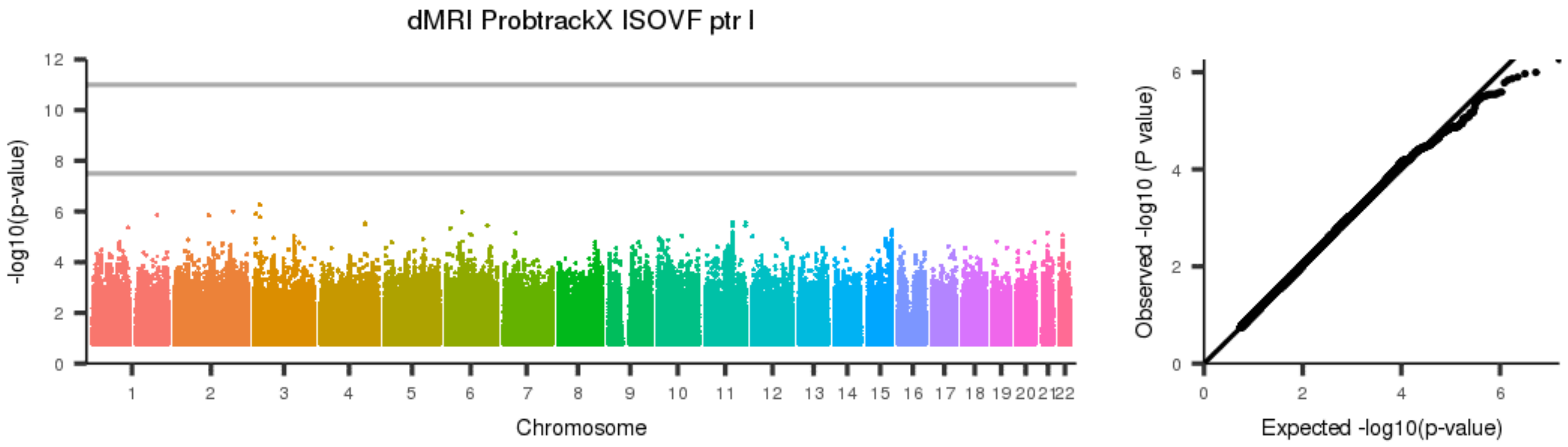

back to list of plots

Figure 864: dMRI ProbtrackX ISOVF ptr r

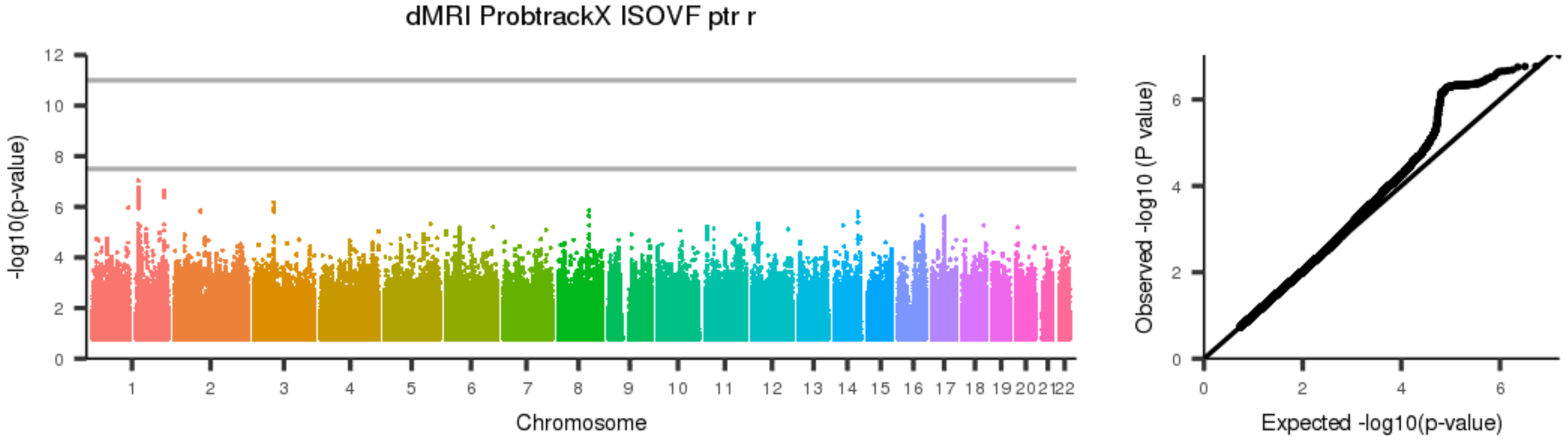

back to list of plots

Figure 865: dMRI ProbtrackX ISOVF slf l

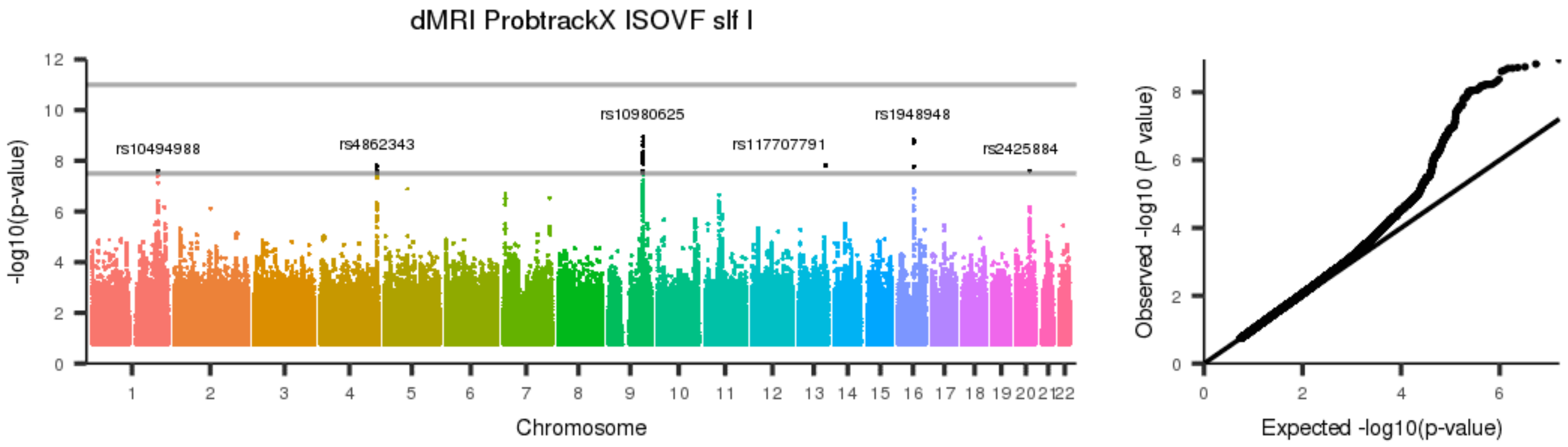

back to list of plots

Figure 866: dMRI ProbtrackX ISOVF slf r

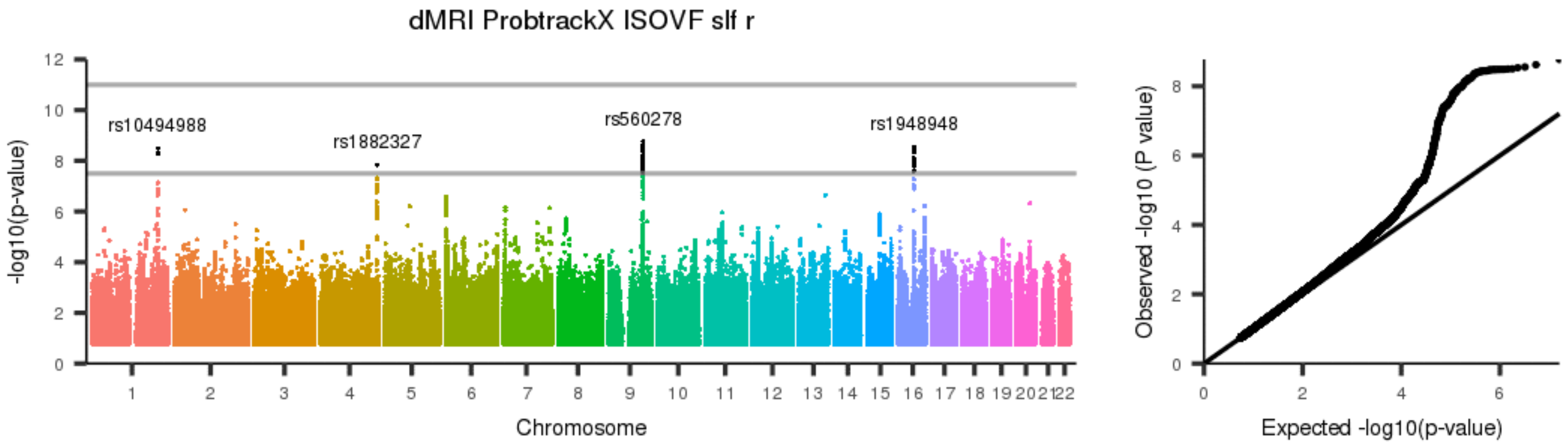

back to list of plots

Figure 867: dMRI ProbtrackX ISOVF str l

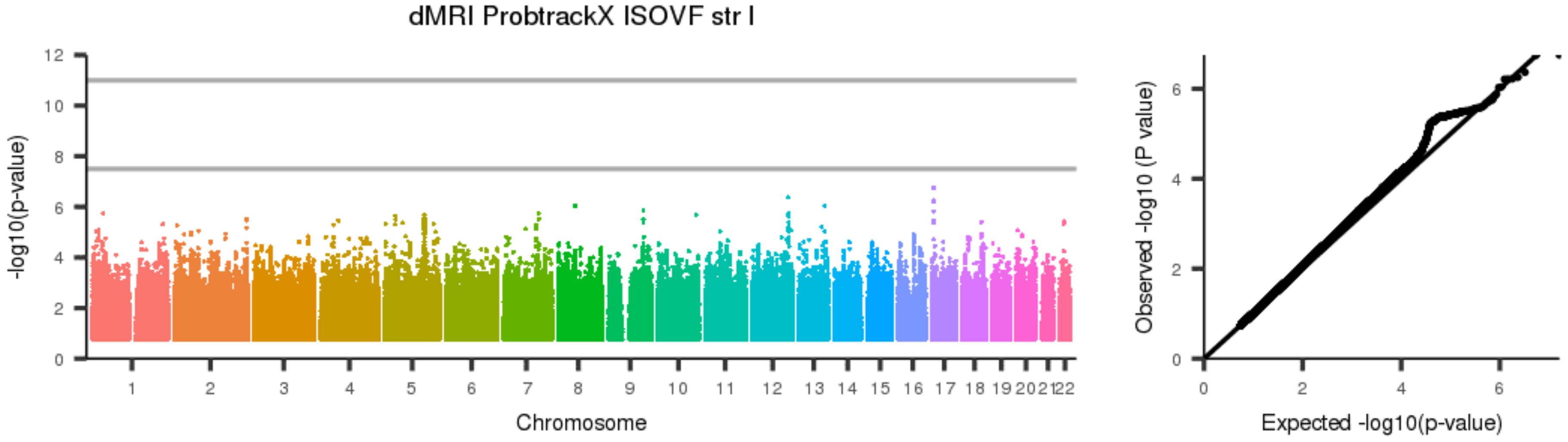

back to list of plots

Figure 868: dMRI ProbtrackX ISOVF str r

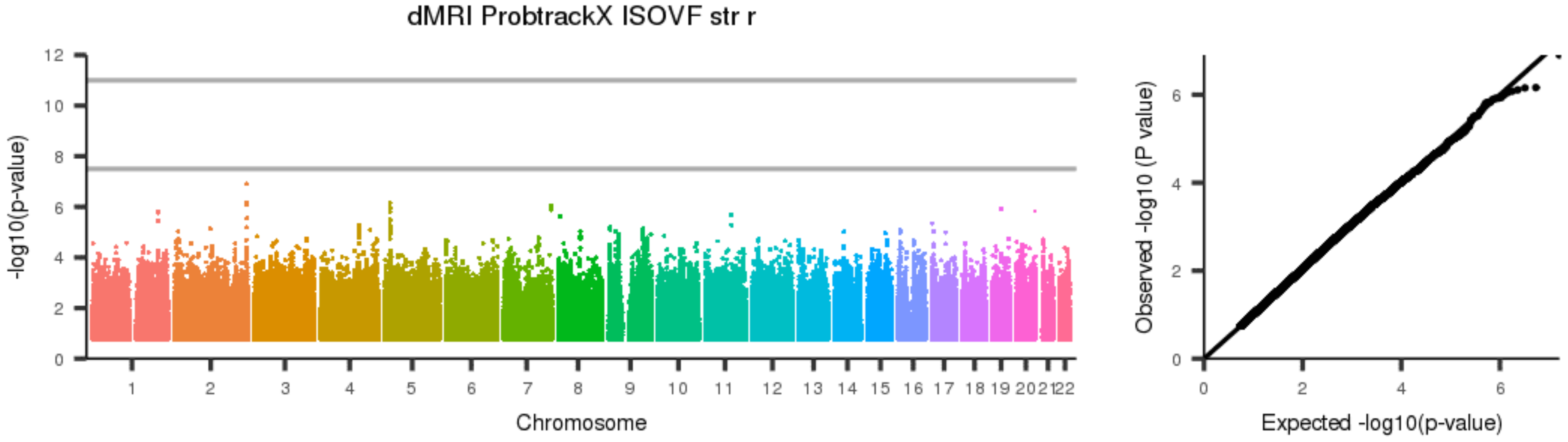

back to list of plots

Figure 869: dMRI ProbtrackX ISOVF unc l

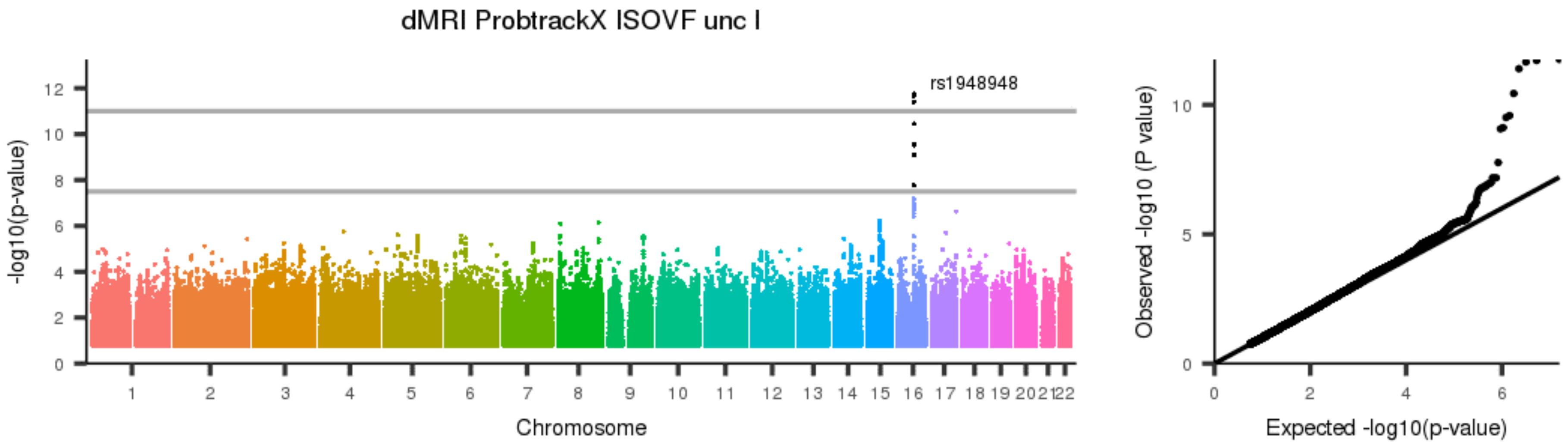

back to list of plots

Figure 870: dMRI ProbtrackX ISOVF unc r

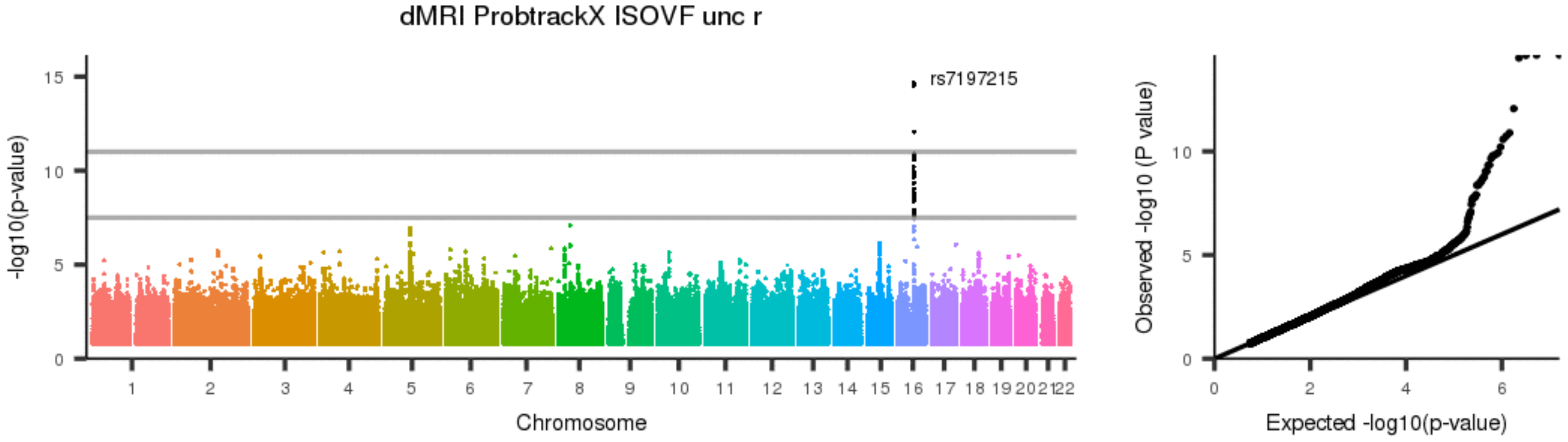

back to list of plots

Figure 871: NETMAT amplitudes (25) 01

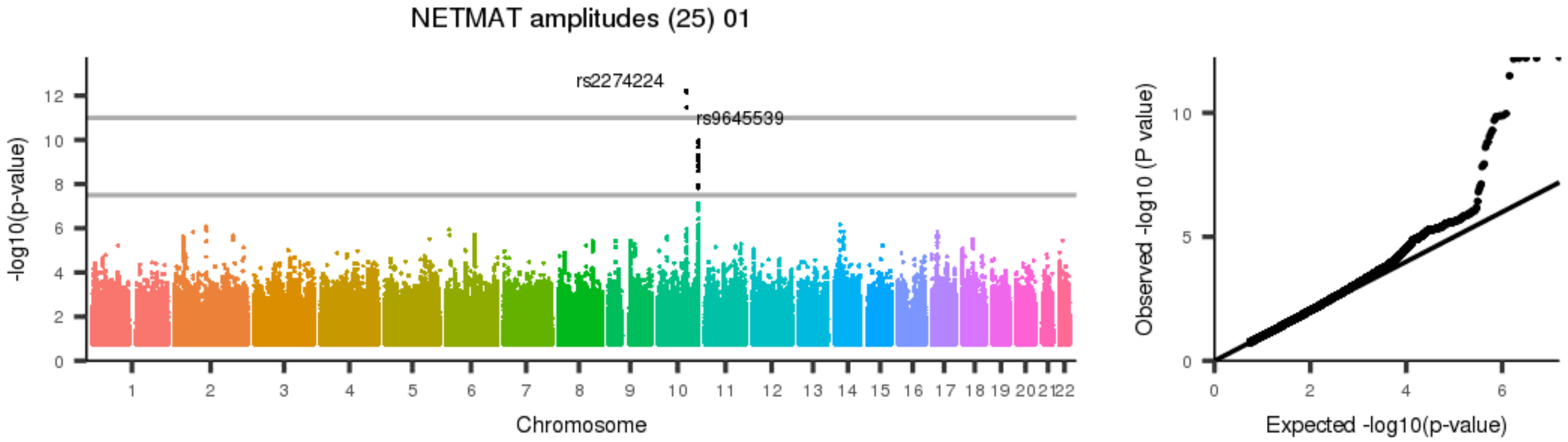

back to list of plots

Figure 872: NETMAT amplitudes (25) 02

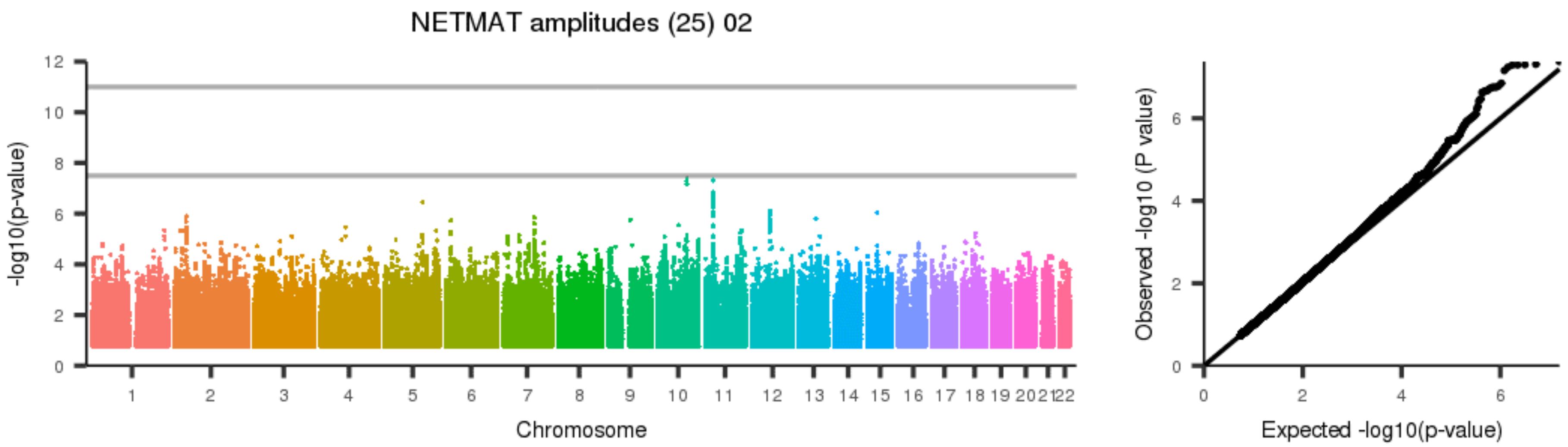

back to list of plots

Figure 873: NETMAT amplitudes (25) 03

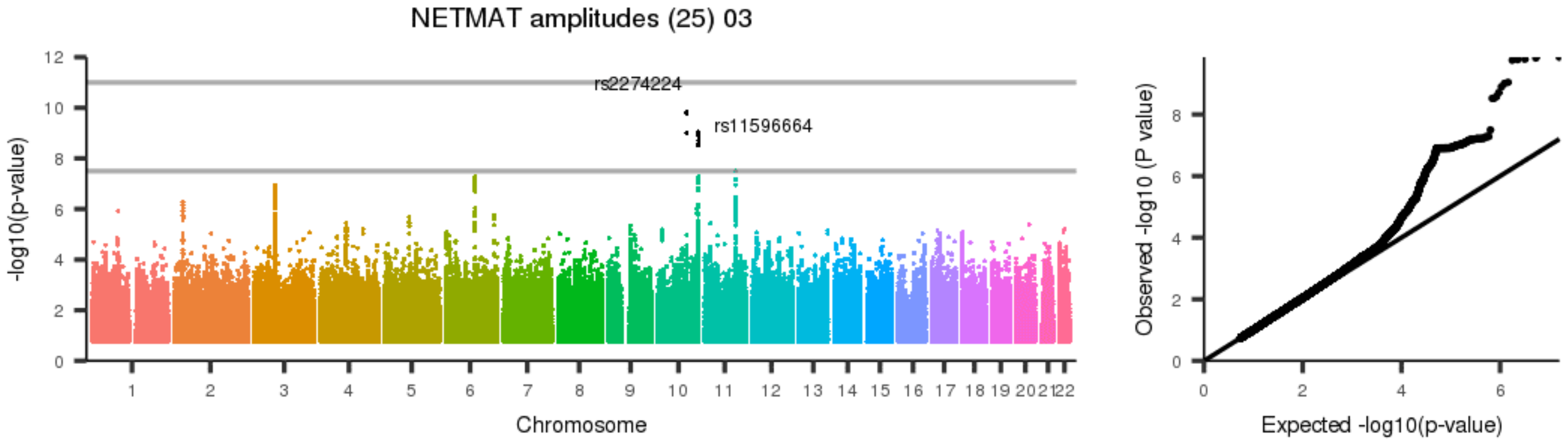

back to list of plots

Figure 874: NETMAT amplitudes (25) 04

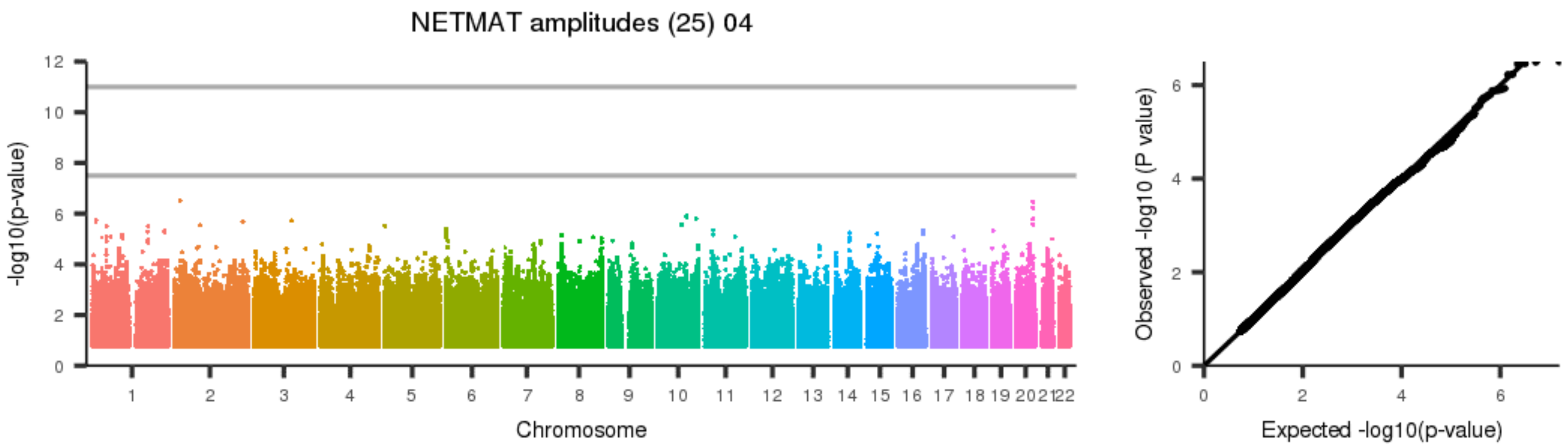

back to list of plots

Figure 875: NETMAT amplitudes (25) 05

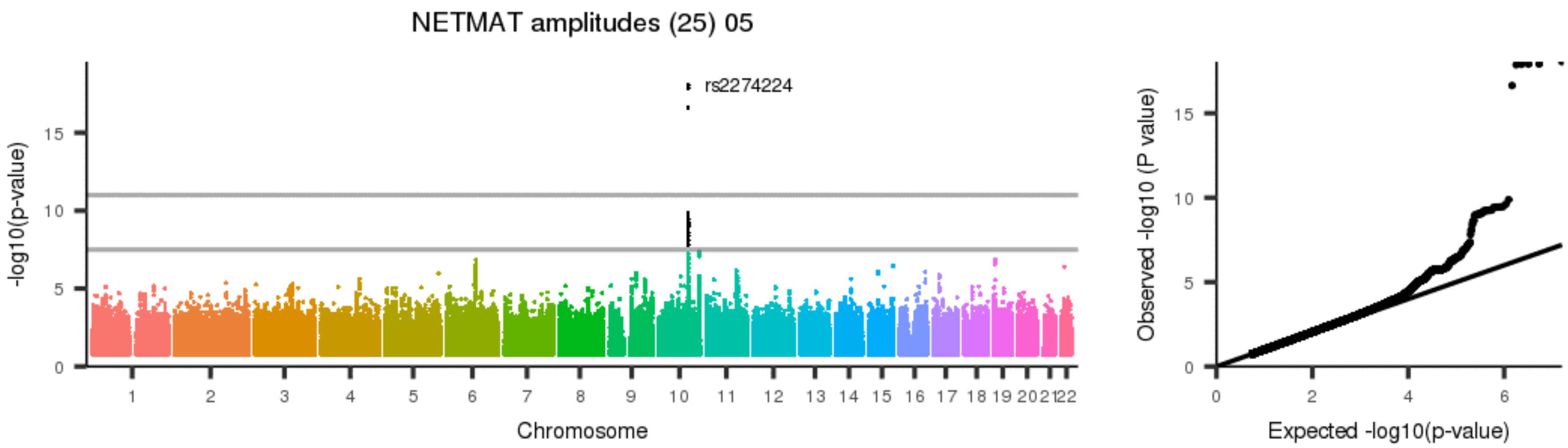

back to list of plots

Figure 876: NETMAT amplitudes (25) 06

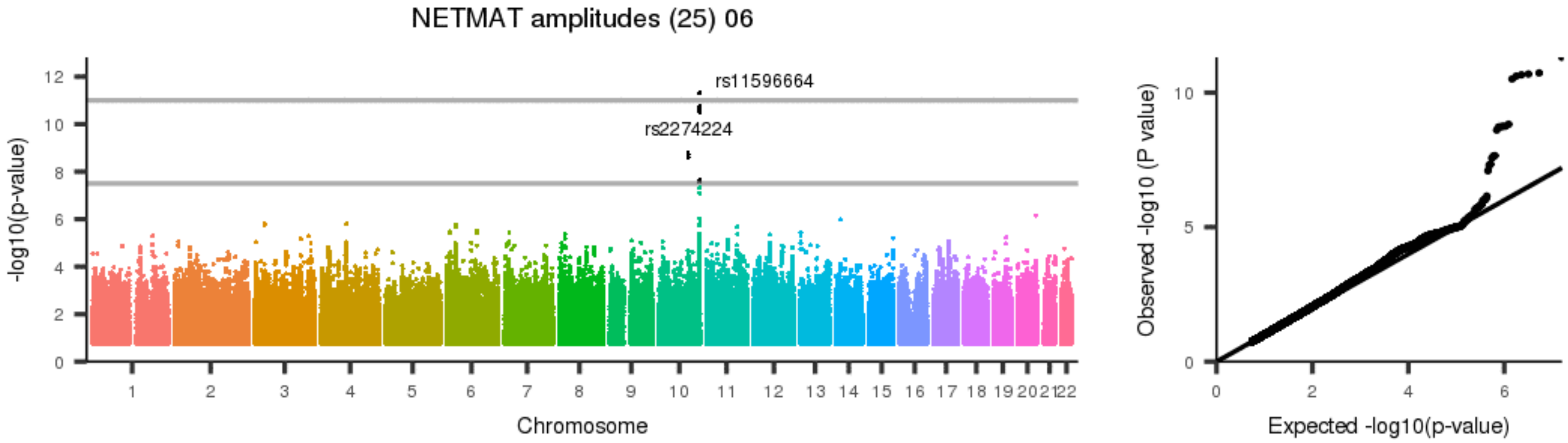

back to list of plots

Figure 877: NETMAT amplitudes (25) 07

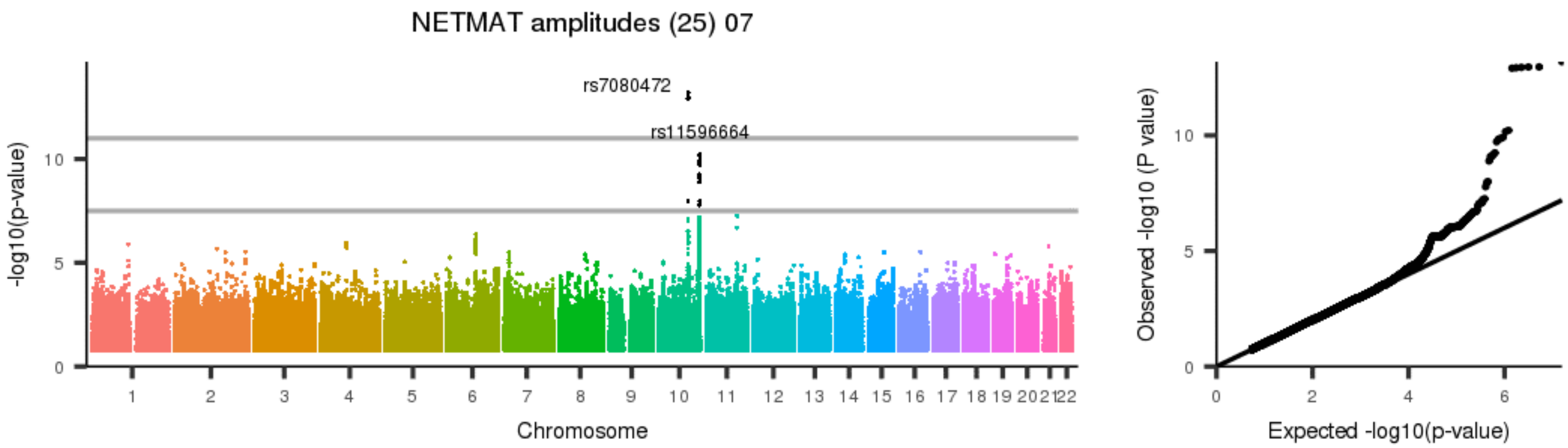

back to list of plots

Figure 878: NETMAT amplitudes (25) 08

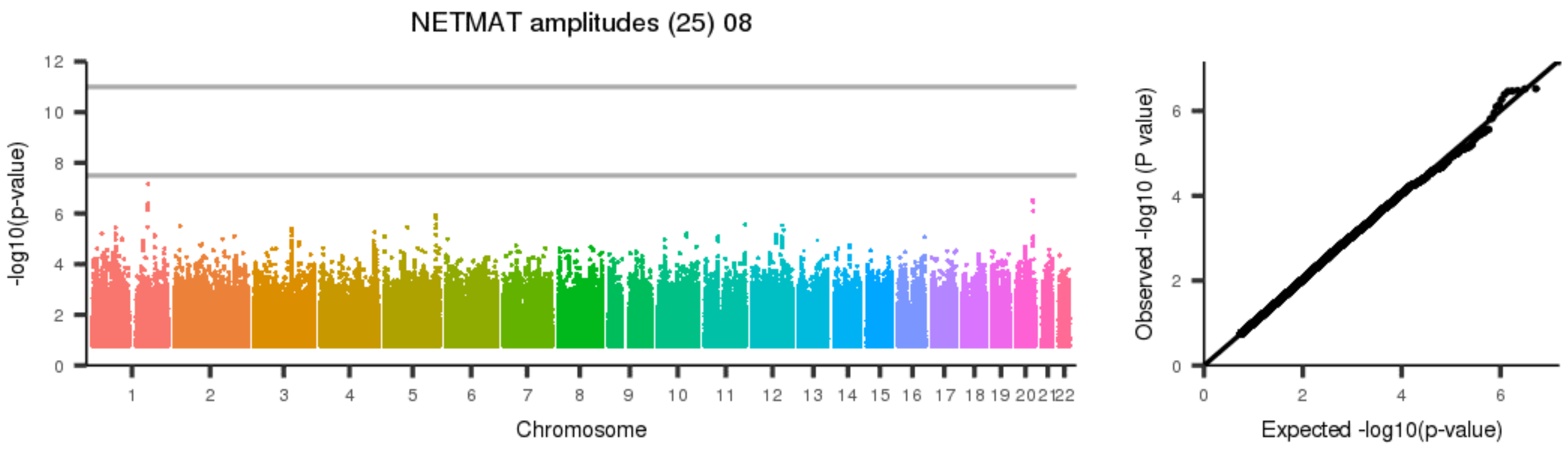

back to list of plots

Figure 879: NETMAT amplitudes (25) 09

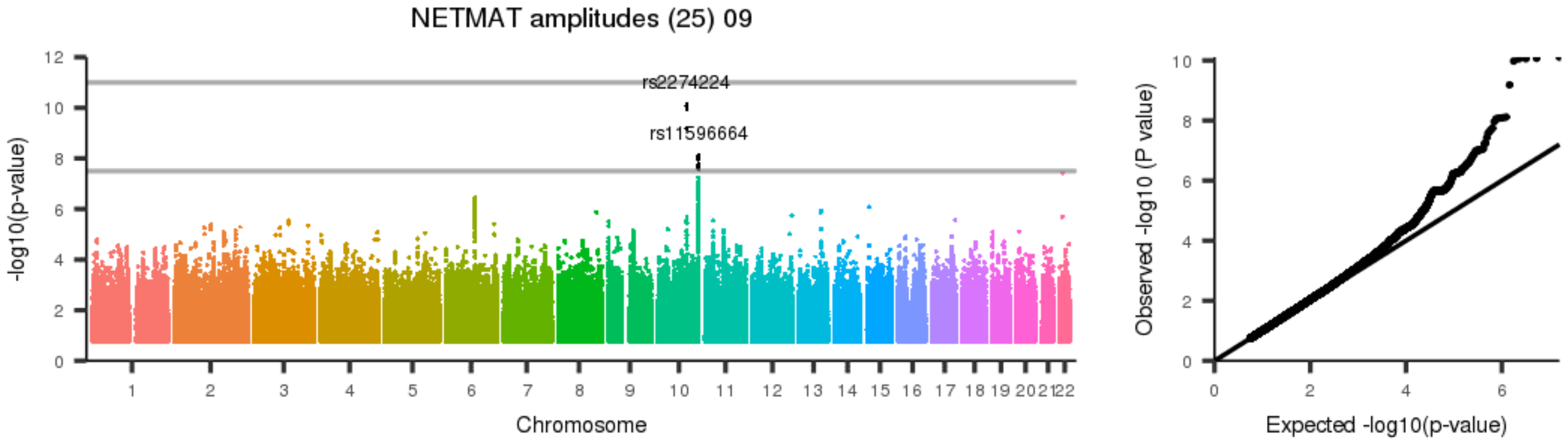

back to list of plots

Figure 880: NETMAT amplitudes (25) 10

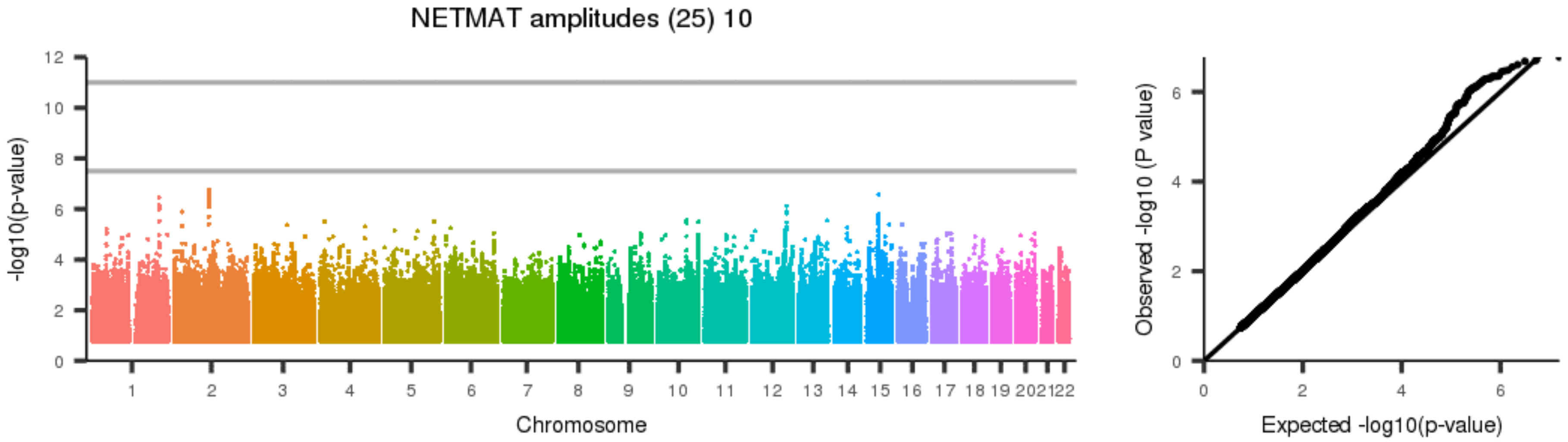

back to list of plots

Figure 881: NETMAT amplitudes (25) 11

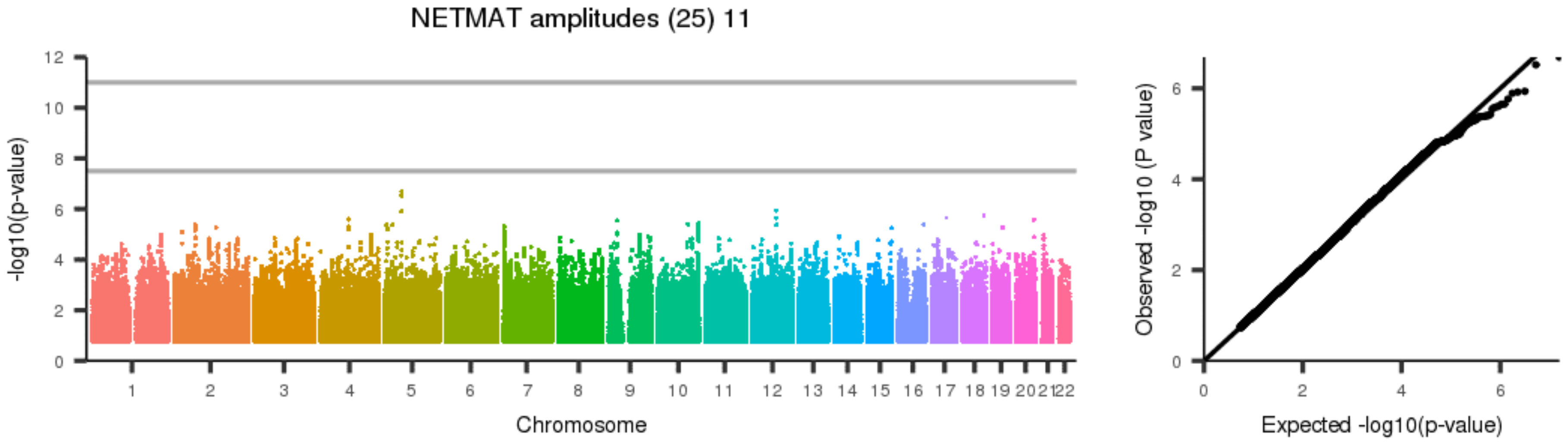

back to list of plots

Figure 882: NETMAT amplitudes (25) 12

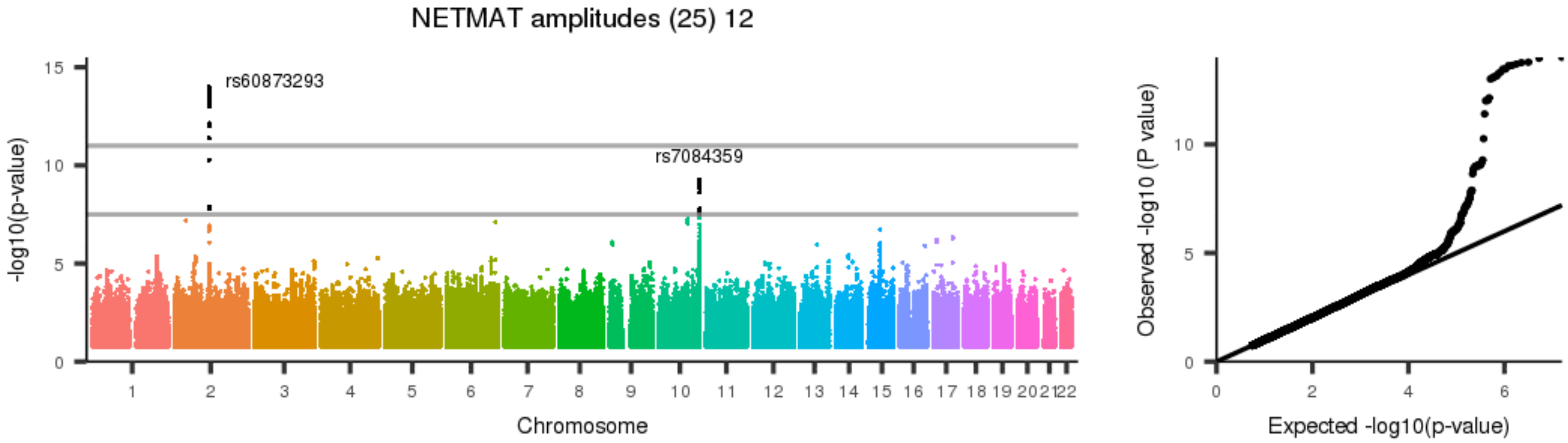

back to list of plots

Figure 883: NETMAT amplitudes (25) 13

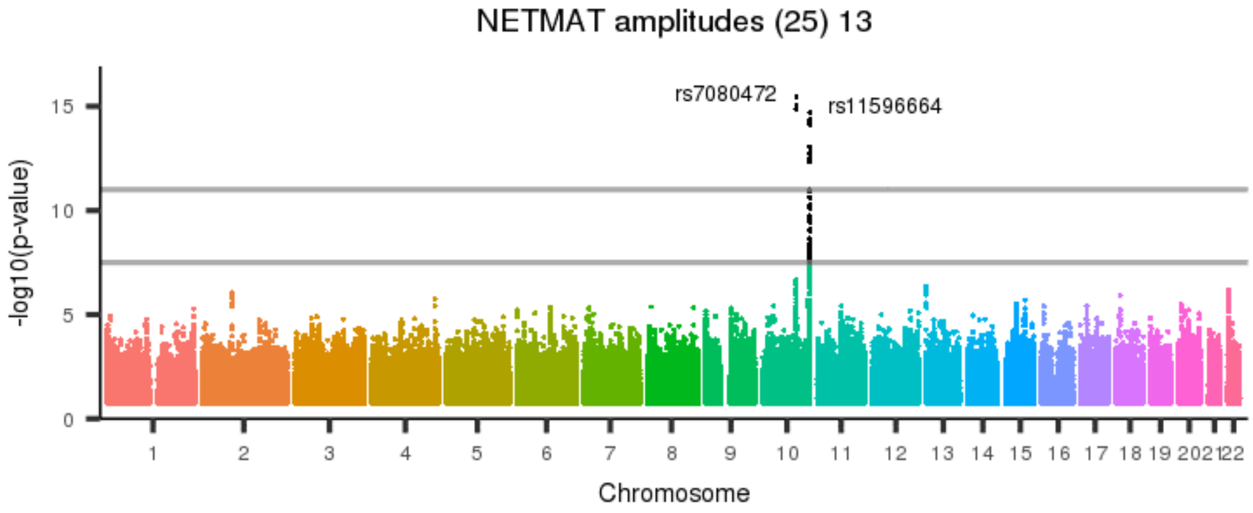

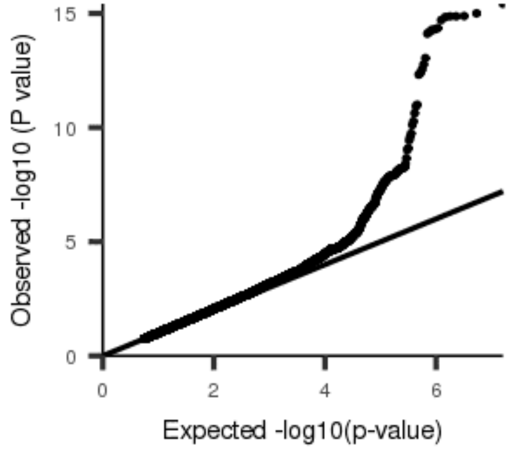

back to list of plots

Figure 884: NETMAT amplitudes (25) 14

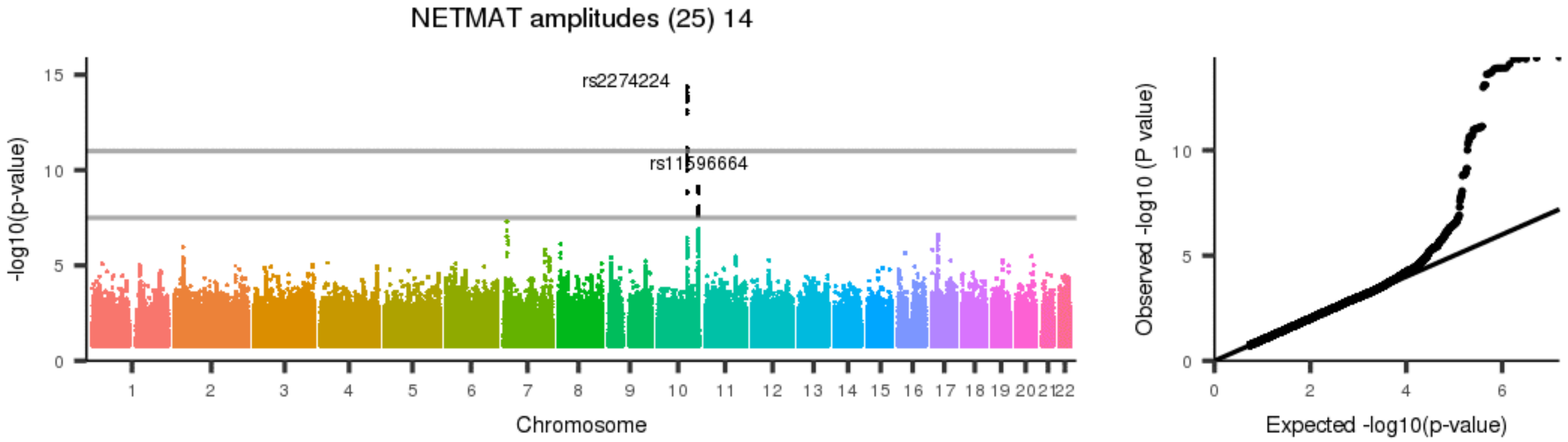

back to list of plots

Figure 885: NETMAT amplitudes (25) 15

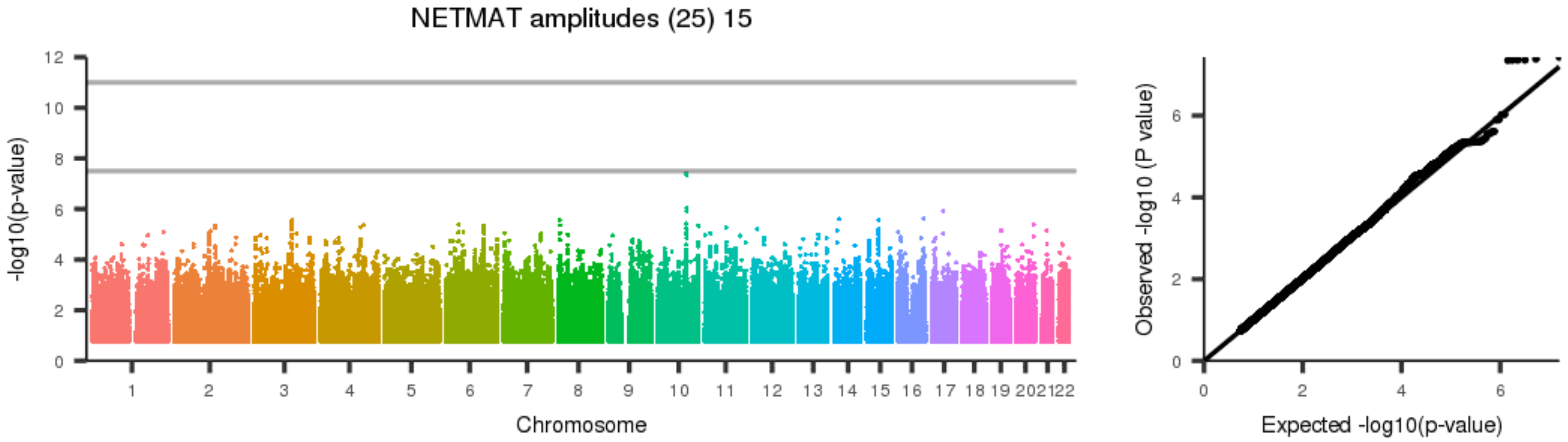

back to list of plots

Figure 886: NETMAT amplitudes (25) 16

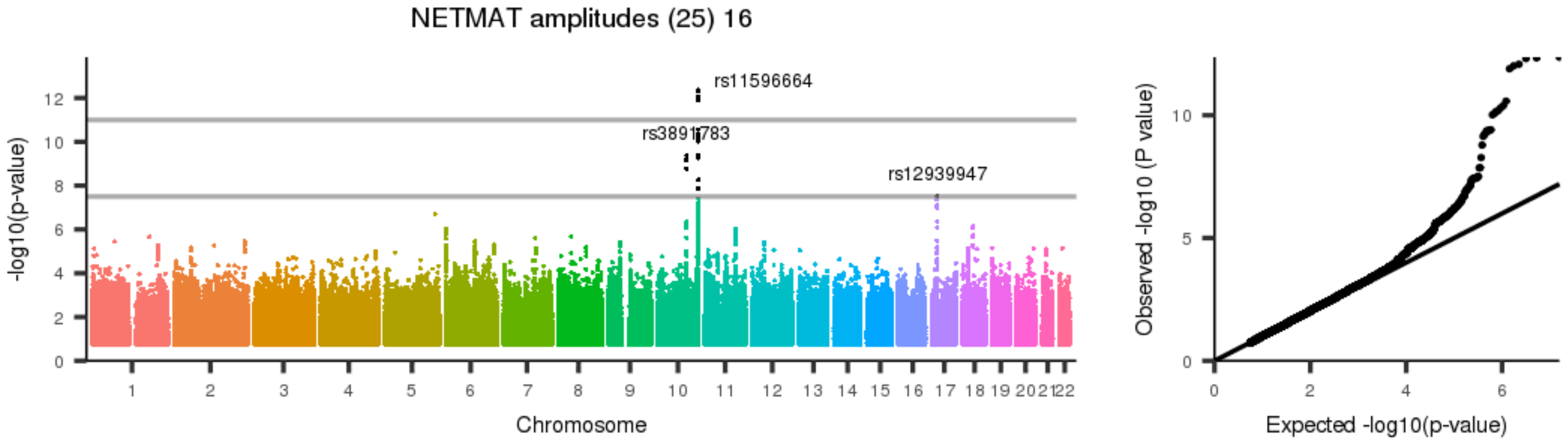

back to list of plots

Figure 887: NETMAT amplitudes (25) 17

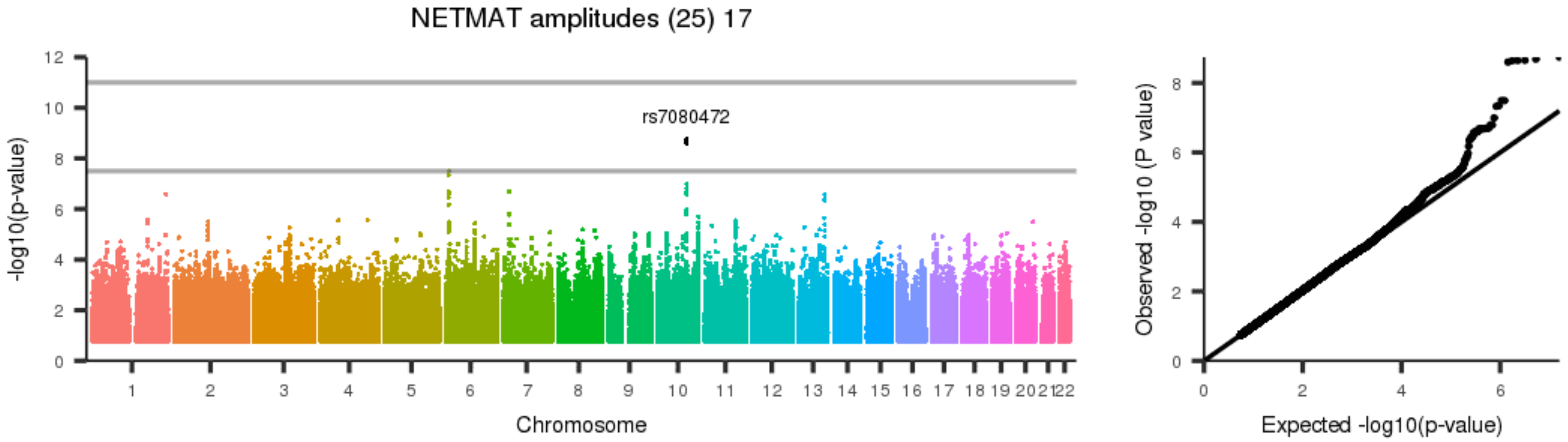

back to list of plots

Figure 888: NETMAT amplitudes (25) 18

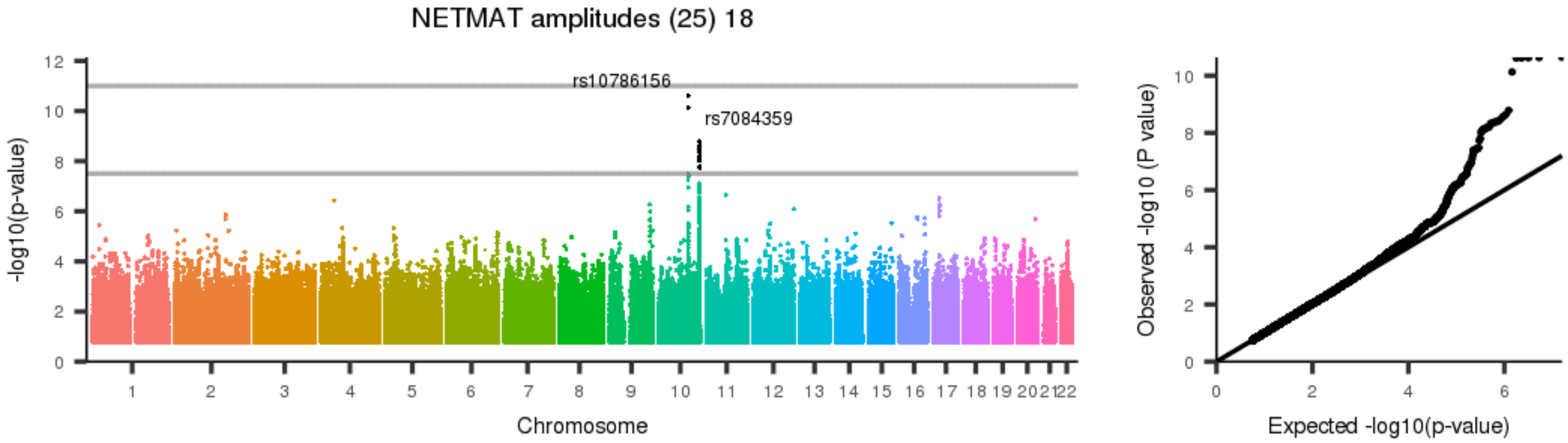

back to list of plots

Figure 889: NETMAT amplitudes (25) 19

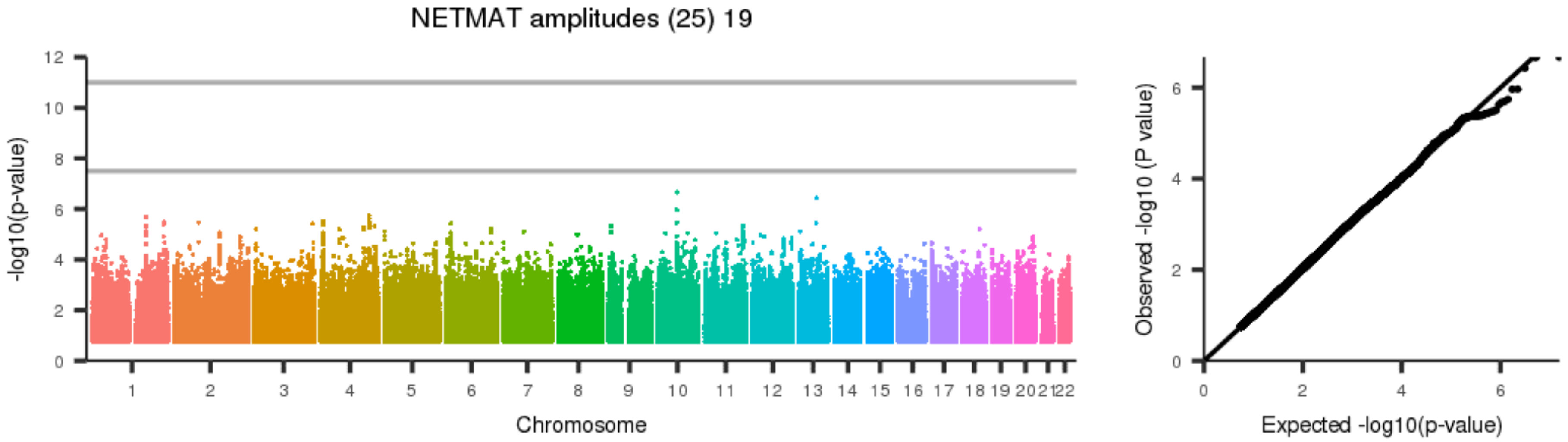

back to list of plots

Figure 890: NETMAT amplitudes (25) 20

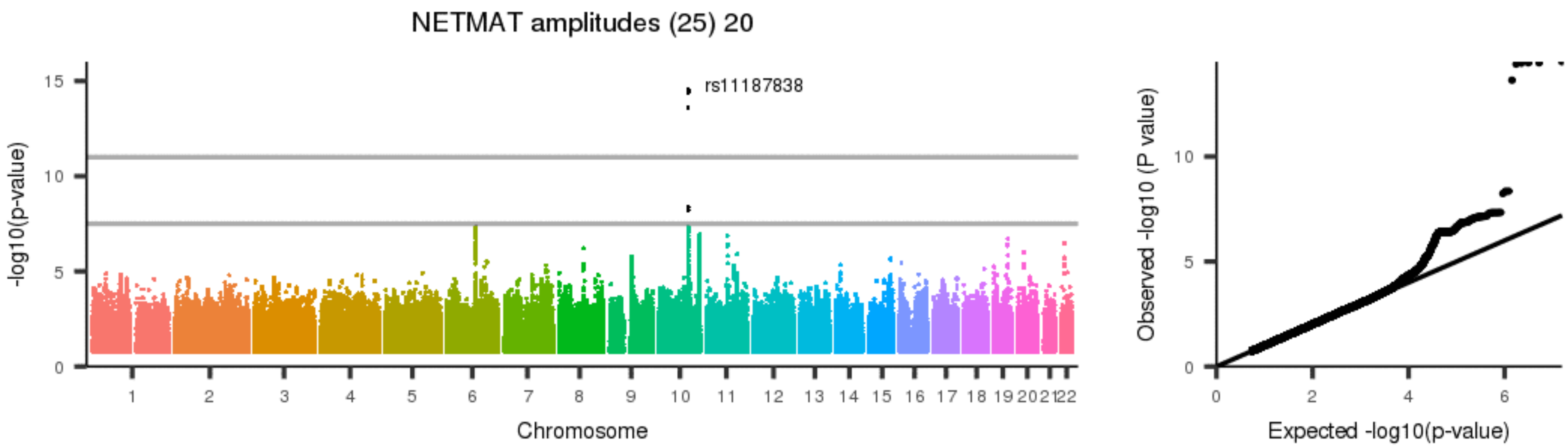

back to list of plots

Figure 891: NETMAT amplitudes (25) 21

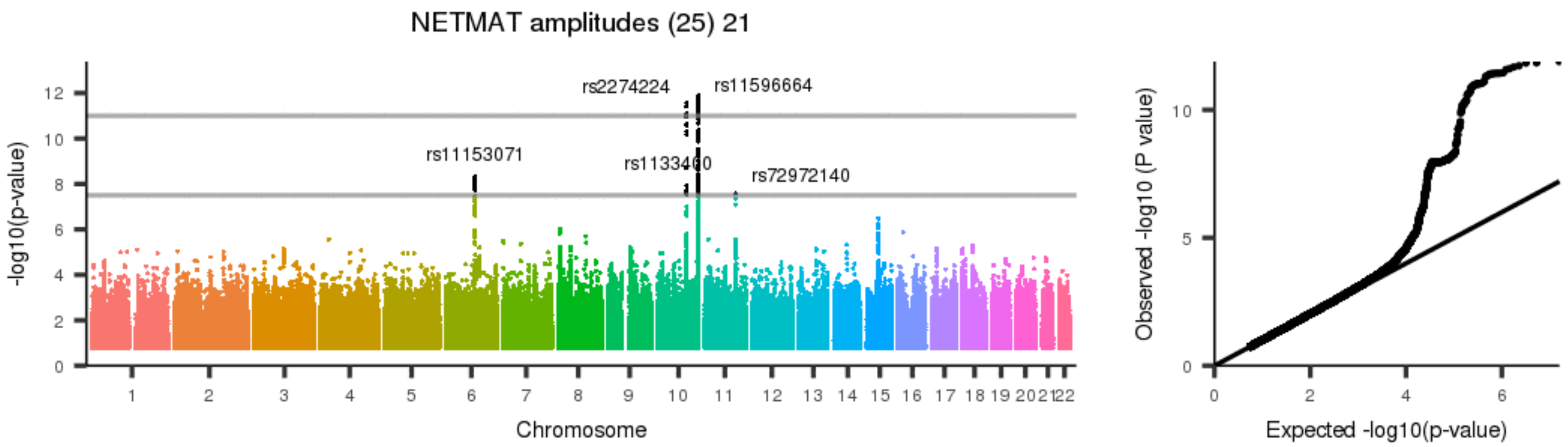

back to list of plots

Figure 892: NETMAT amplitudes (100) 01

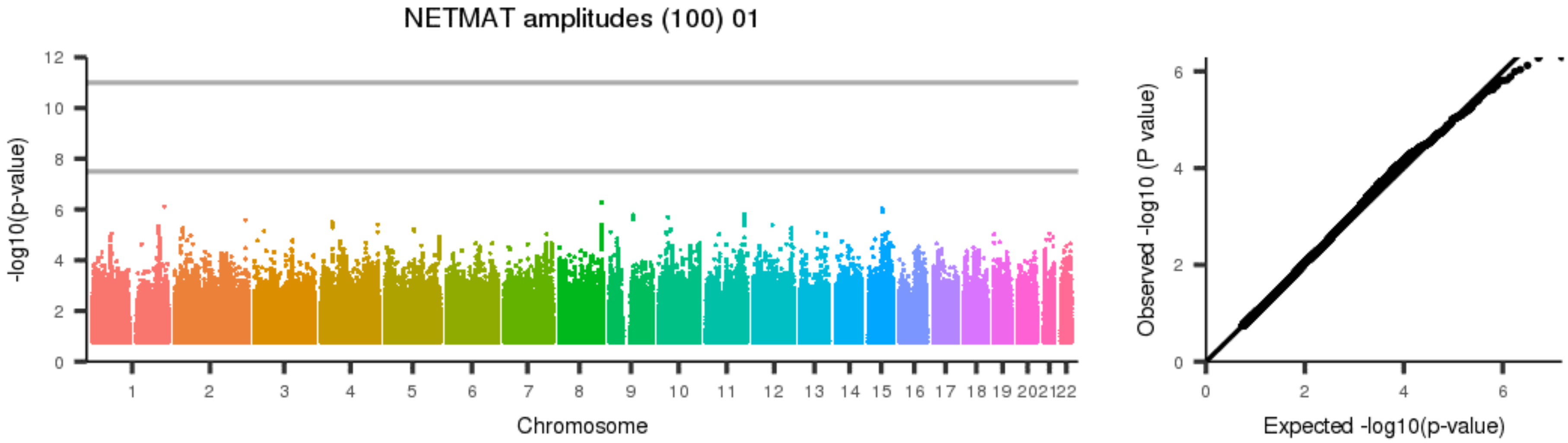

back to list of plots

Figure 893: NETMAT amplitudes (100) 02

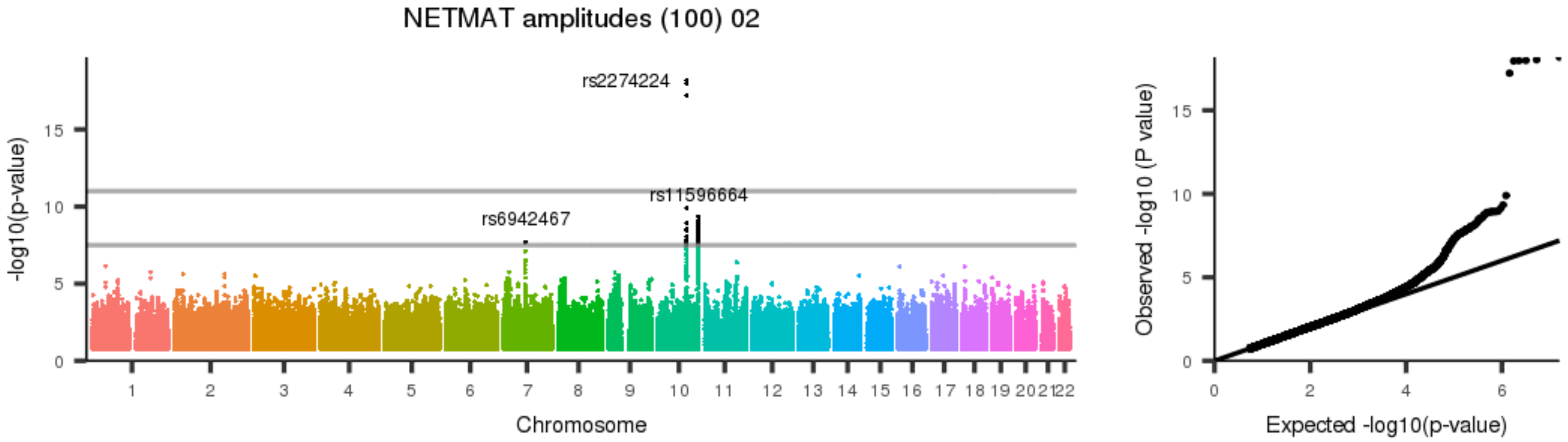

back to list of plots

Figure 894: NETMAT amplitudes (100) 03

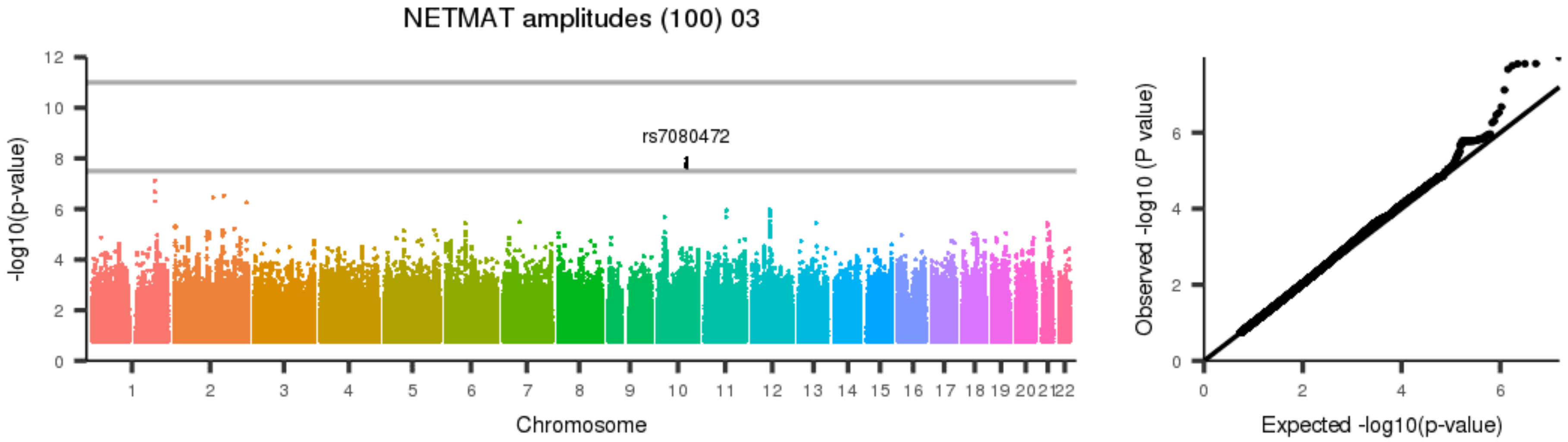

back to list of plots

Figure 895: NETMAT amplitudes (100) 04

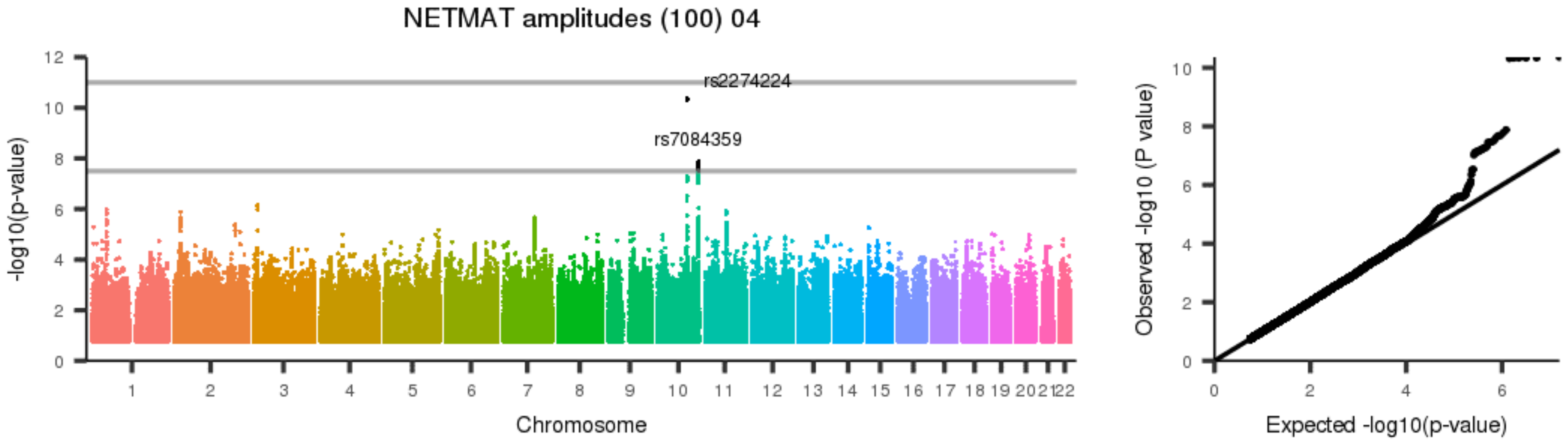

back to list of plots

Figure 896: NETMAT amplitudes (100) 05

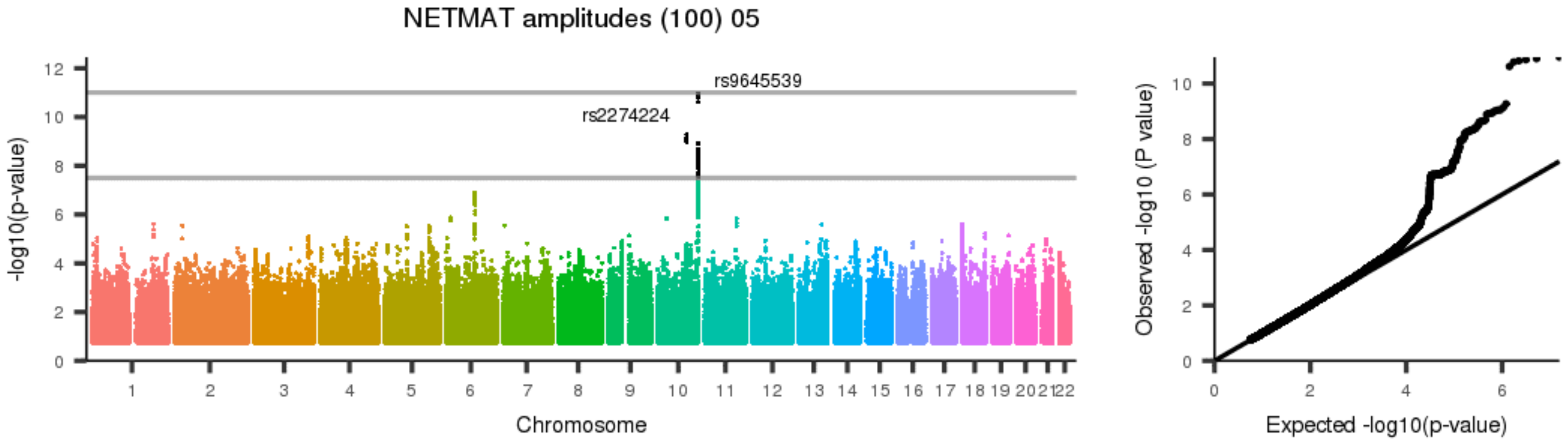

back to list of plots

Figure 897: NETMAT amplitudes (100) 06

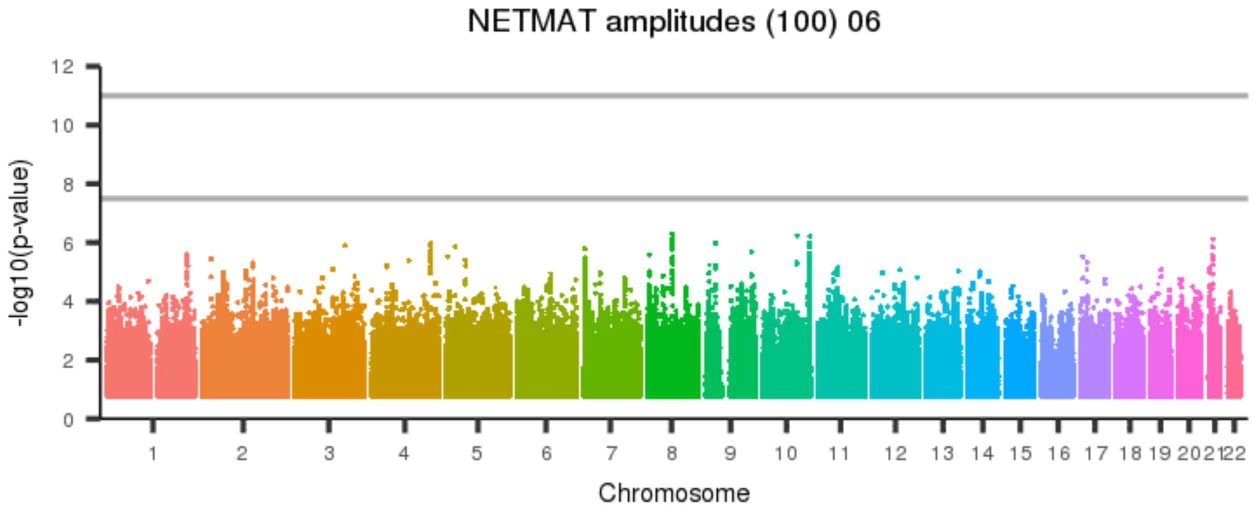

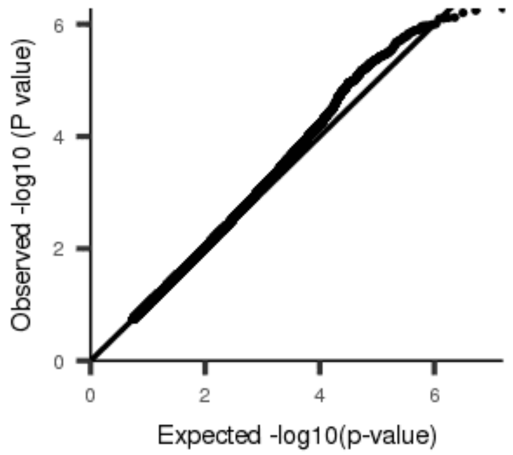

back to list of plots

Figure 898: NETMAT amplitudes (100) 07

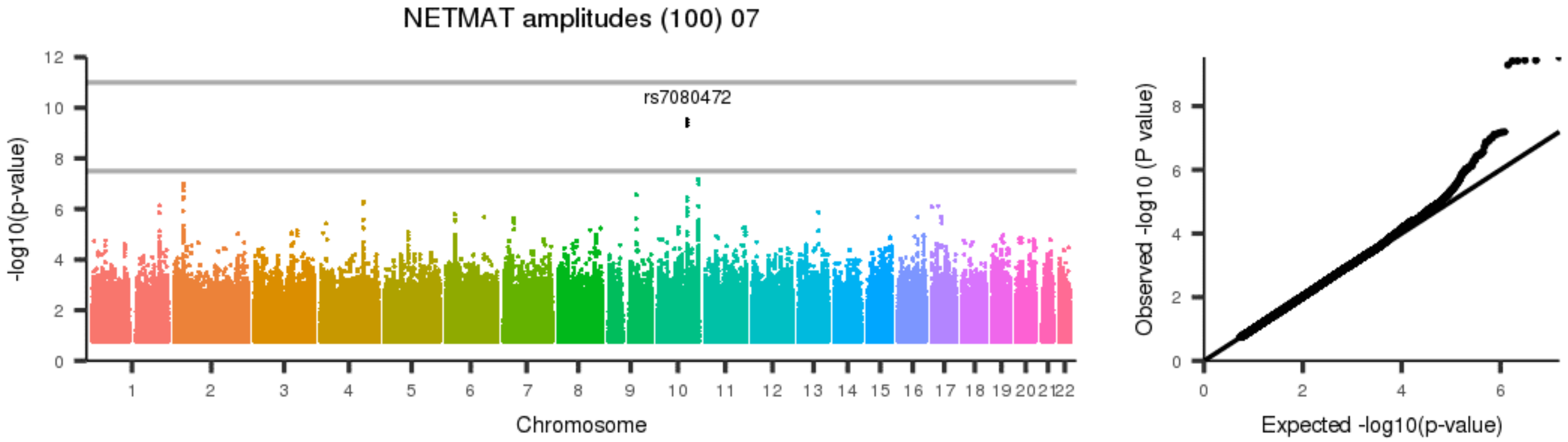

back to list of plots

Figure 899: NETMAT amplitudes (100) 08

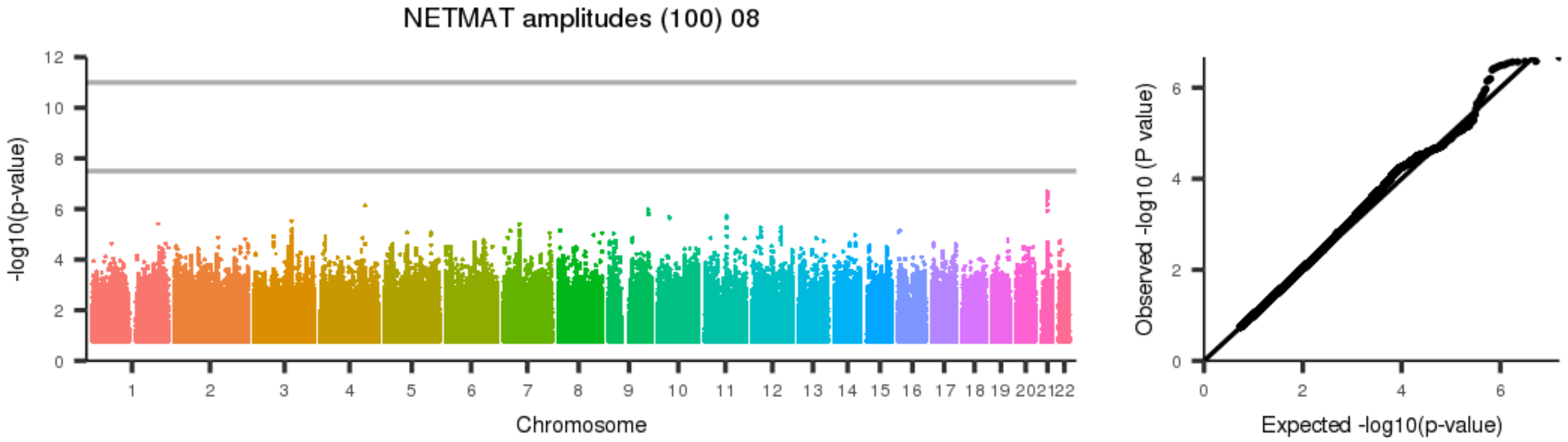

back to list of plots

Figure 900: NETMAT amplitudes (100) 09

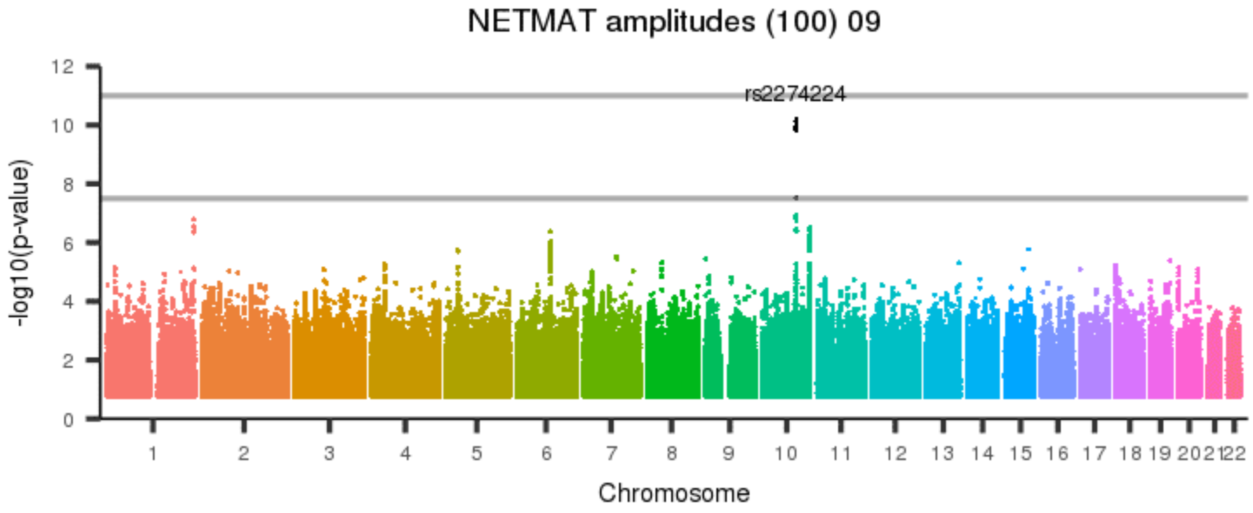

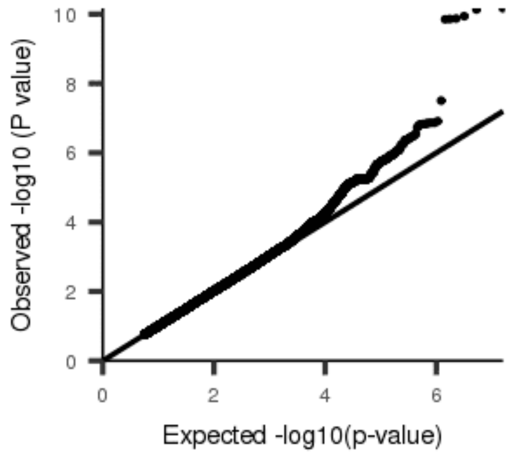

back to list of plots

Figure 901: NETMAT amplitudes (100) 10

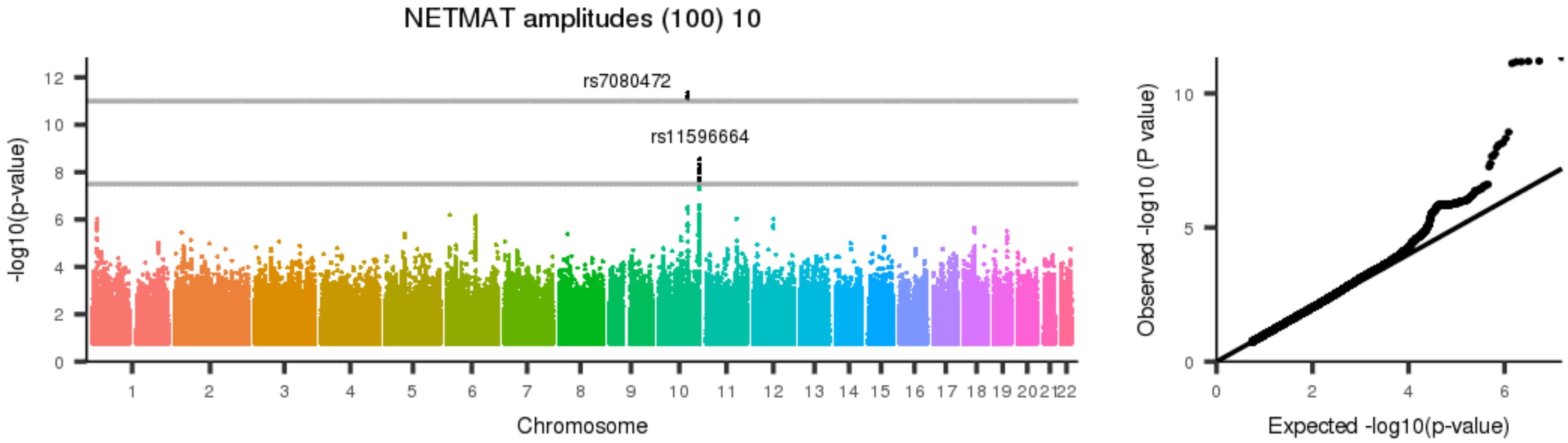

back to list of plots

Figure 902: NETMAT amplitudes (100) 11

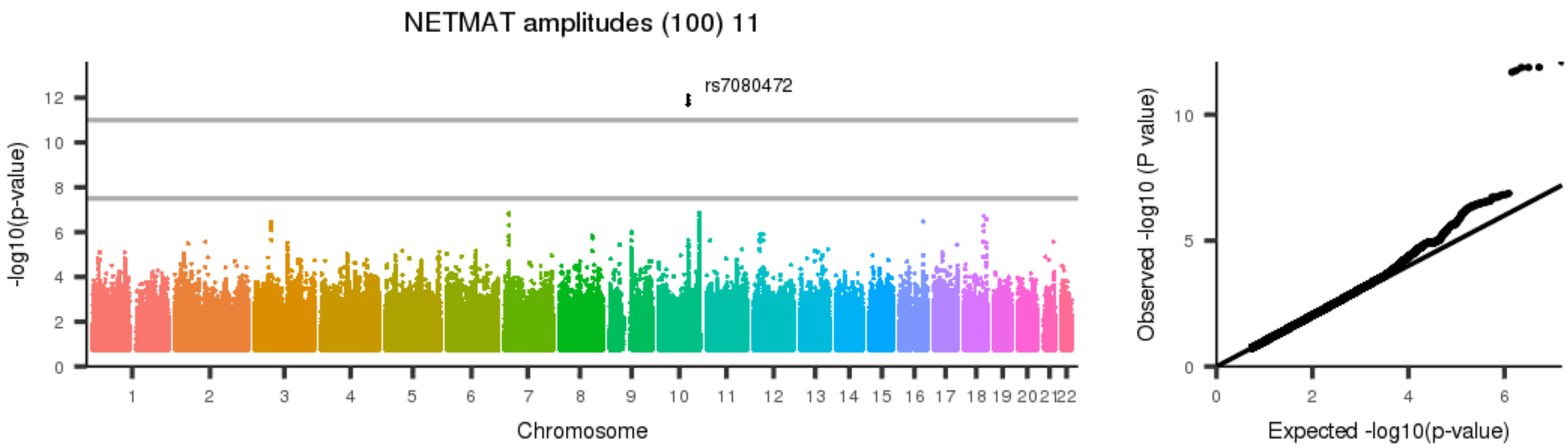

back to list of plots

Figure 903: NETMAT amplitudes (100) 12

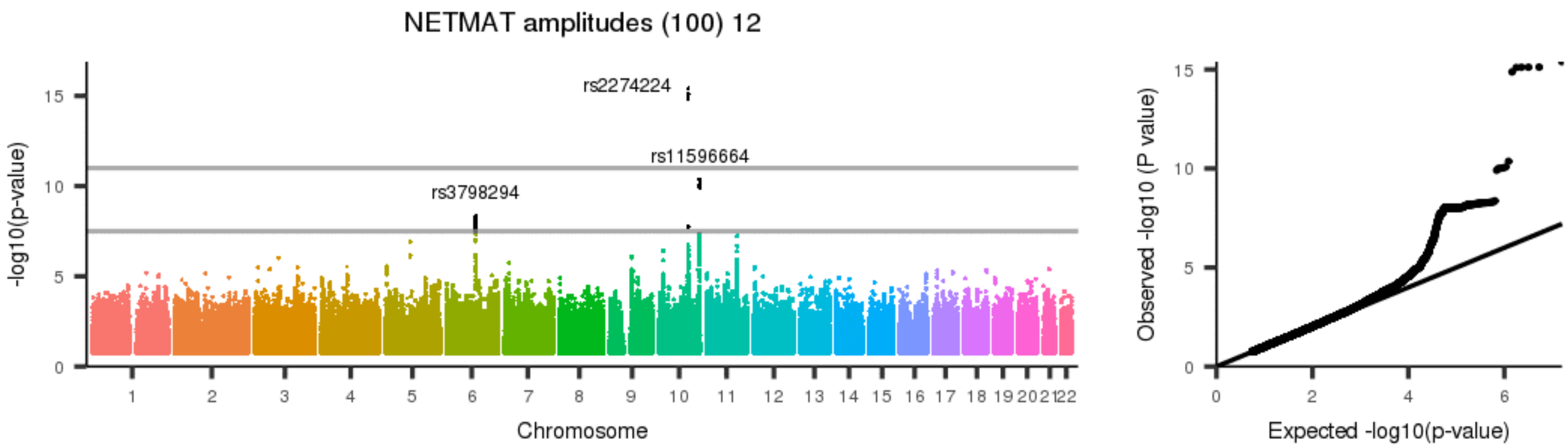

back to list of plots

Figure 904: NETMAT amplitudes (100) 13

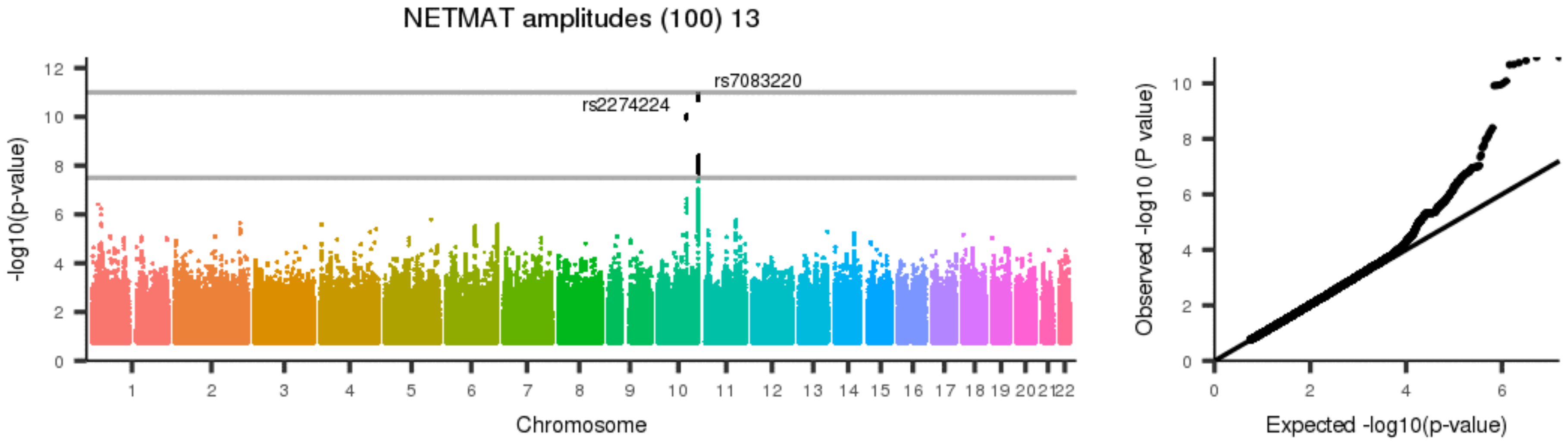

back to list of plots

Figure 905: NETMAT amplitudes (100) 14

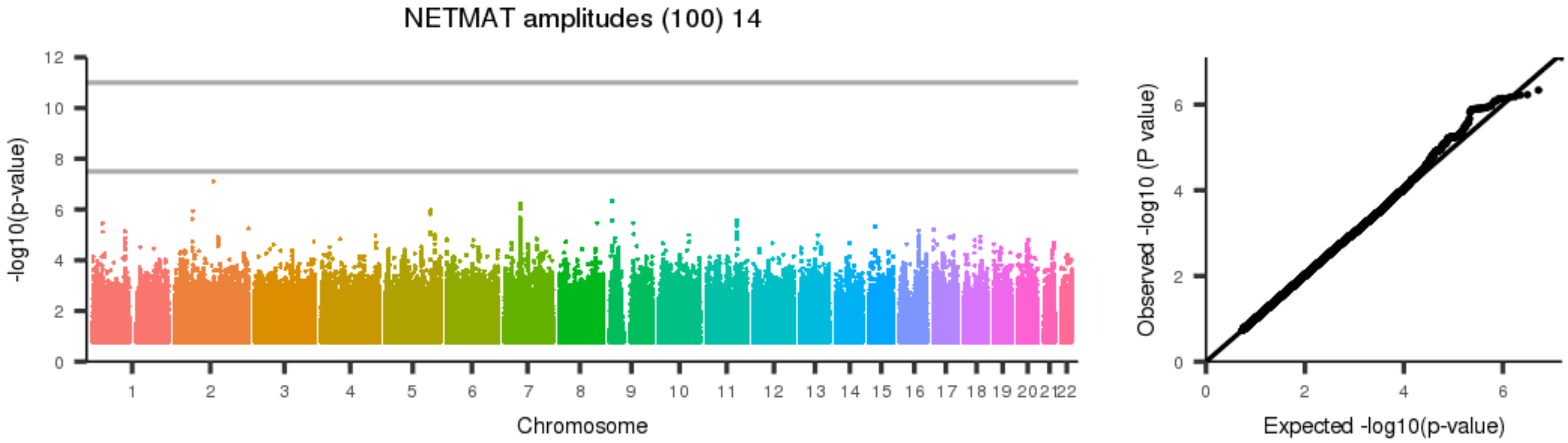

back to list of plots

Figure 906: NETMAT amplitudes (100) 15

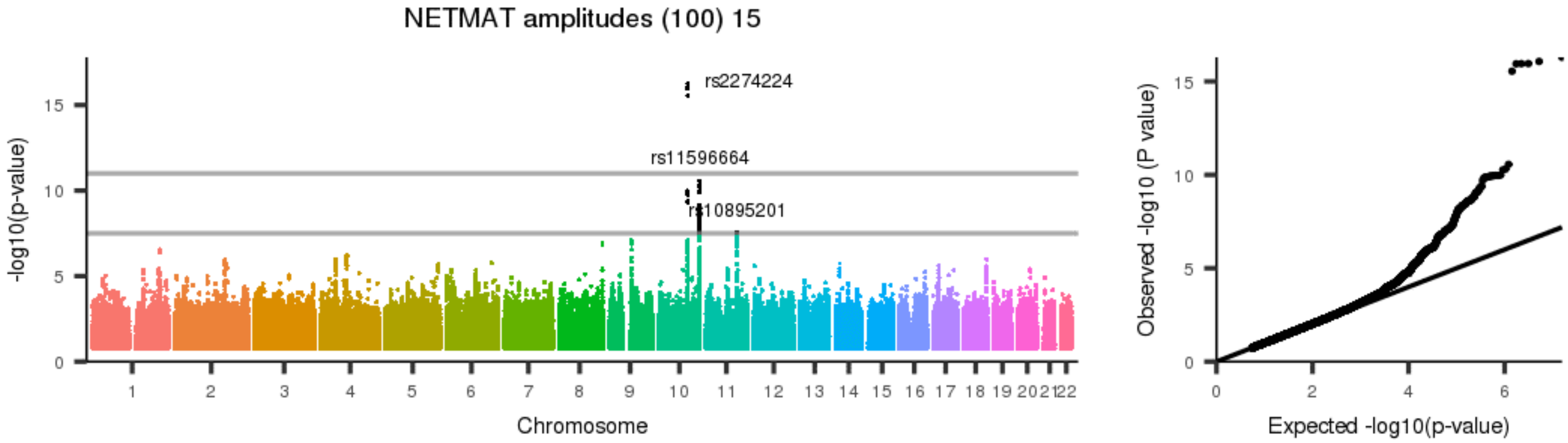

back to list of plots

Figure 907: NETMAT amplitudes (100) 16

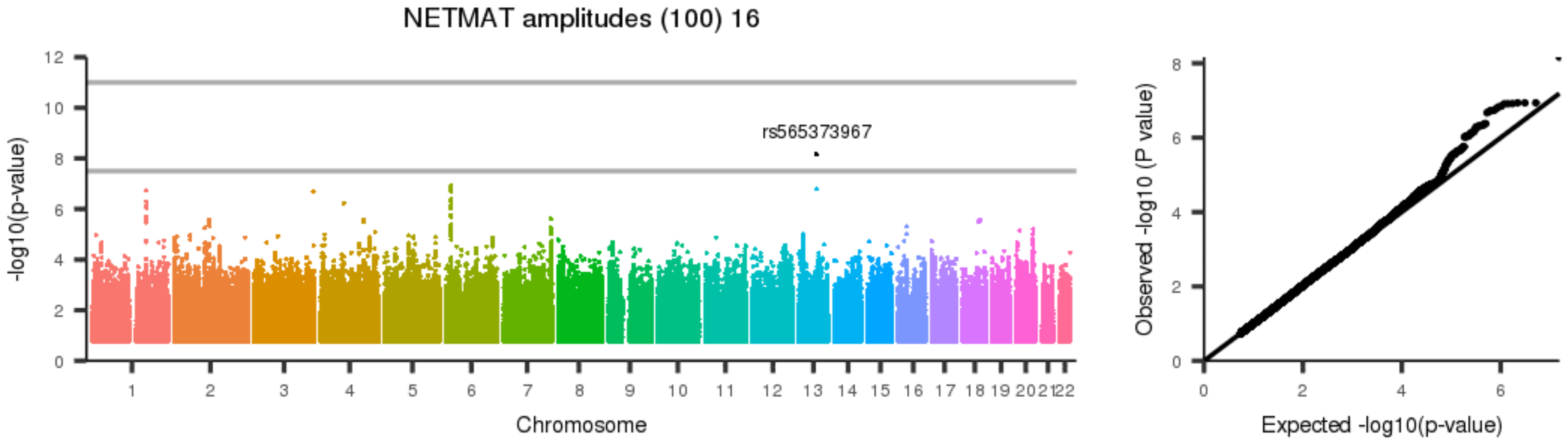

back to list of plots

Figure 908: NETMAT amplitudes (100) 17

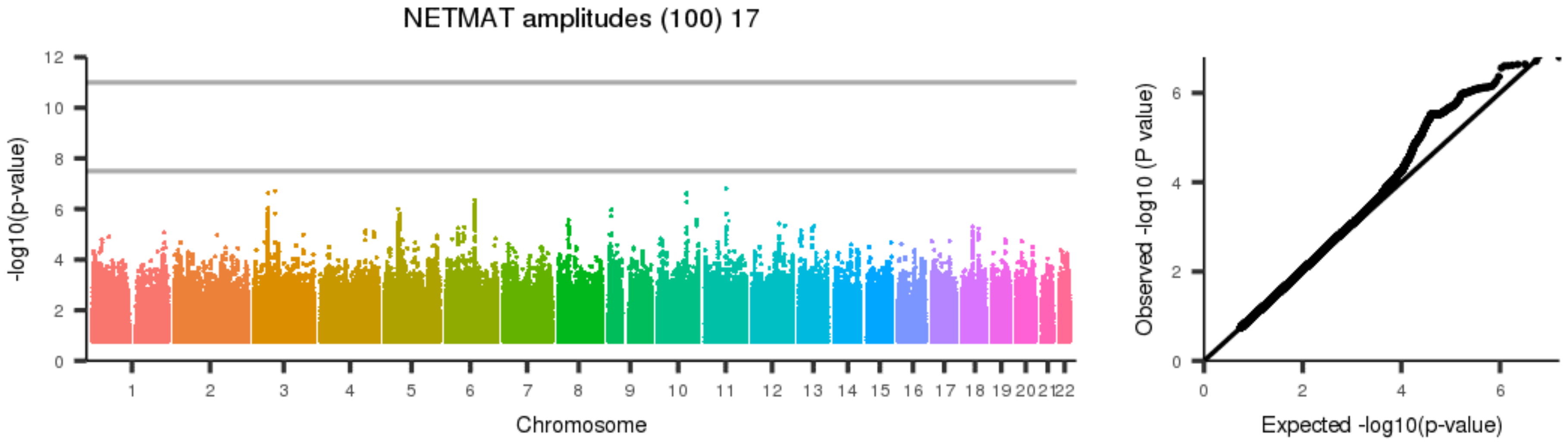

back to list of plots

Figure 909: NETMAT amplitudes (100) 18

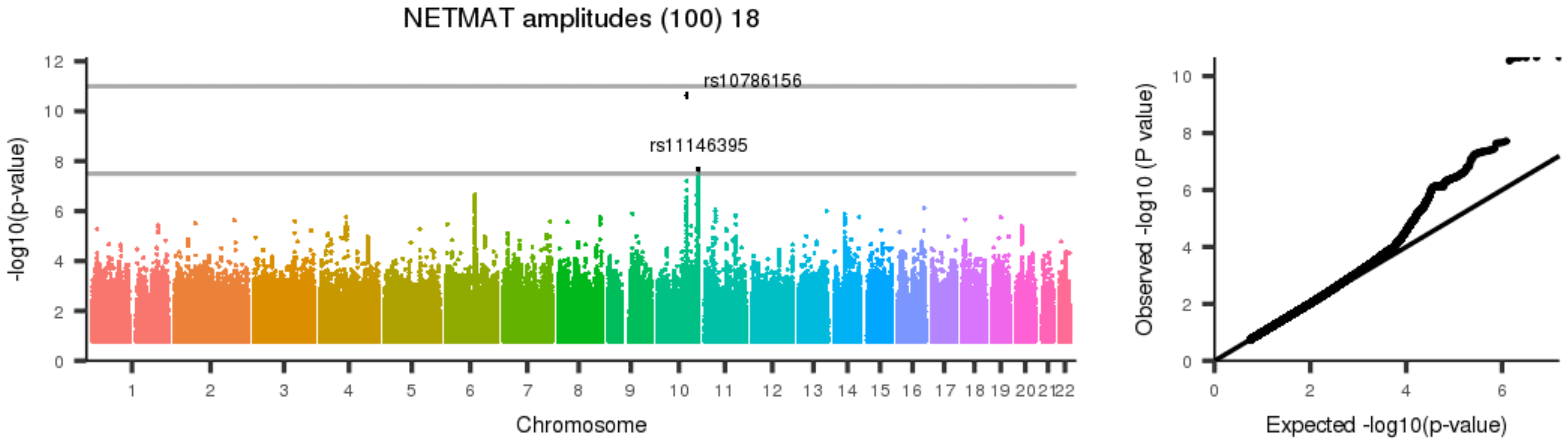

back to list of plots

Figure 910: NETMAT amplitudes (100) 19

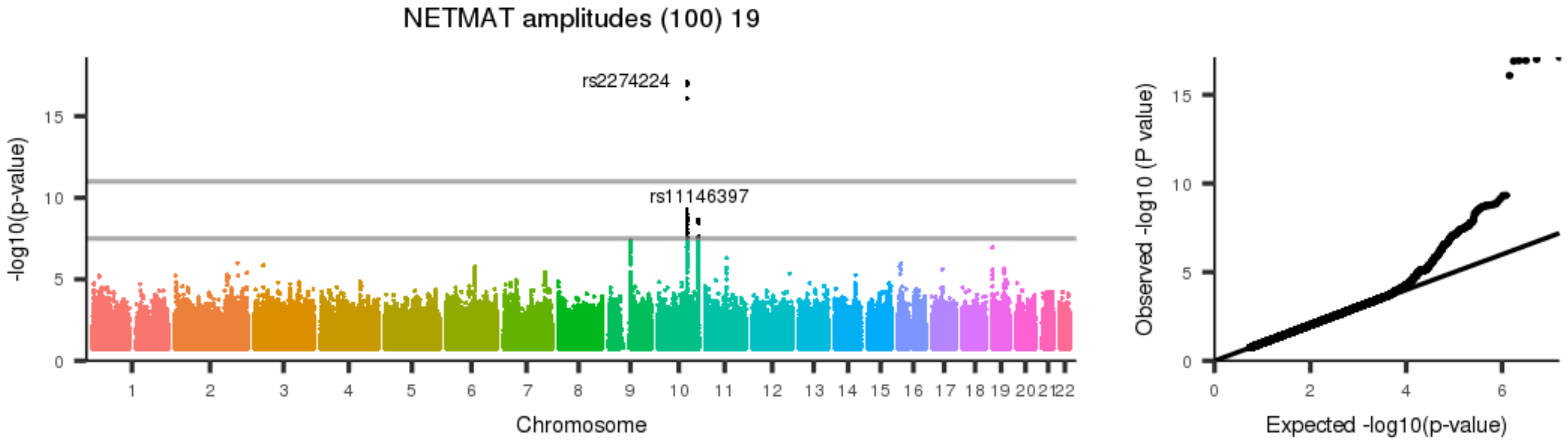

back to list of plots

Figure 911: NETMAT amplitudes (100) 20

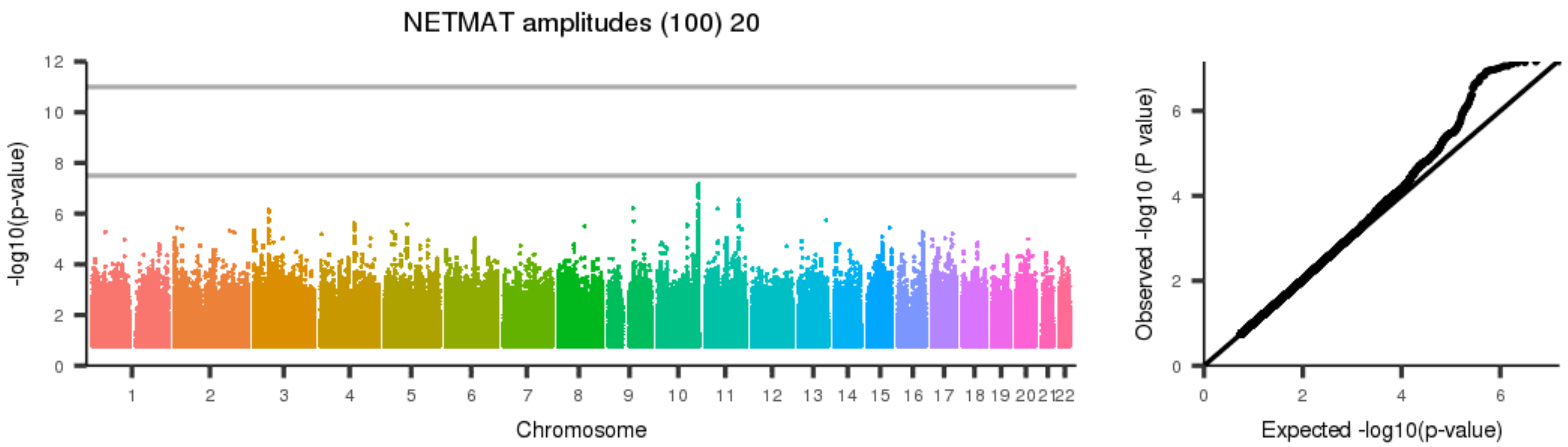

back to list of plots

Figure 912: NETMAT amplitudes (100) 21

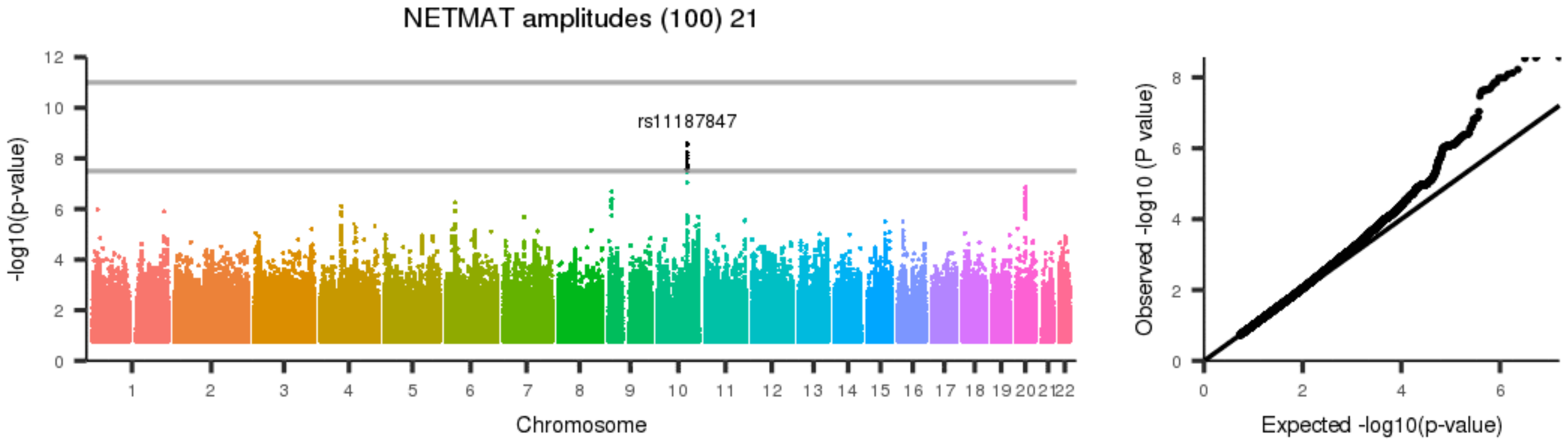

back to list of plots

Figure 913: NETMAT amplitudes (100) 22

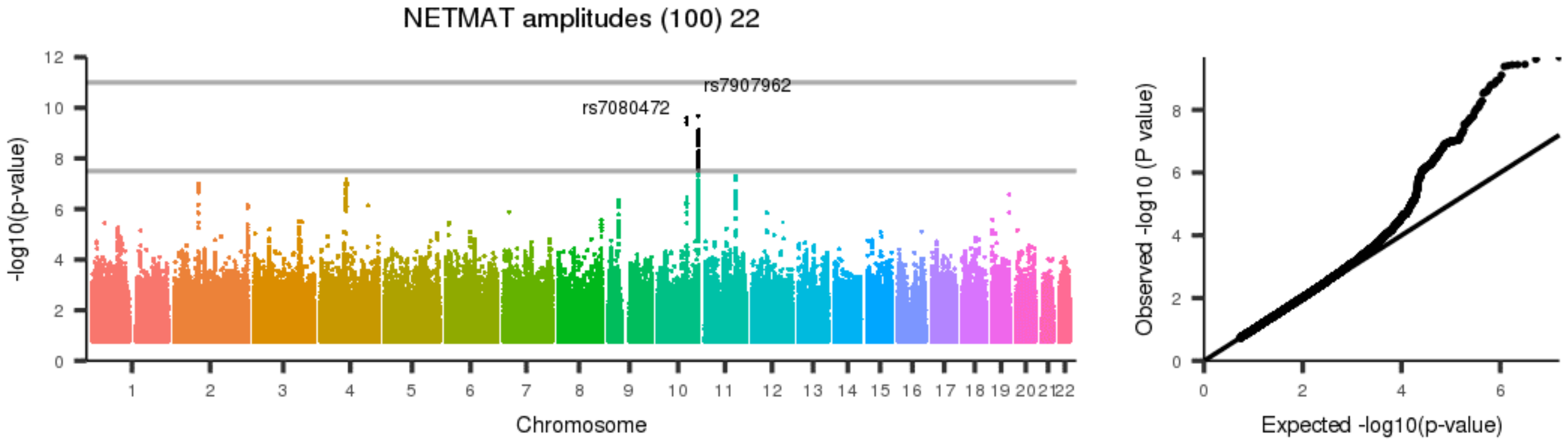

back to list of plots

Figure 914: NETMAT amplitudes (100) 23

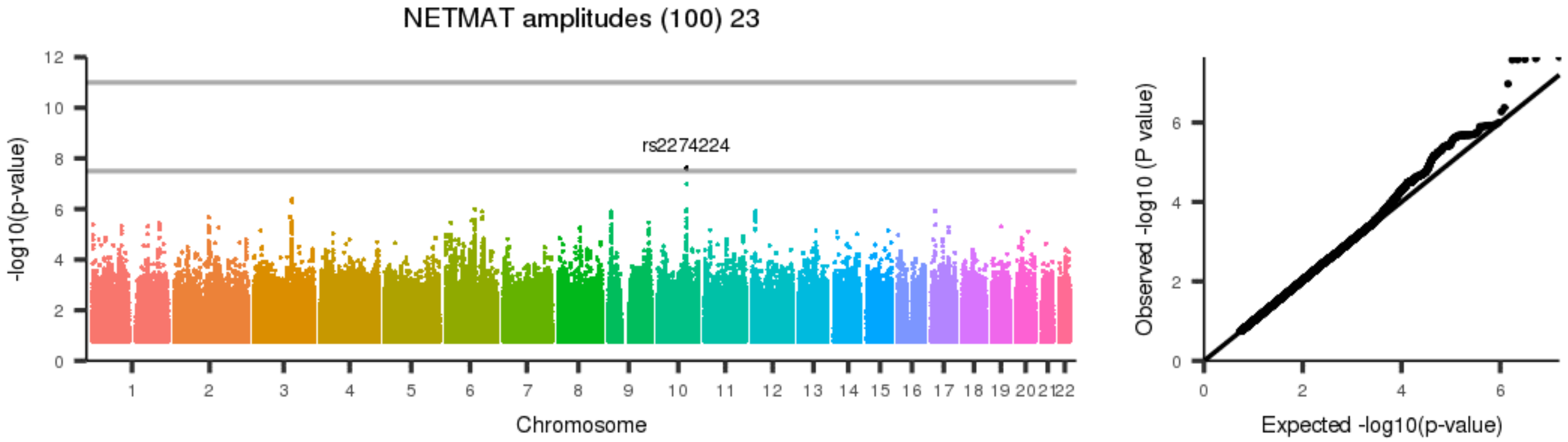

back to list of plots

Figure 915: NETMAT amplitudes (100) 24

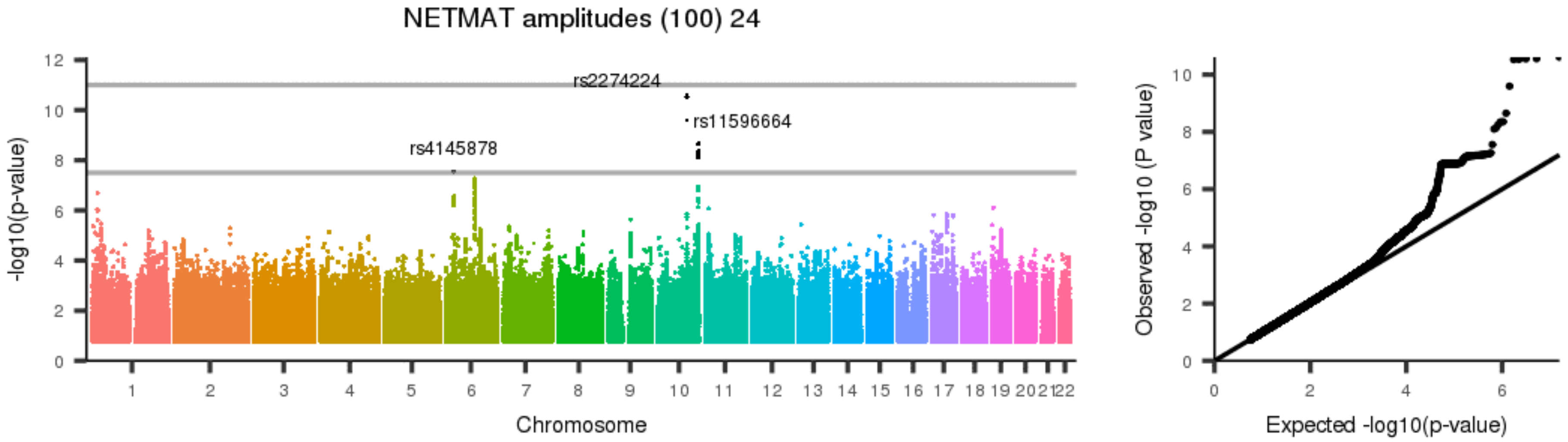

back to list of plots

Figure 916: NETMAT amplitudes (100) 25

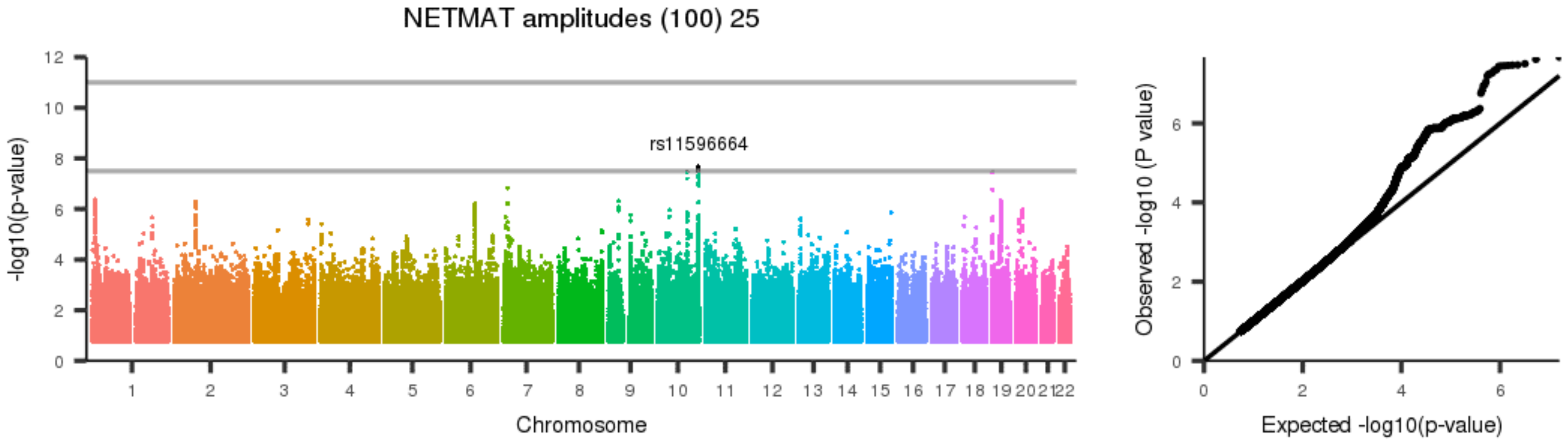

back to list of plots

Figure 917: NETMAT amplitudes (100) 26

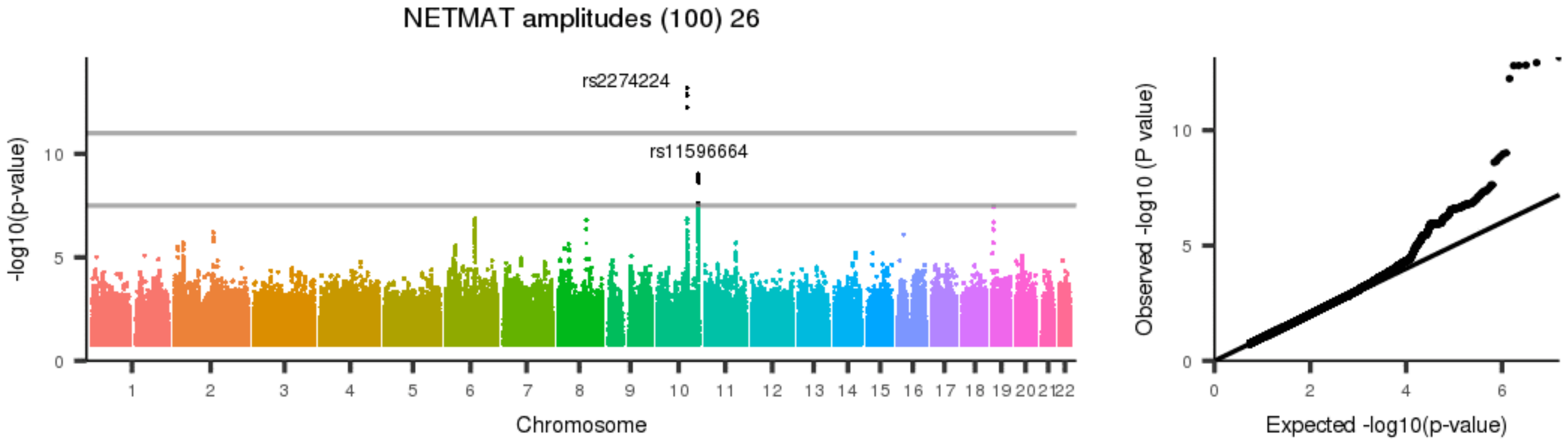

back to list of plots

Figure 918: NETMAT amplitudes (100) 27

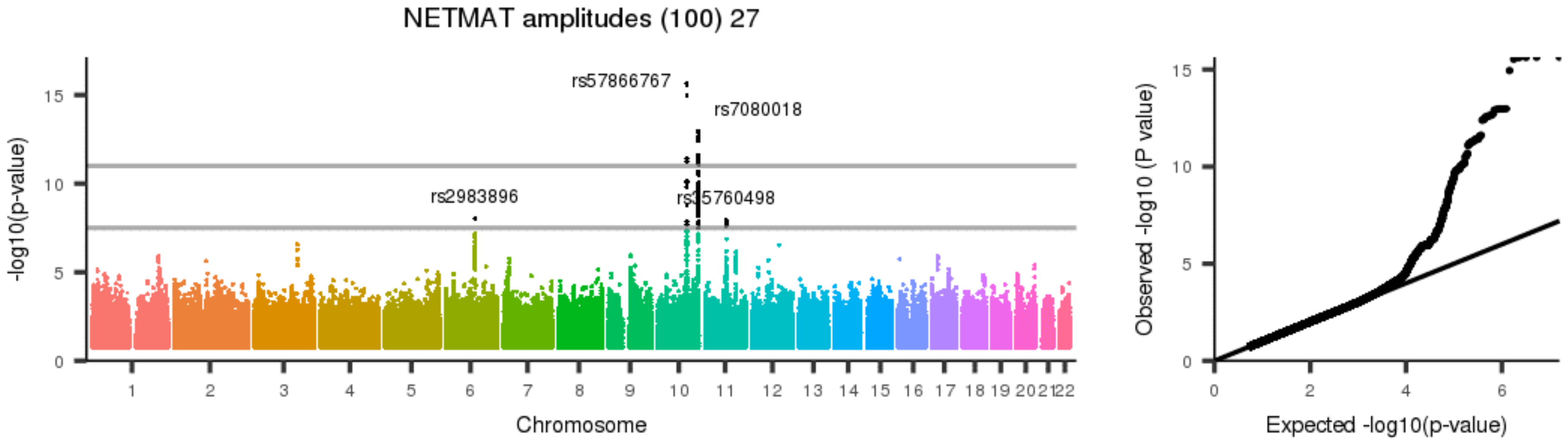

back to list of plots

Figure 919: NETMAT amplitudes (100) 28

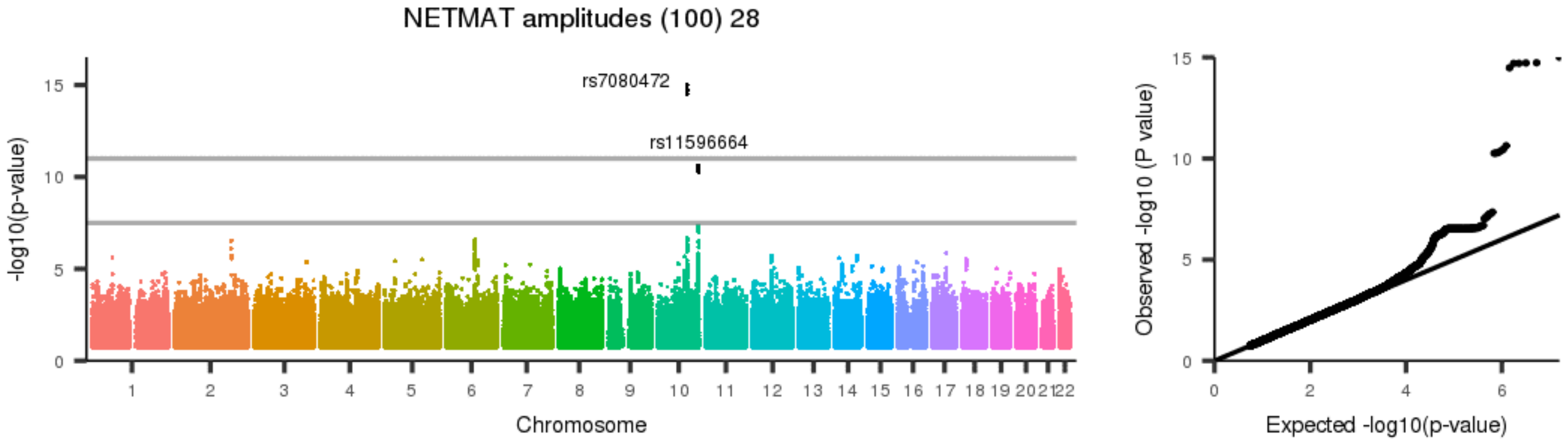

back to list of plots

Figure 920: NETMAT amplitudes (100) 29

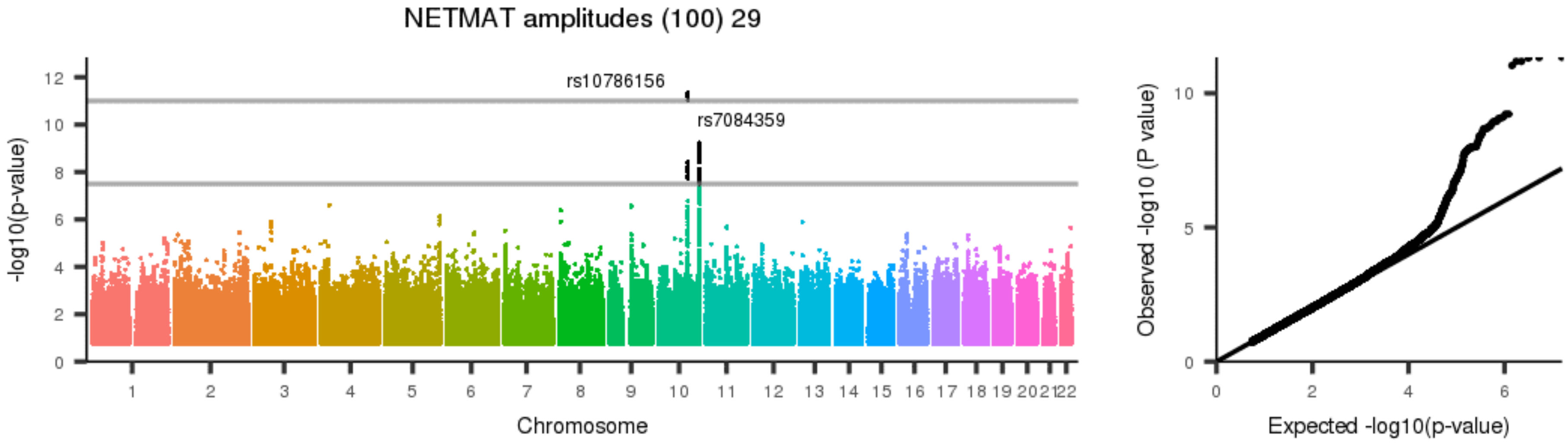

back to list of plots

Figure 921: NETMAT amplitudes (100) 30

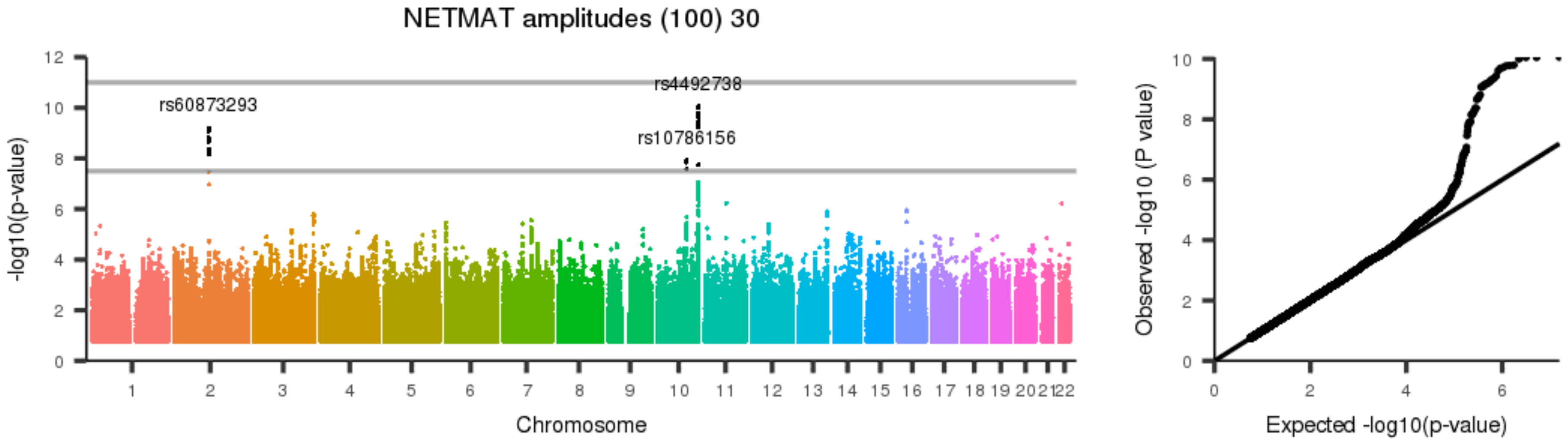

back to list of plots

Figure 922: NETMAT amplitudes (100) 31

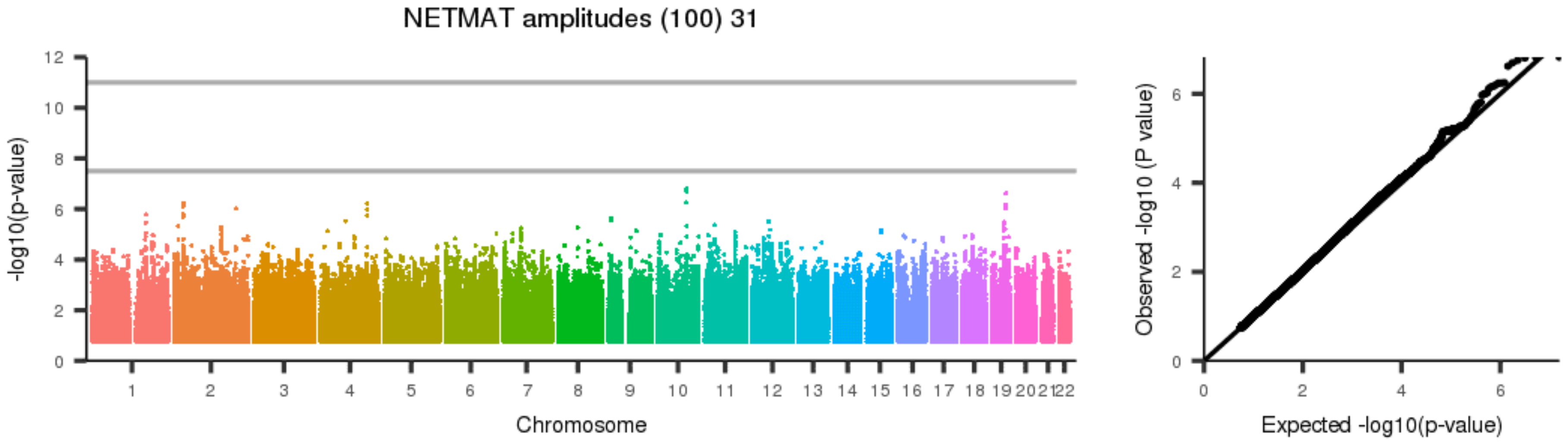

back to list of plots

Figure 923: NETMAT amplitudes (100) 32

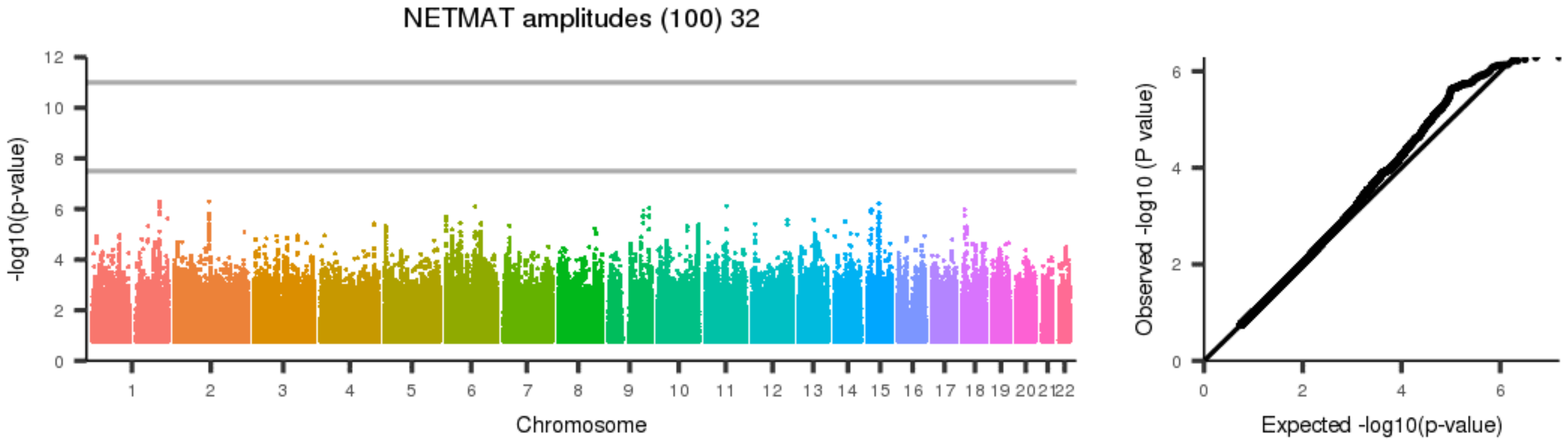

back to list of plots

Figure 924: NETMAT amplitudes (100) 33

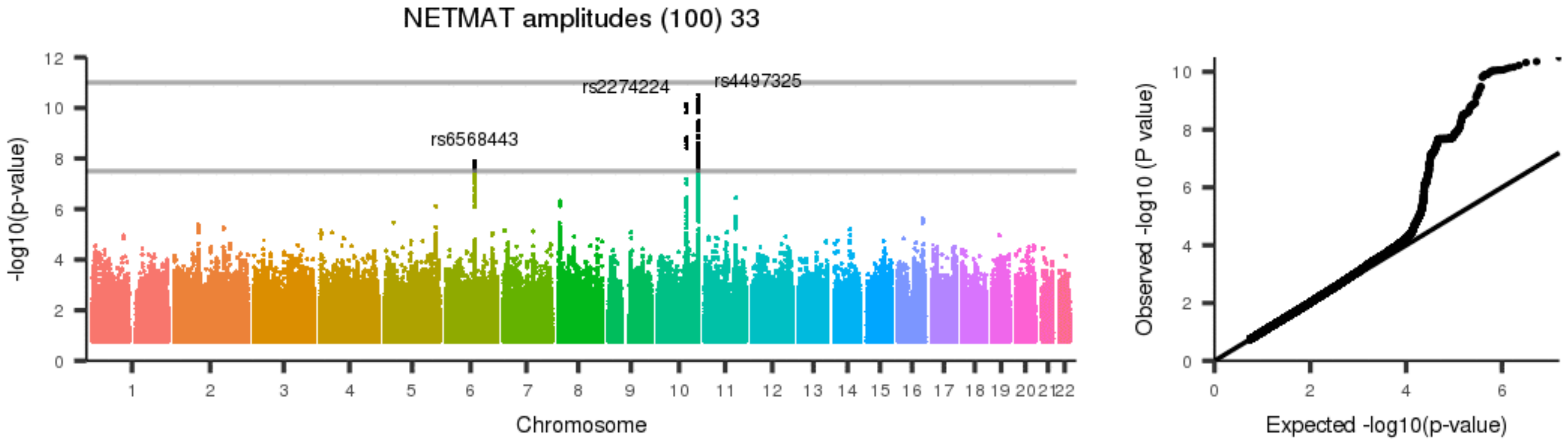

back to list of plots

Figure 925: NETMAT amplitudes (100) 34

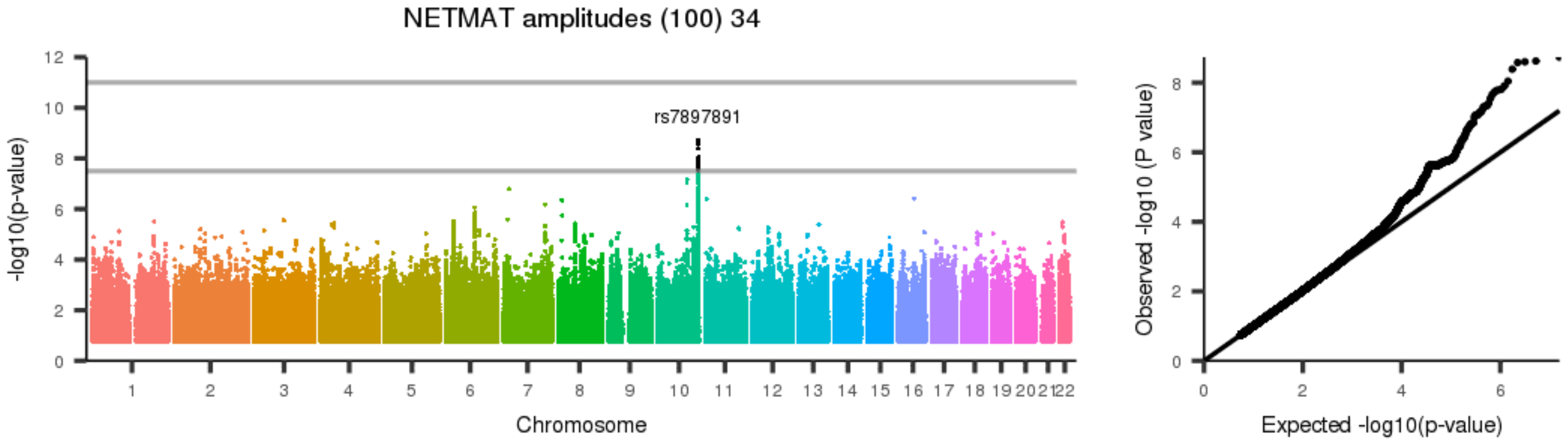

back to list of plots

Figure 926: NETMAT amplitudes (100) 35

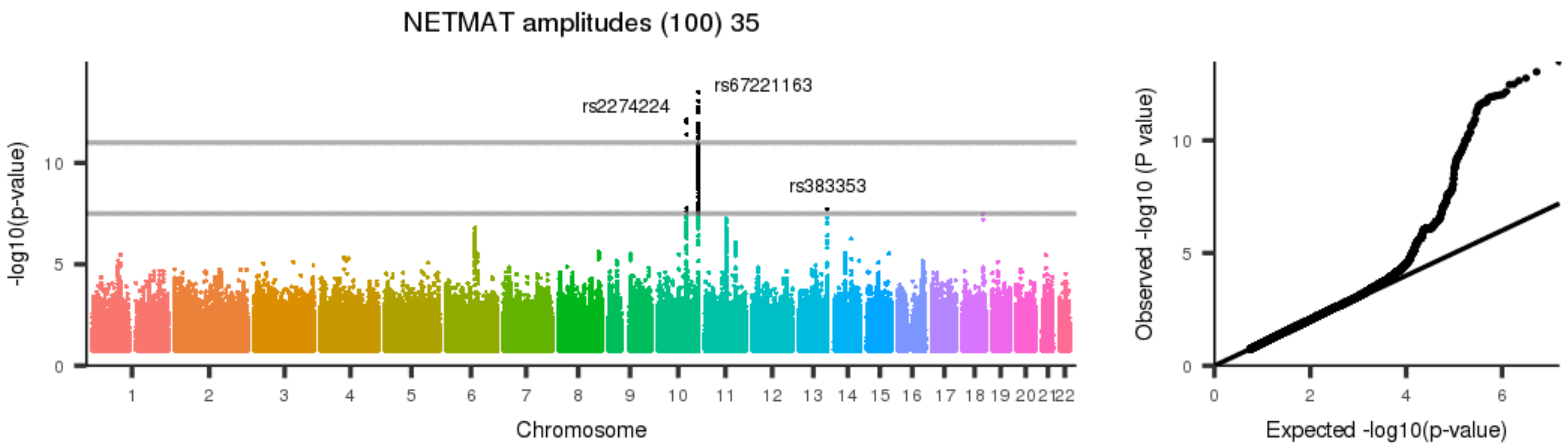

back to list of plots

Figure 927: NETMAT amplitudes (100) 36

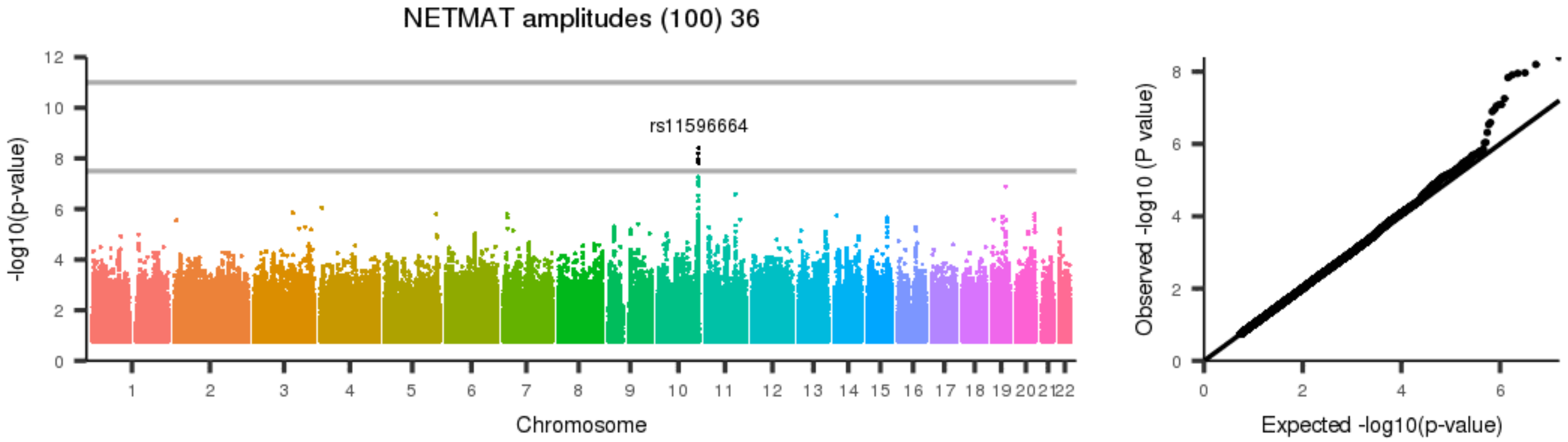

back to list of plots

Figure 928: NETMAT amplitudes (100) 37

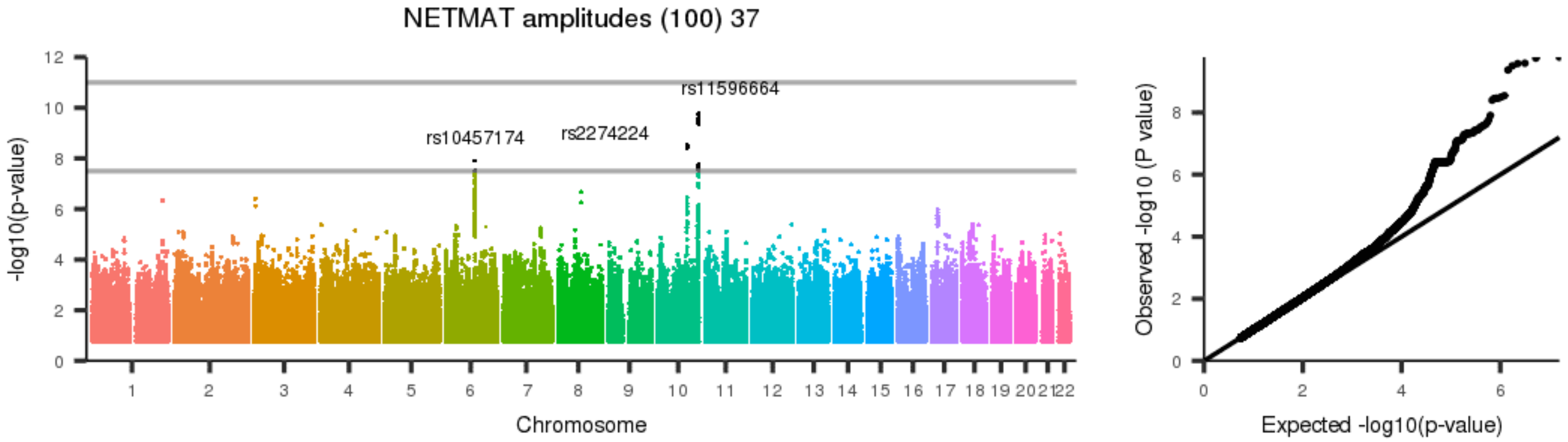

back to list of plots

Figure 929: NETMAT amplitudes (100) 38

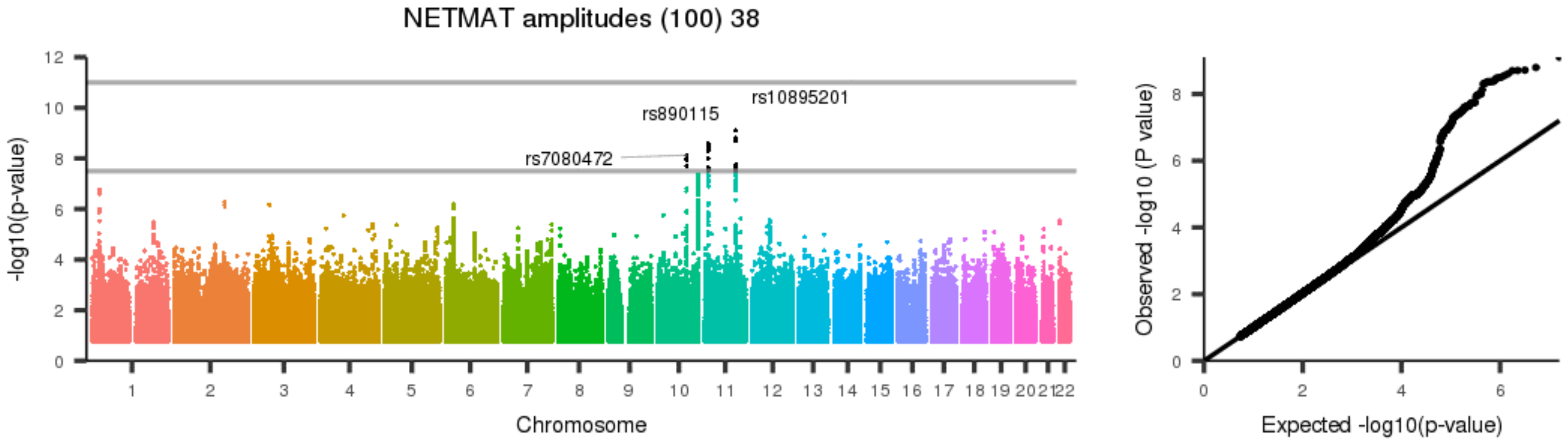

back to list of plots

Figure 930: NETMAT amplitudes (100) 39

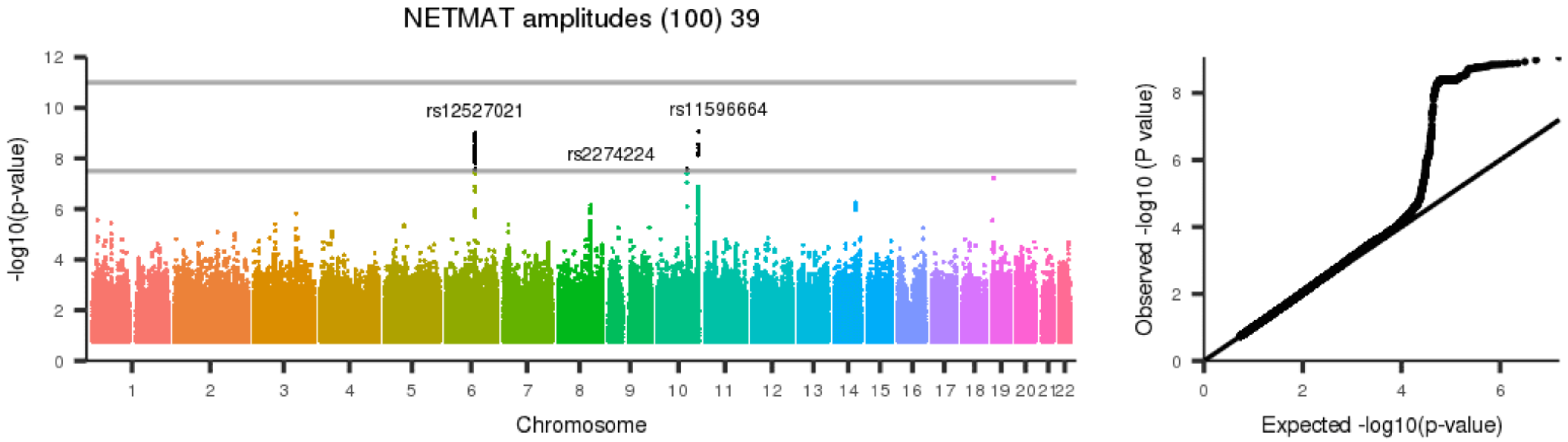

back to list of plots

Figure 931: NETMAT amplitudes (100) 40

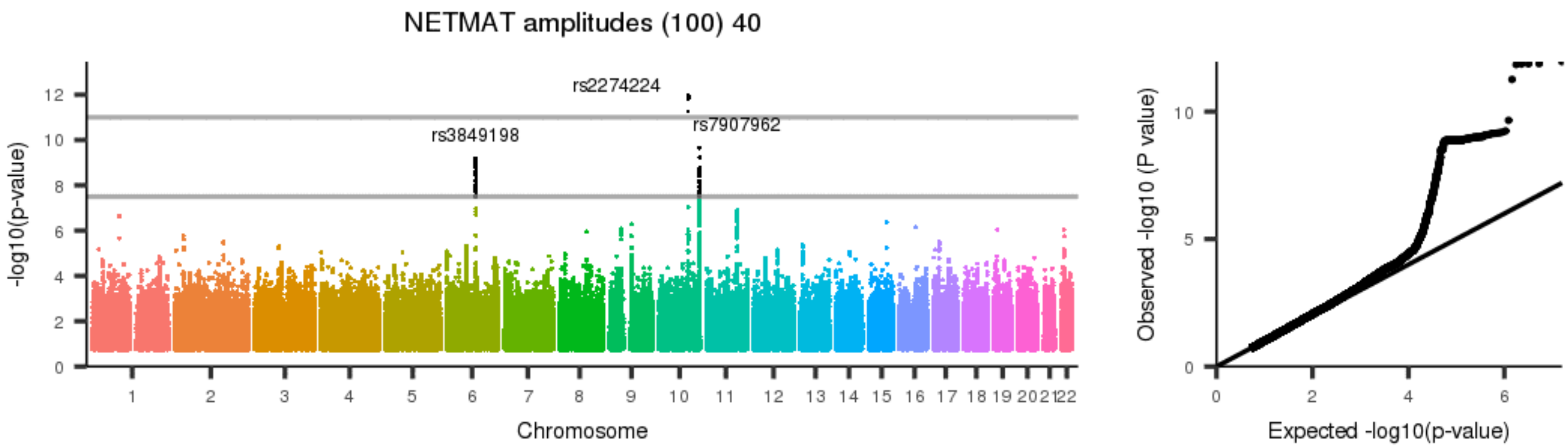

back to list of plots

Figure 932: NETMAT amplitudes (100) 41

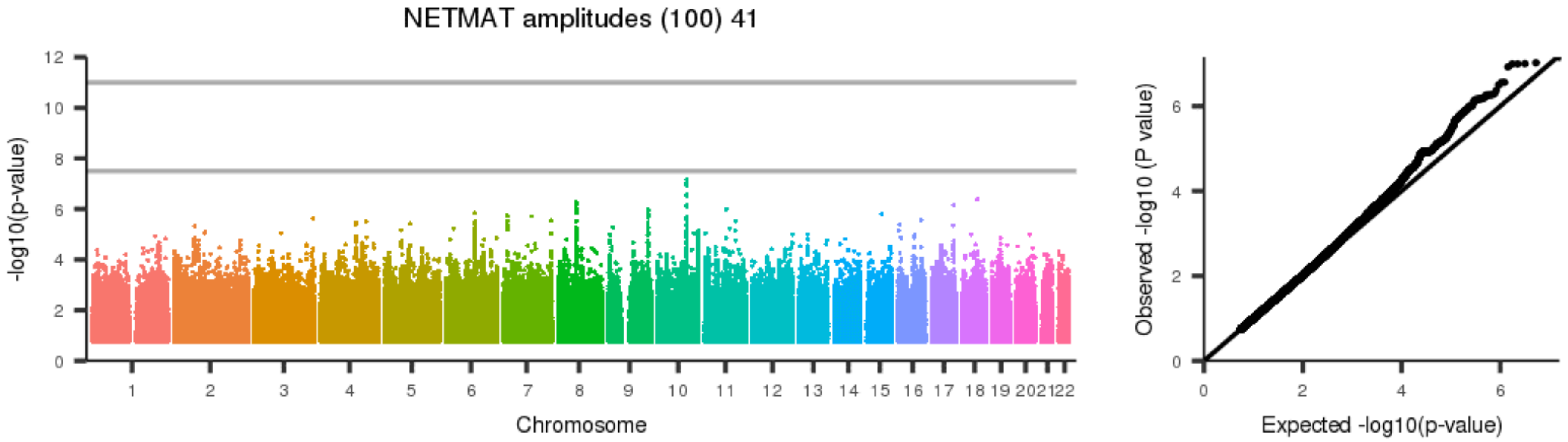

back to list of plots

Figure 933: NETMAT amplitudes (100) 42

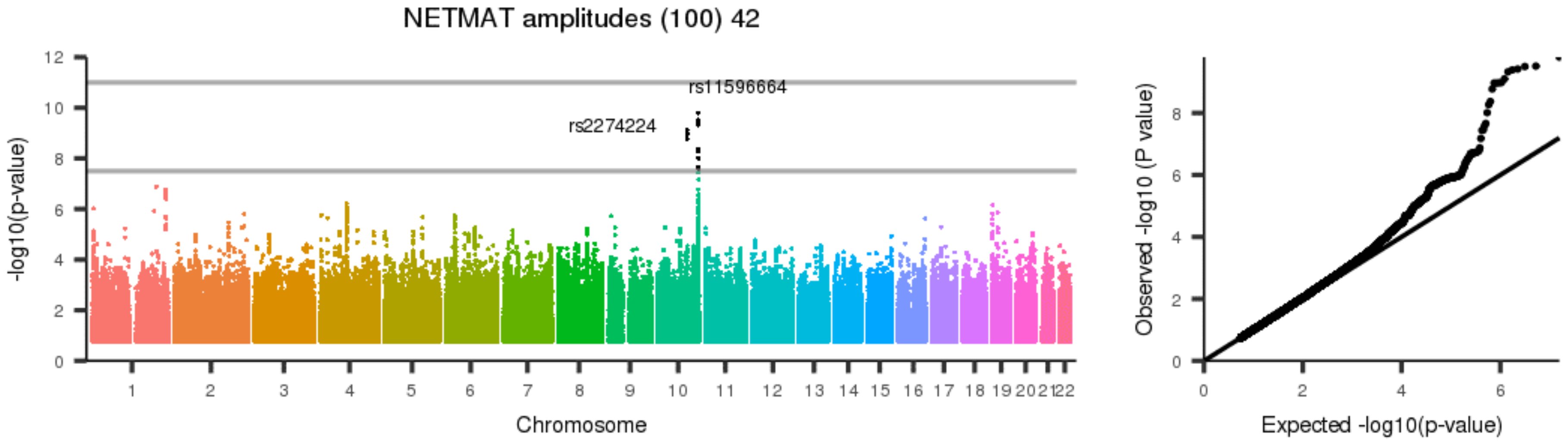

back to list of plots

Figure 934: NETMAT amplitudes (100) 43

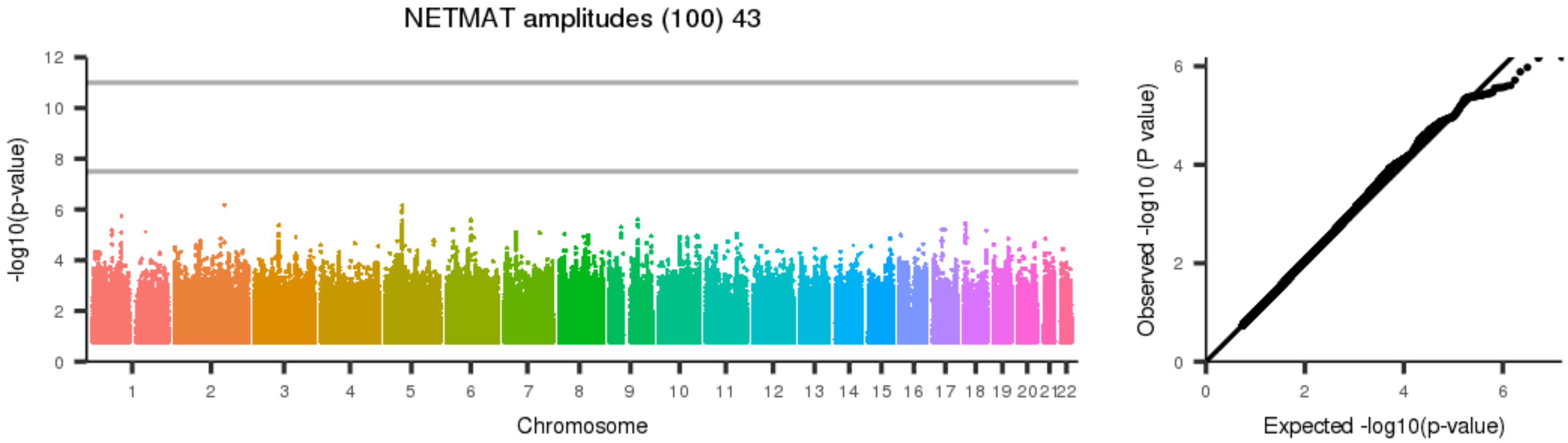

back to list of plots

Figure 935: NETMAT amplitudes (100) 44

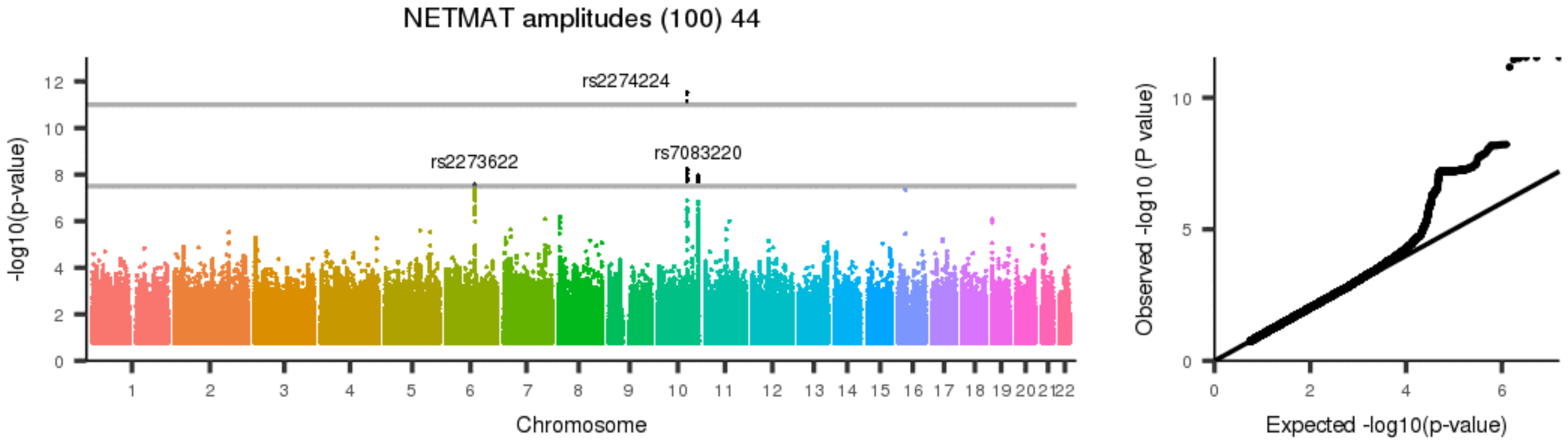

back to list of plots

Figure 936: NETMAT amplitudes (100) 45

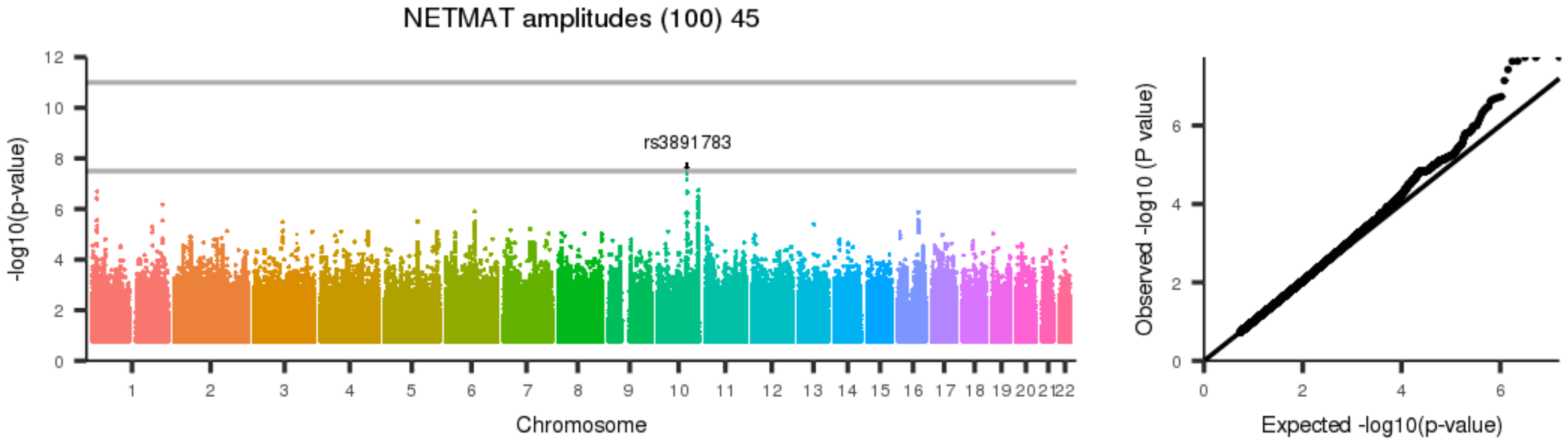

back to list of plots

Figure 937: NETMAT amplitudes (100) 46

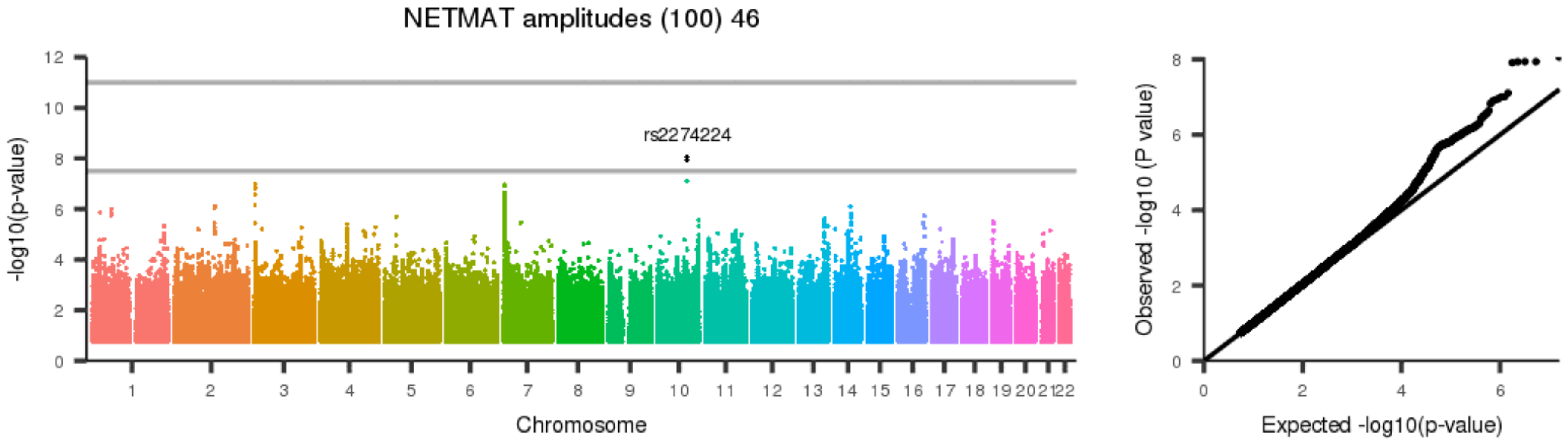

back to list of plots

Figure 938: NETMAT amplitudes (100) 47

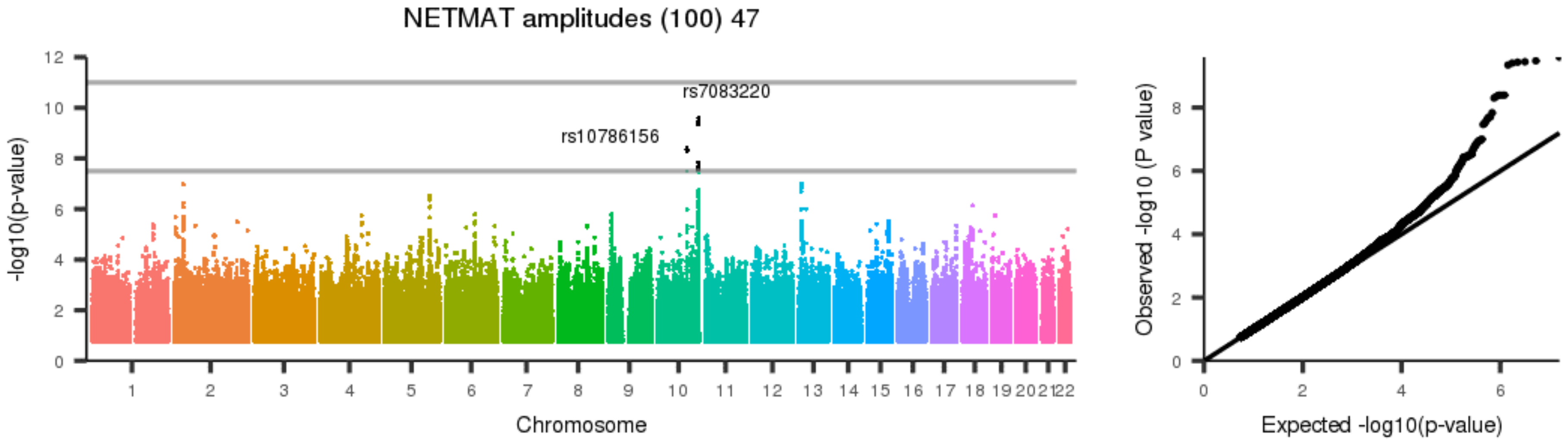

back to list of plots

Figure 939: NETMAT amplitudes (100) 48

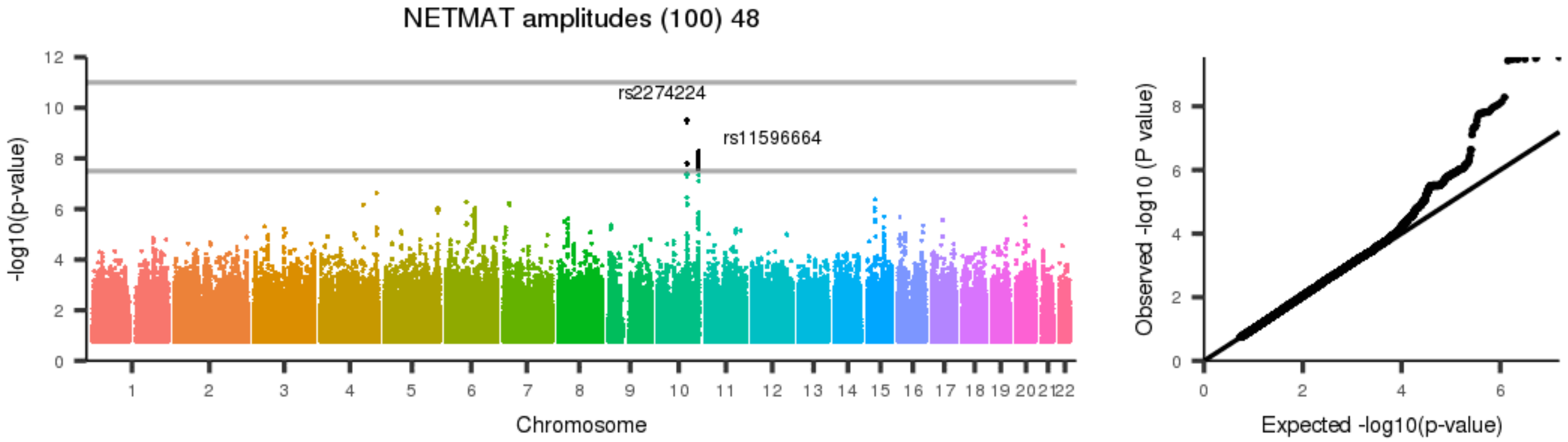

back to list of plots

Figure 940: NETMAT amplitudes (100) 49

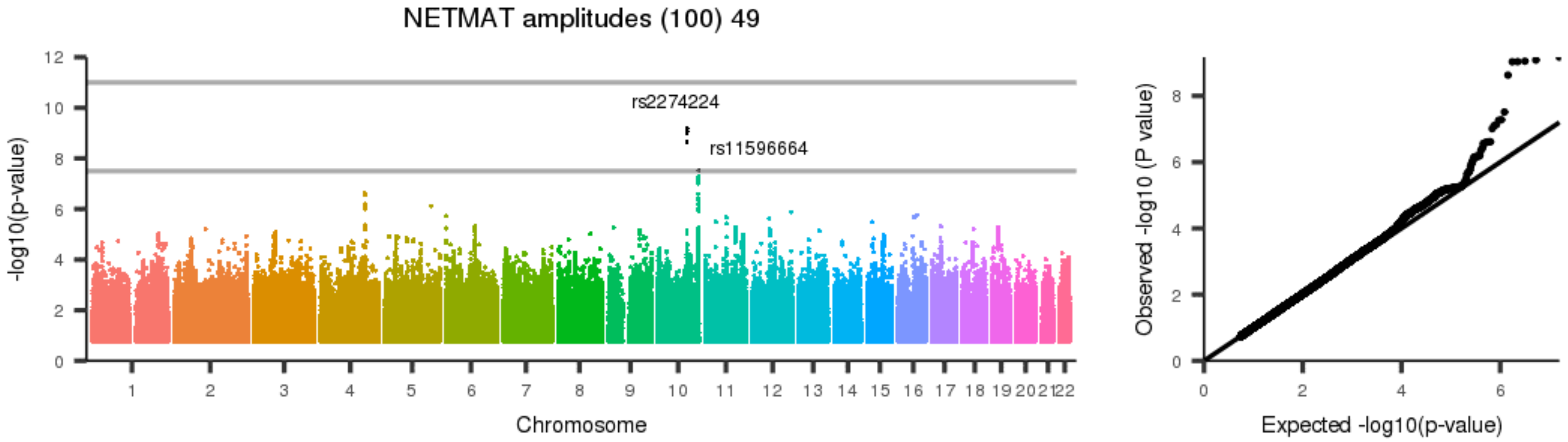

back to list of plots

Figure 941: NETMAT amplitudes (100) 50

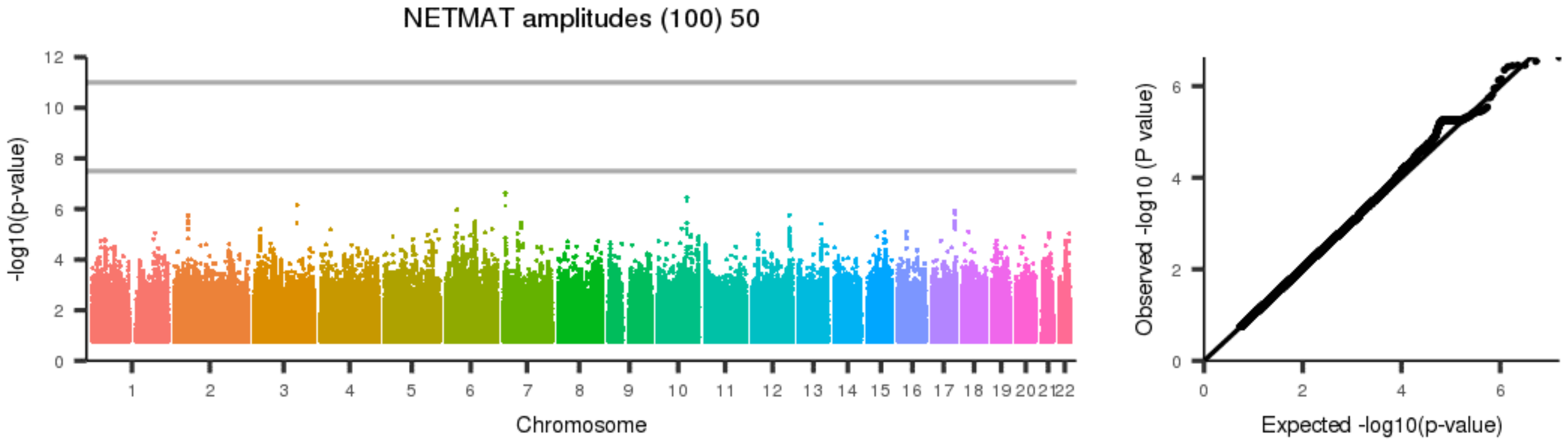

back to list of plots

Figure 942: NETMAT amplitudes (100) 51

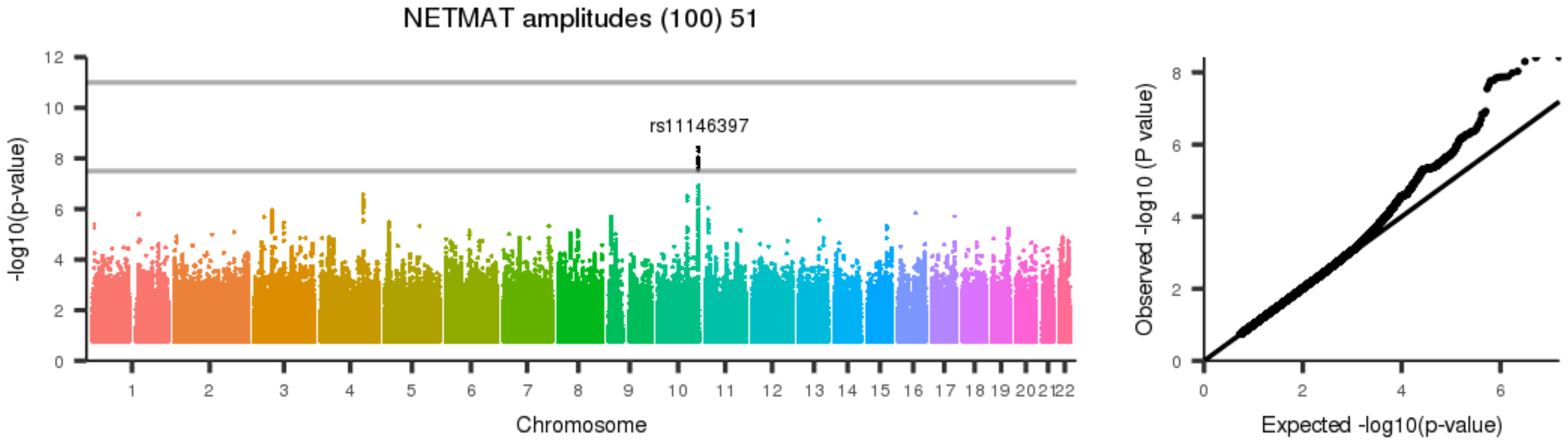

back to list of plots

Figure 943: NETMAT amplitudes (100) 52

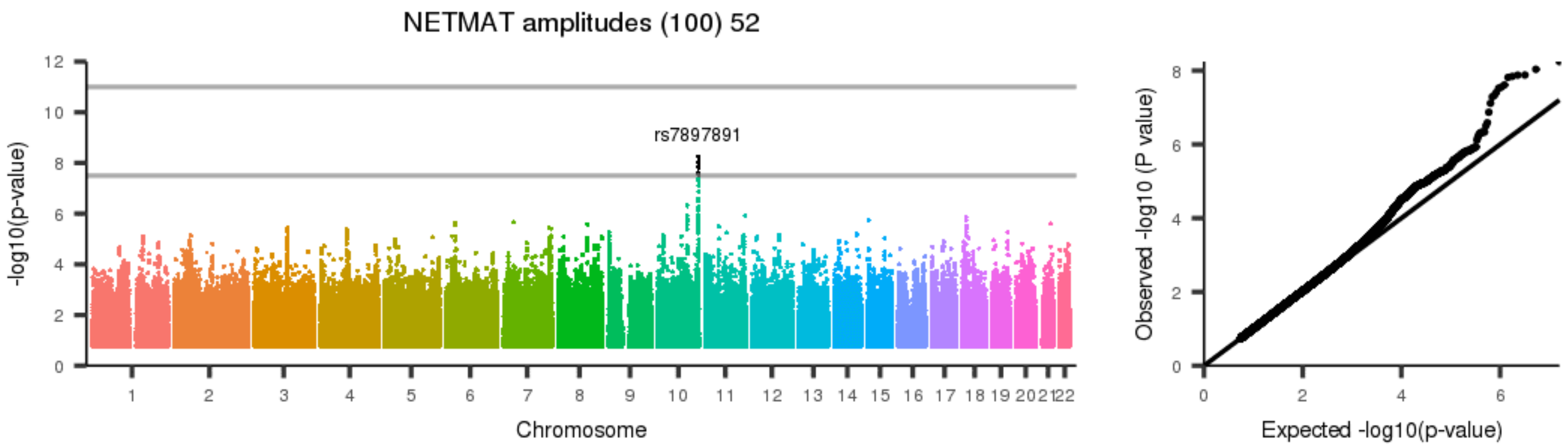

back to list of plots

Figure 944: NETMAT amplitudes (100) 53

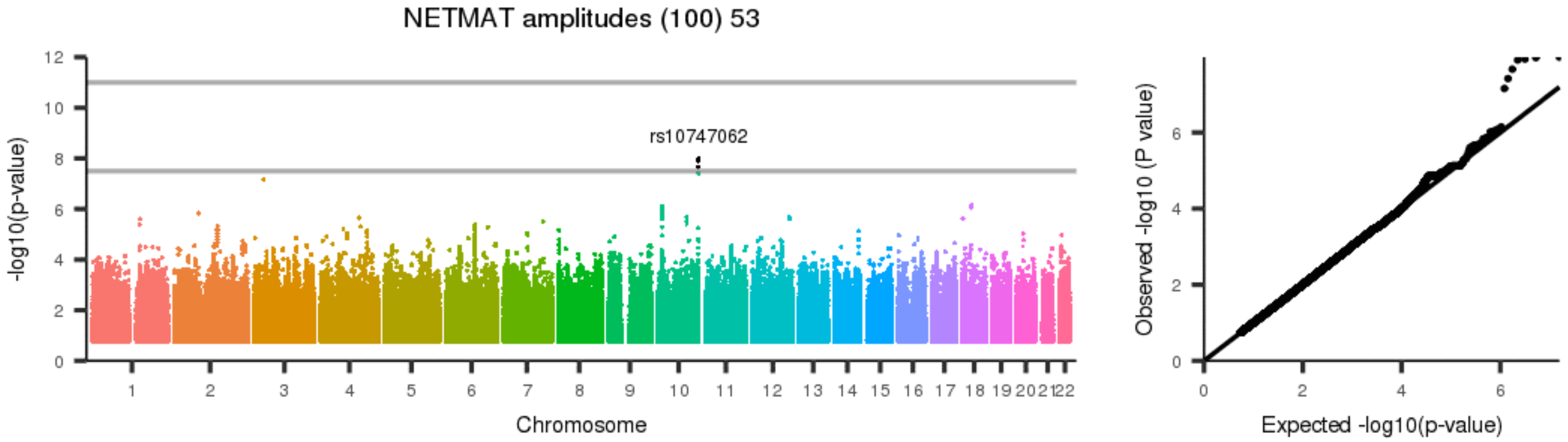

back to list of plots

Figure 945: NETMAT amplitudes (100) 54

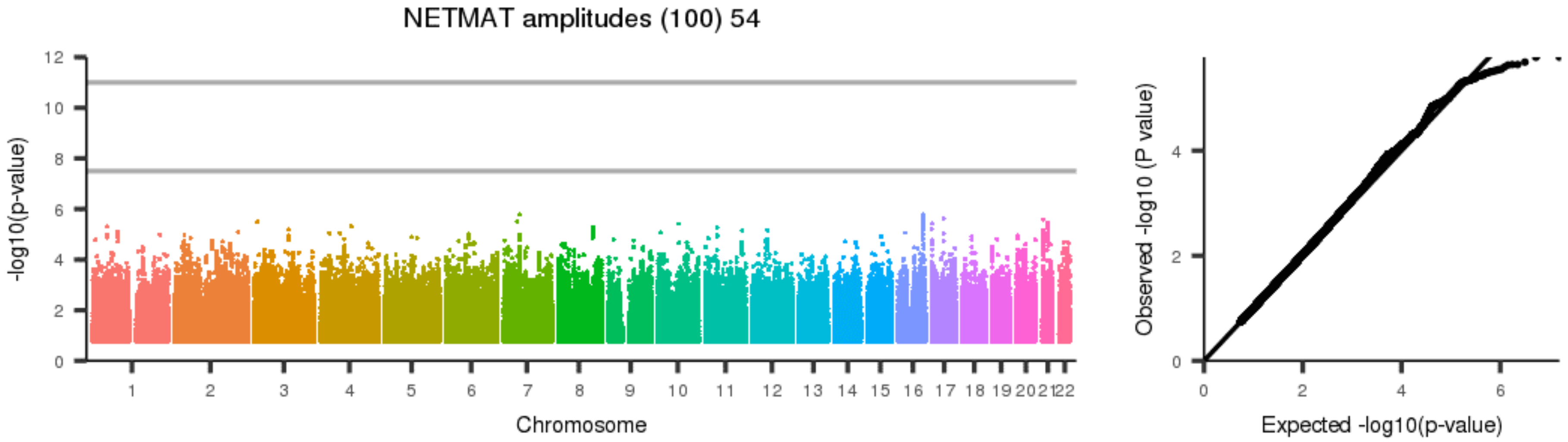

back to list of plots

Figure 946: NETMAT amplitudes (100) 55

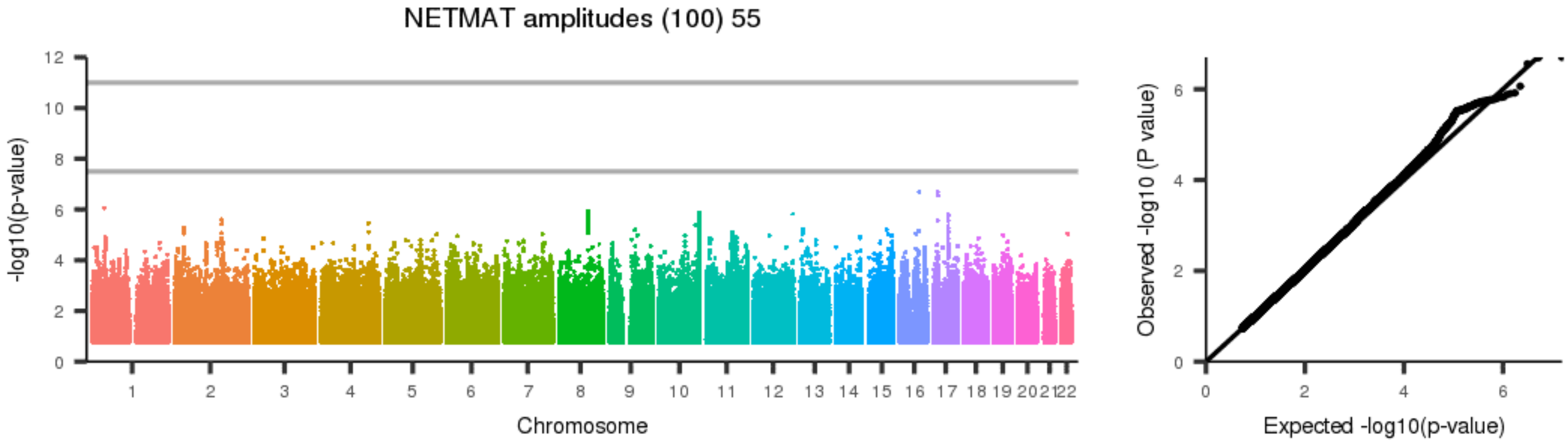

back to list of plots

Figure 947: T1 FIRST left plus right thalamus volume

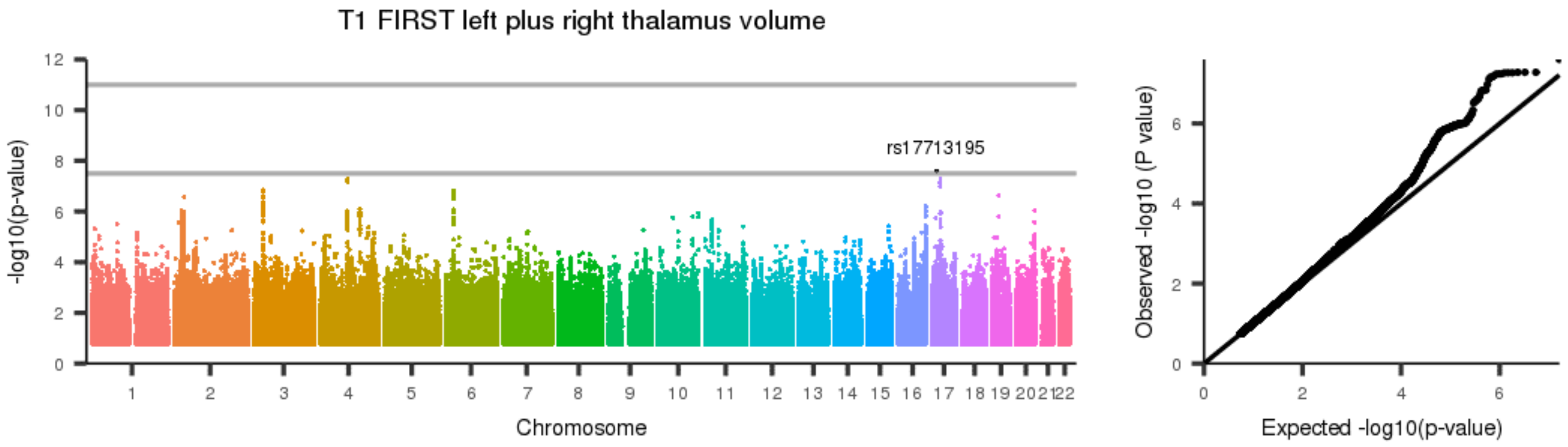

back to list of plots

Figure 948: T1 FIRST left plus right caudate volume

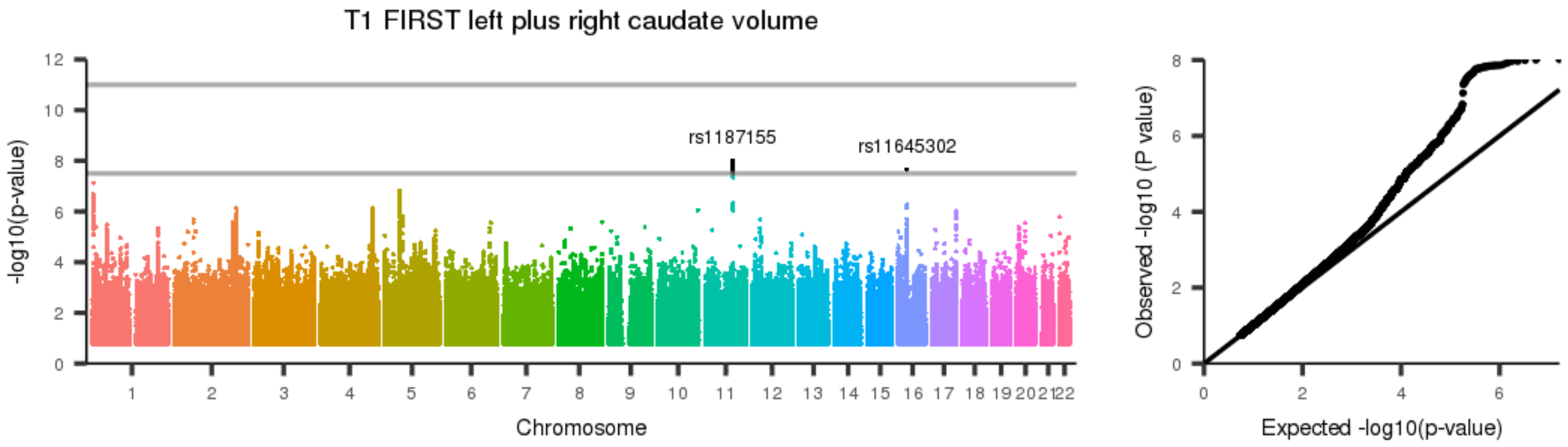

back to list of plots

Figure 949: T1 FIRST left plus right putamen volume

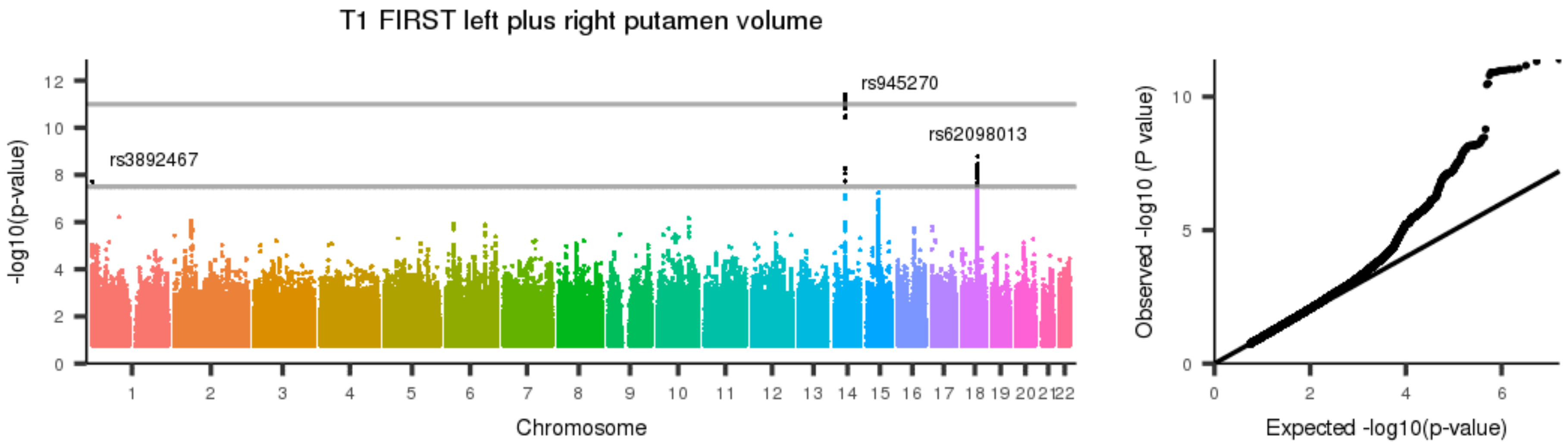

back to list of plots

Figure 950: T1 FIRST left plus right pallidum volume

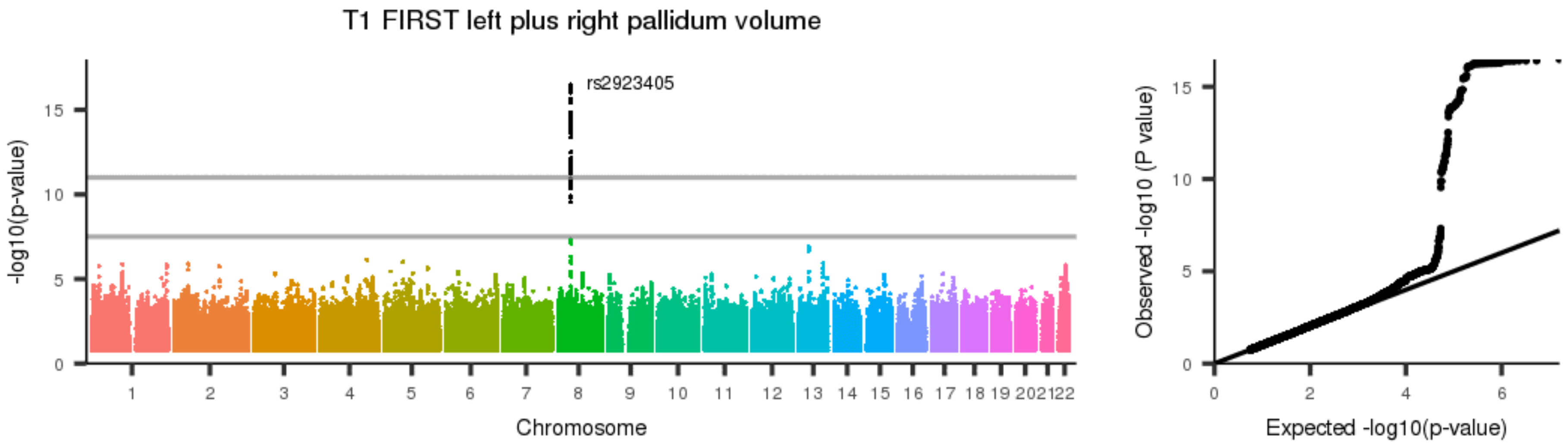

back to list of plots

Figure 951: T1 FIRST left plus right hippocampus volume

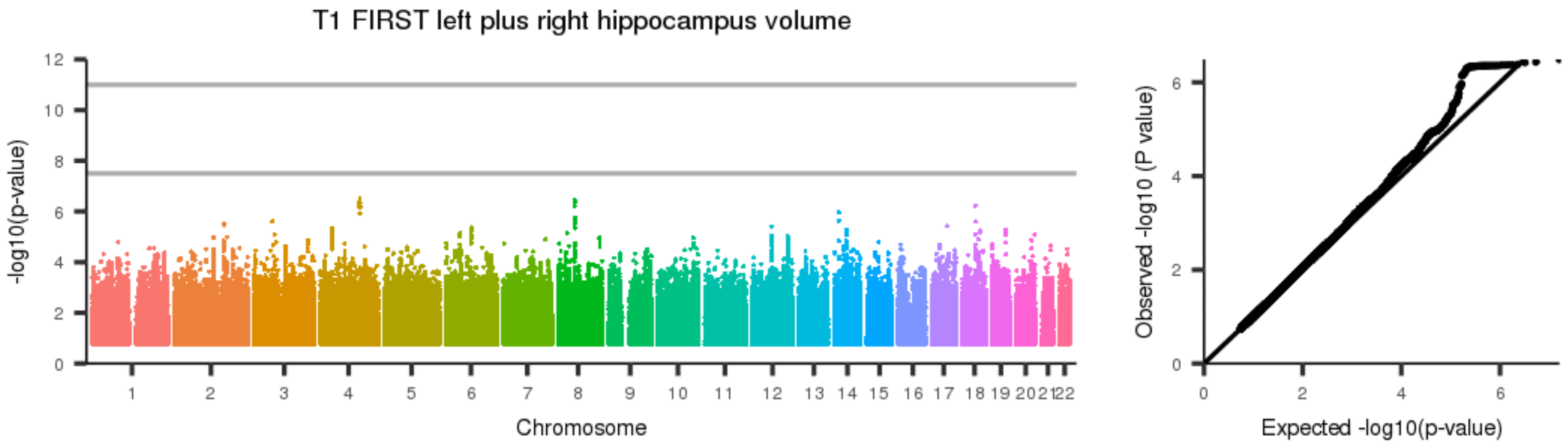

back to list of plots

Figure 952: T1 FIRST left plus right amygdala volume

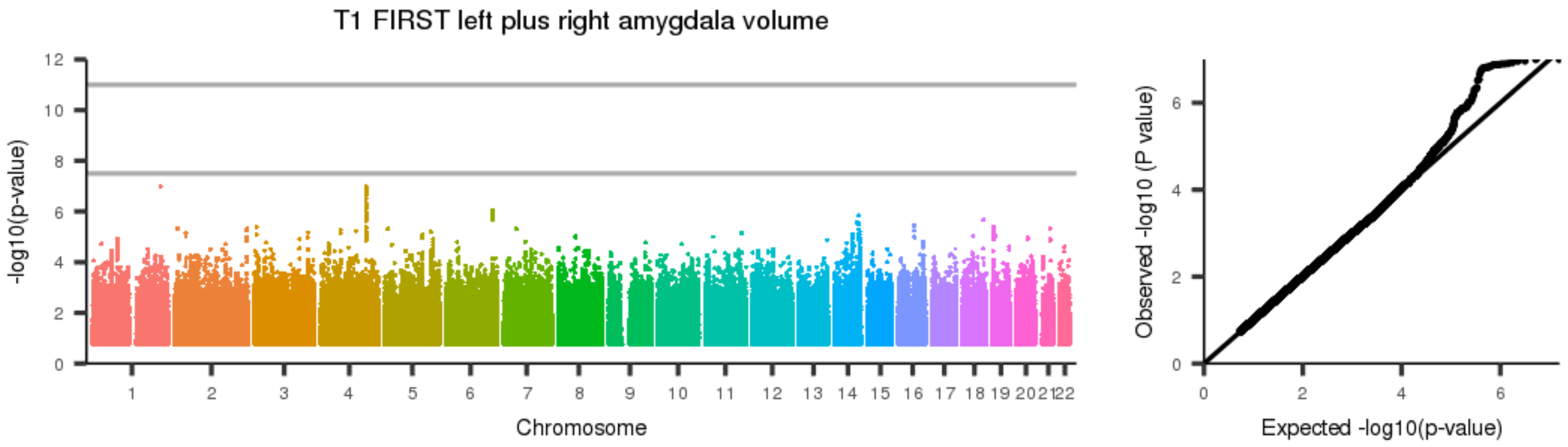

back to list of plots

Figure 953: T1 FIRST left plus right accumbens volume

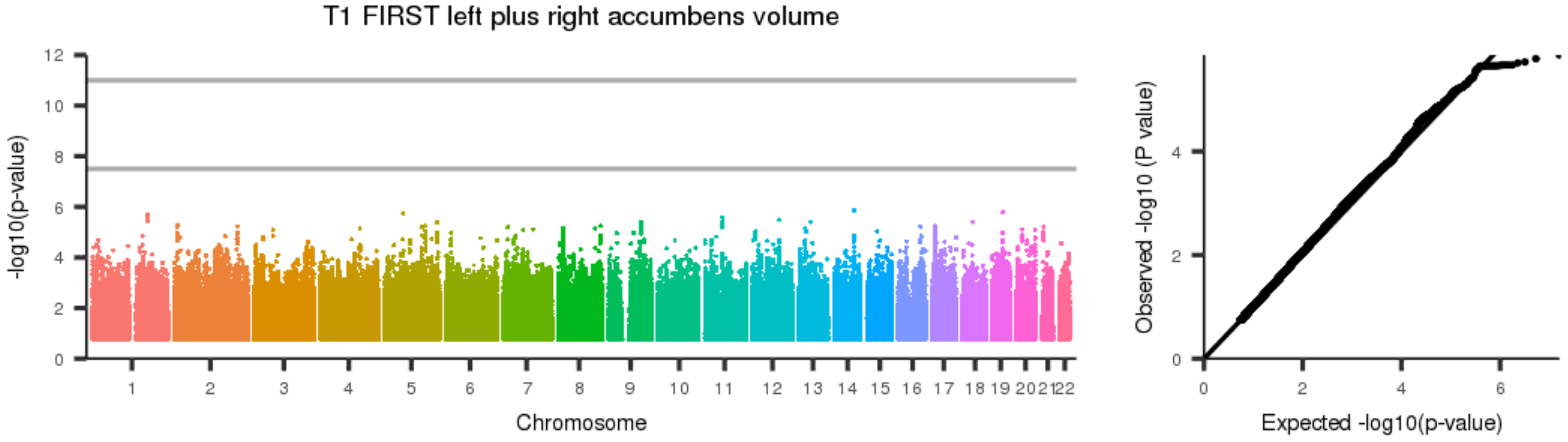

back to list of plots

Figure 954: SWI T2star left plus right thalamus

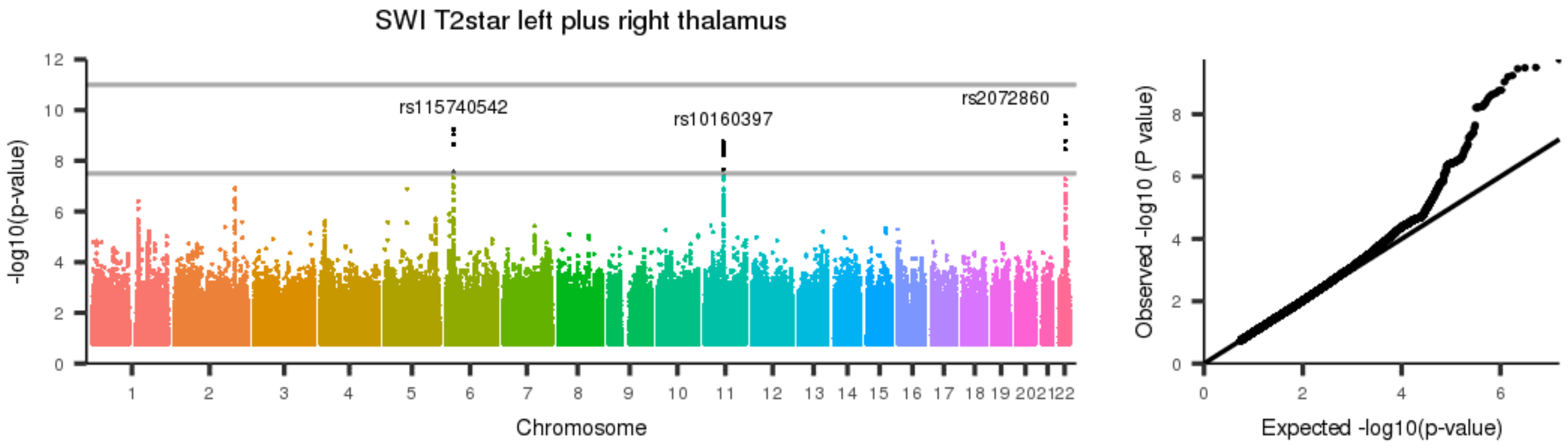

back to list of plots

Figure 955: SWI T2star left plus right caudate

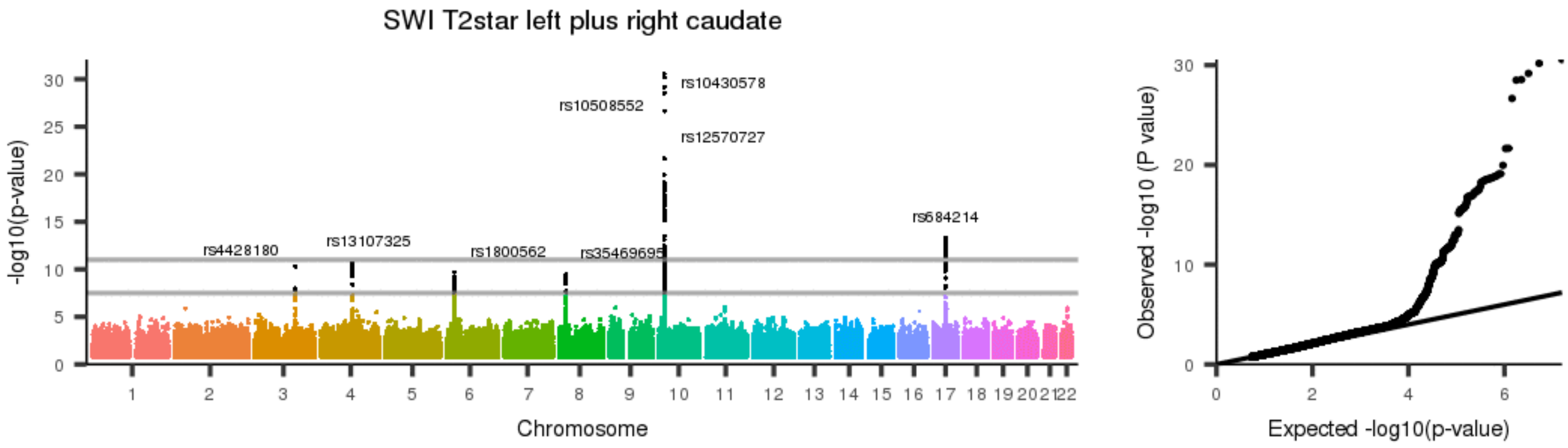

back to list of plots

Figure 956: SWI T2star left plus right putamen

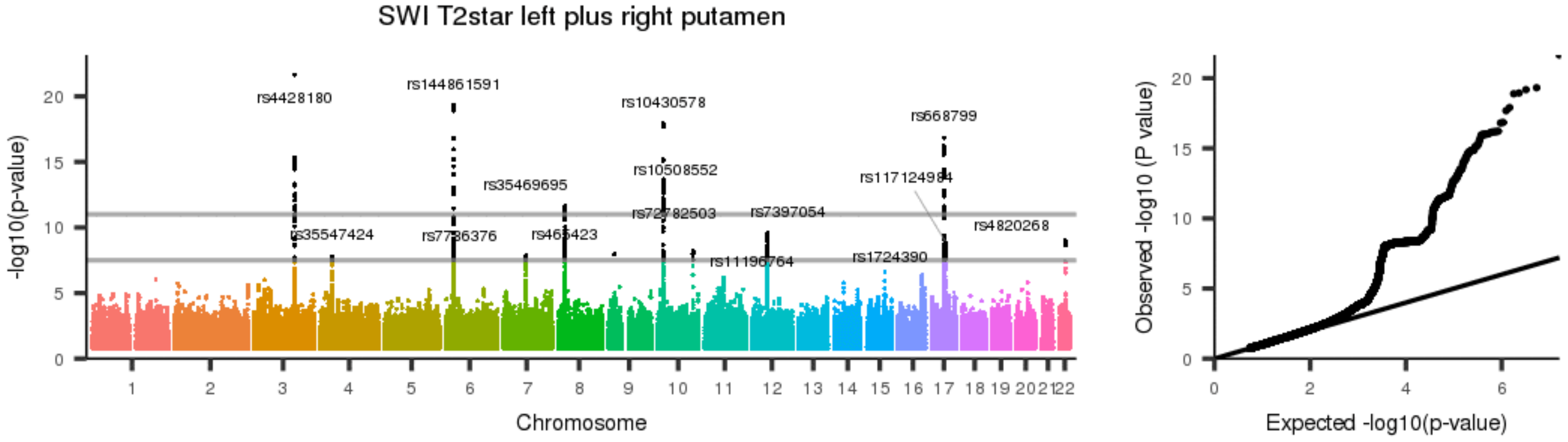

back to list of plots

Figure 957: SWI T2star left plus right pallidum

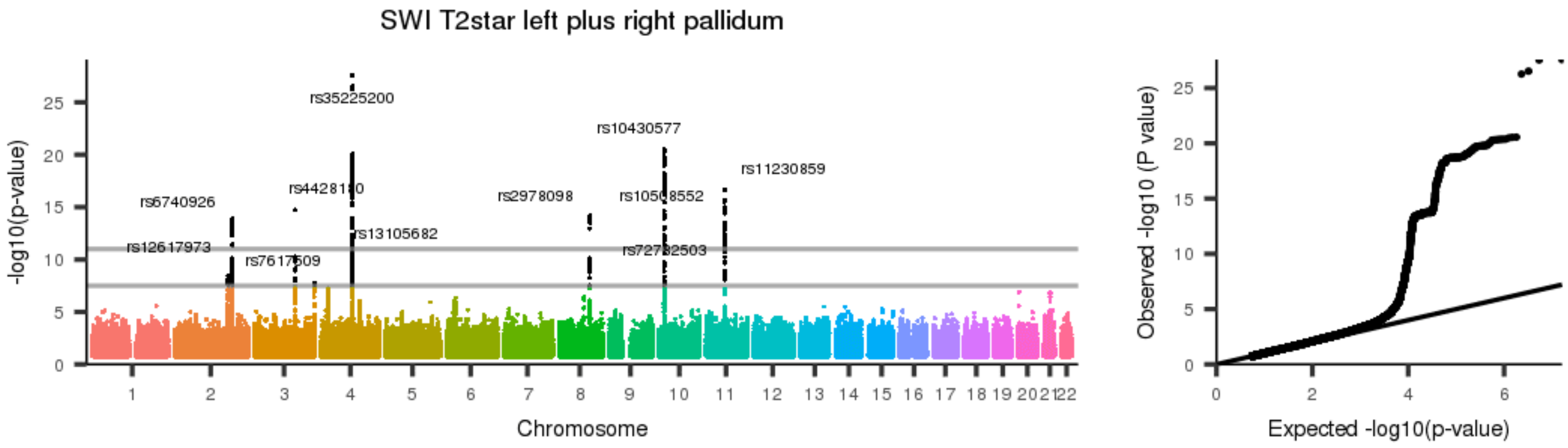

back to list of plots

Figure 958: SWI T2star left plus right hippocampus

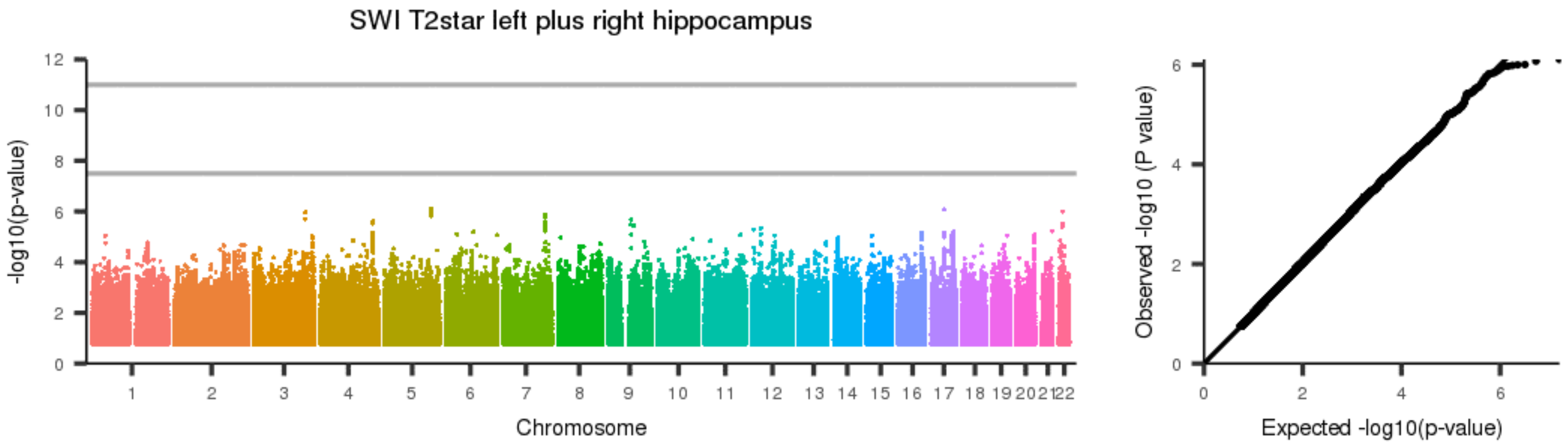

back to list of plots

Figure 959: SWI T2star left plus right amygdala

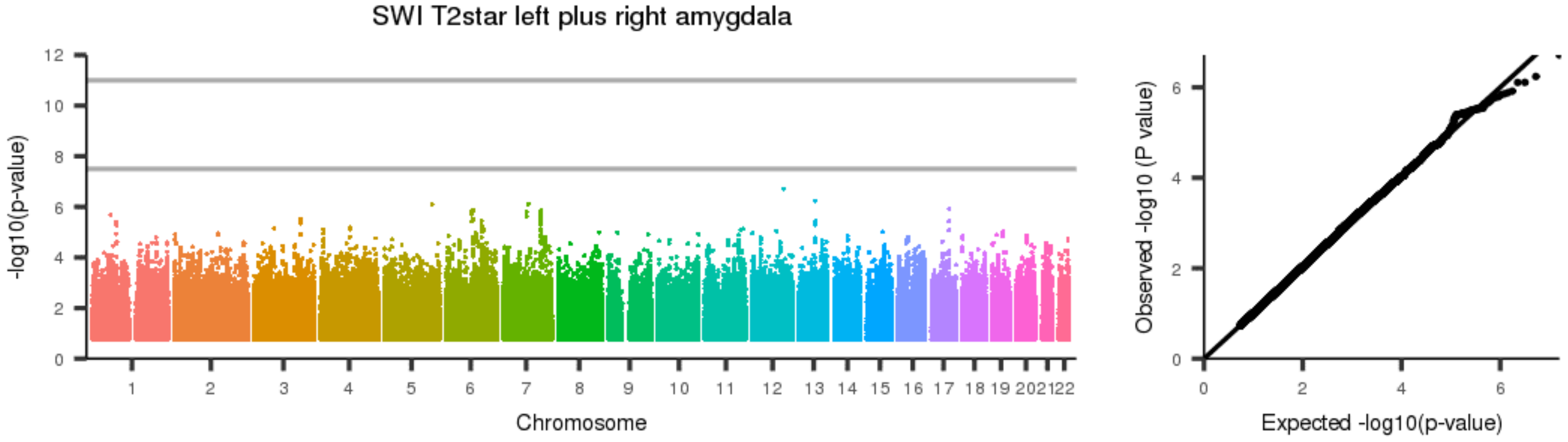

back to list of plots

Figure 960: SWI T2star left plus right accumbens

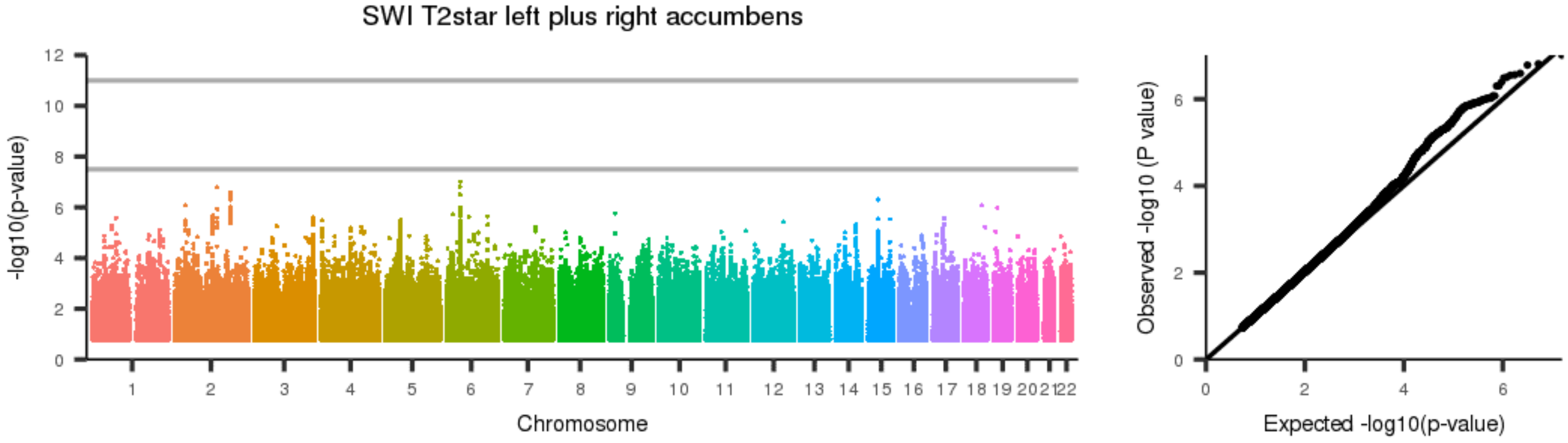

back to list of plots

Figure 961: FreeSurfer volume Left-Lateral-Ventricle

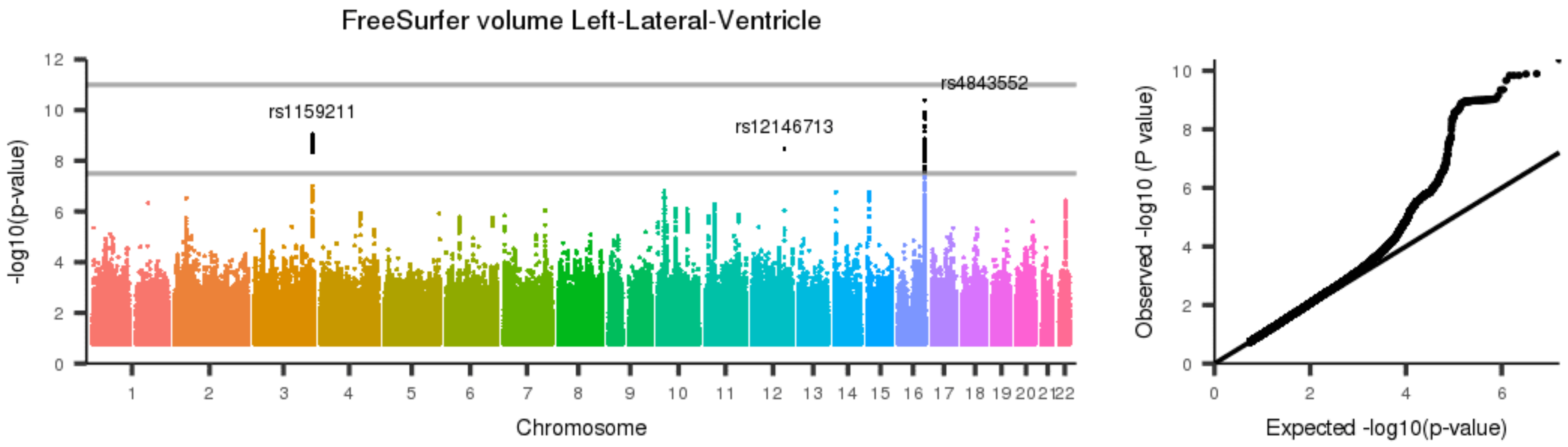

back to list of plots

Figure 962: FreeSurfer volume Left-Inf-Lat-Vent

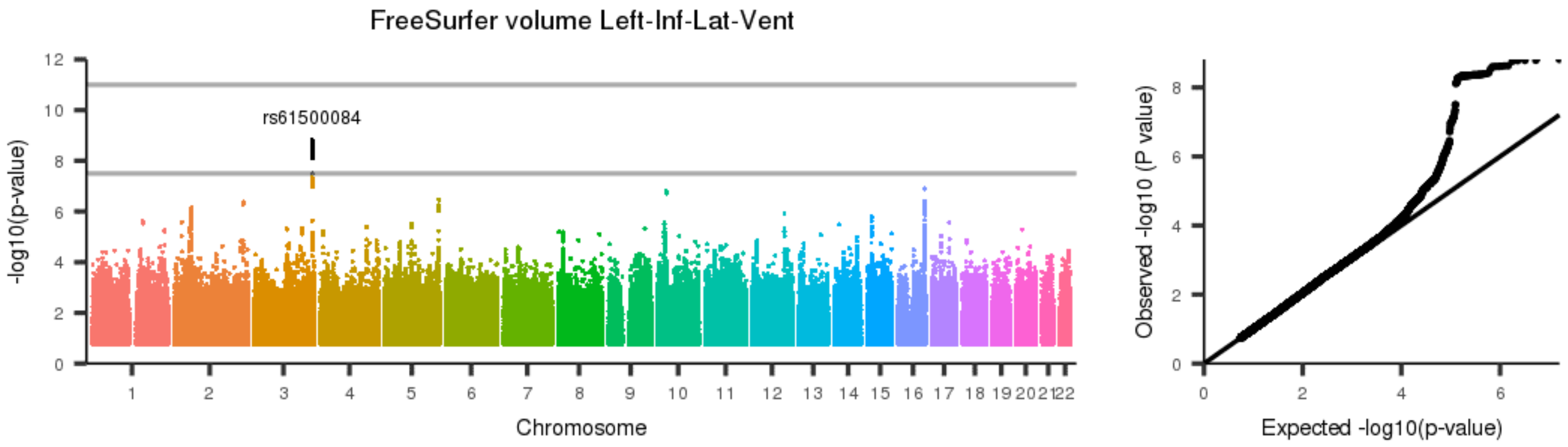

back to list of plots

Figure 963: FreeSurfer volume Left-Cerebellum-White-Matter

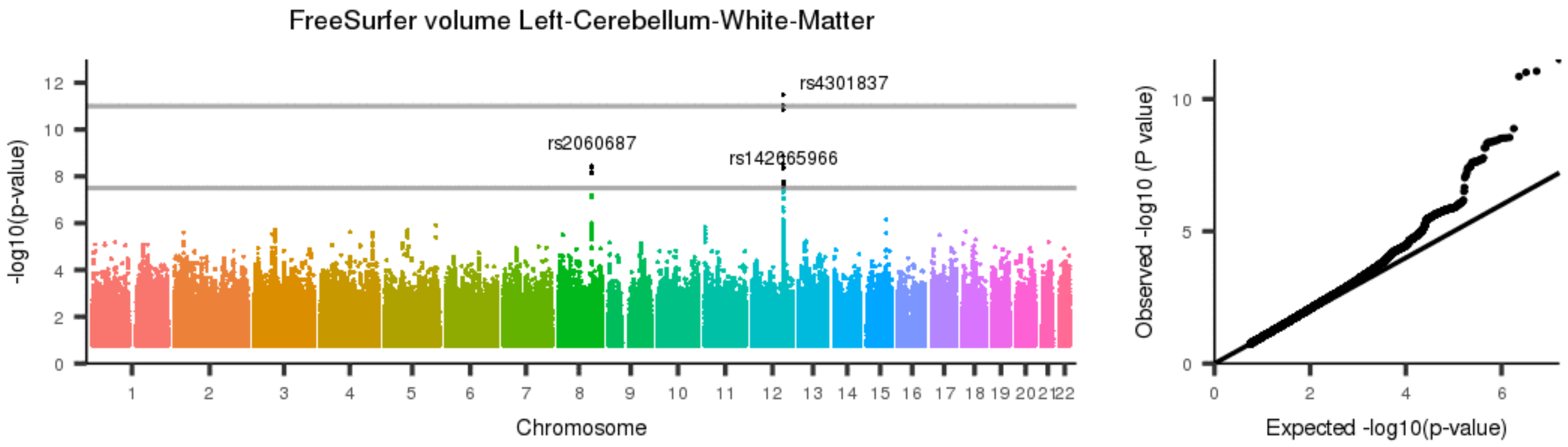

back to list of plots

Figure 964: FreeSurfer volume Left-Cerebellum-Cortex

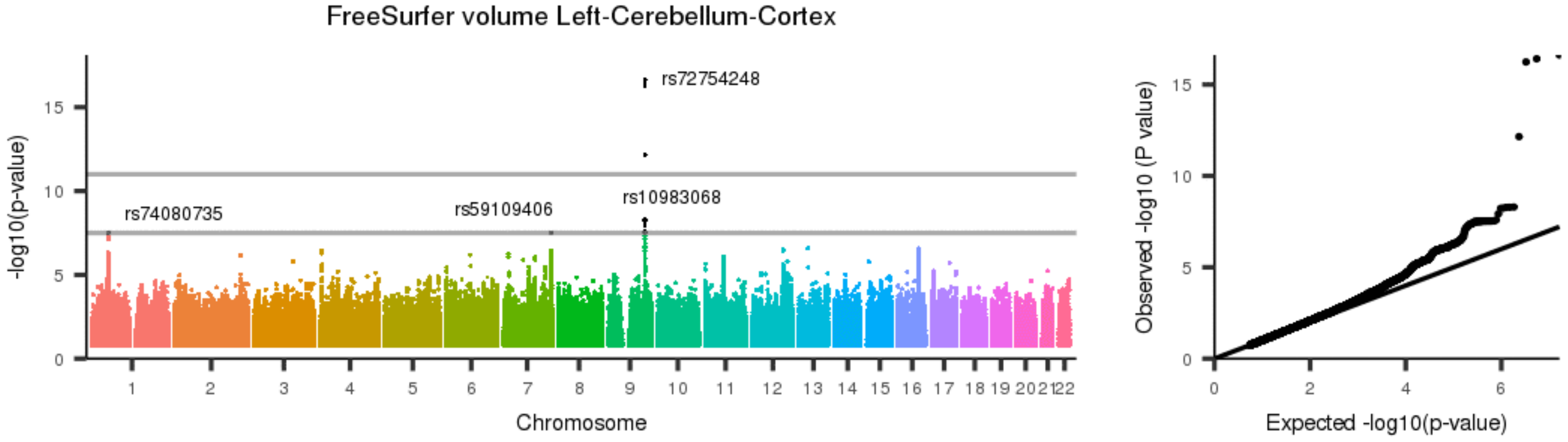

back to list of plots

Figure 965: FreeSurfer volume Left-Thalamus-Proper

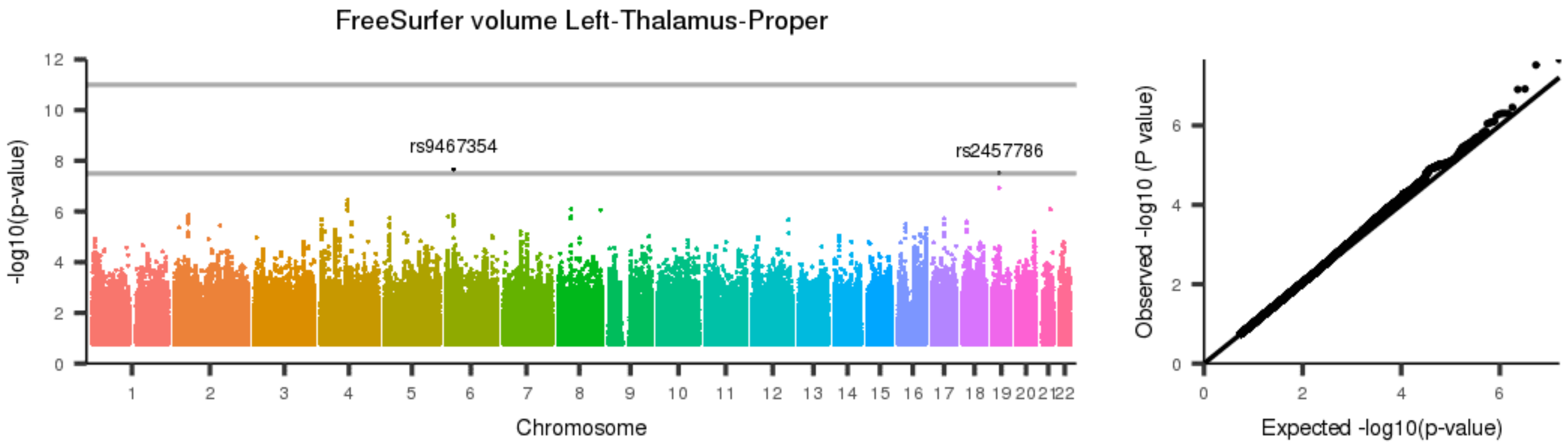

back to list of plots

Figure 966: FreeSurfer volume Left-Caudate

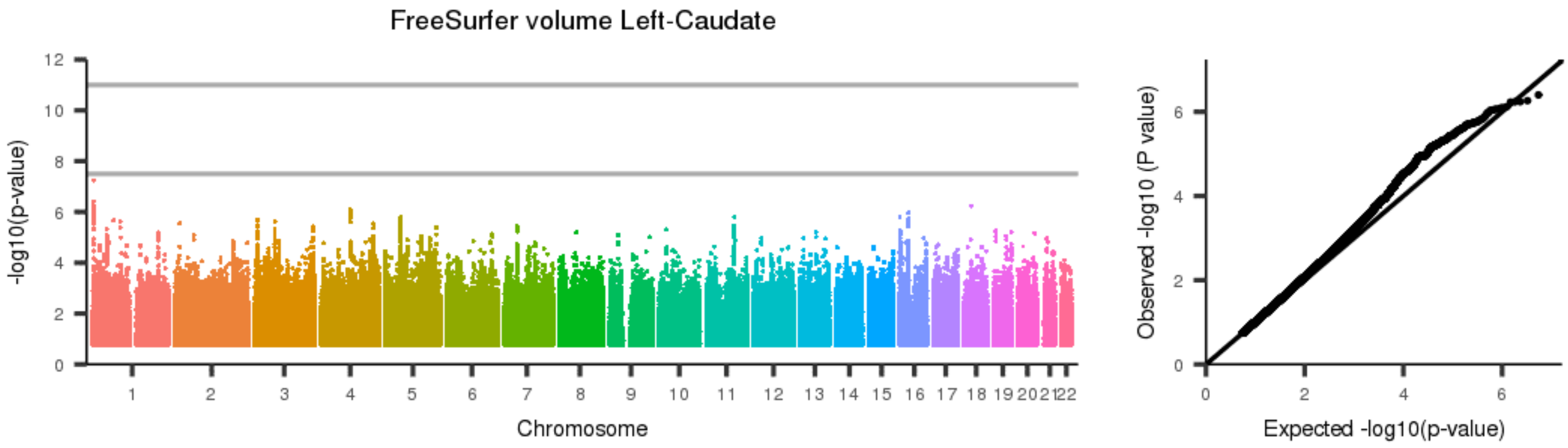

back to list of plots

Figure 967: FreeSurfer volume Left-Putamen

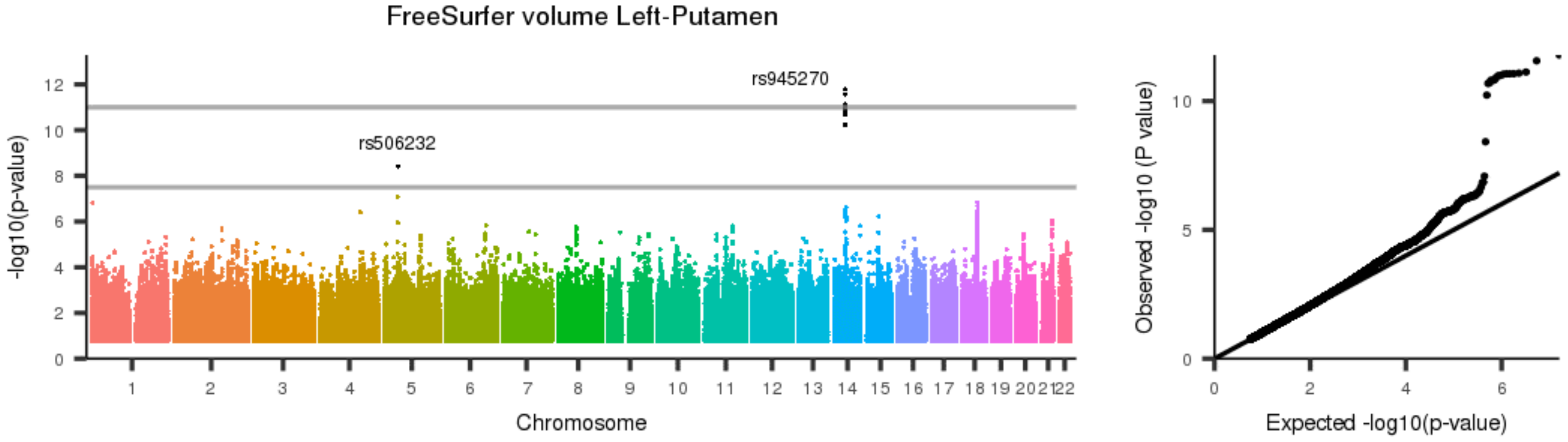

back to list of plots

Figure 968: FreeSurfer volume Left-Pallidum

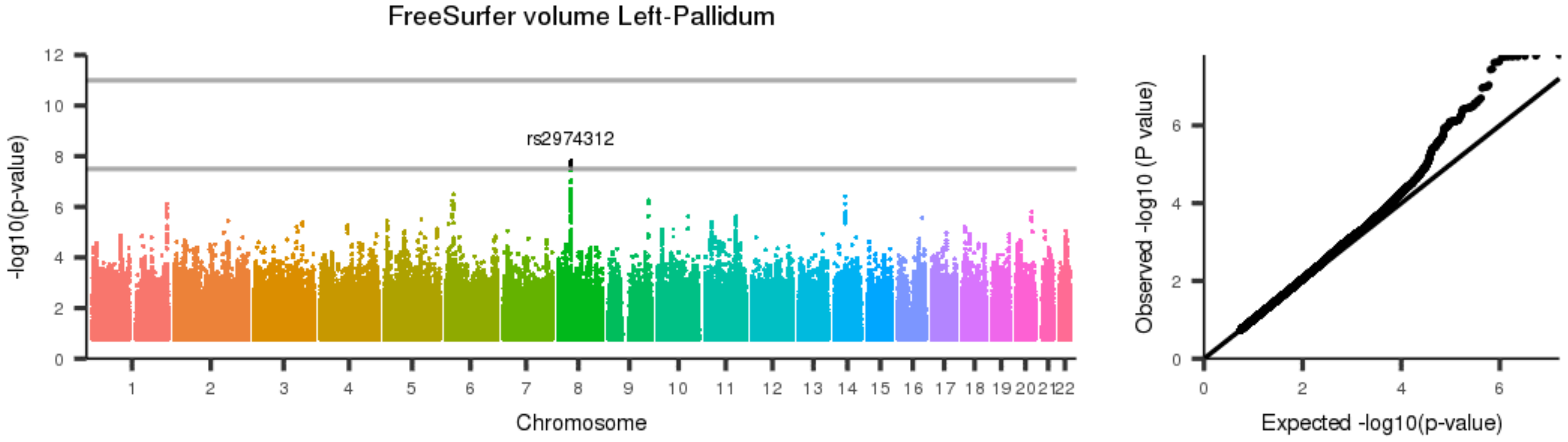

back to list of plots

Figure 969: FreeSurfer volume 3rd-Ventricle

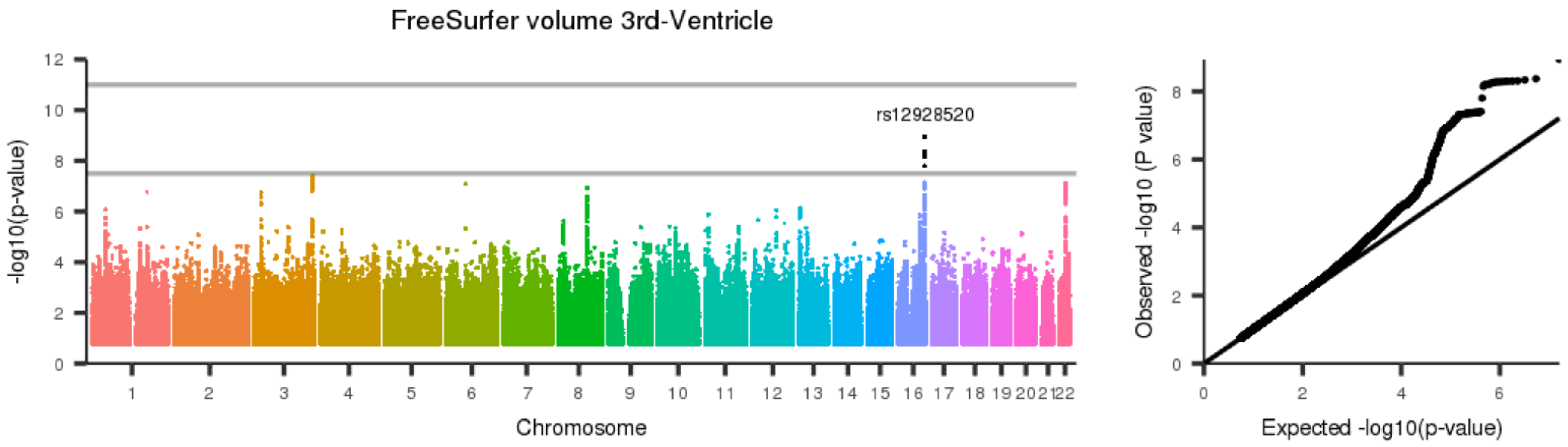

back to list of plots

Figure 970: FreeSurfer volume 4th-Ventricle

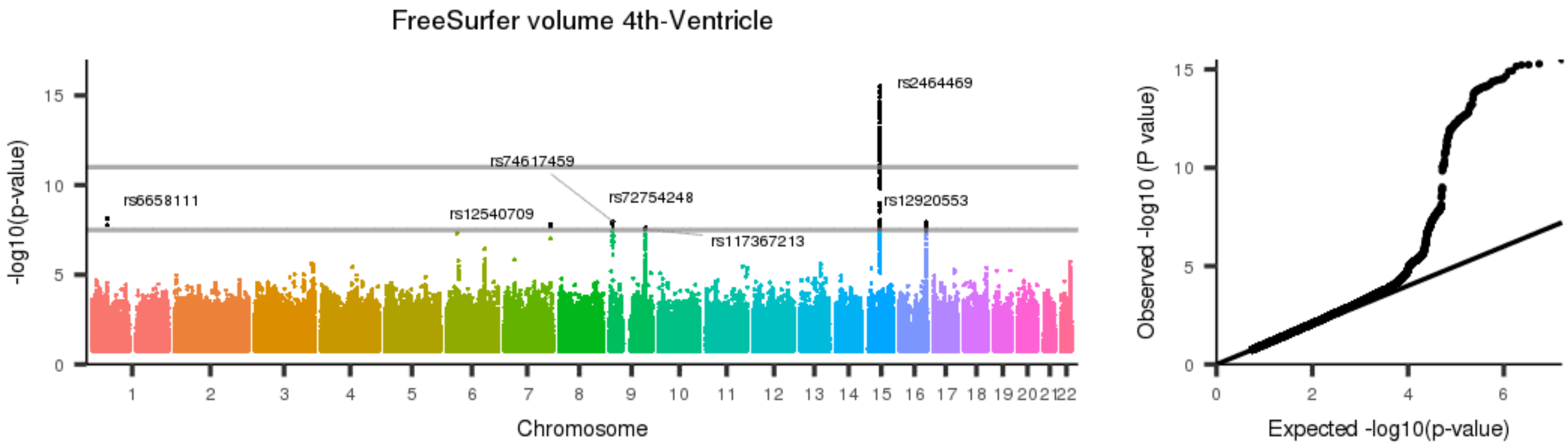

back to list of plots

Figure 971: FreeSurfer volume Brain-Stem

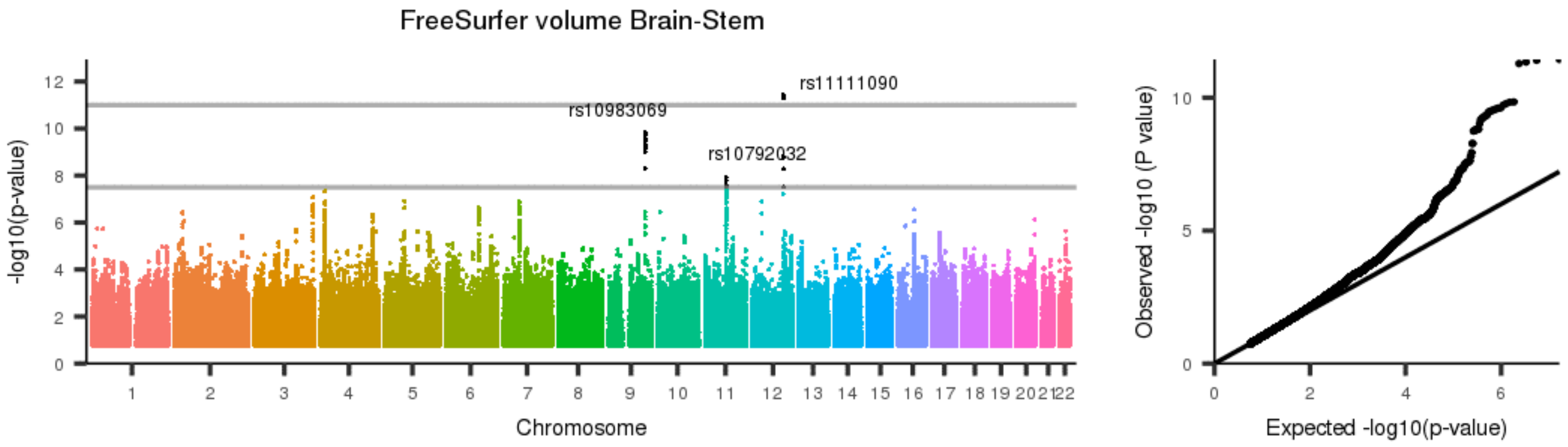

back to list of plots

Figure 972: FreeSurfer volume Left-Hippocampus

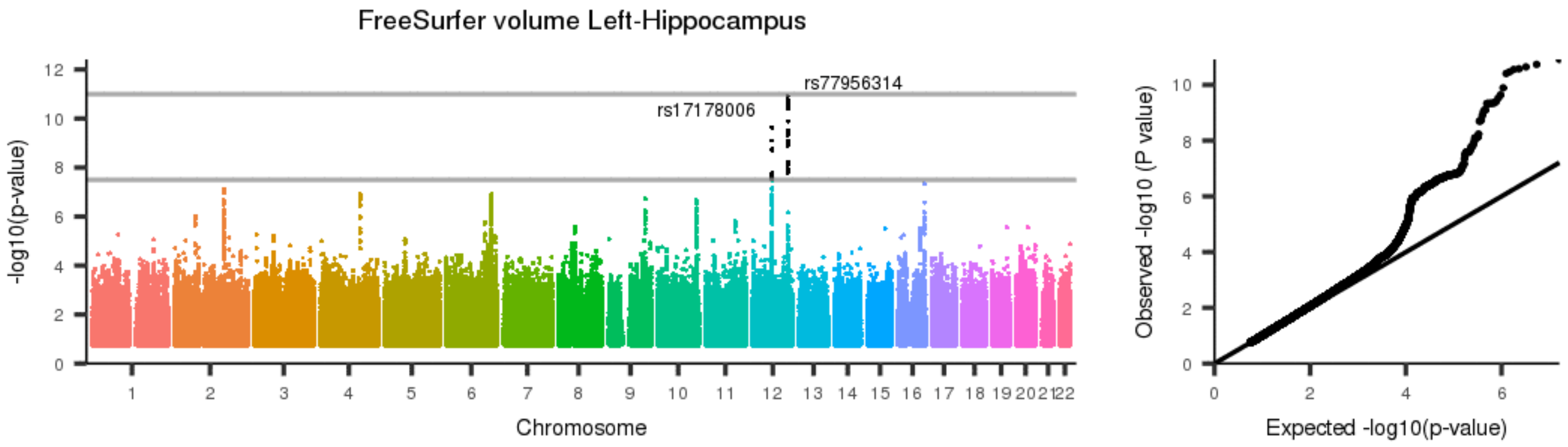

back to list of plots

Figure 973: FreeSurfer volume Left-Amygdala

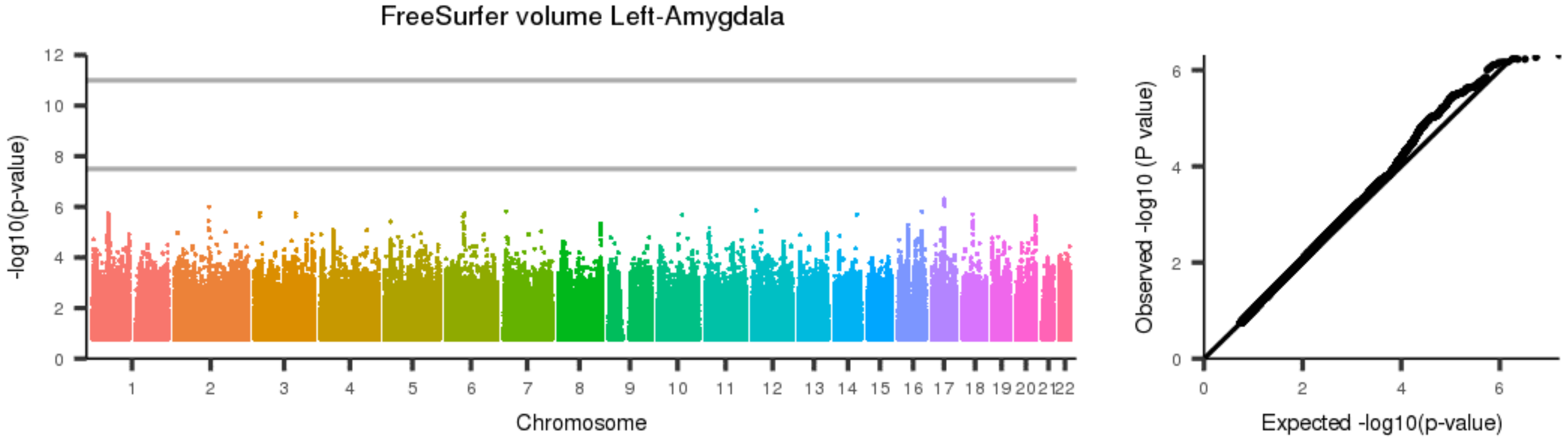

back to list of plots

Figure 974: FreeSurfer volume CSF

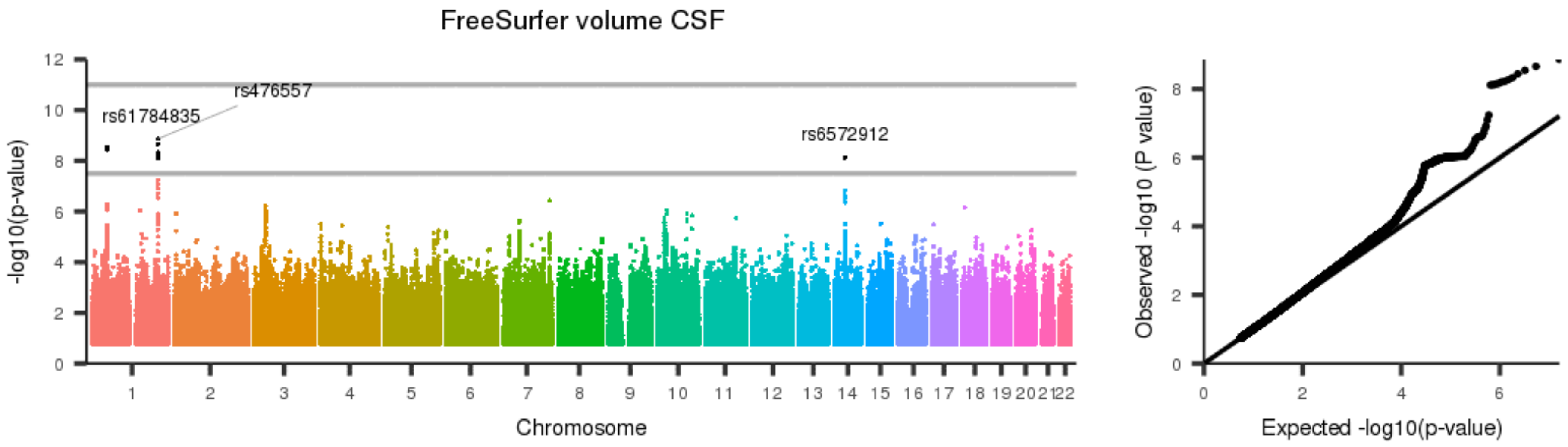

back to list of plots

Figure 975: FreeSurfer volume Left-Accumbens-area

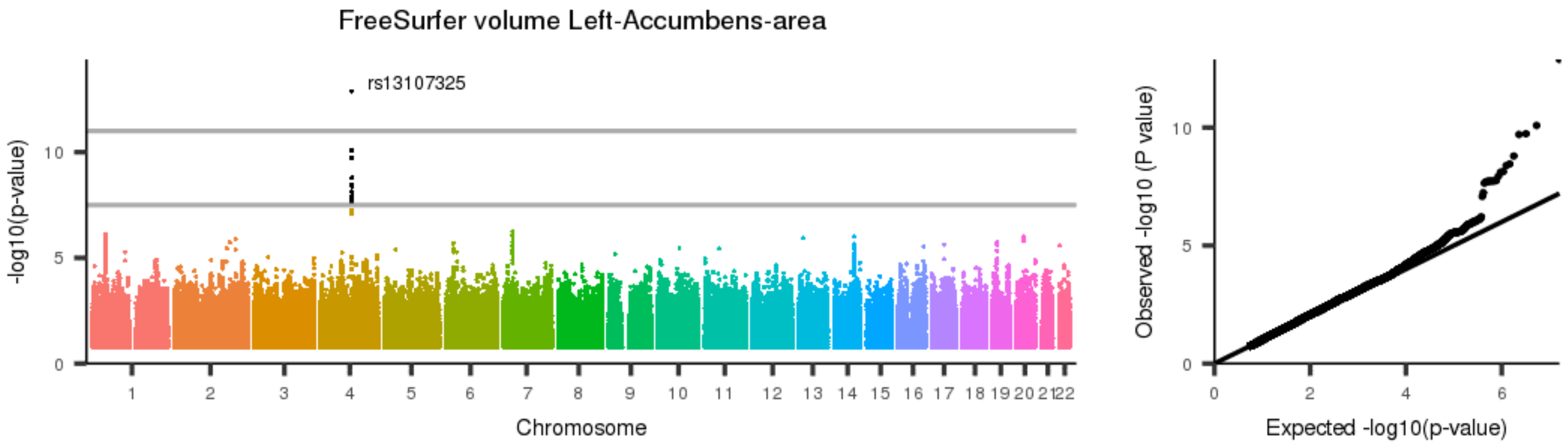

back to list of plots

Figure 976: FreeSurfer volume Left-VentralDC

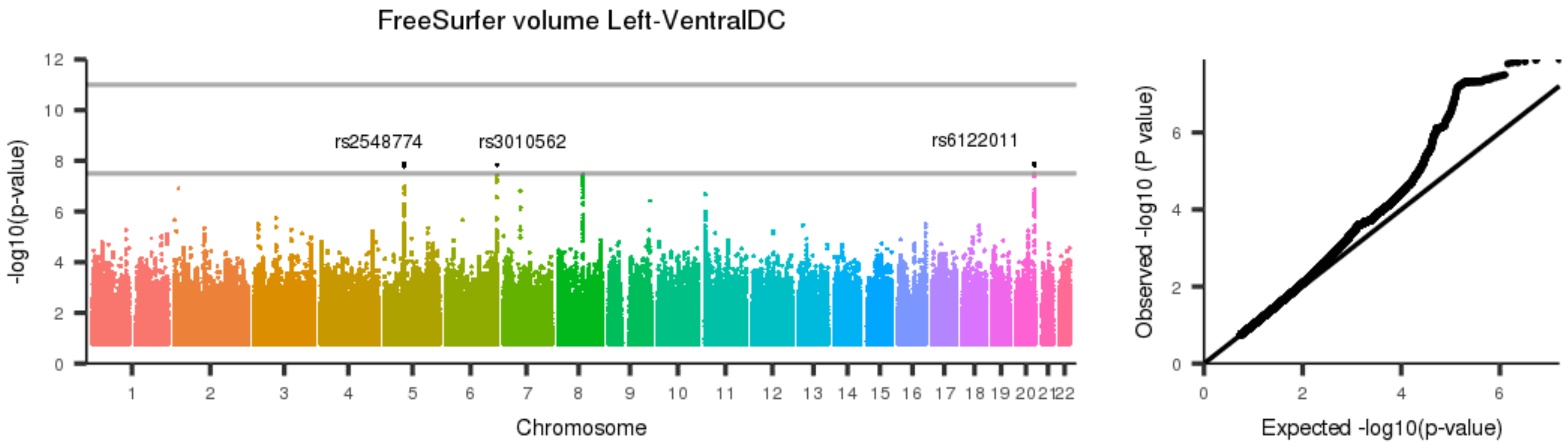

back to list of plots

Figure 977: FreeSurfer volume Left-vessel

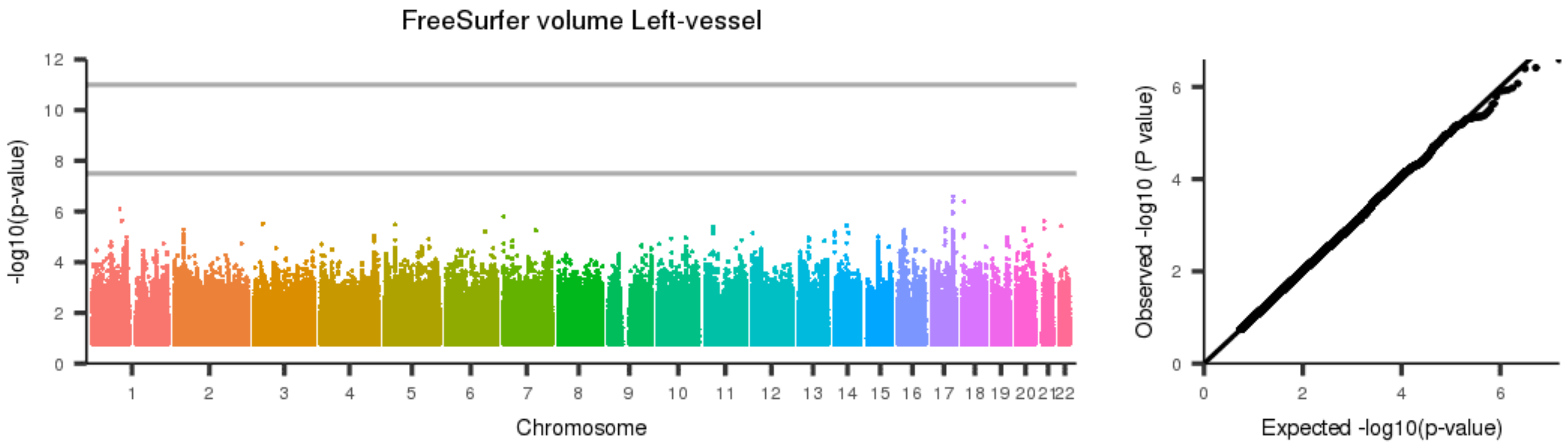

back to list of plots

Figure 978: FreeSurfer volume Left-choroid-plexus

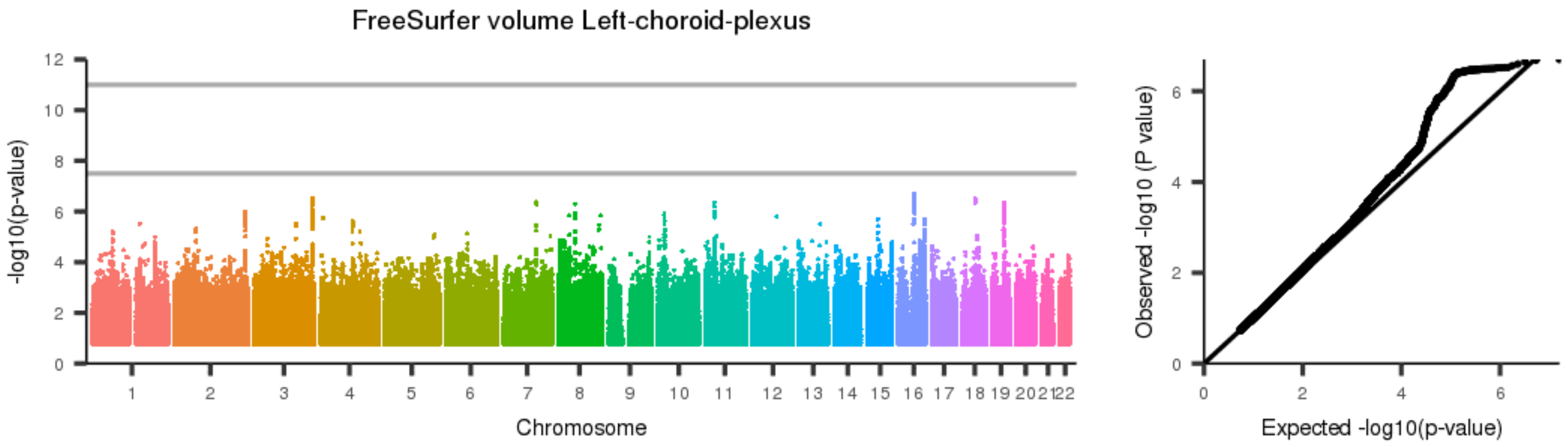

back to list of plots

Figure 979: FreeSurfer volume Right-Lateral-Ventricle

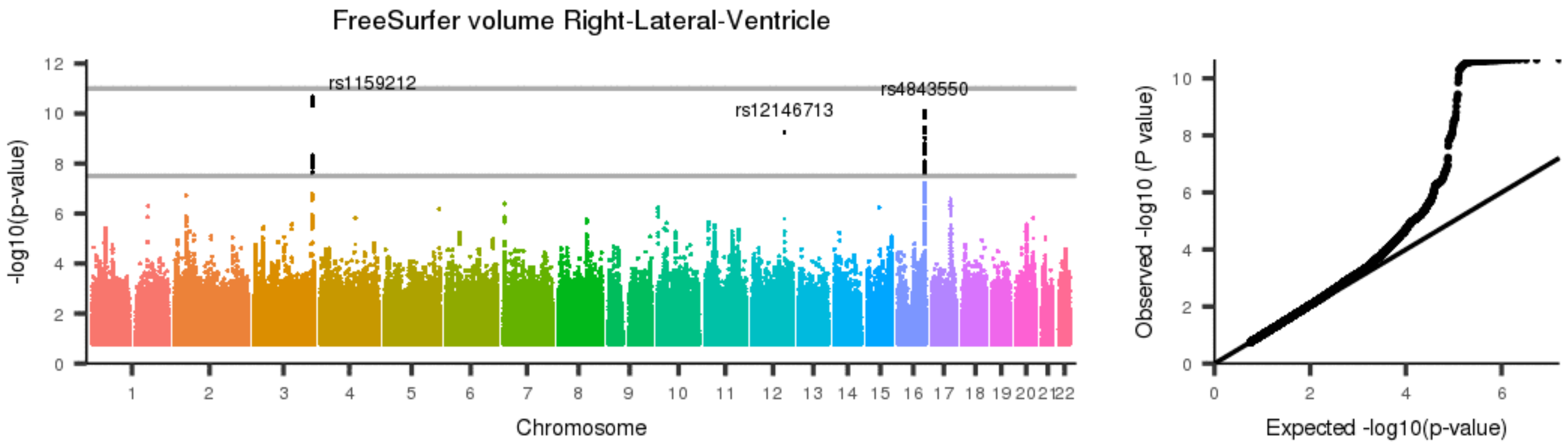

back to list of plots

Figure 980: FreeSurfer volume Right-Inf-Lat-Vent

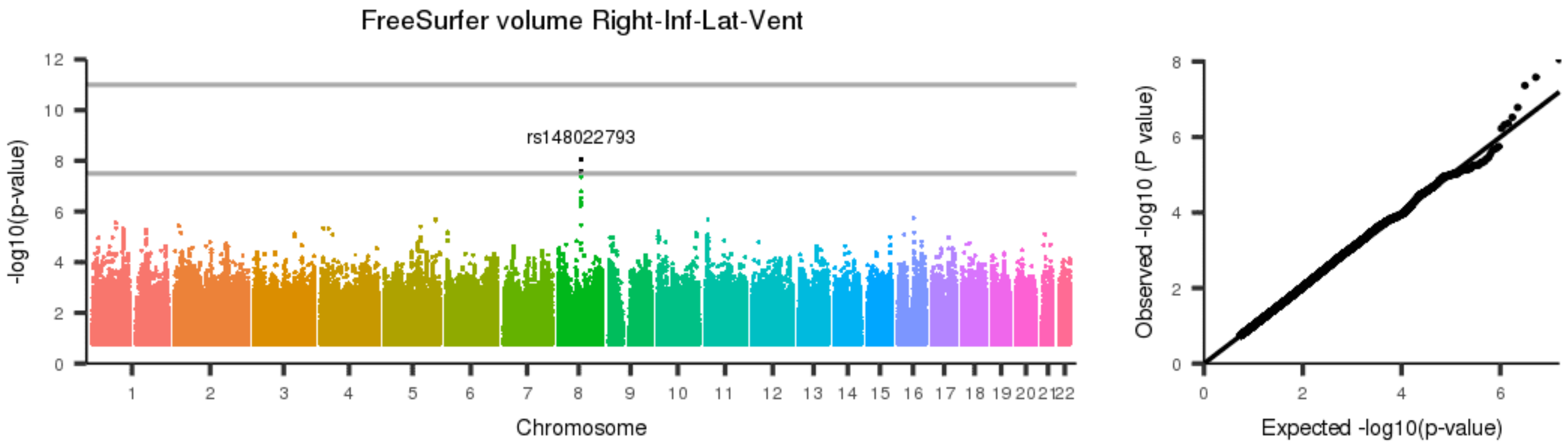

back to list of plots

Figure 981: FreeSurfer volume Right-Cerebellum-White-Matter

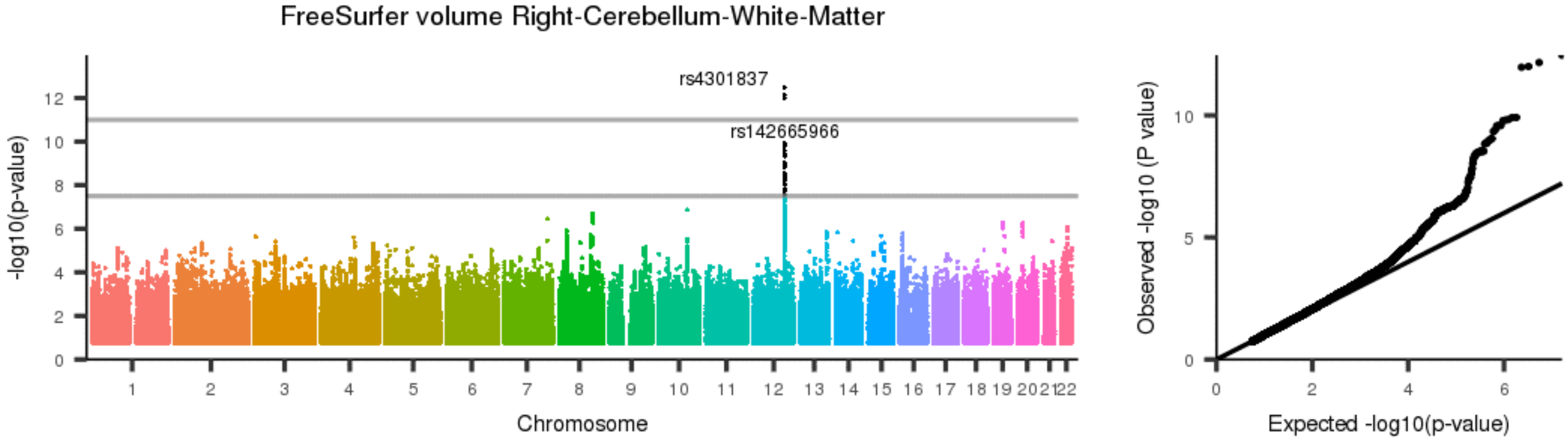

back to list of plots

Figure 982: FreeSurfer volume Right-Cerebellum-Cortex

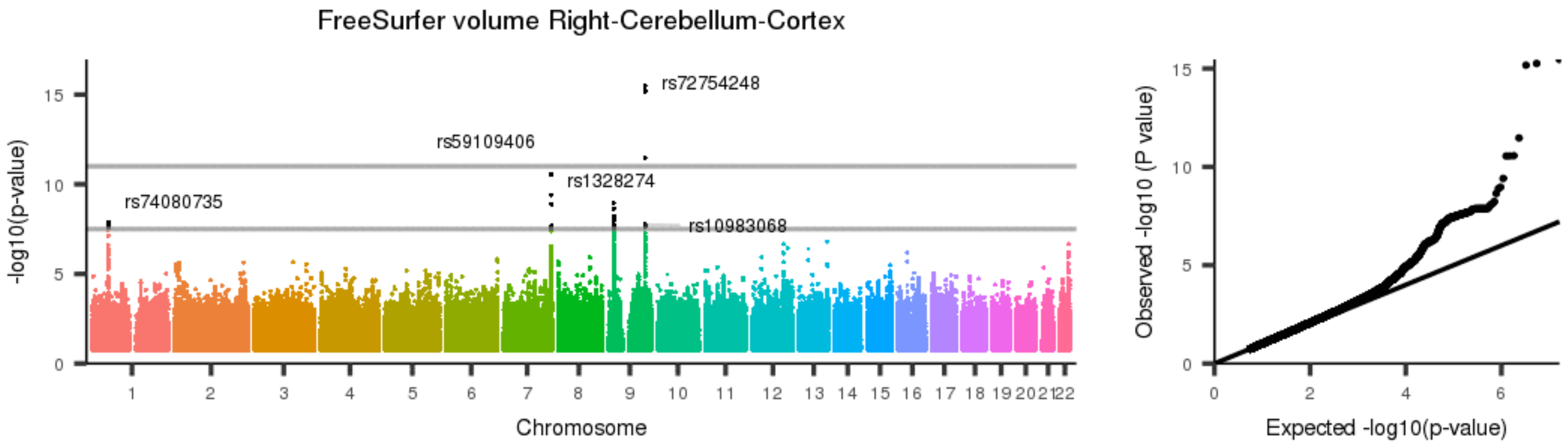

back to list of plots

Figure 983: FreeSurfer volume Right-Thalamus-Proper

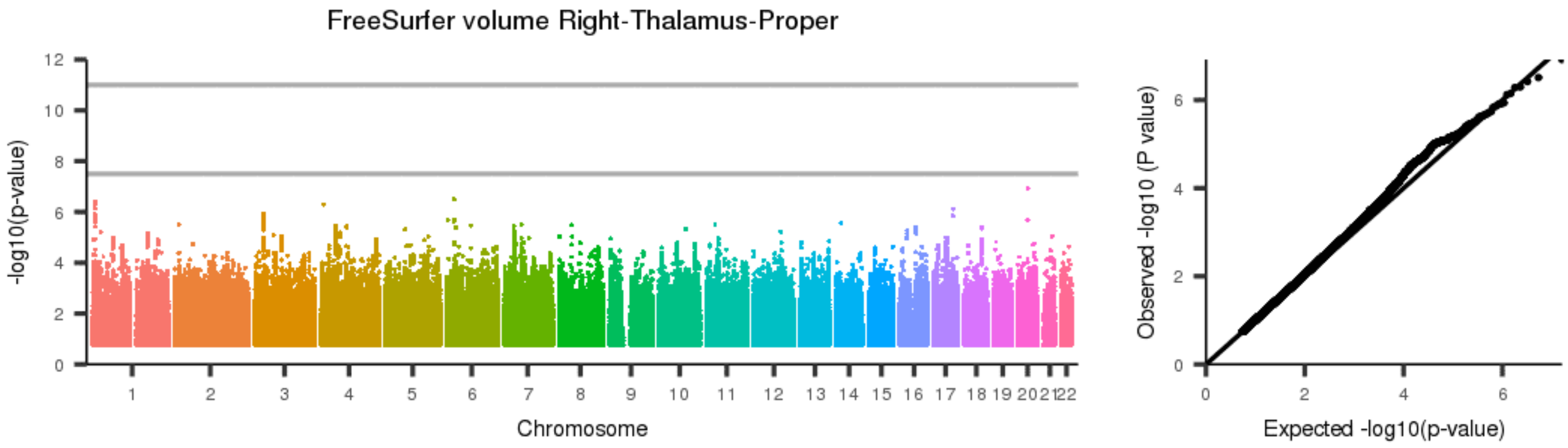

back to list of plots

Figure 984: FreeSurfer volume Right-Caudate

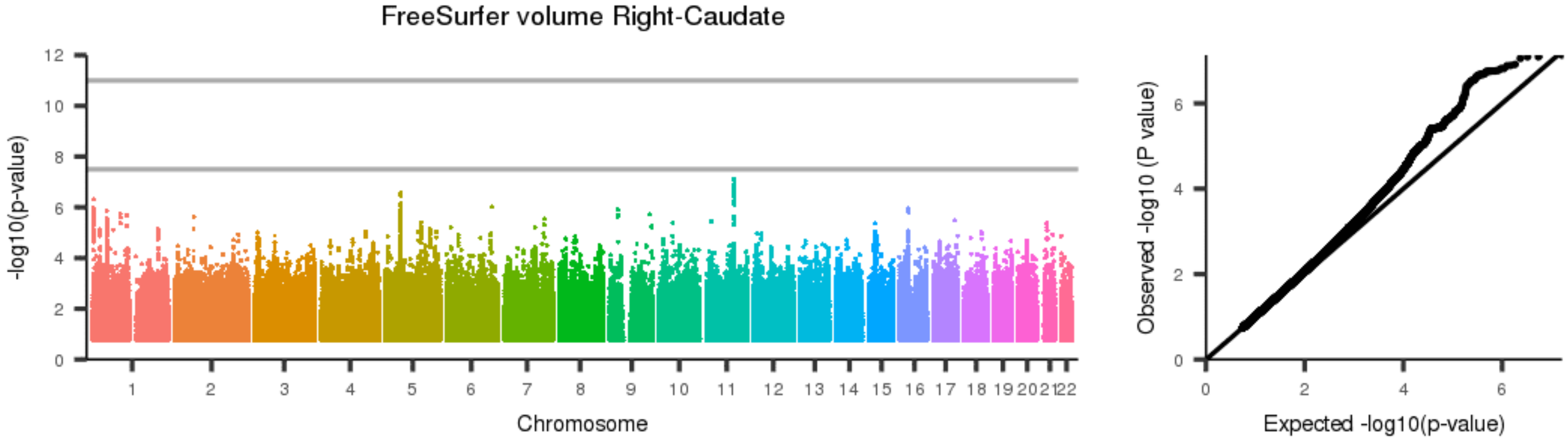

back to list of plots

Figure 985: FreeSurfer volume Right-Putamen

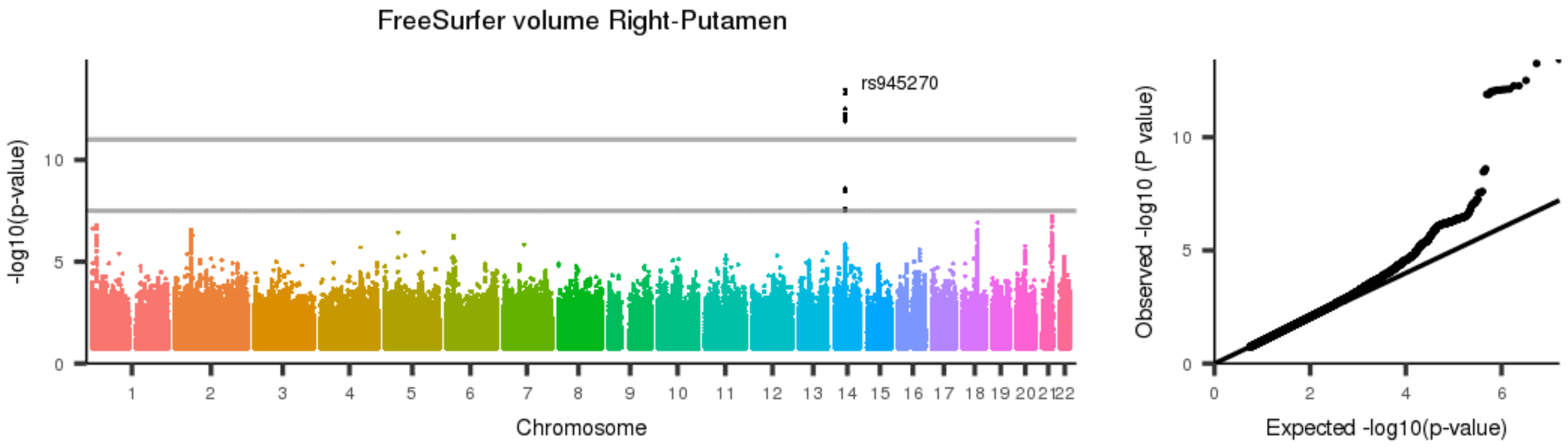

back to list of plots

Figure 986: FreeSurfer volume Right-Pallidum

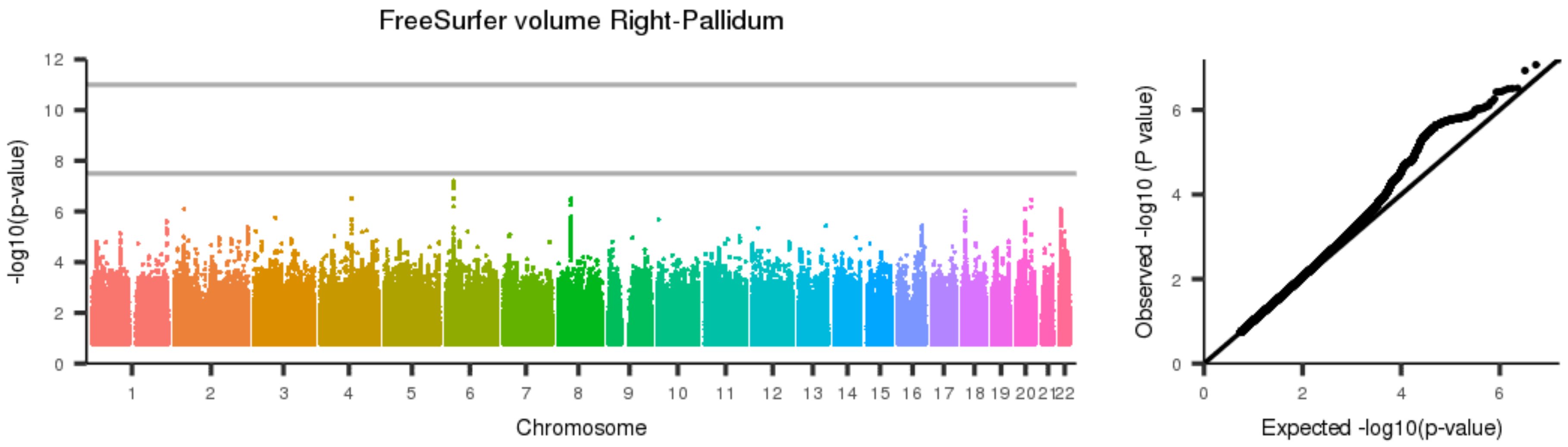

back to list of plots

Figure 987: FreeSurfer volume Right-Hippocampus

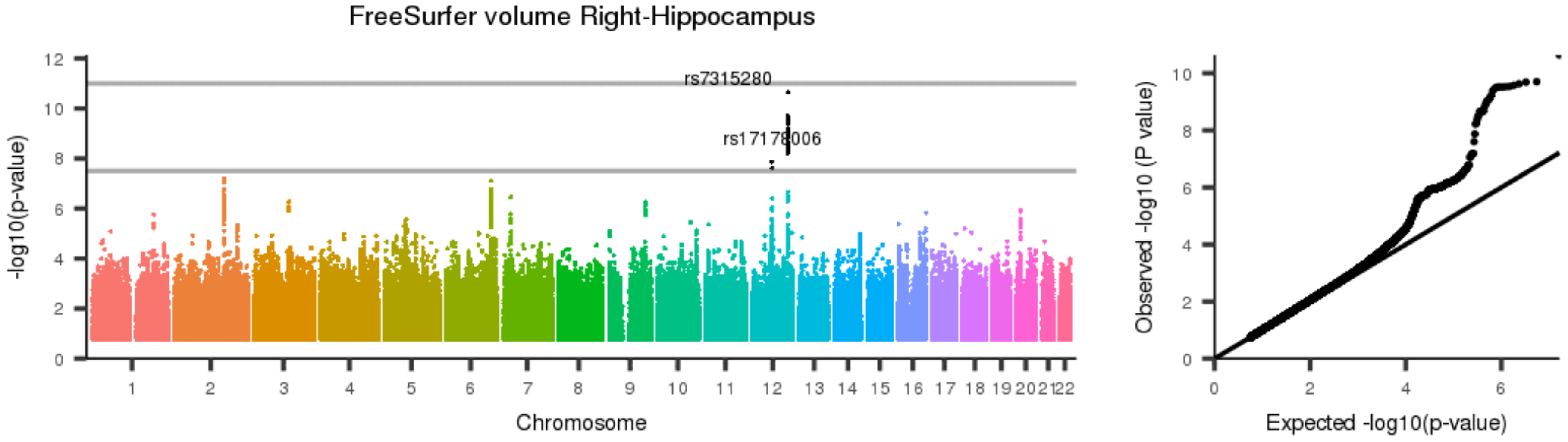

back to list of plots

Figure 988: FreeSurfer volume Right-Amygdala

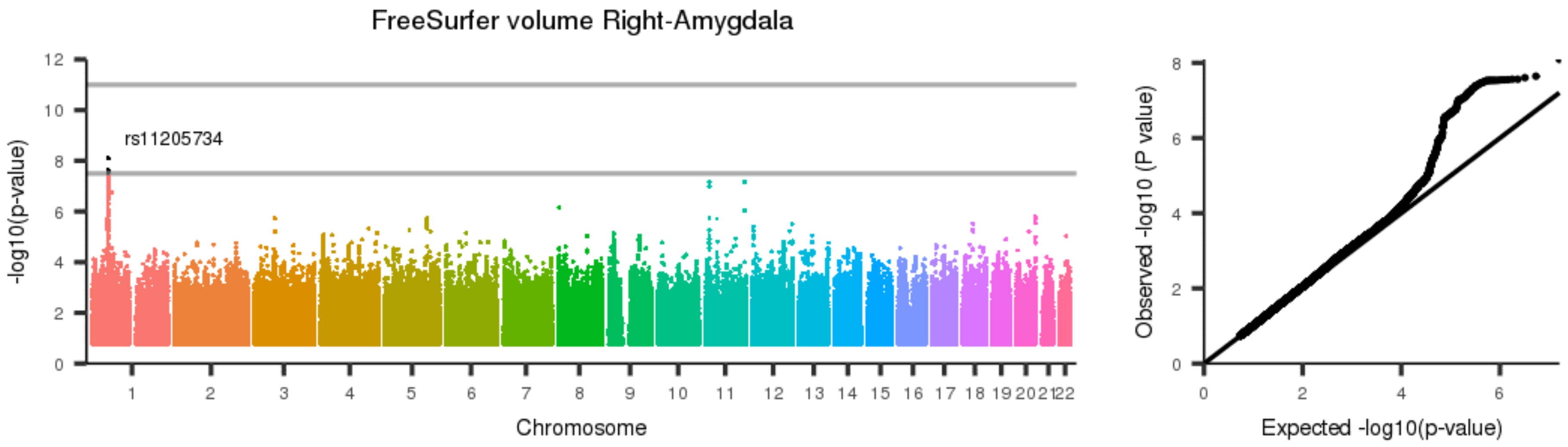

back to list of plots

Figure 989: FreeSurfer volume Right-Accumbens-area

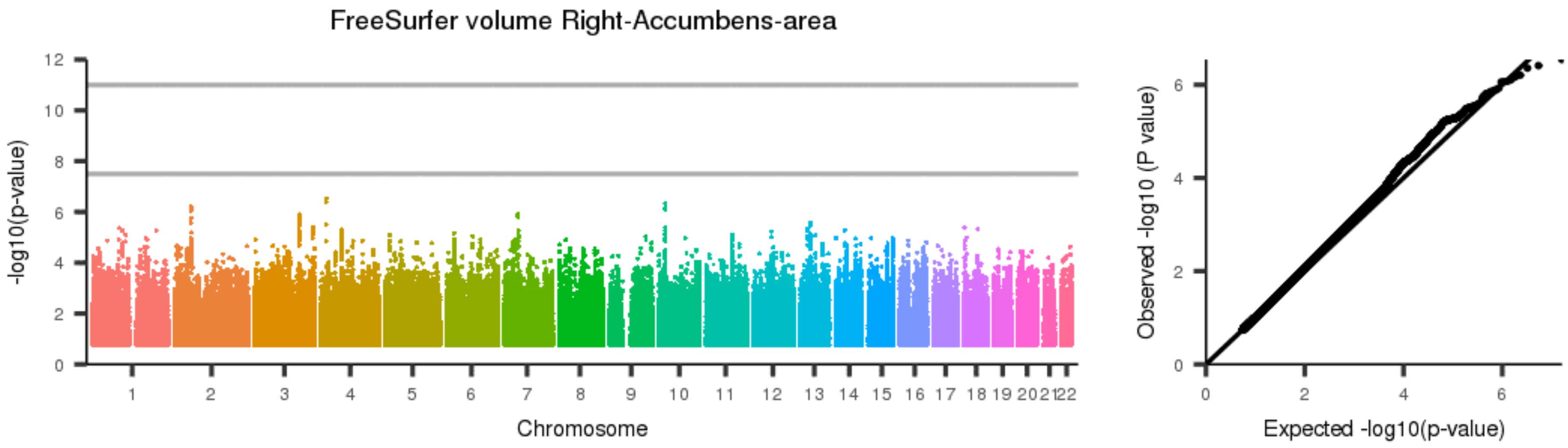

back to list of plots

Figure 990: FreeSurfer volume Right-VentralDC

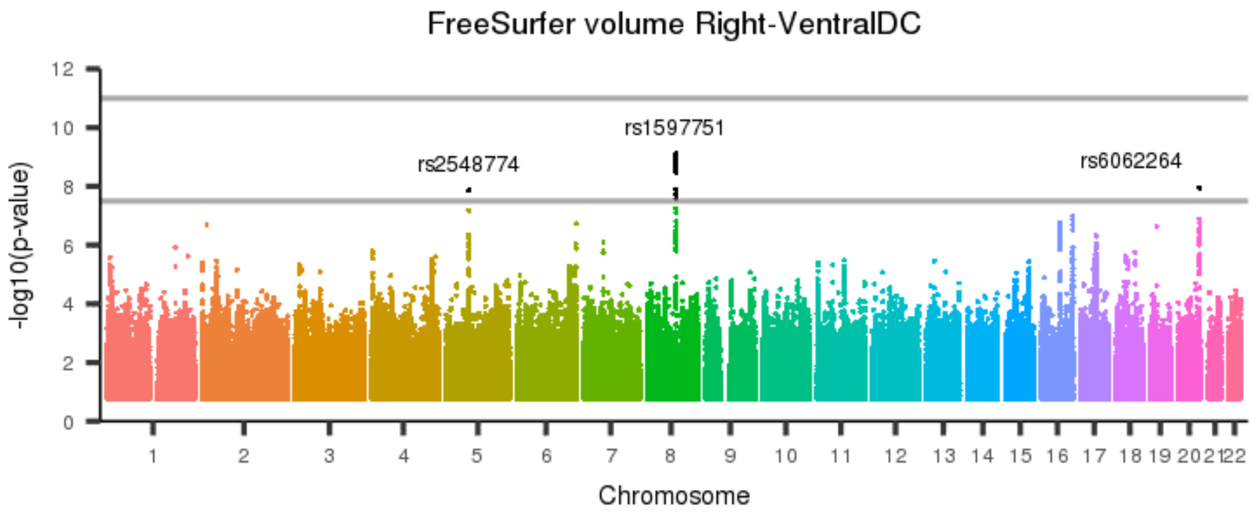

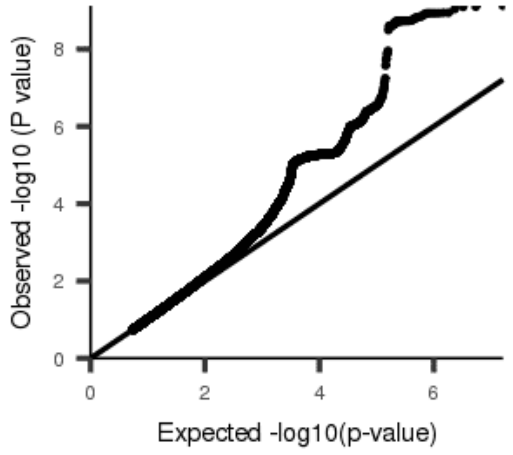

back to list of plots

Figure 991: FreeSurfer volume Right-vessel

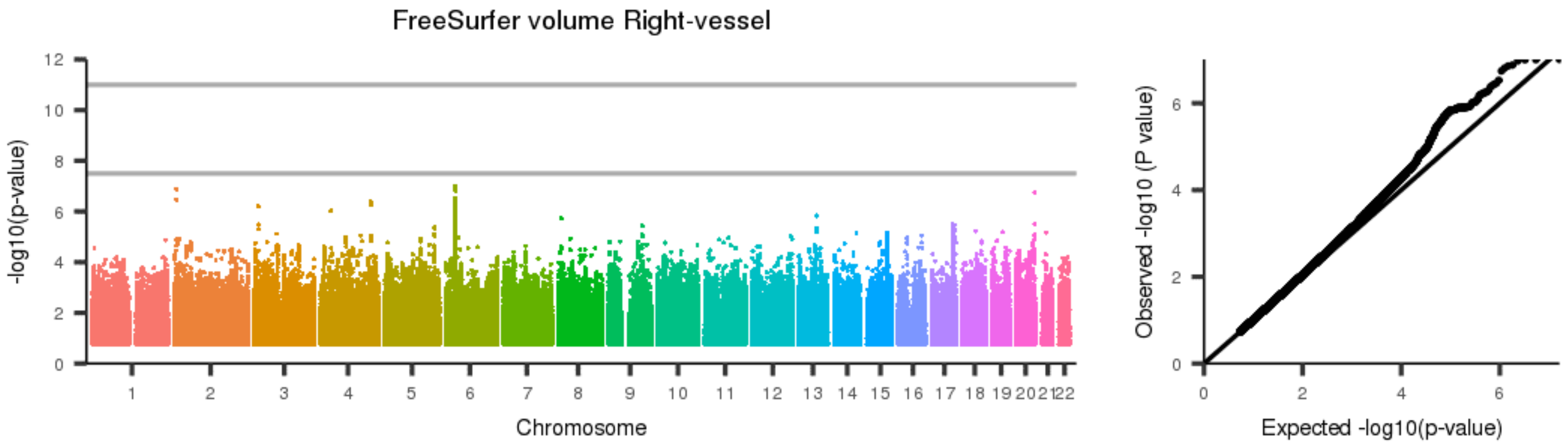

back to list of plots

Figure 992: FreeSurfer volume Right-choroid-plexus

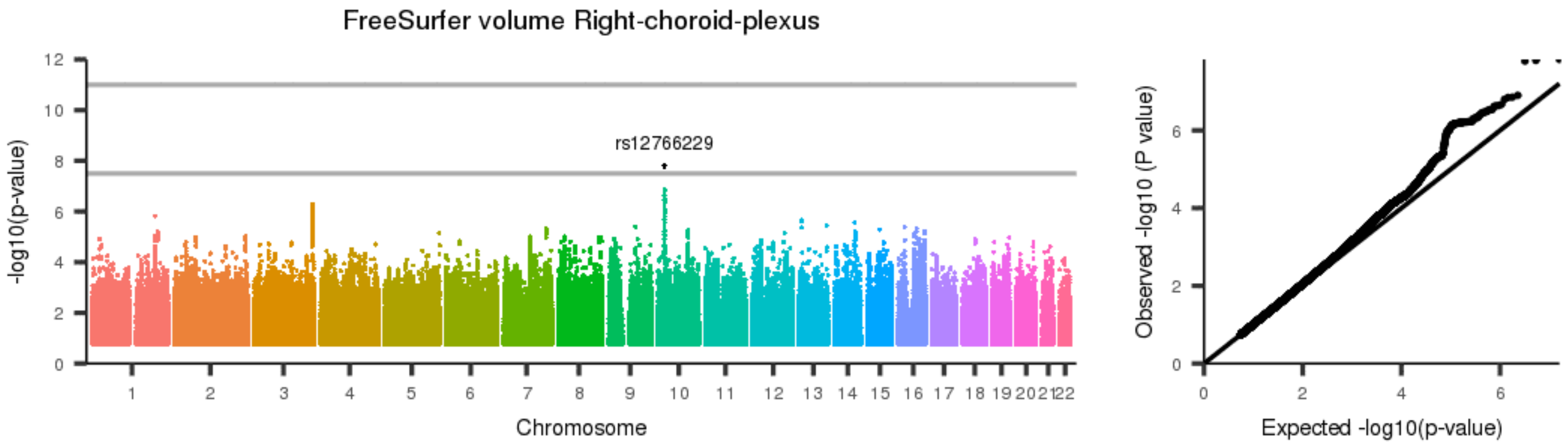

back to list of plots

Figure 993: FreeSurfer volume Optic-Chiasm

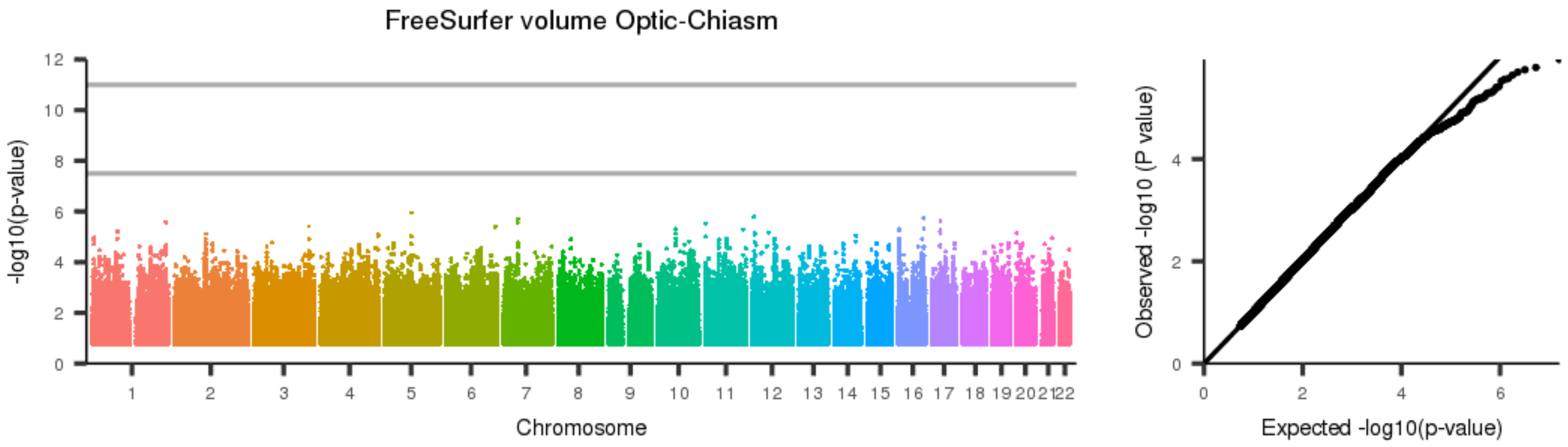

back to list of plots

Figure 994: FreeSurfer volume CC Posterior

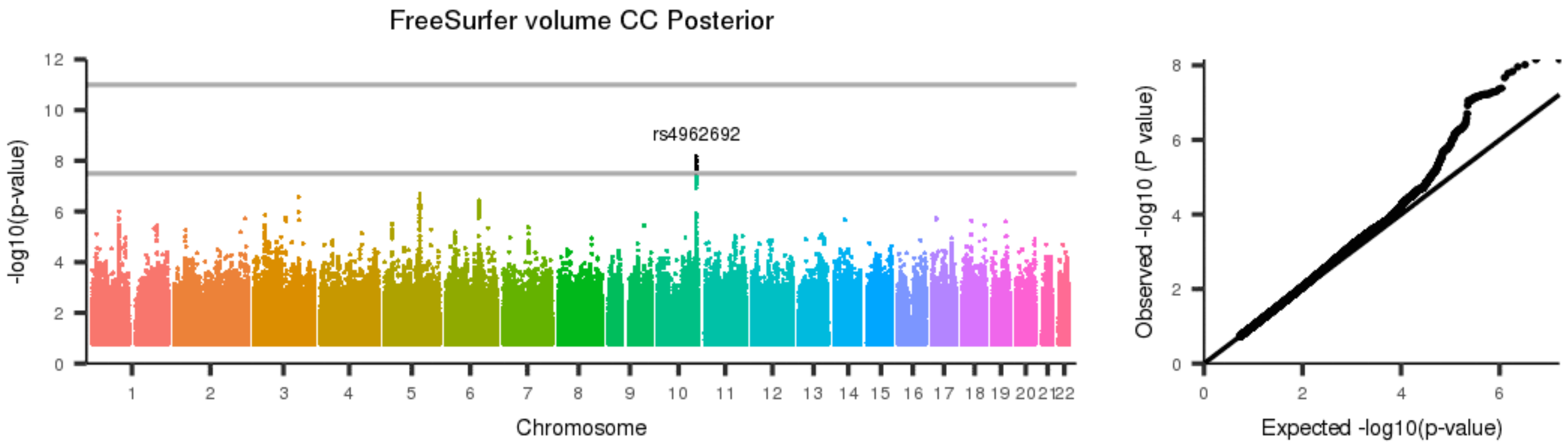

back to list of plots

Figure 995: FreeSurfer volume CC Mid Posterior

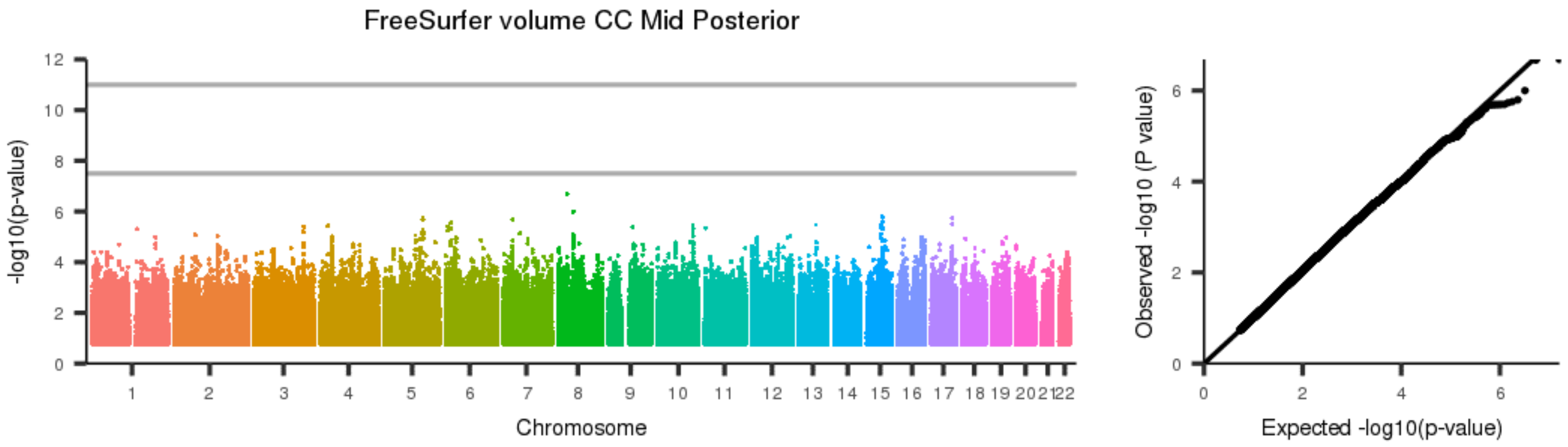

back to list of plots

Figure 996: FreeSurfer volume CC Central

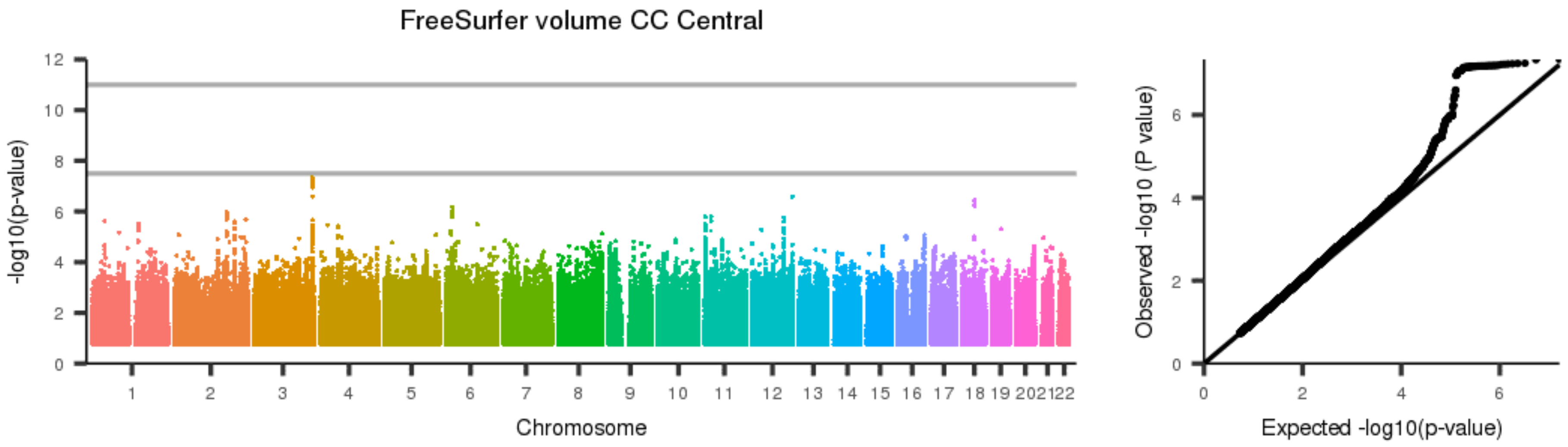

back to list of plots

Figure 997: FreeSurfer volume CC Mid Anterior

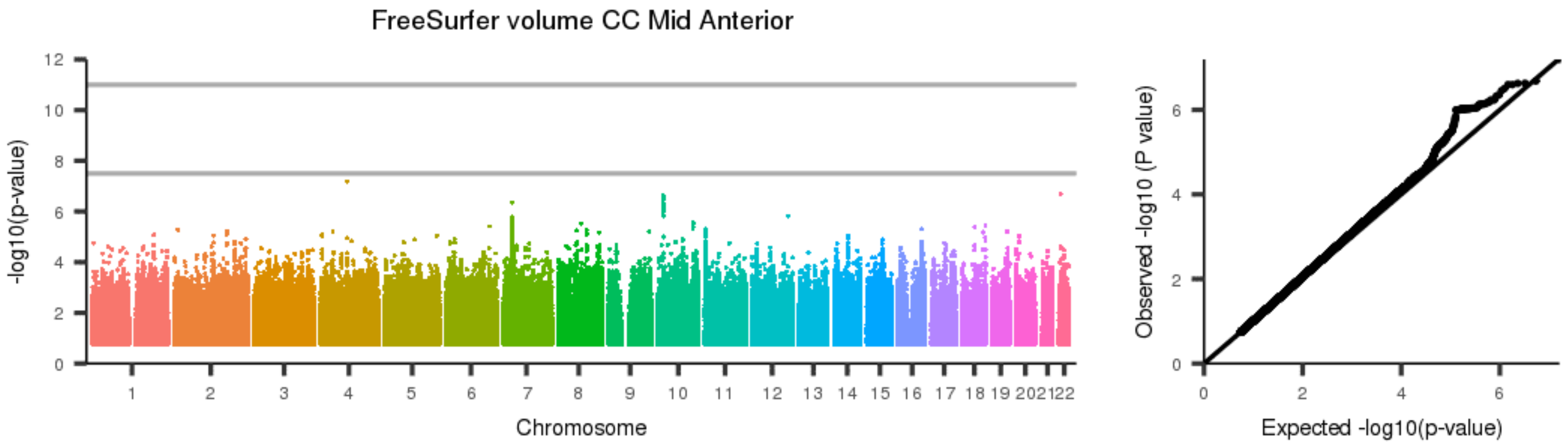

back to list of plots

Figure 998: FreeSurfer volume CC Anterior

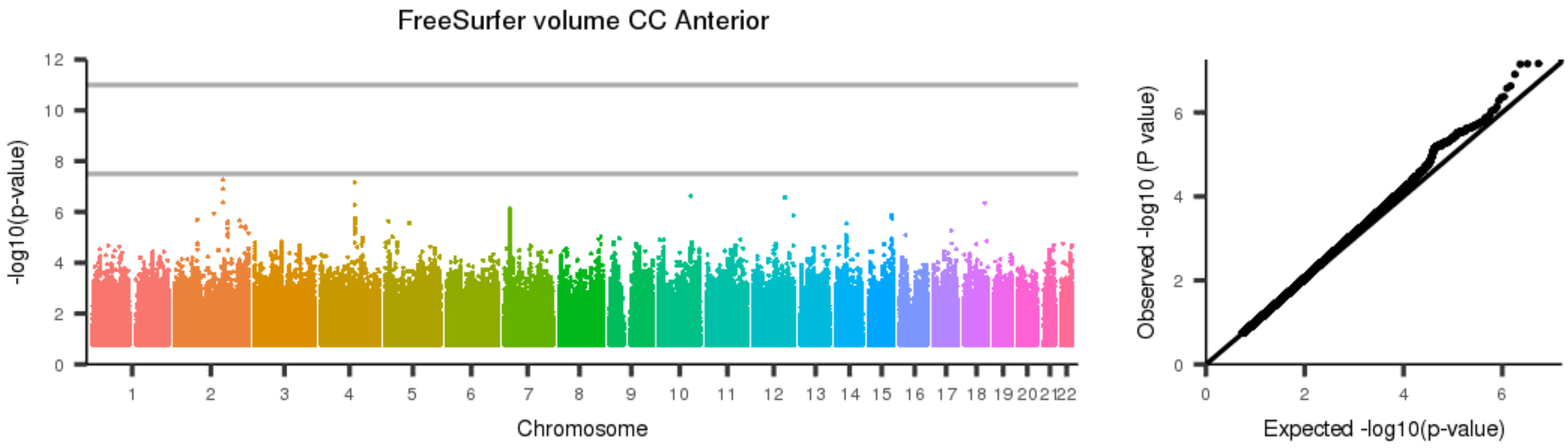

back to list of plots

Figure 999: FreeSurfer volume BrainSegVol

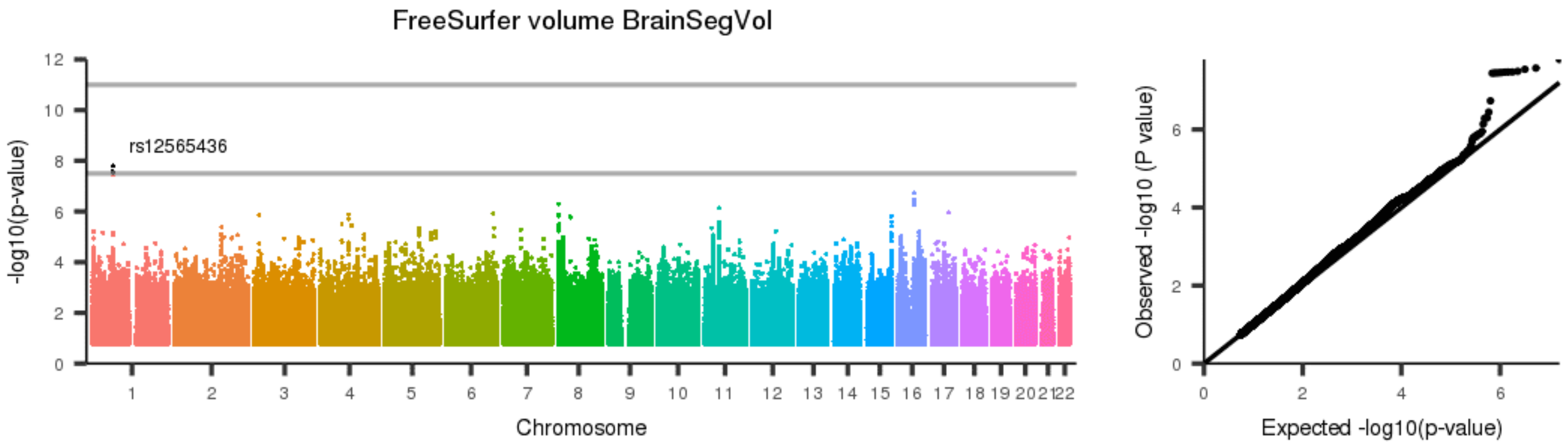

back to list of plots

Figure 1000: FreeSurfer volume BrainSegVolNotVent

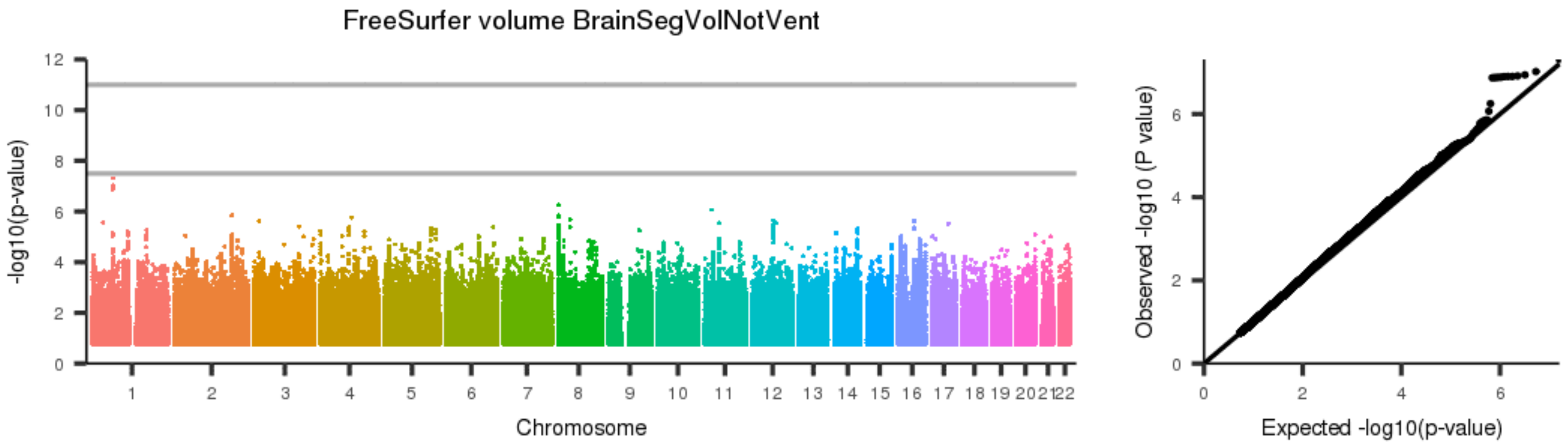

back to list of plots

Figure 1001: FreeSurfer volume BrainSegVolNotVentSurf

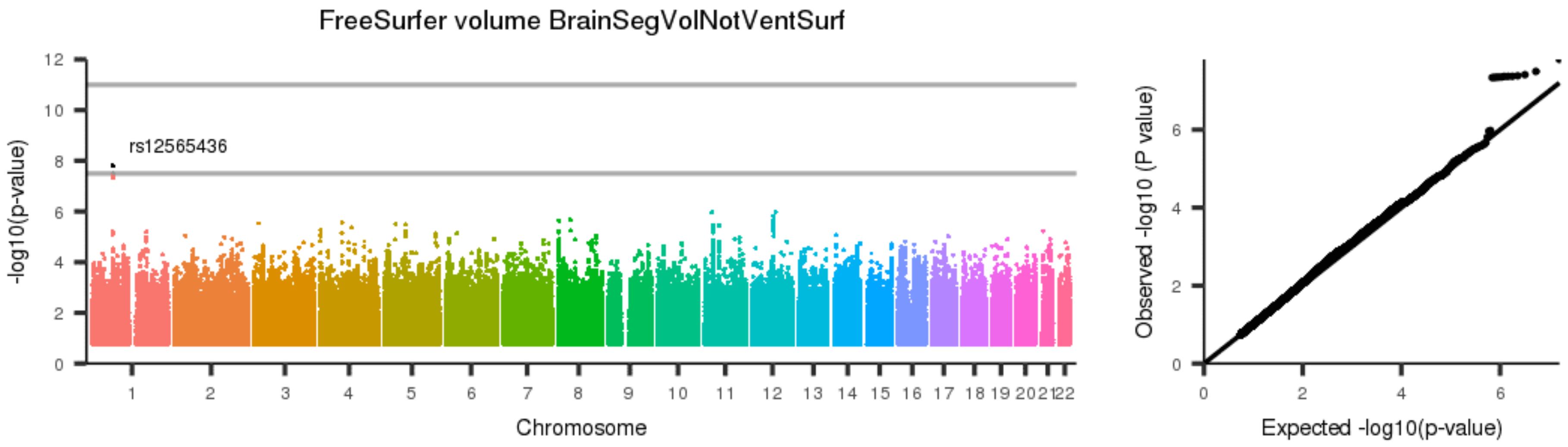

back to list of plots

Figure 1002: FreeSurfer volume lhCortexVol

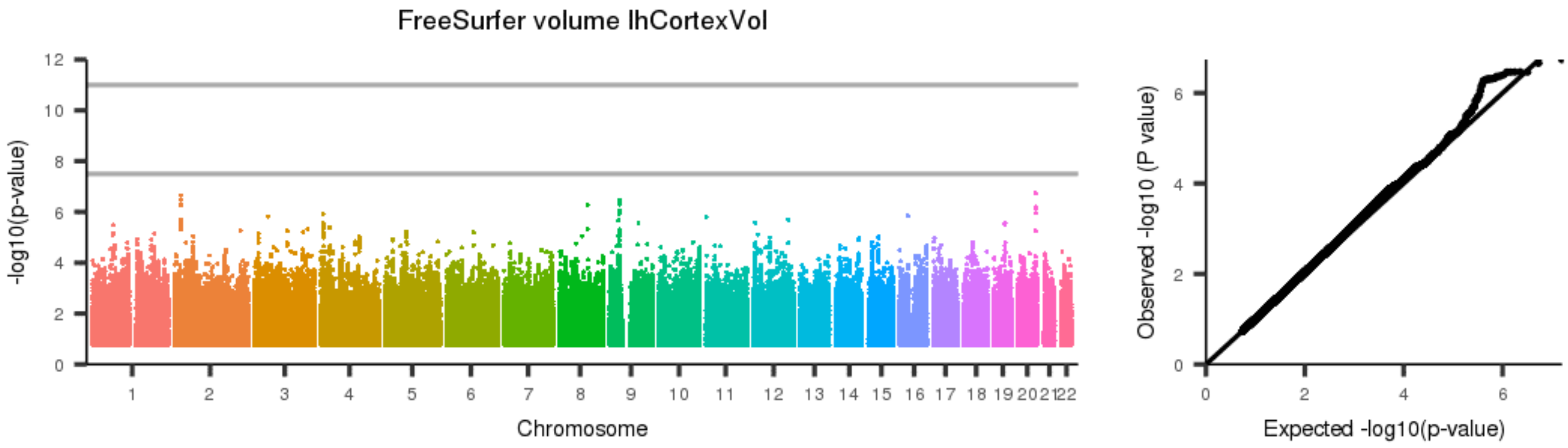

back to list of plots

Figure 1003: FreeSurfer volume rhCortexVol

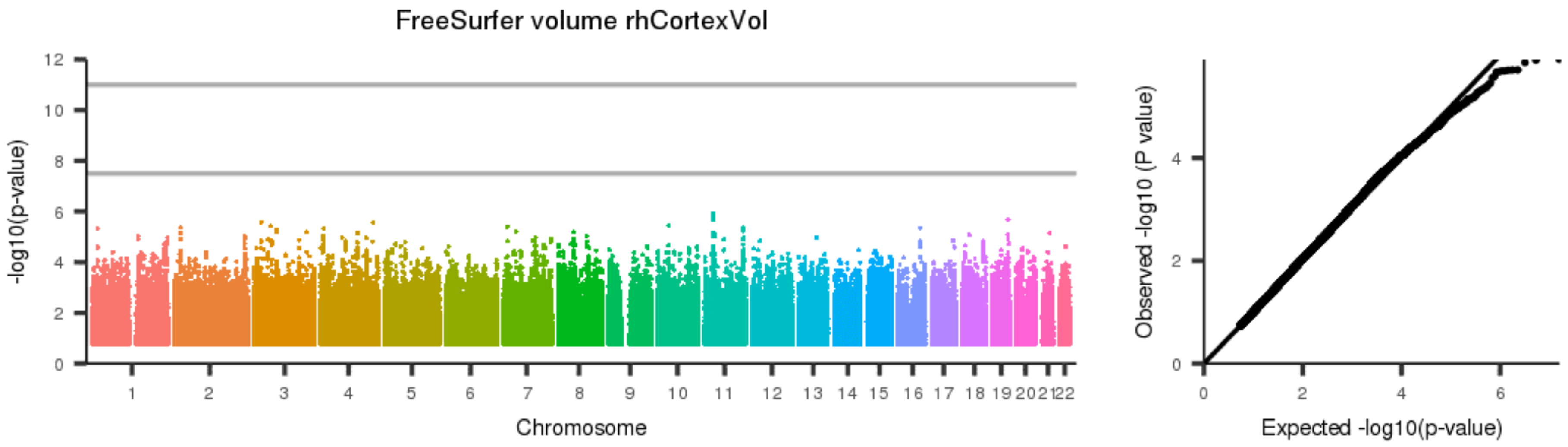

back to list of plots

Figure 1004: FreeSurfer volume CortexVol

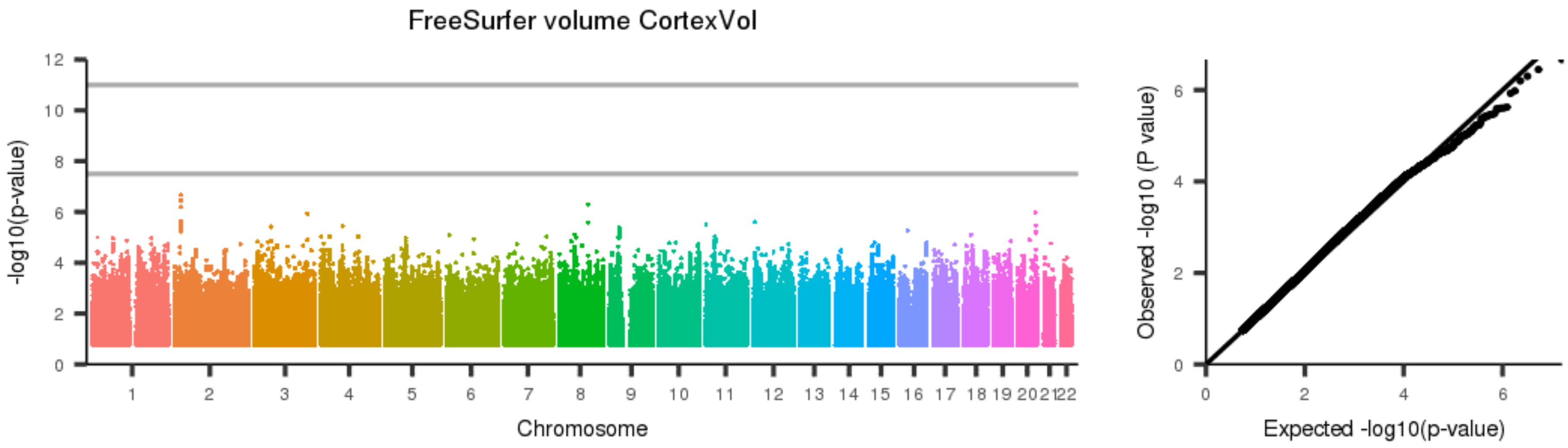

back to list of plots

Figure 1005: FreeSurfer volume lhCerebralWhiteMatterVol

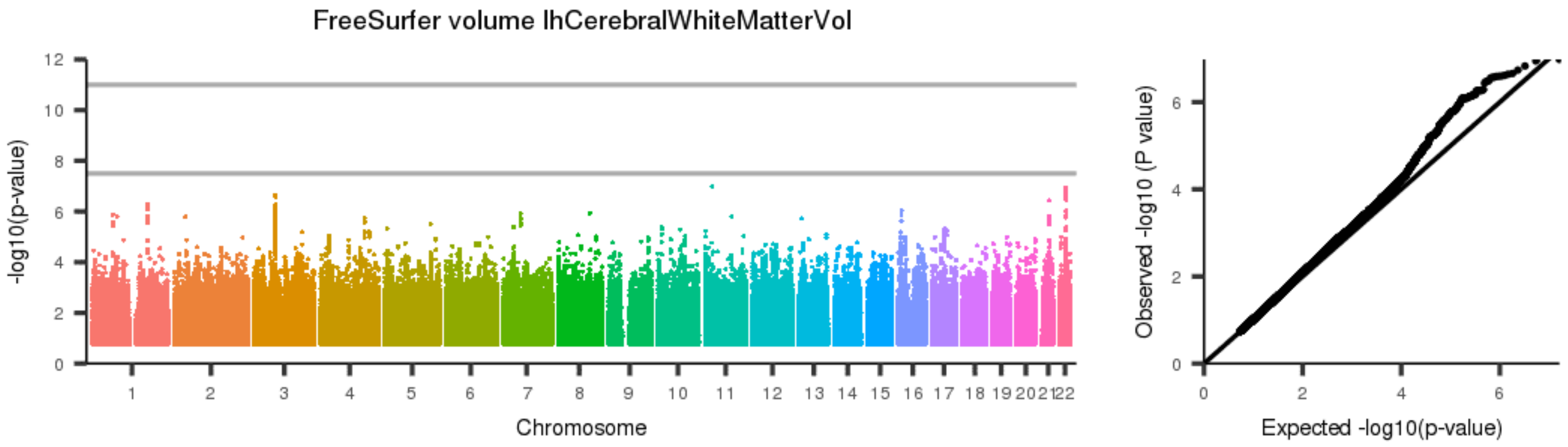

back to list of plots

Figure 1006: FreeSurfer volume rhCerebralWhiteMatterVol

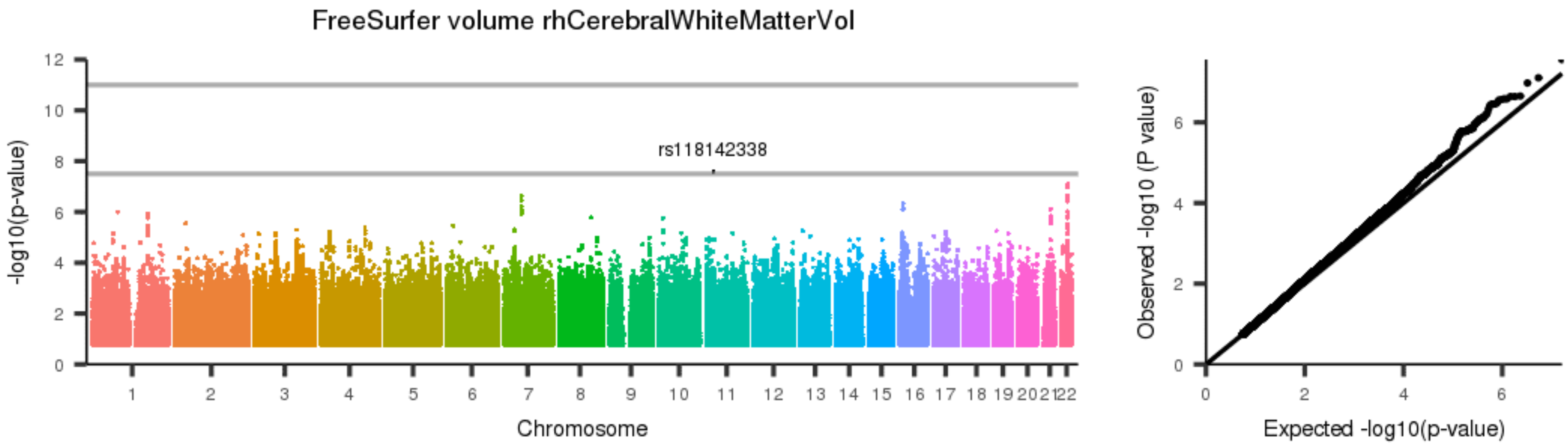

back to list of plots

Figure 1007: FreeSurfer volume CerebralWhiteMatterVol

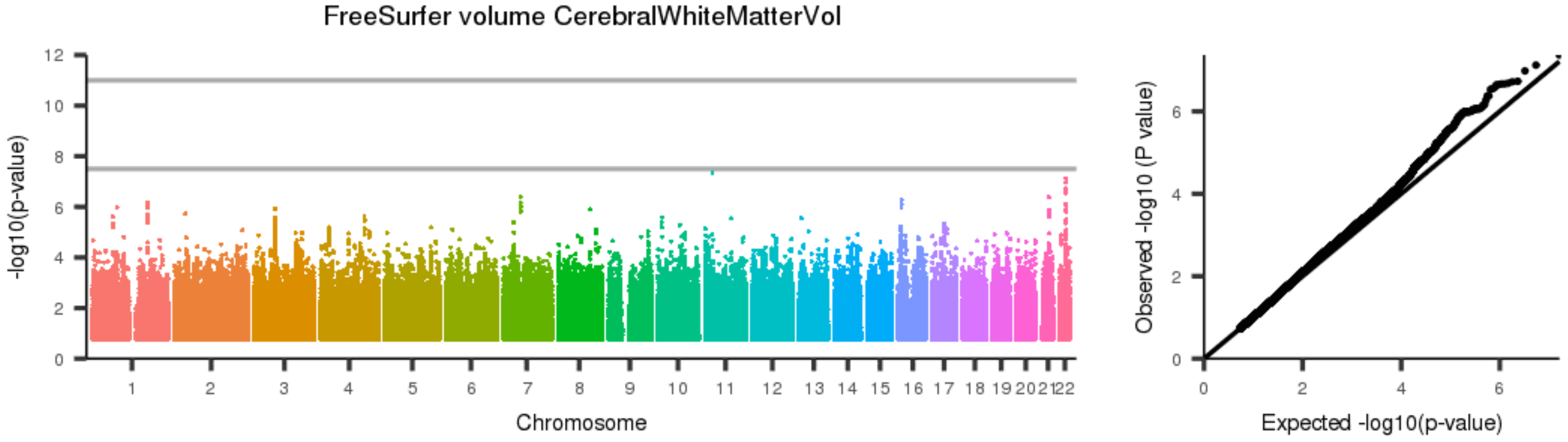

back to list of plots

Figure 1008: FreeSurfer volume SubCortGrayVol

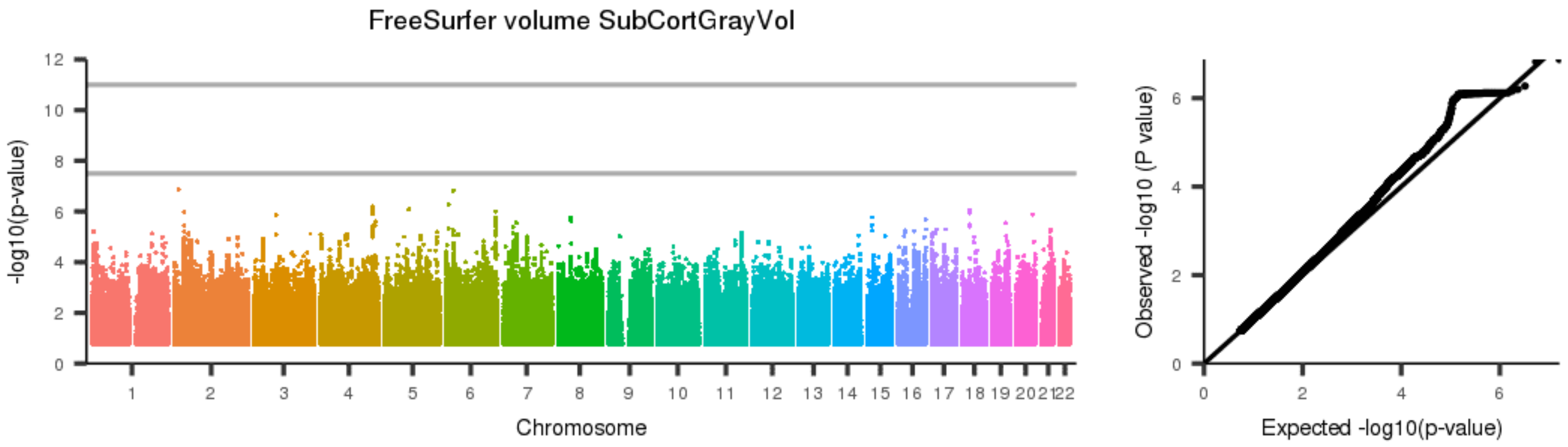

back to list of plots

Figure 1009: FreeSurfer volume TotalGrayVol

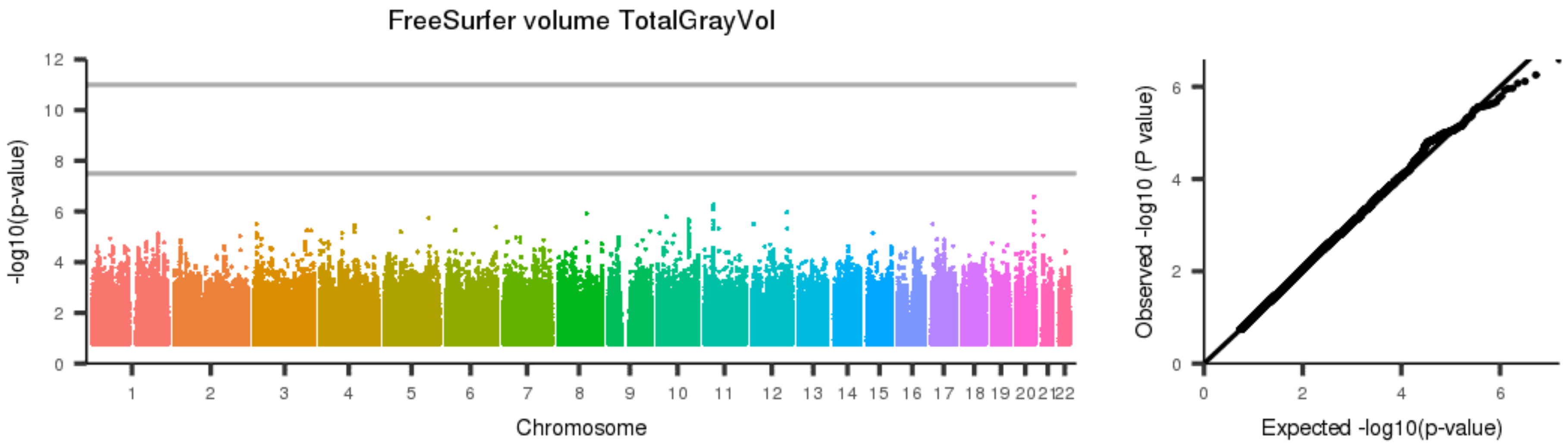

back to list of plots

Figure 1010: FreeSurfer volume SupraTentorialVol

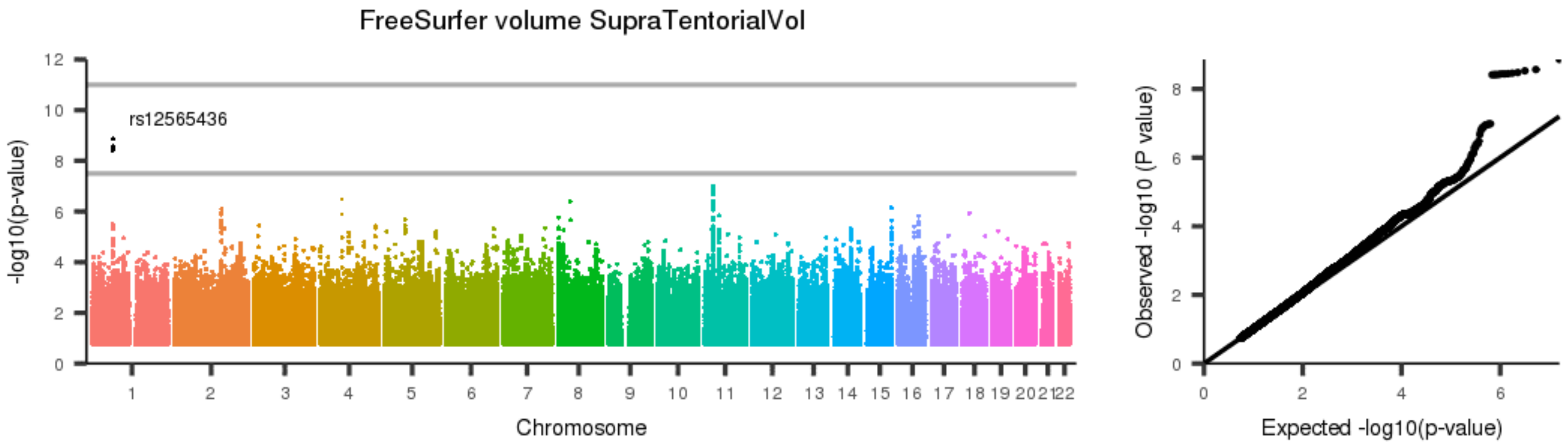

back to list of plots

Figure 1011: FreeSurfer volume SupraTentorialVolNotVent

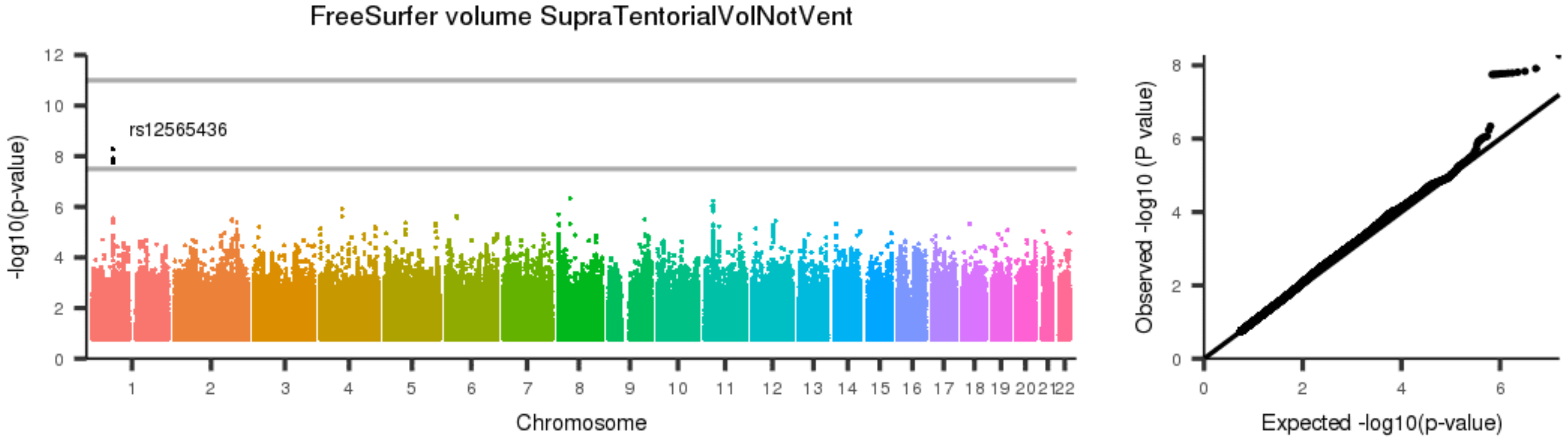

back to list of plots

Figure 1012: FreeSurfer volume SupraTentorialVolNotVentVox

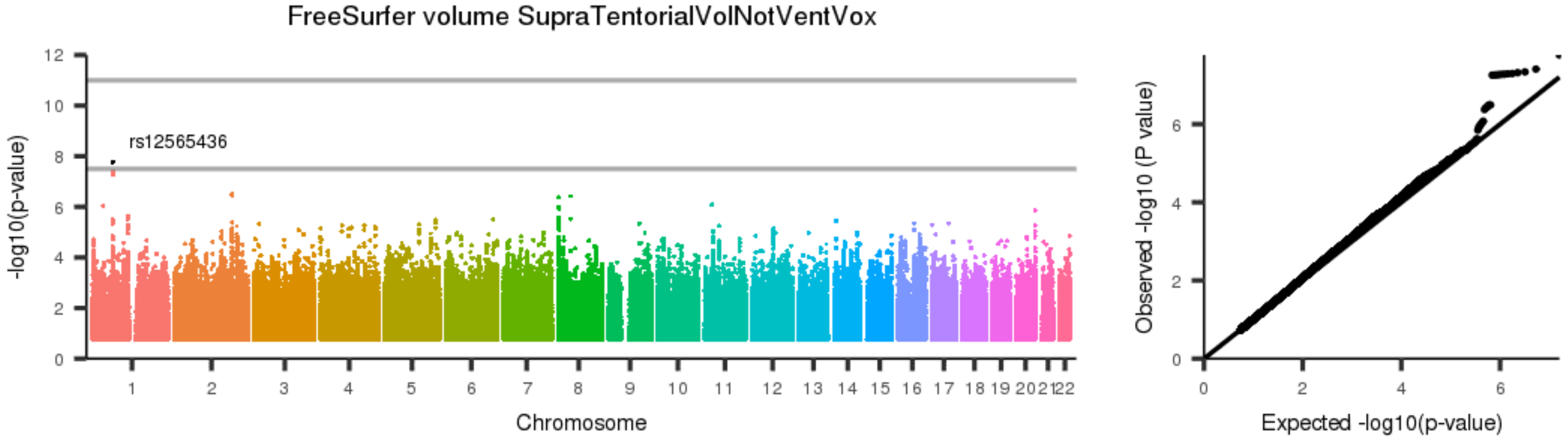

back to list of plots

Figure 1013: FreeSurfer volume MaskVol

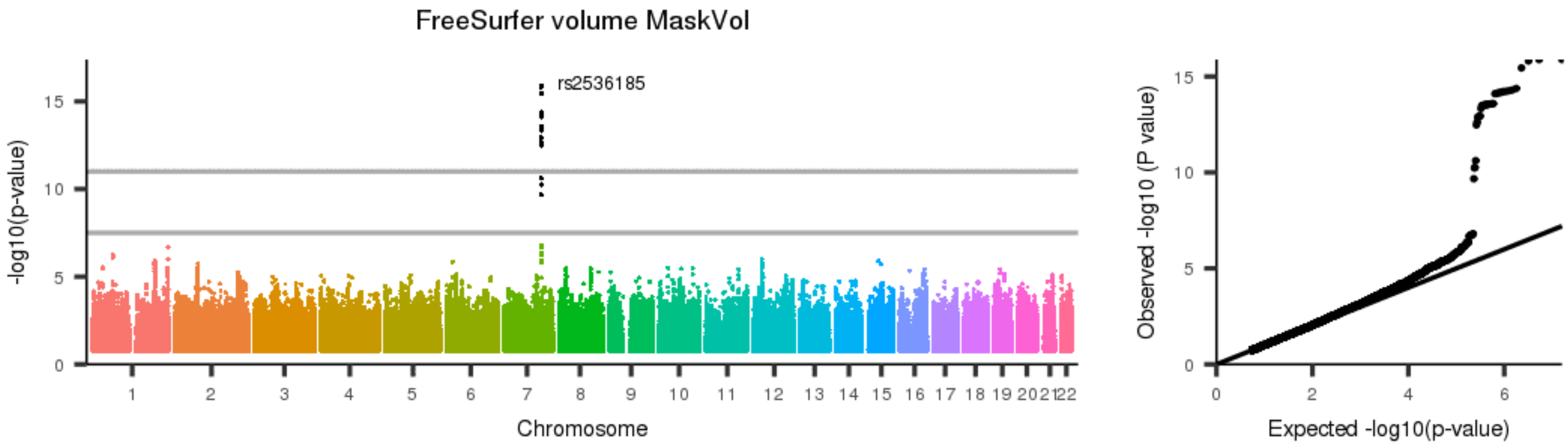

back to list of plots

Figure 1014: FreeSurfer volume BrainSegVol-to-eTIV

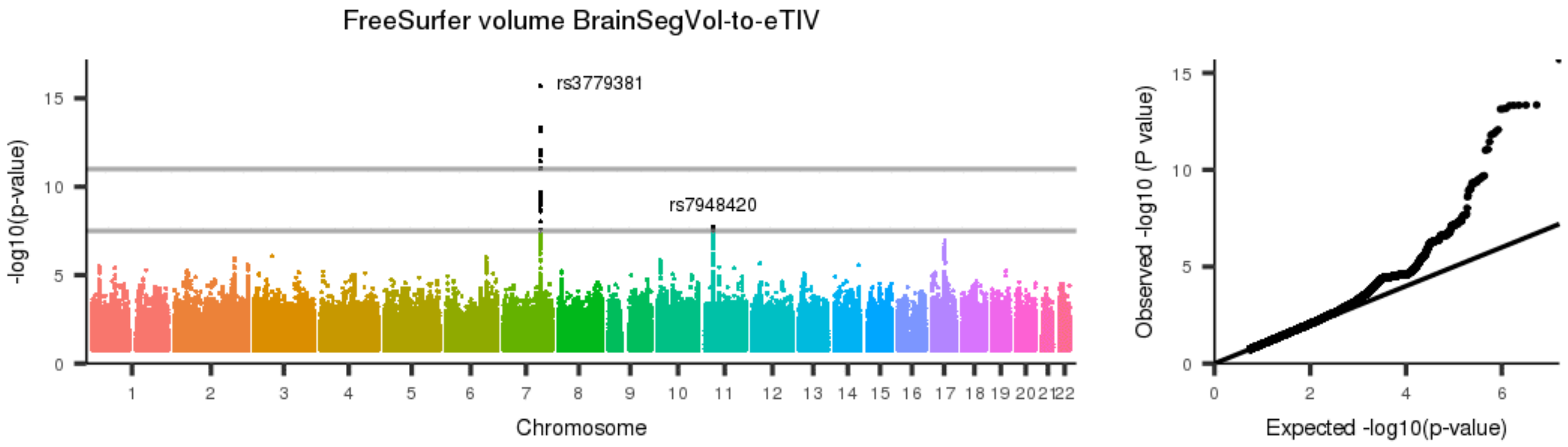

back to list of plots

Figure 1015: FreeSurfer volume MaskVol-to-eTIV

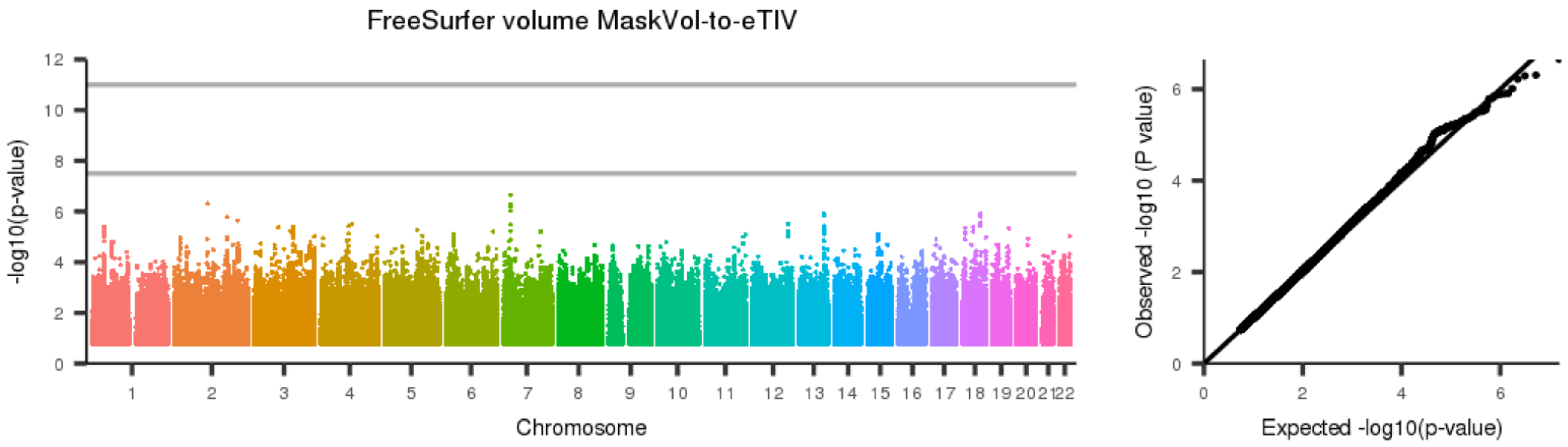

back to list of plots

Figure 1016: FreeSurfer volume lhSurfaceHoles

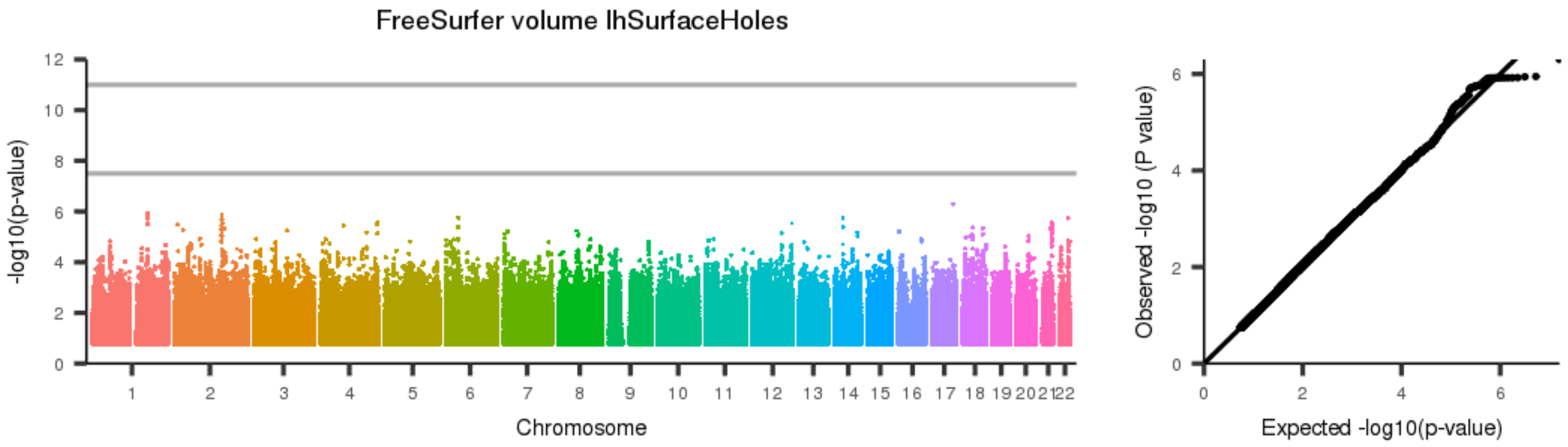

back to list of plots

Figure 1017: FreeSurfer volume rhSurfaceHoles

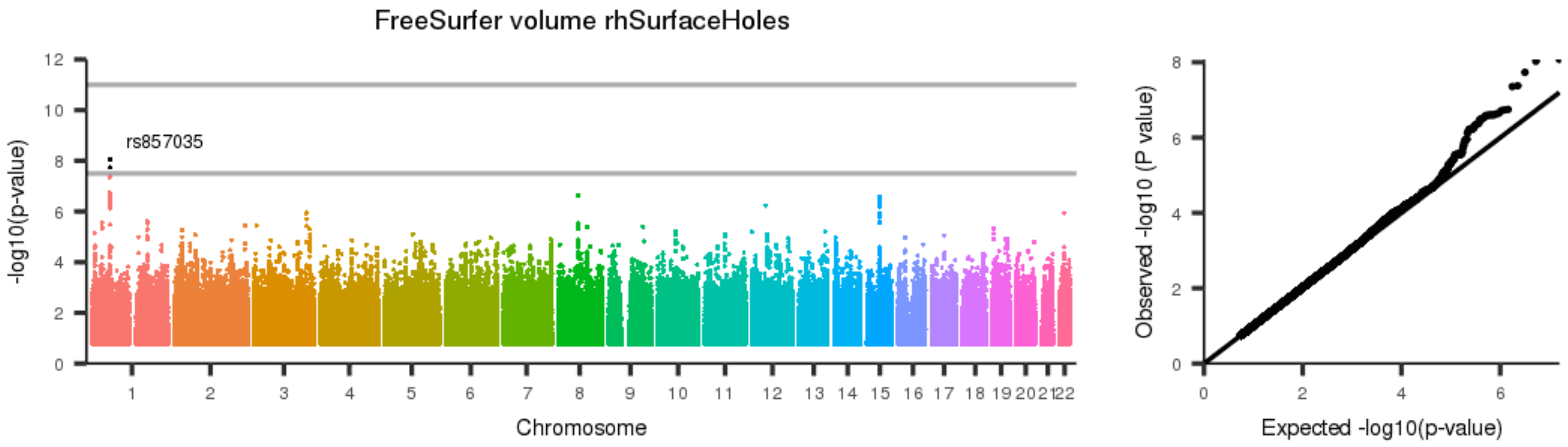

back to list of plots

Figure 1018: FreeSurfer volume SurfaceHoles

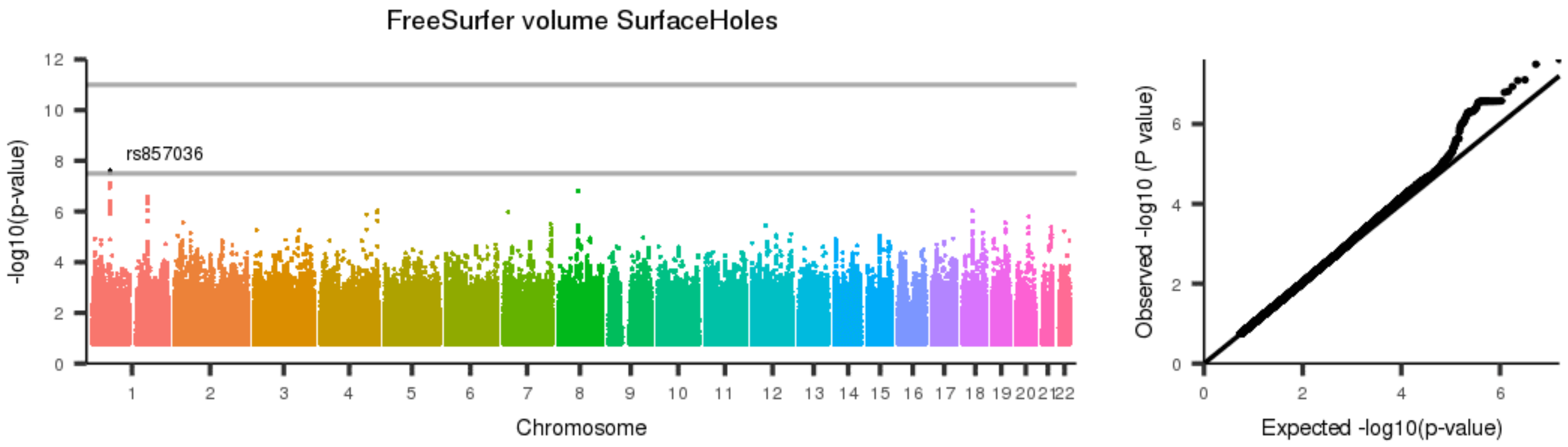

back to list of plots

Figure 1019: FreeSurfer volume EstimatedTotalIntraCranialVol

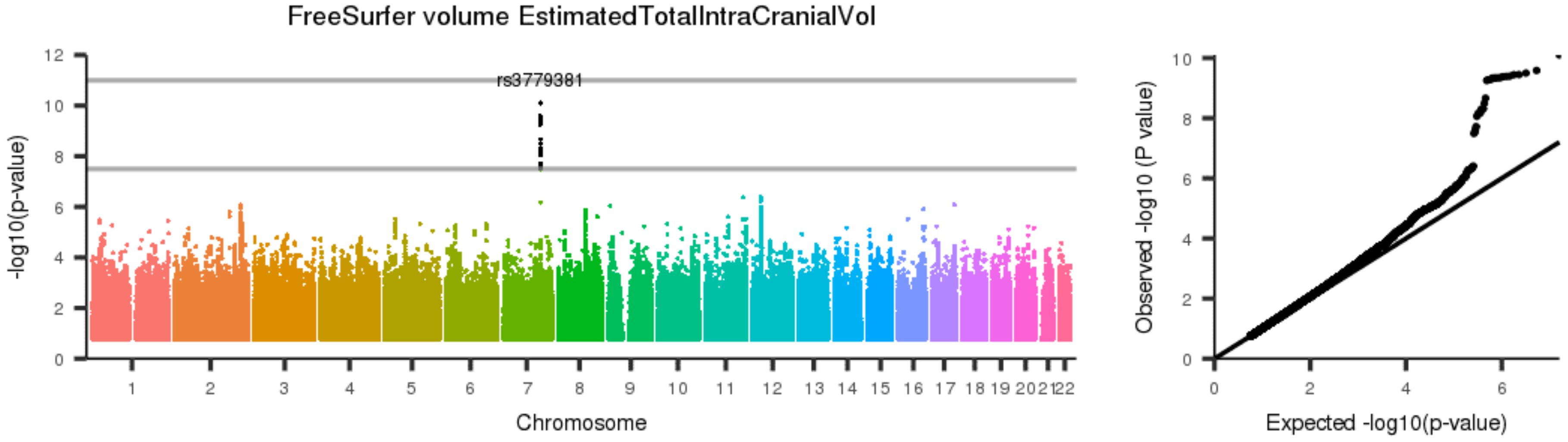

back to list of plots

Figure 1020: FreeSurfer DKTatlas lh caudalanteriorcingulate area

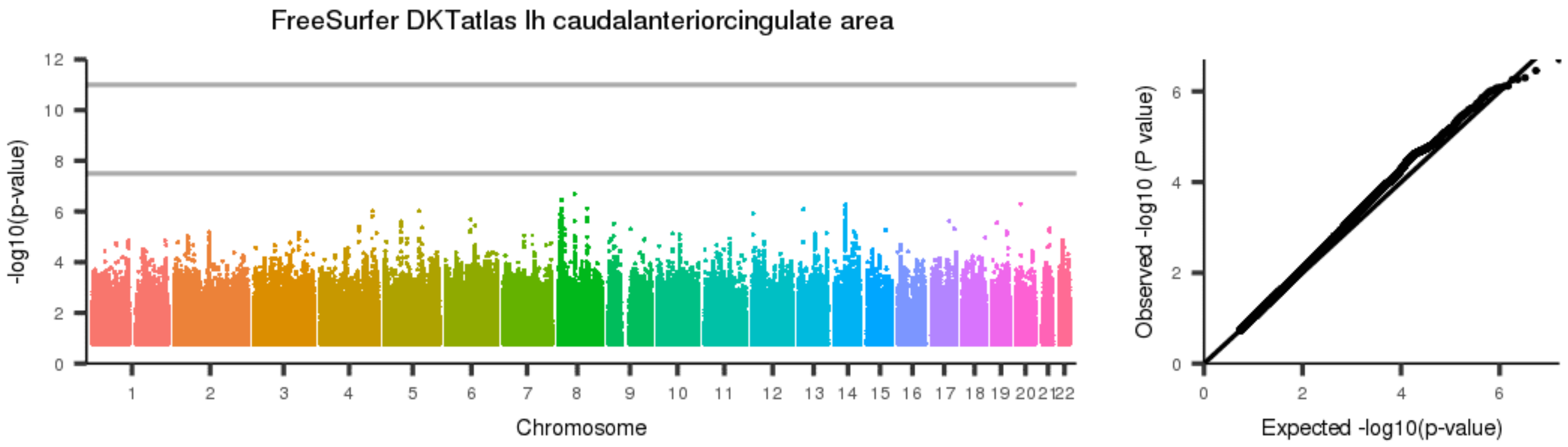

back to list of plots

Figure 1021: FreeSurfer DKTatlas lh caudalmiddlefrontal area

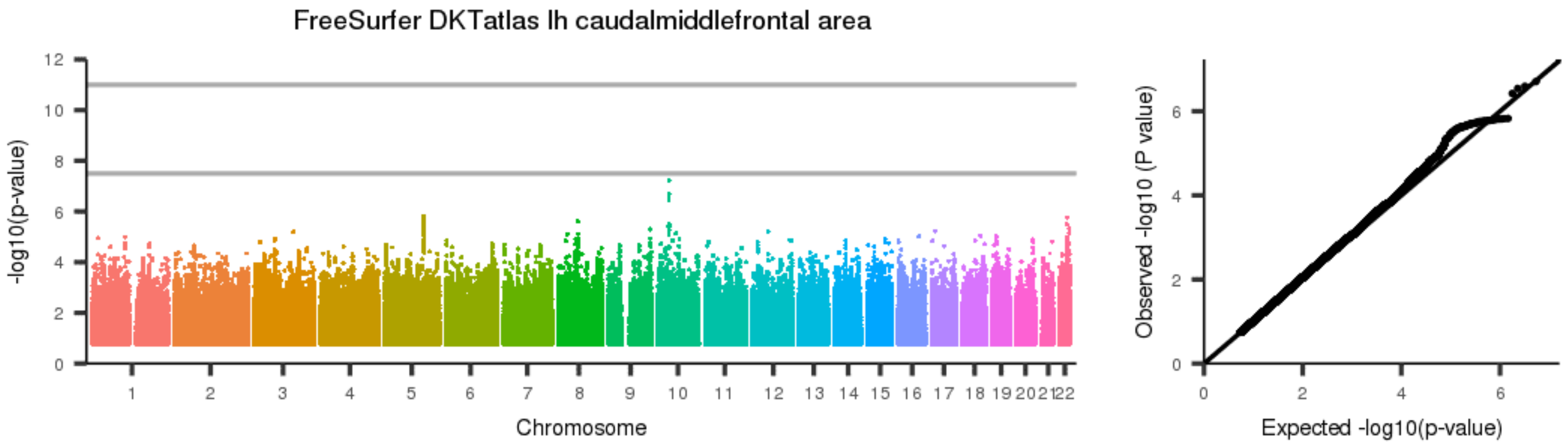

back to list of plots

Figure 1022: FreeSurfer DKTatlas lh cuneus area

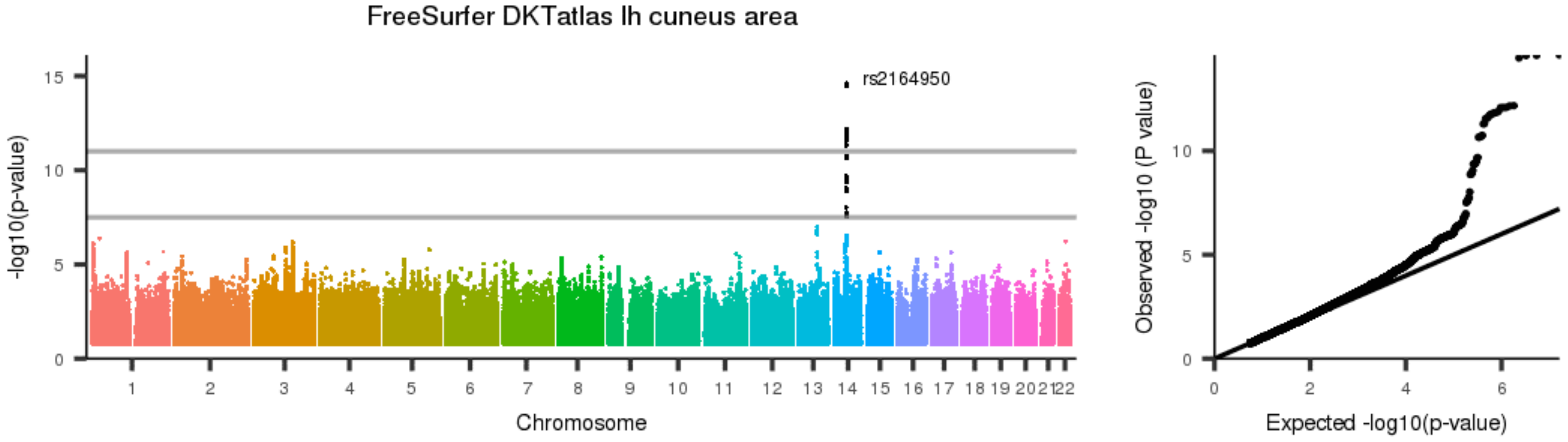

back to list of plots

Figure 1023: FreeSurfer DKTatlas lh entorhinal area

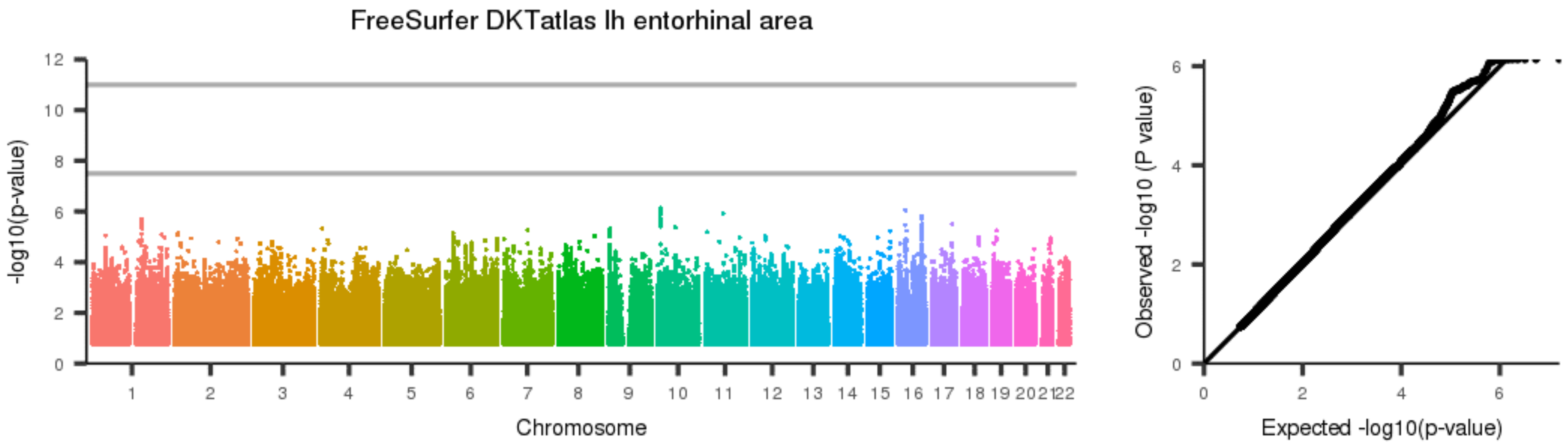

back to list of plots

Figure 1024: FreeSurfer DKTatlas lh fusiform area

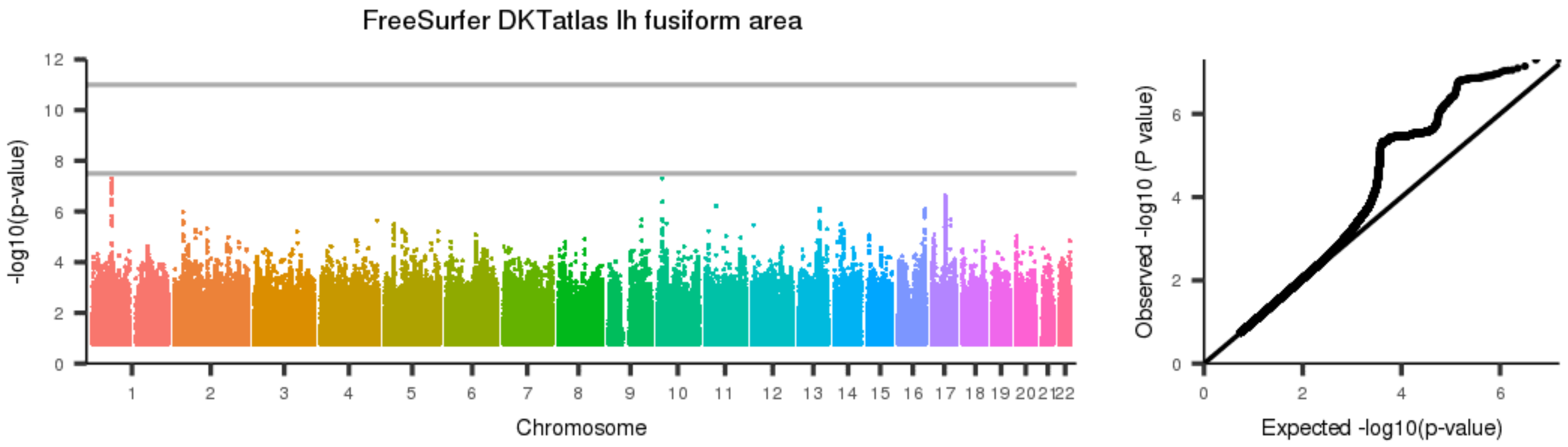

back to list of plots

Figure 1025: FreeSurfer DKTatlas lh inferiorparietal area

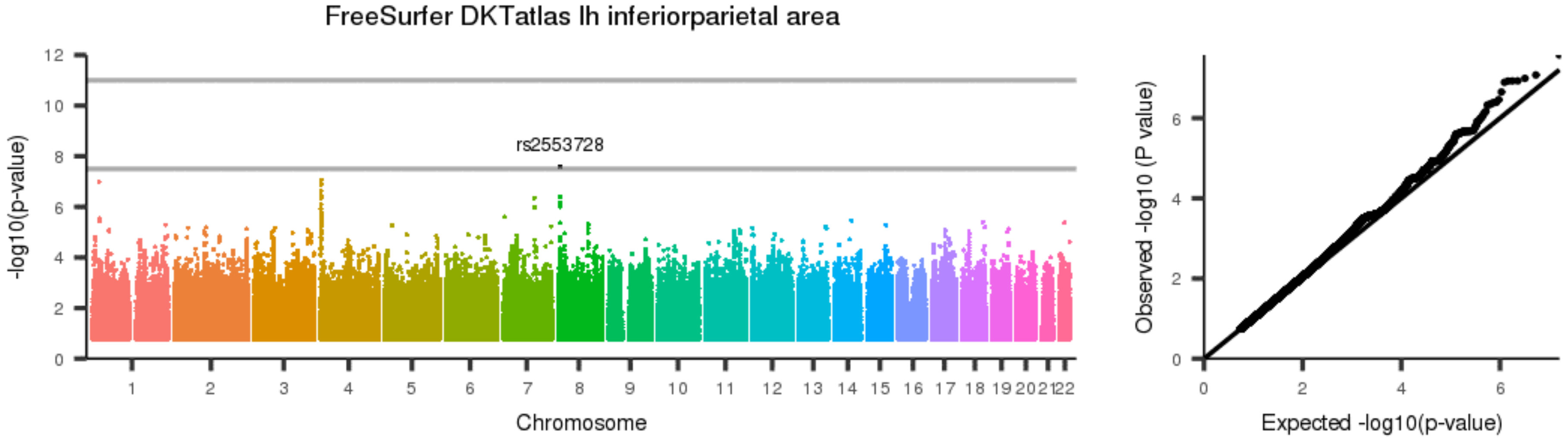

back to list of plots

Figure 1026: FreeSurfer DKTatlas lh inferiortemporal area

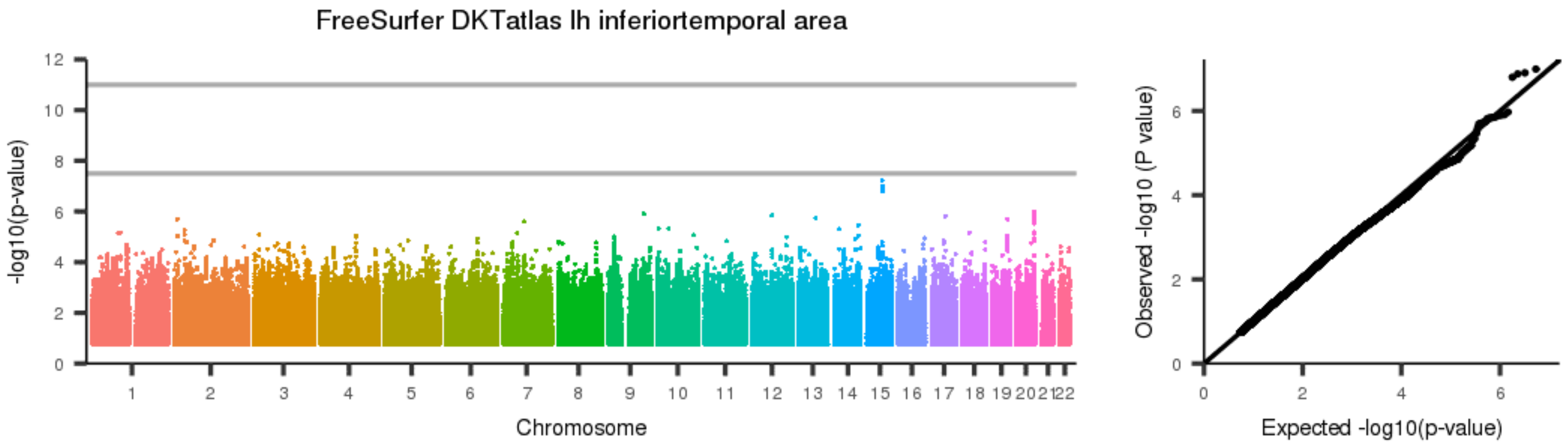

back to list of plots

Figure 1027: FreeSurfer DKTatlas lh isthmuscingulate area

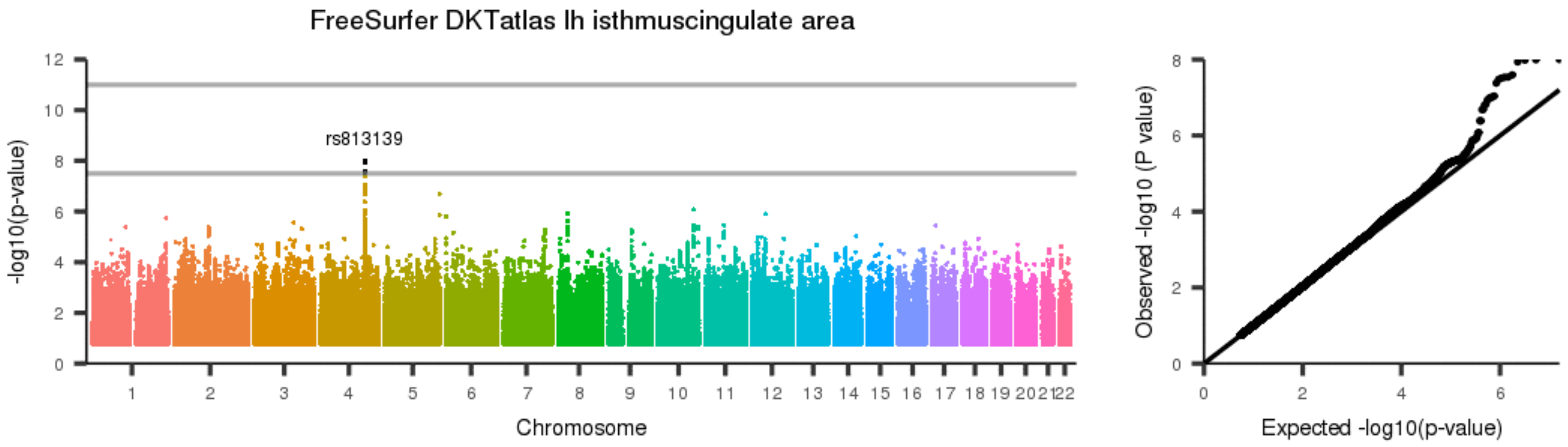

back to list of plots

Figure 1028: FreeSurfer DKTatlas lh lateraloccipital area

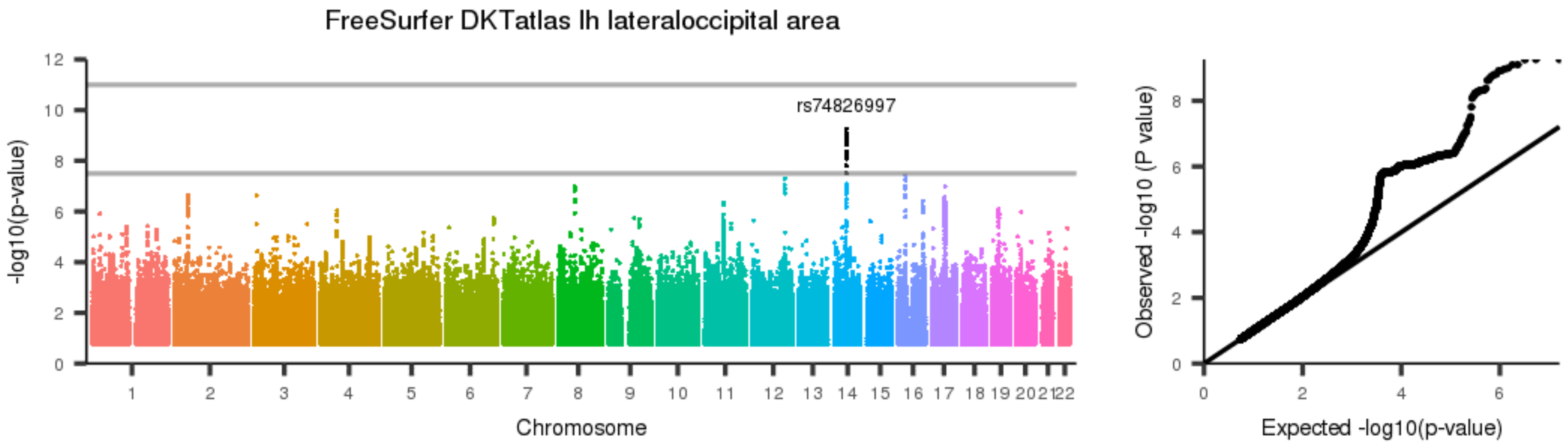

back to list of plots

Figure 1029: FreeSurfer DKTatlas lh lateralorbitofrontal area

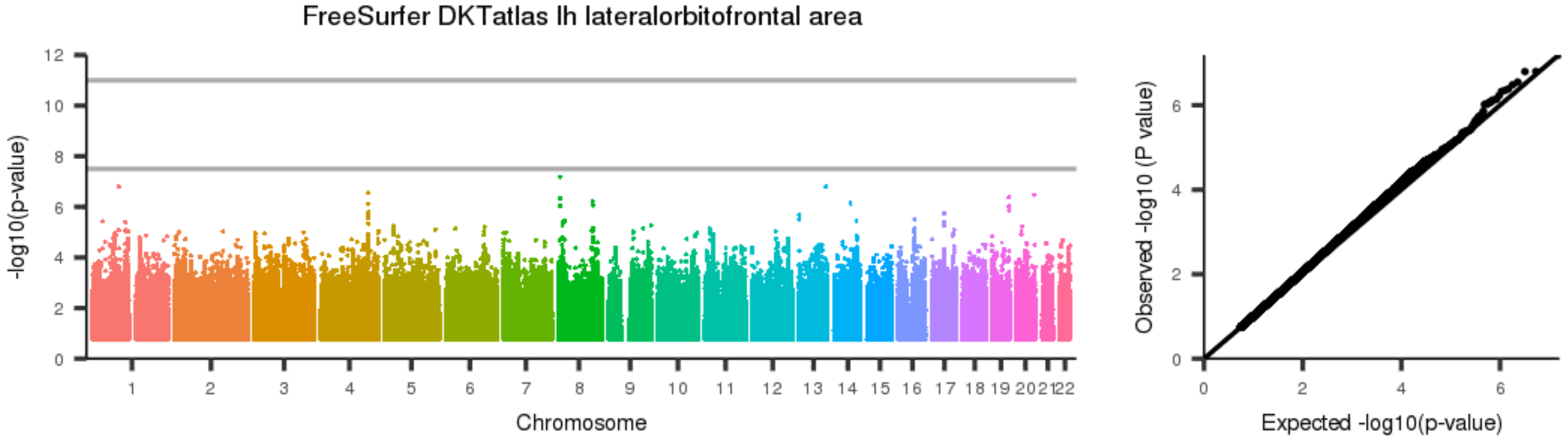

back to list of plots

Figure 1030: FreeSurfer DKTatlas lh lingual area

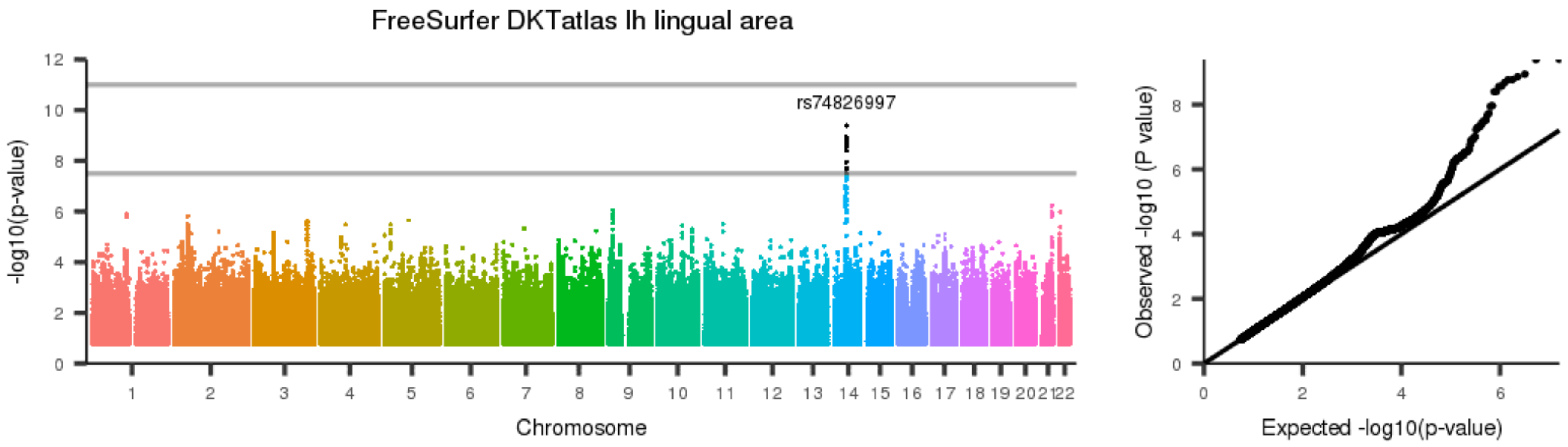

back to list of plots

Figure 1031: FreeSurfer DKTatlas lh medialorbitofrontal area

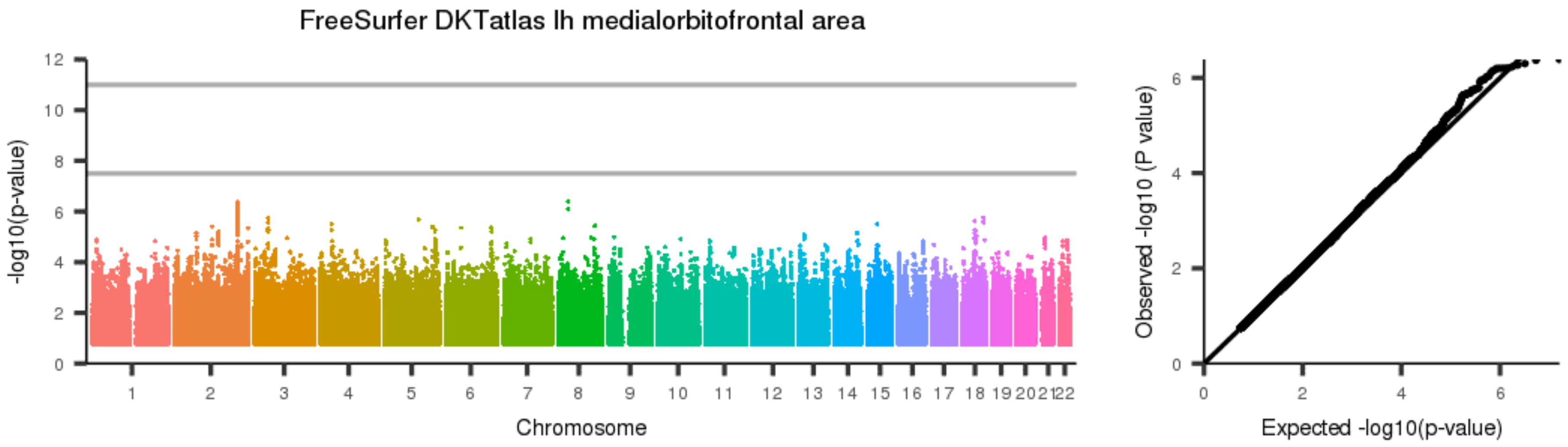

back to list of plots

Figure 1032: FreeSurfer DKTatlas lh middletemporal area

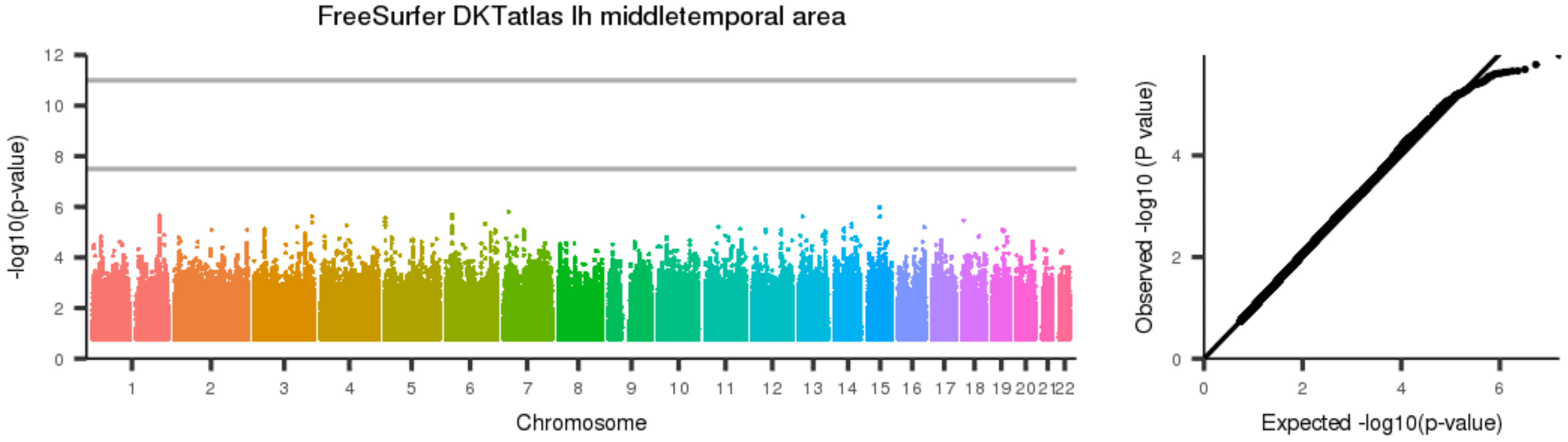

back to list of plots

Figure 1033: FreeSurfer DKTatlas lh parahippocampal area

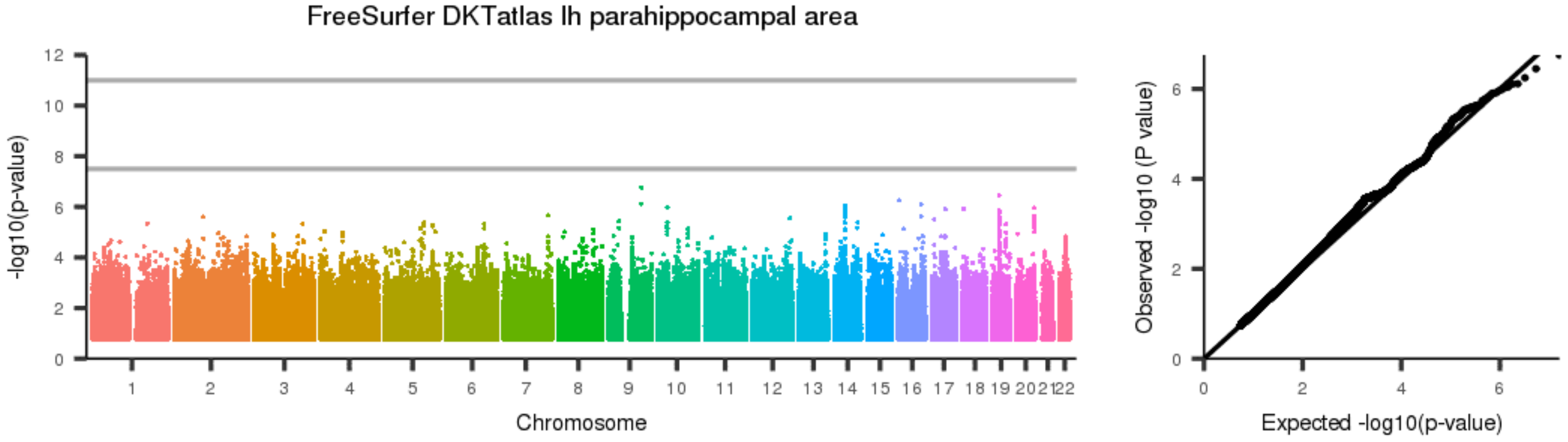

back to list of plots

Figure 1034: FreeSurfer DKTatlas lh paracentral area

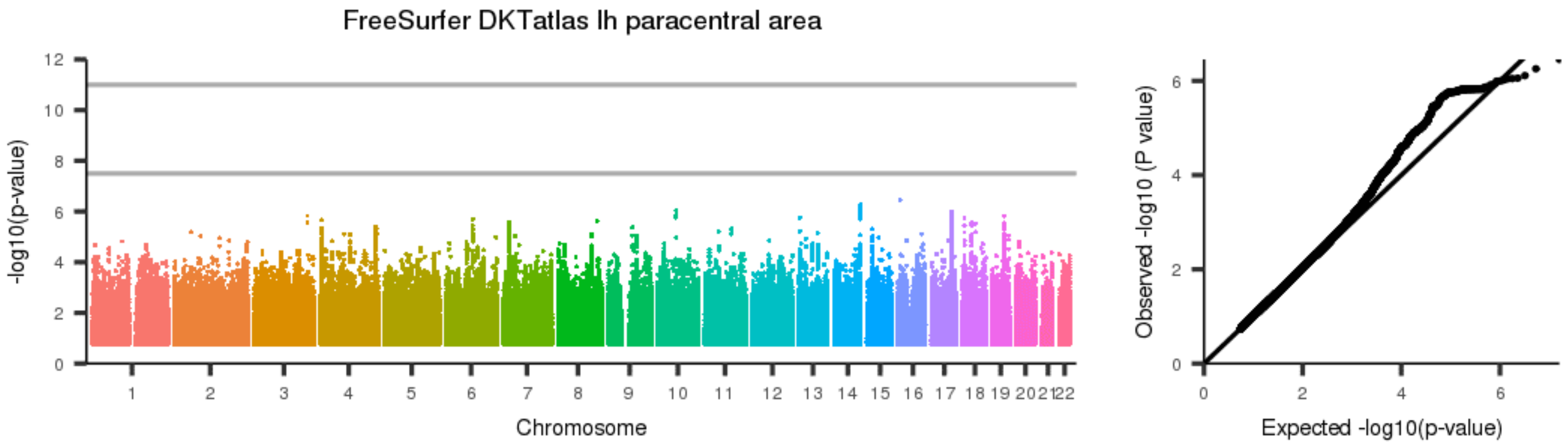

back to list of plots

Figure 1035: FreeSurfer DKTatlas lh parsopercularis area

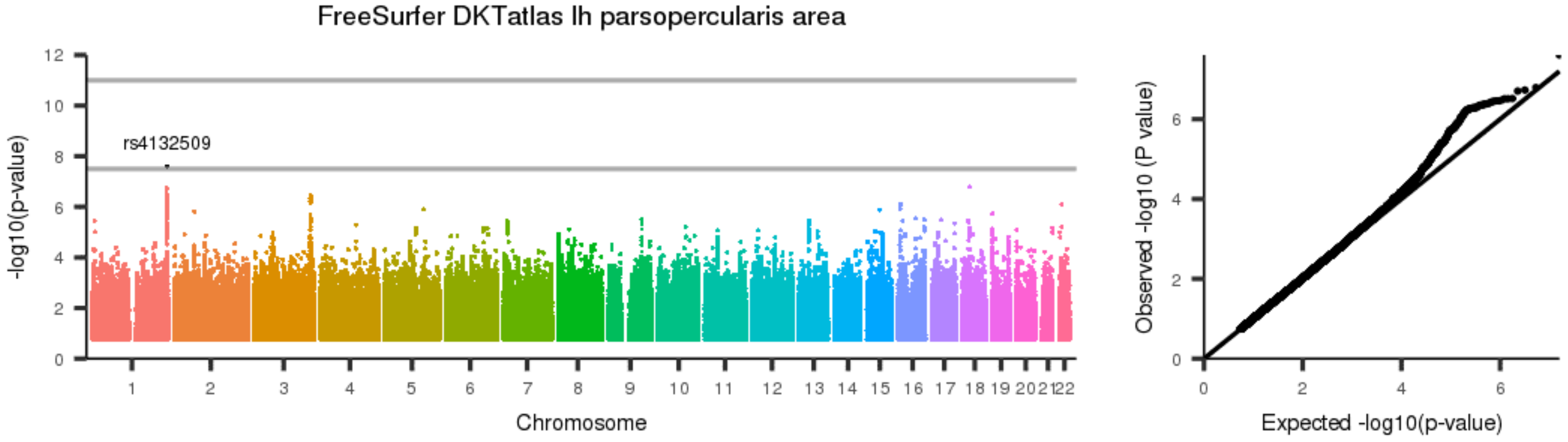

back to list of plots

Figure 1036: FreeSurfer DKTatlas lh parsorbitalis area

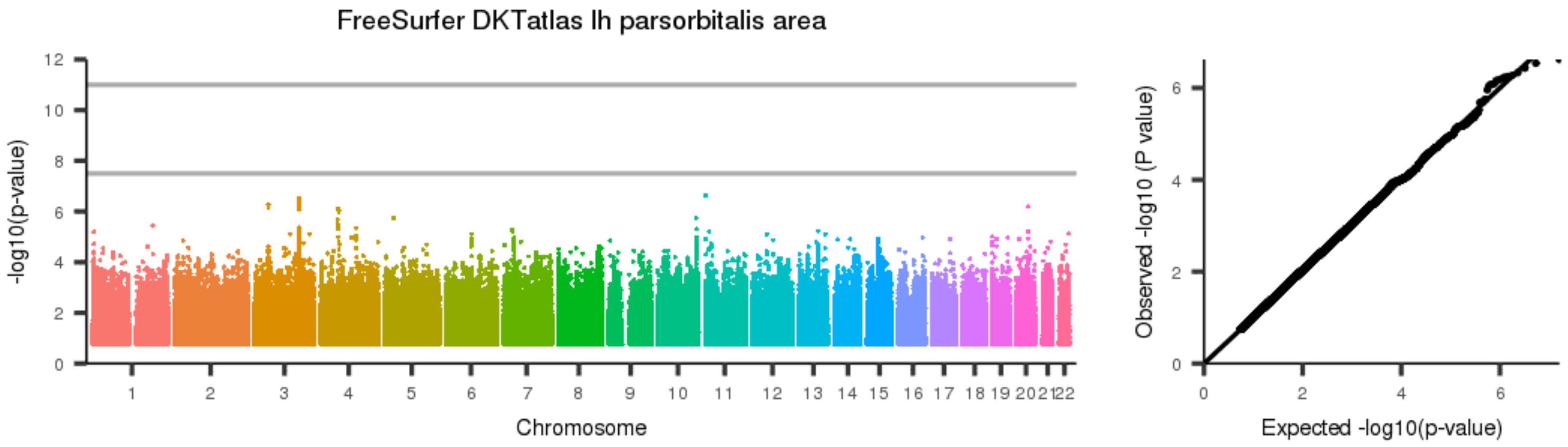

back to list of plots

Figure 1037: FreeSurfer DKTatlas lh parstriangularis area

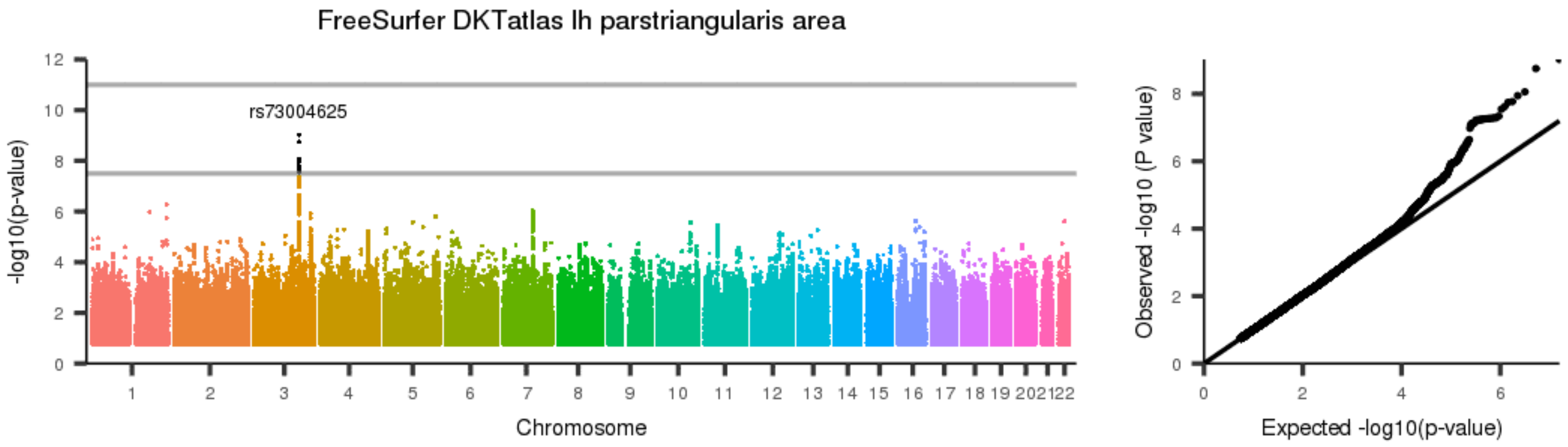

back to list of plots

Figure 1038: FreeSurfer DKTatlas lh pericalcarine area

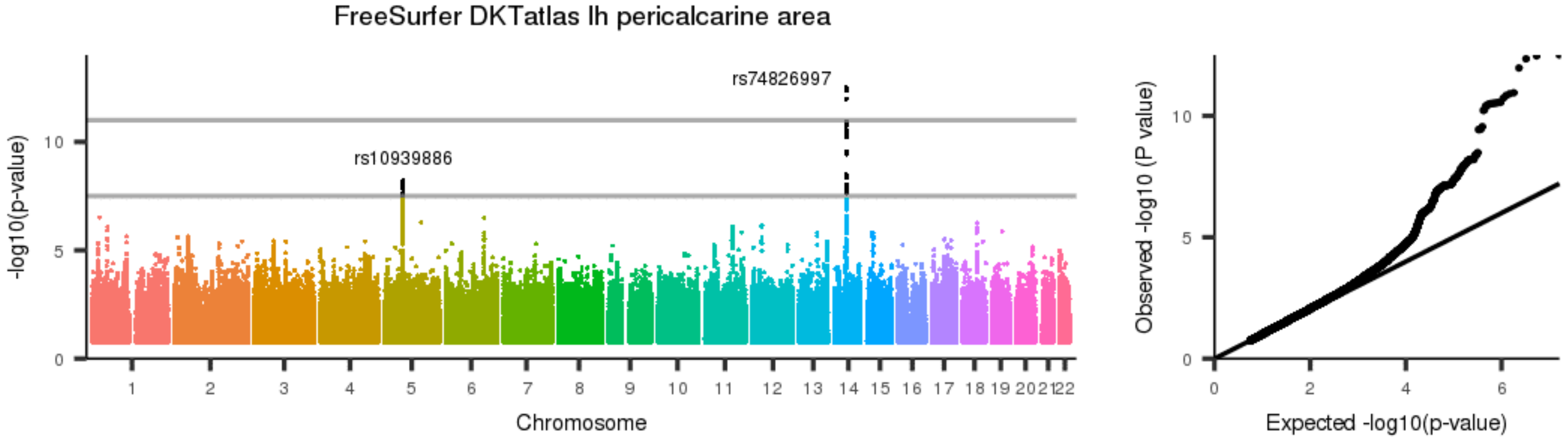

back to list of plots

Figure 1039: FreeSurfer DKTatlas lh postcentral area

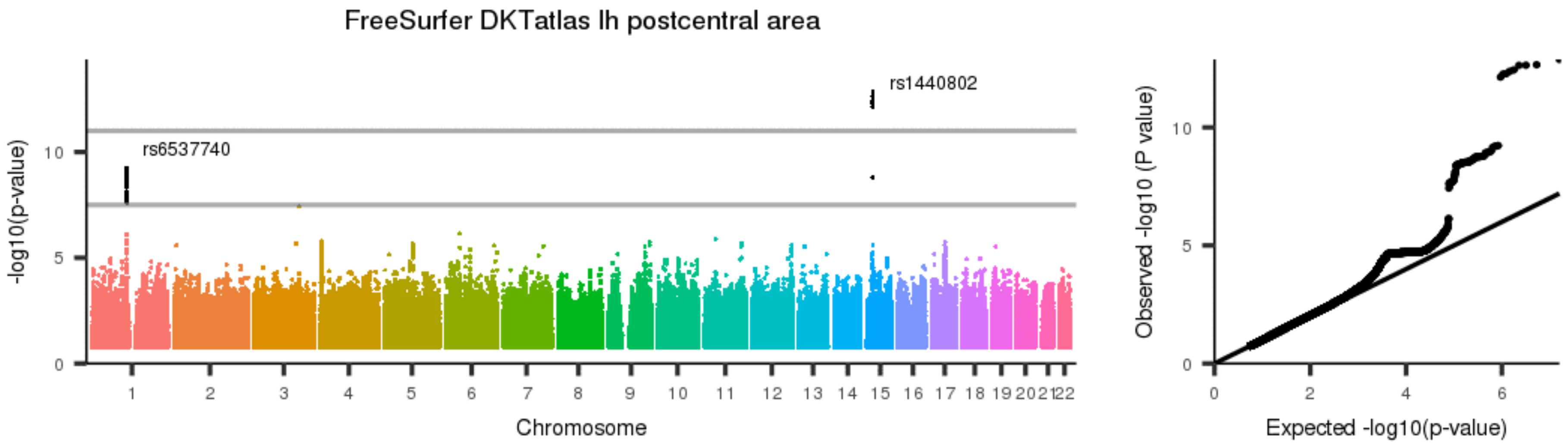

back to list of plots

Figure 1040: FreeSurfer DKTatlas lh posteriorcingulate area

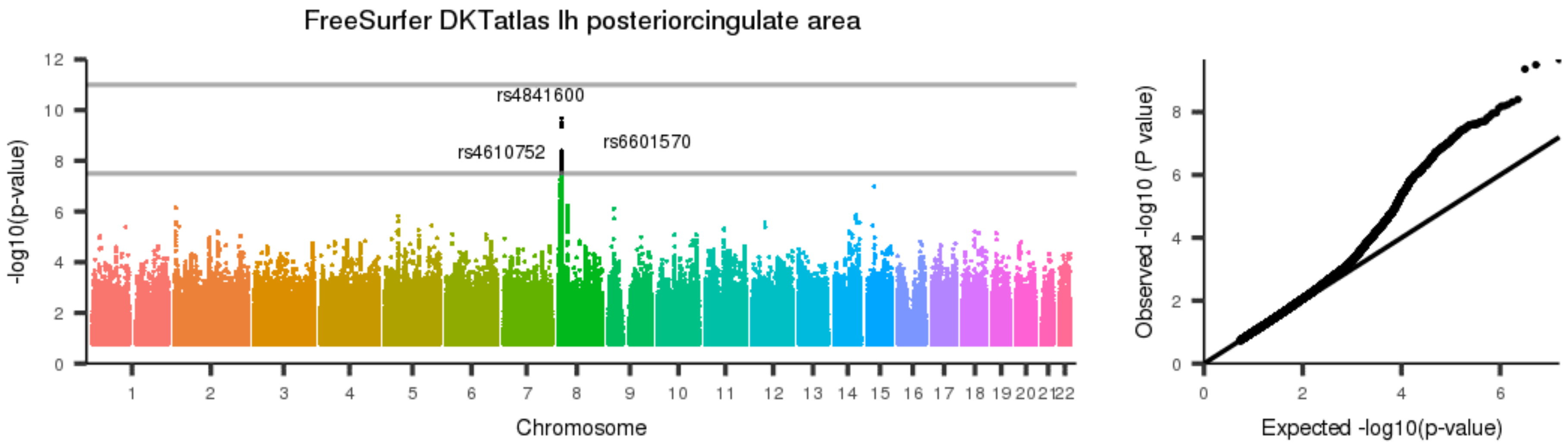

back to list of plots

Figure 1041: FreeSurfer DKTatlas lh precentral area

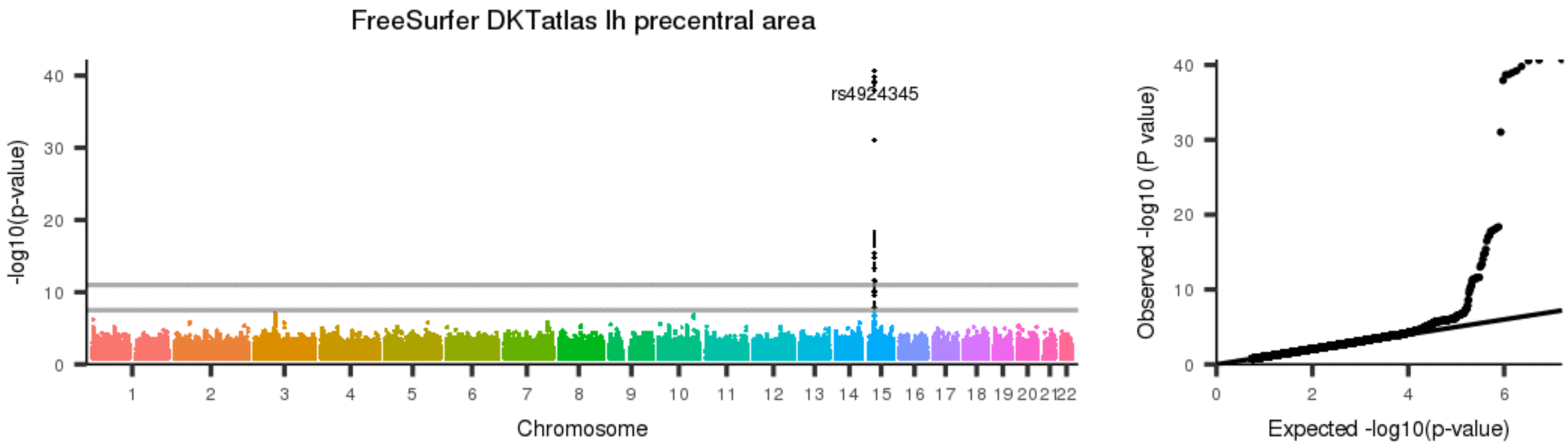

back to list of plots

Figure 1042: FreeSurfer DKTatlas lh precuneus area

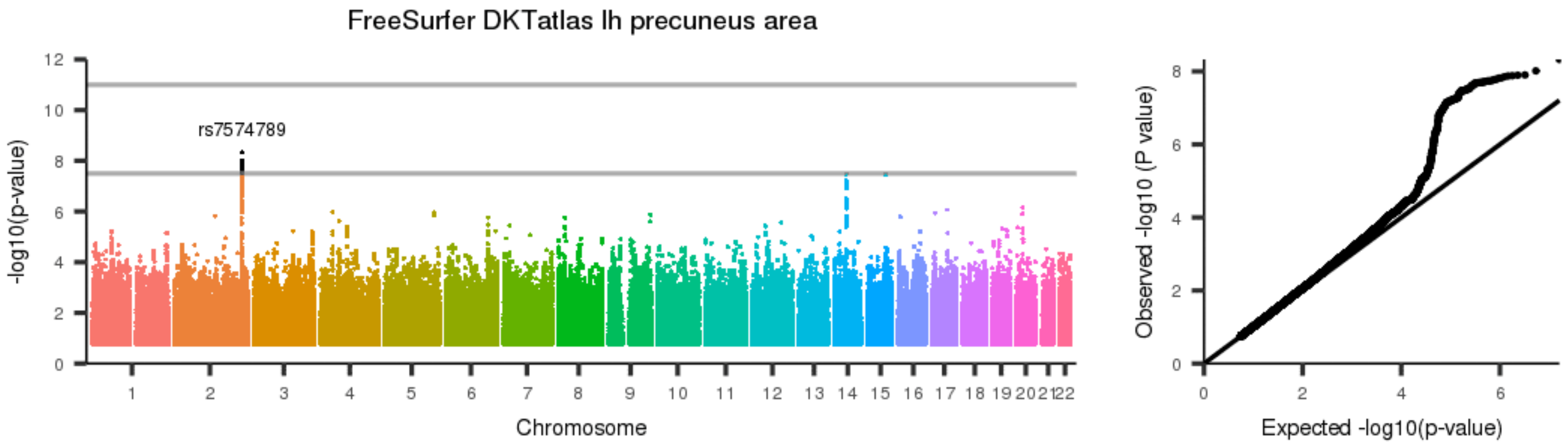

back to list of plots

Figure 1043: FreeSurfer DKTatlas lh rostralanteriorcingulate area

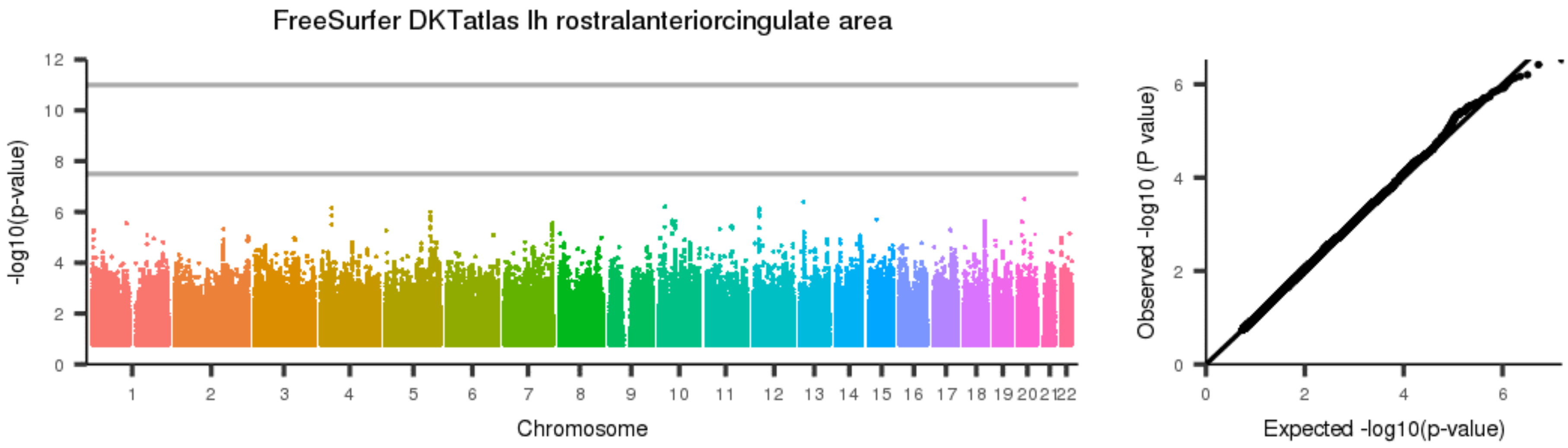

back to list of plots

Figure 1044: FreeSurfer DKTatlas lh rostralmiddlefrontal area

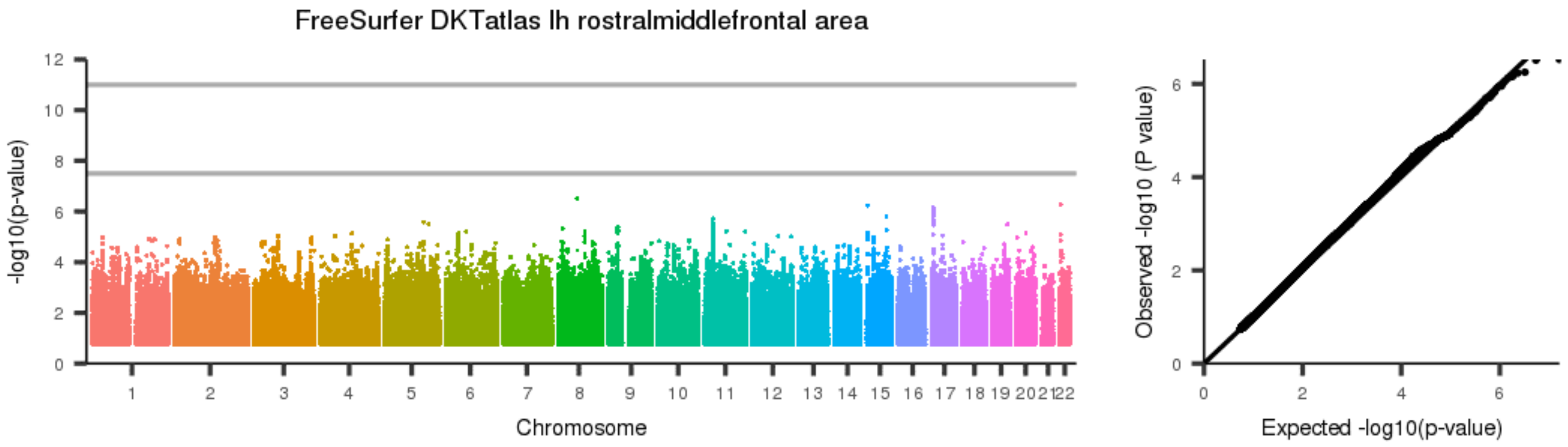

back to list of plots

Figure 1045: FreeSurfer DKTatlas lh superiorfrontal area

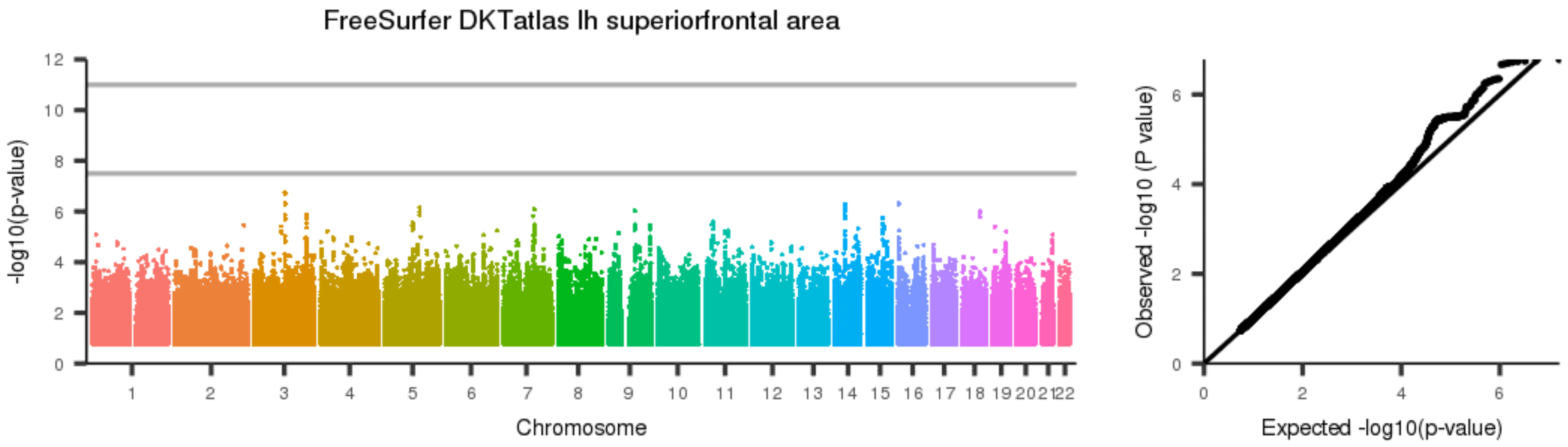

back to list of plots

Figure 1046: FreeSurfer DKTatlas lh superiorparietal area

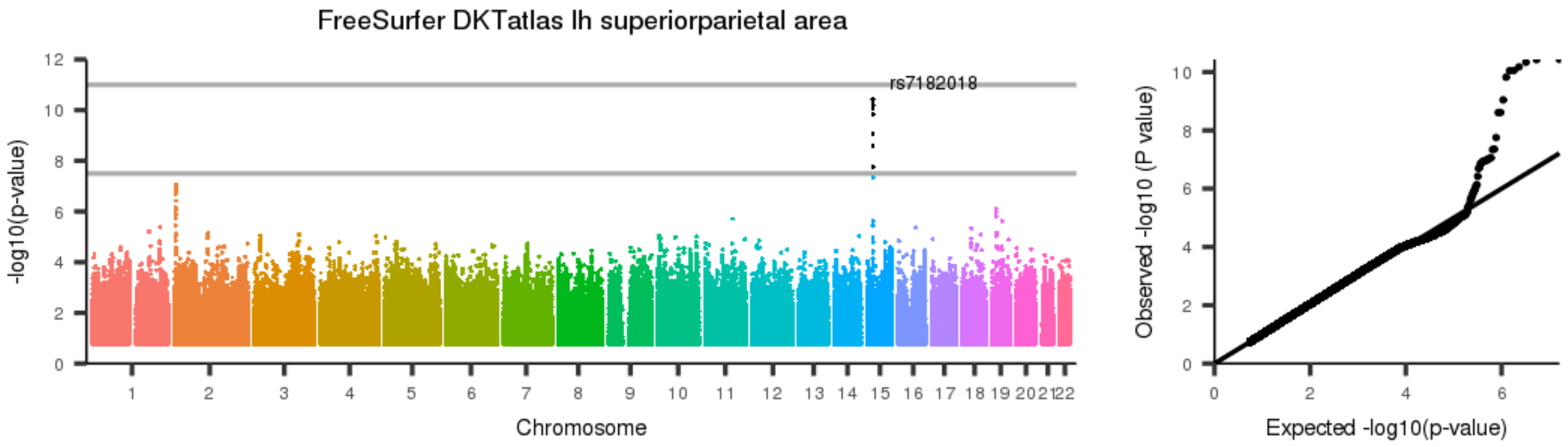

back to list of plots

Figure 1047: FreeSurfer DKTatlas lh superiortemporal area

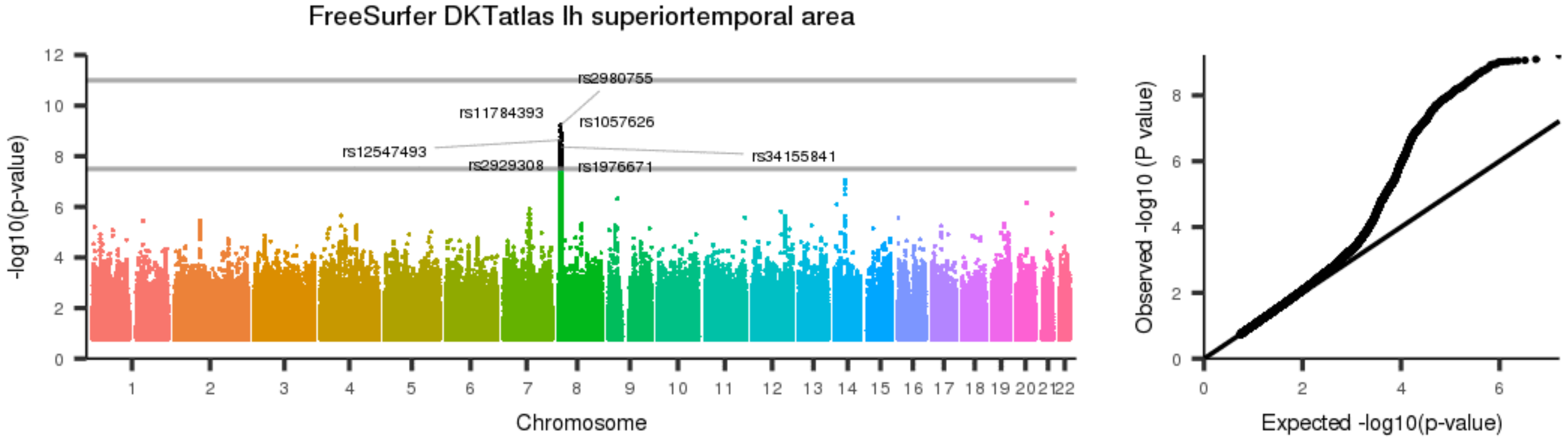

back to list of plots

Figure 1048: FreeSurfer DKTatlas lh supramarginal area

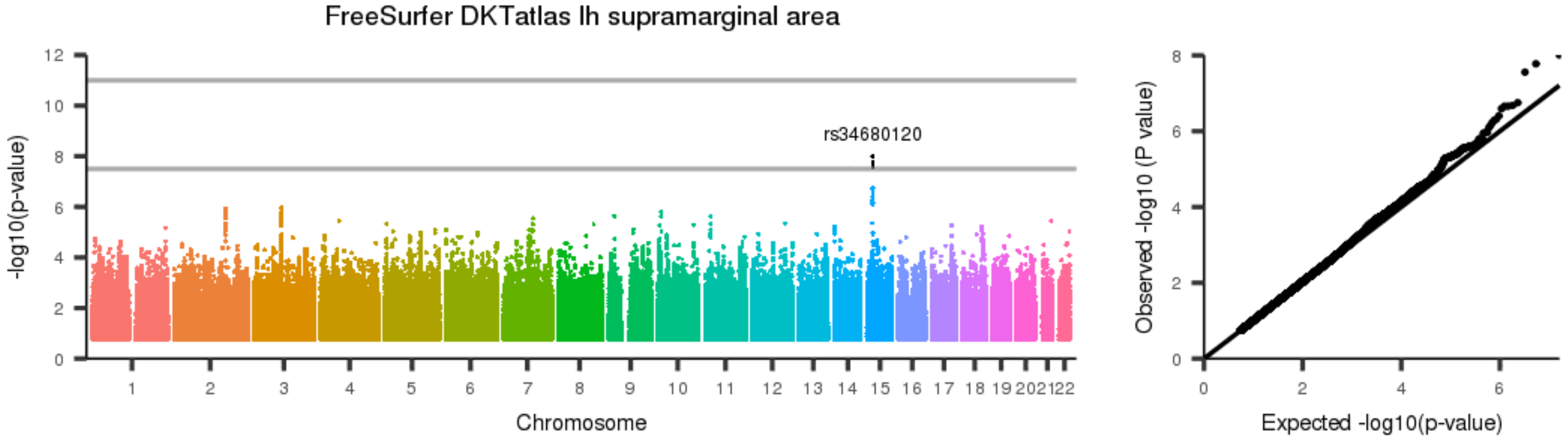

back to list of plots

Figure 1049: FreeSurfer DKTatlas lh transversetemporal area

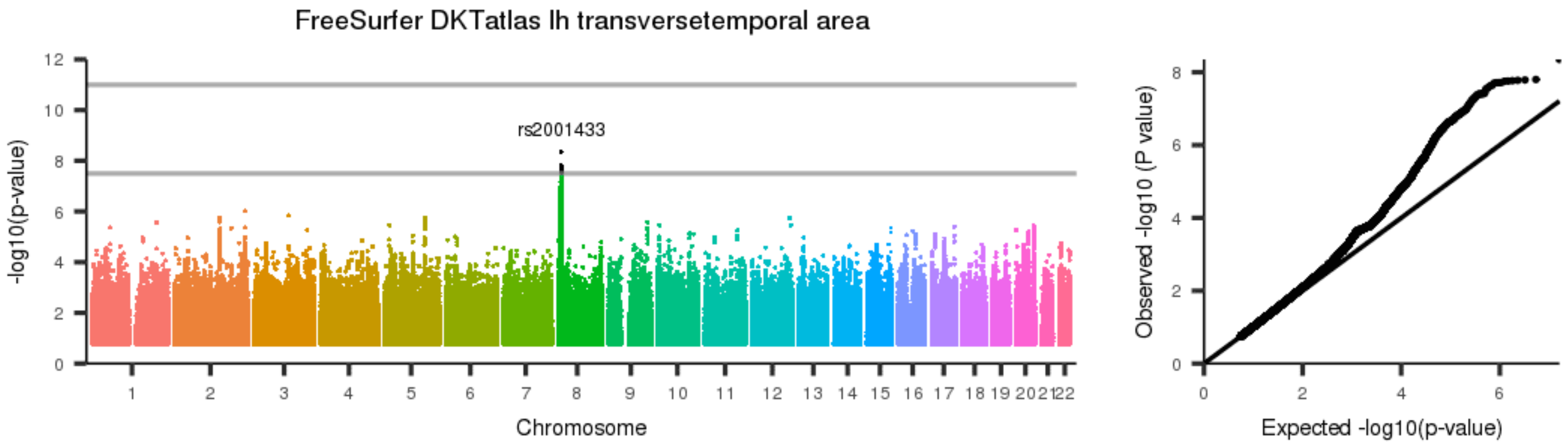

back to list of plots

Figure 1050: FreeSurfer DKTatlas lh insula area

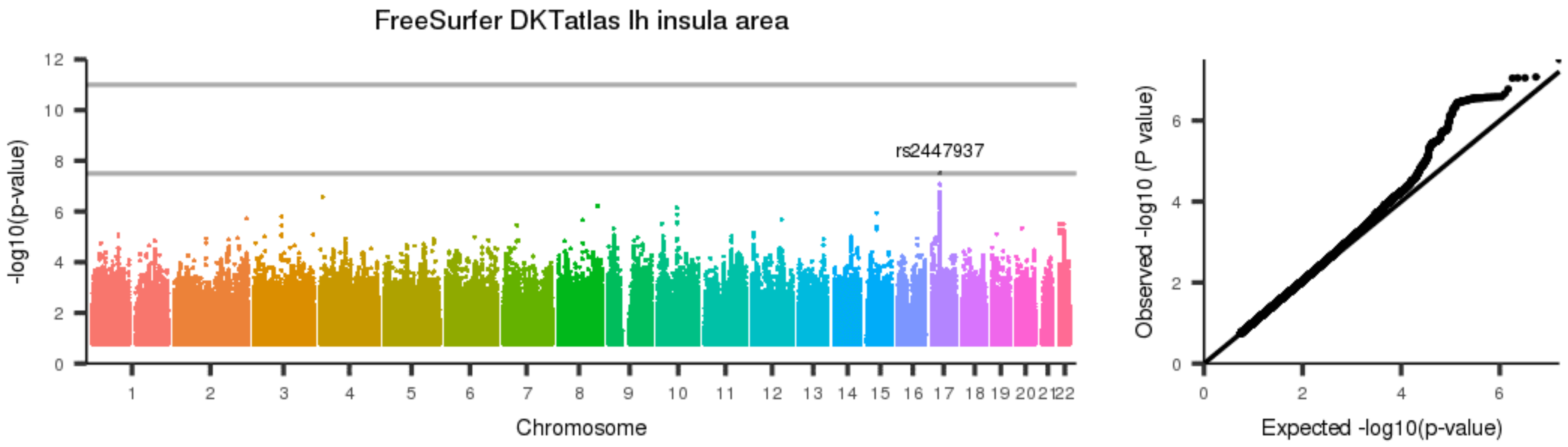

back to list of plots

Figure 1051: FreeSurfer DKTatlas lh WhiteSurfArea area

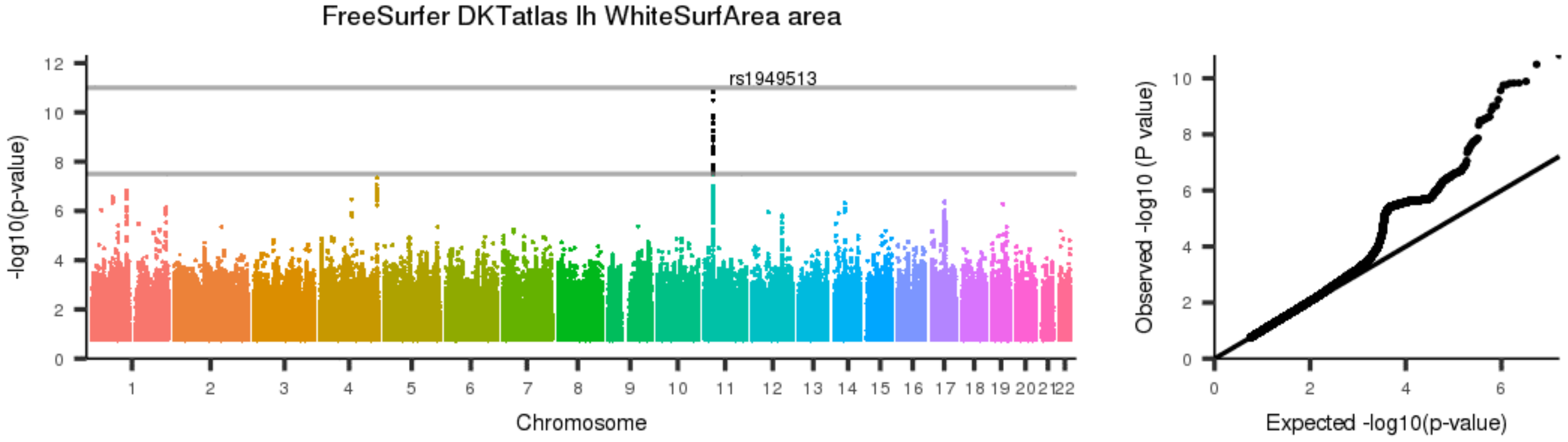

back to list of plots

Figure 1052: FreeSurfer a2009s lh G and S frontomargin area

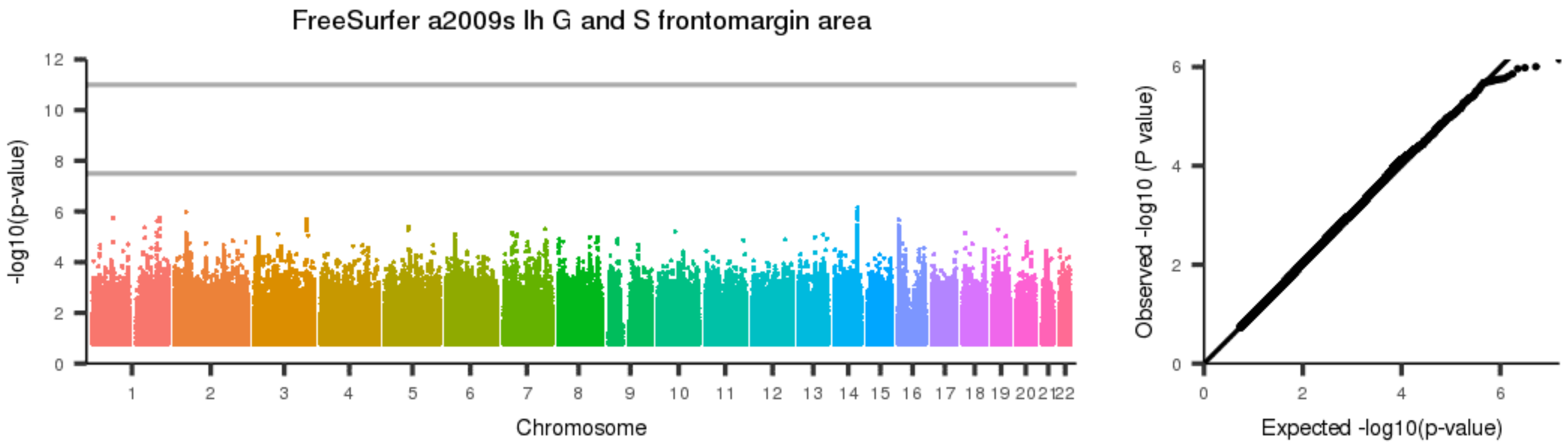

back to list of plots

Figure 1053: FreeSurfer a2009s lh G and S occipital inf area

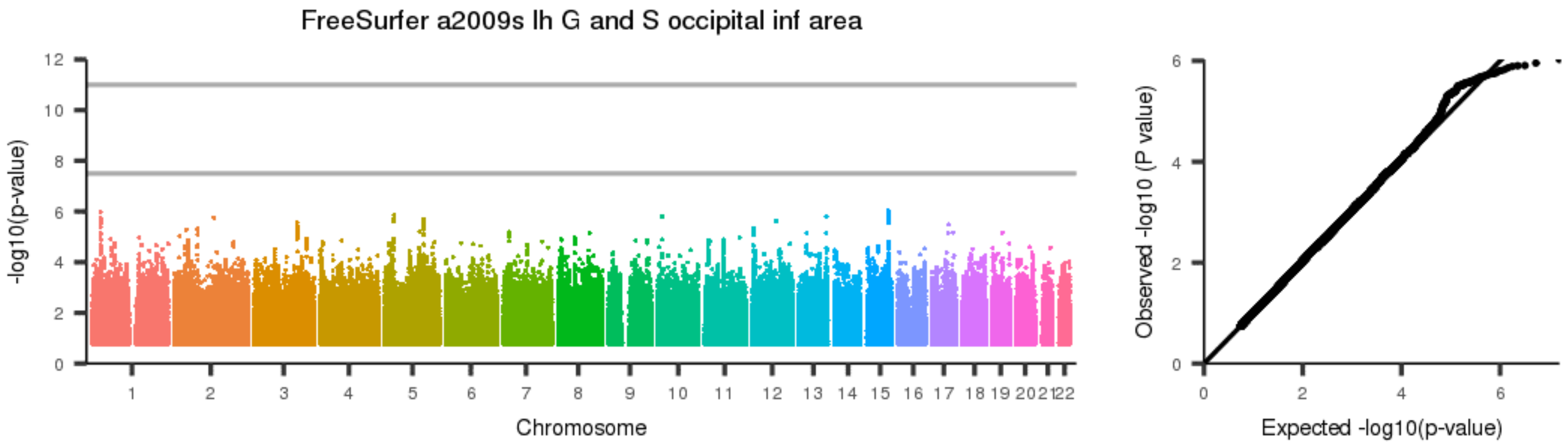

back to list of plots

Figure 1054: FreeSurfer a2009s lh G and S paracentral area

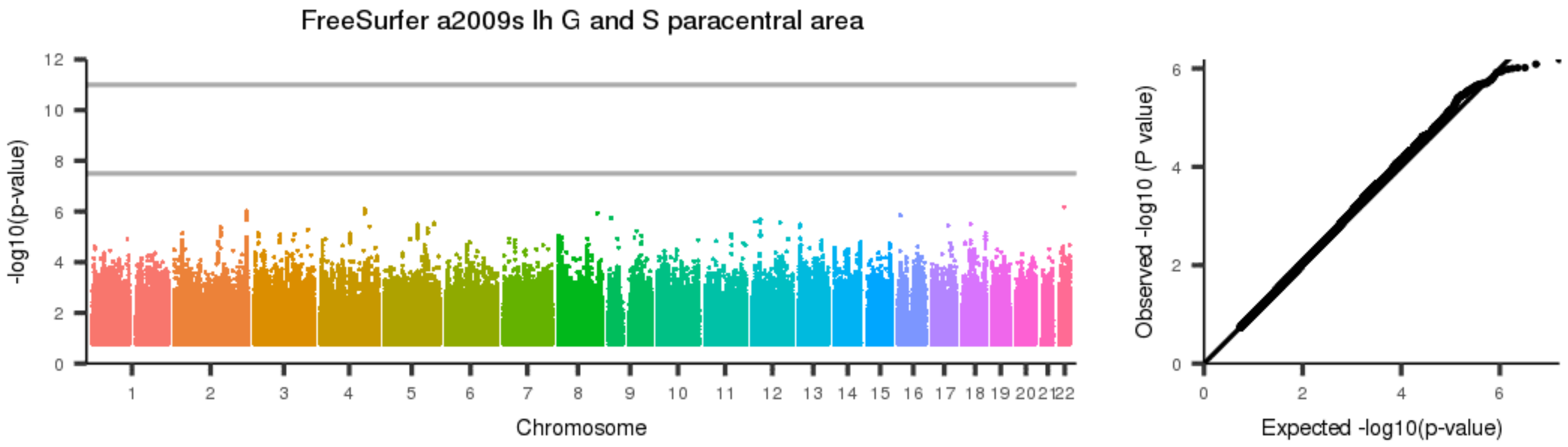

back to list of plots

Figure 1055: FreeSurfer a2009s lh G and S subcentral area

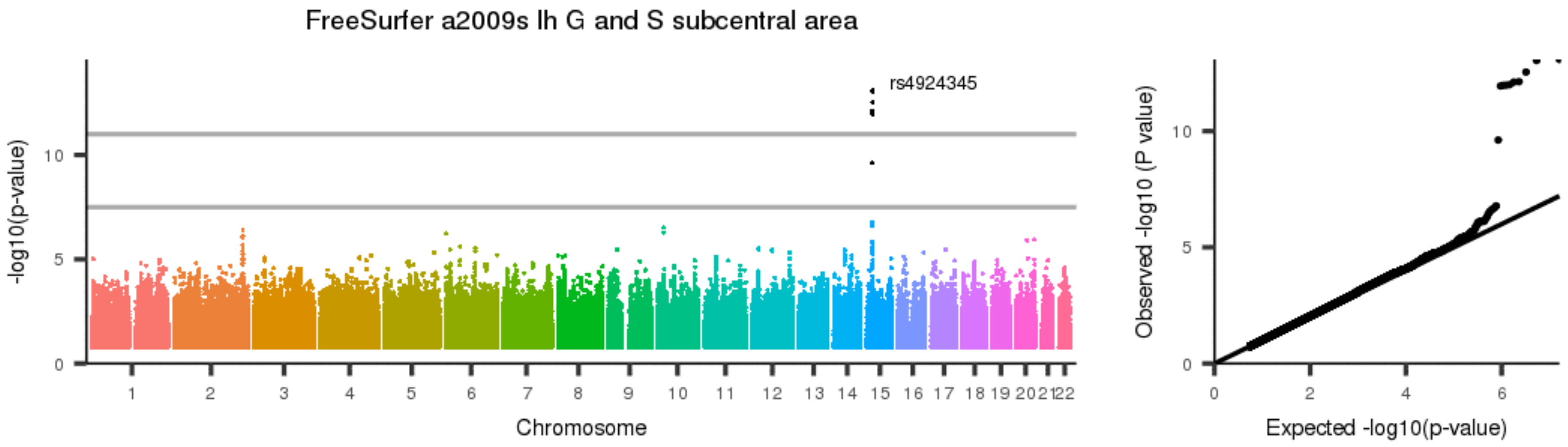

back to list of plots

Figure 1056: FreeSurfer a2009s lh G and S transv frontopol area

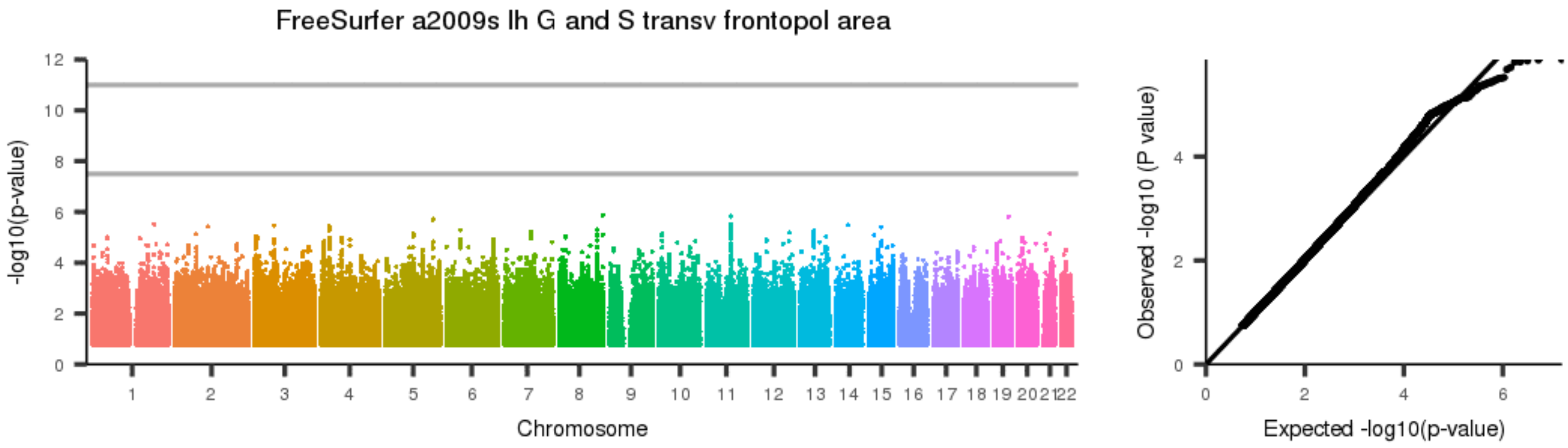

back to list of plots

Figure 1057: FreeSurfer a2009s lh G and S cingul-Ant area

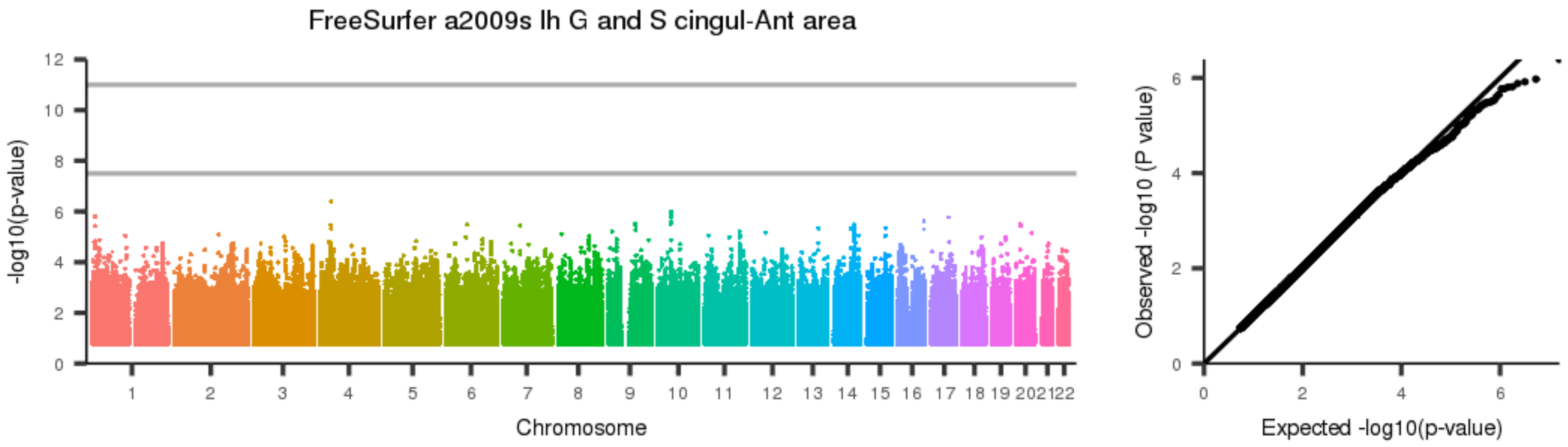

back to list of plots

Figure 1058: FreeSurfer a2009s lh G and S cingul-Mid-Ant area

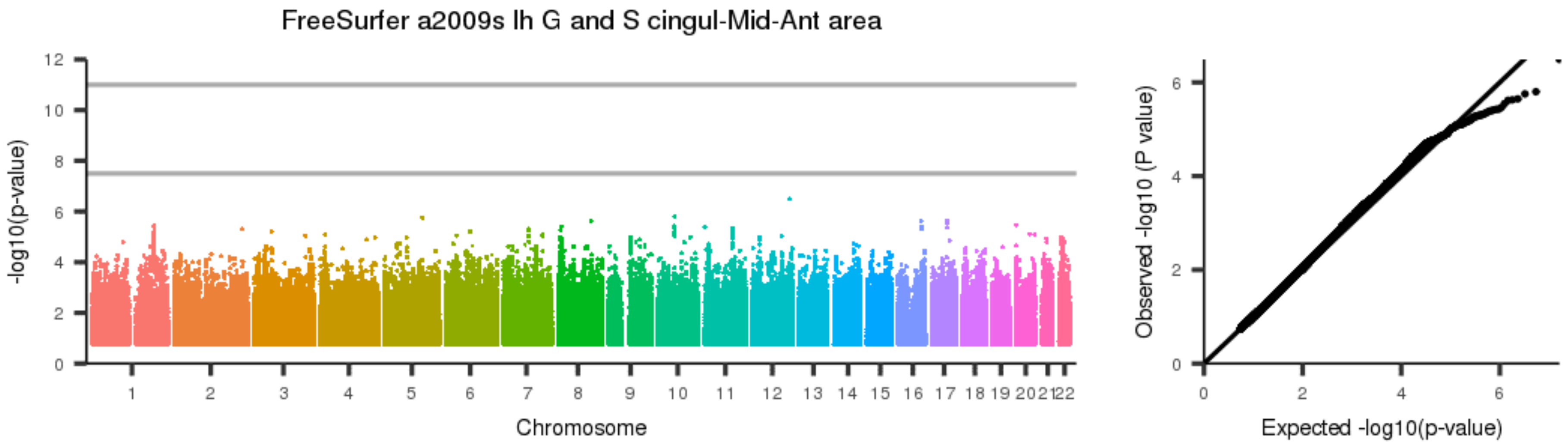

back to list of plots

Figure 1059: FreeSurfer a2009s lh G and S cingul-Mid-Post area

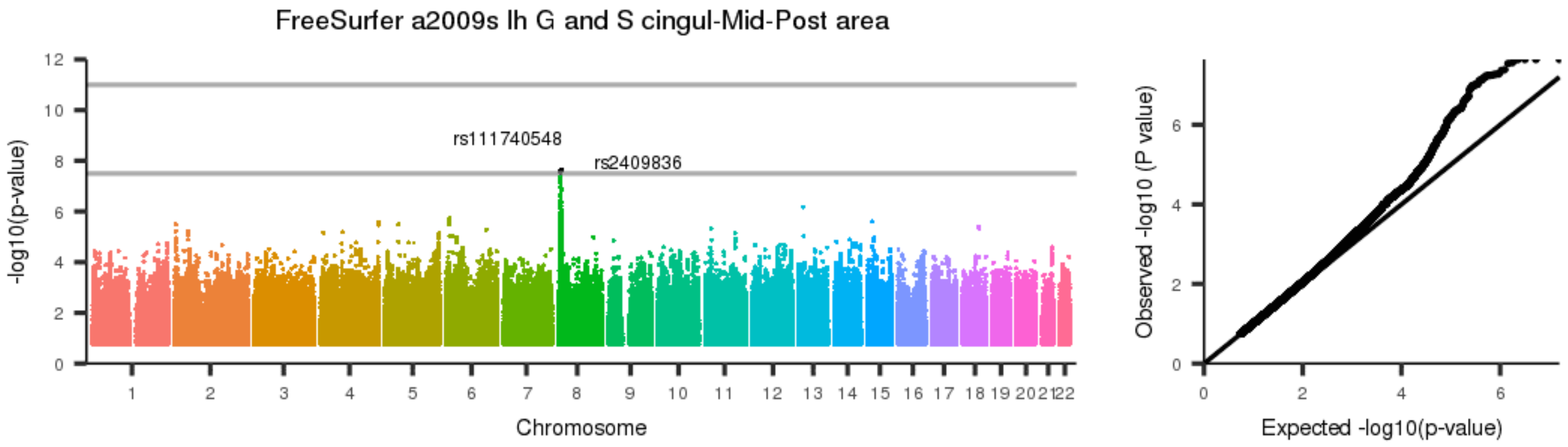

back to list of plots

Figure 1060: FreeSurfer a2009s lh G cingul-Post-dorsal area

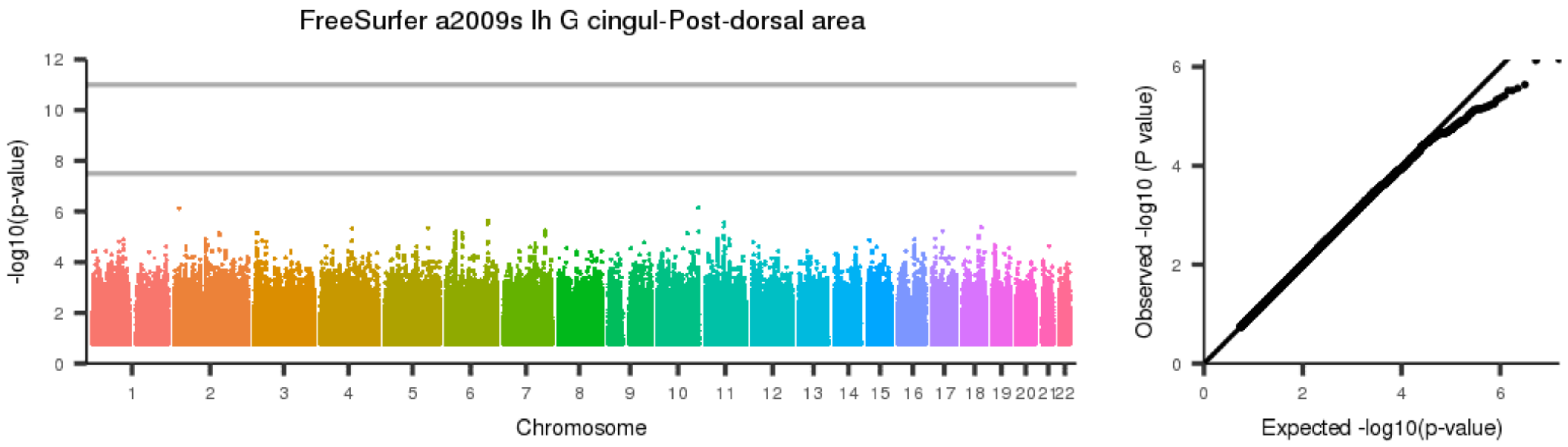

back to list of plots

Figure 1061: FreeSurfer a2009s lh G cingul-Post-ventral area

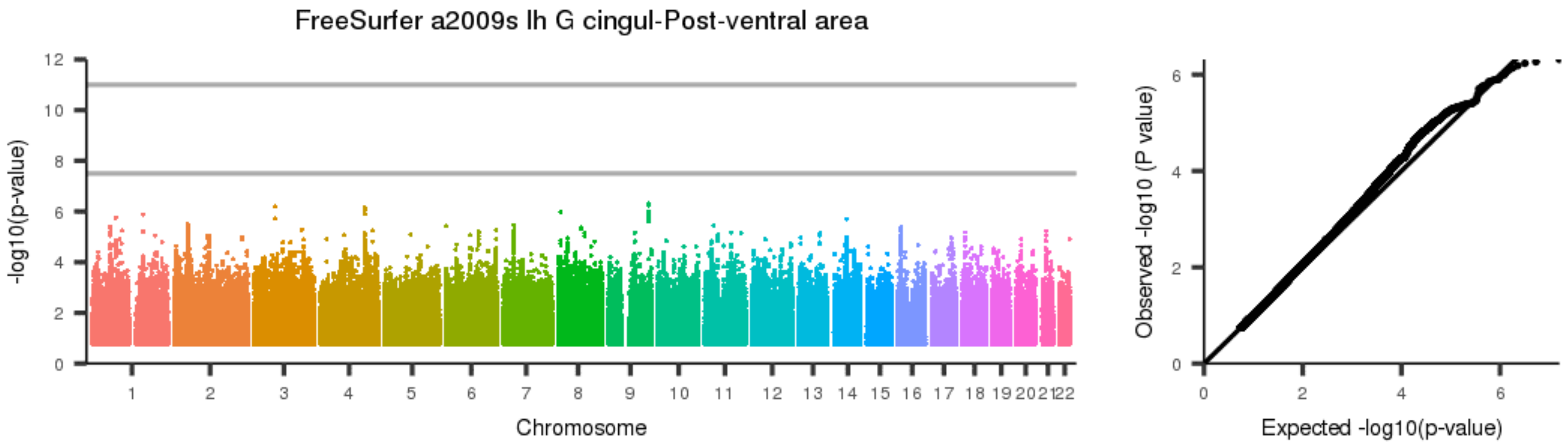

back to list of plots

Figure 1062: FreeSurfer a2009s lh G cuneus area

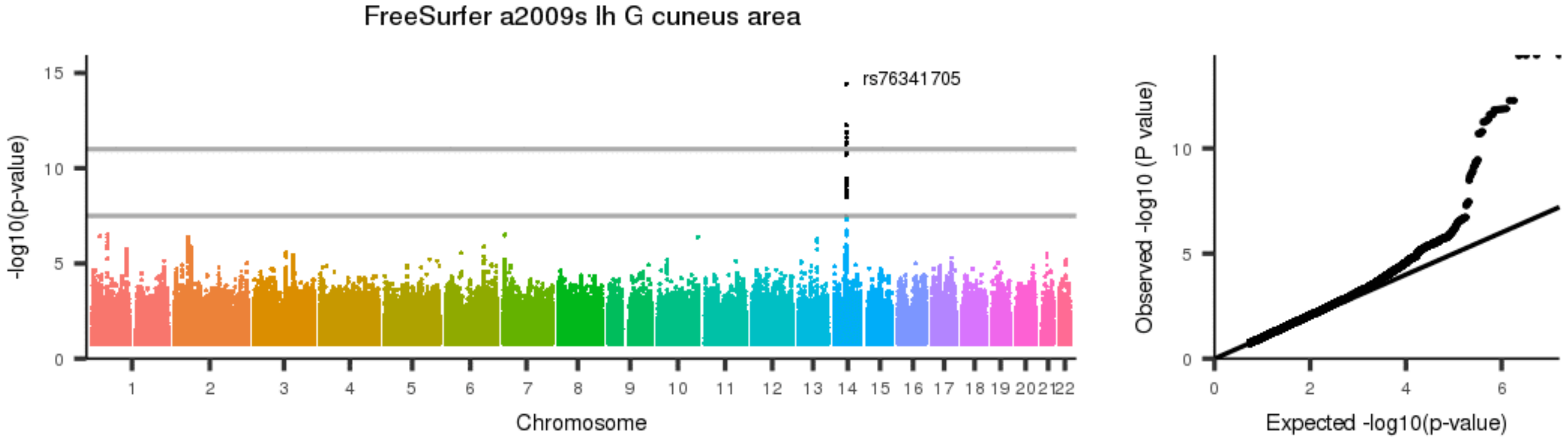

back to list of plots

Figure 1063: FreeSurfer a2009s lh G front inf-Opercular area

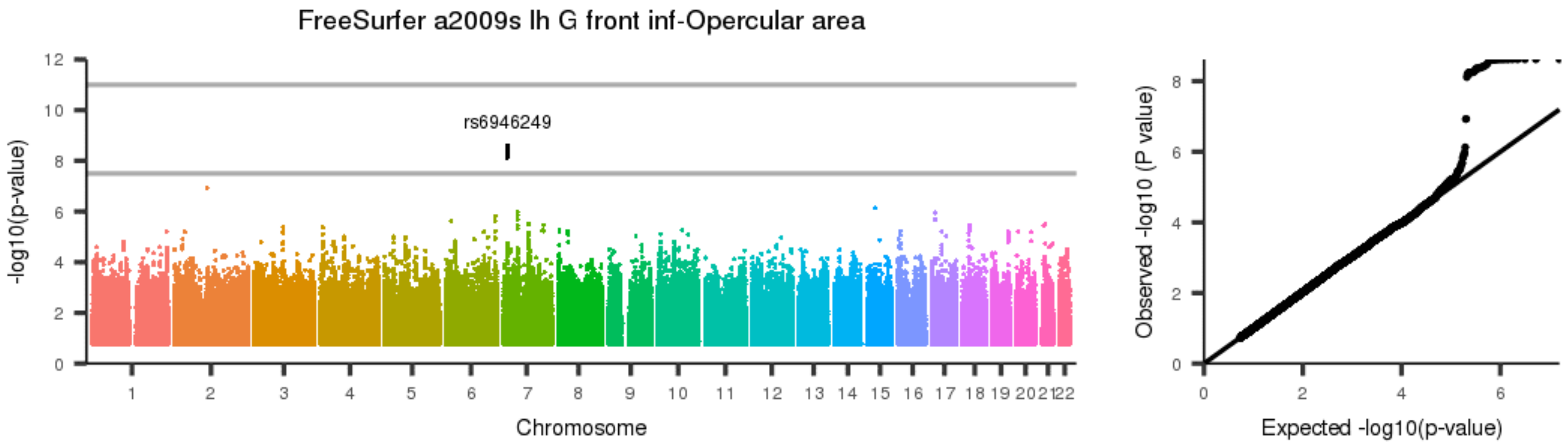

back to list of plots

Figure 1064: FreeSurfer a2009s lh G front inf-Orbital area

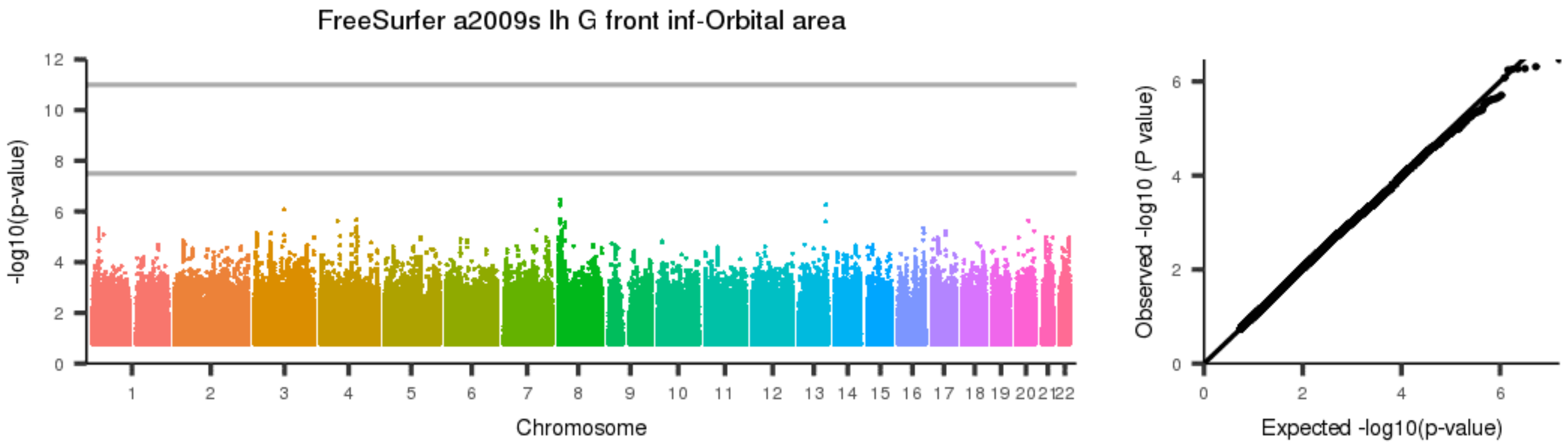

back to list of plots

Figure 1065: FreeSurfer a2009s lh G front inf-Triangul area

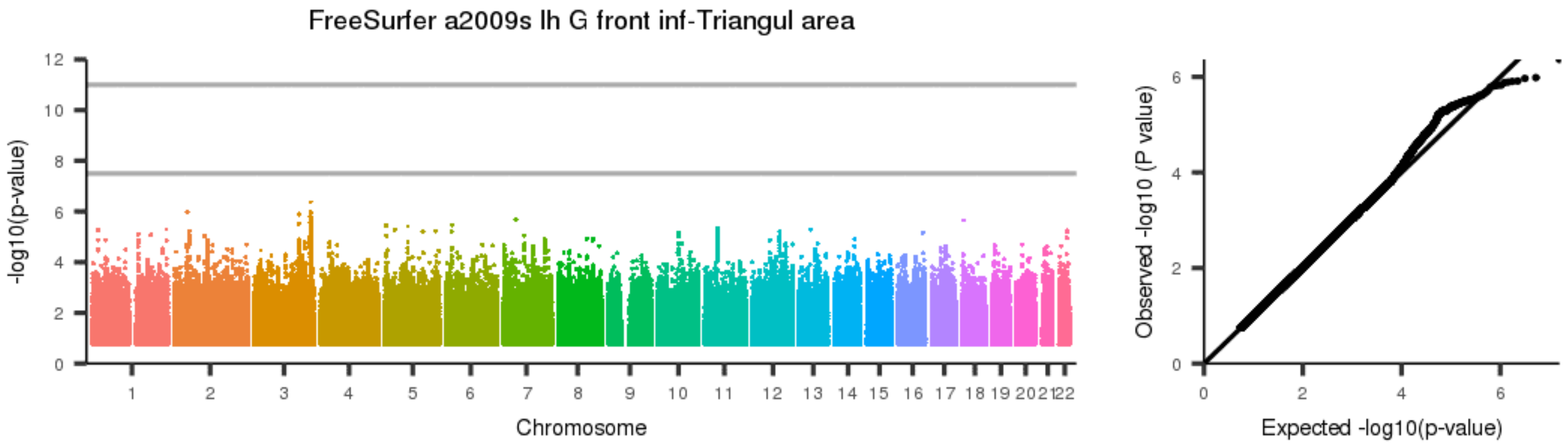

back to list of plots

Figure 1066: FreeSurfer a2009s lh G front middle area

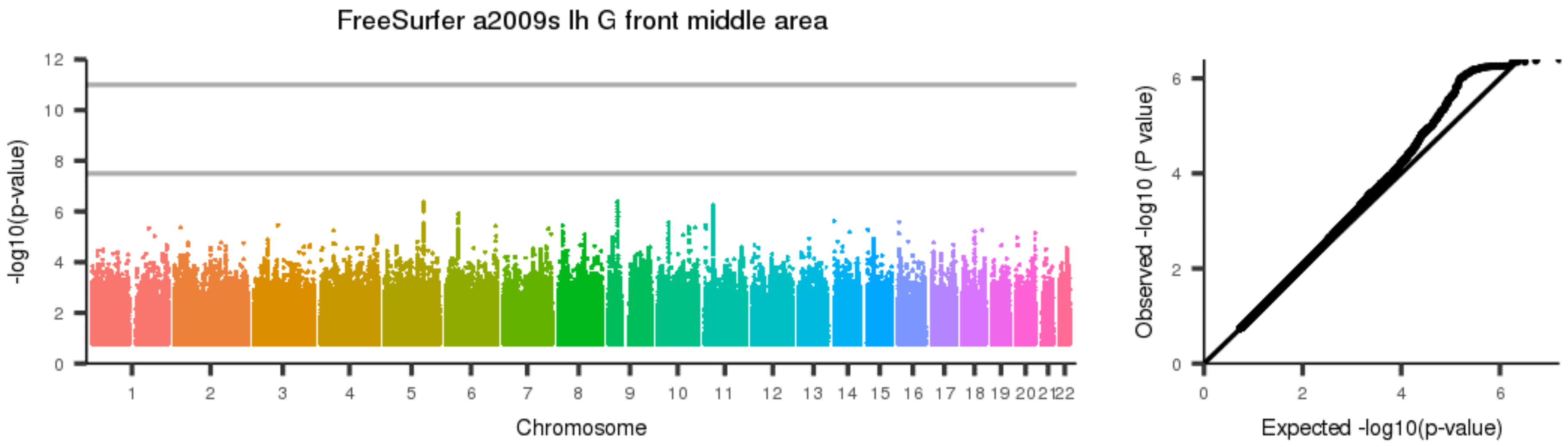

back to list of plots

Figure 1067: FreeSurfer a2009s lh G front sup area

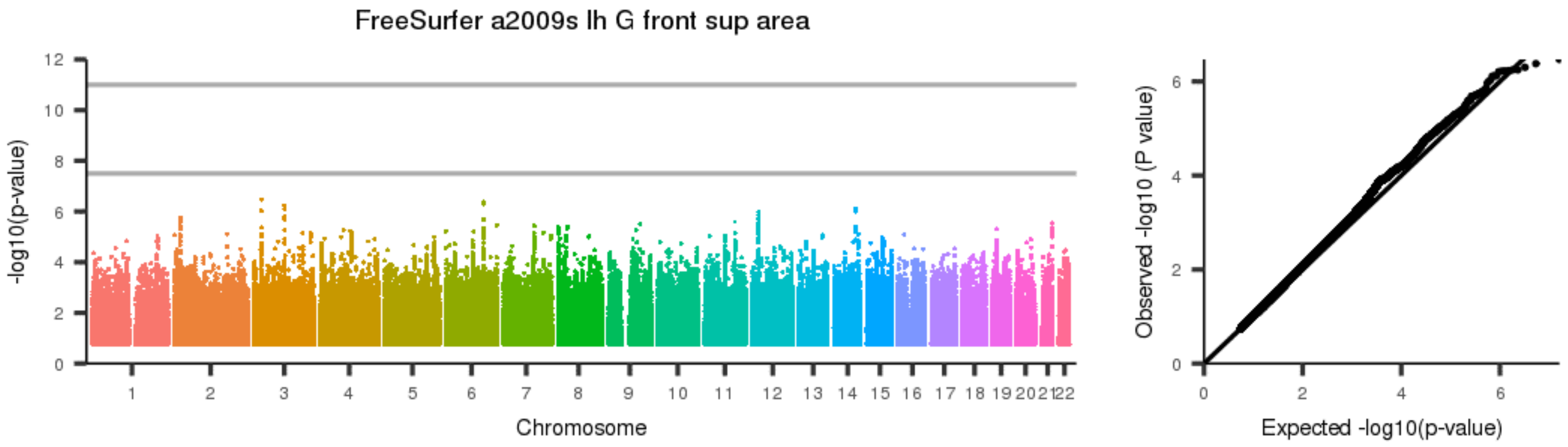

back to list of plots

Figure 1068: FreeSurfer a2009s lh G Ins lg and S cent ins area

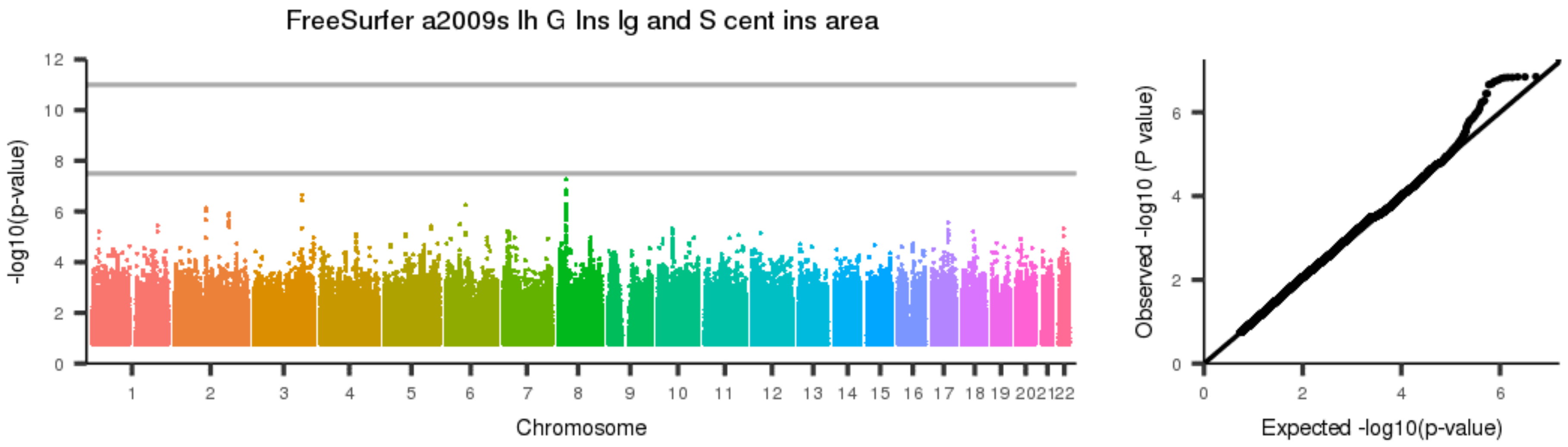

back to list of plots

Figure 1069: FreeSurfer a2009s lh G insular short area

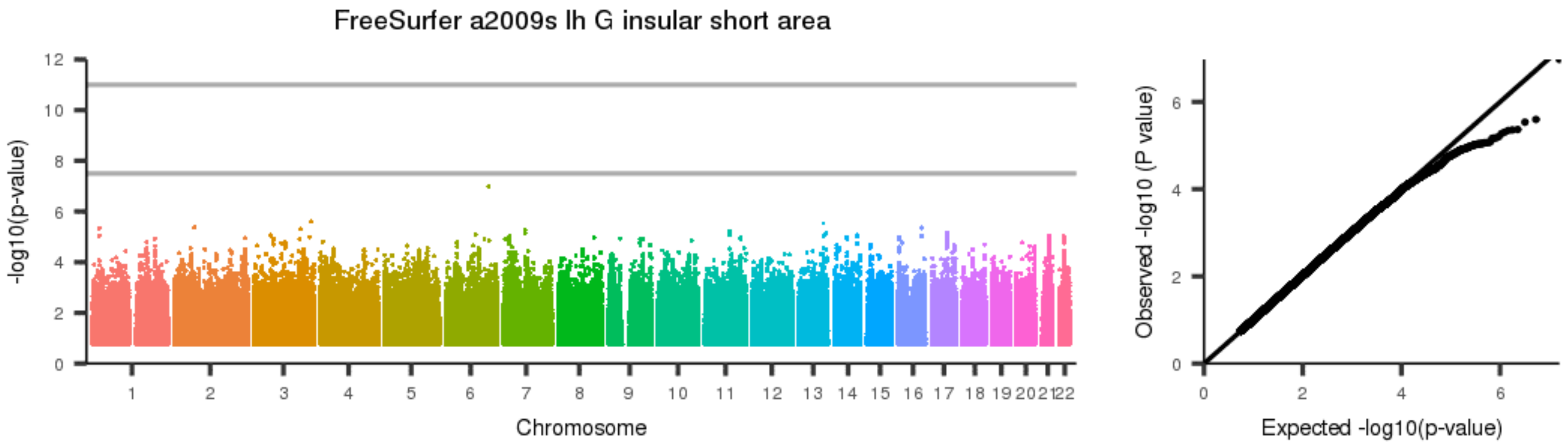

back to list of plots

Figure 1070: FreeSurfer a2009s lh G occipital middle area

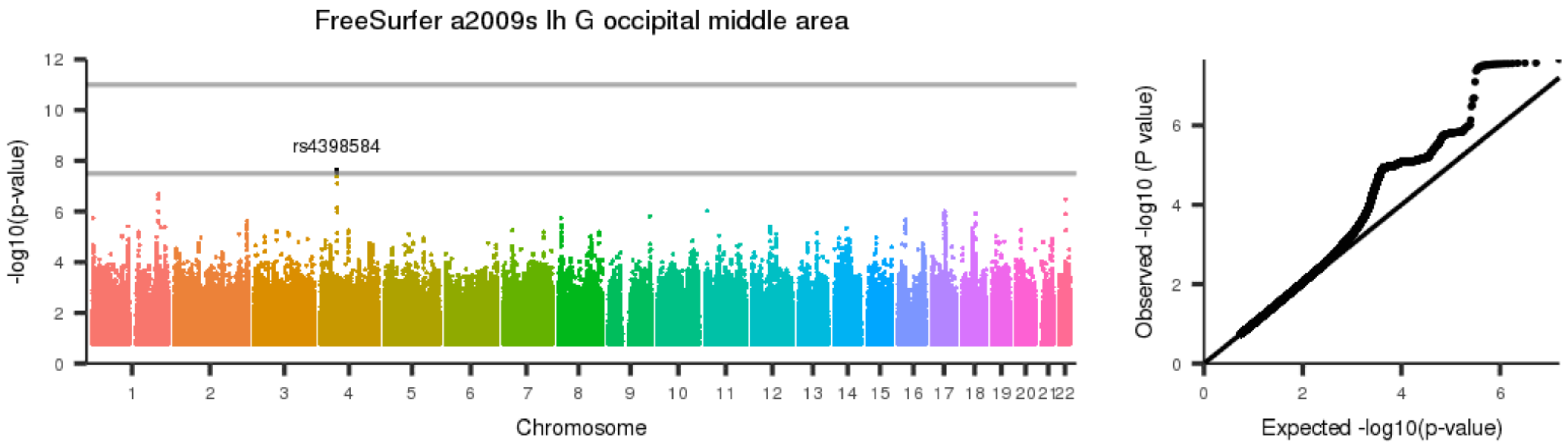

back to list of plots

Figure 1071: FreeSurfer a2009s lh G occipital sup area

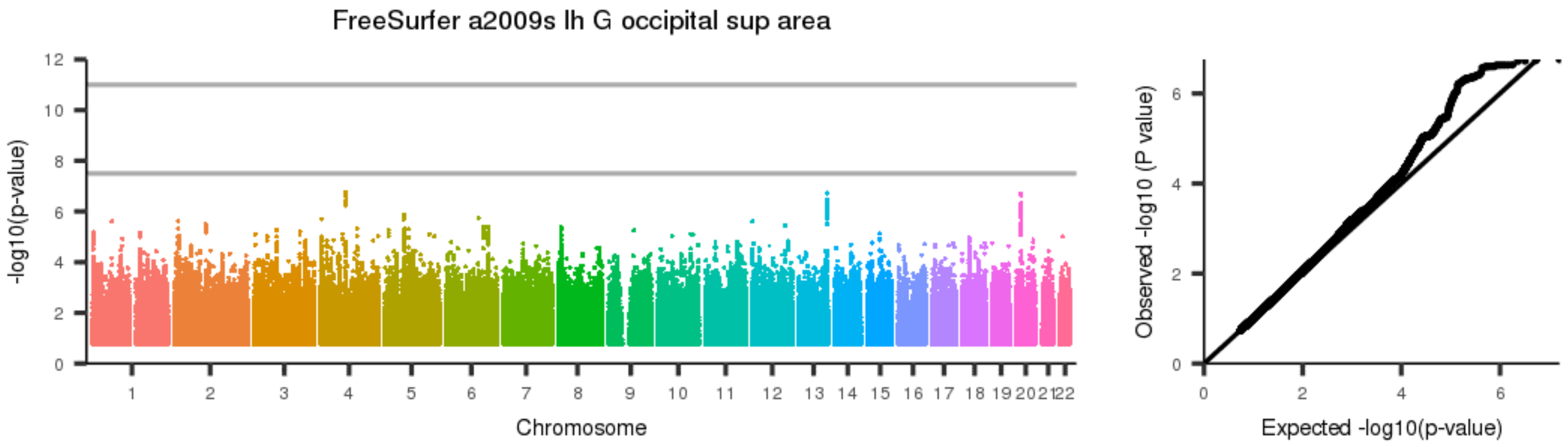

back to list of plots

Figure 1072: FreeSurfer a2009s lh G oc-temp lat-fusifor area

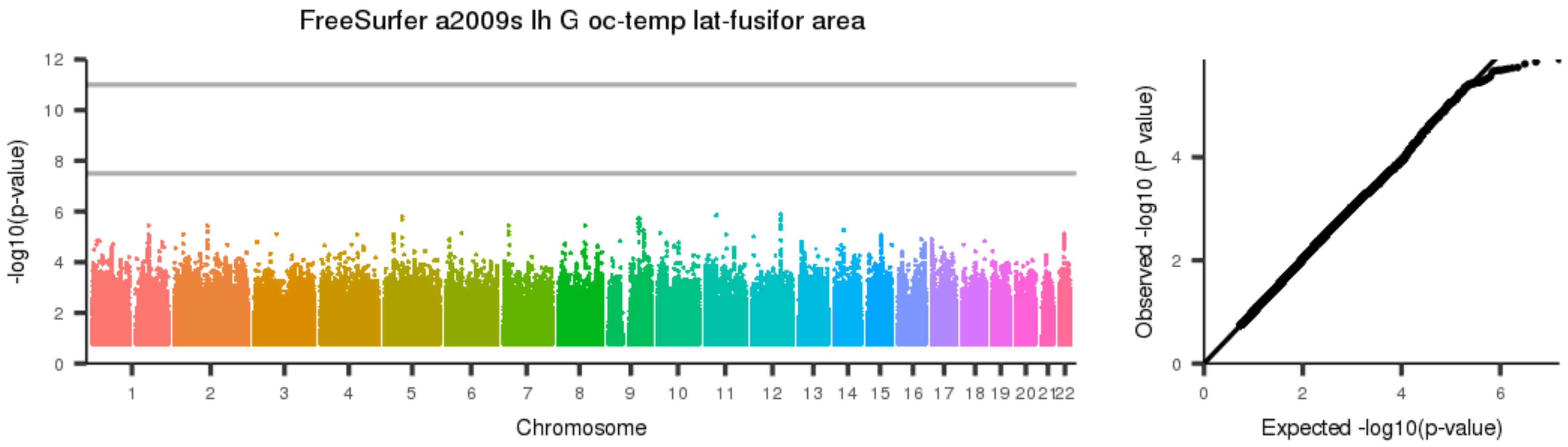

back to list of plots

Figure 1073: FreeSurfer a2009s lh G oc-temp med-Lingual area

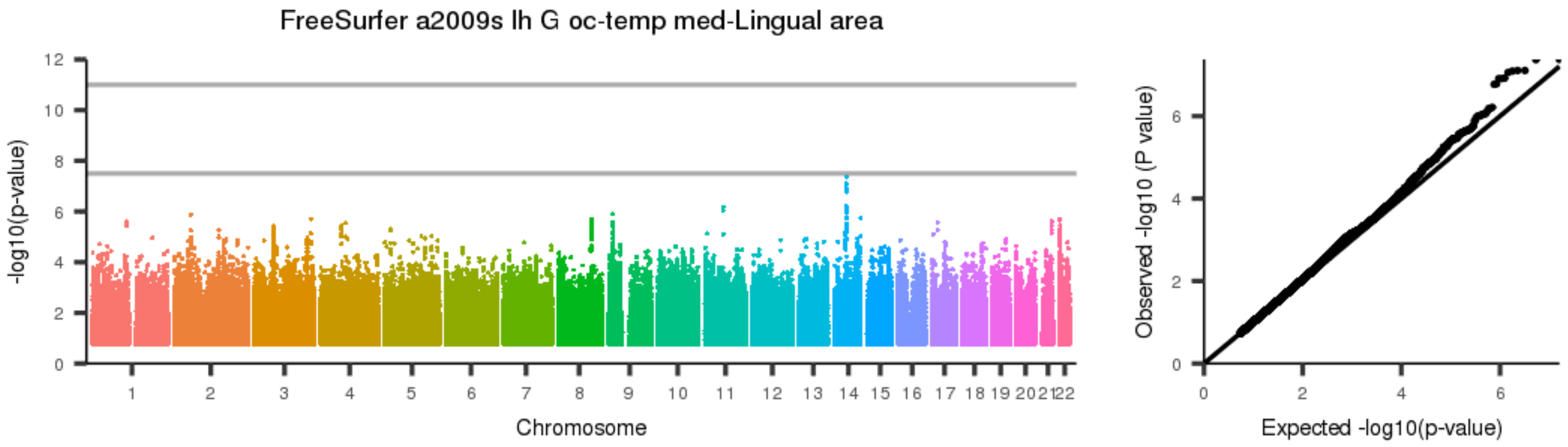

back to list of plots

Figure 1074: FreeSurfer a2009s lh G oc-temp med-Parahip area

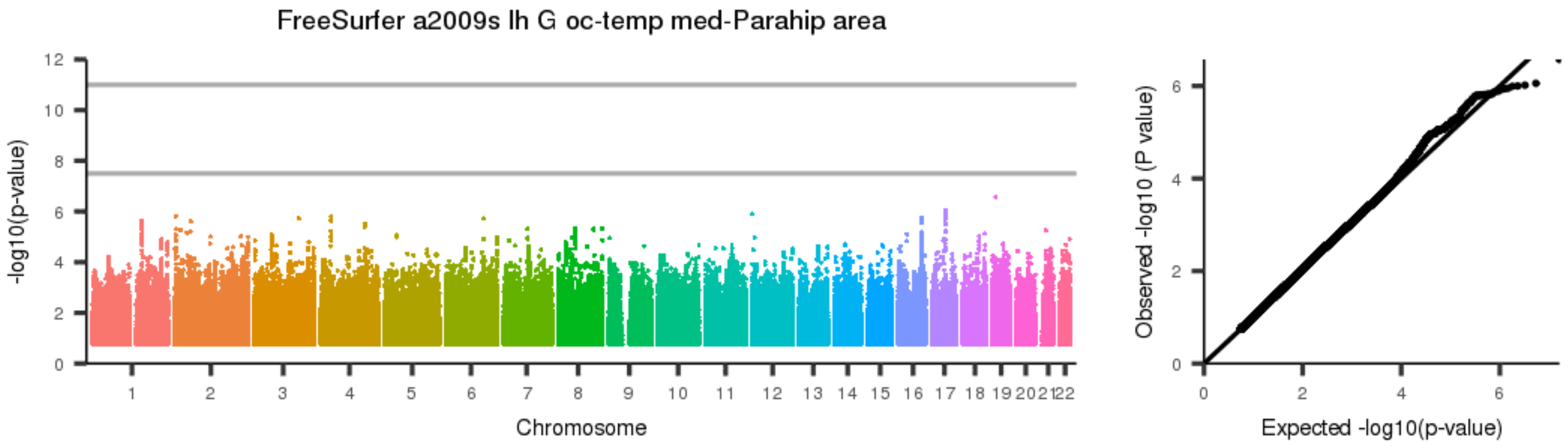

back to list of plots

Figure 1075: FreeSurfer a2009s lh G orbital area

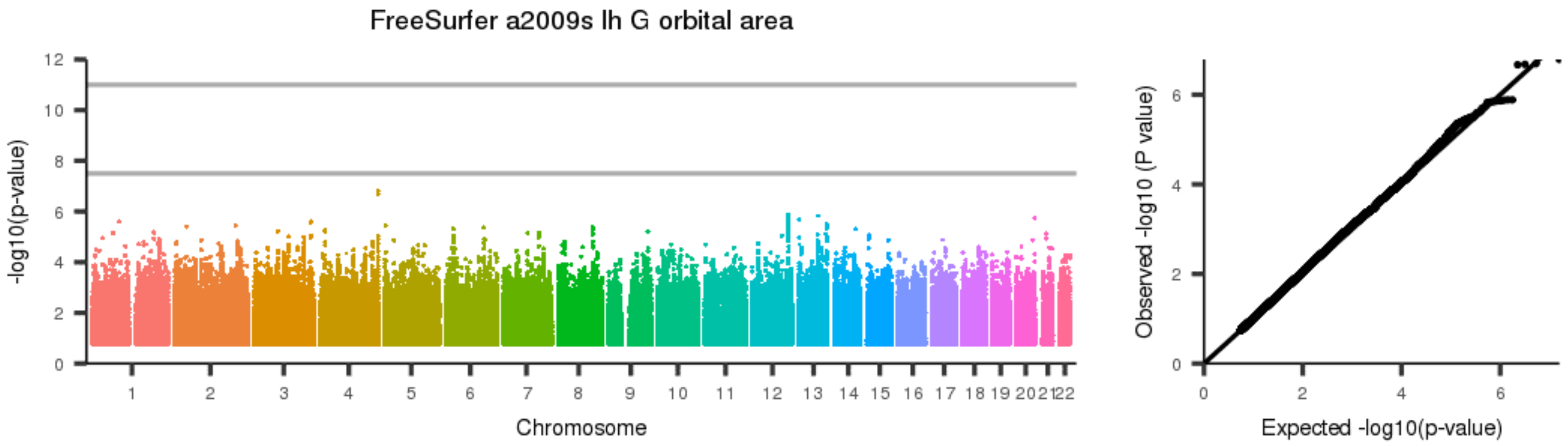

back to list of plots

Figure 1076: FreeSurfer a2009s lh G pariet inf-Angular area

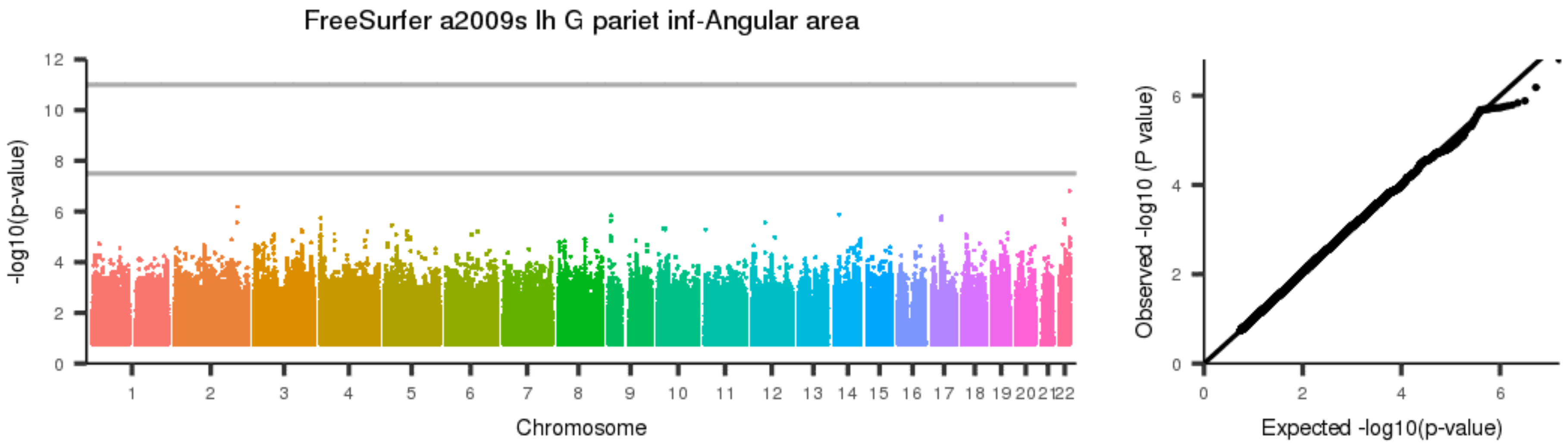

back to list of plots

Figure 1077: FreeSurfer a2009s lh G pariet inf-Supramar area

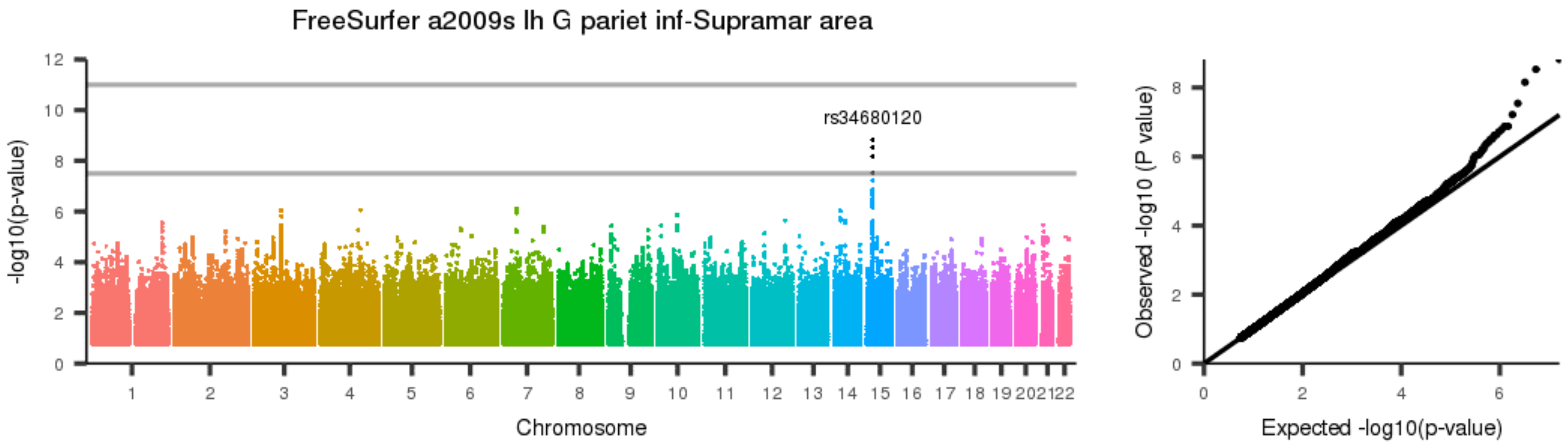

back to list of plots

Figure 1078: FreeSurfer a2009s lh G parietal sup area

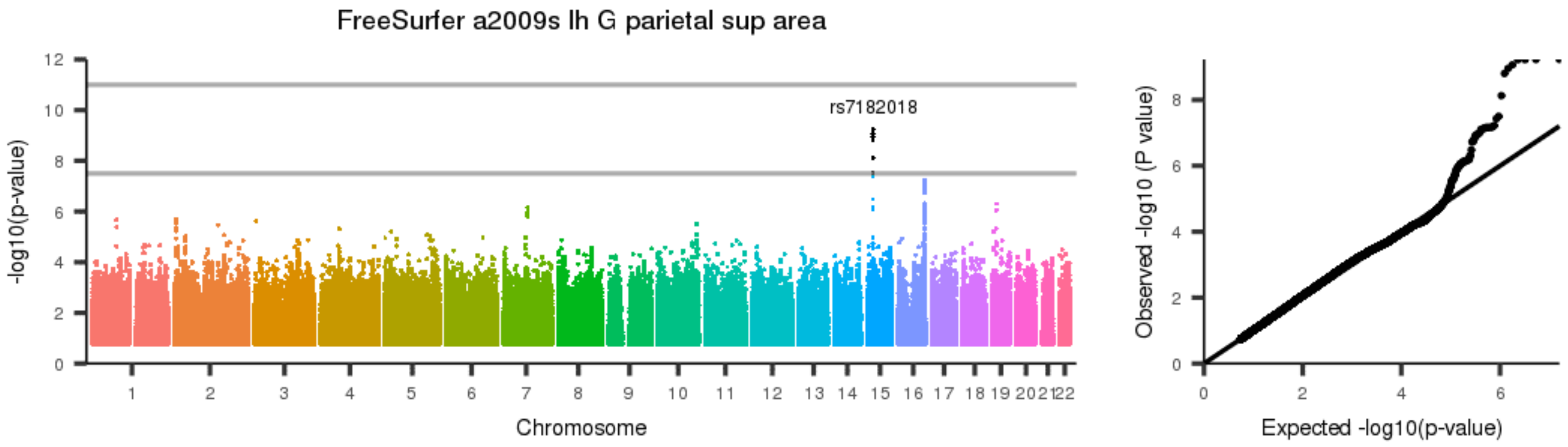

back to list of plots

Figure 1079: FreeSurfer a2009s lh G postcentral area

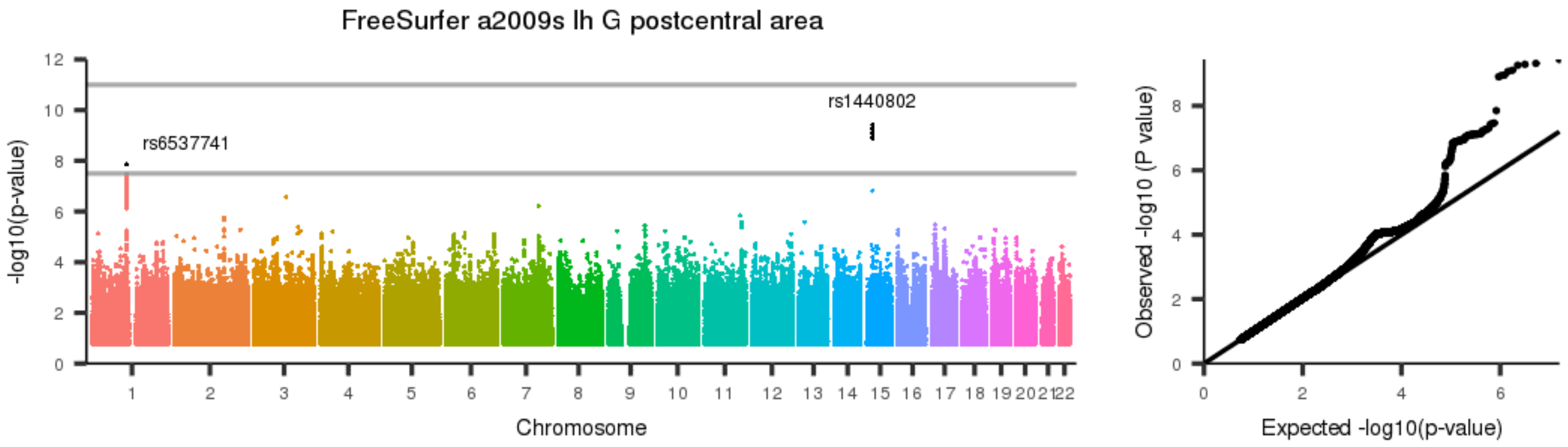

back to list of plots

Figure 1080: FreeSurfer a2009s lh G precentral area

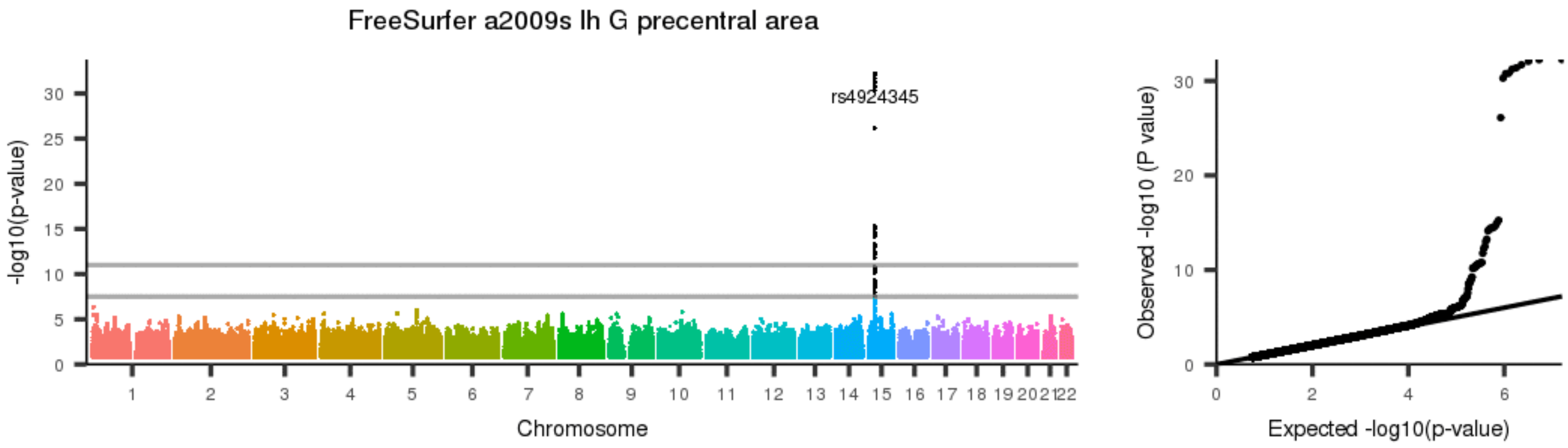

back to list of plots

Figure 1081: FreeSurfer a2009s lh G precuneus area

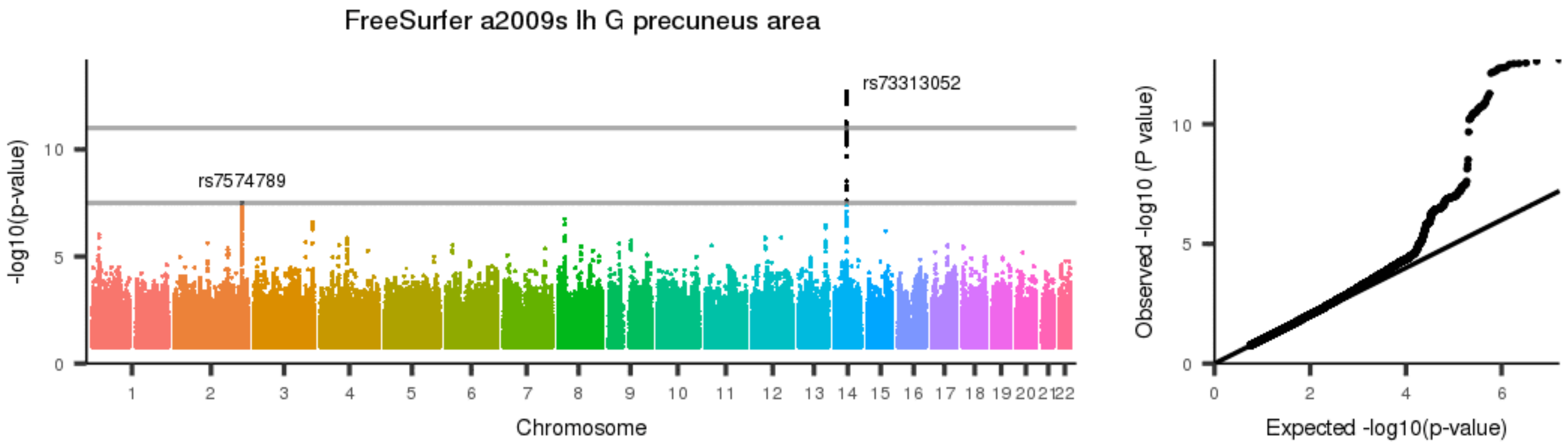

back to list of plots

Figure 1082: FreeSurfer a2009s lh G rectus area

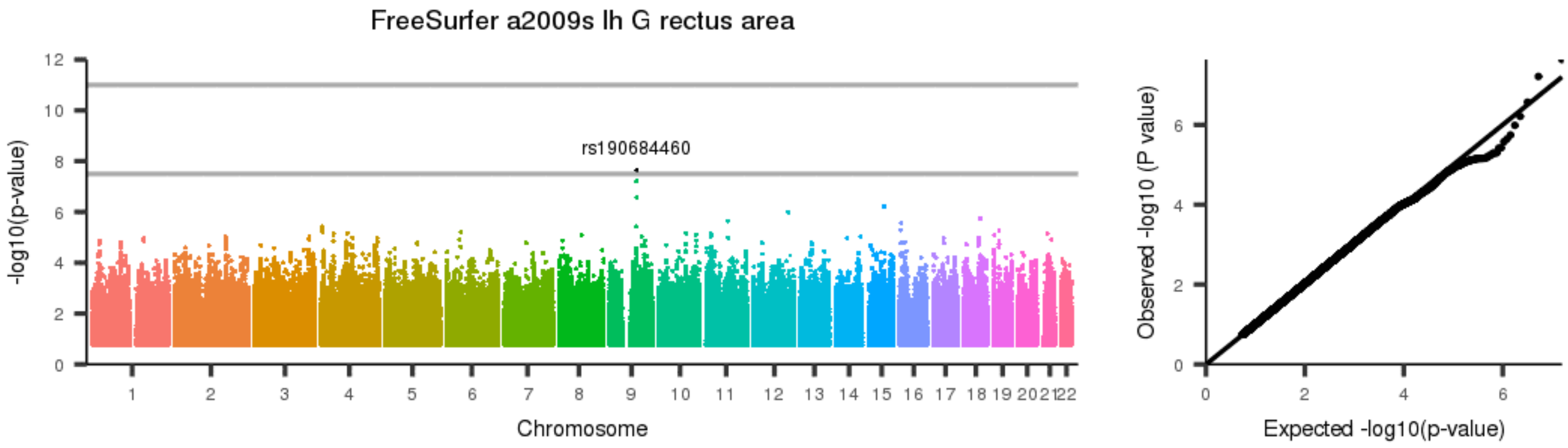

back to list of plots

Figure 1083: FreeSurfer a2009s lh G subcallosal area

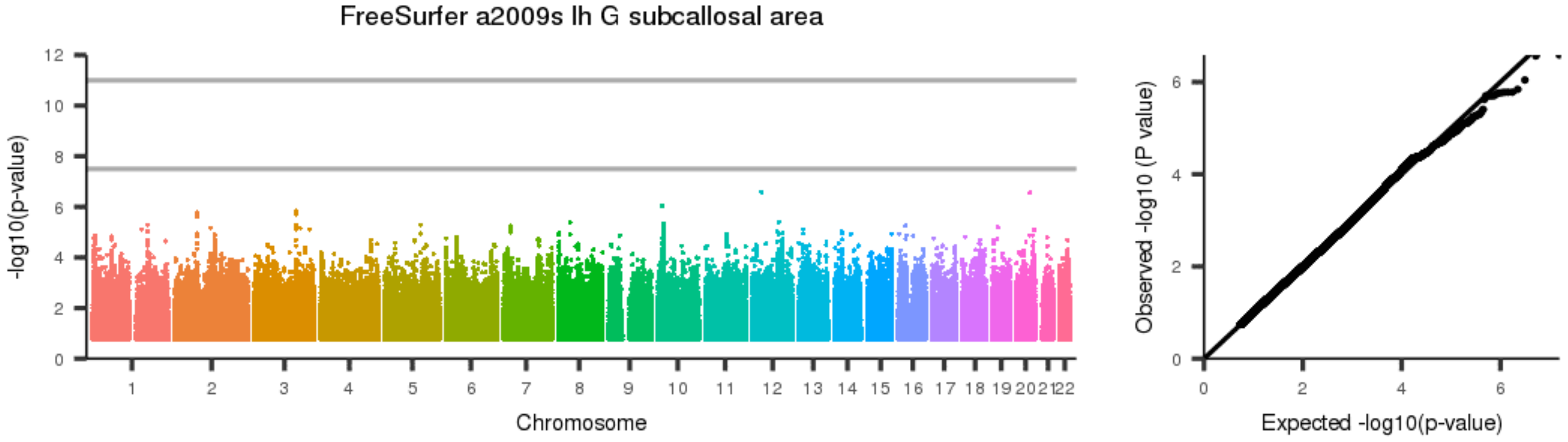

back to list of plots

Figure 1084: FreeSurfer a2009s lh G temp sup-G T transv area

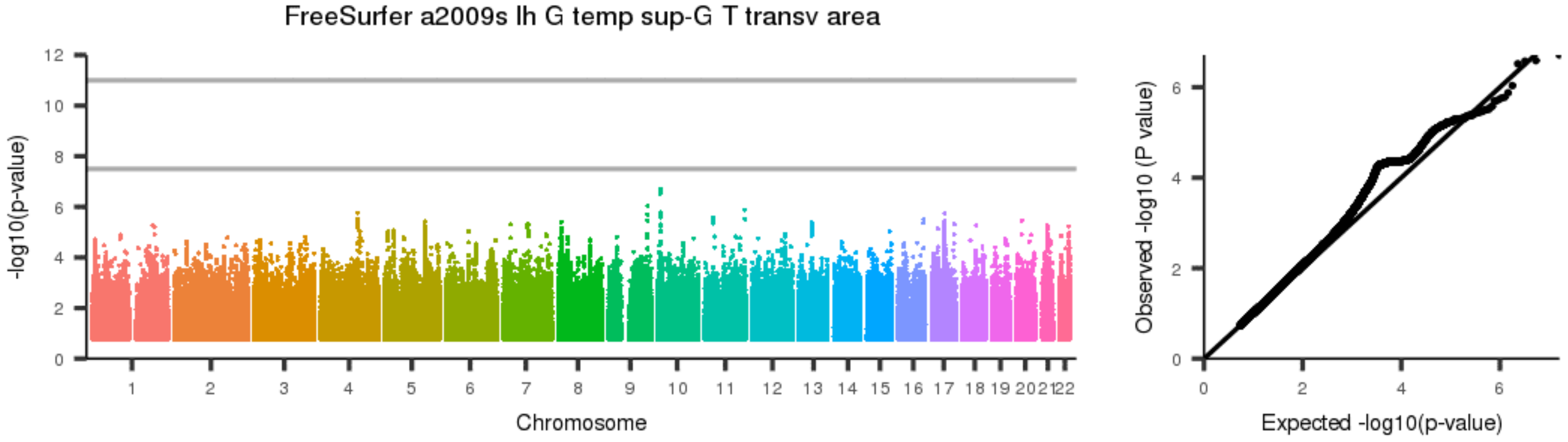

back to list of plots

Figure 1085: FreeSurfer a2009s lh G temp sup-Lateral area

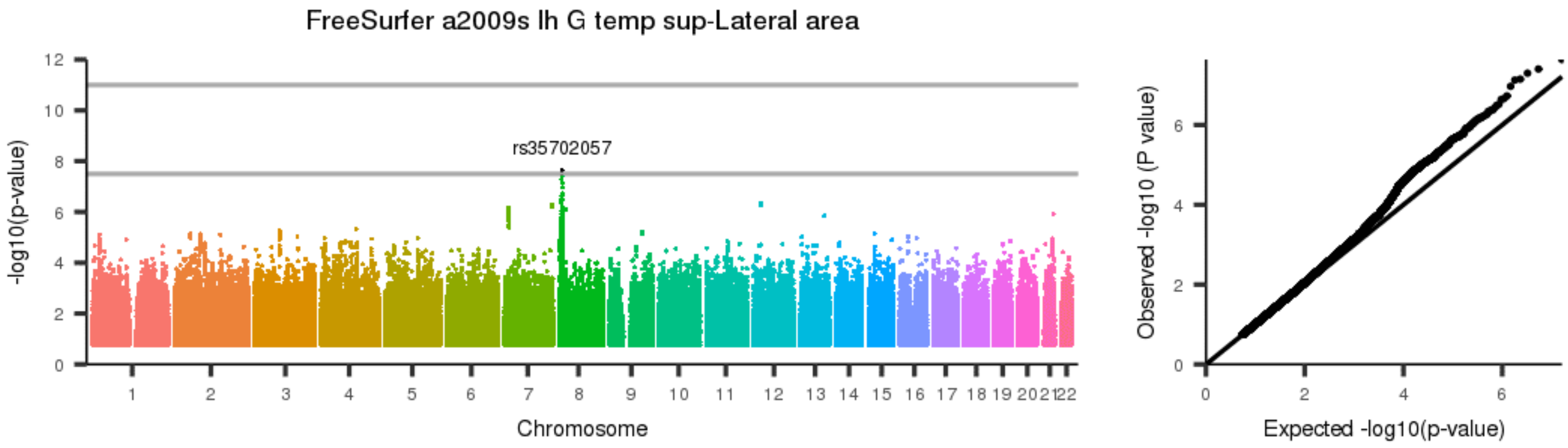

back to list of plots

Figure 1086: FreeSurfer a2009s lh G temp sup-Plan polar area

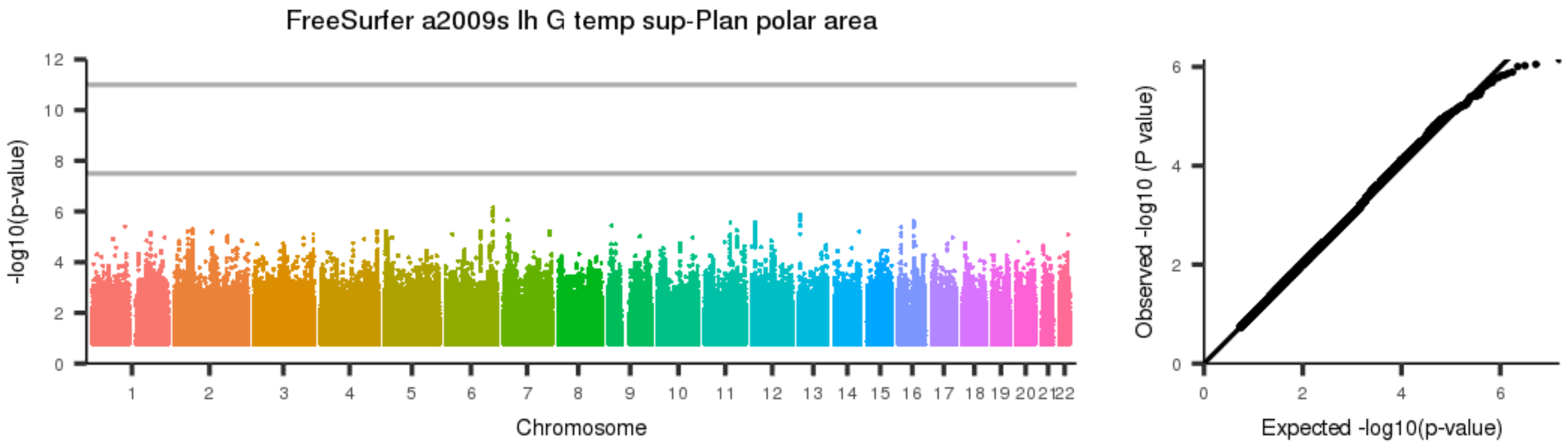

back to list of plots

Figure 1087: FreeSurfer a2009s lh G temp sup-Plan tempo area

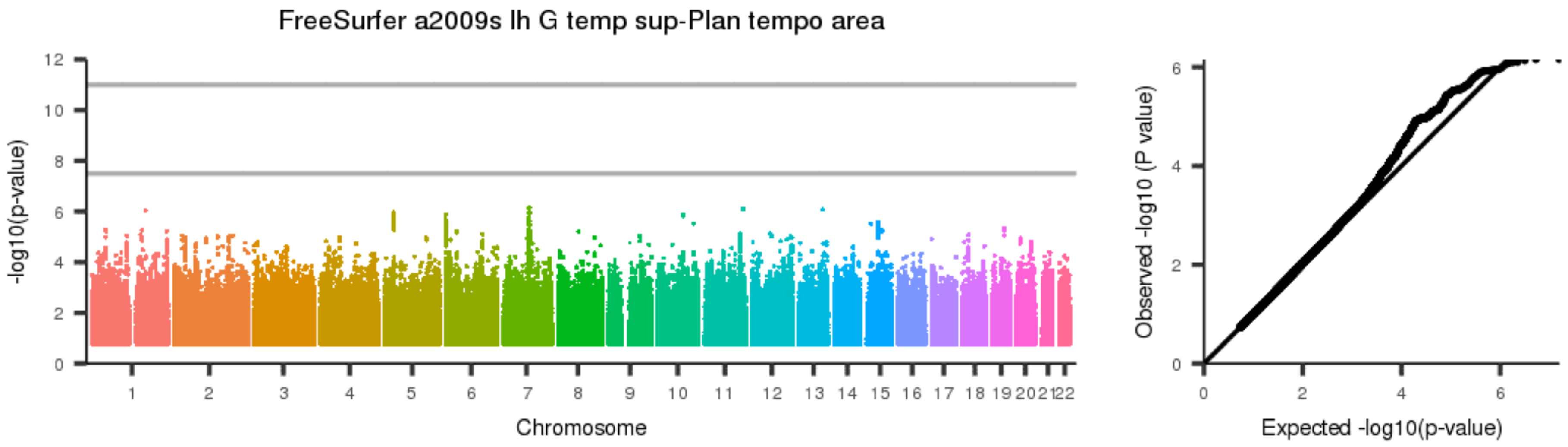

back to list of plots

Figure 1088: FreeSurfer a2009s lh G temporal inf area

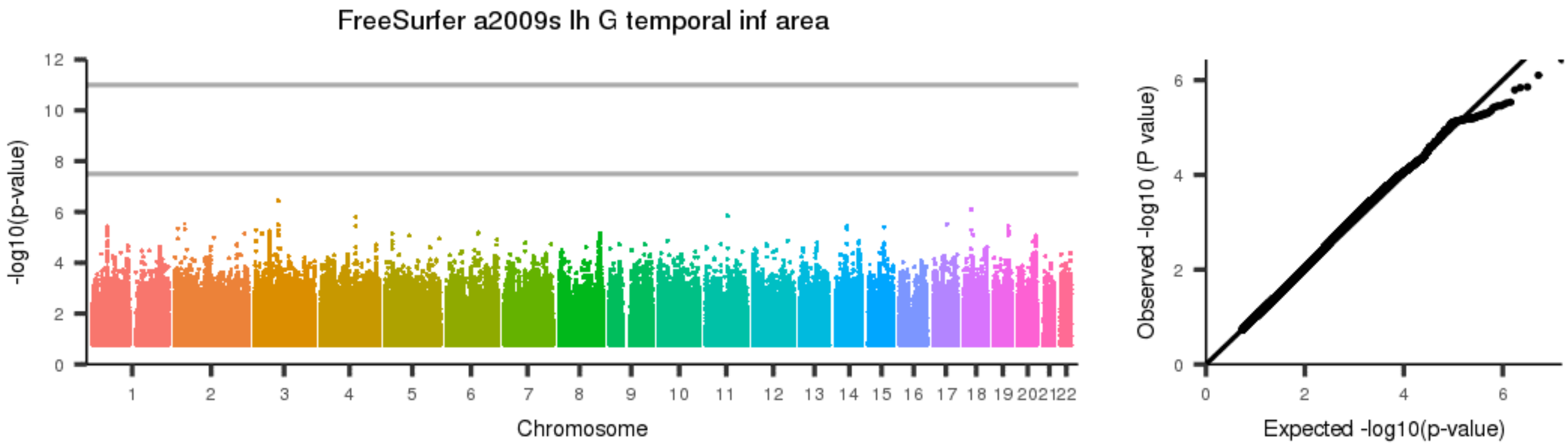

back to list of plots

Figure 1089: FreeSurfer a2009s lh G temporal middle area

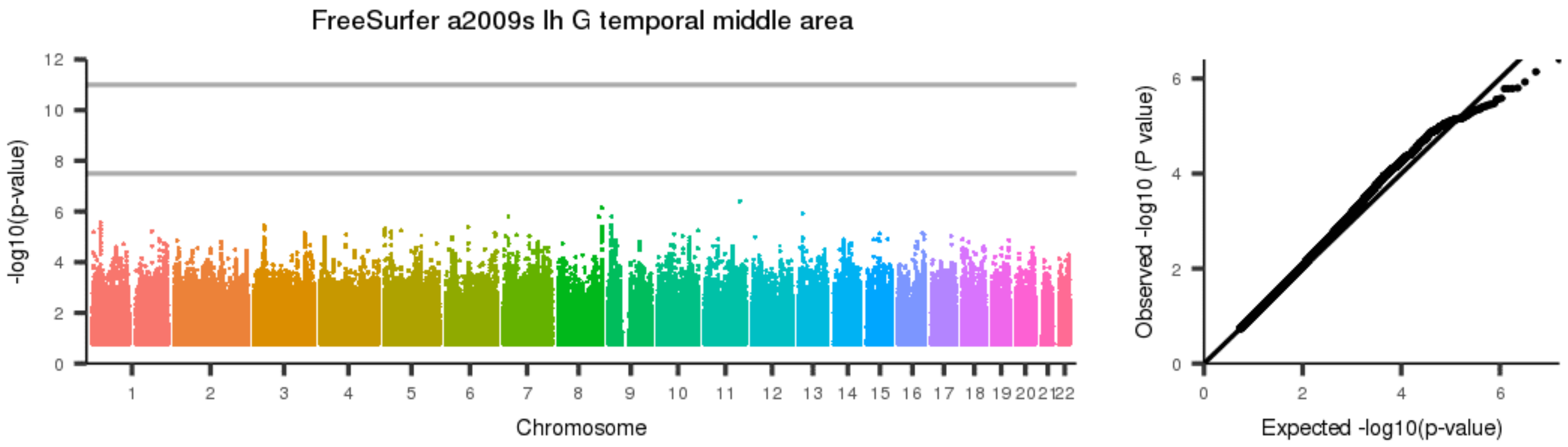

back to list of plots

Figure 1090: FreeSurfer a2009s lh Lat Fis-ant-Horizont area

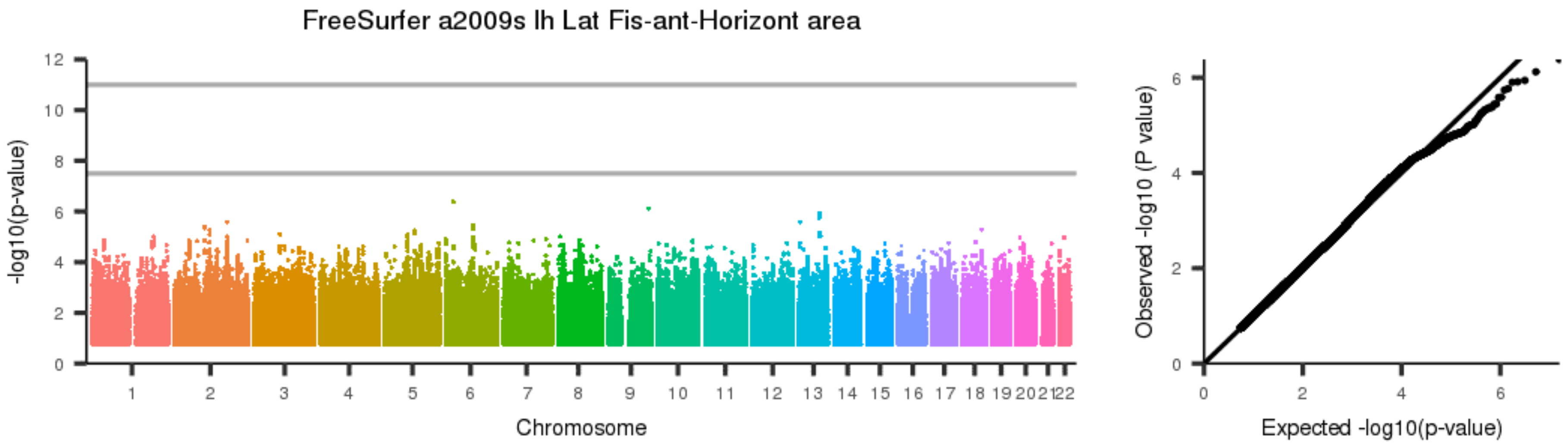

back to list of plots

Figure 1091: FreeSurfer a2009s lh Lat Fis-ant-Vertical area

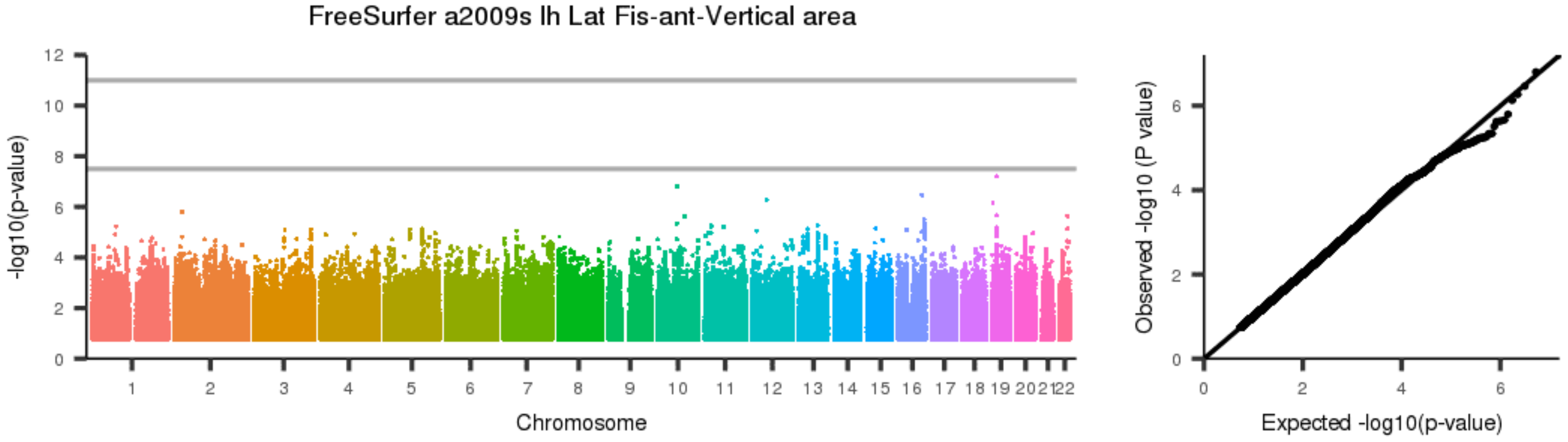

back to list of plots

Figure 1092: FreeSurfer a2009s lh Lat Fis-post area

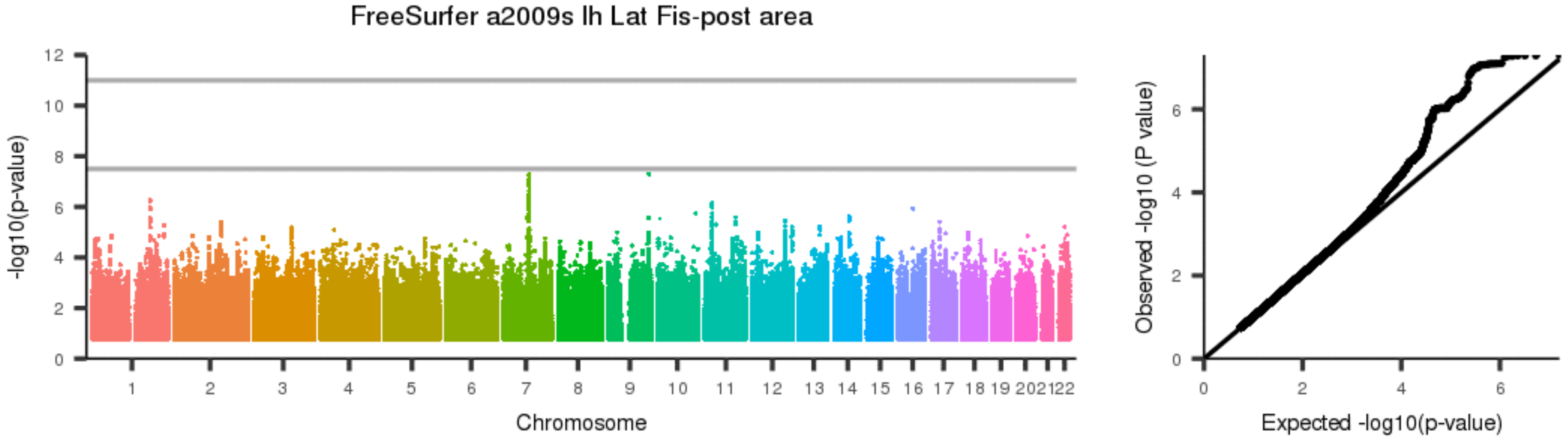

back to list of plots

Figure 1093: FreeSurfer a2009s lh Pole occipital area

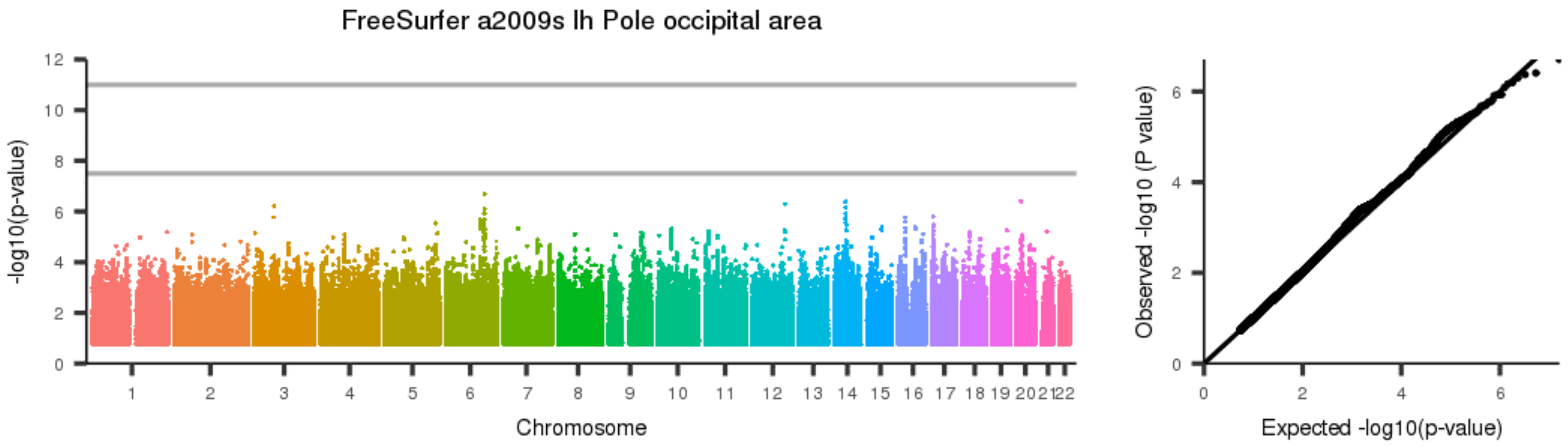

back to list of plots

Figure 1094: FreeSurfer a2009s lh Pole temporal area

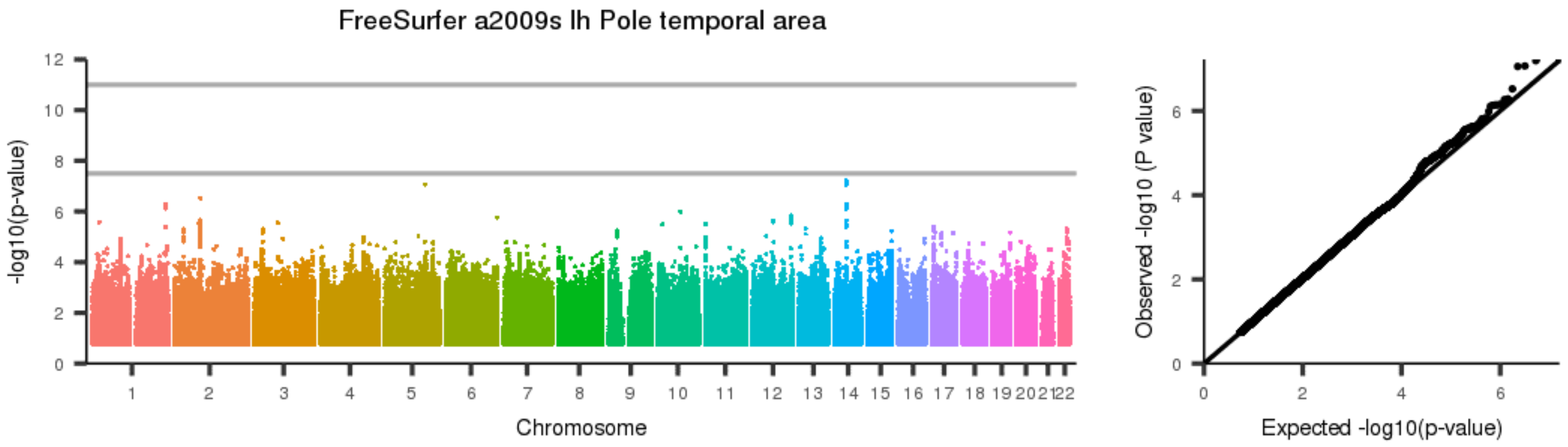

back to list of plots

Figure 1095: FreeSurfer a2009s lh S calcarine area

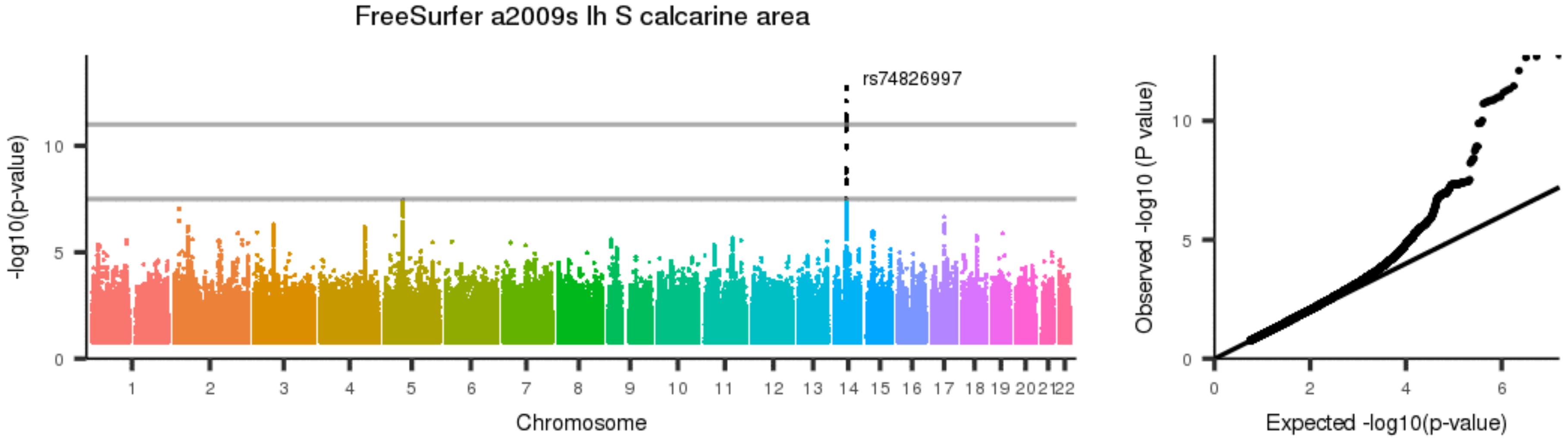

back to list of plots

Figure 1096: FreeSurfer a2009s lh S central area

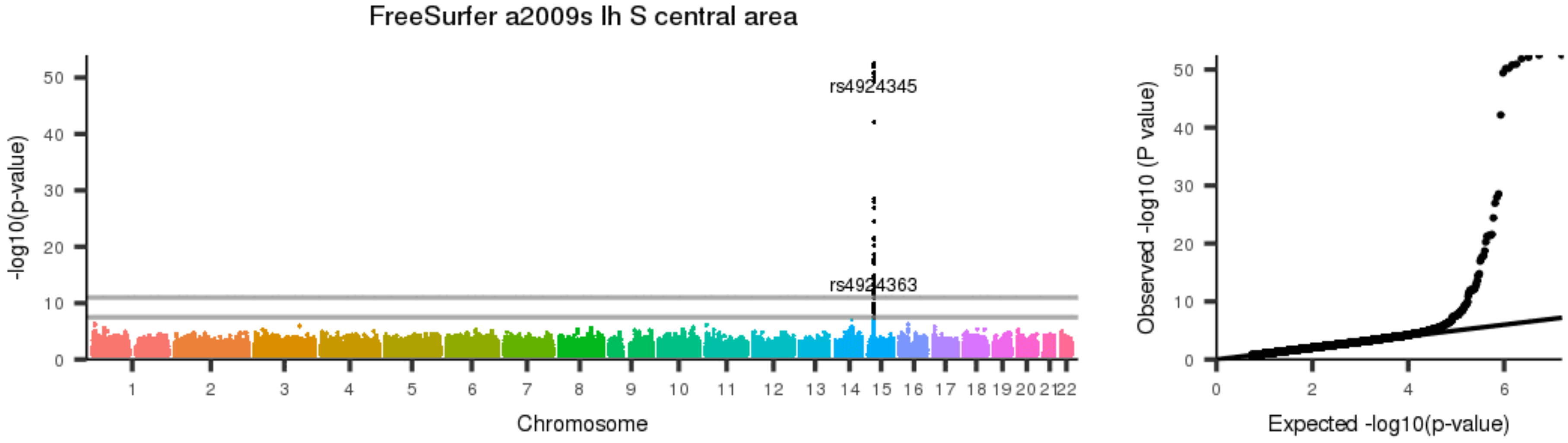

back to list of plots

Figure 1097: FreeSurfer a2009s lh S cingul-Marginalis area

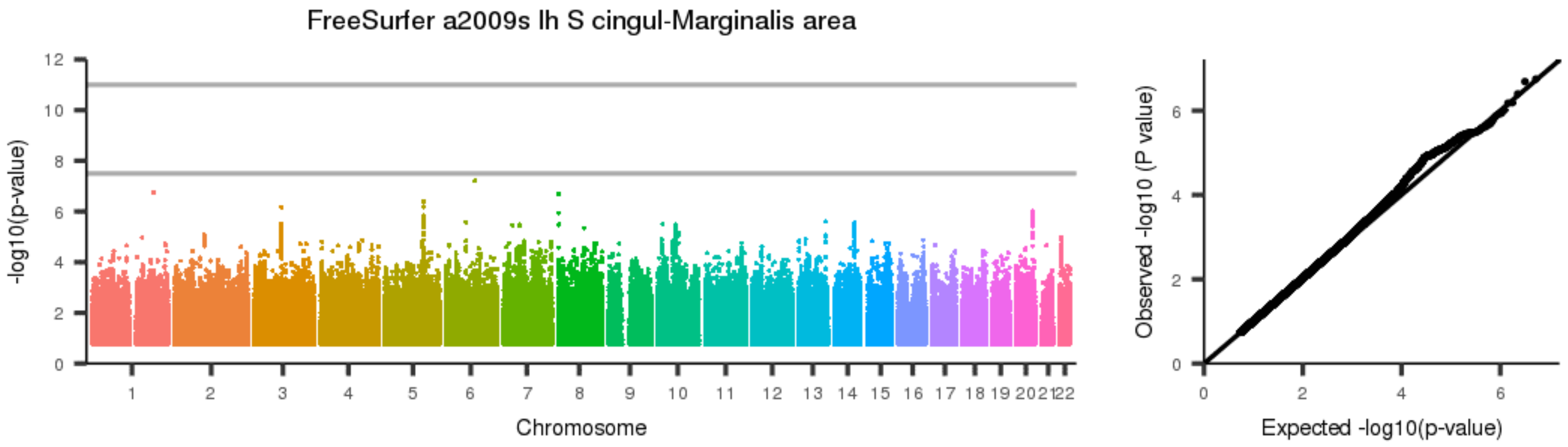

back to list of plots

Figure 1098: FreeSurfer a2009s lh S circular insula ant area

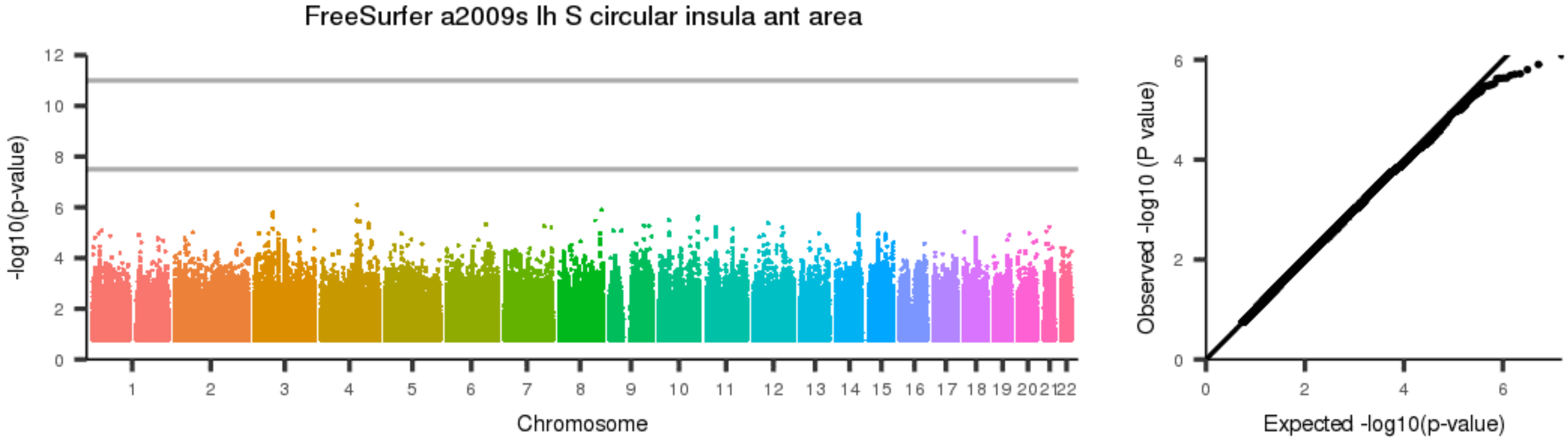

back to list of plots

Figure 1099: FreeSurfer a2009s lh S circular insula inf area

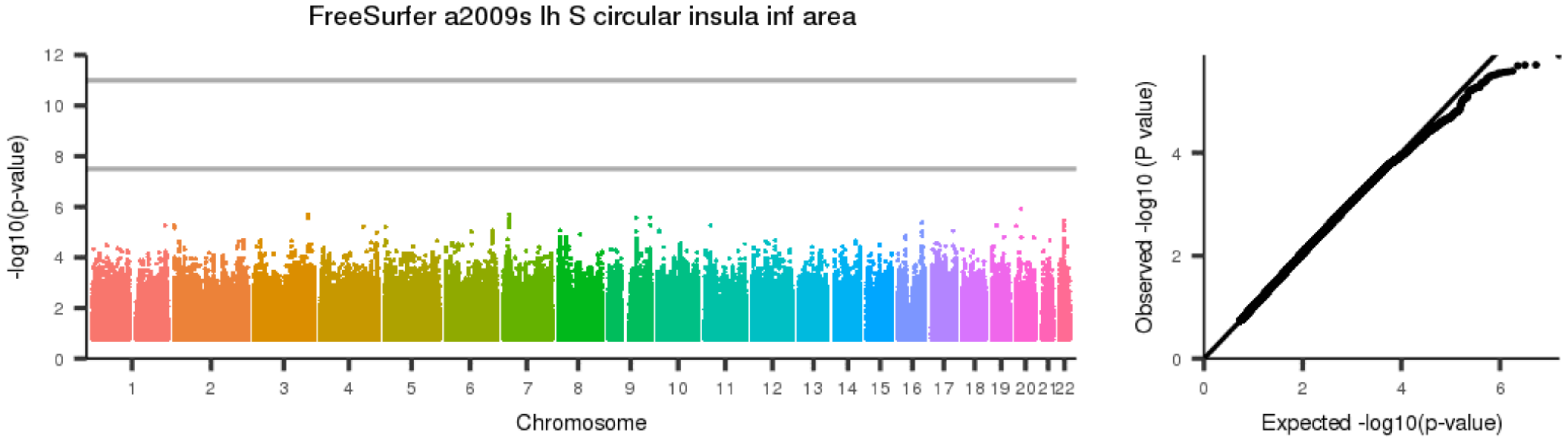

back to list of plots

Figure 1100: FreeSurfer a2009s lh S circular insula sup area

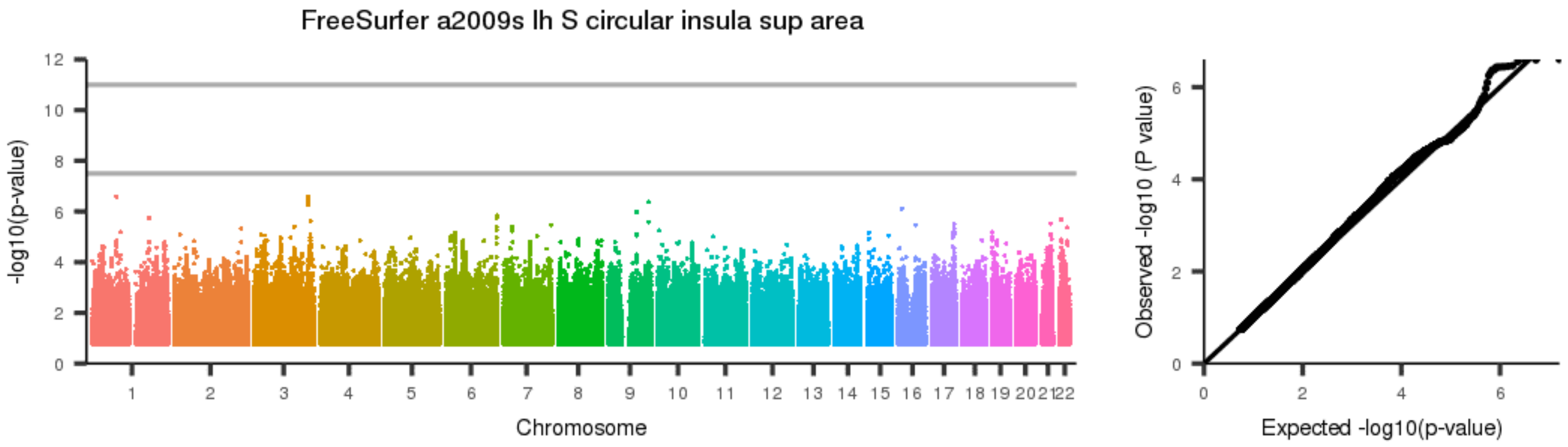

back to list of plots

Figure 1101: FreeSurfer a2009s lh S collat transv ant area

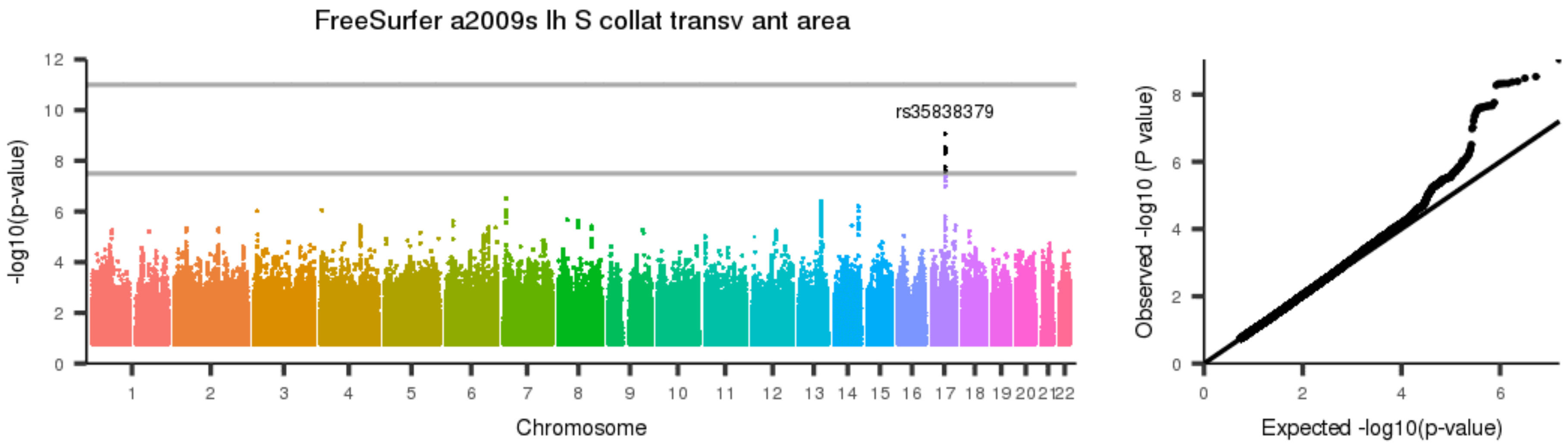

back to list of plots

Figure 1102: FreeSurfer a2009s lh S collat transv post area

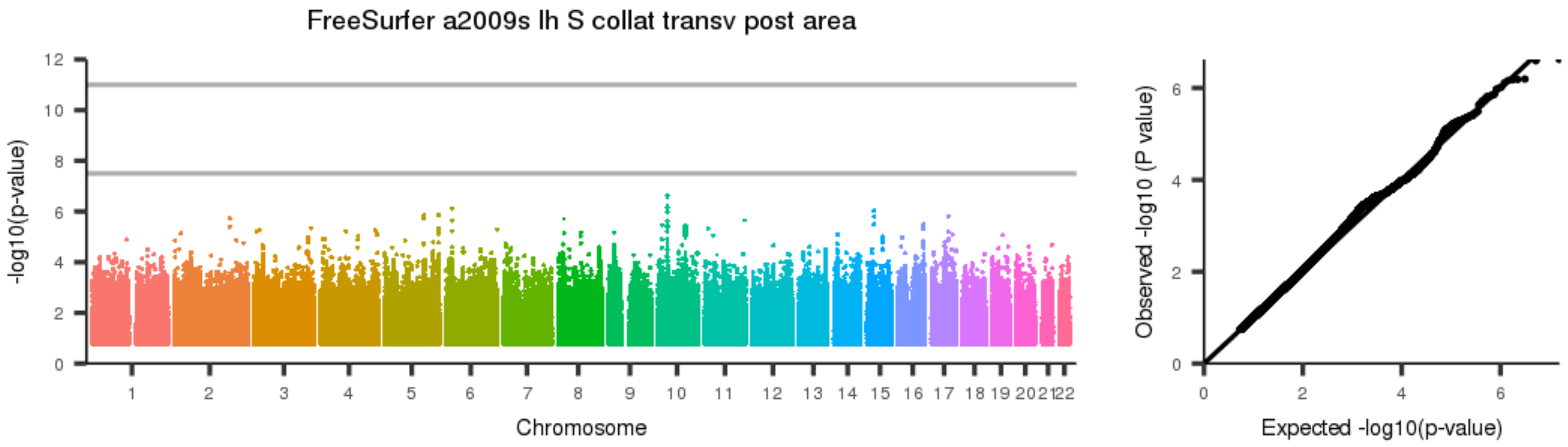

back to list of plots

Figure 1103: FreeSurfer a2009s lh S front inf area

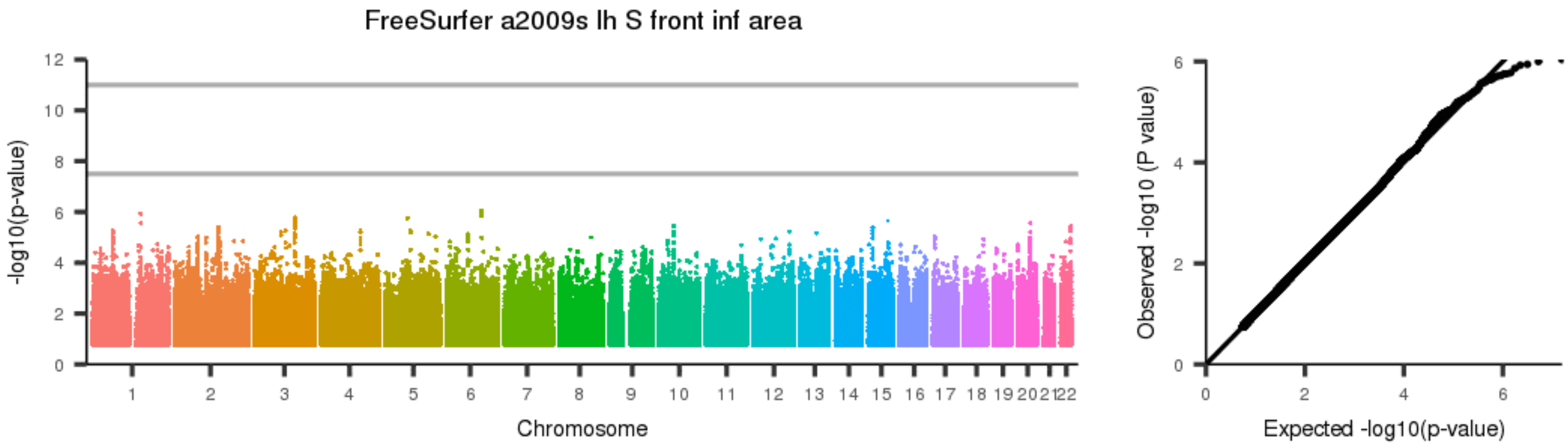

back to list of plots

Figure 1104: FreeSurfer a2009s lh S front middle area

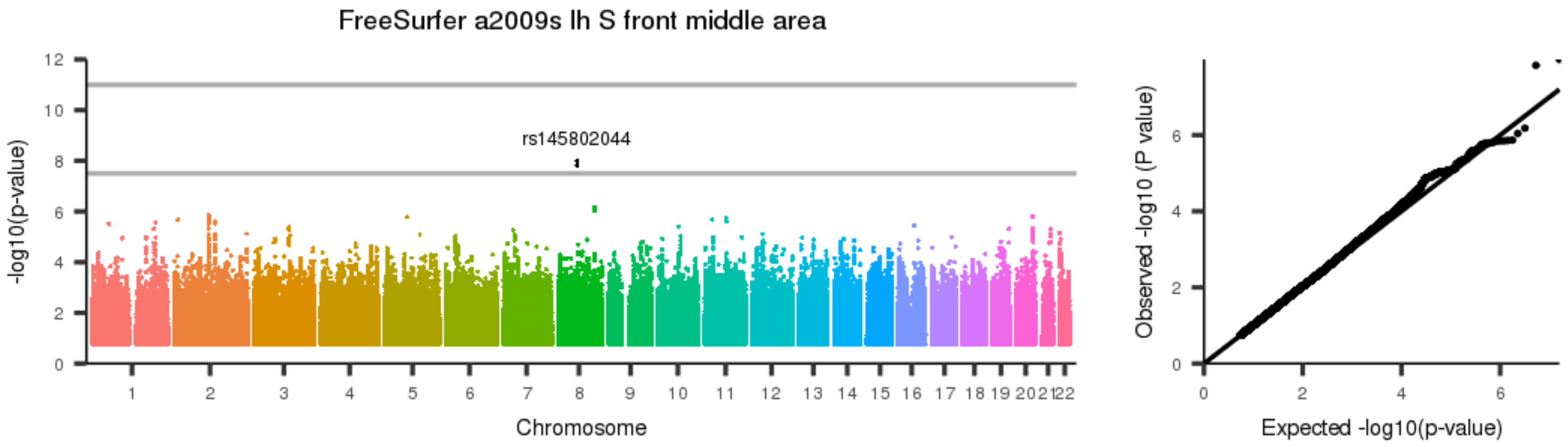

back to list of plots

Figure 1105: FreeSurfer a2009s lh S front sup area

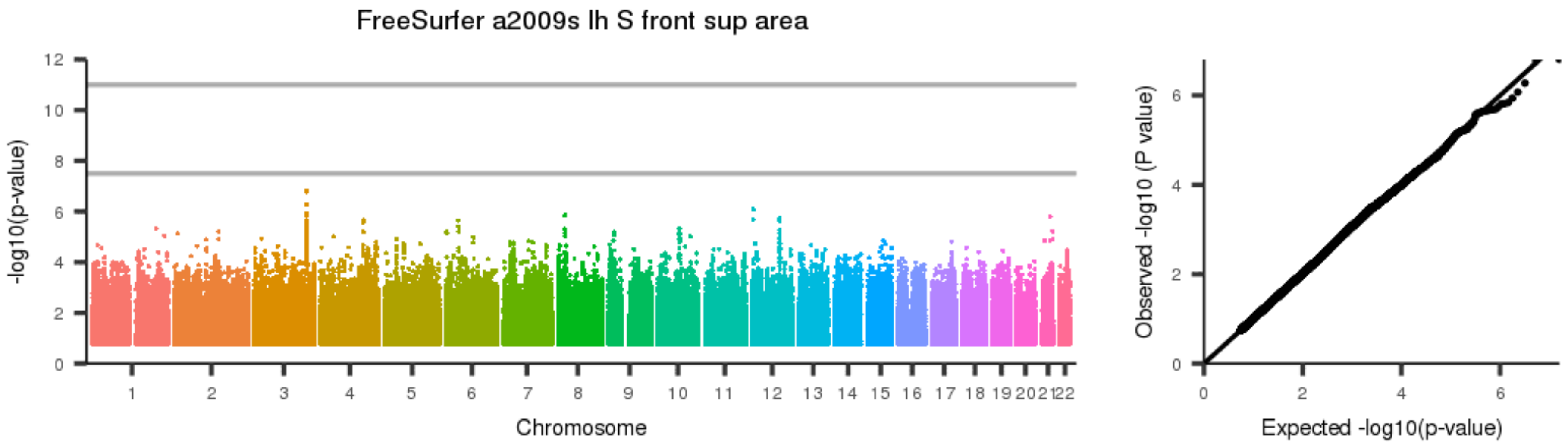

back to list of plots

Figure 1106: FreeSurfer a2009s lh S interm prim-Jensen area

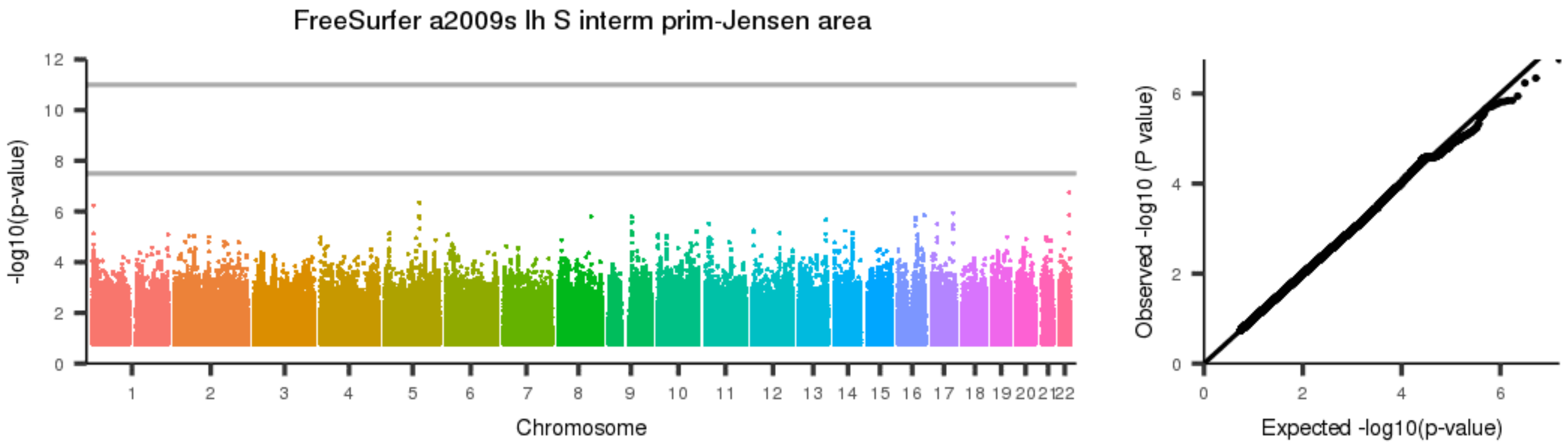

back to list of plots

Figure 1107: FreeSurfer a2009s lh S intrapariet and P trans area

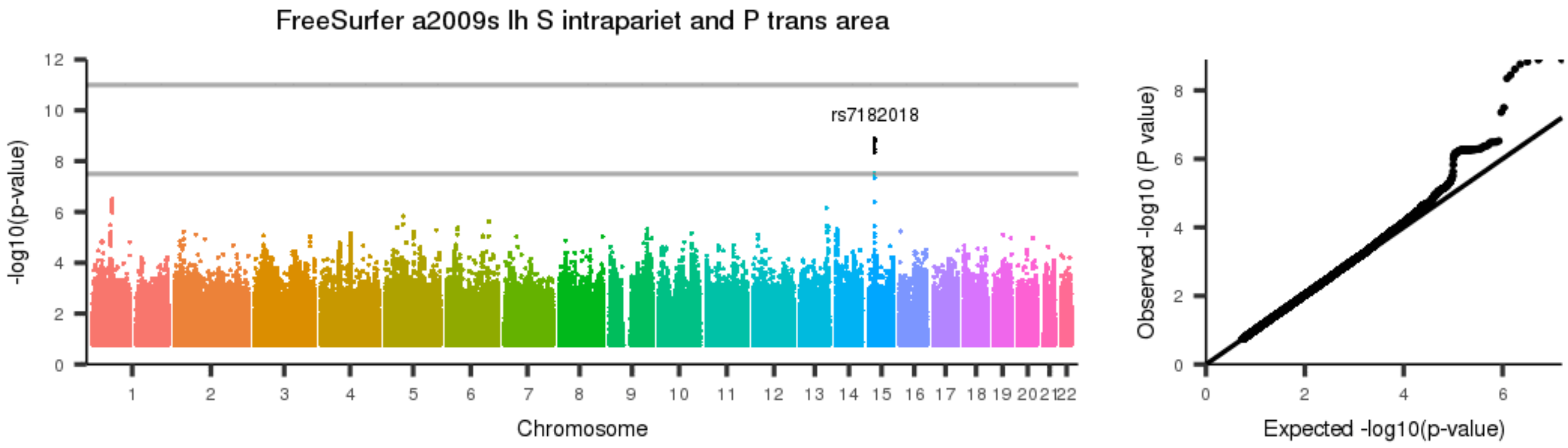

back to list of plots

Figure 1108: FreeSurfer a2009s lh S oc middle and Lunatus area

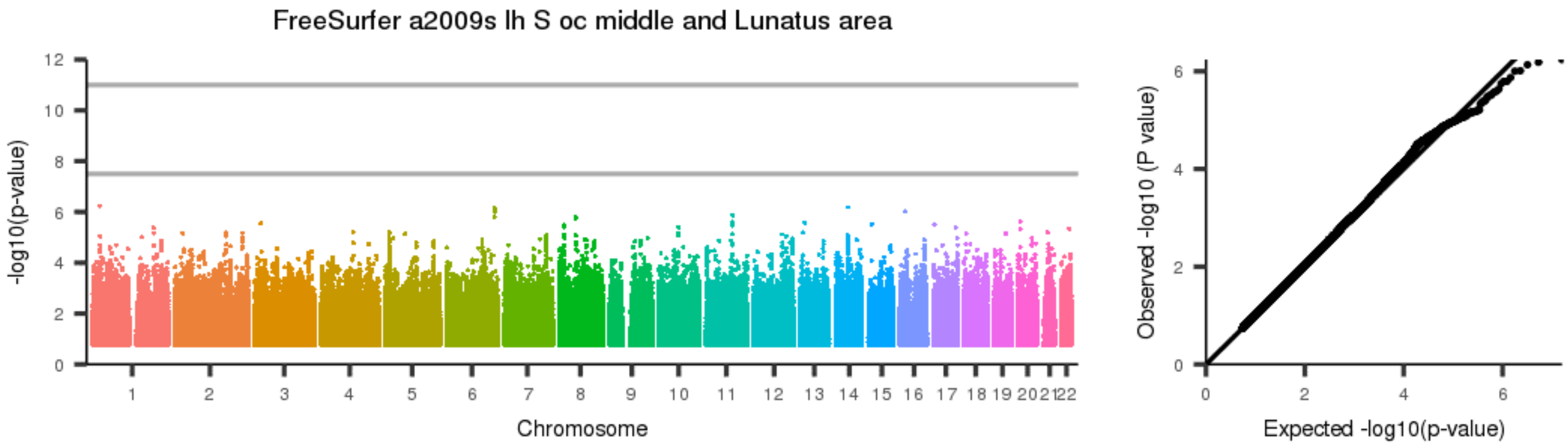

back to list of plots

Figure 1109: FreeSurfer a2009s lh S oc sup and transversal area

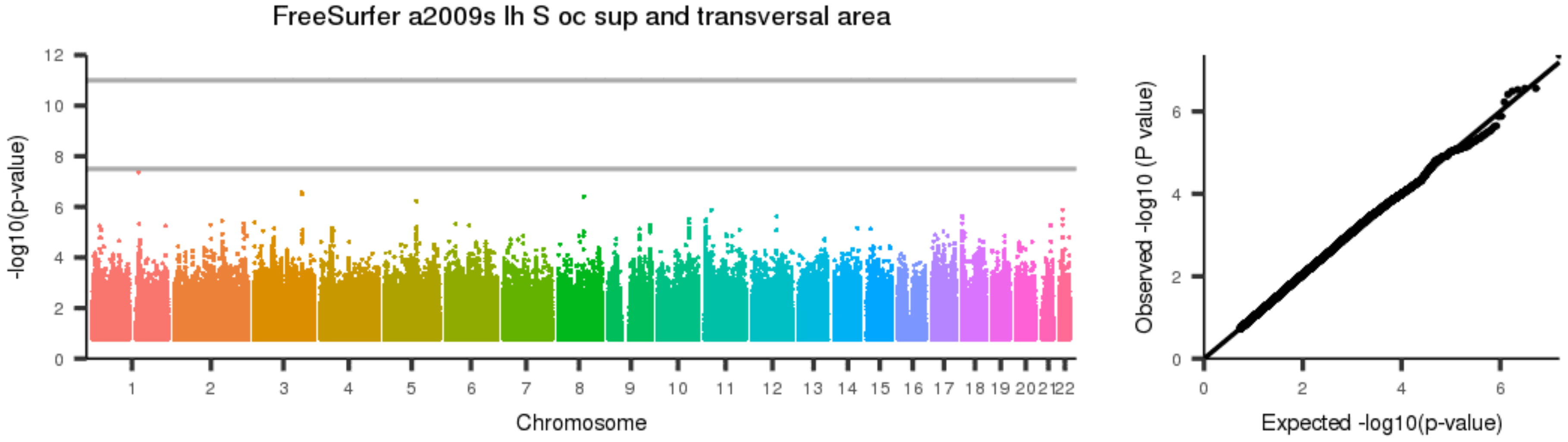

back to list of plots

Figure 1110: FreeSurfer a2009s lh S occipital ant area

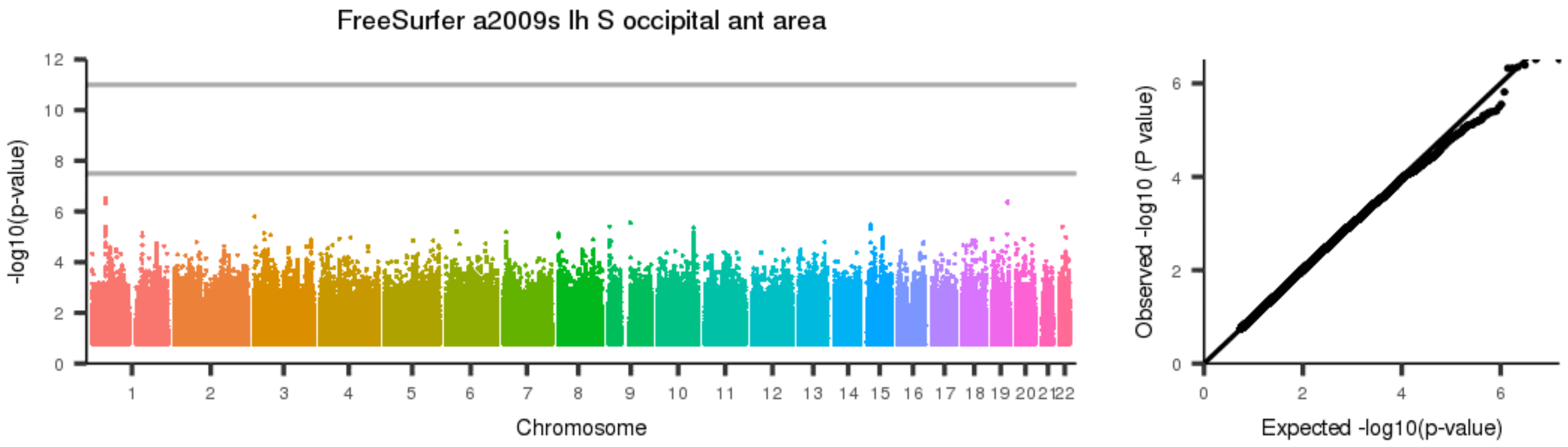

back to list of plots

Figure 1111: FreeSurfer a2009s lh S oc-temp lat area

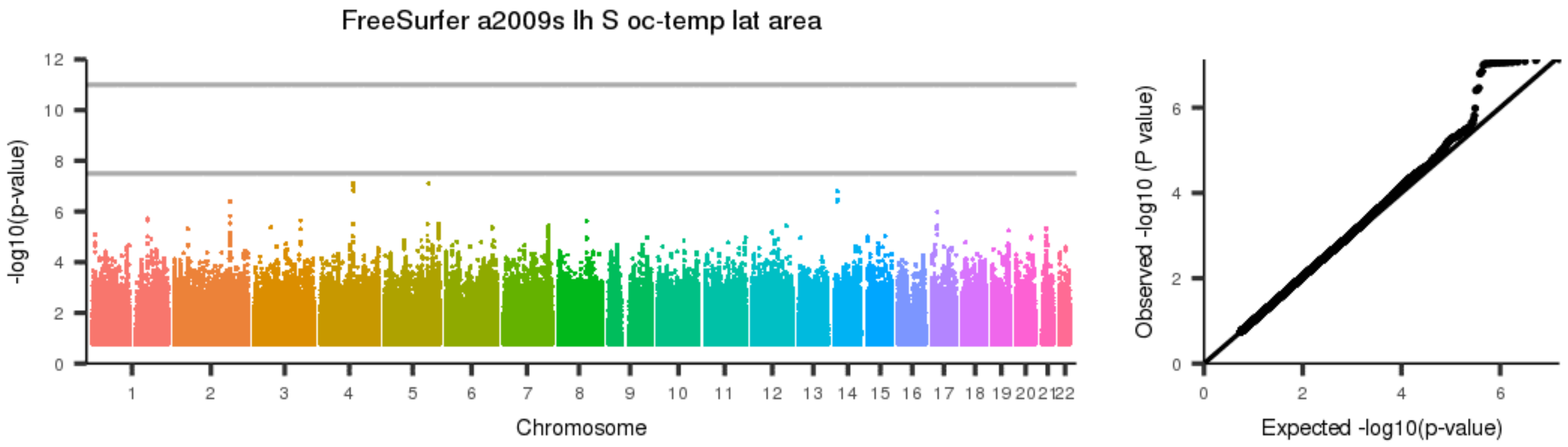

back to list of plots

Figure 1112: FreeSurfer a2009s lh S oc-temp med and Lingual area

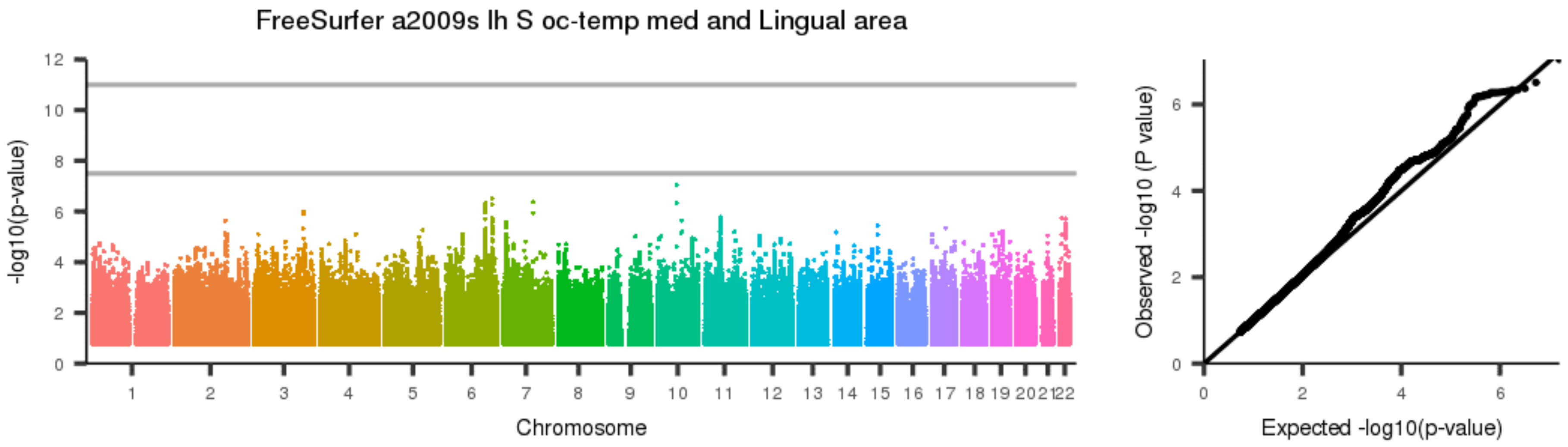

back to list of plots

Figure 1113: FreeSurfer a2009s lh S orbital lateral area

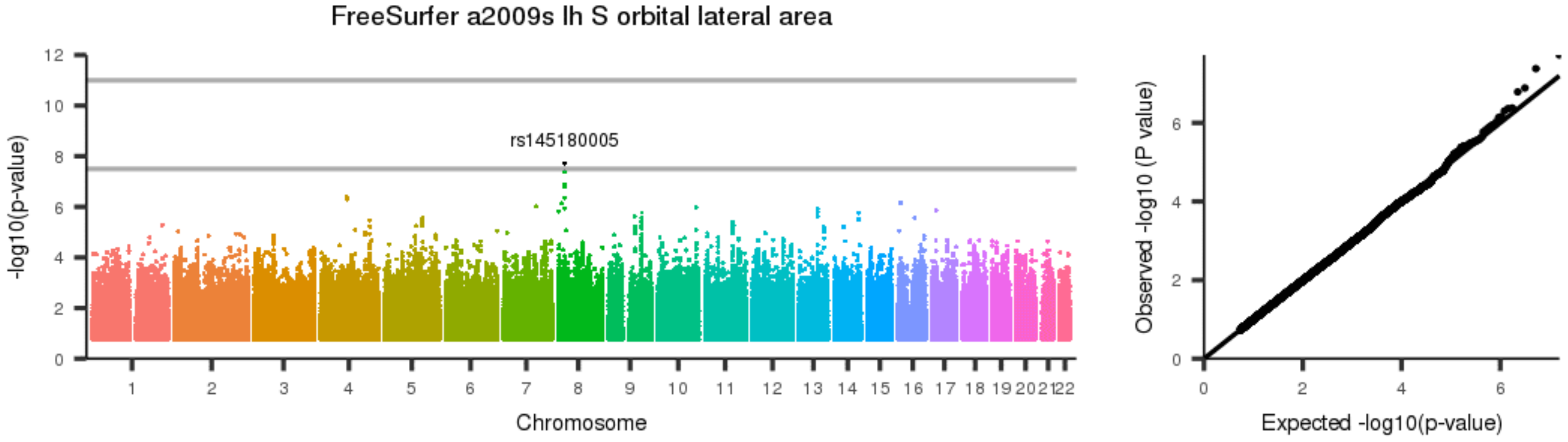

back to list of plots

Figure 1114: FreeSurfer a2009s lh S orbital med-olfact area

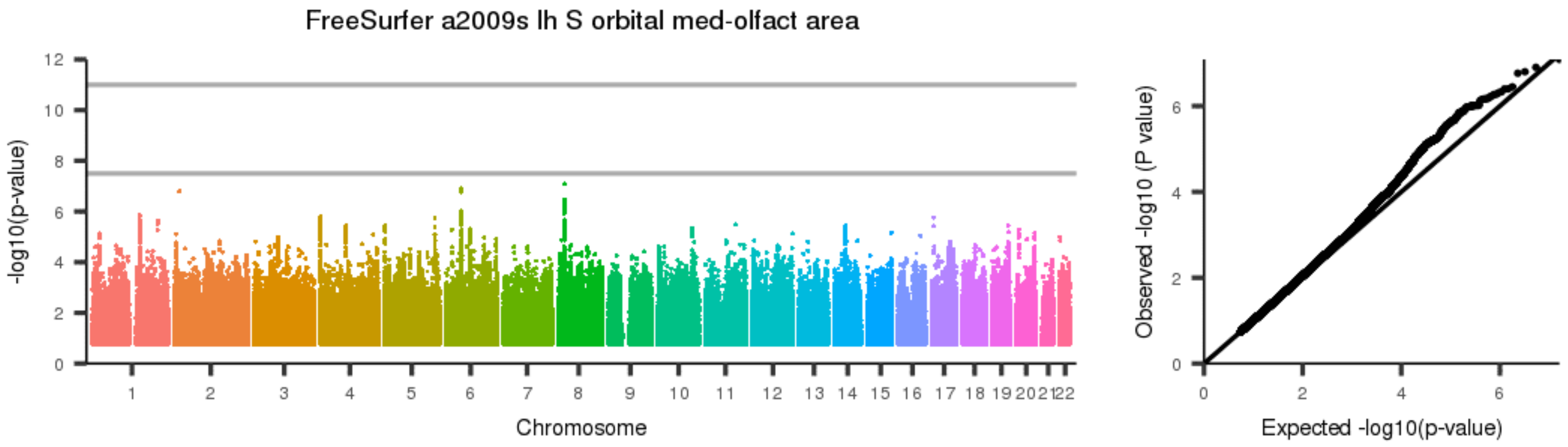

back to list of plots

Figure 1115: FreeSurfer a2009s lh S orbital-H Shaped area

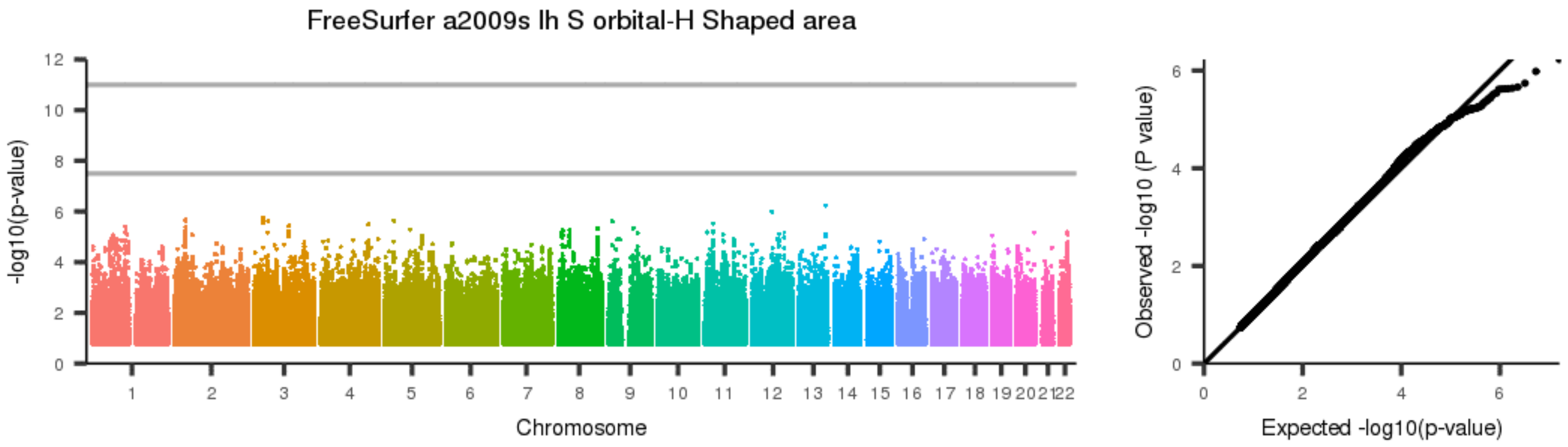

back to list of plots

Figure 1116: FreeSurfer a2009s lh S parieto occipital area

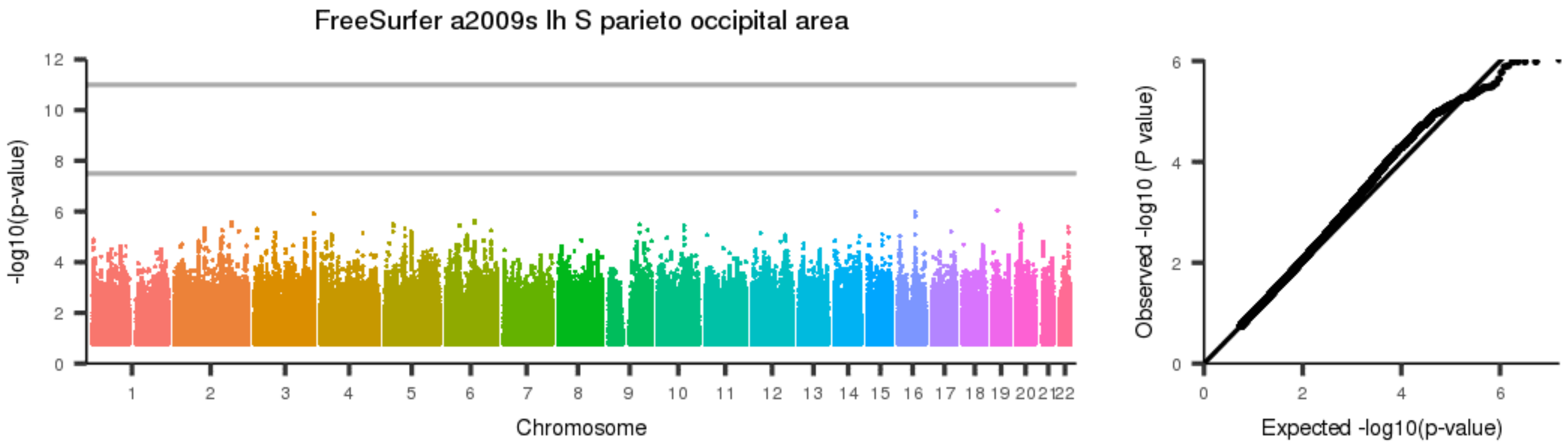

back to list of plots

Figure 1117: FreeSurfer a2009s lh S pericallosal area

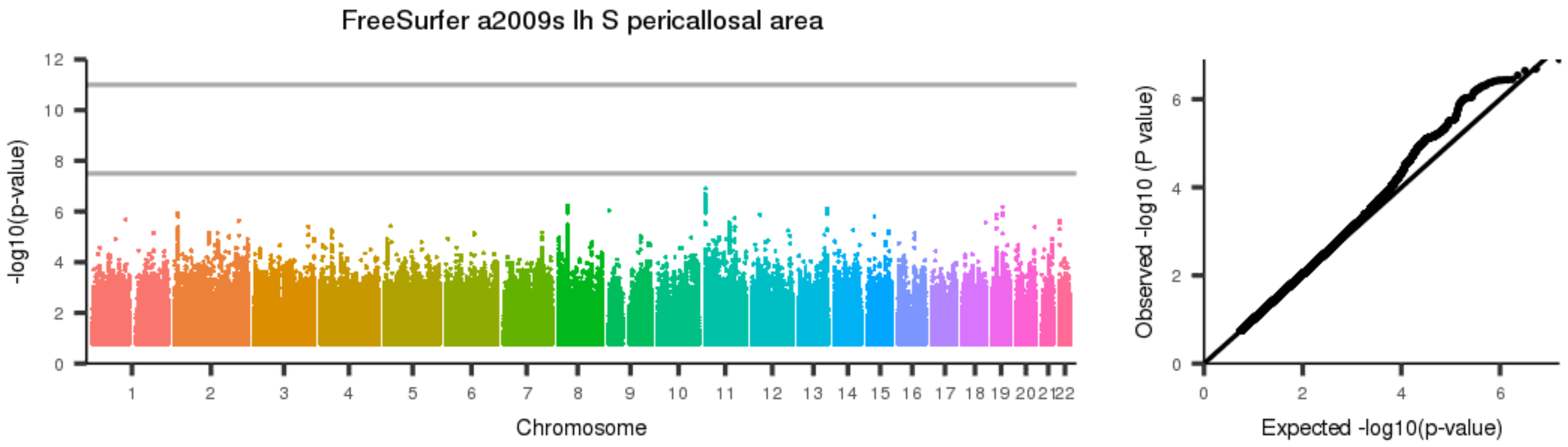

back to list of plots

Figure 1118: FreeSurfer a2009s lh S postcentral area

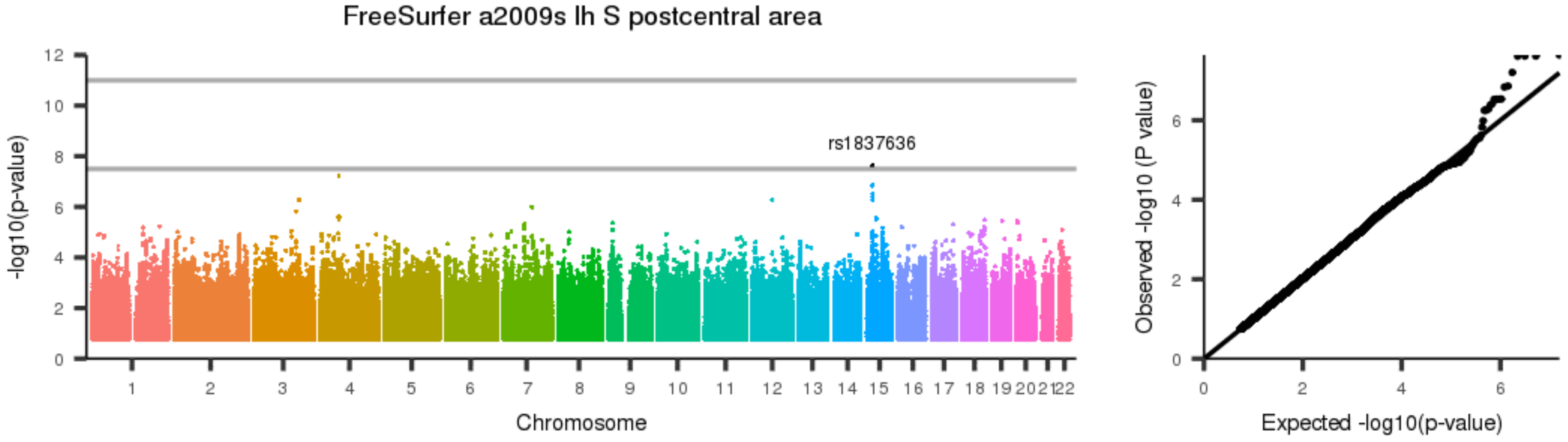

back to list of plots

Figure 1119: FreeSurfer a2009s lh S precentral-inf-part area

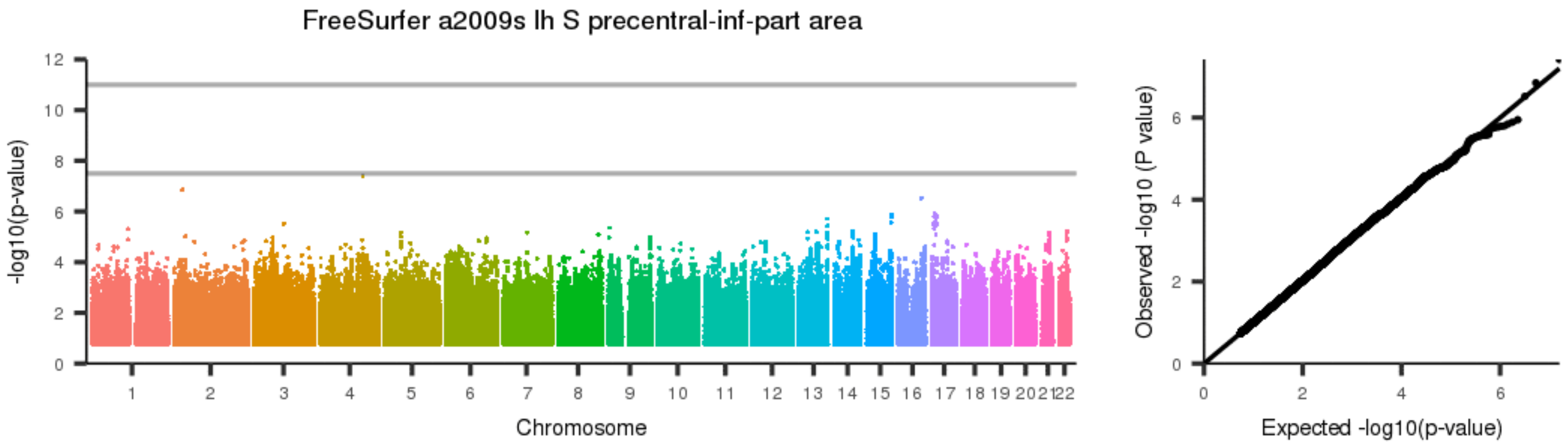

back to list of plots

Figure 1120: FreeSurfer a2009s lh S precentral-sup-part area

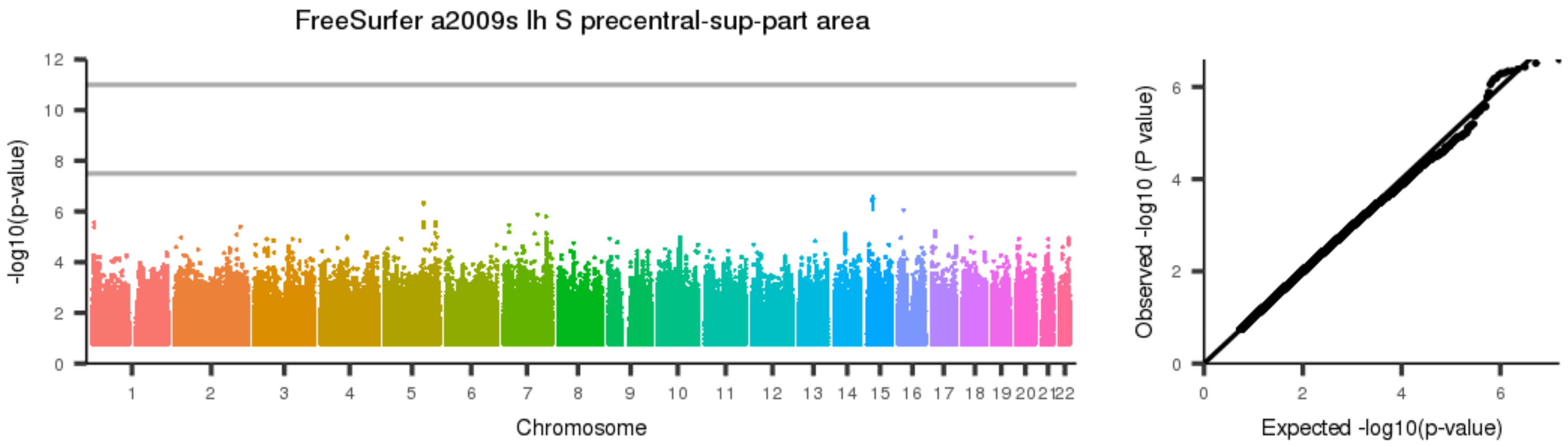

back to list of plots

Figure 1121: FreeSurfer a2009s lh S suborbital area

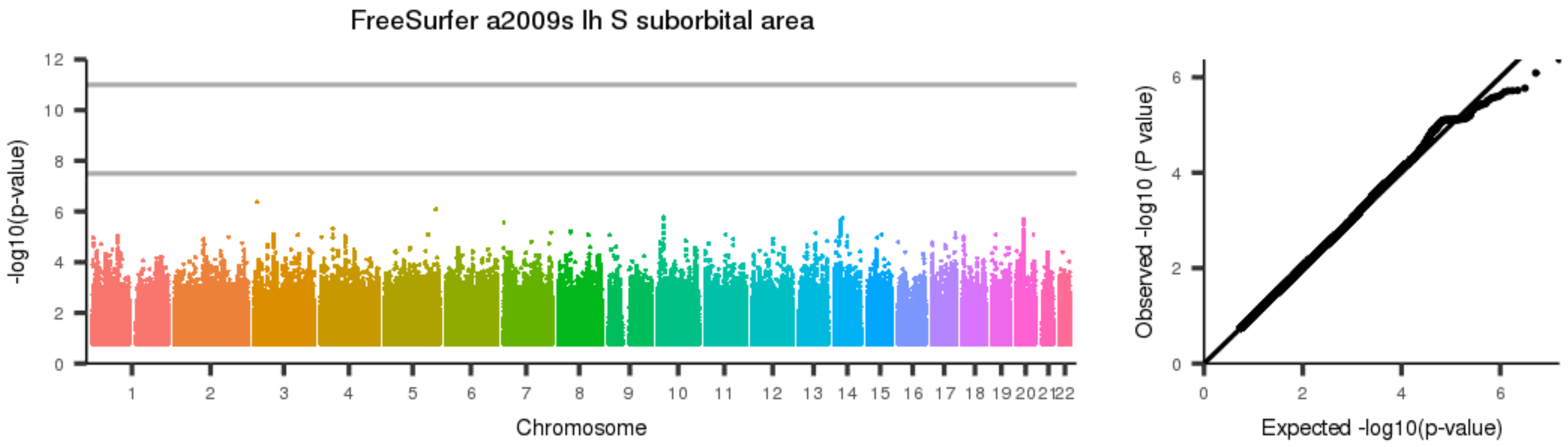

back to list of plots

Figure 1122: FreeSurfer a2009s lh S subparietal area

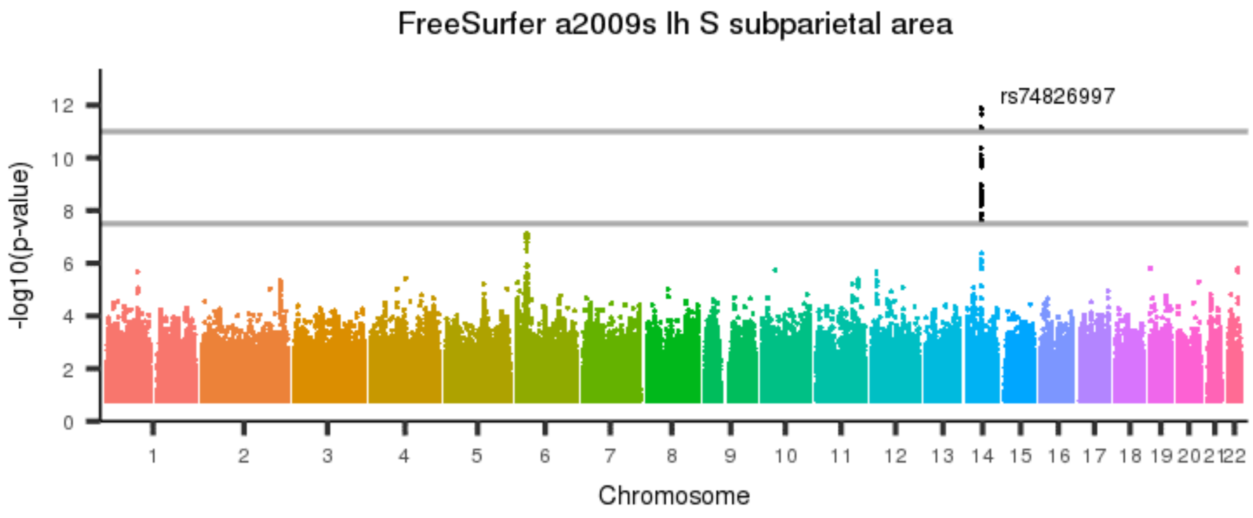

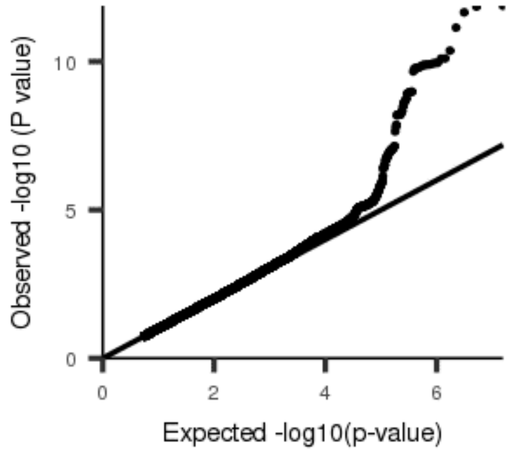

back to list of plots

Figure 1123: FreeSurfer a2009s lh S temporal inf area

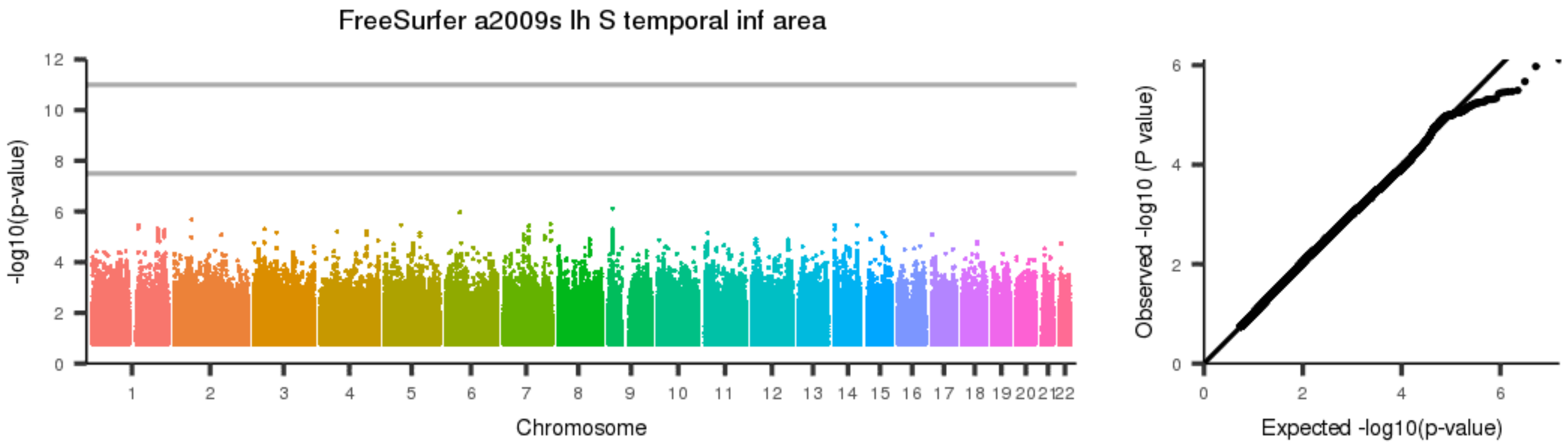

back to list of plots

Figure 1124: FreeSurfer a2009s lh S temporal sup area

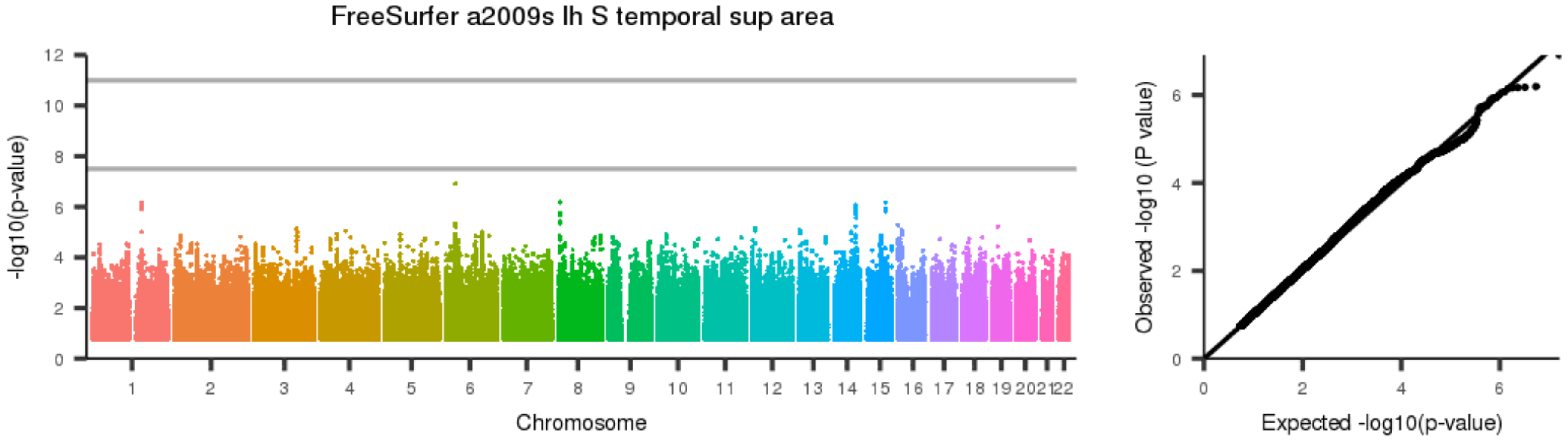

back to list of plots

Figure 1125: FreeSurfer a2009s lh S temporal transverse area

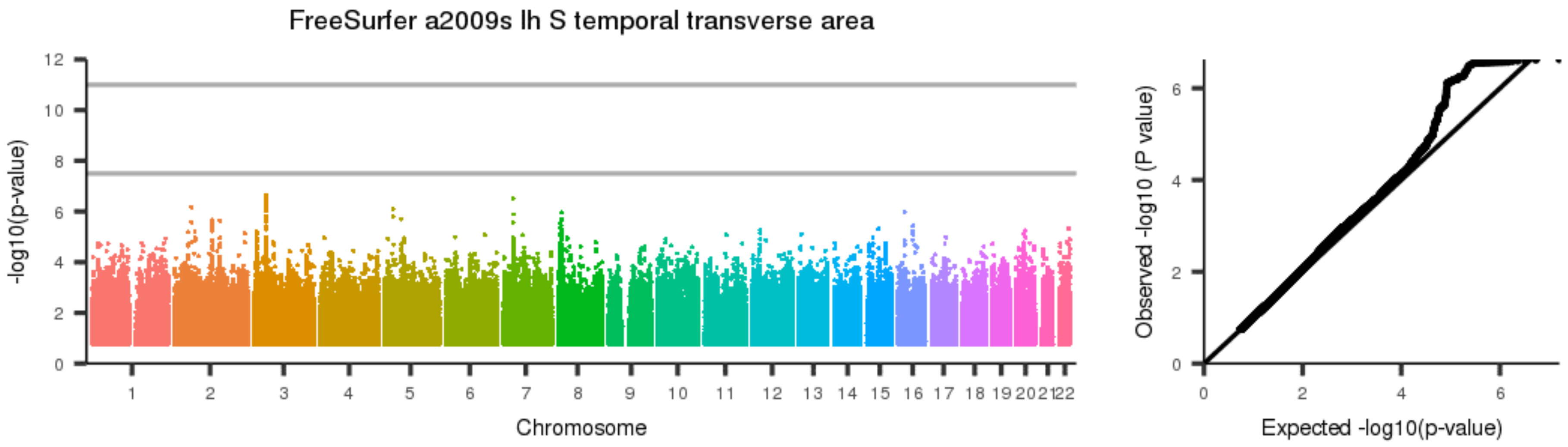

back to list of plots

Figure 1126: FreeSurfer DKTatlas rh caudalanteriorcingulate area

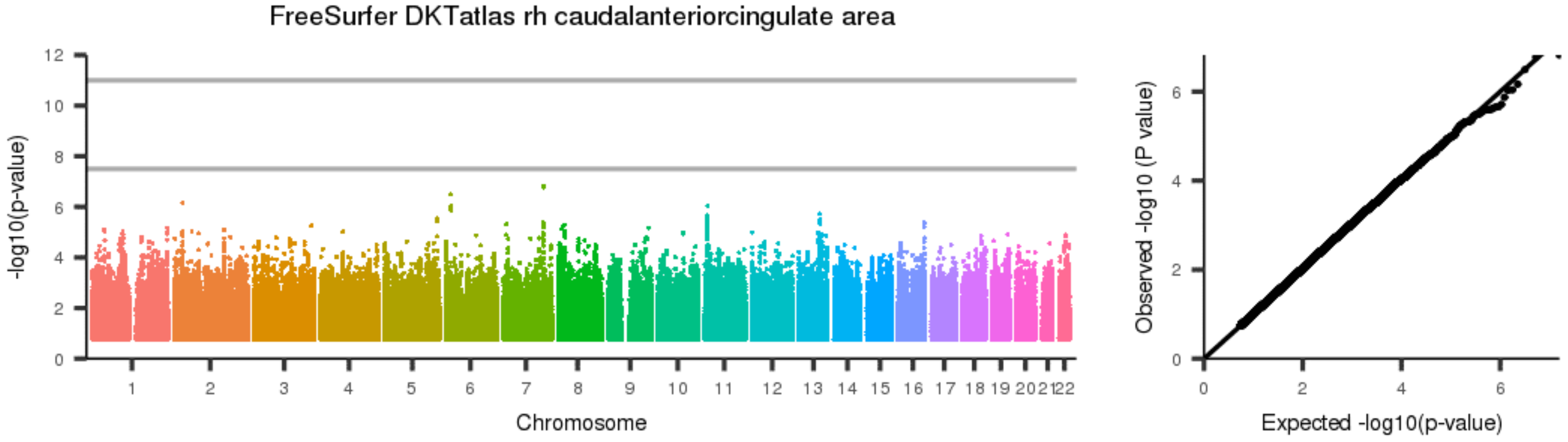

back to list of plots

Figure 1127: FreeSurfer DKTatlas rh caudalmiddlefrontal area

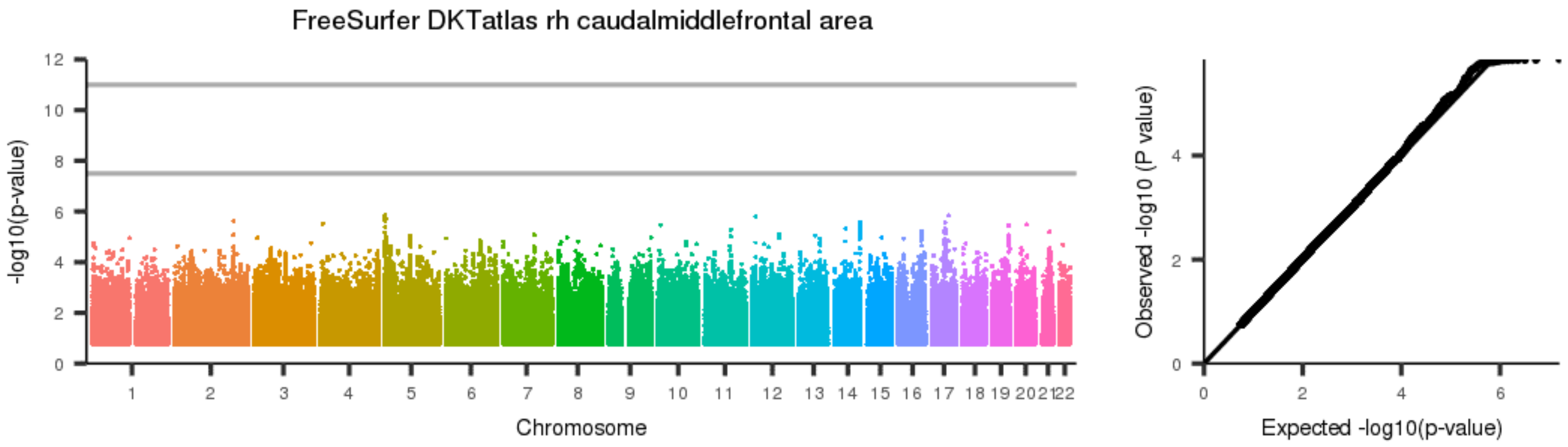

back to list of plots

Figure 1128: FreeSurfer DKTatlas rh cuneus area

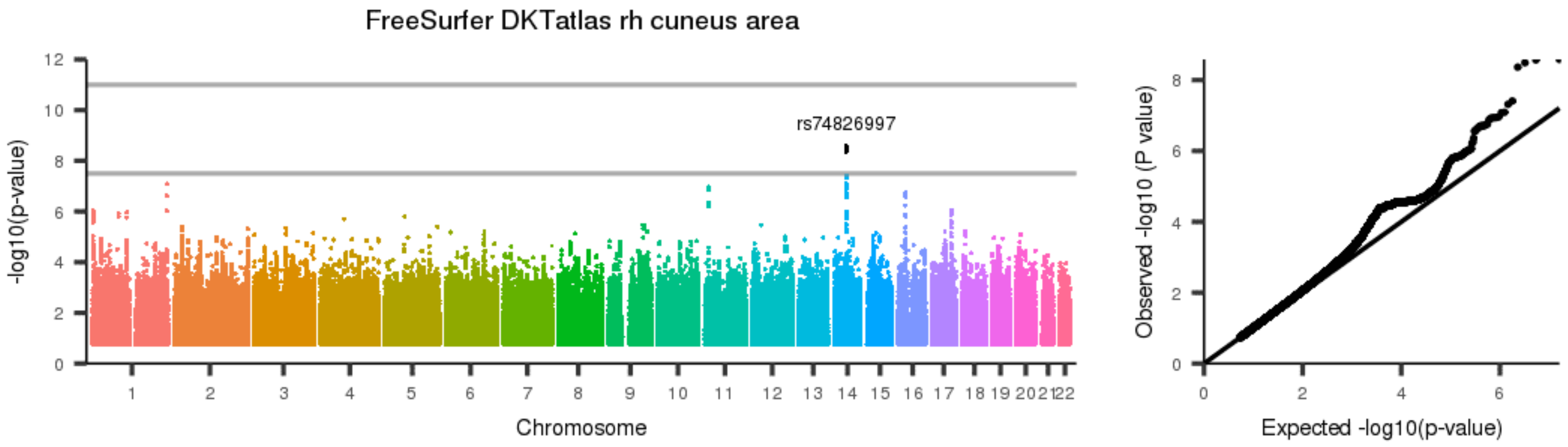

back to list of plots

Figure 1129: FreeSurfer DKTatlas rh entorhinal area

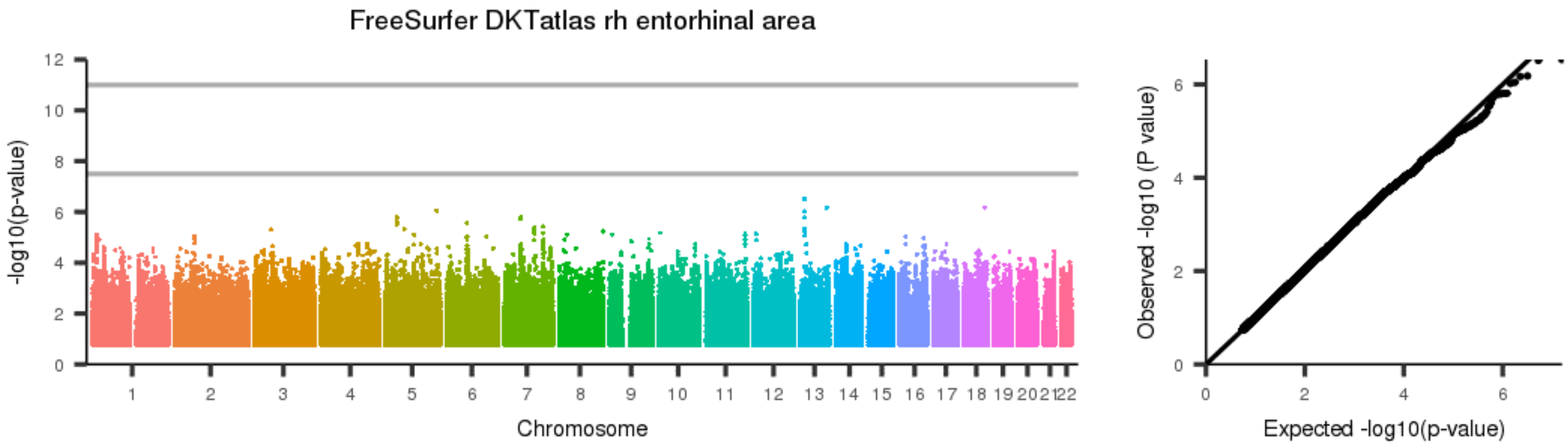

back to list of plots

Figure 1130: FreeSurfer DKTatlas rh fusiform area

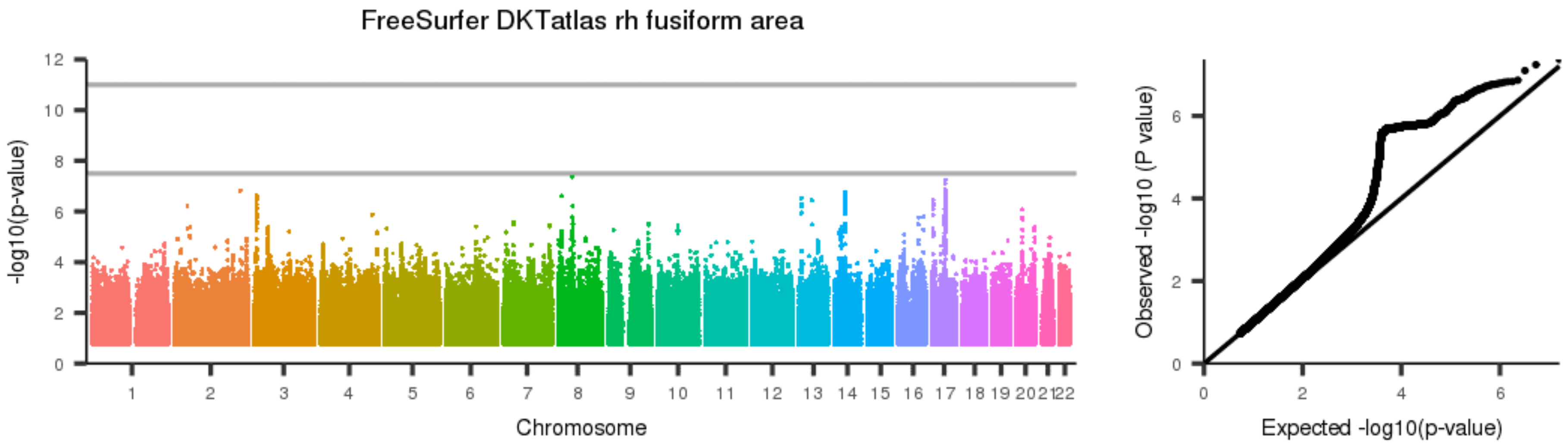

back to list of plots

Figure 1131: FreeSurfer DKTatlas rh inferiorparietal area

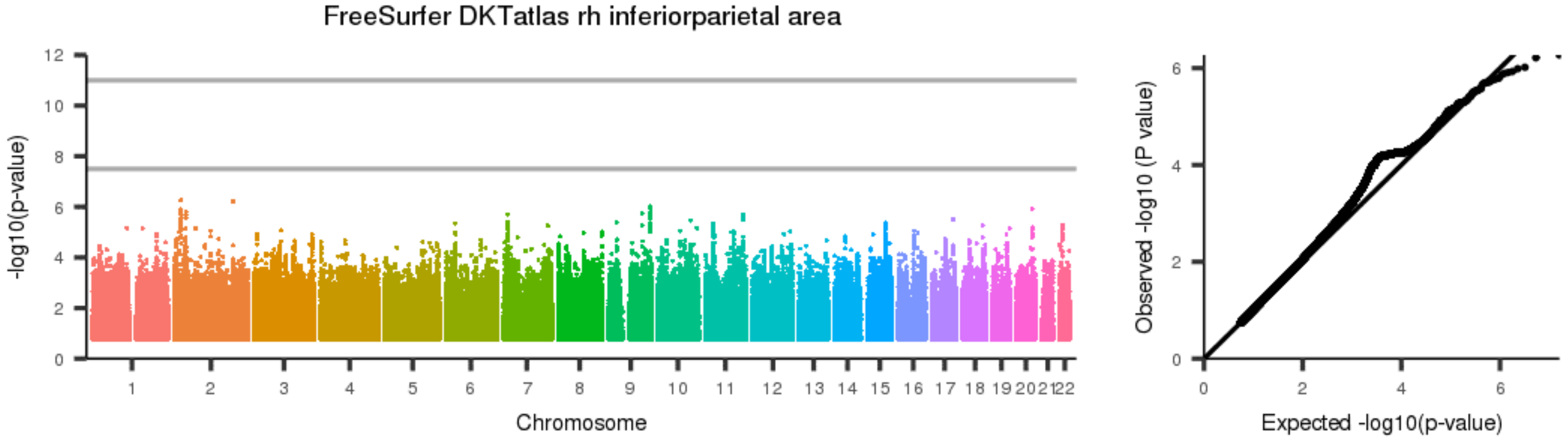

back to list of plots

Figure 1132: FreeSurfer DKTatlas rh inferiortemporal area

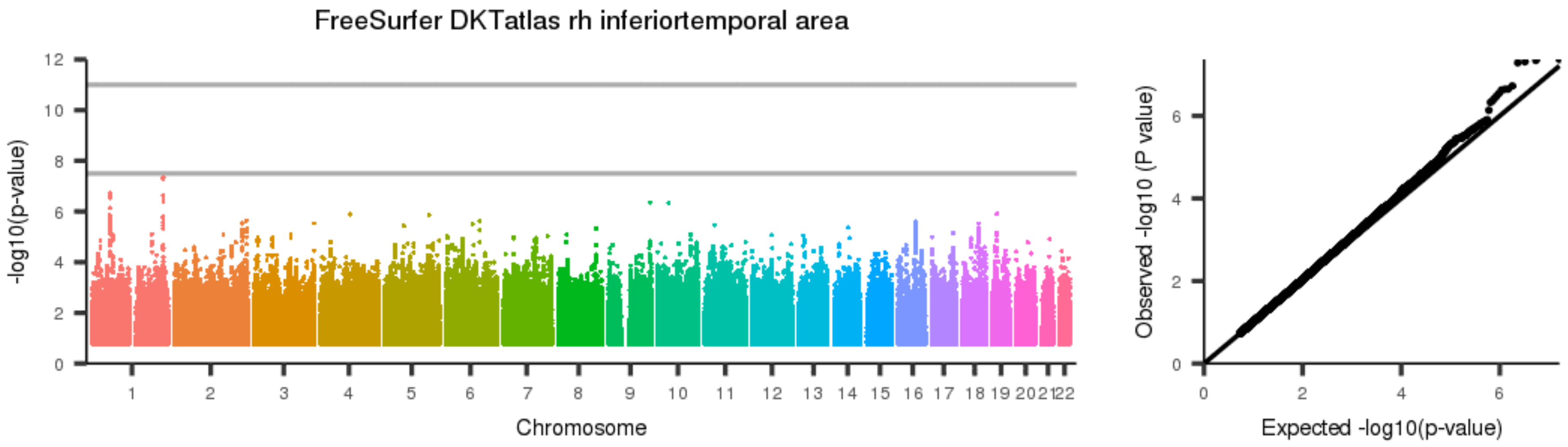

back to list of plots

Figure 1133: FreeSurfer DKTatlas rh isthmuscingulate area

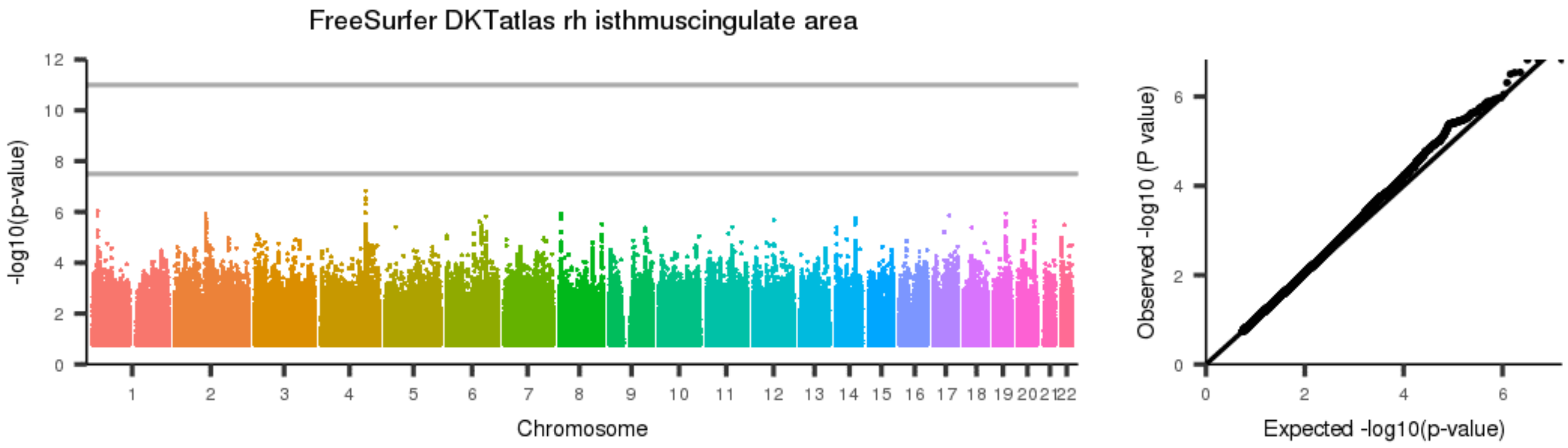

back to list of plots

Figure 1134: FreeSurfer DKTatlas rh lateraloccipital area

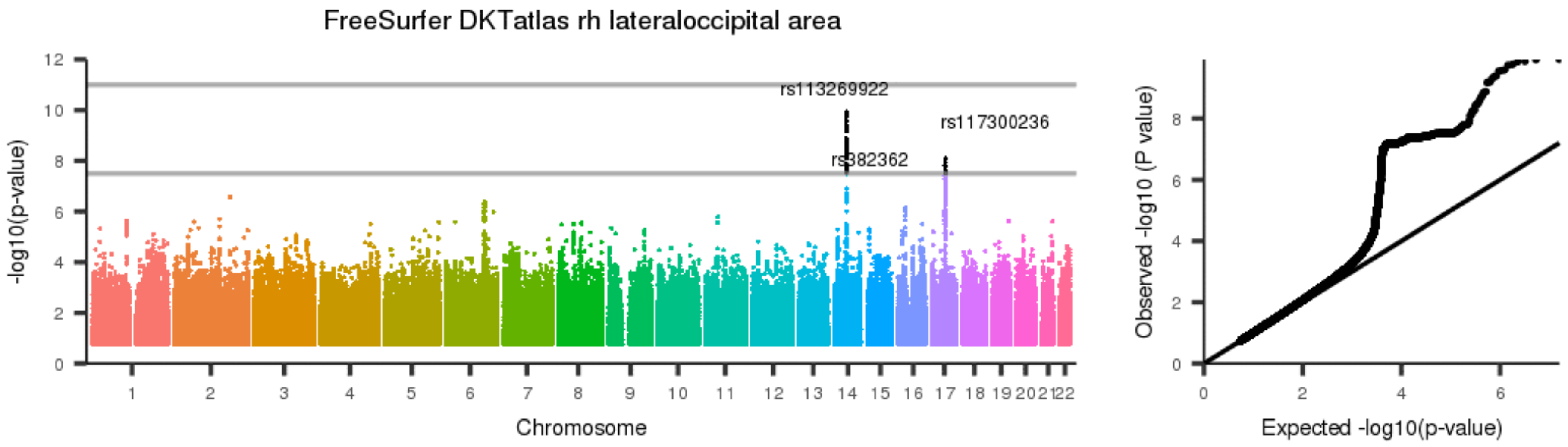

back to list of plots

Figure 1135: FreeSurfer DKTatlas rh lateralorbitofrontal area

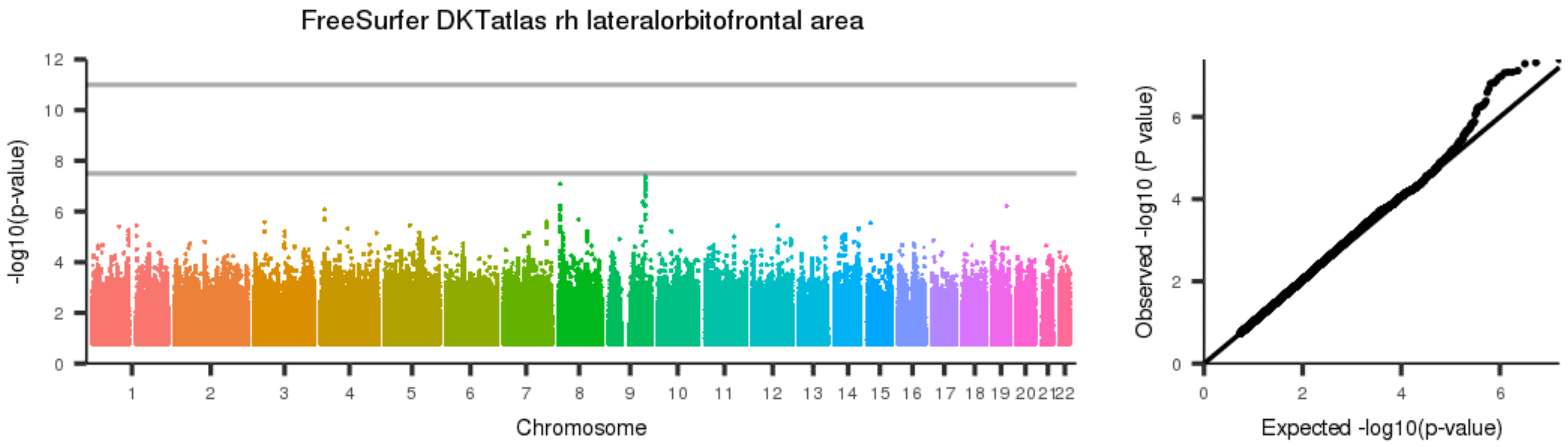

back to list of plots

Figure 1136: FreeSurfer DKTatlas rh lingual area

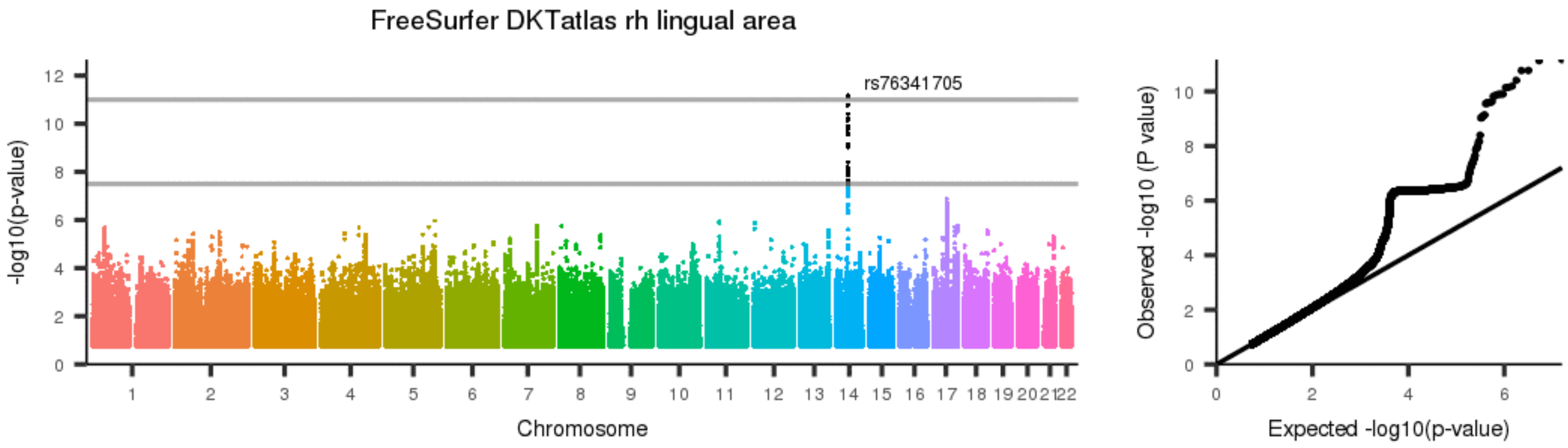

back to list of plots

Figure 1137: FreeSurfer DKTatlas rh medialorbitofrontal area

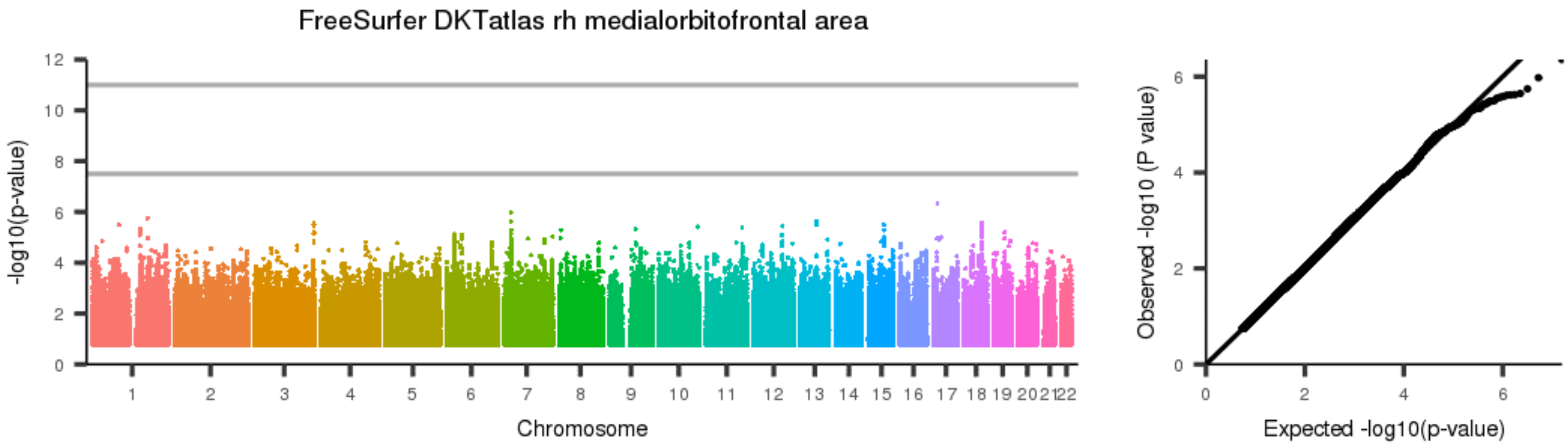

back to list of plots

Figure 1138: FreeSurfer DKTatlas rh middletemporal area

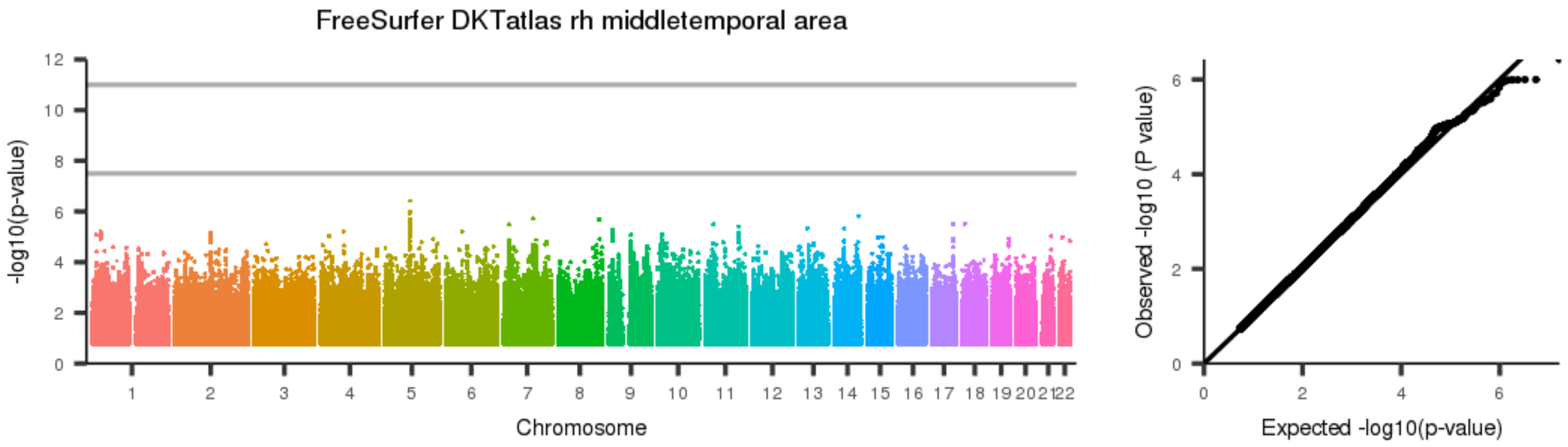

back to list of plots

Figure 1139: FreeSurfer DKTatlas rh parahippocampal area

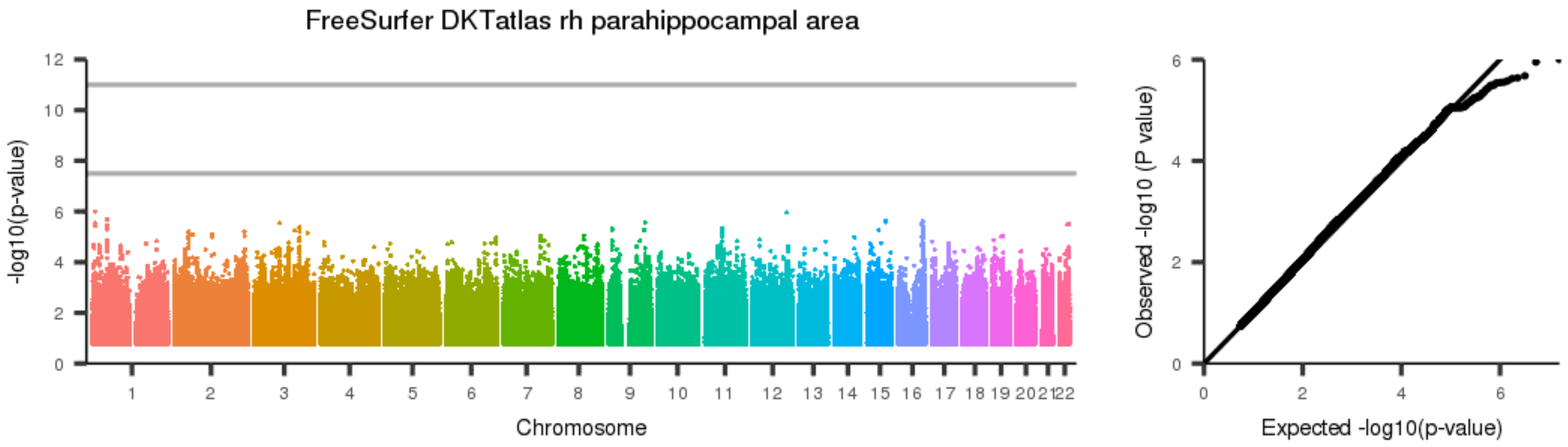

back to list of plots

Figure 1140: FreeSurfer DKTatlas rh paracentral area

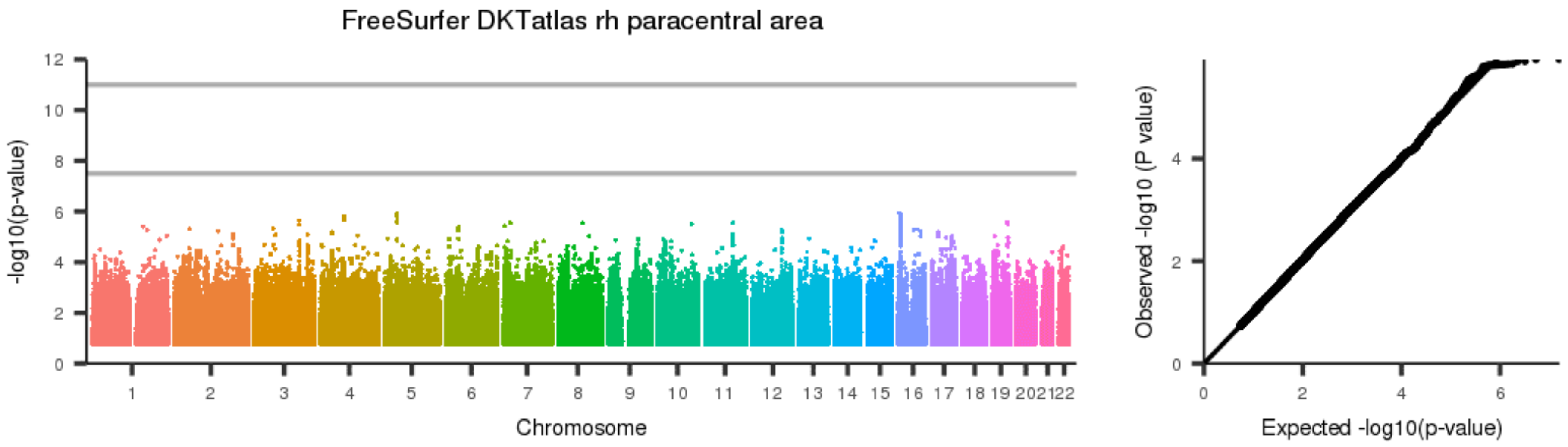

back to list of plots

Figure 1141: FreeSurfer DKTatlas rh parsopercularis area

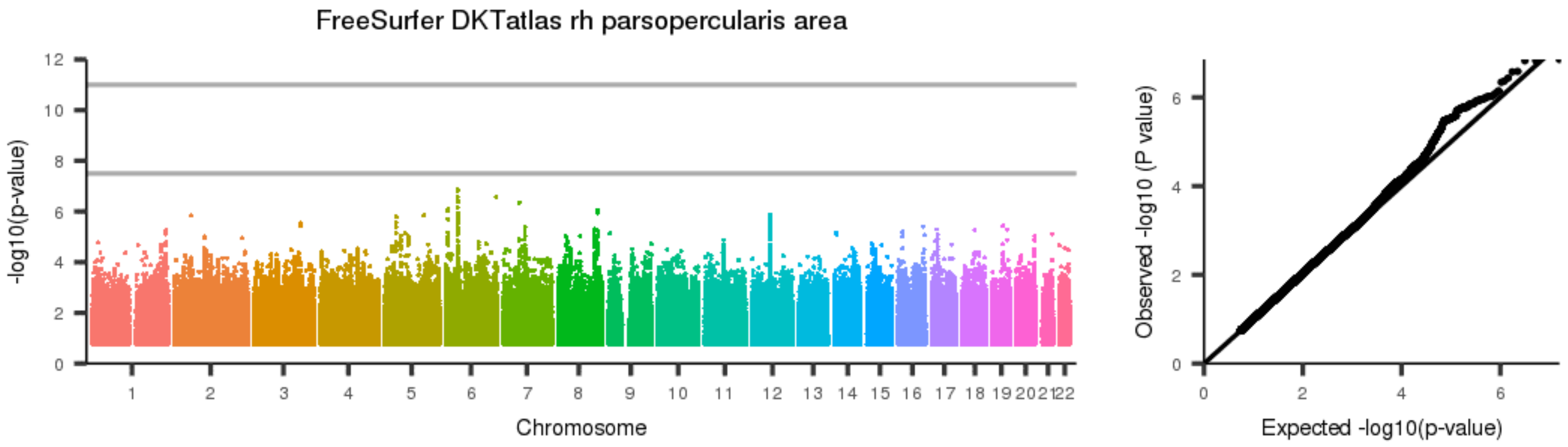

back to list of plots

Figure 1142: FreeSurfer DKTatlas rh parsorbitalis area

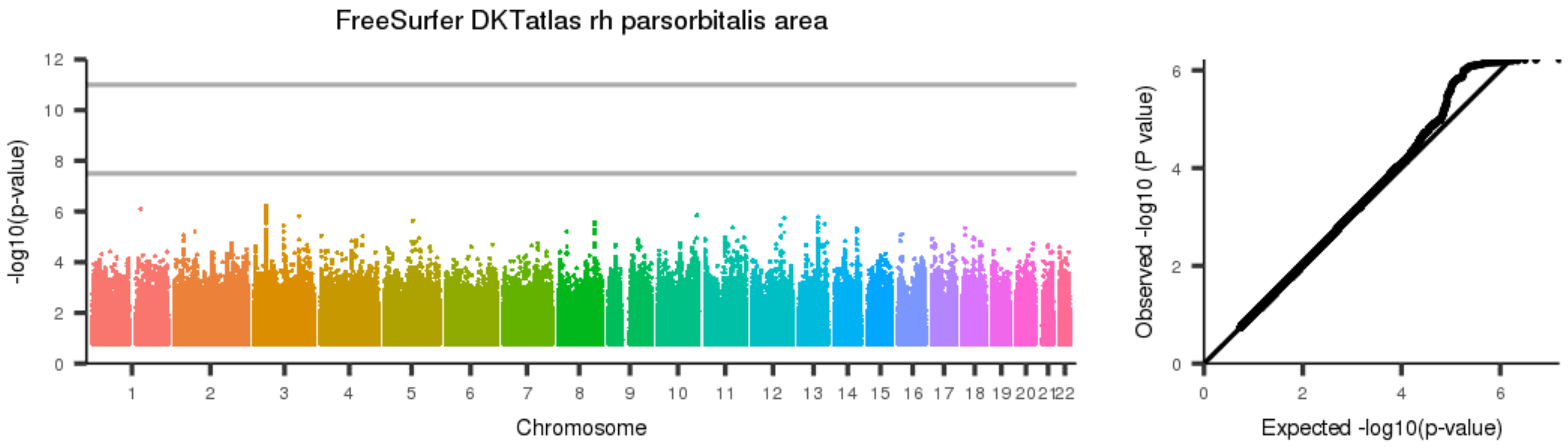

back to list of plots

Figure 1143: FreeSurfer DKTatlas rh parstriangularis area

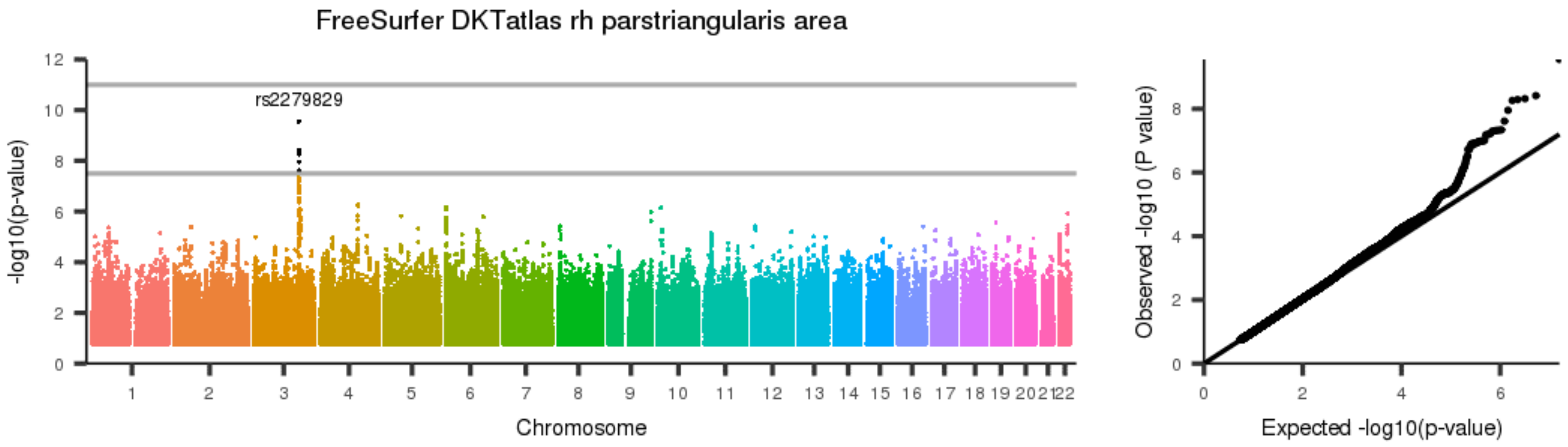

back to list of plots

Figure 1144: FreeSurfer DKTatlas rh pericalcarine area

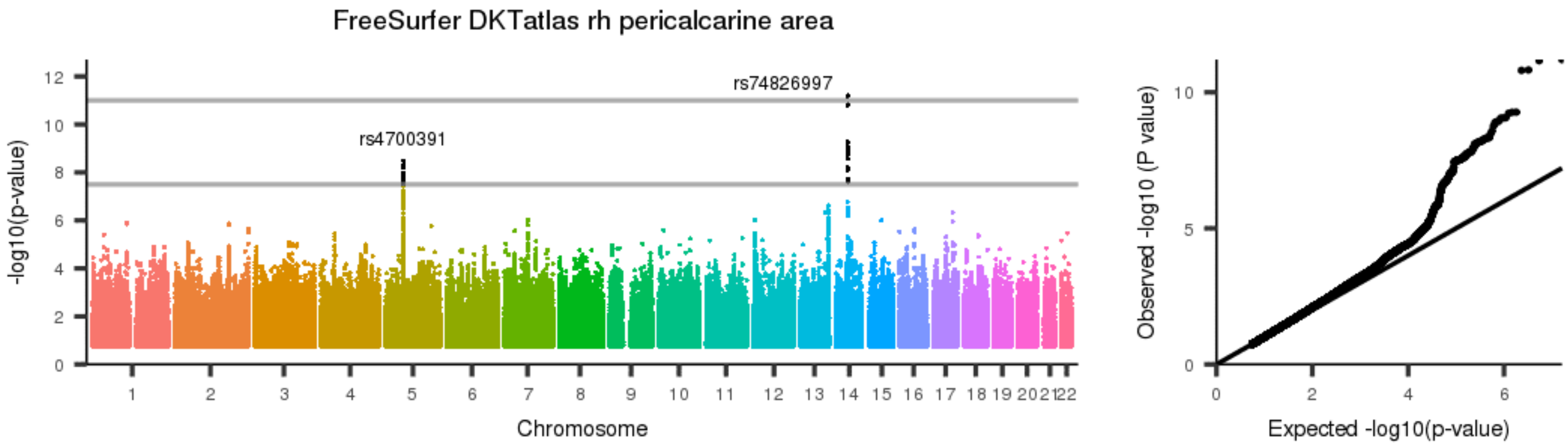

back to list of plots

Figure 1145: FreeSurfer DKTatlas rh postcentral area

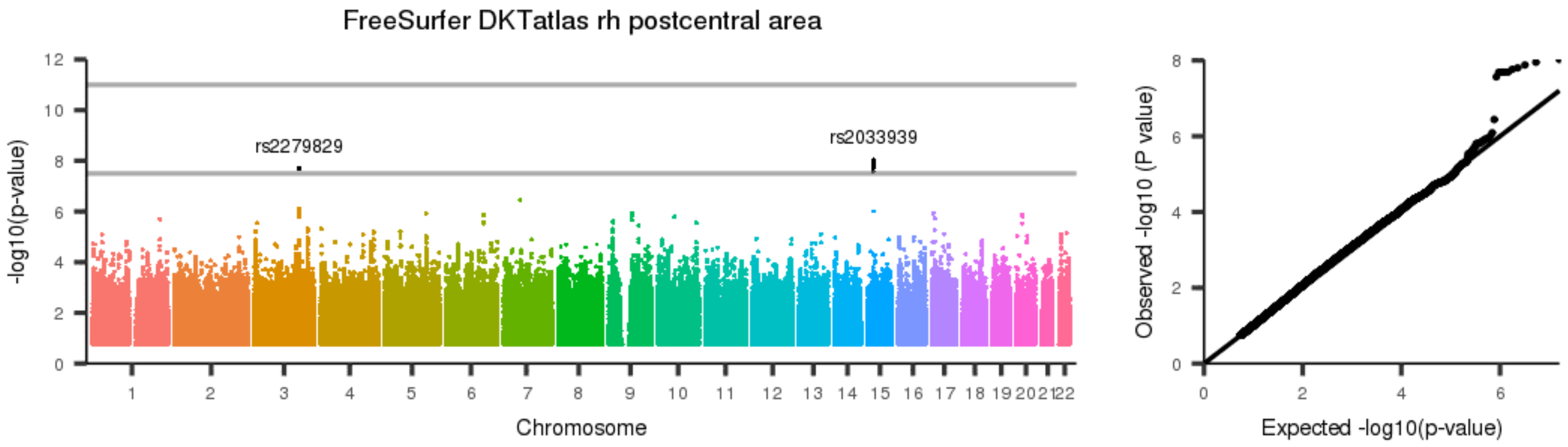

back to list of plots

Figure 1146: FreeSurfer DKTatlas rh posteriorcingulate area

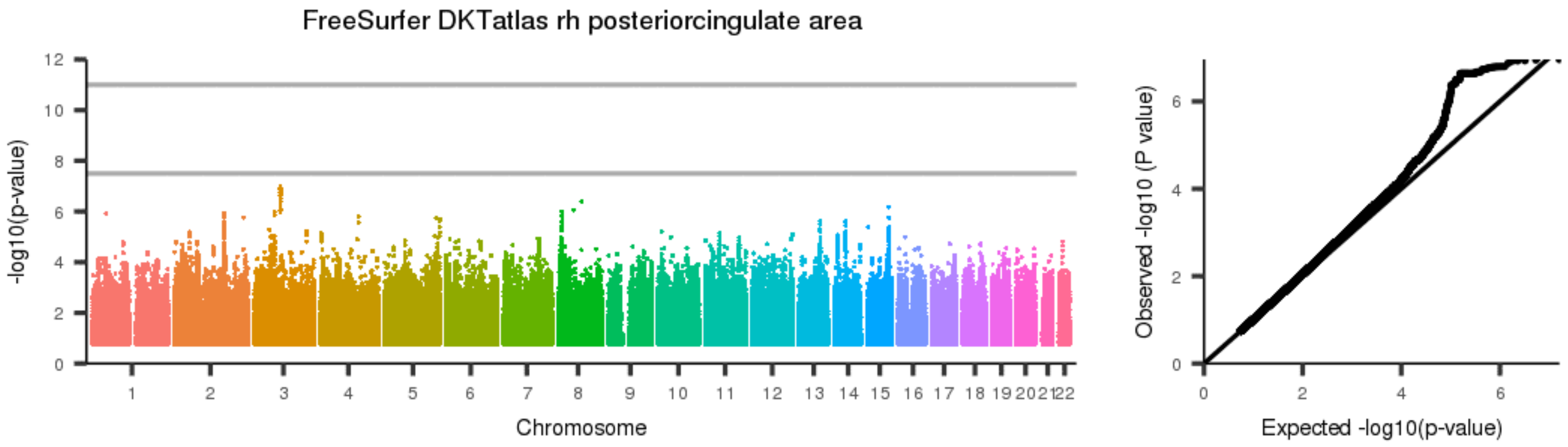

back to list of plots

Figure 1147: FreeSurfer DKTatlas rh precentral area

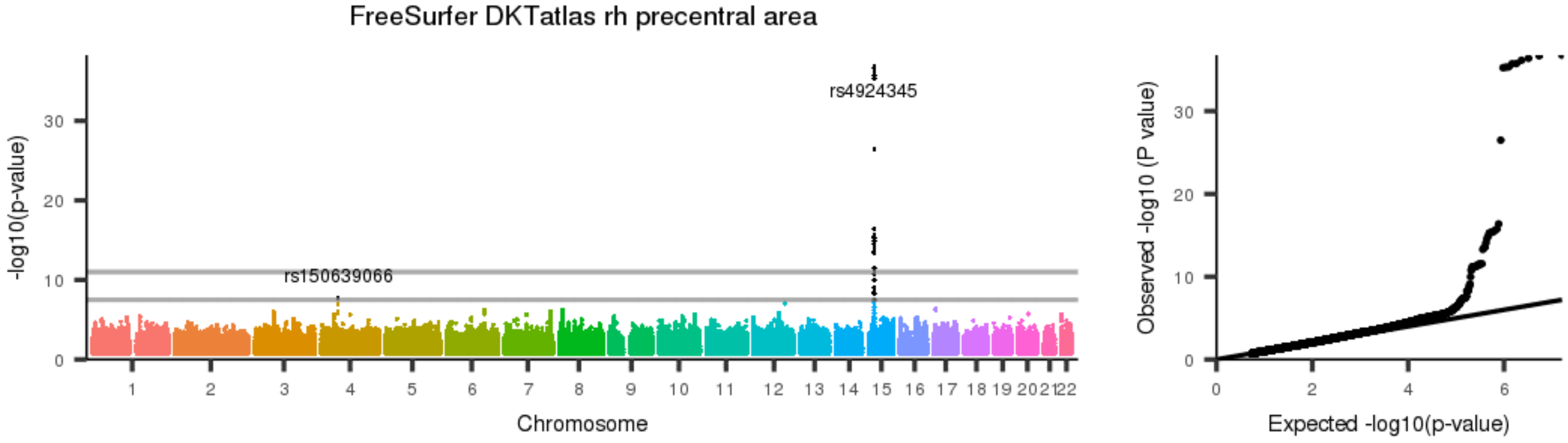

back to list of plots

Figure 1148: FreeSurfer DKTatlas rh precuneus area

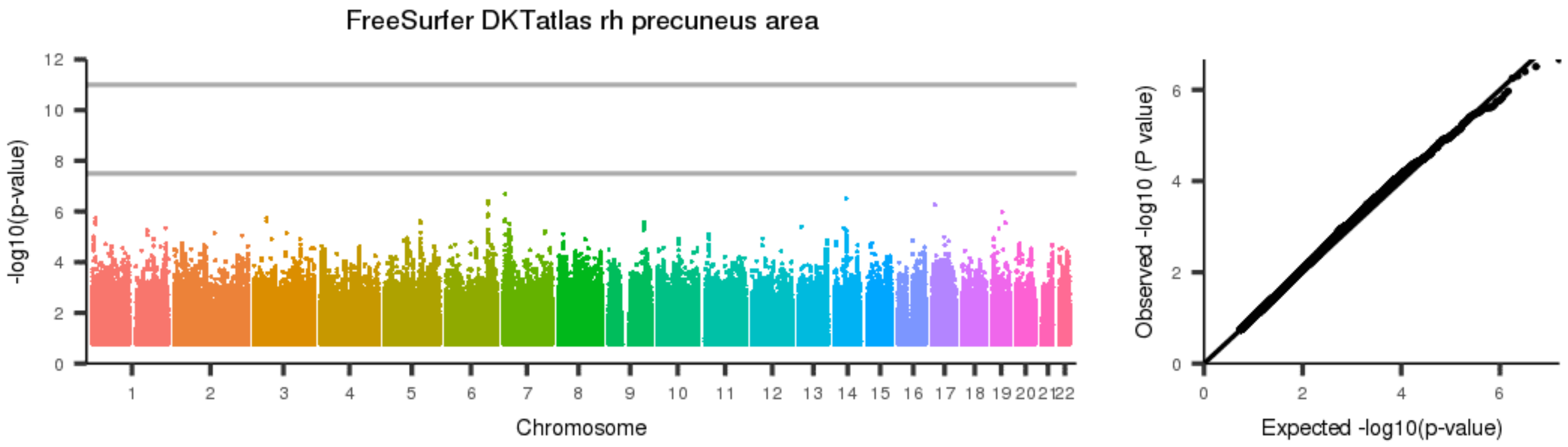

back to list of plots

Figure 1149: FreeSurfer DKTatlas rh rostralanteriorcingulate area

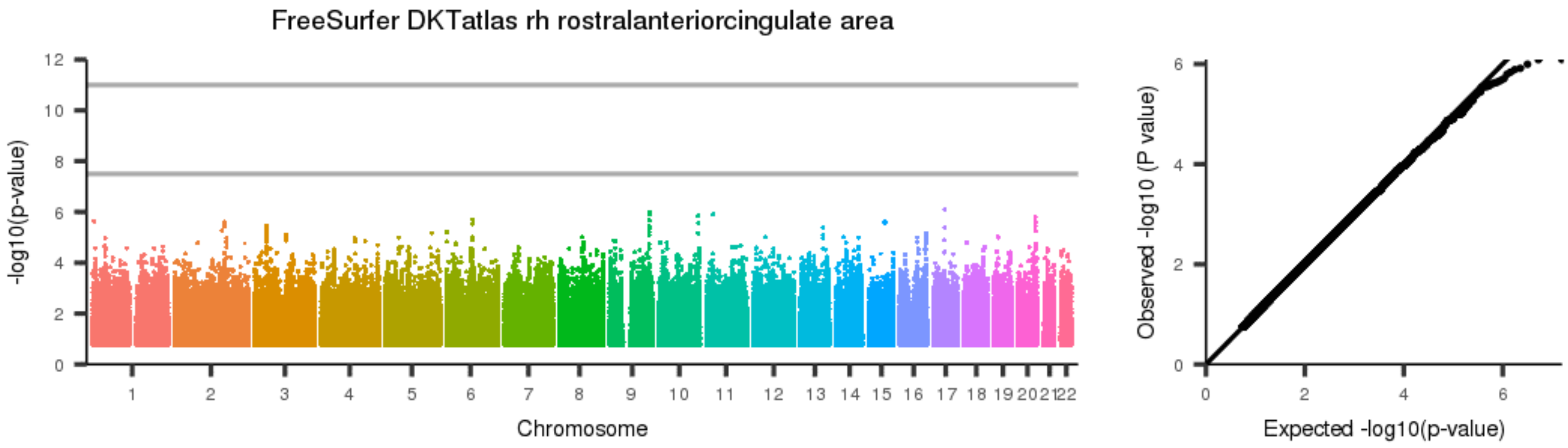

back to list of plots

Figure 1150: FreeSurfer DKTatlas rh rostralmiddlefrontal area

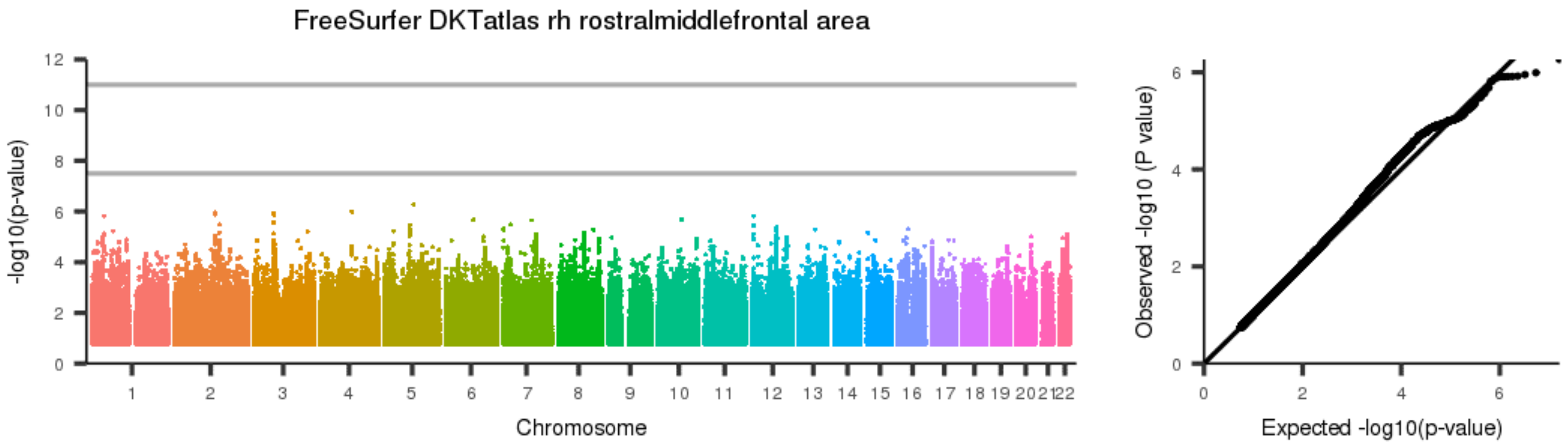

back to list of plots

Figure 1151: FreeSurfer DKTatlas rh superiorfrontal area

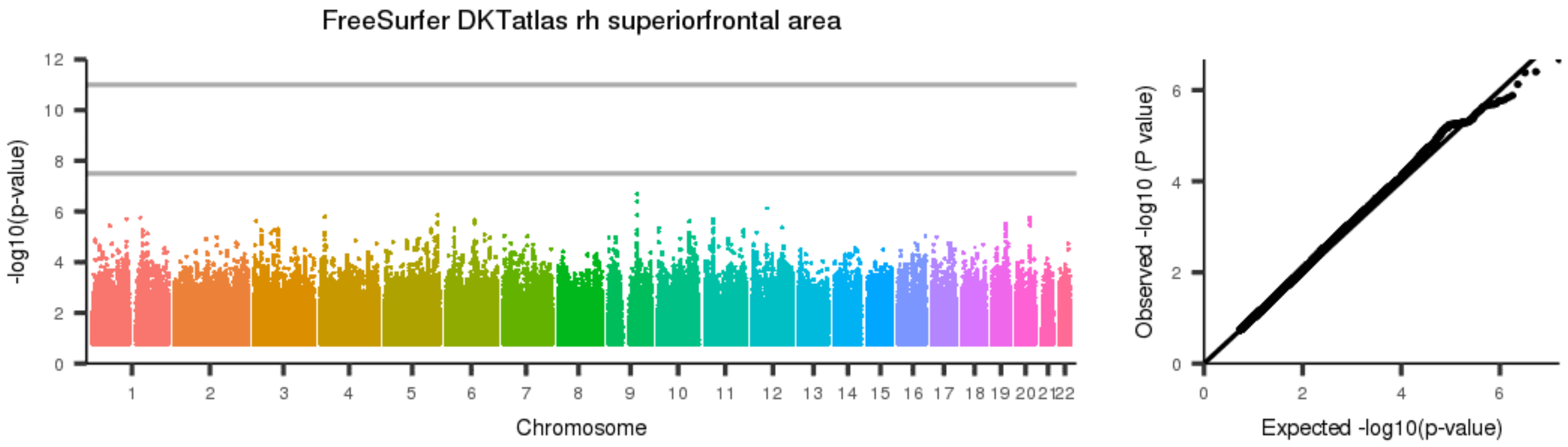

back to list of plots

Figure 1152: FreeSurfer DKTatlas rh superiorparietal area

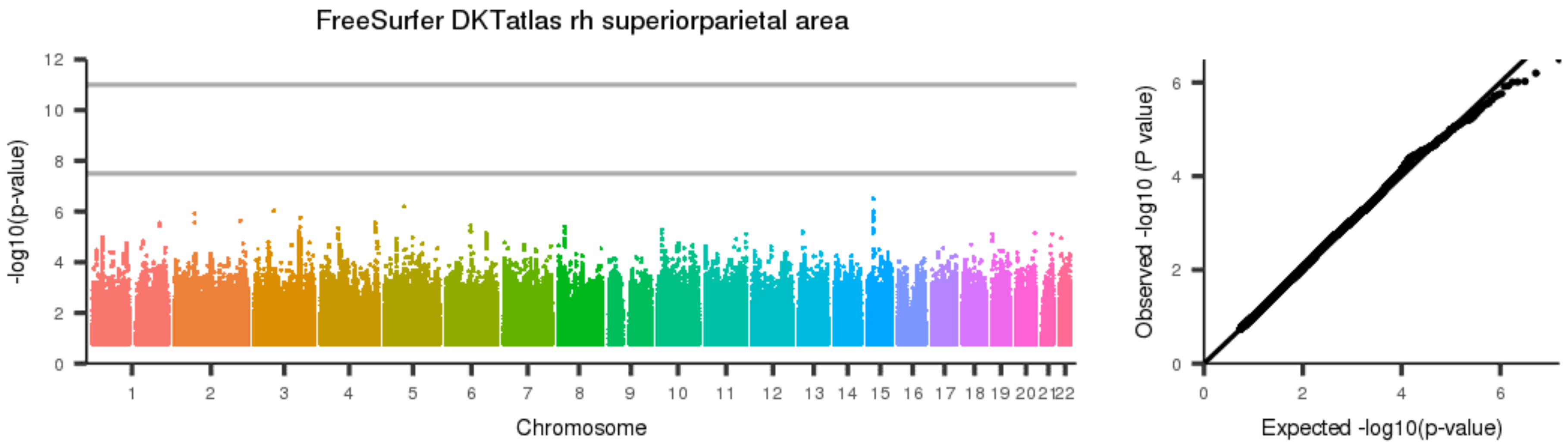

back to list of plots

Figure 1153: FreeSurfer DKTatlas rh superiortemporal area

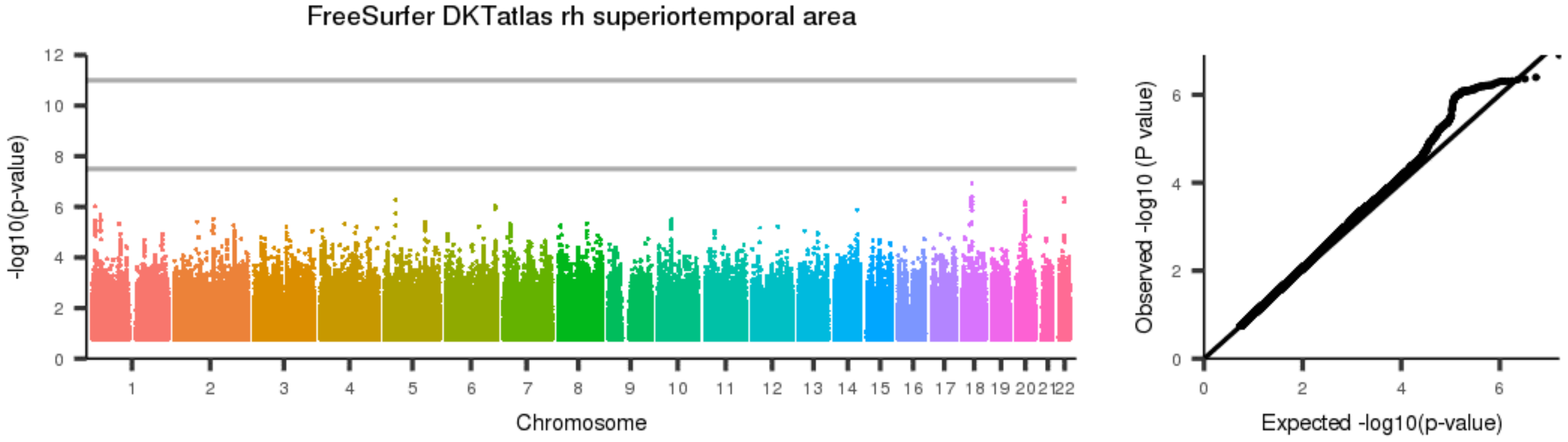

back to list of plots

Figure 1154: FreeSurfer DKTatlas rh supramarginal area

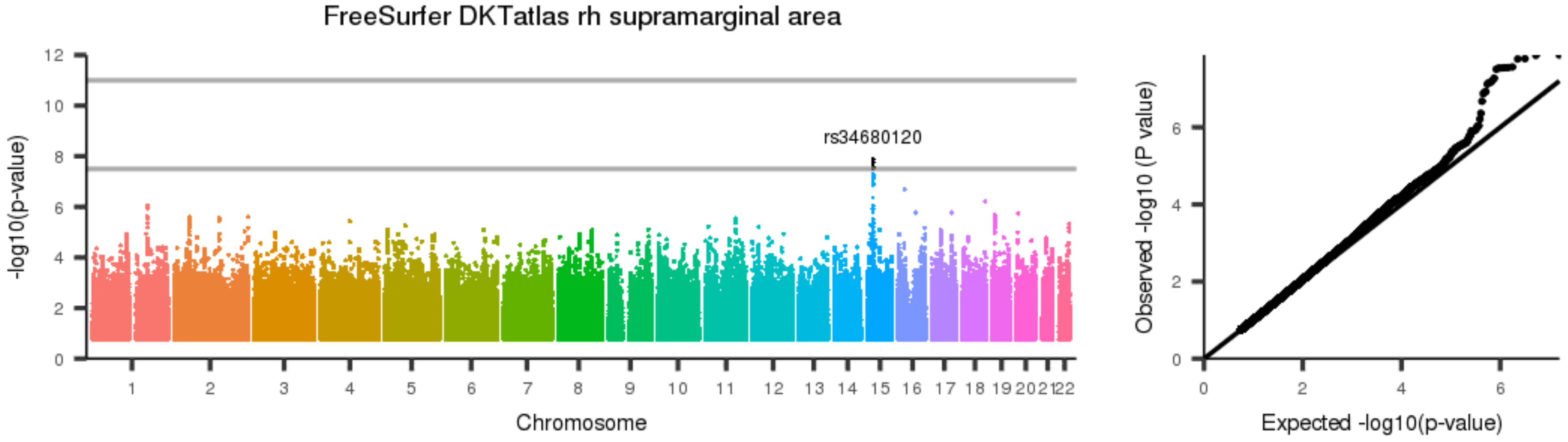

back to list of plots

Figure 1155: FreeSurfer DKTatlas rh transversetemporal area

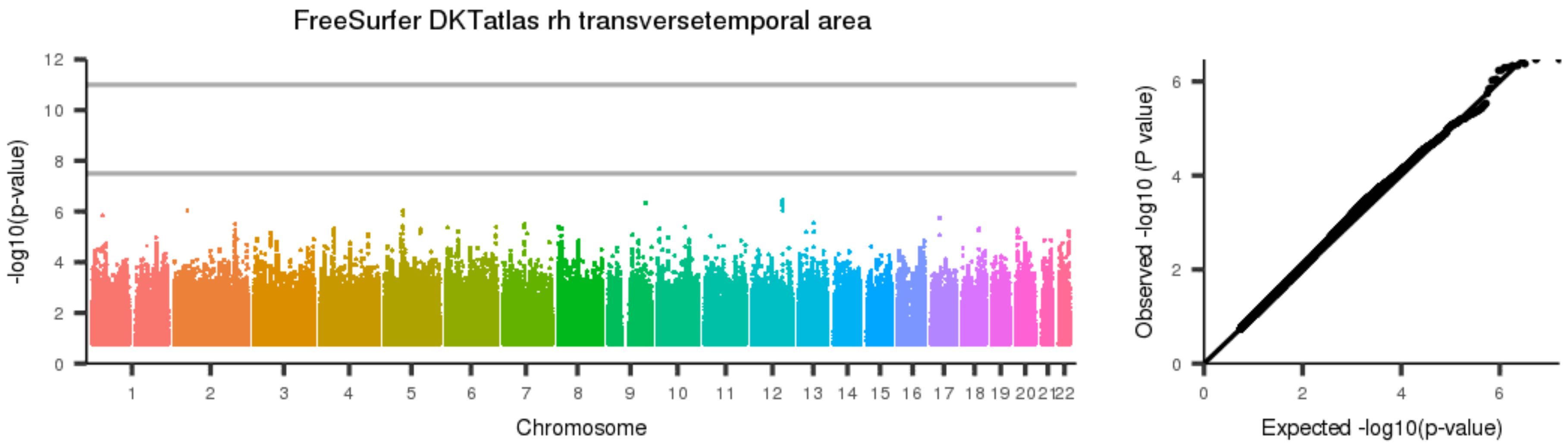

back to list of plots

Figure 1156: FreeSurfer DKTatlas rh insula area

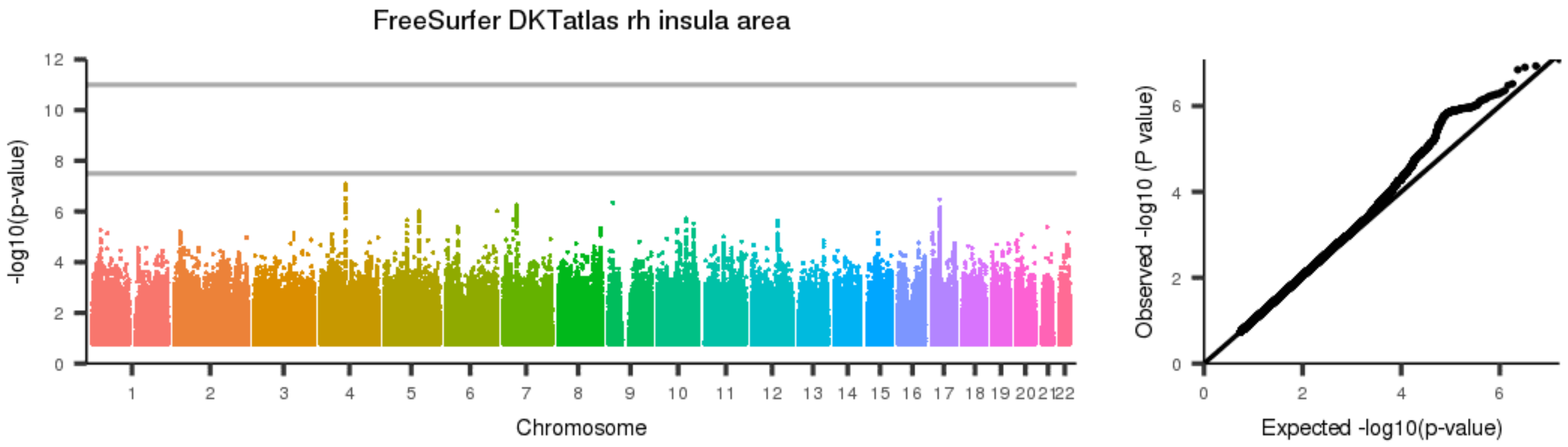

back to list of plots

Figure 1157: FreeSurfer DKTatlas rh WhiteSurfArea area

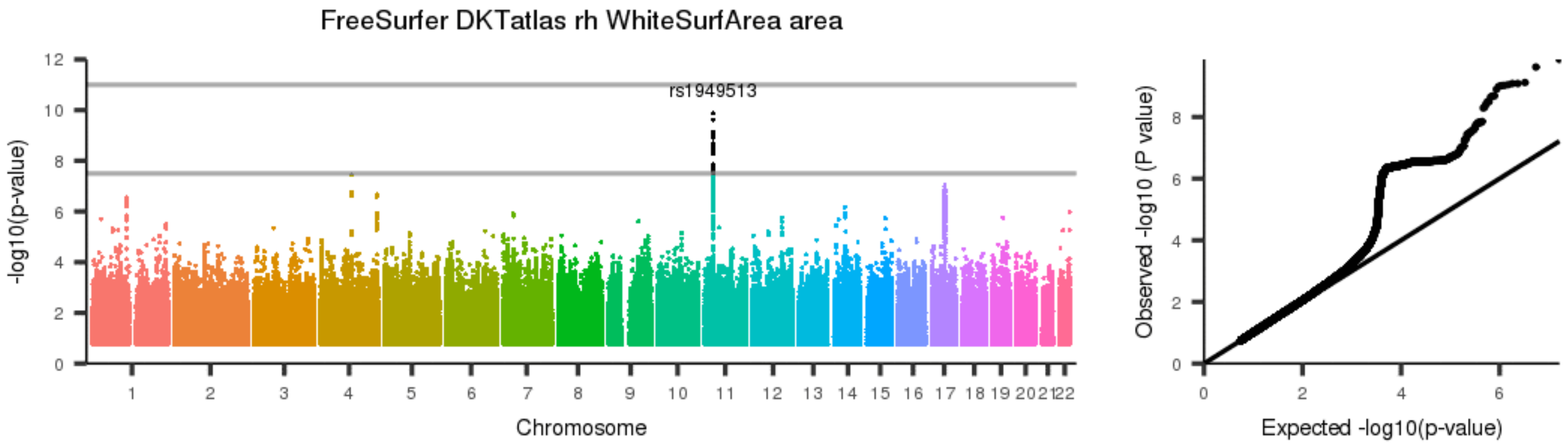

back to list of plots

Figure 1158: FreeSurfer a2009s rh G and S frontomargin area

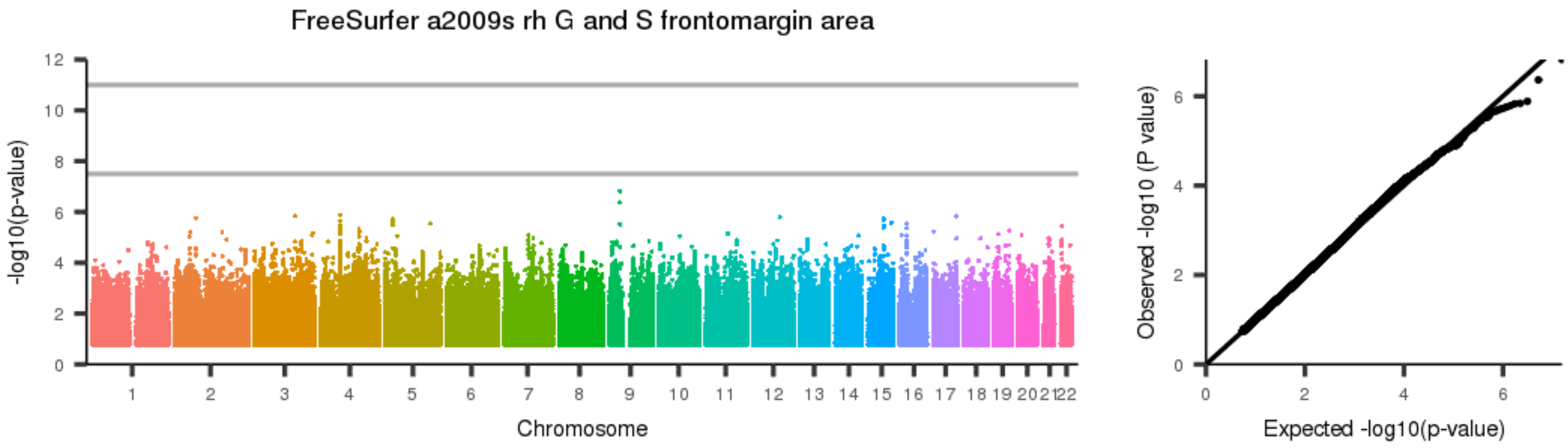

back to list of plots

Figure 1159: FreeSurfer a2009s rh G and S occipital inf area

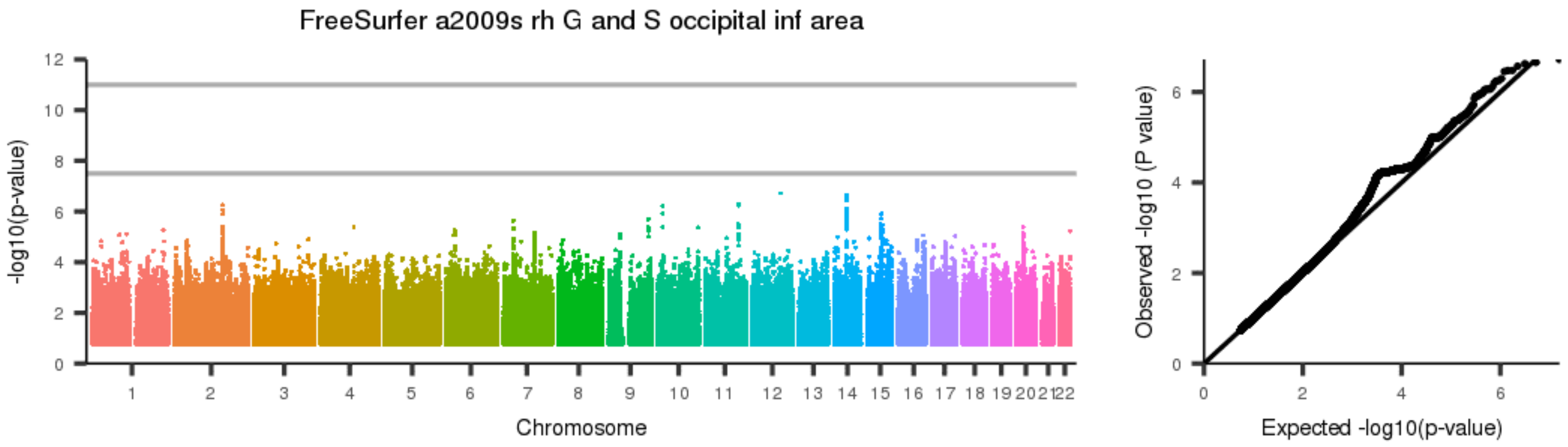

back to list of plots

Figure 1160: FreeSurfer a2009s rh G and S paracentral area

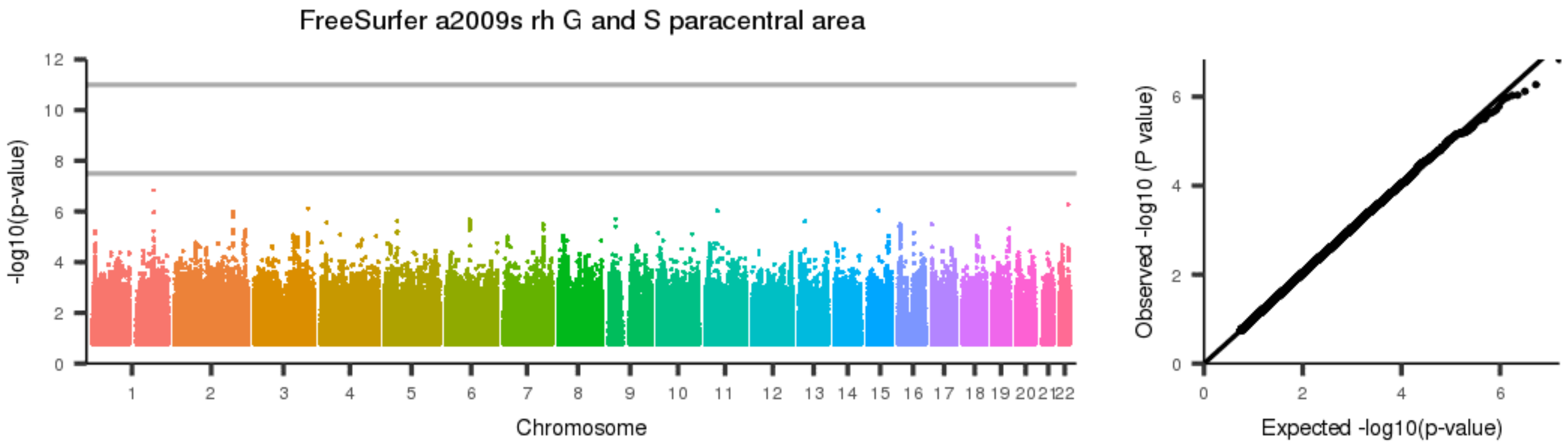

back to list of plots

Figure 1161: FreeSurfer a2009s rh G and S subcentral area

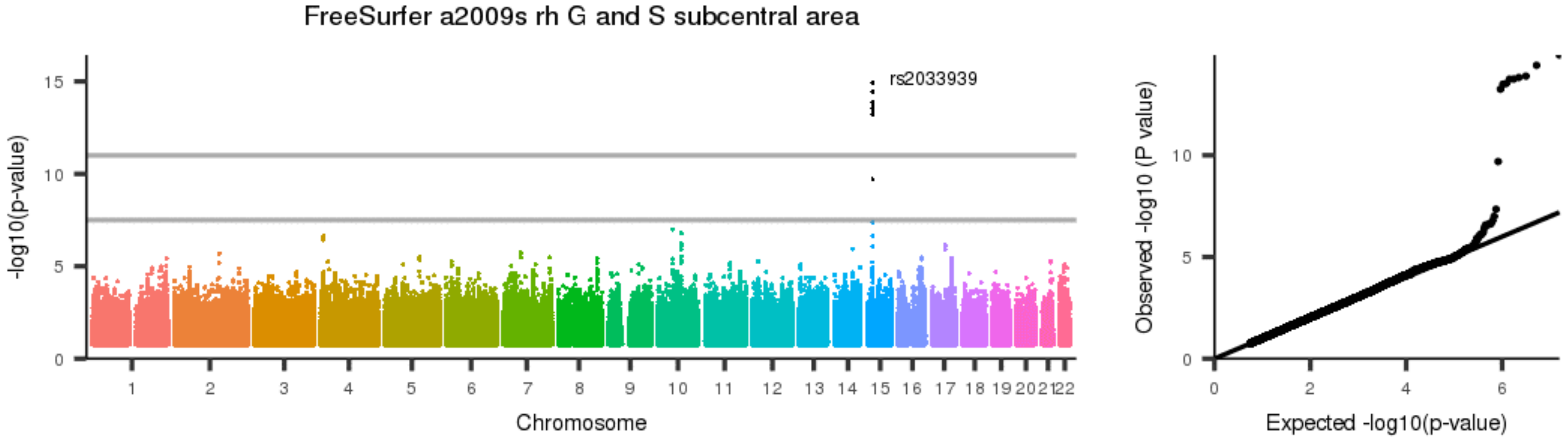

back to list of plots

Figure 1162: FreeSurfer a2009s rh G and S transv frontopol area

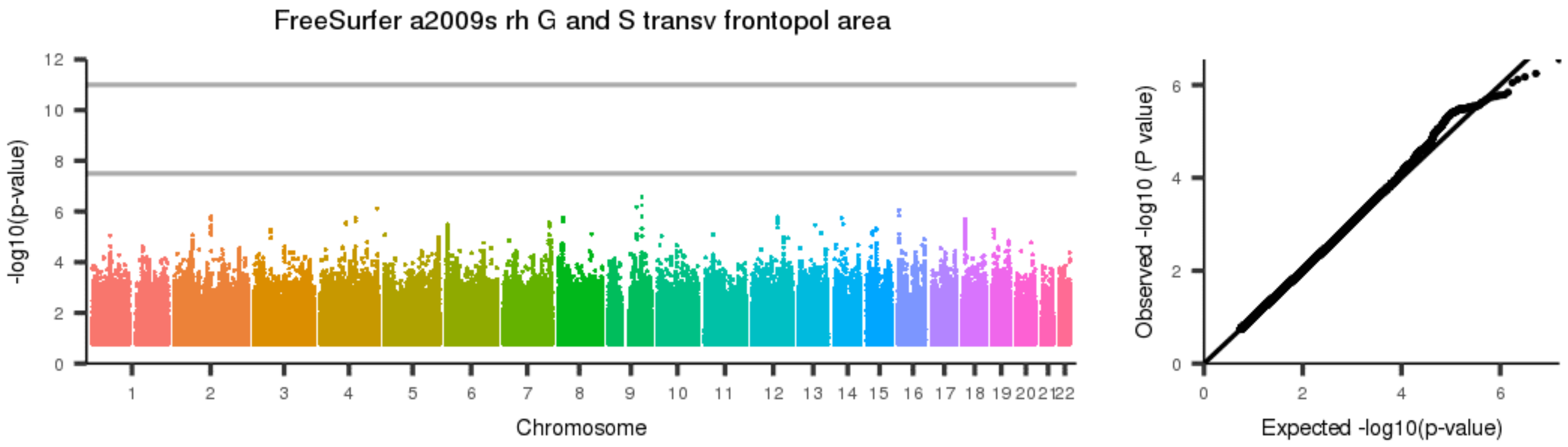

back to list of plots

Figure 1163: FreeSurfer a2009s rh G and S cingul-Ant area

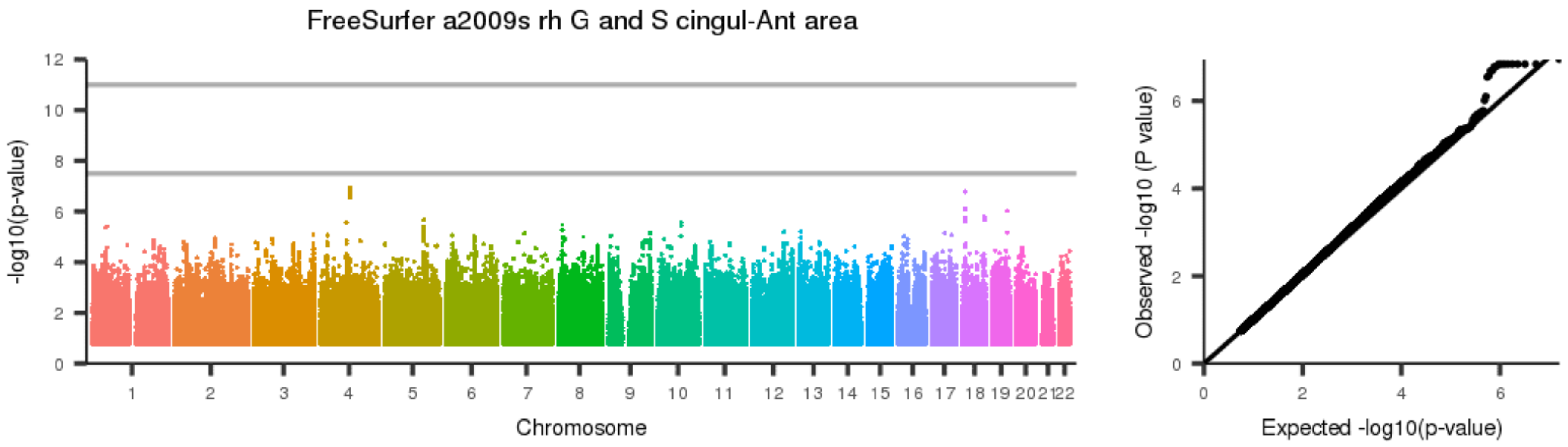

back to list of plots

Figure 1164: FreeSurfer a2009s rh G and S cingul-Mid-Ant area

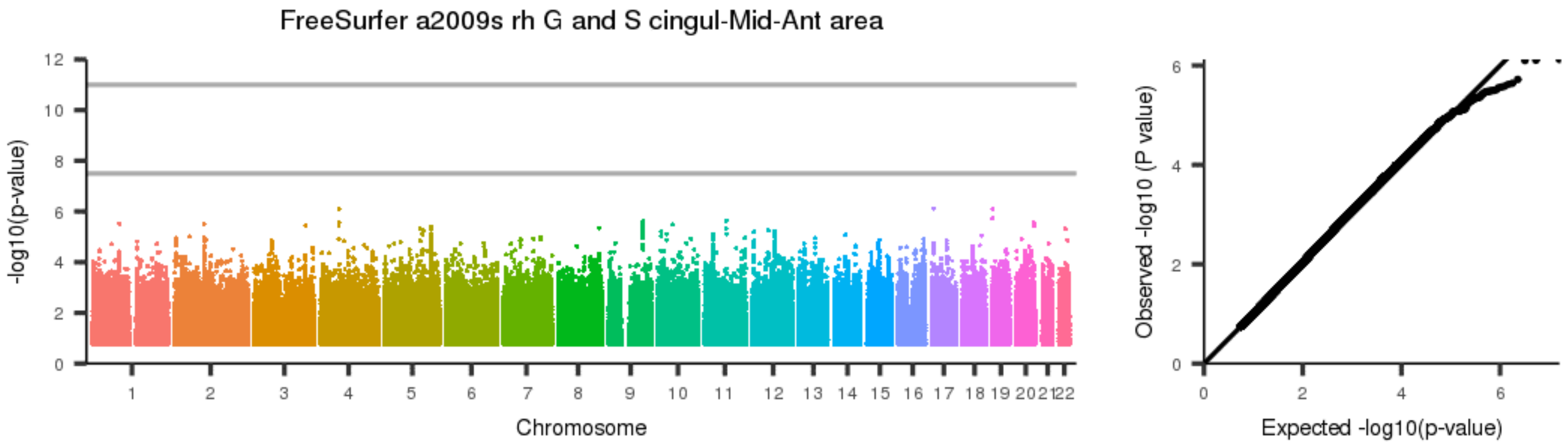

back to list of plots

Figure 1165: FreeSurfer a2009s rh G and S cingul-Mid-Post area

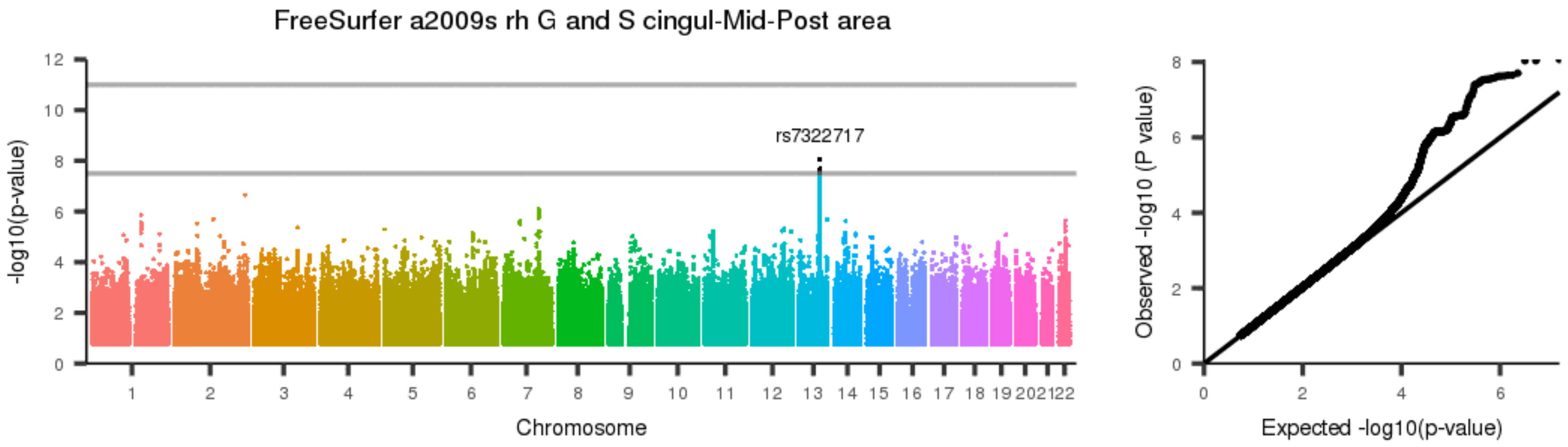

back to list of plots

Figure 1166: FreeSurfer a2009s rh G cingul-Post-dorsal area

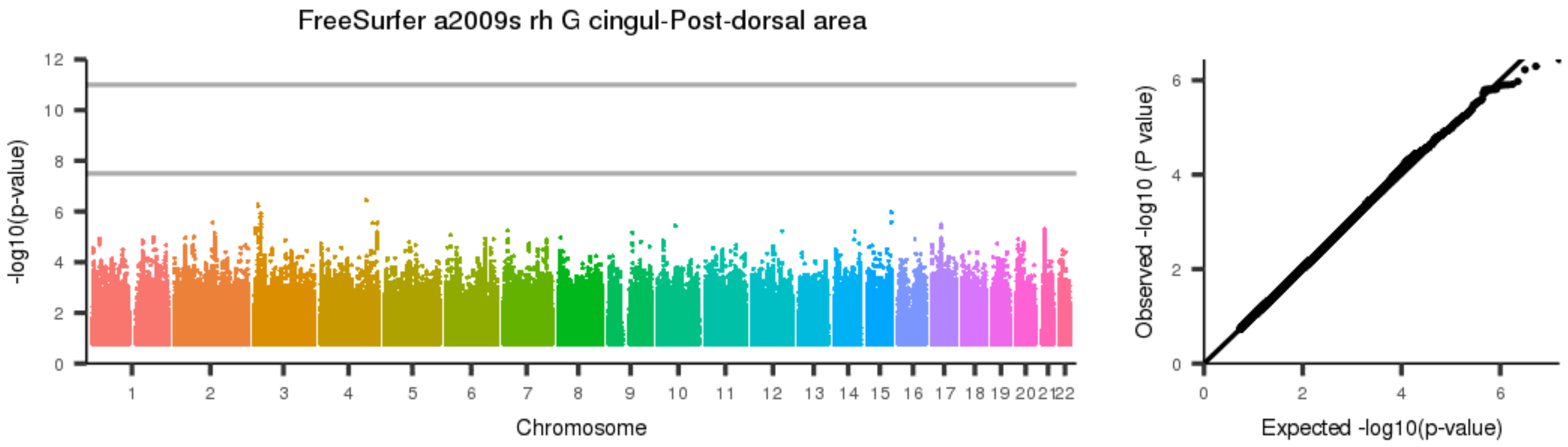

back to list of plots

Figure 1167: FreeSurfer a2009s rh G cingul-Post-ventral area

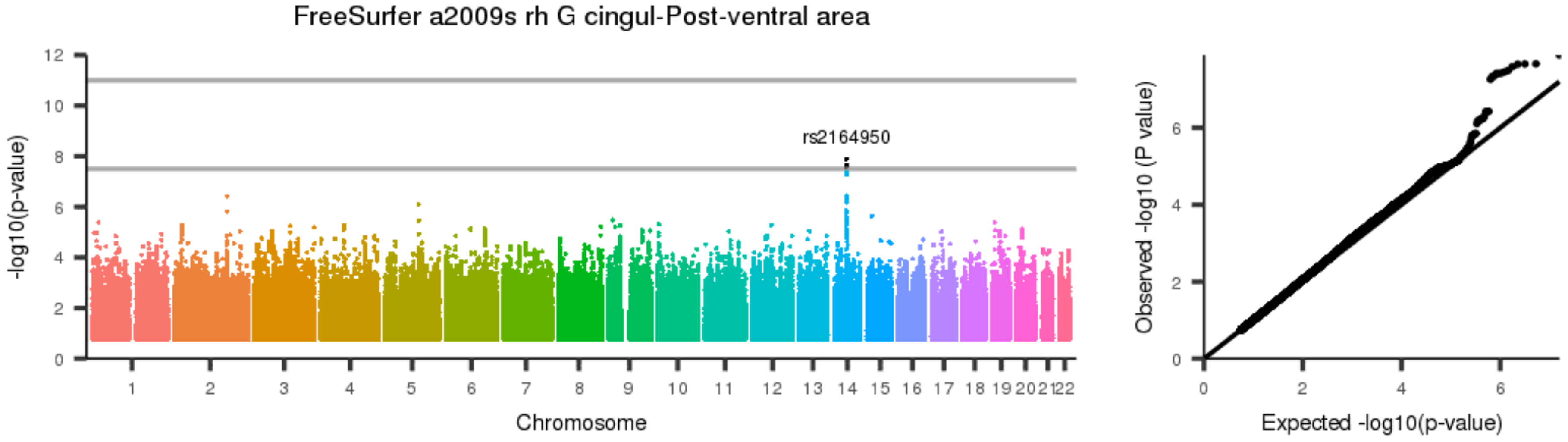

back to list of plots

Figure 1168: FreeSurfer a2009s rh G cuneus area

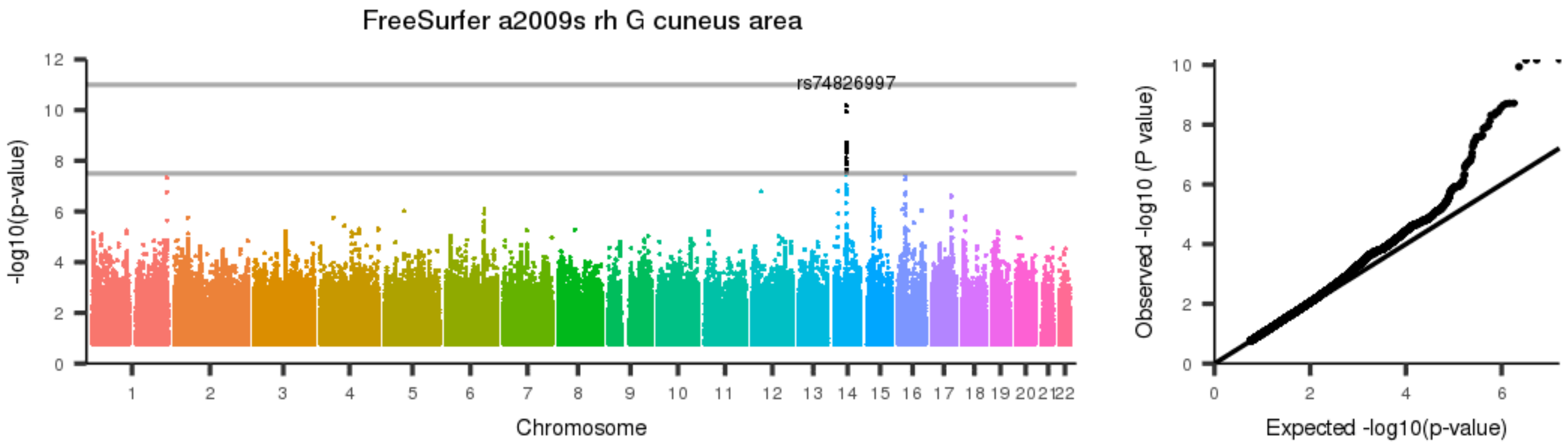

back to list of plots

Figure 1169: FreeSurfer a2009s rh G front inf-Opercular area

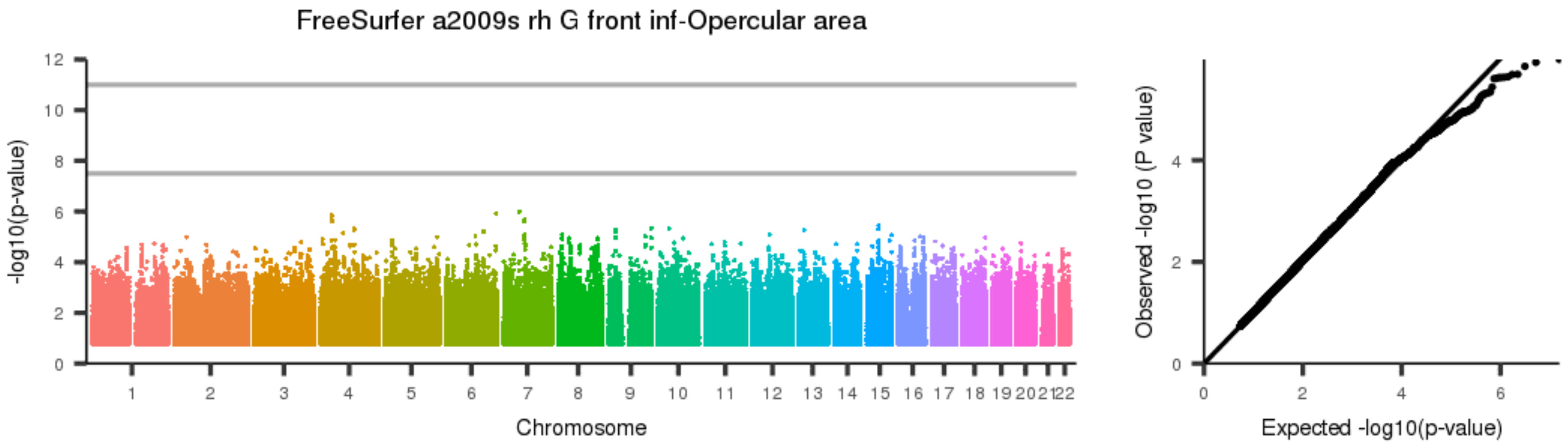

back to list of plots

Figure 1170: FreeSurfer a2009s rh G front inf-Orbital area

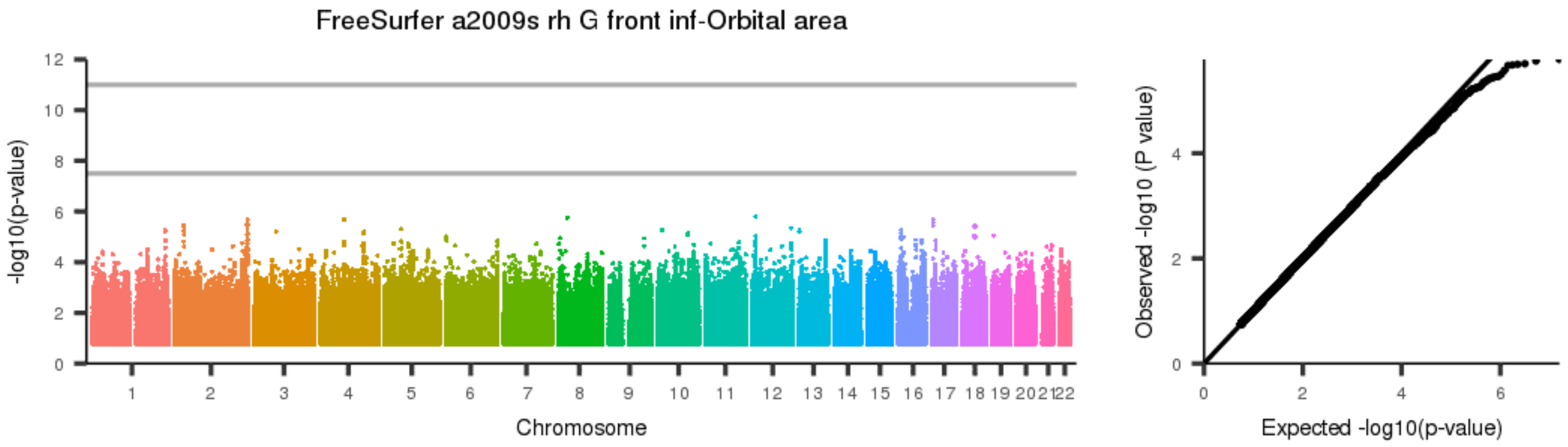

back to list of plots

Figure 1171: FreeSurfer a2009s rh G front inf-Triangul area

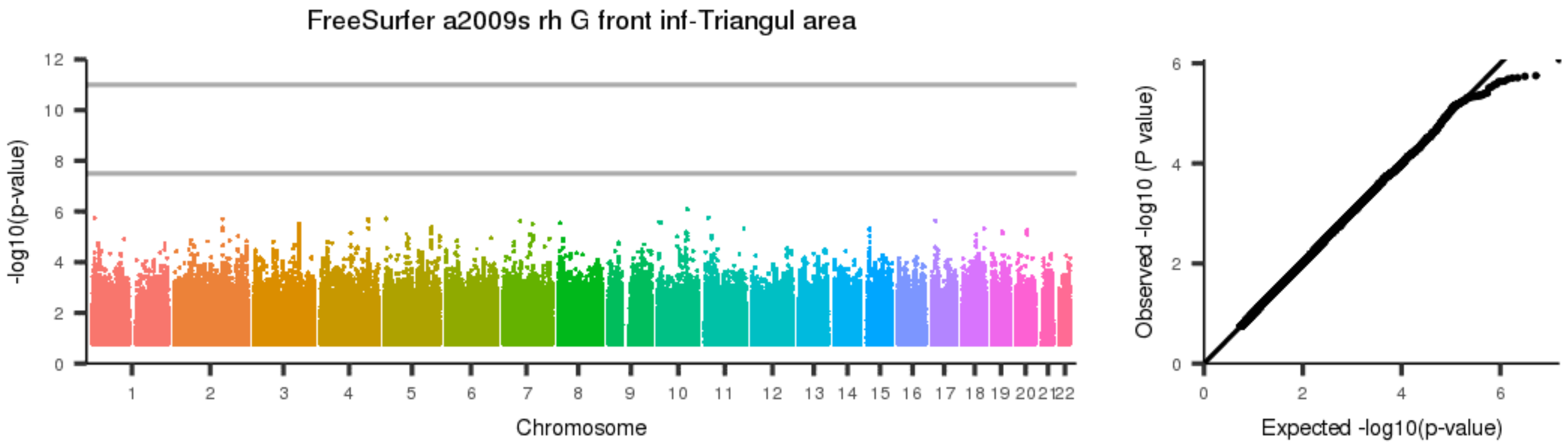

back to list of plots

Figure 1172: FreeSurfer a2009s rh G front middle area

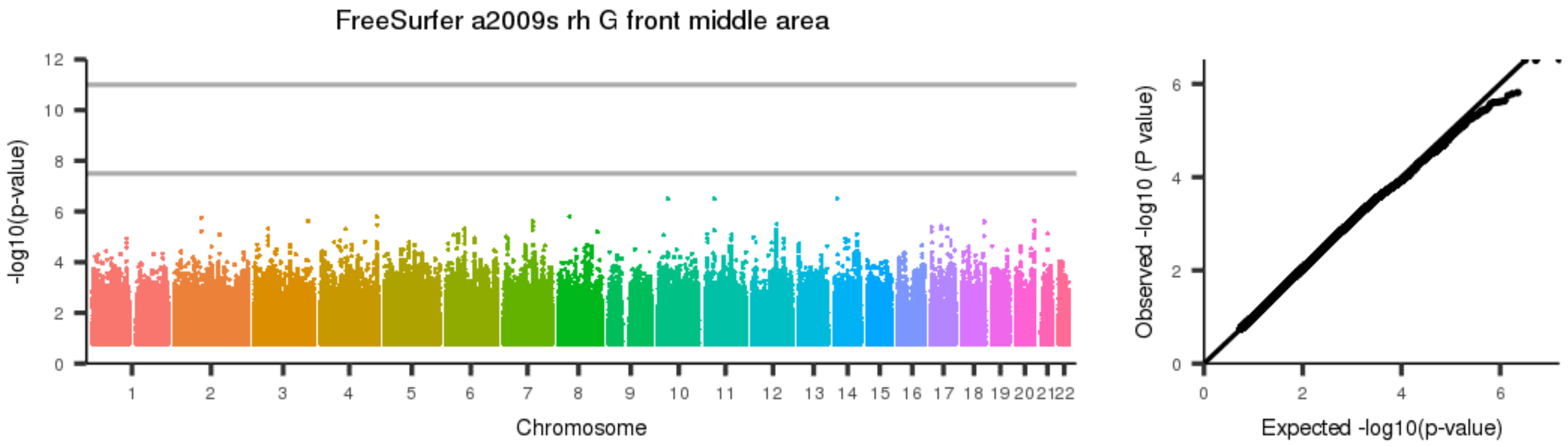

back to list of plots

Figure 1173: FreeSurfer a2009s rh G front sup area

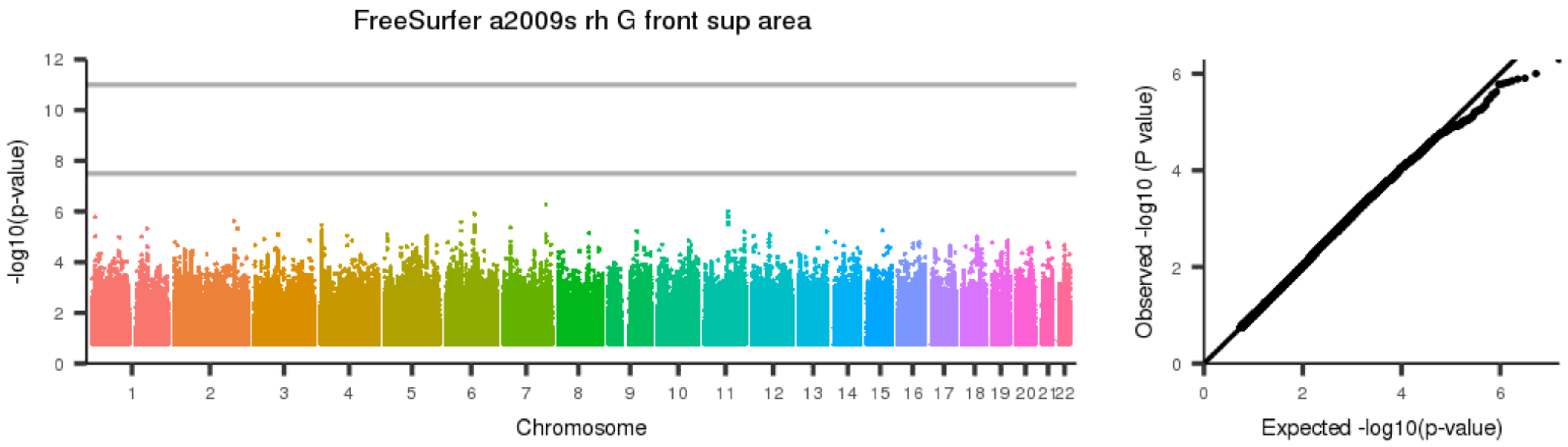

back to list of plots

Figure 1174: FreeSurfer a2009s rh G Ins lg and S cent ins area

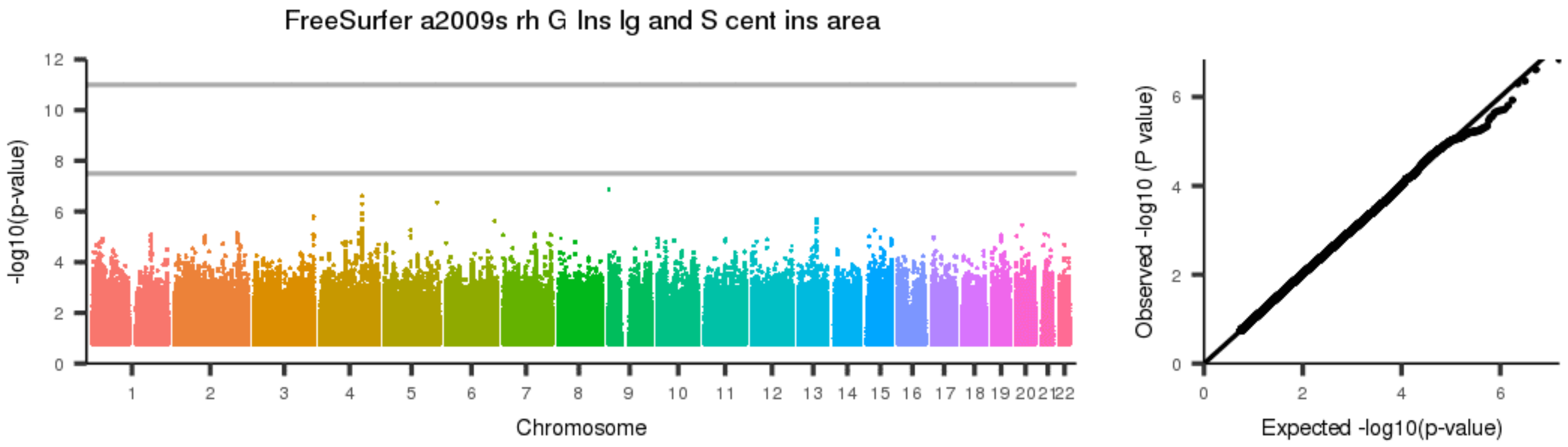

back to list of plots

Figure 1175: FreeSurfer a2009s rh G insular short area

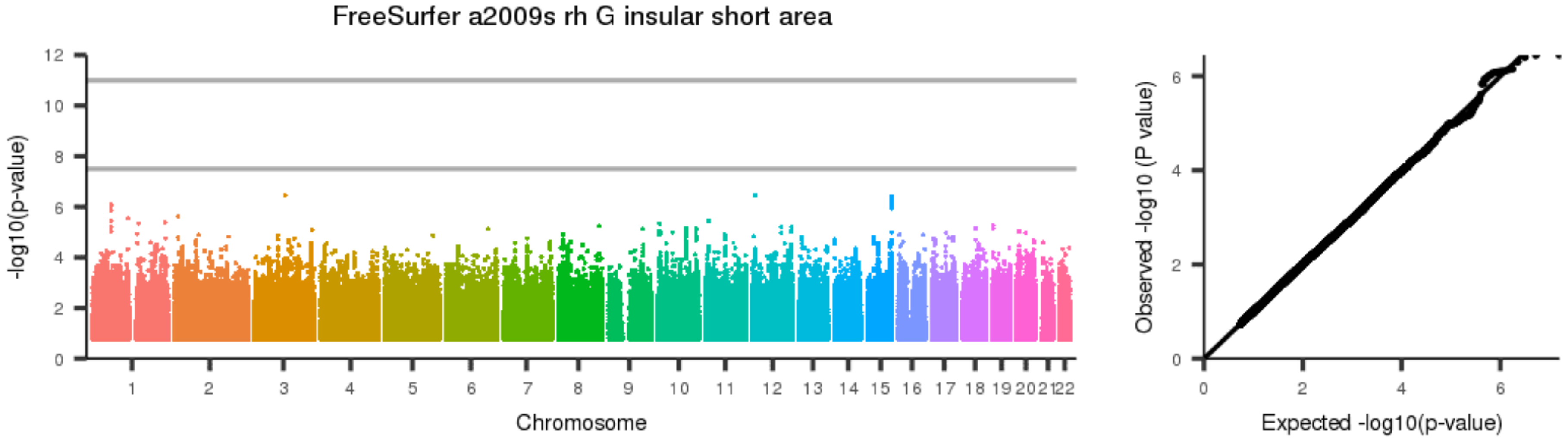

back to list of plots

Figure 1176: FreeSurfer a2009s rh G occipital middle area

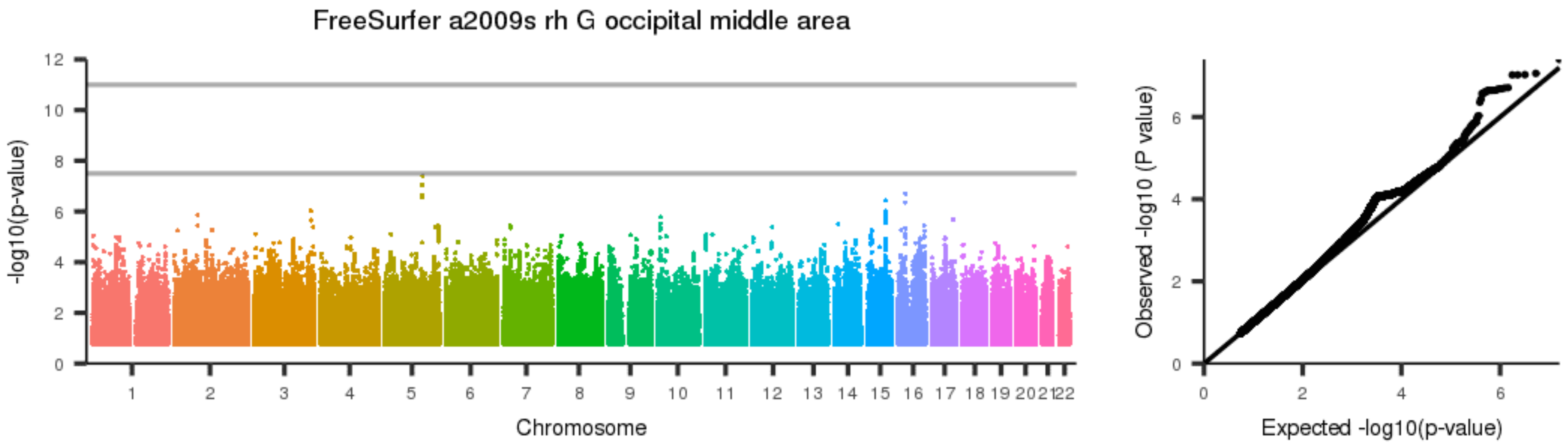

back to list of plots

Figure 1177: FreeSurfer a2009s rh G occipital sup area

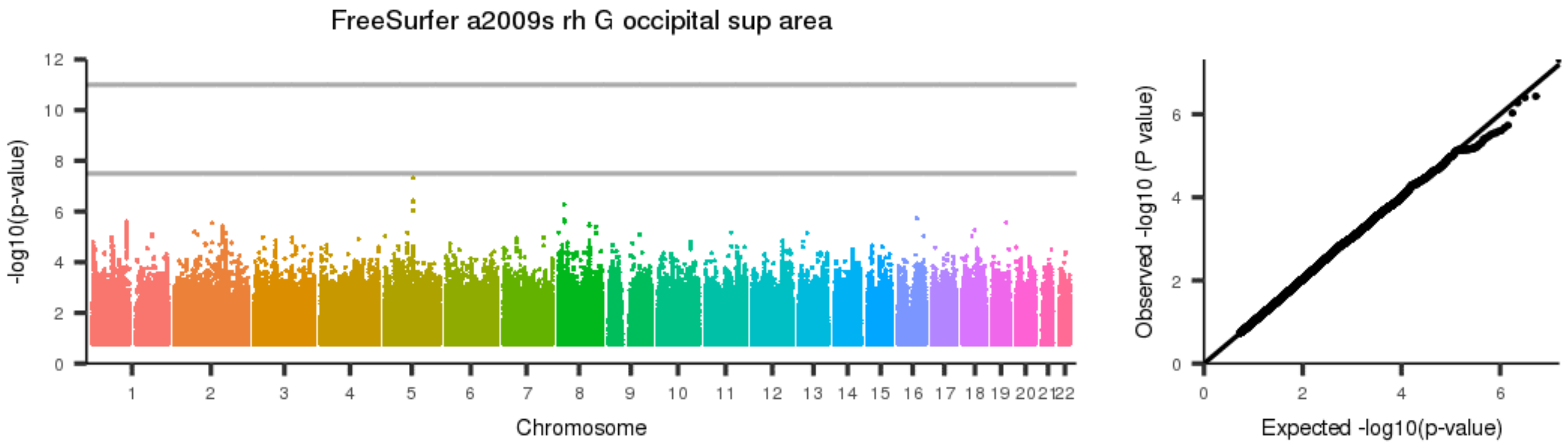

back to list of plots

Figure 1178: FreeSurfer a2009s rh G oc-temp lat-fusifor area

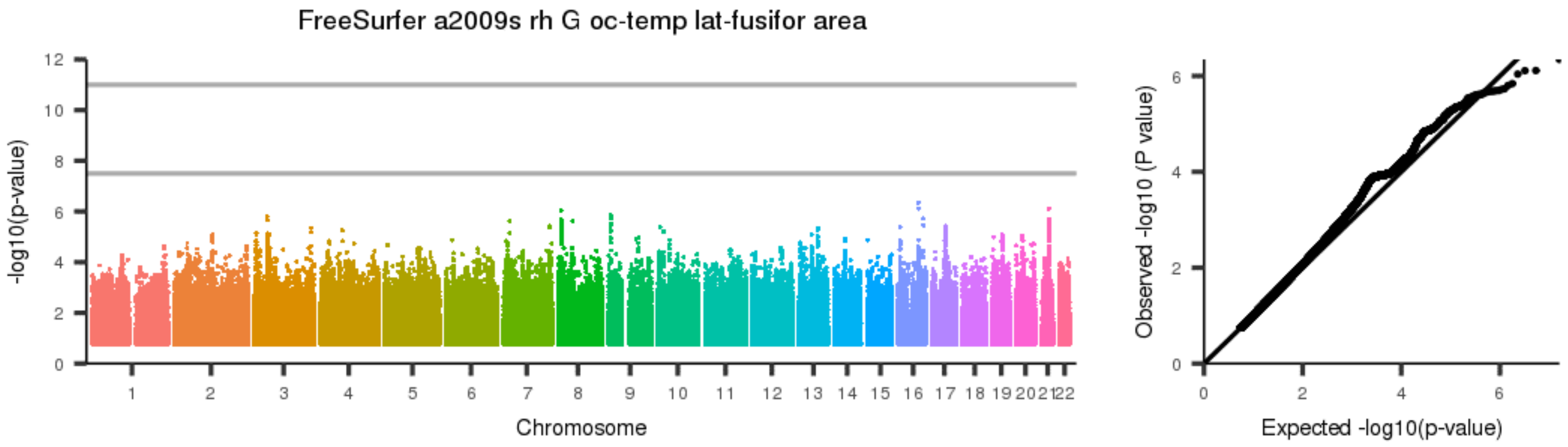

back to list of plots

Figure 1179: FreeSurfer a2009s rh G oc-temp med-Lingual area

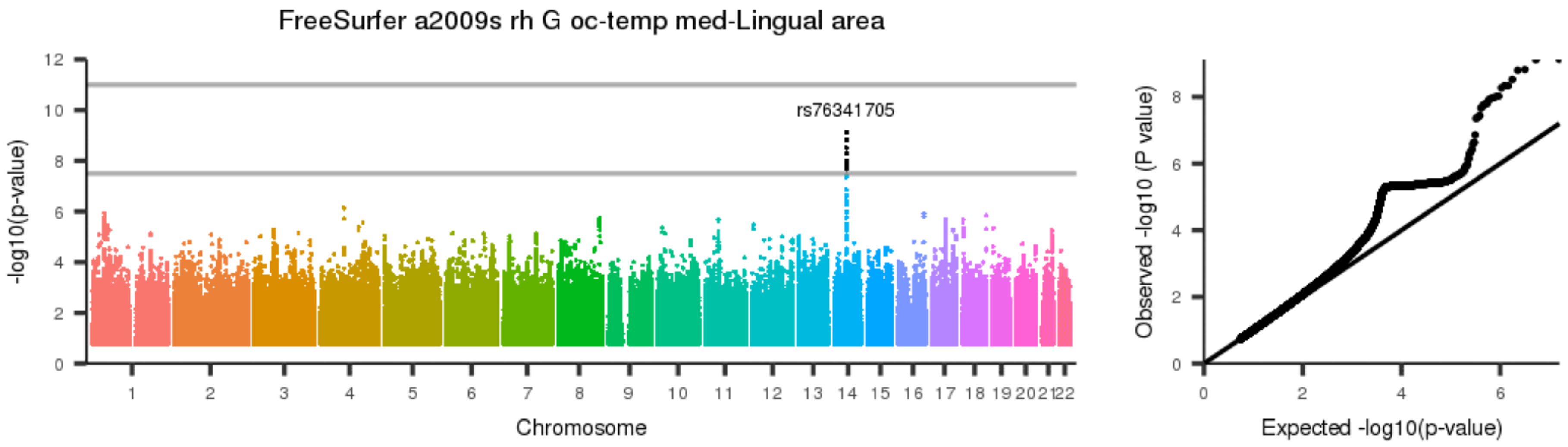

back to list of plots

Figure 1180: FreeSurfer a2009s rh G oc-temp med-Parahip area

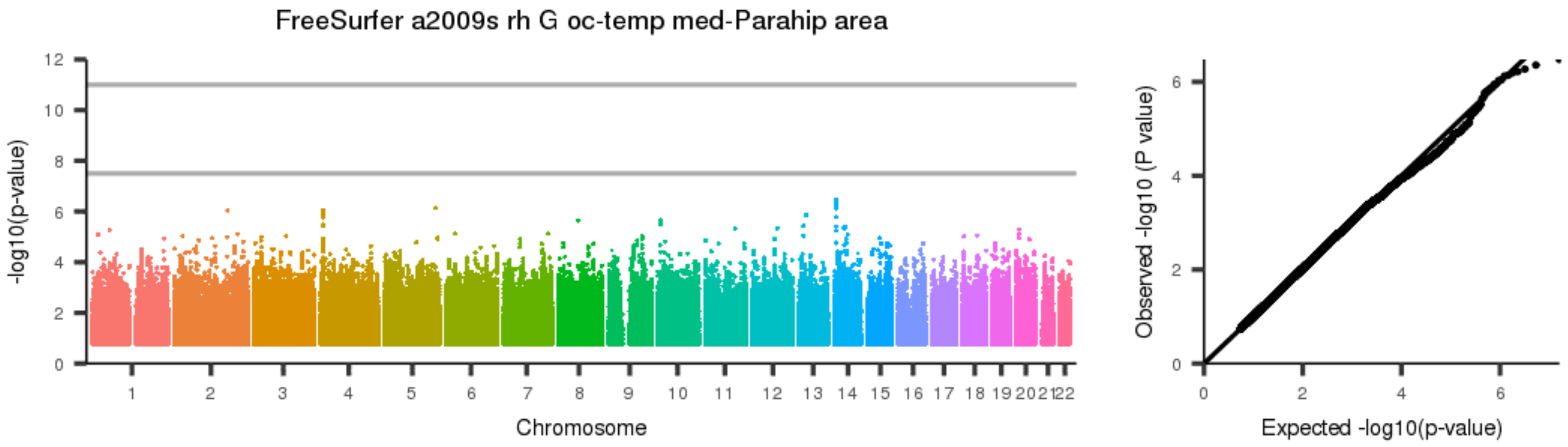

back to list of plots

Figure 1181: FreeSurfer a2009s rh G orbital area

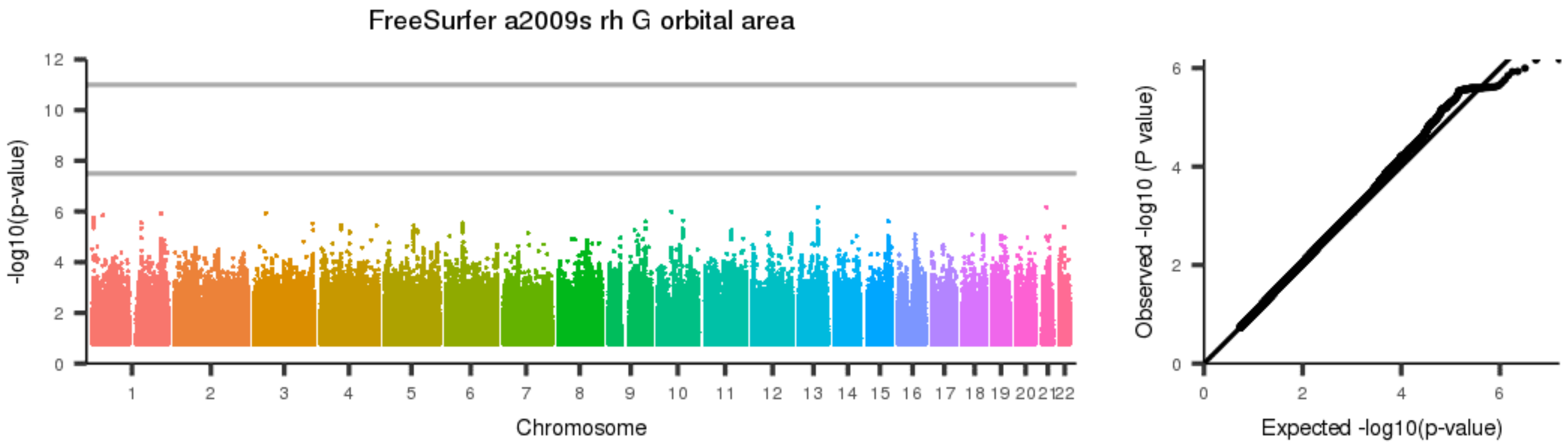

back to list of plots

Figure 1182: FreeSurfer a2009s rh G pariet inf-Angular area

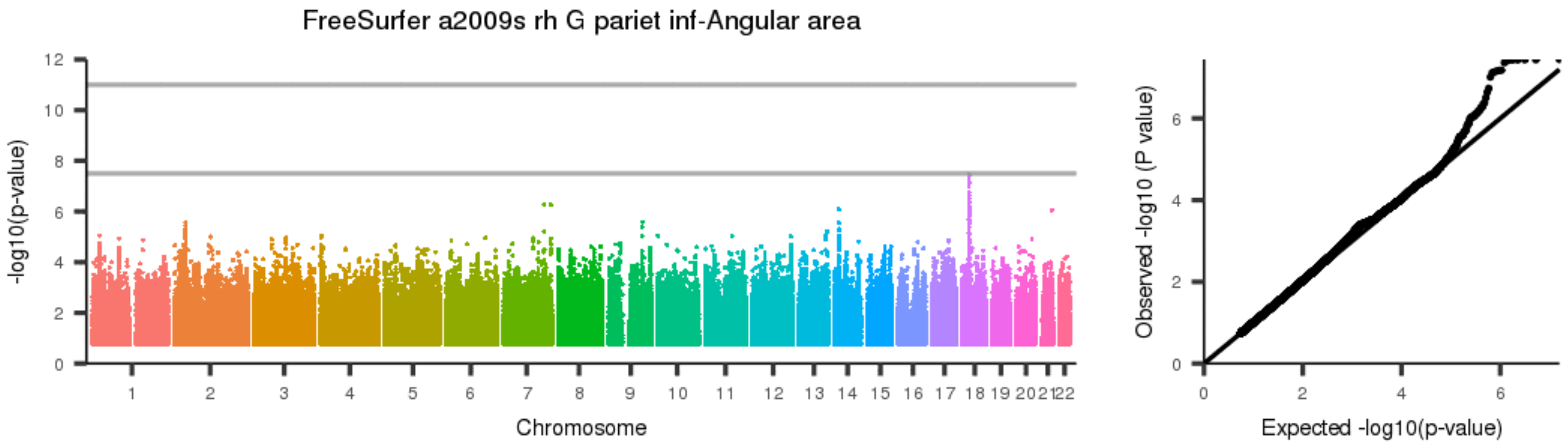

back to list of plots

Figure 1183: FreeSurfer a2009s rh G pariet inf-Supramar area

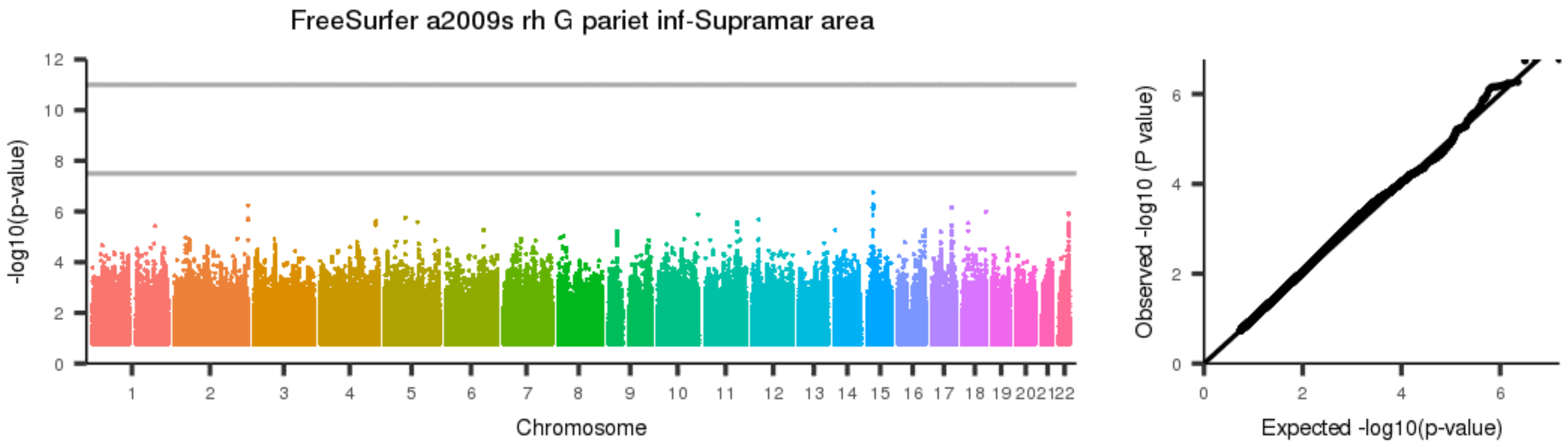

back to list of plots

Figure 1184: FreeSurfer a2009s rh G parietal sup area

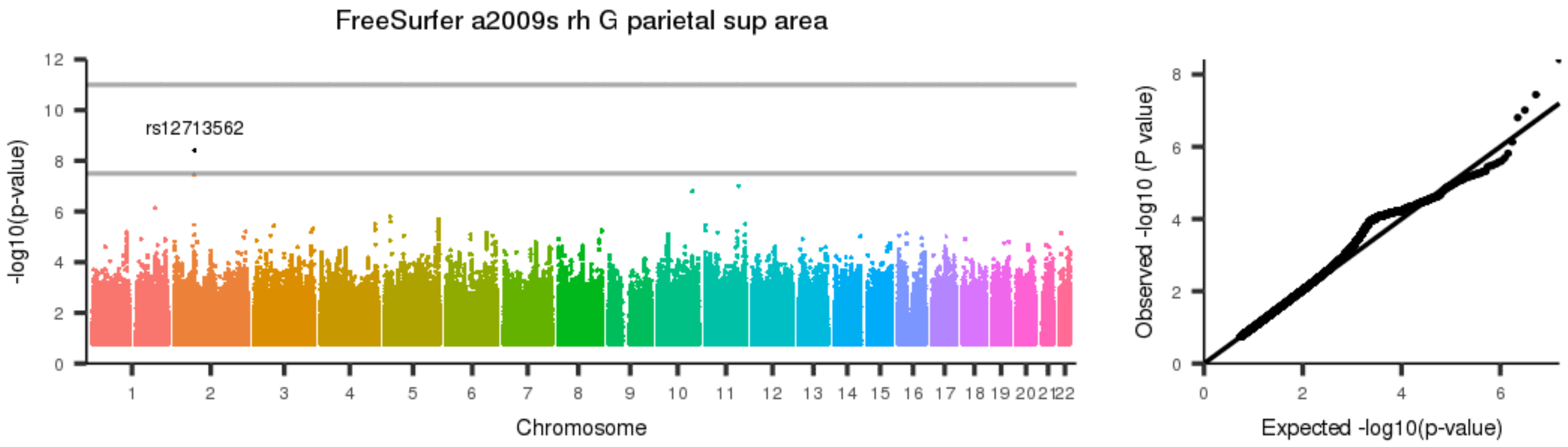

back to list of plots

Figure 1185: FreeSurfer a2009s rh G postcentral area

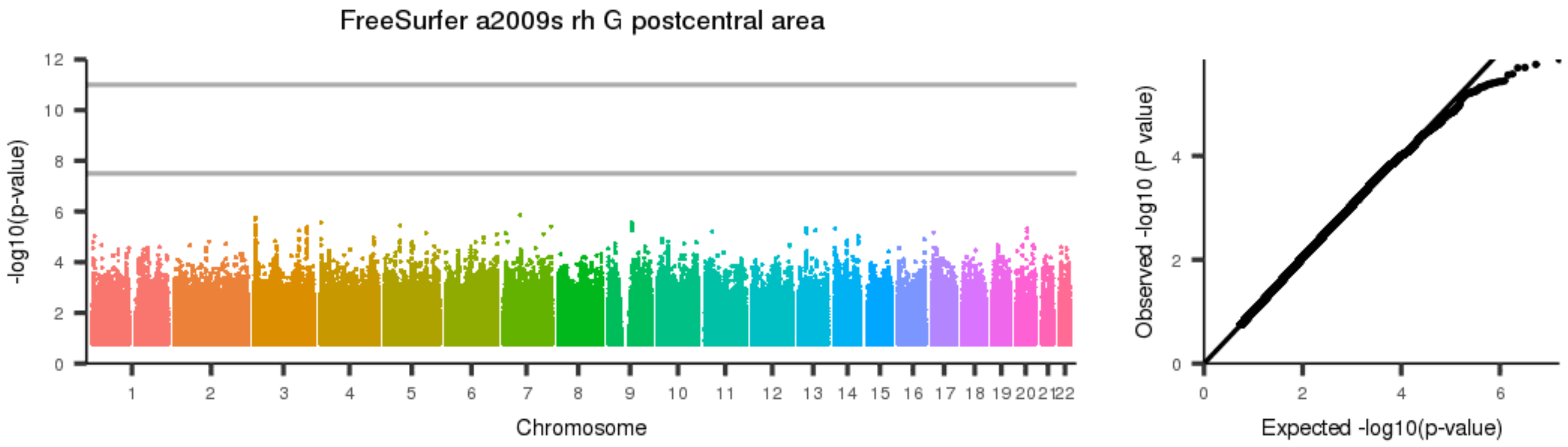

back to list of plots

Figure 1186: FreeSurfer a2009s rh G precentral area

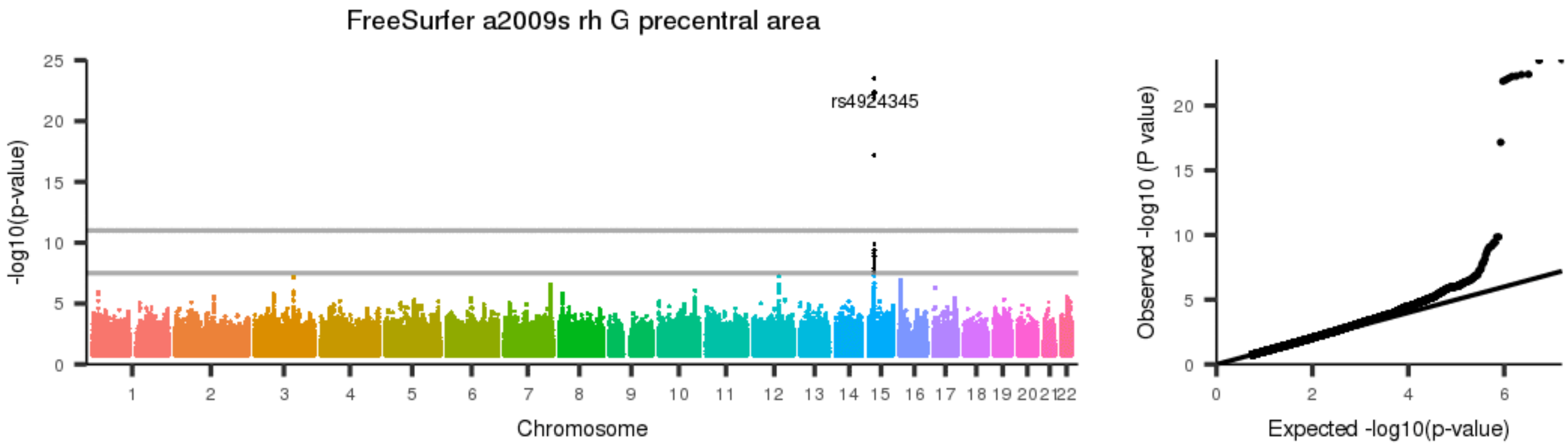

back to list of plots

Figure 1187: FreeSurfer a2009s rh G precuneus area

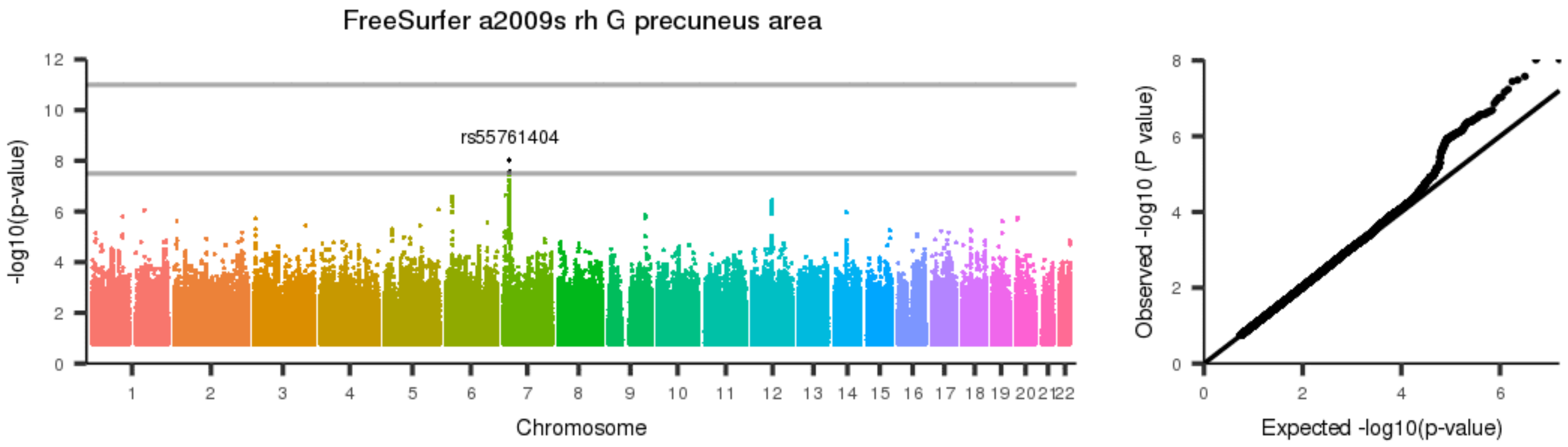

back to list of plots

Figure 1188: FreeSurfer a2009s rh G rectus area

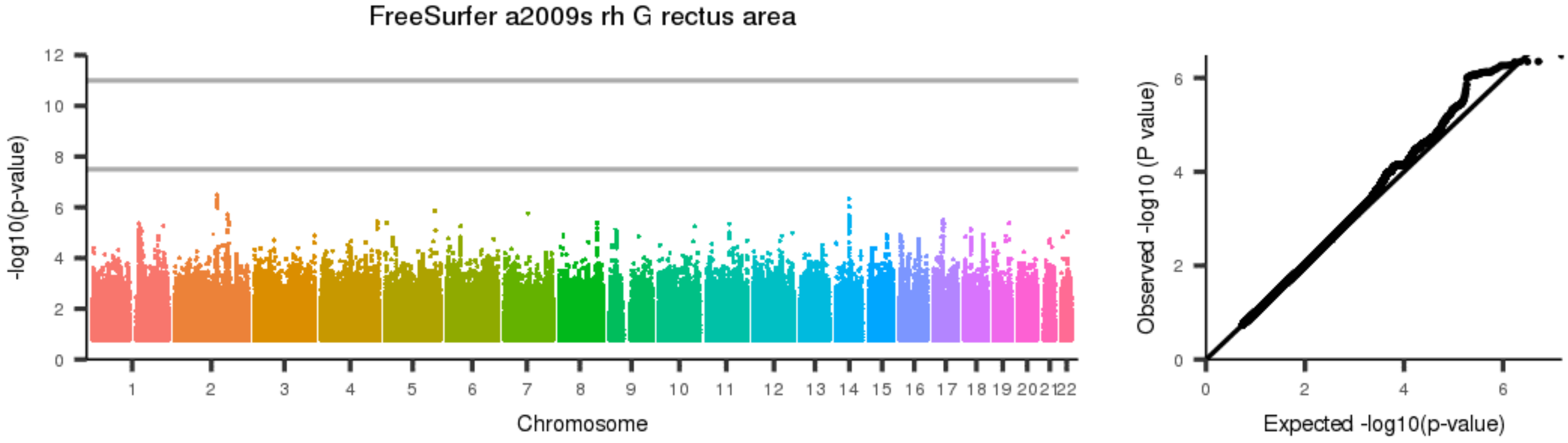

back to list of plots

Figure 1189: FreeSurfer a2009s rh G subcallosal area

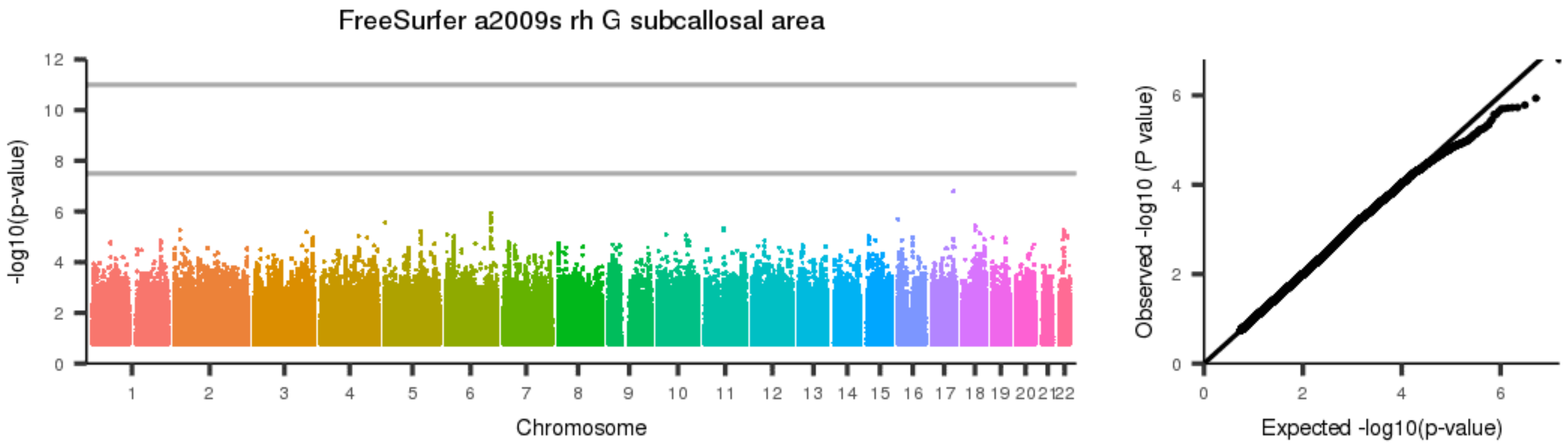

back to list of plots

Figure 1190: FreeSurfer a2009s rh G temp sup-G T transv area

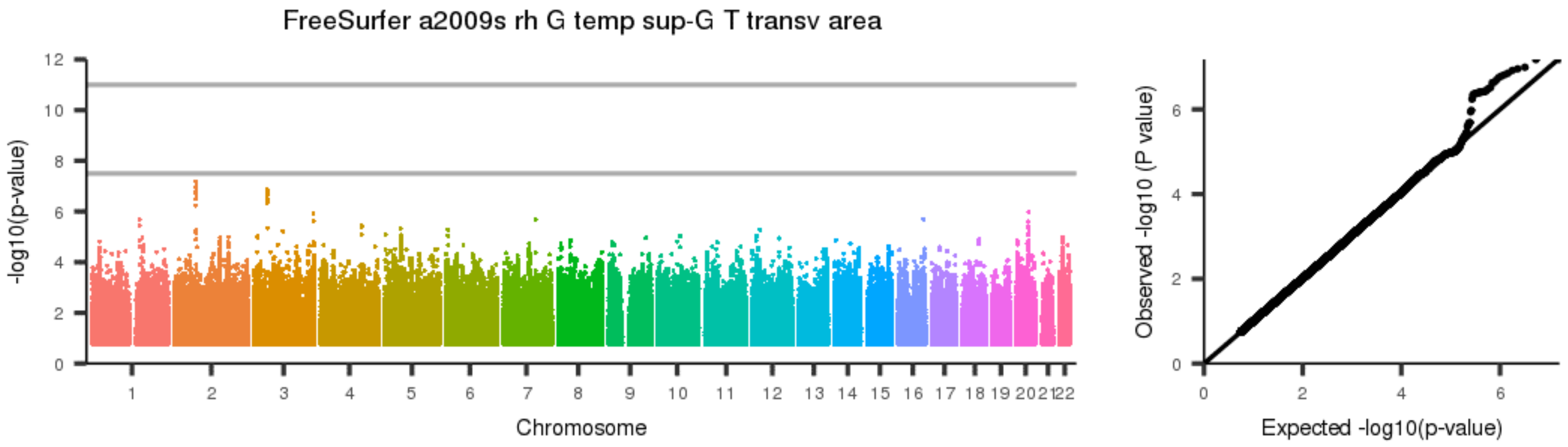

back to list of plots

Figure 1191: FreeSurfer a2009s rh G temp sup-Lateral area

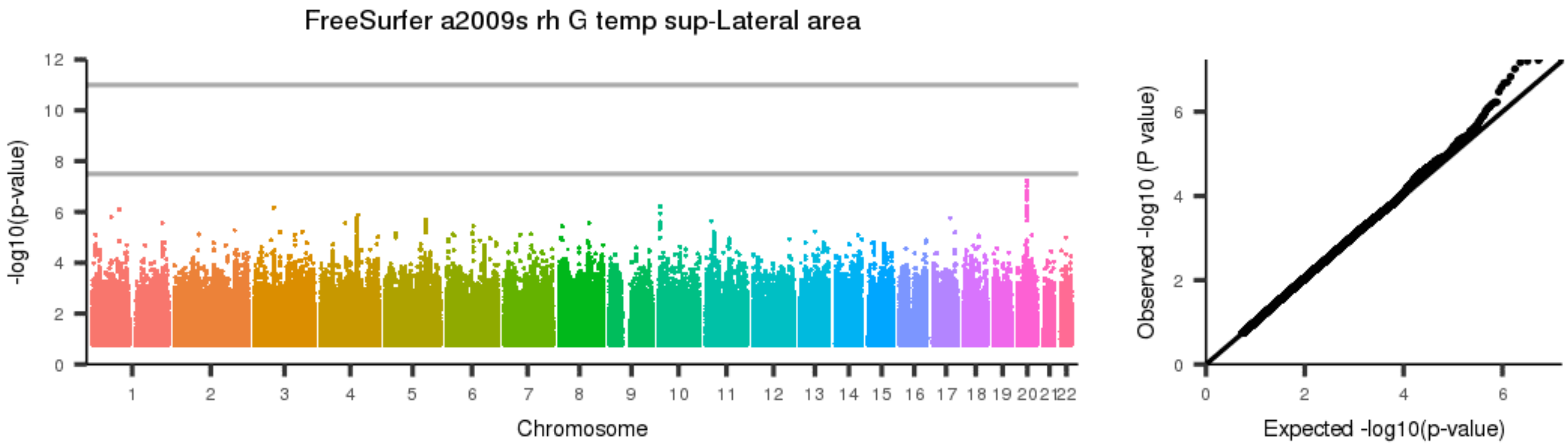

back to list of plots

Figure 1192: FreeSurfer a2009s rh G temp sup-Plan polar area

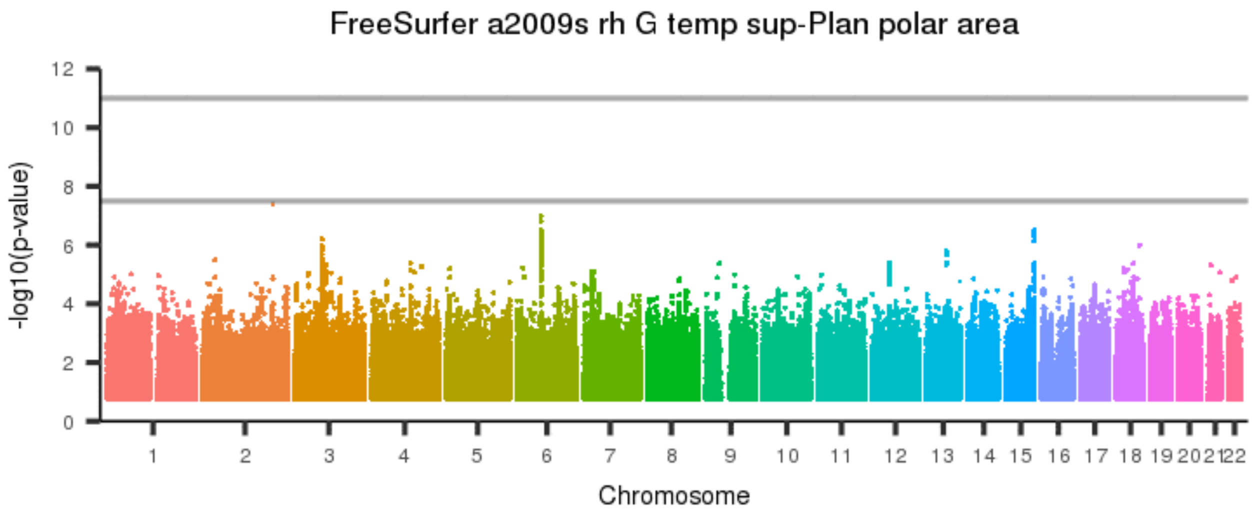

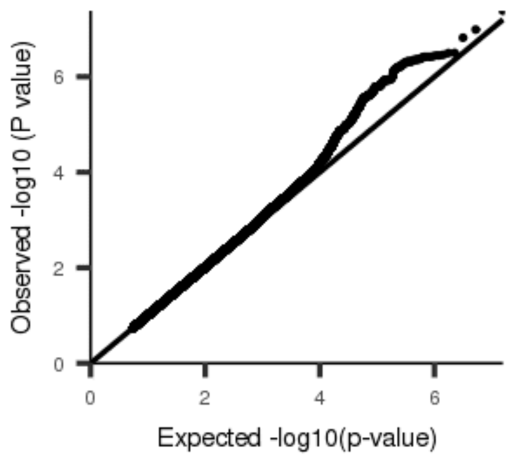

back to list of plots

Figure 1193: FreeSurfer a2009s rh G temp sup-Plan tempo area

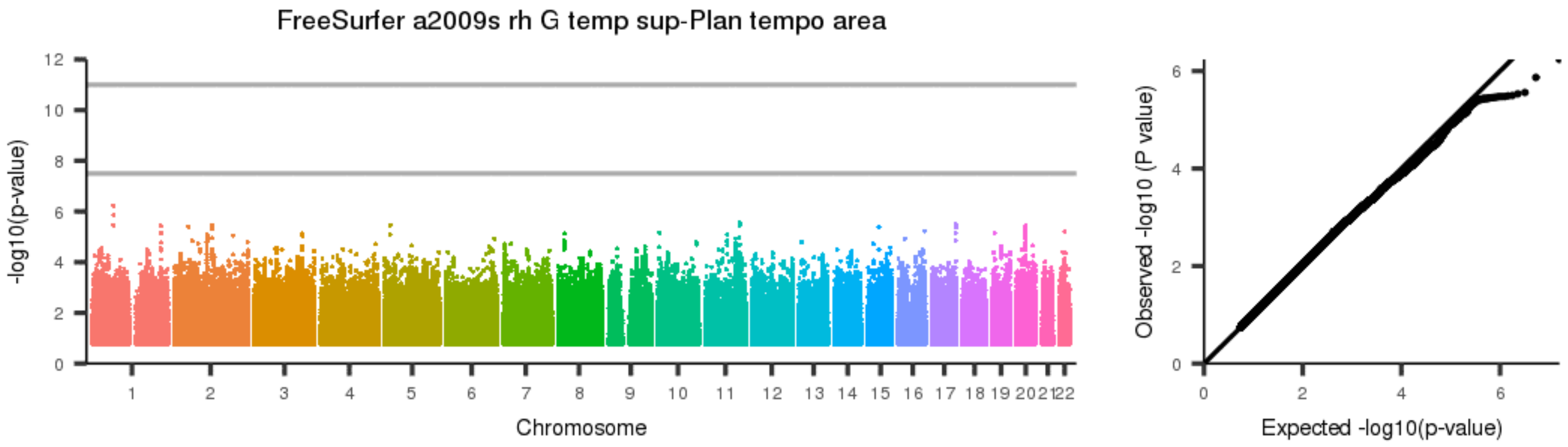

back to list of plots

Figure 1194: FreeSurfer a2009s rh G temporal inf area

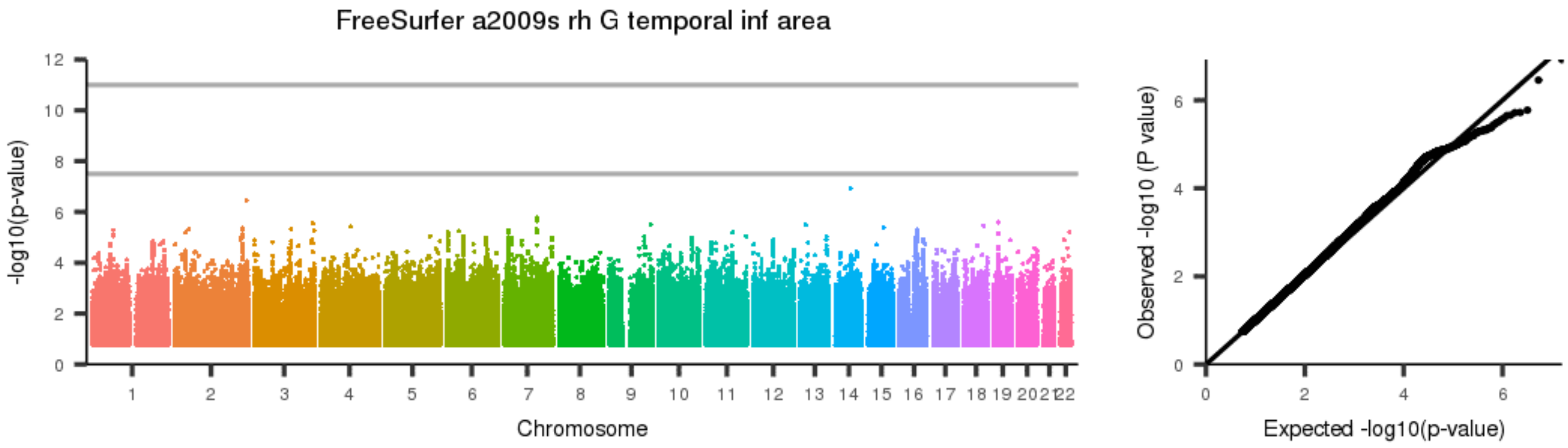

back to list of plots

Figure 1195: FreeSurfer a2009s rh G temporal middle area

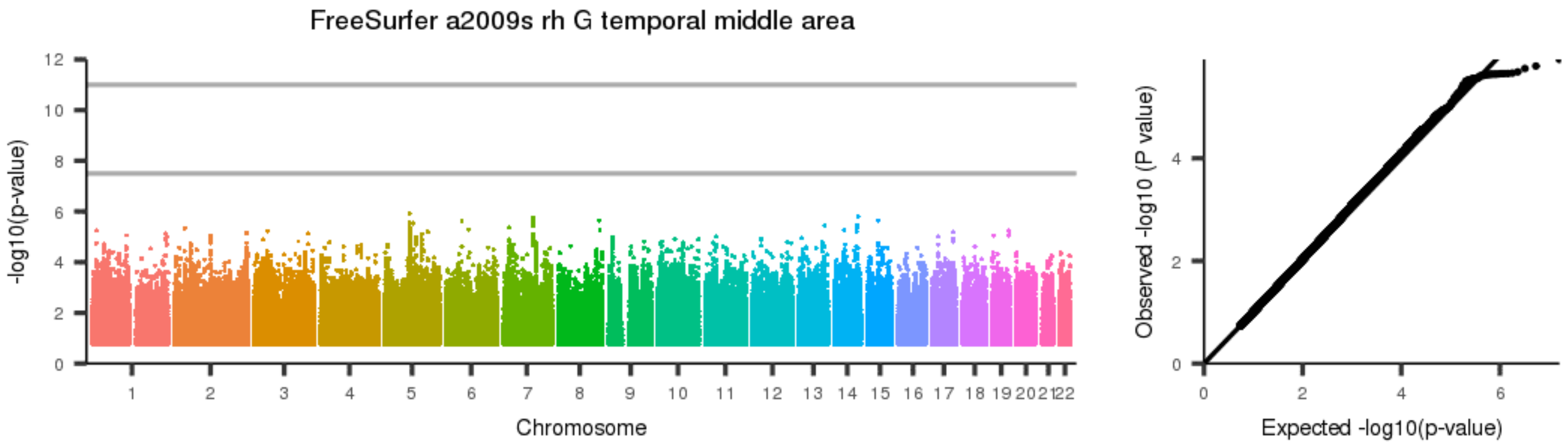

back to list of plots

Figure 1196: FreeSurfer a2009s rh Lat Fis-ant-Horizont area

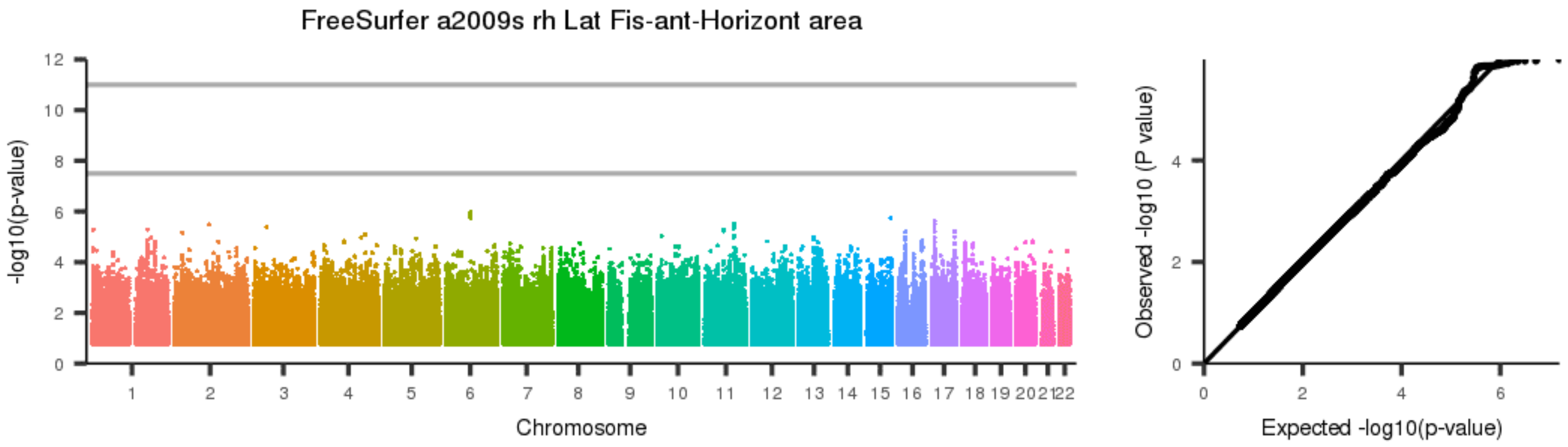

back to list of plots

Figure 1197: FreeSurfer a2009s rh Lat Fis-ant-Vertical area

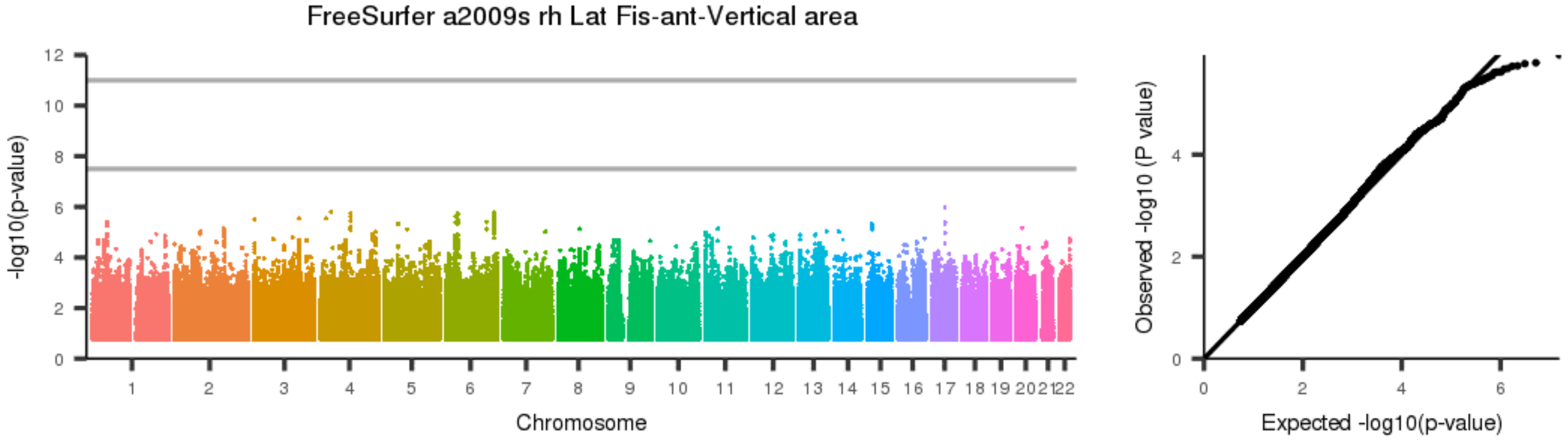

back to list of plots

Figure 1198: FreeSurfer a2009s rh Lat Fis-post area

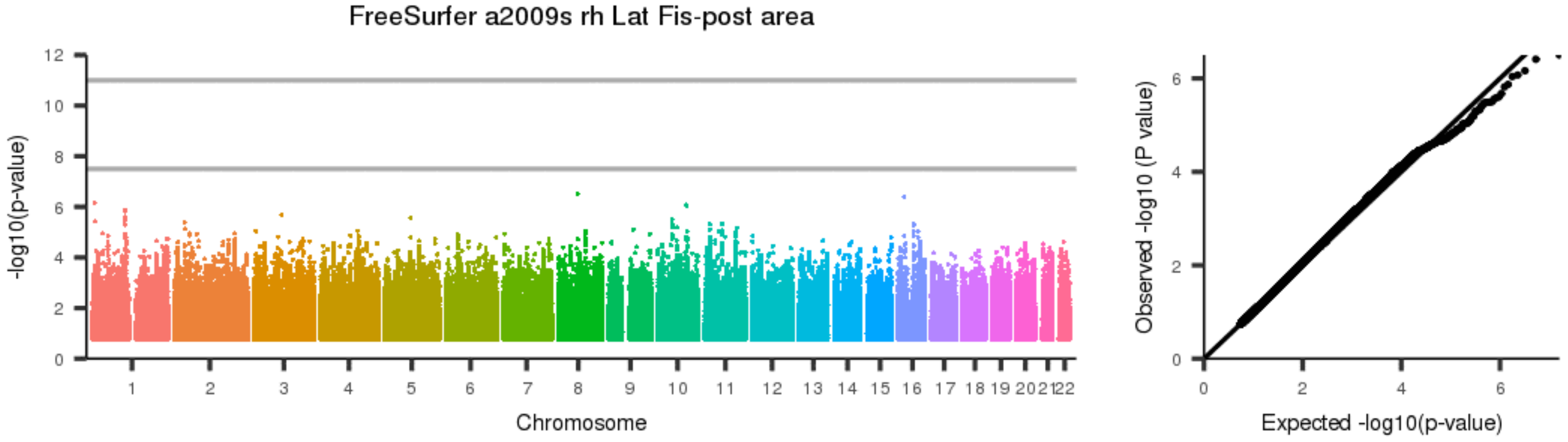

back to list of plots

Figure 1199: FreeSurfer a2009s rh Pole occipital area

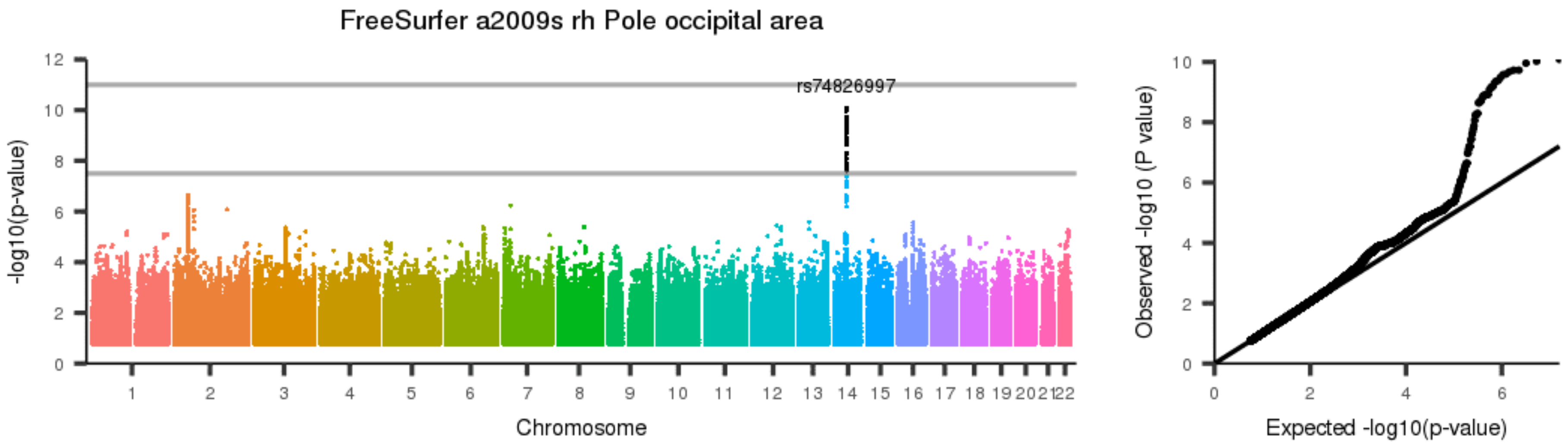

back to list of plots

Figure 1200: FreeSurfer a2009s rh Pole temporal area

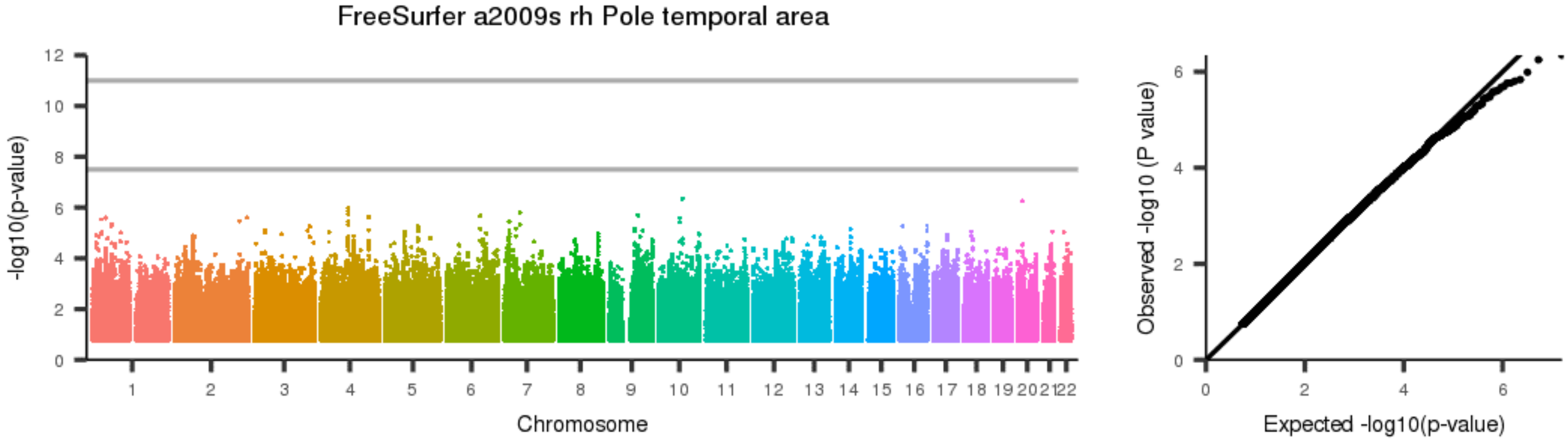

back to list of plots

Figure 1201: FreeSurfer a2009s rh S calcarine area

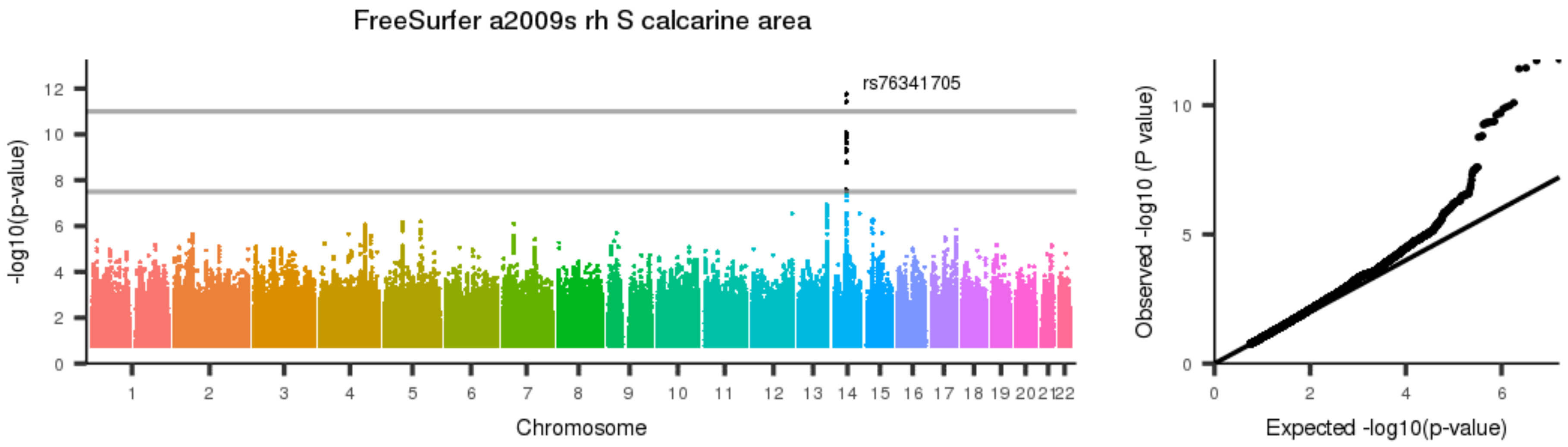

back to list of plots

Figure 1202: FreeSurfer a2009s rh S central area

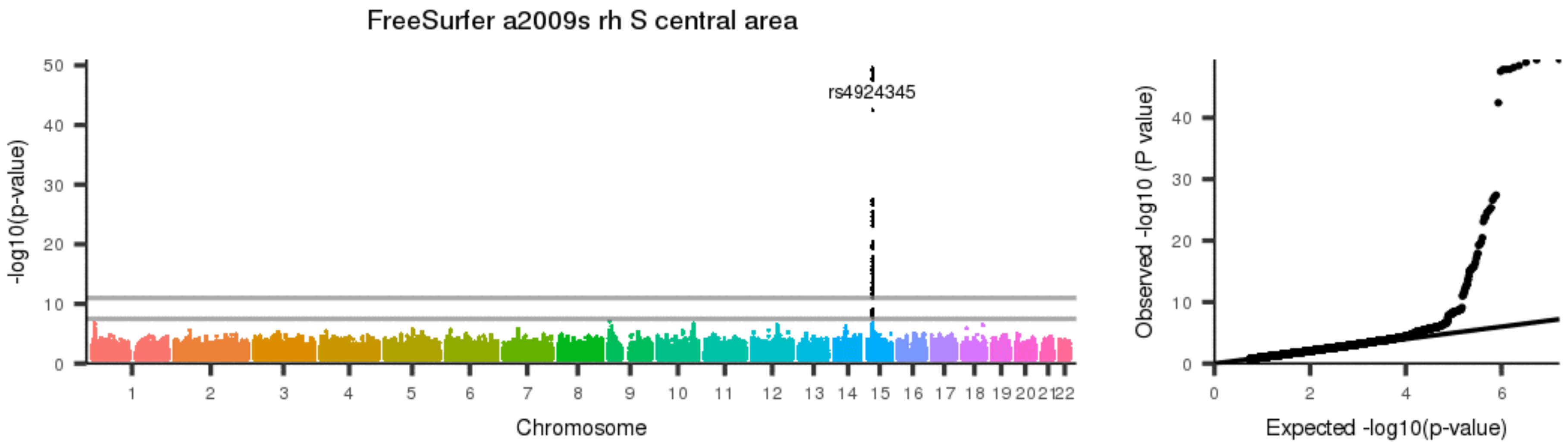

back to list of plots

Figure 1203: FreeSurfer a2009s rh S cingul-Marginalis area

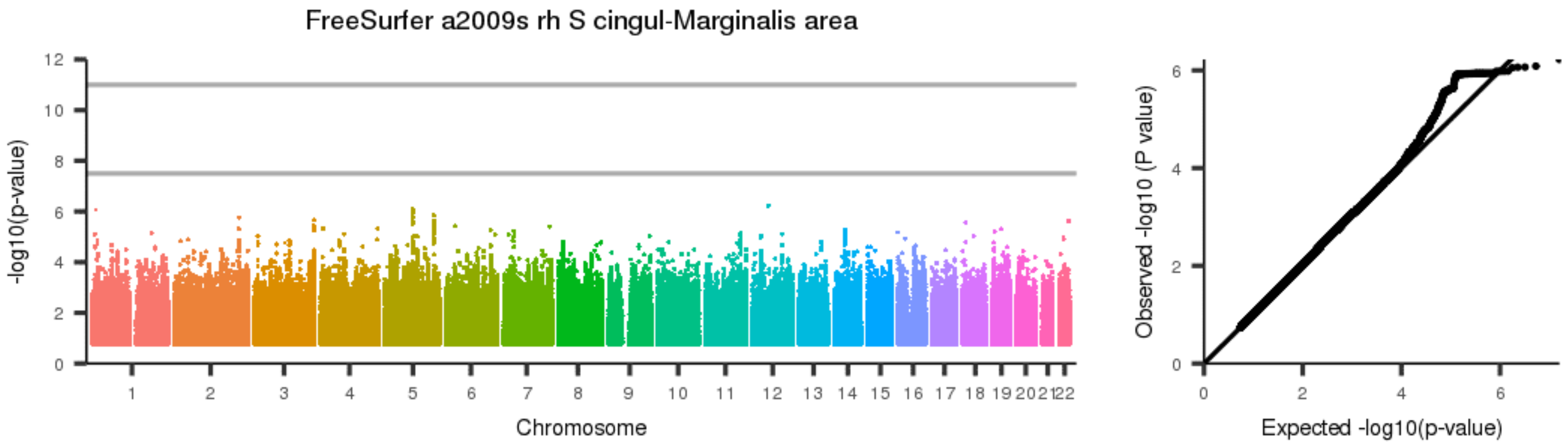

back to list of plots

Figure 1204: FreeSurfer a2009s rh S circular insula ant area

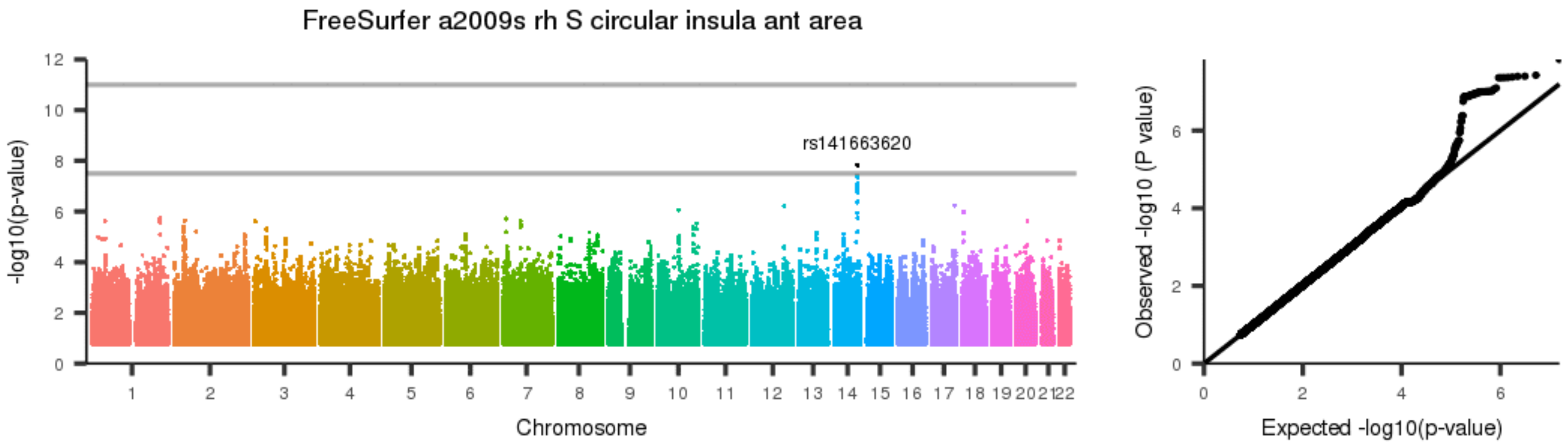

back to list of plots

Figure 1205: FreeSurfer a2009s rh S circular insula inf area

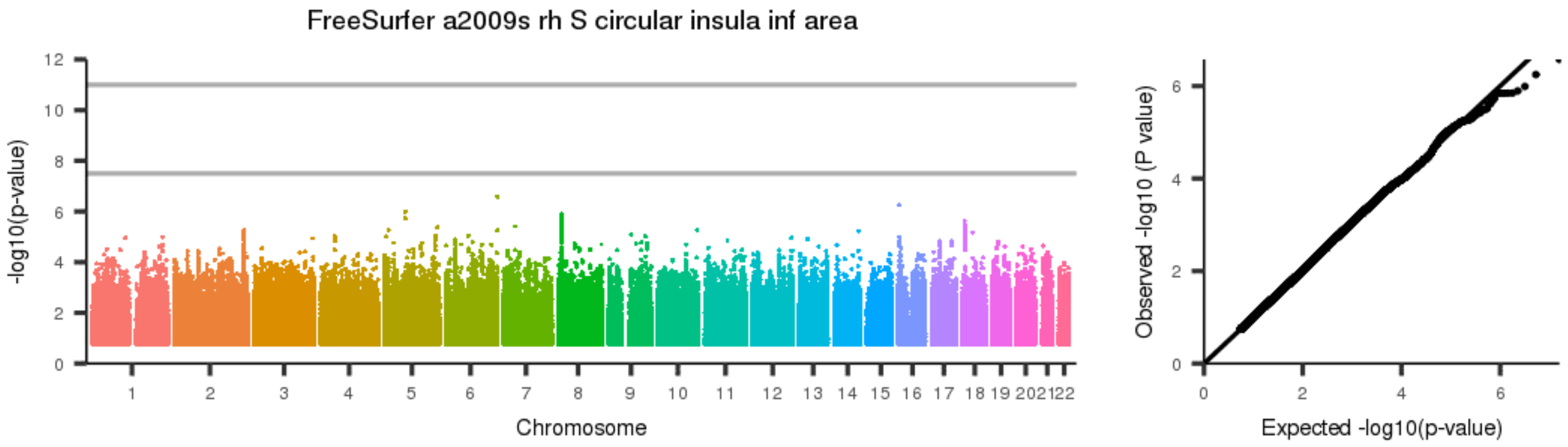

back to list of plots

Figure 1206: FreeSurfer a2009s rh S circular insula sup area

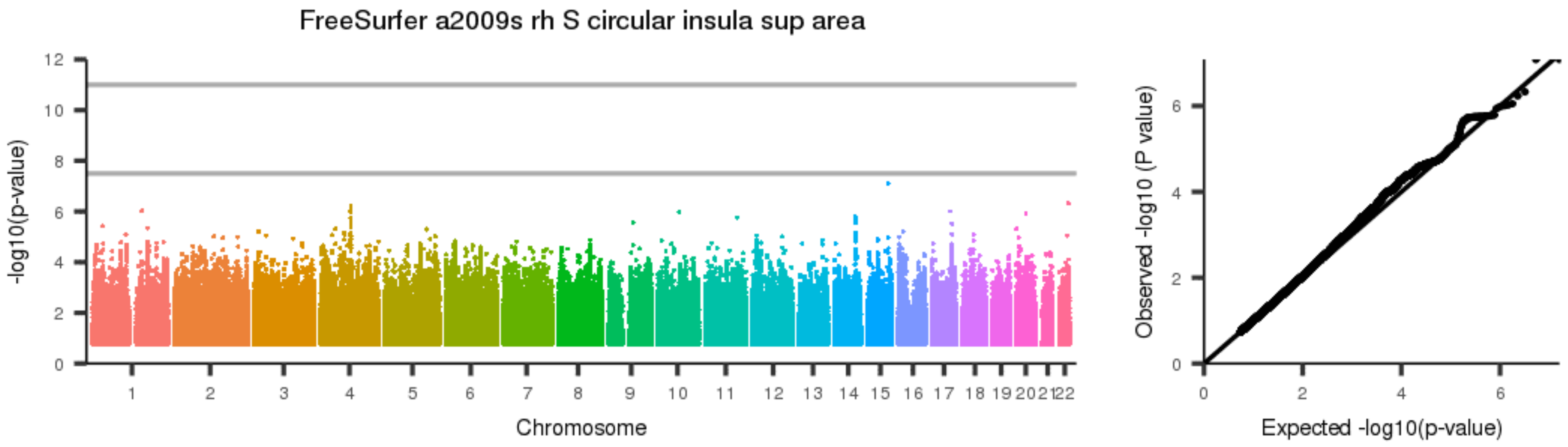

back to list of plots

Figure 1207: FreeSurfer a2009s rh S collat transv ant area

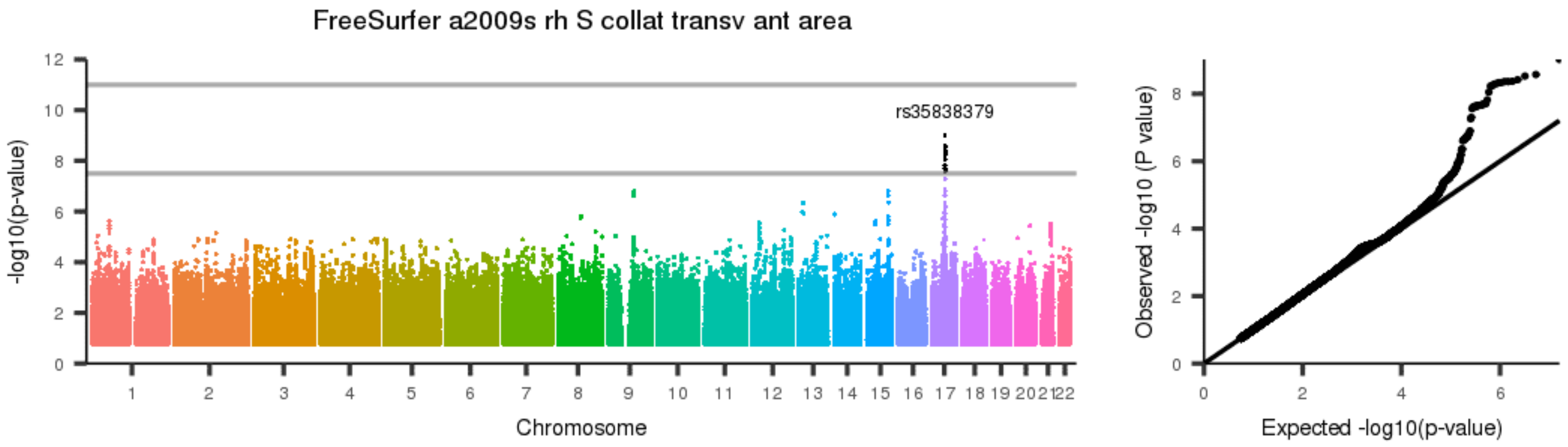

back to list of plots

Figure 1208: FreeSurfer a2009s rh S collat transv post area

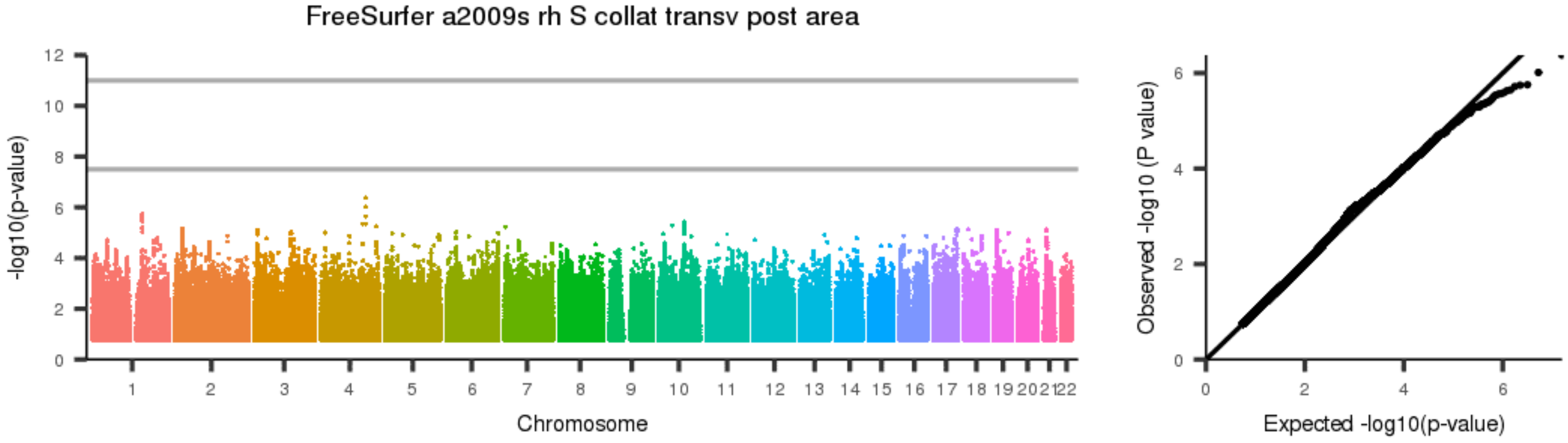

back to list of plots

Figure 1209: FreeSurfer a2009s rh S front inf area

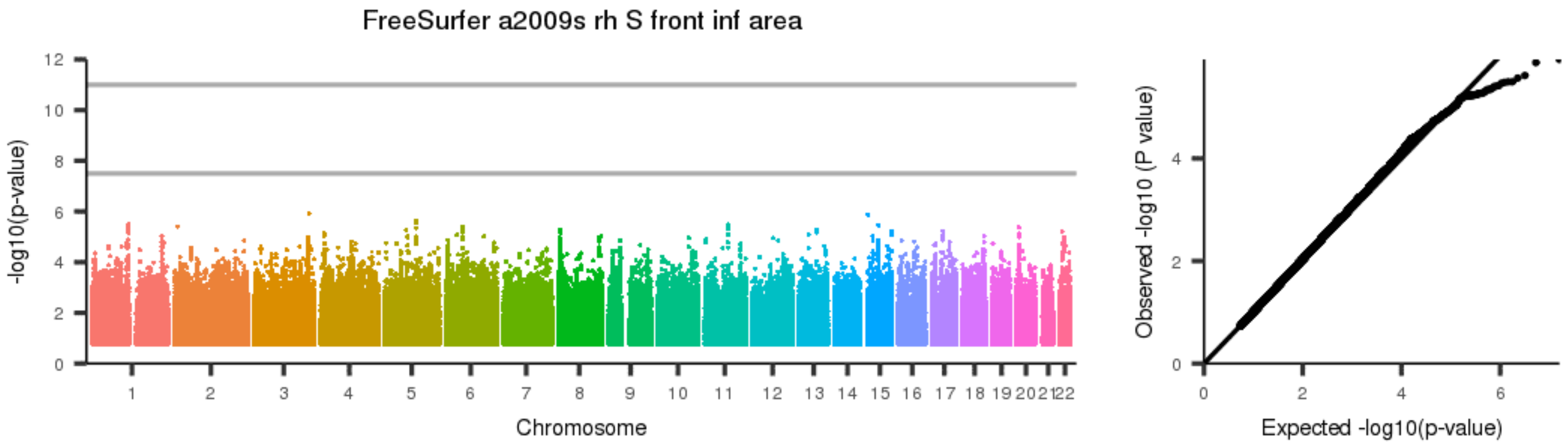

back to list of plots

Figure 1210: FreeSurfer a2009s rh S front middle area

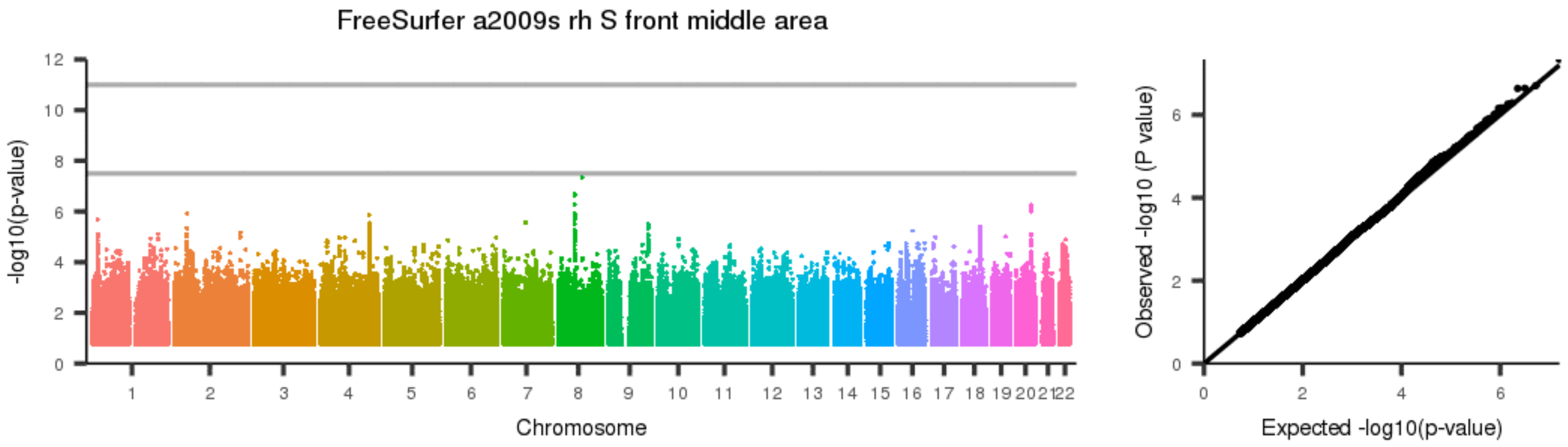

back to list of plots

Figure 1211: FreeSurfer a2009s rh S front sup area

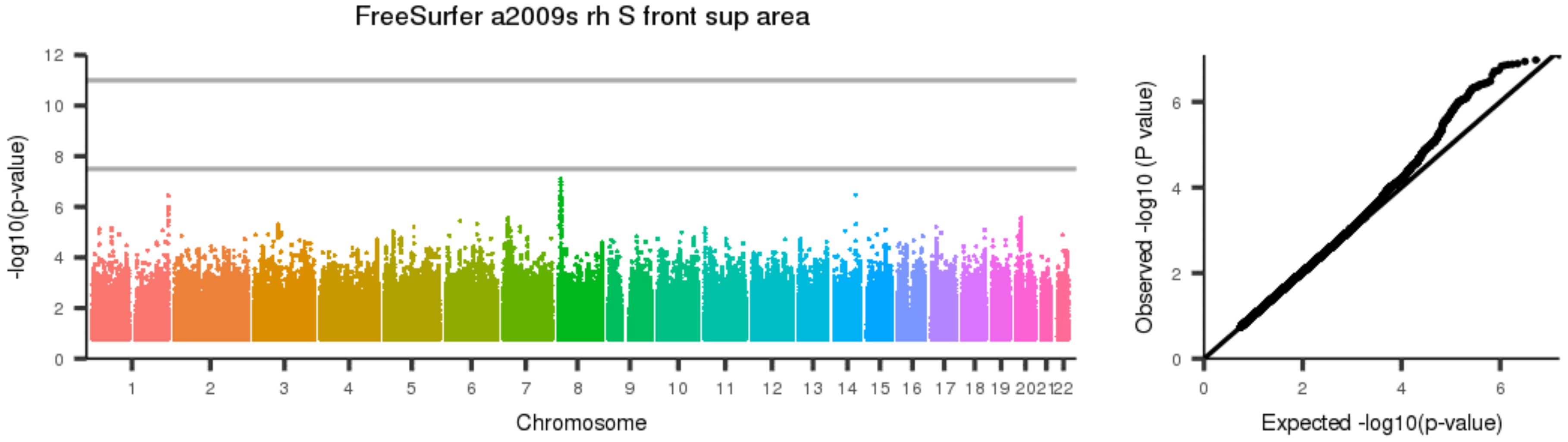

back to list of plots

Figure 1212: FreeSurfer a2009s rh S interm prim-Jensen area

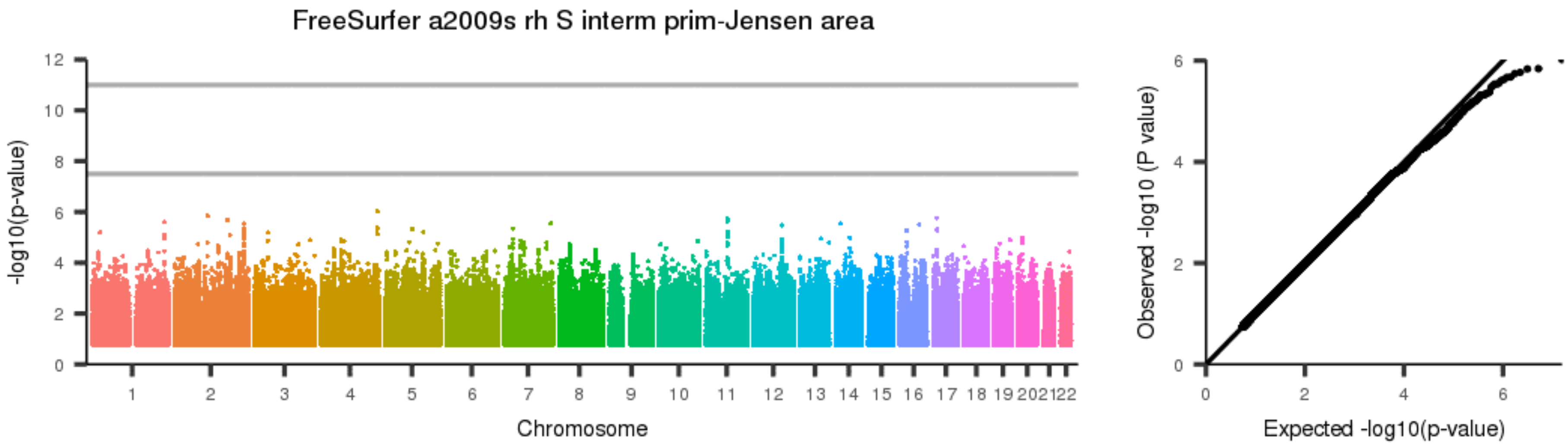

back to list of plots

Figure 1213: FreeSurfer a2009s rh S intrapariet and P trans area

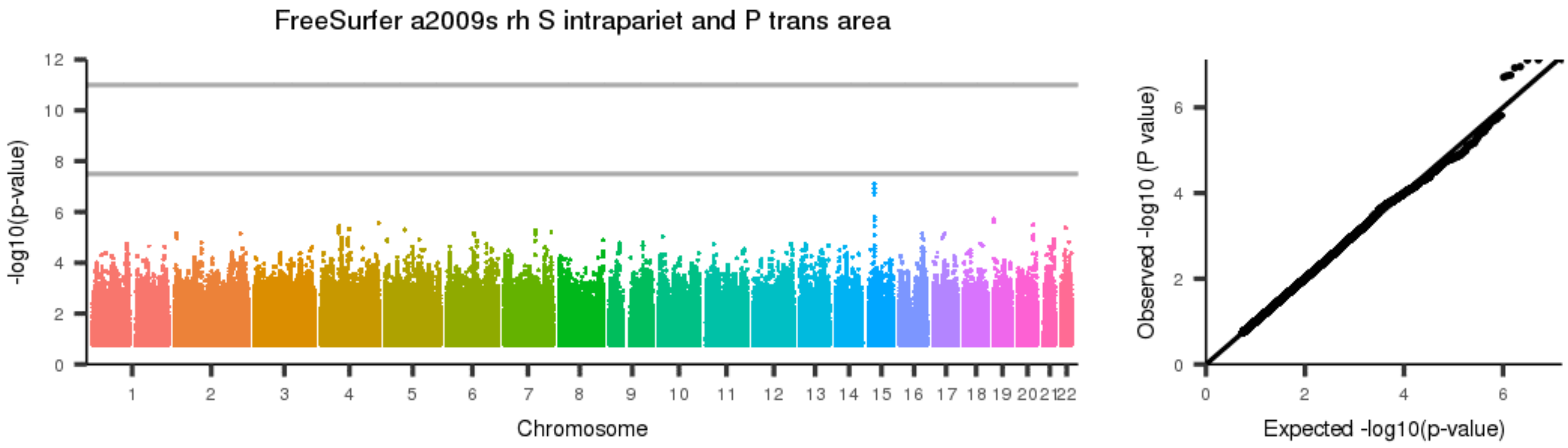

back to list of plots

Figure 1214: FreeSurfer a2009s rh S oc middle and Lunatus area

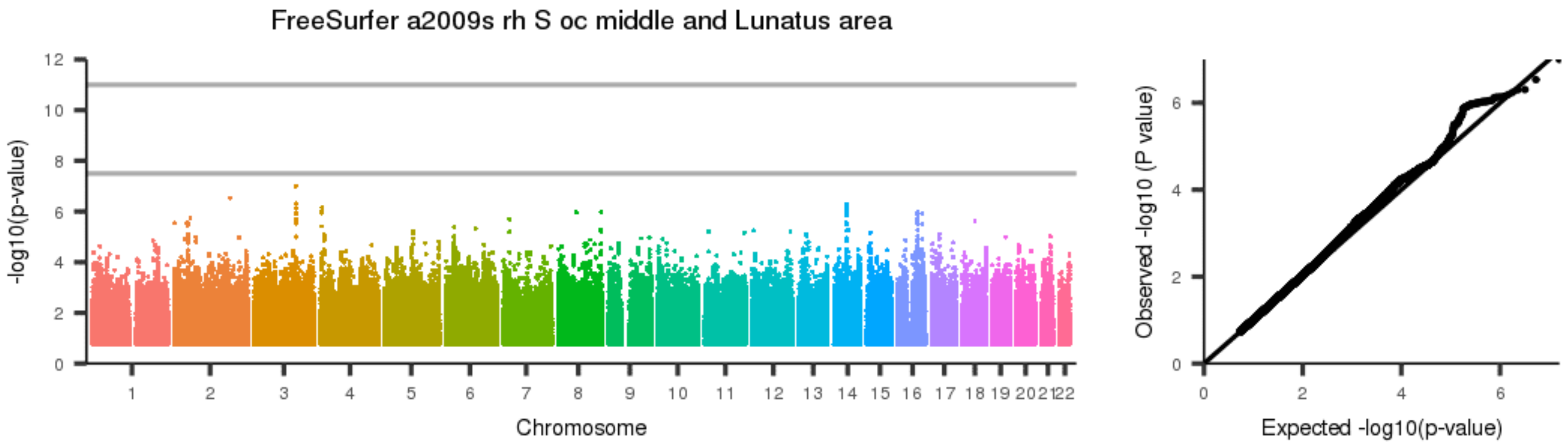

back to list of plots

Figure 1215: FreeSurfer a2009s rh S oc sup and transversal area

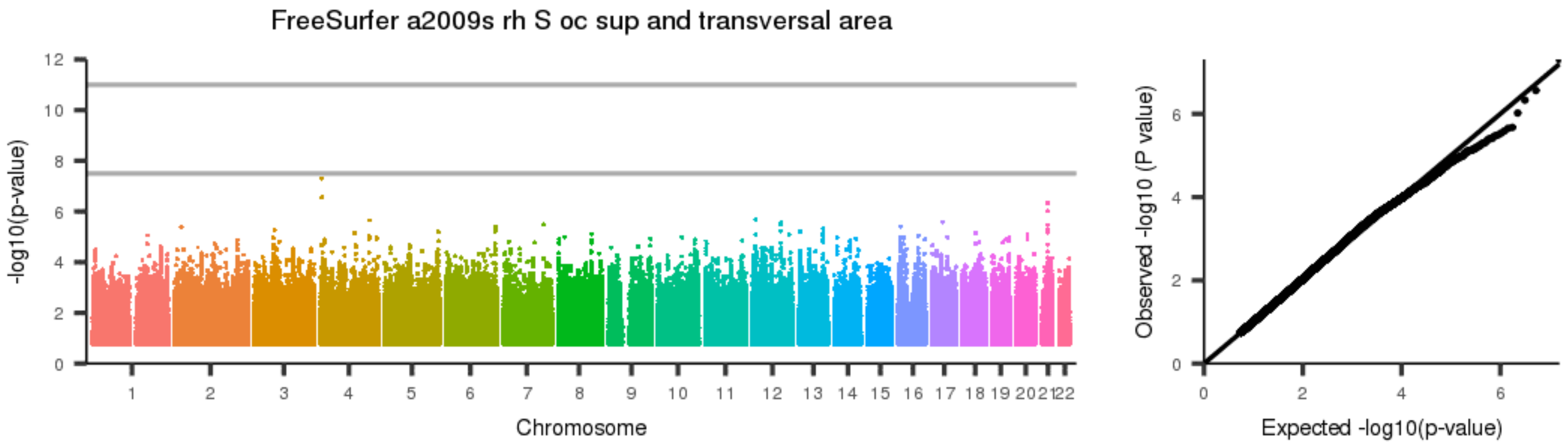

back to list of plots

Figure 1216: FreeSurfer a2009s rh S occipital ant area

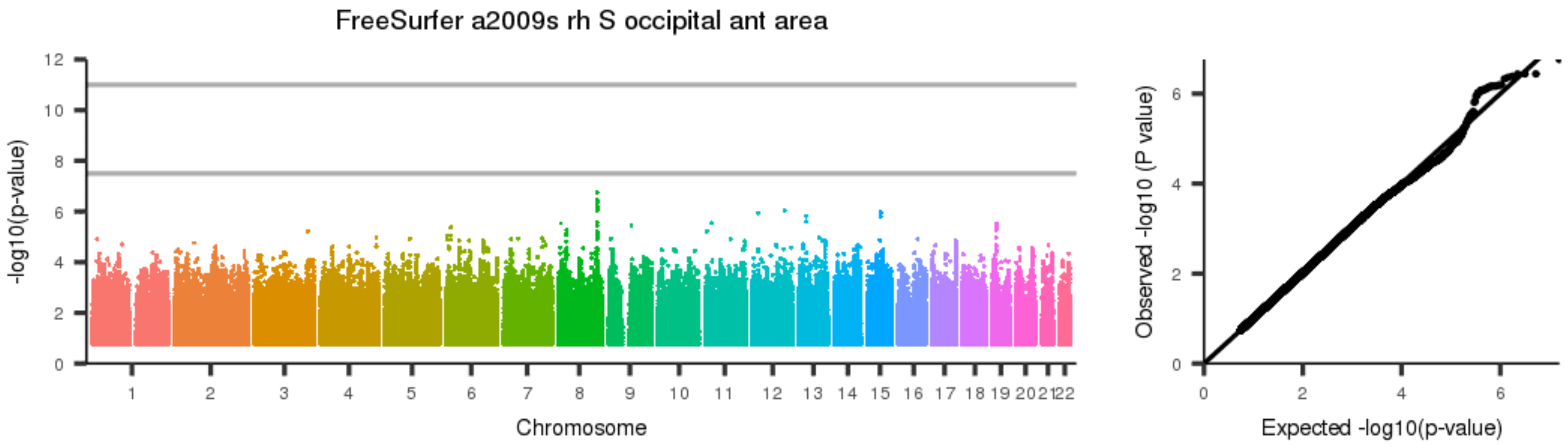

back to list of plots

Figure 1217: FreeSurfer a2009s rh S oc-temp lat area

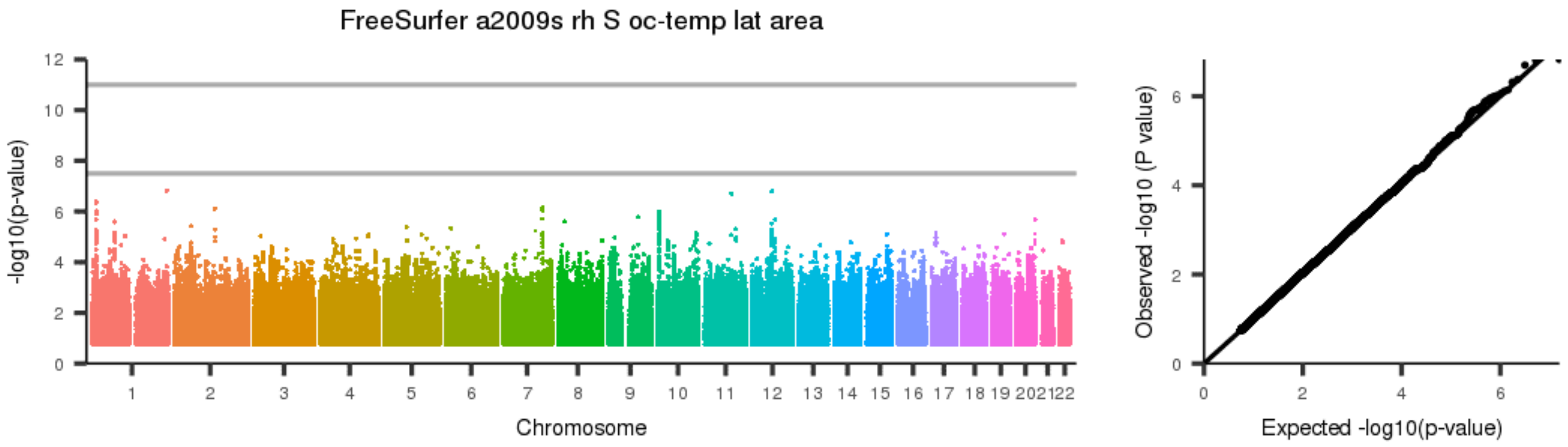

back to list of plots

Figure 1218: FreeSurfer a2009s rh S oc-temp med and Lingual area

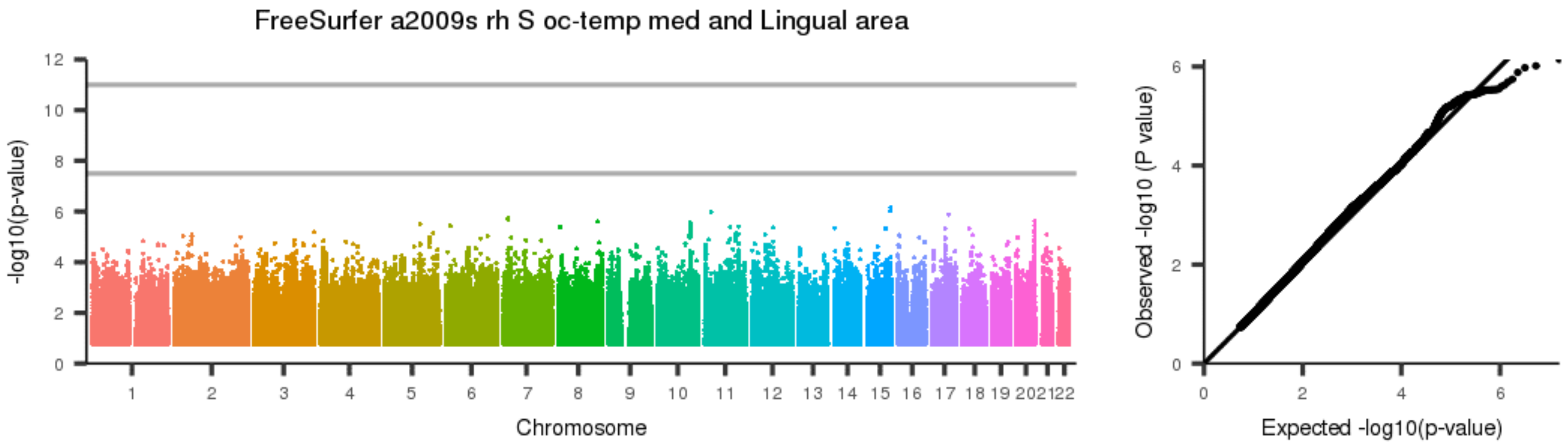

back to list of plots

Figure 1219: FreeSurfer a2009s rh S orbital lateral area

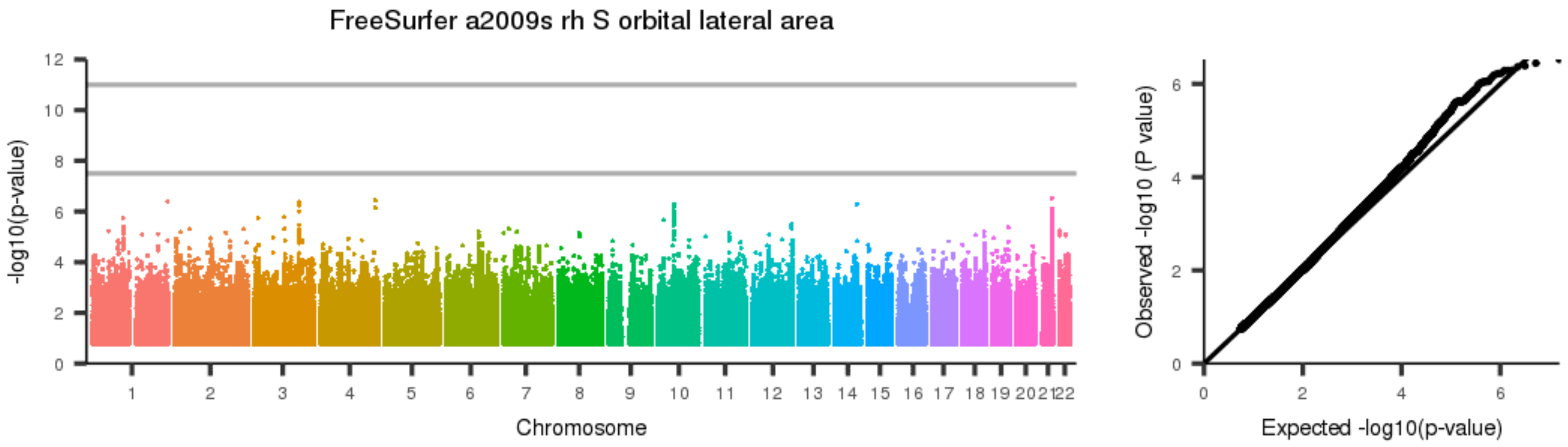

back to list of plots

Figure 1220: FreeSurfer a2009s rh S orbital med-olfact area

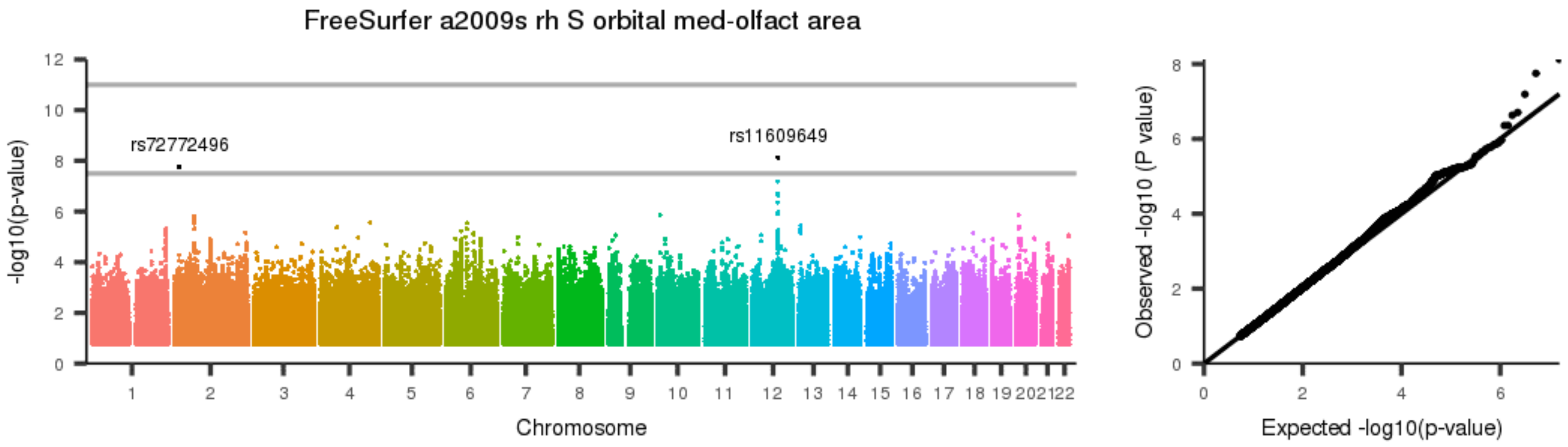

back to list of plots

Figure 1221: FreeSurfer a2009s rh S orbital-H Shaped area

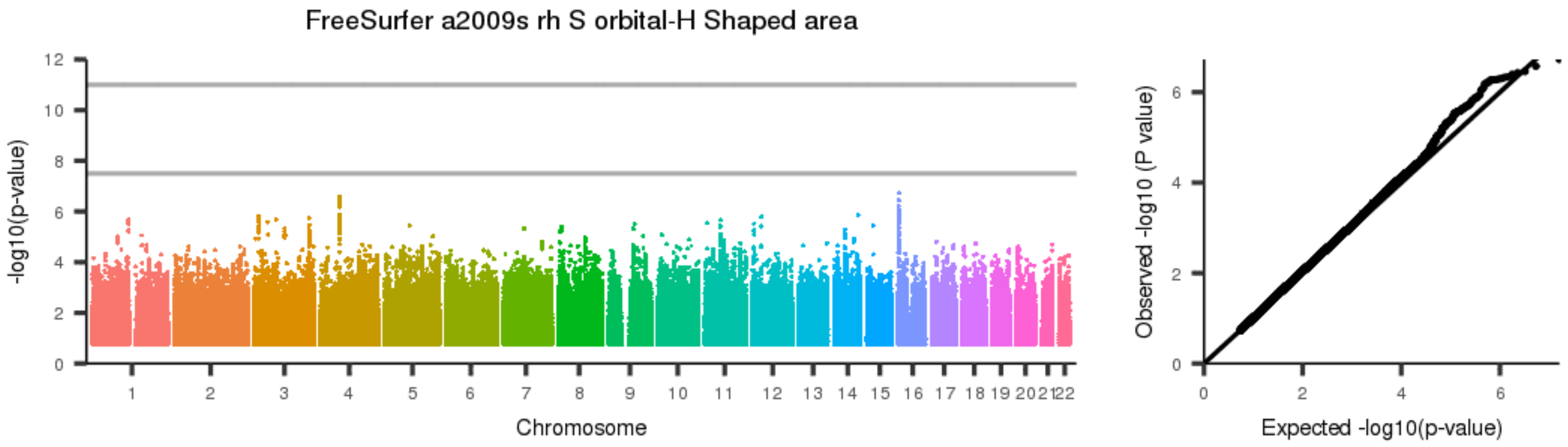

back to list of plots

Figure 1222: FreeSurfer a2009s rh S parieto occipital area

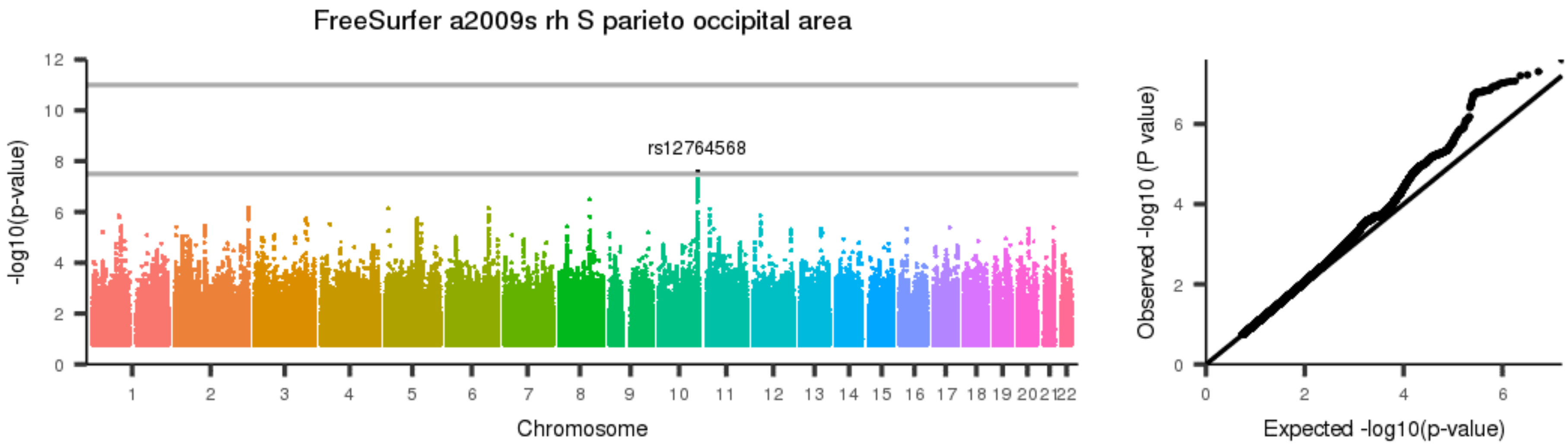

back to list of plots

Figure 1223: FreeSurfer a2009s rh S pericallosal area

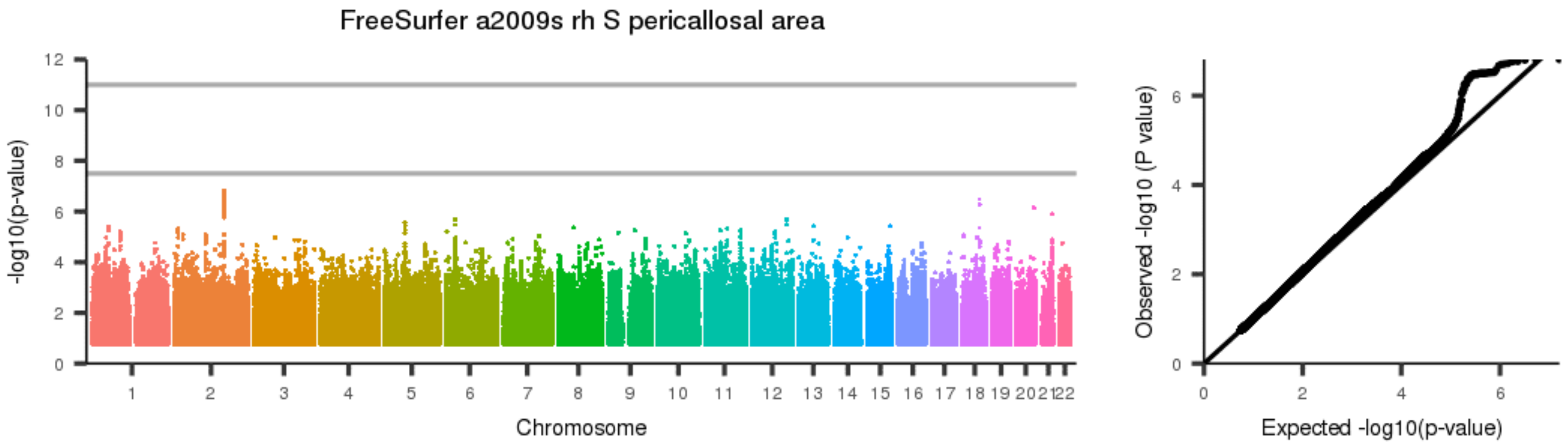

back to list of plots

Figure 1224: FreeSurfer a2009s rh S postcentral area

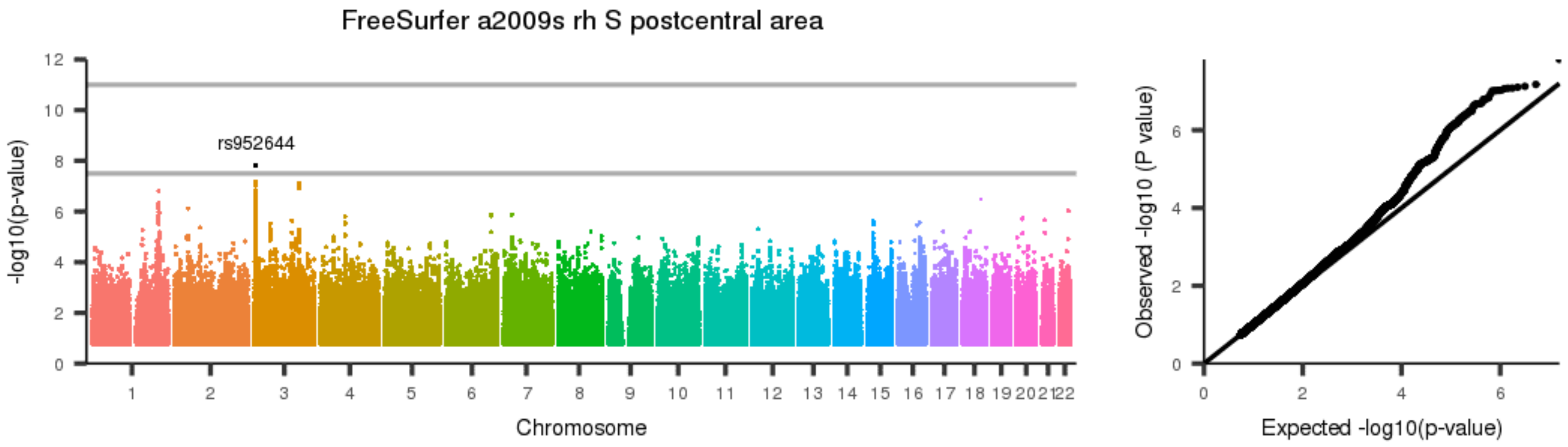

back to list of plots

Figure 1225: FreeSurfer a2009s rh S precentral-inf-part area

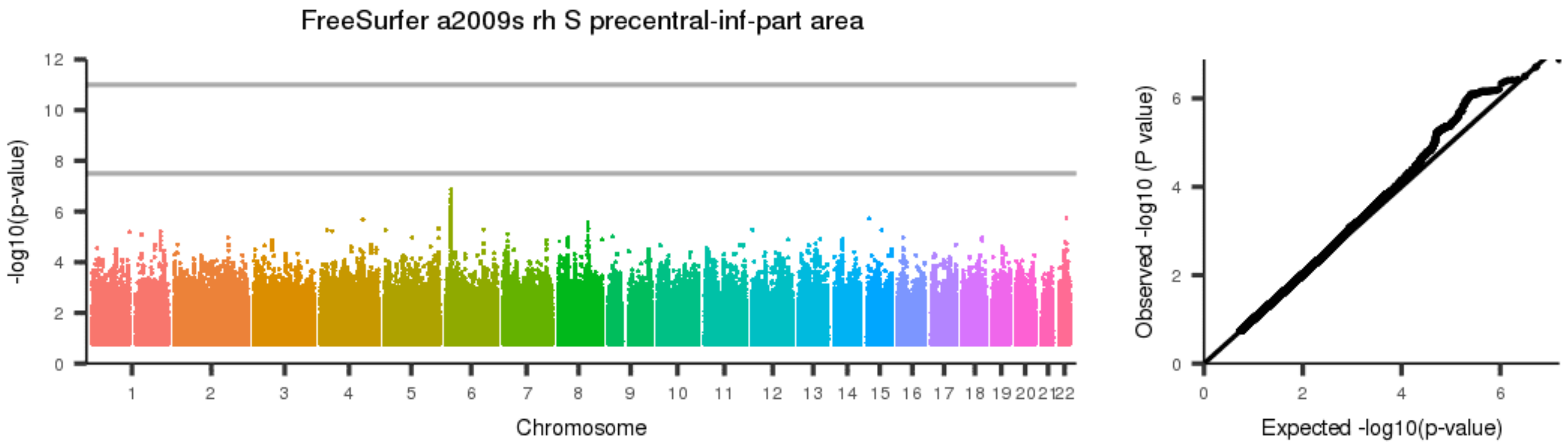

back to list of plots

Figure 1226: FreeSurfer a2009s rh S precentral-sup-part area

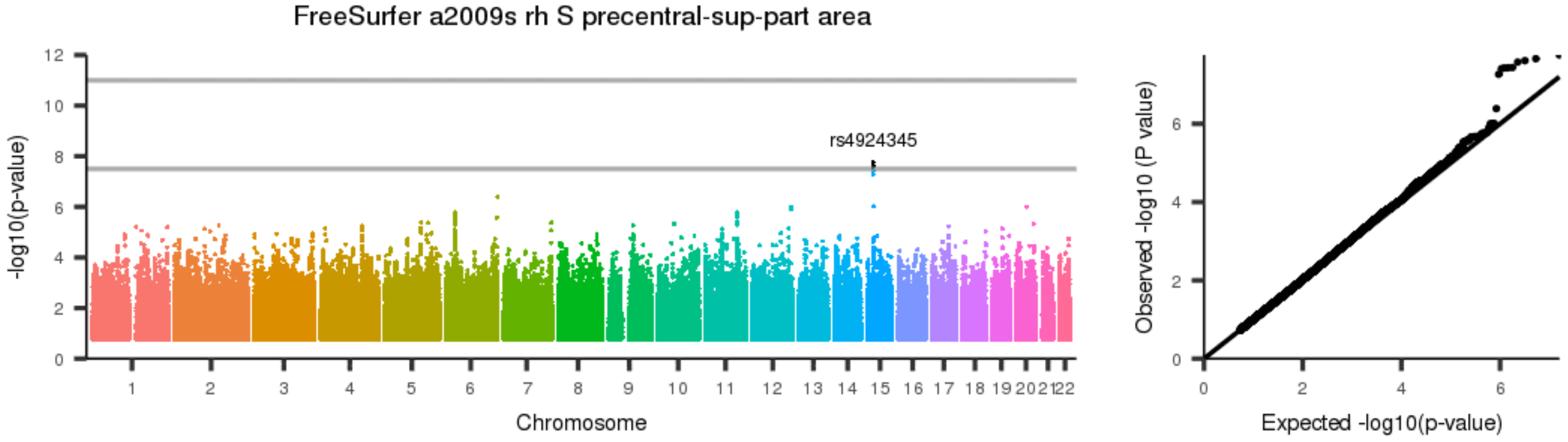

back to list of plots

Figure 1227: FreeSurfer a2009s rh S suborbital area

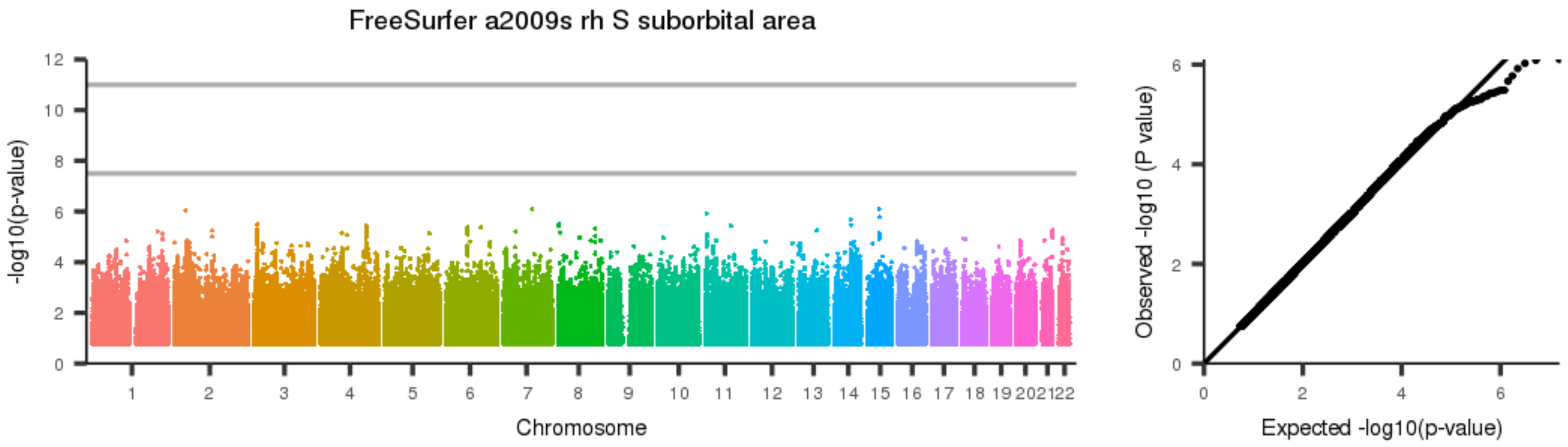

back to list of plots

Figure 1228: FreeSurfer a2009s rh S subparietal area

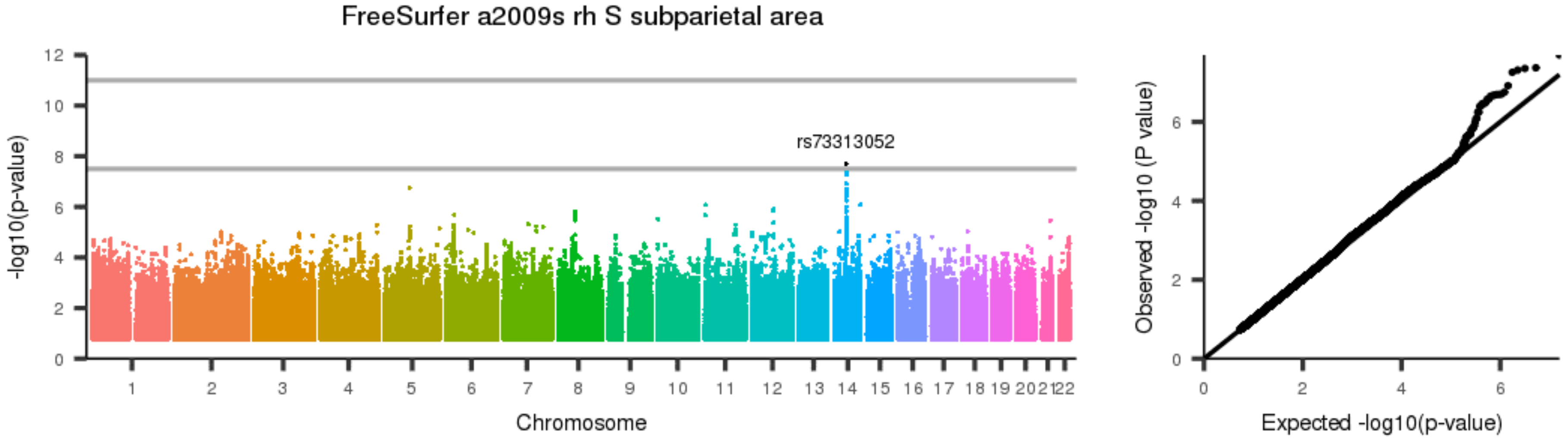

back to list of plots

Figure 1229: FreeSurfer a2009s rh S temporal inf area

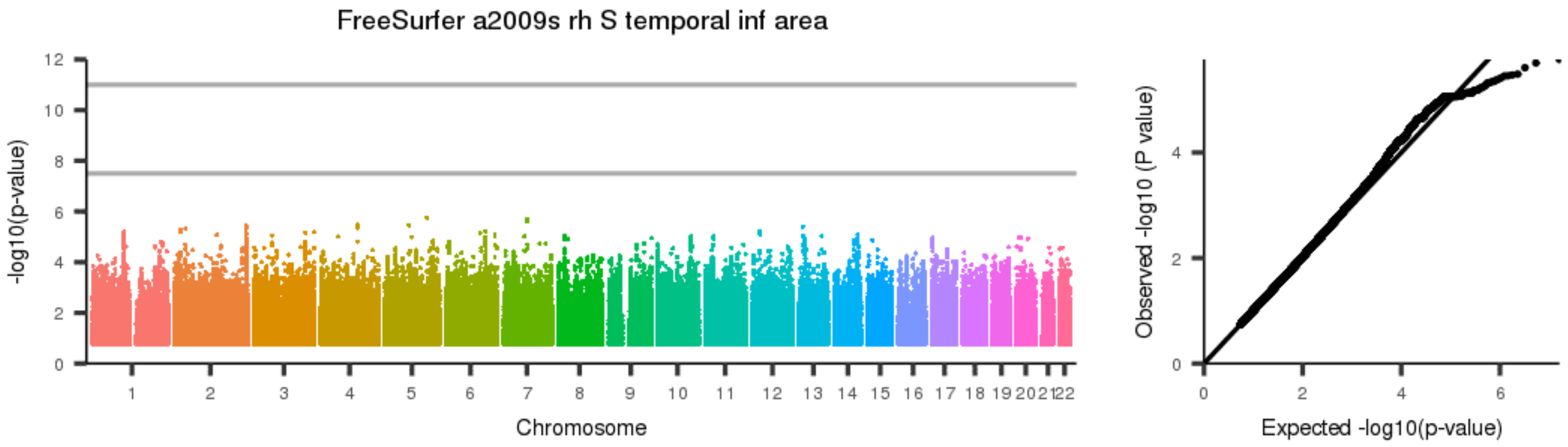

back to list of plots

Figure 1230: FreeSurfer a2009s rh S temporal sup area

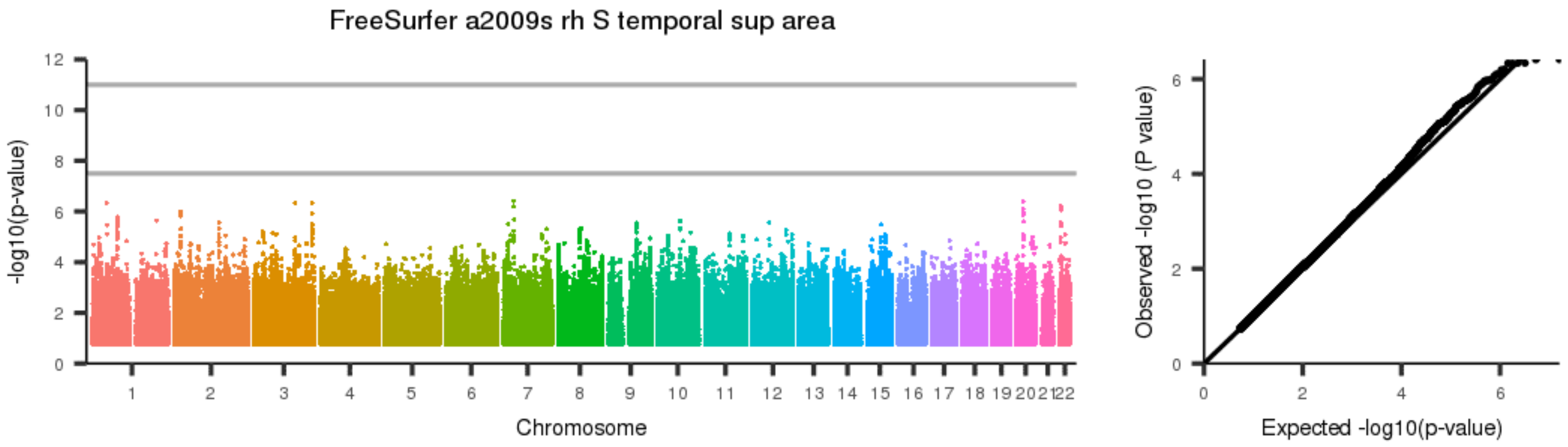

back to list of plots

Figure 1231: FreeSurfer a2009s rh S temporal transverse area

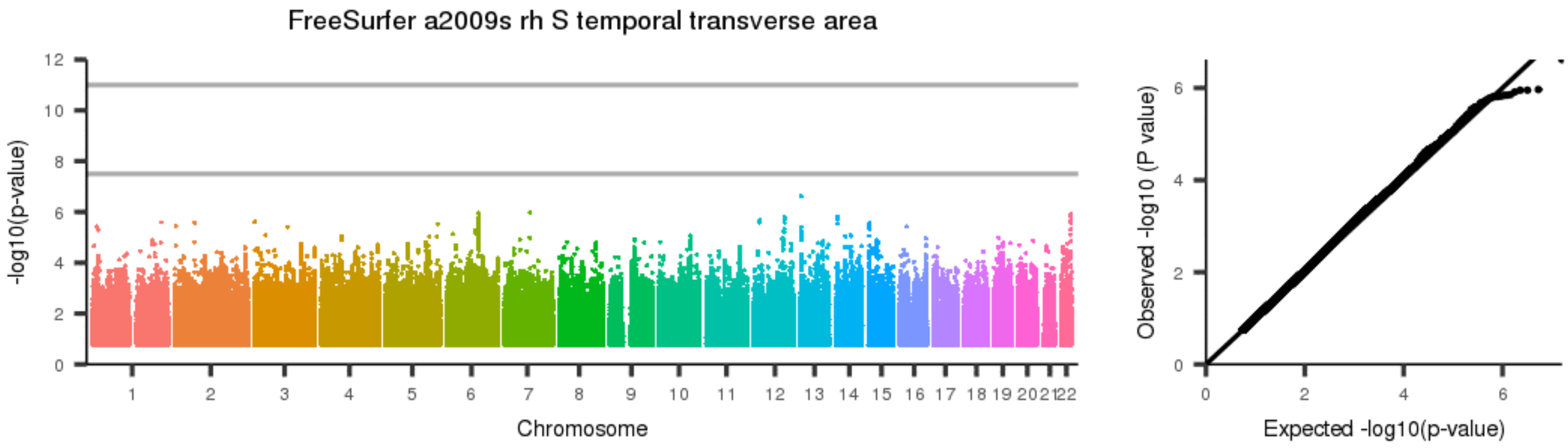

back to list of plots

Figure 1232: FreeSurfer DKTatlas lh caudalanteriorcingulate thickness

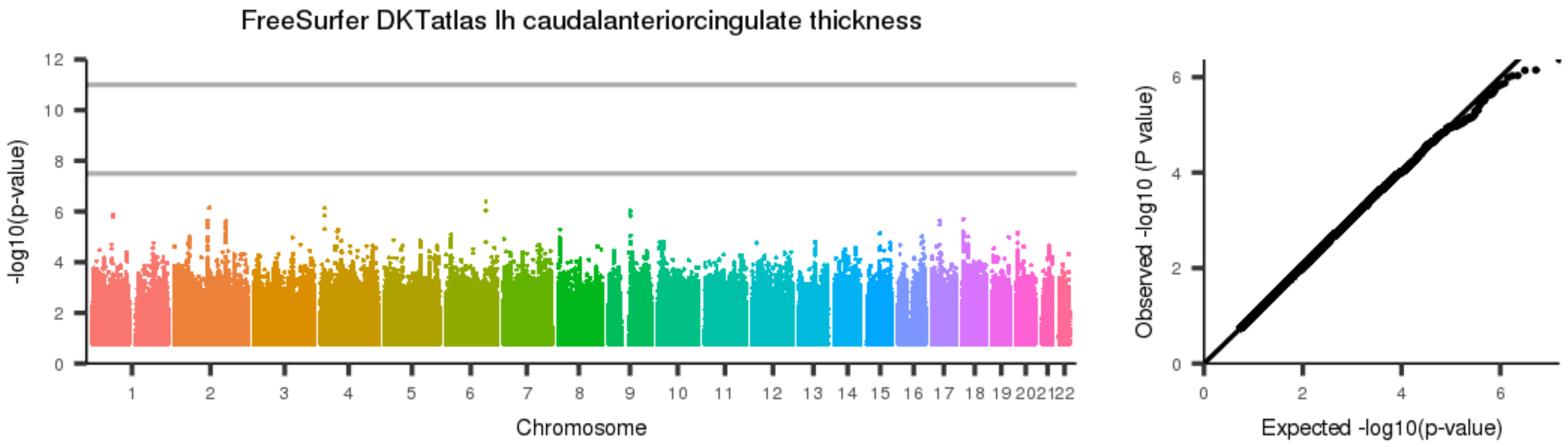

back to list of plots

Figure 1233: FreeSurfer DKTatlas lh caudalmiddlefrontal thickness

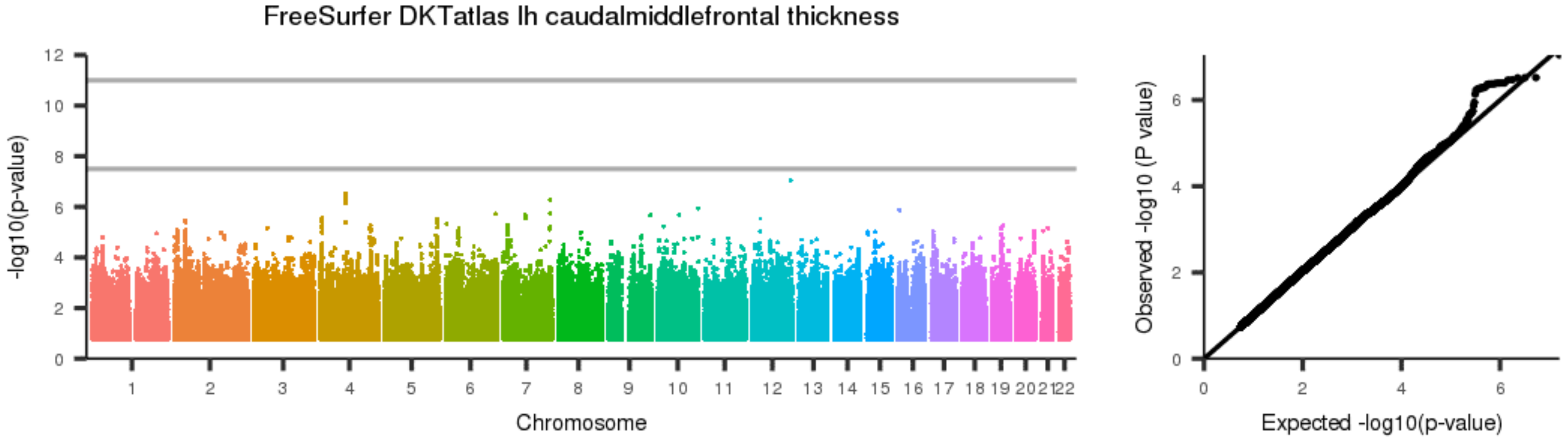

back to list of plots

Figure 1234: FreeSurfer DKTatlas lh cuneus thickness

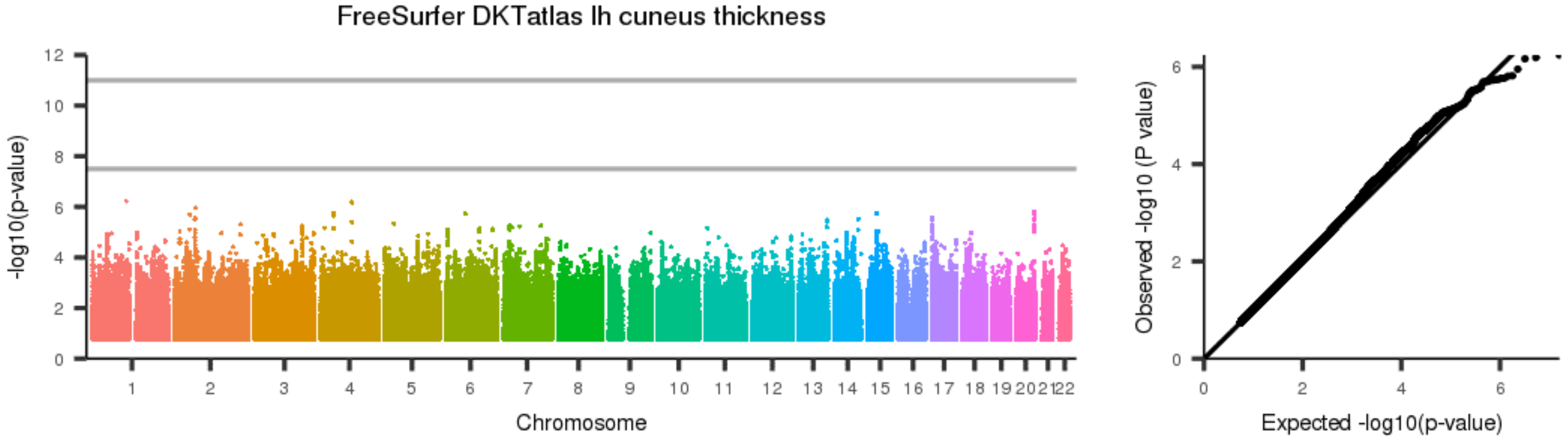

back to list of plots

Figure 1235: FreeSurfer DKTatlas lh entorhinal thickness

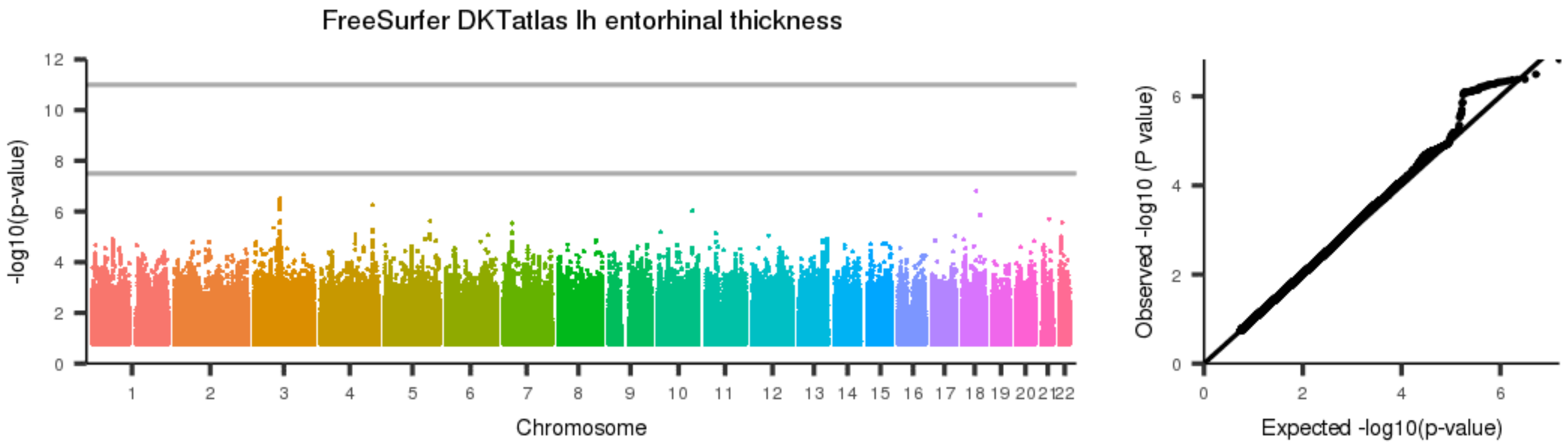

back to list of plots

Figure 1236: FreeSurfer DKTatlas lh fusiform thickness

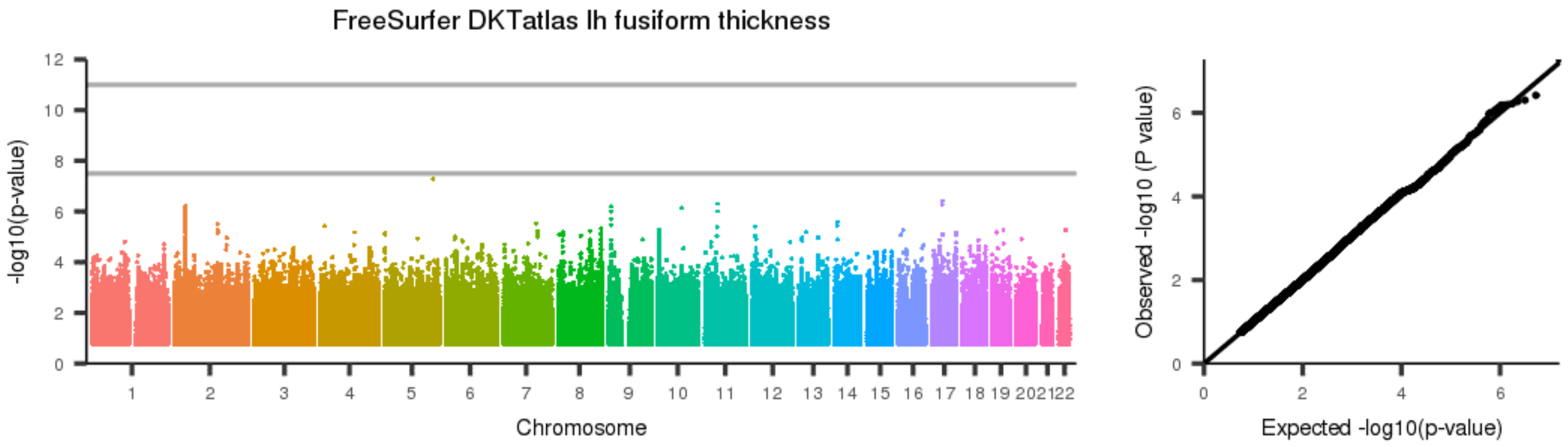

back to list of plots

Figure 1237: FreeSurfer DKTatlas lh inferiorparietal thickness

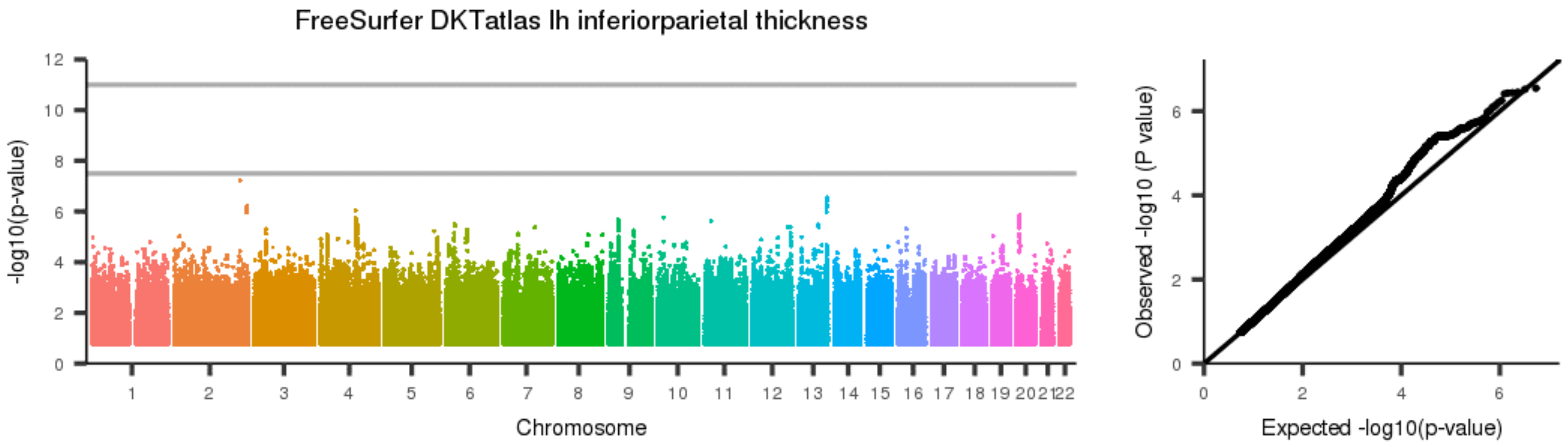

back to list of plots

Figure 1238: FreeSurfer DKTatlas lh inferiortemporal thickness

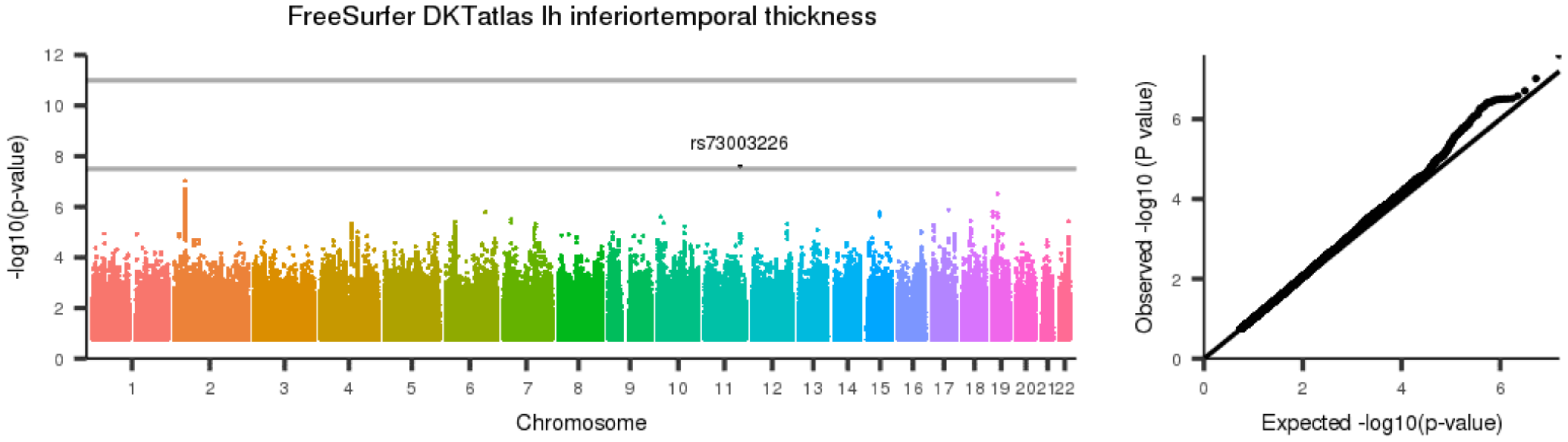

back to list of plots

Figure 1239: FreeSurfer DKTatlas lh isthmuscingulate thickness

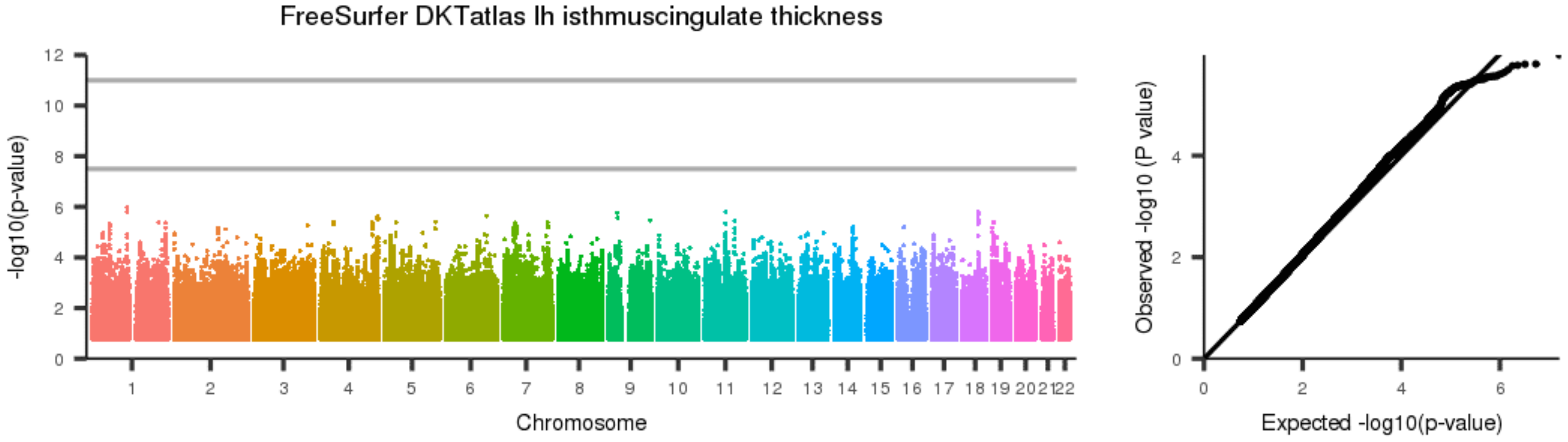

back to list of plots

Figure 1240: FreeSurfer DKTatlas lh lateraloccipital thickness

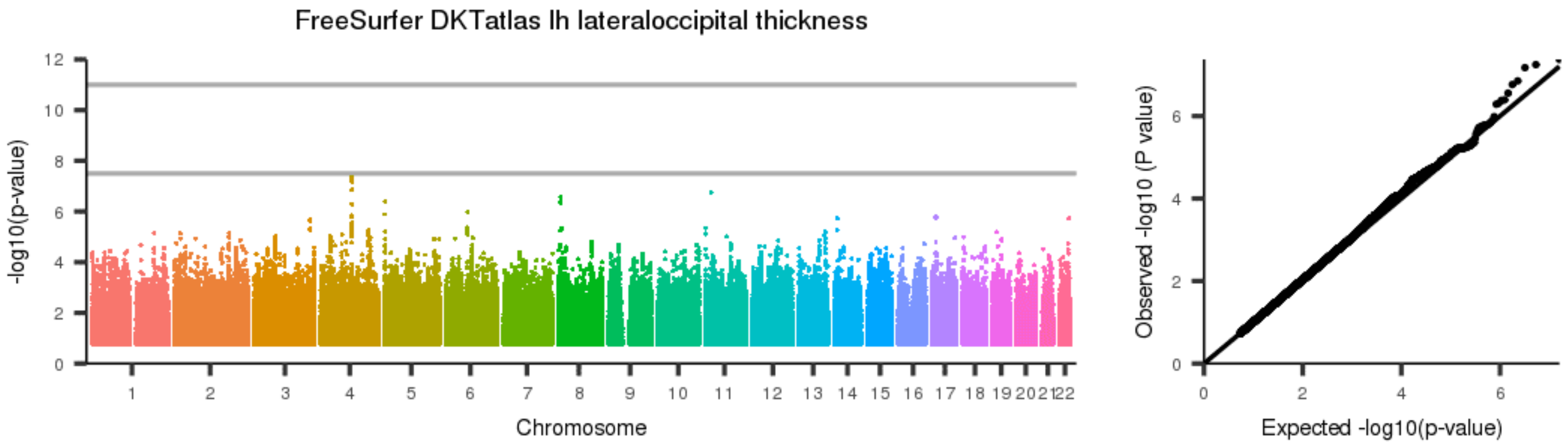

back to list of plots

Figure 1241: FreeSurfer DKTatlas lh lateralorbitofrontal thickness

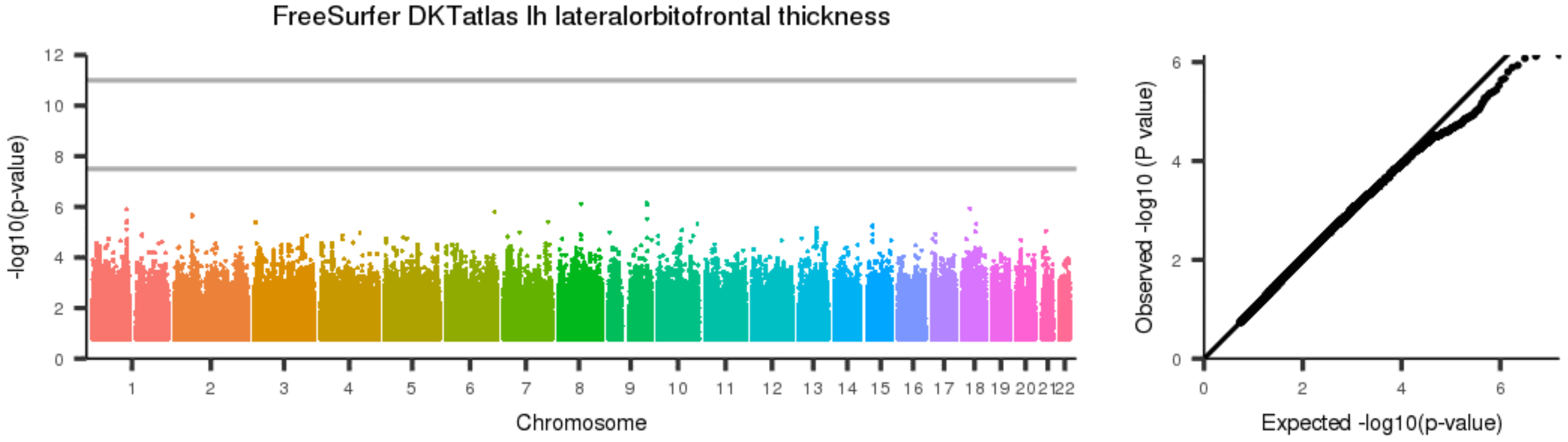

back to list of plots

Figure 1242: FreeSurfer DKTatlas lh lingual thickness

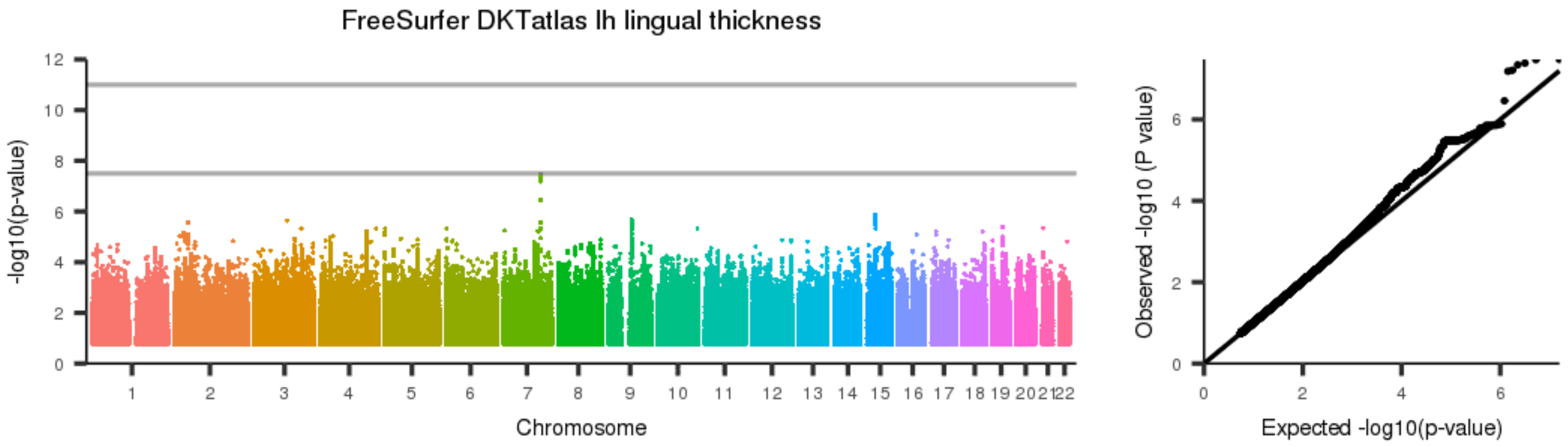

back to list of plots

Figure 1243: FreeSurfer DKTatlas lh medialorbitofrontal thickness

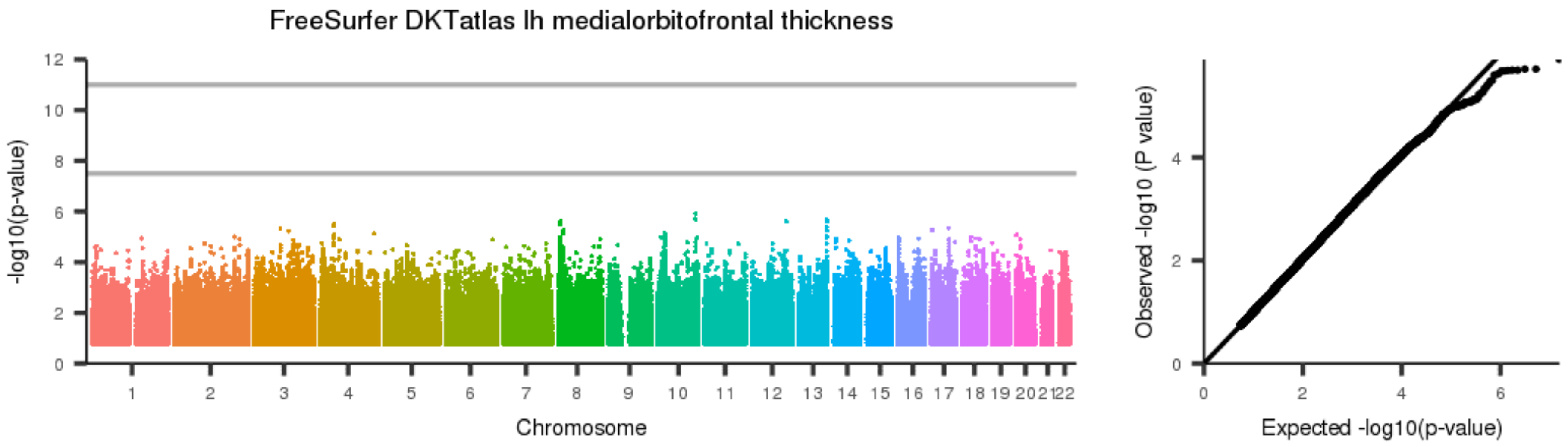

back to list of plots

Figure 1244: FreeSurfer DKTatlas lh middletemporal thickness

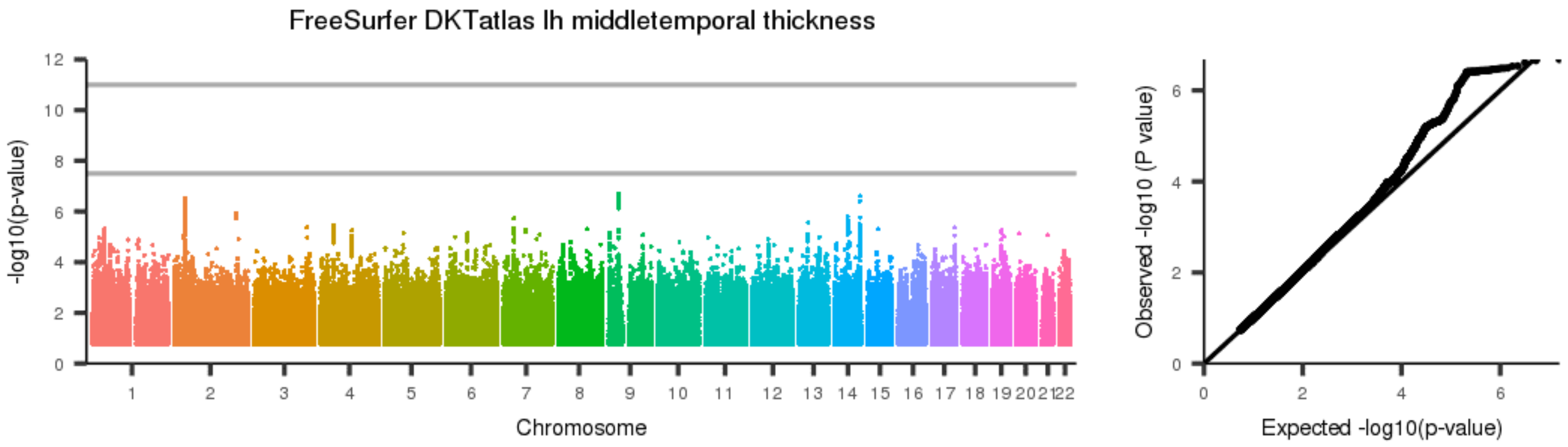

back to list of plots

Figure 1245: FreeSurfer DKTatlas lh parahippocampal thickness

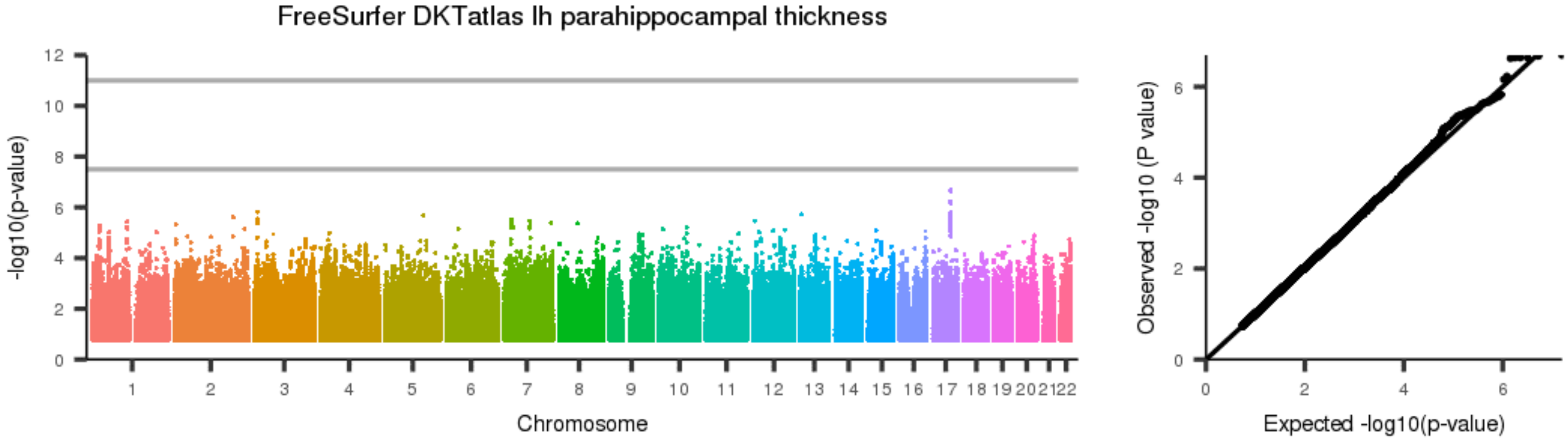

back to list of plots

Figure 1246: FreeSurfer DKTatlas lh paracentral thickness

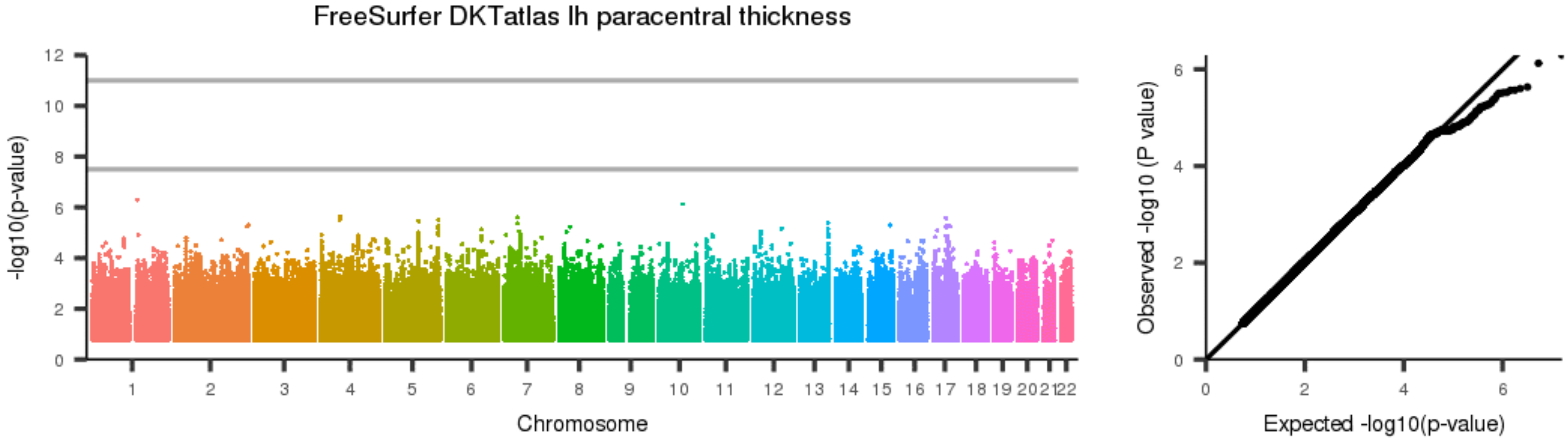

back to list of plots

Figure 1247: FreeSurfer DKTatlas lh parsopercularis thickness

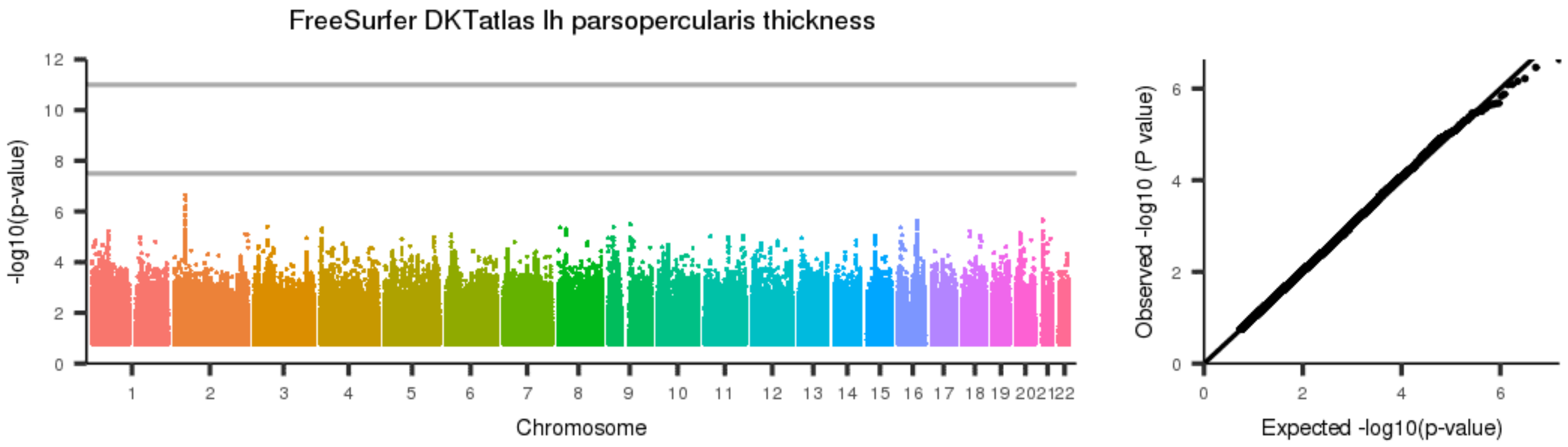

back to list of plots

Figure 1248: FreeSurfer DKTatlas lh parsorbitalis thickness

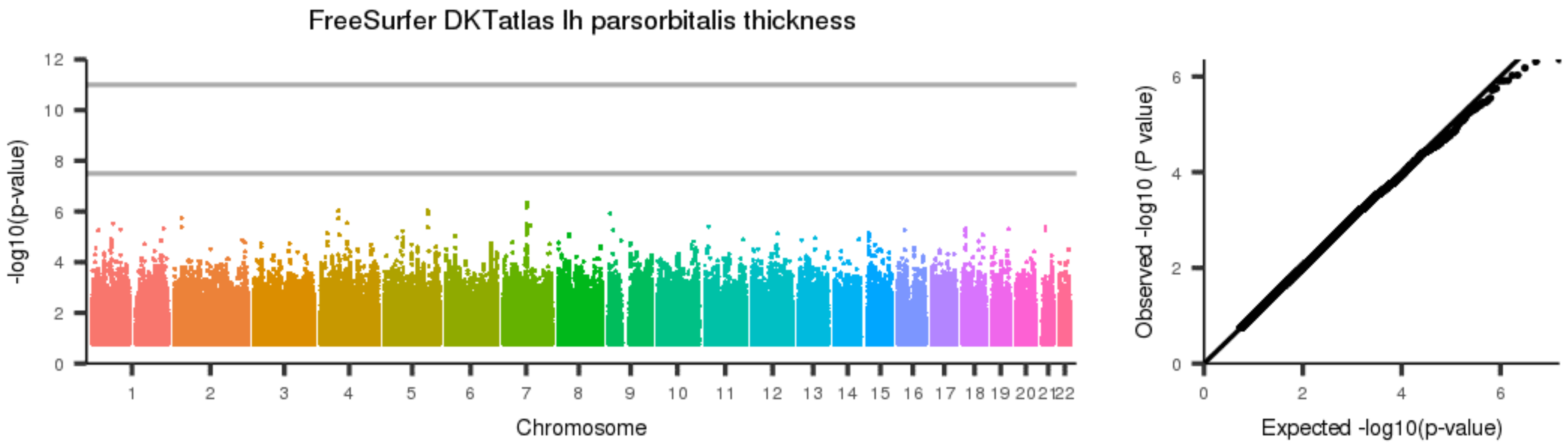

back to list of plots

Figure 1249: FreeSurfer DKTatlas lh parstriangularis thickness

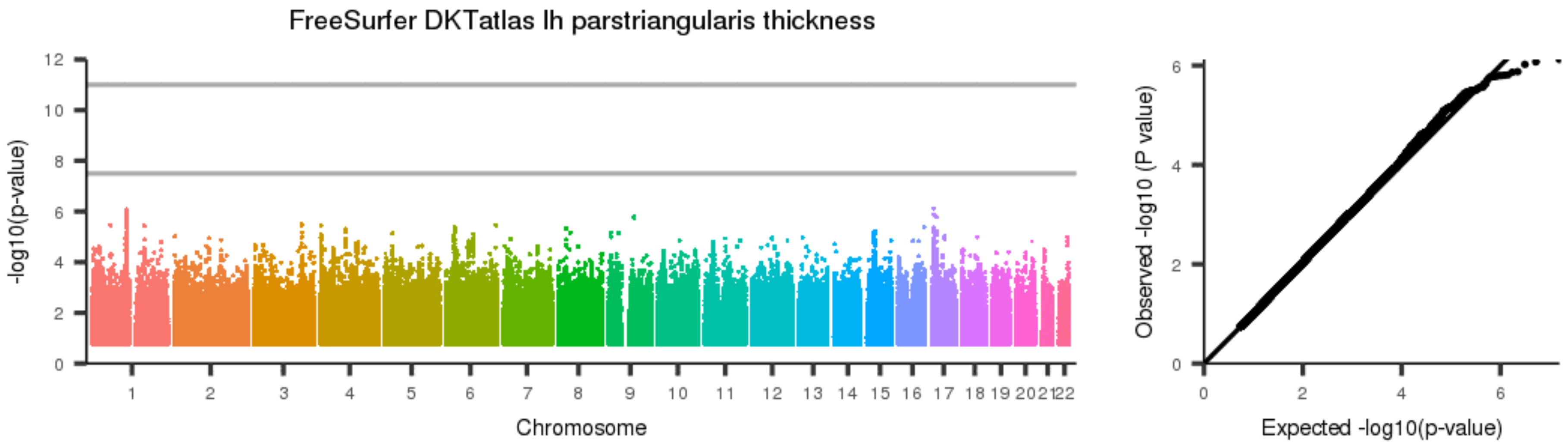

back to list of plots

Figure 1250: FreeSurfer DKTatlas lh pericalcarine thickness

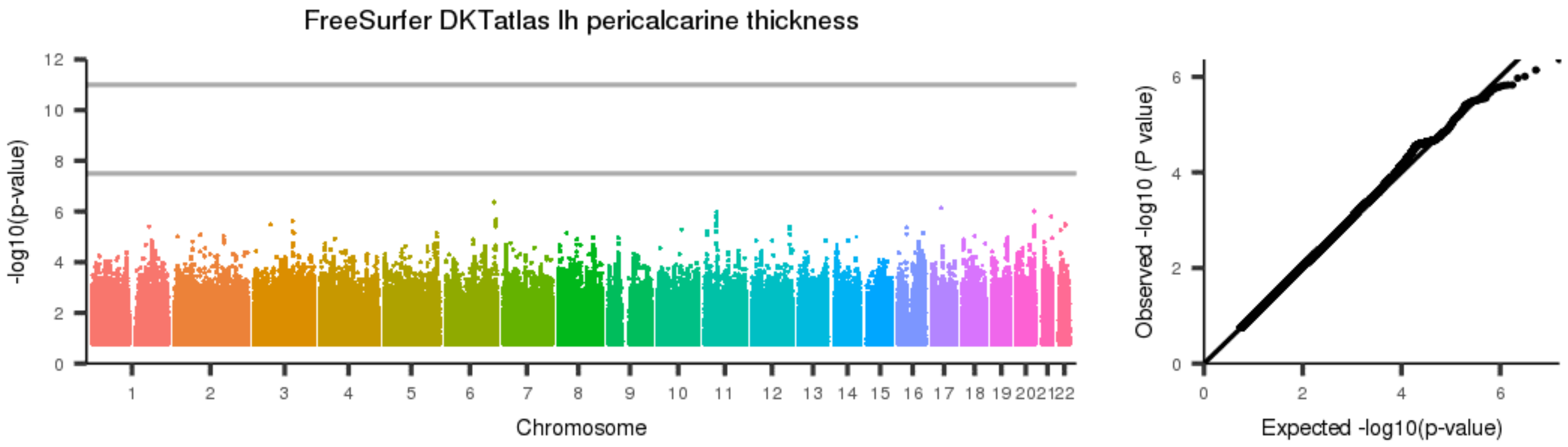

back to list of plots

Figure 1251: FreeSurfer DKTatlas lh postcentral thickness

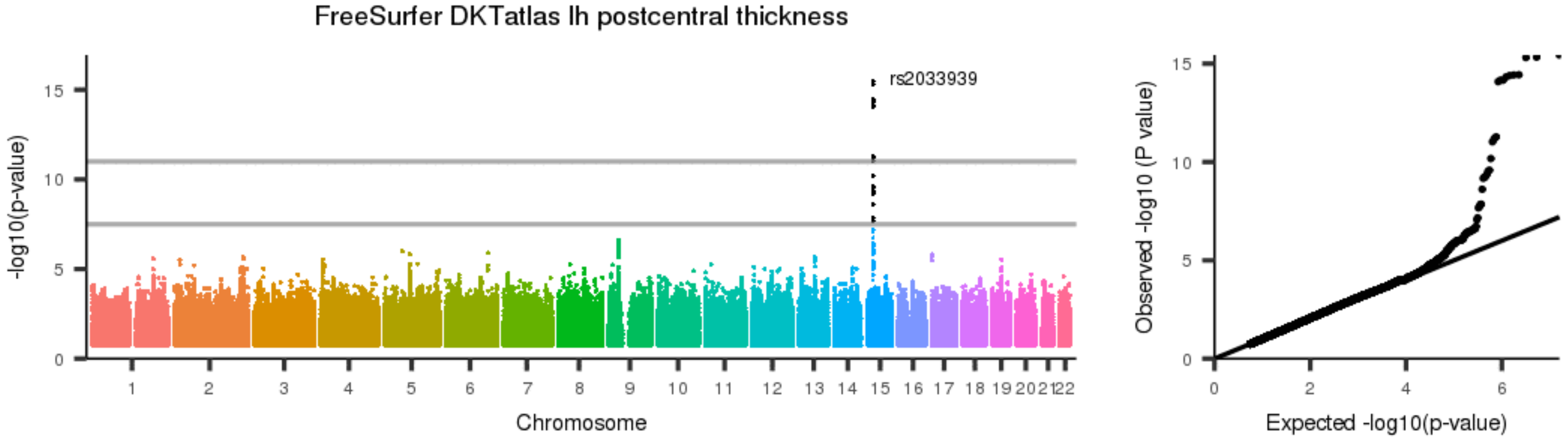

back to list of plots

Figure 1252: FreeSurfer DKTatlas lh posteriorcingulate thickness

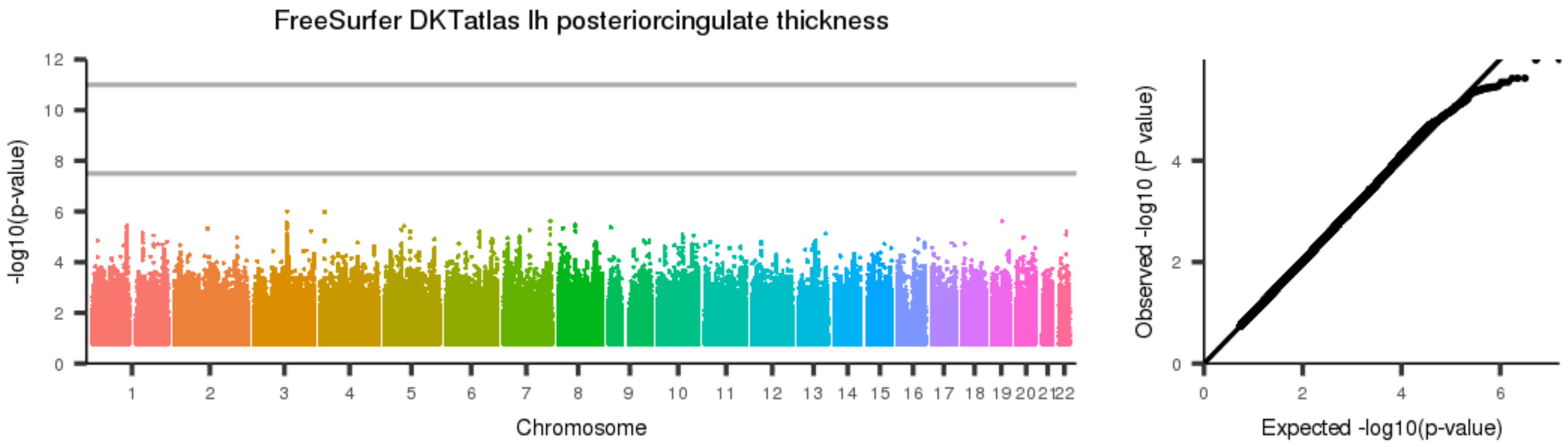

back to list of plots

Figure 1253: FreeSurfer DKTatlas lh precentral thickness

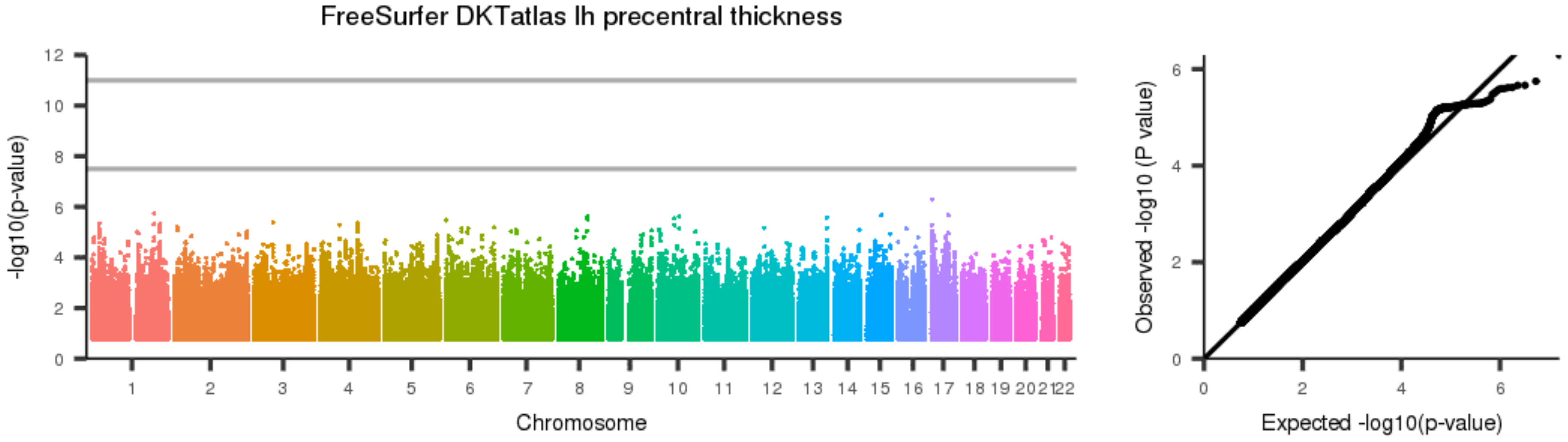

back to list of plots

Figure 1254: FreeSurfer DKTatlas lh precuneus thickness

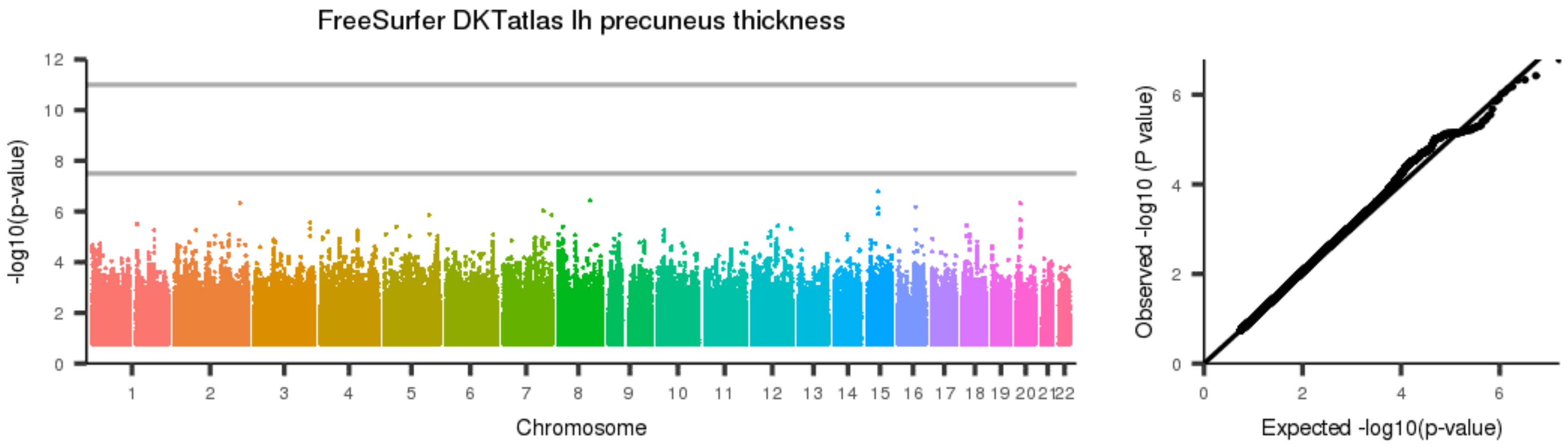

back to list of plots

Figure 1255: FreeSurfer DKTatlas lh rostralanteriorcingulate thickness

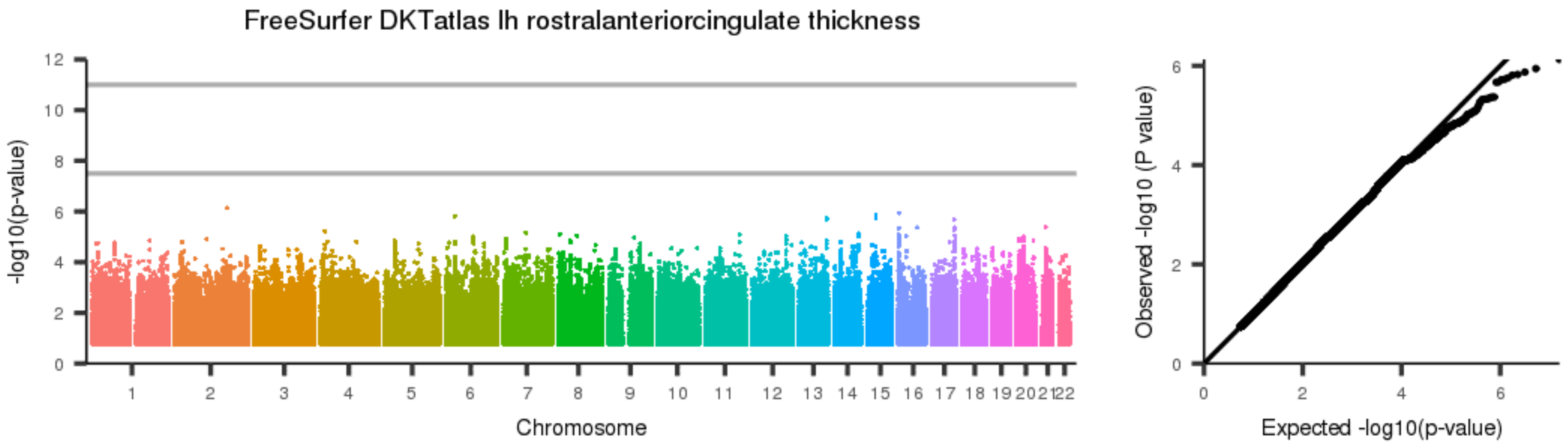

back to list of plots

Figure 1256: FreeSurfer DKTatlas lh rostralmiddlefrontal thickness

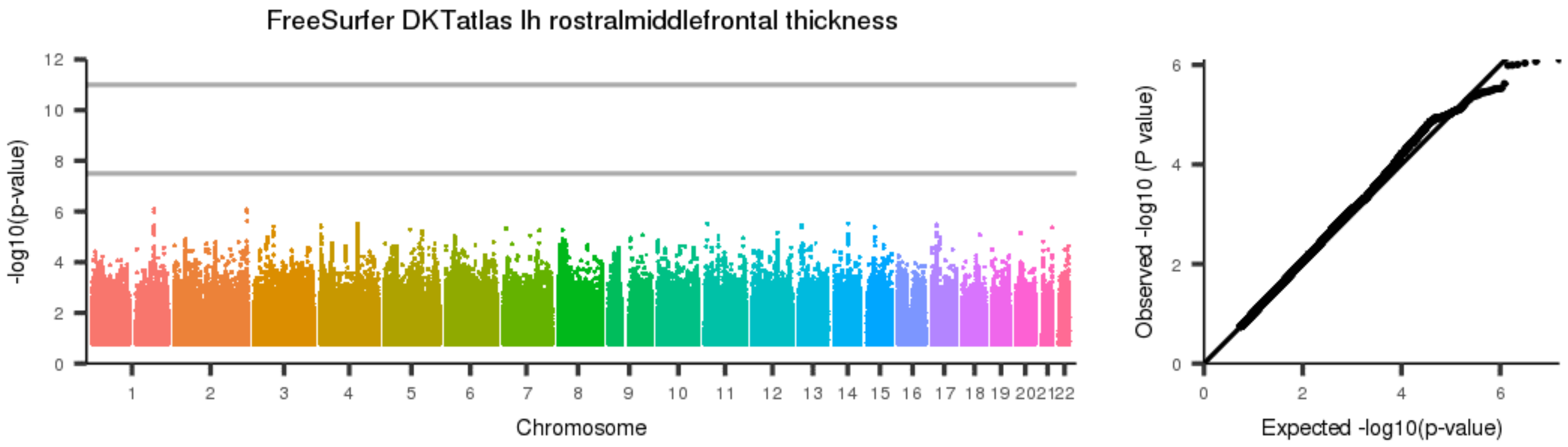

back to list of plots

Figure 1257: FreeSurfer DKTatlas lh superiorfrontal thickness

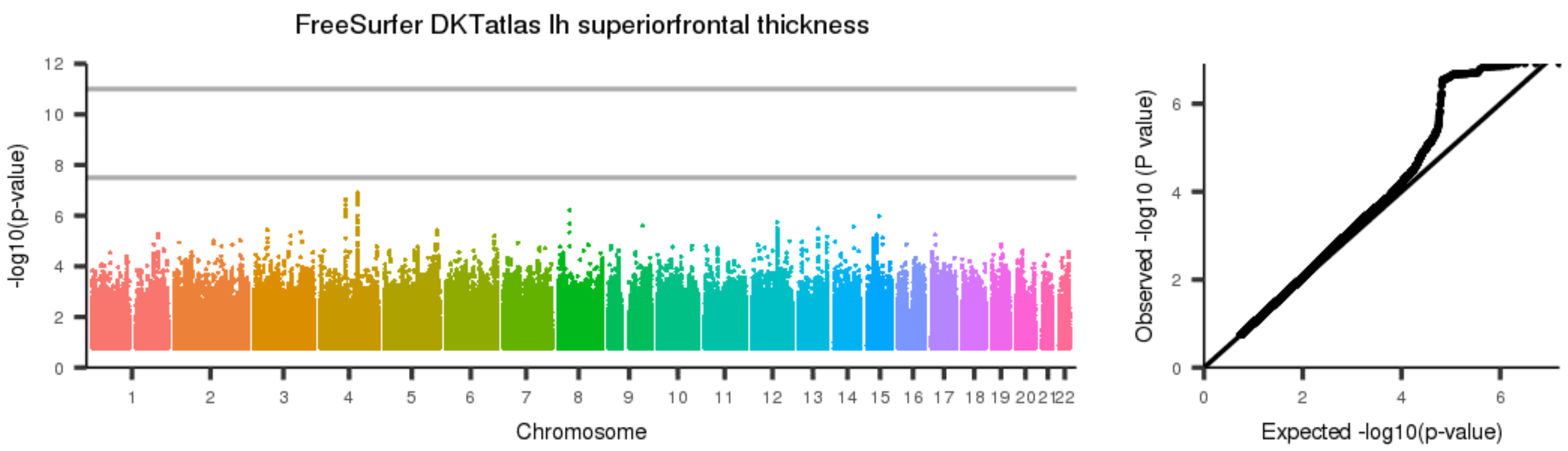

back to list of plots

Figure 1258: FreeSurfer DKTatlas lh superiorparietal thickness

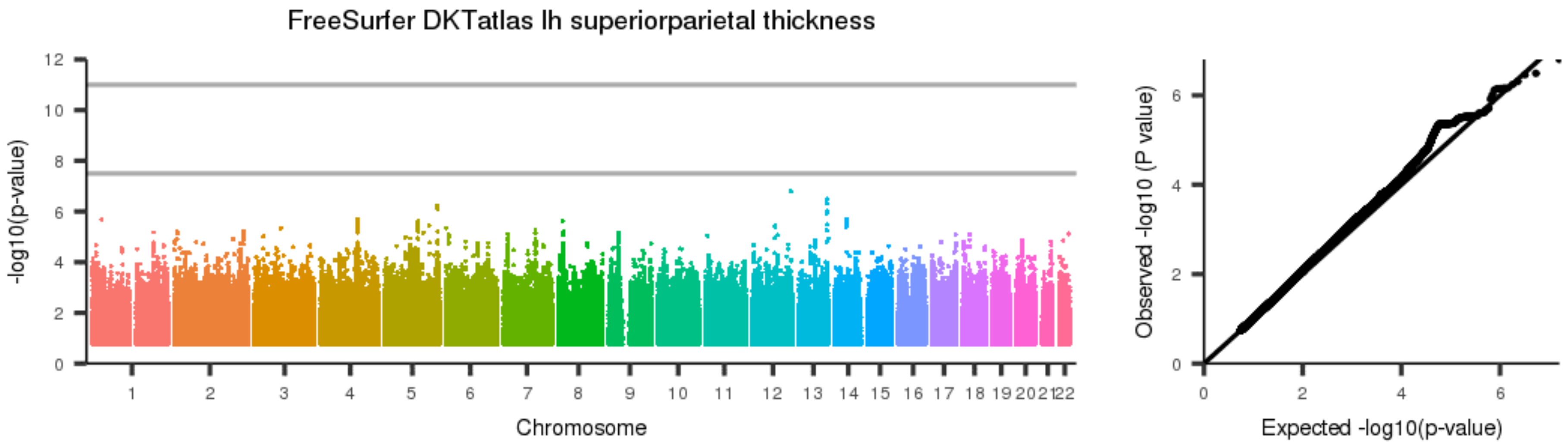

back to list of plots

Figure 1259: FreeSurfer DKTatlas lh superiortemporal thickness

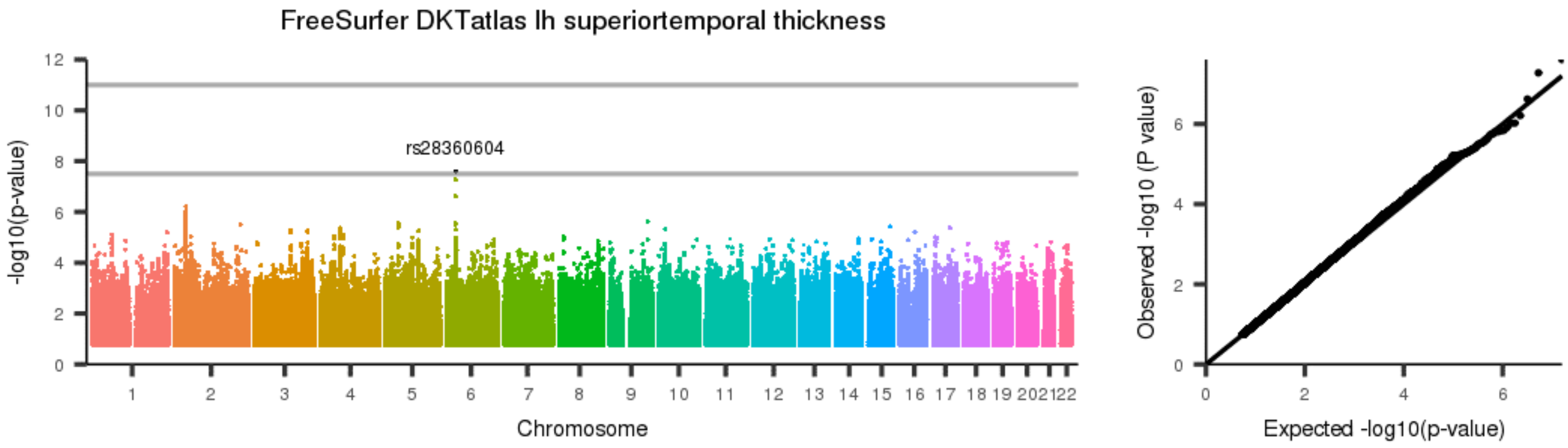

back to list of plots

Figure 1260: FreeSurfer DKTatlas lh supramarginal thickness

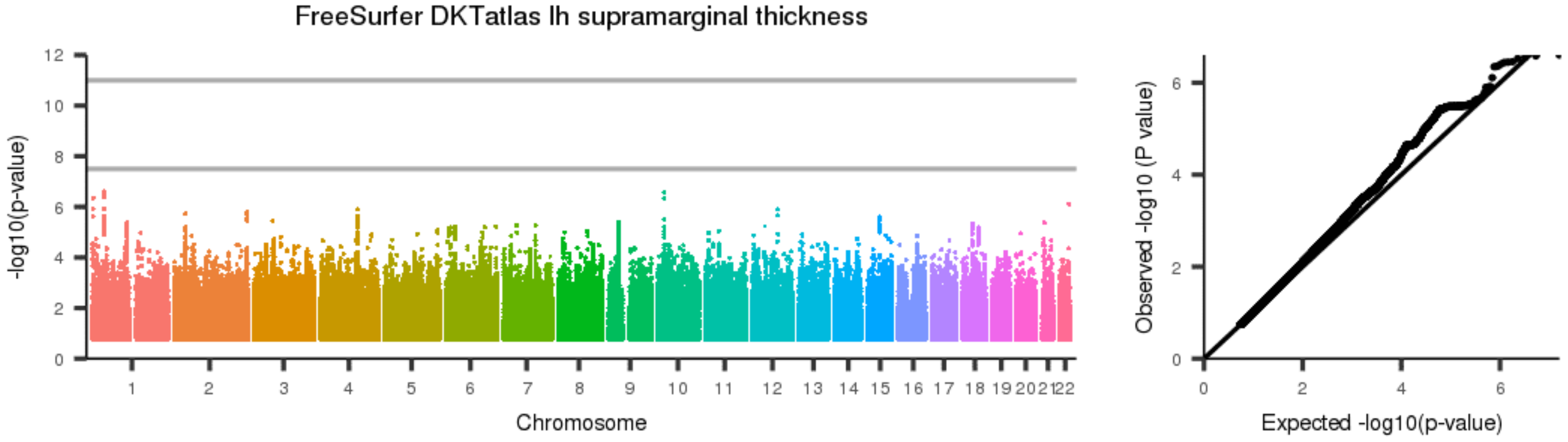

back to list of plots

Figure 1261: FreeSurfer DKTatlas lh transversetemporal thickness

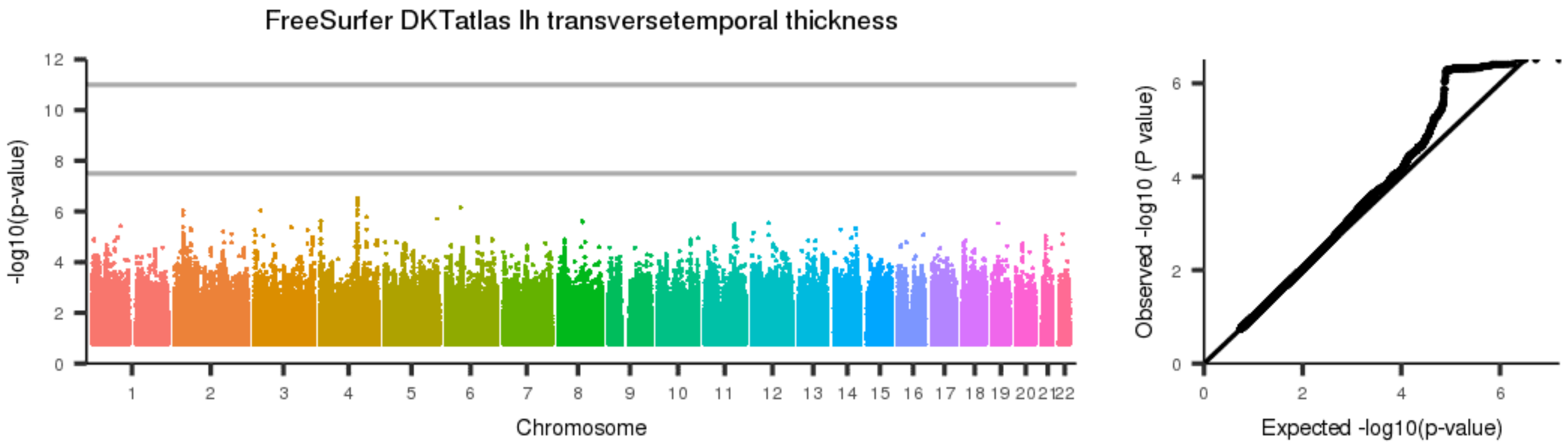

back to list of plots

Figure 1262: FreeSurfer DKTatlas lh insula thickness

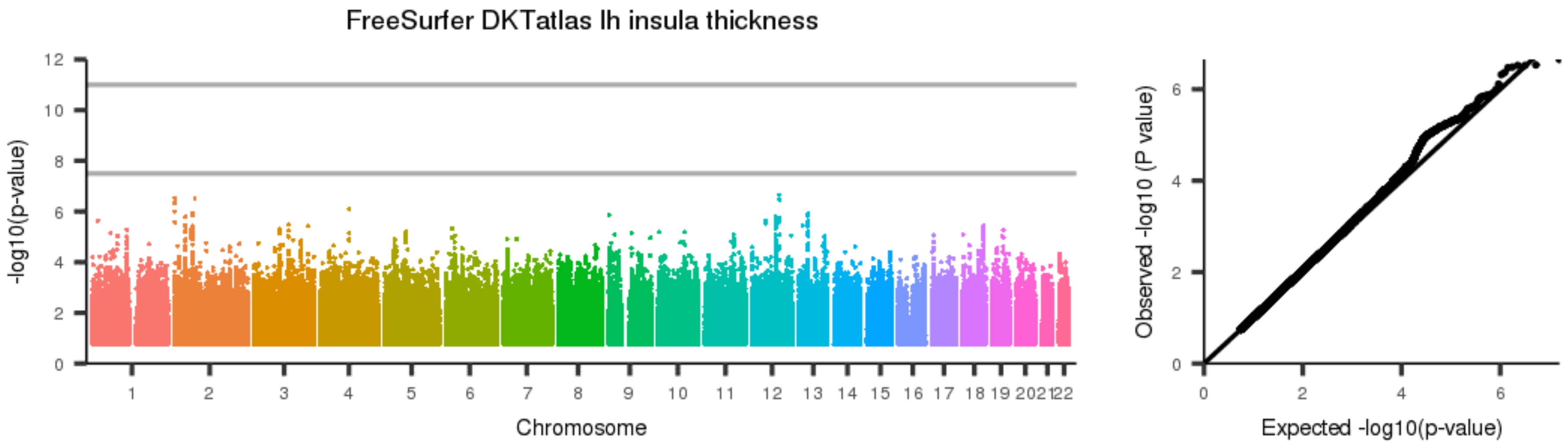

back to list of plots

Figure 1263: FreeSurfer DKTatlas lh MeanThickness thickness

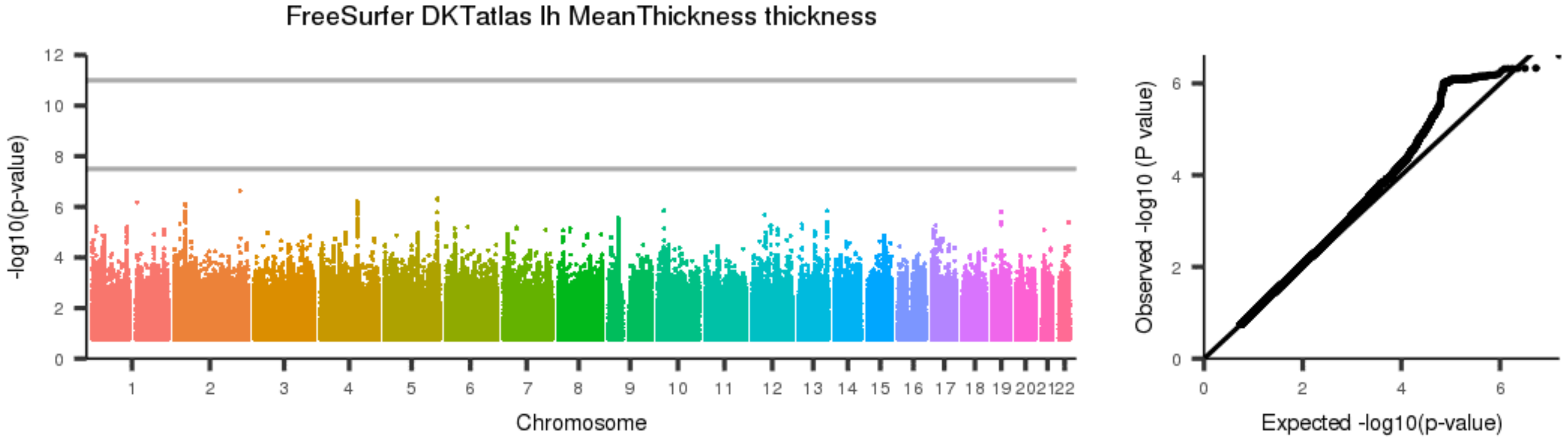

back to list of plots

Figure 1264: FreeSurfer a2009s lh G and S frontomargin thickness

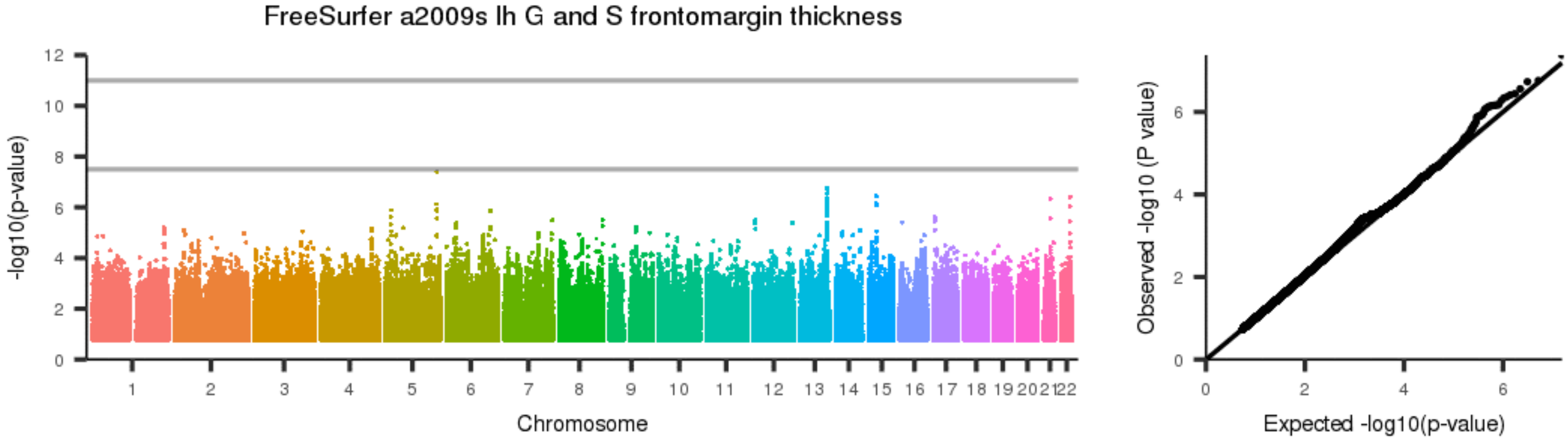

back to list of plots

Figure 1265: FreeSurfer a2009s lh G and S occipital inf thickness

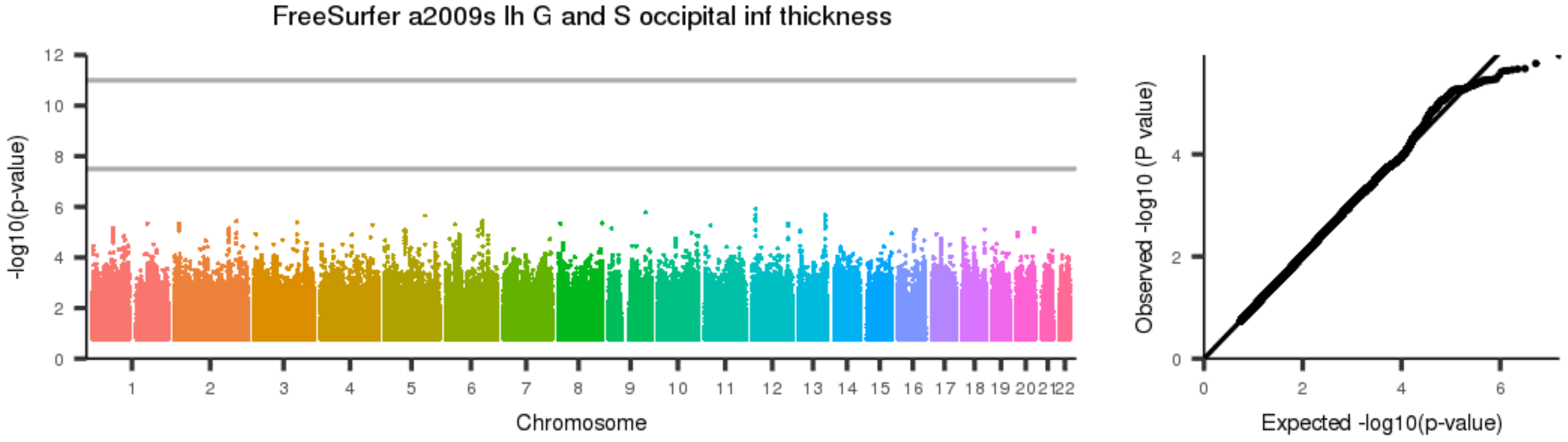

back to list of plots

Figure 1266: FreeSurfer a2009s lh G and S paracentral thickness

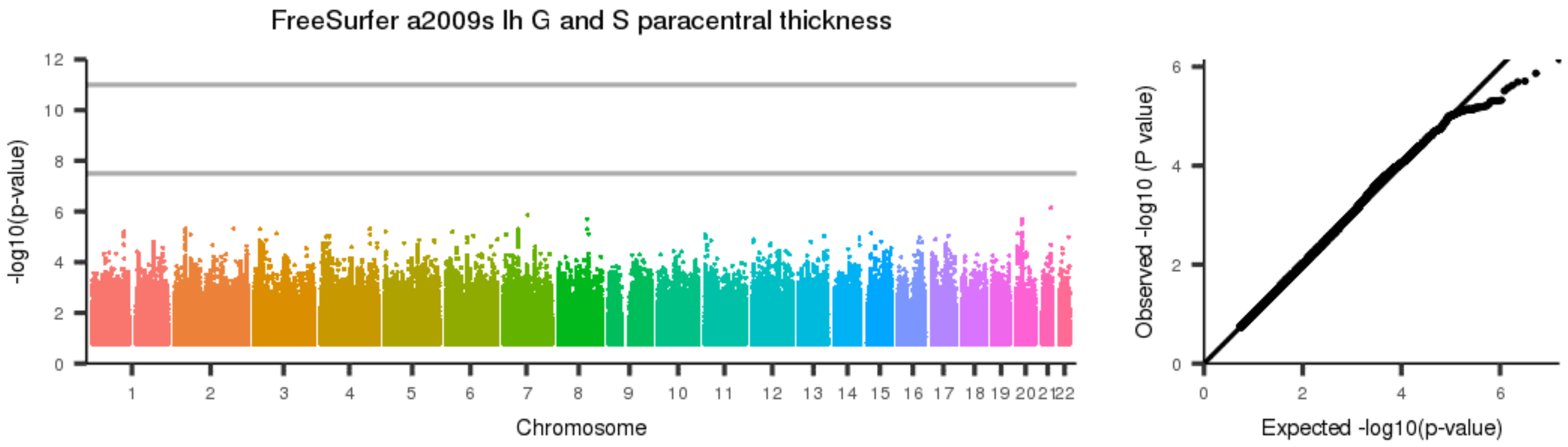

back to list of plots

Figure 1267: FreeSurfer a2009s lh G and S subcentral thickness

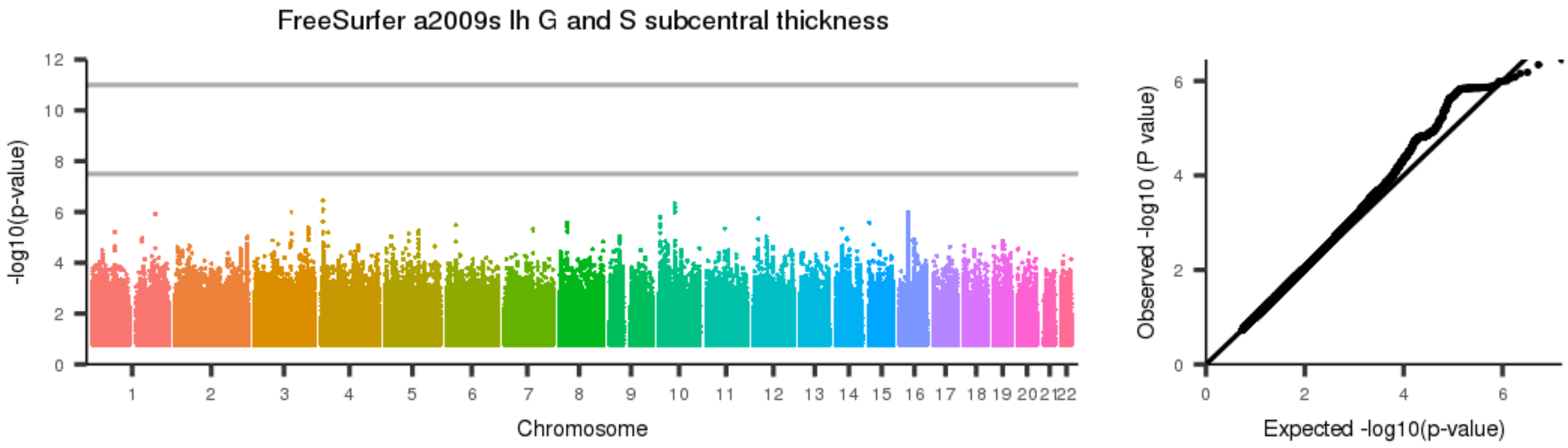

back to list of plots

Figure 1268: FreeSurfer a2009s lh G and S transv frontopol thickness

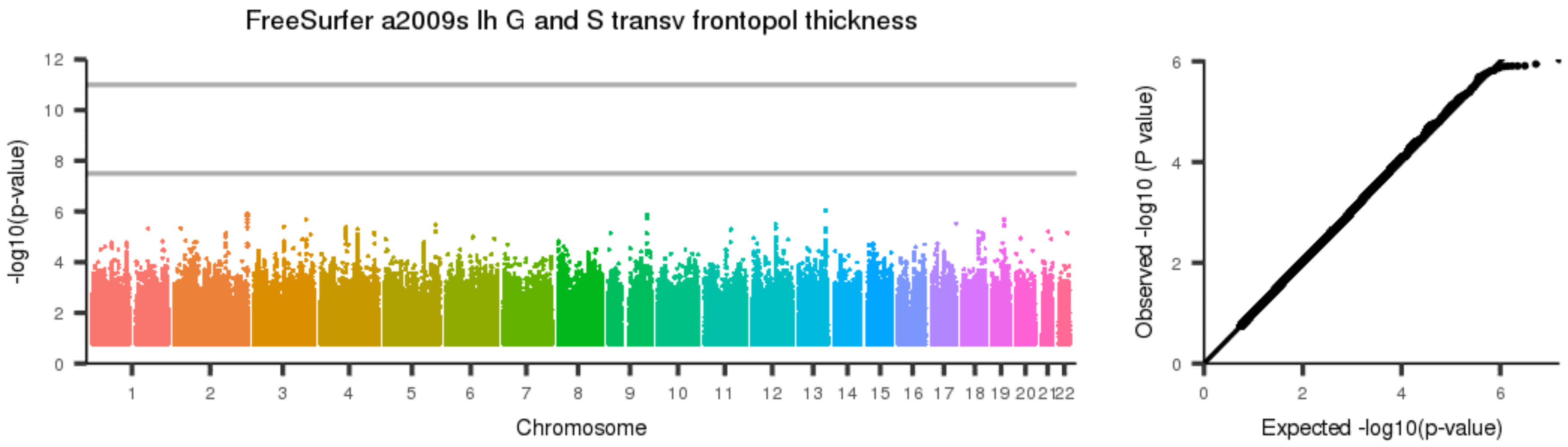

back to list of plots

Figure 1269: FreeSurfer a2009s lh G and S cingul-Ant thickness

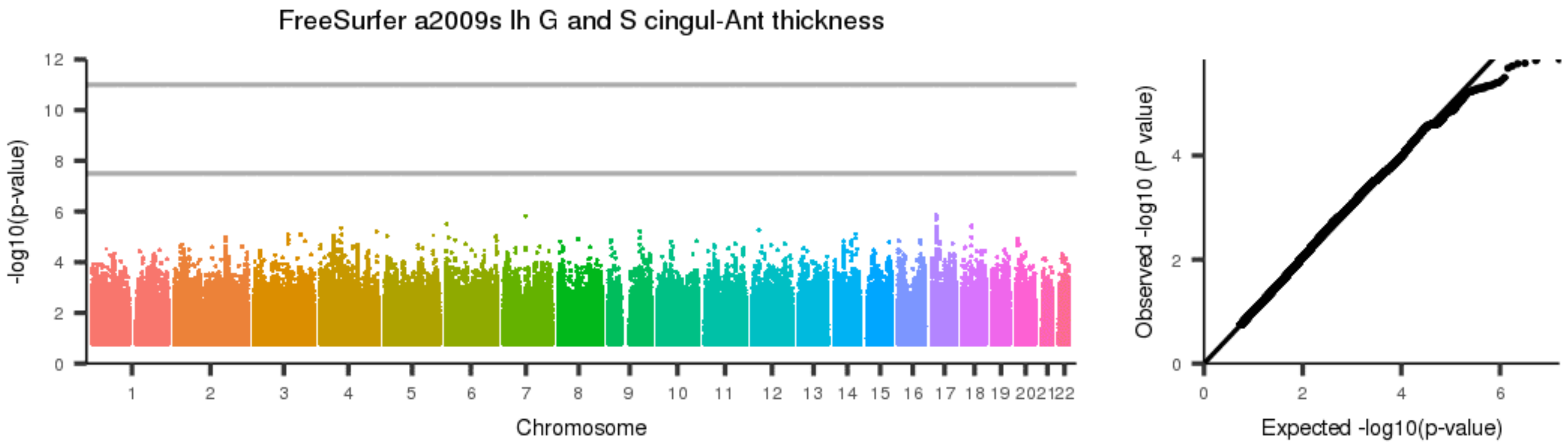

back to list of plots

Figure 1270: FreeSurfer a2009s lh G and S cingul-Mid-Ant thickness

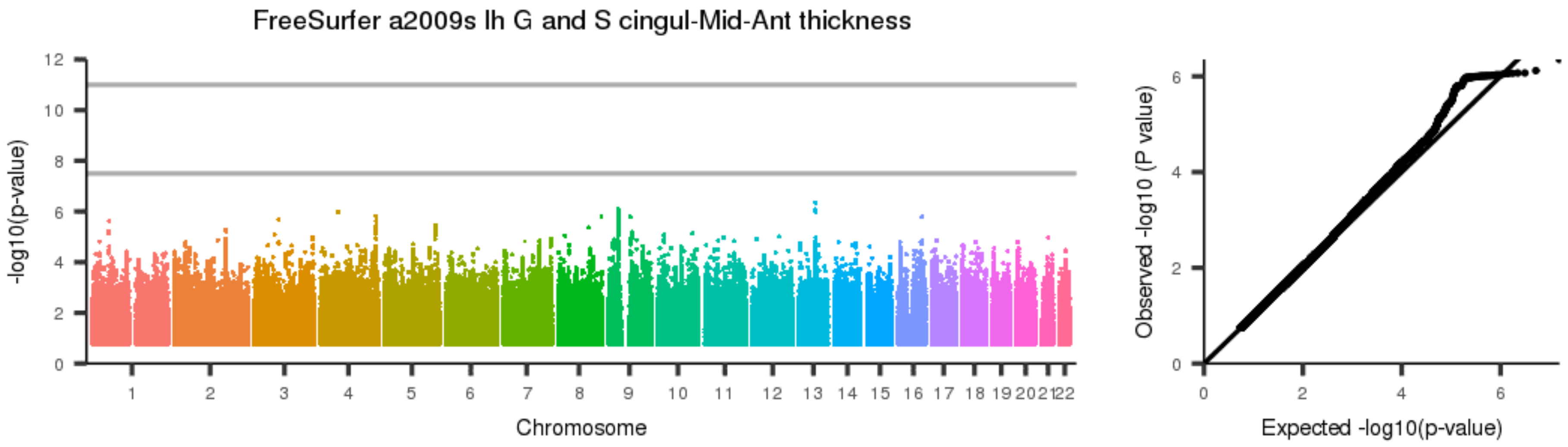

back to list of plots

Figure 1271: FreeSurfer a2009s lh G and S cingul-Mid-Post thickness

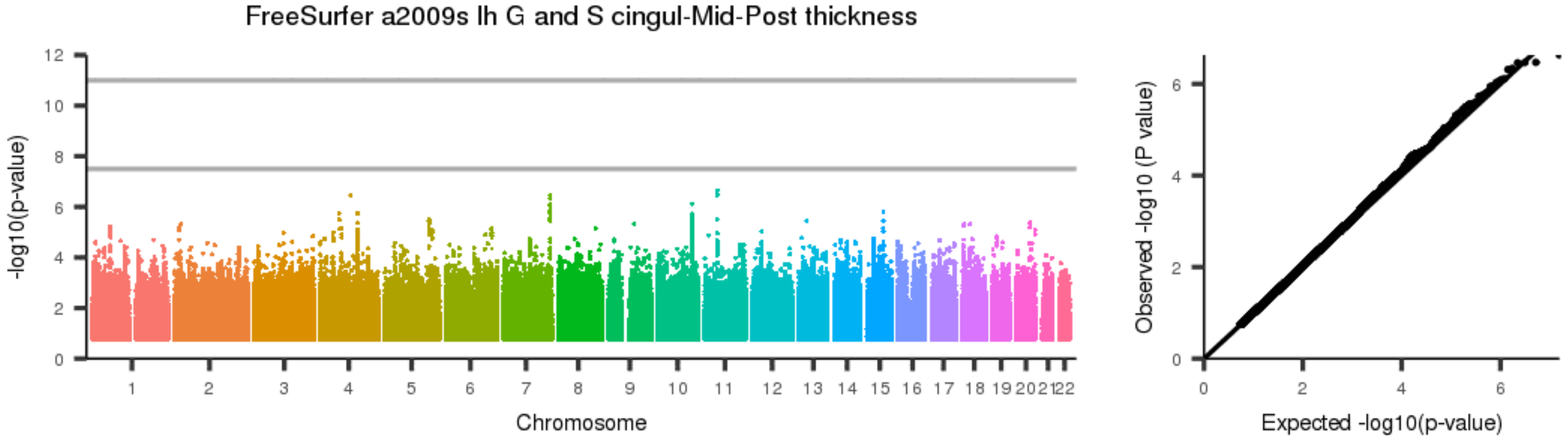

back to list of plots

Figure 1272: FreeSurfer a2009s lh G cingul-Post-dorsal thickness

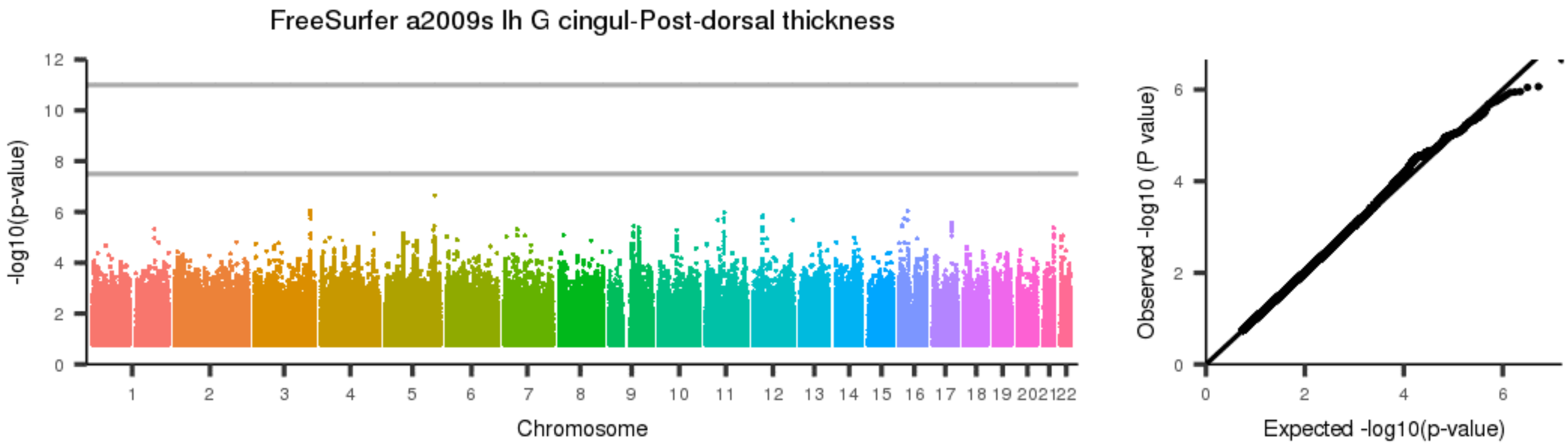

back to list of plots

Figure 1273: FreeSurfer a2009s lh G cingul-Post-ventral thickness

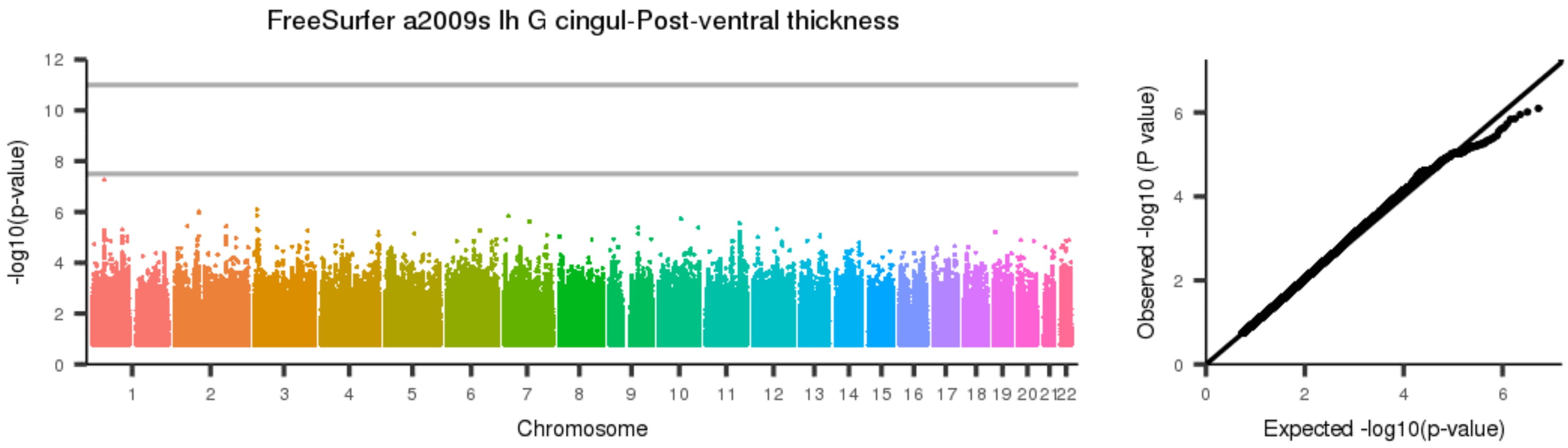

back to list of plots

Figure 1274: FreeSurfer a2009s lh G cuneus thickness

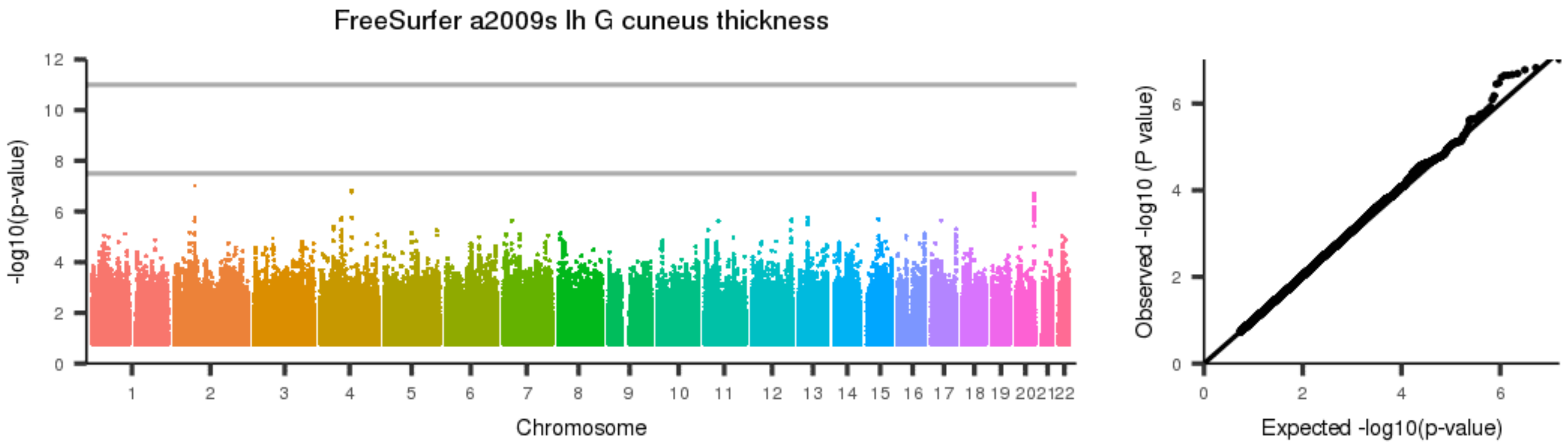

back to list of plots

Figure 1275: FreeSurfer a2009s lh G front inf-Opercular thickness

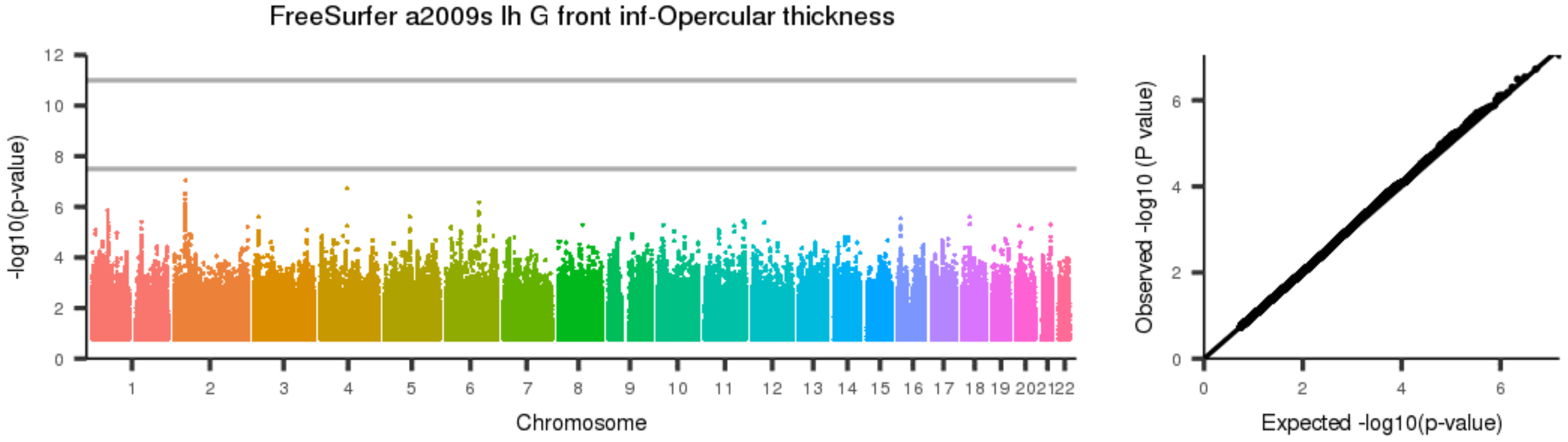

back to list of plots

Figure 1276: FreeSurfer a2009s lh G front inf-Orbital thickness

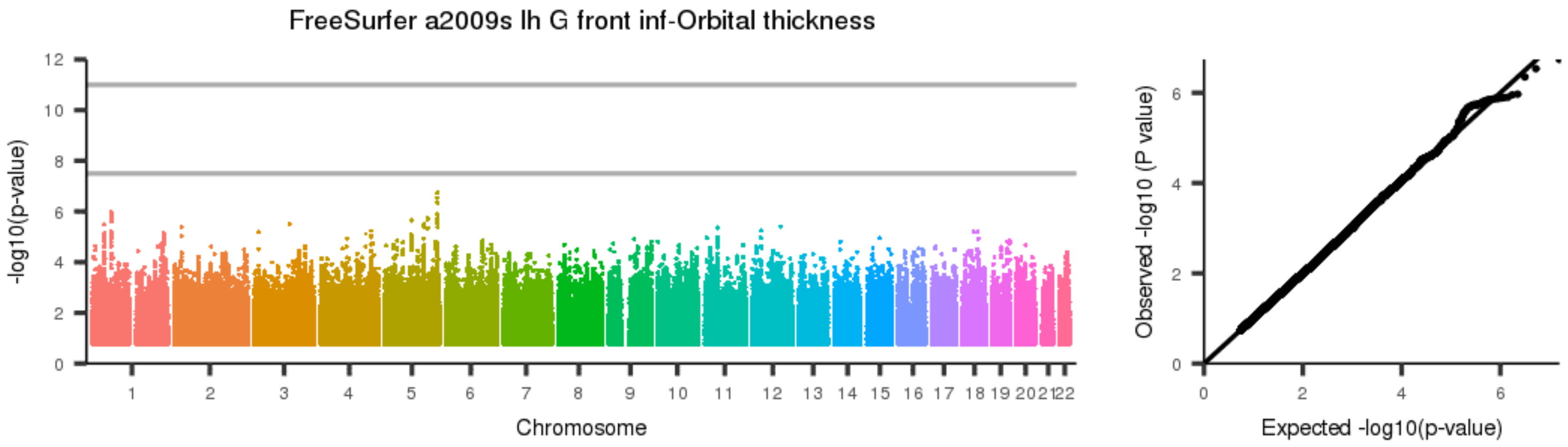

back to list of plots

Figure 1277: FreeSurfer a2009s lh G front inf-Triangul thickness

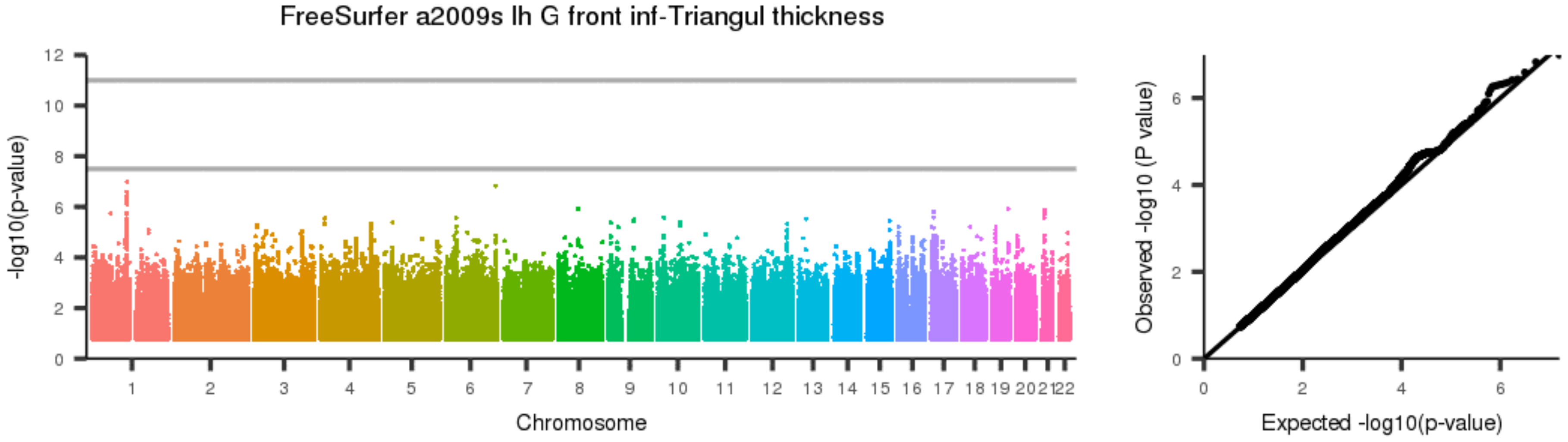

back to list of plots

Figure 1278: FreeSurfer a2009s lh G front middle thickness

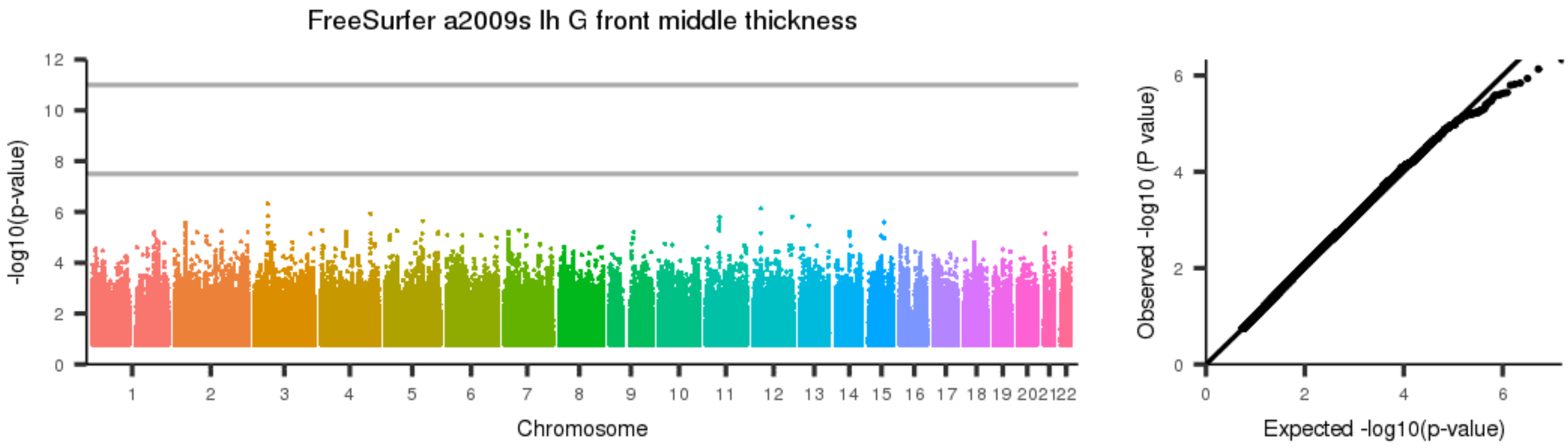

back to list of plots

Figure 1279: FreeSurfer a2009s lh G front sup thickness

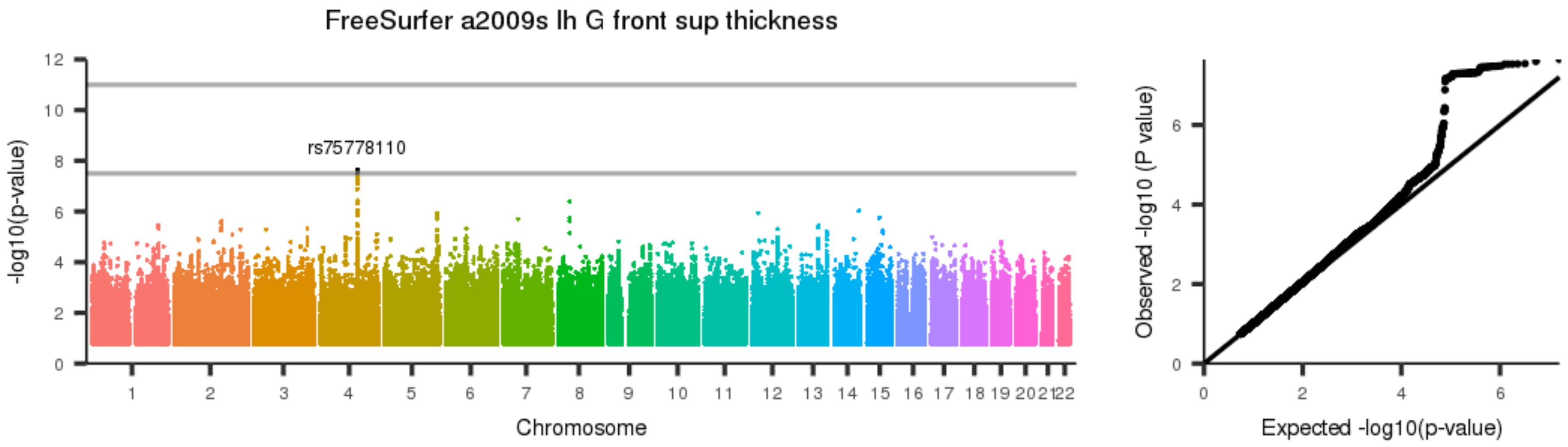

back to list of plots

Figure 1280: FreeSurfer a2009s lh G Ins lg and S cent ins thickness

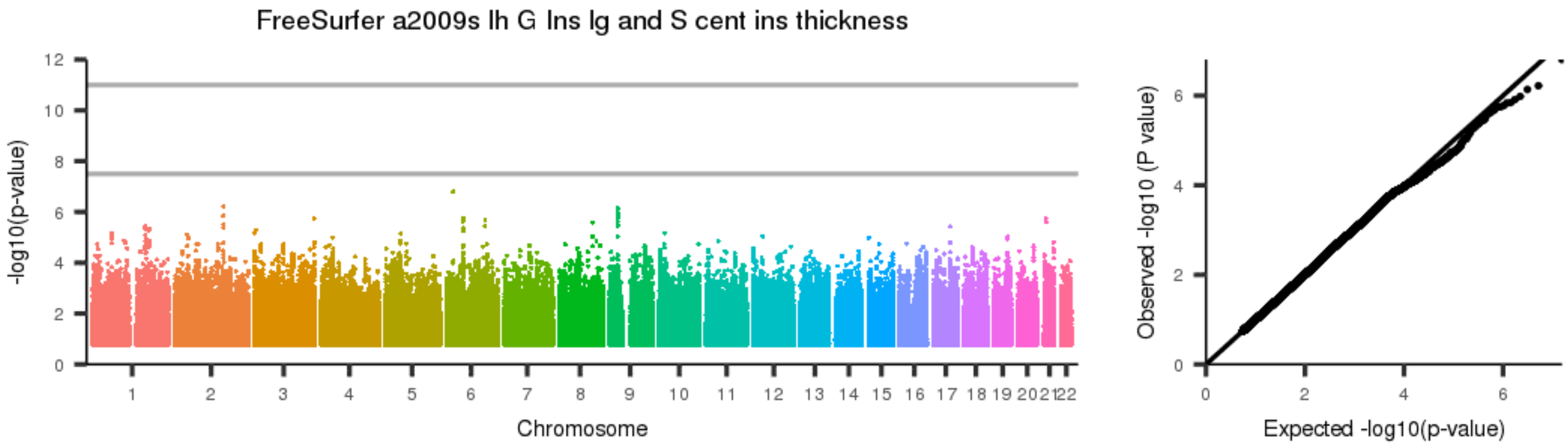

back to list of plots

Figure 1281: FreeSurfer a2009s lh G insular short thickness

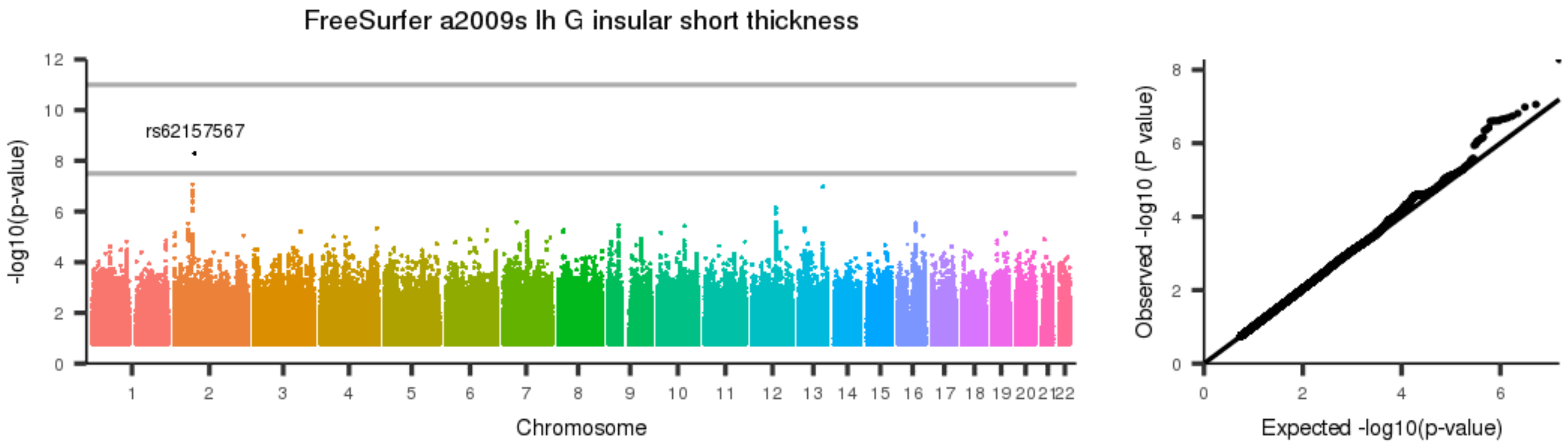

back to list of plots

Figure 1282: FreeSurfer a2009s lh G occipital middle thickness

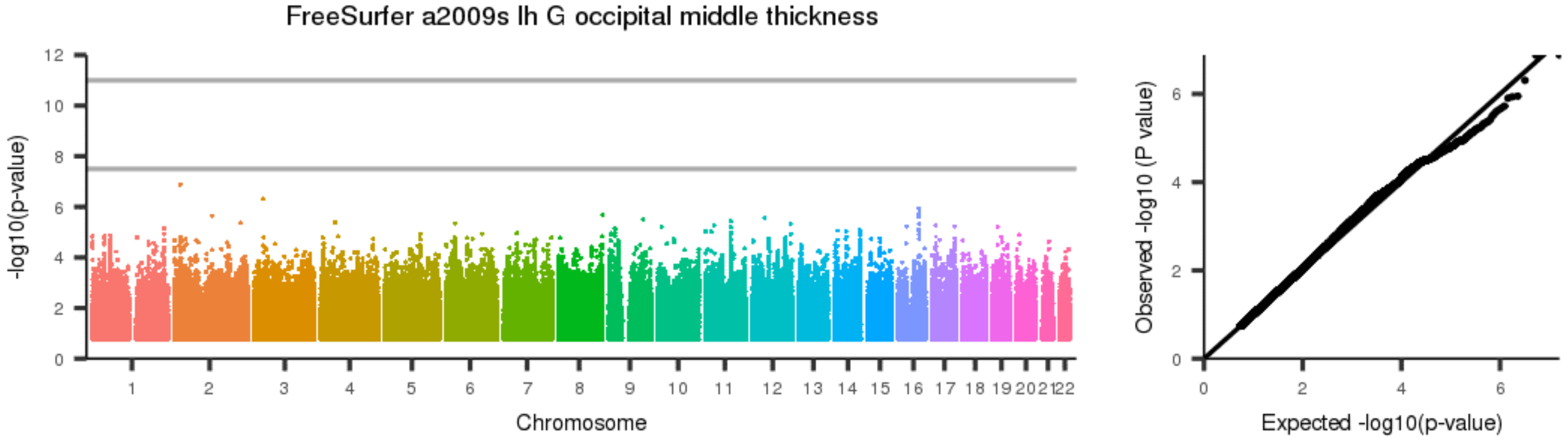

back to list of plots

Figure 1283: FreeSurfer a2009s lh G occipital sup thickness

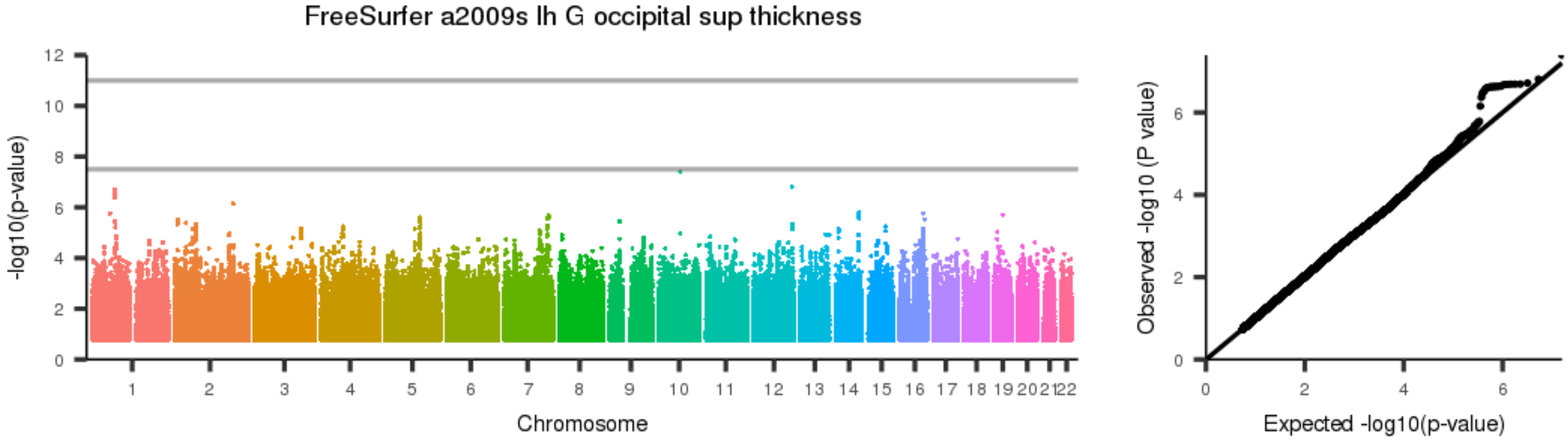

back to list of plots

Figure 1284: FreeSurfer a2009s lh G oc-temp lat-fusifor thickness

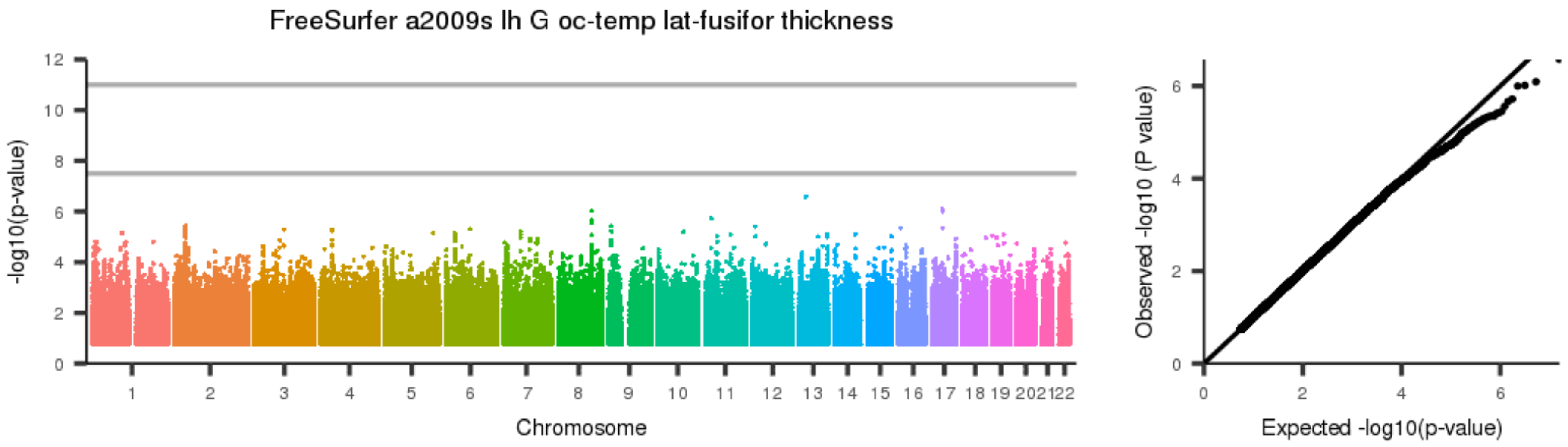

back to list of plots

Figure 1285: FreeSurfer a2009s lh G oc-temp med-Lingual thickness

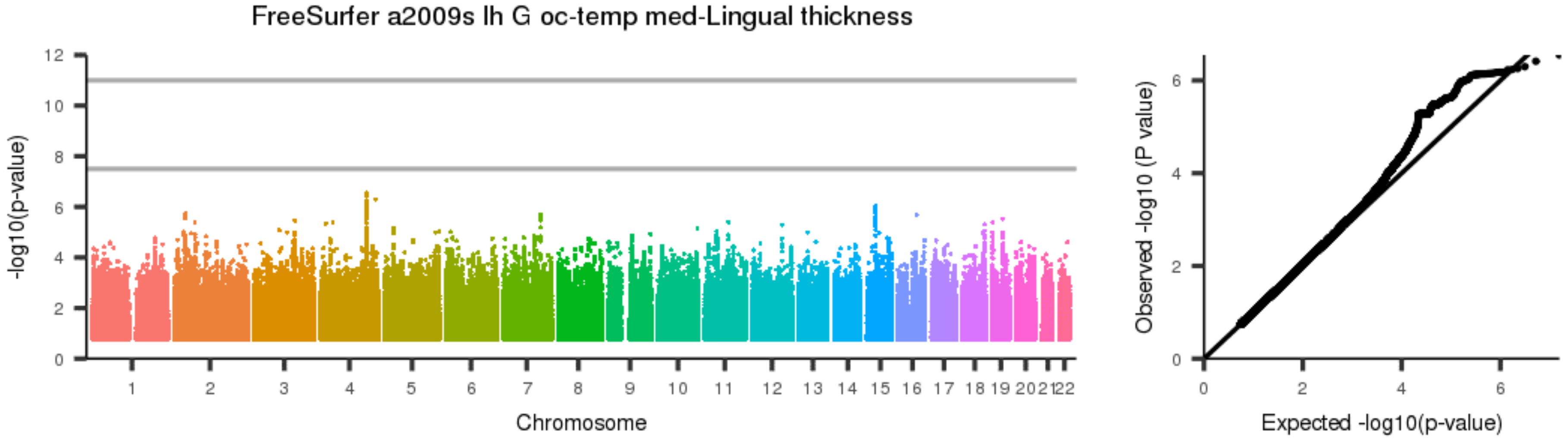

back to list of plots

Figure 1286: FreeSurfer a2009s lh G oc-temp med-Parahip thickness

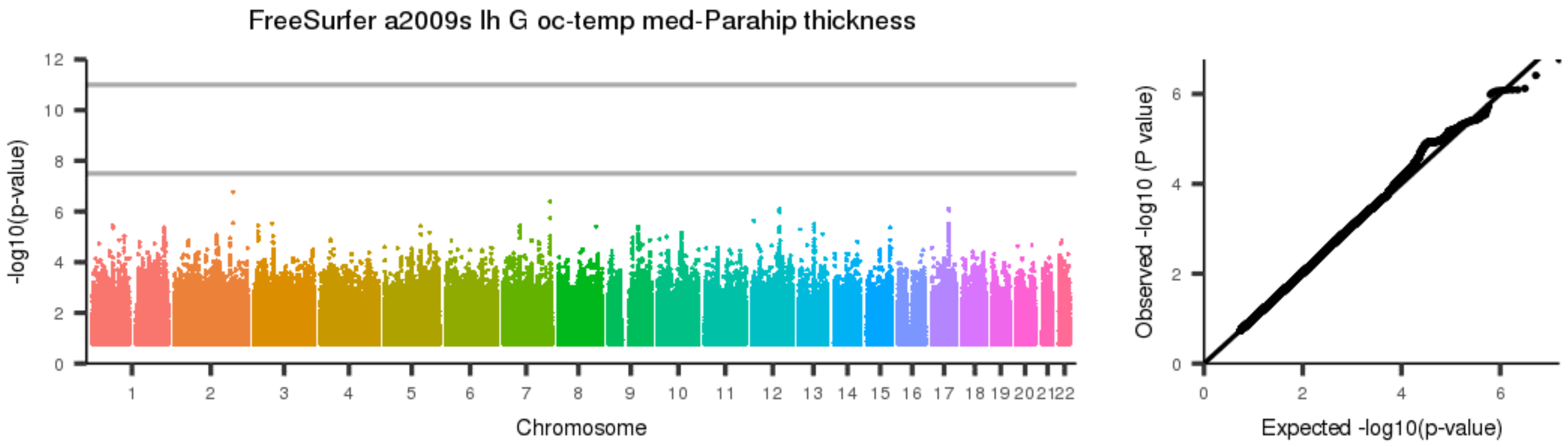

back to list of plots

Figure 1287: FreeSurfer a2009s lh G orbital thickness

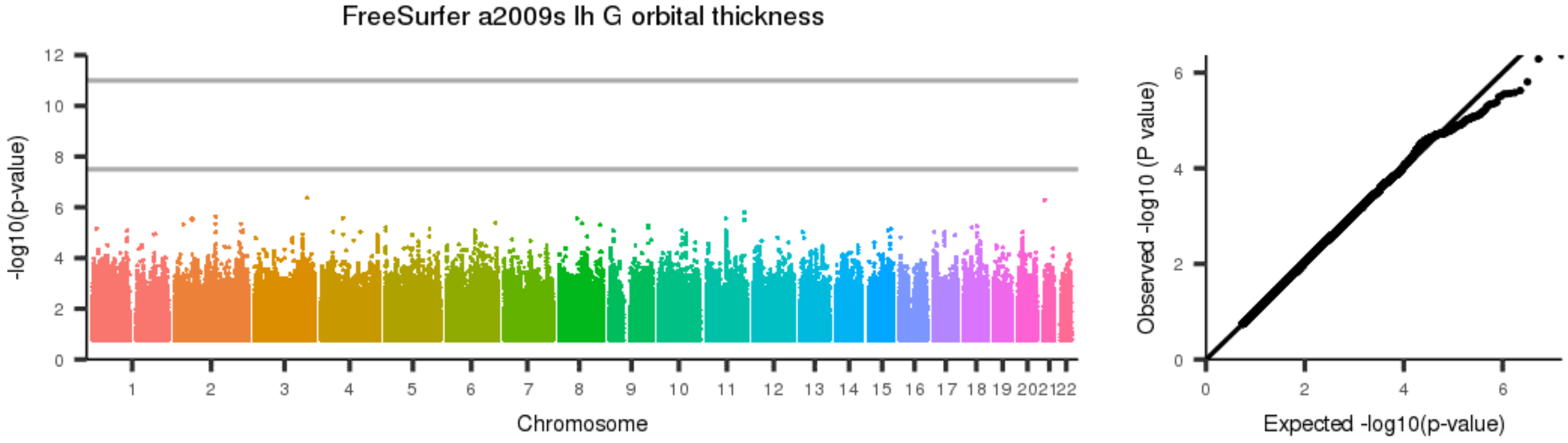

back to list of plots

Figure 1288: FreeSurfer a2009s lh G pariet inf-Angular thickness

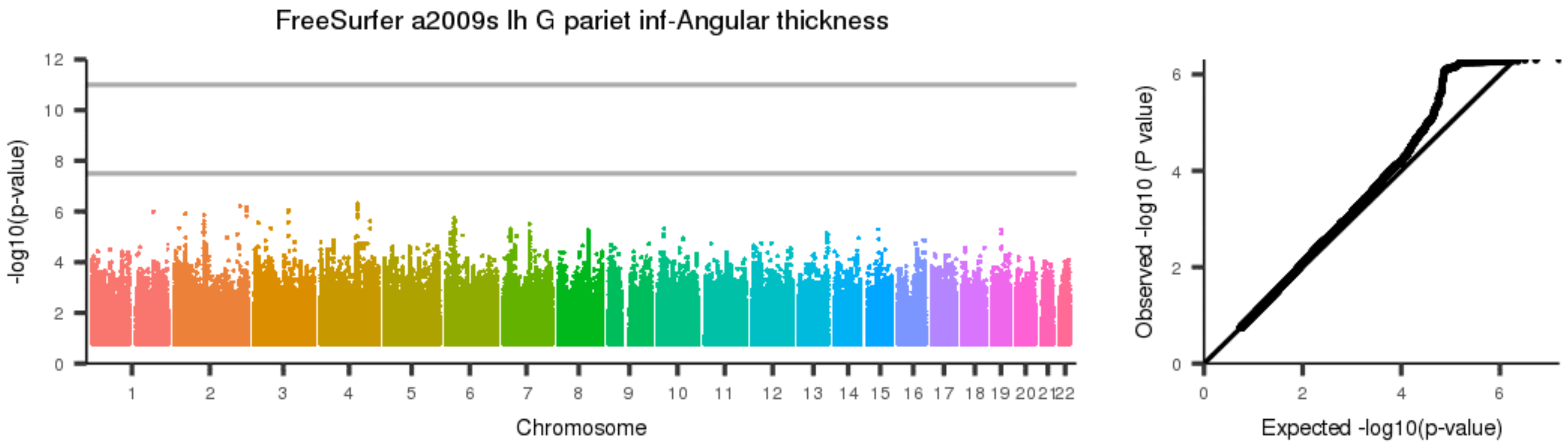

back to list of plots

Figure 1289: FreeSurfer a2009s lh G pariet inf-Supramar thickness

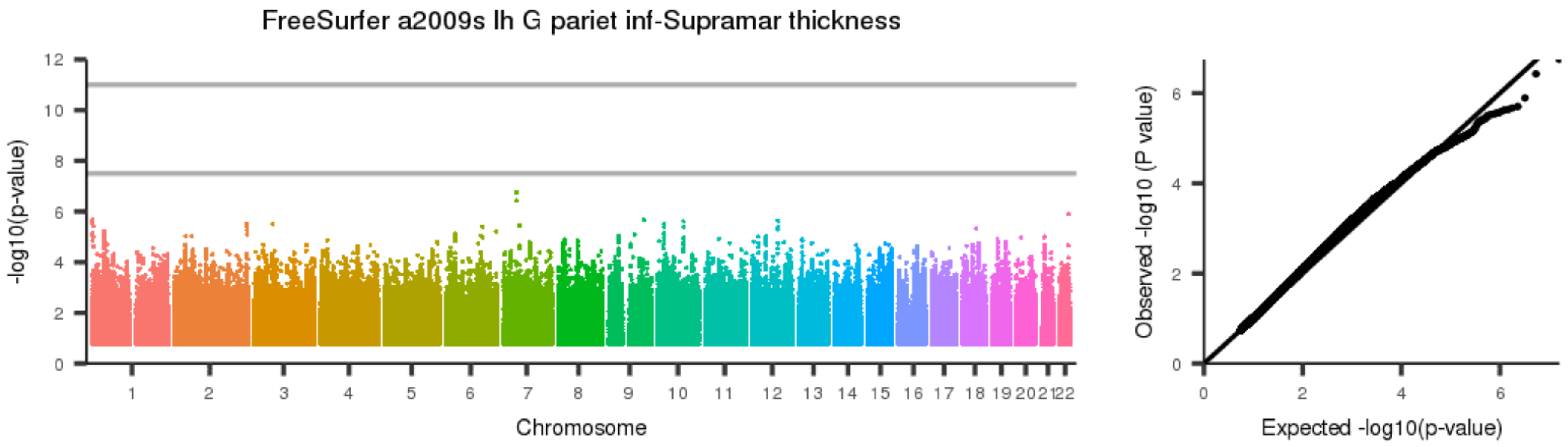

back to list of plots

Figure 1290: FreeSurfer a2009s lh G parietal sup thickness

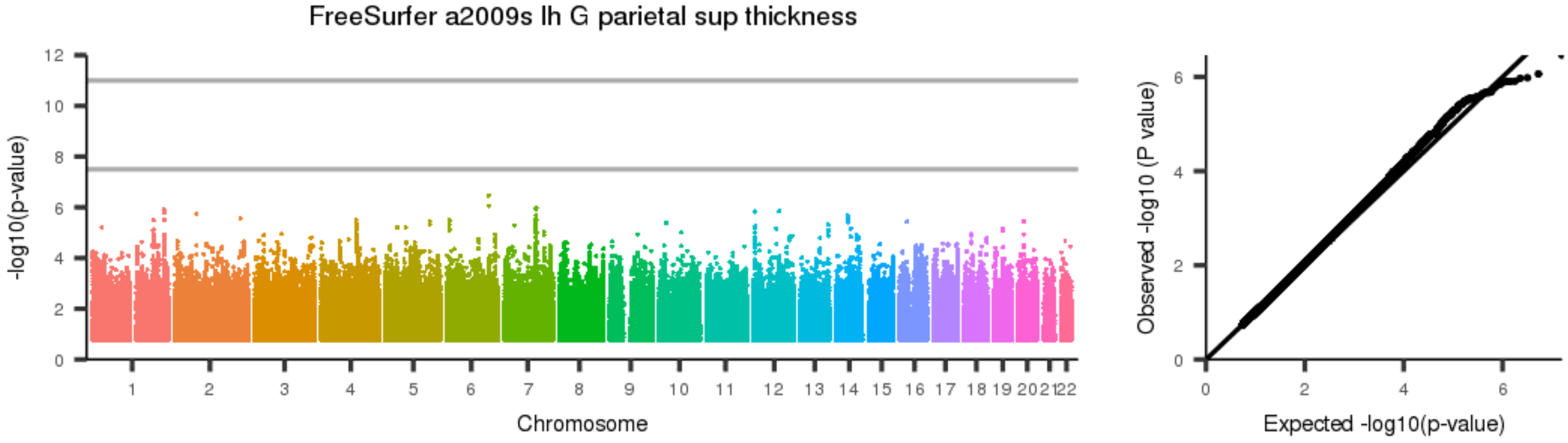

back to list of plots

Figure 1291: FreeSurfer a2009s lh G postcentral thickness

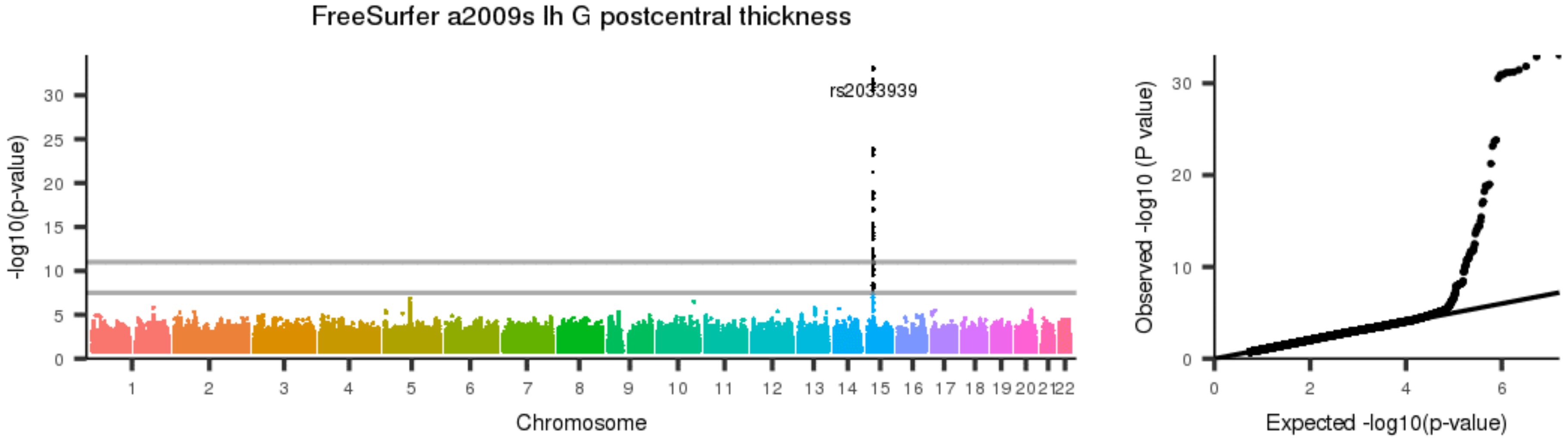

back to list of plots

Figure 1292: FreeSurfer a2009s lh G precentral thickness

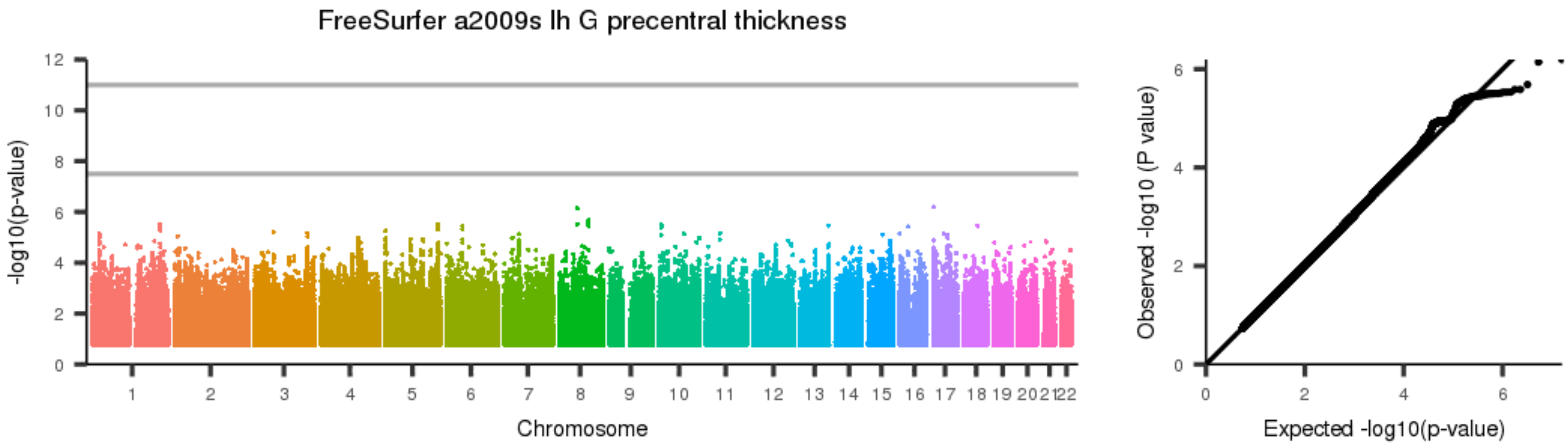

back to list of plots

Figure 1293: FreeSurfer a2009s lh G precuneus thickness

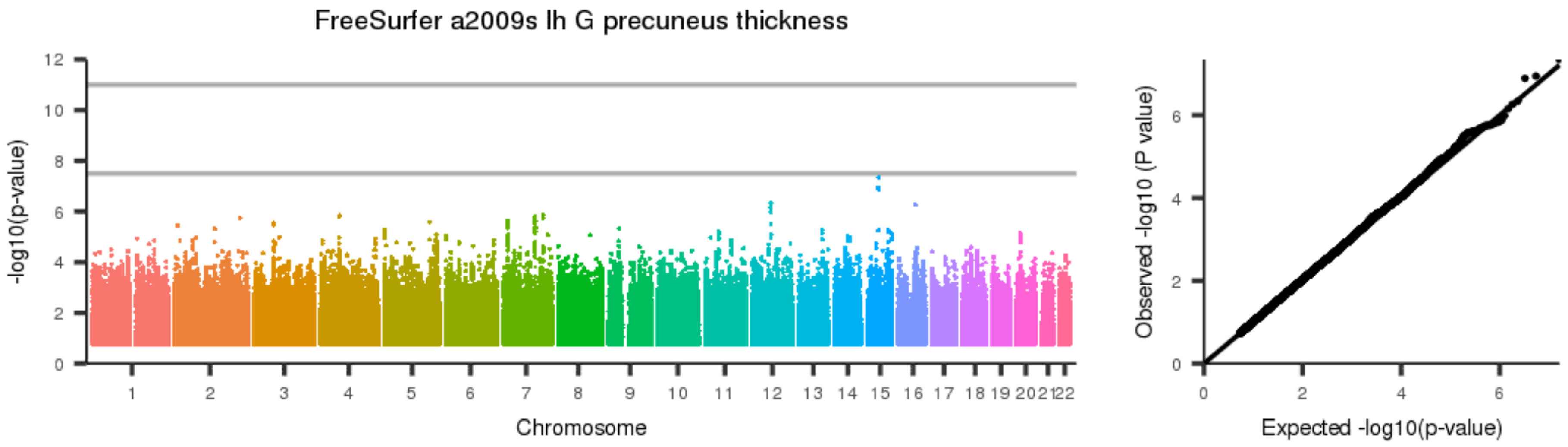

back to list of plots

Figure 1294: FreeSurfer a2009s lh G rectus thickness

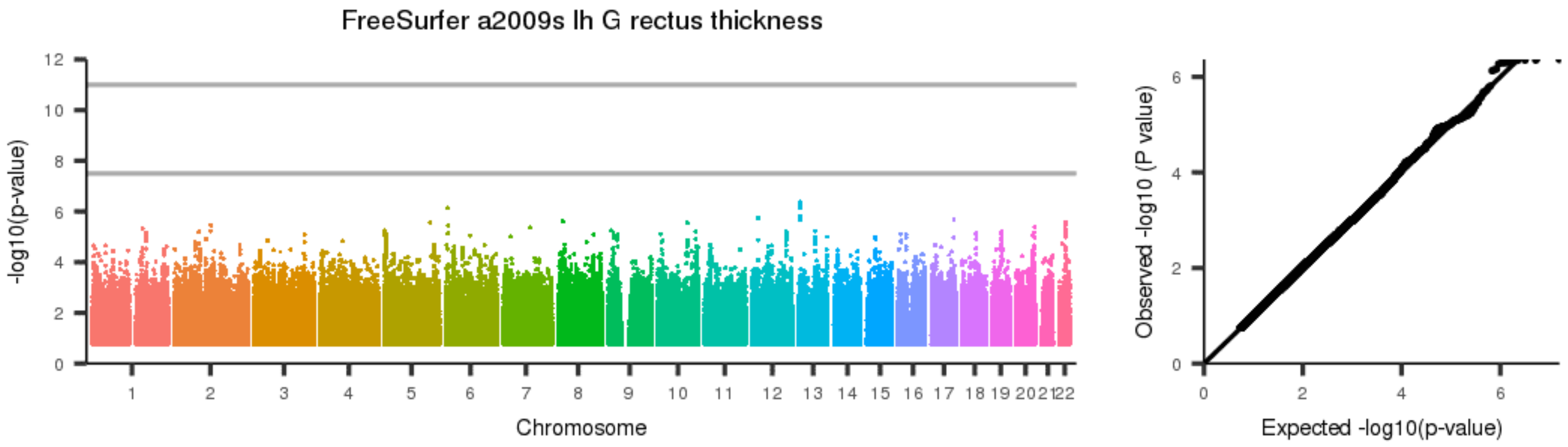

back to list of plots

Figure 1295: FreeSurfer a2009s lh G subcallosal thickness

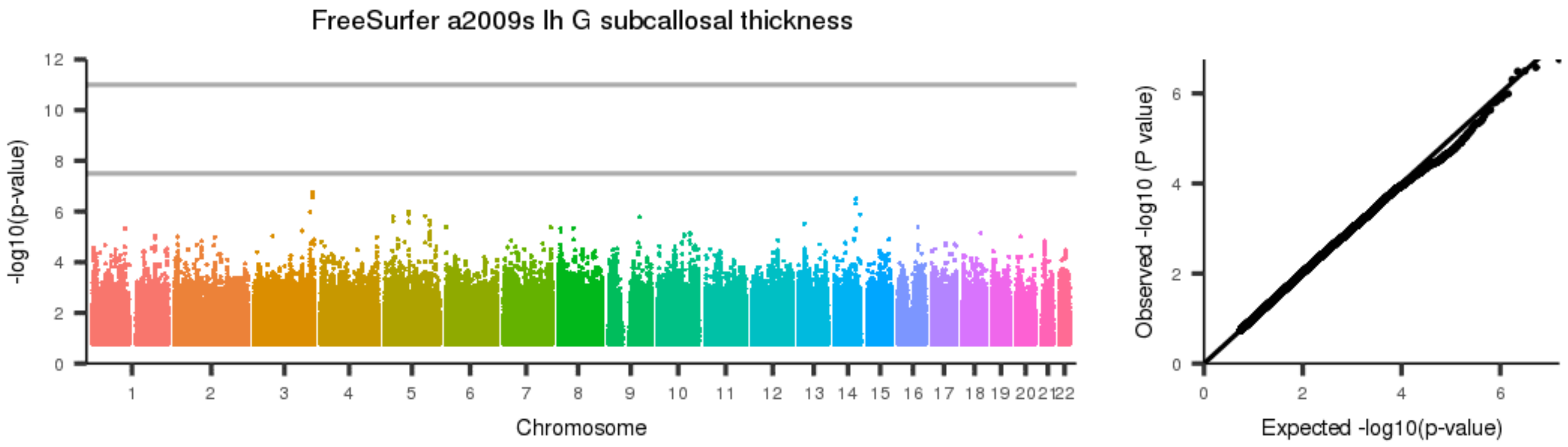

back to list of plots

Figure 1296: FreeSurfer a2009s lh G temp sup-G T transv thickness

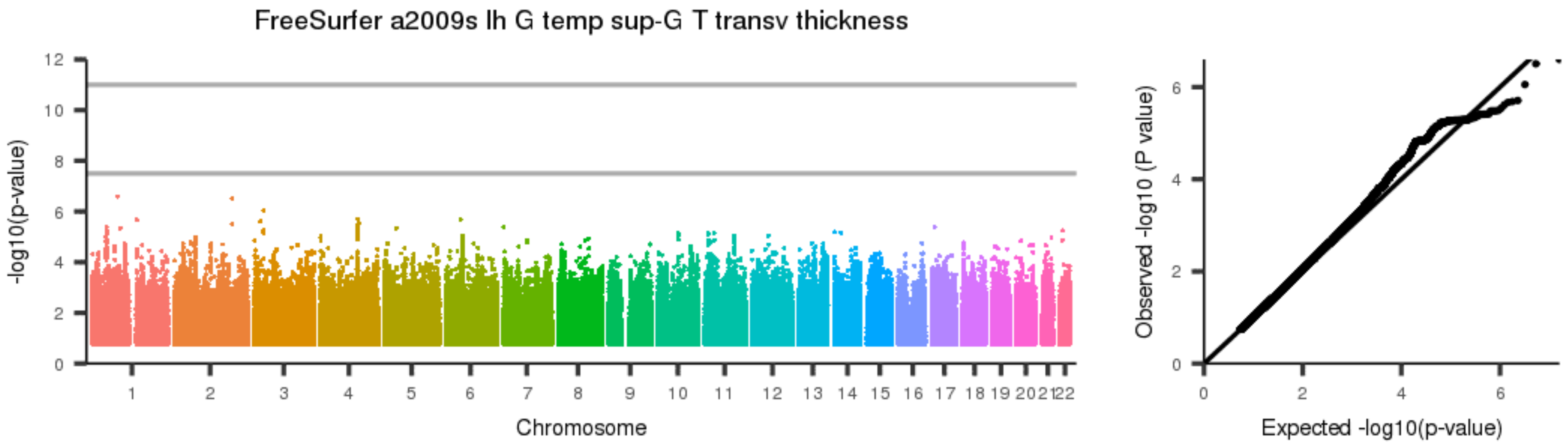

back to list of plots

Figure 1297: FreeSurfer a2009s lh G temp sup-Lateral thickness

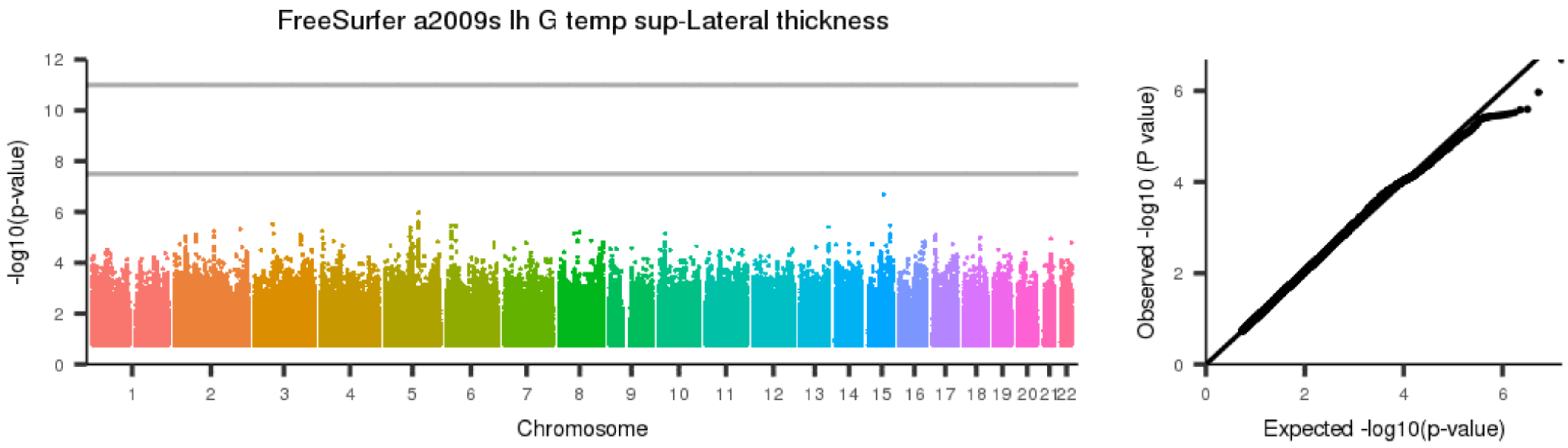

back to list of plots

Figure 1298: FreeSurfer a2009s lh G temp sup-Plan polar thickness

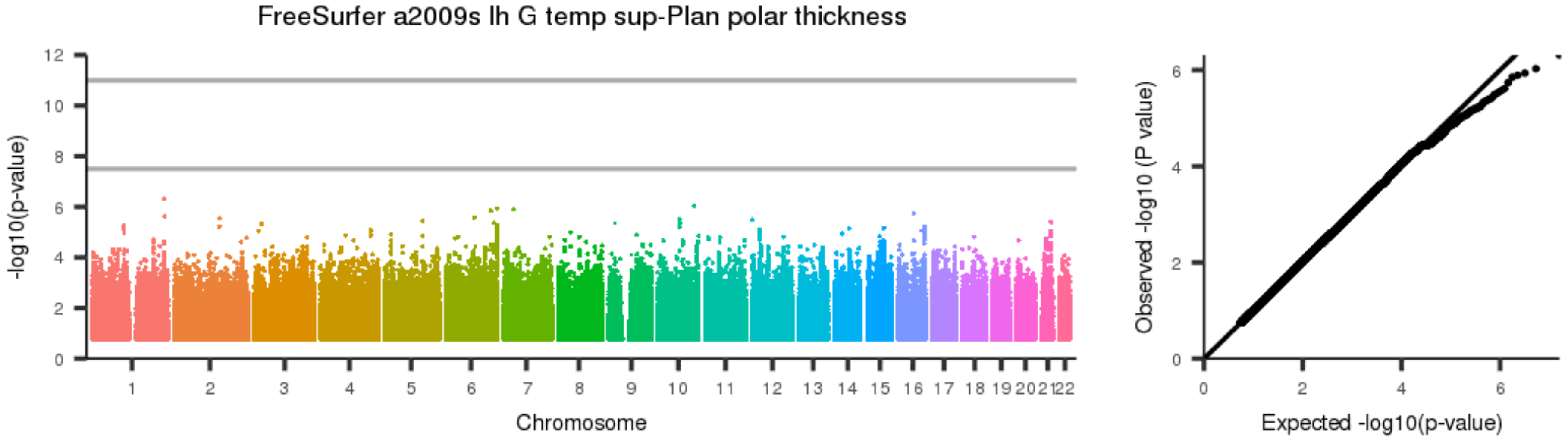

back to list of plots

Figure 1299: FreeSurfer a2009s lh G temp sup-Plan tempo thickness

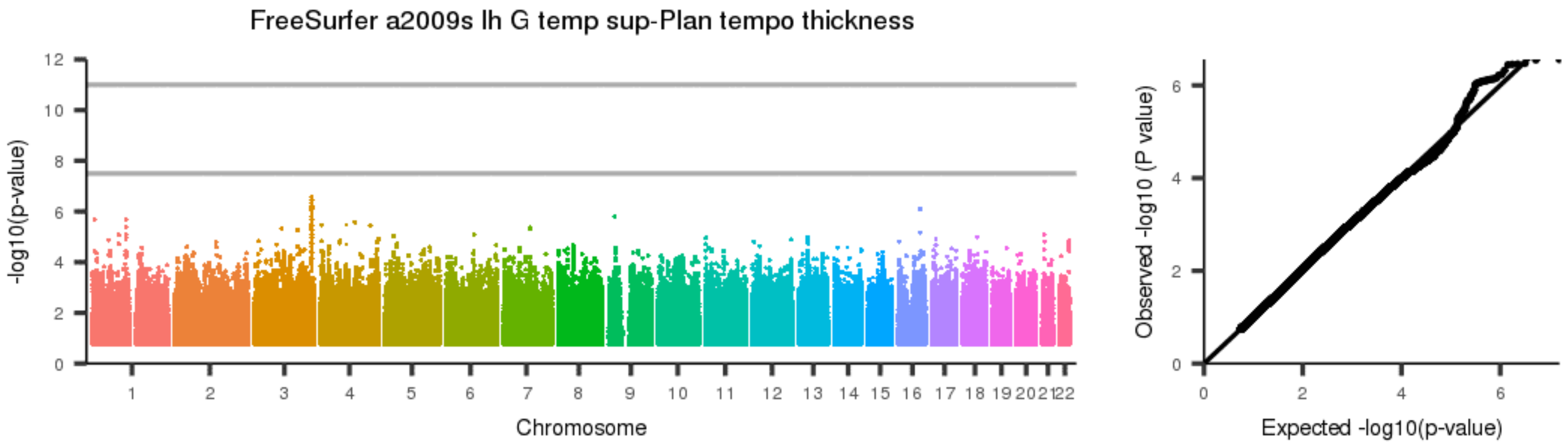

back to list of plots

Figure 1300: FreeSurfer a2009s lh G temporal inf thickness

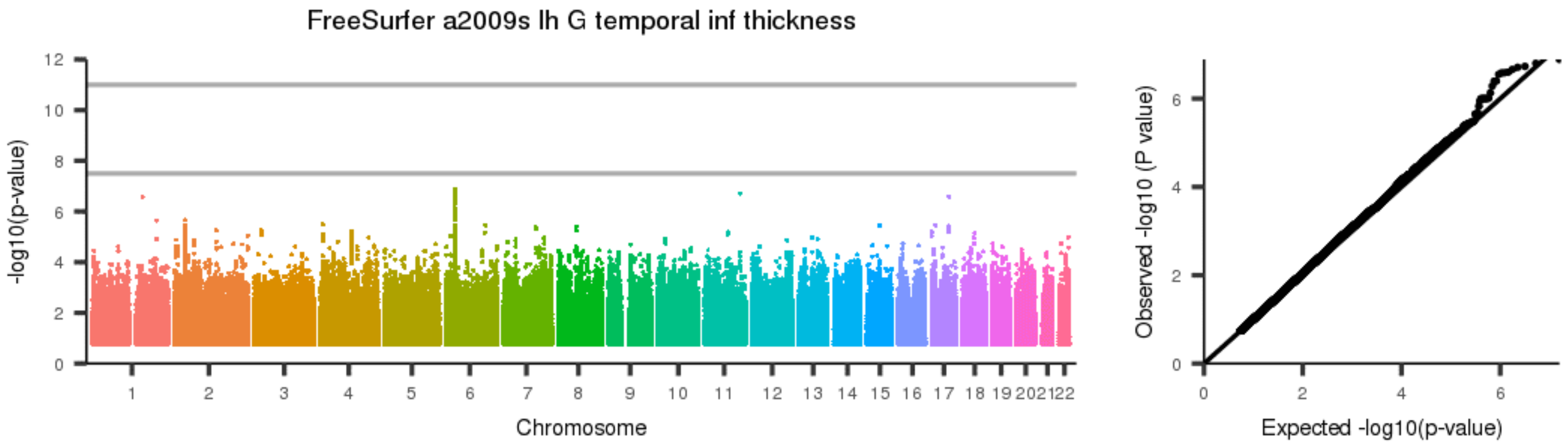

back to list of plots

Figure 1301: FreeSurfer a2009s lh G temporal middle thickness

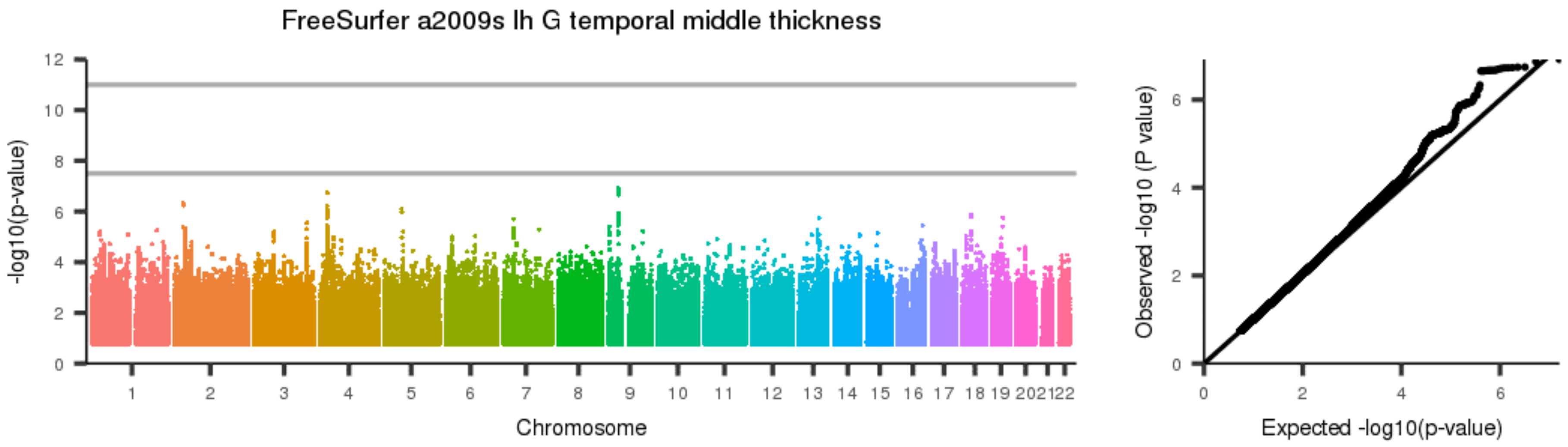

back to list of plots

Figure 1302: FreeSurfer a2009s lh Lat Fis-ant-Horizont thickness

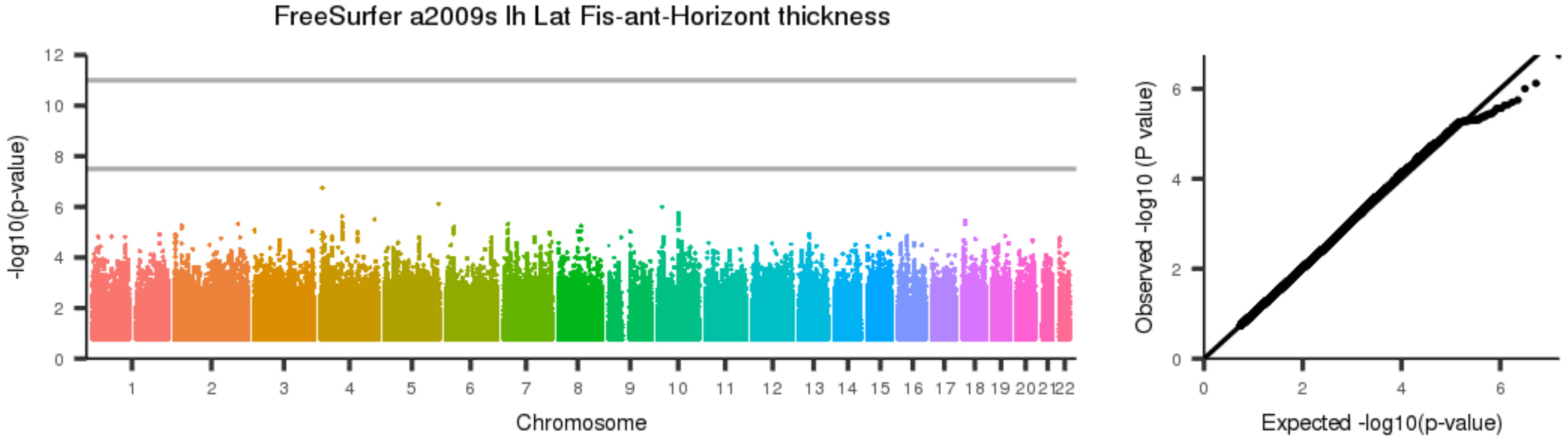

back to list of plots

Figure 1303: FreeSurfer a2009s lh Lat Fis-ant-Vertical thickness

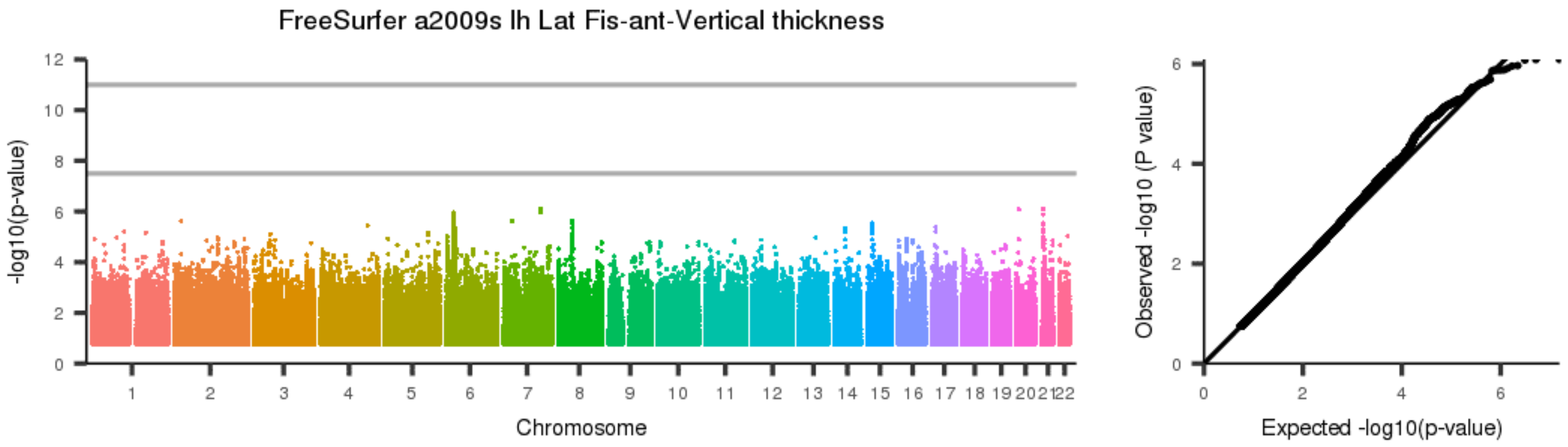

back to list of plots

Figure 1304: FreeSurfer a2009s lh Lat Fis-post thickness

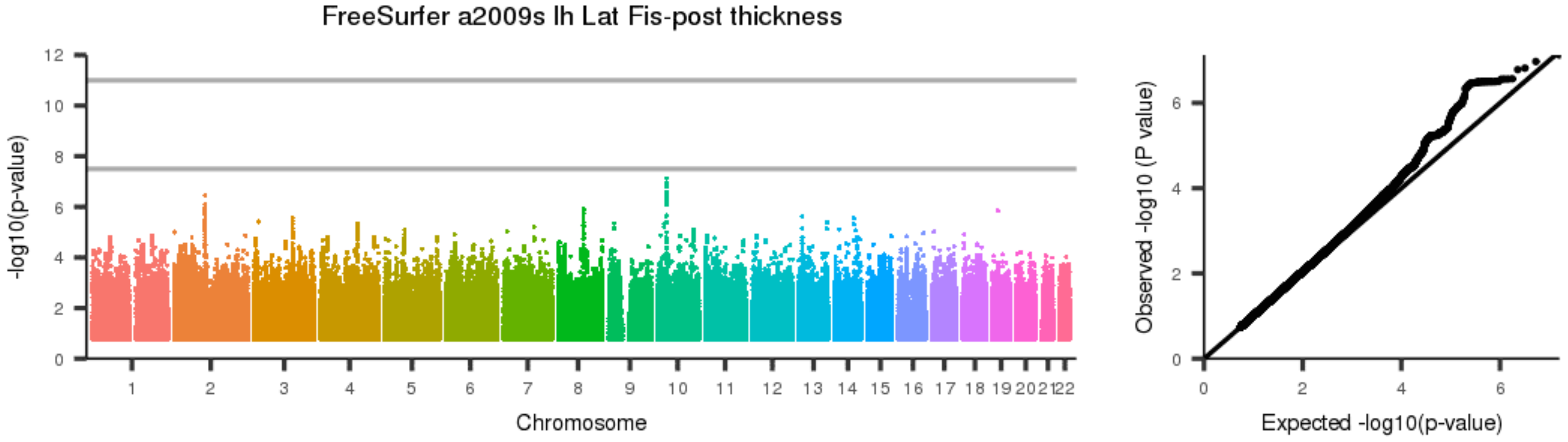

back to list of plots

Figure 1305: FreeSurfer a2009s lh Pole occipital thickness

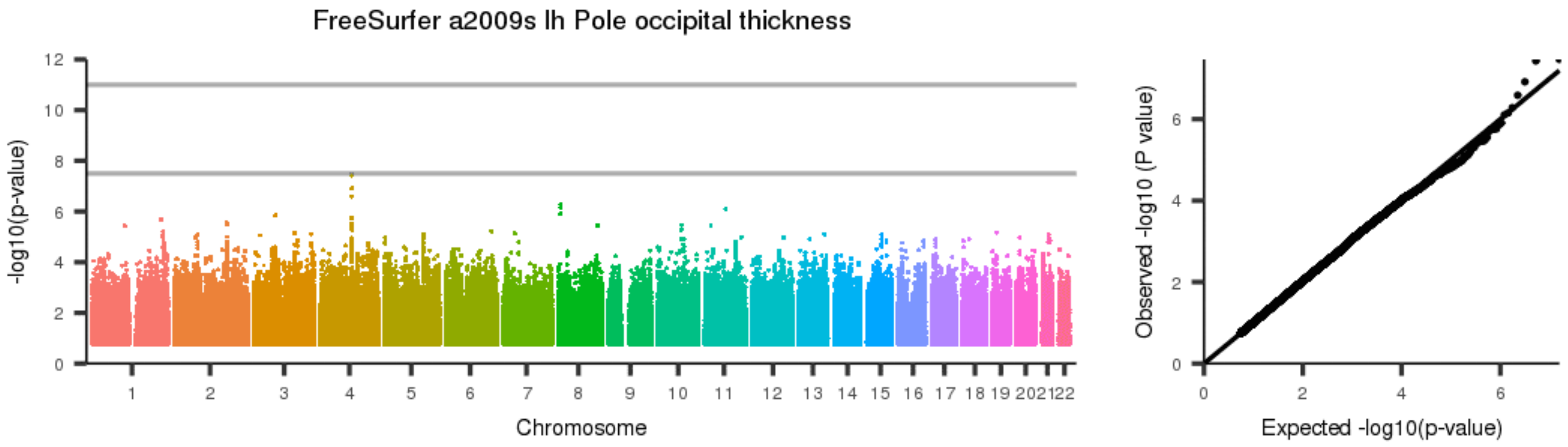

back to list of plots

Figure 1306: FreeSurfer a2009s lh Pole temporal thickness

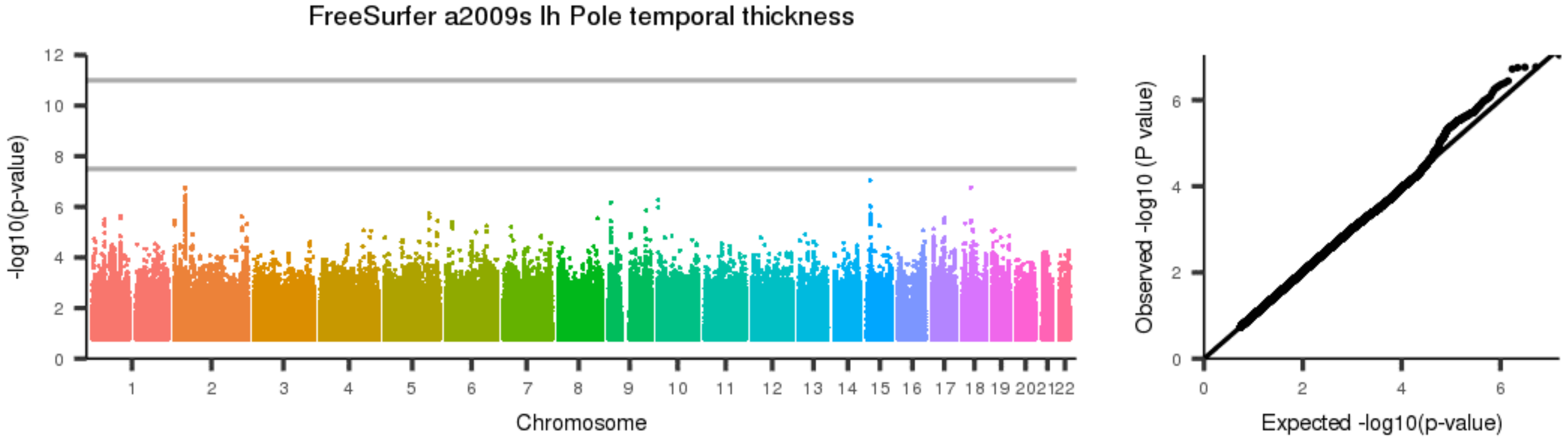

back to list of plots

Figure 1307: FreeSurfer a2009s lh S calcarine thickness

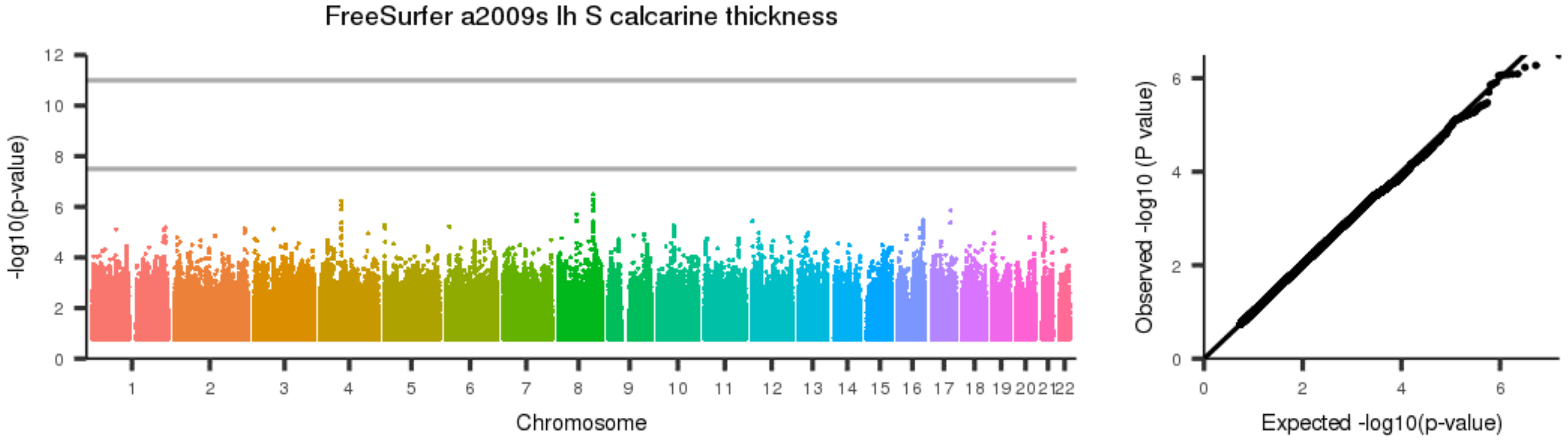

back to list of plots

Figure 1308: FreeSurfer a2009s lh S central thickness

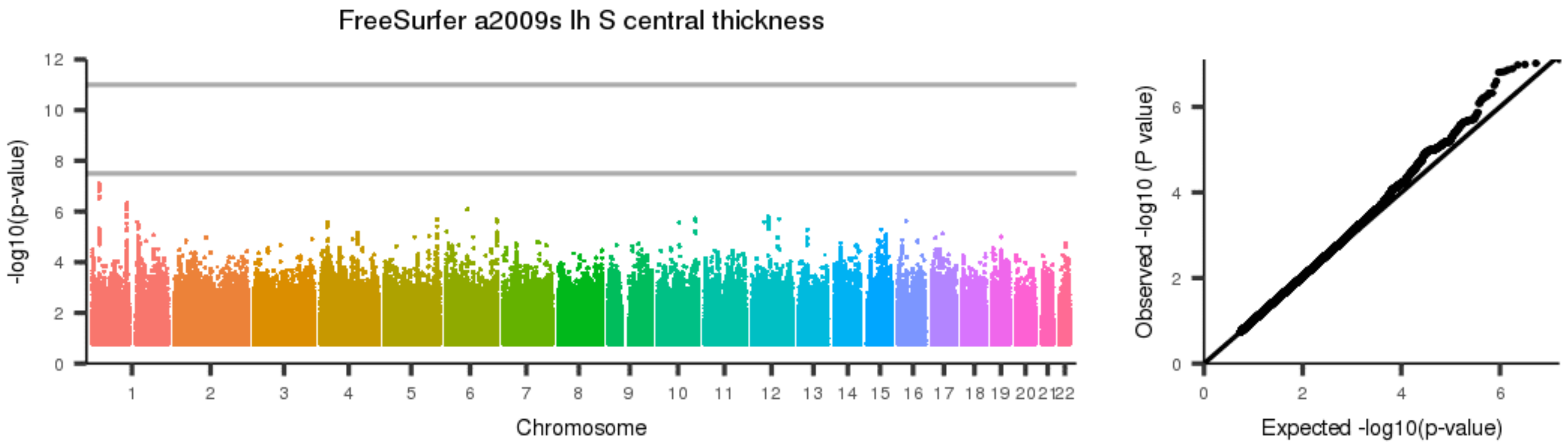

back to list of plots

Figure 1309: FreeSurfer a2009s lh S cingul-Marginalis thickness

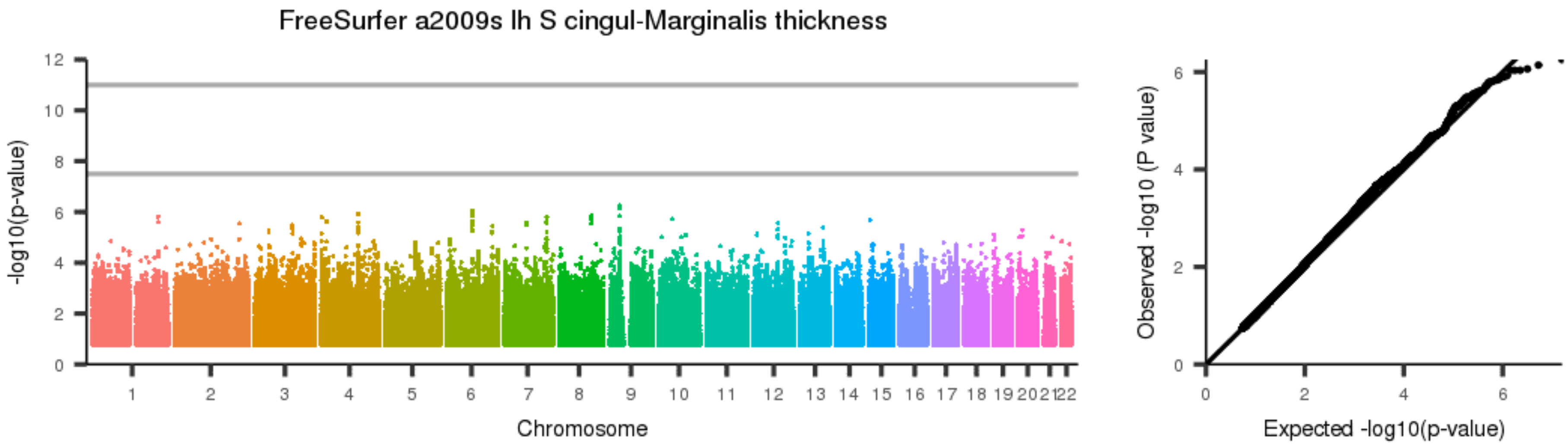

back to list of plots

Figure 1310: FreeSurfer a2009s lh S circular insula ant thickness

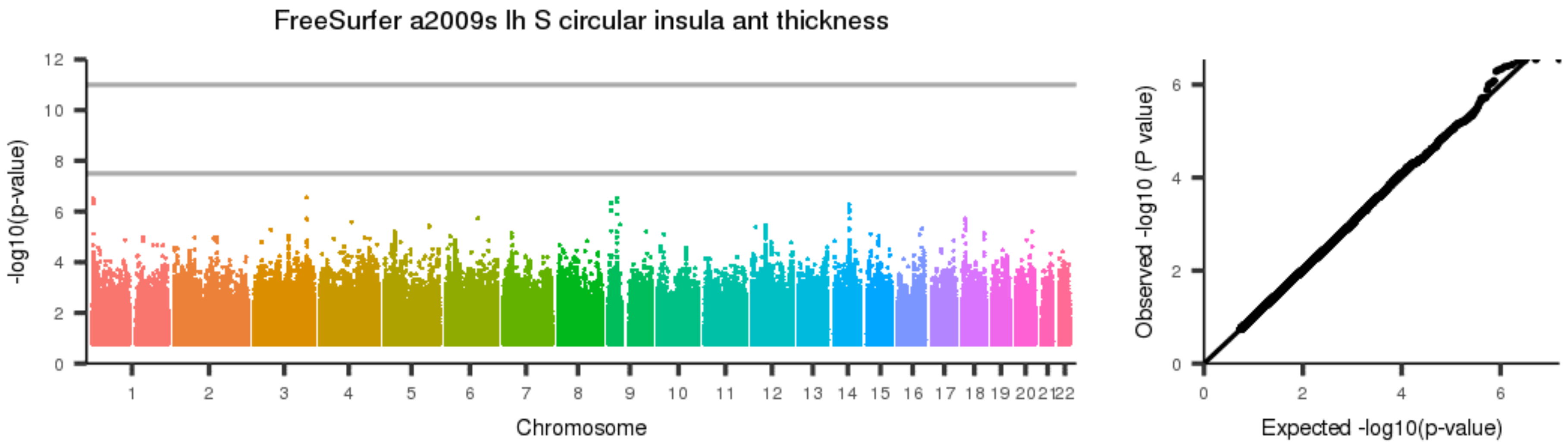

back to list of plots

Figure 1311: FreeSurfer a2009s lh S circular insula inf thickness

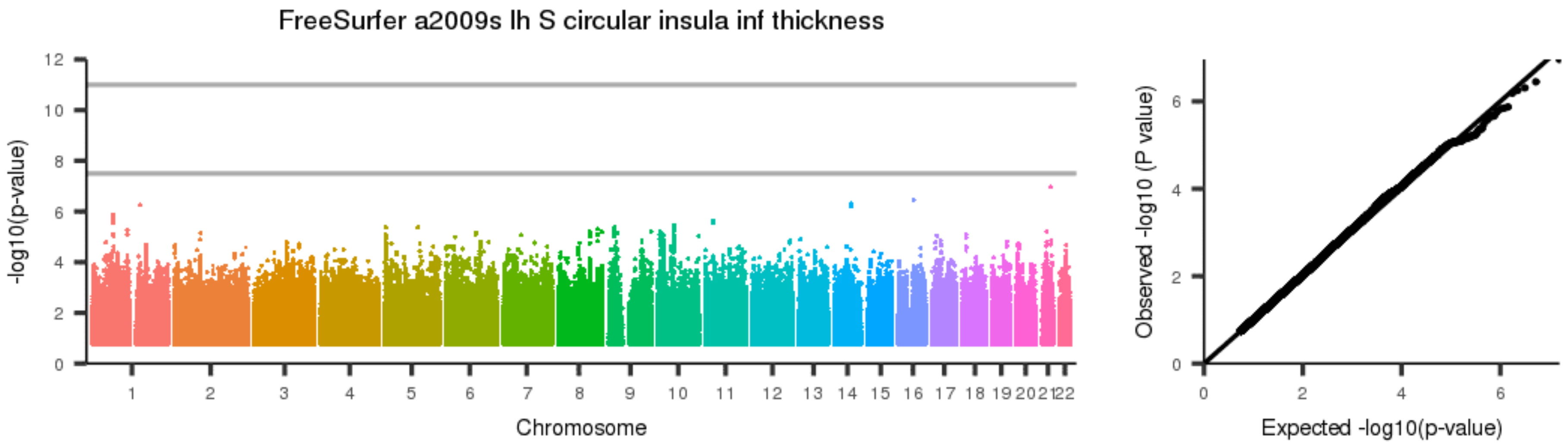

back to list of plots

Figure 1312: FreeSurfer a2009s lh S circular insula sup thickness

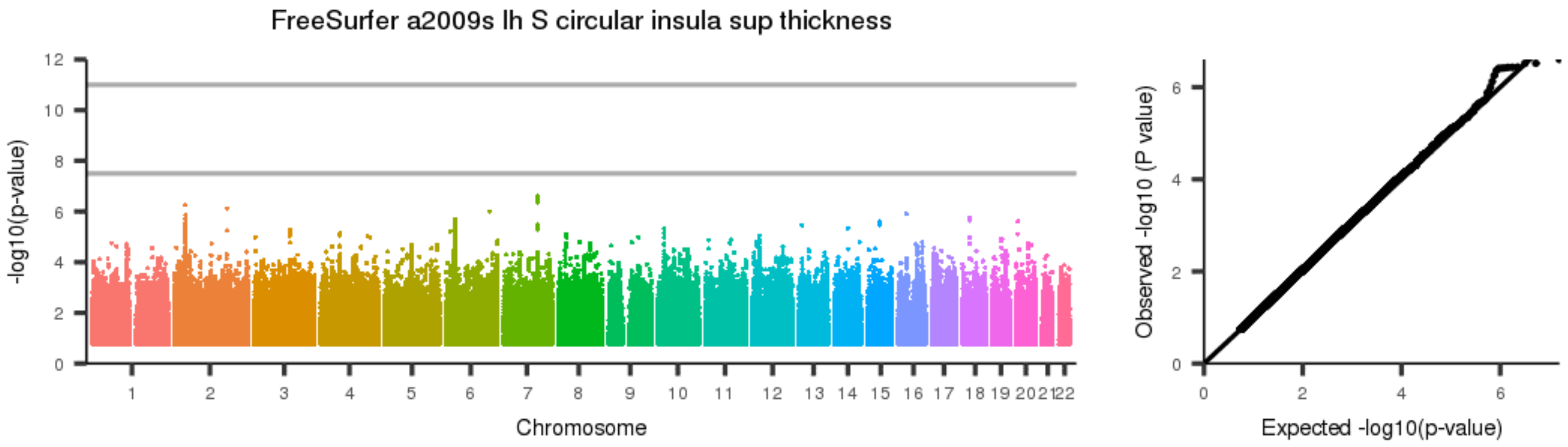

back to list of plots

Figure 1313: FreeSurfer a2009s lh S collat transv ant thickness

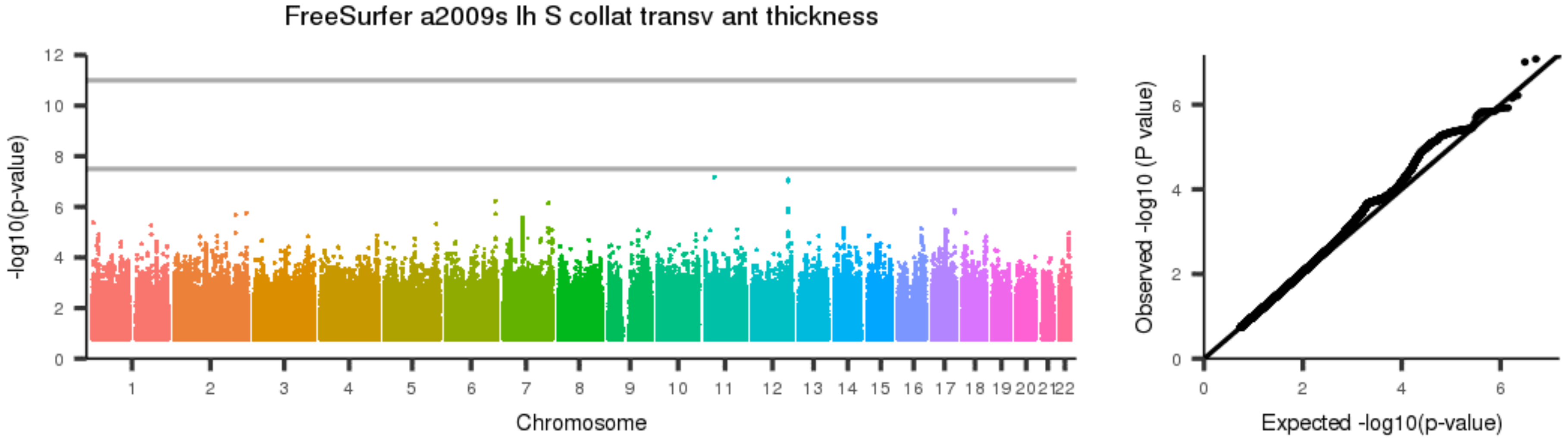

back to list of plots

Figure 1314: FreeSurfer a2009s lh S collat transv post thickness

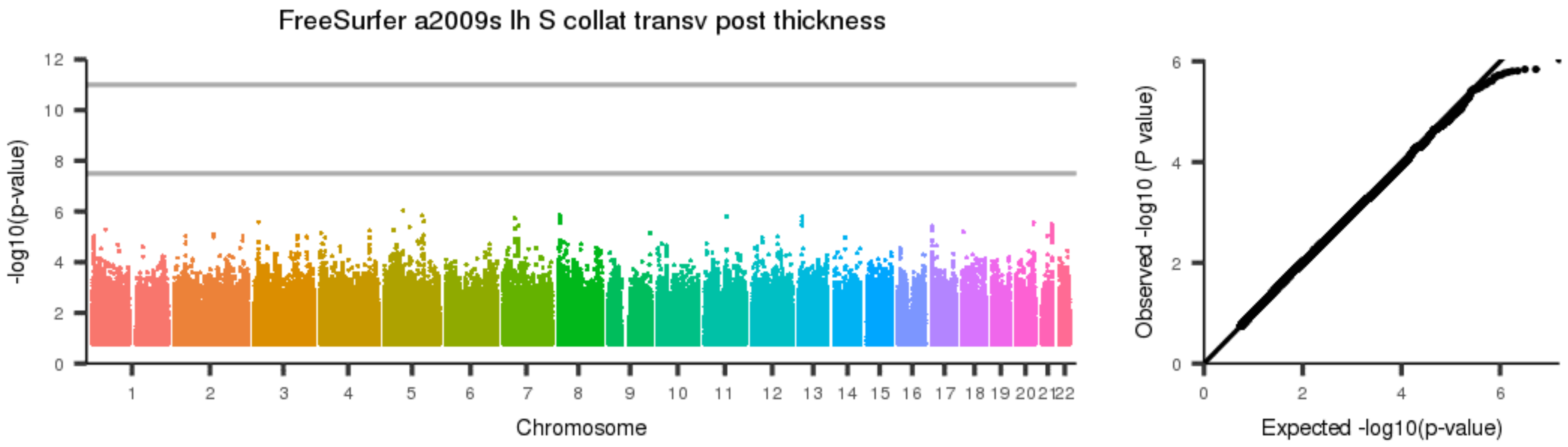

back to list of plots

Figure 1315: FreeSurfer a2009s lh S front inf thickness

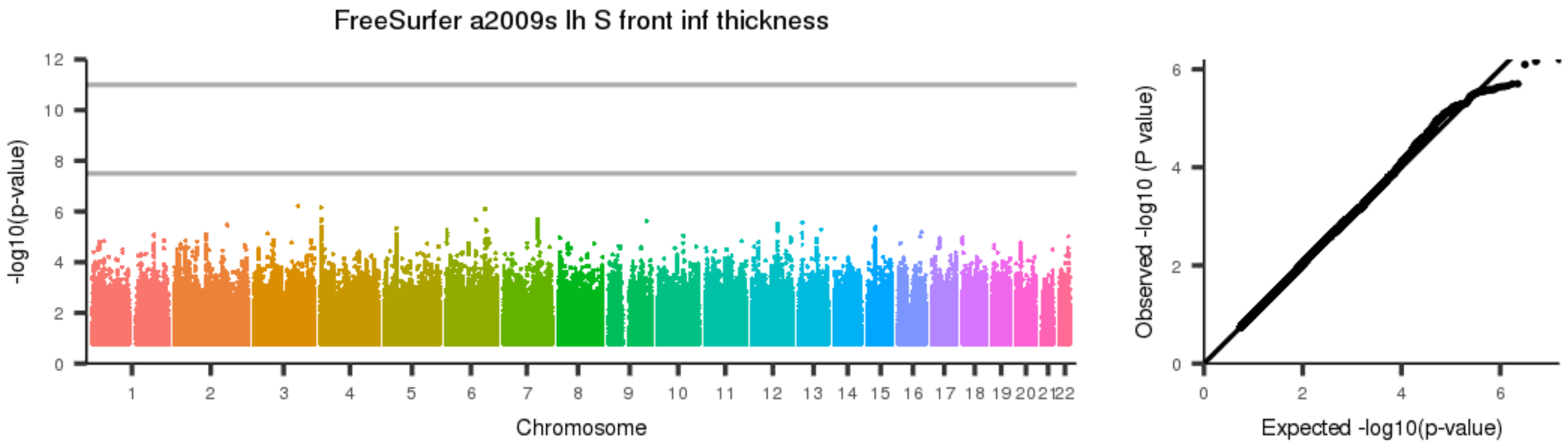

back to list of plots

Figure 1316: FreeSurfer a2009s lh S front middle thickness

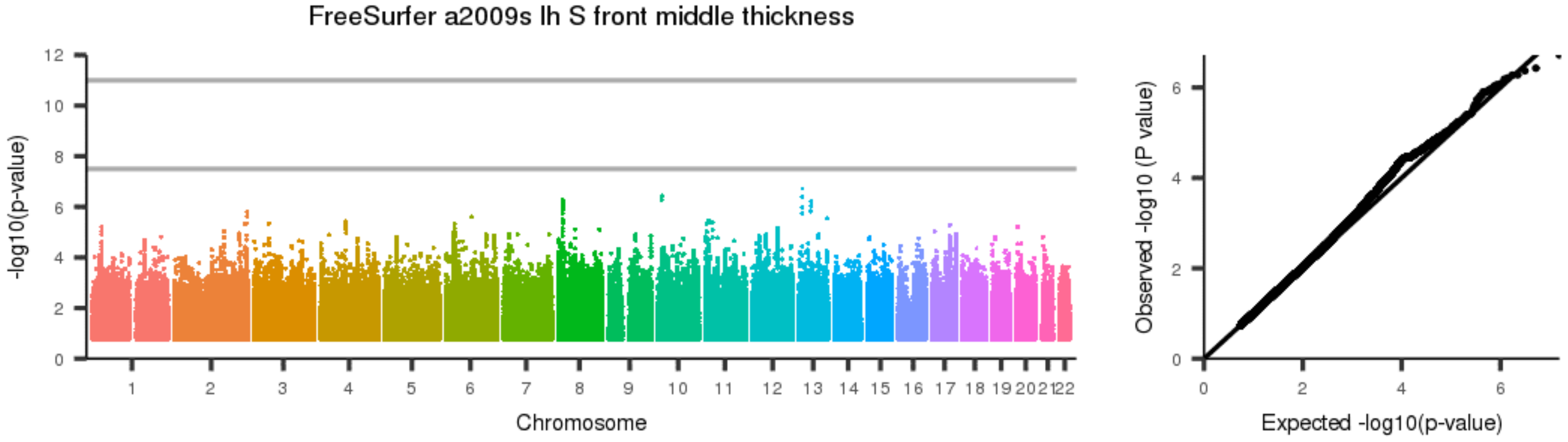

back to list of plots

Figure 1317: FreeSurfer a2009s lh S front sup thickness

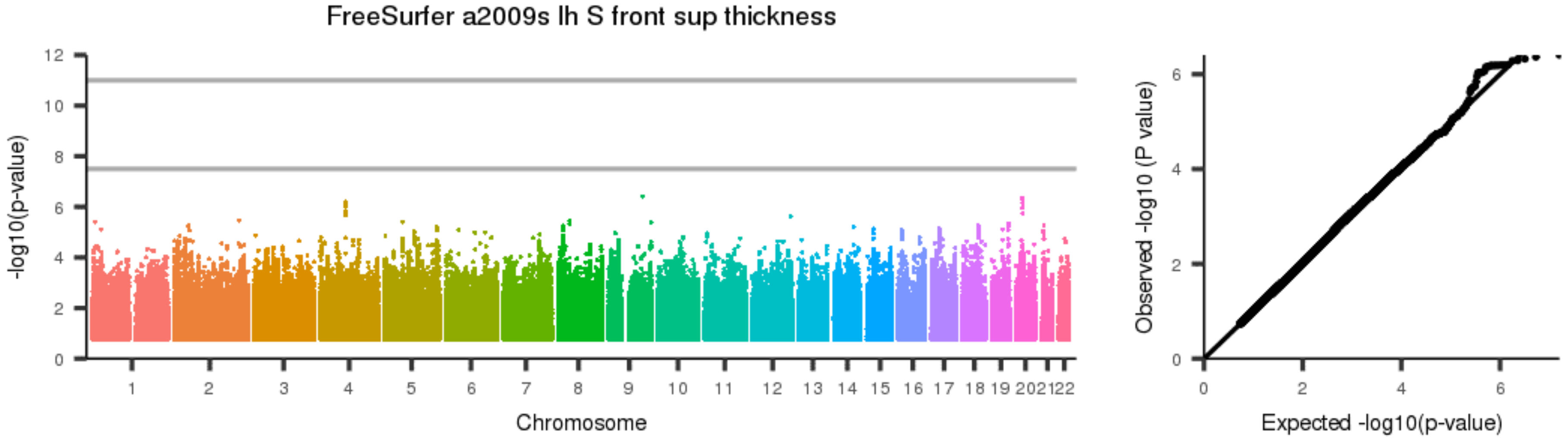

back to list of plots

Figure 1318: FreeSurfer a2009s lh S interm prim-Jensen thickness

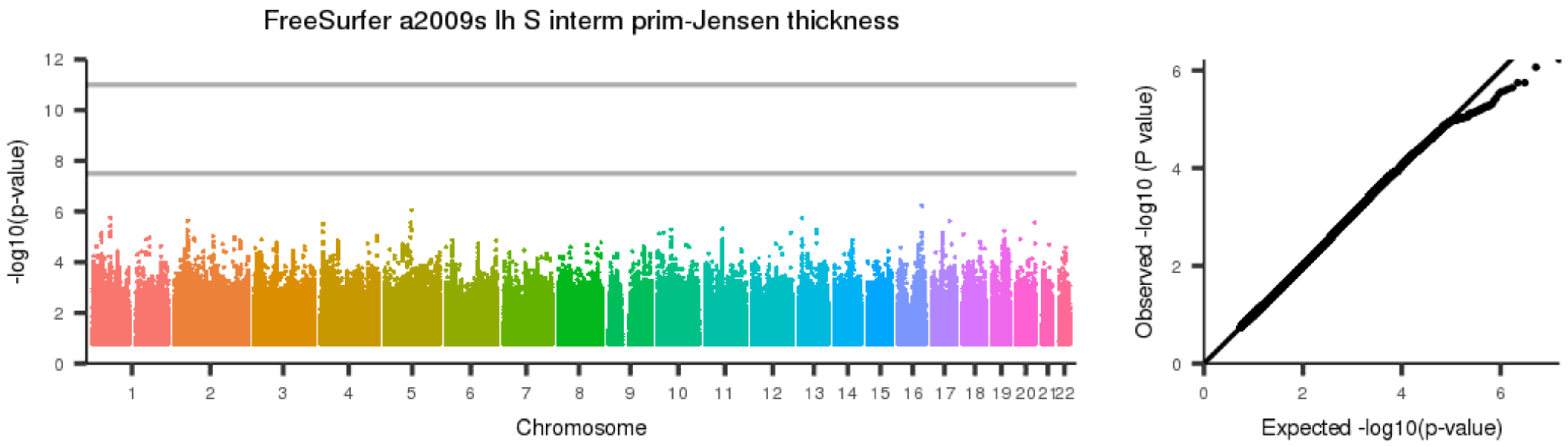

back to list of plots

Figure 1319: FreeSurfer a2009s lh S intrapariet and P trans thickness

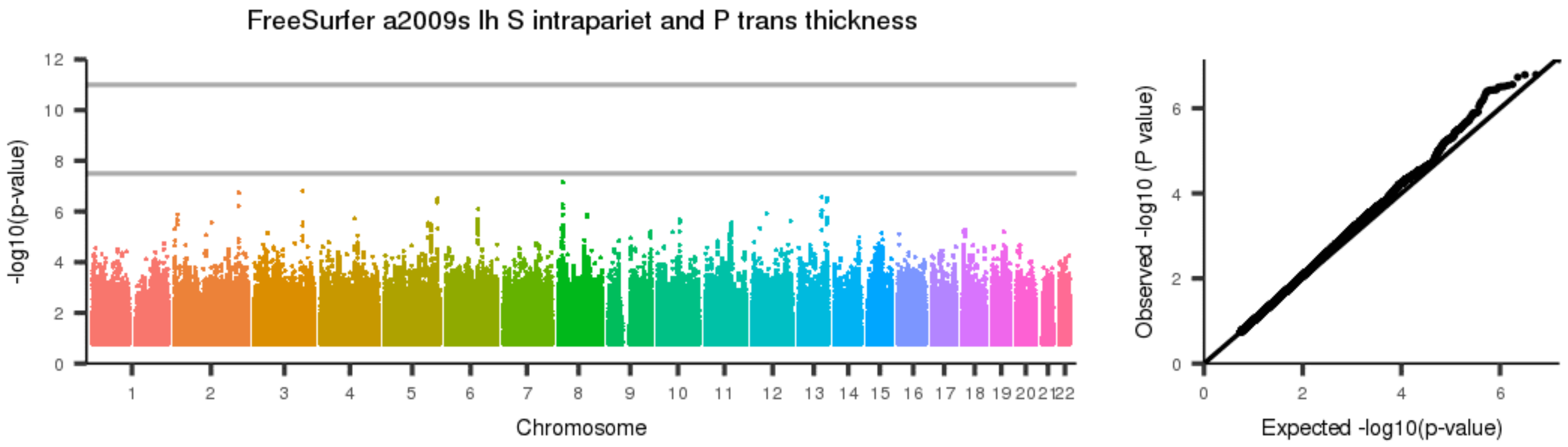

back to list of plots

Figure 1320: FreeSurfer a2009s lh S oc middle and Lunatus thickness

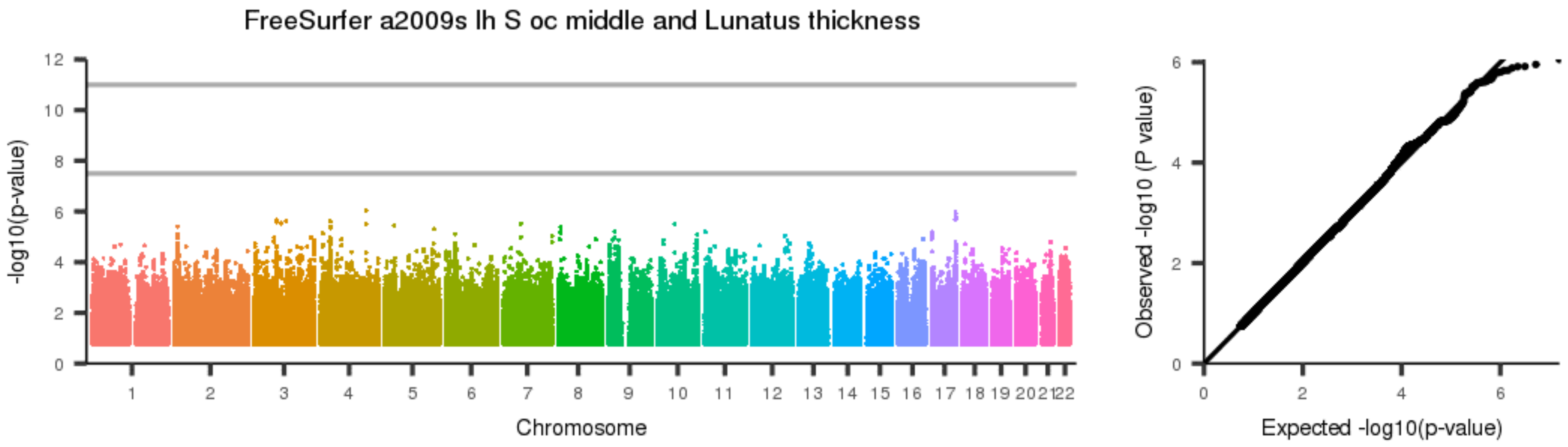

back to list of plots

Figure 1321: FreeSurfer a2009s lh S oc sup and transversal thickness

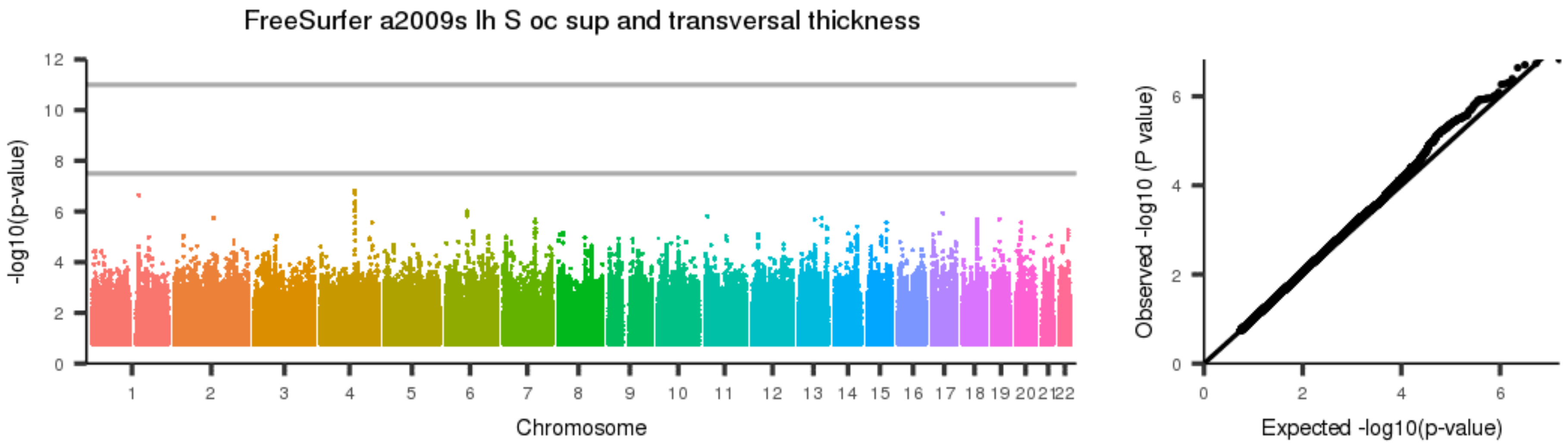

back to list of plots

Figure 1322: FreeSurfer a2009s lh S occipital ant thickness

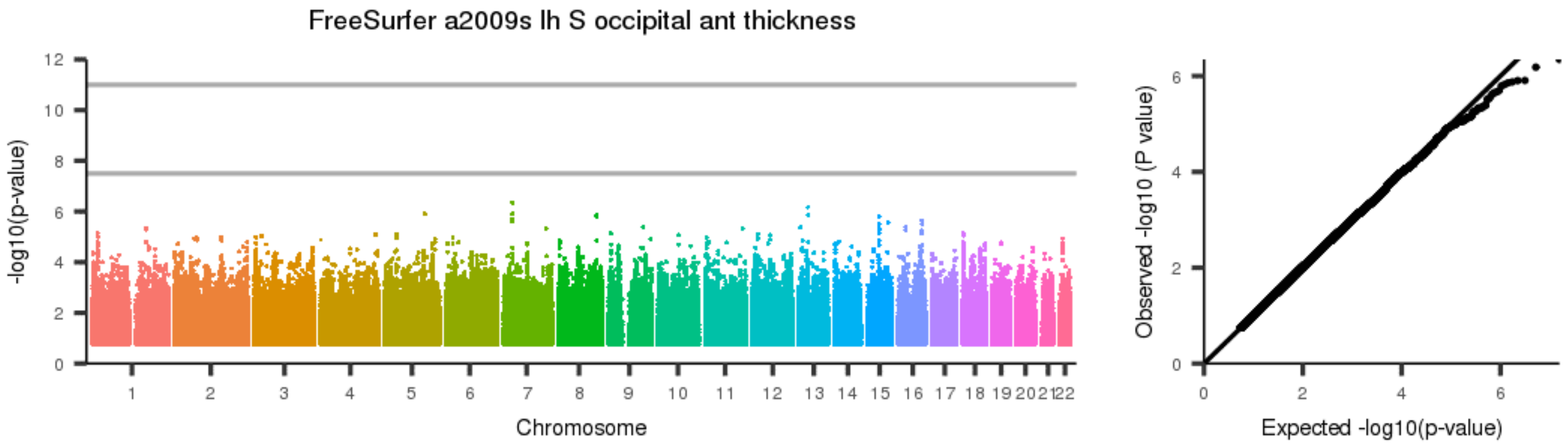

back to list of plots

Figure 1323: FreeSurfer a2009s lh S oc-temp lat thickness

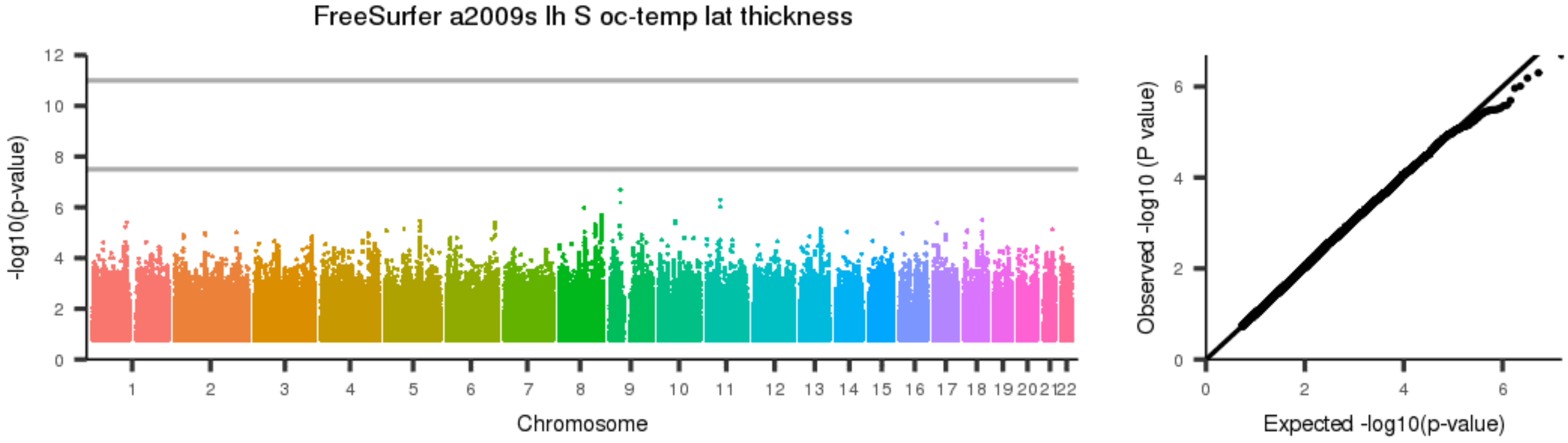

back to list of plots

Figure 1324: FreeSurfer a2009s lh S oc-temp med and Lingual thickness

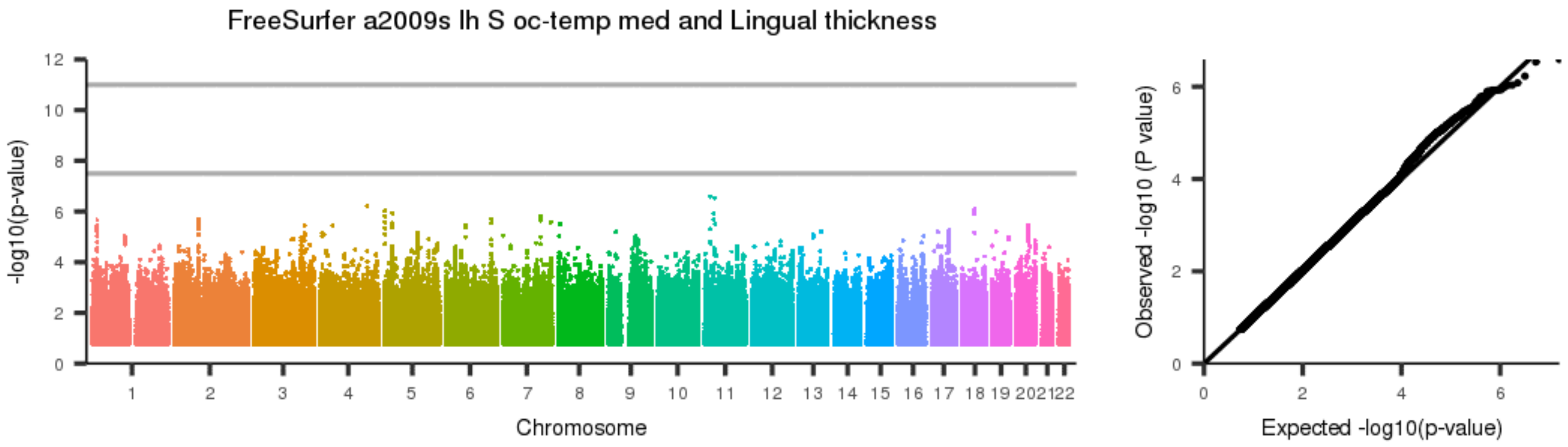

back to list of plots

Figure 1325: FreeSurfer a2009s lh S orbital lateral thickness

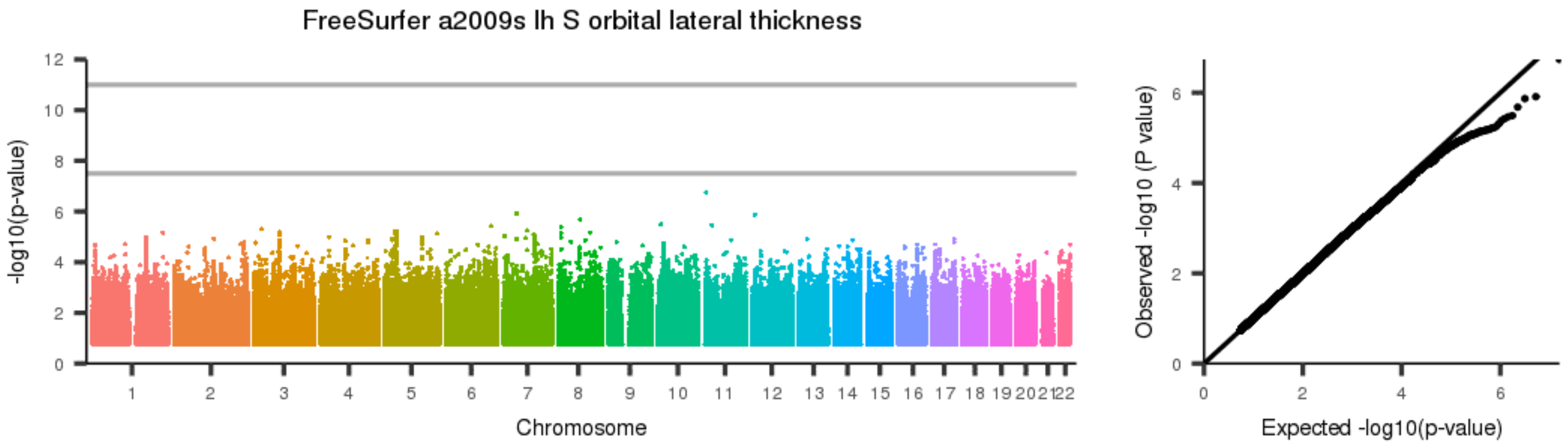

back to list of plots

Figure 1326: FreeSurfer a2009s lh S orbital med-olfact thickness

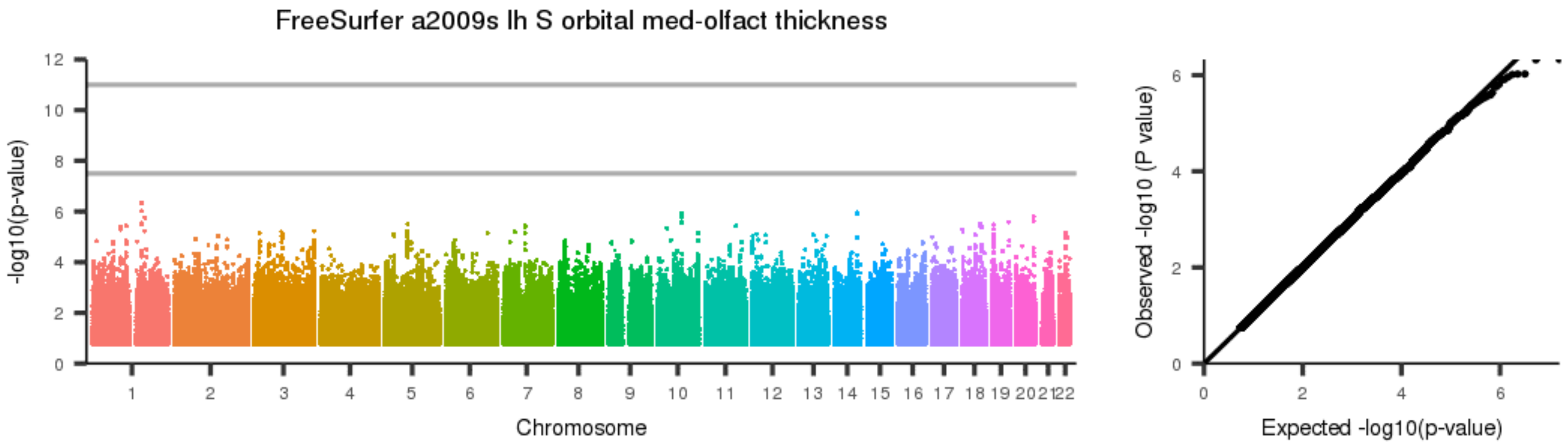

back to list of plots

Figure 1327: FreeSurfer a2009s lh S orbital-H Shaped thickness

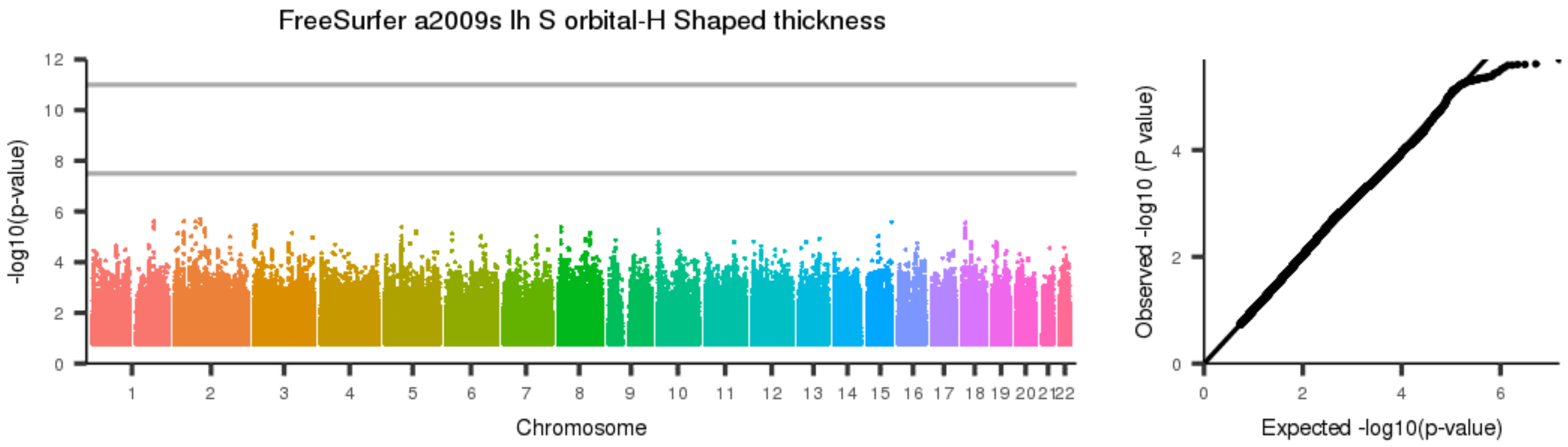

back to list of plots

Figure 1328: FreeSurfer a2009s lh S parieto occipital thickness

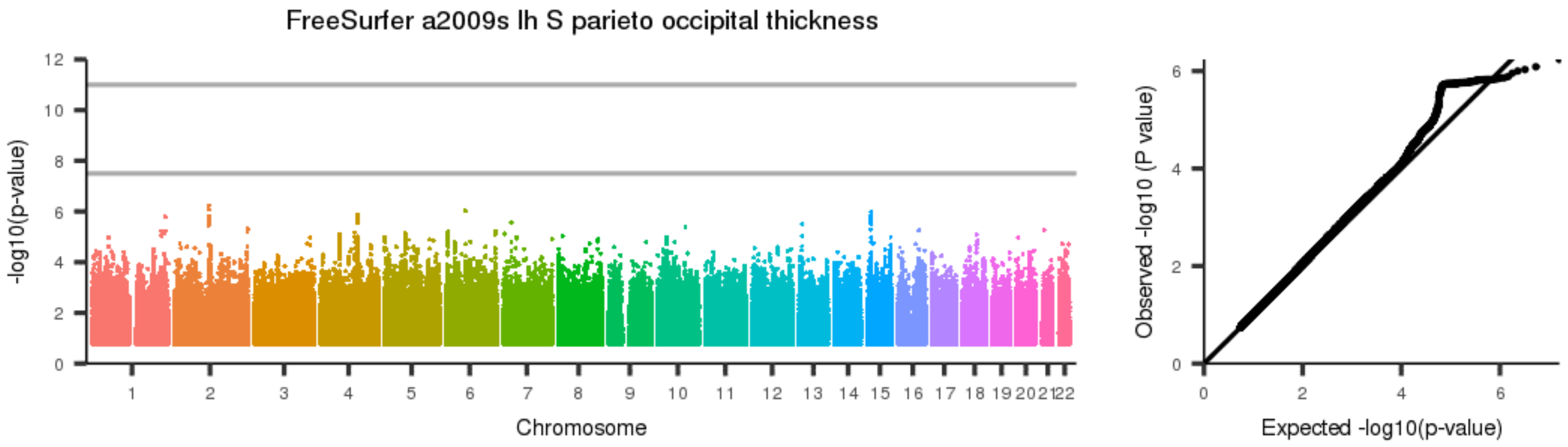

back to list of plots

Figure 1329: FreeSurfer a2009s lh S pericallosal thickness

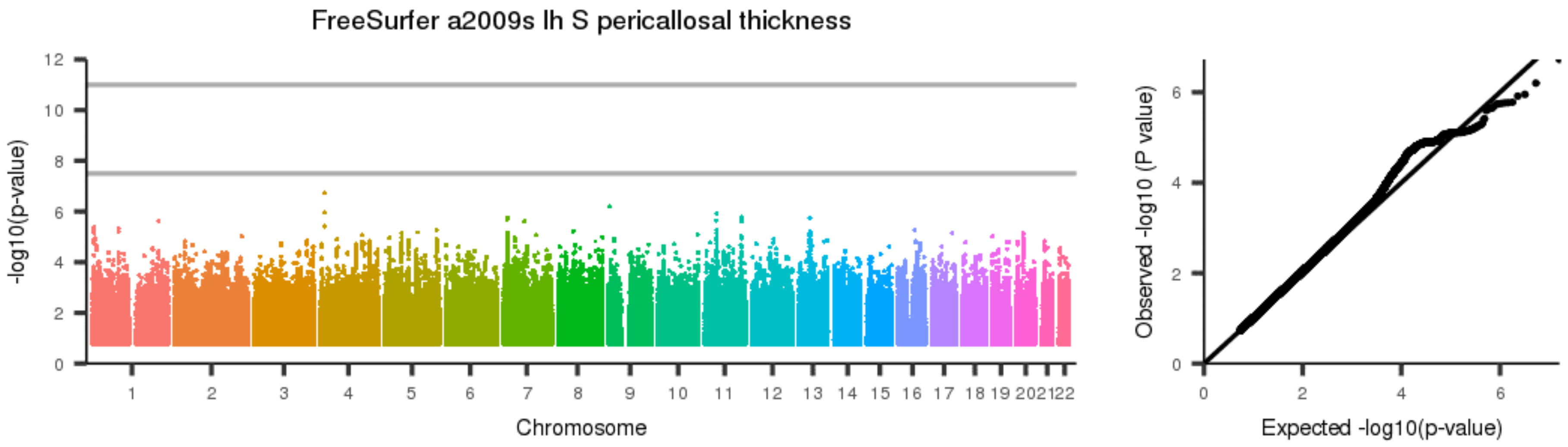

back to list of plots

Figure 1330: FreeSurfer a2009s lh S postcentral thickness

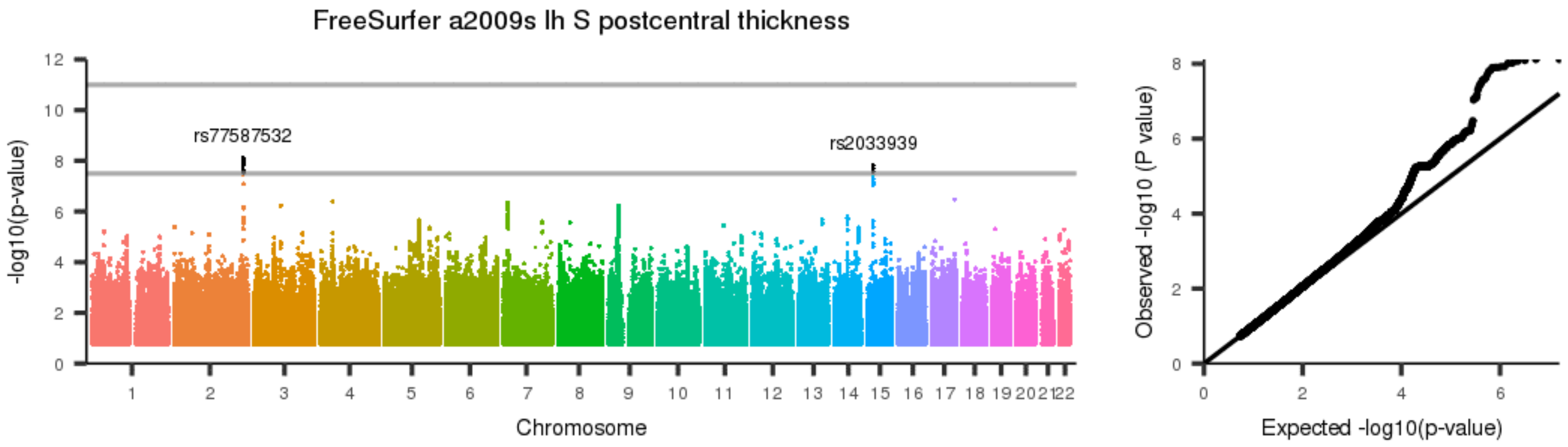

back to list of plots

Figure 1331: FreeSurfer a2009s lh S precentral-inf-part thickness

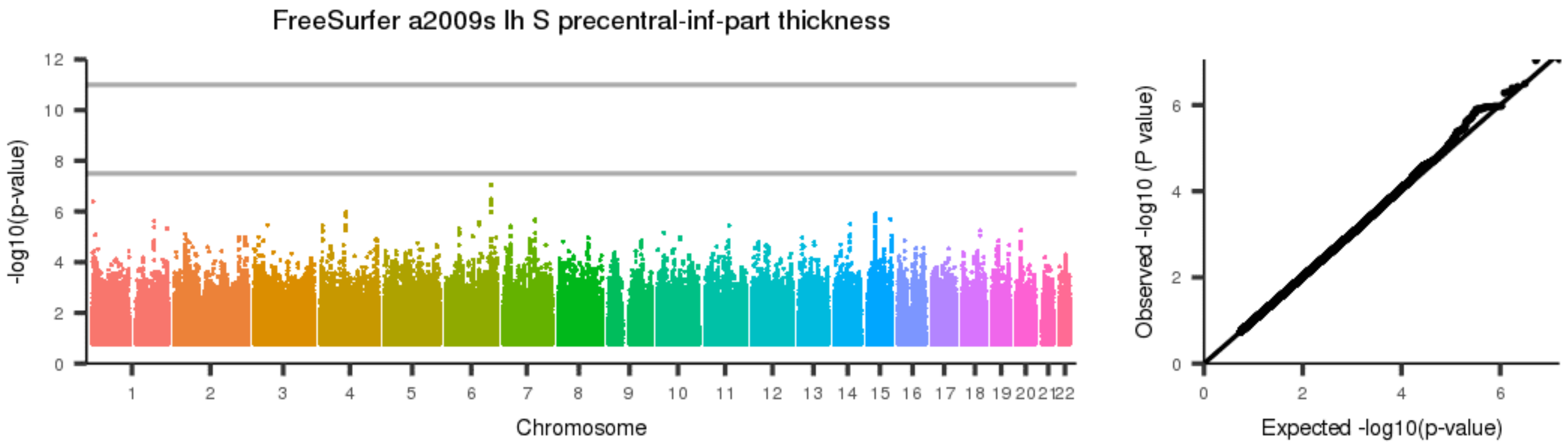

back to list of plots

Figure 1332: FreeSurfer a2009s lh S precentral-sup-part thickness

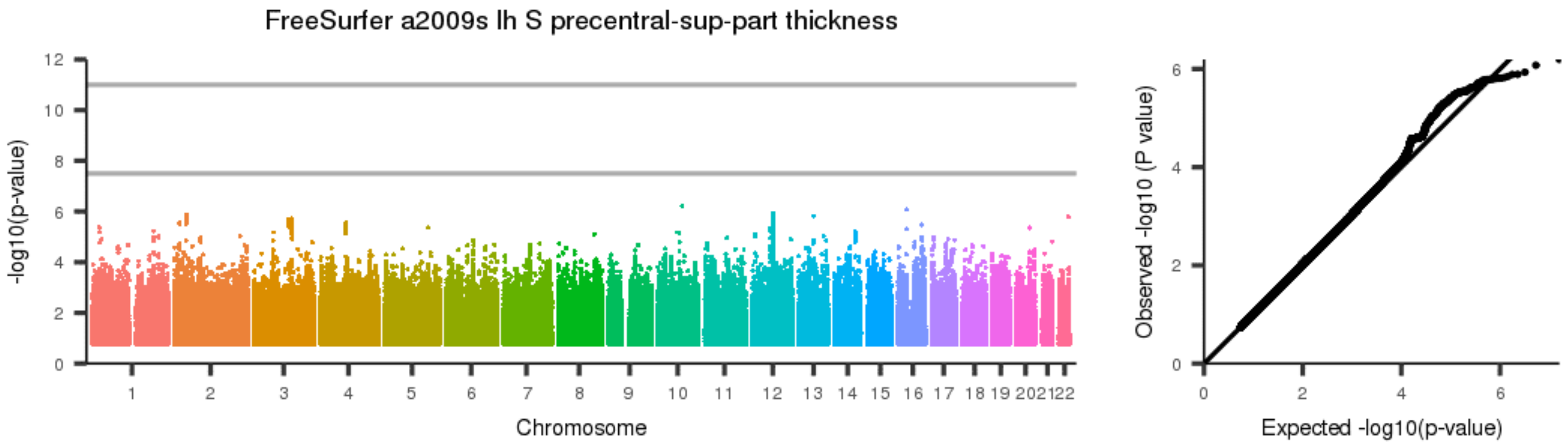

back to list of plots

Figure 1333: FreeSurfer a2009s lh S suborbital thickness

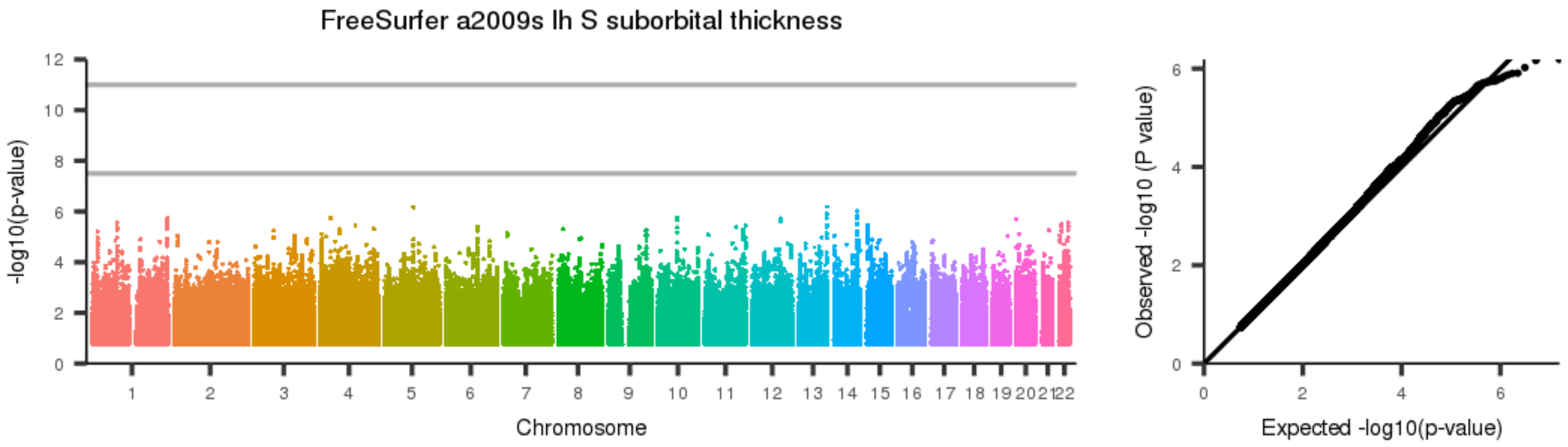

back to list of plots

Figure 1334: FreeSurfer a2009s lh S subparietal thickness

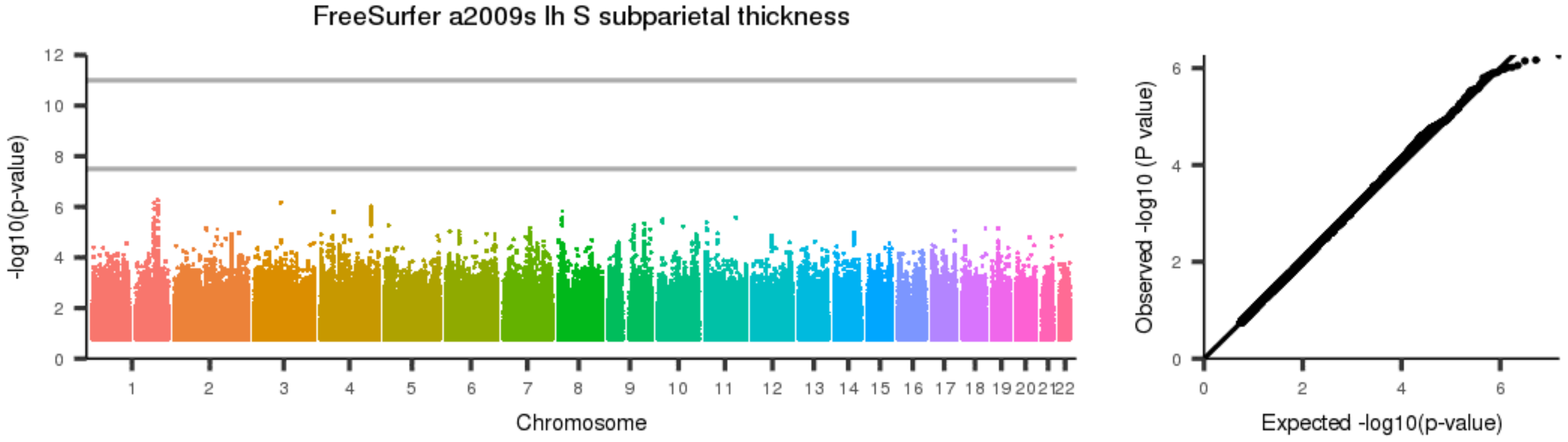

back to list of plots

Figure 1335: FreeSurfer a2009s lh S temporal inf thickness

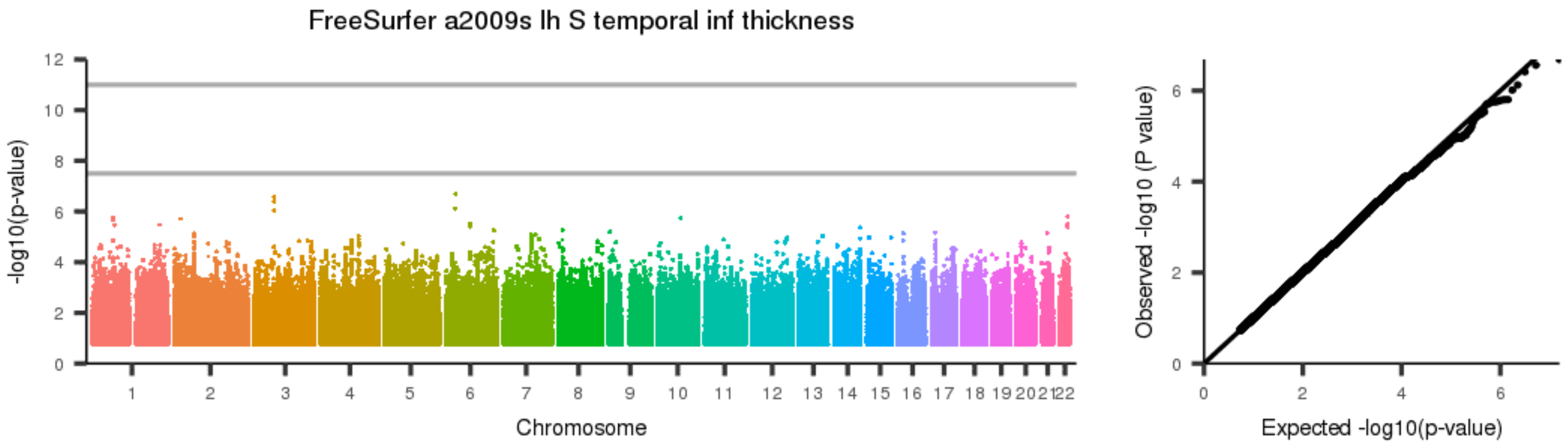

back to list of plots

Figure 1336: FreeSurfer a2009s lh S temporal sup thickness

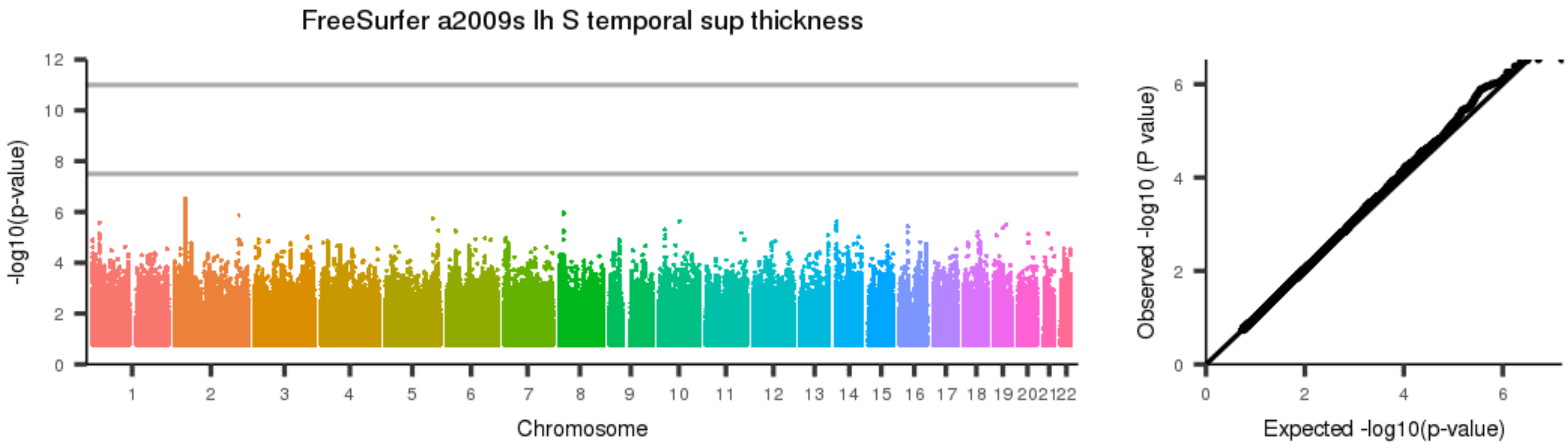

back to list of plots

Figure 1337: FreeSurfer a2009s lh S temporal transverse thickness

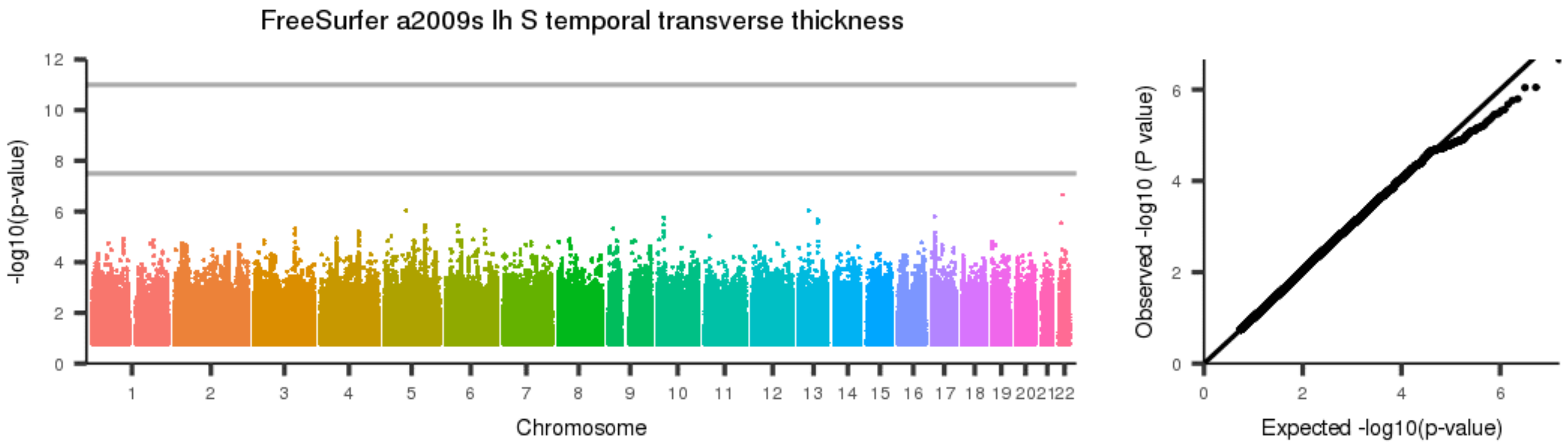

back to list of plots

Figure 1338: FreeSurfer DKTatlas rh caudalanteriorcingulate thickness

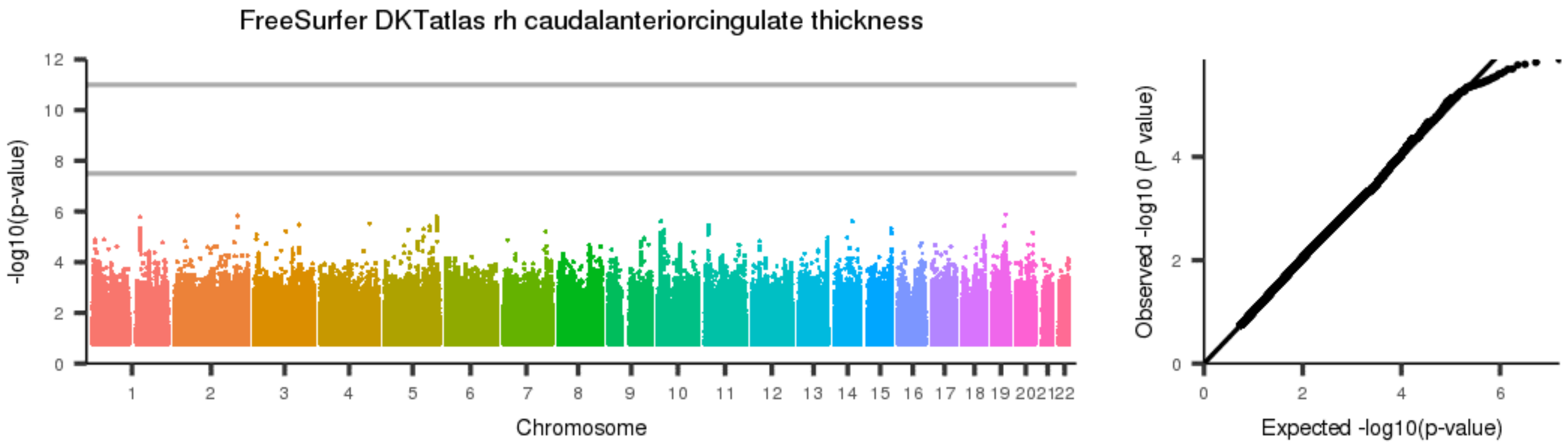

back to list of plots

Figure 1339: FreeSurfer DKTatlas rh caudalmiddlefrontal thickness

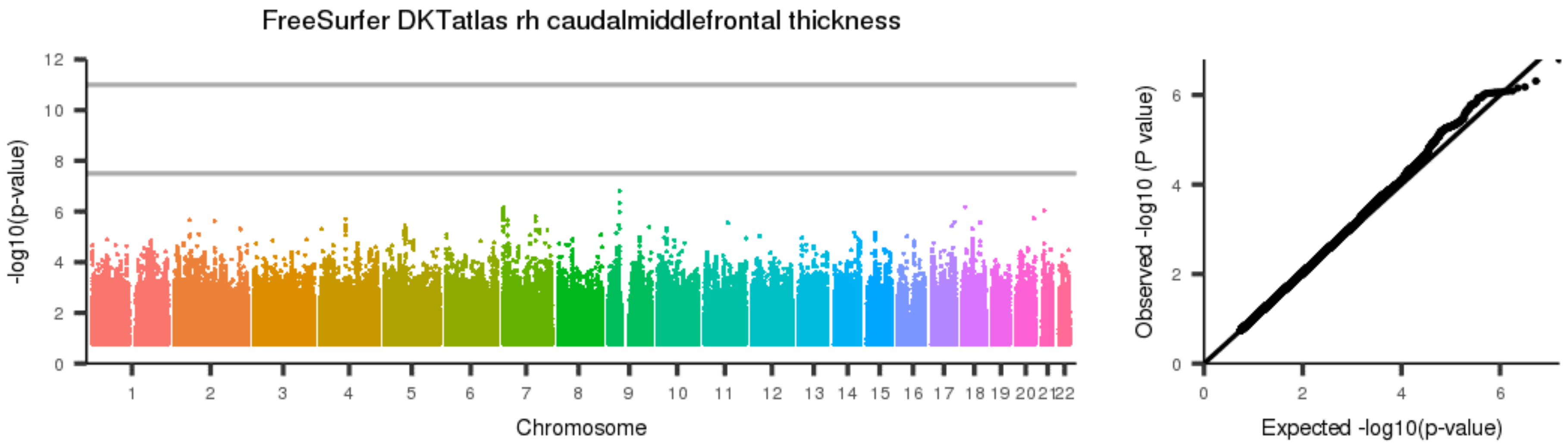

back to list of plots

Figure 1340: FreeSurfer DKTatlas rh cuneus thickness

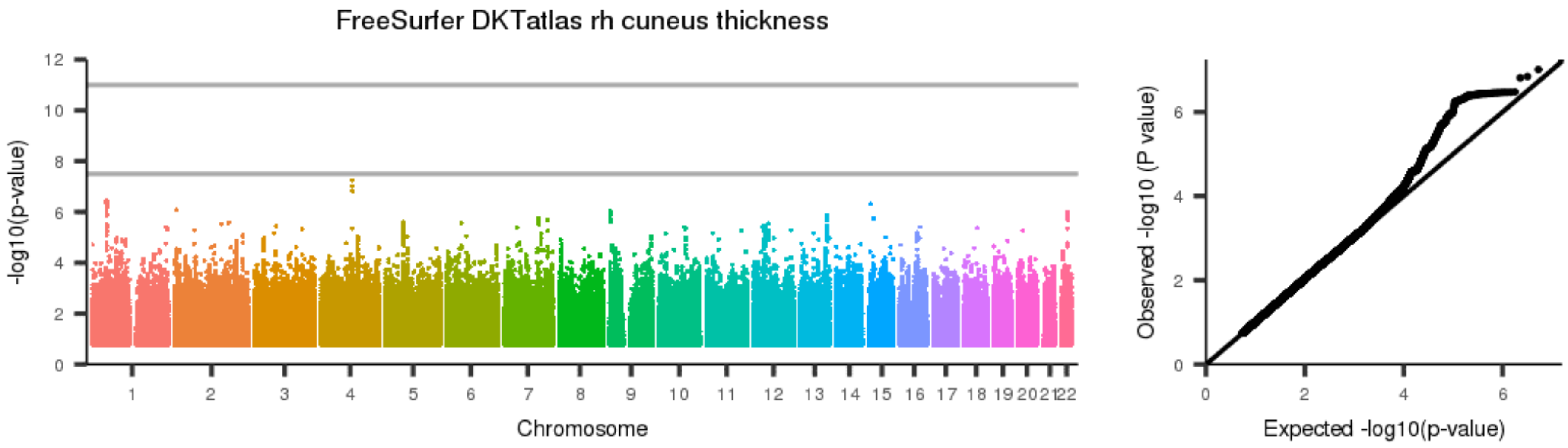

back to list of plots

Figure 1341: FreeSurfer DKTatlas rh entorhinal thickness

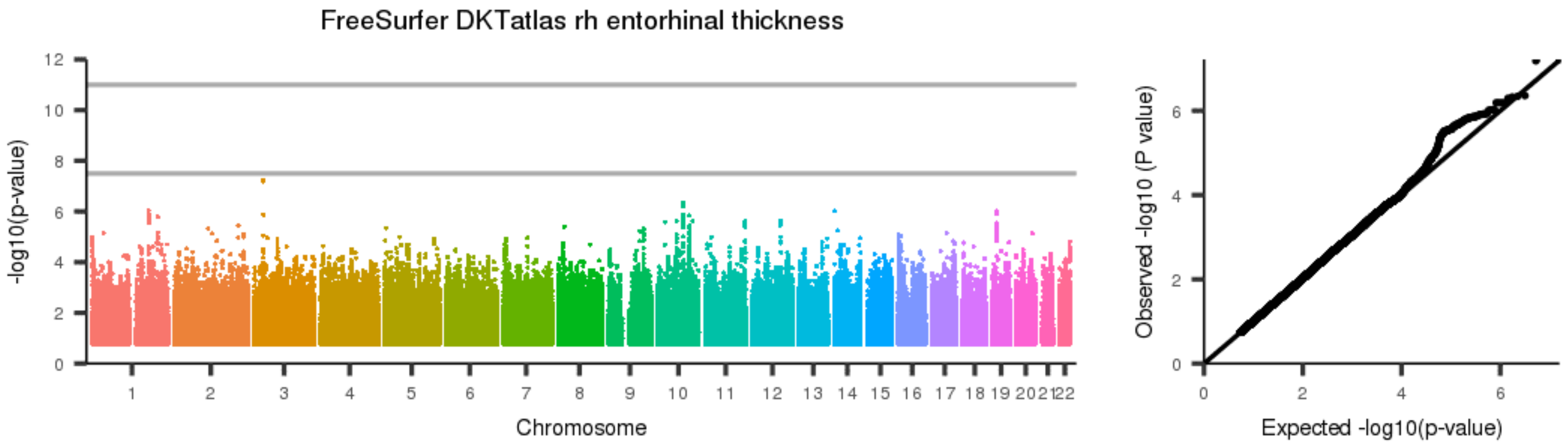

back to list of plots

Figure 1342: FreeSurfer DKTatlas rh fusiform thickness

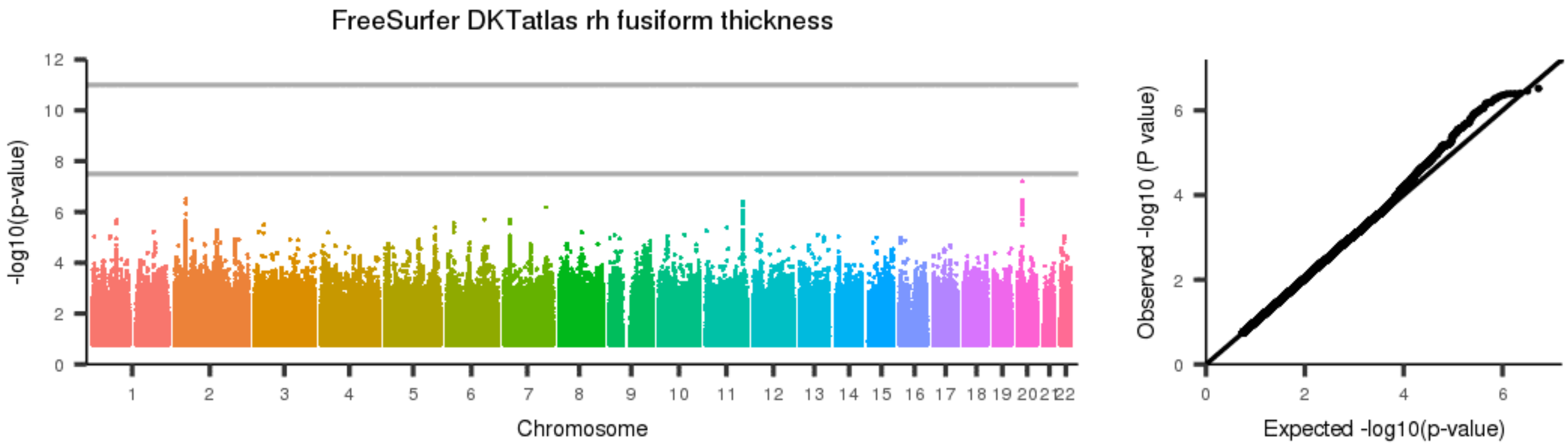

back to list of plots

Figure 1343: FreeSurfer DKTatlas rh inferiorparietal thickness

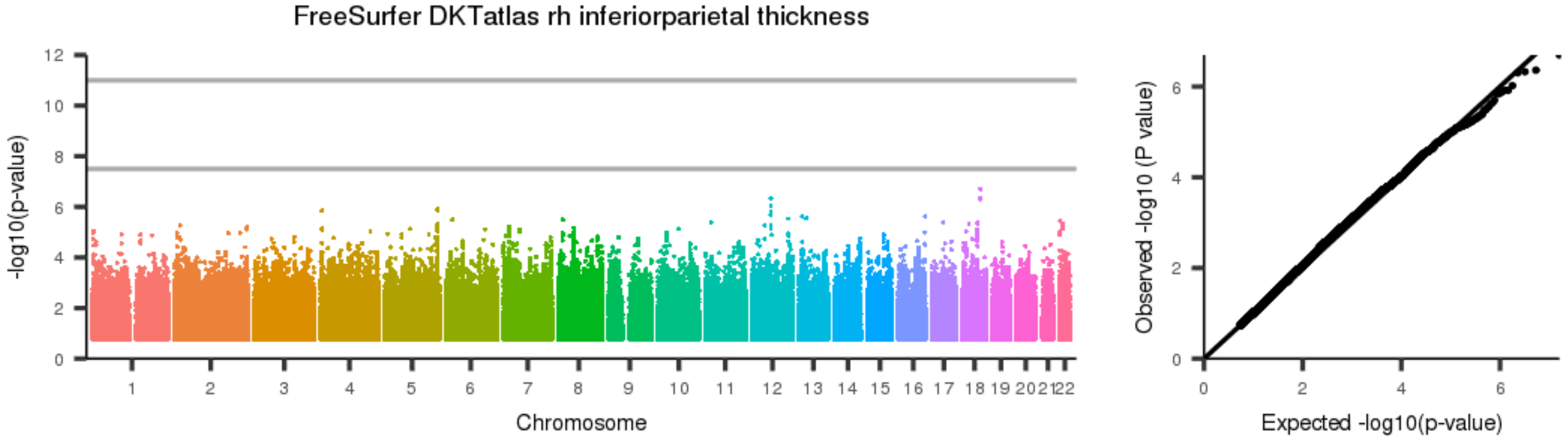

back to list of plots

Figure 1344: FreeSurfer DKTatlas rh inferiortemporal thickness

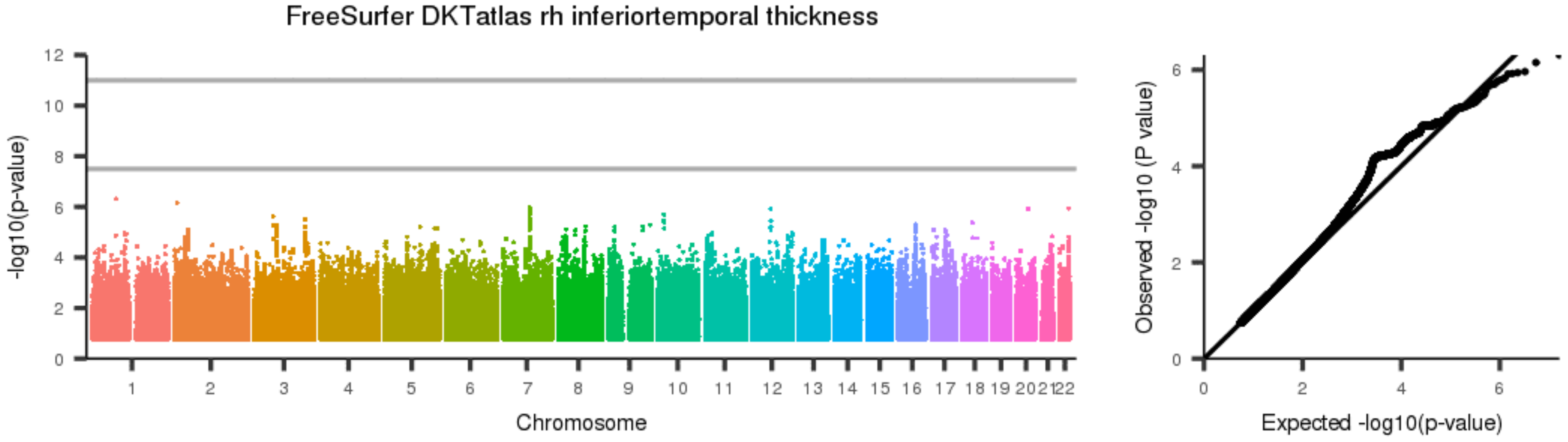

back to list of plots

Figure 1345: FreeSurfer DKTatlas rh isthmuscingulate thickness

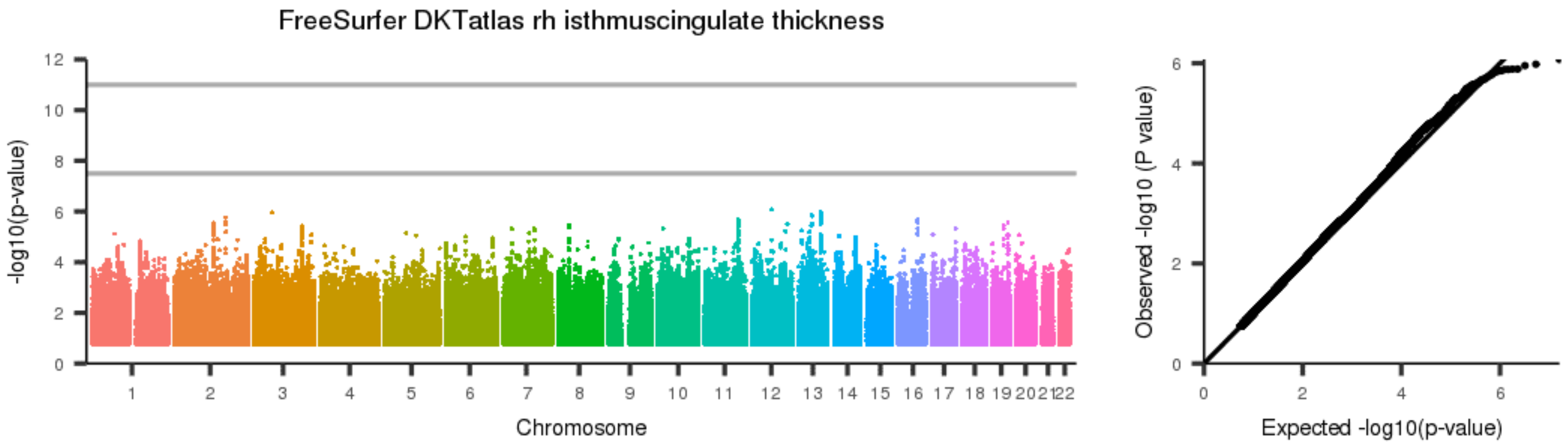

back to list of plots

Figure 1346: FreeSurfer DKTatlas rh lateraloccipital thickness

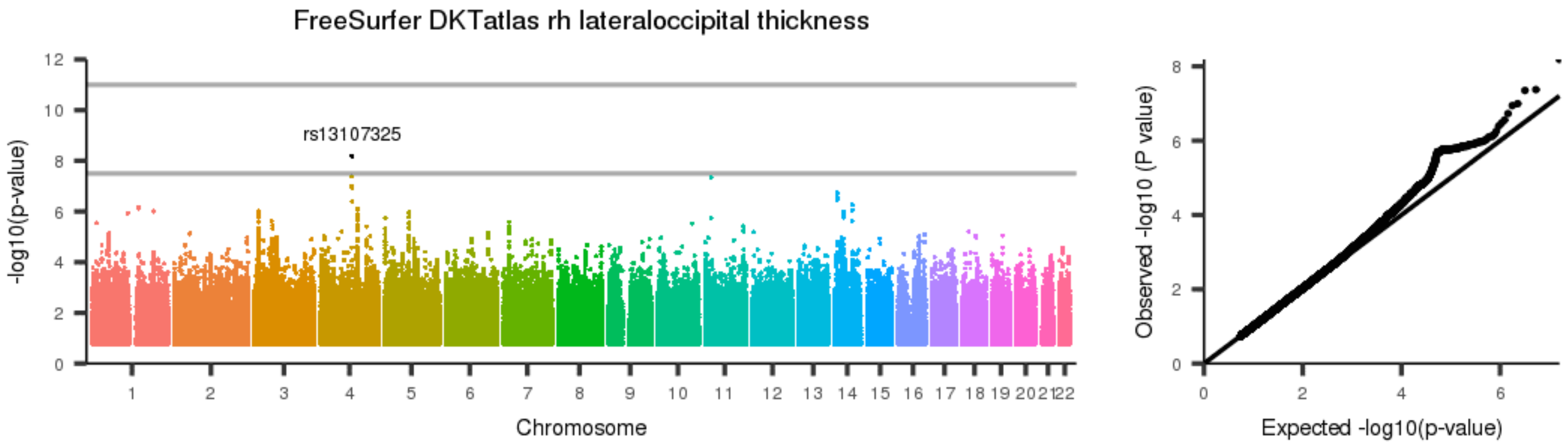

back to list of plots

Figure 1347: FreeSurfer DKTatlas rh lateralorbitofrontal thickness

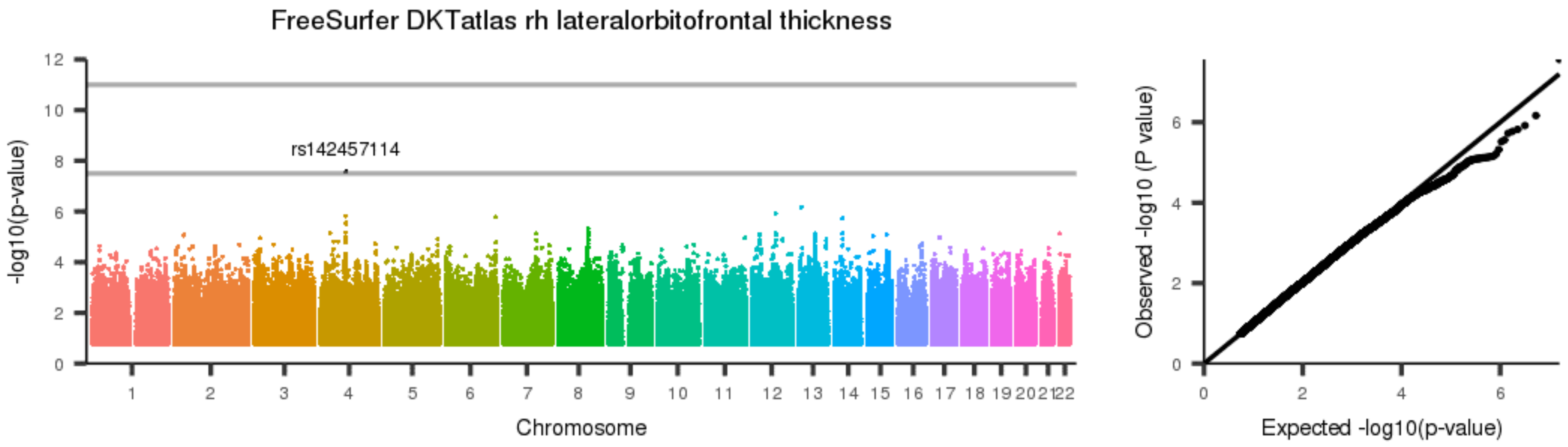

back to list of plots

Figure 1348: FreeSurfer DKTatlas rh lingual thickness

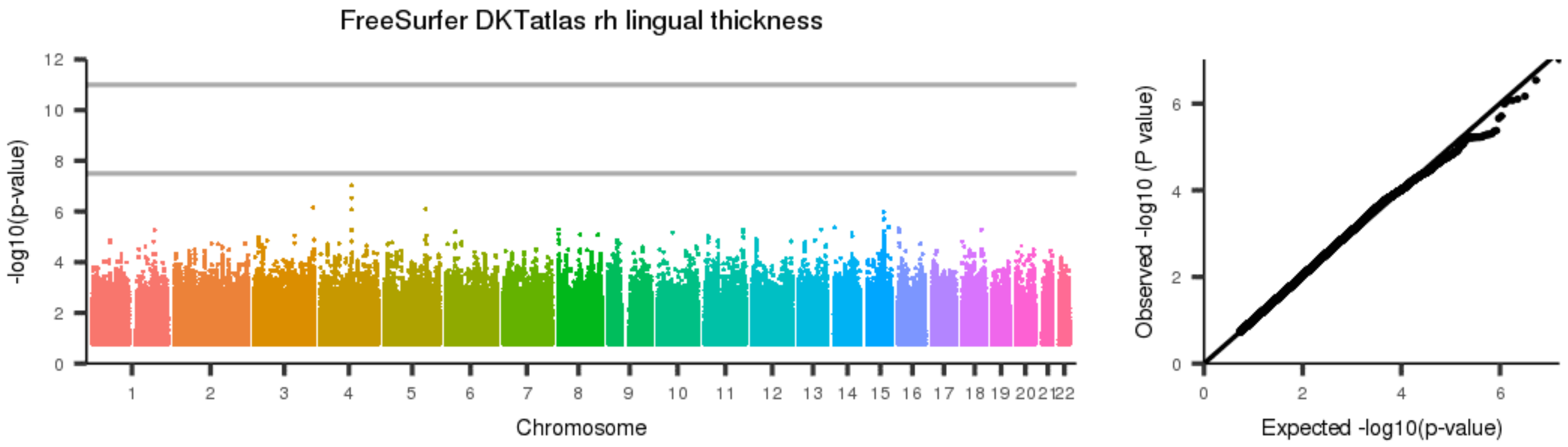

back to list of plots

Figure 1349: FreeSurfer DKTatlas rh medialorbitofrontal thickness

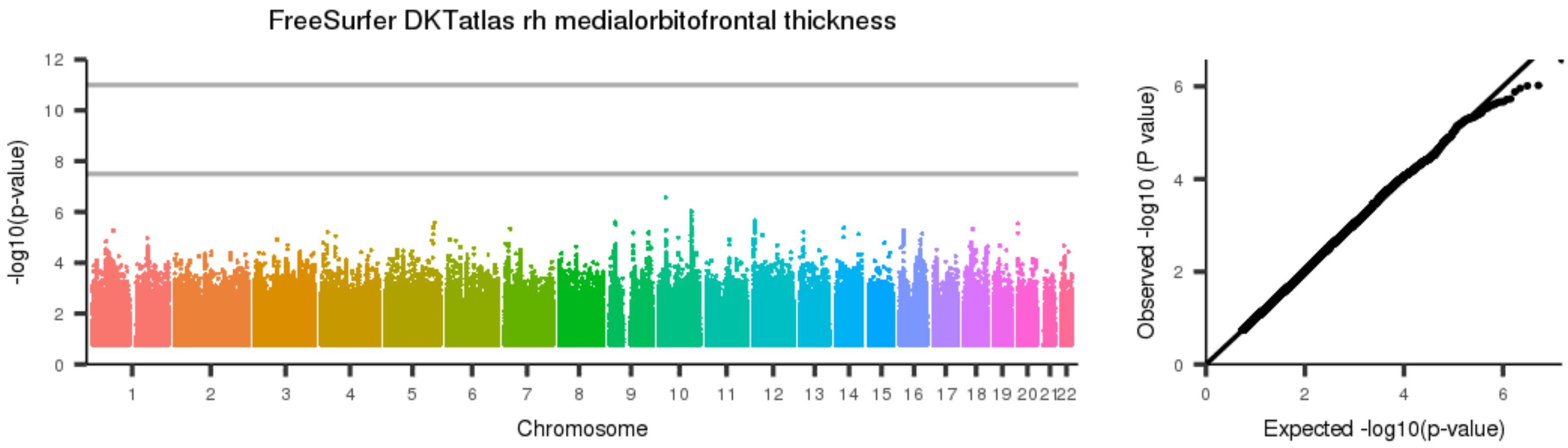

back to list of plots

Figure 1350: FreeSurfer DKTatlas rh middletemporal thickness

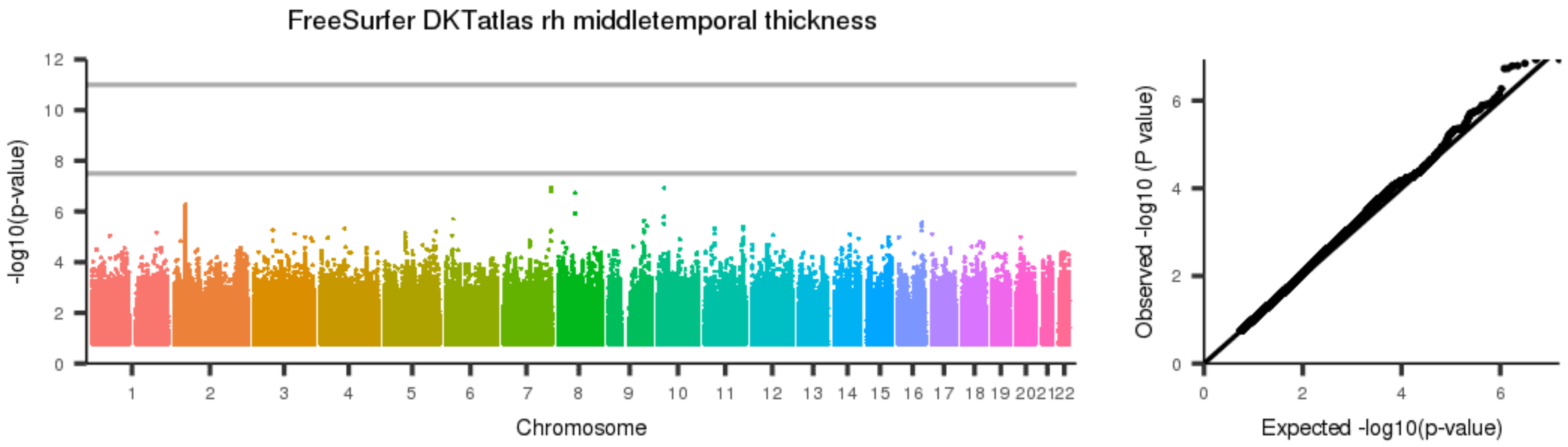

back to list of plots

Figure 1351: FreeSurfer DKTatlas rh parahippocampal thickness

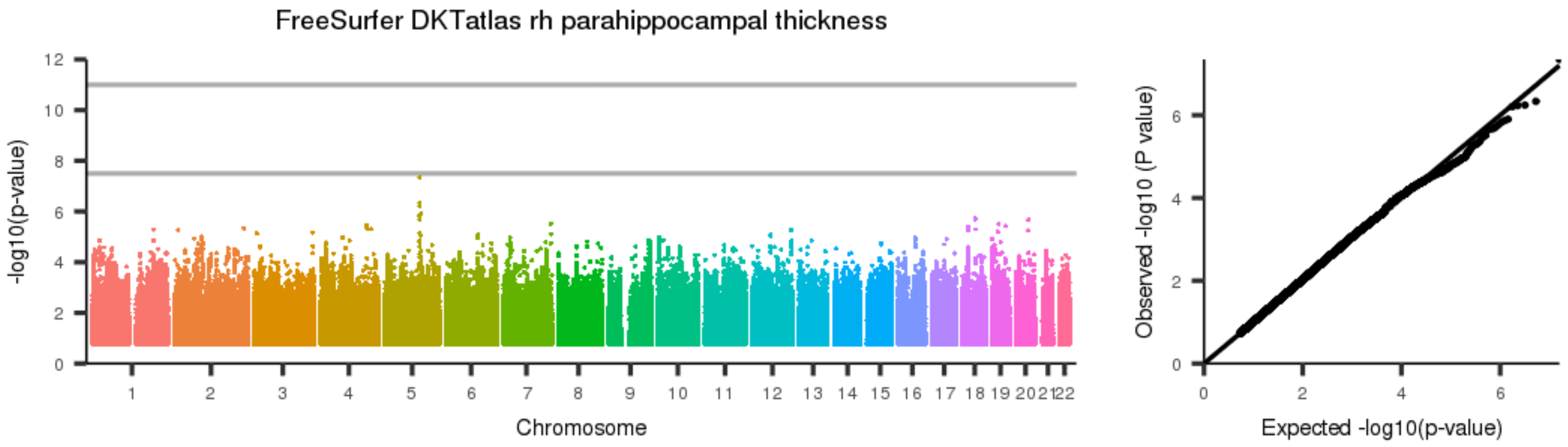

back to list of plots

Figure 1352: FreeSurfer DKTatlas rh paracentral thickness

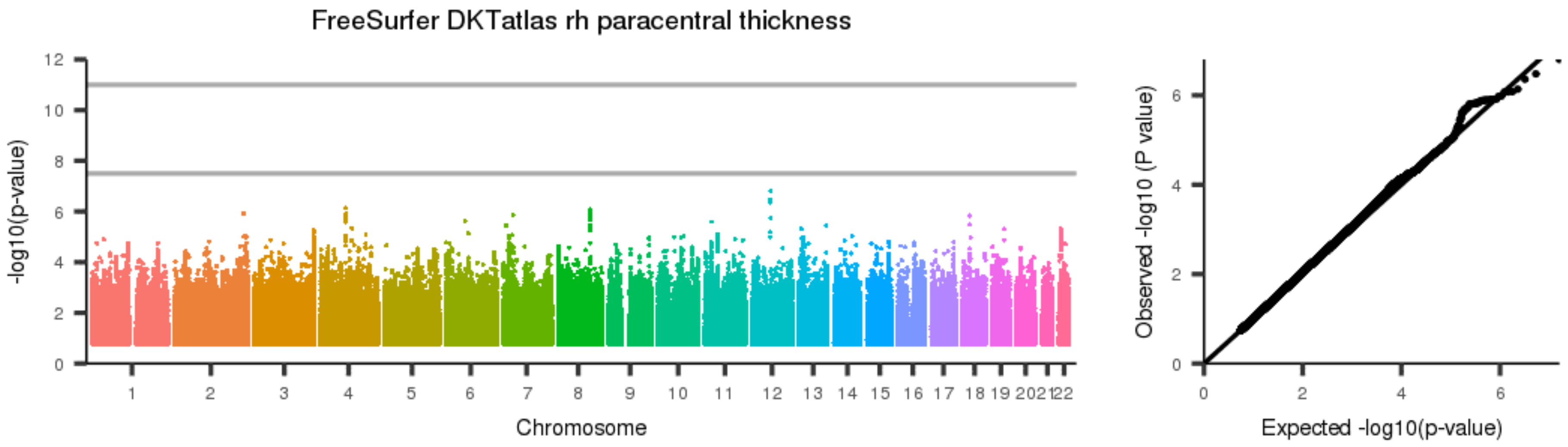

back to list of plots

Figure 1353: FreeSurfer DKTatlas rh parsopercularis thickness

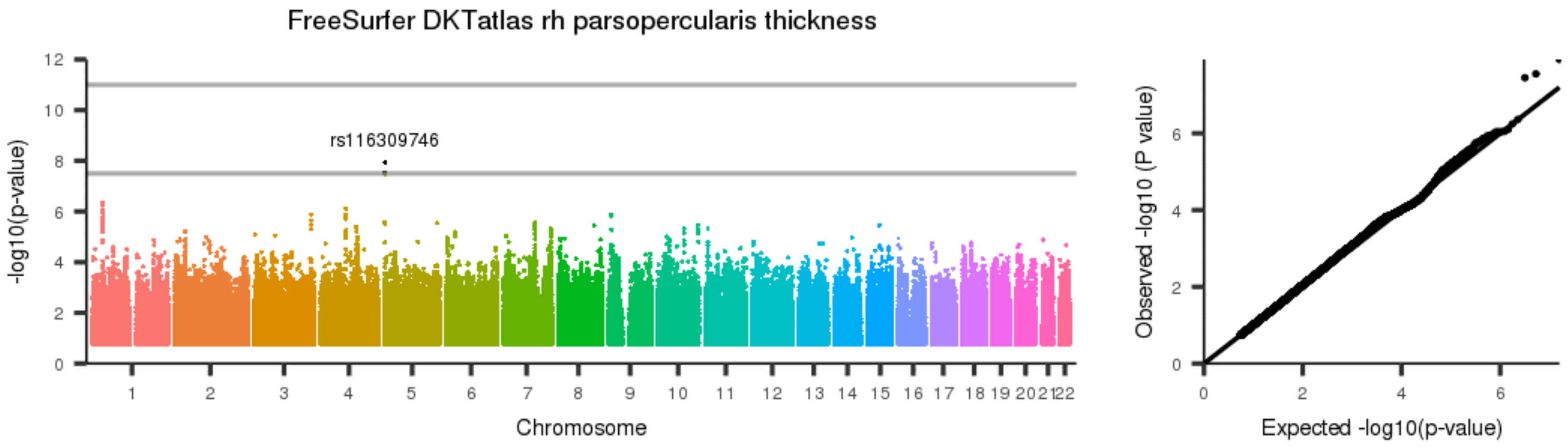

back to list of plots

Figure 1354: FreeSurfer DKTatlas rh parsorbitalis thickness

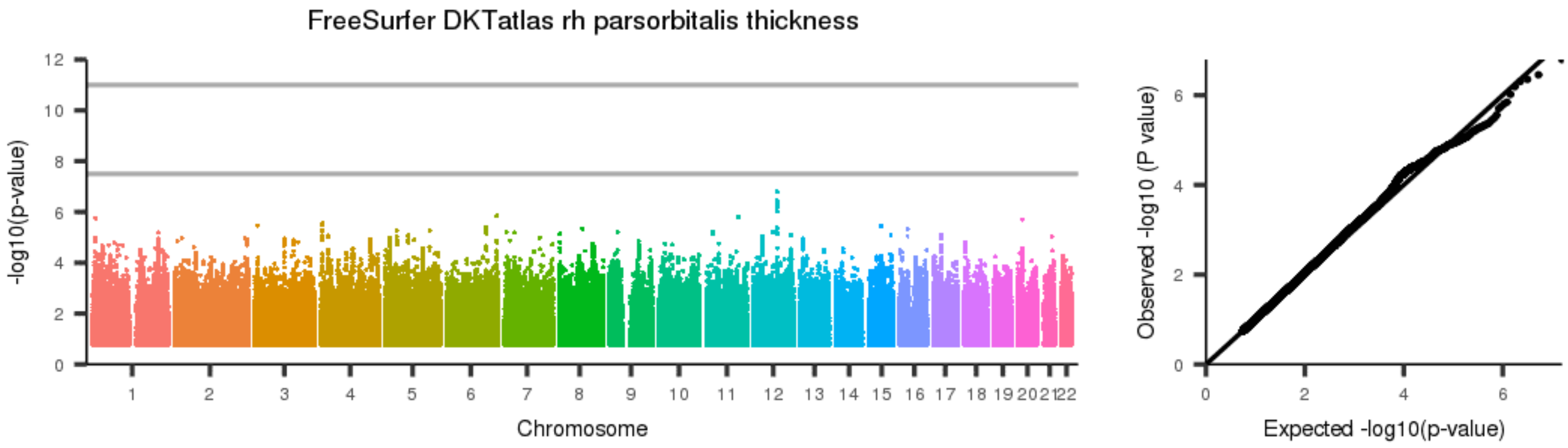

back to list of plots

Figure 1355: FreeSurfer DKTatlas rh parstriangularis thickness

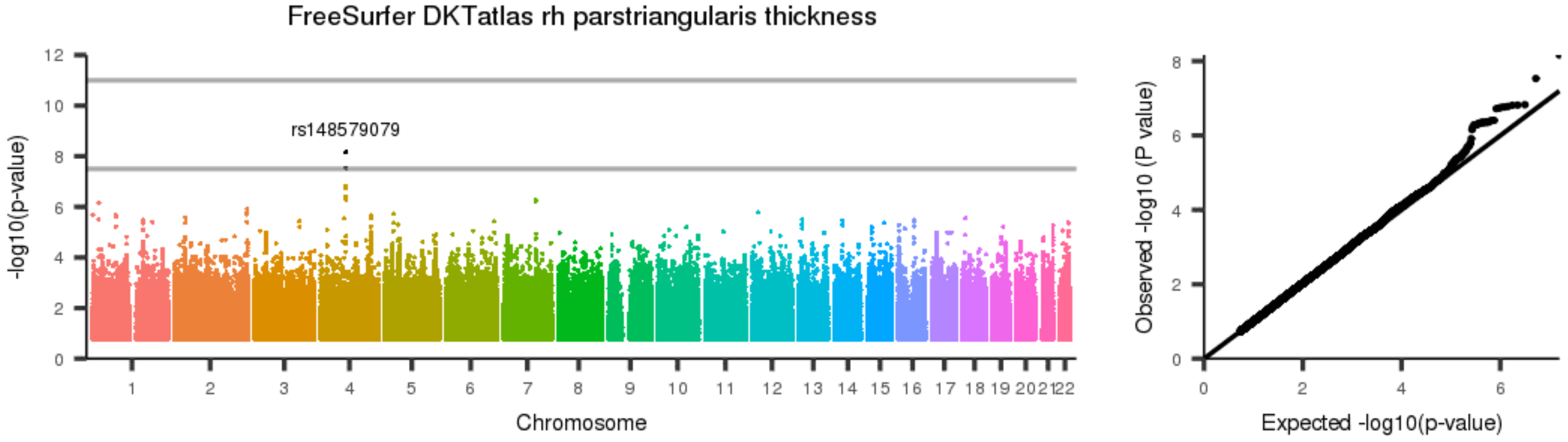

back to list of plots

Figure 1356: FreeSurfer DKTatlas rh pericalcarine thickness

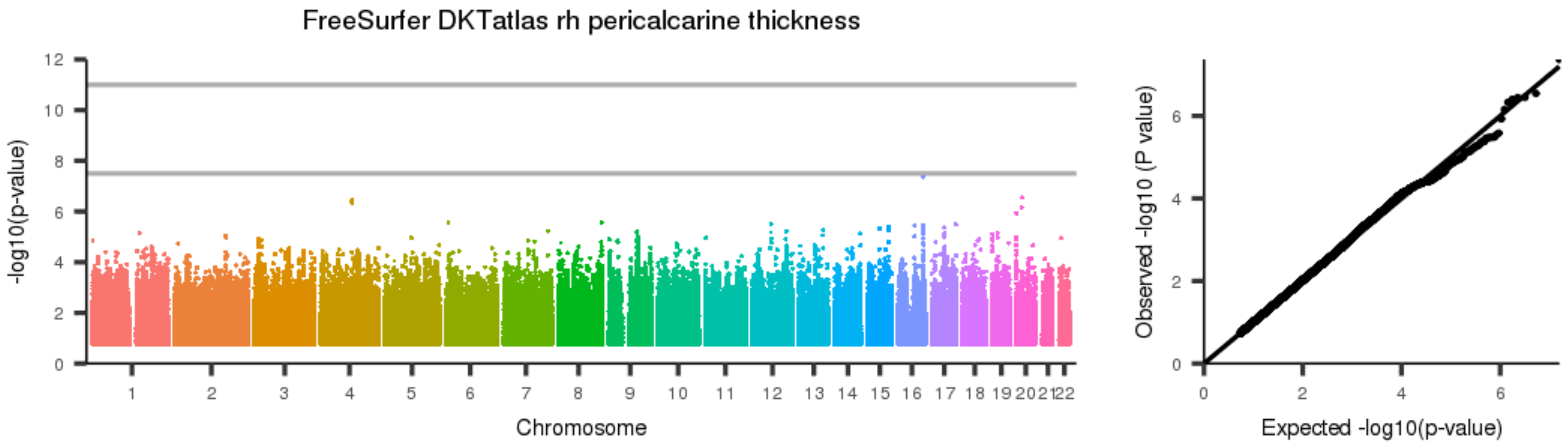

back to list of plots

Figure 1357: FreeSurfer DKTatlas rh postcentral thickness

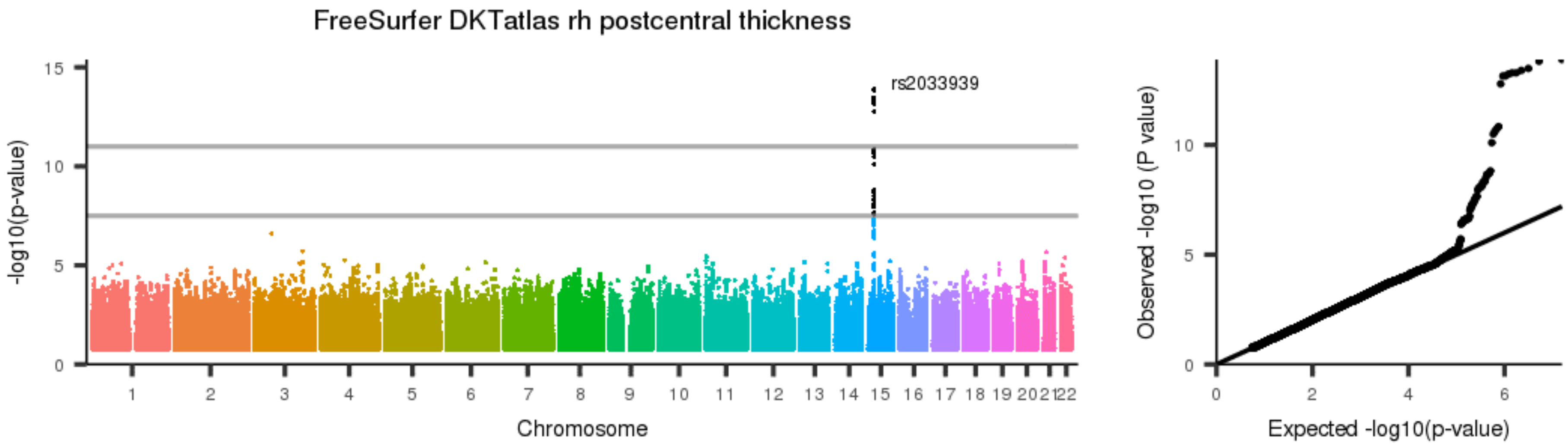

back to list of plots

Figure 1358: FreeSurfer DKTatlas rh posteriorcingulate thickness

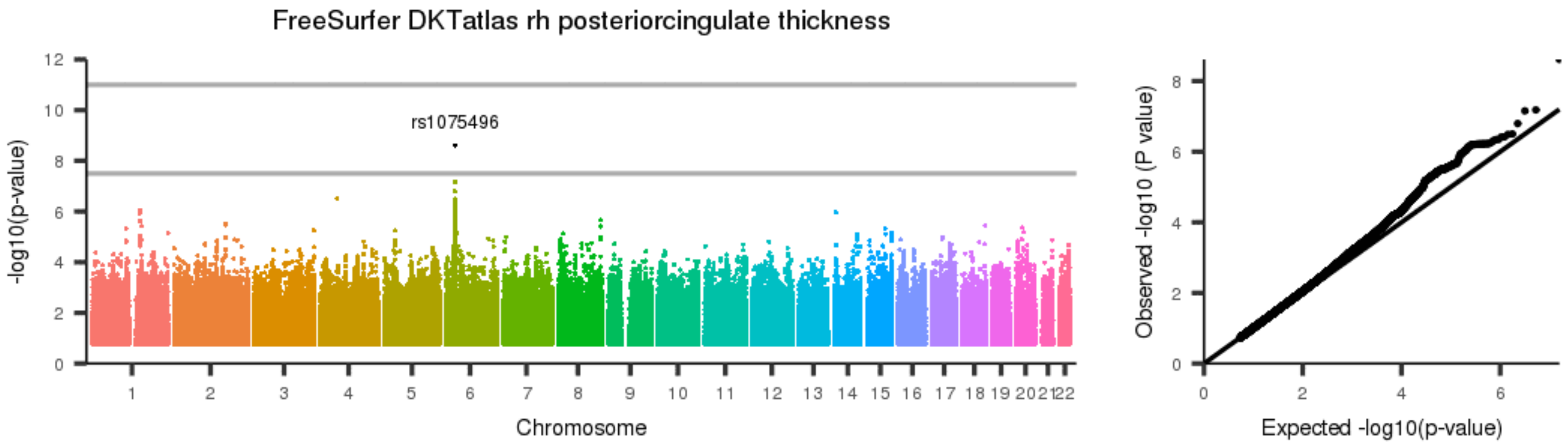

back to list of plots

Figure 1359: FreeSurfer DKTatlas rh precentral thickness

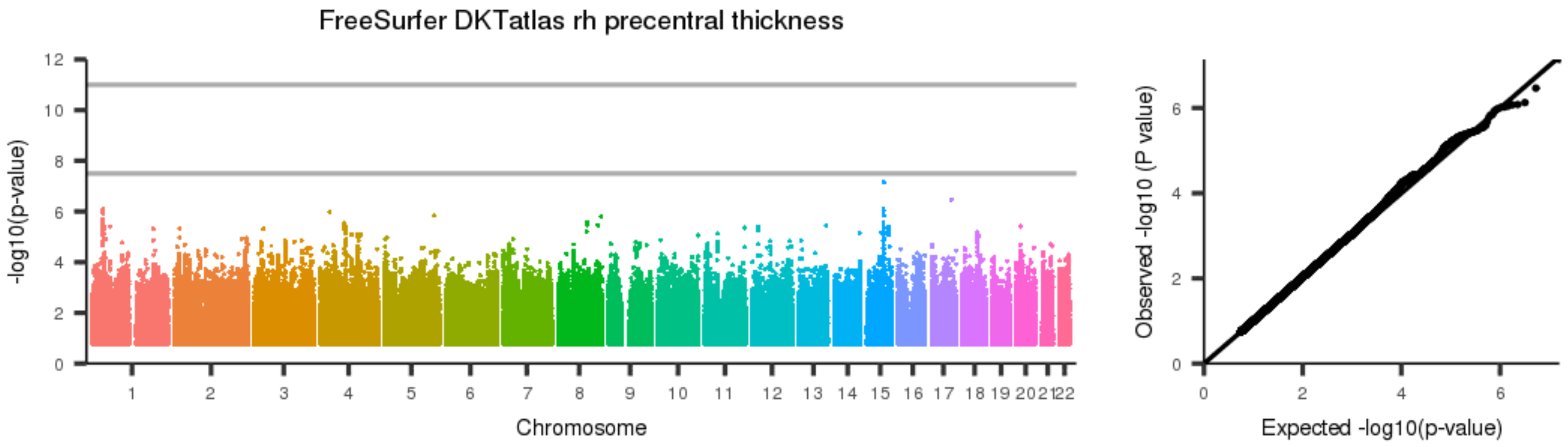

back to list of plots

Figure 1360: FreeSurfer DKTatlas rh precuneus thickness

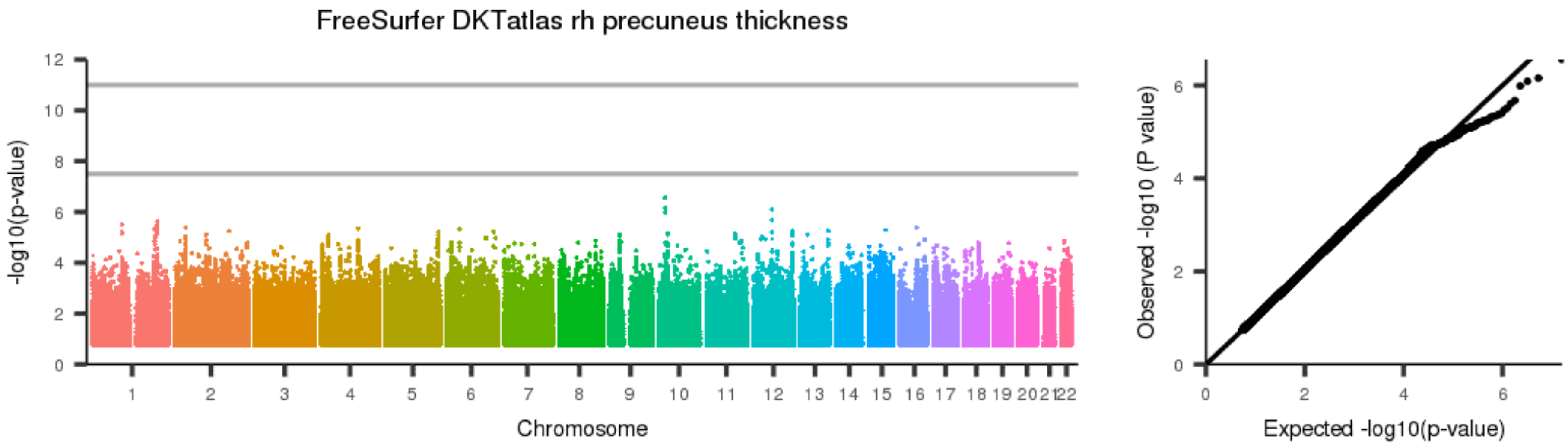

back to list of plots

Figure 1361: FreeSurfer DKTatlas rh rostralanteriorcingulate thickness

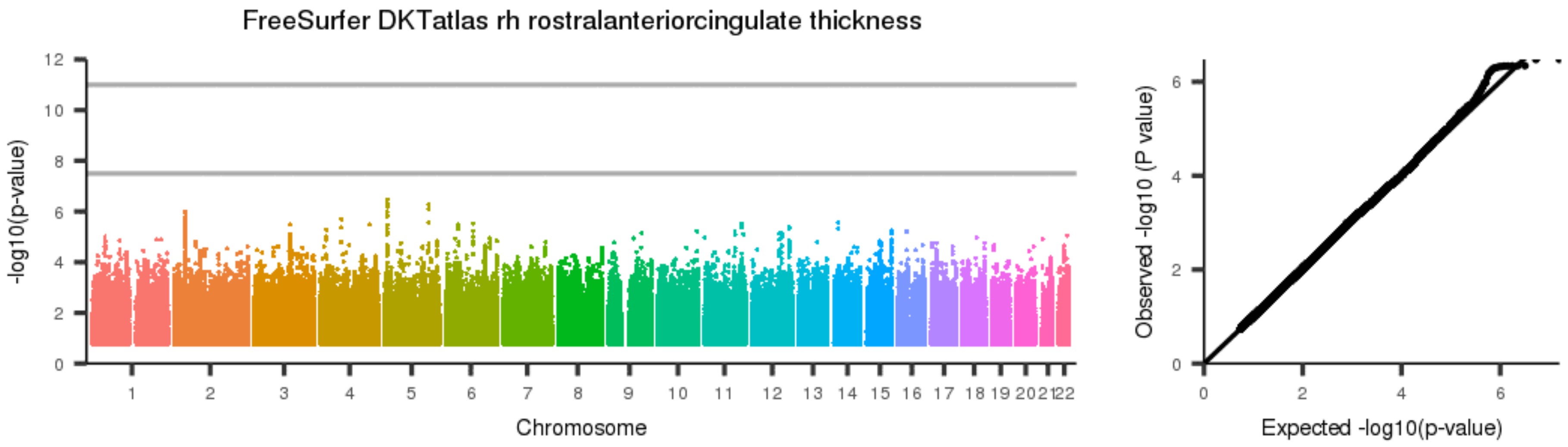

back to list of plots

Figure 1362: FreeSurfer DKTatlas rh rostralmiddlefrontal thickness

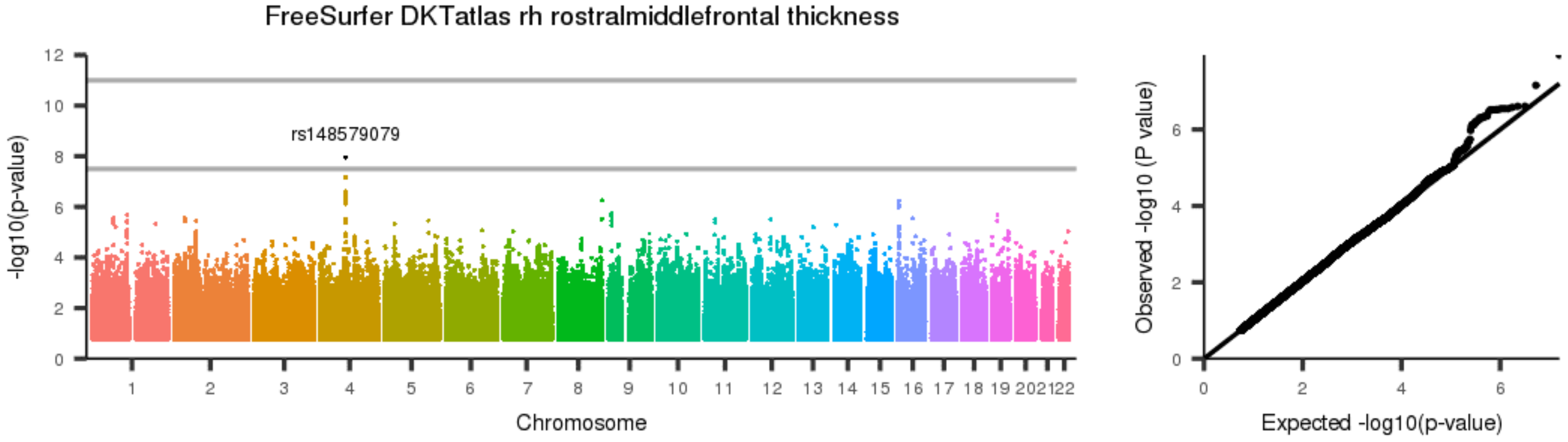

back to list of plots

Figure 1363: FreeSurfer DKTatlas rh superiorfrontal thickness

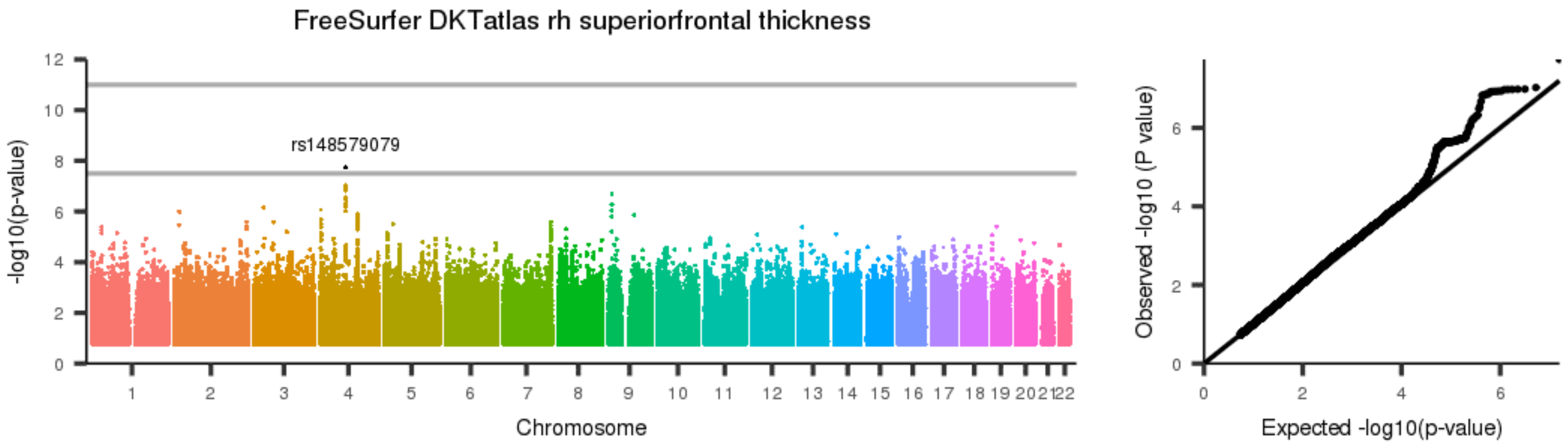

back to list of plots

Figure 1364: FreeSurfer DKTatlas rh superiorparietal thickness

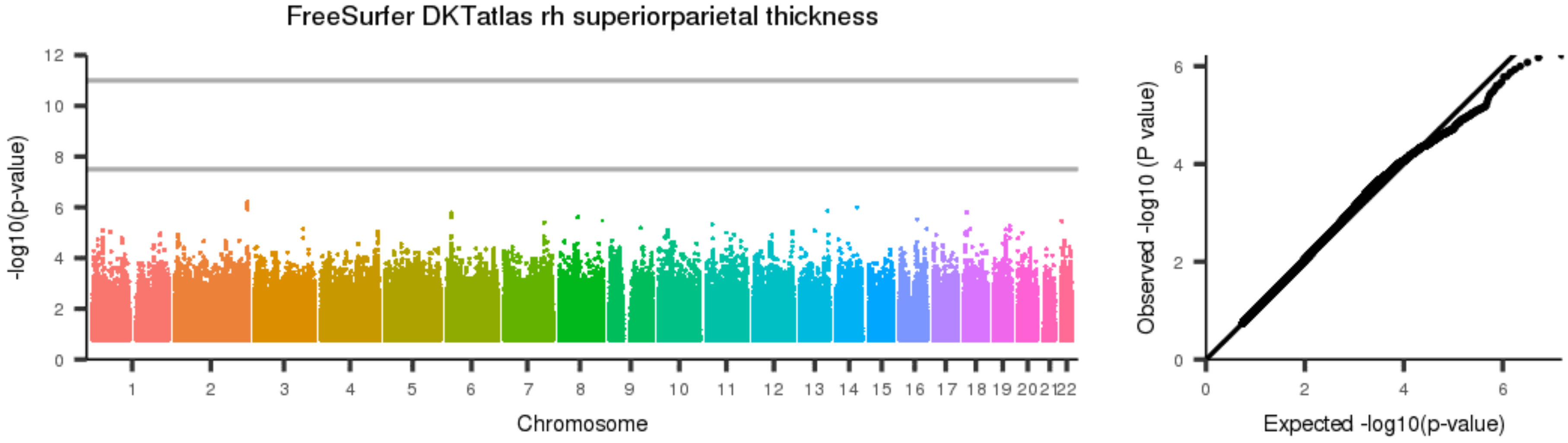

back to list of plots

Figure 1365: FreeSurfer DKTatlas rh superiortemporal thickness

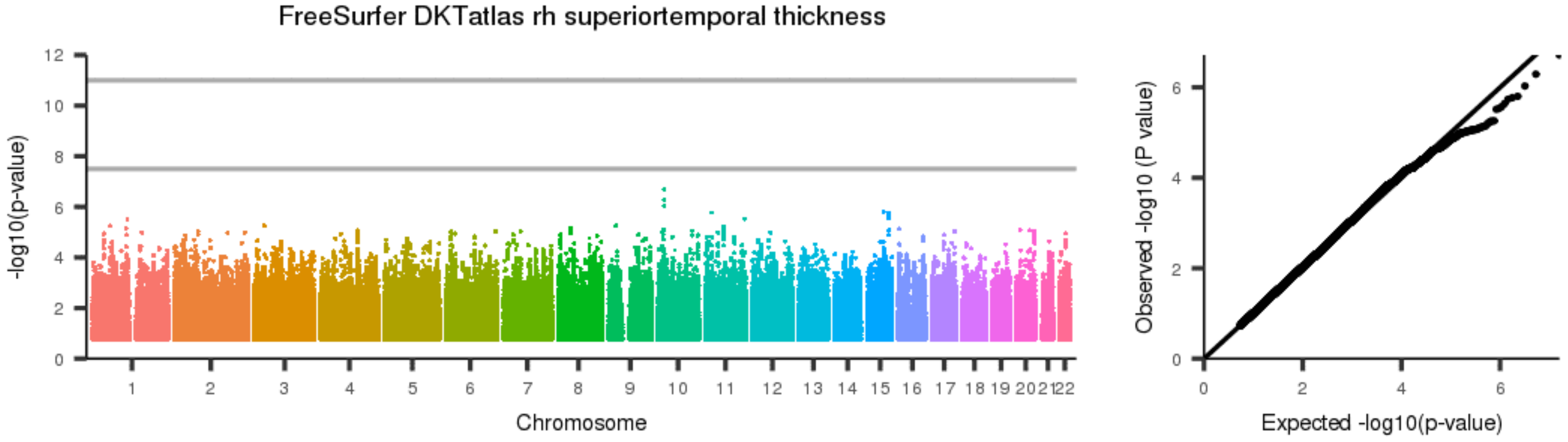

back to list of plots

Figure 1366: FreeSurfer DKTatlas rh supramarginal thickness

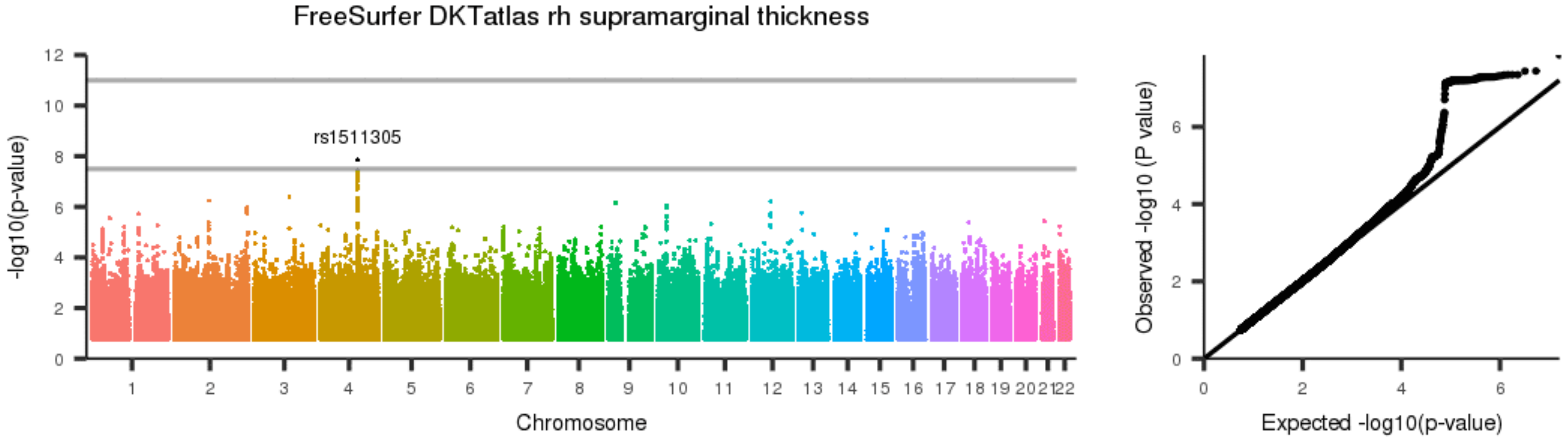

back to list of plots

Figure 1367: FreeSurfer DKTatlas rh transversetemporal thickness

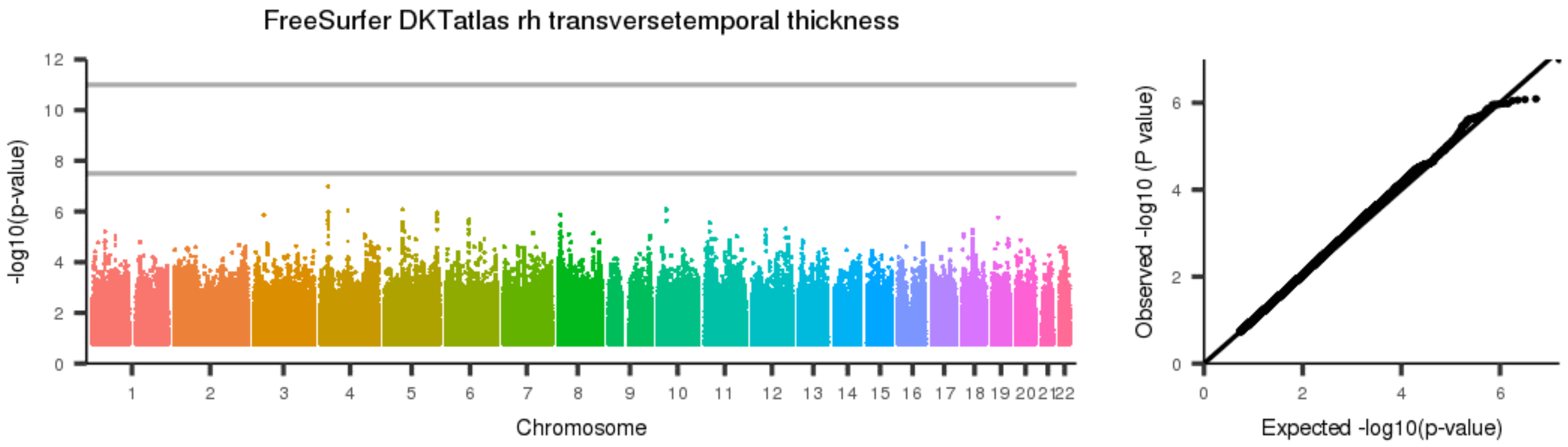

back to list of plots

Figure 1368: FreeSurfer DKTatlas rh insula thickness

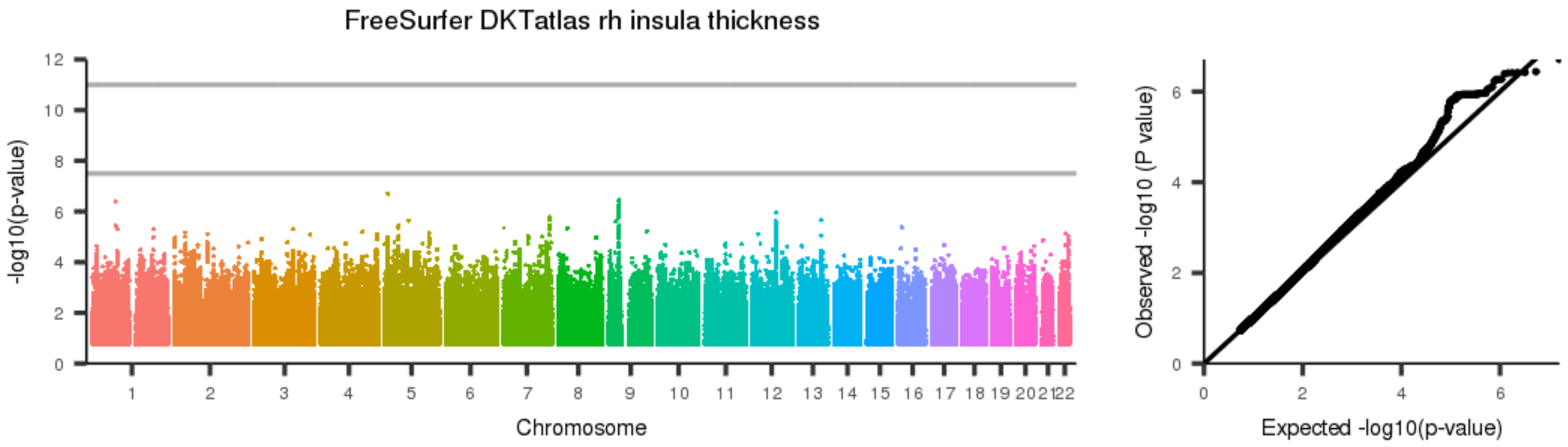

back to list of plots

Figure 1369: FreeSurfer DKTatlas rh MeanThickness thickness

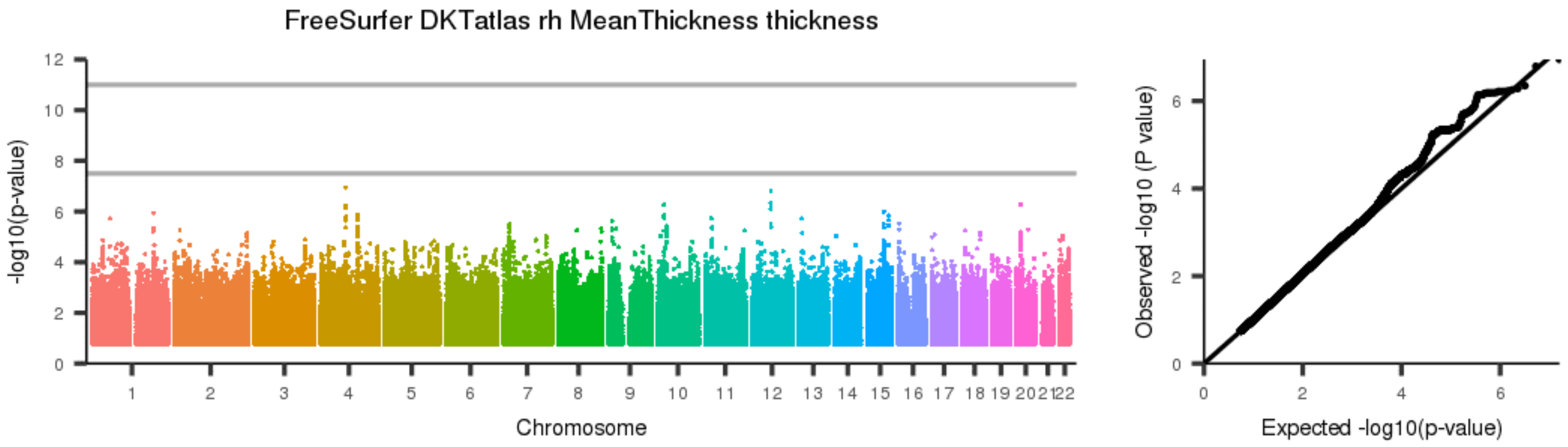

back to list of plots

Figure 1370: FreeSurfer a2009s rh G and S frontomargin thickness

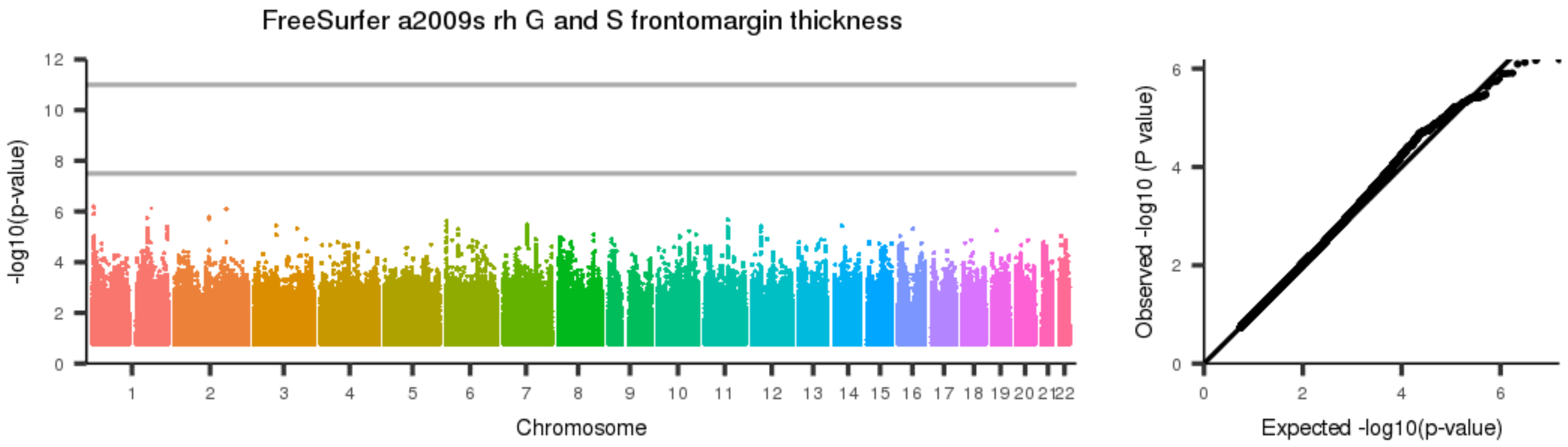

back to list of plots

Figure 1371: FreeSurfer a2009s rh G and S occipital inf thickness

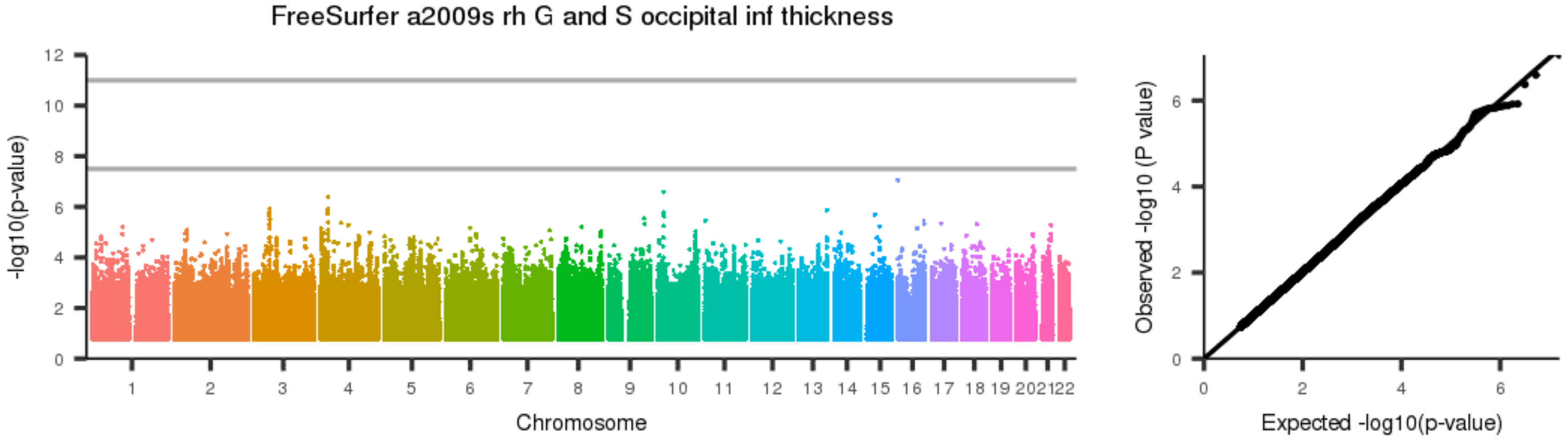

back to list of plots

Figure 1372: FreeSurfer a2009s rh G and S paracentral thickness

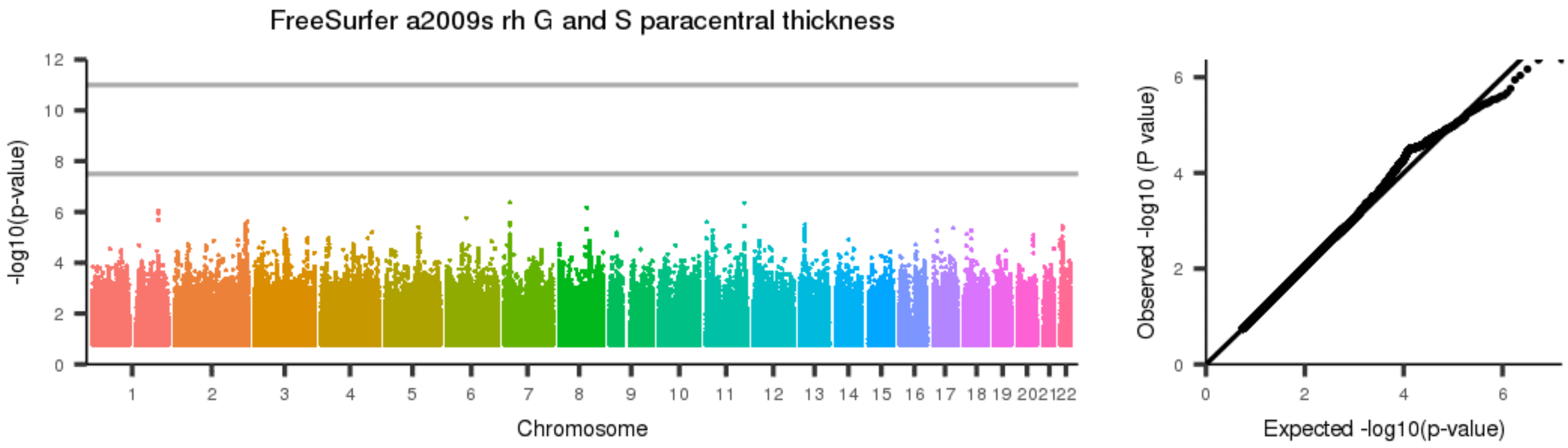

back to list of plots

Figure 1373: FreeSurfer a2009s rh G and S subcentral thickness

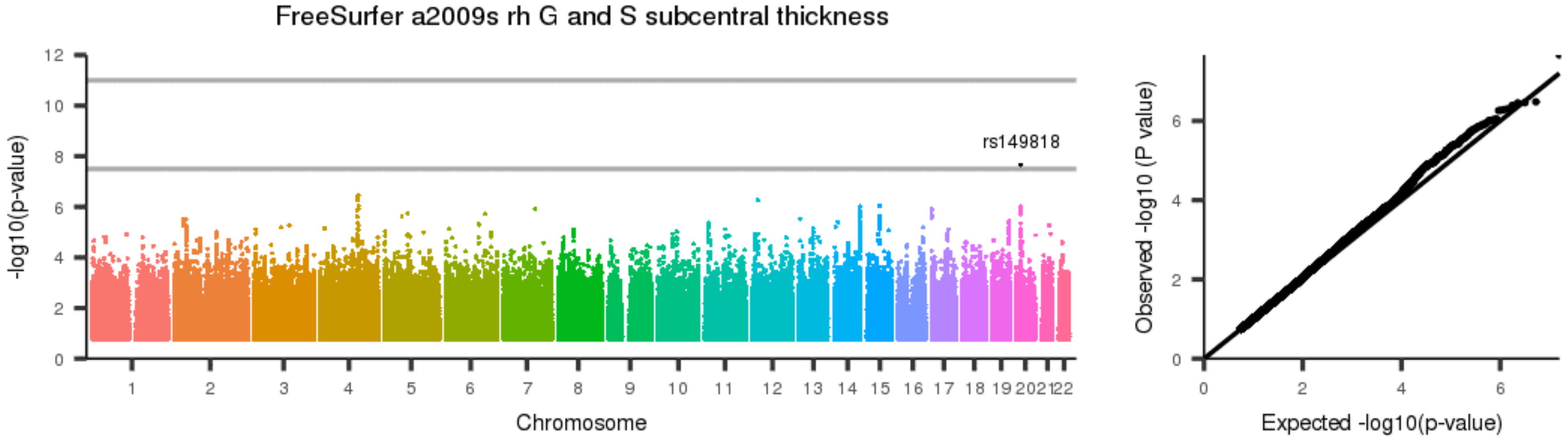

back to list of plots

Figure 1374: FreeSurfer a2009s rh G and S transv frontopol thickness

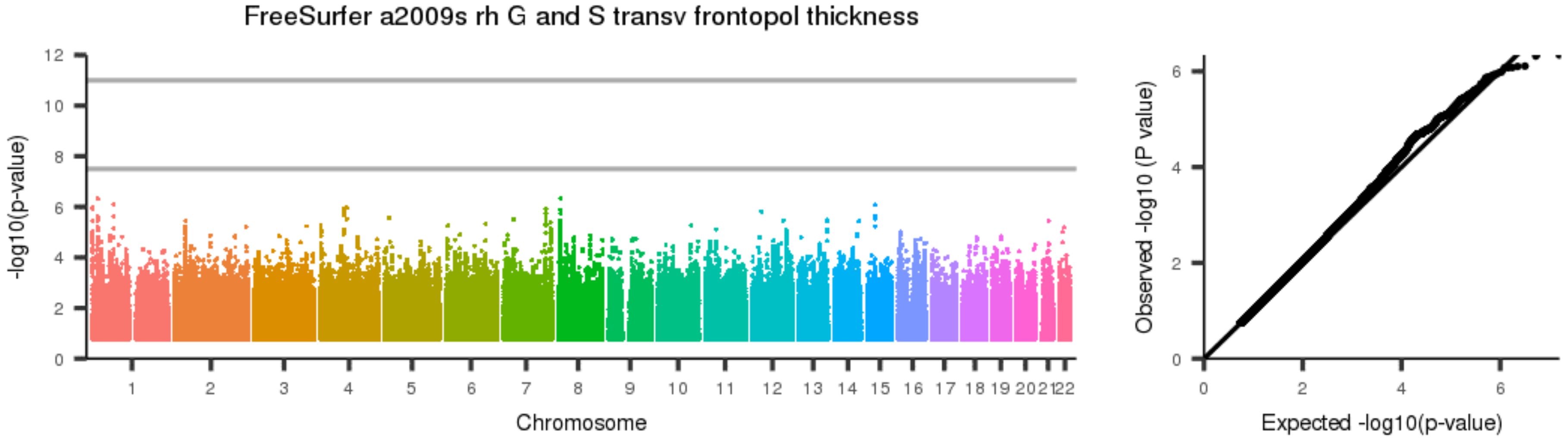

back to list of plots

Figure 1375: FreeSurfer a2009s rh G and S cingul-Ant thickness

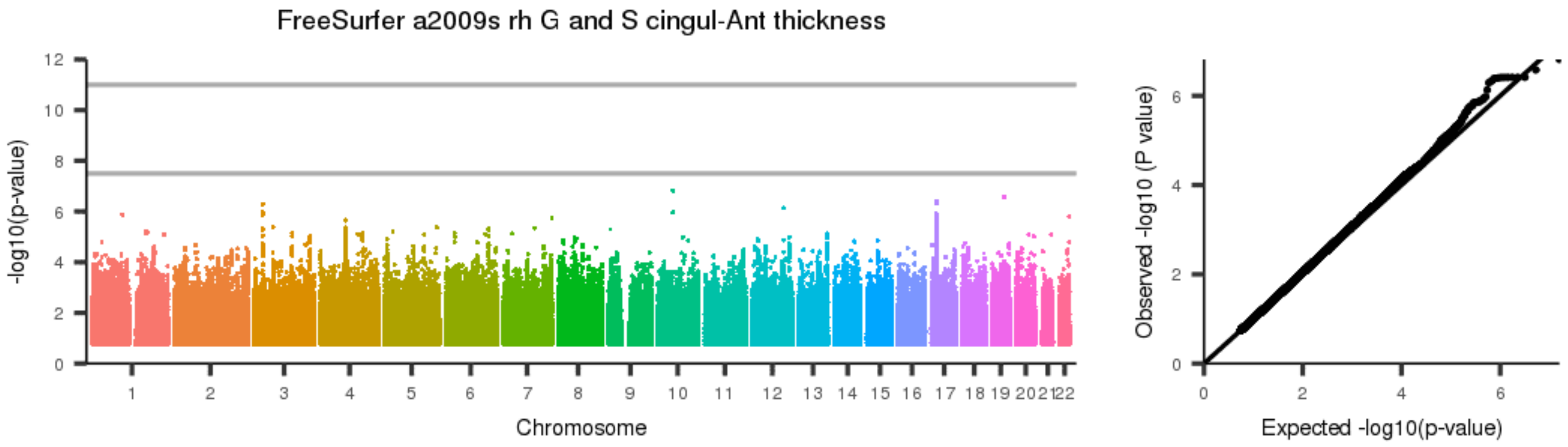

back to list of plots

Figure 1376: FreeSurfer a2009s rh G and S cingul-Mid-Ant thickness

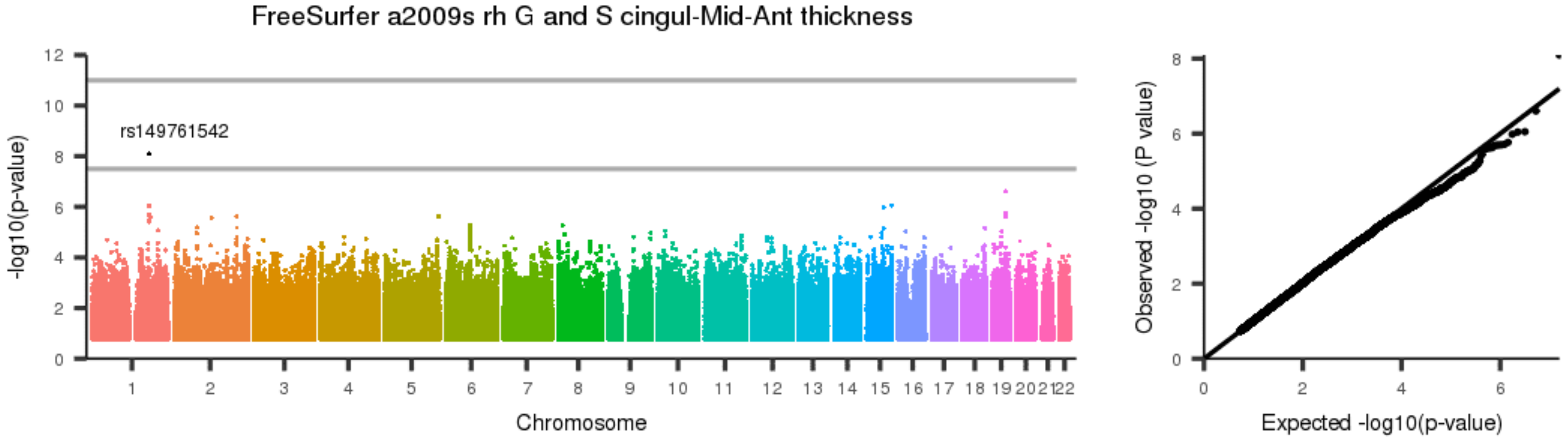

back to list of plots

Figure 1377: FreeSurfer a2009s rh G and S cingul-Mid-Post thickness

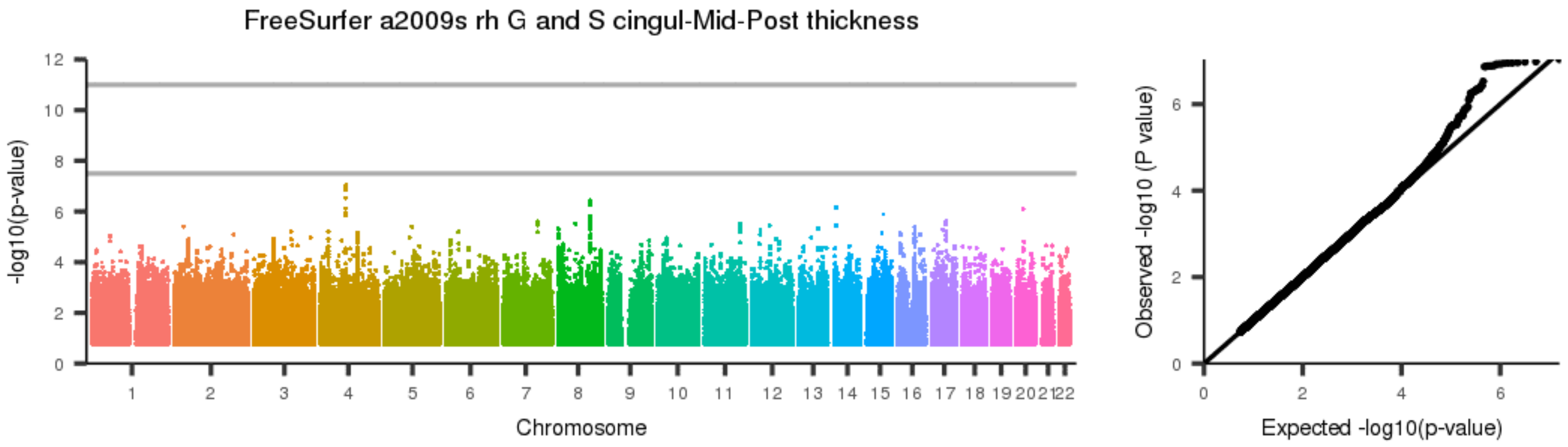

back to list of plots

Figure 1378: FreeSurfer a2009s rh G cingul-Post-dorsal thickness

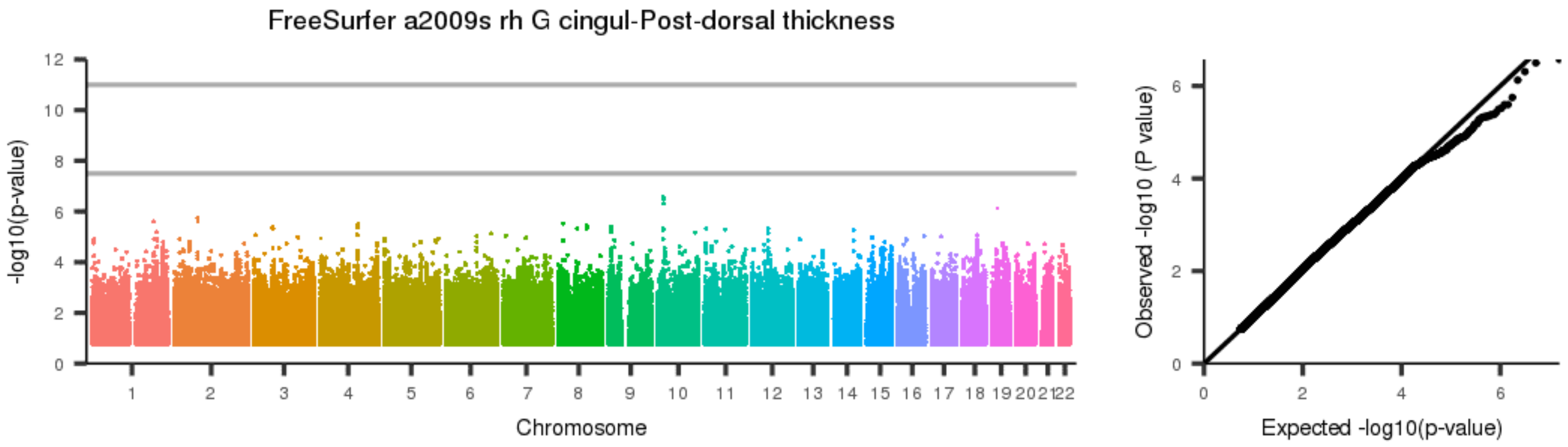

back to list of plots

Figure 1379: FreeSurfer a2009s rh G cingul-Post-ventral thickness

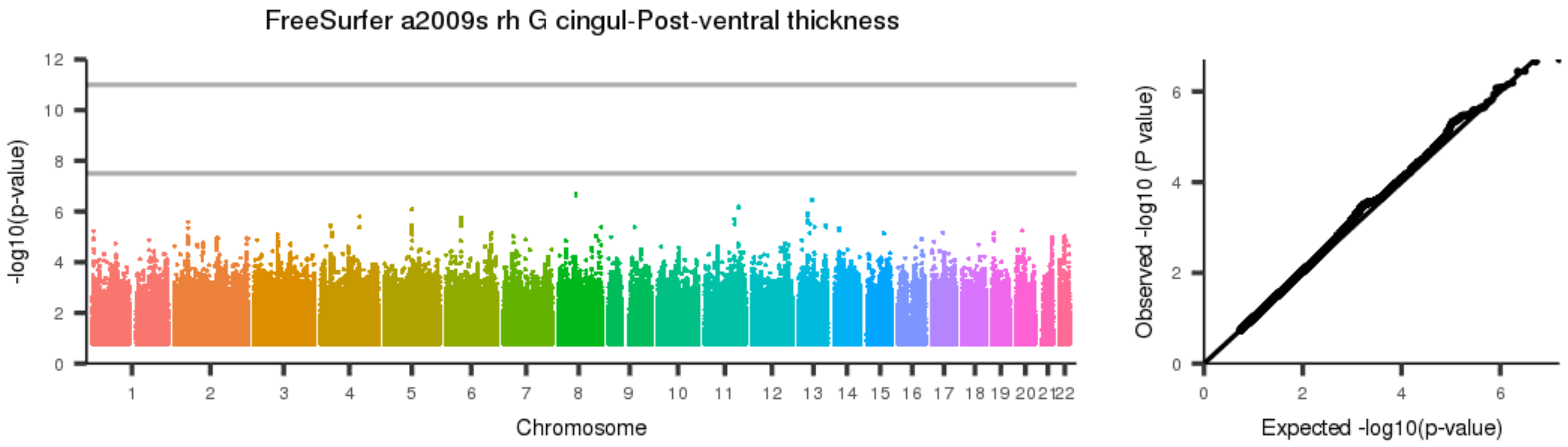

back to list of plots

Figure 1380: FreeSurfer a2009s rh G cuneus thickness

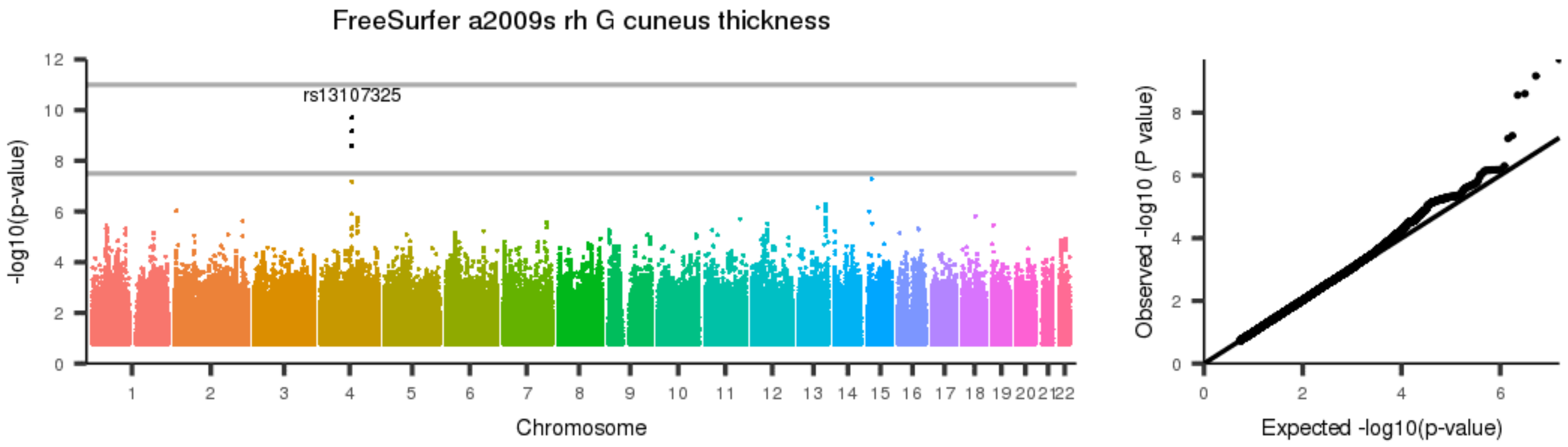

back to list of plots

Figure 1381: FreeSurfer a2009s rh G front inf-Opercular thickness

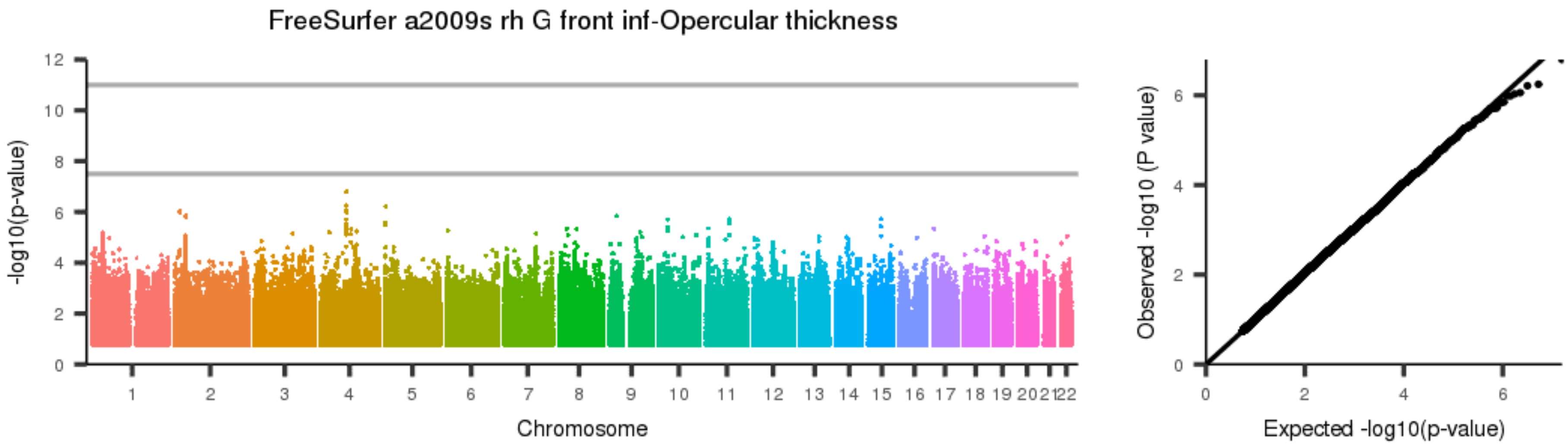

back to list of plots

Figure 1382: FreeSurfer a2009s rh G front inf-Orbital thickness

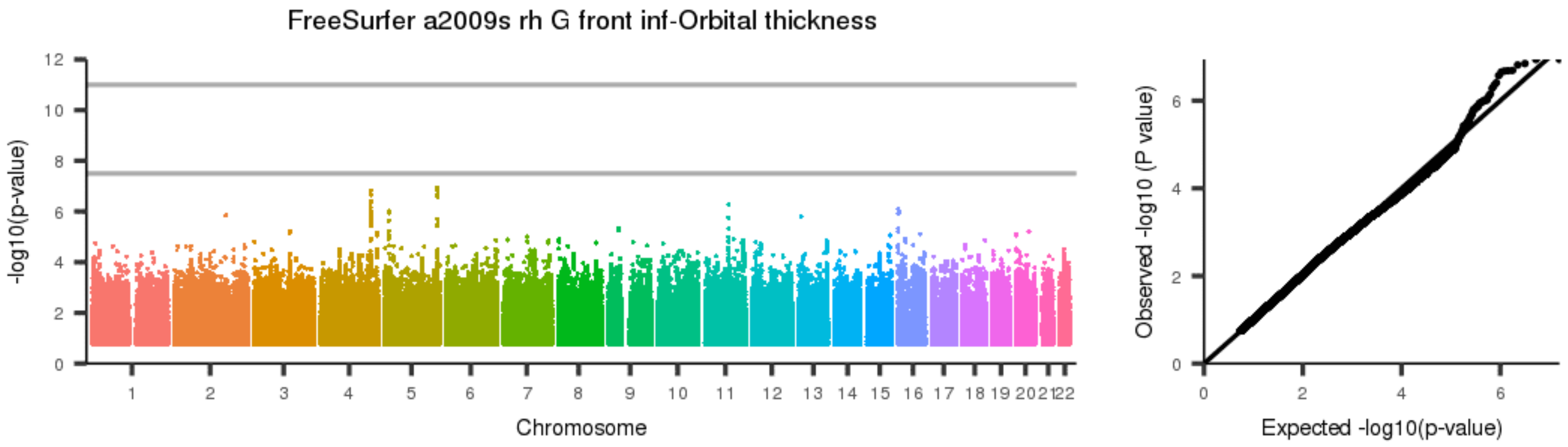

back to list of plots

Figure 1383: FreeSurfer a2009s rh G front inf-Triangul thickness

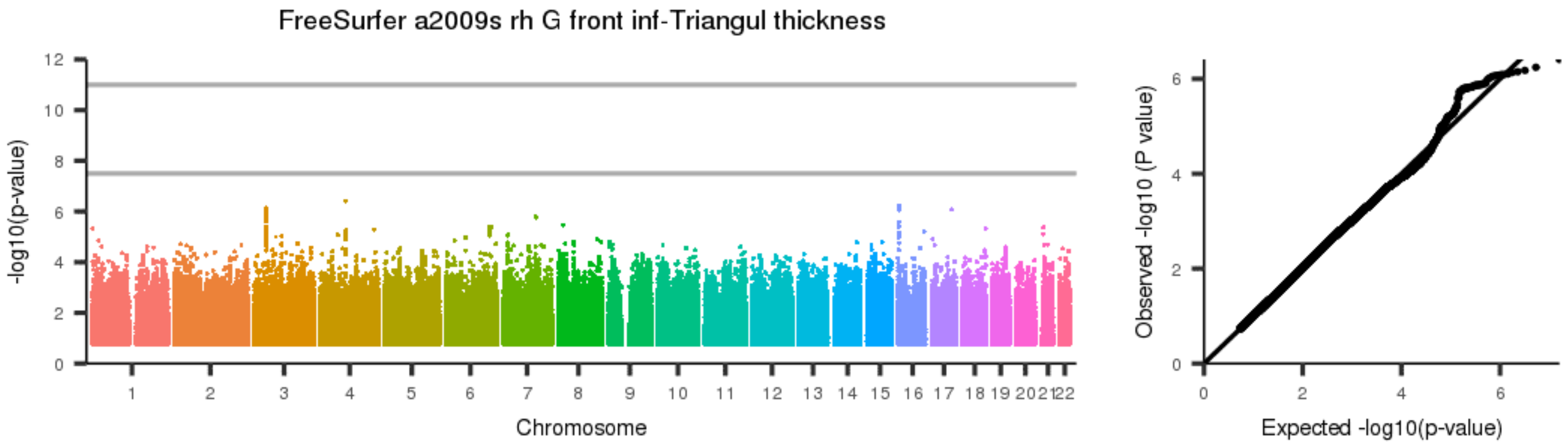

back to list of plots

Figure 1384: FreeSurfer a2009s rh G front middle thickness

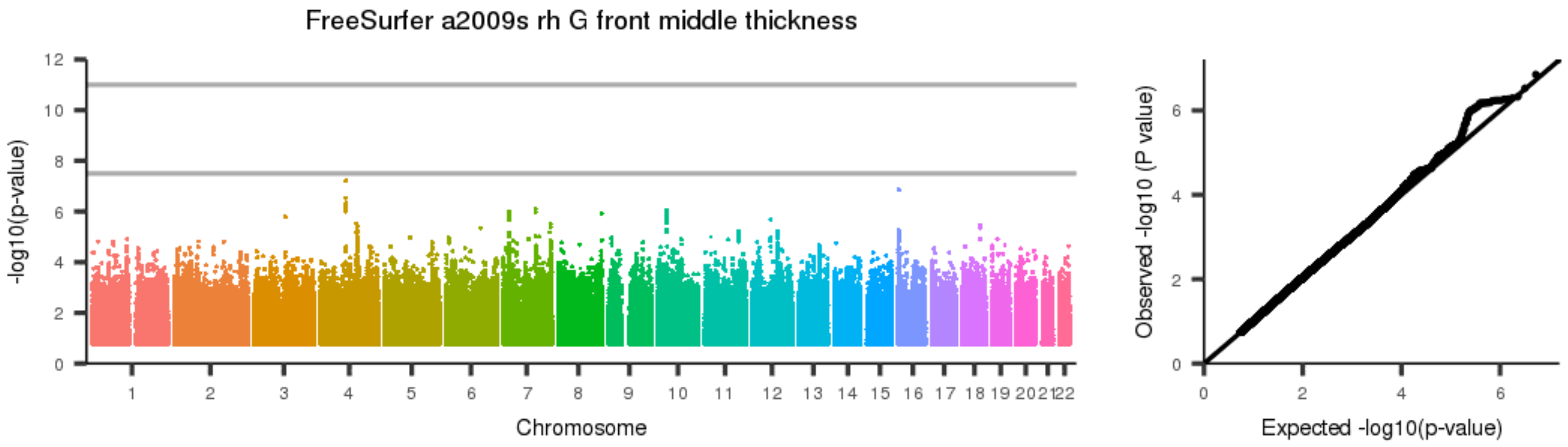

back to list of plots

Figure 1385: FreeSurfer a2009s rh G front sup thickness

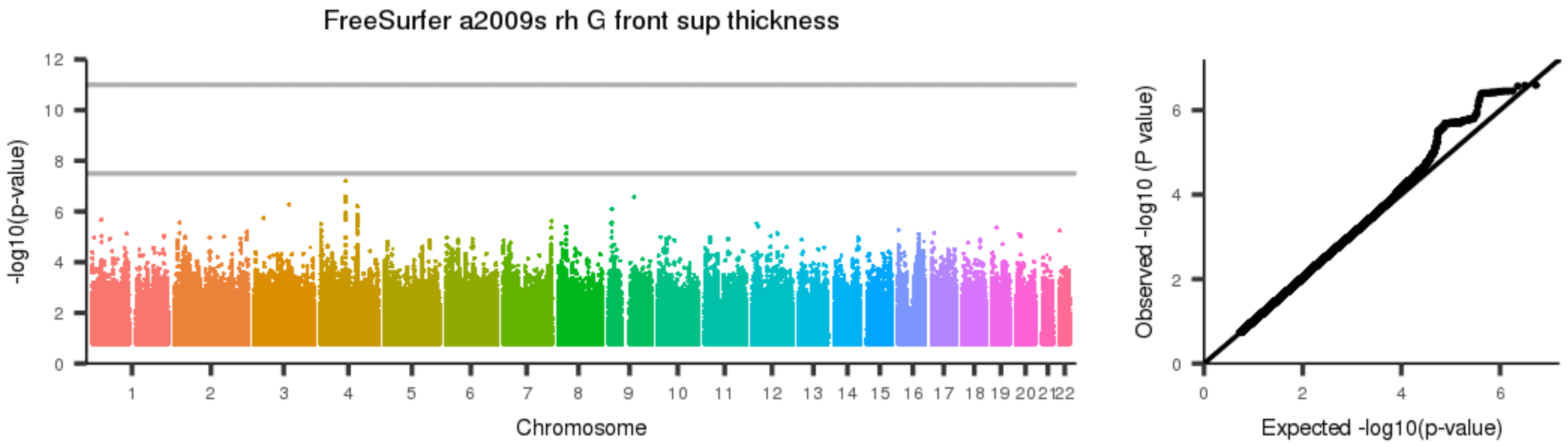

back to list of plots

Figure 1386: FreeSurfer a2009s rh G Ins lg and S cent ins thickness

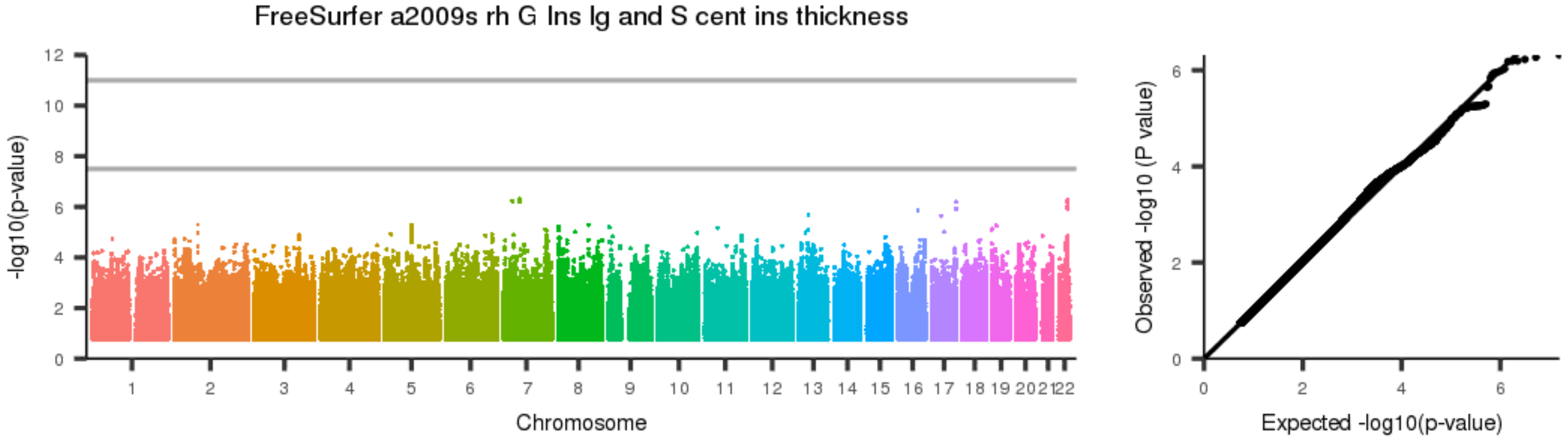

back to list of plots

Figure 1387: FreeSurfer a2009s rh G insular short thickness

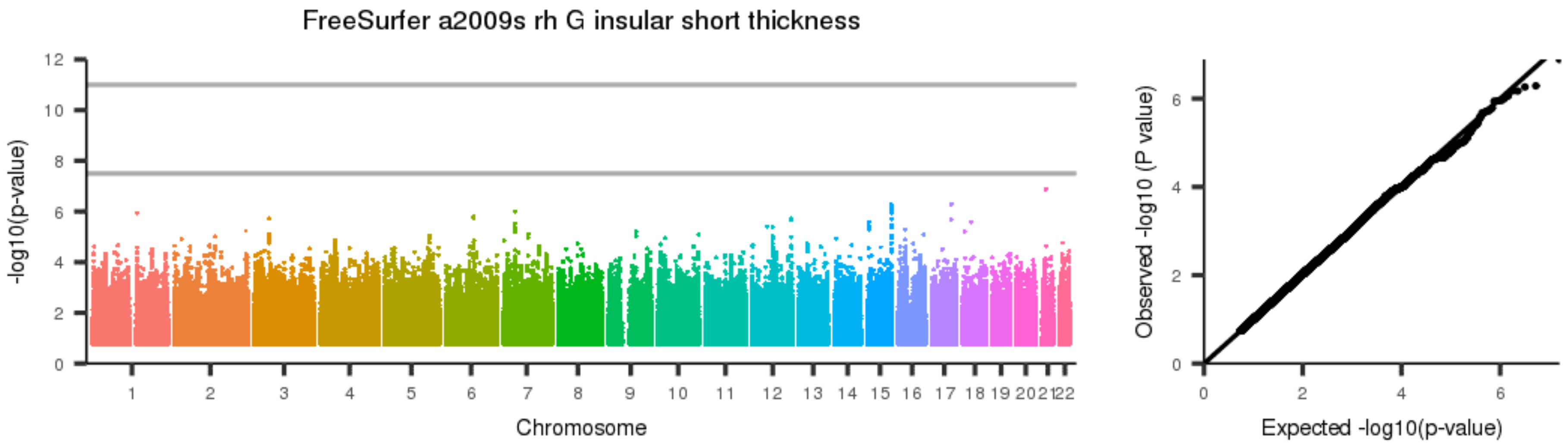

back to list of plots

Figure 1388: FreeSurfer a2009s rh G occipital middle thickness

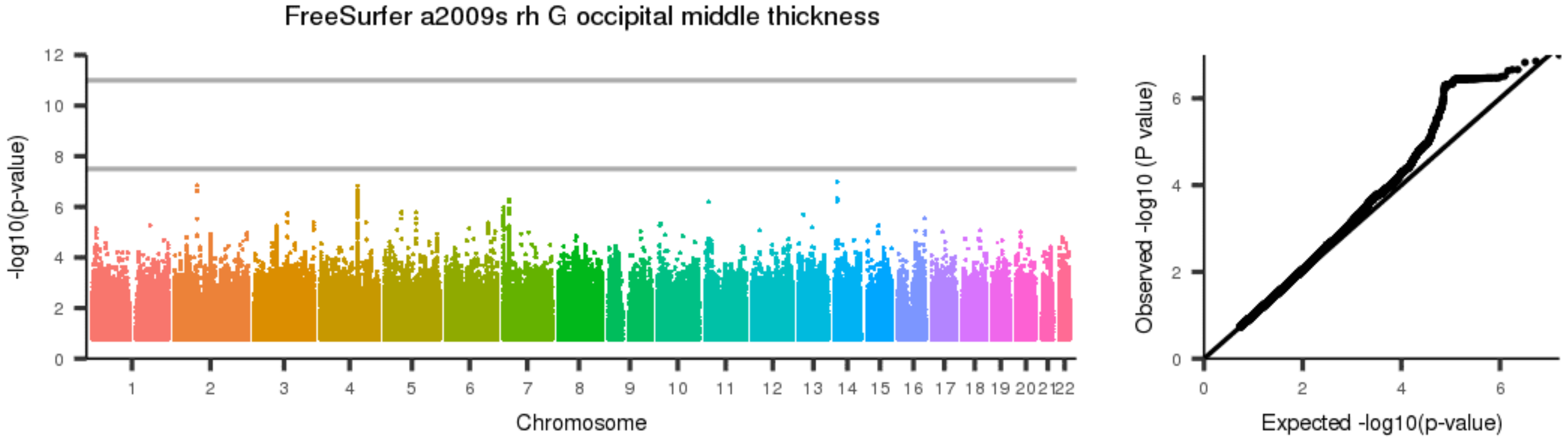

back to list of plots

Figure 1389: FreeSurfer a2009s rh G occipital sup thickness

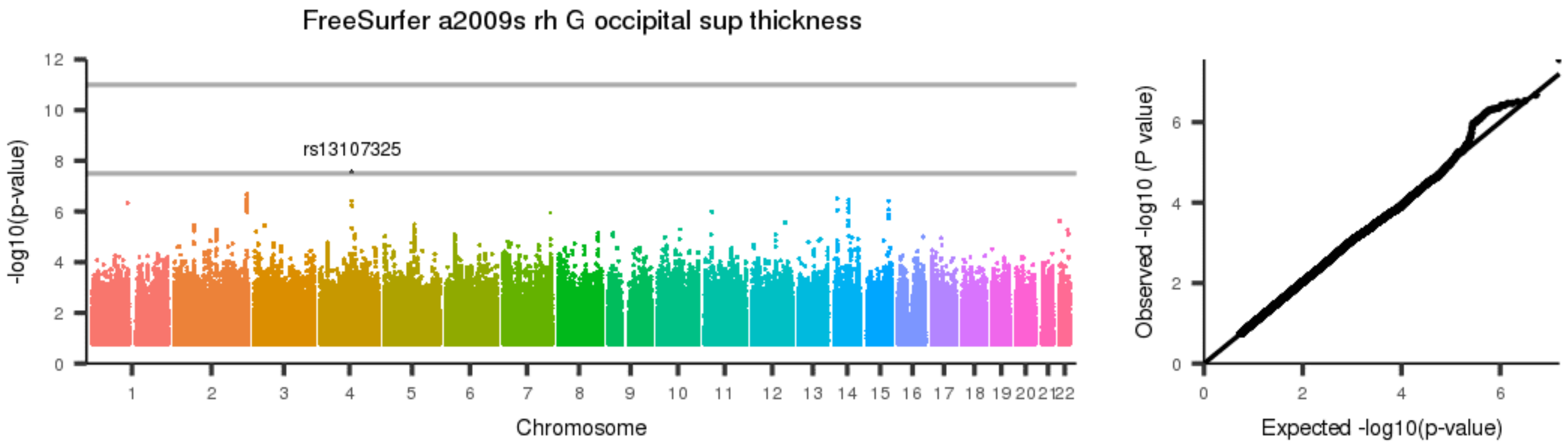

back to list of plots

Figure 1390: FreeSurfer a2009s rh G oc-temp lat-fusifor thickness

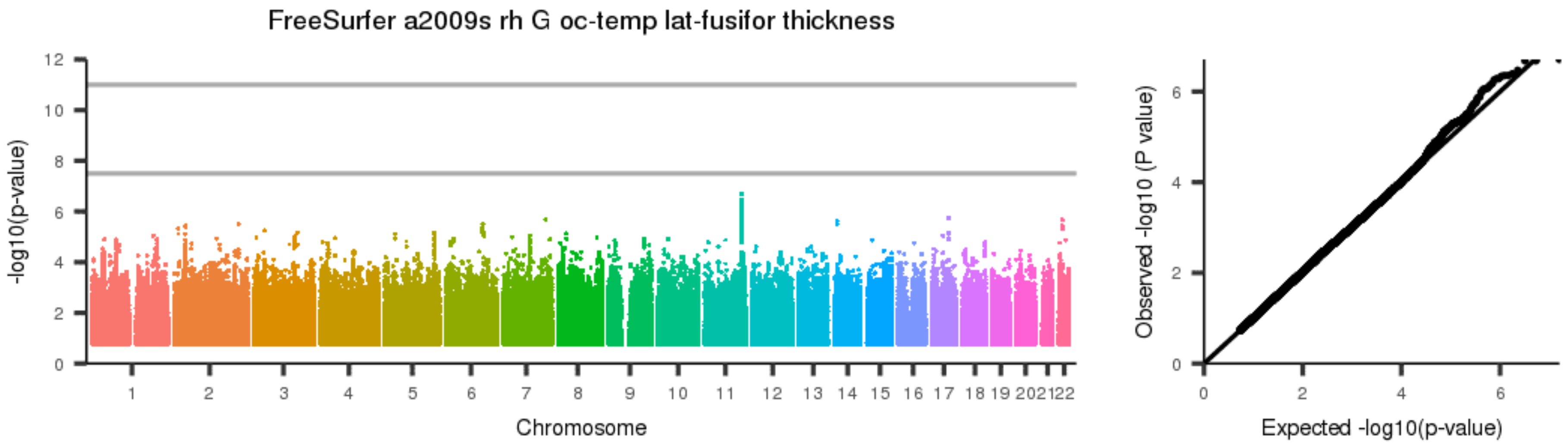

back to list of plots

Figure 1391: FreeSurfer a2009s rh G oc-temp med-Lingual thickness

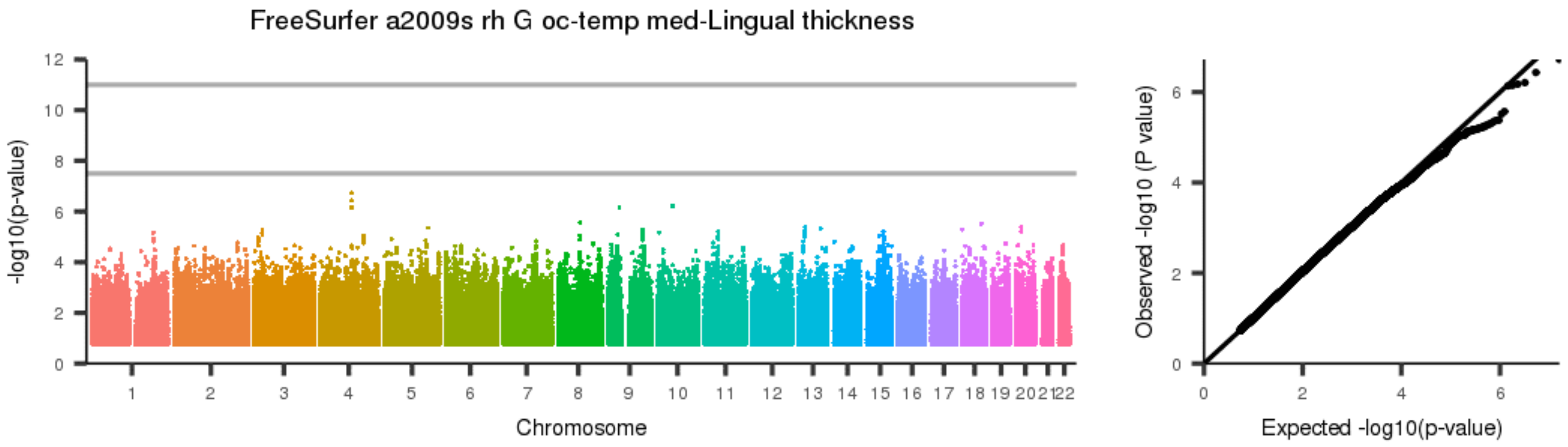

back to list of plots

Figure 1392: FreeSurfer a2009s rh G oc-temp med-Parahip thickness

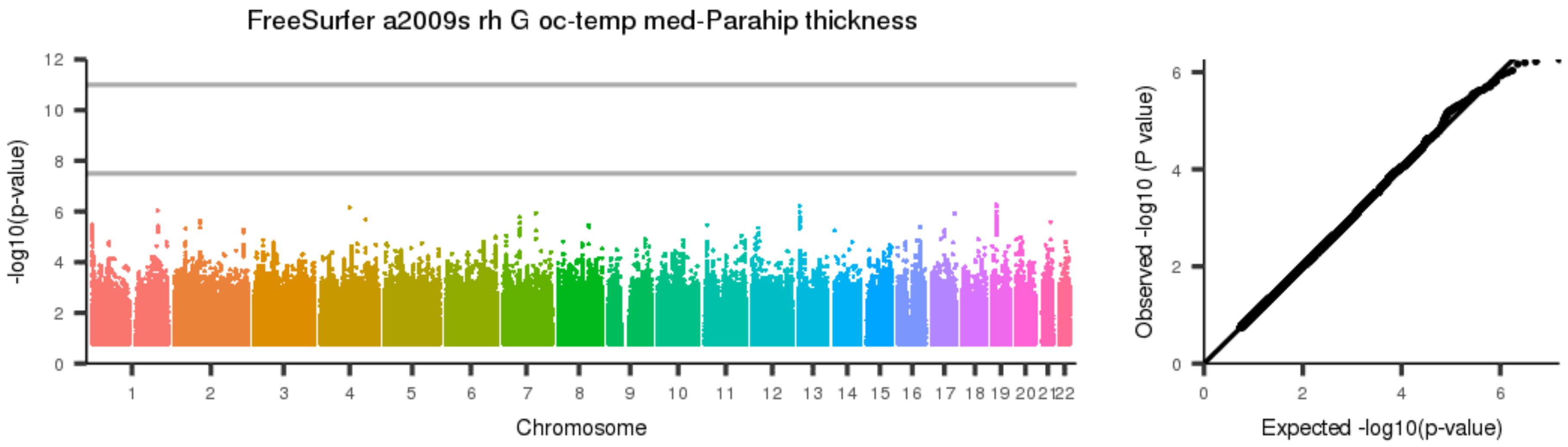

back to list of plots

Figure 1393: FreeSurfer a2009s rh G orbital thickness

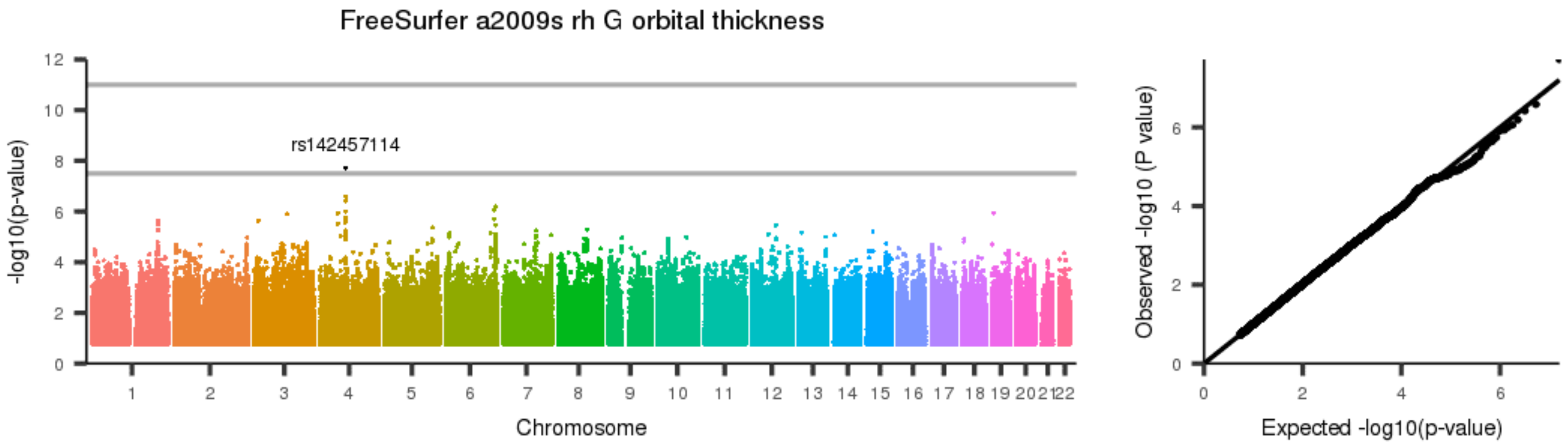

back to list of plots

Figure 1394: FreeSurfer a2009s rh G pariet inf-Angular thickness

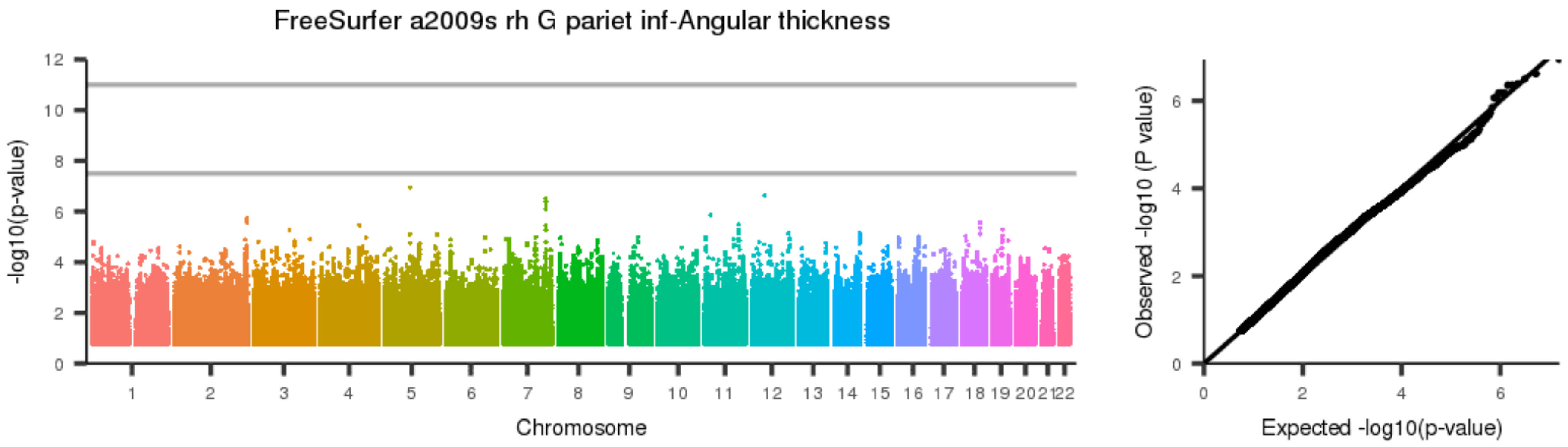

back to list of plots

Figure 1395: FreeSurfer a2009s rh G pariet inf-Supramar thickness

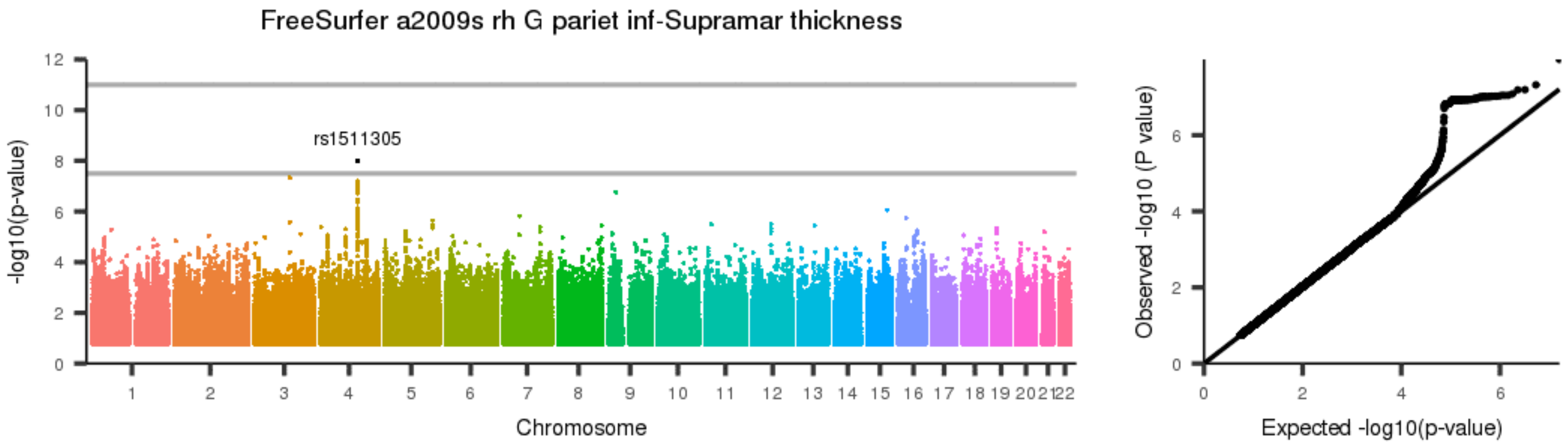

back to list of plots

Figure 1396: FreeSurfer a2009s rh G parietal sup thickness

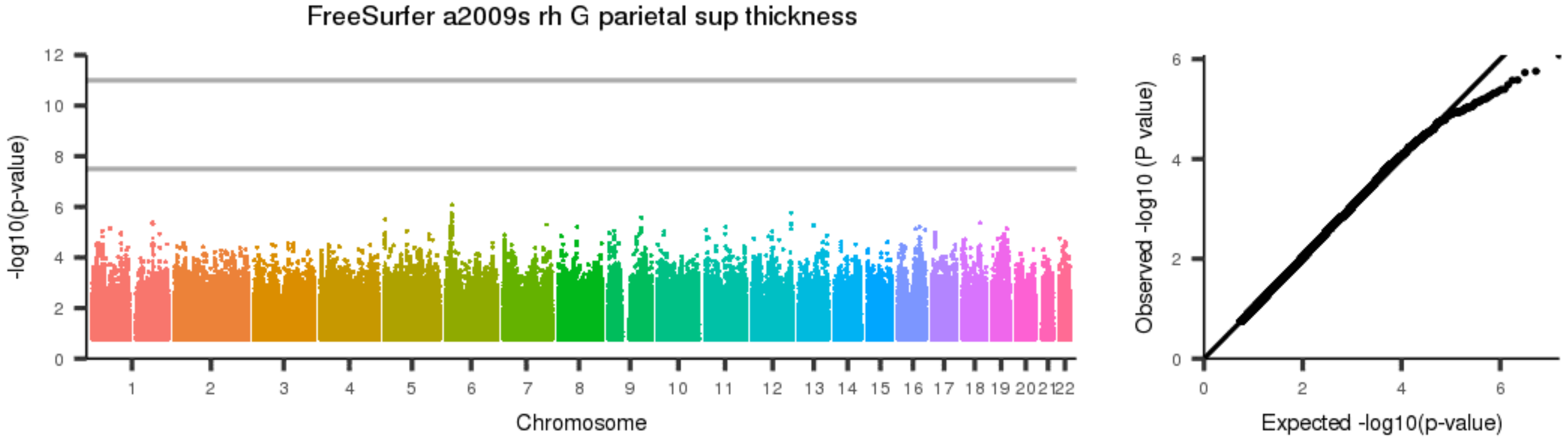

back to list of plots

Figure 1397: FreeSurfer a2009s rh G postcentral thickness

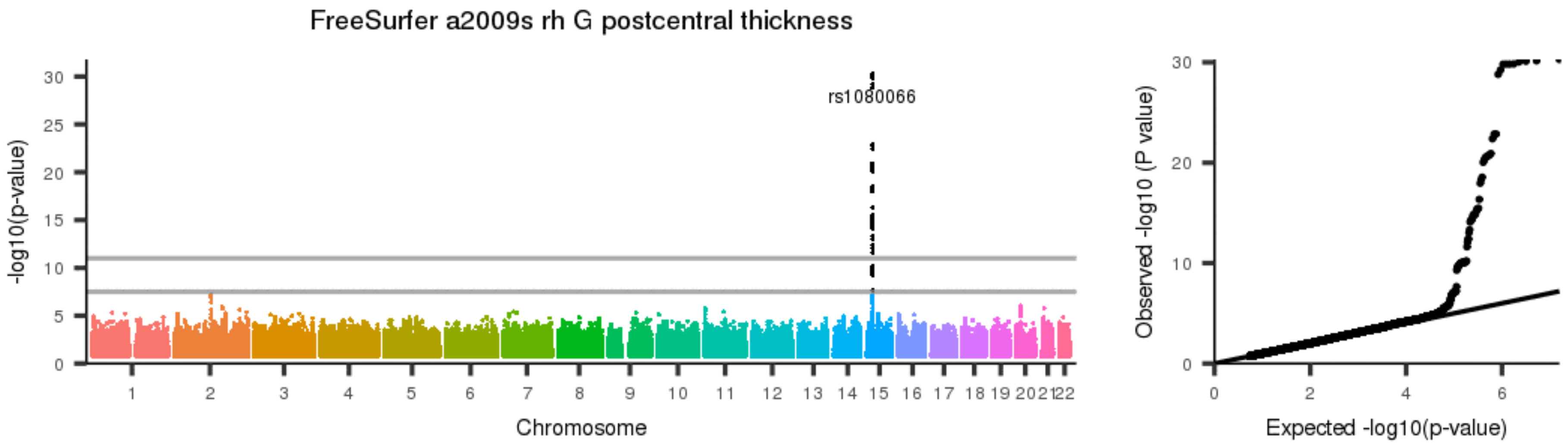

back to list of plots

Figure 1398: FreeSurfer a2009s rh G precentral thickness

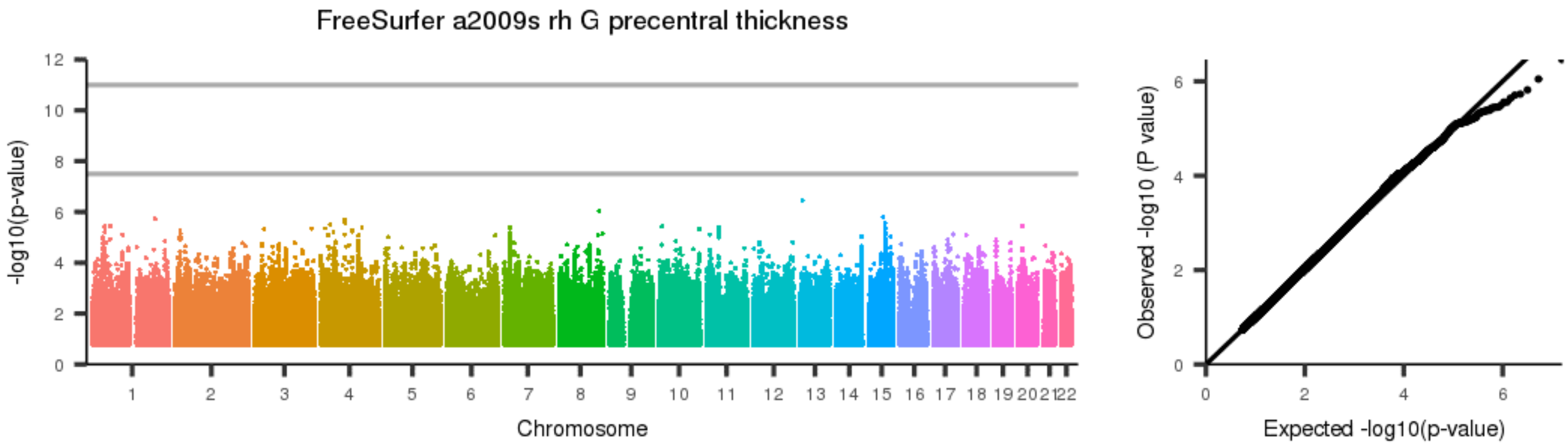

back to list of plots

Figure 1399: FreeSurfer a2009s rh G precuneus thickness

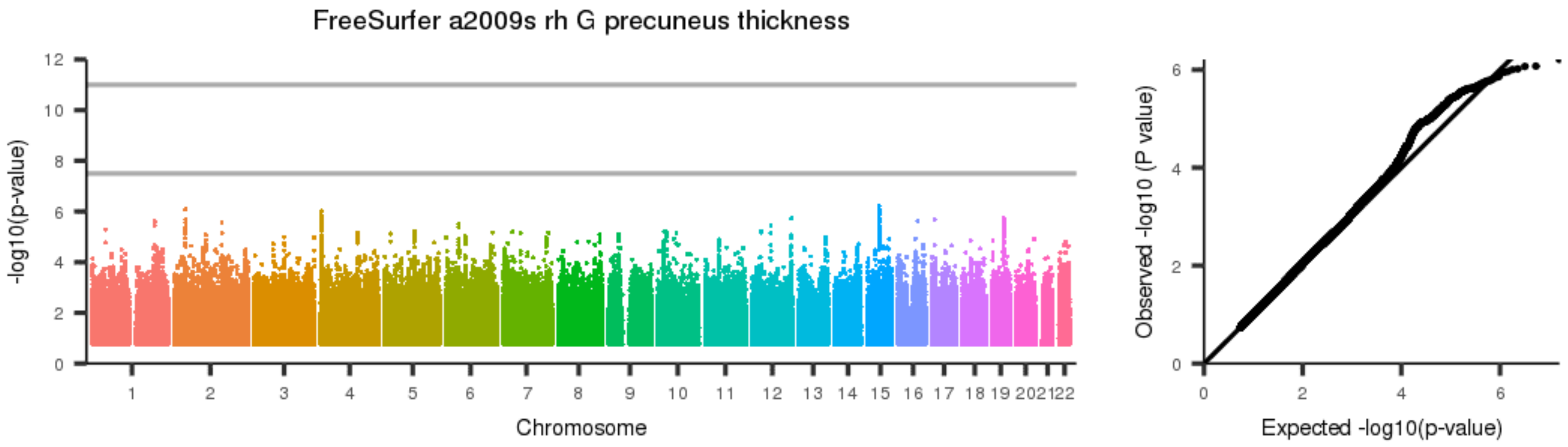

back to list of plots

Figure 1400: FreeSurfer a2009s rh G rectus thickness

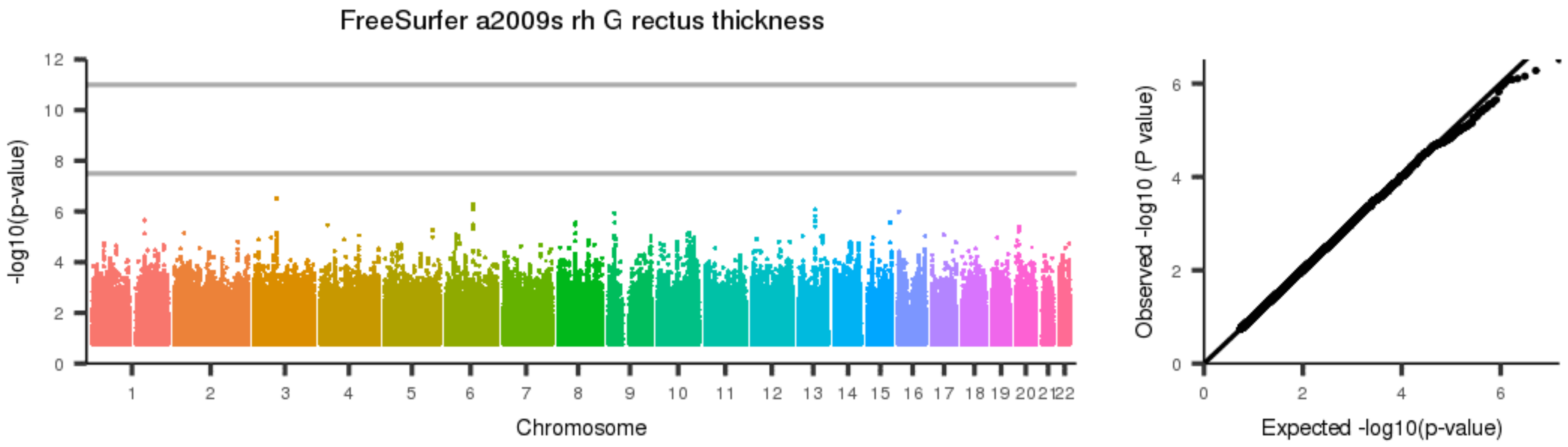

back to list of plots

Figure 1401: FreeSurfer a2009s rh G subcallosal thickness

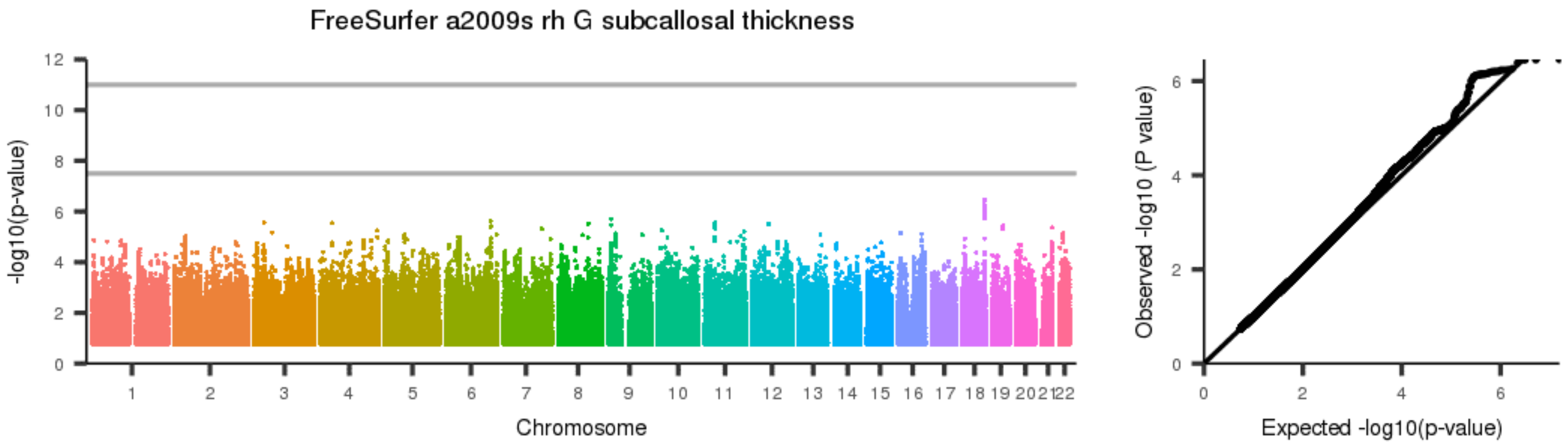

back to list of plots

Figure 1402: FreeSurfer a2009s rh G temp sup-G T transv thickness

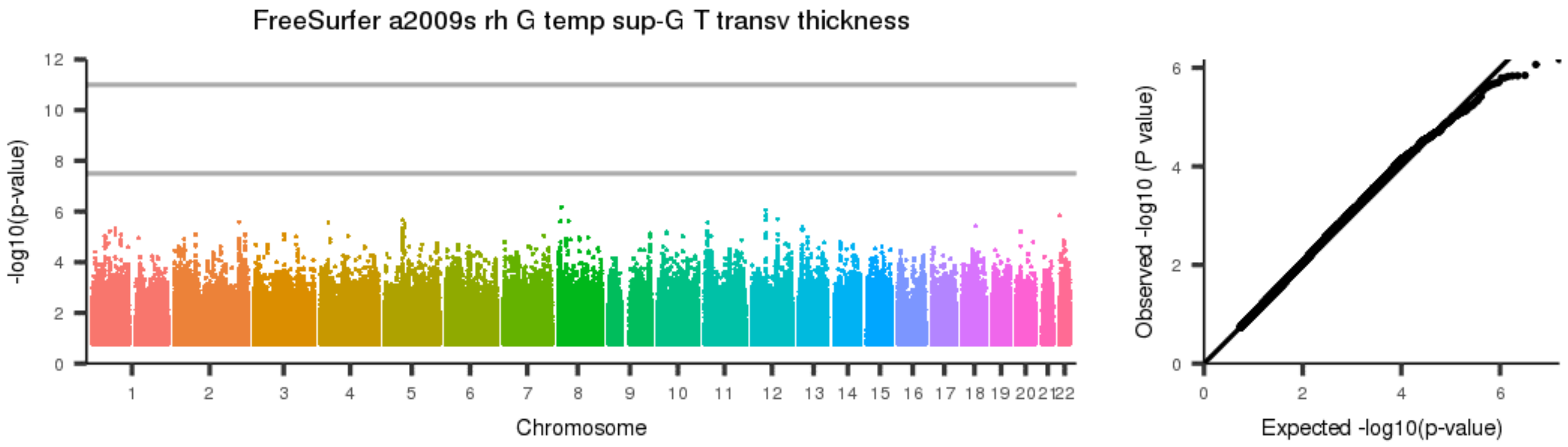

back to list of plots

Figure 1403: FreeSurfer a2009s rh G temp sup-Lateral thickness

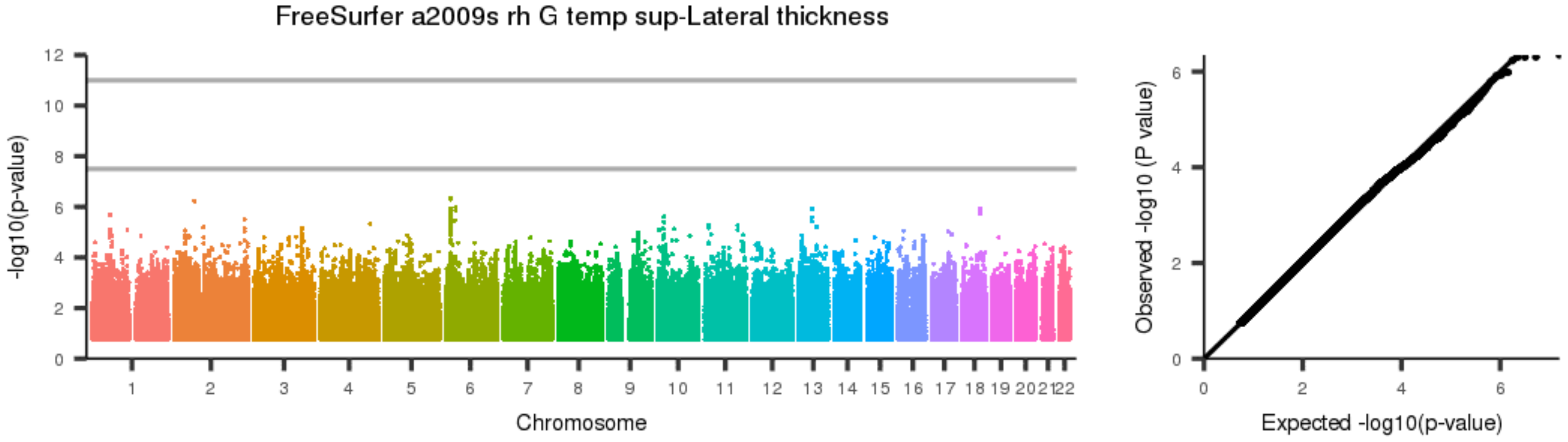

back to list of plots

Figure 1404: FreeSurfer a2009s rh G temp sup-Plan polar thickness

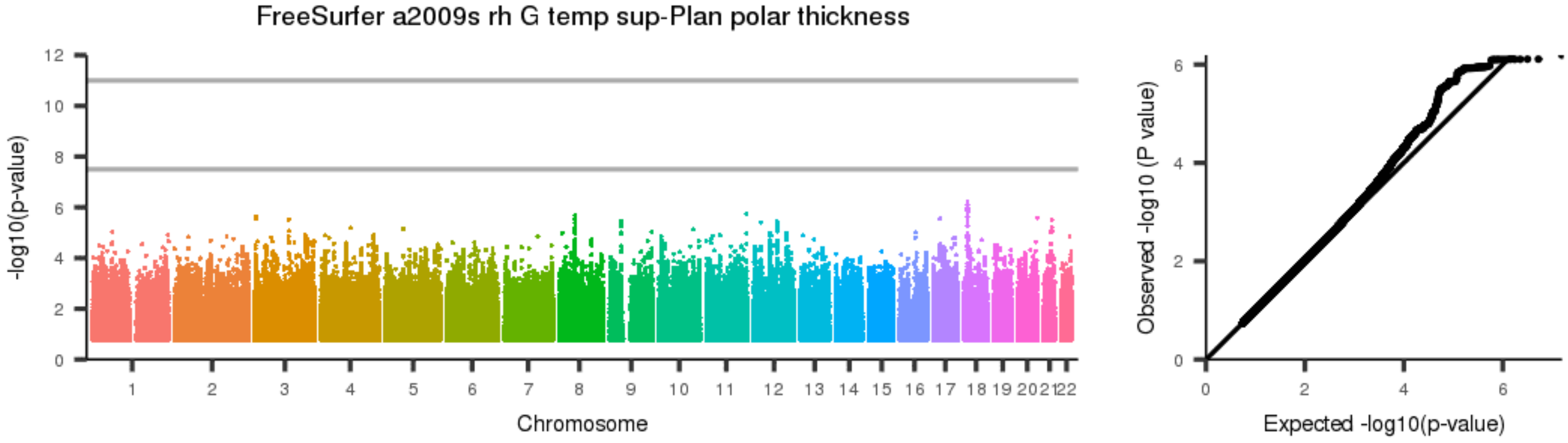

back to list of plots

Figure 1405: FreeSurfer a2009s rh G temp sup-Plan tempo thickness

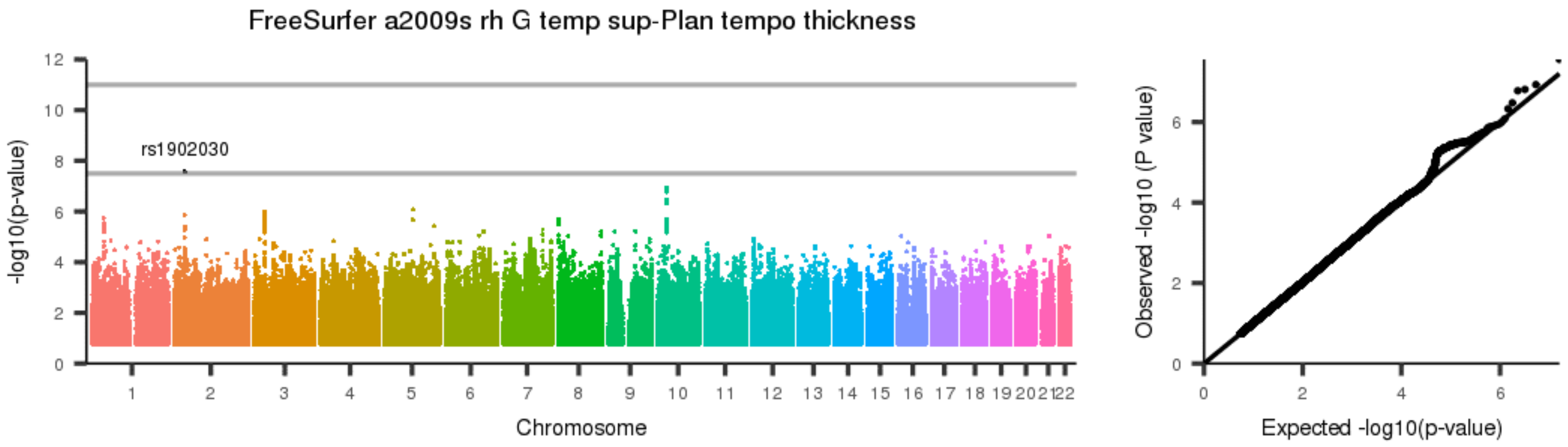

back to list of plots

Figure 1406: FreeSurfer a2009s rh G temporal inf thickness

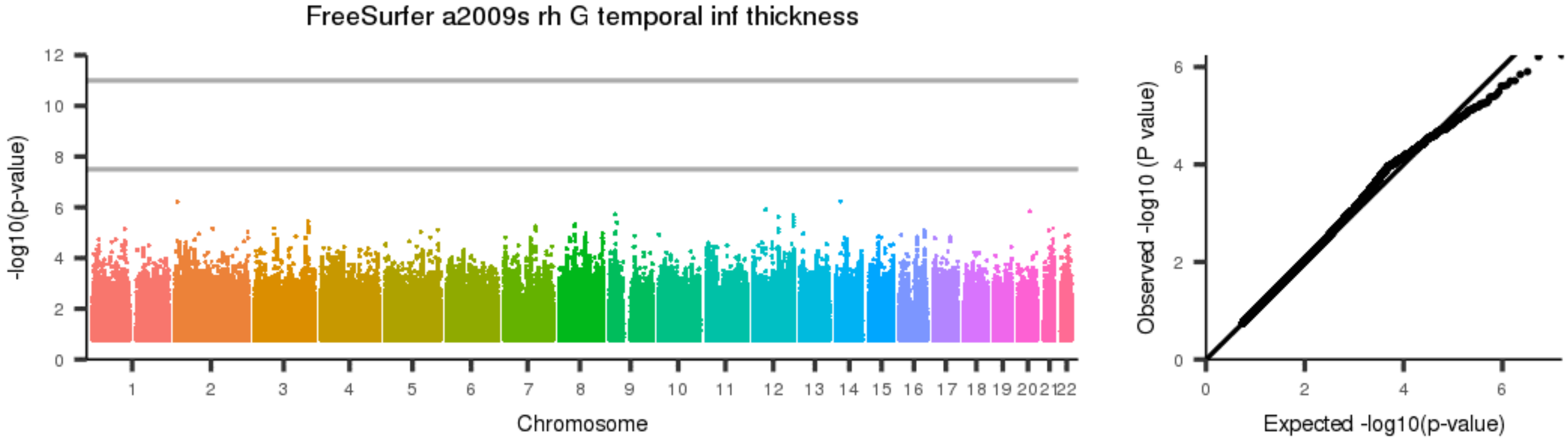

back to list of plots

Figure 1407: FreeSurfer a2009s rh G temporal middle thickness

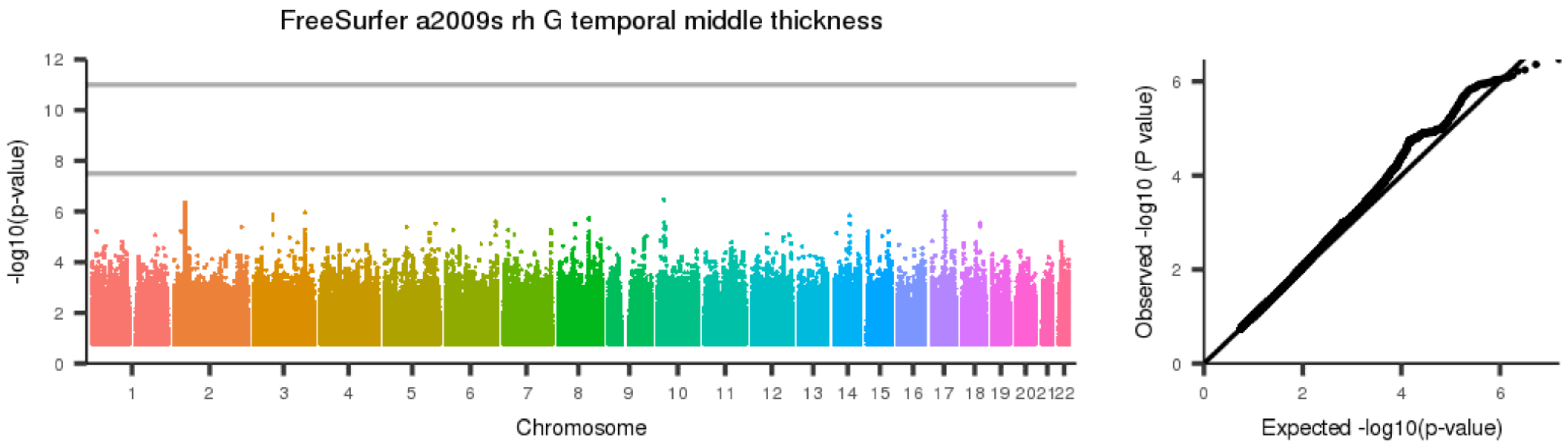

back to list of plots

Figure 1408: FreeSurfer a2009s rh Lat Fis-ant-Horizont thickness

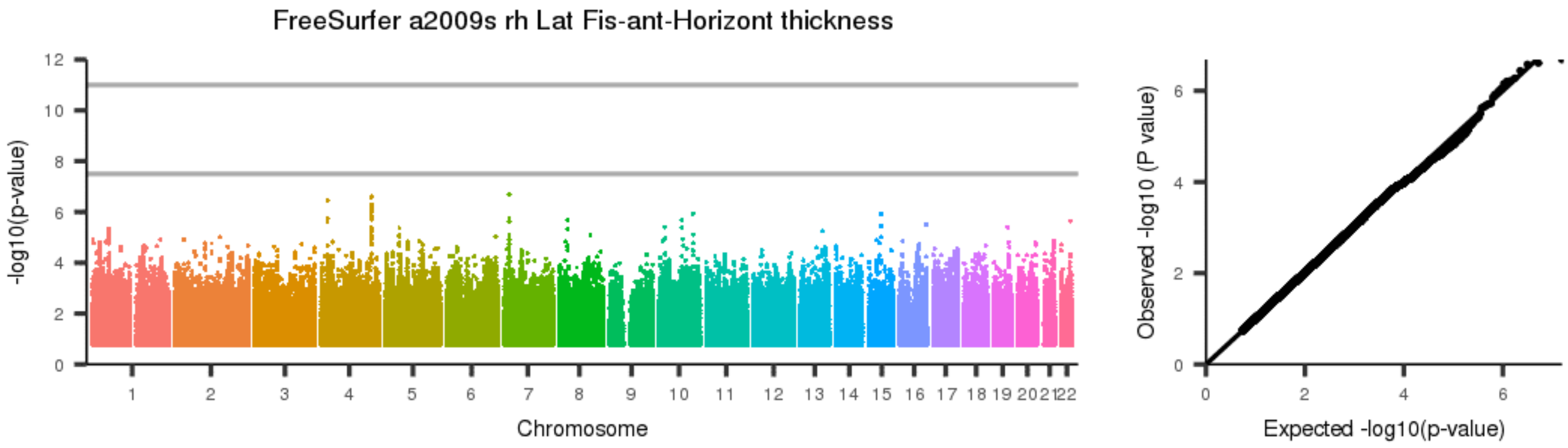

back to list of plots

Figure 1409: FreeSurfer a2009s rh Lat Fis-ant-Vertical thickness

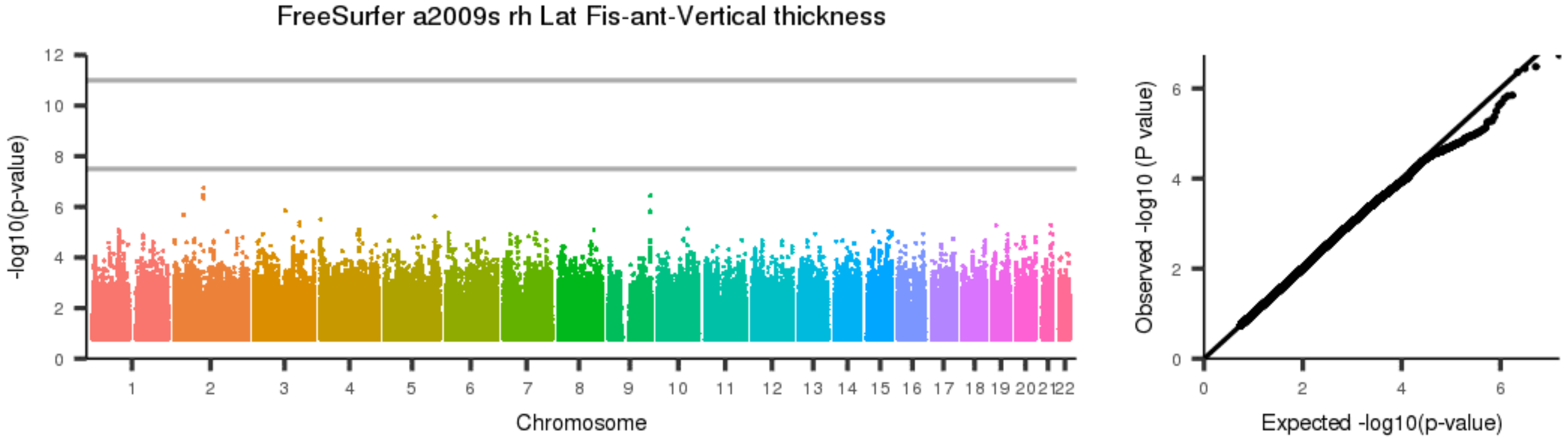

back to list of plots

Figure 1410: FreeSurfer a2009s rh Lat Fis-post thickness

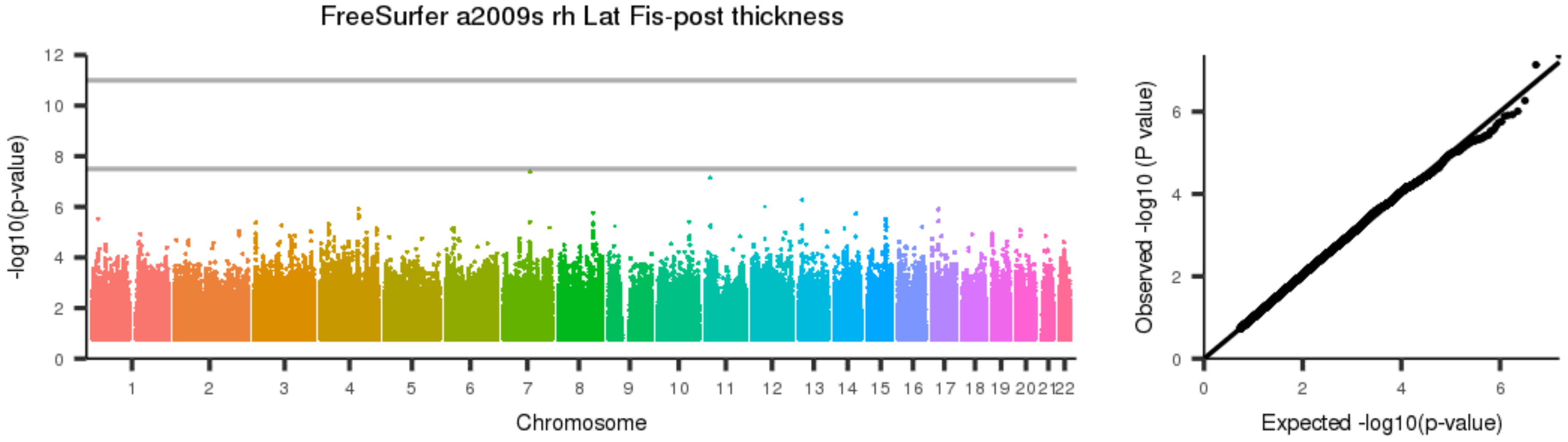

back to list of plots

Figure 1411: FreeSurfer a2009s rh Pole occipital thickness

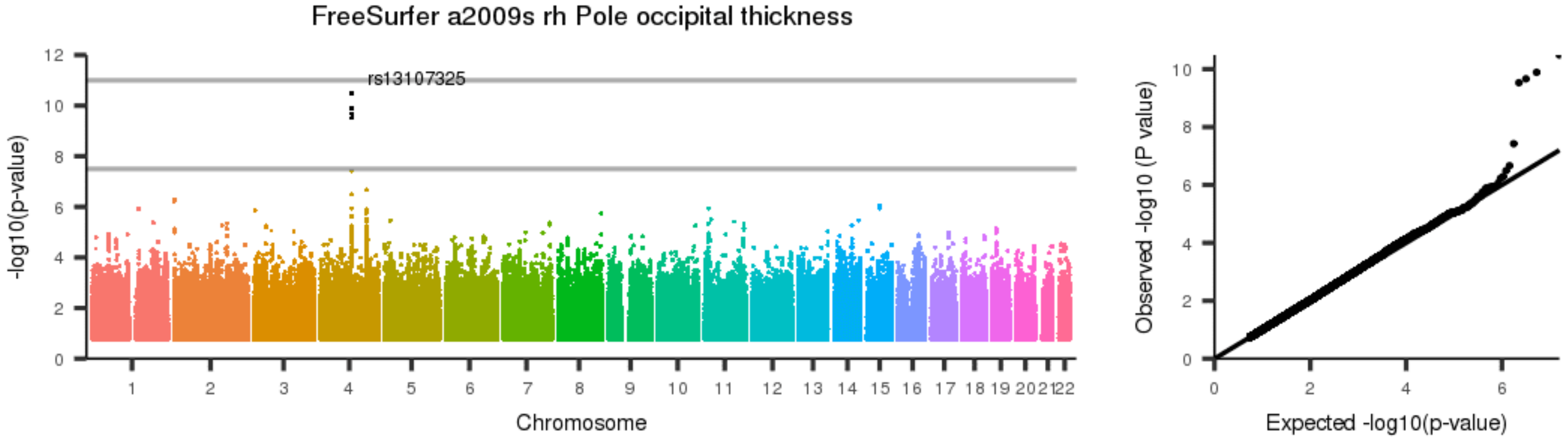

back to list of plots

Figure 1412: FreeSurfer a2009s rh Pole temporal thickness

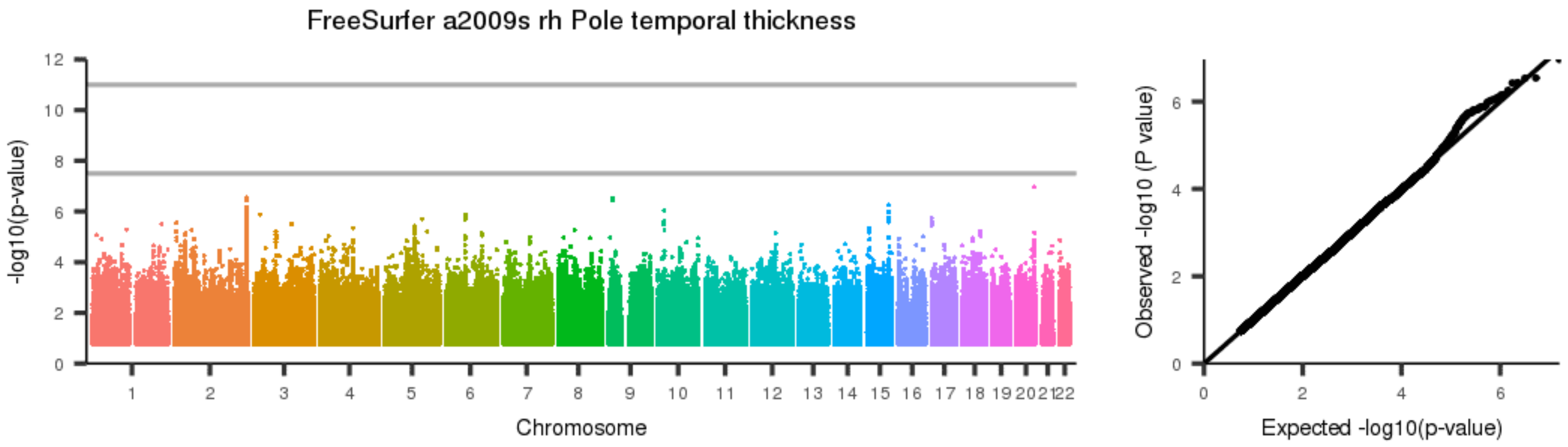

back to list of plots

Figure 1413: FreeSurfer a2009s rh S calcarine thickness

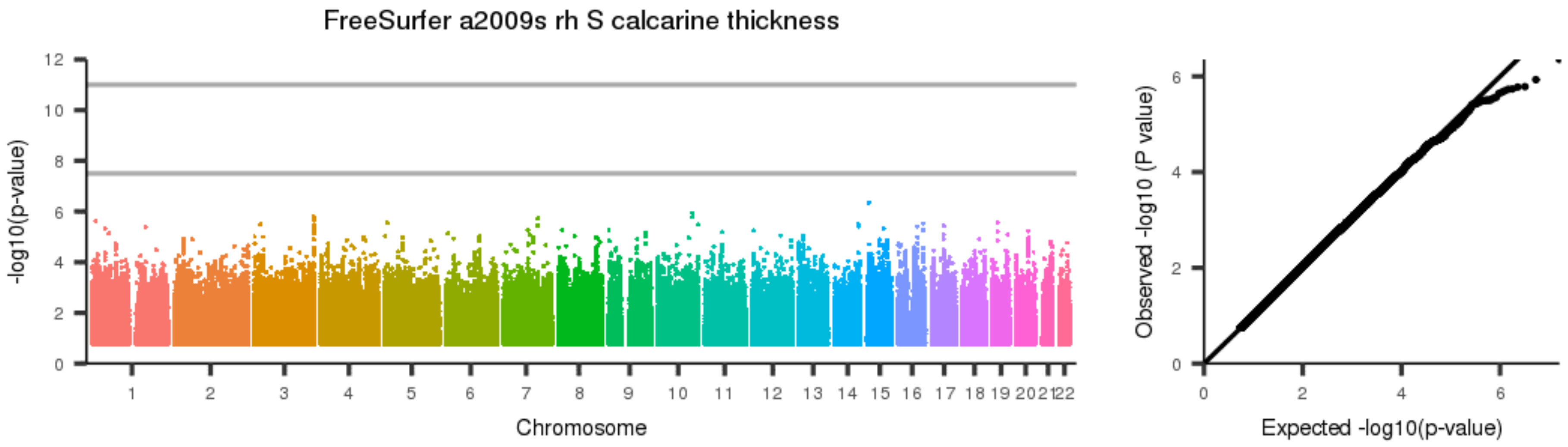

back to list of plots

Figure 1414: FreeSurfer a2009s rh S central thickness

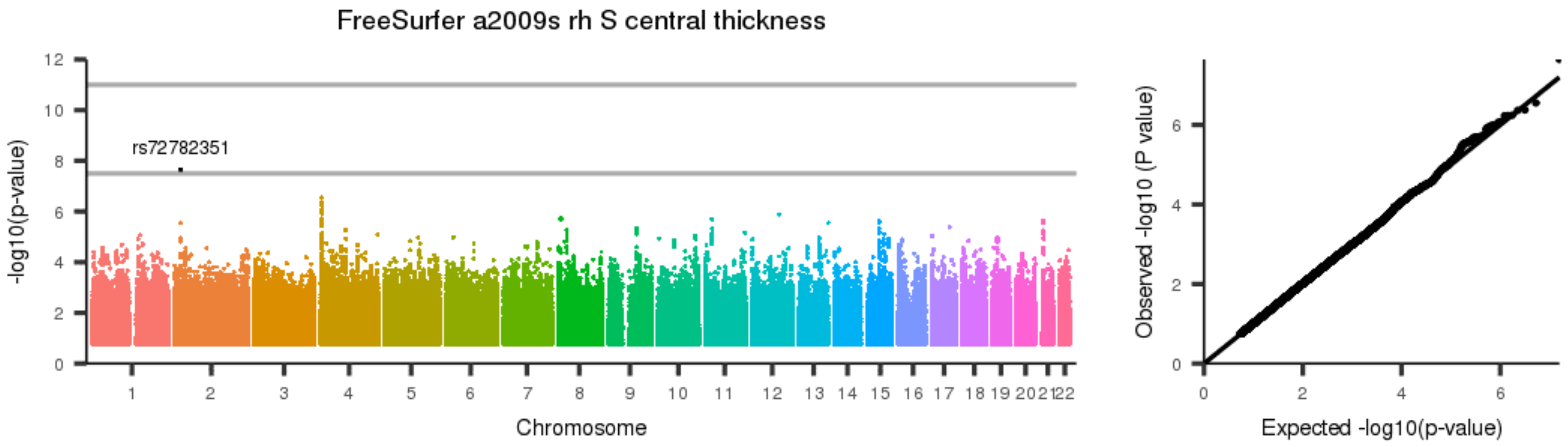

back to list of plots

Figure 1415: FreeSurfer a2009s rh S cingul-Marginalis thickness

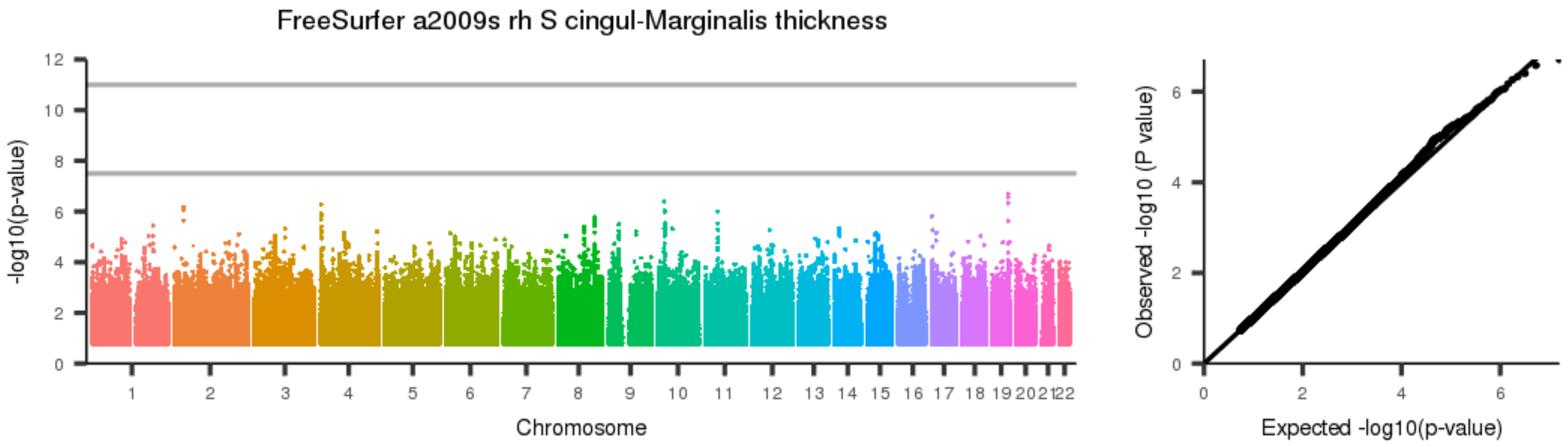

back to list of plots

Figure 1416: FreeSurfer a2009s rh S circular insula ant thickness

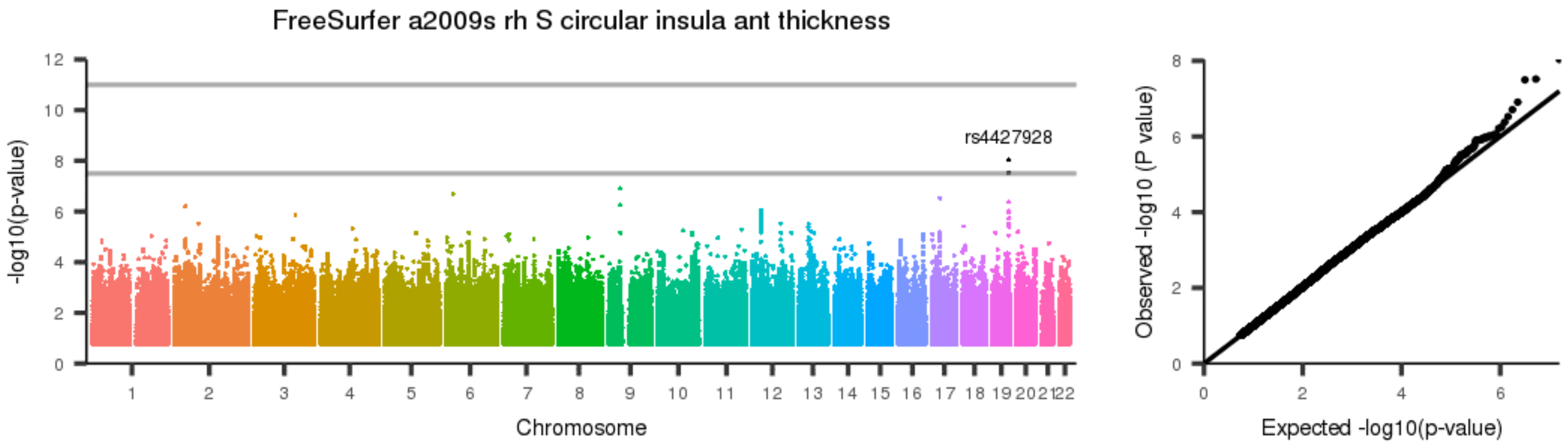

back to list of plots

Figure 1417: FreeSurfer a2009s rh S circular insula inf thickness

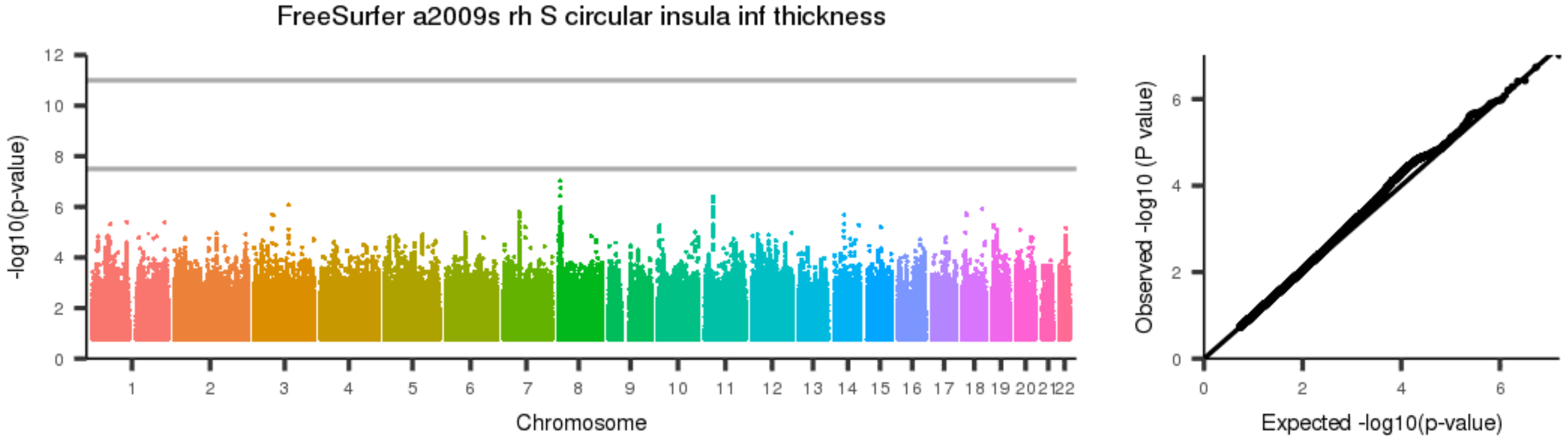

back to list of plots

Figure 1418: FreeSurfer a2009s rh S circular insula sup thickness

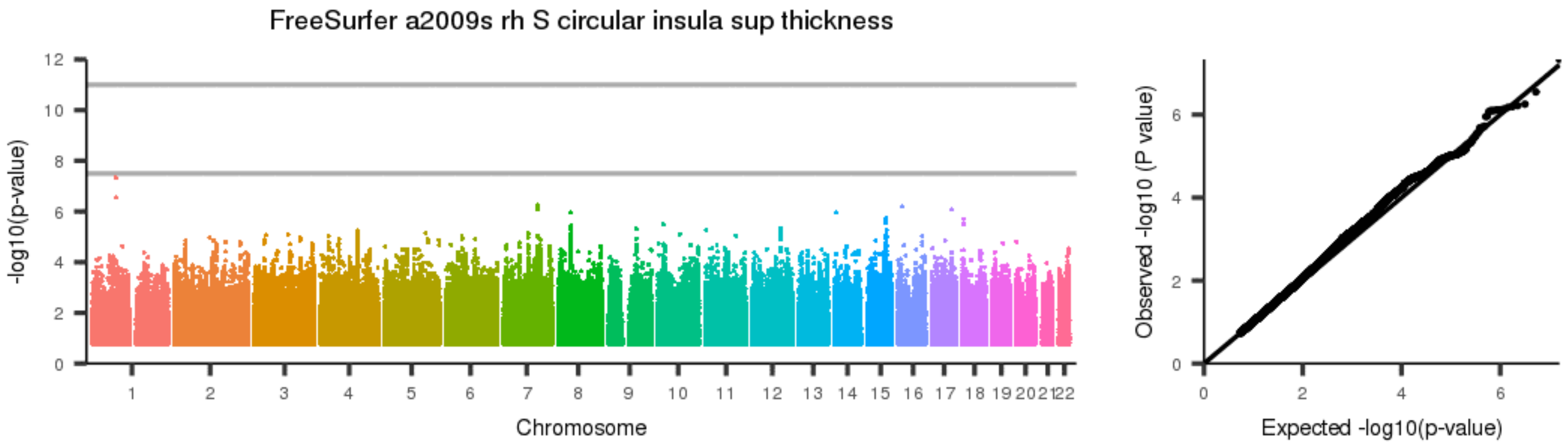

back to list of plots

Figure 1419: FreeSurfer a2009s rh S collat transv ant thickness

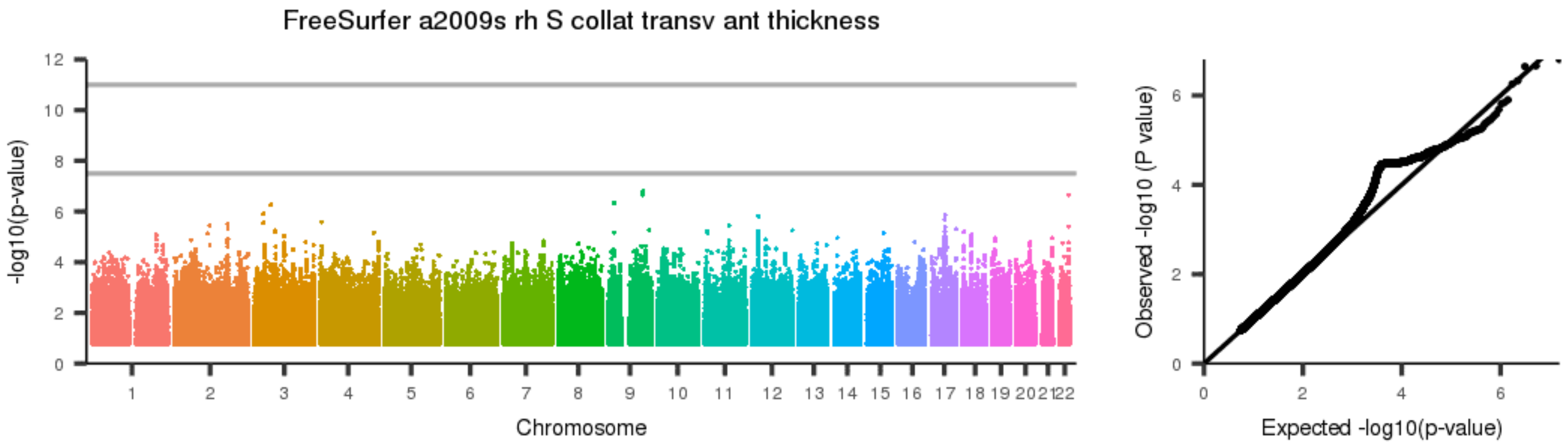

back to list of plots

Figure 1420: FreeSurfer a2009s rh S collat transv post thickness

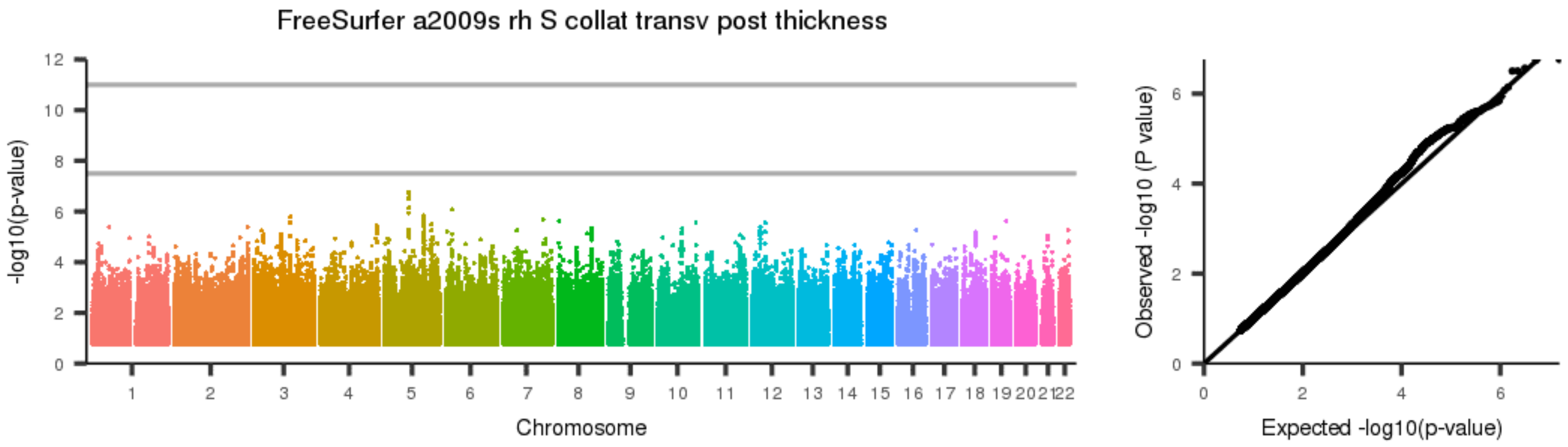

back to list of plots

Figure 1421: FreeSurfer a2009s rh S front inf thickness

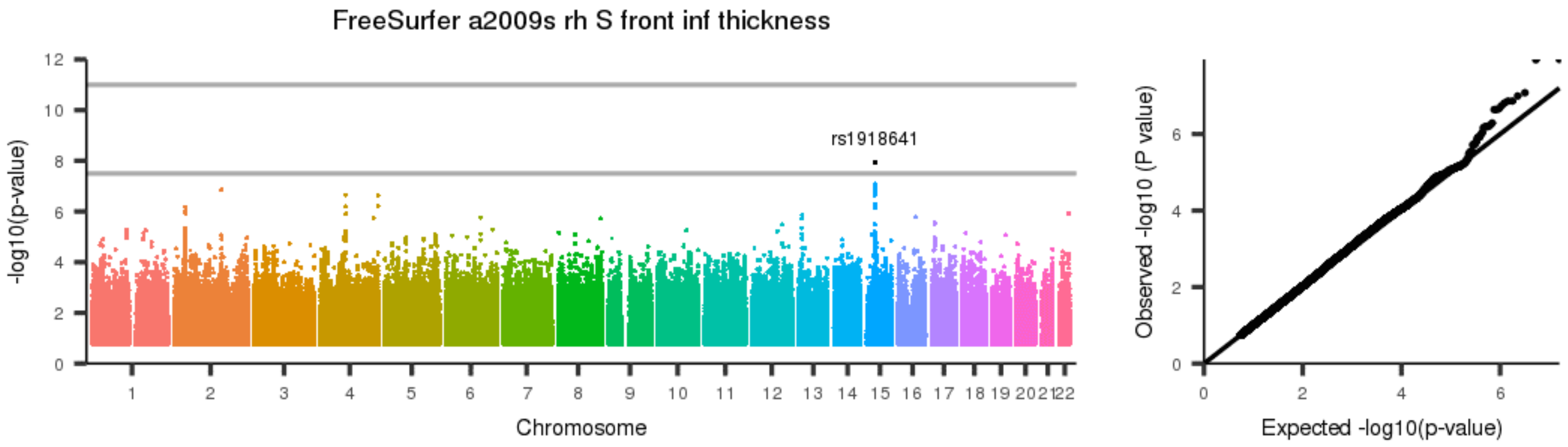

back to list of plots

Figure 1422: FreeSurfer a2009s rh S front middle thickness

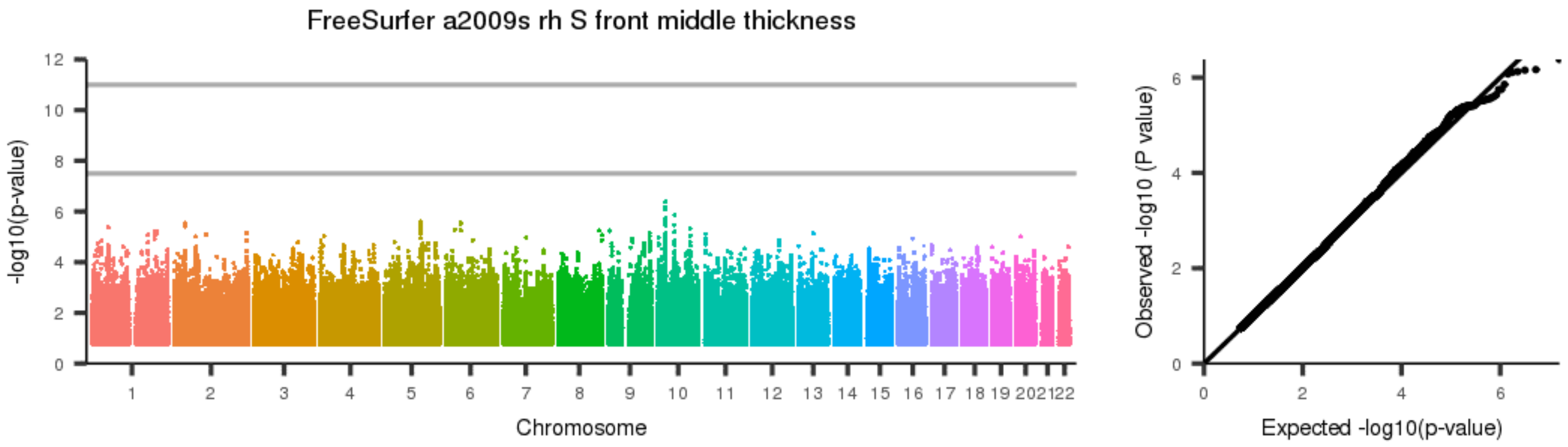

back to list of plots

Figure 1423: FreeSurfer a2009s rh S front sup thickness

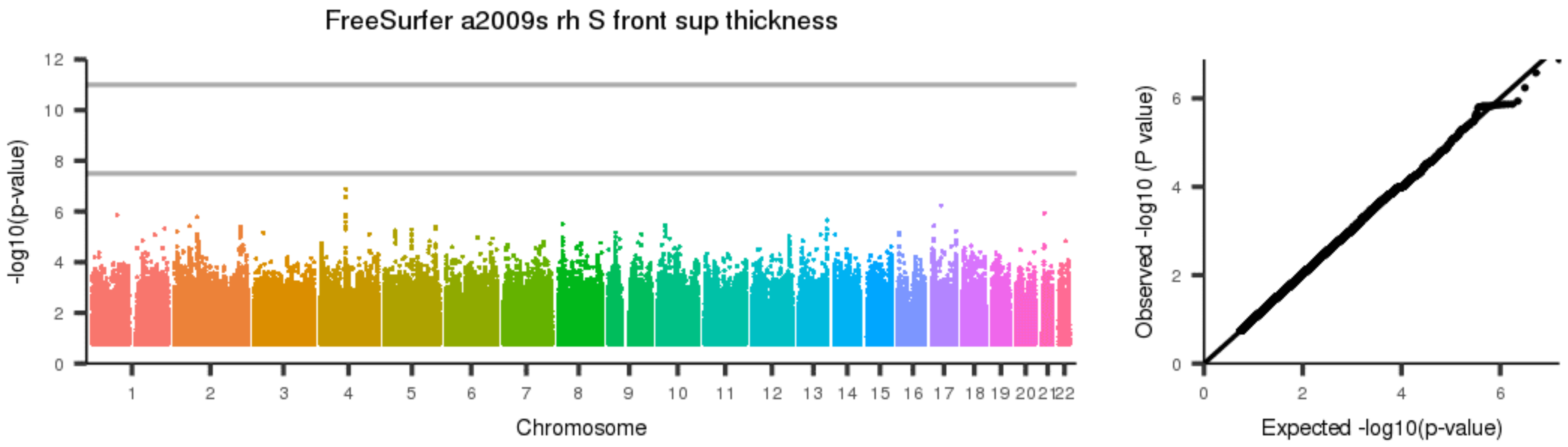

back to list of plots

Figure 1424: FreeSurfer a2009s rh S interm prim-Jensen thickness

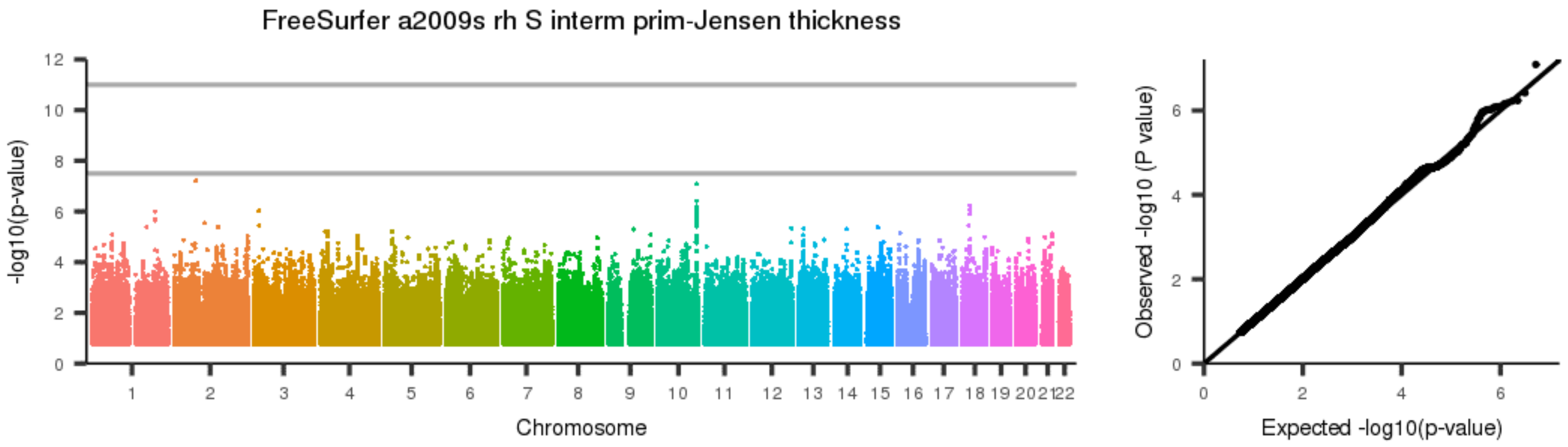

back to list of plots

Figure 1425: FreeSurfer a2009s rh S intrapariet and P trans thickness

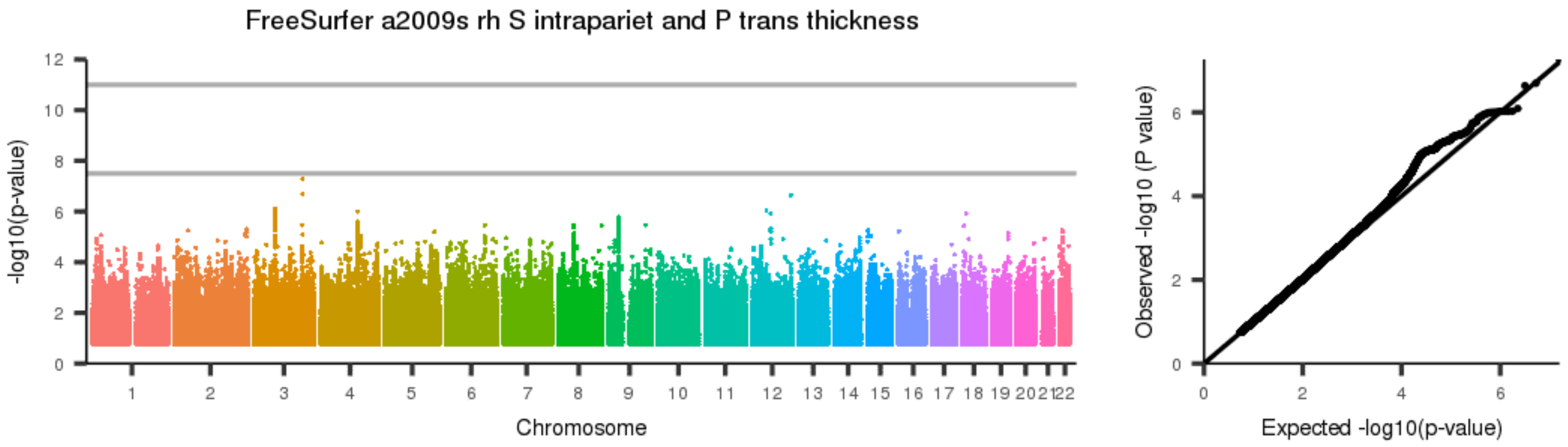

back to list of plots

Figure 1426: FreeSurfer a2009s rh S oc middle and Lunatus thickness

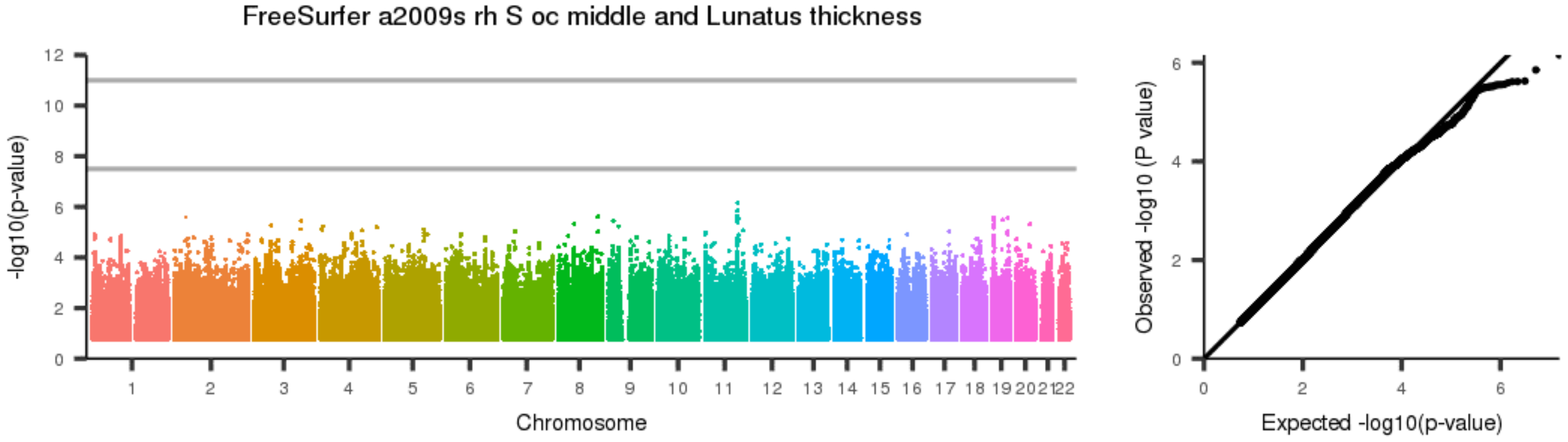

back to list of plots

Figure 1427: FreeSurfer a2009s rh S oc sup and transversal thickness

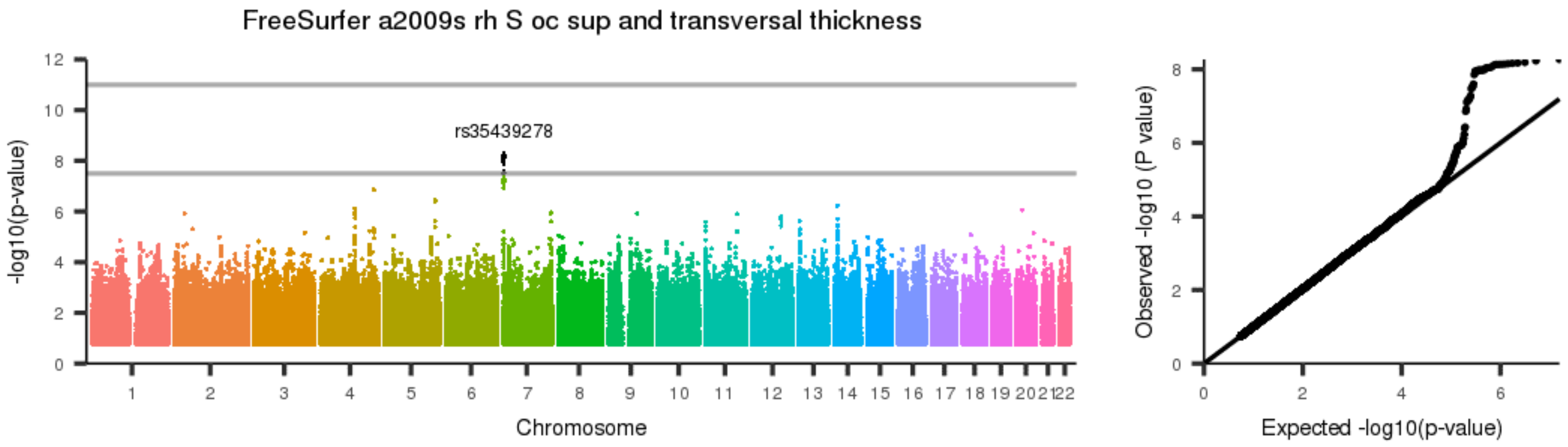

back to list of plots

Figure 1428: FreeSurfer a2009s rh S occipital ant thickness

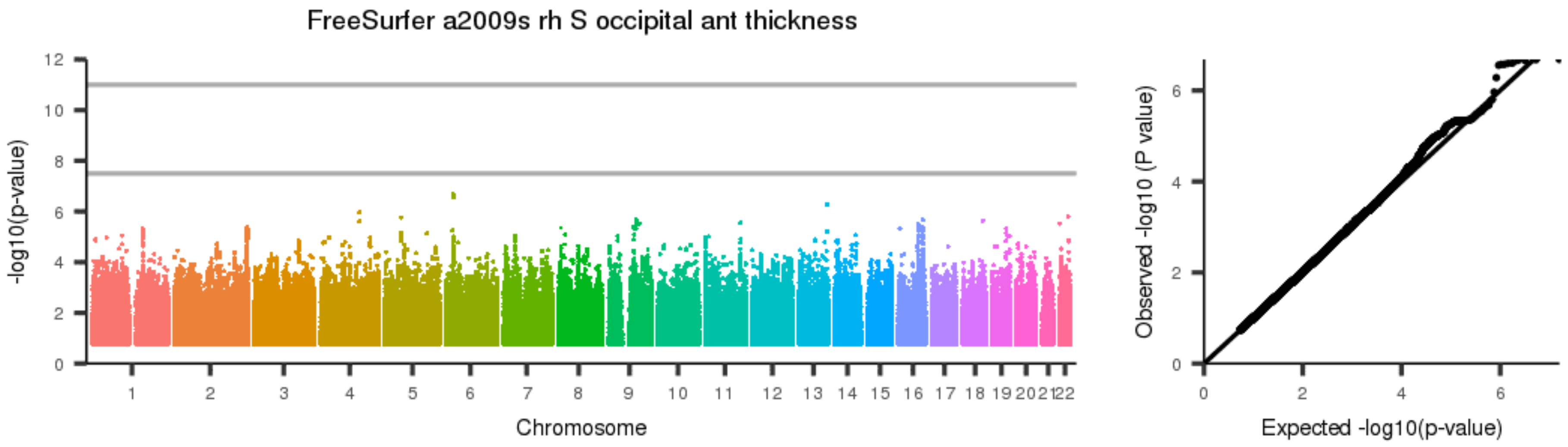

back to list of plots

Figure 1429: FreeSurfer a2009s rh S oc-temp lat thickness

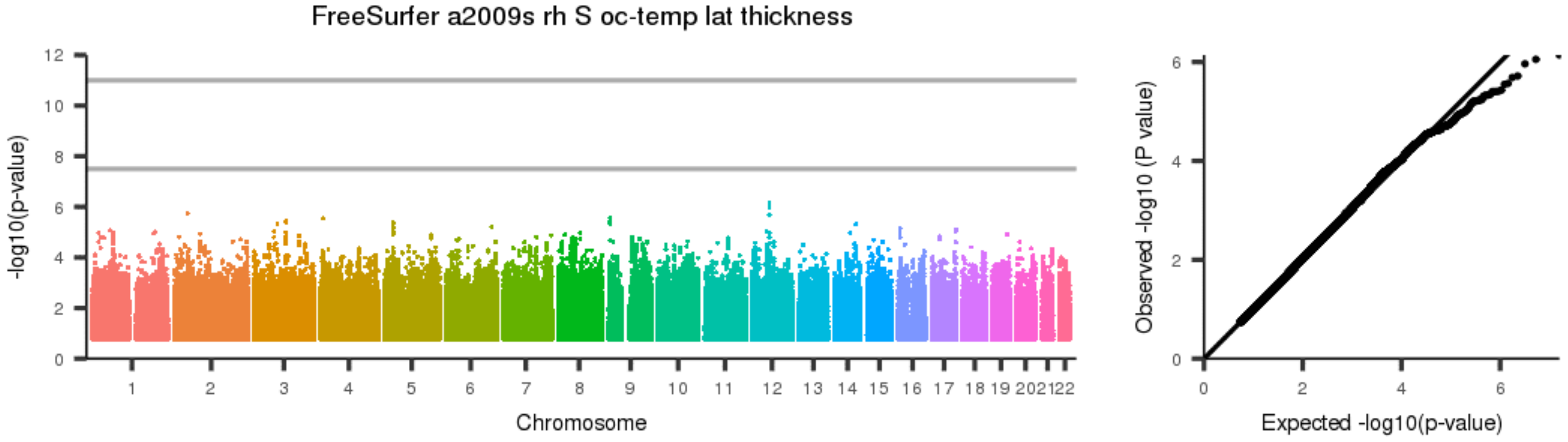

back to list of plots

Figure 1430: FreeSurfer a2009s rh S oc-temp med and Lingual thickness

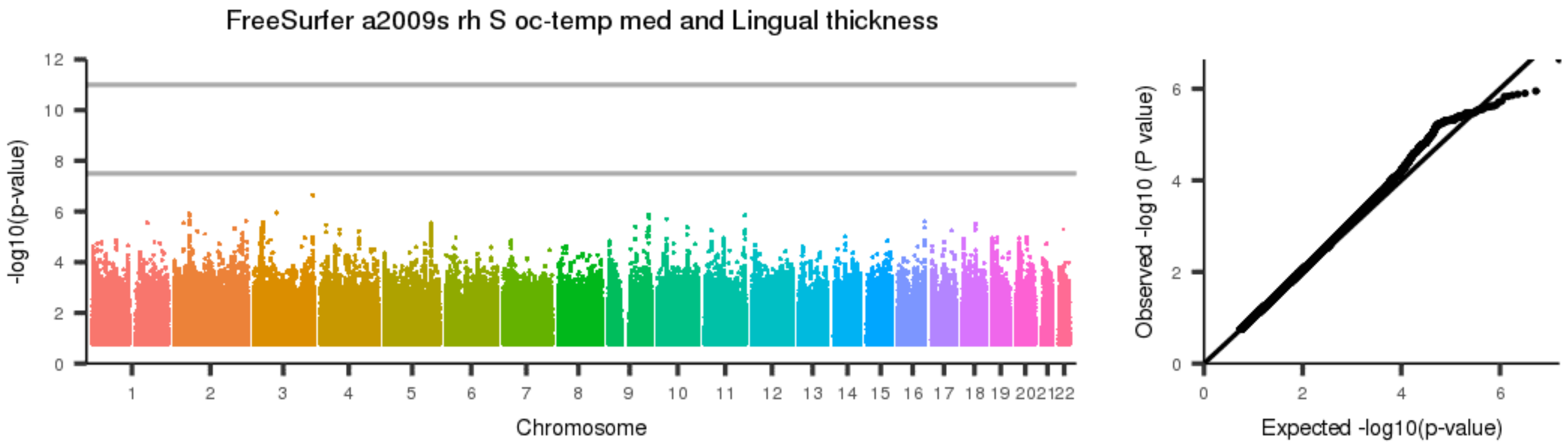

back to list of plots

Figure 1431: FreeSurfer a2009s rh S orbital lateral thickness

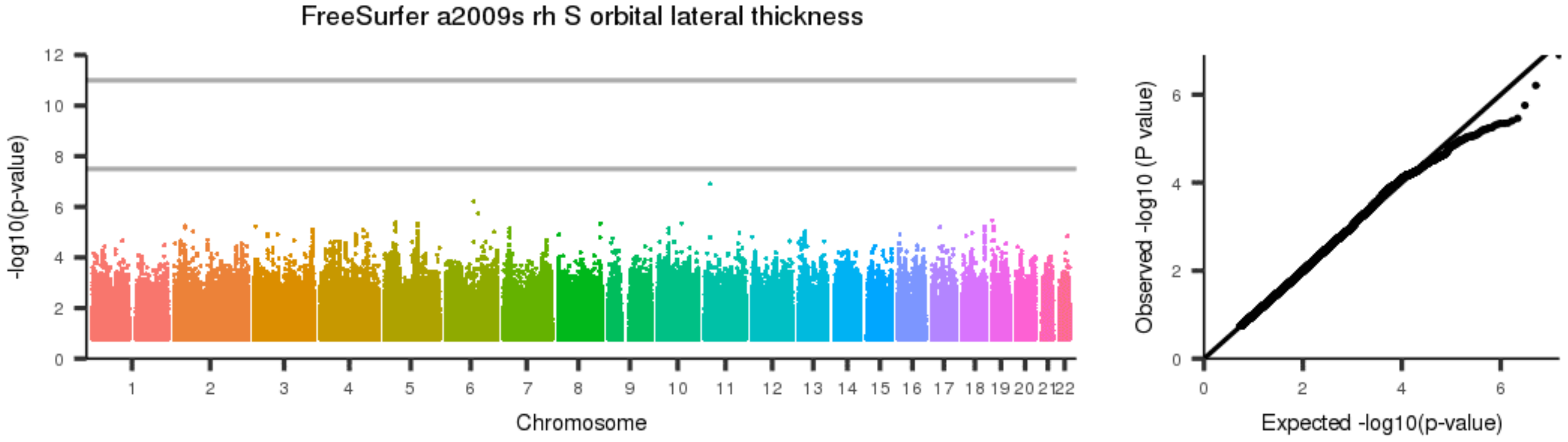

back to list of plots

Figure 1432: FreeSurfer a2009s rh S orbital med-olfact thickness

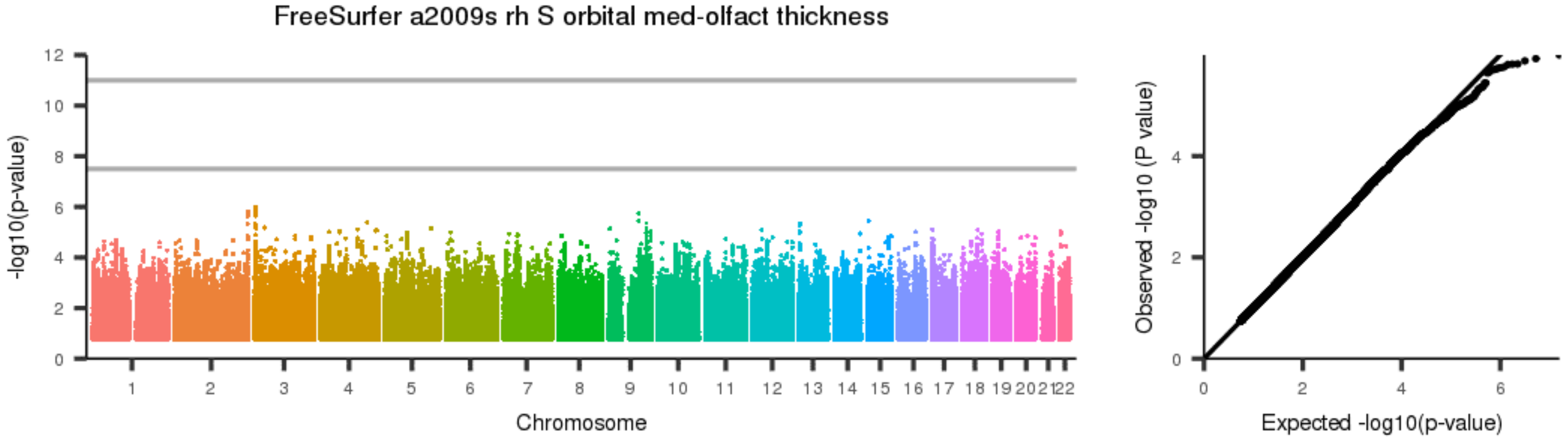

back to list of plots

Figure 1433: FreeSurfer a2009s rh S orbital-H Shaped thickness

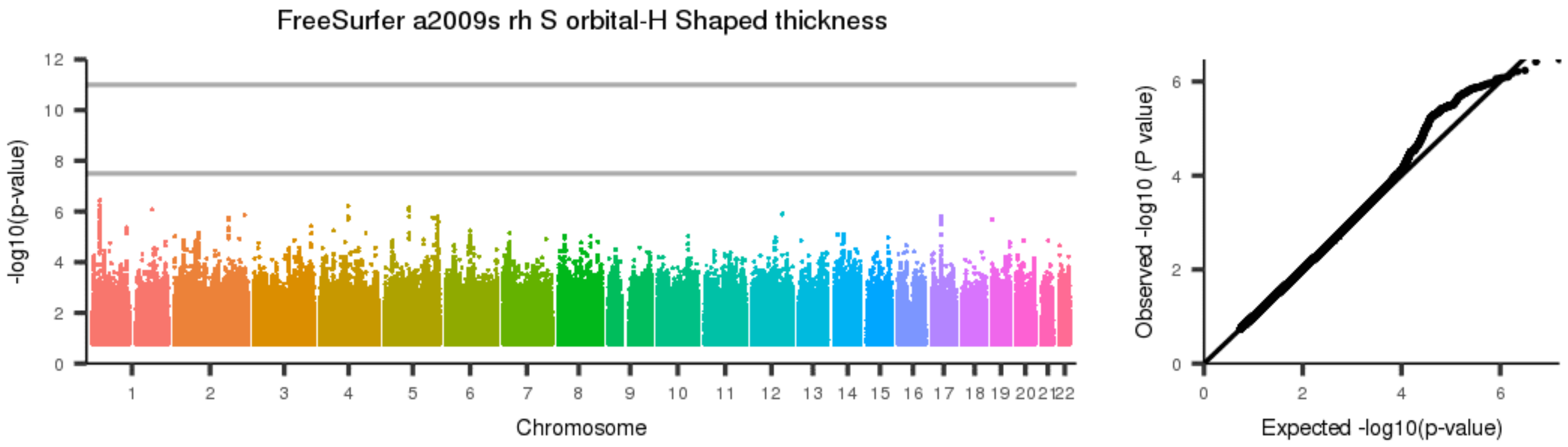

back to list of plots

Figure 1434: FreeSurfer a2009s rh S parieto occipital thickness

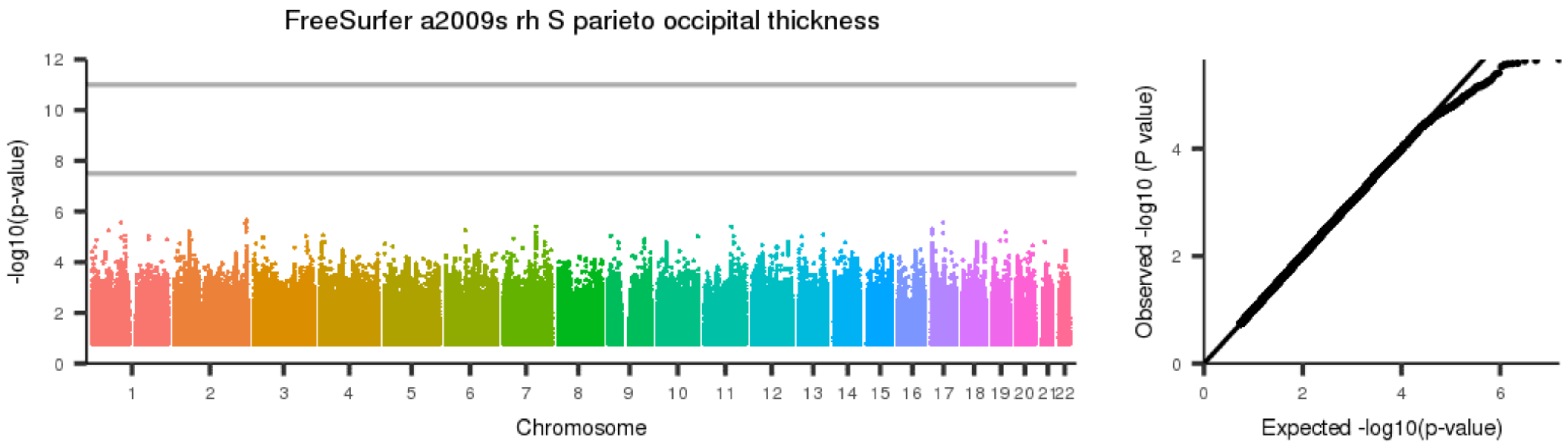

back to list of plots

Figure 1435: FreeSurfer a2009s rh S pericallosal thickness

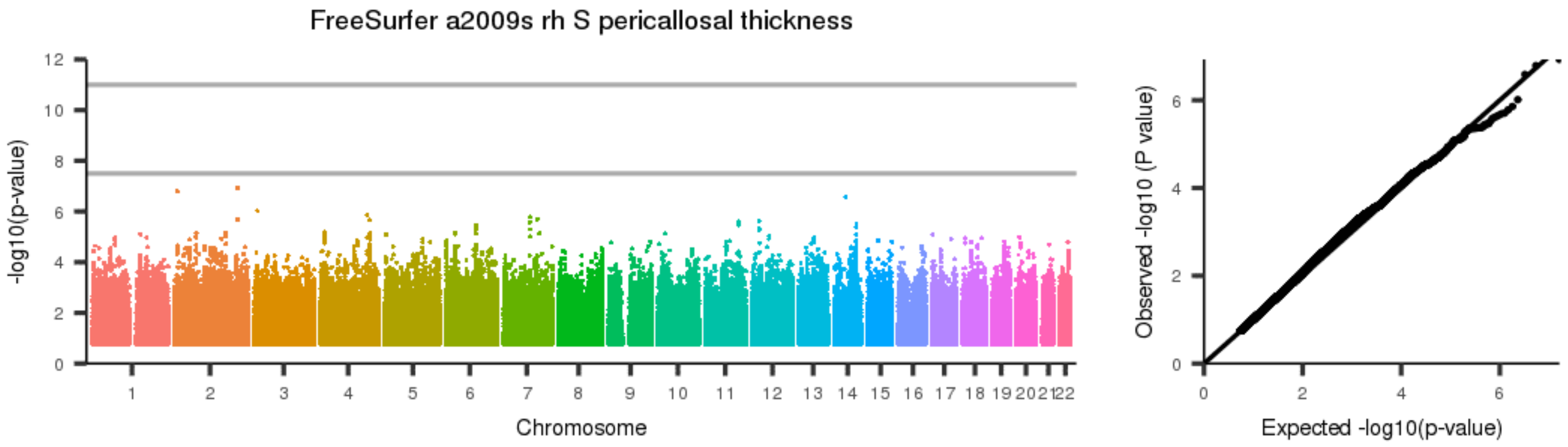

back to list of plots

Figure 1436: FreeSurfer a2009s rh S postcentral thickness

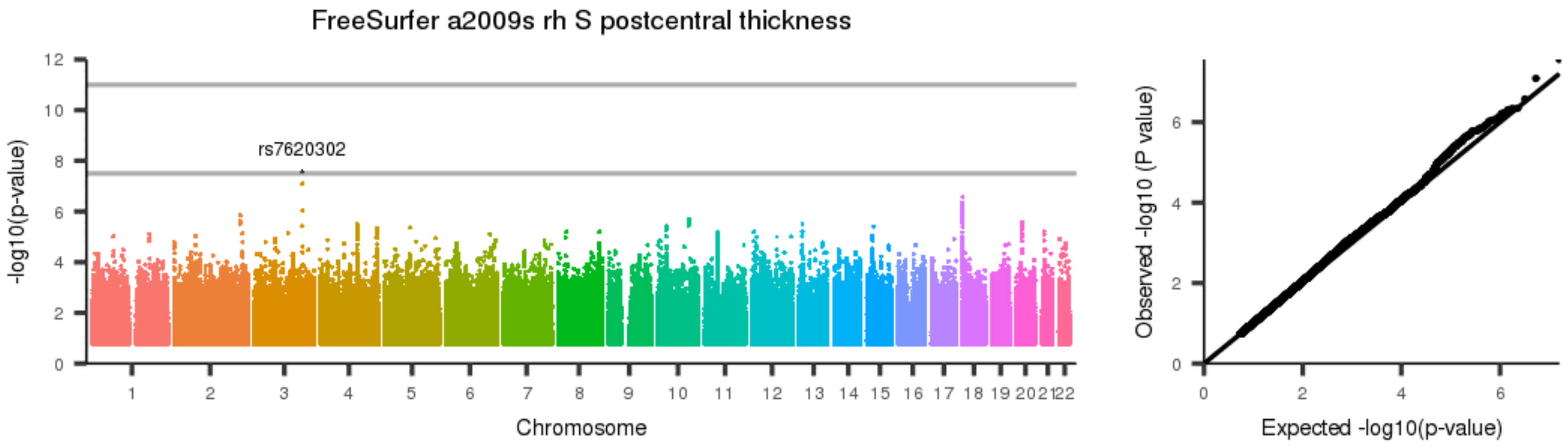

back to list of plots

Figure 1437: FreeSurfer a2009s rh S precentral-inf-part thickness

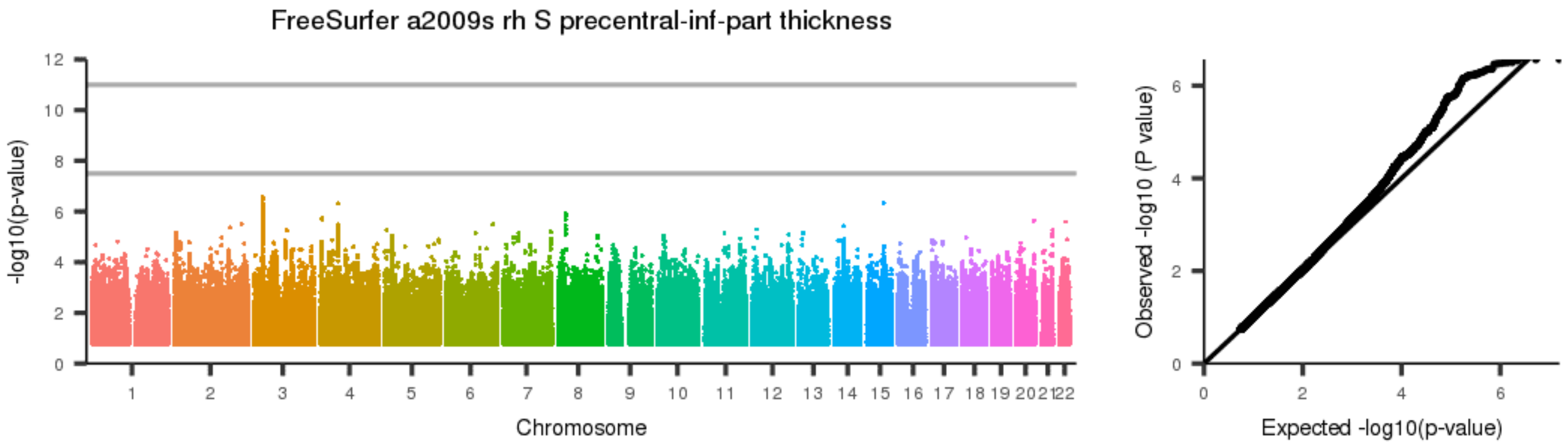

back to list of plots

Figure 1438: FreeSurfer a2009s rh S precentral-sup-part thickness

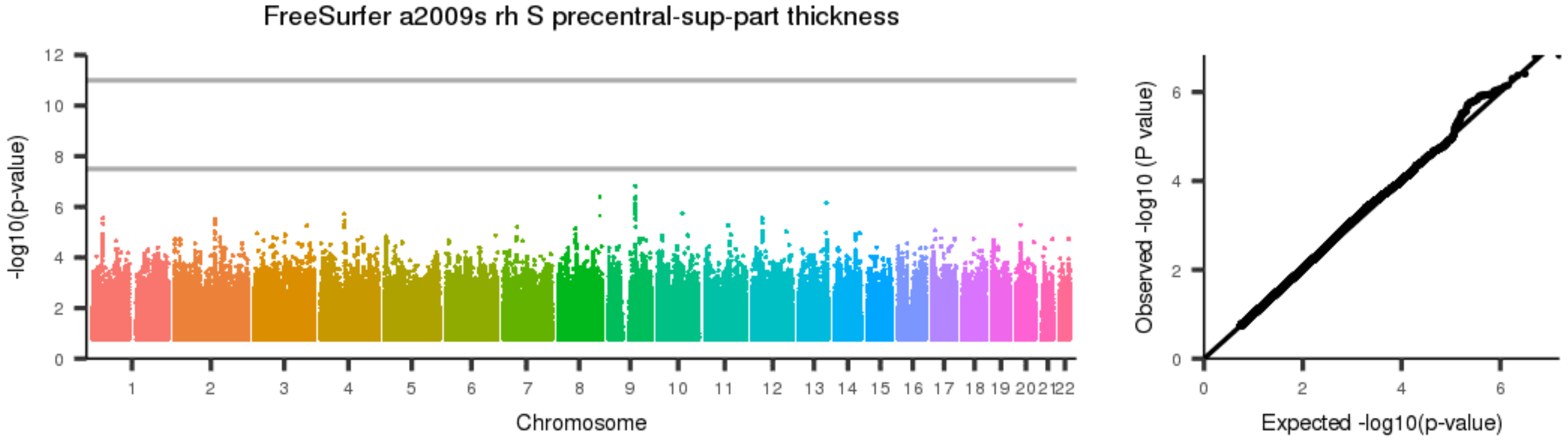

back to list of plots

Figure 1439: FreeSurfer a2009s rh S suborbital thickness

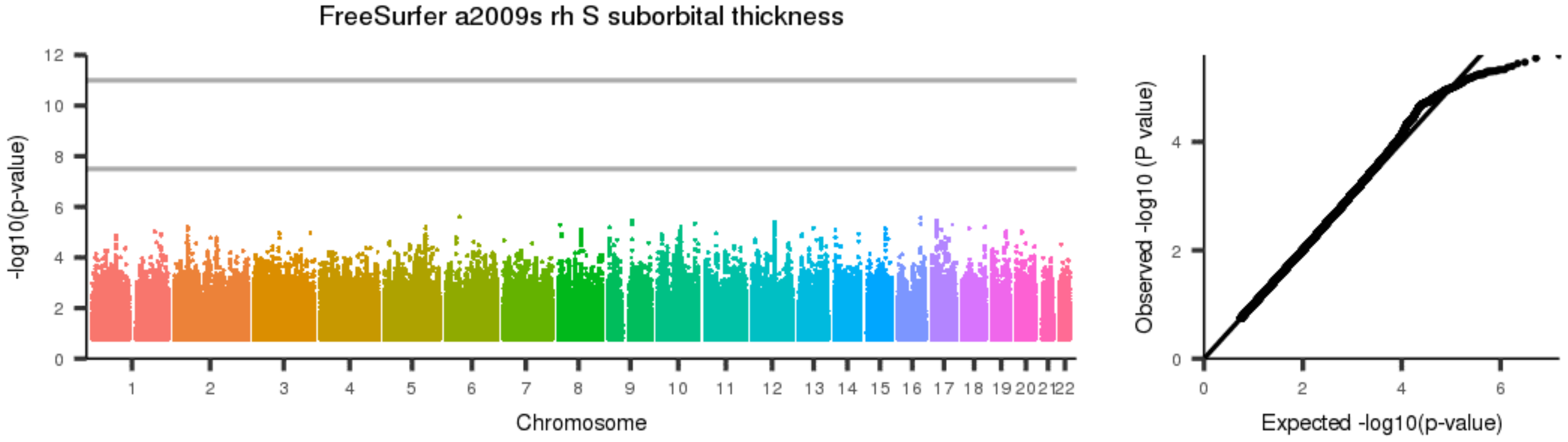

back to list of plots

Figure 1440: FreeSurfer a2009s rh S subparietal thickness

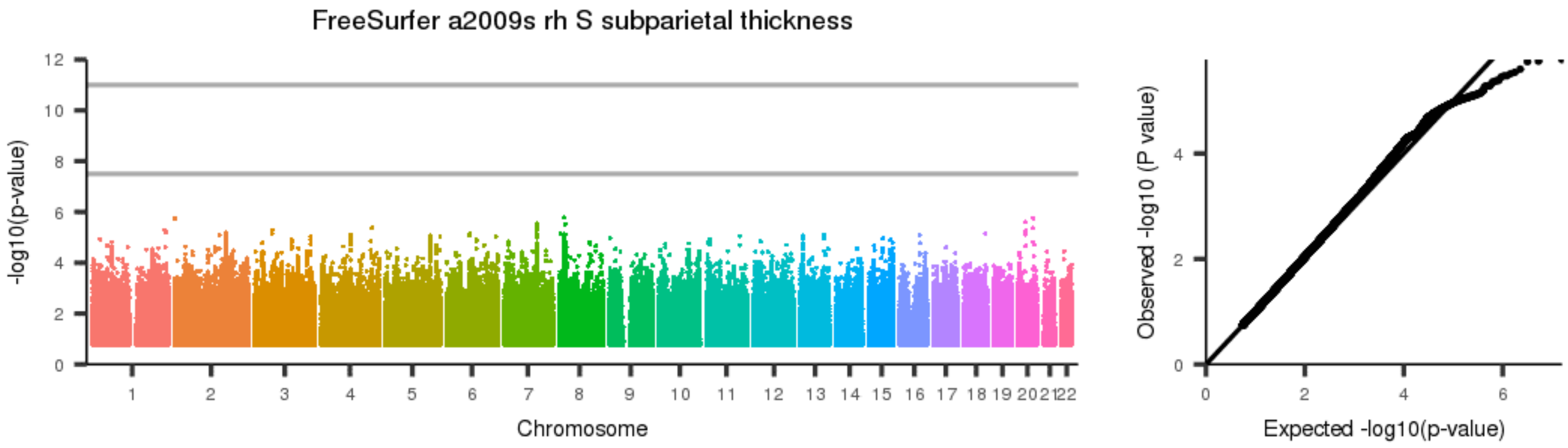

back to list of plots

Figure 1441: FreeSurfer a2009s rh S temporal inf thickness

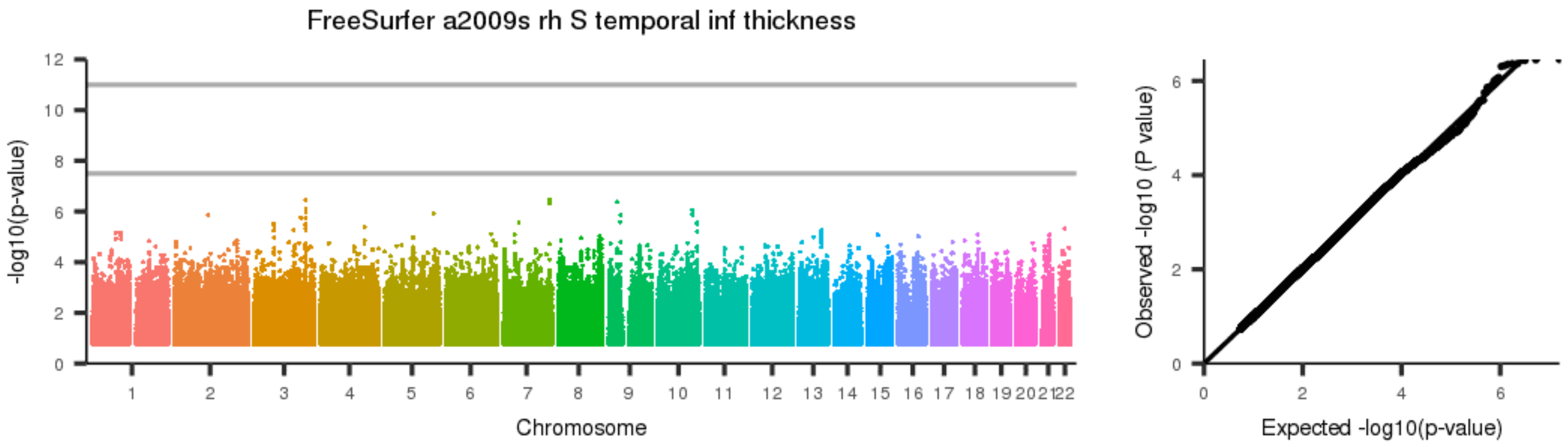

back to list of plots

Figure 1442: FreeSurfer a2009s rh S temporal sup thickness

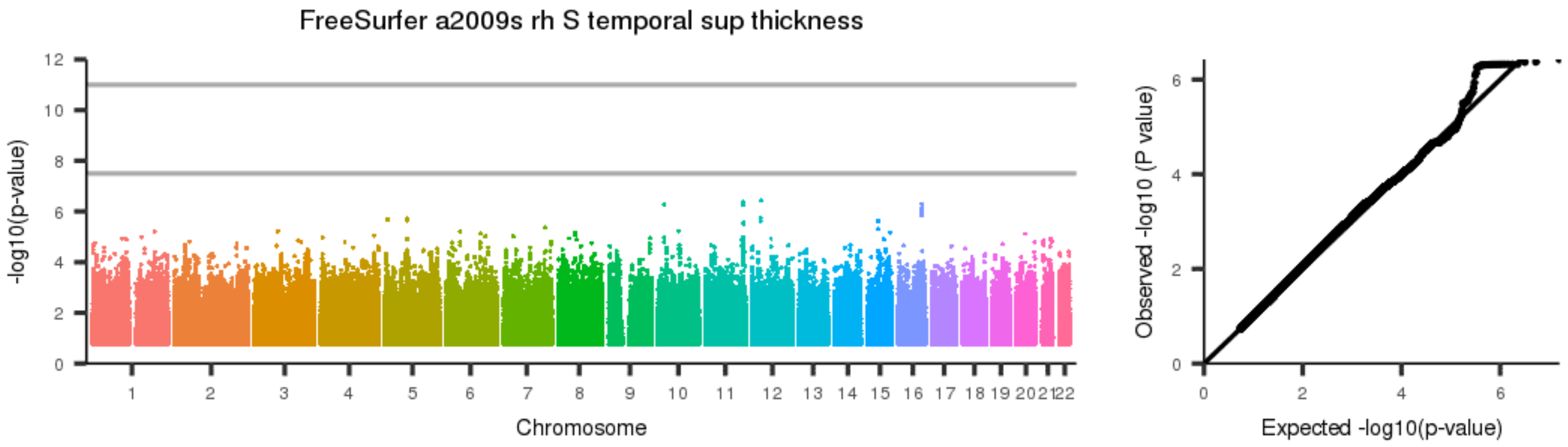

back to list of plots

Figure 1443: FreeSurfer a2009s rh S temporal transverse thickness

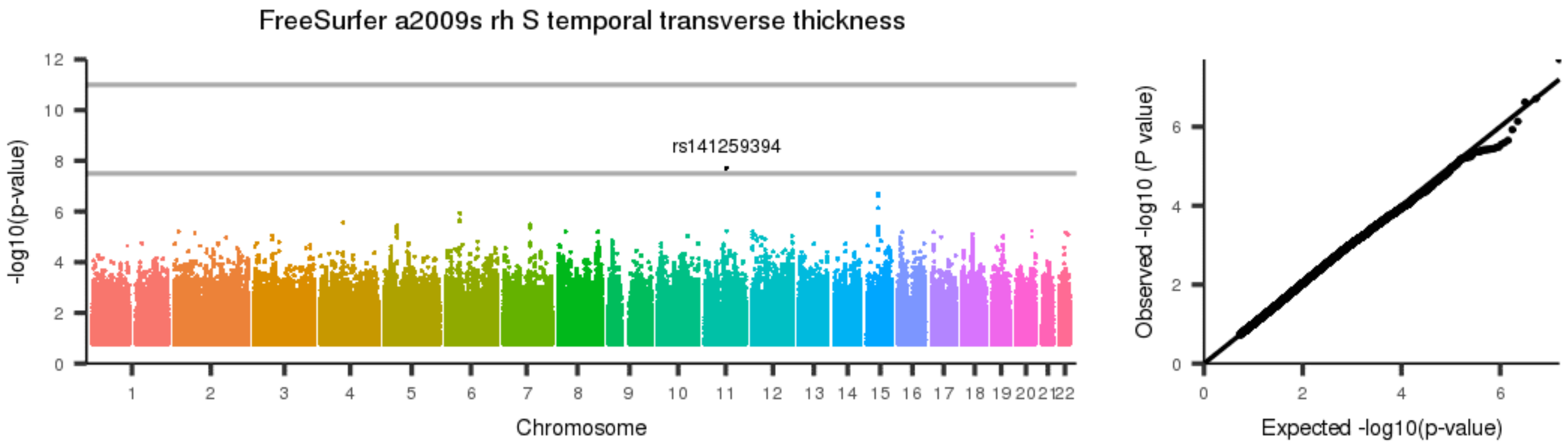

back to list of plots

Figure 1444: NETMAT edge ICA 1

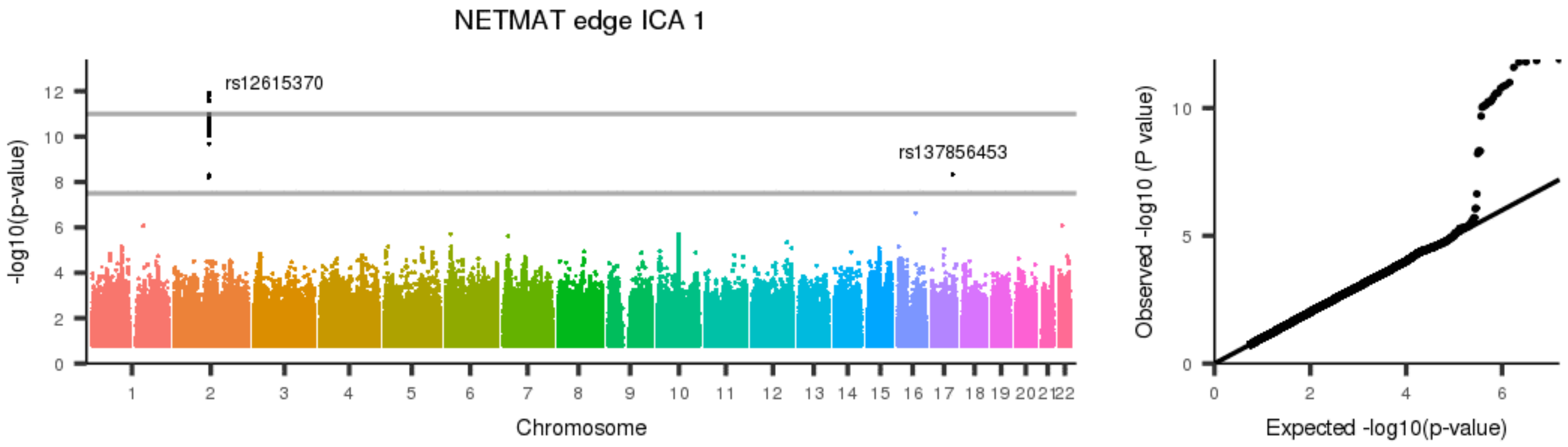

back to list of plots

Figure 1445: NETMAT edge ICA 2

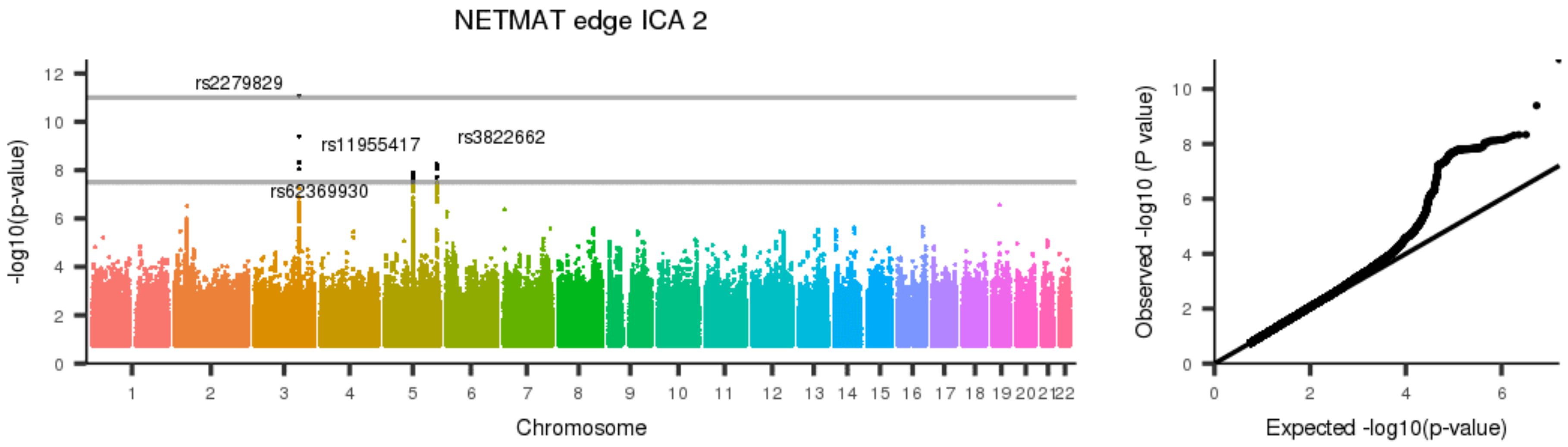

back to list of plots

Figure 1446: NETMAT edge ICA 3

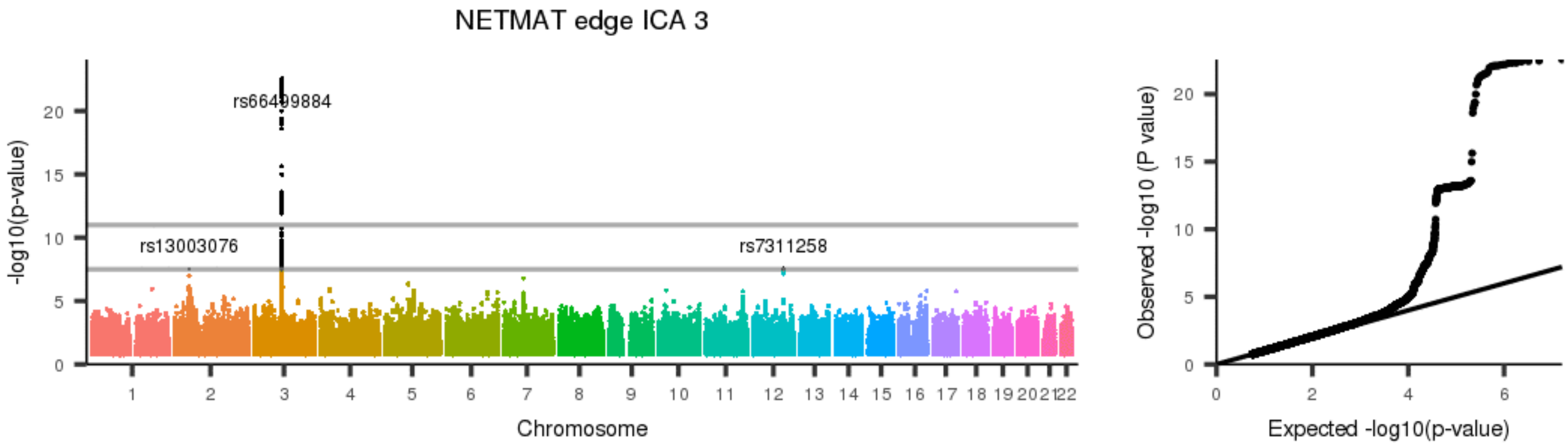

back to list of plots

Figure 1447: NETMAT edge ICA 4

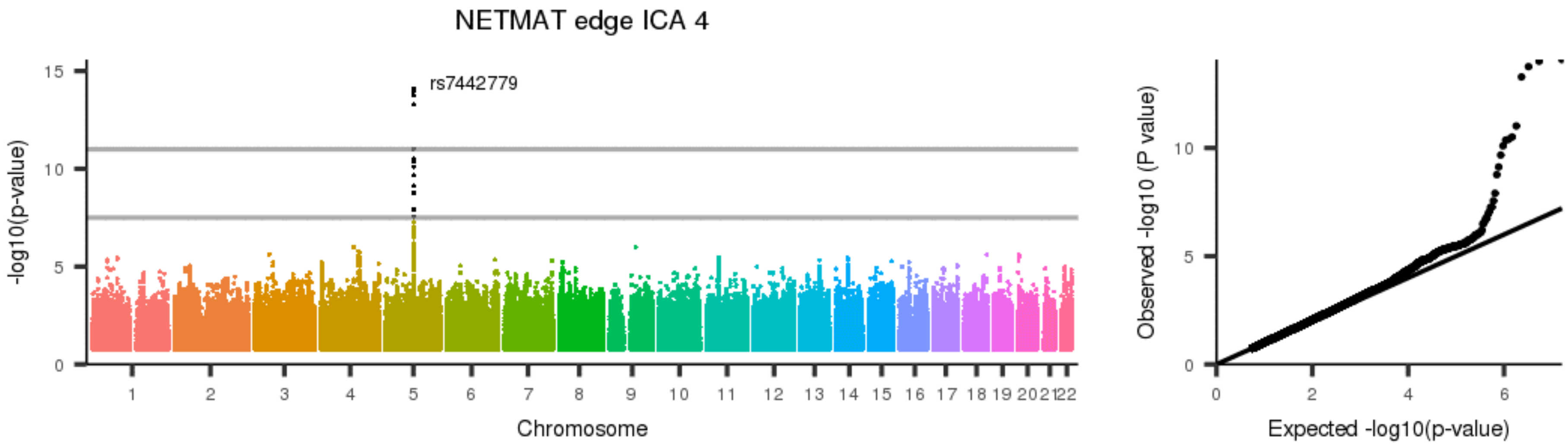

back to list of plots

Figure 1448: NETMAT edge ICA 5

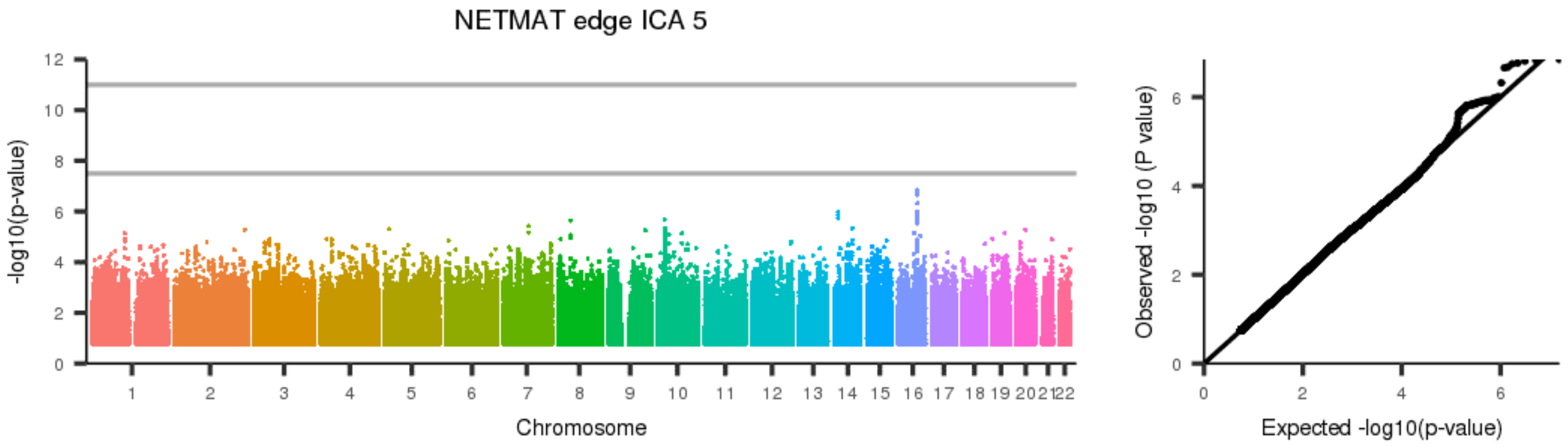

back to list of plots

Figure 1449: NETMAT edge ICA 6

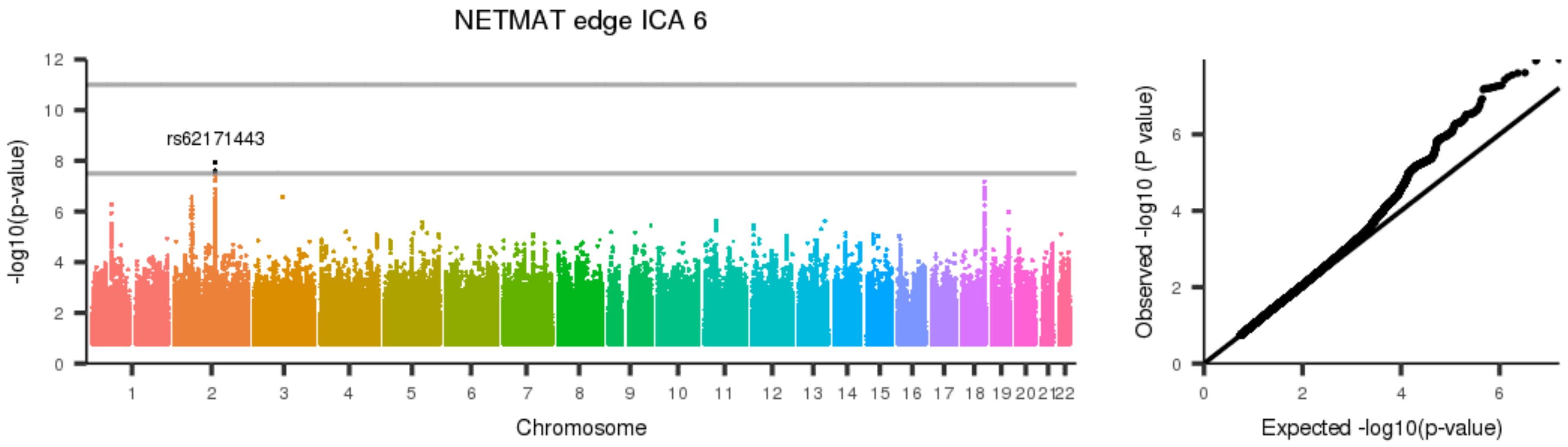

back to list of plots
